# Plant necrotrophic bacterial disease resistance phenotypes, QTL, and metabolites identified through integrated genetic mapping and metabolomics in *Solanum* species

**DOI:** 10.3389/fpls.2024.1336513

**Published:** 2024-03-05

**Authors:** Janak R. Joshi, Dev Paudel, Ethan Eddy, Amy O. Charkowski, Adam L. Heuberger

**Affiliations:** ^1^ Department of Plant Sciences & Plant Pathology, Montana State University, Bozeman, MT, United States; ^2^ Department of Horticulture & Landscape Architecture, Colorado State University, Fort Collins, CO, United States; ^3^ Department of Environmental Horticulture, University of Florida Gulf Coast Research and Education Center, Wimauma, FL, United States; ^4^ Department of Agricultural Biology, Colorado State University, Fort Collins, CO, United States

**Keywords:** potato, soft rot and blackleg resistance, virulence screening, QTL, metabolomics, genetic mapping, pectobacterium

## Abstract

Most food crops are susceptible to necrotrophic bacteria that cause rotting and wilting diseases in fleshy organs and foods. All varieties of cultivated potato (*Solanum tuberosum* L.) are susceptible to diseases caused by *Pectobacterium* species, but resistance has been demonstrated in wild potato relatives including *S. chacoense*. Previous studies demonstrated that resistance is in part mediated by antivirulence activity of phytochemicals in stems and tubers. Little is known about the genetic basis of antivirulence traits, and the potential for inheritance and introgression into cultivated potato is unclear. Here, the metabolites and genetic loci associated with antivirulence traits in *S. chacoense* were elucidated by screening a sequenced *S. tuberosum* x *S. chacoense* recombinant inbred line (RIL) population for antivirulence traits of its metabolite extracts. Metabolite extracts from the RILs exhibited a quantitative distribution for two antivirulence traits that were positively correlated: quorum sensing inhibition and exo-protease inhibition, with some evidence of transgressive segregation, supporting the role of multiple loci and metabolites regulating these resistance-associated systems. Metabolomics was performed on the highly resistant and susceptible RILs that revealed 30 metabolites associated with resistance, including several alkaloids and terpenes. Specifically, several prenylated metabolites were more abundant in resistant RILs. We constructed a high-density linkage map with 795 SNPs mapped to 12 linkage groups, spanning a length of 1,507 cM and a density of 1 marker per 1.89 cM. Genetic mapping of the antivirulence and metabolite data identified five quantitative trait loci (QTLs) related to quorum sensing inhibition that explained 8-28% of the phenotypic variation and two QTLs for protease activity inhibition that explained 14-19% of the phenotypic variation. Several candidate genes including alkaloid, and secondary metabolite biosynthesis that are related to disease resistance were identified within these QTLs. Taken together, these data support that quorum sensing inhibition and exo-protease inhibition assays may serve as breeding targets to improve resistance to nectrotrophic bacterial pathogens in potato and other plants. The identified candidate genes and metabolites can be utilized in marker assisted selection and genomic selection to improve soft- rot and blackleg disease resistance.

## 1 Introduction

Many cultivated plants are susceptible to necrotrophic bacterial pathogens that infect stems and nutrient-rich organs. These bacteria can survive in the natural environment in soil, alternative hosts, irrigation water, and plant debris. Bacteria including *Pectobacterium* and *Dickeya* spp., enter plants through wounds or natural openings and these pathogens may stay latent until favorable conditions occur, at which time the bacteria shift from latent to virulent states. This shift is mediated by acyl homoserine lactone mediated quorum sensing (Põllumaa et al., 2012). During pathogenesis, these bacteria colonize interior parts of the host plant and synthesize exo-enzymes, including pectate lyases, polygalacturonases, exo-proteases, and cellulases, that break down plant cell walls to obtain nutrients from the plant cell walls and from within plant cells (Davidsson et al., 2013). This results in a total collapse of the host tissue leading to fleshy organ decay and stem necrosis that cause major pre- and post-harvest losses. These necrotrophic pathogens are especially destructive due to their ability to remain latent, and infect a wide range of hosts (Ma et al., 2007). Notable, effective management strategies for these diseases are still lacking. Sanitation, quarantine, and exclusion practices are often ineffective as *Pectobacterium* and *Dickeya* spp. are prevalent in the environment. Moreover chemical management options can be phytotoxic, are not systemic, further limiting their efficacy. Consequently, the development of resistant cultivars is critical for improved disease management (Vyska et al., 2016).

Potato (*Solanum tuberosum* L.) is an integral part of the world’s agriculture ranking as third most important food crop. Potato has been cultivated for more than 8,000 years and during this time the crop has been continuously selected for many agronomic and nutritional traits through breeding. Despite these efforts, including disease-resistance breeding cultivated, potatoes remain vulnerable to necrotrophic bacterial pathogens like *Pectobacterium* and *Dickeya* spp. (known to cause soft rot, aerial stem rot, and blackleg in the potatoes).

In contrast, several wild *Solanum* relatives of cultivated potato are resistant to these diseases. Recent phenotyping studies support several independent mechanisms of resistance in these related species. For example, some wild potatoes have a rapid form of wound healing correlated with soft rot resistance (Chung et al., 2017). We recently characterized a different resistance mechanism that is driven by antimicrobial metabolites and proteins extracted from wild potato stems and tubers. These molecules, found in the wild potato *S. chacoense*, may inhibit virulence of *Pectobacterium* by reducing exo-enzyme activity, swimming motility, and quorum sensing (Joshi et al., 2021b, 2022). In these studies, chemical extracts were isolated from a single *S. chacoense* line M6. However, the genetics of *S. chacoense* that control these resistance phenotypes remain uncharacterized, as well as the plant metabolic processes that enable this type of biochemical resistance is understudied.

While cultivated potato shares many morphological and physiological traits with its wild diploid relatives, the tetraploid genetics and excessive heterozygosity of *S. tuberosum* makes it extremely difficult to map loci associated with disease resistance and to introgress traits from wild species. For necrotrophic bacteria, wild species do exhibit some variation in quantitative measures of resistance and the germplasm tends to be highly heterozygous (McCauley, 2021; Lebecka et al., 2021b). Subsequent genetic mapping within wild potato species shows that many necrotrophic bacterial resistance traits are quantitative (Jansky et al., 2014; Chung et al., 2017; Lebecka et al., 2021a; Ma et al., 2022), showing that bacterial disease resistance can be improved via introgression breeding. Currently, the potato industry is experiencing a revolutionary shift in breeding towards diploid genetics, and double monoploid lines (DM) are being used for research (Xu et al., 2011; Jansky et al., 2016; Bethke et al., 2022). These DM lines are still susceptible to pathogens, but they can be readily crossed with diploid wild potato species as an intermediate step in introgression breeding. While these genetic mapping populations tend to be small due to poor tuberization and fruit formation attributed to the mixing of very diverse genetics (*i.e.*, wild x domesticated species crosses), they can still be studied to understand the distribution of resistance traits, their qualitative or quantitative nature, and to begin to understand the integrative relationship of *Solanum* genes, metabolites, and resistance traits. Some studies have performed quantitative trait loci (QTL) analysis of DM x wild diploid *Solanum* populations and identified soft rot resistance across multiple chromosomes (Zimnoch-Guzowska et al., 2000; Lebecka et al., 2021a), although the links between these QTL and biochemical resistance traits such as with *S. chacoense* are unknown.

Here, we utilized a recombinant inbred line (RIL) population derived from a cross of *S. chacoense* M6 x *S. tuberosum* DM1 and evaluated the disease resistance performance of the population. This population is segregated for tuberization, tuber morphology, and stem thickness leading to no tubers, degenerative tubers, and thin stem, making typical virulence assays unreliable or impossible. Previously, we identified metabolite-based resistance in *S. chacoense* M6 that directly affects virulence of bacteria i.e quorum sensing and exo-protease (Joshi et al., 2021a). Therefore traits of interest in this study were the ability of metabolites from RIL population to inhibit quorum sensing (QS-I) and exo-protease activity (EP-I). Our investigation focused on determining whether QS-I and EP-I are quantitative or qualitative traits. Additionally, we explored the inheritance pattern of these traits in a biparental mapping population. Finally, we performed a non-targeted metabolomic analyses on the parents alongside transgressive segregants for the QS-I and EP-I traits to identify components of the potato metabolome that co-vary with the QS-I and EP-I resistance traits.

## 2 Materials and methods

### 2.1 Plant materials, bacterial strains, and chemicals

We generated a recombinant inbred line (RIL) population of potato by crossing *S. tuberosum* DM1-3 (as a female) with *S. chacoense* M6 (male) (McCauley, 2021; Jansky et al., 2024). For this population, a single F_1_ plant was self-pollinated to generate a large F_2_ population. Then the fertile individuals in this population were self-pollinated for 5 or 6 (F_5_/F_6_) generations to develop a potato RIL population consisting of approximately 100 inbred lines. Plants were obtained from the Jansky group and tubers from the F_5_/F_6_ RILs were planted in a greenhouse at the Plant Growth Facilities at Colorado State University, U.S.A. The temperature was set to 24°C and 18°C for day and night cycles respectively with a 16 h day length. Plants were grown in ProMix Bx General Purpose mix, fertilized with Osmocote Plus 15-9-12 (Scotts-MiracleGro, U.S.A.), and irrigated to saturation every other day until used for assays. Aphids and other pests were managed with Botaniguard ES and Molt-X (BioWorks, U.S.A.), Entrust SC (Corteva, U.S.A.), Distance IGR (Valent Biosicences, U.S.A.), Judo and Azatin (OHP Inc., U.S.A.), Avid 0.15EC (Syngenta, U.S.A.), and Compass (Bayer, U.S.A.) according to the manufacturer’s recommendations. Under greenhouse conditions, approximately half of the RILs had deleterious phenotypes including poor growth, no tuber formation, and self-degeneration of tubers for multiplying and continuing the population. Therefore, only 53 RILs were considered suitable for comparative phenotypic analysis. LC-MS-grade water, analytical-grade methanol, acetonitrile (ACN), and hydrochloric acid were purchased from Fisher Chemicals (Thermo Fisher Scientific, U.S.A.) for metabolite extractions. *P. brasiliense* strain Pb1692 was used for all resistance experiments in this study. Nutrient broth (NB), agar, and skim milk powder were purchased from Difco Laboratories (Thermo Fisher Scientific, U.S.A.). Bacteria were grown at 30°C under continuous shaking conditions.

### 2.2 Extraction of metabolites

Stems from six-week-old potato plants from each RIL and parent line were harvested, flash frozen in liquid nitrogen, and ground to a crude powder using mortar and pestle. We performed two biological replicates of extraction from two different plant lots. Ground samples were lyophilized for at least 12 h (HarvestRight, U.S.A.). The freeze-dried stem tissues were then ground to a fine powder using a coffee grinder. For metabolite extraction, 2 ml of 70:30 methanol/water (vol/vol) was added to a 100 mg of tissue, agitated for 2 h at 4°C using a vortex, and then sonicated for 5 min at room temperature. The mixture was then centrifuged at 6,000 × g for 20 min at 4°C and the supernatant was transferred to a new vial and dried under a stream of nitrogen gas (Organomation Associates Inc., U.S.A.). The dry matter (the metabolite extract) was weighed and resuspended in sterile dH_2_O.

### 2.3 Quorum sensing and exo-protease activity assays

Bacterial cultures were grown overnight in NB at 30°C under continuous shaking at 220 rpm. The cultures were centrifuged, and the cells were resuspended into sterile water. This cell suspension was used as a source of inoculum to test bacterial responses to metabolite extracts. Ten-milligrams of each metabolite extract were resuspended in 1 ml of sterile water and the metabolite extract was then inoculated with ~10^6^ CFU of bacteria (calculated using optical density, O.D., measurements – OD value of 1 at 600 nm equivalent to 10^9^ CFU). The metabolite plus bacteria suspensions were incubated for 15 h at 30°C under continuous shaking at 220 rpm. No difference in bacterial multiplication was observed (evaluated using O.D. measurements) (data not shown). These cultures were then centrifuged (8,000 × g, 5 min) to separate the supernatant from the Pb1692 cells. The supernatant was filter sterilized and used to measure quorum sensing activity (QS-A) via acyl homoserine lactone (AHL, using the reporter strain *Chromobacterium violaceum* - CV026) and exo-protease activity (EP-A). The quantitative metrics of these systems were performed in plate assays as previously described (Joshi et al., 2021b). Here, activity (A) is a unit of measure specific to each assay: pigmentation area for QS, and milk powder degradation area for EP. QS and EP inhibitory (I) activity was calculated as: I (%) = (DM1-A – Line-A)/DM1-A x 100, with DM1 as a susceptible control.

### 2.4 Non-targeted metabolomics

The metabolite extracts were analyzed with two independent platforms run in positive and negative mode for a total of four metabolomics data sets: reverse phase ultra high-performance liquid chromatography mass spectrometry (UHPLC-MS), and hydrophilic interaction chromatography HILIC-MS). Together these platforms capture a wide range of molecules with different chemistry. For UHPLC-MS, samples were injected in a randomized order using 1 µl injection volume into a Waters Acquity UPLC system. Separation was achieved using a Waters Acquity UPLC CSH Phenyl Hexyl column (1.7 µM, 1.0 x 100 mm, part number 186009478), using a gradient from solvent A (water, 0.1% formic acid, 2 mM ammonium hydroxide) to solvent B (acetonitrile, 0.1% formic acid). Injections were made in 99% A, held at 99% A for 1 min, ramped to 98% B over 12 minutes, held at 98% B for 3 minutes, and then returned to starting conditions over 0.05 minutes and allowed to re-equilibrate for 3.95 minutes, with a 200 µl/min constant flow rate. The column and samples were held at 65°C and 6°C, respectively. The column eluent was infused into a Waters Xevo G2-XS Q-TOF-MS with an electrospray source in negative and positive mode (as independent runs), scanning 50-1200 m/z at 0.1 seconds per scan, alternating between MS (6 V collision energy) and MSE mode (15-30 V ramp). Calibration was performed using sodium formate with 1 ppm mass accuracy. The capillary voltage was held at 700 V, source temperature at 150°C, and nitrogen desolvation temperature at 600°C with a flow rate of 1000 L/hr. Quality control was performed in both phases and modes by running quality control samples after every 4 or 5 experimental samples. For HILIC-MS, separation was achieved using a Waters Acquity Premier BEH Amide column with built-in fit guard column (1.7 µM, 2.1 x 100 mm, part number 186009508), using a gradient from solvent B (95% acetonitrile, 5% water, 0.1% formic acid, 10 mM ammonium hydroxide) to solvent A (water, 0.1% formic acid, 10mM ammonium hydroxide). Injections were made in 90% B, held at 90% B for 0.5 minutes, ramped to 25% A over 6.50 minutes, ramped to 50% A over 2 minutes, ramped to 85% A over one minute, held at 85% A for 0.50 minutes, returned to starting conditions over one minute, and allowed to re-equilibrate for 3.50 minutes, with a 500 µl/min constant flow rate. The column and samples were held at 30°C and 6°C, respectively. The column eluent was infused into a Waters Xevo G2-XS Q-TOF-MS with an electrospray source in positive and negative ionization mode (as independent runs), scanning 50-1200 m/z at 0.1 seconds per scan, alternating between MS (6 V collision energy) and MSE mode (15-30 V ramp). Calibration was performed using sodium formate with 1 ppm mass accuracy. The capillary voltage was held at 700 V (positive ionization mode) or 1800 V (negative ionization mode), source temperature at 150°C, and nitrogen desolvation temperature at 600°C with a flow rate of 1000 L/hr.

For processing, data were converted from Waters.RAW to.mzML using Proteowizard MSConvert version 3.0.20154, and peak detection, detection, alignment, grouping, retention time correction, and peak filling was performed using XCMS in R (Smith et al., 2006). Deconvolution and normalization were performed using the RAMClust package in R (Broeckling et al., 2014). Interpretation of spectra was done using the R package Interpret MS Spectrum (Lai et al., 2018). Spectral clusters with their spectral abundance were exported to MS Excel for further analysis. The information was used to match the external databases and tools such as MS-Finder (Tsugawa et al., 2016), SIRIUS (Dührkop et al., 2019), HMDB (Wishart et al., 2022) and MassBank of North America for annotation.

### 2.5 Construction of linkage map and QTL mapping

Genotyping by sequencing (Elshire et al., 2011) was done on the genomic DNA of the parents and the RIL digested with restriction enzyme as mentioned in Jansky et al (Jansky et al., 2024). Raw reads were trimmed for quality and adapter sequences were trimmed using Trimmomatic (Bolger et al., 2014). High quality reads were aligned to the potato genome using BWA-MEM and SNPs were called using Freebayes. SNPs were filtered with a minimum quality of 20. Filtered SNP data was utilized for genetic linkage analysis using OneMap R for inbred based populations (Margarido et al., 2007). The marker positions were based on the reference genome DM_1-3_516_R44 – v6.1 (Pham et al., 2020). QTL identification was done with composite interval mapping in Windows QTL Cartographer v 2.5 011 (Wang et al., 2012). Mean values of QS-A, QS-I, EP-A, and EP-I across 8 replicates were used for QTL analysis. The sliding window for all traits was 1 cM. A forward and backward stepwise regression method with a probability of 0.1 and a window size of 5 cM were utilized to determine cofactors. LOD thresholds for significance was selected by using the thousand-permutation test to each data set (*p ≤* 0.05) (Churchill and Doerge, 1994). For each QTL, the 95% confidence interval was calculated using a 2-LOD support interval (van Ooijen, 1992).

### 2.6 Mapping genes associated with metabolites/metabolic pathways

Metabolites strongly associated with disease resistance were traced for their metabolic pathways and chromosomal location of pathway genes using plant metabolic pathway database PotatoCyc (SolCyc Biochemical Pathways) (Fernandez-Pozo et al., 2015). Associated genes in metabolic pathways were mapped to chromosomes of *Solanum tuberosum* group phujera DM1-3 (v4.03, id52025) using a visualization tool Phenogram (https://visualization.ritchielab.org/ Ritchie Lab, University of Pennsylvania).

### 2.7 Statistical analysis

RIL phenotypic data were analyzed with GraphPad Prism v10 (Dotmatics, Boston, MA, U.S.A.) including histograms by frequency distribution binning and establishing nonlinear fit lines. One-way Analysis of Variance (ANOVA) tests were conducted to compare lines with Tukey *post-hoc* tests on QS-A and EP-A data with a *p* threshold of 0.05, and Spearman’s rank correlation was performed in Graphpad Prism v10. Data was tested for normality using the Kolmogorov-Smirnov test with a *p* threshold of 0.05. QS-I and EP-I were calculated as [DM1 activity – Line activity]/[DM1 activity] x 100%. Metabolomics data was analyzed using SIMCA v17.0 (Sartorius AG, Gottingen, Germany). Orthogonal Partial Least Square (OPLS) models were developed using the two-way Orthogonal Partial Least Square (O2PLS) workflow (Bouhaddani et al., 2016) by regressing the QS-A and EP-A data (two independent y’s) against the metabolite data (x) in a single model and establishing two components to explain joint (Component 1) and unique variation (Component 2). All multivariate data was z scaled. Cross-validation was performed using the 1/7^th^ leave out approach and reported as predictive power ranging between 0-100% (Q^2^). Univariate metabolite z transformations were performed by comparing metabolite abundances of lines to the mean of lines classified as “susceptible” based on QS-A and EP-A values that were not different from DM1, with a significance threshold of z > 1.96 corresponding to p < 0.05 indicating an association to resistance. All graphs were illustrated using Graphpad Prism.

## 3 Results

### 3.1 Exo-enzyme and quorum sensing inhibition were distributed as quantitative traits in the M6 x DM1 RIL population

In the M6 x DM1 RIL population generated from a cross of *S. tuberosum* line DM1-3 (diploid potato) to M6 (a diploid potato inbred line) we evaluated two specific metabolite-based mechanisms correlated with disease resistance, quorum sensing inhibition (QS-I) and exo-protease inhibition (EP-I) and compared them among RILs. These assays are compatible with tissues of varying morphologies because the assay is normalized with metabolite extractions.

**Figure 1:**
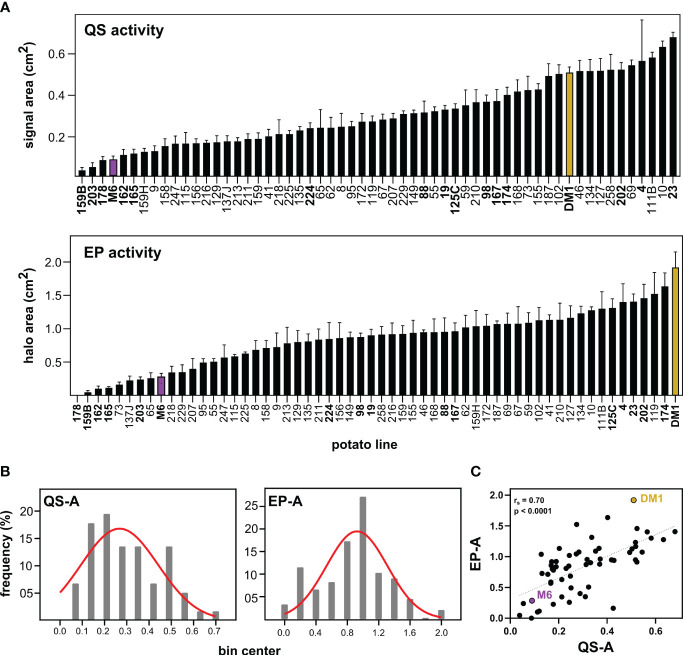
Effects of *Solanum* metabolite extracts on virulence-associated traits of *Pectobacterium*. **(A)** Metabolites were extracted from *S. chacoense* M6 (resistant), *S. tuberosum* DM1 (susceptible), or M6 x DM1 recombinant inbred lines (RILs) and tested for effects on quorum sensing activity (QS-A) and exo-protease activity (EP-A) of *Pectobacterium brasiliense* Pb1692. **(B)** Histogram distribution of activity data in the population fitted to a Gaussian curve. **(C)** Spearman’s rank correlation of QS and EP activity, with regression line added as a visual aid. QS and EP activity data is presented as the mean ± SEM with n=8 replicates, with M6 (purple) and DM1 (brown) used as resistant and susceptible controls, respectively. RILs in bold text were chosen for metabolomics analysis.

Screening of parental and progeny metabolites for QS and EP activity (QS-A and EP-A, **Figure 1A**) showed that for QS-A, M6 and DM1 had 0.09 and 0.51 activity units respectively, with the QS-I of M6 at 82% of DM1. Of the 56 RIL lines, 3 lines exhibited transgressive segregation in the resistant direction (i.e., lower QS-A than the parent M6, QS-I between 83-92%), although these were not statistically different than M6 (ANOVA, Tukey *Post-hoc* p > 0.05). Ten RILs had greater QS_a_ than DM1, however, most of them were equally distributed between DM1 and M6. For EP-A, M6 and DM1 were at 0.09 and 0.51, corresponding to an EP-I of M6 at 85% of DM1. Eight RILs exhibited transgressive segregation in the resistant direction for EP, (EP-I between 87-100%) while no lines varieties transgressed DM1 in EP-related susceptibility.

Both QS-A and EP-A values were normally distributed (Kolmogorov-Smirnov tests, p > 0.10) and support QS-I and EP-I as quantitative resistance traits (**Figure 1B**). The relationship between QS-I and EP-I was evaluated using correlation analysis of the activity data (**Figure 1C**). The two traits were moderately correlated with Spearman’s rank correlation r_s_ = 0.70 (p < 0.0001, n = 52), indicating that some, but not all metabolites affecting both QS and EP were either identical or were in the same extracts. For the remainder of this study, the RIL lines that were high in both QS-I and EP-I were denoted as “resistant lines”, and all other lines were classified as “intermediate” or “susceptible”.

### 3.2 QTL analysis identified genomic regions associated with disease resistance phenotypes

**Figure 2:**
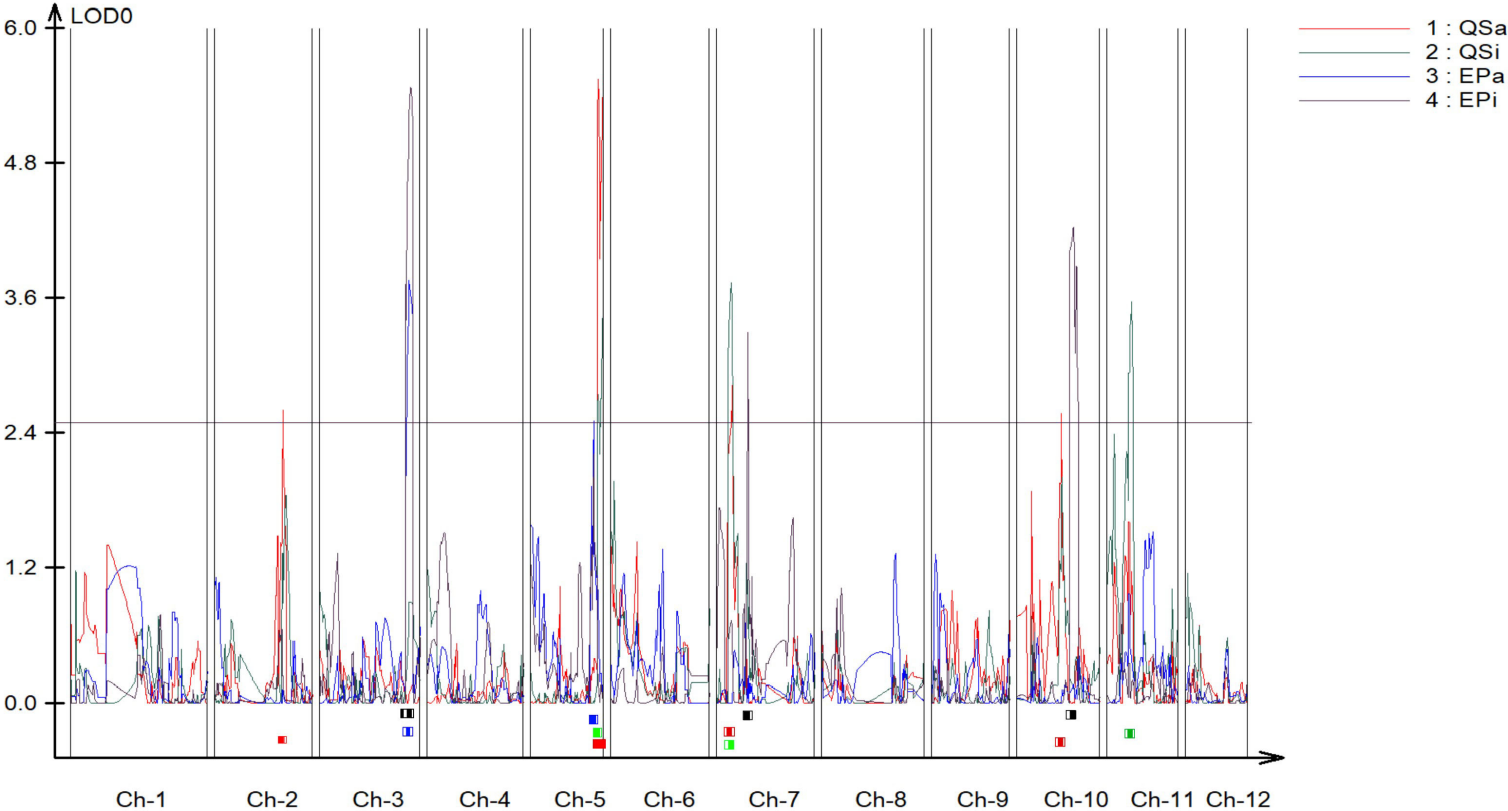
Quantitative trait locus (QTL) mapping of biochemical antivirulence traits in *S. tuberosum.* Resistance-associated traits included quorum sensing and exo-protease activity and inhibition data (QS-A, QS-I, EP-A, EP-I) each mapped independently using logarithm of the odds (LOD) scores as associations to genomic regions. Chr = linkage group (chromosome). QS-A (red): Quorum sensing activity, QS-I (green): % quorum sensing inhibition compared to susceptible DM1, EP-A (blue): Exo-protease activity, EP-I (black): % of exo-protease inhibition to susceptible DM1.

A genetic linkage map was constructed covering all the 12 chromosomes of potato (**Figure S1**). The genetic map had a total length of 1507 cM with an average marker interval of 0.72 cM. The largest linkage group was linkage group 1 which spanned 188.63 cM and contained 109 markers (**Table S1**). The length of each linkage group ranged from 85.04 cM to 188.63 cM and density ranged from 0.31 to 0.698 markers per cM. We identified 5 QTLs for QS-I (**Table 1**) that explained 8-28% of the variation. Two QTLs were identified in chromosome 5, while 1 QTL each was identified on chromosomes 2, 7, and 10 respectively (**Figure 2**). For protease activity, two QTLs were identified in chromosomes 3 and 5 that explained 14-19% of the phenotypic variation. Interestingly, the analysis of QS-I (vs. QS-A) identified two new QTLs including one on chromosome 11 that explained 15% of the phenotypic variation (**Table 1**). Similarly, for EP-I, we identified one QTL previously identified for EP-A and two new QTLs on chromosome 7 and 10 that explained 13-18% of the phenotypic variation. The 10,000 bp flanking region for each significant QTL was searched in the potato genome. For qQS-2-1 there were 54 genes, for qQS-5-1&2 there were 217 genes, for qQS-7-1 there were 39 genes, for qQS-10-1 there were 31 genes, for qPA-3-1 there were 249 genes, for qPA-5-1 there were 217 genes.

**Table 1:** Summary of QTLs identified for quorum sensing and exo-protease activity/inhibition.

Trait	QTL code	Chromosome	Peak marker position (cM)	Peak LOD*	PVE (R^2^)*
QS-A	qQS-A-2-1	2	93.11	2.61	0.10
QS-A	qQS-A-5-1	5	92.11	5.55	0.28
QS-A	qQS-A-5-2	5	98.11	5.39	0.25
QS-A	qQS-A-7-1	7	21.11	2.82	0.11
QS-A	qQS-A-10-1	10	60.48	2.58	0.09
QS-I	qQS-I-5-1	5	92.11	2.75	0.13
QS-I	qQS-I-7-1	7	19.51	3.75	0.16
QS-I	qQS-I-11-1	11	32.81	3.57	0.15
EP-A	qEP-A-3-1	3	122.49	3.76	0.19
EP-A	qEP-A-5-1	5	86.00	2.51	0.14
EP-I	qEP-I-3-2	3	124.51	5.48	0.26
EP-I	qEP-I-7-1	7	42.51	3.30	0.13
EP-I	qEP-I-10-1	10	77.21	4.24	0.18

*LOD, logarithm of the odds; PVE, variation explained.

### 3.3 Metabolites associated with QS-I and EP-I

**Figure 3:**
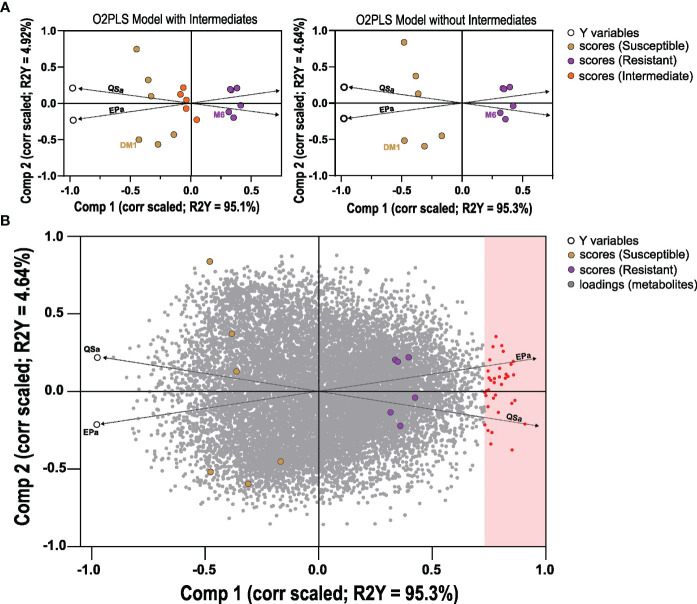
Orthogonal Projection to Latent Structures analysis of metabolites detected in potato RILs. Data was generated based on 15,460 metabolites (x) regressed against QS-A and EP-A (y) data in two O2PLS models: with 15 lines that includes intermediates, or 10 lines of only resistant and susceptible. **(A)** O2PLS scores plots, correlation scaled for 15 (left) or 10 lines (right), with the data points for the two Y variables QS-A and EP-A noted as a black circle. **(B)** O2PLS scores and loadings plot model of the 10 lines (scores) with metabolite loadings shown (gray dots), correlation scaled. Metabolites that met the joint variation (Component 1) threshold of 0.70 were considered associated with resistance. Dashed lines were manually added to highlight the direction of the Y variable vectors.

A comparative metabolomics experiment was performed to identify metabolites associated with QS-I and/or EP-I in RILs that inhibit these virulence traits. Because of the positive correlation between QS-I and EP-I, and that these traits are positively associated with disease resistance (Joshi et al., 2021b), a single subset of 15 lines was evaluated for metabolomics analysis with classifications of highly resistant, moderately resistant, or susceptible (5 lines from each group), along with the two parents. The four metabolomics platforms (UHPLC-MS, HILIC-MS, positive and negative mode) detected approximately 15,460 metabolites (**Table S2**). The 15,460 metabolites were regressed against QS-A or EP-A using orthogonal project to latent structures analysis, with both y variables included the same model (OPLS method, as an O2PLS design). The first model that included intermediates had a cumulative R^2^Y = 99%, however the model failed cross-validation with Q^2 ^= 48% (**Figure 3A**). A second OPLS model was constructed without data from lines without intermediate phenotypes achieved R^2^Y = 99% and Q^2 ^= 61%, supporting that metabolites profiles could sufficiently predict QS_a_ or EP_a_ (**Figure 3B**). In both models, two predictive components were generated that were linked to overall resistance (e.g. both QS_a_ and EP_a_, joint variation, Component 1, ~95%) and a subset of resistance (e.g. either QS-A or EP-A, unique variation, Component 2, ~5%). The second model was subsequently analyzed for metabolites that met a correlated loadings threshold of 0.70 for Component 1, and these were determined to be metabolites associated with resistance. For Component 2, loadings values of > 0.10 were determined to be more associated with EP-A than QS-A and indicate trait bias, and loadings of < -0.10 indicate bias towards QS_a_. Component 2 values in between -0.10 and 0.10 were considered associated with both traits.

**Figure 4:**
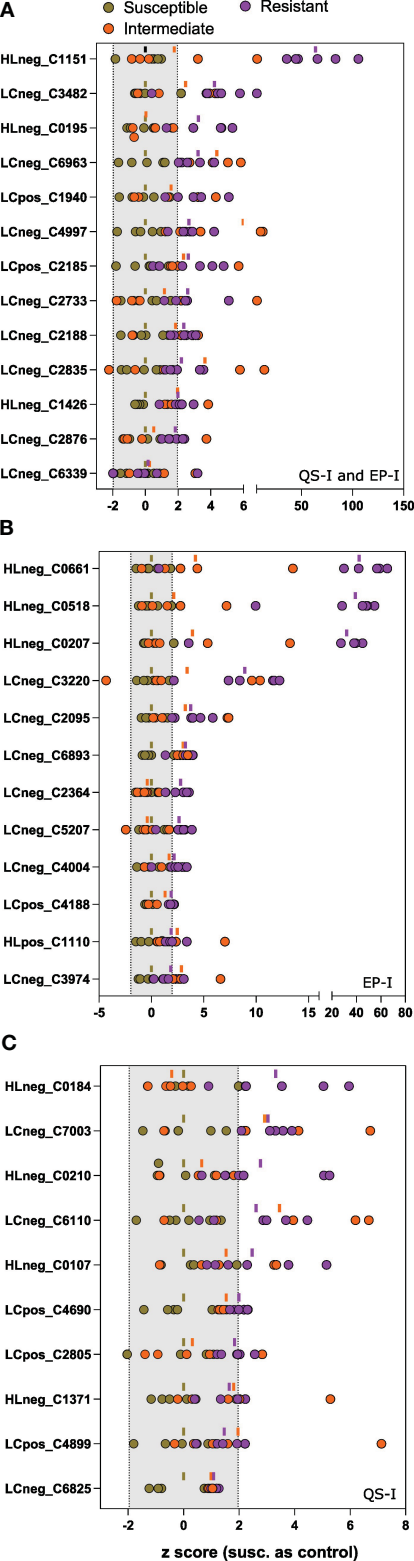
Univariate analysis of metabolites associated with inhibition of virulence traits. Z scores of each metabolite for each of the 15 lines (as dots) compared to the susceptible group as the control. Metabolites were determined to be associated with resistance based on an analysis of the OPLS loadings for **(A)** joint variation, QS-I and EP-I, or unique variation indicating trait bias for **(B)** EP-I and **(C)** QS-I. The dash above each z row indicates the mean z for the resistant (purple), intermediate (orange), or susceptible group (brown) for that metabolite. Z scores outside of the shaded region (-1.96 to 1.96) support statistical significance compared to the susceptible controls. Metabolites are reported as platform detected_compound C number.

In total, 35 metabolites met the Component 1 threshold and were classified as associated with virulence inhibition. Of the 35 metabolites, the Component 2 analysis denoted that 13 metabolites were associated with both QS-I and EP-I, 10 metabolites with QS-I, and 12 metabolites with EP-I. The metabolite abundances were normalized using z transformation to analyze trends among each individual resistance and susceptible lines, with the intermediates added back to the analysis, and with DM1 set to 0 as the susceptible control (**Figures 4A–C**). All metabolites were confirmed to have higher mean z scores in the resistant lines compared to susceptible, supporting the OPLS model successfully characterized metabolites associated with QS-I and EP-I in this population. Interestingly, no metabolites exhibited a clear trend of high levels in resistant lines, medium levels in intermediates, and low levels in susceptible, confirming the OPLS model with intermediates that failed cross-validation. Some metabolites were higher in intermediates than resistant (e.g. LCneg_C4997), but often the intermediates were grouped with the susceptible (e.g. LCneg_2364). Further, only a few metabolites exhibited a presence/absence type of pattern in the data, denoted by excessively high z scores (e.g. HLneg_C0661, HLneg_C0518, HLneg_C0207), with most metabolites existing in both the resistant and susceptible lines, but at higher levels in the resistant lines. Two metabolites were higher in the resistant RILs than DM1, except for the parent M6 that was equal to or below DM1 (HLneg_C0184, LCneg_C2835).

The metabolites were annotated based on interpretation of mass spectra and cross-listing spectral data with metabolite databases (**Table 2**). Of the 35 metabolites, 19 were annotated and 16 were classified as unknowns with the inability to match to databases, or the inability to determine elemental formulas from the spectral data. Two alkaloids that are rather notable in *Solanum* spp. were the glycoalkaloid solacauline (HLneg_C0195) and the kukoamine N1,N5,N10-Tris-trans-p-coumaroylspermine (LCneg_C4997), both in the QS-I/EP-I classification. Three metabolites were annotated as terpenes and all trended in the EP-I direction, with no terpenes being associated with only QS-I: piperochromenoic acid (LCneg_C6339), demissine (HLneg_C0207), and parasiloxanthin (HLpos_C1110). Additionally, 2-C-methyl-D-erythritol-2,4-cyclodiphosphate (LCneg_C3974) is a terpene precursor metabolite and was biased towards EP-I. Two compounds were annotated as terpene conjugates, being alternate classes with a prenyl group attached to the base structure: isopentyl gentiobioside (two metabolite signals, LCneg_C6893, LCneg_6963, EP-I) and N6-(delta2-isopentenyl)-adenosine 5’-diphosphate (LCneg_C3220, a putative prenylated purine, EP-I). A third prenylated flavonoid was identified (albanin H, LCposC4899), although this was linked to QS-I, as well as the non-prenylated polyphenolic feruloylquinic acid (HLneg_C0184).

**Table 2:** Metabolites associated with QS-I and EP-I in wild potato and resistant RILs.

Class	Sub-class	Metabolite	ID^a^	Mass^b^	error (ppm)	Corr^c^	Trait Bias^c^	Trait Corr^c^
Alkaloids	glycoalkaloids	solacauline	HLneg_C0195	M = 826.4706	1	0.77	-0.04	QS-I/EP-I
		demissine	HLneg_C0207	[M+F]^-^= 1036.5593	10	0.85	0.18	EP-I
	kukoamines	N1,N5,N10-Tris-trans-p-coumaroylspermine	LCneg_C4997	M = 640.3067	30	0.83	0.10	QS-I/EP-I
Terpenes	intermediates	2-C-methyl-D-erythritol-2,4-cyclodiphosphate	LCneg_C3974	[M+FA-H]^-^ = 320.9796	1	0.72	0.28	EP-I
	tetraterpenes	parasiloxanthin	HLpos_C1110	[M+H]^+^ = 571.4609	17	0.77	0.29	EP-I
Saccharides	prenylated	isopentyl gentiobioside	LCneg_C6893	M = 412.1908	9	0.81	0.30	EP-I
		isopentyl gentiobioside	LCneg_C6963	M = 412.1908	9	0.86	0.11	EP-I
Phenolics	polyphenolics	feruloylquinic acid	HLneg_C0184	M = 368.1121	4	0.75*	-0.1	QS-I
	prenylated	albanin H	LCpos_C4899	[M+2H]^2+^ = 421.1674	5	0.70	-0.34	QS-I
		piperochromenoic acid	LCneg_C6339	M = 340.2066	8	0.8*	0.05	QS-I/EP-I
Nucleotides	purines	diadenosine pentaphosphate	LCneg_C6110	[M-H]^-^ = 914.9778	32	0.73	-0.21	QS-I
	prenylated	N6-(delta2-isopentenyl)-adenosine 5’-diphosphate	LCneg_C3220	[M-H]^-^ = 491.038	51	0.81	0.26	EP-I
	pyrimidines	UDP-alpha-D-xylose	LCneg_C2876	M = 533.9952	65	.082	0.09	QS-I/EP-I
Lipids	glycerolipids	TG(62:0)	LCneg_C2188	[M-H_2_0-H]^-^ = 969.9628	5	0.77	0.08	QS-I/EP-I
		TG(63:0)	LCpos_C1940	[M+H+NH_4_]^2+^ = 517.9981	15	0.75	0.09	QS-I/EP-I
		TG(58:0)	LCneg_C4004	M = 932.9485	37	0.75	0.13	EP-I
		DG-3-OH(40:5)	LCpos_C2185	[M+Na]^+^ = 741.5279	0	0.76	0.07	QS-I/EP-I
		PE-NMe2(42:2)	HLneg_C0518	M = 855.6676	5	0.86	0.19	EP-I
		PE-NMe2(38:6)	LCpos_C2805	M = 791.5465	0	0.79	-0.13	QS-I
Unknown	phenolics	unknown phenolic	HLneg_C1151	M = 232.0754	1	0.86	-0.06	QS-I/EP-I
		unknown phenolic	HLneg_C1426	[M-H]^-^ = 401.0705		0.85	-0.08	QS-I/EP-I
		unknown flavonoid glycoside	LCpos_C4690	[M + K]^+^ = 821.1921	2	0.85	-0.38	QS-I
	glycosides	unknown glycoside	HLneg_C0210	M = 396.8958	9	0.76	-0.26	QS-I
	lipids	unknown lipid	LCneg_C3482	[M-H]^-^ = 162.8387		0.77	0.06	QS-I/EP-I
	unknown	unknown	HLneg_C0107	M = 350.8118	5	0.75	-0.25	QS-I
		unknown	LCneg_C2364	M = 660.9885	2	0.83	0.11	EP-I
		unknown	LCneg_C2733	M = 806.9983	8	0.80	0.09	QS-I/EP-I
		unknown	HLneg_C1371	[M-H]^-^ = 280.9837		0.76	-0.34	QS-I
		unknown	HLneg_C0661	[M-H]^-^ = 448.8301		0.80	0.15	EP-I
		unknown	LCpos_C4188	[M+H]^+^ = 515.036		0.75	0.20	EP-I
		unknown	LCneg_C2835	[M-H]^-^ = 633.9719		0.78*	-0.02	QS-I/EP-I
		unknown	LCneg_C6825	[M-H]^-^ = 644.9649		0.69	-0.32	QS-I
		unknown	LCneg_C7003	[M-H]^-^ = 703.9523		0.91	-0.21	QS-I
		unknown	LCneg_C2095	[M-H]^-^ = 769.9478		0.78	0.35	EP-I
		unknown	LCneg_C5207	[M-H]^-^ = 937.9673		0.72	0.3	EP-I

a: metabolomics platform (HILIC or reverse phase LC; positive or negative mode); b: inferred mass M based on InterpretMS algorithm or parent ion used for annotation, with unknowns reporting one assumed ion in the spectrum (e.g.[M-H]^-^); c: corr = O2PLS Component 1 loading representing positive correlation value to QS-I/EP-I traits, known as joint predictive variation; trait bias = O2PLS Component 2 loading of bias correlation to QS-I (negative) or EP-I (positive) traits, with bias thresholds set at > or < 0.10; * = DM1 z score greater than M6.

### 3.4 Association of disease resistance related metabolites with genetic variants

**Figure 5:**
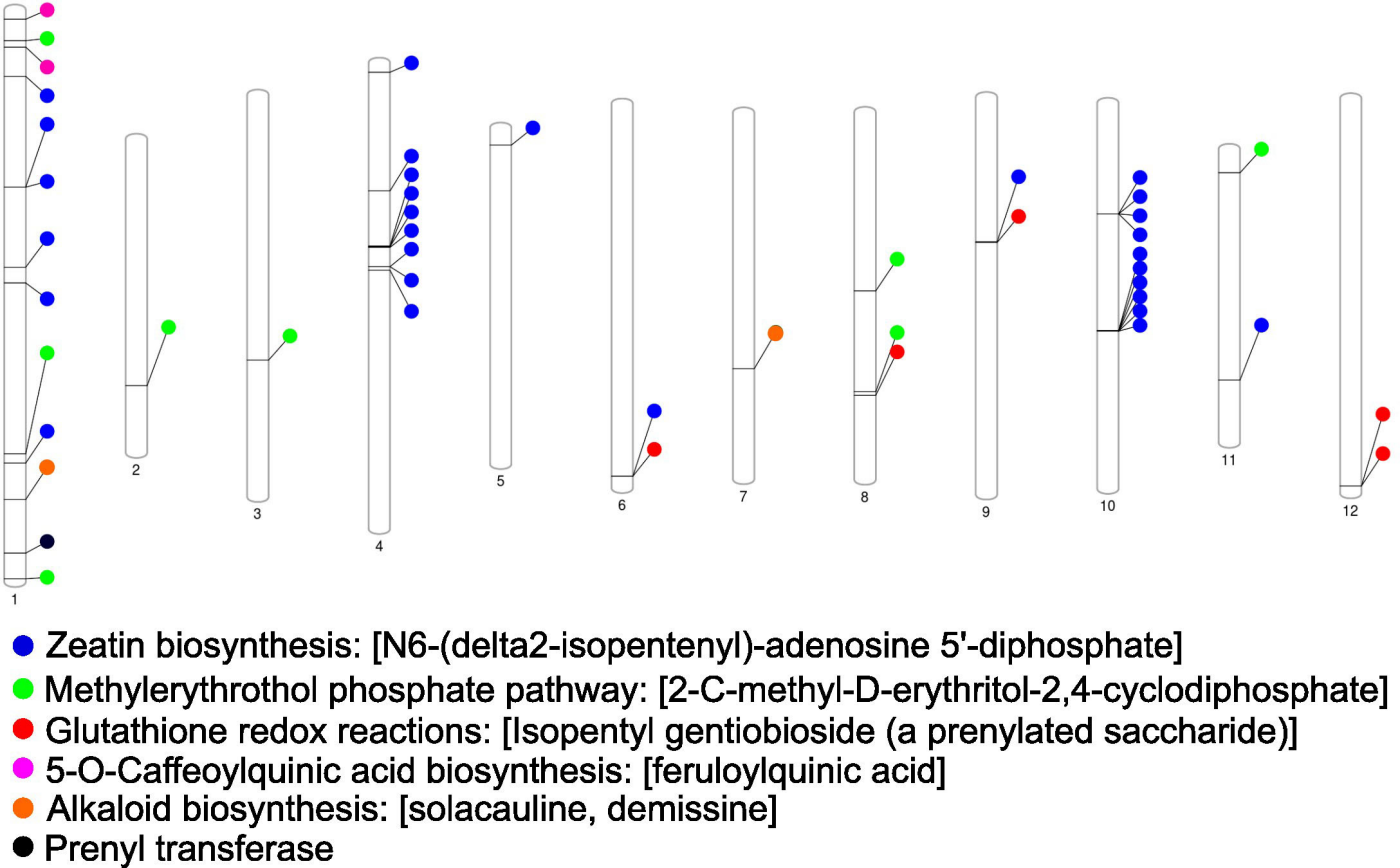
Genetic map of the chromosomes from the *Solanum tuberosum* group phujera DM1-3 (v4.03, id52025). The location of genes associated with soft rot and blackleg resistant metabolites is shown across potato chromosomes with individual color denoting specified biochemical pathways.

Candidate resistance metabolites that were consistently higher across all tested resistant lines were annotated and EP-I assigned to specific metabolic pathways and aligned to the chromosomal locations of pathway genes (**Figure 5**). These prominent pathways include alkaloid biosynthesis, zeatin biosynthesis, methylerythritol phosphate pathway (non-MVA terpene synthesis), glutathione redox reactions, 5-O-caffeoylquinic acid (phenolic) biosynthesis, and prenyl transferases. Notably, genes associated with these pathways were distributed across 12 distinct potato chromosomes. A comparative analysis revealed overlaps between these genomic regions and the identified QTL regions, which encompass genes related to alkaloid synthesis, phenolic biosynthesis, peroxidases, and acylsugar acyltransferase.

## 4 Discussion

The resistance to necrotrophic bacterial diseases observed in wild *Solanum* species is multifactorial, with biomolecules playing a direct and indirect role in combating the disease. For chemical resistance, *S. chacoense* M6 has several sub-classes of compounds that work together to collectively reduce bacterial virulence and slow or completely prevent the development of disease. Therefore, there are several anti-virulence properties of plant molecules found on wild *Solanum*. We describe two sub-classes of these as antivirulence proteins (Joshi et al., 2022) and antivirulence metabolites (Joshi et al., 2021b). For proteins, these have been classified as protease inhibitors that affect bacterial exo-protease activity, motility, and cell morphology, but not quorum sensing. For metabolites, these molecules affect quorum sensing, motility, and exo-protease inhibition, but not morphology. This supports that the wild potato *S. chacoense* contains a mixture of molecules individually affecting sub-components of bacterial virulence, possibly with a synergy that culminates in broad phenotypic measures of host resistance, or less decay of plant tissues.

The phenomenon that multiple molecular compounds interact with multiple processes is consistent with the observation that resistance in *S. chacoense* is multi-genic (Zimnoch-Guzowska et al., 2000; Chung et al., 2017). Recently, RILs were developed by crossing DM1×M6, that allows use of genetic and high throughput metabolomic tools to explore soft rot and blackleg resistance in M6 potato (McCauley, 2021; Jansky et al., 2024). The RILs from the DM1×M6 were impossible to effectively compare for resistance in later generations (e.g., F_5_/F_6_) due to high phenotypic variability that may affect virulence assays, such as varying stem thickness, self-degenerating tubers, or inability to tuberize. In this study, we worked with a sub-set of the RIL population that allowed the study of metabolites, genes, and anti-virulence traits correlated with resistance. While the power of genetic associations is relatively low compared to a typical plant genetics study, we were still able to describe the distribution of resistance traits in this population.

Specifically, the two antivirulence traits were explored: the ability for a metabolite extract to inhibit quorum sensing or exo-protease activity (QS-I, EP-I). Importantly, these are quantitative assays and therefore inhibition can be inferred as a percent reduction compared to a control, in this experiment with DM1 as the control. The distribution of the QS and EP activity data was normal among the RILs, with some evidence of potential transgressive segregation (**Figure 1B**). This supports that QS-I and EP-I are quantitatively inherited traits and therefore linked to multiple genes and/or metabolites, and that QS-I and EP-I are new traits that can be bred for in plants. Further, QS-I and EP-I were moderately positively correlated, indicating some metabolites or regulators impact both processes, but there are also metabolites that uniquely affect these sub-components of virulence. This is consistent with our previous observation that protease inhibitors affected some, but not all virulence processes in this system (Joshi et al., 2022).

The metabolomics analysis was performed on a subset of the RIL population. While screening the full RIL population would be a stronger design to associate metabolites with QS-I and EP-I, we were still able to create a predictive model that provided significant hits of metabolites associated with resistance. The OPLS method was used for its ability to work with mass spectrometry (x) data, and the normally distributed phenotypic (y) data as shown in **Figure 1A**. Further, we could integrate QS-I and EP-I concepts into a single model (similar to O2PLS (Bouhaddani et al., 2016)), in which the data could be divided into two subsets: joint variation (metabolites affecting both QS-I and EP-I), and individual variation (metabolites affecting either QS-I or EP-I, a virulence trait bias components). The output was a two-dimensional space that provided a score to each metabolite for each of the four options (**Figure 3B**). Interestingly, the model resulted in approximately 95% of the variation being explained by the joint component, and 5% for the trait bias component. This is inconsistent with the Spearman rank correlation data, which would support more variation on the trait bias component (r_s_ = 0.70), although the metabolomics data may be skewed with the 15 lines chosen for their effectiveness for both the QS-I and EP-I traits. This may also be due to the lack of intermediates in the final OPLS model; intermediates were a major component to decipher the distribution of QS-I or EP-I phenotypes in this population (**Figure 1**), but metabolomics analysis that included these intermediates resulted in poor models (**Figure 3**). One explanation is that the difficulty in the metabolomics analysis to decipher very minor differences in metabolites may lead to major differences in phenotypes in the intermediates. Therefore, these metabolomics data are most confident in the ability to discriminate the highly resistant RILs from the highly susceptible RILs, rather than subtle differences that occur in the intermediate RILs.

The metabolomics data showed that the highly resistant RILs were associated with a set of 35 metabolites that included a mixture of lipids, alkaloids, terpenes, phenolics, and several unknowns. Most lipids were determined to be membrane glycolipids or storage lipids. As lipid profiles are inherited, this class of compounds is expected to be indirectly related to resistance, where this profile is inherited from M6 to the resistant RILs, however not associated with resistance. Of the 35 resistance-related metabolites, 16 were ‘known unknowns’ (MSI levels 3-4 (Sumner et al., 2007)), and these are expected to occur in an experimental design that includes a wild plant species and progeny crossed with diploid potato. Regardless, this report includes mass spectral data for these compounds to continue to be tracked in subsequent studies.

Several compounds that were associated with resistance have known or predicted roles in resistance and virulence, and specifically for QS-I or EP-I. Three alkaloids were found to be associated with resistance, solacauline, demissine, and N1,N5,N10-Tris-trans-p-coumaroylspermine. Solacauline and demissine are steroidal alkaloids, a class of compounds largely unique to *Solanum* and *Liliaceae*, of which approximately 300 structures have been reported (Morillo et al., 2020). Steroidal alkaloids are nitrogenous sterols (a solanidane group) that are usually conjugated to 2-4 saccharides, with the saccharide component being critical to their bioactivity (Delbrouck et al., 2023). These compounds provide protection by disrupting cell membranes of pests and pathogens (Niño et al., 2009). The two main potato glycoalkaloids, chaconine and solanine, were not associated with resistance in this experiment. Solacauline and demissine have a similar solanidane backbone as chaconine and solanine, but both have unique saccharide components. Solacauline has a linear trisaccharide chain, compared to branched chain trisaccharides (chacotriose and solatriose) found in most glycoalkaloids (Shakya and Navarre, 2008). Glycoalkaloids may affect bacterial membranes (Yan et al., 2021), however given *S. chacoense* M6 extracts do not exhibit bactericidal activity (Joshi et al., 2021b), it is more likely to interact with bacterial proteins involved in virulence, and this interaction may be improved with the linear chain saccharide component. Further, demissine has a branched tetrasaccharide component, and this was biased towards EP-I, further supporting that the saccharide component of glycoalkaloids may be critical in determining their direct effects on bacterial processes and overall impact on resistance to these pathogens.

A second major class of metabolites associated with QS-I and EP-I are terpenes and terpene conjugates of other specialized metabolites. Terpene synthesis is linked to glycoalkaloid synthesis via the synthesis of sterols (glycoalkaloids are based on triterpenes). The terpene biosynthesis precursor 2-C-methyl-D-erythritol-2,4-cyclodiphosphate was associated with resistance in the RIL population, although biased toward EP-I. In these data, the common terpene-related trend was the prenylation of specialized metabolites. Prenylation is a modification to secondary metabolites that results in changes to the compounds solubility, and usually enhances function including antibacterial activity and enzyme inhibition, for example docking of the prenyl group in protein active sites (Botta et al., 2005; Yazaki et al., 2009; Chen et al., 2014; Wang et al., 2017). Therefore, the presence of prenyl side chains on specialized, exogenous plant molecules could directly affect bacterial proteins that regulate virulence pathways. The M6 resistance related metabolites included two prenylated phenolics (albanin H, piperochromenoic acid), a prenylated saccharide (isopentyl gentiobioside), and a prenylated purine (N6-(delta2-isopentenyl)-adenosine 5’-diphosphate, putative annotation). Gentiobiose is a saccharide common in unripe (green) tomato fruit (Dumville and Fry, 2003) and would therefore be expected in other *Solanum* spp. Prenylated gentiobiose has further been detected in tomato cell culture (De Rosa et al., 1996), and isopentyl gentiobioside was previously found to be associated with resistance in *S. chacoense* (Joshi et al., 2021b). For effects on QS, some bacteria such as *Bacillus* have prenylated pheromones (e.g. ComX), with the prenyl group being essential to protein-binding function and overall diversity (Ansaldi et al., 2002; Okada et al., 2017). Prenylated phenolics have demonstrated QS-I properties (Paguigan et al., 2019), and in other metabolite-protein interactions, prenylated forms have stronger activity (Osorio et al., 2021). While *Pectobacterium* AHL pheromones are acylated and not prenylated, the side chains have similar molecular sizes, shapes, and lipophilic chemical properties to support this system a potential resistance mechanism by *S. chacoense.* Further, as with prenylated proteins, the prenylation of water-soluble metabolites such as saccharides and phenolics may focus their activity on cell membranes, where significant regulation of QS occurs (Joshi et al., 2021b).

Several associations between QS-I and EP-I resistance traits and individual antivirulence metabolites and metabolic pathways in *S.* chacoense were observed with genetic mapping. The overall SNP-QTL mapping approach was conducted using exo-protease activity inhibition (EP-A, EP-I), and quorum sensing inhibition (QS-A, QS-I) as a phenotype to pinpoint markers associated with these traits. Notably, protease inhibition demonstrated associations with markers located on chromosome 3, 5, 7, and 10. Similarly, AHL inhibition was linked to markers found on chromosome 2, 5, 7, 10, and 11. These markers encompass a wide range of regions within the potato chromosome, many of which are correlated with disease resistance metabolites and associated pathways, including acylsugar acyltransferase, alkaloid biosynthesis, phenolic biosynthesis, and peroxidases. Our findings are in line with recent findings where acylsugar metabolism, alkaloid biosynthesis, and phenolic biosynthesis is shown to be associated with plant-microbe interaction and disease resistance (Mandal et al., 2020; Song et al., 2023; Yuan et al., 2023). In summary, the integration of two robust approaches, genetic analysis, and metabolomics, has shed light on the biochemical pathways associated with soft rot and blackleg diseases resistance.

## Data availability statement

The raw datasets presented in this study has been deposited in the online repository Metabolights: https://www.ebi.ac.uk/metabolights/, accession number MTBLS8906. Data will be made available after review by the resource.

## Author contributions

JJ: Writing – original draft, Writing – review & editing. DP: Writing – review & editing. AC: Writing – review & editing. EE: Writing – review & editing. AH: Writing – original draft, Writing – review & editing.
