# Supplementary figures and images for: Plant necrotrophic bacterial disease resistance phenotypes, QTL, and metabolites identified through integrated genetic mapping and metabolomics in Solanum species
Source: Front Plant Sci. 2024 Mar 5;15:1336513. doi: 10.3389/fpls.2024.1336513 (PMC10949924; doi:10.3389/fpls.2024.1336513)

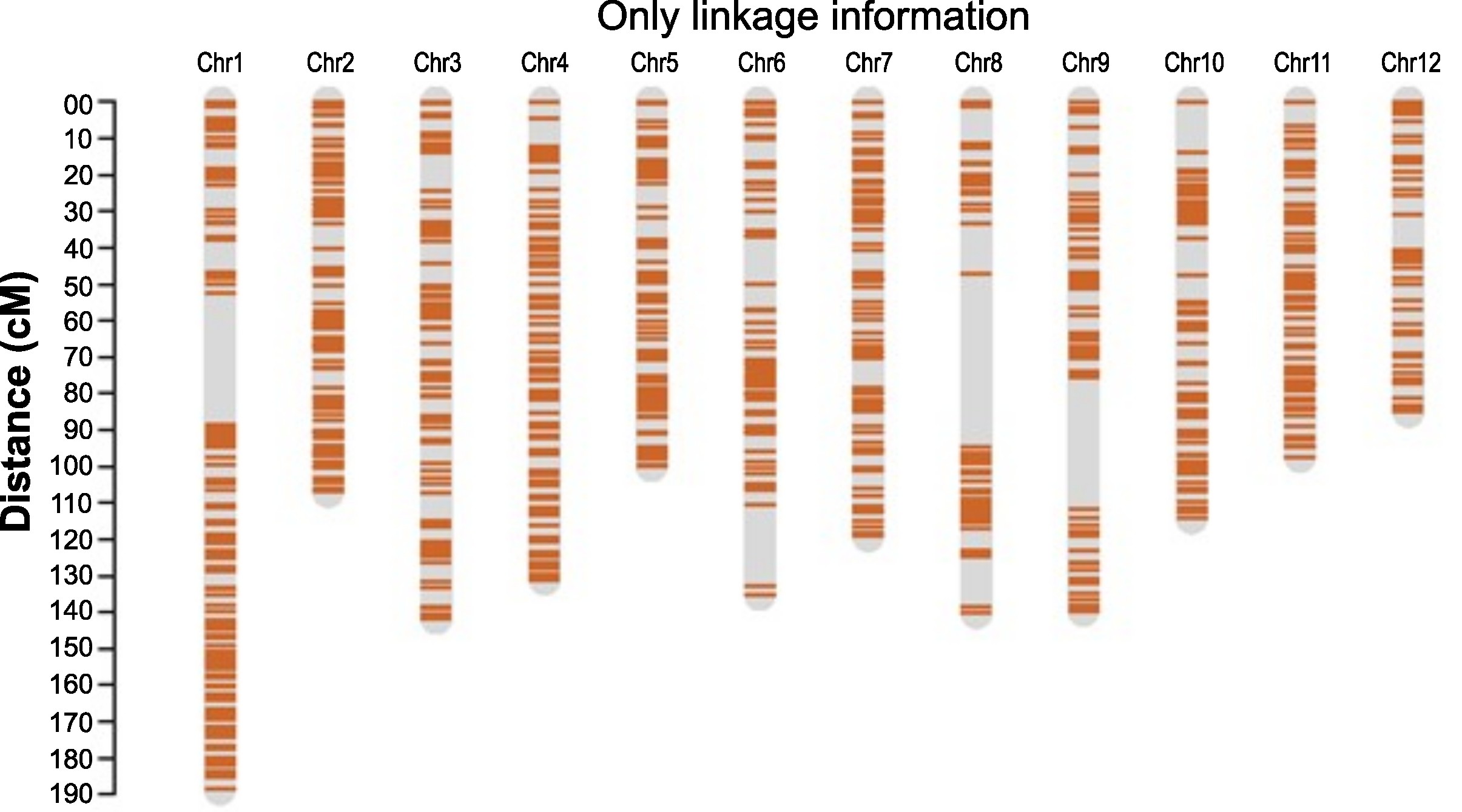

Supplement: Supplementary file 5 [file Image_1.jpeg]
