# Supplementary material for: Plant necrotrophic bacterial disease resistance phenotypes, QTL, and metabolites identified through integrated genetic mapping and metabolomics in Solanum species
Source: Front Plant Sci. 2024 Mar 5;15:1336513. doi: 10.3389/fpls.2024.1336513 (PMC10949924; doi:10.3389/fpls.2024.1336513)
Supplement: Supplementary file 1 [file DataSheet_1.pdf]

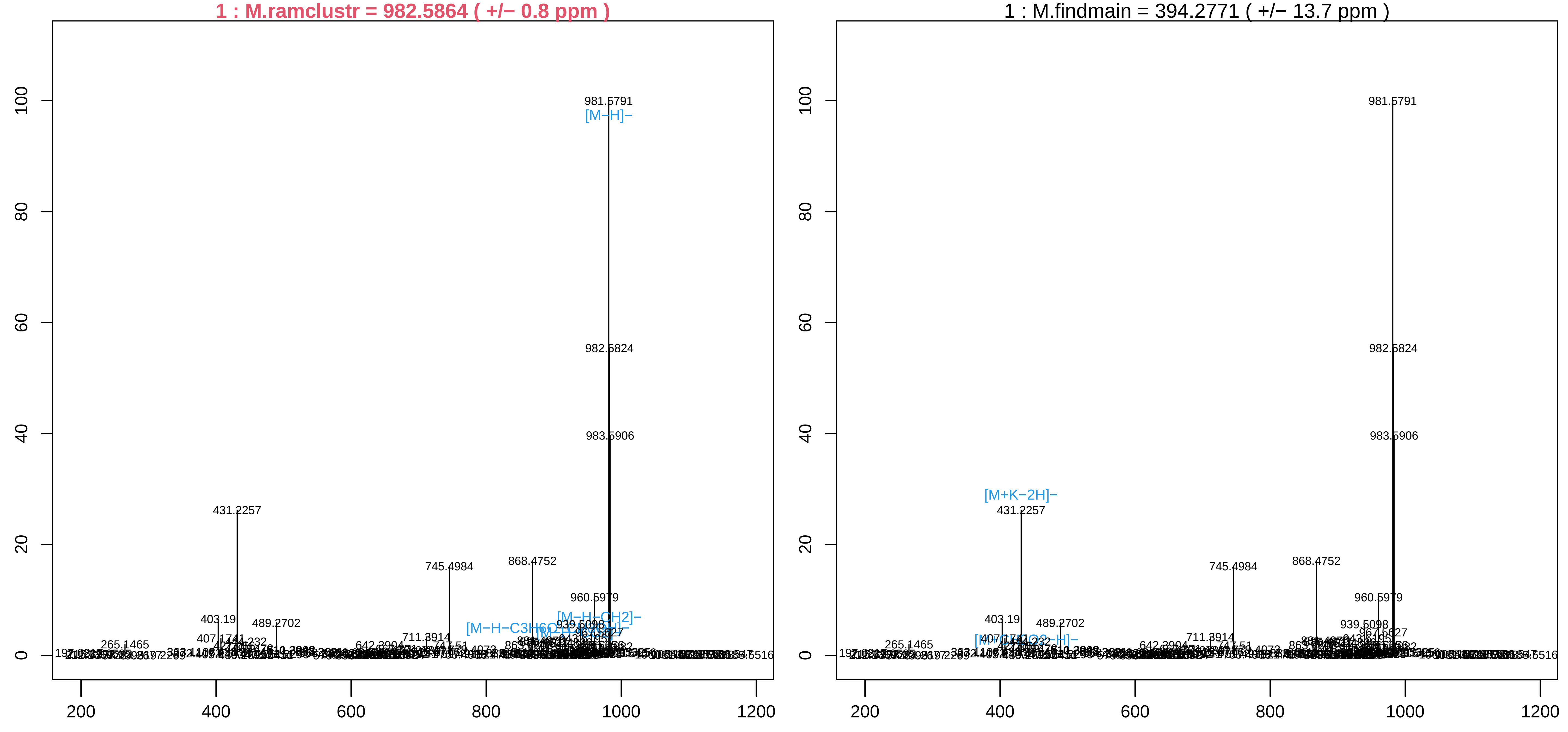
1 : M.ramclustr = 982.5864 (+/− 0.8 ppm)
1 : M.findmain = 394.2771 (+/− 13.7 ppm)
981.5791
[M−H]−
982.5824
983.5906
431.2257
868.4752
745.4984
960.5979
[M−H−CH2]−
939.5098
403.19
489.2702
711.3914
265.1465
642.2904
[M+K−2H]−
967.5627
0
20
40
60
80
100
200
400
600
800
1000
1200

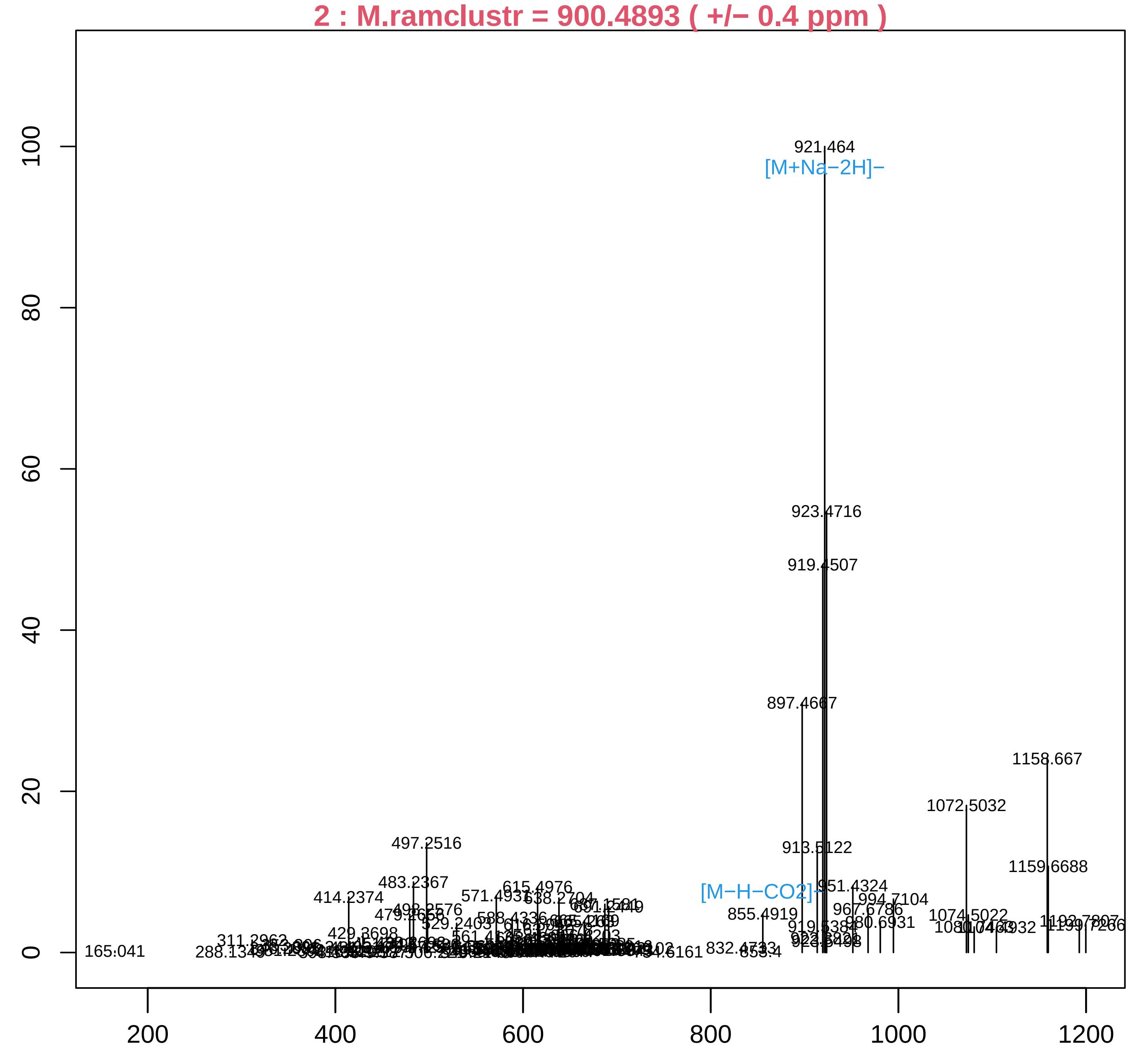
2 : M.ramclustr = 900.4893 (+/− 0.4 ppm)
921.464
[M+Na−2H]−
923.4716
919.4507
897.4667
1158.667
1072.5032
913.5122
1159.6688
497.2516
483.2367
414.2374
571.4931
615.4976
951.4324
994.7104
967.6786
980.6931
855.4919
1074.5022
[M−H−CO2]−
429.3698
311.2962
165.041
288.1343
832.4733
0
20
40
60
80
100
200
400
600
800
1000
1200
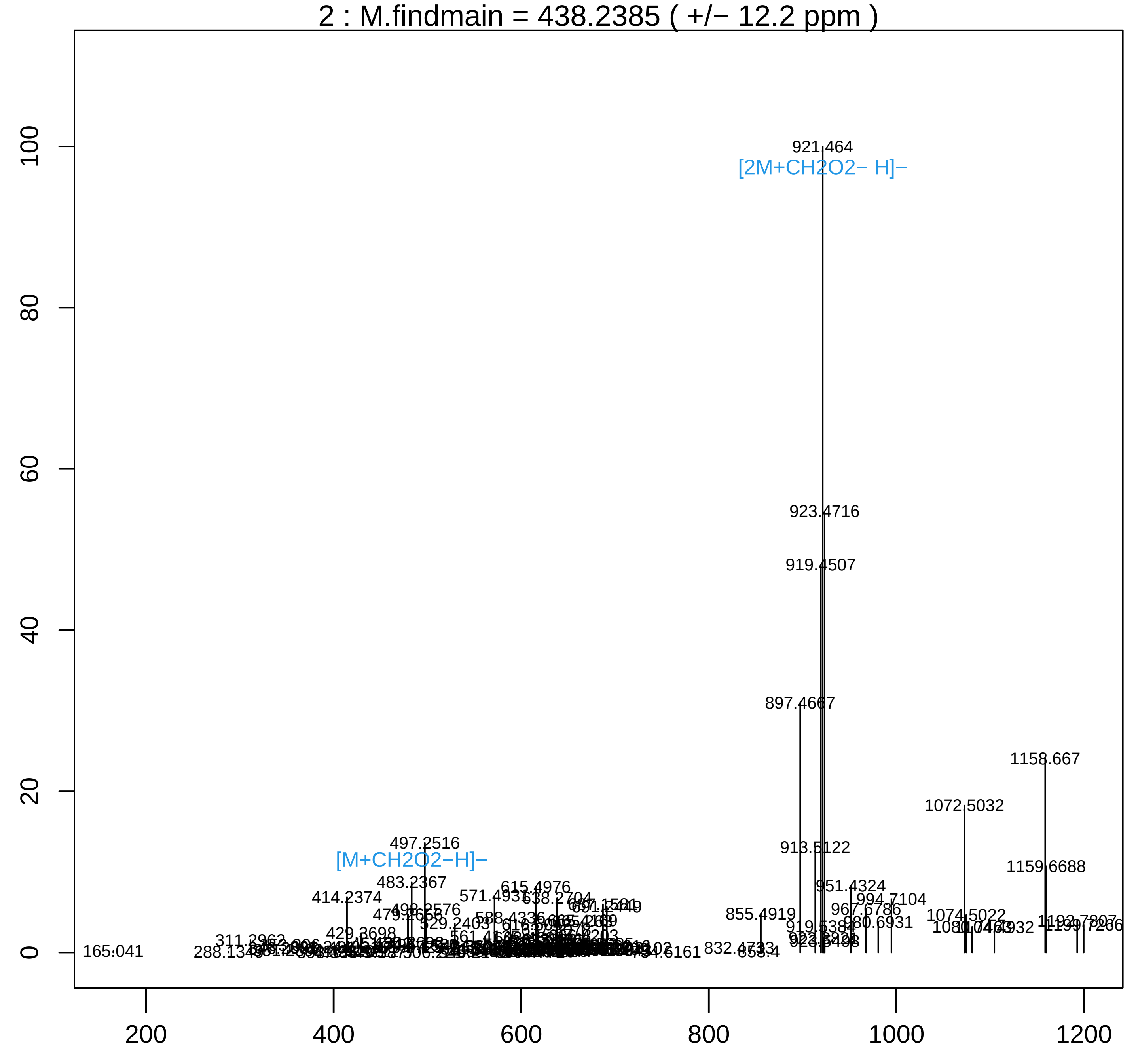
2 : M.findmain = 438.2385 (+/− 12.2 ppm)
921.464
[2M+CH2O2− H]−
923.4716
919.4507
897.4667
1158.667
1072.5032
913.5122
1159.6688
497.2516
[M+CH2O2−H]−
483.2367
414.2374
571.4931
615.4976
951.4324
994.7104
967.6786
980.6931
855.4919
1074.5022
429.3698
311.2962
165.041
288.1343
832.4733
0
20
40
60
80
100
200
400
600
800
1000
1200

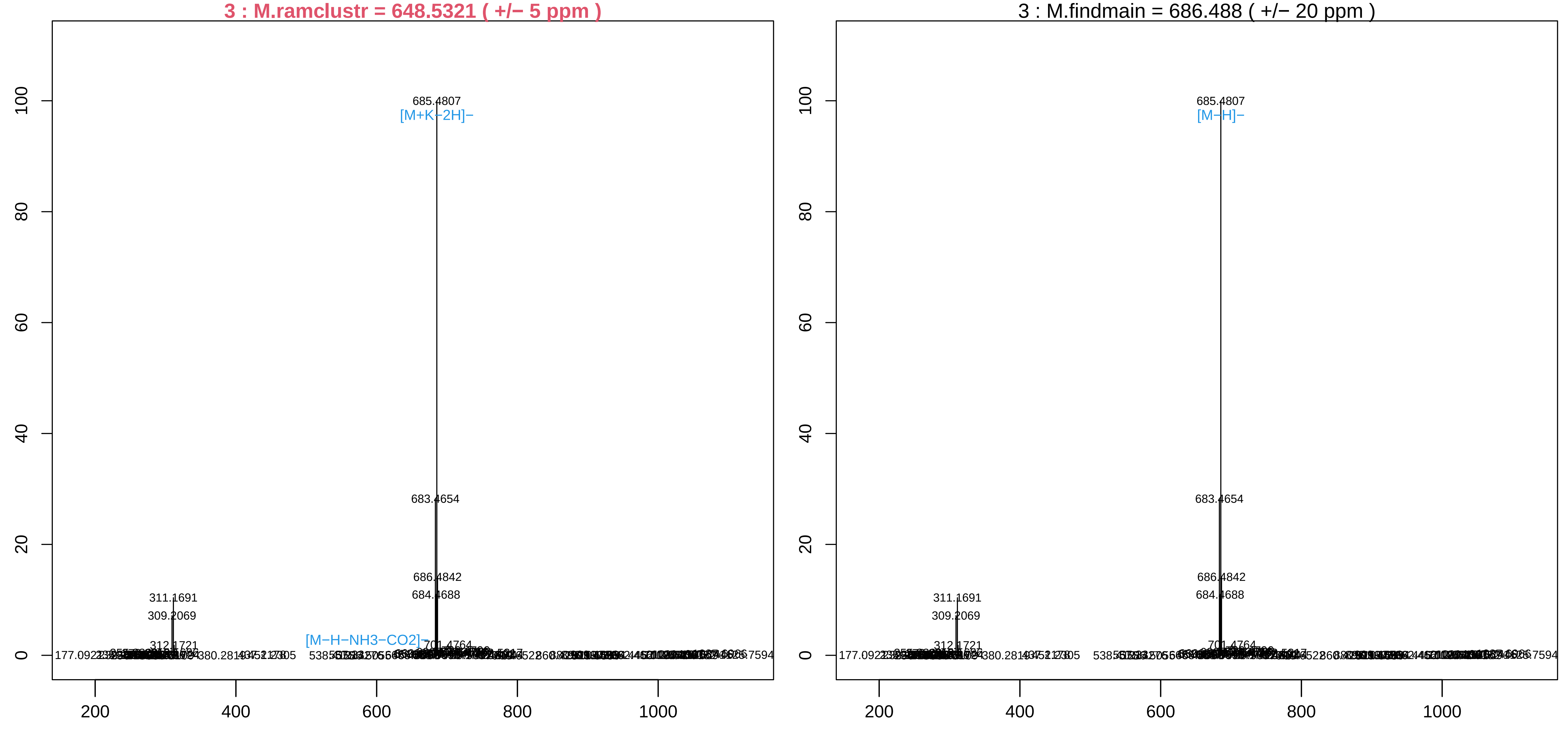
3 : M.ramclustr = 648.5321 (+/− 5 ppm)
685.4807
[M+K−2H]−
683.4654
686.4842
684.4688
311.1691
309.2069
312.1721
701.4764
[M−H−NH3−CO2]−
3 : M.findmain = 686.488 (+/− 20 ppm)
685.4807
[M−H]−
683.4654
686.4842
684.4688
311.1691
309.2069
312.1721
701.4764
0
20
40
60
80
100
200
400
600
800
1000

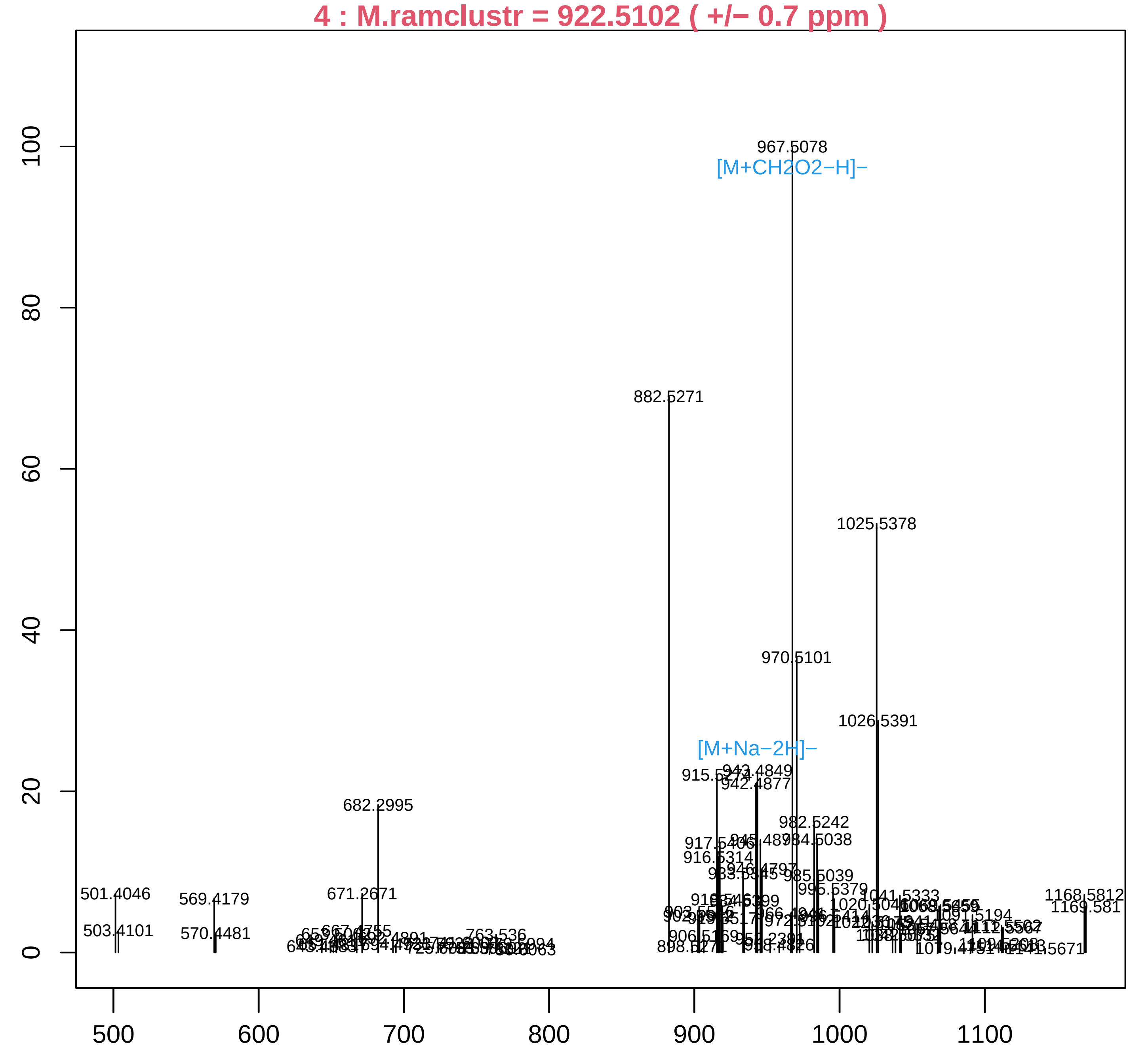
4 : M.ramclustr = 922.5102 (+/− 0.7 ppm)
967.5078
[M+CH2O2−H]−
882.5271
1025.5378
970.5101
1026.5391
[M+Na−2H]−
682.2995
501.4046
569.4179
671.2671
503.4101
570.4481
1168.5812
1169.581
0
20
40
60
80
100
500
600
700
800
900
1000
1100

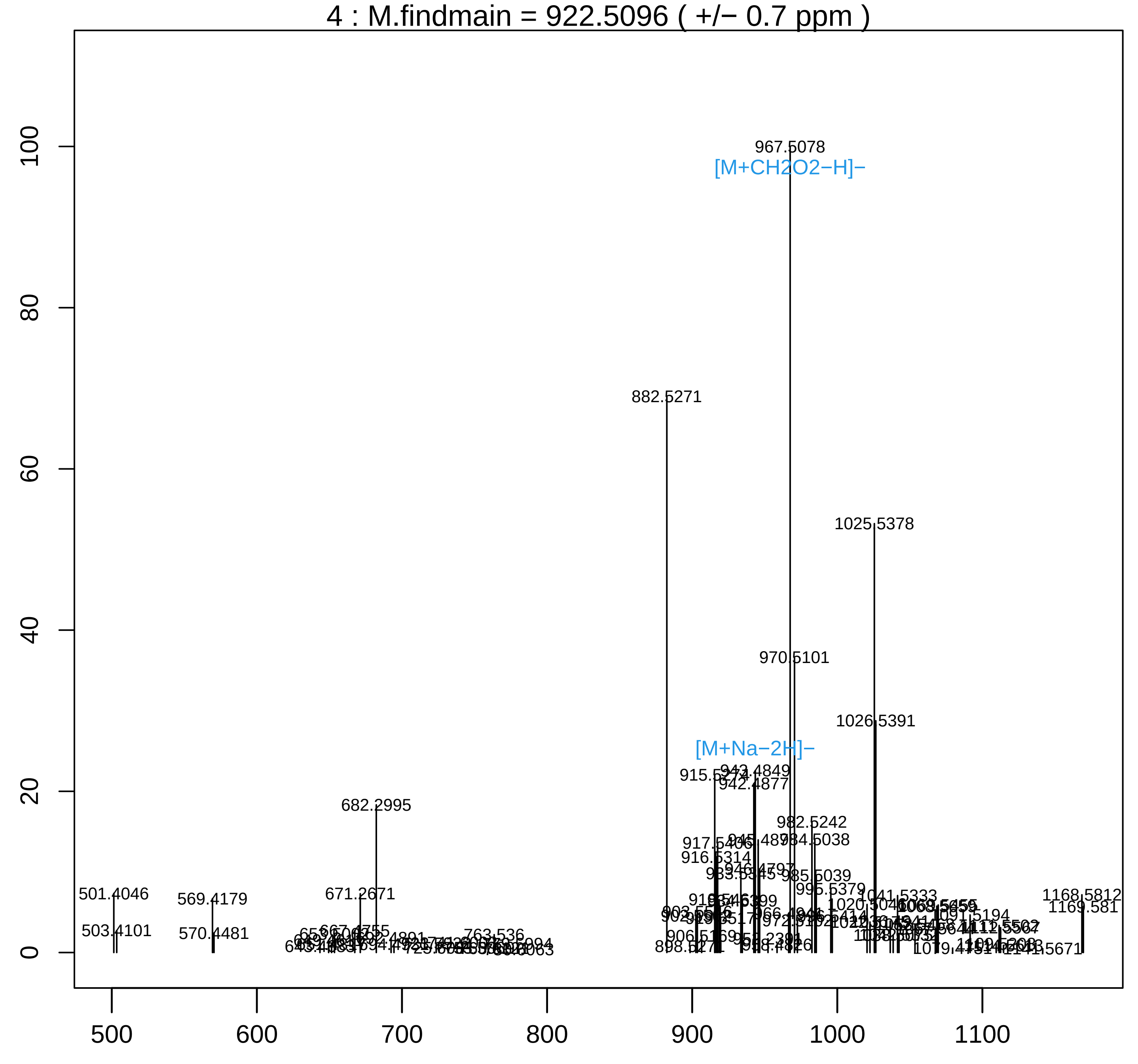
4 : M.findmain = 922.5096 (+/− 0.7 ppm)
967.5078
[M+CH2O2−H]−
882.5271
1025.5378
970.5101
1026.5391
[M+Na−2H]−
682.2995
501.4046
569.4179
671.2671
503.4101
570.4481
1168.5812
1169.581
0
20
40
60
80
100
500
600
700
800
900
1000
1100

5 : M.ramclustr = 744.4944 (+/− 0.5 ppm)

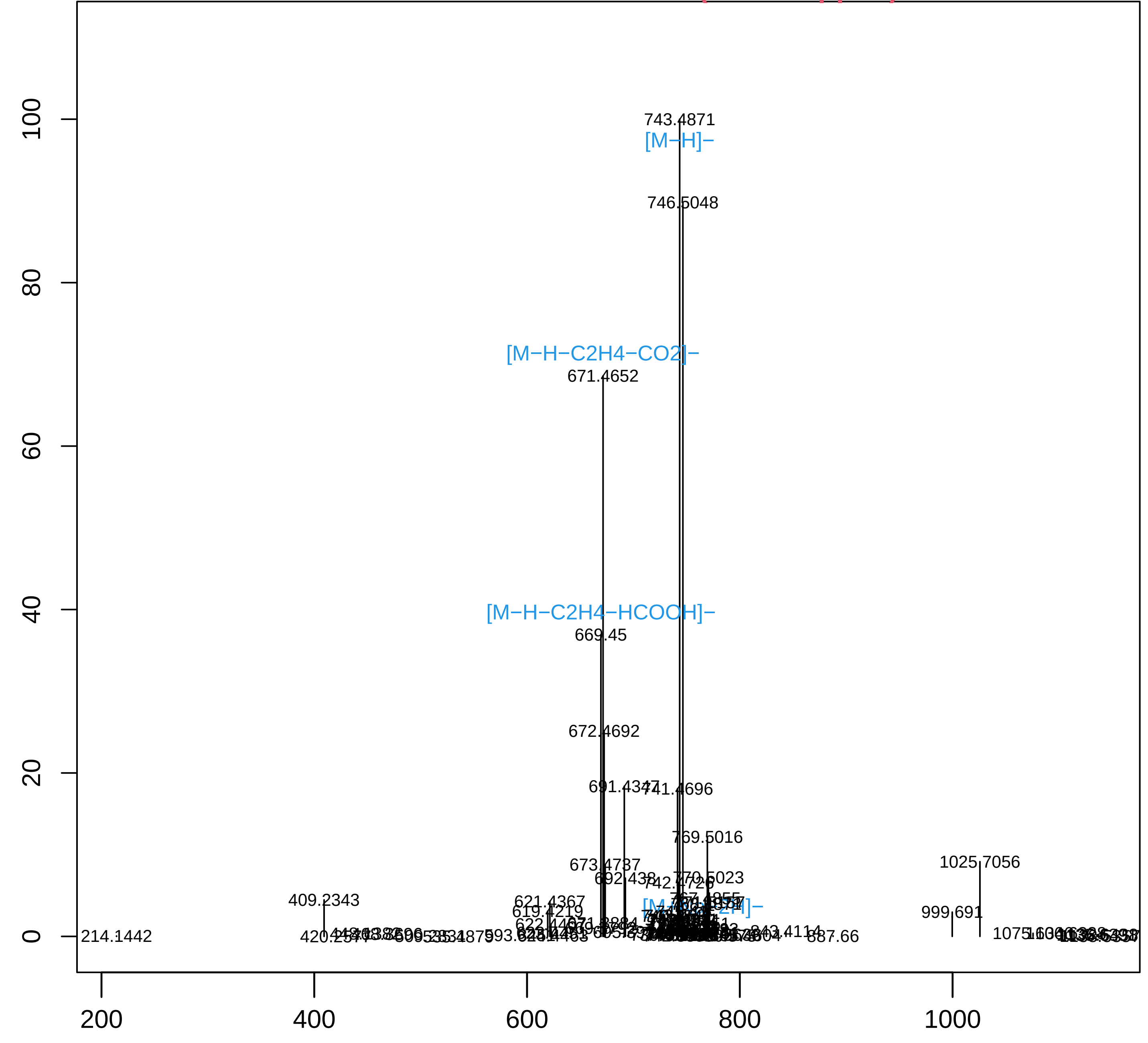

5 : M.findmain = 744.4944 (+/− 0.3 ppm)

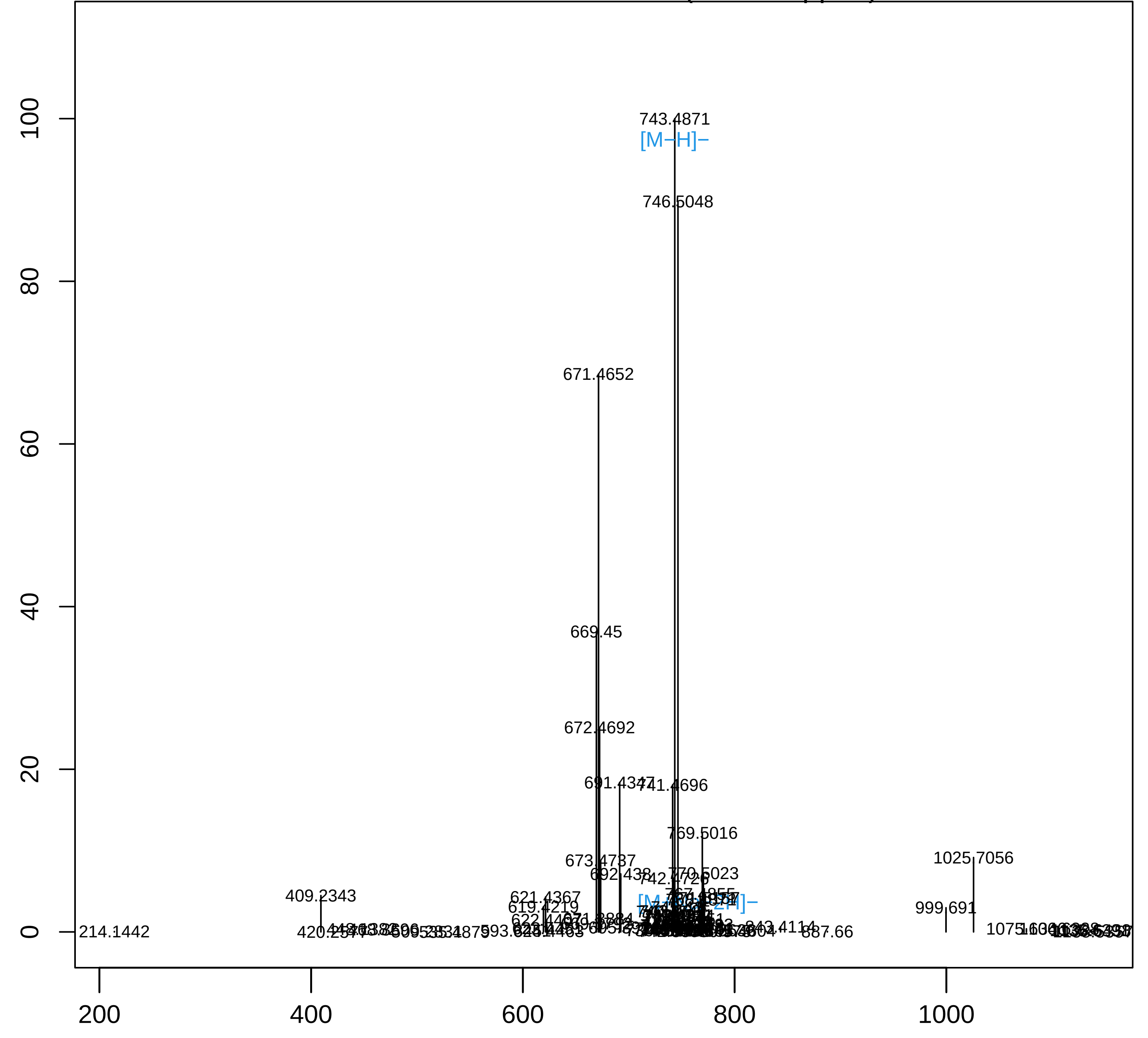

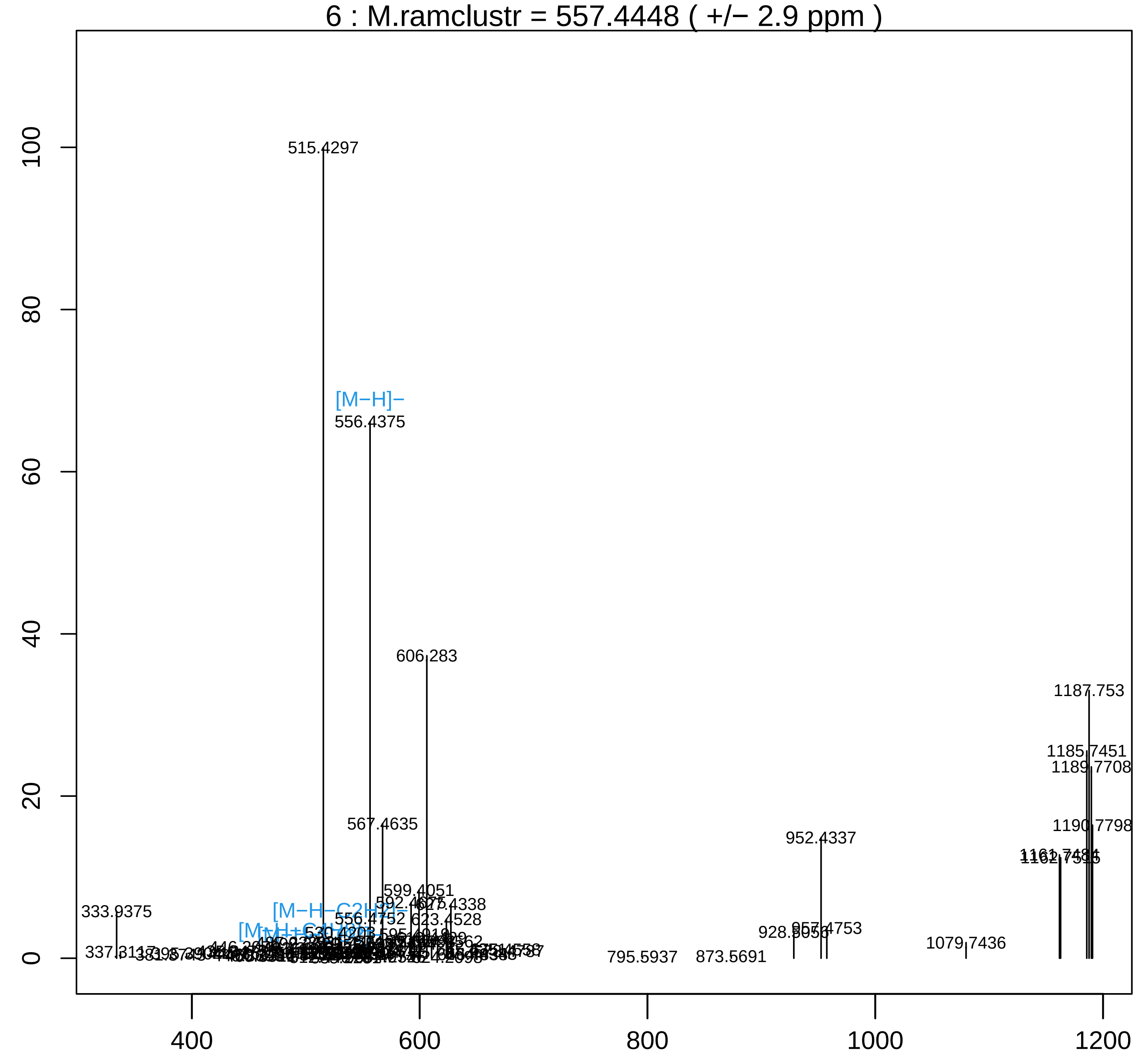
6 : M.ramclustr = 557.4448 (+/− 2.9 ppm)
515.4297
[M−H]−
556.4375
606.283
567.4635
599.4051
333.9375
795.5937
873.5691
952.4337
1079.7436
1187.753
1185.7451
1189.7708
1190.7798
0
20
40
60
80
100
400
600
800
1000
1200

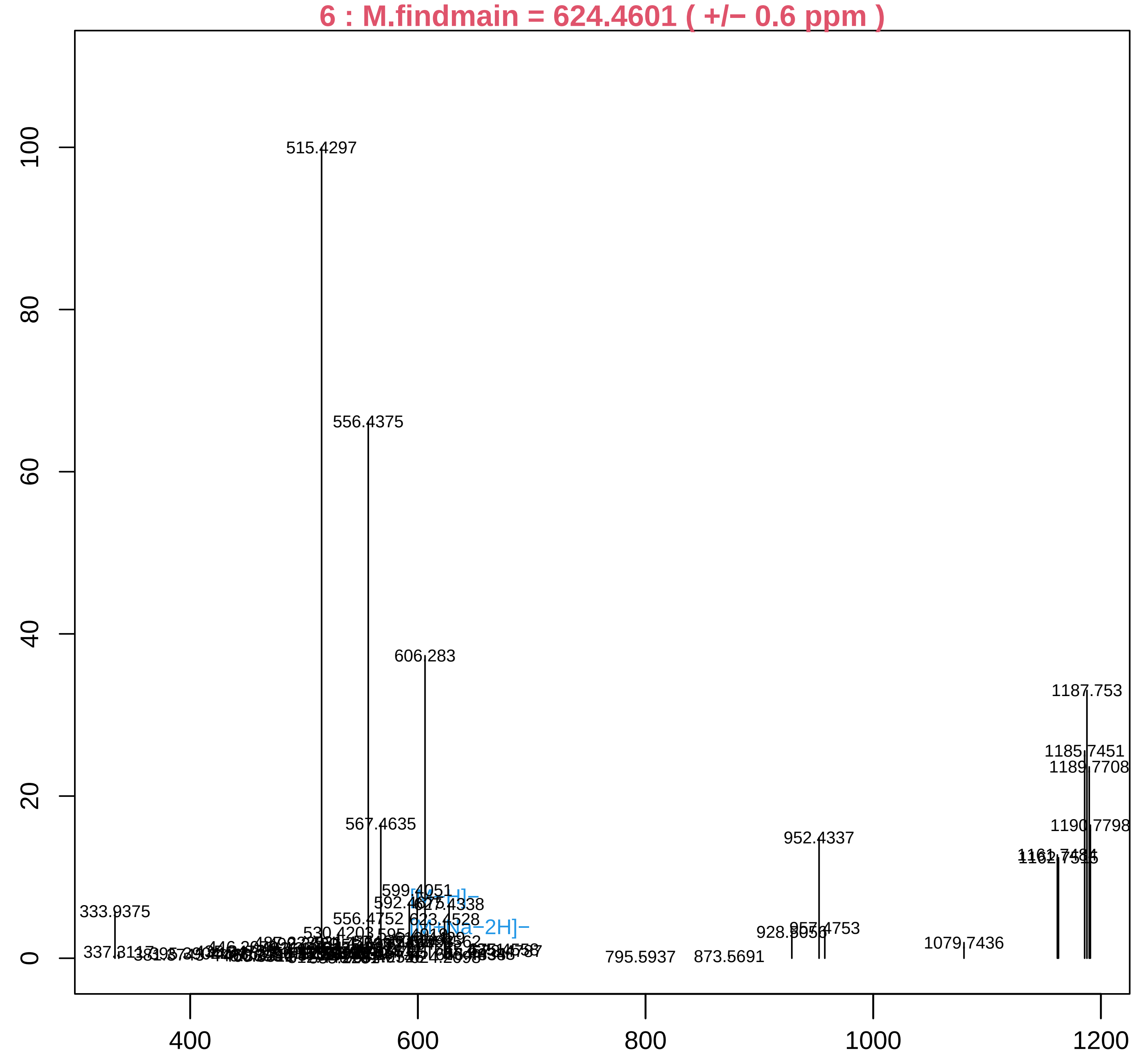
6 : M.findmain = 624.4601 (+/− 0.6 ppm)
515.4297
556.4375
606.283
567.4635
599.4051
333.9375
795.5937
873.5691
952.4337
1079.7436
1187.753
1185.7451
1189.7708
1190.7798
0
20
40
60
80
100
400
600
800
1000
1200

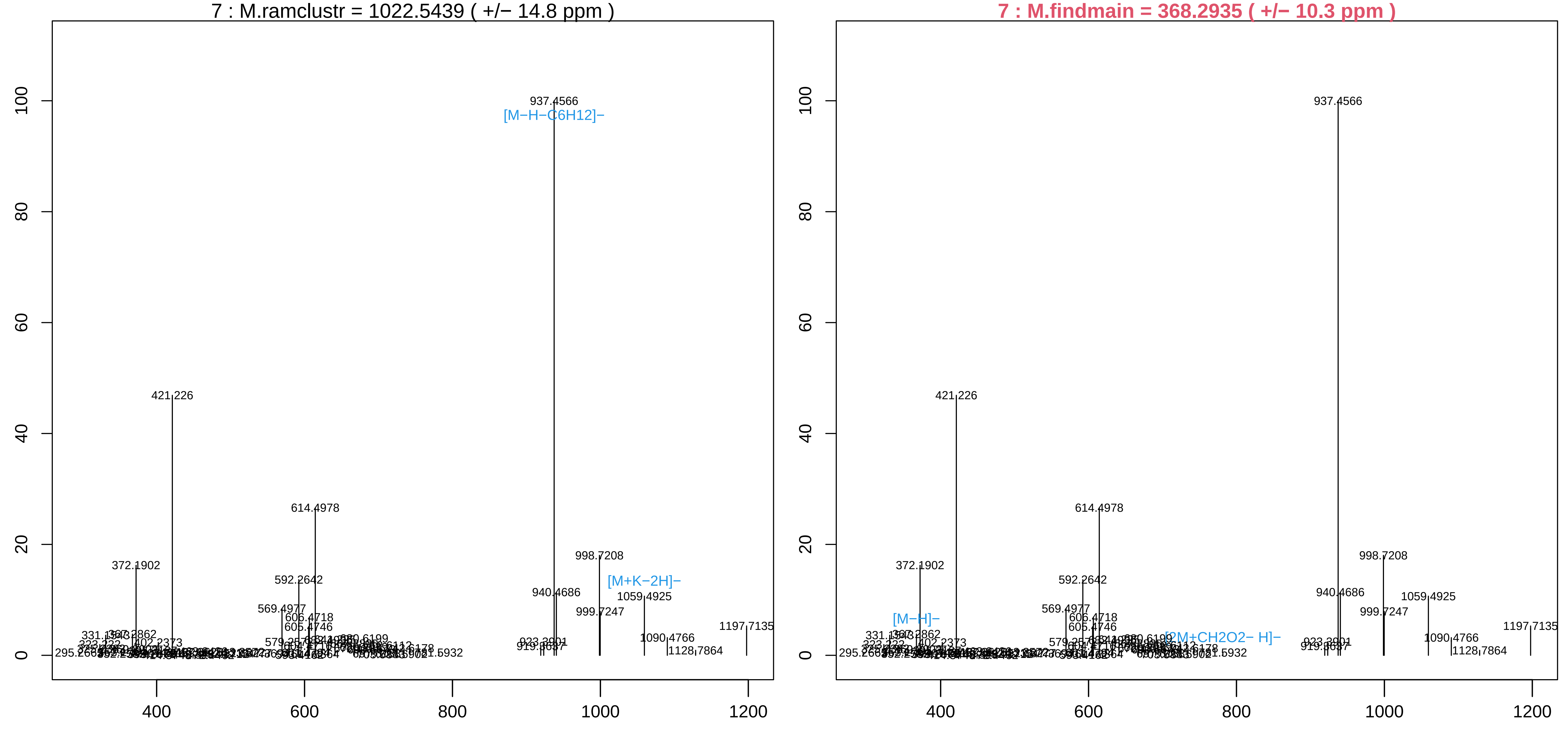

7 : M.ramclustr = 1022.5439 (+/− 14.8 ppm)
[M−H−C6H12]−
[M+K−2H]−
937.4566
421.226
614.4978
372.1902
592.2642
569.4977
606.4718
605.4746
998.7208
940.4686
999.7247
1059.4925
1090.4766
1128.7864
1197.7135
923.2901
7 : M.findmain = 368.2935 (+/− 10.3 ppm)
[M−H]−
[2M+CH2O2− H]−
0
20
40
60
80
100
400
600
800
1000
1200

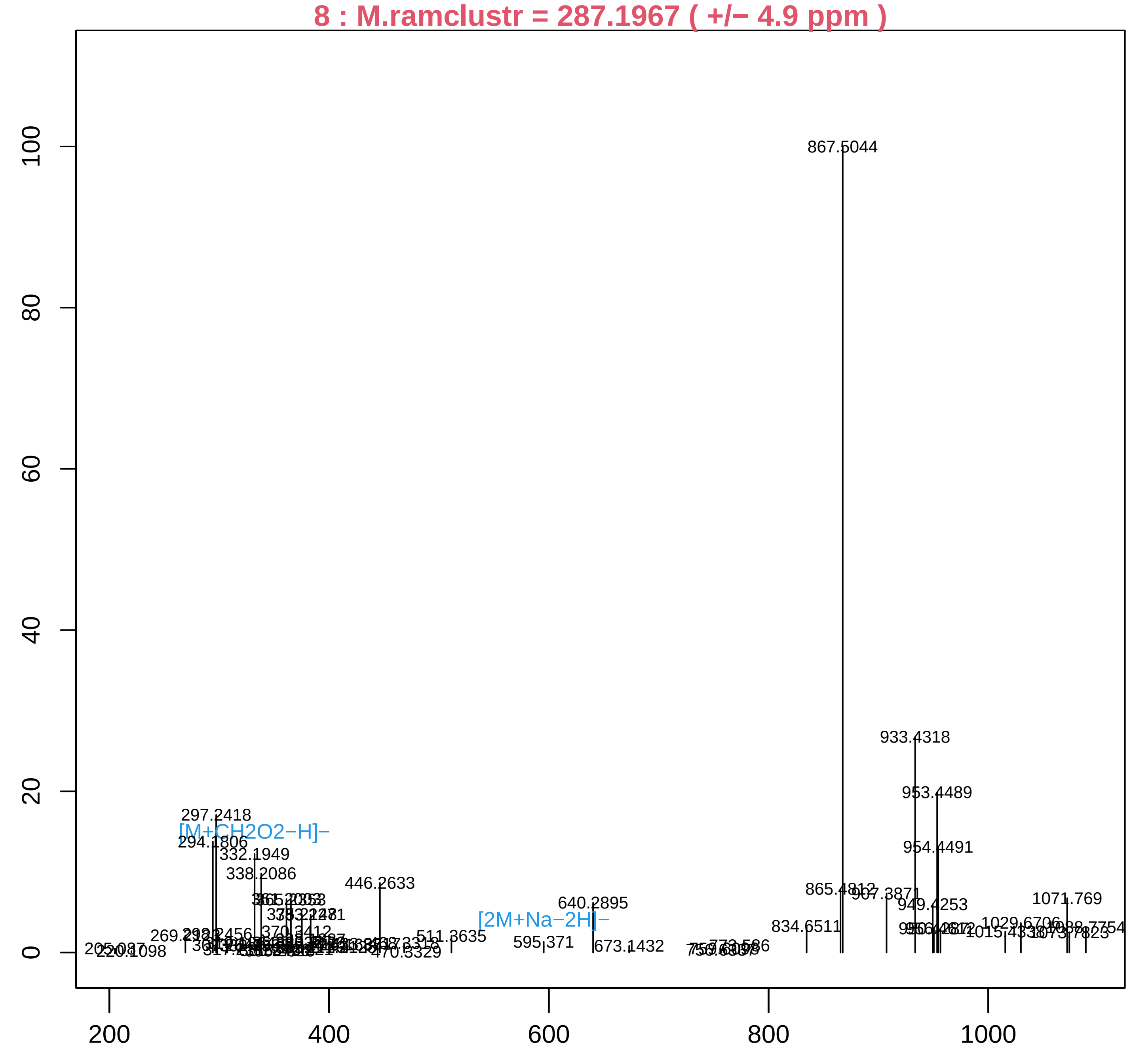
8 : M.ramclustr = 287.1967 (+/− 4.9 ppm)
867.5044
933.4318
953.4489
954.4491
297.2418
[M+CH2O2−H]−
294.1806
332.1949
338.2086
446.2633
865.4812
907.3871
949.4253
1071.769
640.2895
[2M+Na−2H]−
834.6511
1029.6706
511.3635
595.371
673.1432
470.5329
220.1098
0
20
40
60
80
100
200
400
600
800
1000

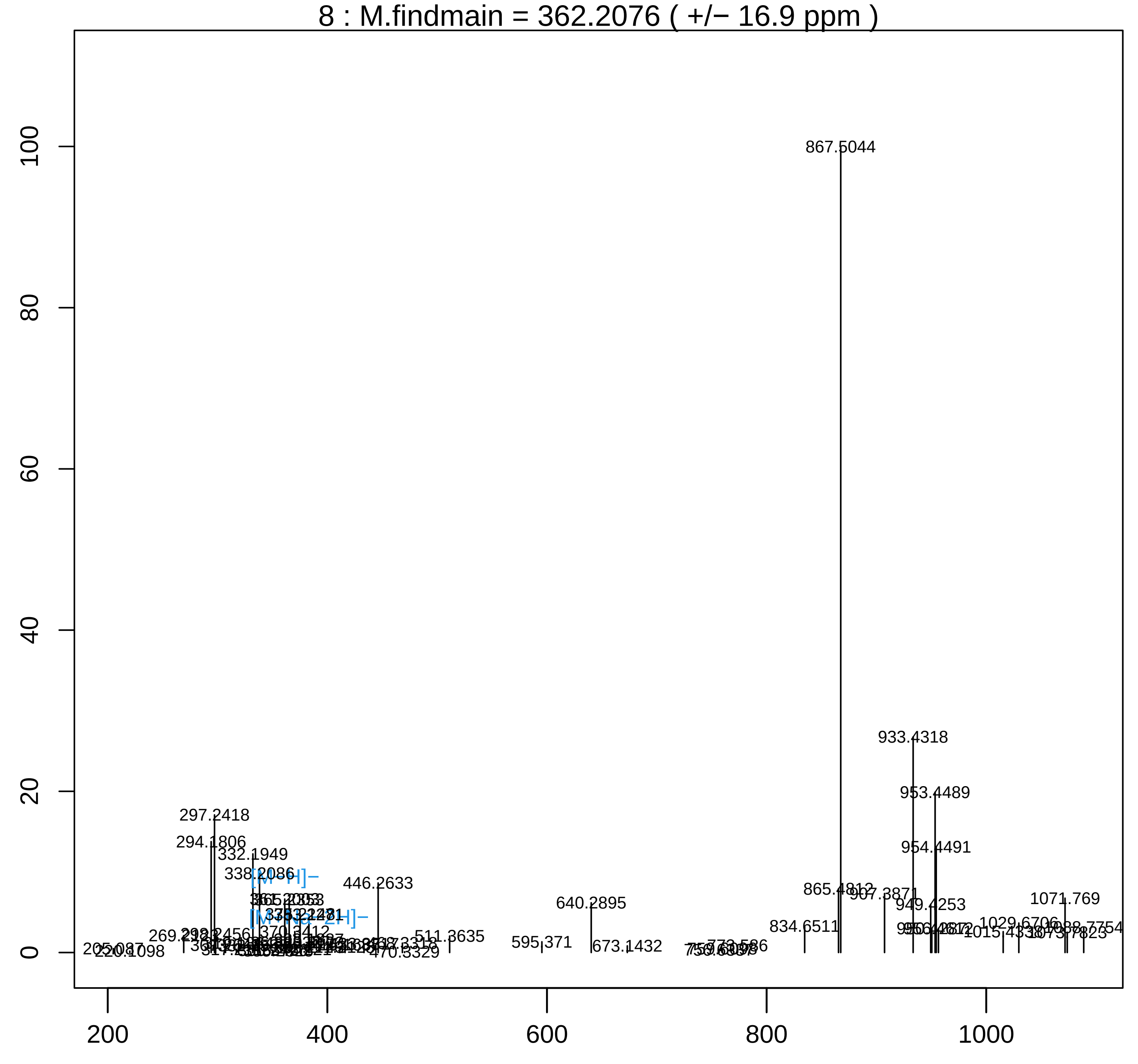
8 : M.findmain = 362.2076 (+/− 16.9 ppm)
867.5044
933.4318
953.4489
954.4491
297.2418
294.1806
332.1949
[M−H]−
446.2633
865.4812
907.3871
949.4253
1071.769
640.2895
834.6511
1029.6706
511.3635
595.371
673.1432
470.5329
220.1098
0
20
40
60
80
100
200
400
600
800
1000

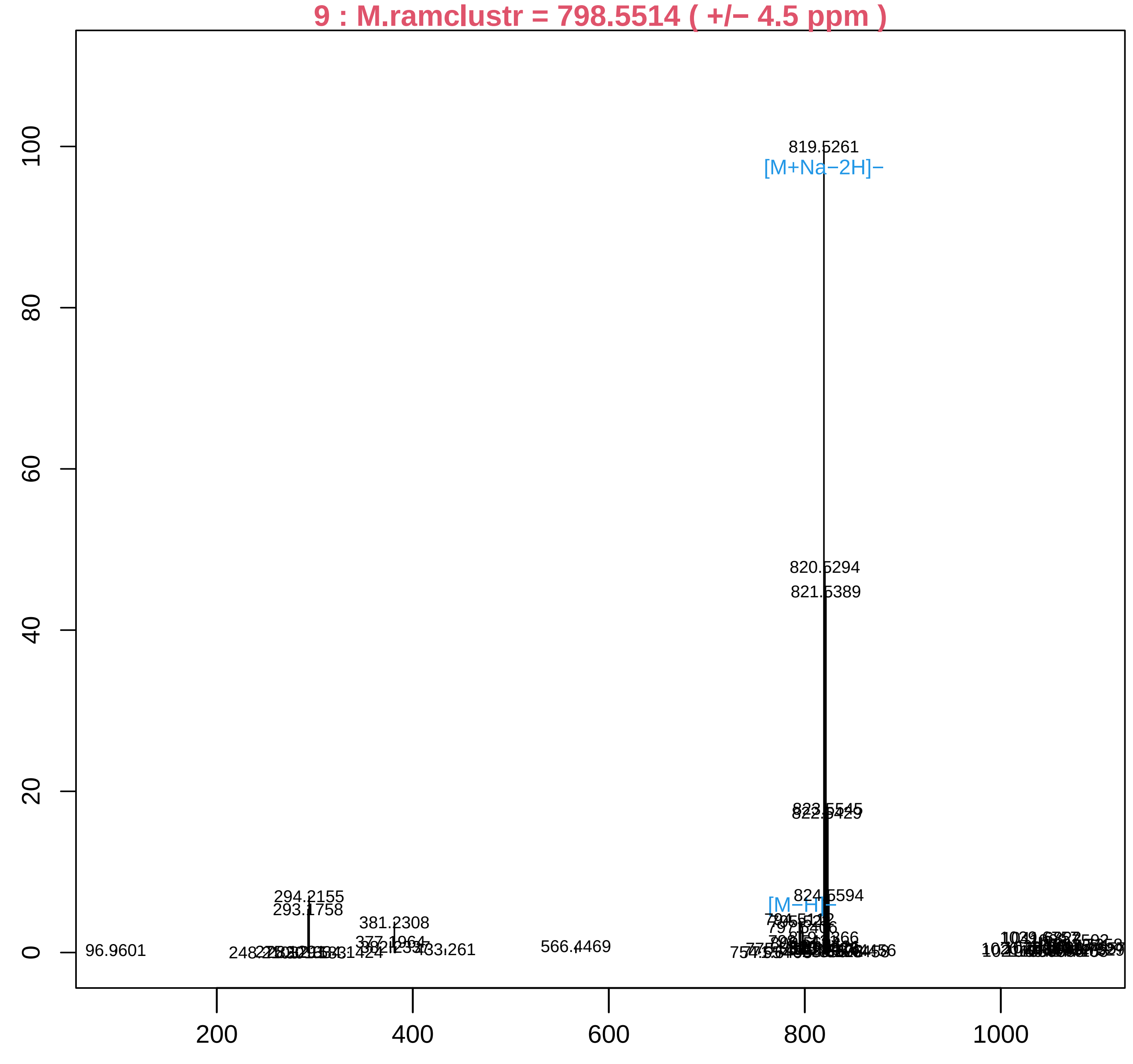
9 : M.ramclustr = 798.5514 (+/− 4.5 ppm)
819.5261
[M+Na−2H]−
820.5294
821.5389
824.5594
[M−H]−
294.2155
293.1758
381.2308
566.4469
96.9601
433.261
0
20
40
60
80
100
200
400
600
800
1000

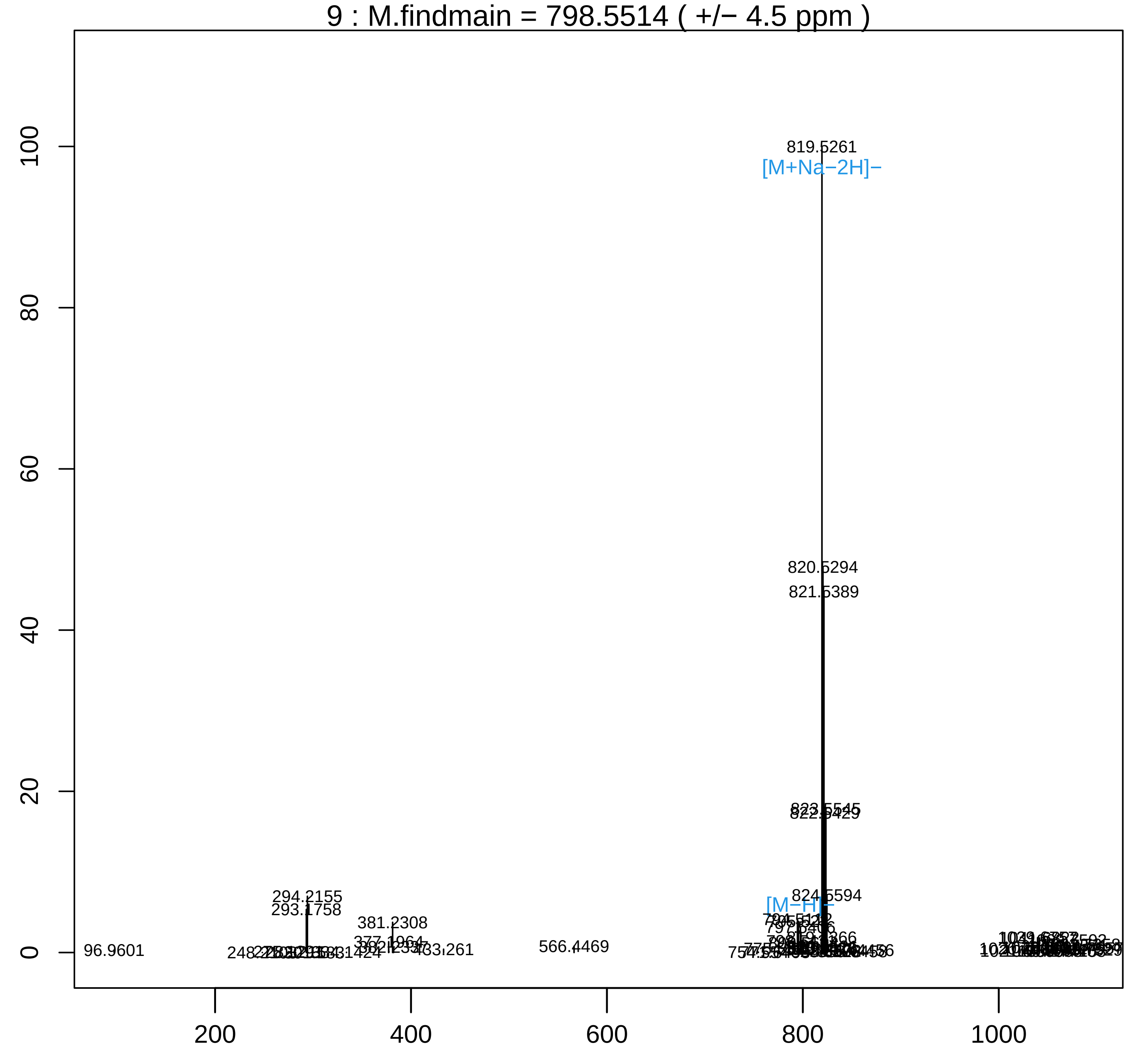
9 : M.findmain = 798.5514 (+/− 4.5 ppm)
819.5261
[M+Na−2H]−
820.5294
821.5389
824.5594
[M−H]−
294.2155
293.1758
381.2308
566.4469
96.9601
433.261
0
20
40
60
80
100
200
400
600
800
1000

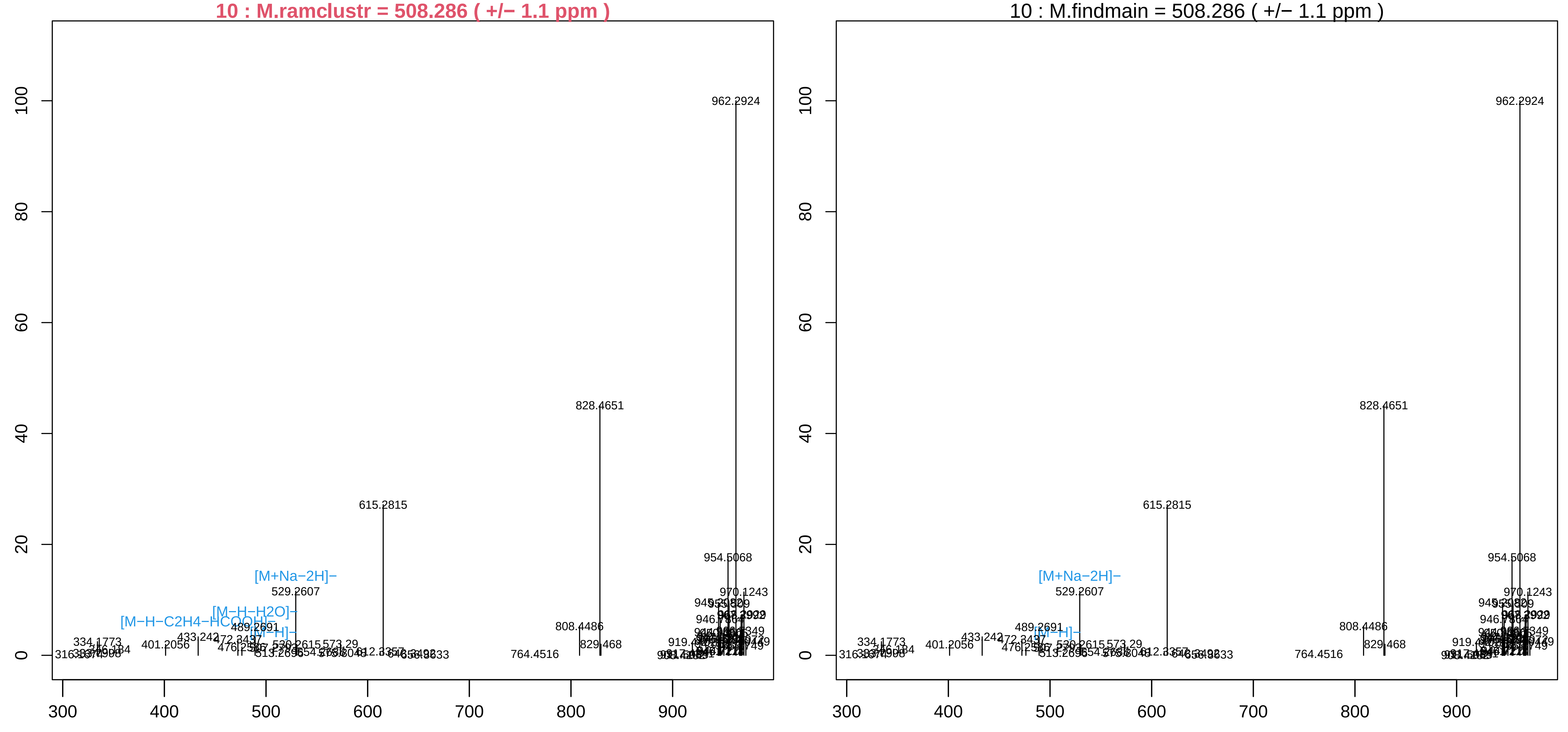
10 : M.ramclustr = 508.286 (+/− 1.1 ppm)
10 : M.findmain = 508.286 (+/− 1.1 ppm)
0
20
40
60
80
100
300
400
500
600
700
800
900
962.2924
828.4651
615.2815
954.5068
970.1243
[M+Na−2H]−
529.2607
[M−H−H2O]−
[M−H−C2H4−HCOOH]−
489.2691
[M−H]−
808.4486
829.468
334.1773
401.2056
433.242
472.3437
573.29
764.4516
919.4

11 : M.ramclustr = 816.5051 (+/− 2.1 ppm)

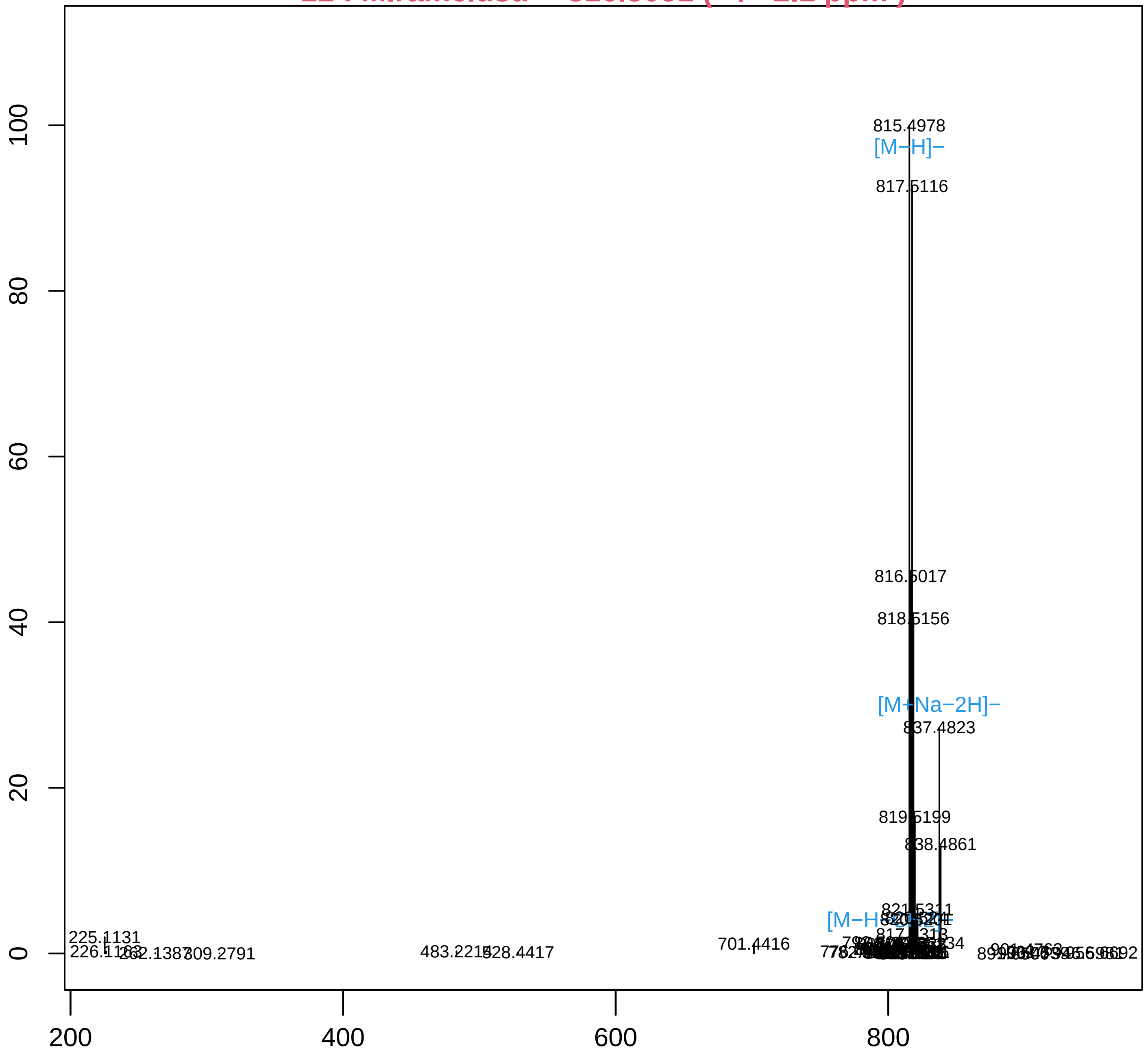

11 : M.findmain = 816.5051 (+/− 3 ppm)

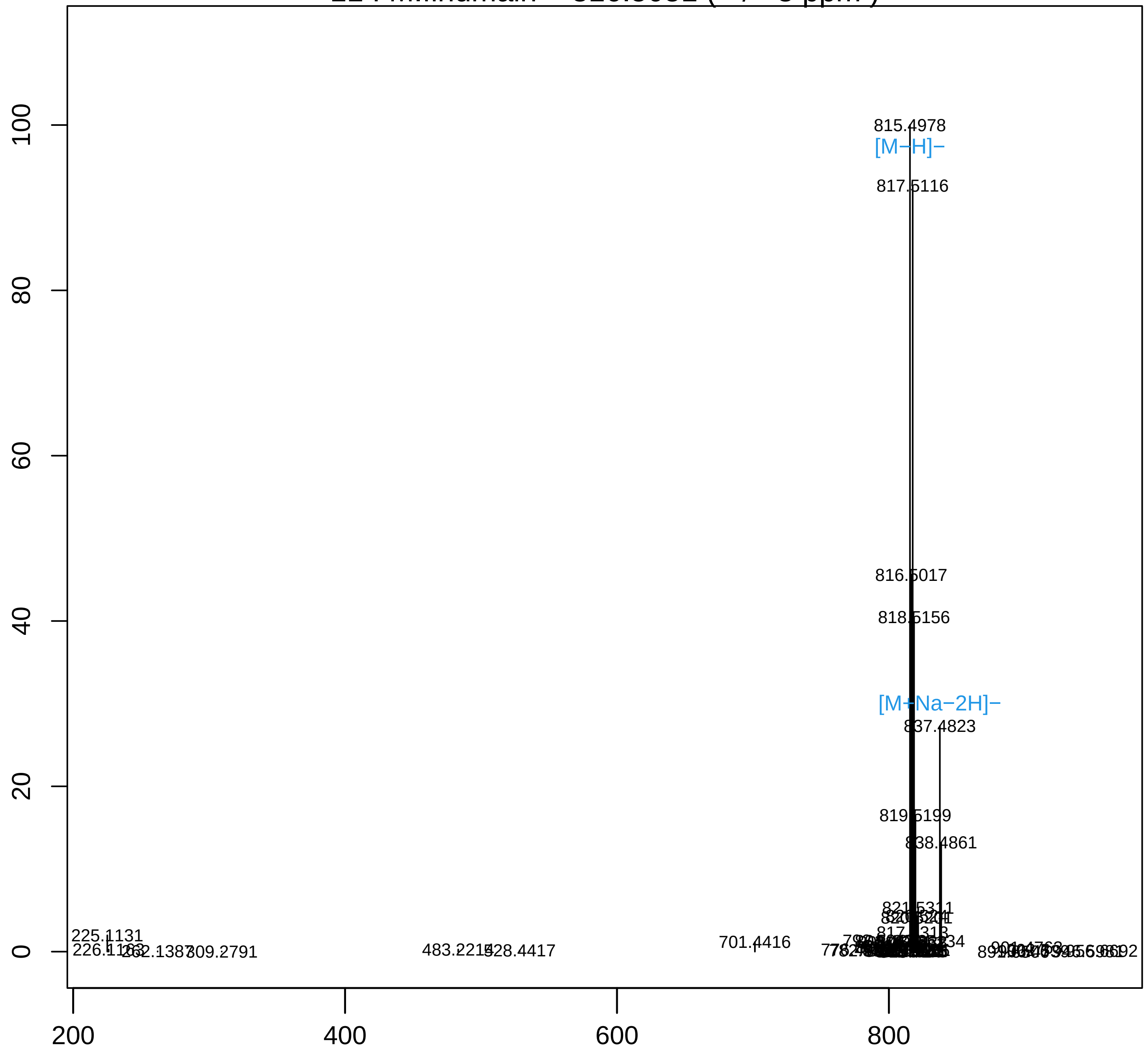

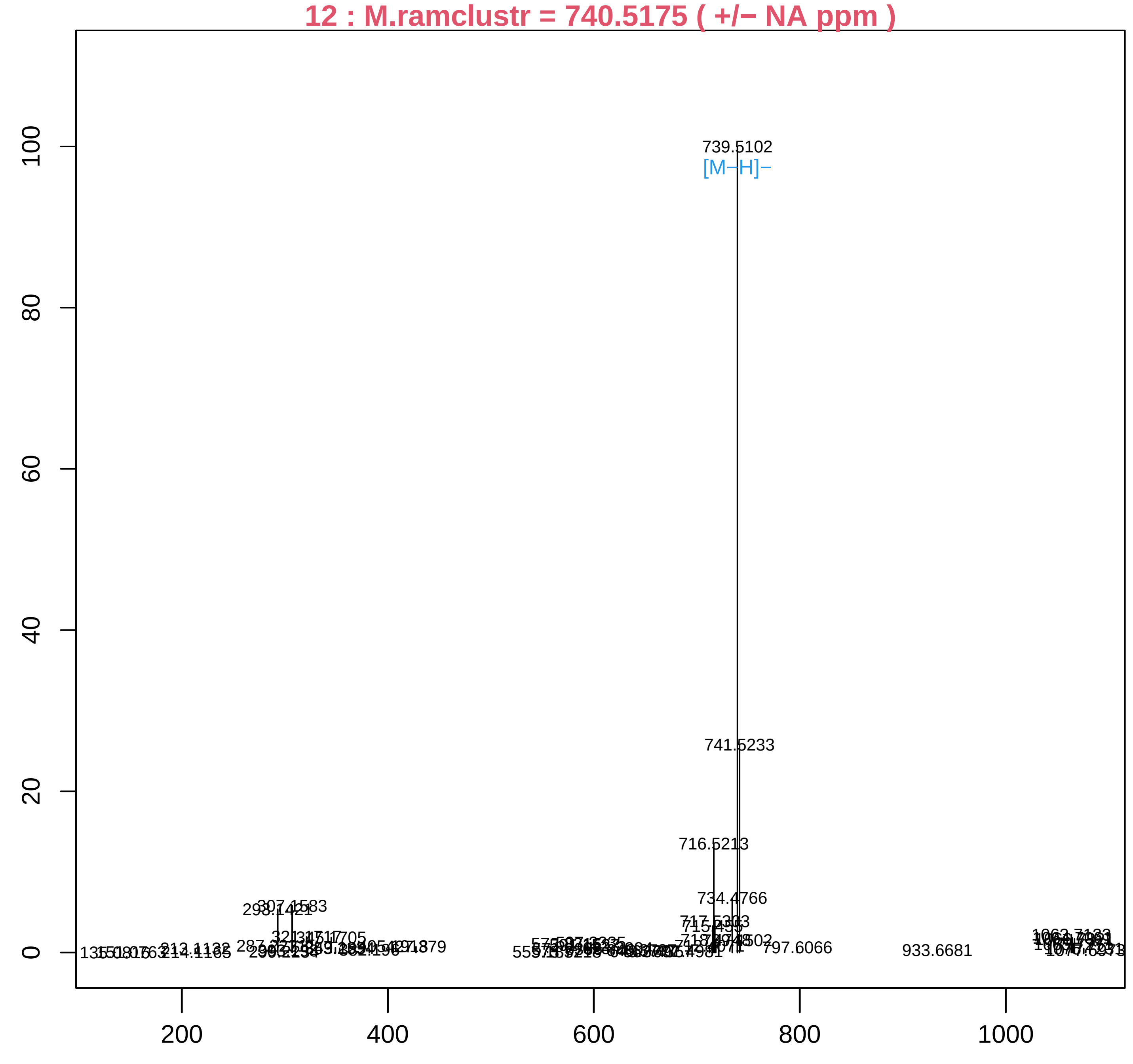
12 : M.ramclustr = 740.5175 (+/− NA ppm)
739.5102
[M−H]−
741.5233
716.5213
734.4766
797.6066
933.6681
0
20
40
60
80
100
200
400
600
800
1000

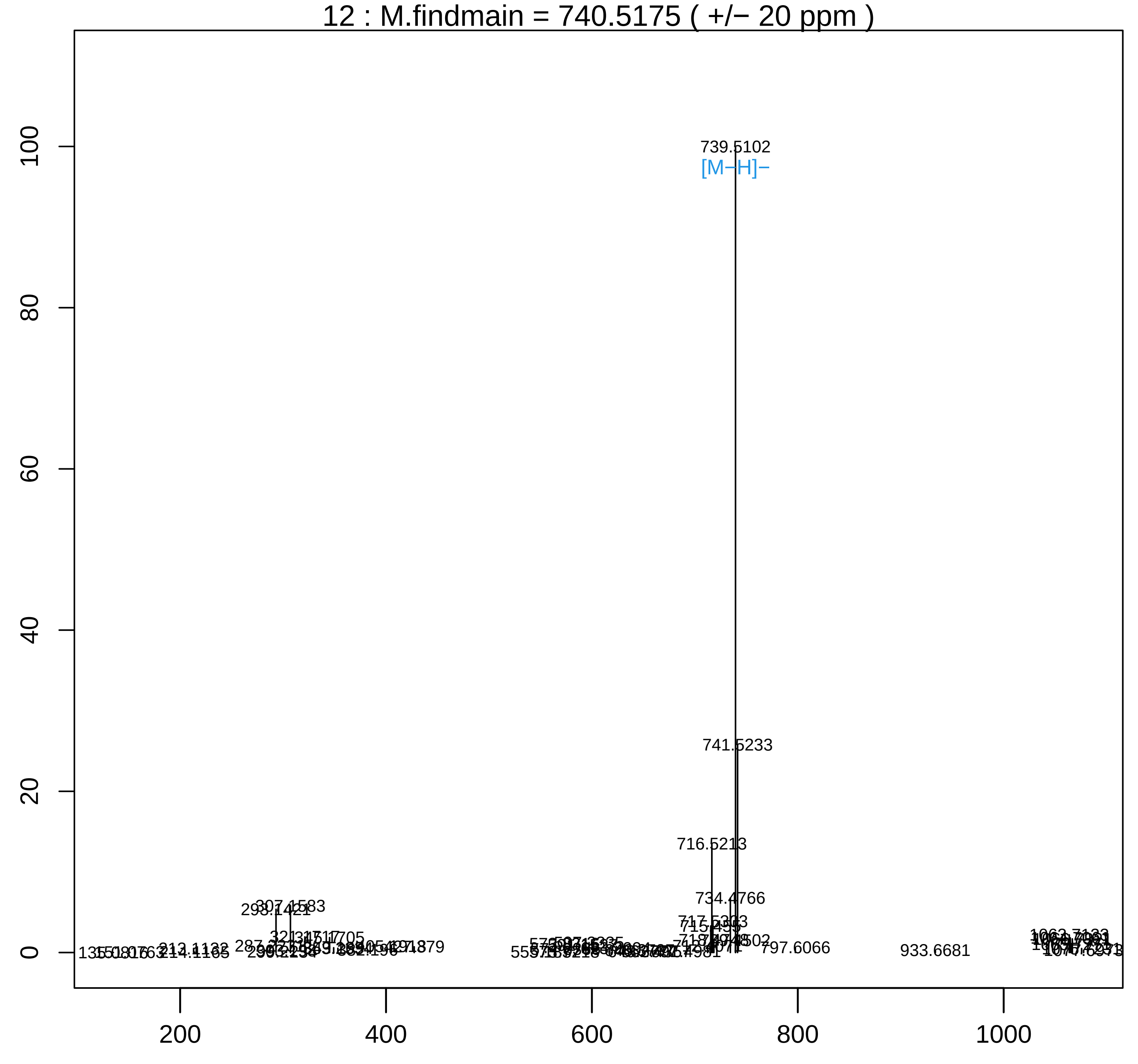
12 : M.findmain = 740.5175 (+/− 20 ppm)
739.5102
[M−H]−
741.5233
716.5213
734.4766
797.6066
933.6681
0
20
40
60
80
100
200
400
600
800
1000

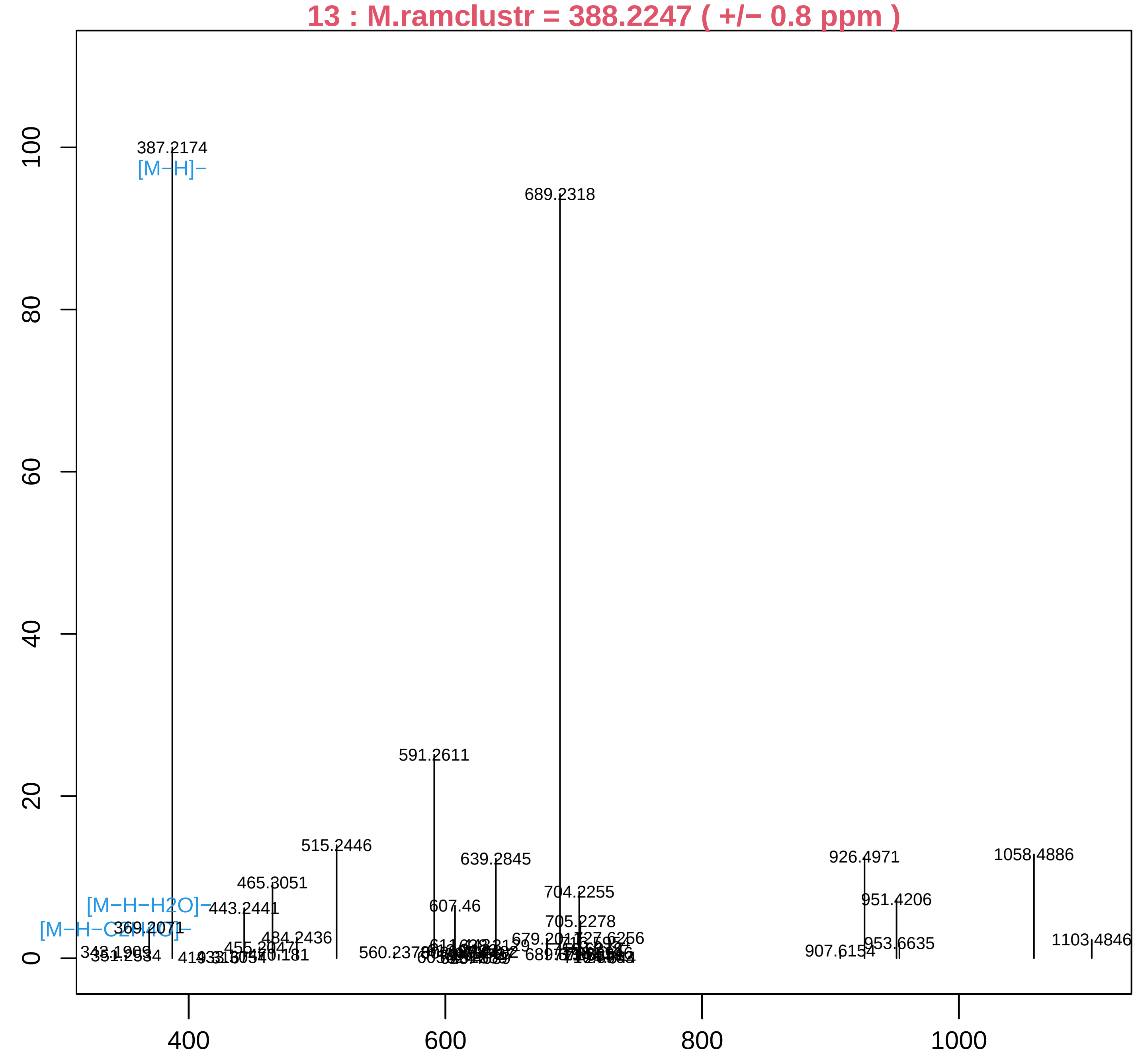
13 : M.ramclustr = 388.2247 (+/− 0.8 ppm)
387.2174
[M−H]−
689.2318
591.2611
515.2446
639.2845
465.3051
443.2441
607.46
704.2255
705.2278
434.2436
369.2071
[M−H−H2O]−
926.4971
951.4206
953.6635
907.6154
1058.4886
1103.4846
0
20
40
60
80
100
400
600
800
1000
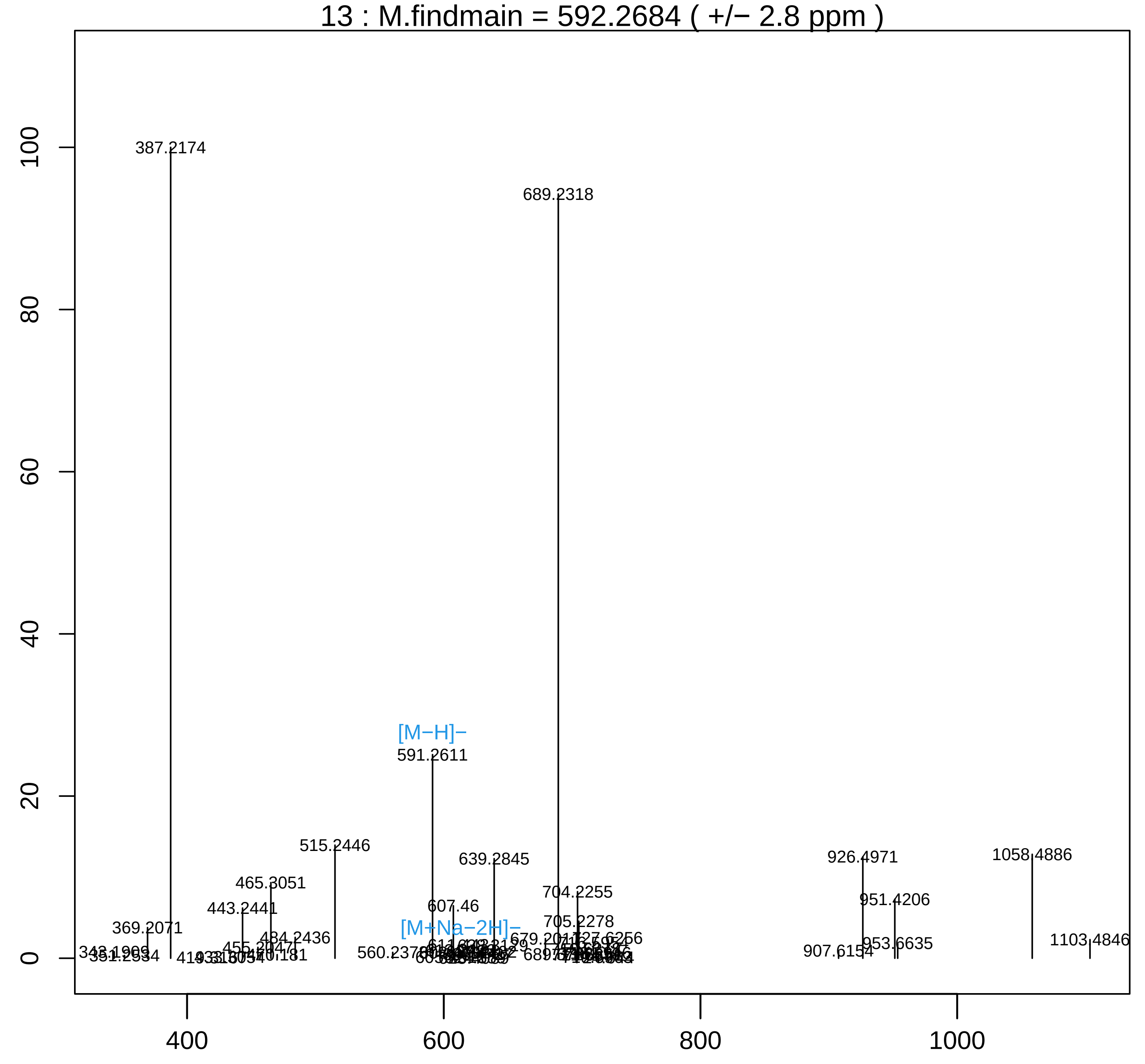
13 : M.findmain = 592.2684 (+/− 2.8 ppm)
387.2174
689.2318
[M−H]−
591.2611
515.2446
639.2845
465.3051
443.2441
607.46
[M+Na−2H]−
704.2255
705.2278
434.2436
369.2071
926.4971
951.4206
953.6635
907.6154
1058.4886
1103.4846
0
20
40
60
80
100
400
600
800
1000

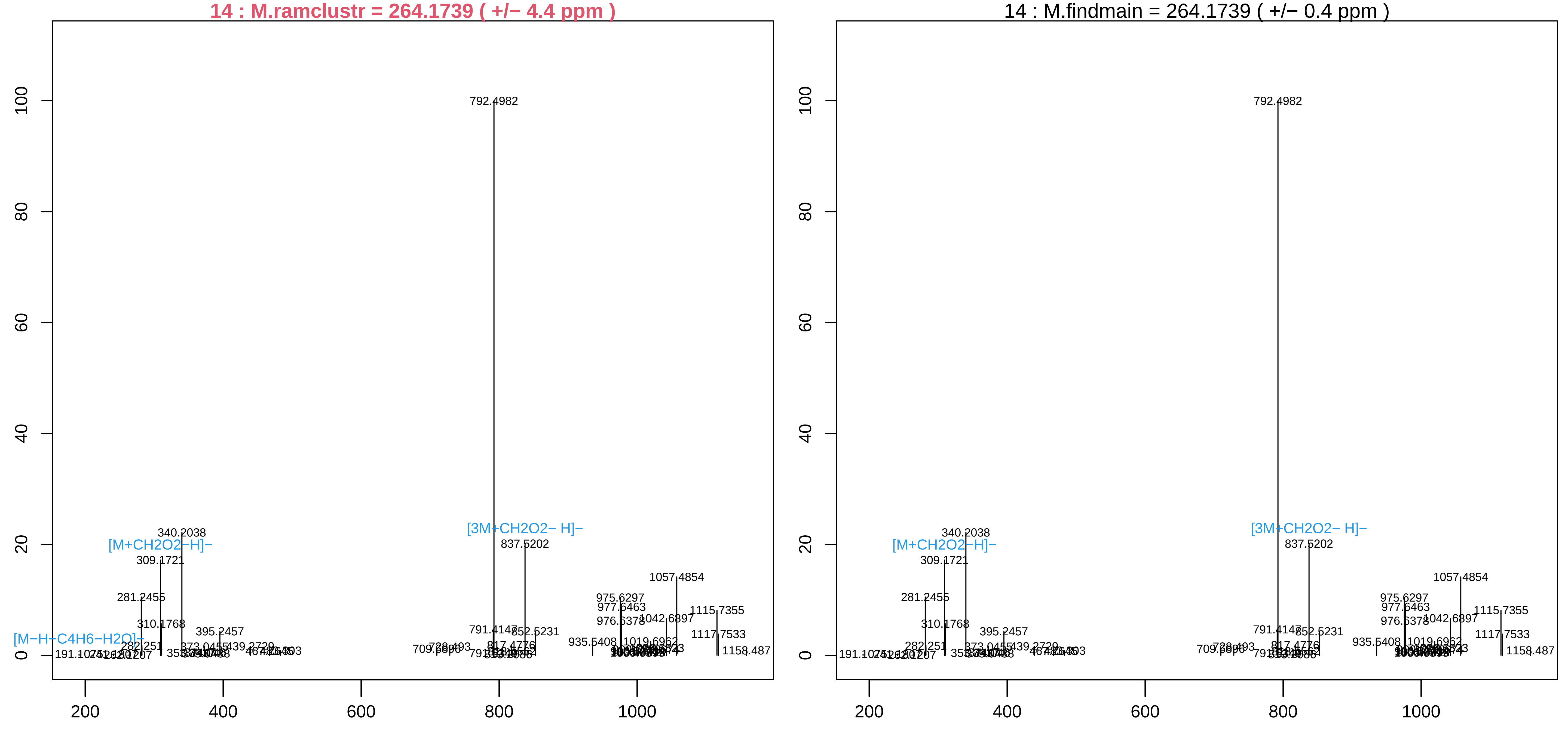
14 : M.ramclustr = 264.1739 (+/− 4.4 ppm)
14 : M.findmain = 264.1739 (+/− 0.4 ppm)
792.4982
[3M+CH2O2− H]−
837.5202
[M+CH2O2−H]−
340.2038
309.1721
281.2455
310.1768
395.2457
[M−H−C4H6−H2O]−
282.251
1057.4854
975.6297
977.6463
976.6378
1042.6897
1115.7355
1117.7533
1158.487
791.4147
852.5231
935.5408
1019.6962
817.4776
439.2729
373.0455
191.1025
709.6606
0
20
40
60
80
100
200
400
600
800
1000

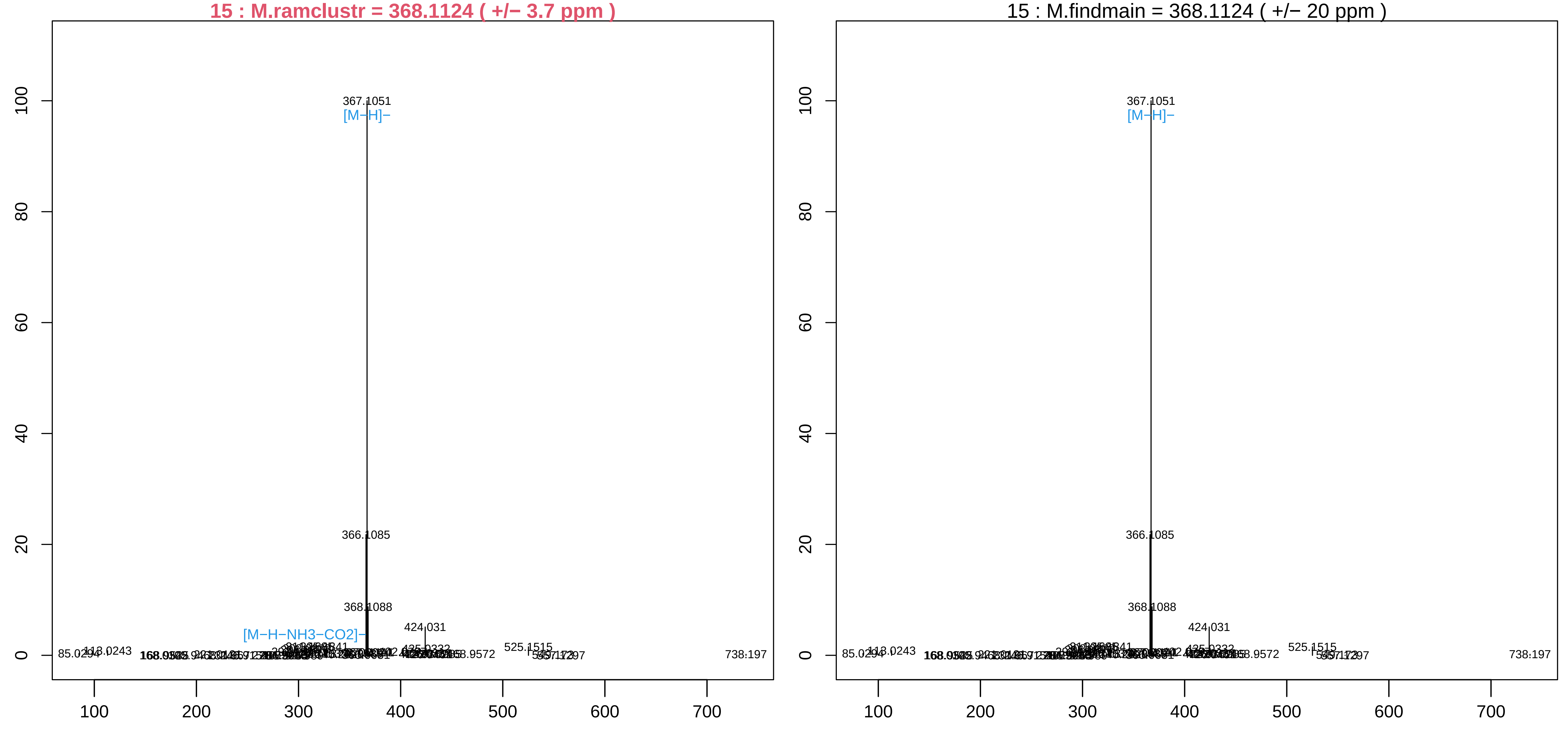
15 : M.ramclustr = 368.1124 (+/− 3.7 ppm)
367.1051
[M−H]−
366.1085
368.1088
424.031
[M−H−NH3−CO2]−
525.1515
85.0294
113.0243
738.197
15 : M.findmain = 368.1124 (+/− 20 ppm)
367.1051
[M−H]−
366.1085
368.1088
424.031
525.1515
85.0294
113.0243
738.197
0
20
40
60
80
100
100
200
300
400
500
600
700

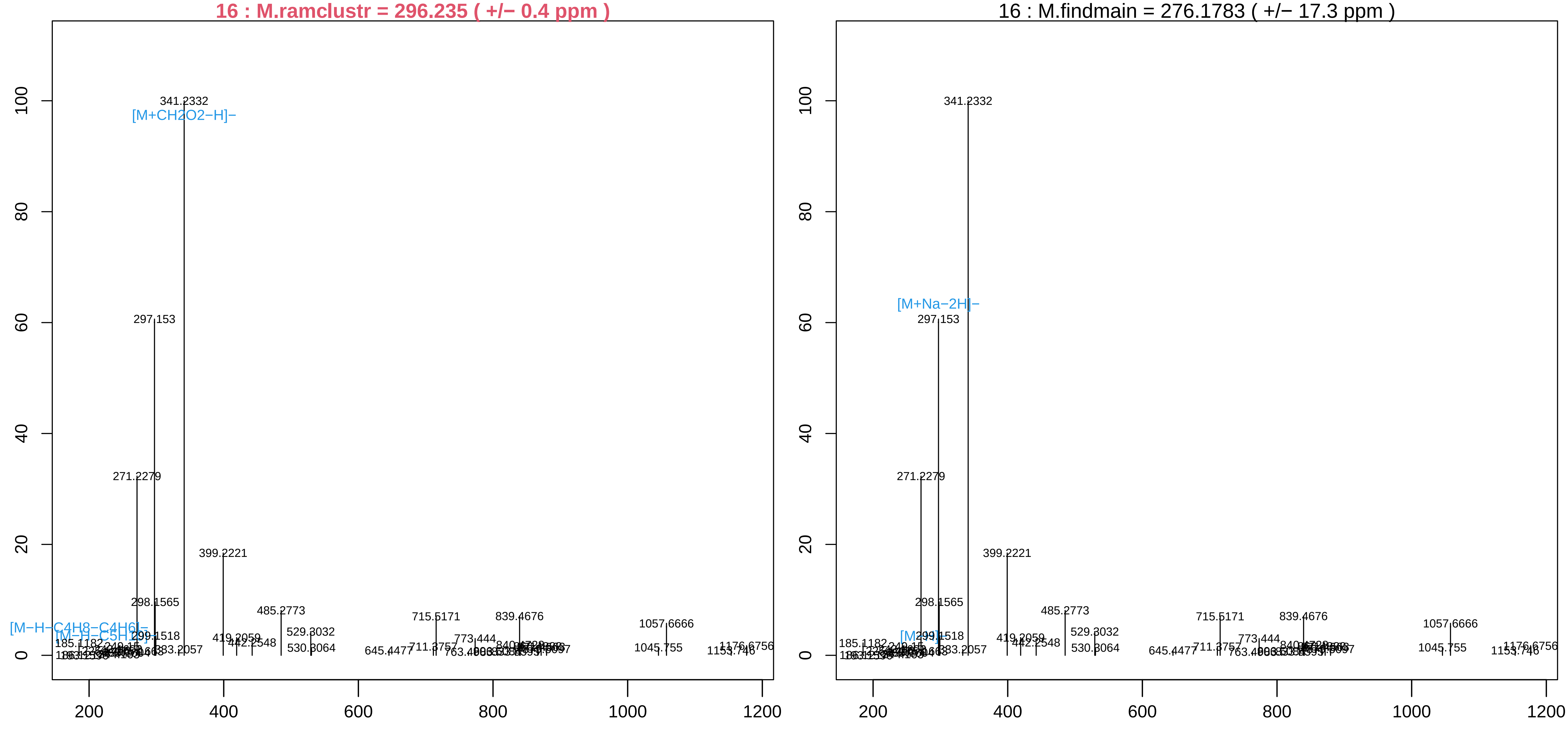

16 : M.ramclustr = 296.235 (+/− 0.4 ppm)
341.2332
[M+CH2O2−H]−
297.153
271.2279
399.2221
298.1565
485.2773
715.5171
839.4676
1057.6666
529.3032
299.1518
419.2059
442.2548
773.444
530.3064
645.4477
711.3757
1045.755
1153.746
1176.6756
185.1182
383.2057
16 : M.findmain = 276.1783 (+/− 17.3 ppm)
341.2332
[M+Na−2H]−
297.153
271.2279
399.2221
298.1565
485.2773
715.5171
839.4676
1057.6666
529.3032
299.1518
419.2059
442.2548
773.444
530.3064
645.4477
711.3757
1045.755
1153.746
1176.6756
185.1182
383.2057
0
20
40
60
80
100
200
400
600
800
1000
1200

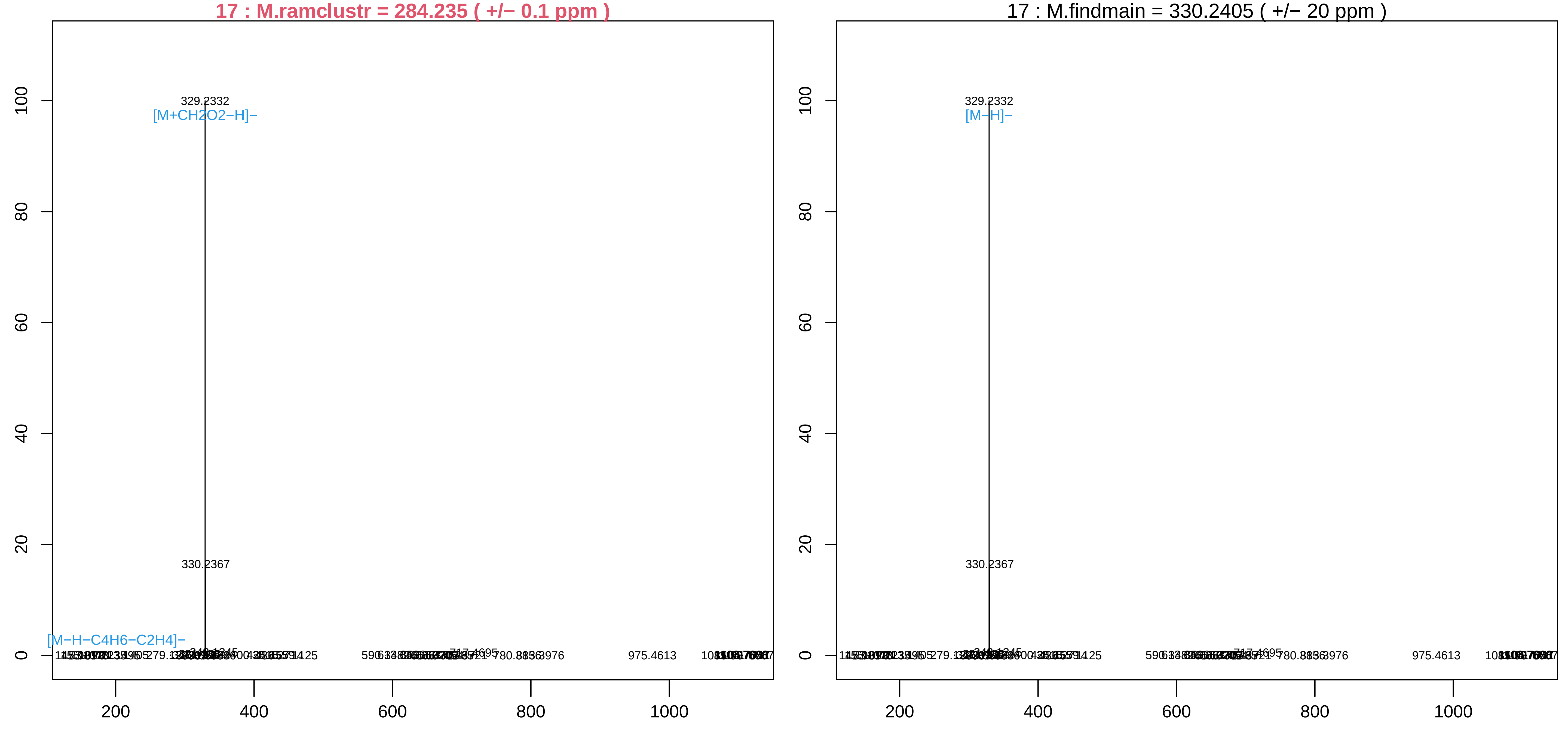
17 : M.ramclustr = 284.235 (+/− 0.1 ppm)
329.2332
[M+CH2O2−H]−
330.2367
[M−H−C4H6−C2H4]−
975.4613
17 : M.findmain = 330.2405 (+/− 20 ppm)
329.2332
[M−H]−
330.2367
975.4613

18 : M.ramclustr = 710.4877 (+/− NA ppm)

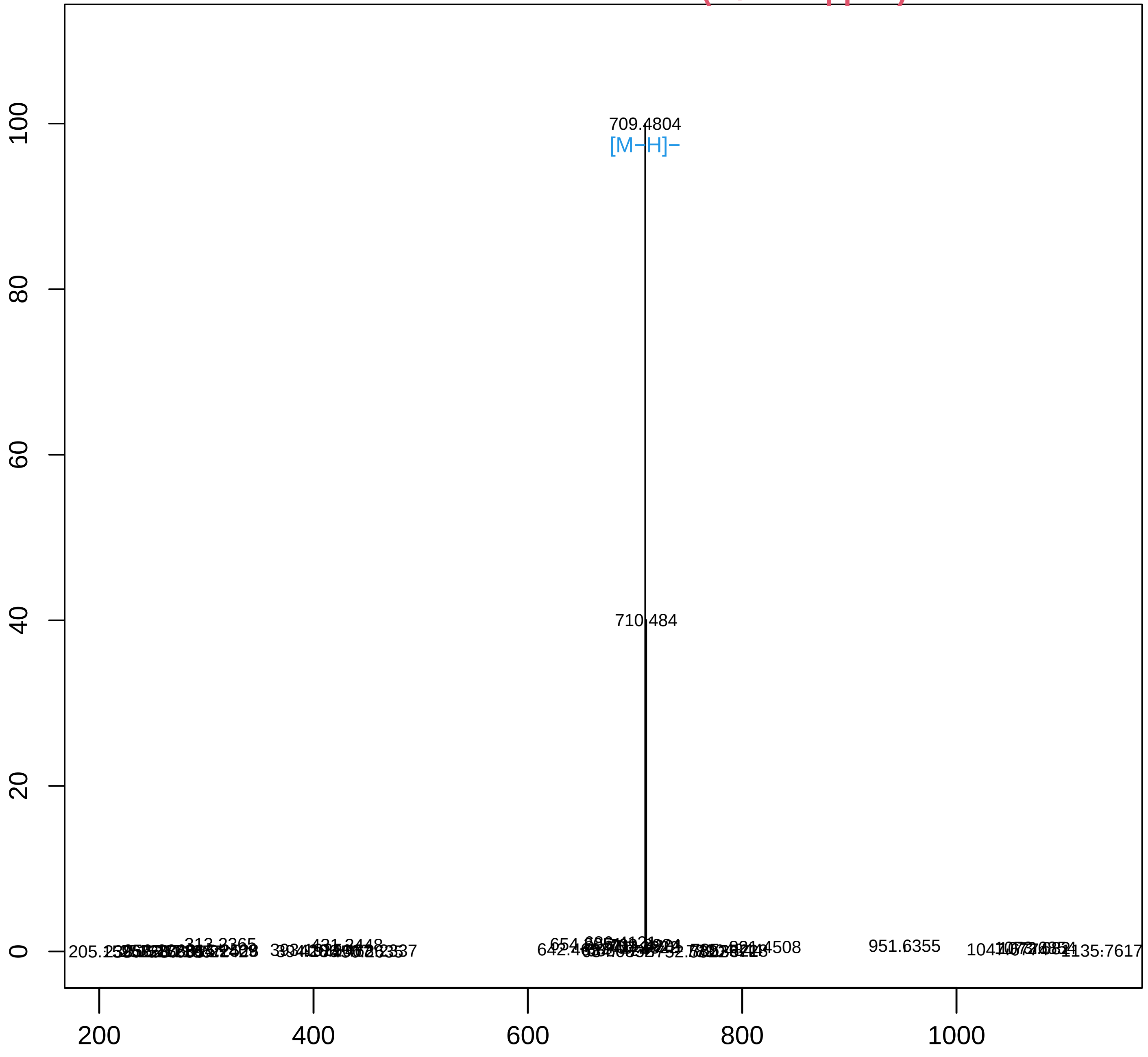

18 : M.findmain = 710.4877 (+/− 20 ppm)

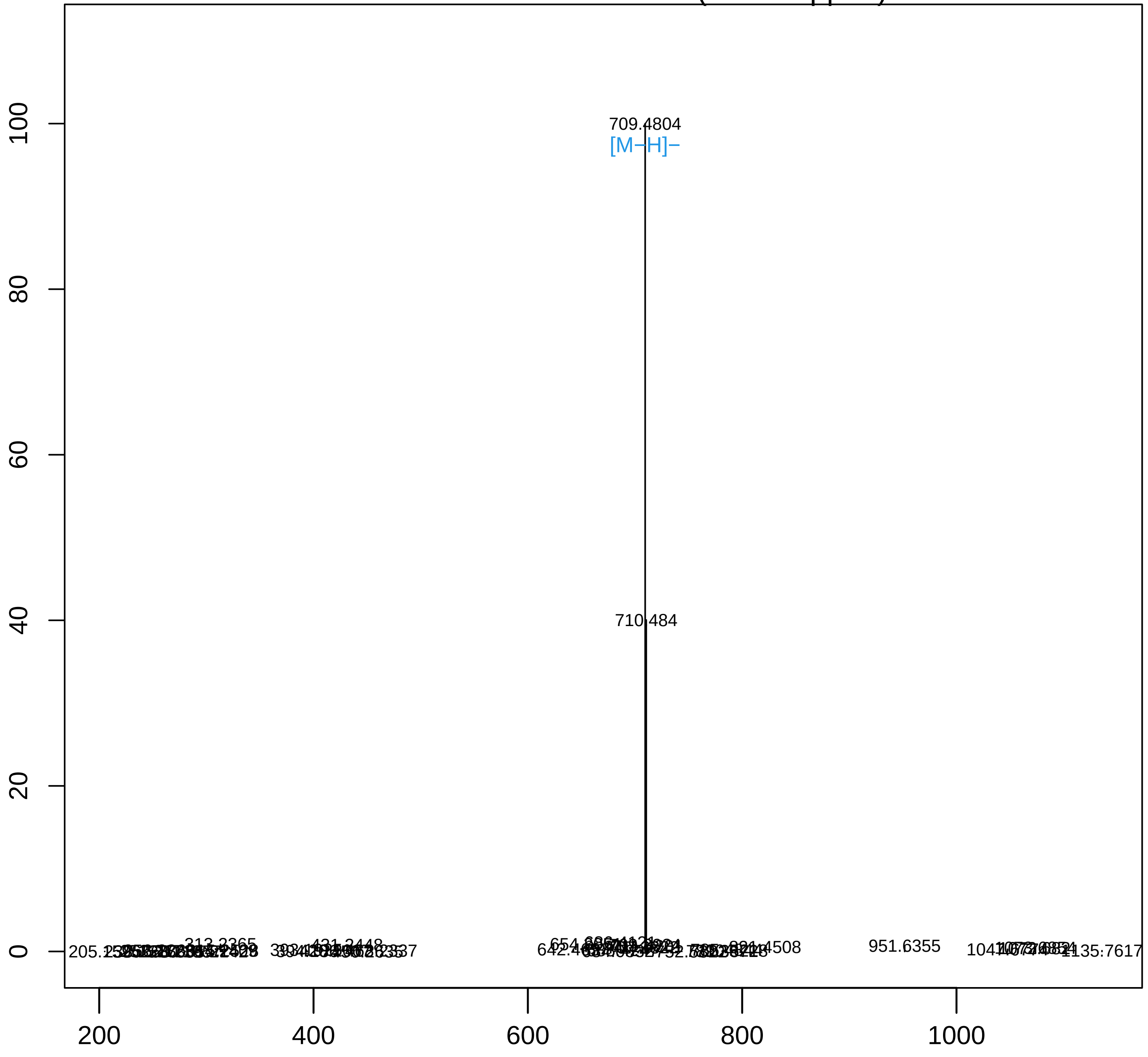

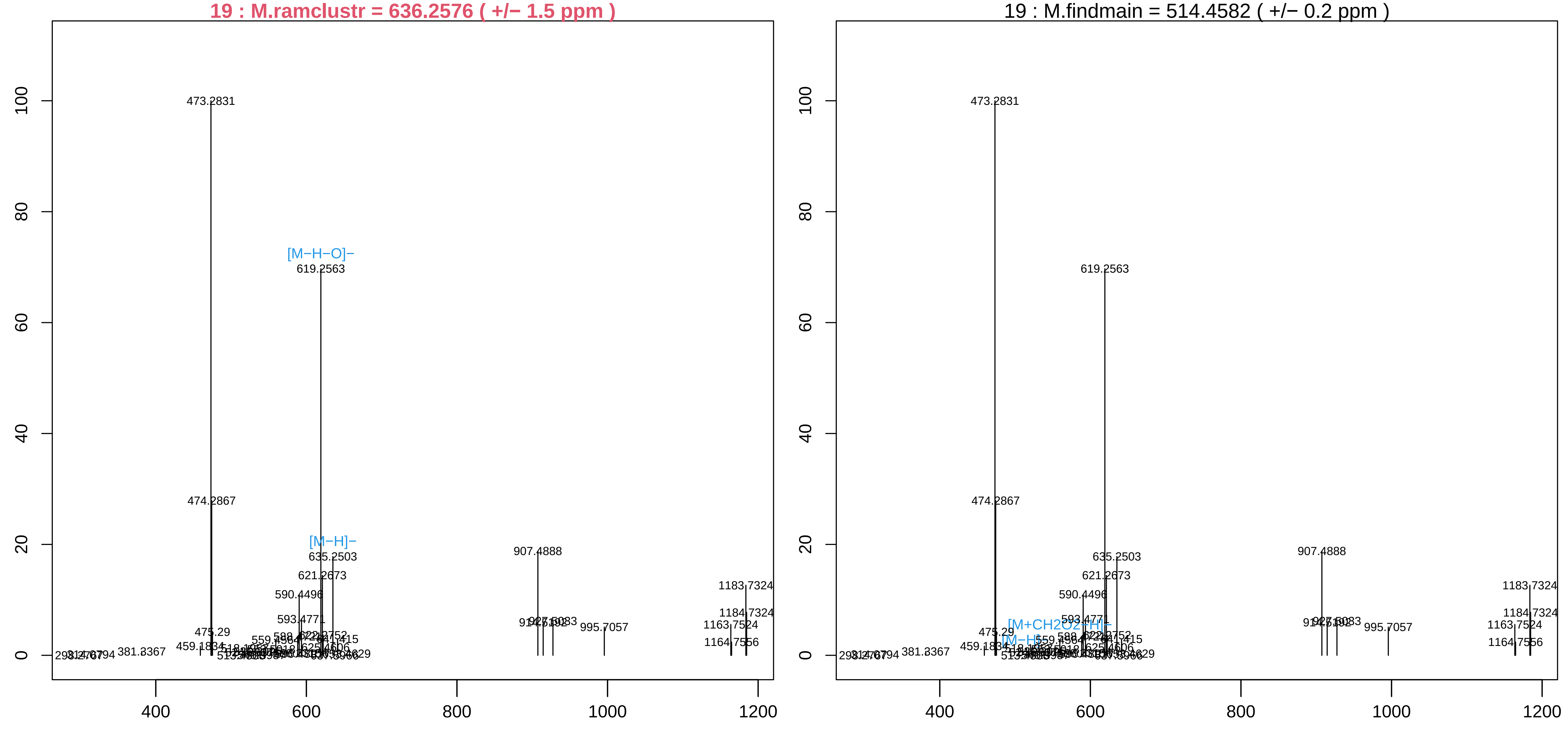
19 : M.ramclustr = 636.2576 (+/− 1.5 ppm)
473.2831
[M−H−O]−
619.2563
474.2867
[M−H]−
635.2503
621.2673
590.4496
593.4771
475.29
459.1834
381.3367
907.4888
995.7057
1183.7324
1184.7324
1163.7524
1164.7556
19 : M.findmain = 514.4582 (+/− 0.2 ppm)
473.2831
619.2563
474.2867
635.2503
621.2673
590.4496
593.4771
[M+CH2O2−H]−
[M−H]−
475.29
459.1834
381.3367
907.4888
995.7057
1183.7324
1184.7324
1163.7524
1164.7556
0
20
40
60
80
100
400
600
800
1000
1200

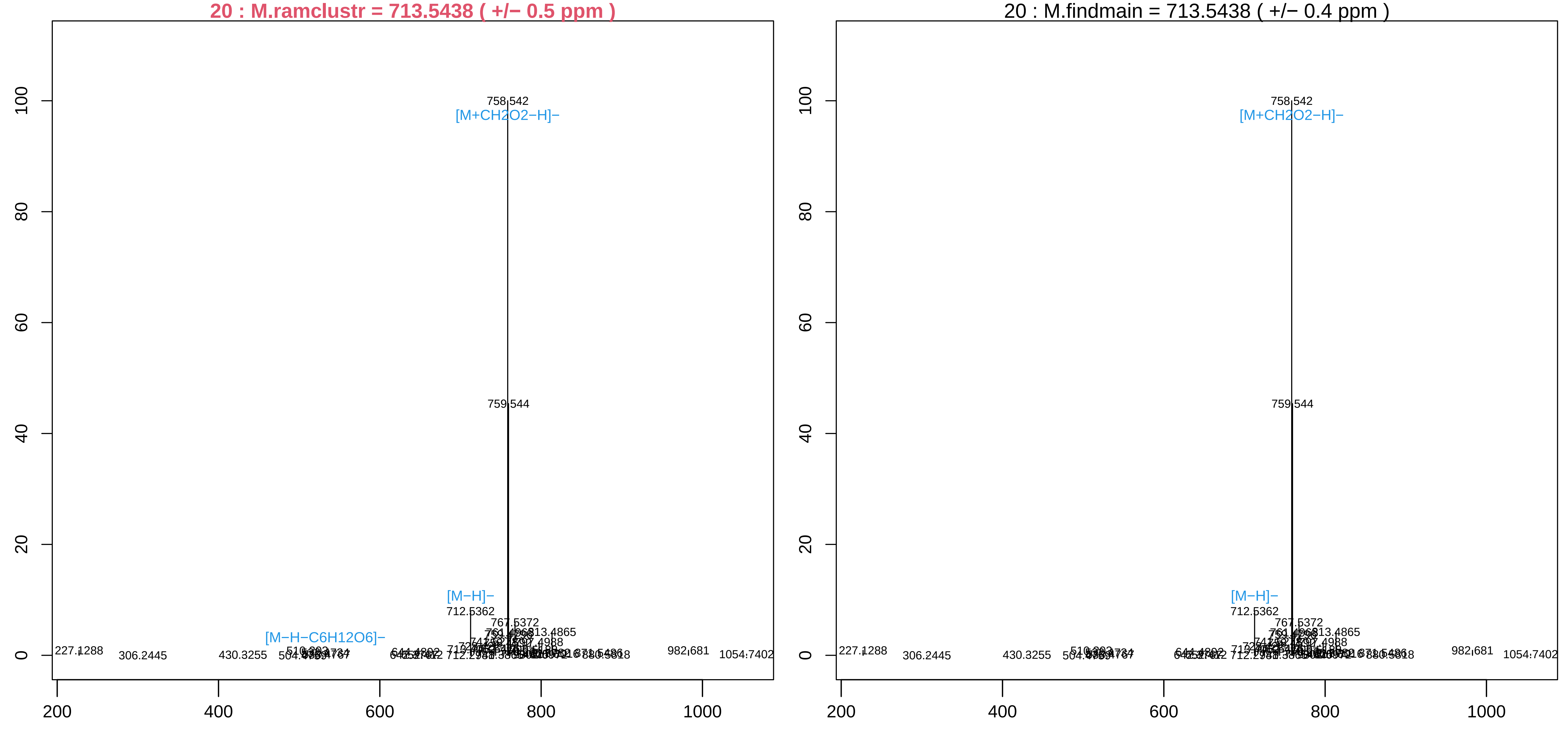

20 : M.ramclustr = 713.5438 (+/− 0.5 ppm)
20 : M.findmain = 713.5438 (+/− 0.4 ppm)
758.542
[M+CH2O2−H]−
759.544
[M−H]−
712.5362
767.5372
813.4865
797.4988
[M−H−C6H12O6]−
227.1288
306.2445
430.3255
644.4292
982.681
1054.7402
880.5818
0
20
40
60
80
100
200
400
600
800
1000

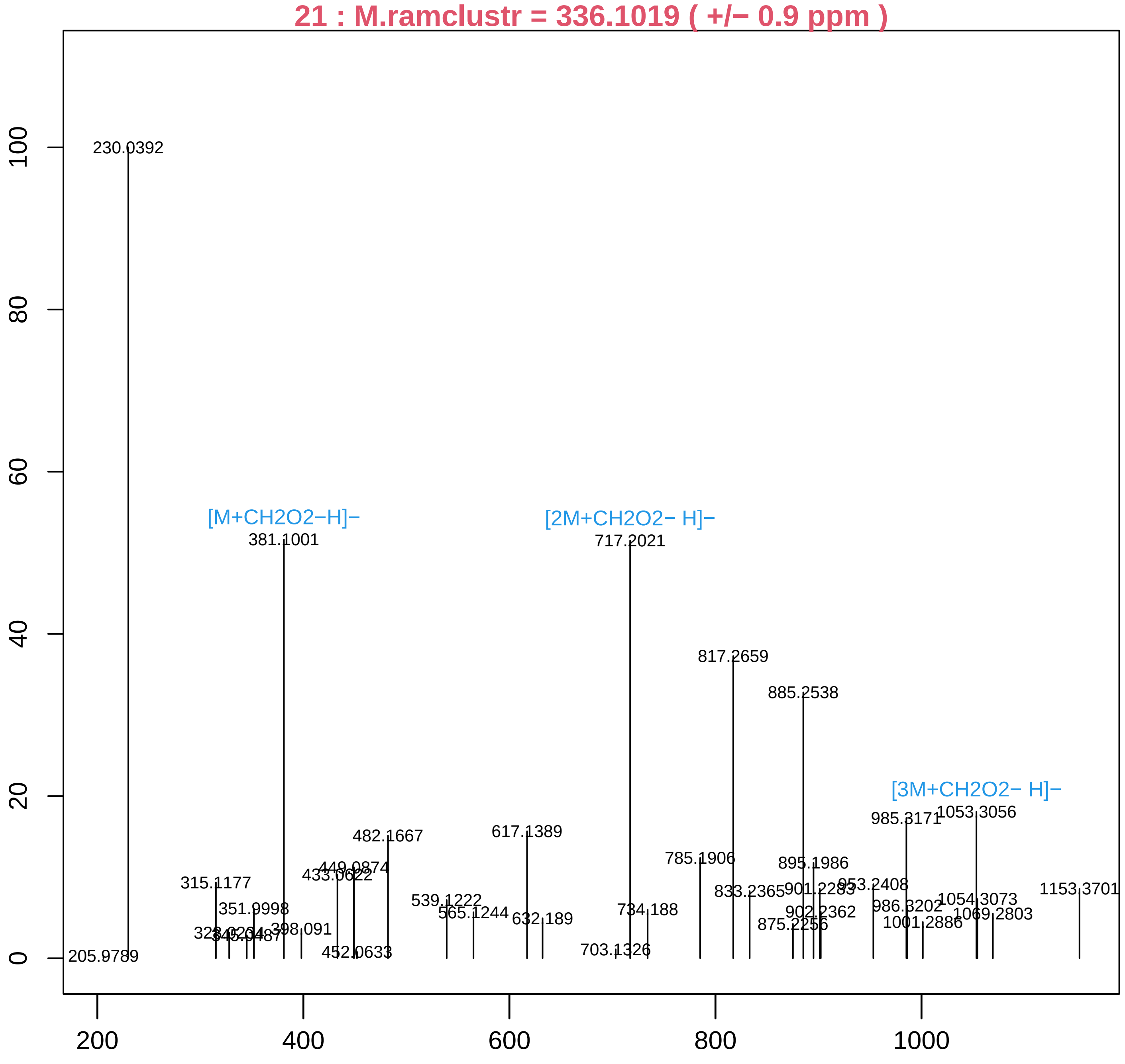
21 : M.ramclustr = 336.1019 (+/− 0.9 ppm)
230.0392
[M+CH2O2−H]−
381.1001
[2M+CH2O2− H]−
717.2021
817.2659
885.2538
[3M+CH2O2− H]−
985.3171
1053.3056
482.1667
617.1389
785.1906
895.1986
315.1177
449.0874
433.0622
953.2408
833.2365
901.2283
1153.3701
539.1222
1054.3073
351.9998
734.188
986.3202
565.1244
902.2362
1069.2803
632.189
1001.2886
398.091
875.2256
703.1326
452.0633
205.9789
0
20
40
60
80
100
200
400
600
800
1000

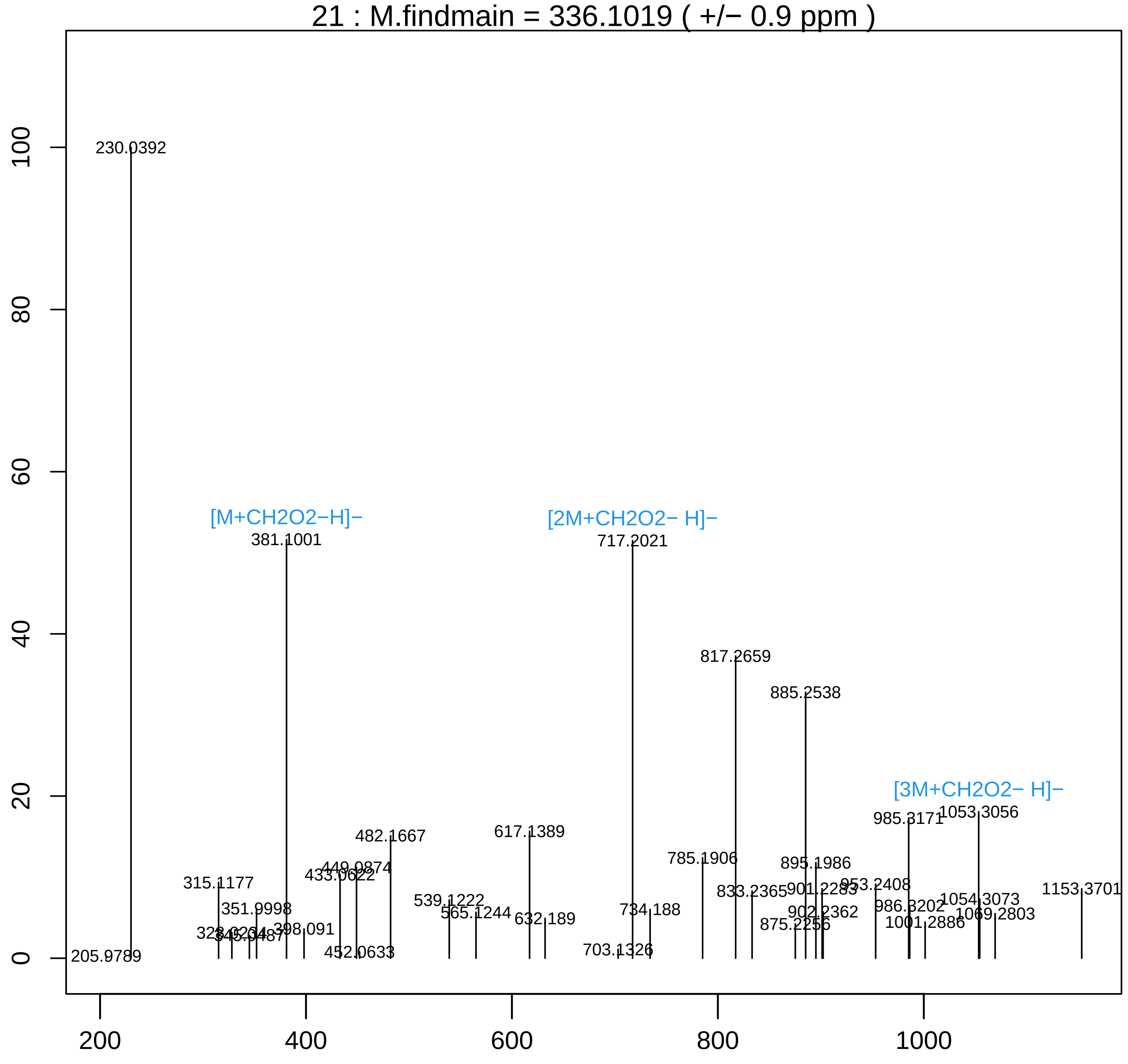
21 : M.findmain = 336.1019 (+/− 0.9 ppm)
230.0392
[M+CH2O2−H]−
381.1001
[2M+CH2O2− H]−
717.2021
817.2659
885.2538
[3M+CH2O2− H]−
985.3171
1053.3056
482.1667
617.1389
785.1906
895.1986
315.1177
449.0874
433.0622
953.2408
833.2365
901.2283
1153.3701
539.1222
1054.3073
351.9998
734.188
986.3202
565.1244
902.2362
1069.2803
632.189
1001.2886
398.091
875.2256
703.1326
452.0633
205.9789
0
20
40
60
80
100
200
400
600
800
1000

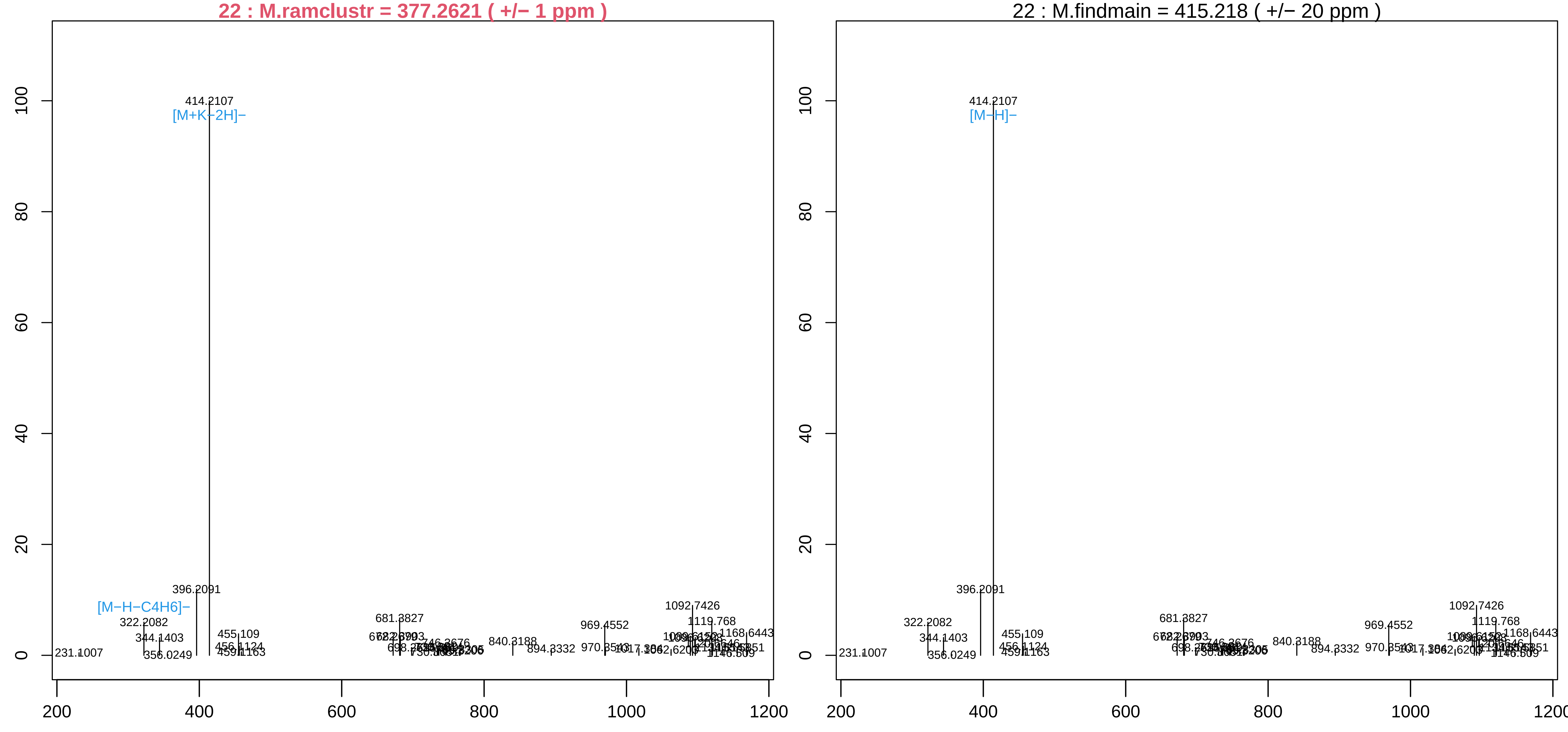

22 : M.ramclustr = 377.2621 (+/− 1 ppm)
414.2107
[M+K−2H]−
396.2091
[M−H−C4H6]−
322.2082
344.1403
231.1007
356.0249
455.109
456.1124
459.1163
681.3827
840.3188
894.3332
969.4552
1092.7426
1119.768
1168.6443
1146.609
22 : M.findmain = 415.218 (+/− 20 ppm)
414.2107
[M−H]−
396.2091
322.2082
344.1403
231.1007
356.0249
455.109
456.1124
459.1163
681.3827
840.3188
894.3332
969.4552
1092.7426
1119.768
1168.6443
1146.609
0
20
40
60
80
100
200
400
600
800
1000
1200

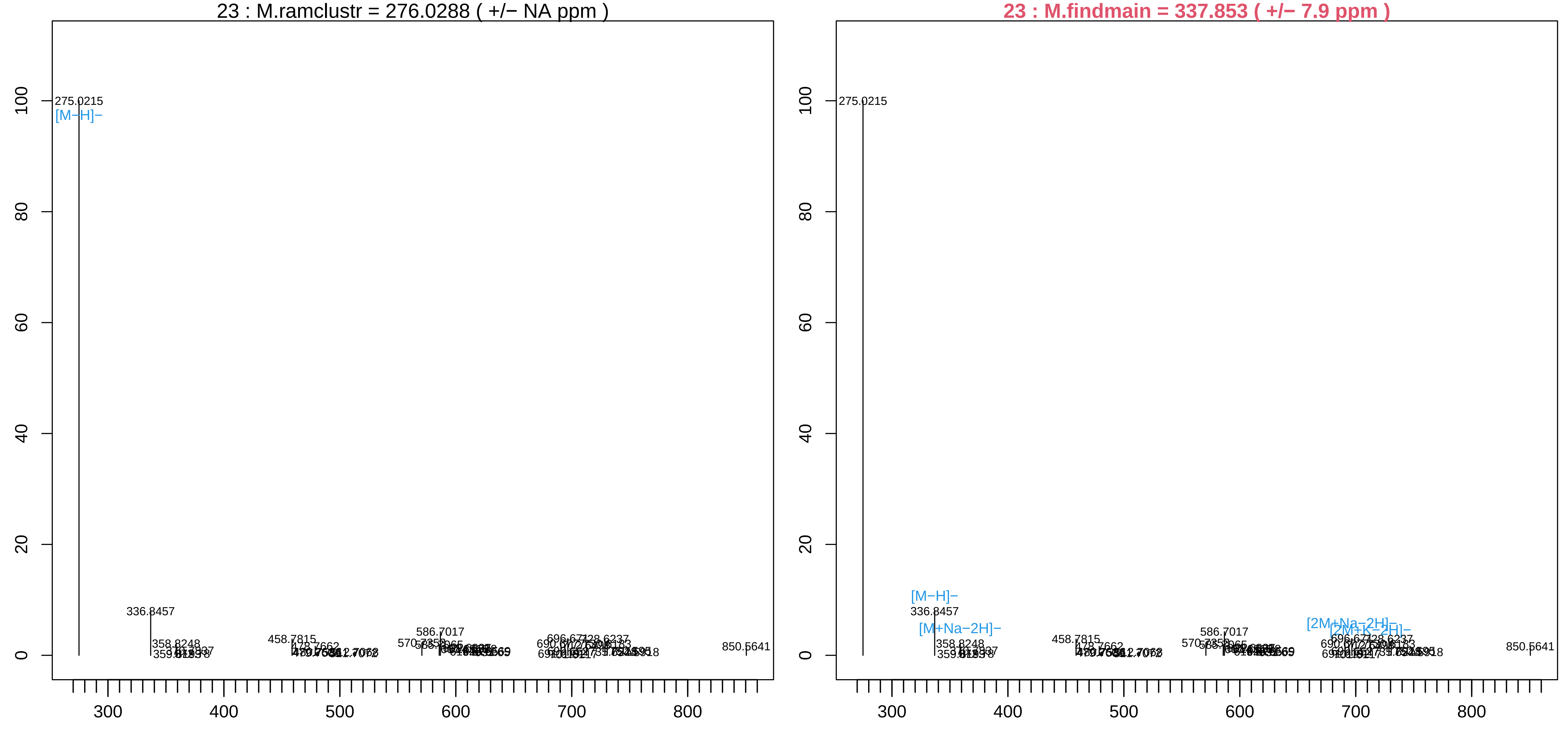
23 : M.ramclustr = 276.0288 (+/− NA ppm)
23 : M.findmain = 337.853 (+/− 7.9 ppm)
275.0215
[M−H]−
336.8457
[M+Na−2H]−
[2M+Na−2H]−
[2M+K−2H]−
358.8248
458.7815
586.7017
850.5641
0
20
40
60
80
100
300
400
500
600
700
800

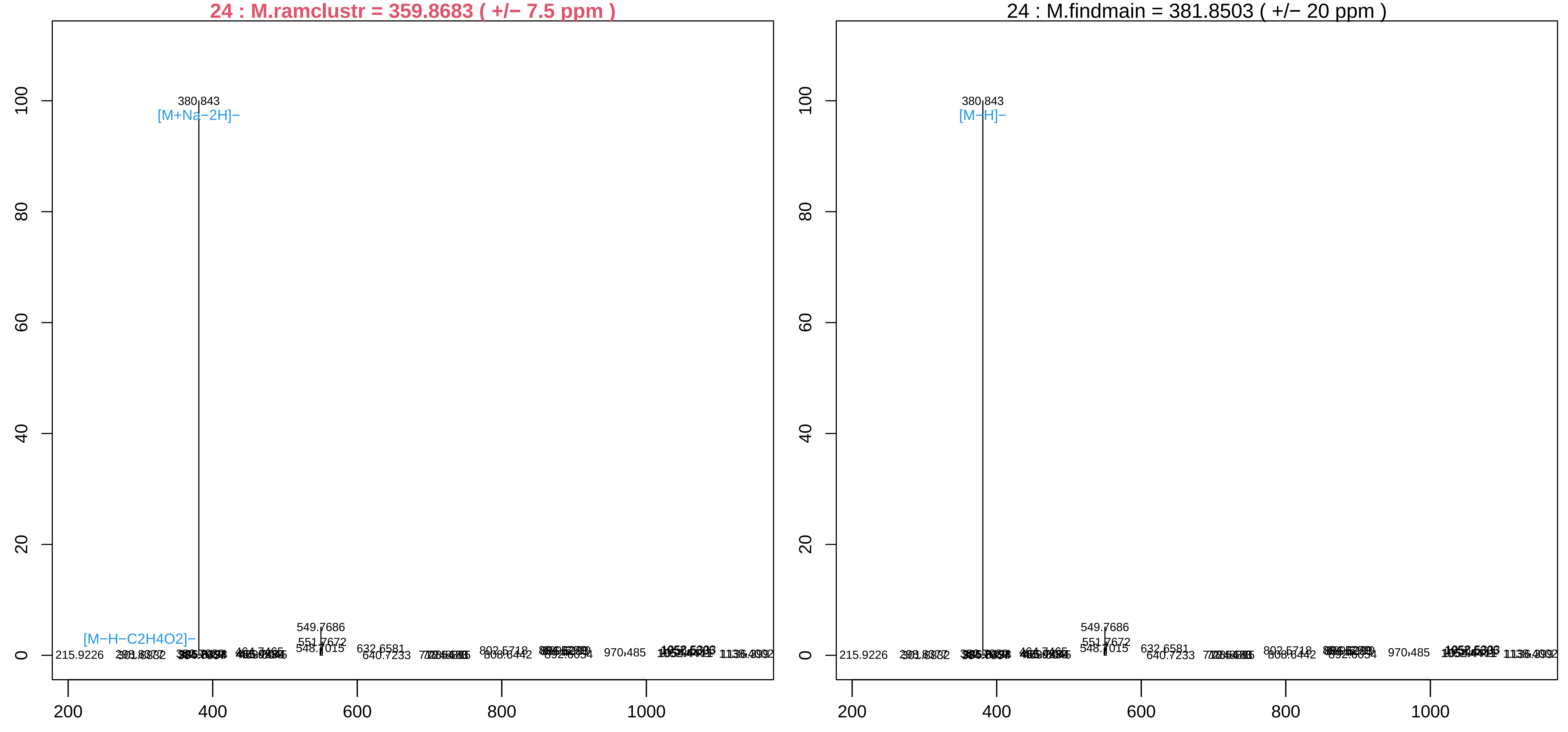

24 : M.ramclustr = 359.8683 (+/− 7.5 ppm)
24 : M.findmain = 381.8503 (+/− 20 ppm)
380.843
[M+Na−2H]−
[M−H−C2H4O2]−
[M−H]−
549.7686
551.7672
548.7015
632.6581
640.7233
802.5718
808.6442
970.485
215.9226
1136.4992

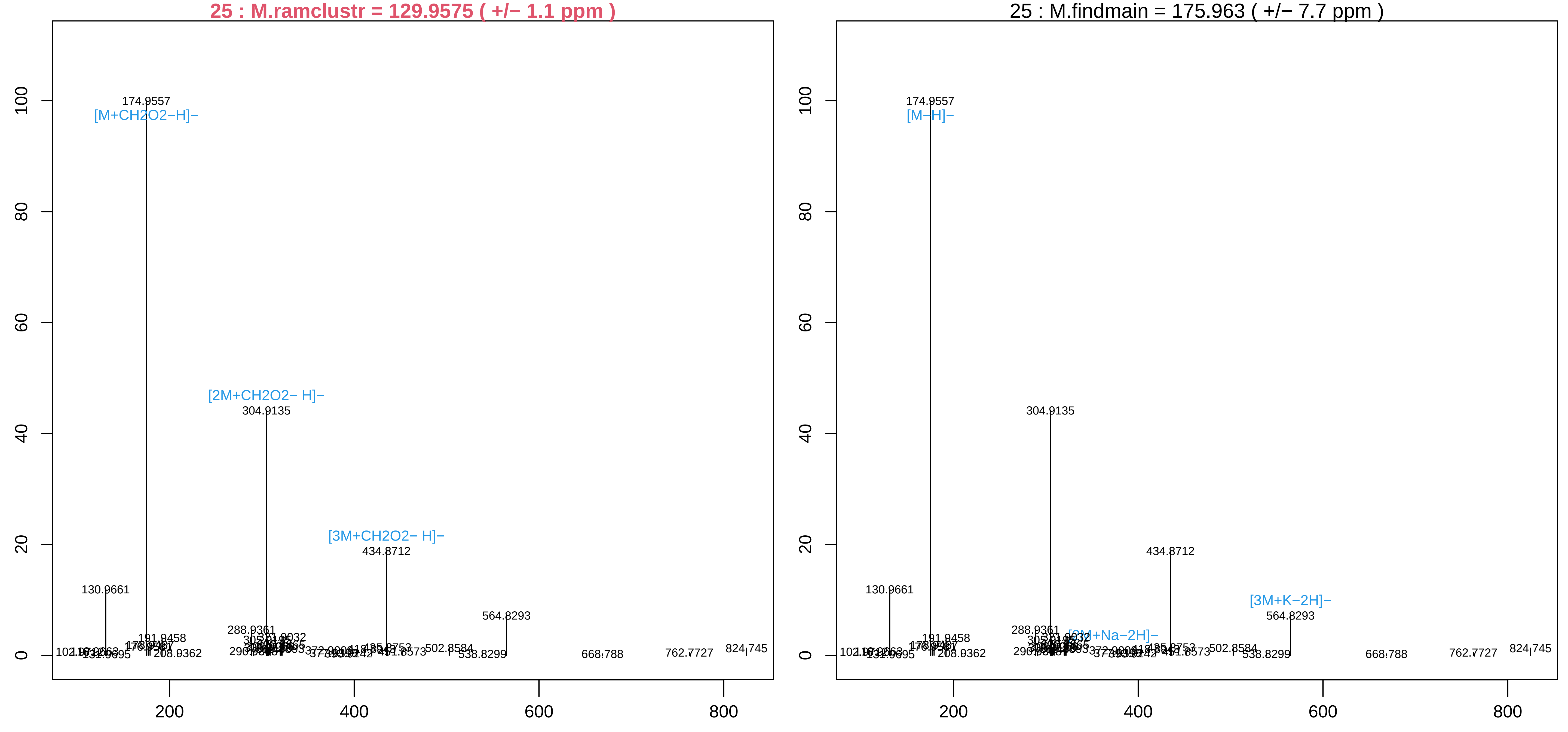
25 : M.ramclustr = 129.9575 (+/− 1.1 ppm)
174.9557
[M+CH2O2−H]−
[2M+CH2O2− H]−
304.9135
[3M+CH2O2− H]−
434.8712
130.9661
564.8293
288.9361
191.9458
208.9362
502.8584
538.8299
668.788
762.7727
824.745
25 : M.findmain = 175.963 (+/− 7.7 ppm)
174.9557
[M−H]−
304.9135
434.8712
[3M+K−2H]−
564.8293
130.9661
288.9361
191.9458
208.9362
502.8584
538.8299
668.788
762.7727
824.745

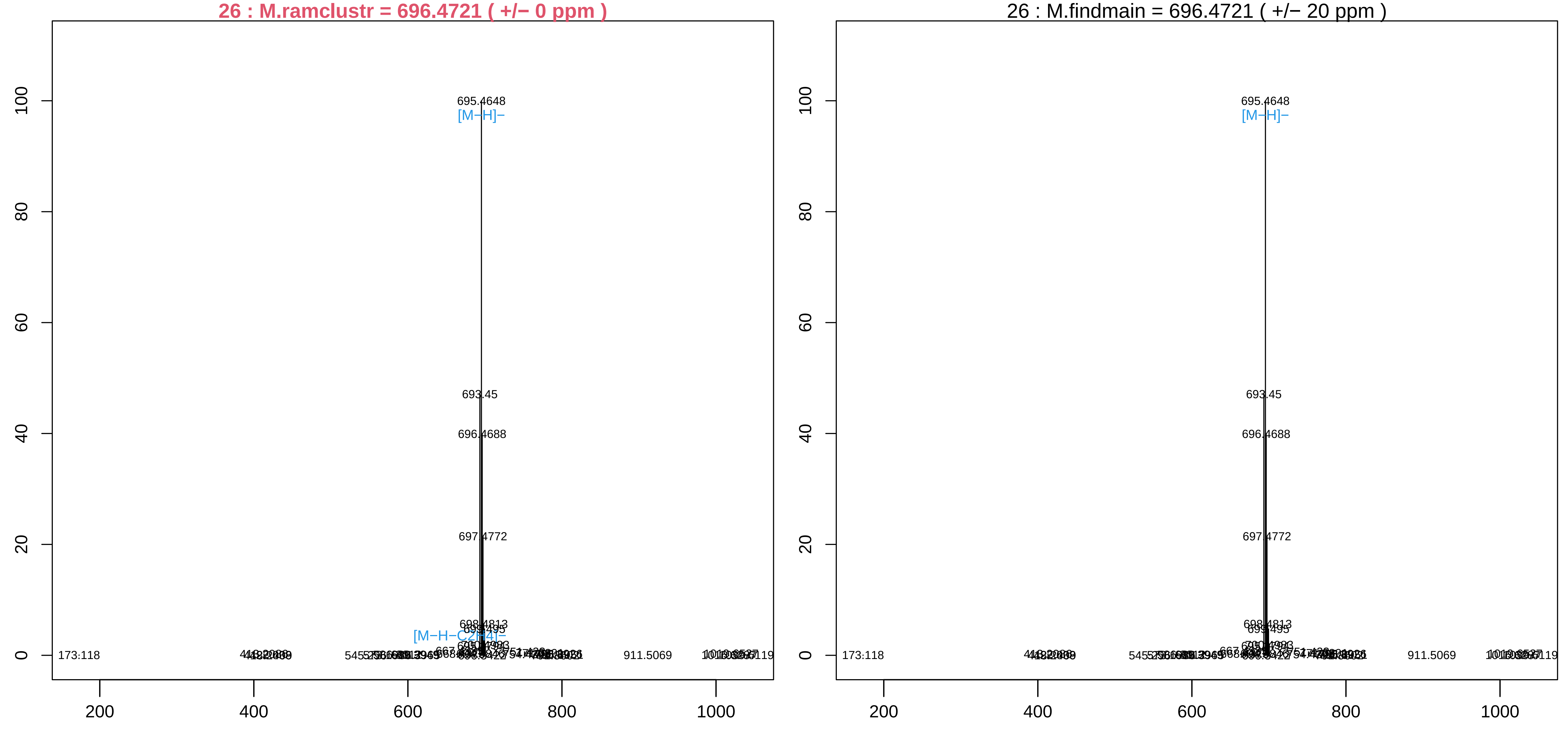

26 : M.ramclustr = 696.4721 (+/− 0 ppm)
695.4648
[M−H]−
693.45
696.4688
697.4772
698.4813
[M−H−C2H4]−
173:118
911.5069
26 : M.findmain = 696.4721 (+/− 20 ppm)
695.4648
[M−H]−
693.45
696.4688
697.4772
698.4813
173:118
911.5069

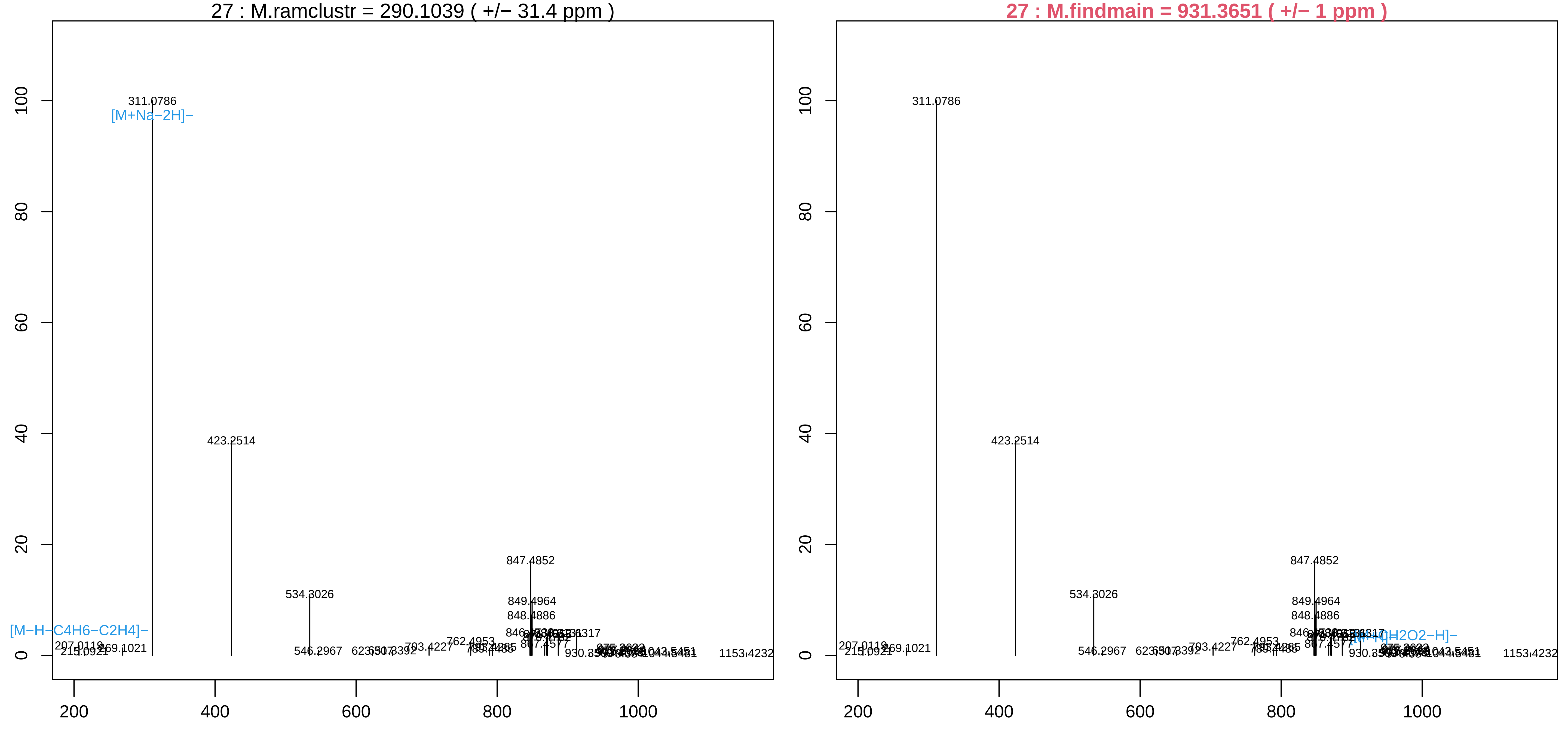
27 : M.ramclustr = 290.1039 (+/− 31.4 ppm)
311.0786
[M+Na−2H]−
423.2514
534.3026
847.4852
849.4964
848.4886
[M−H−C4H6−C2H4]−
207.0119
215.0921
269.1021
546.2967
703.4227
762.4953
867.4577
930.3
1042.5451
1153.4232
27 : M.findmain = 931.3651 (+/− 1 ppm)
311.0786
423.2514
534.3026
847.4852
849.4964
848.4886
0
20
40
60
80
100
200
400
600
800
1000

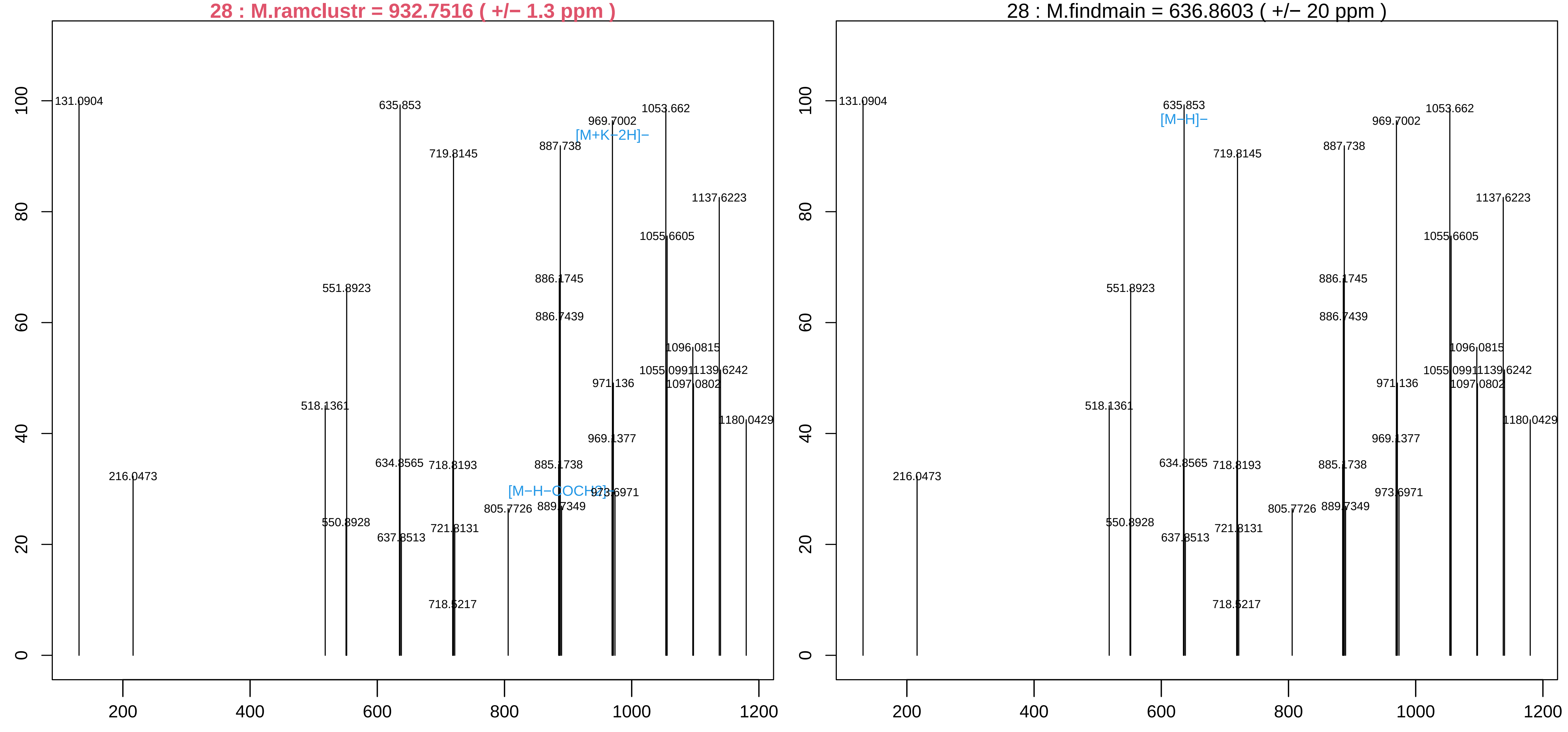
28 : M.ramclustr = 932.7516 (+/− 1.3 ppm)
131.0904
216.0473
518.1361
551.8923
550.8928
635.853
634.8565
637.8513
719.8145
718.8193
721.8131
718.5217
805.7726
887.738
886.1745
886.7439
885.1738
889.7349
969.7002
[M+K−2H]−
971.136
969.1377
973.6971
[M−H−COCH2]−
1053.662
1055.6605
1055.0991
1096.0815
1097.0802
1137.6223
1139.6242
1180.0429
28 : M.findmain = 636.8603 (+/− 20 ppm)
131.0904
216.0473
518.1361
551.8923
550.8928
635.853
[M−H]−
634.8565
637.8513
719.8145
718.8193
721.8131
718.5217
805.7726
887.738
886.1745
886.7439
885.1738
889.7349
969.7002
971.136
969.1377
973.6971
1053.662
1055.6605
1055.0991
1096.0815
1097.0802
1137.6223
1139.6242
1180.0429
0
20
40
60
80
100
200
400
600
800
1000
1200

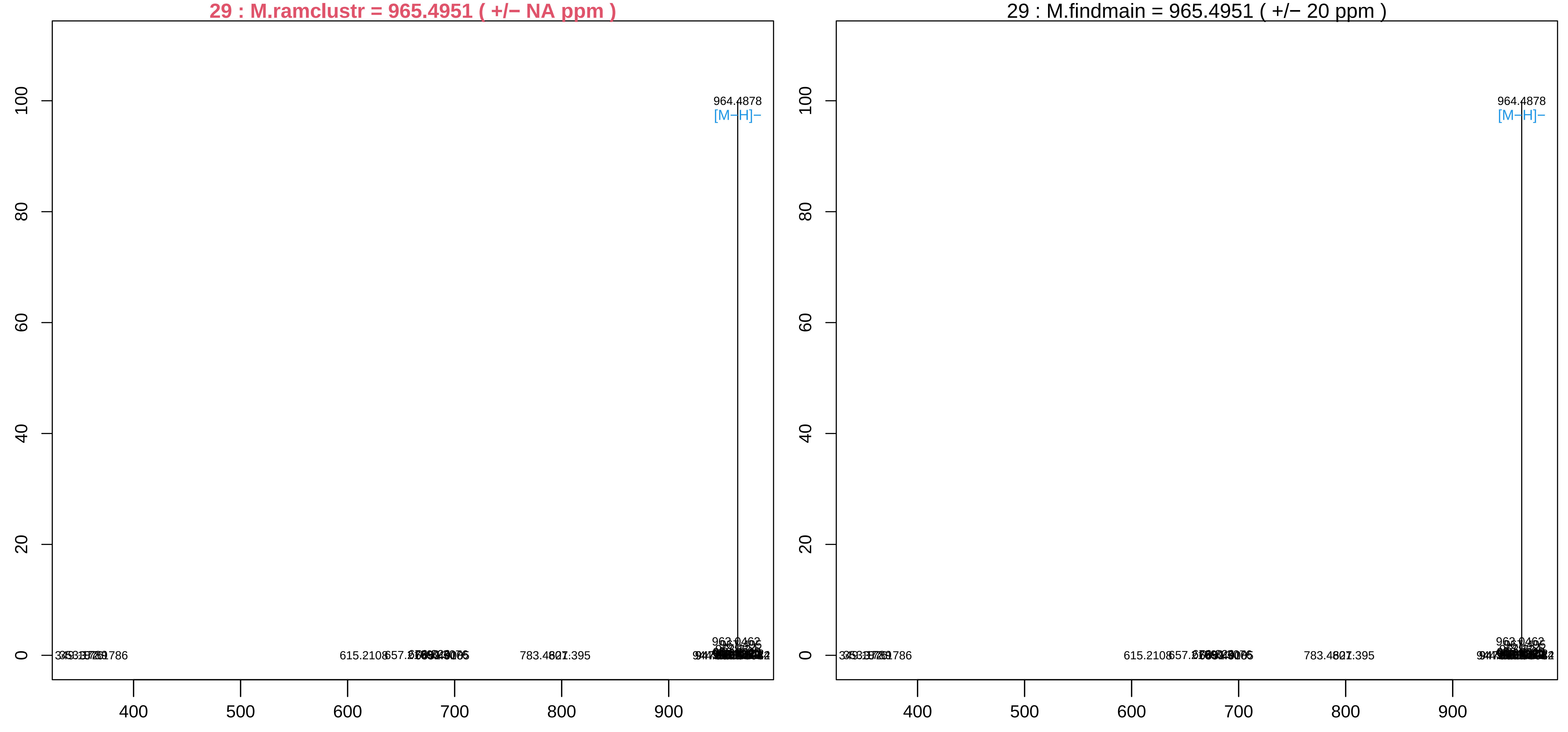
29 : M.ramclustr = 965.4951 (+/− NA ppm)
29 : M.findmain = 965.4951 (+/− 20 ppm)
964.4878
[M−H]−
615.2108
783.4627
100
80
60
40
20
0
400
500
600
700
800
900

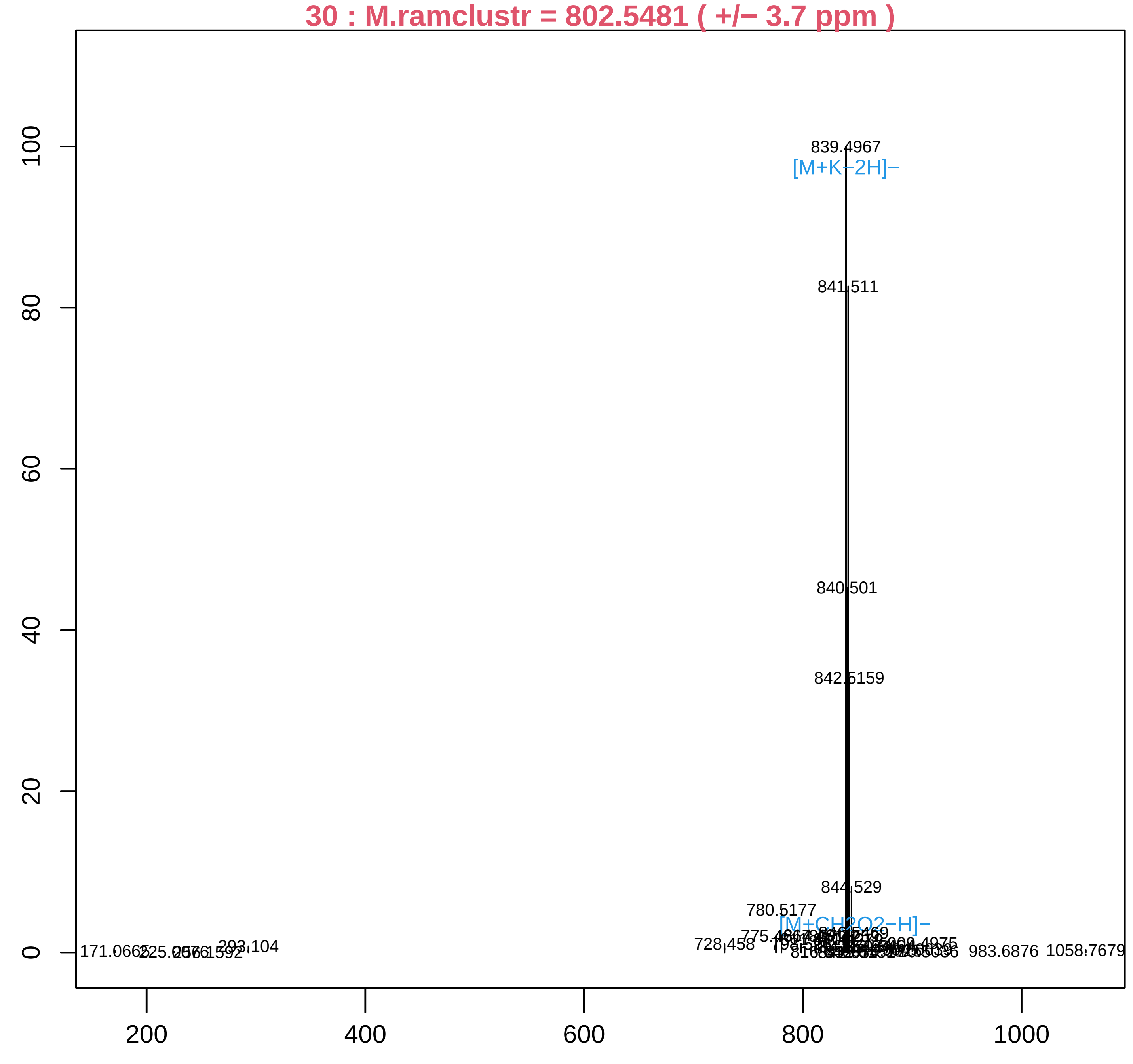
30 : M.ramclustr = 802.5481 (+/− 3.7 ppm)
839.4967
[M+K−2H]−
841.511
840.501
842.5159
844.529
780.5177
[M+CH2O2−H]−
728.458
171.0662
293.104
983.6876
1058.7679
0
20
40
60
80
100
200
400
600
800
1000

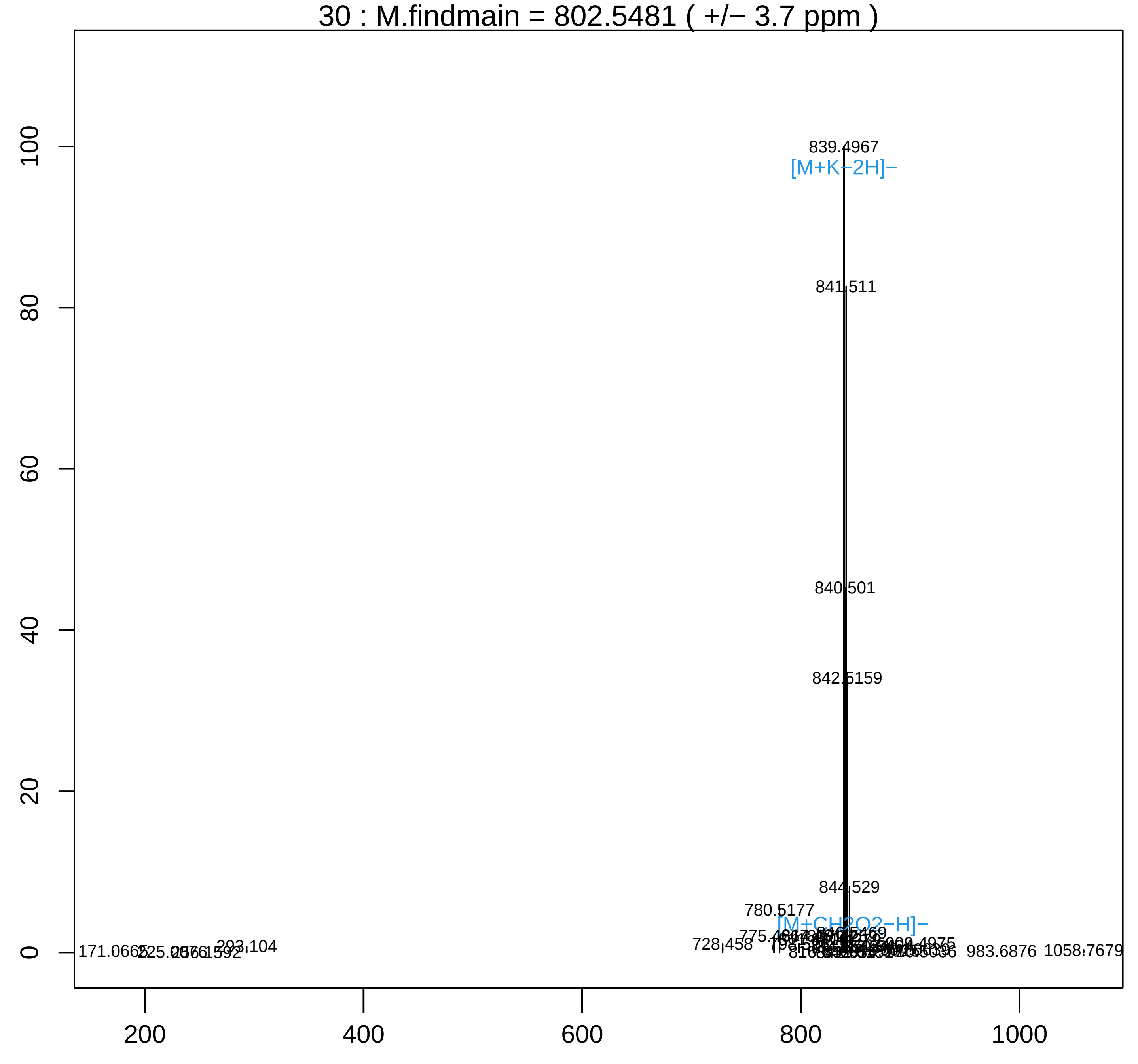
30 : M.findmain = 802.5481 (+/− 3.7 ppm)
839.4967
[M+K−2H]−
841.511
840.501
842.5159
844.529
780.5177
[M+CH2O2−H]−
728.458
171.0662
293.104
983.6876
1058.7679
0
20
40
60
80
100
200
400
600
800
1000

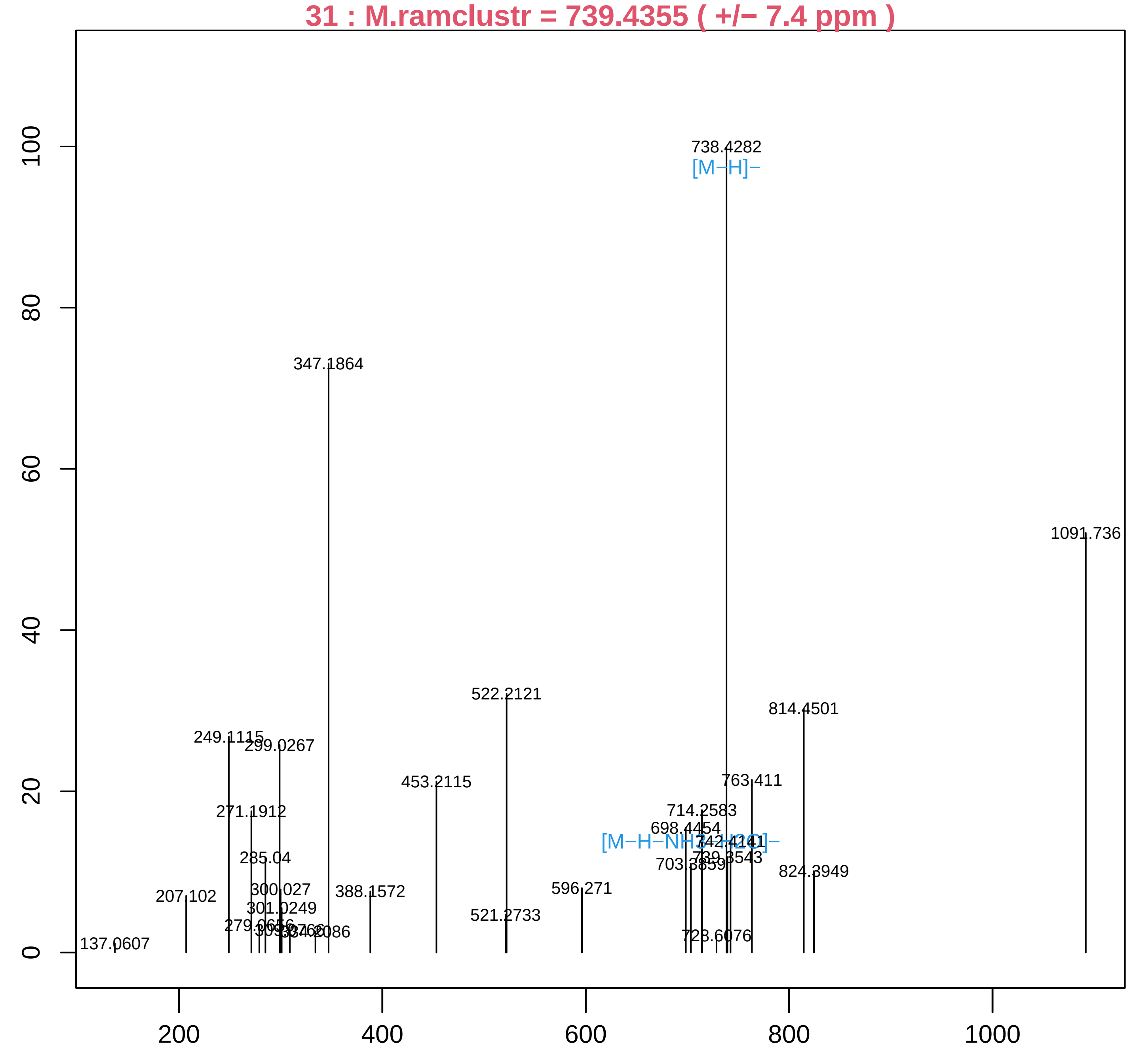
31 : M.ramclustr = 739.4355 (+/− 7.4 ppm)
738.4282
[M−H]−
347.1864
1091.736
522.2121
814.4501
249.1115
299.0267
453.2115
763.411
271.1912
714.2583
698.4454
[M−H−NH3+K+Cl]−
742.4141
285.04
703.3859
739.3543
824.3949
300.027
388.1572
596.271
207.102
301.0249
521.2733
279.0656
723.6076
137.0607

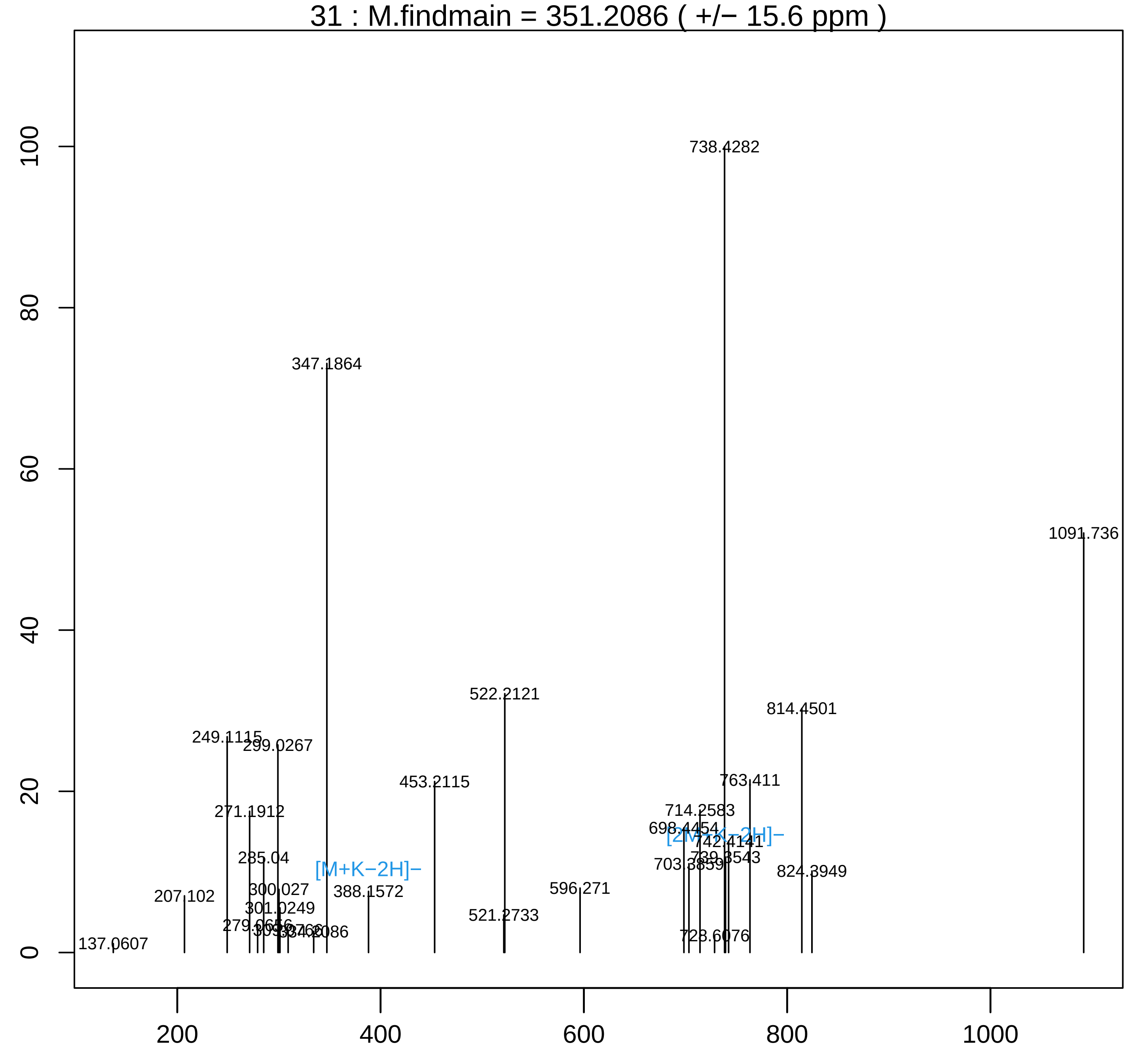
31 : M.findmain = 351.2086 (+/− 15.6 ppm)
738.4282
347.1864
1091.736
522.2121
814.4501
249.1115
299.0267
453.2115
763.411
271.1912
714.2583
698.4454
[2M+K−2H]−
742.4141
285.04
703.3859
739.3543
[M+K−2H]−
824.3949
300.027
388.1572
596.271
207.102
301.0249
521.2733
279.0656
723.6076
137.0607

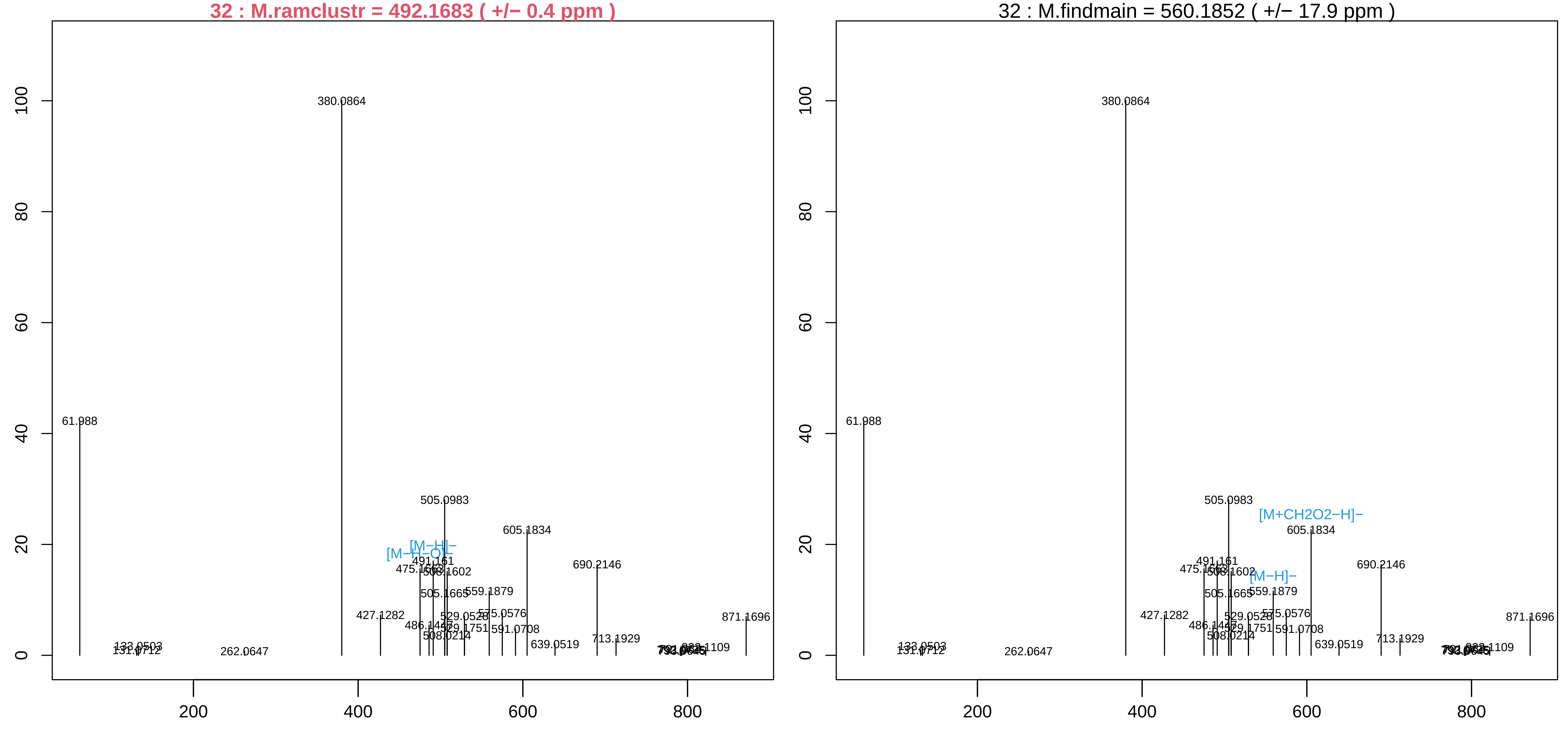
32 : M.ramclustr = 492.1683 (+/− 0.4 ppm)
32 : M.findmain = 560.1852 (+/− 17.9 ppm)
380.0864
61.988
505.0983
605.1834
690.2146
491.161
508.1602
505.1665
559.1879
575.0576
529.0528
591.0708
427.1282
639.0519
713.1929
871.1696
262.0647
133.0503
131.0712
508.0214
[M−H]−
[M+CH2O2−H]−
0
20
40
60
80
100
200
400
600
800

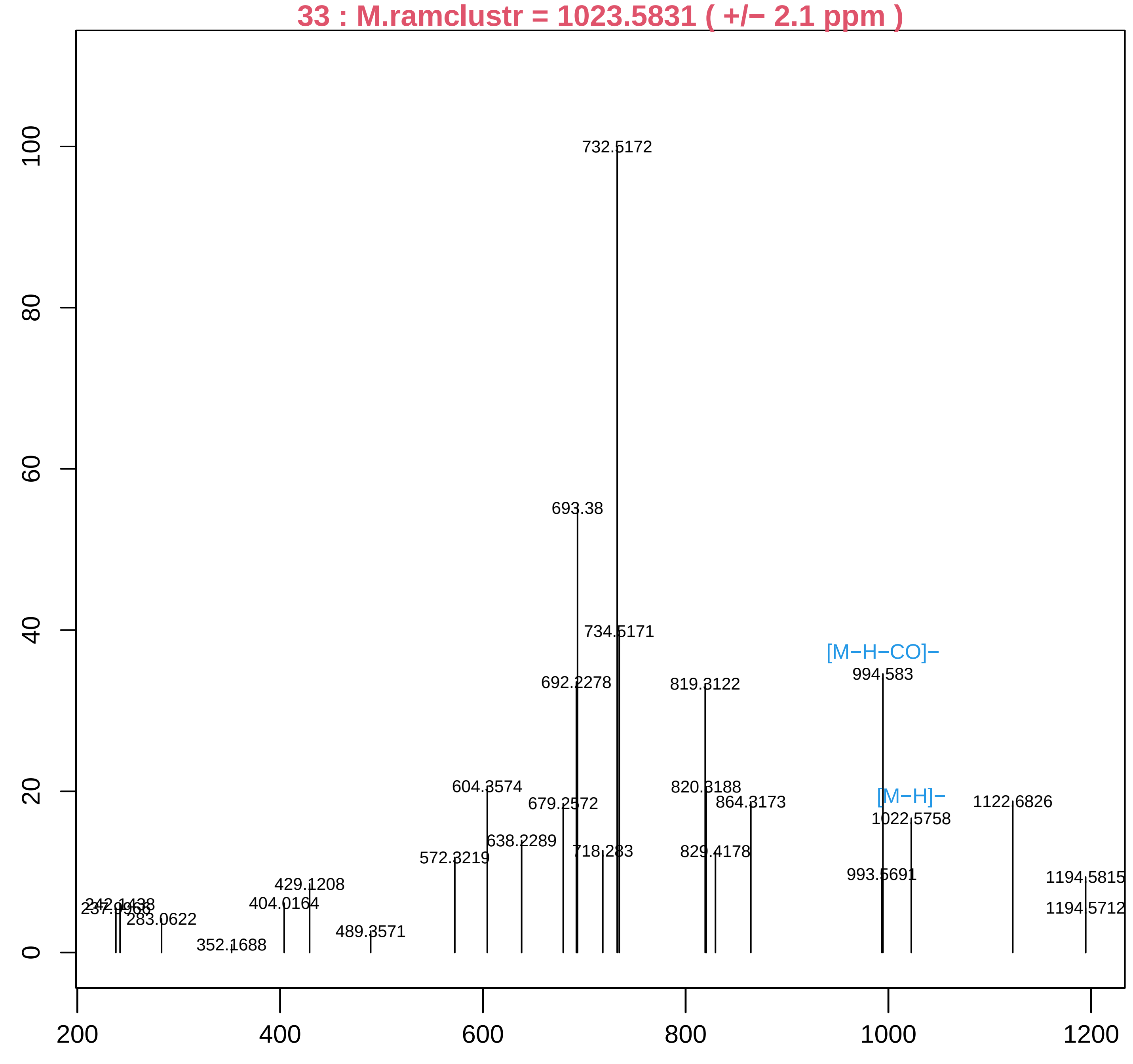
33 : M.ramclustr = 1023.5831 (+/− 2.1 ppm)
732.5172
693.38
734.5171
[M−H−CO]−
994.583
692.2278
819.3122
604.3574
820.3188
679.2572
864.3173
[M−H]−
1122.6826
1022.5758
638.2289
572.3219
718.283
829.4178
993.5691
1194.5815
429.1208
242.1438
404.0164
1194.5712
283.0622
489.3571
352.1688
0
20
40
60
80
100
200
400
600
800
1000
1200

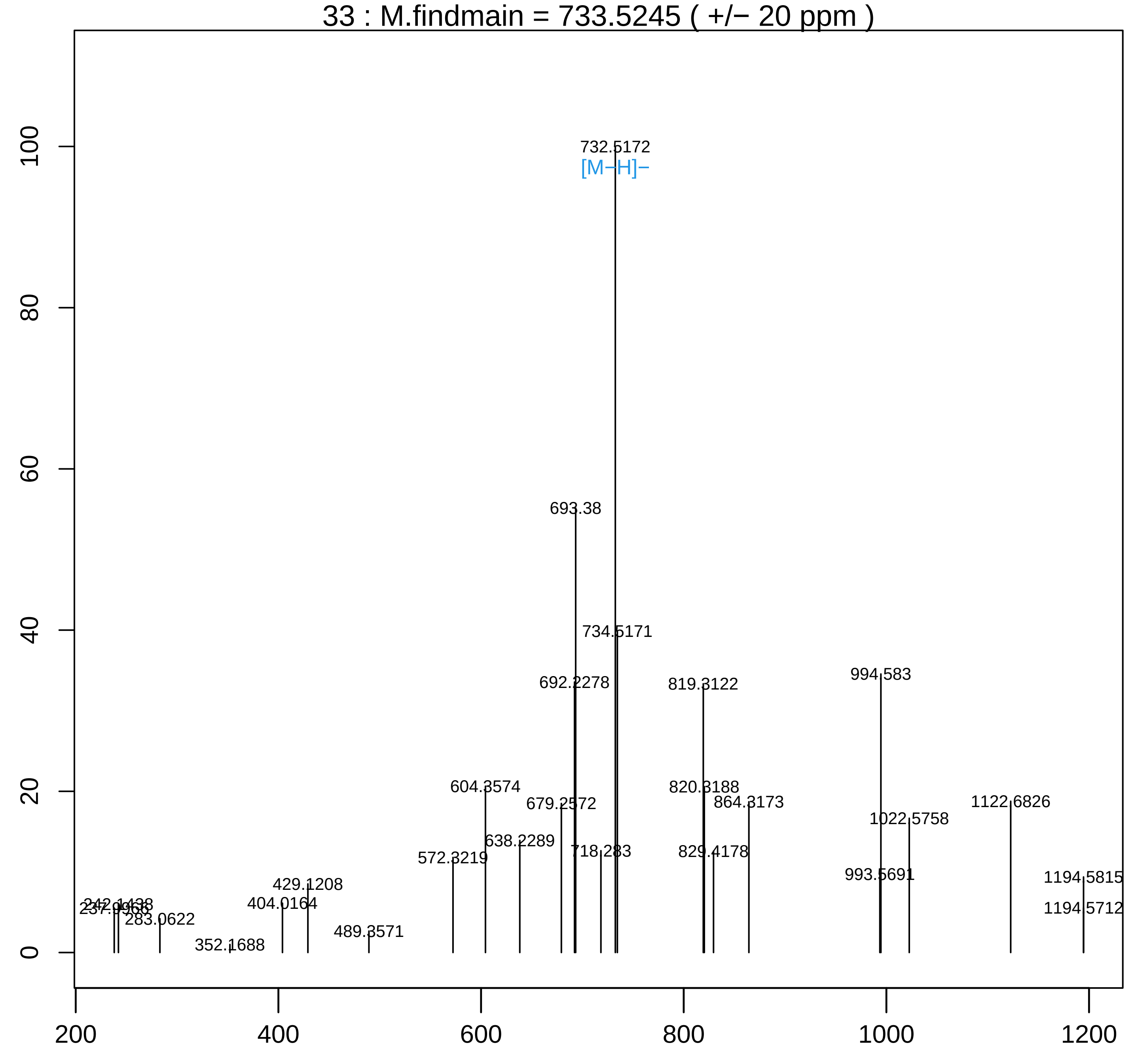
33 : M.findmain = 733.5245 (+/− 20 ppm)
732.5172
[M−H]−
693.38
734.5171
994.583
692.2278
819.3122
604.3574
820.3188
679.2572
864.3173
1122.6826
1022.5758
638.2289
572.3219
718.283
829.4178
993.5691
1194.5815
429.1208
242.1438
404.0164
1194.5712
283.0622
489.3571
352.1688
0
20
40
60
80
100
200
400
600
800
1000
1200

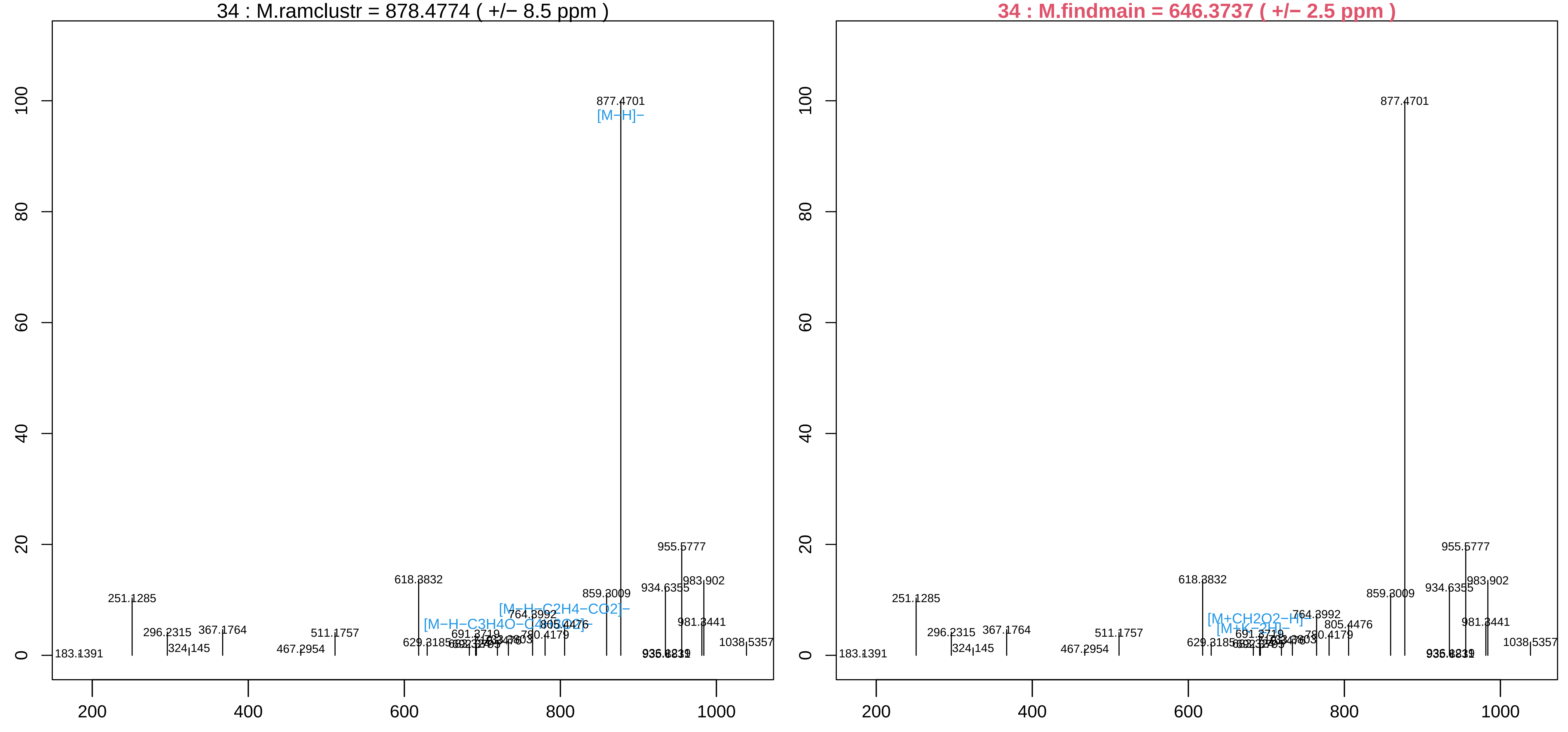
34 : M.ramclustr = 878.4774 (+/− 8.5 ppm)
877.4701
[M−H]−
955.5777
983.902
934.6355
859.3009
981.3441
1038.5357
618.3832
764.3992
805.4476
780.4179
[M−H−C2H4−CO2]−
251.1285
296.2315
367.1764
324.145
511.1757
467.2954
183.1391
629.3185
691.3719
100
80
60
40
20
0
200
400
600
800
1000
34 : M.findmain = 646.3737 (+/− 2.5 ppm)
877.4701
955.5777
983.902
934.6355
859.3009
981.3441
1038.5357
618.3832
764.3992
805.4476
780.4179
[M+CH2O2−H]−
251.1285
296.2315
367.1764
324.145
511.1757
467.2954
183.1391
629.3185
691.3719
100
80
60
40
20
0
200
400
600
800
1000

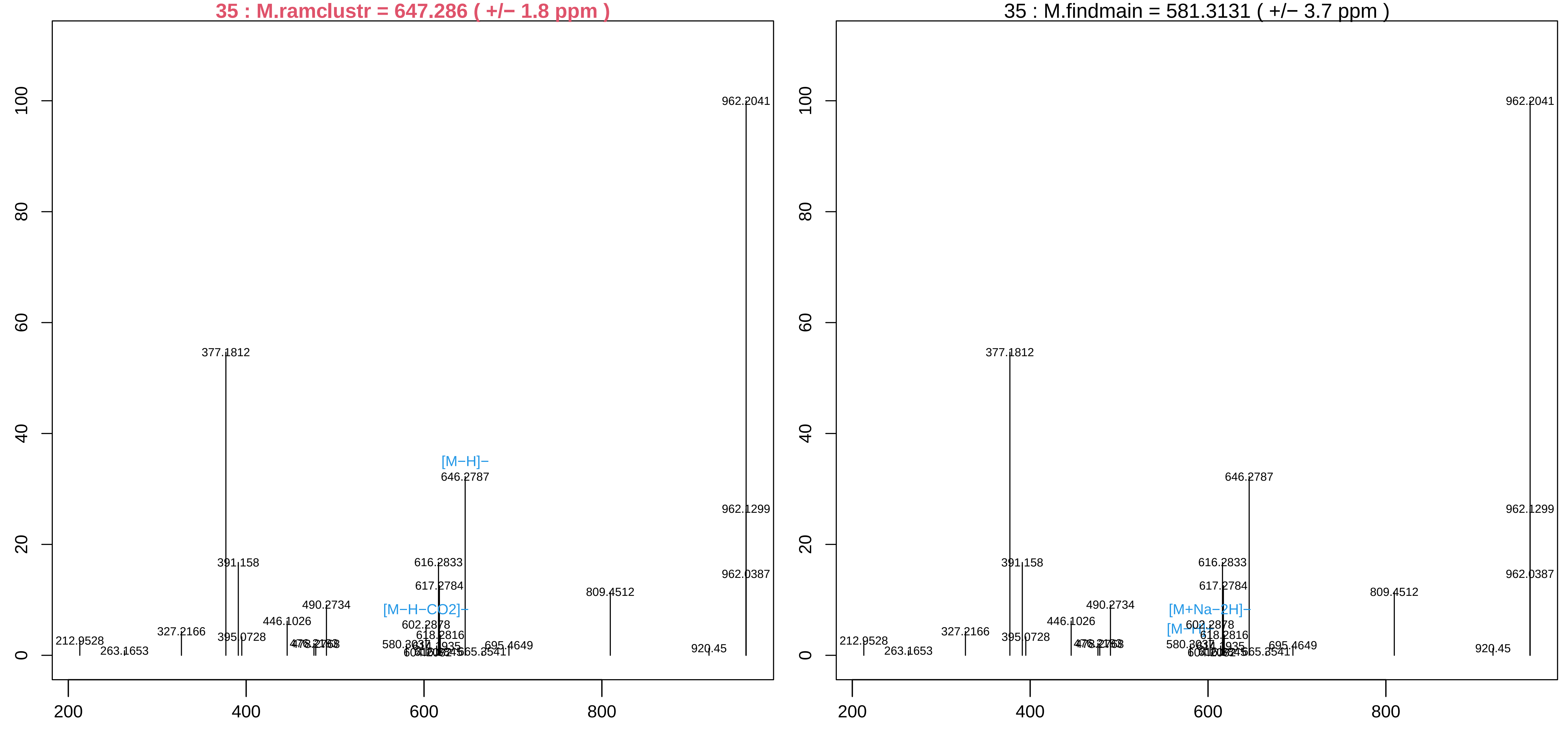
35 : M.ramclustr = 647.286 (+/− 1.8 ppm)
35 : M.findmain = 581.3131 (+/− 3.7 ppm)
[M−H]−
[M−H−CO2]−
[M+Na−2H]−
962.2041
377.1812
646.2787
962.1299
391.158
616.2833
962.0387
617.2784
809.4512
490.2734
446.1026
602.2878
327.2166
618.2816
395.0728
212.9528
695.4649
263.1653
920.45
0
20
40
60
80
100
200
400
600
800

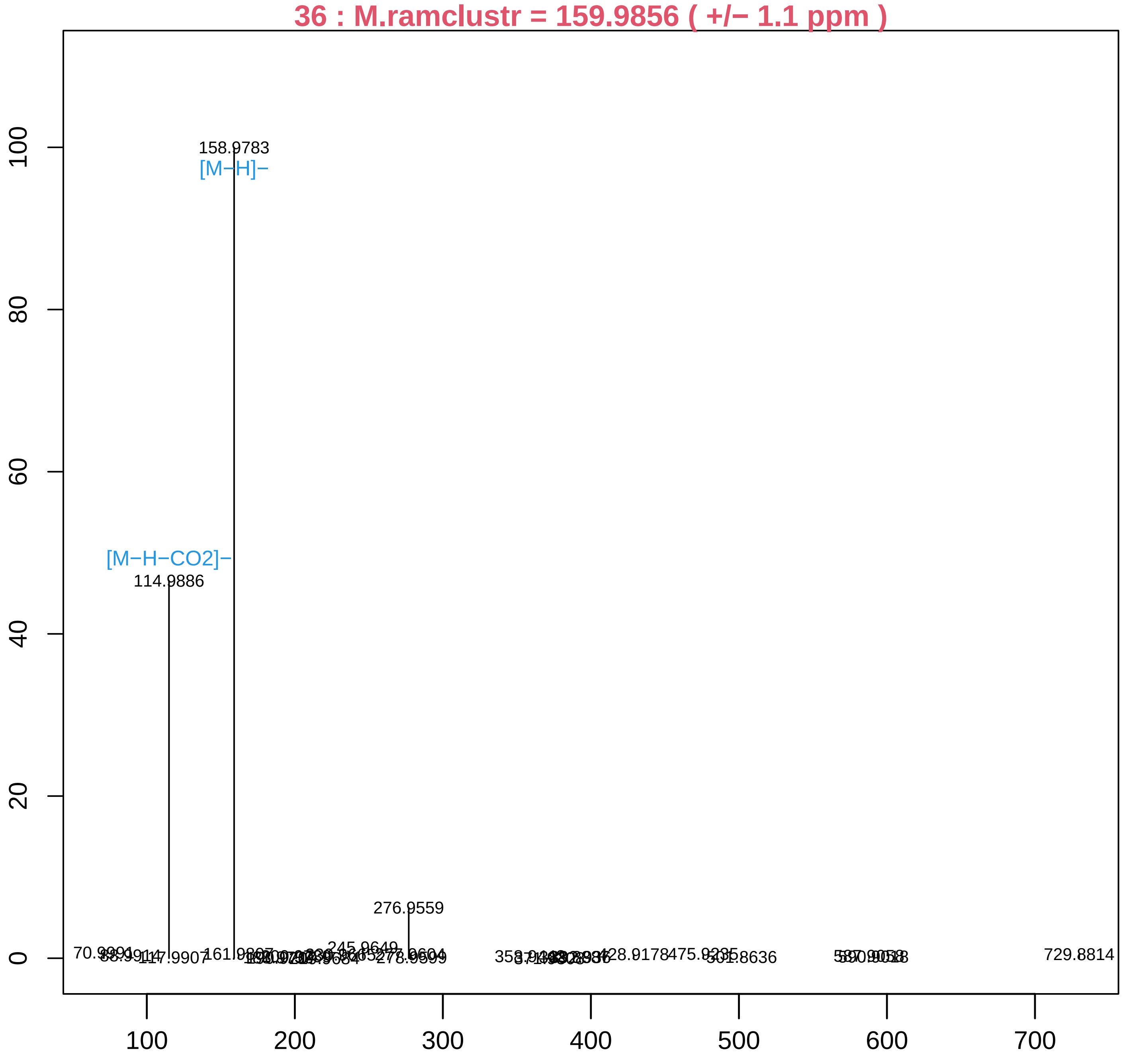
36 : M.ramclustr = 159.9856 (+/− 1.1 ppm)
158.9783
[M−H]−
[M−H−CO2]−
114.9886
276.9559
70.9891
117.9907
245.9649
428.9178
475.9235
501.8636
729.8814
0
20
40
60
80
100
100
200
300
400
500
600
700

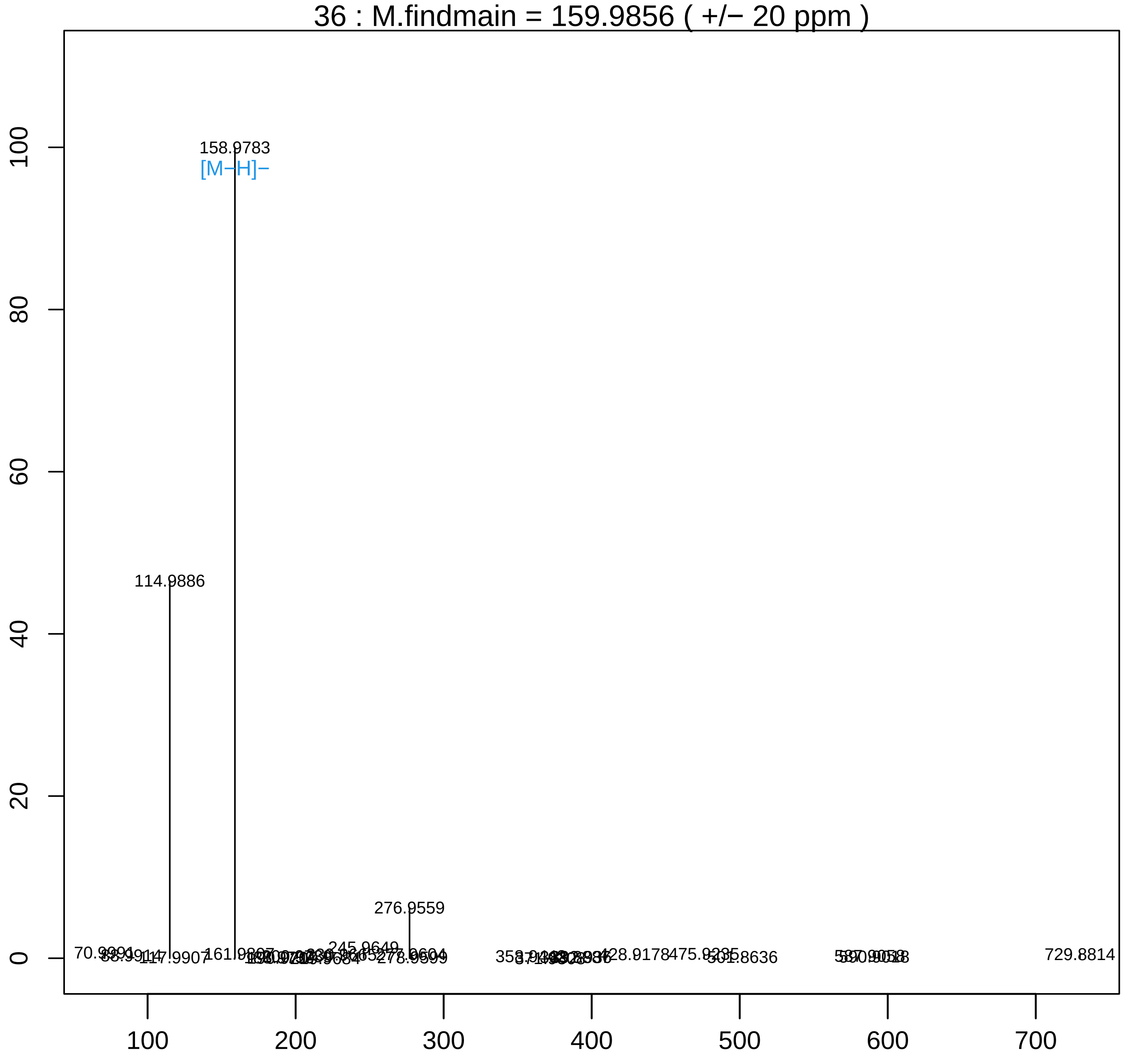
36 : M.findmain = 159.9856 (+/− 20 ppm)
158.9783
[M−H]−
114.9886
276.9559
70.9891
117.9907
245.9649
428.9178
475.9235
501.8636
729.8814
0
20
40
60
80
100
100
200
300
400
500
600
700

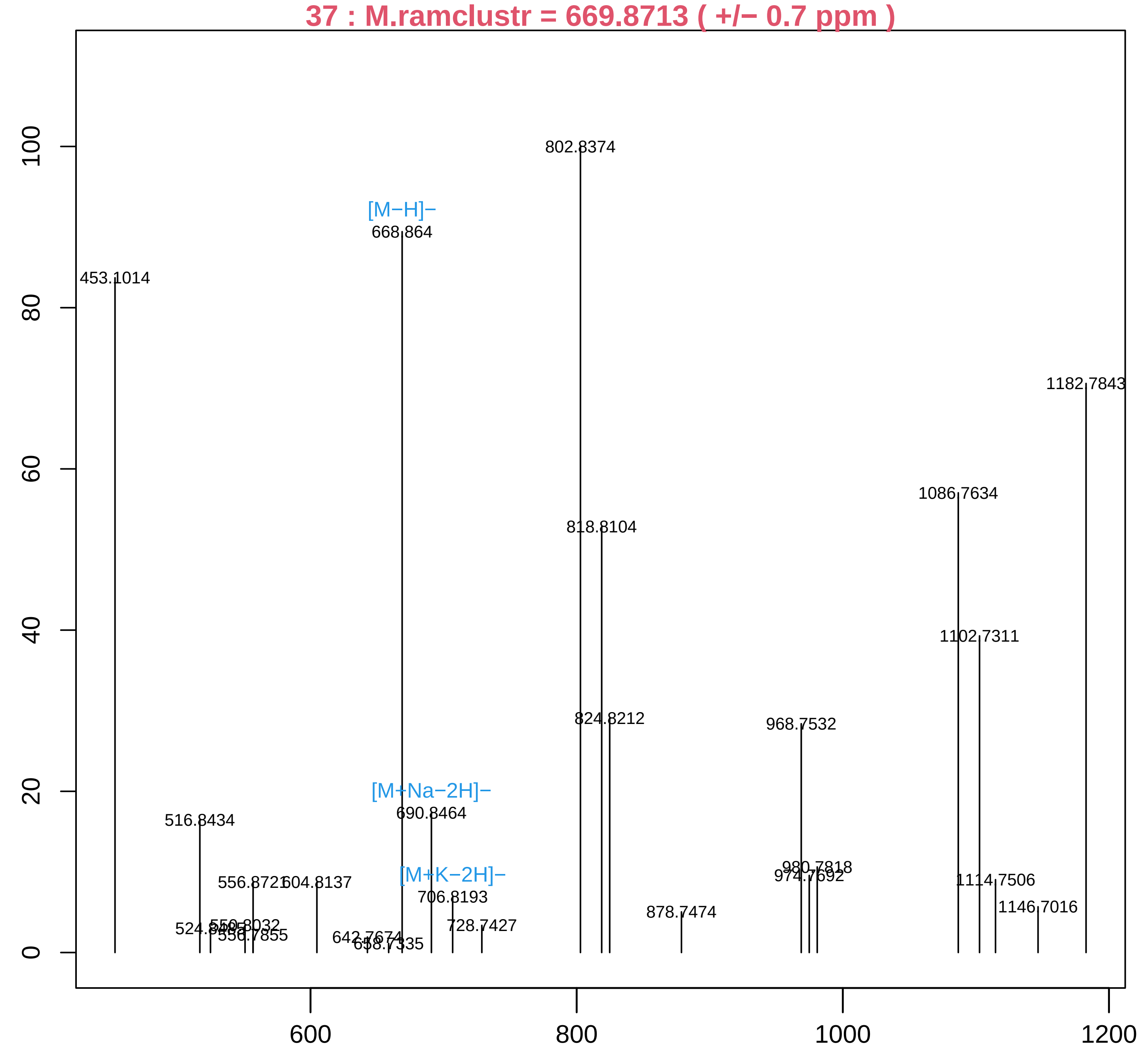
37 : M.ramclustr = 669.8713 (+/− 0.7 ppm)
[M−H]−
[M+Na−2H]−
[M+K−2H]−
453.1014
516.8434
550.8032
556.7855
556.8721
604.8137
642.7674
658.7335
668.864
690.8464
706.8193
728.7427
802.8374
818.8104
824.8212
878.7474
968.7532
974.7692
980.7818
1086.7634
1102.7311
1114.7506
1146.7016
1182.7843
0
20
40
60
80
100
600
800
1000
1200
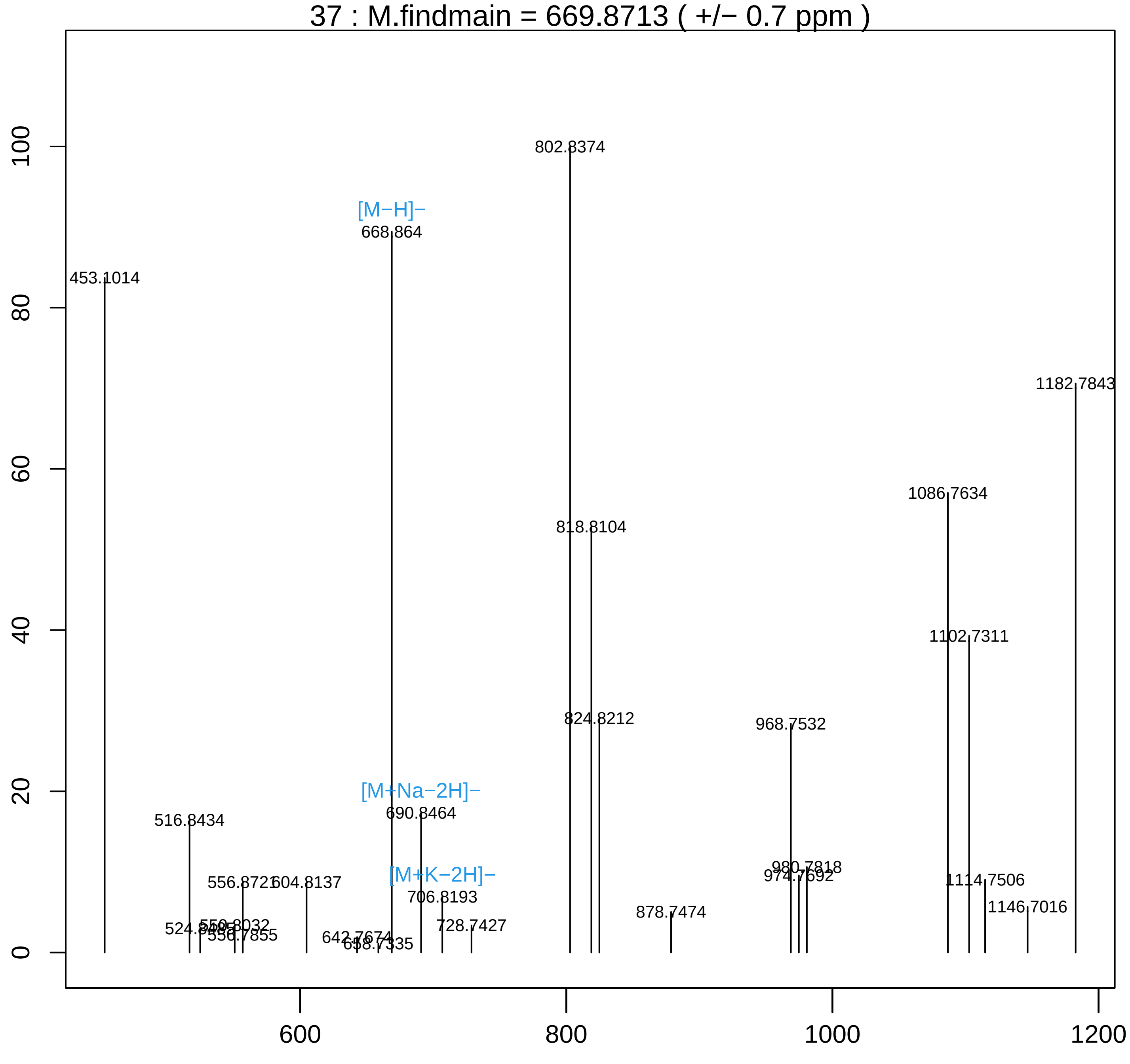
37 : M.findmain = 669.8713 (+/− 0.7 ppm)
[M−H]−
[M+Na−2H]−
[M+K−2H]−
453.1014
516.8434
550.8032
556.7855
556.8721
604.8137
642.7674
658.7335
668.864
690.8464
706.8193
728.7427
802.8374
818.8104
824.8212
878.7474
968.7532
974.7692
980.7818
1086.7634
1102.7311
1114.7506
1146.7016
1182.7843
0
20
40
60
80
100
600
800
1000
1200

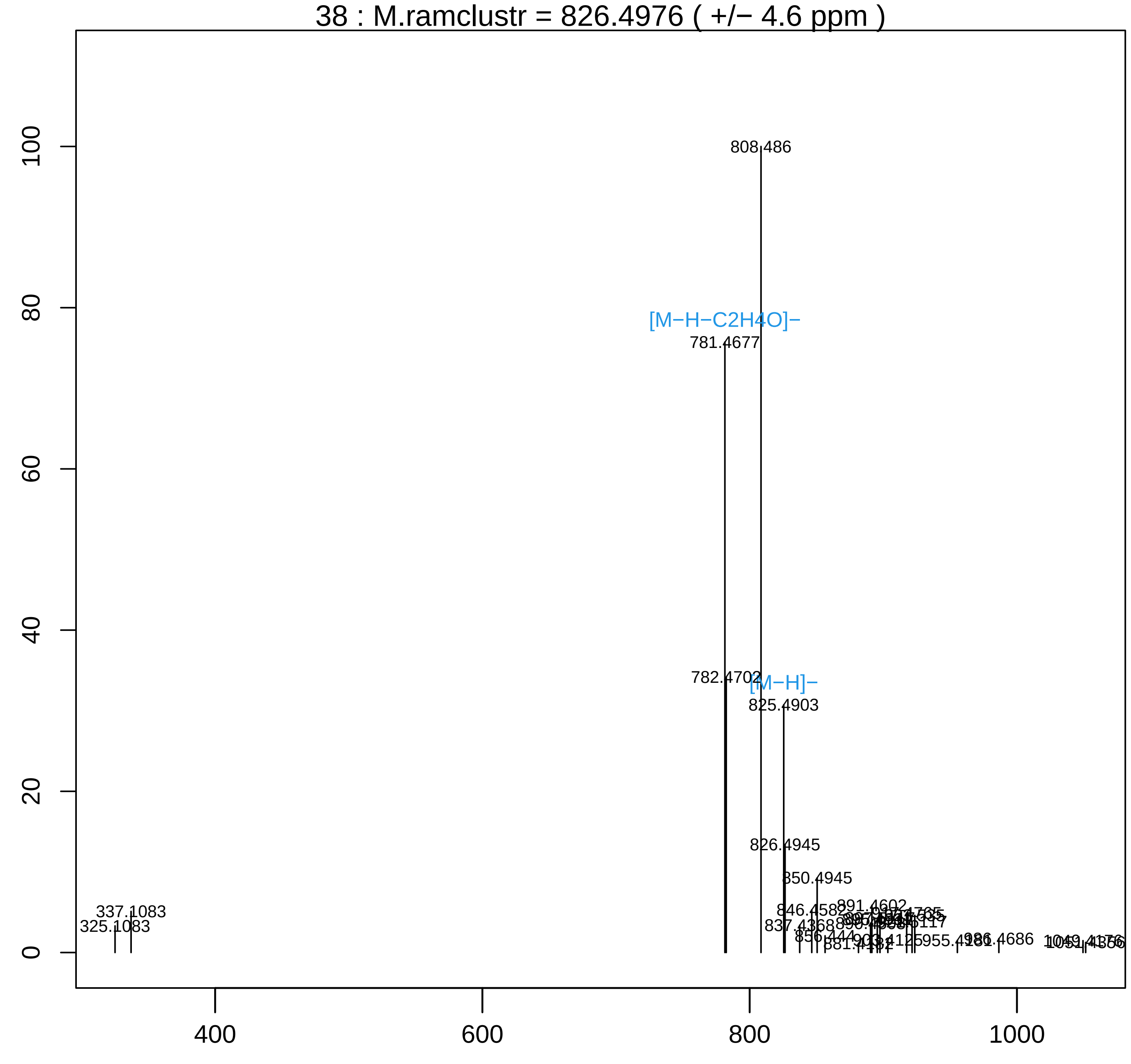
38 : M.ramclustr = 826.4976 (+/− 4.6 ppm)
808.486
[M−H−C2H4O]−
781.4677
782.4702
[M−H]−
825.4903
826.4945
850.4945
337.1083
325.1083
0
20
40
60
80
100
400
600
800
1000

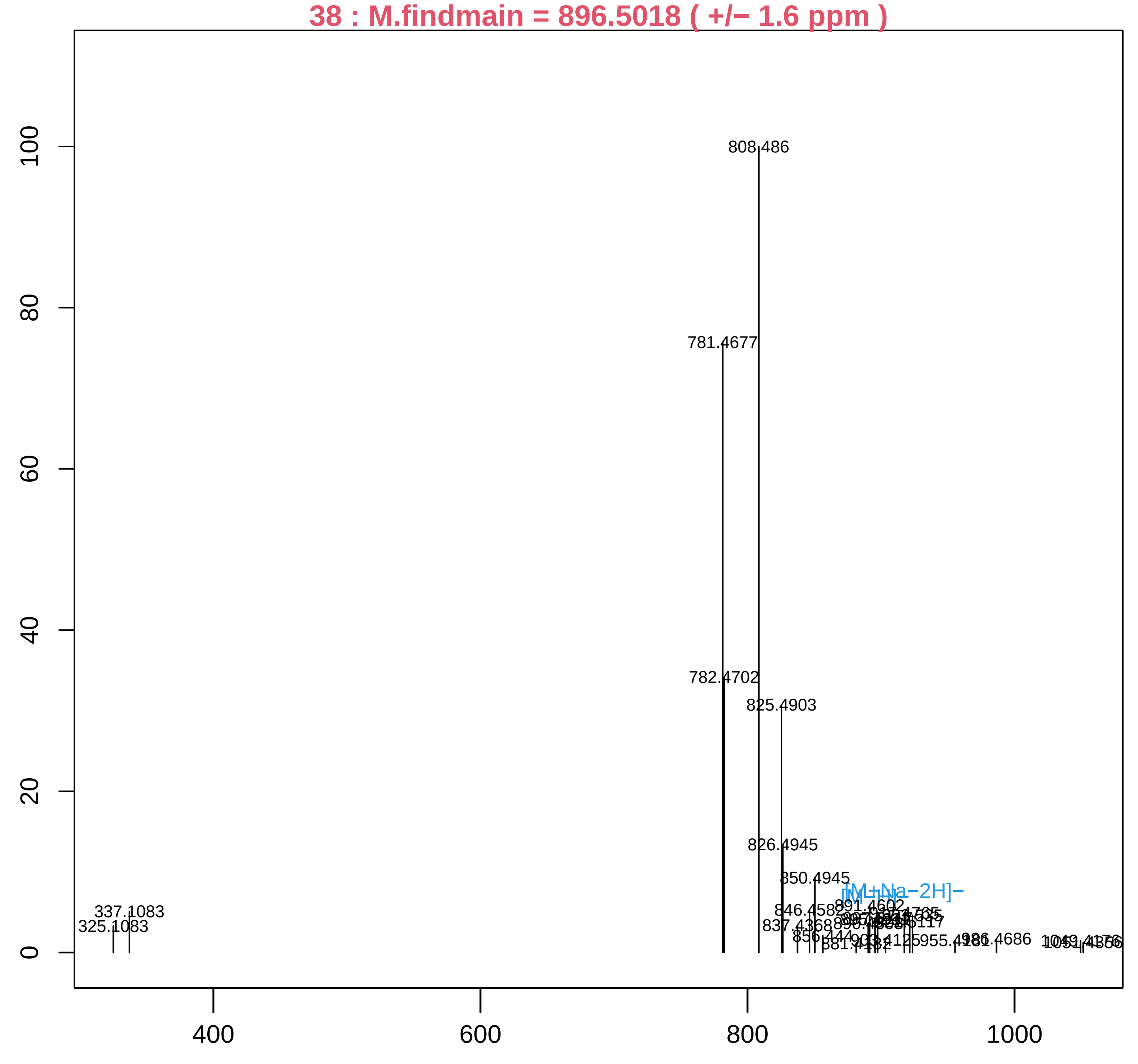
38 : M.findmain = 896.5018 (+/− 1.6 ppm)
808.486
781.4677
782.4702
825.4903
826.4945
850.4945
[M+Na−2H]−
337.1083
325.1083
0
20
40
60
80
100
400
600
800
1000

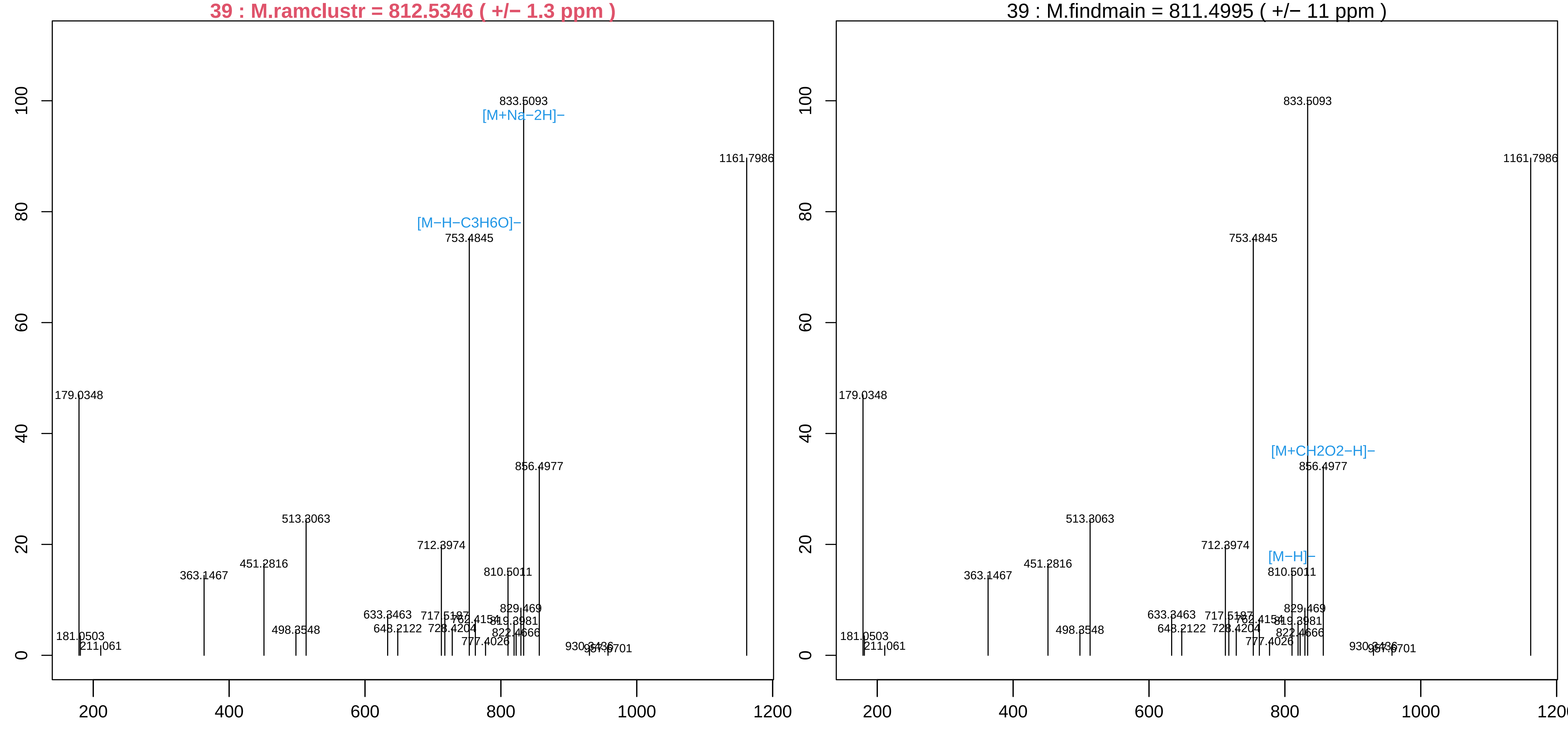

39 : M.ramclustr = 812.5346 (+/− 1.3 ppm)
39 : M.findmain = 811.4995 (+/− 11 ppm)
833.5093
[M+Na−2H]−
1161.7986
[M−H−C3H6O]−
753.4845
179.0348
856.4977
[M+CH2O2−H]−
513.3063
712.3974
[M−H]−
451.2816
363.1467
810.5011
829.469
633.3463
717.5187
762.4154
819.3981
643.2122
728.4204
498.3548
822.4666
181.0503
777.4026
211.061
930.3436
957.6701
0
20
40
60
80
100
200
400
600
800
1000
1200

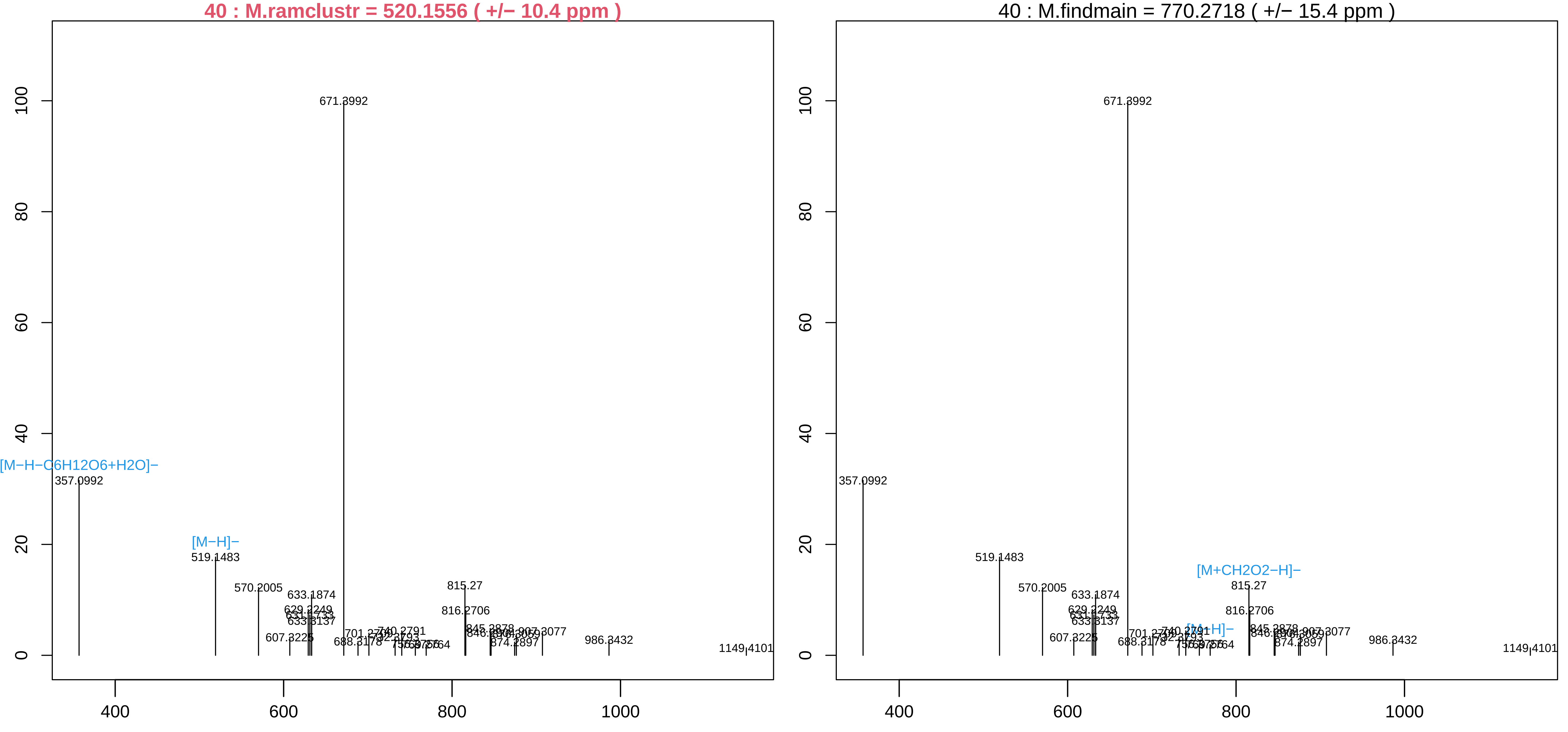
40 : M.ramclustr = 520.1556 (+/− 10.4 ppm)
[M−H−C6H12O6+H2O]−
357.0992
[M−H]−
519.1483
570.2005
671.3992
815.27
816.2706
986.3432
1149.4101
40 : M.findmain = 770.2718 (+/− 15.4 ppm)
[M+CH2O2−H]−
[M−H]−

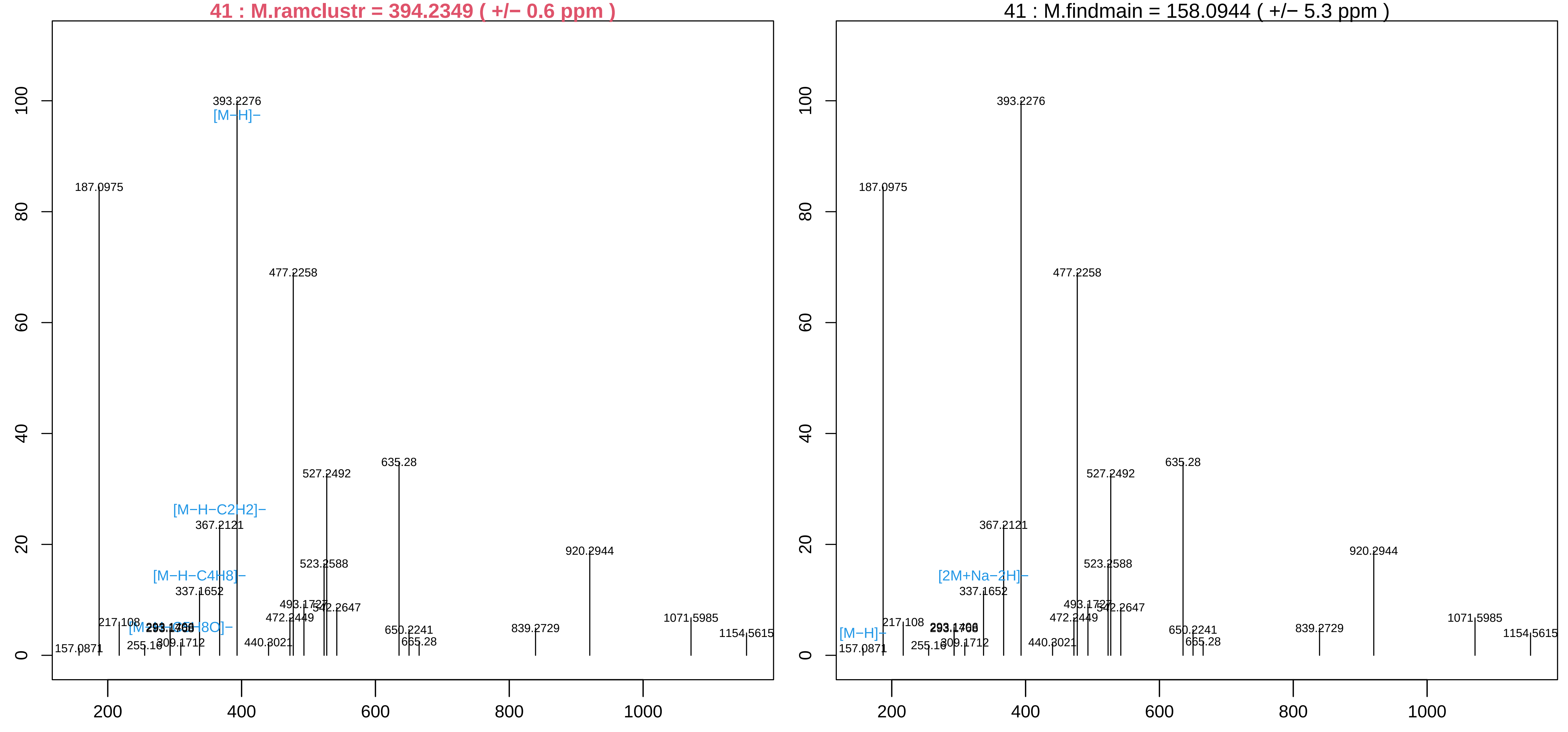

41 : M.ramclustr = 394.2349 (+/− 0.6 ppm)
393.2276
[M−H]−
187.0975
477.2258
635.28
527.2492
[M−H−C2H2]−
367.2121
920.2944
523.2588
[M−H−C4H8]−
337.1652
493.1727
542.2647
217.108
472.2449
1071.5985
650.2241
839.2729
1154.5615
255.16
309.1712
440.3021
665.28
157.0871
41 : M.findmain = 158.0944 (+/− 5.3 ppm)
[2M+Na−2H]−
[M−H]−

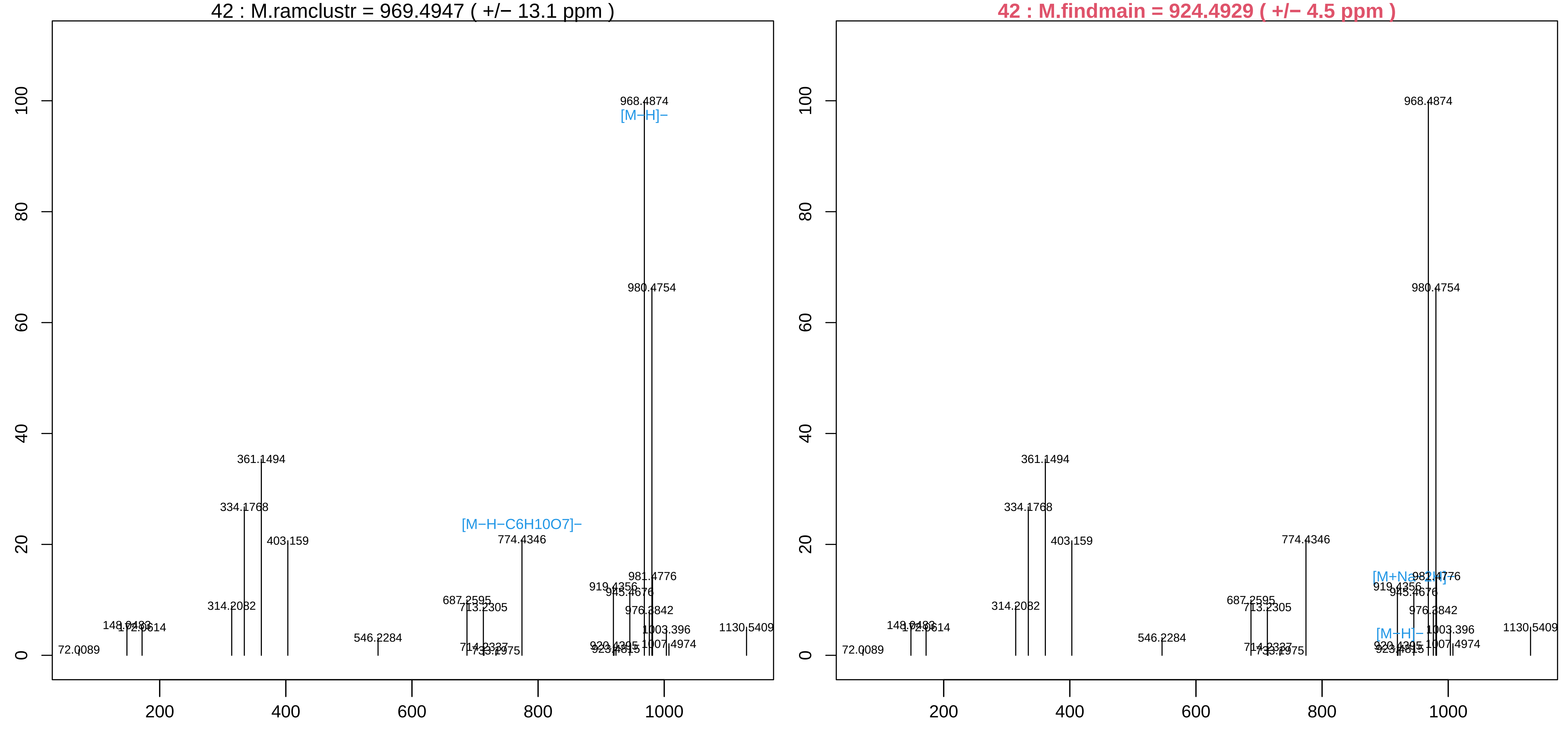

42 : M.ramclustr = 969.4947 (+/− 13.1 ppm)
42 : M.findmain = 924.4929 (+/− 4.5 ppm)
968.4874
[M−H]−
980.4754
361.1494
334.1768
403.159
[M−H−C6H10O7]−
774.4346
981.4776
919.4356
945.4676
976.3842
314.2082
687.2595
713.2305
1003.396
1130.5409
148.0482
172.0614
546.2284
714.2327
733.1975
920.4395
923.4815
1007.4974
72.0089
[M+Na−2H]−
0
20
40
60
80
100
200
400
600
800
1000

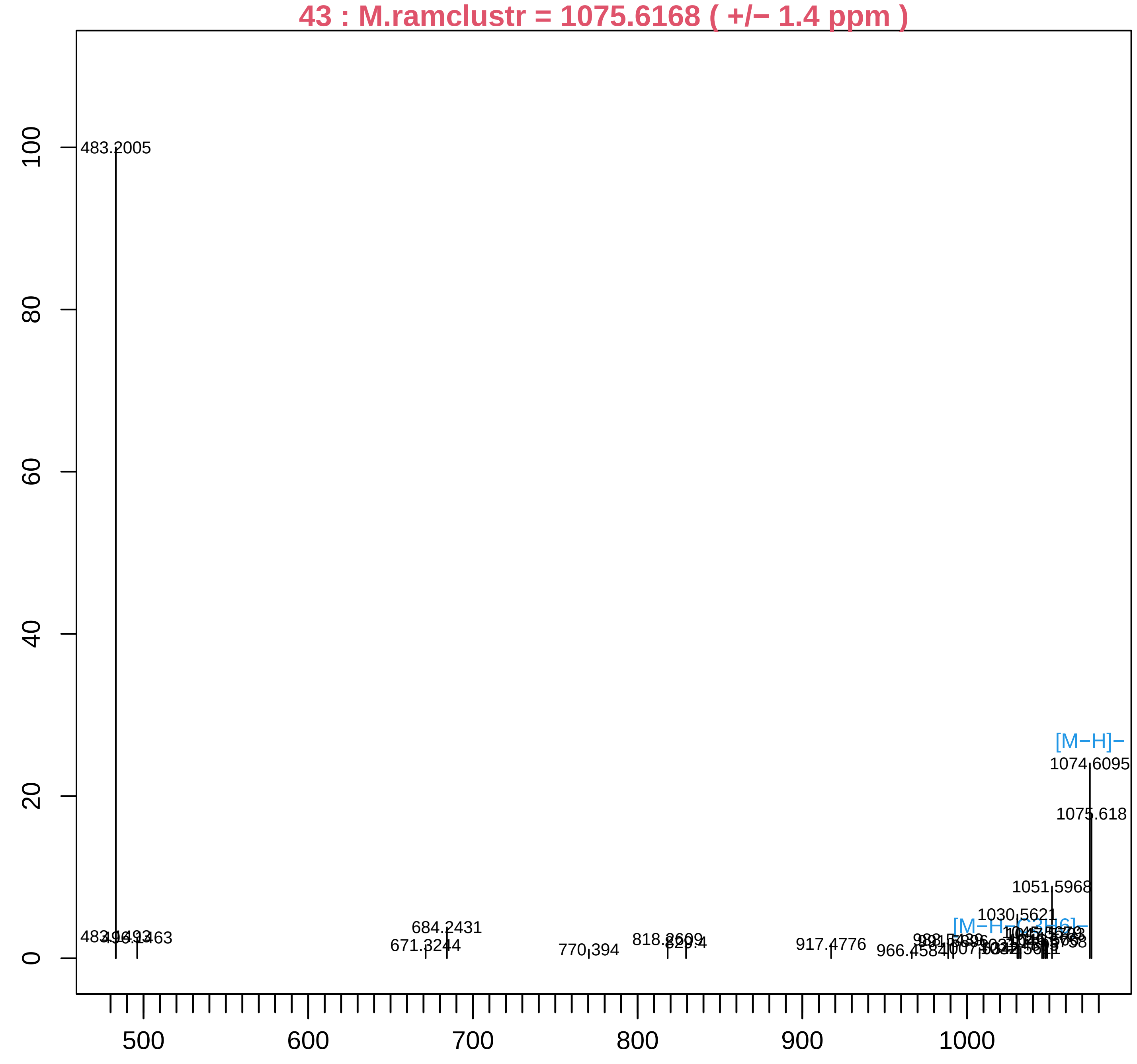

43 : M.ramclustr = 1075.6168 (+/− 1.4 ppm)
483.2005
[M−H]−
1074.6095
1075.618
1051.5968
1030.5621
684.2431
671.3244
770.394
818.8609
917.4776
966.4584

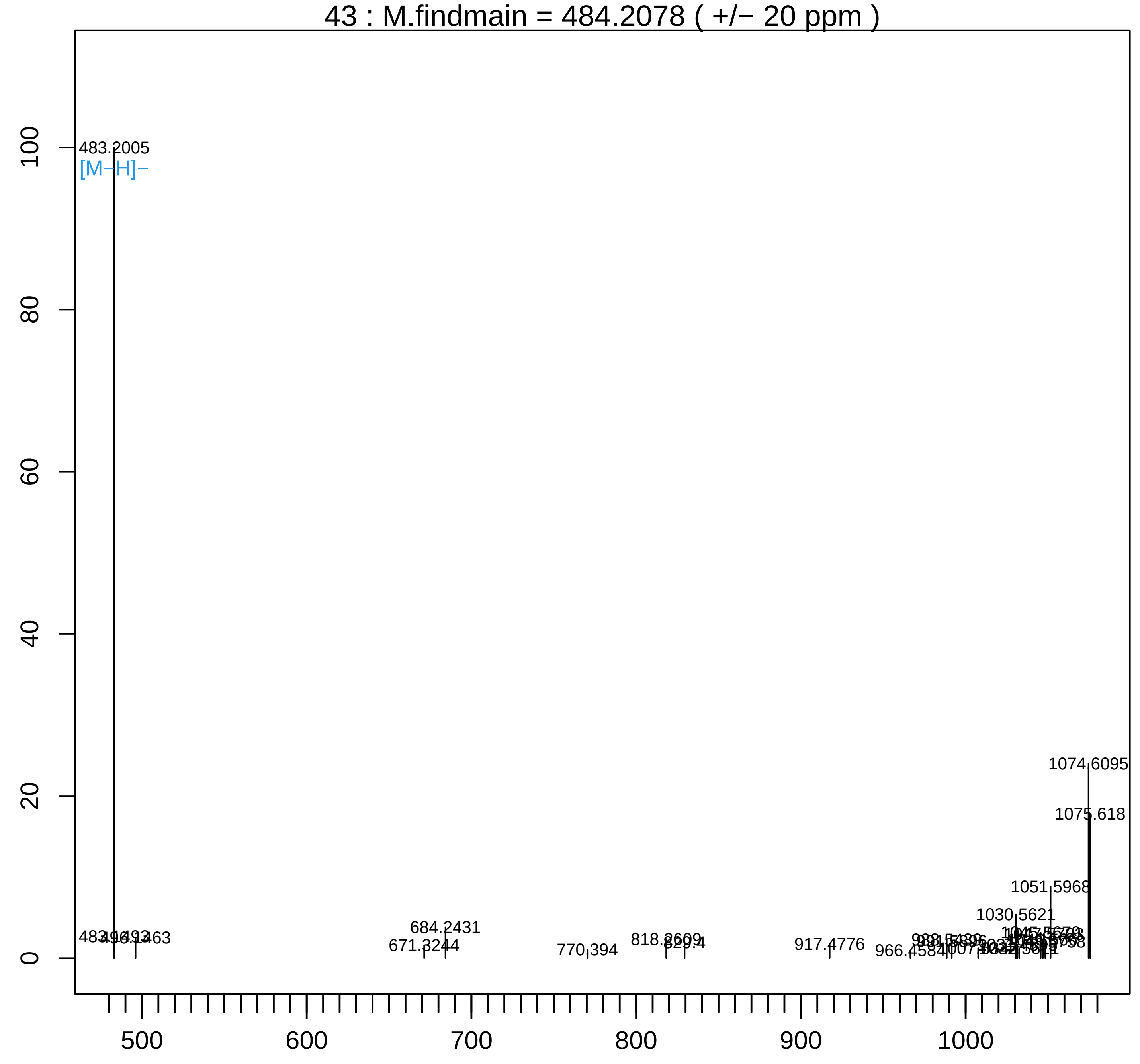

43 : M.findmain = 484.2078 (+/− 20 ppm)
483.2005
[M−H]−
1074.6095
1075.618
1051.5968
1030.5621
684.2431
671.3244
770.394
818.8609
917.4776
966.4584

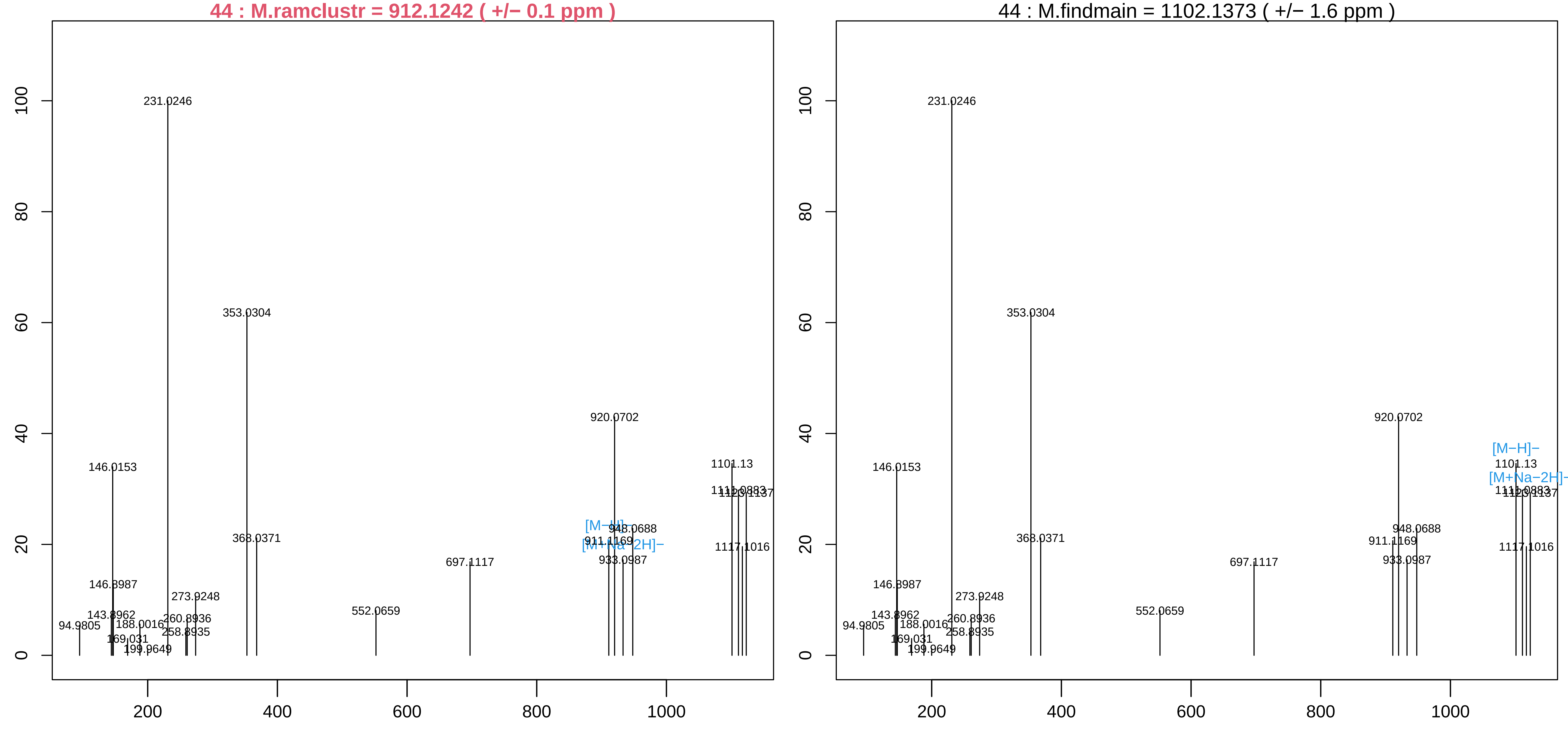
44 : M.ramclustr = 912.1242 (+/− 0.1 ppm)
44 : M.findmain = 1102.1373 (+/− 1.6 ppm)
231.0246
353.0304
920.0702
146.0153
1101.13
1111.0883
1123.1137
[M−H]−
948.0688
911.1169
[M+Na−2H]−
368.0371
933.0987
1117.1016
697.1117
146.8987
273.9248
552.0659
143.8962
188.0016
260.8936
94.9805
258.8935
169.031
199.9649
0
20
40
60
80
100
200
400
600
800
1000

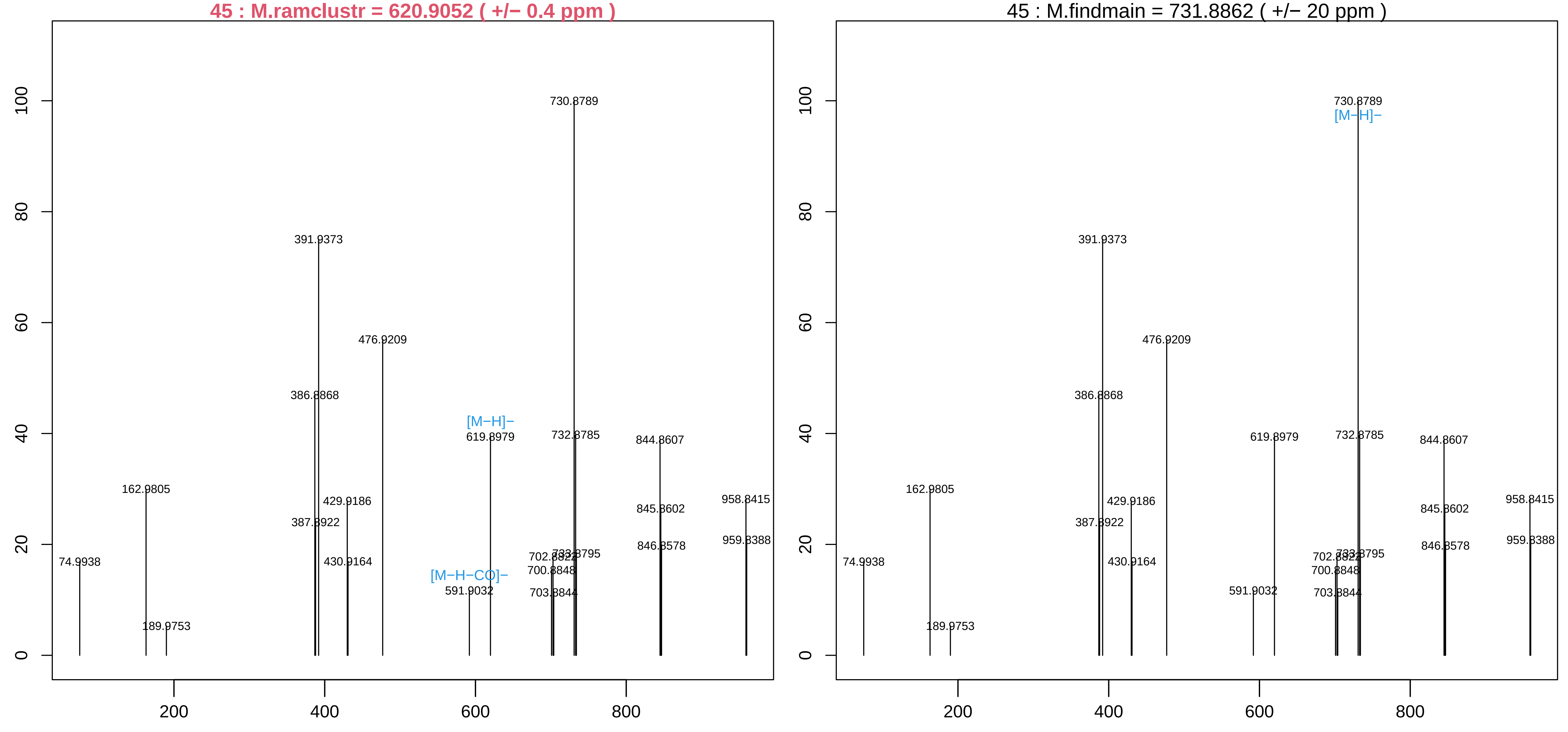

45 : M.ramclustr = 620.9052 (+/− 0.4 ppm)
45 : M.findmain = 731.8862 (+/− 20 ppm)
730.8789
[M−H]−
391.9373
476.9209
386.8868
[M−H]−
619.8979
732.8785
844.8607
162.9805
429.9186
845.8602
958.8415
387.8922
846.8578
959.8388
74.9938
430.9164
702.8822
733.8795
700.8848
[M−H−CO]−
591.9032
703.8844
189.9753
0
20
40
60
80
100
200
400
600
800

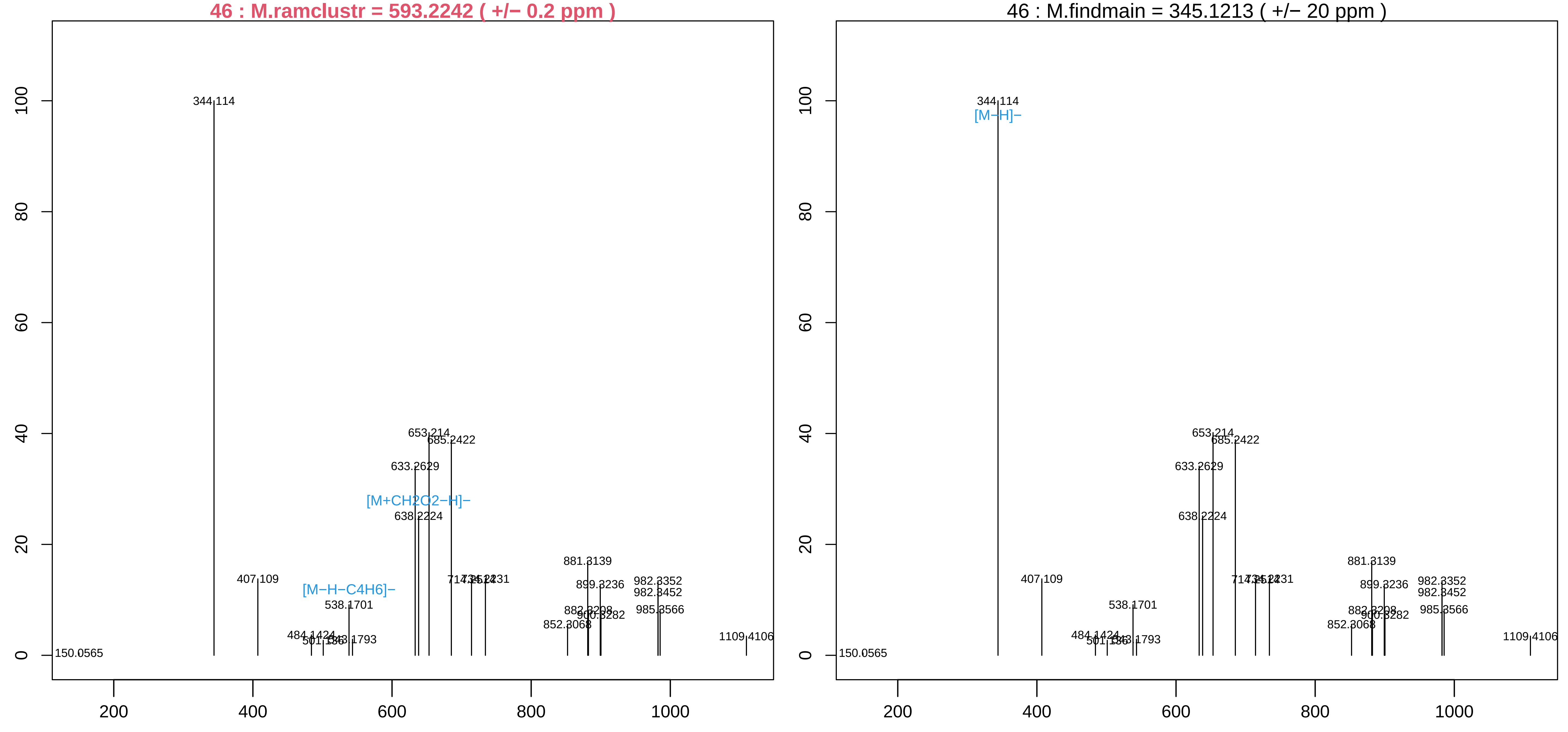
46 : M.ramclustr = 593.2242 (+/− 0.2 ppm)
46 : M.findmain = 345.1213 (+/− 20 ppm)
344.114
[M−H]−
407.109
484.1424
501.136
538.1701
543.1793
633.2629
638.2224
653.214
685.2422
714.2524
734.2231
852.3068
881.3139
882.3208
899.3236
900.3282
982.3352
982.3452
985.3566
1109.4106
150.0565
[M+CH2O2−H]−
[M−H−C4H6]−
0
20
40
60
80
100
200
400
600
800
1000

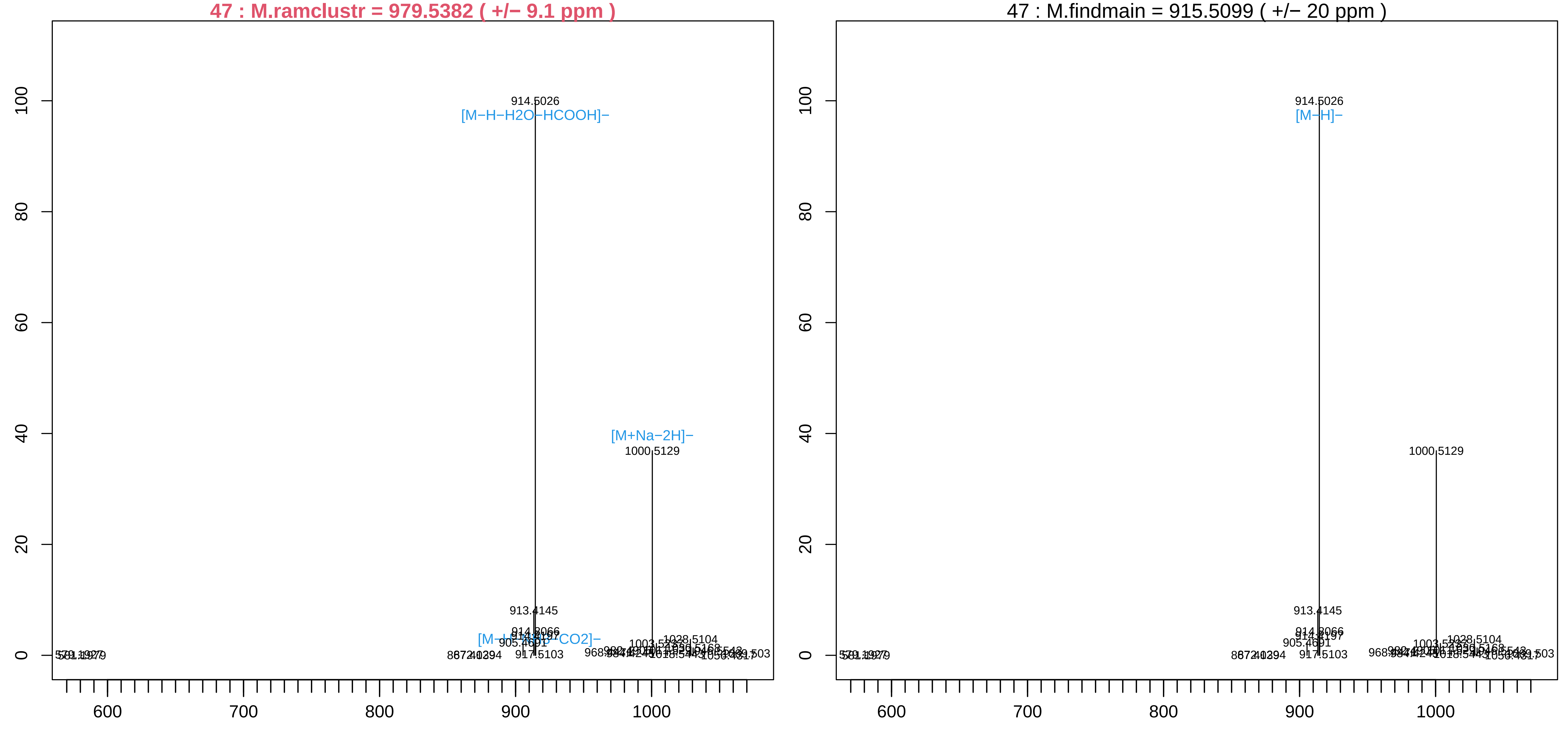

47 : M.ramclustr = 979.5382 (+/− 9.1 ppm)
914.5026
[M−H−H2O−HCOOH]−
[M+Na−2H]−
1000.5129
913.4145
905.4691
917.5103
1028.5104
47 : M.findmain = 915.5099 (+/− 20 ppm)
[M−H]−
0
20
40
60
80
100
600
700
800
900
1000

48 : M.ramclustr = 342.1158 (+/− 0.3 ppm)

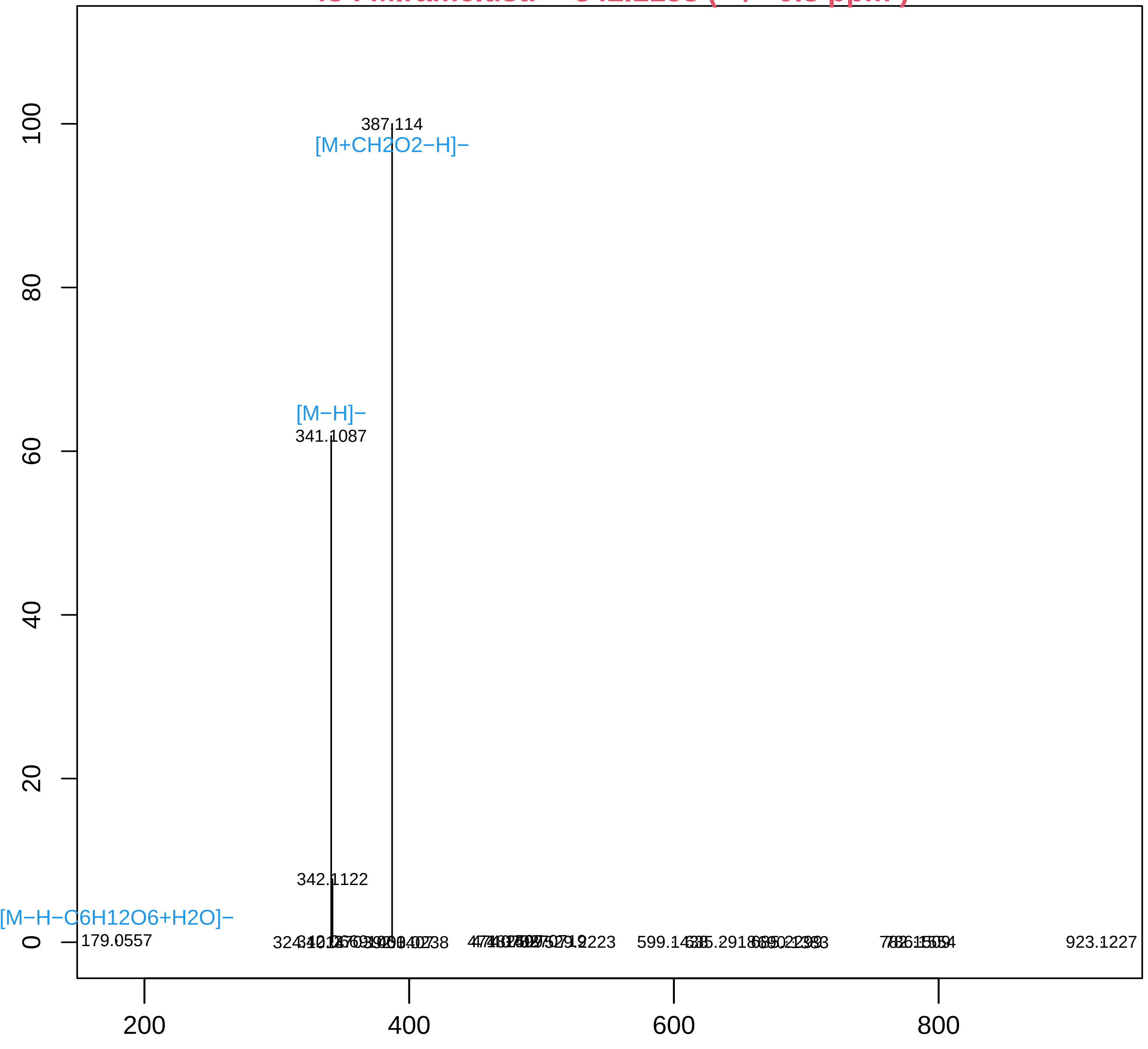

48 : M.findmain = 342.1158 (+/− 0.5 ppm)

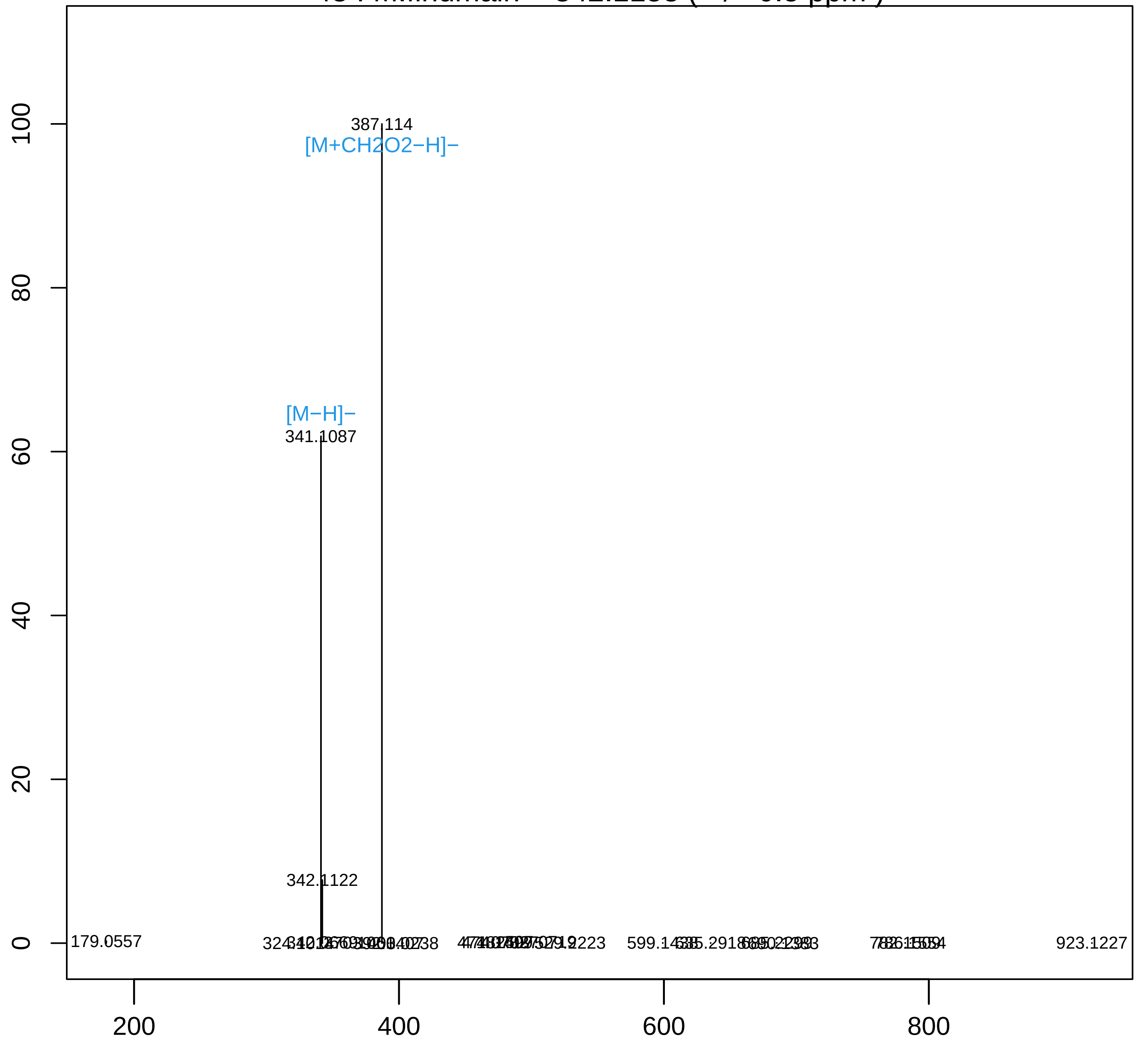

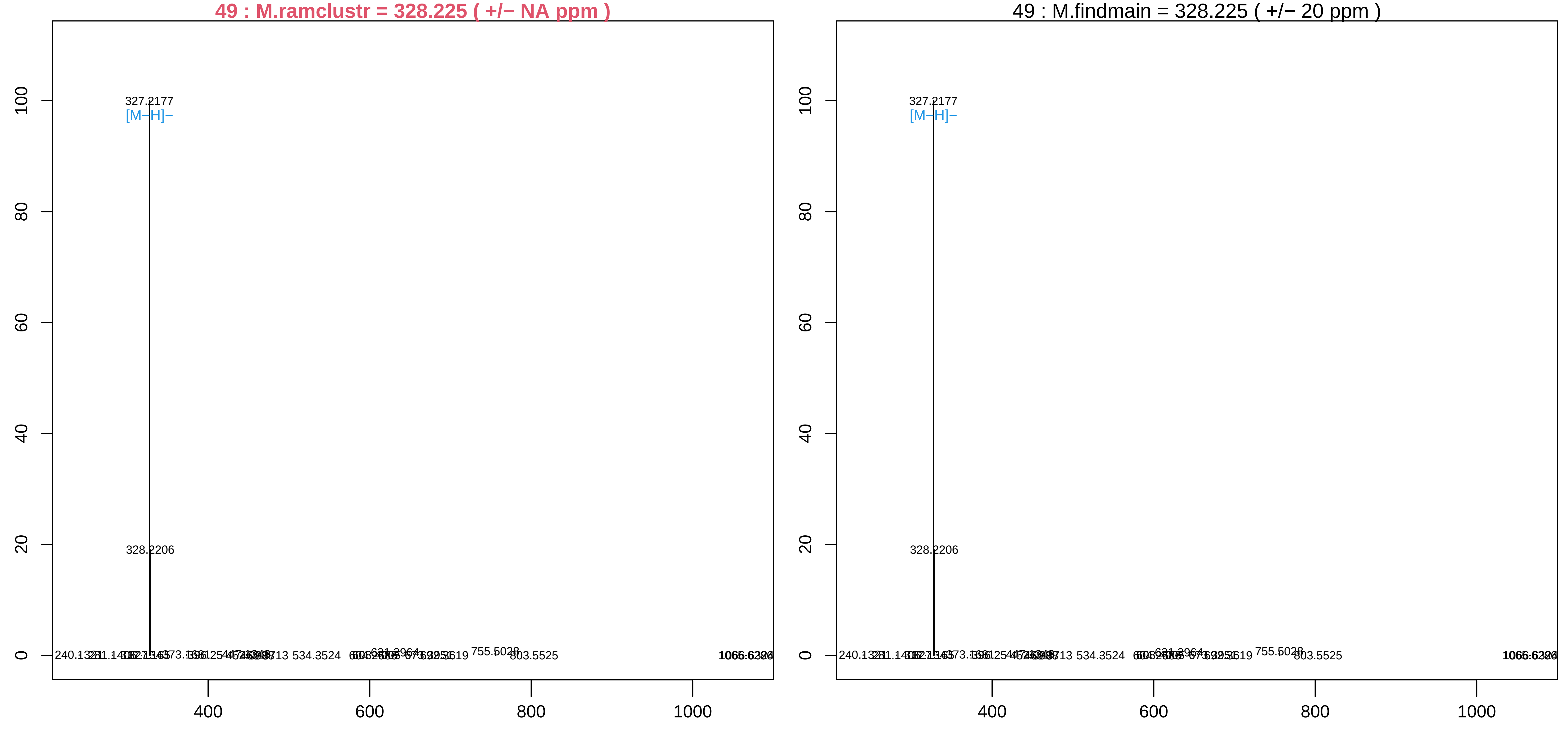

49 : M.ramclustr = 328.225 (+/− NA ppm)
327.2177
[M−H]−
328.2206
755.5028
603.5525
534.3524
240.1321
1066.6324
49 : M.findmain = 328.225 (+/− 20 ppm)
327.2177
[M−H]−
328.2206
755.5028
603.5525
534.3524
240.1321
1066.6324

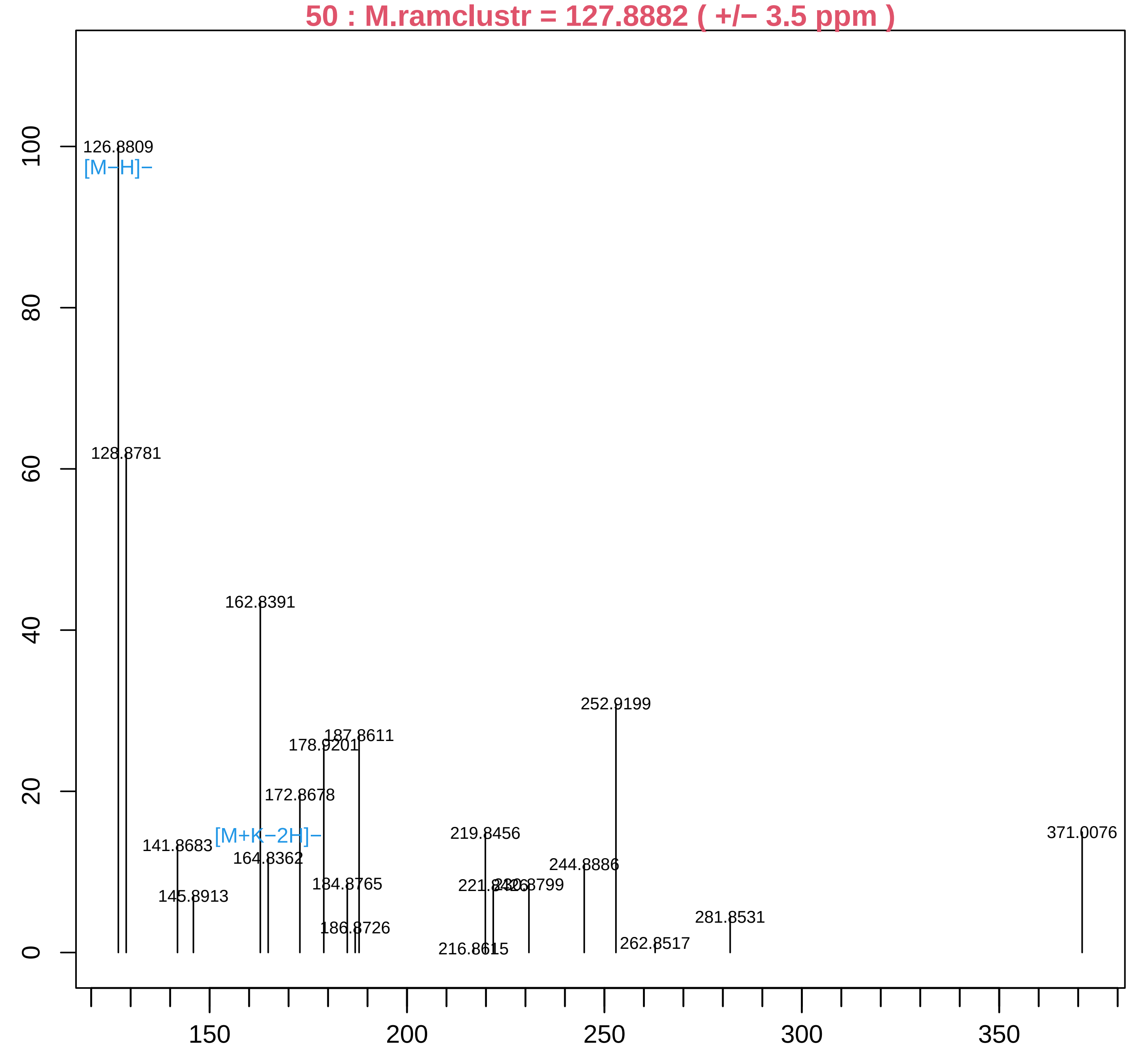
50 : M.ramclustr = 127.8882 (+/− 3.5 ppm)
126.8809
[M−H]−
128.8781
162.8391
252.9199
187.8611
178.9201
172.8678
[M+K−2H]−
141.8683
164.8362
219.8456
371.0076
244.8886
145.8913
184.8765
281.8531
186.8726
262.8517
216.8615
0
20
40
60
80
100
150
200
250
300
350

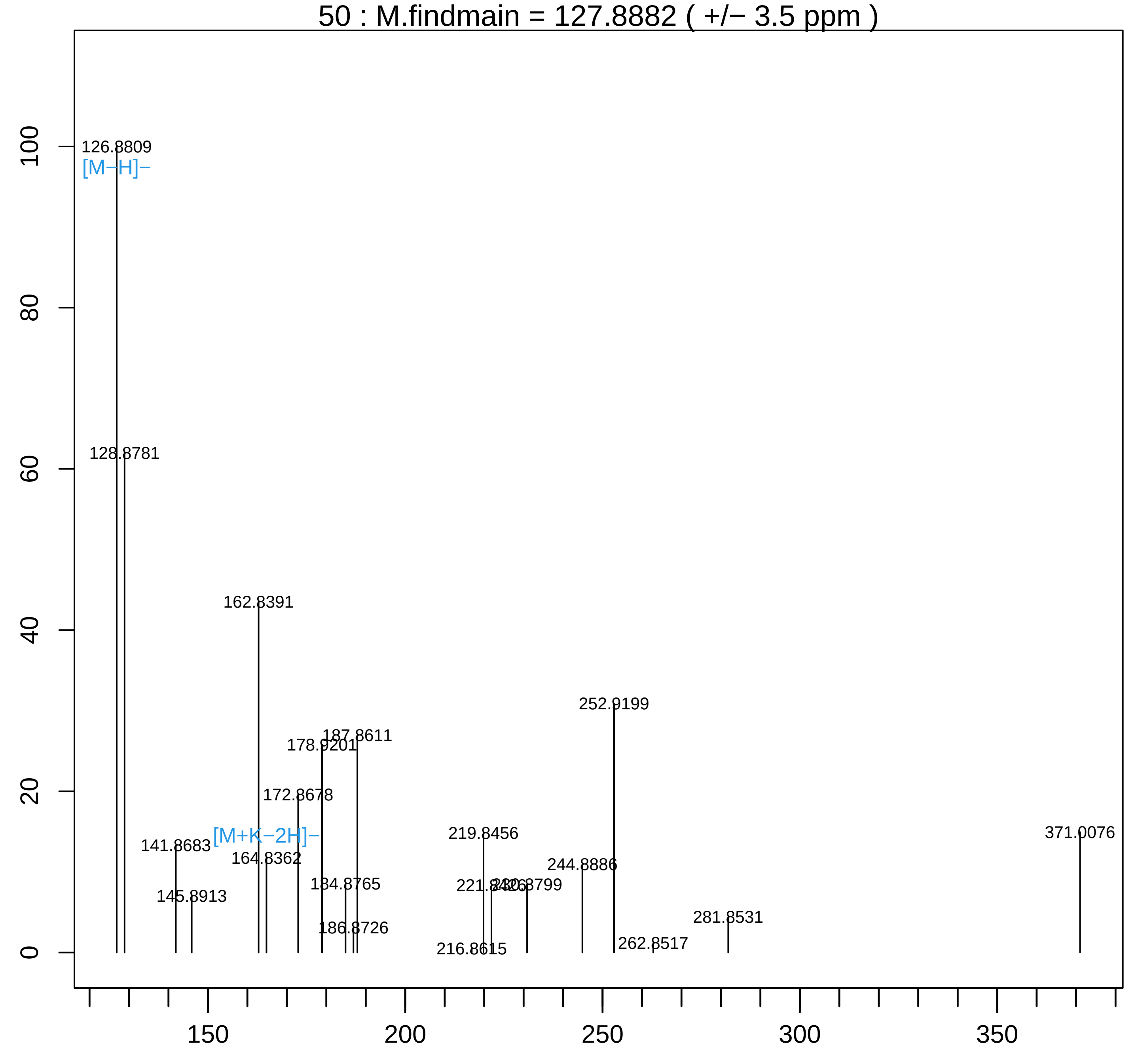
50 : M.findmain = 127.8882 (+/− 3.5 ppm)
126.8809
[M−H]−
128.8781
162.8391
252.9199
187.8611
178.9201
172.8678
[M+K−2H]−
141.8683
164.8362
219.8456
371.0076
244.8886
145.8913
184.8765
281.8531
186.8726
262.8517
216.8615
0
20
40
60
80
100
150
200
250
300
350

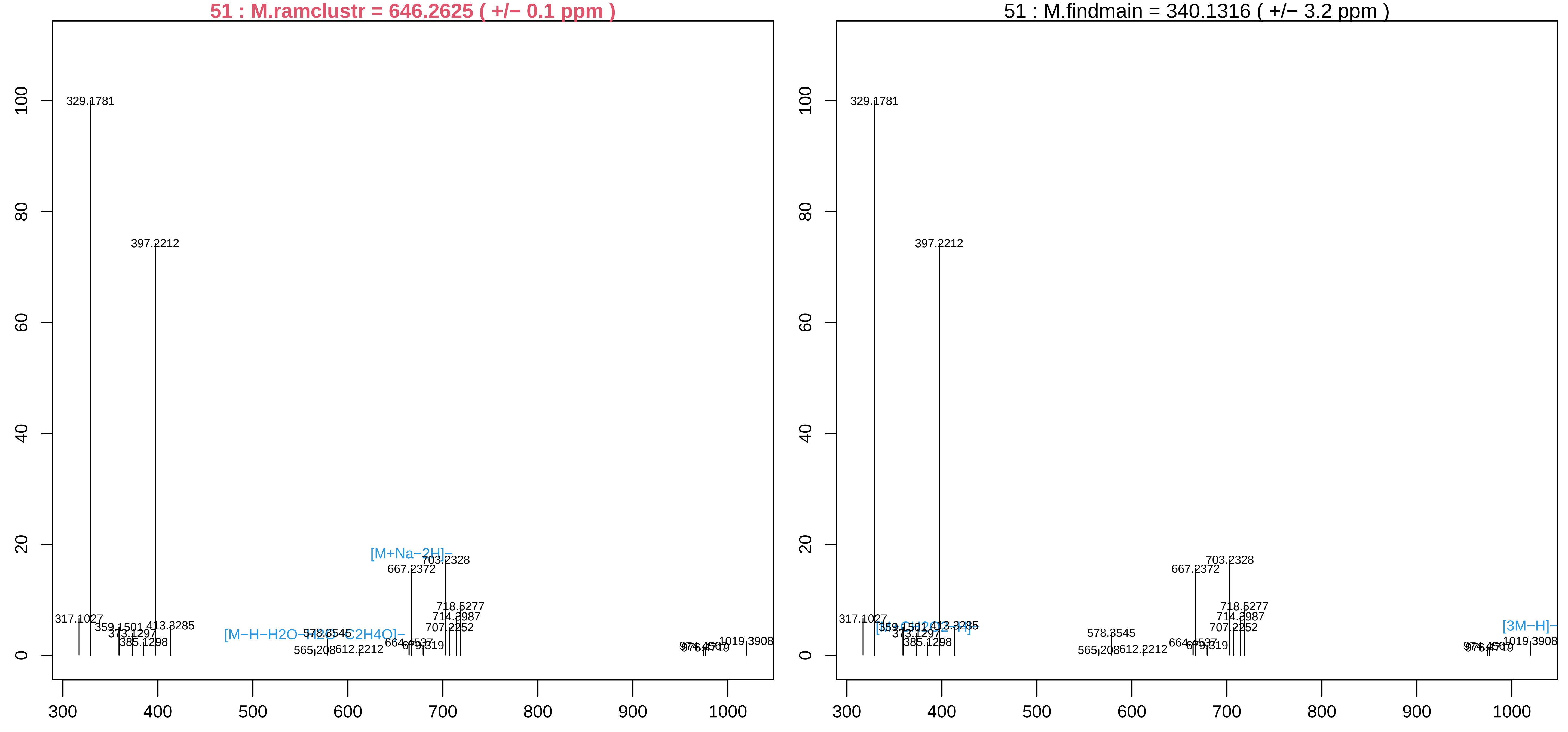

51 : M.ramclustr = 646.2625 (+/− 0.1 ppm)
51 : M.findmain = 340.1316 (+/− 3.2 ppm)
329.1781
397.2212
317.1027
359.1501
373.1297
385.1298
413.3285
[M−H−H2O−H2S−C2H4O]−
578.3545
565.208
612.2212
[M+Na−2H]−
667.2372
703.2328
664.4537
679.319
718.5277
714.3987
707.2252
974.4501
976.4719
1019.3908
[3M−H]−

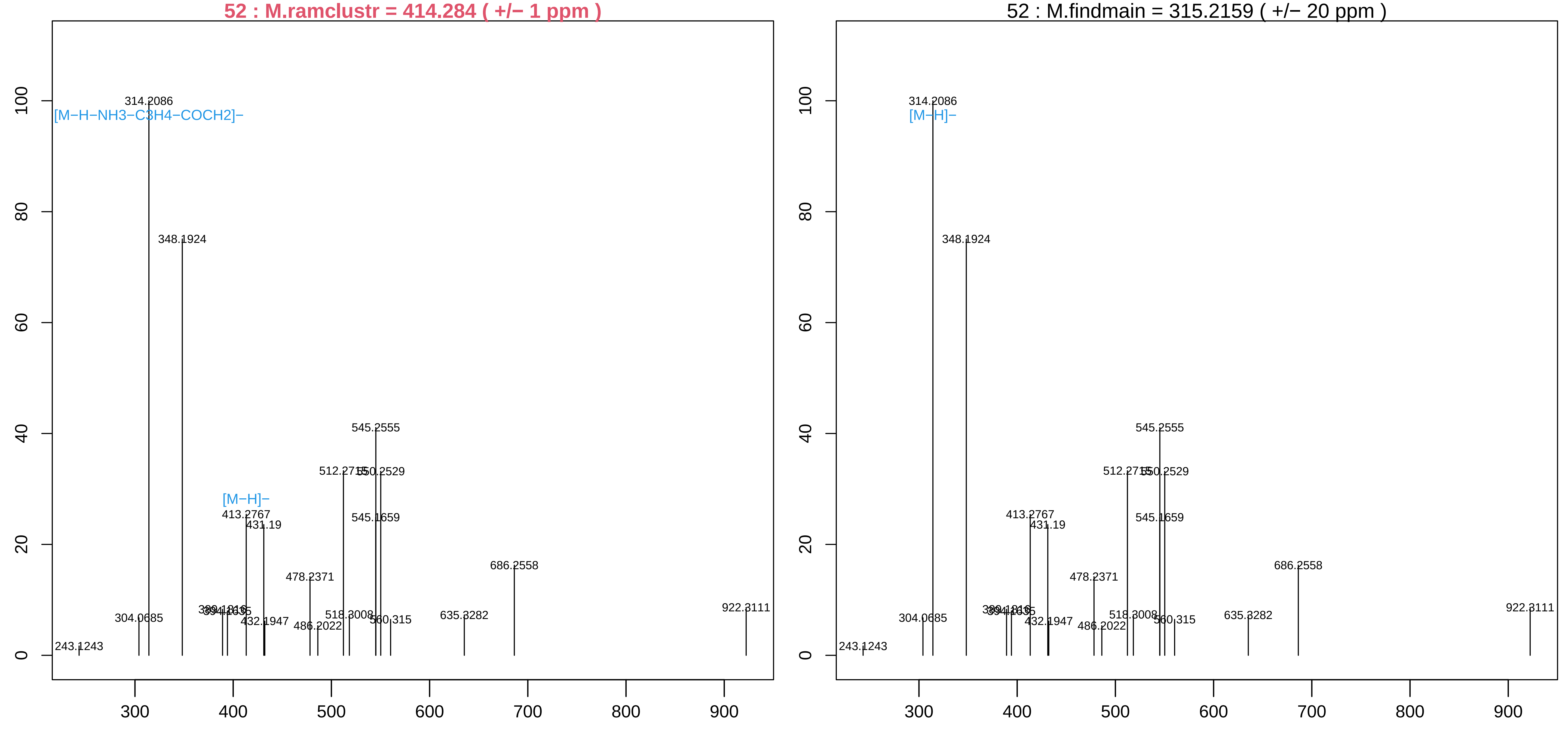
52 : M.ramclustr = 414.284 (+/− 1 ppm)
314.2086
[M−H−NH3−C3H4−COCH2]−
348.1924
545.2555
512.2715
550.2529
[M−H]−
413.2767
431.19
545.1659
686.2558
478.2371
922.3111
304.0685
243.1243
518.3008
560.315
635.3282
432.1947
486.2022
52 : M.findmain = 315.2159 (+/− 20 ppm)
314.2086
[M−H]−
348.1924
545.2555
512.2715
550.2529
413.2767
431.19
545.1659
686.2558
478.2371
922.3111
304.0685
243.1243
518.3008
560.315
635.3282
432.1947
486.2022
0
20
40
60
80
100
300
400
500
600
700
800
900

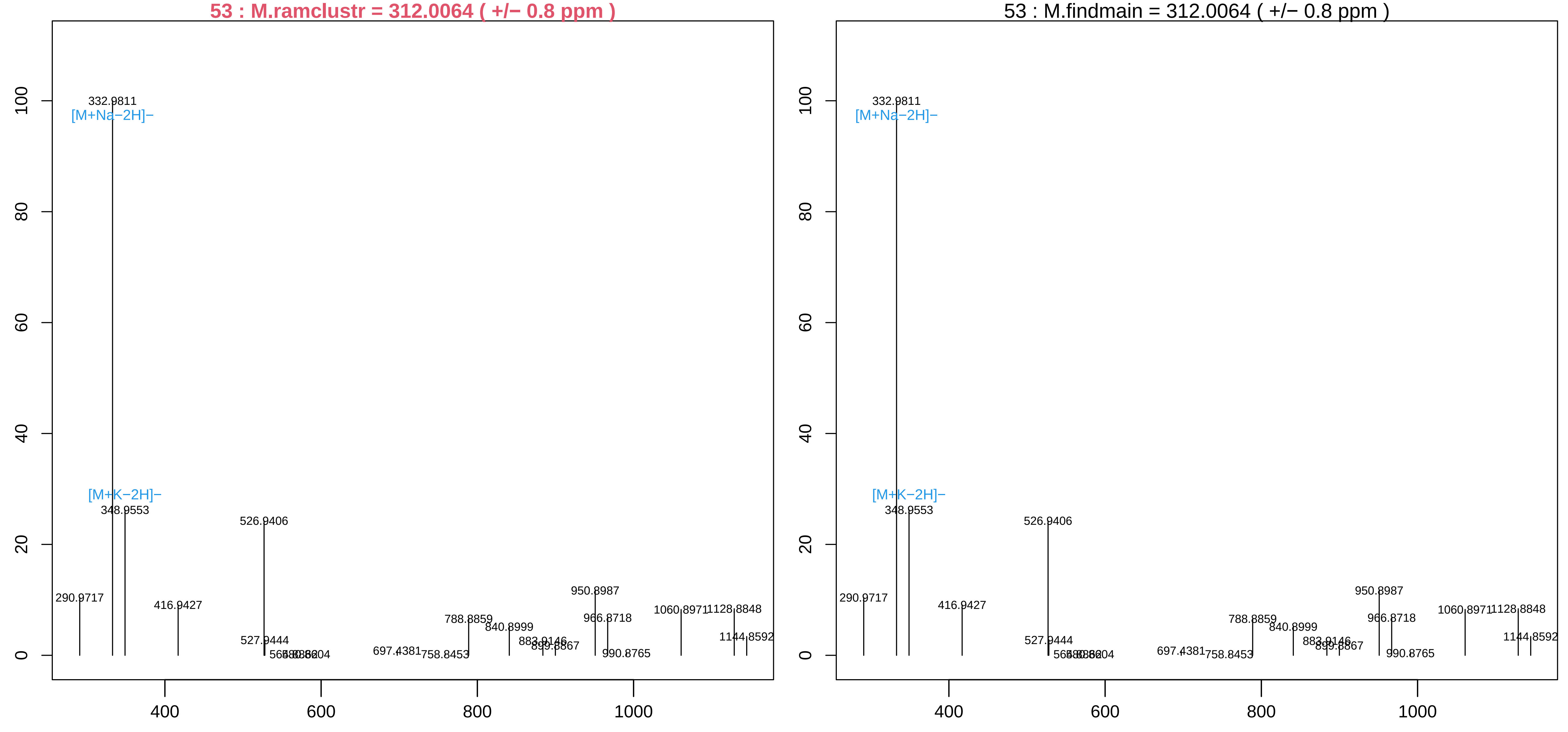
53 : M.ramclustr = 312.0064 (+/− 0.8 ppm)
332.9811
[M+Na−2H]−
[M+K−2H]−
348.9553
526.9406
290.9717
416.9427
527.9444
697.4381
758.8453
788.8859
840.8999
883.9146
899.8867
950.8987
966.8718
990.8765
1060.8971
1128.8848
1144.8592
0
20
40
60
80
100
400
600
800
1000
53 : M.findmain = 312.0064 (+/− 0.8 ppm)
332.9811
[M+Na−2H]−
[M+K−2H]−
348.9553
526.9406
290.9717
416.9427
527.9444
697.4381
758.8453
788.8859
840.8999
883.9146
899.8867
950.8987
966.8718
990.8765
1060.8971
1128.8848
1144.8592
0
20
40
60
80
100
400
600
800
1000

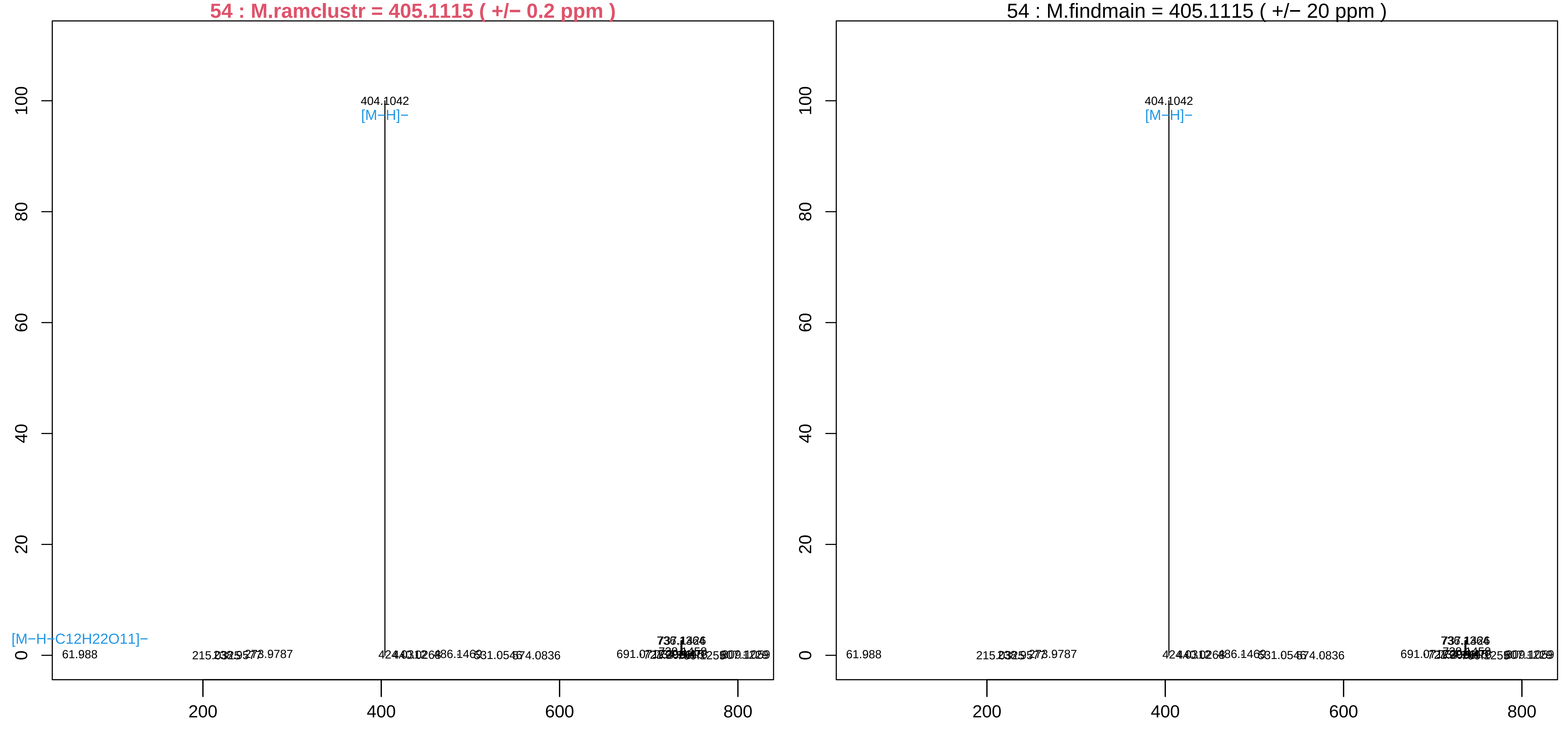

54 : M.ramclustr = 405.1115 (+/− 0.2 ppm)
54 : M.findmain = 405.1115 (+/− 20 ppm)
404.1042
[M−H]−
[M−H−C12H22O11]−
61.988
273.9787
486.1465
574.0836
0
20
40
60
80
100
200
400
600
800

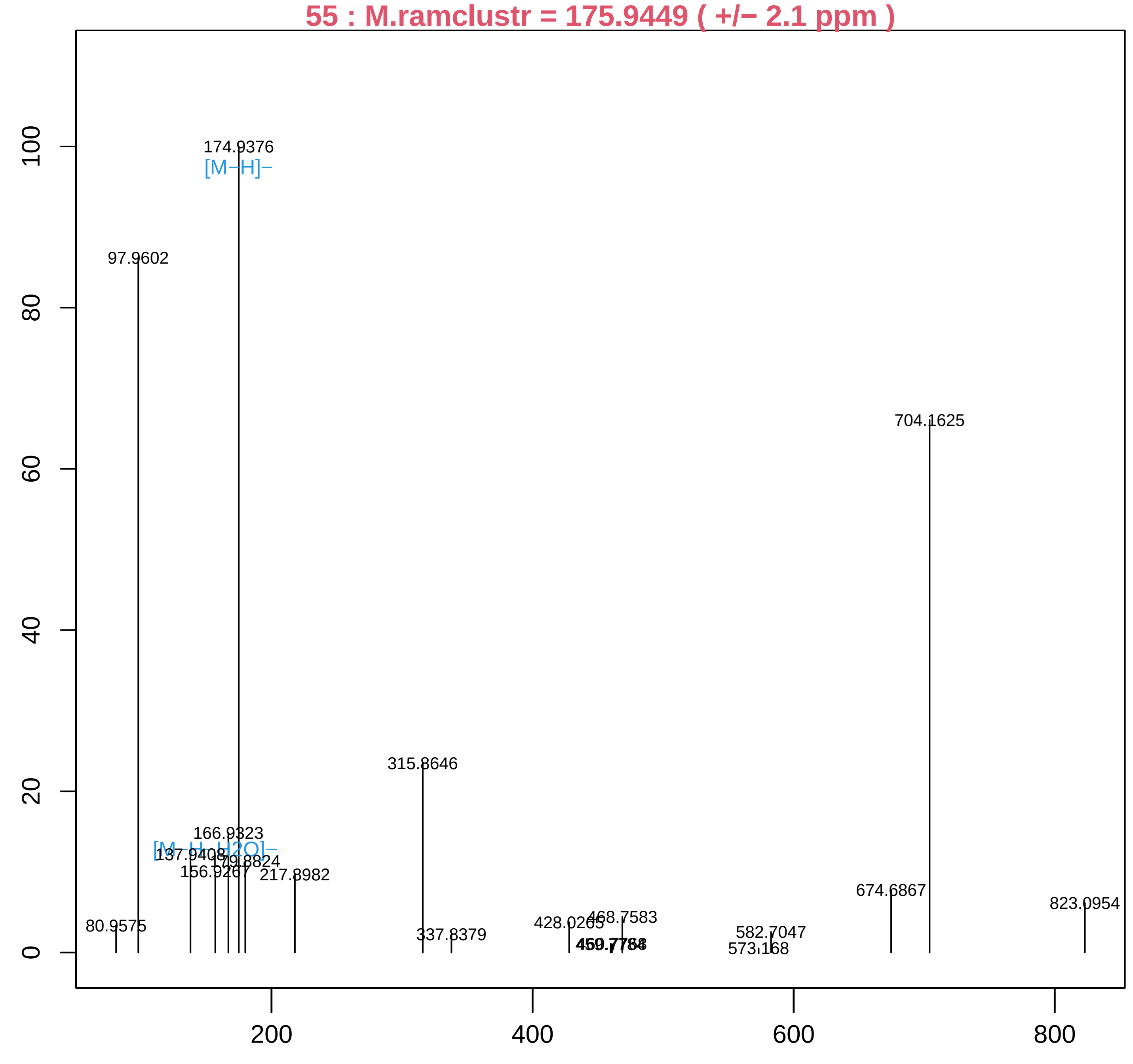
55 : M.ramclustr = 175.9449 (+/− 2.1 ppm)
174.9376
[M−H]−
97.9602
704.1625
315.8646
166.9323
137.9408
179.8824
156.9267
217.8982
674.6867
823.0954
80.9575
337.8379
428.0265
468.7583
582.7047
573.168
0
20
40
60
80
100
200
400
600
800

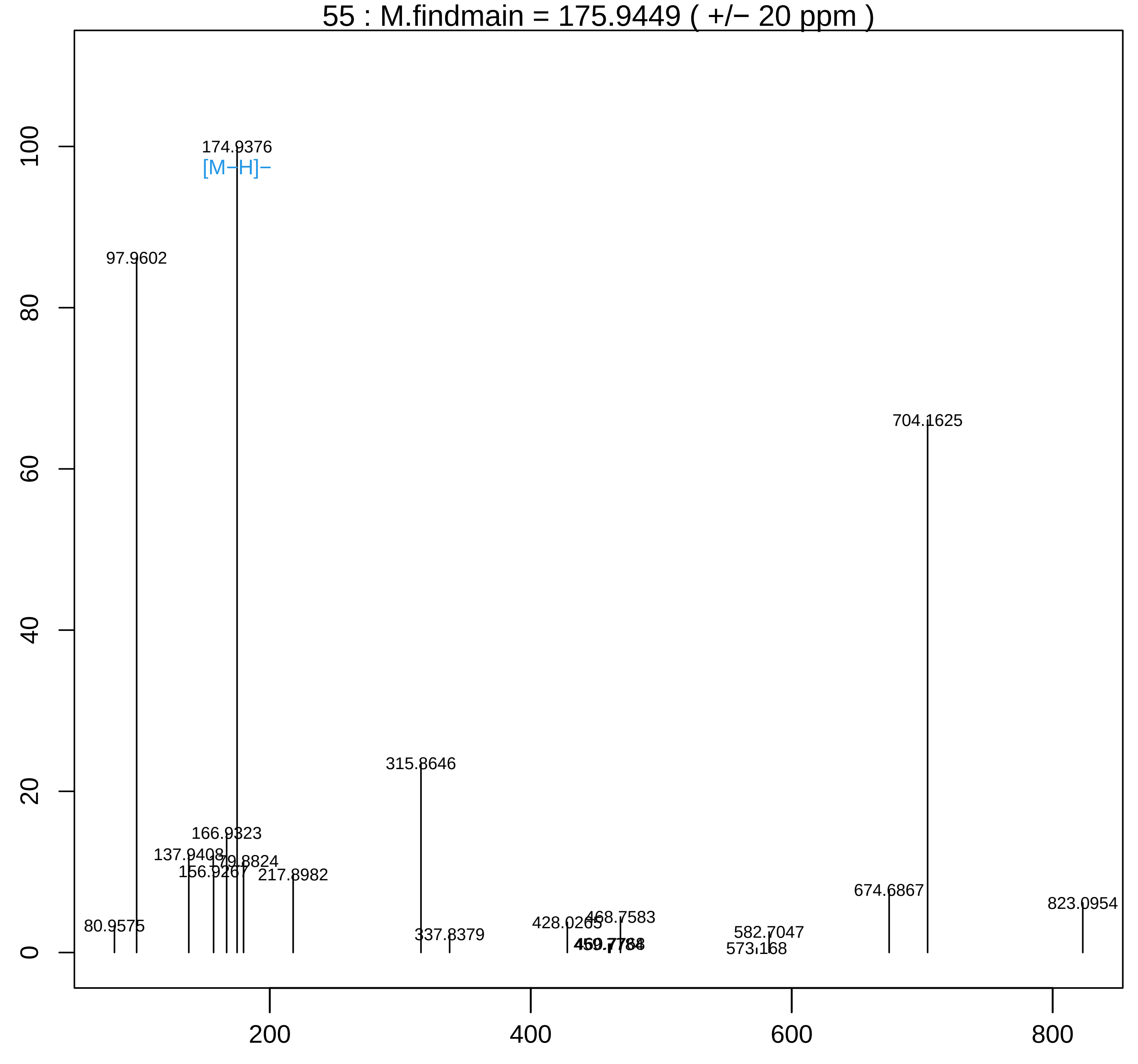
55 : M.findmain = 175.9449 (+/− 20 ppm)
174.9376
[M−H]−
97.9602
704.1625
315.8646
166.9323
137.9408
179.8824
156.9267
217.8982
674.6867
823.0954
80.9575
337.8379
428.0265
468.7583
582.7047
573.168
0
20
40
60
80
100
200
400
600
800

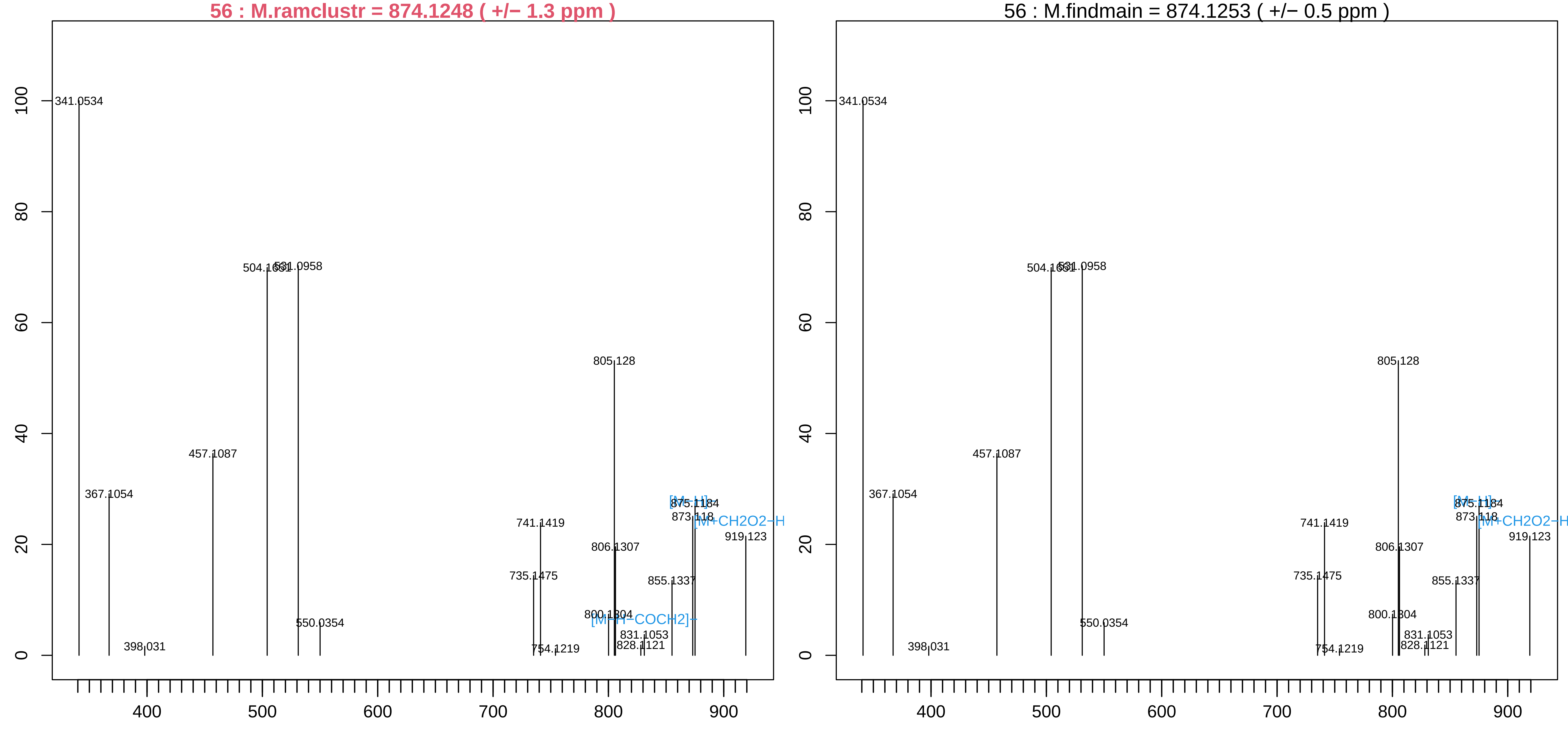

56 : M.ramclustr = 874.1248 (+/− 1.3 ppm)
56 : M.findmain = 874.1253 (+/− 0.5 ppm)
341.0534
367.1054
398.031
457.1087
504.1631
531.0958
550.0354
735.1475
741.1419
754.1219
800.1304
805.128
806.1307
828.1121
831.1053
855.1337
873.118
875.1184
919.123
[M-H]-
[M+CH2O2-H
[M-H-COCH2]-
0
20
40
60
80
100
400
500
600
700
800
900

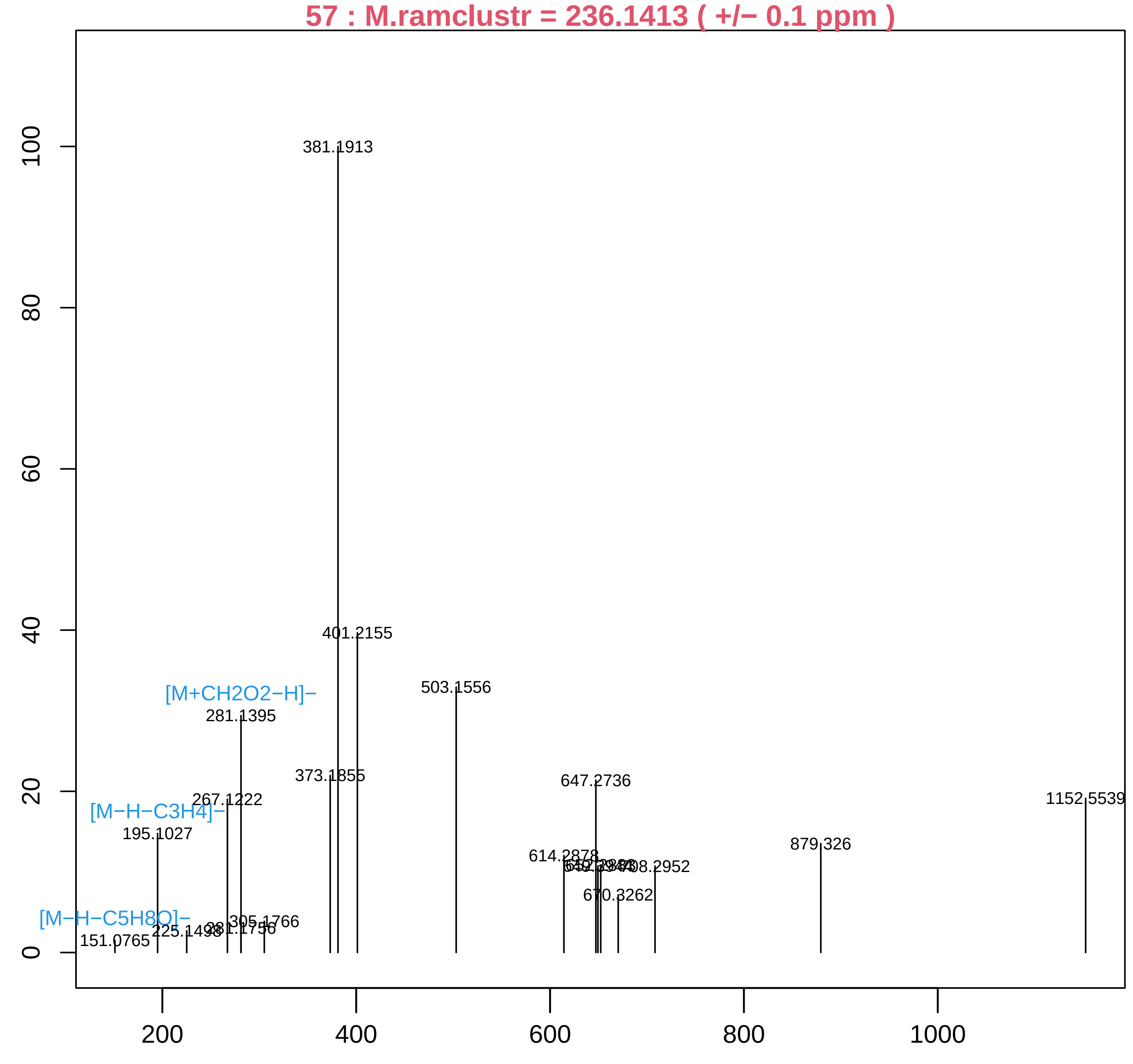
57 : M.ramclustr = 236.1413 (+/− 0.1 ppm)
381.1913
401.2155
503.1556
[M+CH2O2−H]−
281.1395
373.1855
647.2736
267.1222
[M−H−C3H4]−
195.1027
614.2878
879.326
1152.5539
708.2952
670.3262
[M−H−C5H8O]−
305.1766
225.1498
281.1756
151.0765
0
20
40
60
80
100
200
400
600
800
1000

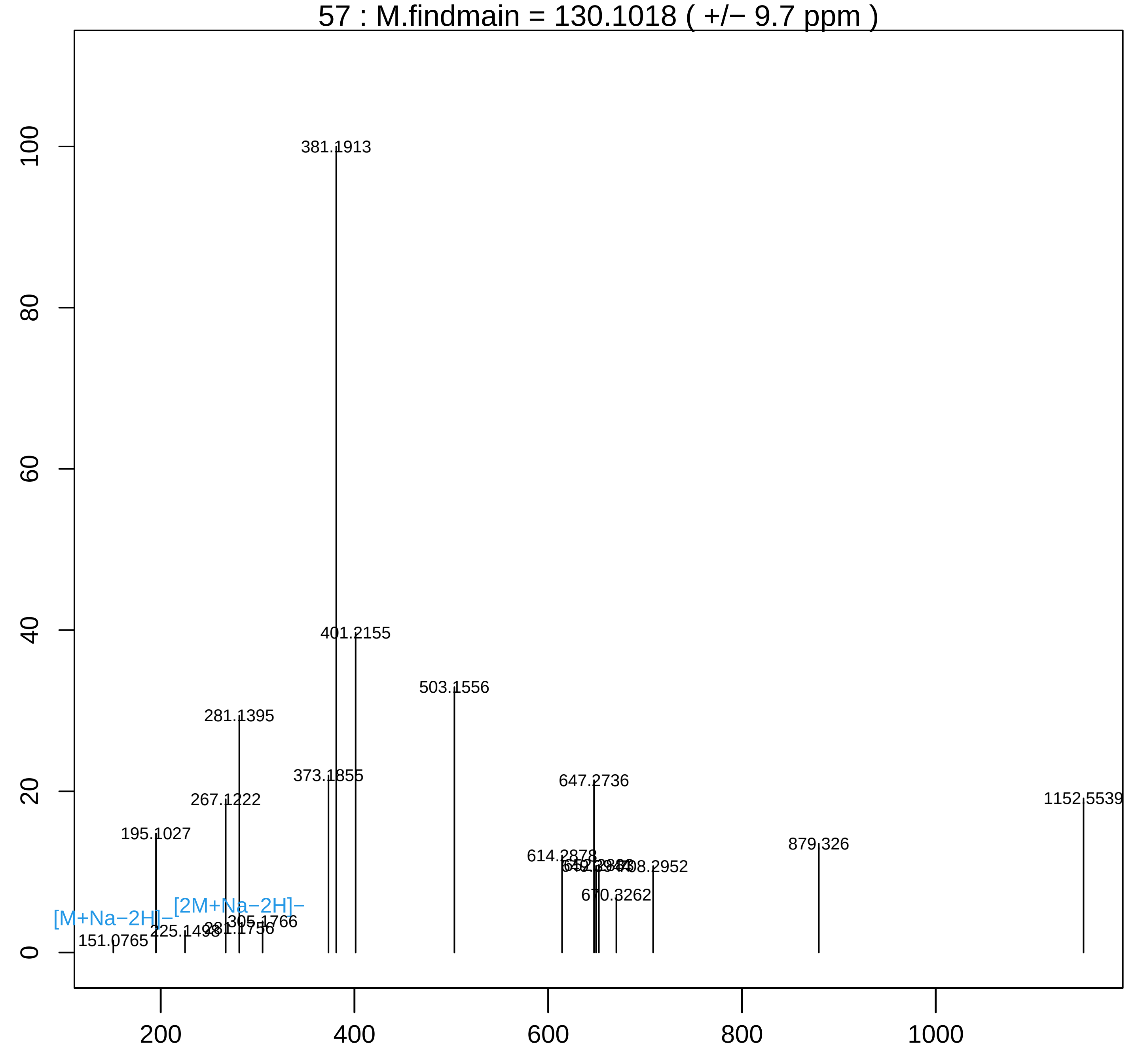
57 : M.findmain = 130.1018 (+/− 9.7 ppm)
381.1913
401.2155
503.1556
281.1395
373.1855
647.2736
267.1222
195.1027
614.2878
879.326
1152.5539
708.2952
670.3262
[2M+Na−2H]−
[M+Na−2H]−
305.1766
225.1498
281.1756
151.0765
0
20
40
60
80
100
200
400
600
800
1000

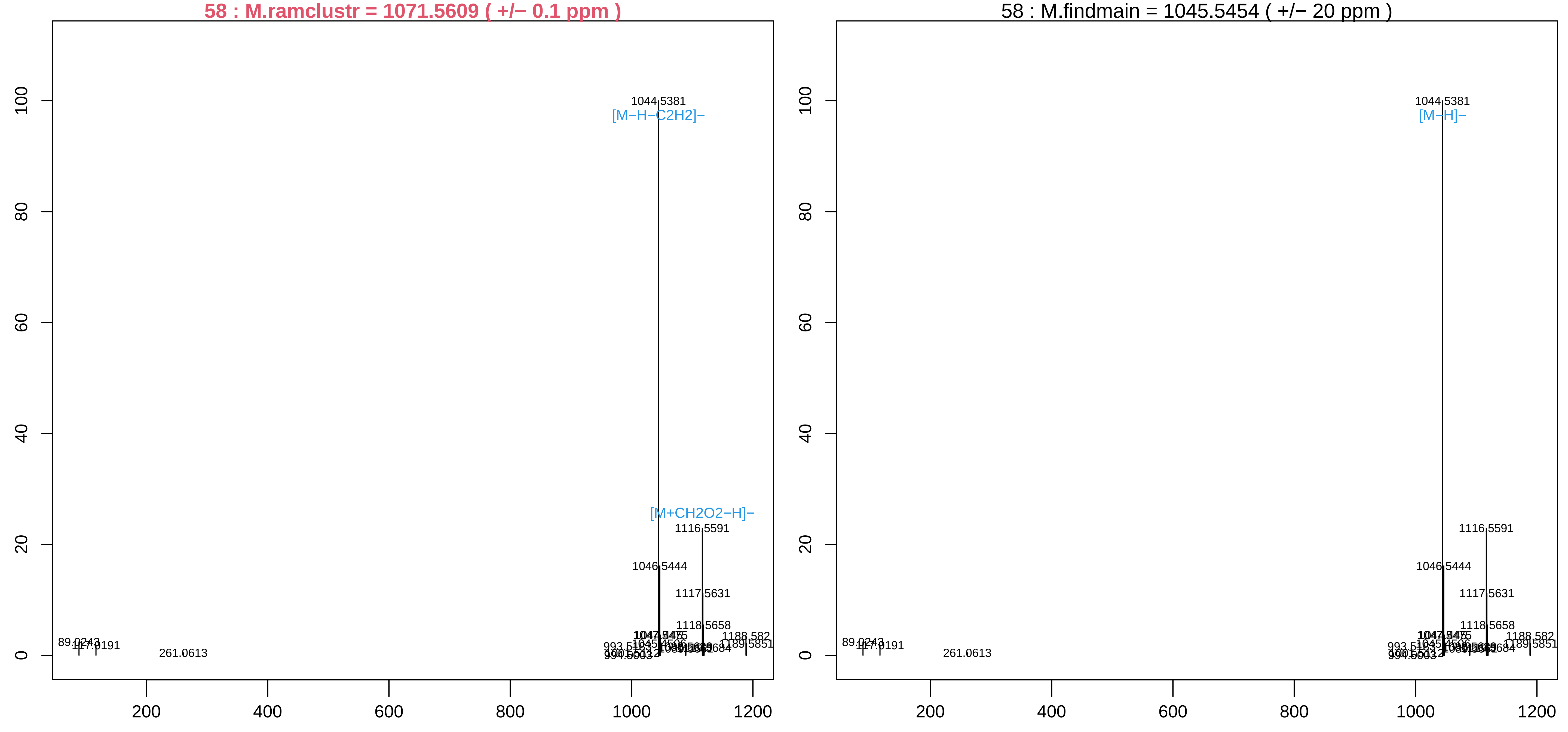
58 : M.ramclustr = 1071.5609 (+/− 0.1 ppm)
1044.5381
[M−H−C2H2]−
[M+CH2O2−H]−
1116.5591
1046.5444
1117.5631
1118.5658
1188.582
1189.5851
89.0243
117.0191
261.0613
58 : M.findmain = 1045.5454 (+/− 20 ppm)
1044.5381
[M−H]−
1116.5591
1046.5444
1117.5631
1118.5658
1188.582
1189.5851
89.0243
117.0191
261.0613

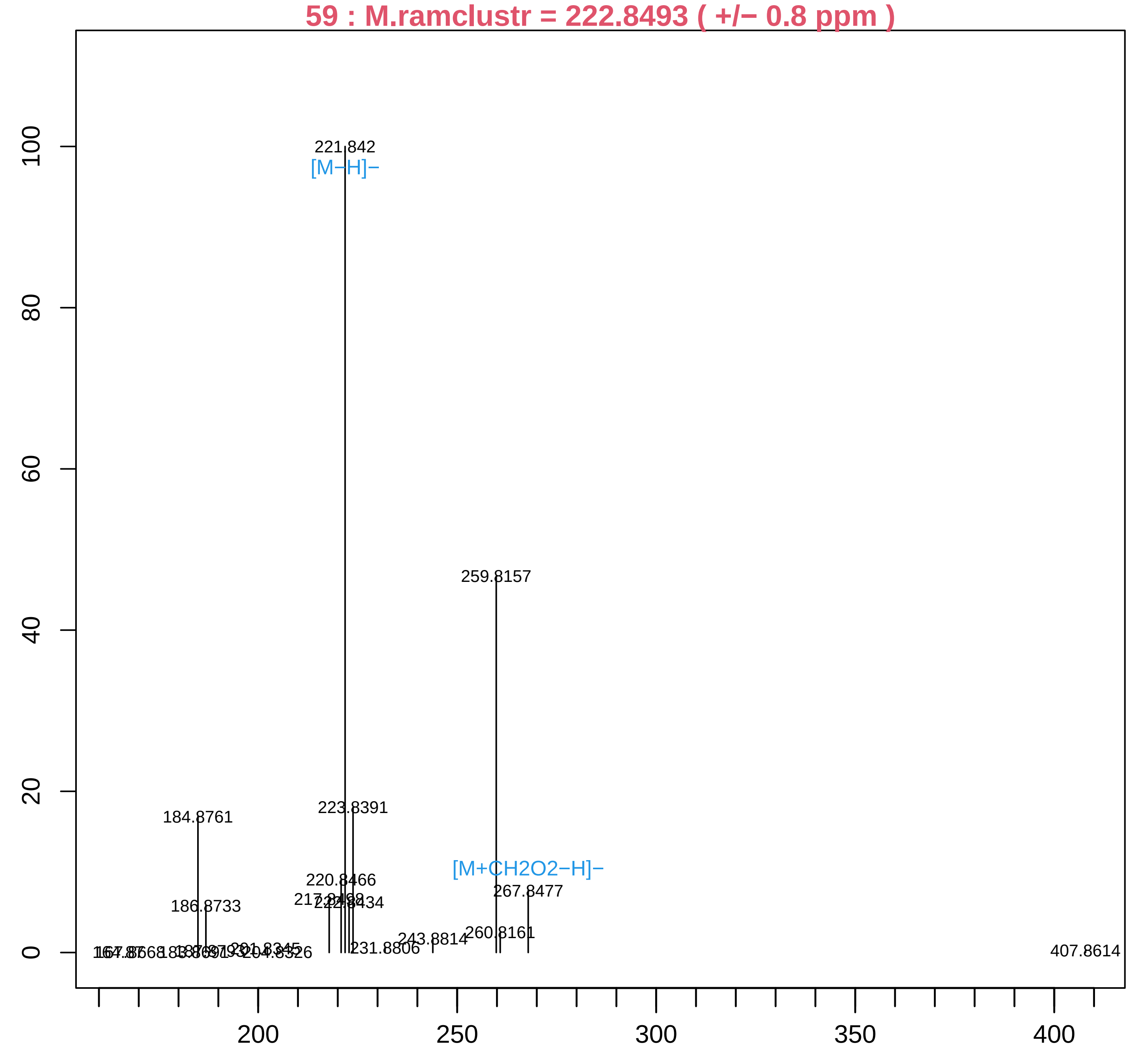
59 : M.ramclustr = 222.8493 (+/− 0.8 ppm)
221.842
[M−H]−
259.8157
184.8761
223.8391
[M+CH2O2−H]−
220.8466
267.8477
186.8733
222.8434
231.8806
243.8814
260.8161
407.8614

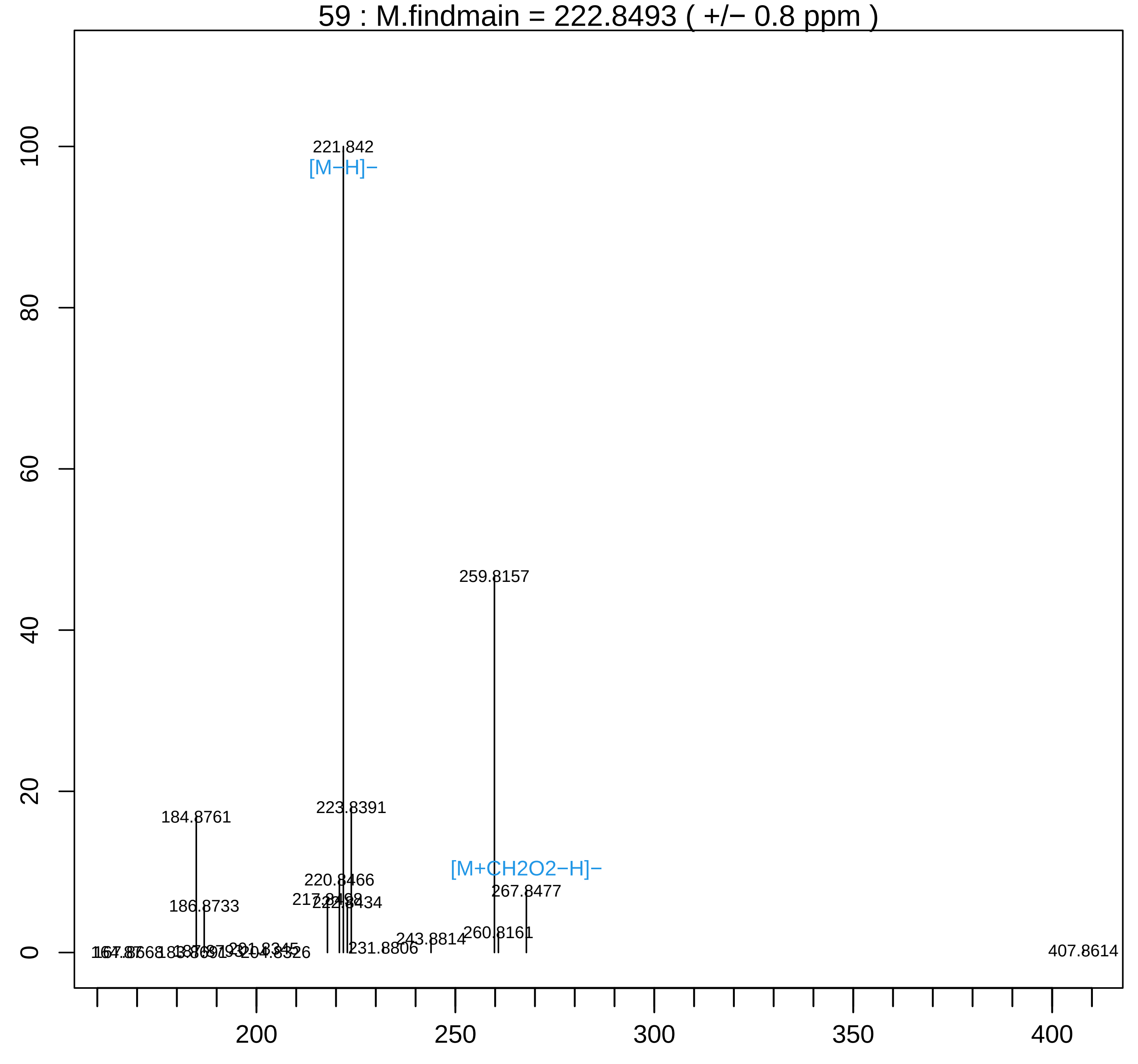
59 : M.findmain = 222.8493 (+/− 0.8 ppm)
221.842
[M−H]−
259.8157
184.8761
223.8391
[M+CH2O2−H]−
220.8466
267.8477
186.8733
222.8434
231.8806
243.8814
260.8161
407.8614

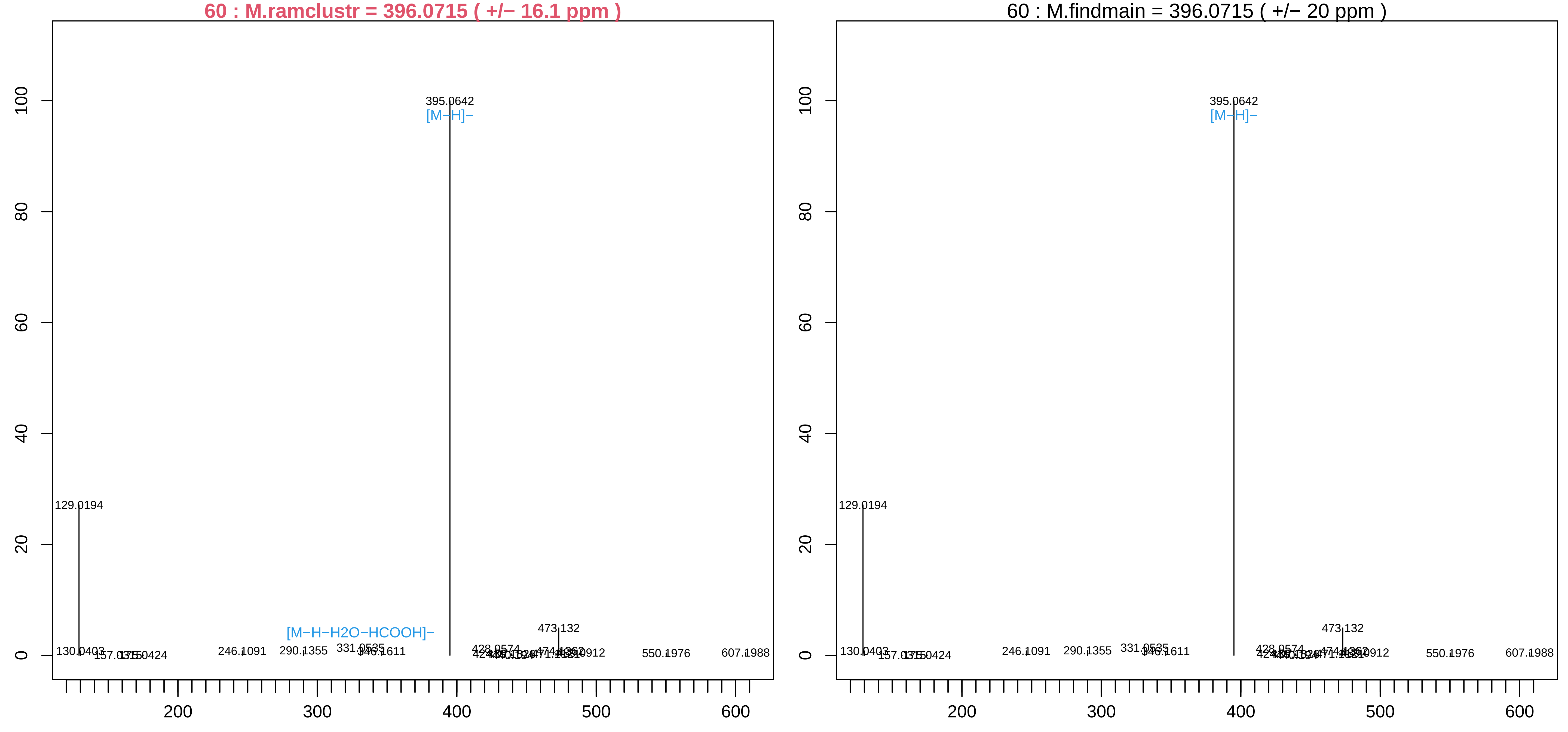
60 : M.ramclustr = 396.0715 (+/− 16.1 ppm)
395.0642
[M−H]−
129.0194
[M−H−H2O−HCOOH]−
473.132
130.0403
246.1091
290.1355
331.0535
346.1611
428.0574
550.1976
607.1988
0
20
40
60
80
100
200
300
400
500
600
60 : M.findmain = 396.0715 (+/− 20 ppm)
395.0642
[M−H]−
129.0194
473.132
130.0403
246.1091
290.1355
331.0535
346.1611
428.0574
550.1976
607.1988
0
20
40
60
80
100
200
300
400
500
600

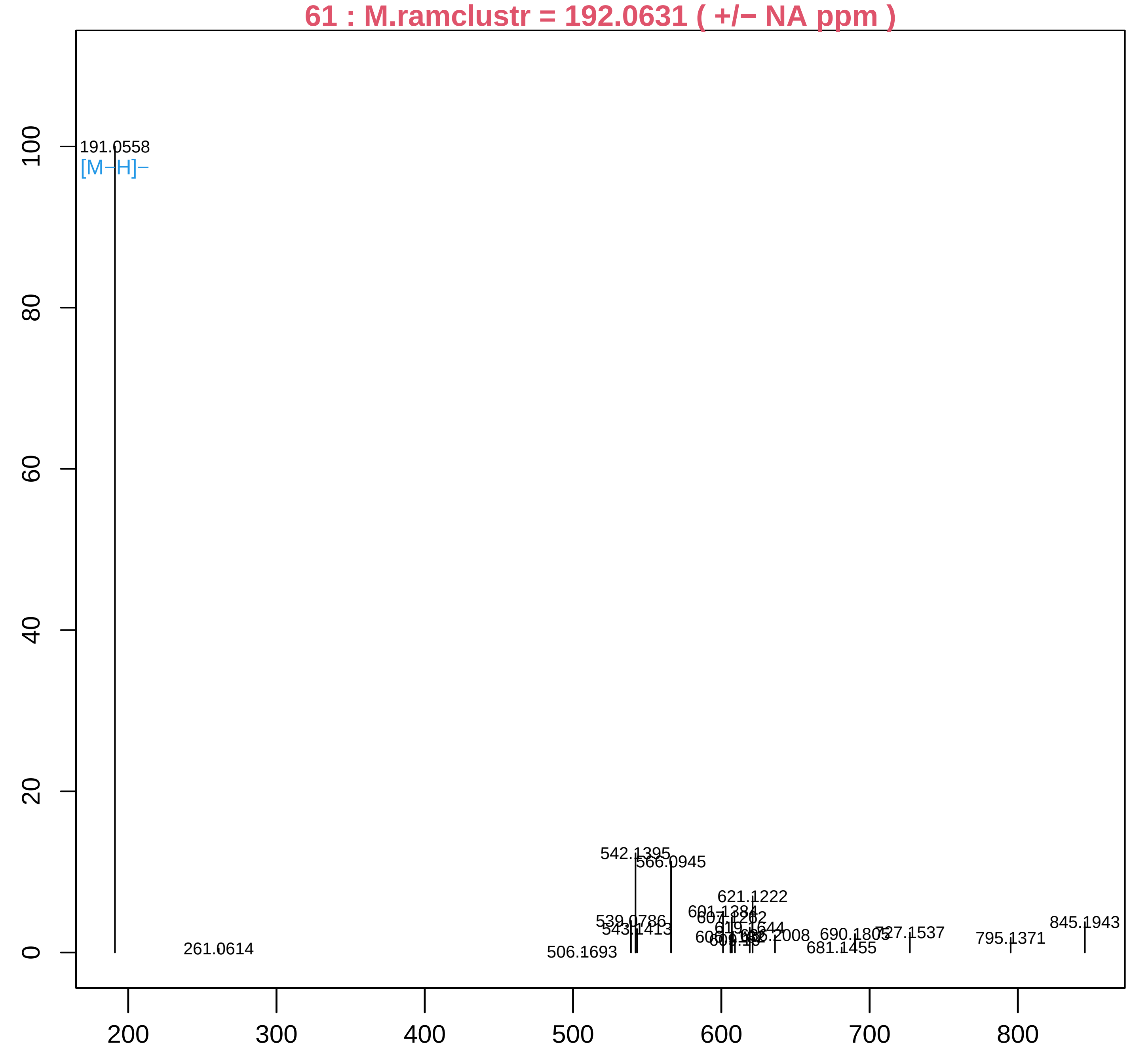
61 : M.ramclustr = 192.0631 (+/− NA ppm)
191.0558
[M−H]−
261.0614
506.1693
539.0786
543.1413
542.1395
566.0945
621.1222
601.1384
681.1455
690.1805
727.1537
795.1371
845.1943

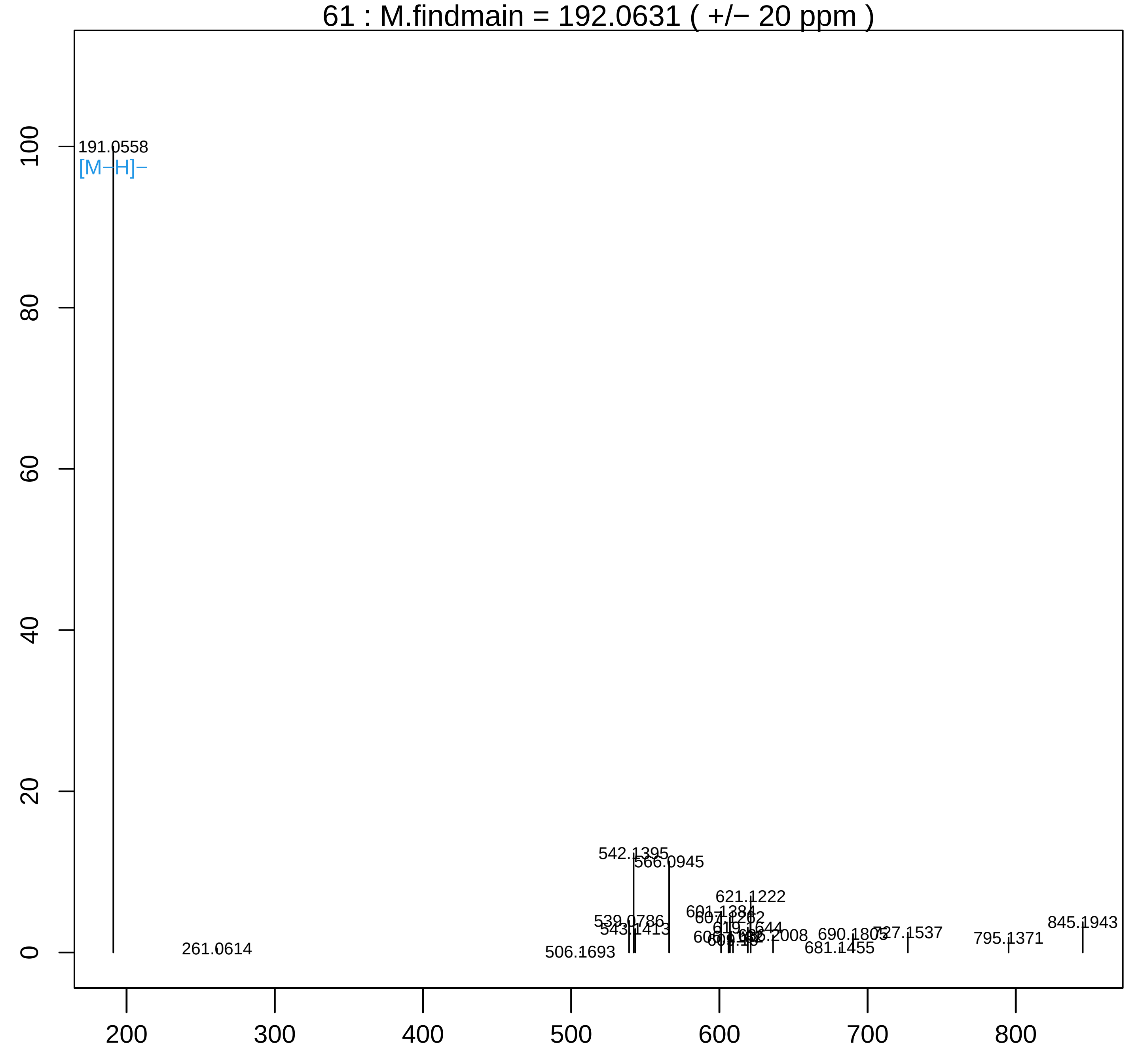
61 : M.findmain = 192.0631 (+/− 20 ppm)
191.0558
[M−H]−
261.0614
506.1693
539.0786
543.1413
542.1395
566.0945
621.1222
601.1384
681.1455
690.1805
727.1537
795.1371
845.1943

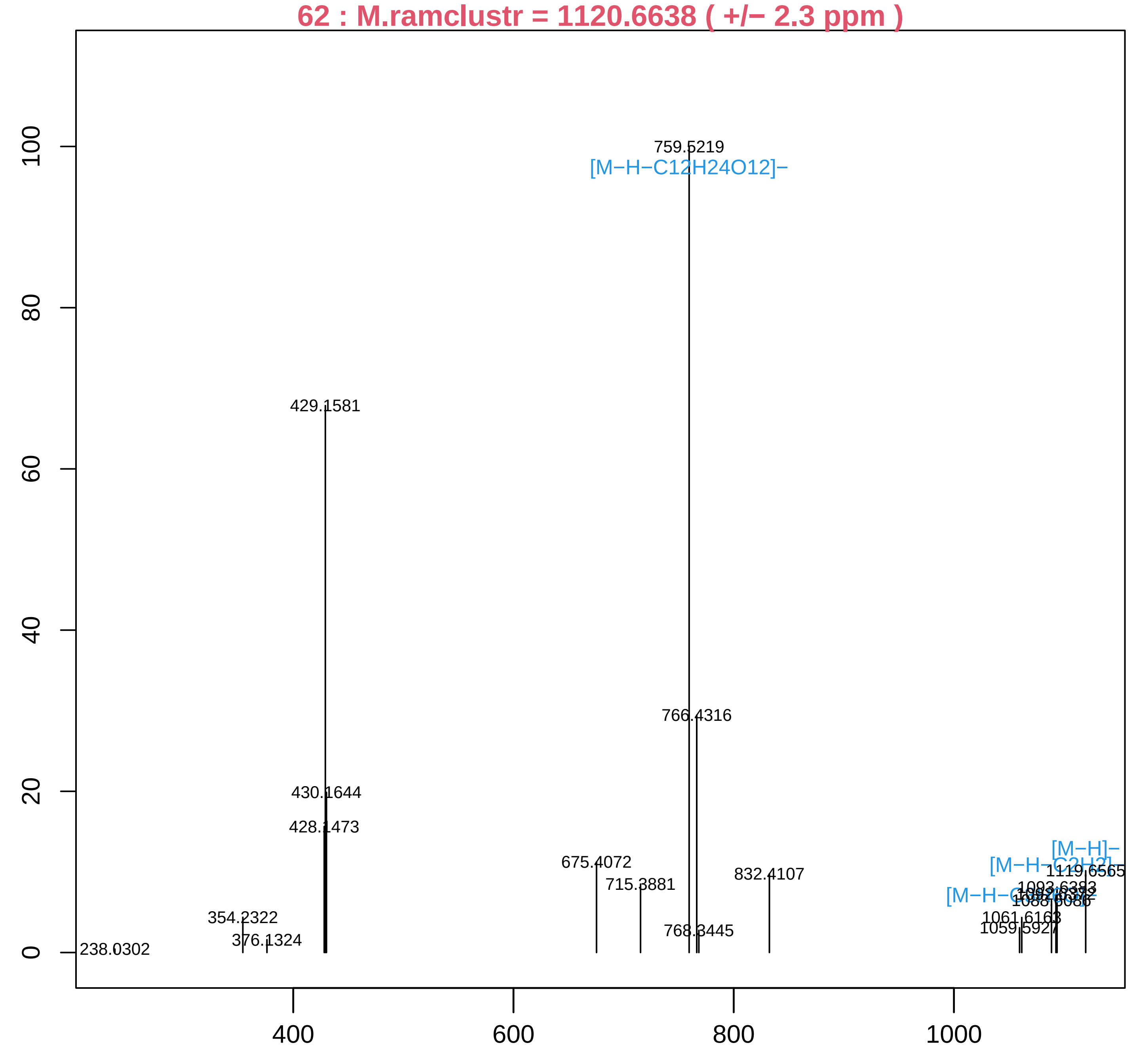
62 : M.ramclustr = 1120.6638 (+/− 2.3 ppm)
759.5219
[M−H−C12H24O12]−
429.1581
766.4316
430.1644
428.1473
675.4072
715.3881
832.4107
768.3445
354.2322
376.1324
238.0302
[M−H]−
1119.6565
1061.6163
1059.5927
0
20
40
60
80
100
400
600
800
1000

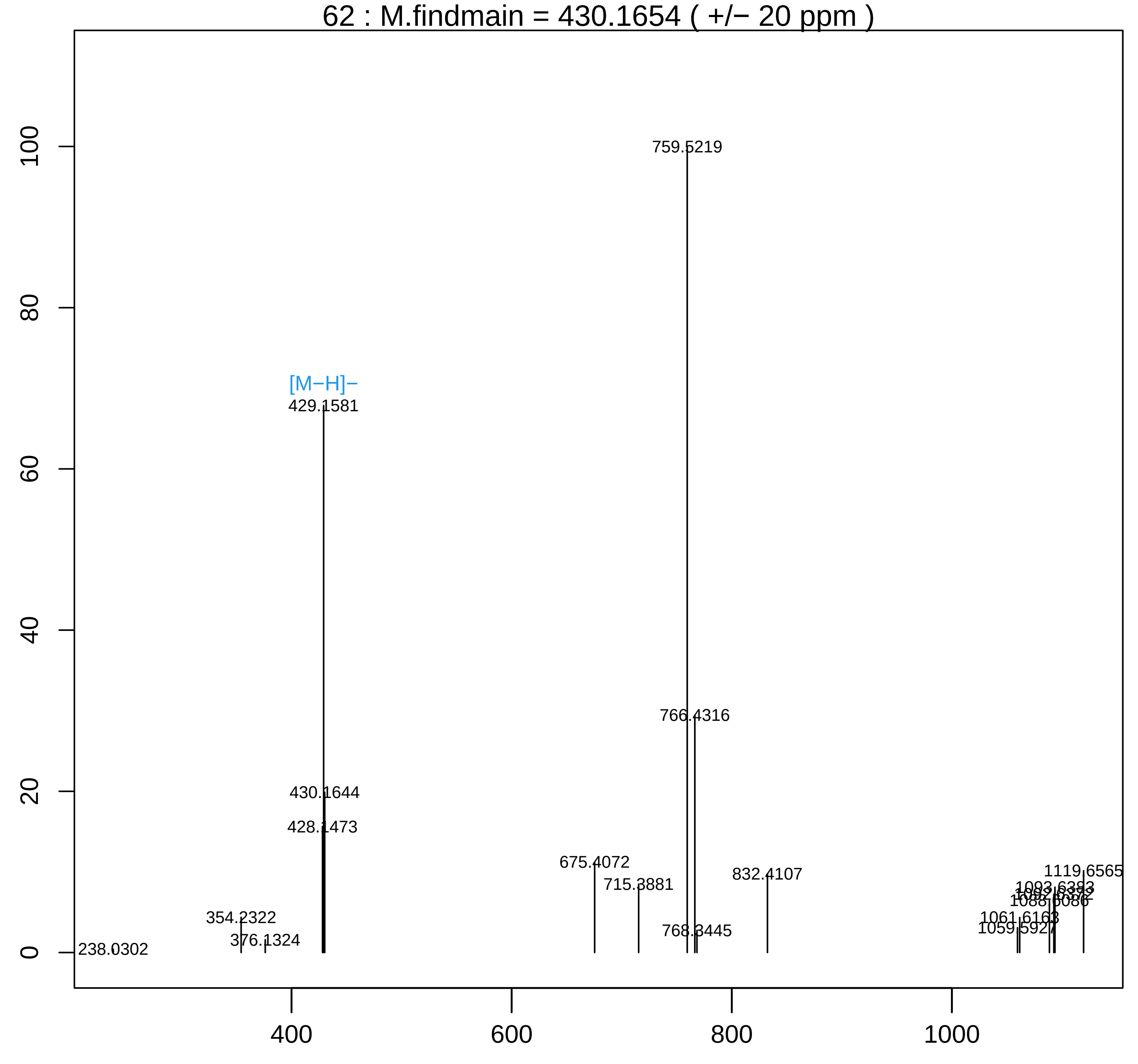
62 : M.findmain = 430.1654 (+/− 20 ppm)
759.5219
[M−H]−
429.1581
766.4316
430.1644
428.1473
675.4072
715.3881
832.4107
768.3445
354.2322
376.1324
238.0302
1119.6565
1093.6393
1088.5086
1061.6163
1059.5927
0
20
40
60
80
100
400
600
800
1000

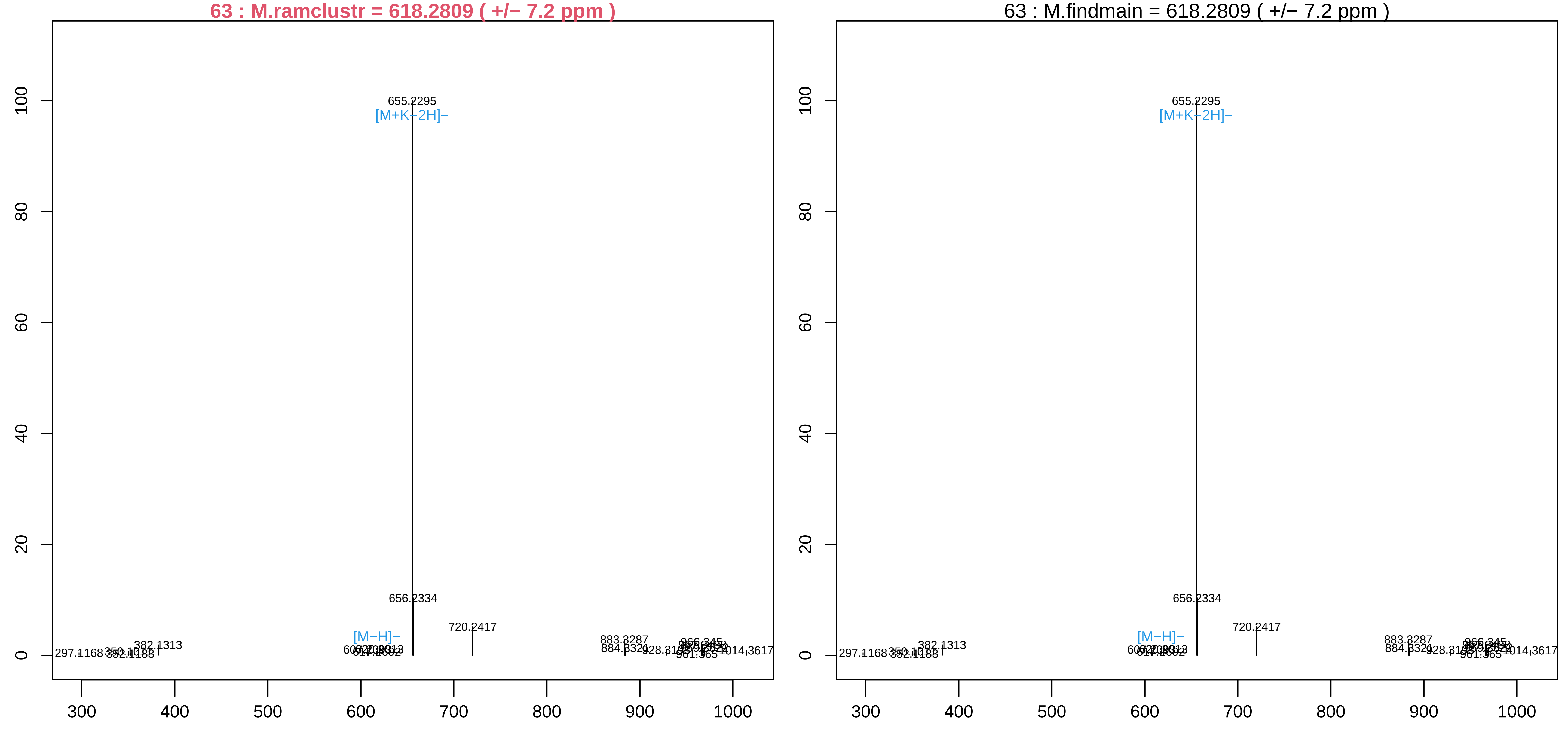

63 : M.ramclustr = 618.2809 (+/− 7.2 ppm)
63 : M.findmain = 618.2809 (+/− 7.2 ppm)
655.2295
[M+K−2H]−
656.2334
720.2417
[M−H]−
382.1313
297.1168
883.3287
884.3321
966.345
1014.3617
961.365
0
20
40
60
80
100
300
400
500
600
700
800
900
1000

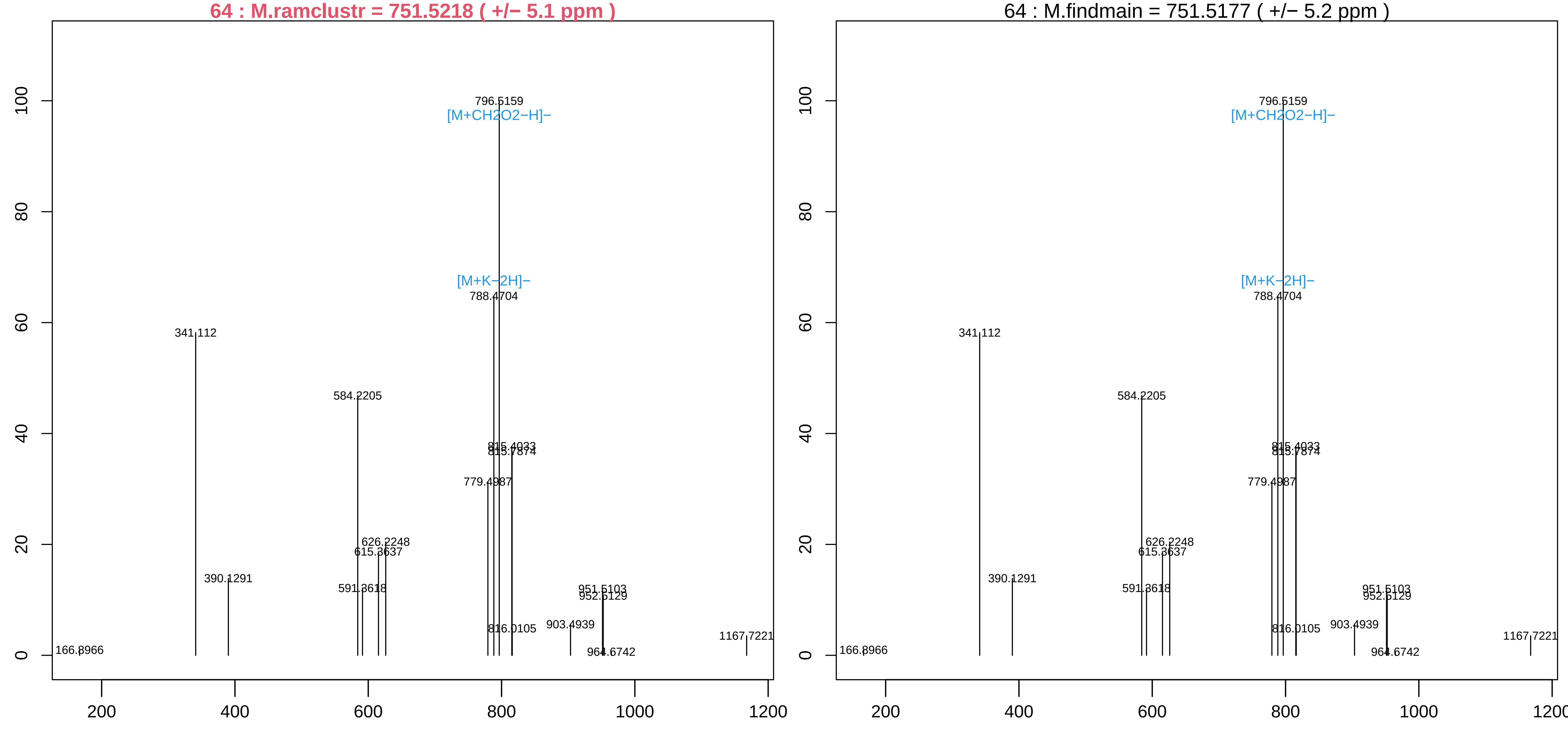

64 : M.ramclustr = 751.5218 (+/− 5.1 ppm)
64 : M.findmain = 751.5177 (+/− 5.2 ppm)
796.5159
[M+CH2O2−H]−
[M+K−2H]−
788.4704
341.112
584.2205
815.4933
815.7874
779.4987
626.2248
615.3637
390.1291
591.3618
951.5103
952.5129
903.4939
816.0105
1167.7221
166.8966
964.6742
0
20
40
60
80
100
200
400
600
800
1000
1200

65 : M.ramclustr = 776.5393 (+/− NA ppm)

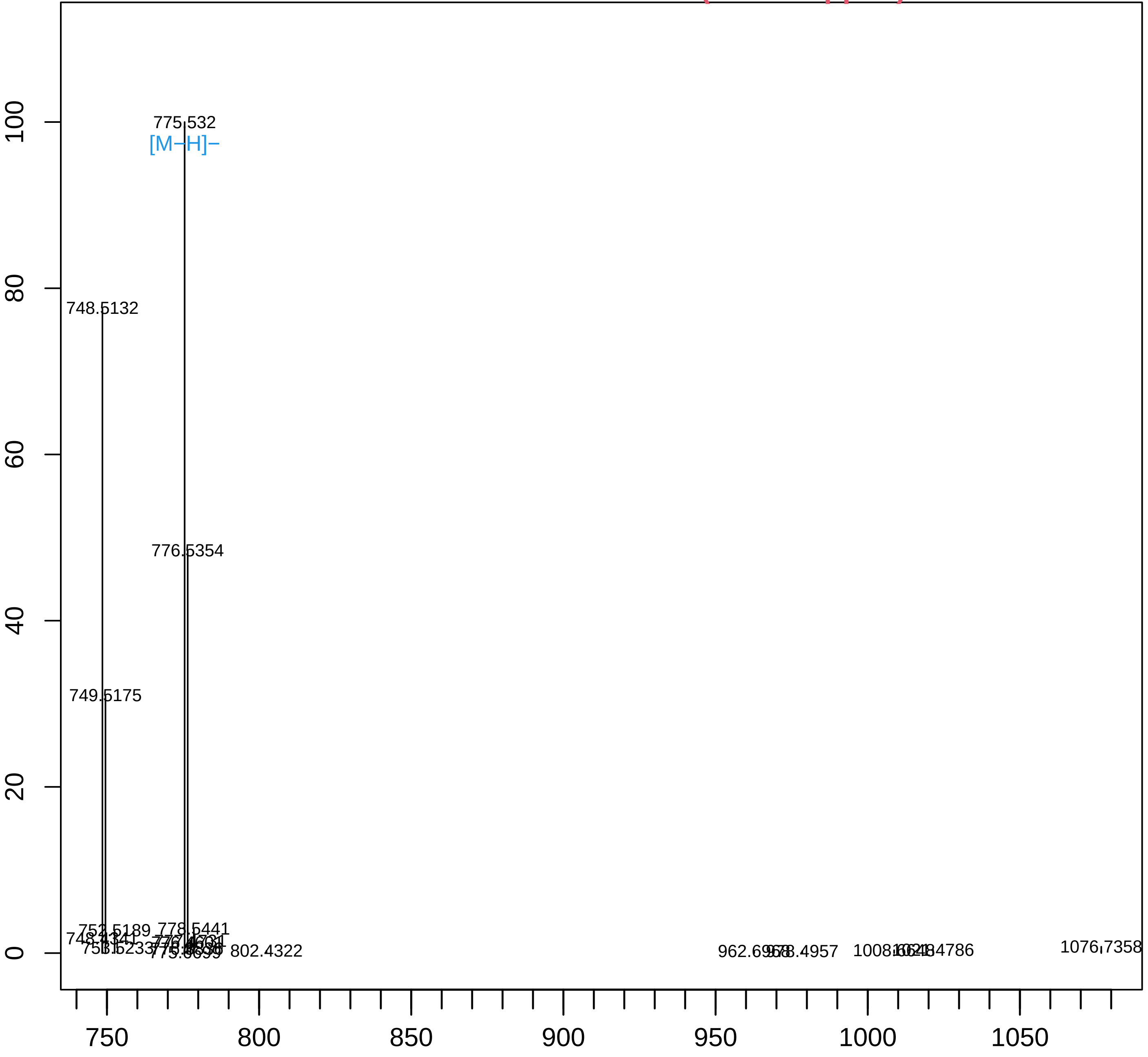

65 : M.findmain = 776.5393 (+/− 20 ppm)

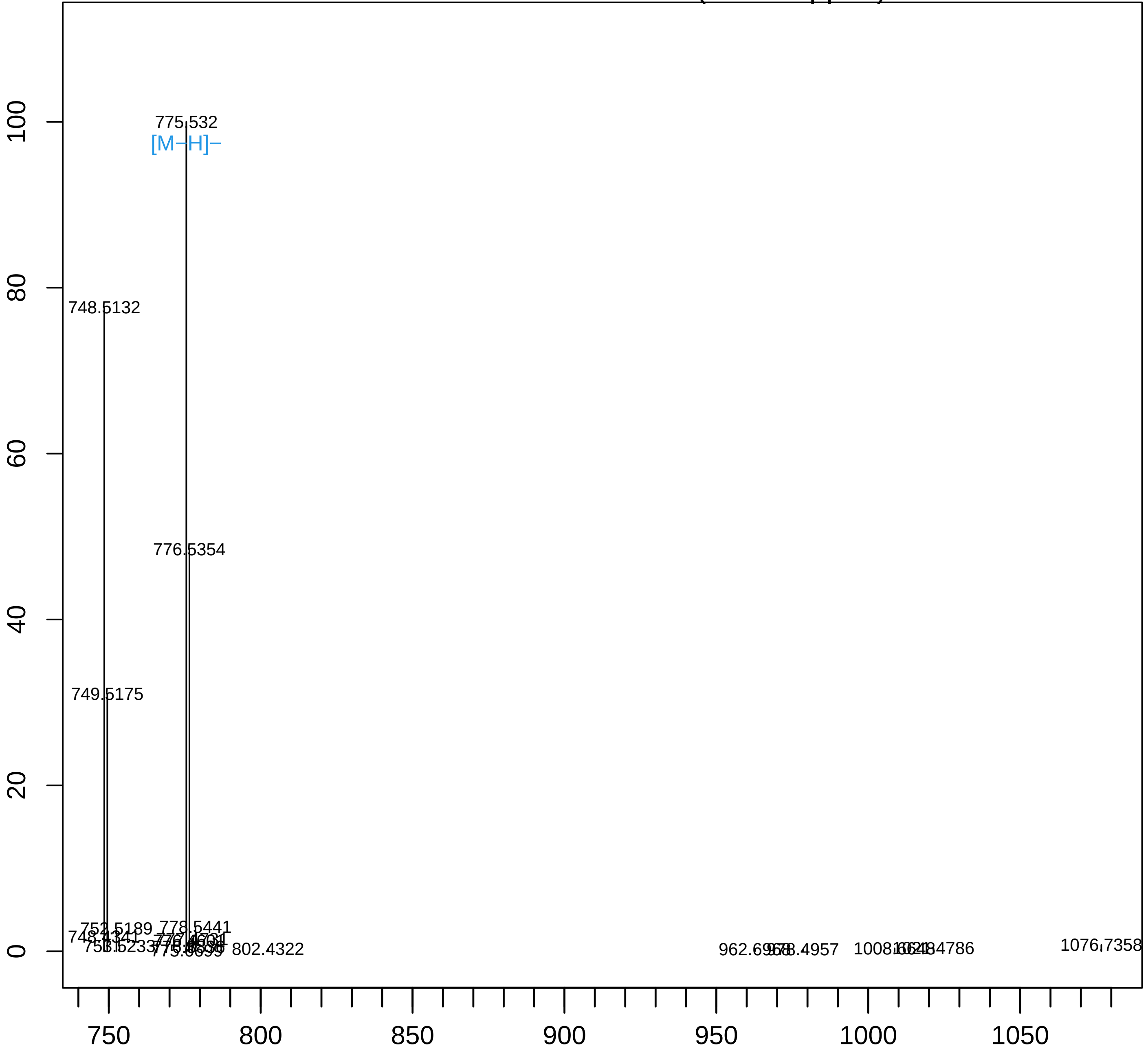

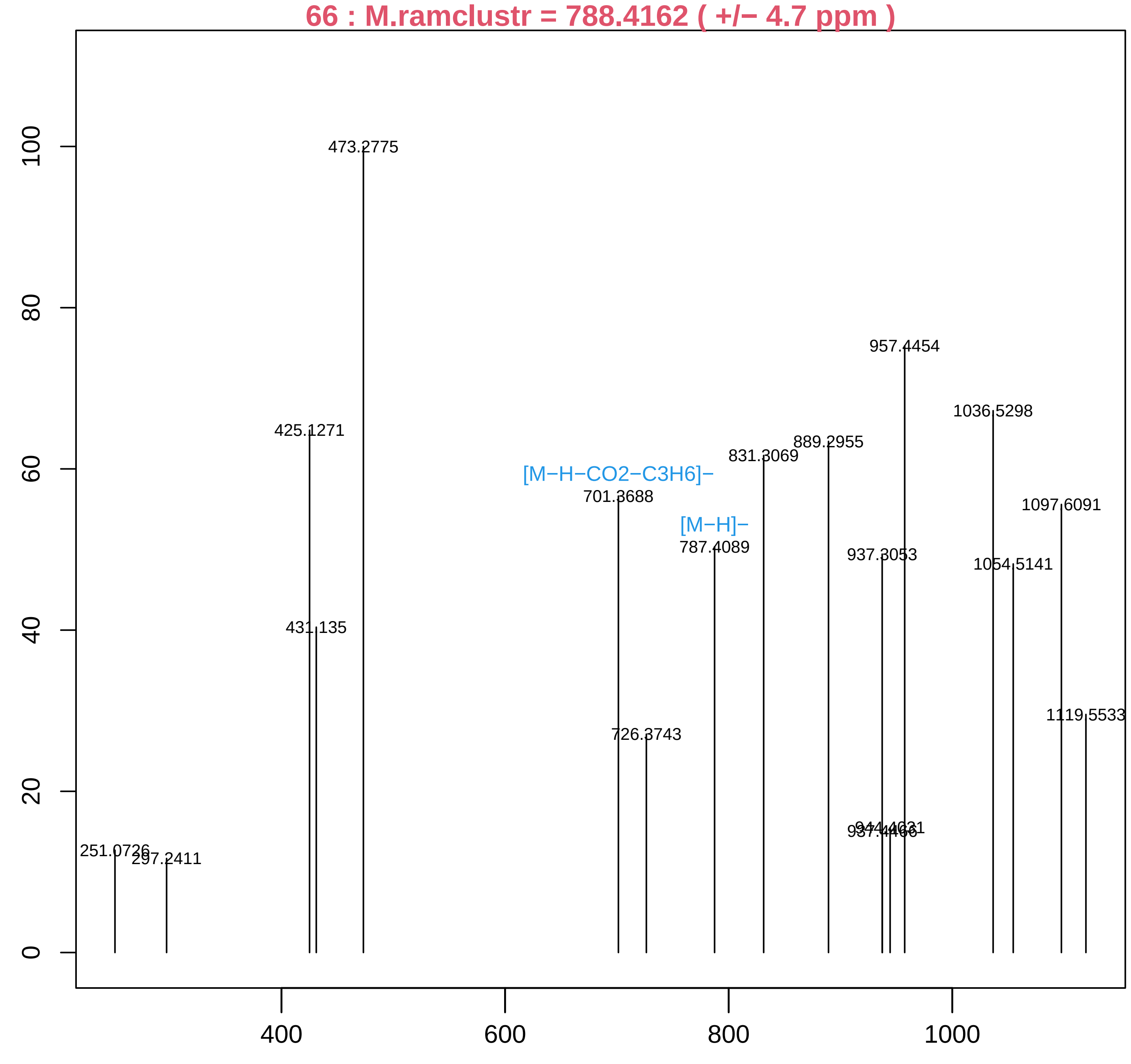
66 : M.ramclustr = 788.4162 (+/− 4.7 ppm)
473.2775
425.1271
431.135
251.0726
297.2411
[M−H−CO2−C3H6]−
701.3688
726.3743
[M−H]−
787.4089
831.3069
889.2955
957.4454
937.3053
1036.5298
1054.5141
1097.6091
1119.5533
0
20
40
60
80
100
400
600
800
1000

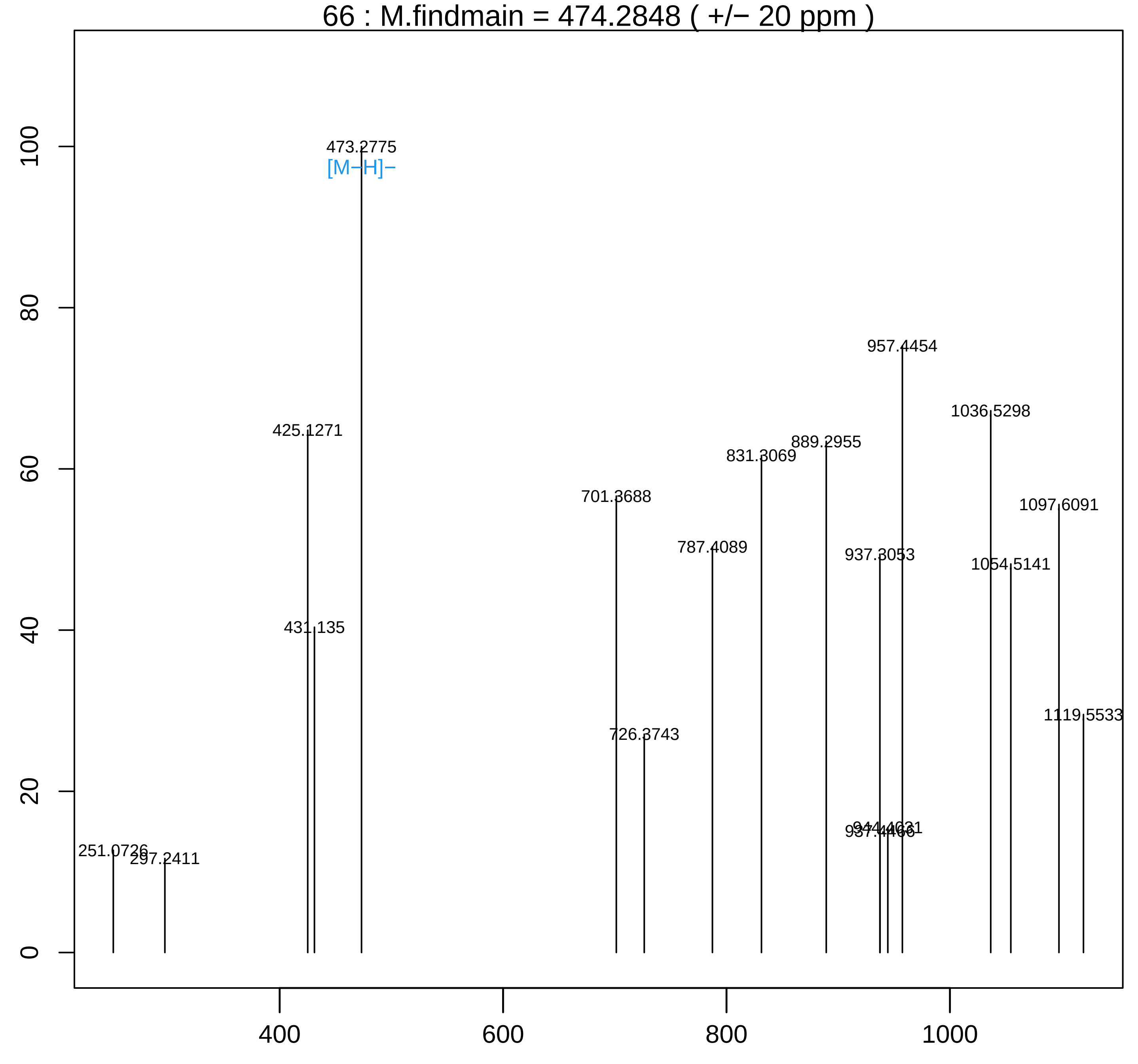
66 : M.findmain = 474.2848 (+/− 20 ppm)
473.2775
[M−H]−
425.1271
431.135
251.0726
297.2411
701.3688
726.3743
787.4089
831.3069
889.2955
957.4454
937.3053
1036.5298
1054.5141
1097.6091
1119.5533
0
20
40
60
80
100
400
600
800
1000

67 : M.ramclustr = 135.0541 (+/− NA ppm)

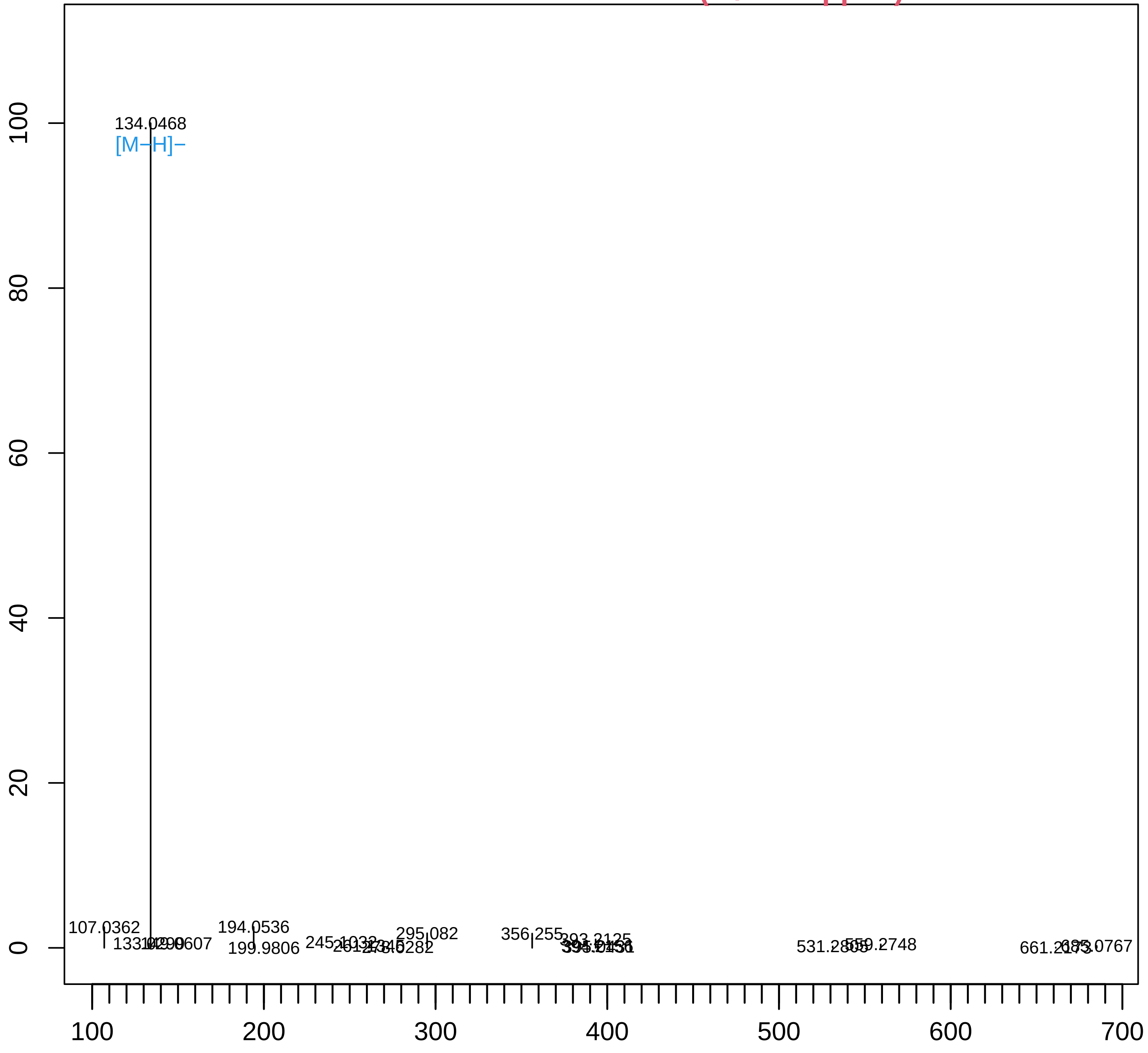

67 : M.findmain = 135.0541 (+/− 20 ppm)

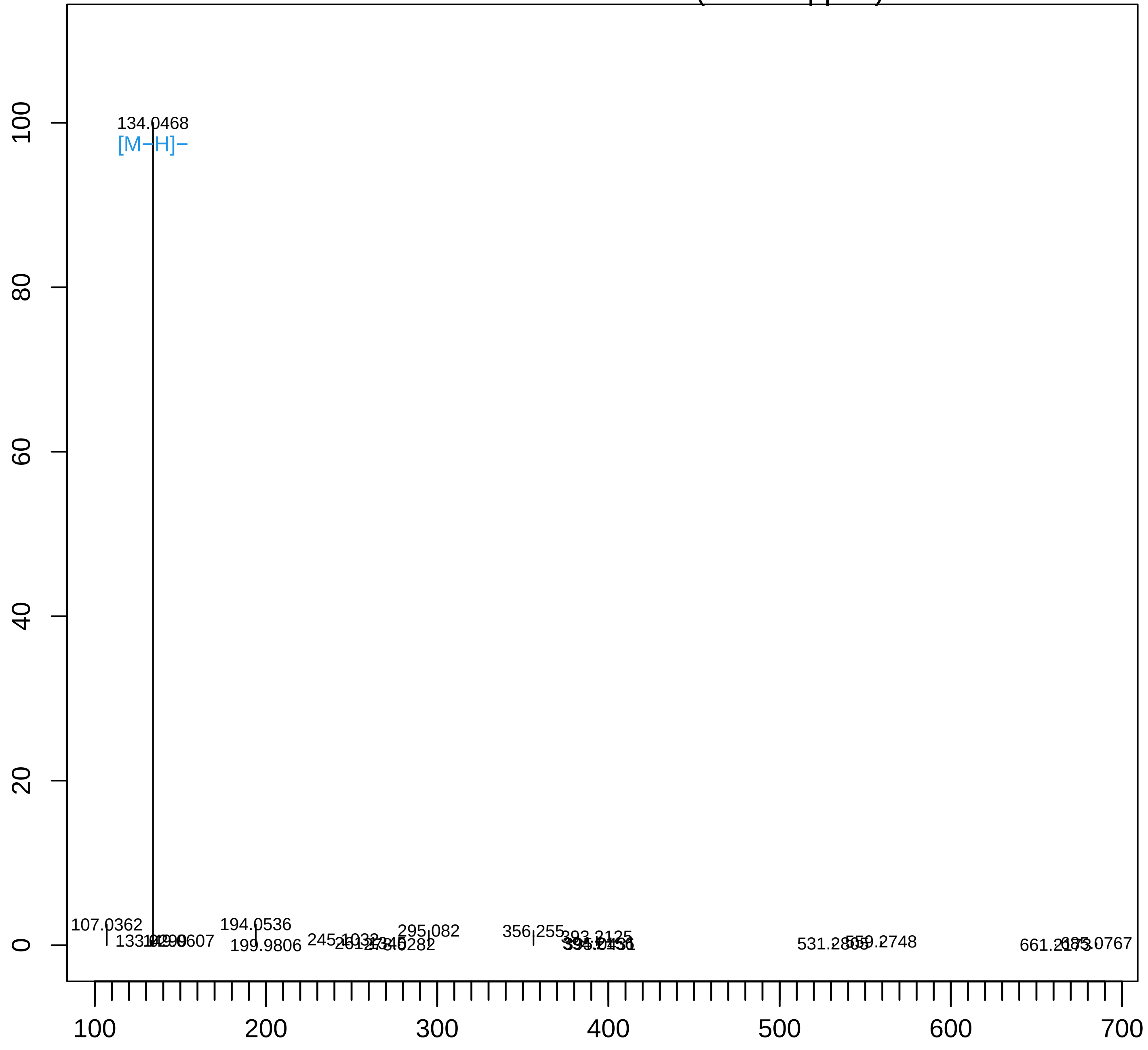

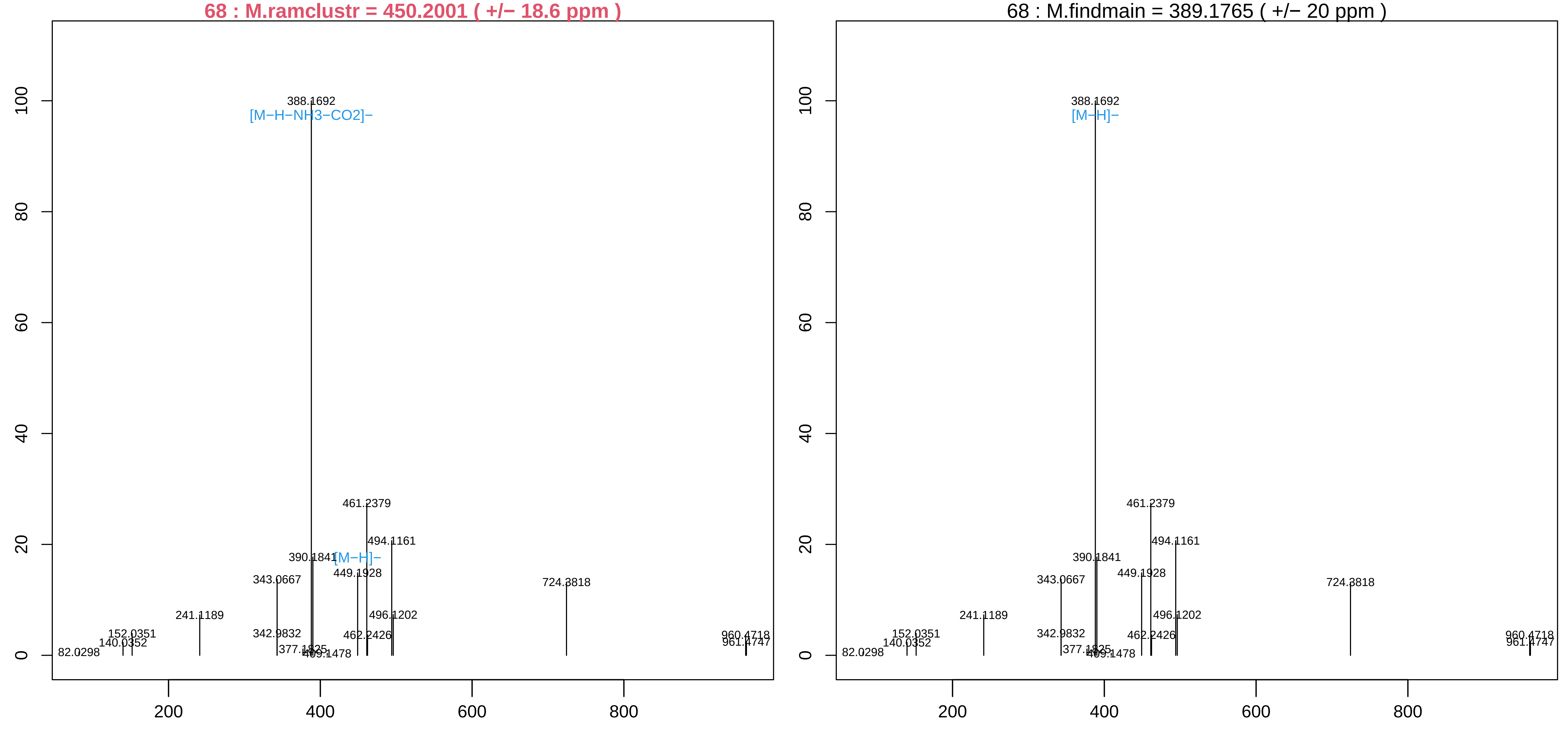
68 : M.ramclustr = 450.2001 (+/− 18.6 ppm)
388.1692
[M−H−NH3−CO2]−
390.1841
[M−H]−
461.2379
494.1161
449.1928
343.0667
724.3818
241.1189
496.1202
152.0351
140.0352
342.9832
462.2426
960.4718
961.4747
82.0298
377.1825
409.1478
68 : M.findmain = 389.1765 (+/− 20 ppm)
388.1692
[M−H]−
0
20
40
60
80
100
200
400
600
800

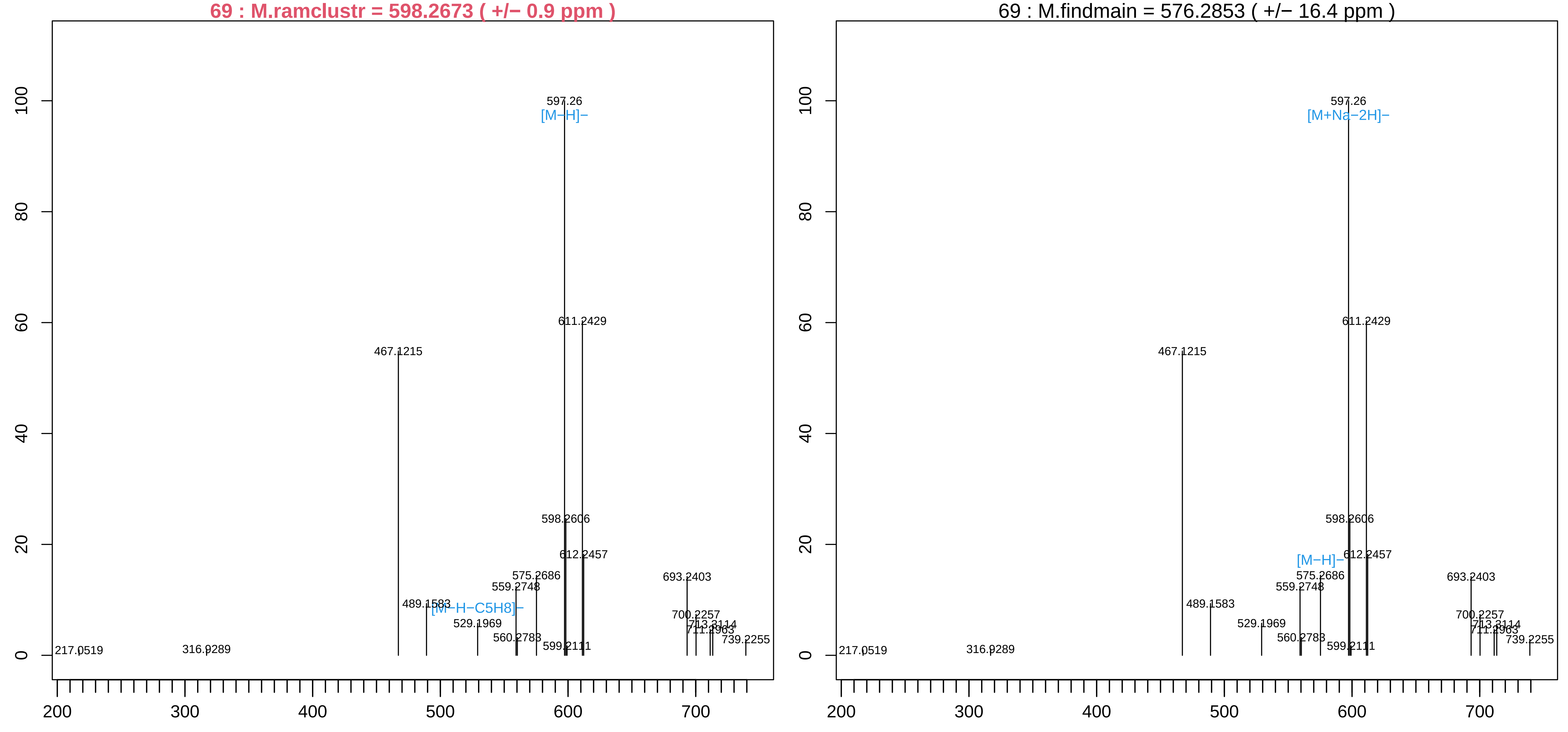

69 : M.ramclustr = 598.2673 (+/− 0.9 ppm)
597.26
[M−H]−
611.2429
467.1215
598.2606
612.2457
575.2686
559.2748
693.2403
489.1583
[M−H−C5H8]−
529.1969
700.2257
713.3114
711.2963
560.2783
739.2255
599.2111
217.0519
316.9289
69 : M.findmain = 576.2853 (+/− 16.4 ppm)
[M+Na−2H]−
[M−H]−

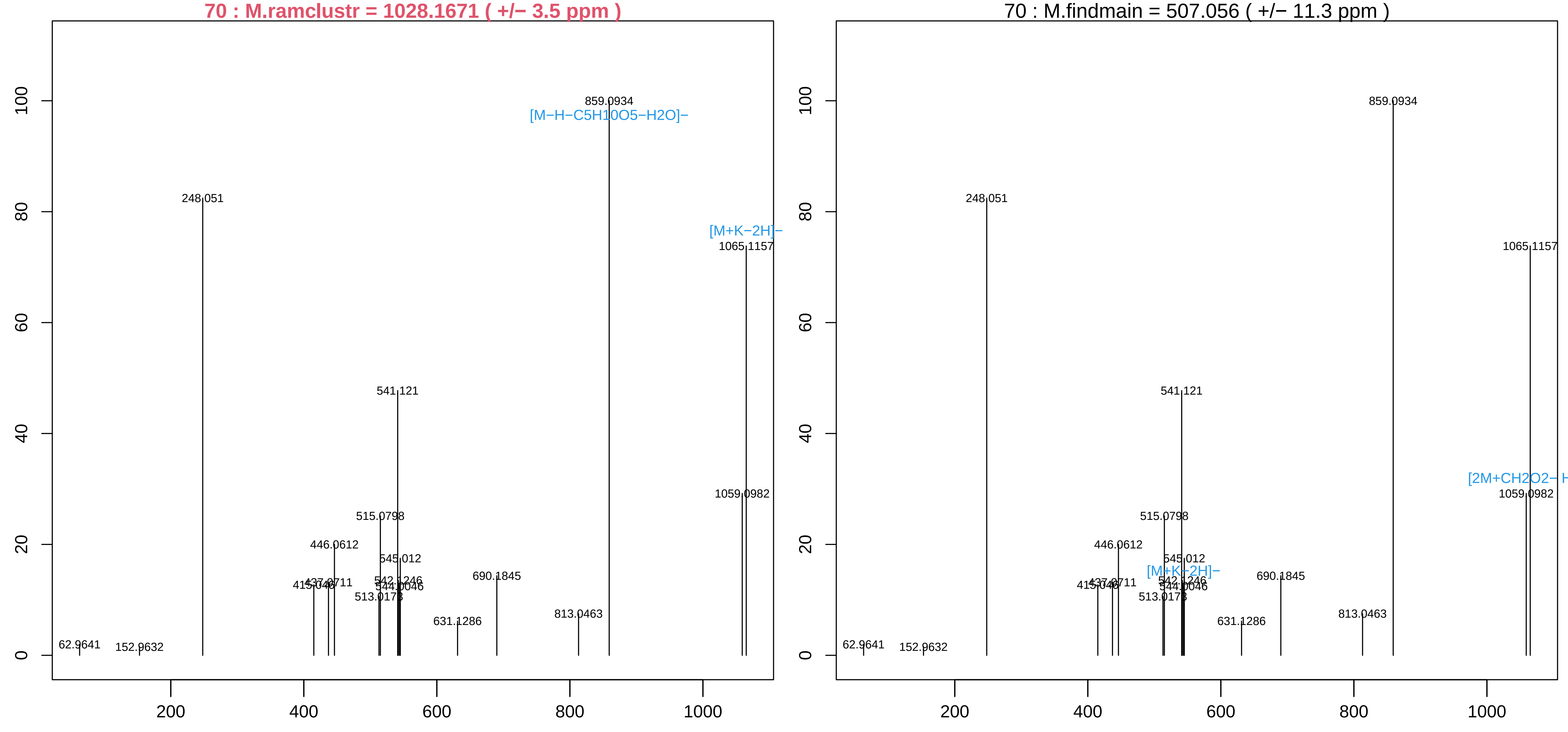
70 : M.ramclustr = 1028.1671 (+/− 3.5 ppm)
[M−H−C5H10O5−H2O]−
859.0934
248.051
[M+K−2H]−
1065.1157
541.121
1059.0982
515.0798
446.0612
545.012
415.046
437.0711
542.1246
544.0046
513.0173
690.1845
631.1286
813.0463
62.9641
152.9632
70 : M.findmain = 507.056 (+/− 11.3 ppm)
859.0934
248.051
1065.1157
541.121
[2M+CH2O2−H
1059.0982
515.0798
446.0612
545.012
[M+K−2H]−
415.046
437.0711
542.1246
544.0046
513.0173
690.1845
631.1286
813.0463
62.9641
152.9632
0
20
40
60
80
100
200
400
600
800
1000

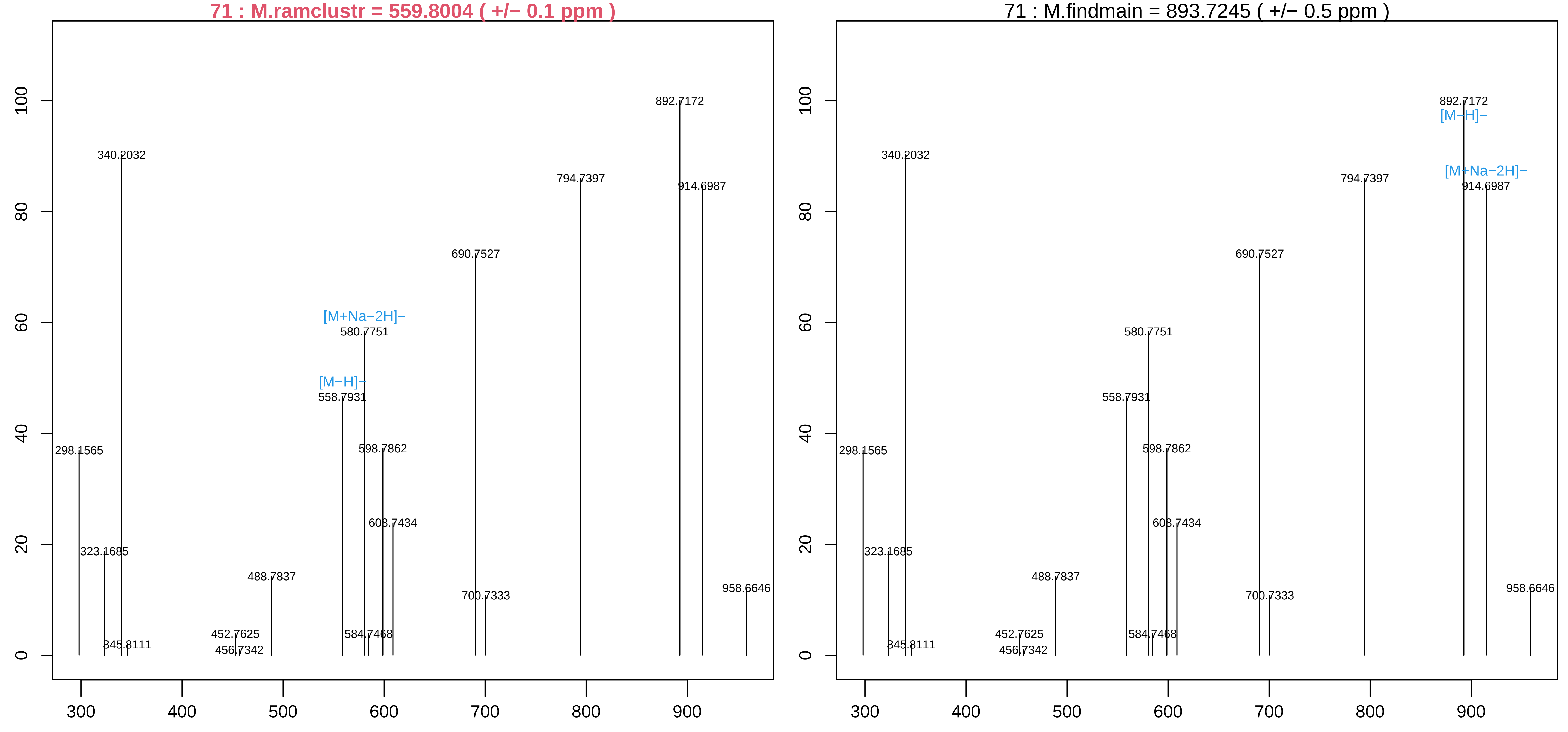

71 : M.ramclustr = 559.8004 (+/− 0.1 ppm)
71 : M.findmain = 893.7245 (+/− 0.5 ppm)
298.1565
323.1685
340.2032
345.8111
452.7625
456.7342
488.7837
558.7931
[M−H]−
580.7751
[M+Na−2H]−
584.7468
598.7862
608.7434
690.7527
700.7333
794.7397
892.7172
914.6987
958.6646
0
20
40
60
80
100
300
400
500
600
700
800
900

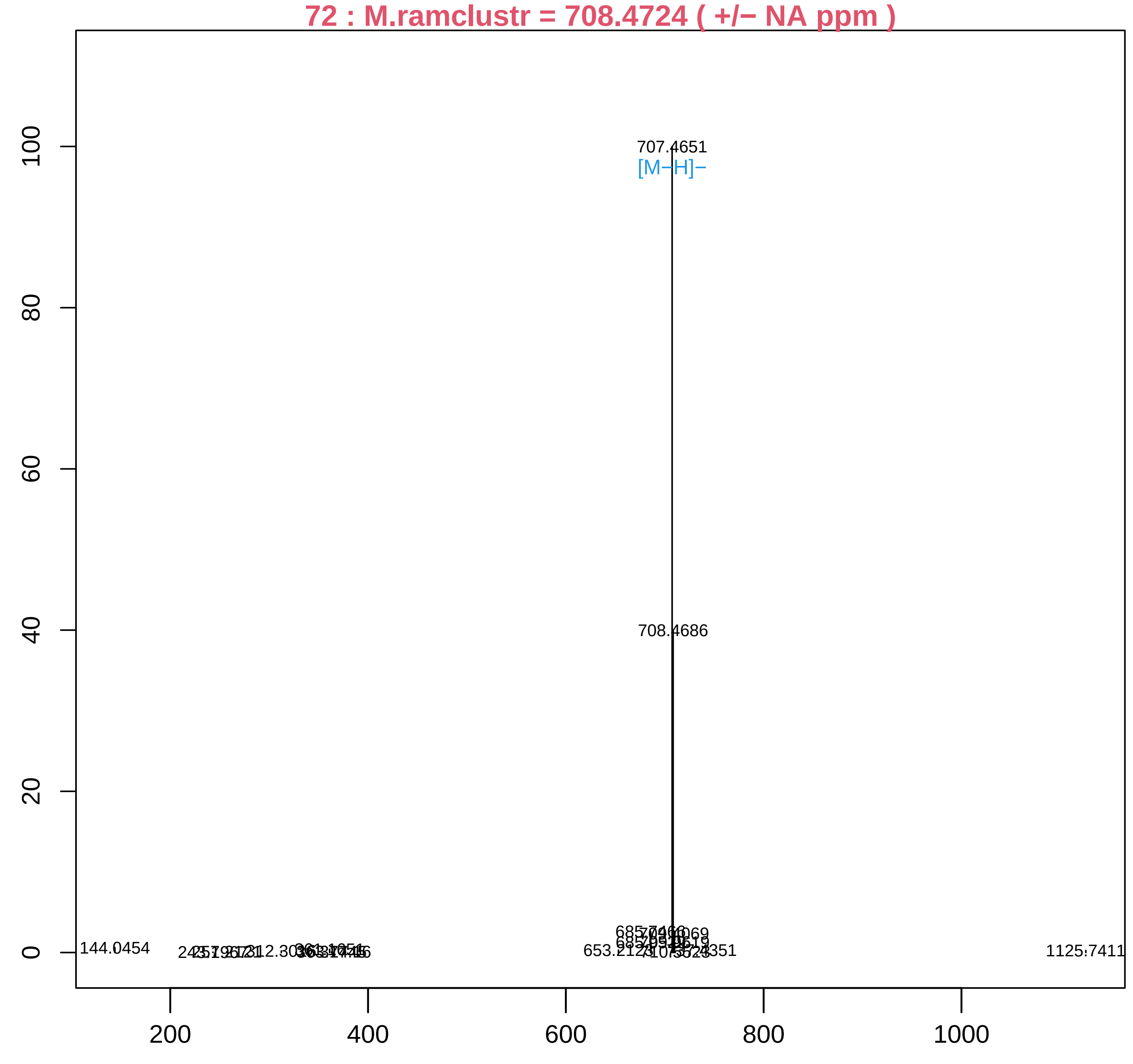
72 : M.ramclustr = 708.4724 (+/− NA ppm)
707.4651
[M−H]−
708.4686
144.0454
1125.7411
0
20
40
60
80
100
200
400
600
800
1000
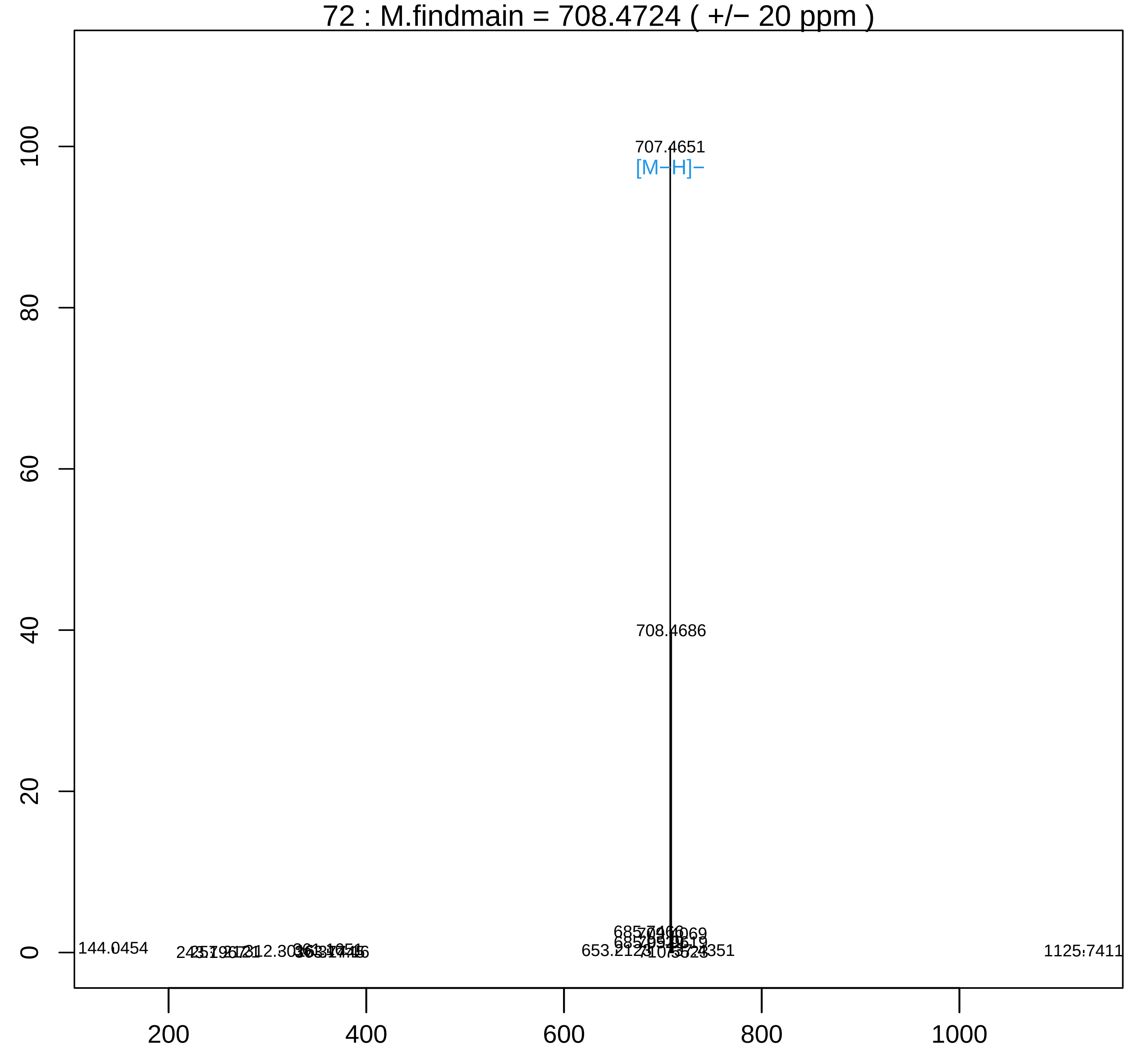
72 : M.findmain = 708.4724 (+/− 20 ppm)
707.4651
[M−H]−
708.4686
144.0454
1125.7411
0
20
40
60
80
100
200
400
600
800
1000

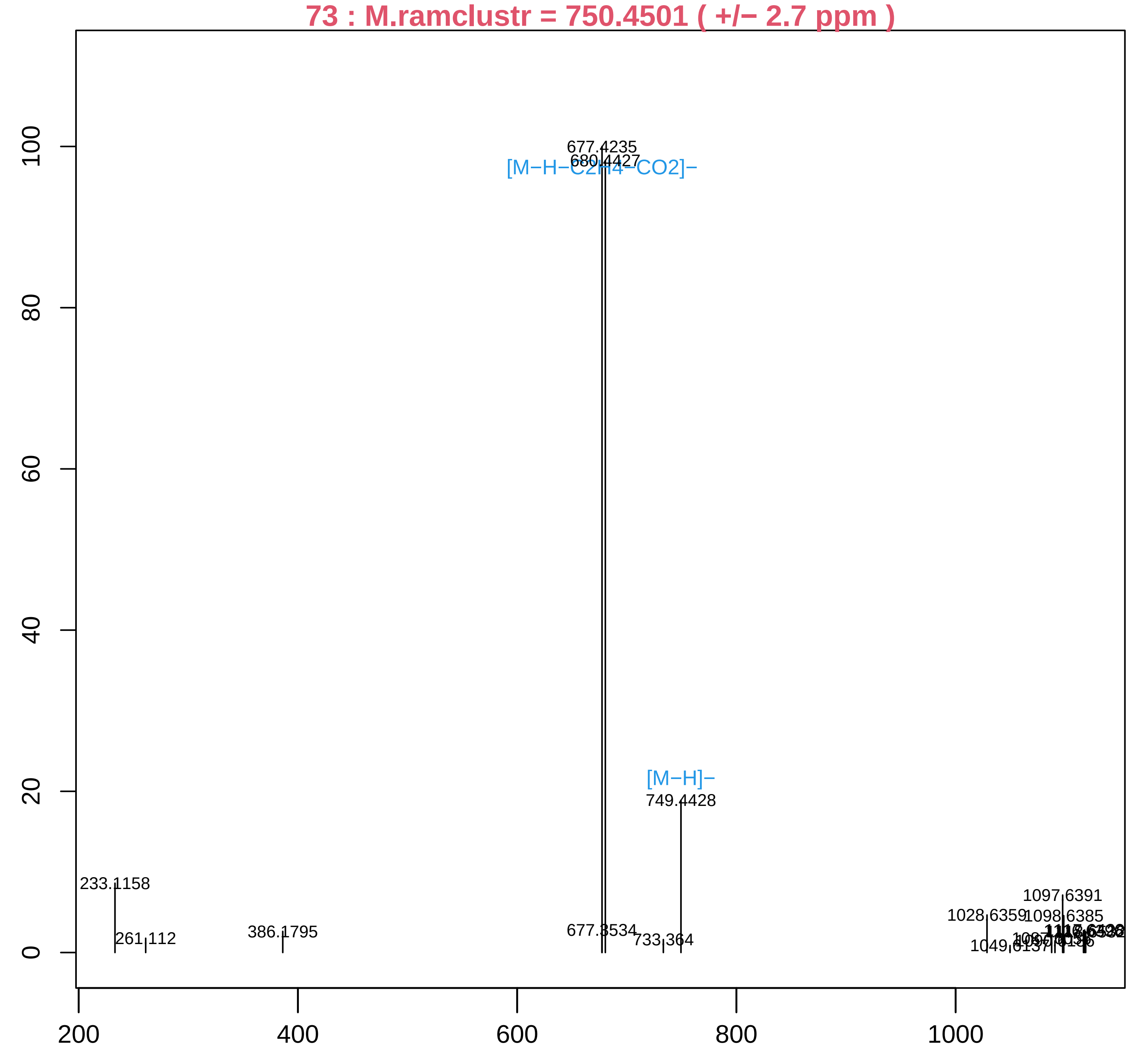
73 : M.ramclustr = 750.4501 (+/− 2.7 ppm)
677.4235
680.4427
[M−H−C2H4−CO2]−
[M−H]−
749.4428
233.1158
261.112
386.1795
677.3534
733.364
1097.6391
1028.6359
1098.6385
1049.6137
0
20
40
60
80
100
200
400
600
800
1000

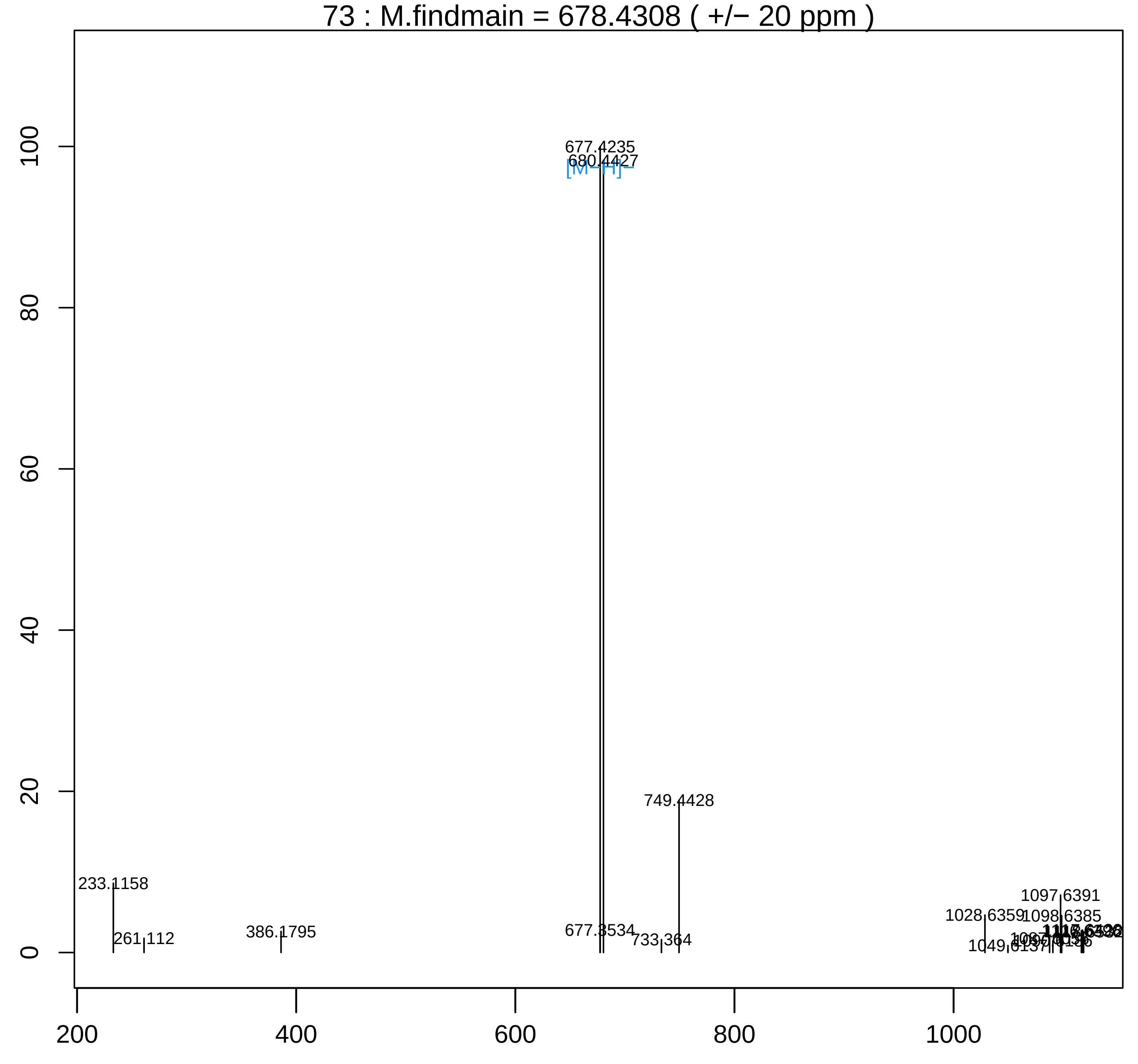
73 : M.findmain = 678.4308 (+/− 20 ppm)
677.4235
680.4427
[M−H]−
749.4428
233.1158
261.112
386.1795
677.3534
733.364
1097.6391
1028.6359
1098.6385
1049.6137
0
20
40
60
80
100
200
400
600
800
1000

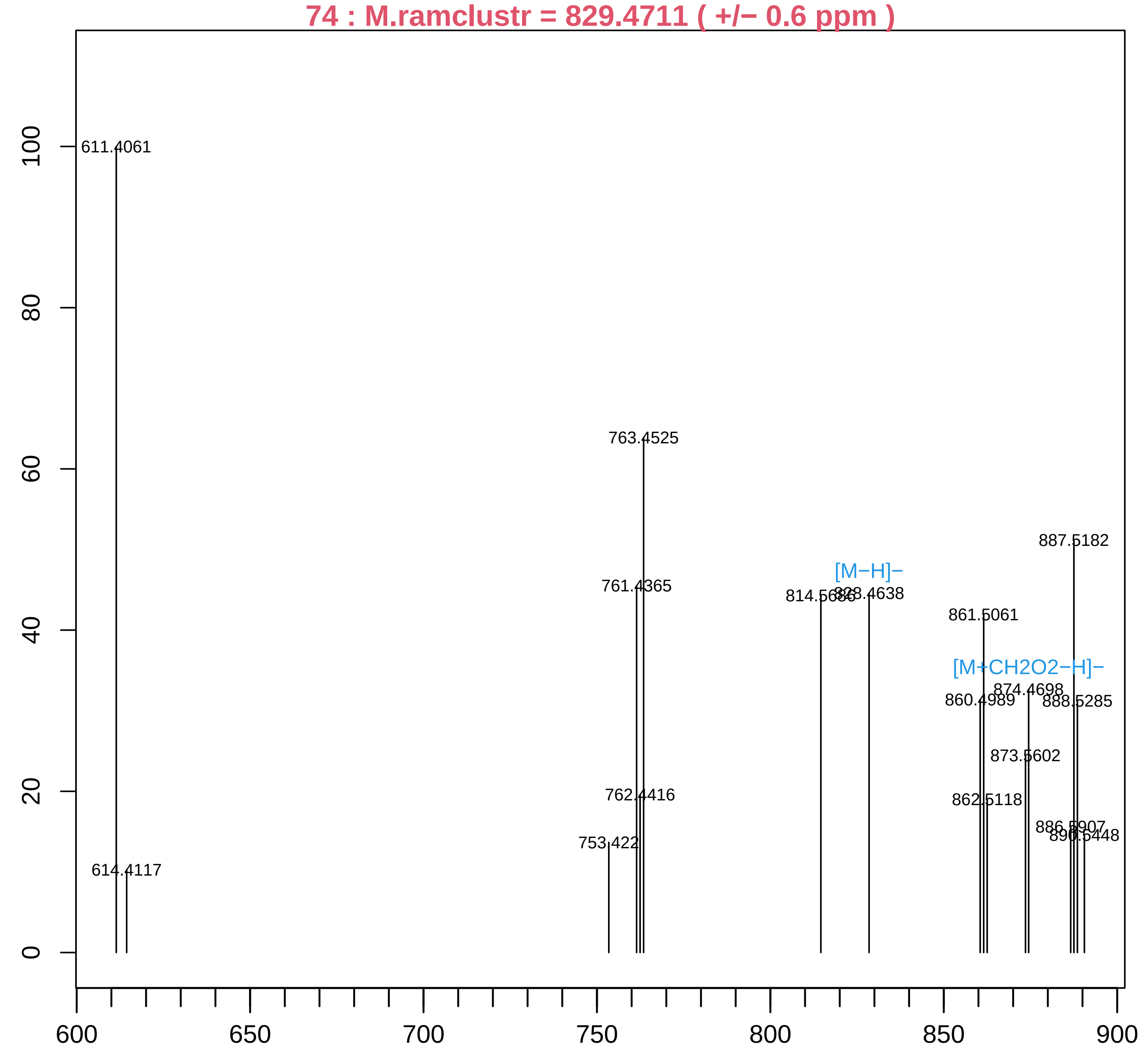
74 : M.ramclustr = 829.4711 (+/− 0.6 ppm)
611.4061
614.4117
753.422
761.4365
762.4416
763.4525
814.5686
828.4638
[M−H]−
860.4989
861.5061
862.5118
873.5602
874.4698
[M+CH2O2−H]−
886.5907
887.5182
888.5285
890.5448

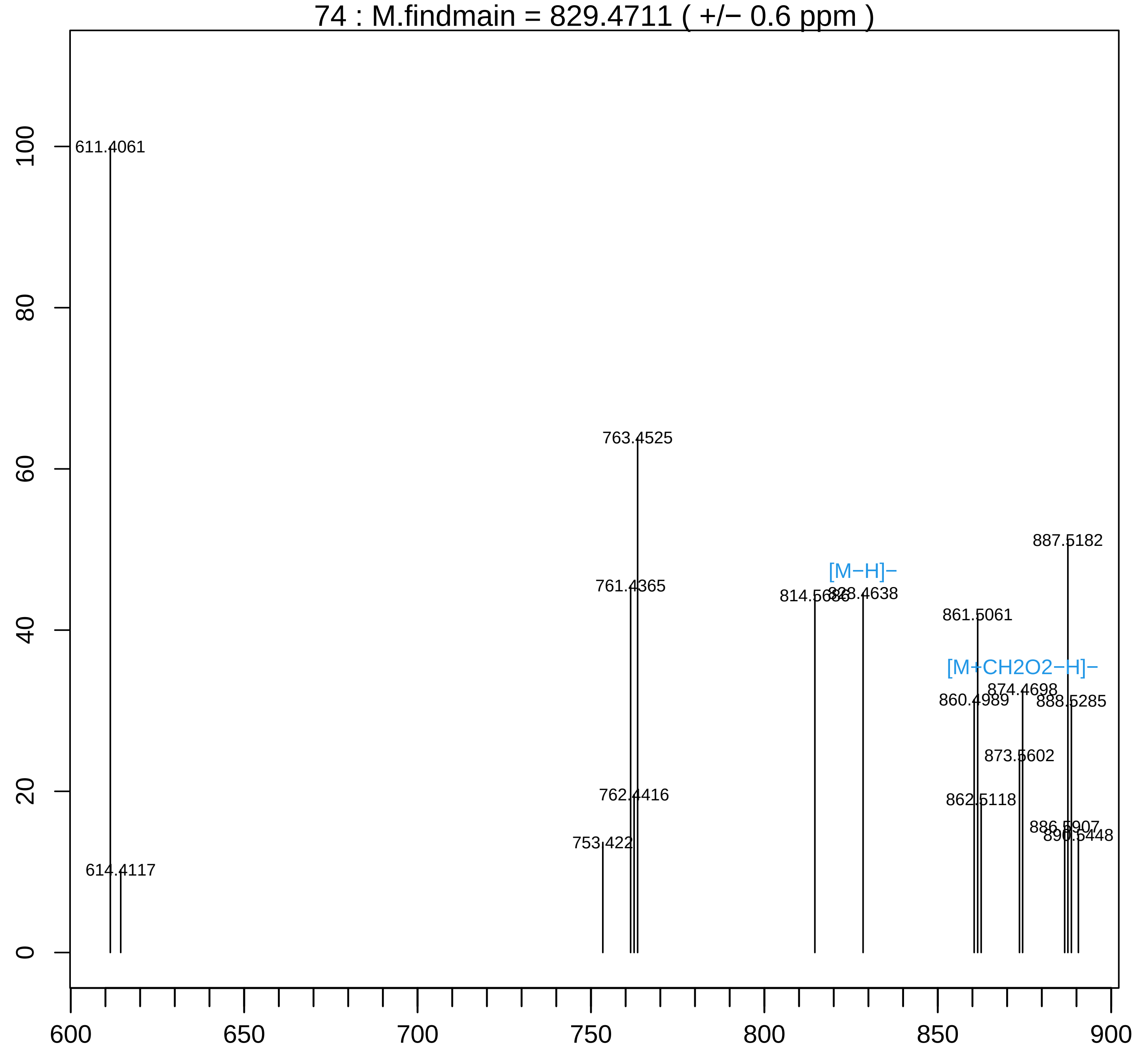
74 : M.findmain = 829.4711 (+/− 0.6 ppm)
611.4061
614.4117
753.422
761.4365
762.4416
763.4525
814.5686
828.4638
[M−H]−
860.4989
861.5061
862.5118
873.5602
874.4698
[M+CH2O2−H]−
886.5907
887.5182
888.5285
890.5448

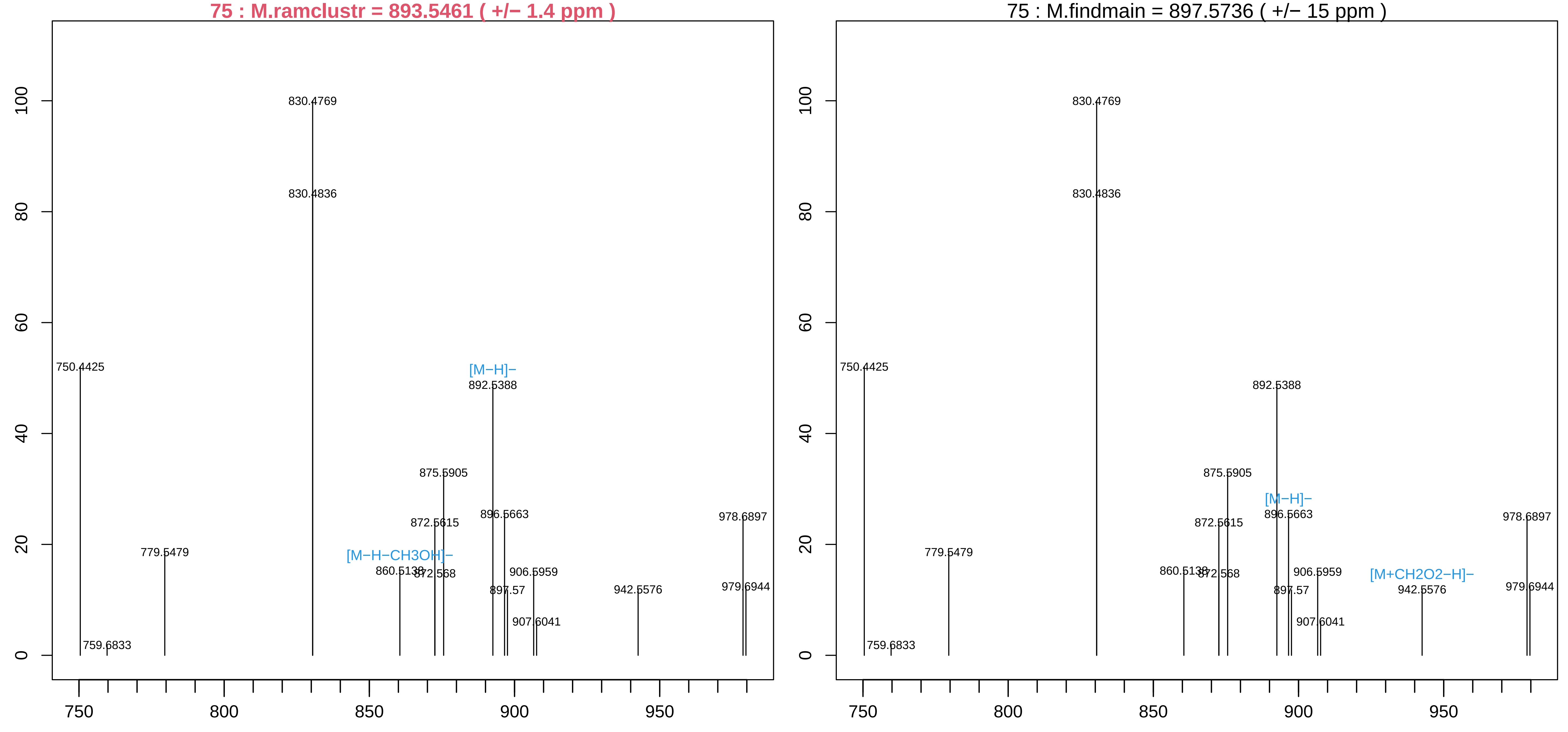

75 : M.ramclustr = 893.5461 (+/− 1.4 ppm)
830.4769
830.4836
750.4425
[M−H]−
892.5388
875.5905
872.5615
896.5663
978.6897
779.5479
[M−H−CH3OH]−
860.5138
872.568
906.5959
897.57
942.5576
979.6944
907.6041
759.6833
75 : M.findmain = 897.5736 (+/− 15 ppm)
[M+CH2O2−H]−
0
20
40
60
80
100
750
800
850
900
950

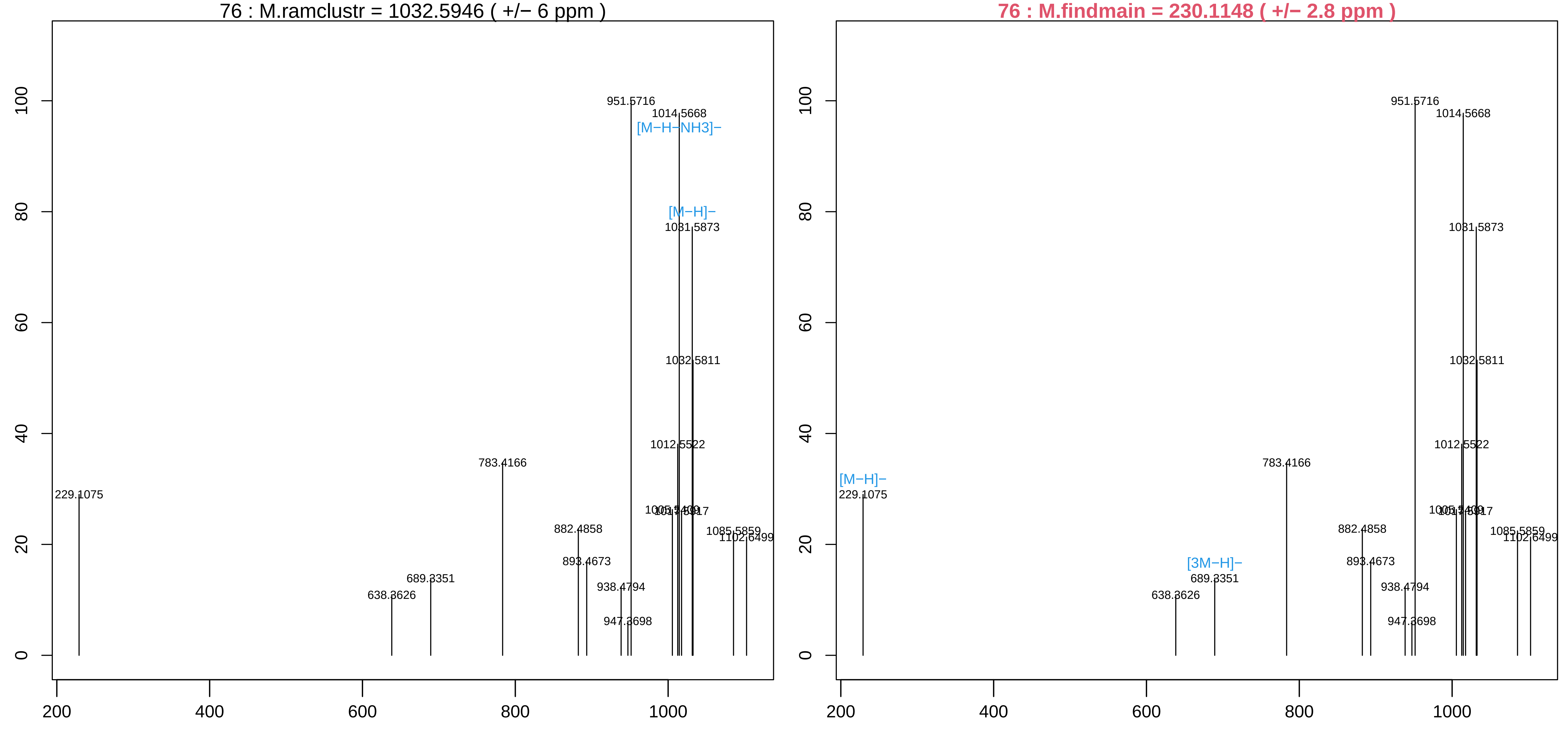

76 : M.ramclustr = 1032.5946 (+/− 6 ppm)
76 : M.findmain = 230.1148 (+/− 2.8 ppm)
951.5716
1014.5668
[M−H−NH3]−
[M−H]−
1031.5873
1032.5811
1012.5522
783.4166
229.1075
1005.5409
1017.5917
882.4858
1085.5859
1102.6499
893.4673
689.3351
938.4794
638.3626
947.3698
[3M−H]−
0
20
40
60
80
100
200
400
600
800
1000

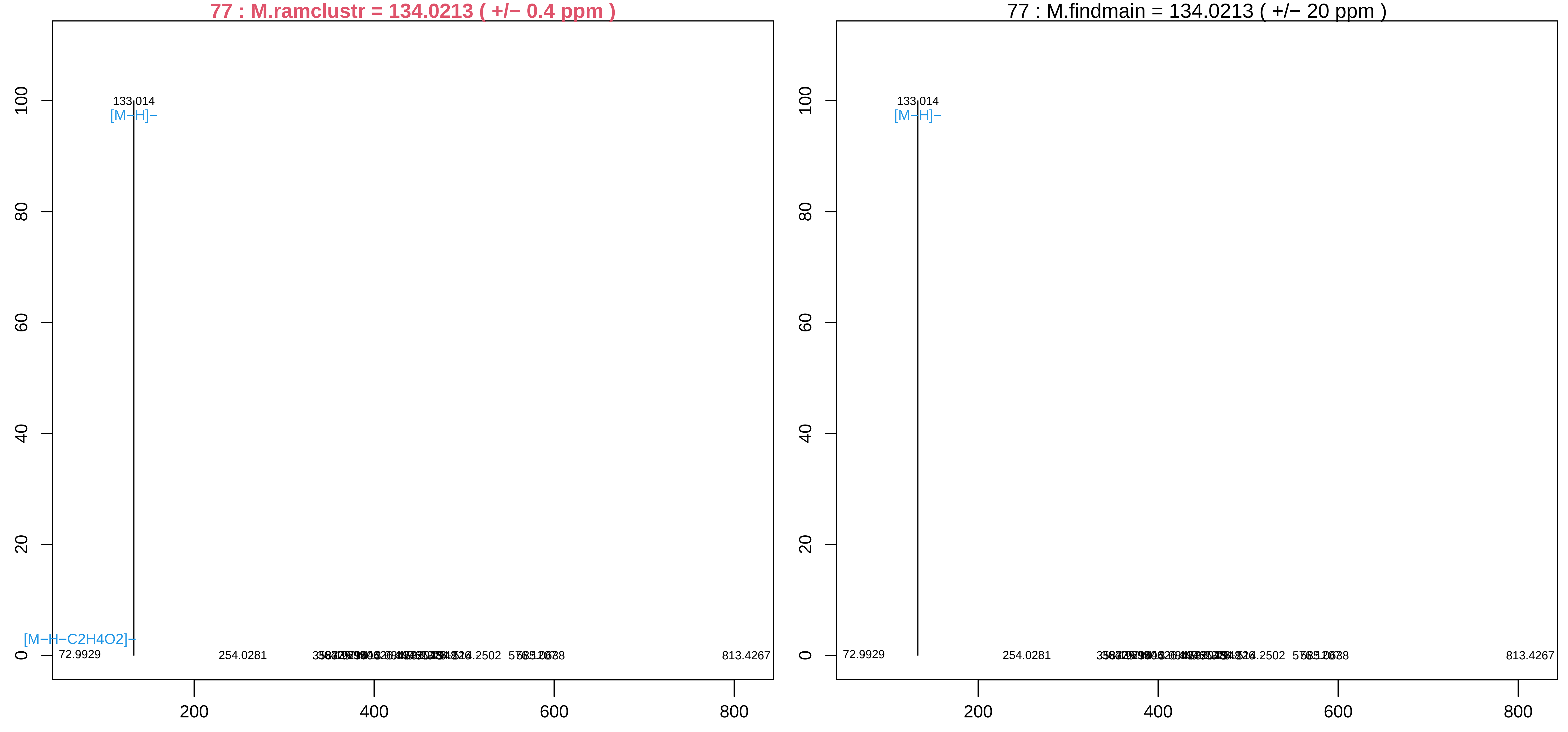
77 : M.ramclustr = 134.0213 (+/− 0.4 ppm)
133.014
[M−H]−
[M−H−C2H4O2]−
72.9929
254.0281
813.4267
77 : M.findmain = 134.0213 (+/− 20 ppm)
133.014
[M−H]−
72.9929
254.0281
813.4267
0
20
40
60
80
100
200
400
600
800

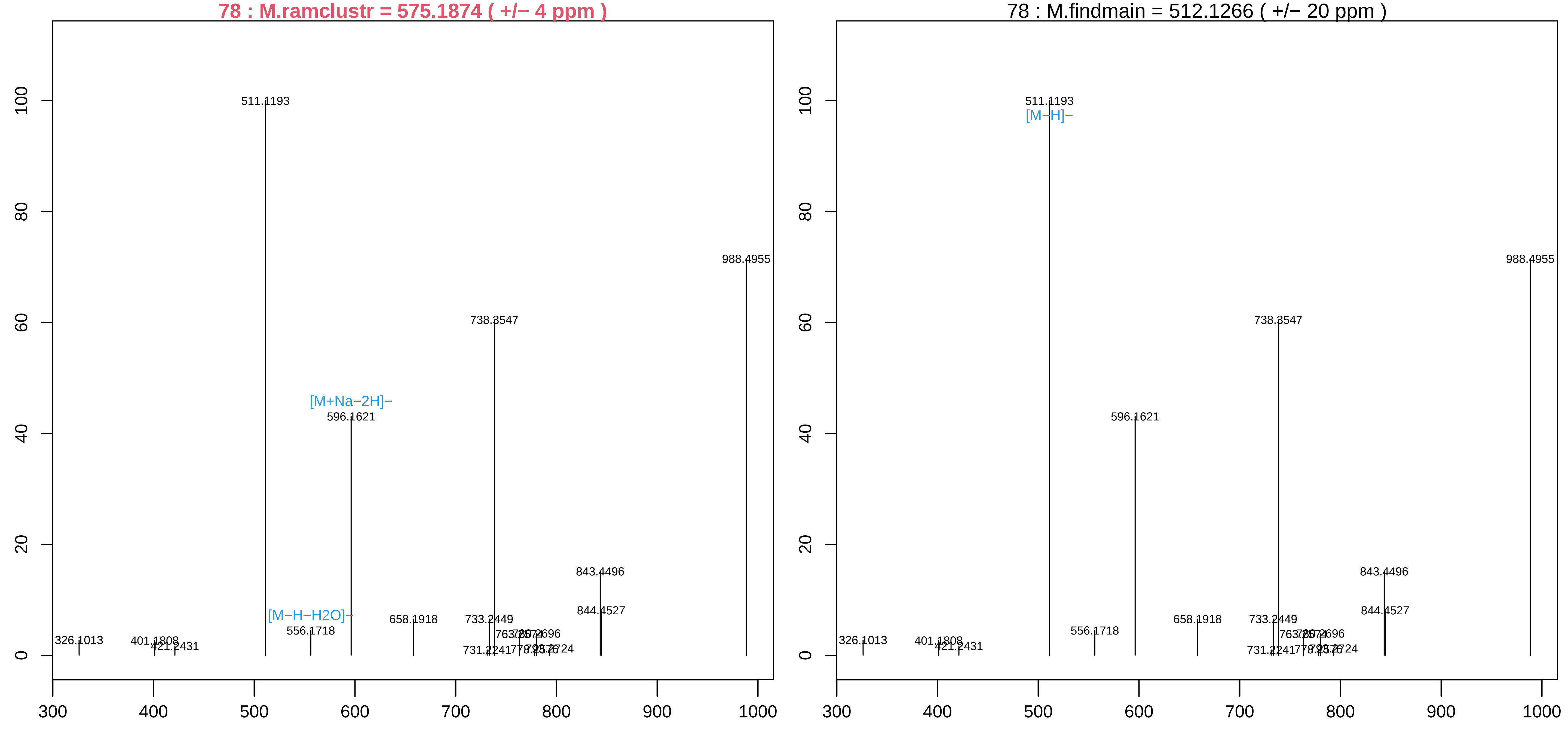

78 : M.ramclustr = 575.1874 (+/− 4 ppm)
511.1193
988.4955
738.3547
[M+Na−2H]−
596.1621
843.4496
844.4527
[M−H−H2O]−
556.1718
658.1918
733.2449
326.1013
401.1808
421.2431
731.2241
0
20
40
60
80
100
300
400
500
600
700
800
900
1000
78 : M.findmain = 512.1266 (+/− 20 ppm)
511.1193
[M−H]−
988.4955
738.3547
596.1621
843.4496
844.4527
556.1718
658.1918
733.2449
326.1013
401.1808
421.2431
731.2241
0
20
40
60
80
100
300
400
500
600
700
800
900
1000

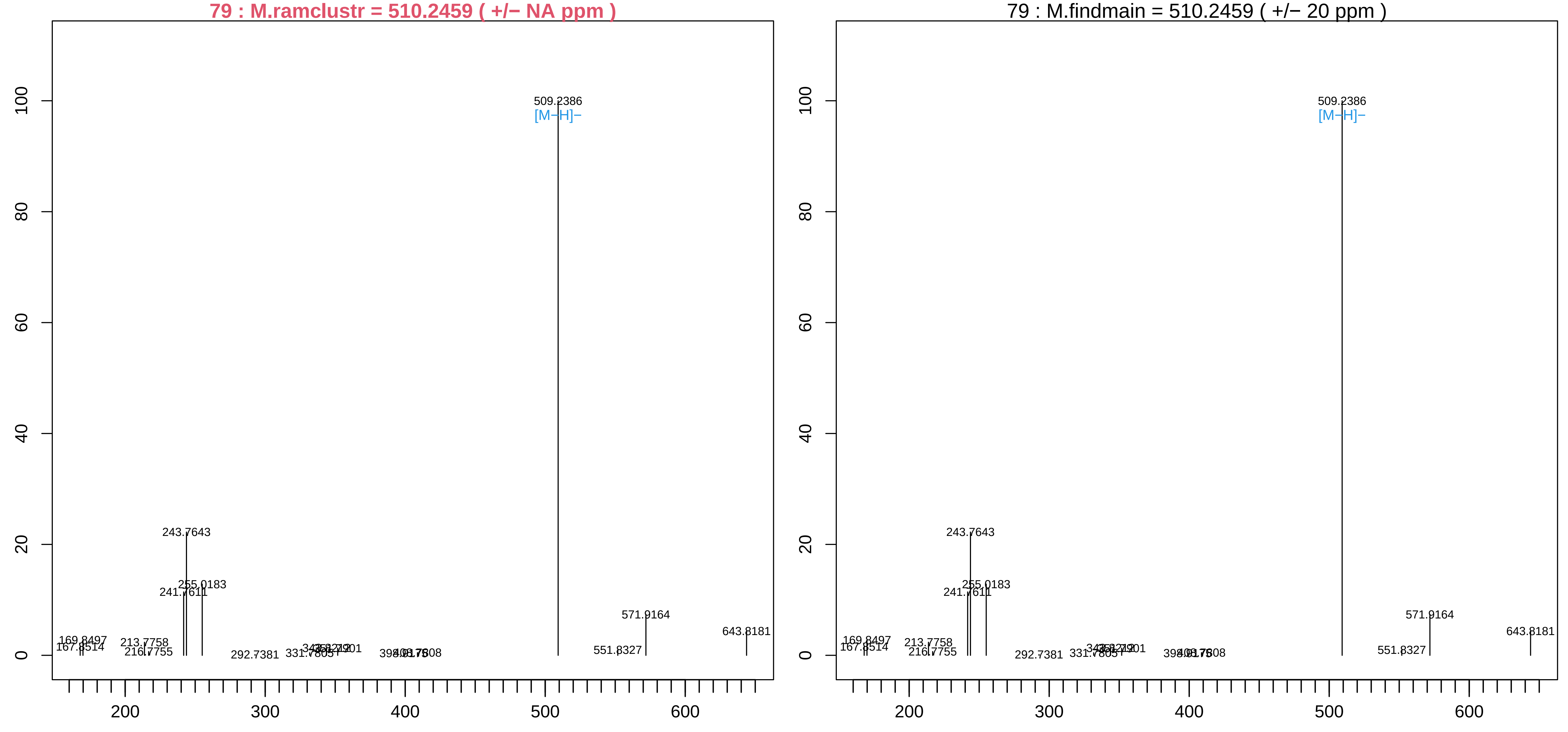
79 : M.ramclustr = 510.2459 (+/− NA ppm)
79 : M.findmain = 510.2459 (+/− 20 ppm)
509.2386
[M−H]−
243.7643
255.0183
241.7611
571.9164
643.8181
169.8497
167.8514
213.7758
216.7755
292.7381
331.7805
551.8327
0
20
40
60
80
100
200
300
400
500
600

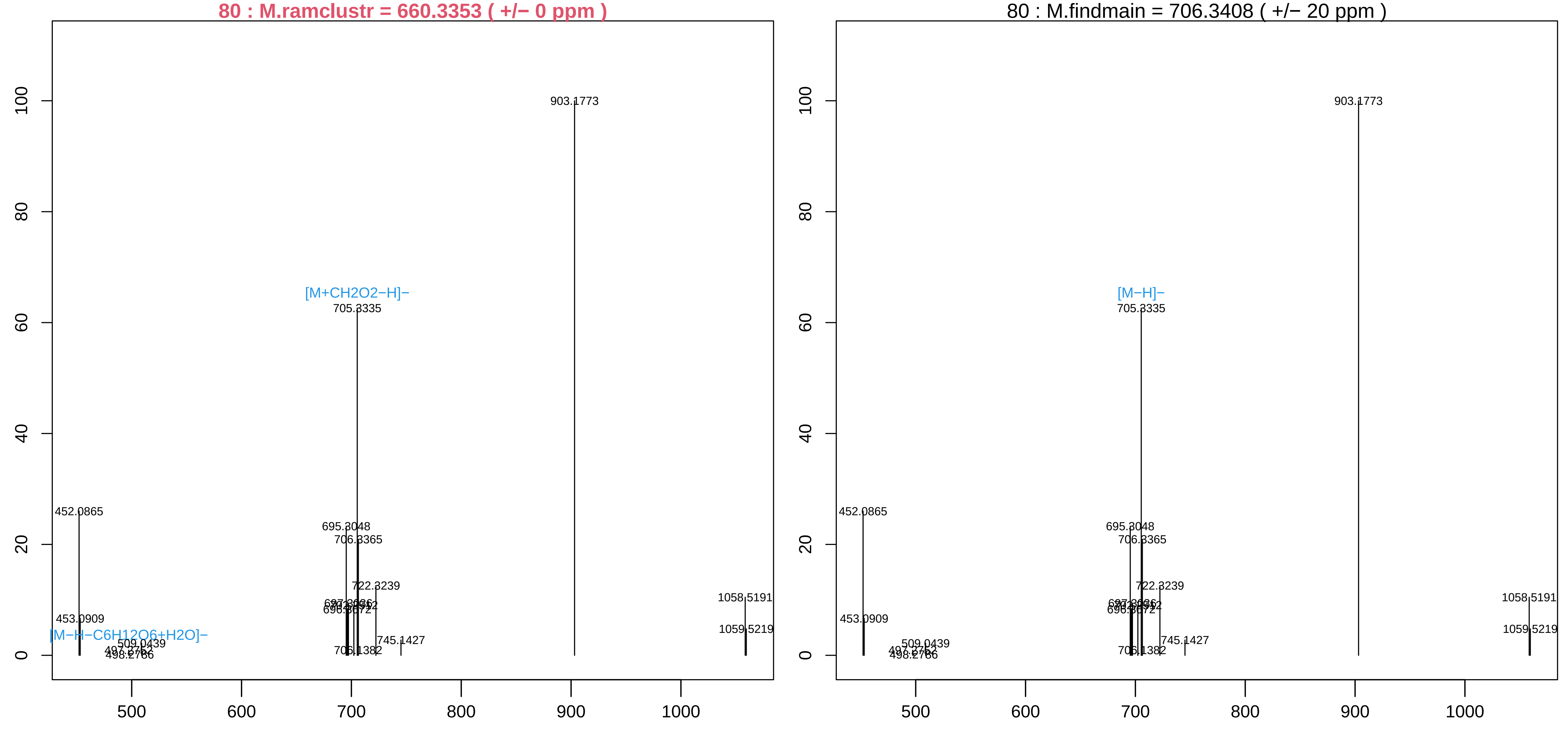
80 : M.ramclustr = 660.3353 (+/− 0 ppm)
[M+CH2O2−H]−
705.3335
903.1773
452.0865
695.3048
706.3365
722.3239
453.0909
[M−H−C6H12O6+H2O]−
509.0439
745.1427
1058.5191
1059.5219
80 : M.findmain = 706.3408 (+/− 20 ppm)
[M−H]−
705.3335
903.1773
452.0865
695.3048
706.3365
722.3239
453.0909
509.0439
745.1427
1058.5191
1059.5219
0
20
40
60
80
100
500
600
700
800
900
1000

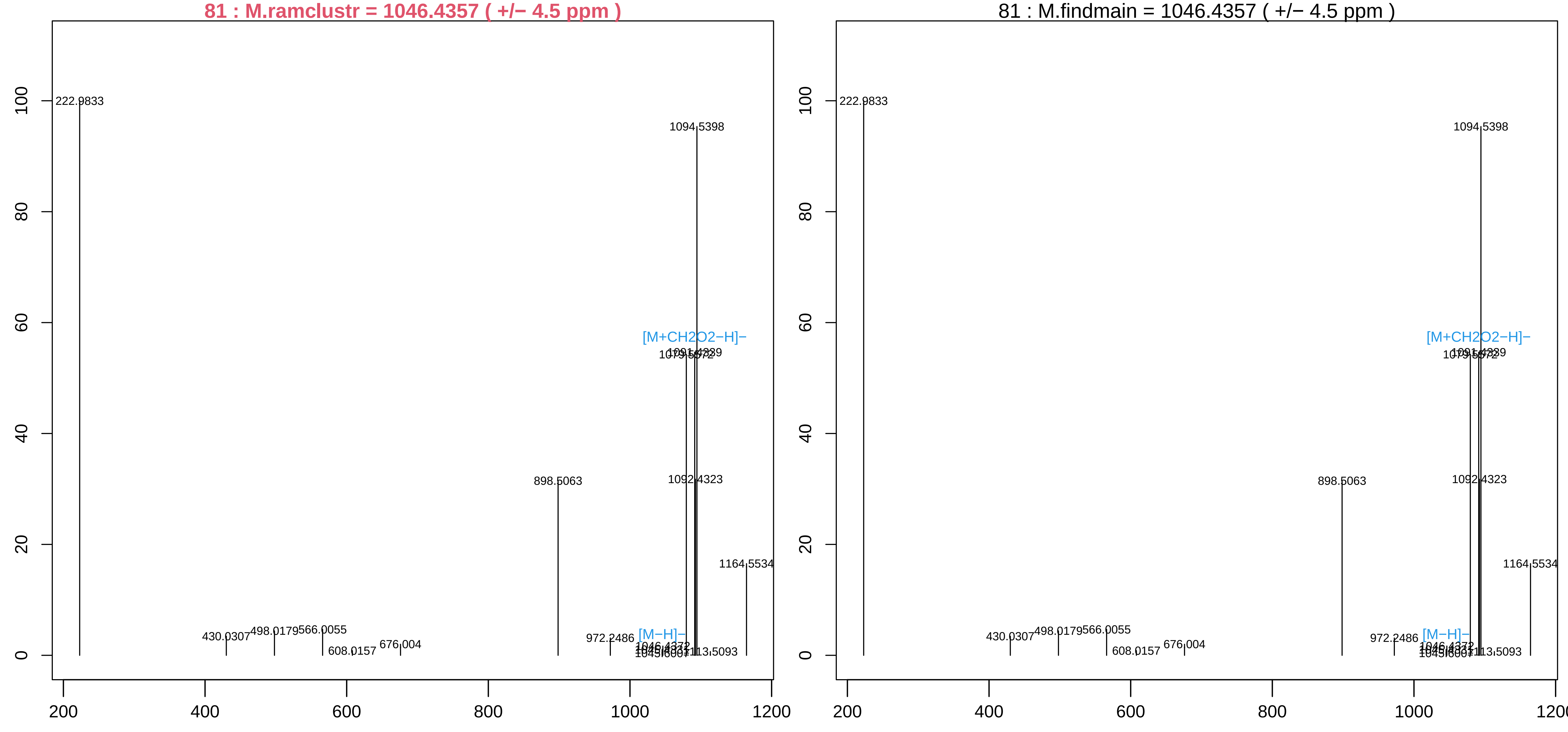

81 : M.ramclustr = 1046.4357 (+/− 4.5 ppm)
81 : M.findmain = 1046.4357 (+/− 4.5 ppm)
222.9833
1094.5398
[M+CH2O2−H]−
1091.4229
1079.5572
898.5063
1092.4323
1164.5534
430.0307
498.0179
566.0055
608.0157
676.004
972.2486
[M−H]−
1046.4372
1113.5093
0
20
40
60
80
100
200
400
600
800
1000
1200

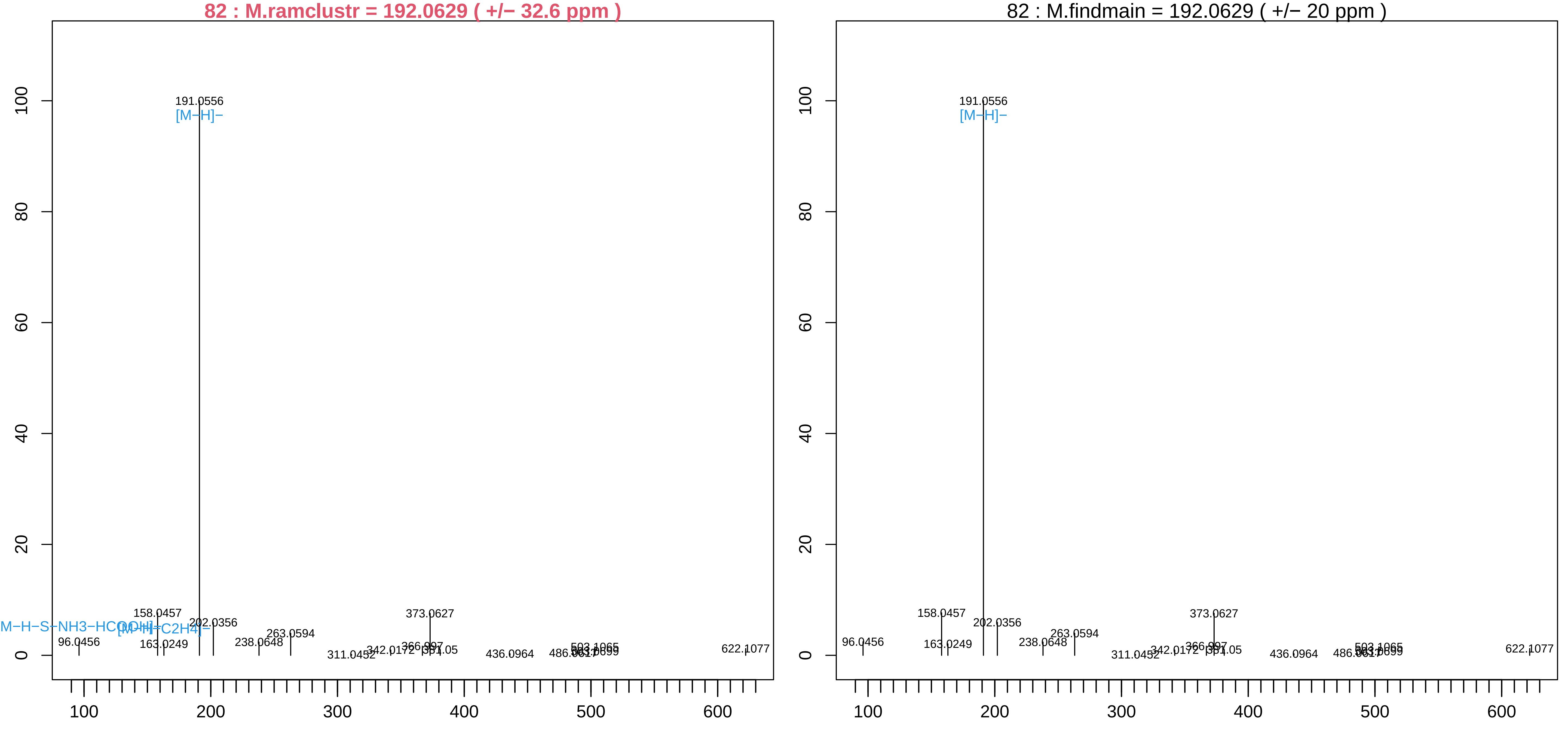

82 : M.ramclustr = 192.0629 (+/− 32.6 ppm)
82 : M.findmain = 192.0629 (+/− 20 ppm)
191.0556
[M−H]−
158.0457
202.0356
373.0627
M−H−S−NH3−HCOOH]
[M−H−C2H4]−
96.0456
163.0249
238.0648
263.0594
311.0452
342.0172
366.997
391.05
436.0964
486.0617
503.1065
622.1077
0
20
40
60
80
100
200
300
400
500
600

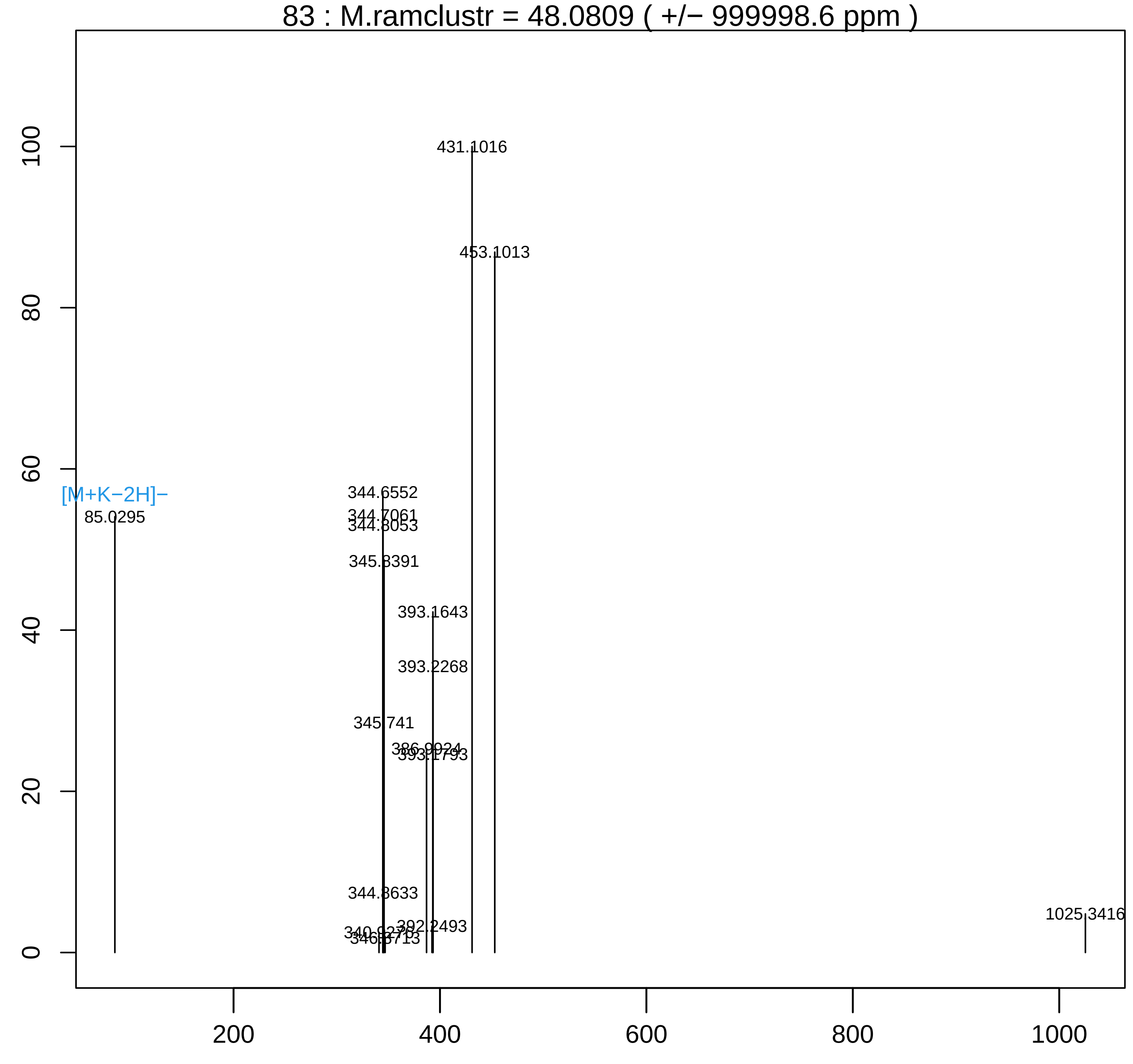

83 : M.ramclustr = 48.0809 (+/− 999998.6 ppm)
[M+K−2H]−
85.0295
344.6552
344.7061
344.8053
345.3391
393.1643
393.2268
345.741
386.9024
393.1793
344.3633
340.9237
346.3713
392.2493
431.1016
453.1013
1025.3416

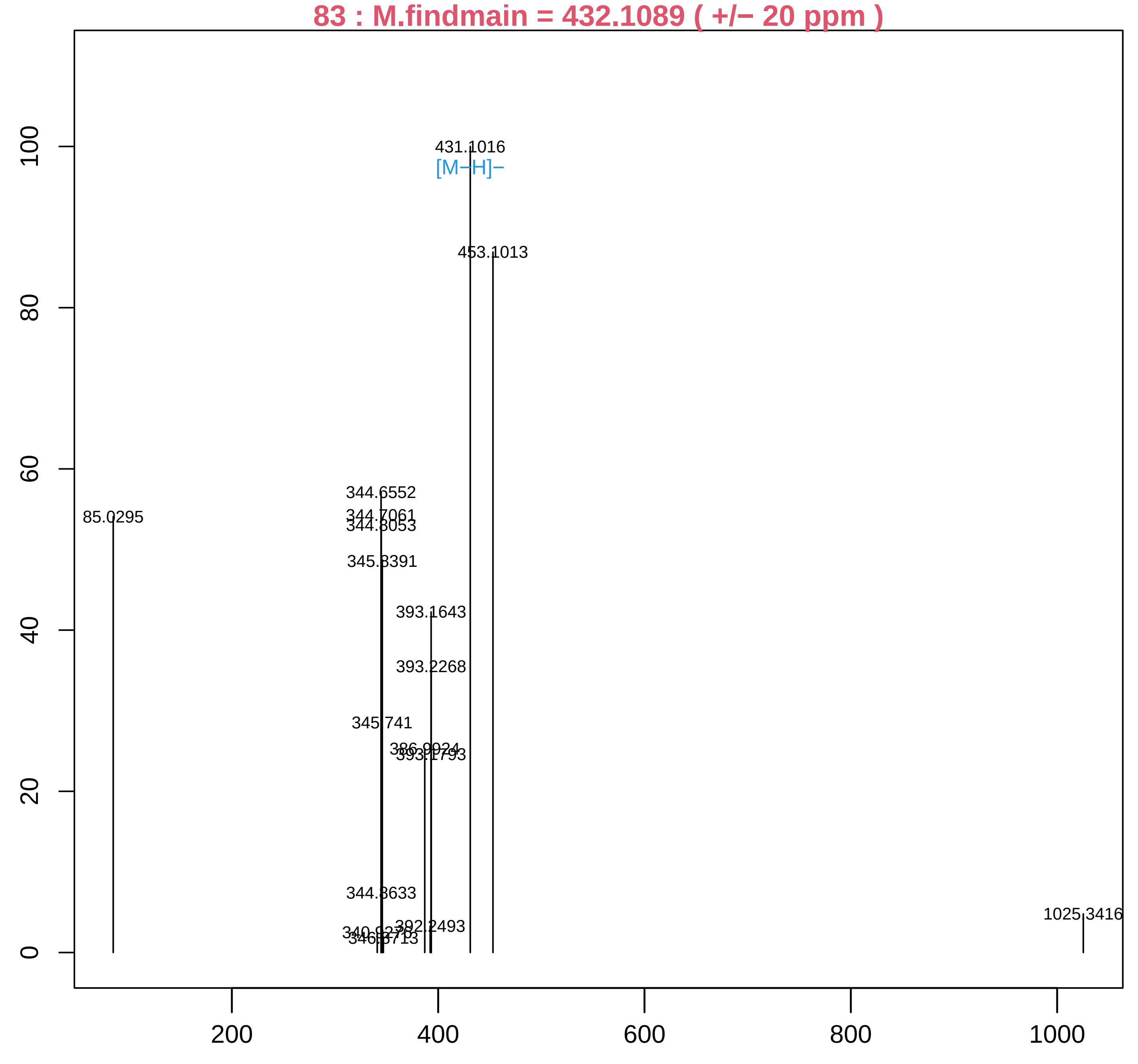

83 : M.findmain = 432.1089 (+/− 20 ppm)
431.1016
[M−H]−
453.1013
85.0295
344.6552
344.7061
344.8053
345.3391
393.1643
393.2268
345.741
386.9024
393.1793
344.3633
340.9237
346.3713
392.2493
1025.3416

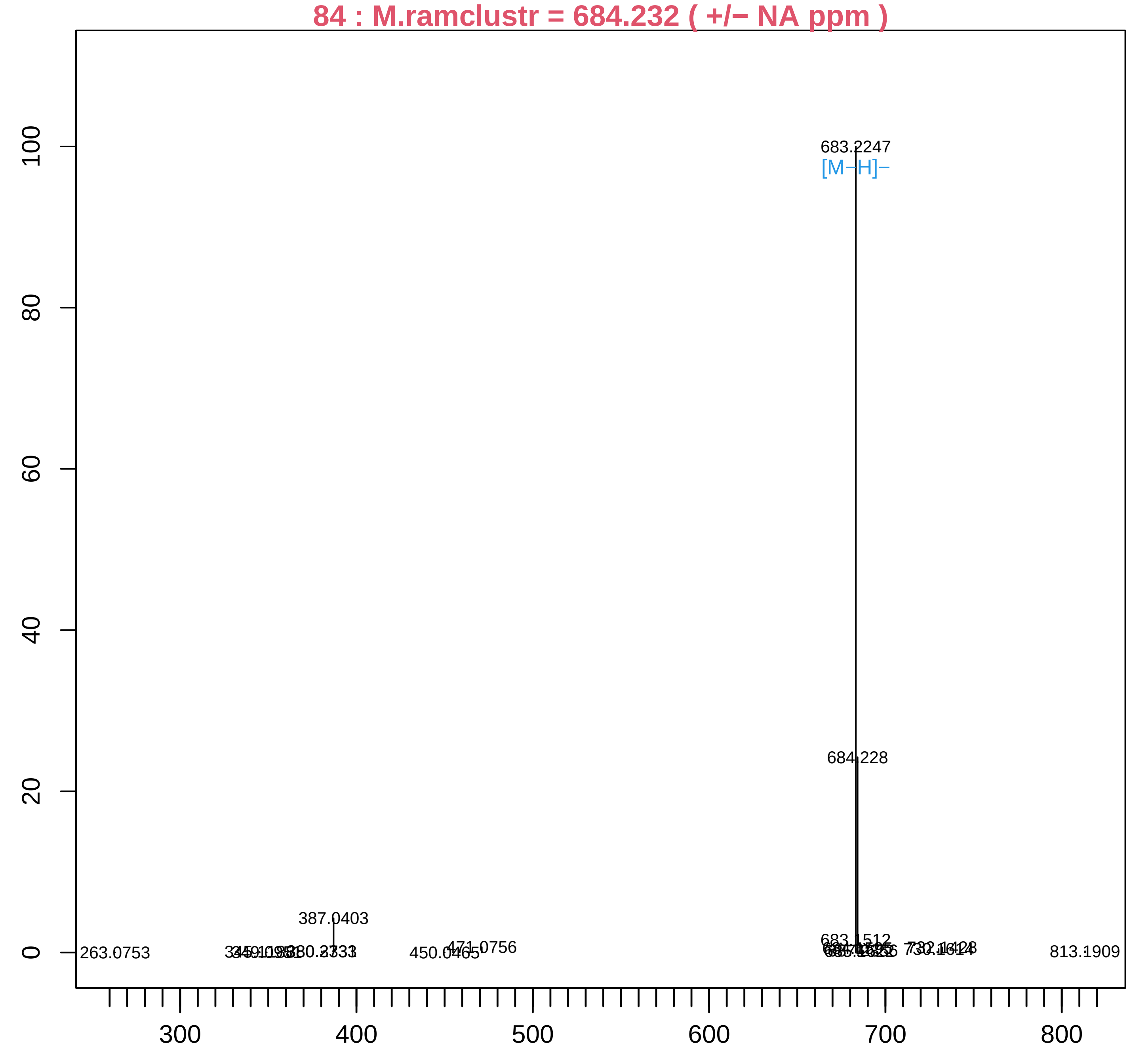
84 : M.ramclustr = 684.232 (+/− NA ppm)
683.2247
[M−H]−
684.228
387.0403
683.1512
471.0756
263.0753
450.0465
813.1909
0
20
40
60
80
100
300
400
500
600
700
800

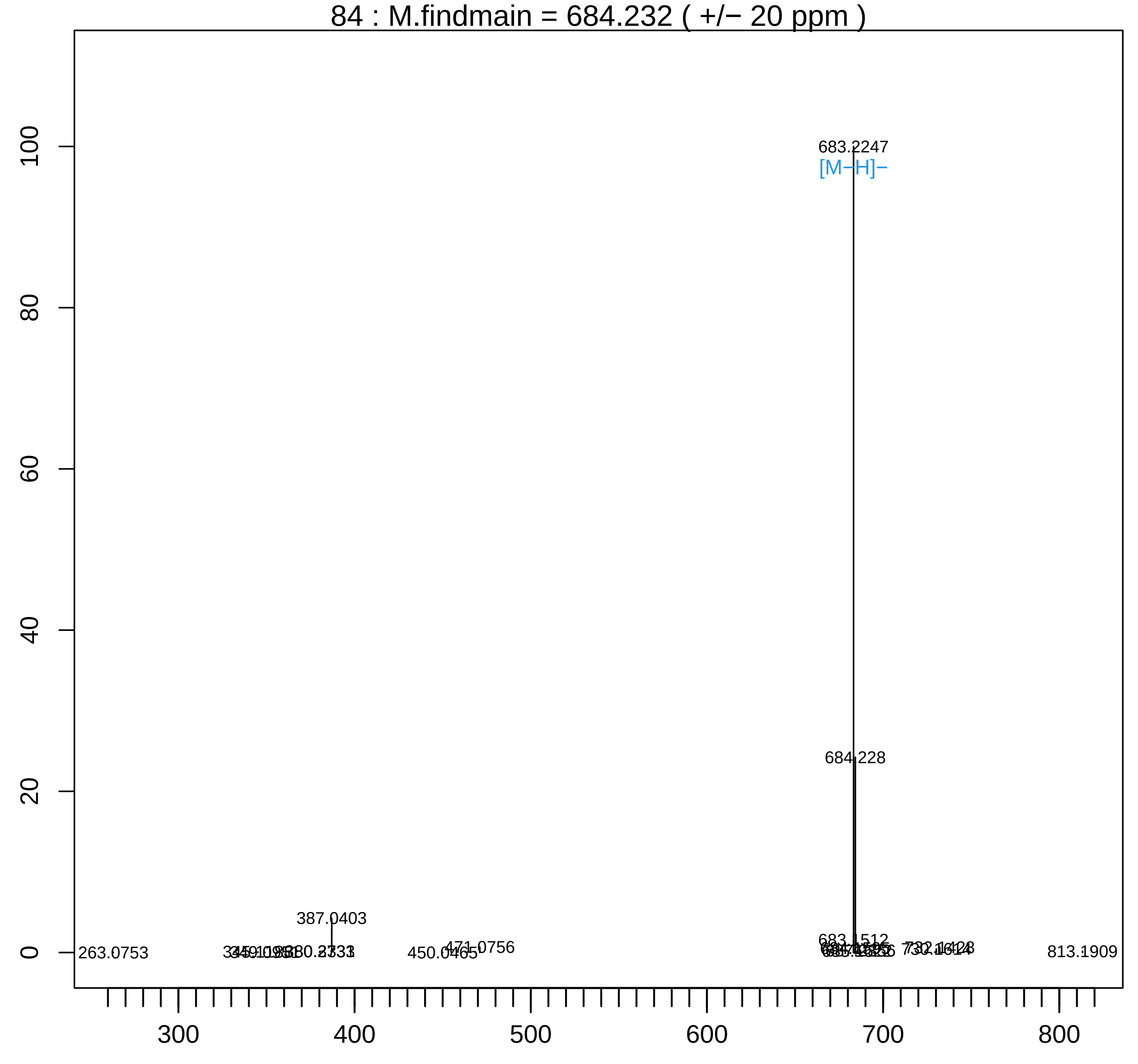
84 : M.findmain = 684.232 (+/− 20 ppm)
683.2247
[M−H]−
684.228
387.0403
683.1512
471.0756
263.0753
450.0465
813.1909
0
20
40
60
80
100
300
400
500
600
700
800

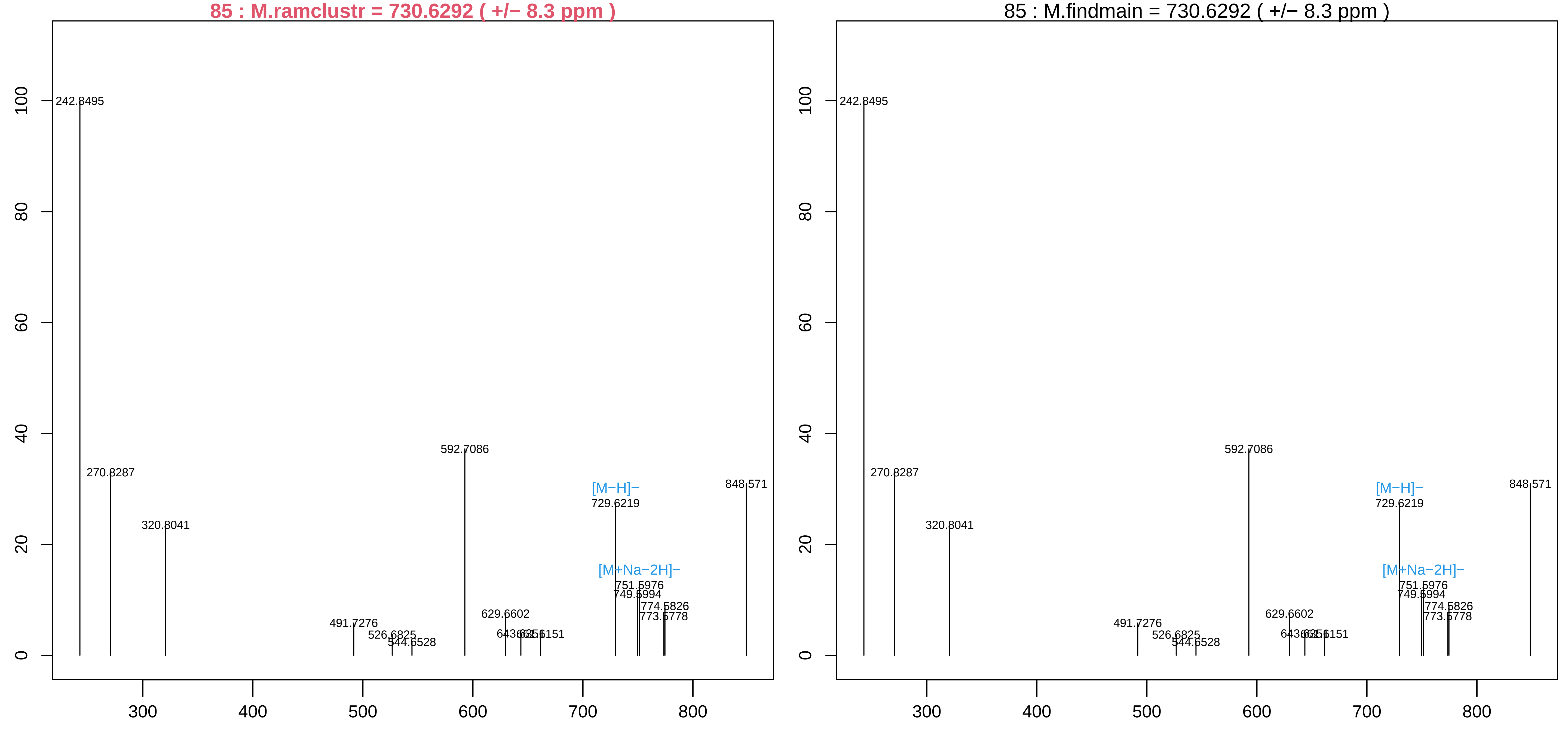

85 : M.ramclustr = 730.6292 (+/− 8.3 ppm)
85 : M.findmain = 730.6292 (+/− 8.3 ppm)
242.8495
270.8287
320.8041
491.7276
526.6825
544.6528
592.7086
629.6602
661.6151
[M−H]−
729.6219
[M+Na−2H]−
751.5976
749.5994
774.5826
773.5778
848.571
0
20
40
60
80
100
300
400
500
600
700
800

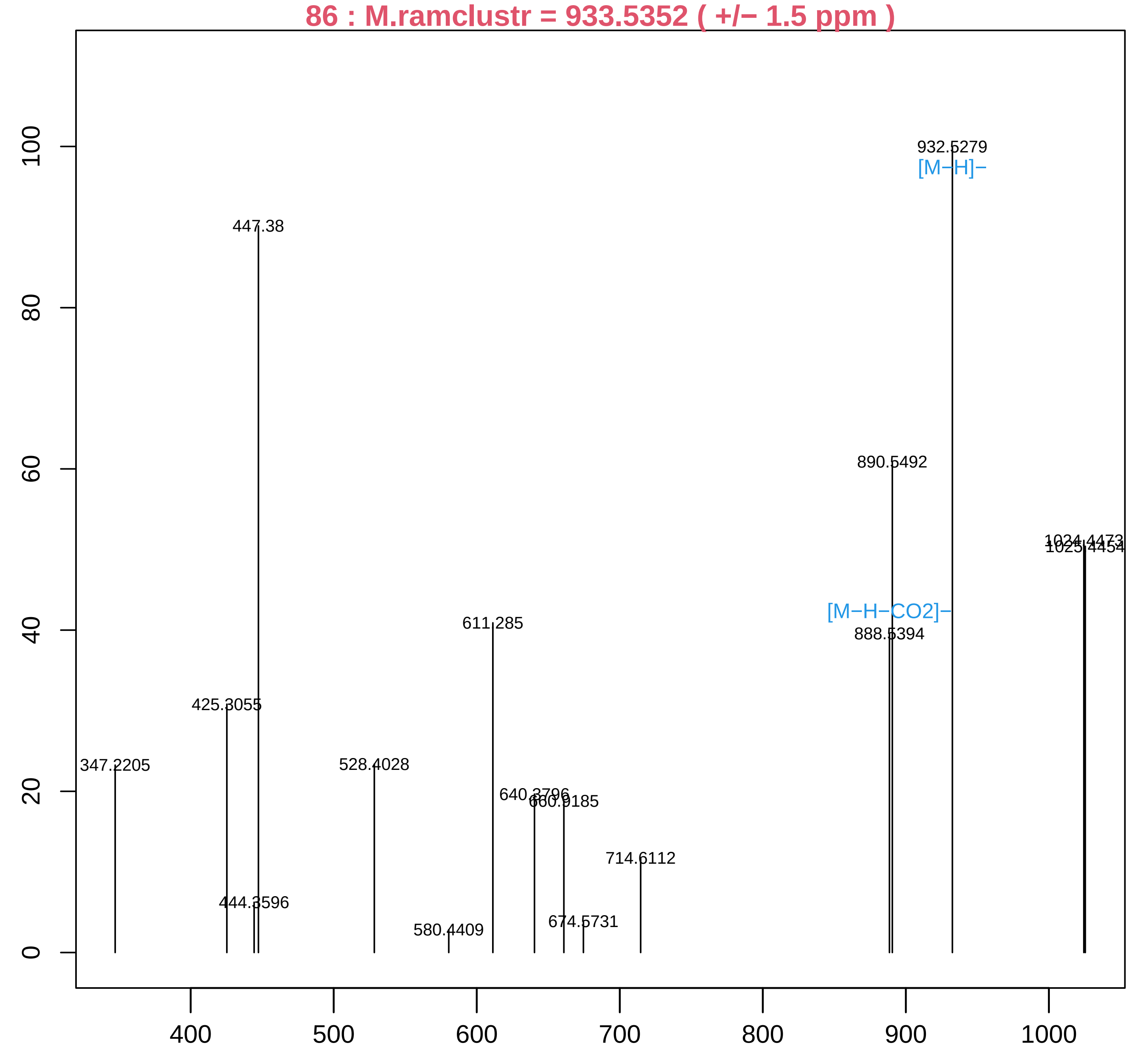
86 : M.ramclustr = 933.5352 (+/− 1.5 ppm)
932.5279
[M−H]−
447.38
890.5492
1024.4473
1025.4454
[M−H−CO2]−
888.5394
611.285
425.3055
347.2205
528.4028
640.3796
660.9185
714.6112
444.3596
580.4409
674.5731
0
20
40
60
80
100
400
500
600
700
800
900
1000

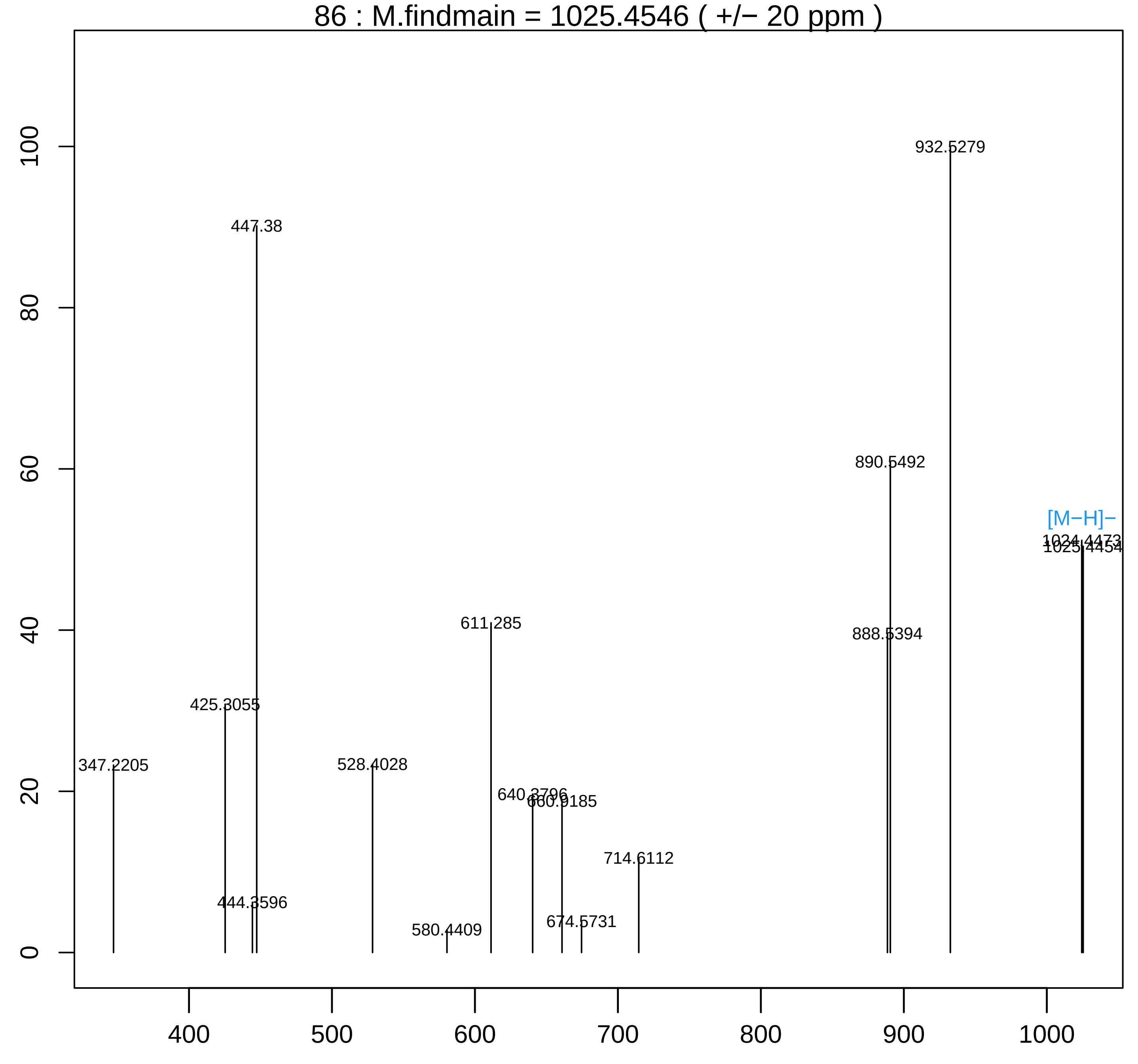
86 : M.findmain = 1025.4546 (+/− 20 ppm)
932.5279
447.38
890.5492
[M−H]−
1024.4473
1025.4454
611.285
888.5394
425.3055
347.2205
528.4028
640.3796
660.9185
714.6112
444.3596
580.4409
674.5731
0
20
40
60
80
100
400
500
600
700
800
900
1000

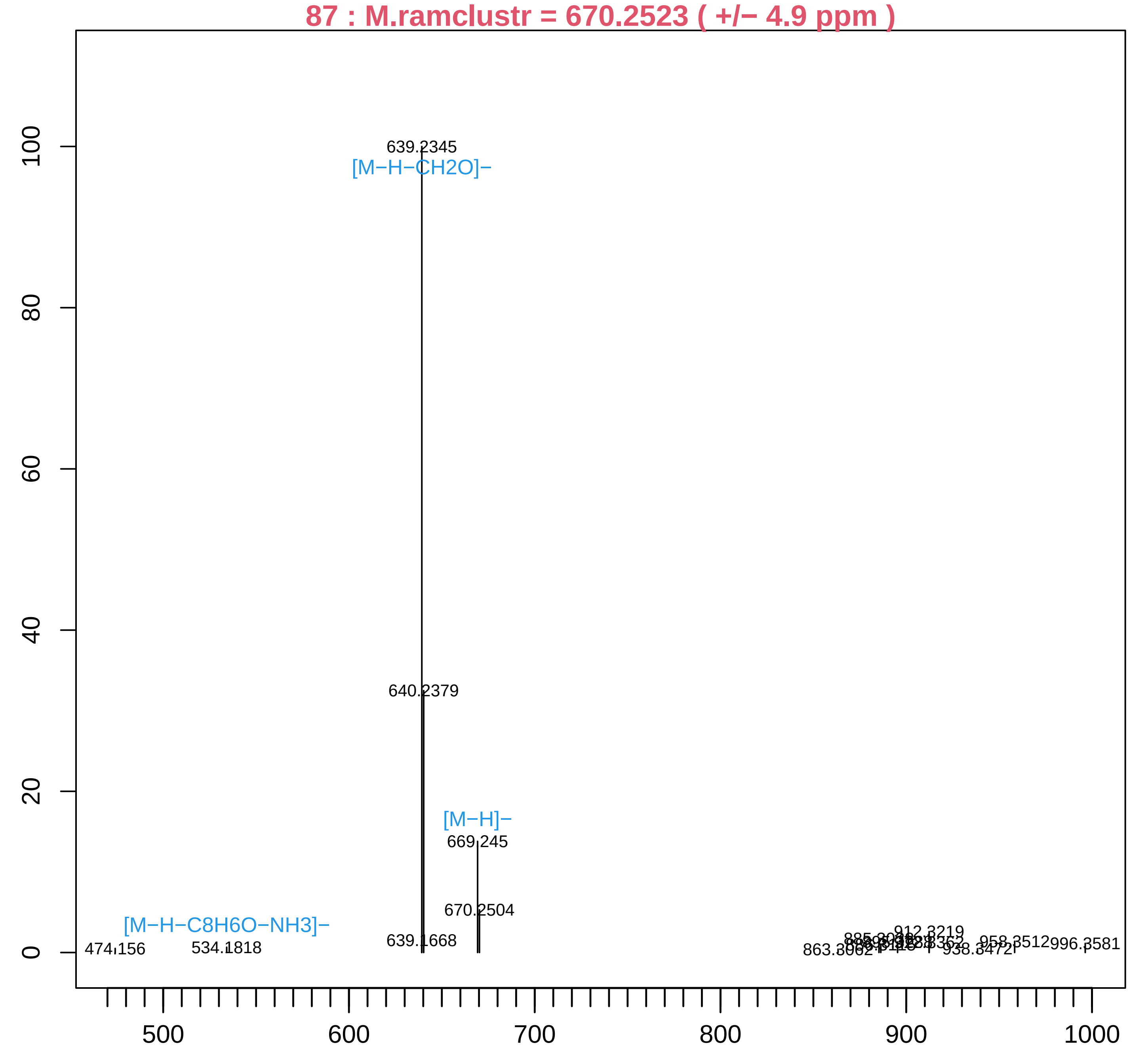

87 : M.ramclustr = 670.2523 (+/− 4.9 ppm)
639.2345
[M−H−CH2O]−
640.2379
[M−H]−
669.245
670.2504
[M−H−C8H6O−NH3]−
474.156
534.1818
639.1668
912.2219
958.3512
996.3581
938.3472
863.3082
500
600
700
800
900
1000
0
20
40
60
80
100

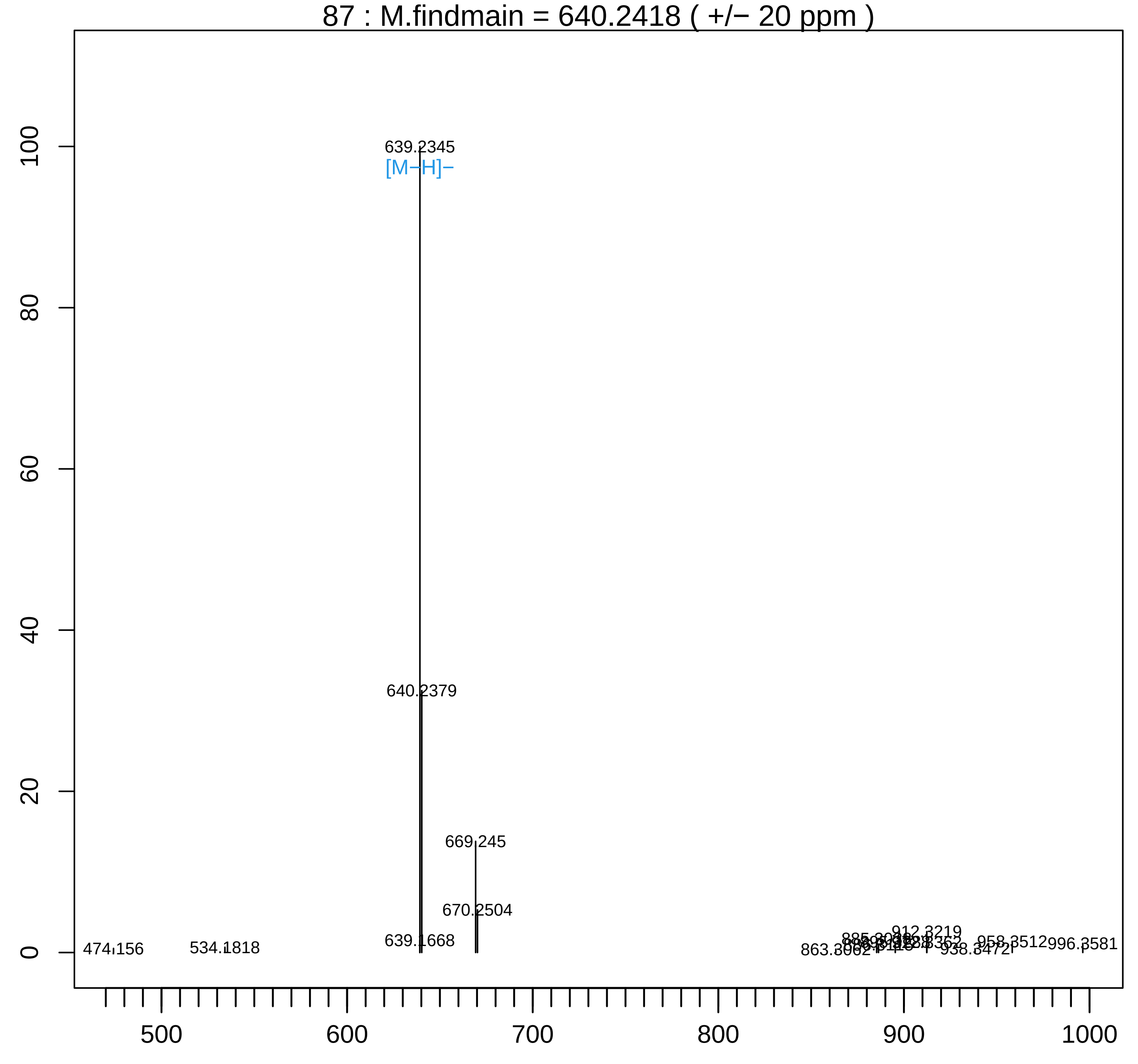

87 : M.findmain = 640.2418 (+/− 20 ppm)
639.2345
[M−H]−
640.2379
669.245
670.2504
474.156
534.1818
639.1668
912.2219
958.3512
996.3581
938.3472
863.3082
500
600
700
800
900
1000
0
20
40
60
80
100

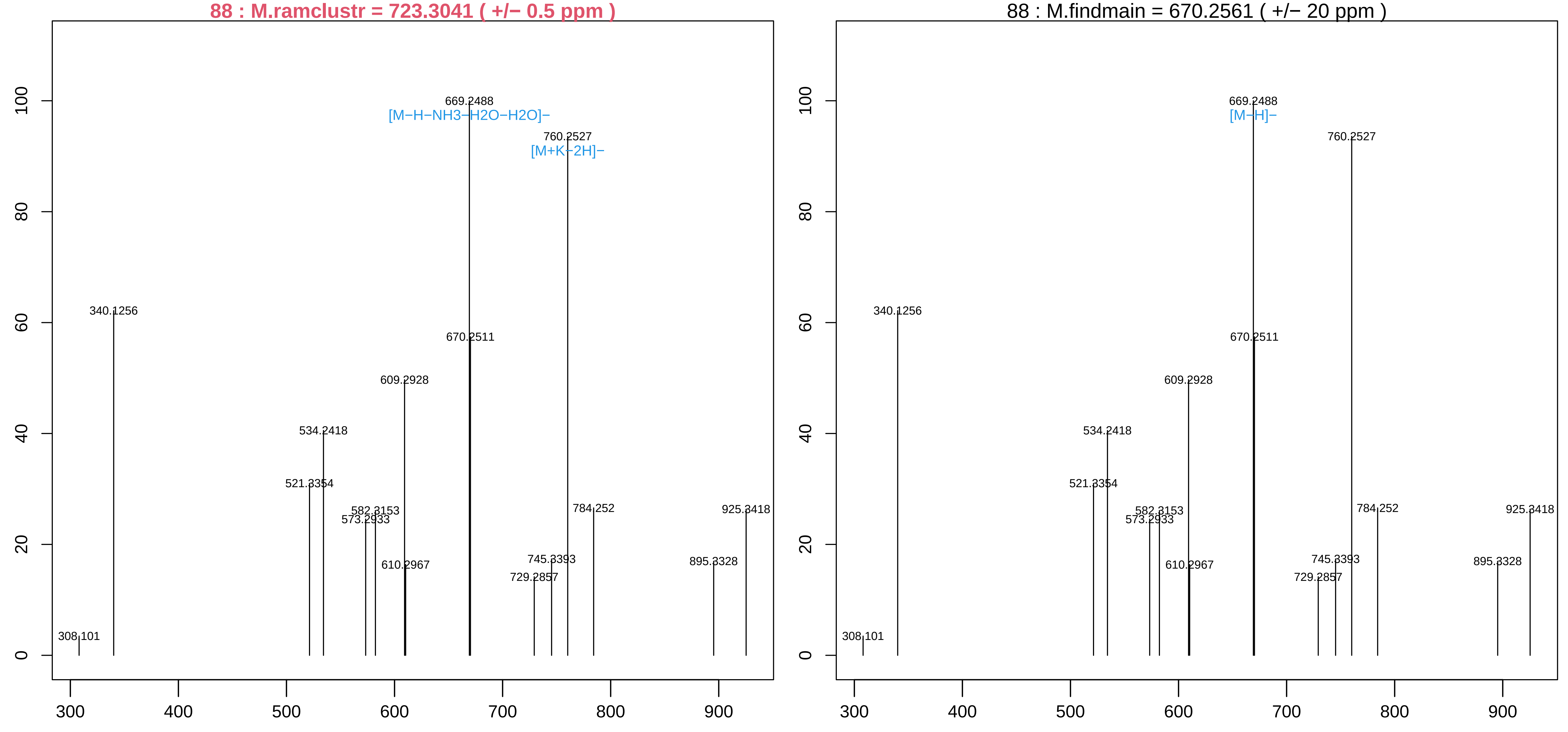

88 : M.ramclustr = 723.3041 (+/− 0.5 ppm)
669.2488
[M−H−NH3−H2O−H2O]−
760.2527
[M+K−2H]−
340.1256
670.2511
609.2928
534.2418
521.3354
582.3153
573.2933
784.252
925.3418
745.3393
610.2967
729.2857
895.3328
308.101
88 : M.findmain = 670.2561 (+/− 20 ppm)
669.2488
[M−H]−
760.2527
340.1256
670.2511
609.2928
534.2418
521.3354
582.3153
573.2933
784.252
925.3418
745.3393
610.2967
729.2857
895.3328
308.101
0
20
40
60
80
100
300
400
500
600
700
800
900

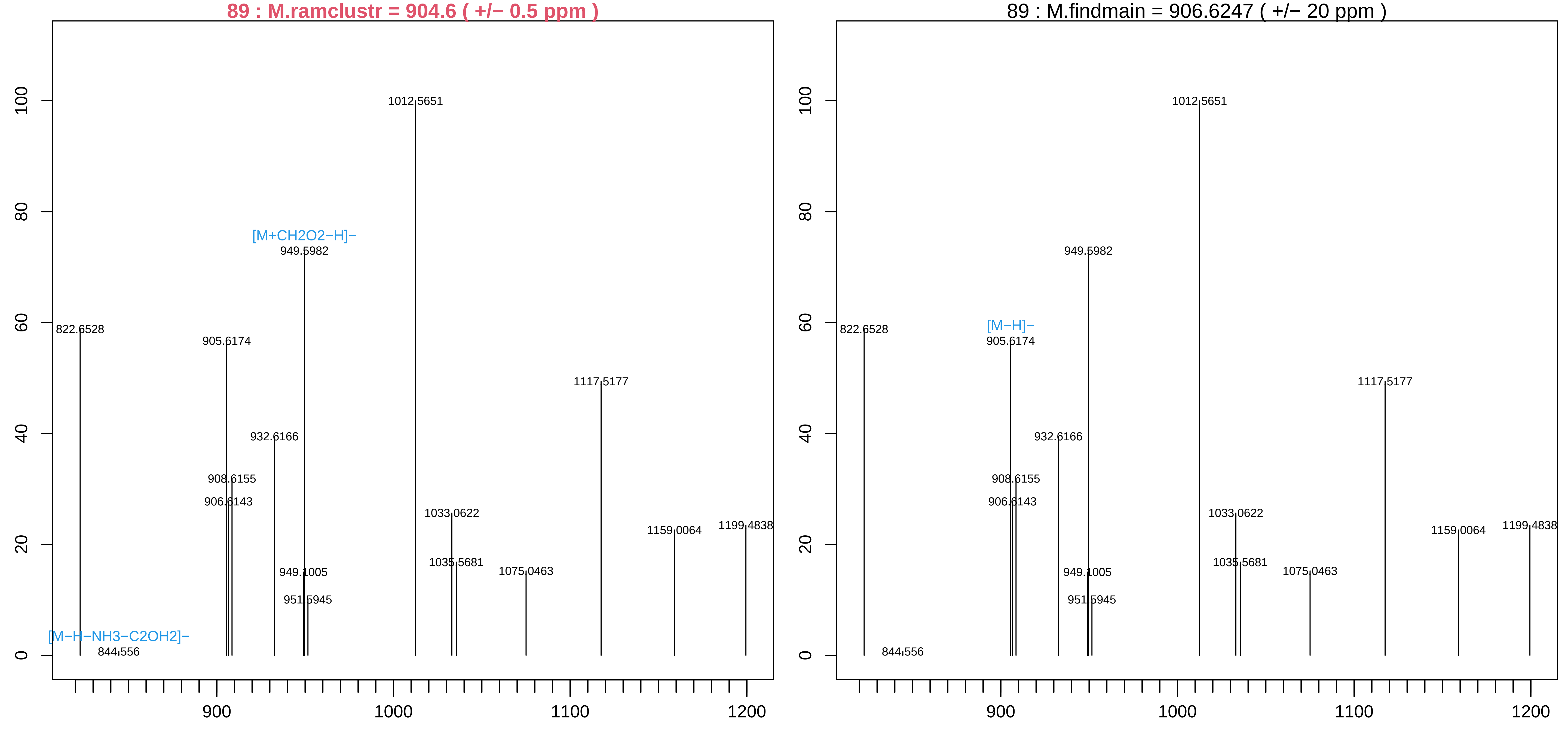

89 : M.ramclustr = 904.6 (+/− 0.5 ppm)
89 : M.findmain = 906.6247 (+/− 20 ppm)
[M+CH2O2−H]−
[M−H−NH3−C2OH2]−
[M−H]−
1012.5651
949.5982
822.6528
905.6174
932.6166
908.6155
906.6143
1033.0622
1035.5681
1075.0463
1117.5177
1159.0064
1199.4838
949.1005
951.5945
844.556
0
20
40
60
80
100
900
1000
1100
1200

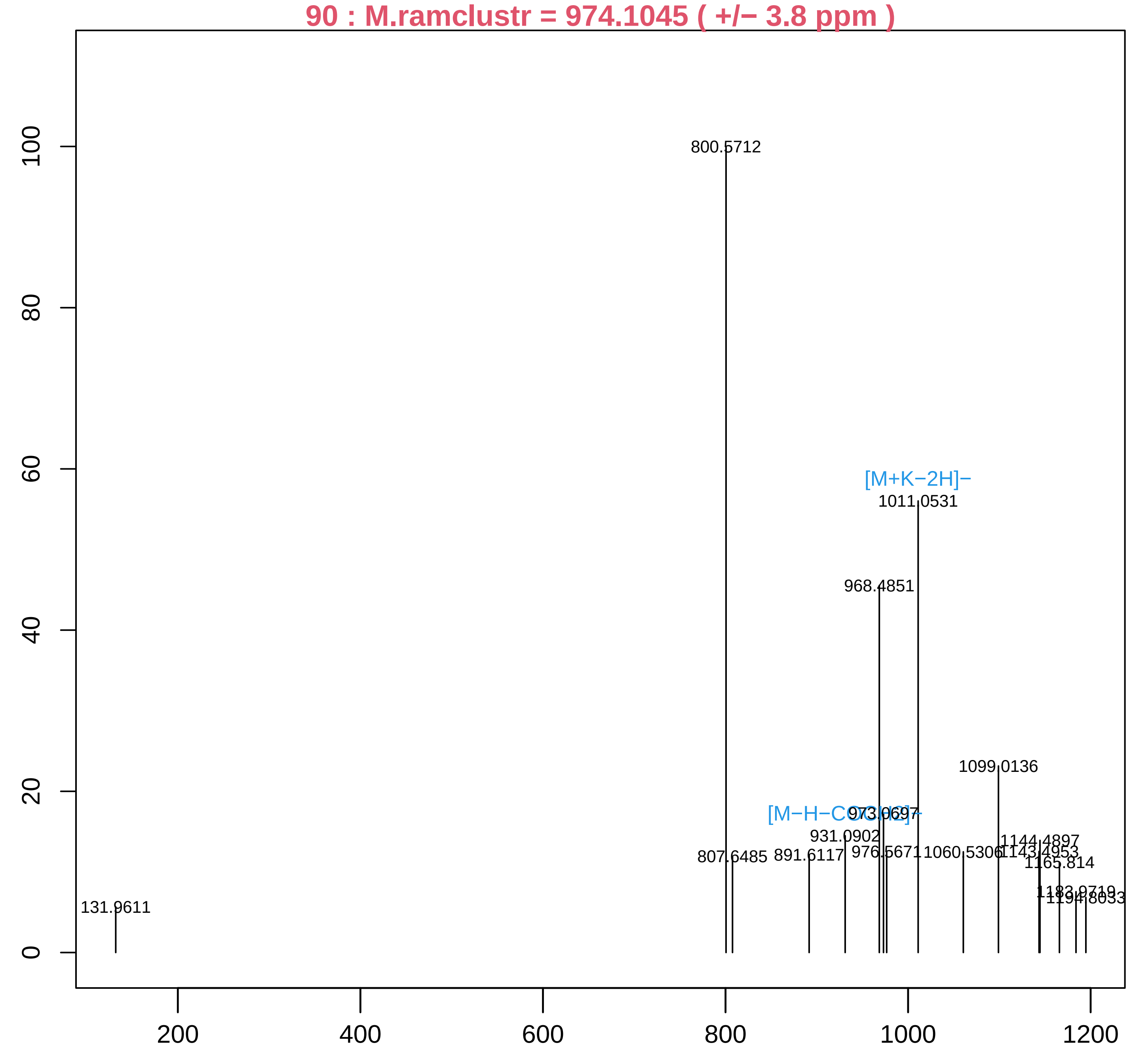

90 : M.ramclustr = 974.1045 (+/− 3.8 ppm)
800.5712
[M+K−2H]−
1011.0531
968.4851
1099.0136
[M−H−C3H6O2]−
973.0697
931.0902
1144.4897
807.6485
891.6117
976.5671
1060.5306
1143.4953
1165.814
1183.9719
1194.8033
131.9611

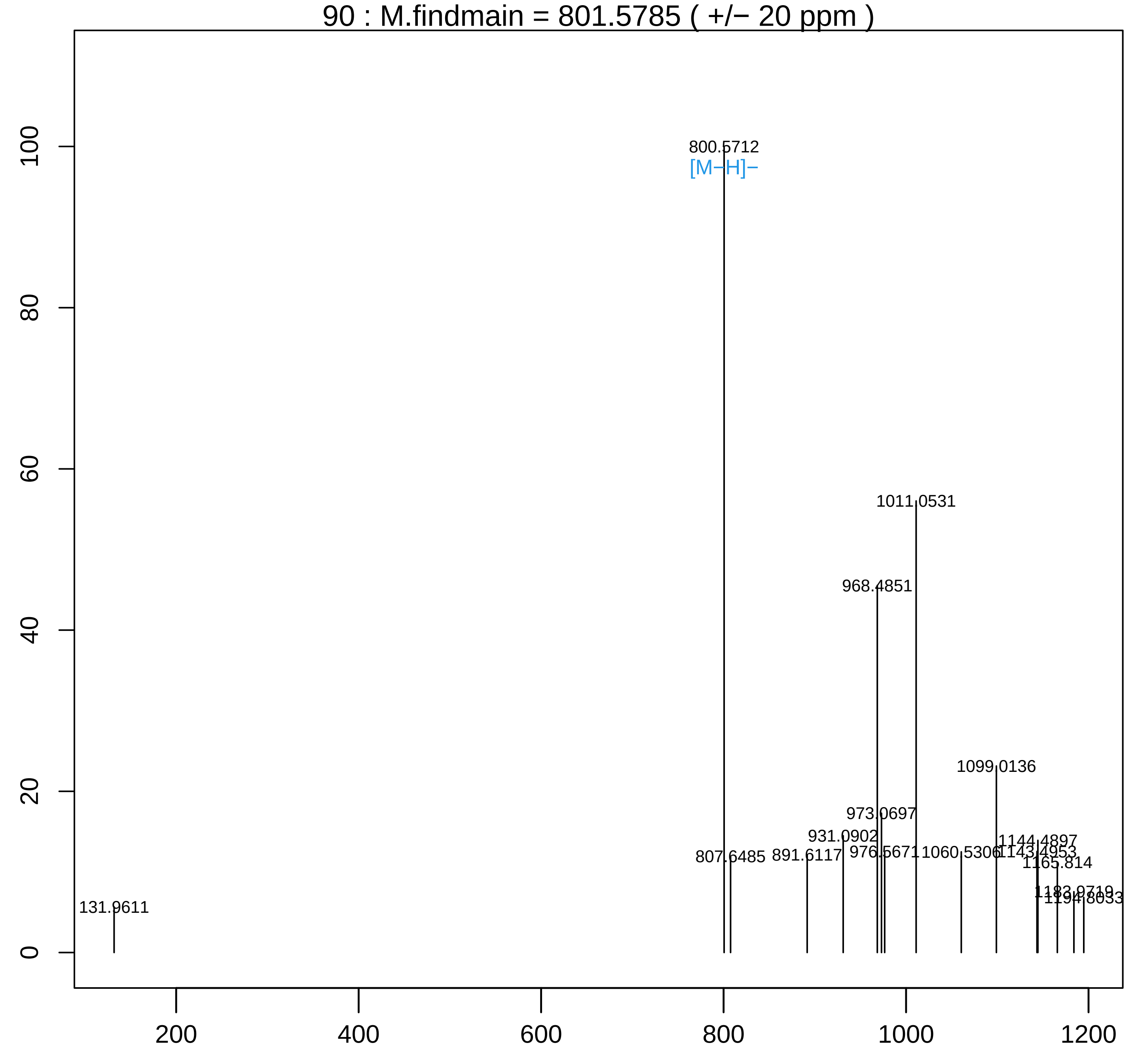

90 : M.findmain = 801.5785 (+/− 20 ppm)
800.5712
[M−H]−
1011.0531
968.4851
1099.0136
973.0697
931.0902
1144.4897
807.6485
891.6117
976.5671
1060.5306
1143.4953
1165.814
1183.9719
1194.8033
131.9611

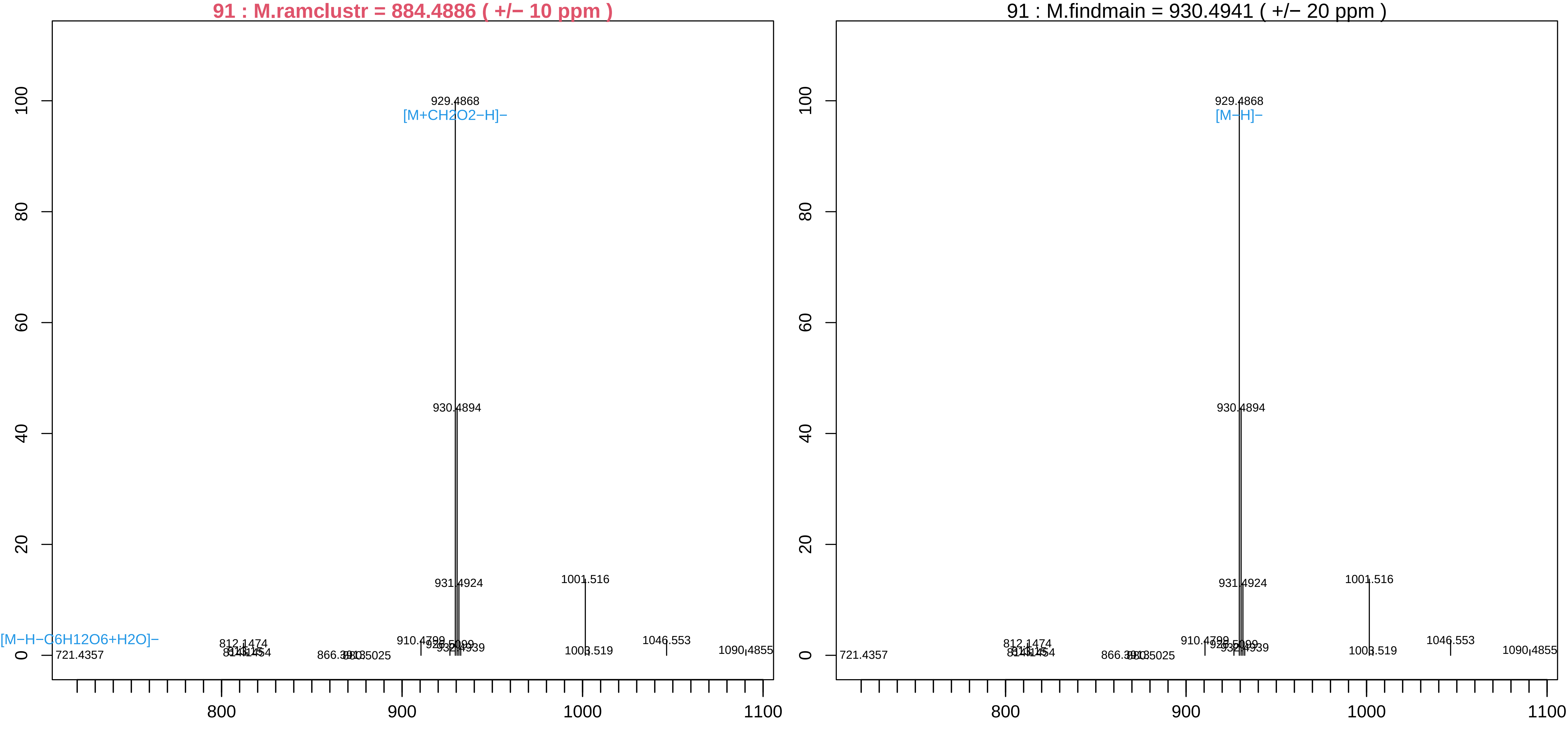
91 : M.ramclustr = 884.4886 (+/− 10 ppm)
929.4868
[M+CH2O2−H]−
930.4894
931.4924
1001.516
[M−H−C6H12O6+H2O]−
721.4357
812.1474
910.4799
1046.553
1003.519
1090.4855
91 : M.findmain = 930.4941 (+/− 20 ppm)
929.4868
[M−H]−
930.4894
931.4924
1001.516
721.4357
812.1474
910.4799
1046.553
1003.519
1090.4855
0
20
40
60
80
100
800
900
1000
1100

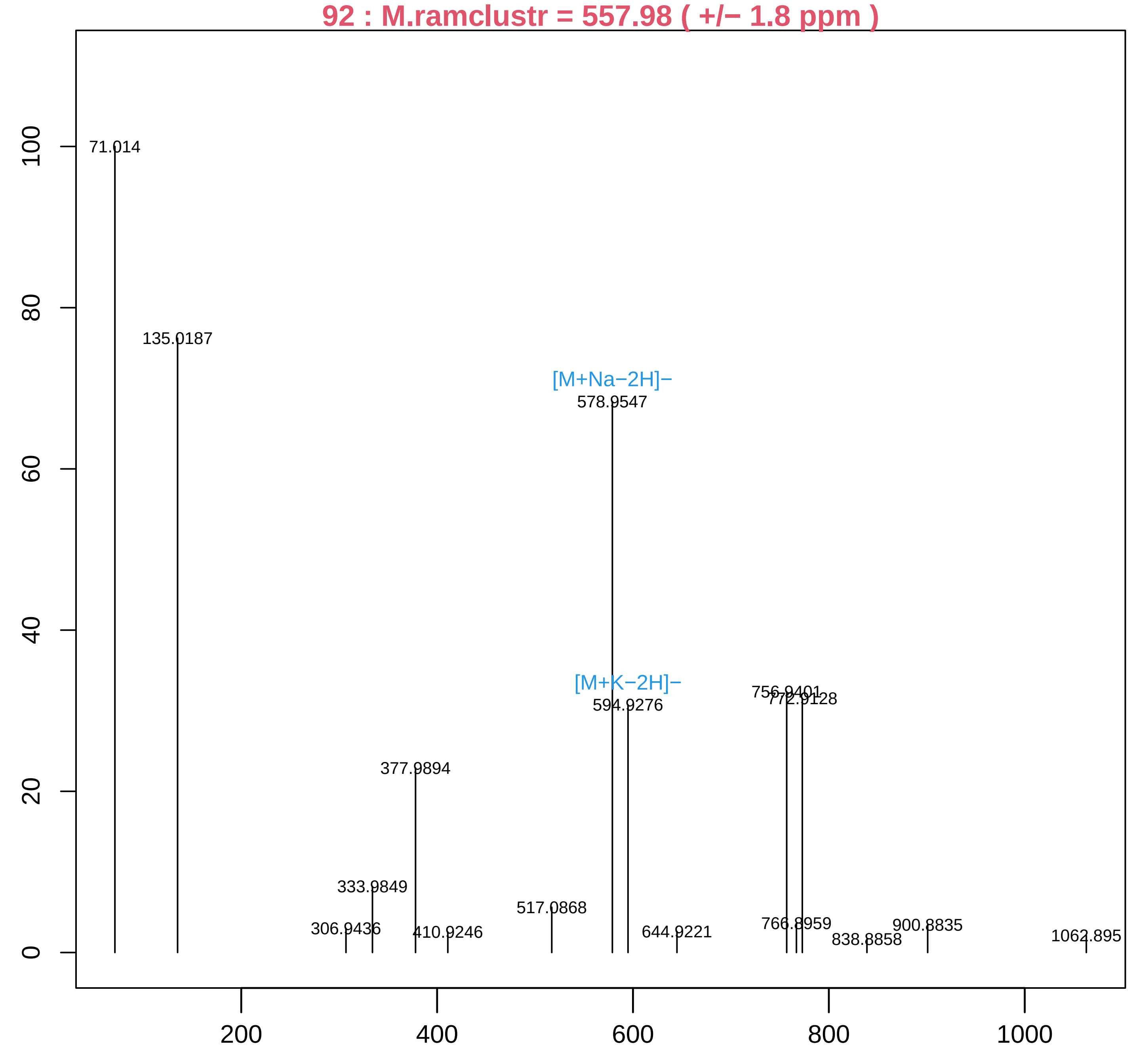

92 : M.ramclustr = 557.98 (+/− 1.8 ppm)
71.014
135.0187
[M+Na−2H]−
578.9547
[M+K−2H]−
594.9276
756.9401
772.9128
377.9894
333.9849
306.9436
410.9246
517.0868
644.9221
766.8959
838.8858
900.8835
1062.895
0
20
40
60
80
100
200
400
600
800
1000

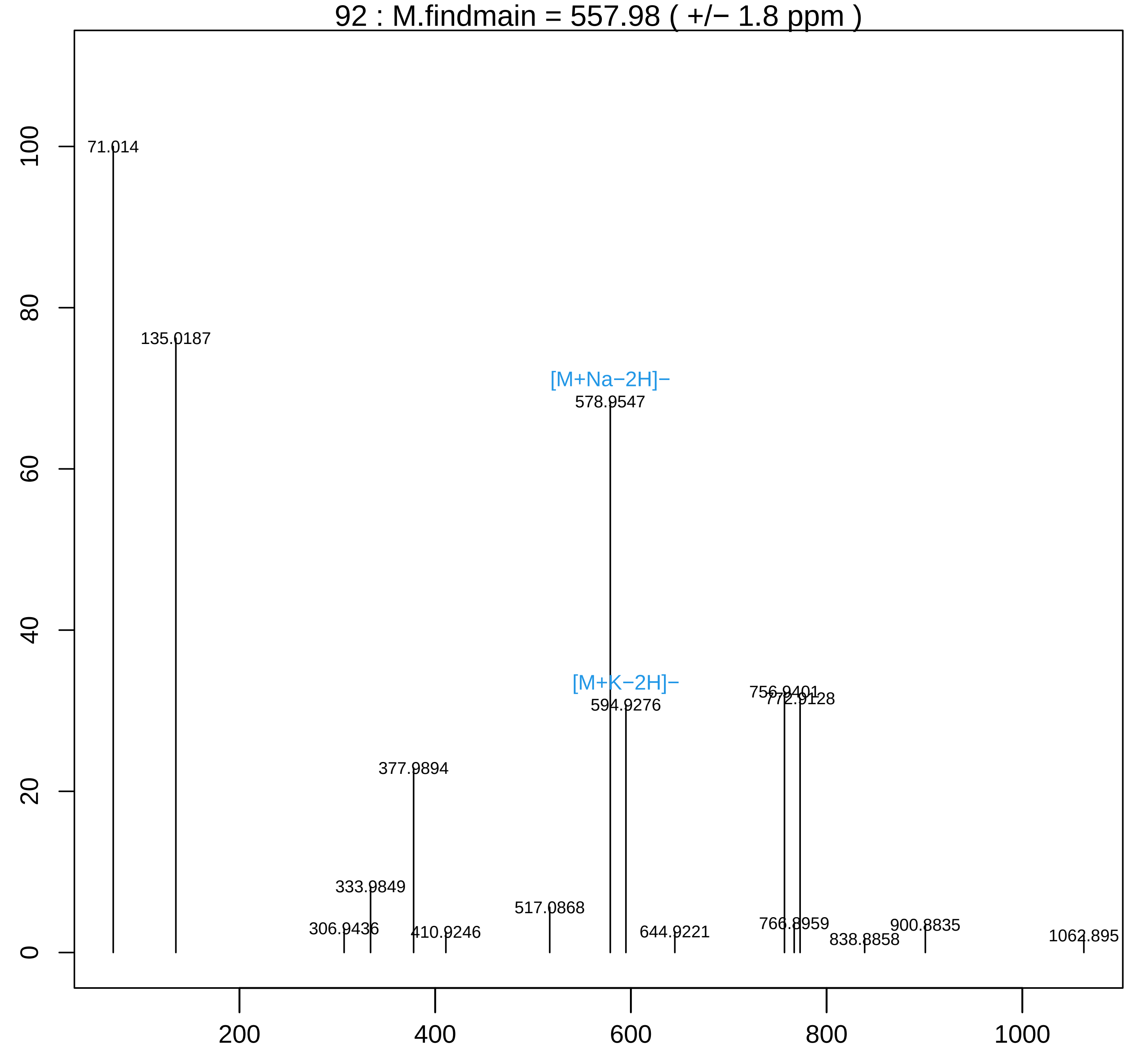

92 : M.findmain = 557.98 (+/− 1.8 ppm)
71.014
135.0187
[M+Na−2H]−
578.9547
[M+K−2H]−
594.9276
756.9401
772.9128
377.9894
333.9849
306.9436
410.9246
517.0868
644.9221
766.8959
838.8858
900.8835
1062.895
0
20
40
60
80
100
200
400
600
800
1000

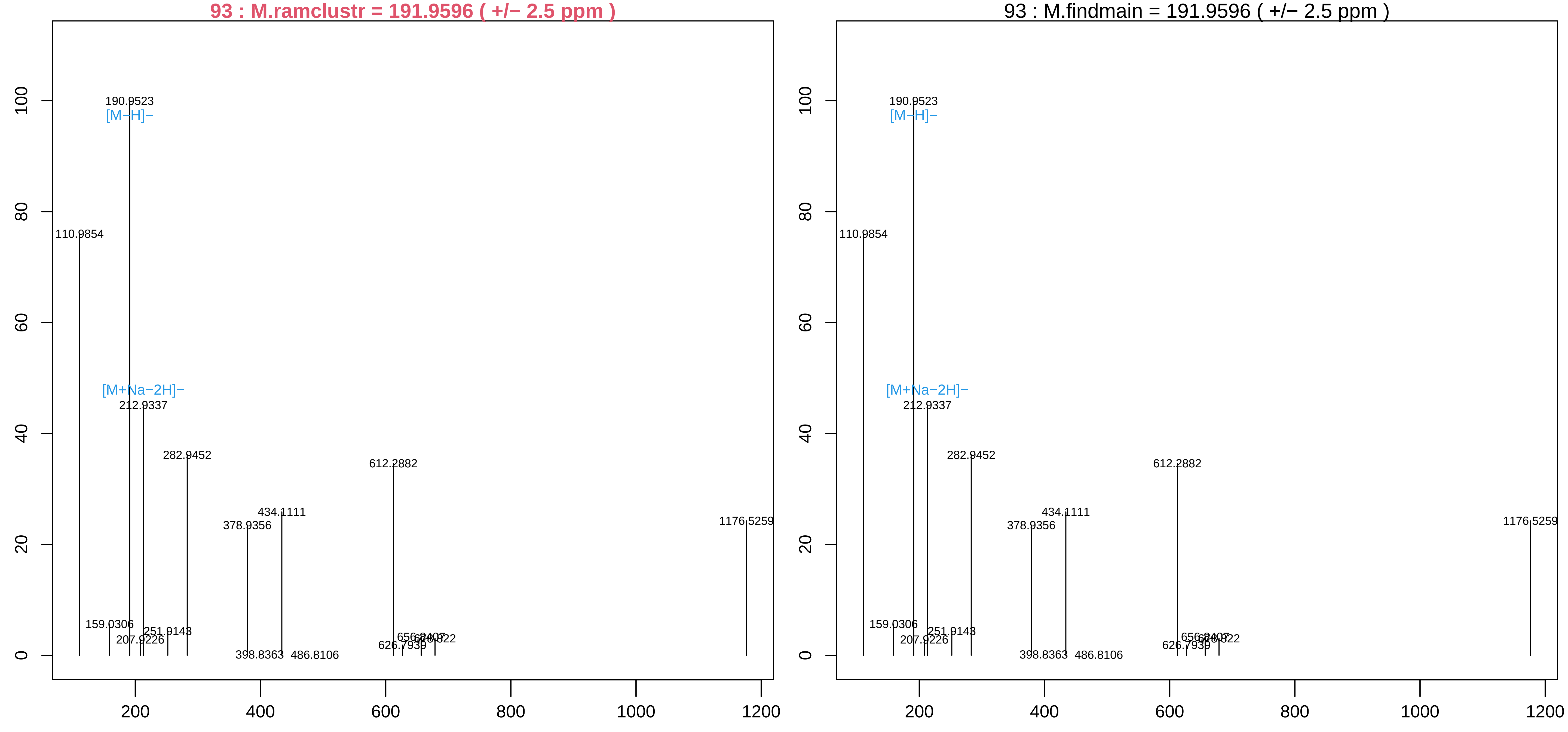
93 : M.ramclustr = 191.9596 (+/− 2.5 ppm)
93 : M.findmain = 191.9596 (+/− 2.5 ppm)
190.9523
[M−H]−
110.9854
[M+Na−2H]−
212.9337
282.9452
612.2882
434.1111
378.9356
1176.5259
159.0306
251.9143
207.9226
656.9407
678.8822
626.7939
398.8363
486.8106
0
20
40
60
80
100
200
400
600
800
1000
1200

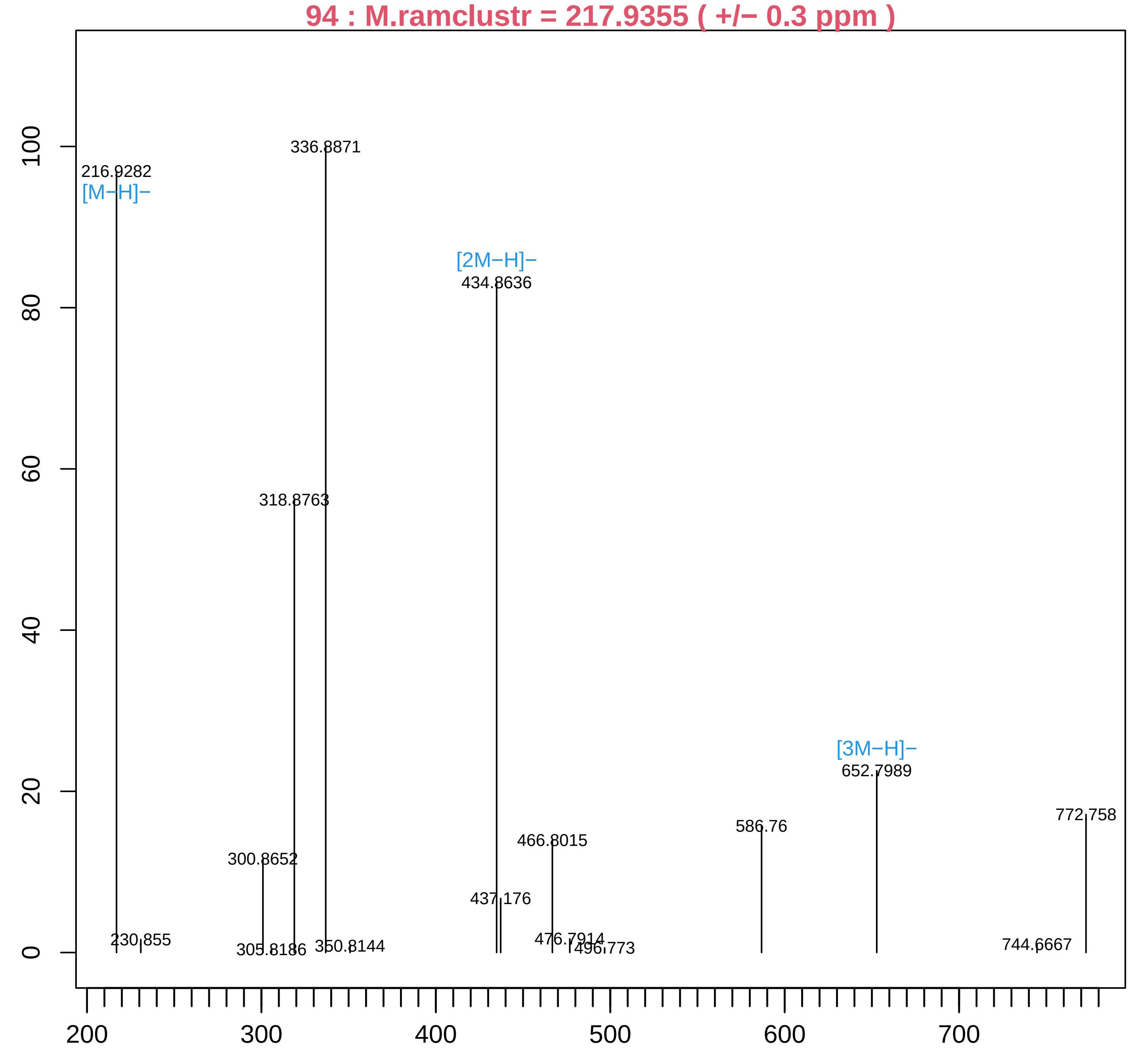

94 : M.ramclustr = 217.9355 (+/− 0.3 ppm)
216.9282
[M−H]−
336.8871
318.8763
[2M−H]−
434.8636
300.8652
230.855
305.8186
350.8144
437.176
466.8015
476.7914
496.773
586.76
[3M−H]−
652.7989
744.6667
772.758
0
20
40
60
80
100
200
300
400
500
600
700

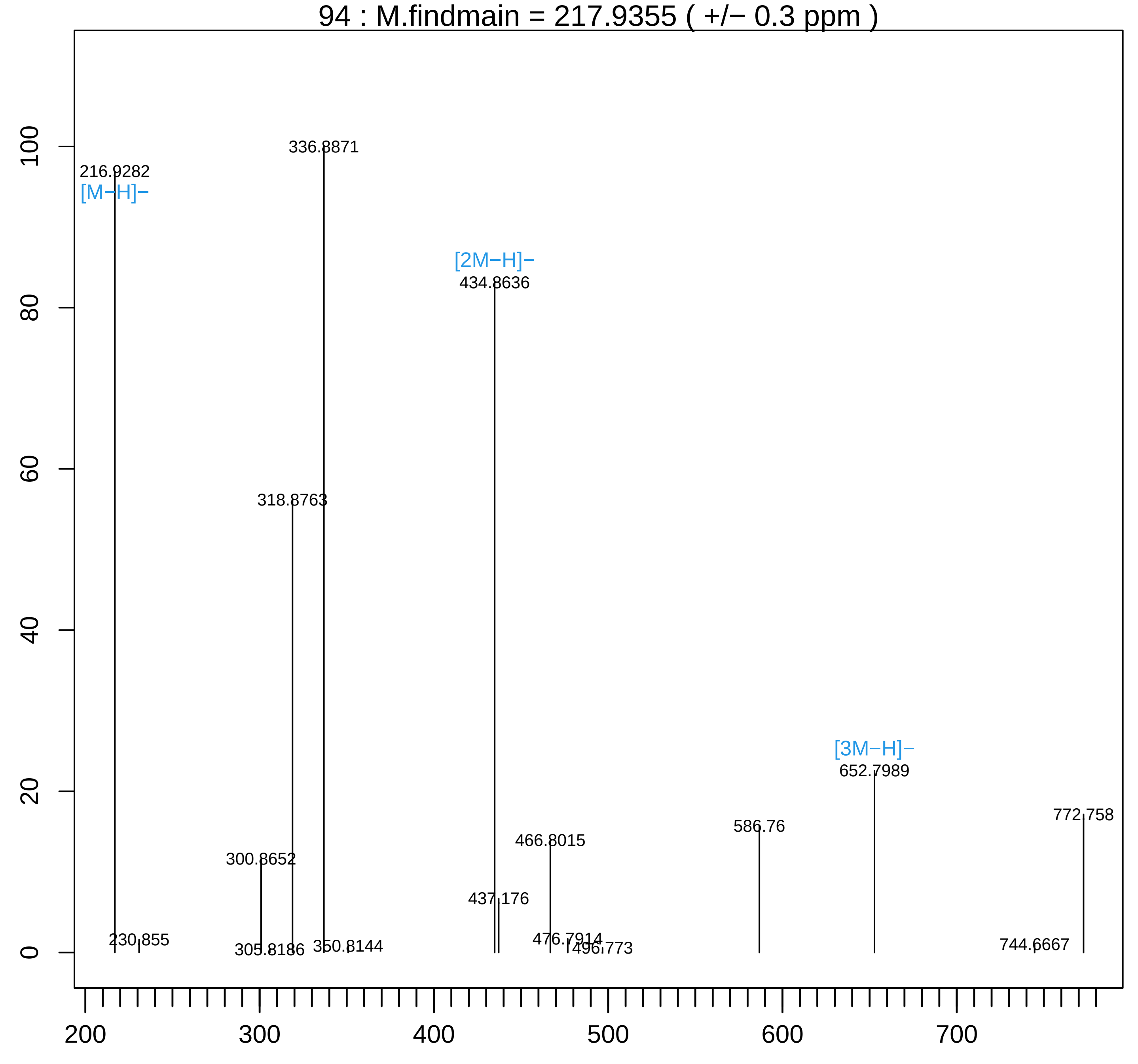

94 : M.findmain = 217.9355 (+/− 0.3 ppm)
216.9282
[M−H]−
336.8871
318.8763
[2M−H]−
434.8636
300.8652
230.855
305.8186
350.8144
437.176
466.8015
476.7914
496.773
586.76
[3M−H]−
652.7989
744.6667
772.758
0
20
40
60
80
100
200
300
400
500
600
700

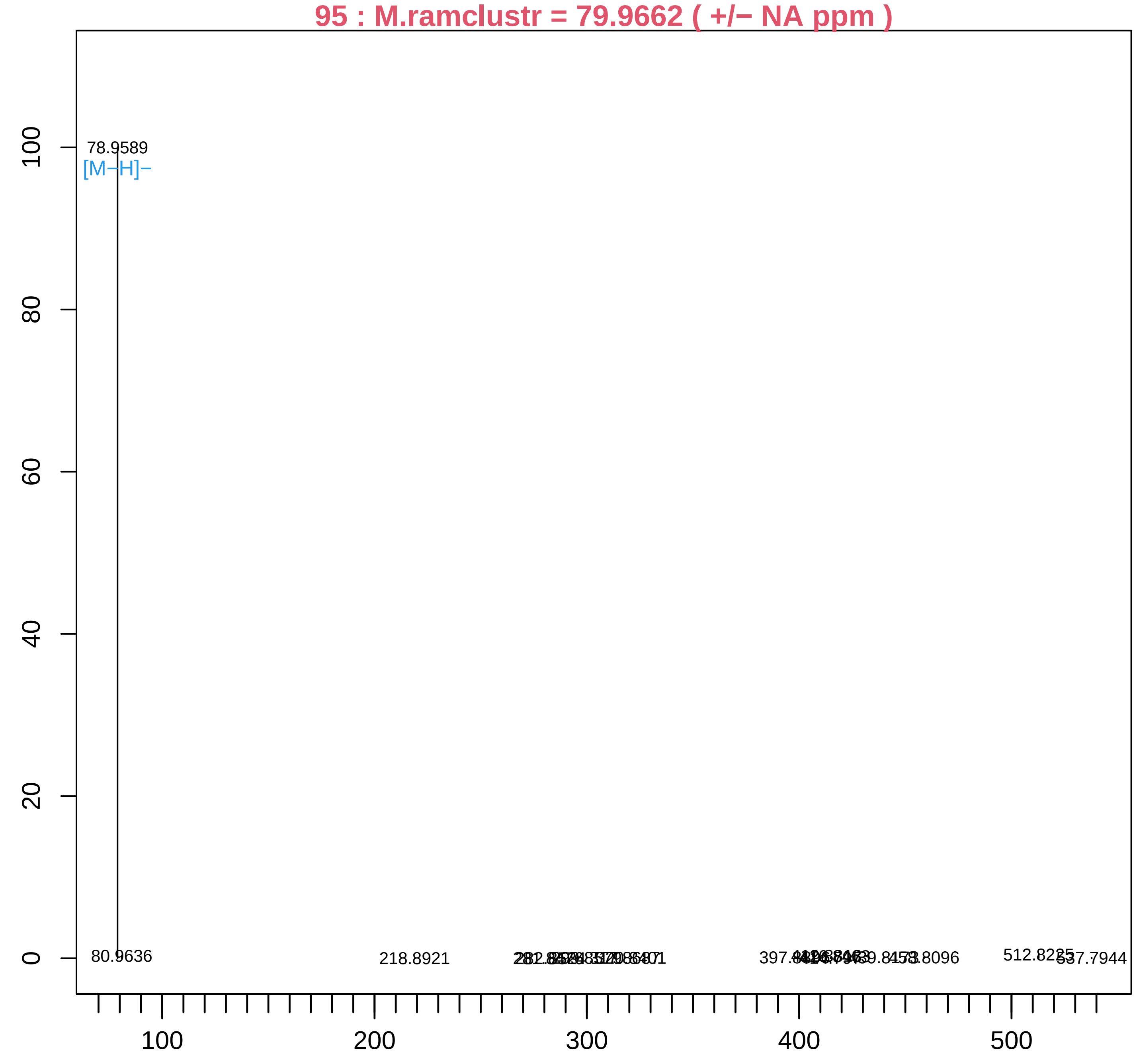
95 : M.ramclustr = 79.9662 (+/− NA ppm)
78.9589
[M−H]−
80.9636
218.8921
397.
453.8096
512.8225
537.7944
0
20
40
60
80
100
100
200
300
400
500

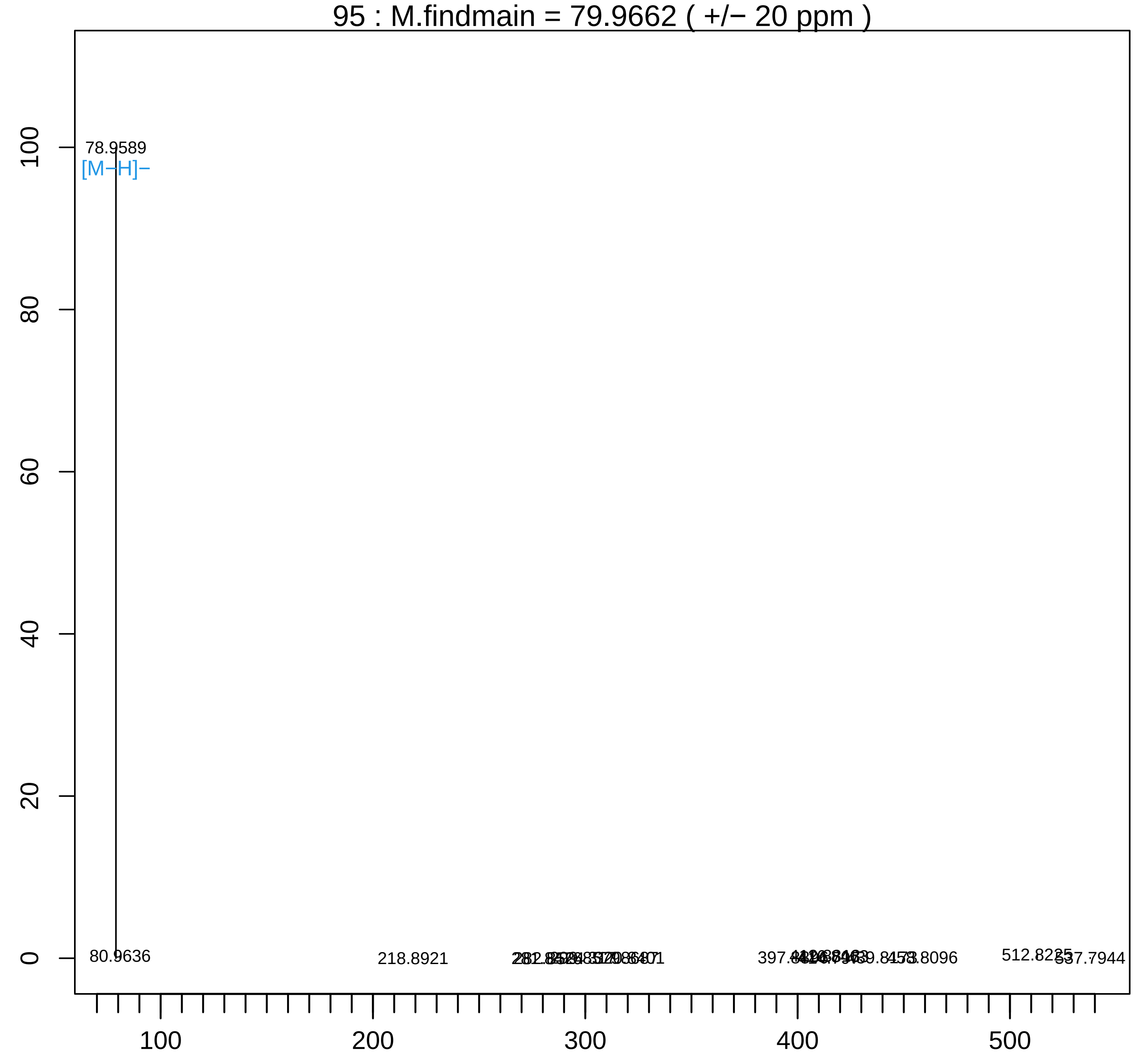
95 : M.findmain = 79.9662 (+/− 20 ppm)
78.9589
[M−H]−
80.9636
218.8921
397.
453.8096
512.8225
537.7944
0
20
40
60
80
100
100
200
300
400
500

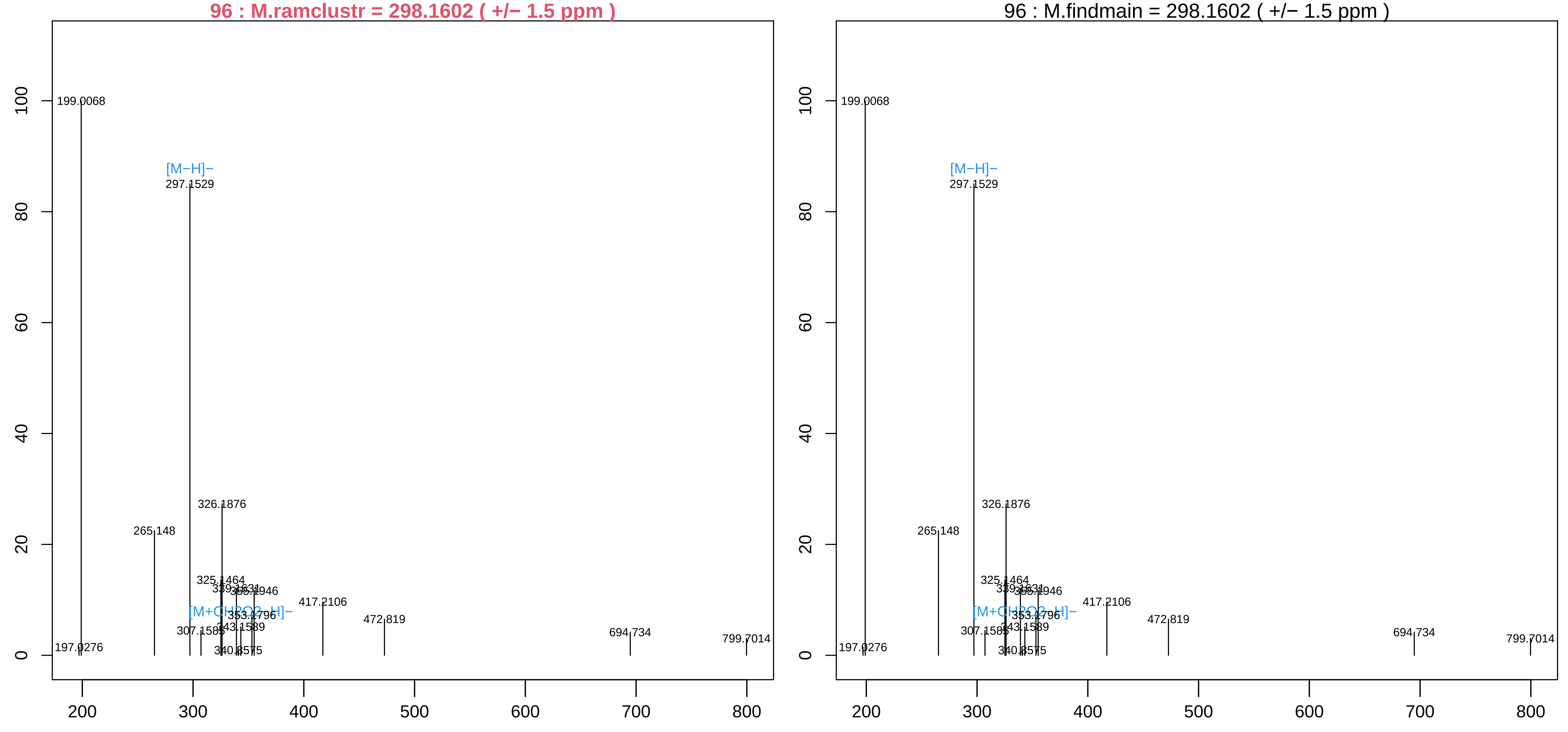

96 : M.ramclustr = 298.1602 (+/− 1.5 ppm)
96 : M.findmain = 298.1602 (+/− 1.5 ppm)
199.0068
[M−H]−
297.1529
326.1876
265.148
325.1464
339.1631
355.1946
[M+CH2O2−H]−
353.1796
417.2106
472.819
307.1585
343.1589
197.0276
340.8575
694.734
799.7014
0
20
40
60
80
100
200
300
400
500
600
700
800

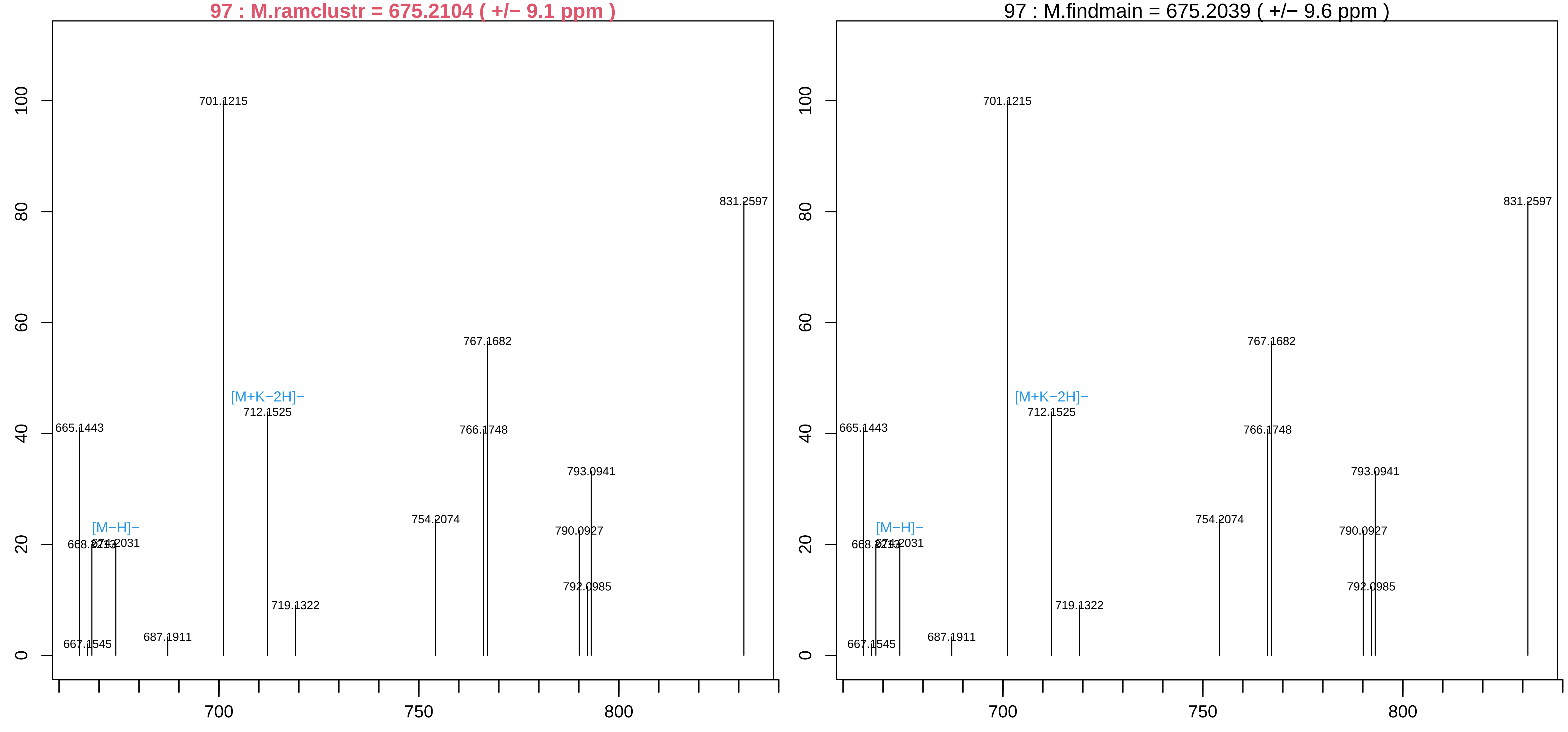

97 : M.ramclustr = 675.2104 (+/− 9.1 ppm)
97 : M.findmain = 675.2039 (+/− 9.6 ppm)
701.1215
831.2597
767.1682
[M+K−2H]−
712.1525
665.1443
766.1748
793.0941
[M−H]−
668.2213
671.2031
754.2074
790.0927
792.0985
719.1322
687.1911
667.1545
0
20
40
60
80
100
700
750
800

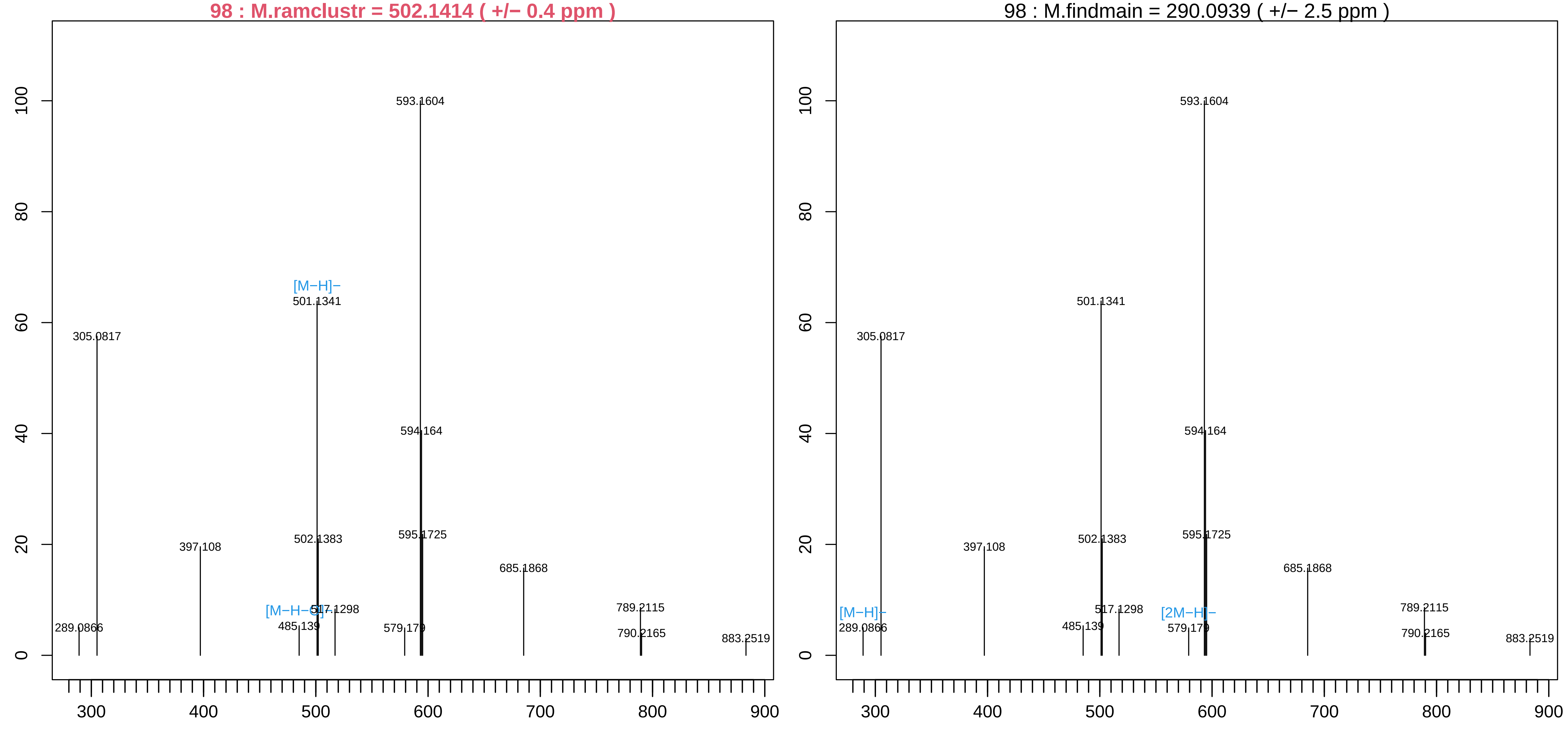
98 : M.ramclustr = 502.1414 (+/− 0.4 ppm)
[M−H]−
501.1341
593.1604
305.0817
594.164
397.108
502.1383
595.1725
685.1868
[M−H−O]−
517.1298
789.2115
289.0866
485.139
579.179
790.2165
883.2519
98 : M.findmain = 290.0939 (+/− 2.5 ppm)
[M−H]−
[2M−H]−
0
20
40
60
80
100
300
400
500
600
700
800
900

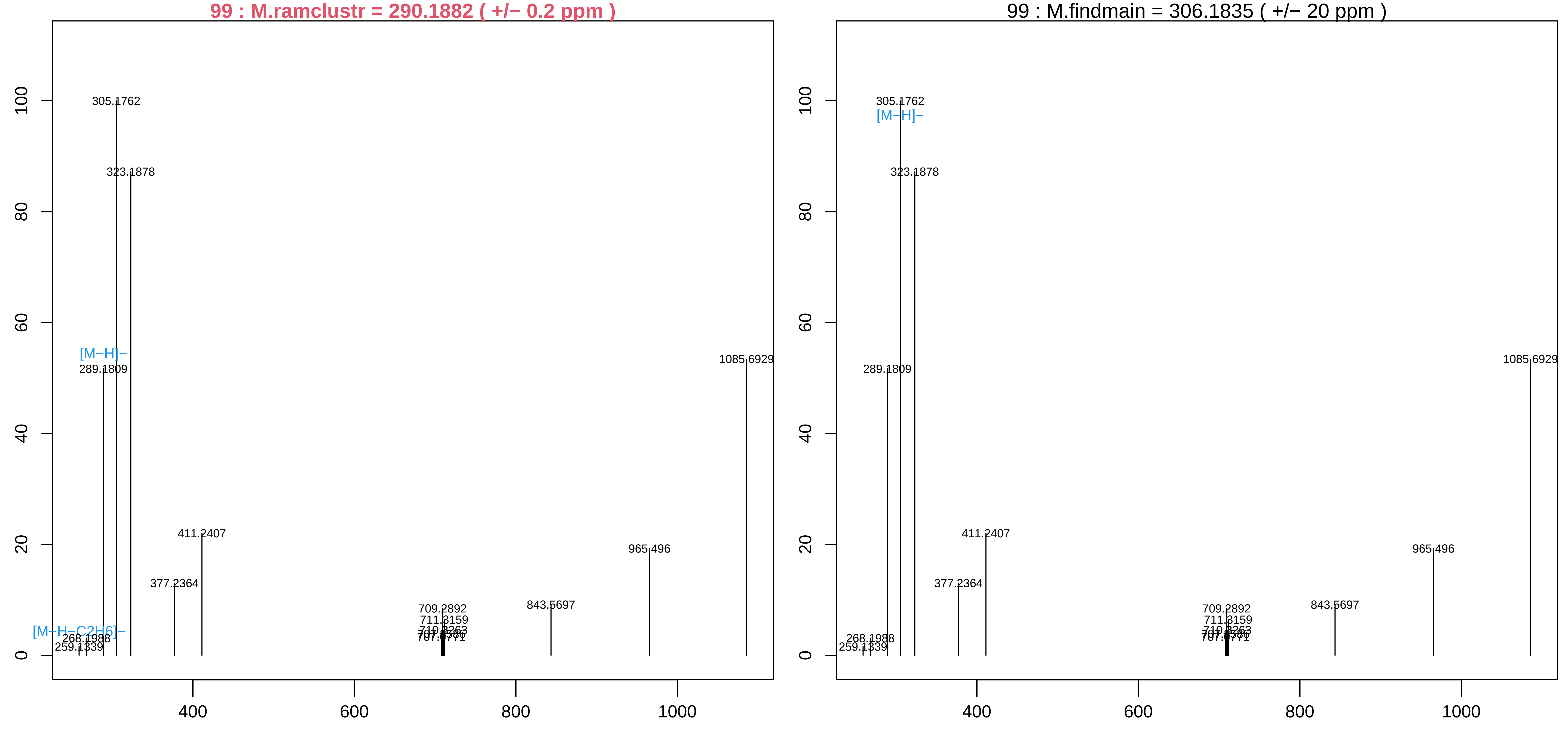
99 : M.ramclustr = 290.1882 (+/− 0.2 ppm)
305.1762
323.1878
[M−H]−
289.1809
411.2407
377.2364
709.2892
711.3159
843.5697
965.496
1085.6929
[M−H−C2H6]−
268.1988
259.1339
99 : M.findmain = 306.1835 (+/− 20 ppm)
305.1762
[M−H]−
323.1878
289.1809
411.2407
377.2364
709.2892
711.3159
843.5697
965.496
1085.6929
268.1988
259.1339
0
20
40
60
80
100
400
600
800
1000

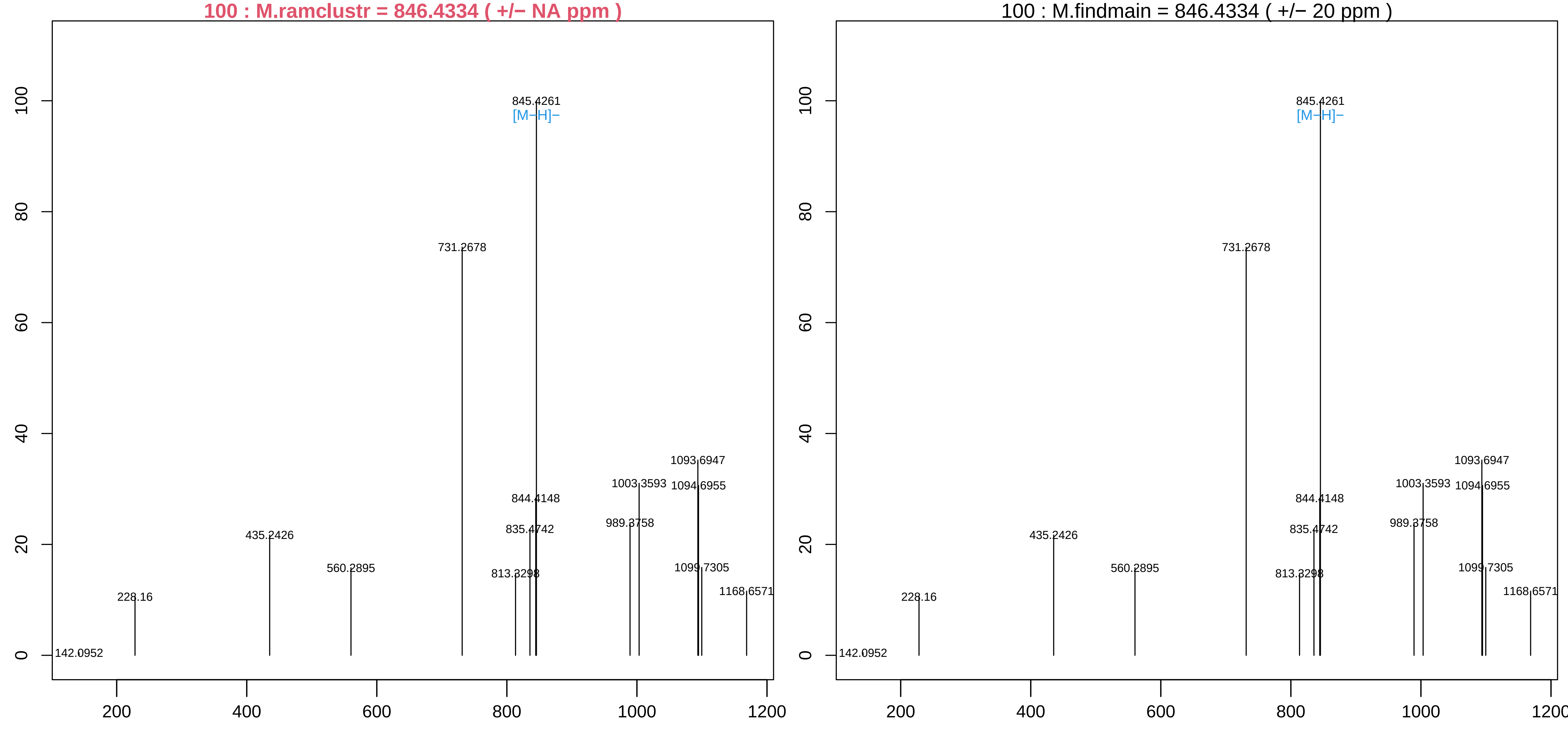
100 : M.ramclustr = 846.4334 (+/− NA ppm)
845.4261
[M−H]−
731.2678
1093.6947
1003.3593
1094.6955
844.4148
989.3758
435.2426
835.4742
560.2895
1099.7305
813.3298
1168.6571
228.16
142.0952
100 : M.findmain = 846.4334 (+/− 20 ppm)
845.4261
[M−H]−
731.2678
1093.6947
1003.3593
1094.6955
844.4148
989.3758
435.2426
835.4742
560.2895
1099.7305
813.3298
1168.6571
228.16
142.0952

101 : M.ramclustr = 903.5909 (+/− 3.7 ppm)

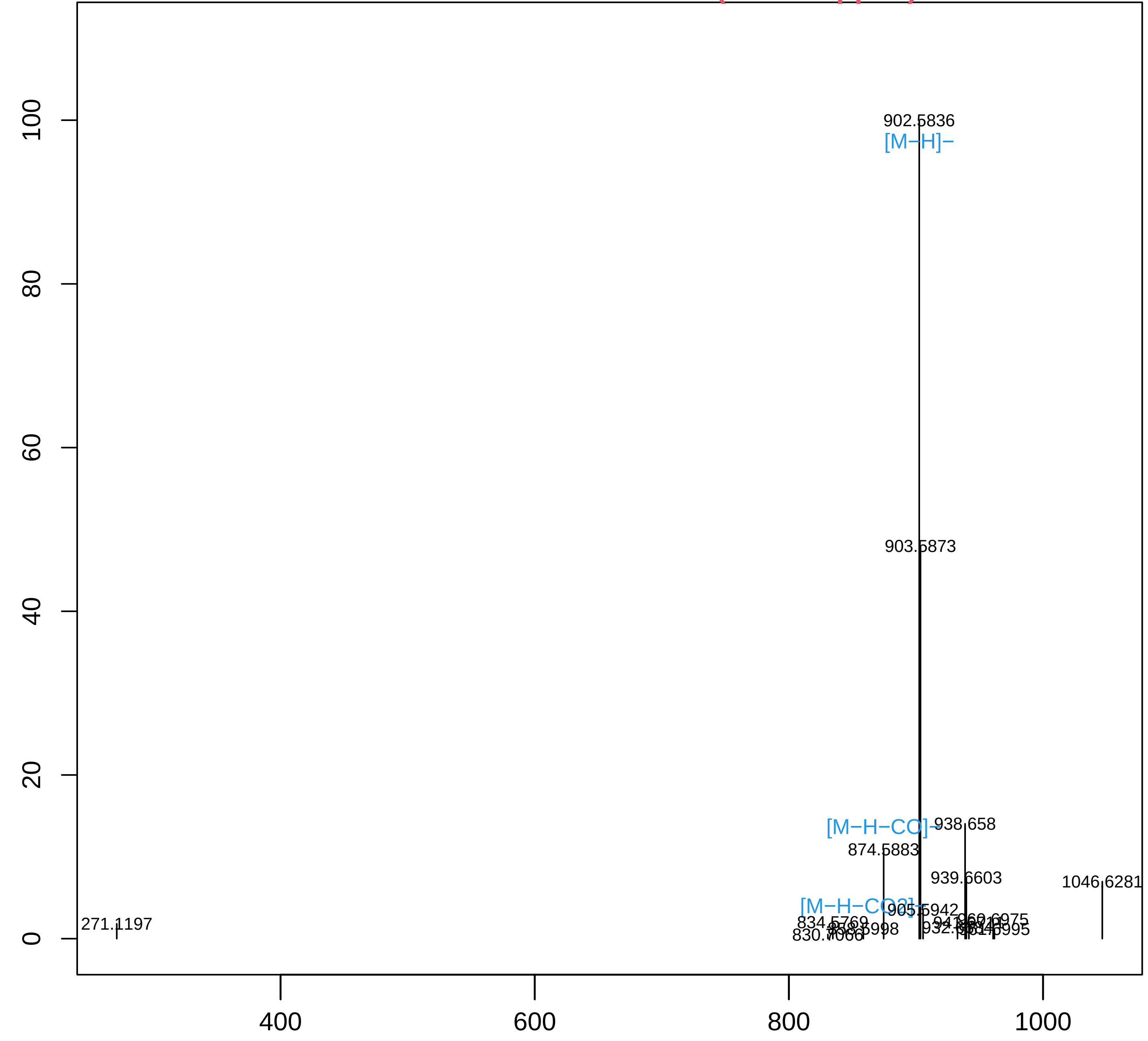

101 : M.findmain = 903.5909 (+/− 20 ppm)

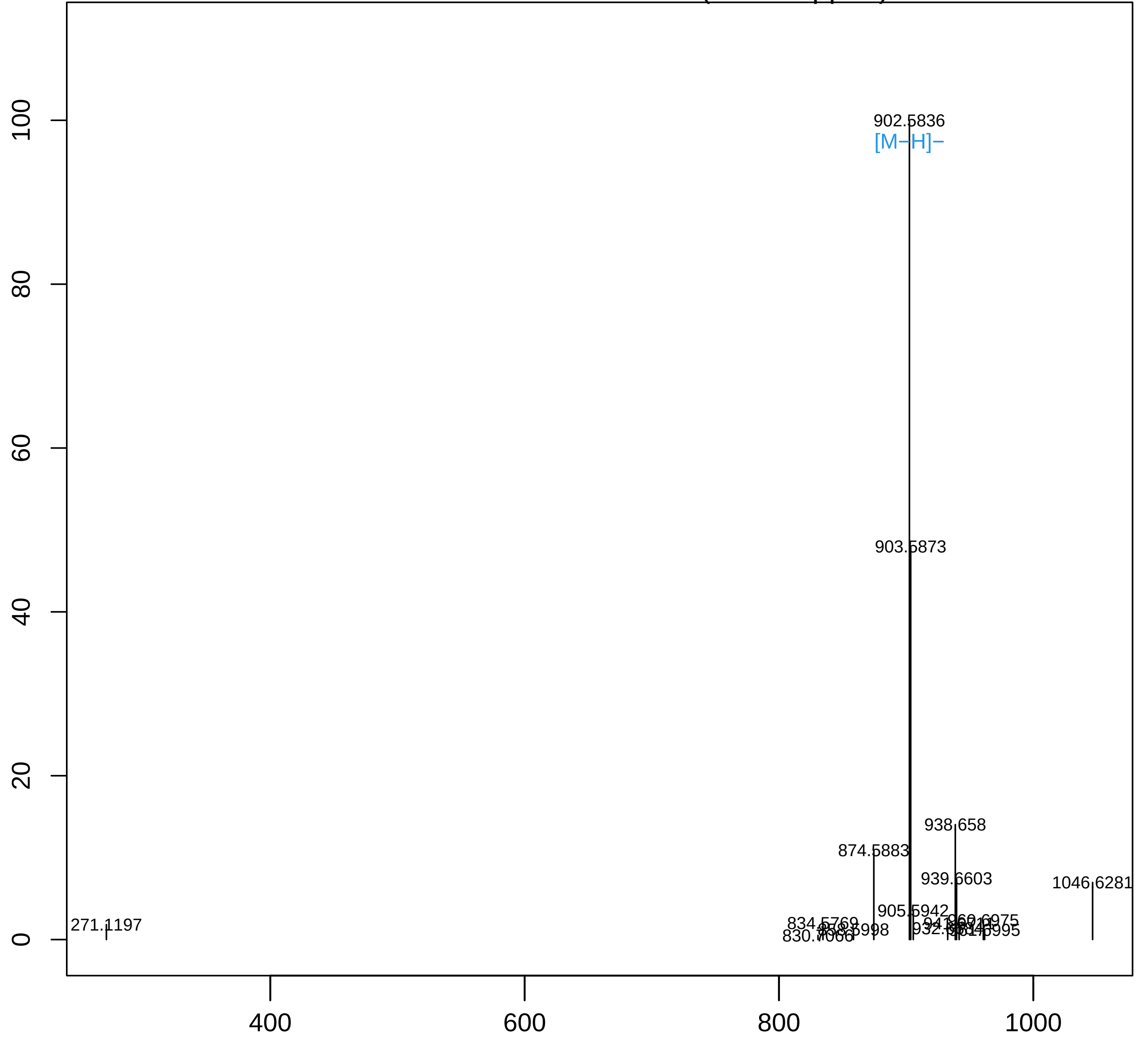

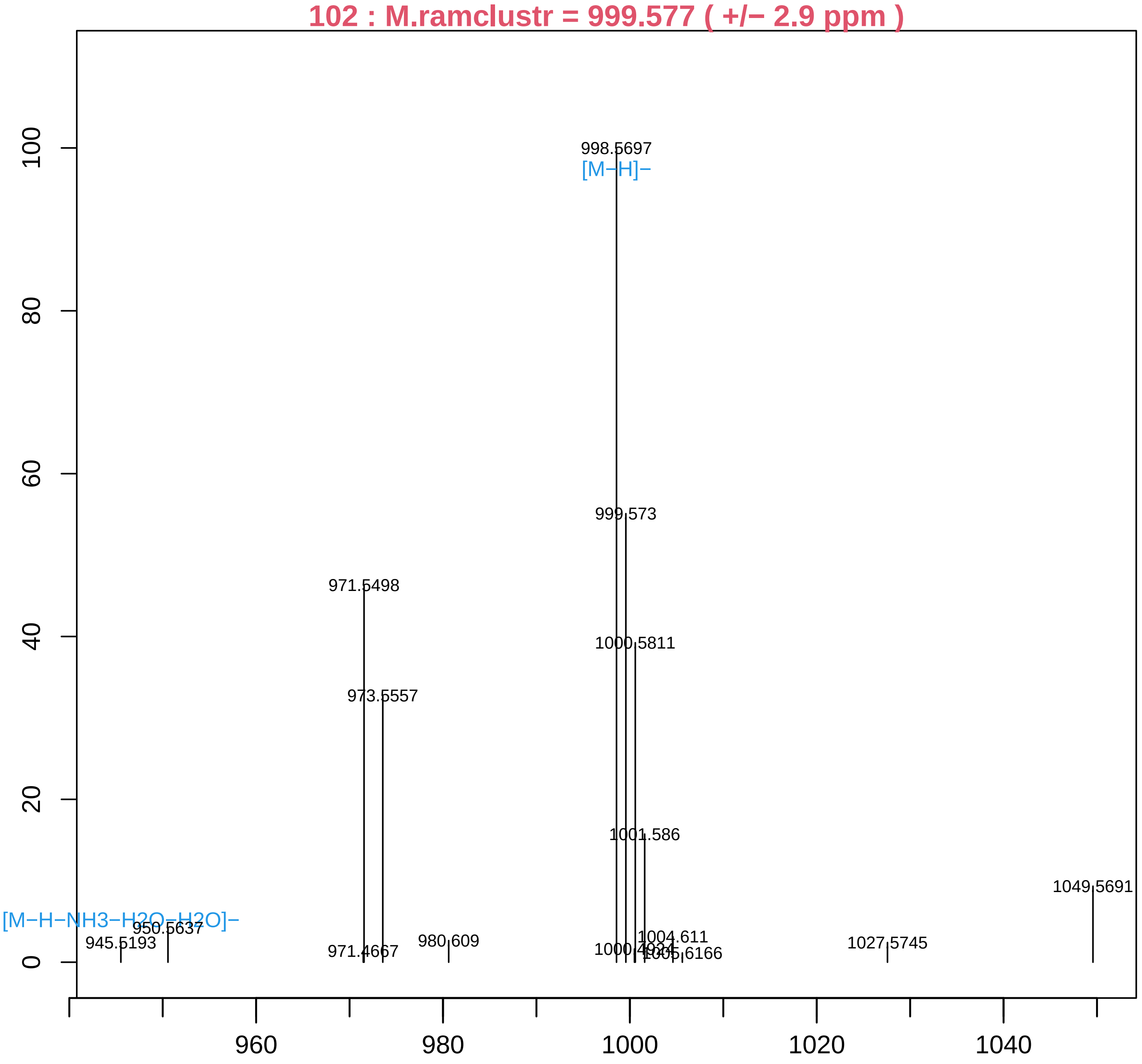
102 : M.ramclustr = 999.577 (+/− 2.9 ppm)
998.5697
[M−H]−
999.573
971.5498
1000.5811
973.5557
1001.586
1049.5691
[M−H−NH3−H2O−H2O]−
950.5637
945.5193
971.4667
980.609
1000.4924
1004.611
1005.6166
1027.5745
0
20
40
60
80
100
960
980
1000
1020
1040

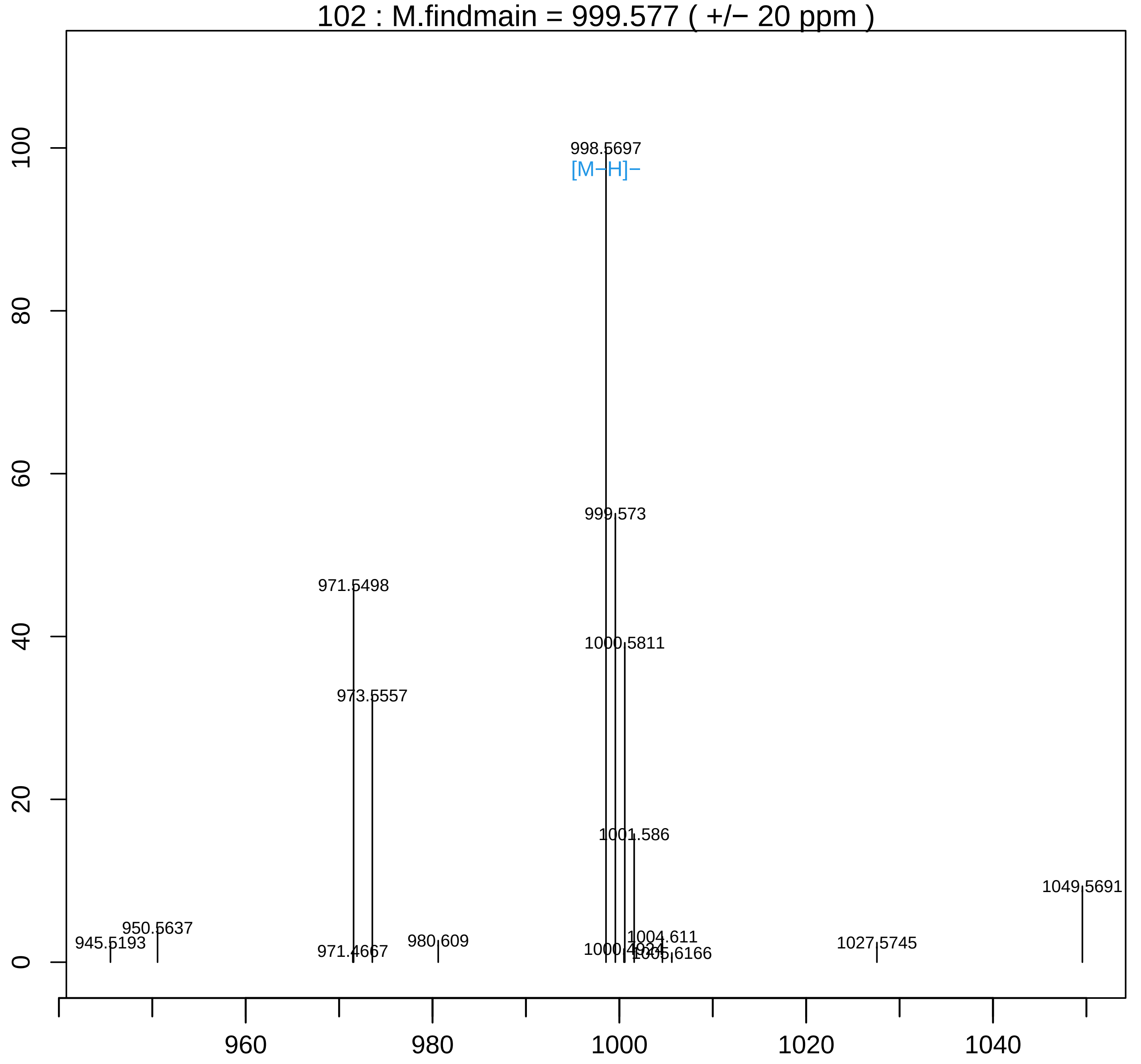
102 : M.findmain = 999.577 (+/− 20 ppm)
998.5697
[M−H]−
999.573
971.5498
1000.5811
973.5557
1001.586
1049.5691
950.5637
945.5193
971.4667
980.609
1000.4924
1004.611
1005.6166
1027.5745
0
20
40
60
80
100
960
980
1000
1020
1040

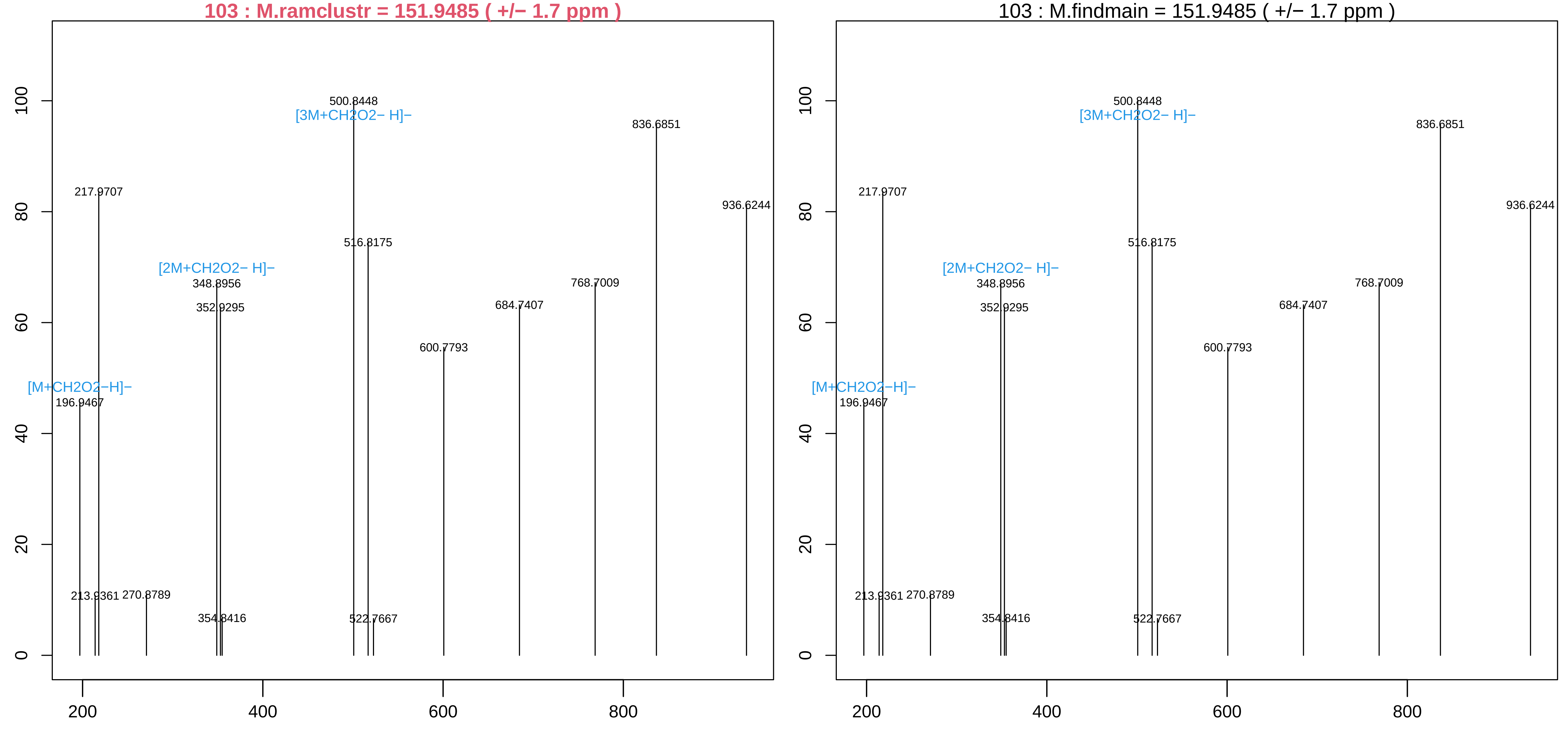

103 : M.ramclustr = 151.9485 (+/− 1.7 ppm)
103 : M.findmain = 151.9485 (+/− 1.7 ppm)
[M+CH2O2−H]−
[2M+CH2O2− H]−
[3M+CH2O2− H]−
196.9467
213.9361
217.9707
270.8789
348.8956
352.9295
354.8416
500.8448
516.8175
522.7667
600.7793
684.7407
768.7009
836.6851
936.6244
0
20
40
60
80
100
200
400
600
800

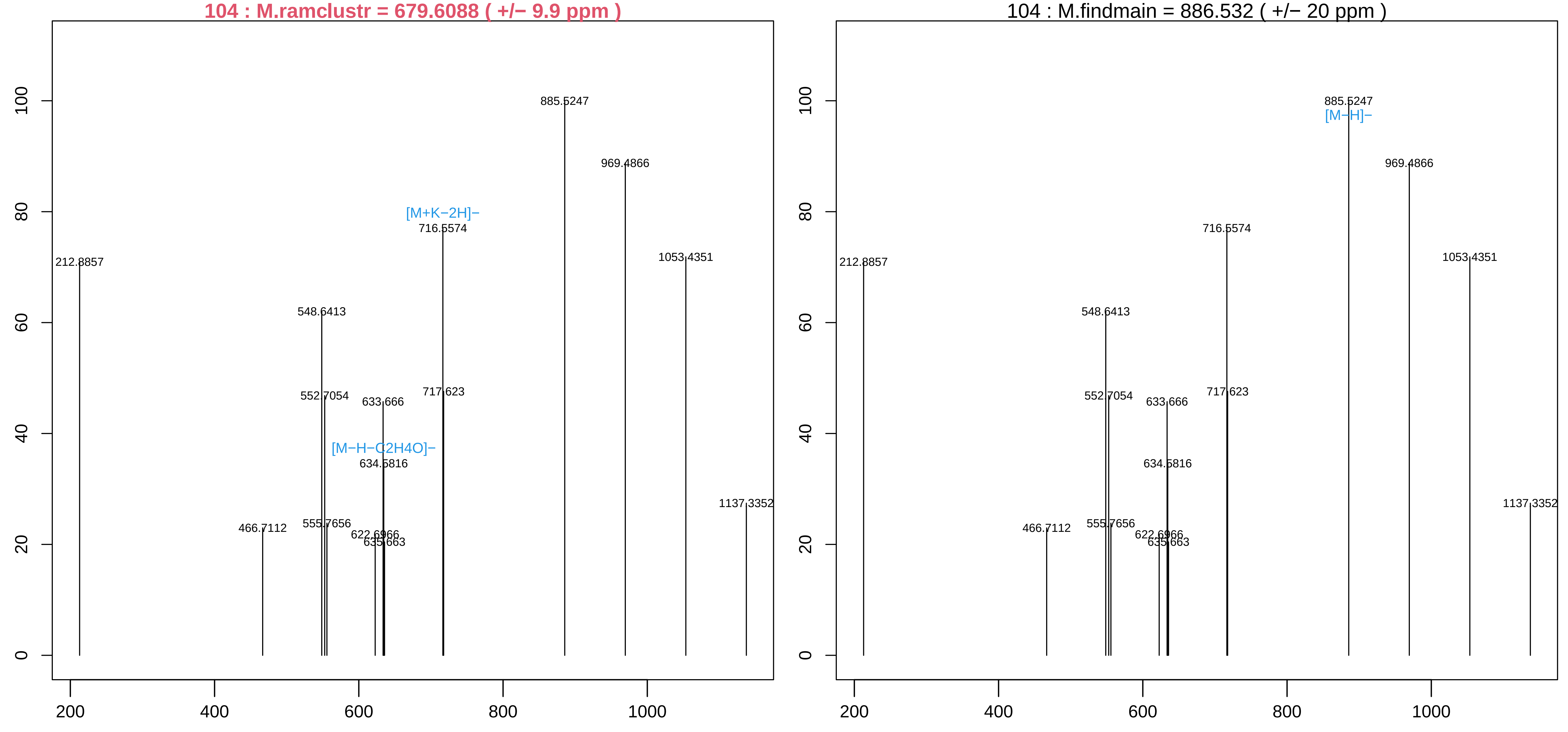
104 : M.ramclustr = 679.6088 (+/− 9.9 ppm)
[M+K−2H]−
[M−H−C2H4O]−
212.8857
466.7112
548.6413
552.7054
555.7656
622.6966
633.666
634.5816
635.663
716.5574
717.623
885.5247
969.4866
1053.4351
1137.3352
104 : M.findmain = 886.532 (+/− 20 ppm)
[M−H]−
0
20
40
60
80
100
200
400
600
800
1000

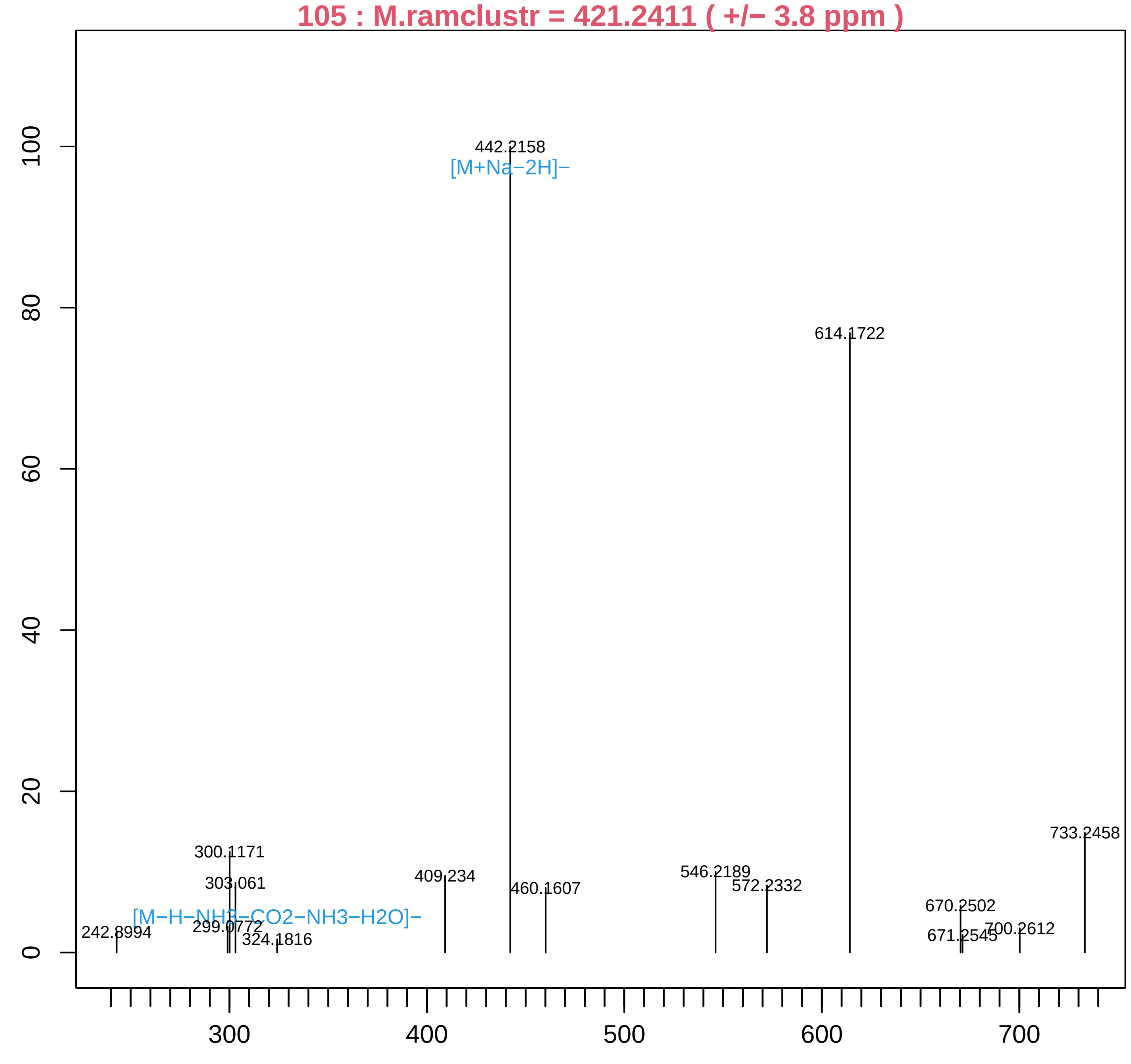

105 : M.ramclustr = 421.2411 (+/− 3.8 ppm)
442.2158
[M+Na−2H]−
614.1722
300.1171
303.061
[M−H−NH3−CO2−NH3−H2O]−
242.8994
299.0772
324.1816
409.234
460.1607
546.2189
572.2332
670.2502
671.2545
700.2612
733.2458
0
20
40
60
80
100
300
400
500
600
700

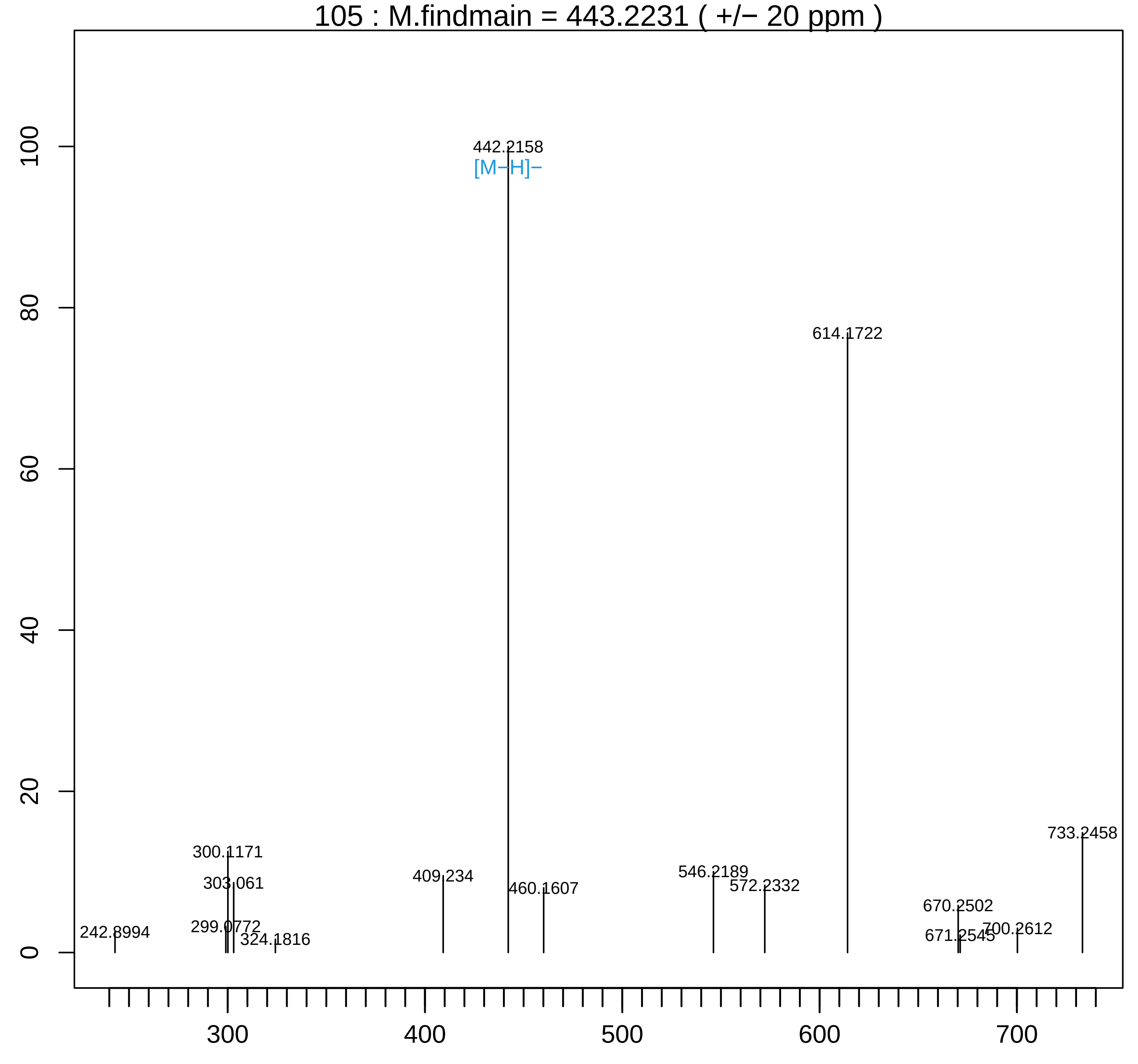

105 : M.findmain = 443.2231 (+/− 20 ppm)
442.2158
[M−H]−
614.1722
300.1171
303.061
242.8994
299.0772
324.1816
409.234
460.1607
546.2189
572.2332
670.2502
671.2545
700.2612
733.2458
0
20
40
60
80
100
300
400
500
600
700

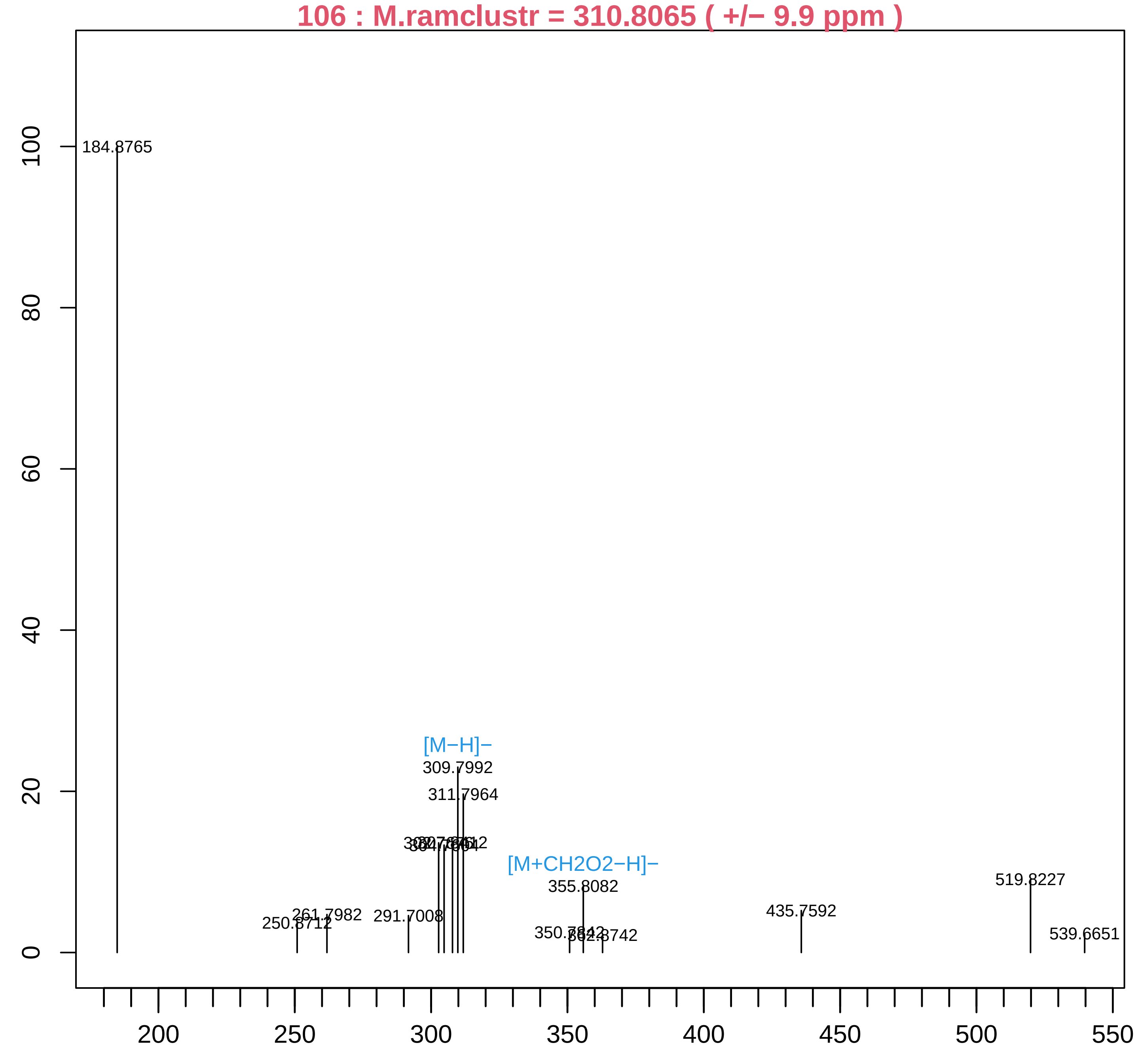

106 : M.ramclustr = 310.8065 (+/− 9.9 ppm)
184.8765
[M−H]−
309.7992
311.7964
[M+CH2O2−H]−
355.8082
519.8227
435.7592
261.7982
291.7008
250.8712
539.6651
0
20
40
60
80
100
200
250
300
350
400
450
500
550

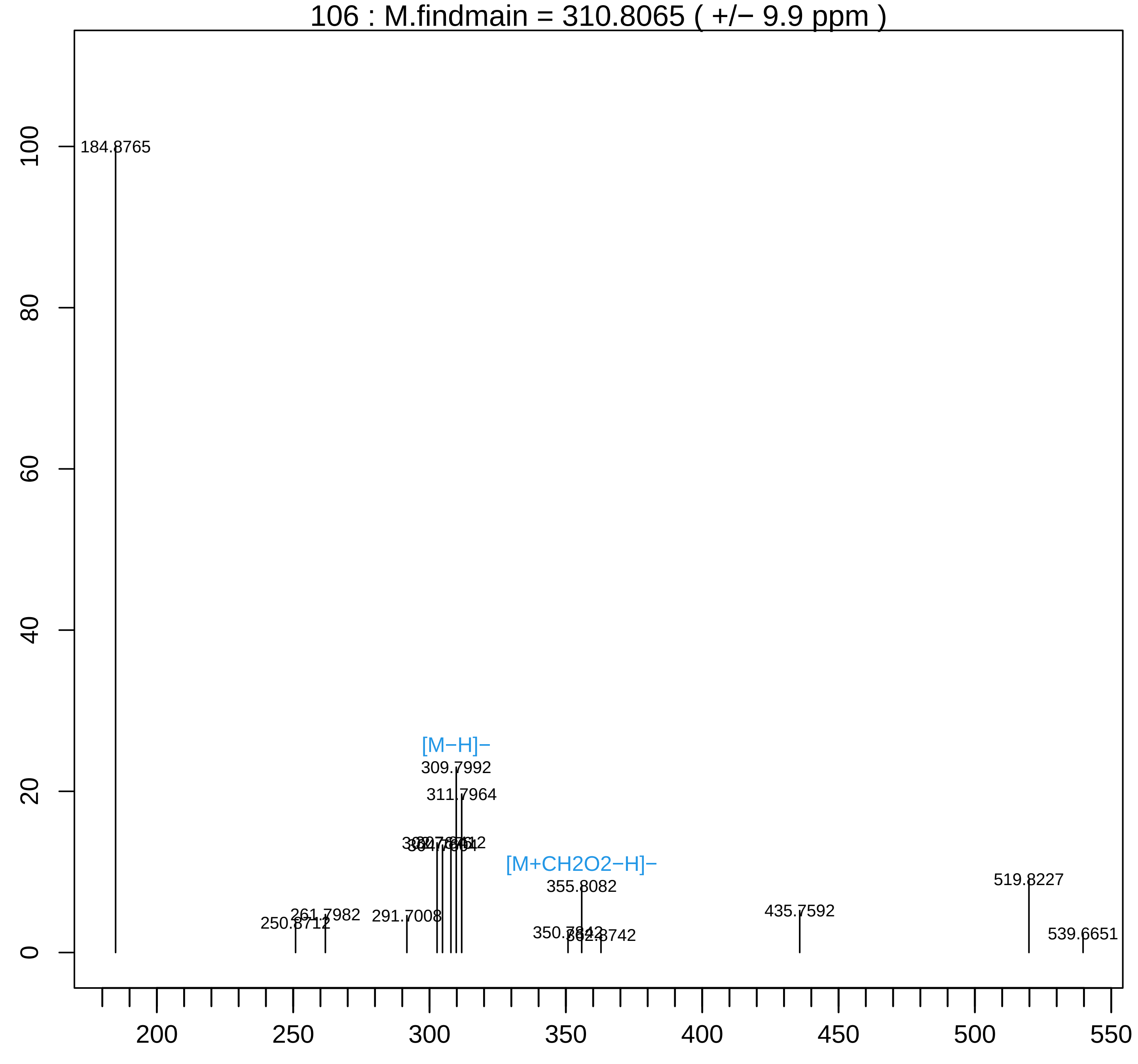

106 : M.findmain = 310.8065 (+/− 9.9 ppm)
184.8765
[M−H]−
309.7992
311.7964
[M+CH2O2−H]−
355.8082
519.8227
435.7592
261.7982
291.7008
250.8712
539.6651
0
20
40
60
80
100
200
250
300
350
400
450
500
550

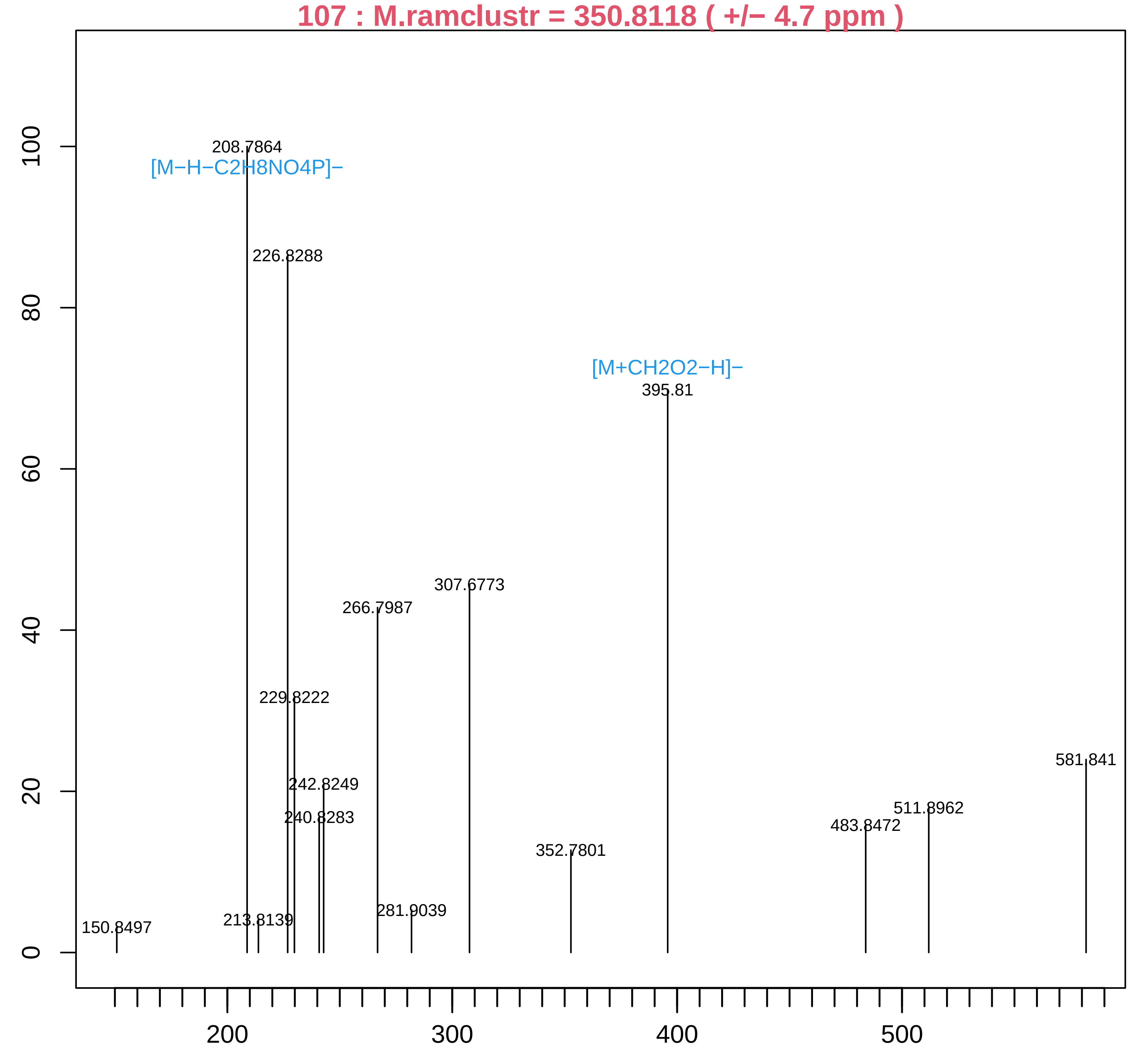

107 : M.ramclustr = 350.8118 (+/− 4.7 ppm)
208.7864
[M−H−C2H8NO4P]−
226.8288
[M+CH2O2−H]−
395.81
307.6773
266.7987
229.8222
242.8249
240.8283
511.8962
483.8472
581.841
352.7801
281.9039
213.8139
150.8497
0
20
40
60
80
100
200
300
400
500

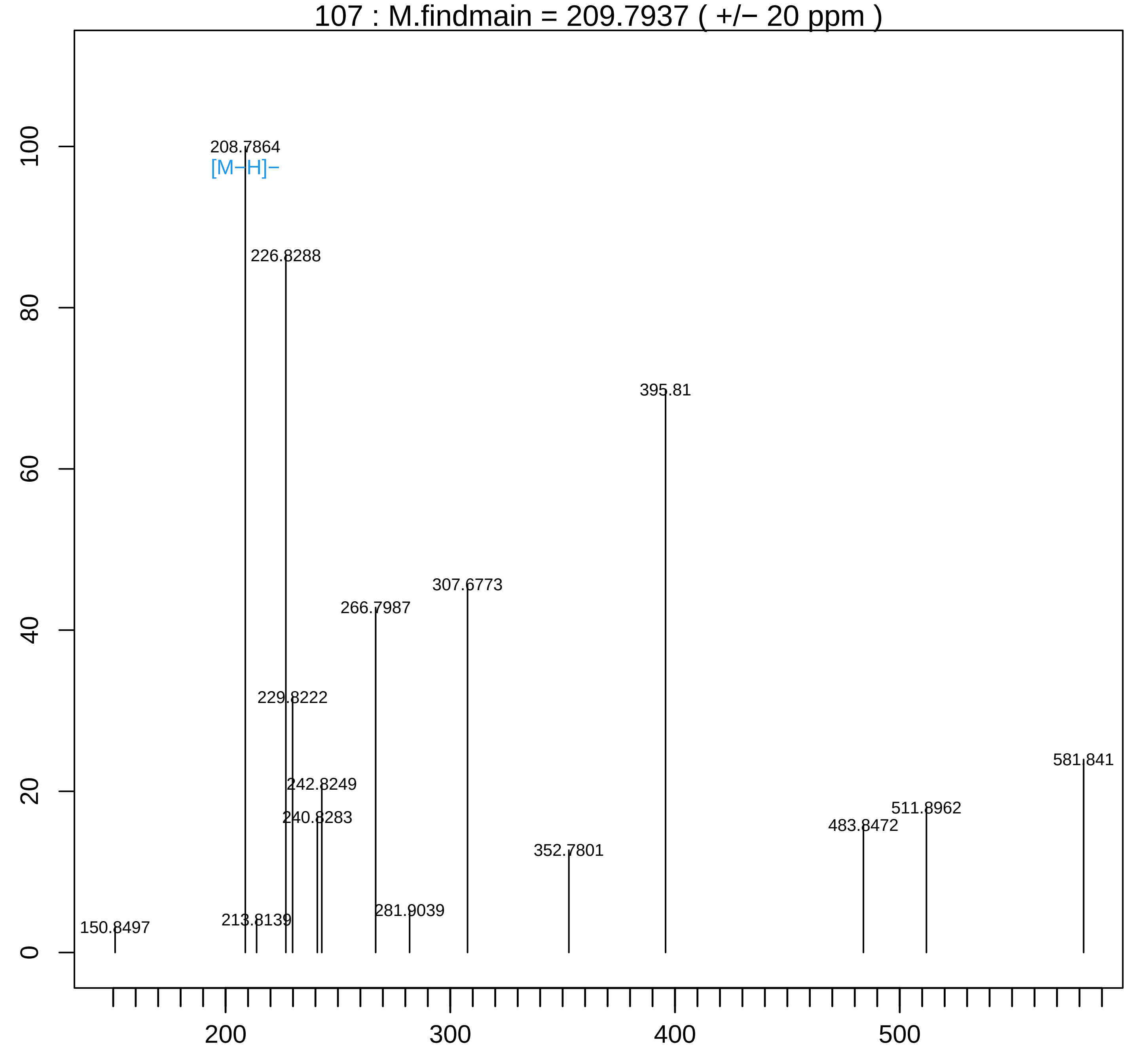

107 : M.findmain = 209.7937 (+/− 20 ppm)
208.7864
[M−H]−
226.8288
395.81
307.6773
266.7987
229.8222
242.8249
240.8283
511.8962
483.8472
581.841
352.7801
281.9039
213.8139
150.8497
0
20
40
60
80
100
200
300
400
500

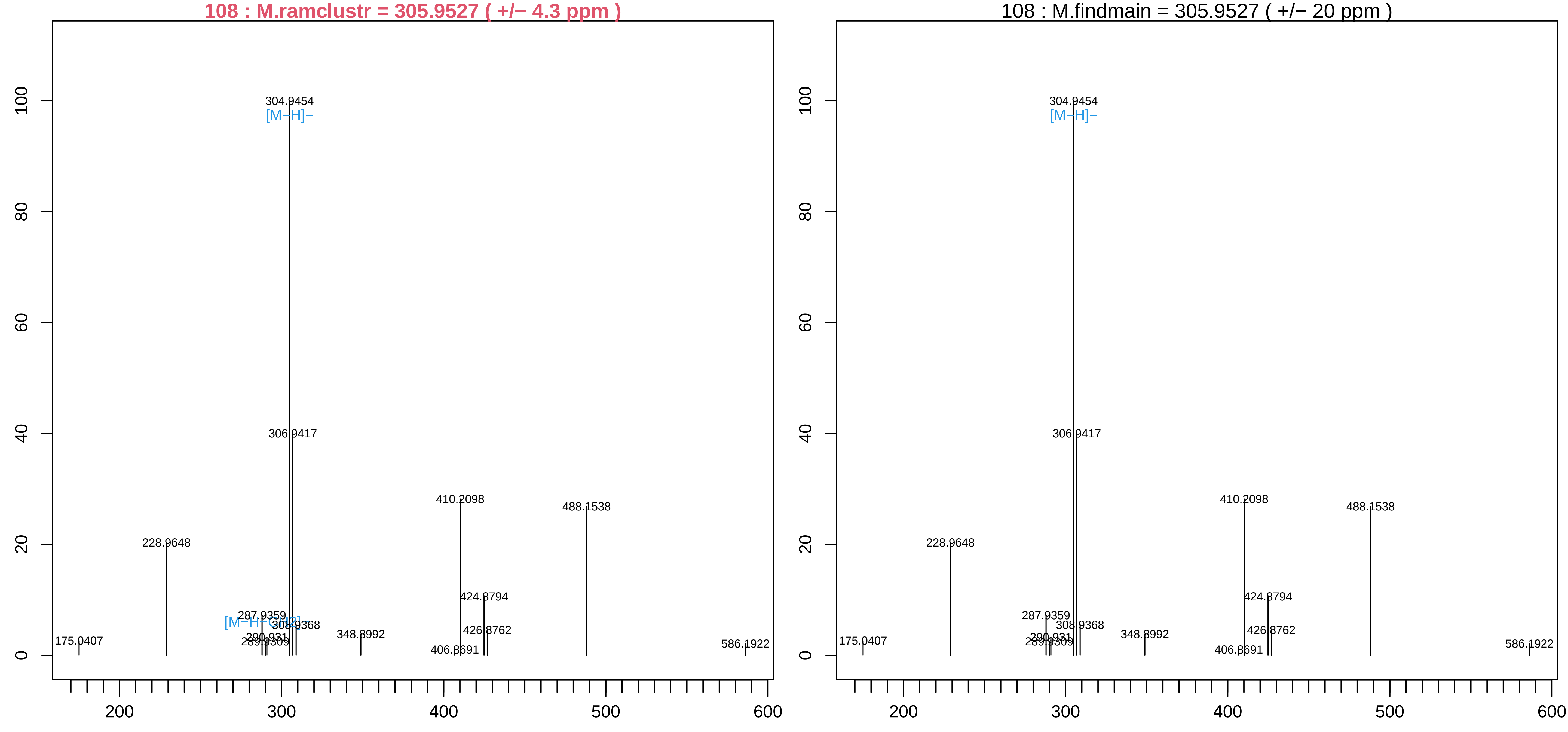

108 : M.ramclustr = 305.9527 (+/− 4.3 ppm)
304.9454
[M−H]−
306.9417
410.2098
488.1538
228.9648
424.8794
287.9359
308.9368
426.8762
348.8992
290.931
289.9309
175.0407
586.1922
406.8691
0
20
40
60
80
100
200
300
400
500
600
108 : M.findmain = 305.9527 (+/− 20 ppm)
304.9454
[M−H]−
306.9417
410.2098
488.1538
228.9648
424.8794
287.9359
308.9368
426.8762
348.8992
290.931
289.9309
175.0407
586.1922
406.8691
0
20
40
60
80
100
200
300
400
500
600

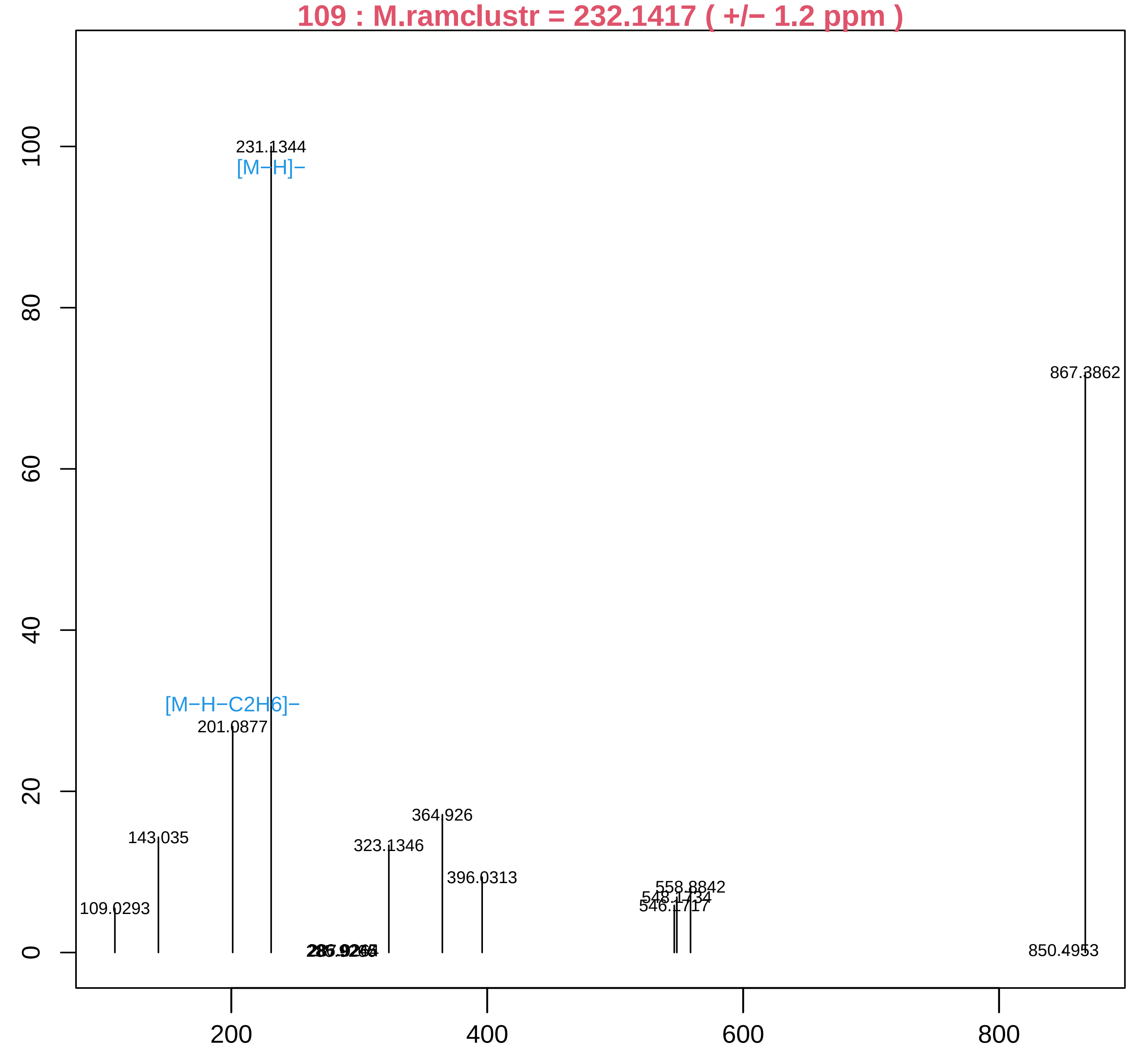
109 : M.ramclustr = 232.1417 (+/− 1.2 ppm)
231.1344
[M−H]−
867.3862
[M−H−C2H6]−
201.0877
143.035
364.926
323.1346
396.0313
558.8842
548.1734
546.1717
109.0293
850.4953

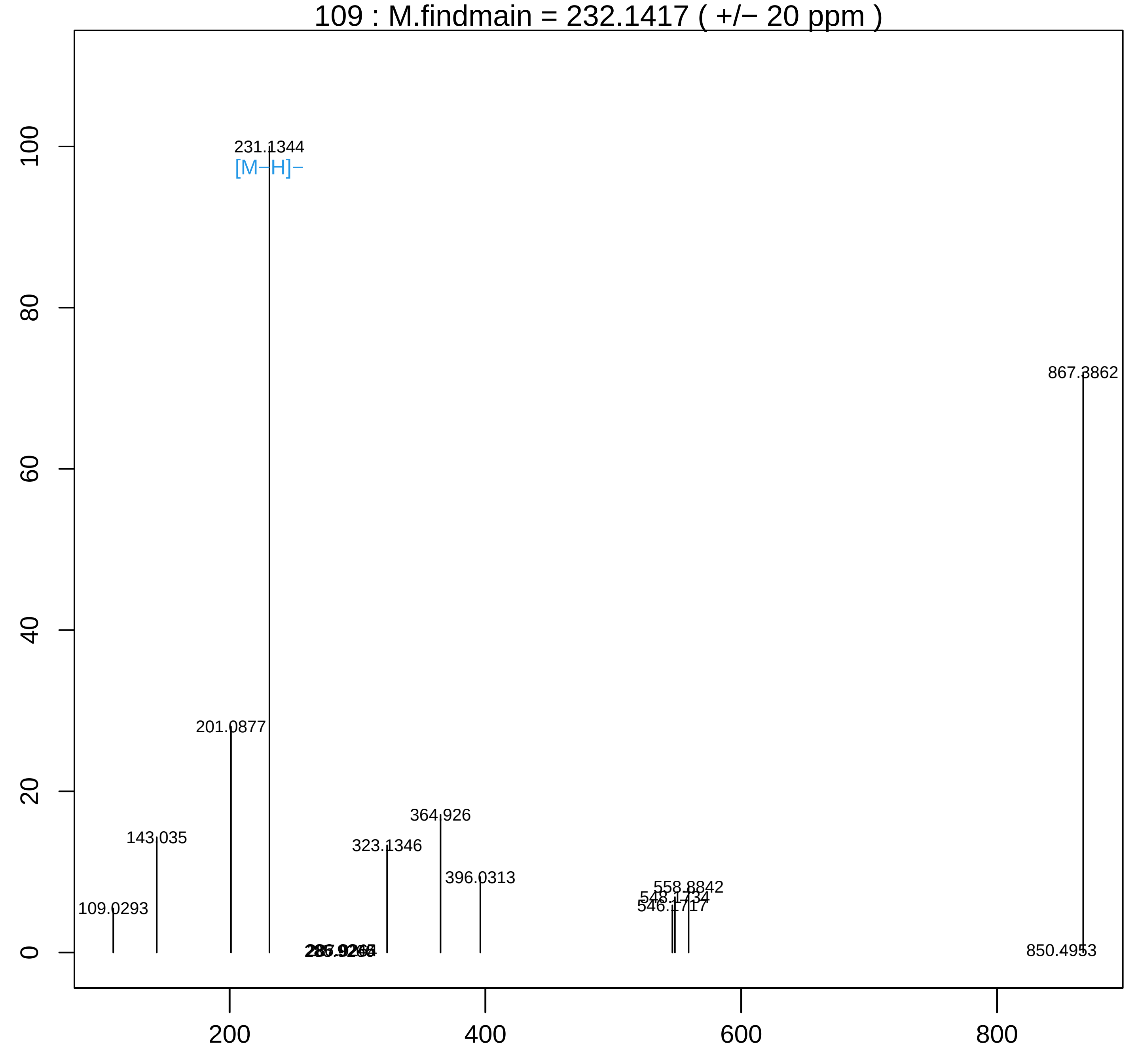
109 : M.findmain = 232.1417 (+/− 20 ppm)
231.1344
[M−H]−
867.3862
201.0877
143.035
364.926
323.1346
396.0313
558.8842
548.1734
546.1717
109.0293
850.4953

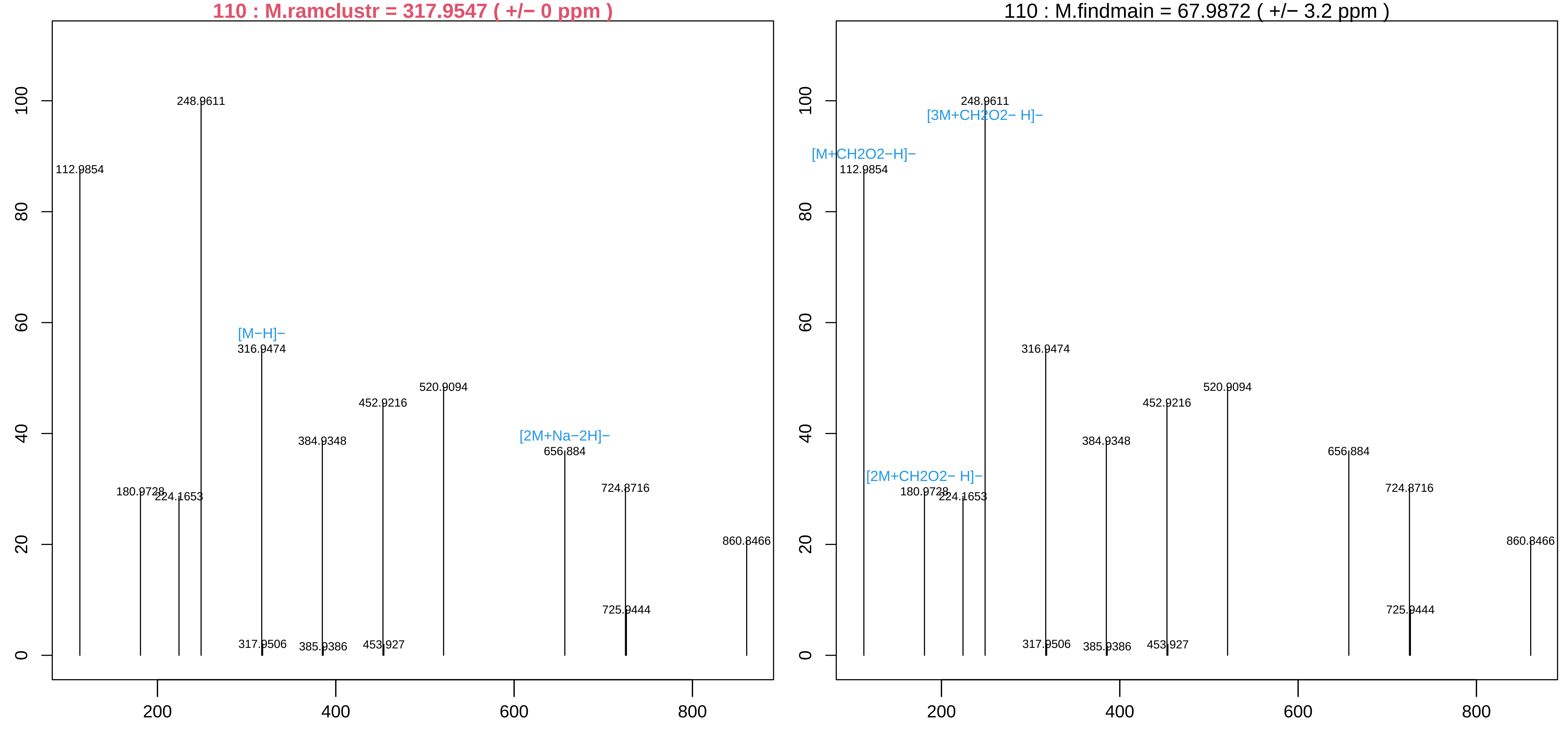

110 : M.ramclustr = 317.9547 (+/− 0 ppm)
112.9854
180.9728
224.1653
248.9611
[M−H]−
316.9474
317.9506
384.9348
385.9386
452.9216
453.927
520.9094
[2M+Na−2H]−
656.884
724.8716
725.9444
860.8466
110 : M.findmain = 67.9872 (+/− 3.2 ppm)
[M+CH2O2−H]−
[3M+CH2O2− H]−
[2M+CH2O2− H]−
0
20
40
60
80
100
200
400
600
800

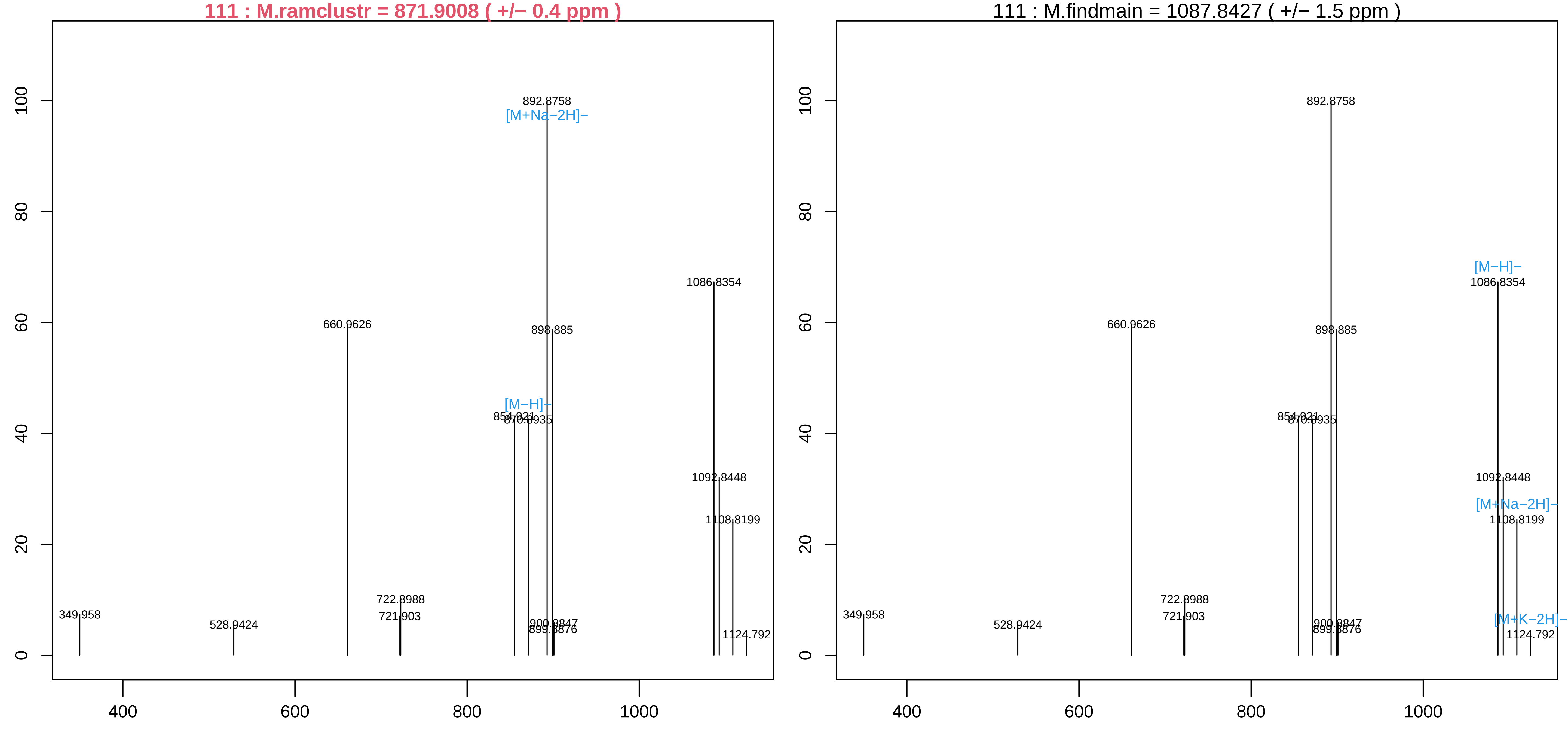
111 : M.ramclustr = 871.9008 (+/− 0.4 ppm)
111 : M.findmain = 1087.8427 (+/− 1.5 ppm)
892.8758
[M+Na−2H]−
1086.8354
660.9626
898.885
[M−H]−
854.921
870.8935
1092.8448
1108.8199
722.8988
349.958
721.903
528.9424
900.8847
899.8876
1124.792
[M+K−2H]−
0
20
40
60
80
100
400
600
800
1000

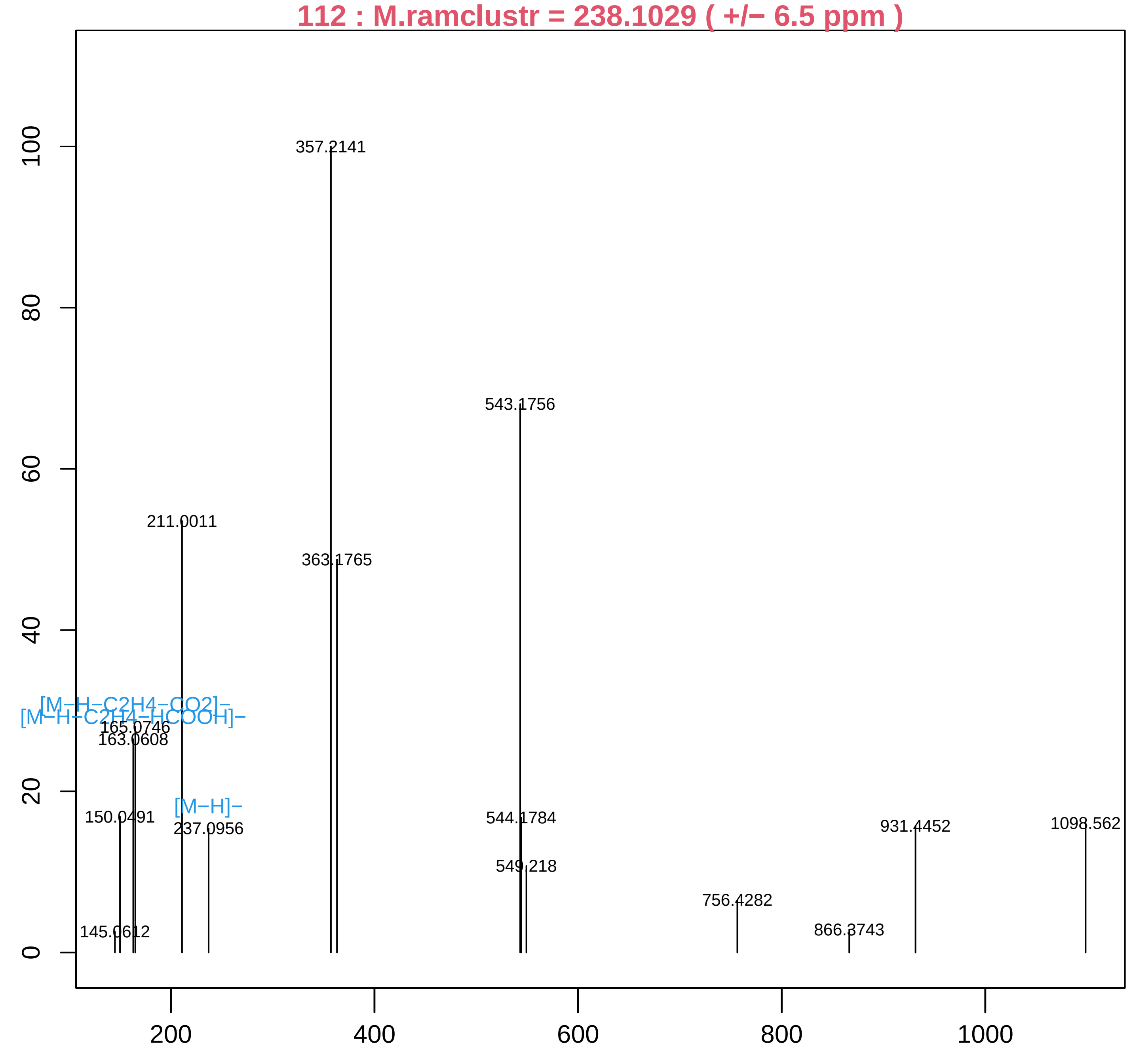
112 : M.ramclustr = 238.1029 (+/− 6.5 ppm)
357.2141
543.1756
211.0011
363.1765
[M−H−C2H4−CO2]−
[M−H−C2H4−HCOOH]−
165.0746
163.0608
150.0491
[M−H]−
237.0956
544.1784
549.218
931.4452
1098.562
756.4282
866.3743
145.0612
0
20
40
60
80
100
200
400
600
800
1000

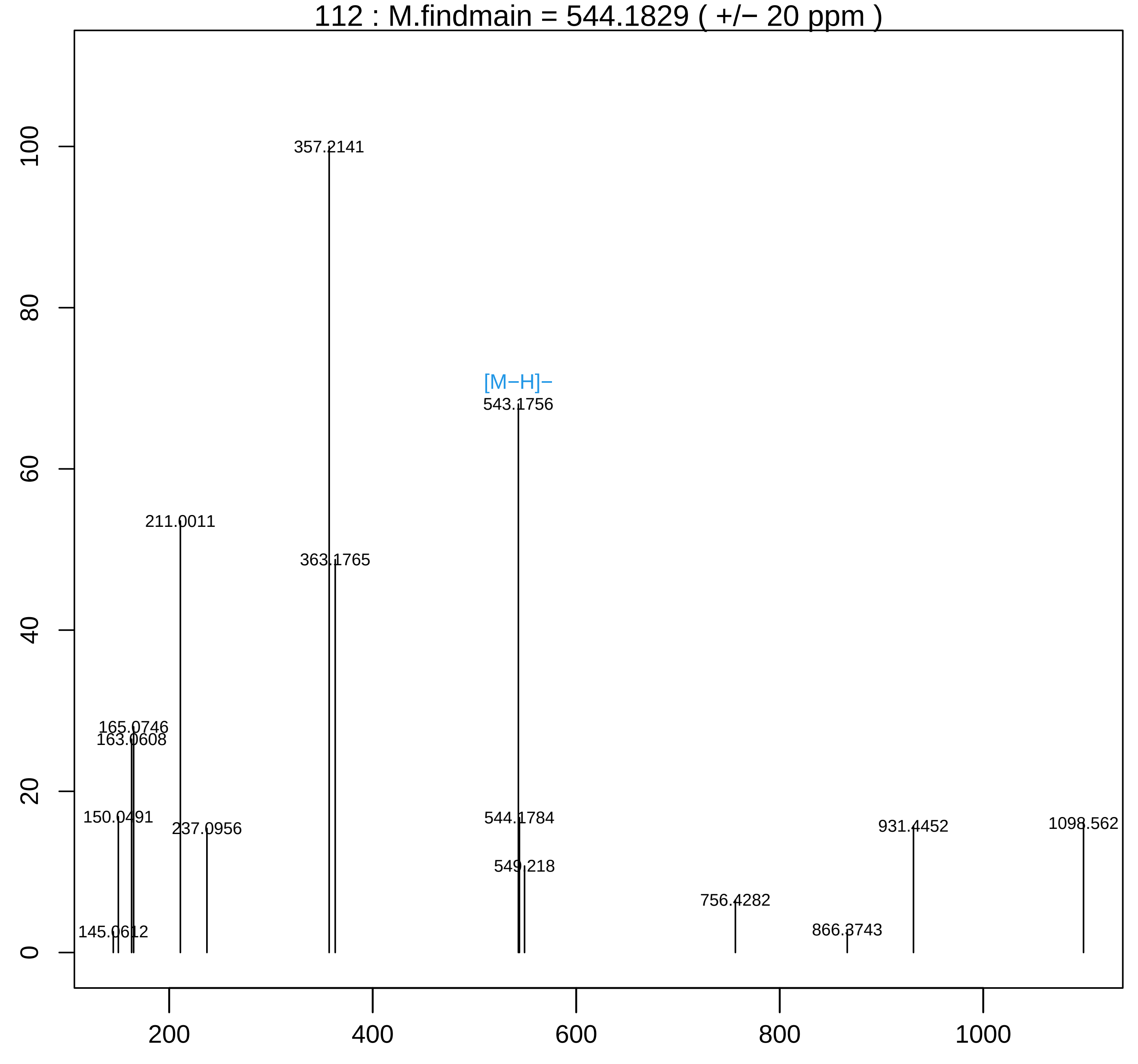
112 : M.findmain = 544.1829 (+/− 20 ppm)
357.2141
[M−H]−
543.1756
211.0011
363.1765
165.0746
163.0608
150.0491
237.0956
544.1784
549.218
931.4452
1098.562
756.4282
866.3743
145.0612
0
20
40
60
80
100
200
400
600
800
1000

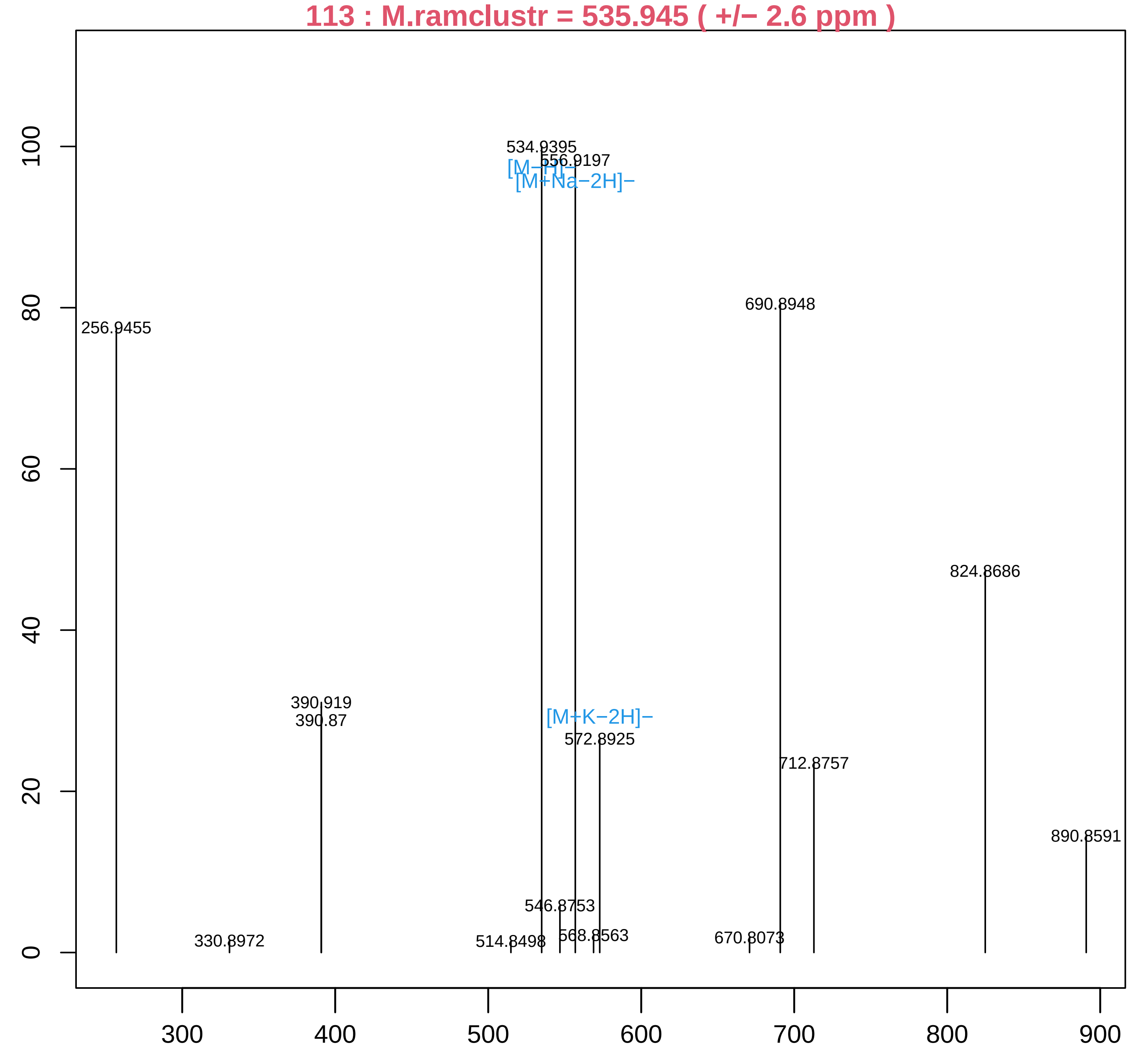
113 : M.ramclustr = 535.945 (+/− 2.6 ppm)
534.9395
[M−H]−
556.9197
[M+Na−2H]−
256.9455
690.8948
824.8686
390.919
390.87
[M+K−2H]−
572.8925
712.8757
890.8591
546.8753
568.8563
330.8972
514.8498
670.8073
0
20
40
60
80
100
300
400
500
600
700
800
900

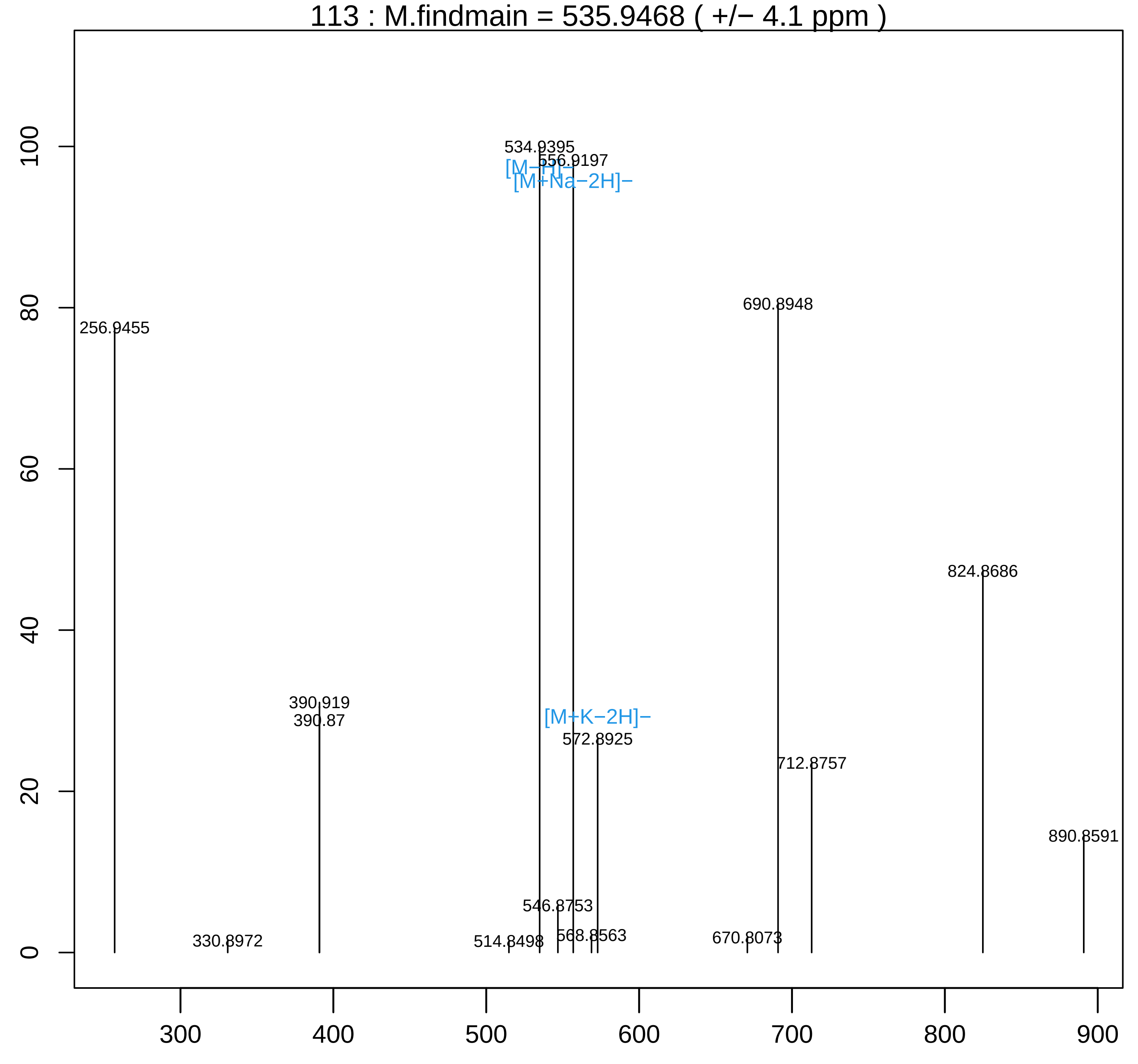
113 : M.findmain = 535.9468 (+/− 4.1 ppm)
534.9395
[M−H]−
556.9197
[M+Na−2H]−
256.9455
690.8948
824.8686
390.919
390.87
[M+K−2H]−
572.8925
712.8757
890.8591
546.8753
568.8563
330.8972
514.8498
670.8073
0
20
40
60
80
100
300
400
500
600
700
800
900

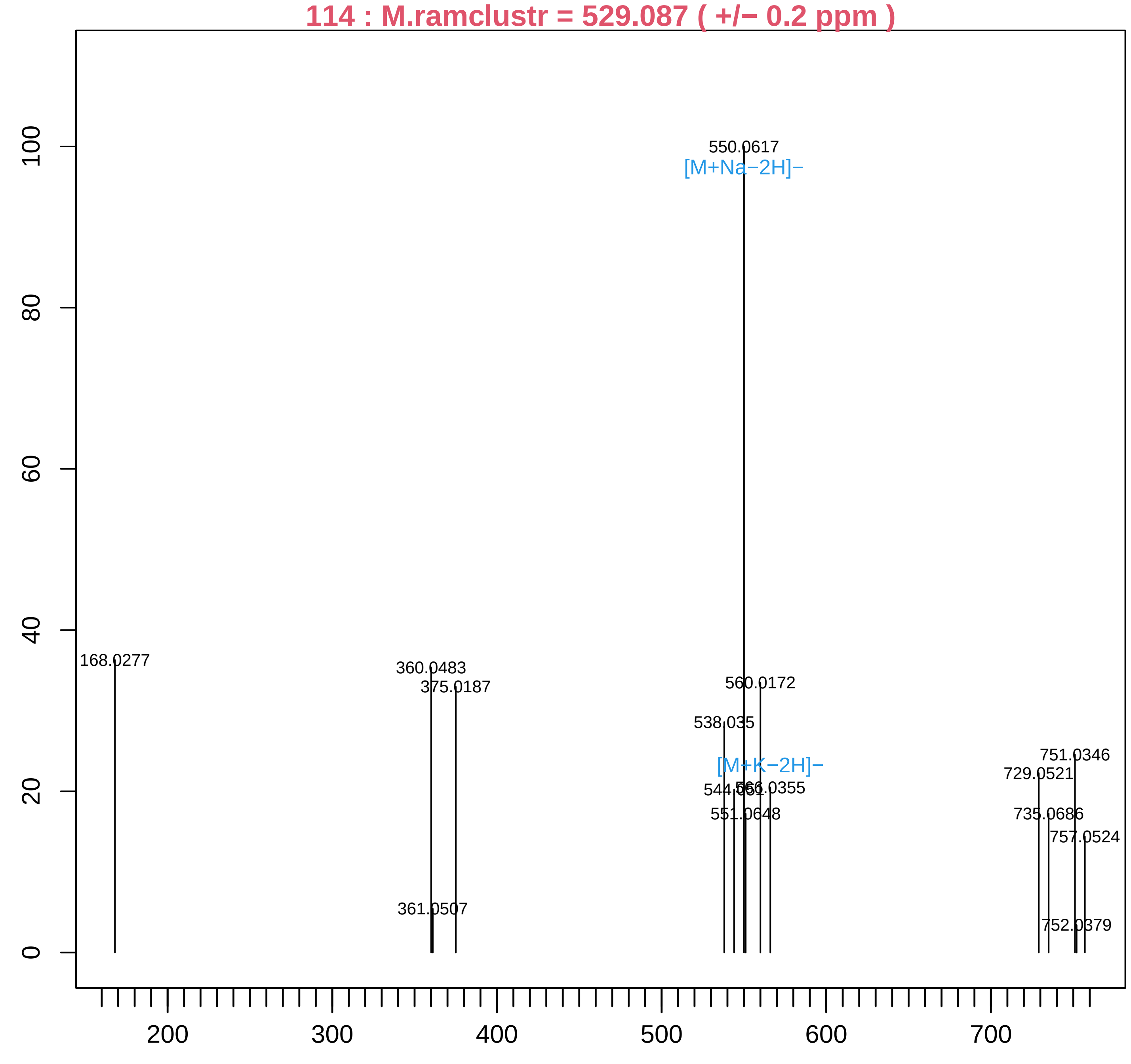

114 : M.ramclustr = 529.087 (+/− 0.2 ppm)
550.0617
[M+Na−2H]−
168.0277
360.0483
375.0187
560.0172
538.035
[M+K−2H]−
551.0648
566.0355
361.0507
729.0521
751.0346
735.0686
757.0524
752.0379

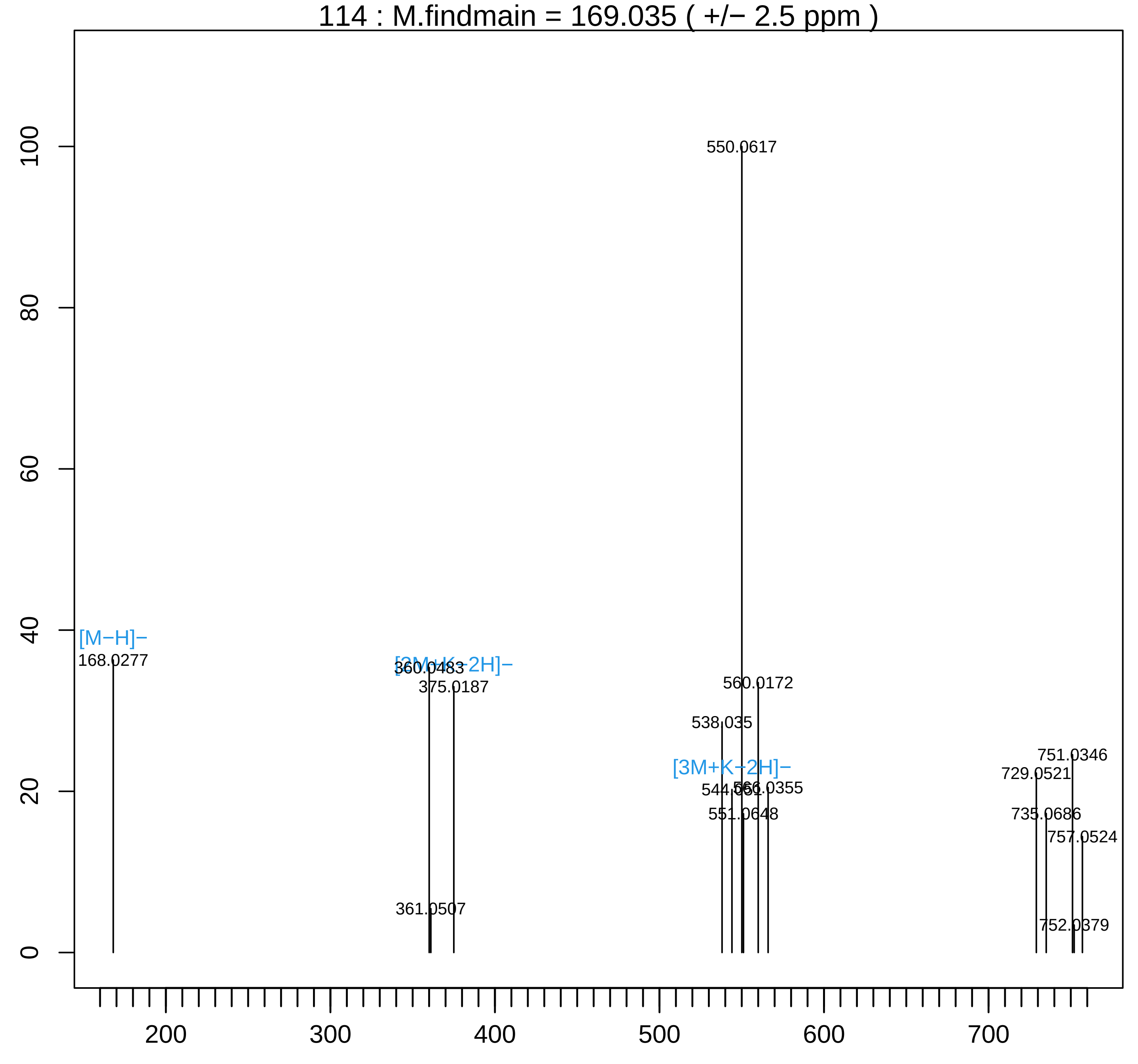

114 : M.findmain = 169.035 (+/− 2.5 ppm)
550.0617
[M−H]−
168.0277
360.0483
375.0187
560.0172
538.035
[3M+K−2H]−
551.0648
566.0355
361.0507
729.0521
751.0346
735.0686
757.0524
752.0379

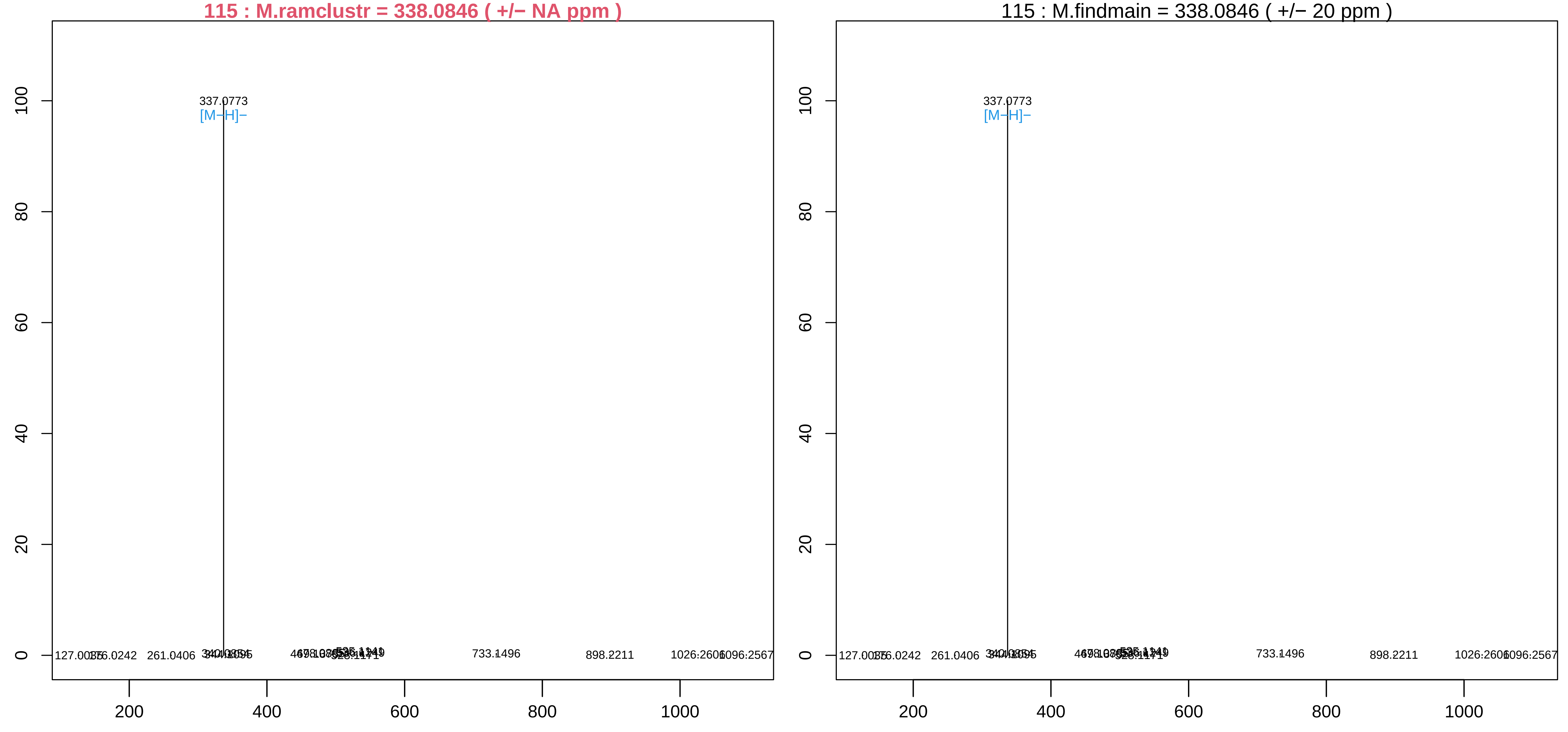
115 : M.ramclustr = 338.0846 (+/− NA ppm)
337.0773
[M−H]−
127.0036
261.0406
733.1496
898.2211
1096:2567
115 : M.findmain = 338.0846 (+/− 20 ppm)
337.0773
[M−H]−
127.0036
261.0406
733.1496
898.2211
1096:2567

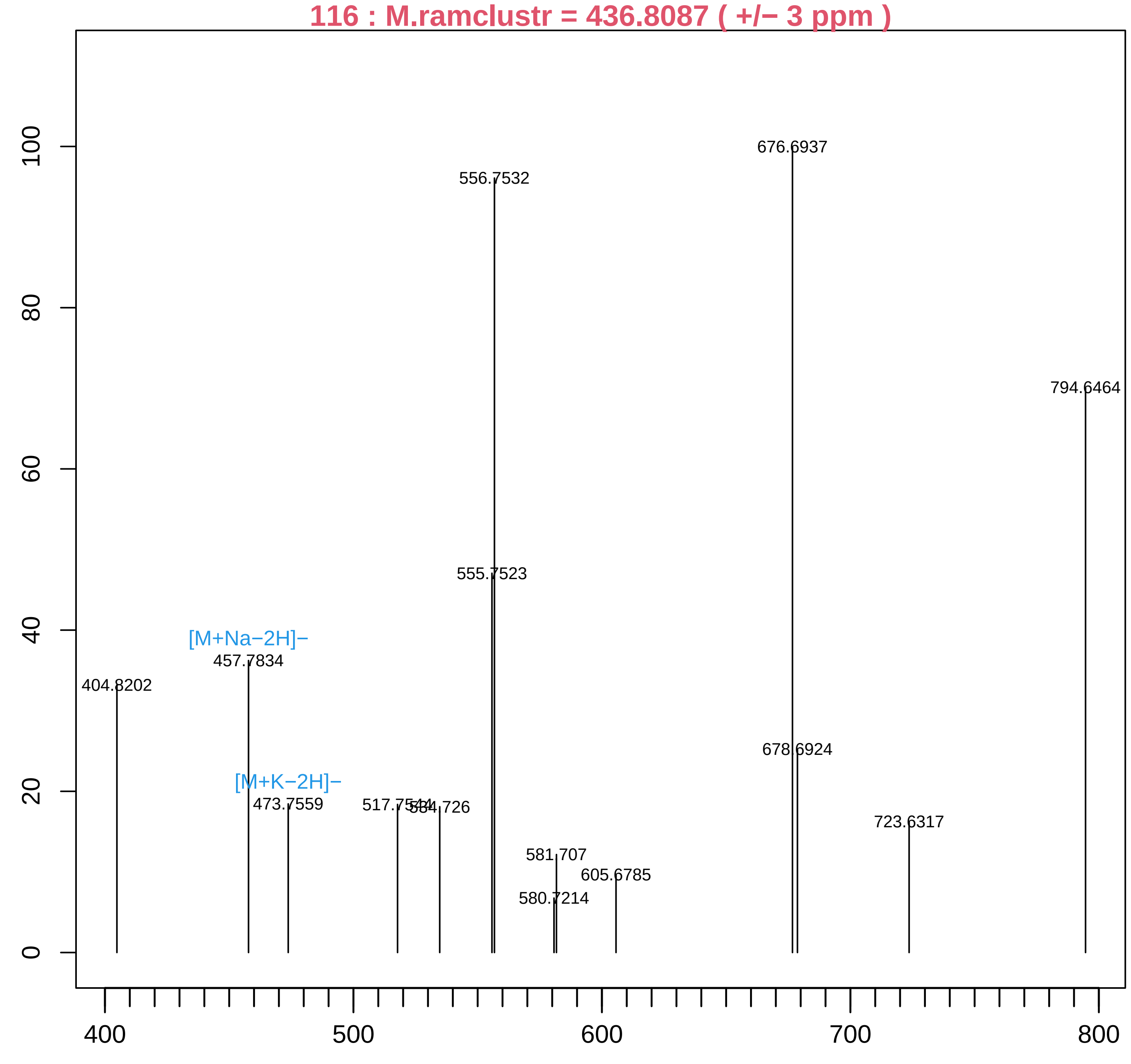
116 : M.ramclustr = 436.8087 (+/− 3 ppm)
676.6937
556.7532
794.6464
555.7523
[M+Na−2H]−
457.7834
404.8202
678.6924
[M+K−2H]−
473.7559
517.7544
534.726
723.6317
581.707
605.6785
580.7214
0
20
40
60
80
100
400
500
600
700
800

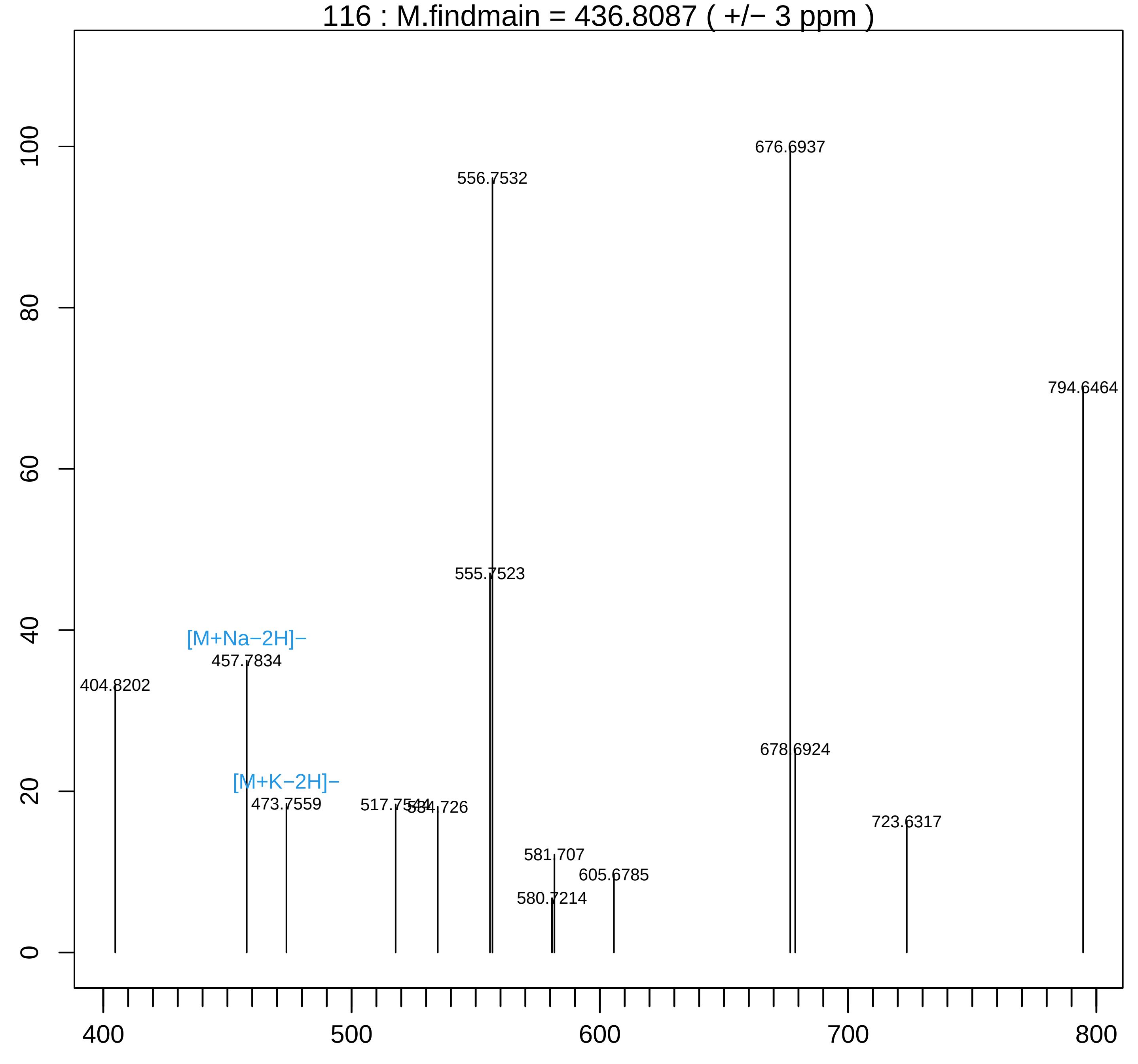
116 : M.findmain = 436.8087 (+/− 3 ppm)
676.6937
556.7532
794.6464
555.7523
[M+Na−2H]−
457.7834
404.8202
678.6924
[M+K−2H]−
473.7559
517.7544
534.726
723.6317
581.707
605.6785
580.7214
0
20
40
60
80
100
400
500
600
700
800

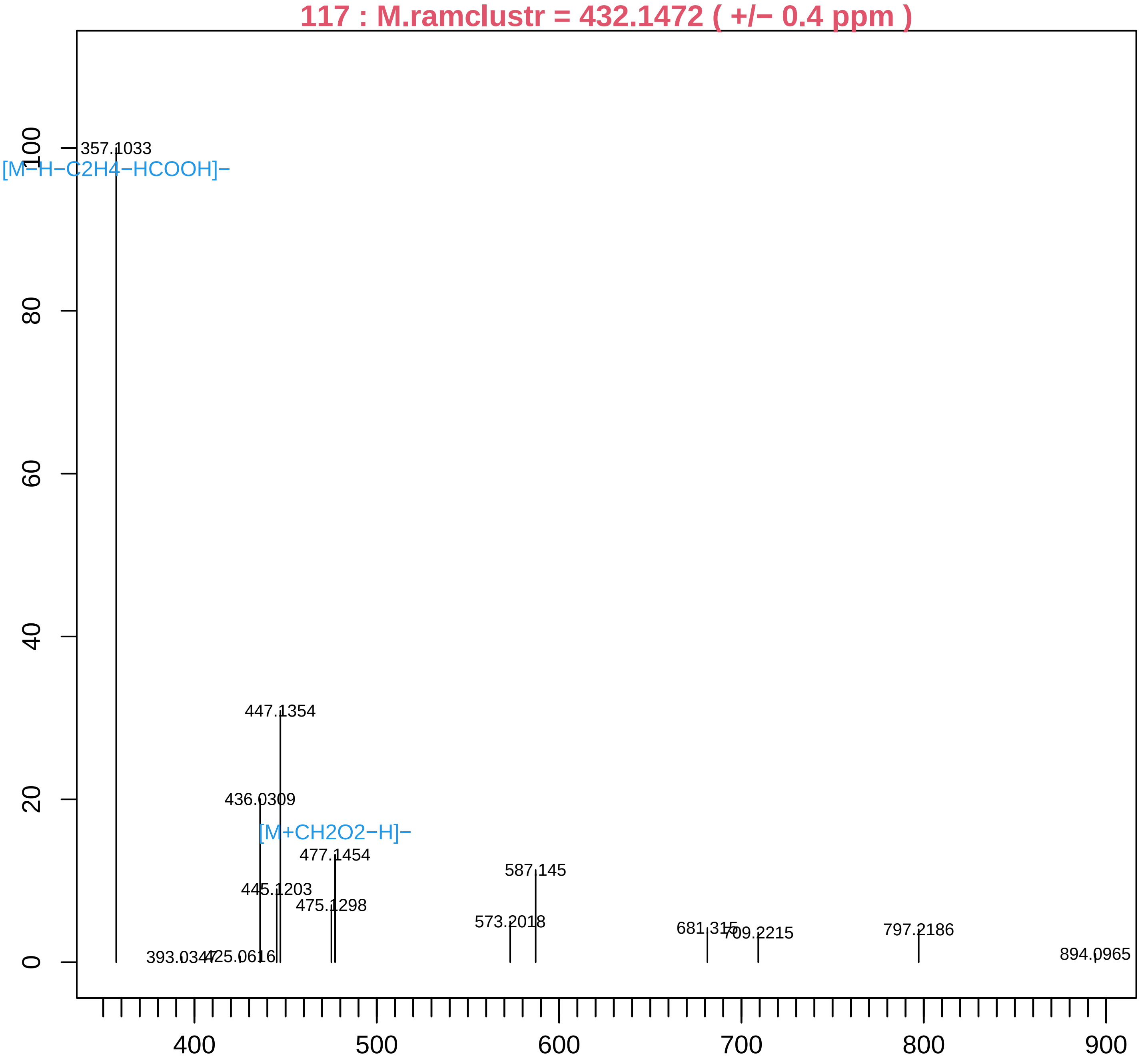
117 : M.ramclustr = 432.1472 (+/− 0.4 ppm)
357.1033
[M−H−C2H4−HCOOH]−
447.1354
436.0309
[M+CH2O2−H]−
477.1454
445.1203
475.1298
587.145
573.2018
681.315
709.2215
797.2186
894.0965
393.0347
425.0616
0
20
40
60
80
100
400
500
600
700
800
900

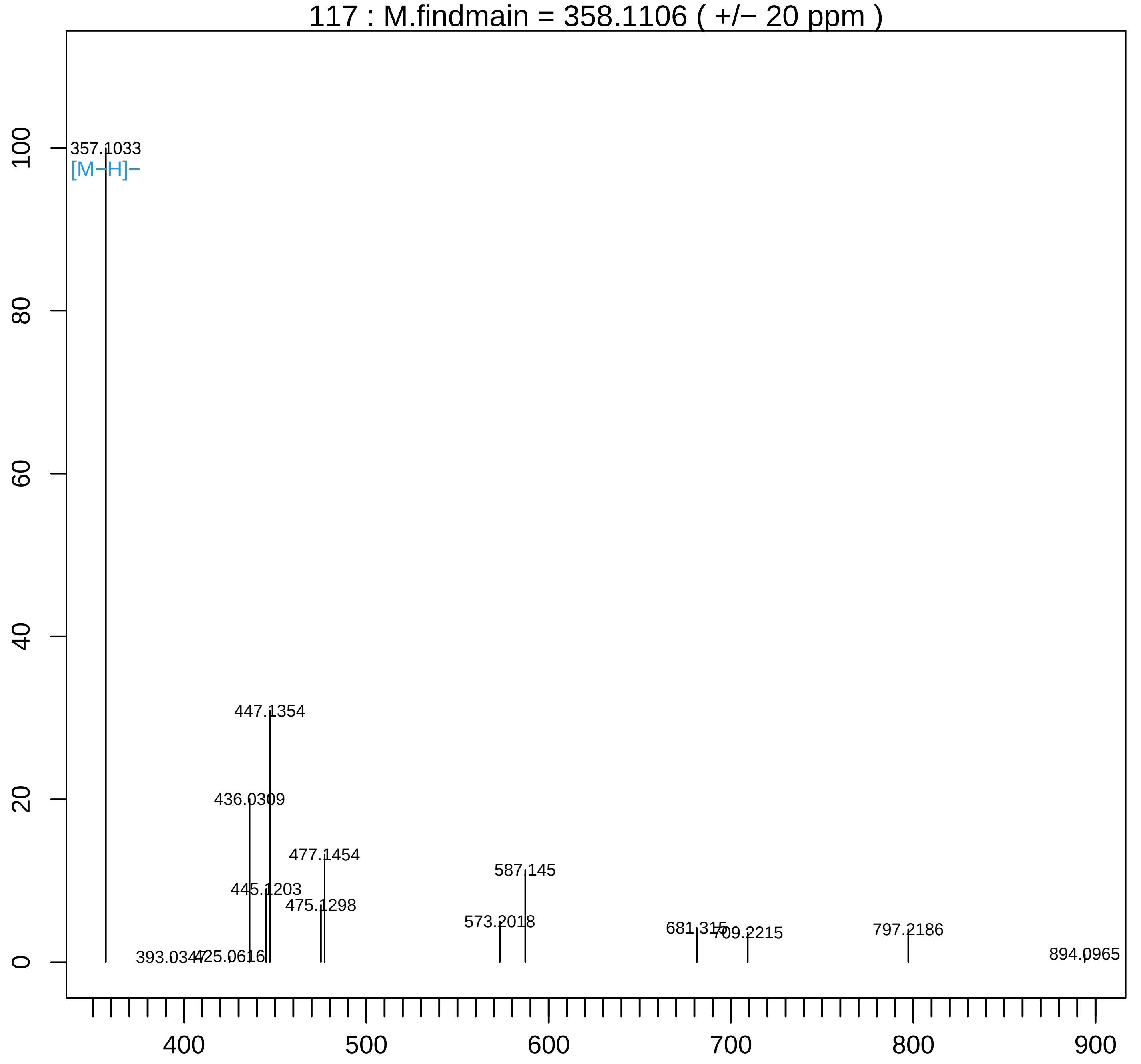
117 : M.findmain = 358.1106 (+/− 20 ppm)
357.1033
[M−H]−
447.1354
436.0309
477.1454
445.1203
475.1298
587.145
573.2018
681.315
709.2215
797.2186
894.0965
393.0347
425.0616
0
20
40
60
80
100
400
500
600
700
800
900

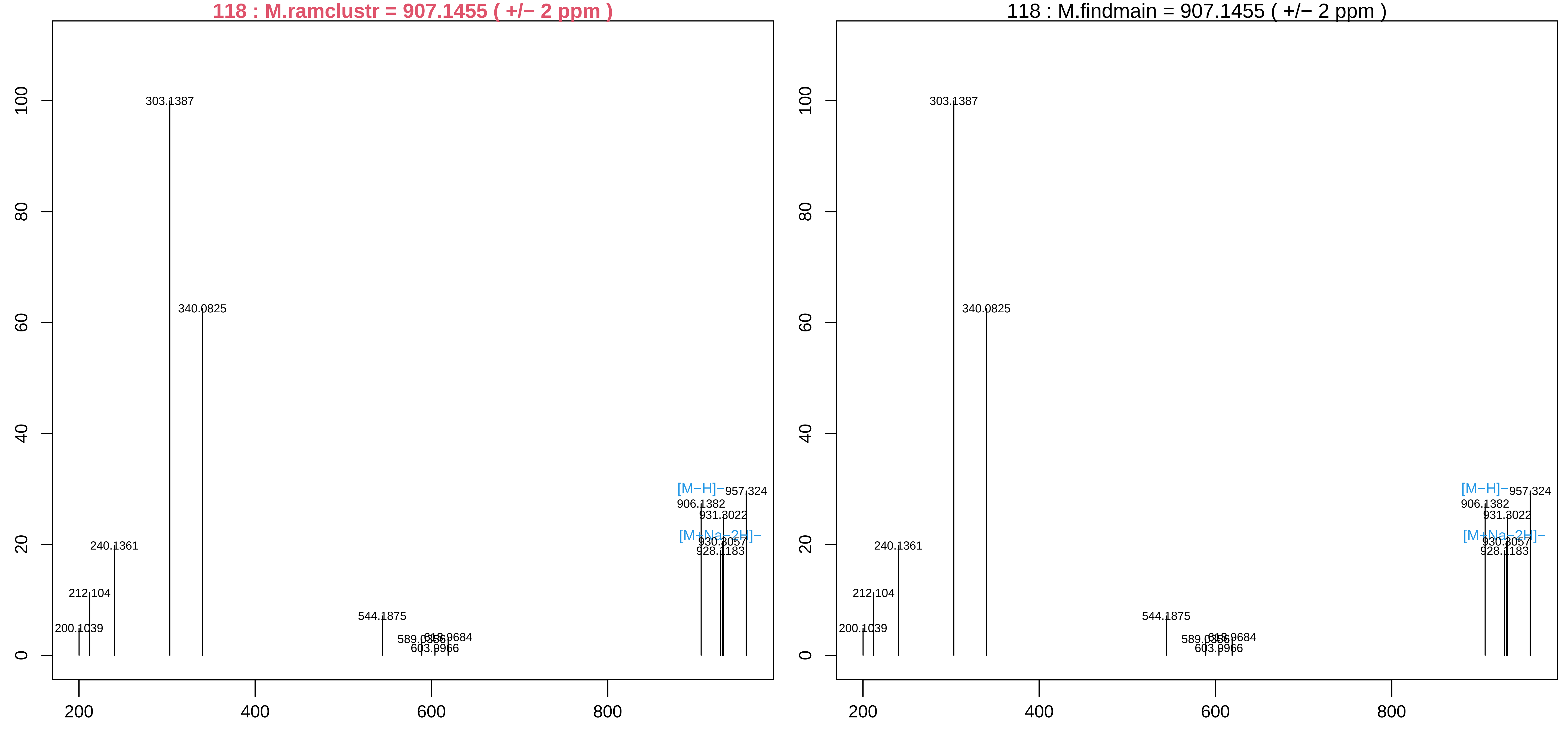
118 : M.ramclustr = 907.1455 (+/− 2 ppm)
118 : M.findmain = 907.1455 (+/− 2 ppm)
303.1387
340.0825
240.1361
212.104
200.1039
544.1875
589.0886
618.9684
603.9966
[M−H]−
957.324
906.1382
931.3022
[M+Na−2H]−
930.3057
928.1183
0
20
40
60
80
100
200
400
600
800

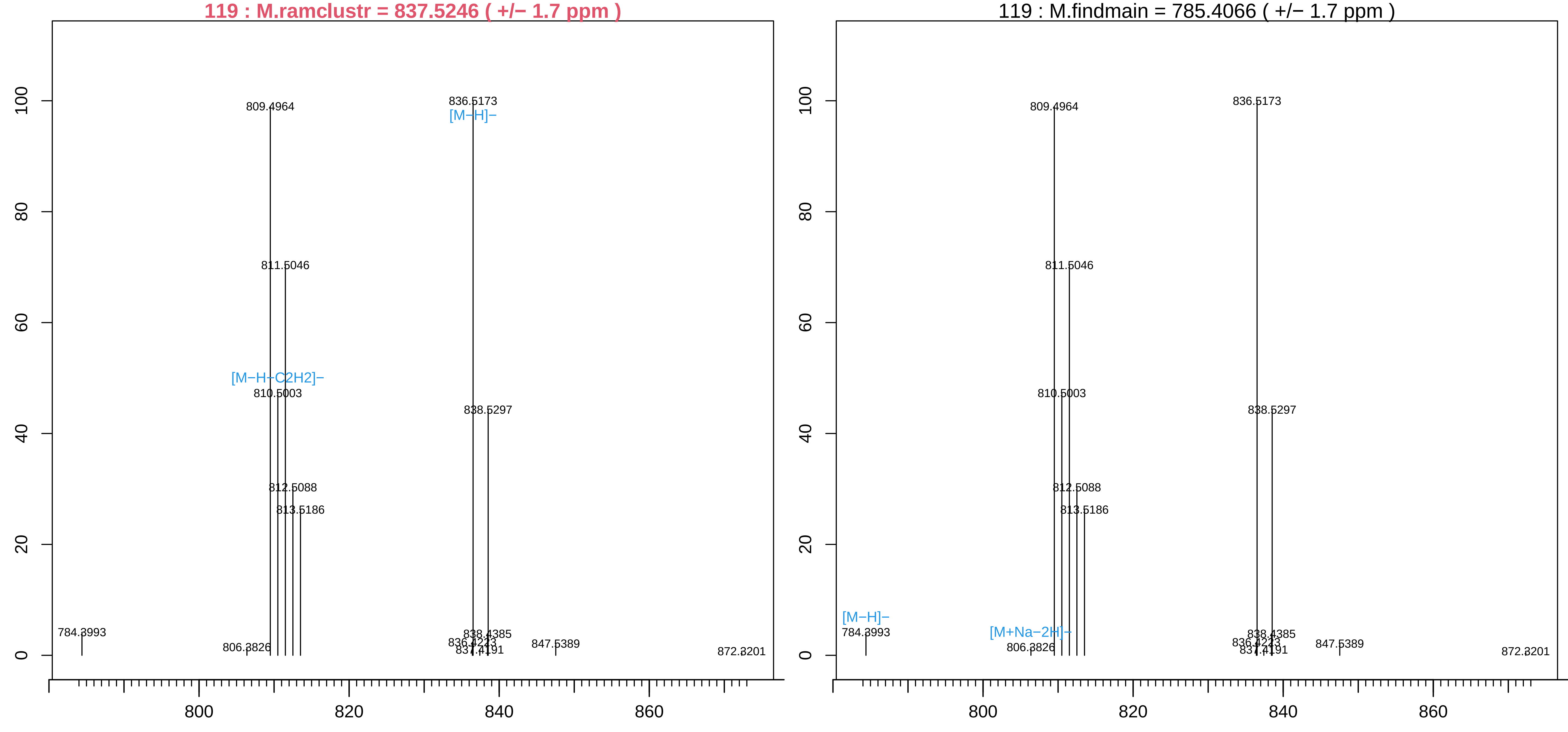

119 : M.ramclustr = 837.5246 (+/− 1.7 ppm)
119 : M.findmain = 785.4066 (+/− 1.7 ppm)
809.4964
836.5173
[M−H]−
811.5046
[M−H−C2H2]−
810.5003
838.5297
812.5088
813.5186
784.3993
806.3826
838.4385
836.4223
837.4191
847.5389
872.3201
[M+Na−2H]−
0
20
40
60
80
100
800
820
840
860

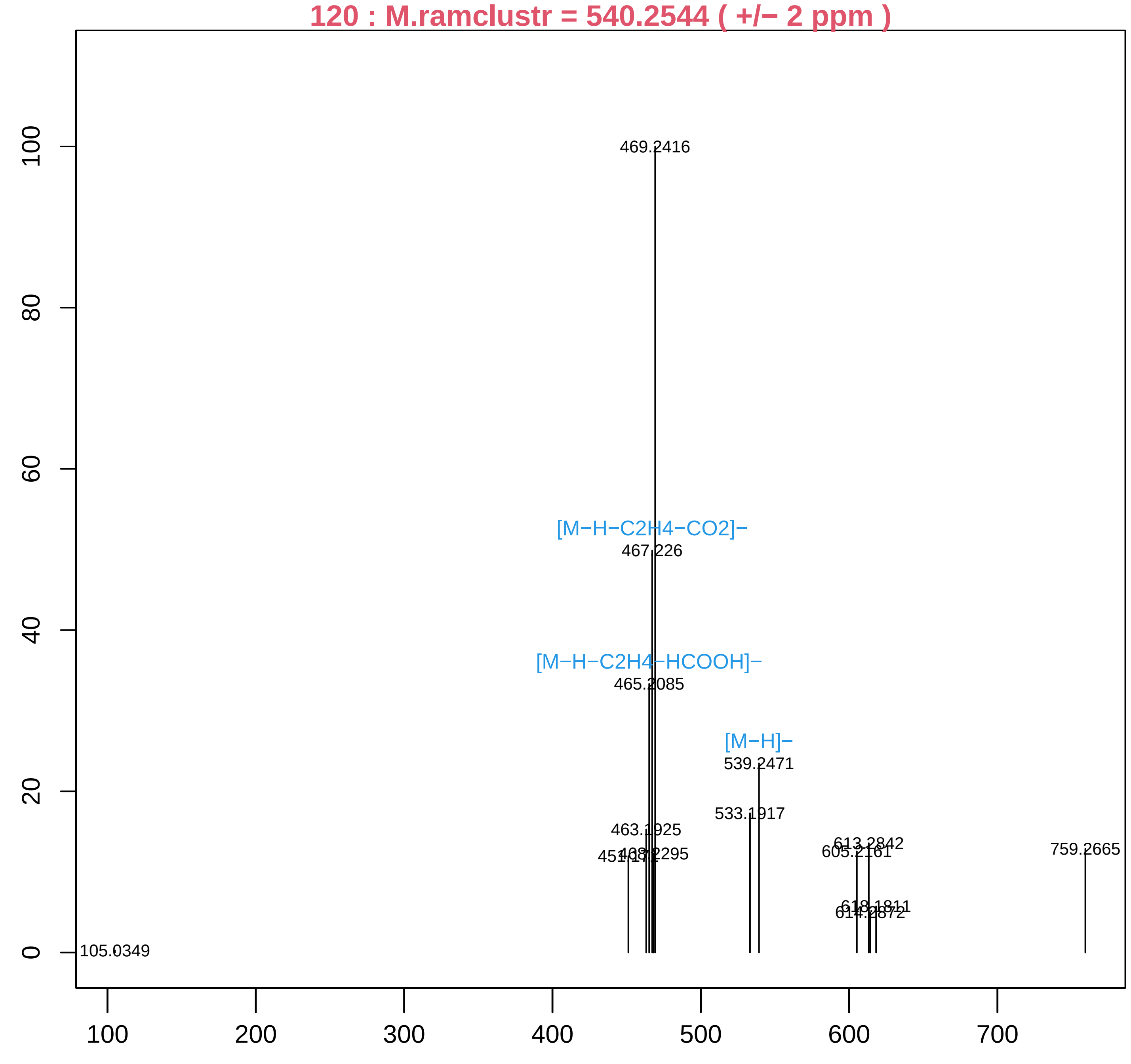
120 : M.ramclustr = 540.2544 (+/− 2 ppm)
469.2416
[M−H−C2H4−CO2]−
467.226
[M−H−C2H4−HCOOH]−
465.2085
[M−H]−
539.2471
533.1917
463.1925
451.1171
468.2295
613.2842
605.2161
759.2665
618.1811
614.2872
105.0349
0
20
40
60
80
100
100
200
300
400
500
600
700

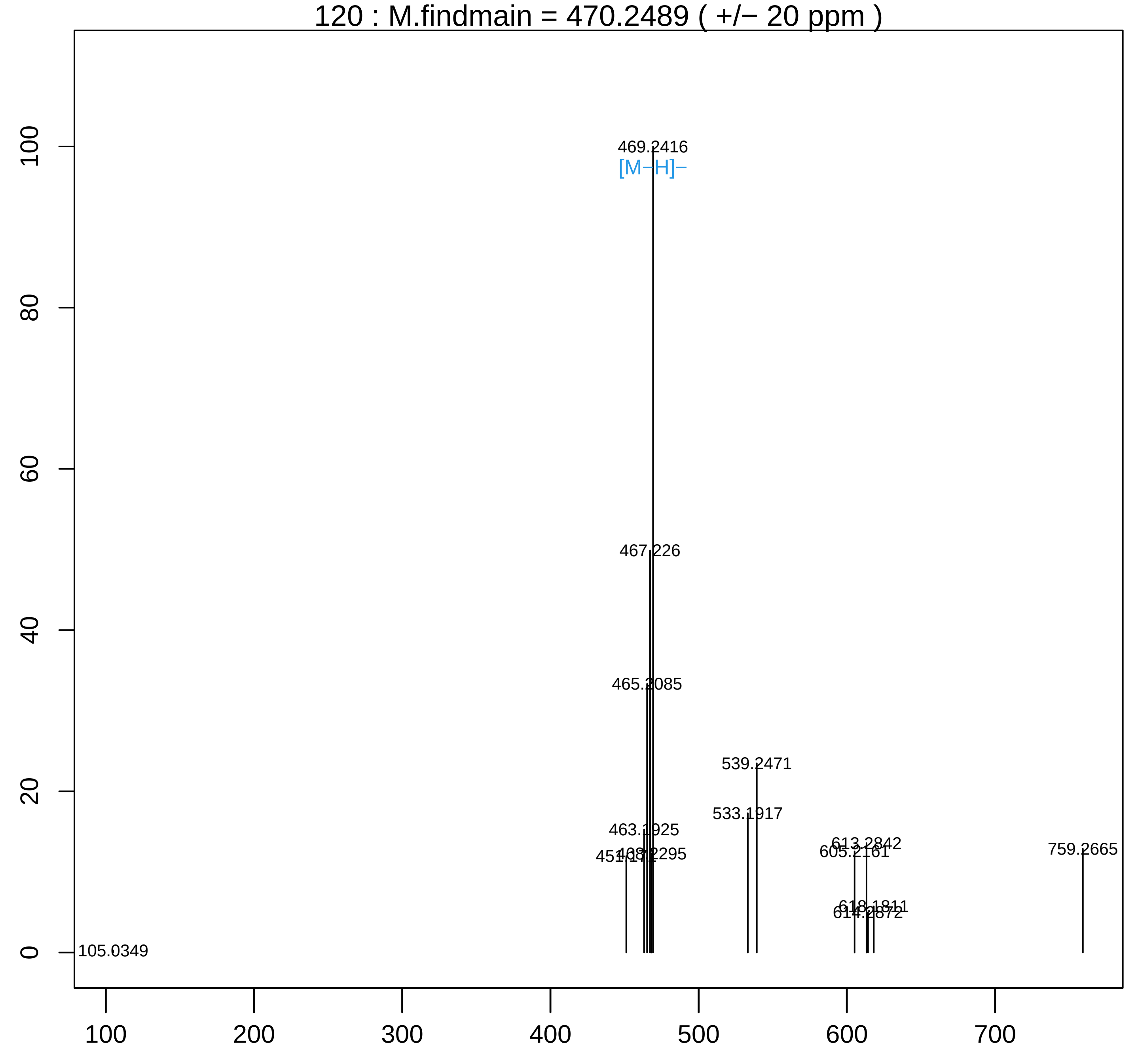
120 : M.findmain = 470.2489 (+/− 20 ppm)
469.2416
[M−H]−
467.226
465.2085
539.2471
533.1917
463.1925
451.1171
468.2295
613.2842
605.2161
759.2665
618.1811
614.2872
105.0349
0
20
40
60
80
100
100
200
300
400
500
600
700

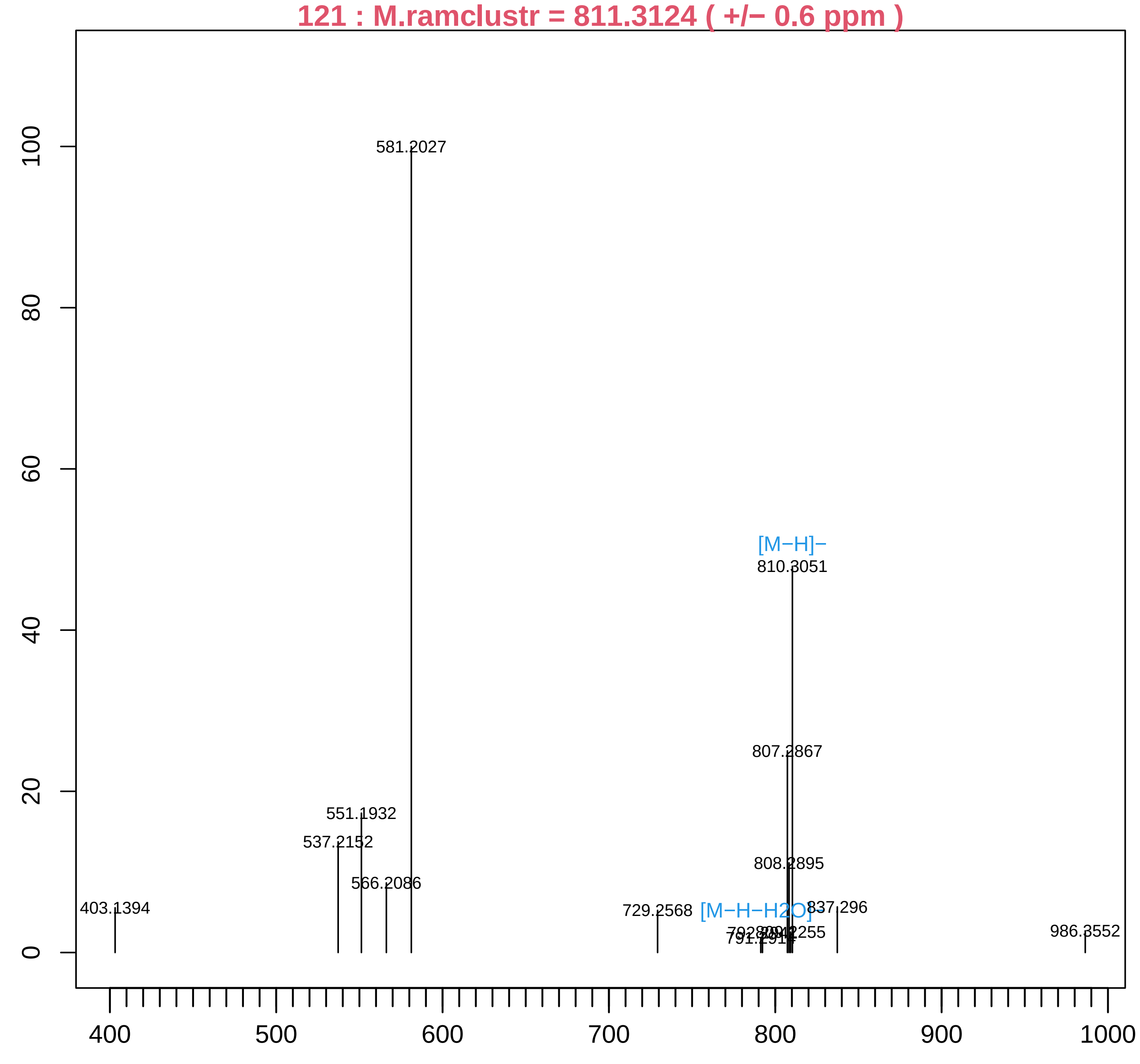

121 : M.ramclustr = 811.3124 (+/− 0.6 ppm)
581.2027
[M−H]−
810.3051
807.2867
551.1932
537.2152
808.2895
566.2086
403.1394
729.2568
[M−H−H2O]−
837.296
986.3552
0
20
40
60
80
100
400
500
600
700
800
900
1000

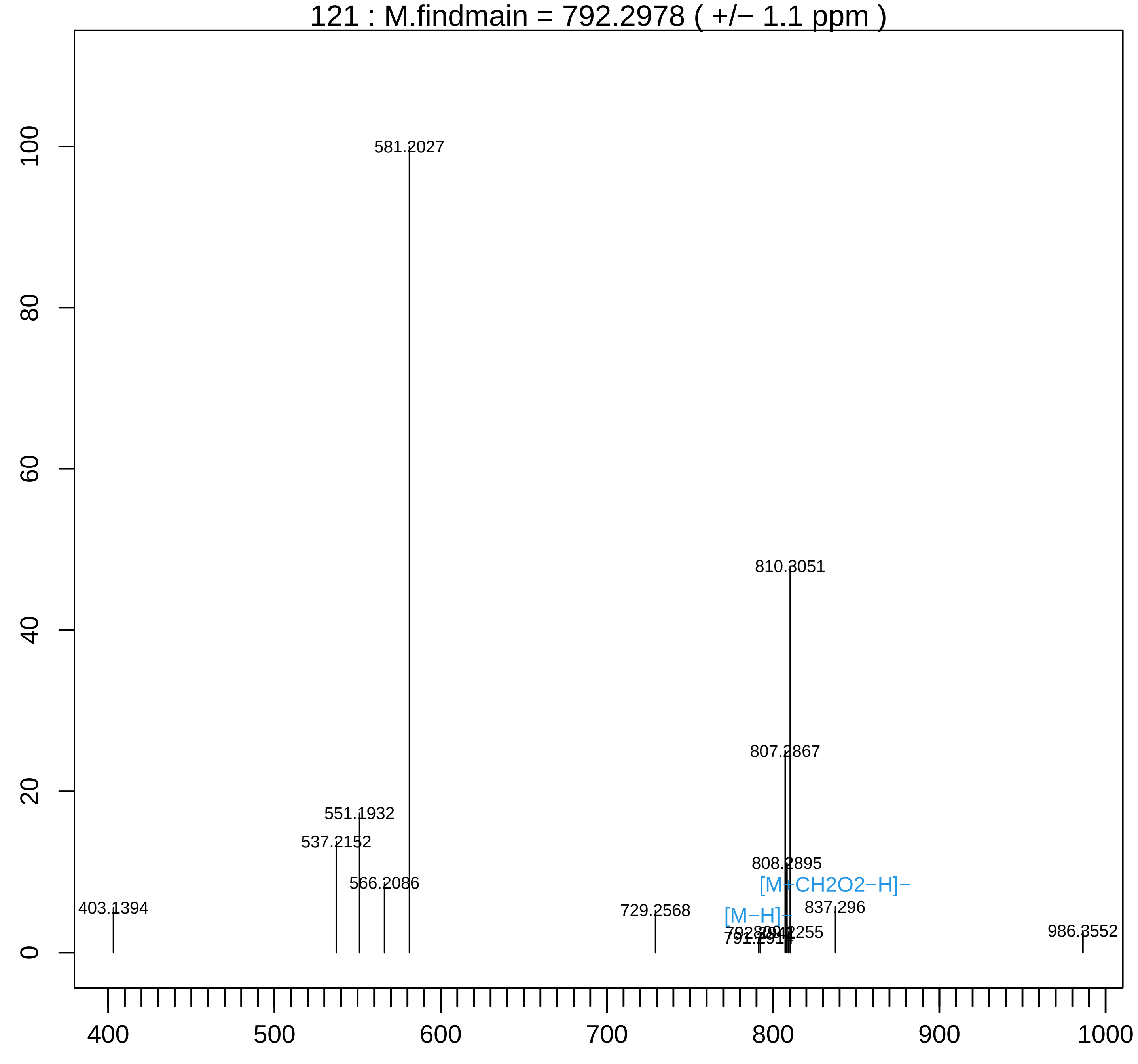

121 : M.findmain = 792.2978 (+/− 1.1 ppm)
581.2027
810.3051
807.2867
551.1932
537.2152
808.2895
566.2086
[M+CH2O2−H]−
403.1394
729.2568
[M−H]−
837.296
986.3552
0
20
40
60
80
100
400
500
600
700
800
900
1000

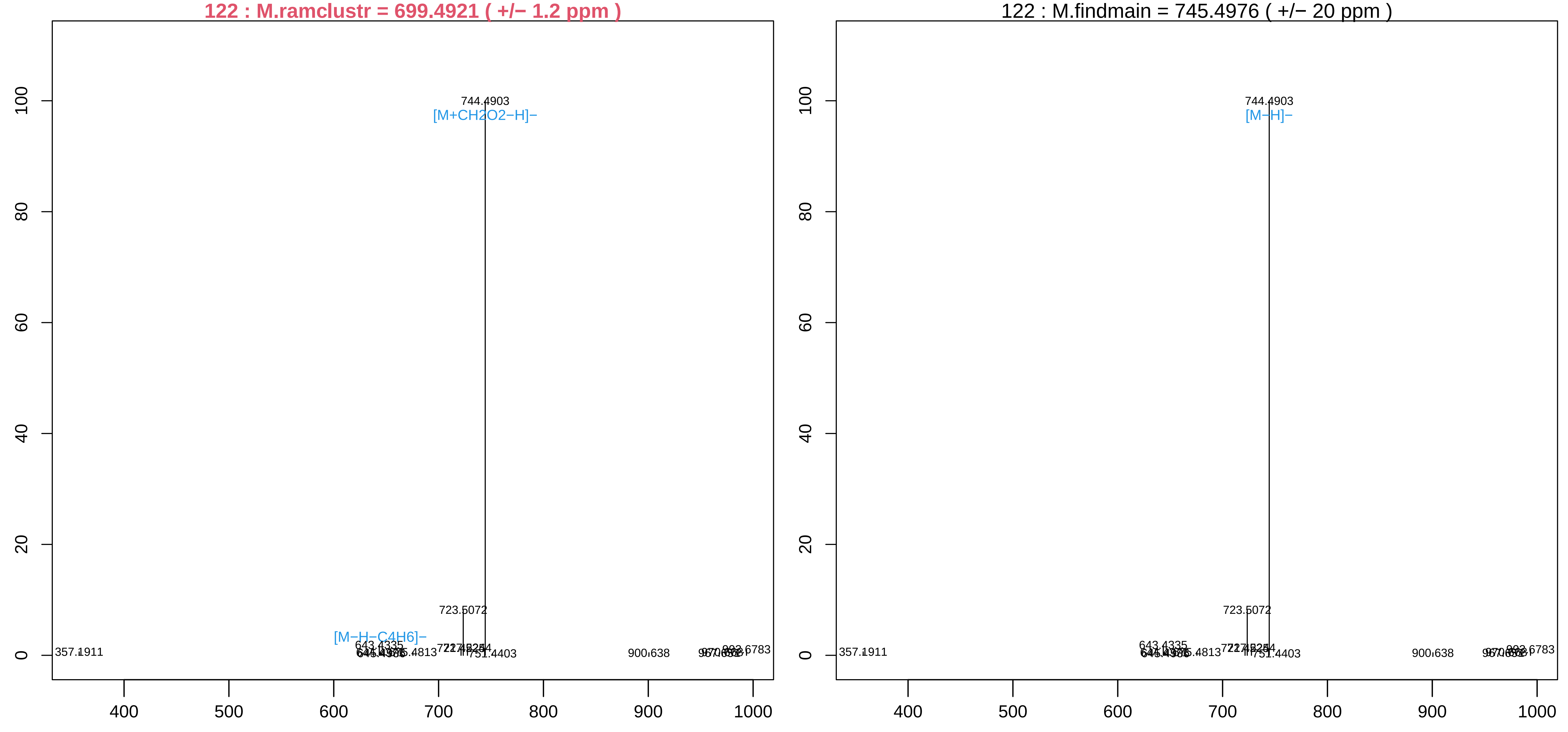
122 : M.ramclustr = 699.4921 (+/− 1.2 ppm)
744.4903
[M+CH2O2−H]−
723.5072
[M−H−C4H6]−
643.4335
357.1911
900.638
993.6783
751.4403
122 : M.findmain = 745.4976 (+/− 20 ppm)
744.4903
[M−H]−
723.5072
643.4335
357.1911
900.638
993.6783
751.4403
0
20
40
60
80
100
400
500
600
700
800
900
1000

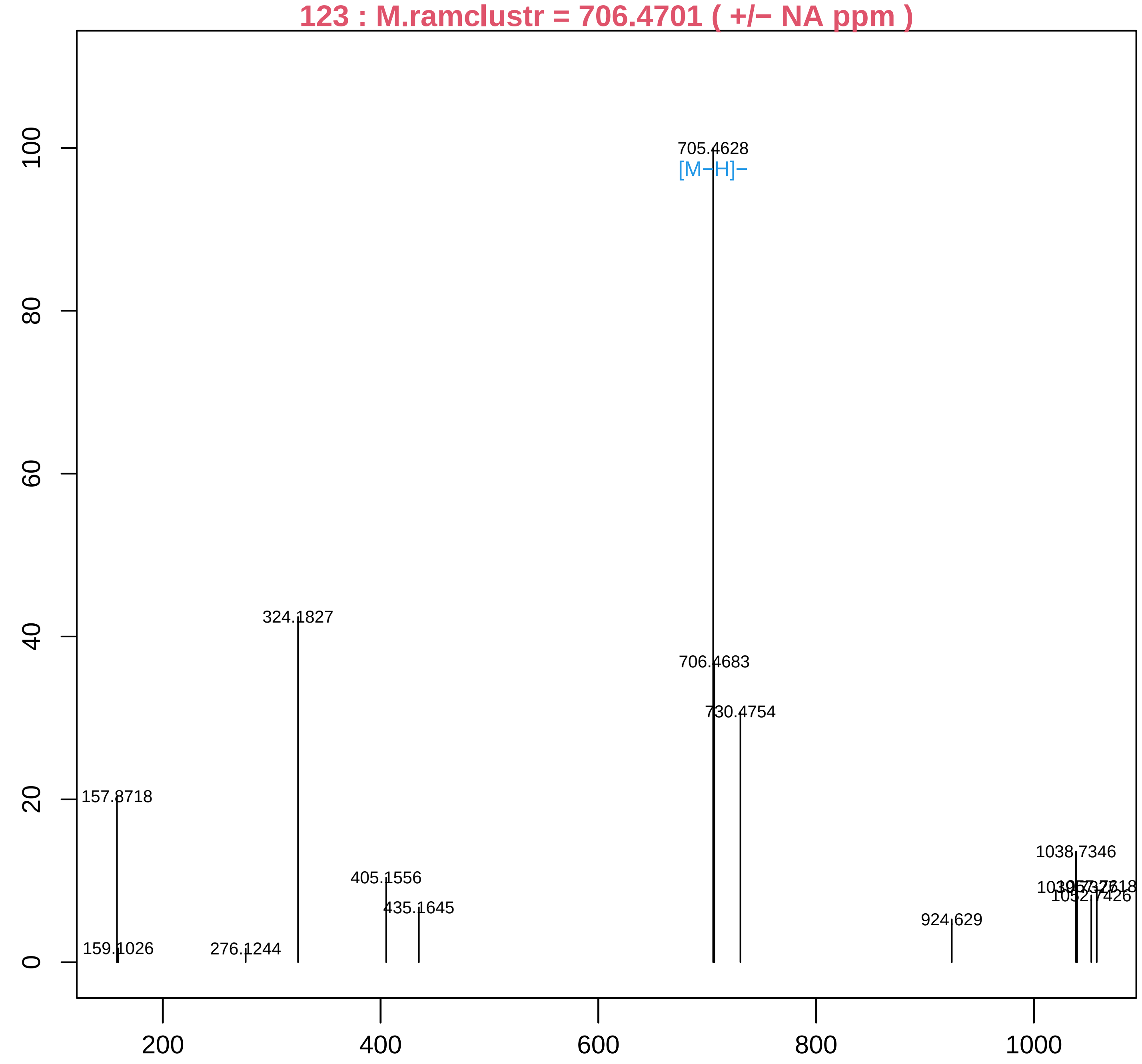

123 : M.ramclustr = 706.4701 (+/− NA ppm)
705.4628
[M−H]−
324.1827
706.4683
730.4754
157.8718
405.1556
435.1645
1038.7346
924.629
159.1026
276.1244
0
20
40
60
80
100
200
400
600
800
1000

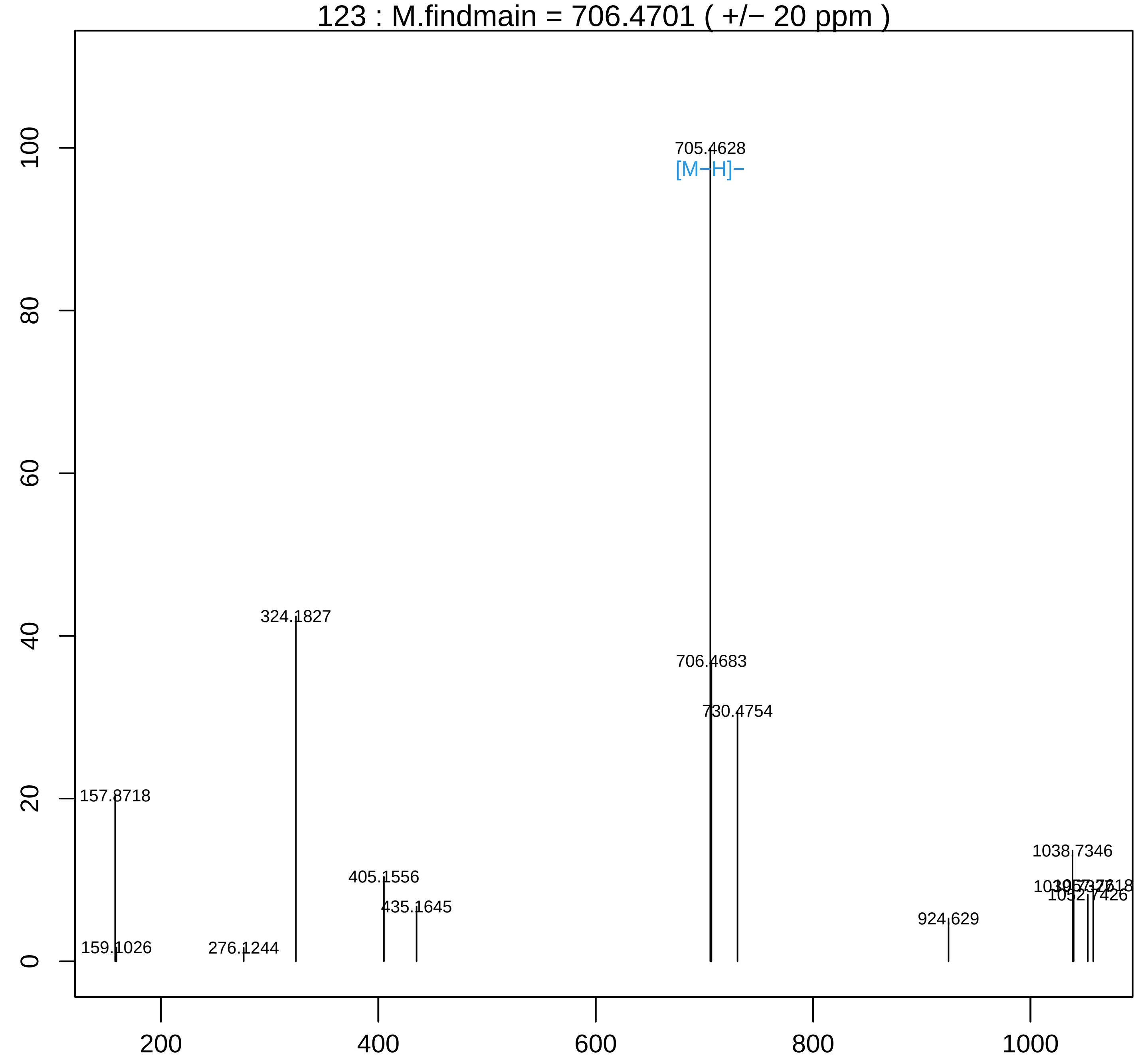

123 : M.findmain = 706.4701 (+/− 20 ppm)
705.4628
[M−H]−
324.1827
706.4683
730.4754
157.8718
405.1556
435.1645
1038.7346
924.629
159.1026
276.1244
0
20
40
60
80
100
200
400
600
800
1000

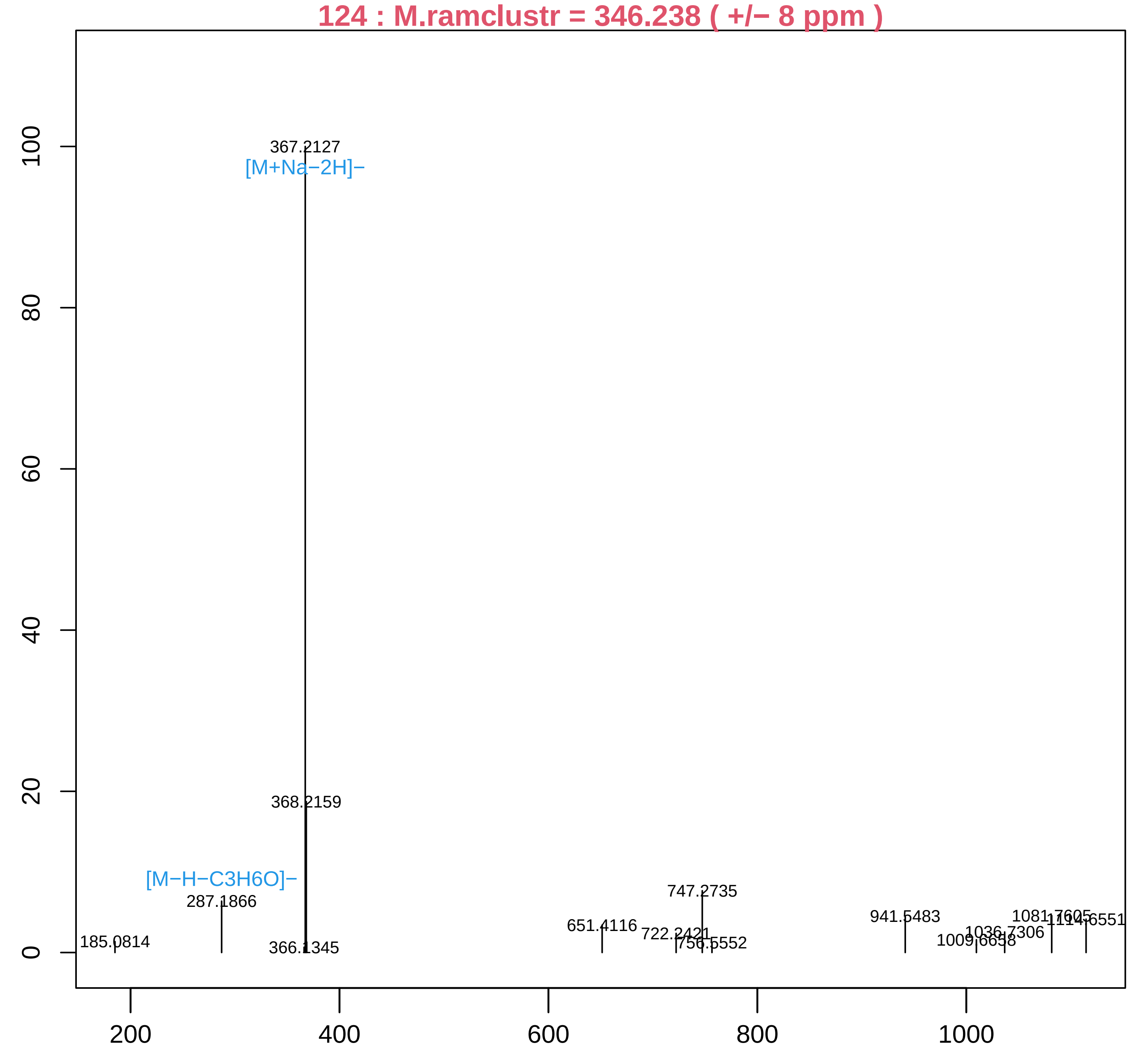
124 : M.ramclustr = 346.238 (+/− 8 ppm)
367.2127
[M+Na−2H]−
368.2159
[M−H−C3H6O]−
287.1866
185.0814
366.1345
651.4116
747.2735
722.2421
756.5552
941.5483
1009.6658
1036.7306
1081.7605
1114.6551
0
20
40
60
80
100
200
400
600
800
1000

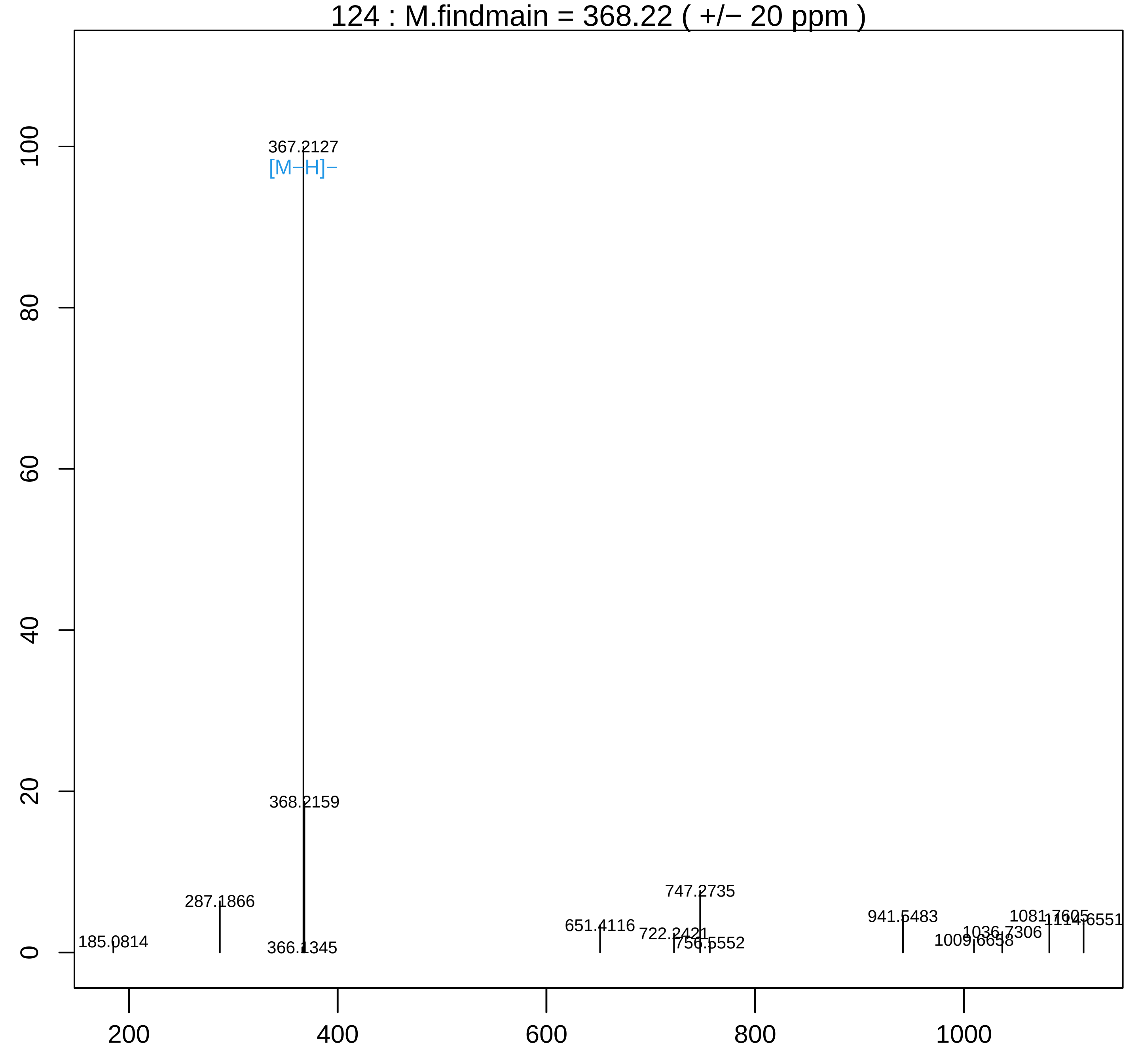
124 : M.findmain = 368.22 (+/− 20 ppm)
367.2127
[M−H]−
368.2159
287.1866
185.0814
366.1345
651.4116
747.2735
722.2421
756.5552
941.5483
1009.6658
1036.7306
1081.7605
1114.6551
0
20
40
60
80
100
200
400
600
800
1000

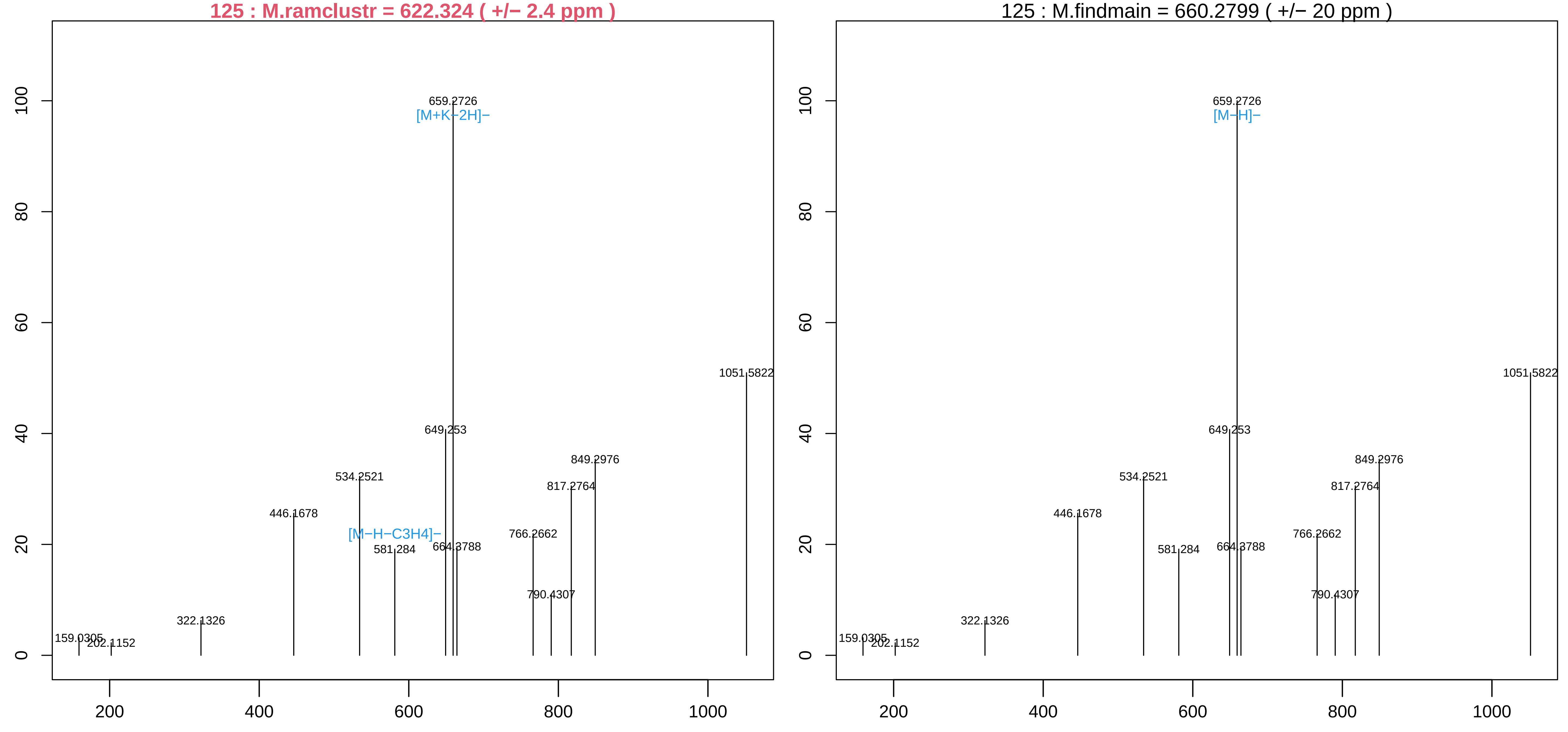
125 : M.ramclustr = 622.324 (+/− 2.4 ppm)
[M+K−2H]−
[M−H−C3H4]−
659.2726
649.253
534.2521
446.1678
581.284
664.3788
766.2662
790.4307
817.2764
849.2976
1051.5822
322.1326
159.0305
202.1152
125 : M.findmain = 660.2799 (+/− 20 ppm)
[M−H]−
0
20
40
60
80
100
200
400
600
800
1000

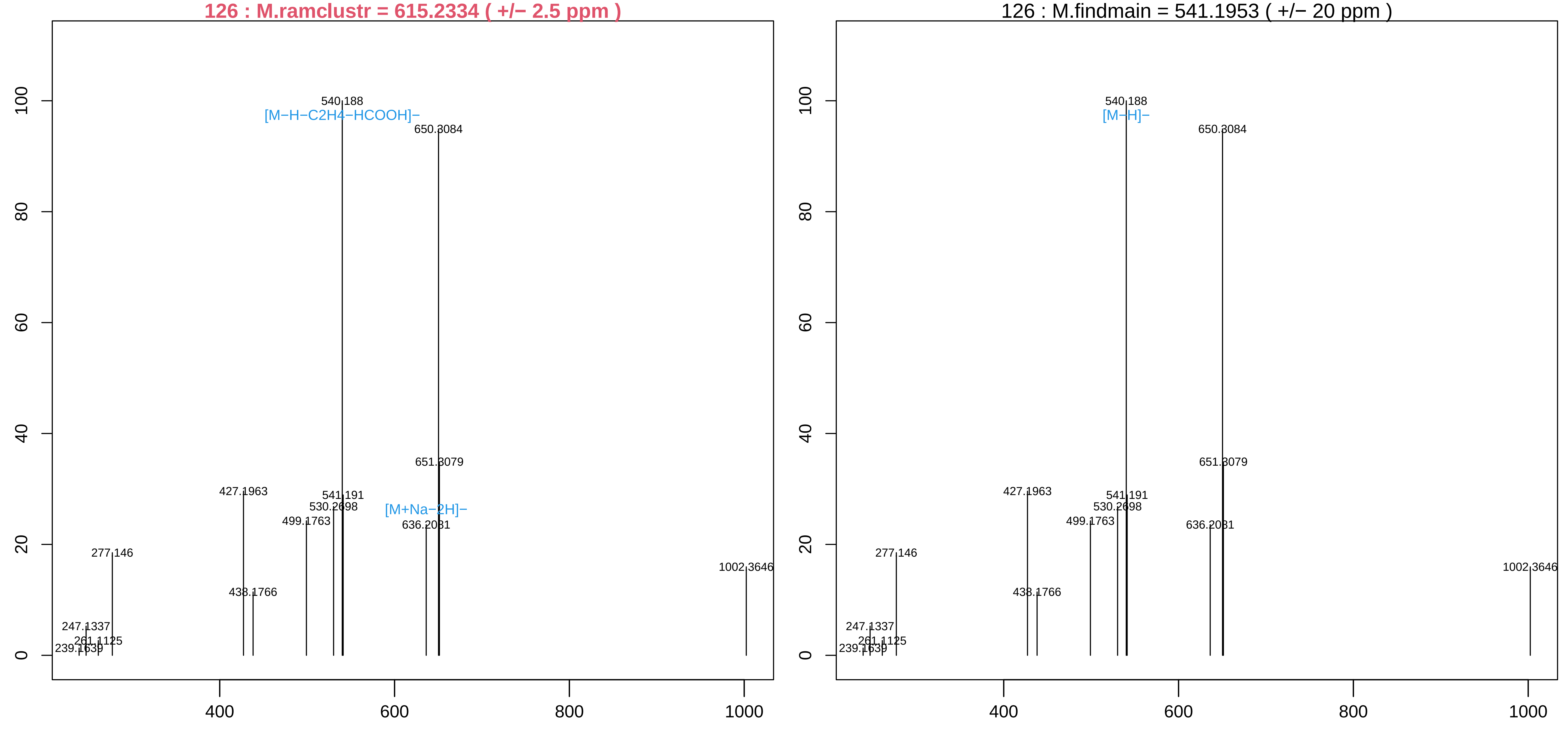
126 : M.ramclustr = 615.2334 (+/− 2.5 ppm)
126 : M.findmain = 541.1953 (+/− 20 ppm)
540.188
[M−H−C2H4−HCOOH]−
650.3084
651.3079
427.1963
541.191
530.2698
[M+Na−2H]−
499.1763
636.2031
277.146
1002.3646
438.1766
247.1337
261.1125
239.1639
[M−H]−
0
20
40
60
80
100
400
600
800
1000

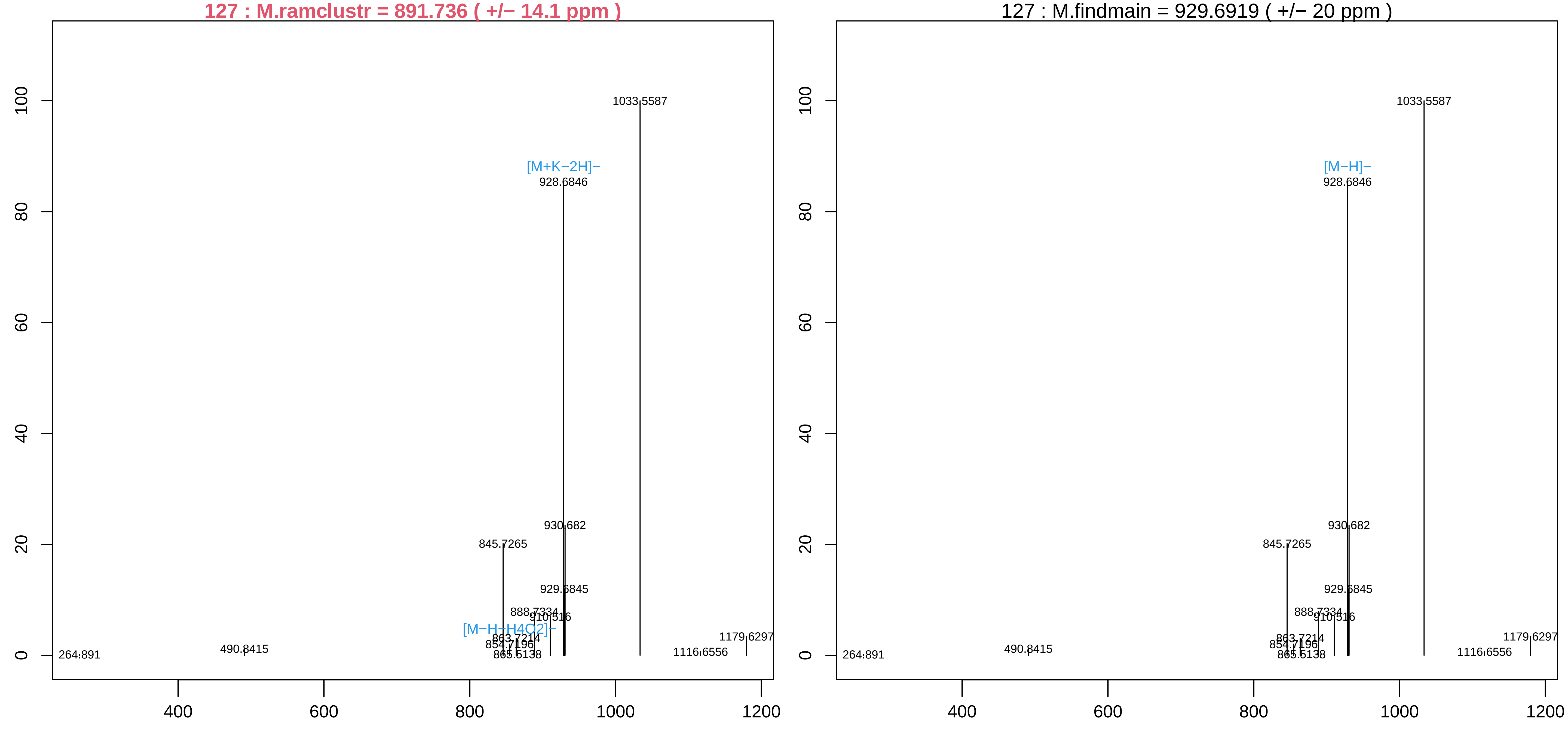
127 : M.ramclustr = 891.736 (+/− 14.1 ppm)
127 : M.findmain = 929.6919 (+/− 20 ppm)
1033.5587
[M+K−2H]−
[M−H]−
928.6846
930.682
845.7265
929.5845
888.7334
910.516
[M−H−H4O2]−
863.7214
854.7196
865.5138
490.8415
264.891
1116.6556
1179.6297
0
20
40
60
80
100
400
600
800
1000
1200

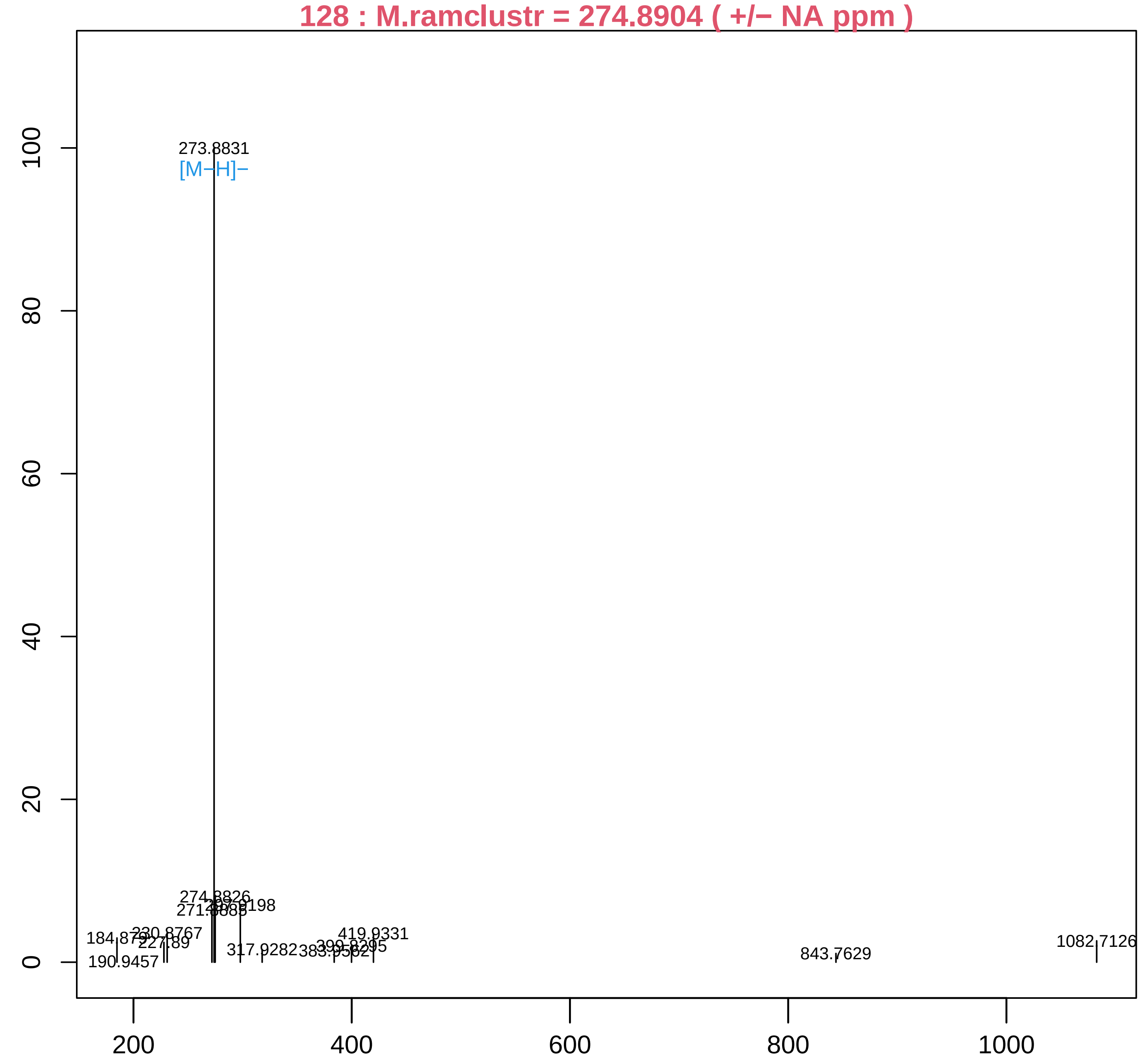
128 : M.ramclustr = 274.8904 (+/− NA ppm)
273.8831
[M−H]−
274.8826
271.8085
184.8799
230.8767
227.89
190.9457
317.9282
383.9562
399.9295
419.9331
843.7629
1082.7126
0
20
40
60
80
100
200
400
600
800
1000

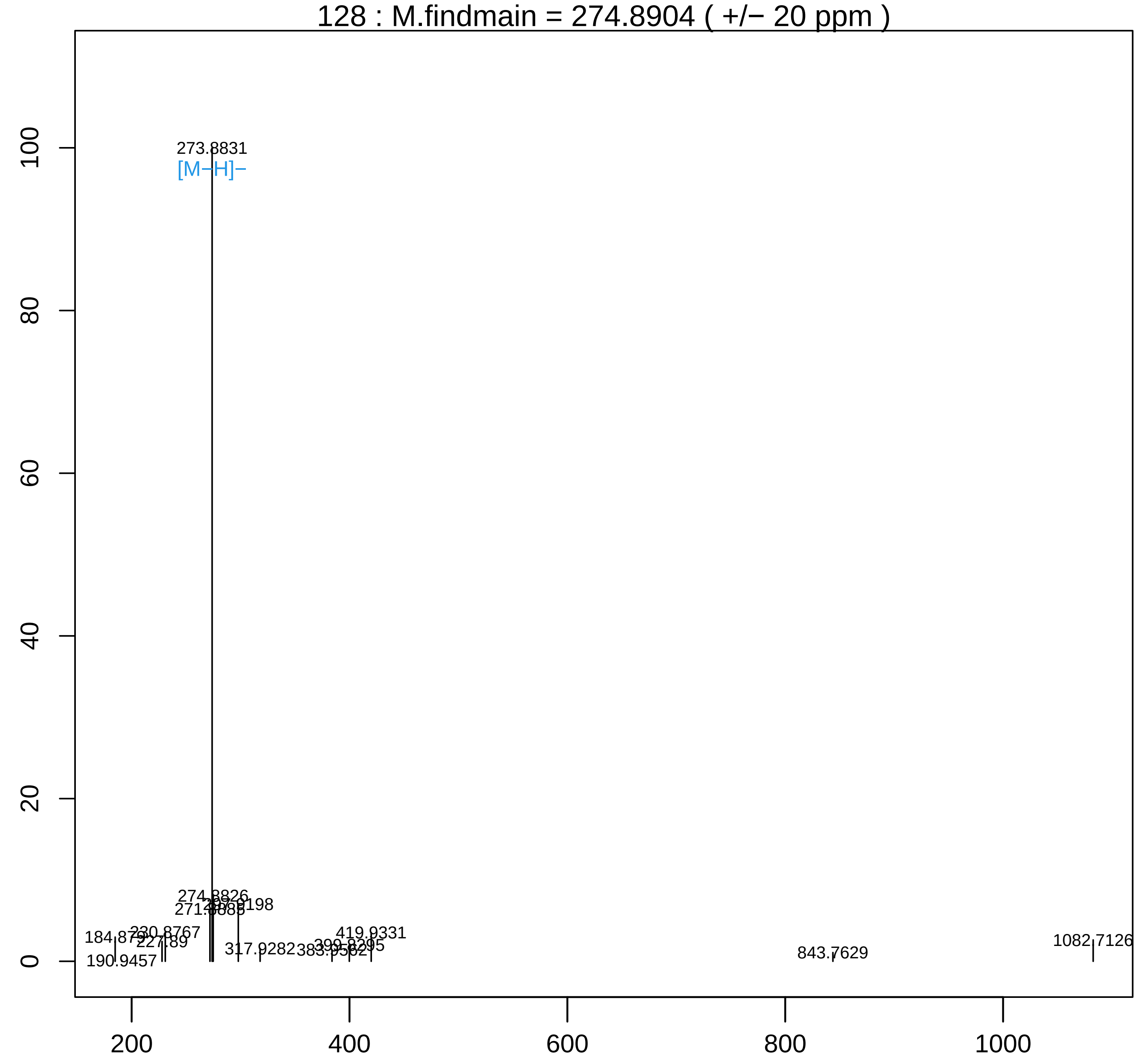
128 : M.findmain = 274.8904 (+/− 20 ppm)
273.8831
[M−H]−
274.8826
271.8085
184.8799
230.8767
227.89
190.9457
317.9282
383.9562
399.9295
419.9331
843.7629
1082.7126
0
20
40
60
80
100
200
400
600
800
1000

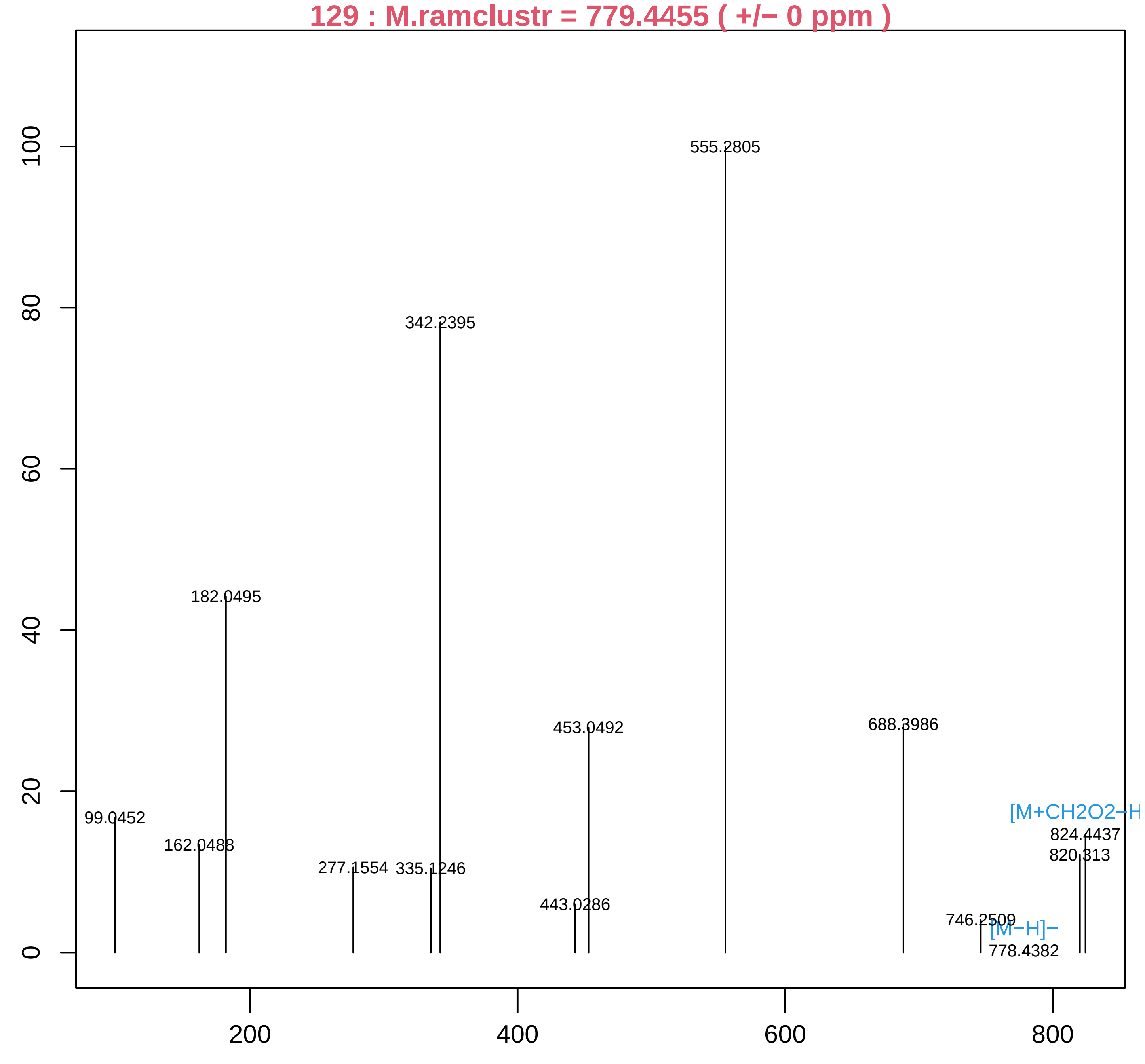

129 : M.ramclustr = 779.4455 (+/− 0 ppm)
0
20
40
60
80
100
200
400
600
800
99.0452
162.0488
182.0495
277.1554
335.1246
342.2395
443.0286
453.0492
555.2805
688.3986
746.2509
[M−H]−
778.4382
820.313
824.4437
[M+CH2O2−H

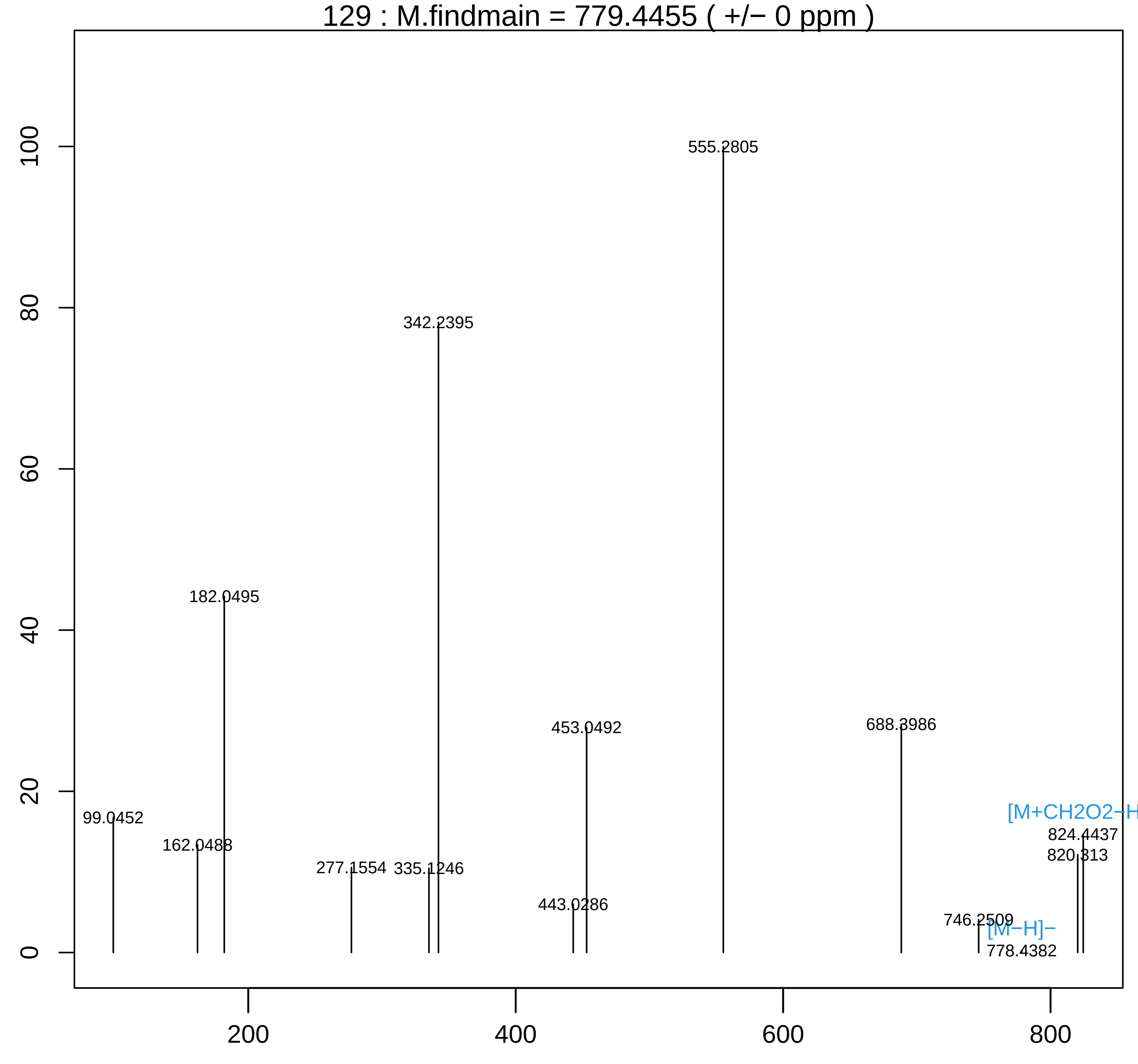

129 : M.findmain = 779.4455 (+/− 0 ppm)
0
20
40
60
80
100
200
400
600
800
99.0452
162.0488
182.0495
277.1554
335.1246
342.2395
443.0286
453.0492
555.2805
688.3986
746.2509
[M−H]−
778.4382
820.313
824.4437
[M+CH2O2−H

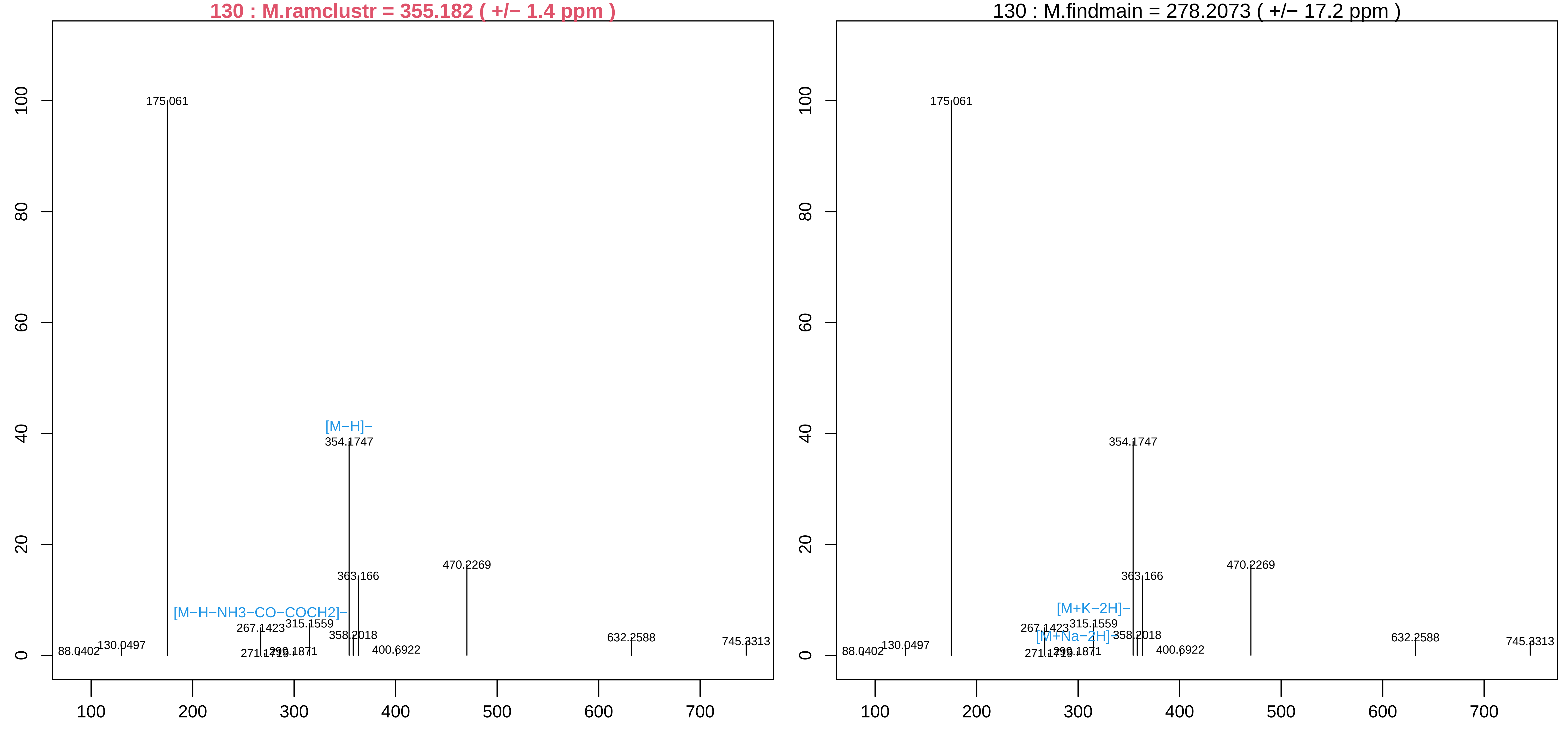

130 : M.ramclustr = 355.182 (+/− 1.4 ppm)
[M−H]−
354.1747
175.061
363.166
470.2269
[M−H−NH3−CO−COCH2]−
267.1423
315.1559
358.2018
88.0402
130.0497
271.1719
299.1871
400.6922
632.2588
745.3313
130 : M.findmain = 278.2073 (+/− 17.2 ppm)
[M+K−2H]−
[M+Na−2H]−

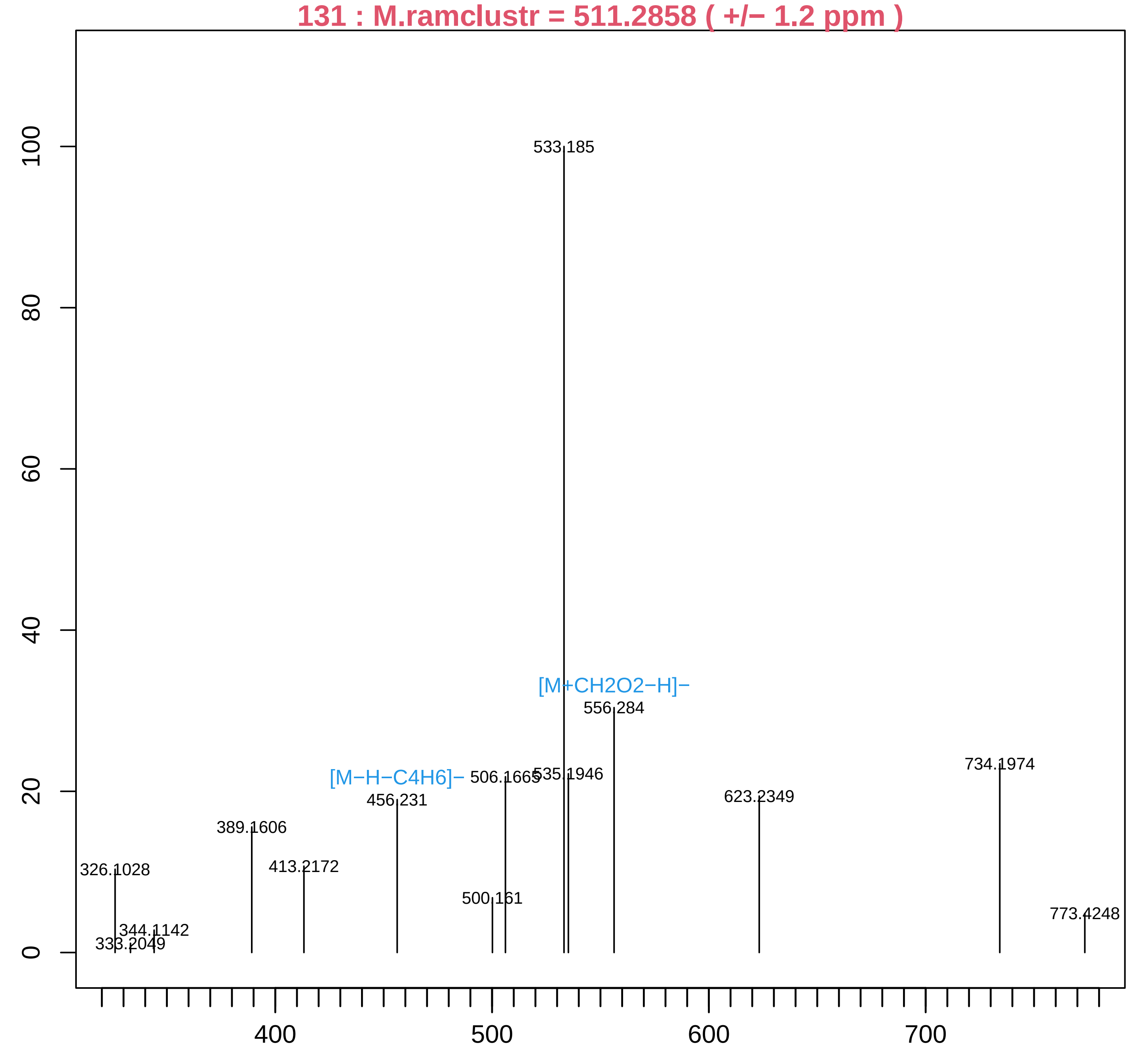
131 : M.ramclustr = 511.2858 (+/− 1.2 ppm)
533.185
[M+CH2O2−H]−
556.284
[M−H−C4H6]−
506.1665
535.1946
734.1974
456.231
623.2349
389.1606
326.1028
413.2172
500.161
773.4248
344.1142
333.2049
0
20
40
60
80
100
400
500
600
700

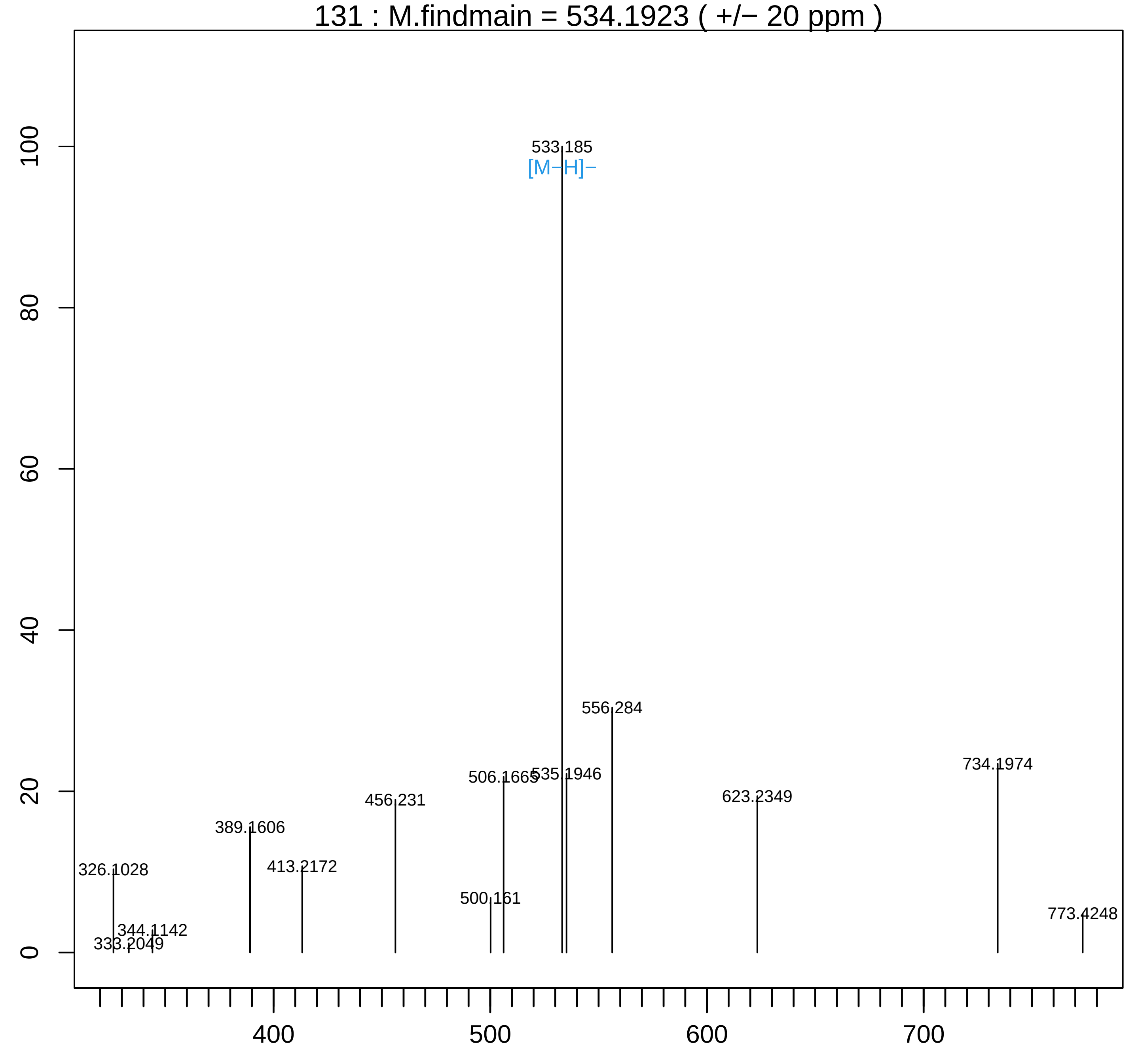
131 : M.findmain = 534.1923 (+/− 20 ppm)
533.185
[M−H]−
556.284
506.1665
535.1946
734.1974
456.231
623.2349
389.1606
326.1028
413.2172
500.161
773.4248
344.1142
333.2049
0
20
40
60
80
100
400
500
600
700

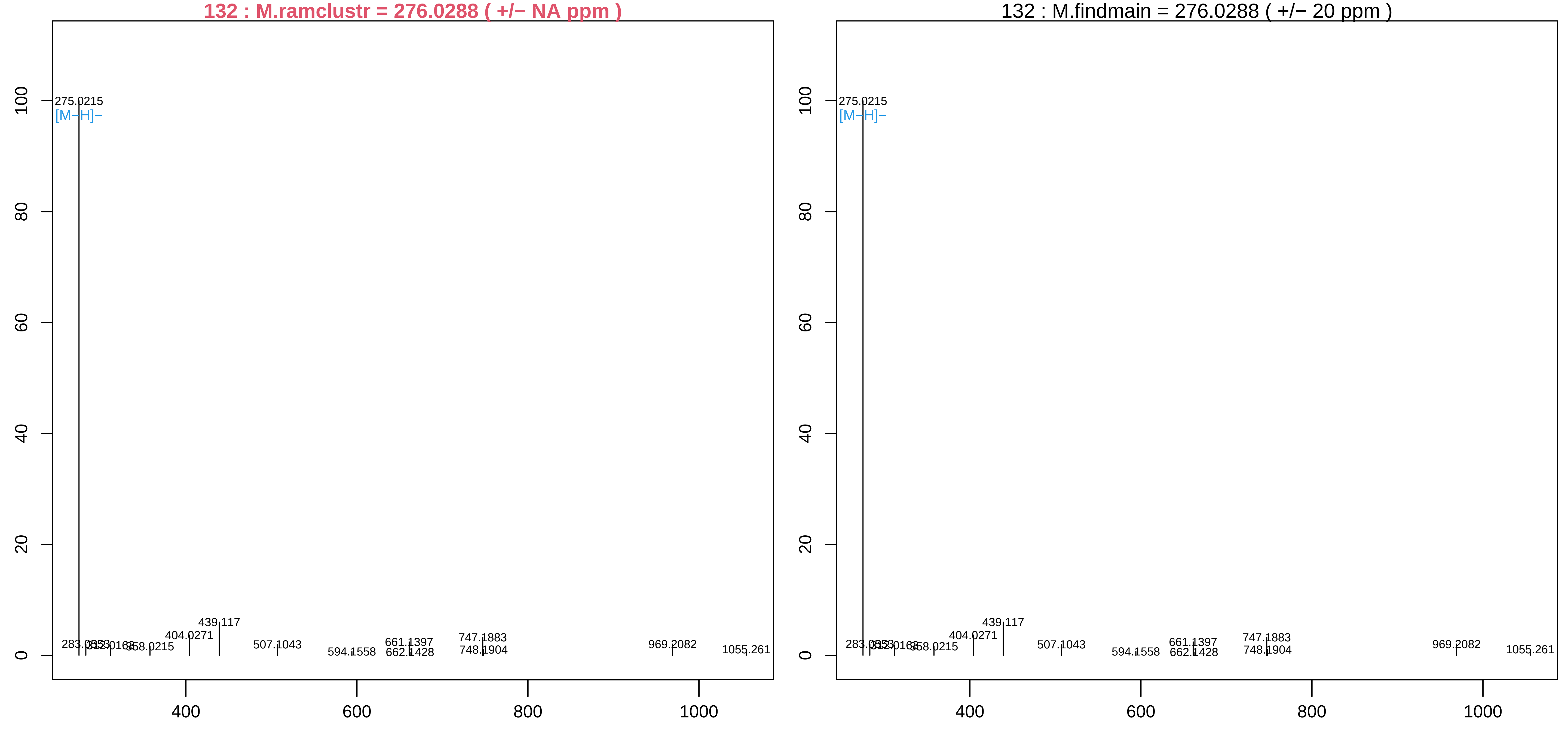

132 : M.ramclustr = 276.0288 (+/− NA ppm)
275.0215
[M−H]−
283.0553
313.0168
358.0215
404.0271
439.117
507.1043
594.1558
661.1397
662.1428
747.1883
748.1904
969.2082
1055.261
132 : M.findmain = 276.0288 (+/− 20 ppm)
275.0215
[M−H]−
283.0553
313.0168
358.0215
404.0271
439.117
507.1043
594.1558
661.1397
662.1428
747.1883
748.1904
969.2082
1055.261

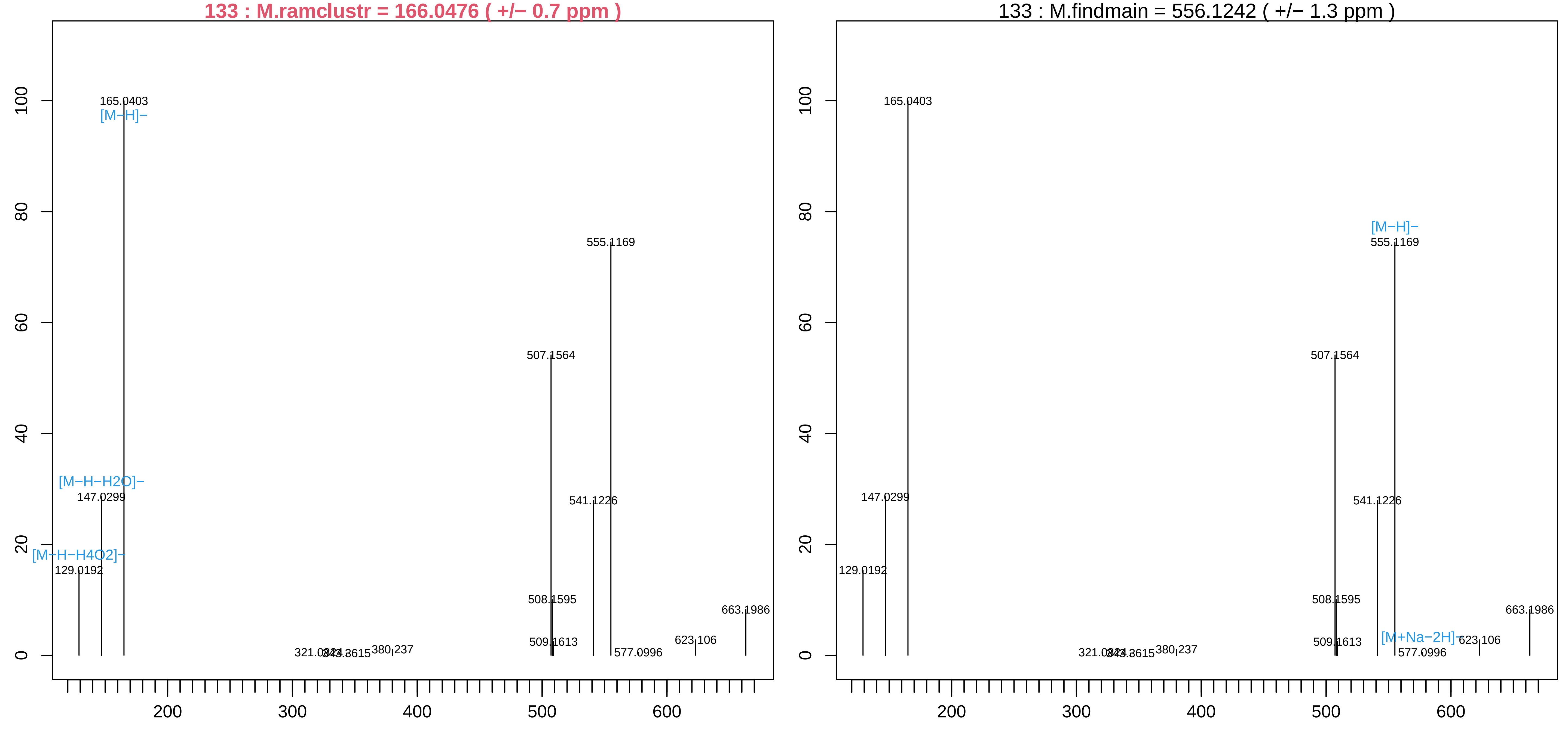
133 : M.ramclustr = 166.0476 (+/− 0.7 ppm)
165.0403
[M−H]−
[M−H−H2O]−
147.0299
[M−H−H4O2]−
129.0192
321.0824
343.3615
380.237
507.1564
508.1595
509.1613
541.1226
555.1169
577.0996
623.106
663.1986
0
20
40
60
80
100
200
300
400
500
600
133 : M.findmain = 556.1242 (+/− 1.3 ppm)
165.0403
147.0299
129.0192
321.0824
343.3615
380.237
507.1564
508.1595
509.1613
541.1226
[M−H]−
555.1169
[M+Na−2H]−
577.0996
623.106
663.1986

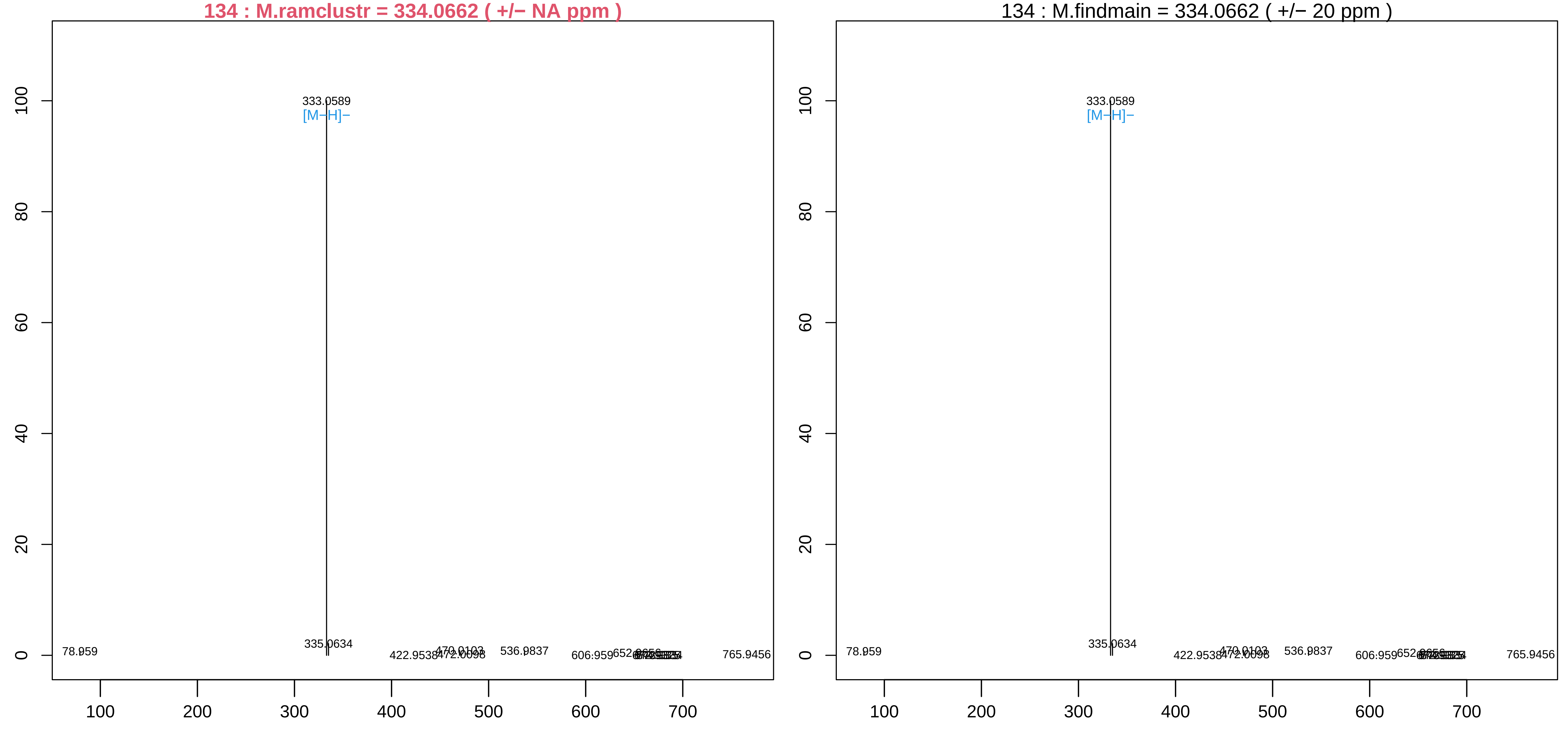
134 : M.ramclustr = 334.0662 (+/− NA ppm)
134 : M.findmain = 334.0662 (+/− 20 ppm)
333.0589
[M−H]−
335.0634
78.959
422.9538
470.0103
472.0098
536.9837
606.959
765.9456
0
20
40
60
80
100
200
300
400
500
600
700

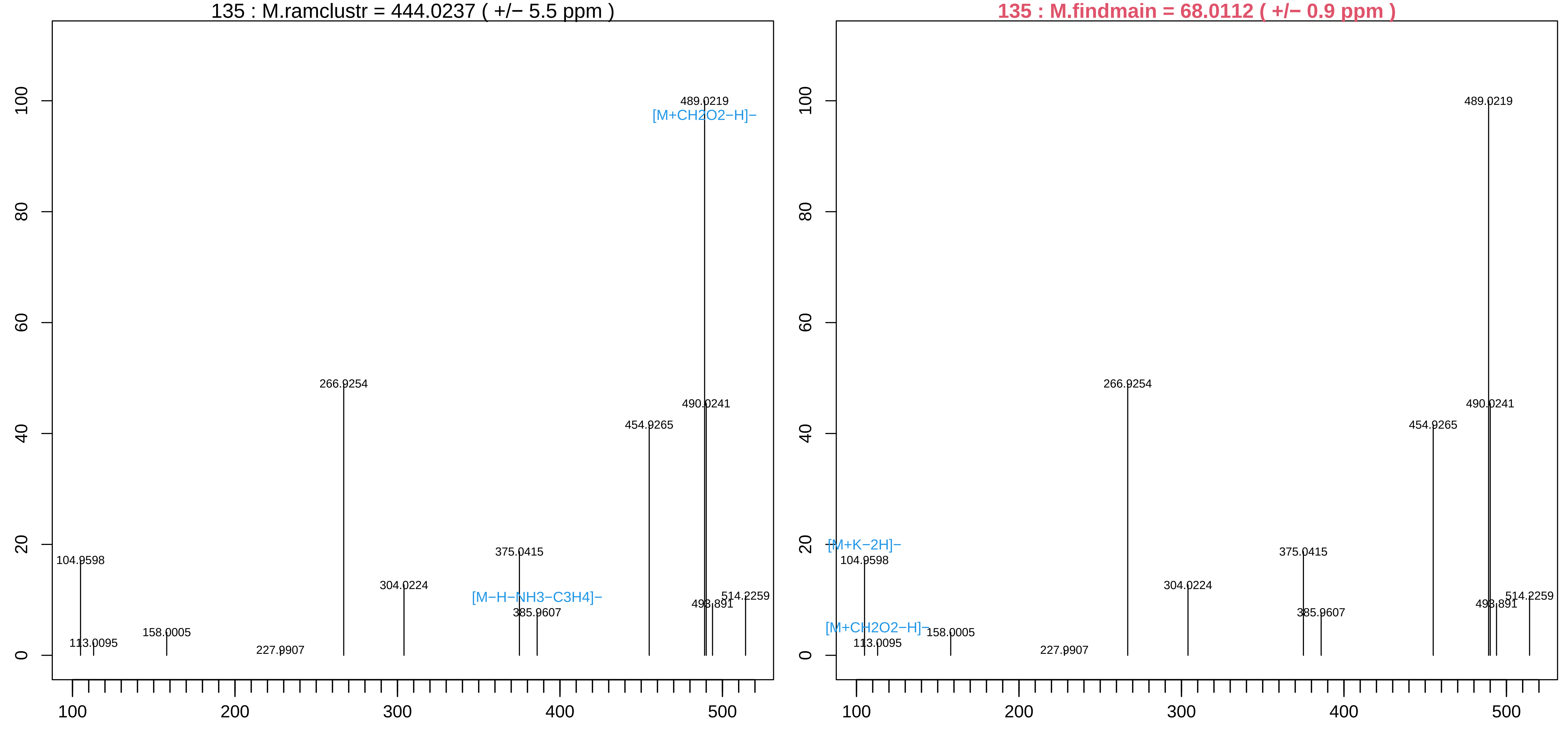
135 : M.ramclustr = 444.0237 (+/− 5.5 ppm)
135 : M.findmain = 68.0112 (+/− 0.9 ppm)
489.0219
[M+CH2O2−H]−
266.9254
490.0241
454.9265
375.0415
104.9598
304.0224
[M−H−NH3−C3H4]−
385.9607
493.891
514.2259
158.0005
113.0095
227.9907
[M+K−2H]−
[M+CH2O2−H]−
0
20
40
60
80
100
200
300
400
500

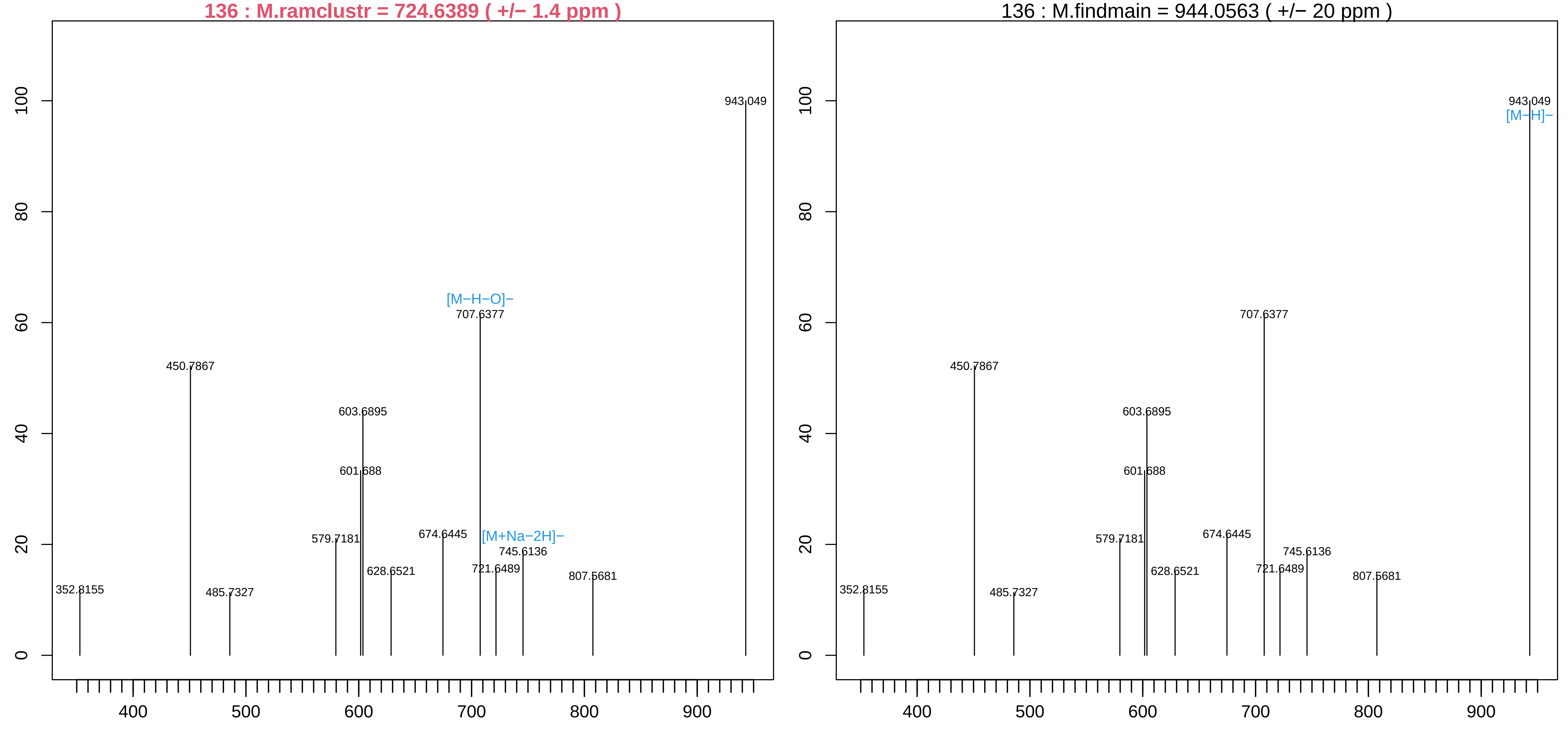
136 : M.ramclustr = 724.6389 (+/− 1.4 ppm)
943.049
450.7867
603.6895
601.688
579.7181
674.6445
628.6521
[M−H−O]−
707.6377
[M+Na−2H]−
745.6136
721.6489
807.5681
352.8155
485.7327
136 : M.findmain = 944.0563 (+/− 20 ppm)
943.049
[M−H]−
0
20
40
60
80
100
400
500
600
700
800
900

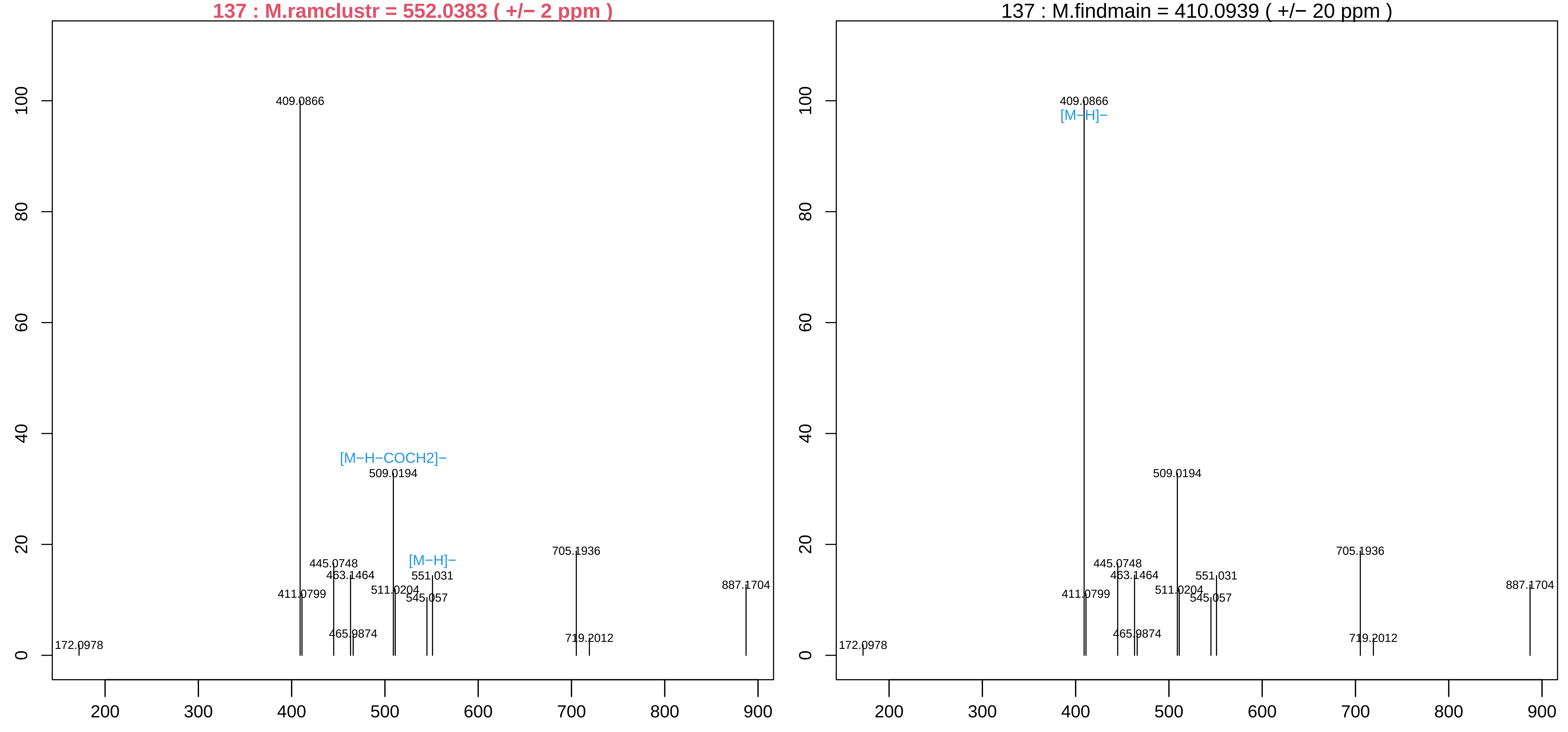
137 : M.ramclustr = 552.0383 (+/− 2 ppm)
137 : M.findmain = 410.0939 (+/− 20 ppm)
409.0866
[M−H]−
[M−H−COCH2]−
509.0194
445.0748
463.1464
411.0799
511.0204
551.031
545.057
465.9874
705.1936
719.2012
887.1704
172.0978
0
20
40
60
80
100
200
300
400
500
600
700
800
900

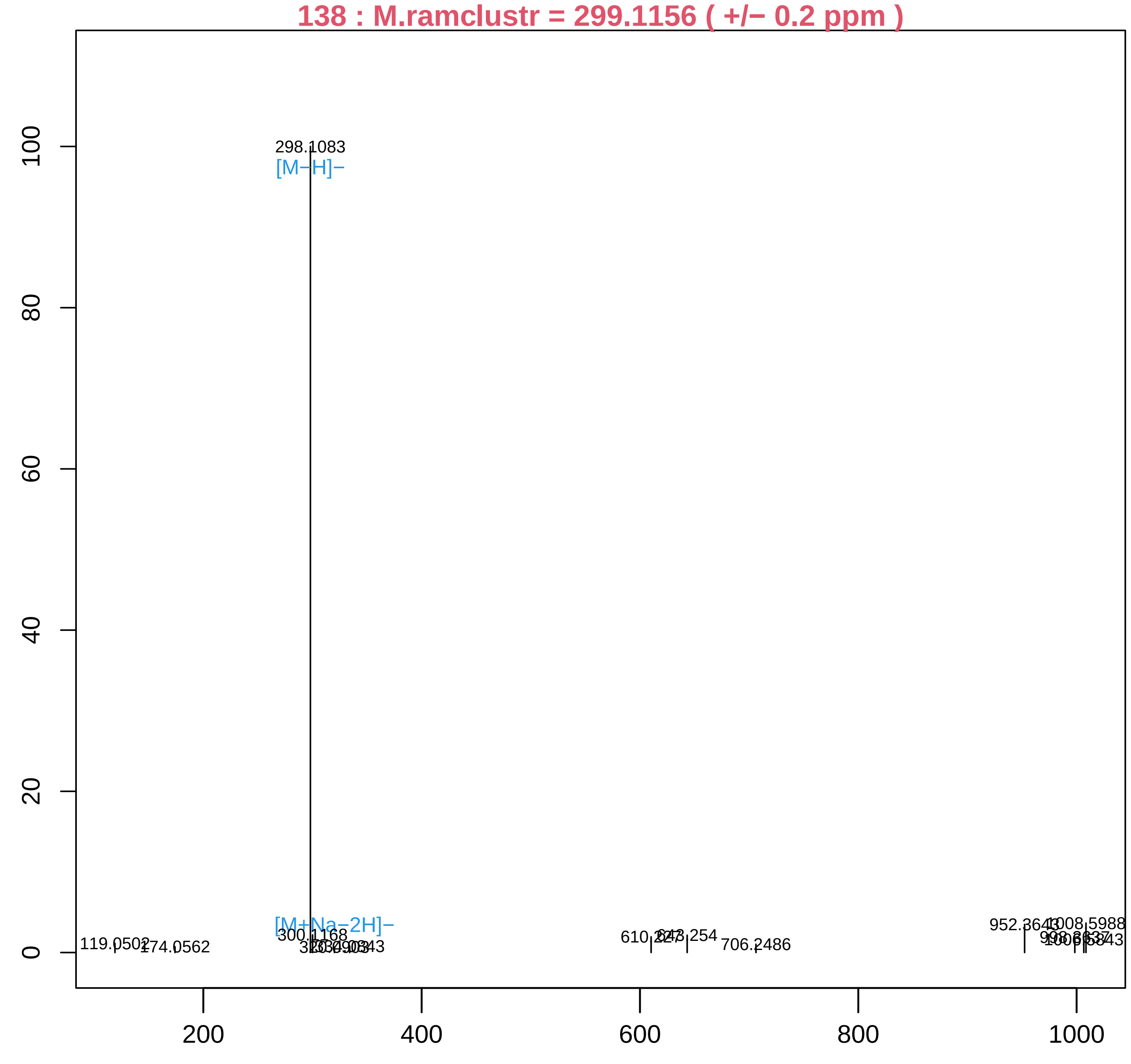
138 : M.ramclustr = 299.1156 (+/− 0.2 ppm)
298.1083
[M−H]−
[M+Na−2H]−
300.1168
119.0502
174.0562
610.227
643.254
706.2486
952.3643
1008.5988
0
20
40
60
80
100
200
400
600
800
1000

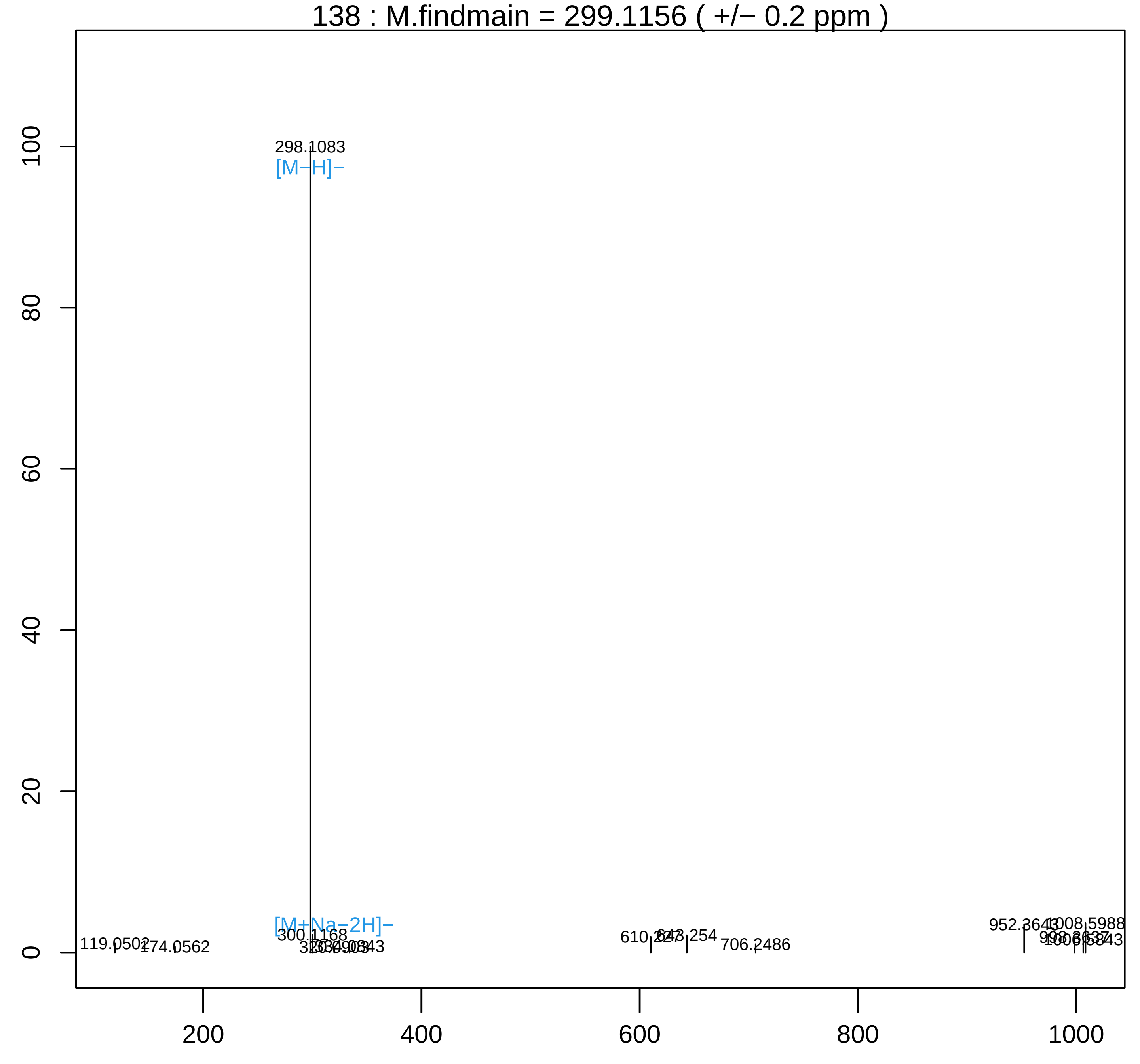
138 : M.findmain = 299.1156 (+/− 0.2 ppm)
298.1083
[M−H]−
[M+Na−2H]−
300.1168
119.0502
174.0562
610.227
643.254
706.2486
952.3643
1008.5988
0
20
40
60
80
100
200
400
600
800
1000

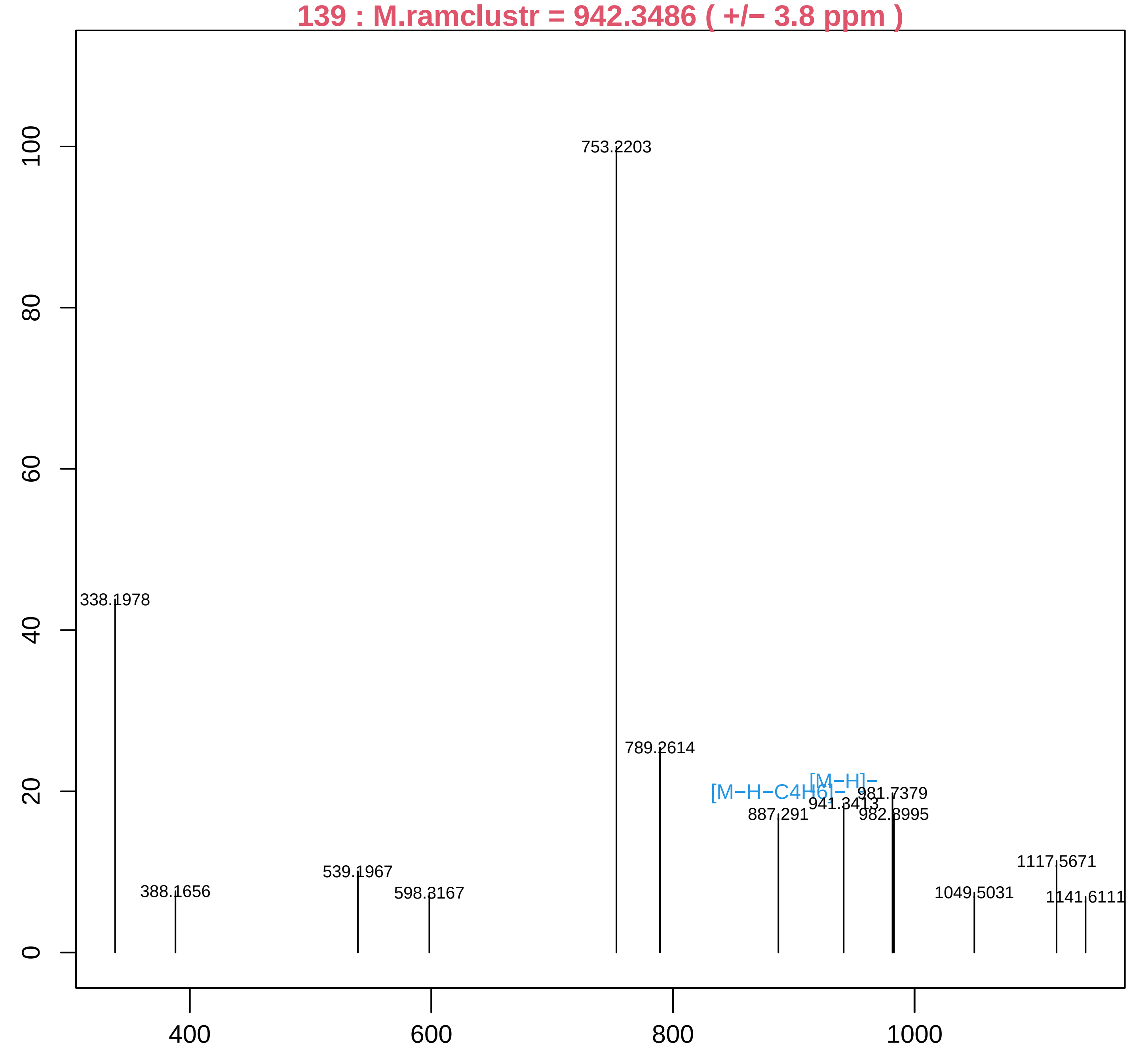
139 : M.ramclustr = 942.3486 (+/− 3.8 ppm)
753.2203
338.1978
789.2614
[M−H−C4H6]−
[M−H]−
981.7379
941.3413
982.3995
887.291
1117.5671
539.1967
388.1656
598.3167
1049.5031
1141.6111
0
20
40
60
80
100
400
600
800
1000

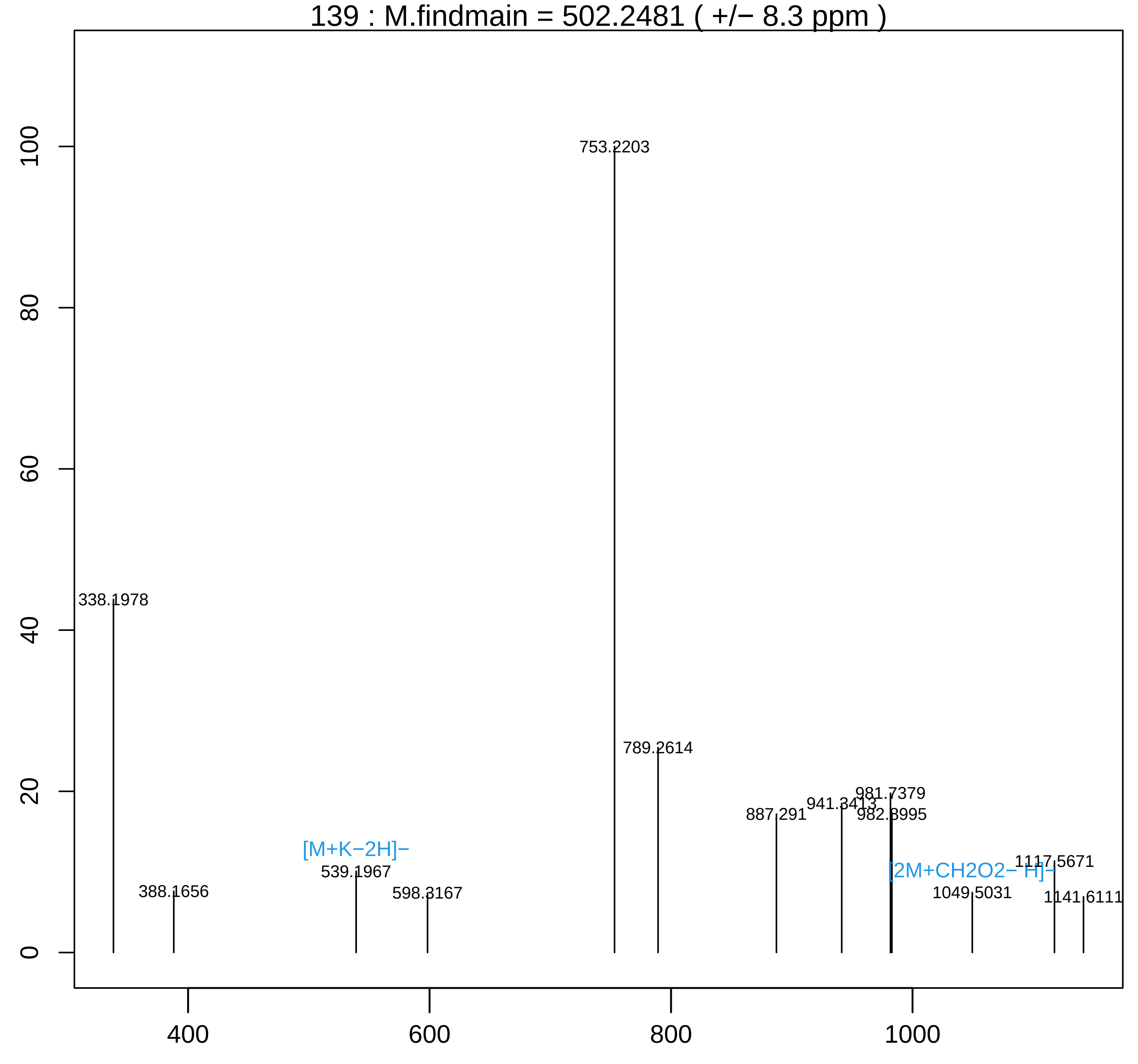
139 : M.findmain = 502.2481 (+/− 8.3 ppm)
753.2203
338.1978
789.2614
981.7379
941.3413
982.3995
887.291
[M+K−2H]−
539.1967
[2M+CH2O2−H]−
1117.5671
388.1656
598.3167
1049.5031
1141.6111
0
20
40
60
80
100
400
600
800
1000

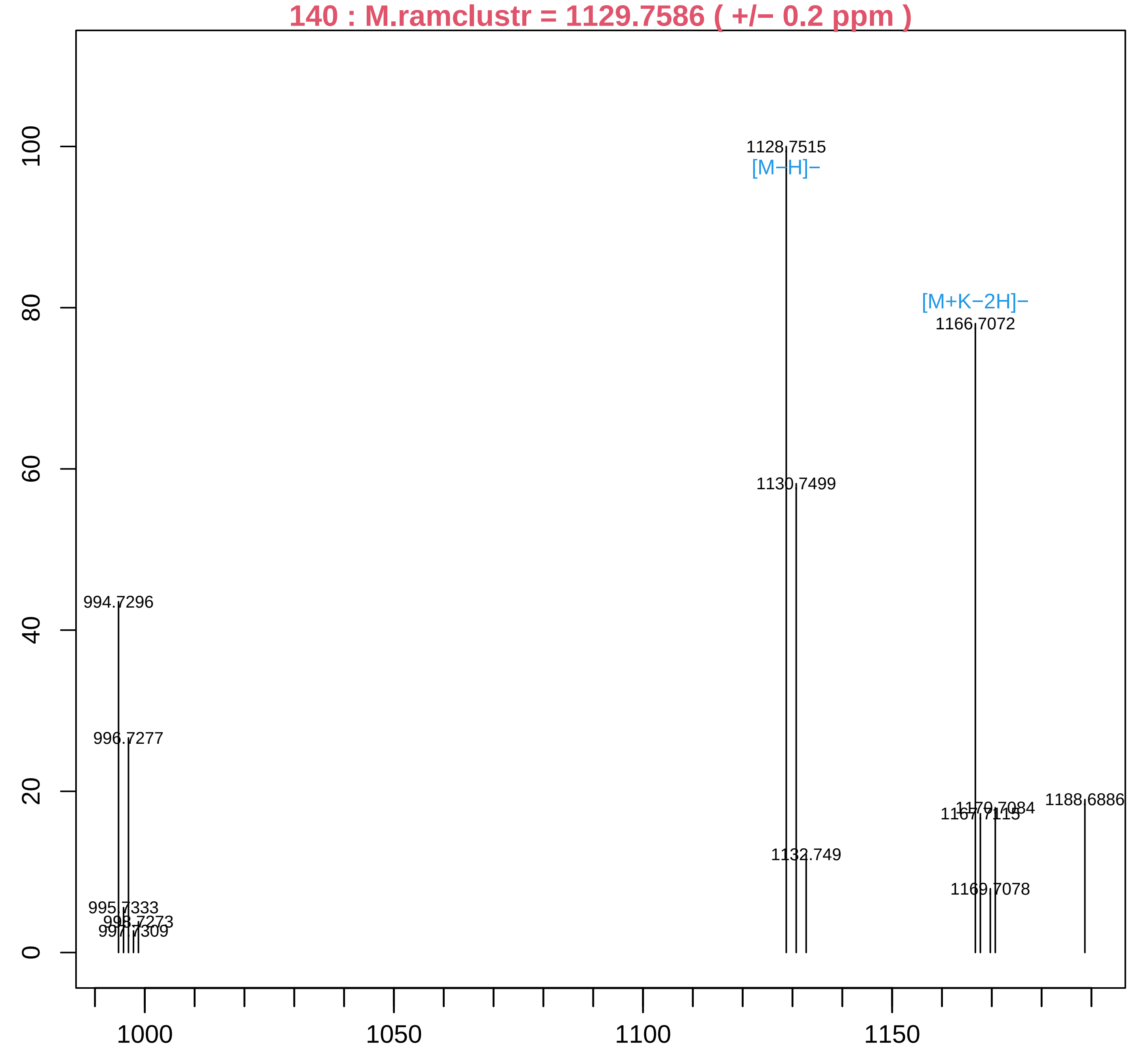
140 : M.ramclustr = 1129.7586 (+/− 0.2 ppm)
1128.7515
[M−H]−
1130.7499
1132.749
[M+K−2H]−
1166.7072
1167.7115
1169.7078
1170.7084
1188.6886
994.7296
995.7333
996.7277
997.7309
998.7273
0
20
40
60
80
100
1000
1050
1100
1150

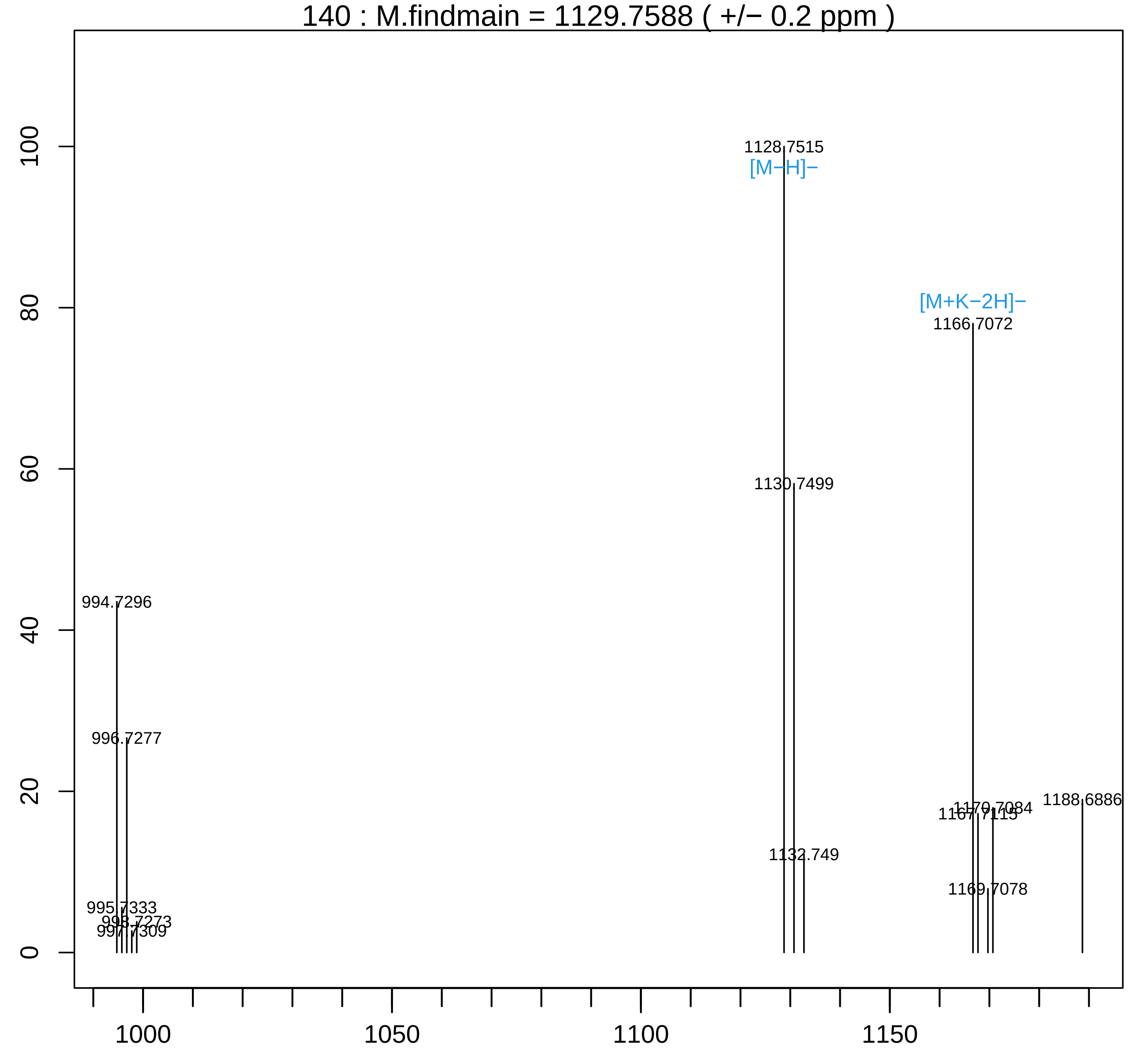
140 : M.findmain = 1129.7588 (+/− 0.2 ppm)
1128.7515
[M−H]−
1130.7499
1132.749
[M+K−2H]−
1166.7072
1167.7115
1169.7078
1170.7084
1188.6886
994.7296
995.7333
996.7277
997.7309
998.7273
0
20
40
60
80
100
1000
1050
1100
1150

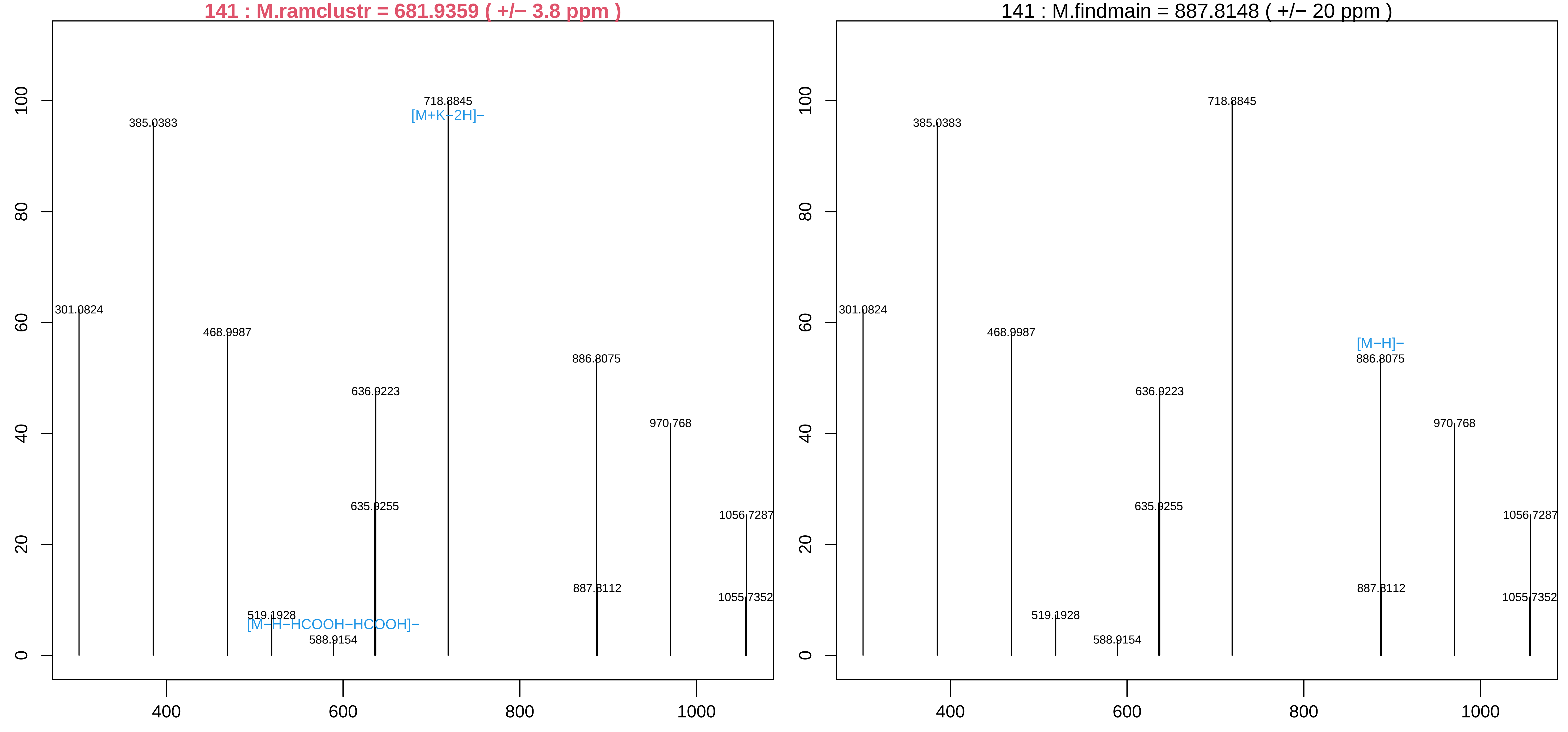

141 : M.ramclustr = 681.9359 (+/− 3.8 ppm)
141 : M.findmain = 887.8148 (+/− 20 ppm)
301.0824
385.0383
468.9987
519.1928
[M−H−HCOOH−HCOOH]−
588.9154
635.9255
636.9223
718.8845
[M+K−2H]−
886.8075
[M−H]−
887.8112
970.768
1055.7352
1056.7287
0
20
40
60
80
100
400
600
800
1000

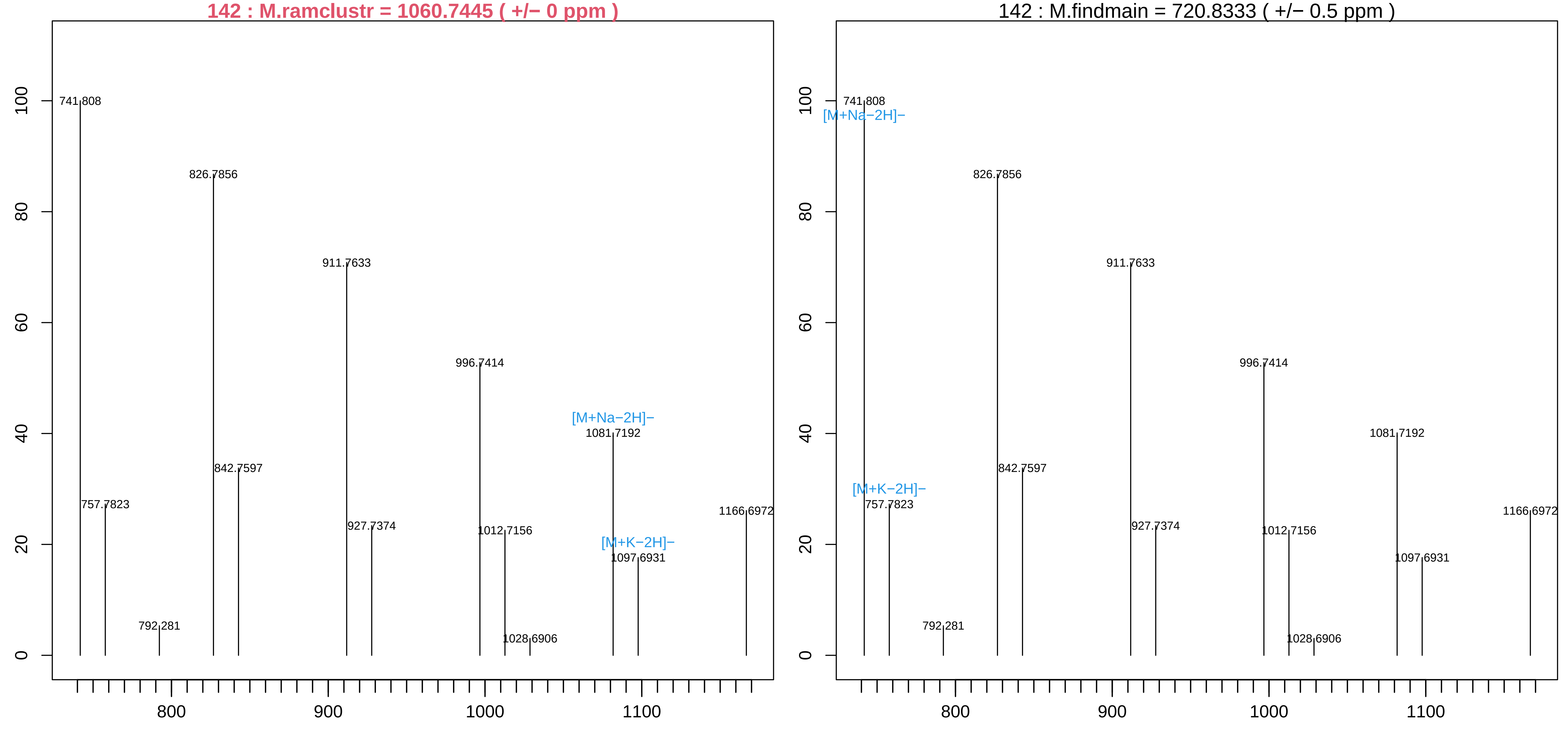

142 : M.ramclustr = 1060.7445 (+/− 0 ppm)
741.808
826.7856
911.7633
996.7414
[M+Na−2H]−
1081.7192
842.7597
757.7823
927.7374
1012.7156
[M+K−2H]−
1097.6931
1166.6972
792.281
1028.6906
0
20
40
60
80
100
800
900
1000
1100
142 : M.findmain = 720.8333 (+/− 0.5 ppm)
741.808
[M+Na−2H]−
826.7856
911.7633
996.7414
1081.7192
842.7597
[M+K−2H]−
757.7823
927.7374
1012.7156
1166.6972
1097.6931
792.281
1028.6906
0
20
40
60
80
100
800
900
1000
1100

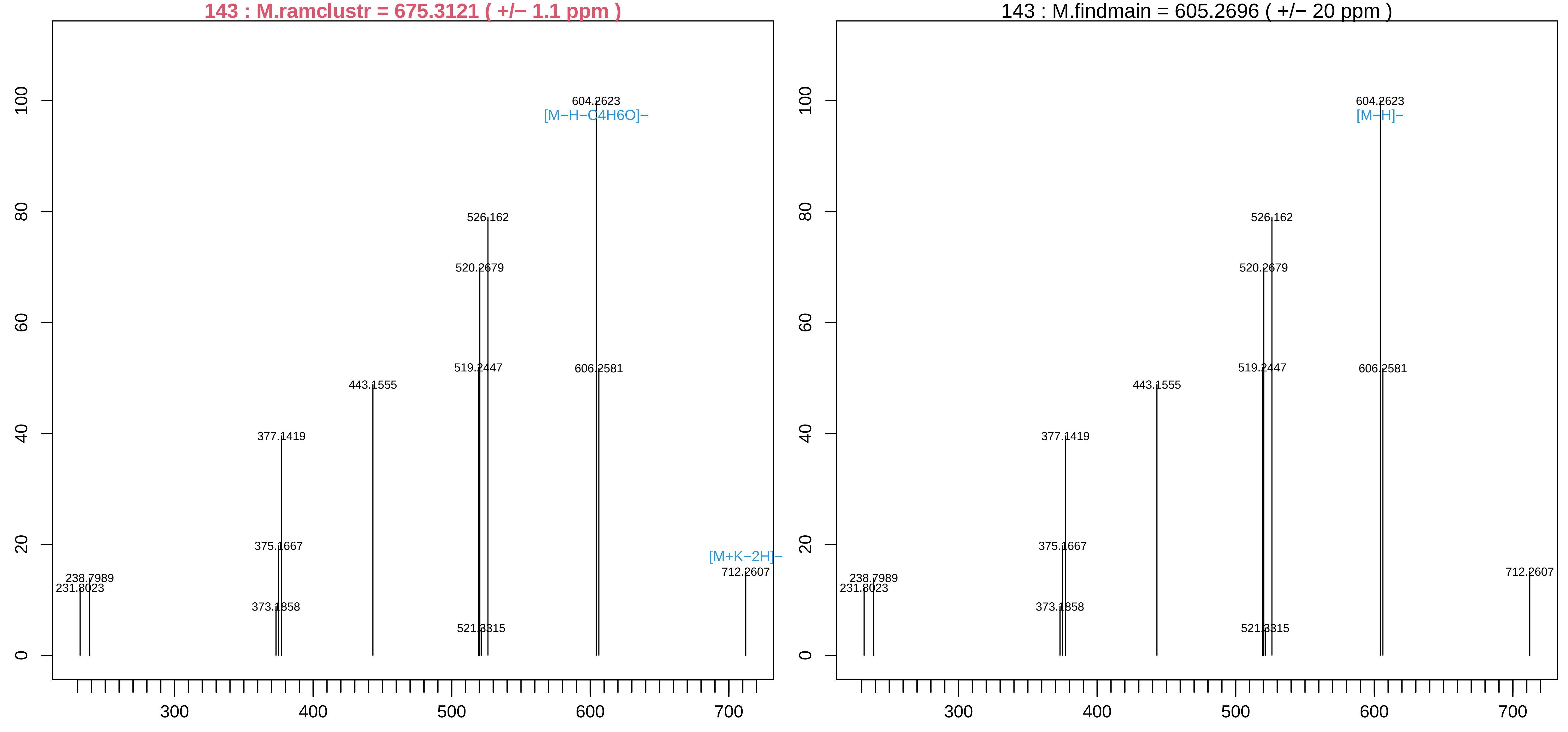
143 : M.ramclustr = 675.3121 (+/− 1.1 ppm)
604.2623
[M−H−C4H6O]−
526.162
520.2679
519.2447
606.2581
443.1555
377.1419
375.1667
373.1858
521.3315
238.7989
231.8023
[M+K−2H]−
712.2607
143 : M.findmain = 605.2696 (+/− 20 ppm)
604.2623
[M−H]−
526.162
520.2679
519.2447
606.2581
443.1555
377.1419
375.1667
373.1858
521.3315
238.7989
231.8023
712.2607
0
20
40
60
80
100
300
400
500
600
700

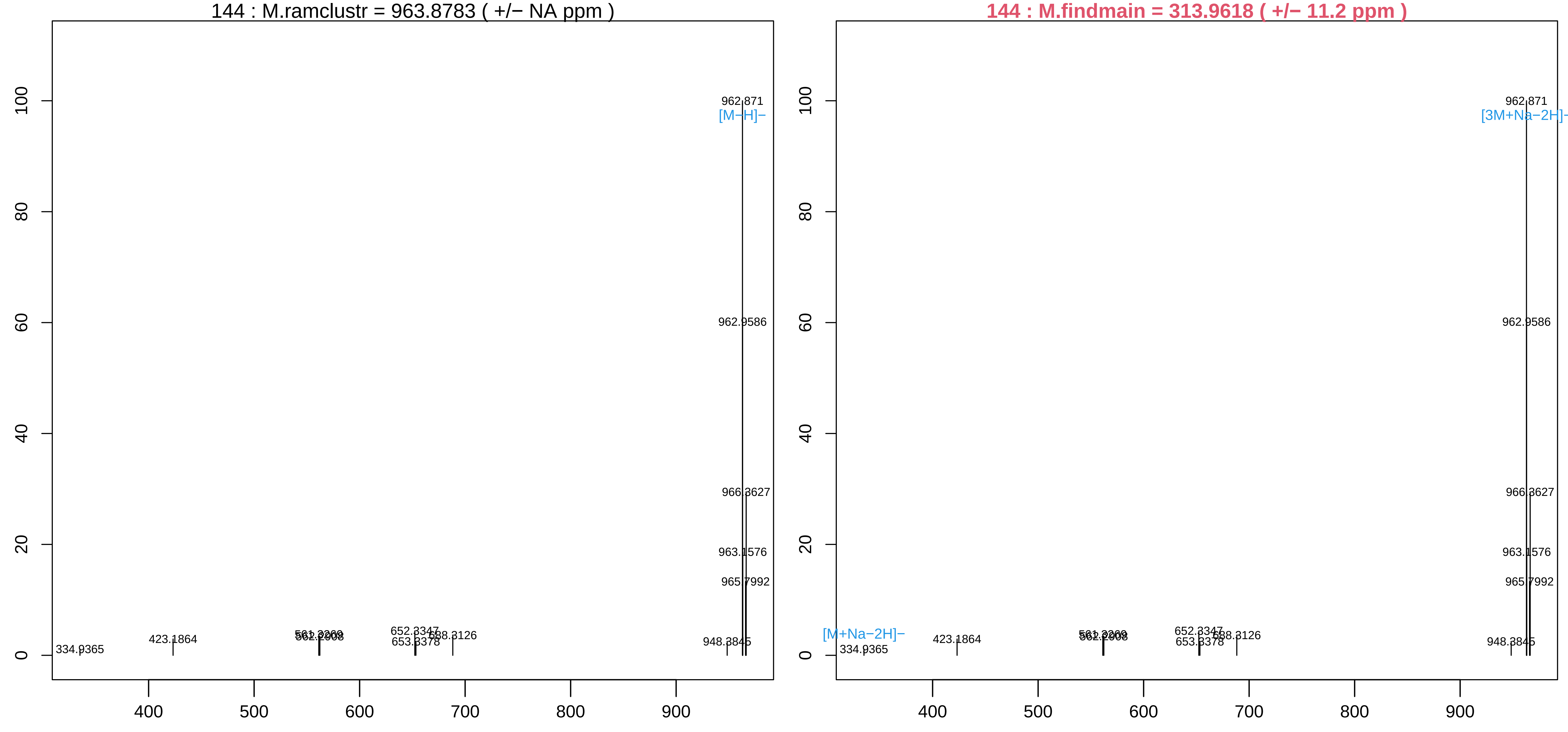

144 : M.ramclustr = 963.8783 (+/− NA ppm)
962.871
[M−H]−
962.9586
966.3627
963.1576
965.7992
948.3845
688.3126
652.3347
653.3378
561.3269
562.3308
423.1864
334.9365
144 : M.findmain = 313.9618 (+/− 11.2 ppm)
962.871
[3M+Na−2H]−
962.9586
966.3627
963.1576
965.7992
948.3845
688.3126
652.3347
653.3378
561.3269
562.3308
423.1864
[M+Na−2H]−
334.9365

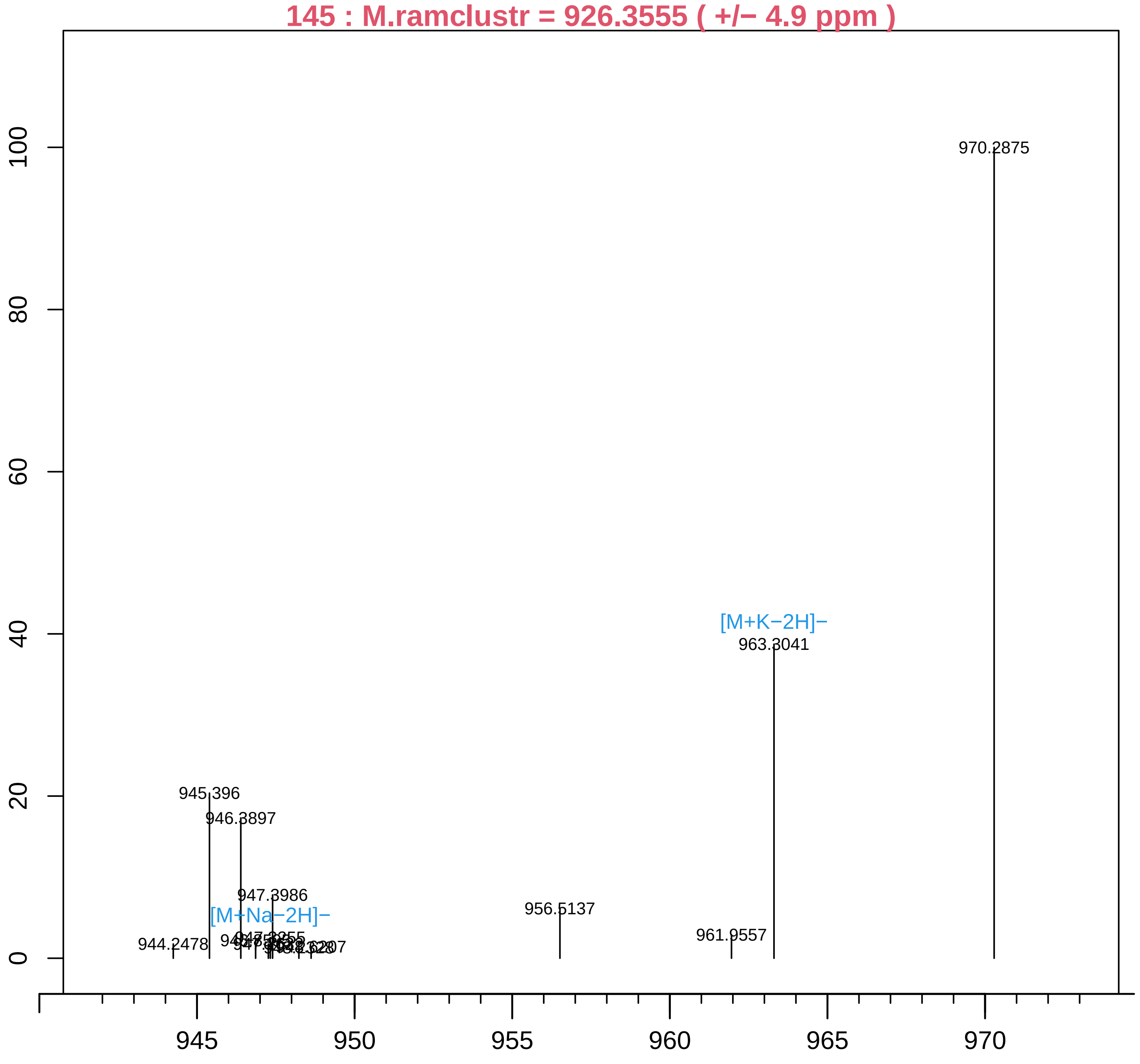
145 : M.ramclustr = 926.3555 (+/− 4.9 ppm)
970.2875
[M+K−2H]−
963.3041
961.9557
956.5137
947.3986
[M+Na−2H]−
946.3897
945.396
944.2478
0
20
40
60
80
100
945
950
955
960
965
970

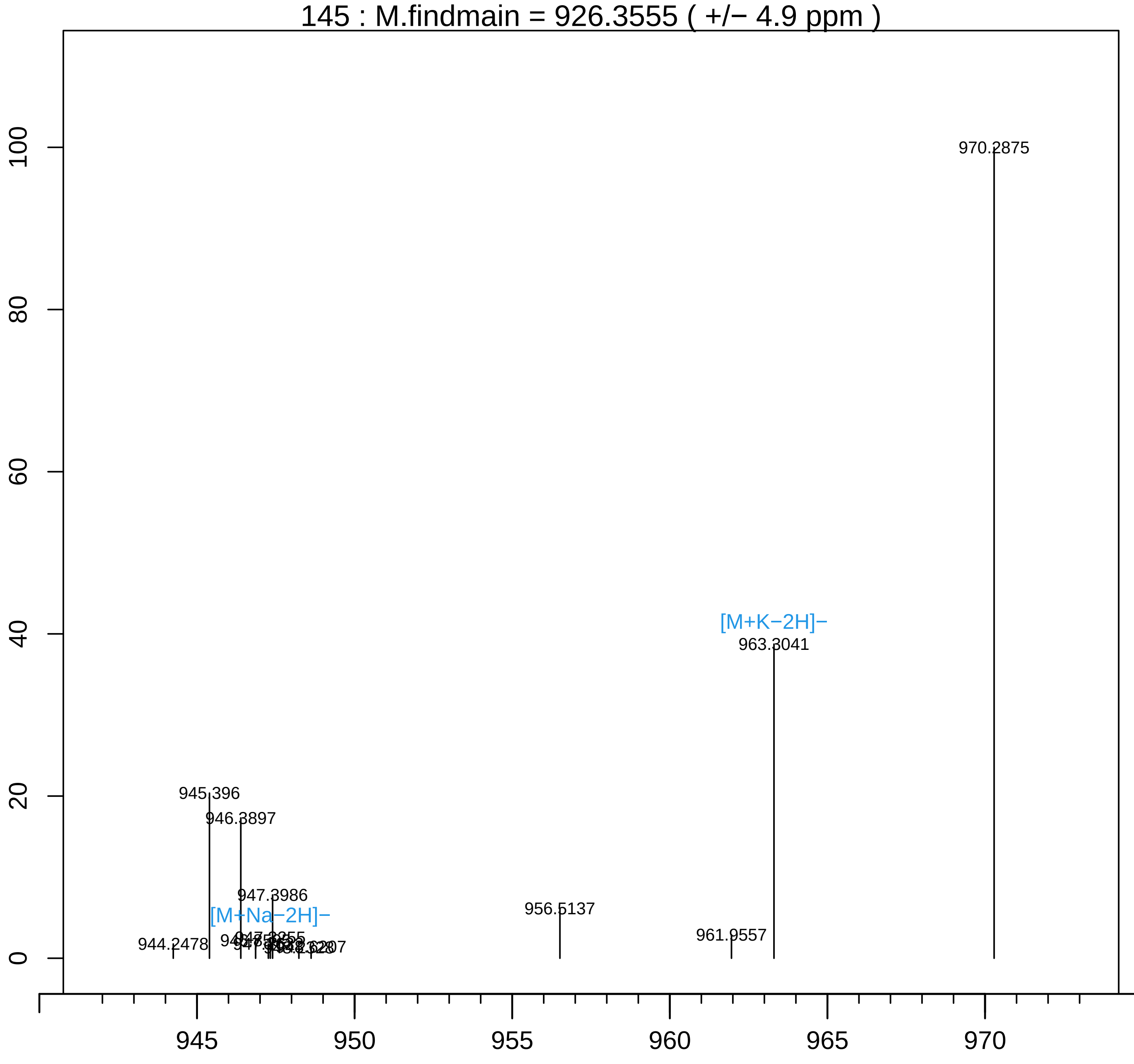
145 : M.findmain = 926.3555 (+/− 4.9 ppm)
970.2875
[M+K−2H]−
963.3041
961.9557
956.5137
947.3986
[M+Na−2H]−
946.3897
945.396
944.2478
0
20
40
60
80
100
945
950
955
960
965
970

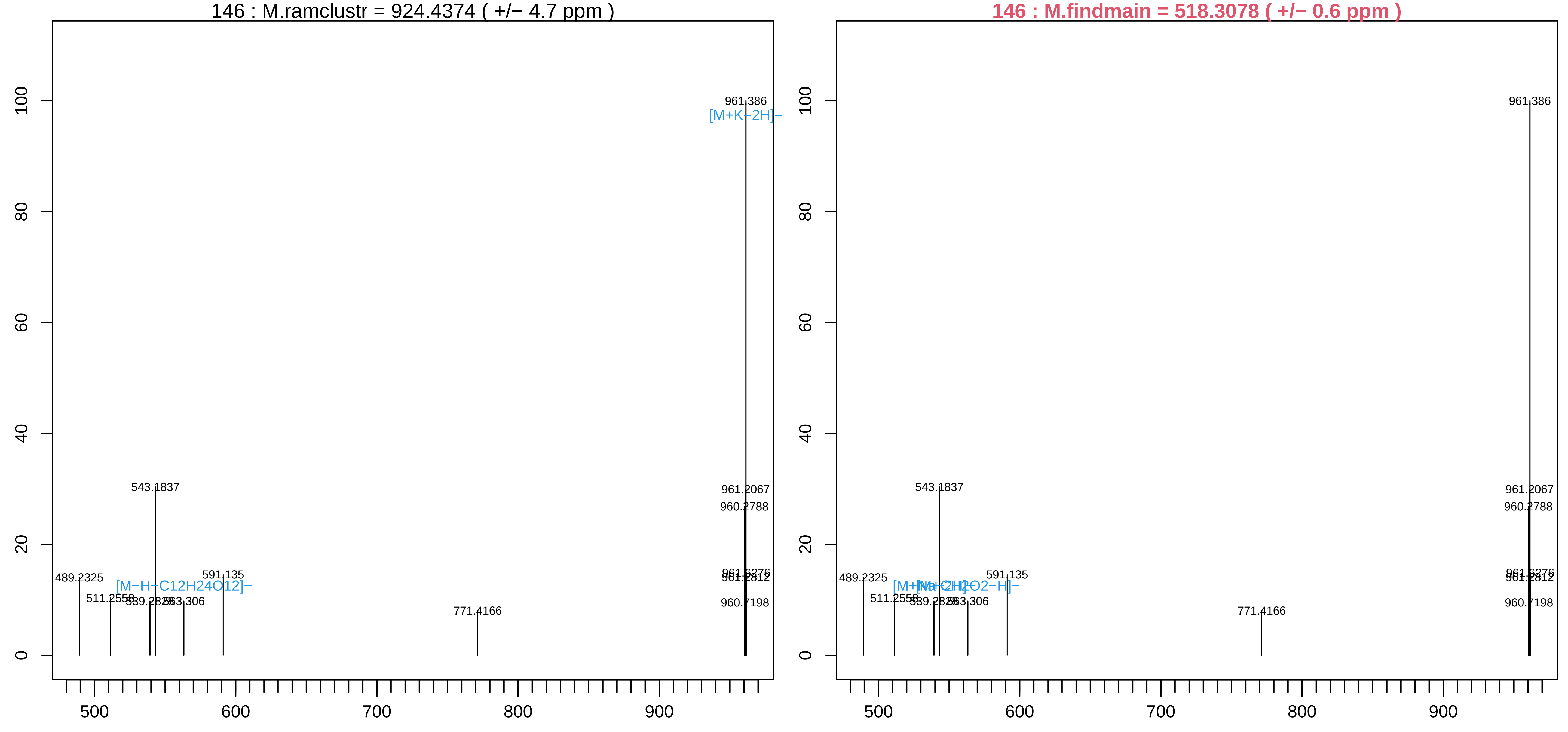

146 : M.ramclustr = 924.4374 (+/− 4.7 ppm)
961.386
[M+K−2H]−
543.1837
961.2067
960.2788
961.6276
961.2812
489.2325
591.135
[M−H−C12H24O12]−
511.2558
539.2828
563.306
960.7198
771.4166
0
20
40
60
80
100
500
600
700
800
900
146 : M.findmain = 518.3078 (+/− 0.6 ppm)
961.386
543.1837
961.2067
960.2788
961.6276
961.2812
489.2325
591.135
511.2558
539.2828
563.306
960.7198
771.4166
0
20
40
60
80
100
500
600
700
800
900

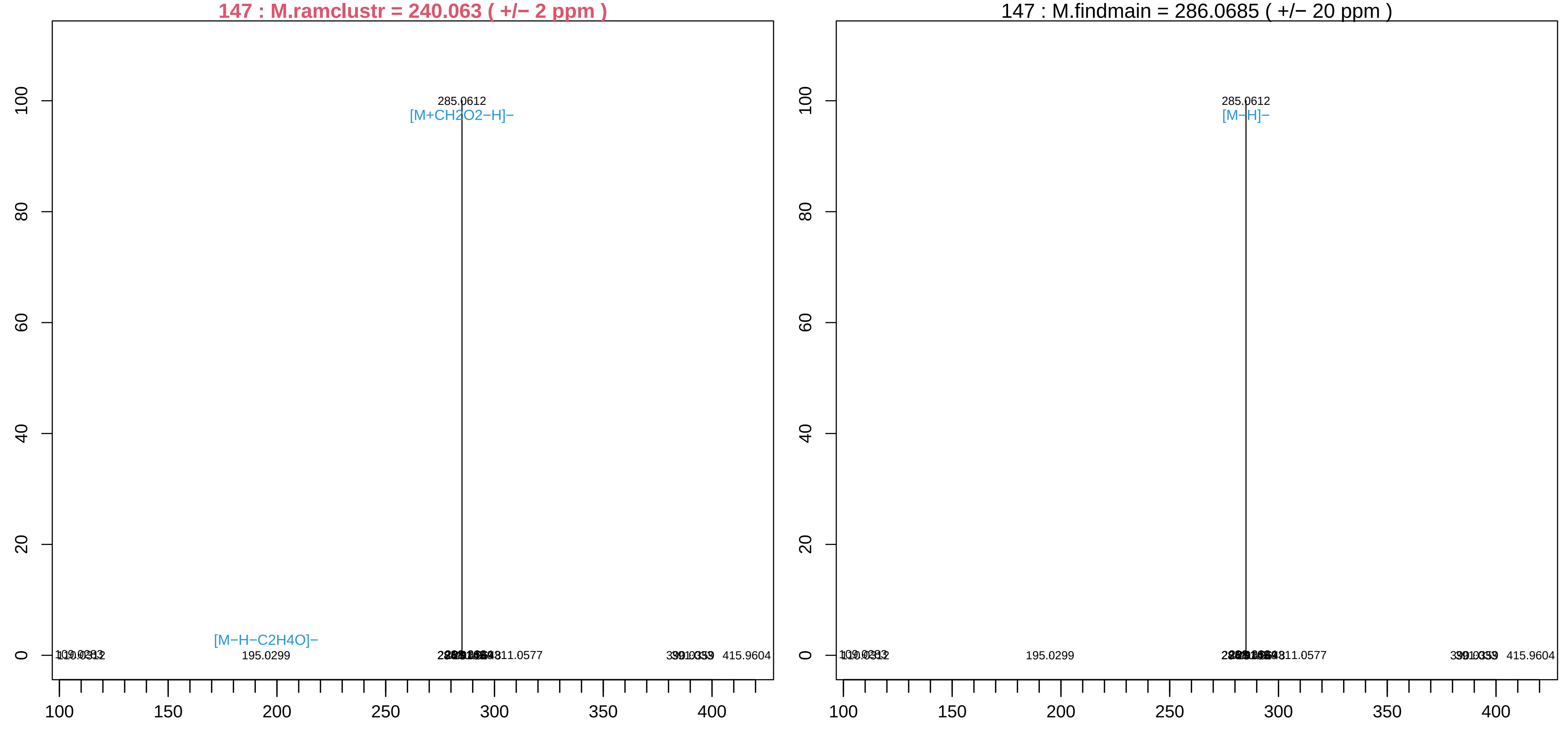
147 : M.ramclustr = 240.063 (+/− 2 ppm)
285.0612
[M+CH2O2−H]−
[M−H−C2H4O]−
195.0299
311.0577
415.9604
147 : M.findmain = 286.0685 (+/− 20 ppm)
285.0612
[M−H]−
195.0299
311.0577
415.9604
0
20
40
60
80
100
100
150
200
250
300
350
400

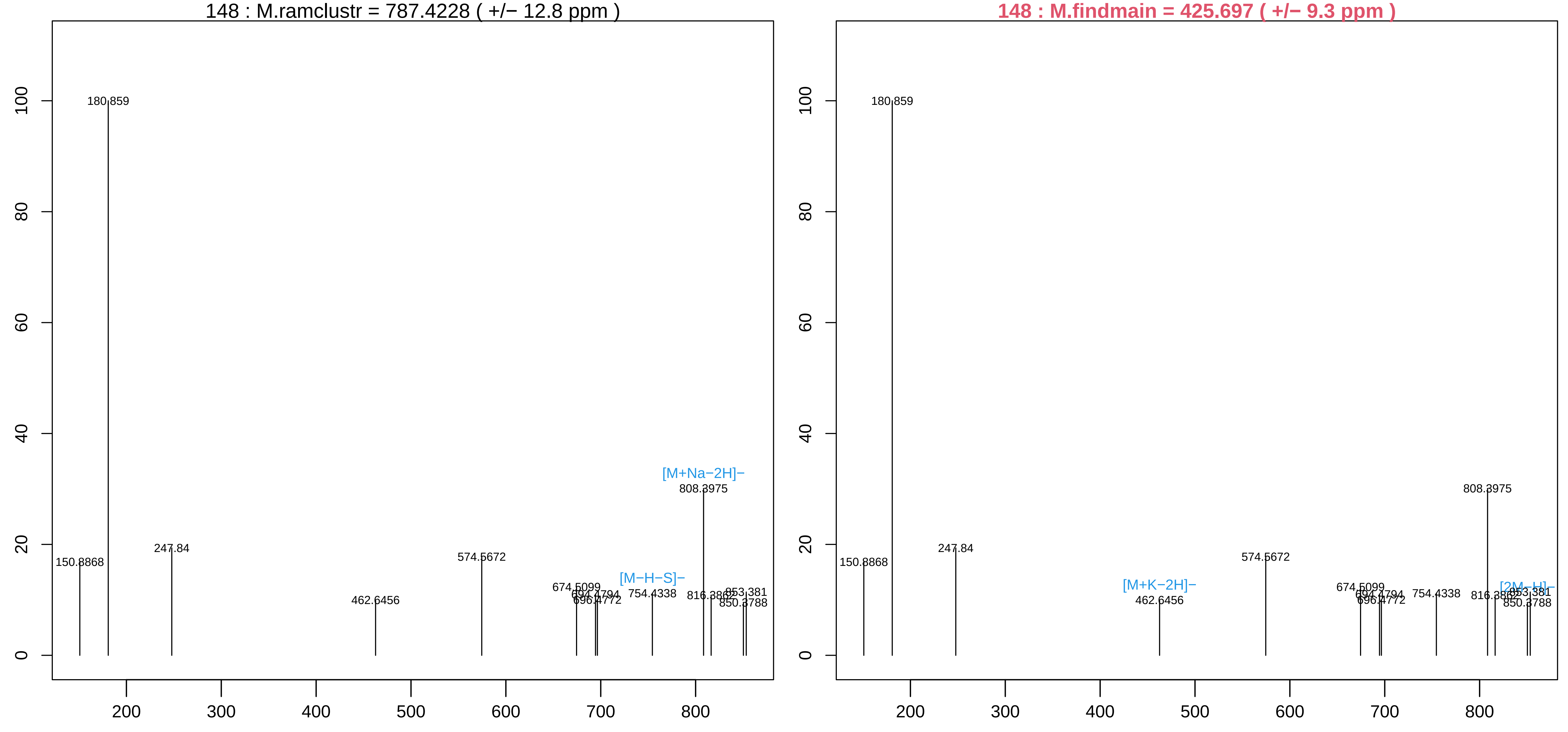

148 : M.ramclustr = 787.4228 (+/− 12.8 ppm)
148 : M.findmain = 425.697 (+/− 9.3 ppm)
180.859
150.8868
247.84
462.6456
574.5672
674.5099
694.4794
696.4772
754.4338
808.3975
816.3862
850.3788
853.381
[M+Na−2H]−
[M−H−S]−
[M+K−2H]−
[2M−H]−
0
20
40
60
80
100
200
300
400
500
600
700
800

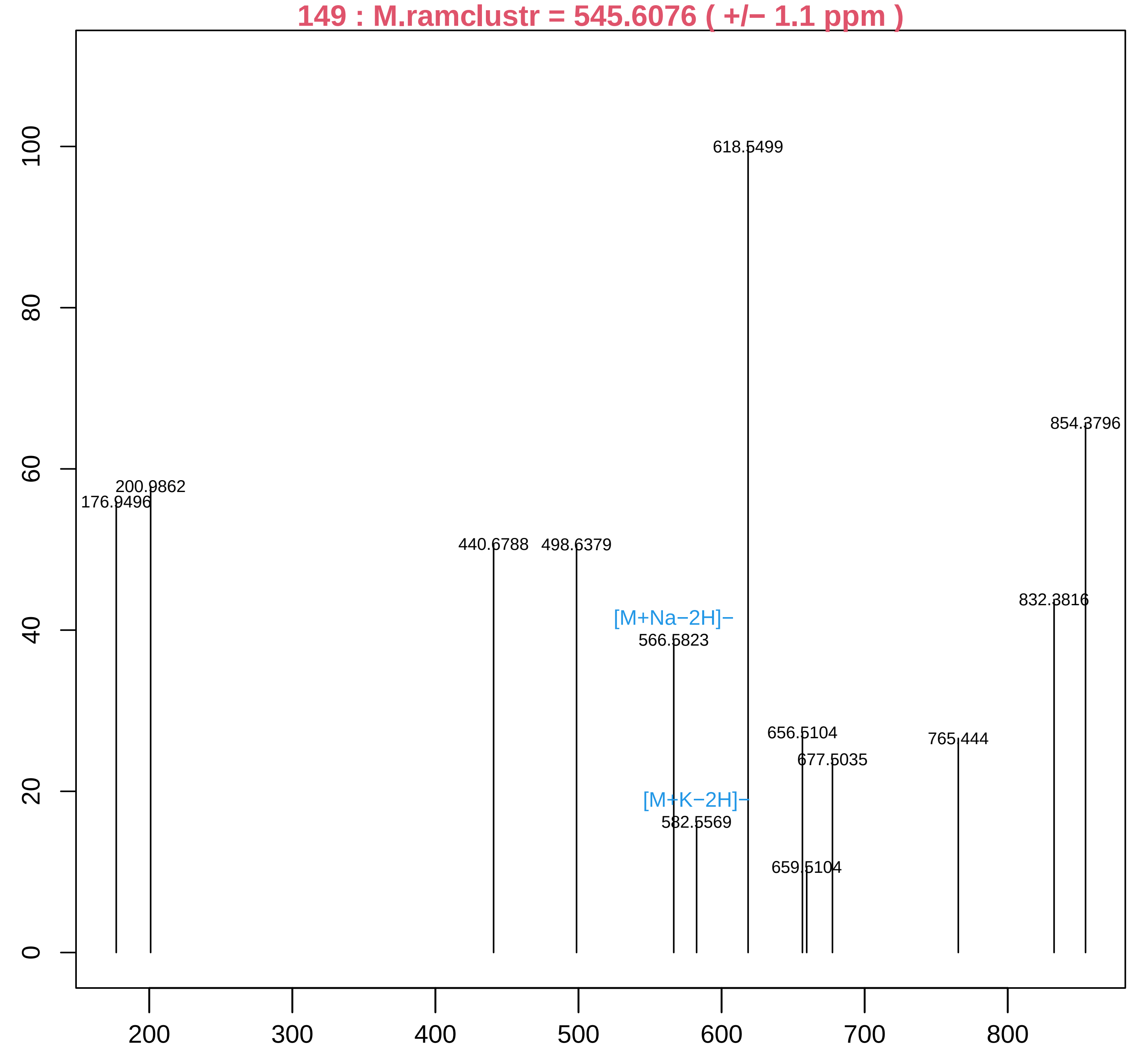
149 : M.ramclustr = 545.6076 (+/− 1.1 ppm)
618.5499
854.3796
200.9862
176.9496
440.6788
498.6379
[M+Na−2H]−
566.5823
832.3816
656.5104
677.5035
765.444
[M+K−2H]−
582.5569
659.5104
0
20
40
60
80
100
200
300
400
500
600
700
800

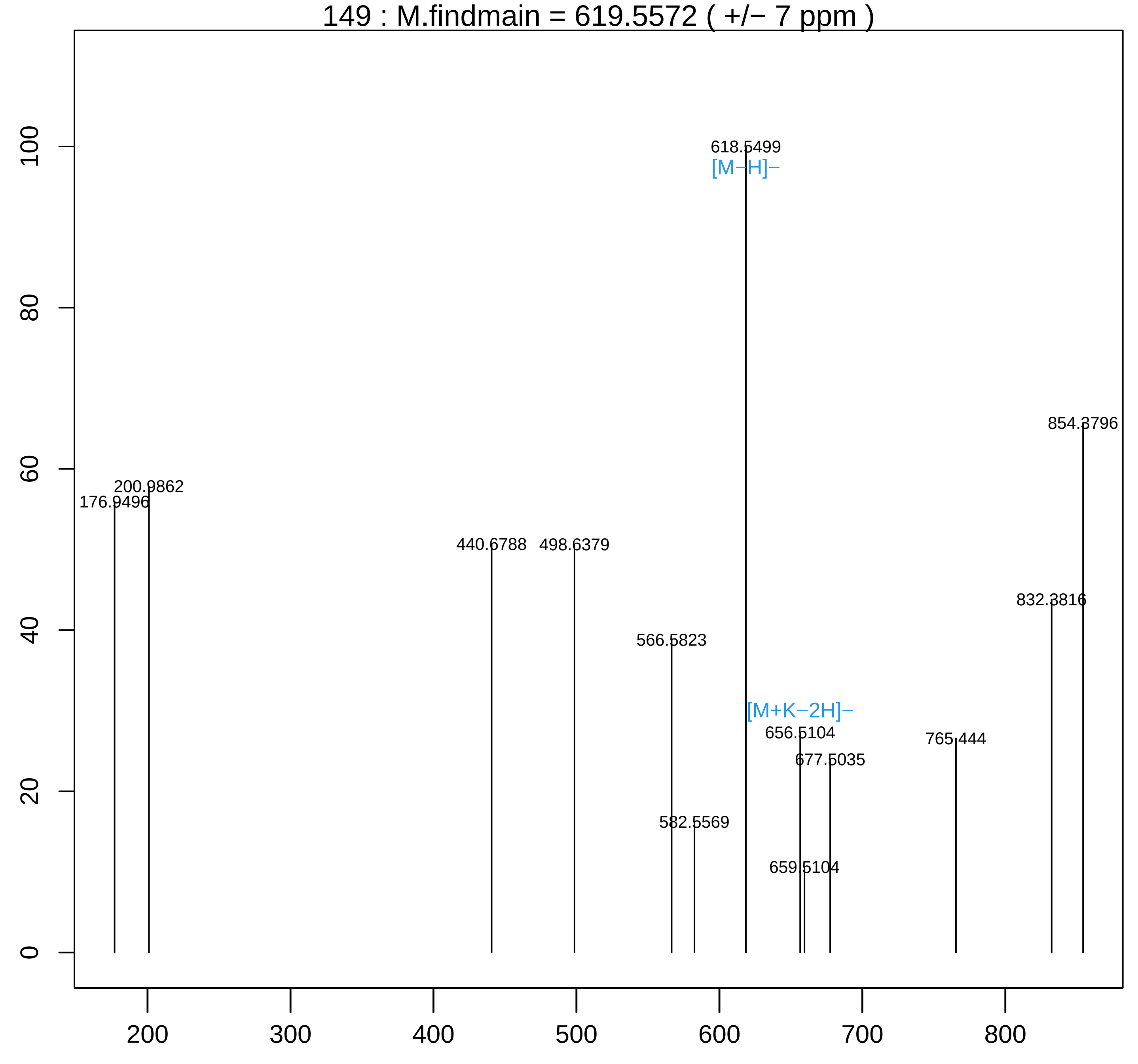
149 : M.findmain = 619.5572 (+/− 7 ppm)
618.5499
[M−H]−
854.3796
200.9862
176.9496
440.6788
498.6379
566.5823
832.3816
[M+K−2H]−
656.5104
677.5035
765.444
582.5569
659.5104
0
20
40
60
80
100
200
300
400
500
600
700
800

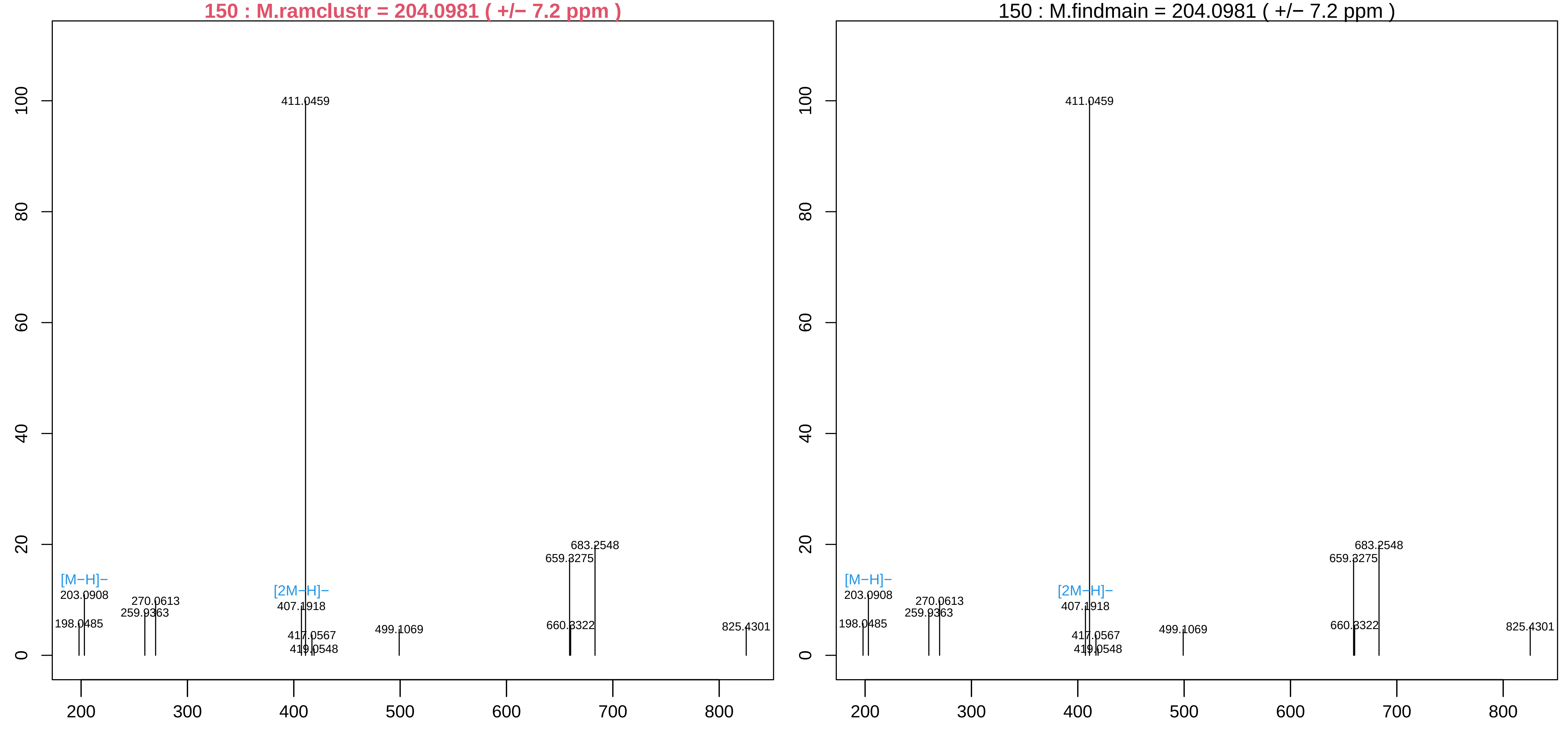
150 : M.ramclustr = 204.0981 (+/− 7.2 ppm)
150 : M.findmain = 204.0981 (+/− 7.2 ppm)
411.0459
683.2548
659.3275
[M−H]−
203.0908
270.0613
259.9363
198.0485
[2M−H]−
407.1918
417.0567
419.0548
499.1069
660.3322
825.4301
0
20
40
60
80
100
200
300
400
500
600
700
800

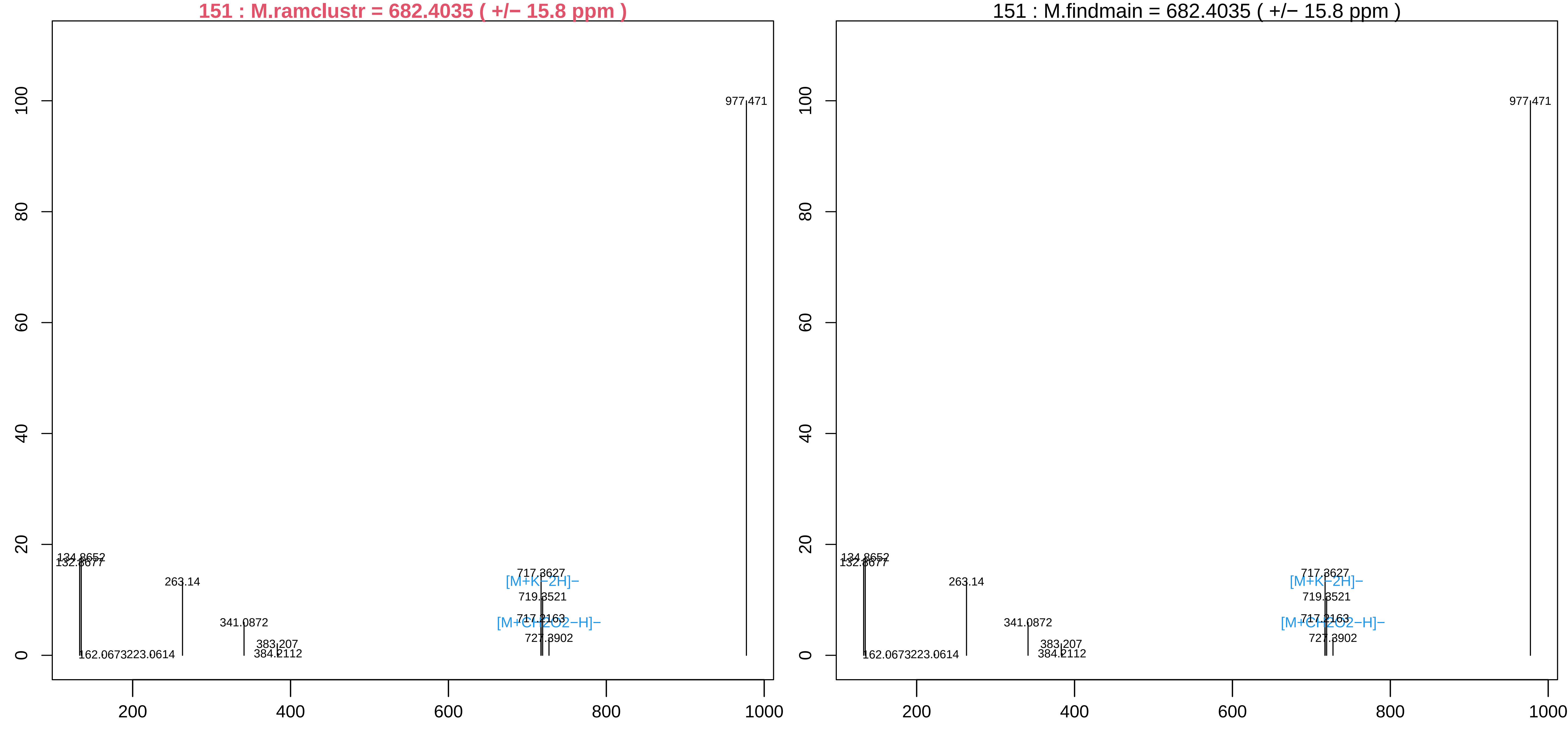

151 : M.ramclustr = 682.4035 (+/− 15.8 ppm)
151 : M.findmain = 682.4035 (+/− 15.8 ppm)
977.471
134.8652
132.8677
263.14
341.0872
383.207
384.2112
162.0673223.0614
717.3627
[M+K−2H]−
719.3521
717.2163
[M+CH2O2−H]−
727.3902
0
20
40
60
80
100
200
400
600
800
1000

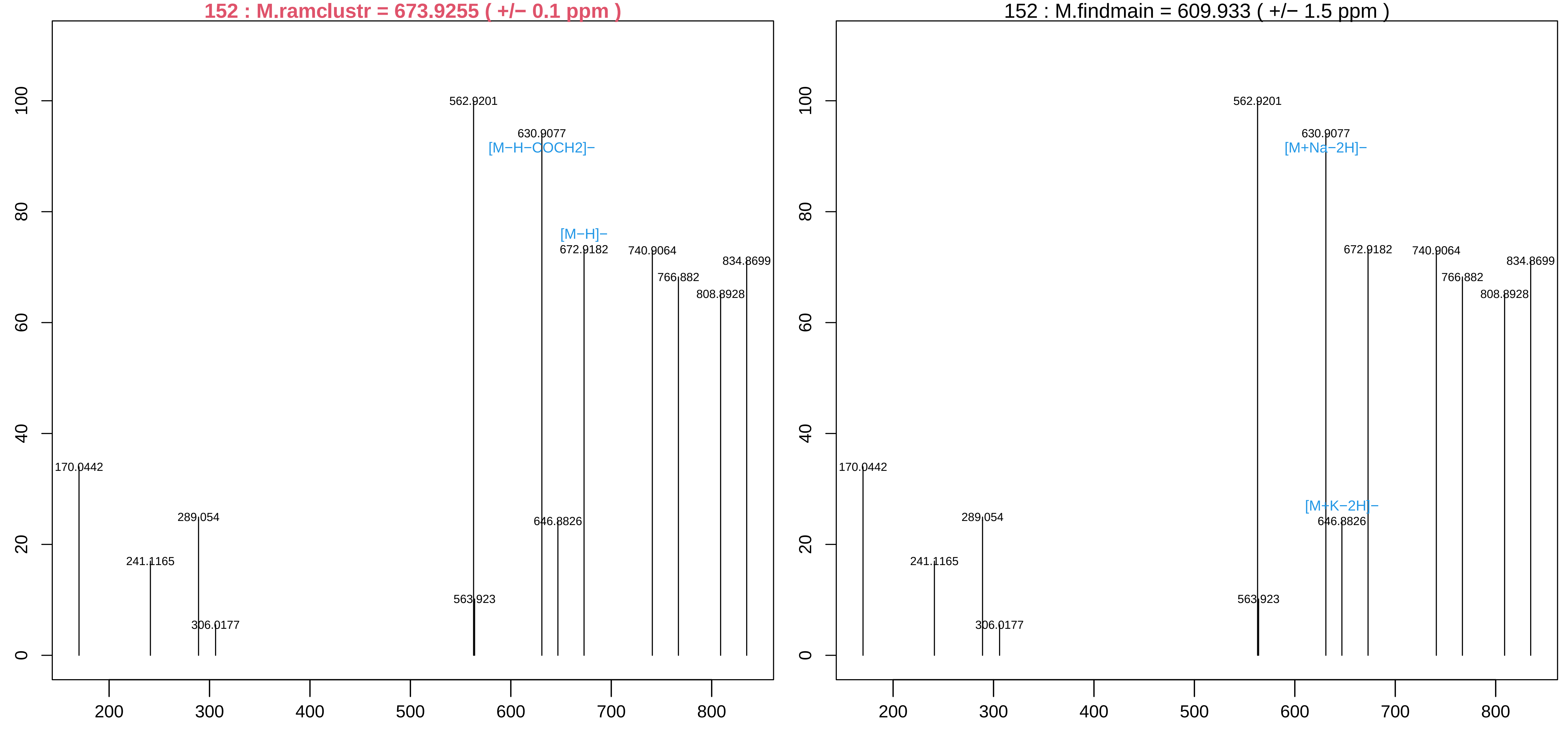

152 : M.ramclustr = 673.9255 (+/− 0.1 ppm)
562.9201
630.9077
[M−H−COCH2]−
[M−H]−
672.9182
740.9064
834.8699
766.882
808.8928
170.0442
289.054
646.8826
241.1165
563.923
306.0177
152 : M.findmain = 609.933 (+/− 1.5 ppm)
[M+Na−2H]−
[M+K−2H]−
0
20
40
60
80
100
200
300
400
500
600
700
800

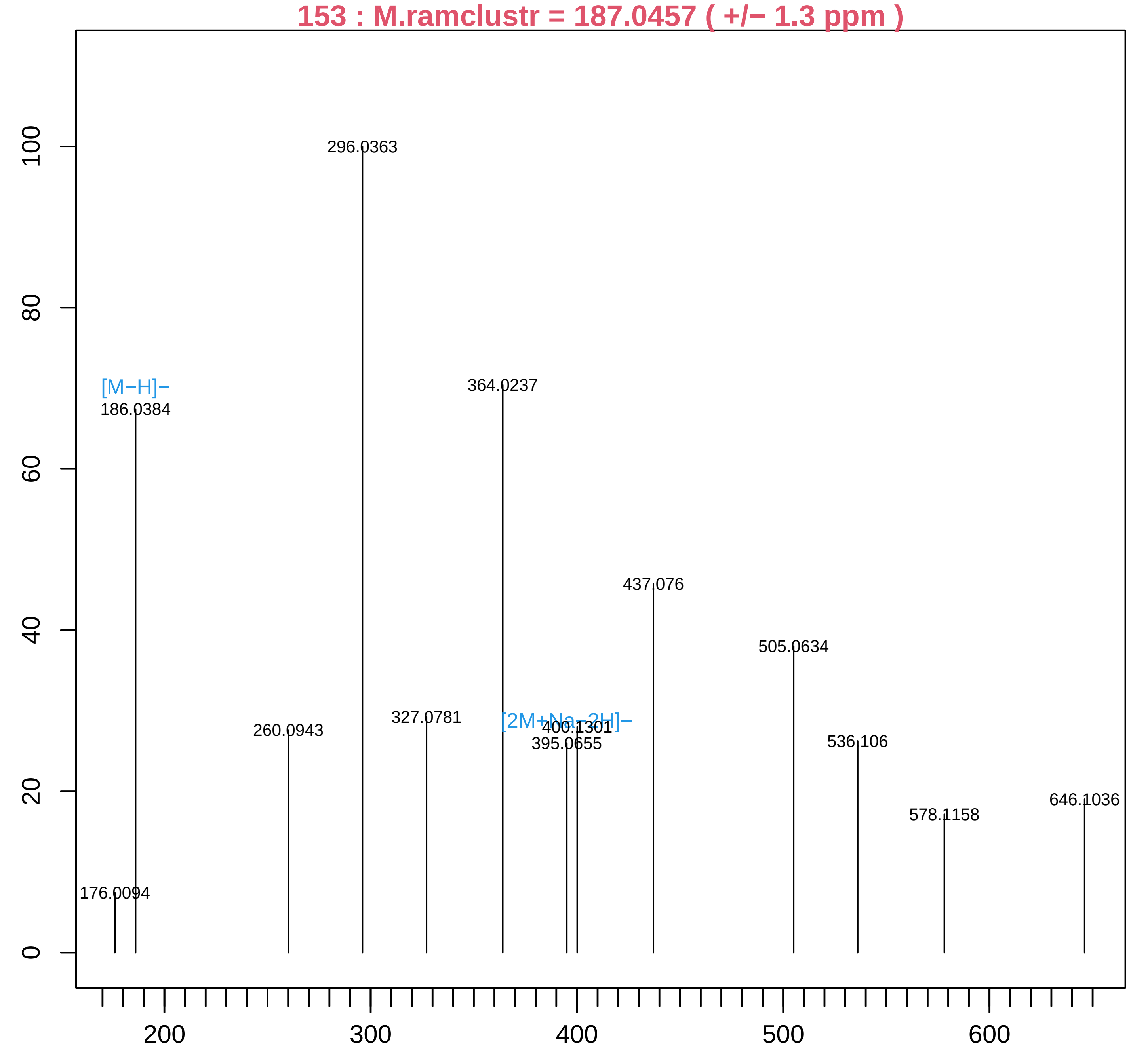
153 : M.ramclustr = 187.0457 (+/− 1.3 ppm)
[M−H]−
[2M+Na−2H]−
176.0094
186.0384
260.0943
296.0363
327.0781
364.0237
395.0655
400.1301
437.076
505.0634
536.106
578.1158
646.1036
0
20
40
60
80
100
200
300
400
500
600

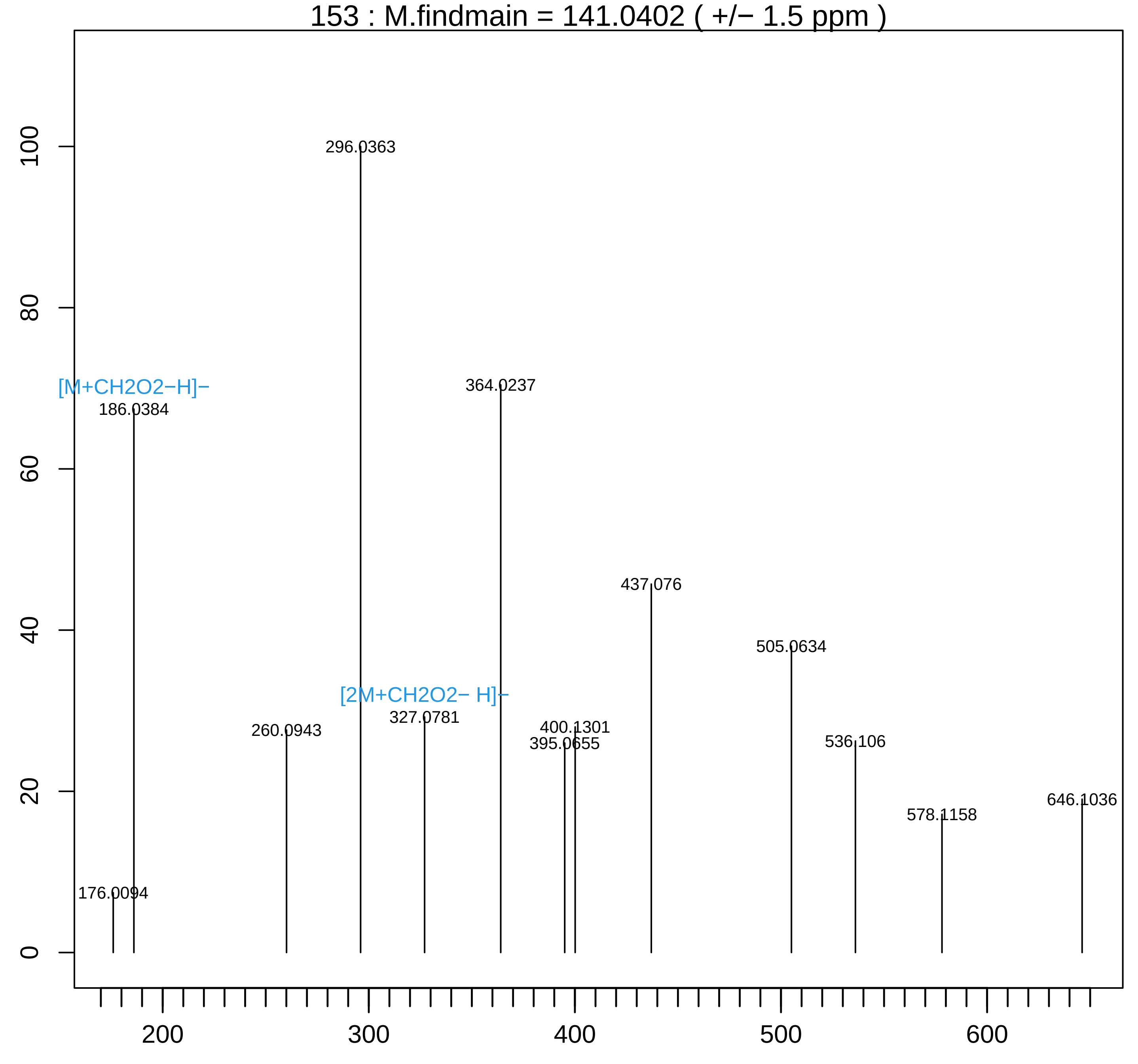
153 : M.findmain = 141.0402 (+/− 1.5 ppm)
[M+CH2O2−H]−
[2M+CH2O2− H]−
176.0094
186.0384
260.0943
296.0363
327.0781
364.0237
395.0655
400.1301
437.076
505.0634
536.106
578.1158
646.1036
0
20
40
60
80
100
200
300
400
500
600

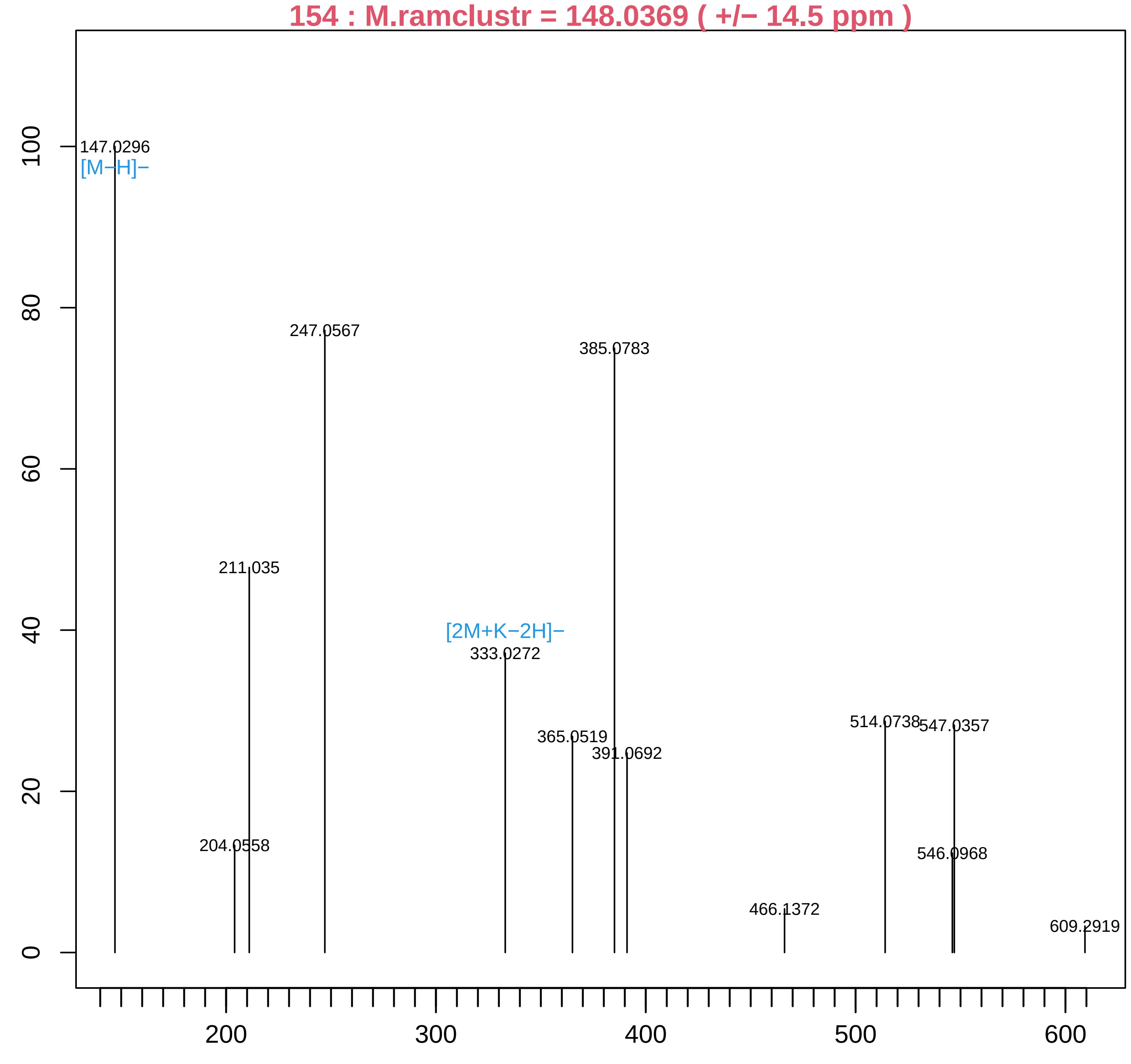
154 : M.ramclustr = 148.0369 (+/− 14.5 ppm)
147.0296
[M−H]−
247.0567
385.0783
211.035
[2M+K−2H]−
333.0272
365.0519
391.0692
514.0738
547.0357
204.0558
546.0968
466.1372
609.2919
0
20
40
60
80
100
200
300
400
500
600

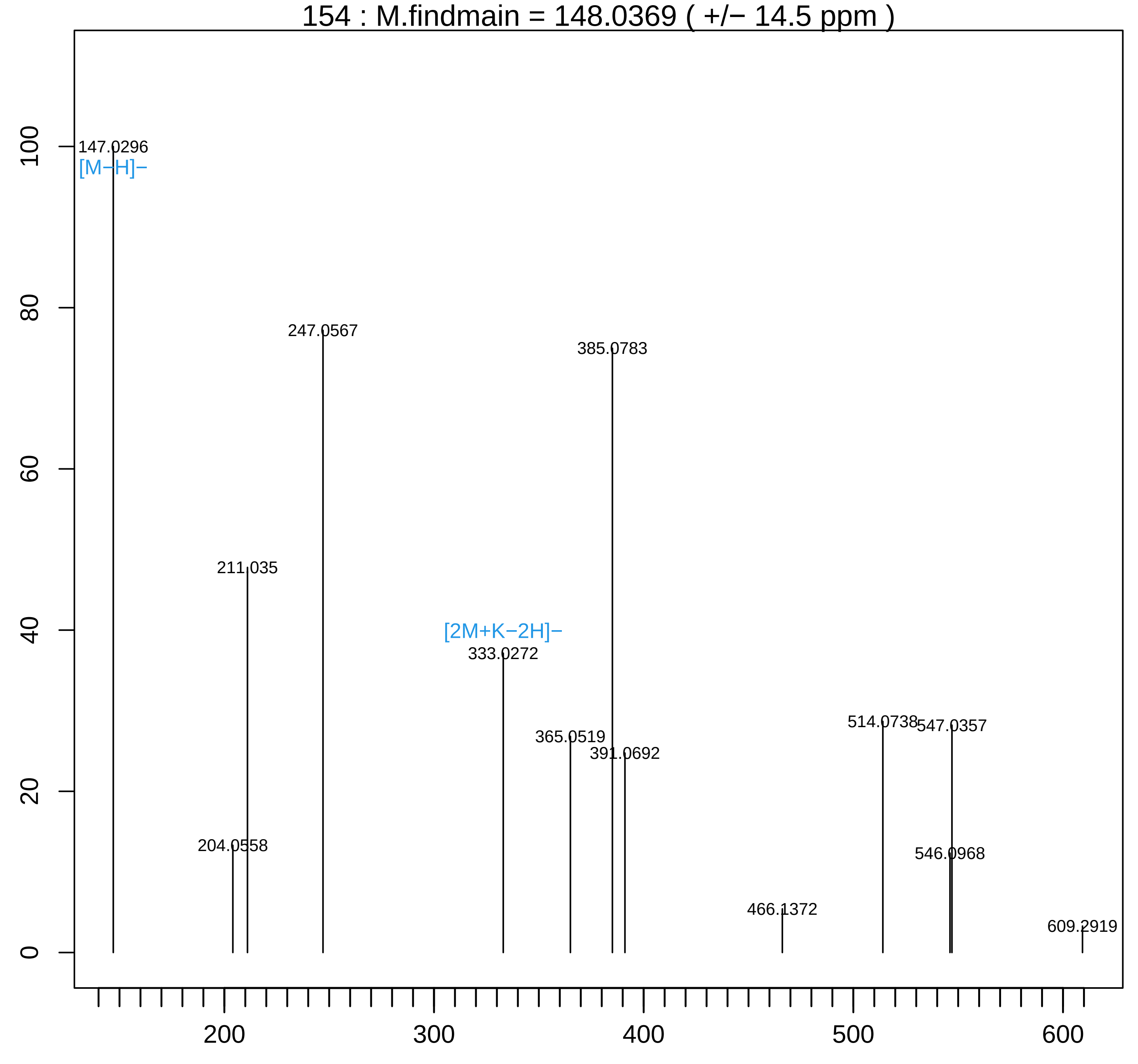
154 : M.findmain = 148.0369 (+/− 14.5 ppm)
147.0296
[M−H]−
247.0567
385.0783
211.035
[2M+K−2H]−
333.0272
365.0519
391.0692
514.0738
547.0357
204.0558
546.0968
466.1372
609.2919
0
20
40
60
80
100
200
300
400
500
600

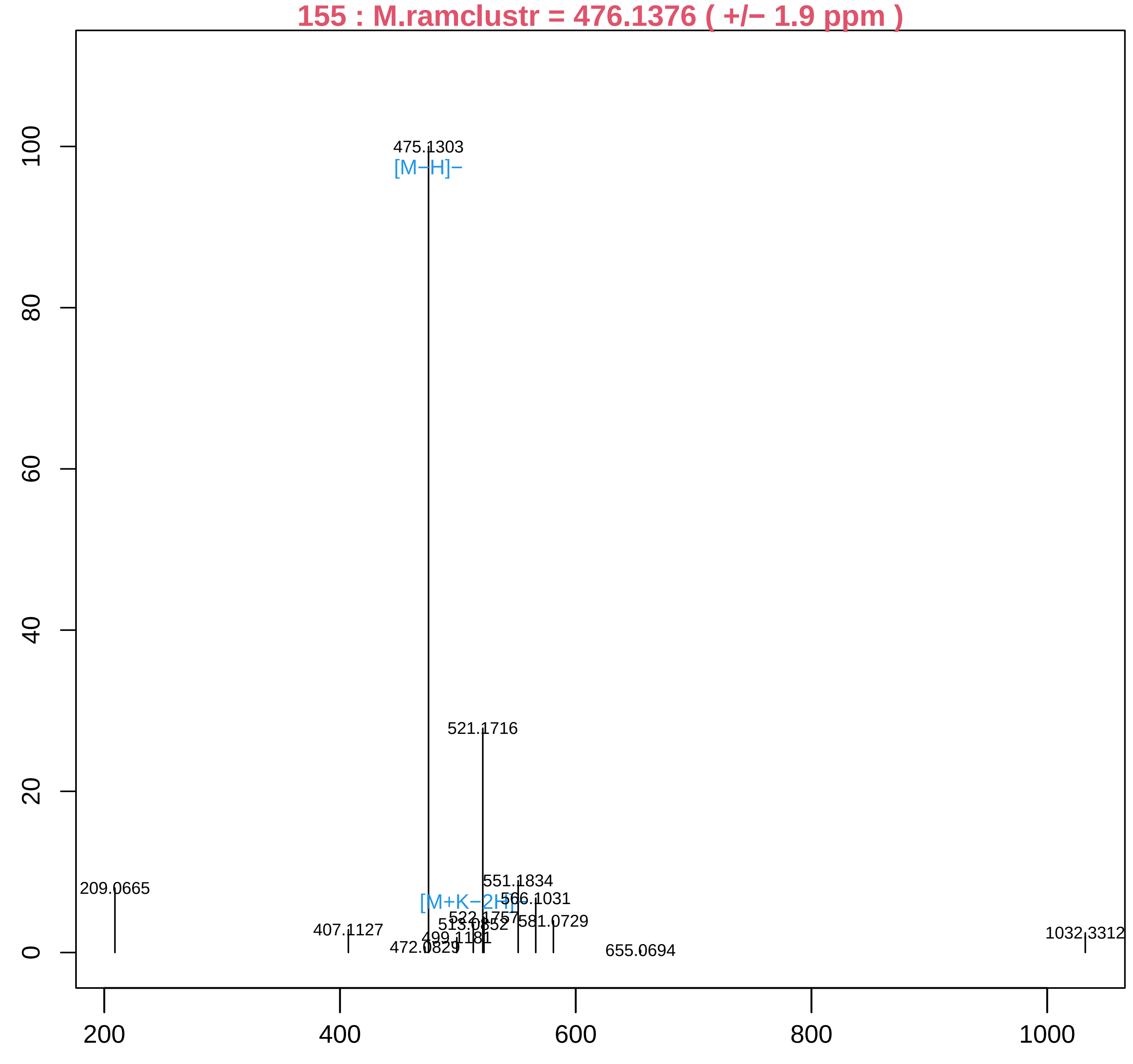

155 : M.ramclustr = 476.1376 (+/− 1.9 ppm)
475.1303
[M−H]−
521.1716
209.0665
407.1127
551.1834
566.1031
[M+K−2H]−
522.1757
513.0852
581.0729
499.1181
472.0829
655.0694
1032.3312
0
20
40
60
80
100
200
400
600
800
1000

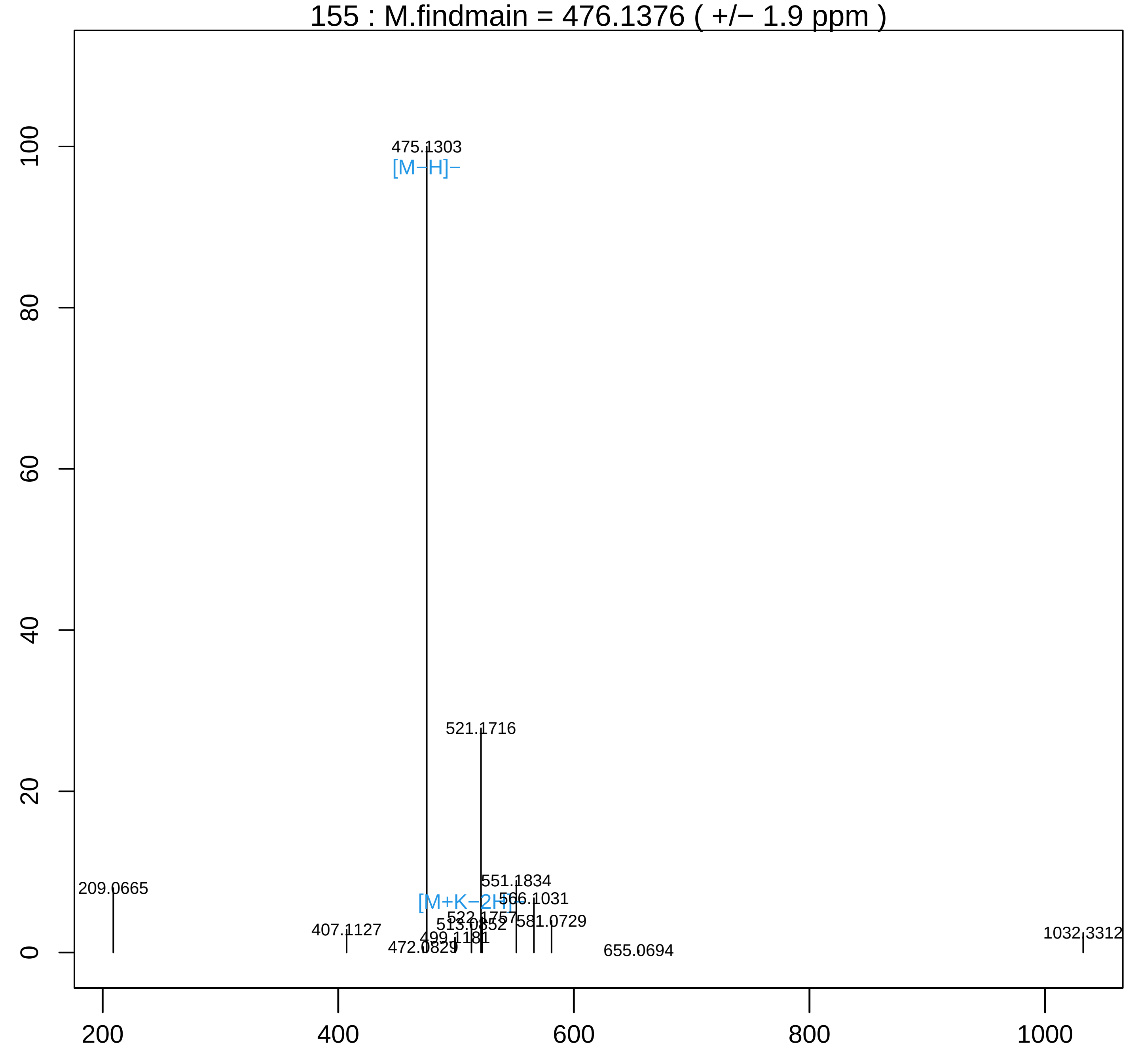

155 : M.findmain = 476.1376 (+/− 1.9 ppm)
475.1303
[M−H]−
521.1716
209.0665
407.1127
551.1834
566.1031
[M+K−2H]−
522.1757
513.0852
581.0729
499.1181
472.0829
655.0694
1032.3312
0
20
40
60
80
100
200
400
600
800
1000

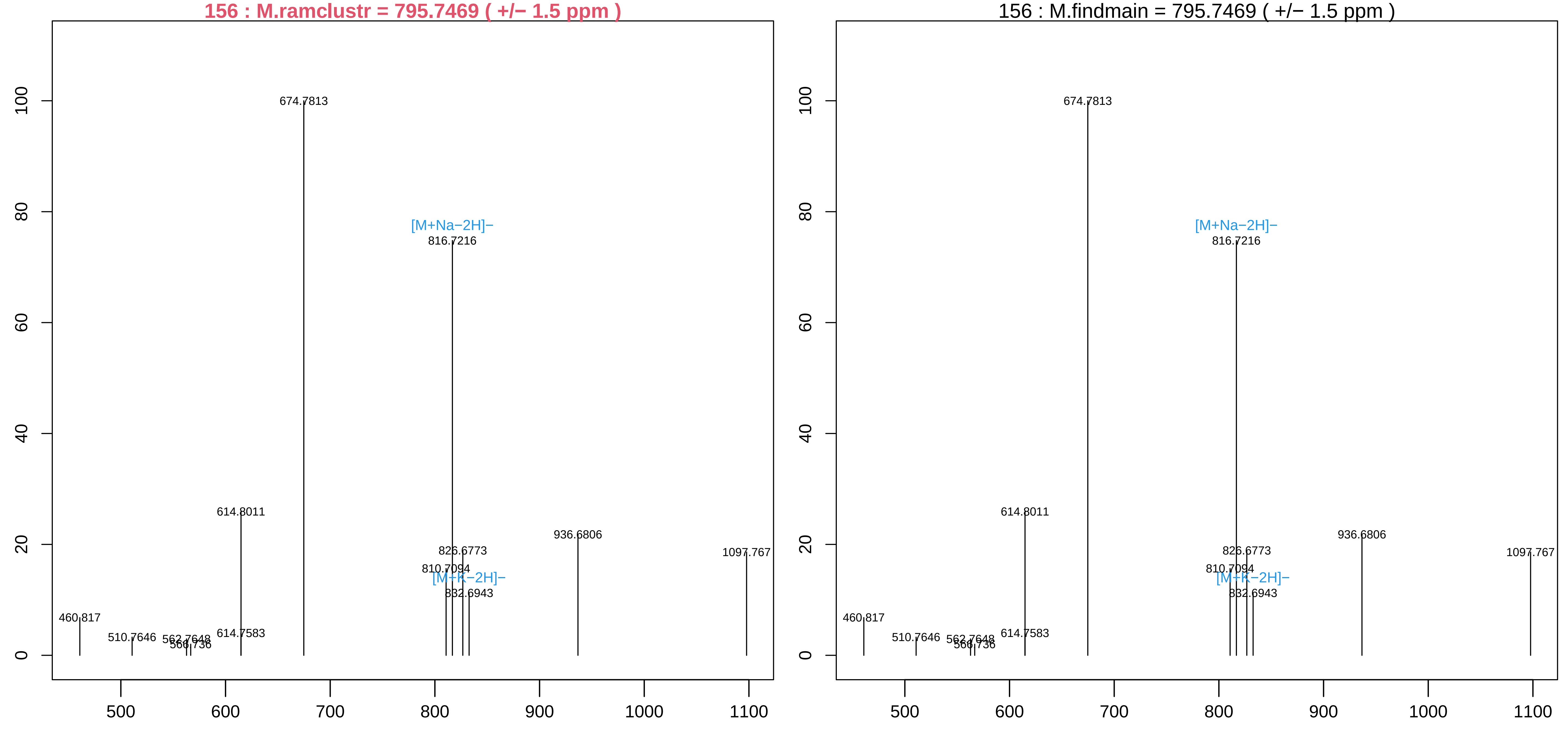
156 : M.ramclustr = 795.7469 (+/− 1.5 ppm)
156 : M.findmain = 795.7469 (+/− 1.5 ppm)
674.7813
[M+Na−2H]−
816.7216
614.8011
936.6806
826.6773
810.7094
[M+K−2H]−
832.6943
1097.767
460.817
510.7646
562.7648
566.736
614.7583
0
20
40
60
80
100
500
600
700
800
900
1000
1100

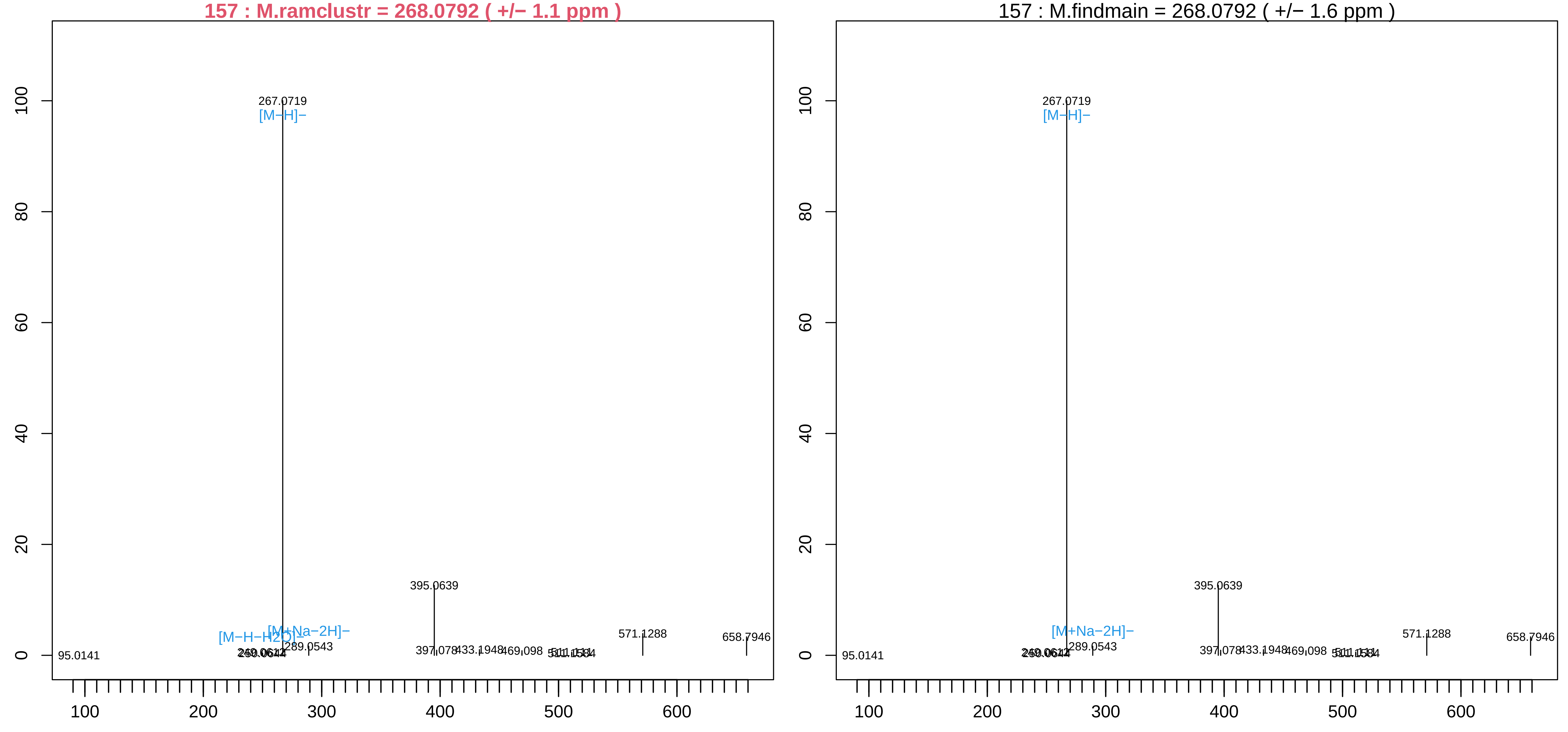
157 : M.ramclustr = 268.0792 (+/− 1.1 ppm)
267.0719
[M−H]−
395.0639
[M+Na−2H]−
[M−H−H2O]−
289.0543
95.0141
397.078
433.1948
469.098
571.1288
658.7946
157 : M.findmain = 268.0792 (+/− 1.6 ppm)
267.0719
[M−H]−
395.0639
[M+Na−2H]−
289.0543
95.0141
397.078
433.1948
469.098
571.1288
658.7946

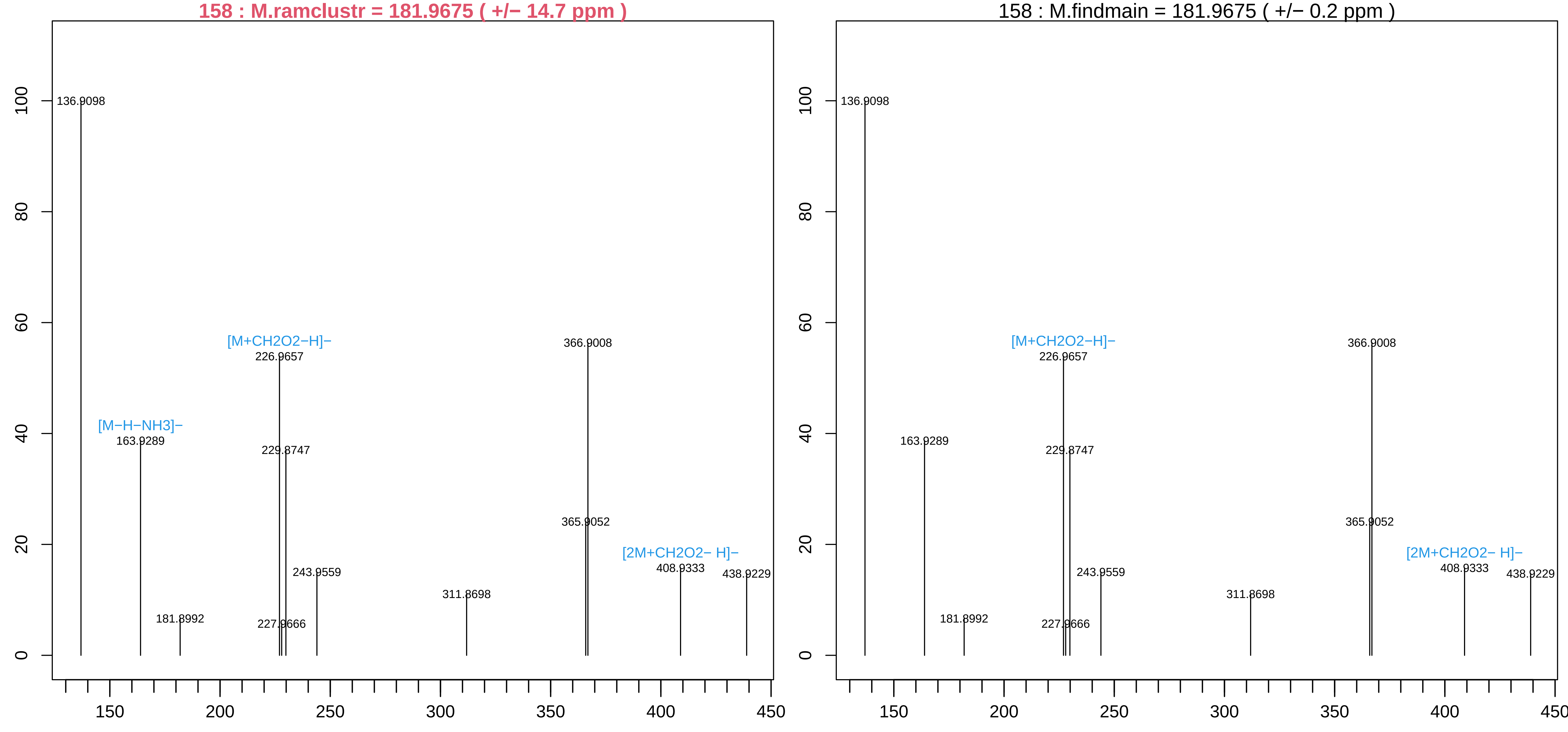
158 : M.ramclustr = 181.9675 (+/− 14.7 ppm)
136.9098
[M−H−NH3]−
163.9289
181.8992
[M+CH2O2−H]−
226.9657
229.8747
227.9666
243.9559
311.8698
366.9008
365.9052
[2M+CH2O2− H]−
408.9333
438.9229
158 : M.findmain = 181.9675 (+/− 0.2 ppm)
136.9098
163.9289
181.8992
[M+CH2O2−H]−
226.9657
229.8747
227.9666
243.9559
311.8698
366.9008
365.9052
[2M+CH2O2− H]−
408.9333
438.9229
0
20
40
60
80
100
150
200
250
300
350
400
450

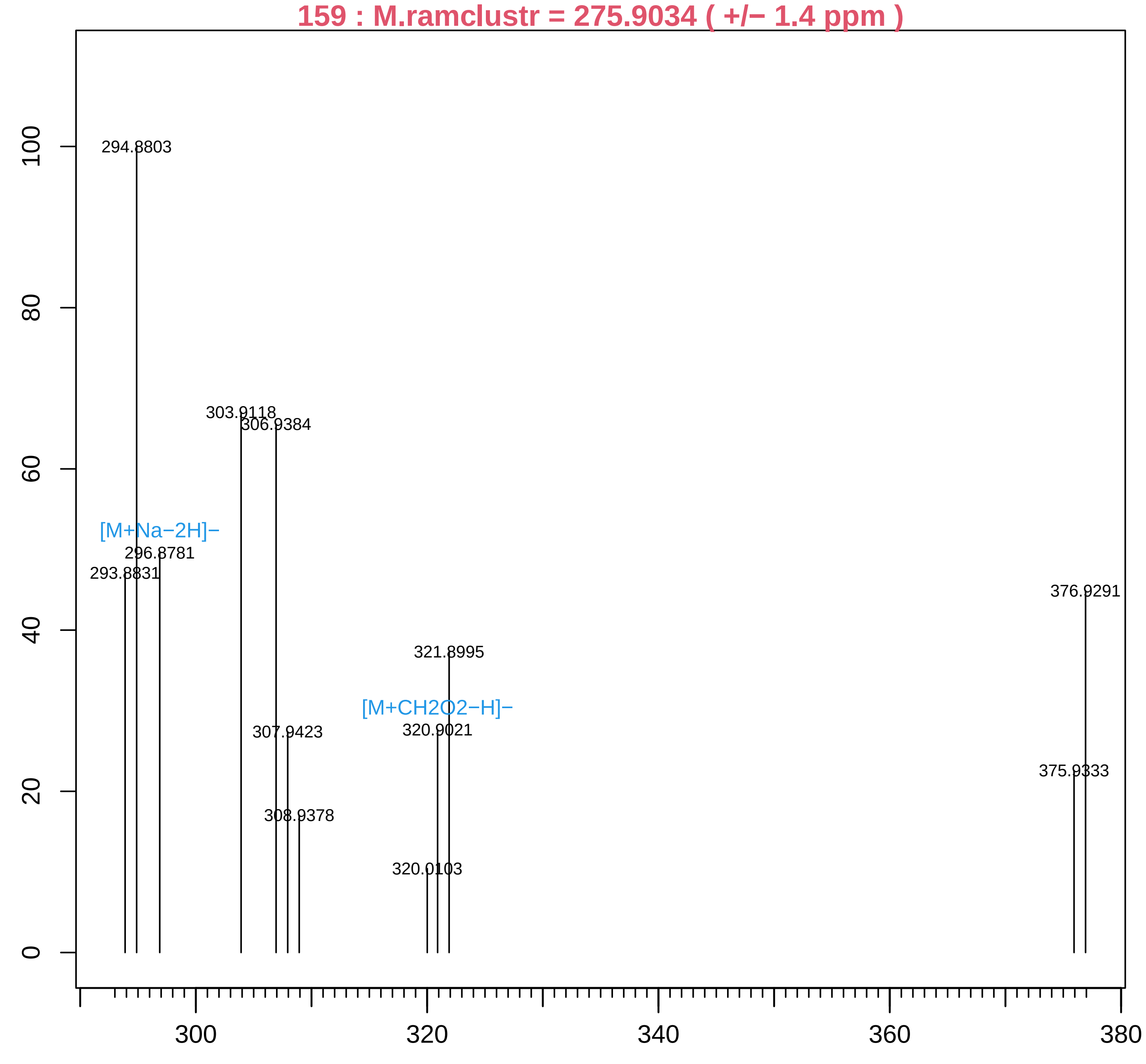

159 : M.ramclustr = 275.9034 (+/− 1.4 ppm)
294.8803
303.9118
306.9384
[M+Na−2H]−
296.8781
293.8831
376.9291
321.8995
[M+CH2O2−H]−
307.9423
320.9021
375.9333
308.9378
320.0103

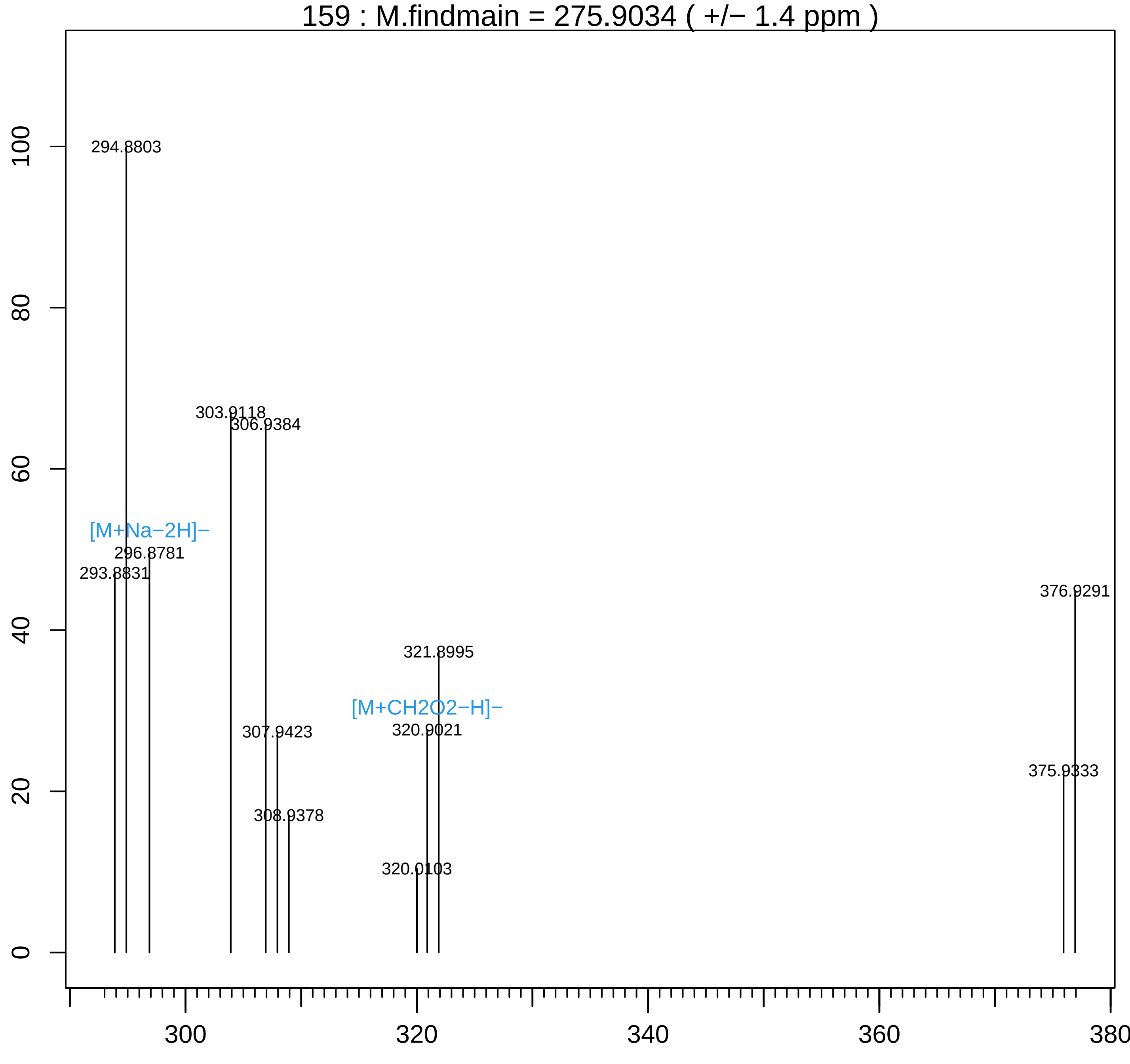

159 : M.findmain = 275.9034 (+/− 1.4 ppm)
294.8803
303.9118
306.9384
[M+Na−2H]−
296.8781
293.8831
376.9291
321.8995
[M+CH2O2−H]−
307.9423
320.9021
375.9333
308.9378
320.0103

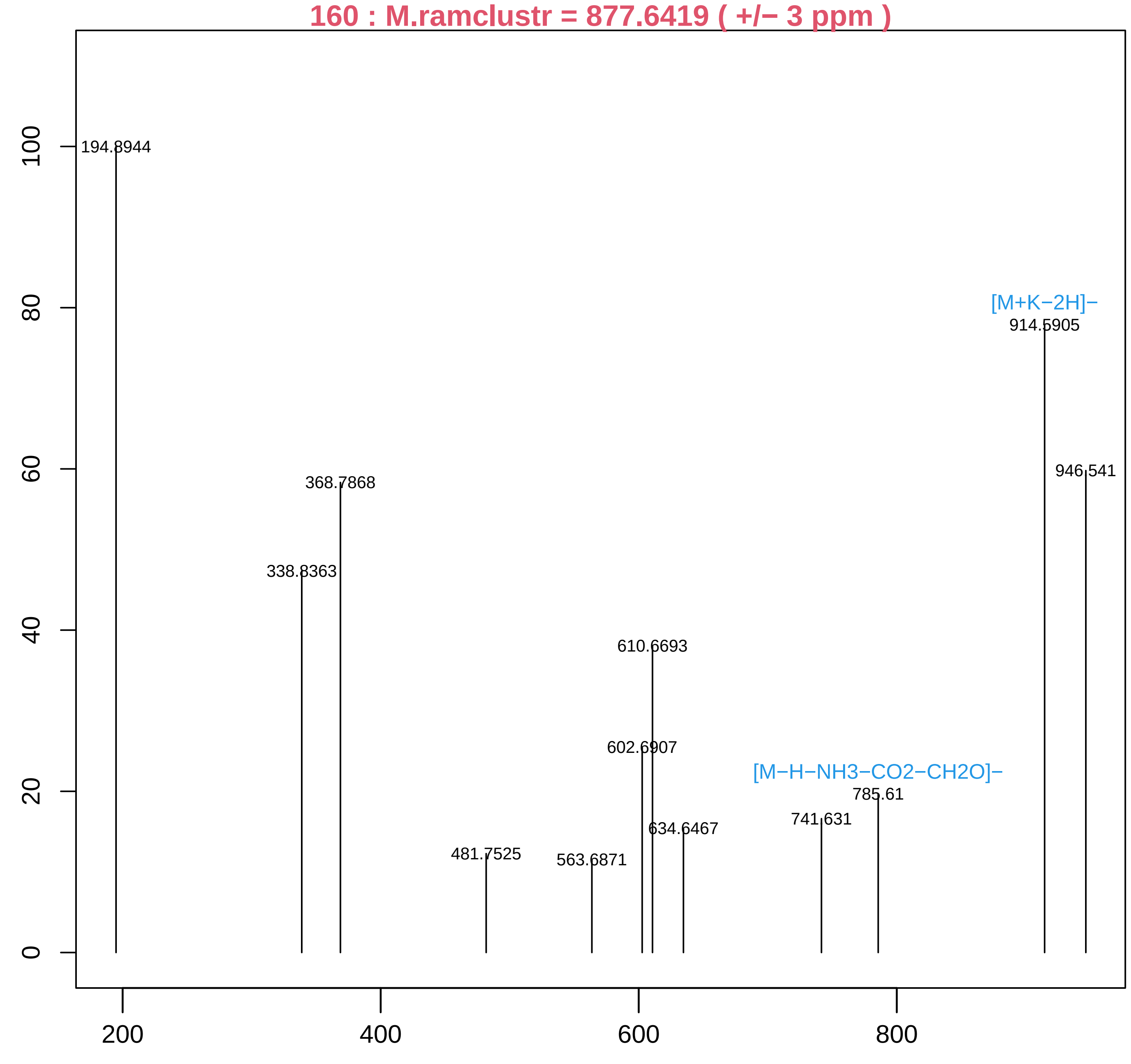
160 : M.ramclustr = 877.6419 (+/− 3 ppm)
194.8944
338.8363
368.7868
481.7525
563.6871
602.6907
610.6693
634.6467
741.631
785.61
[M−H−NH3−CO2−CH2O]−
914.5905
[M+K−2H]−
946.541
0
20
40
60
80
100
200
400
600
800

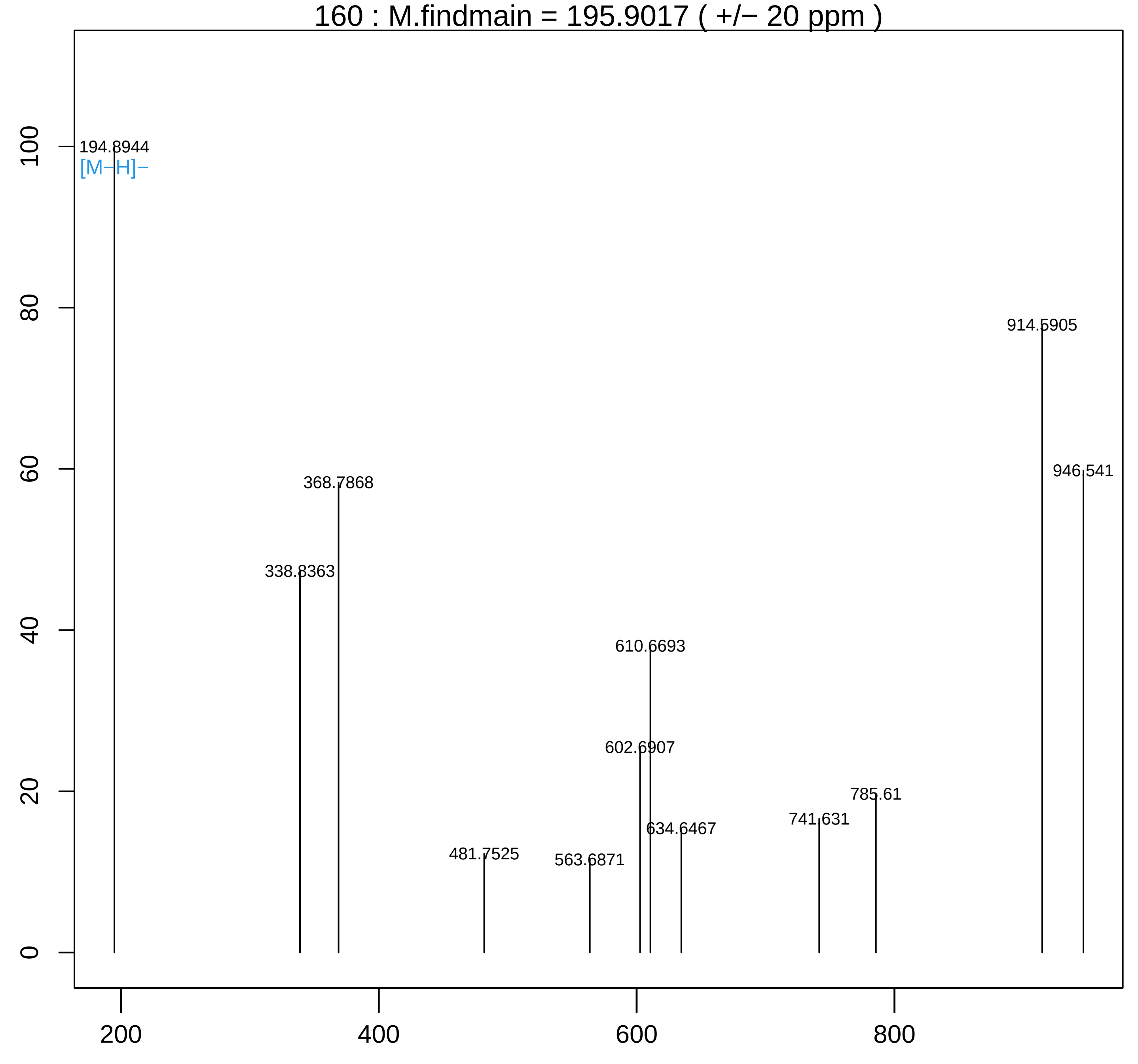
160 : M.findmain = 195.9017 (+/− 20 ppm)
194.8944
[M−H]−
338.8363
368.7868
481.7525
563.6871
602.6907
610.6693
634.6467
741.631
785.61
914.5905
946.541
0
20
40
60
80
100
200
400
600
800

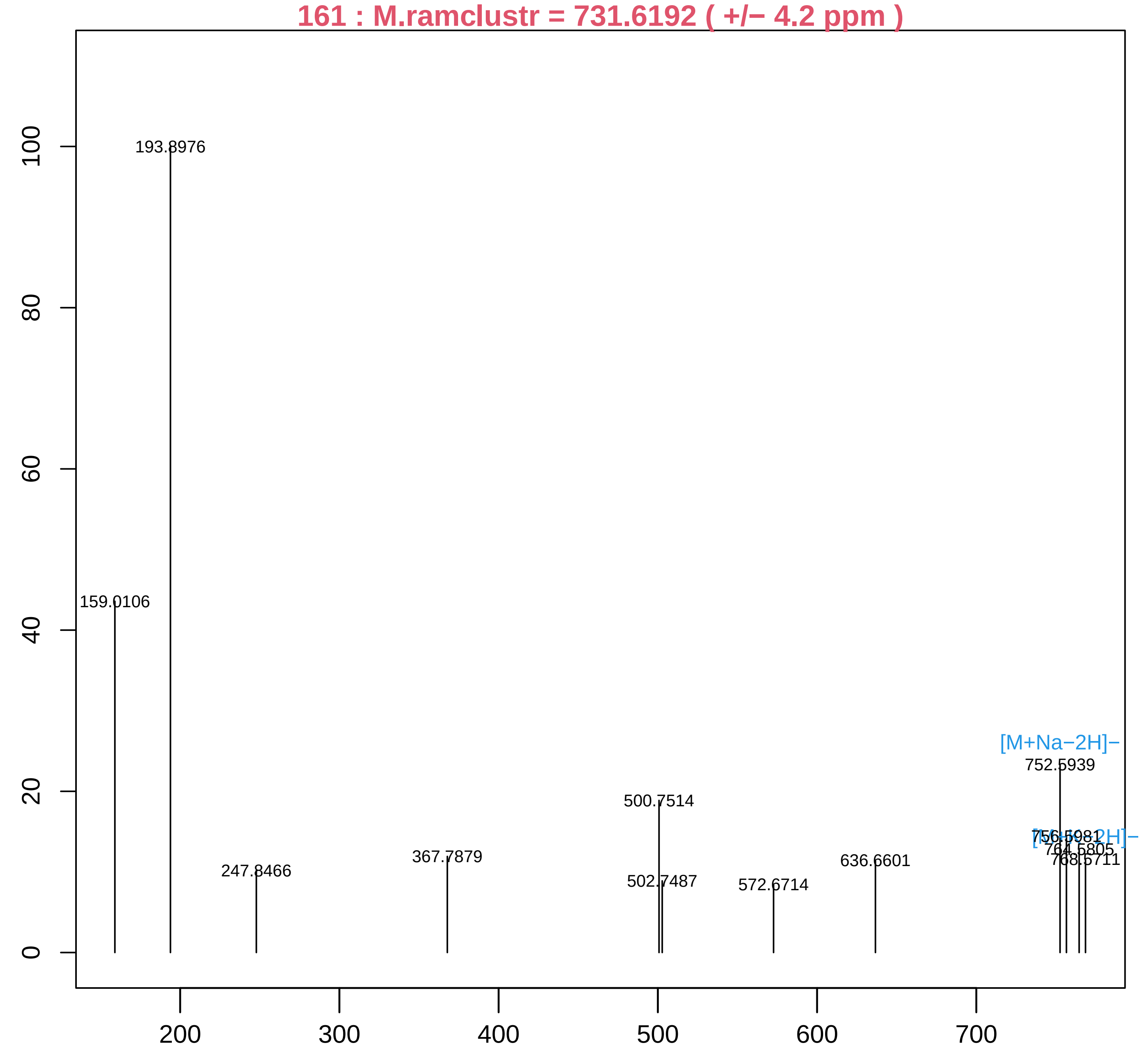
161 : M.ramclustr = 731.6192 (+/− 4.2 ppm)
0
20
40
60
80
100
200
300
400
500
600
700
193.8976
159.0106
247.8466
367.7879
500.7514
502.7487
572.6714
636.6601
[M+Na−2H]−
752.5939
764.5805
768.5711

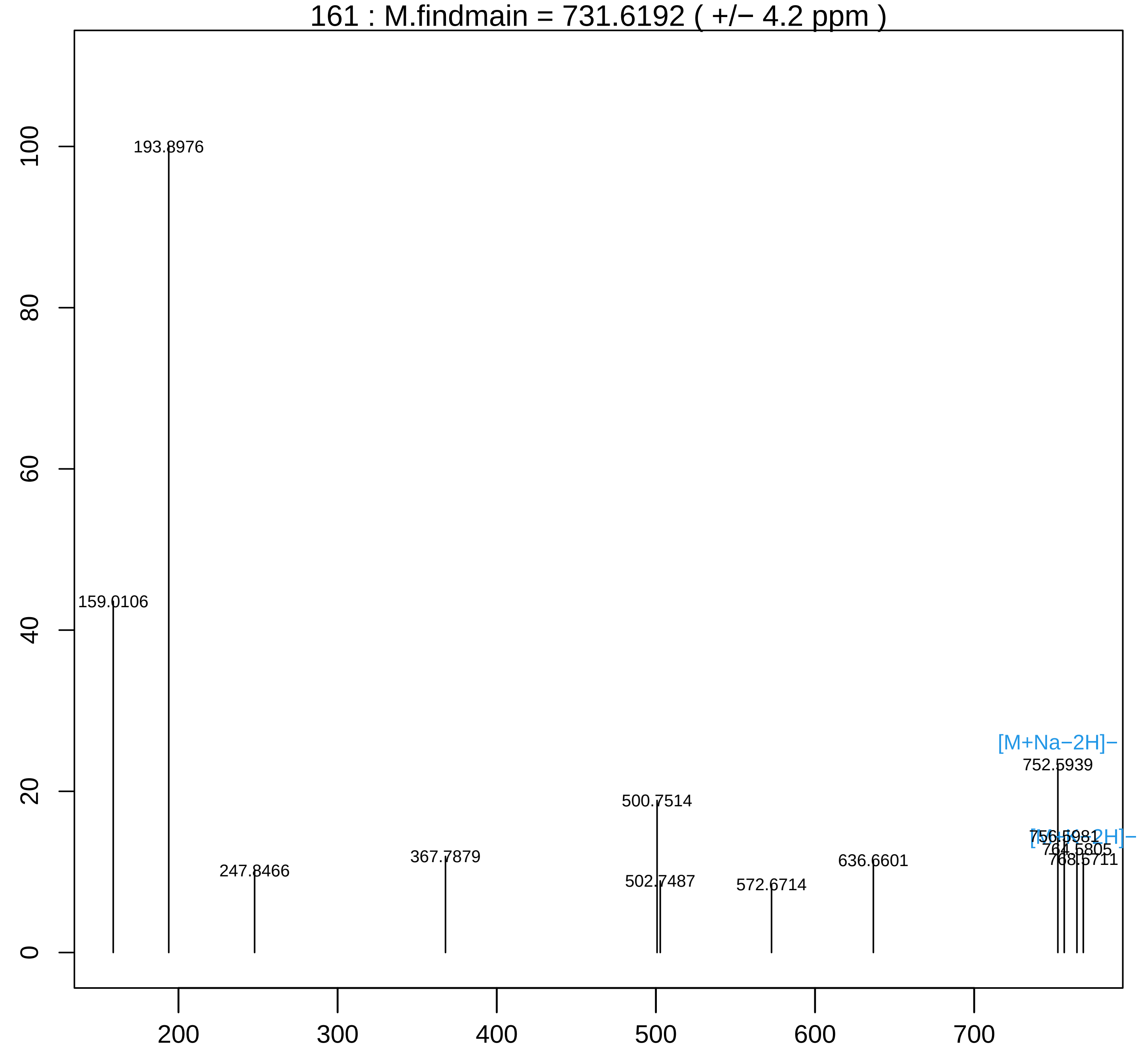
161 : M.findmain = 731.6192 (+/− 4.2 ppm)
0
20
40
60
80
100
200
300
400
500
600
700
193.8976
159.0106
247.8466
367.7879
500.7514
502.7487
572.6714
636.6601
[M+Na−2H]−
752.5939
764.5805
768.5711

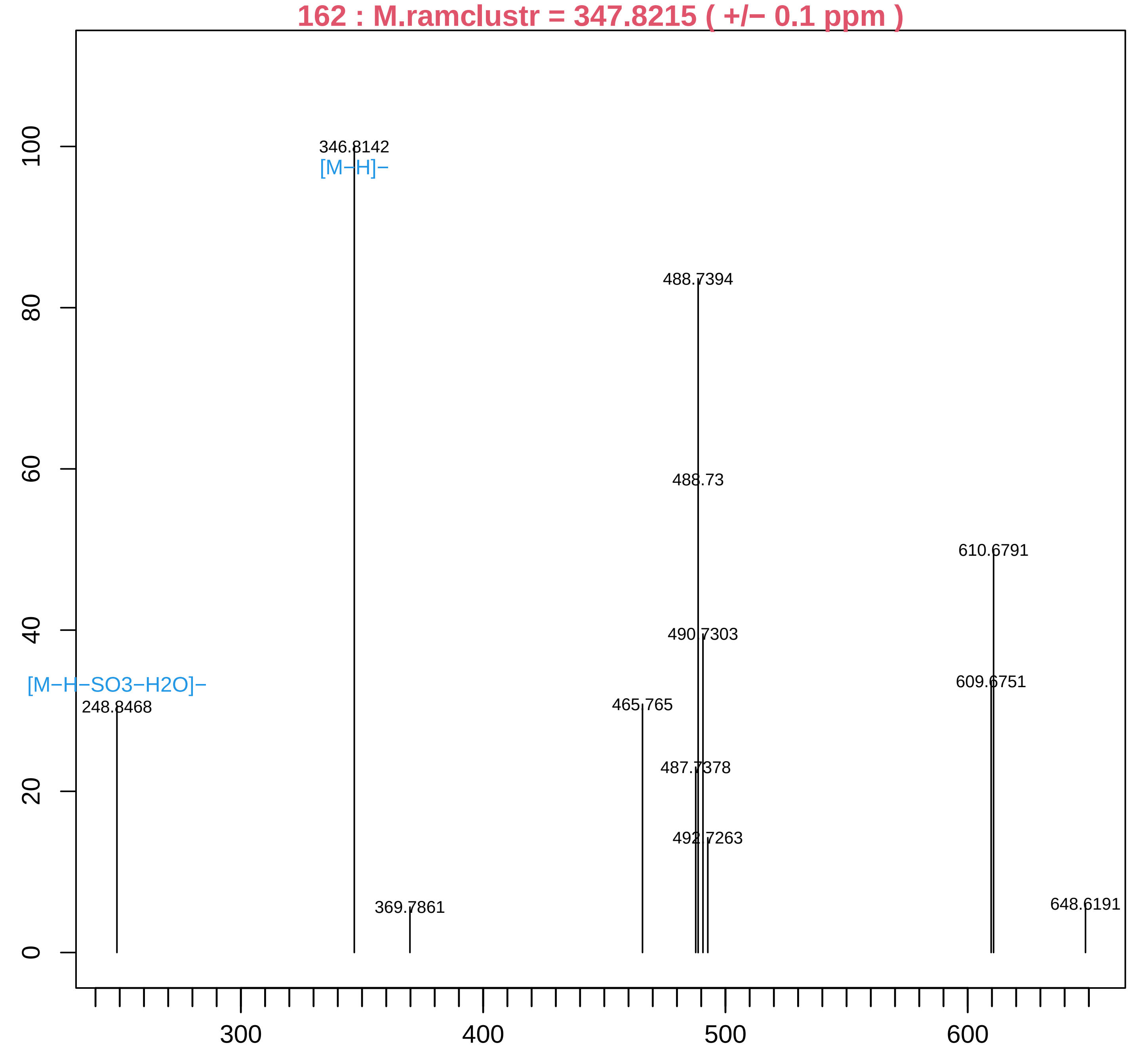
162 : M.ramclustr = 347.8215 (+/− 0.1 ppm)
346.8142
[M−H]−
488.7394
488.73
610.6791
490.7303
[M−H−SO3−H2O]−
248.8468
465.765
609.6751
487.7378
492.7263
369.7861
648.6191
0
20
40
60
80
100
300
400
500
600

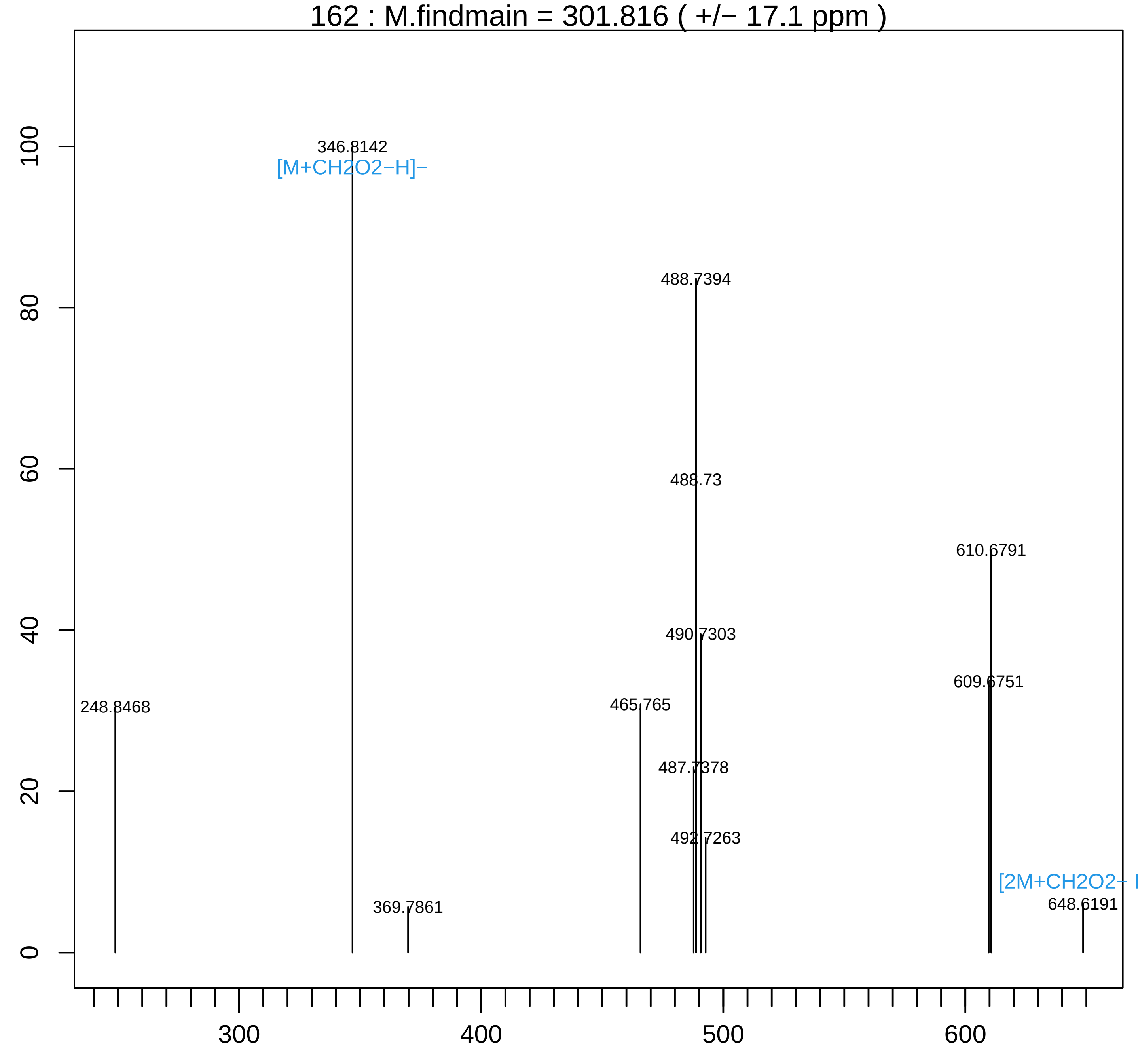
162 : M.findmain = 301.816 (+/− 17.1 ppm)
346.8142
[M+CH2O2−H]−
488.7394
488.73
610.6791
490.7303
248.8468
465.765
609.6751
487.7378
492.7263
369.7861
648.6191
0
20
40
60
80
100
300
400
500
600

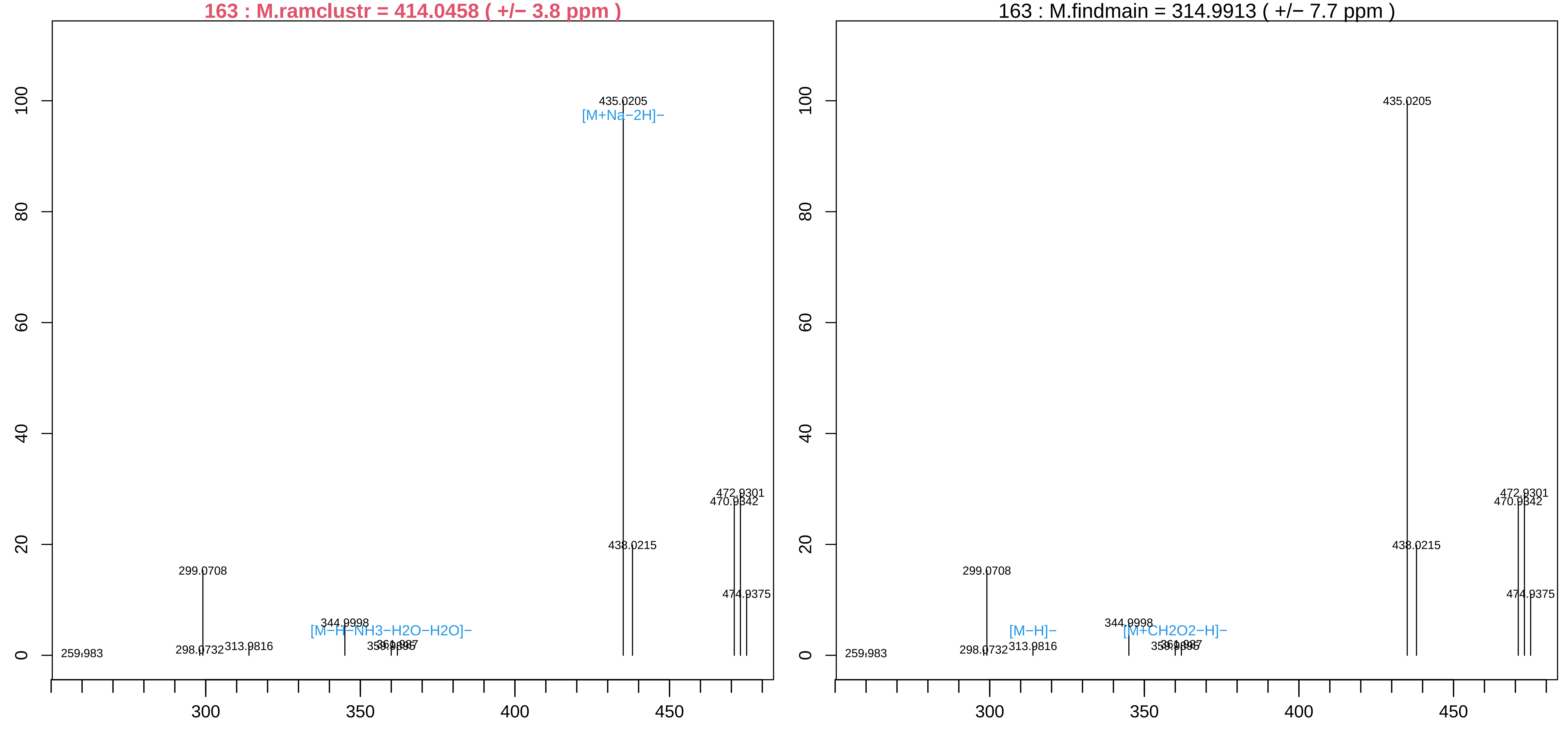

163 : M.ramclustr = 414.0458 (+/− 3.8 ppm)
435.0205
[M+Na−2H]−
472.9301
470.9342
438.0215
299.0708
474.9375
344.9998
[M−H−NH3−H2O−H2O]−
361.987
359.9895
259.983
298.0732
313.9816
163 : M.findmain = 314.9913 (+/− 7.7 ppm)
435.0205
472.9301
470.9342
438.0215
299.0708
474.9375
344.9998
[M−H]−
[M+CH2O2−H]−
361.987
359.9895
259.983
298.0732
313.9816
0
20
40
60
80
100
300
350
400
450

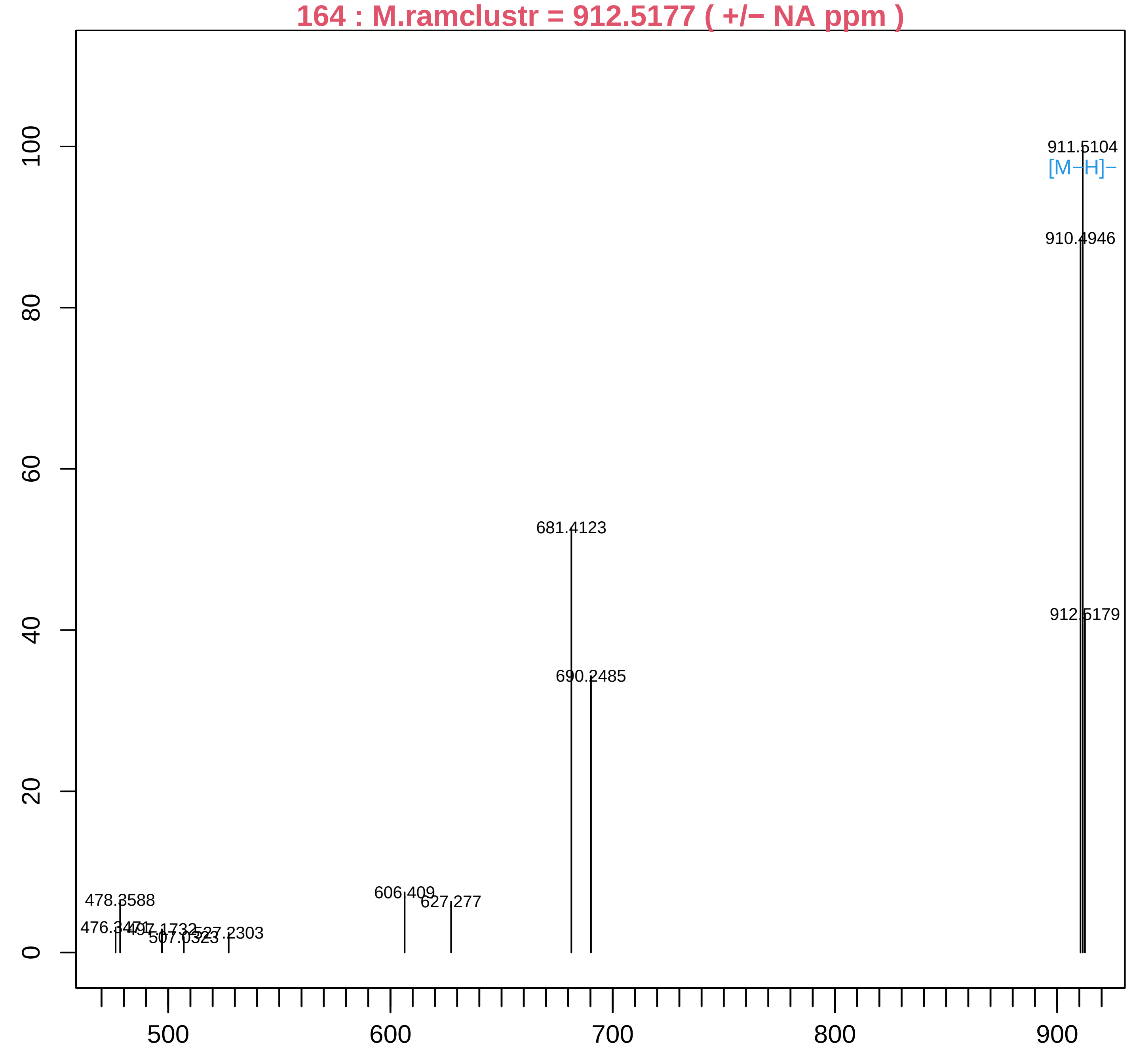
164 : M.ramclustr = 912.5177 (+/− NA ppm)
911.5104
[M−H]−
910.4946
681.4123
912.5179
690.2485
478.3588
606.409
627.277
476.3471
497.1732
507.0323
527.2303
0
20
40
60
80
100
500
600
700
800
900

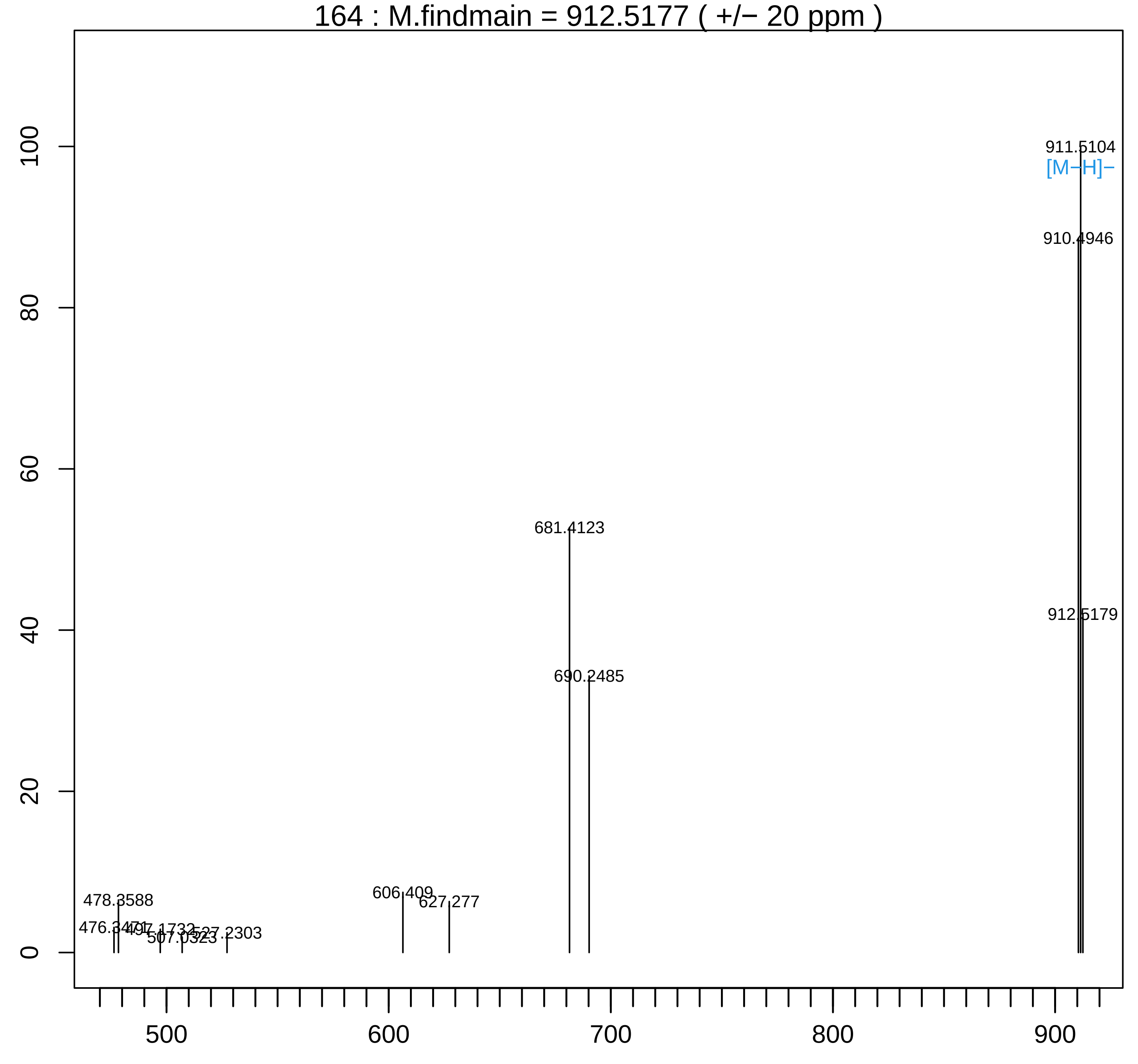
164 : M.findmain = 912.5177 (+/− 20 ppm)
911.5104
[M−H]−
910.4946
681.4123
912.5179
690.2485
478.3588
606.409
627.277
476.3471
497.1732
507.0323
527.2303
0
20
40
60
80
100
500
600
700
800
900

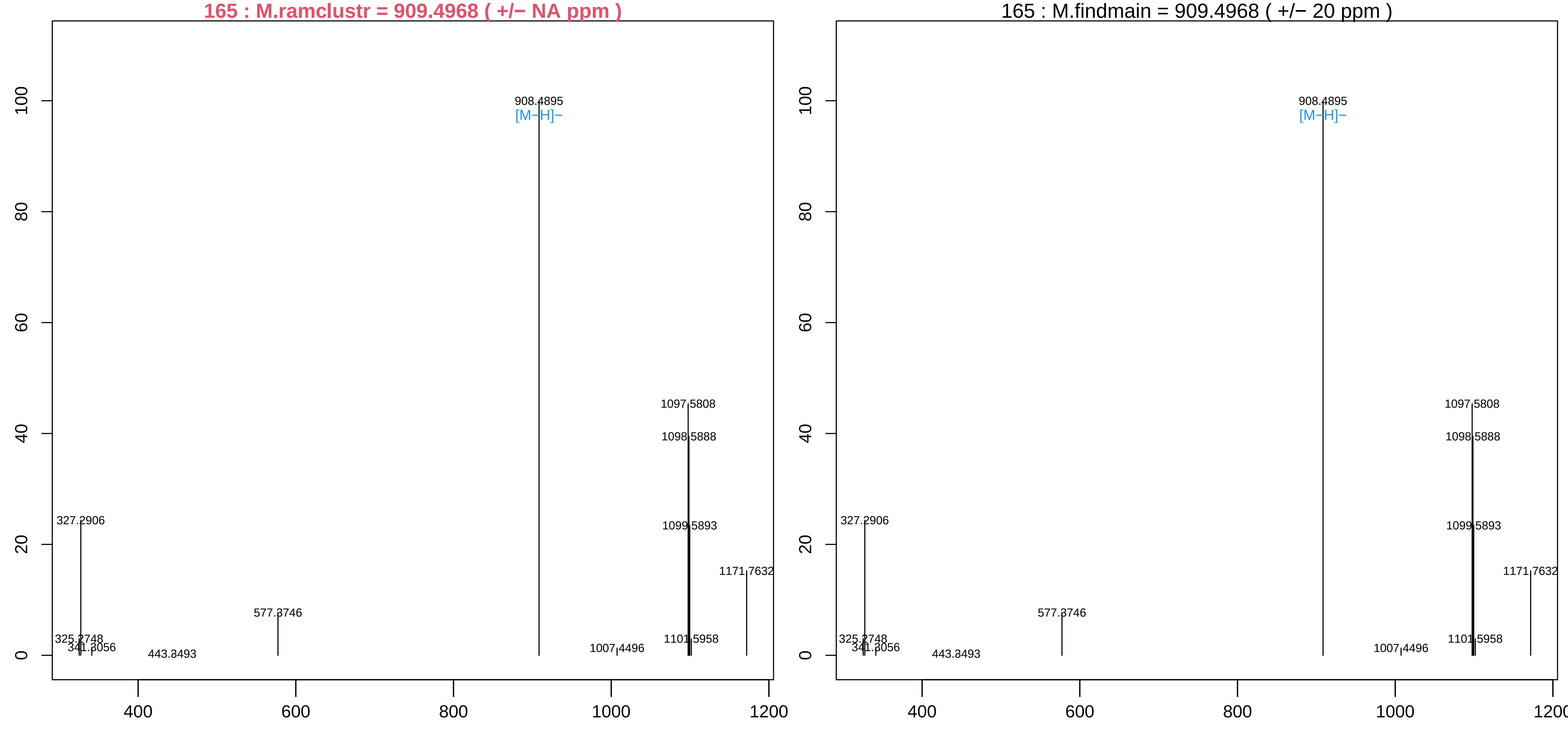

165 : M.ramclustr = 909.4968 (+/− NA ppm)
165 : M.findmain = 909.4968 (+/− 20 ppm)
908.4895
[M−H]−
1097.5808
1098.5888
1099.5893
1101.5958
1171.7632
1007.4496
327.2906
325.2748
341.3056
443.3493
577.3746
0
20
40
60
80
100
400
600
800
1000
1200

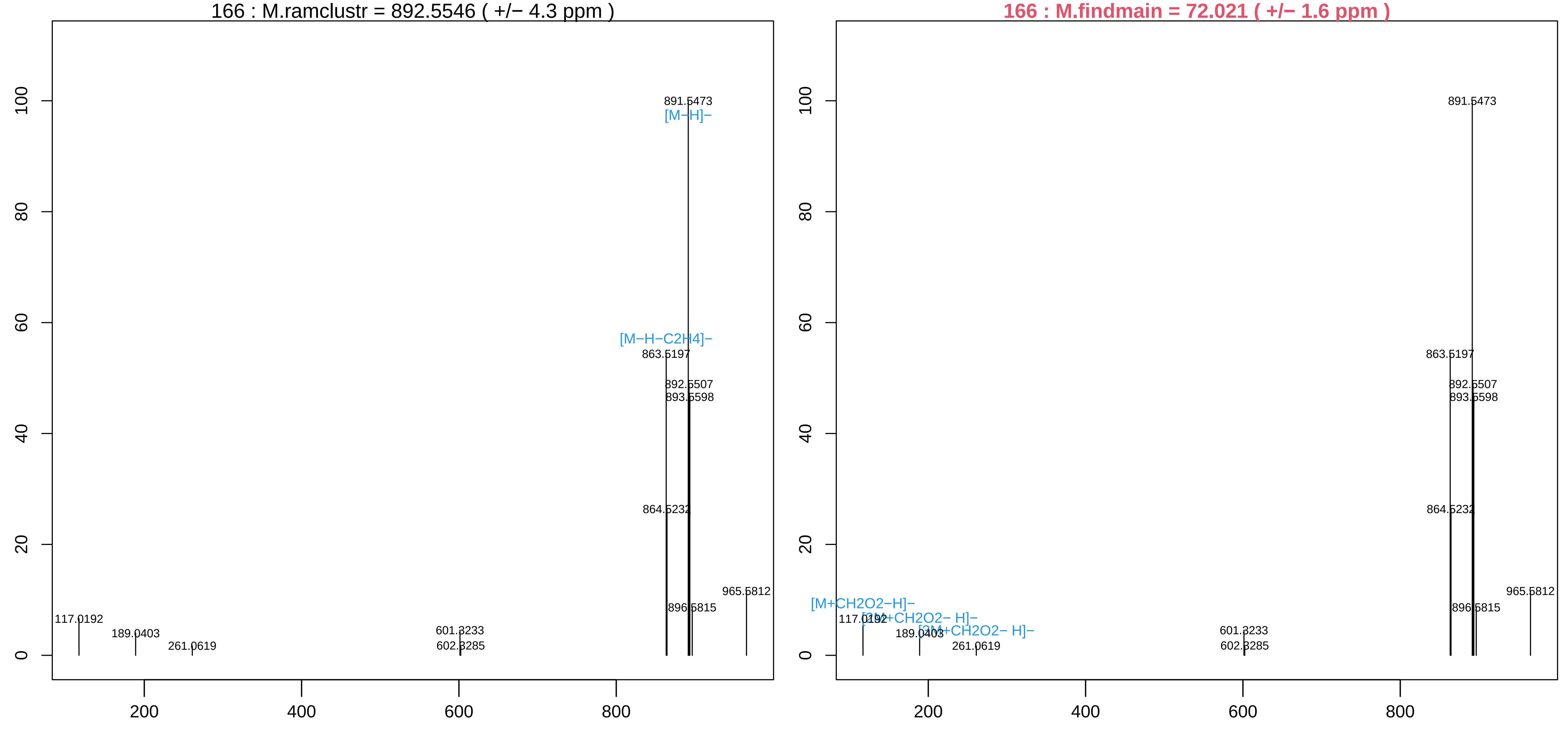

166 : M.ramclustr = 892.5546 (+/− 4.3 ppm)
166 : M.findmain = 72.021 (+/− 1.6 ppm)
891.5473
[M−H]−
[M−H−C2H4]−
863.5197
892.5507
893.5598
864.5232
896.5815
965.5812
117.0192
189.0403
261.0619
601.3233
602.3285
[M+CH2O2−H]−
[2M+CH2O2− H]−
[3M+CH2O2− H]−
0
20
40
60
80
100
200
400
600
800

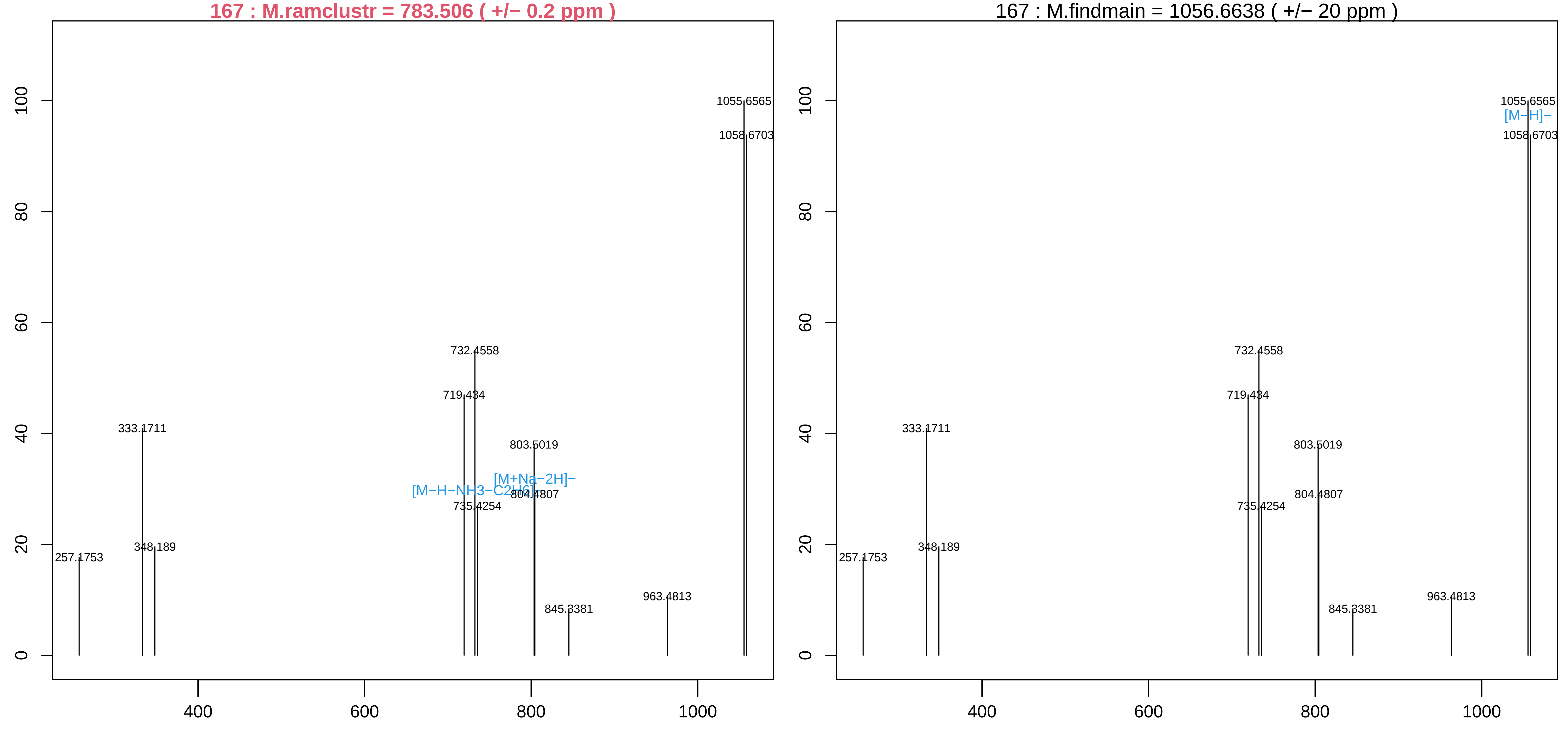
167 : M.ramclustr = 783.506 (+/− 0.2 ppm)
1055.6565
1058.6703
732.4558
719.434
333.1711
803.5019
[M+Na−2H]−
[M−H−NH3−C2H6]−
804.4807
735.4254
348.189
257.1753
963.4813
845.3381
167 : M.findmain = 1056.6638 (+/− 20 ppm)
1055.6565
[M−H]−
1058.6703
732.4558
719.434
333.1711
803.5019
804.4807
735.4254
348.189
257.1753
963.4813
845.3381
0
20
40
60
80
100
400
600
800
1000

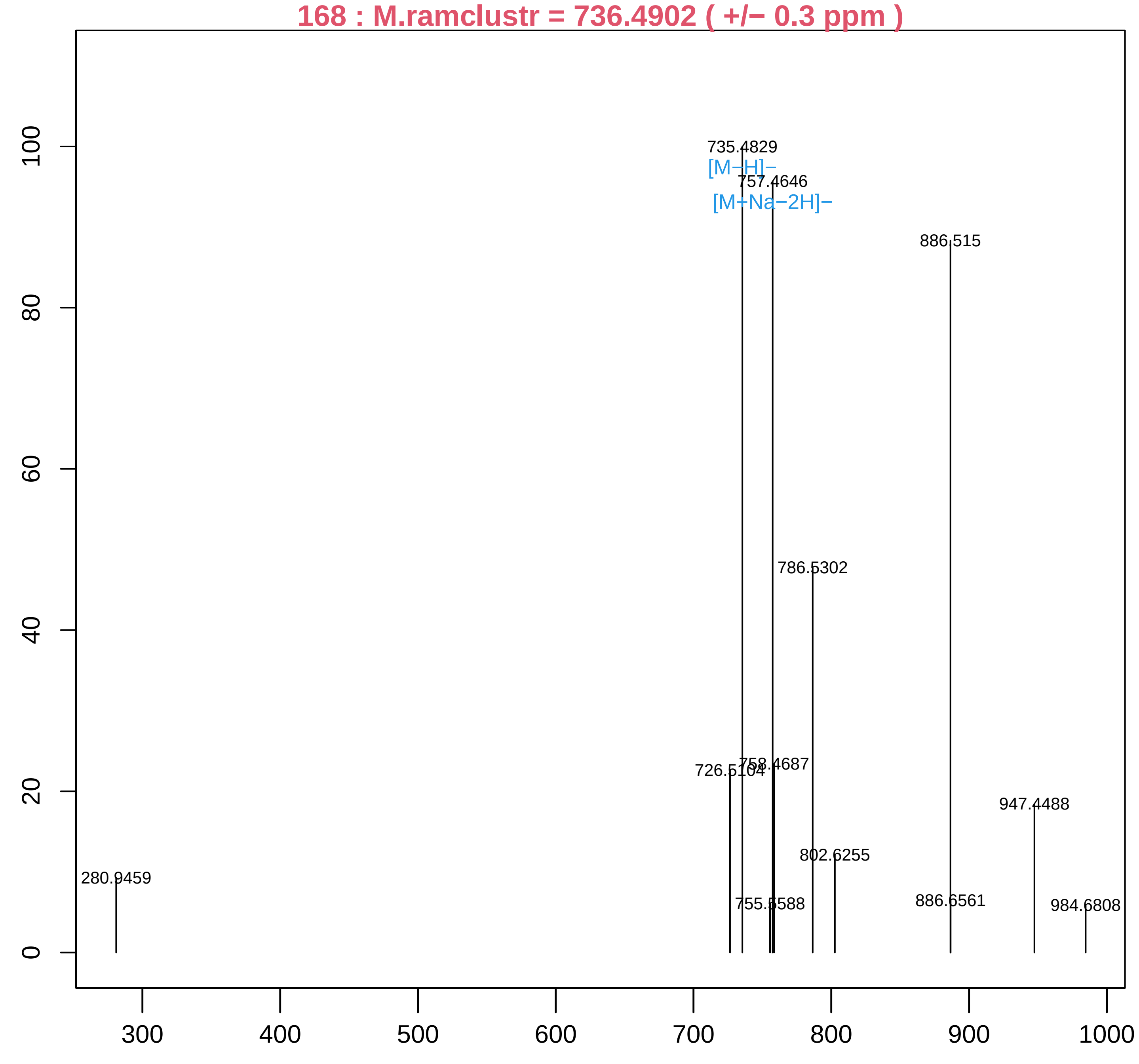
168 : M.ramclustr = 736.4902 (+/− 0.3 ppm)
735.4829
[M−H]−
757.4646
[M+Na−2H]−
886.515
786.5302
726.5104
758.4687
947.4488
802.6255
280.9459
755.5588
886.6561
984.6808
0
20
40
60
80
100
300
400
500
600
700
800
900
1000

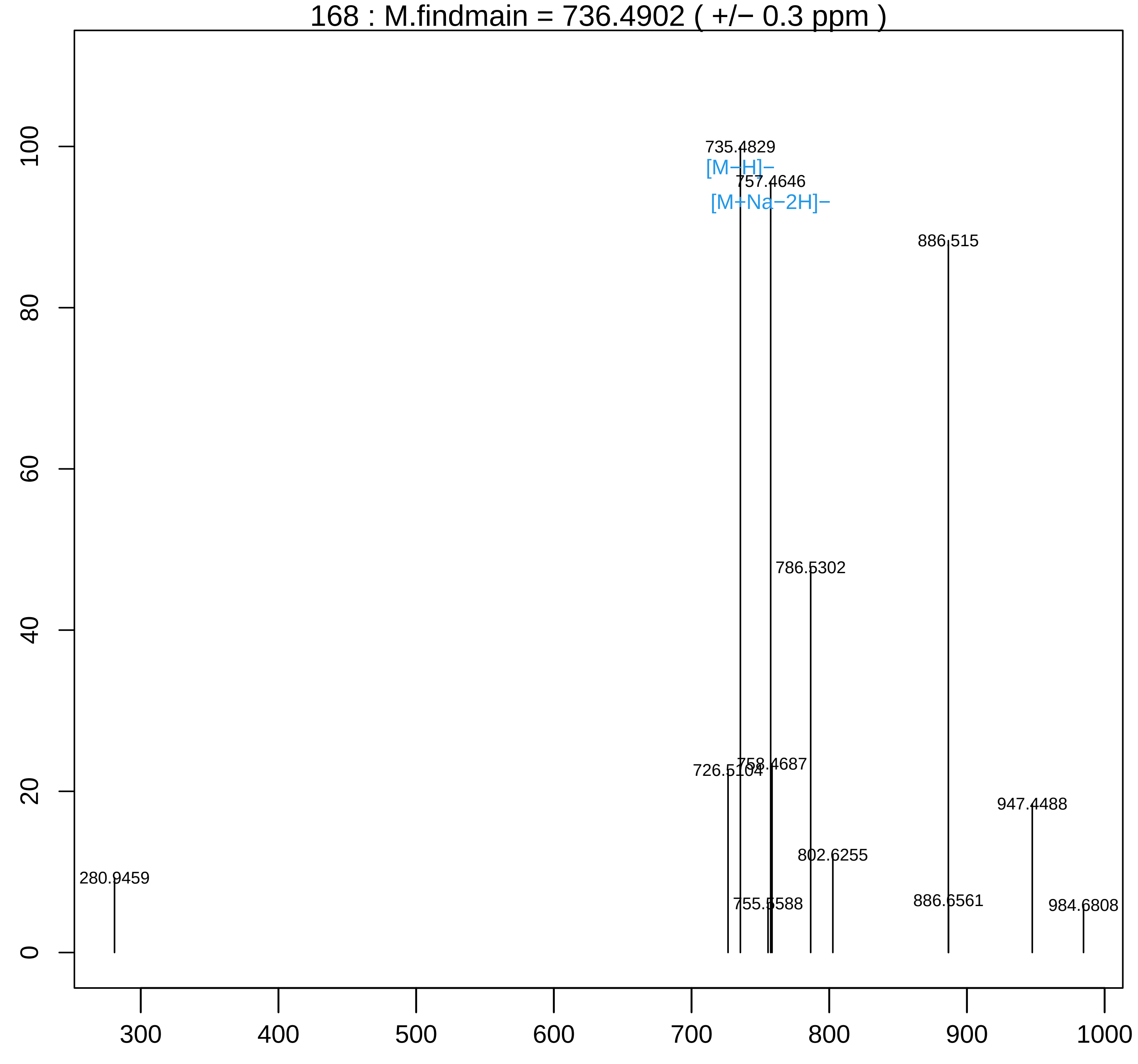
168 : M.findmain = 736.4902 (+/− 0.3 ppm)
735.4829
[M−H]−
757.4646
[M+Na−2H]−
886.515
786.5302
726.5104
758.4687
947.4488
802.6255
280.9459
755.5588
886.6561
984.6808
0
20
40
60
80
100
300
400
500
600
700
800
900
1000

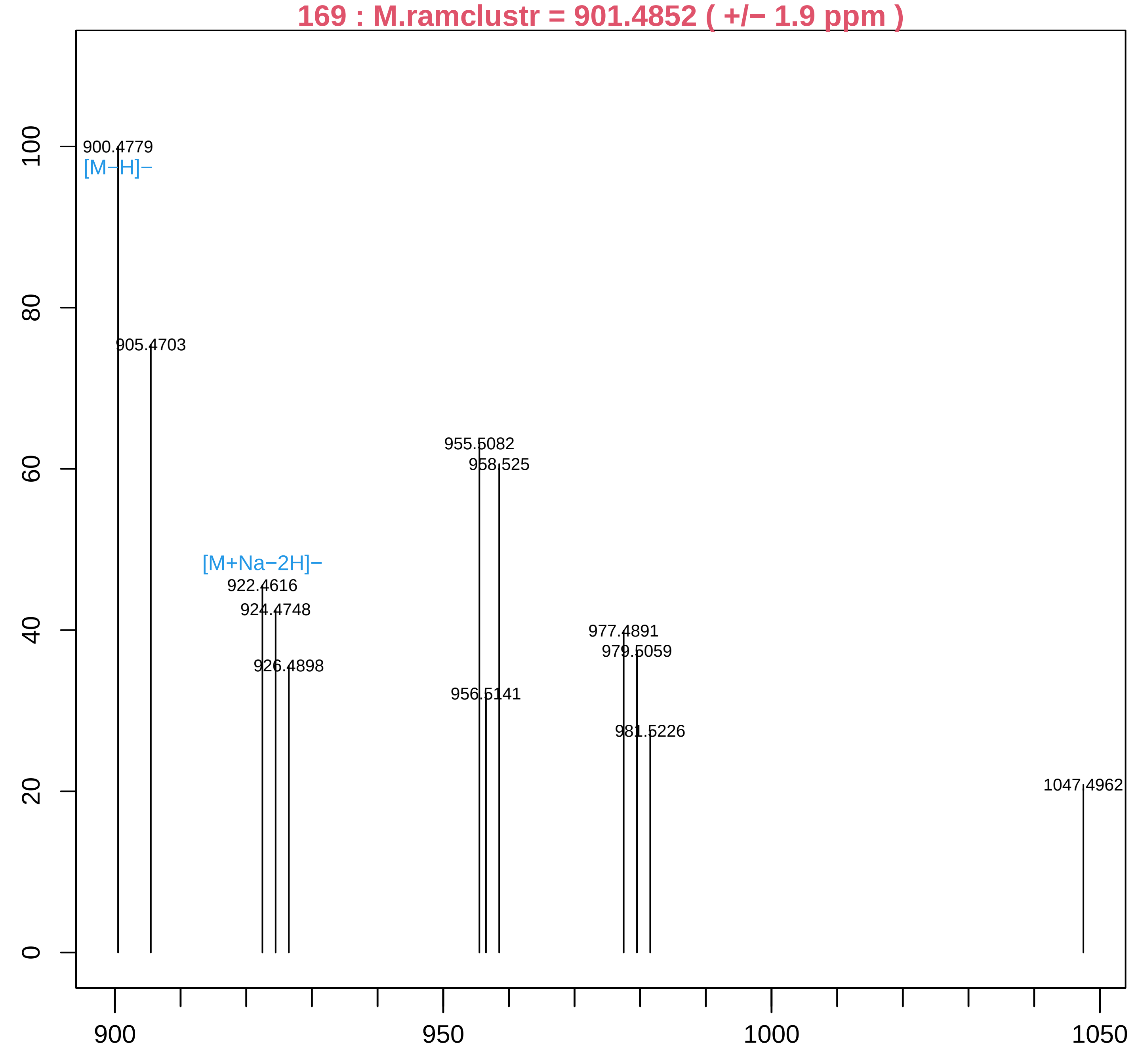

169 : M.ramclustr = 901.4852 (+/− 1.9 ppm)
900.4779
[M−H]−
905.4703
[M+Na−2H]−
922.4616
924.4748
926.4898
955.5082
958.525
956.5141
977.4891
979.5059
981.5226
1047.4962
0
20
40
60
80
100
900
950
1000
1050

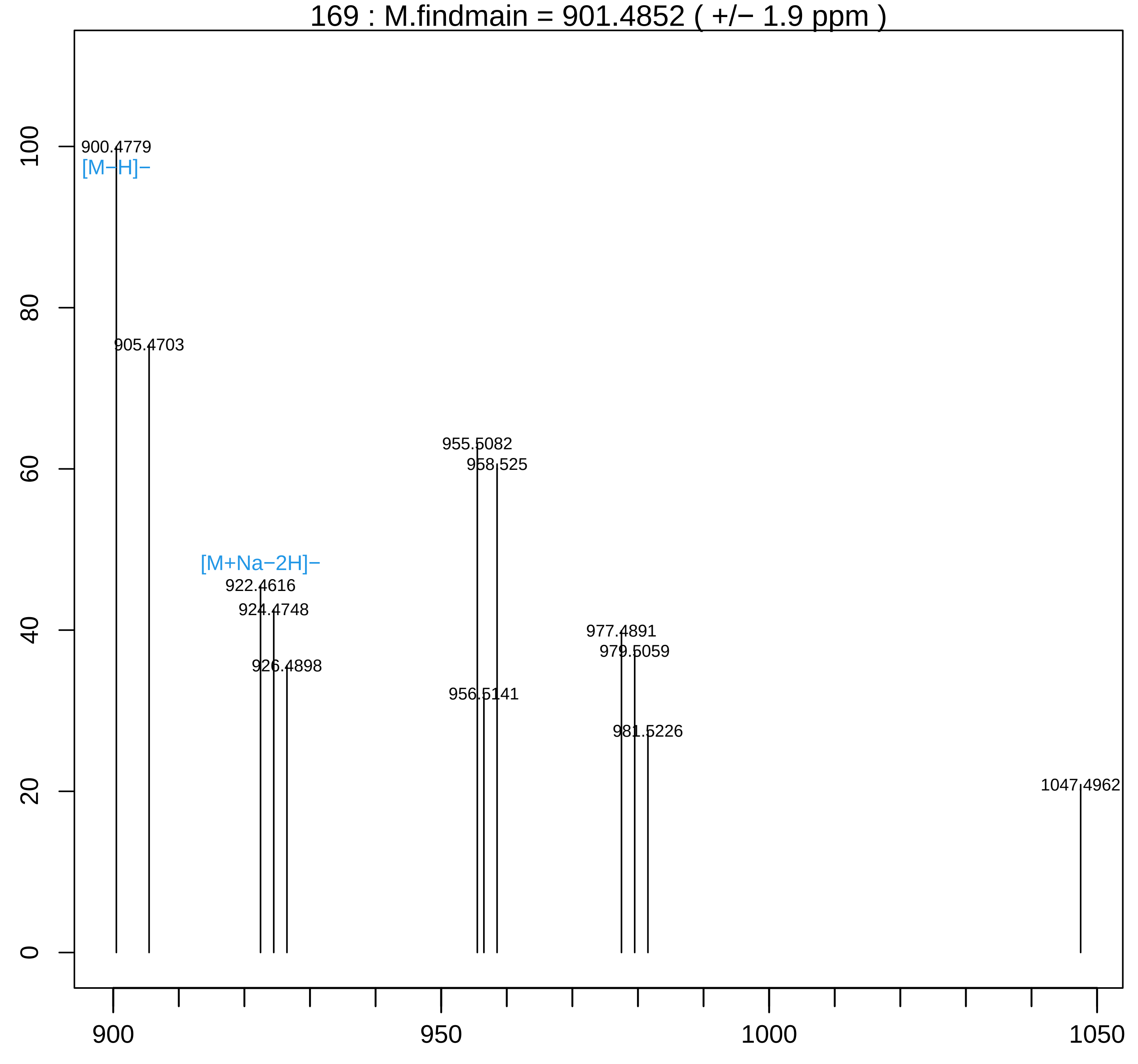

169 : M.findmain = 901.4852 (+/− 1.9 ppm)
900.4779
[M−H]−
905.4703
[M+Na−2H]−
922.4616
924.4748
926.4898
955.5082
958.525
956.5141
977.4891
979.5059
981.5226
1047.4962
0
20
40
60
80
100
900
950
1000
1050

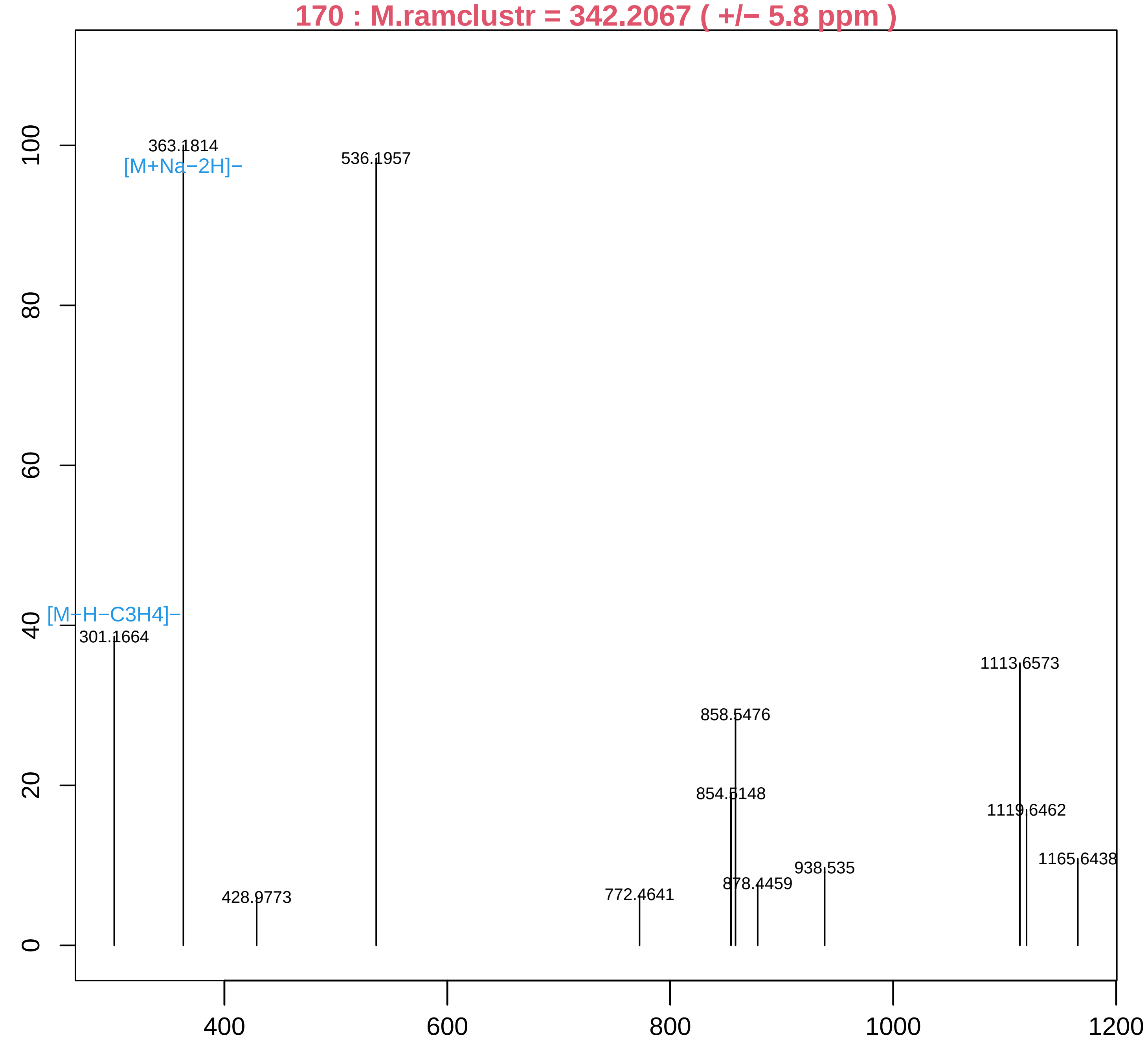

170 : M.ramclustr = 342.2067 (+/− 5.8 ppm)
363.1814
[M+Na−2H]−
536.1957
[M−H−C3H4]−
301.1664
428.9773
772.4641
858.5476
854.5148
878.4459
938.535
1113.6573
1119.6462
1165.6438
0
20
40
60
80
100
400
600
800
1000
1200

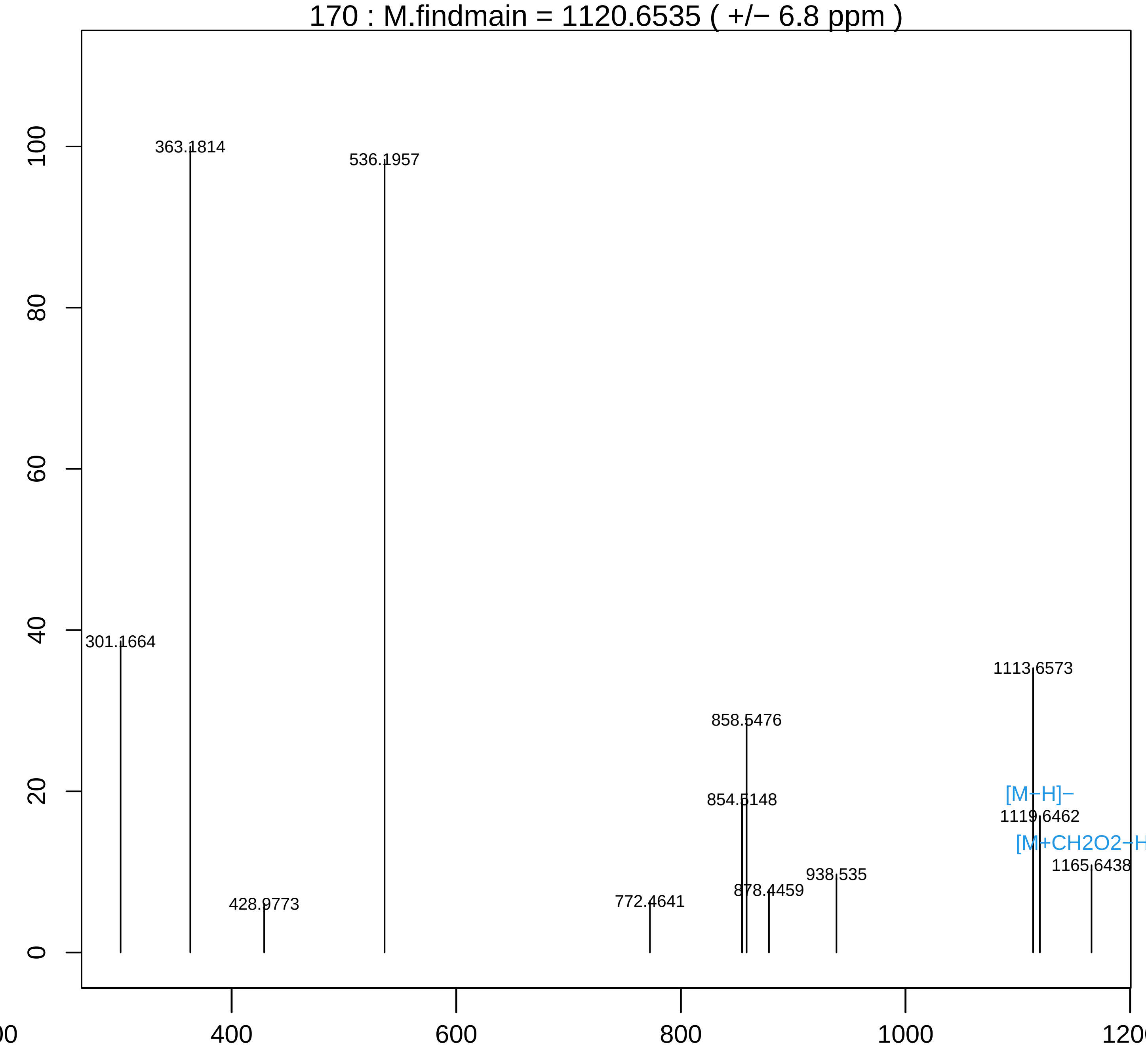

170 : M.findmain = 1120.6535 (+/− 6.8 ppm)
363.1814
536.1957
301.1664
428.9773
772.4641
858.5476
854.5148
878.4459
938.535
1113.6573
[M−H]−
1119.6462
1165.6438
0
20
40
60
80
100
400
600
800
1000

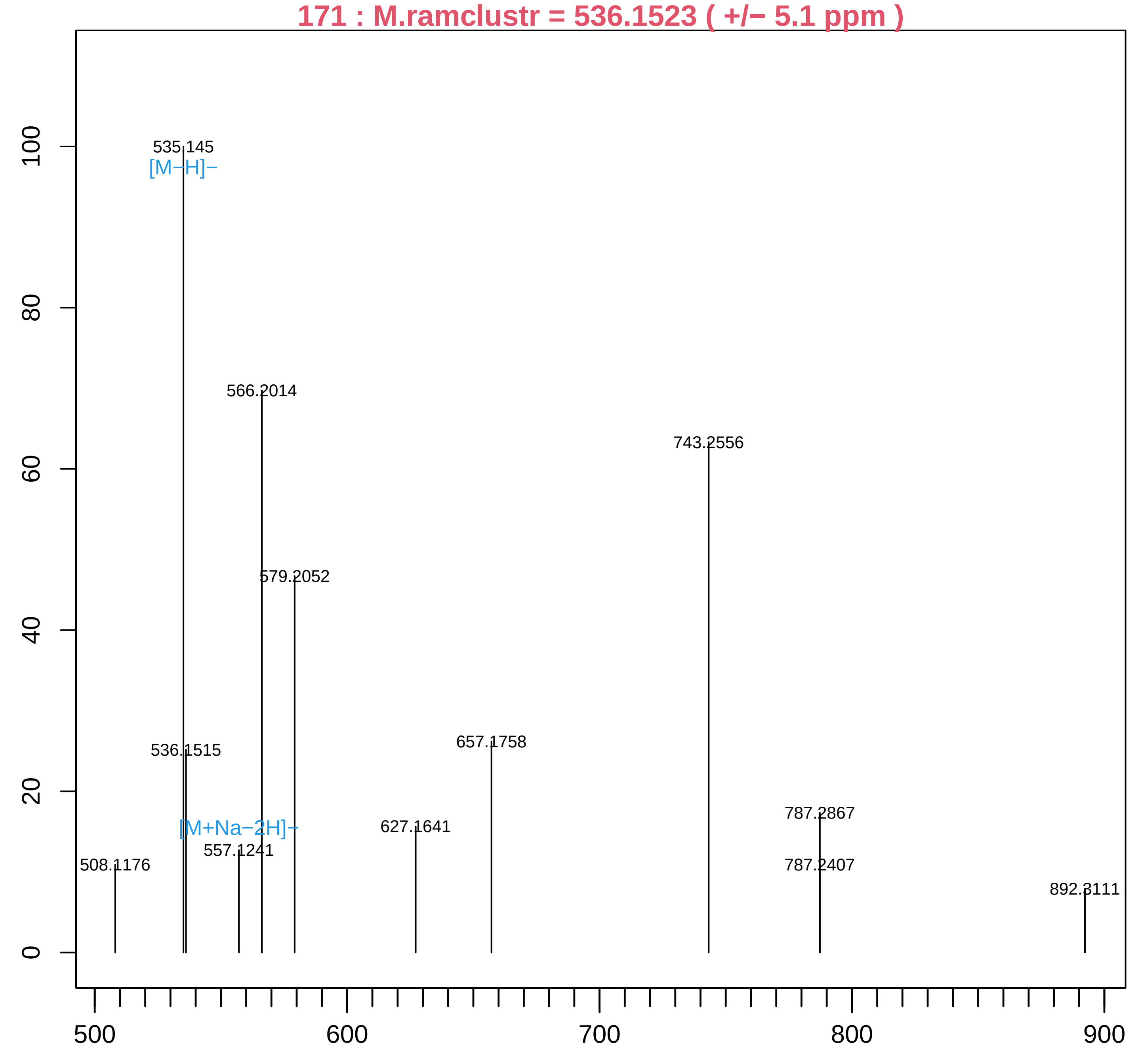
171 : M.ramclustr = 536.1523 (+/− 5.1 ppm)
535.145
[M−H]−
566.2014
743.2556
579.2052
536.1515
657.1758
787.2867
[M+Na−2H]−
627.1641
557.1241
508.1176
787.2407
892.3111
0
20
40
60
80
100
500
600
700
800
900

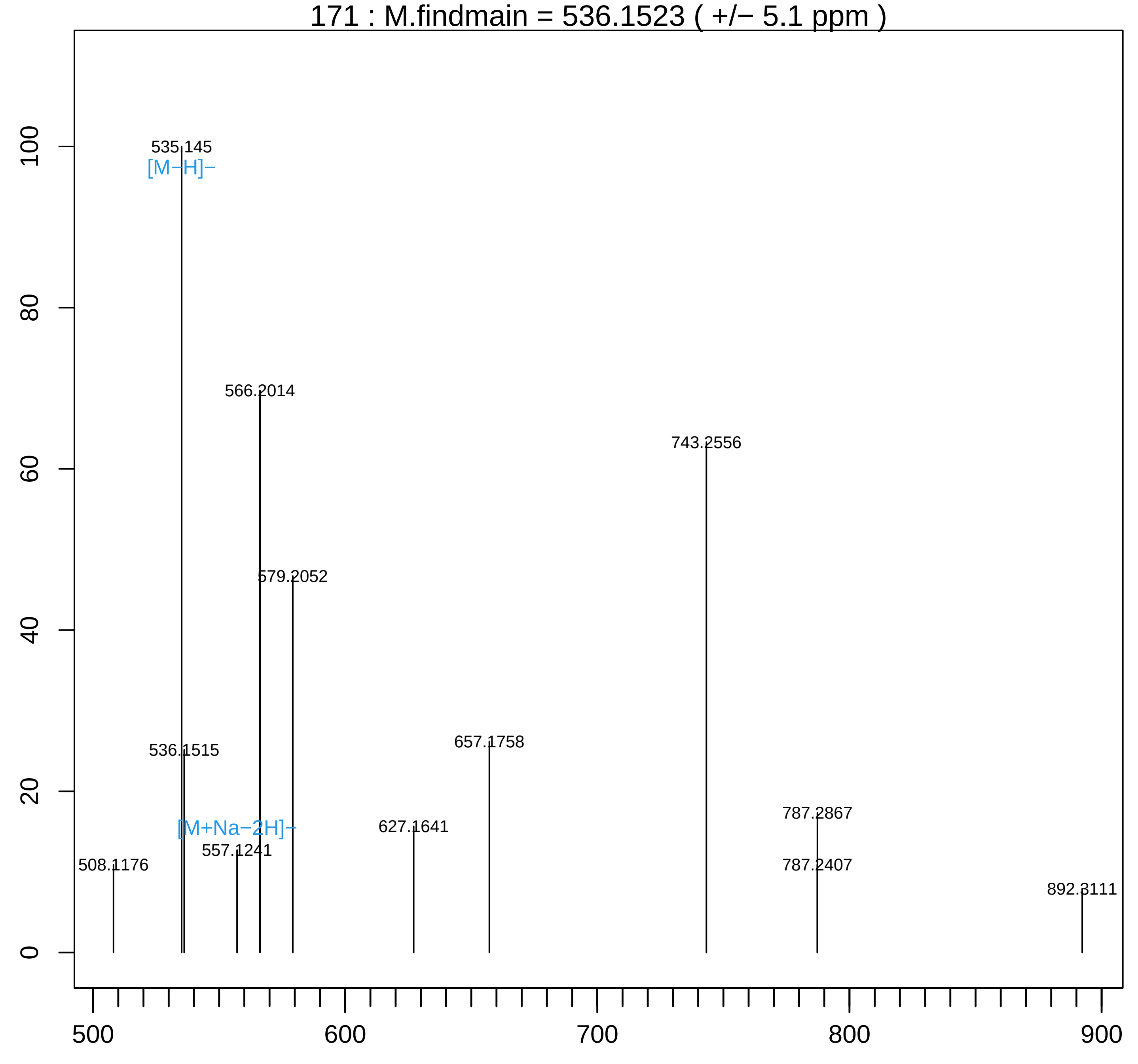
171 : M.findmain = 536.1523 (+/− 5.1 ppm)
535.145
[M−H]−
566.2014
743.2556
579.2052
536.1515
657.1758
787.2867
[M+Na−2H]−
627.1641
557.1241
508.1176
787.2407
892.3111
0
20
40
60
80
100
500
600
700
800
900

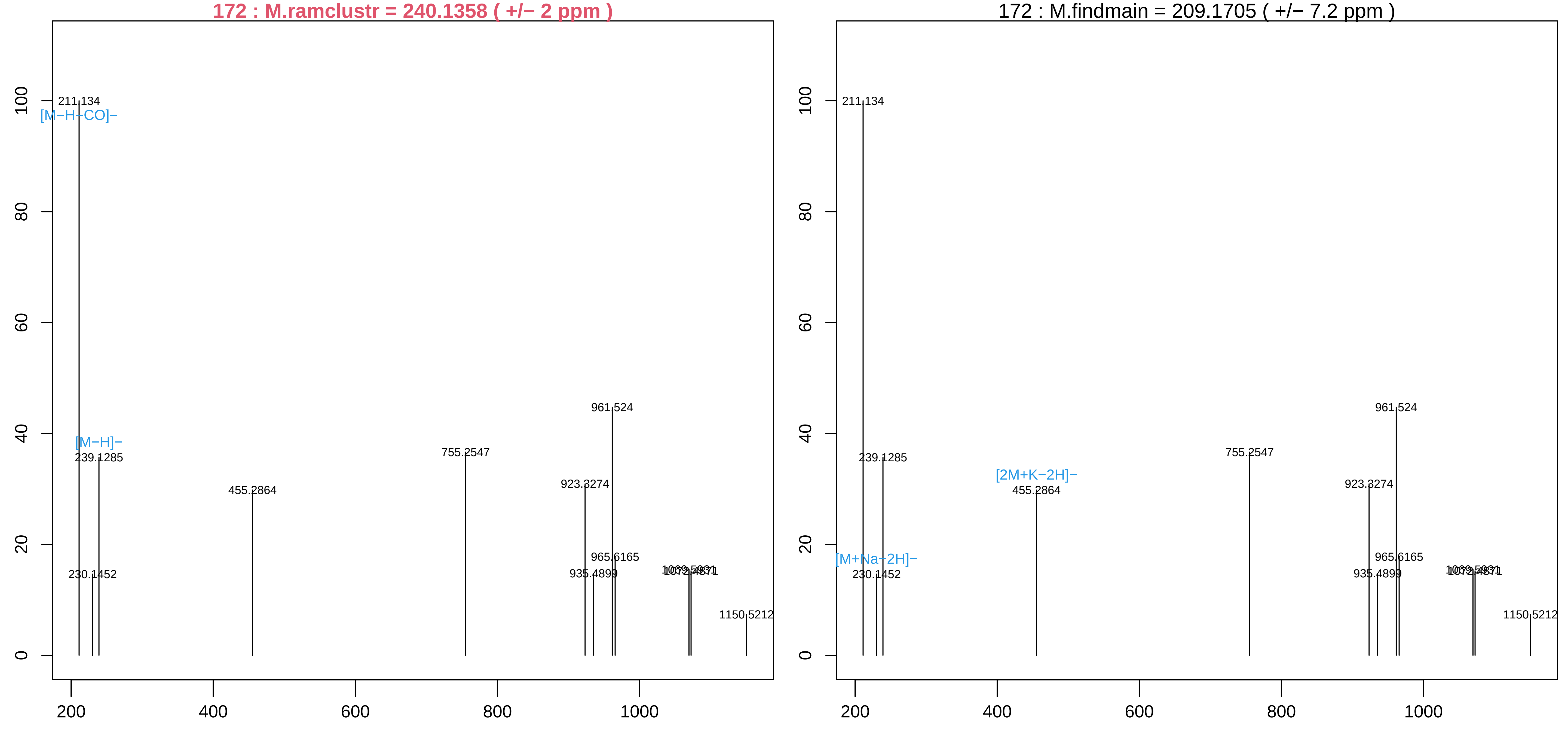
172 : M.ramclustr = 240.1358 (+/− 2 ppm)
211.134
[M−H−CO]−
[M−H]−
239.1285
230.1452
455.2864
755.2547
923.3274
935.4899
961.524
965.6165
1069.5931
1072.4871
1150.5212
172 : M.findmain = 209.1705 (+/− 7.2 ppm)
[2M+K−2H]−
[M+Na−2H]−

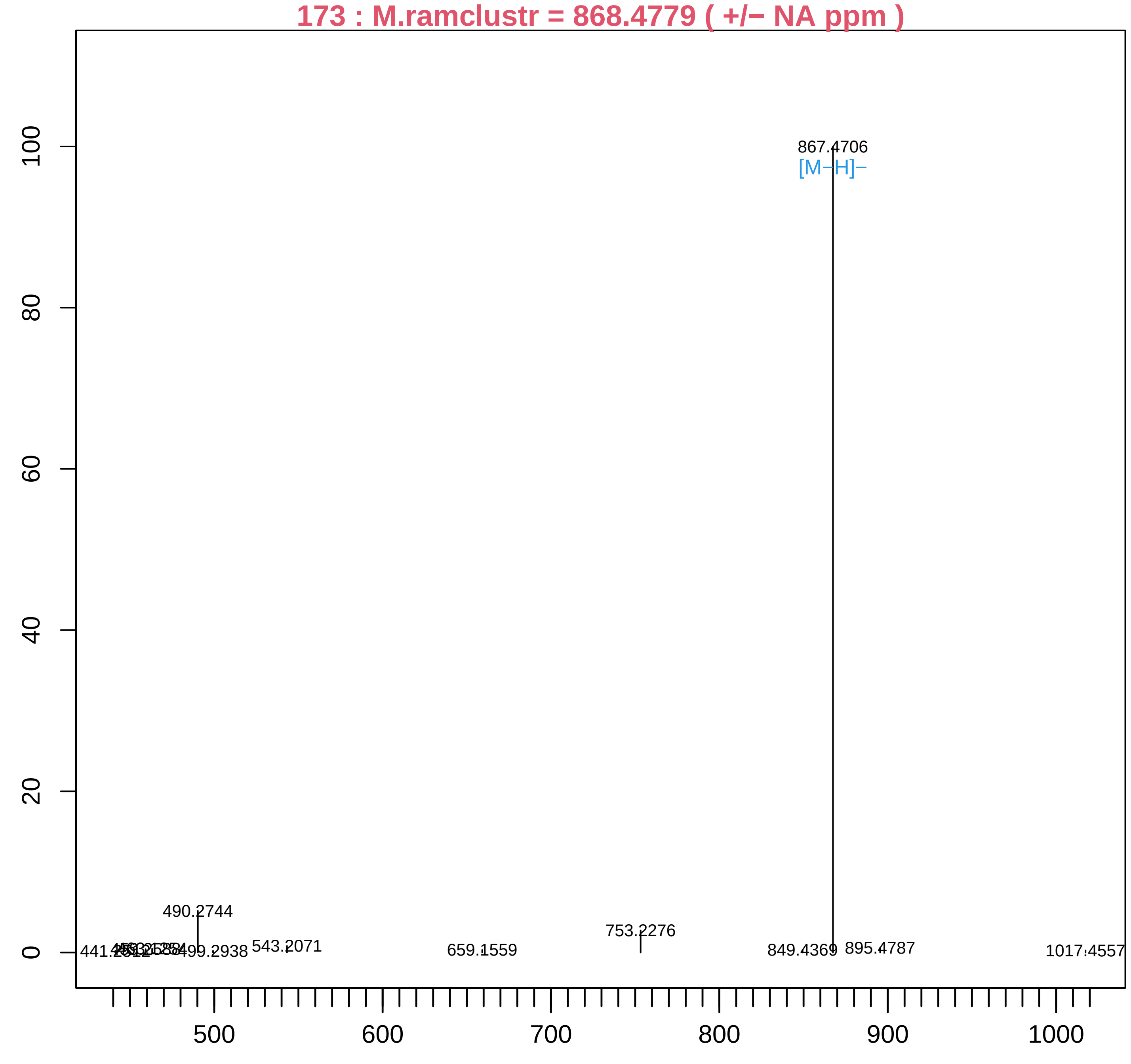
173 : M.ramclustr = 868.4779 (+/− NA ppm)
867.4706
[M−H]−
490.2744
753.2276
441.2512
499.2938
543.2071
659.1559
849.4369
895.4787
1017.4557

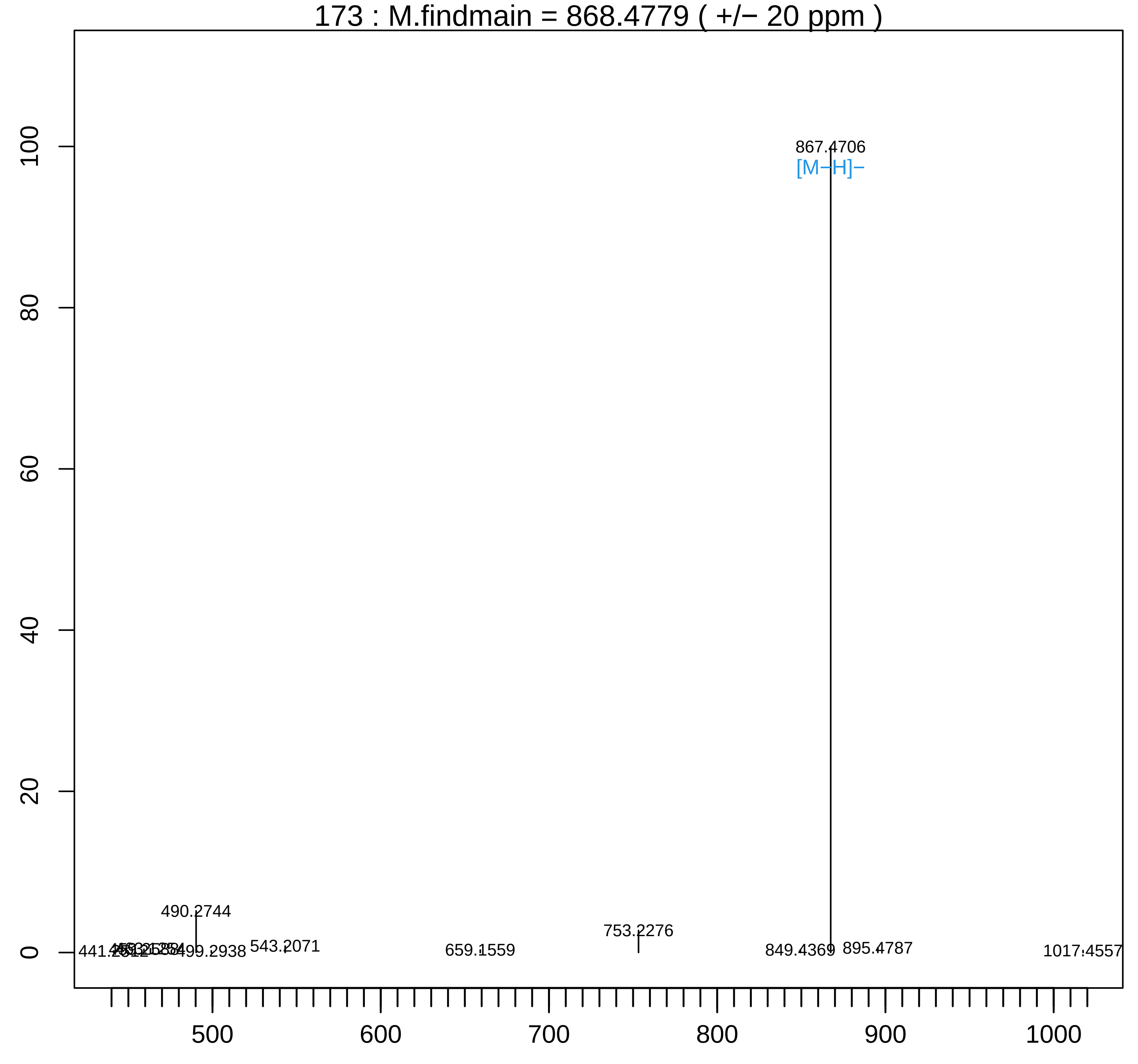
173 : M.findmain = 868.4779 (+/− 20 ppm)
867.4706
[M−H]−
490.2744
753.2276
441.2512
499.2938
543.2071
659.1559
849.4369
895.4787
1017.4557

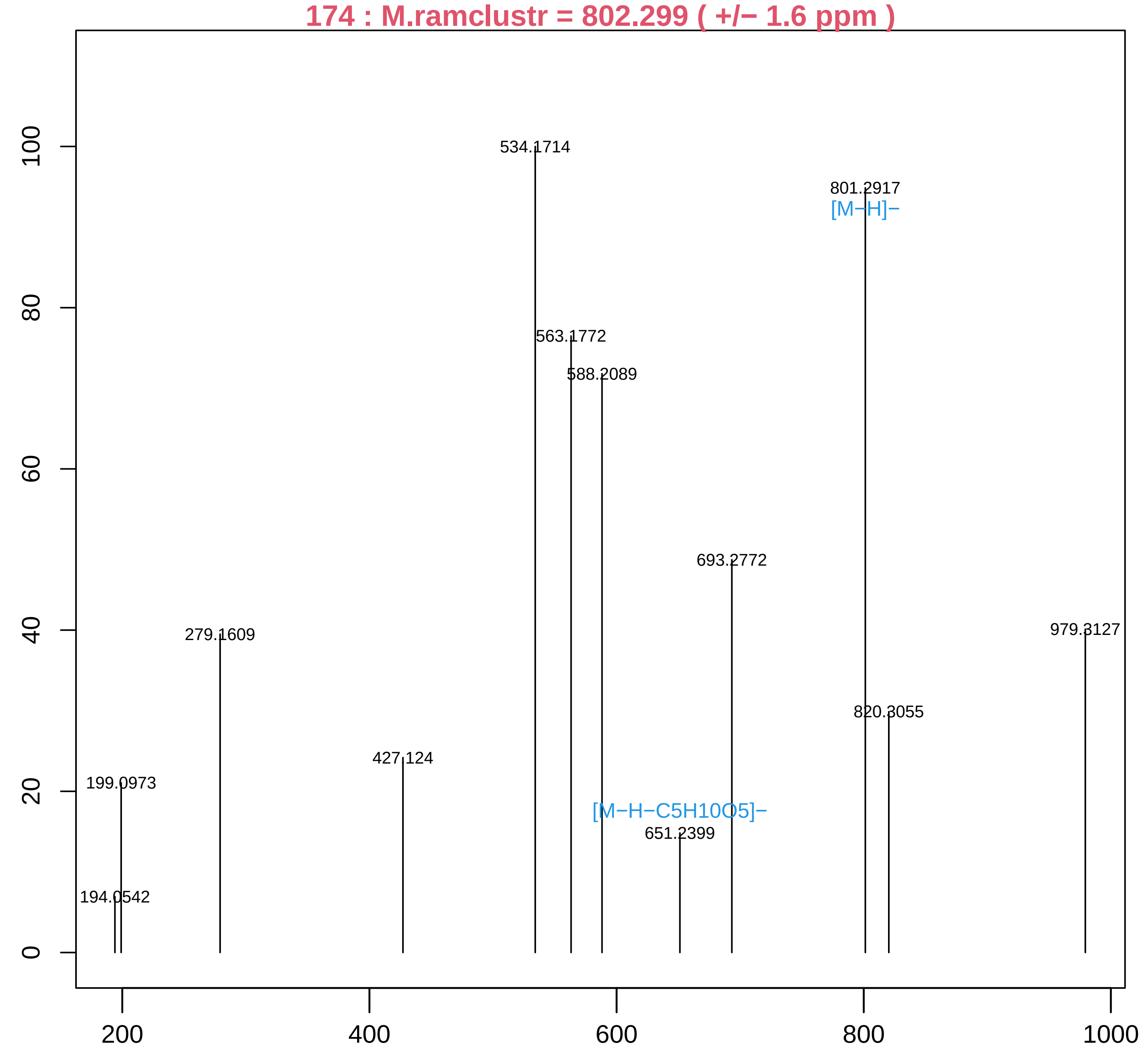

174 : M.ramclustr = 802.299 (+/− 1.6 ppm)
534.1714
801.2917
[M−H]−
563.1772
588.2089
693.2772
279.1609
979.3127
820.3055
427.124
199.0973
[M−H−C5H10O5]−
651.2399
194.0542
0
20
40
60
80
100
200
400
600
800
1000

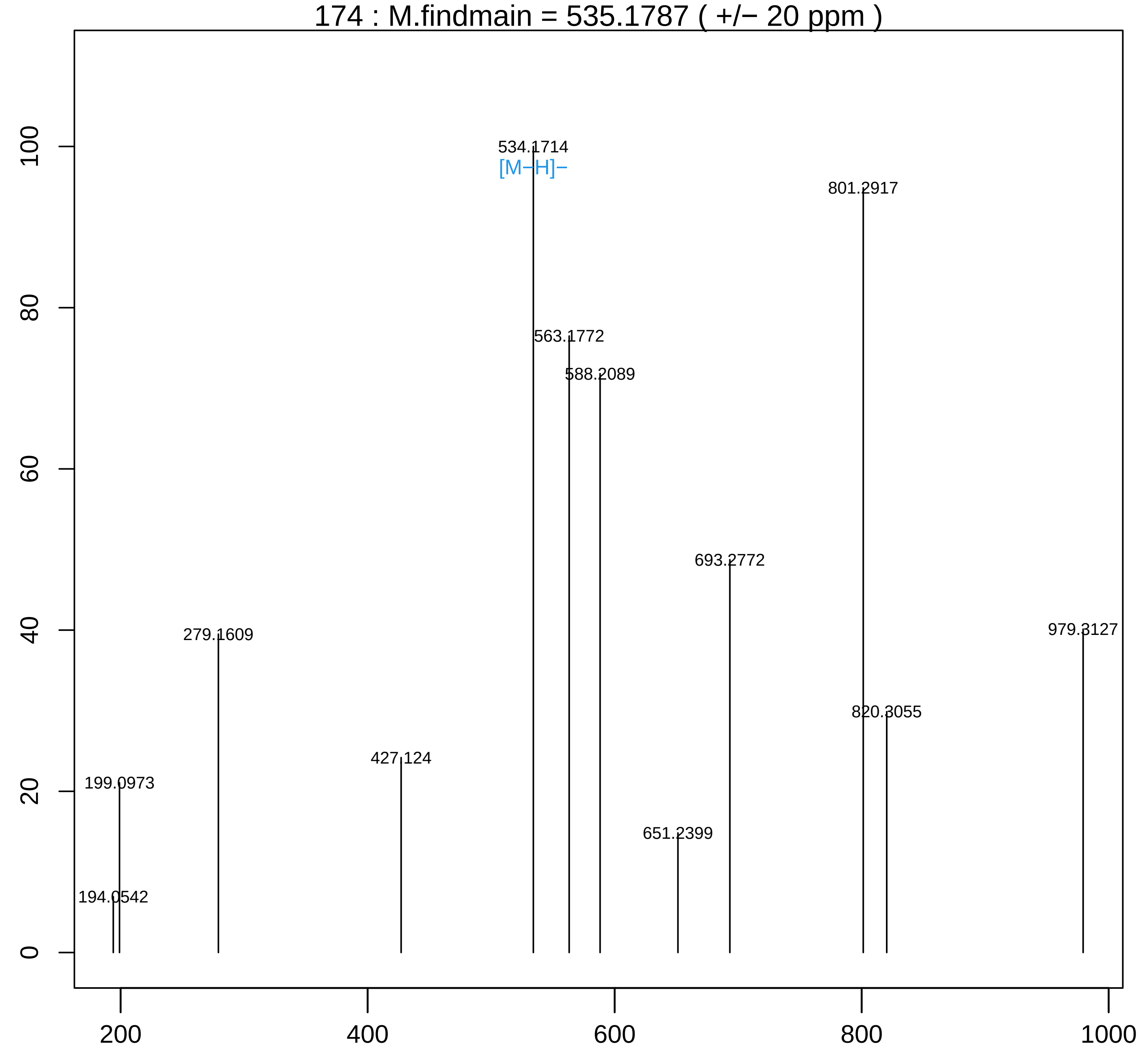

174 : M.findmain = 535.1787 (+/− 20 ppm)
534.1714
[M−H]−
801.2917
563.1772
588.2089
693.2772
279.1609
979.3127
820.3055
427.124
199.0973
651.2399
194.0542
0
20
40
60
80
100
200
400
600
800
1000

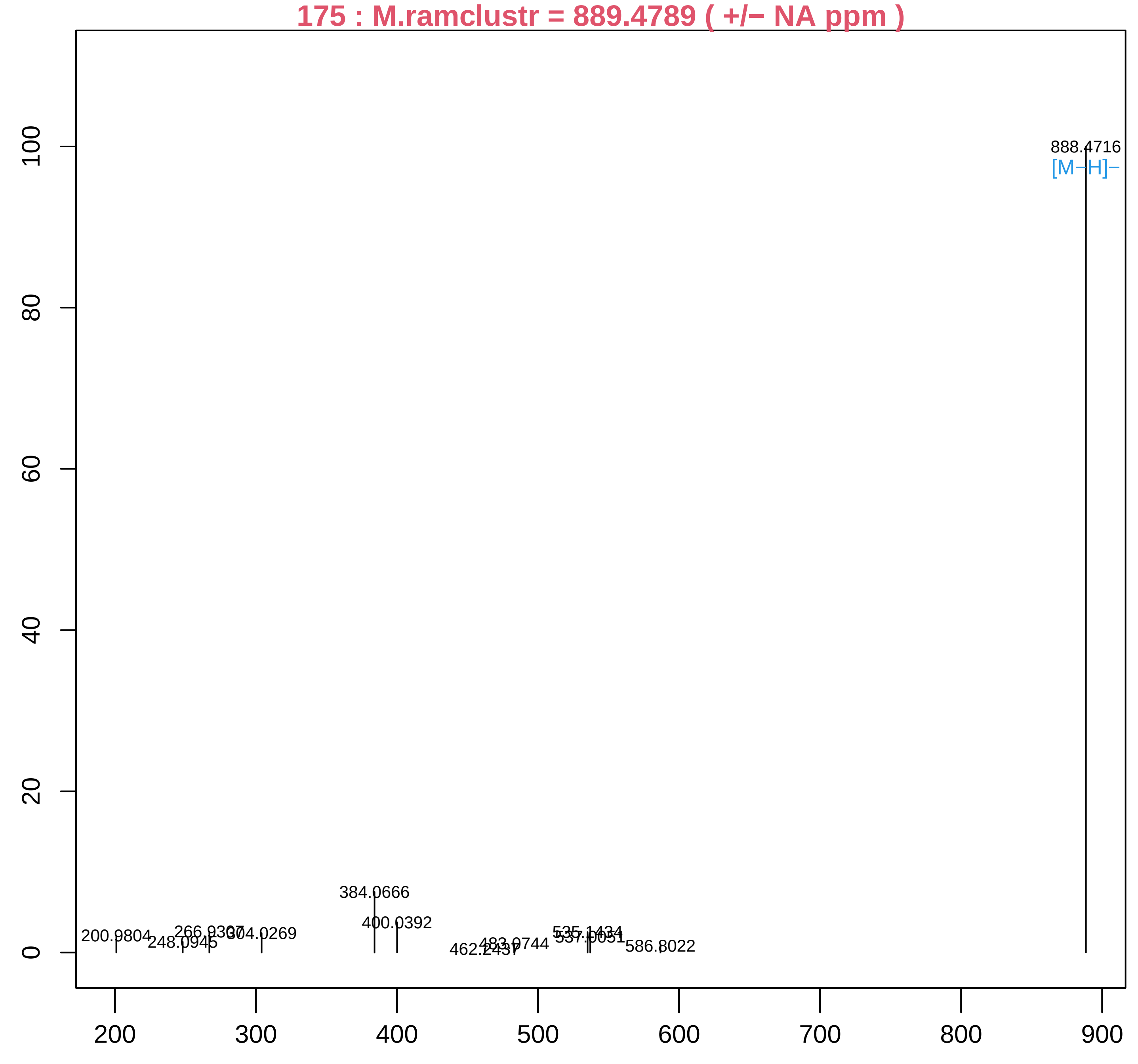
175 : M.ramclustr = 889.4789 (+/− NA ppm)
888.4716
[M−H]−
384.0666
400.0392
200.9804
248.0946
266.9307
304.0269
462.2437
483.0744
535.1434
537.0051
586.8022
0
20
40
60
80
100
200
300
400
500
600
700
800
900

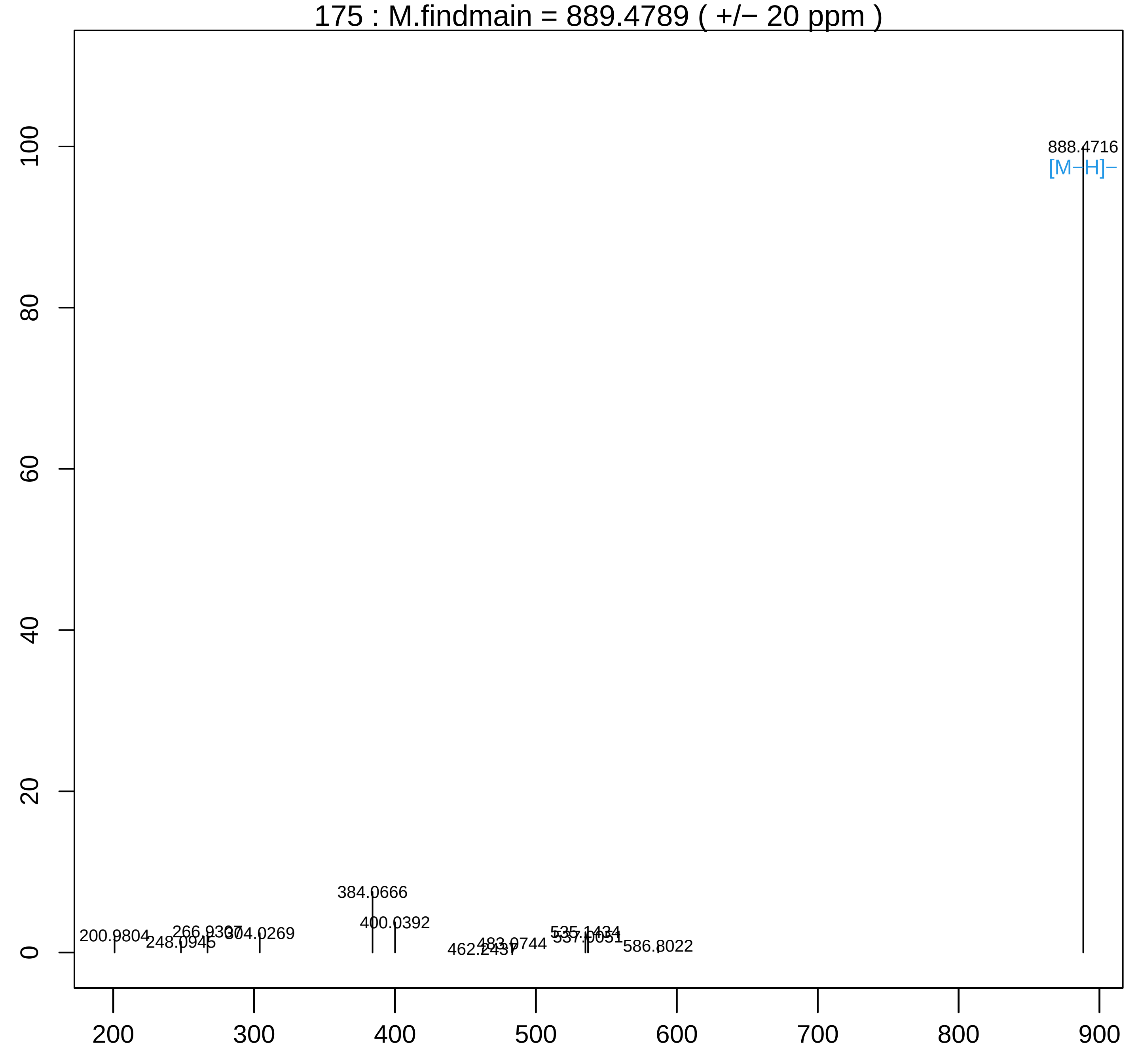
175 : M.findmain = 889.4789 (+/− 20 ppm)
888.4716
[M−H]−
384.0666
400.0392
200.9804
248.0946
266.9307
304.0269
462.2437
483.0744
535.1434
537.0051
586.8022
0
20
40
60
80
100
200
300
400
500
600
700
800
900

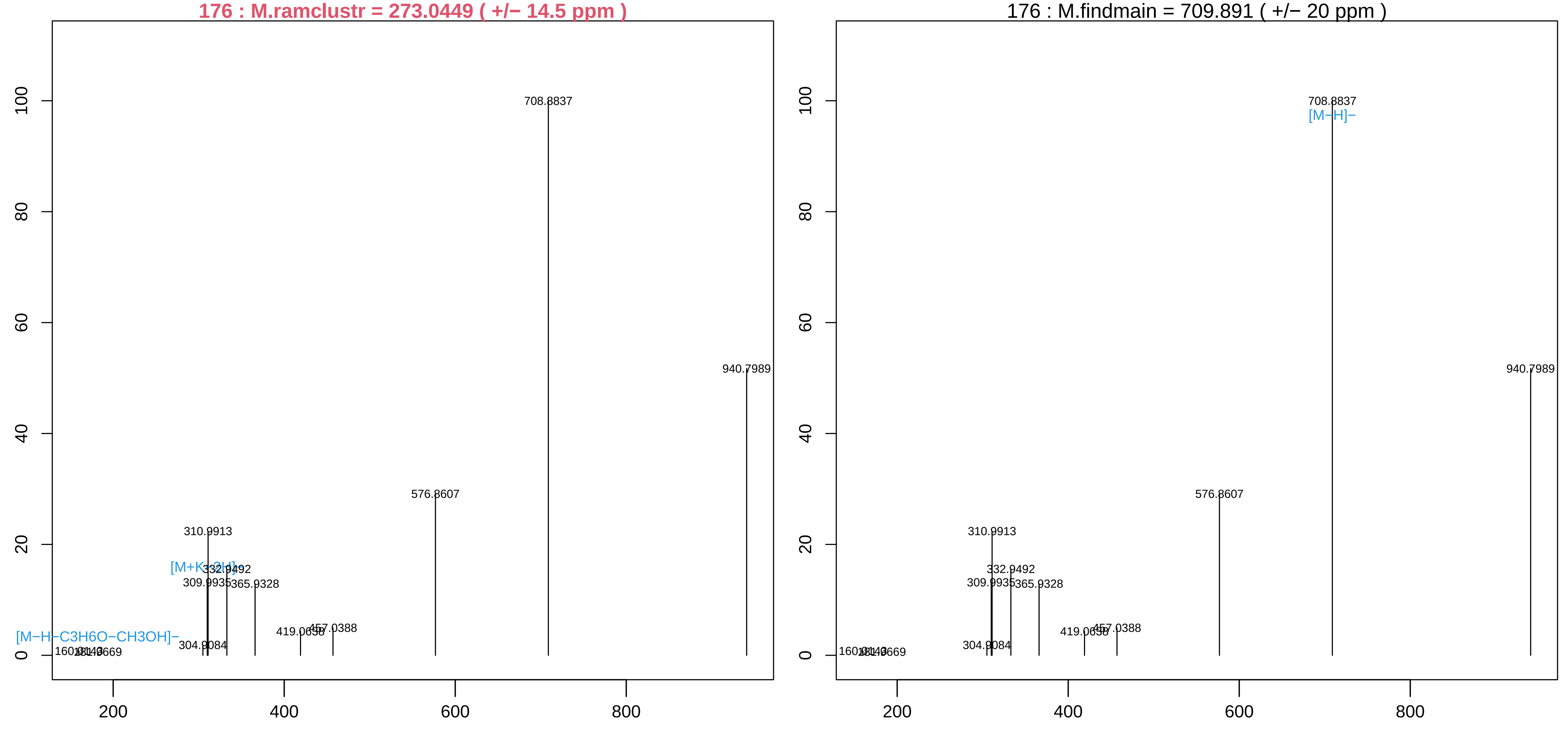

176 : M.ramclustr = 273.0449 (+/− 14.5 ppm)
708.8837
940.7989
576.8607
310.9913
[M+K−2H]−
332.9492
309.9935
365.9328
419.0658
457.0388
[M−H−C3H6O−CH3OH]−
304.9084
160.0149
181.9669
176 : M.findmain = 709.891 (+/− 20 ppm)
708.8837
[M−H]−
940.7989
576.8607
310.9913
332.9492
309.9935
365.9328
419.0658
457.0388
304.9084
160.0149
181.9669
0
20
40
60
80
100
200
400
600
800

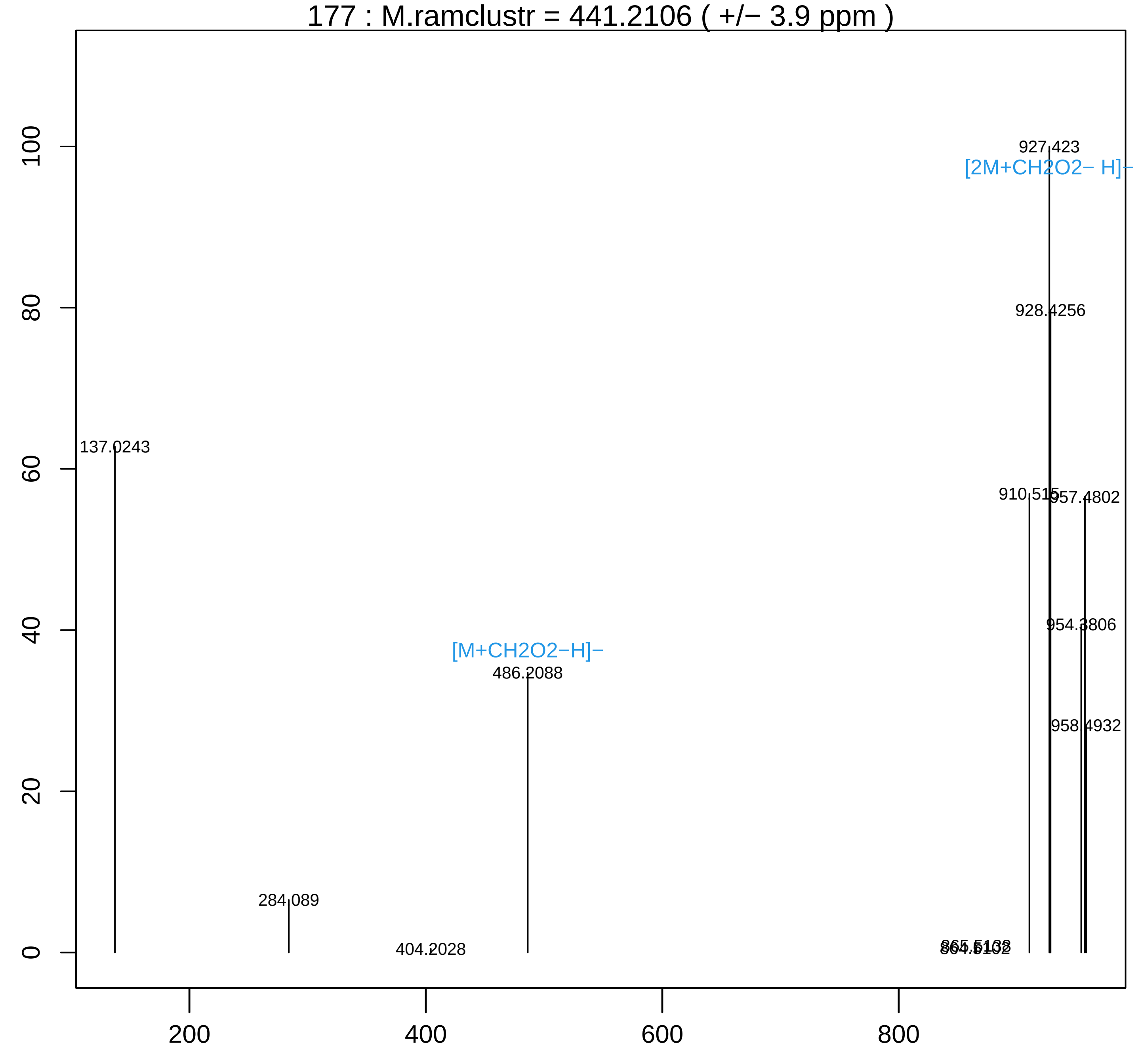
177 : M.ramclustr = 441.2106 (+/− 3.9 ppm)
927.423
[2M+CH2O2− H]−
928.4256
137.0243
910.515
957.4802
954.3806
[M+CH2O2−H]−
486.2088
958.4932
284.089
404.2028
865.5132
0
20
40
60
80
100
200
400
600
800

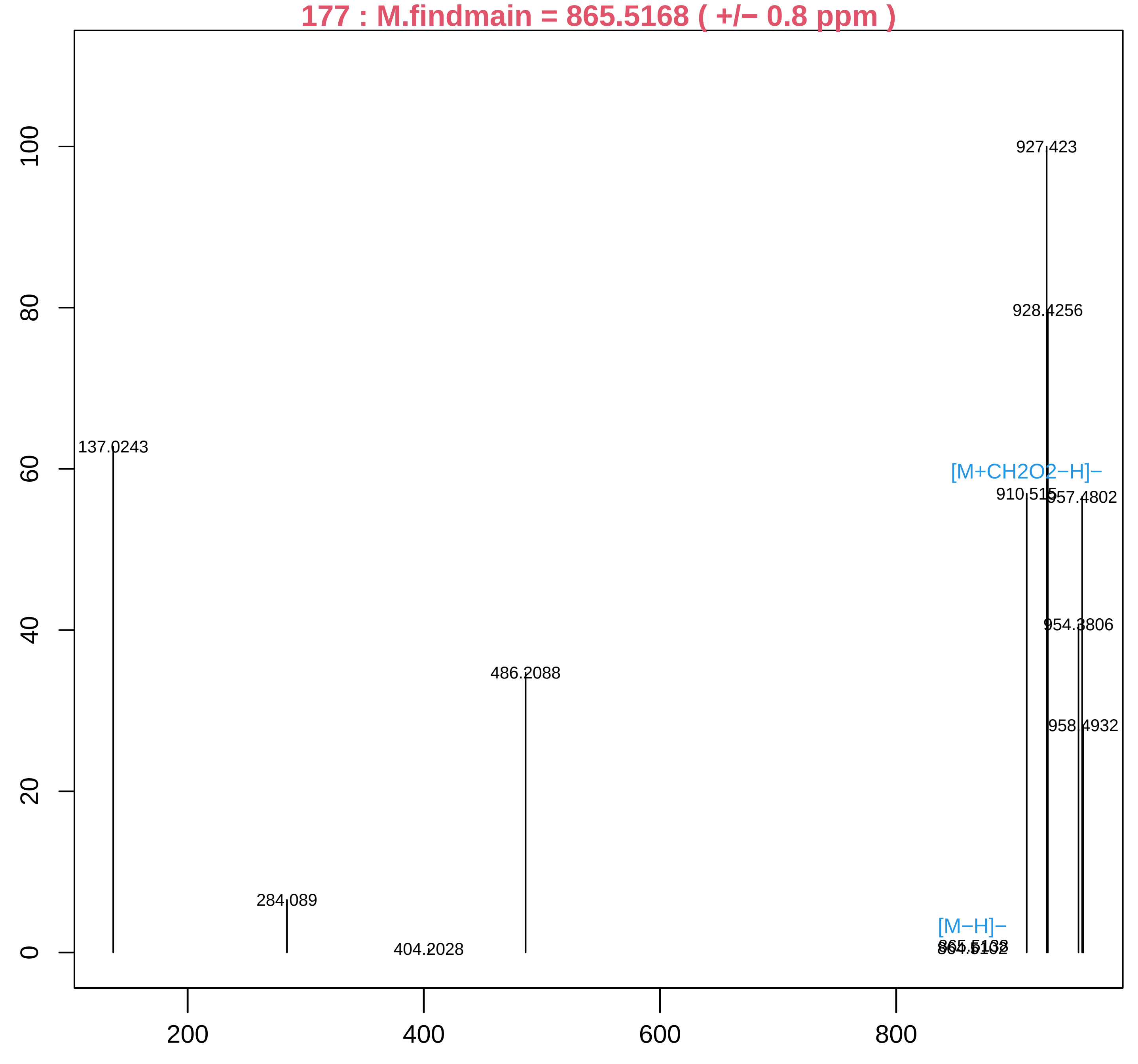
177 : M.findmain = 865.5168 (+/− 0.8 ppm)
927.423
928.4256
137.0243
[M+CH2O2−H]−
910.515
957.4802
954.3806
486.2088
958.4932
284.089
[M−H]−
404.2028
865.5132
0
20
40
60
80
100
200
400
600
800

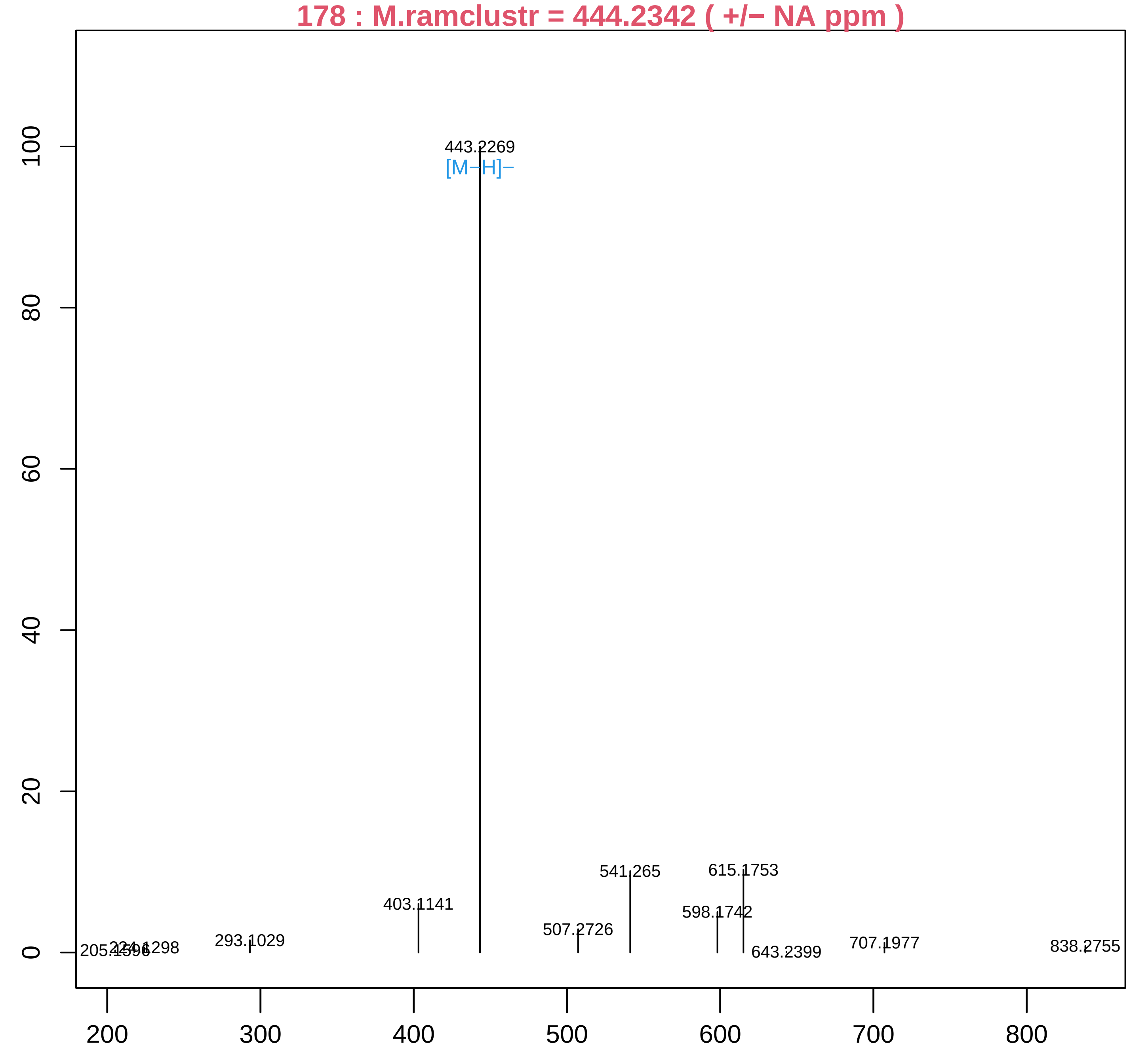

178 : M.ramclustr = 444.2342 (+/− NA ppm)
443.2269
[M−H]−
205.1596
224.1298
293.1029
403.1141
507.2726
541.265
598.1742
615.1753
643.2399
707.1977
838.2755

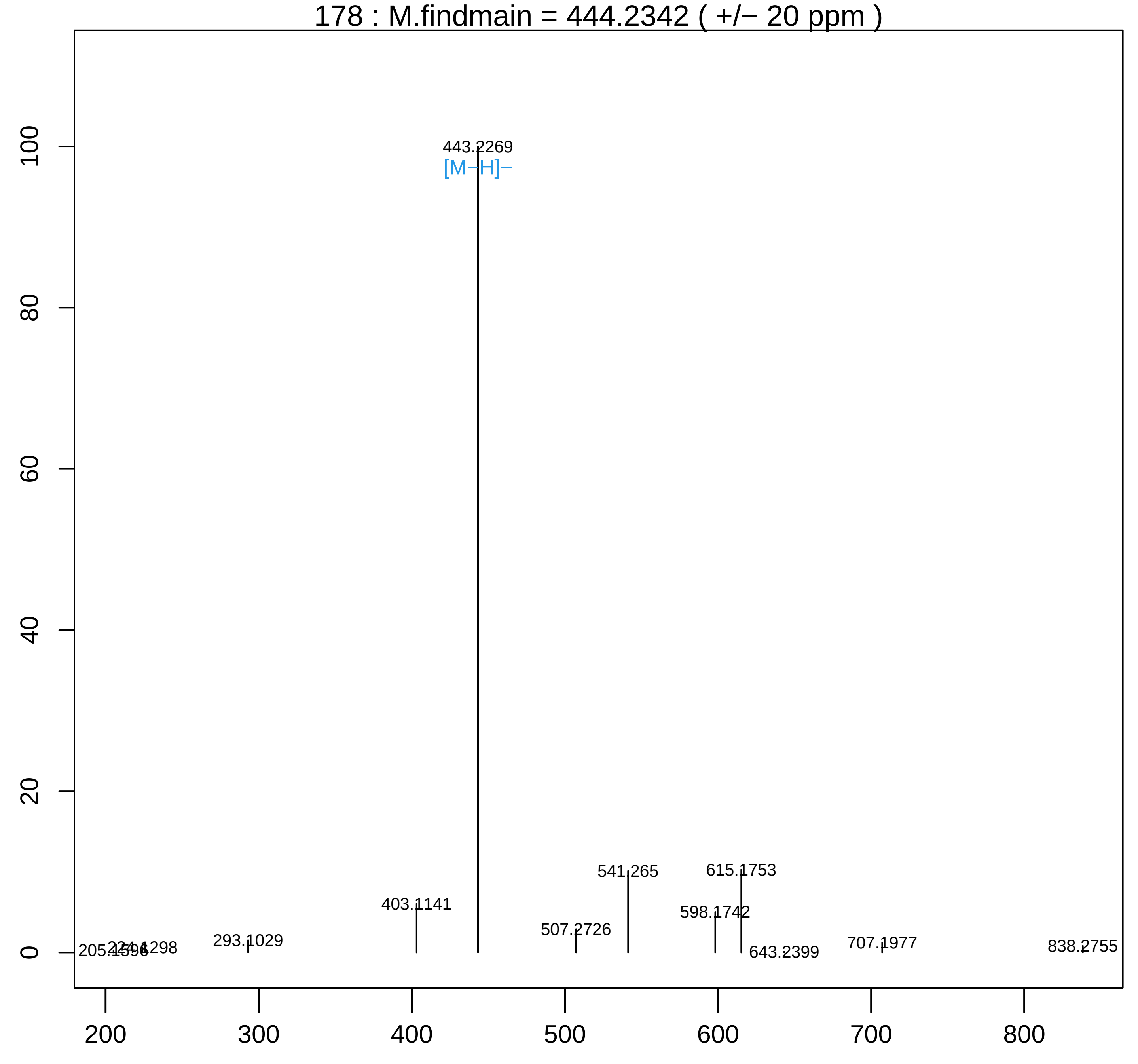

178 : M.findmain = 444.2342 (+/− 20 ppm)
443.2269
[M−H]−
205.1596
224.1298
293.1029
403.1141
507.2726
541.265
598.1742
615.1753
643.2399
707.1977
838.2755

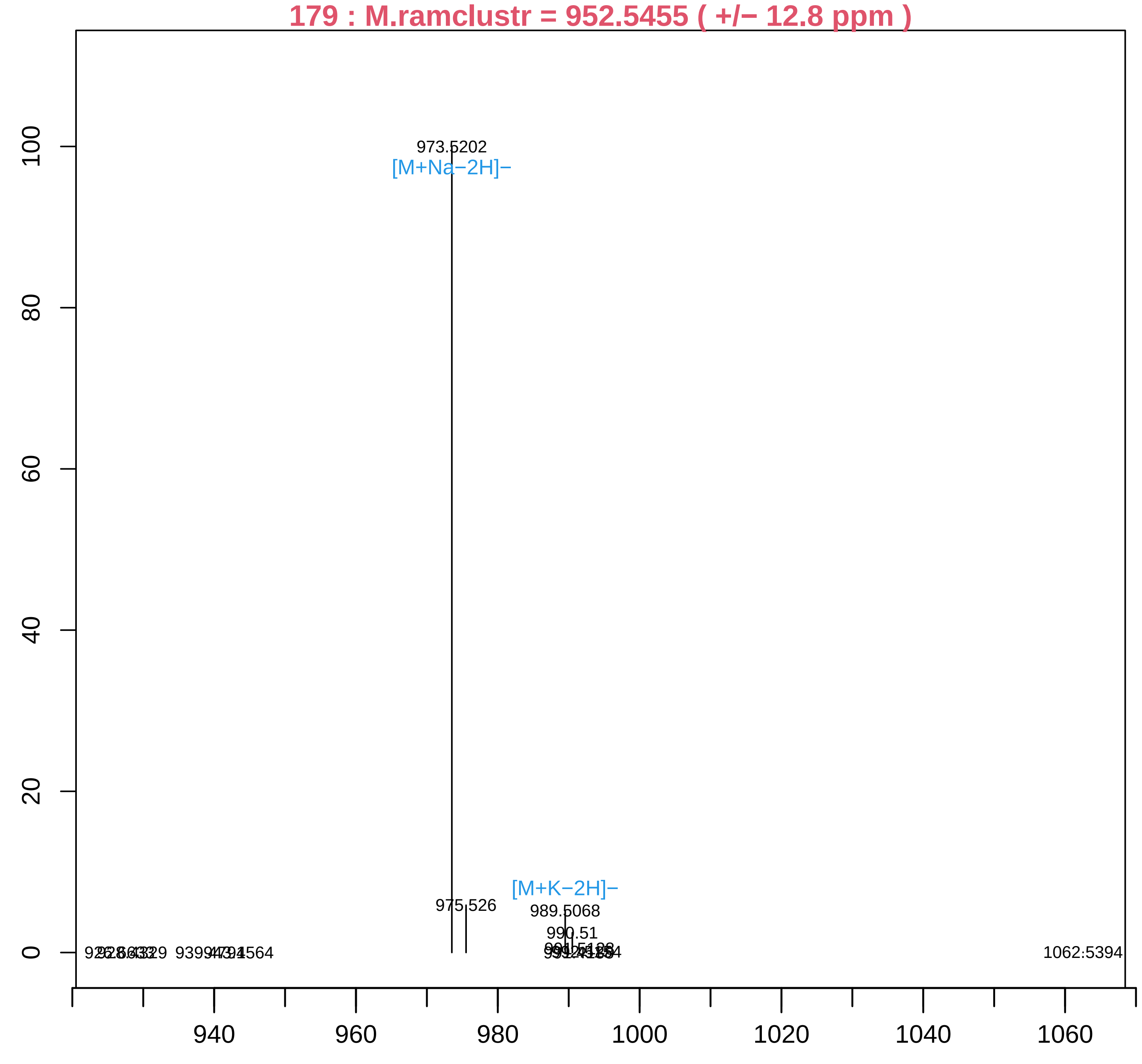
179 : M.ramclustr = 952.5455 (+/− 12.8 ppm)
973.5202
[M+Na−2H]−
975.526
[M+K−2H]−
989.5068
990.51
1062.5394
0
20
40
60
80
100
940
960
980
1000
1020
1040
1060

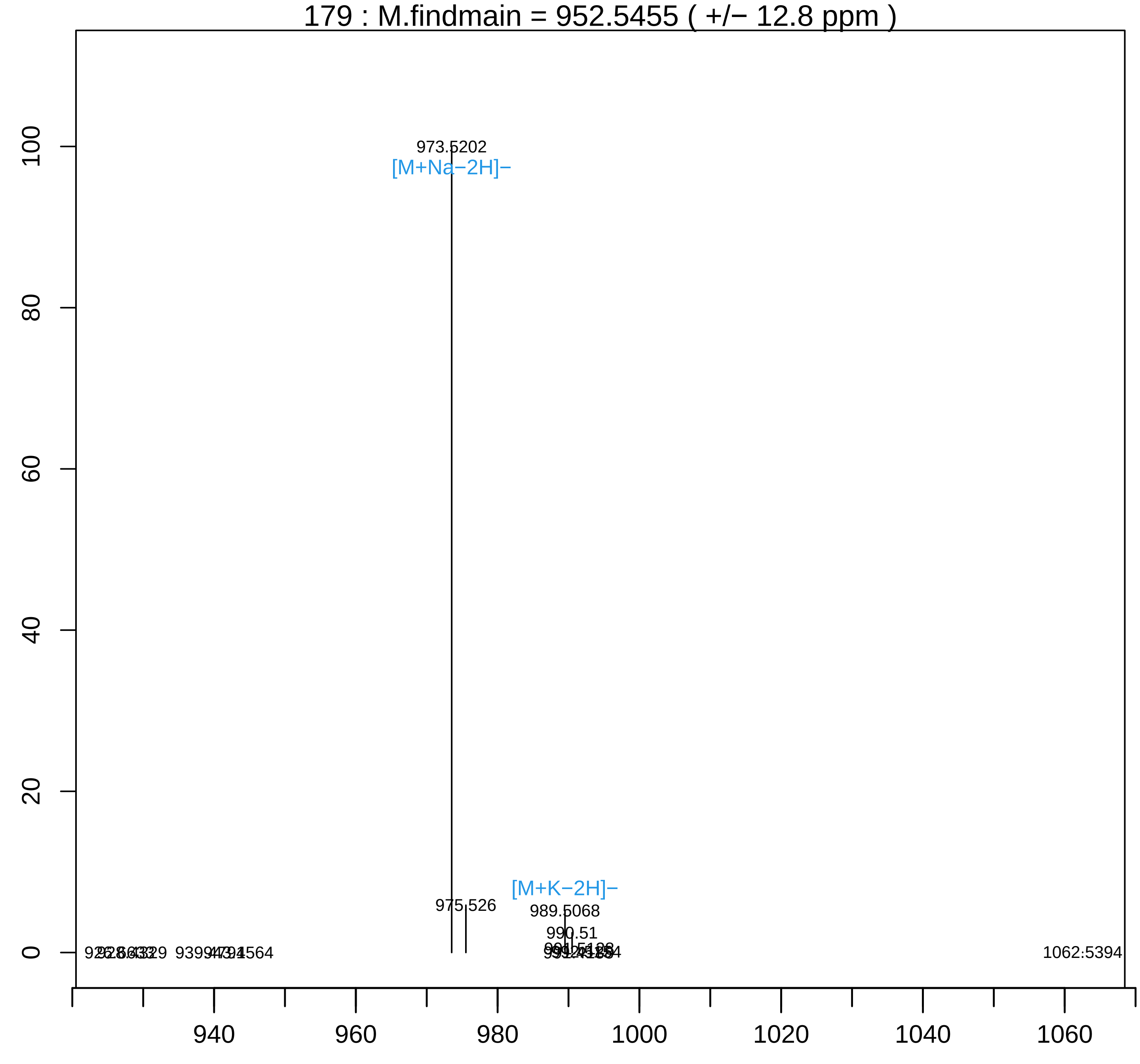
179 : M.findmain = 952.5455 (+/− 12.8 ppm)
973.5202
[M+Na−2H]−
975.526
[M+K−2H]−
989.5068
990.51
1062.5394
0
20
40
60
80
100
940
960
980
1000
1020
1040
1060

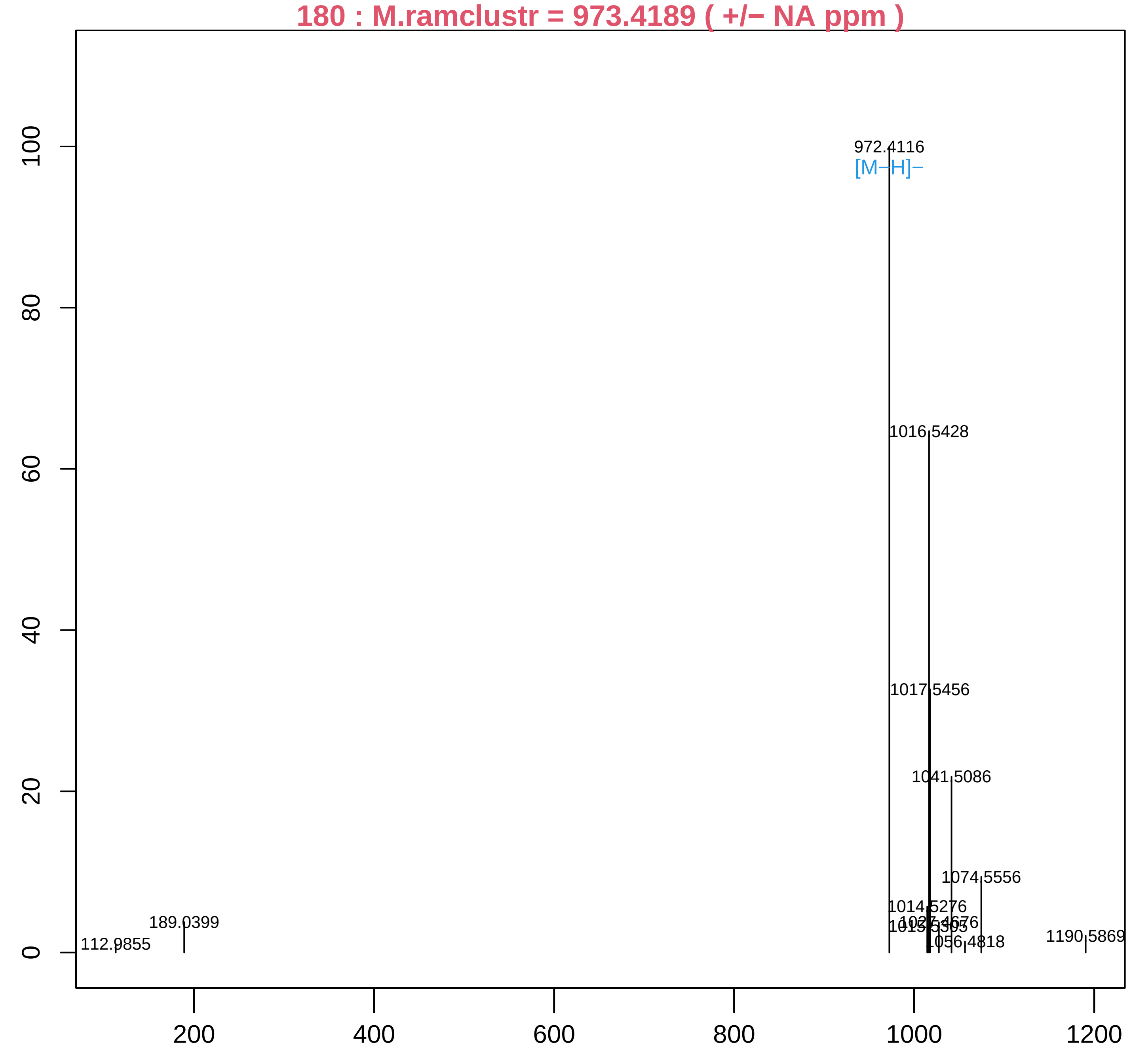
180 : M.ramclustr = 973.4189 (+/− NA ppm)
972.4116
[M−H]−
1016.5428
1017.5456
1041.5086
1074.5556
1014.5276
189.0399
112.9855
1056.4818
1190.5869
0
20
40
60
80
100
200
400
600
800
1000
1200

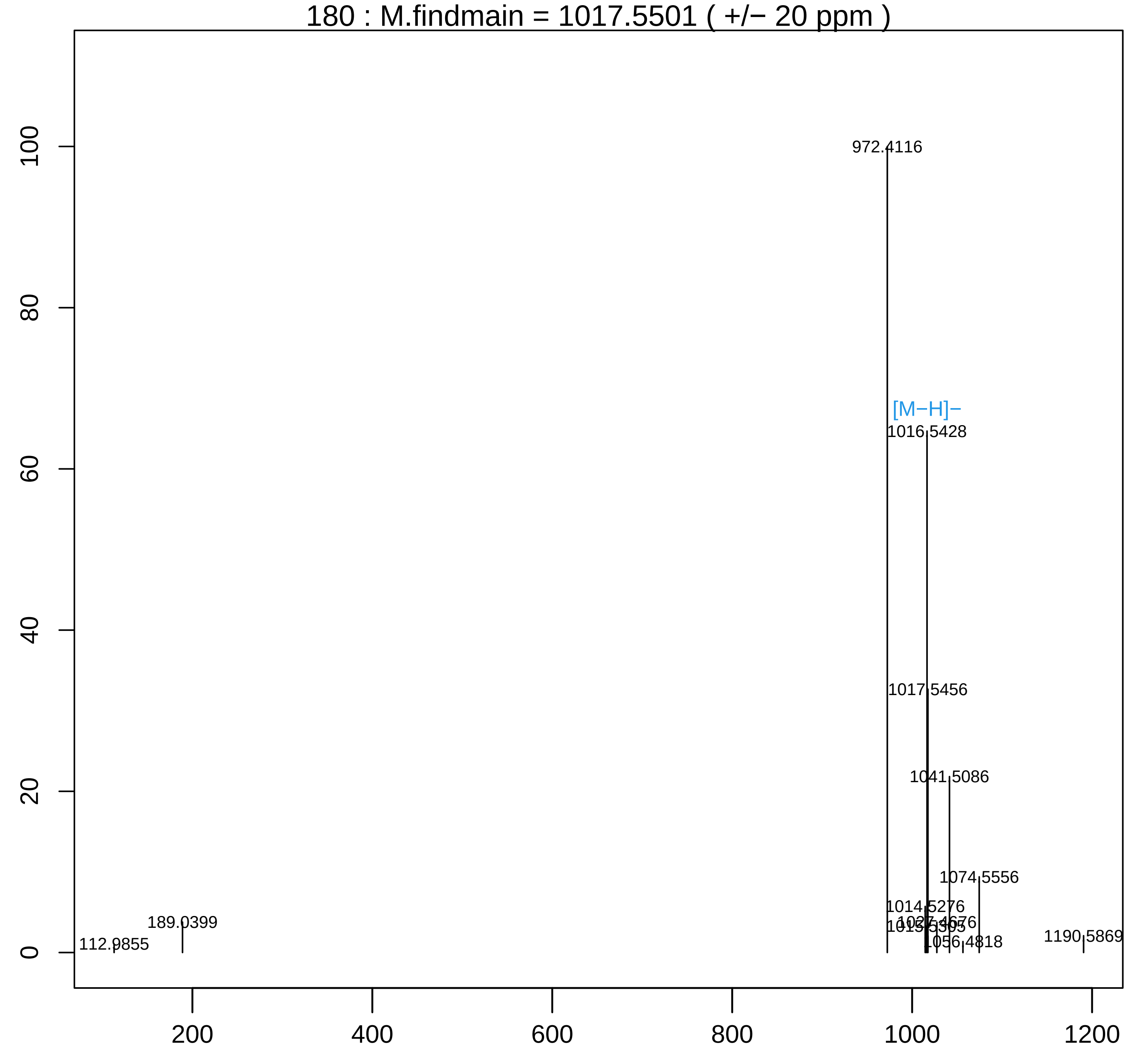
180 : M.findmain = 1017.5501 (+/− 20 ppm)
972.4116
[M−H]−
1016.5428
1017.5456
1041.5086
1074.5556
1014.5276
189.0399
112.9855
1056.4818
1190.5869
0
20
40
60
80
100
200
400
600
800
1000
1200

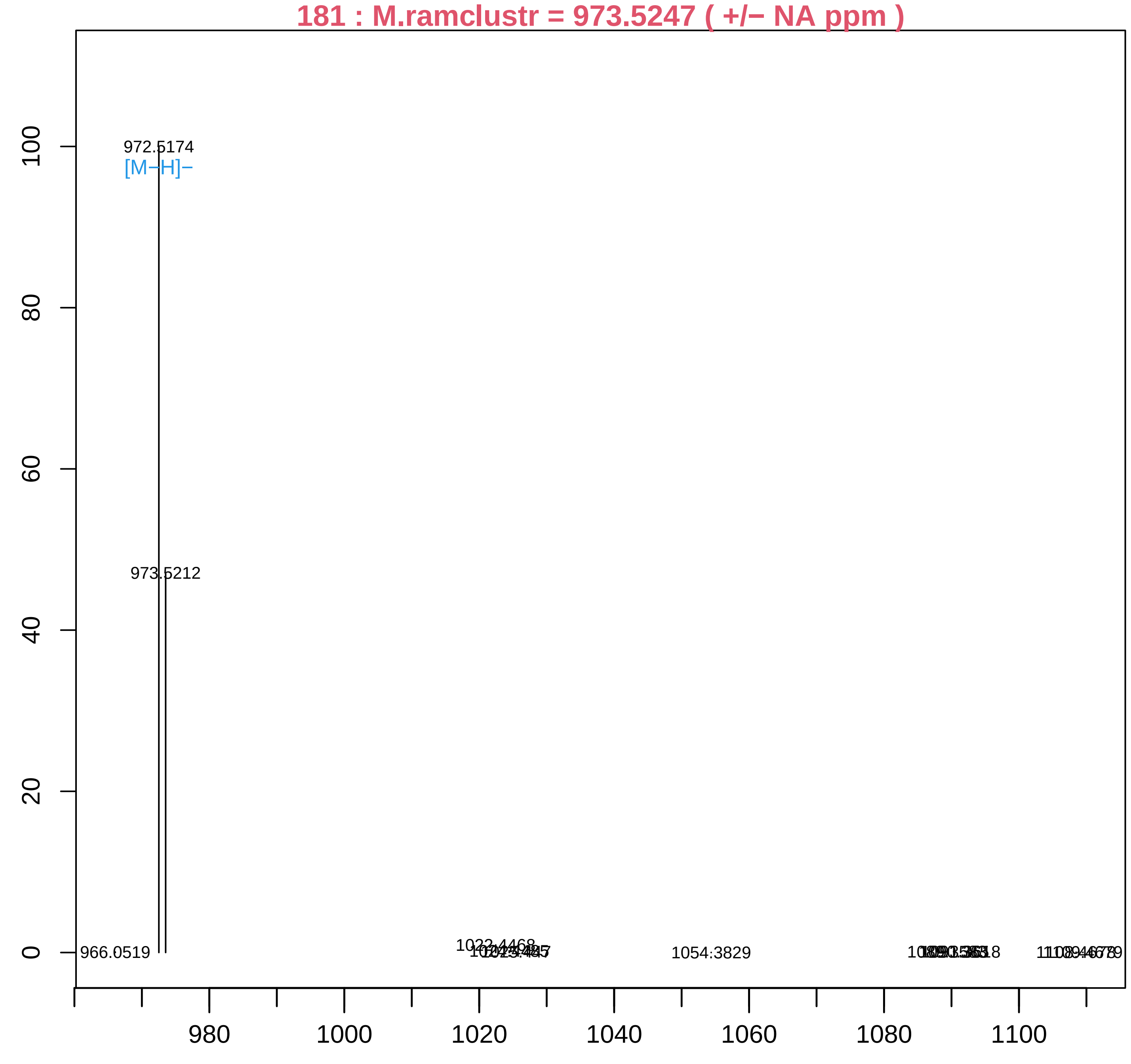
181 : M.ramclustr = 973.5247 (+/− NA ppm)
972.5174
[M−H]−
973.5212
966.0519
1054.3829
0
20
40
60
80
100
980
1000
1020
1040
1060
1080
1100

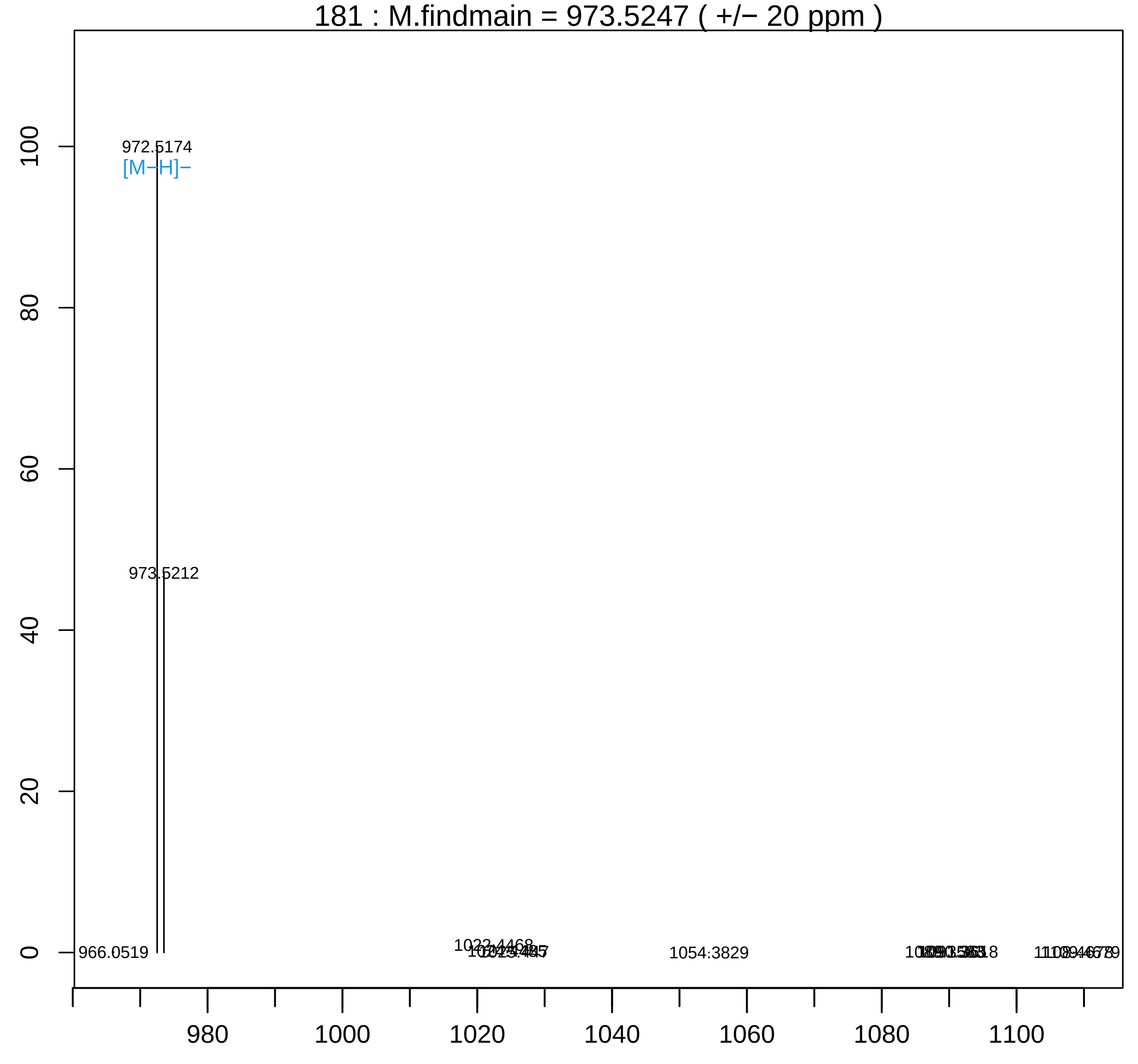
181 : M.findmain = 973.5247 (+/− 20 ppm)
972.5174
[M−H]−
973.5212
966.0519
1054.3829
0
20
40
60
80
100
980
1000
1020
1040
1060
1080
1100

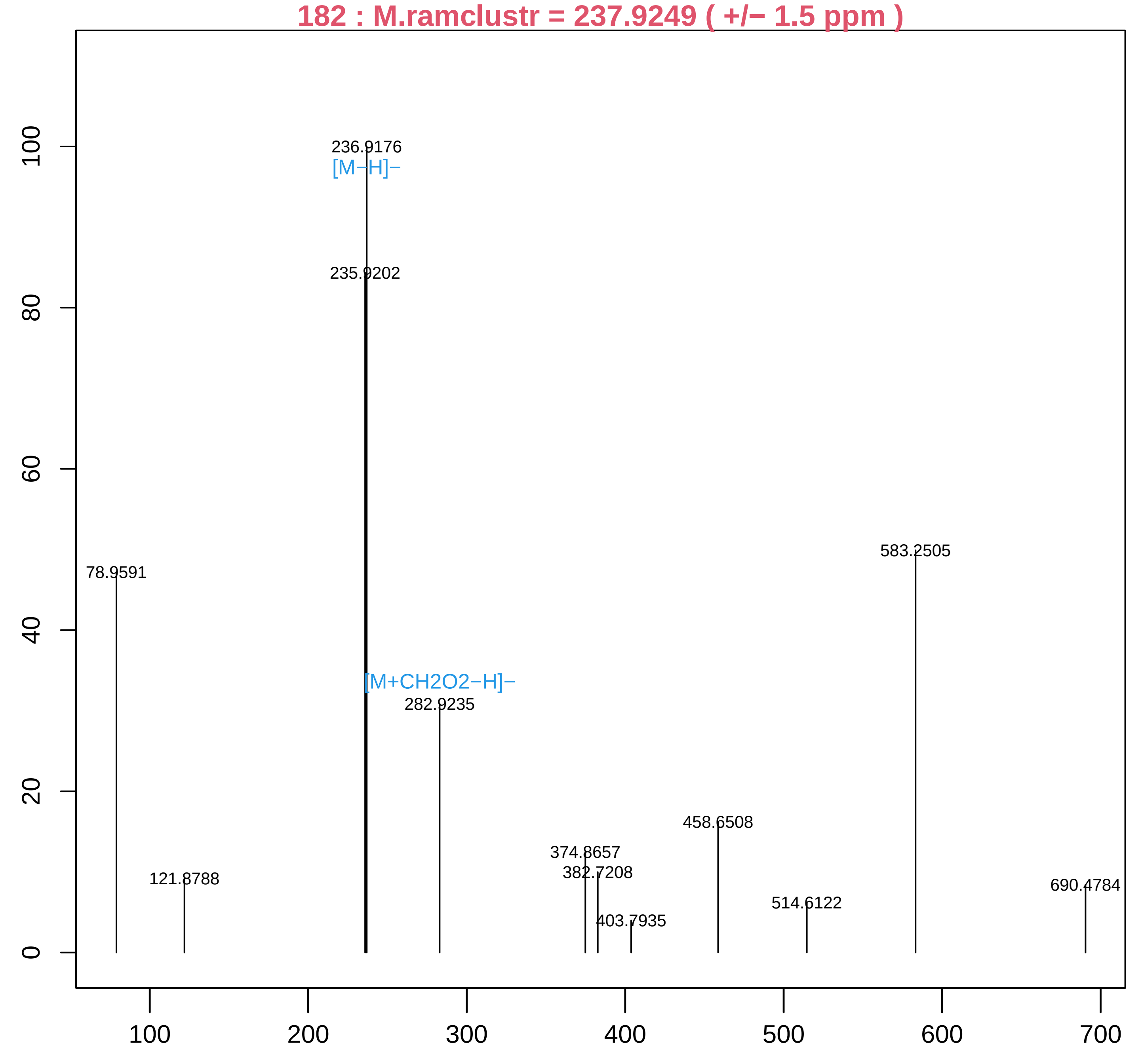
182 : M.ramclustr = 237.9249 (+/− 1.5 ppm)
236.9176
[M−H]−
235.9202
78.9591
583.2505
[M+CH2O2−H]−
282.9235
458.6508
374.8657
382.7208
121.8788
690.4784
514.6122
403.7935
0
20
40
60
80
100
100
200
300
400
500
600
700

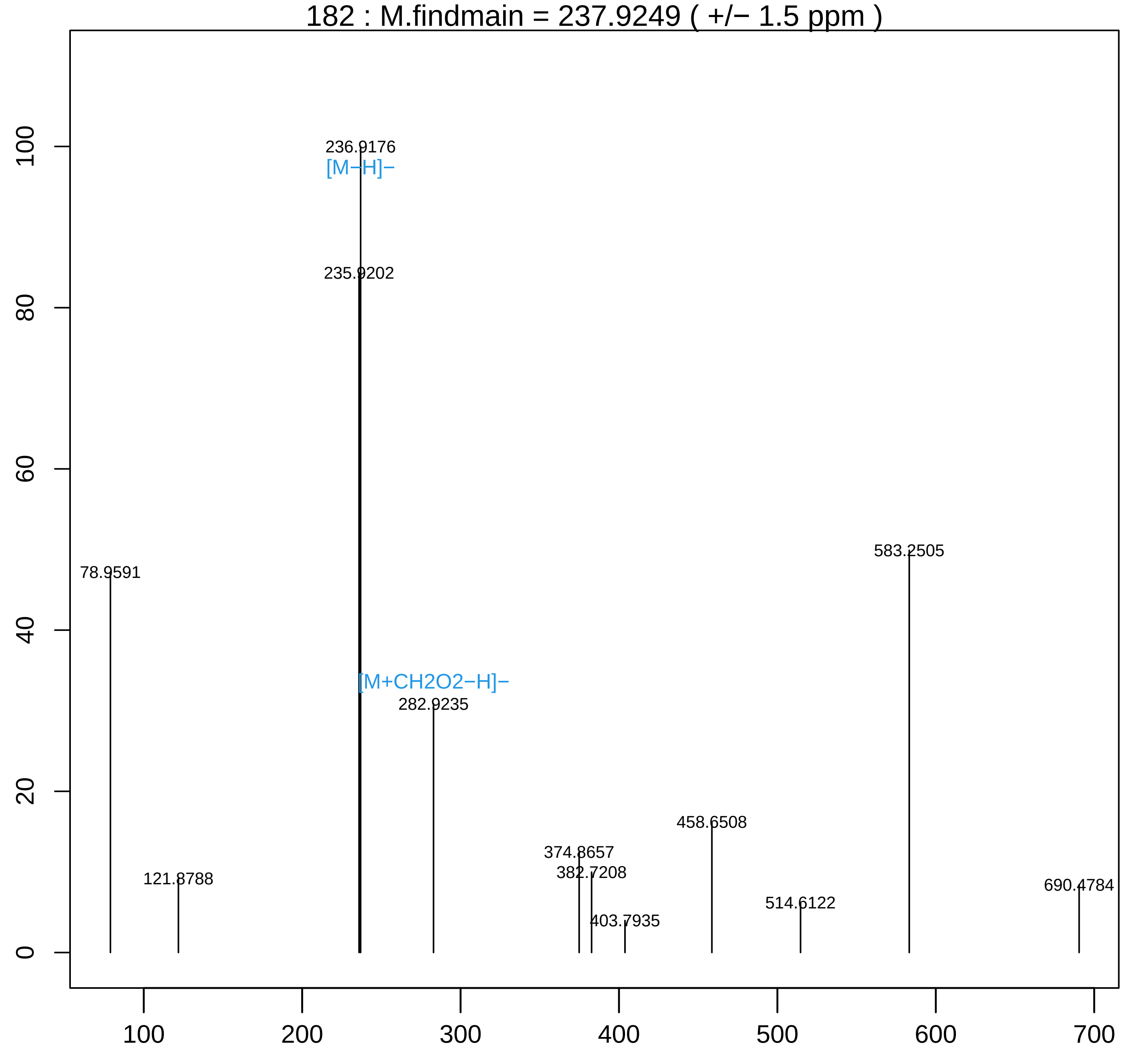
182 : M.findmain = 237.9249 (+/− 1.5 ppm)
236.9176
[M−H]−
235.9202
78.9591
583.2505
[M+CH2O2−H]−
282.9235
458.6508
374.8657
382.7208
121.8788
690.4784
514.6122
403.7935
0
20
40
60
80
100
100
200
300
400
500
600
700

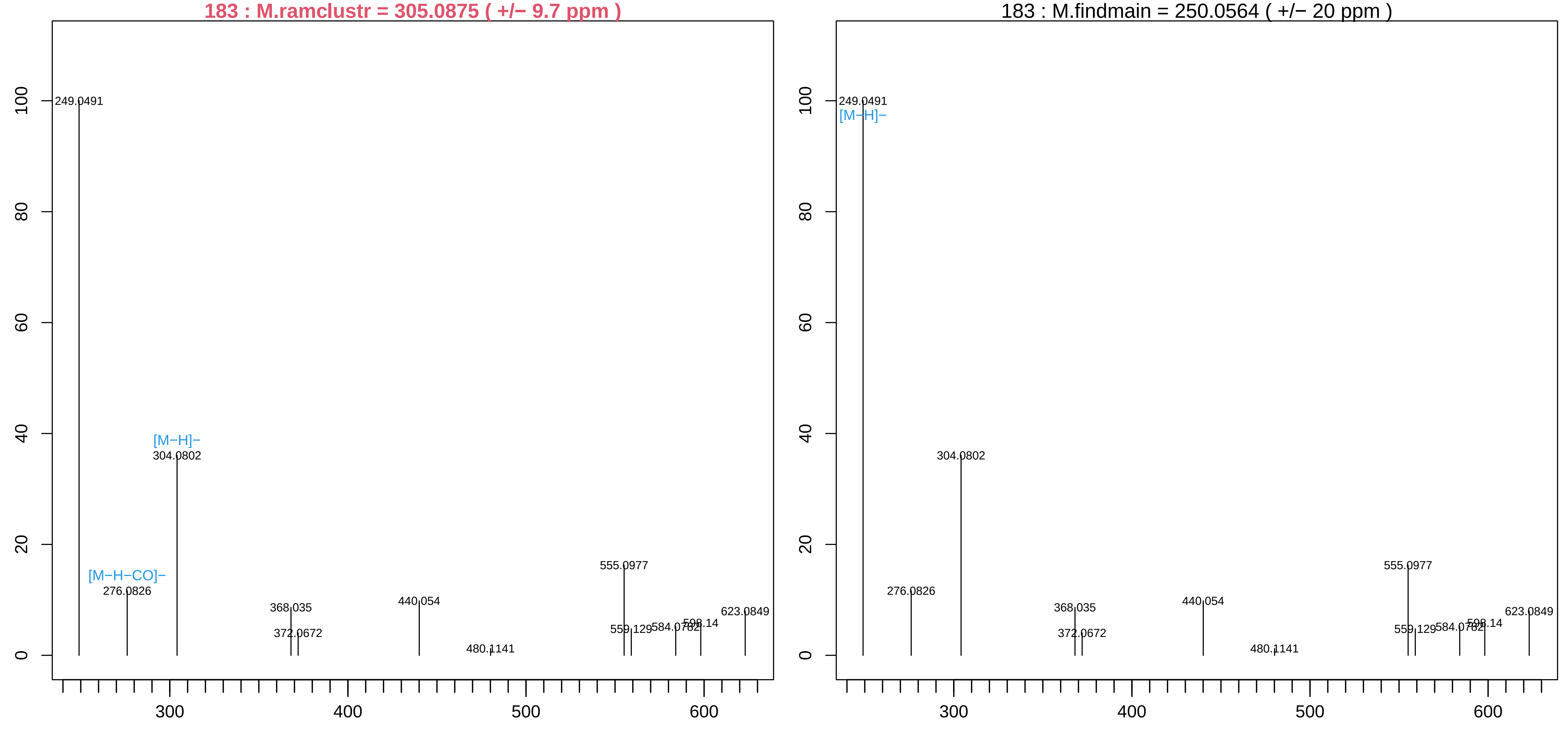

183 : M.ramclustr = 305.0875 (+/− 9.7 ppm)
249.0491
[M−H−CO]−
276.0826
[M−H]−
304.0802
368.035
372.0672
440.054
480.1141
555.0977
559.129
584.0782
598.14
623.0849
183 : M.findmain = 250.0564 (+/− 20 ppm)
249.0491
[M−H]−
276.0826
304.0802
368.035
372.0672
440.054
480.1141
555.0977
559.129
584.0782
598.14
623.0849

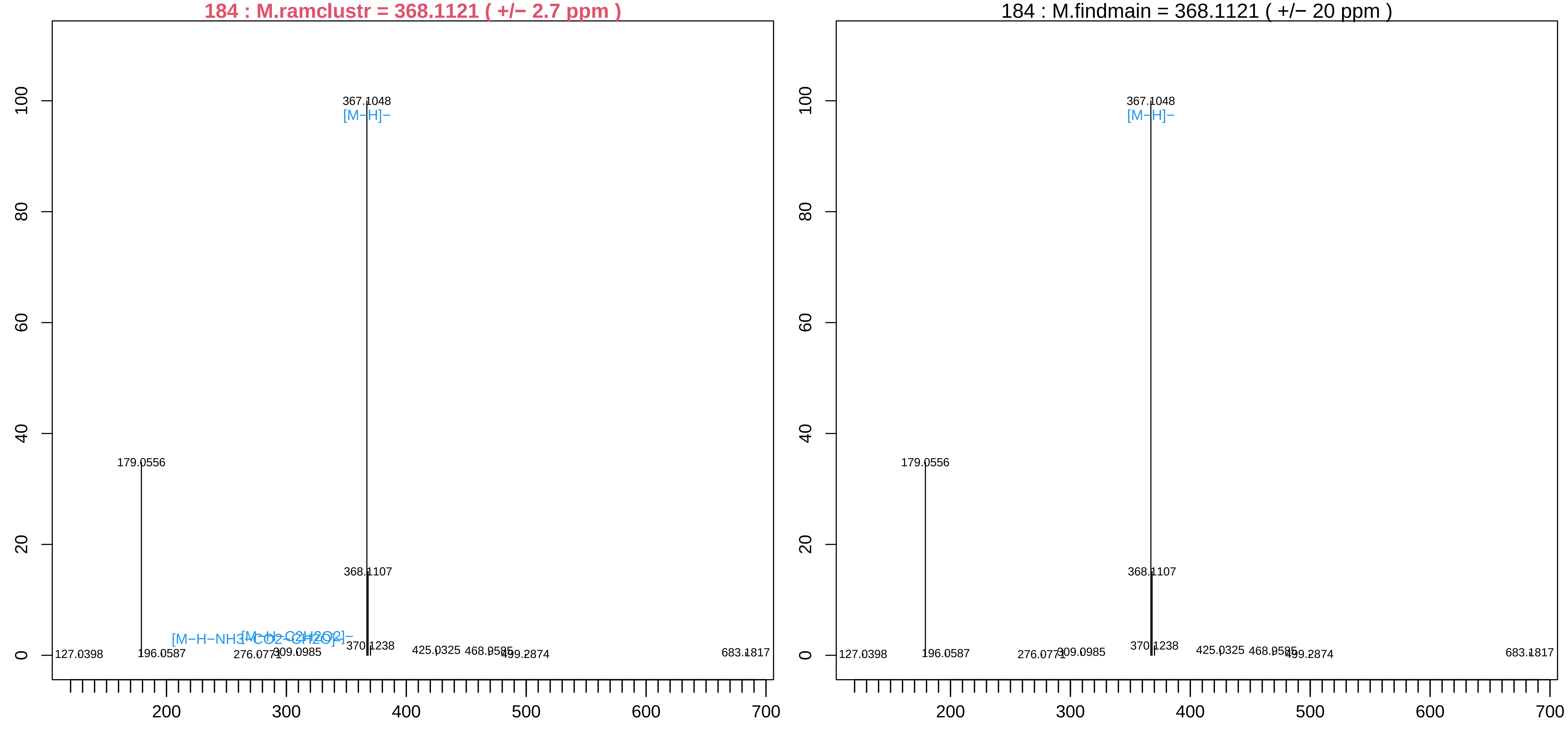

184 : M.ramclustr = 368.1121 (+/− 2.7 ppm)
367.1048
[M−H]−
179.0556
368.1107
[M−H−NH3−CO2−CH2O]−
[M−H−C2H2O2]−
370.1238
127.0398
196.0587
276.0771
309.0985
425.0325
468.9525
499.2874
683.1817
184 : M.findmain = 368.1121 (+/− 20 ppm)
367.1048
[M−H]−
179.0556
368.1107
370.1238
127.0398
196.0587
276.0771
309.0985
425.0325
468.9525
499.2874
683.1817

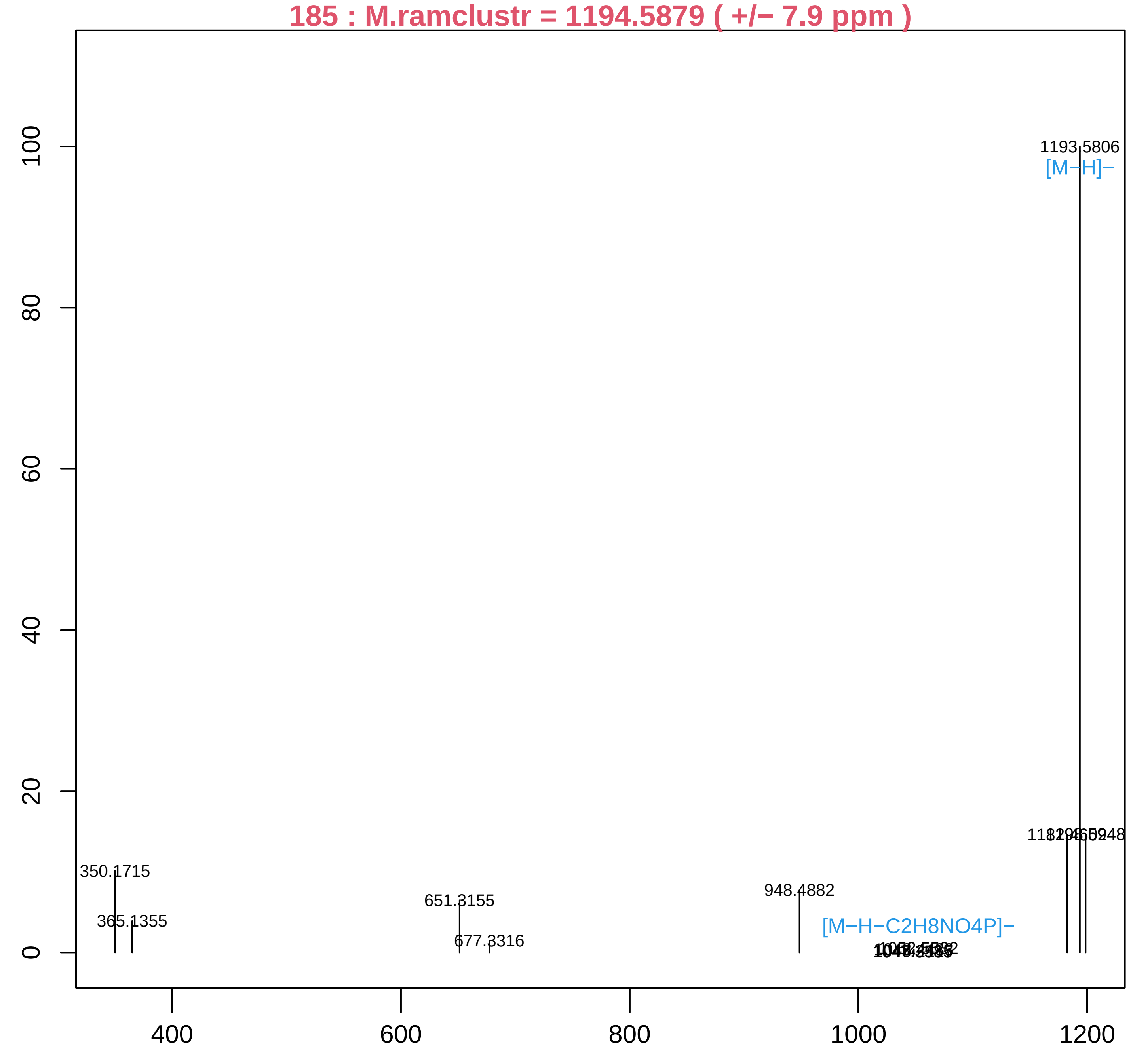
185 : M.ramclustr = 1194.5879 (+/− 7.9 ppm)
1193.5806
[M−H]−
350.1715
365.1355
651.3155
677.3316
948.4882
[M−H−C2H8NO4P]−
0
20
40
60
80
100
400
600
800
1000
1200
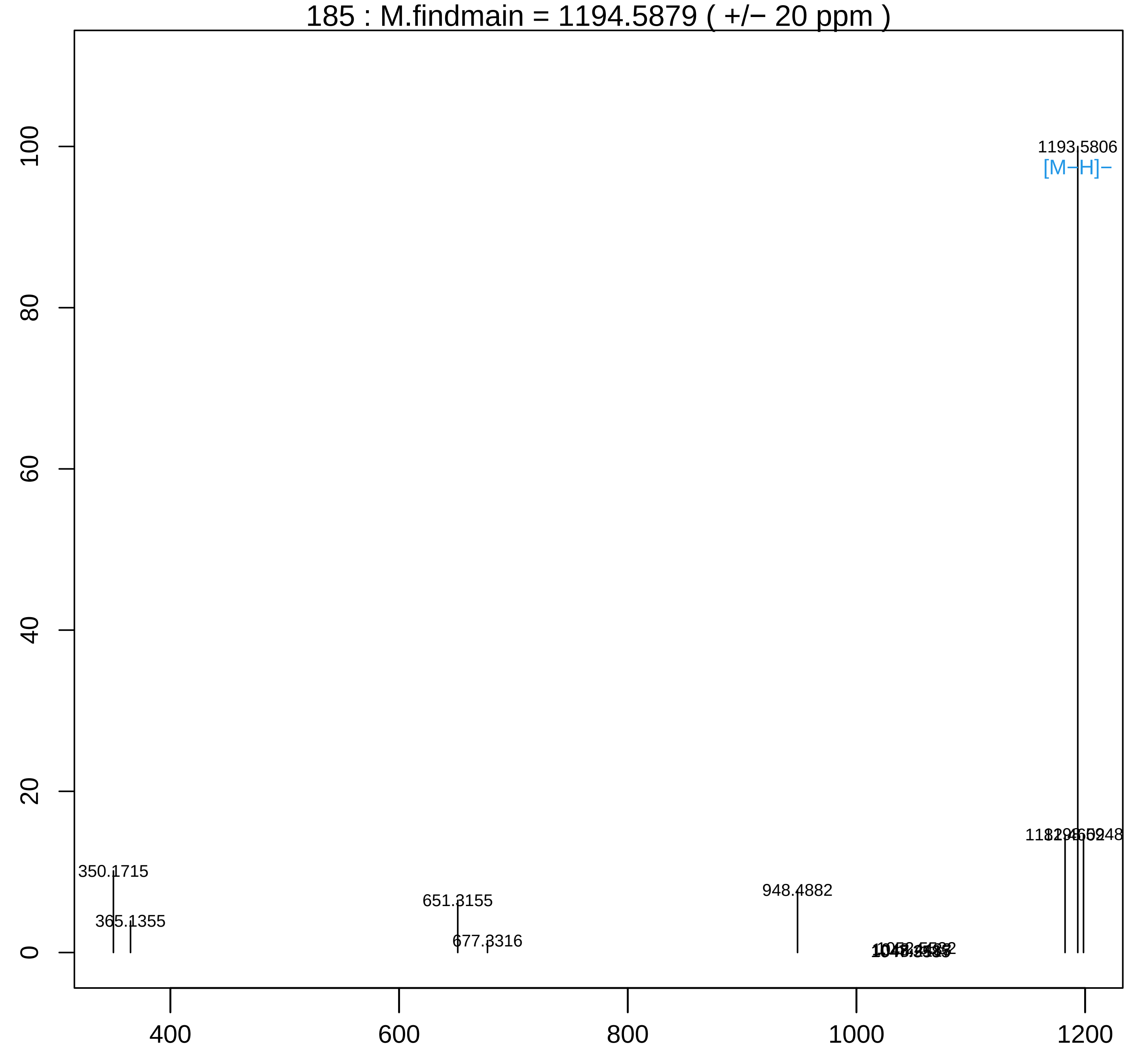
185 : M.findmain = 1194.5879 (+/− 20 ppm)
1193.5806
[M−H]−
350.1715
365.1355
651.3155
677.3316
948.4882
0
20
40
60
80
100
400
600
800
1000
1200

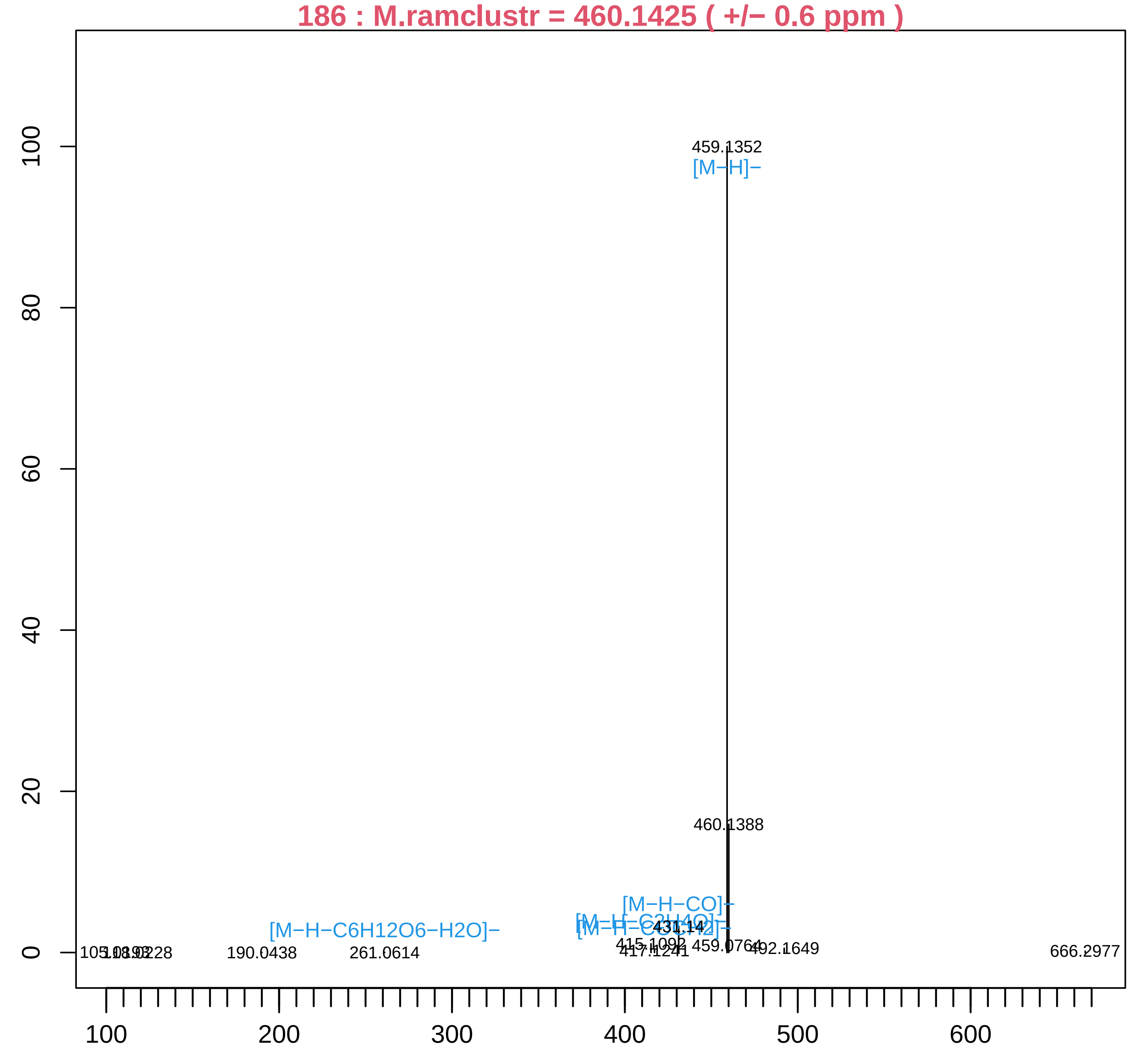
186 : M.ramclustr = 460.1425 (+/− 0.6 ppm)
459.1352
[M−H]−
460.1388
[M−H−CO]−
431.14
[M−H−C6H12O6−H2O]−
415.1092
417.1241
459.0764
492.1649
190.0438
261.0614
666.2977
100
200
300
400
500
600
0
20
40
60
80
100

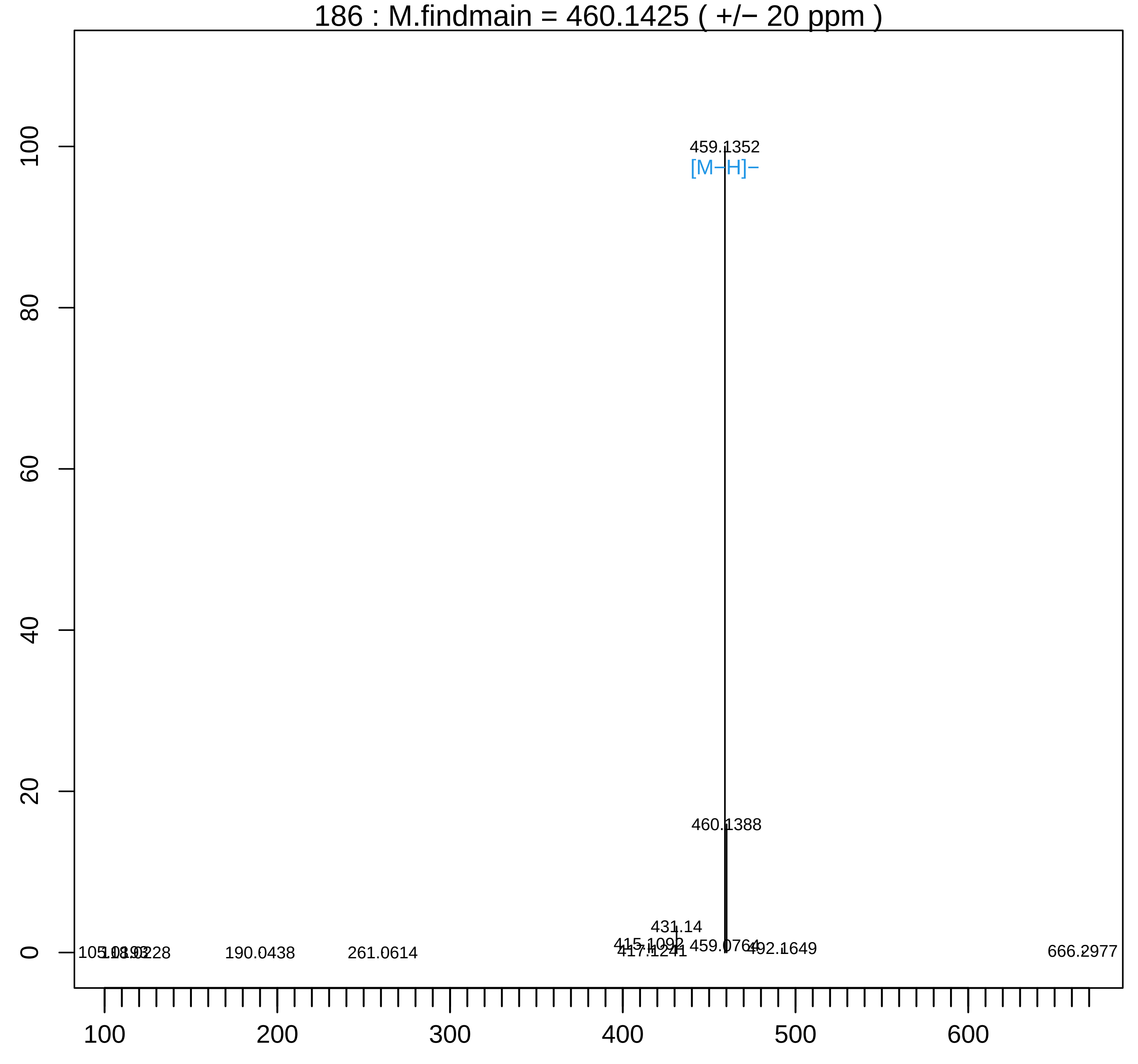
186 : M.findmain = 460.1425 (+/− 20 ppm)
459.1352
[M−H]−
460.1388
431.14
415.1092
417.1241
459.0764
492.1649
190.0438
261.0614
666.2977
100
200
300
400
500
600
0
20
40
60
80
100

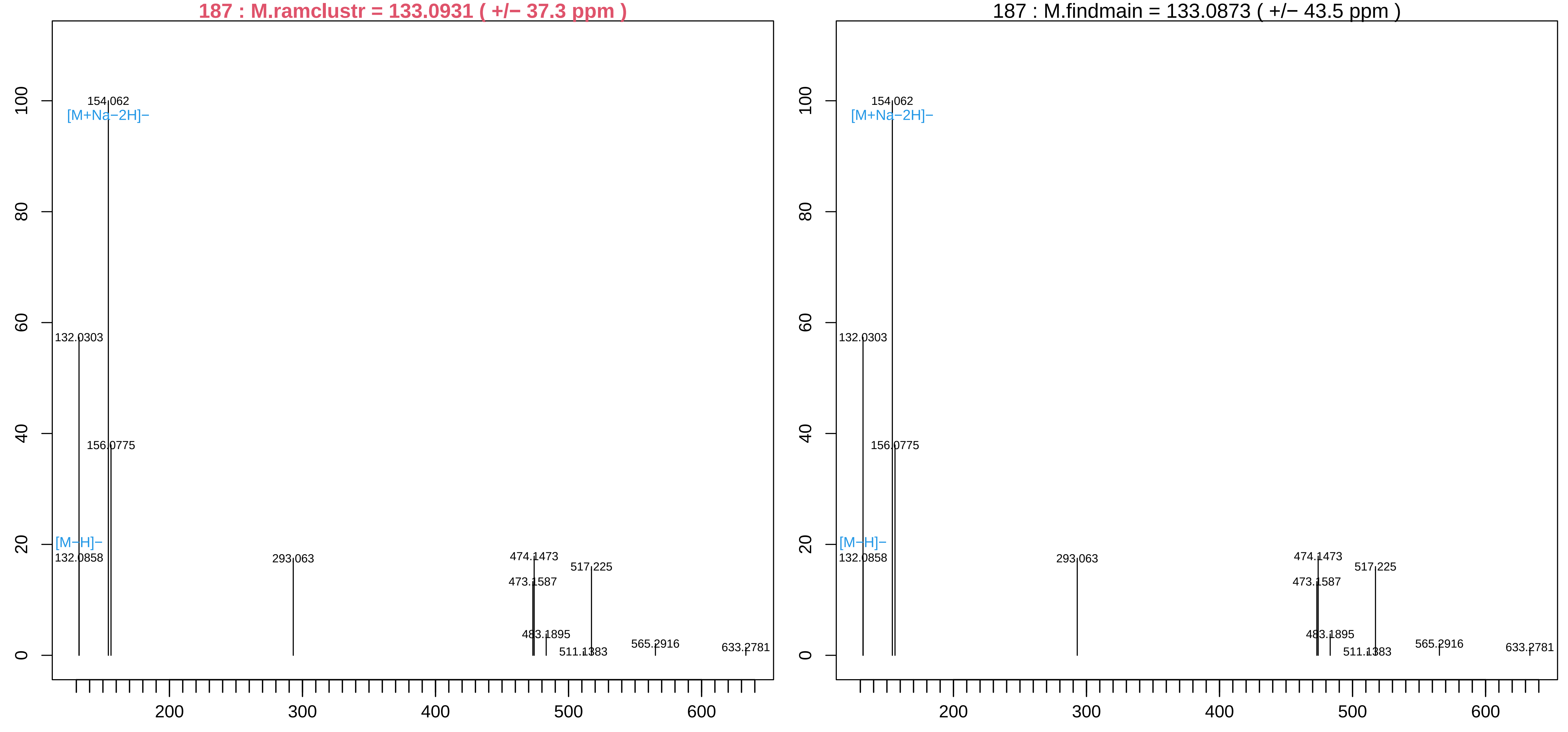
187 : M.ramclustr = 133.0931 (+/− 37.3 ppm)
154.062
[M+Na−2H]−
132.0303
156.0775
[M−H]−
132.0858
293.063
474.1473
473.1587
517.225
483.1895
511.1383
565.2916
633.2781
187 : M.findmain = 133.0873 (+/− 43.5 ppm)
0
20
40
60
80
100
200
300
400
500
600

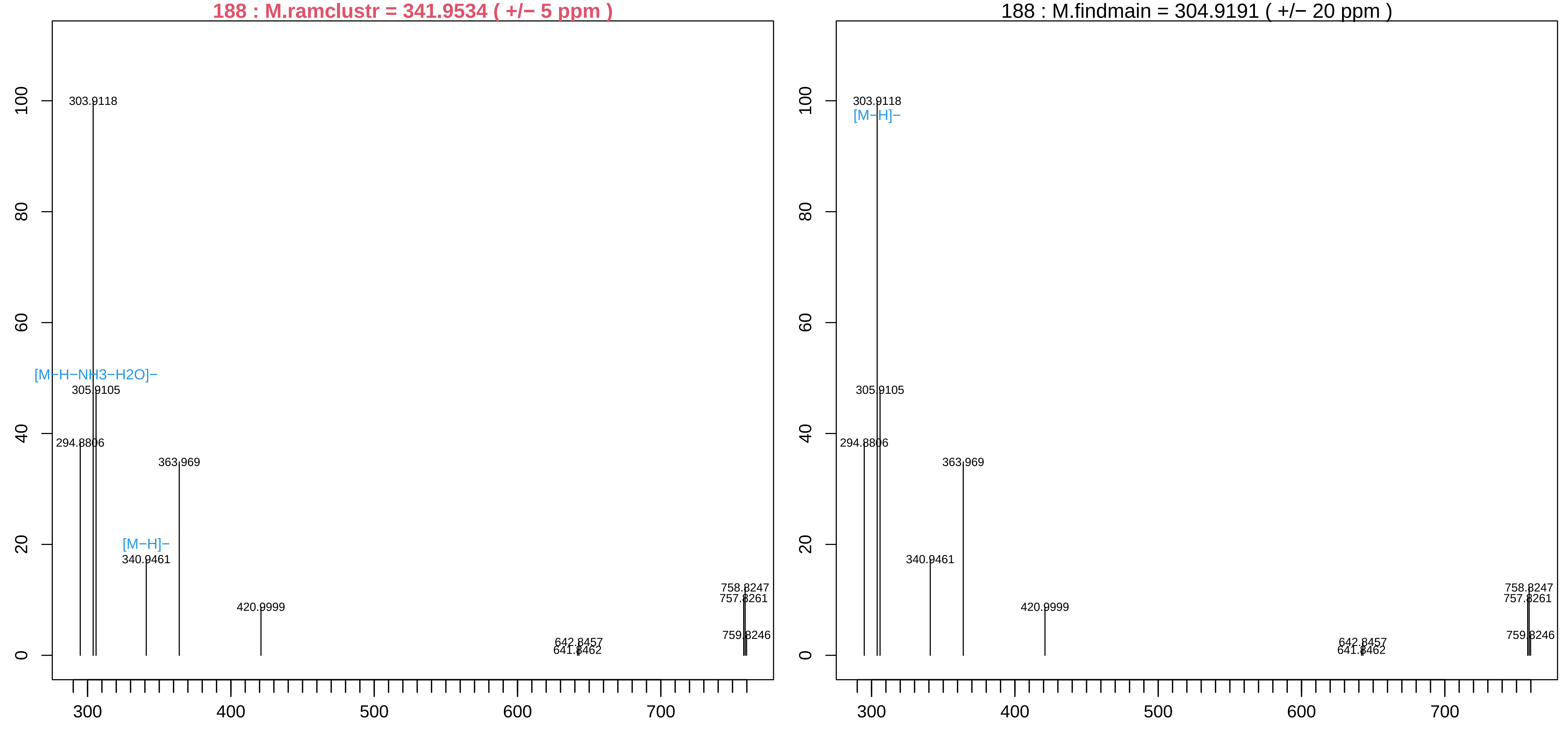
188 : M.ramclustr = 341.9534 (+/− 5 ppm)
303.9118
[M−H−NH3−H2O]−
305.9105
294.8806
363.969
[M−H]−
340.9461
420.9999
758.8247
757.8261
759.8246
642.8457
641.8462
188 : M.findmain = 304.9191 (+/− 20 ppm)
303.9118
[M−H]−
305.9105
294.8806
363.969
340.9461
420.9999
758.8247
757.8261
759.8246
642.8457
641.8462

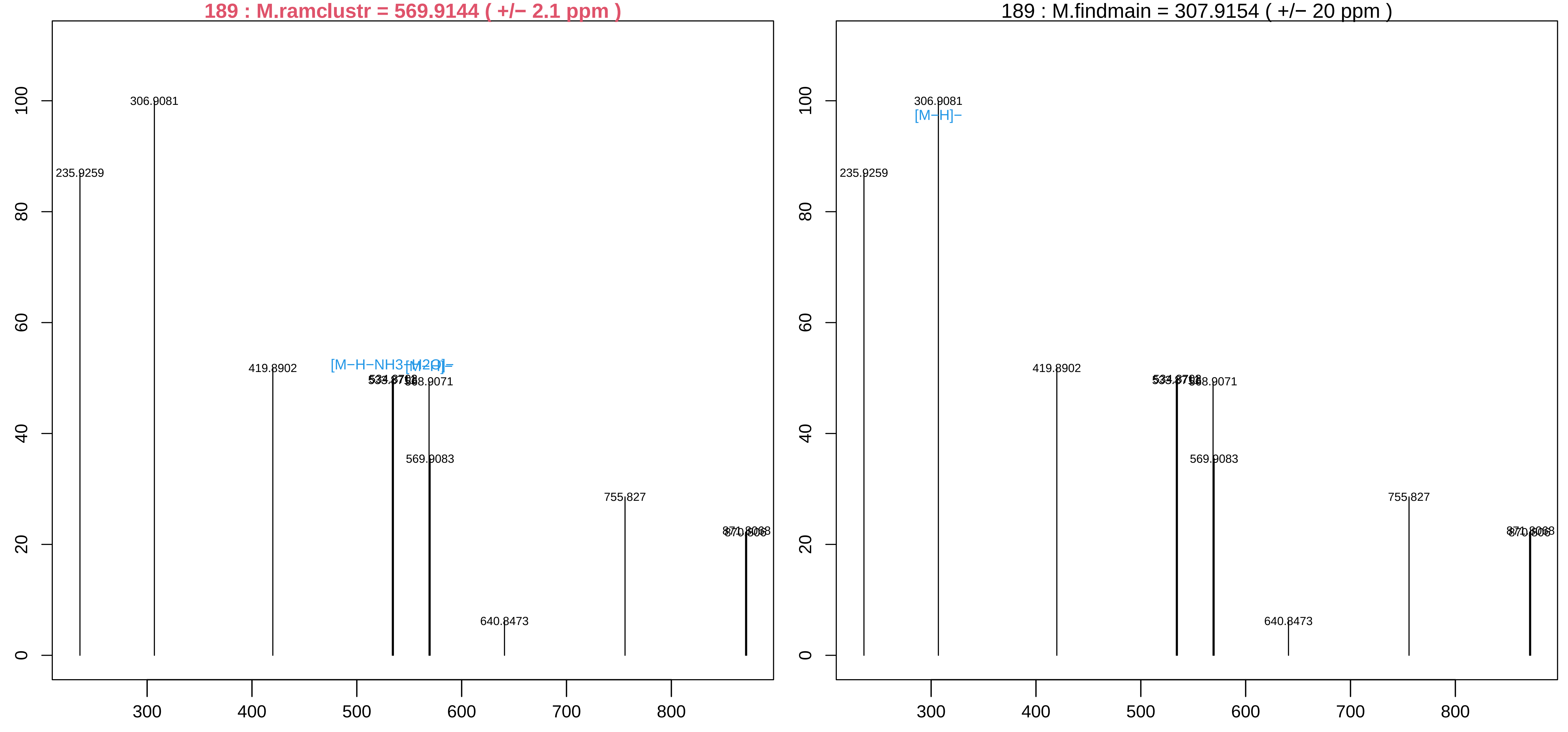
189 : M.ramclustr = 569.9144 (+/− 2.1 ppm)
189 : M.findmain = 307.9154 (+/− 20 ppm)
306.9081
235.9259
419.8902
[M−H−NH3+H2O]−
[M−H]−
568.9071
569.9083
640.8473
755.827
[M−H]−
0
20
40
60
80
100
300
400
500
600
700
800

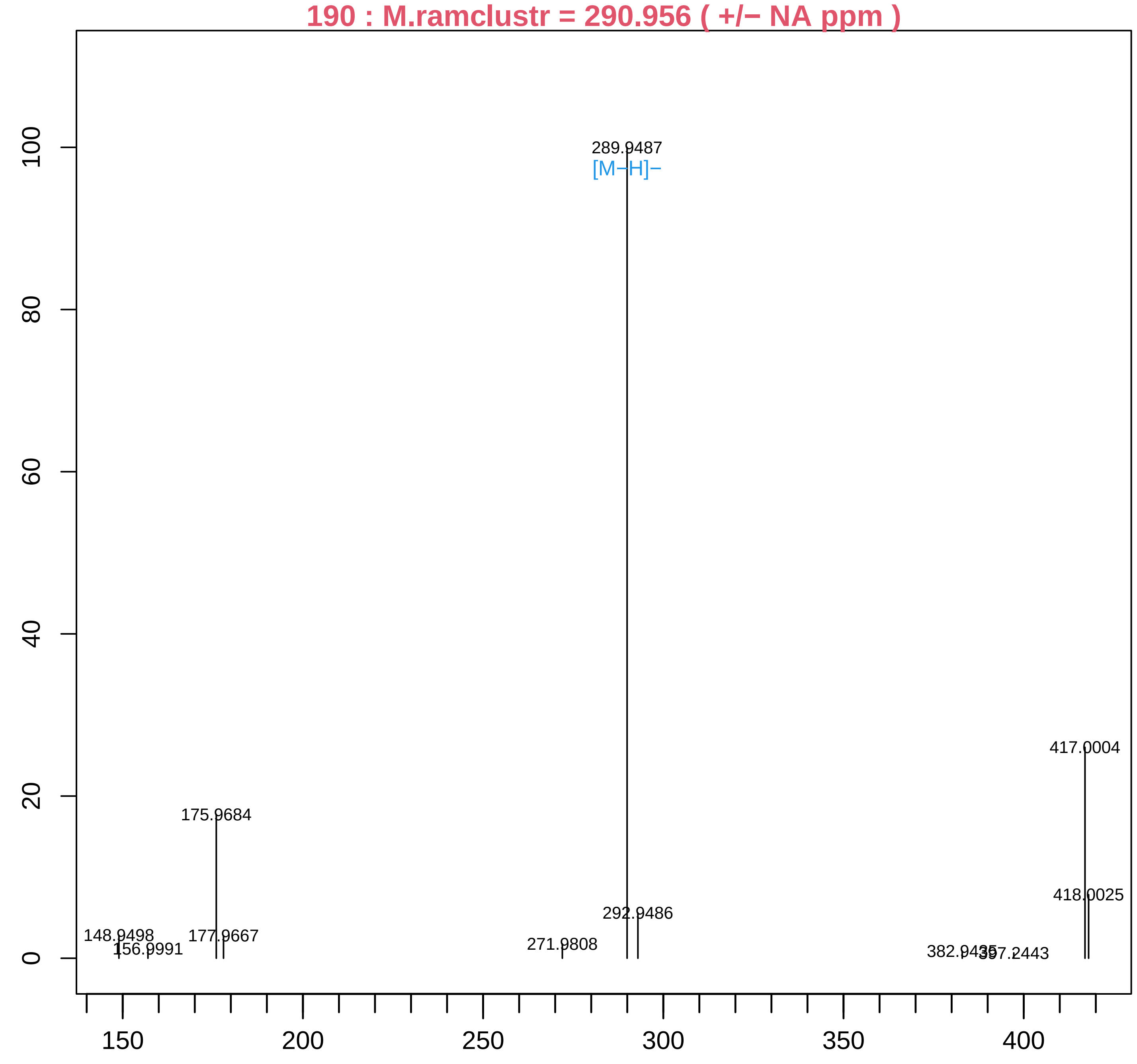
190 : M.ramclustr = 290.956 (+/− NA ppm)
289.9487
[M−H]−
175.9684
148.9498
156.9991
177.9667
271.9808
292.9486
382.9435
397.2443
417.0004
418.0025
0
20
40
60
80
100
150
200
250
300
350
400

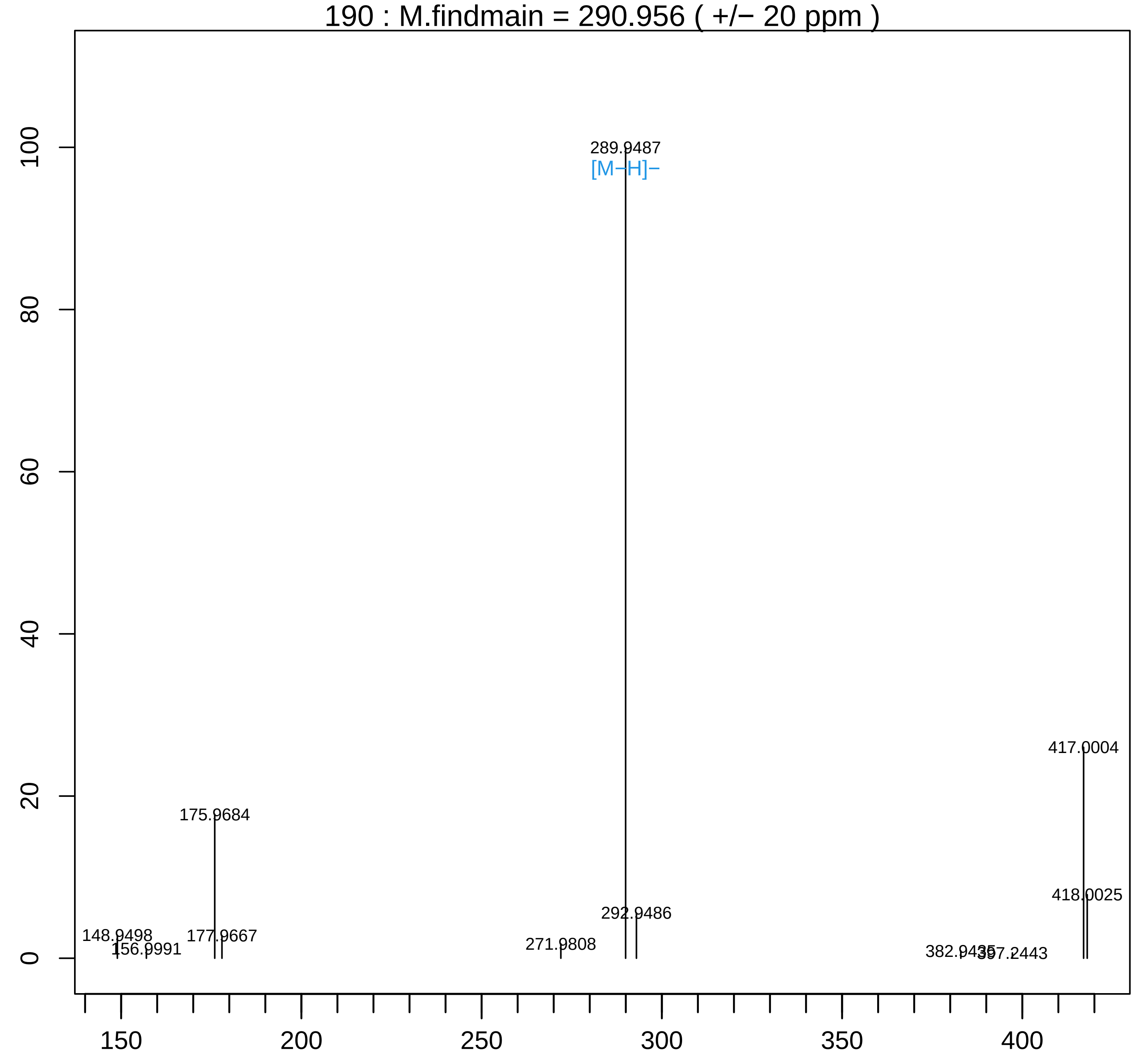
190 : M.findmain = 290.956 (+/− 20 ppm)
289.9487
[M−H]−
175.9684
148.9498
156.9991
177.9667
271.9808
292.9486
382.9435
397.2443
417.0004
418.0025
0
20
40
60
80
100
150
200
250
300
350
400

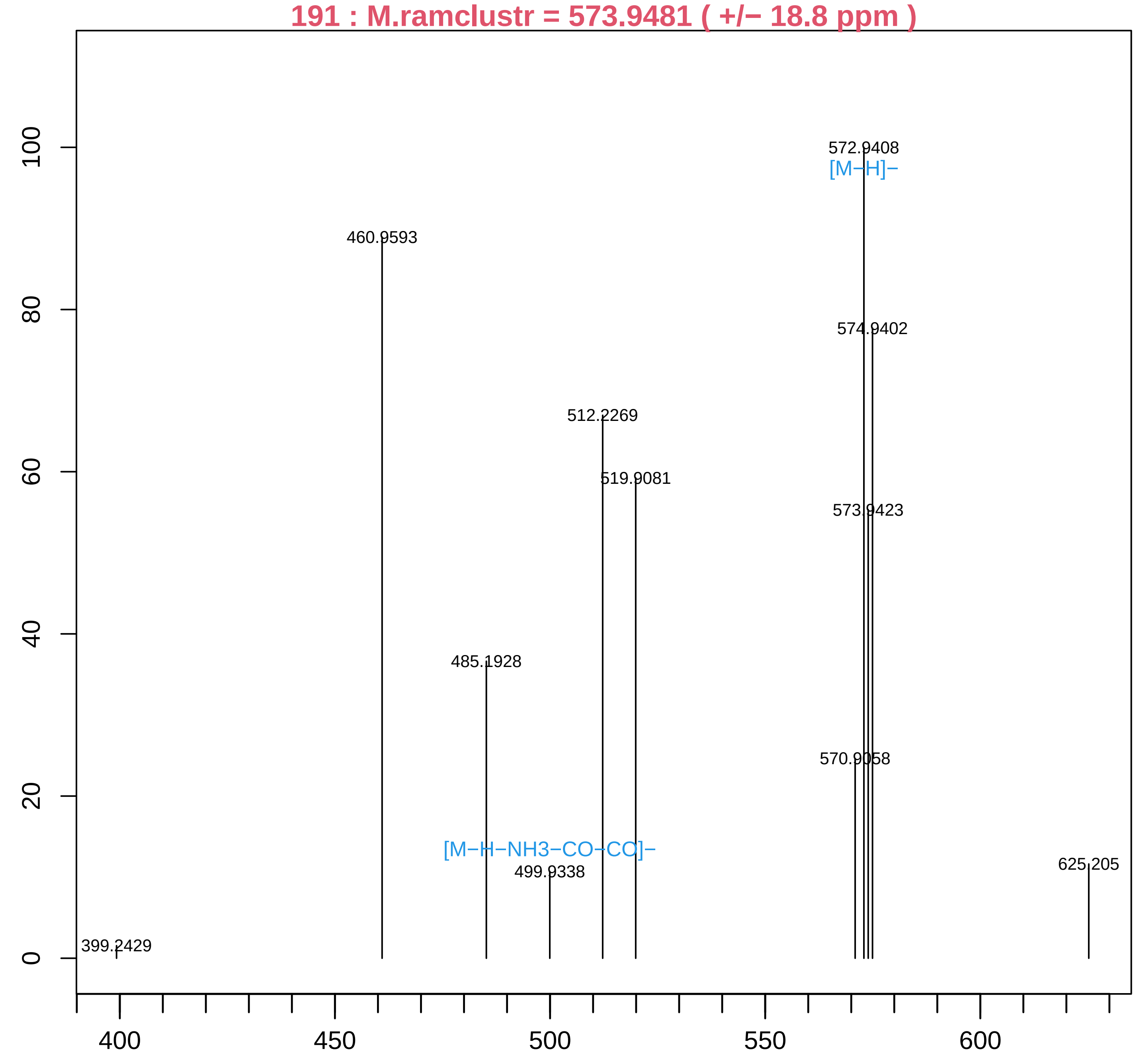
191 : M.ramclustr = 573.9481 (+/− 18.8 ppm)
572.9408
[M−H]−
460.9593
574.9402
512.2269
519.9081
573.9423
485.1928
570.9058
[M−H−NH3−CO−CO]−
499.9338
625.205
399.2429
0
20
40
60
80
100
400
450
500
550
600

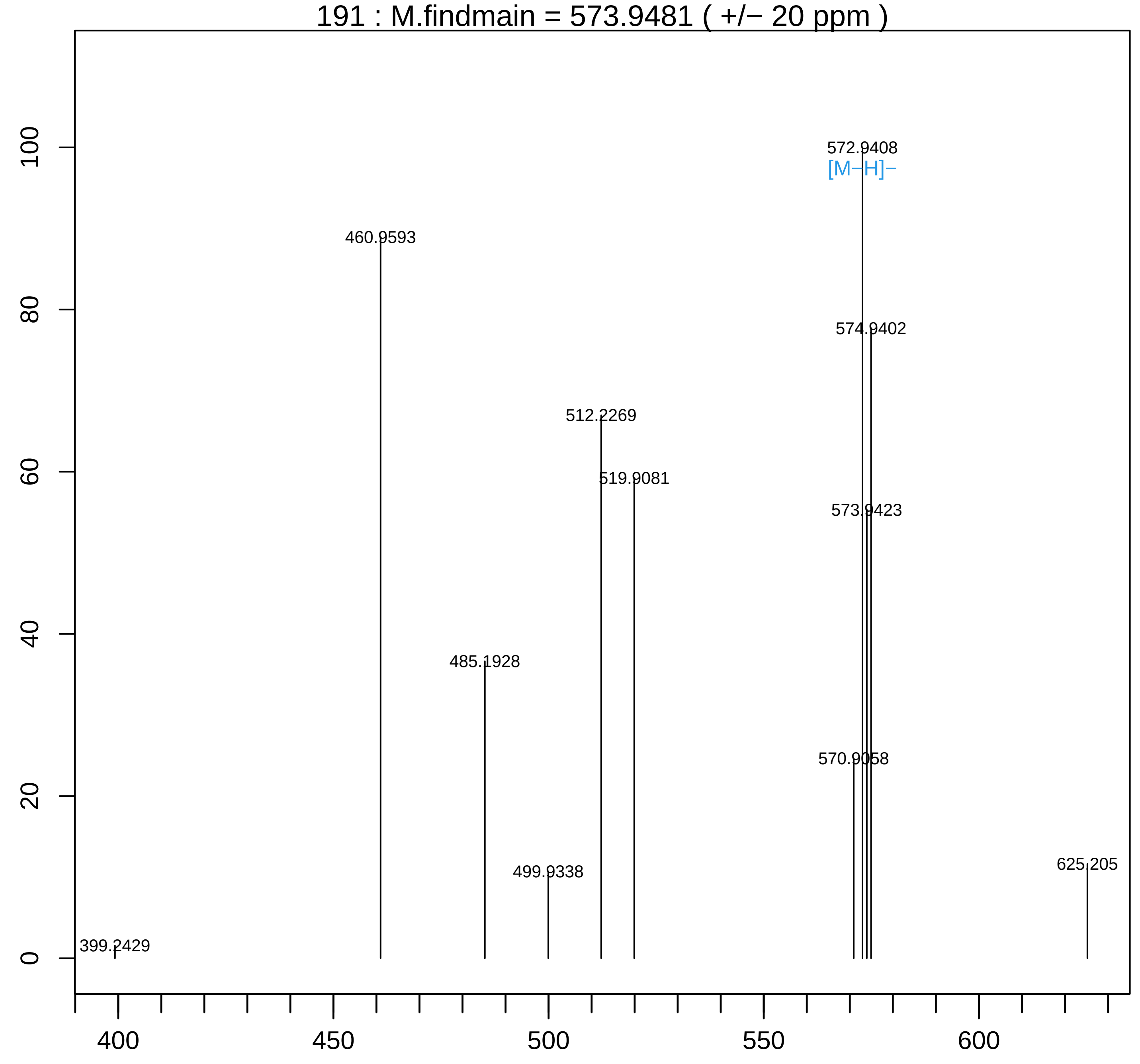
191 : M.findmain = 573.9481 (+/− 20 ppm)
572.9408
[M−H]−
460.9593
574.9402
512.2269
519.9081
573.9423
485.1928
570.9058
499.9338
625.205
399.2429
0
20
40
60
80
100
400
450
500
550
600

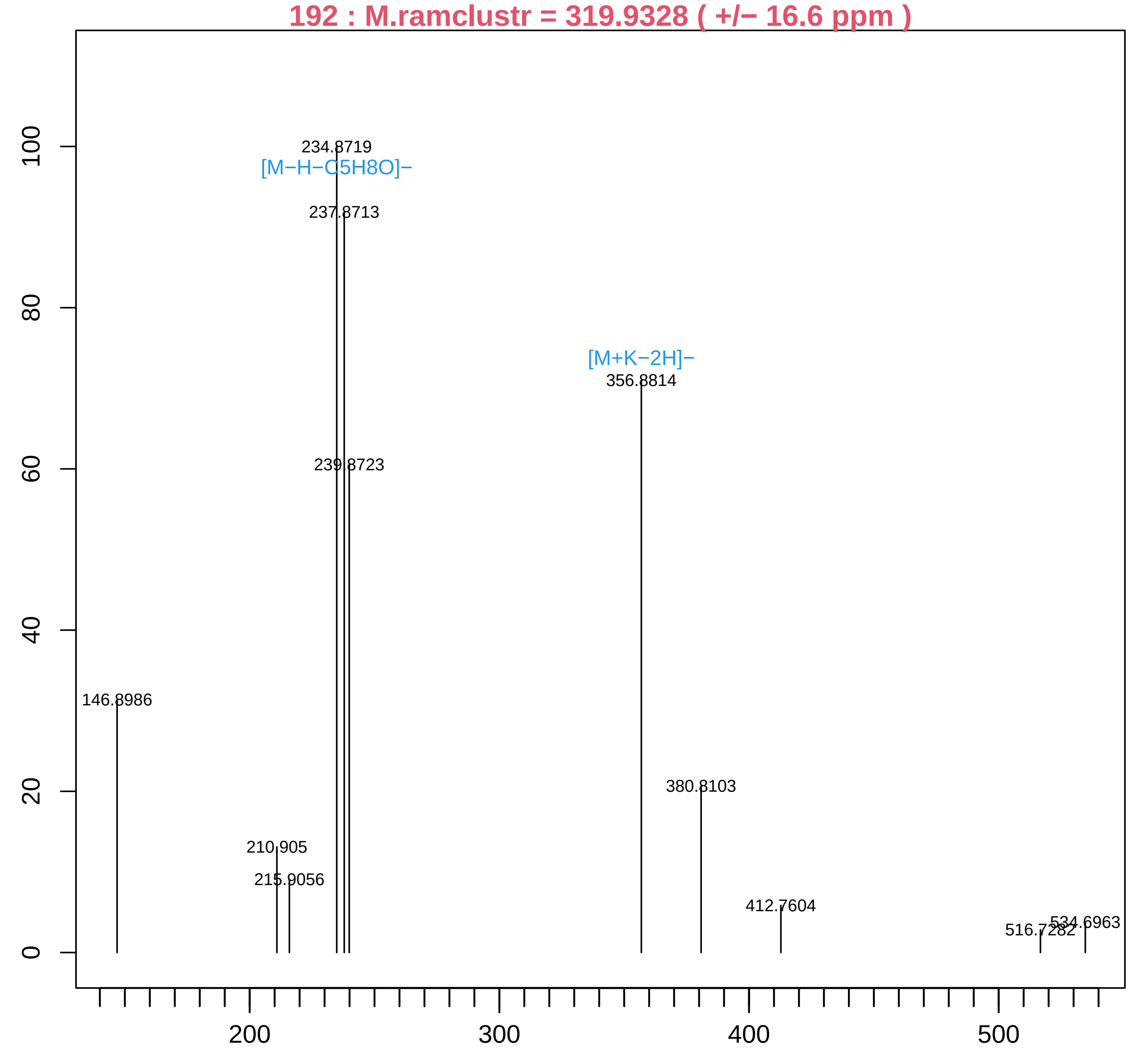
192 : M.ramclustr = 319.9328 (+/− 16.6 ppm)
234.8719
[M−H−C5H8O]−
237.8713
[M+K−2H]−
356.8814
239.8723
146.8986
380.8103
210.905
215.9056
412.7604
516.7282
534.6963
0
20
40
60
80
100
200
300
400
500
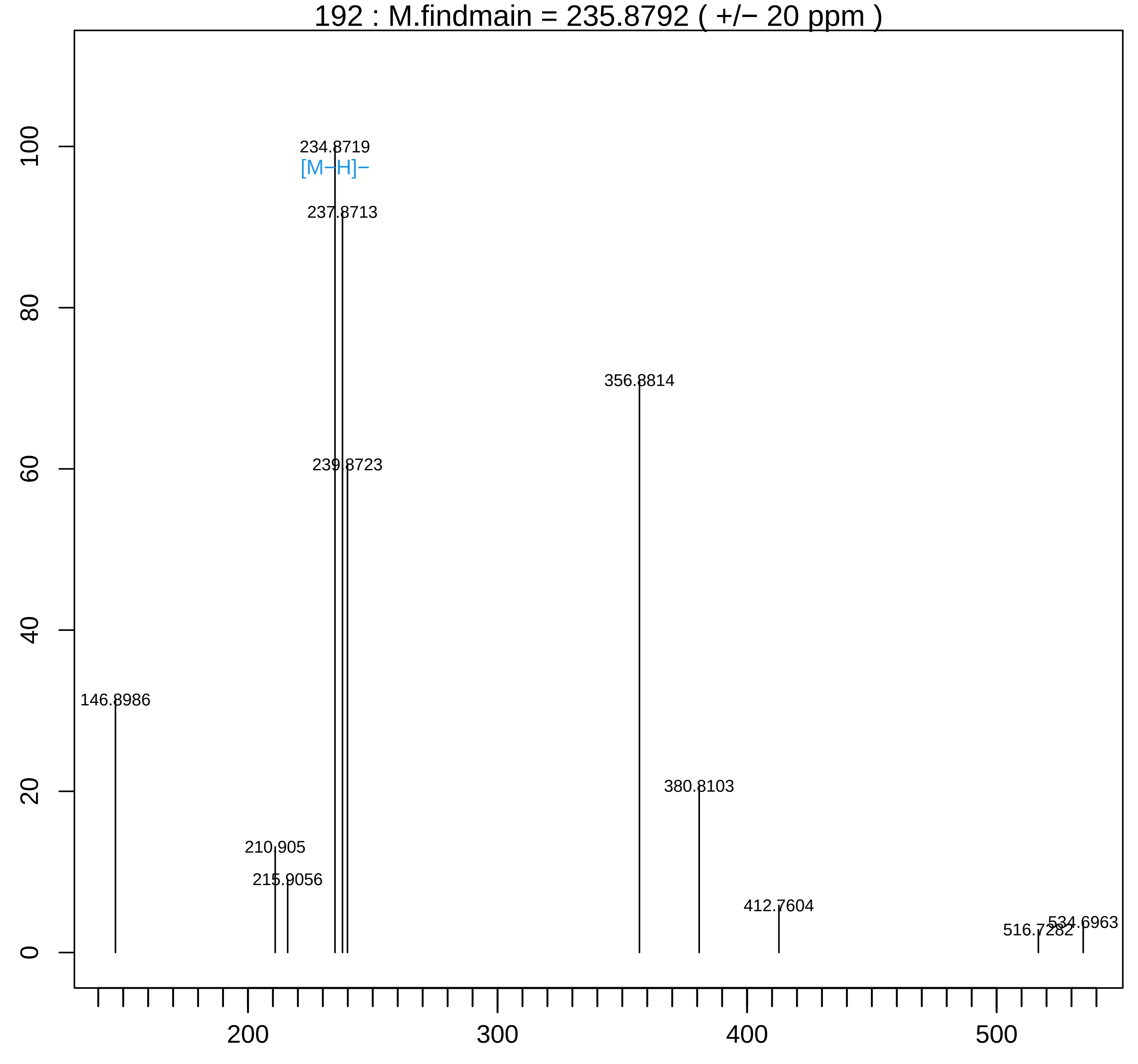
192 : M.findmain = 235.8792 (+/− 20 ppm)
234.8719
[M−H]−
237.8713
356.8814
239.8723
146.8986
380.8103
210.905
215.9056
412.7604
516.7282
534.6963
0
20
40
60
80
100
200
300
400
500

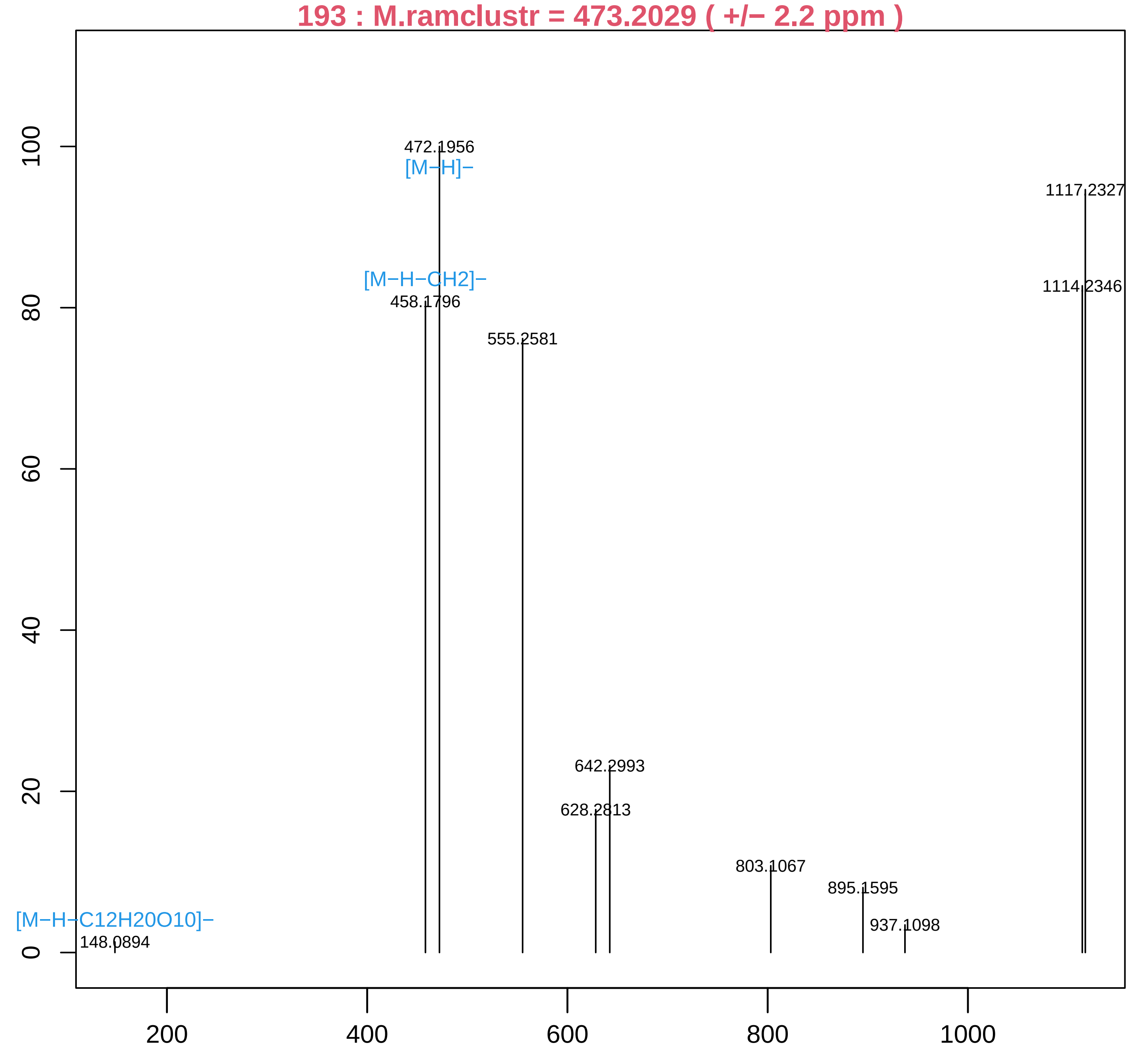
193 : M.ramclustr = 473.2029 (+/− 2.2 ppm)
472.1956
[M−H]−
1117.2327
[M−H−CH2]−
458.1796
1114.2346
555.2581
642.2993
628.2813
803.1067
895.1595
937.1098
[M−H−C12H20O10]−
148.0894
0
20
40
60
80
100
200
400
600
800
1000

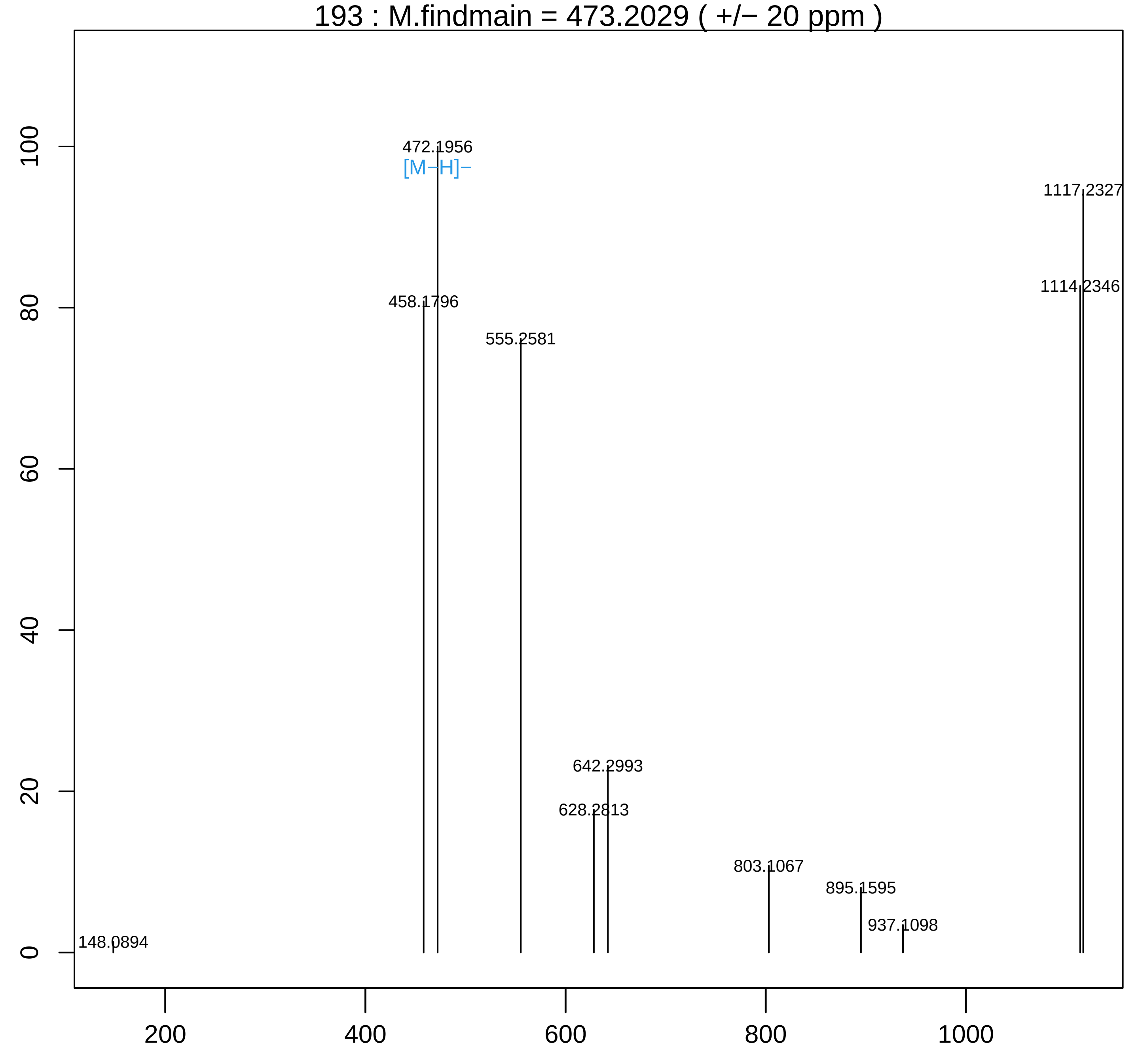
193 : M.findmain = 473.2029 (+/− 20 ppm)
472.1956
[M−H]−
1117.2327
1114.2346
458.1796
555.2581
642.2993
628.2813
803.1067
895.1595
937.1098
148.0894
0
20
40
60
80
100
200
400
600
800
1000

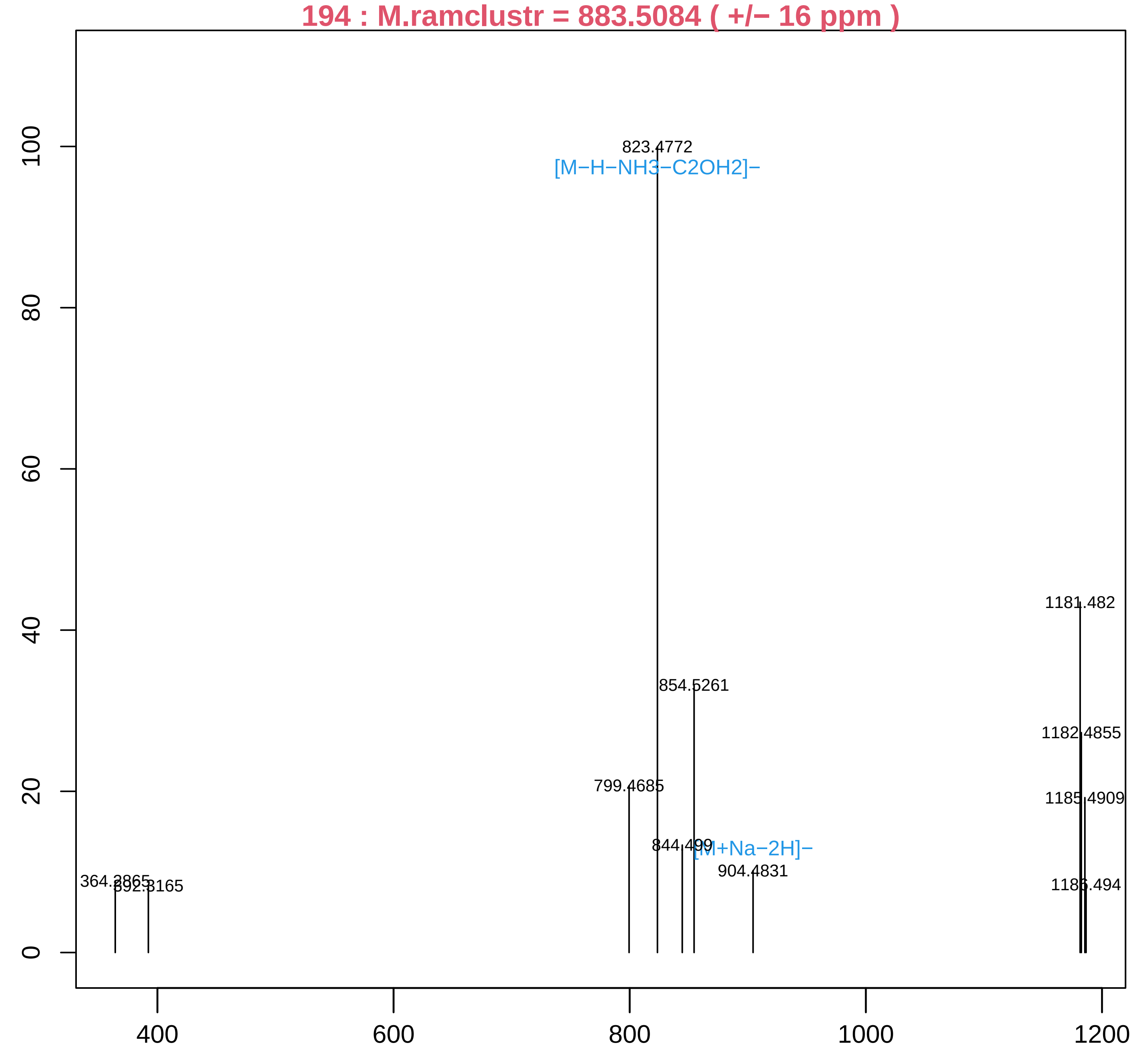
194 : M.ramclustr = 883.5084 (+/− 16 ppm)
823.4772
[M−H−NH3−C2OH2]−
1181.482
854.5261
1182.4855
799.4685
1185.4909
844.499
[M+Na−2H]−
904.4831
364.2865
392.3165
1186.494
0
20
40
60
80
100
400
600
800
1000
1200

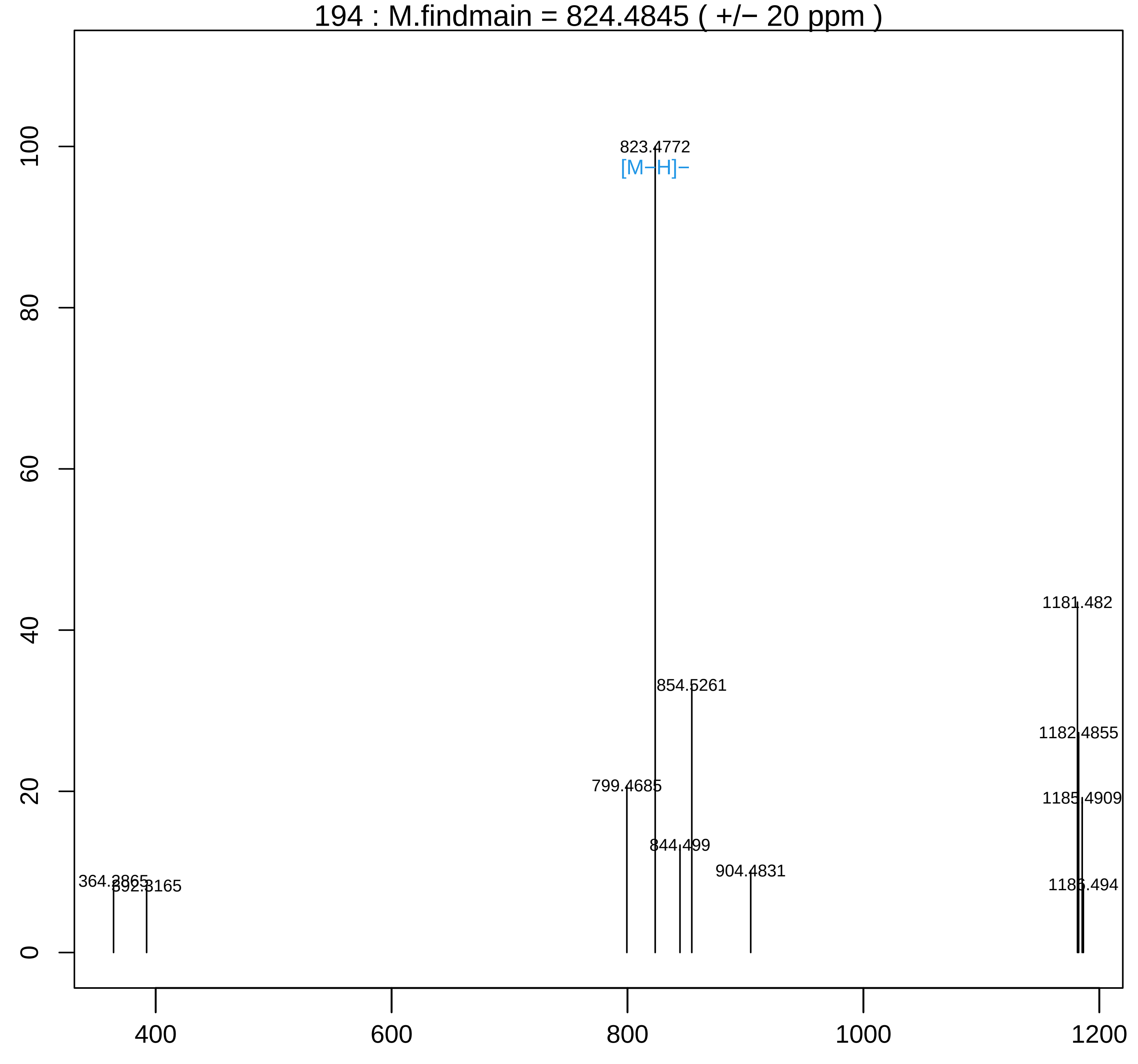
194 : M.findmain = 824.4845 (+/− 20 ppm)
823.4772
[M−H]−
1181.482
854.5261
1182.4855
799.4685
1185.4909
844.499
904.4831
364.2865
392.3165
1186.494
0
20
40
60
80
100
400
600
800
1000
1200

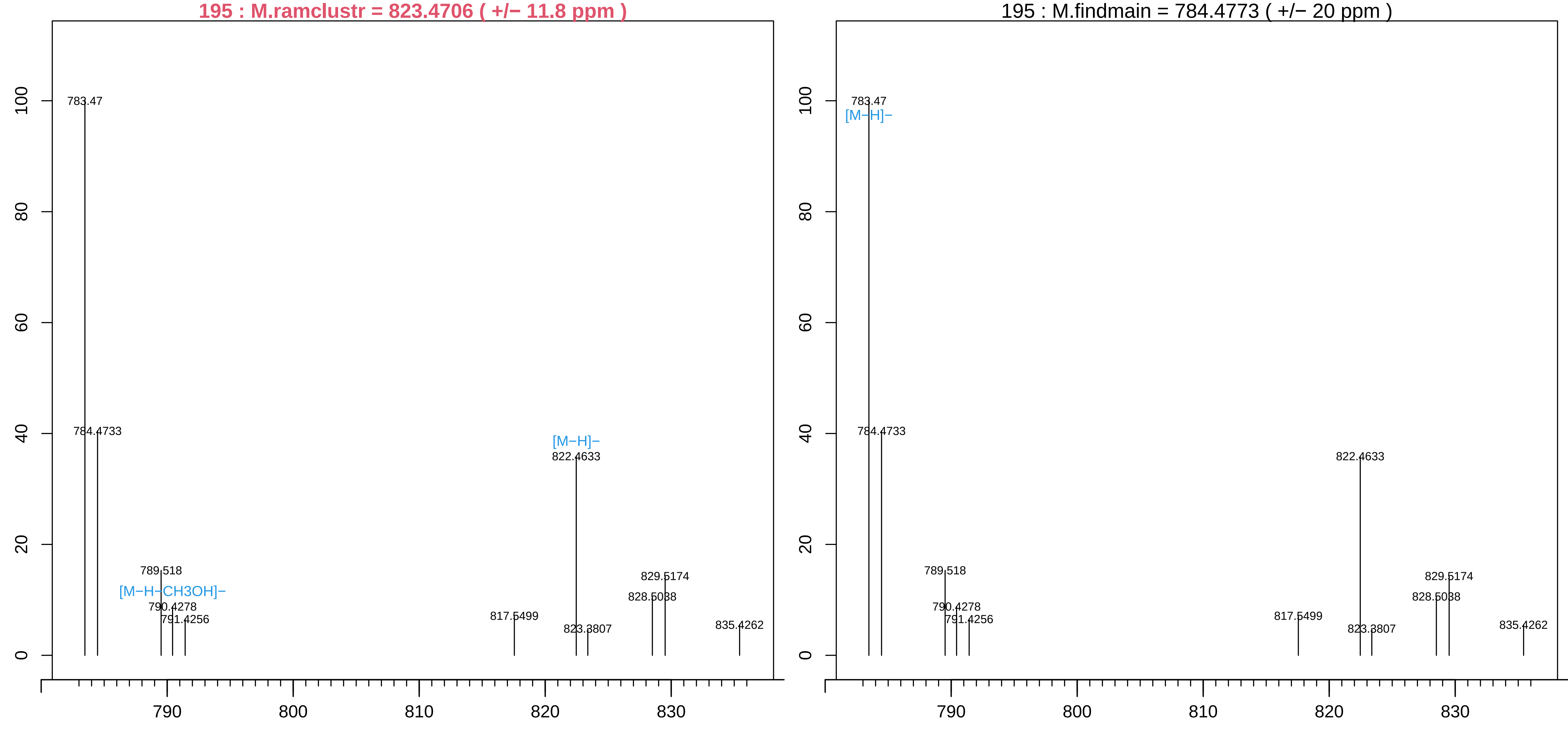
195 : M.ramclustr = 823.4706 (+/− 11.8 ppm)
783.47
784.4733
789.518
[M−H−CH3OH]−
790.4278
791.4256
817.5499
[M−H]−
822.4633
823.3807
828.5038
829.5174
835.4262
195 : M.findmain = 784.4773 (+/− 20 ppm)
783.47
[M−H]−
784.4733
789.518
790.4278
791.4256
817.5499
822.4633
823.3807
828.5038
829.5174
835.4262
0
20
40
60
80
100
790
800
810
820
830

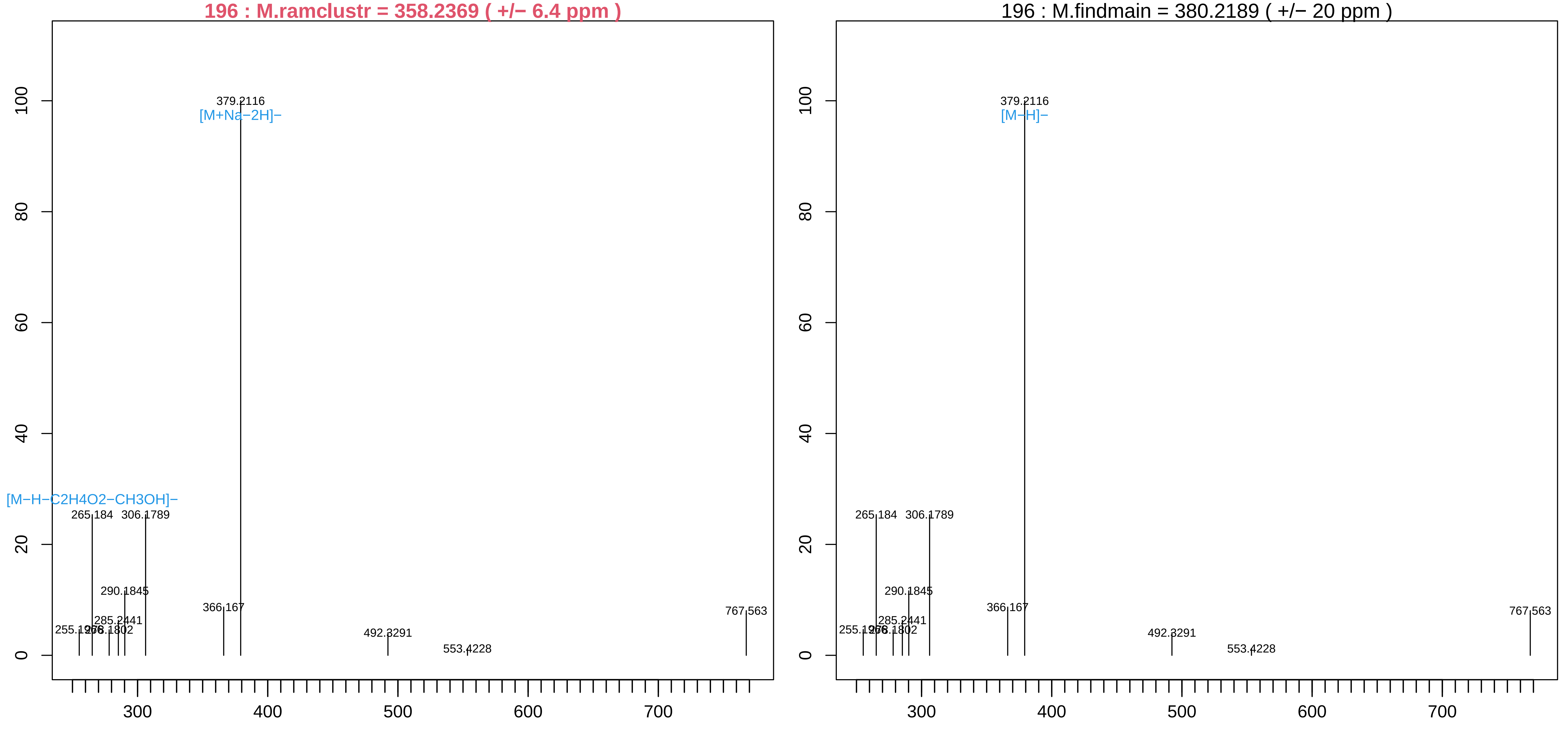

196 : M.ramclustr = 358.2369 (+/− 6.4 ppm)
379.2116
[M+Na−2H]−
[M−H−C2H4O2−CH3OH]−
265.184
306.1789
290.1845
366.167
767.563
285.2441
255.1976
278.1802
492.3291
553.4228
196 : M.findmain = 380.2189 (+/− 20 ppm)
379.2116
[M−H]−
265.184
306.1789
290.1845
366.167
767.563
285.2441
255.1976
278.1802
492.3291
553.4228
0
20
40
60
80
100
300
400
500
600
700

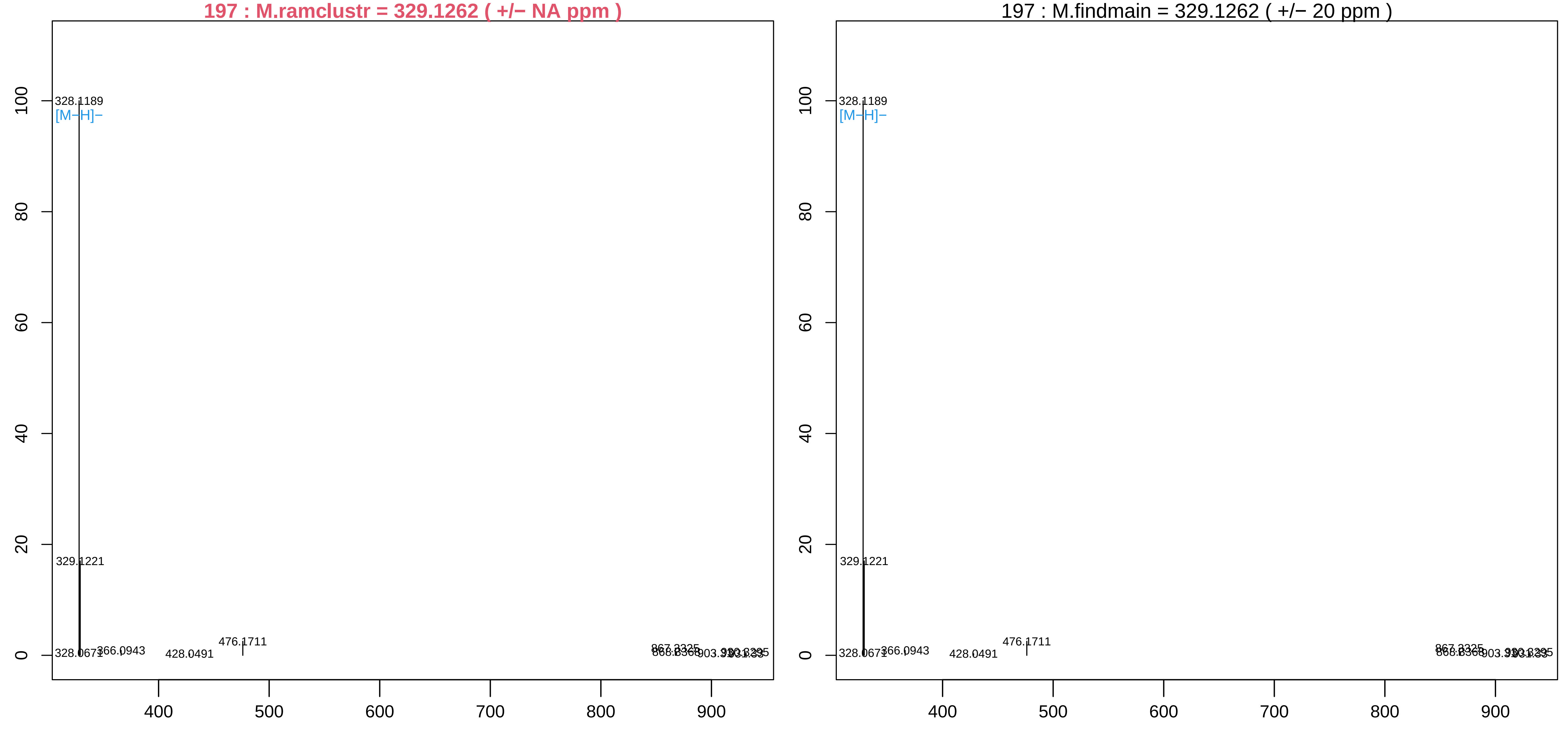

197 : M.ramclustr = 329.1262 (+/− NA ppm)
328.1189
[M−H]−
329.1221
328.0671
366.0943
428.0491
476.1711
867.3325
197 : M.findmain = 329.1262 (+/− 20 ppm)
328.1189
[M−H]−
329.1221
328.0671
366.0943
428.0491
476.1711
867.3325

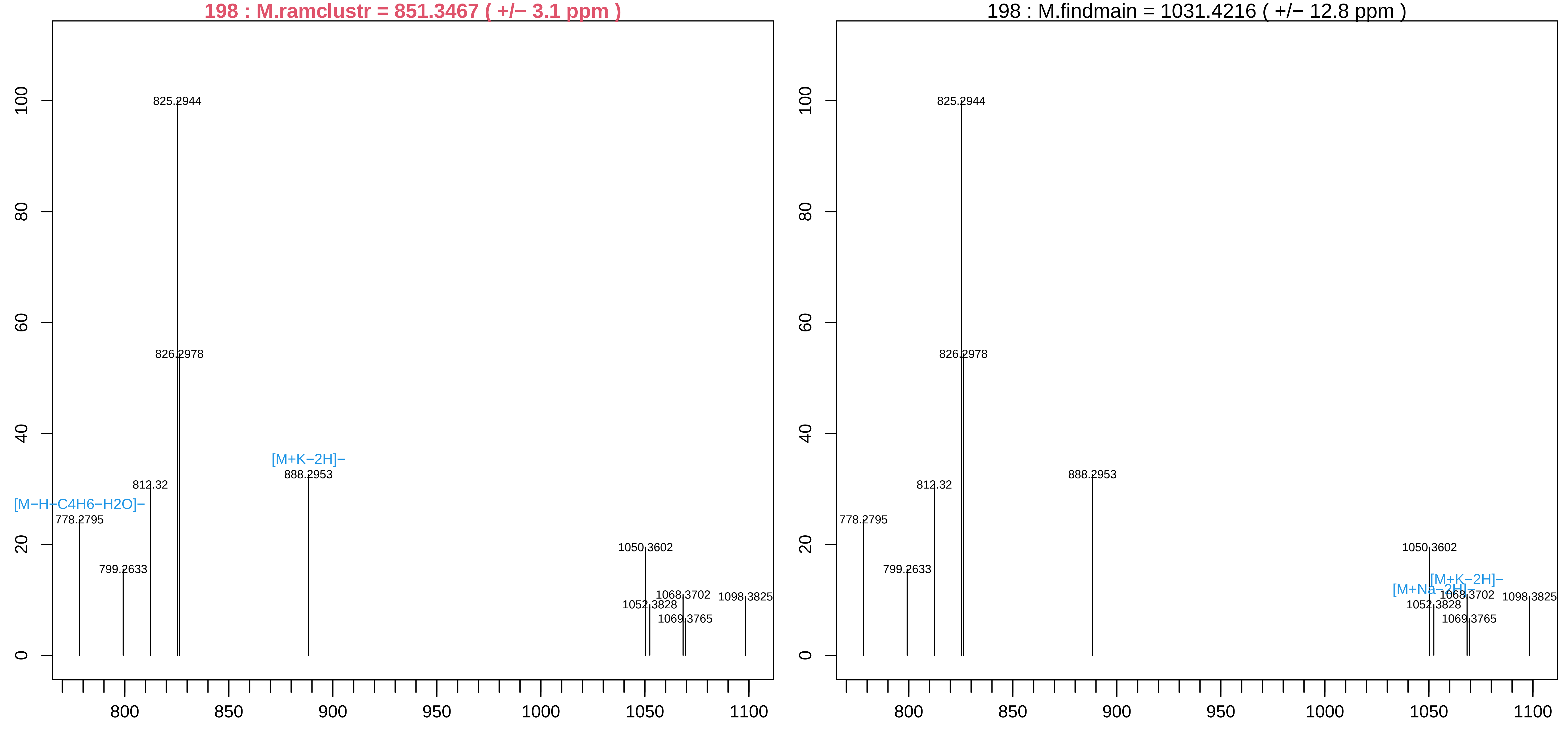
198 : M.ramclustr = 851.3467 (+/− 3.1 ppm)
825.2944
826.2978
[M+K−2H]−
888.2953
812.32
[M−H−C4H6−H2O]−
778.2795
799.2633
1050.3602
1068.3702
1098.3825
1052.3828
1069.3765
198 : M.findmain = 1031.4216 (+/− 12.8 ppm)
825.2944
826.2978
888.2953
812.32
778.2795
799.2633
1050.3602
[M+K−2H]−
[M+Na−2H]−
1068.3702
1098.3825
1052.3828
1069.3765

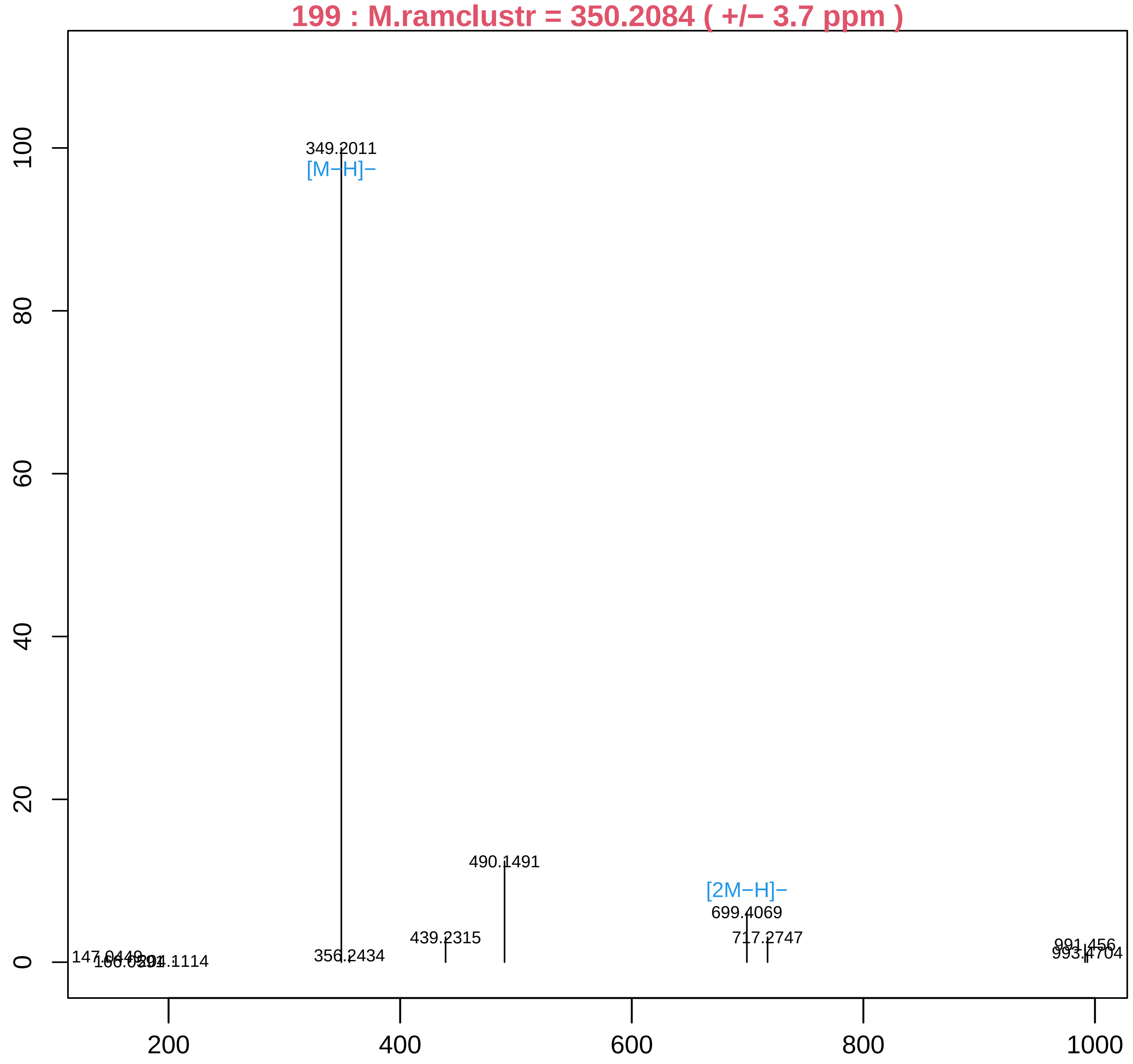
199 : M.ramclustr = 350.2084 (+/− 3.7 ppm)
349.2011
[M−H]−
490.1491
[2M−H]−
699.4069
717.2747
439.2315
356.2434
147.0448
160.0291
201.1114
991.456
993.4704
0
20
40
60
80
100
200
400
600
800
1000

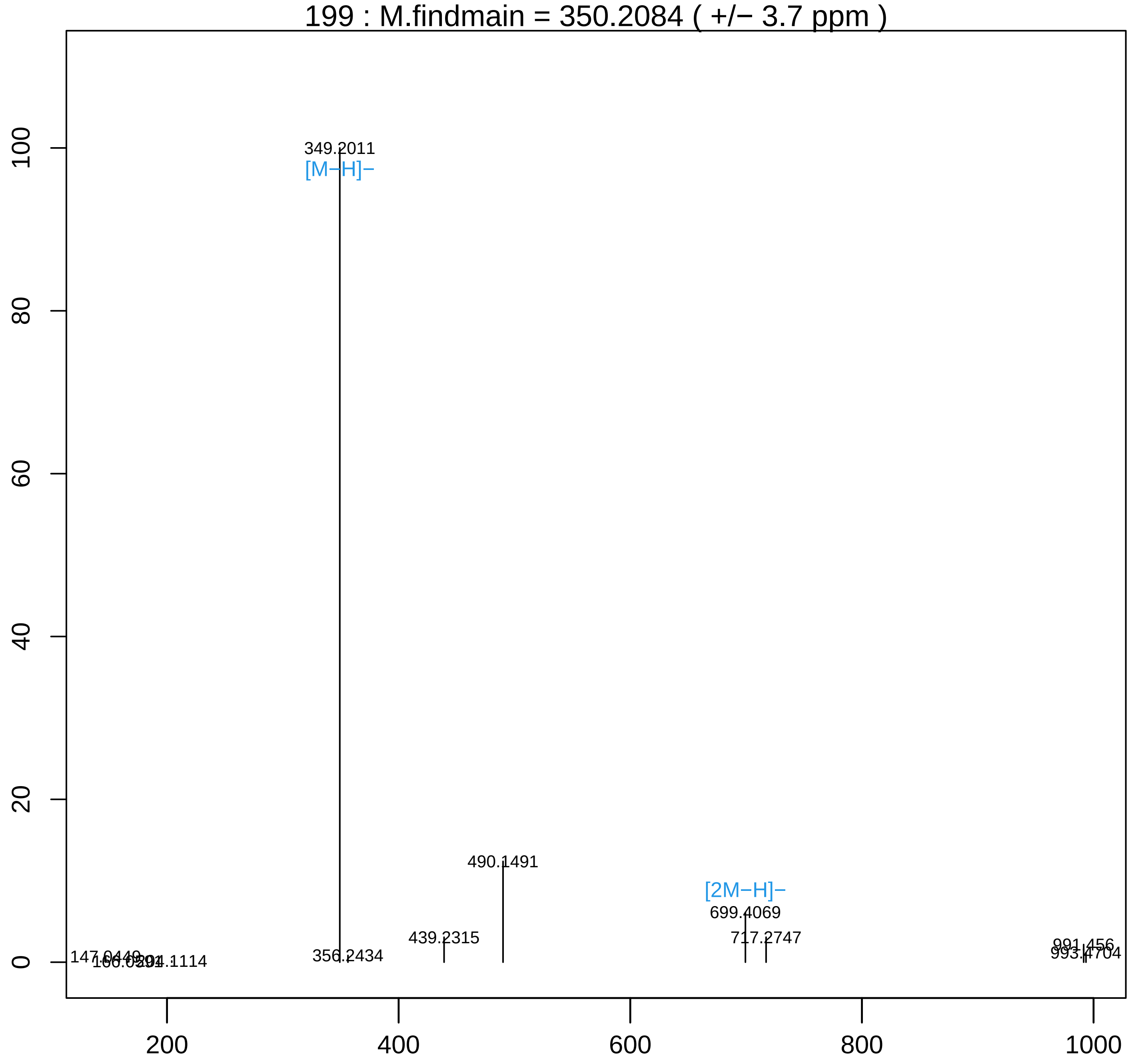
199 : M.findmain = 350.2084 (+/− 3.7 ppm)
349.2011
[M−H]−
490.1491
[2M−H]−
699.4069
717.2747
439.2315
356.2434
147.0448
160.0291
201.1114
991.456
993.4704
0
20
40
60
80
100
200
400
600
800
1000

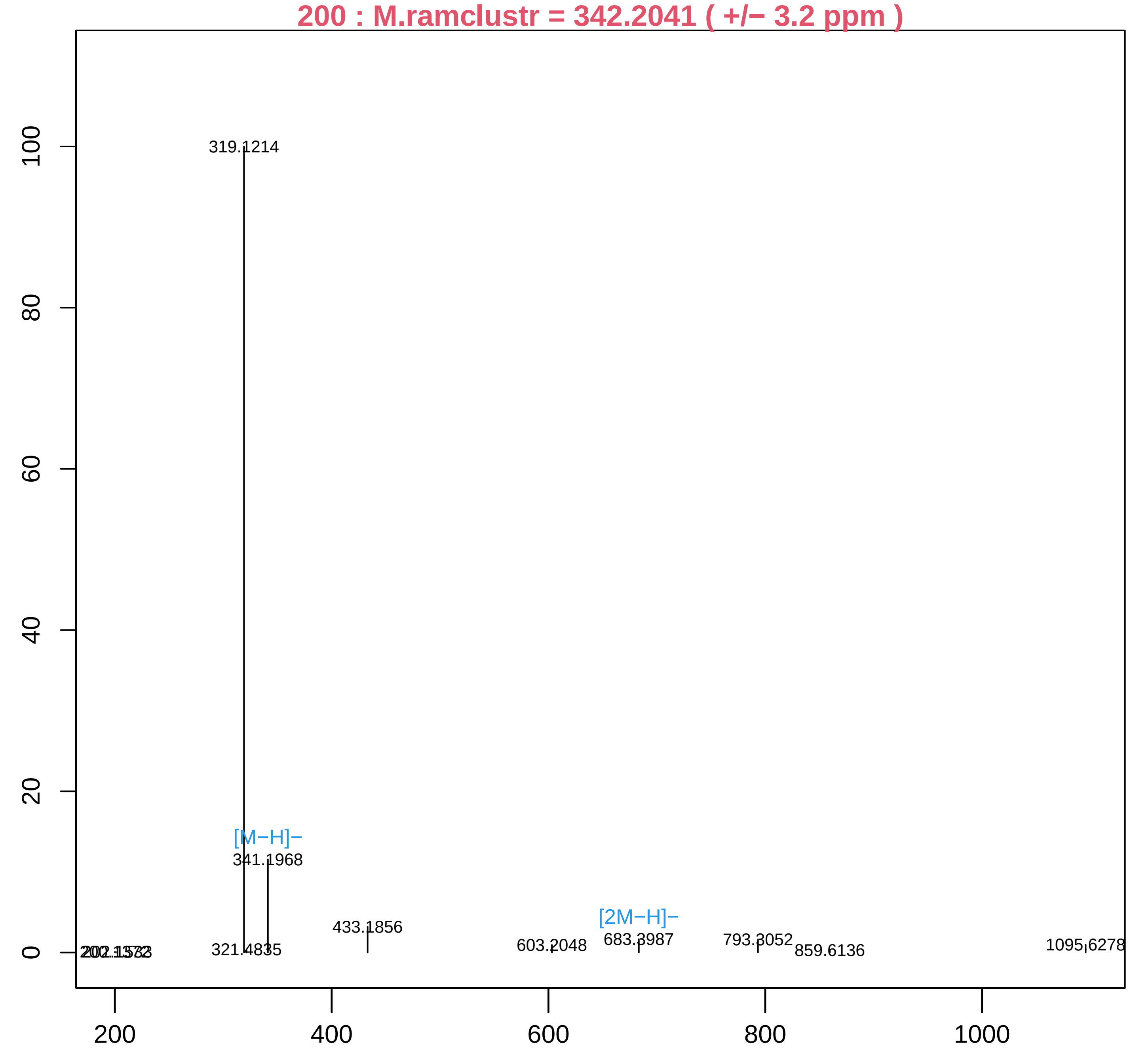
200 : M.ramclustr = 342.2041 (+/− 3.2 ppm)
319.1214
[M−H]−
341.1968
433.1856
[2M−H]−
683.3987
603.2048
793.3052
859.6136
1095.6278
321.4835
0
20
40
60
80
100
200
400
600
800
1000

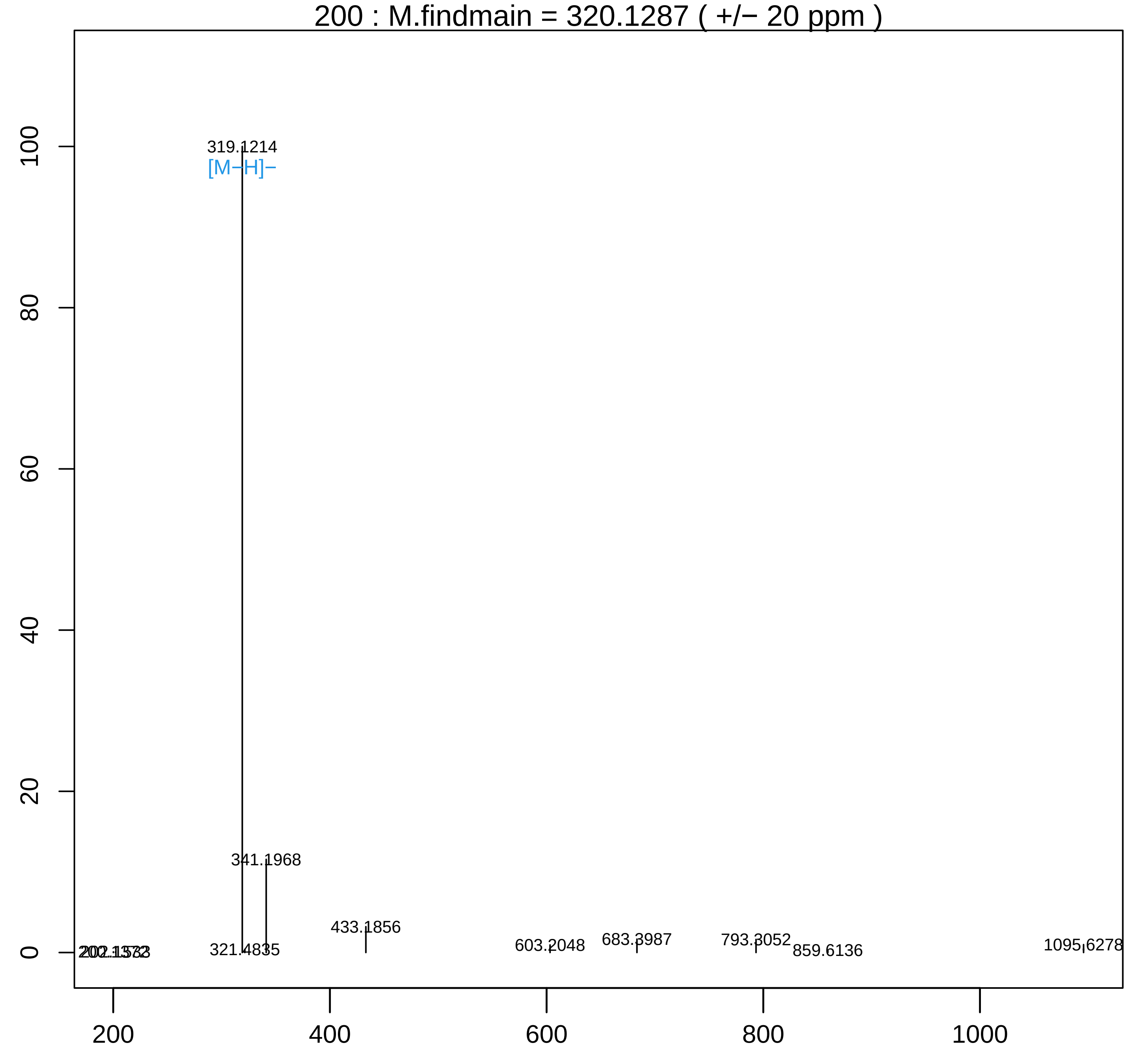
200 : M.findmain = 320.1287 (+/− 20 ppm)
319.1214
[M−H]−
341.1968
433.1856
603.2048
683.3987
793.3052
859.6136
1095.6278
321.4835
0
20
40
60
80
100
200
400
600
800
1000

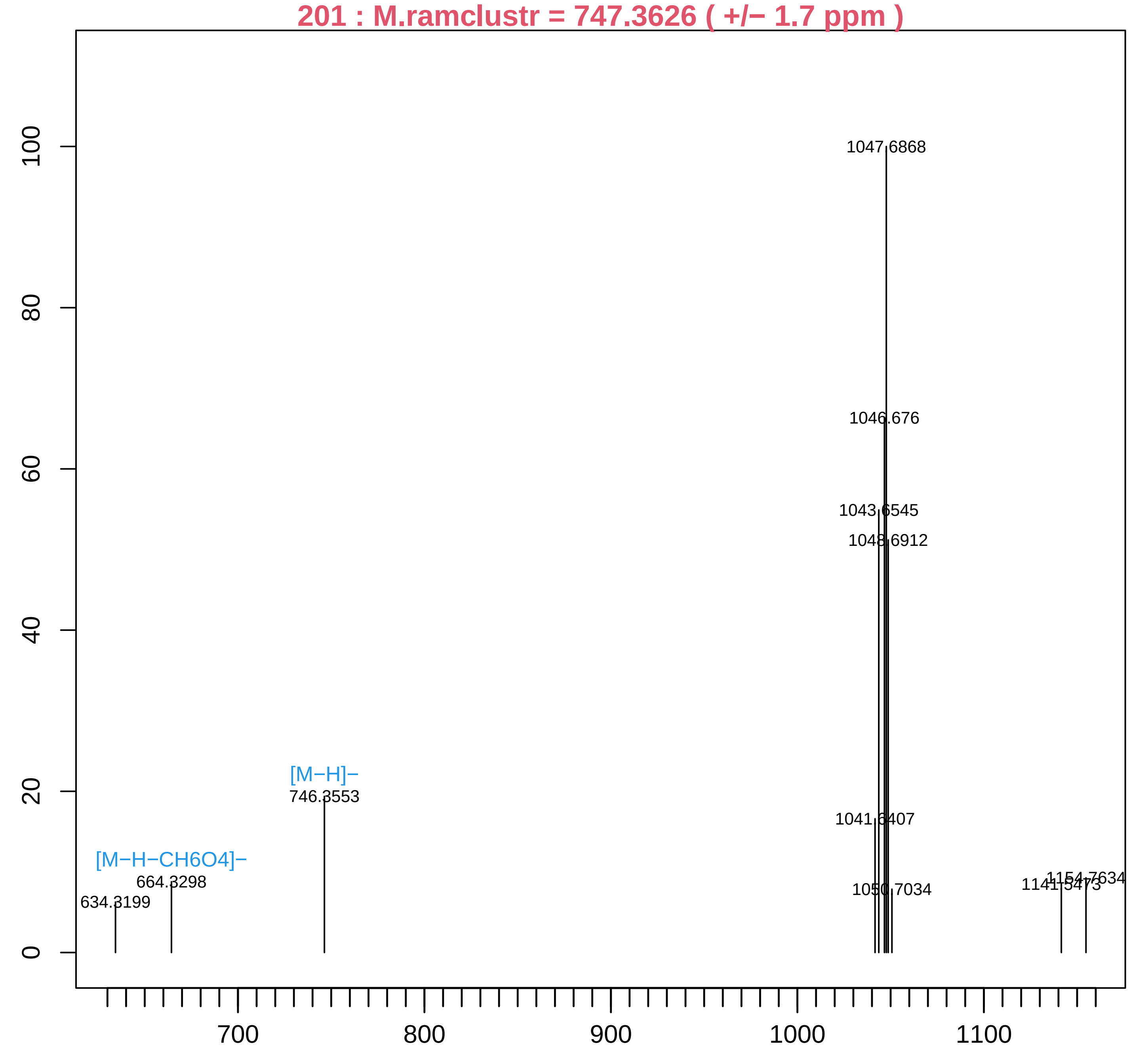
201 : M.ramclustr = 747.3626 (+/− 1.7 ppm)
1047.6868
1046.676
1043.6545
1048.6912
[M−H]−
746.3553
1041.6407
[M−H−CH6O4]−
664.3298
634.3199
1050.7034
1141.5473
1154.7634
0
20
40
60
80
100
700
800
900
1000
1100

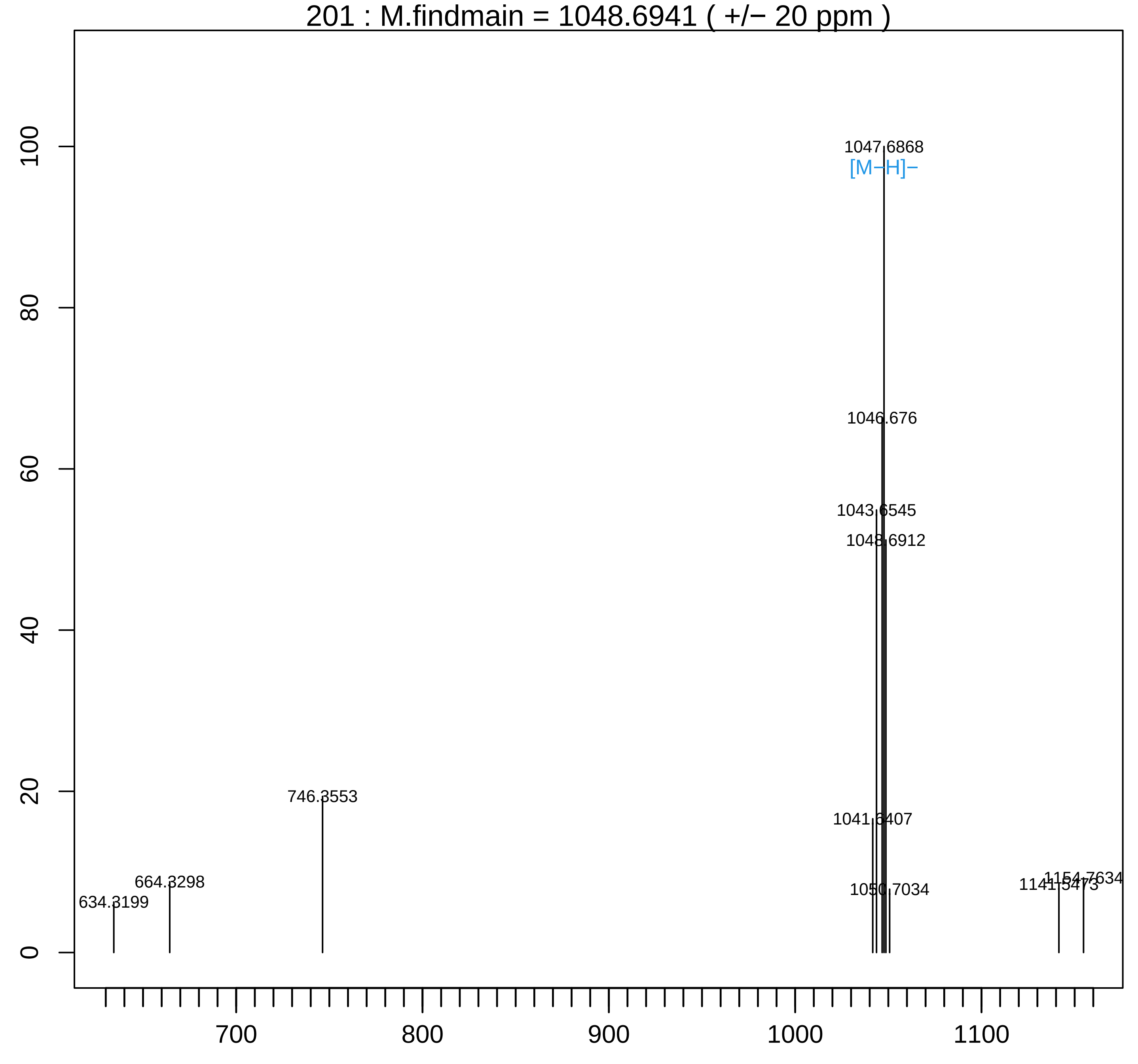
201 : M.findmain = 1048.6941 (+/− 20 ppm)
1047.6868
[M−H]−
1046.676
1043.6545
1048.6912
746.3553
1041.6407
664.3298
634.3199
1050.7034
1141.5473
1154.7634
0
20
40
60
80
100
700
800
900
1000
1100

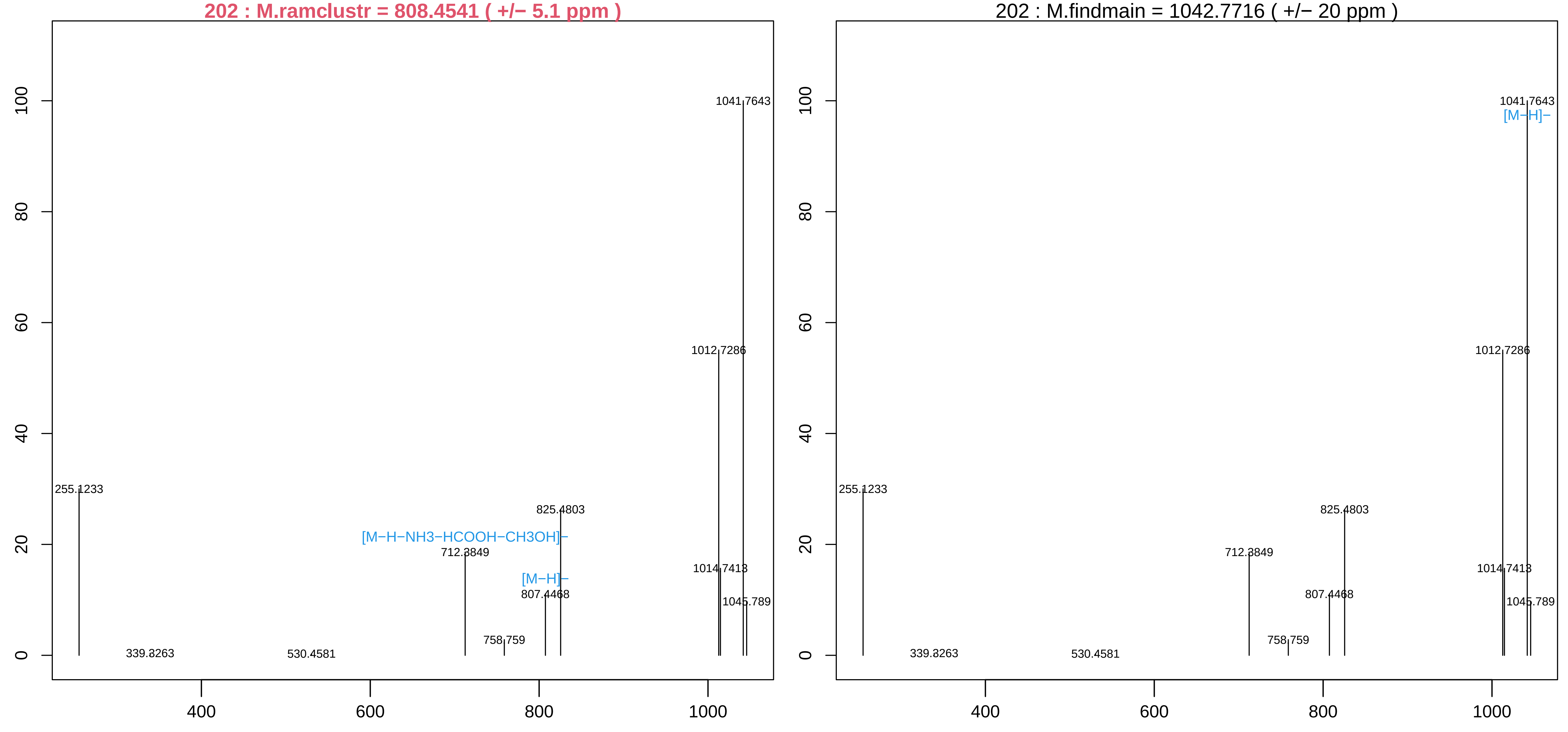
202 : M.ramclustr = 808.4541 (+/− 5.1 ppm)
202 : M.findmain = 1042.7716 (+/− 20 ppm)
1041.7643
[M−H]−
1012.7286
1014.7413
1045.789
255.1233
825.4803
[M−H−NH3−HCOOH−CH3OH]−
712.3849
807.4468
758.759
339.8263
530.4581
0
20
40
60
80
100
400
600
800
1000

203 : M.ramclustr = 979.7035 (+/− 1.3 ppm)

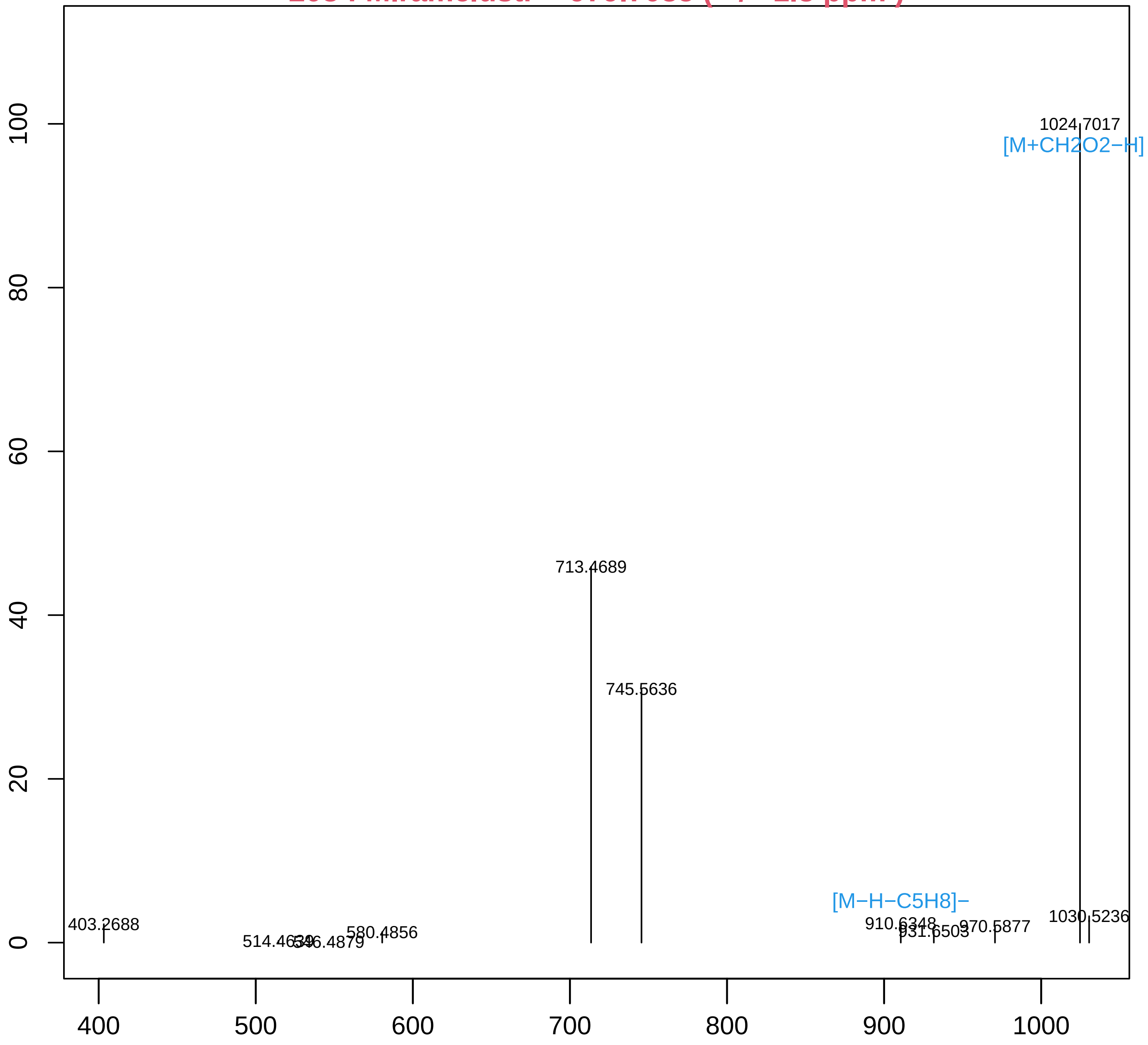

203 : M.findmain = 1025.709 (+/− 20 ppm)

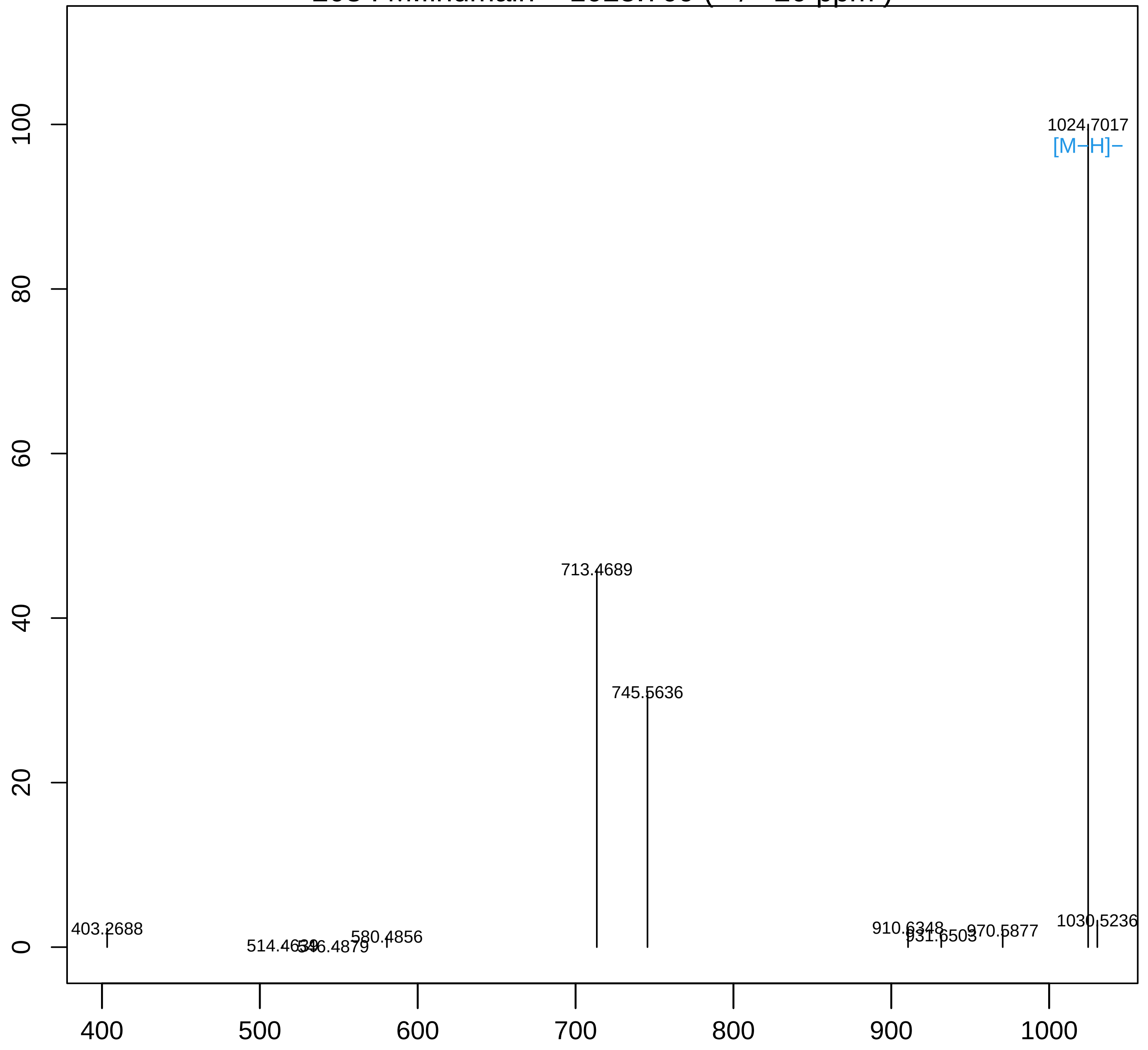

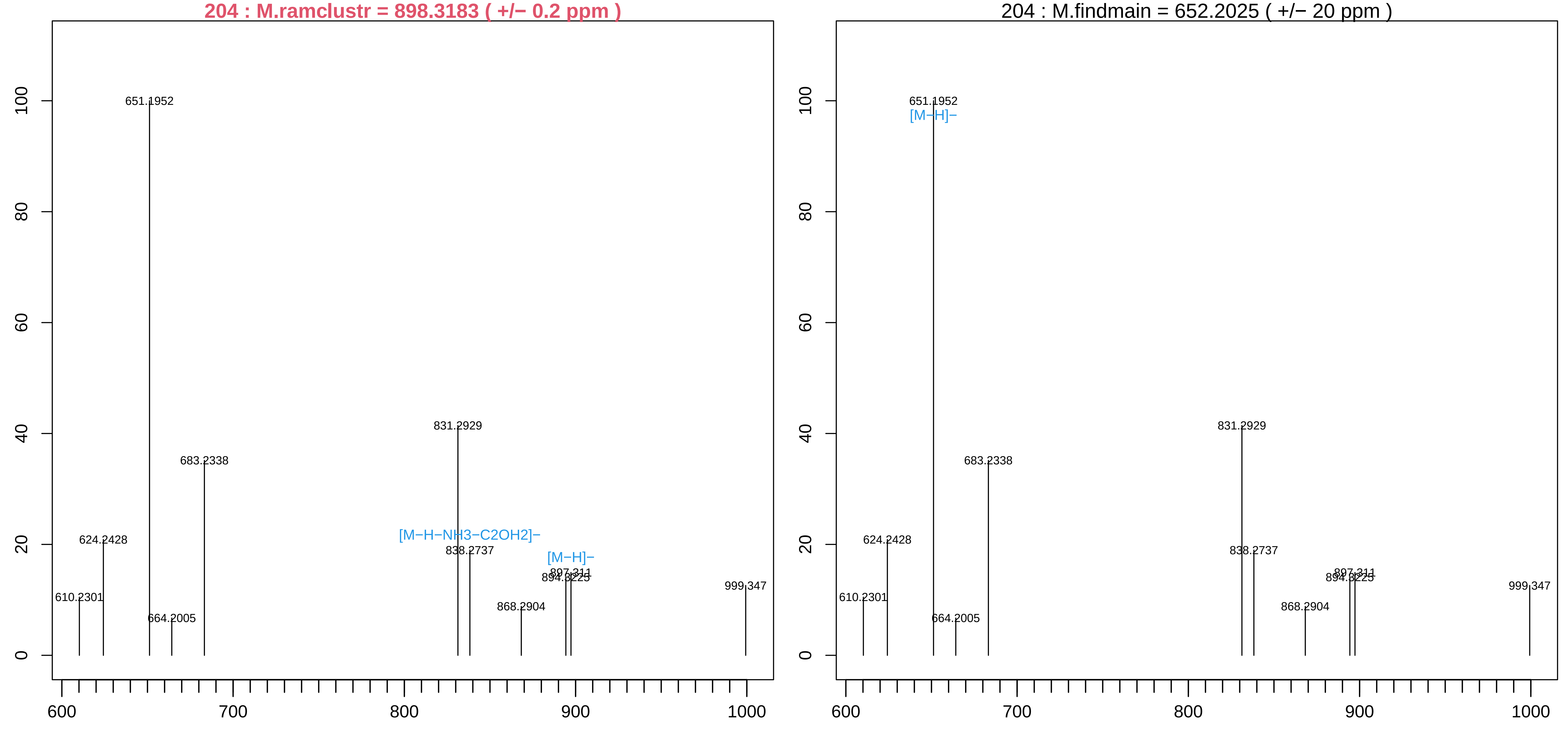
204 : M.ramclustr = 898.3183 (+/− 0.2 ppm)
651.1952
831.2929
683.2338
624.2428
[M−H−NH3−C2OH2]−
838.2737
[M−H]−
897.311
894.3225
999.347
610.2301
868.2904
664.2005
0
20
40
60
80
100
600
700
800
900
1000
204 : M.findmain = 652.2025 (+/− 20 ppm)
651.1952
[M−H]−
831.2929
683.2338
624.2428
838.2737
897.311
894.3225
999.347
610.2301
868.2904
664.2005
0
20
40
60
80
100
600
700
800
900
1000

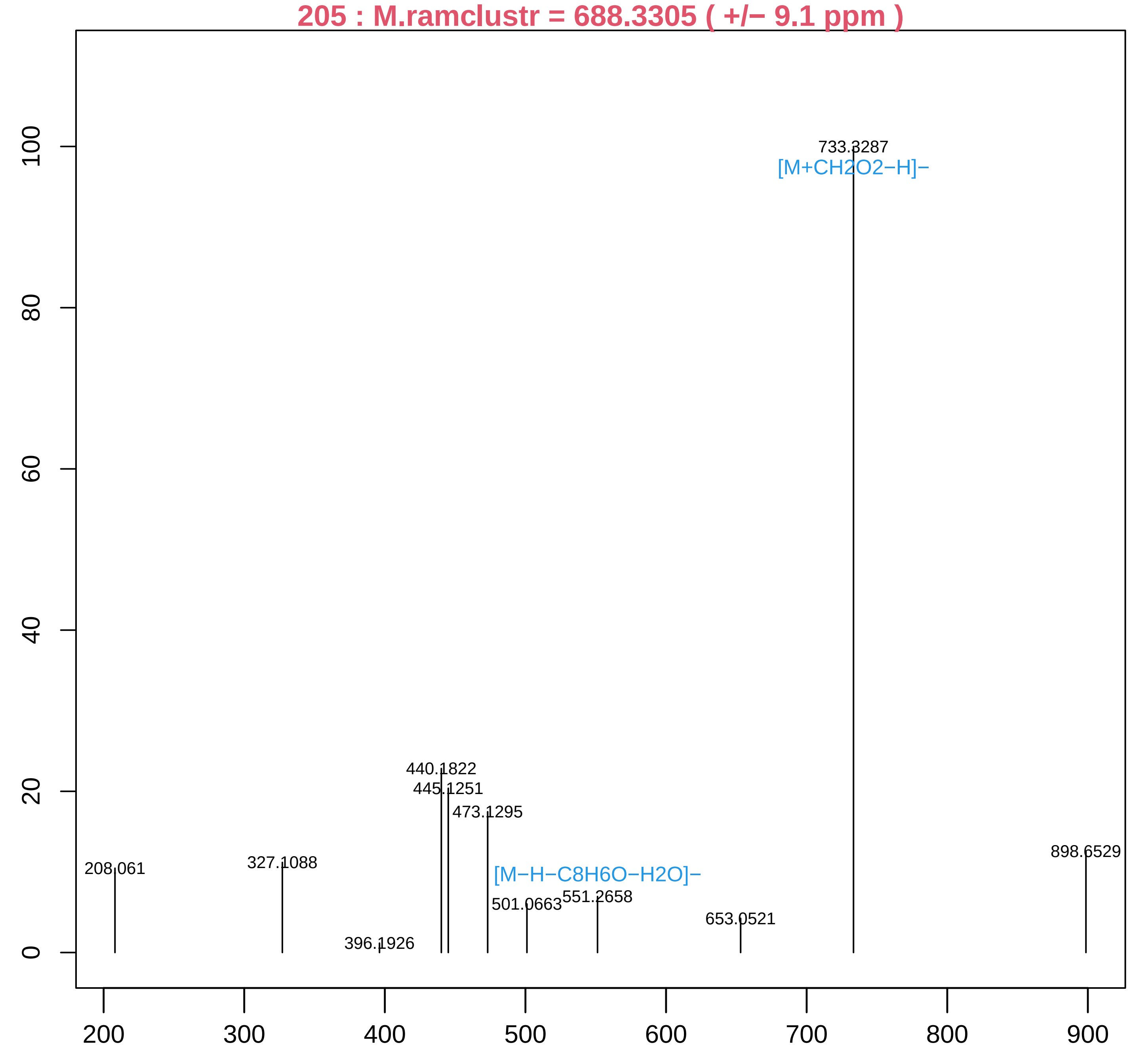

205 : M.ramclustr = 688.3305 (+/− 9.1 ppm)
733.3287
[M+CH2O2−H]−
440.1822
445.1251
473.1295
[M−H−C8H6O−H2O]−
208.061
327.1088
396.1926
501.0663
551.2658
653.0521
898.6529
0
20
40
60
80
100
200
300
400
500
600
700
800
900

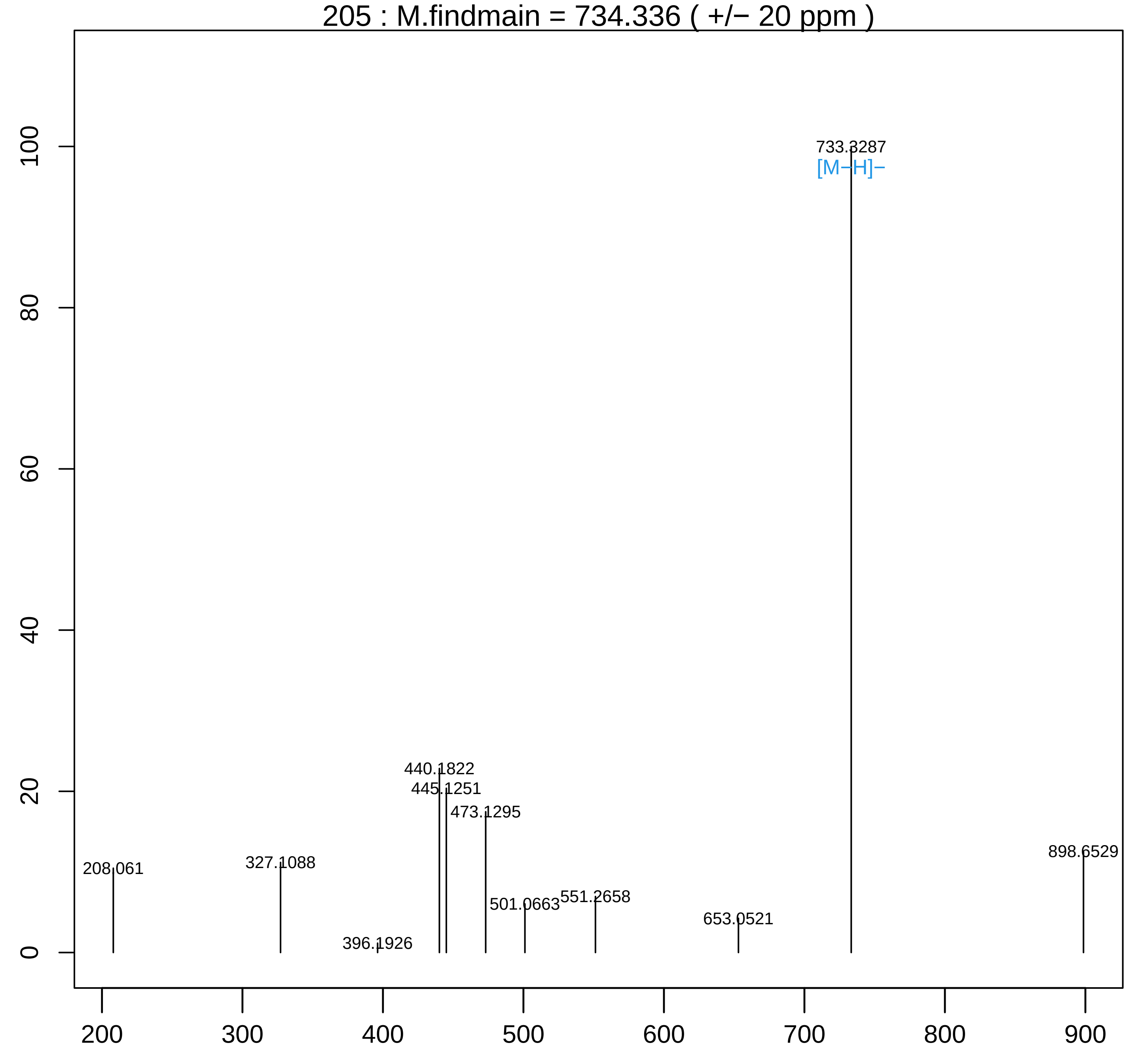

205 : M.findmain = 734.336 (+/− 20 ppm)
733.3287
[M−H]−
440.1822
445.1251
473.1295
208.061
327.1088
396.1926
501.0663
551.2658
653.0521
898.6529
0
20
40
60
80
100
200
300
400
500
600
700
800
900

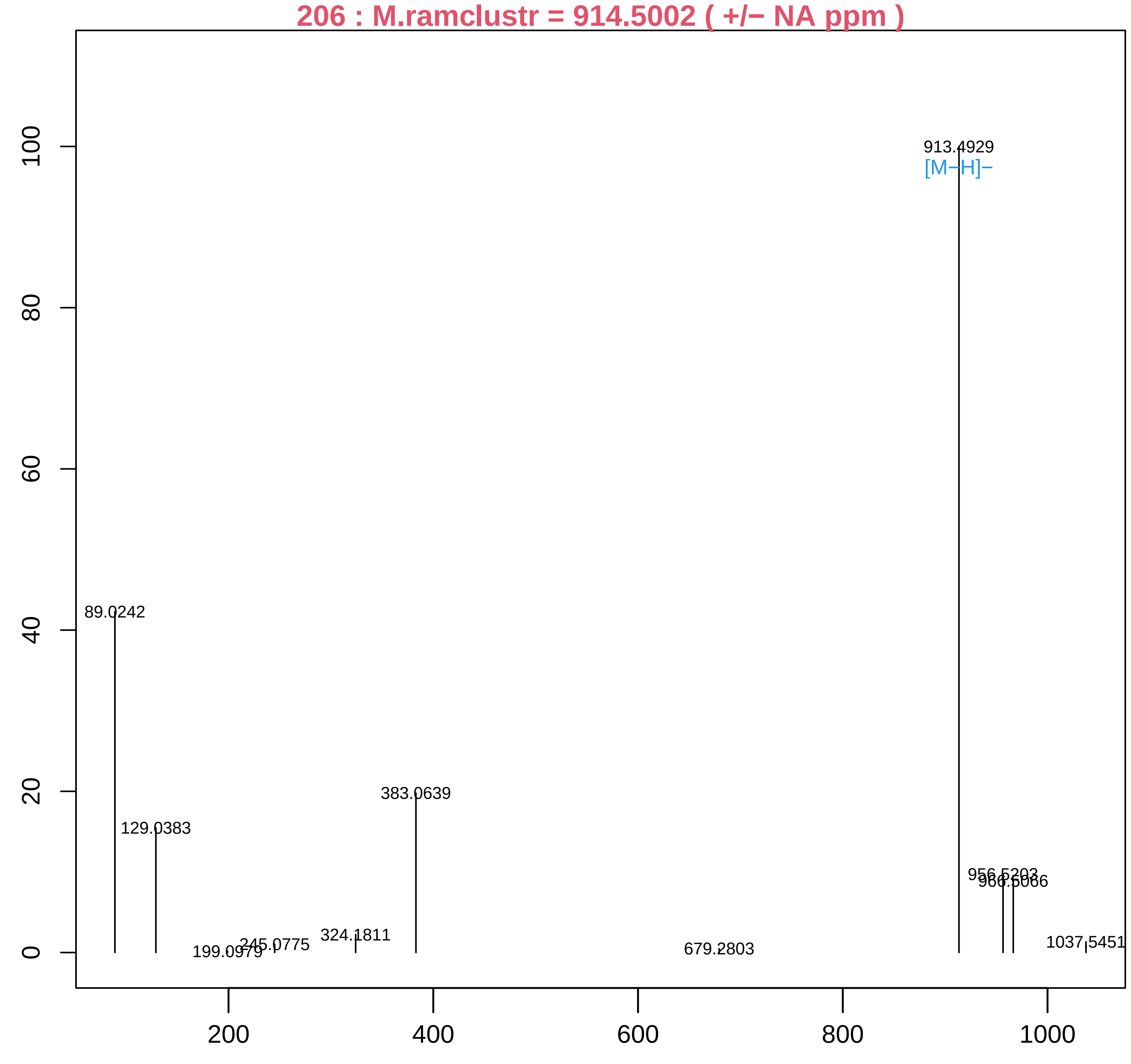

206 : M.ramclustr = 914.5002 (+/− NA ppm)
913.4929
[M−H]−
89.0242
129.0383
199.0979
245.0775
324.1811
383.0639
679.2803
956.5203
966.5066
1037.5451
0
20
40
60
80
100
200
400
600
800
1000

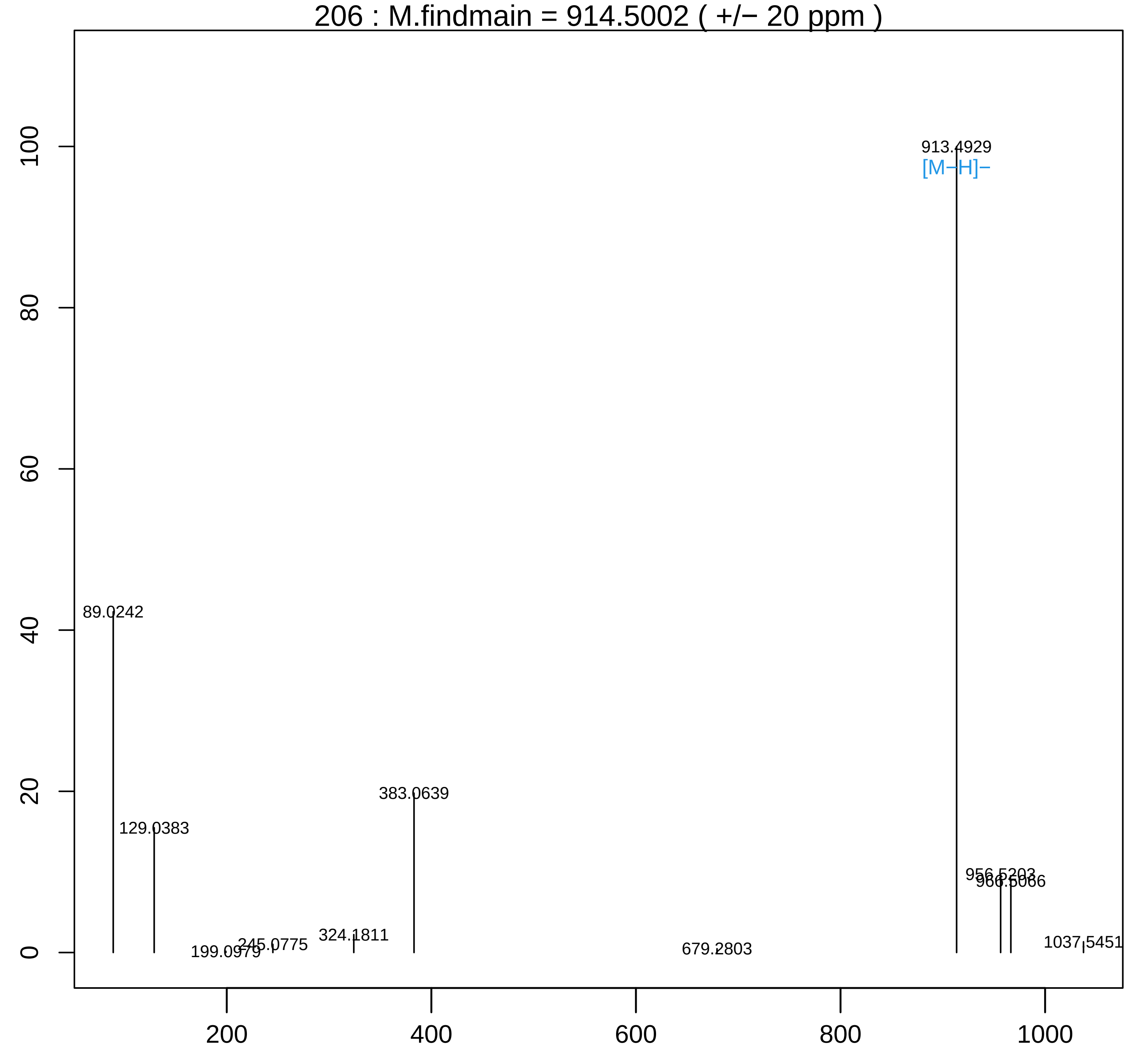

206 : M.findmain = 914.5002 (+/− 20 ppm)
913.4929
[M−H]−
89.0242
129.0383
199.0979
245.0775
324.1811
383.0639
679.2803
956.5203
966.5066
1037.5451
0
20
40
60
80
100
200
400
600
800
1000

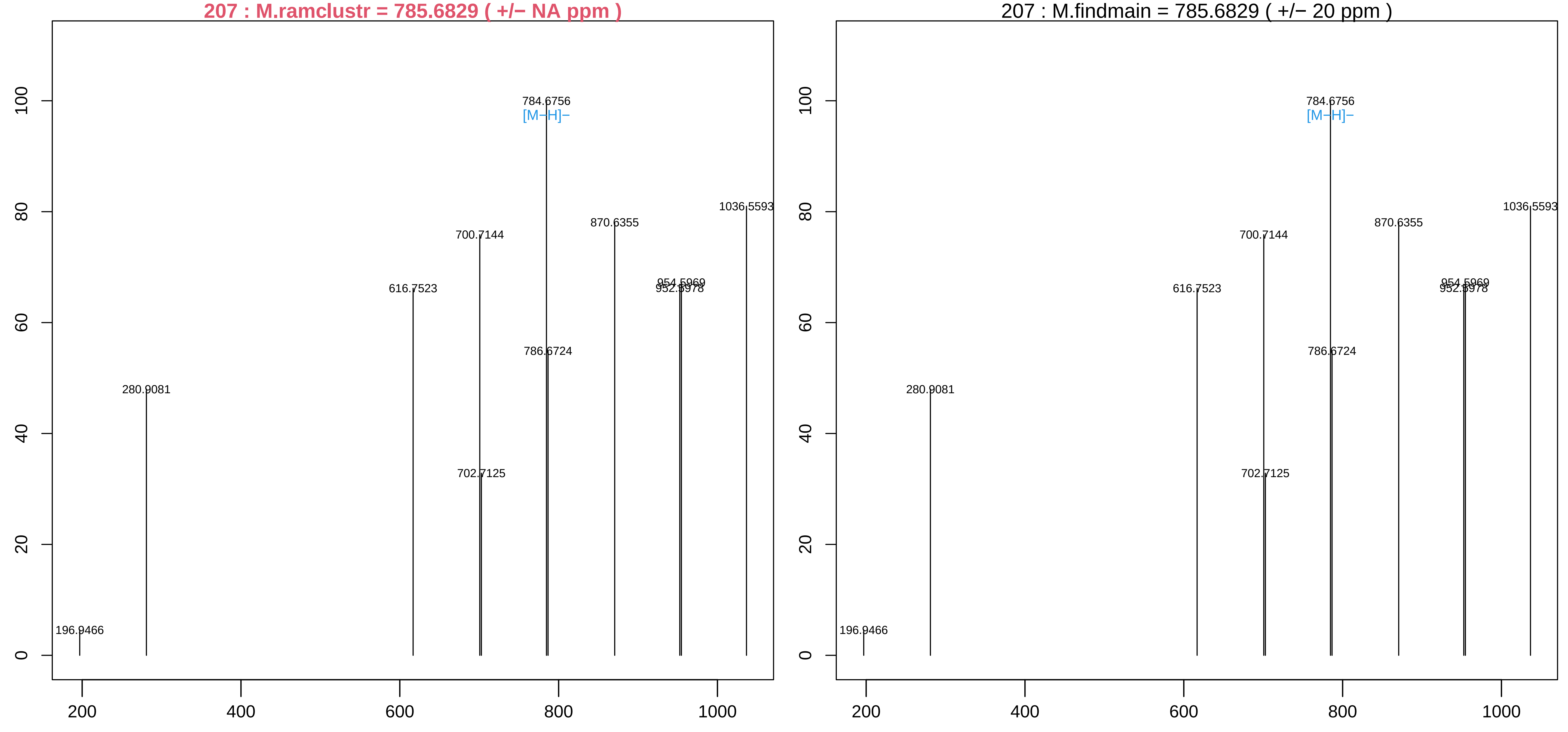
207 : M.ramclustr = 785.6829 (+/− NA ppm)
207 : M.findmain = 785.6829 (+/− 20 ppm)
784.6756
[M−H]−
1036.5593
870.6355
700.7144
616.7523
954.5969
952.5978
786.6724
280.9081
702.7125
196.9466
0
20
40
60
80
100
200
400
600
800
1000

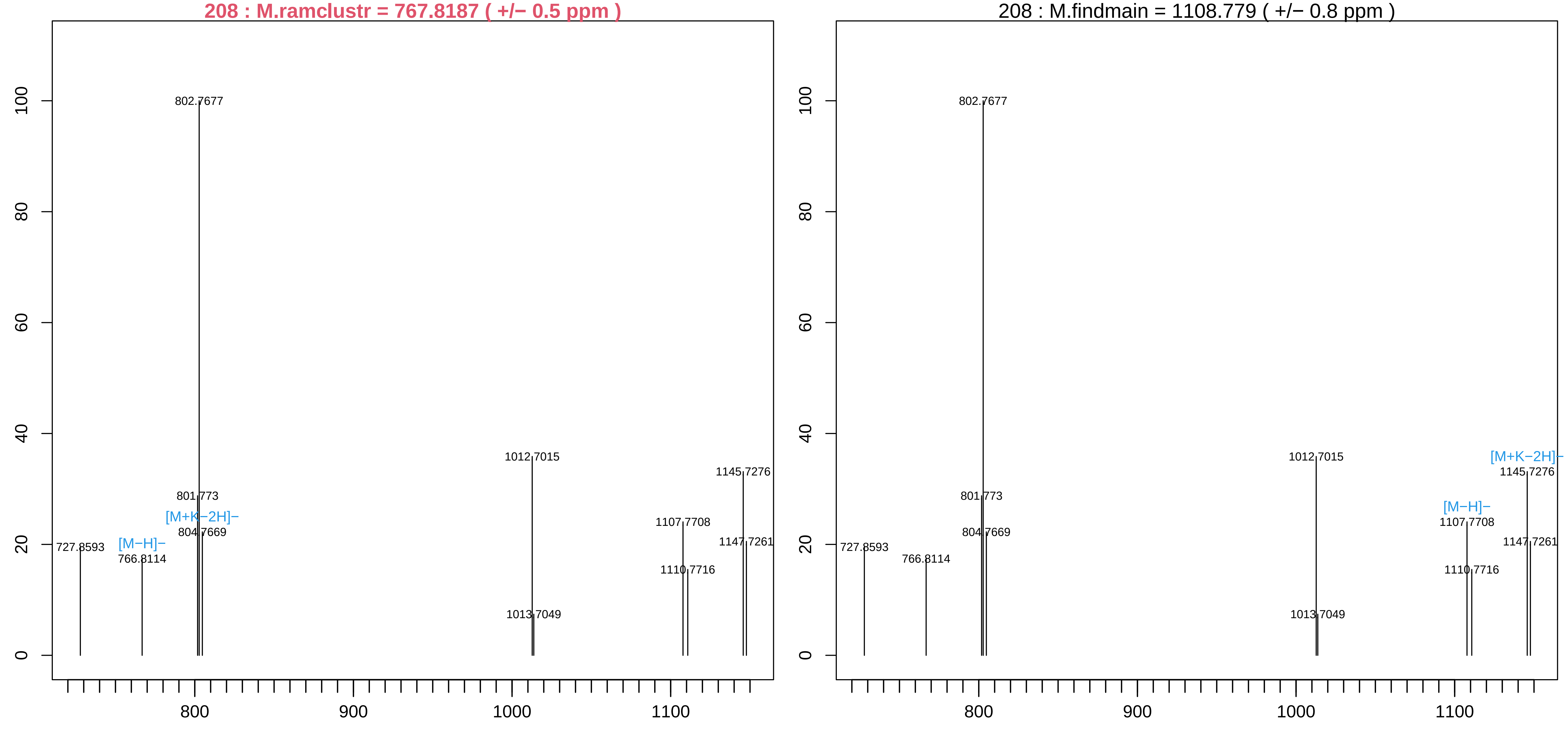

208 : M.ramclustr = 767.8187 (+/− 0.5 ppm)
802.7677
801.773
[M+K−2H]−
804.7669
727.8593
[M−H]−
766.8114
1012.7015
1013.7049
1107.7708
1110.7716
1145.7276
1147.7261
0
20
40
60
80
100
800
900
1000
1100
208 : M.findmain = 1108.779 (+/− 0.8 ppm)
802.7677
801.773
804.7669
727.8593
766.8114
1012.7015
1013.7049
[M−H]−
1107.7708
1110.7716
[M+K−2H]−
1145.7276
1147.7261
0
20
40
60
80
100
800
900
1000
1100

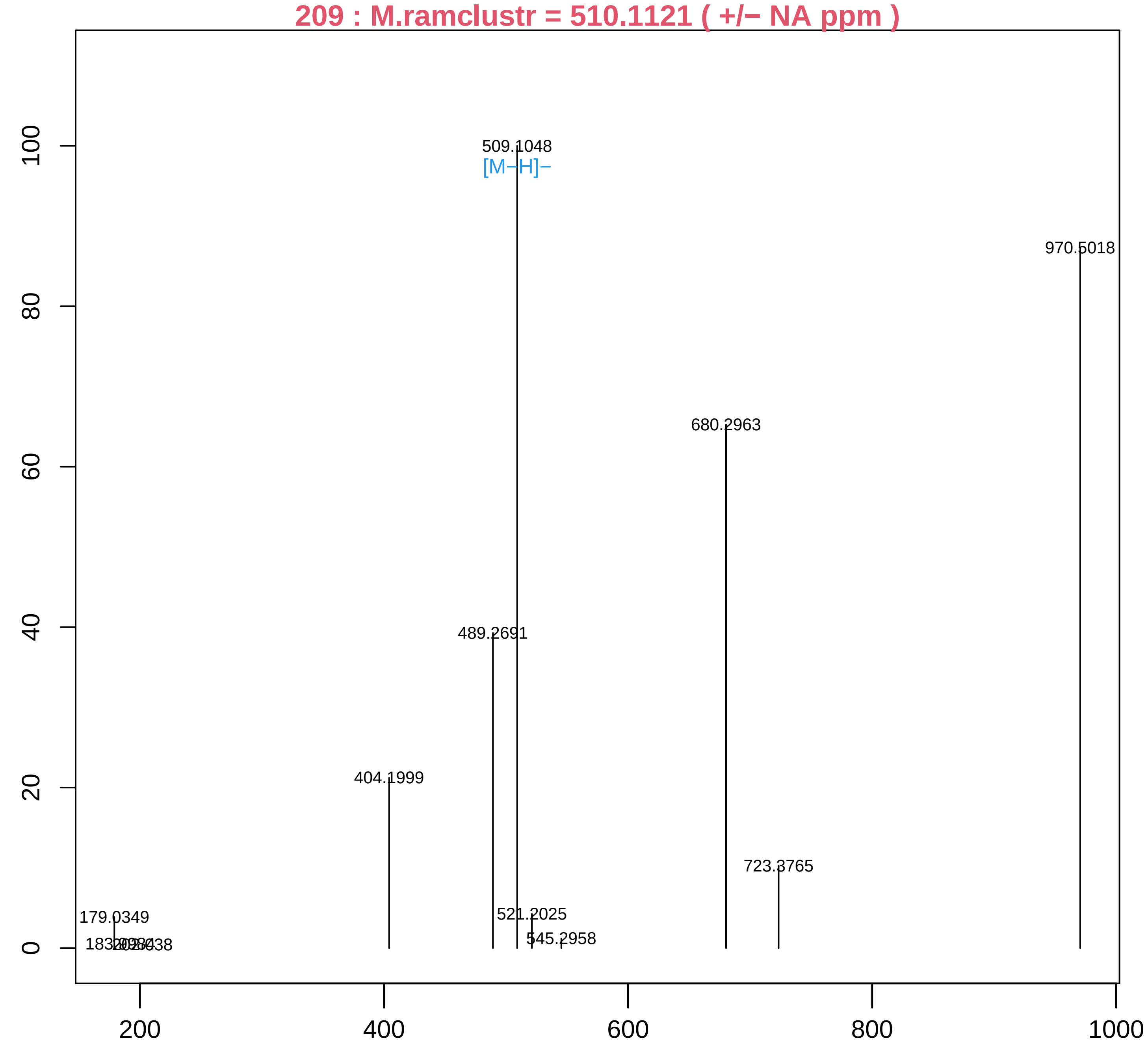
209 : M.ramclustr = 510.1121 (+/− NA ppm)
509.1048
[M−H]−
970.5018
680.2963
489.2691
404.1999
723.3765
179.0349
521.2025
545.2958
0
20
40
60
80
100
200
400
600
800
1000

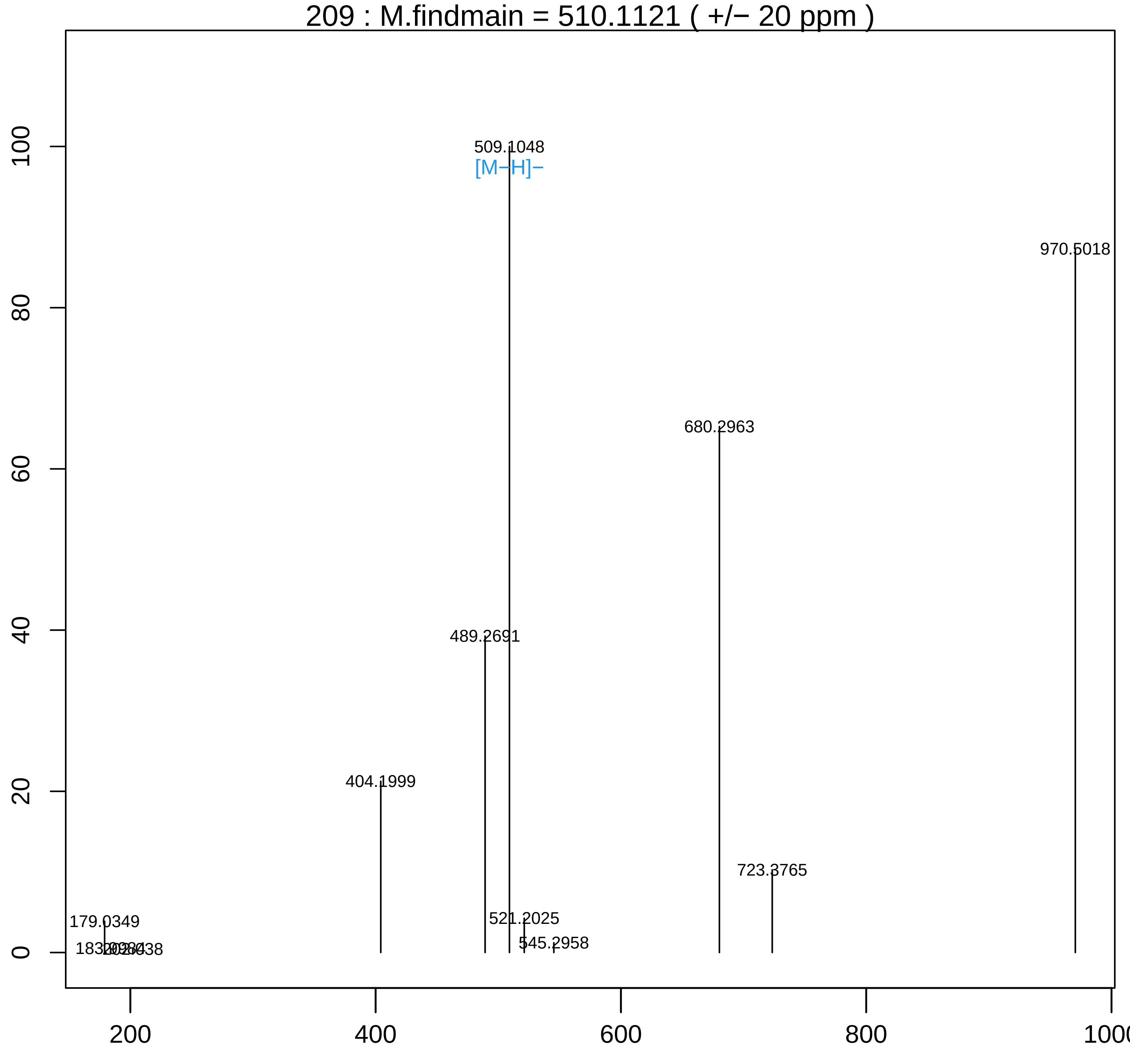
209 : M.findmain = 510.1121 (+/− 20 ppm)
509.1048
[M−H]−
970.5018
680.2963
489.2691
404.1999
723.3765
179.0349
521.2025
545.2958
0
20
40
60
80
100
200
400
600
800
1000

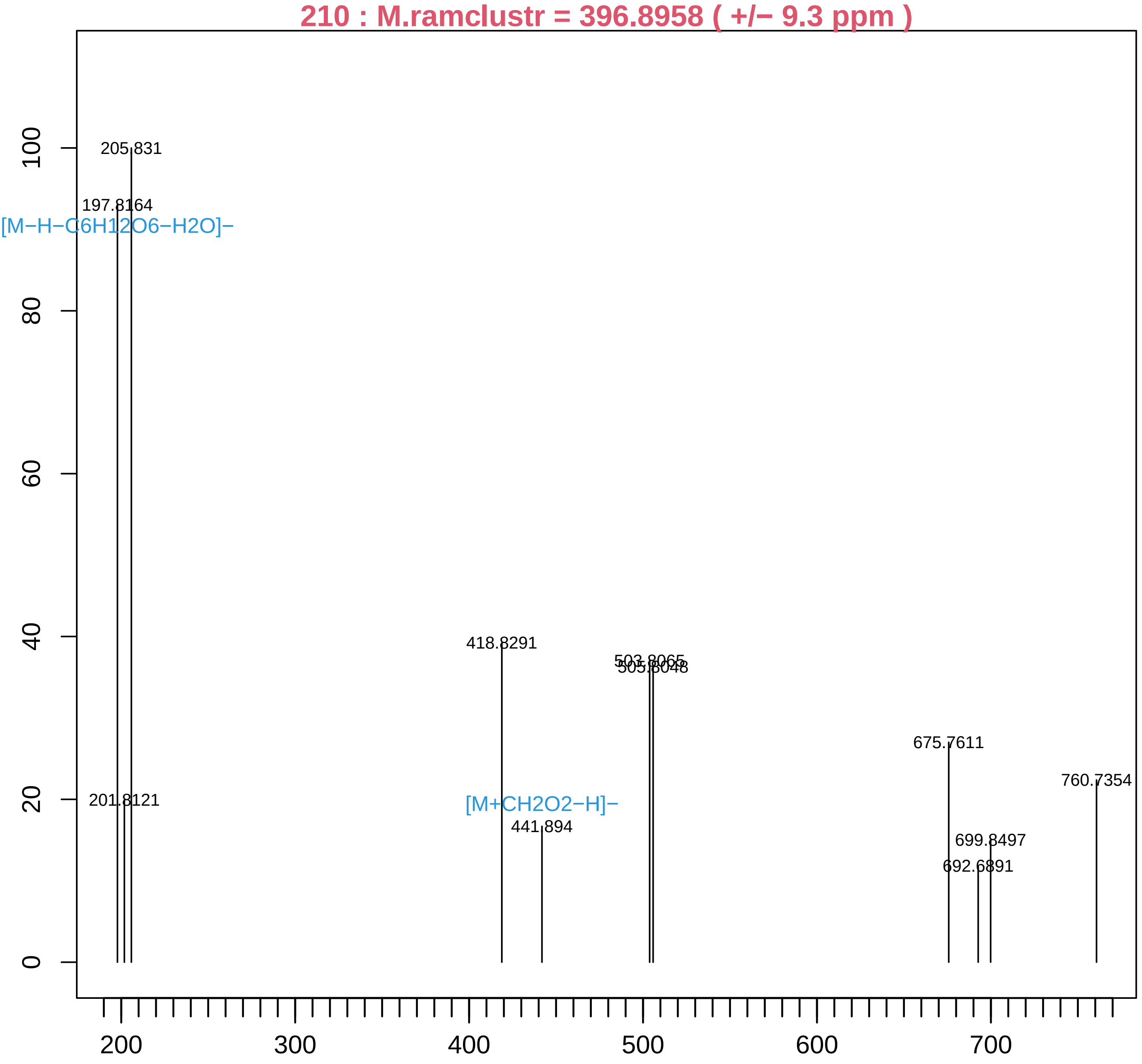
210 : M.ramclustr = 396.8958 (+/− 9.3 ppm)
205.831
197.8164
[M−H−C6H12O6−H2O]−
201.8121
418.8291
[M+CH2O2−H]−
441.894
503.8065
505.8048
675.7611
699.8497
692.6891
760.7354
0
20
40
60
80
100
200
300
400
500
600
700

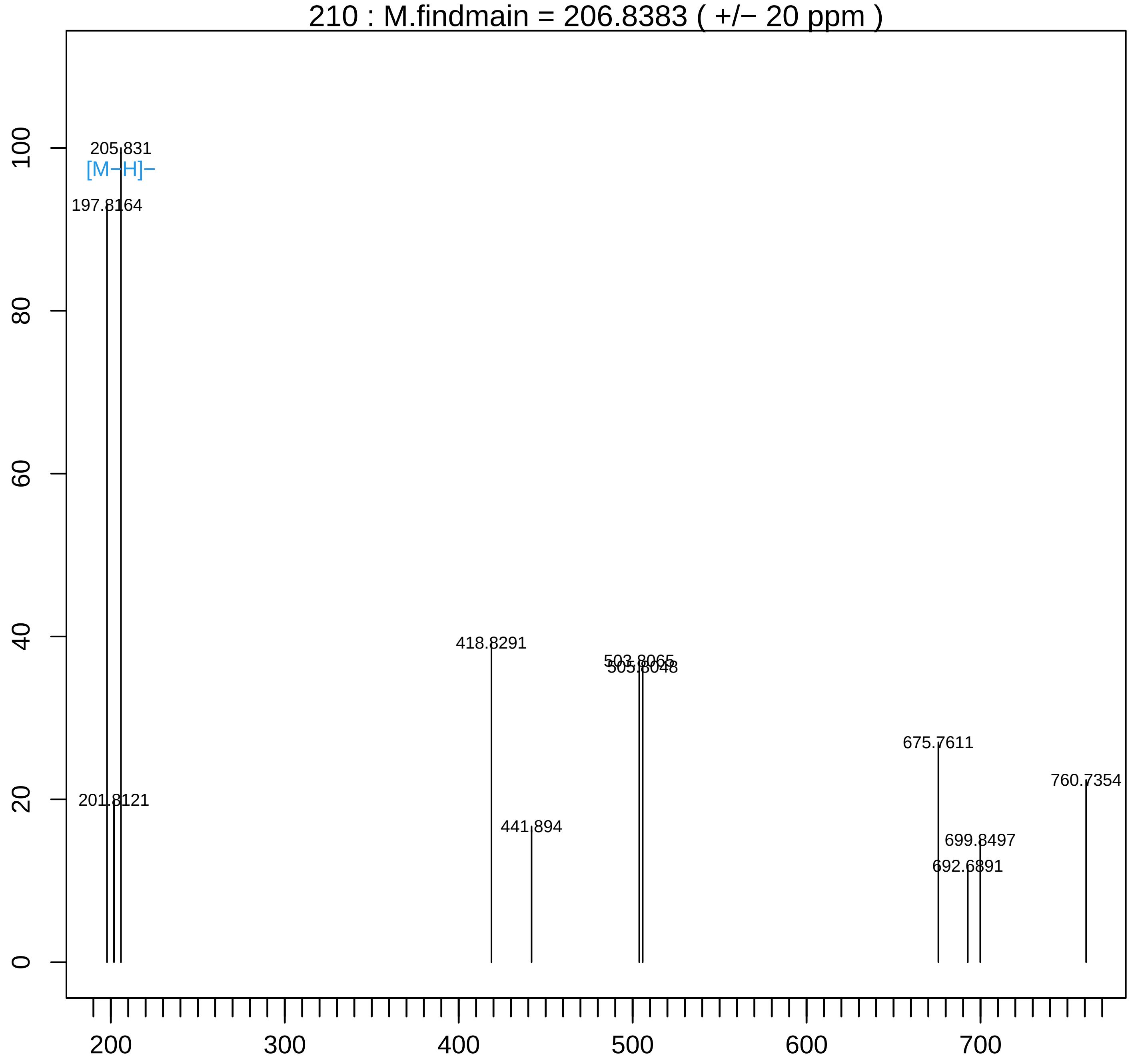
210 : M.findmain = 206.8383 (+/− 20 ppm)
205.831
[M−H]−
197.8164
201.8121
418.8291
441.894
503.8065
505.8048
675.7611
699.8497
692.6891
760.7354
0
20
40
60
80
100
200
300
400
500
600
700

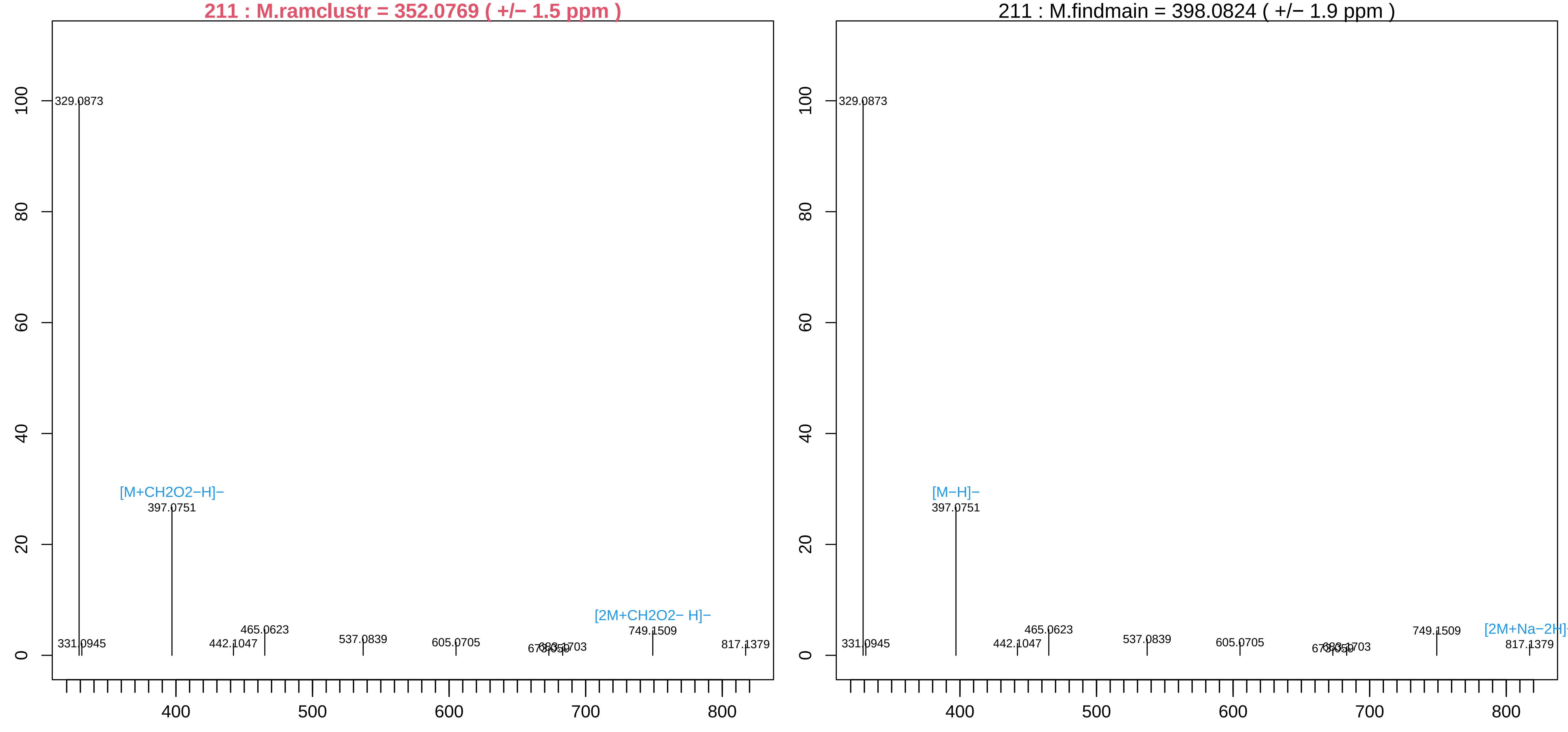
211 : M.ramclustr = 352.0769 (+/− 1.5 ppm)
329.0873
[M+CH2O2−H]−
397.0751
331.0945
442.1047
465.0623
537.0839
605.0705
673.0959
683.1703
[2M+CH2O2− H]−
749.1509
817.1379
211 : M.findmain = 398.0824 (+/− 1.9 ppm)
329.0873
[M−H]−
397.0751
331.0945
442.1047
465.0623
537.0839
605.0705
673.0959
683.1703
749.1509
[2M+Na−2H]−
817.1379
0
20
40
60
80
100
400
500
600
700
800

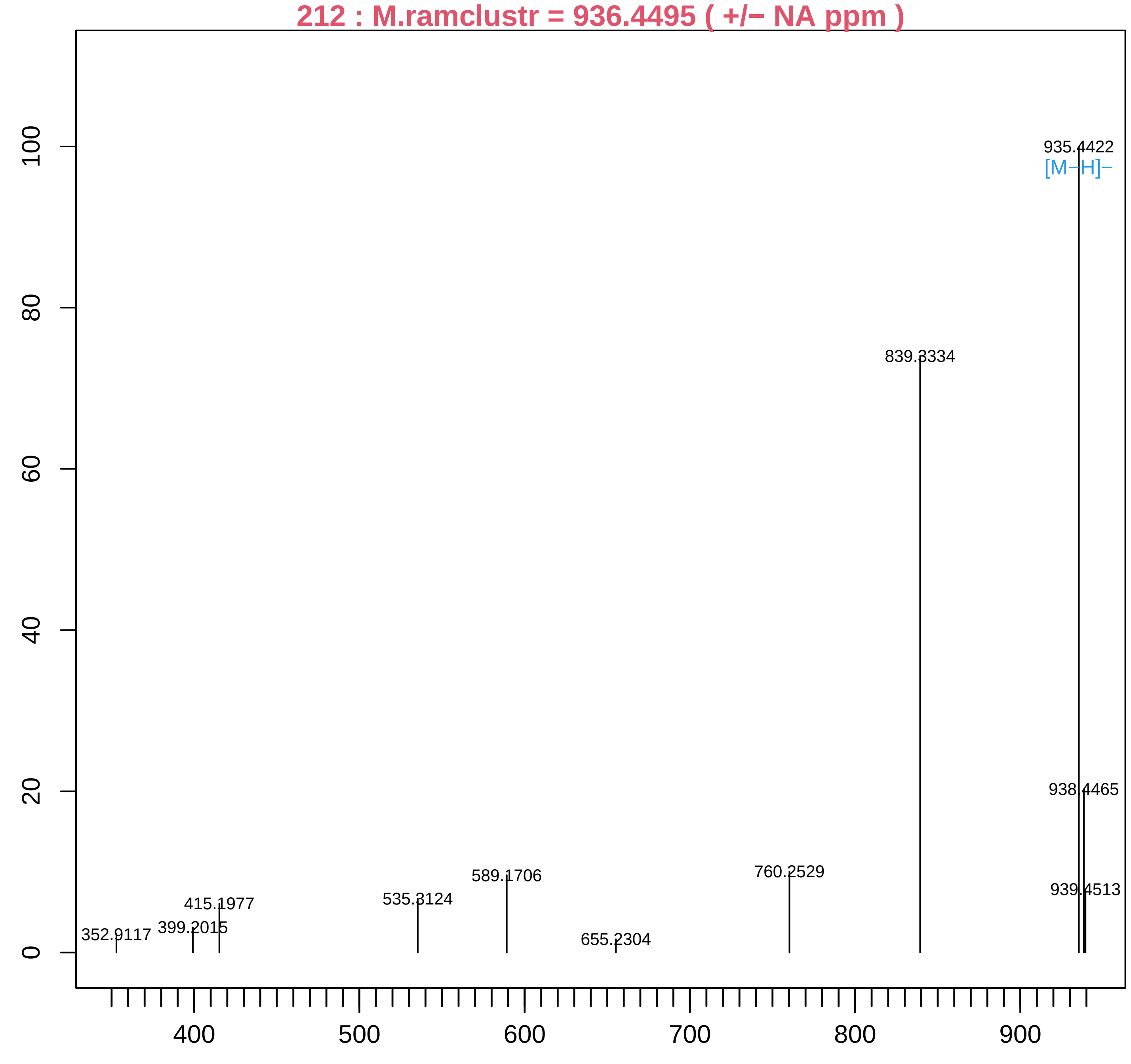
212 : M.ramclustr = 936.4495 (+/− NA ppm)
935.4422
[M−H]−
839.3334
938.4465
939.4513
760.2529
589.1706
535.3124
415.1977
399.2015
352.9117
655.2304
0
20
40
60
80
100
400
500
600
700
800
900

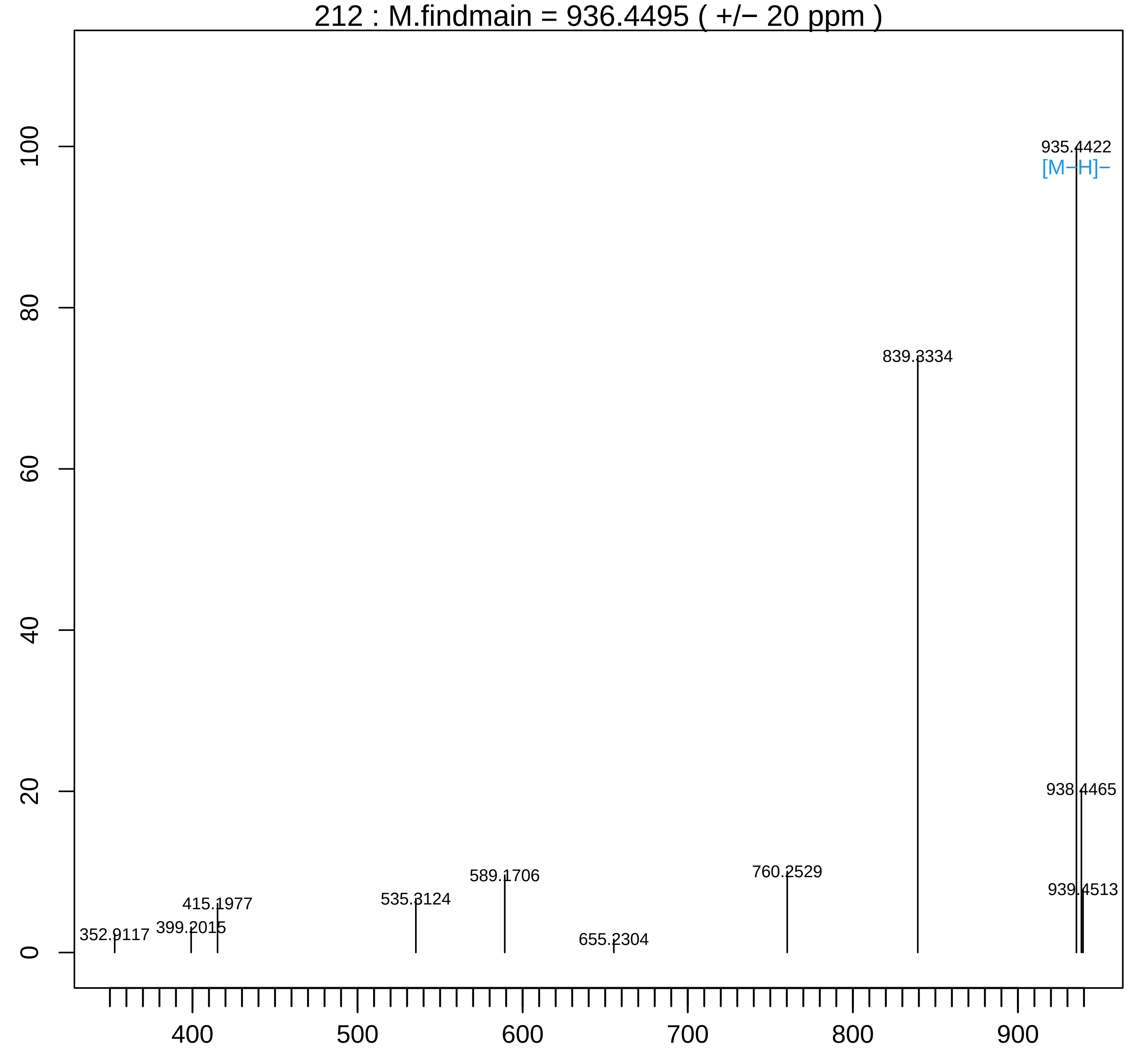
212 : M.findmain = 936.4495 (+/− 20 ppm)
935.4422
[M−H]−
839.3334
938.4465
939.4513
760.2529
589.1706
535.3124
415.1977
399.2015
352.9117
655.2304
0
20
40
60
80
100
400
500
600
700
800
900

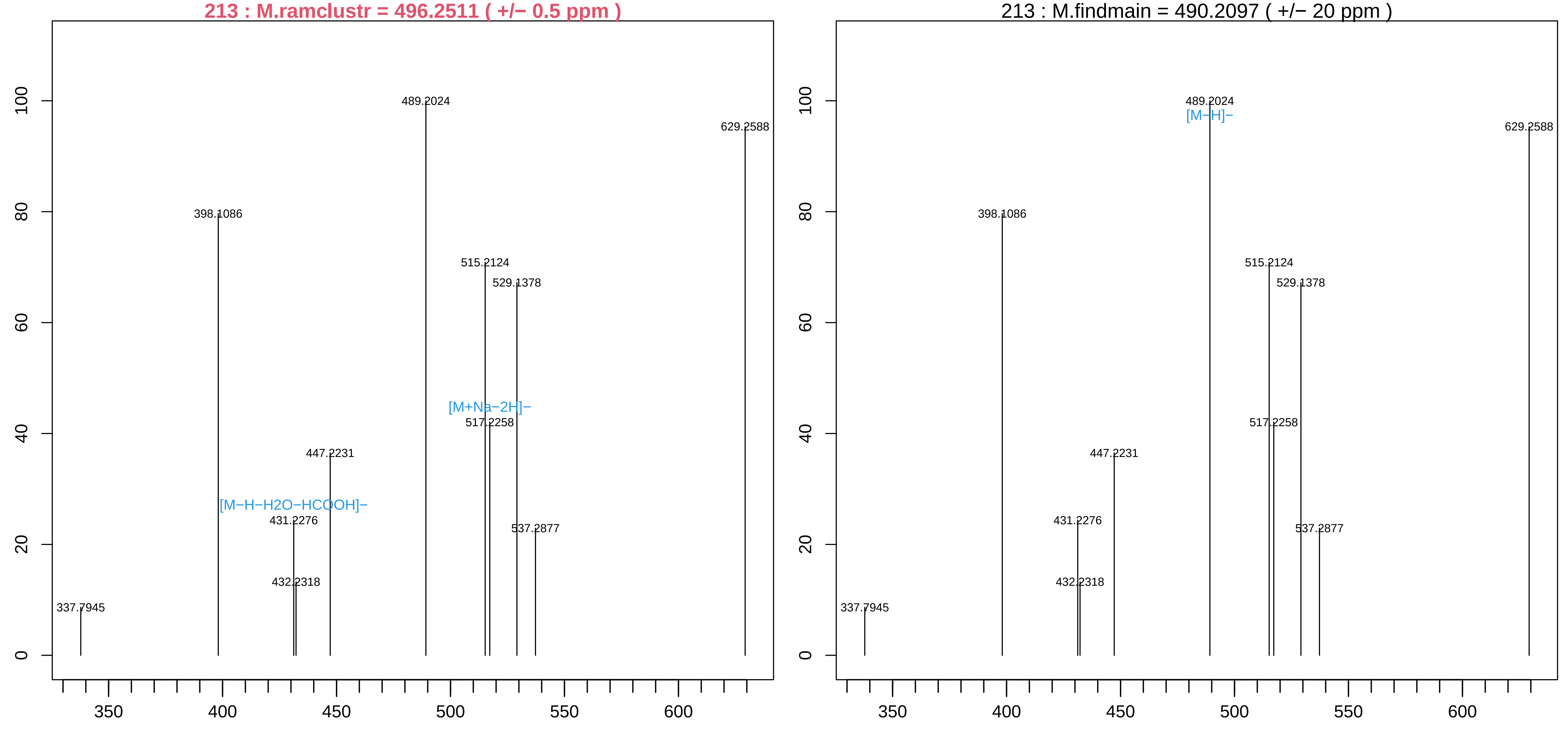

213 : M.ramclustr = 496.2511 (+/− 0.5 ppm)
213 : M.findmain = 490.2097 (+/− 20 ppm)
489.2024
[M−H]−
629.2588
398.1086
515.2124
529.1378
[M+Na−2H]−
517.2258
447.2231
[M−H−H2O−HCOOH]−
431.2276
537.2877
432.2318
337.7945
0
20
40
60
80
100
350
400
450
500
550
600

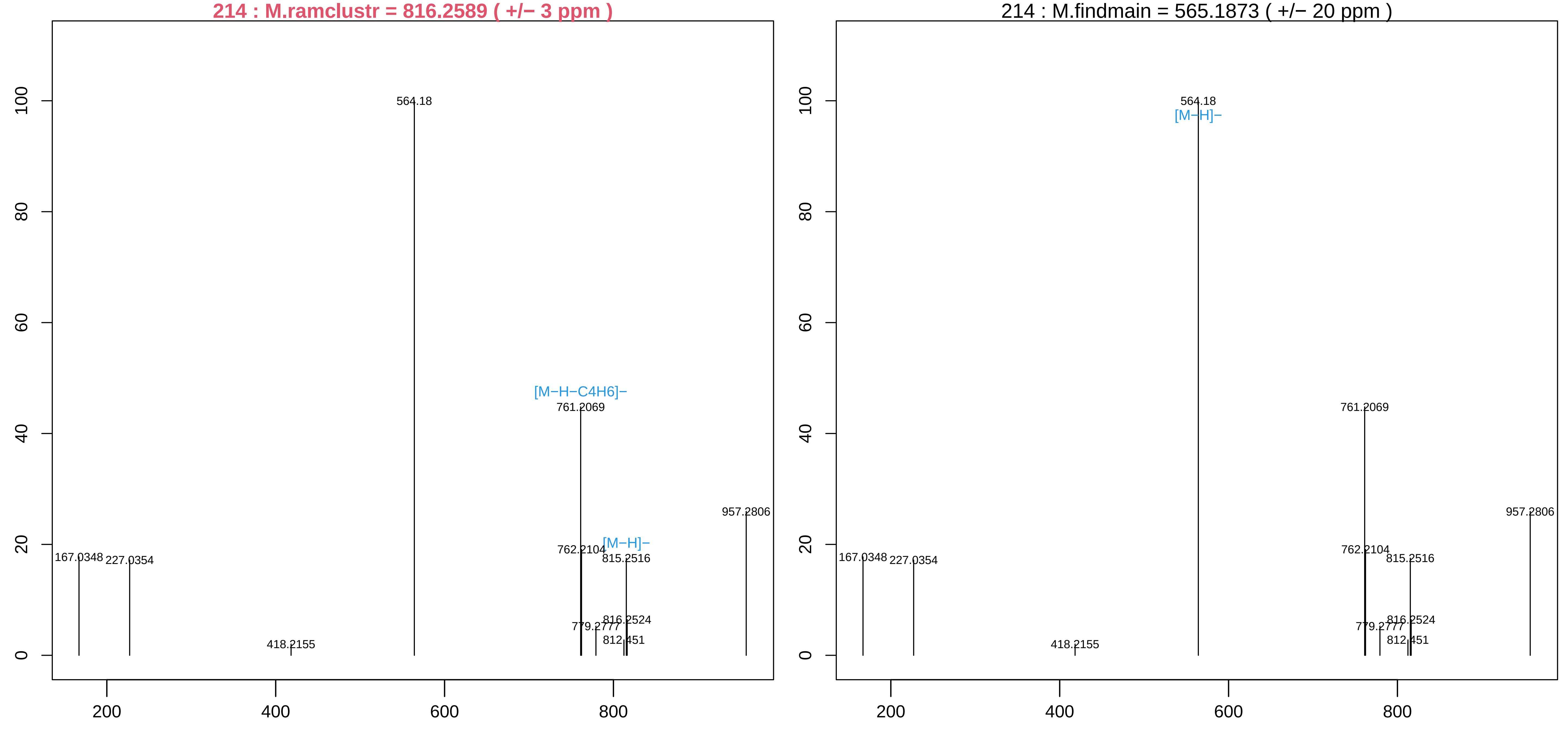
214 : M.ramclustr = 816.2589 (+/− 3 ppm)
564.18
[M−H−C4H6]−
761.2069
957.2806
762.2104
[M−H]−
815.2516
167.0348
227.0354
816.2524
779.2777
812.451
418.2155
214 : M.findmain = 565.1873 (+/− 20 ppm)
564.18
[M−H]−
761.2069
957.2806
762.2104
815.2516
167.0348
227.0354
816.2524
779.2777
812.451
418.2155
0
20
40
60
80
100
200
400
600
800

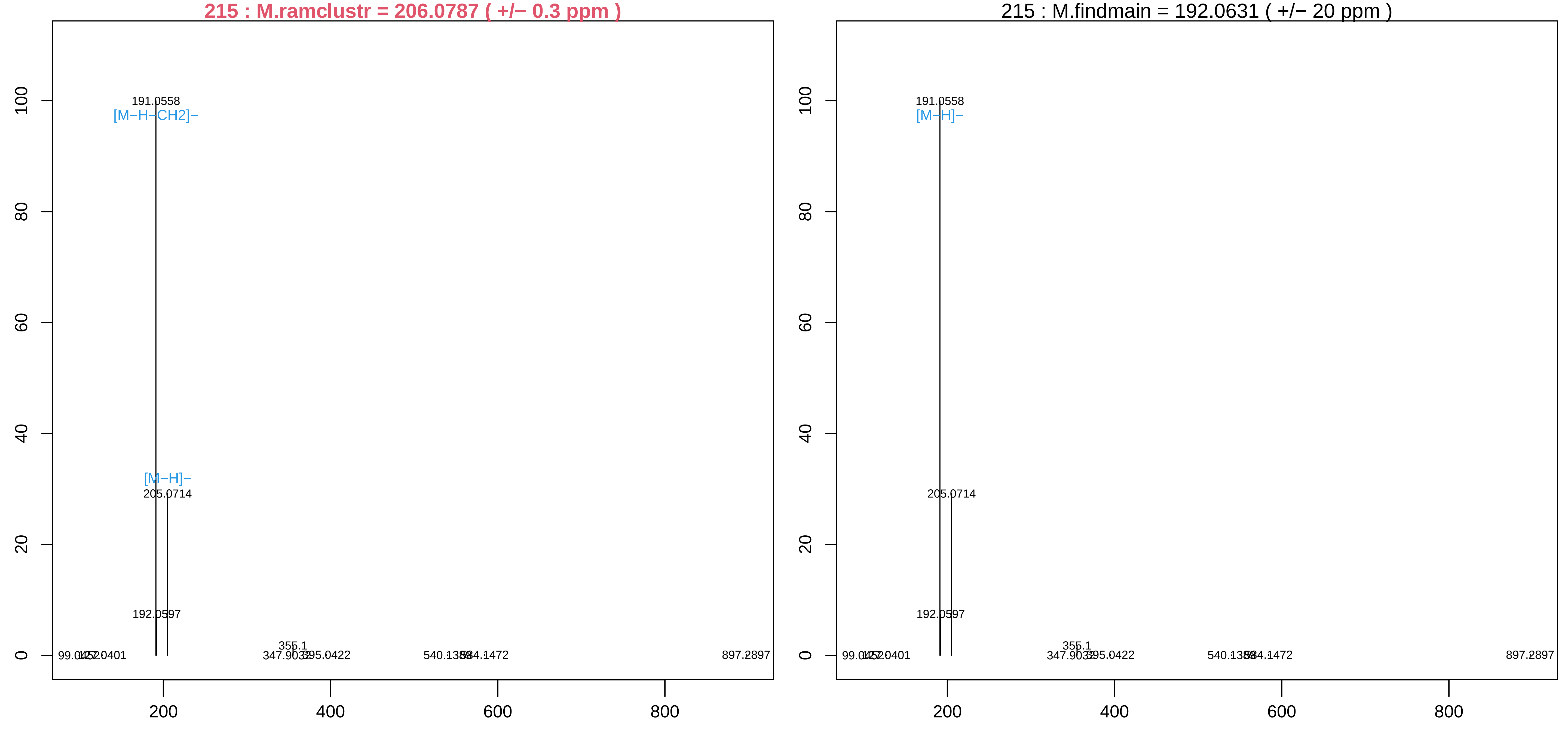

215 : M.ramclustr = 206.0787 (+/− 0.3 ppm)
191.0558
[M−H−CH2]−
[M−H]−
205.0714
192.0597
355.1
347.9032
395.0422
540.1358
584.1472
897.2897
215 : M.findmain = 192.0631 (+/− 20 ppm)
191.0558
[M−H]−
205.0714
192.0597
355.1
347.9032
395.0422
540.1358
584.1472
897.2897

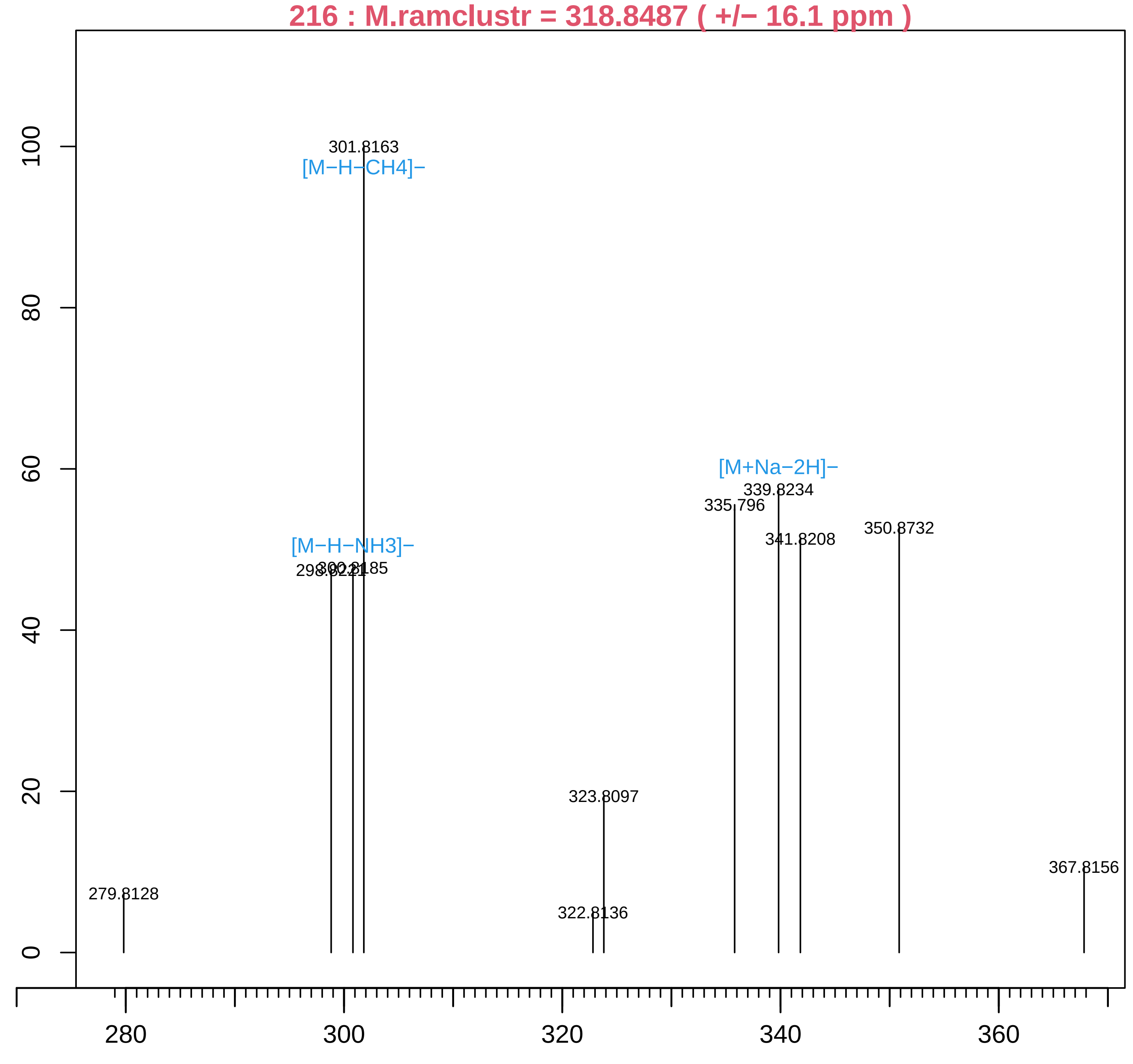
216 : M.ramclustr = 318.8487 (+/− 16.1 ppm)
301.8163
[M−H−CH4]−
[M−H−NH3]−
298.8221
300.8185
[M+Na−2H]−
339.8234
335.796
341.8208
350.8732
323.8097
322.8136
279.8128
367.8156
0
20
40
60
80
100
280
300
320
340
360

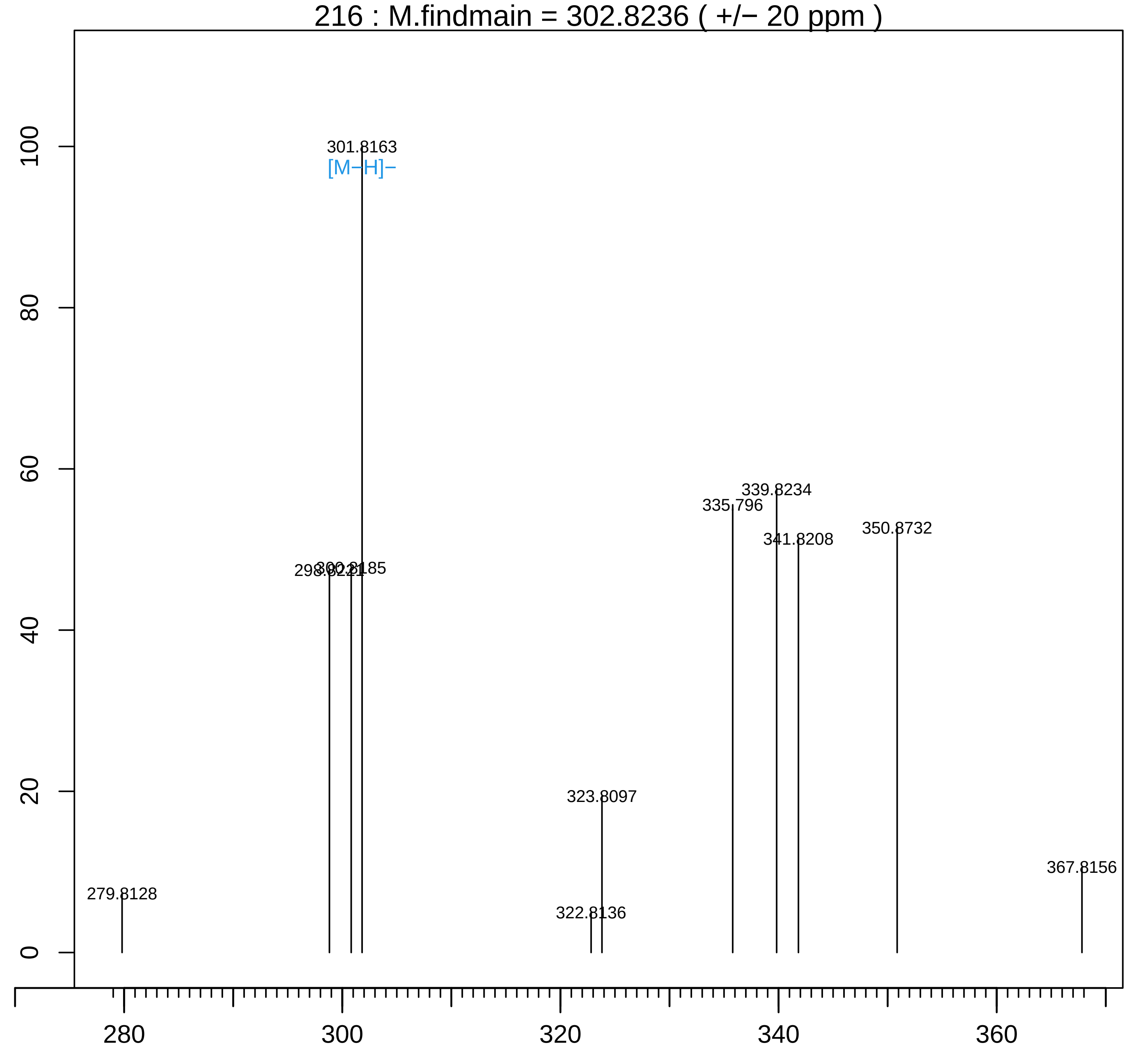
216 : M.findmain = 302.8236 (+/− 20 ppm)
301.8163
[M−H]−
298.8221
300.8185
339.8234
335.796
341.8208
350.8732
323.8097
322.8136
279.8128
367.8156
0
20
40
60
80
100
280
300
320
340
360

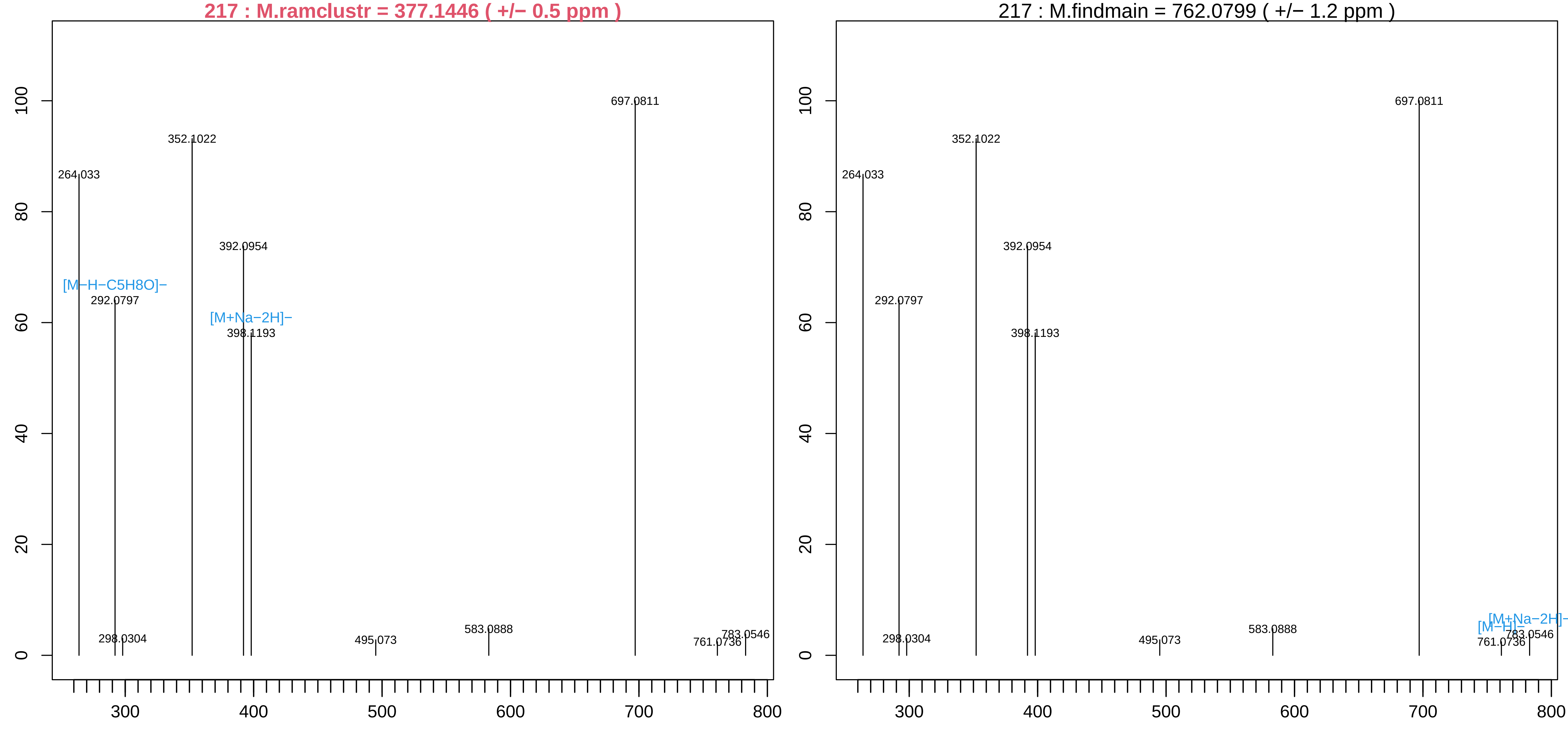

217 : M.ramclustr = 377.1446 (+/− 0.5 ppm)
264.033
352.1022
392.0954
[M−H−C5H8O]−
292.0797
[M+Na−2H]−
398.1193
697.0811
298.0304
495.073
583.0888
761.0736
783.0546
217 : M.findmain = 762.0799 (+/− 1.2 ppm)
264.033
352.1022
392.0954
292.0797
398.1193
697.0811
298.0304
495.073
583.0888
[M+Na−2H]−
[M−H]−
761.0736
783.0546
0
20
40
60
80
100
300
400
500
600
700
800

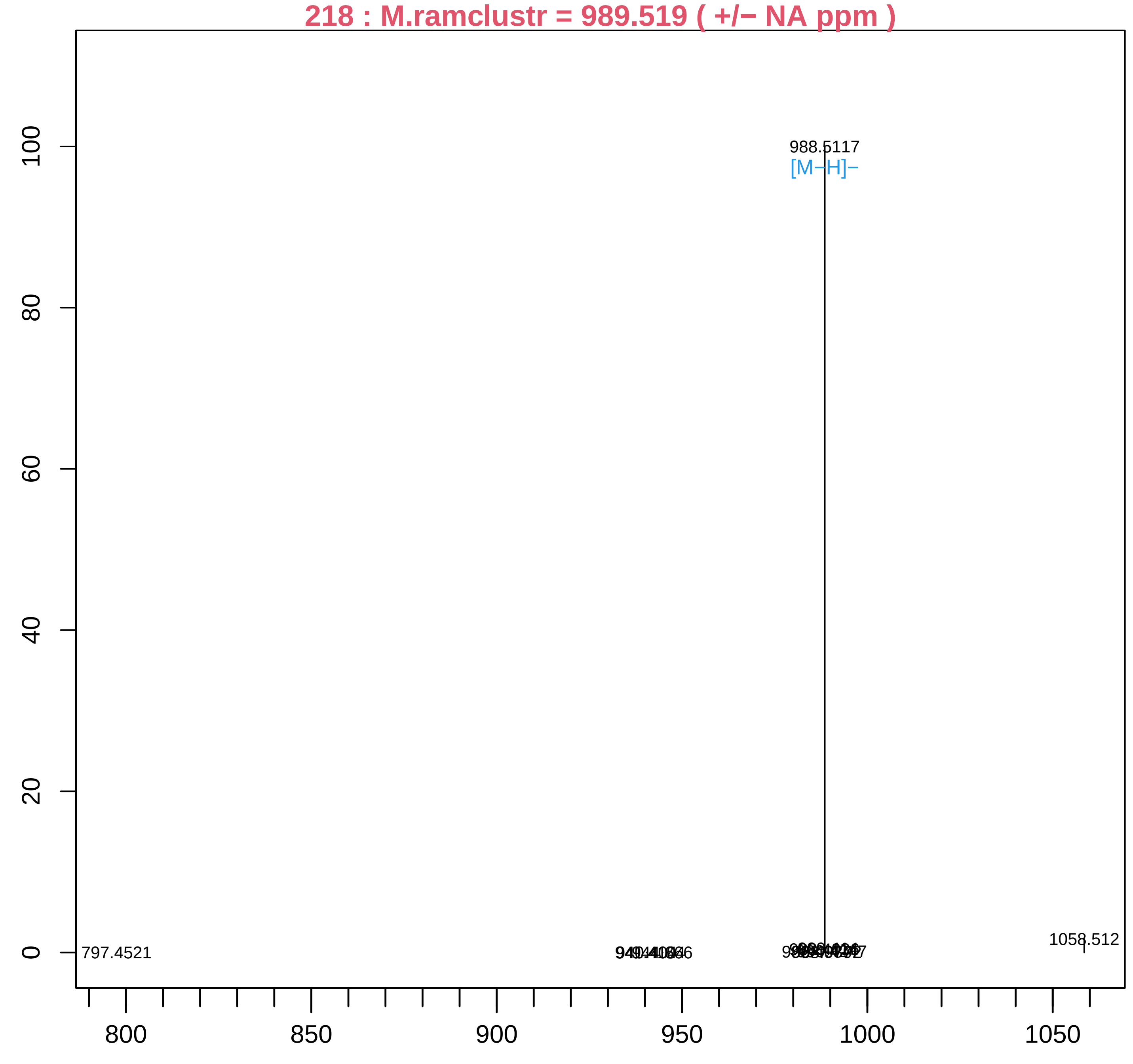

218 : M.ramclustr = 989.519 (+/− NA ppm)
988.5117
[M−H]−
797.4521
1058.512

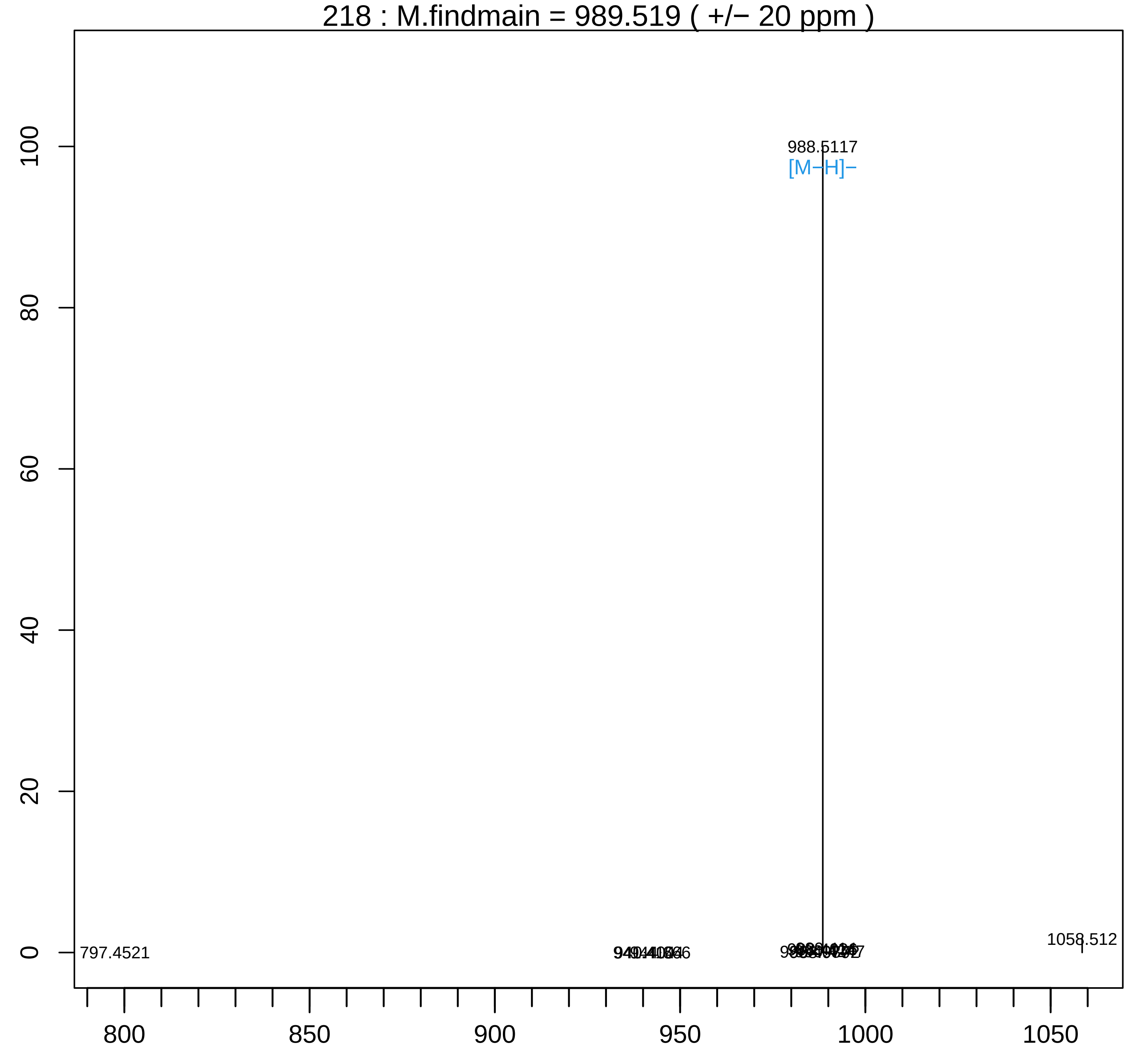

218 : M.findmain = 989.519 (+/− 20 ppm)
988.5117
[M−H]−
797.4521
1058.512

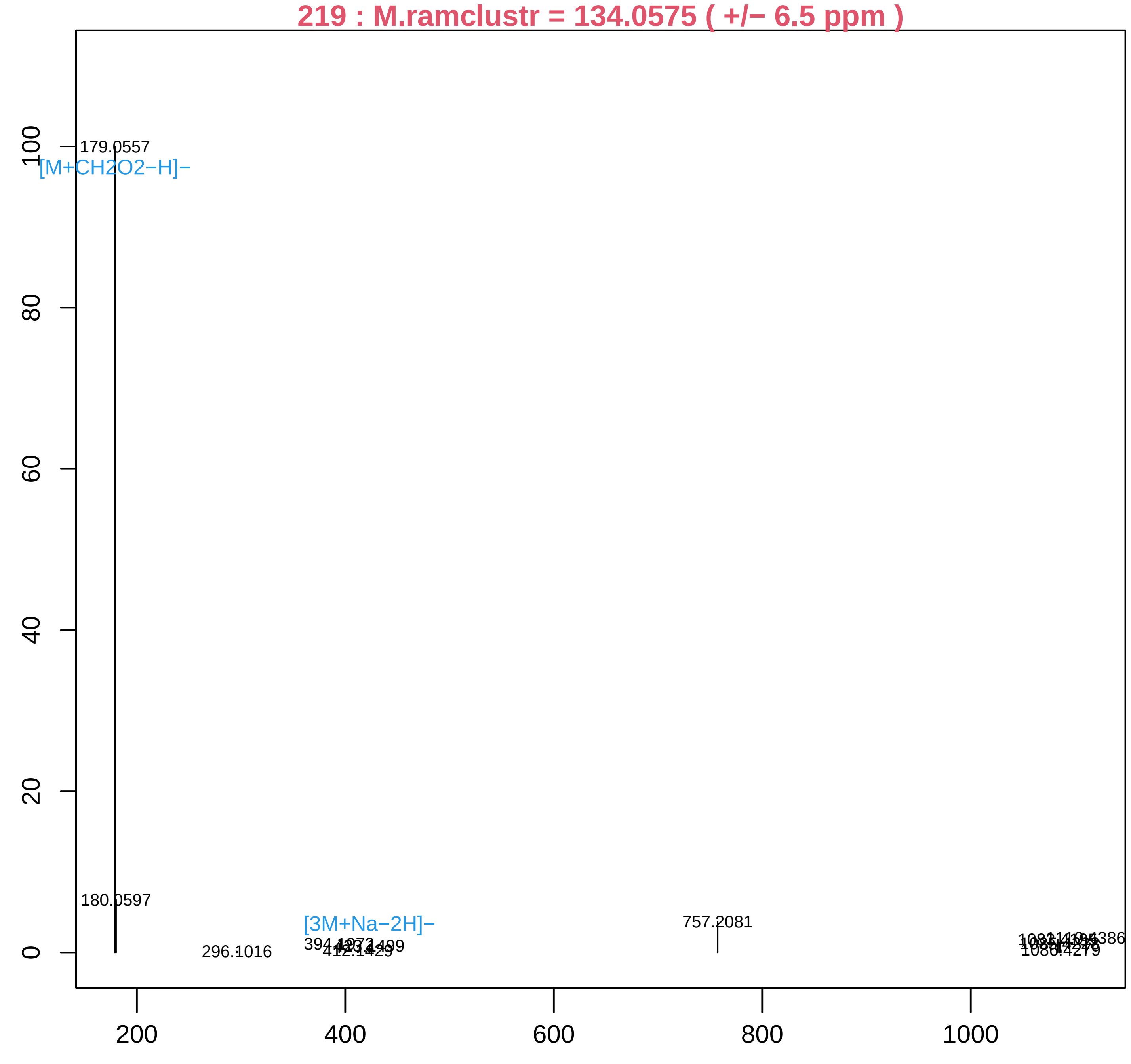

219 : M.ramclustr = 134.0575 (+/− 6.5 ppm)
179.0557
[M+CH2O2−H]−
180.0597
296.1016
[3M+Na−2H]−
412.1429
757.2081
1080.4279
0
20
40
60
80
100
200
400
600
800
1000

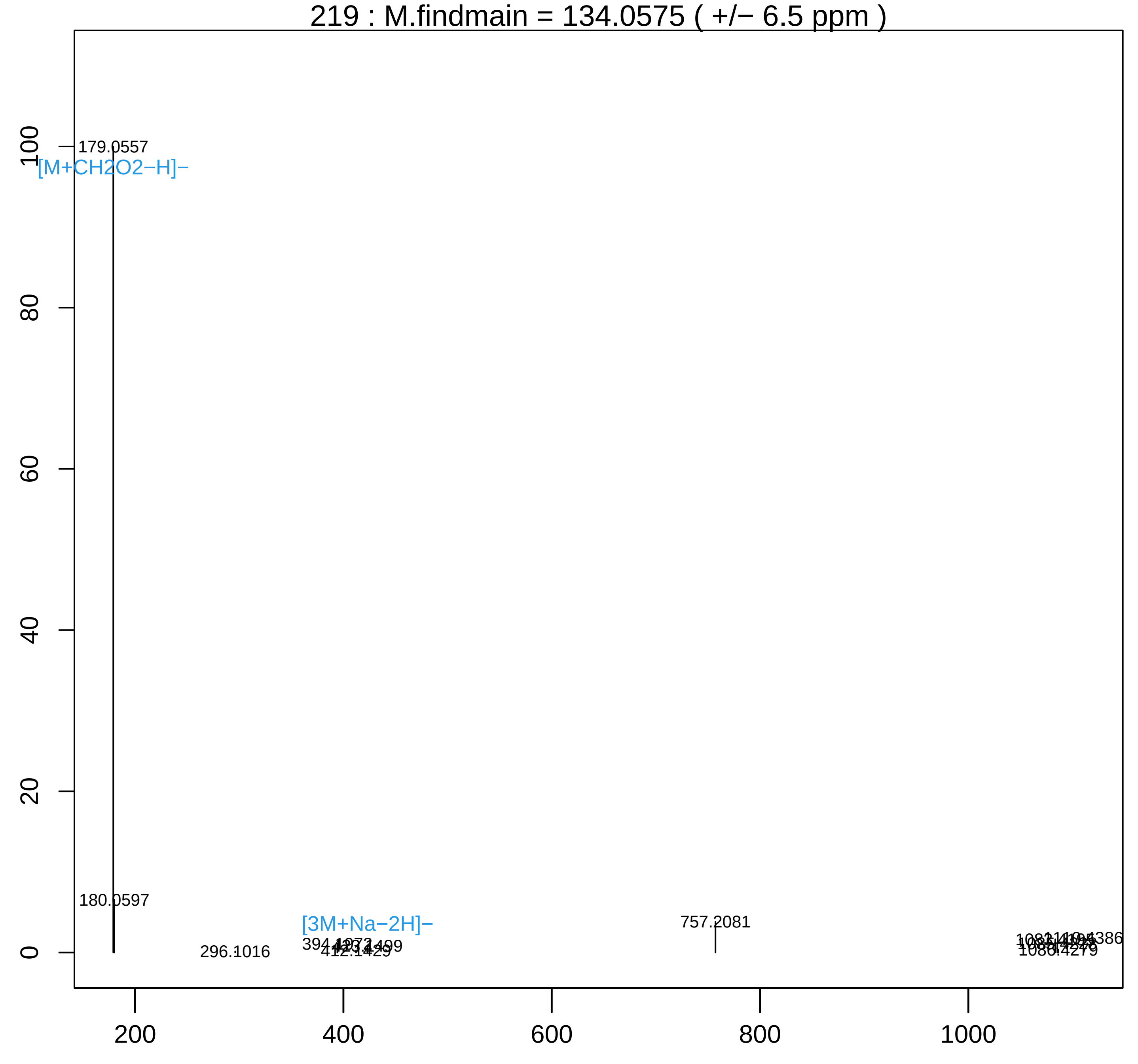

219 : M.findmain = 134.0575 (+/− 6.5 ppm)
179.0557
[M+CH2O2−H]−
180.0597
296.1016
[3M+Na−2H]−
412.1429
757.2081
1080.4279
0
20
40
60
80
100
200
400
600
800
1000

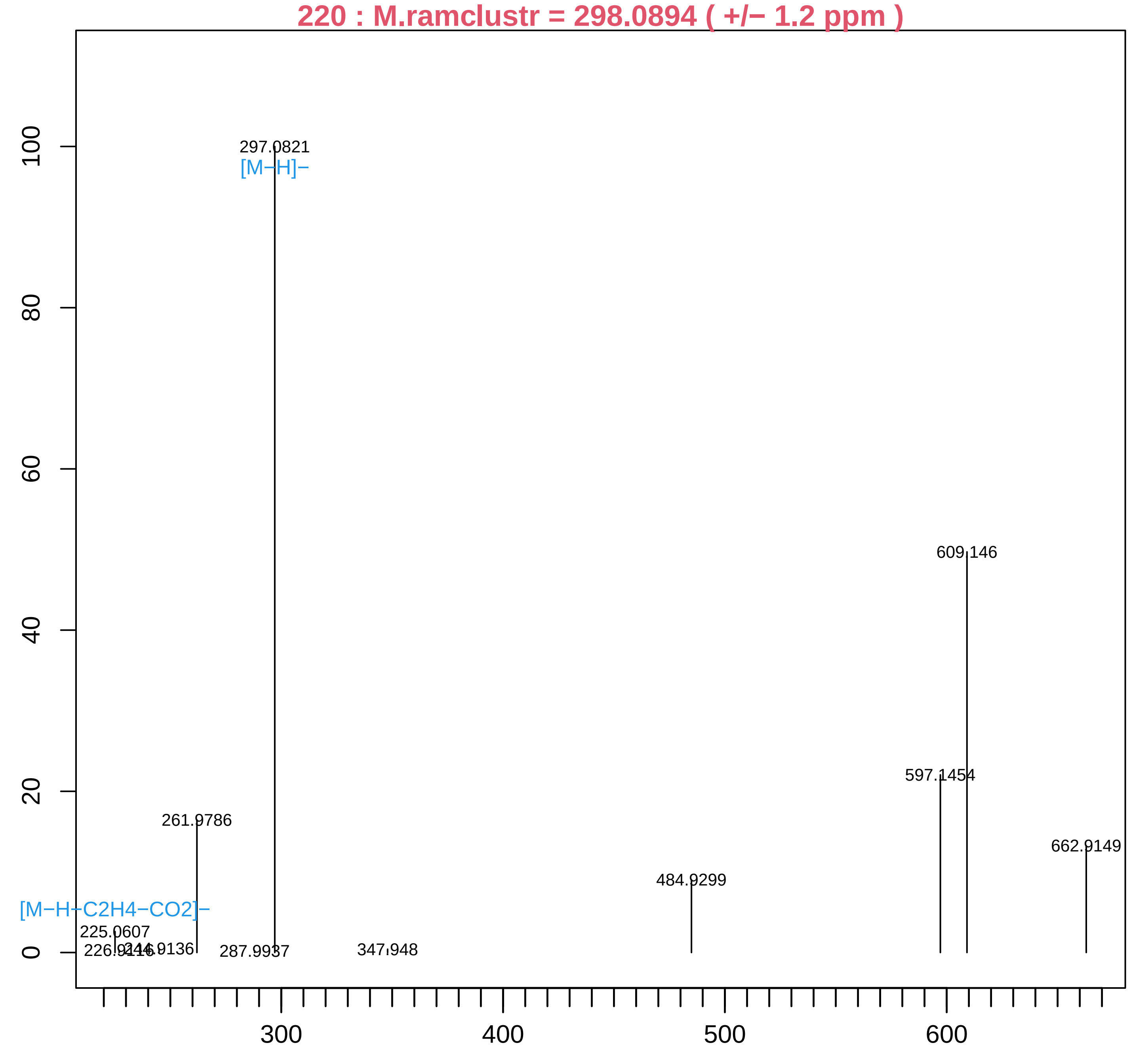

220 : M.ramclustr = 298.0894 (+/− 1.2 ppm)
297.0821
[M−H]−
609.146
597.1454
261.9786
662.9149
484.9299
[M−H−C2H4−CO2]−
225.0607
226.9116
244.9136
287.9937
347.948
0
20
40
60
80
100
300
400
500
600

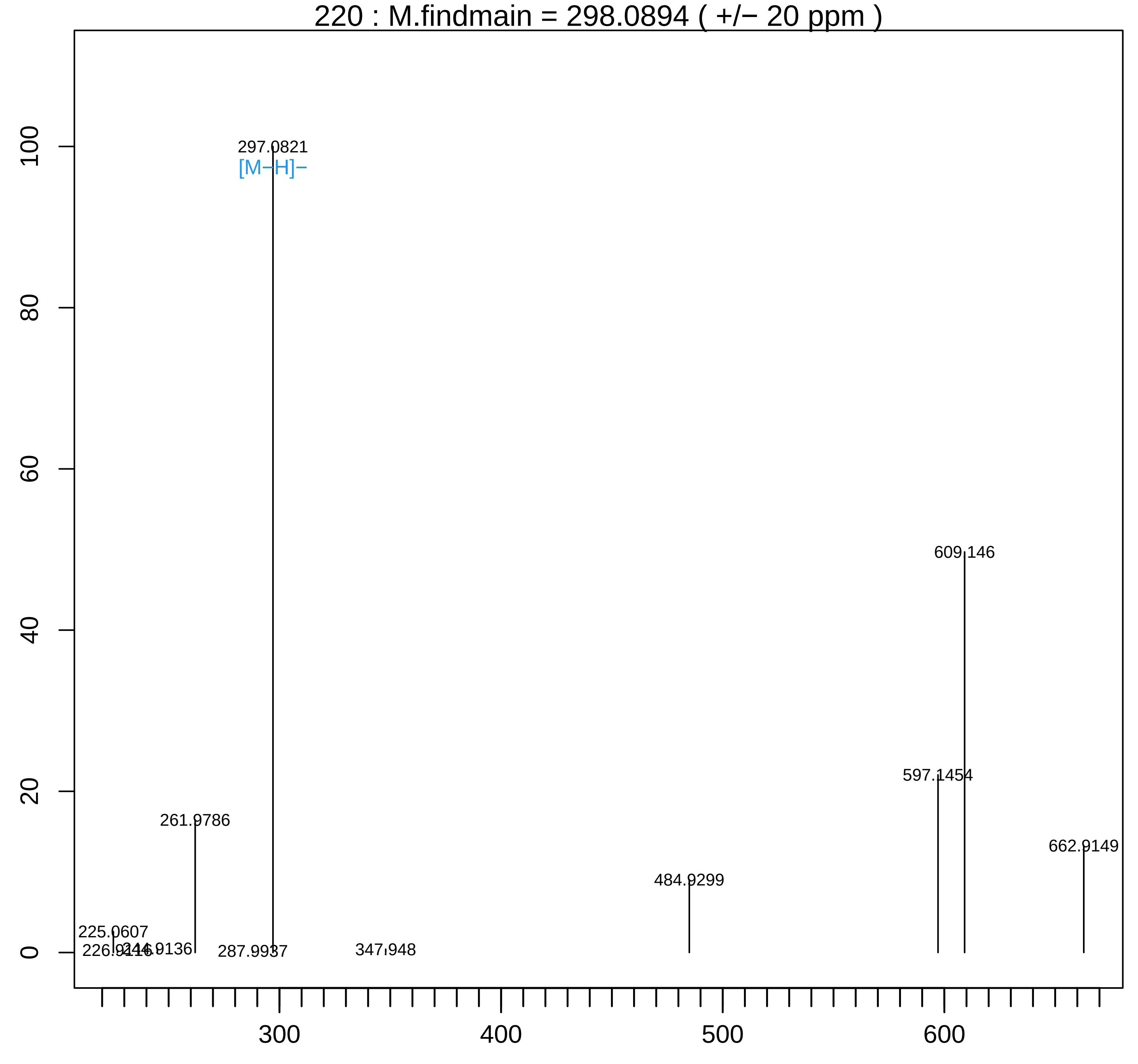

220 : M.findmain = 298.0894 (+/− 20 ppm)
297.0821
[M−H]−
609.146
597.1454
261.9786
662.9149
484.9299
225.0607
226.9116
244.9136
287.9937
347.948
0
20
40
60
80
100
300
400
500
600

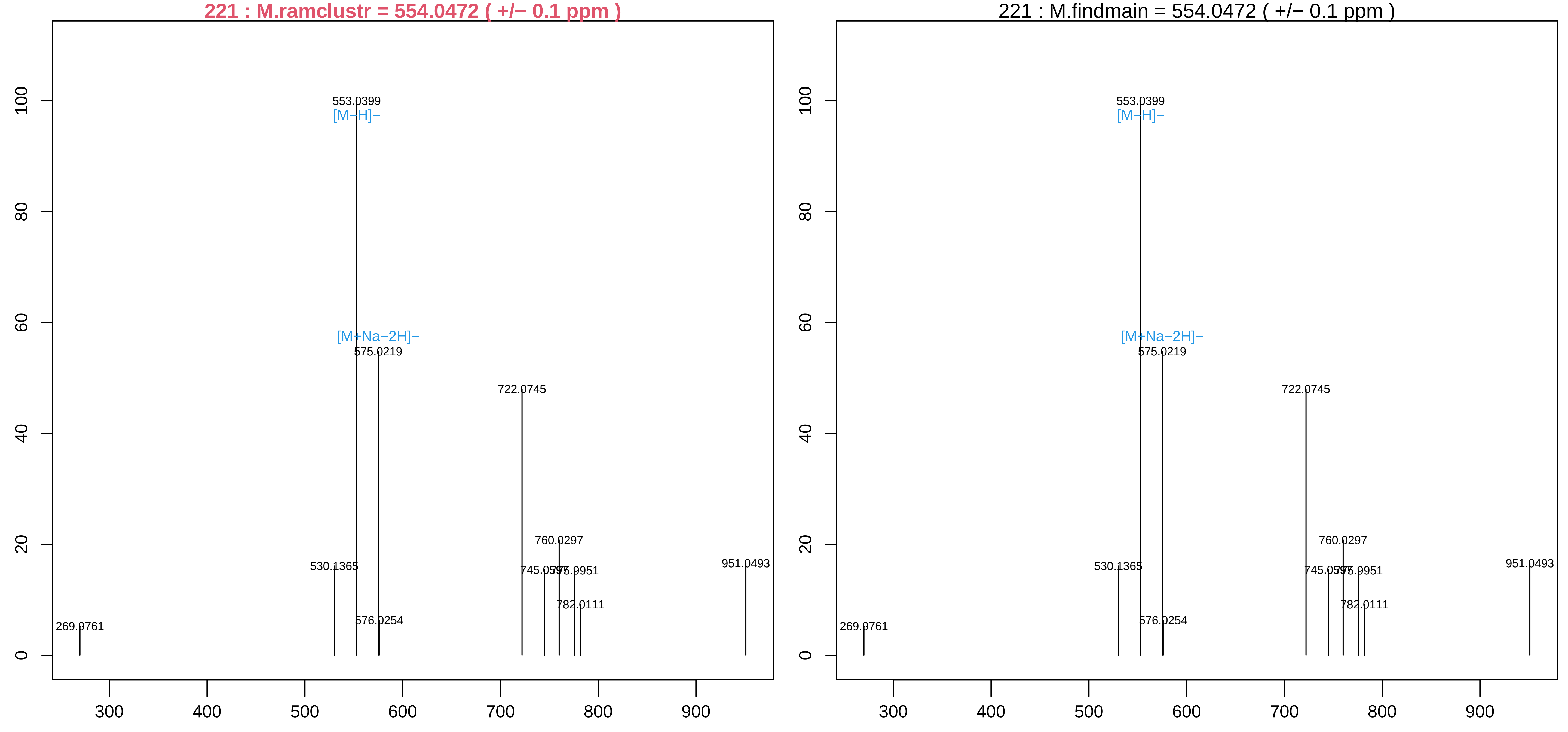
221 : M.ramclustr = 554.0472 (+/− 0.1 ppm)
553.0399
[M−H]−
[M+Na−2H]−
575.0219
722.0745
760.0297
530.1365
745.0597
775.9951
951.0493
782.0111
576.0254
269.9761
221 : M.findmain = 554.0472 (+/− 0.1 ppm)
553.0399
[M−H]−
[M+Na−2H]−
575.0219
722.0745
760.0297
530.1365
745.0597
775.9951
951.0493
782.0111
576.0254
269.9761

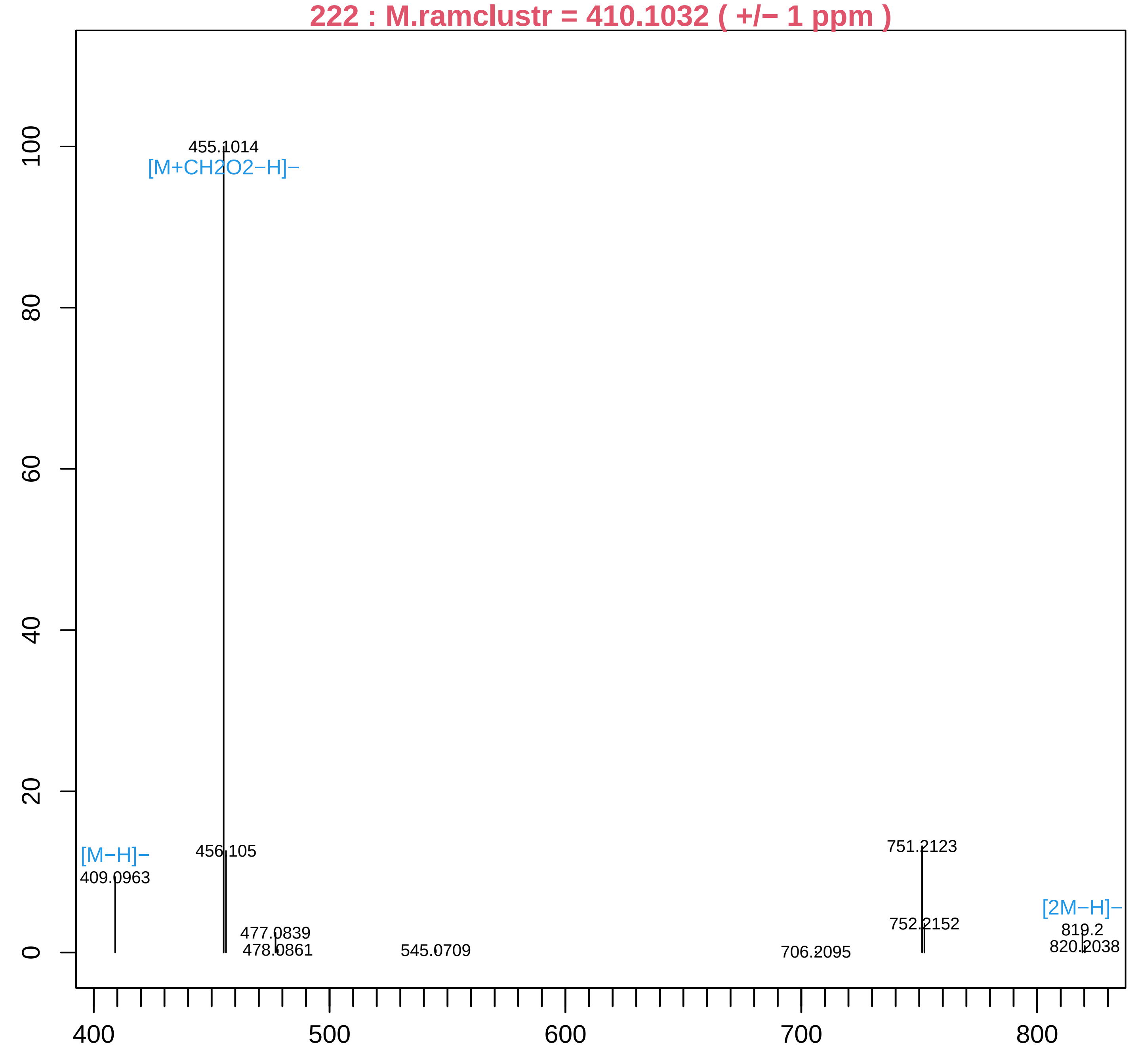
222 : M.ramclustr = 410.1032 (+/− 1 ppm)
455.1014
[M+CH2O2−H]−
[M−H]−
409.0963
456.105
477.0839
478.0861
545.0709
706.2095
751.2123
752.2152
[2M−H]−
819.2
820.2038
0
20
40
60
80
100
400
500
600
700
800
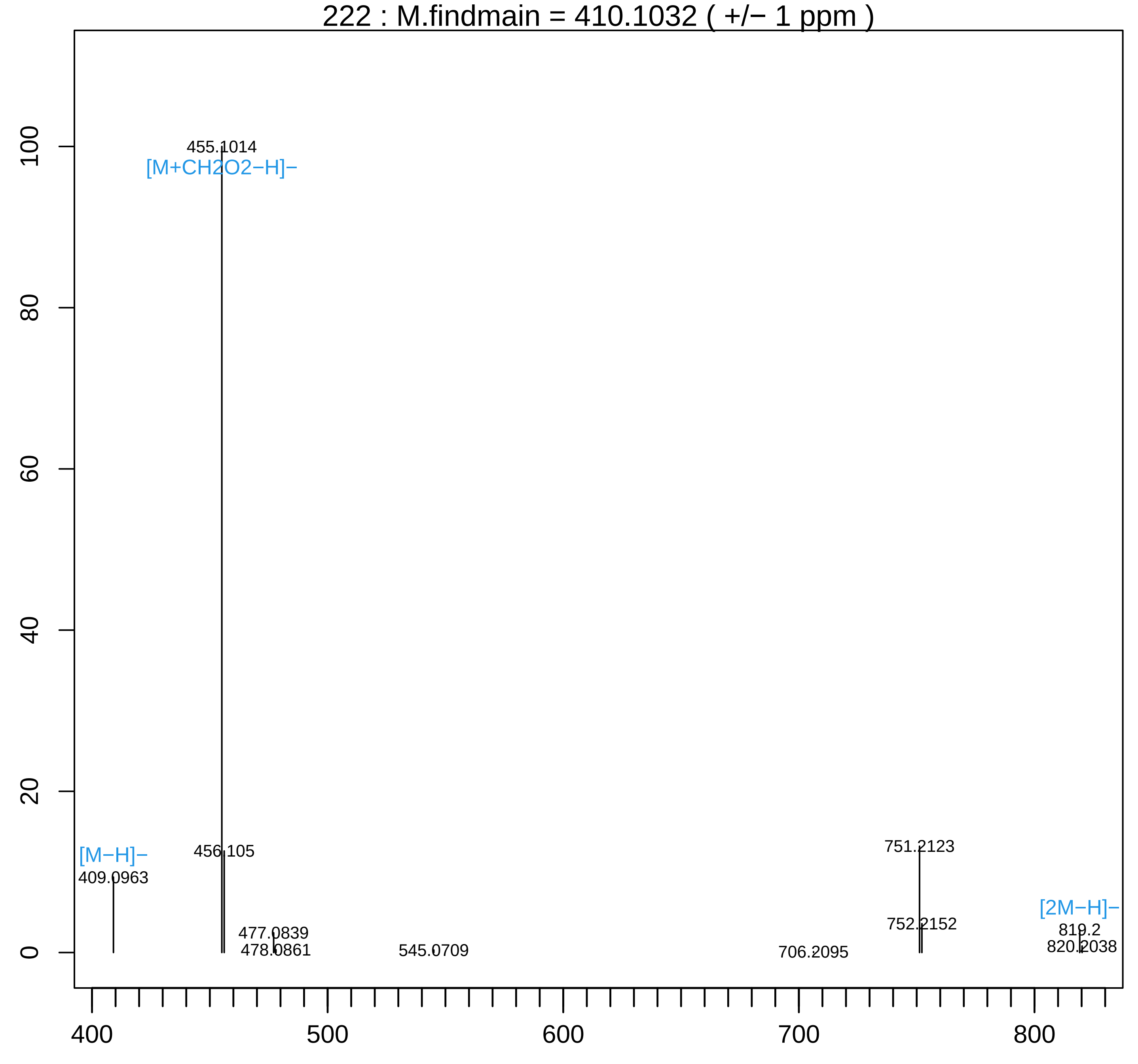
222 : M.findmain = 410.1032 (+/− 1 ppm)
455.1014
[M+CH2O2−H]−
[M−H]−
409.0963
456.105
477.0839
478.0861
545.0709
706.2095
751.2123
752.2152
[2M−H]−
819.2
820.2038
0
20
40
60
80
100
400
500
600
700
800

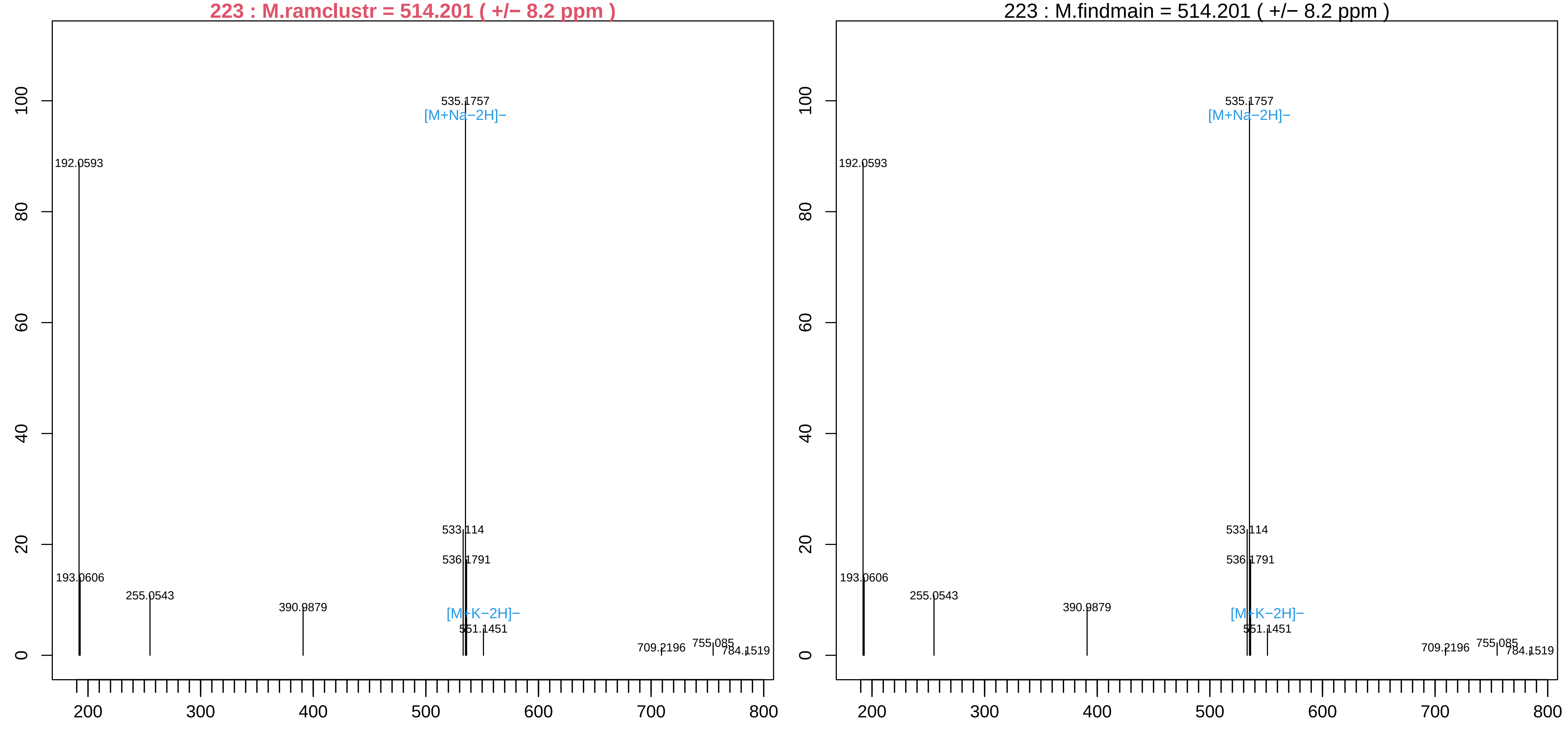
223 : M.ramclustr = 514.201 (+/− 8.2 ppm)
223 : M.findmain = 514.201 (+/− 8.2 ppm)
535.1757
[M+Na−2H]−
192.0593
533.114
536.1791
193.0606
255.0543
390.9879
[M+K−2H]−
551.1451
709.2196
755.085
784.1519
0
20
40
60
80
100
200
300
400
500
600
700
800

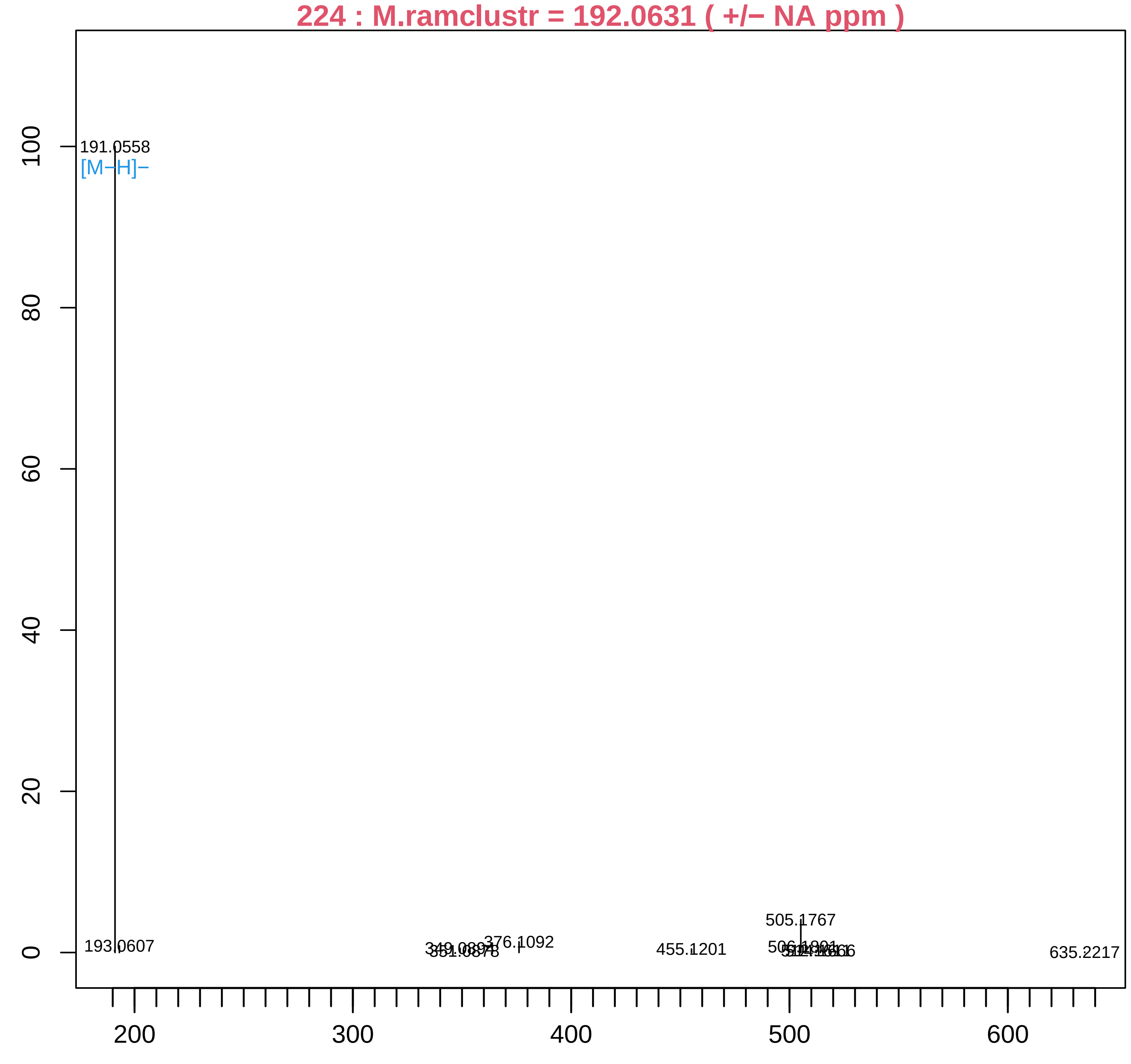

224 : M.ramclustr = 192.0631 (+/− NA ppm)
191.0558
[M−H]−
193.0607
349.0894
351.0878
376.1092
455.1201
505.1767
506.1801
635.2217

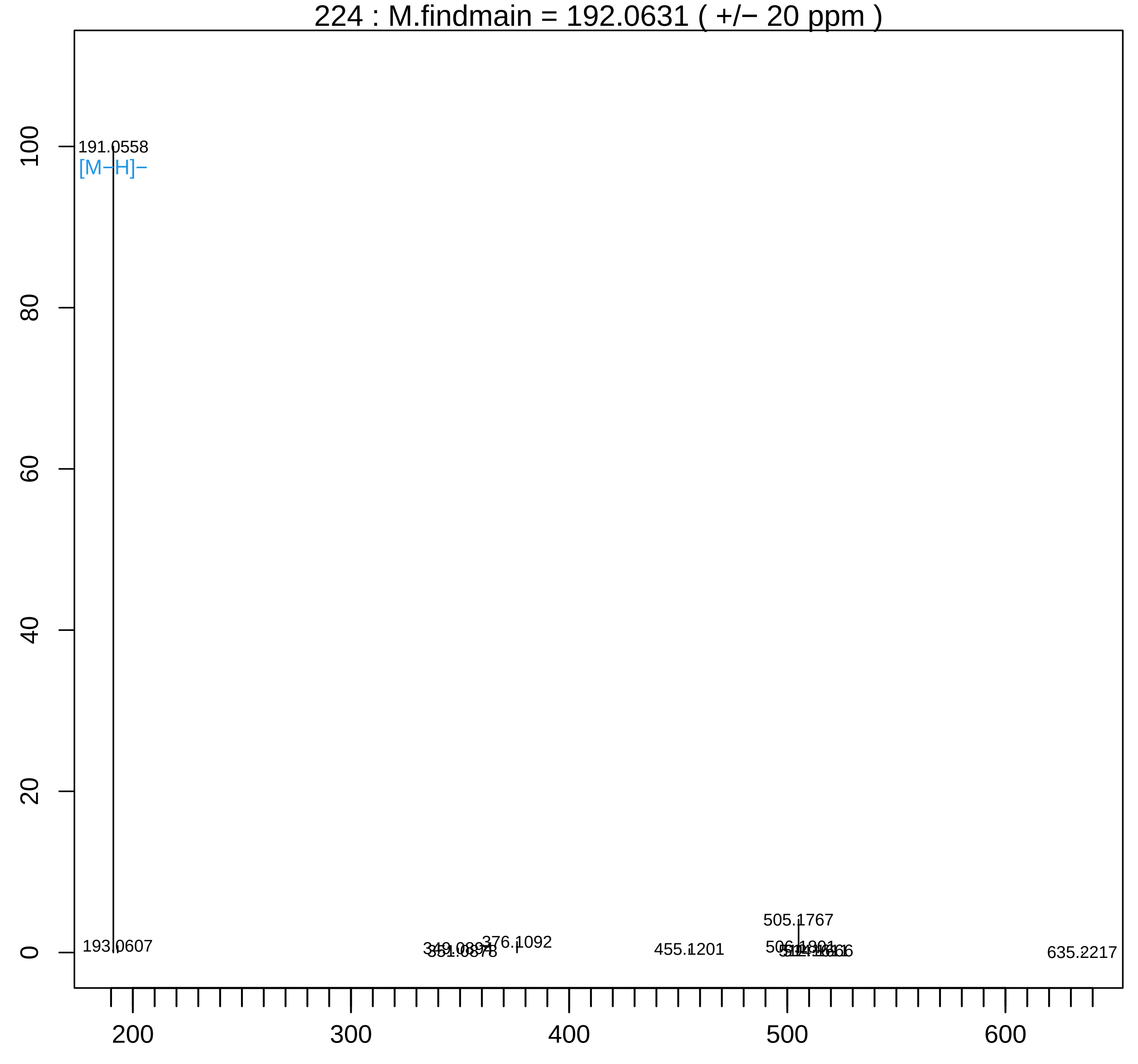

224 : M.findmain = 192.0631 (+/− 20 ppm)
191.0558
[M−H]−
193.0607
349.0894
351.0878
376.1092
455.1201
505.1767
506.1801
635.2217

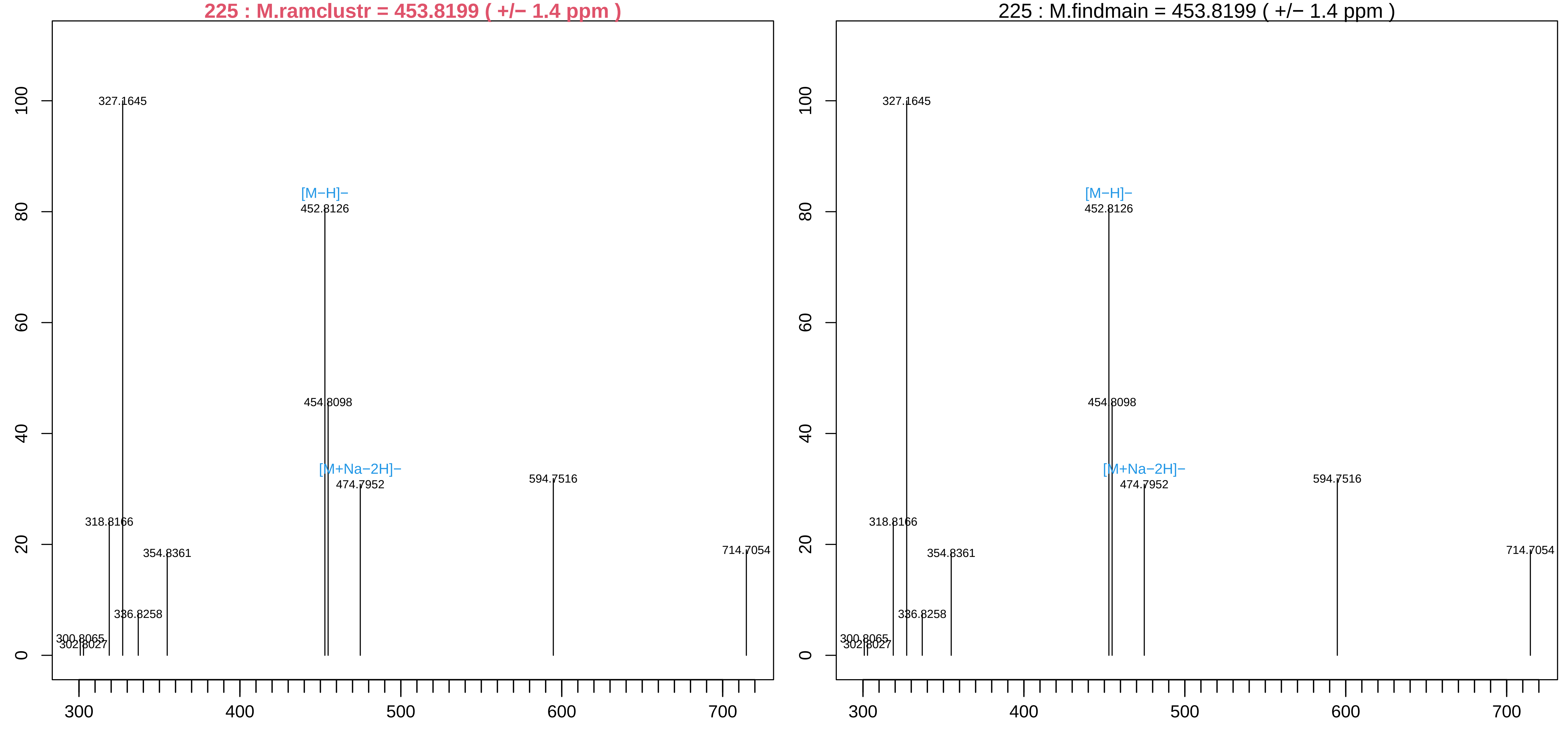
225 : M.ramclustr = 453.8199 (+/− 1.4 ppm)
327.1645
[M−H]−
452.8126
454.8098
[M+Na−2H]−
474.7952
594.7516
318.8166
354.8361
714.7054
336.8258
300.8065
302.8027
225 : M.findmain = 453.8199 (+/− 1.4 ppm)
327.1645
[M−H]−
452.8126
454.8098
[M+Na−2H]−
474.7952
594.7516
318.8166
354.8361
714.7054
336.8258
300.8065
302.8027
0
20
40
60
80
100
300
400
500
600
700

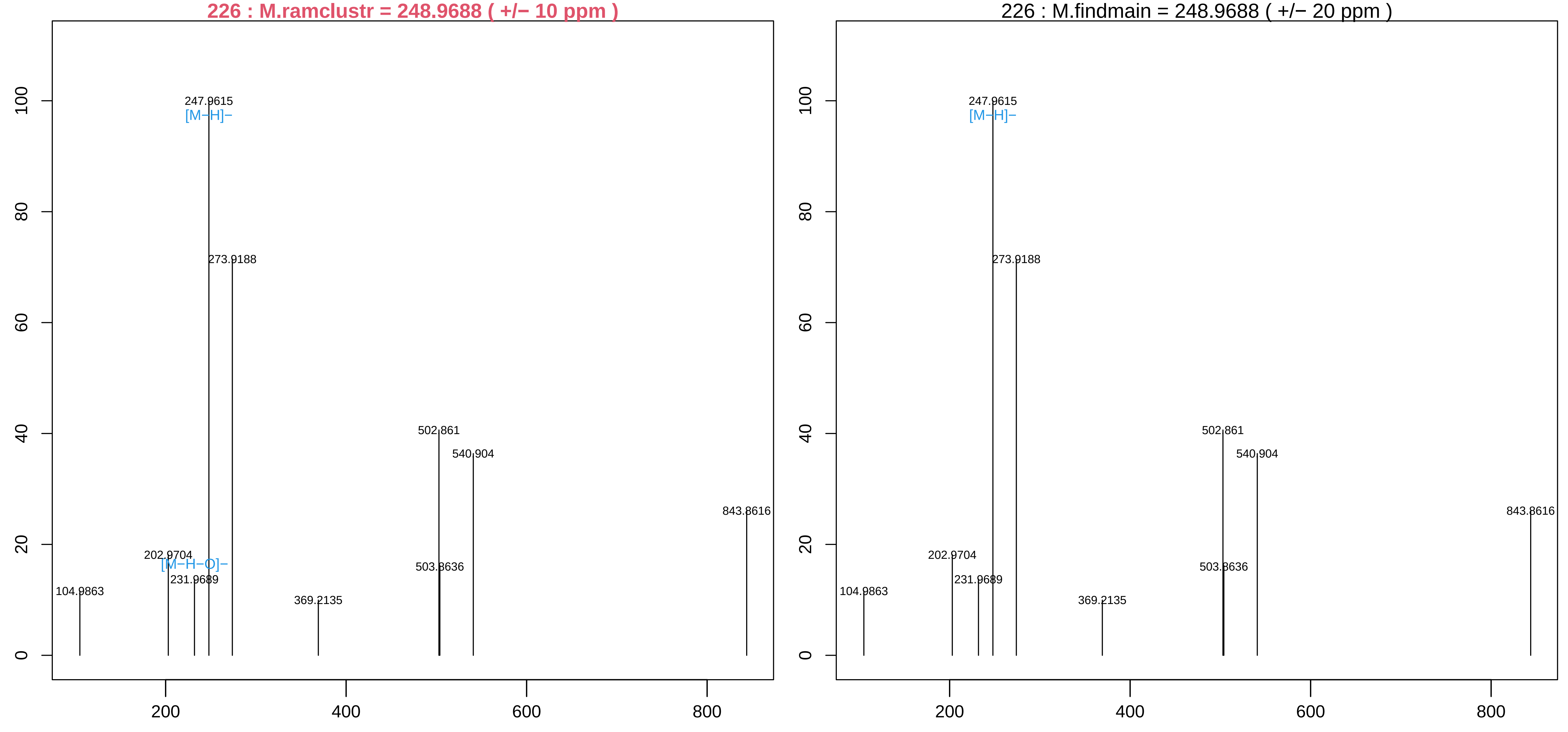
226 : M.ramclustr = 248.9688 (+/− 10 ppm)
226 : M.findmain = 248.9688 (+/− 20 ppm)
247.9615
[M−H]−
273.9188
502.861
540.904
843.8616
202.9704
[M−H−O]−
231.9689
104.9863
369.2135
503.8636
0
20
40
60
80
100
200
400
600
800

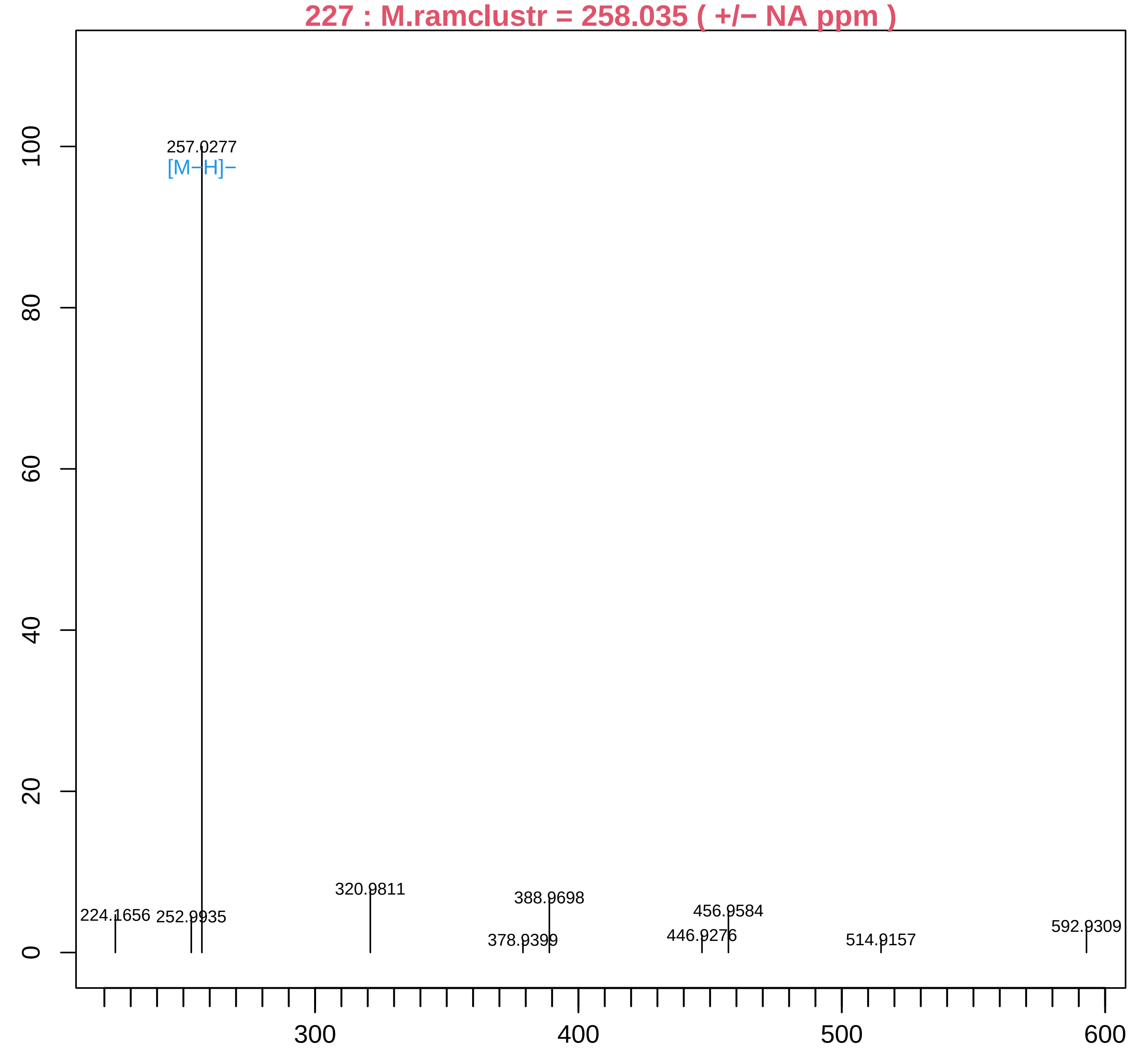
227 : M.ramclustr = 258.035 (+/− NA ppm)
257.0277
[M−H]−
224.1656
252.9935
320.9811
388.9698
378.9399
456.9584
446.9276
514.9157
592.9309
0
20
40
60
80
100
300
400
500
600

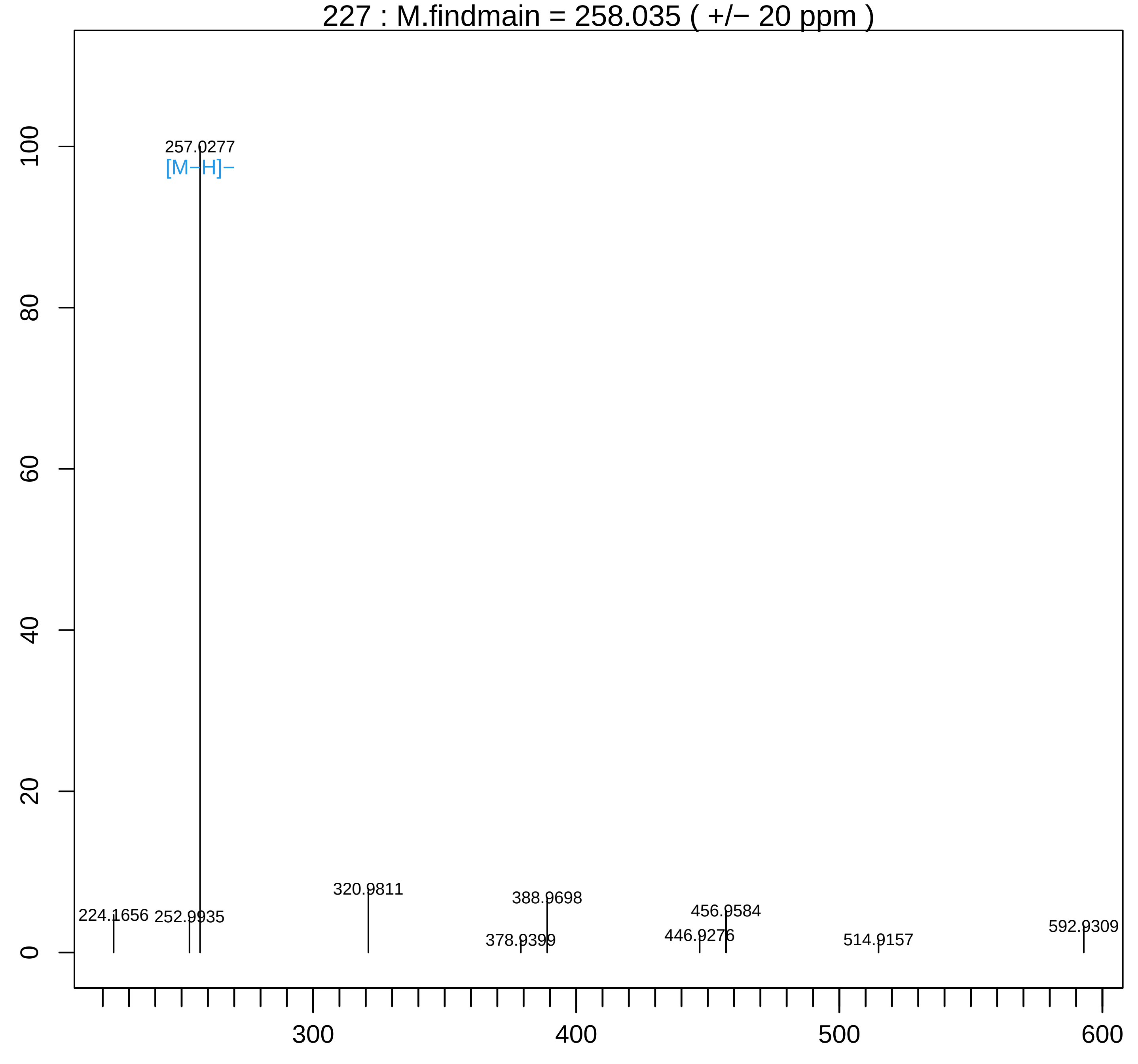
227 : M.findmain = 258.035 (+/− 20 ppm)
257.0277
[M−H]−
224.1656
252.9935
320.9811
388.9698
378.9399
456.9584
446.9276
514.9157
592.9309
0
20
40
60
80
100
300
400
500
600

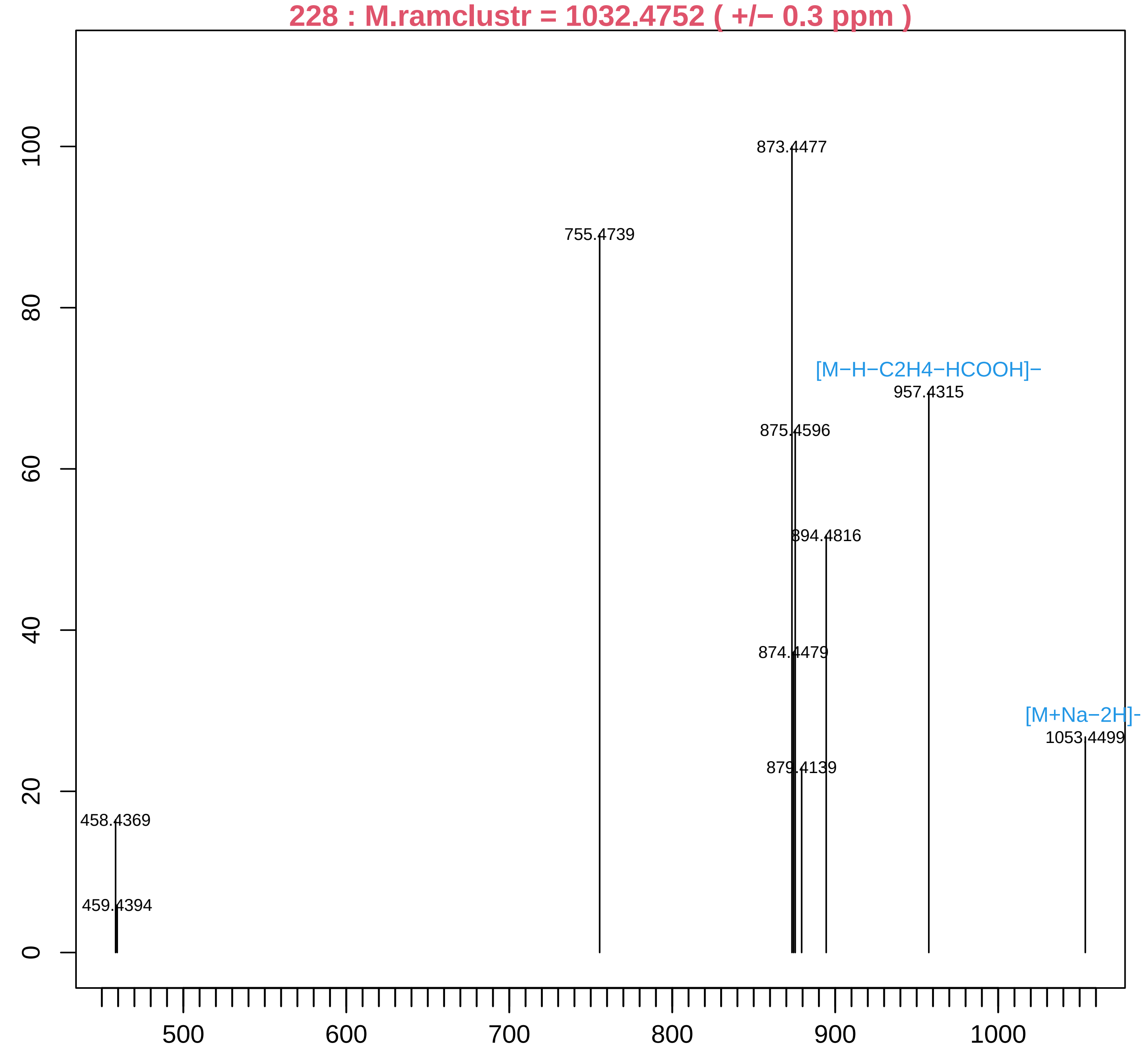

228 : M.ramclustr = 1032.4752 (+/− 0.3 ppm)
873.4477
755.4739
[M−H−C2H4−HCOOH]−
957.4315
875.4596
894.4816
874.4479
[M+Na−2H]-
1053.4499
879.4139
458.4369
459.4394
0
20
40
60
80
100
500
600
700
800
900
1000

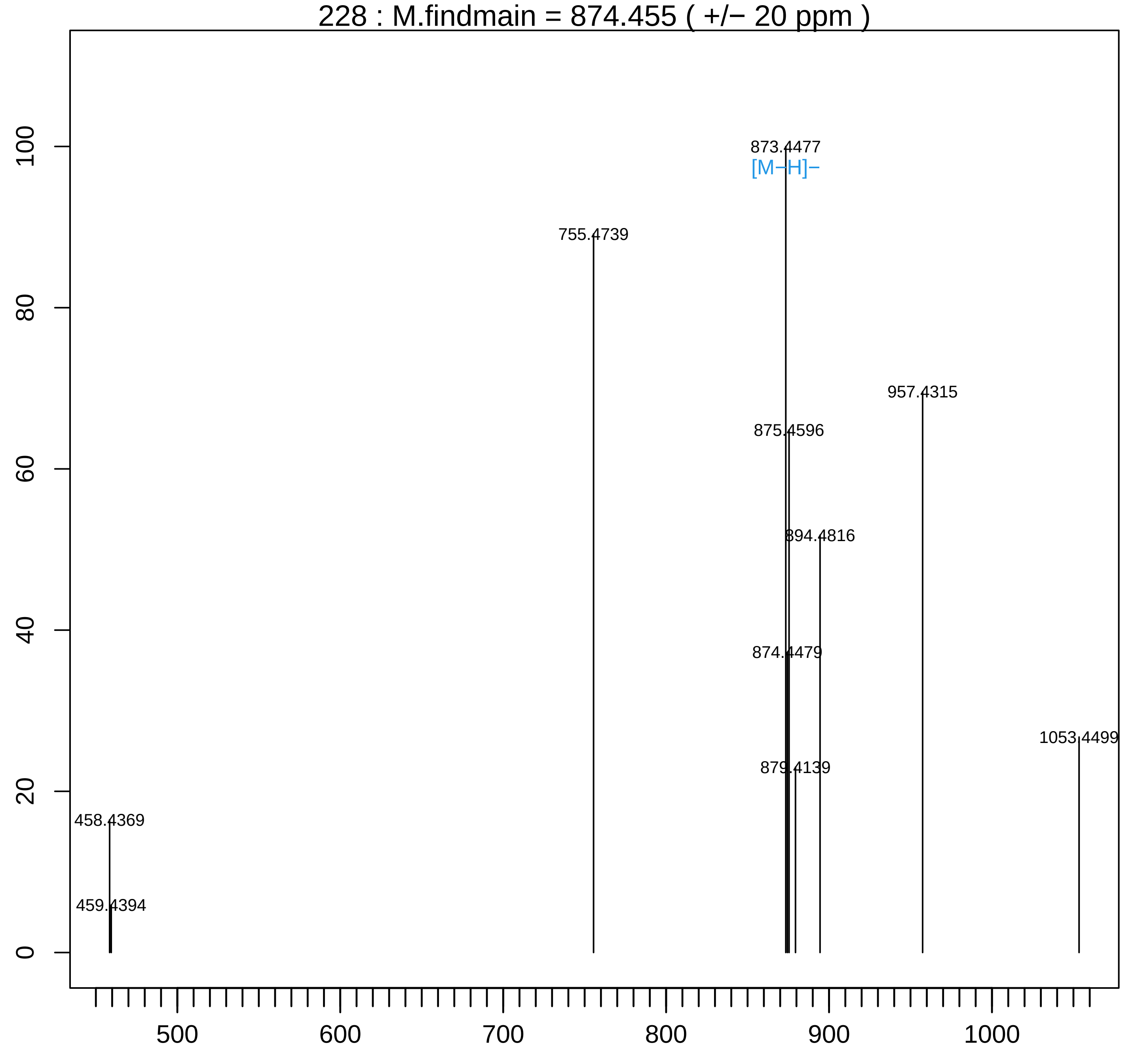

228 : M.findmain = 874.455 (+/− 20 ppm)
873.4477
[M−H]−
755.4739
957.4315
875.4596
894.4816
874.4479
1053.4499
879.4139
458.4369
459.4394
0
20
40
60
80
100
500
600
700
800
900
1000

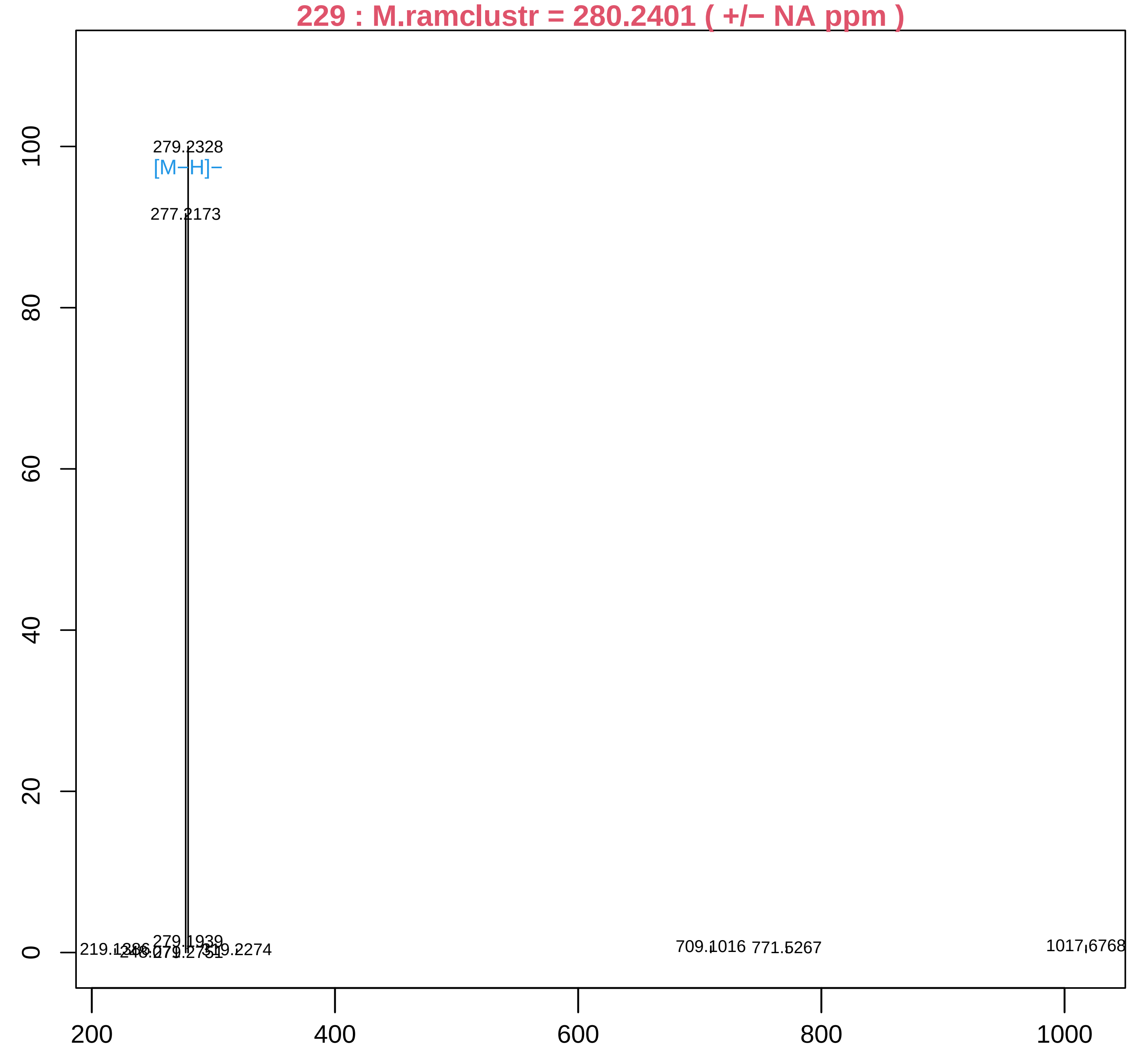
229 : M.ramclustr = 280.2401 (+/− NA ppm)
279.2328
[M−H]−
277.2173
279.1939
219.1286
709.1016
771.5267
1017.6768
0
20
40
60
80
100
200
400
600
800
1000

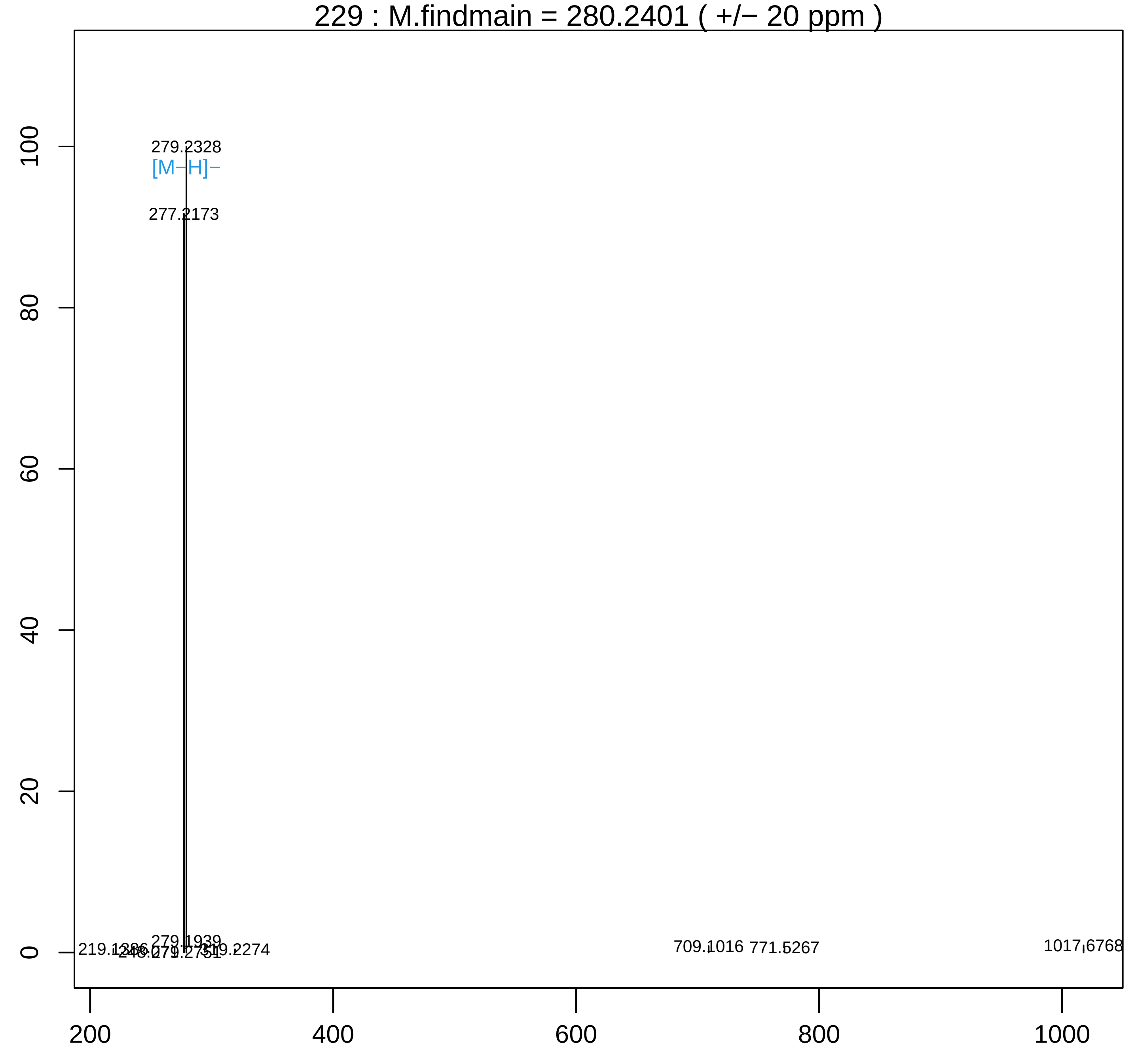
229 : M.findmain = 280.2401 (+/− 20 ppm)
279.2328
[M−H]−
277.2173
279.1939
219.1286
709.1016
771.5267
1017.6768
0
20
40
60
80
100
200
400
600
800
1000

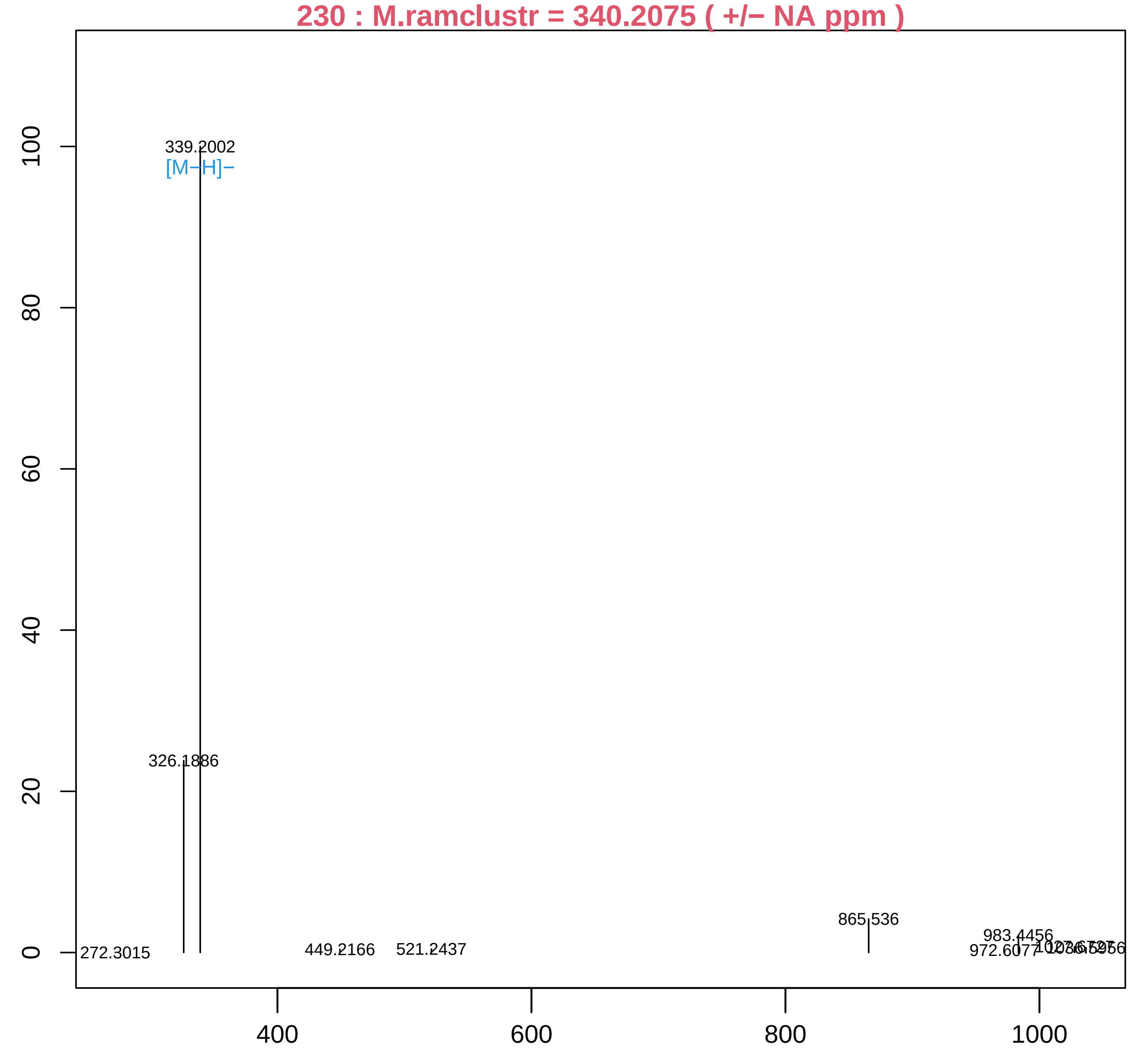
230 : M.ramclustr = 340.2075 (+/− NA ppm)
339.2002
[M−H]−
326.1886
272.3015
449.2166
521.2437
865.536
983.4456
972.6077
0
20
40
60
80
100
400
600
800
1000

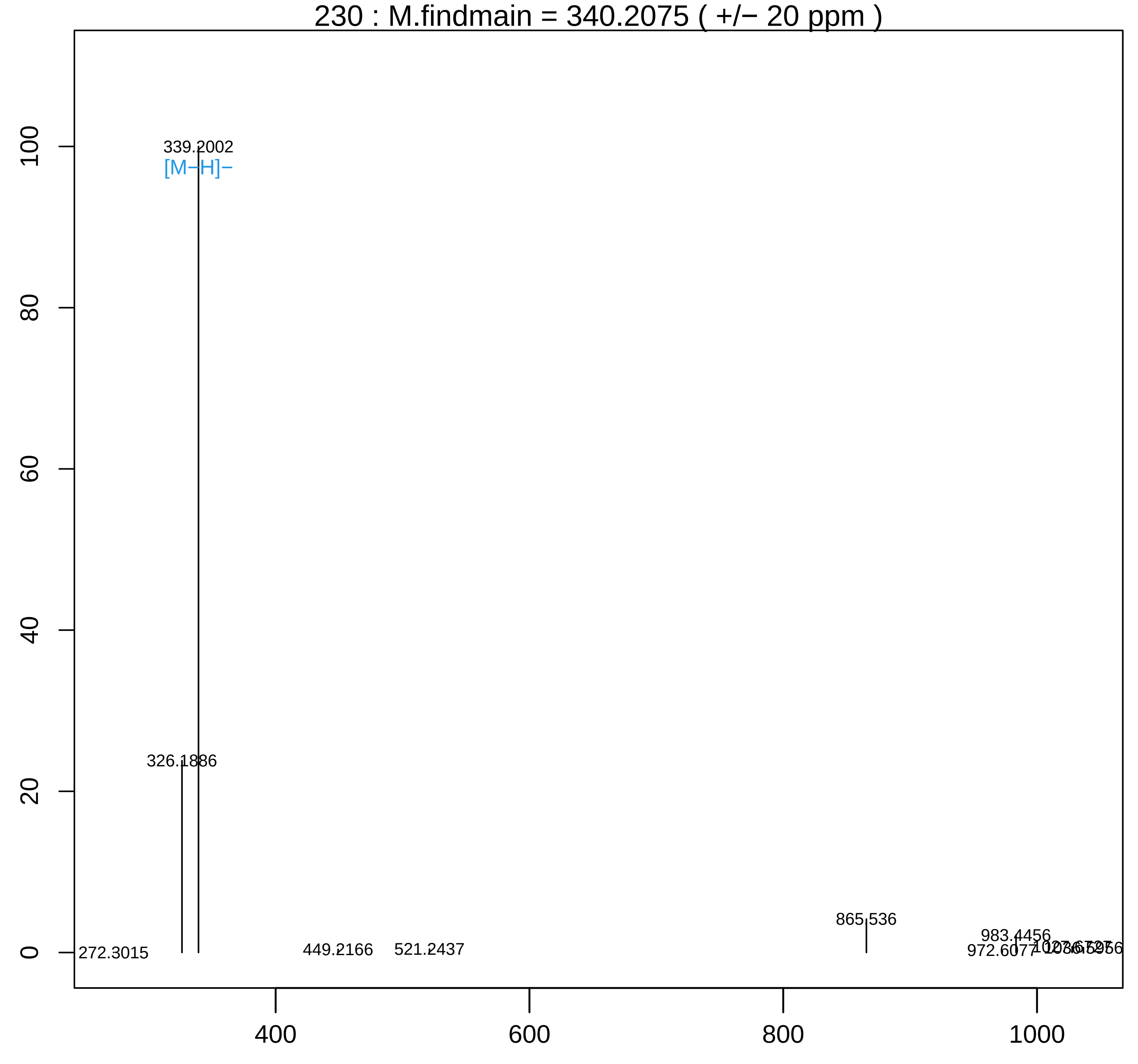
230 : M.findmain = 340.2075 (+/− 20 ppm)
339.2002
[M−H]−
326.1886
272.3015
449.2166
521.2437
865.536
983.4456
972.6077
0
20
40
60
80
100
400
600
800
1000

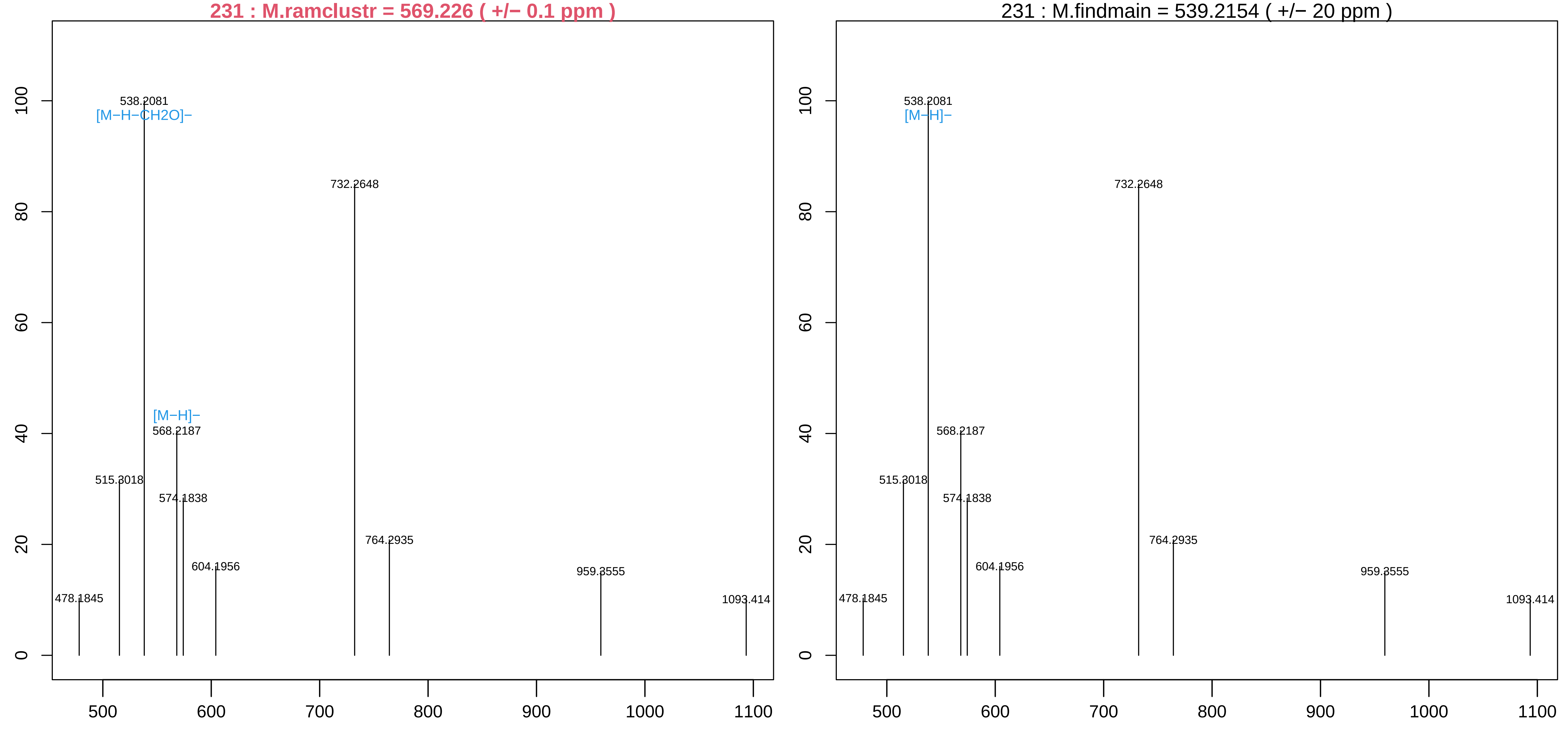
231 : M.ramclustr = 569.226 (+/− 0.1 ppm)
538.2081
[M−H−CH2O]−
732.2648
[M−H]−
568.2187
515.3018
574.1838
764.2935
604.1956
959.3555
478.1845
1093.414
0
20
40
60
80
100
500
600
700
800
900
1000
1100
231 : M.findmain = 539.2154 (+/− 20 ppm)
538.2081
[M−H]−
732.2648
568.2187
515.3018
574.1838
764.2935
604.1956
959.3555
478.1845
1093.414

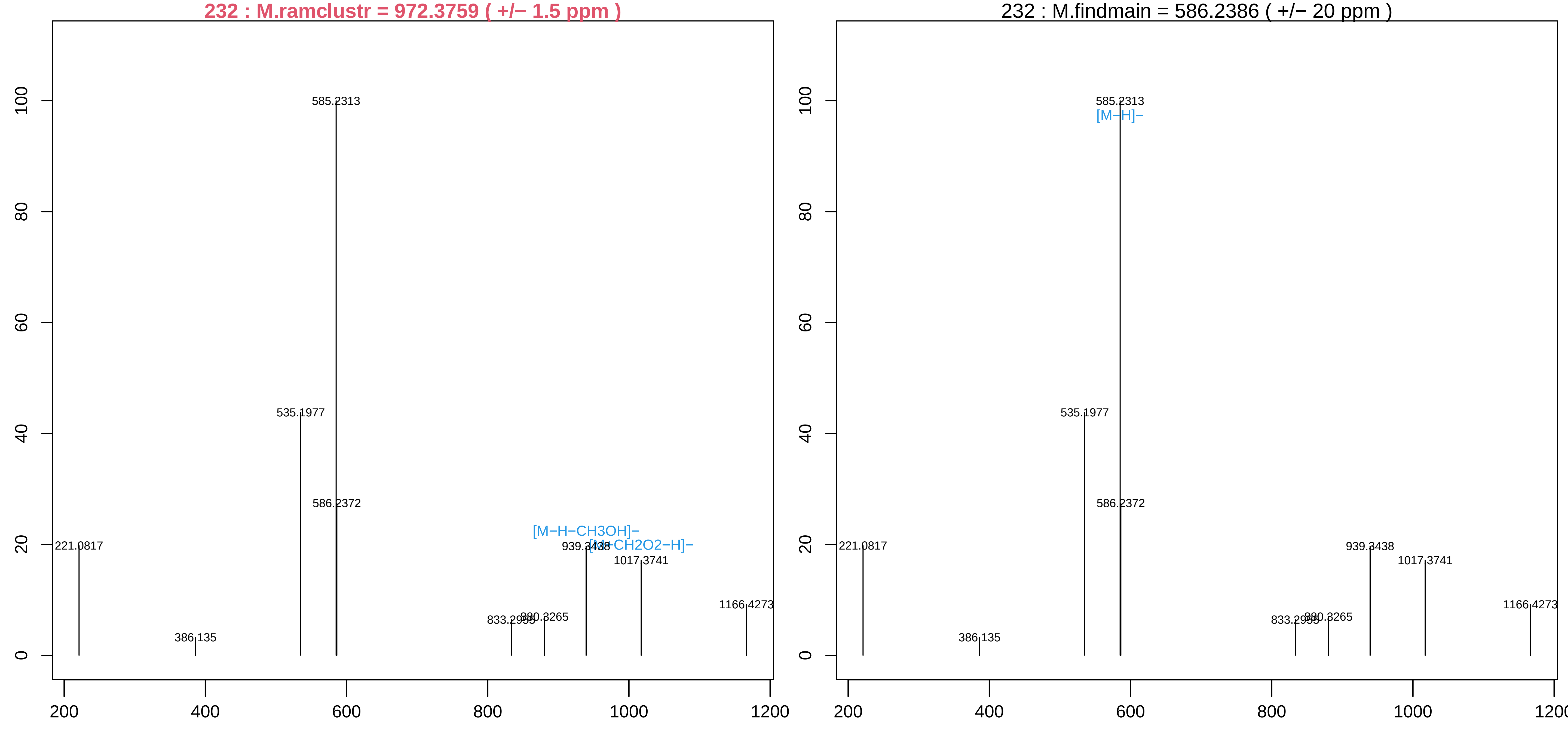
232 : M.ramclustr = 972.3759 (+/− 1.5 ppm)
585.2313
535.1977
586.2372
221.0817
386.135
833.2955
880.3265
939.3438
[M−H−CH3OH]−
[M−CH2O2−H]−
1017.3741
1166.4273
232 : M.findmain = 586.2386 (+/− 20 ppm)
585.2313
[M−H]−
535.1977
586.2372
221.0817
386.135
833.2955
880.3265
939.3438
1017.3741
1166.4273
0
20
40
60
80
100
200
400
600
800
1000
1200

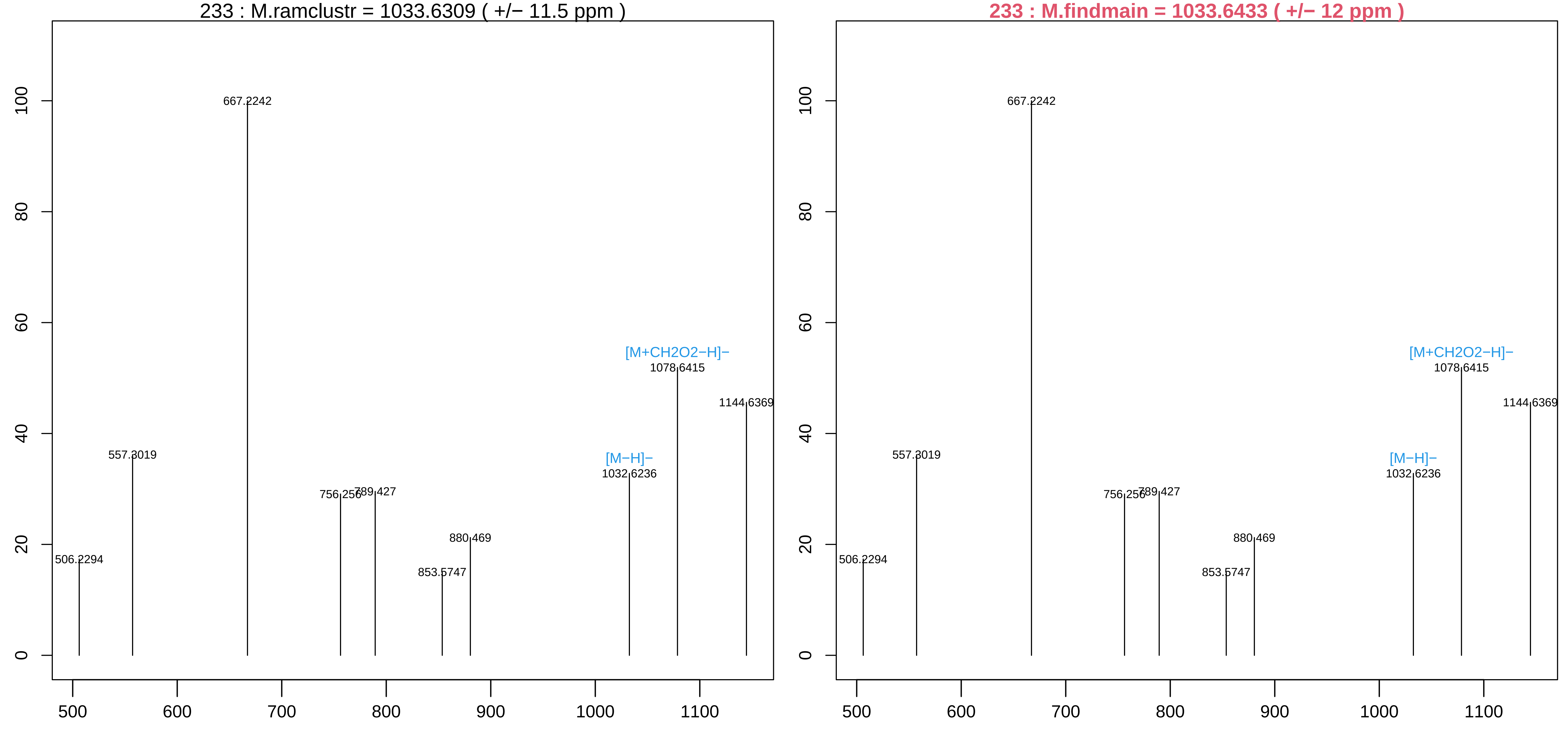
233 : M.ramclustr = 1033.6309 (+/− 11.5 ppm)
667.2242
557.3019
506.2294
756.256
789.427
853.5747
880.469
[M−H]−
1032.6236
[M+CH2O2−H]−
1078.6415
1144.6369
233 : M.findmain = 1033.6433 (+/− 12 ppm)
667.2242
557.3019
506.2294
756.256
789.427
853.5747
880.469
[M−H]−
1032.6236
[M+CH2O2−H]−
1078.6415
1144.6369

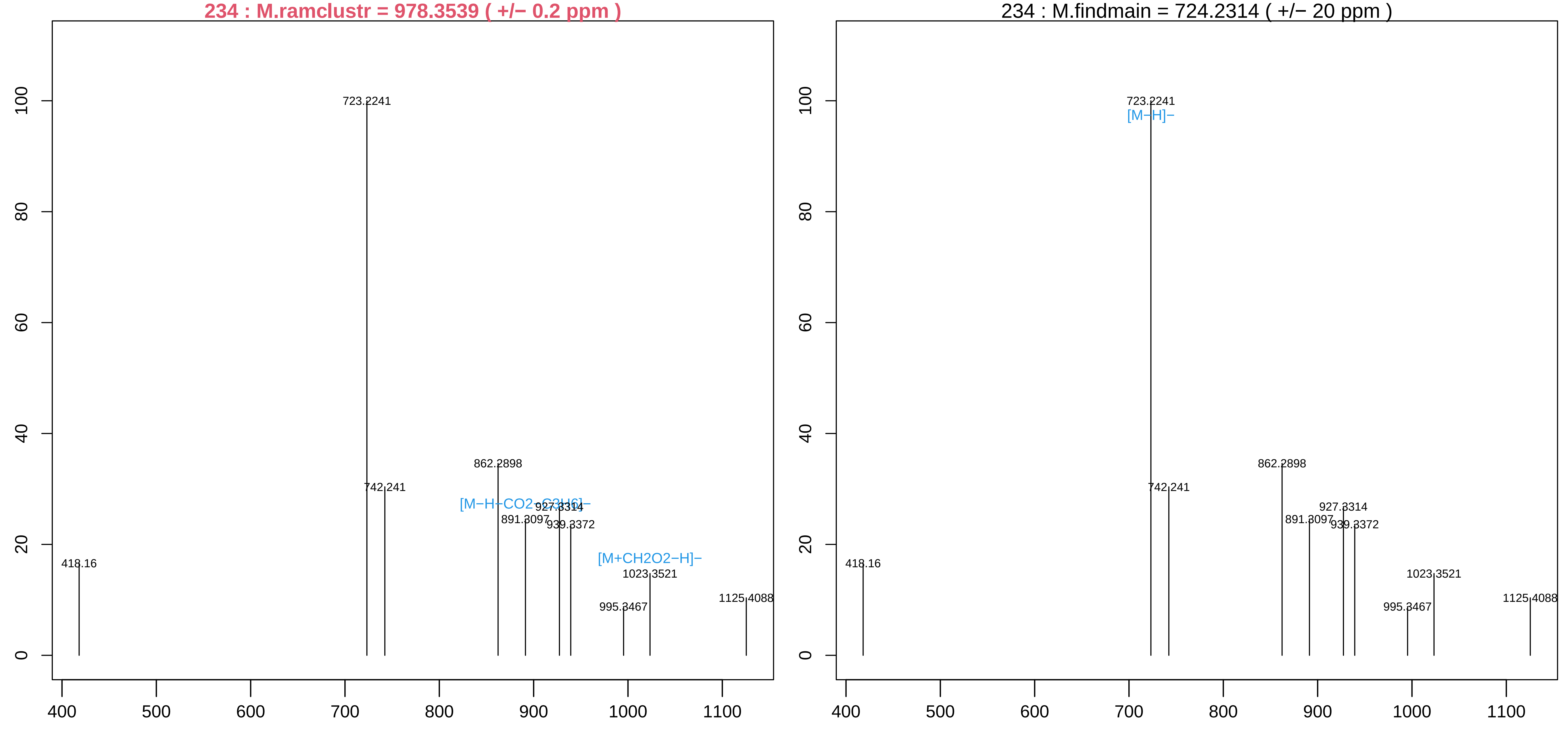
234 : M.ramclustr = 978.3539 (+/− 0.2 ppm)
723.2241
742.241
862.2898
[M−H−CO2−C3H6]−
927.3314
891.3097
939.3372
[M+CH2O2−H]−
1023.3521
995.3467
1125.4088
418.16
234 : M.findmain = 724.2314 (+/− 20 ppm)
723.2241
[M−H]−
742.241
862.2898
927.3314
891.3097
939.3372
1023.3521
995.3467
1125.4088
418.16
0
20
40
60
80
100
400
500
600
700
800
900
1000
1100

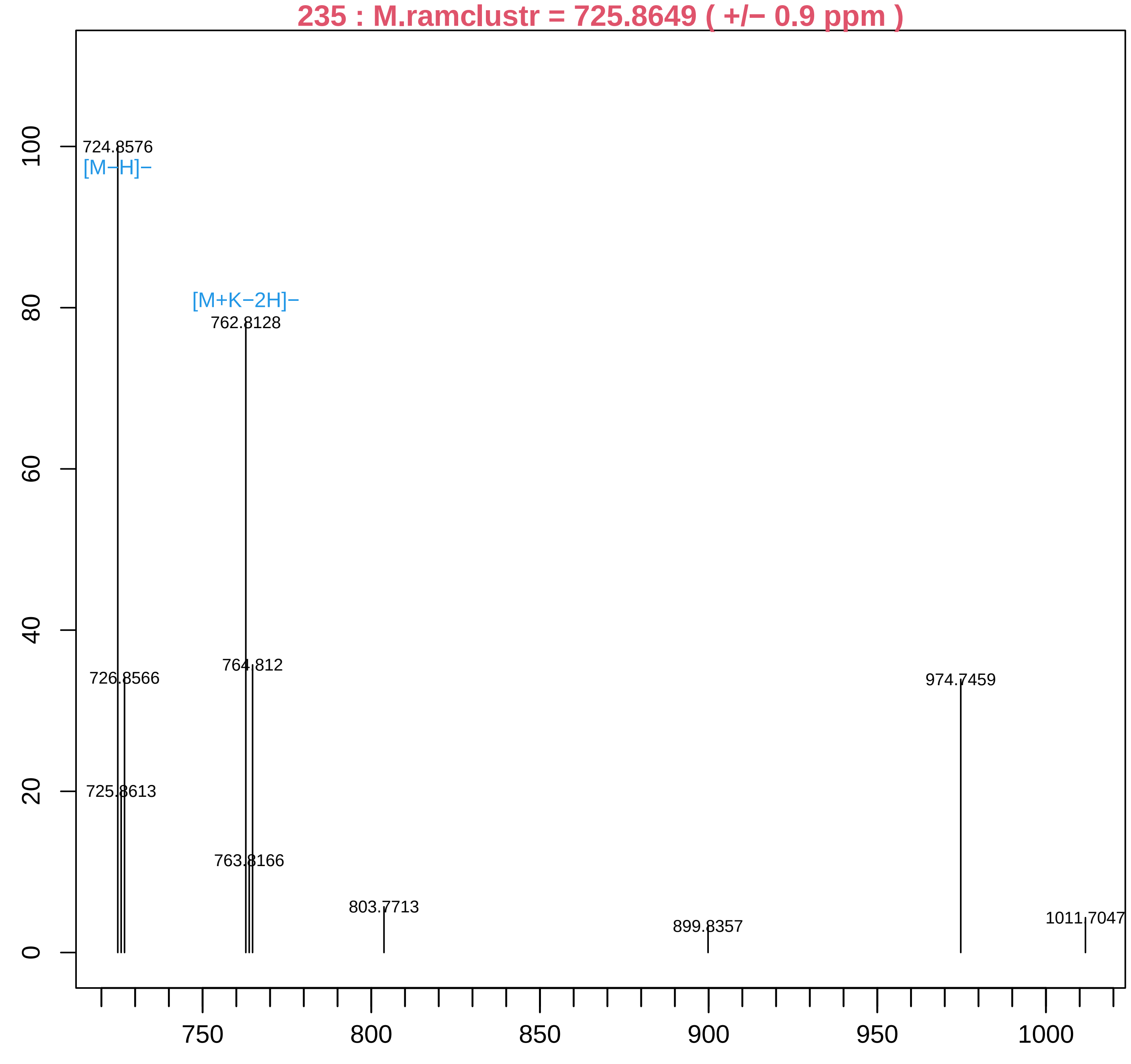
235 : M.ramclustr = 725.8649 (+/− 0.9 ppm)
724.8576
[M−H]−
[M+K−2H]−
762.8128
726.8566
725.8613
764.812
763.8166
803.7713
899.8357
974.7459
1011.7047
0
20
40
60
80
100
750
800
850
900
950
1000

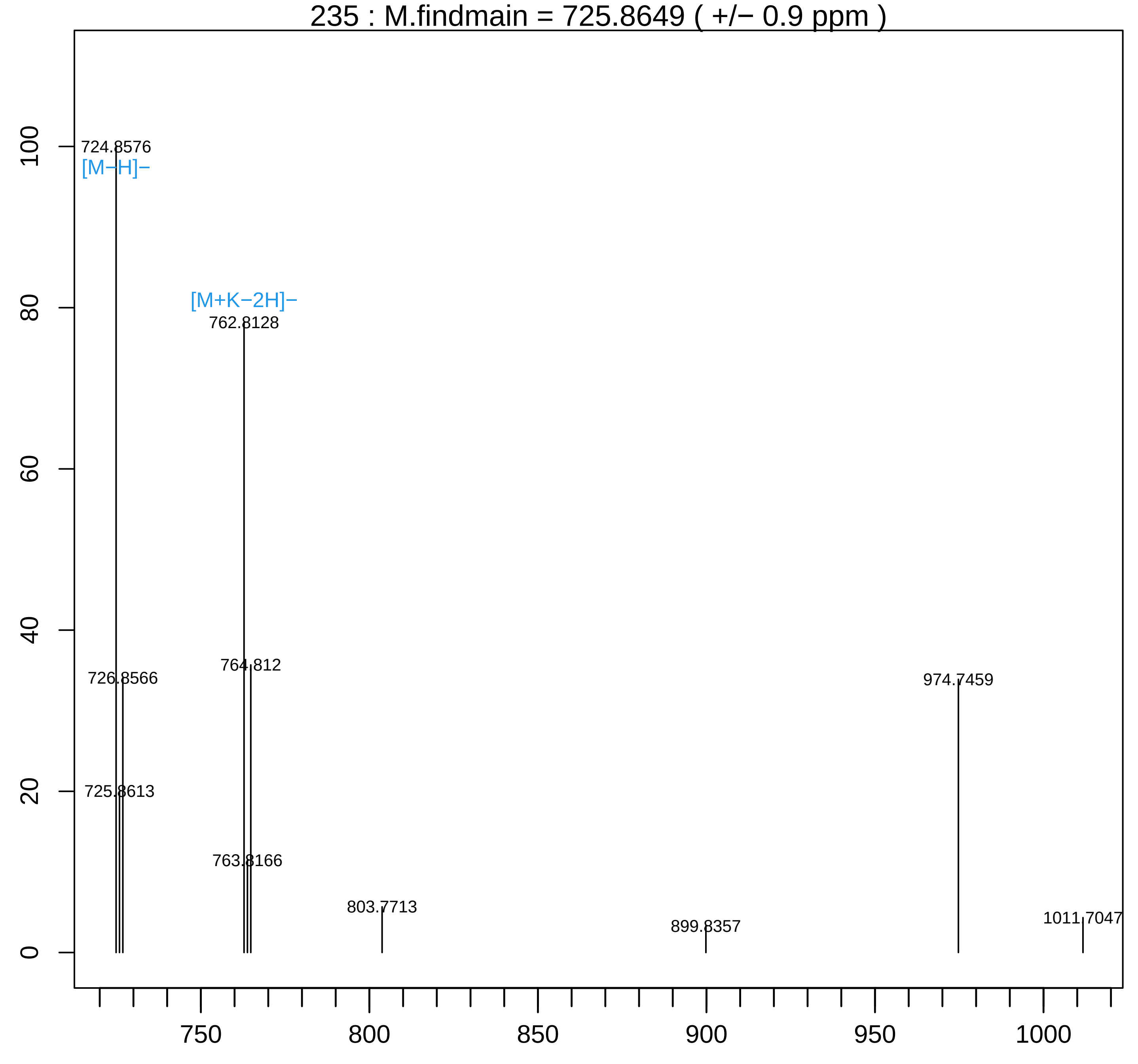
235 : M.findmain = 725.8649 (+/− 0.9 ppm)
724.8576
[M−H]−
[M+K−2H]−
762.8128
726.8566
725.8613
764.812
763.8166
803.7713
899.8357
974.7459
1011.7047
0
20
40
60
80
100
750
800
850
900
950
1000

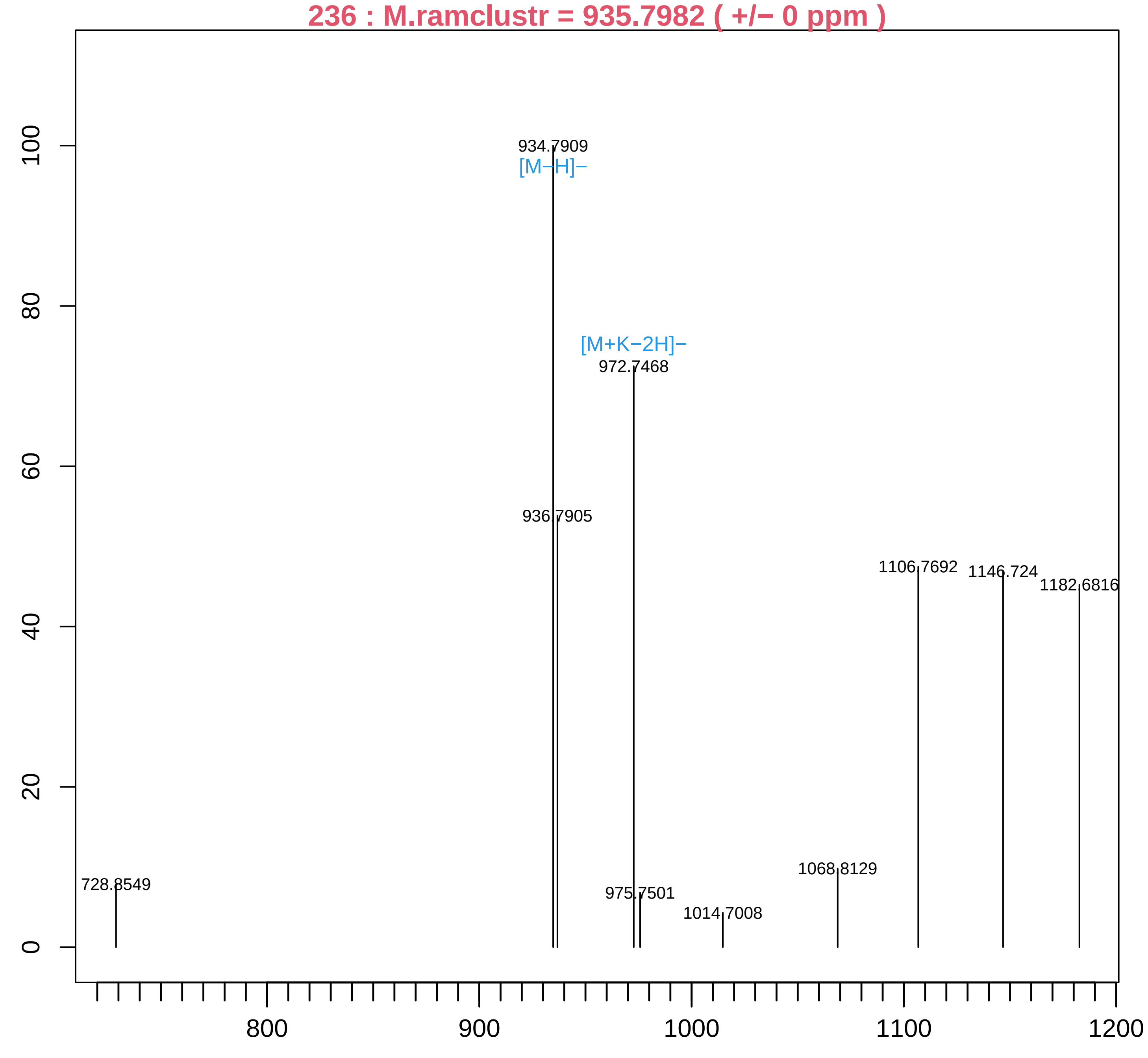
236 : M.ramclustr = 935.7982 (+/− 0 ppm)
934.7909
[M−H]−
[M+K−2H]−
972.7468
936.7905
1106.7692
1146.724
1182.6816
728.8549
975.7501
1014.7008
1068.8129
0
20
40
60
80
100
800
900
1000
1100
1200

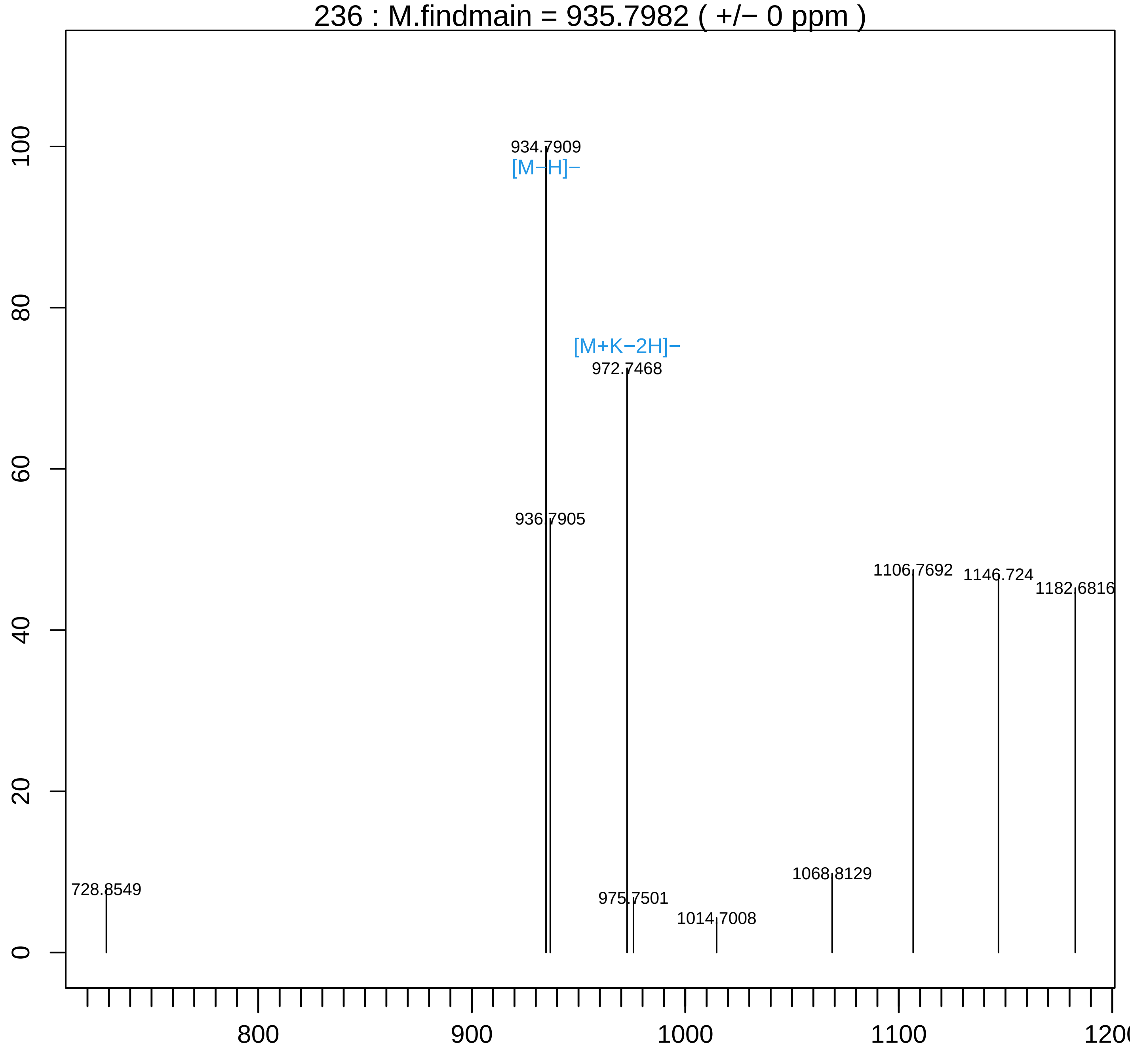
236 : M.findmain = 935.7982 (+/− 0 ppm)
934.7909
[M−H]−
[M+K−2H]−
972.7468
936.7905
1106.7692
1146.724
1182.6816
728.8549
975.7501
1014.7008
1068.8129
0
20
40
60
80
100
800
900
1000
1100

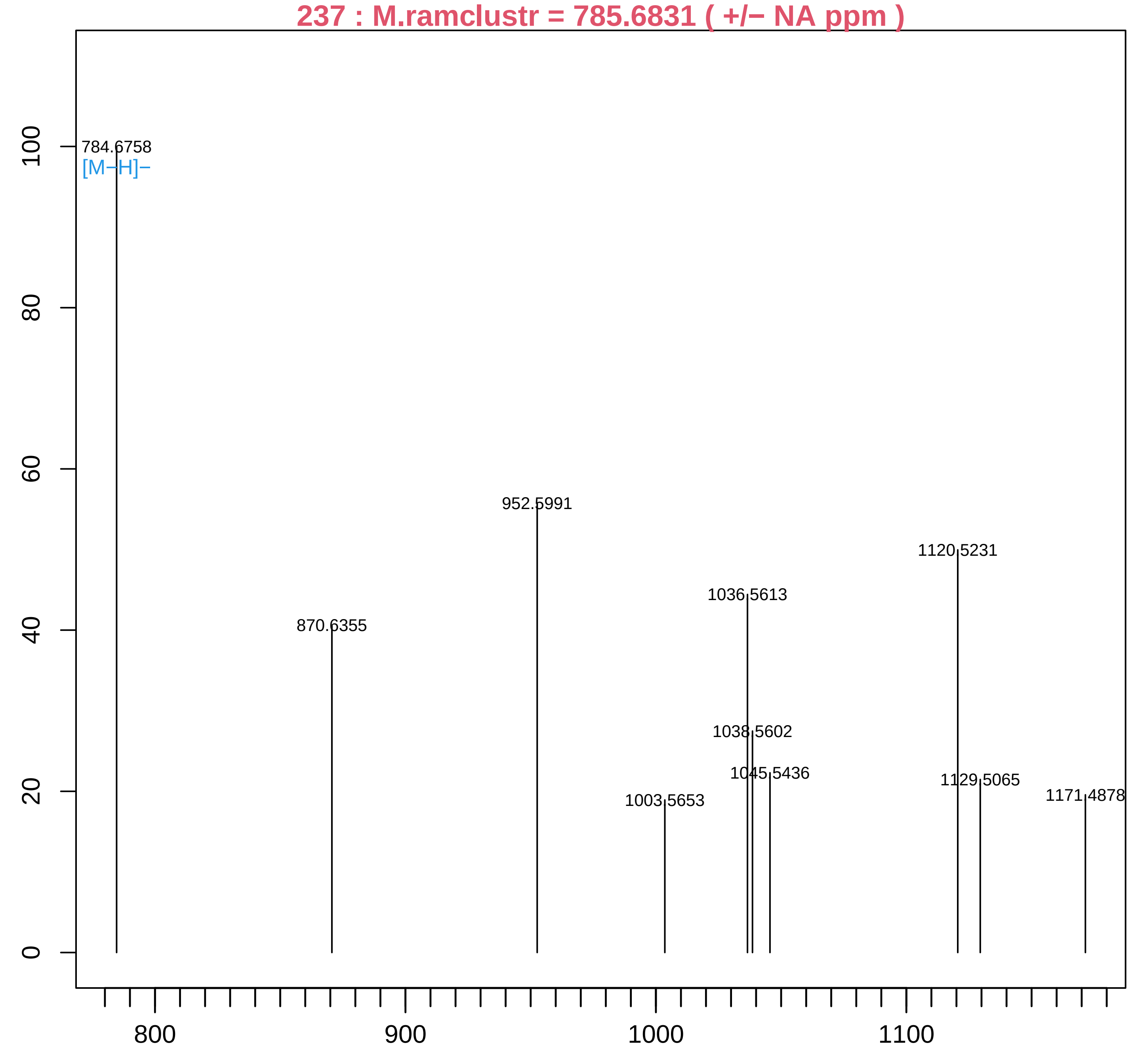
237 : M.ramclustr = 785.6831 (+/− NA ppm)
784.6758
[M−H]−
870.6355
952.5991
1003.5653
1036.5613
1038.5602
1045.5436
1120.5231
1129.5065
1171.4878
0
20
40
60
80
100
800
900
1000
1100

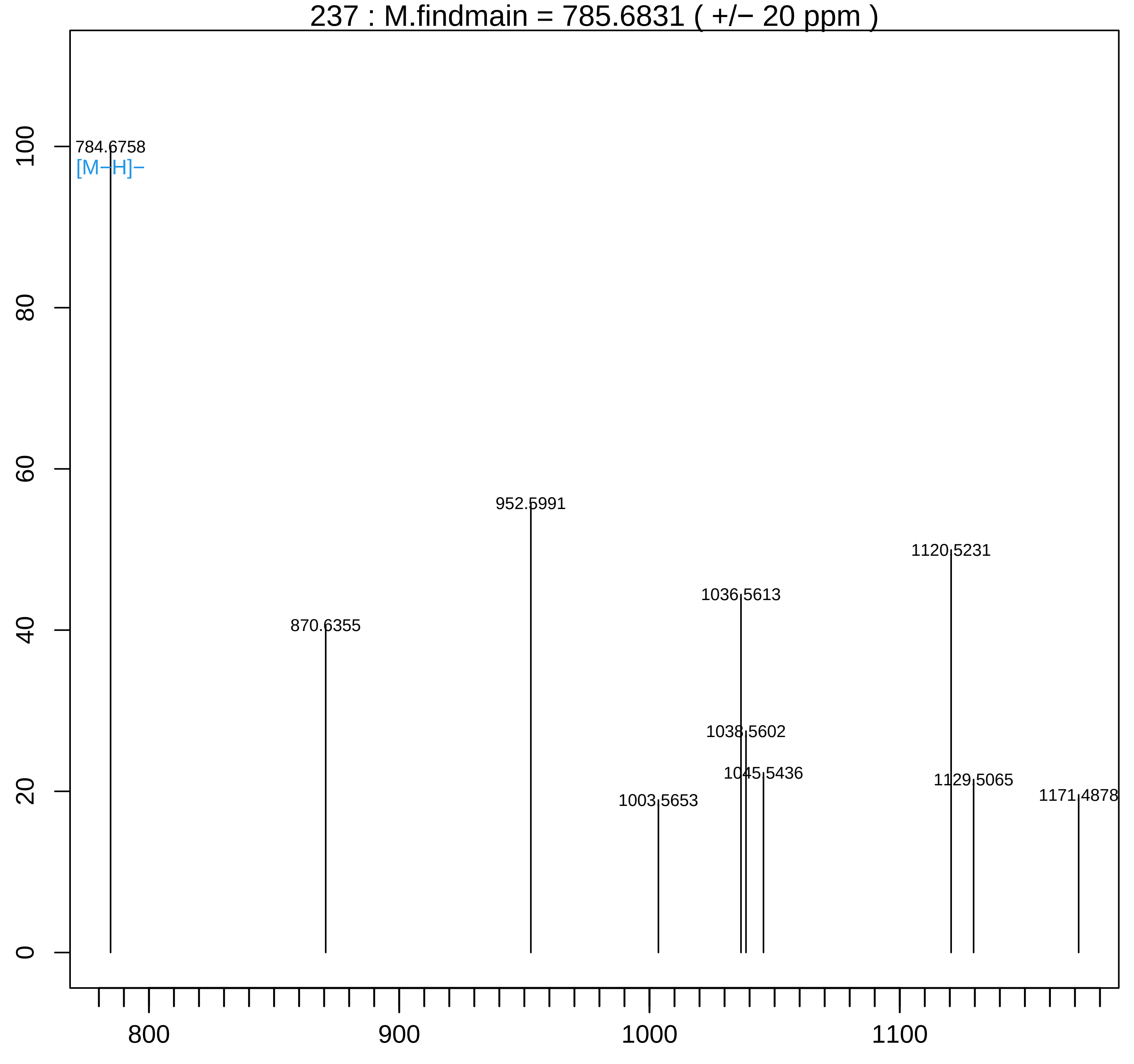
237 : M.findmain = 785.6831 (+/− 20 ppm)
784.6758
[M−H]−
870.6355
952.5991
1003.5653
1036.5613
1038.5602
1045.5436
1120.5231
1129.5065
1171.4878
0
20
40
60
80
100
800
900
1000
1100

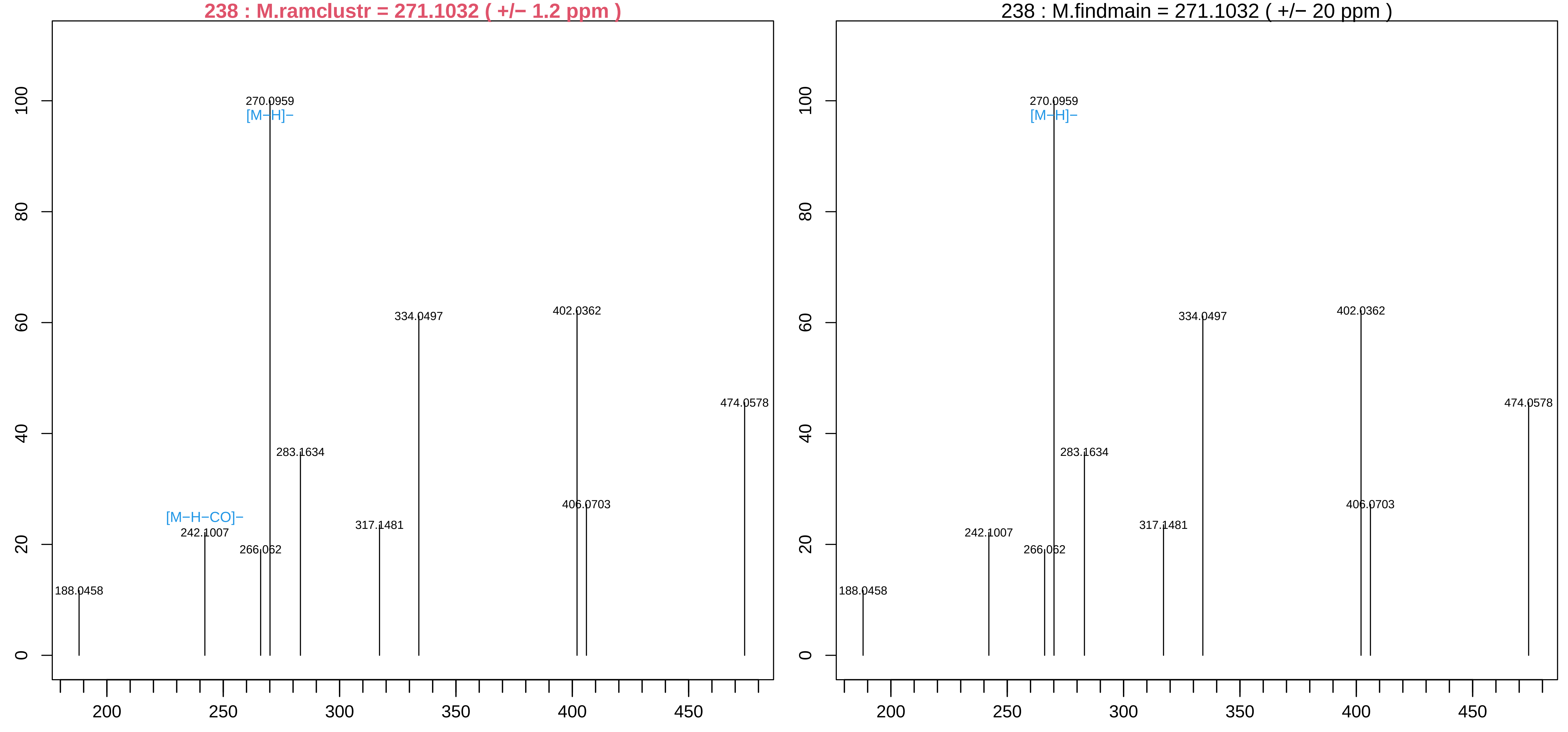
238 : M.ramclustr = 271.1032 (+/− 1.2 ppm)
270.0959
[M−H]−
334.0497
402.0362
474.0578
283.1634
406.0703
[M−H−CO]−
242.1007
317.1481
266.062
188.0458
238 : M.findmain = 271.1032 (+/− 20 ppm)
270.0959
[M−H]−
334.0497
402.0362
474.0578
283.1634
406.0703
242.1007
317.1481
266.062
188.0458
0
20
40
60
80
100
200
250
300
350
400
450

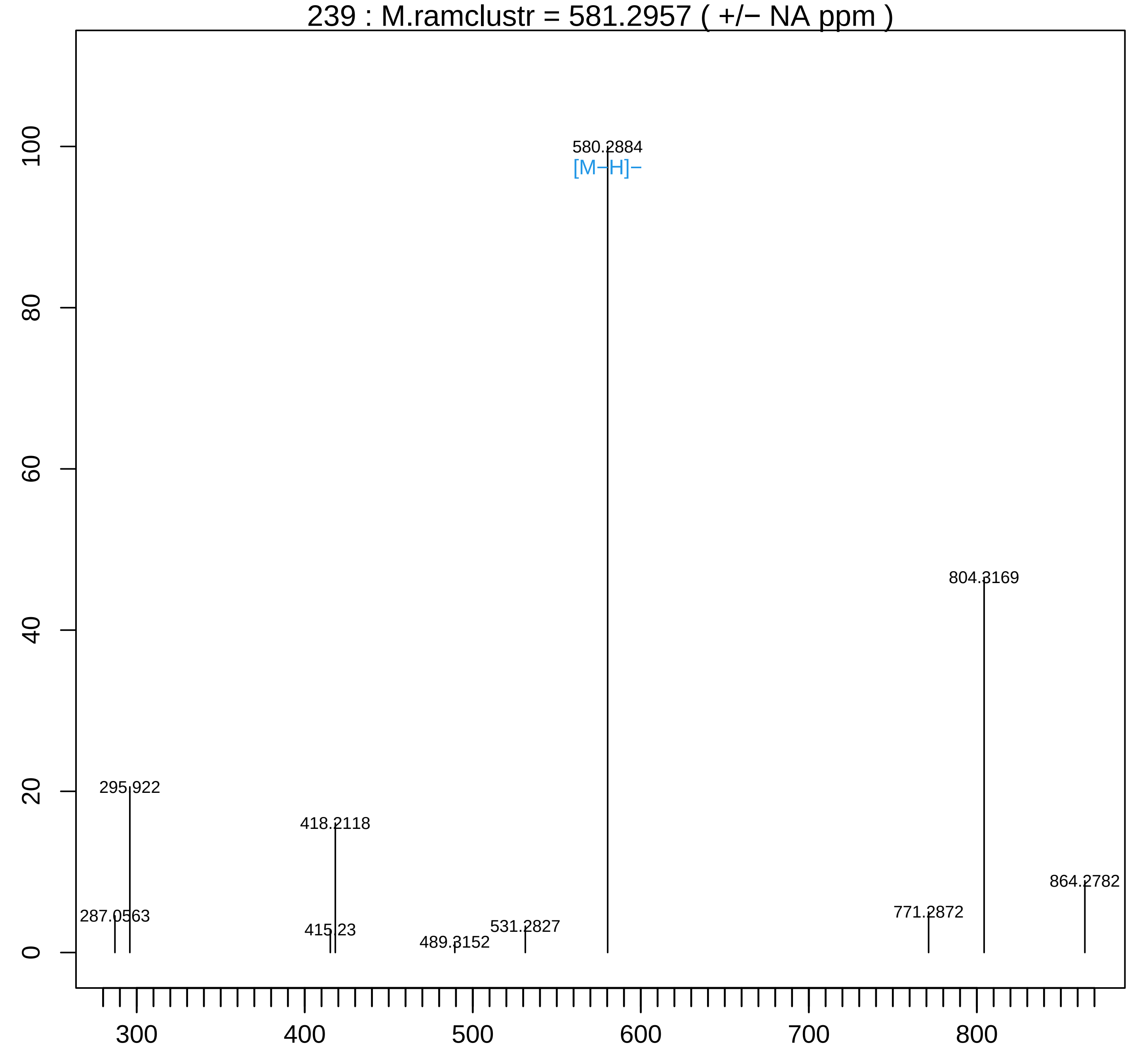

239 : M.ramclustr = 581.2957 (+/− NA ppm)
580.2884
[M−H]−
804.3169
295.922
418.2118
287.0563
415.23
489.3152
531.2827
771.2872
864.2782
0
20
40
60
80
100
300
400
500
600
700
800

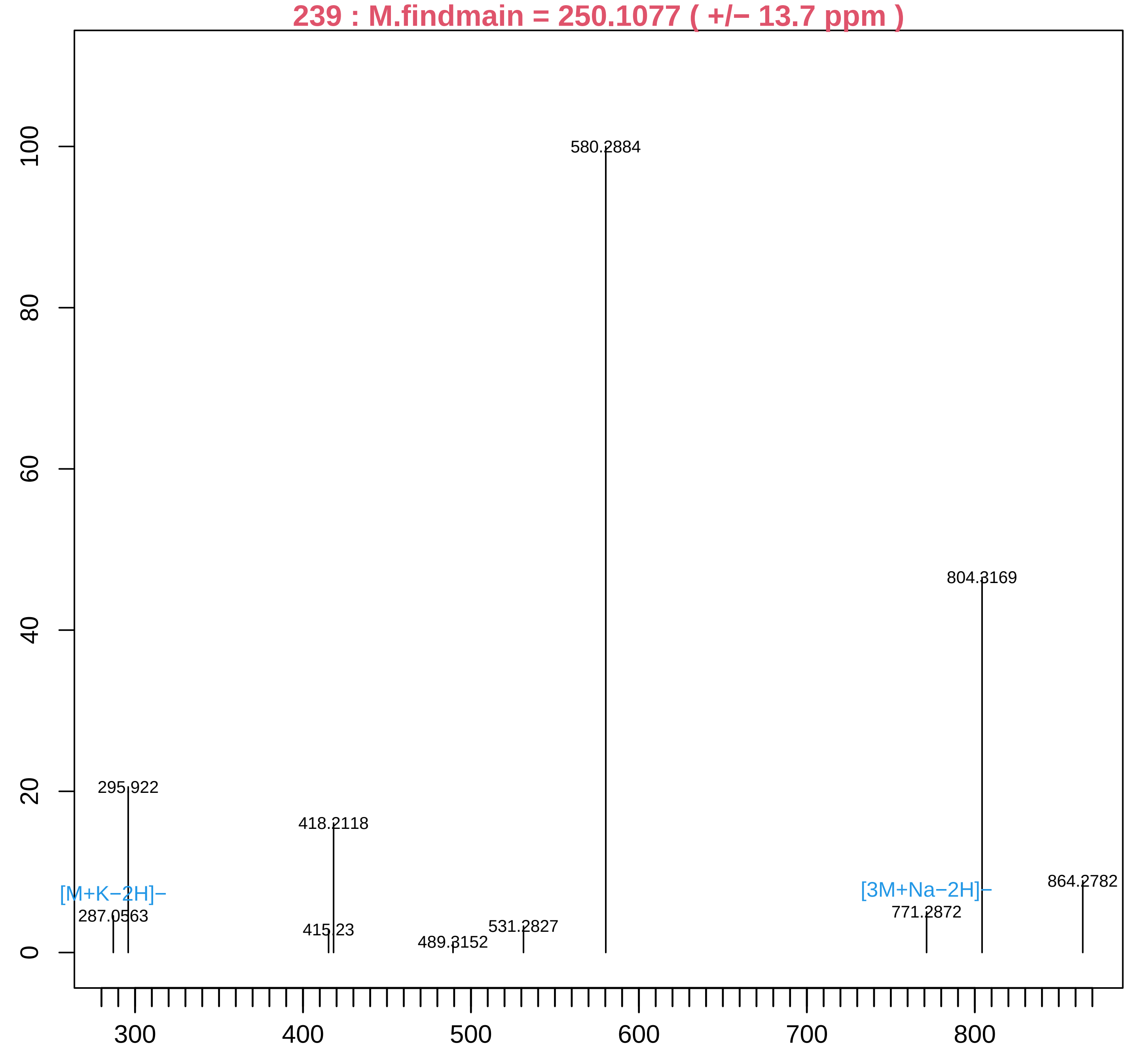

239 : M.findmain = 250.1077 (+/− 13.7 ppm)
580.2884
804.3169
295.922
418.2118
[M+K−2H]−
287.0563
415.23
489.3152
531.2827
[3M+Na−2H]−
771.2872
864.2782
0
20
40
60
80
100
300
400
500
600
700
800

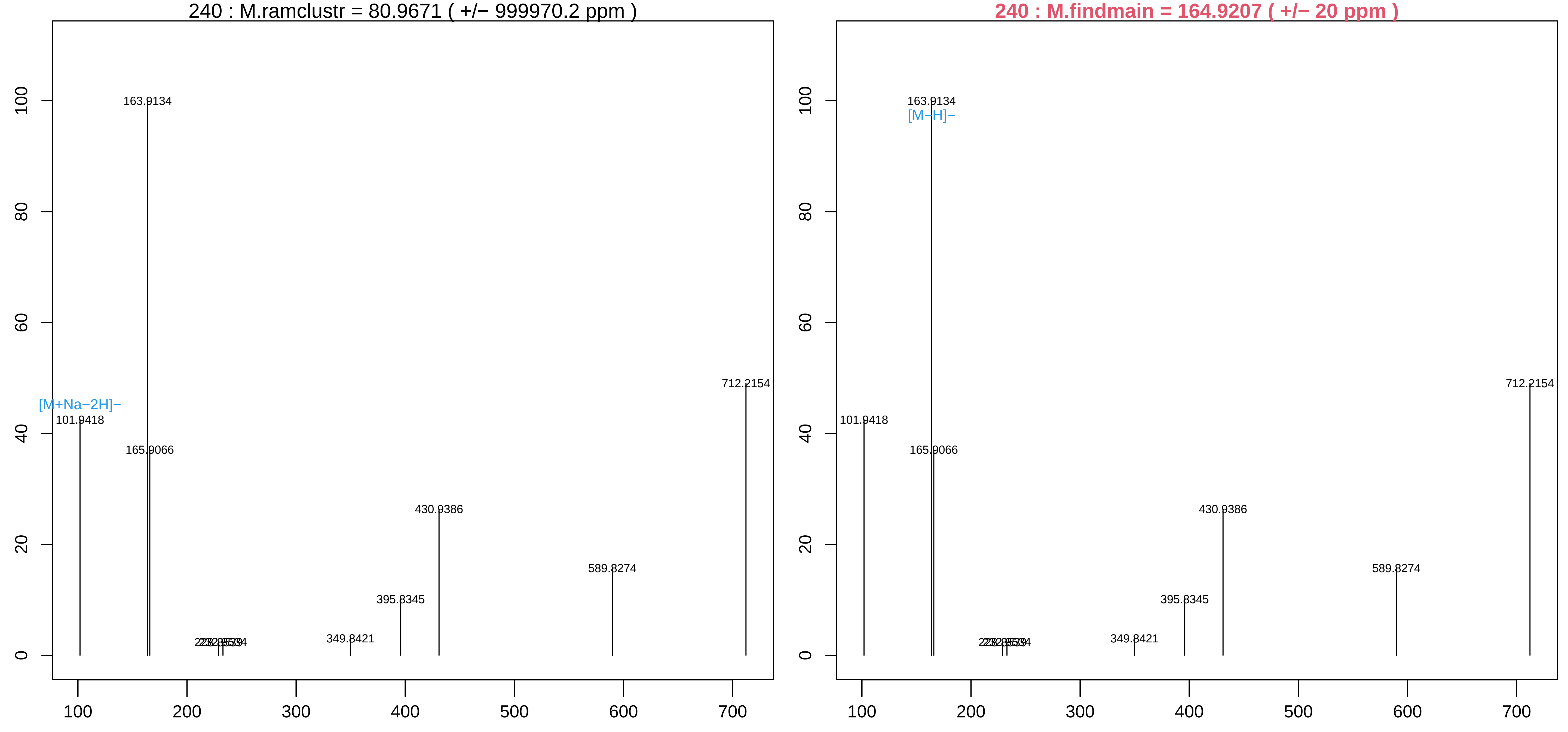
240 : M.ramclustr = 80.9671 (+/− 999970.2 ppm)
163.9134
[M+Na−2H]−
101.9418
165.9066
430.9386
589.8274
395.8345
349.8421
712.2154
240 : M.findmain = 164.9207 (+/− 20 ppm)
163.9134
[M−H]−
101.9418
165.9066
430.9386
589.8274
395.8345
349.8421
712.2154

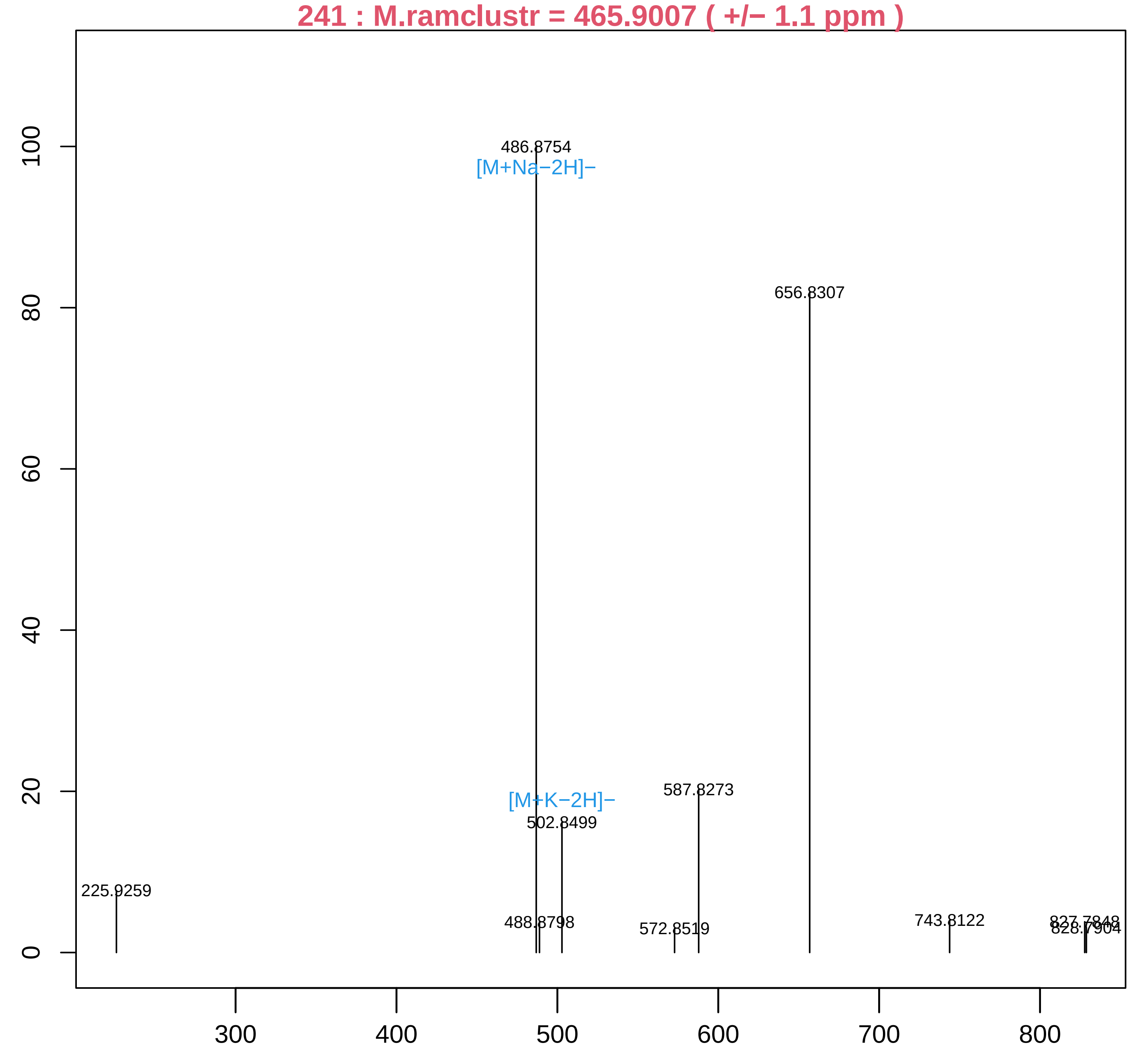
241 : M.ramclustr = 465.9007 (+/− 1.1 ppm)
486.8754
[M+Na−2H]−
656.8307
587.8273
[M+K−2H]−
502.8499
225.9259
488.8798
572.8519
743.8122
827.7848
828.7904
0
20
40
60
80
100
300
400
500
600
700
800

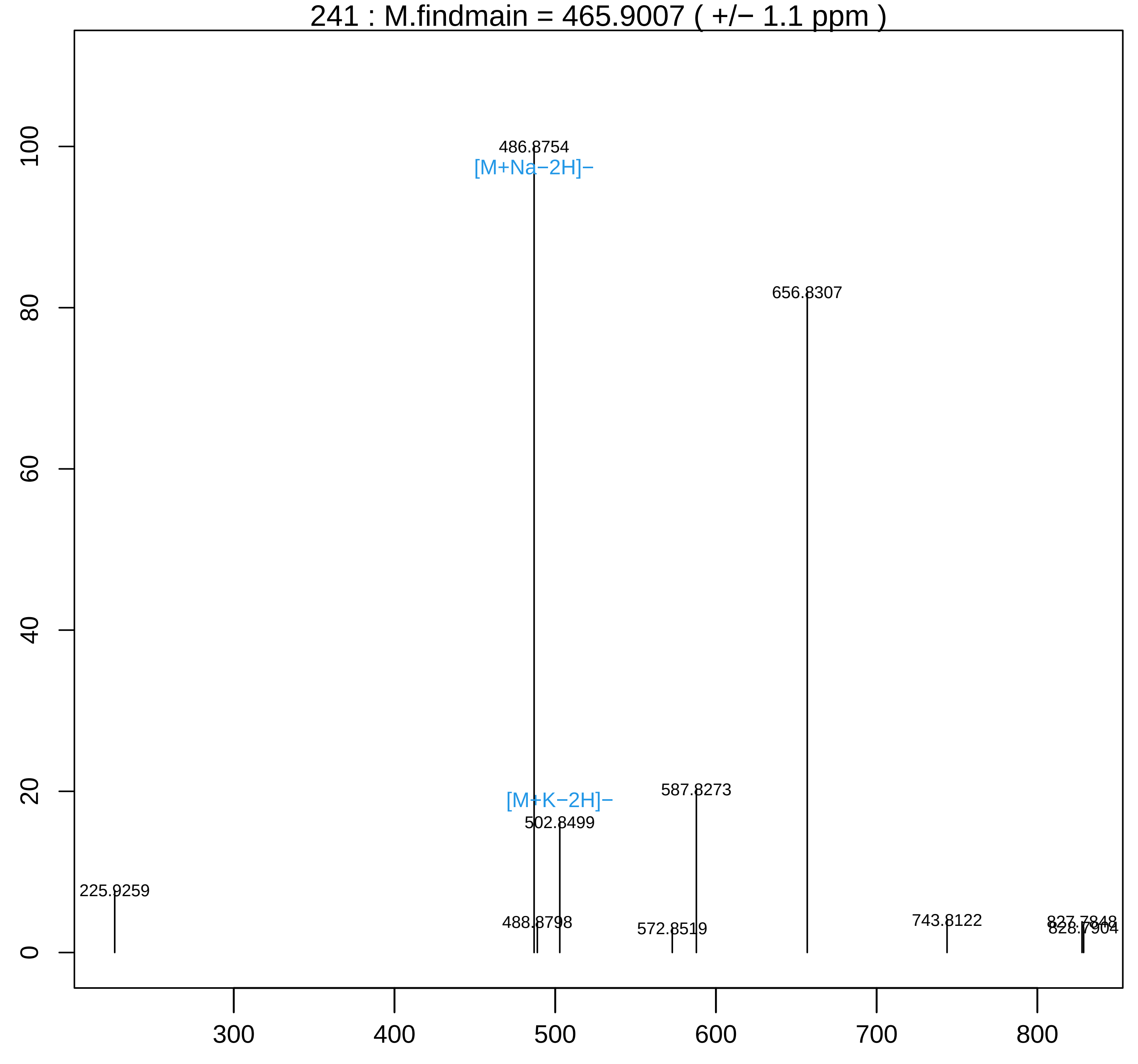
241 : M.findmain = 465.9007 (+/− 1.1 ppm)
486.8754
[M+Na−2H]−
656.8307
587.8273
[M+K−2H]−
502.8499
225.9259
488.8798
572.8519
743.8122
827.7848
828.7904
0
20
40
60
80
100
300
400
500
600
700
800

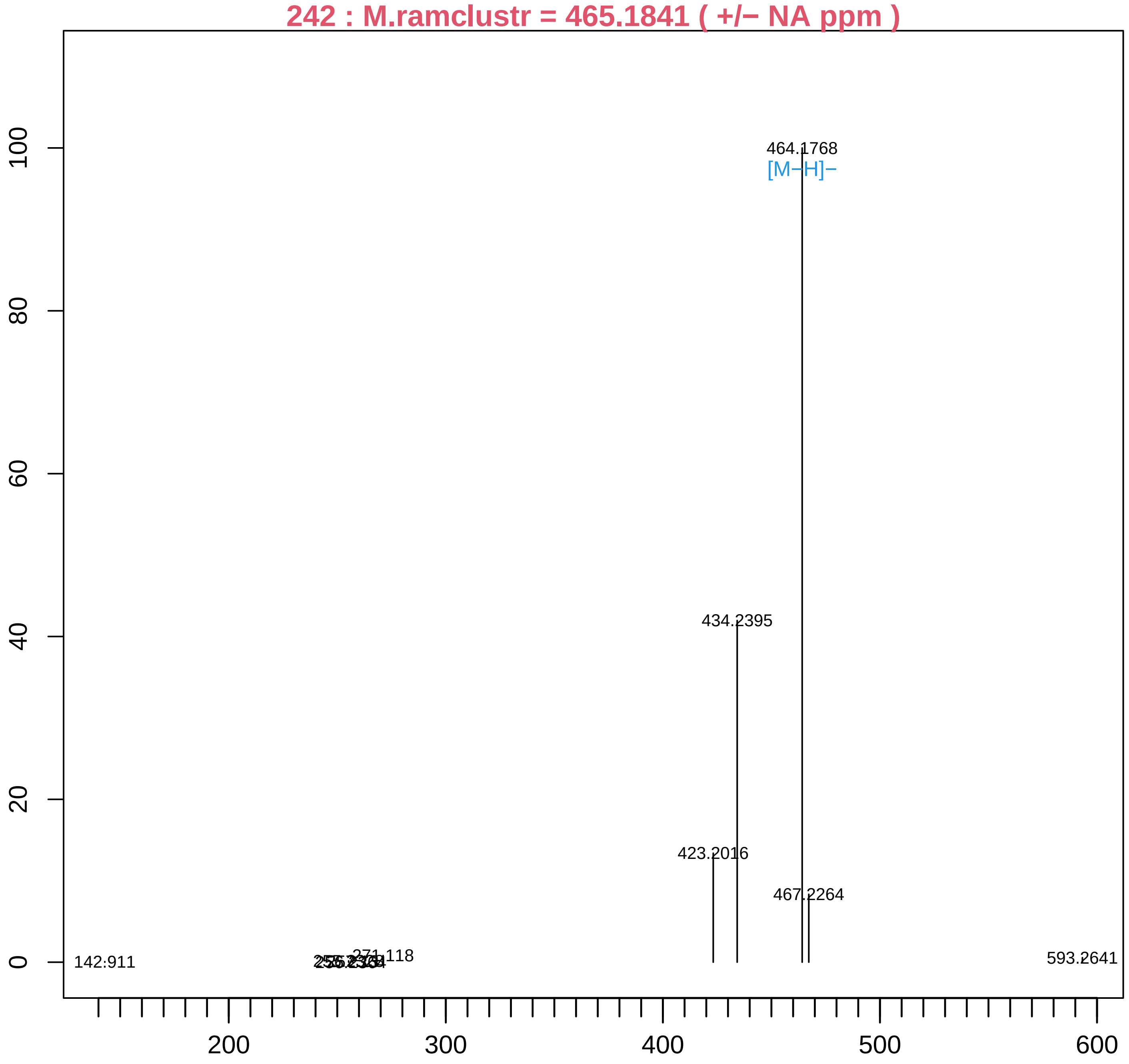

242 : M.ramclustr = 465.1841 (+/− NA ppm)
464.1768
[M−H]−
434.2395
423.2016
467.2264
142.911
271.118
593.2641
0
20
40
60
80
100
200
300
400
500
600

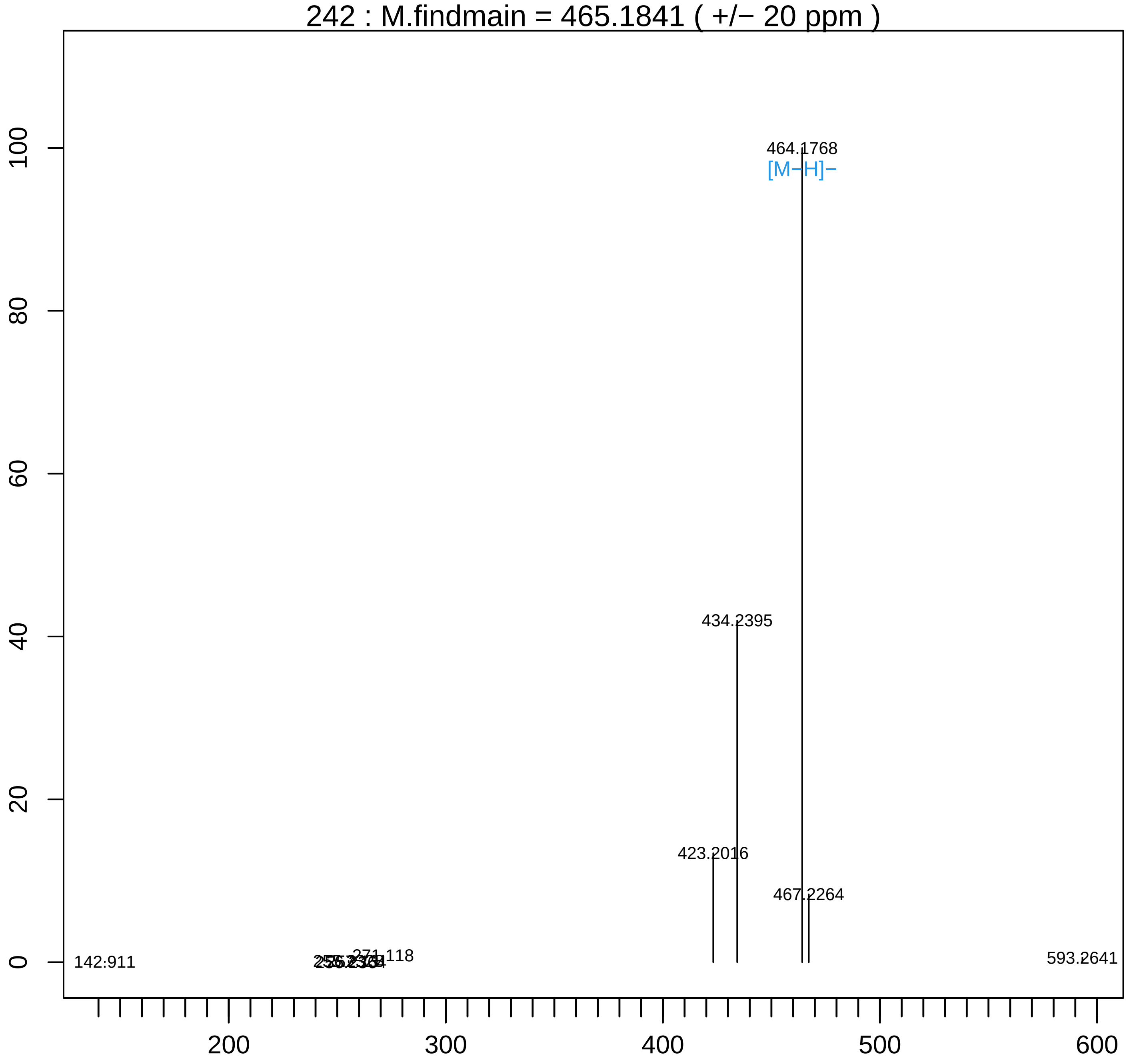

242 : M.findmain = 465.1841 (+/− 20 ppm)
464.1768
[M−H]−
434.2395
423.2016
467.2264
142.911
271.118
593.2641
0
20
40
60
80
100
200
300
400
500
600

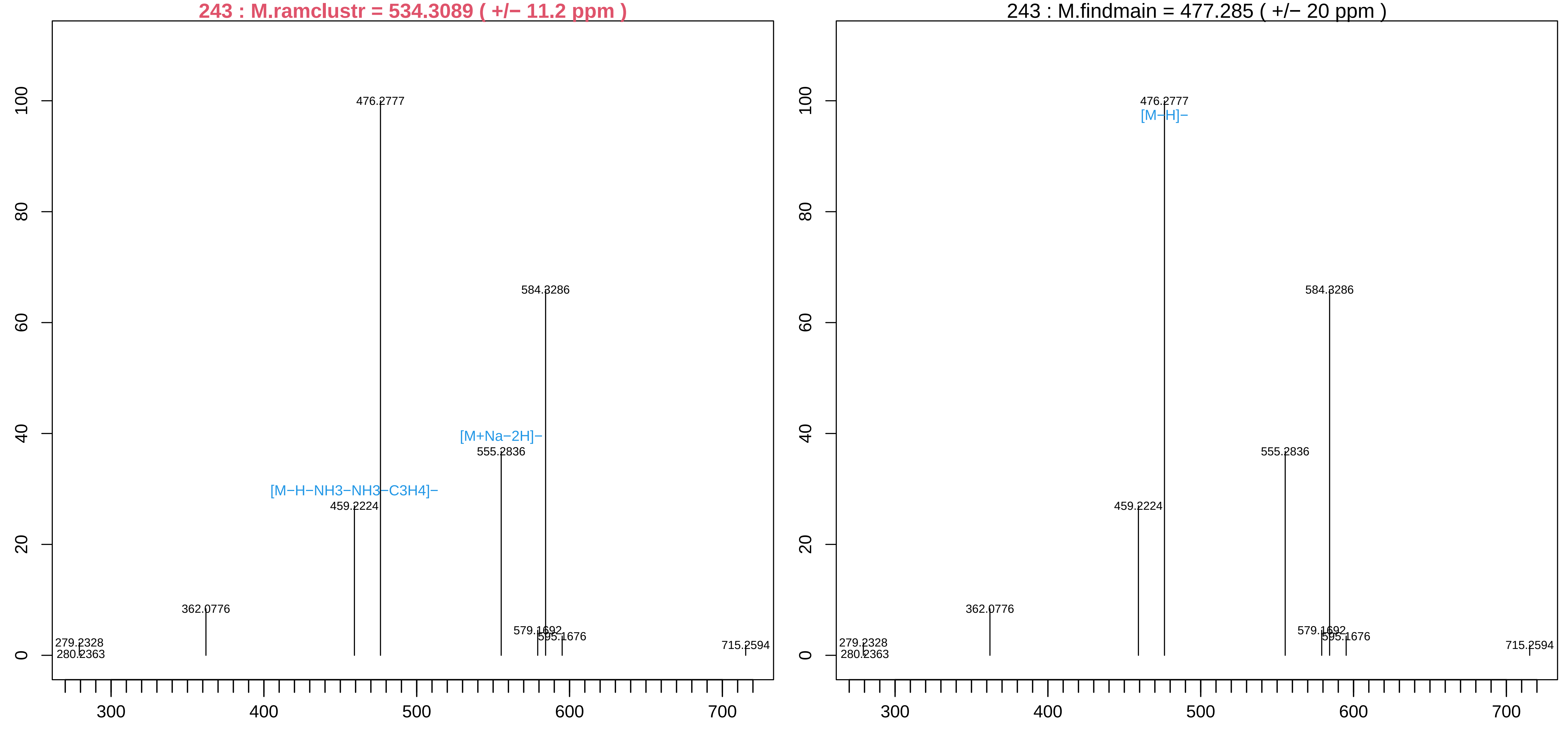
243 : M.ramclustr = 534.3089 (+/− 11.2 ppm)
476.2777
584.3286
[M+Na−2H]−
555.2836
[M−H−NH3−NH3−C3H4]−
459.2224
362.0776
579.1692
595.1676
279.2328
280.2363
715.2594
243 : M.findmain = 477.285 (+/− 20 ppm)
476.2777
[M−H]−
584.3286
555.2836
459.2224
362.0776
579.1692
595.1676
279.2328
280.2363
715.2594
0
20
40
60
80
100
300
400
500
600
700

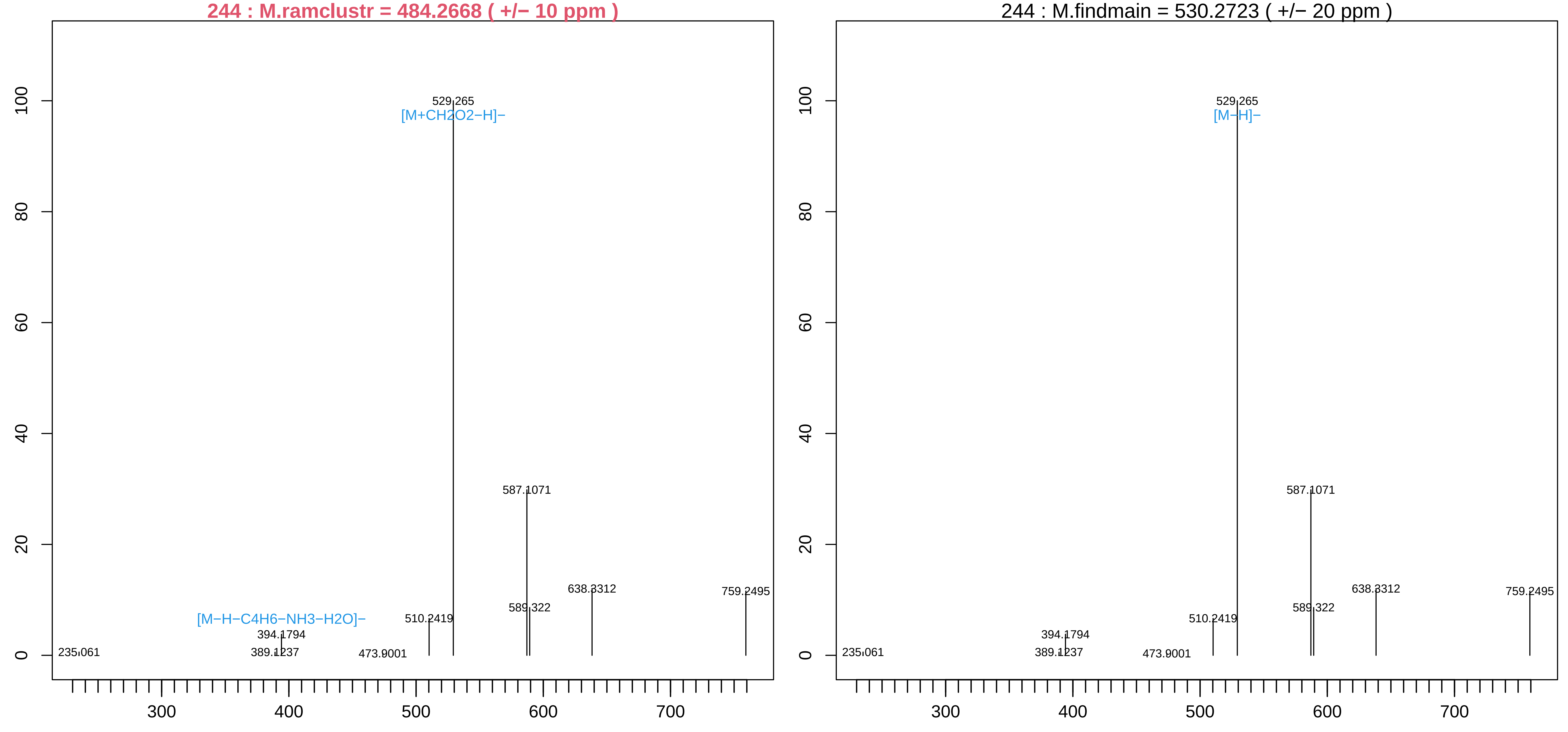

244 : M.ramclustr = 484.2668 (+/− 10 ppm)
529.265
[M+CH2O2−H]−
587.1071
638.3312
759.2495
589.322
510.2419
[M−H−C4H6−NH3−H2O]−
394.1794
389.1237
473.9001
235.061
244 : M.findmain = 530.2723 (+/− 20 ppm)
529.265
[M−H]−
0
20
40
60
80
100
300
400
500
600
700

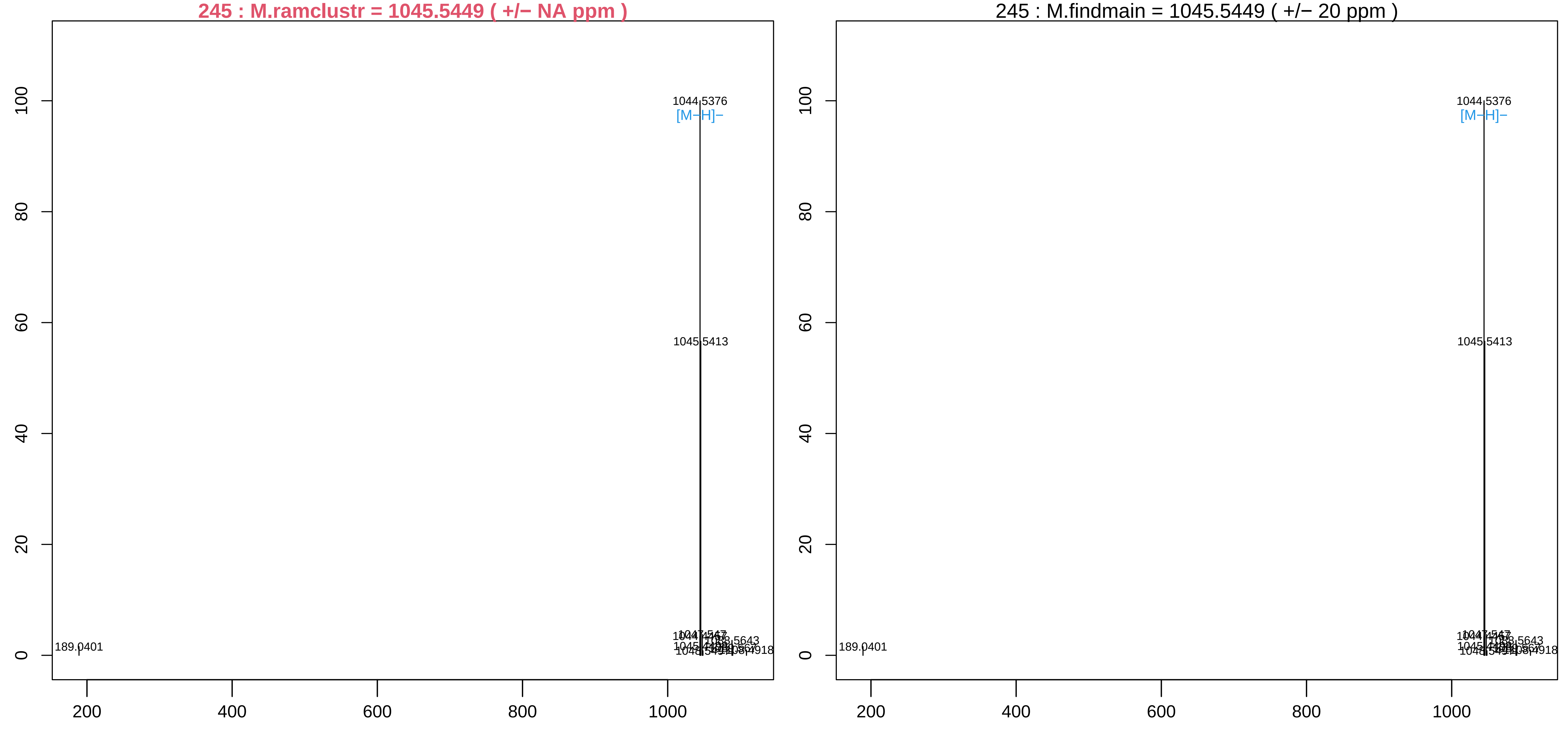
245 : M.ramclustr = 1045.5449 (+/− NA ppm)
1044.5376
[M−H]−
1045.5413
189.0401
245 : M.findmain = 1045.5449 (+/− 20 ppm)
1044.5376
[M−H]−
1045.5413
189.0401
0
20
40
60
80
100
200
400
600
800
1000

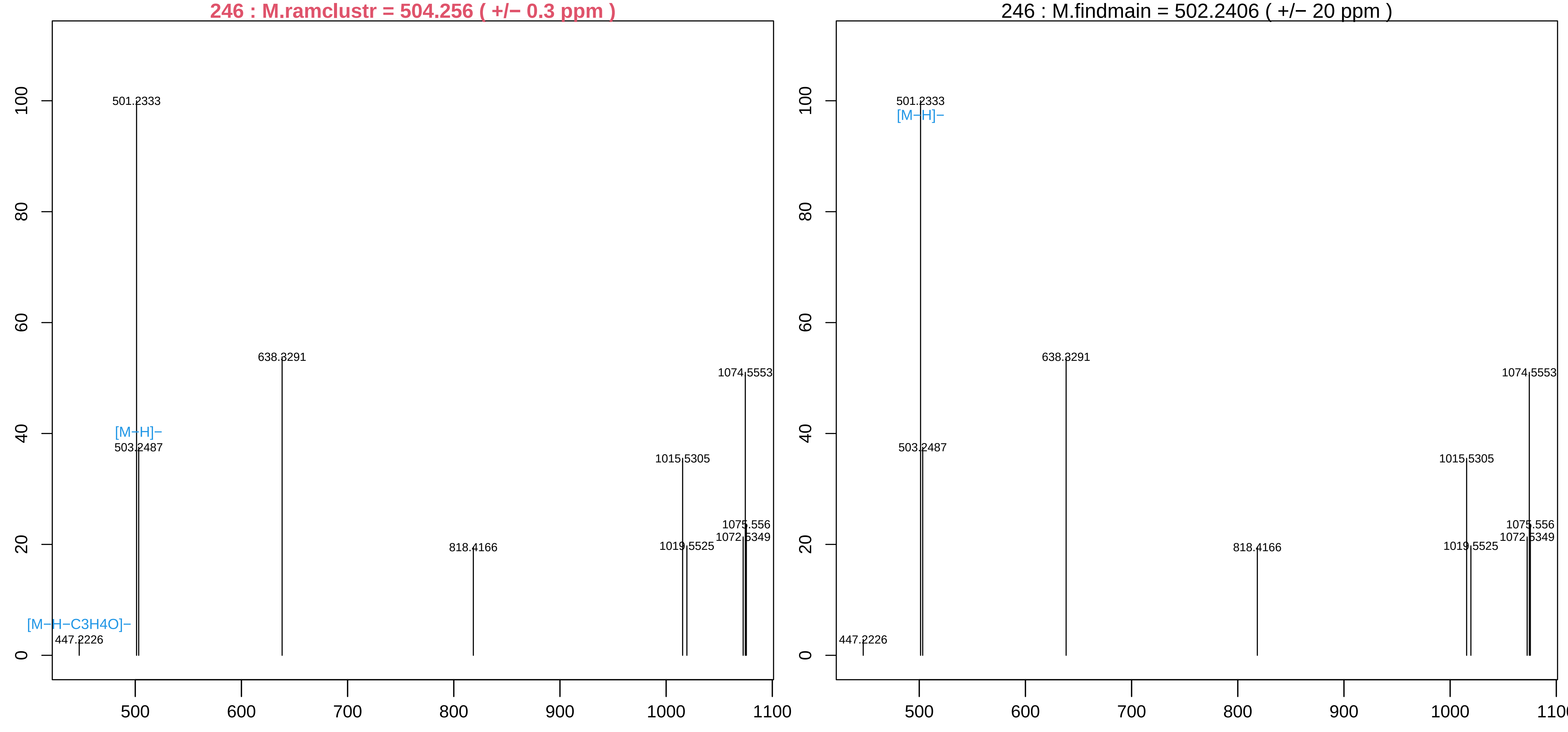

246 : M.ramclustr = 504.256 (+/− 0.3 ppm)
501.2333
[M−H]−
503.2487
638.3291
818.4166
1015.5305
1019.5525
1074.5553
1075.556
1072.5349
[M−H−C3H4O]−
447.2226
246 : M.findmain = 502.2406 (+/− 20 ppm)
501.2333
[M−H]−
503.2487
638.3291
818.4166
1015.5305
1019.5525
1074.5553
1075.556
1072.5349
447.2226
0
20
40
60
80
100
500
600
700
800
900
1000
1100

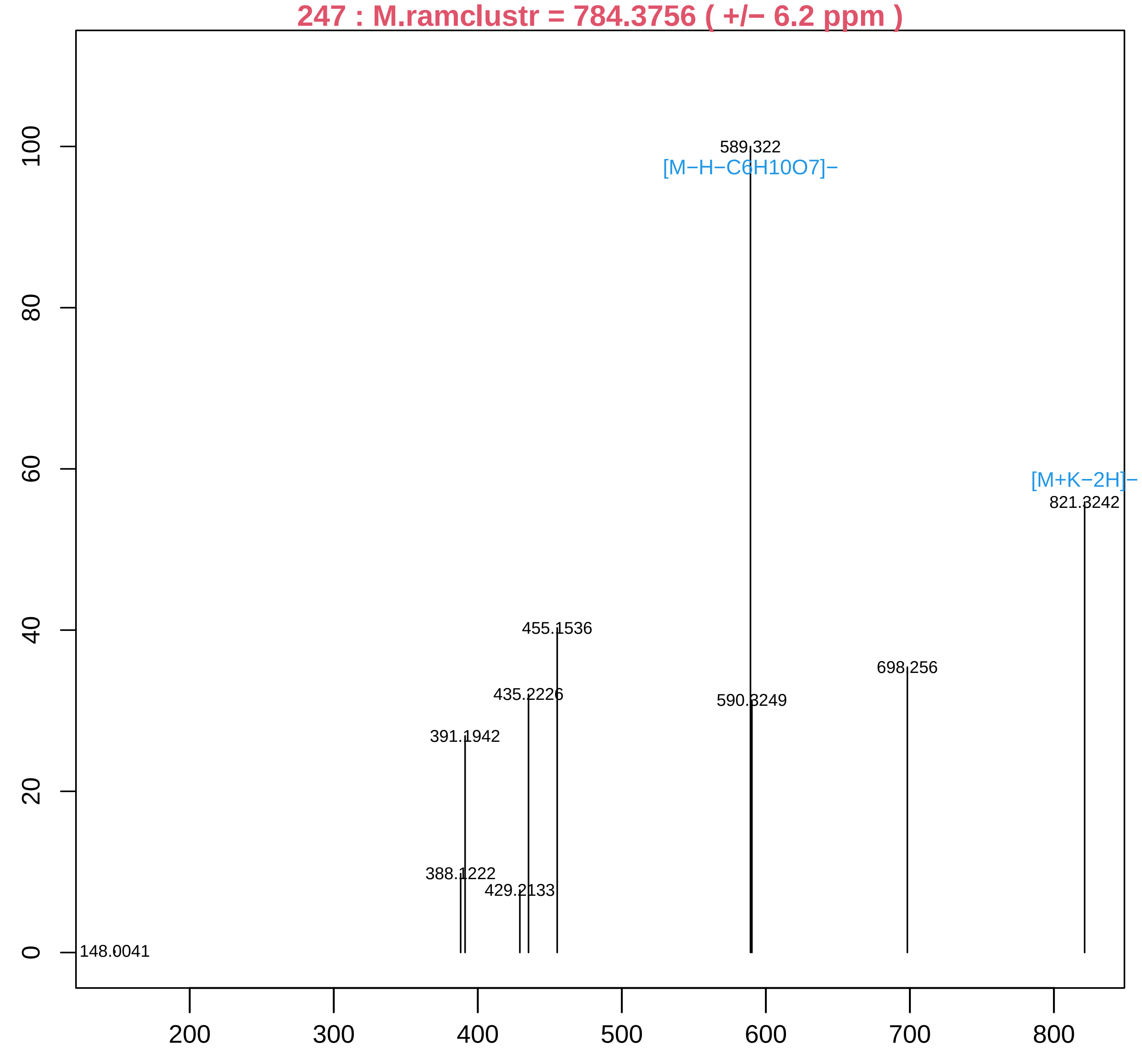

247 : M.ramclustr = 784.3756 (+/− 6.2 ppm)
589.322
[M−H−C6H10O7]−
[M+K−2H]−
821.3242
455.1536
435.2226
391.1942
590.3249
698.256
388.1222
429.2133
148.0041
0
20
40
60
80
100
200
300
400
500
600
700
800

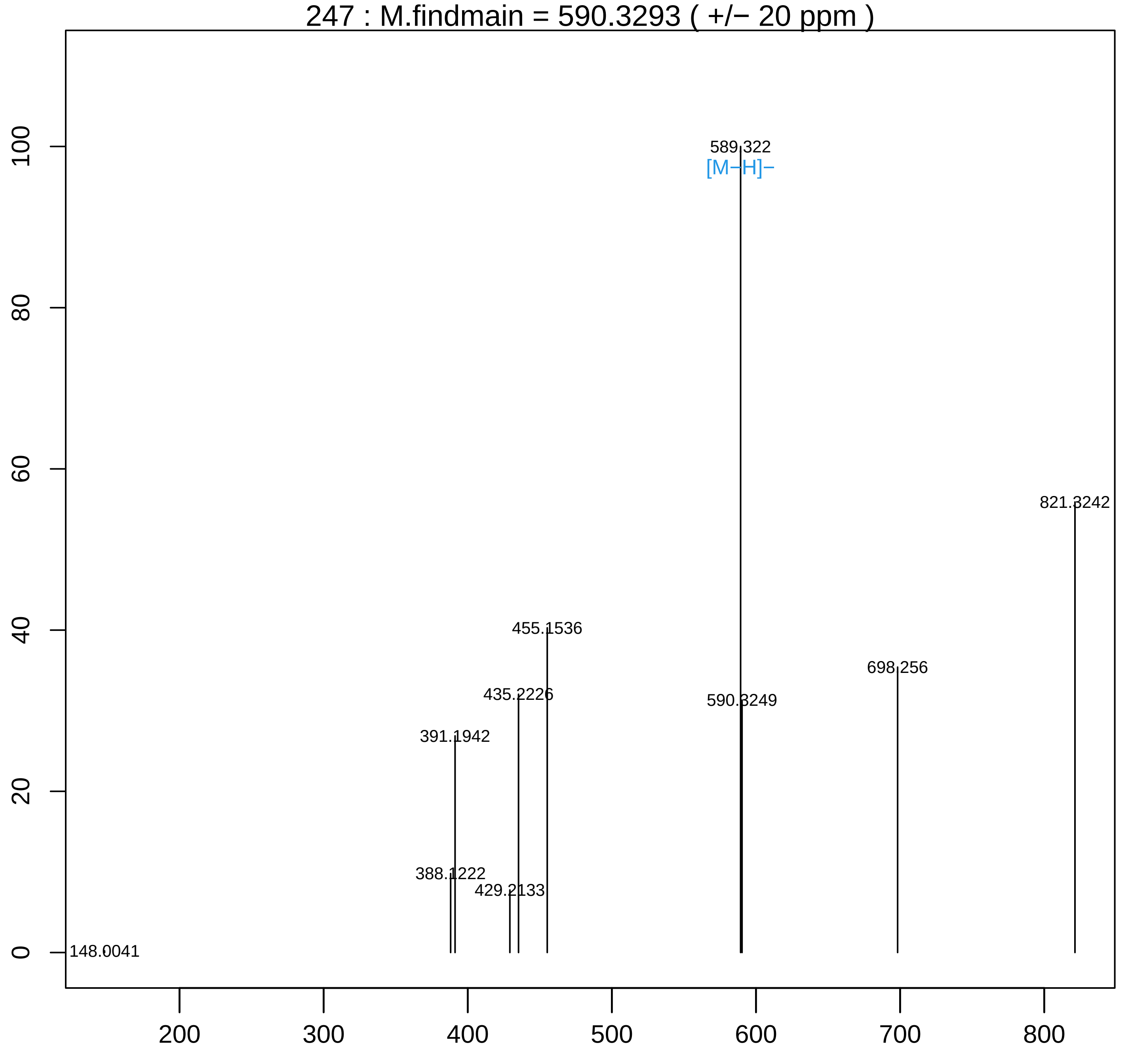

247 : M.findmain = 590.3293 (+/− 20 ppm)
589.322
[M−H]−
821.3242
455.1536
435.2226
391.1942
590.3249
698.256
388.1222
429.2133
148.0041
0
20
40
60
80
100
200
300
400
500
600
700
800

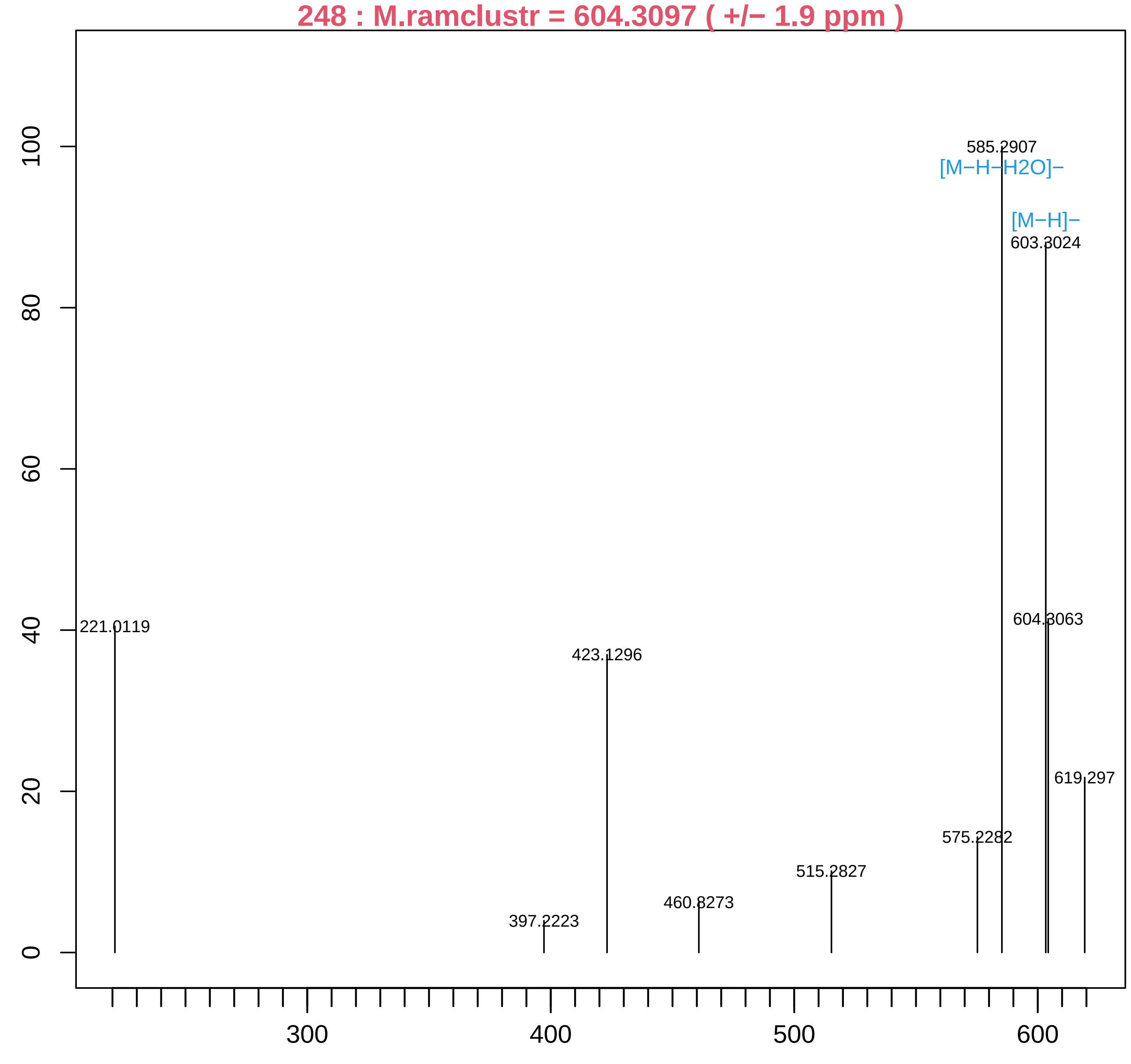
248 : M.ramclustr = 604.3097 (+/− 1.9 ppm)
585.2907
[M−H−H2O]−
[M−H]−
603.3024
604.3063
221.0119
423.1296
619.297
575.2282
515.2827
460.8273
397.2223
0
20
40
60
80
100
300
400
500
600

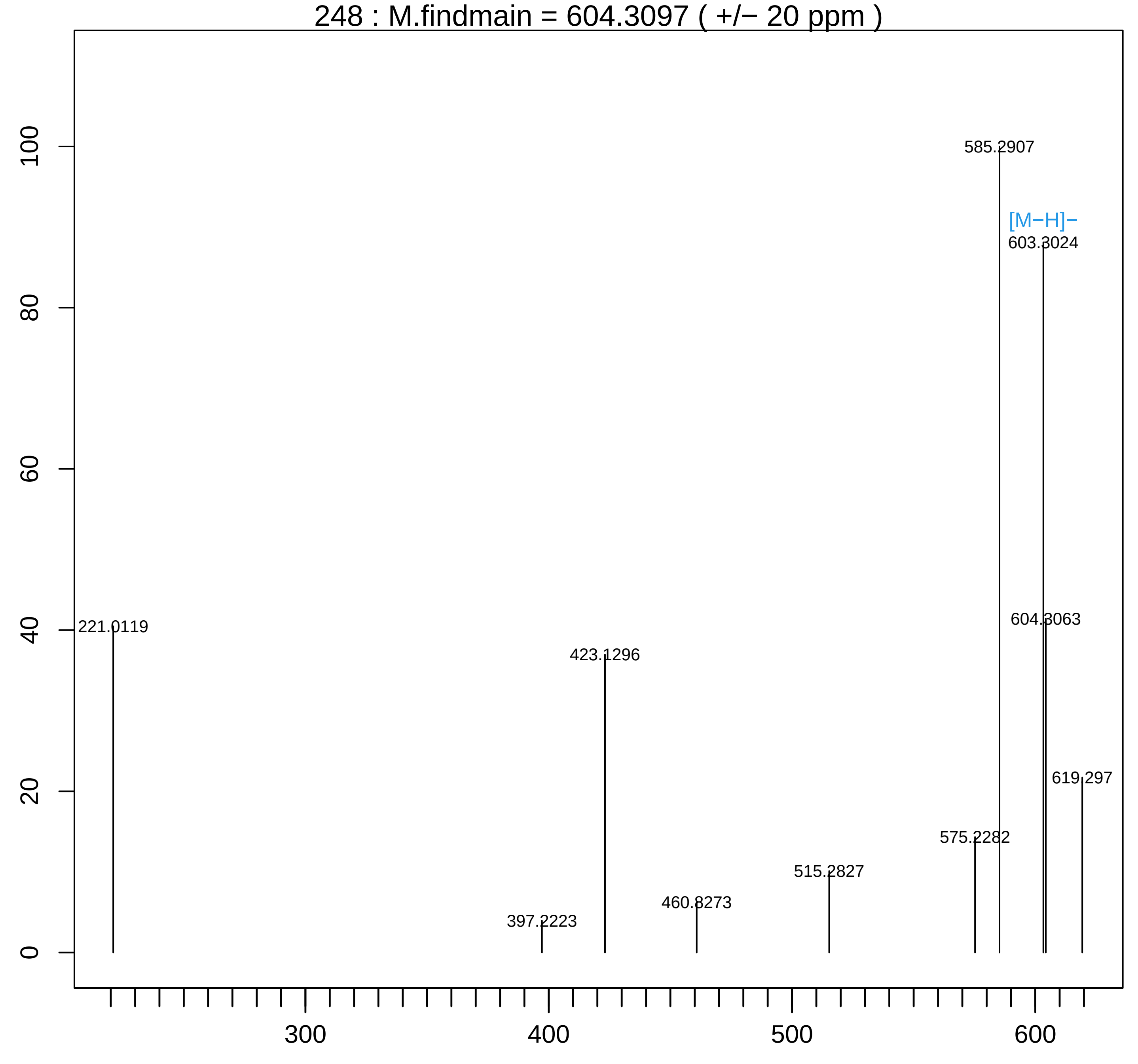
248 : M.findmain = 604.3097 (+/− 20 ppm)
585.2907
[M−H]−
603.3024
604.3063
221.0119
423.1296
619.297
575.2282
515.2827
460.8273
397.2223
0
20
40
60
80
100
300
400
500
600

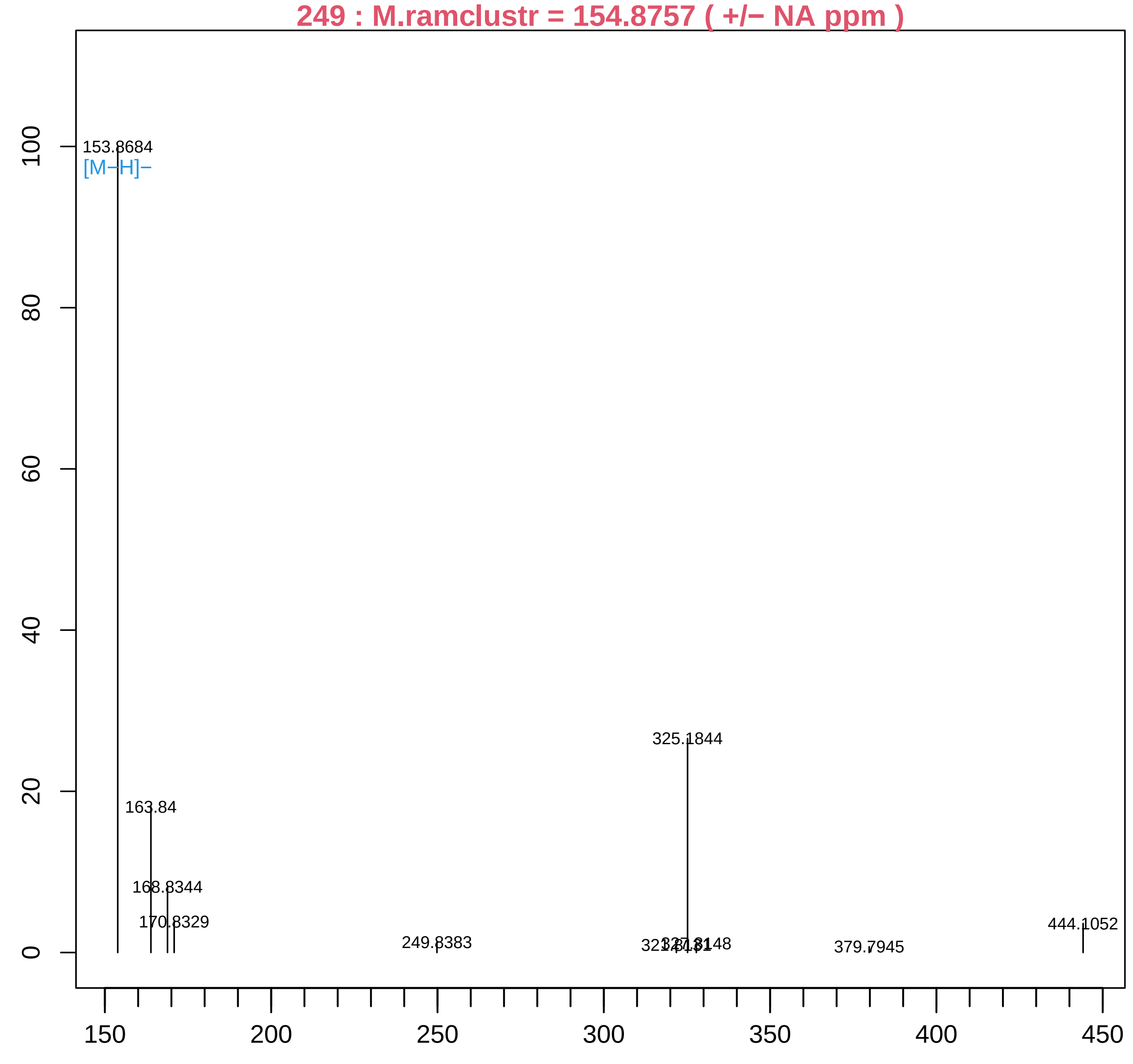

249 : M.ramclustr = 154.8757 (+/− NA ppm)
153.8684
[M−H]−
163.84
168.8344
170.8329
249.8383
325.1844
379.7945
444.1052
0
20
40
60
80
100
150
200
250
300
350
400
450

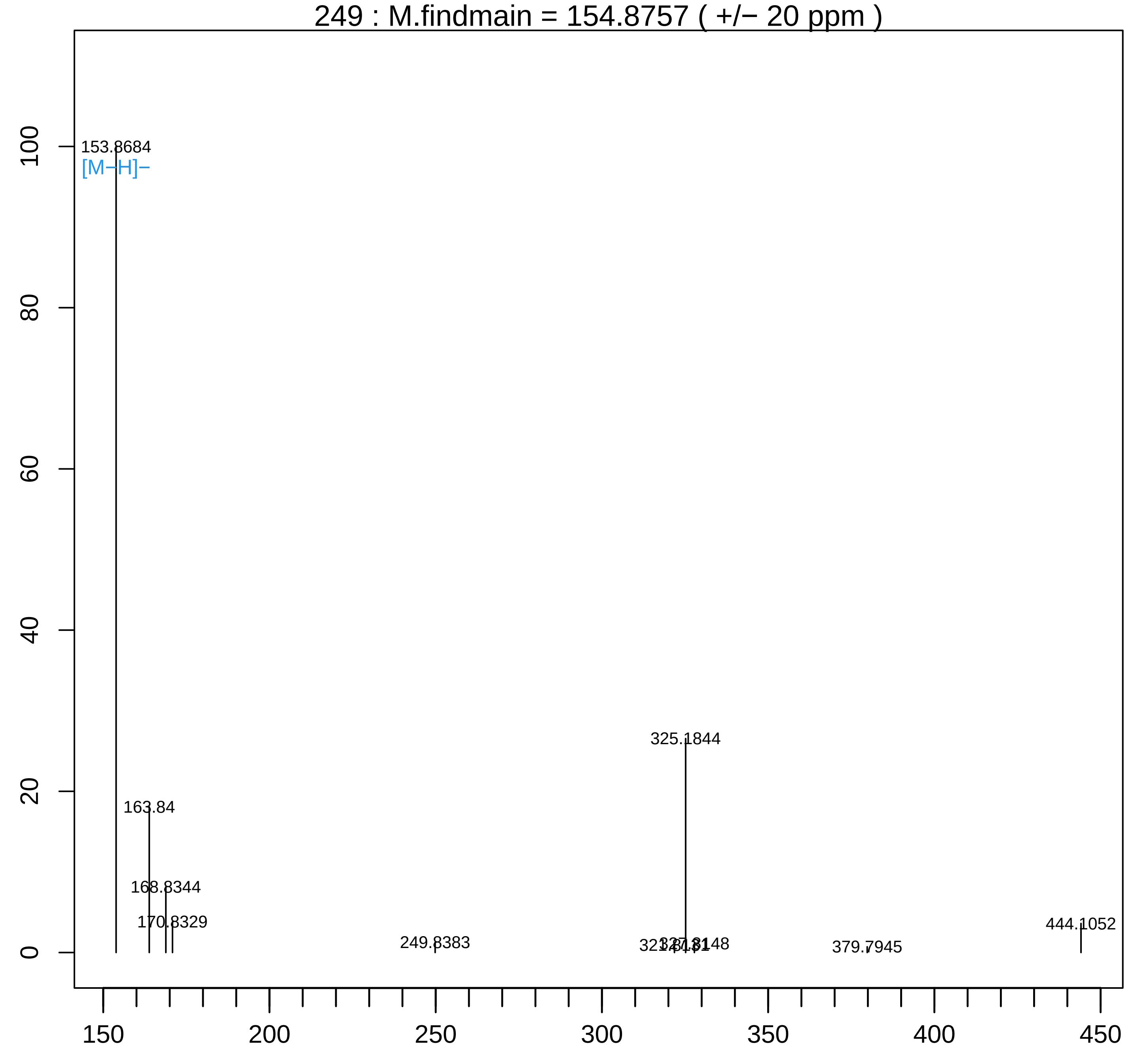

249 : M.findmain = 154.8757 (+/− 20 ppm)
153.8684
[M−H]−
163.84
168.8344
170.8329
249.8383
325.1844
379.7945
444.1052
0
20
40
60
80
100
150
200
250
300
350
400
450

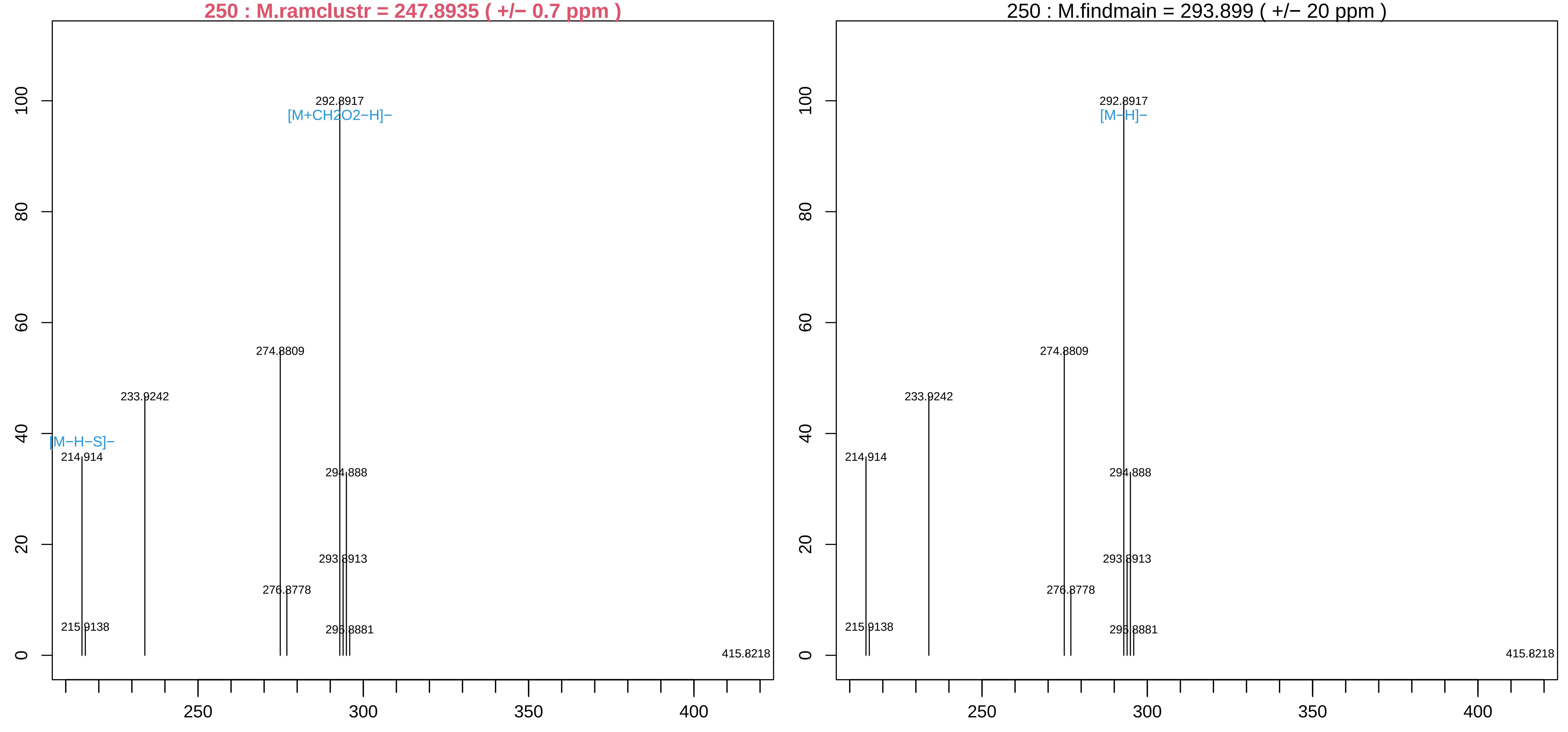
250 : M.ramclustr = 247.8935 (+/− 0.7 ppm)
292.8917
[M+CH2O2−H]−
274.8809
233.9242
[M−H−S]−
214.914
294.888
293.8913
276.8778
215.9138
295.8881
415.8218
250 : M.findmain = 293.899 (+/− 20 ppm)
292.8917
[M−H]−
274.8809
233.9242
214.914
294.888
293.8913
276.8778
215.9138
295.8881
415.8218
0
20
40
60
80
100
250
300
350
400

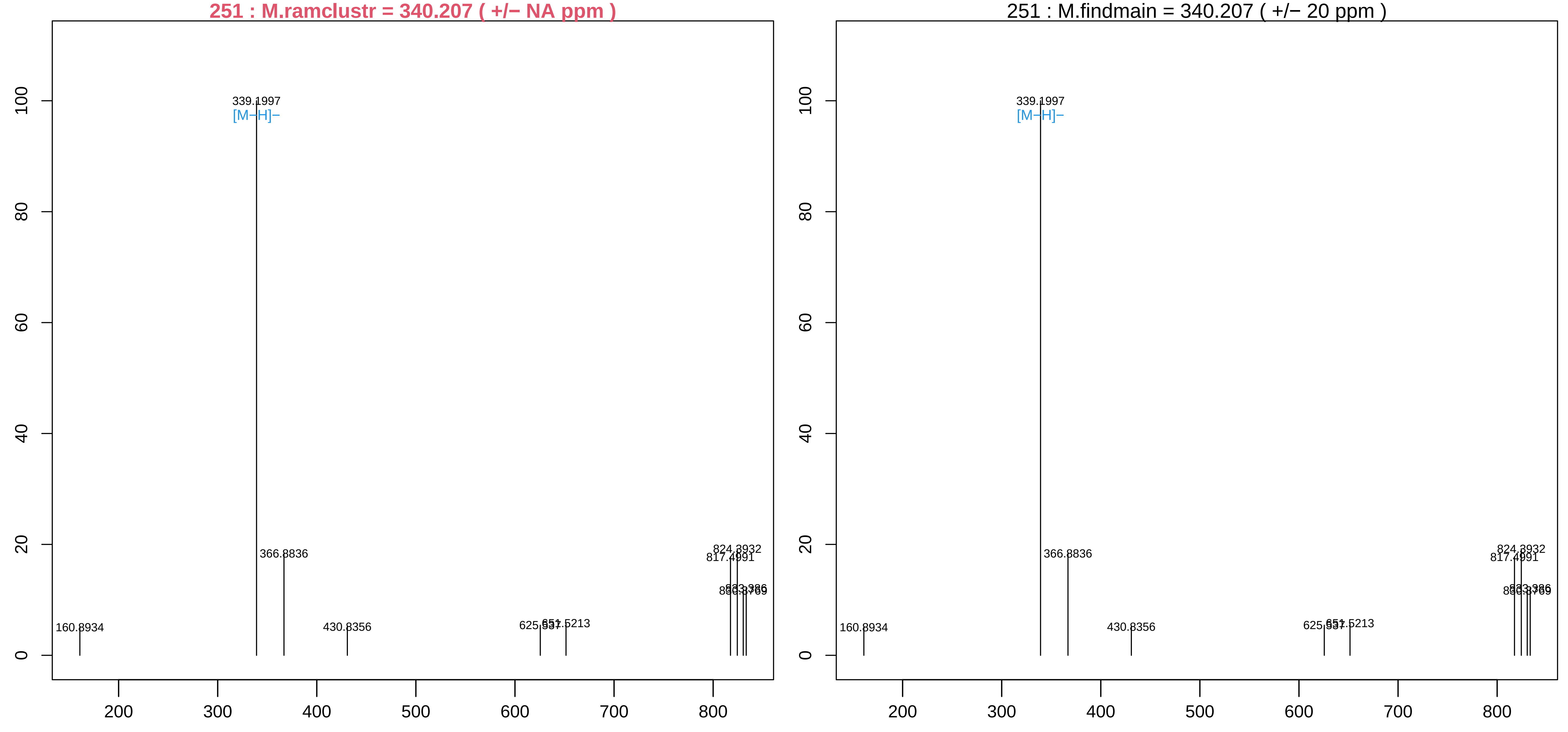
251 : M.ramclustr = 340.207 (+/− NA ppm)
339.1997
[M−H]−
366.8836
160.8934
430.8356
651.5213
824.3932
817.4991
251 : M.findmain = 340.207 (+/− 20 ppm)
339.1997
[M−H]−
366.8836
160.8934
430.8356
651.5213
824.3932
817.4991
0
20
40
60
80
100
200
300
400
500
600
700
800

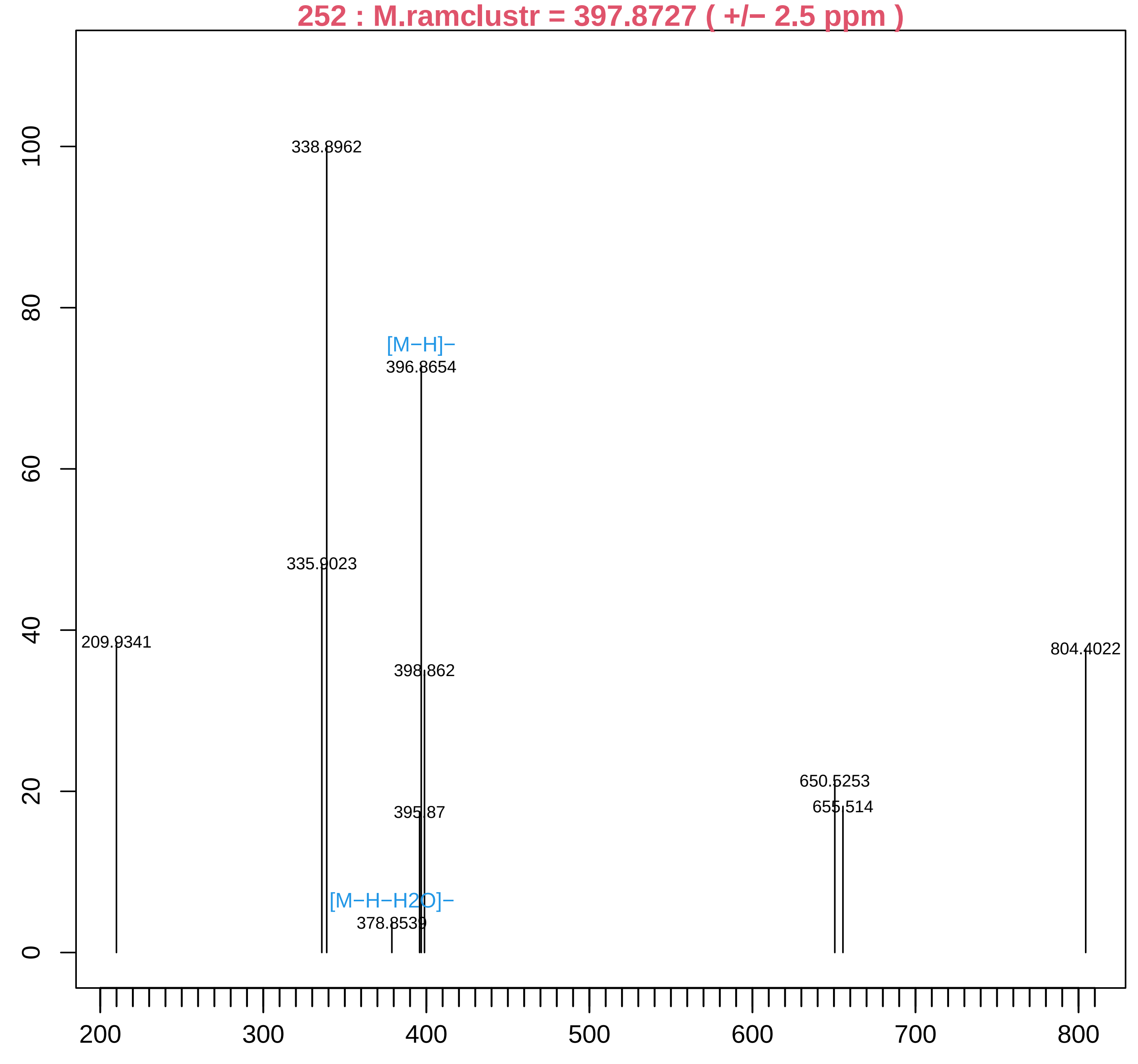
252 : M.ramclustr = 397.8727 (+/− 2.5 ppm)
338.8962
335.9023
209.9341
[M−H]−
396.8654
398.862
395.87
[M−H−H2O]−
378.8539
650.5253
655.514
804.4022
0
20
40
60
80
100
200
300
400
500
600
700
800
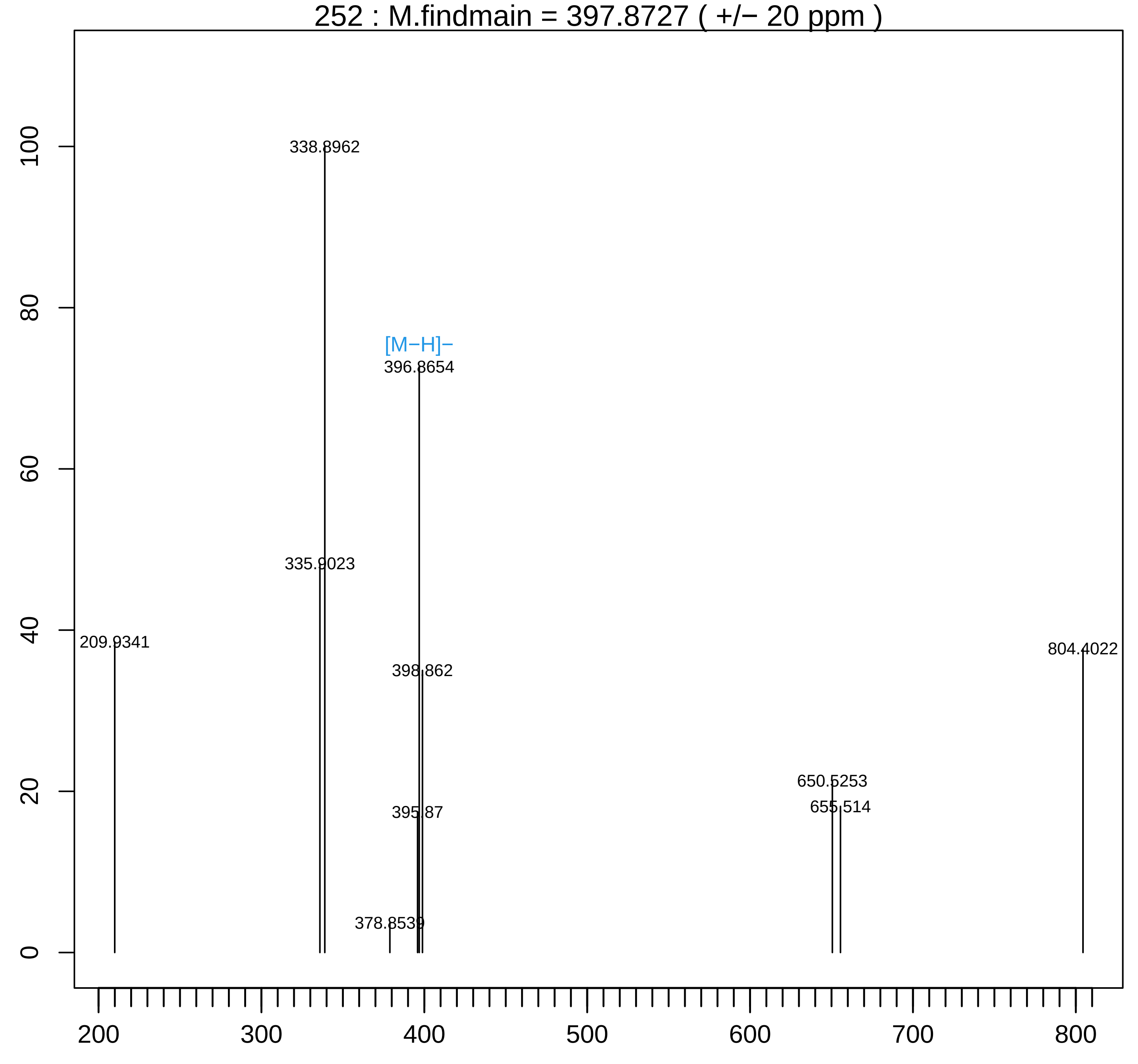
252 : M.findmain = 397.8727 (+/− 20 ppm)
338.8962
335.9023
209.9341
[M−H]−
396.8654
398.862
395.87
378.8539
650.5253
655.514
804.4022
0
20
40
60
80
100
200
300
400
500
600
700
800

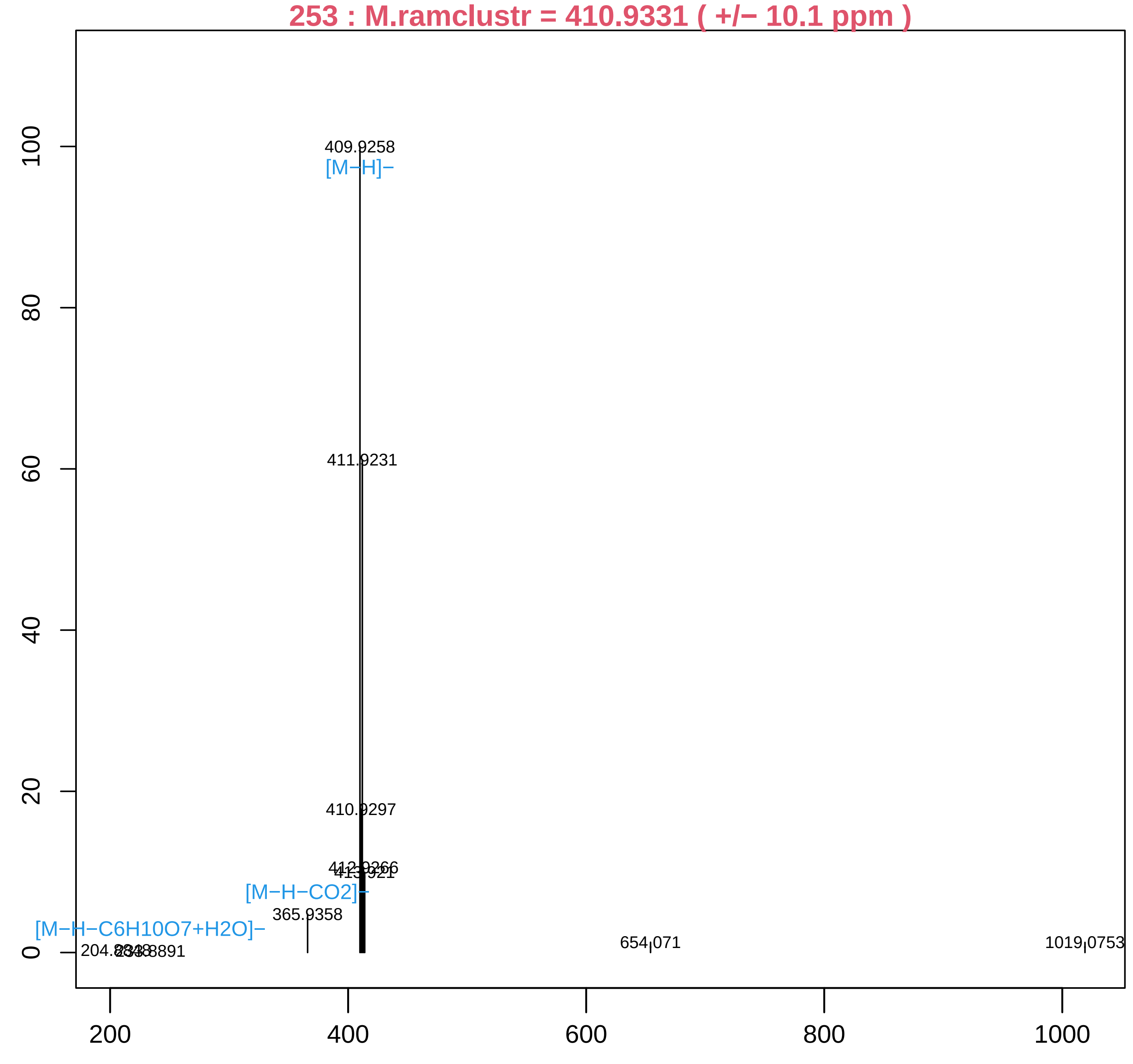
253 : M.ramclustr = 410.9331 (+/− 10.1 ppm)
409.9258
[M−H]−
411.9231
410.9297
412.9266
[M−H−CO2]−
365.9358
[M−H−C6H10O7+H2O]−
654.071
1019.0753
0
20
40
60
80
100
200
400
600
800
1000

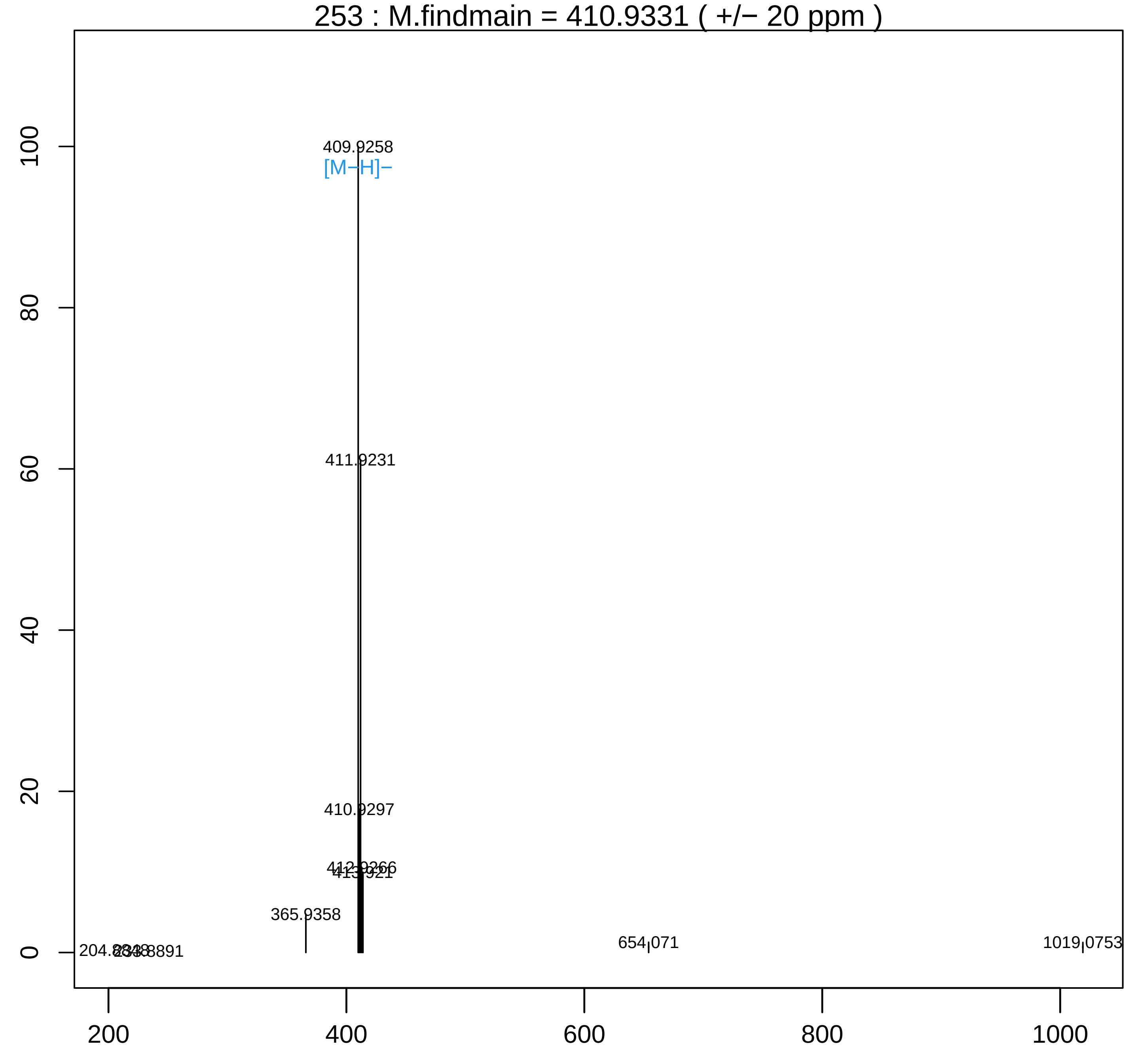
253 : M.findmain = 410.9331 (+/− 20 ppm)
409.9258
[M−H]−
411.9231
410.9297
412.9266
365.9358
654.071
1019.0753
0
20
40
60
80
100
200
400
600
800
1000

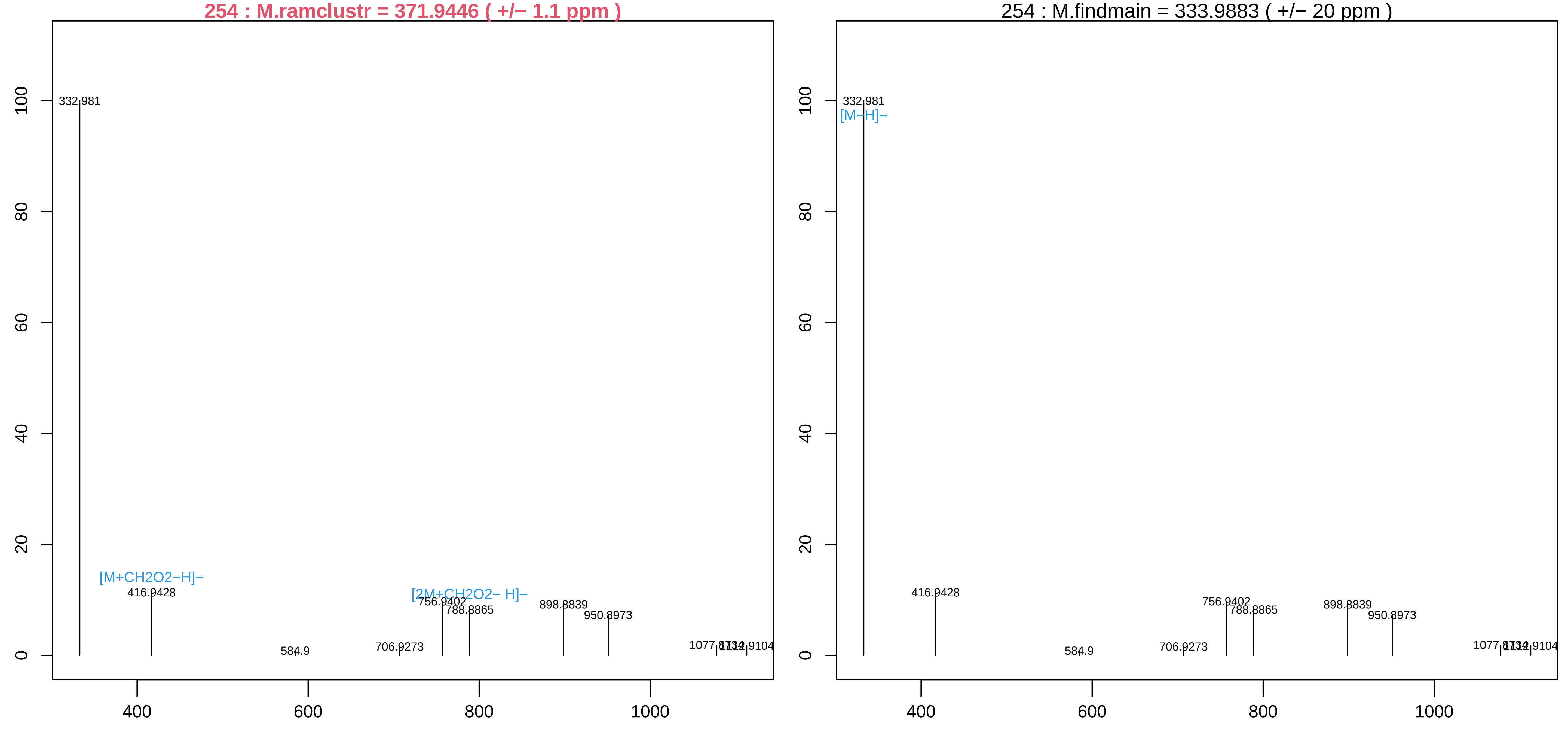
254 : M.ramclustr = 371.9446 (+/− 1.1 ppm)
332.981
[M+CH2O2−H]−
416.9428
584.9
706.9273
[2M+CH2O2− H]−
756.9402
788.8865
898.8839
950.8973
1077.8734
1112.9104
254 : M.findmain = 333.9883 (+/− 20 ppm)
332.981
[M−H]−
416.9428
584.9
706.9273
756.9402
788.8865
898.8839
950.8973
1077.8734
1112.9104

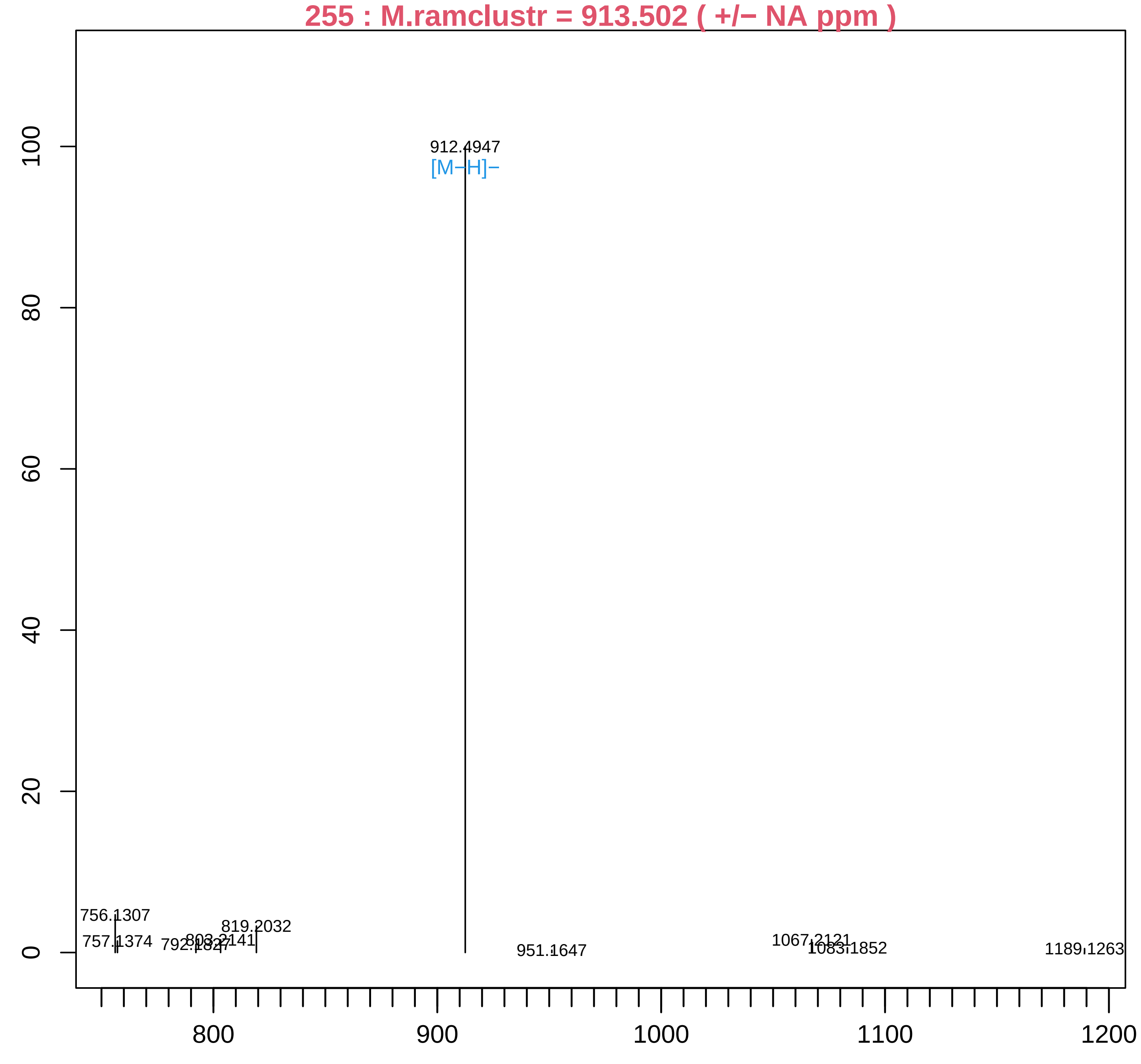

255 : M.ramclustr = 913.502 (+/− NA ppm)
912.4947
[M−H]−
756.1307
757.1374
792.1847
803.2141
819.2032
951.1647
1067.2121
1083.1852
1189.1263
0
20
40
60
80
100
800
900
1000
1100
1200

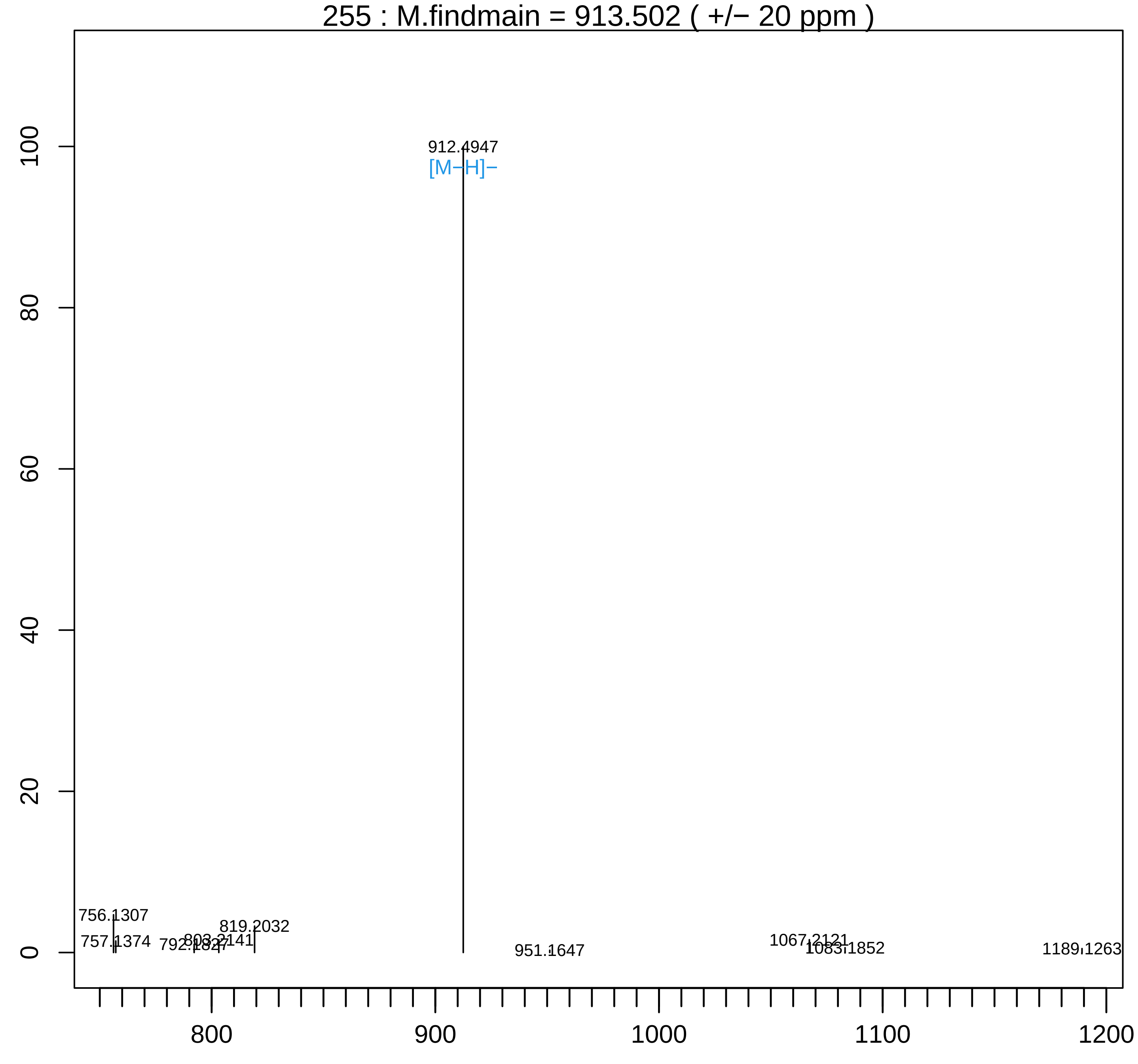

255 : M.findmain = 913.502 (+/− 20 ppm)
912.4947
[M−H]−
756.1307
757.1374
792.1847
803.2141
819.2032
951.1647
1067.2121
1083.1852
1189.1263
0
20
40
60
80
100
800
900
1000
1100
1200

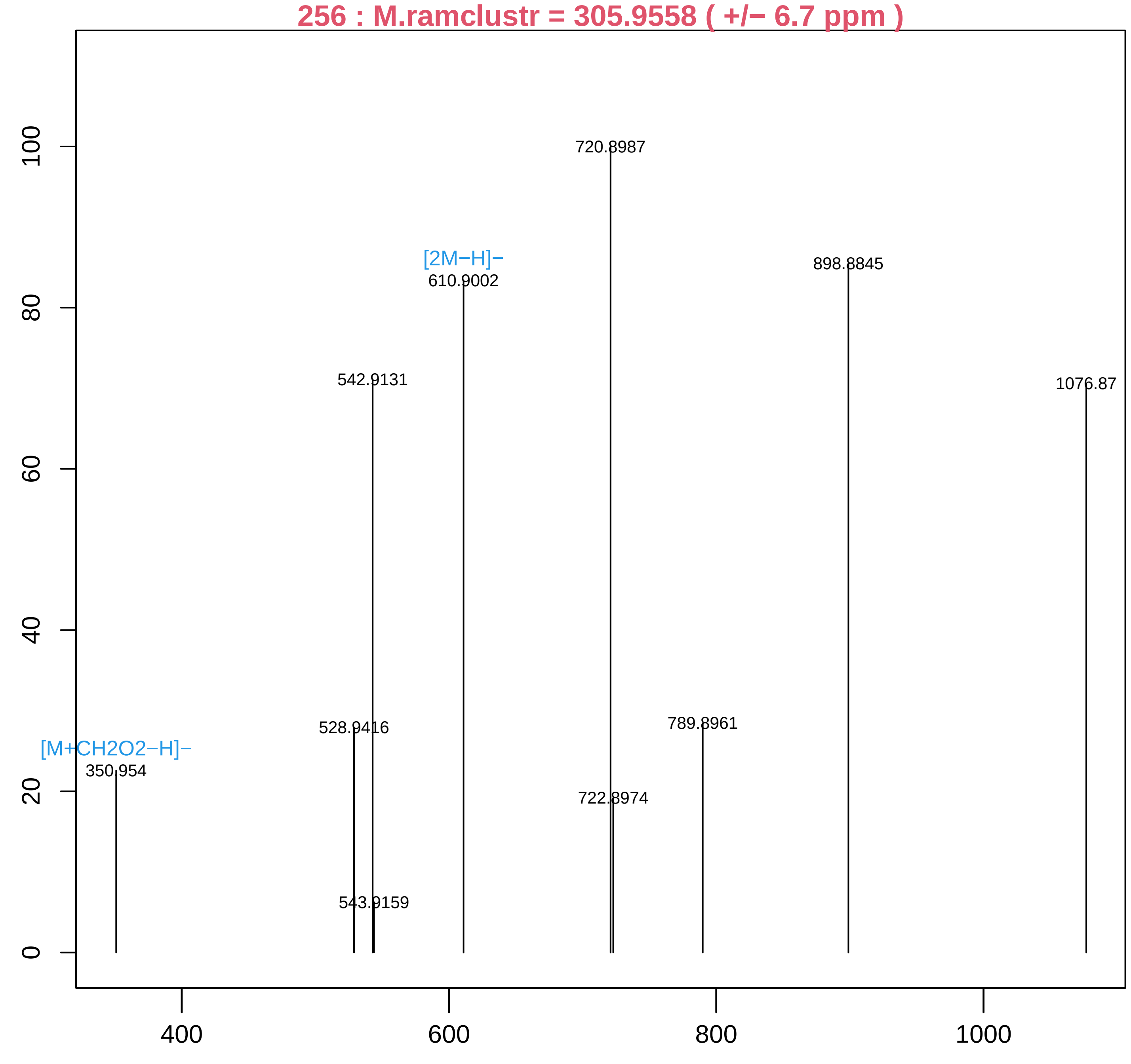
256 : M.ramclustr = 305.9558 (+/− 6.7 ppm)
0
20
40
60
80
100
400
600
800
1000
720.8987
[2M−H]−
610.9002
898.8845
542.9131
1076.87
528.9416
789.8961
[M+CH2O2−H]−
350.954
722.8974
543.9159

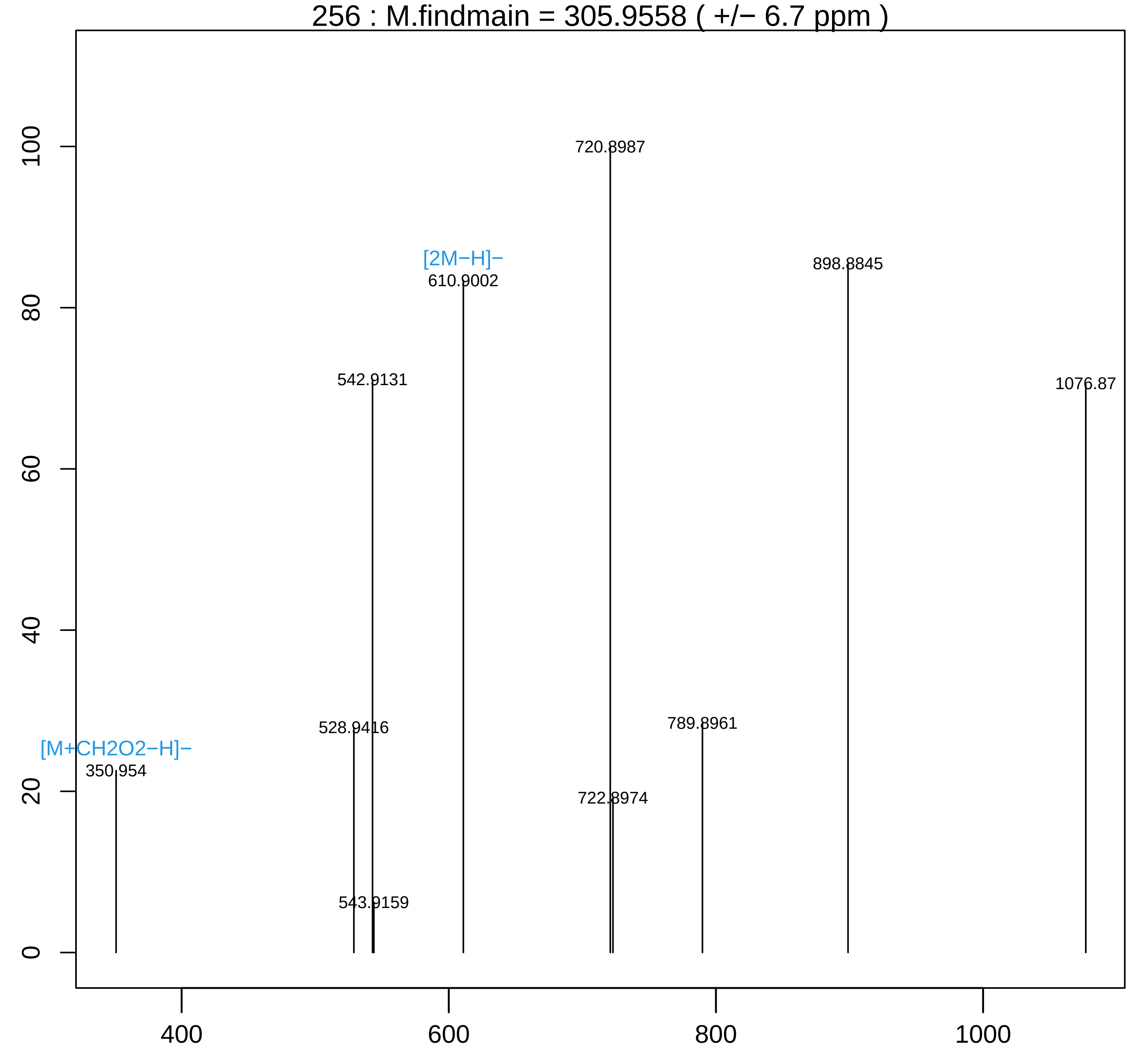
256 : M.findmain = 305.9558 (+/− 6.7 ppm)
0
20
40
60
80
100
400
600
800
1000
720.8987
[2M−H]−
610.9002
898.8845
542.9131
1076.87
528.9416
789.8961
[M+CH2O2−H]−
350.954
722.8974
543.9159

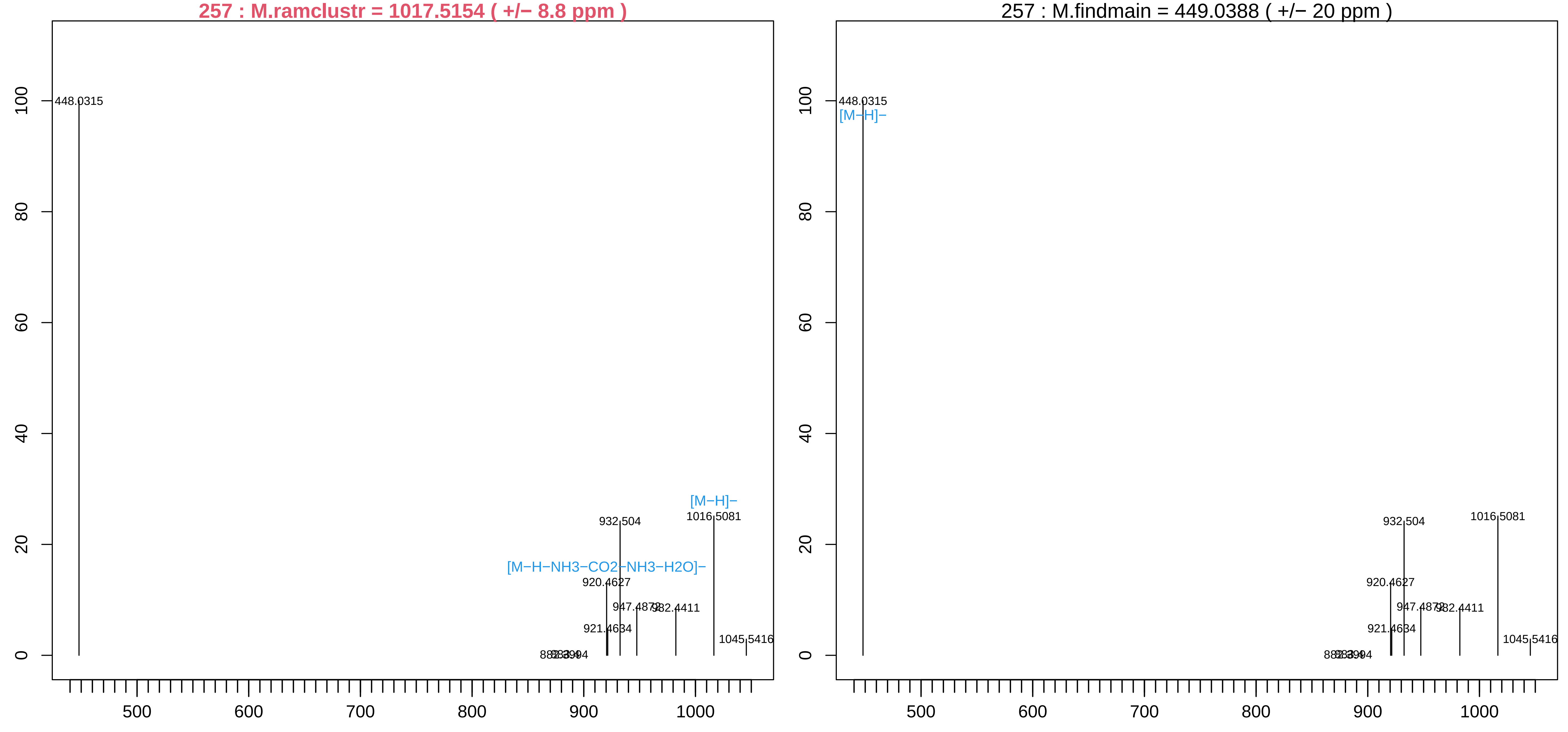
257 : M.ramclustr = 1017.5154 (+/− 8.8 ppm)
257 : M.findmain = 449.0388 (+/− 20 ppm)
448.0315
[M−H]−
1016.5081
932.504
[M−H−NH3−CO2−NH3−H2O]−
920.4627
947.4872
982.4411
921.4634
1045.5416
0
20
40
60
80
100
500
600
700
800
900
1000

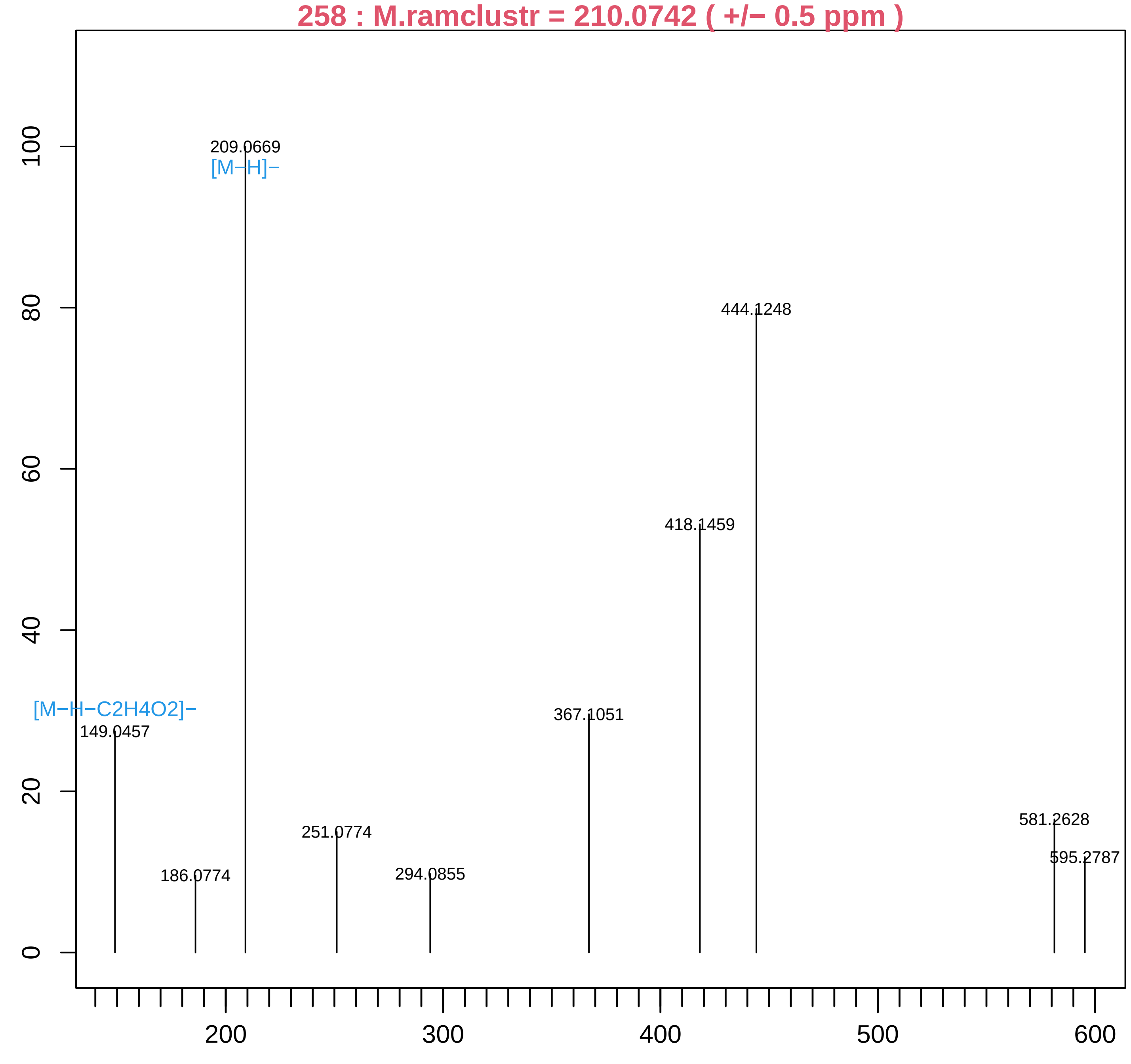
258 : M.ramclustr = 210.0742 (+/− 0.5 ppm)
209.0669
[M−H]−
444.1248
418.1459
[M−H−C2H4O2]−
149.0457
367.1051
251.0774
186.0774
294.0855
581.2628
595.2787
0
20
40
60
80
100
200
300
400
500
600
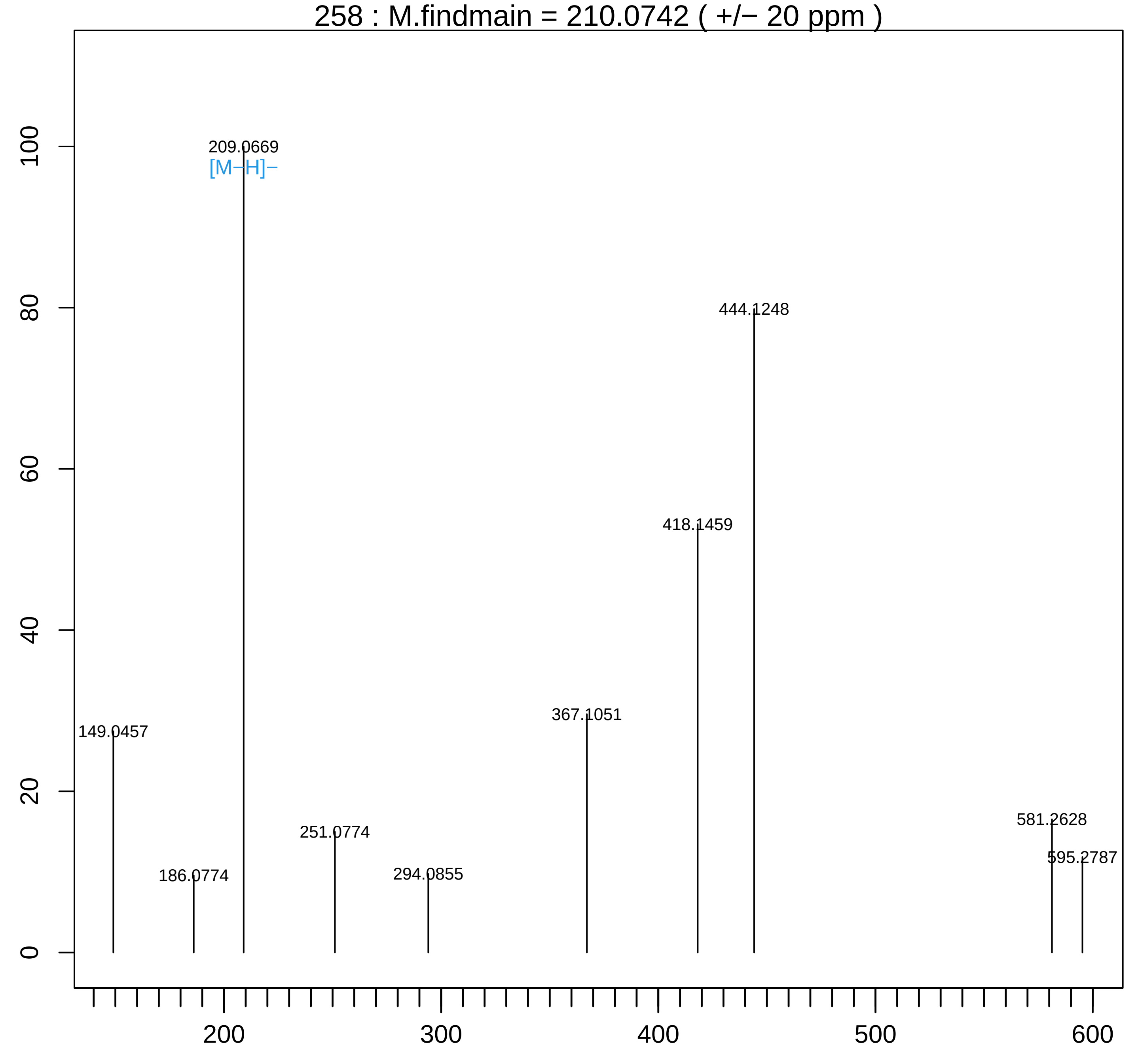
258 : M.findmain = 210.0742 (+/− 20 ppm)
209.0669
[M−H]−
444.1248
418.1459
149.0457
367.1051
251.0774
186.0774
294.0855
581.2628
595.2787
0
20
40
60
80
100
200
300
400
500
600

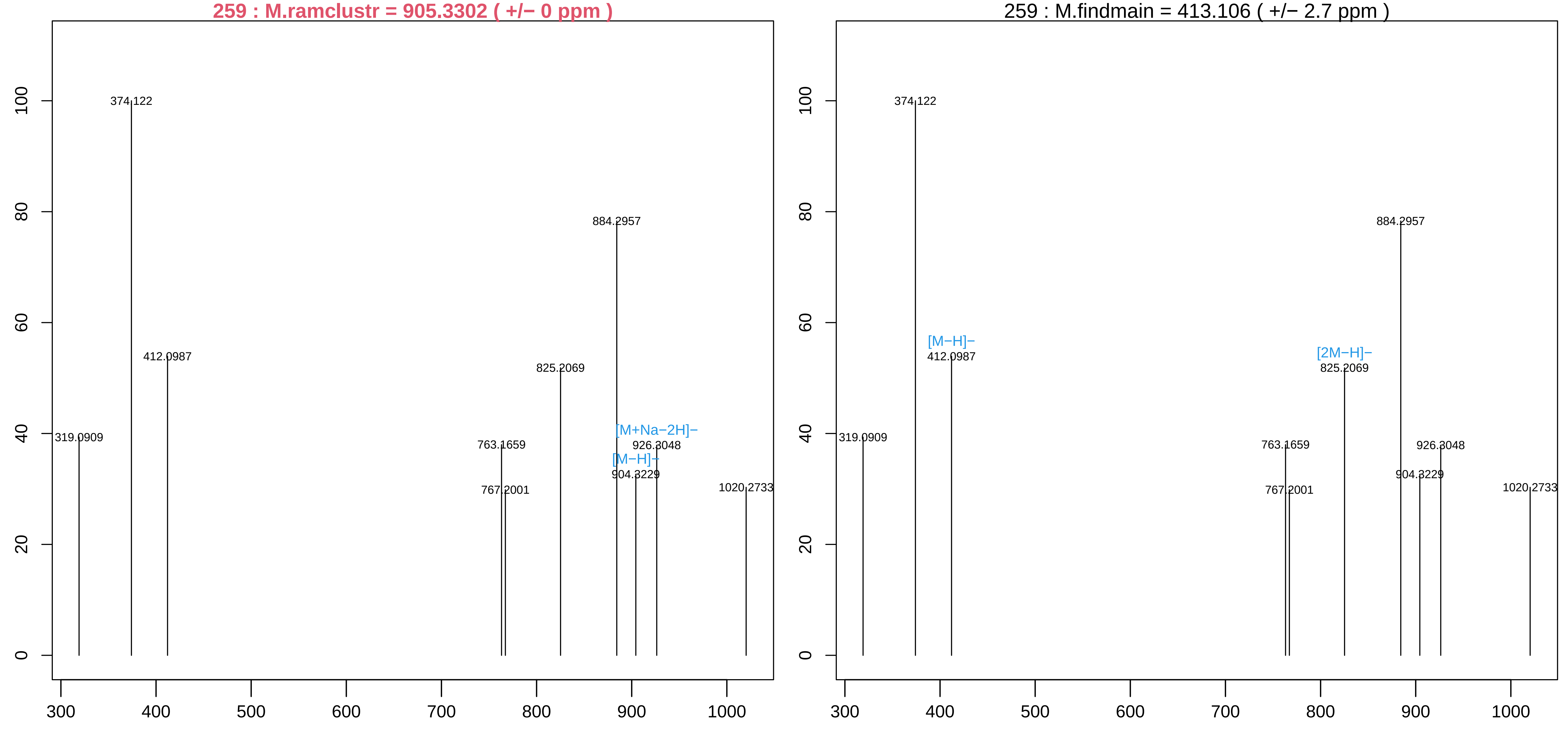
259 : M.ramclustr = 905.3302 (+/− 0 ppm)
374.122
884.2957
412.0987
825.2069
[M+Na−2H]−
319.0909
763.1659
926.3048
[M−H]−
904.3229
767.2001
1020.2733
259 : M.findmain = 413.106 (+/− 2.7 ppm)
374.122
884.2957
[M−H]−
412.0987
[2M−H]−
825.2069
319.0909
763.1659
926.3048
904.3229
767.2001
1020.2733
0
20
40
60
80
100
300
400
500
600
700
800
900
1000

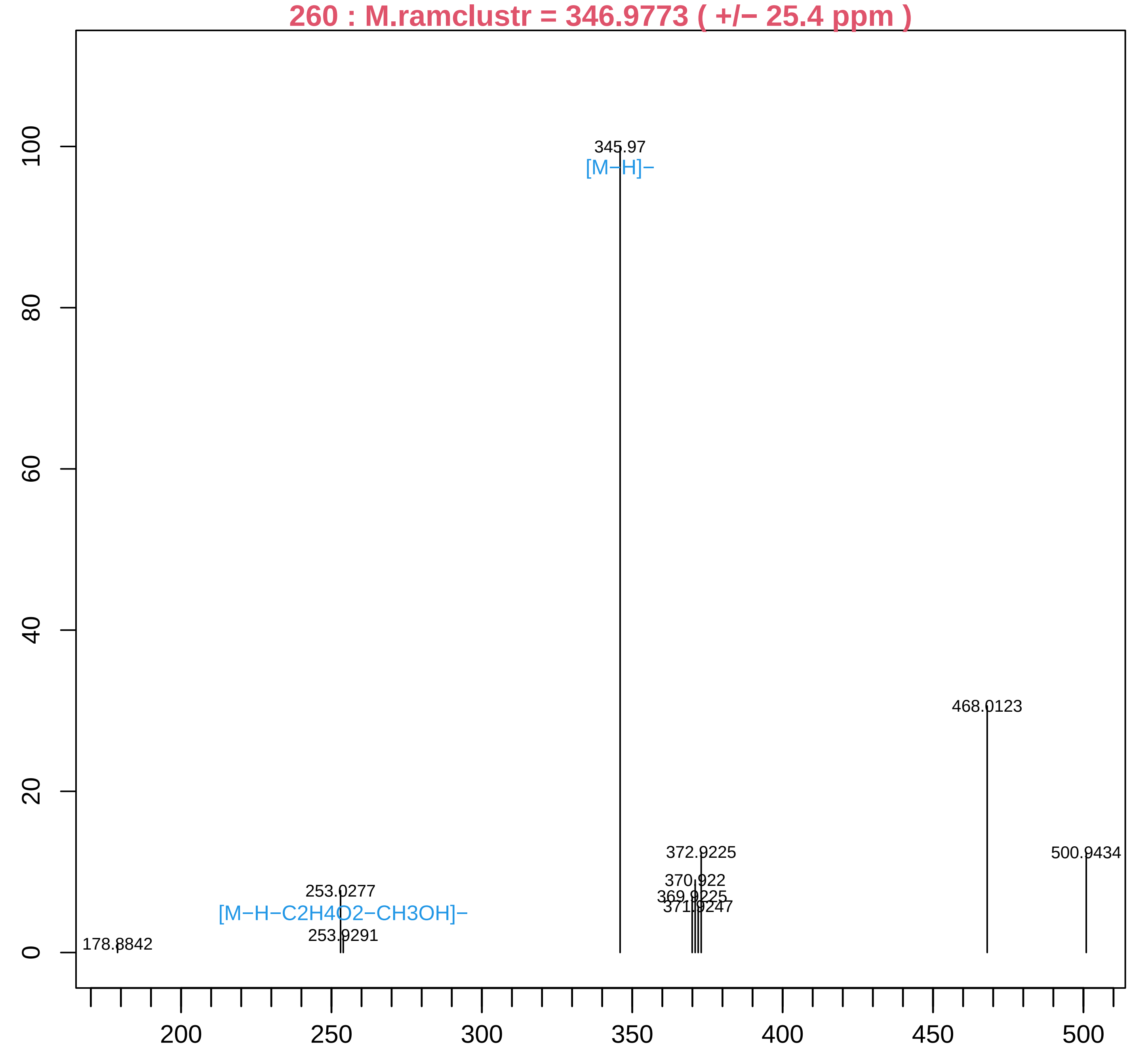
260 : M.ramclustr = 346.9773 (+/− 25.4 ppm)
345.97
[M−H]−
468.0123
372.9225
500.9434
370.922
369.9225
371.9247
253.0277
[M−H−C2H4O2−CH3OH]−
253.9291
178.8842
0
20
40
60
80
100
200
250
300
350
400
450
500

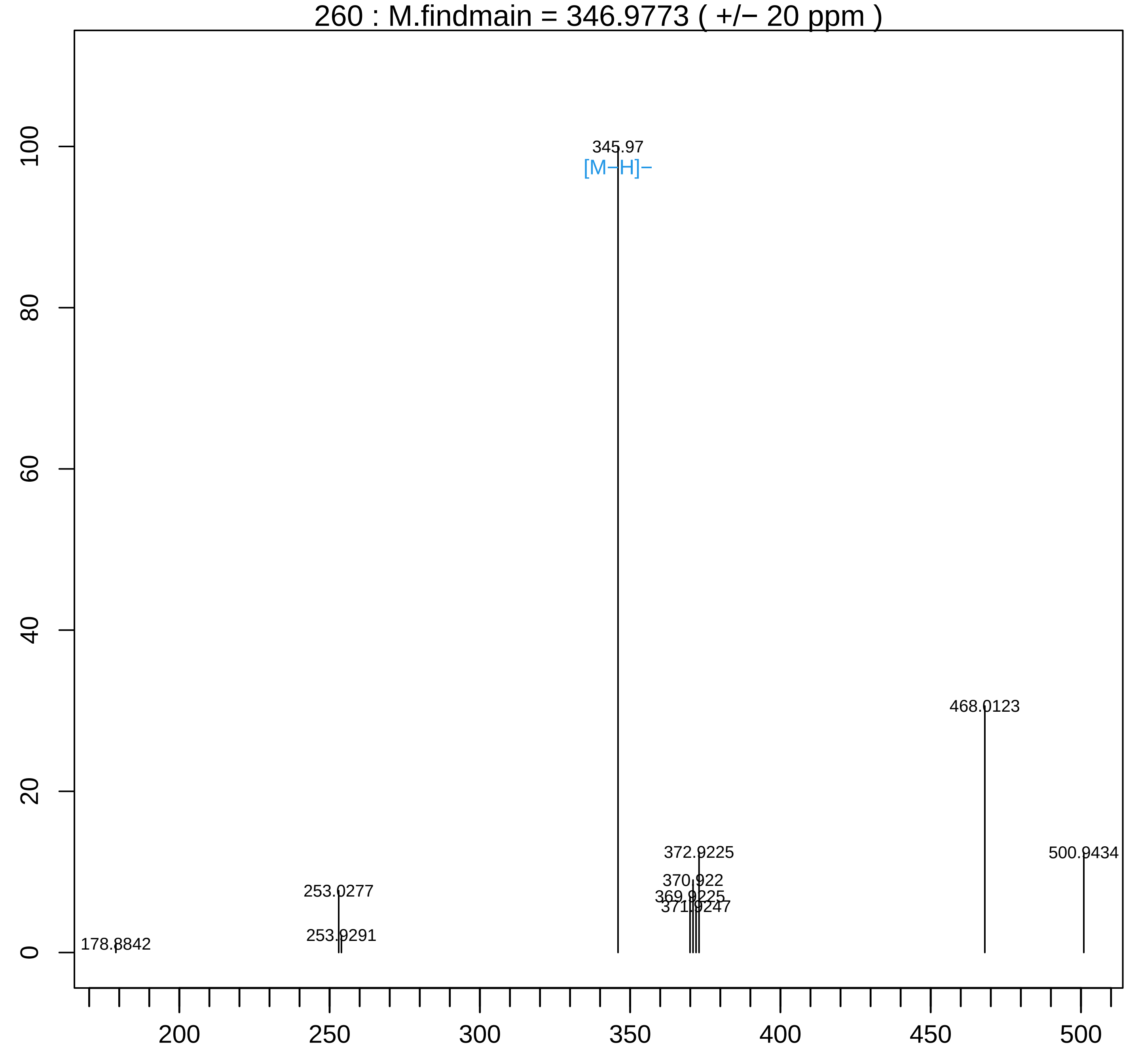
260 : M.findmain = 346.9773 (+/− 20 ppm)
345.97
[M−H]−
468.0123
372.9225
500.9434
370.922
369.9225
371.9247
253.0277
253.9291
178.8842
0
20
40
60
80
100
200
250
300
350
400
450
500

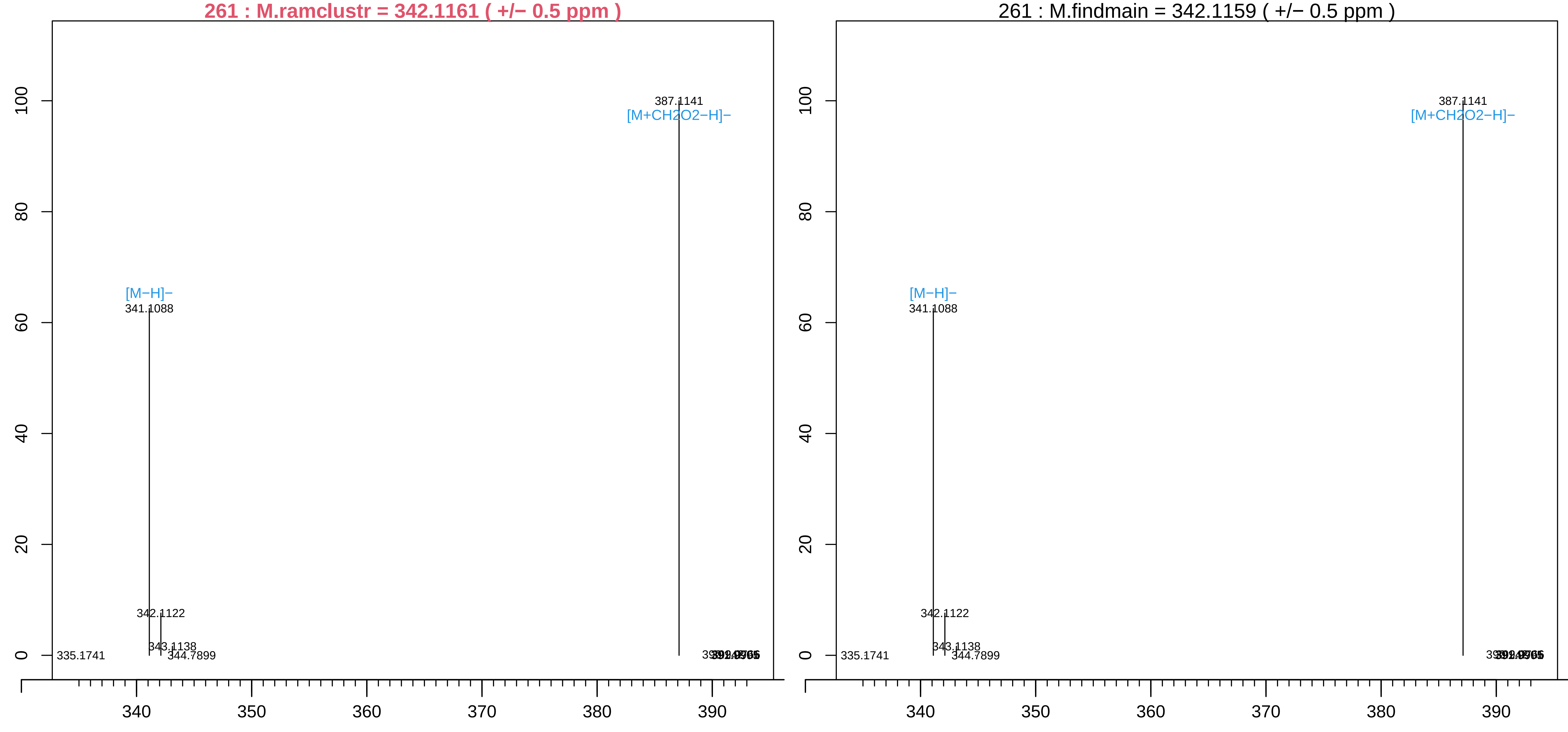
261 : M.ramclustr = 342.1161 (+/− 0.5 ppm)
261 : M.findmain = 342.1159 (+/− 0.5 ppm)
387.1141
[M+CH2O2−H]−
[M−H]−
341.1088
342.1122
343.1138
344.7899
335.1741
0
20
40
60
80
100
340
350
360
370
380
390

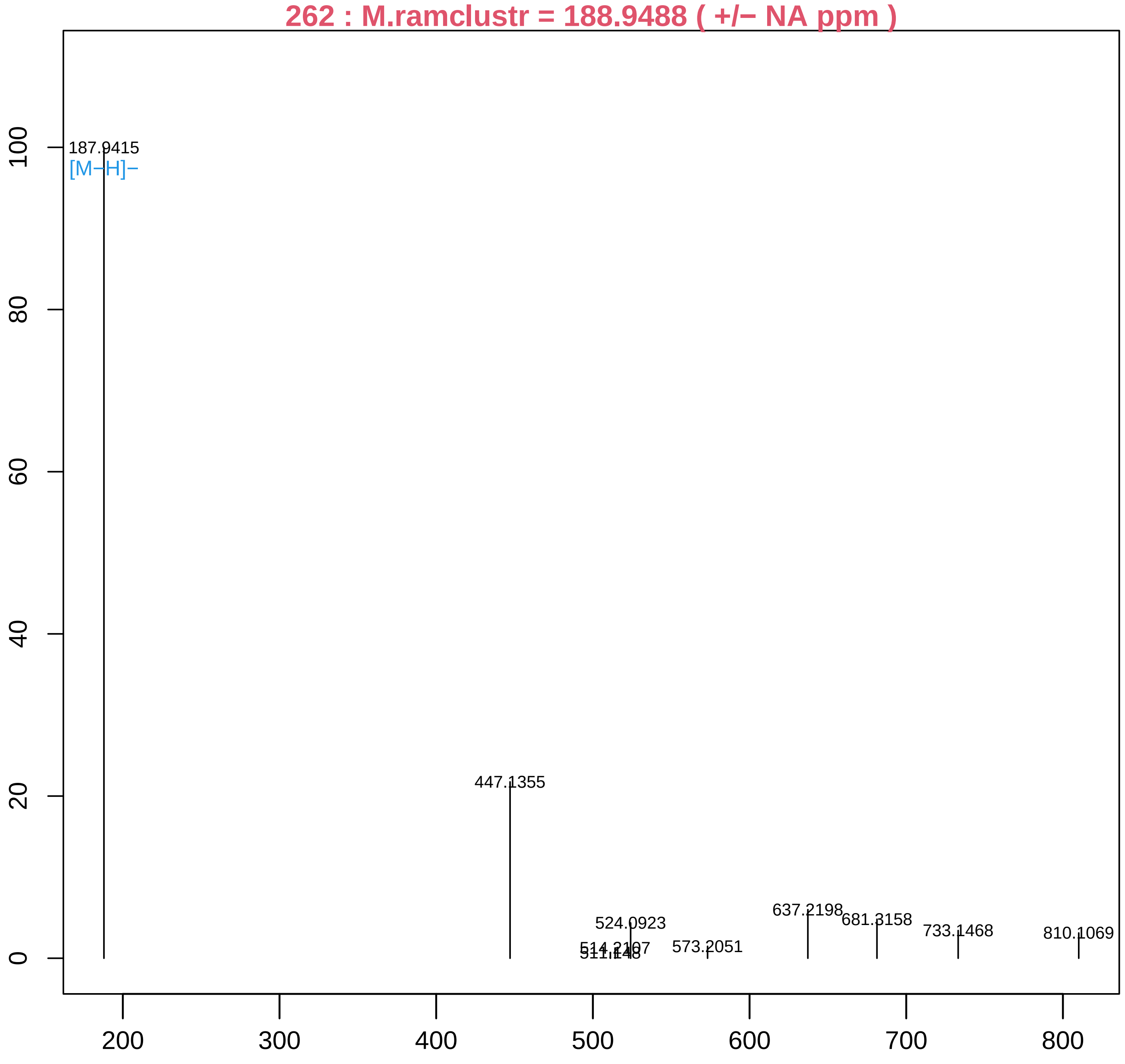
262 : M.ramclustr = 188.9488 (+/− NA ppm)
187.9415
[M−H]−
447.1355
524.0923
573.2051
637.2198
681.3158
733.1468
810.1069
0
20
40
60
80
100
200
300
400
500
600
700
800

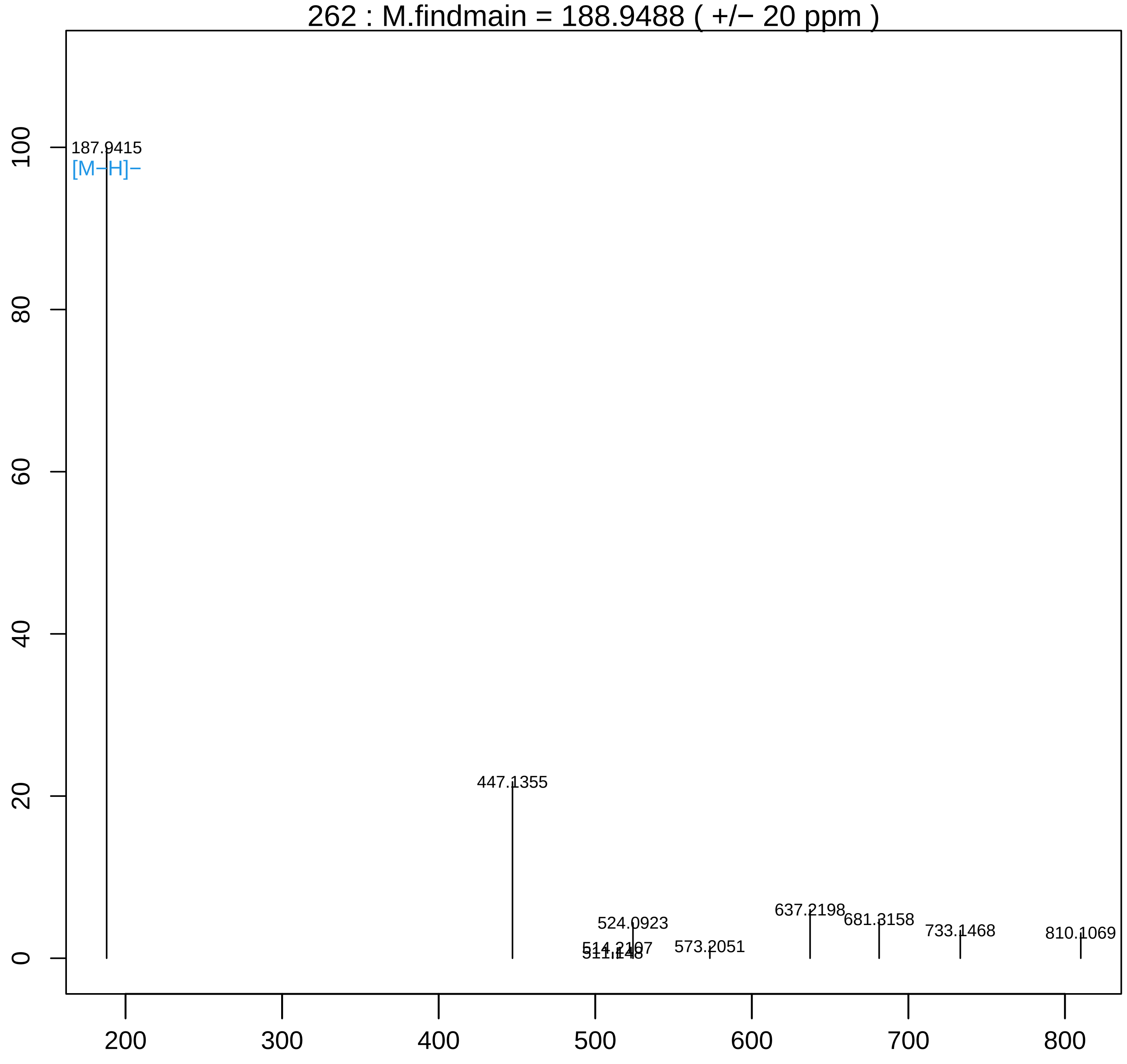
262 : M.findmain = 188.9488 (+/− 20 ppm)
187.9415
[M−H]−
447.1355
524.0923
573.2051
637.2198
681.3158
733.1468
810.1069
0
20
40
60
80
100
200
300
400
500
600
700
800

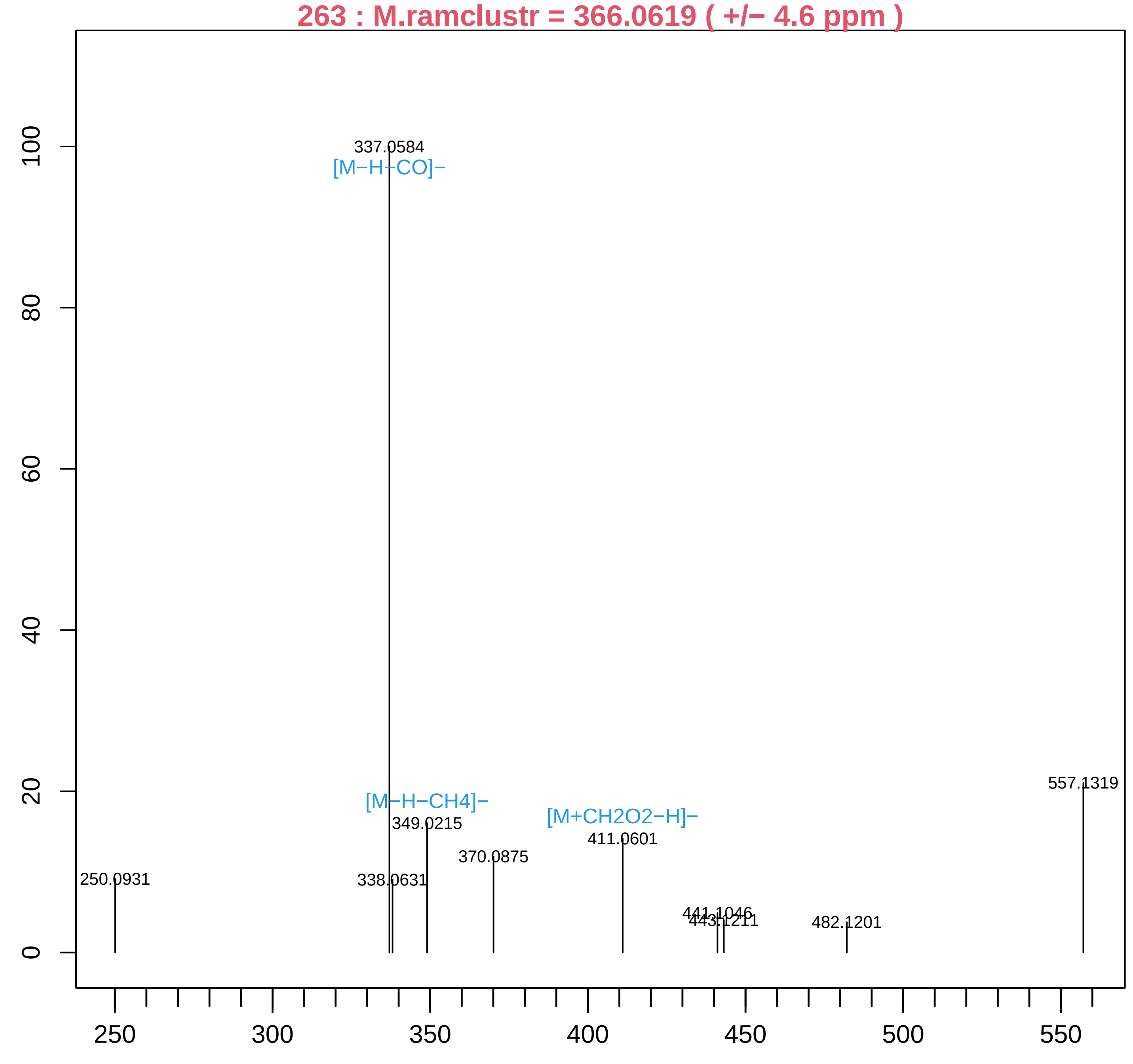
263 : M.ramclustr = 366.0619 (+/− 4.6 ppm)
337.0584
[M−H−CO]−
[M−H−CH4]−
349.0215
[M+CH2O2−H]−
411.0601
370.0875
250.0931
338.0631
441.1046
443.1211
482.1201
557.1319
0
20
40
60
80
100
250
300
350
400
450
500
550

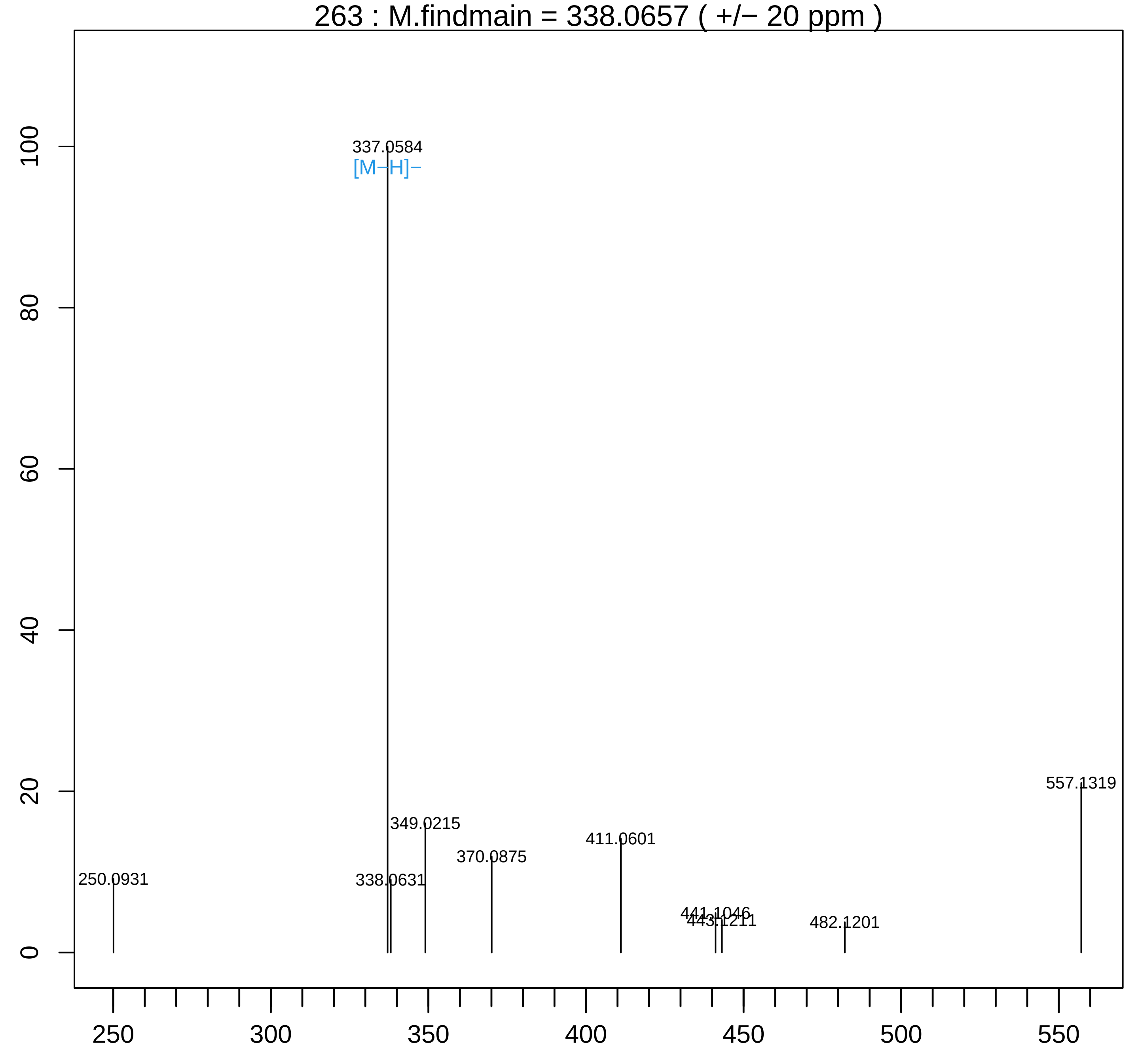
263 : M.findmain = 338.0657 (+/− 20 ppm)
337.0584
[M−H]−
349.0215
411.0601
370.0875
250.0931
338.0631
441.1046
443.1211
482.1201
557.1319
0
20
40
60
80
100
250
300
350
400
450
500
550

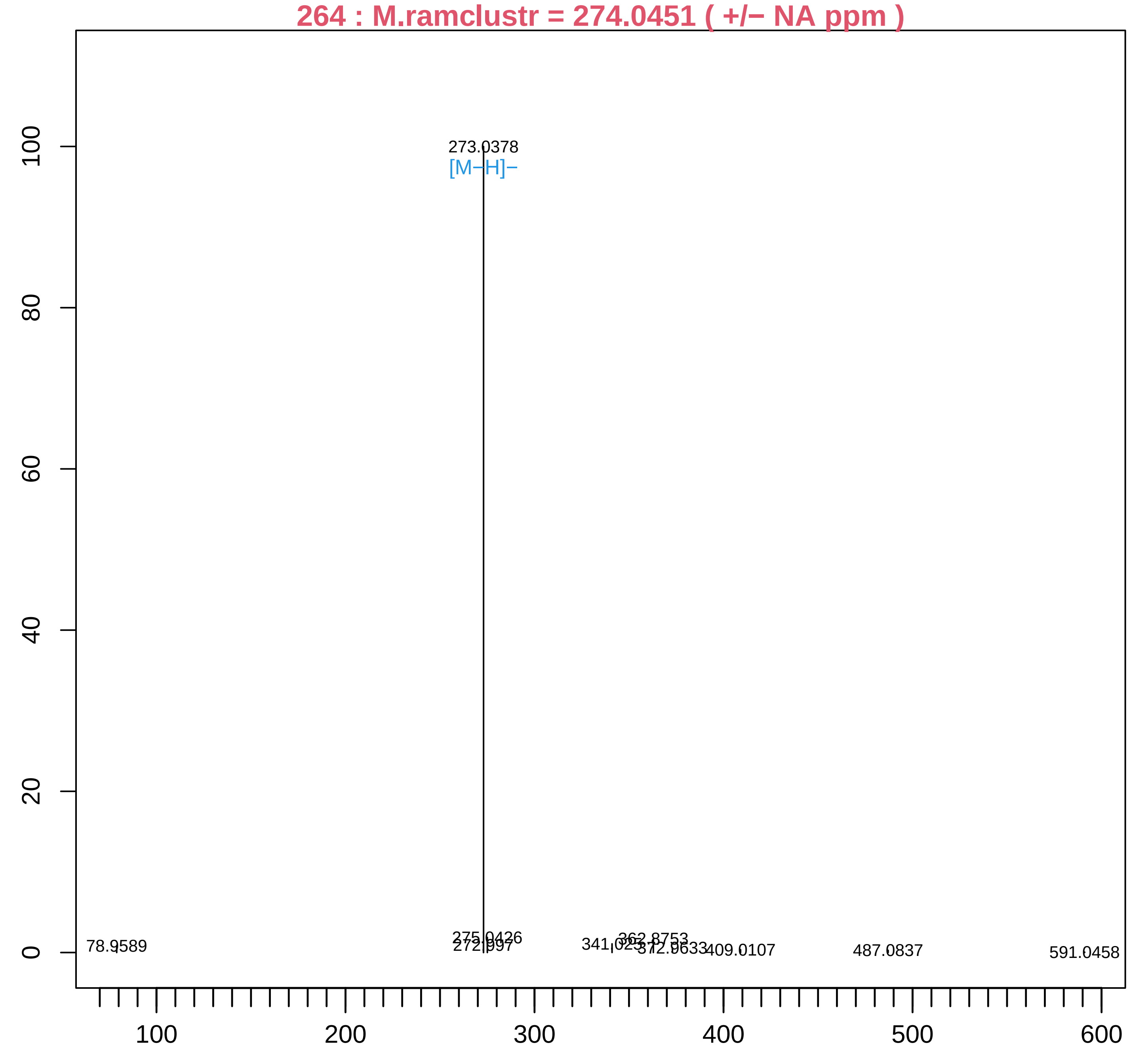
264 : M.ramclustr = 274.0451 (+/− NA ppm)
273.0378
[M−H]−
78.9589
275.0426
362.8753
372.9633
409.0107
487.0837
591.0458
0
20
40
60
80
100
200
300
400
500
600

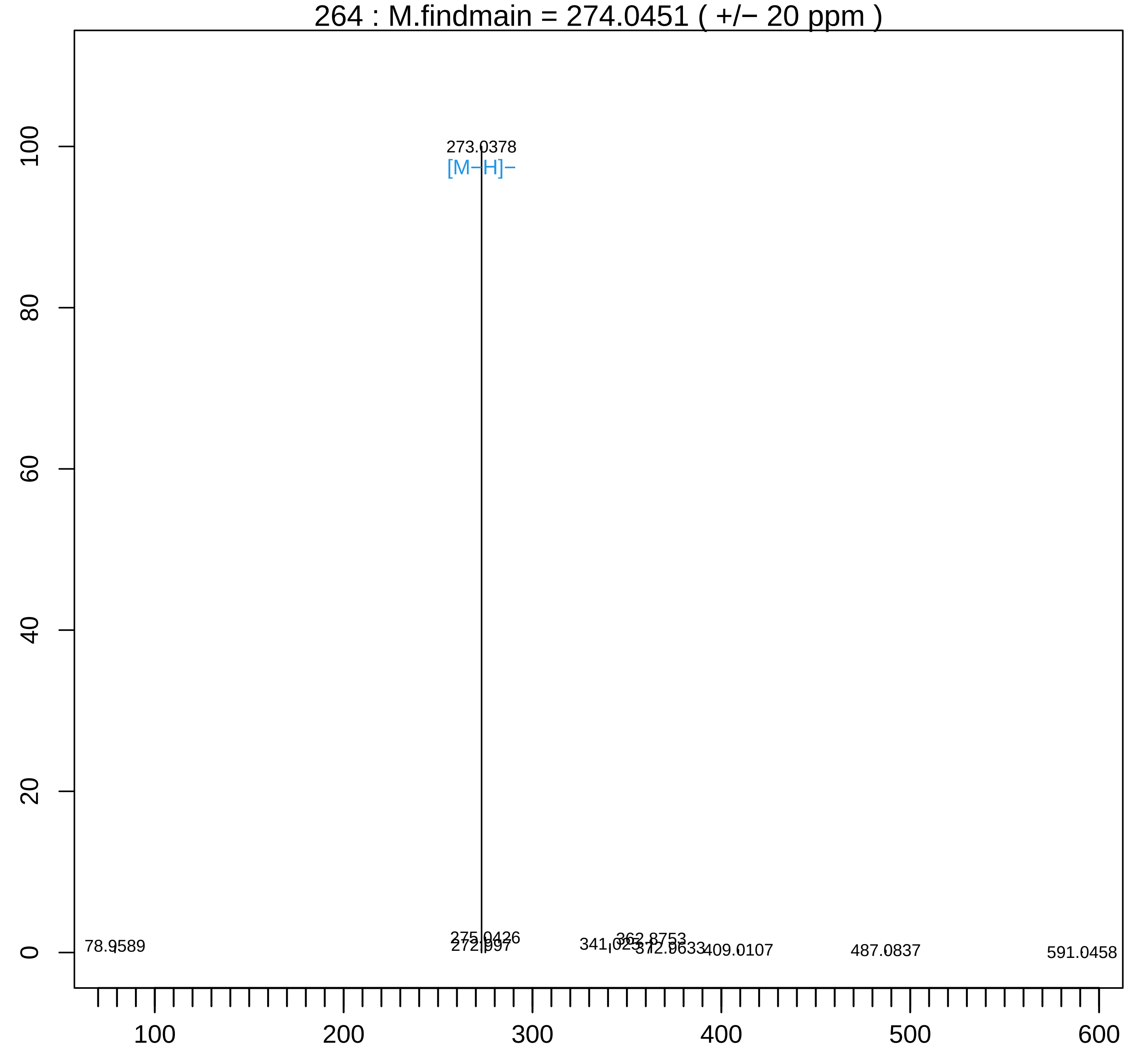
264 : M.findmain = 274.0451 (+/− 20 ppm)
273.0378
[M−H]−
78.9589
275.0426
362.8753
372.9633
409.0107
487.0837
591.0458
0
20
40
60
80
100
200
300
400
500
600

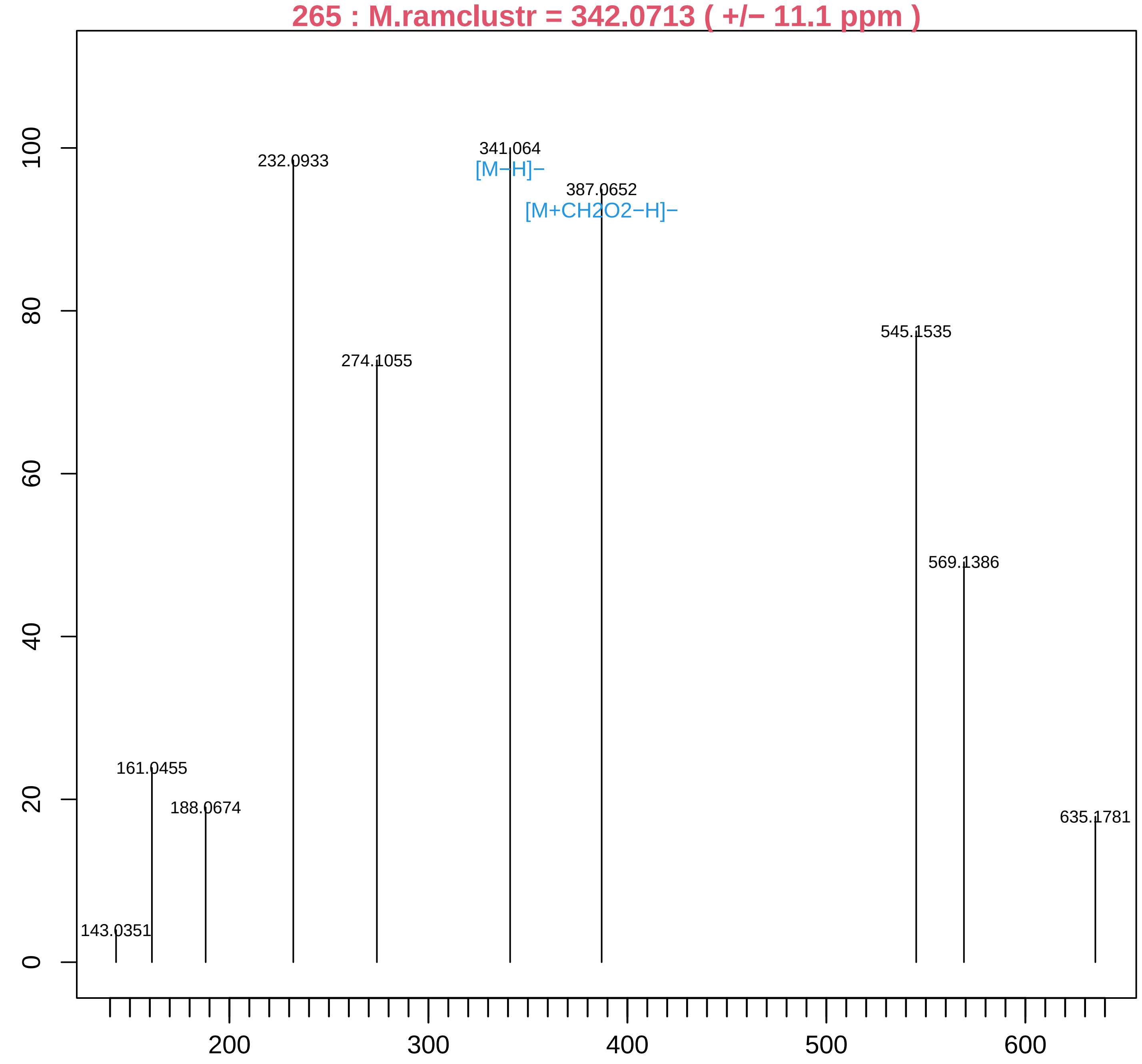
265 : M.ramclustr = 342.0713 (+/− 11.1 ppm)
341.064
[M−H]−
387.0652
[M+CH2O2−H]−
232.0933
274.1055
545.1535
569.1386
635.1781
161.0455
188.0674
143.0351
0
20
40
60
80
100
200
300
400
500
600

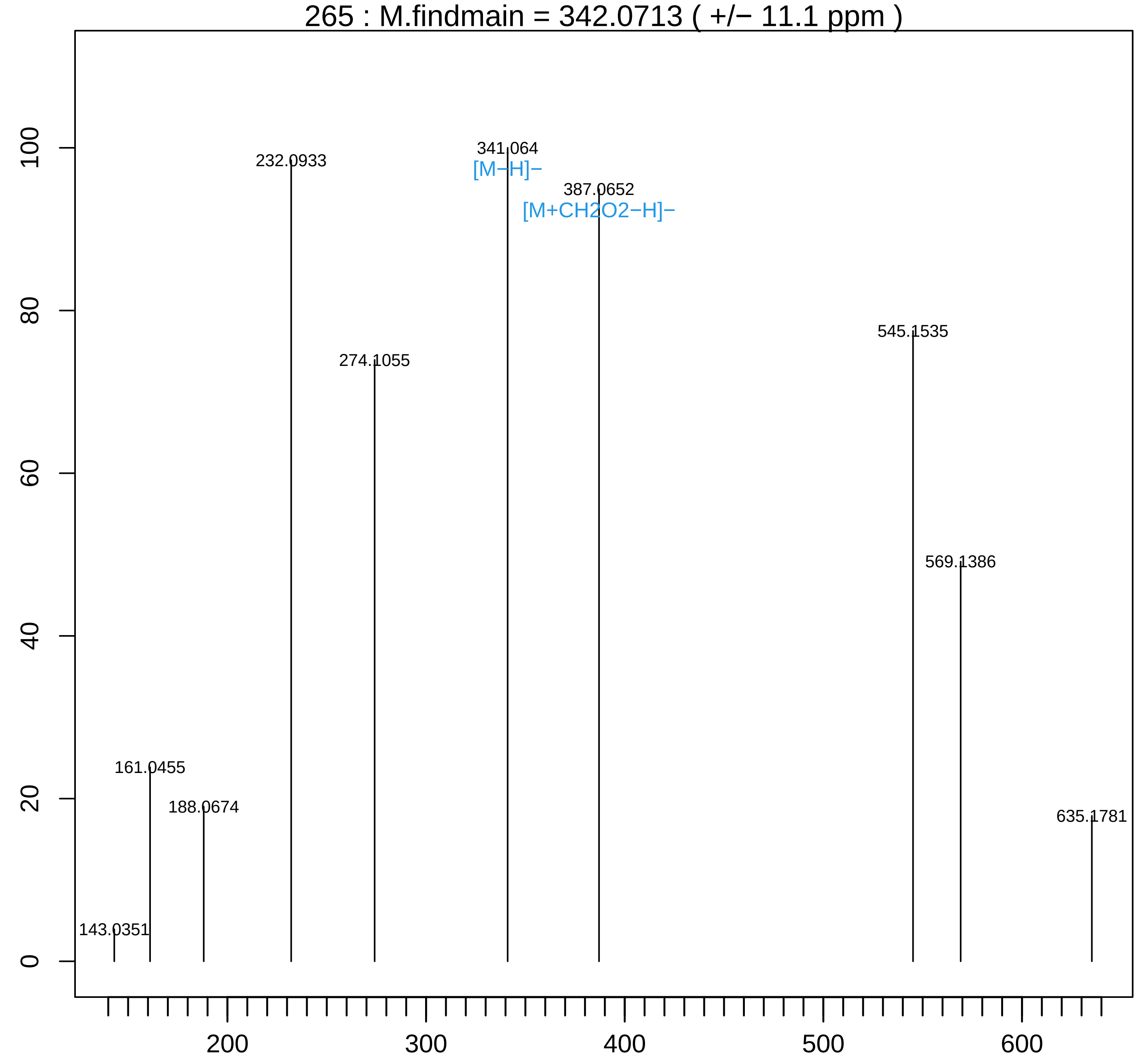
265 : M.findmain = 342.0713 (+/− 11.1 ppm)
341.064
[M−H]−
387.0652
[M+CH2O2−H]−
232.0933
274.1055
545.1535
569.1386
635.1781
161.0455
188.0674
143.0351
0
20
40
60
80
100
200
300
400
500
600

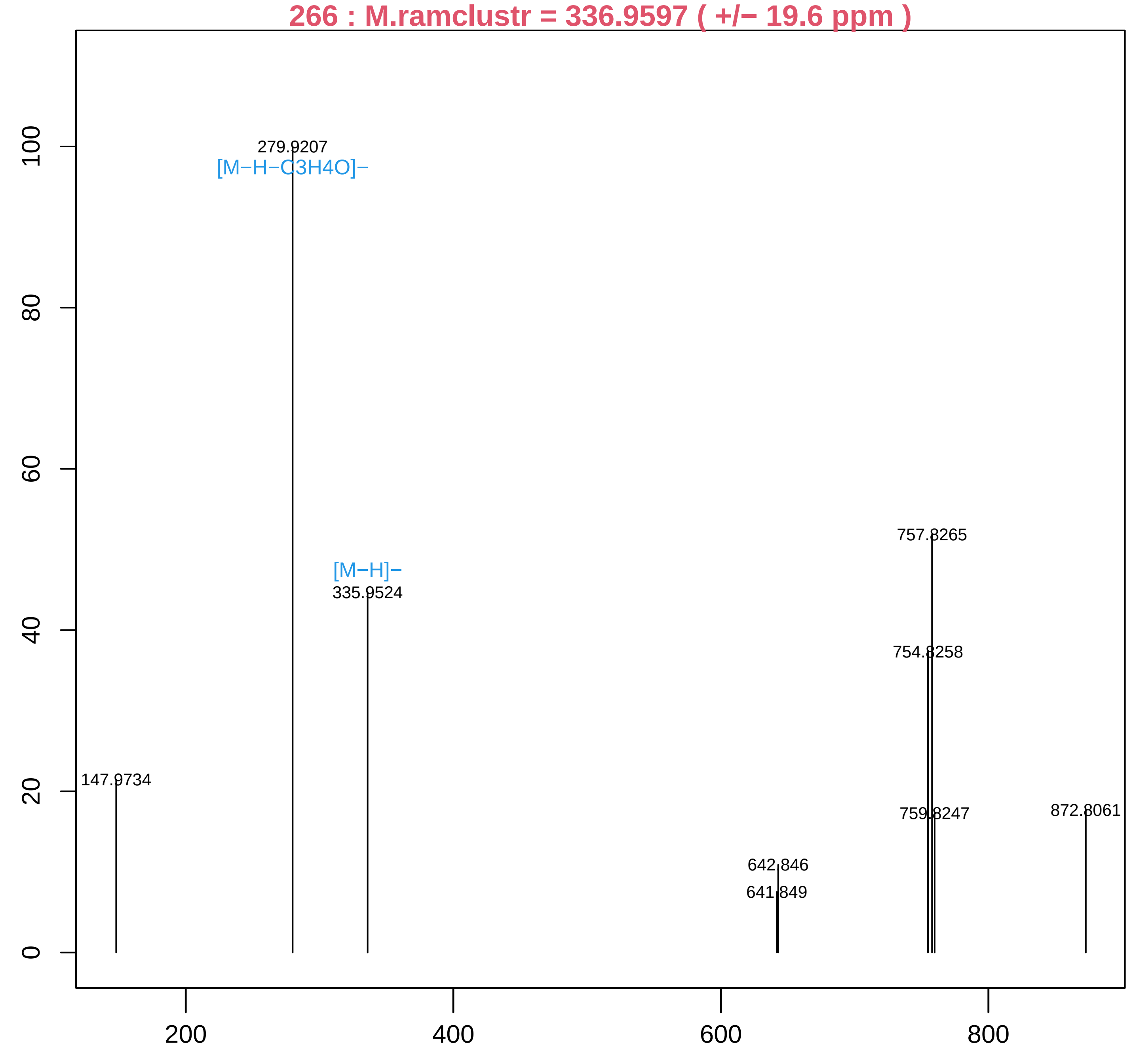
266 : M.ramclustr = 336.9597 (+/− 19.6 ppm)
279.9207
[M−H−C3H4O]−
[M−H]−
335.9524
147.9734
757.8265
754.8258
759.8247
872.8061
642.846
641.849
0
20
40
60
80
100
200
400
600
800

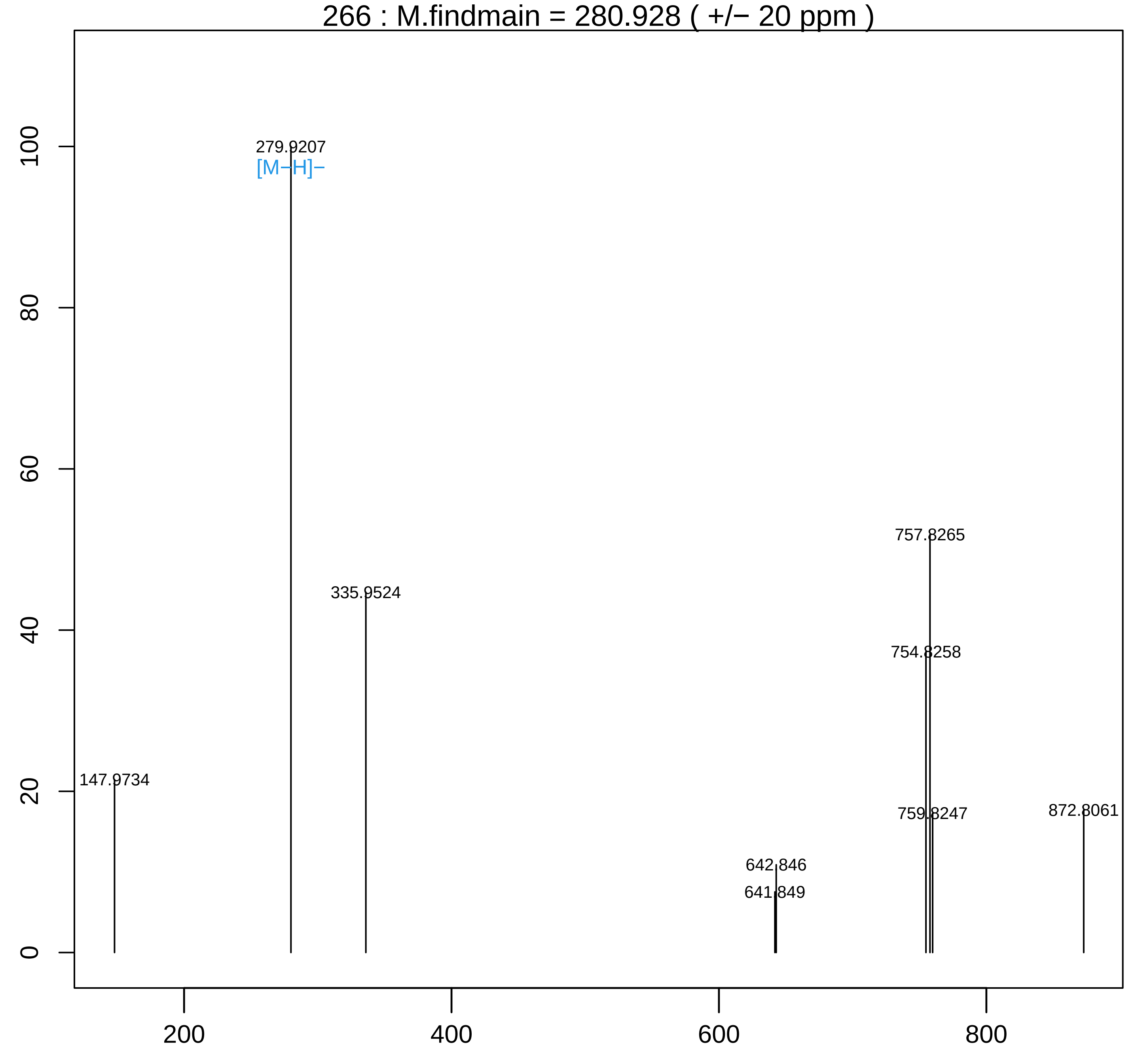
266 : M.findmain = 280.928 (+/− 20 ppm)
279.9207
[M−H]−
335.9524
147.9734
757.8265
754.8258
759.8247
872.8061
642.846
641.849
0
20
40
60
80
100
200
400
600
800

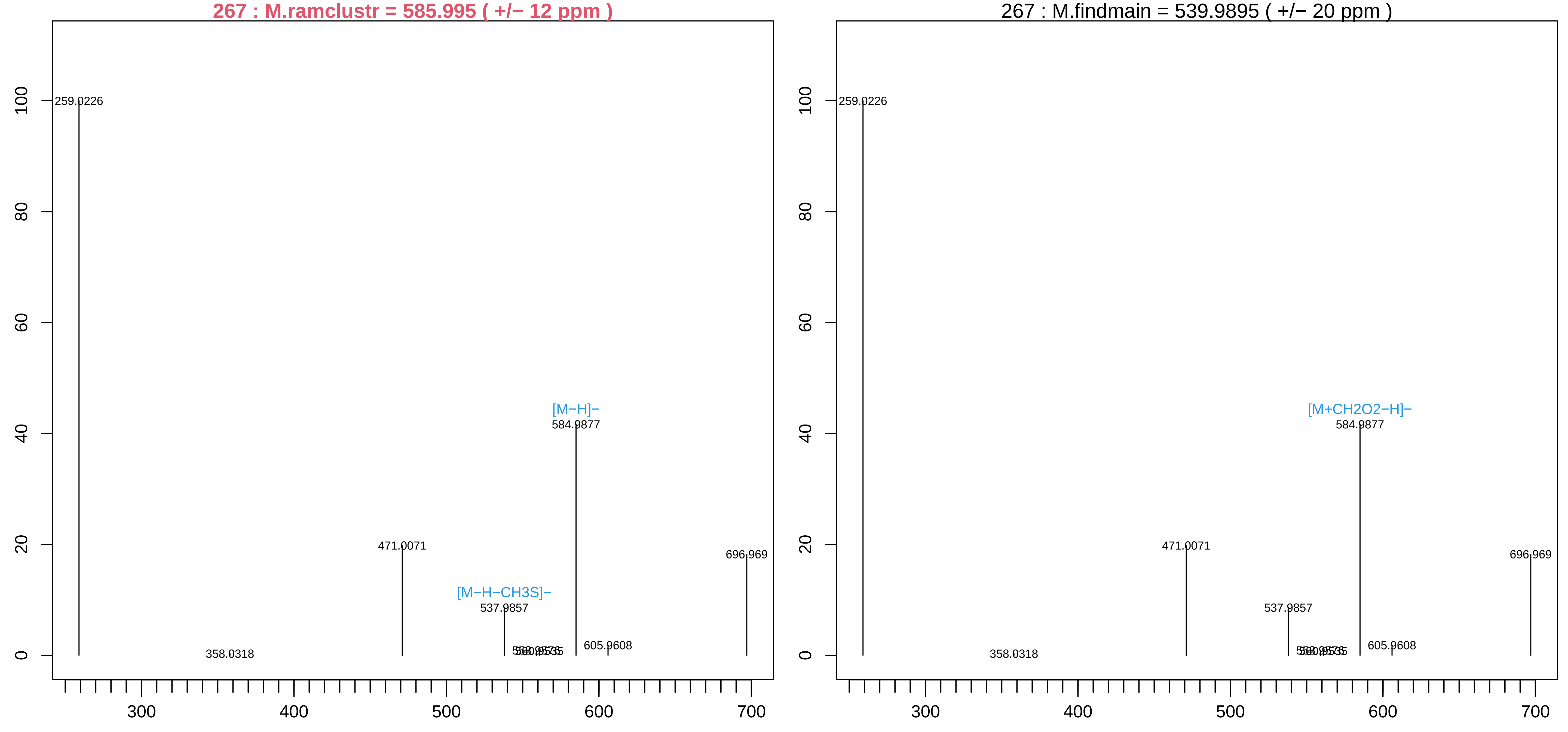

267 : M.ramclustr = 585.995 (+/− 12 ppm)
259.0226
471.0071
[M−H−CH3S]−
537.9857
[M−H]−
584.9877
605.9608
696.969
358.0318
267 : M.findmain = 539.9895 (+/− 20 ppm)
[M+CH2O2−H]−
0
20
40
60
80
100
300
400
500
600
700

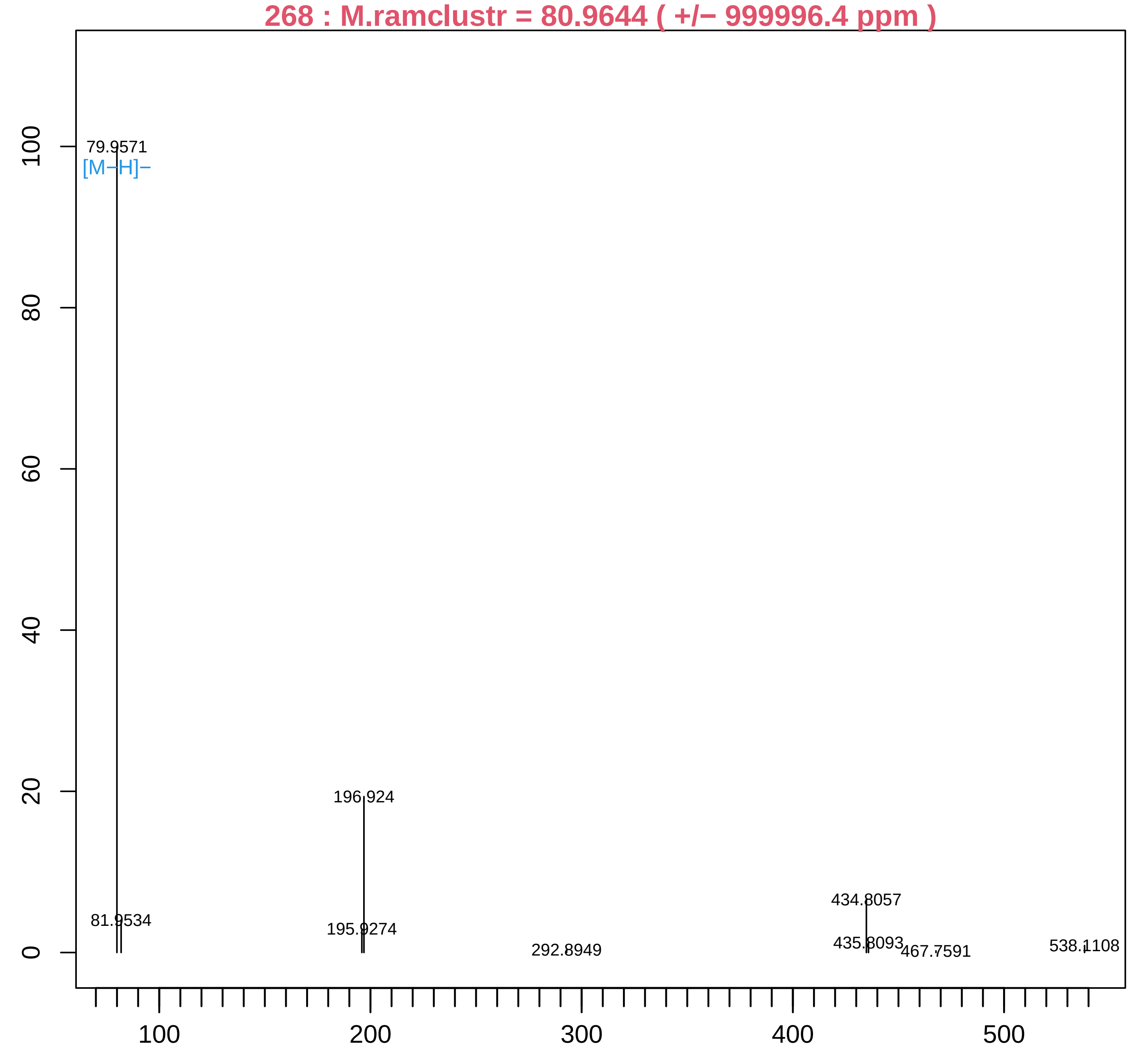

268 : M.ramclustr = 80.9644 (+/− 999996.4 ppm)
79.9571
[M−H]−
196.924
81.9534
195.9274
292.8949
434.8057
435.8093
467.7591
538.1108
0
20
40
60
80
100
100
200
300
400
500

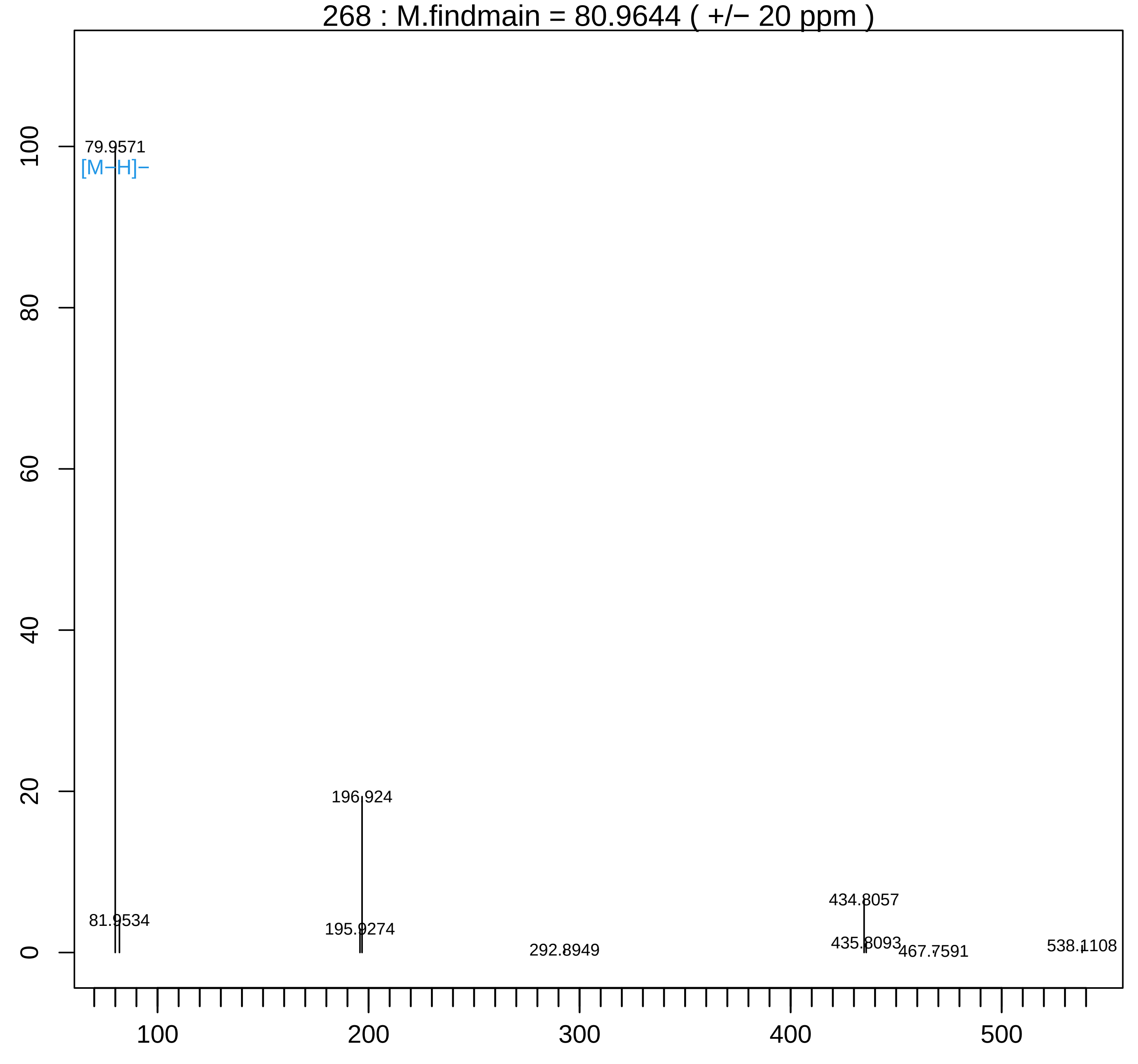

268 : M.findmain = 80.9644 (+/− 20 ppm)
79.9571
[M−H]−
196.924
81.9534
195.9274
292.8949
434.8057
435.8093
467.7591
538.1108
0
20
40
60
80
100
100
200
300
400
500

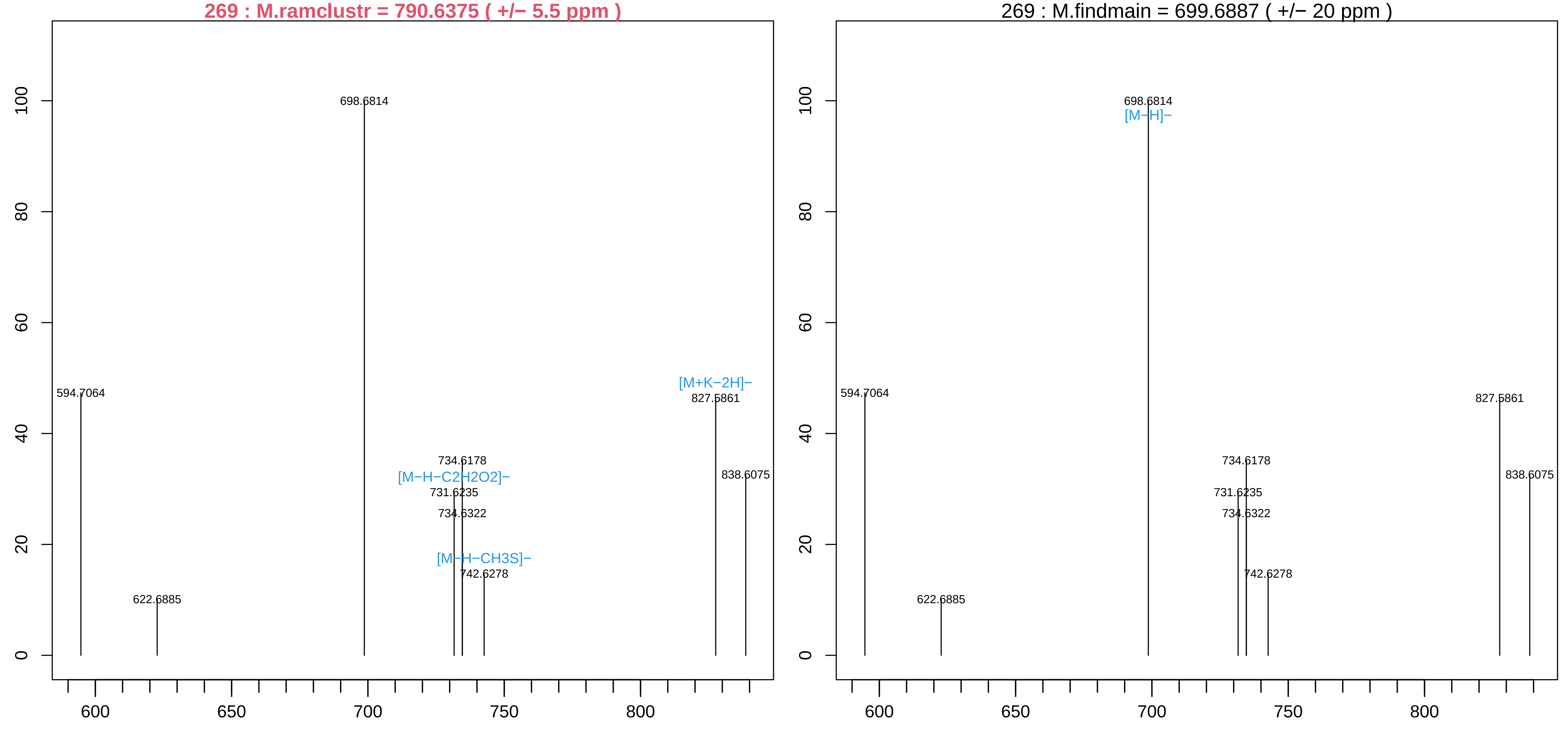

269 : M.ramclustr = 790.6375 (+/− 5.5 ppm)
594.7064
622.6885
698.6814
734.6178
[M−H−C2H2O2]−
731.6235
734.6322
[M−H−CH3S]−
742.6278
[M+K−2H]−
827.5861
838.6075
0
20
40
60
80
100
600
650
700
750
800
269 : M.findmain = 699.6887 (+/− 20 ppm)
698.6814
[M−H]−
594.7064
622.6885
734.6178
731.6235
734.6322
742.6278
827.5861
838.6075
0
20
40
60
80
100
600
650
700
750
800

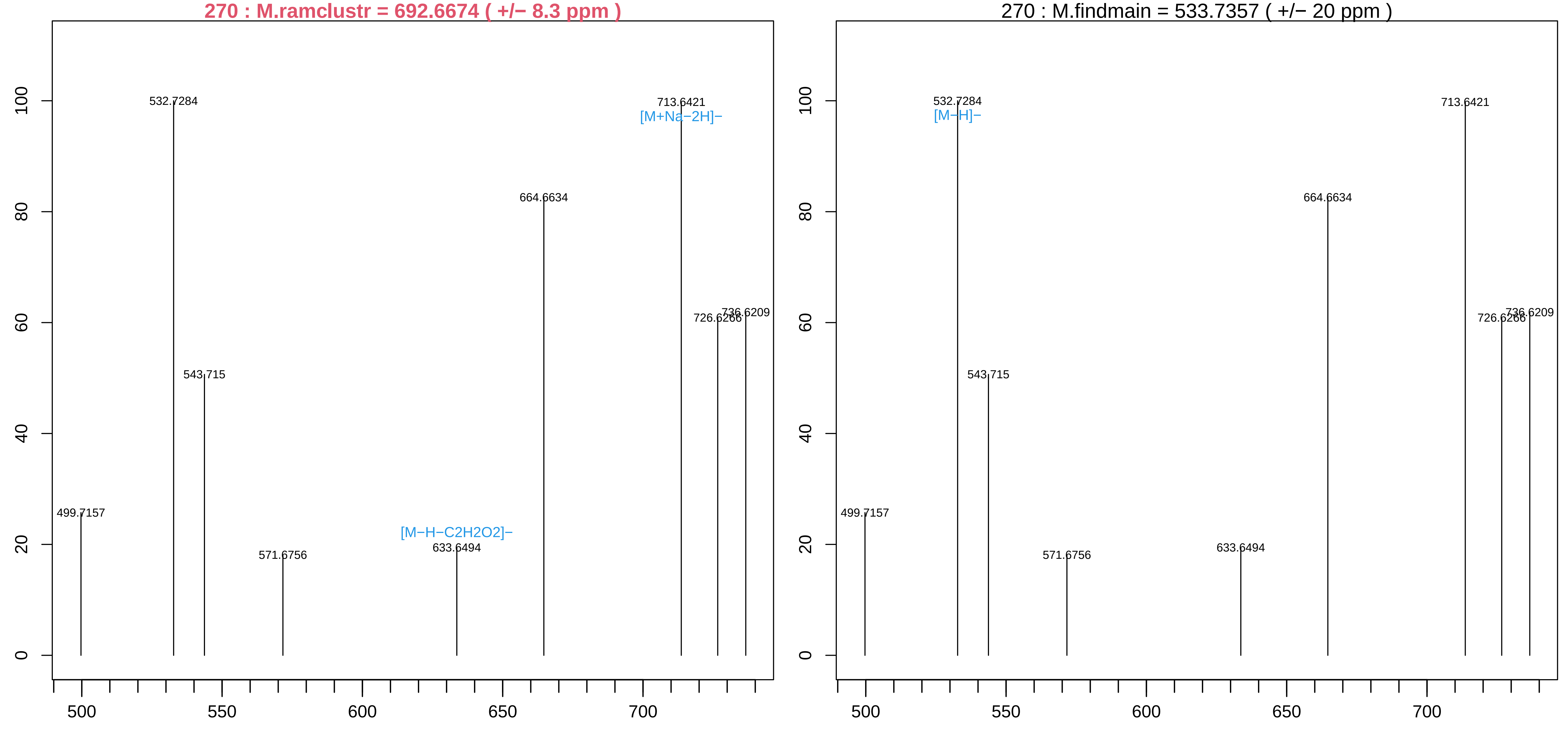
270 : M.ramclustr = 692.6674 (+/− 8.3 ppm)
532.7284
713.6421
[M+Na−2H]−
664.6634
726.6266
736.6209
543.715
499.7157
[M−H−C2H2O2]−
633.6494
571.6756
270 : M.findmain = 533.7357 (+/− 20 ppm)
532.7284
[M−H]−
713.6421
664.6634
726.6266
736.6209
543.715
499.7157
571.6756
633.6494
0
20
40
60
80
100
500
550
600
650
700

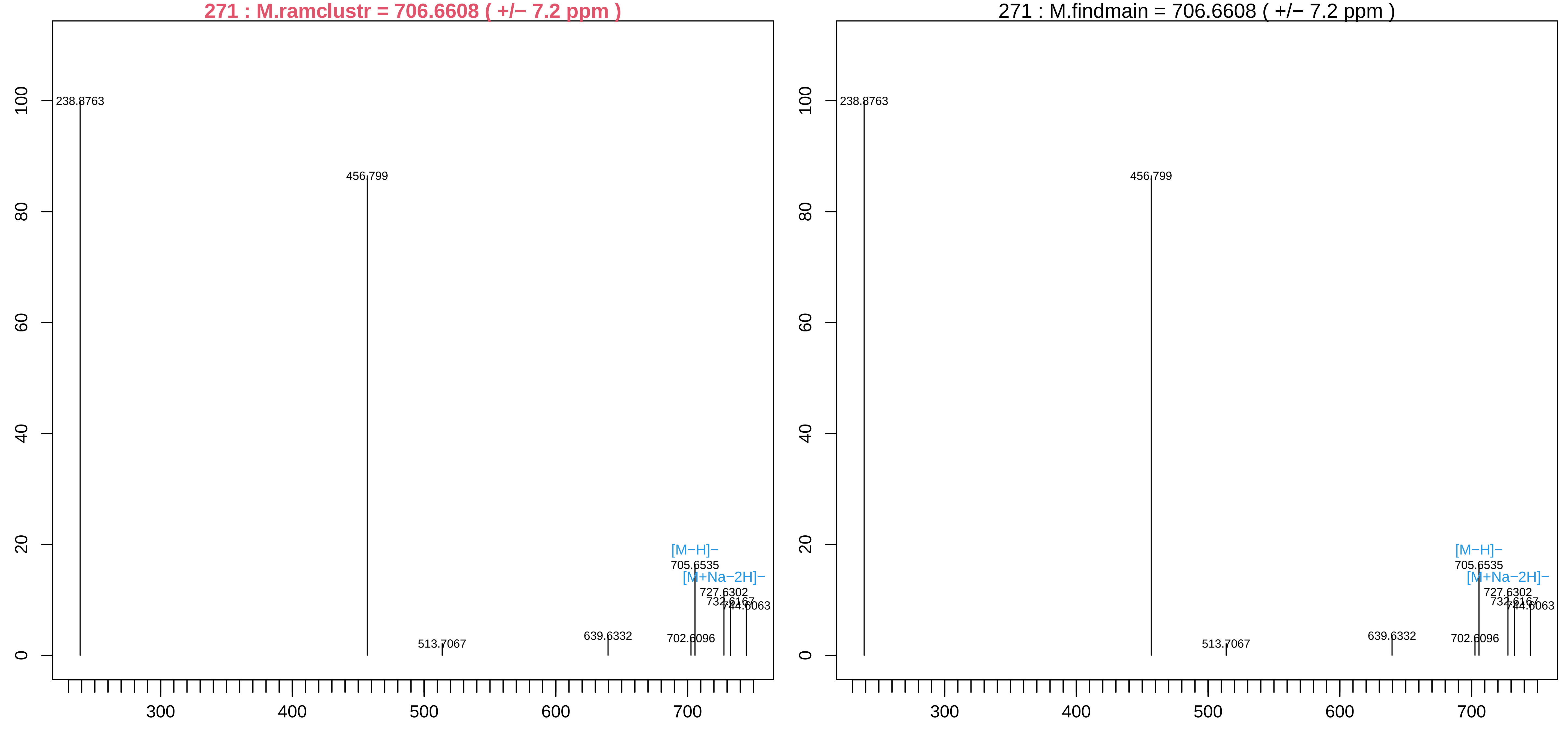
271 : M.ramclustr = 706.6608 (+/− 7.2 ppm)
238.8763
456.799
513.7067
639.6332
702.6096
[M−H]−
705.6535
[M+Na−2H]−
727.6302
732.6167
744.6063
271 : M.findmain = 706.6608 (+/− 7.2 ppm)
238.8763
456.799
513.7067
639.6332
702.6096
[M−H]−
705.6535
[M+Na−2H]−
727.6302
732.6167
744.6063

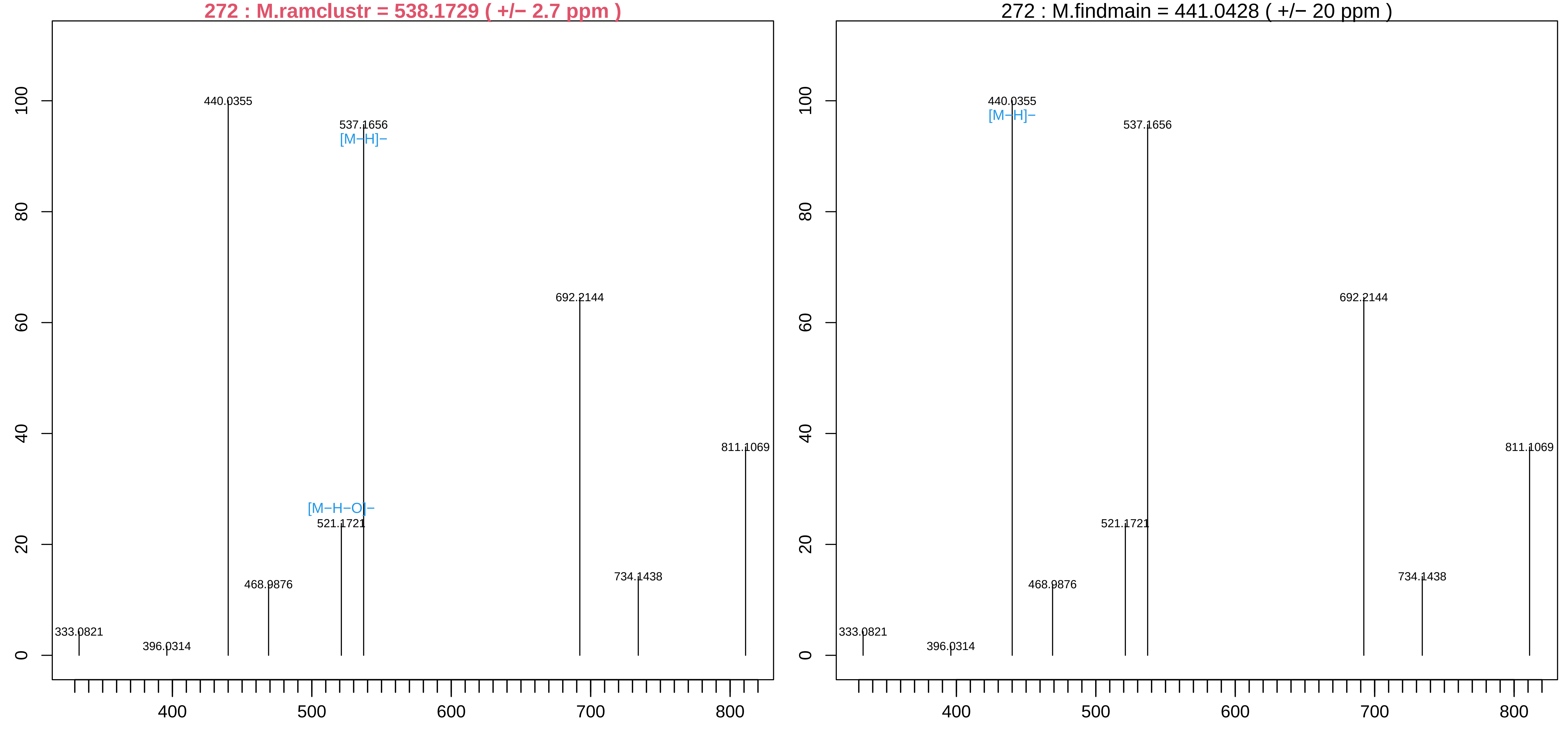

272 : M.ramclustr = 538.1729 (+/− 2.7 ppm)
440.0355
537.1656
[M−H]−
692.2144
811.1069
[M−H−O]−
521.1721
468.9876
734.1438
333.0821
396.0314
272 : M.findmain = 441.0428 (+/− 20 ppm)
440.0355
[M−H]−
537.1656
692.2144
811.1069
521.1721
468.9876
734.1438
333.0821
396.0314

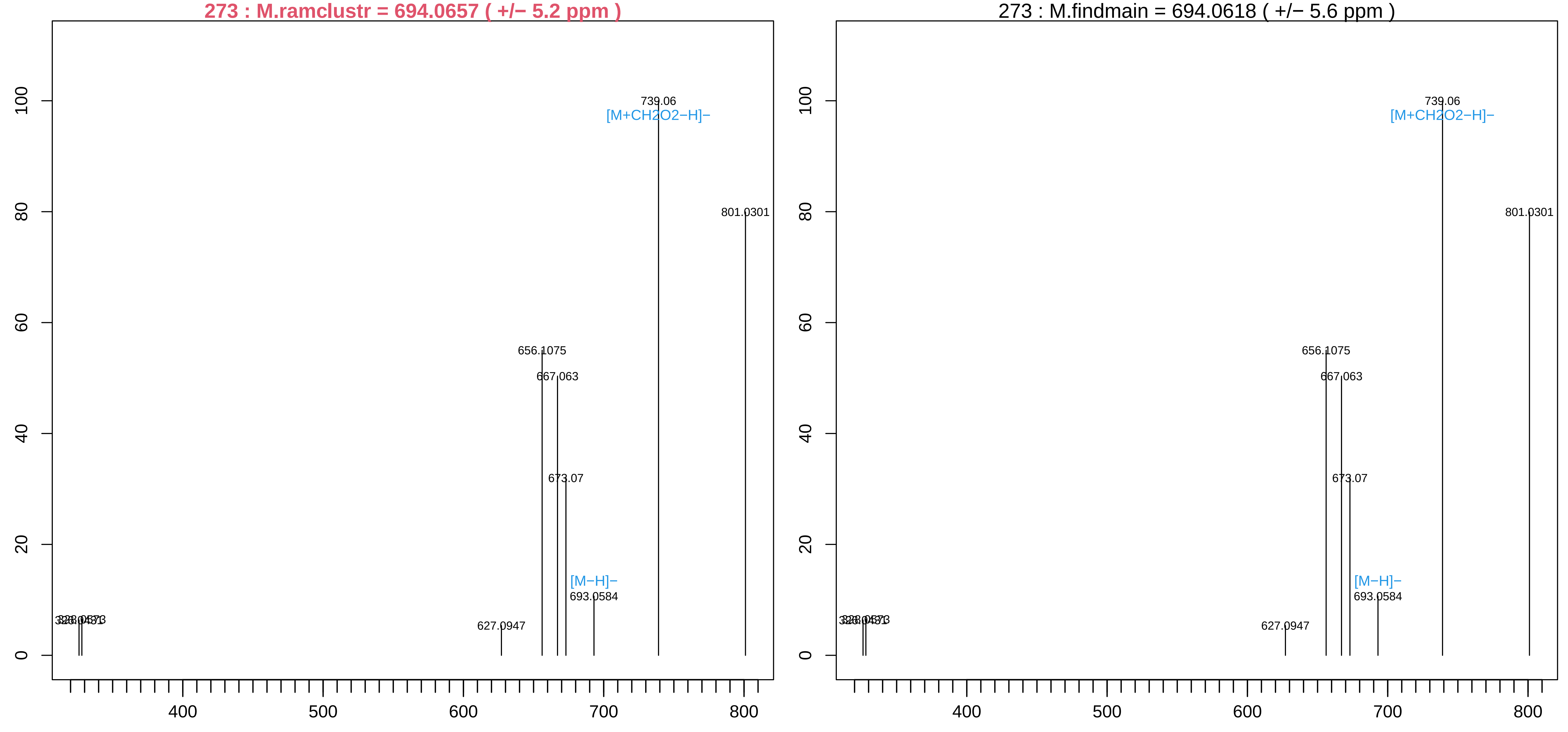

273 : M.ramclustr = 694.0657 (+/− 5.2 ppm)
739.06
[M+CH2O2−H]−
801.0301
656.1075
667.063
673.07
[M−H]−
693.0584
627.0947
0
20
40
60
80
100
400
500
600
700
800
273 : M.findmain = 694.0618 (+/− 5.6 ppm)
739.06
[M+CH2O2−H]−
801.0301
656.1075
667.063
673.07
[M−H]−
693.0584
627.0947
0
20
40
60
80
100
400
500
600
700
800

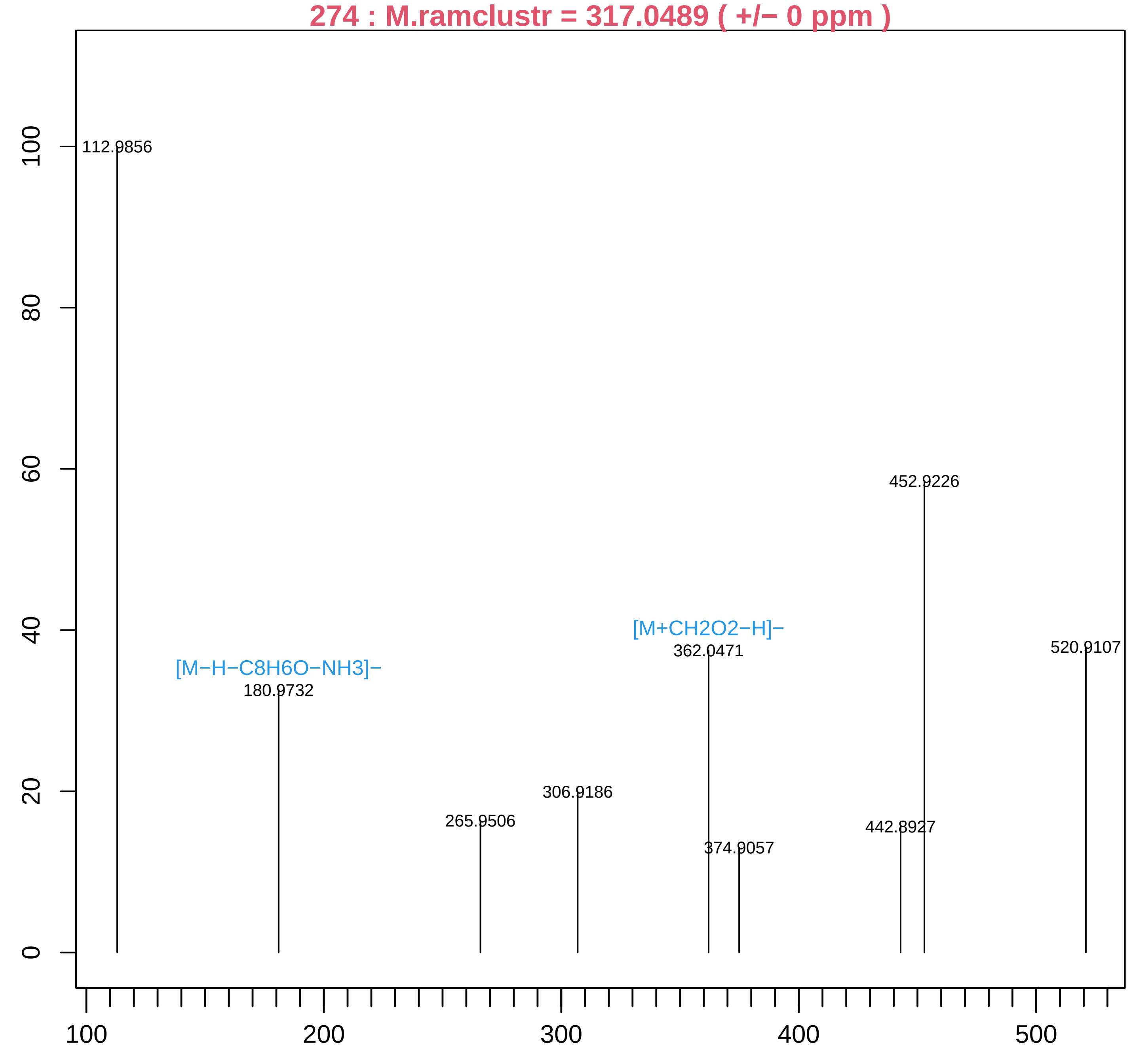

274 : M.ramclustr = 317.0489 (+/− 0 ppm)
112.9856
[M−H−C8H6O−NH3]−
180.9732
265.9506
306.9186
[M+CH2O2−H]−
362.0471
374.9057
442.8927
452.9226
520.9107
0
20
40
60
80
100
100
200
300
400
500

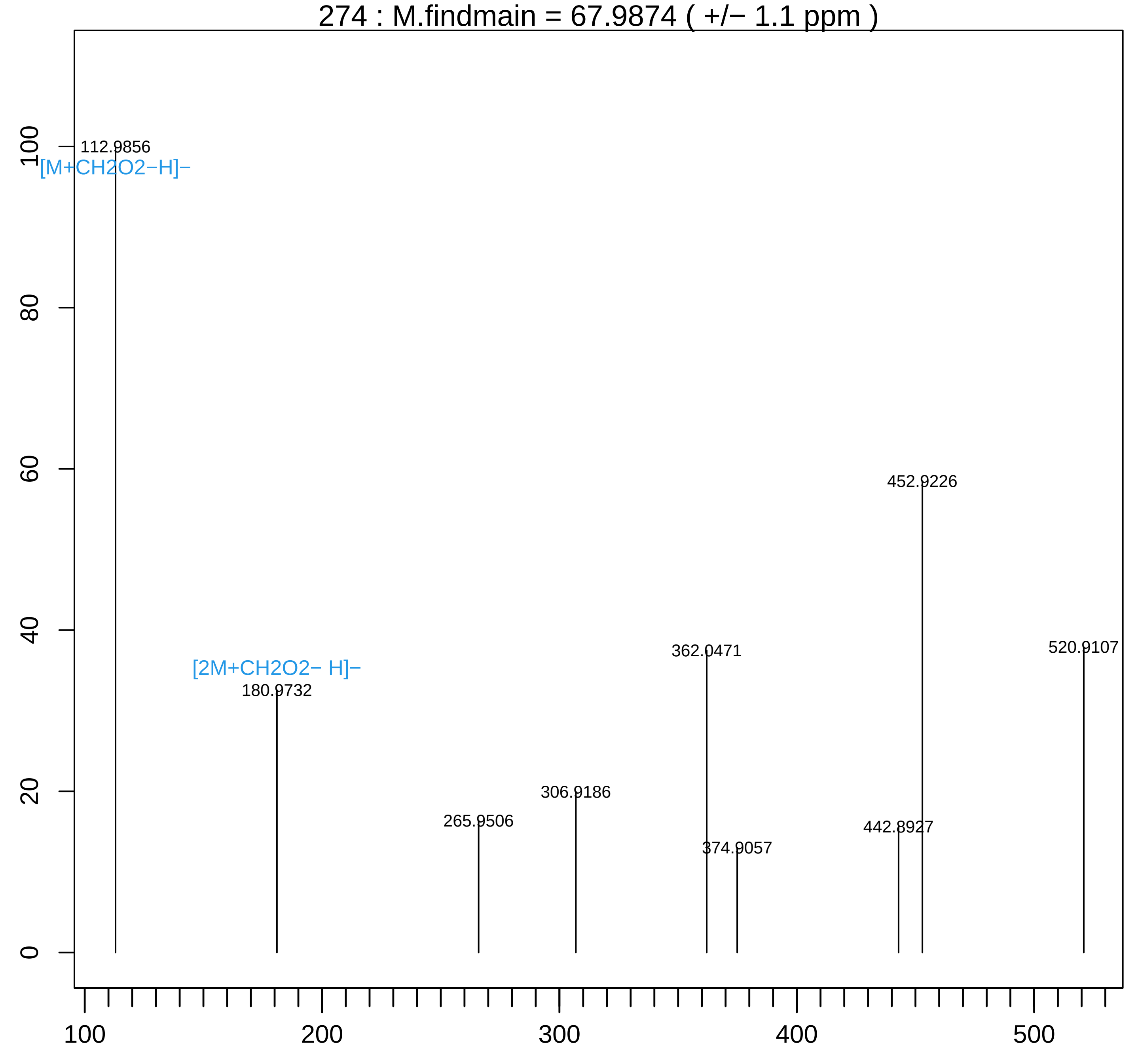

274 : M.findmain = 67.9874 (+/− 1.1 ppm)
112.9856
[M+CH2O2−H]−
[2M+CH2O2− H]−
180.9732
265.9506
306.9186
362.0471
374.9057
442.8927
452.9226
520.9107
0
20
40
60
80
100
100
200
300
400
500

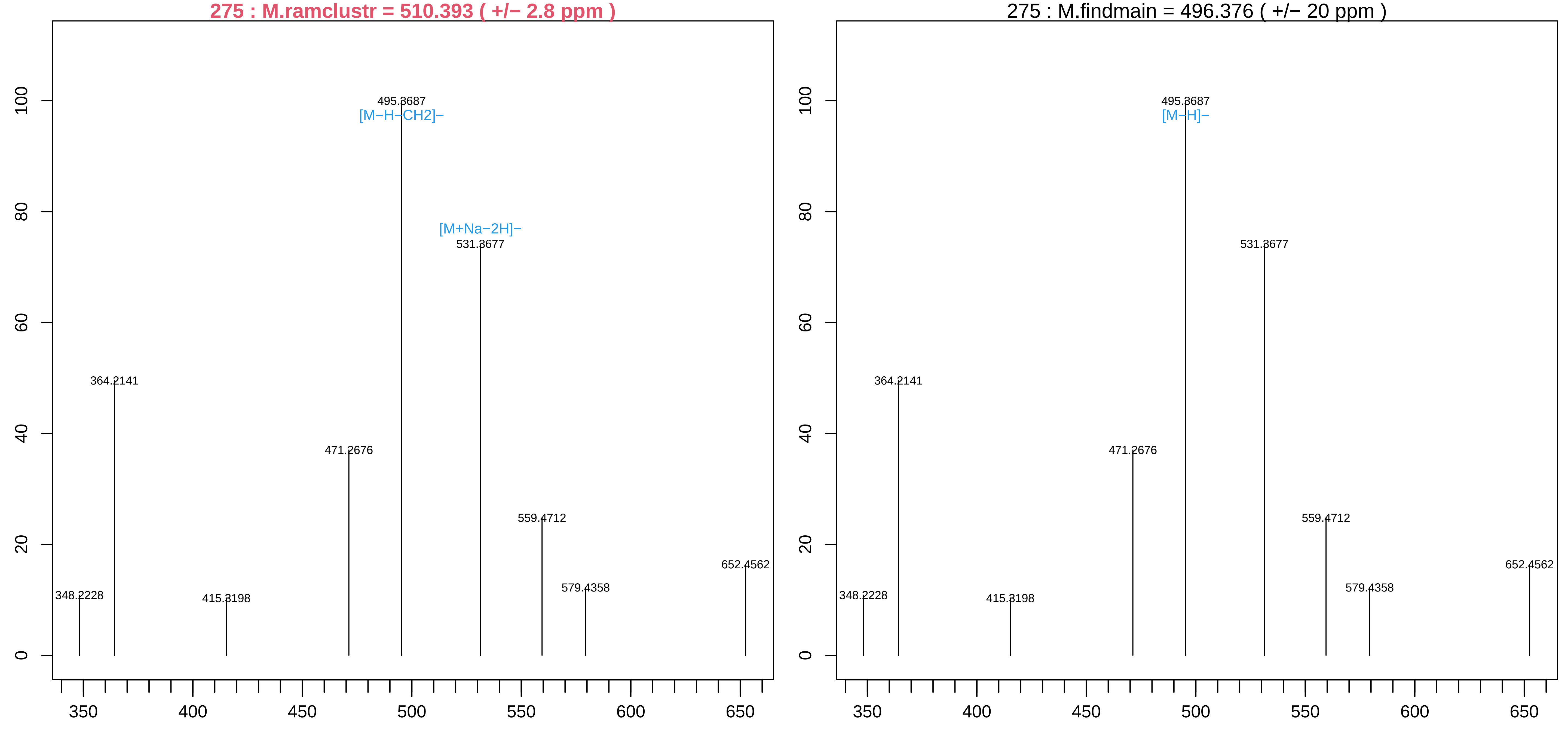
275 : M.ramclustr = 510.393 (+/− 2.8 ppm)
495.3687
[M−H−CH2]−
[M+Na−2H]−
531.3677
364.2141
471.2676
559.4712
652.4562
579.4358
348.2228
415.3198
275 : M.findmain = 496.376 (+/− 20 ppm)
495.3687
[M−H]−
531.3677
364.2141
471.2676
559.4712
652.4562
579.4358
348.2228
415.3198
0
20
40
60
80
100
350
400
450
500
550
600
650

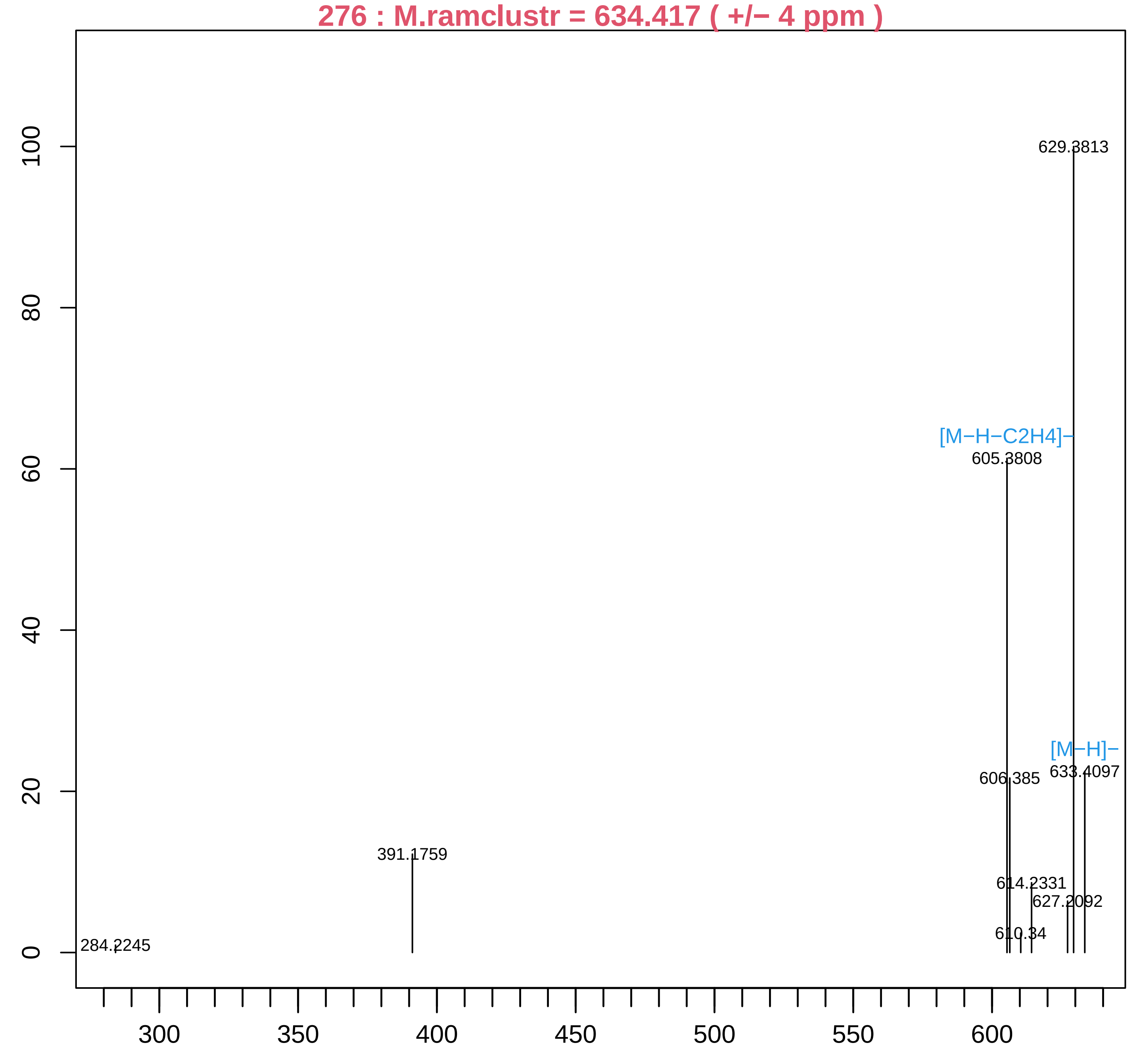

276 : M.ramclustr = 634.417 (+/− 4 ppm)
629.3813
[M−H−C2H4]−
605.3808
[M−H]−
633.4097
606.385
391.1759
614.2331
627.2092
610.34
284.2245
0
20
40
60
80
100
300
350
400
450
500
550
600

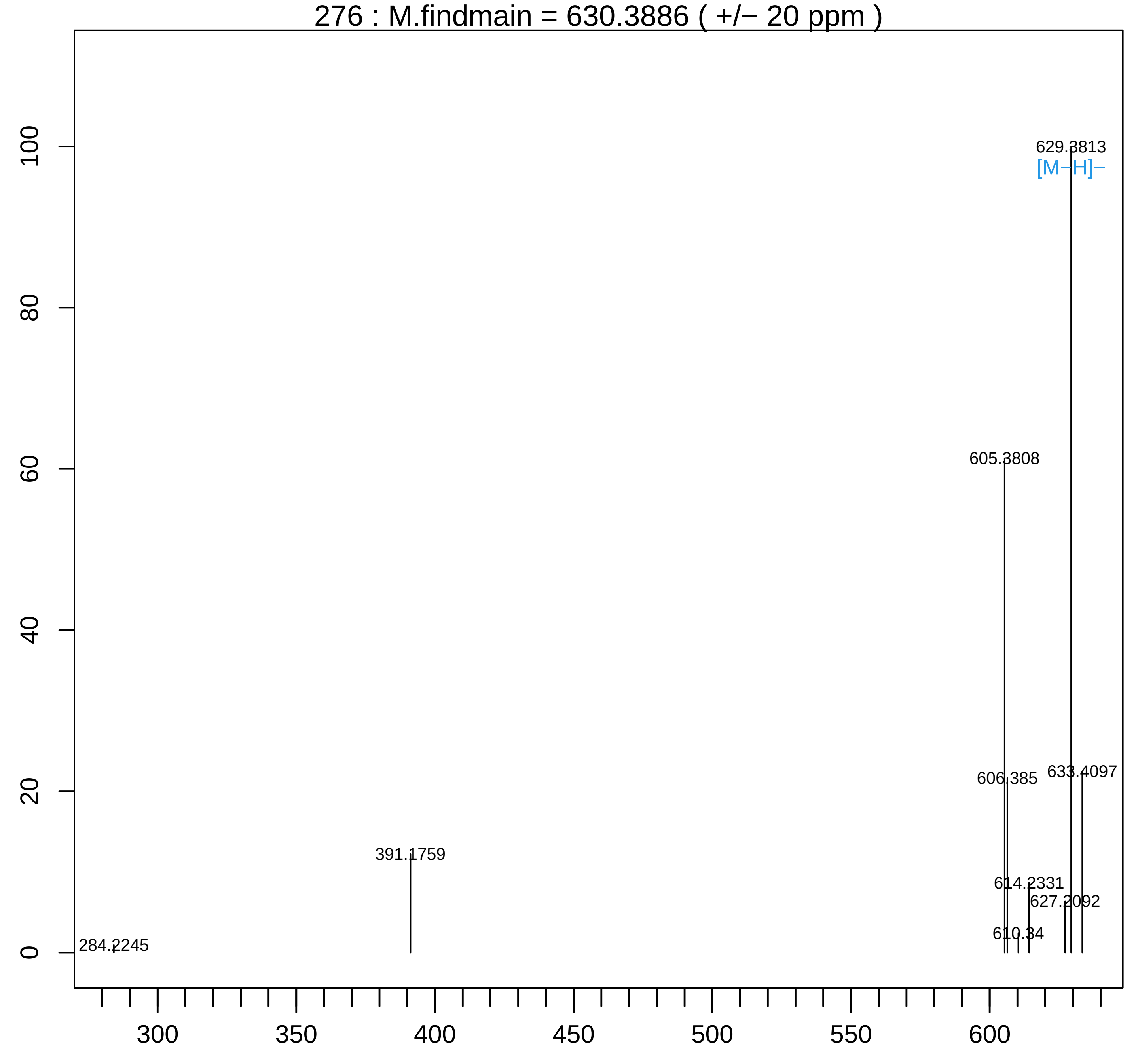

276 : M.findmain = 630.3886 (+/− 20 ppm)
629.3813
[M−H]−
605.3808
606.385
633.4097
391.1759
614.2331
627.2092
610.34
284.2245
0
20
40
60
80
100
300
350
400
450
500
550
600

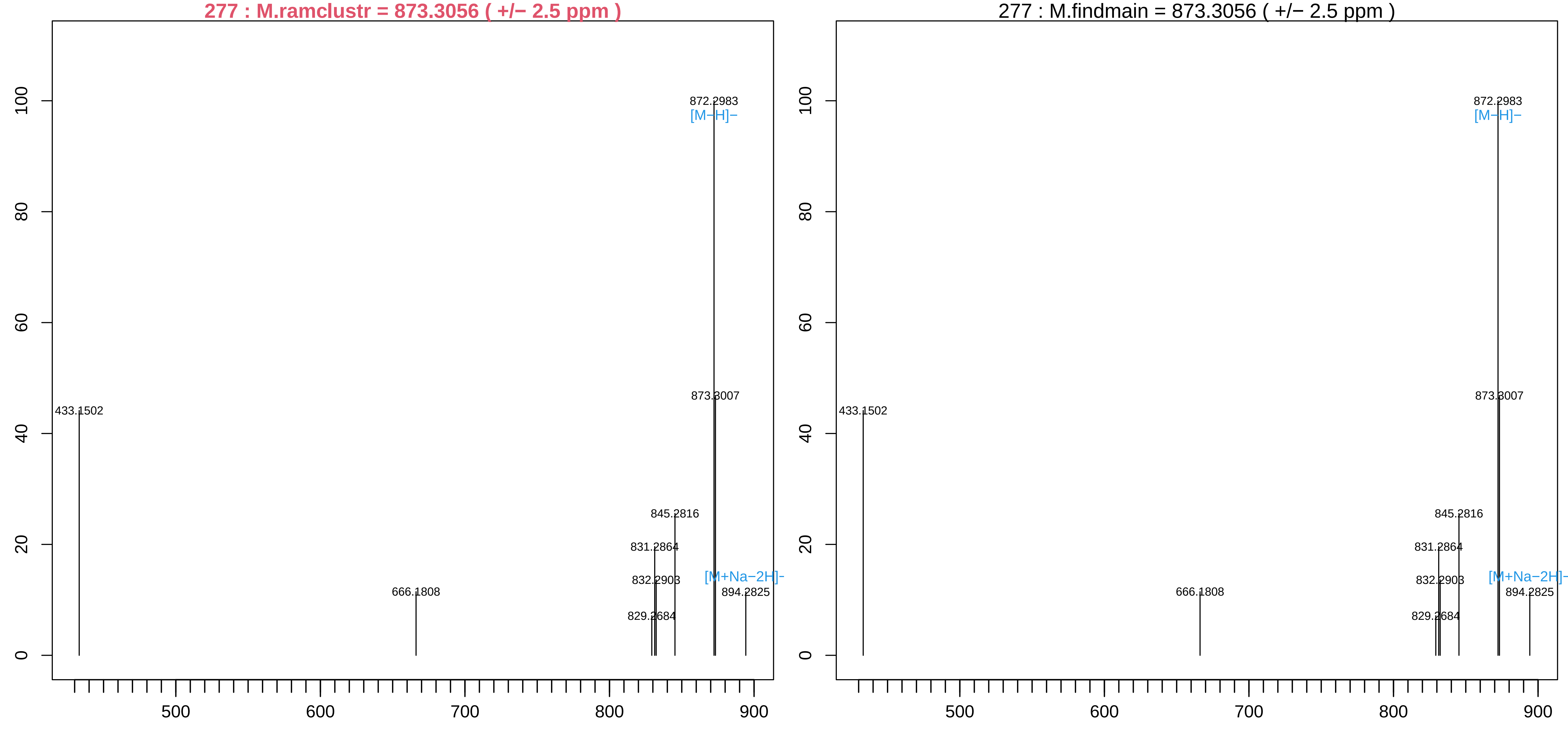

277 : M.ramclustr = 873.3056 (+/− 2.5 ppm)
872.2983
[M−H]−
873.3007
433.1502
845.2816
831.2864
832.2903
[M+Na−2H]−
666.1808
894.2825
829.2684
0
20
40
60
80
100
500
600
700
800
900
277 : M.findmain = 873.3056 (+/− 2.5 ppm)
872.2983
[M−H]−
873.3007
433.1502
845.2816
831.2864
832.2903
[M+Na−2H]−
666.1808
894.2825
829.2684
0
20
40
60
80
100
500
600
700
800
900

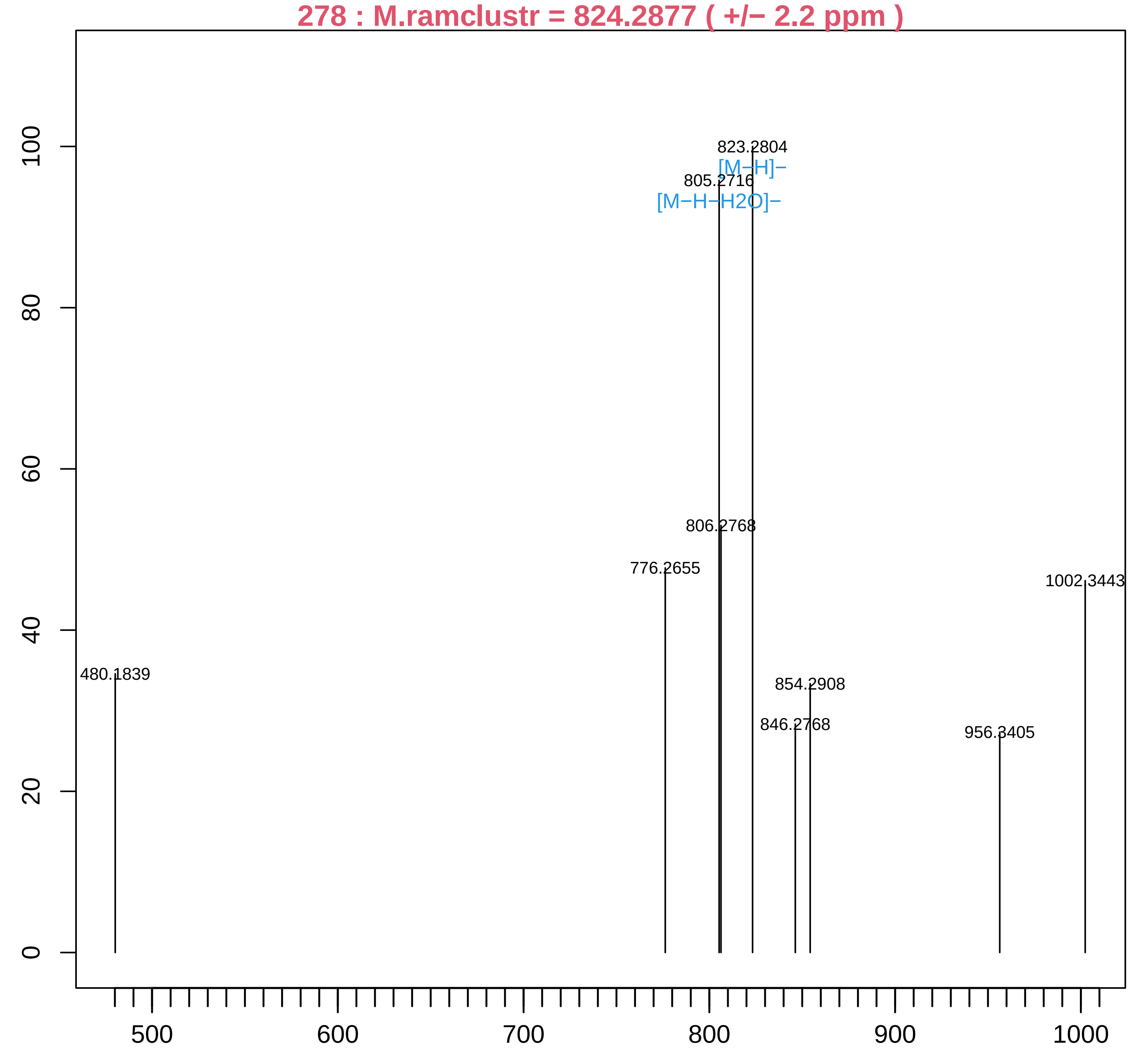

278 : M.ramclustr = 824.2877 (+/− 2.2 ppm)
823.2804
[M−H]−
805.2716
[M−H−H2O]−
806.2768
776.2655
1002.3443
480.1839
854.2908
846.2768
956.3405
0
20
40
60
80
100
500
600
700
800
900
1000

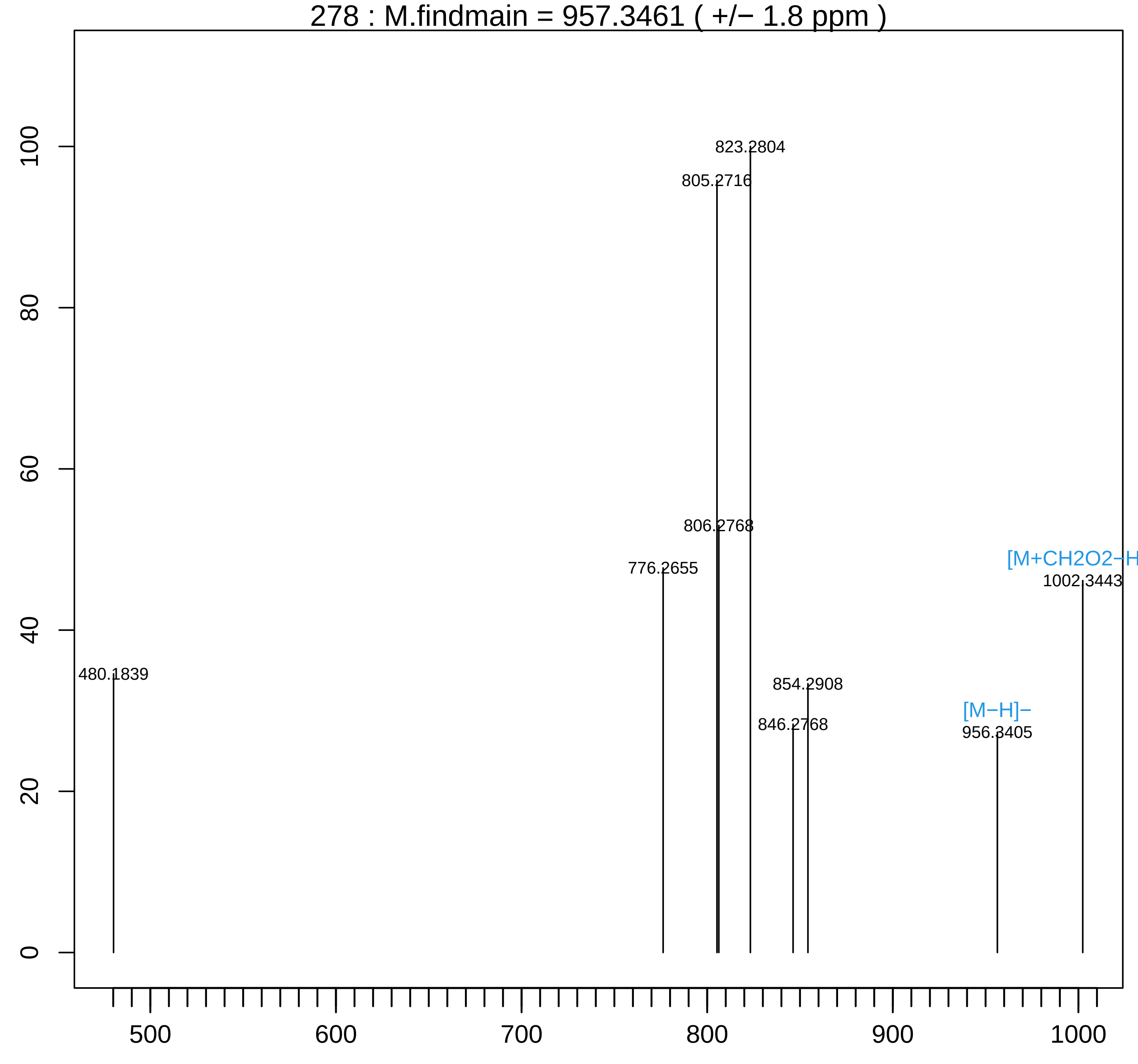

278 : M.findmain = 957.3461 (+/− 1.8 ppm)
823.2804
805.2716
806.2768
776.2655
[M+CH2O2−H
1002.3443
480.1839
854.2908
846.2768
[M−H]−
956.3405
0
20
40
60
80
100
500
600
700
800
900
1000

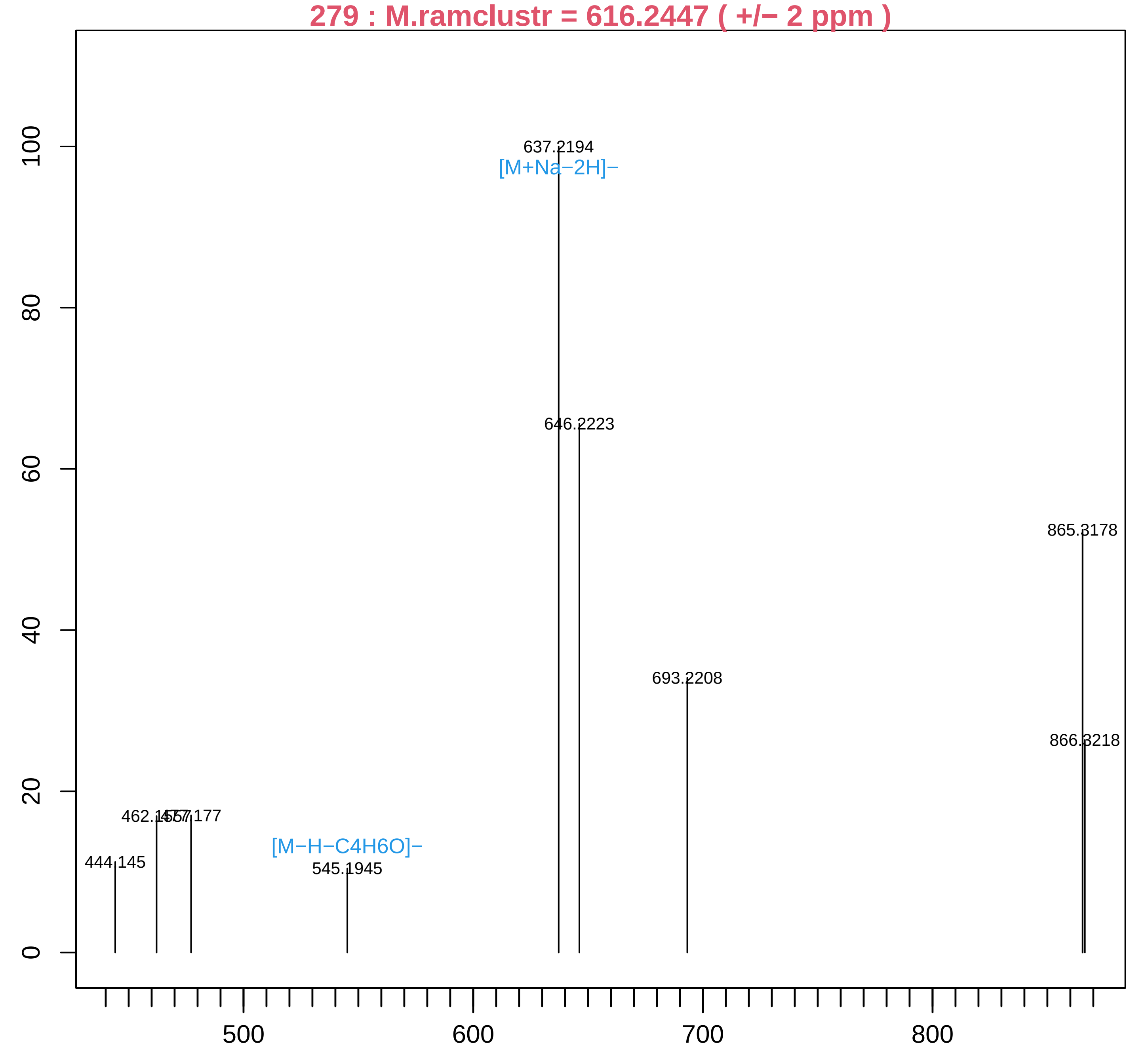
279 : M.ramclustr = 616.2447 (+/− 2 ppm)
637.2194
[M+Na−2H]−
646.2223
865.3178
693.2208
866.3218
462.1457
477.177
444.145
[M−H−C4H6O]−
545.1945
0
20
40
60
80
100
500
600
700
800

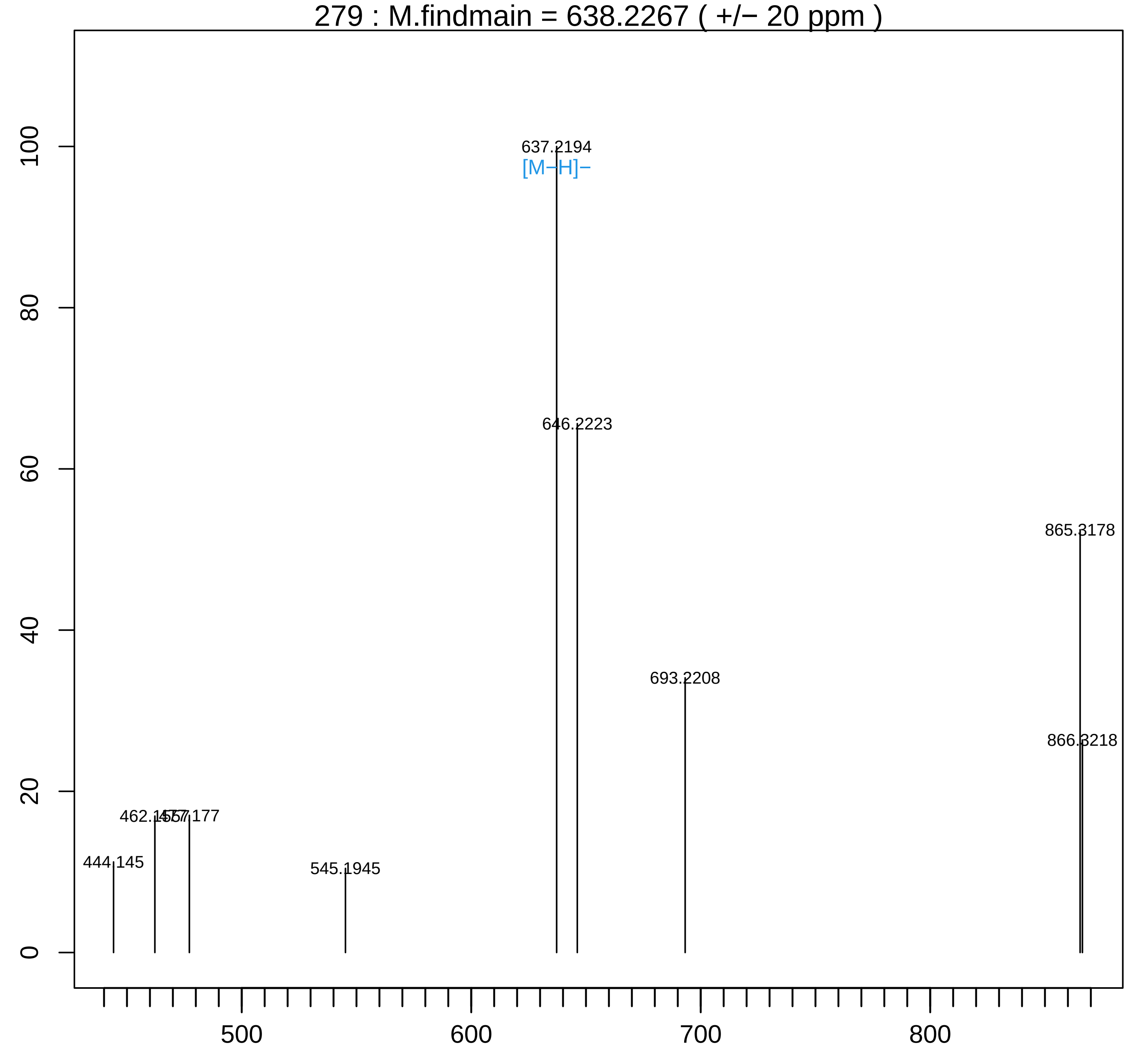
279 : M.findmain = 638.2267 (+/− 20 ppm)
637.2194
[M−H]−
646.2223
865.3178
693.2208
866.3218
462.1457
477.177
444.145
545.1945
0
20
40
60
80
100
500
600
700
800

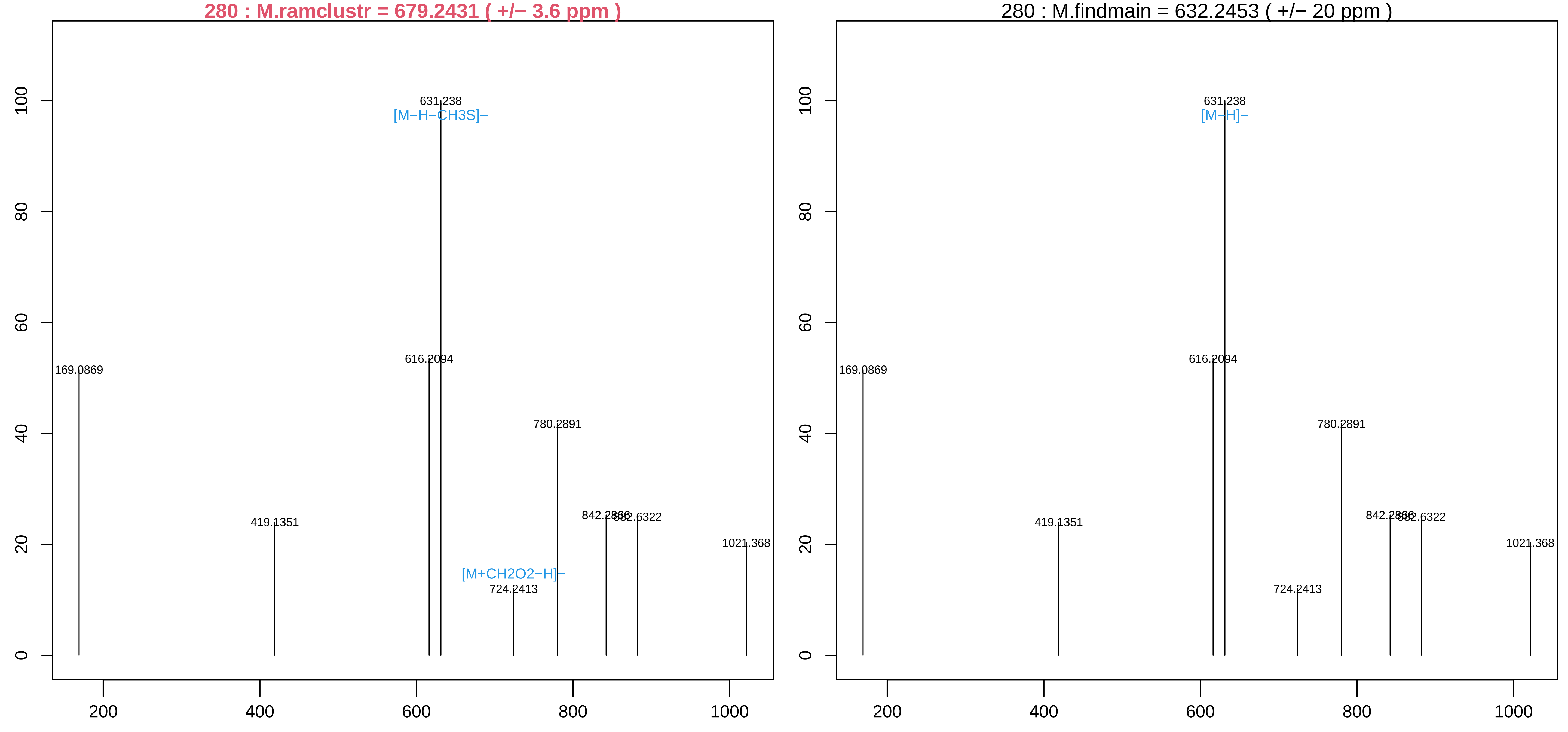
280 : M.ramclustr = 679.2431 (+/− 3.6 ppm)
631.238
[M−H−CH3S]−
616.2094
169.0869
780.2891
842.2863
882.6322
419.1351
1021.368
[M+CH2O2−H]−
724.2413
0
20
40
60
80
100
200
400
600
800
1000
280 : M.findmain = 632.2453 (+/− 20 ppm)
631.238
[M−H]−
616.2094
169.0869
780.2891
842.2863
882.6322
419.1351
1021.368
724.2413
0
20
40
60
80
100
200
400
600
800
1000

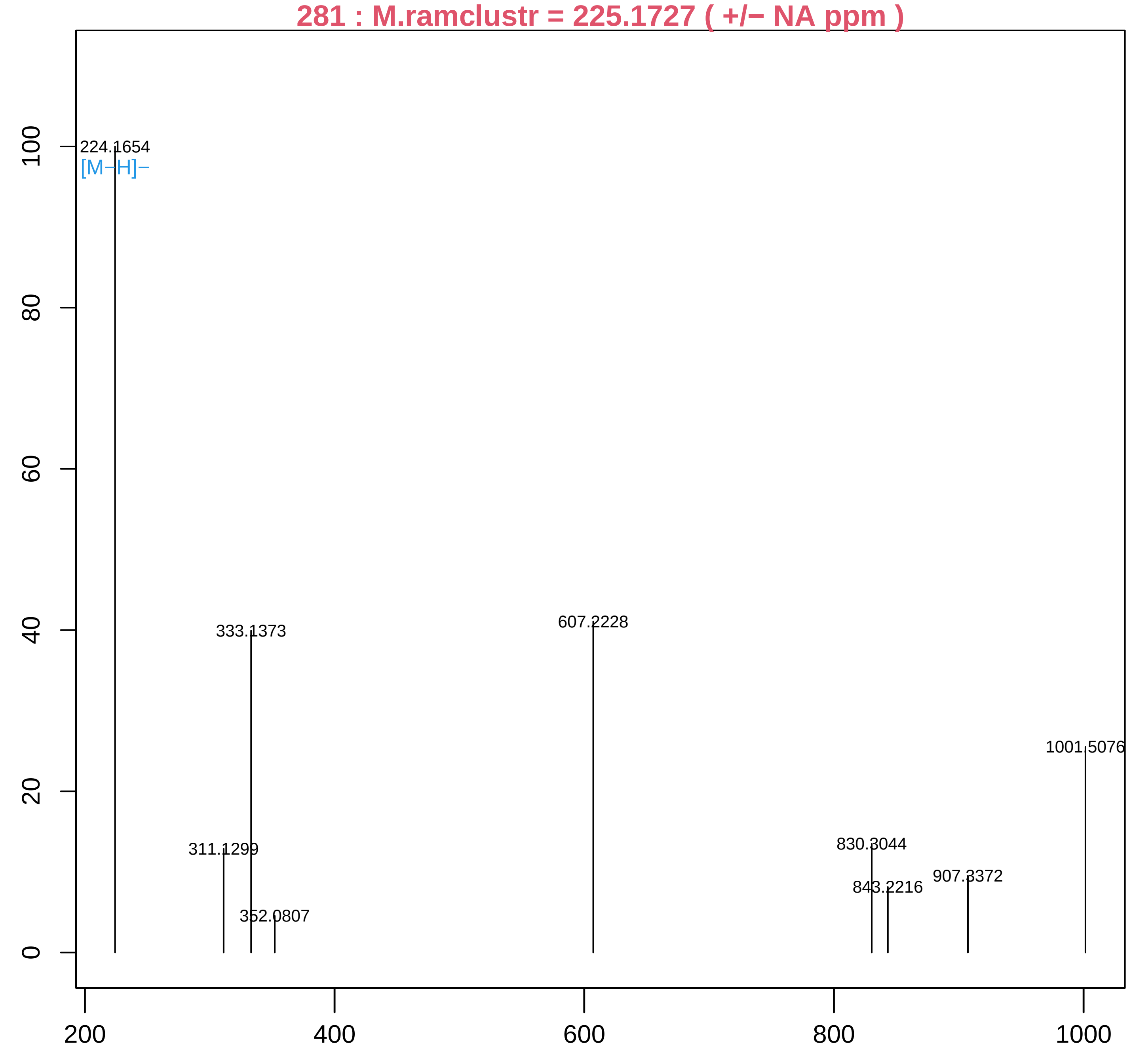

281 : M.ramclustr = 225.1727 (+/− NA ppm)
224.1654
[M−H]−
333.1373
607.2228
311.1299
352.0807
830.3044
843.2216
907.3372
1001.5076
0
20
40
60
80
100
200
400
600
800
1000

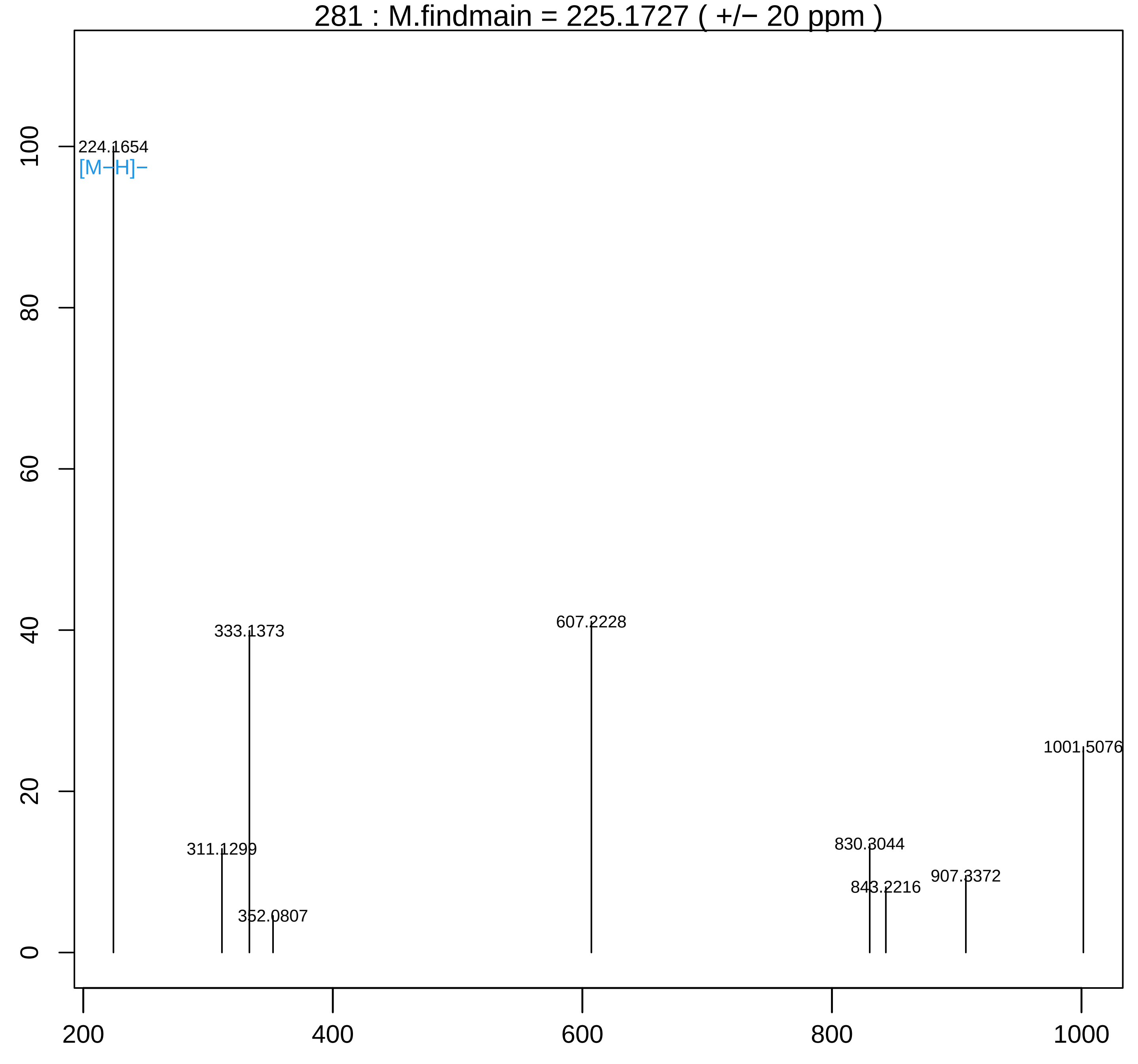

281 : M.findmain = 225.1727 (+/− 20 ppm)
224.1654
[M−H]−
333.1373
607.2228
311.1299
352.0807
830.3044
843.2216
907.3372
1001.5076
0
20
40
60
80
100
200
400
600
800
1000

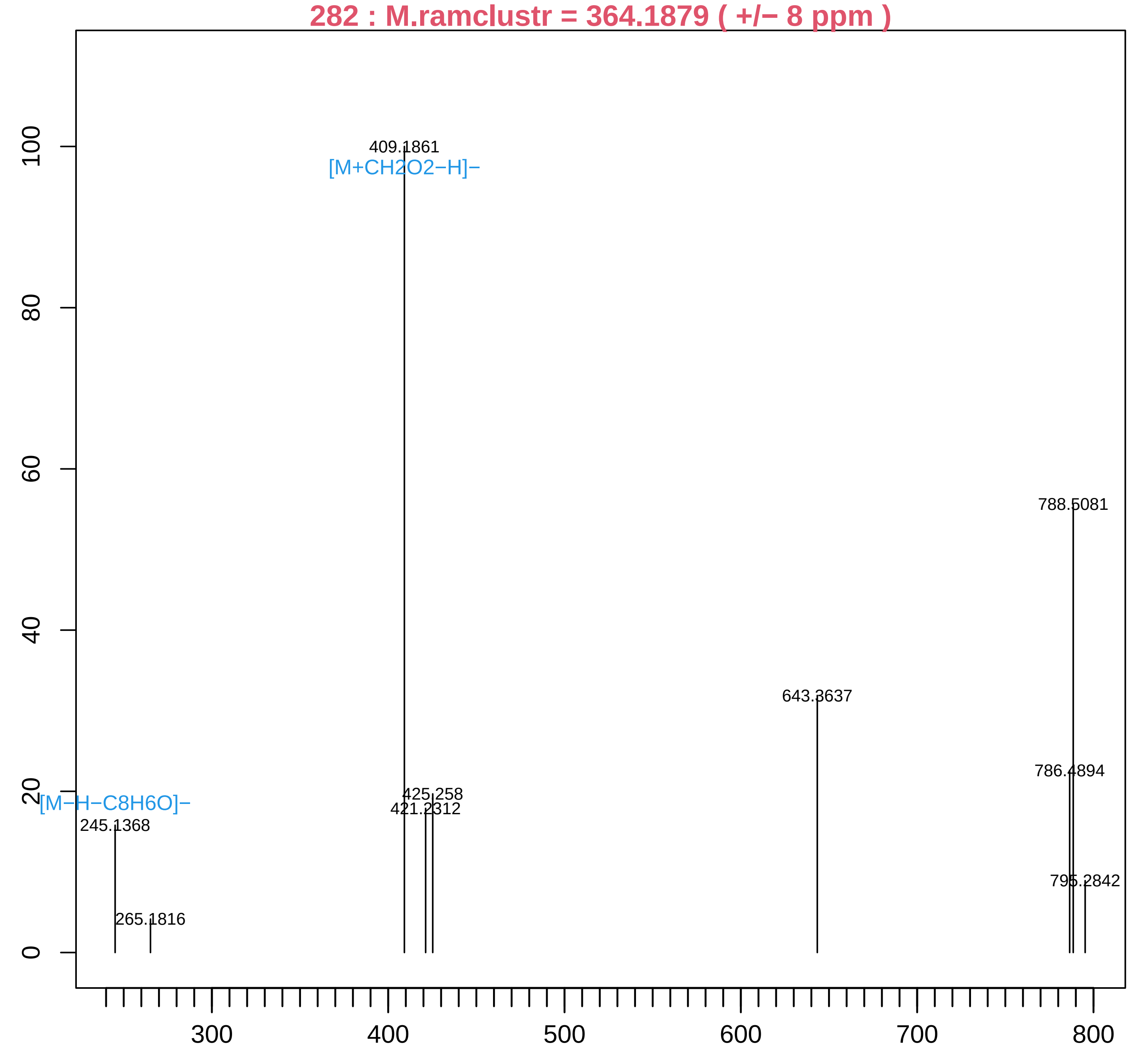
282 : M.ramclustr = 364.1879 (+/− 8 ppm)
409.1861
[M+CH2O2−H]−
788.5081
643.3637
786.4894
425.258
421.2312
[M−H−C8H6O]−
245.1368
795.2842
265.1816
0
20
40
60
80
100
300
400
500
600
700
800

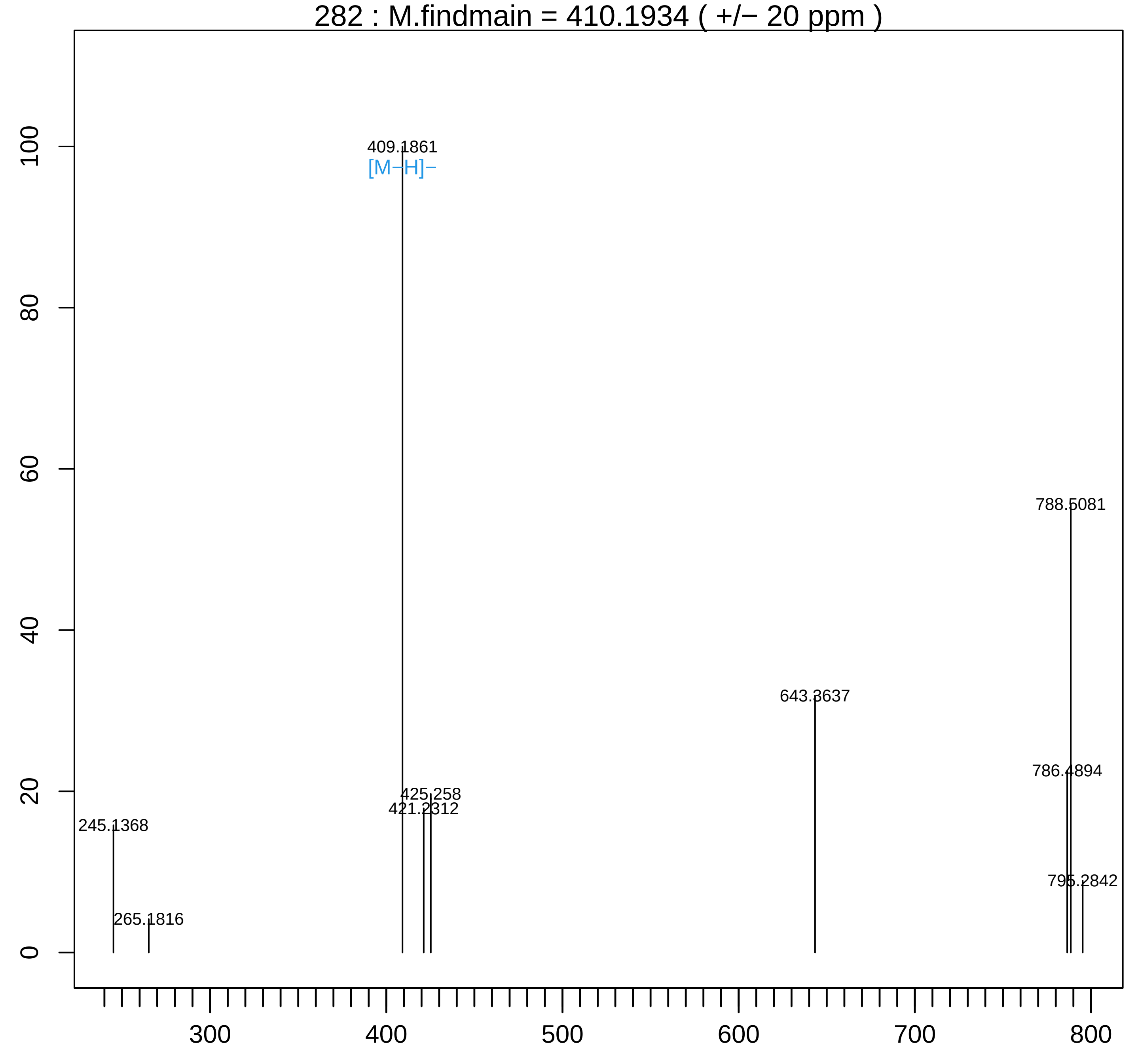
282 : M.findmain = 410.1934 (+/− 20 ppm)
409.1861
[M−H]−
788.5081
643.3637
786.4894
425.258
421.2312
245.1368
795.2842
265.1816
0
20
40
60
80
100
300
400
500
600
700
800

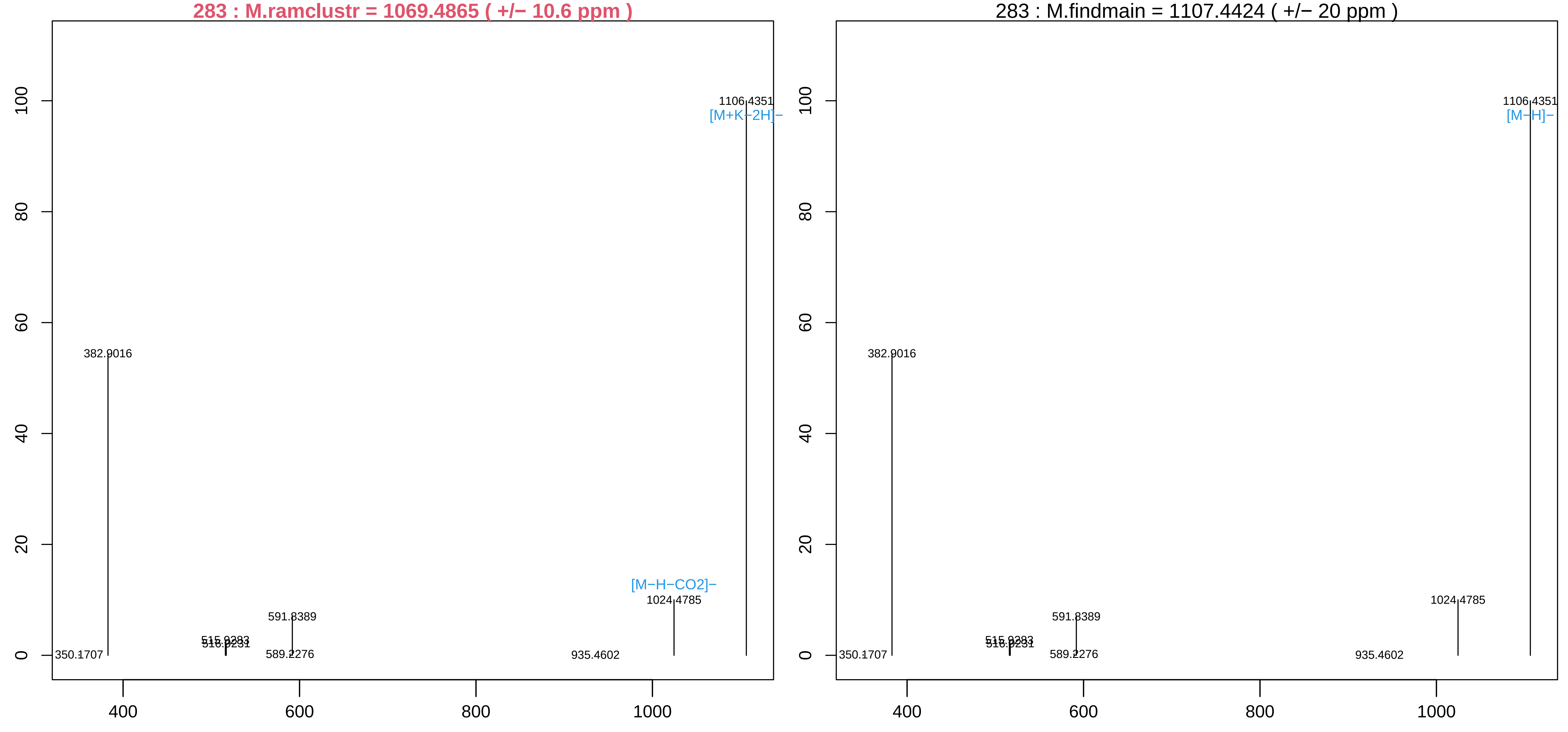
283 : M.ramclustr = 1069.4865 (+/− 10.6 ppm)
283 : M.findmain = 1107.4424 (+/− 20 ppm)
1106.4351
[M+K−2H]−
[M−H]−
382.9016
591.8389
515.9283
516.9231
589.2276
350.1707
935.4602
1024.4785
[M−H−CO2]−
0
20
40
60
80
100
400
600
800
1000

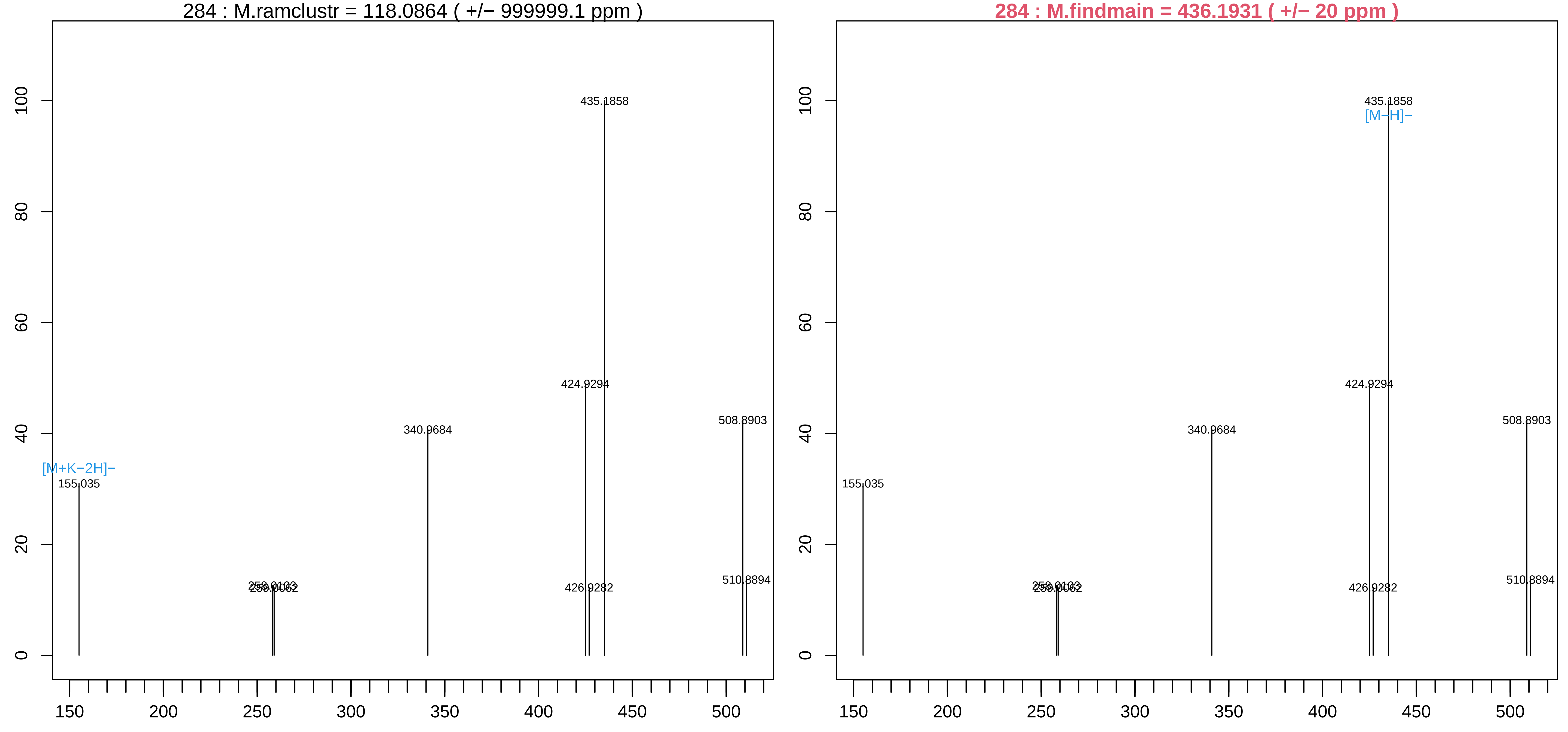
284 : M.ramclustr = 118.0864 (+/− 999999.1 ppm)
435.1858
424.9294
508.8903
340.9684
[M+K−2H]−
155.035
258.0103
259.0062
426.9282
510.8894
284 : M.findmain = 436.1931 (+/− 20 ppm)
435.1858
[M−H]−
424.9294
508.8903
340.9684
155.035
258.0103
259.0062
426.9282
510.8894

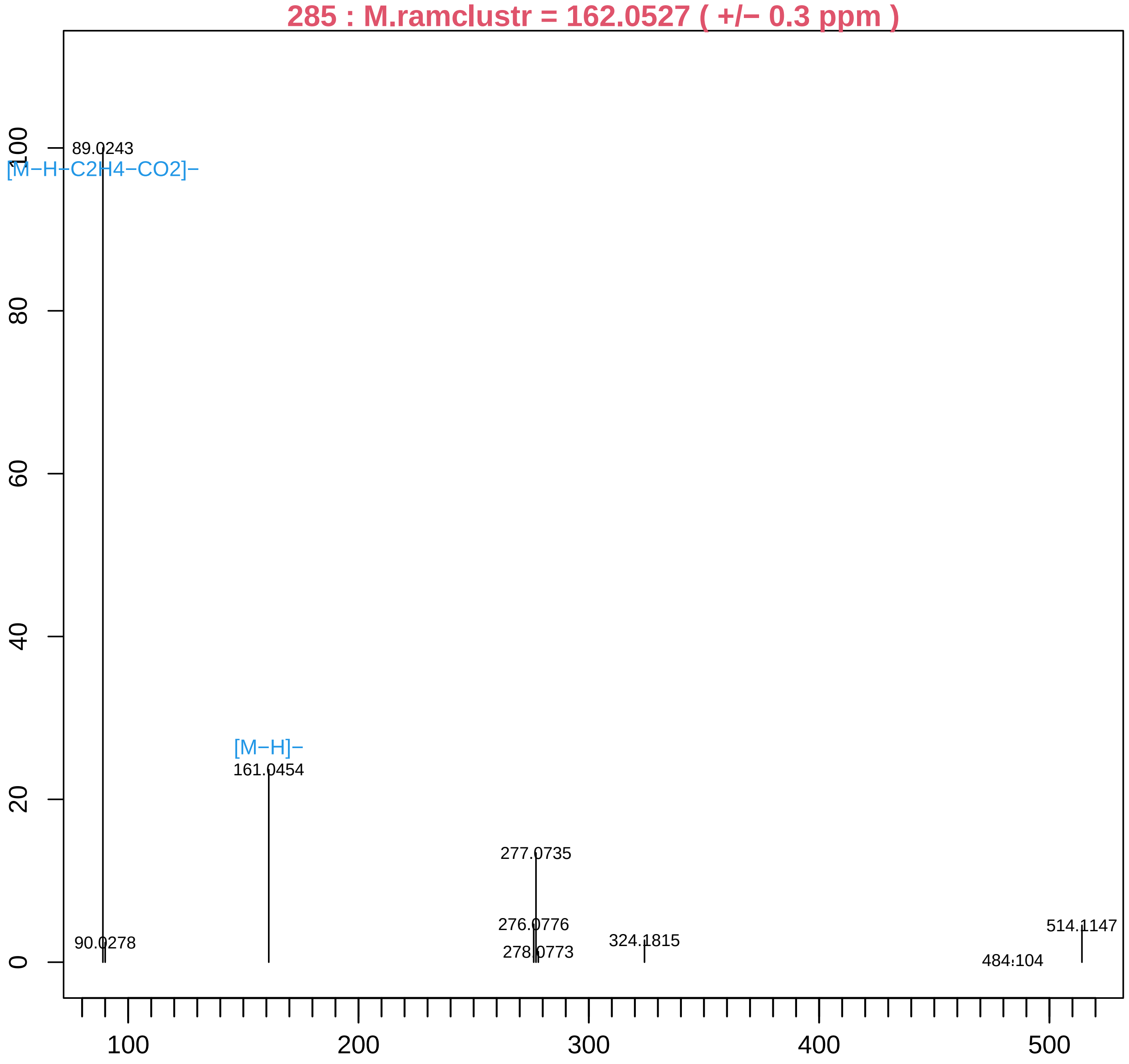

285 : M.ramclustr = 162.0527 (+/− 0.3 ppm)
89.0243
[M−H−C2H4−CO2]−
[M−H]−
161.0454
277.0735
276.0776
278.0773
90.0278
324.1815
484.104
514.1147
0
20
40
60
80
100
200
300
400
500

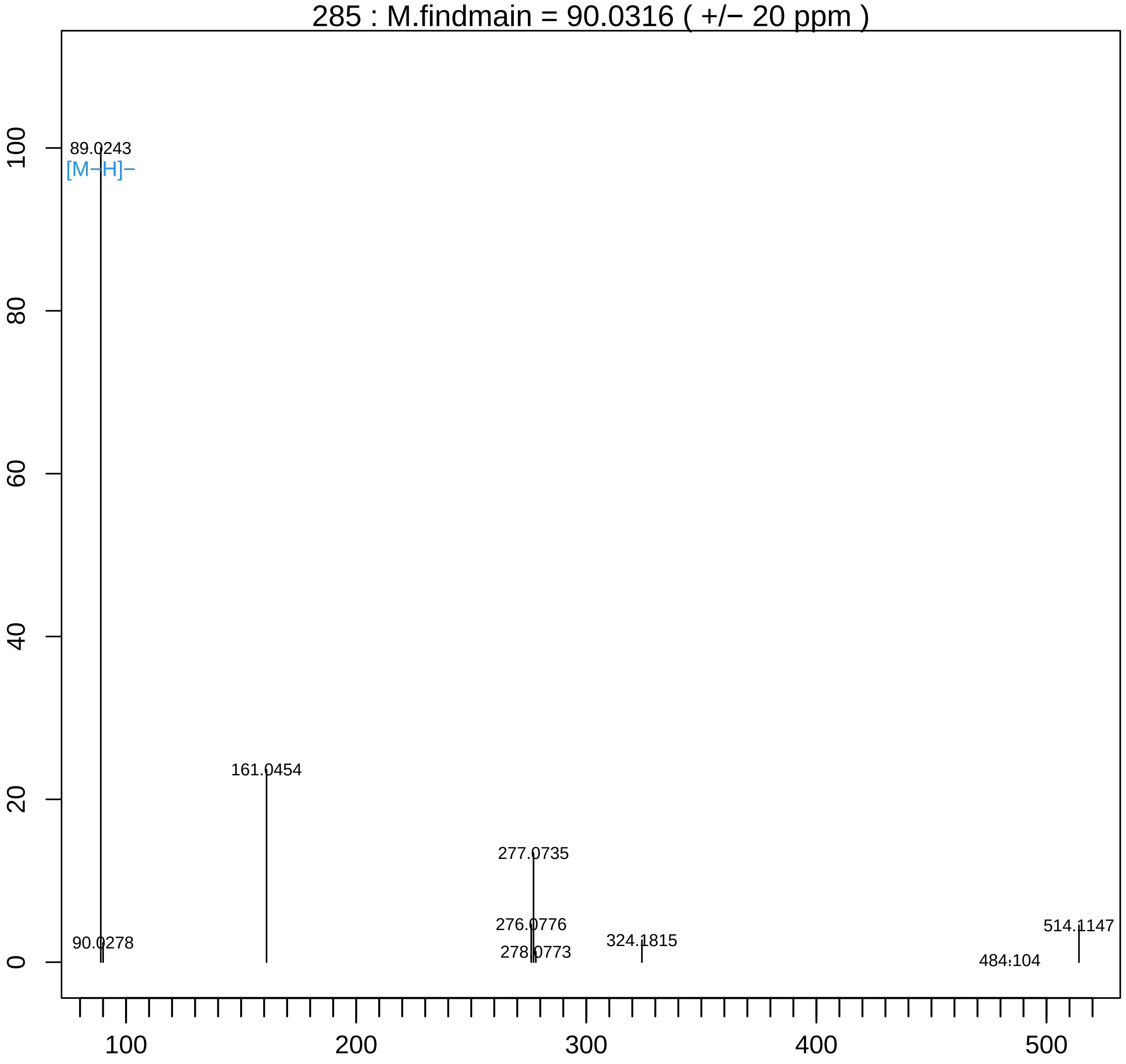

285 : M.findmain = 90.0316 (+/− 20 ppm)
89.0243
[M−H]−
161.0454
277.0735
276.0776
278.0773
90.0278
324.1815
484.104
514.1147
0
20
40
60
80
100
200
300
400
500

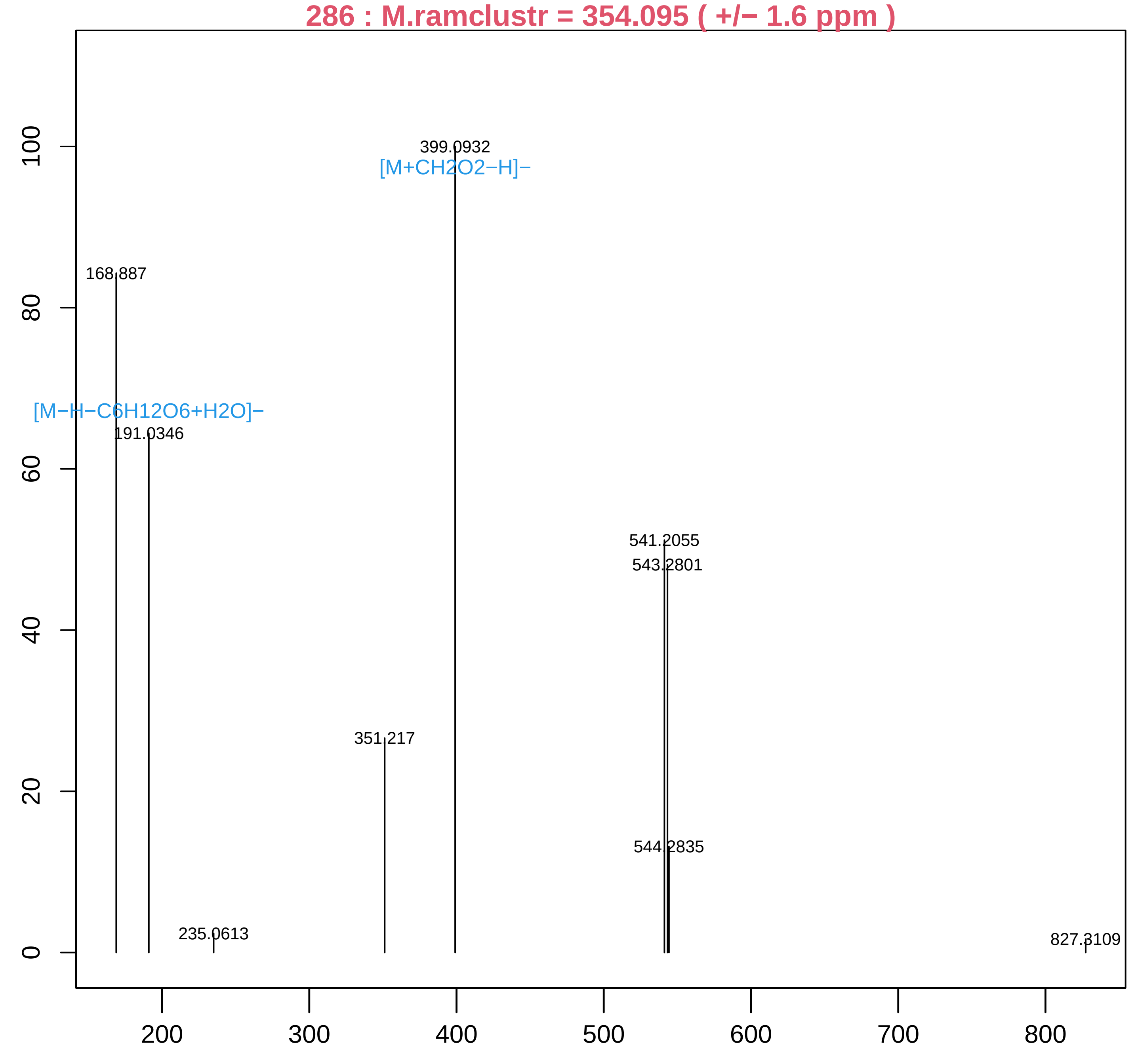
286 : M.ramclustr = 354.095 (+/− 1.6 ppm)
399.0932
[M+CH2O2−H]−
168.887
[M−H−C6H12O6+H2O]−
191.0346
541.2055
543.2801
351.217
544.2835
235.0613
827.3109
0
20
40
60
80
100
200
300
400
500
600
700
800

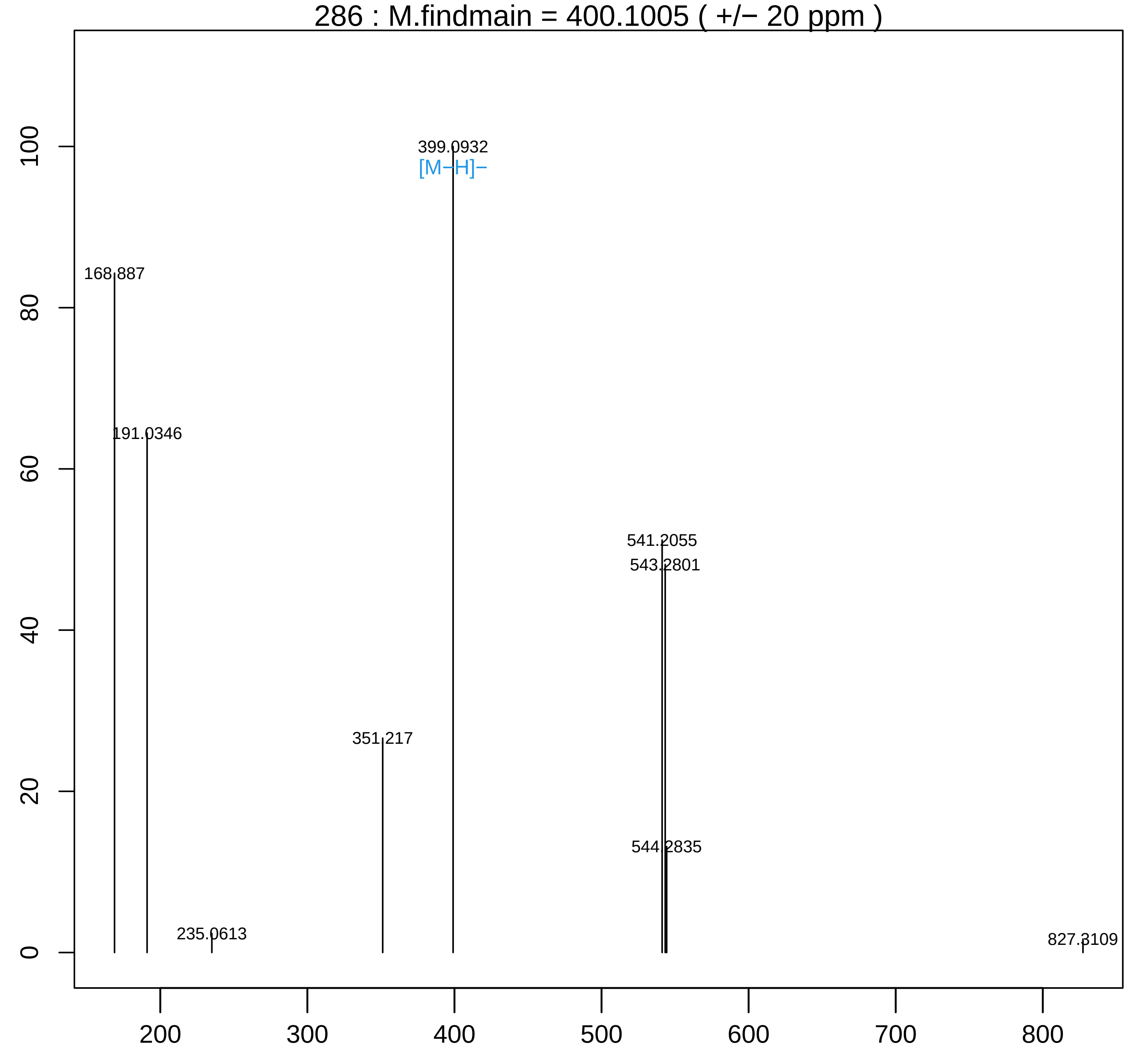
286 : M.findmain = 400.1005 (+/− 20 ppm)
399.0932
[M−H]−
168.887
191.0346
541.2055
543.2801
351.217
544.2835
235.0613
827.3109
0
20
40
60
80
100
200
300
400
500
600
700
800

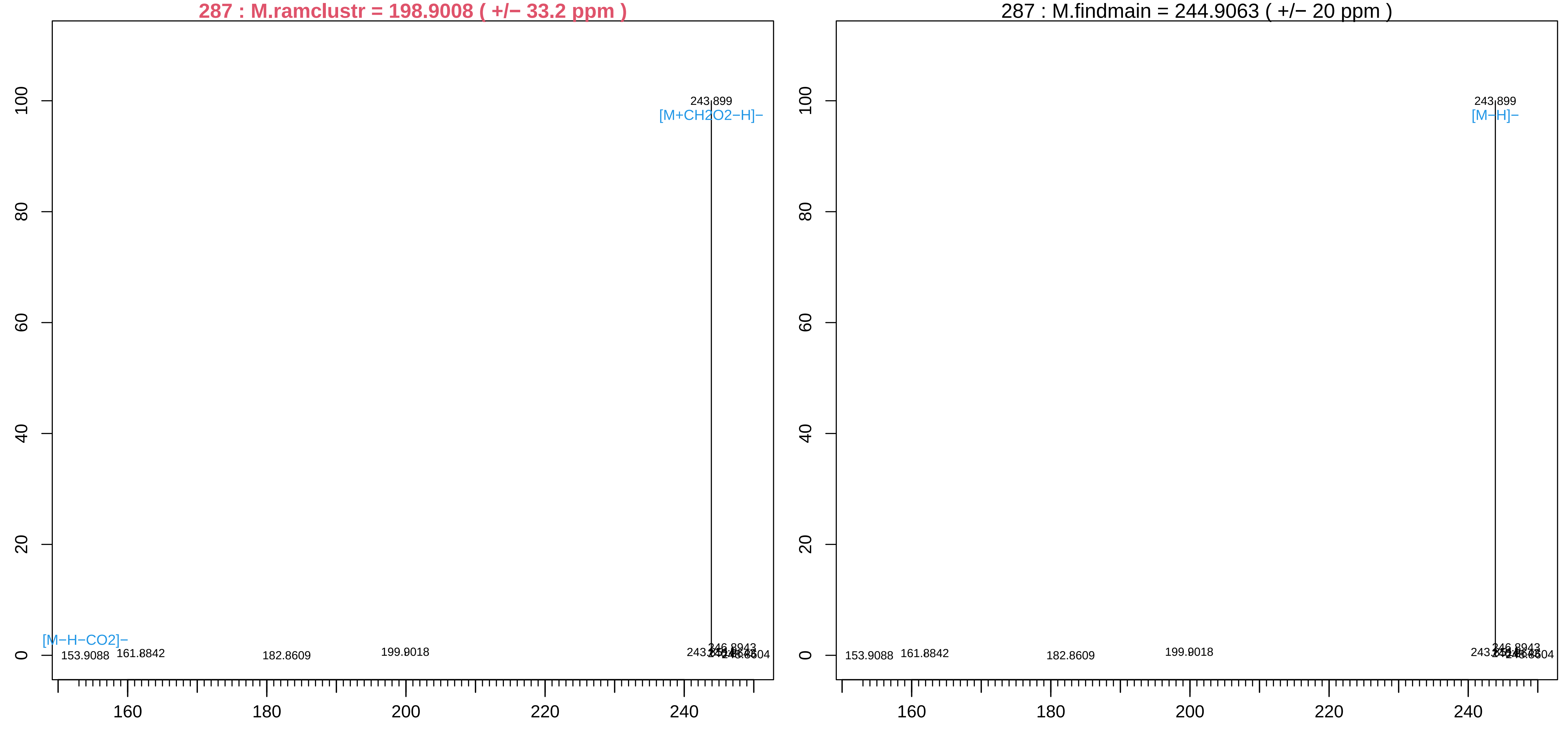

287 : M.ramclustr = 198.9008 (+/− 33.2 ppm)
243.899
[M+CH2O2−H]−
[M−H−CO2]−
153.9088
161.8842
182.8609
199.9018
246.8943
287 : M.findmain = 244.9063 (+/− 20 ppm)
243.899
[M−H]−
153.9088
161.8842
182.8609
199.9018
246.8943

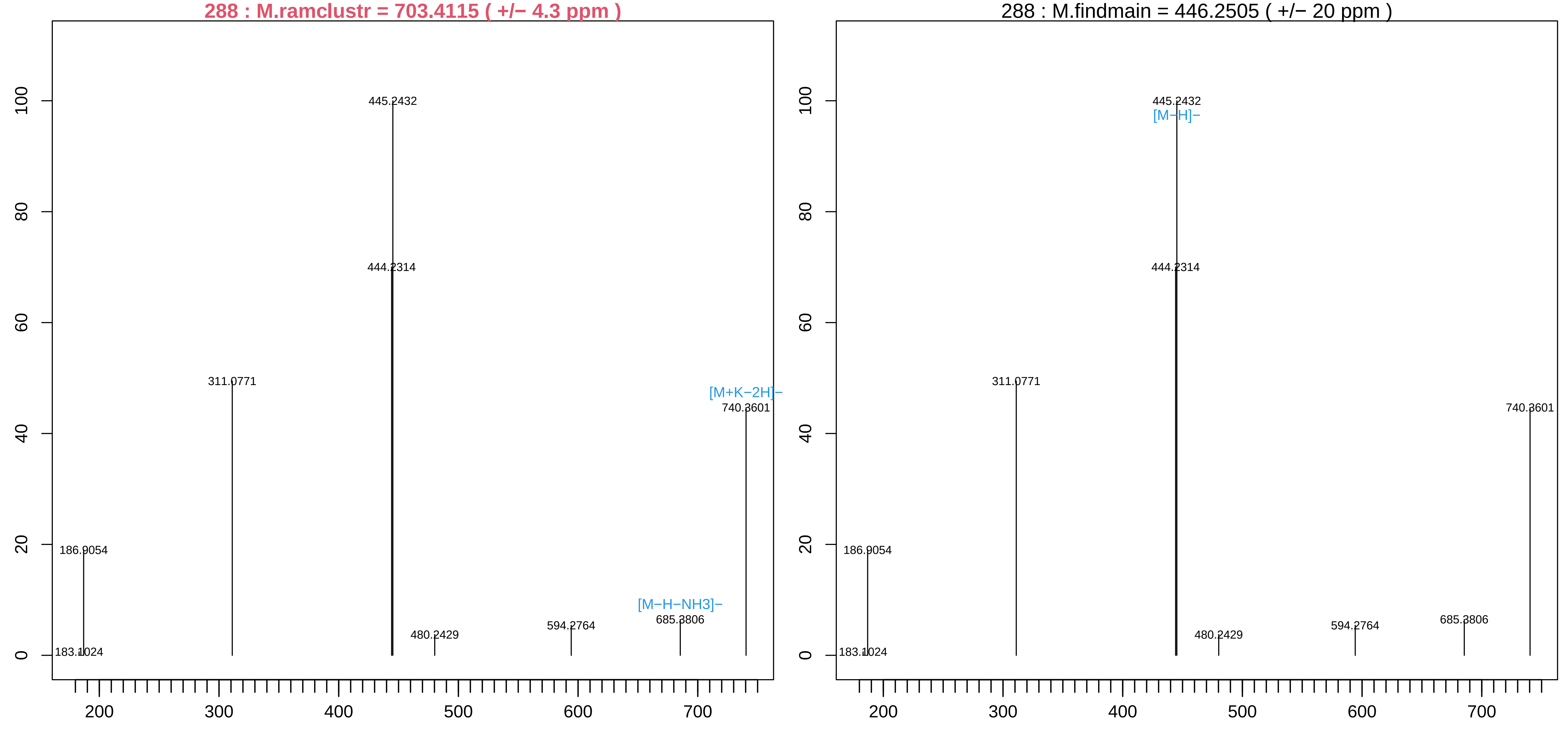
288 : M.ramclustr = 703.4115 (+/− 4.3 ppm)
445.2432
444.2314
311.0771
[M+K−2H]−
740.3601
186.9054
[M−H−NH3]−
685.3806
594.2764
480.2429
183.1024
288 : M.findmain = 446.2505 (+/− 20 ppm)
445.2432
[M−H]−
444.2314
311.0771
740.3601
186.9054
685.3806
594.2764
480.2429
183.1024
0
20
40
60
80
100
200
300
400
500
600
700

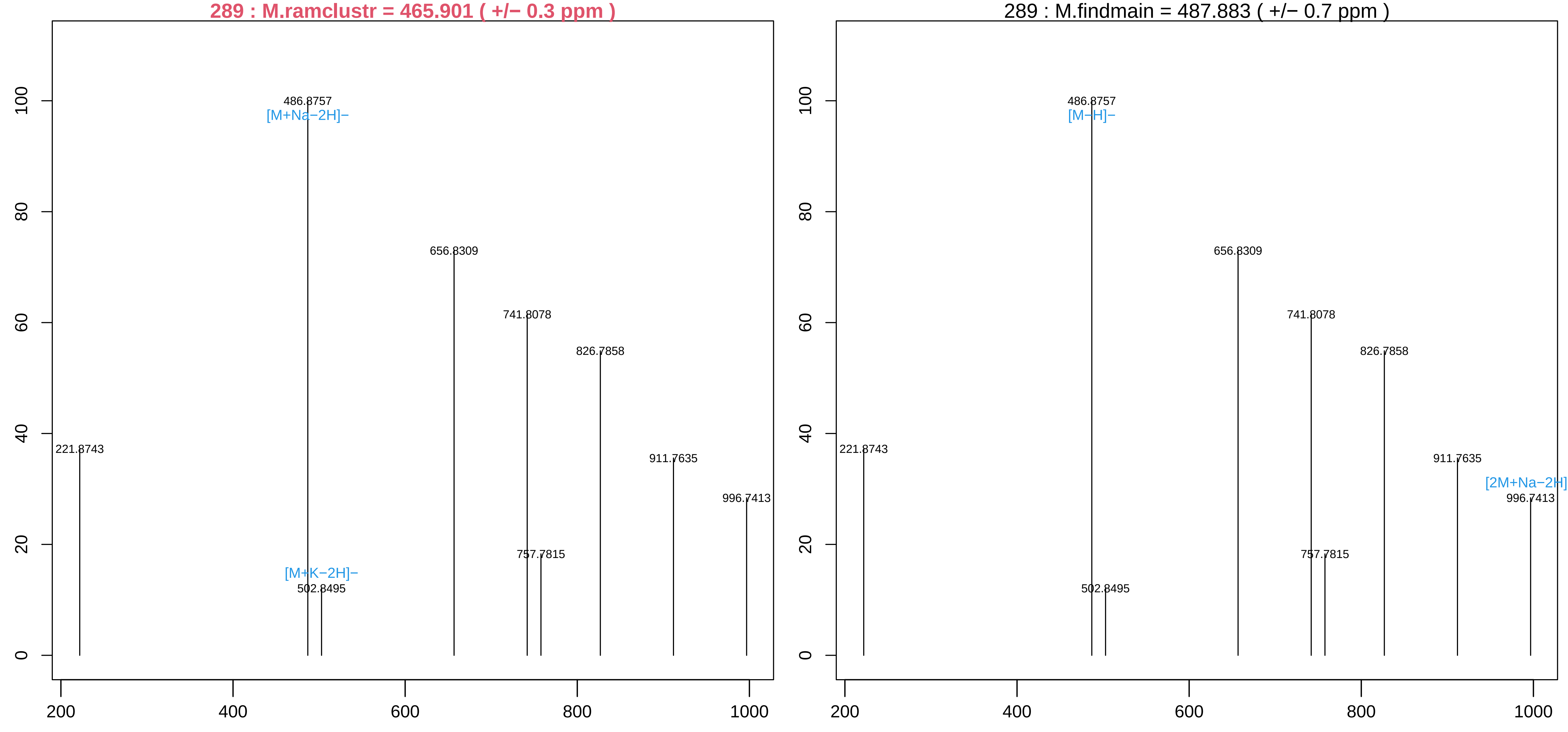
289 : M.ramclustr = 465.901 (+/− 0.3 ppm)
486.8757
[M+Na−2H]−
656.8309
741.8078
826.7858
221.8743
911.7635
996.7413
757.7815
[M+K−2H]−
502.8495
289 : M.findmain = 487.883 (+/− 0.7 ppm)
486.8757
[M−H]−
656.8309
741.8078
826.7858
221.8743
911.7635
[2M+Na−2H]
996.7413
757.7815
502.8495
0
20
40
60
80
100
200
400
600
800
1000

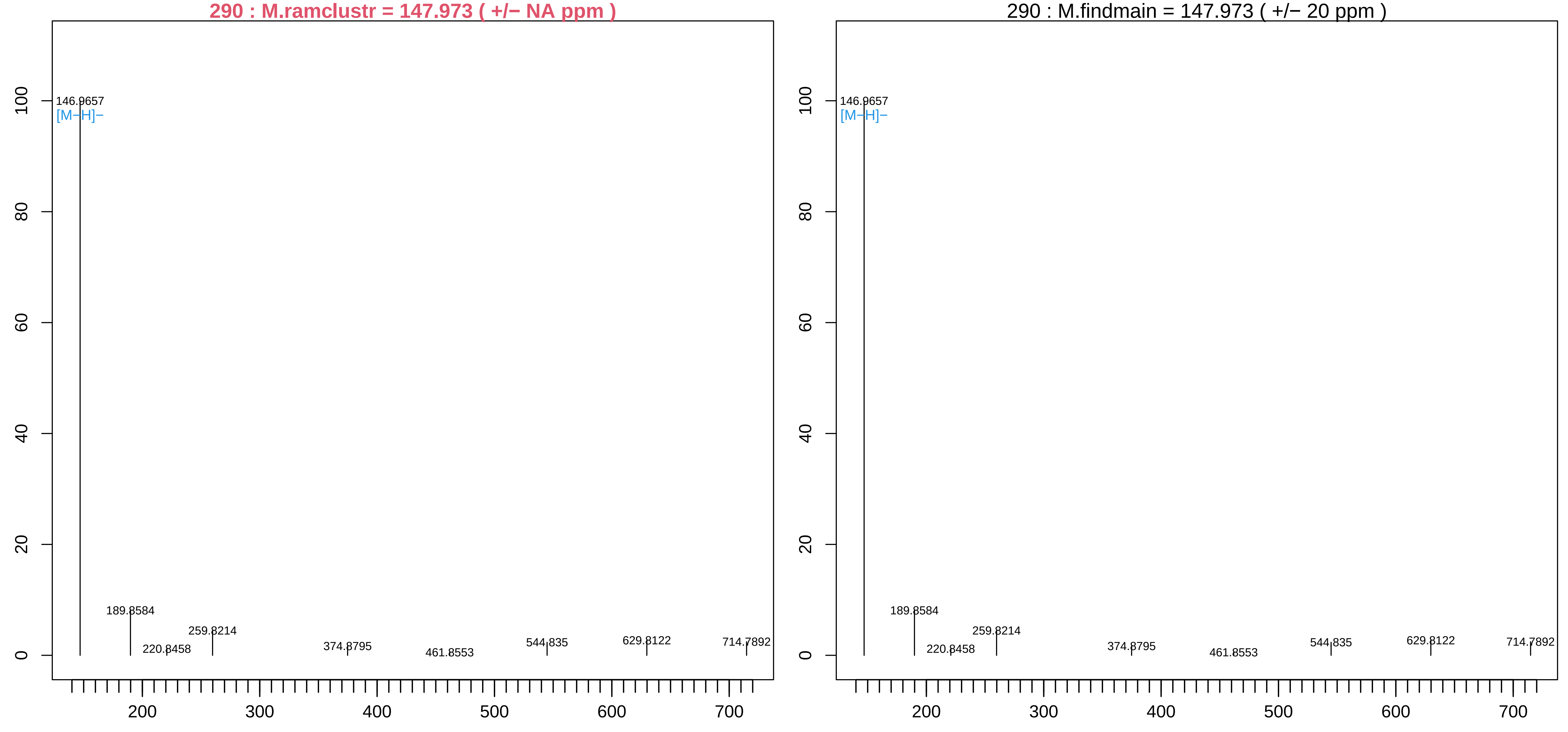
290 : M.ramclustr = 147.973 (+/− NA ppm)
290 : M.findmain = 147.973 (+/− 20 ppm)
0
20
40
60
80
100
200
300
400
500
600
700
146.9657
[M−H]−
189.8584
220.8458
259.8214
374.8795
461.8553
544.835
629.8122
714.7892

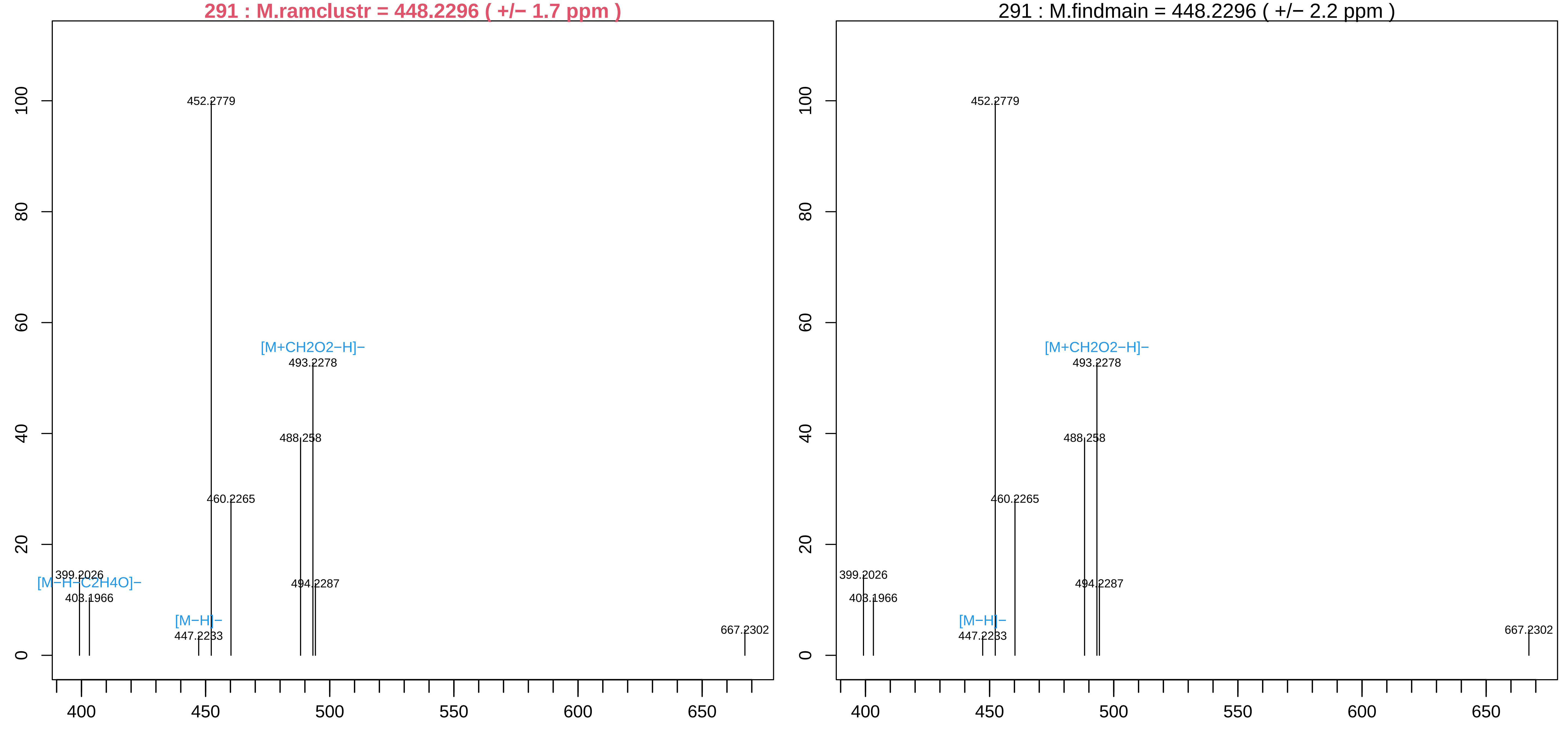
291 : M.ramclustr = 448.2296 (+/− 1.7 ppm)
452.2779
[M+CH2O2−H]−
493.2278
488.258
460.2265
399.2026
[M−H−C2H4O]−
494.2287
403.1966
[M−H]−
447.2233
667.2302
0
20
40
60
80
100
400
450
500
550
600
650
291 : M.findmain = 448.2296 (+/− 2.2 ppm)
452.2779
[M+CH2O2−H]−
493.2278
488.258
460.2265
399.2026
494.2287
403.1966
[M−H]−
447.2233
667.2302
0
20
40
60
80
100
400
450
500
550
600
650

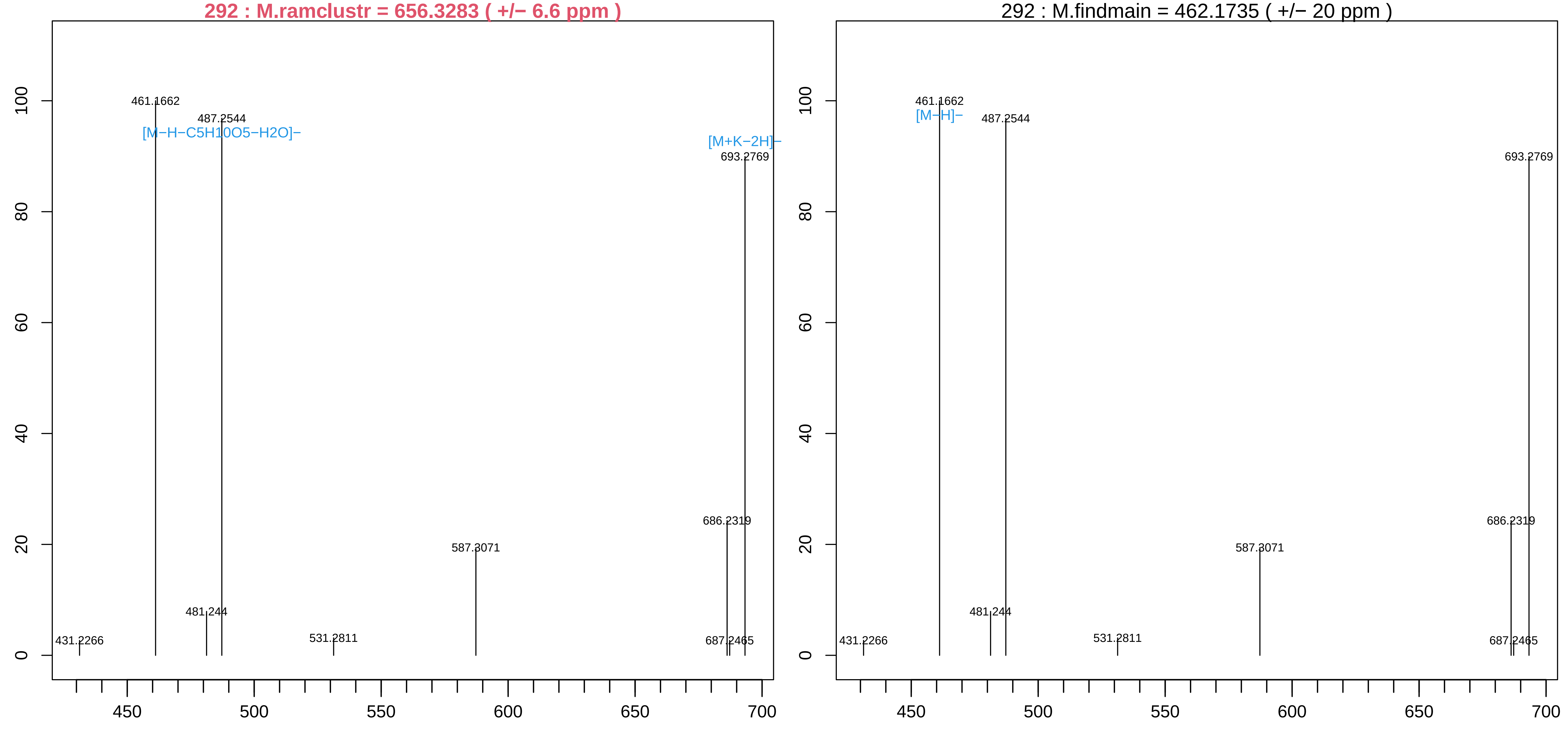

292 : M.ramclustr = 656.3283 (+/− 6.6 ppm)
461.1662
487.2544
[M−H−C5H10O5−H2O]−
[M+K−2H]−
693.2769
686.2319
587.3071
481.244
431.2266
531.2811
687.2465
292 : M.findmain = 462.1735 (+/− 20 ppm)
461.1662
[M−H]−
487.2544
693.2769
686.2319
587.3071
481.244
431.2266
531.2811
687.2465

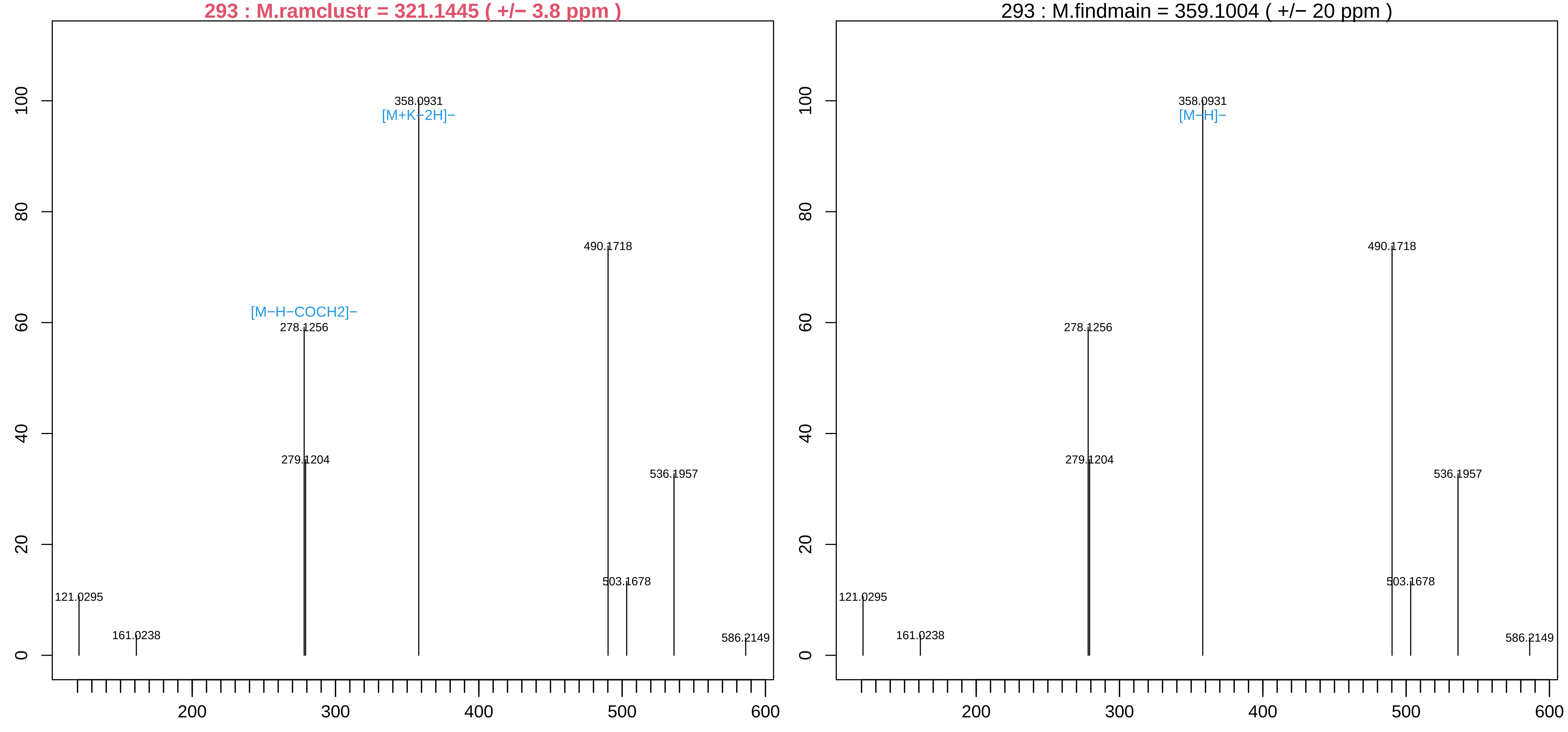

293 : M.ramclustr = 321.1445 (+/− 3.8 ppm)
358.0931
[M+K−2H]−
[M−H−COCH2]−
278.1256
279.1204
490.1718
503.1678
536.1957
121.0295
161.0238
586.2149
293 : M.findmain = 359.1004 (+/− 20 ppm)
[M−H]−
0
20
40
60
80
100
200
300
400
500
600

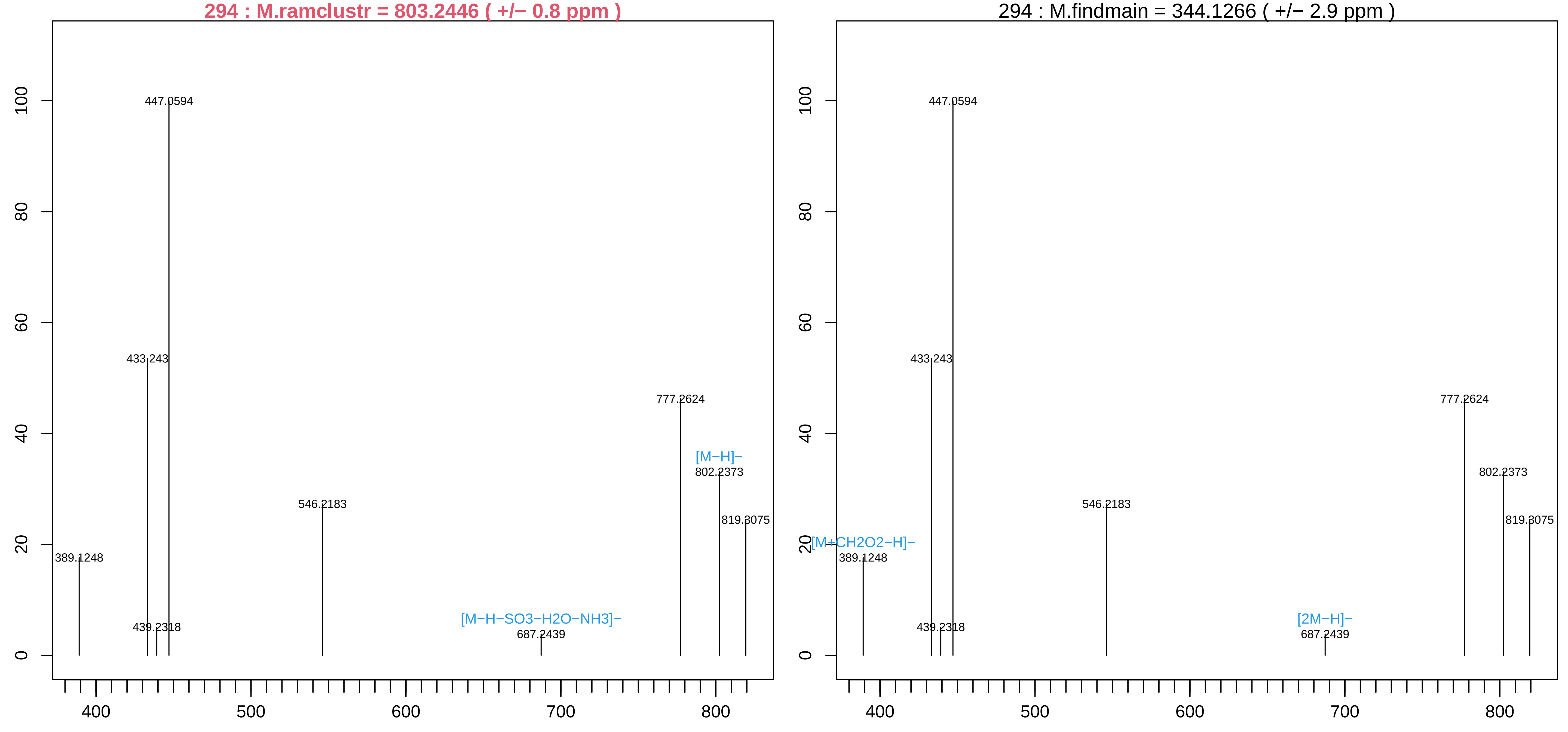

294 : M.ramclustr = 803.2446 (+/− 0.8 ppm)
447.0594
433.243
439.2318
389.1248
546.2183
[M−H−SO3−H2O−NH3]−
687.2439
777.2624
[M−H]−
802.2373
819.3075
0
20
40
60
80
100
400
500
600
700
800
294 : M.findmain = 344.1266 (+/− 2.9 ppm)
447.0594
433.243
439.2318
[M+CH2O2−H]−
389.1248
546.2183
[2M−H]−
687.2439
777.2624
802.2373
819.3075
0
20
40
60
80
100
400
500
600
700
800

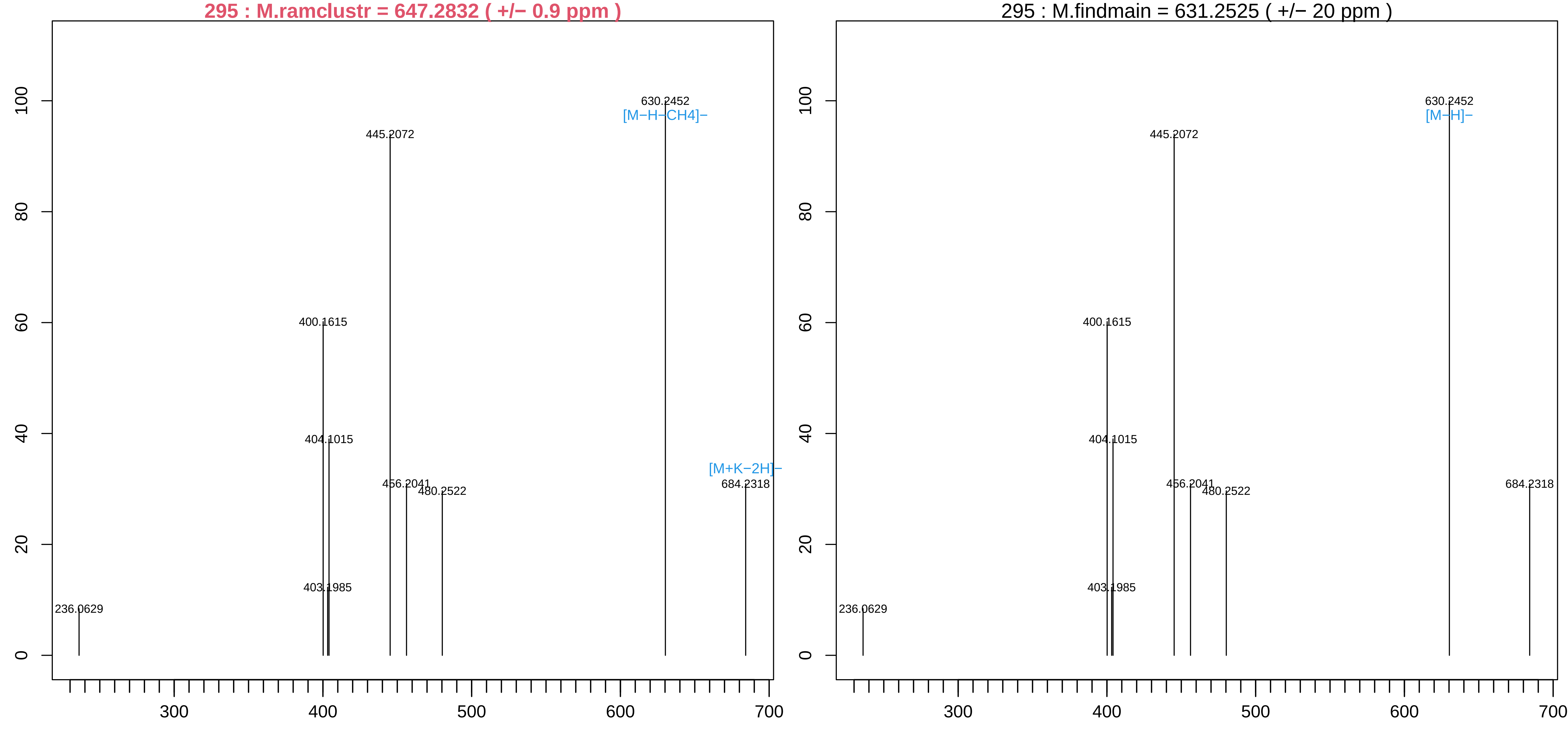
295 : M.ramclustr = 647.2832 (+/− 0.9 ppm)
630.2452
[M−H−CH4]−
445.2072
400.1615
404.1015
[M+K−2H]−
456.2041
480.2522
684.2318
403.1985
236.0629
0
20
40
60
80
100
300
400
500
600
700
295 : M.findmain = 631.2525 (+/− 20 ppm)
630.2452
[M−H]−
445.2072
400.1615
404.1015
456.2041
480.2522
684.2318
403.1985
236.0629

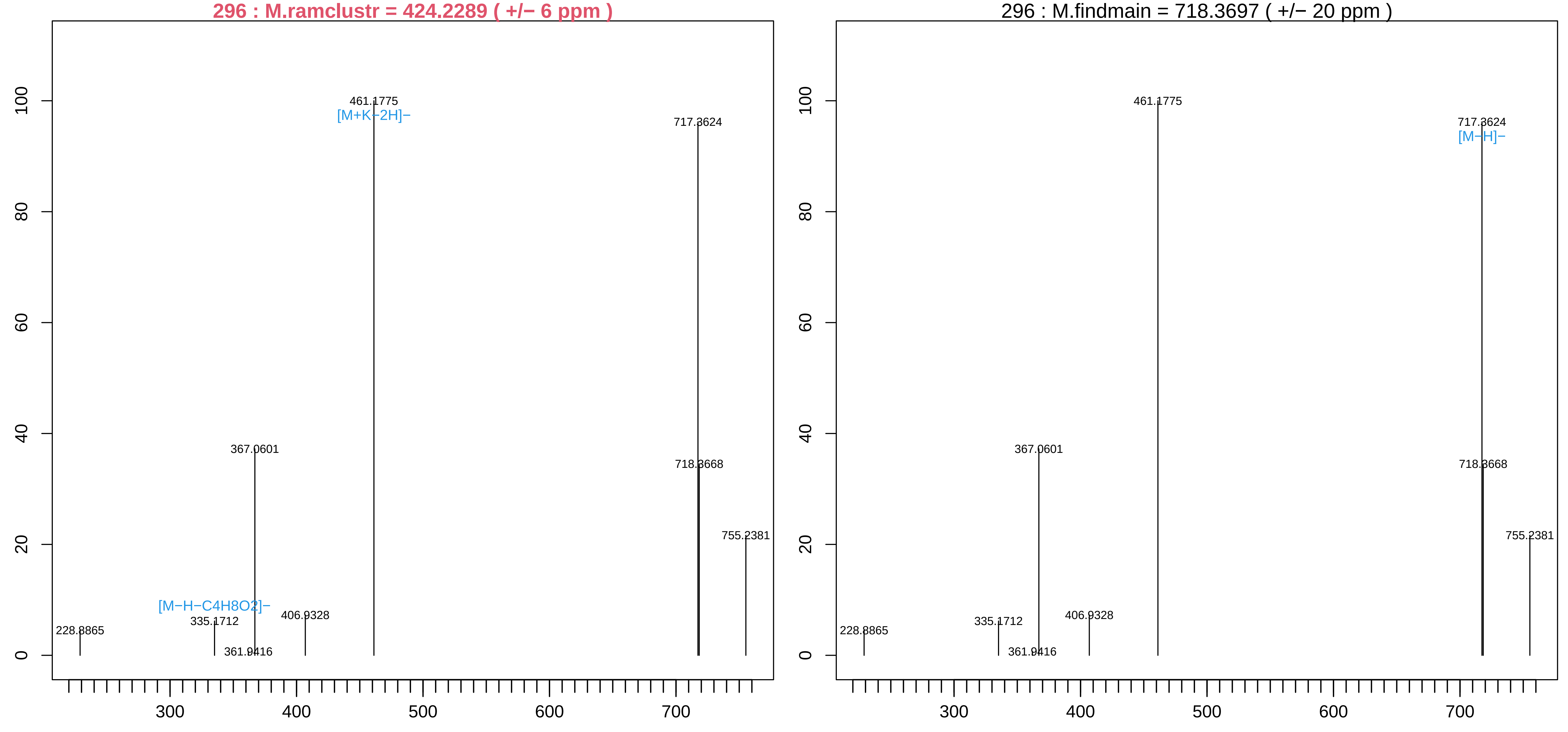

296 : M.ramclustr = 424.2289 (+/− 6 ppm)
461.1775
[M+K−2H]−
717.3624
367.0601
718.3668
755.2381
[M−H−C4H8O2]−
335.1712
406.9328
228.8865
361.9416
296 : M.findmain = 718.3697 (+/− 20 ppm)
461.1775
717.3624
[M−H]−
367.0601
718.3668
755.2381
335.1712
406.9328
228.8865
361.9416
0
20
40
60
80
100
300
400
500
600
700

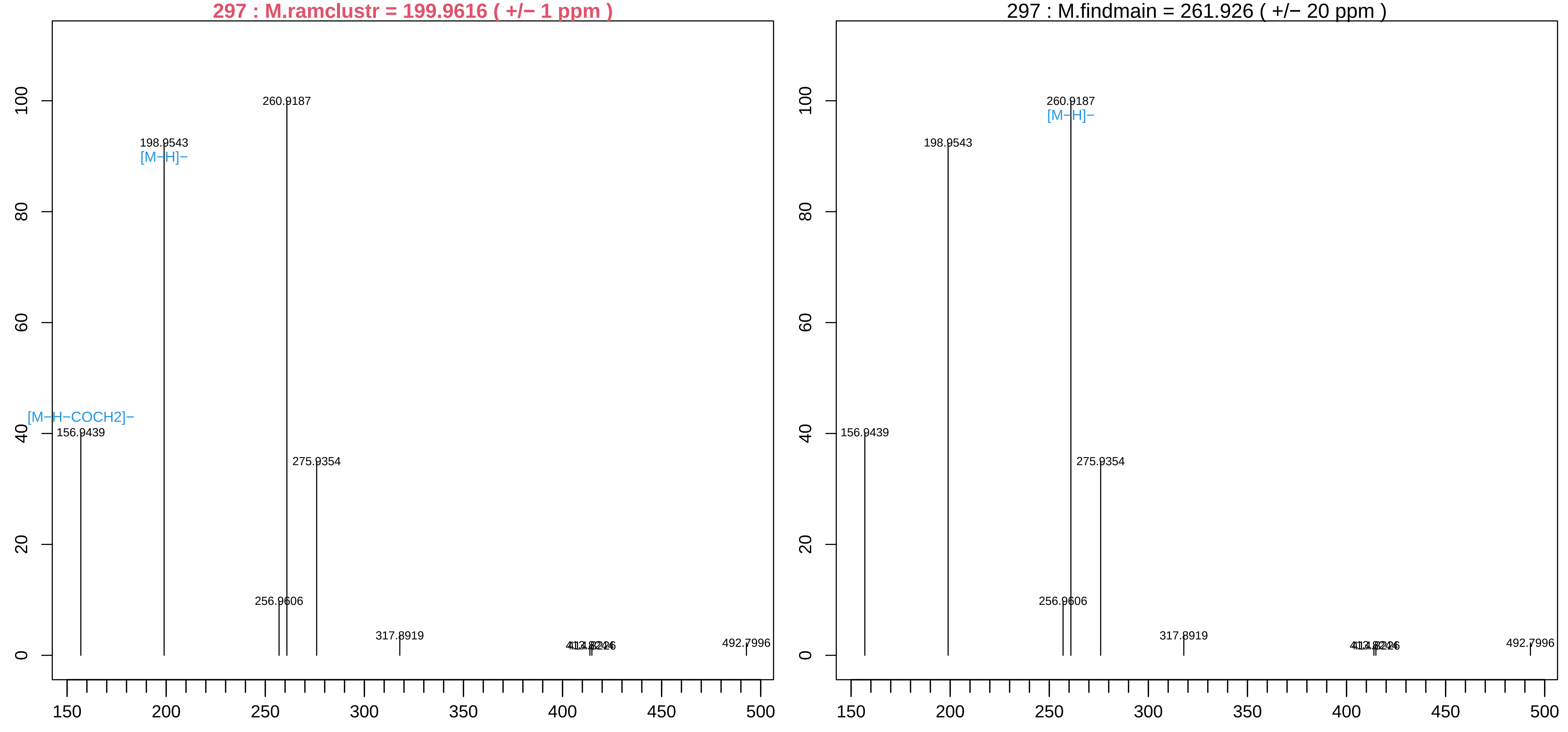
297 : M.ramclustr = 199.9616 (+/− 1 ppm)
260.9187
198.9543
[M−H]−
[M−H−COCH2]−
156.9439
275.9354
256.9606
317.8919
413.8224
492.7996
297 : M.findmain = 261.926 (+/− 20 ppm)
260.9187
[M−H]−
198.9543
156.9439
275.9354
256.9606
317.8919
413.8224
492.7996

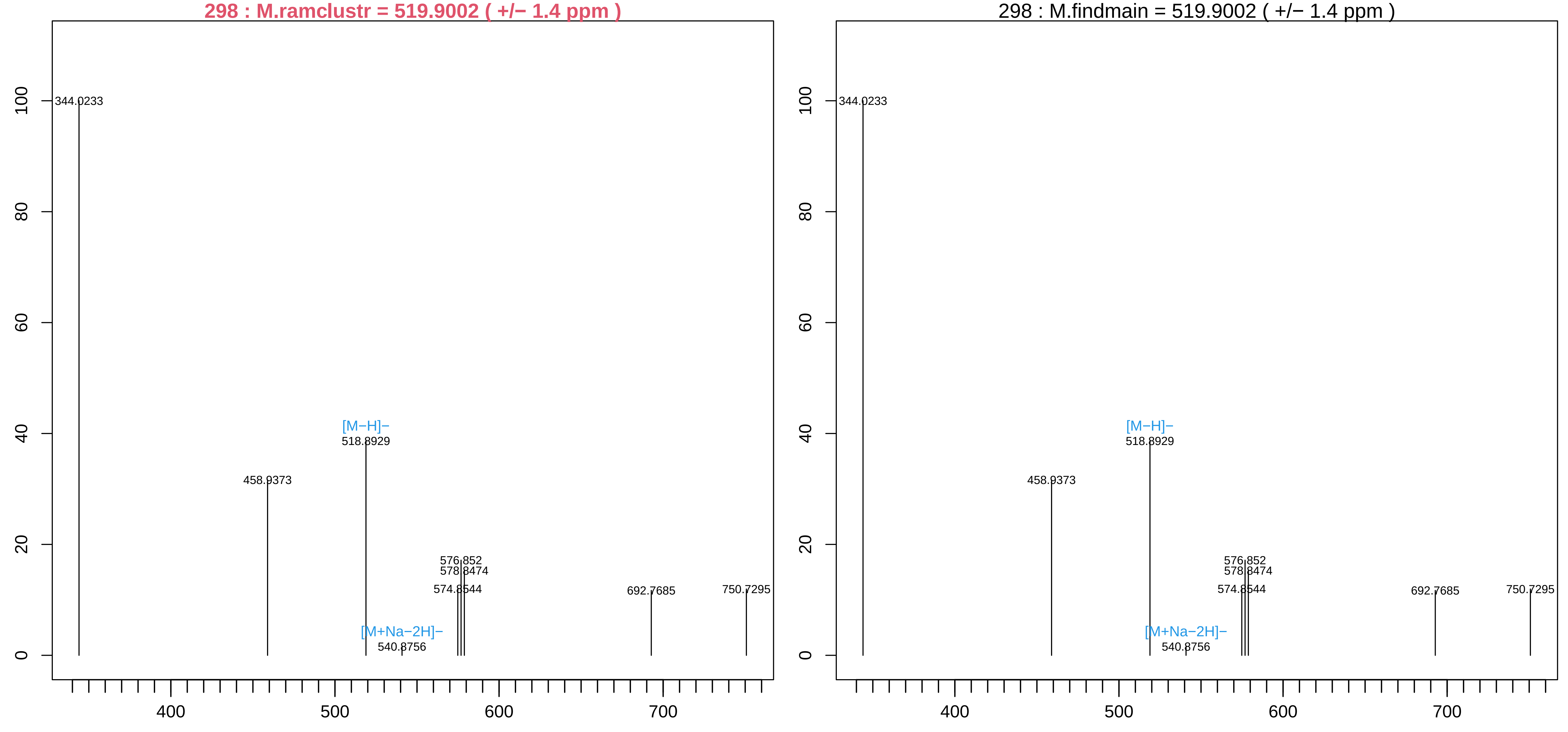

298 : M.ramclustr = 519.9002 (+/− 1.4 ppm)
298 : M.findmain = 519.9002 (+/− 1.4 ppm)
344.0233
458.9373
[M−H]−
518.8929
[M+Na−2H]−
540.8756
574.8544
576.852
578.8474
692.7685
750.7295
0
20
40
60
80
100
400
500
600
700

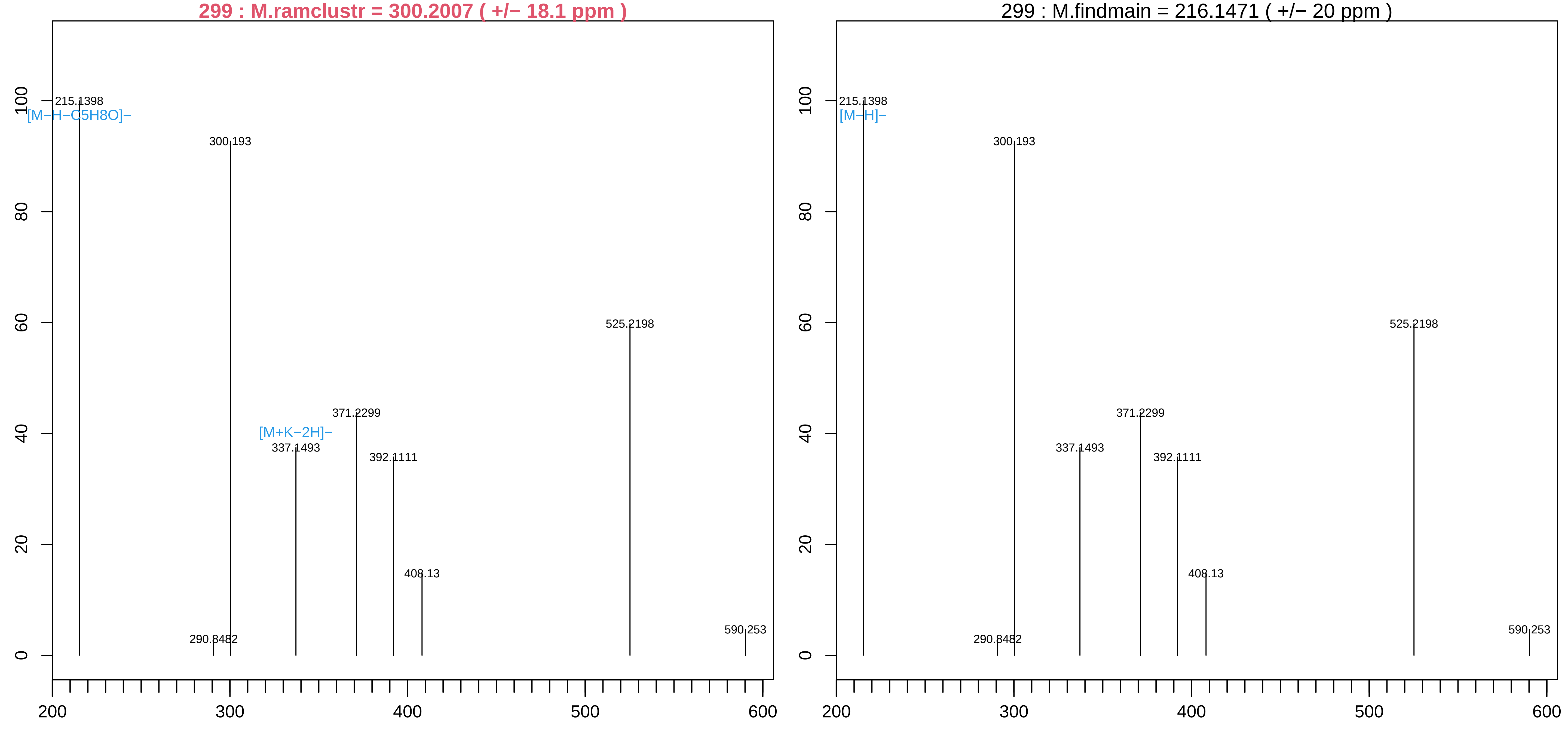

299 : M.ramclustr = 300.2007 (+/− 18.1 ppm)
215.1398
[M−H−C5H8O]−
300.193
[M+K−2H]−
337.1493
371.2299
392.1111
408.13
525.2198
290.8482
590.253
0
20
40
60
80
100
200
300
400
500
600
299 : M.findmain = 216.1471 (+/− 20 ppm)
215.1398
[M−H]−
300.193
337.1493
371.2299
392.1111
408.13
525.2198
290.8482
590.253
0
20
40
60
80
100
200
300
400
500
600

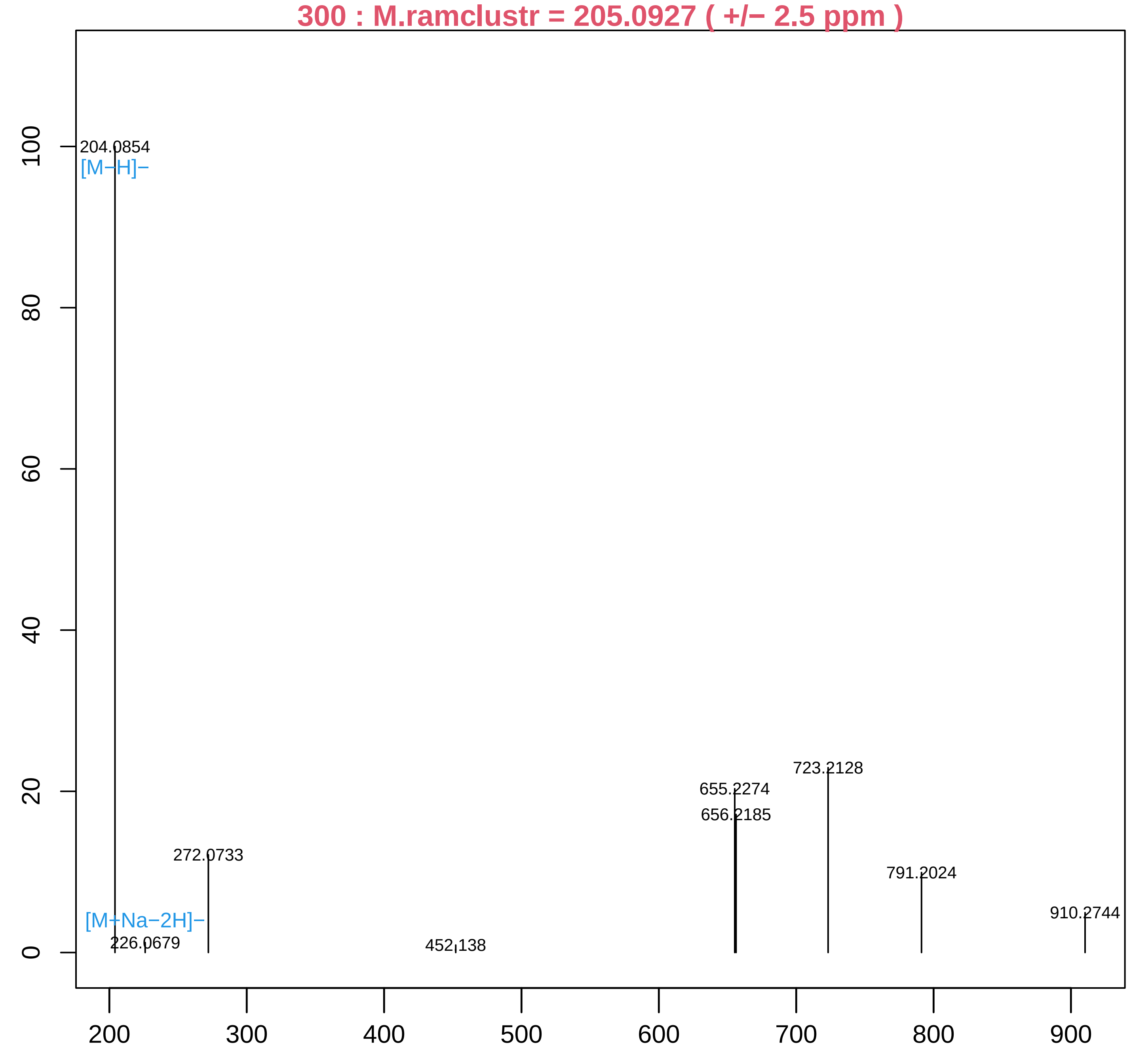
300 : M.ramclustr = 205.0927 (+/− 2.5 ppm)
204.0854
[M−H]−
[M+Na−2H]−
226.0679
272.0733
452.138
655.2274
656.2185
723.2128
791.2024
910.2744
0
20
40
60
80
100
200
300
400
500
600
700
800
900
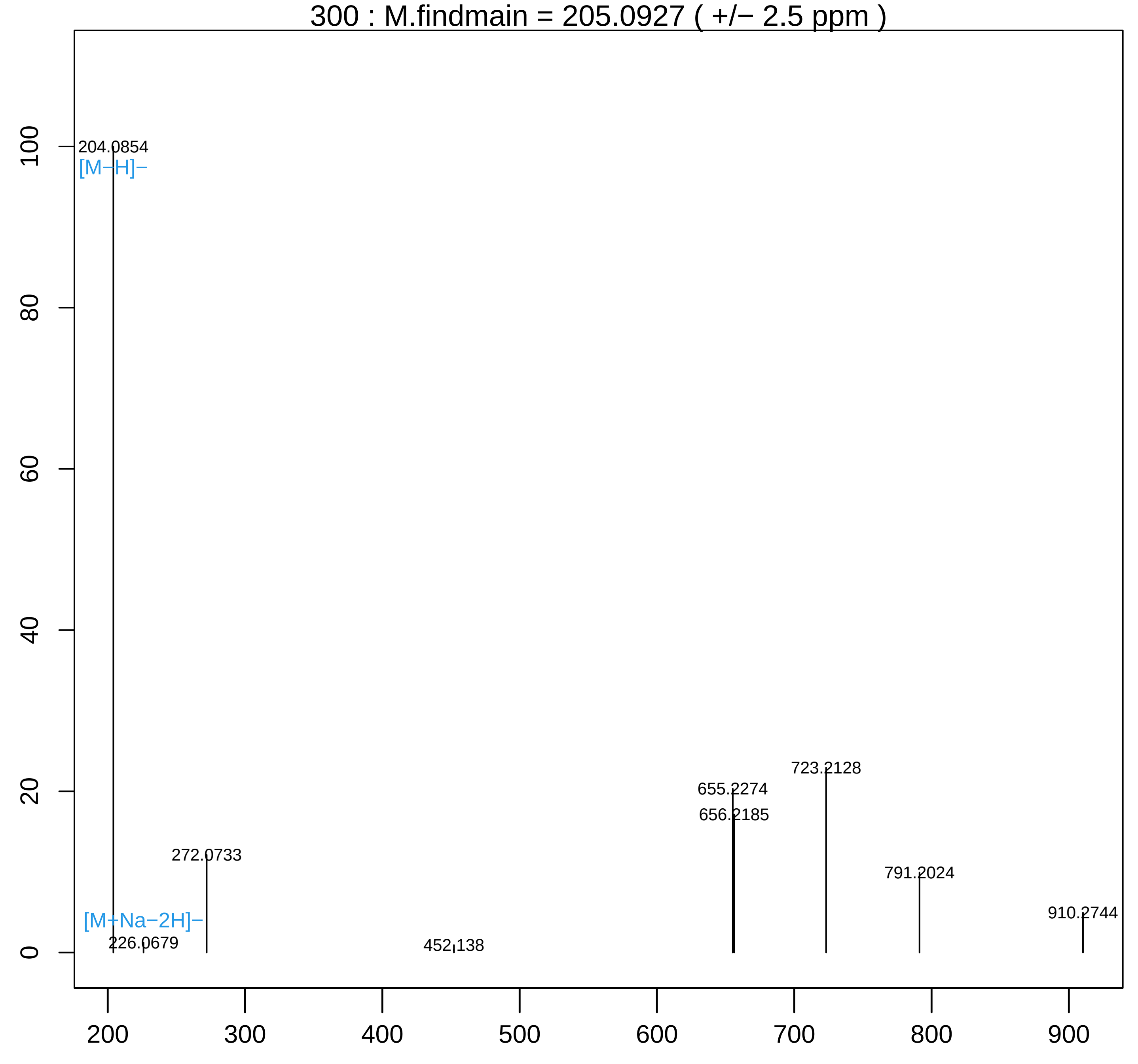
300 : M.findmain = 205.0927 (+/− 2.5 ppm)
204.0854
[M−H]−
[M+Na−2H]−
226.0679
272.0733
452.138
655.2274
656.2185
723.2128
791.2024
910.2744
0
20
40
60
80
100
200
300
400
500
600
700
800
900

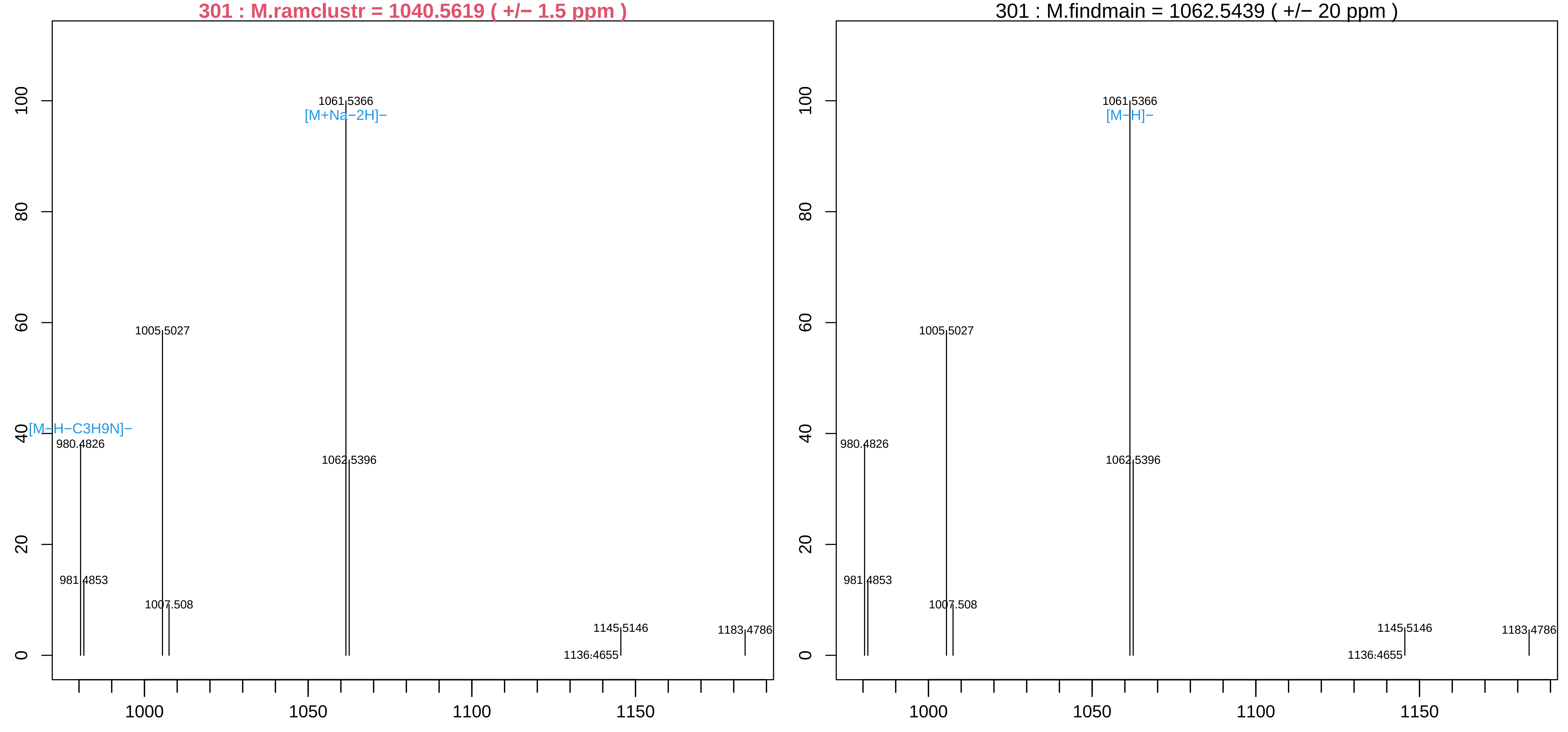

301 : M.ramclustr = 1040.5619 (+/− 1.5 ppm)
1061.5366
[M+Na−2H]−
1005.5027
[M−H−C3H9N]−
980.4826
1062.5396
981.4853
1007.508
1145.5146
1183.4786
1136.4655
301 : M.findmain = 1062.5439 (+/− 20 ppm)
1061.5366
[M−H]−
1005.5027
980.4826
1062.5396
981.4853
1007.508
1145.5146
1183.4786
1136.4655
0
20
40
60
80
100
1000
1050
1100
1150

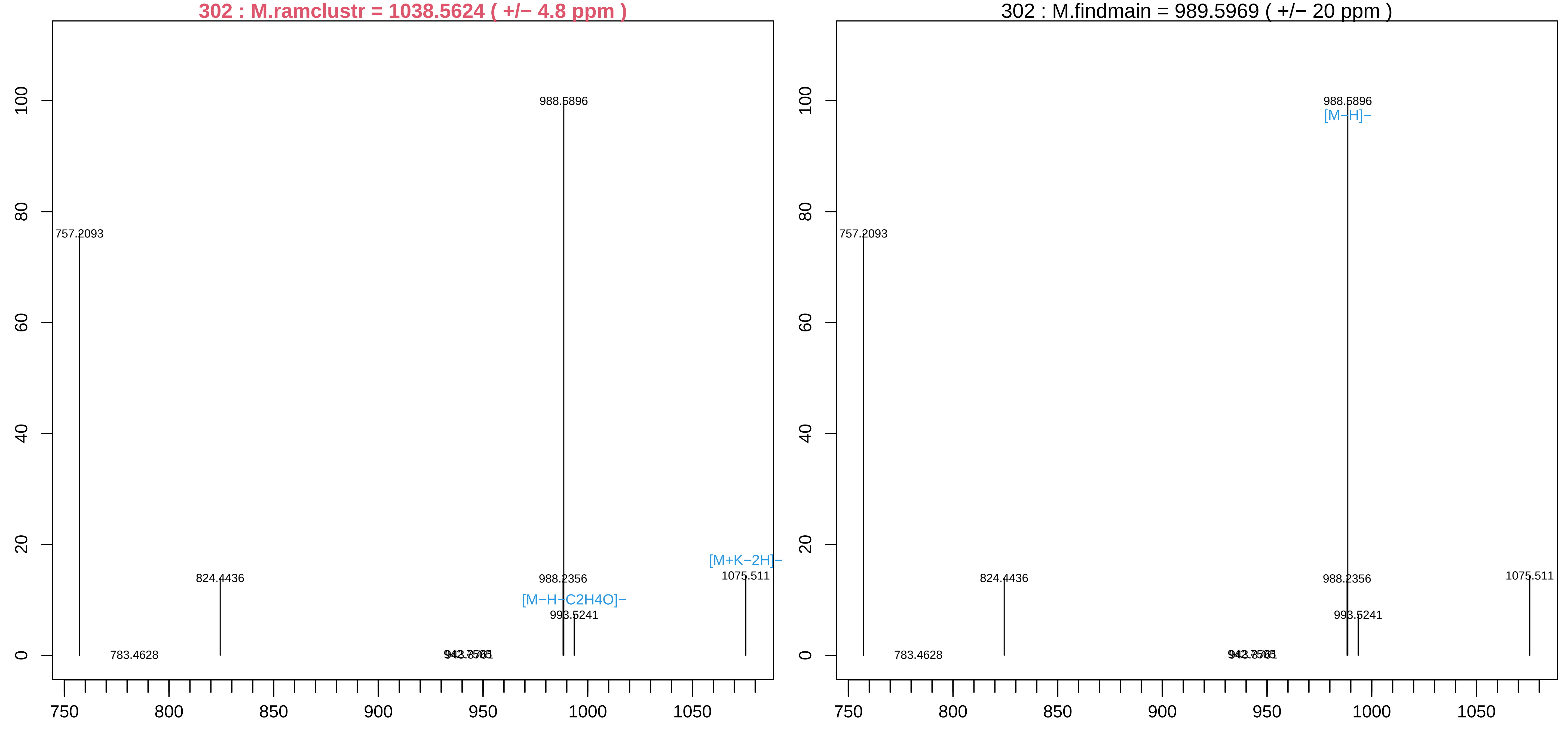
302 : M.ramclustr = 1038.5624 (+/− 4.8 ppm)
988.5896
757.2093
824.4436
783.4628
988.2356
[M−H−C2H4O]−
993.5241
[M+K−2H]−
1075.511
302 : M.findmain = 989.5969 (+/− 20 ppm)
988.5896
[M−H]−
757.2093
824.4436
783.4628
988.2356
993.5241
1075.511

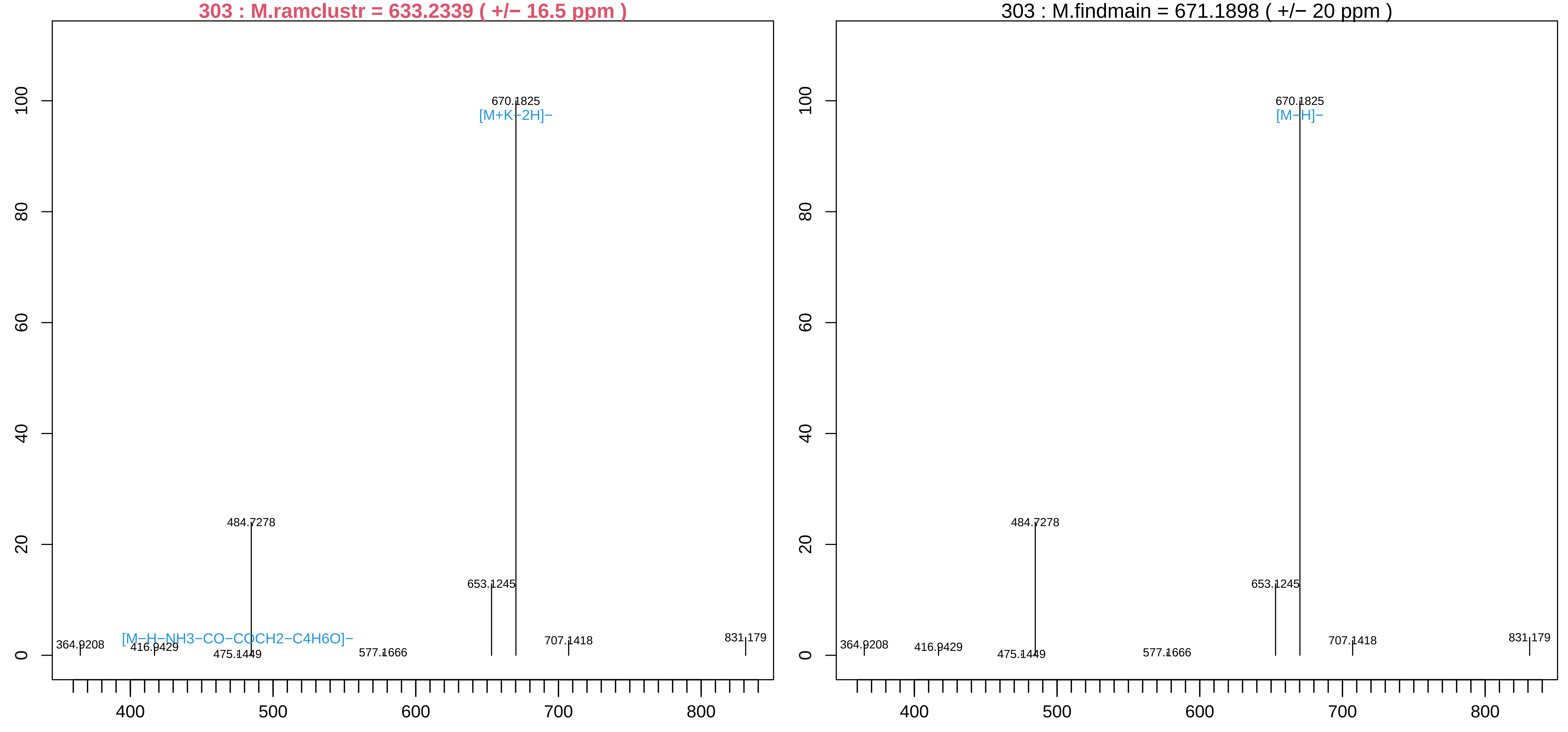
303 : M.ramclustr = 633.2339 (+/− 16.5 ppm)
670.1825
[M+K−2H]−
484.7278
653.1245
[M−H−NH3−CO−COCH2−C4H6O]−
364.9208
416.9429
475.1449
577.1666
707.1418
831.179
0
20
40
60
80
100
400
500
600
700
800
303 : M.findmain = 671.1898 (+/− 20 ppm)
670.1825
[M−H]−
484.7278
653.1245
364.9208
416.9429
475.1449
577.1666
707.1418
831.179
0
20
40
60
80
100
400
500
600
700
800

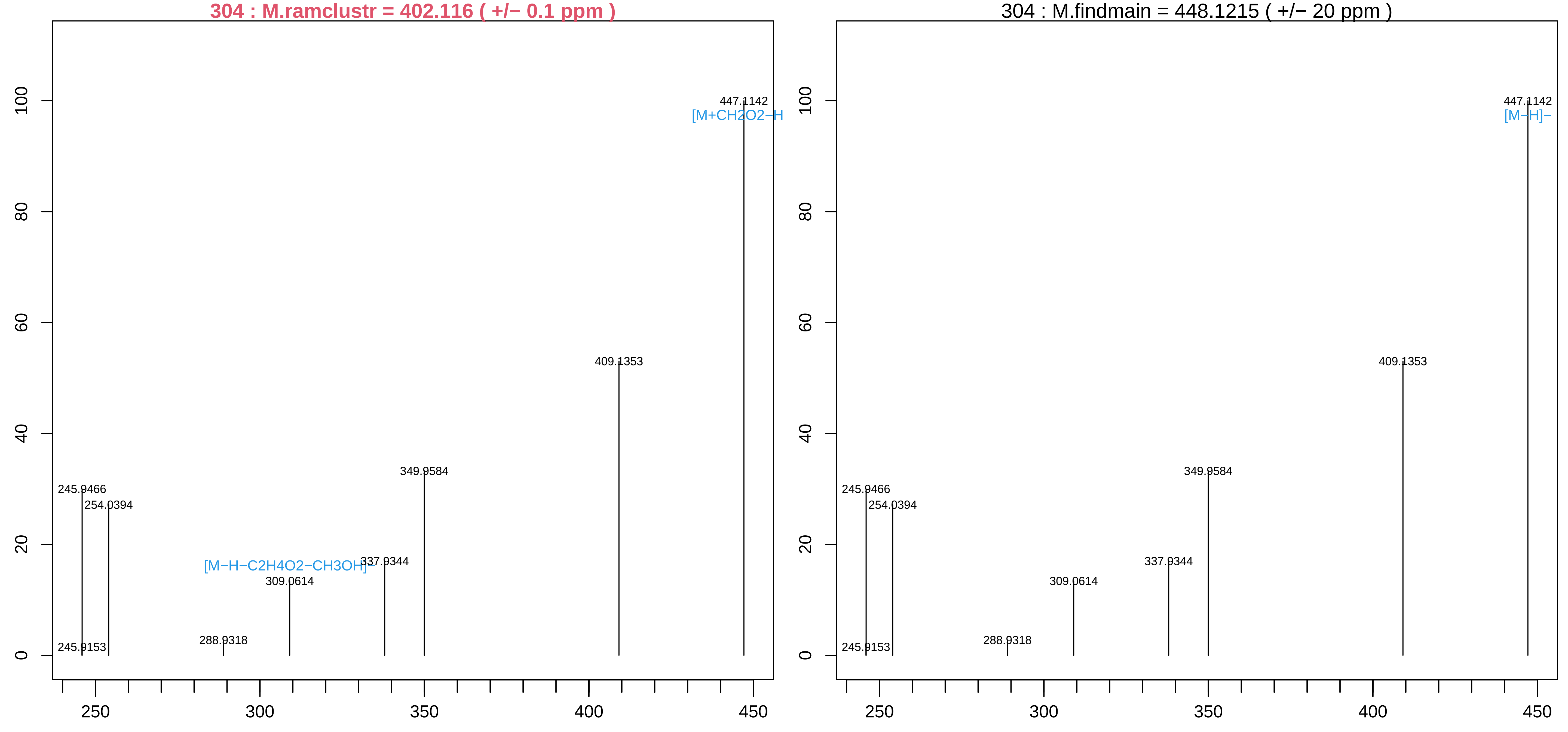
304 : M.ramclustr = 402.116 (+/− 0.1 ppm)
447.1142
[M+CH2O2−H
409.1353
349.9584
245.9466
254.0394
337.9344
[M−H−C2H4O2−CH3OH]−
309.0614
288.9318
245.9153
304 : M.findmain = 448.1215 (+/− 20 ppm)
447.1142
[M−H]−
409.1353
349.9584
245.9466
254.0394
337.9344
309.0614
288.9318
245.9153
0
20
40
60
80
100
250
300
350
400
450

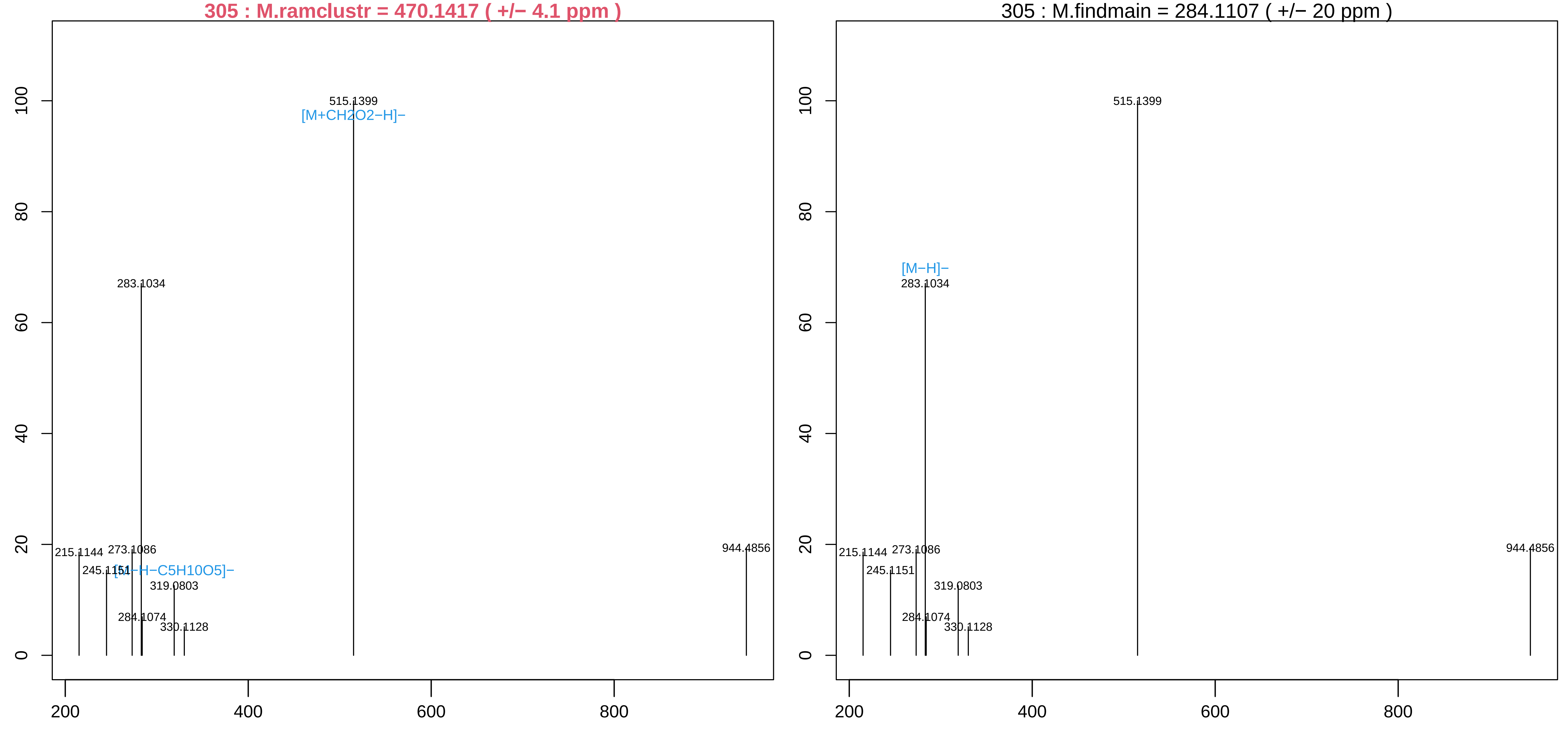
305 : M.ramclustr = 470.1417 (+/− 4.1 ppm)
515.1399
[M+CH2O2−H]−
283.1034
215.1144
273.1086
245.1151
[M−H−C5H10O5]−
319.0803
284.1074
330.1128
944.4856
0
20
40
60
80
100
200
400
600
800
305 : M.findmain = 284.1107 (+/− 20 ppm)
515.1399
[M−H]−
283.1034
215.1144
273.1086
245.1151
319.0803
284.1074
330.1128
944.4856
0
20
40
60
80
100
200
400
600
800

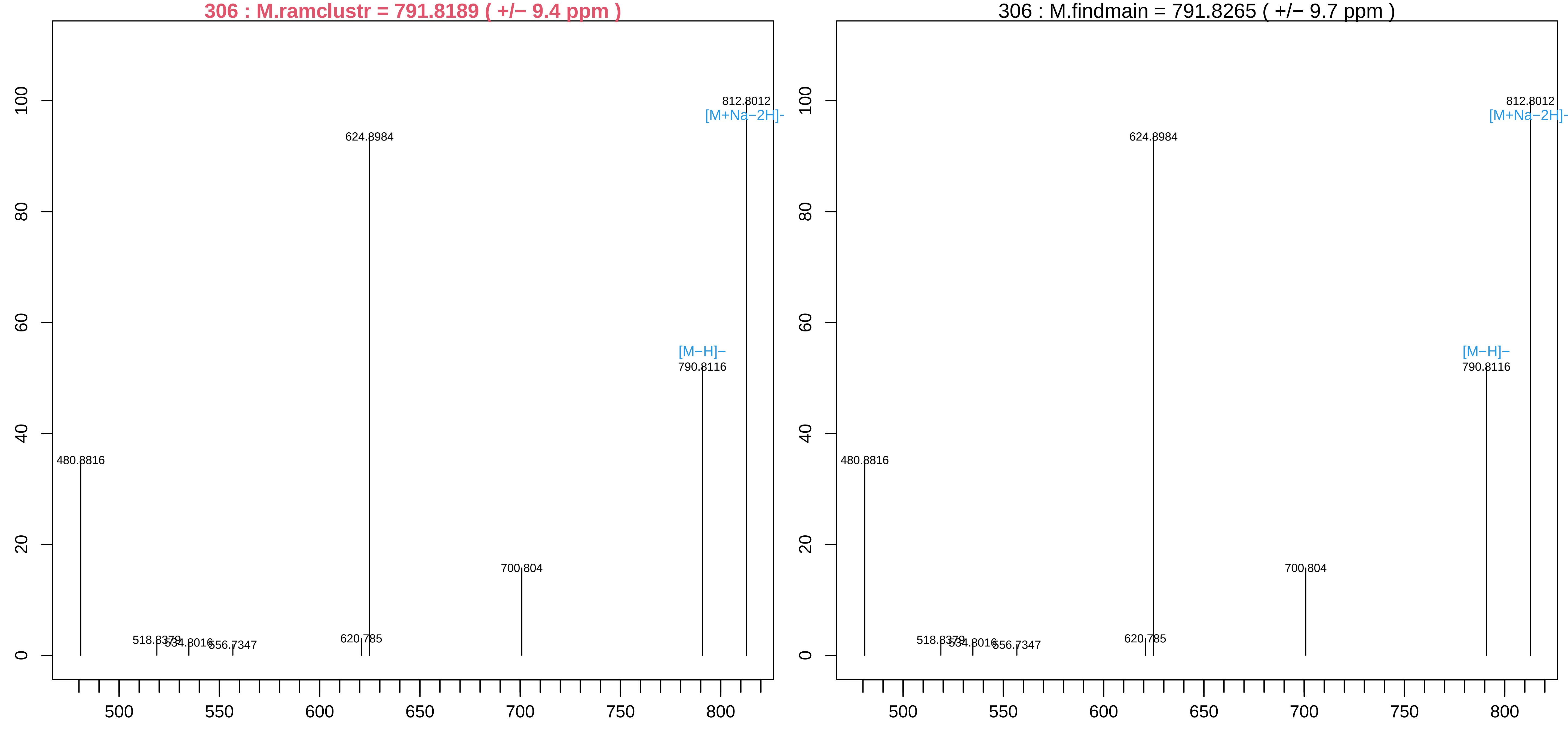
306 : M.ramclustr = 791.8189 (+/− 9.4 ppm)
306 : M.findmain = 791.8265 (+/− 9.7 ppm)
812.8012
[M+Na−2H]-
624.8984
[M−H]−
790.8116
480.8816
700.804
518.8379
534.8016
556.7347
620.785
0
20
40
60
80
100
500
550
600
650
700
750
800

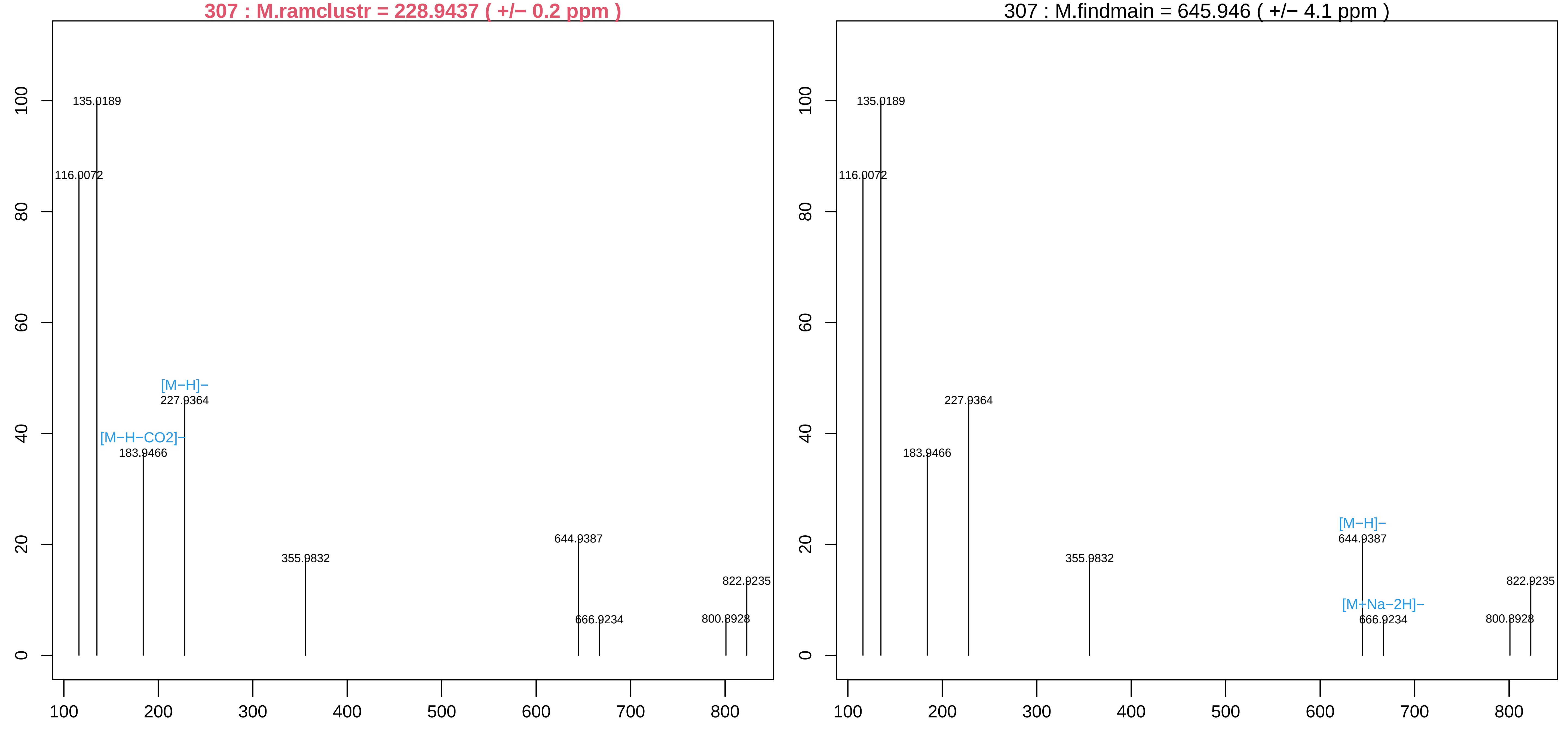

307 : M.ramclustr = 228.9437 (+/− 0.2 ppm)
135.0189
116.0072
[M−H]−
227.9364
[M−H−CO2]−
183.9466
355.9832
644.9387
666.9234
800.8928
822.9235
0
20
40
60
80
100
100
200
300
400
500
600
700
800
307 : M.findmain = 645.946 (+/− 4.1 ppm)
135.0189
116.0072
227.9364
183.9466
355.9832
[M−H]−
644.9387
[M+Na−2H]−
666.9234
800.8928
822.9235

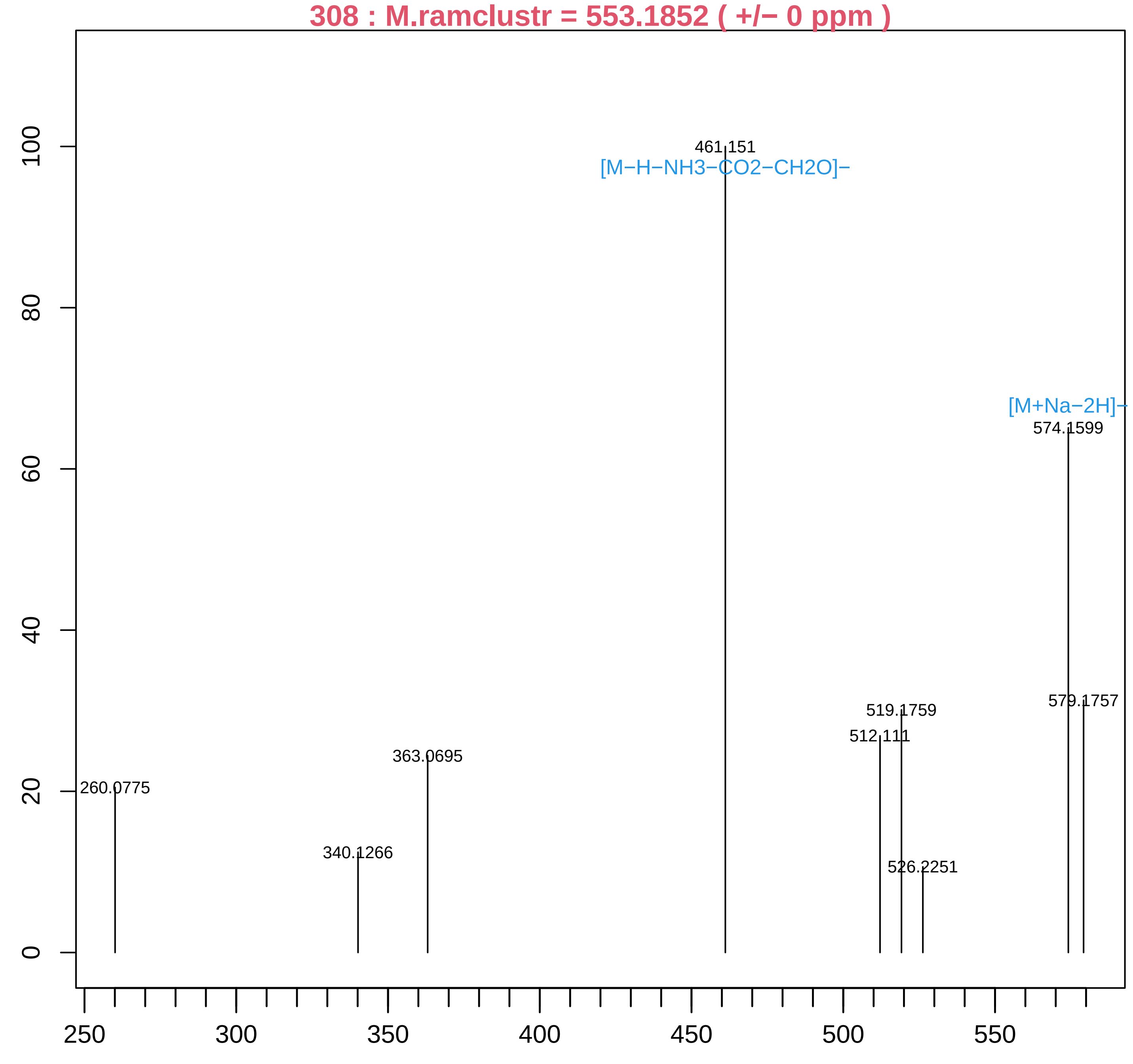
308 : M.ramclustr = 553.1852 (+/− 0 ppm)
461.151
[M−H−NH3−CO2−CH2O]−
[M+Na−2H]−
574.1599
579.1757
519.1759
512.111
363.0695
260.0775
340.1266
526.2251
0
20
40
60
80
100
250
300
350
400
450
500
550

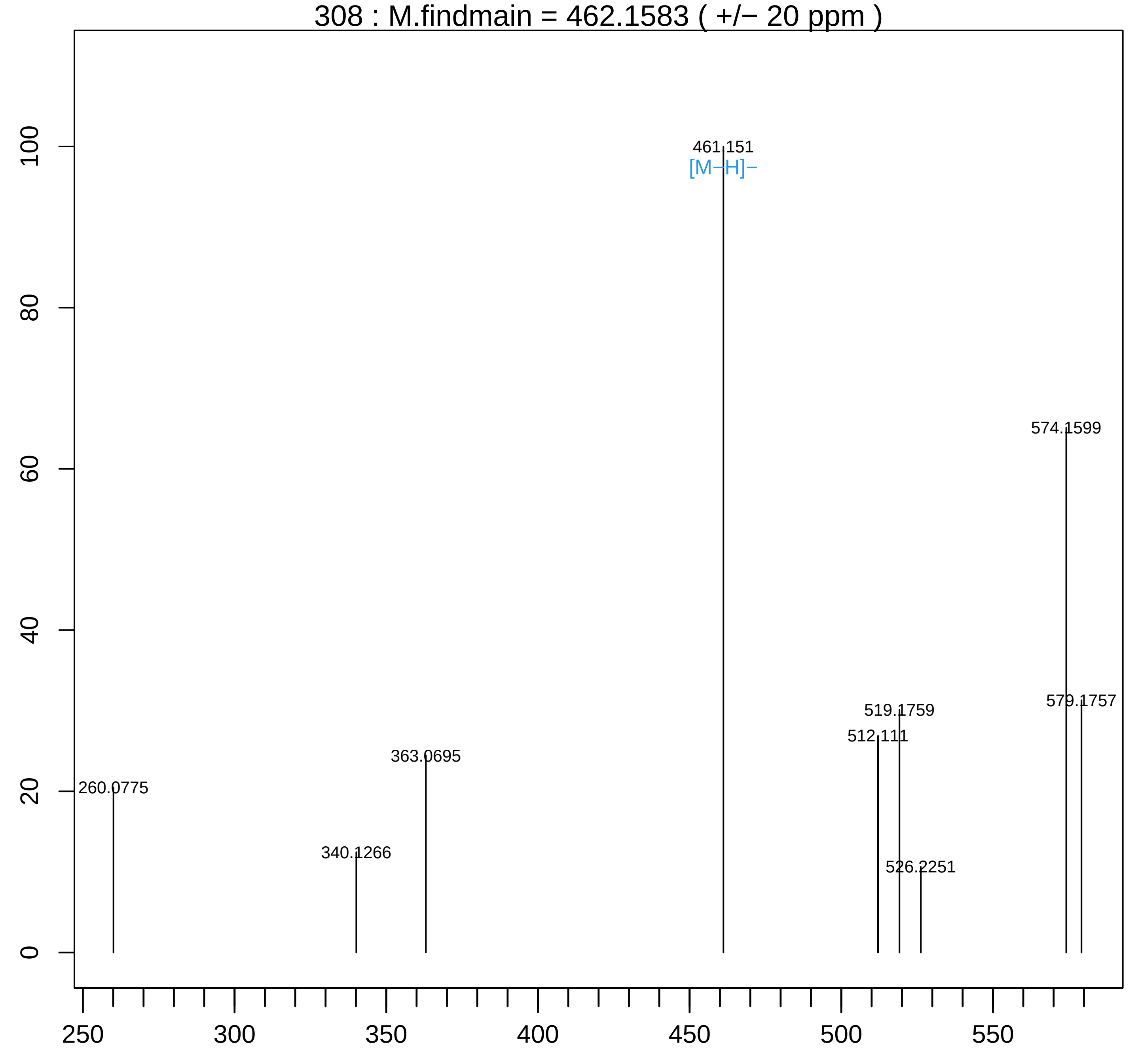
308 : M.findmain = 462.1583 (+/− 20 ppm)
461.151
[M−H]−
574.1599
579.1757
519.1759
512.111
363.0695
260.0775
340.1266
526.2251
0
20
40
60
80
100
250
300
350
400
450
500
550

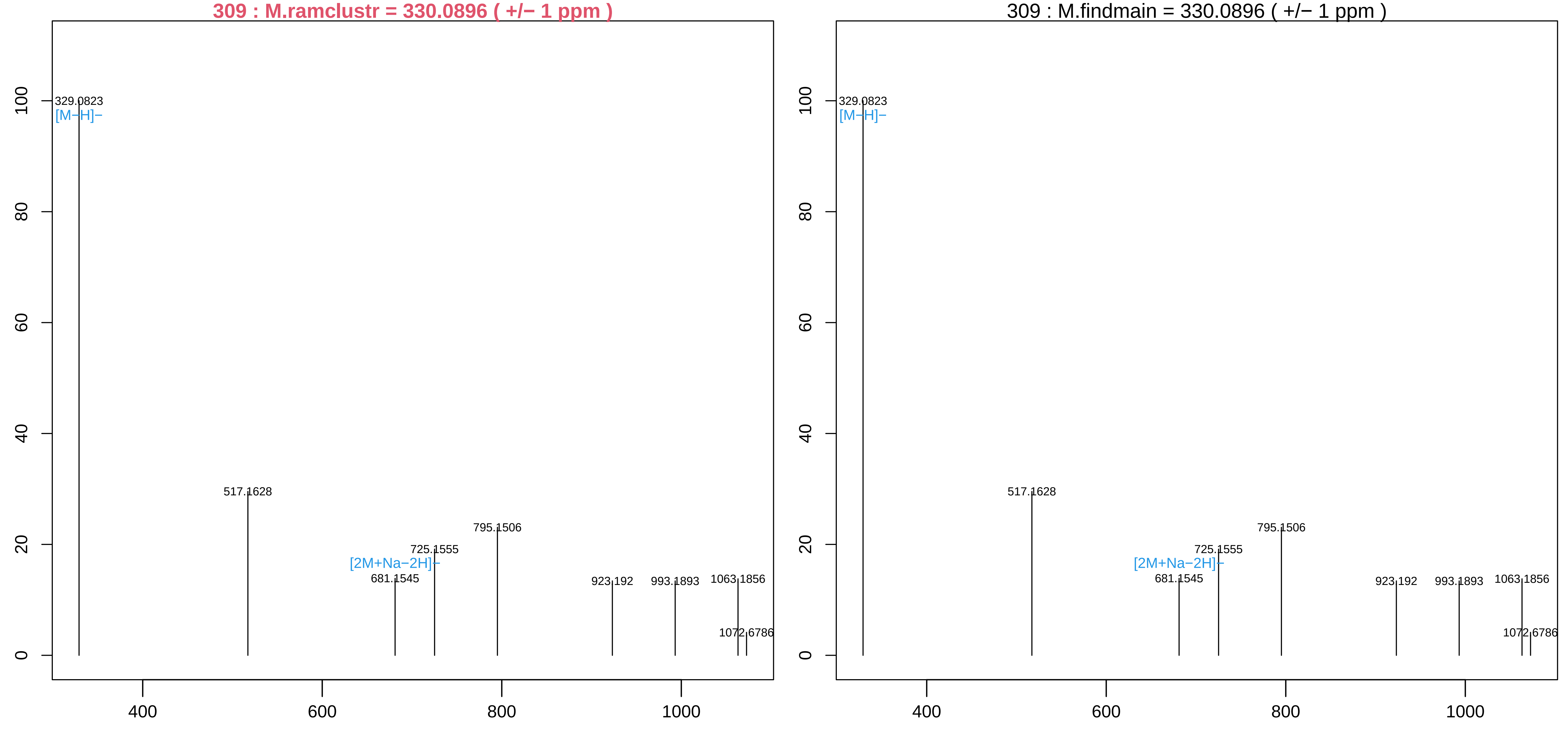
309 : M.ramclustr = 330.0896 (+/− 1 ppm)
329.0823
[M−H]−
517.1628
795.1506
725.1555
[2M+Na−2H]−
681.1545
923.192
993.1893
1063.1856
1072.6786
309 : M.findmain = 330.0896 (+/− 1 ppm)
329.0823
[M−H]−
517.1628
795.1506
725.1555
[2M+Na−2H]−
681.1545
923.192
993.1893
1063.1856
1072.6786
0
20
40
60
80
100
400
600
800
1000

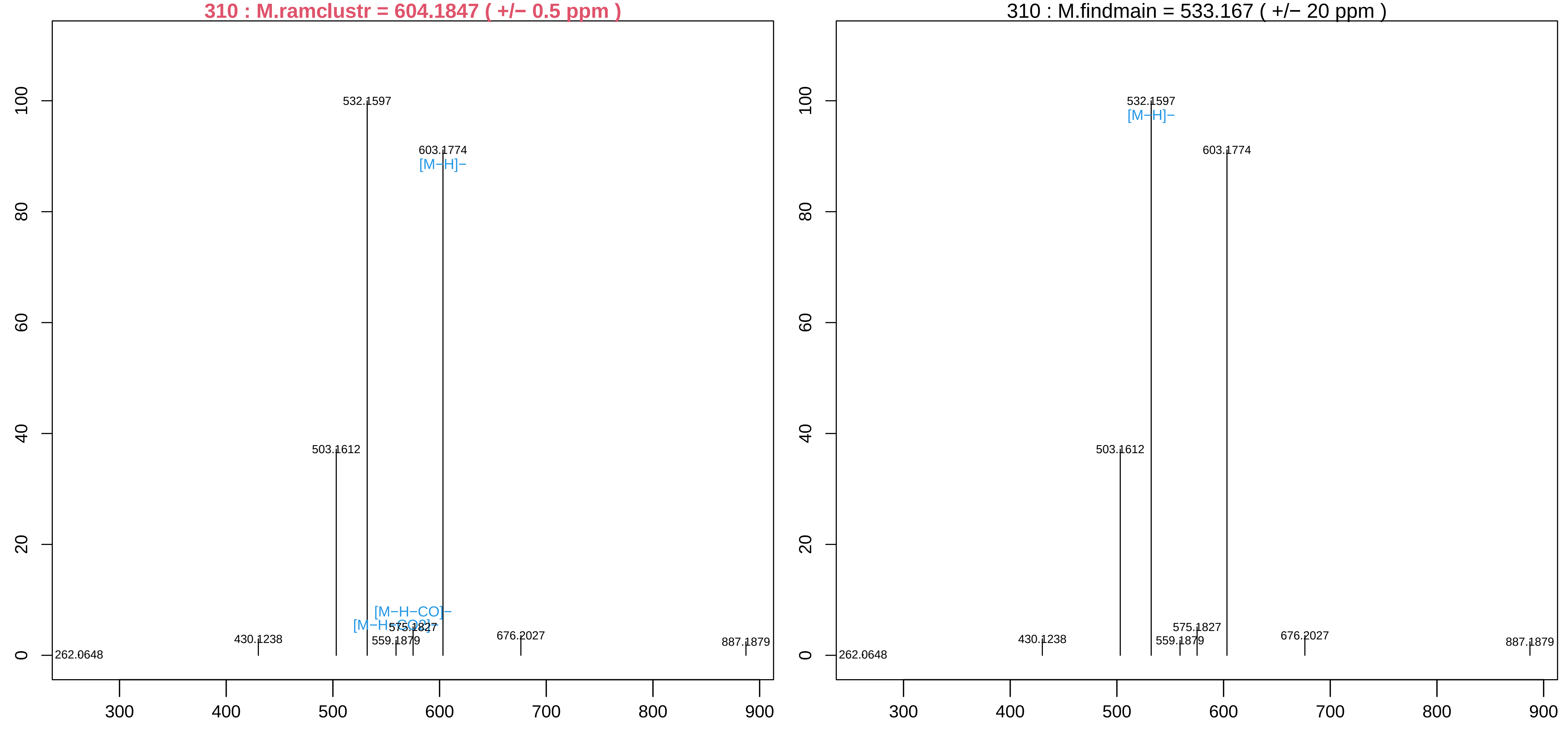
310 : M.ramclustr = 604.1847 (+/− 0.5 ppm)
532.1597
603.1774
[M−H]−
503.1612
[M−H−CO]−
[M−H−CO2]−
575.1827
559.1879
430.1238
676.2027
887.1879
262.0648
310 : M.findmain = 533.167 (+/− 20 ppm)
532.1597
[M−H]−
603.1774
503.1612
575.1827
559.1879
430.1238
676.2027
887.1879
262.0648
0
20
40
60
80
100
300
400
500
600
700
800
900

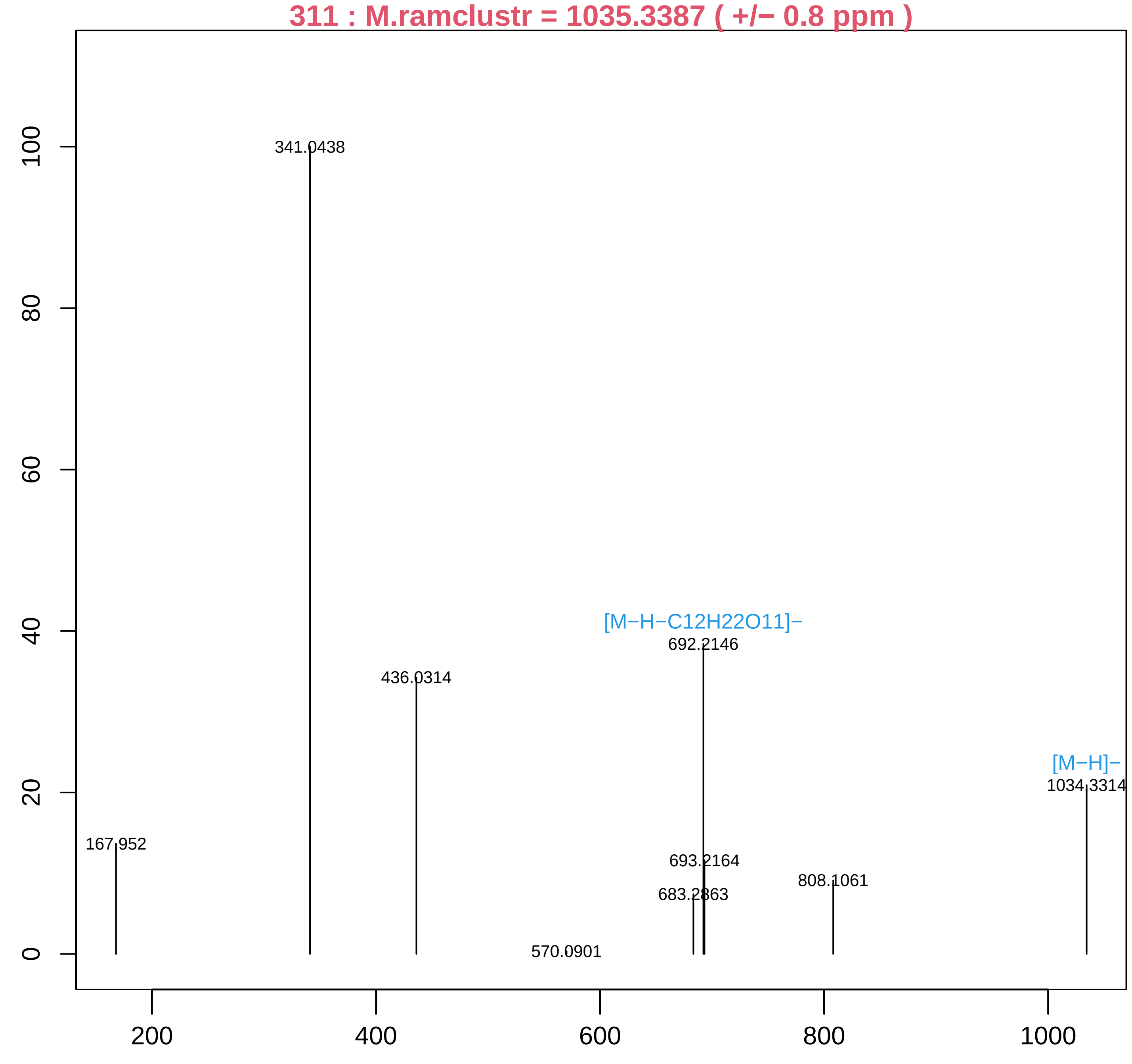

311 : M.ramclustr = 1035.3387 (+/− 0.8 ppm)
341.0438
436.0314
[M−H−C12H22O11]−
692.2146
[M−H]−
1034.3314
167.952
693.2164
808.1061
683.2863
570.0901
0
20
40
60
80
100
200
400
600
800
1000

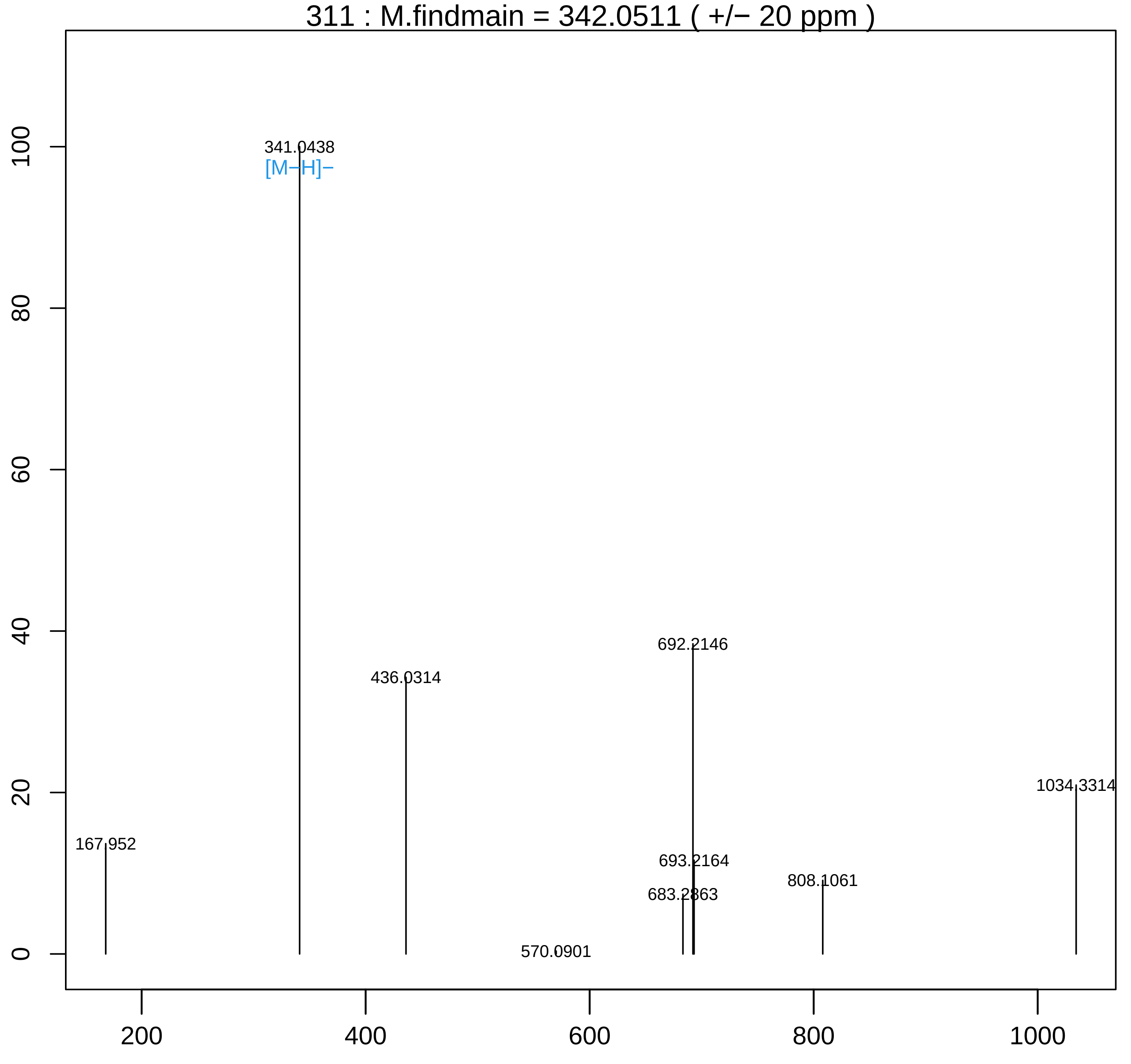

311 : M.findmain = 342.0511 (+/− 20 ppm)
341.0438
[M−H]−
436.0314
692.2146
1034.3314
167.952
693.2164
808.1061
683.2863
570.0901
0
20
40
60
80
100
200
400
600
800
1000

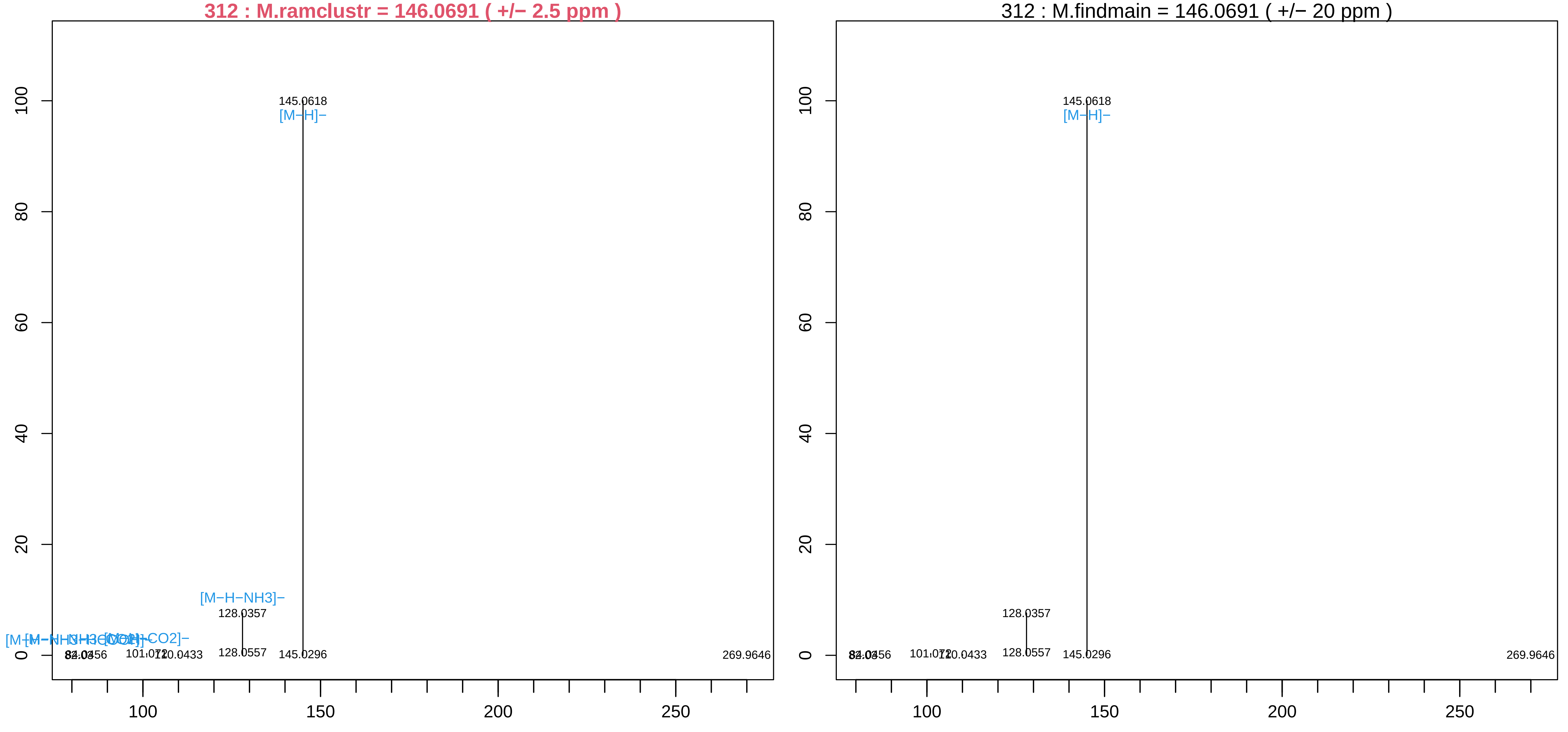
312 : M.ramclustr = 146.0691 (+/− 2.5 ppm)
145.0618
[M−H]−
[M−H−NH3]−
128.0357
[M−H−CO2]−
128.0557
145.0296
269.9646
101.0712
120.0433
0
20
40
60
80
100
150
200
250
312 : M.findmain = 146.0691 (+/− 20 ppm)
145.0618
[M−H]−
128.0357
128.0557
145.0296
269.9646
120.0433

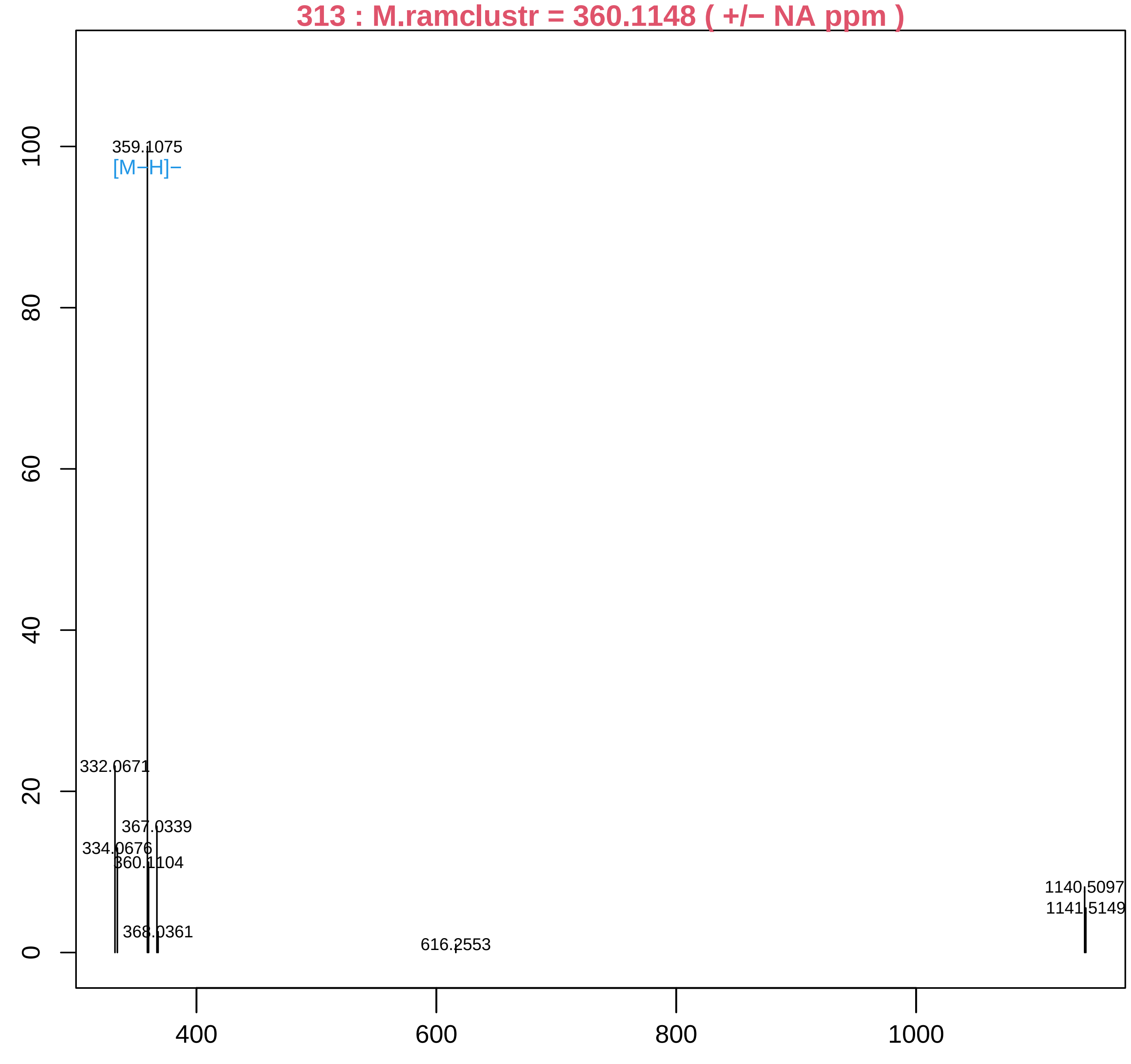
313 : M.ramclustr = 360.1148 (+/− NA ppm)
359.1075
[M−H]−
332.0671
367.0339
334.0676
360.1104
368.0361
616.2553
1140.5097
1141.5149
0
20
40
60
80
100
400
600
800
1000

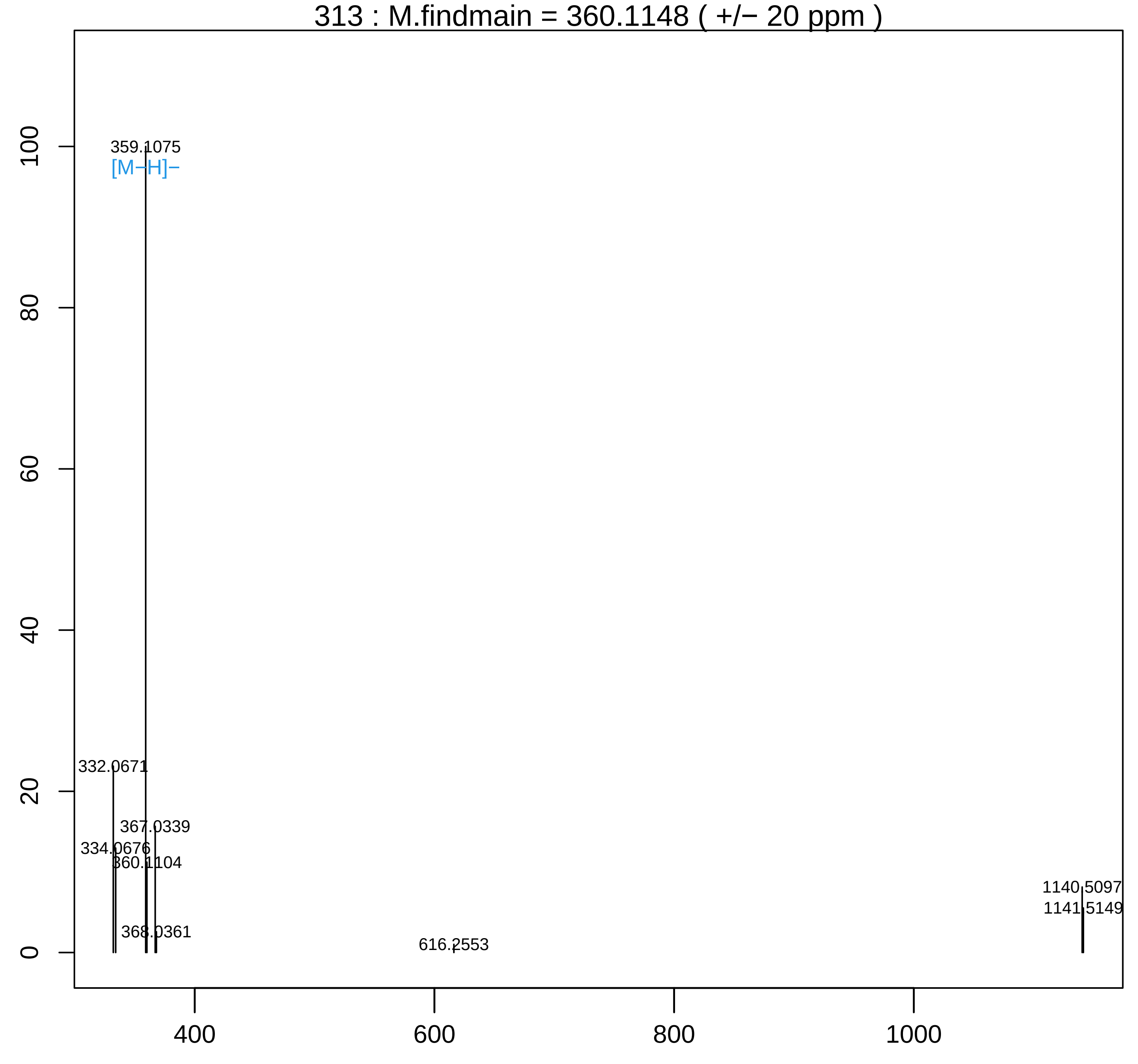
313 : M.findmain = 360.1148 (+/− 20 ppm)
359.1075
[M−H]−
332.0671
367.0339
334.0676
360.1104
368.0361
616.2553
1140.5097
1141.5149
0
20
40
60
80
100
400
600
800
1000

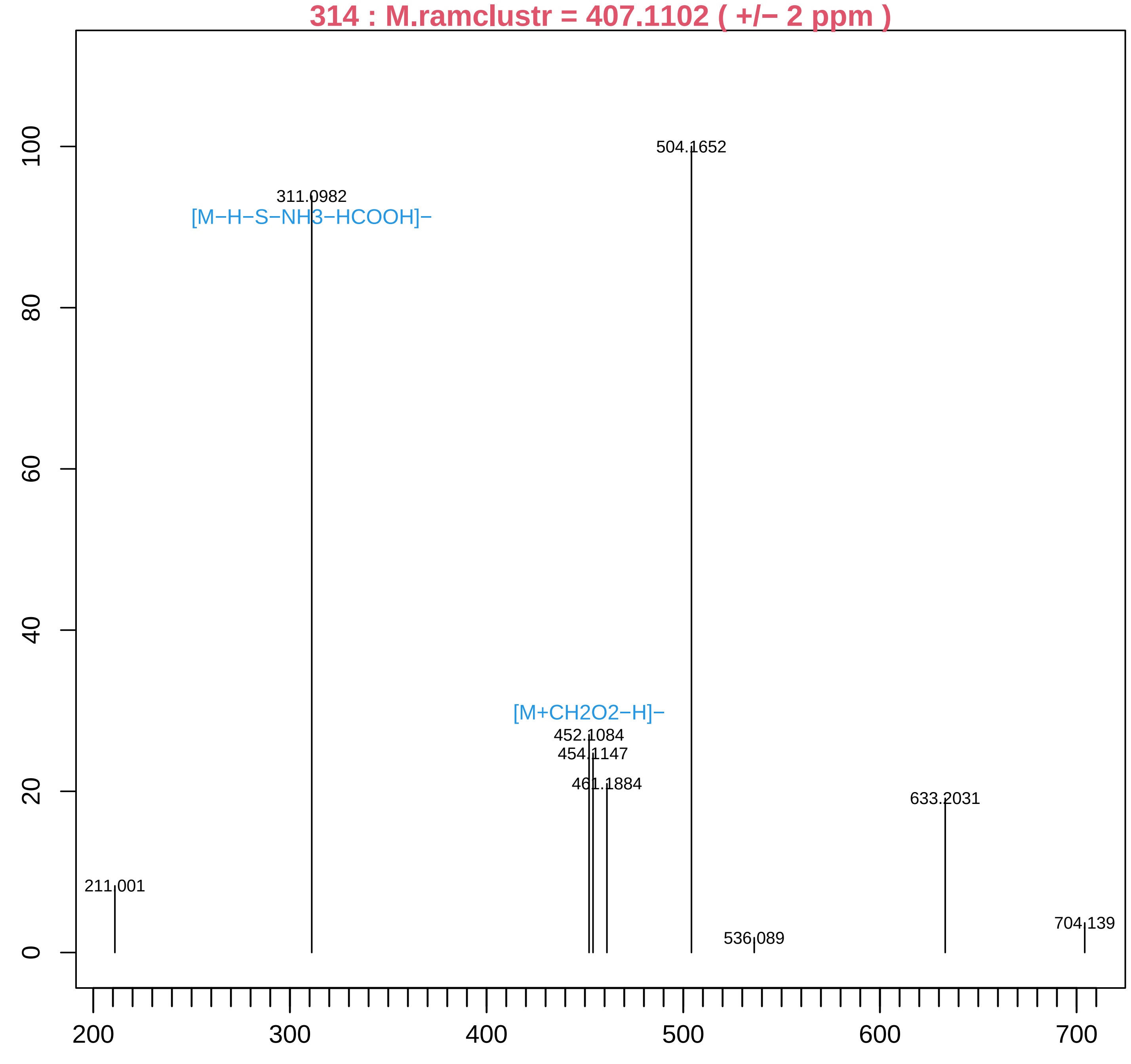

314 : M.ramclustr = 407.1102 (+/− 2 ppm)
504.1652
311.0982
[M−H−S−NH3−HCOOH]−
[M+CH2O2−H]−
452.1084
454.1147
461.1884
633.2031
211.001
536.089
704.139
0
20
40
60
80
100
200
300
400
500
600
700

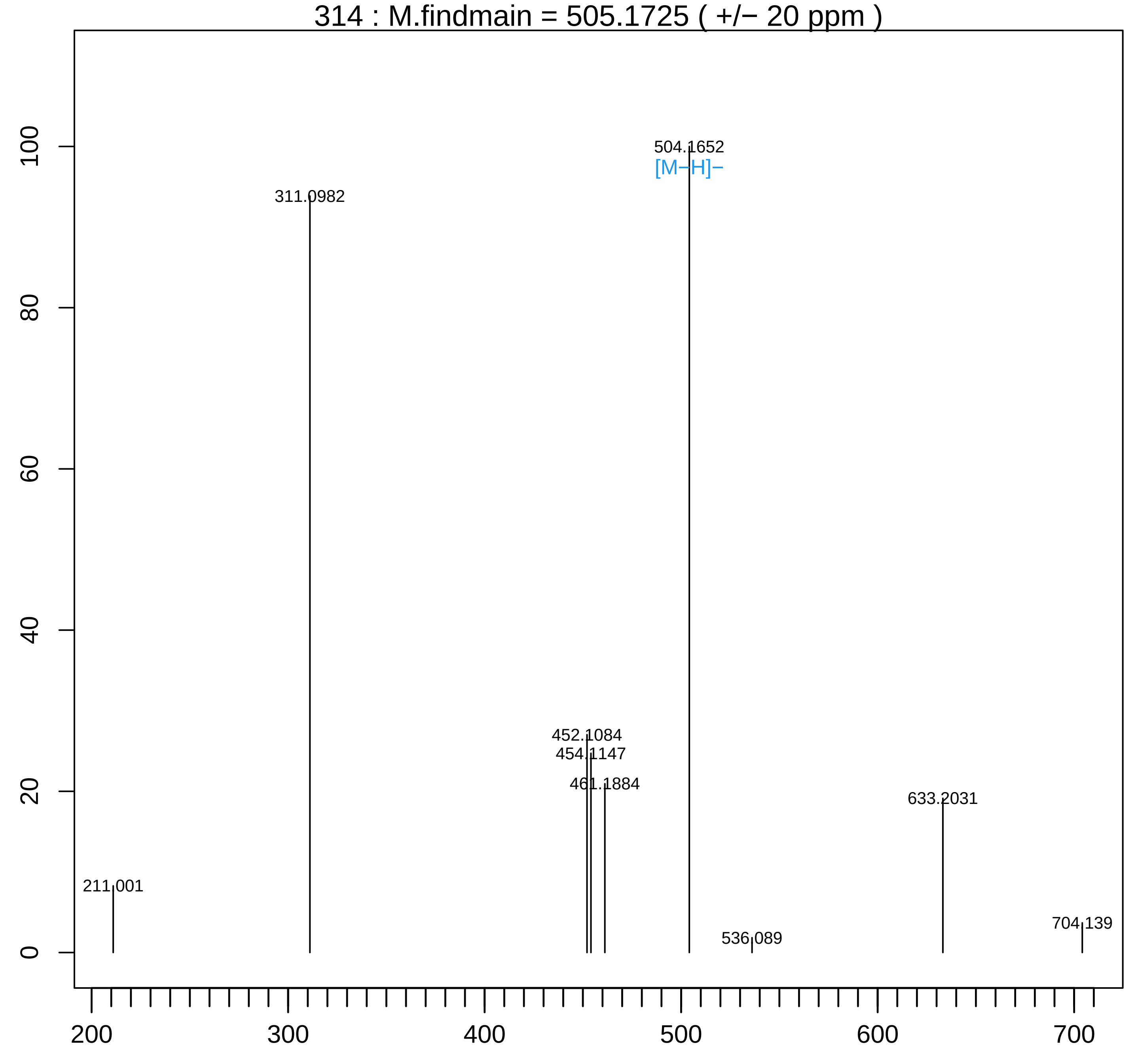

314 : M.findmain = 505.1725 (+/− 20 ppm)
504.1652
[M−H]−
311.0982
452.1084
454.1147
461.1884
633.2031
211.001
536.089
704.139
0
20
40
60
80
100
200
300
400
500
600
700

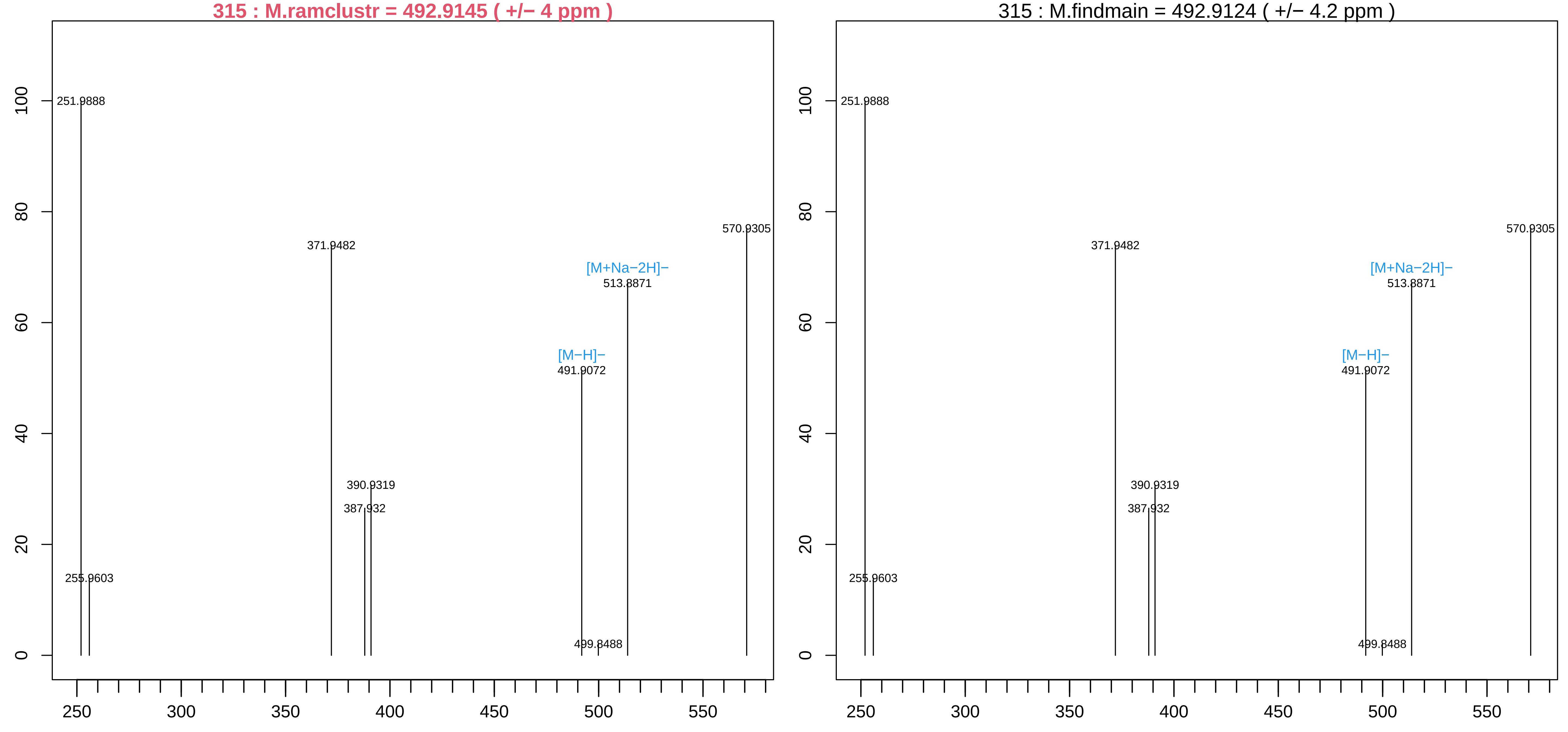

315 : M.ramclustr = 492.9145 (+/− 4 ppm)
315 : M.findmain = 492.9124 (+/− 4.2 ppm)
251.9888
255.9603
371.9482
387.932
390.9319
[M−H]−
491.9072
499.8488
[M+Na−2H]−
513.8871
570.9305
0
20
40
60
80
100
250
300
350
400
450
500
550

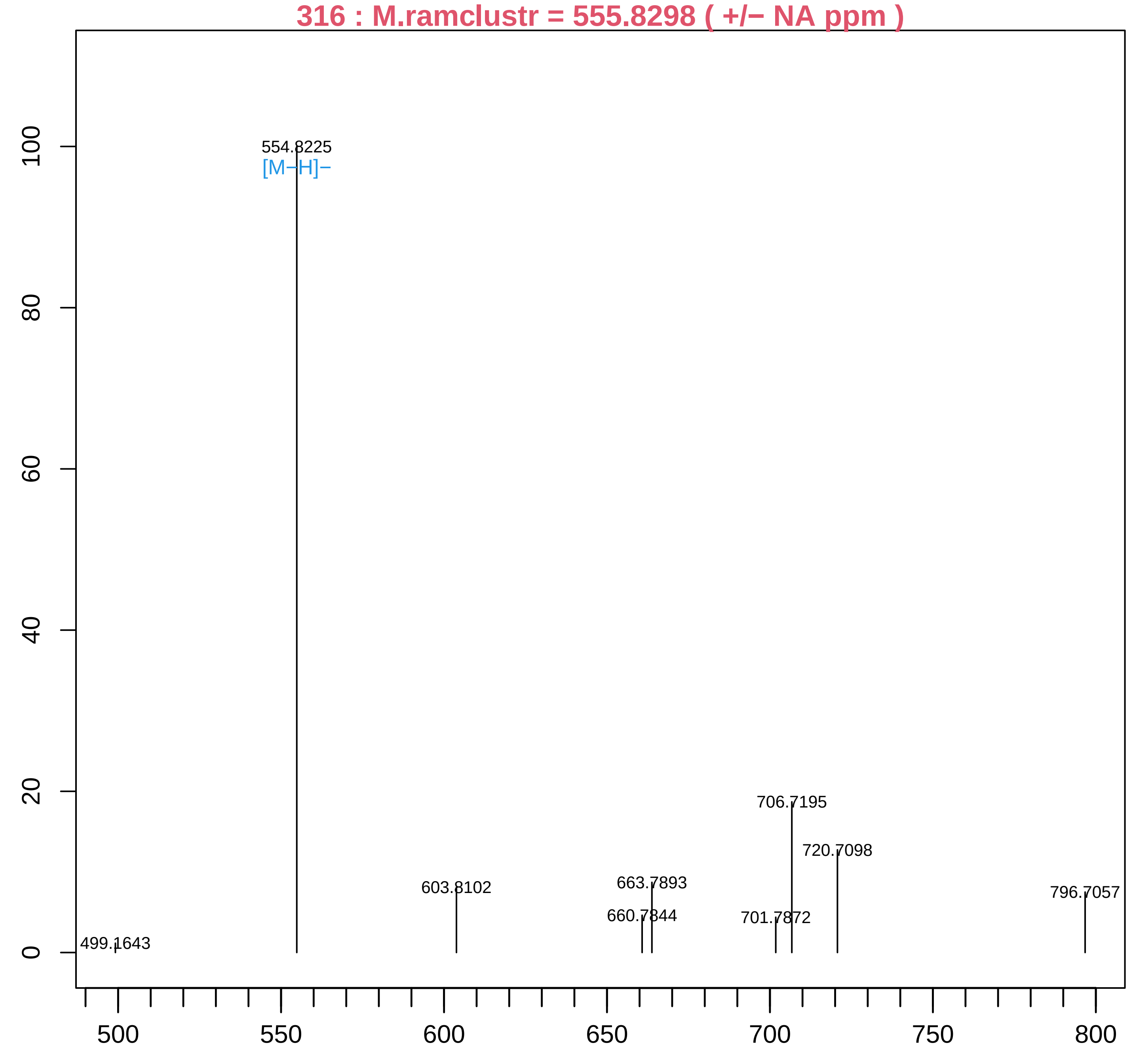
316 : M.ramclustr = 555.8298 (+/− NA ppm)
554.8225
[M−H]−
499.1643
603.8102
660.7844
663.7893
701.7872
706.7195
720.7098
796.7057
0
20
40
60
80
100
500
550
600
650
700
750
800

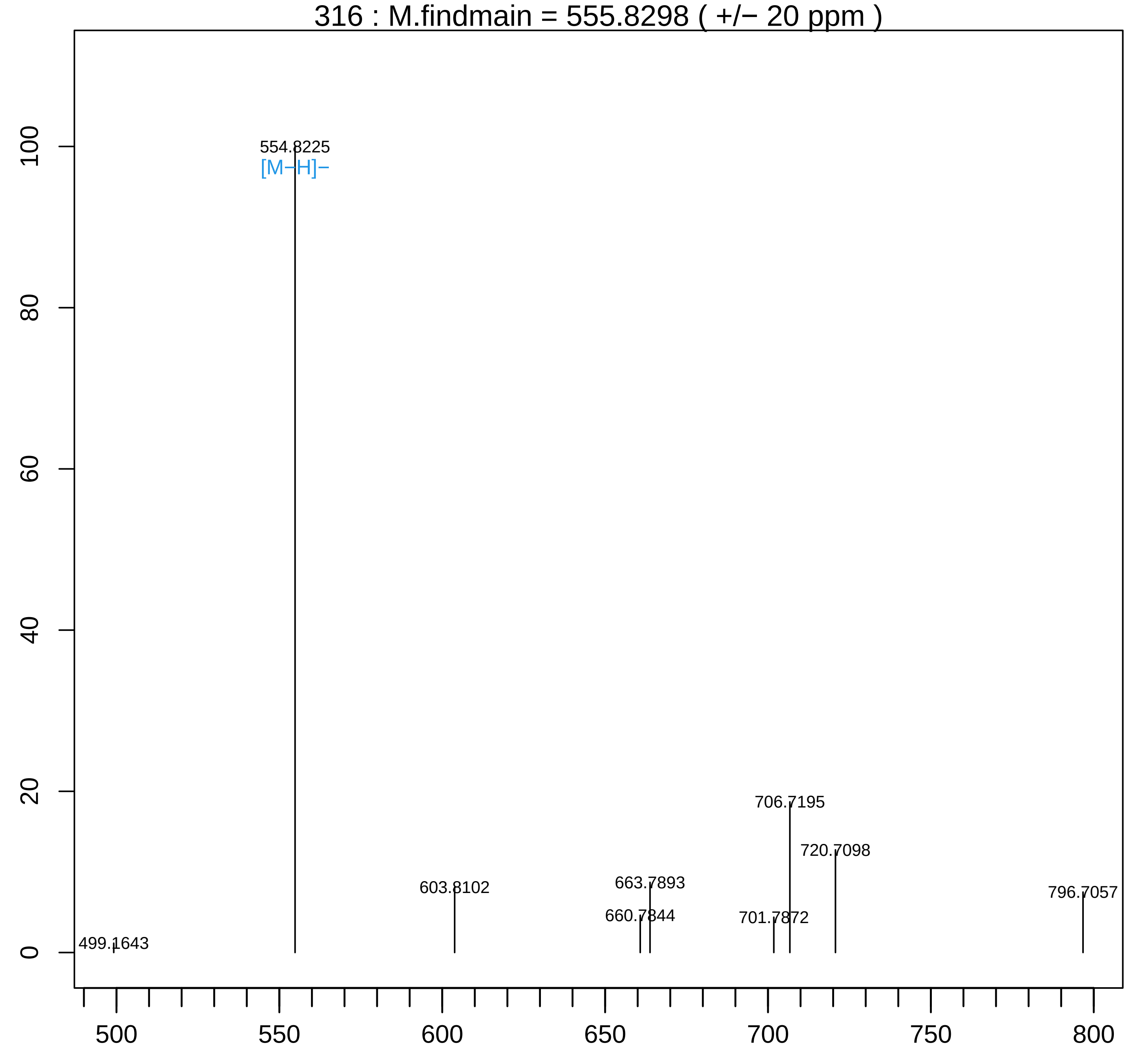
316 : M.findmain = 555.8298 (+/− 20 ppm)
554.8225
[M−H]−
499.1643
603.8102
660.7844
663.7893
701.7872
706.7195
720.7098
796.7057
0
20
40
60
80
100
500
550
600
650
700
750
800

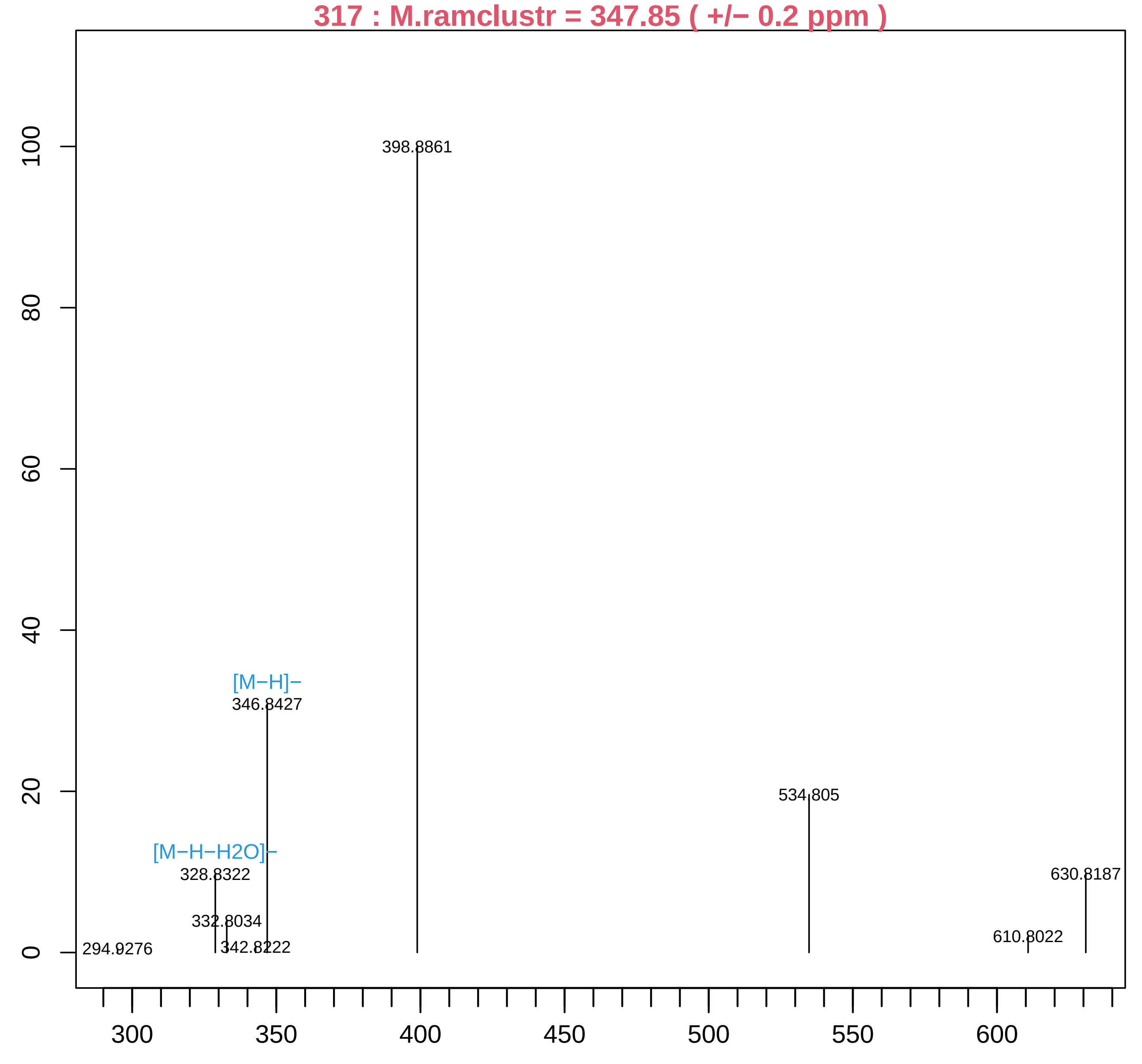
317 : M.ramclustr = 347.85 (+/− 0.2 ppm)
398.8861
[M−H]−
346.8427
534.805
[M−H−H2O]−
328.8322
630.8187
332.8034
610.8022
294.9276
342.8222

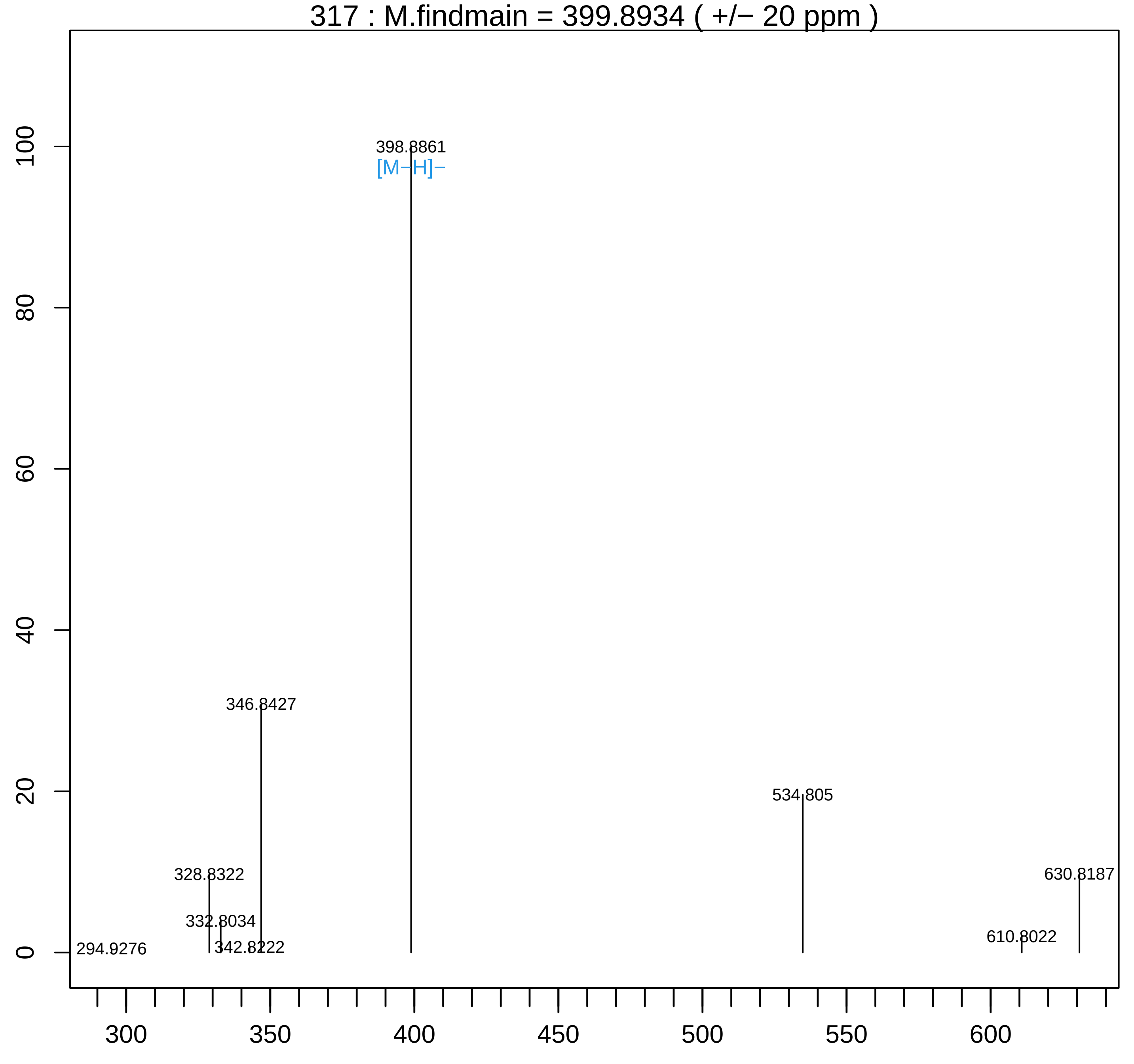
317 : M.findmain = 399.8934 (+/− 20 ppm)
398.8861
[M−H]−
346.8427
534.805
328.8322
630.8187
332.8034
610.8022
294.9276
342.8222

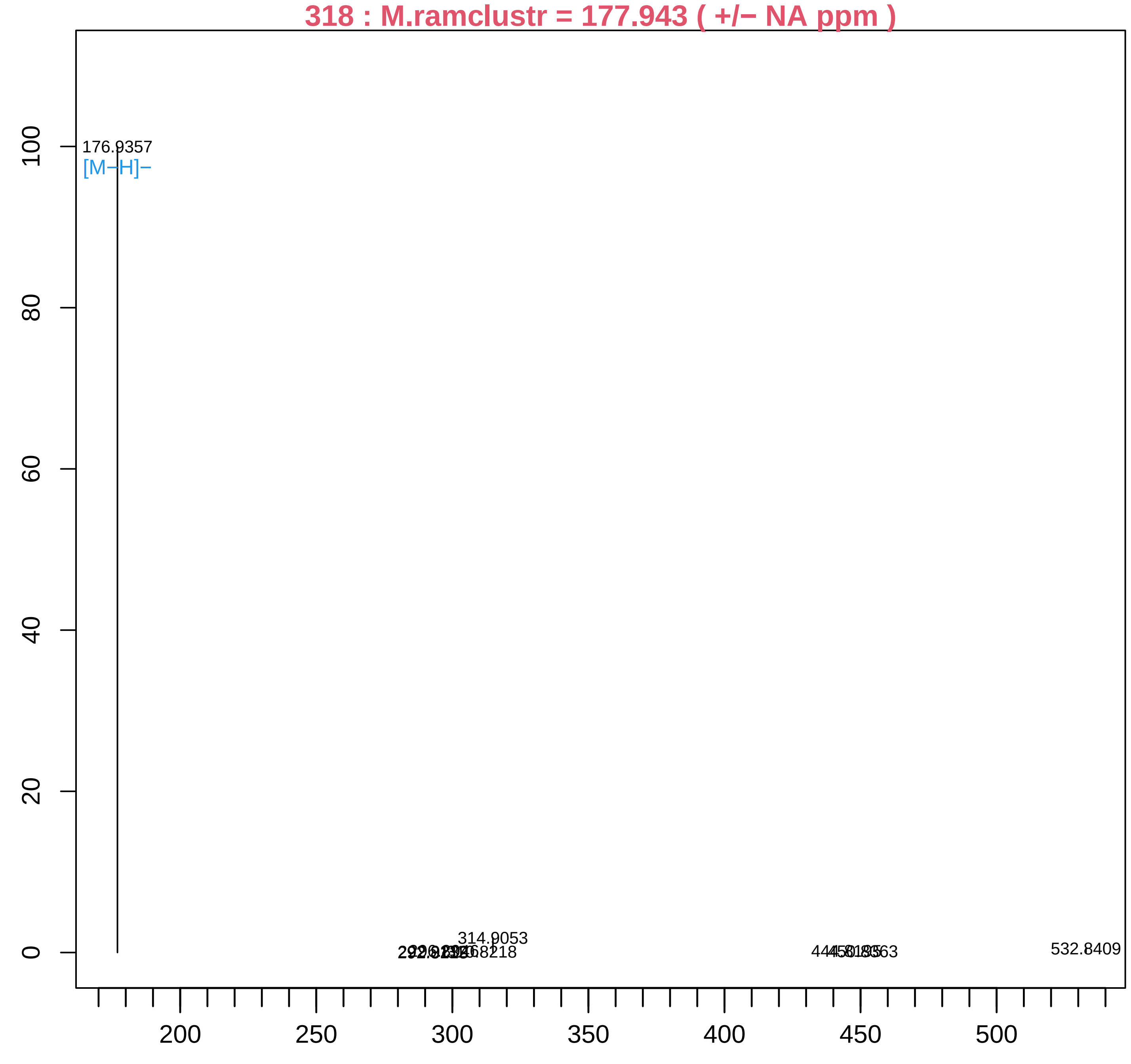
318 : M.ramclustr = 177.943 (+/− NA ppm)
176.9357
[M−H]−
314.9053
532.8409
0
20
40
60
80
100
200
250
300
350
400
450
500

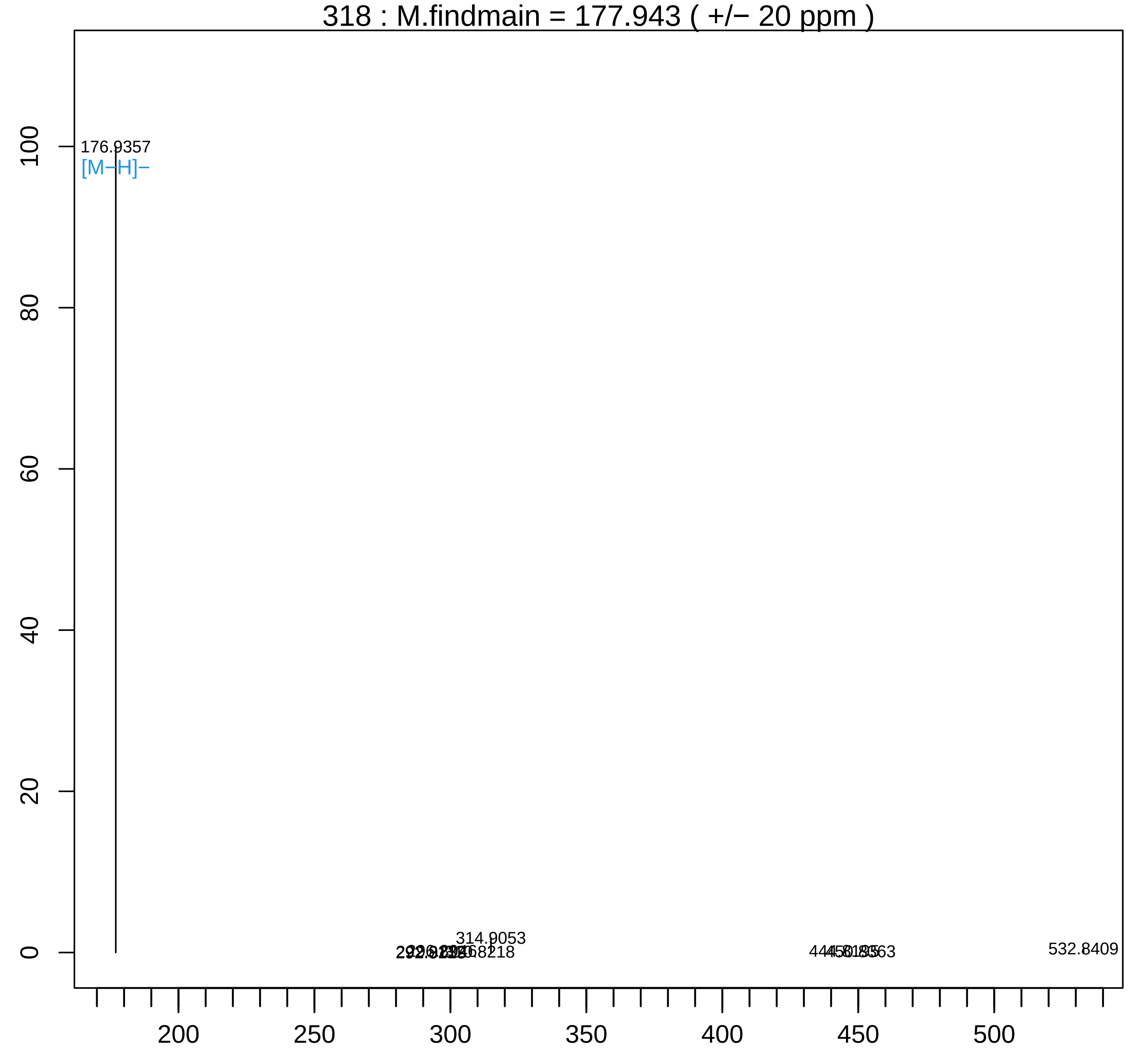
318 : M.findmain = 177.943 (+/− 20 ppm)
176.9357
[M−H]−
314.9053
532.8409
0
20
40
60
80
100
200
250
300
350
400
450
500

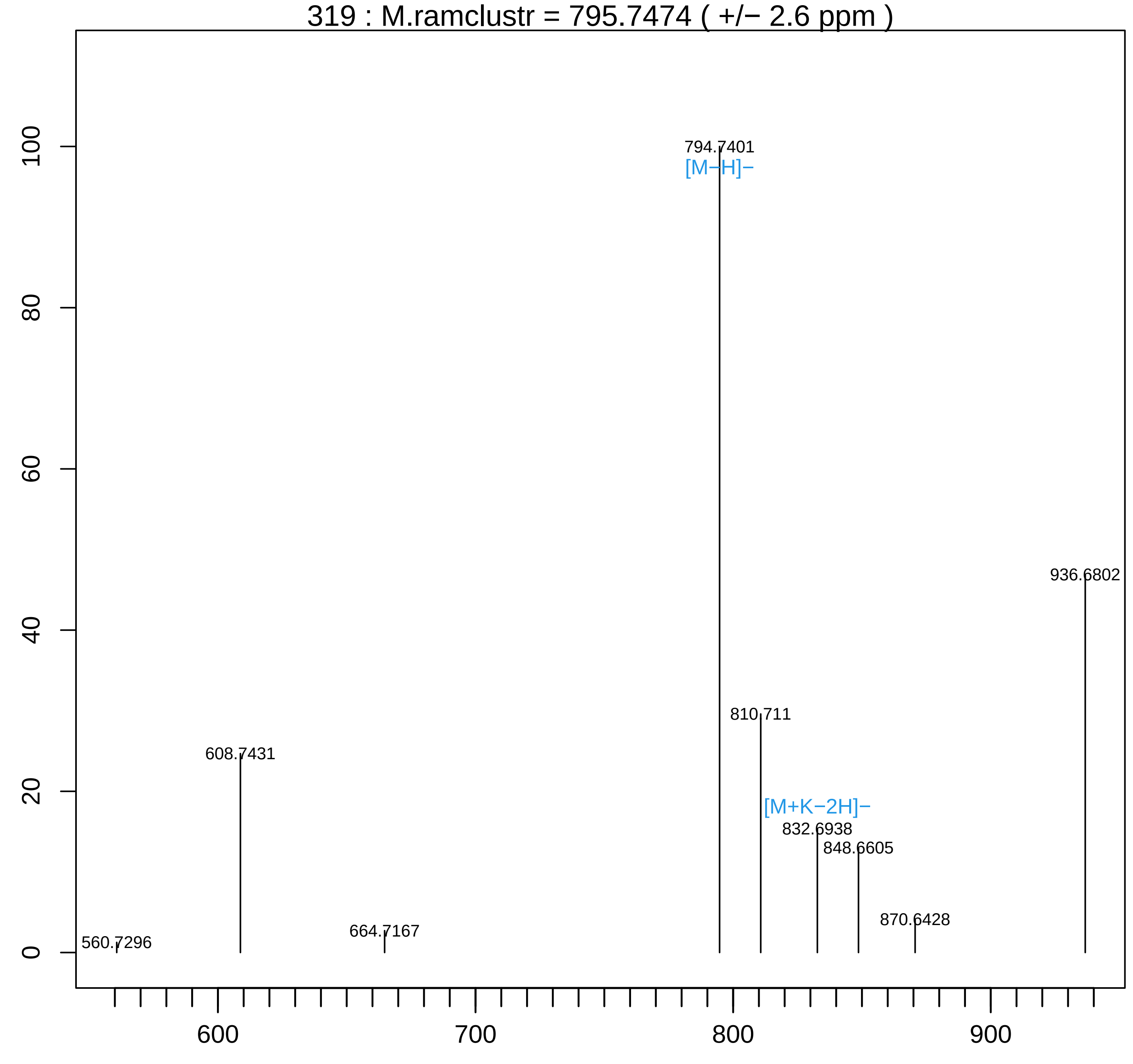
319 : M.ramclustr = 795.7474 (+/− 2.6 ppm)
794.7401
[M−H]−
936.6802
810.711
608.7431
[M+K−2H]−
832.6938
848.6605
870.6428
664.7167
560.7296
0
20
40
60
80
100
600
700
800
900

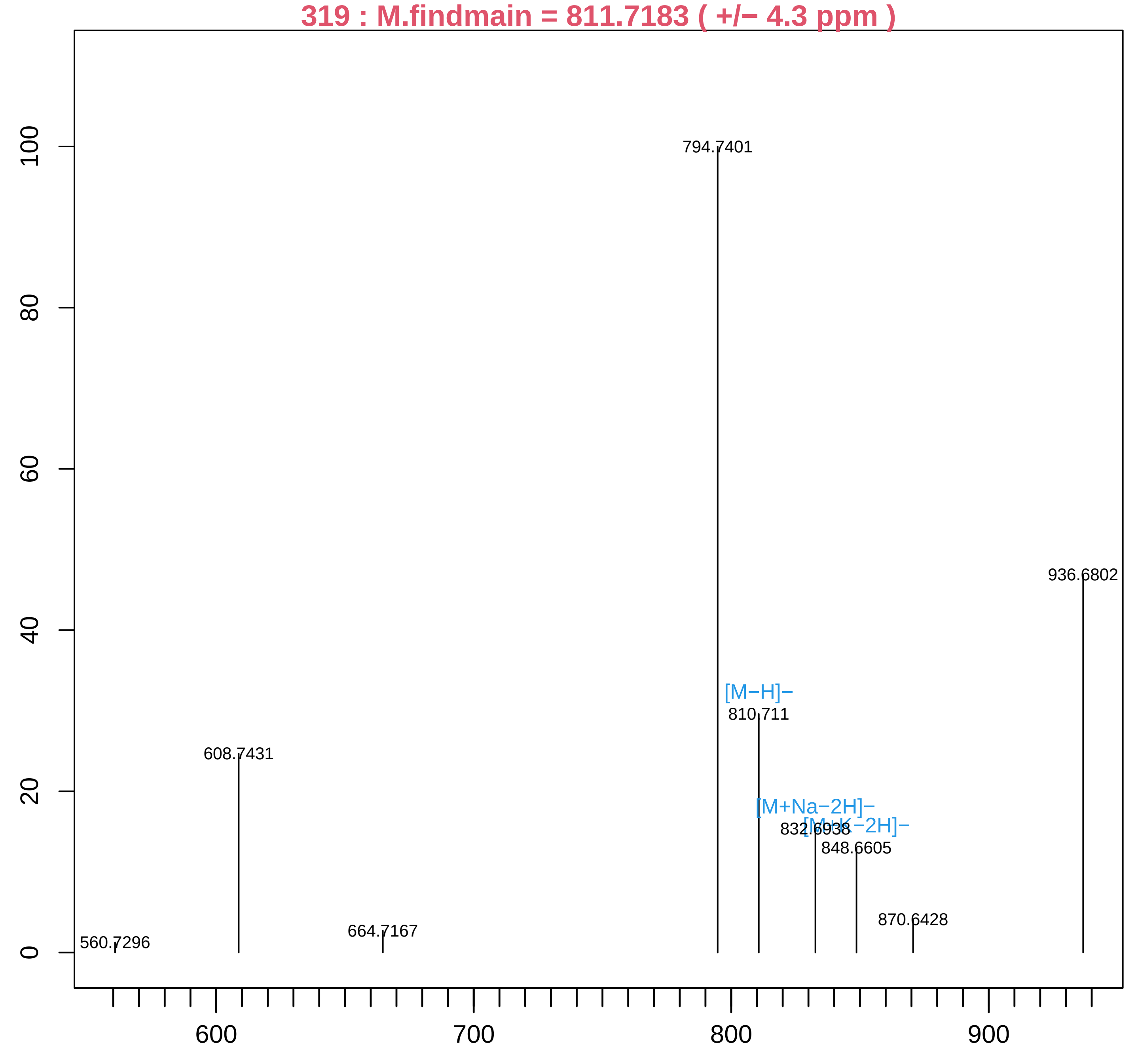
319 : M.findmain = 811.7183 (+/− 4.3 ppm)
794.7401
936.6802
[M−H]−
810.711
608.7431
[M+Na−2H]−
832.6938
[M+K−2H]−
848.6605
870.6428
664.7167
560.7296
0
20
40
60
80
100
600
700
800
900

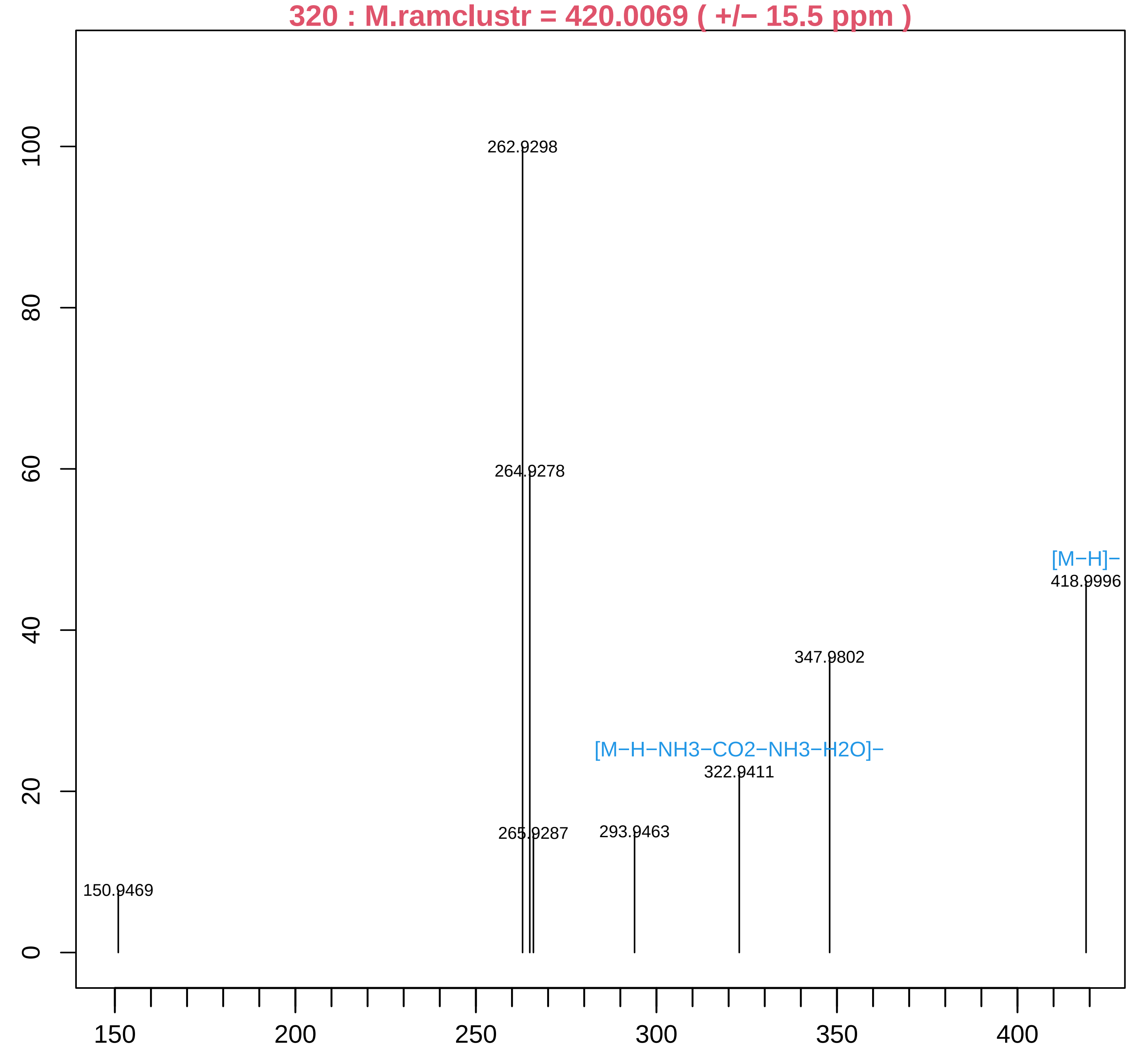
320 : M.ramclustr = 420.0069 (+/− 15.5 ppm)
262.9298
264.9278
265.9287
293.9463
[M−H−NH3−CO2−NH3−H2O]−
322.9411
347.9802
[M−H]−
418.9996
150.9469
0
20
40
60
80
100
150
200
250
300
350
400

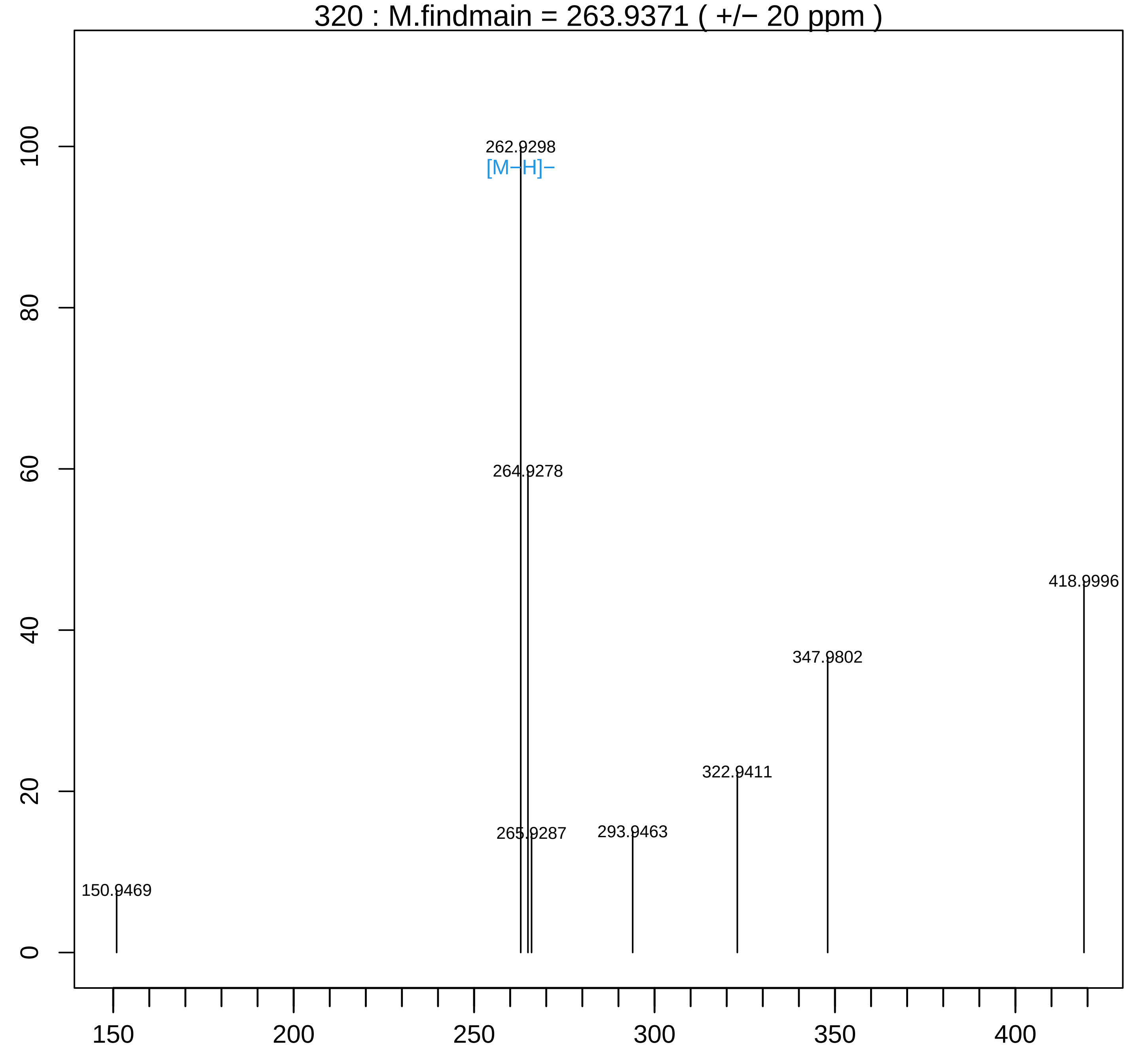
320 : M.findmain = 263.9371 (+/− 20 ppm)
262.9298
[M−H]−
264.9278
265.9287
293.9463
322.9411
347.9802
418.9996
150.9469
0
20
40
60
80
100
150
200
250
300
350
400

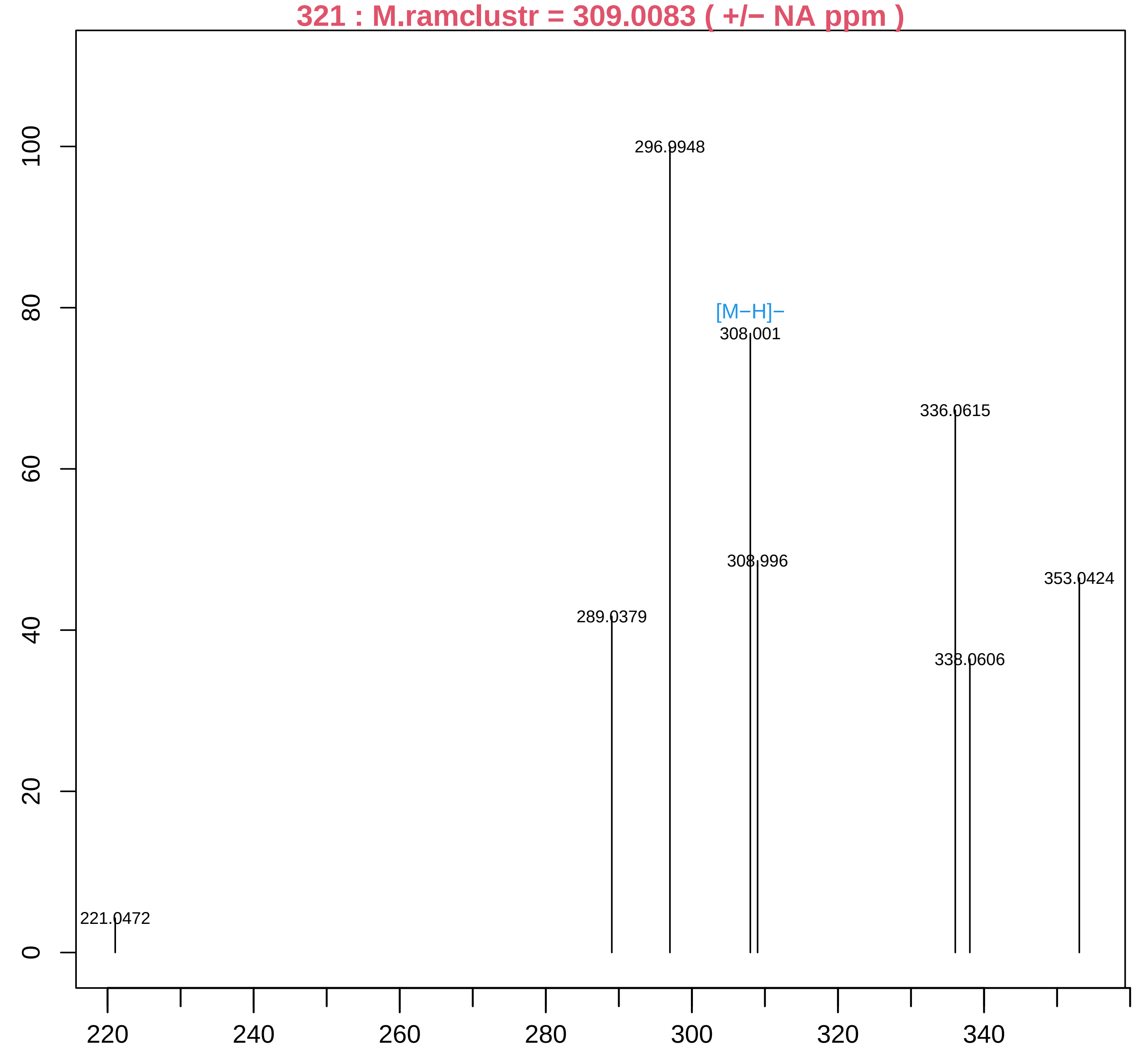
321 : M.ramclustr = 309.0083 (+/− NA ppm)
296.9948
[M−H]−
308.001
336.0615
308.996
353.0424
289.0379
338.0606
221.0472
0
20
40
60
80
100
220
240
260
280
300
320
340

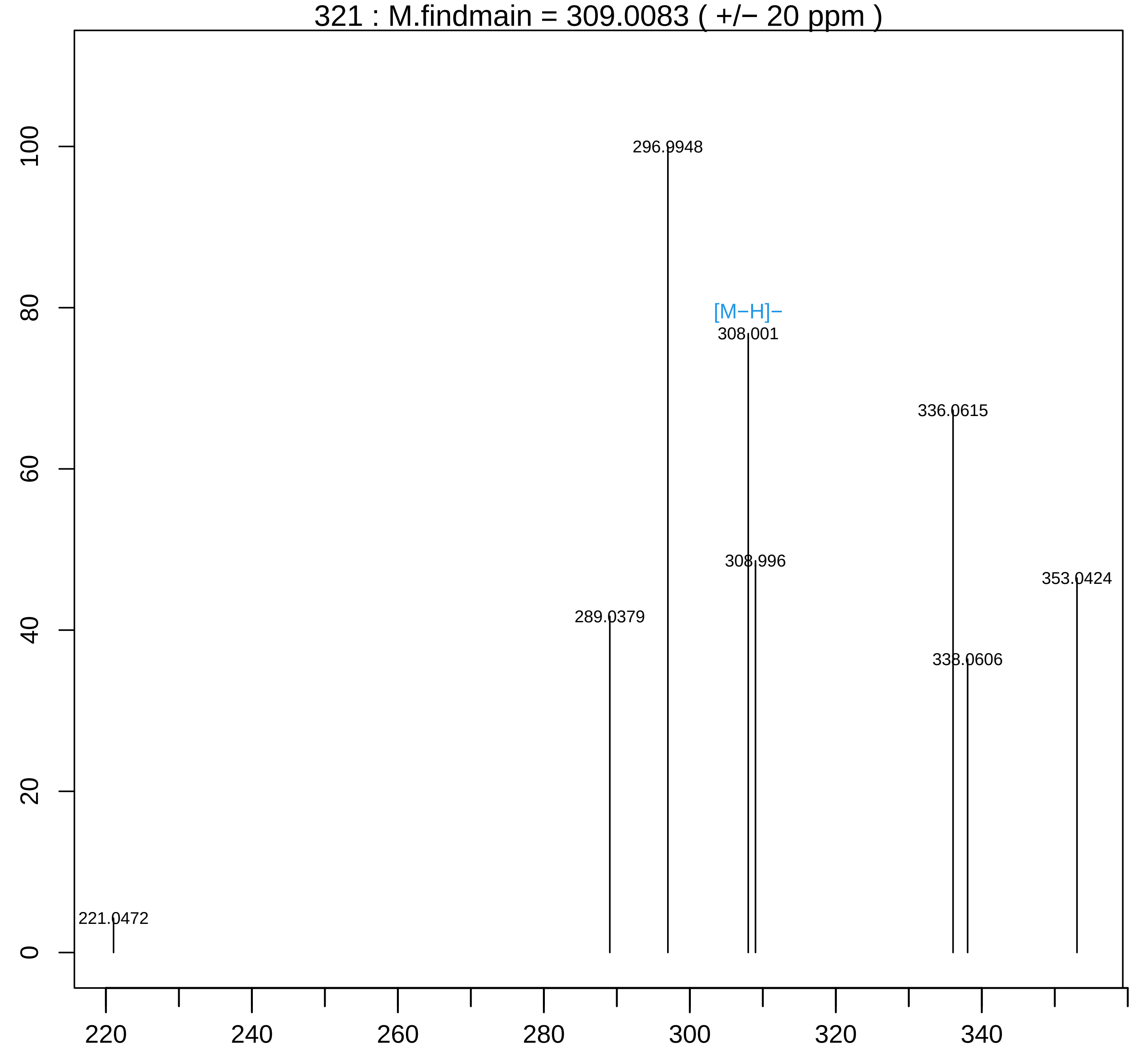
321 : M.findmain = 309.0083 (+/− 20 ppm)
296.9948
[M−H]−
308.001
336.0615
308.996
353.0424
289.0379
338.0606
221.0472
0
20
40
60
80
100
220
240
260
280
300
320
340

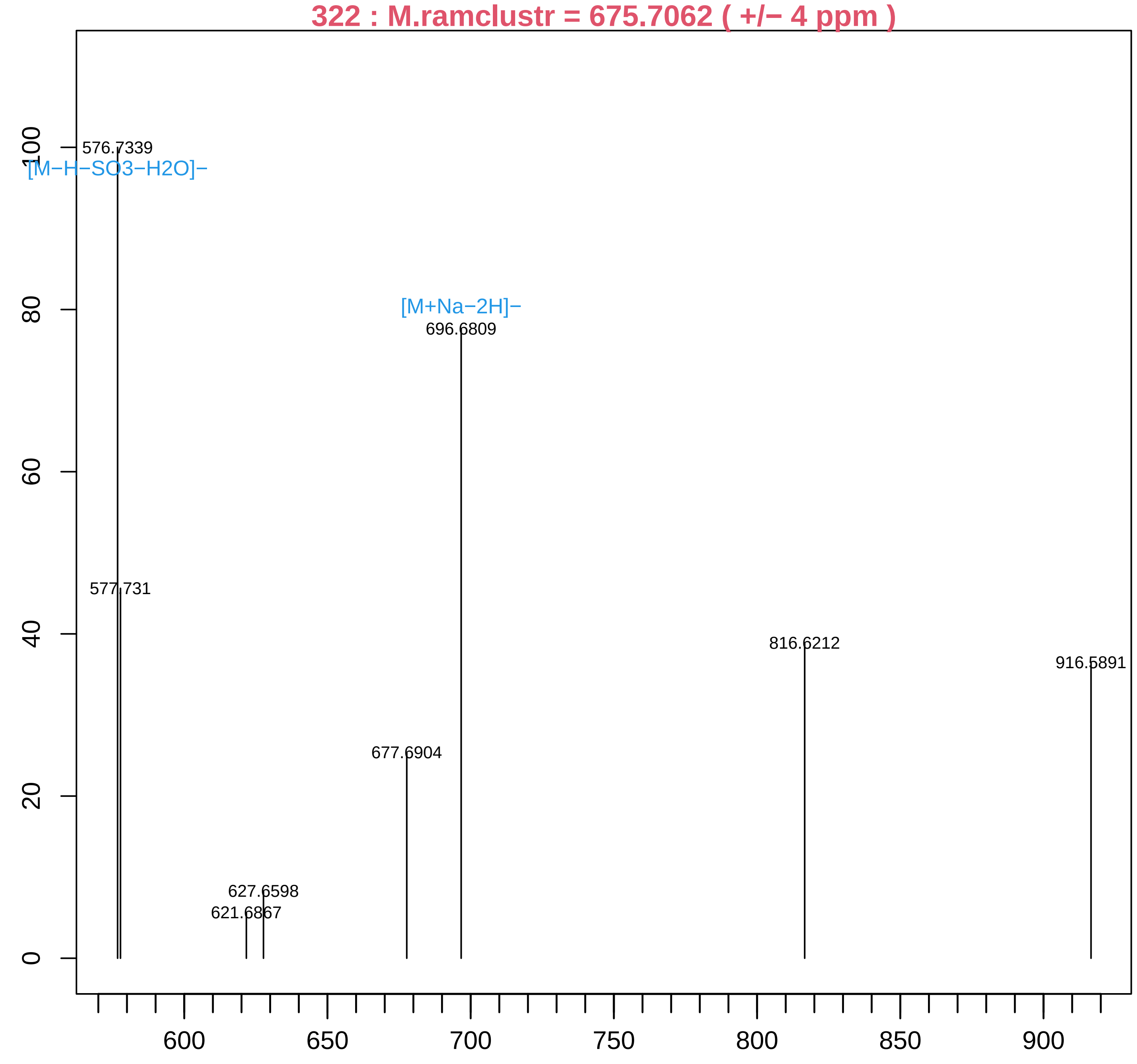
322 : M.ramclustr = 675.7062 (+/− 4 ppm)
576.7339
[M−H−SO3−H2O]−
577.731
621.6867
627.6598
677.6904
[M+Na−2H]−
696.6809
816.6212
916.5891
0
20
40
60
80
100
600
650
700
750
800
850
900

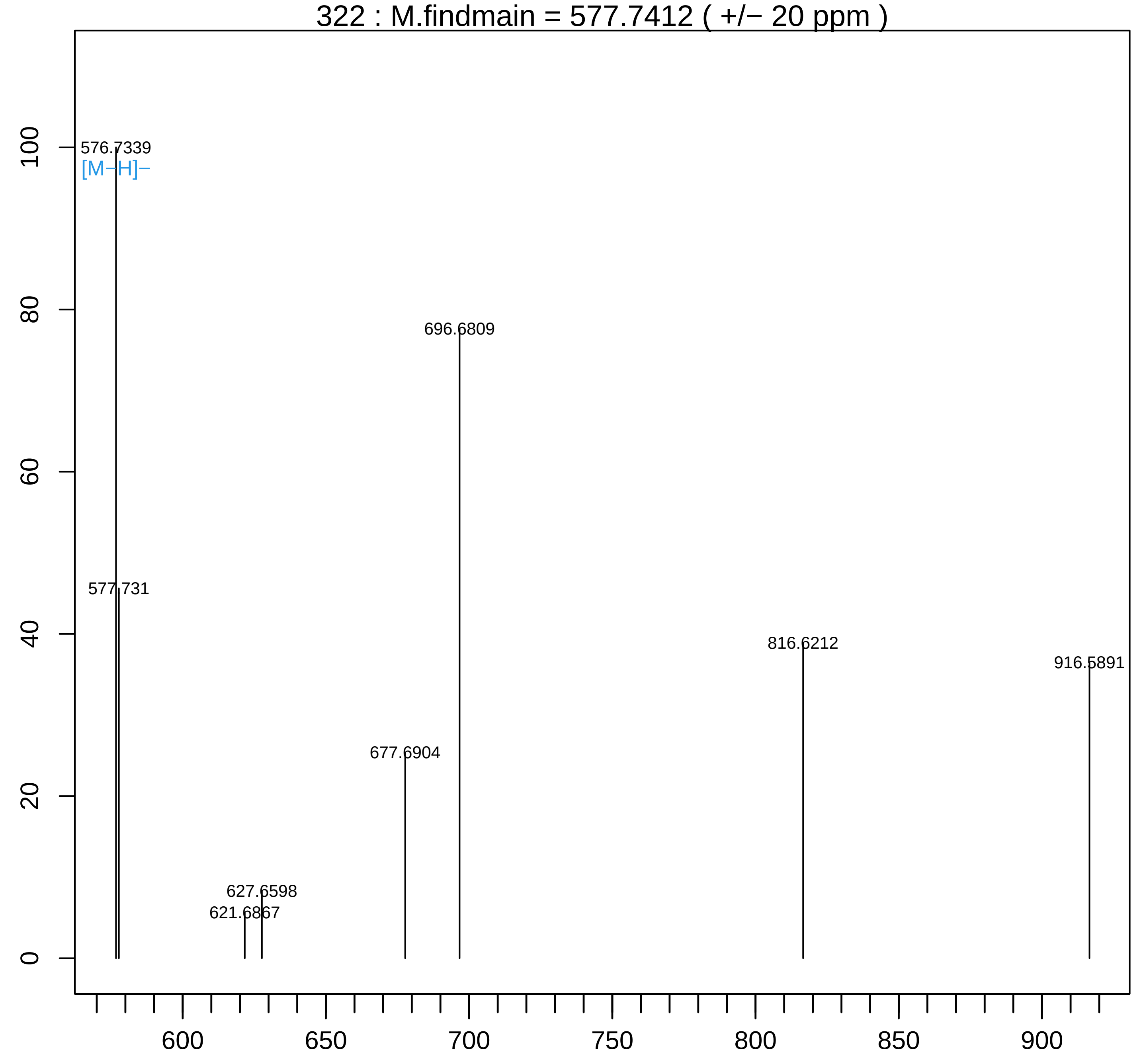
322 : M.findmain = 577.7412 (+/− 20 ppm)
576.7339
[M−H]−
577.731
621.6867
627.6598
677.6904
696.6809
816.6212
916.5891
0
20
40
60
80
100
600
650
700
750
800
850
900

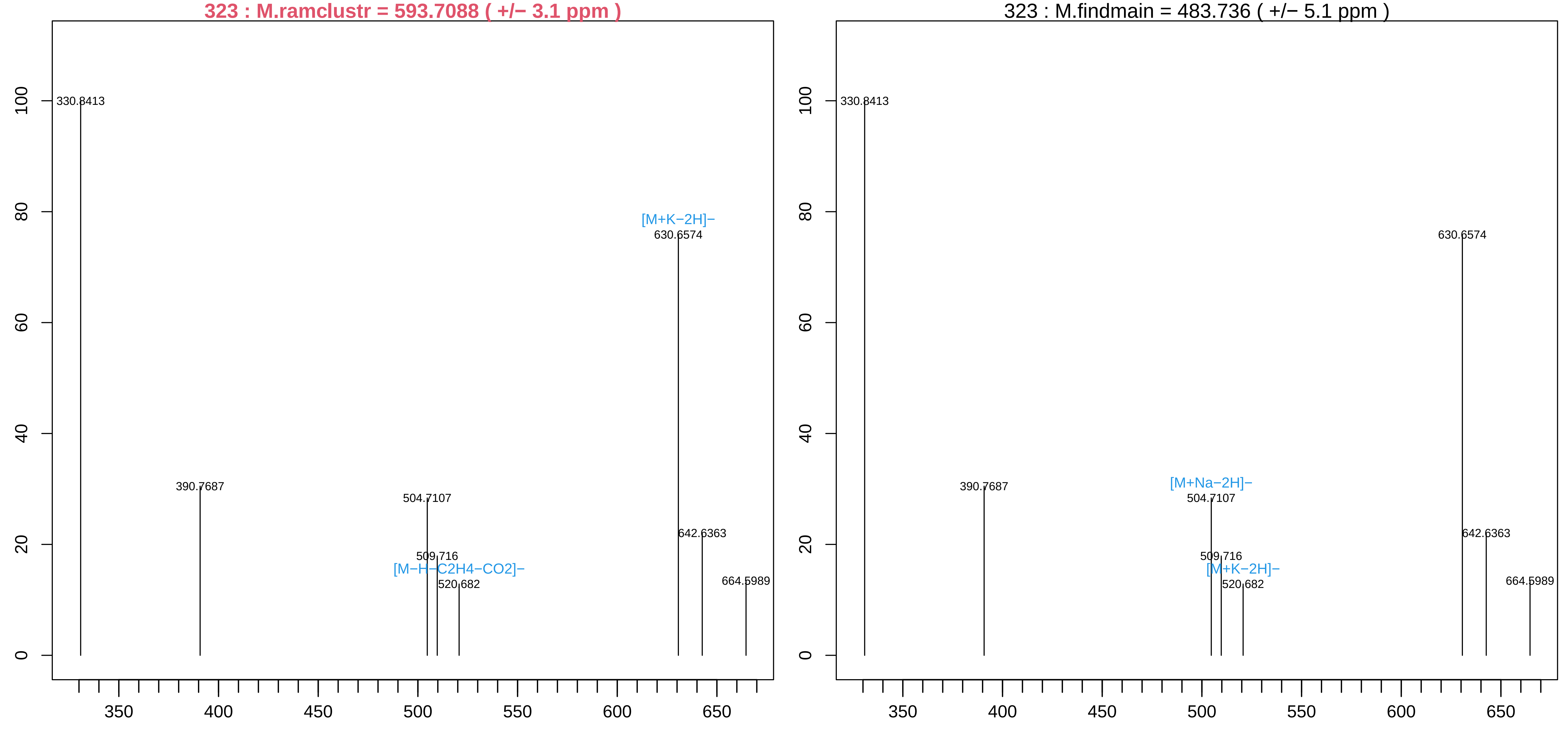

323 : M.ramclustr = 593.7088 (+/− 3.1 ppm)
330.8413
390.7687
504.7107
509.716
[M−H−C2H4−CO2]−
520.682
[M+K−2H]−
630.6574
642.6363
664.5989
323 : M.findmain = 483.736 (+/− 5.1 ppm)
330.8413
390.7687
[M+Na−2H]−
504.7107
509.716
[M+K−2H]−
520.682
630.6574
642.6363
664.5989

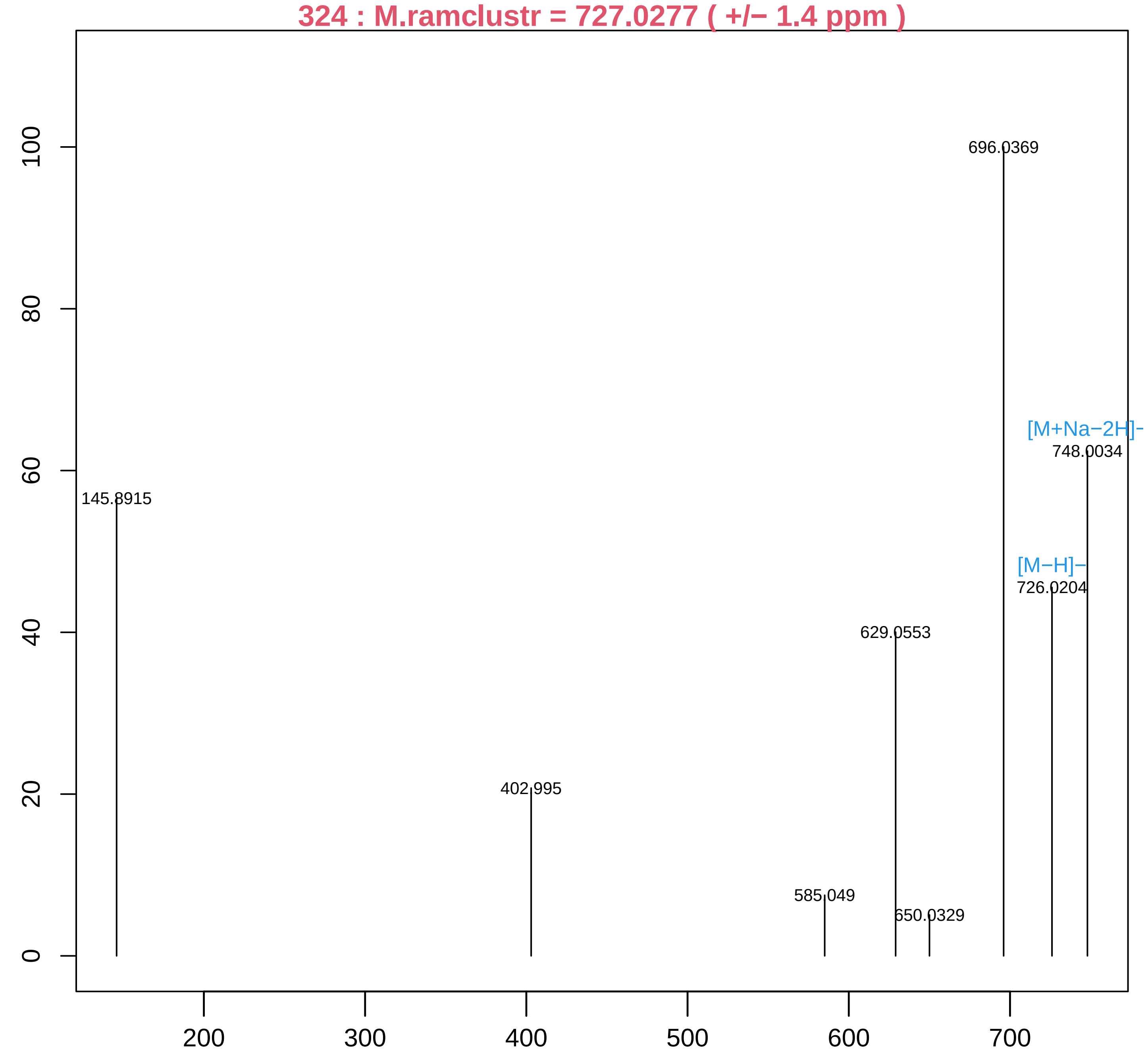
324 : M.ramclustr = 727.0277 (+/− 1.4 ppm)
696.0369
[M+Na−2H]−
748.0034
145.8915
[M−H]−
726.0204
629.0553
402.995
585.049
650.0329
0
20
40
60
80
100
200
300
400
500
600
700

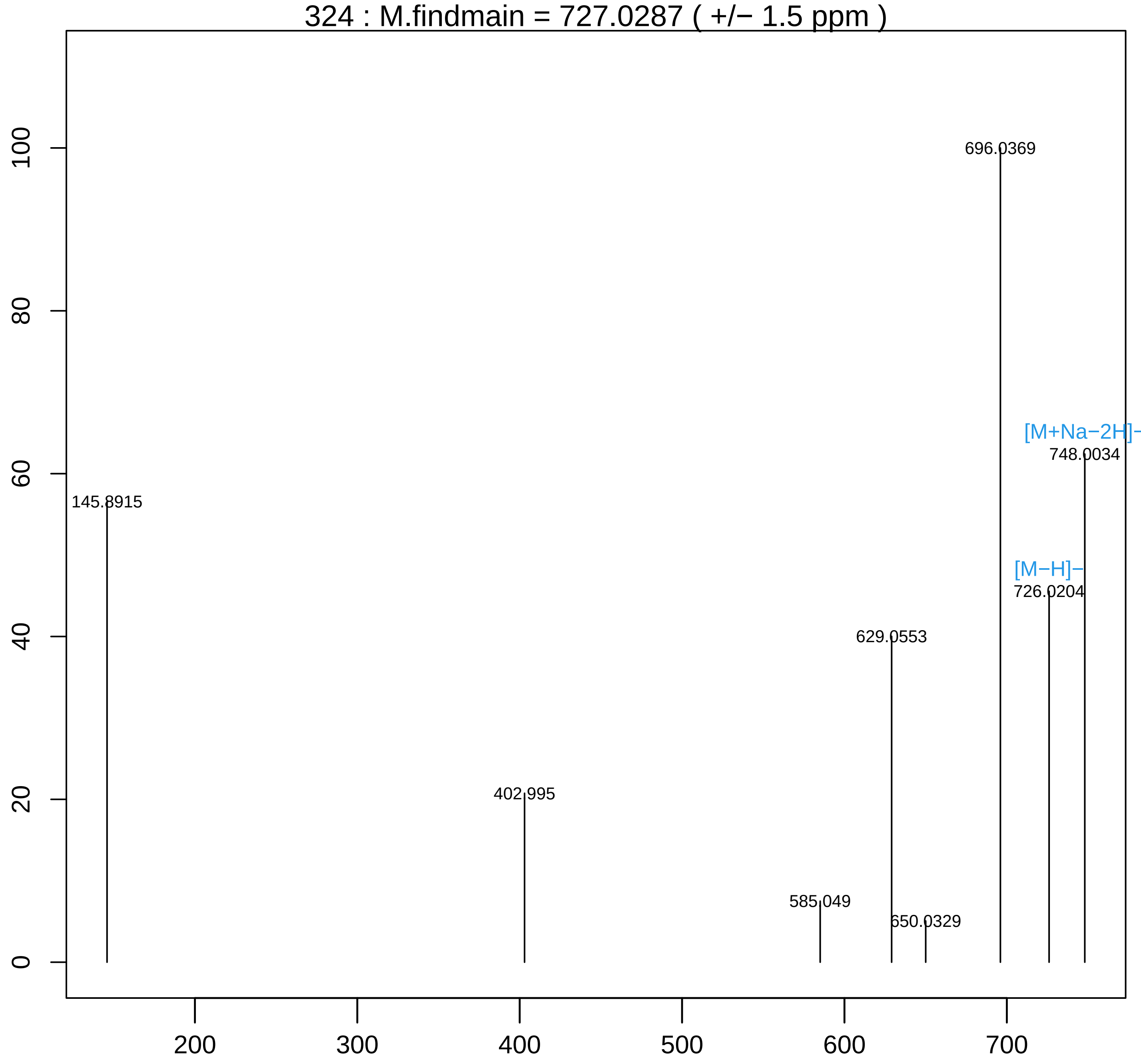
324 : M.findmain = 727.0287 (+/− 1.5 ppm)
696.0369
[M+Na−2H]−
748.0034
145.8915
[M−H]−
726.0204
629.0553
402.995
585.049
650.0329
0
20
40
60
80
100
200
300
400
500
600
700

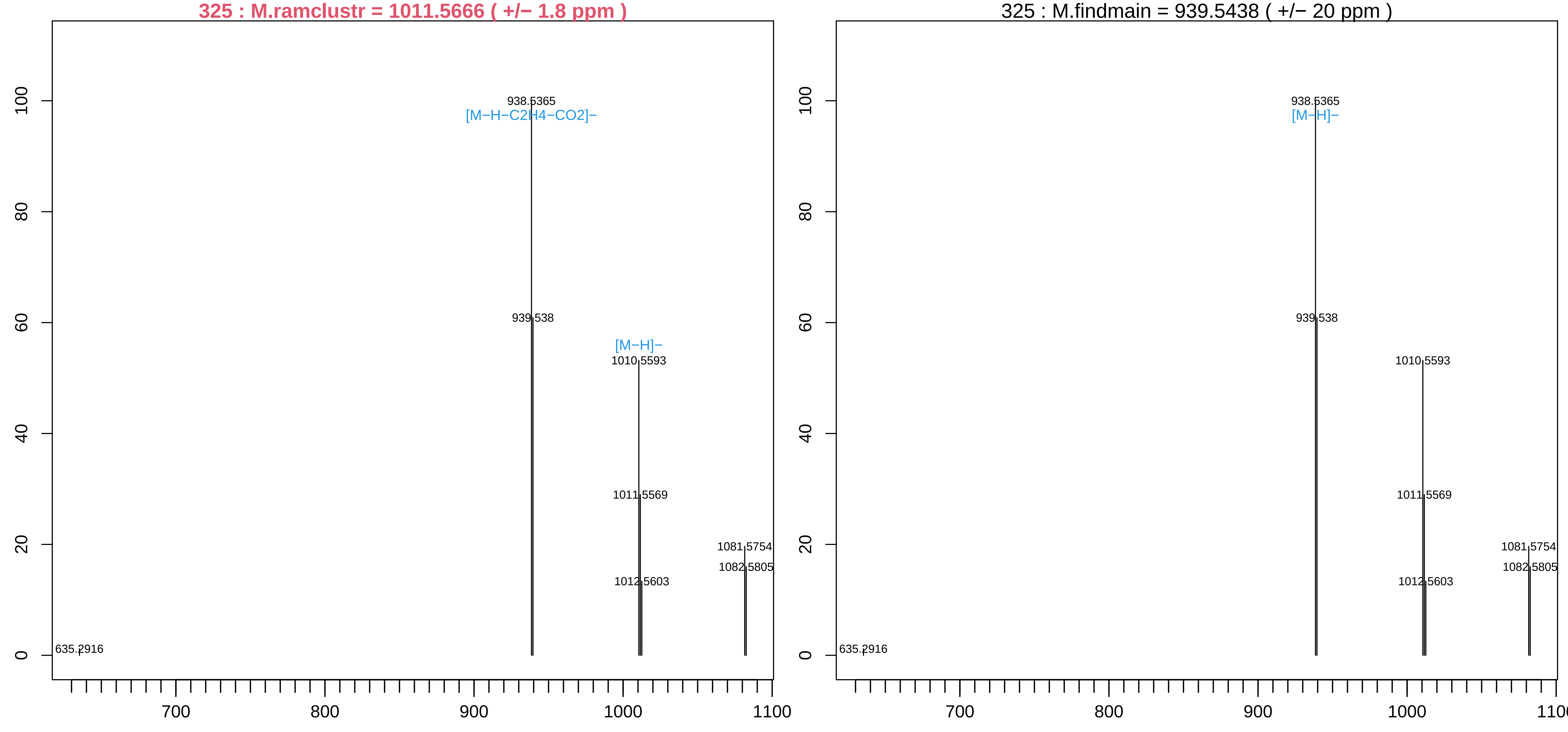
325 : M.ramclustr = 1011.5666 (+/− 1.8 ppm)
938.5365
[M−H−C2H4−CO2]−
939.538
[M−H]−
1010.5593
1011.5569
1012.5603
1081.5754
1082.5805
635.2916
325 : M.findmain = 939.5438 (+/− 20 ppm)
938.5365
[M−H]−
939.538
1010.5593
1011.5569
1012.5603
1081.5754
1082.5805
635.2916
0
20
40
60
80
100
700
800
900
1000
1100

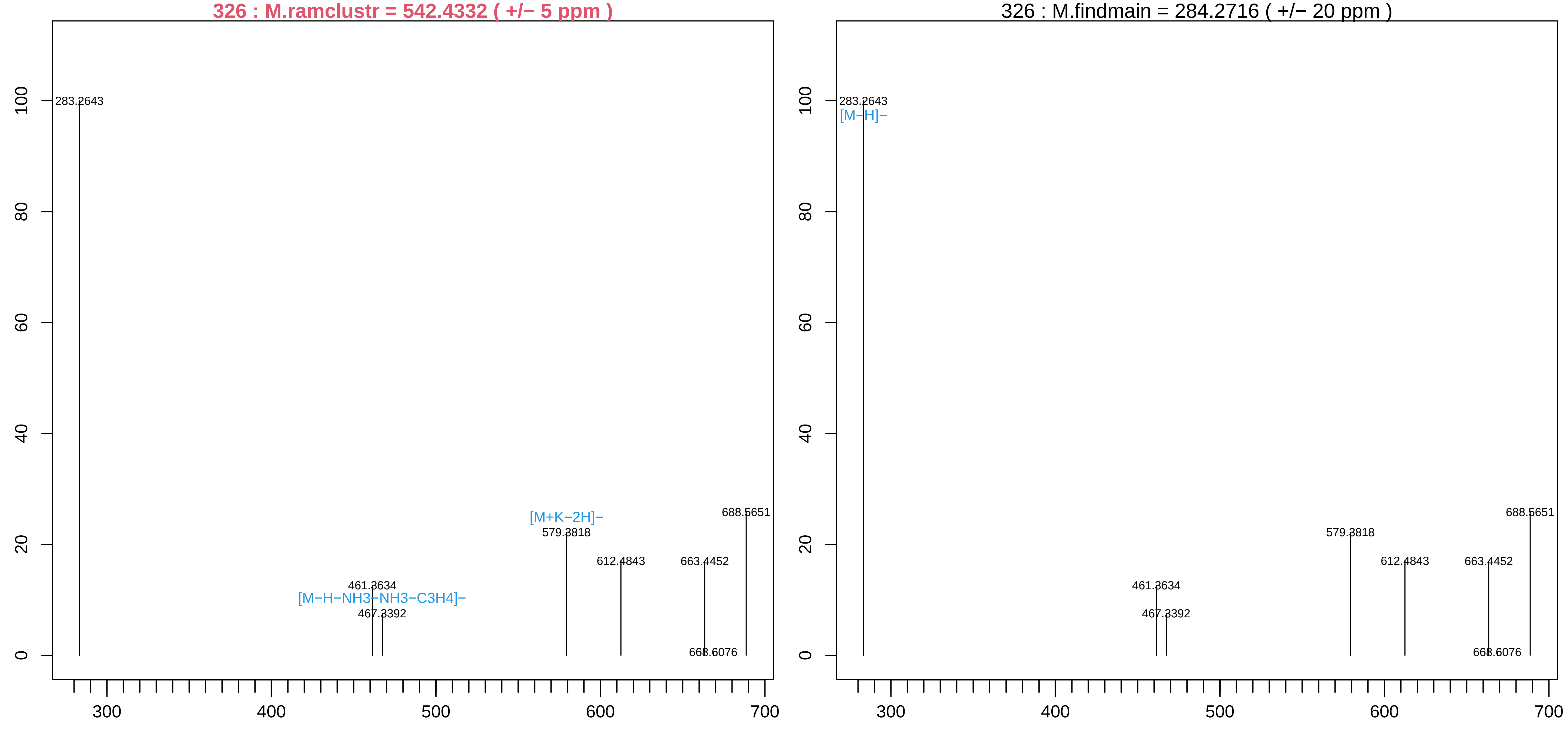

326 : M.ramclustr = 542.4332 (+/− 5 ppm)
283.2643
461.3634
[M−H−NH3−NH3−C3H4]−
467.3392
[M+K−2H]−
579.3818
612.4843
663.4452
668.6076
688.5651
326 : M.findmain = 284.2716 (+/− 20 ppm)
283.2643
[M−H]−
461.3634
467.3392
579.3818
612.4843
663.4452
668.6076
688.5651
0
20
40
60
80
100
300
400
500
600
700

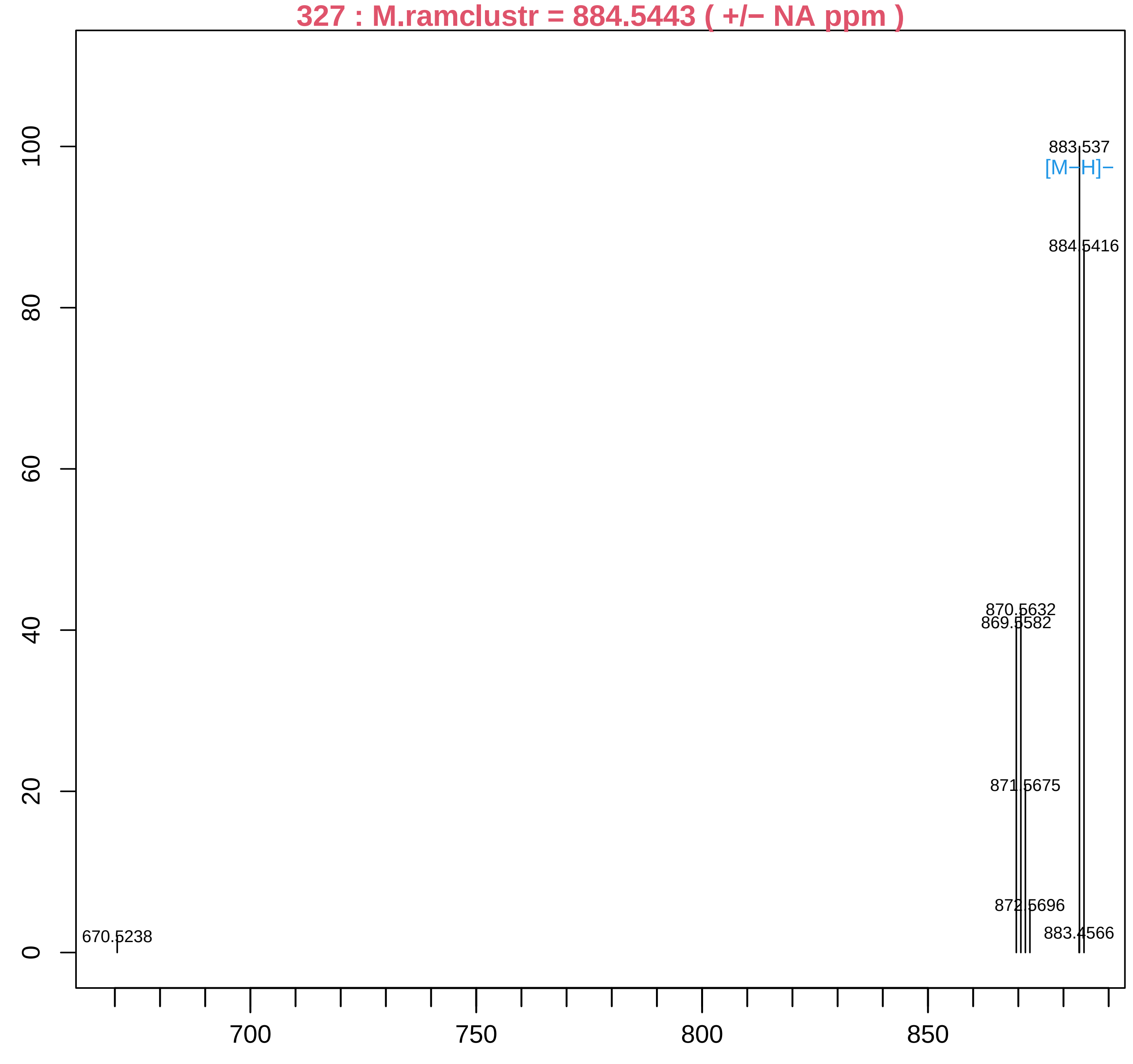
327 : M.ramclustr = 884.5443 (+/− NA ppm)
883.537
[M−H]−
884.5416
870.5632
869.5582
871.5675
872.5696
883.4566
670.5238
0
20
40
60
80
100
700
750
800
850

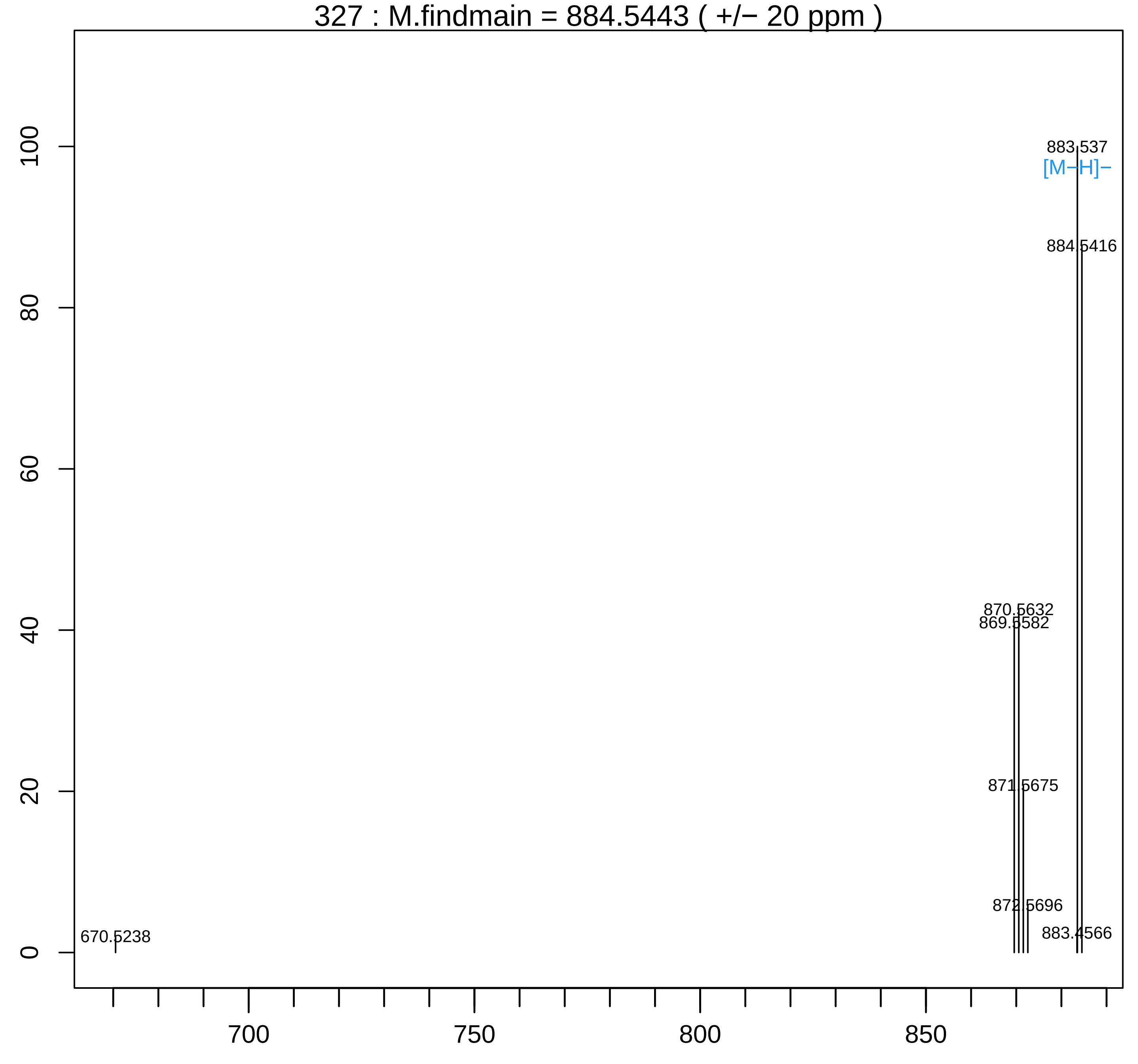
327 : M.findmain = 884.5443 (+/− 20 ppm)
883.537
[M−H]−
884.5416
870.5632
869.5582
871.5675
872.5696
883.4566
670.5238
0
20
40
60
80
100
700
750
800
850

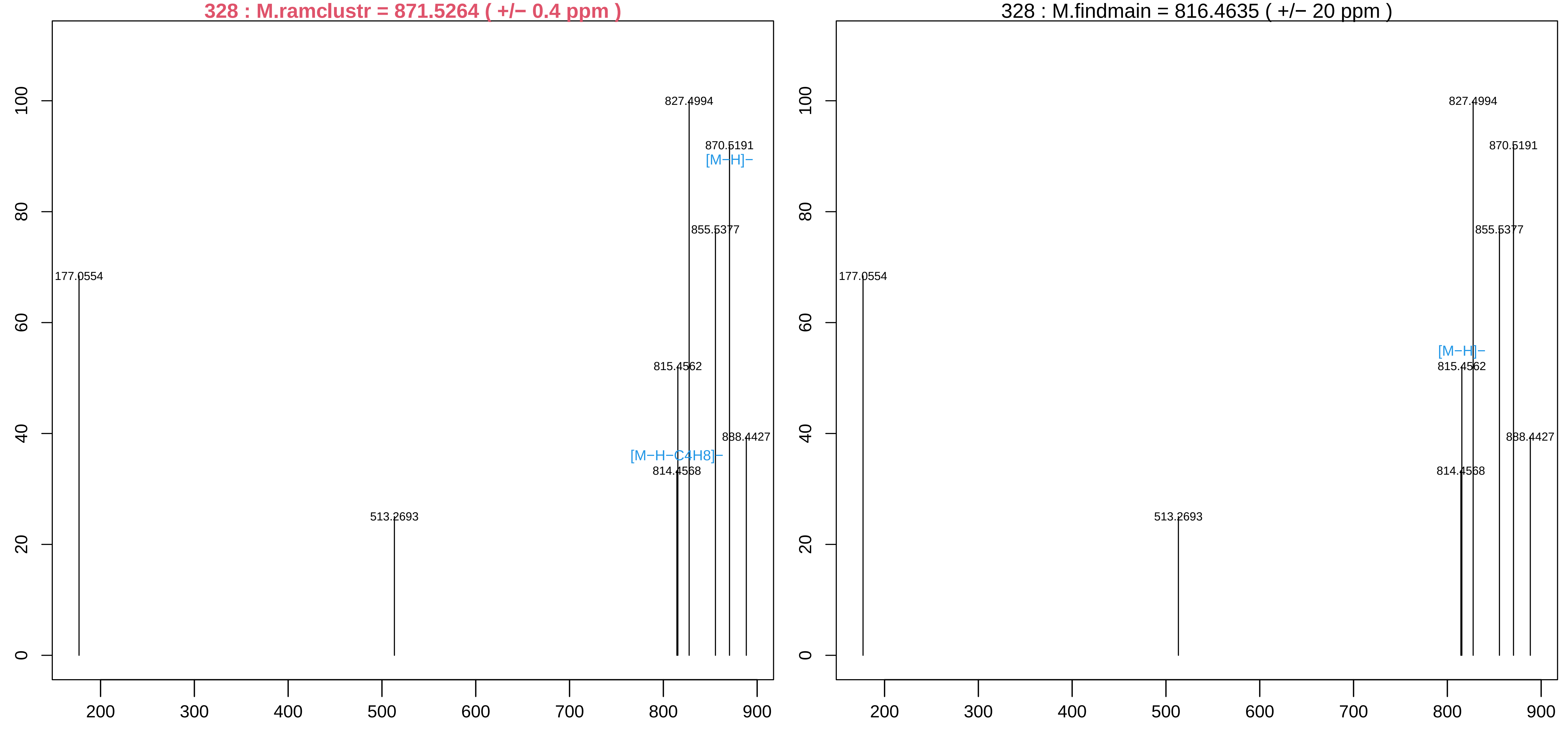

328 : M.ramclustr = 871.5264 (+/− 0.4 ppm)
827.4994
870.5191
[M−H]−
855.5377
177.0554
815.4562
888.4427
[M−H−C4H8]−
814.4568
513.2693
328 : M.findmain = 816.4635 (+/− 20 ppm)
827.4994
870.5191
855.5377
177.0554
[M−H]−
815.4562
888.4427
814.4568
513.2693

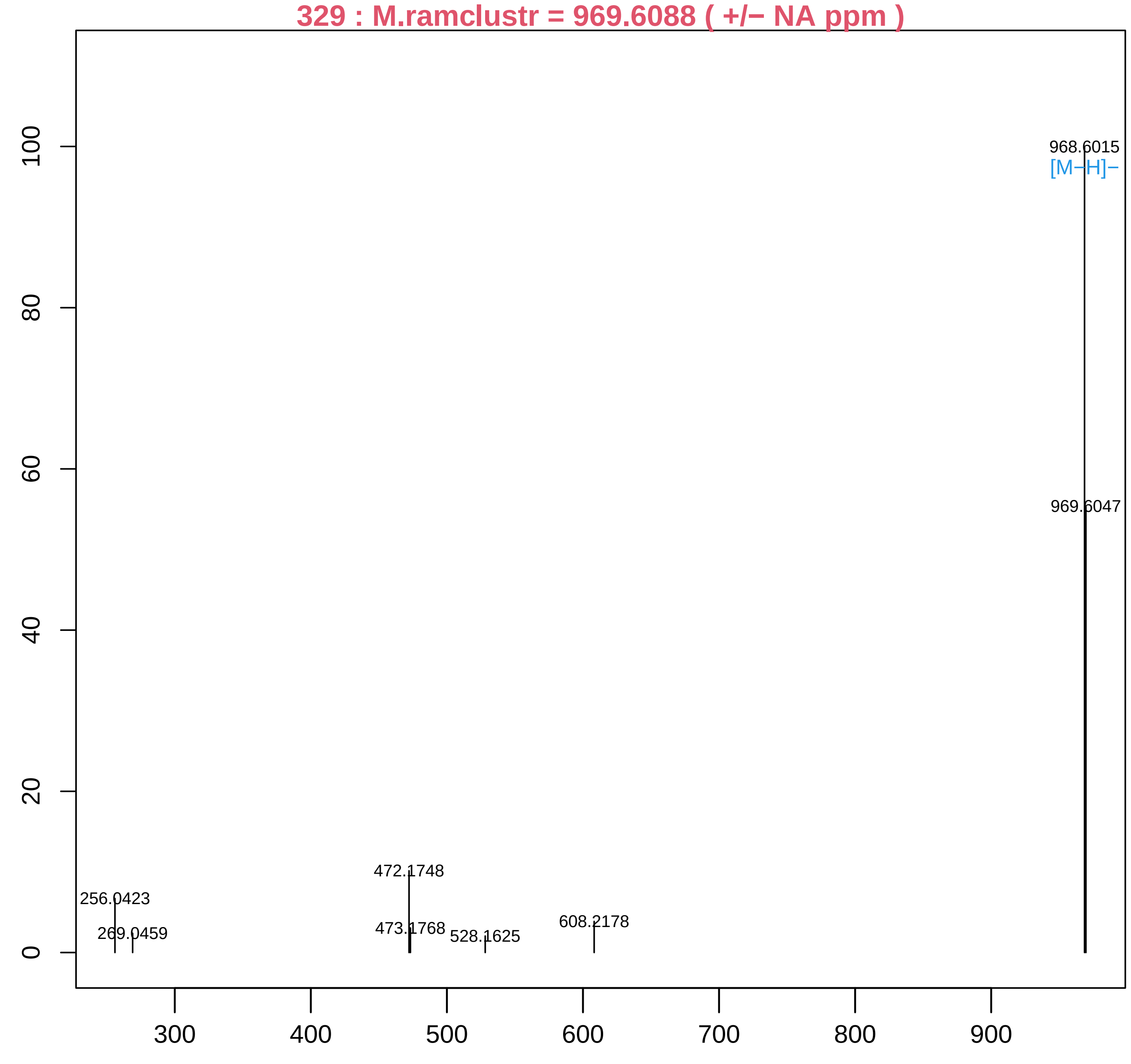

329 : M.ramclustr = 969.6088 (+/− NA ppm)
968.6015
[M−H]−
969.6047
472.1748
256.0423
269.0459
473.1768
528.1625
608.2178
0
20
40
60
80
100
300
400
500
600
700
800
900

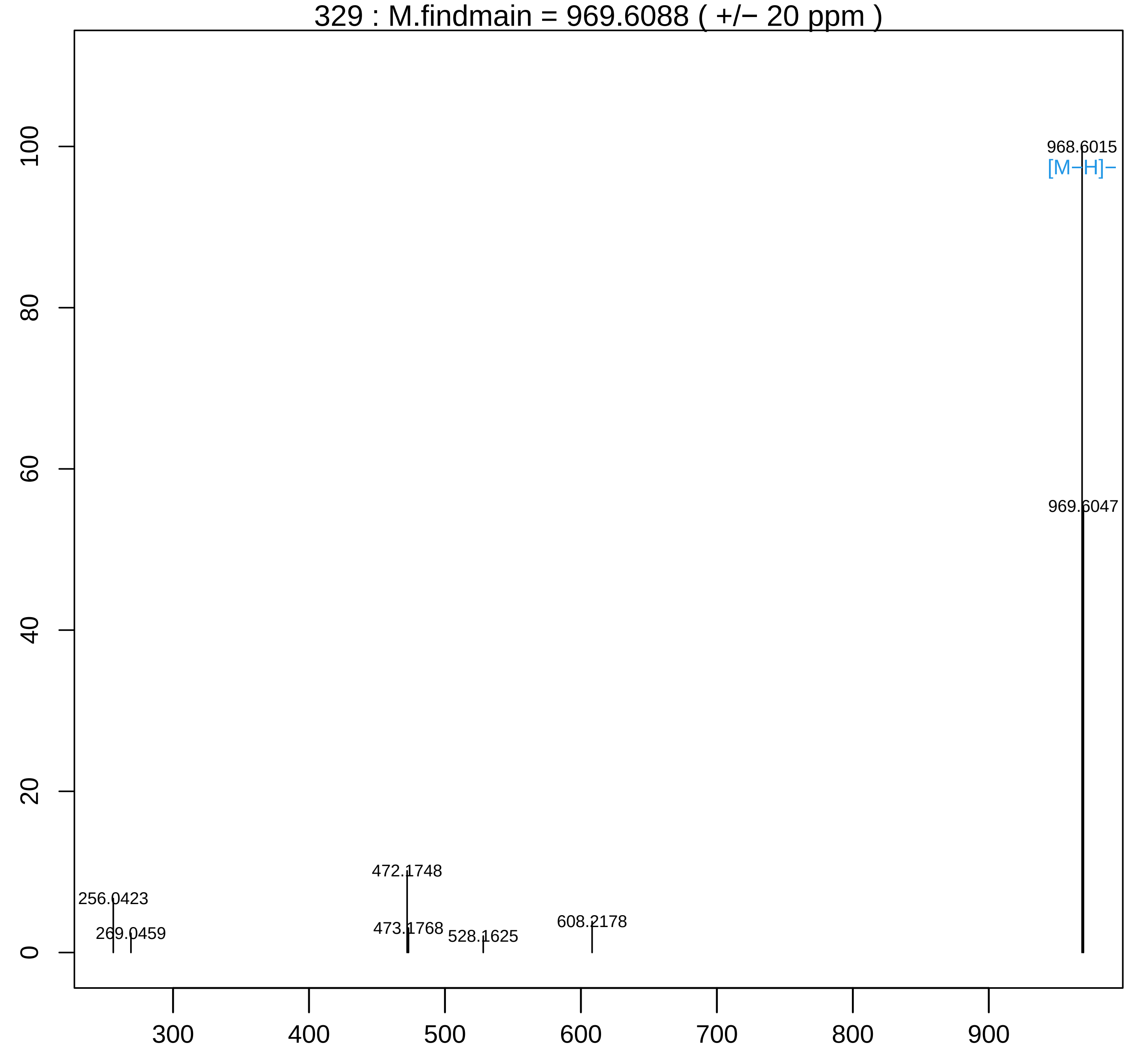

329 : M.findmain = 969.6088 (+/− 20 ppm)
968.6015
[M−H]−
969.6047
472.1748
256.0423
269.0459
473.1768
528.1625
608.2178
0
20
40
60
80
100
300
400
500
600
700
800
900

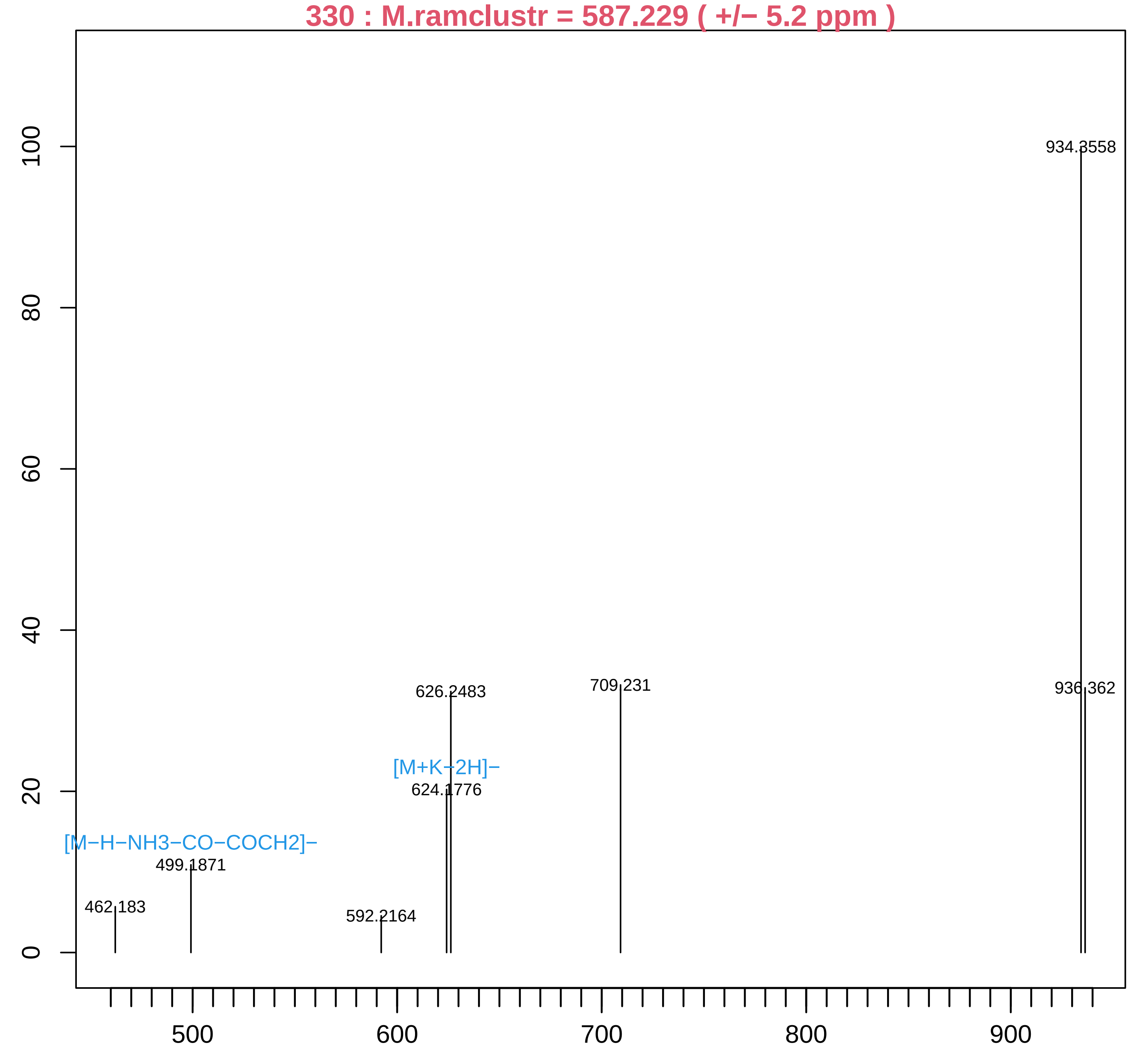
330 : M.ramclustr = 587.229 (+/− 5.2 ppm)
934.3558
936.362
709.231
626.2483
[M+K−2H]−
624.1776
[M−H−NH3−CO−COCH2]−
499.1871
462.183
592.2164
0
20
40
60
80
100
500
600
700
800
900
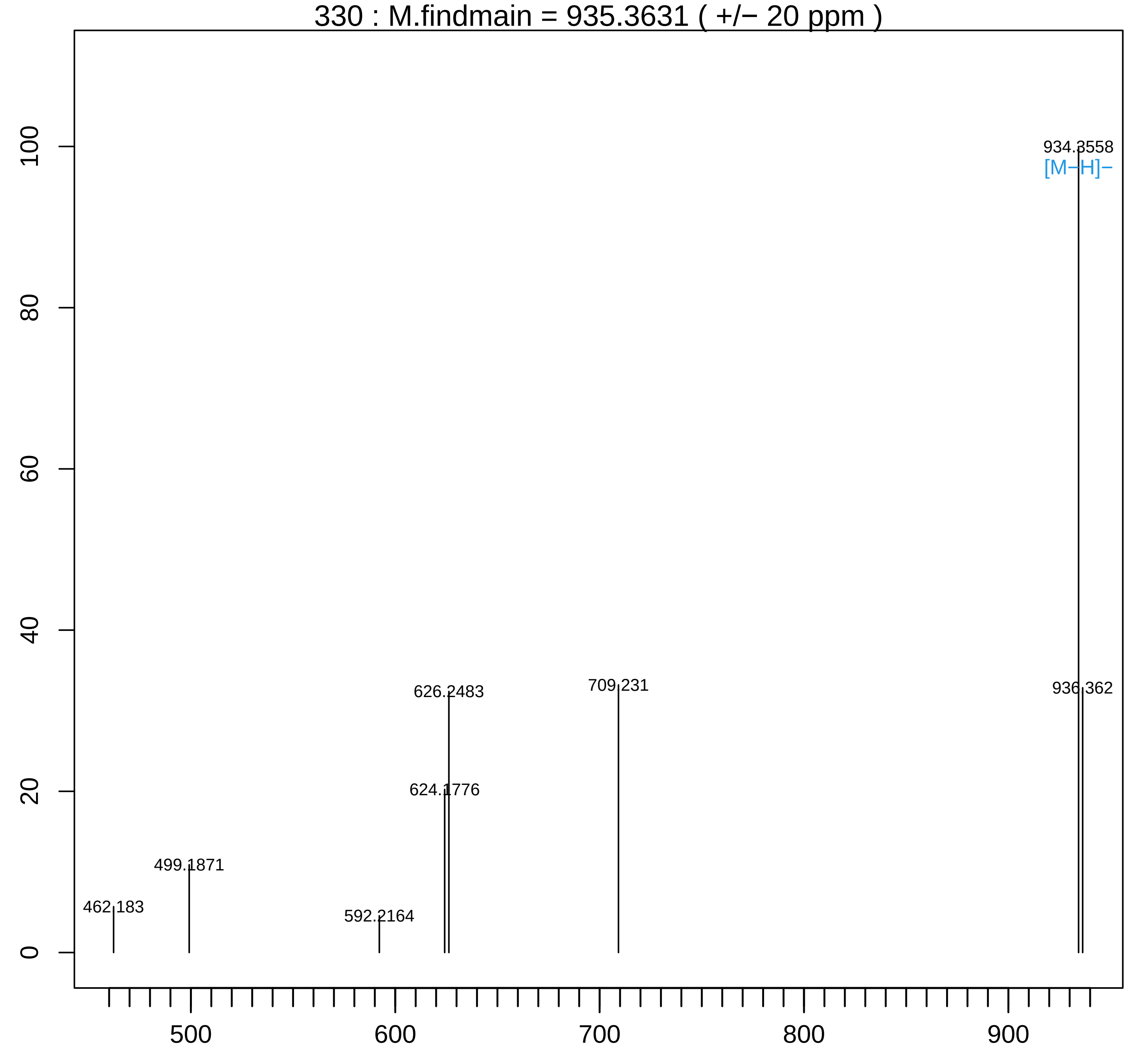
330 : M.findmain = 935.3631 (+/− 20 ppm)
934.3558
[M−H]−
936.362
709.231
626.2483
624.1776
499.1871
462.183
592.2164
0
20
40
60
80
100
500
600
700
800
900

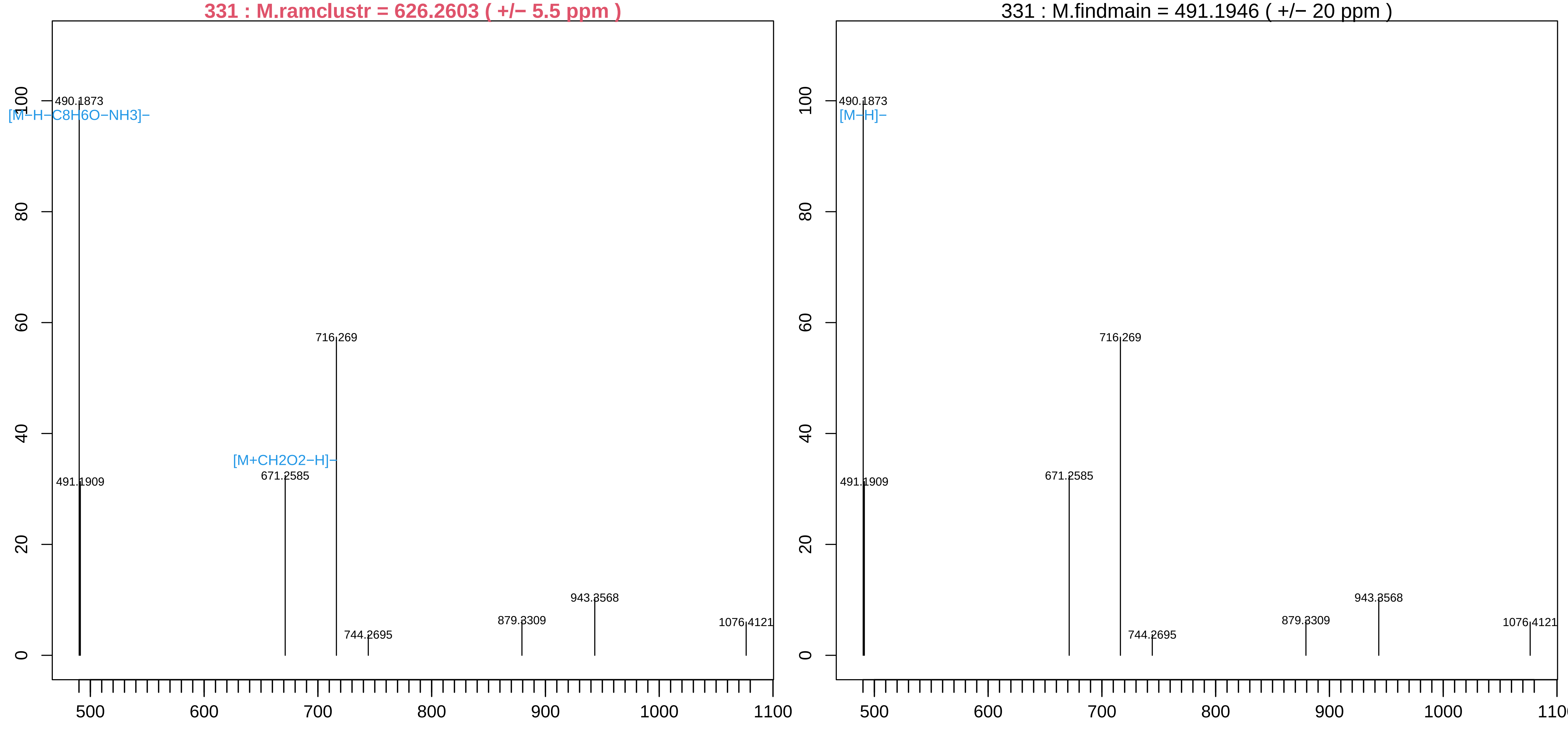
331 : M.ramclustr = 626.2603 (+/− 5.5 ppm)
490.1873
[M−H−C8H6O−NH3]−
491.1909
[M+CH2O2−H]−
671.2585
716.269
744.2695
879.3309
943.3568
1076.4121
0
20
40
60
80
100
500
600
700
800
900
1000
1100
331 : M.findmain = 491.1946 (+/− 20 ppm)
490.1873
[M−H]−
491.1909
671.2585
716.269
744.2695
879.3309
943.3568
1076.4121
0
20
40
60
80
100
500
600
700
800
900
1000
1100

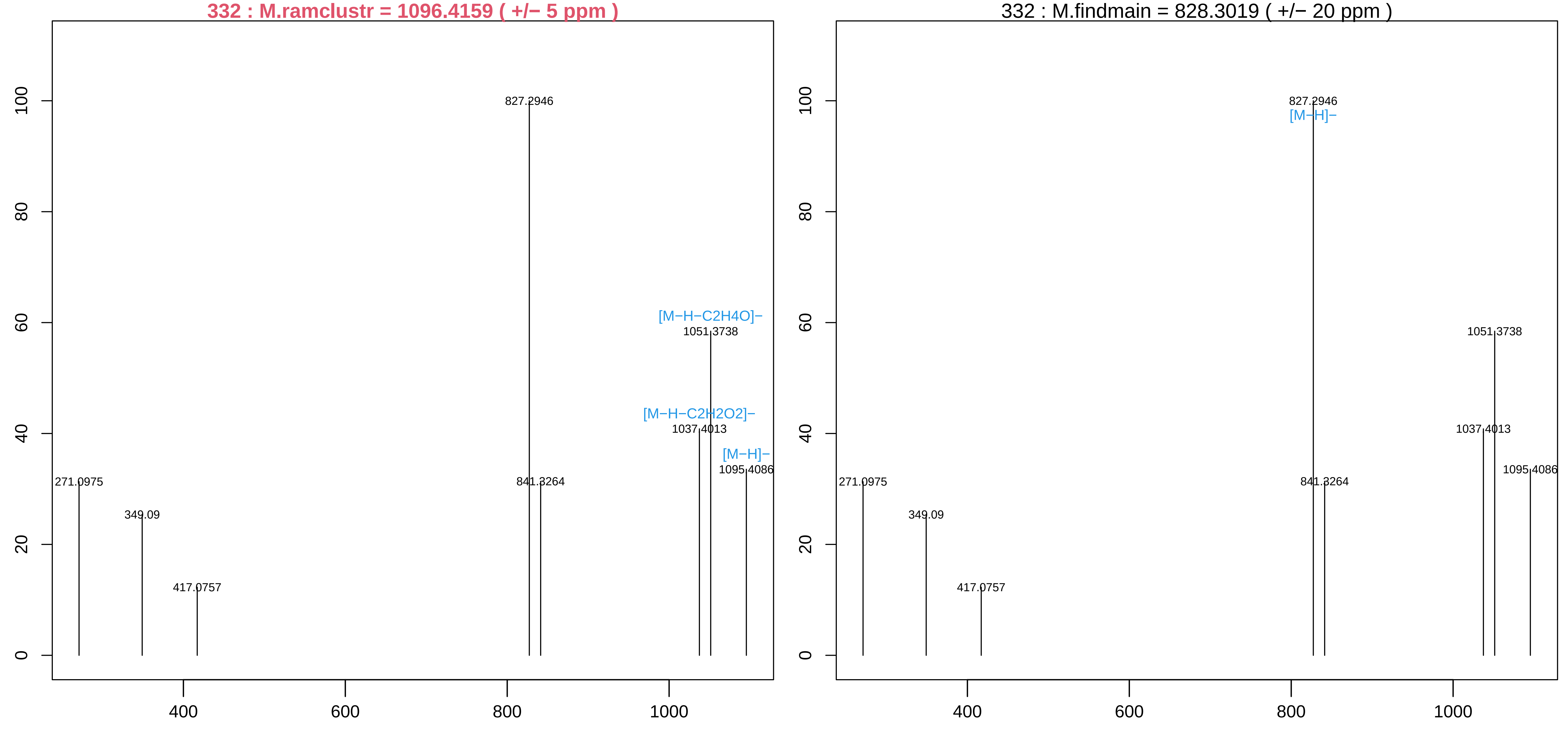
332 : M.ramclustr = 1096.4159 (+/− 5 ppm)
827.2946
[M−H−C2H4O]−
1051.3738
[M−H−C2H2O2]−
1037.4013
[M−H]−
1095.4086
271.0975
841.3264
349.09
417.0757
332 : M.findmain = 828.3019 (+/− 20 ppm)
827.2946
[M−H]−
1051.3738
1037.4013
1095.4086
271.0975
841.3264
349.09
417.0757
0
20
40
60
80
100
400
600
800
1000

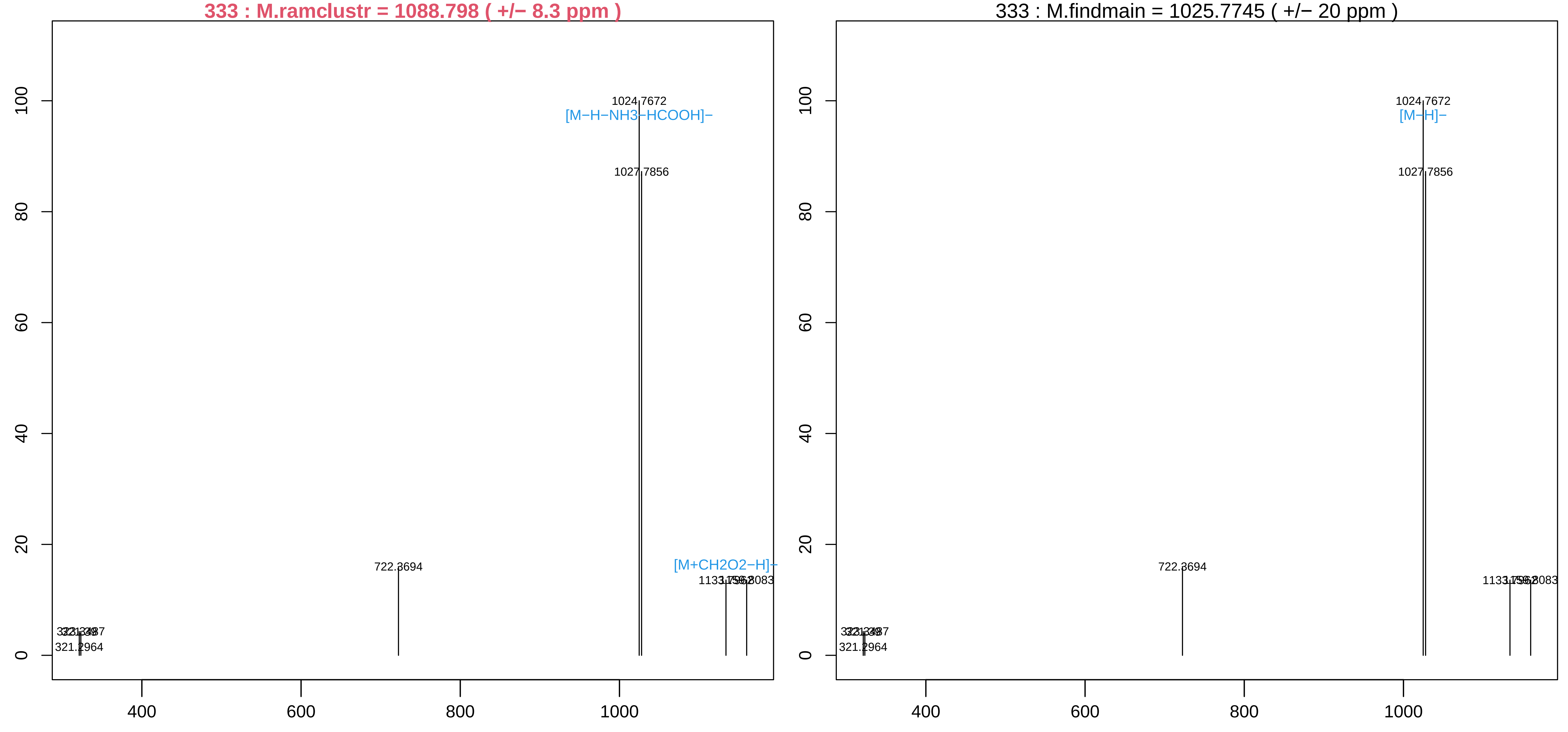

333 : M.ramclustr = 1088.798 (+/− 8.3 ppm)
1024.7672
[M−H−NH3−HCOOH]−
1027.7856
722.3694
[M+CH2O2−H]−
321.2964
333 : M.findmain = 1025.7745 (+/− 20 ppm)
1024.7672
[M−H]−
1027.7856
722.3694
321.2964

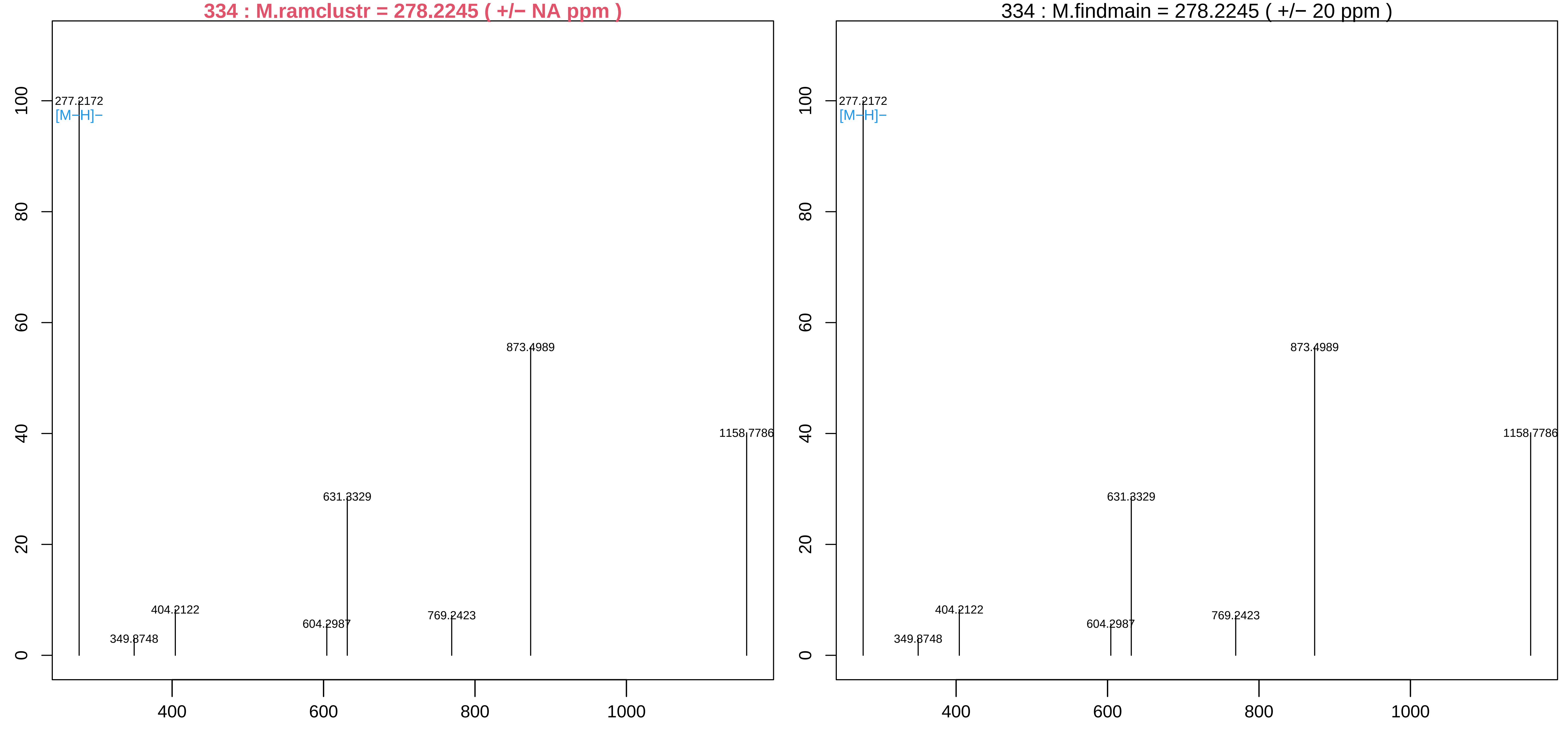

334 : M.ramclustr = 278.2245 (+/− NA ppm)
277.2172
[M−H]−
873.4989
1158.7786
631.3329
404.2122
604.2987
769.2423
349.8748
334 : M.findmain = 278.2245 (+/− 20 ppm)
277.2172
[M−H]−
873.4989
1158.7786
631.3329
404.2122
604.2987
769.2423
349.8748

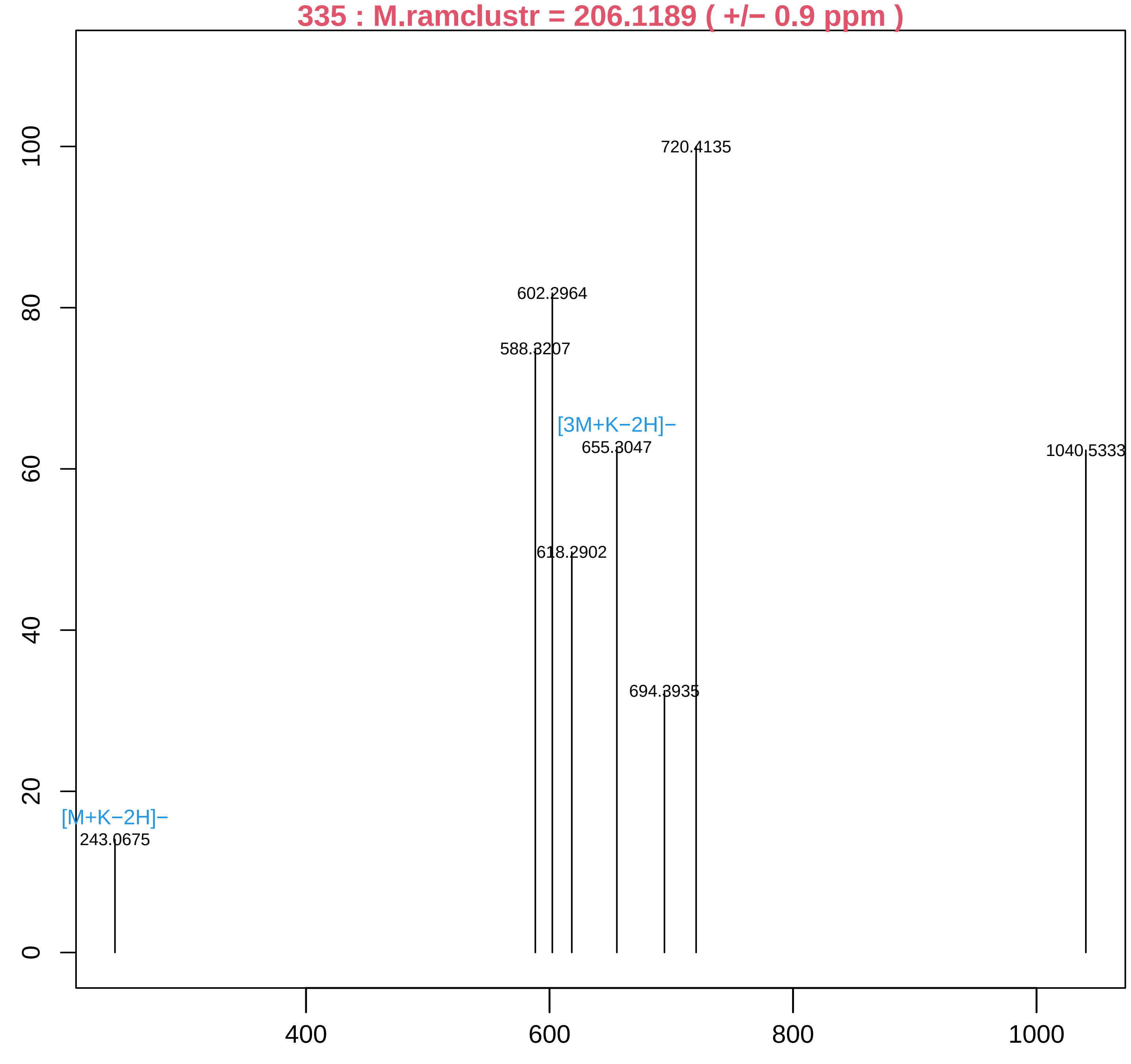
335 : M.ramclustr = 206.1189 (+/− 0.9 ppm)
720.4135
602.2964
588.3207
[3M+K−2H]−
655.3047
1040.5333
618.2902
694.3935
[M+K−2H]−
243.0675
0
20
40
60
80
100
400
600
800
1000

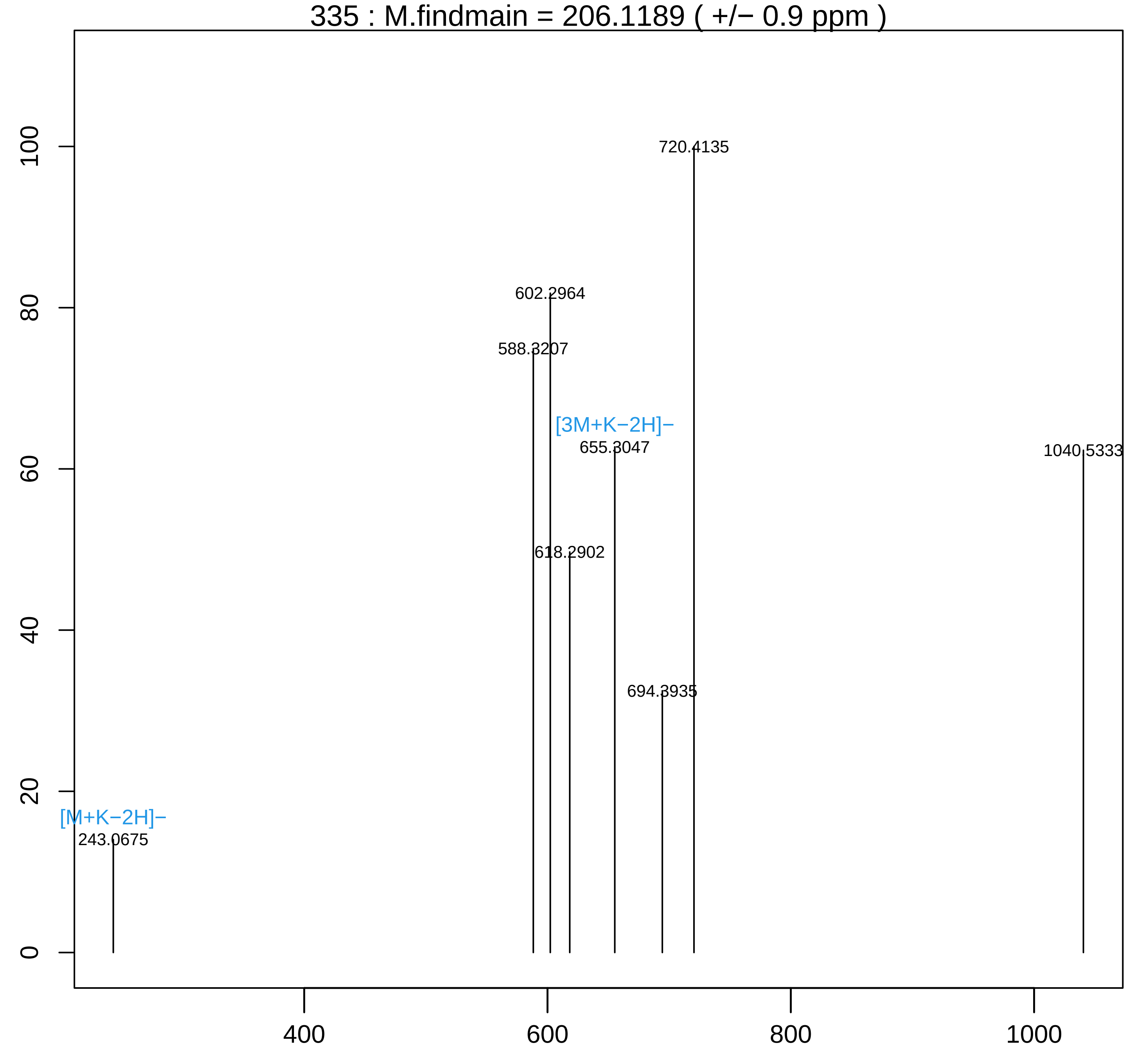
335 : M.findmain = 206.1189 (+/− 0.9 ppm)
720.4135
602.2964
588.3207
[3M+K−2H]−
655.3047
1040.5333
618.2902
694.3935
[M+K−2H]−
243.0675
0
20
40
60
80
100
400
600
800
1000

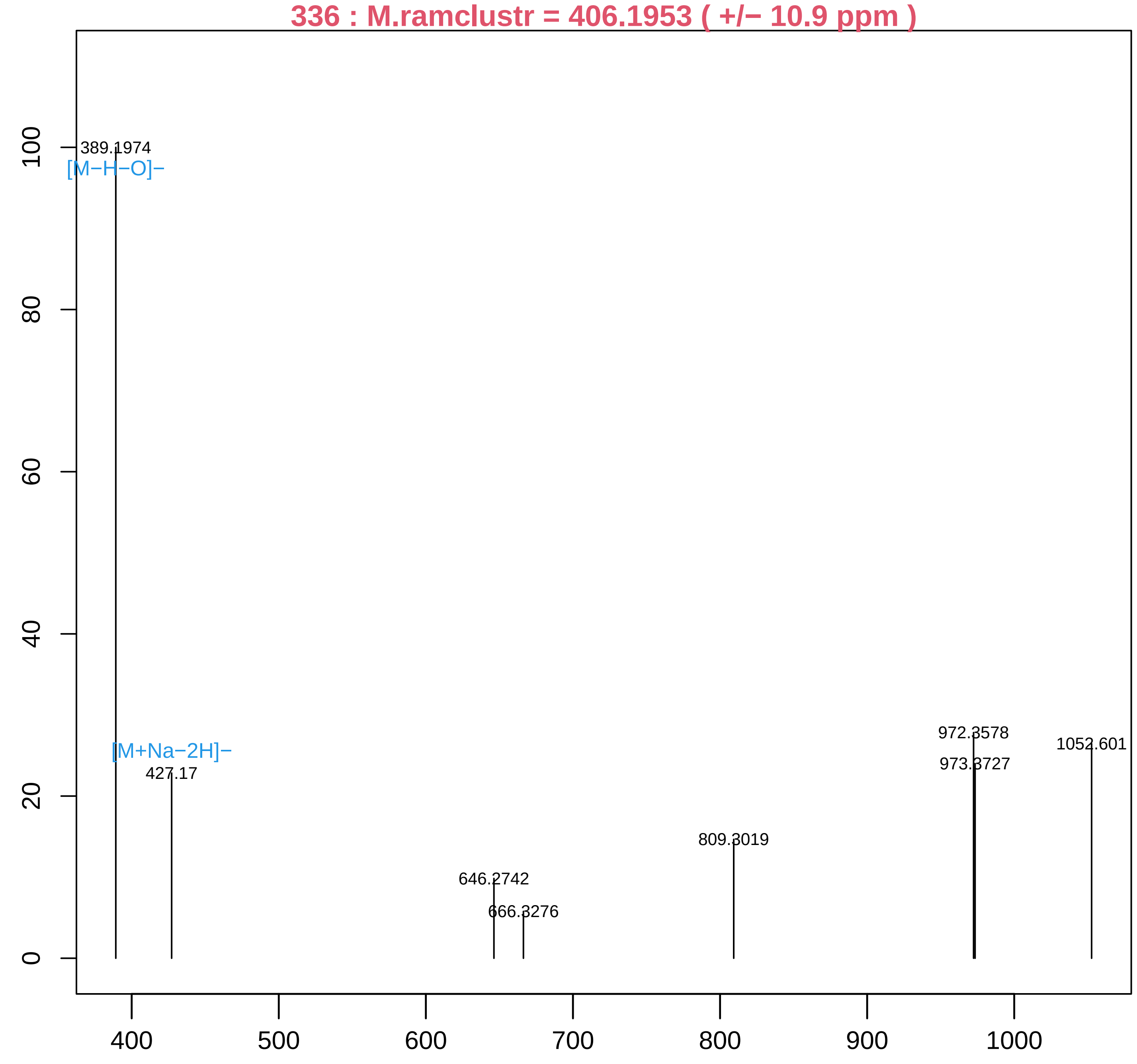
336 : M.ramclustr = 406.1953 (+/− 10.9 ppm)
389.1974
[M−H−O]−
[M+Na−2H]−
427.17
646.2742
666.3276
809.3019
972.3578
973.3727
1052.601
0
20
40
60
80
100
400
500
600
700
800
900
1000

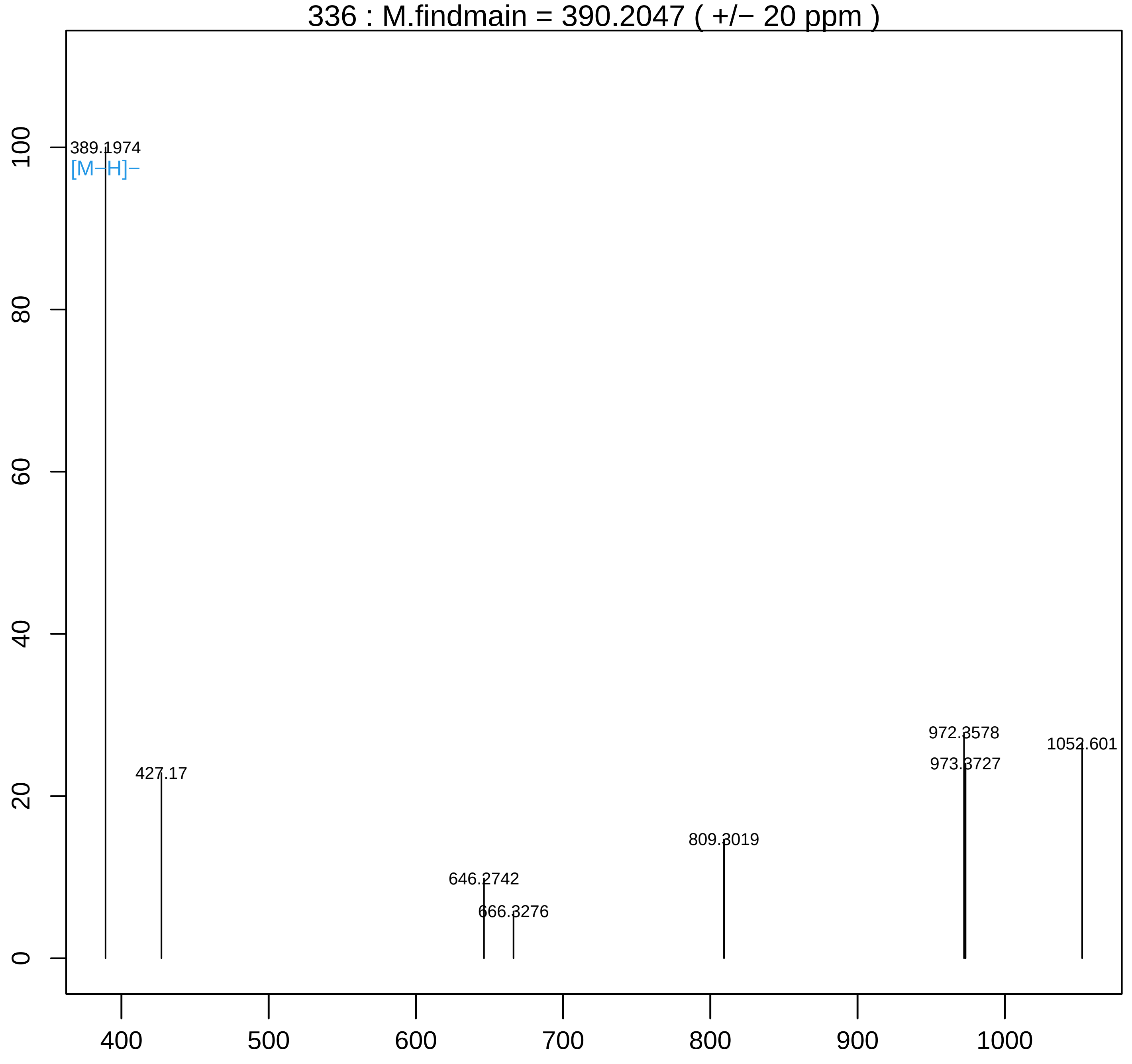
336 : M.findmain = 390.2047 (+/− 20 ppm)
389.1974
[M−H]−
427.17
646.2742
666.3276
809.3019
972.3578
973.3727
1052.601
0
20
40
60
80
100
400
500
600
700
800
900
1000

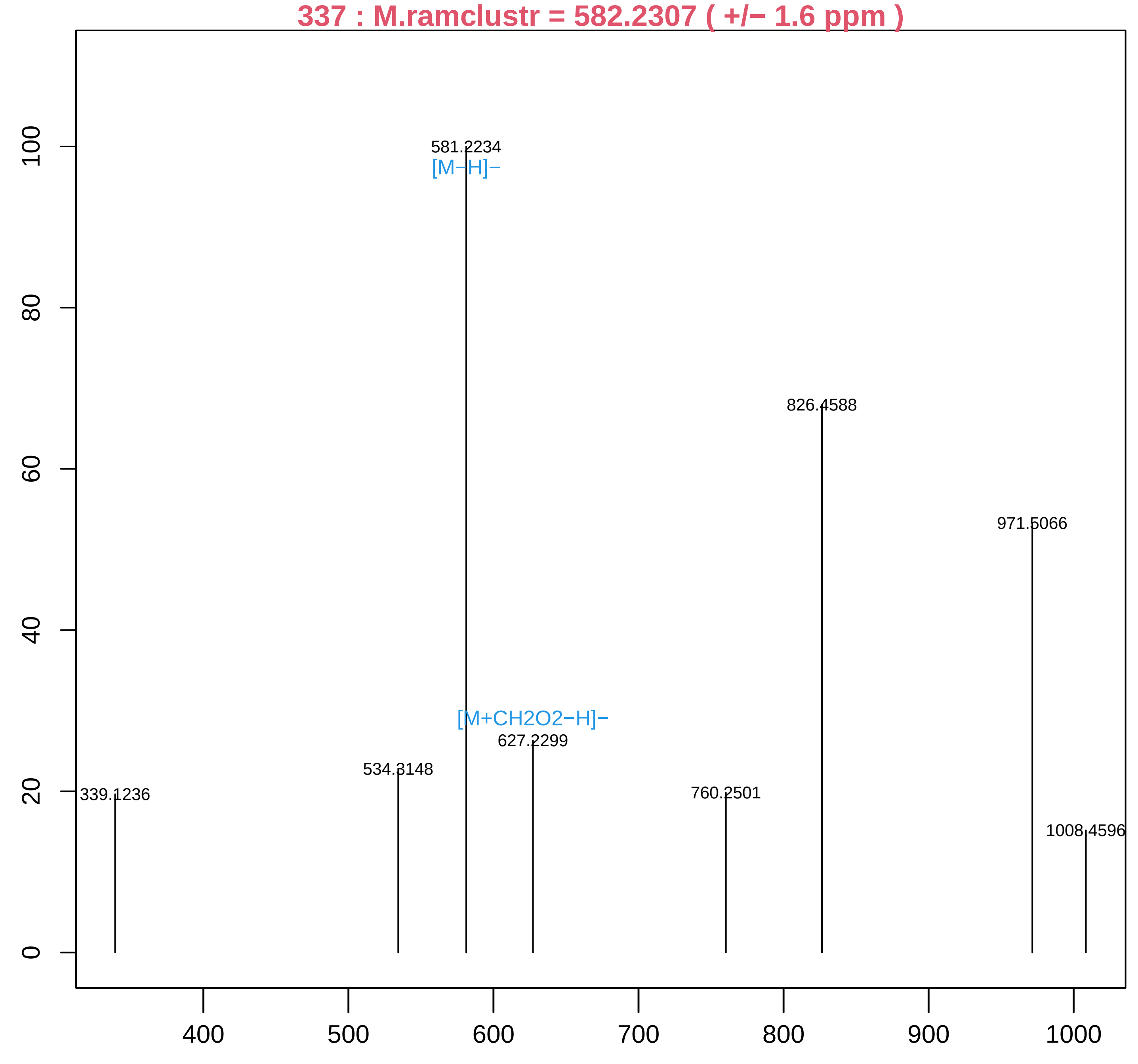
337 : M.ramclustr = 582.2307 (+/− 1.6 ppm)
581.2234
[M−H]−
826.4588
971.5066
[M+CH2O2−H]−
627.2299
534.3148
339.1236
760.2501
1008.4596
0
20
40
60
80
100
400
500
600
700
800
900
1000
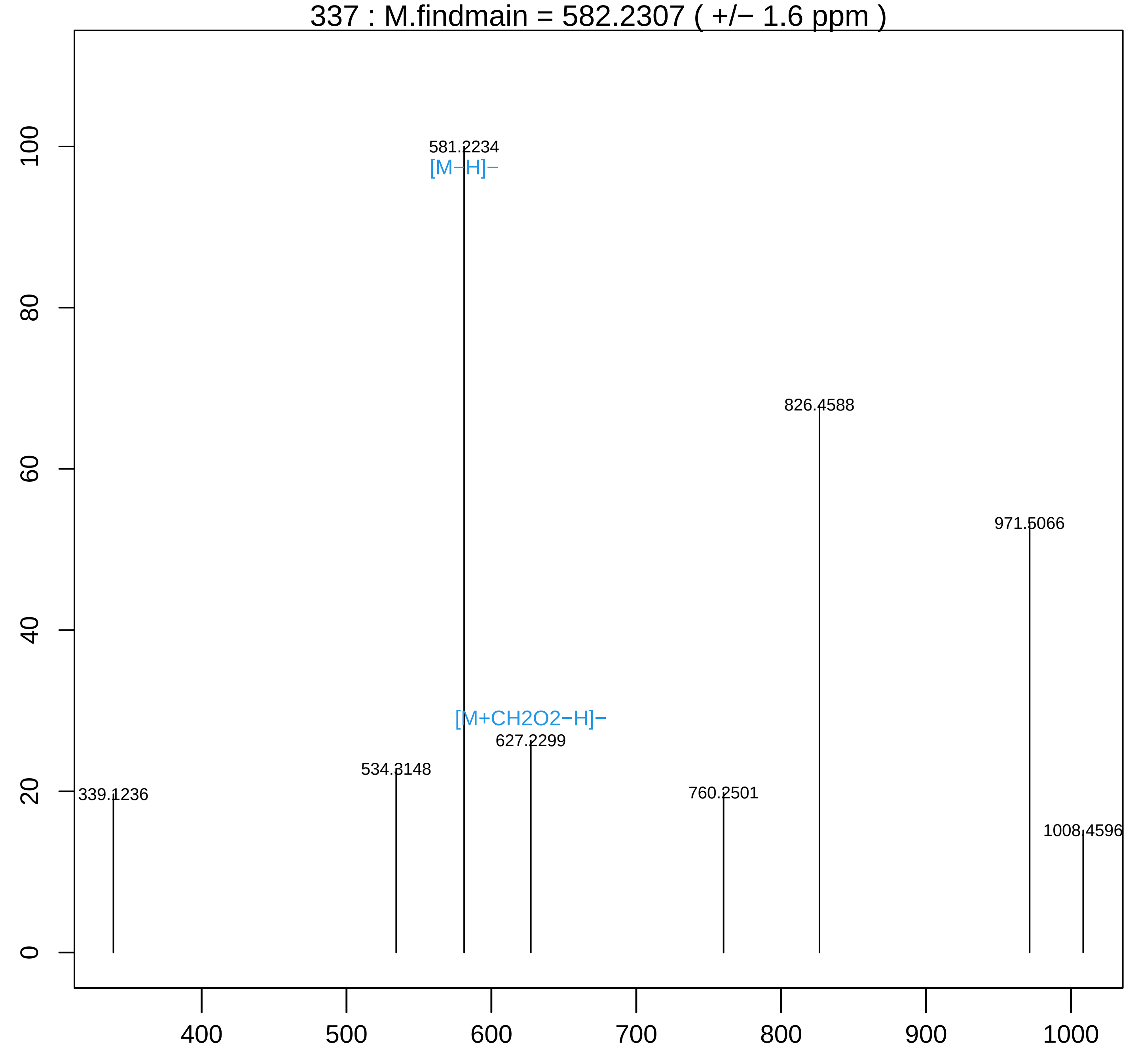
337 : M.findmain = 582.2307 (+/− 1.6 ppm)
581.2234
[M−H]−
826.4588
971.5066
[M+CH2O2−H]−
627.2299
534.3148
339.1236
760.2501
1008.4596
0
20
40
60
80
100
400
500
600
700
800
900
1000

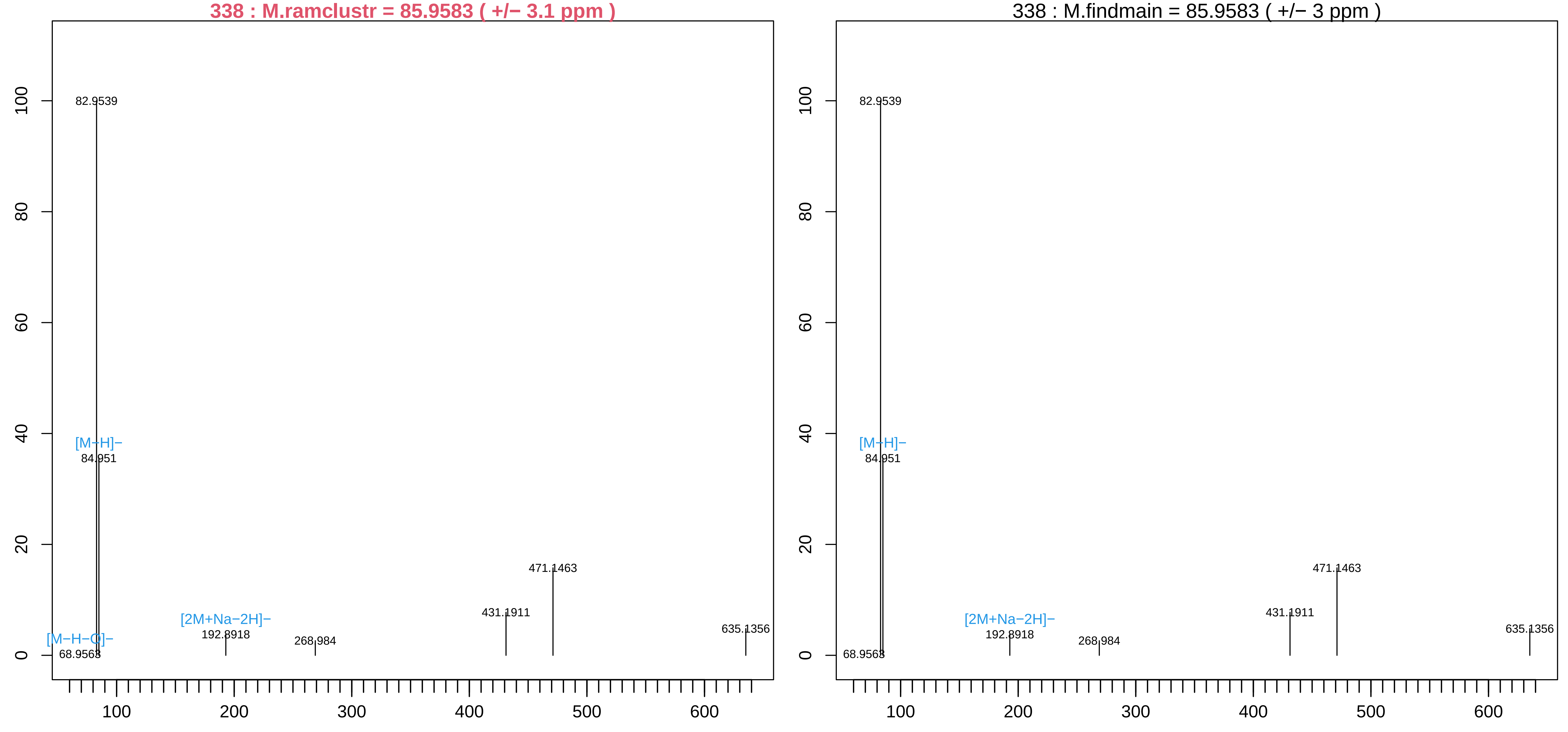

338 : M.ramclustr = 85.9583 (+/− 3.1 ppm)
82.9539
[M−H]−
84.951
[M−H−O]−
68.9563
[2M+Na−2H]−
192.8918
268.984
431.1911
471.1463
635.1356
338 : M.findmain = 85.9583 (+/− 3 ppm)
82.9539
[M−H]−
84.951
68.9563
[2M+Na−2H]−
192.8918
268.984
431.1911
471.1463
635.1356

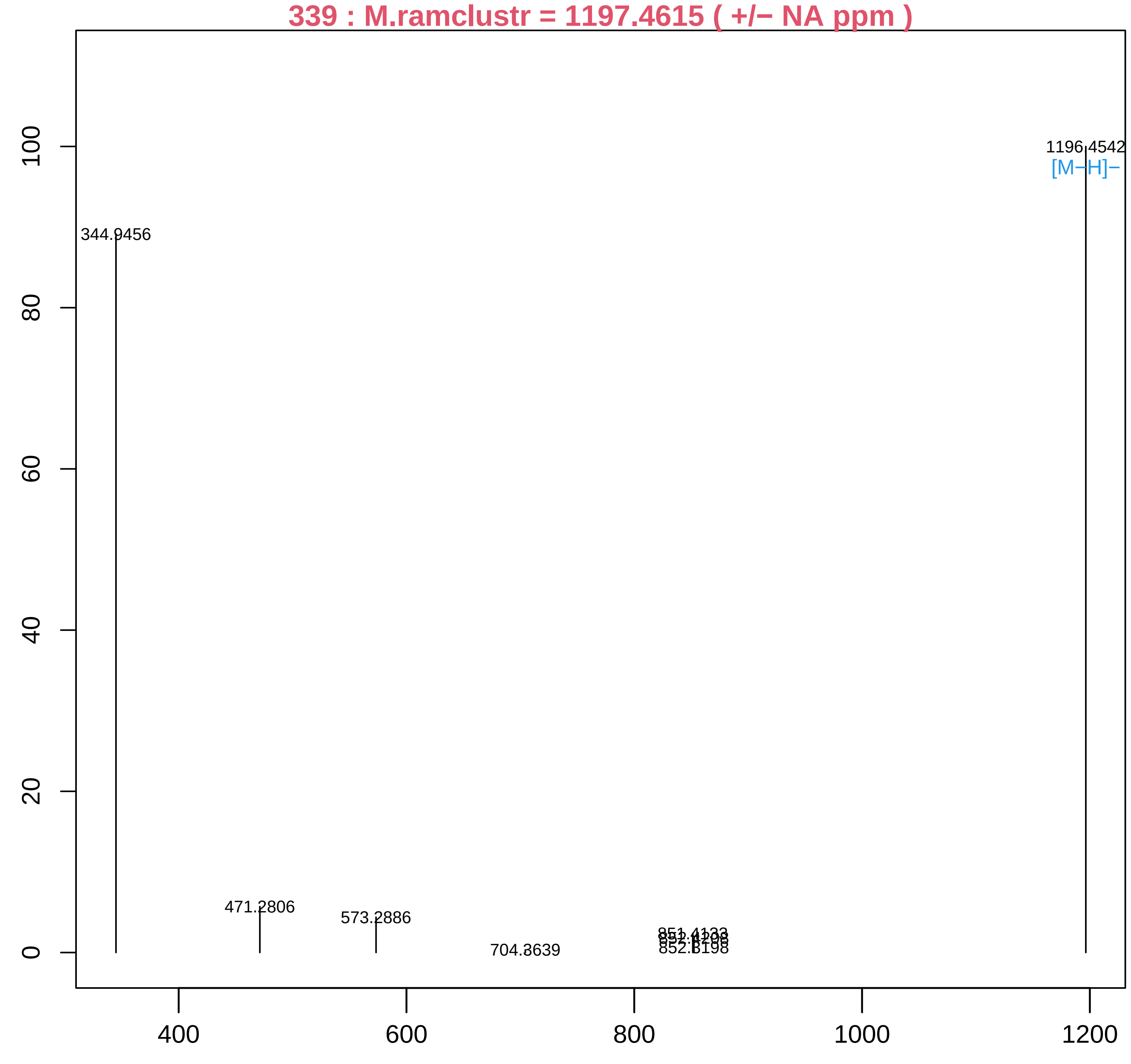
339 : M.ramclustr = 1197.4615 (+/− NA ppm)
1196.4542
[M−H]−
344.9456
471.2806
573.2886
704.3639
851.4133
852.4208
852.5198
0
20
40
60
80
100
400
600
800
1000
1200

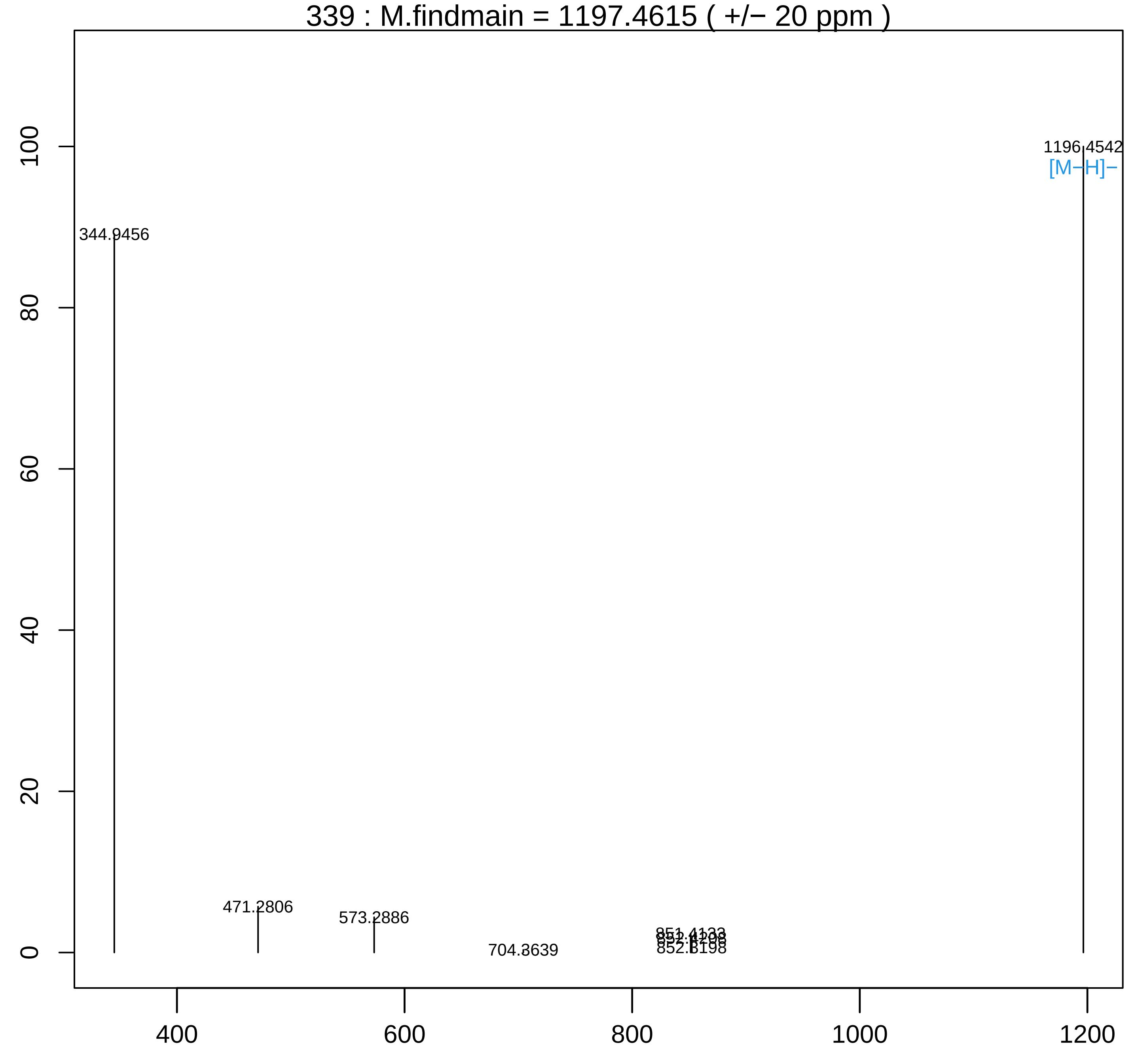
339 : M.findmain = 1197.4615 (+/− 20 ppm)
1196.4542
[M−H]−
344.9456
471.2806
573.2886
704.3639
851.4133
852.4208
852.5198
0
20
40
60
80
100
400
600
800
1000
1200

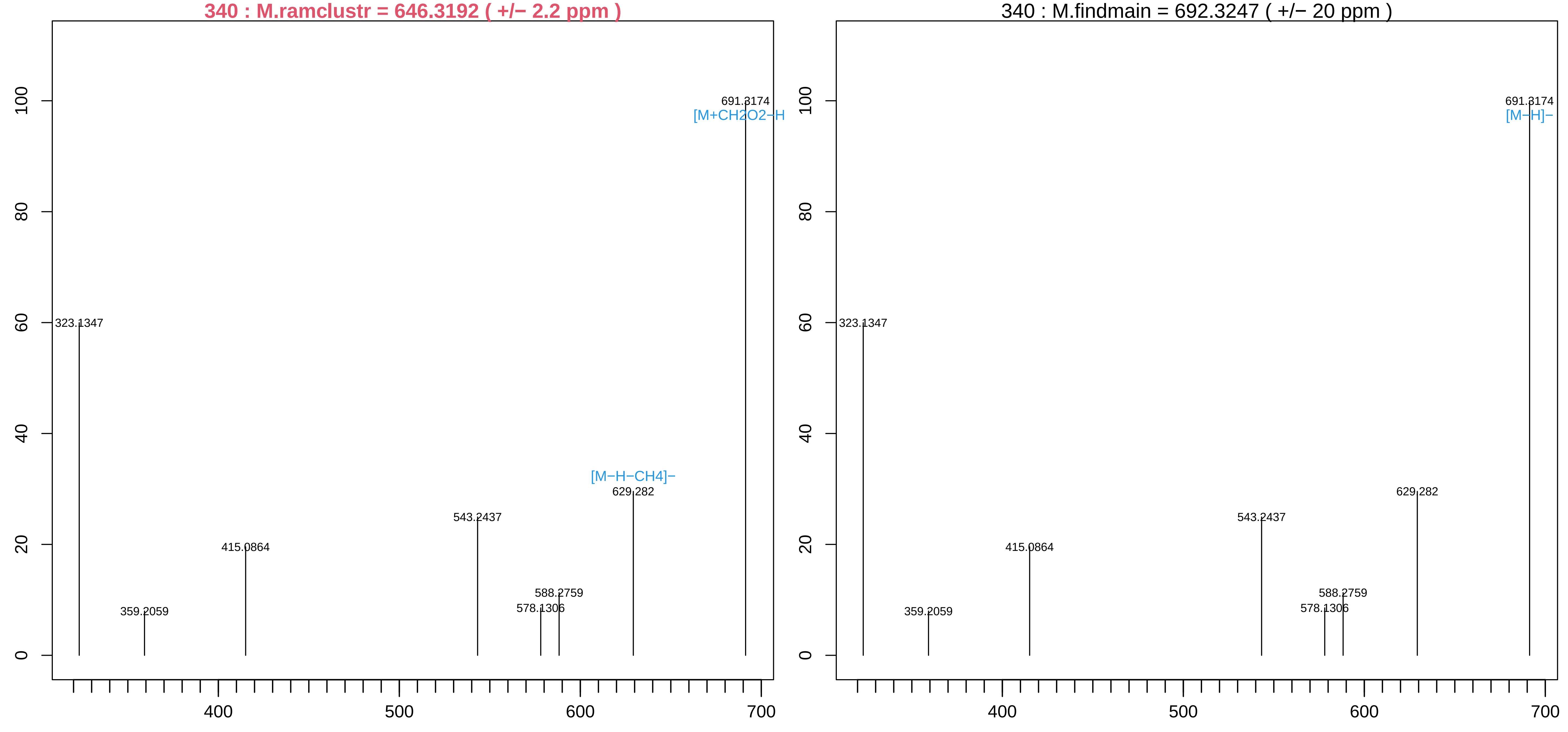
340 : M.ramclustr = 646.3192 (+/− 2.2 ppm)
691.3174
[M+CH2O2−H
323.1347
359.2059
415.0864
543.2437
[M−H−CH4]−
629.282
578.1306
588.2759
340 : M.findmain = 692.3247 (+/− 20 ppm)
691.3174
[M−H]−
0
20
40
60
80
100
400
500
600
700

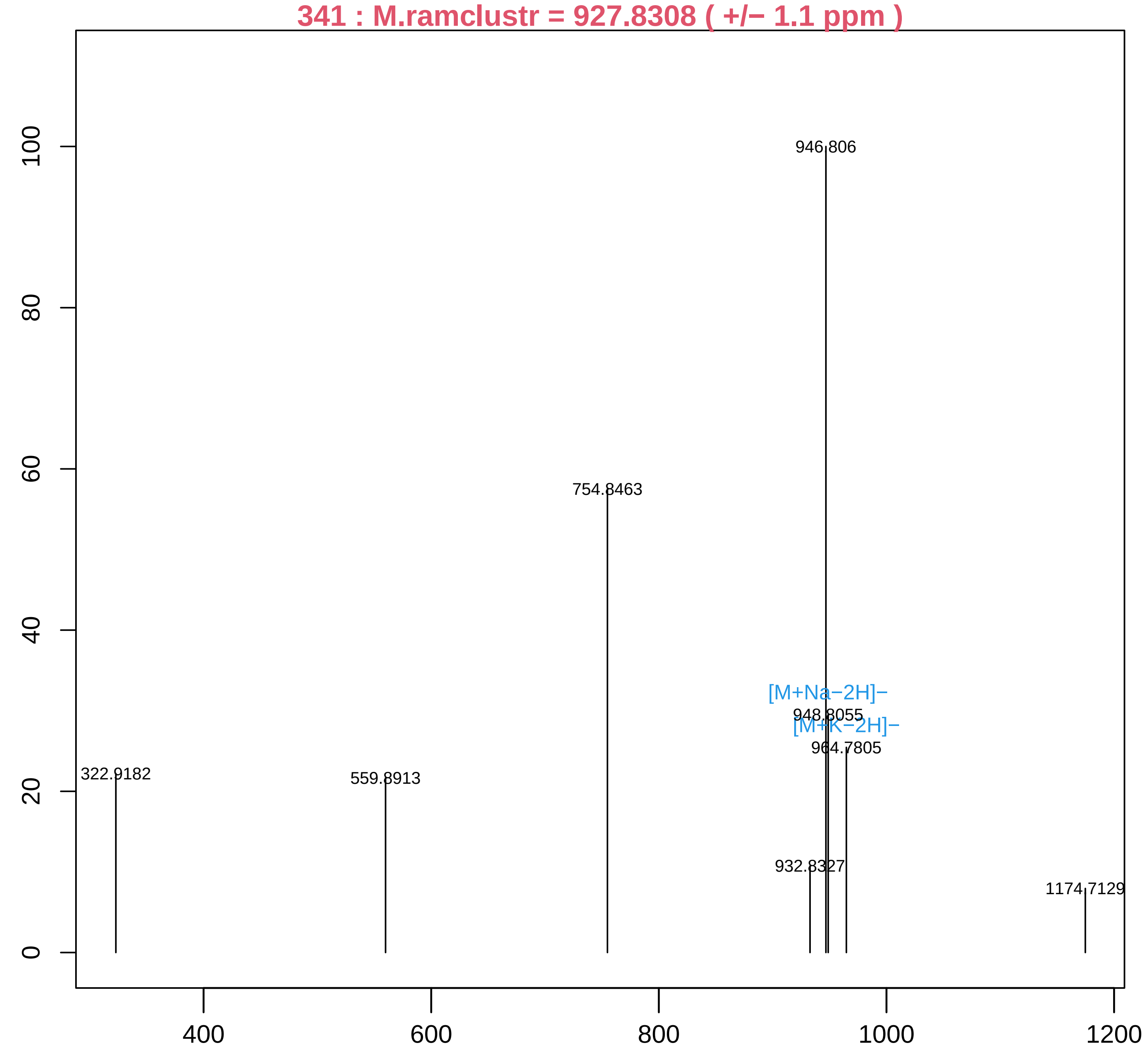
341 : M.ramclustr = 927.8308 (+/− 1.1 ppm)
946.806
754.8463
[M+Na−2H]−
948.8055
[M+K−2H]−
964.7805
322.9182
559.8913
932.8327
1174.7129
0
20
40
60
80
100
400
600
800
1000
1200

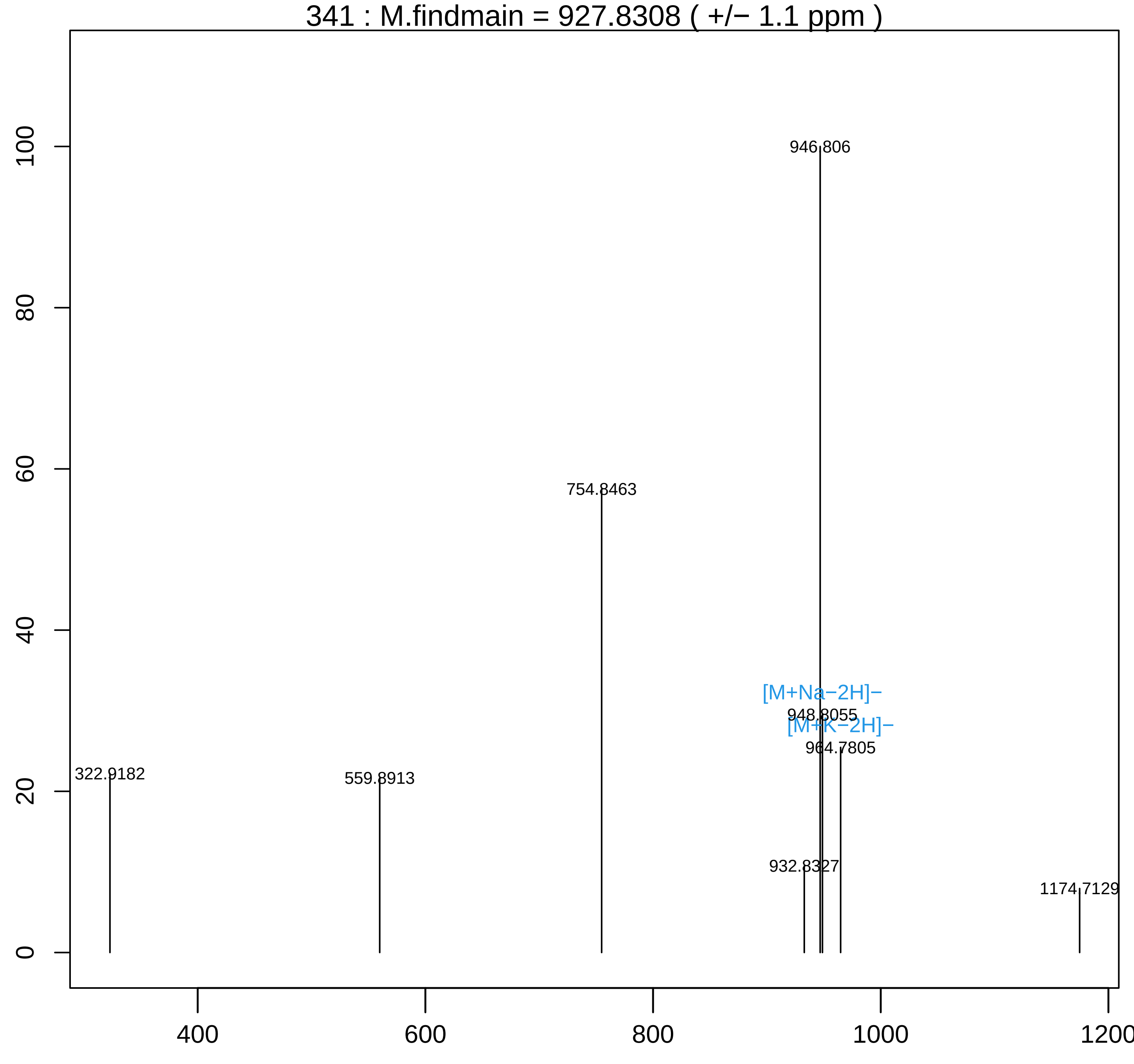
341 : M.findmain = 927.8308 (+/− 1.1 ppm)
946.806
754.8463
[M+Na−2H]−
948.8055
[M+K−2H]−
964.7805
322.9182
559.8913
932.8327
1174.7129
0
20
40
60
80
100
400
600
800
1000
1200

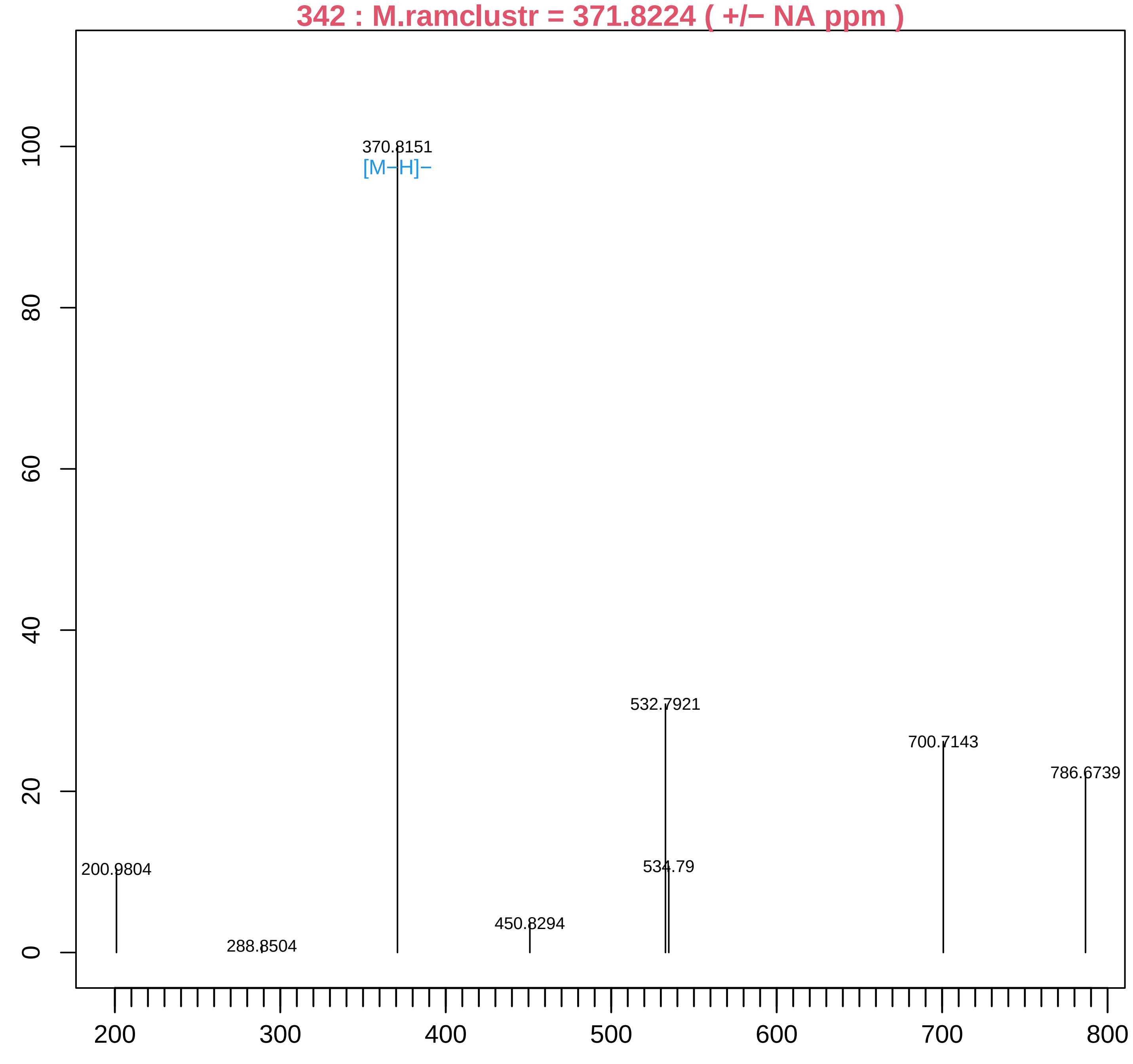
342 : M.ramclustr = 371.8224 (+/− NA ppm)
370.8151
[M−H]−
532.7921
700.7143
786.6739
200.9804
534.79
450.8294
288.8504
0
20
40
60
80
100
200
300
400
500
600
700
800

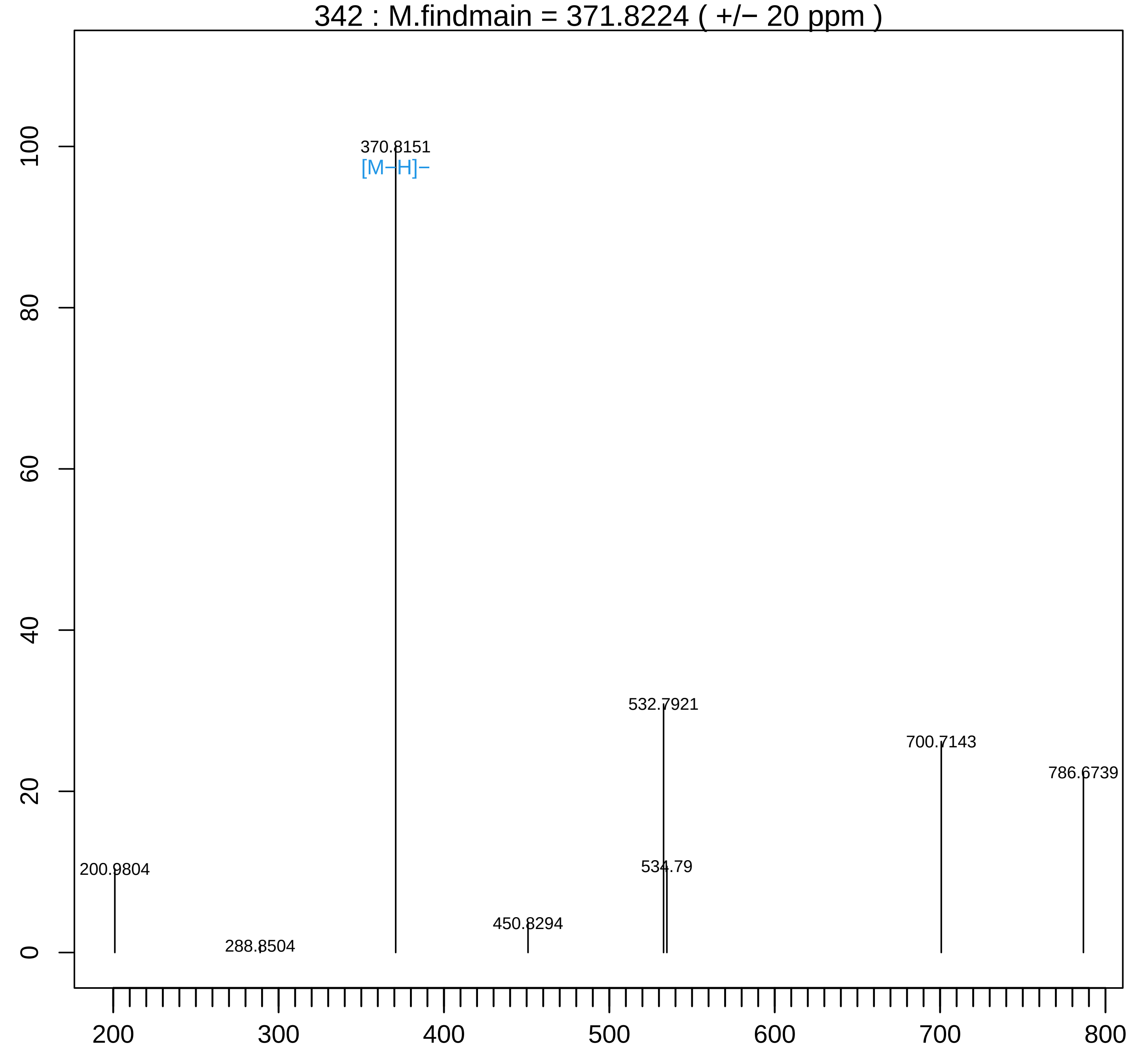
342 : M.findmain = 371.8224 (+/− 20 ppm)
370.8151
[M−H]−
532.7921
700.7143
786.6739
200.9804
534.79
450.8294
288.8504
0
20
40
60
80
100
200
300
400
500
600
700
800

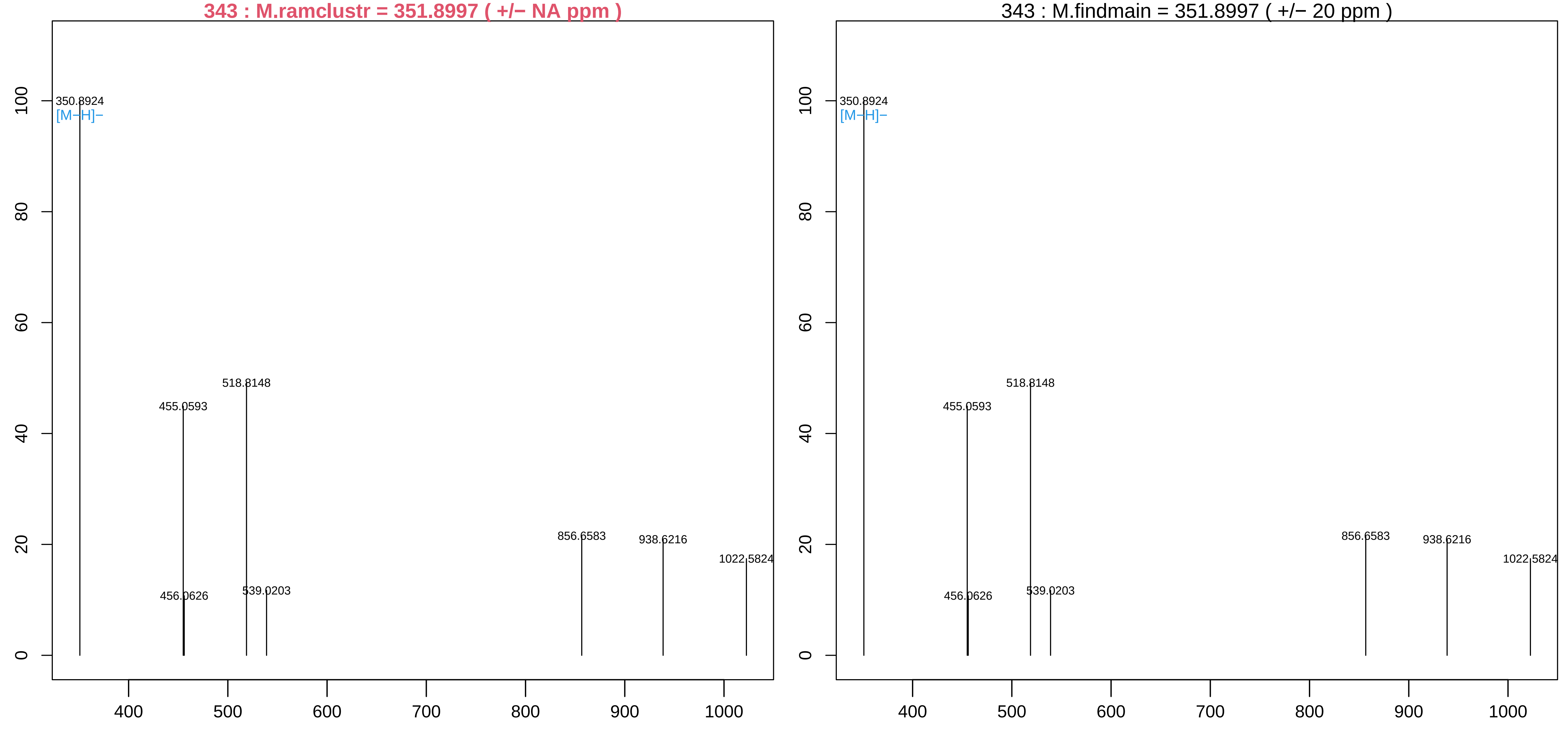
343 : M.ramclustr = 351.8997 (+/− NA ppm)
343 : M.findmain = 351.8997 (+/− 20 ppm)
350.8924
[M−H]−
455.0593
456.0626
518.8148
539.0203
856.6583
938.6216
1022.5824
0
20
40
60
80
100
400
500
600
700
800
900
1000

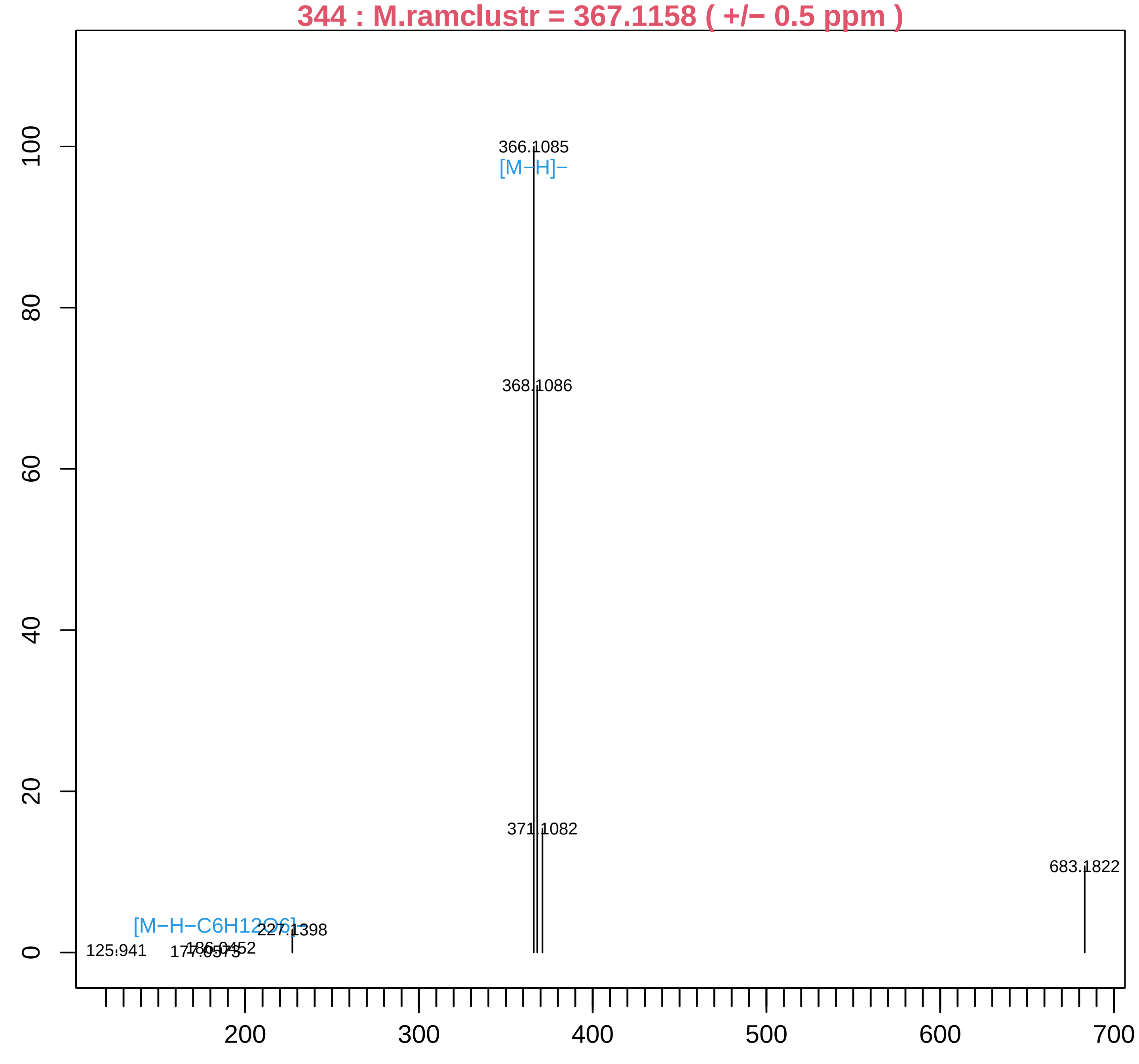
344 : M.ramclustr = 367.1158 (+/− 0.5 ppm)
366.1085
[M−H]−
368.1086
371.1082
683.1822
[M−H−C6H12O6]−
227.1398
125.941
177.0575
186.0452
0
20
40
60
80
100
200
300
400
500
600
700

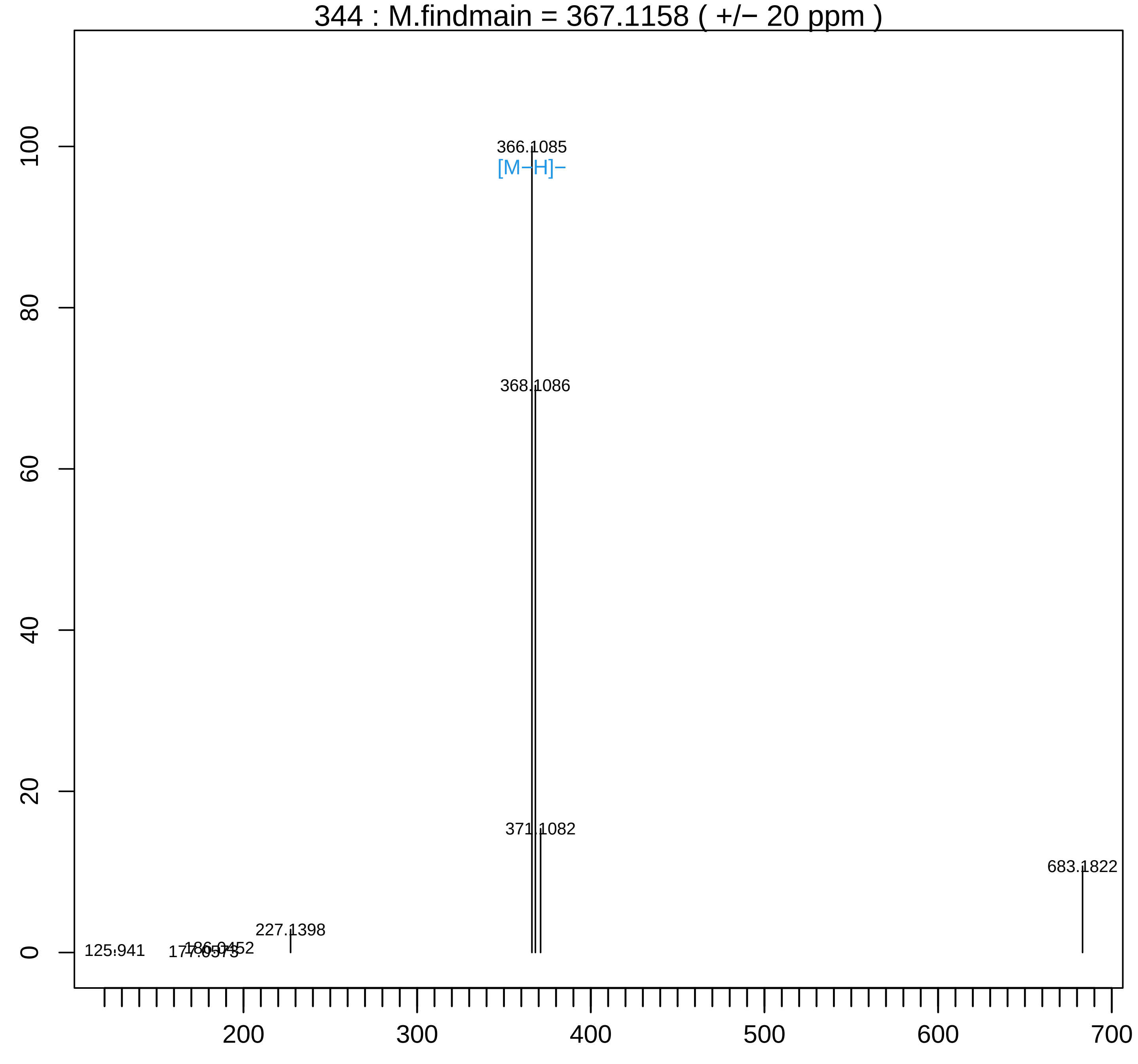
344 : M.findmain = 367.1158 (+/− 20 ppm)
366.1085
[M−H]−
368.1086
371.1082
683.1822
227.1398
125.941
177.0575
186.0452
0
20
40
60
80
100
200
300
400
500
600
700

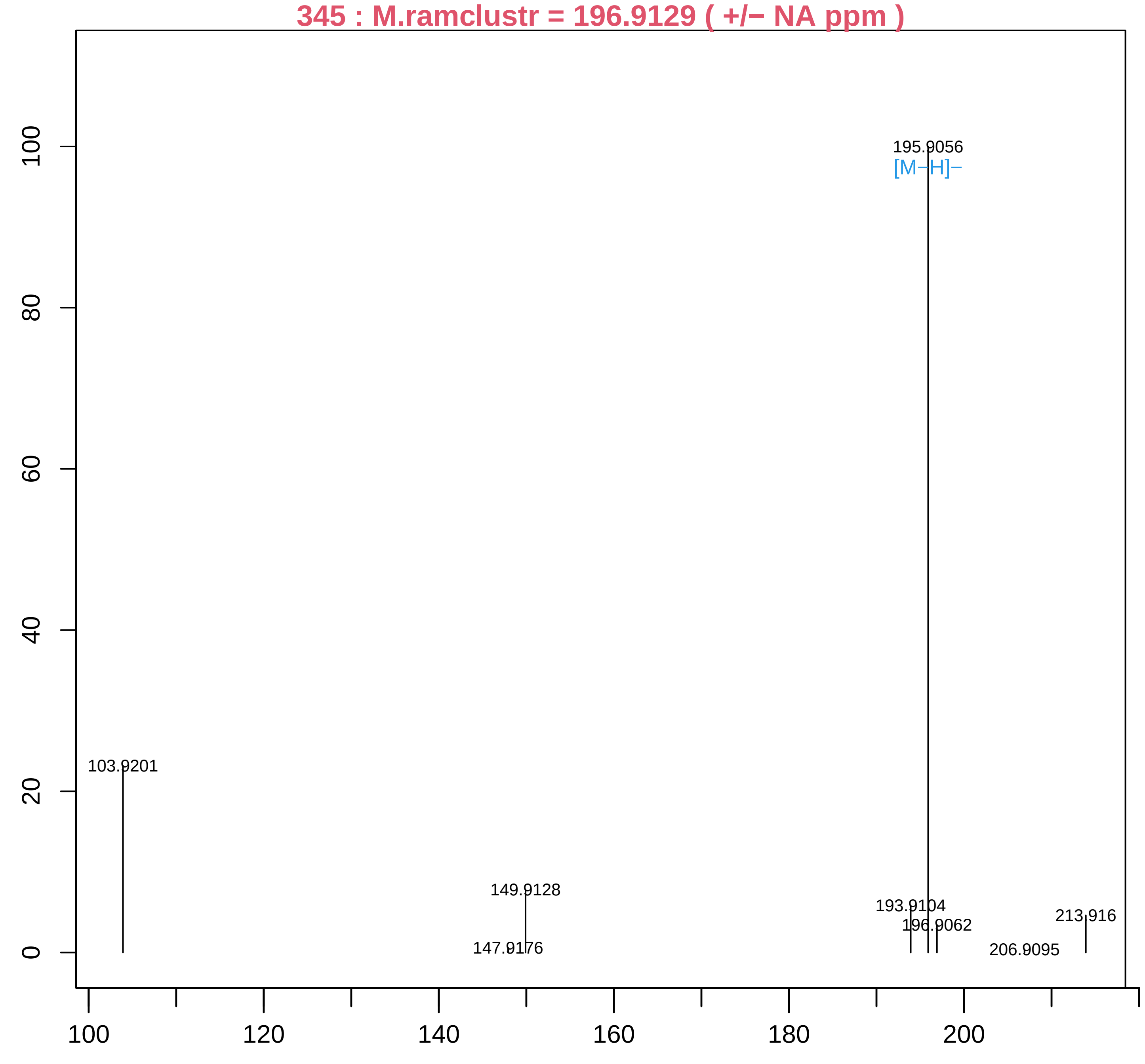
345 : M.ramclustr = 196.9129 (+/− NA ppm)
195.9056
[M−H]−
103.9201
149.9128
193.9104
196.9062
213.916
147.9176
206.9095
0
20
40
60
80
100
120
140
160
180
200

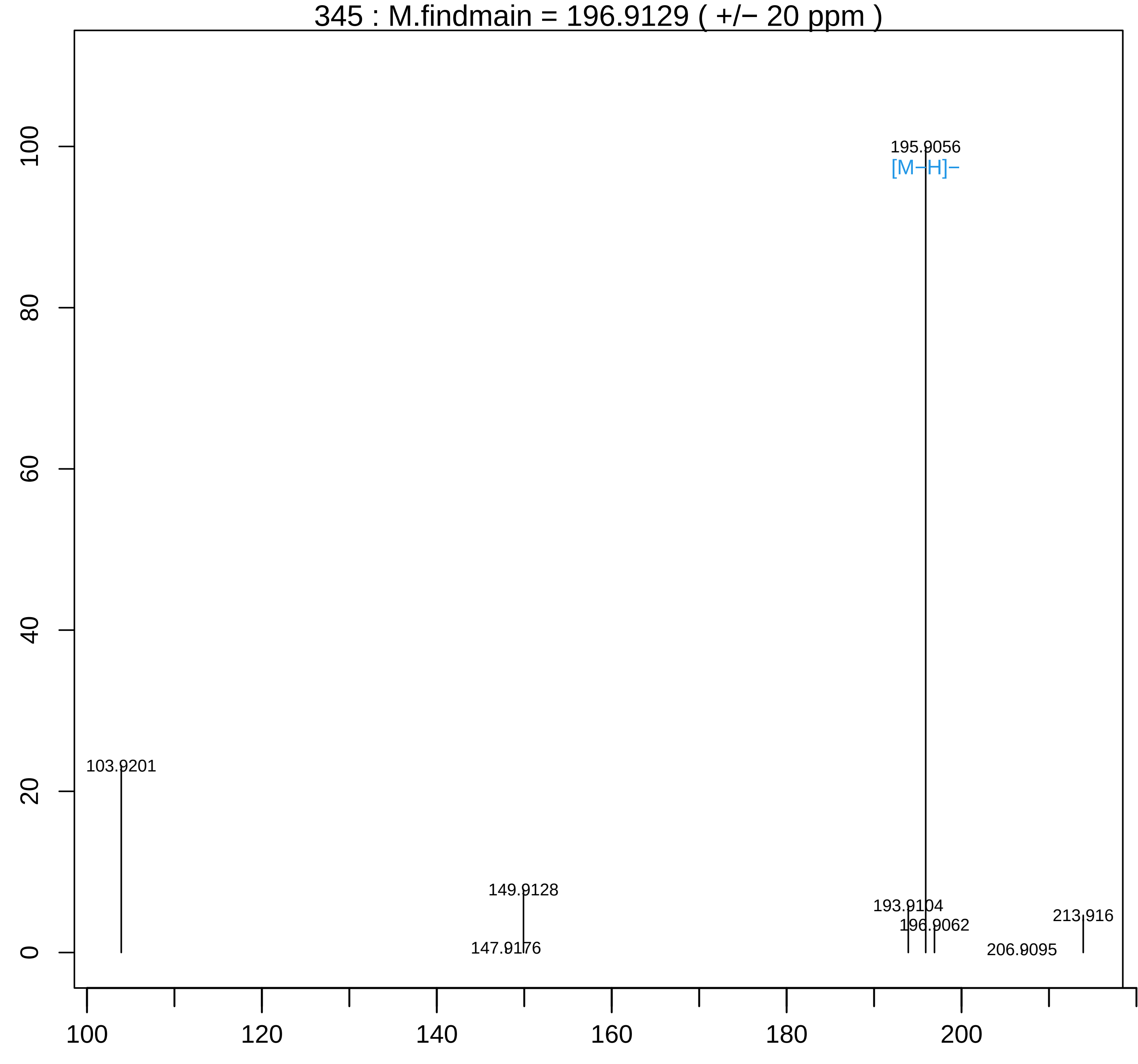
345 : M.findmain = 196.9129 (+/− 20 ppm)
195.9056
[M−H]−
103.9201
149.9128
193.9104
196.9062
213.916
147.9176
206.9095
0
20
40
60
80
100
120
140
160
180
200

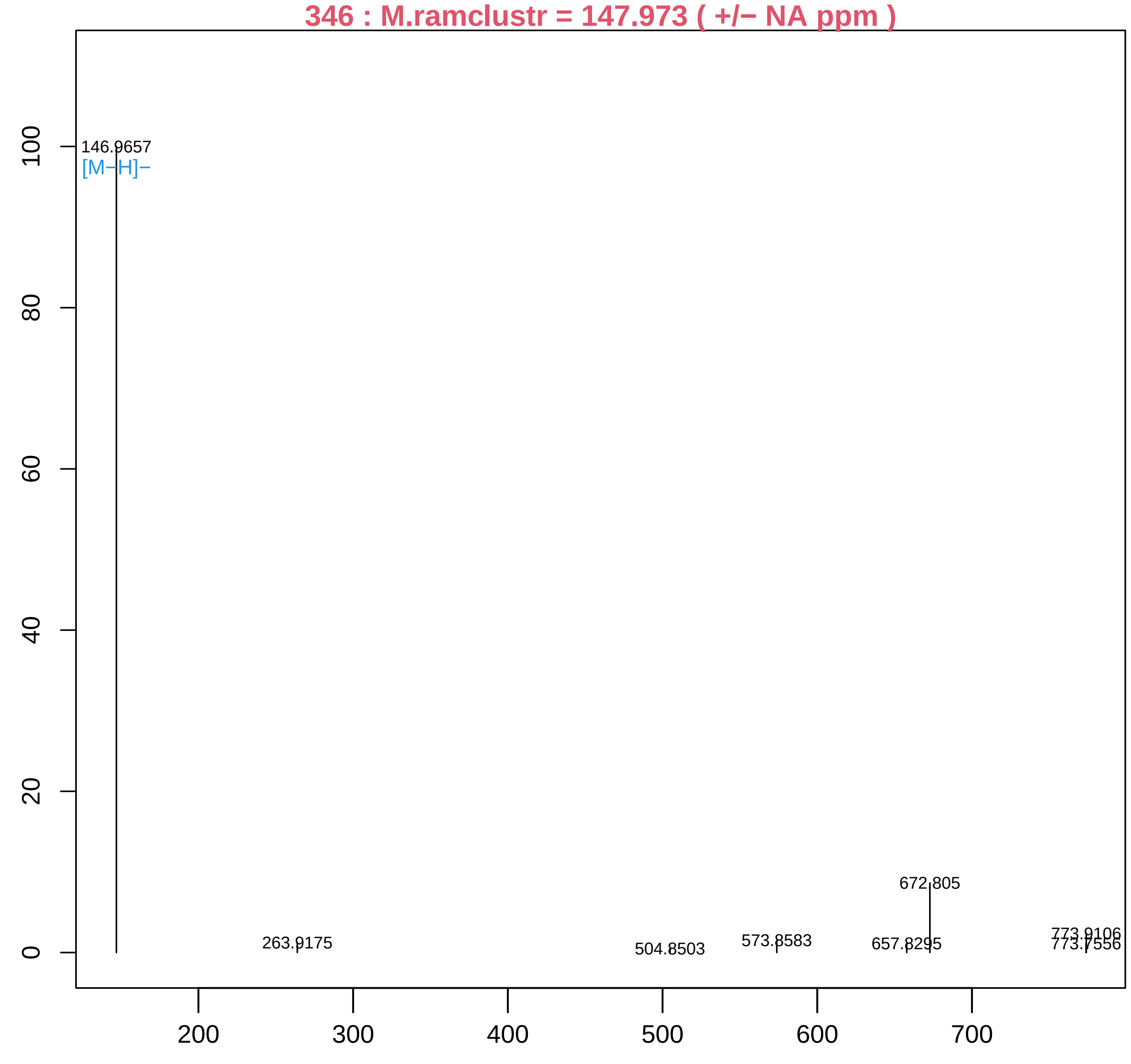
346 : M.ramclustr = 147.973 (+/− NA ppm)
146.9657
[M−H]−
263.9175
504.8503
573.8583
657.8295
672.805
773.9106
773.7556
0
20
40
60
80
100
200
300
400
500
600
700

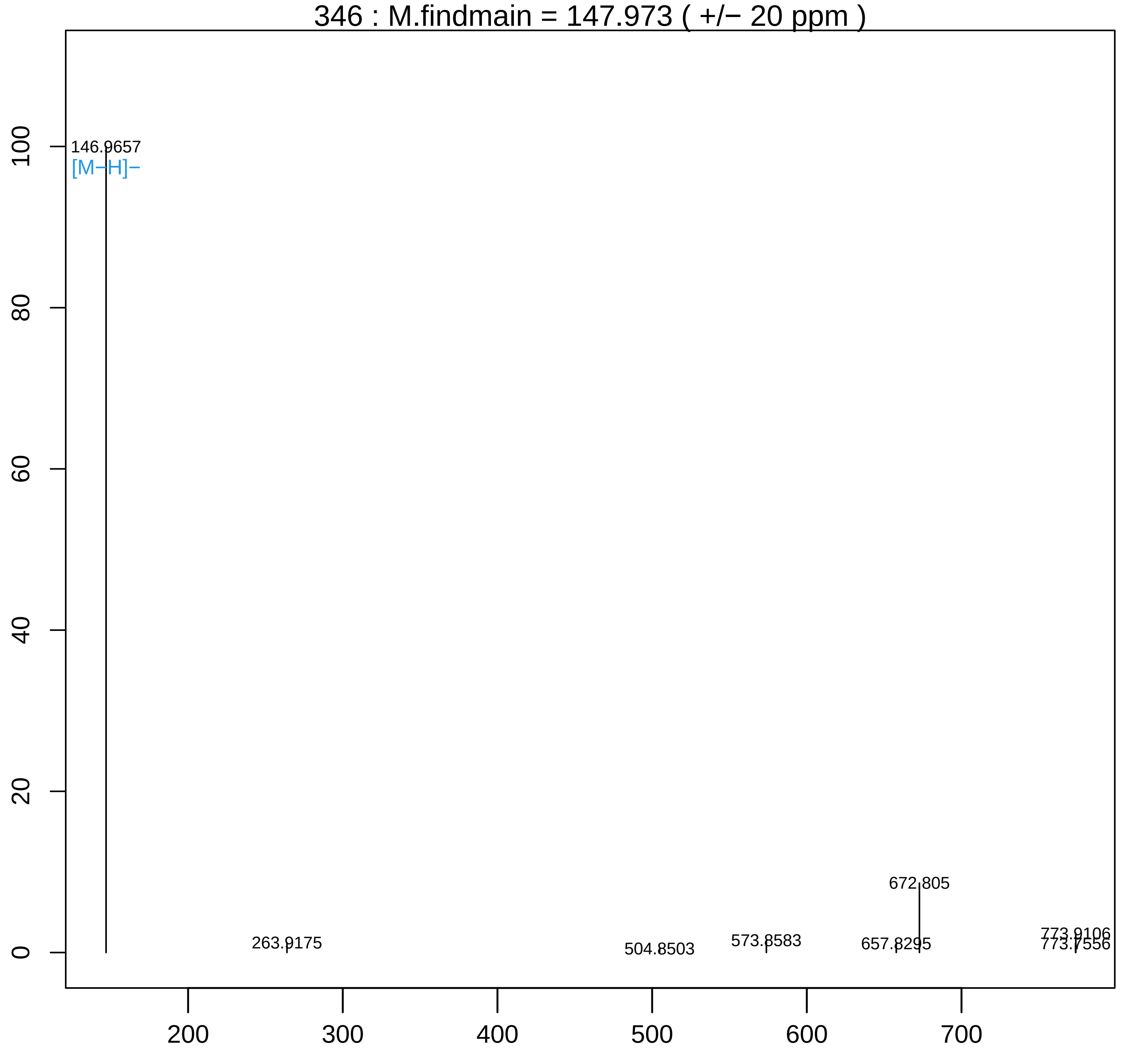
346 : M.findmain = 147.973 (+/− 20 ppm)
146.9657
[M−H]−
263.9175
504.8503
573.8583
657.8295
672.805
773.9106
773.7556
0
20
40
60
80
100
200
300
400
500
600
700

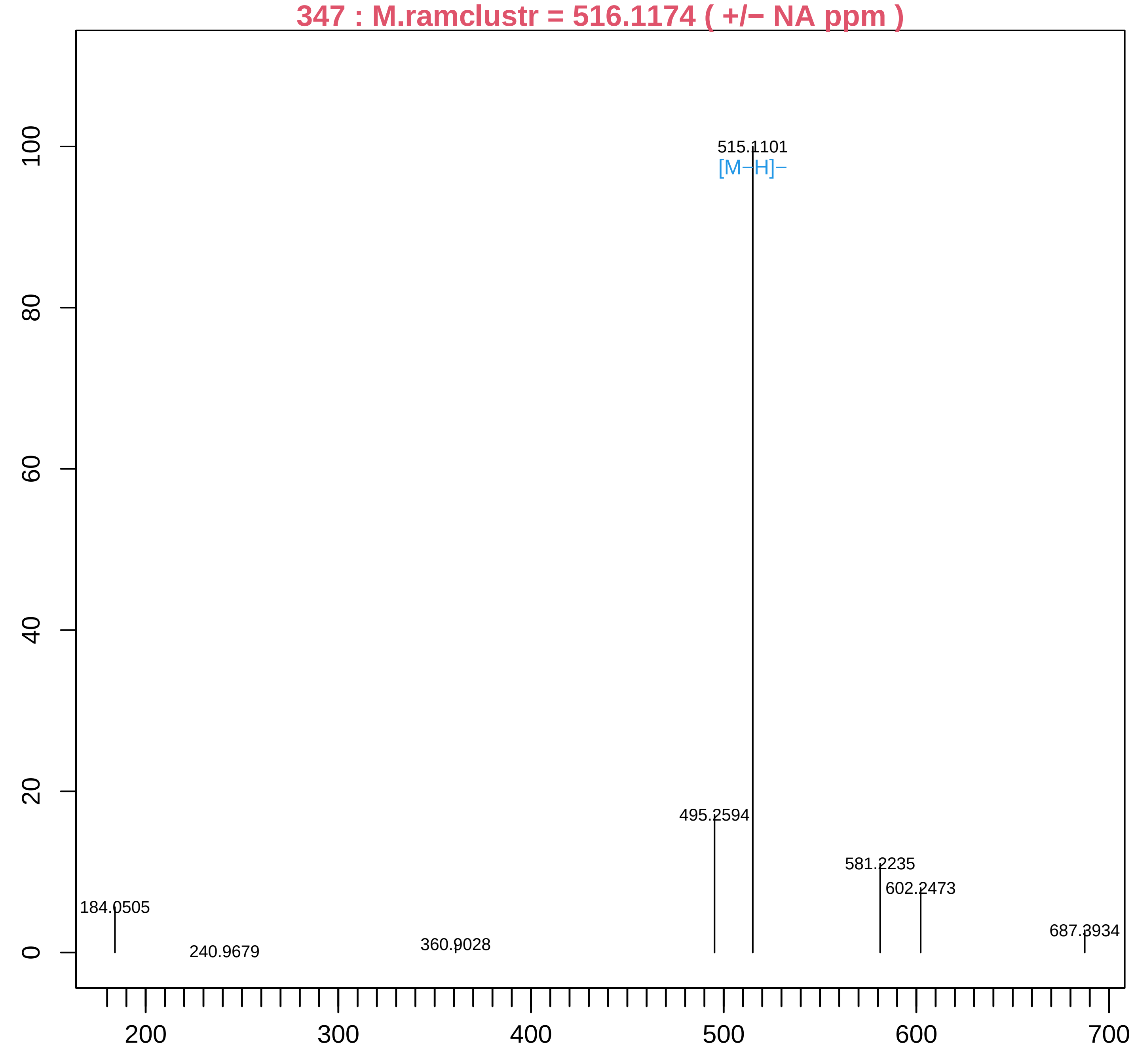
347 : M.ramclustr = 516.1174 (+/− NA ppm)
515.1101
[M−H]−
495.2594
581.2235
602.2473
184.0505
687.3934
360.9028
240.9679
0
20
40
60
80
100
200
300
400
500
600
700

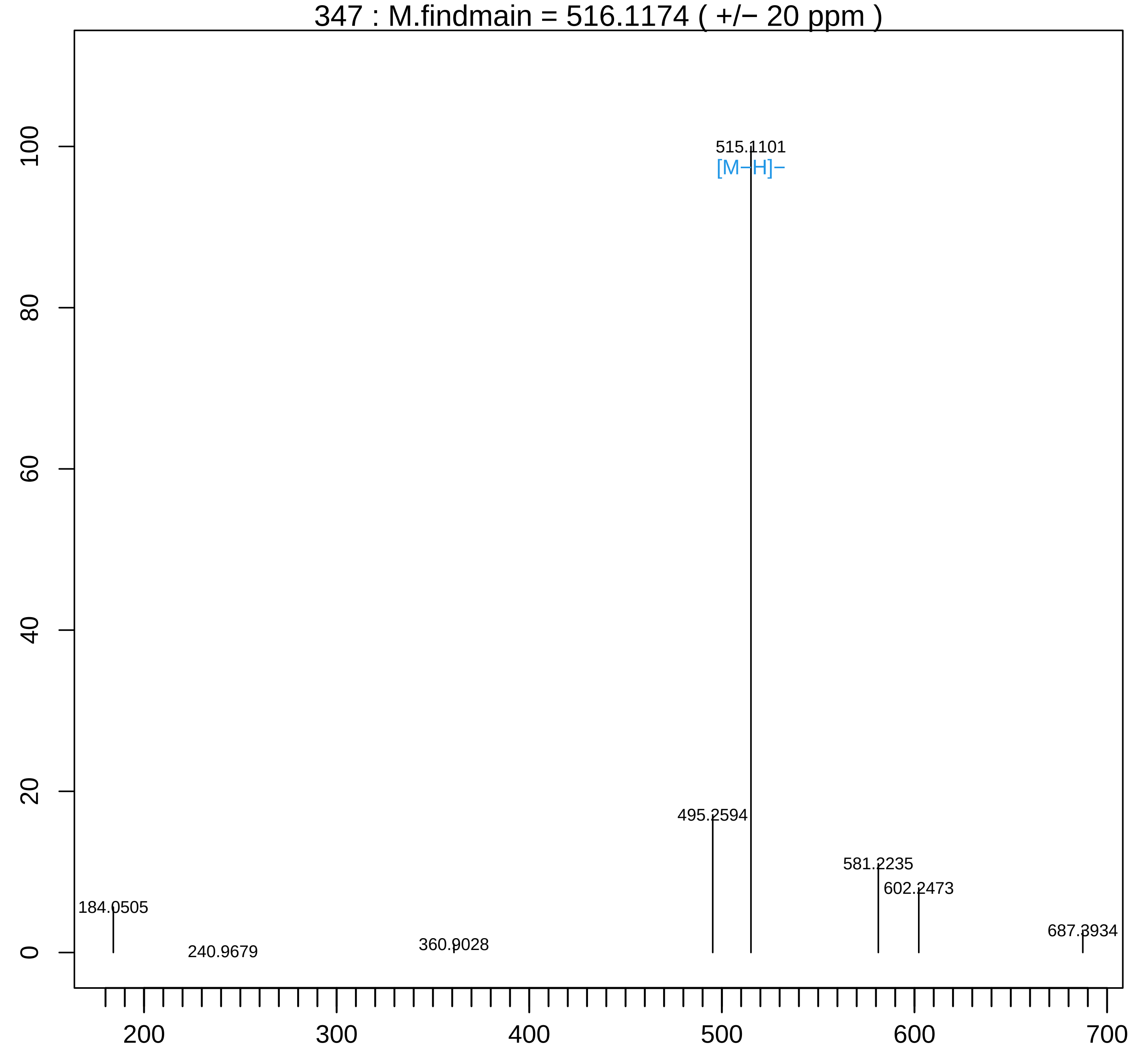
347 : M.findmain = 516.1174 (+/− 20 ppm)
515.1101
[M−H]−
495.2594
581.2235
602.2473
184.0505
687.3934
360.9028
240.9679
0
20
40
60
80
100
200
300
400
500
600
700

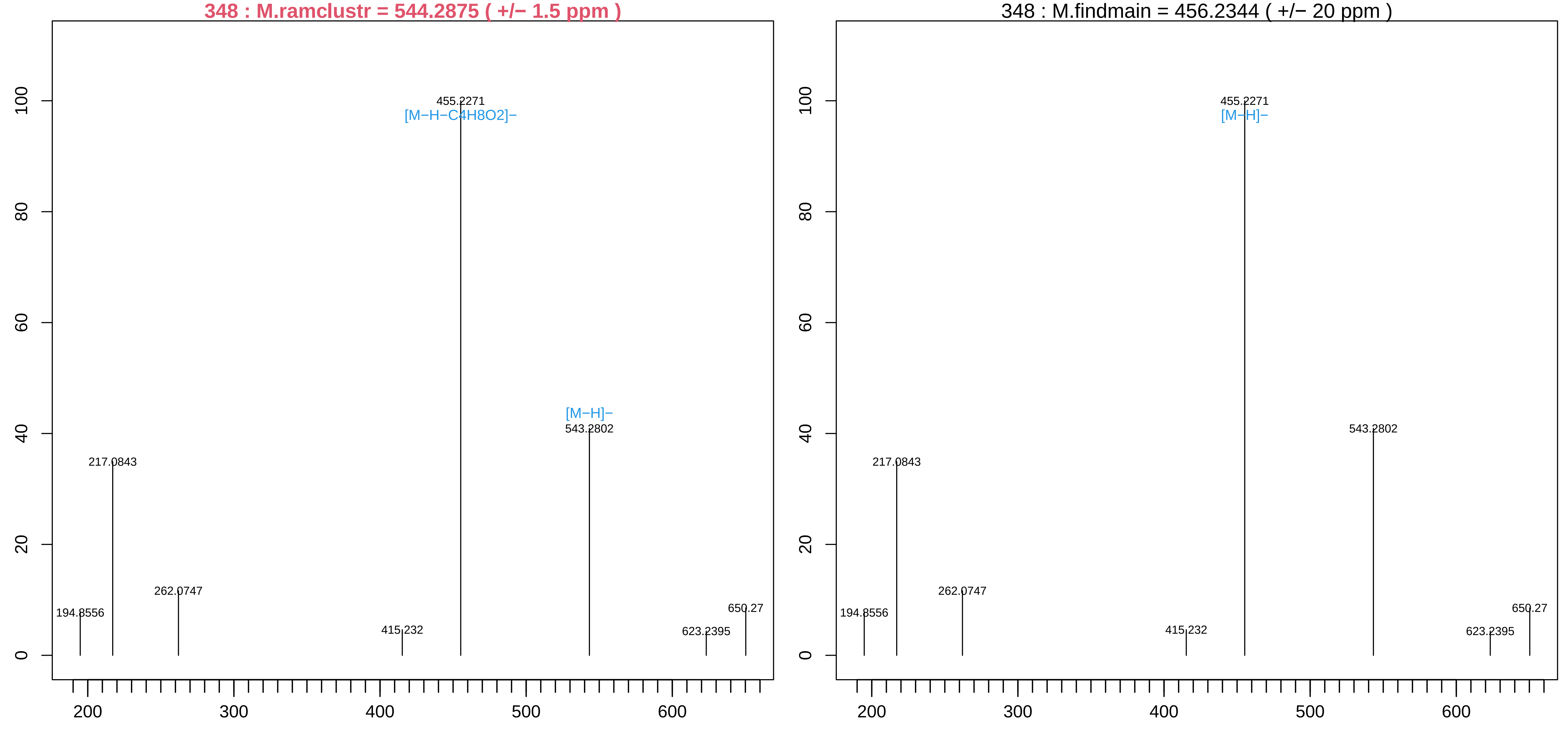

348 : M.ramclustr = 544.2875 (+/− 1.5 ppm)
455.2271
[M−H−C4H8O2]−
[M−H]−
543.2802
217.0843
262.0747
194.8556
415.232
623.2395
650.27
348 : M.findmain = 456.2344 (+/− 20 ppm)
455.2271
[M−H]−
543.2802
217.0843
262.0747
194.8556
415.232
623.2395
650.27

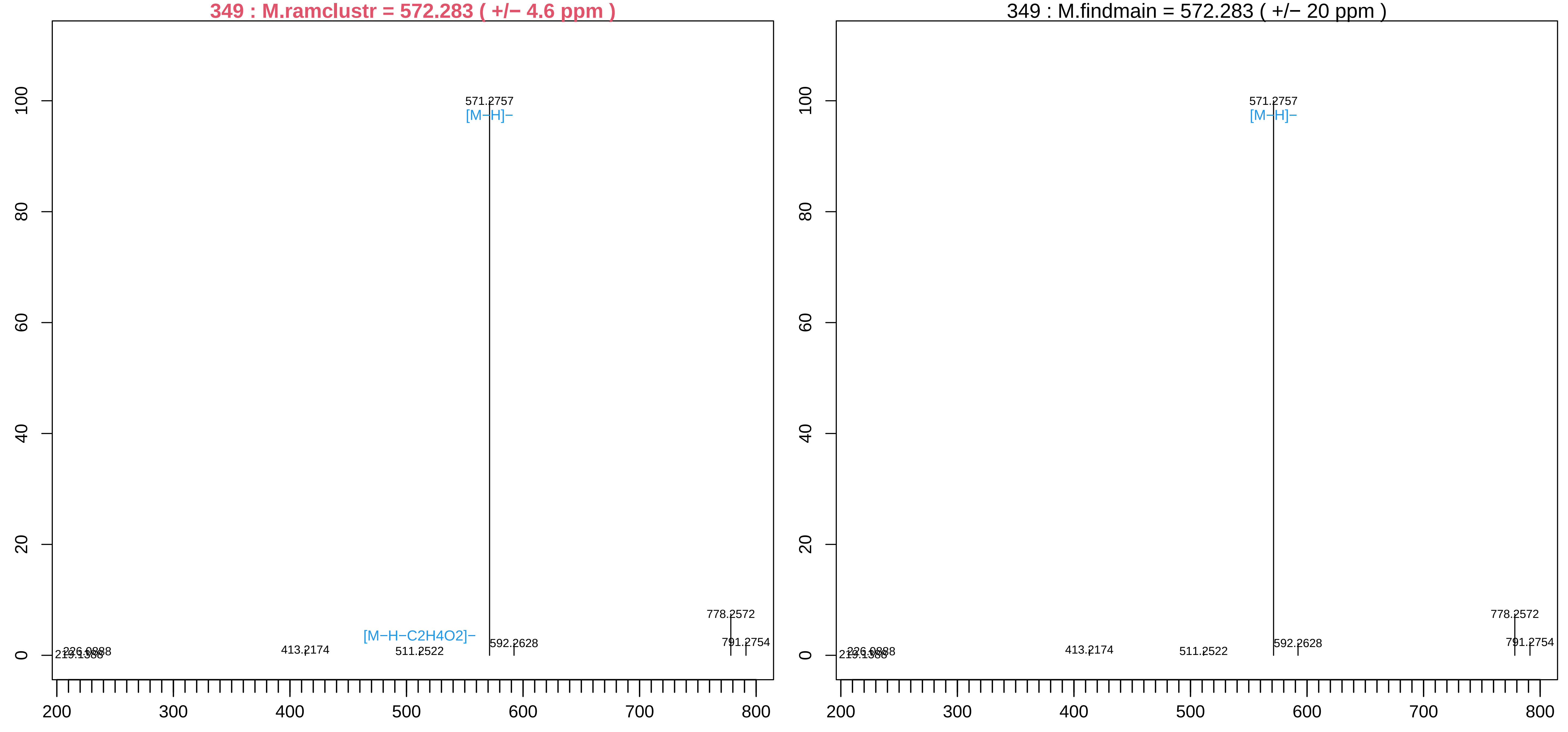
349 : M.ramclustr = 572.283 (+/− 4.6 ppm)
571.2757
[M−H]−
[M−H−C2H4O2]−
778.2572
791.2754
592.2628
413.2174
511.2522
226.0888
219.1388
349 : M.findmain = 572.283 (+/− 20 ppm)
571.2757
[M−H]−
778.2572
791.2754
592.2628
413.2174
511.2522
226.0888
219.1388

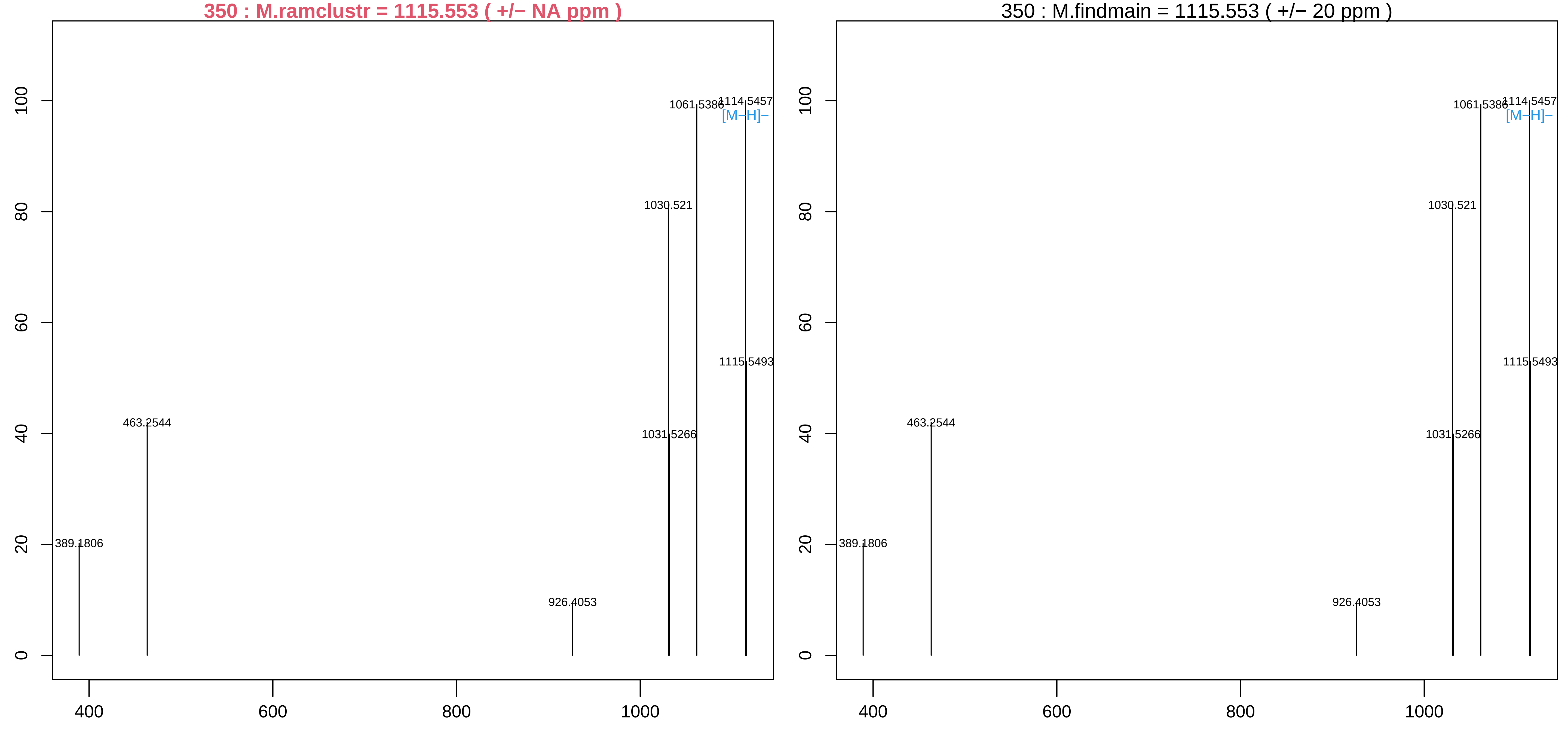

350 : M.ramclustr = 1115.553 (+/− NA ppm)
1114.5457
1061.5386
[M−H]−
1030.521
1115.5493
463.2544
1031.5266
389.1806
926.4053
350 : M.findmain = 1115.553 (+/− 20 ppm)
1114.5457
1061.5386
[M−H]−
1030.521
1115.5493
463.2544
1031.5266
389.1806
926.4053

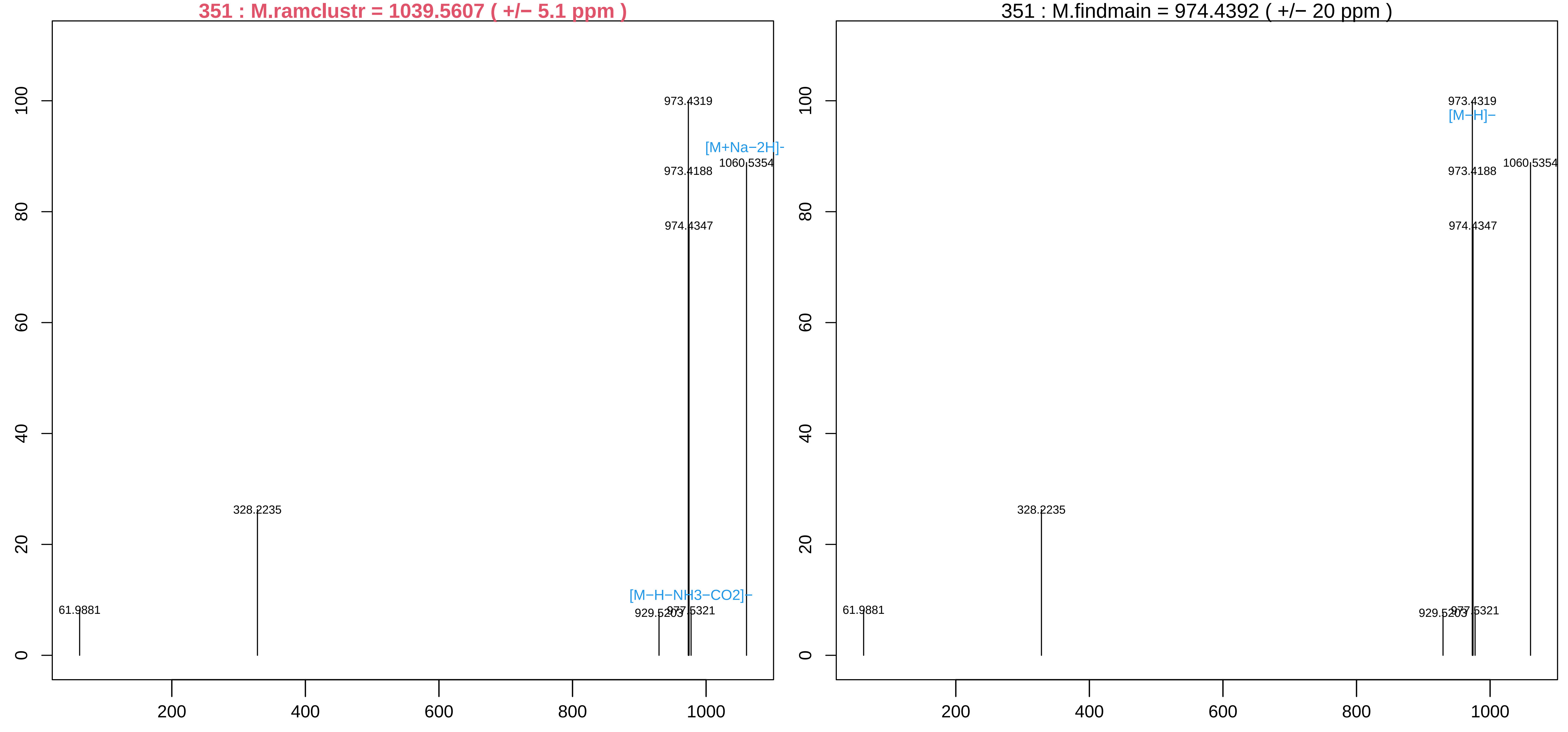
351 : M.ramclustr = 1039.5607 (+/− 5.1 ppm)
973.4319
[M+Na−2H]-
1060.5354
973.4188
974.4347
328.2235
[M−H−NH3−CO2]−
61.9881
929.5203
977.5321
351 : M.findmain = 974.4392 (+/− 20 ppm)
973.4319
[M−H]−
1060.5354
973.4188
974.4347
328.2235
61.9881
929.5203
977.5321
0
20
40
60
80
100
200
400
600
800
1000

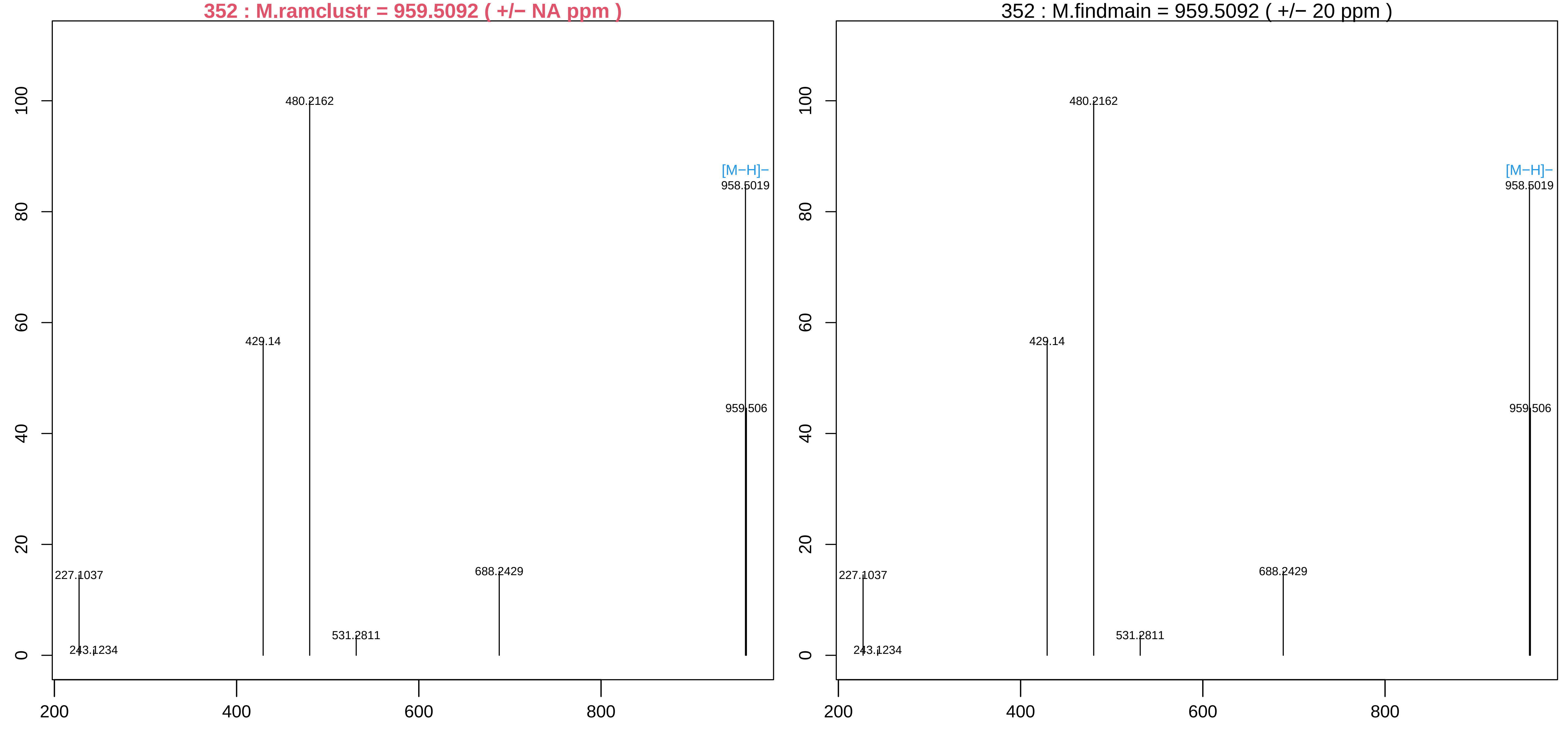
352 : M.ramclustr = 959.5092 (+/− NA ppm)
480.2162
[M−H]−
958.5019
429.14
959.506
227.1037
688.2429
531.2811
243.1234
0
20
40
60
80
100
200
400
600
800
352 : M.findmain = 959.5092 (+/− 20 ppm)
480.2162
[M−H]−
958.5019
429.14
959.506
227.1037
688.2429
531.2811
243.1234
0
20
40
60
80
100
200
400
600
800

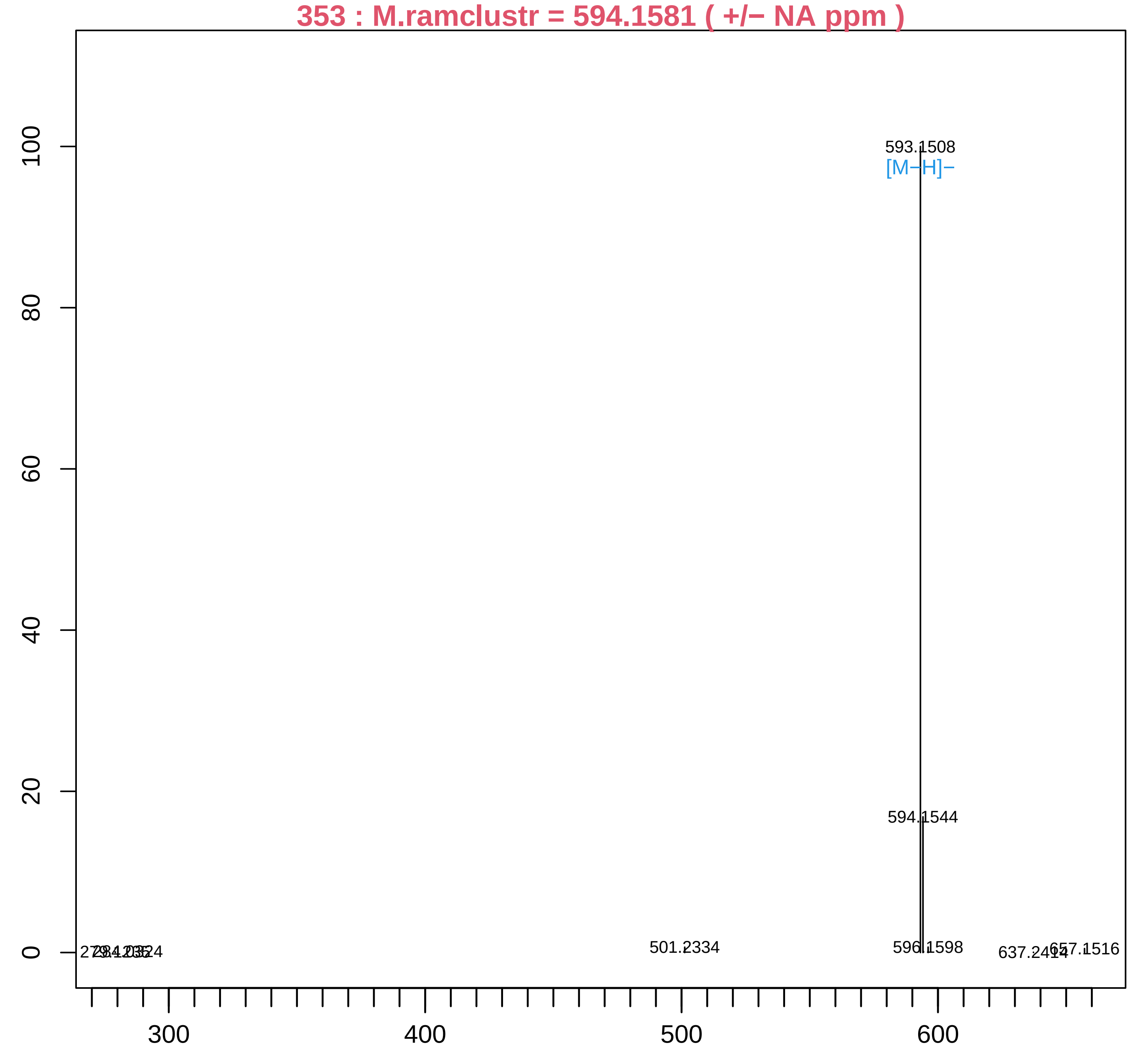

353 : M.ramclustr = 594.1581 (+/− NA ppm)
593.1508
[M−H]−
594.1544
501.2334
596.1598
637.2414
657.1516
0
20
40
60
80
100
300
400
500
600

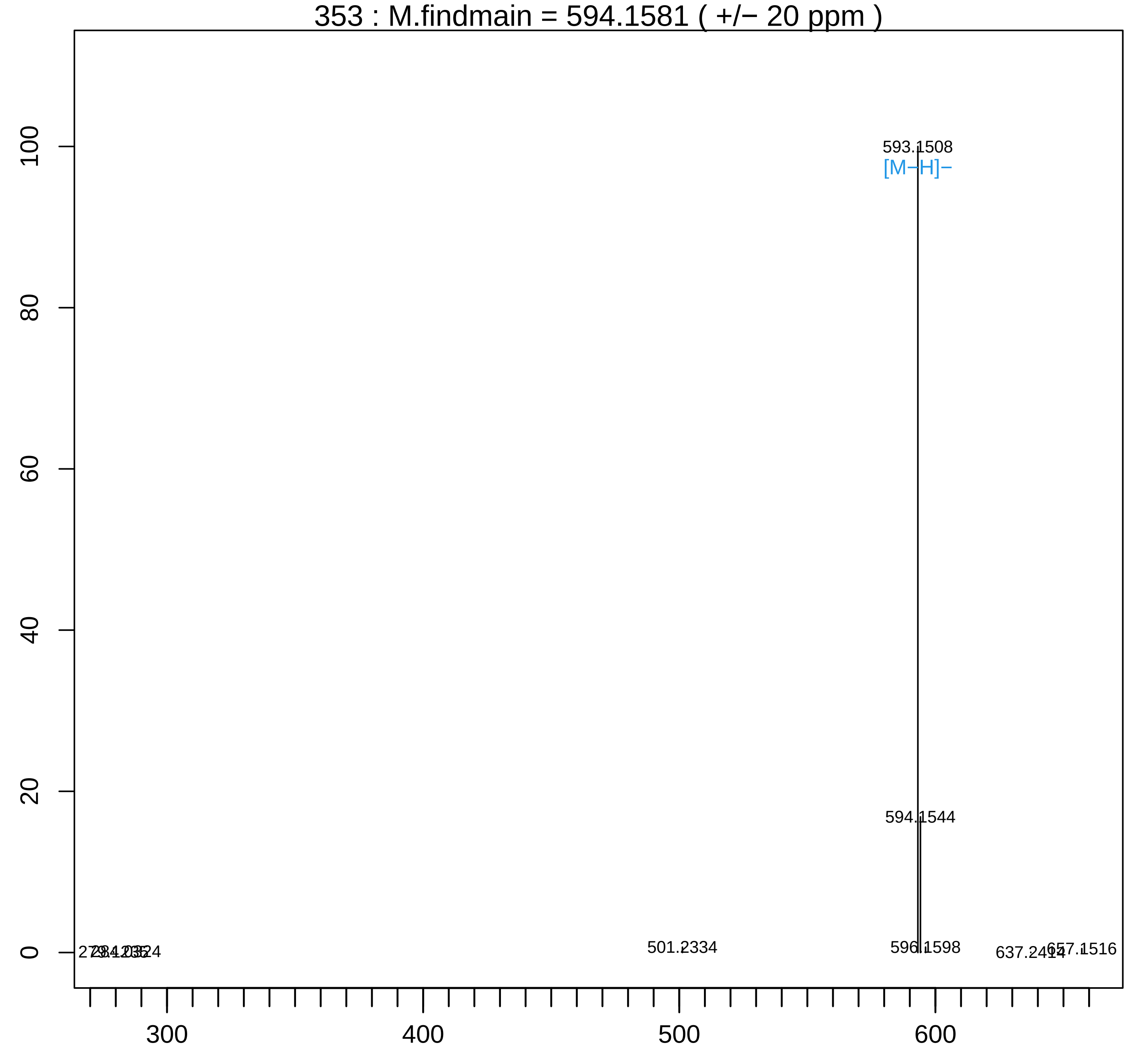

353 : M.findmain = 594.1581 (+/− 20 ppm)
593.1508
[M−H]−
594.1544
501.2334
596.1598
637.2414
657.1516
0
20
40
60
80
100
300
400
500
600

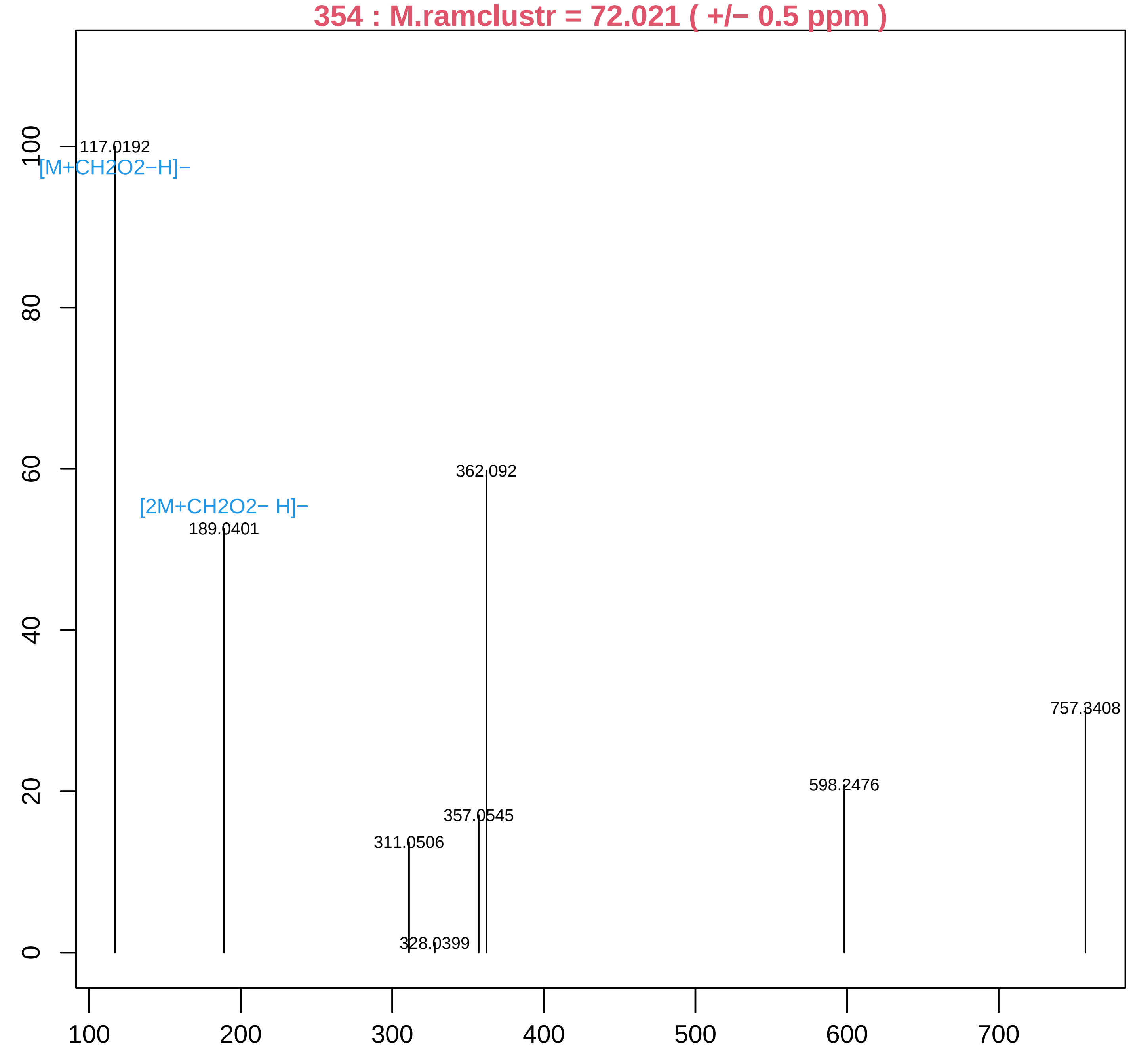

354 : M.ramclustr = 72.021 (+/− 0.5 ppm)
117.0192
[M+CH2O2−H]−
362.092
[2M+CH2O2− H]−
189.0401
757.3408
598.2476
357.0545
311.0506
328.0399
0
20
40
60
80
100
100
200
300
400
500
600
700

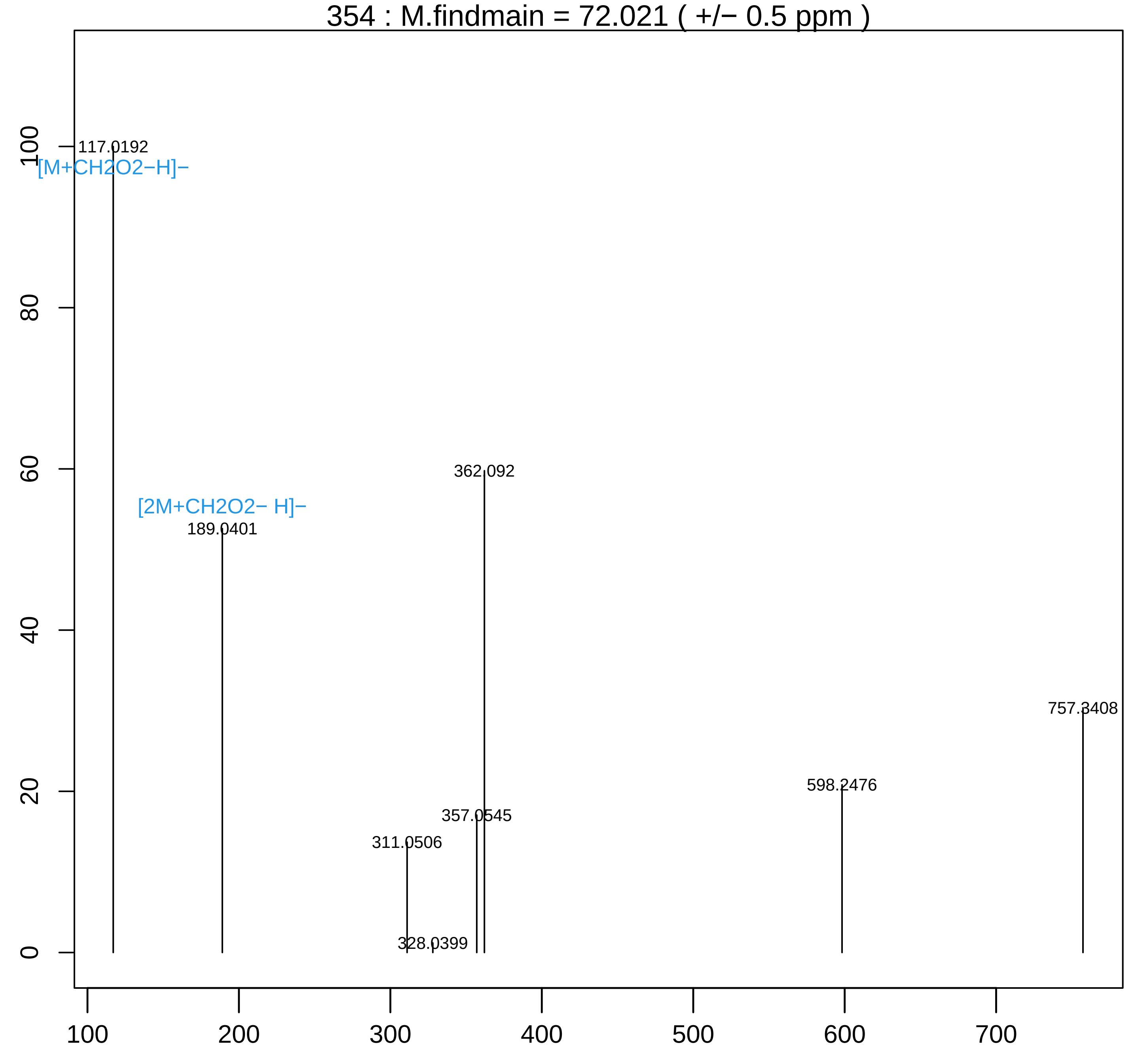

354 : M.findmain = 72.021 (+/− 0.5 ppm)
117.0192
[M+CH2O2−H]−
362.092
[2M+CH2O2− H]−
189.0401
757.3408
598.2476
357.0545
311.0506
328.0399
0
20
40
60
80
100
100
200
300
400
500
600
700

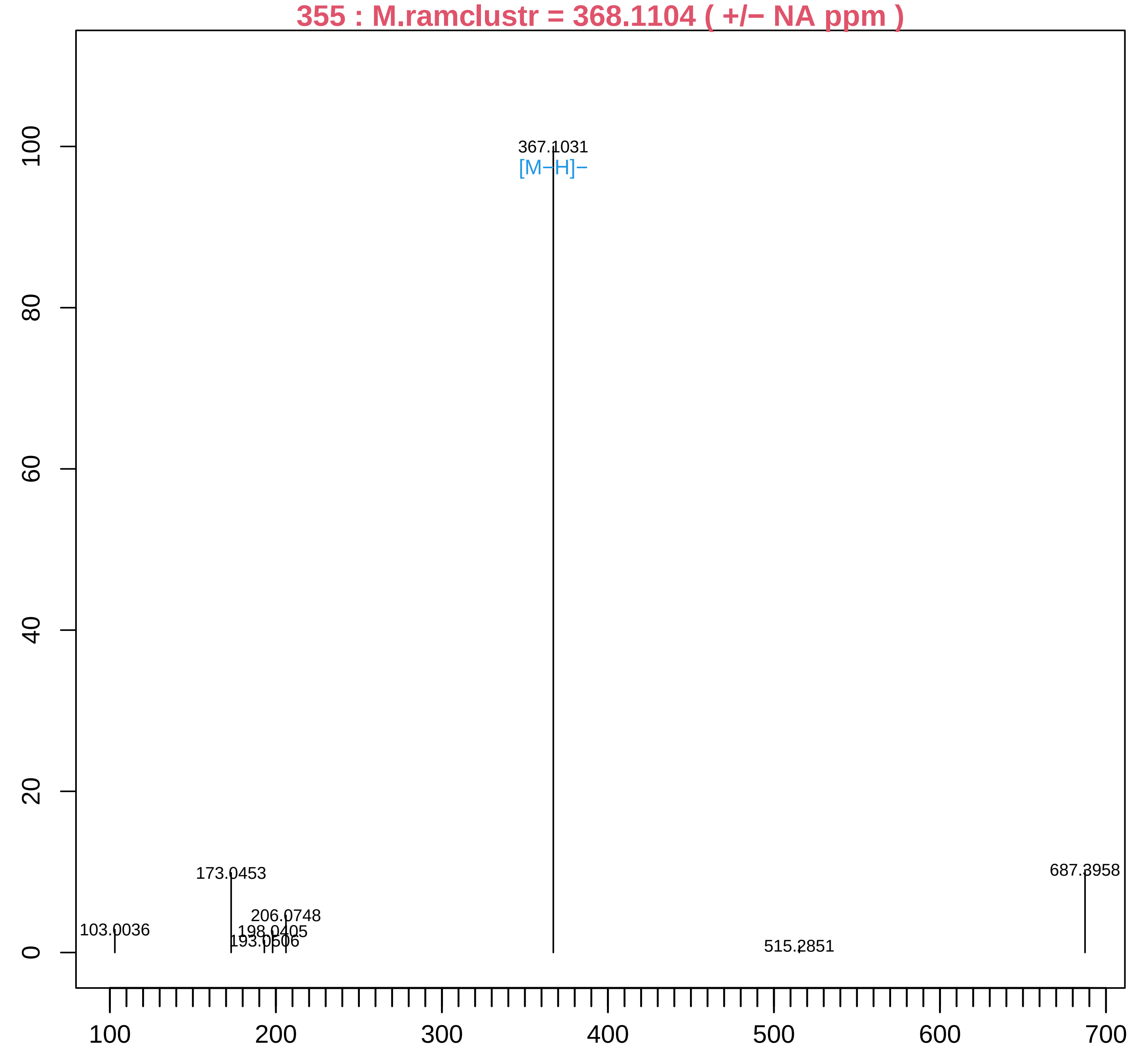
355 : M.ramclustr = 368.1104 (+/− NA ppm)
367.1031
[M−H]−
173.0453
206.0748
198.0405
193.0506
103.0036
515.2851
687.3958
0
20
40
60
80
100
100
200
300
400
500
600
700

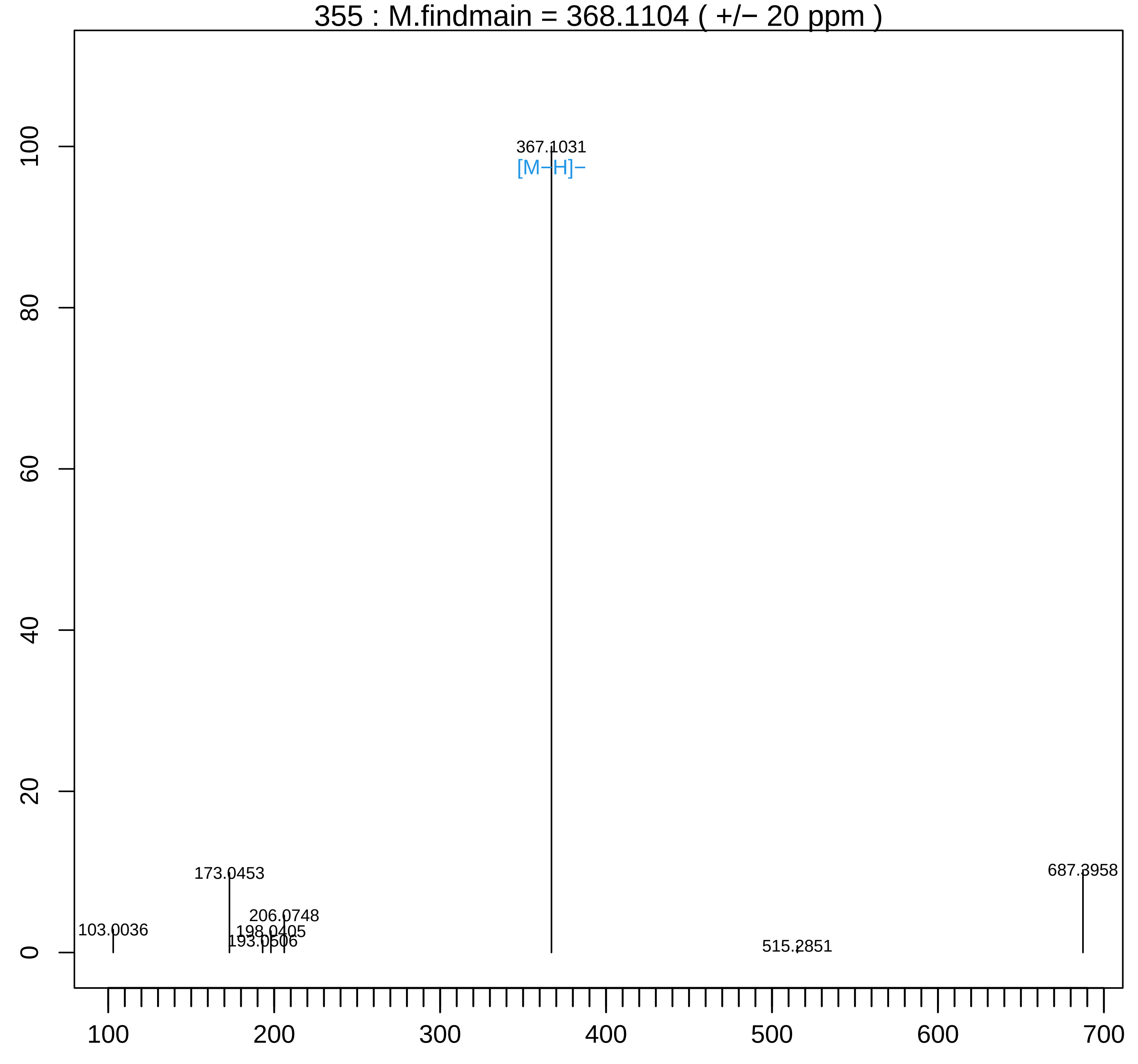
355 : M.findmain = 368.1104 (+/− 20 ppm)
367.1031
[M−H]−
173.0453
206.0748
198.0405
193.0506
103.0036
515.2851
687.3958
0
20
40
60
80
100
100
200
300
400
500
600
700

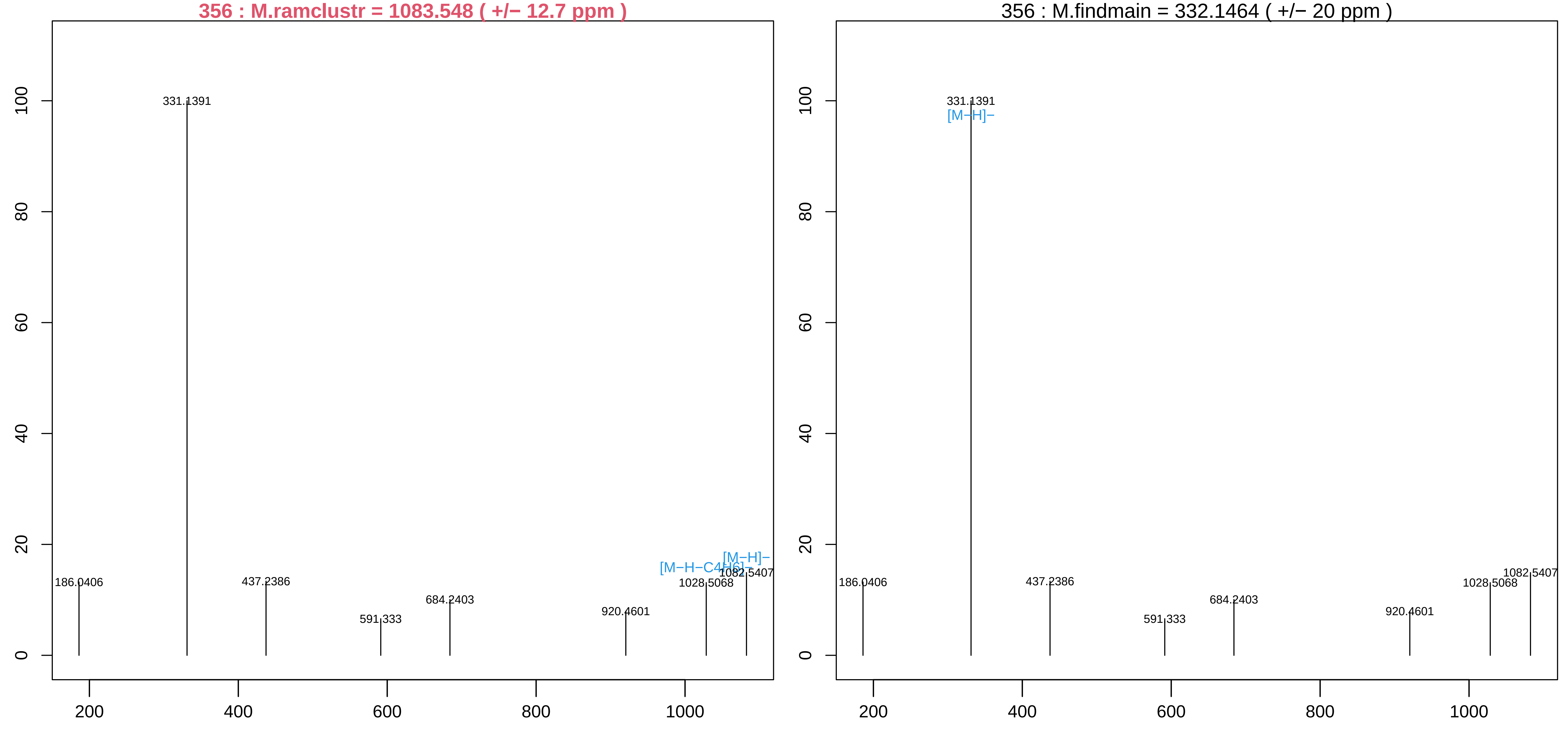

356 : M.ramclustr = 1083.548 (+/− 12.7 ppm)
331.1391
186.0406
437.2386
591.333
684.2403
920.4601
1028.5068
1082.5407
[M−H]−
[M−H−C4H6]−
356 : M.findmain = 332.1464 (+/− 20 ppm)
331.1391
[M−H]−
186.0406
437.2386
591.333
684.2403
920.4601
1028.5068
1082.5407
0
20
40
60
80
100
200
400
600
800
1000

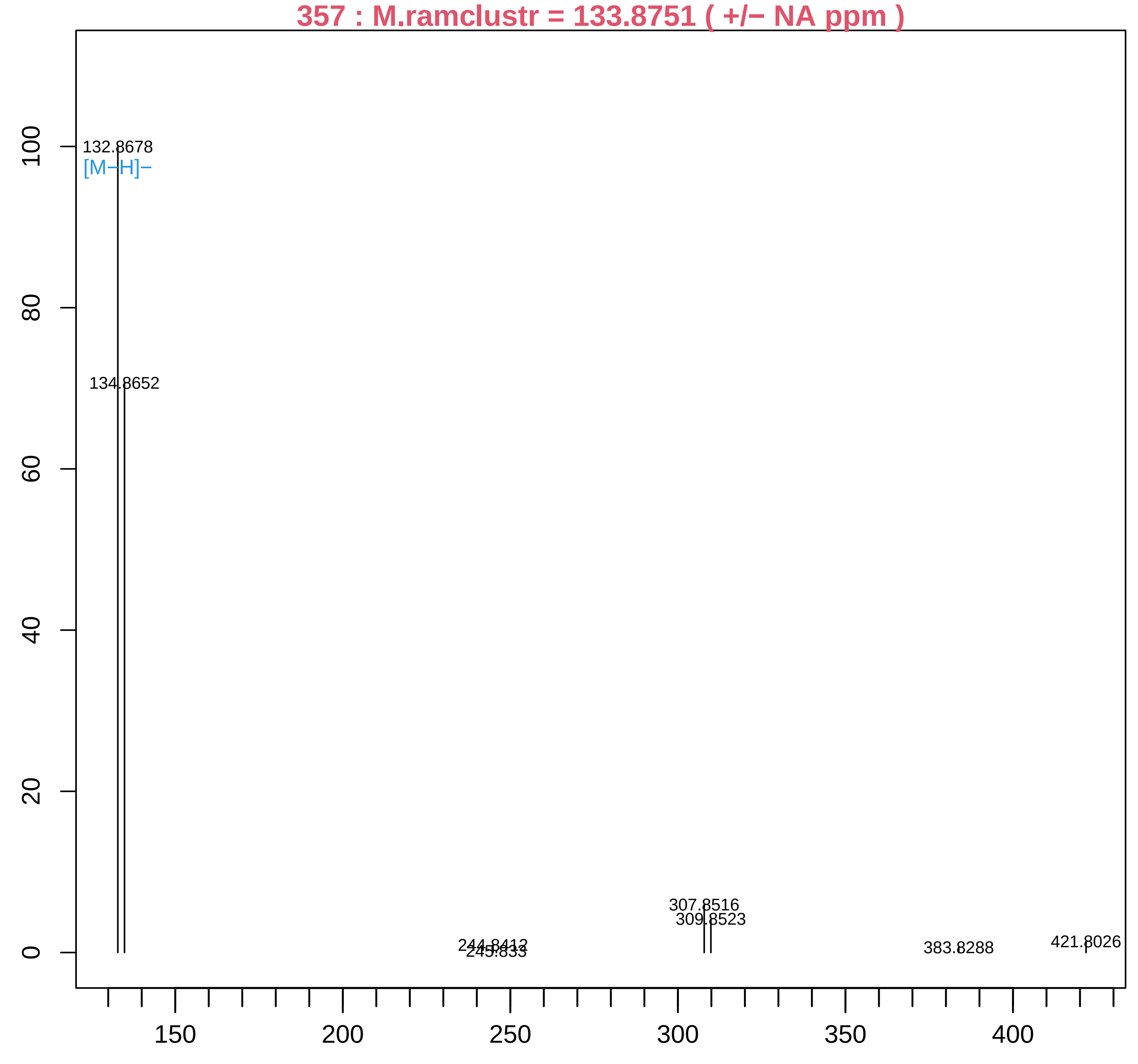

357 : M.ramclustr = 133.8751 (+/− NA ppm)
132.8678
[M−H]−
134.8652
244.8412
245.833
307.8516
309.8523
383.8288
421.8026
0
20
40
60
80
100
150
200
250
300
350
400

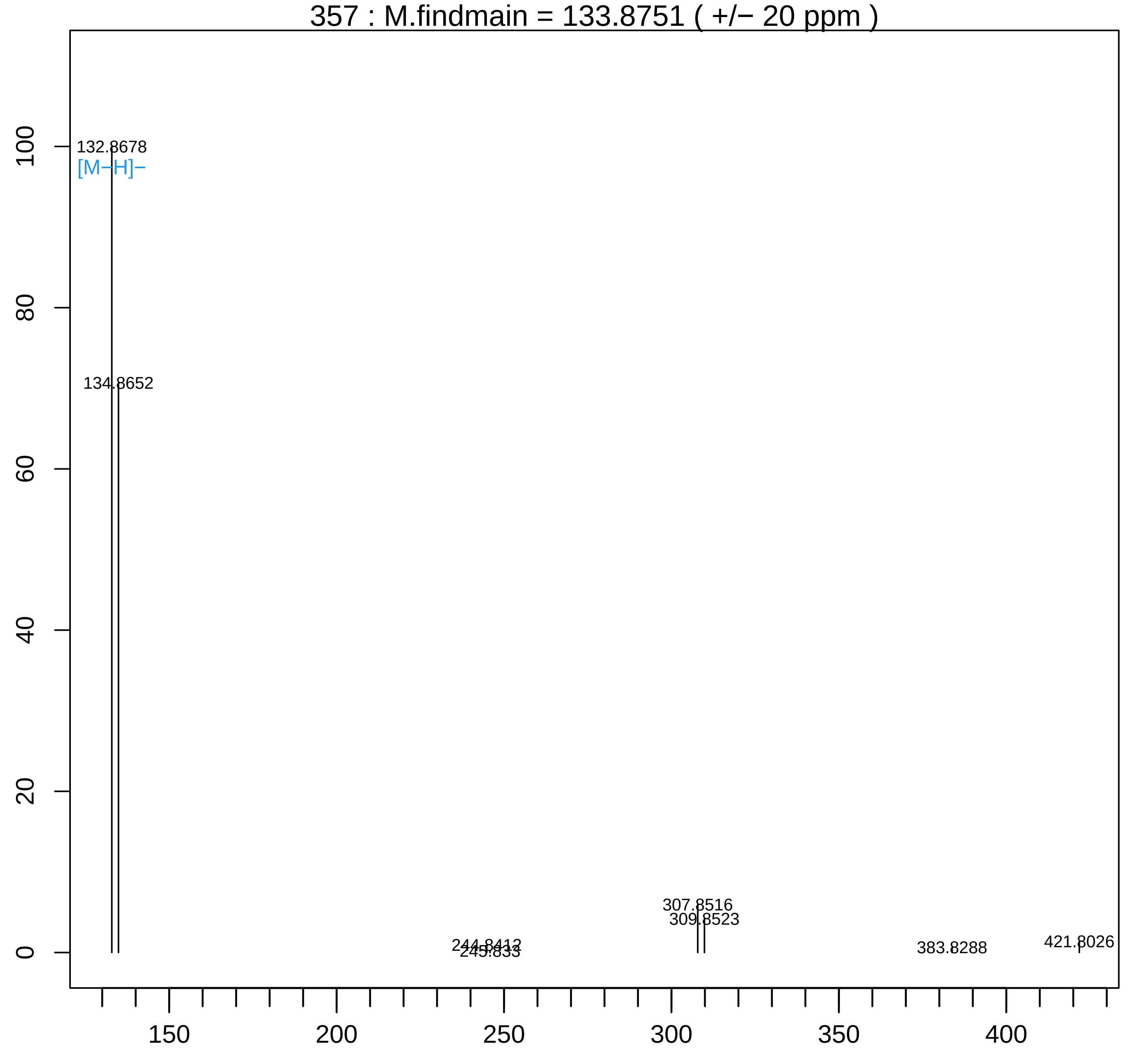

357 : M.findmain = 133.8751 (+/− 20 ppm)
132.8678
[M−H]−
134.8652
244.8412
245.833
307.8516
309.8523
383.8288
421.8026
0
20
40
60
80
100
150
200
250
300
350
400

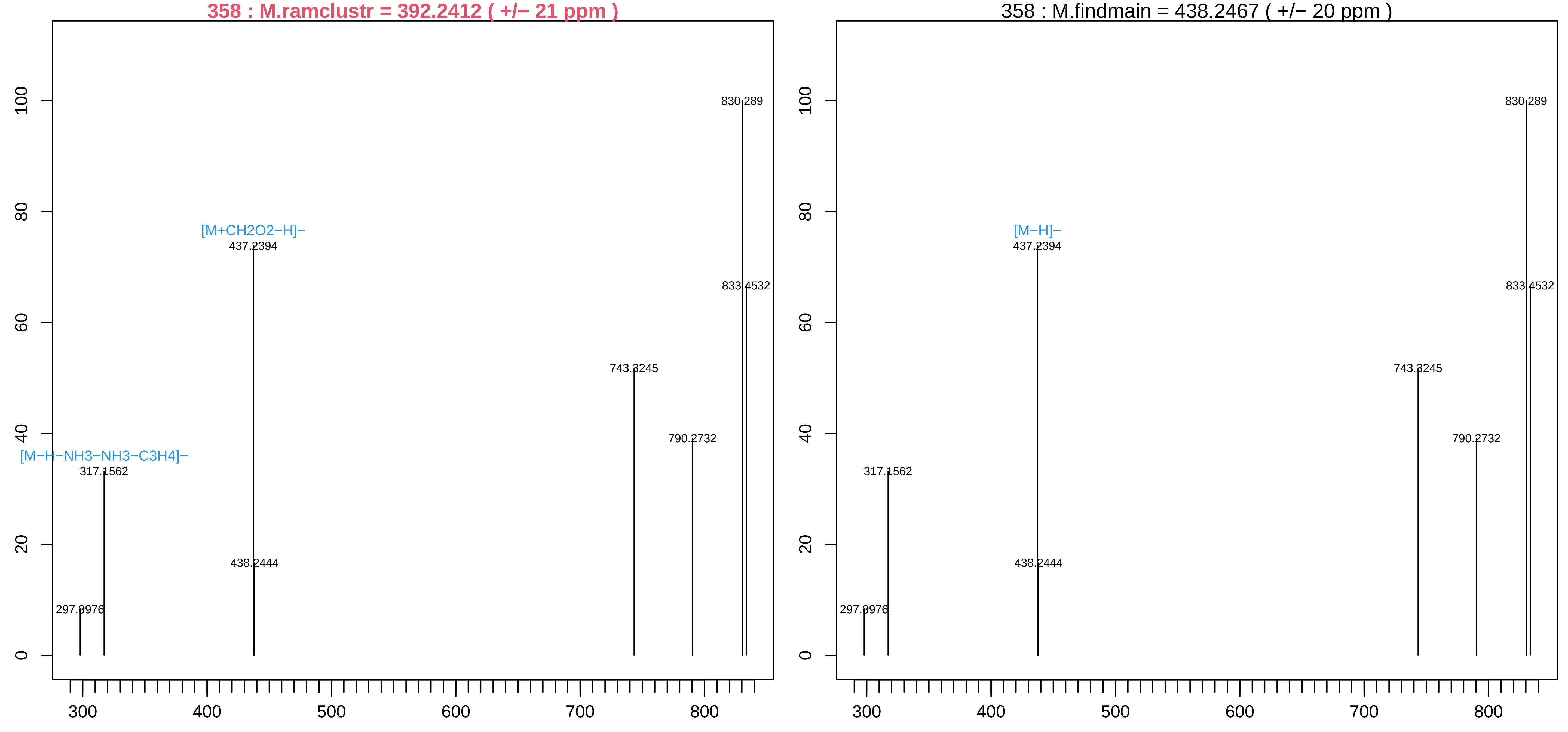
358 : M.ramclustr = 392.2412 (+/− 21 ppm)
[M+CH2O2−H]−
437.2394
[M−H−NH3−NH3−C3H4]−
317.1562
297.8976
438.2444
743.3245
790.2732
830.289
833.4532
358 : M.findmain = 438.2467 (+/− 20 ppm)
[M−H]−
437.2394
317.1562
297.8976
438.2444
743.3245
790.2732
830.289
833.4532
0
20
40
60
80
100
300
400
500
600
700
800

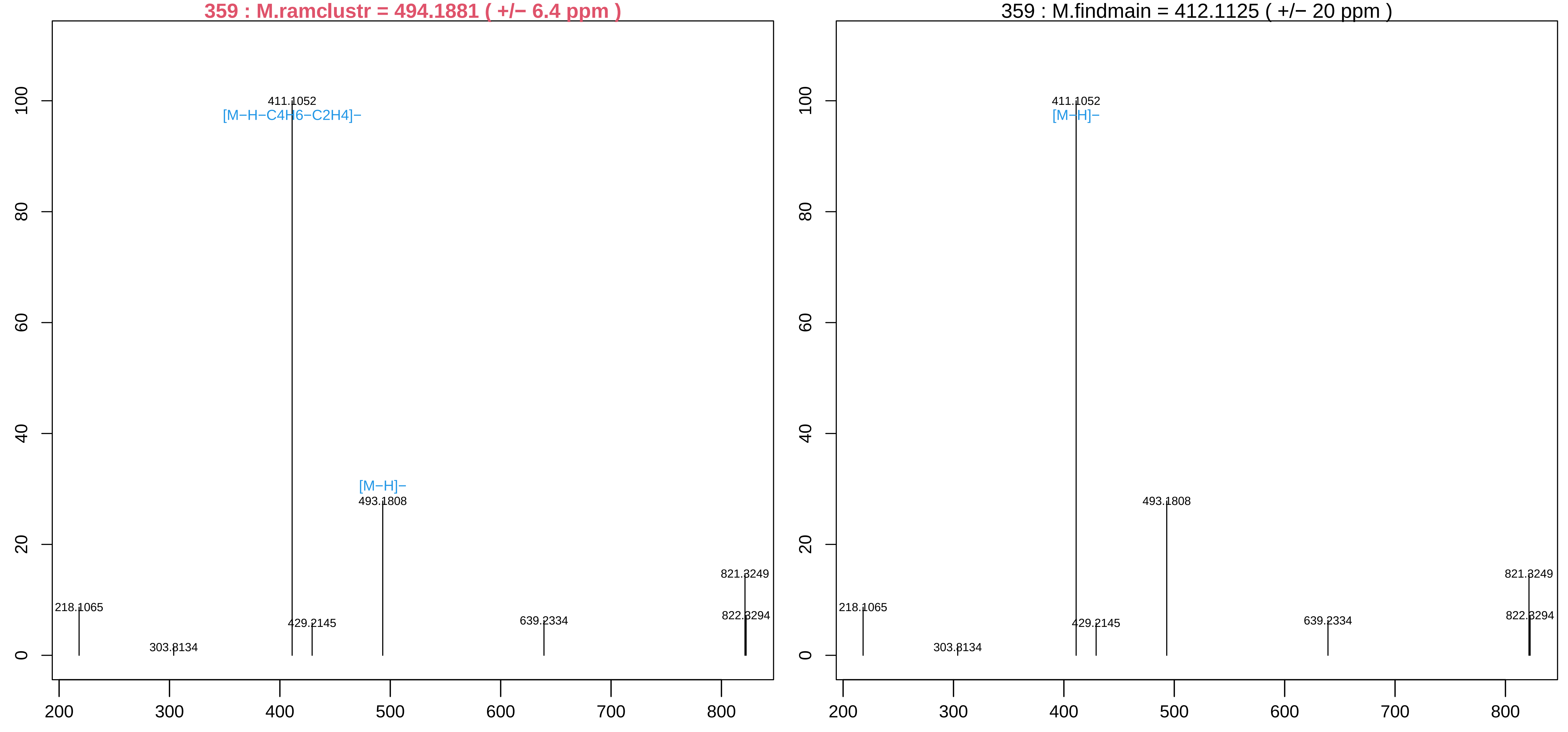
359 : M.ramclustr = 494.1881 (+/− 6.4 ppm)
411.1052
[M−H−C4H6−C2H4]−
[M−H]−
493.1808
218.1065
303.8134
429.2145
639.2334
821.3249
822.3294
359 : M.findmain = 412.1125 (+/− 20 ppm)
411.1052
[M−H]−
493.1808
218.1065
303.8134
429.2145
639.2334
821.3249
822.3294

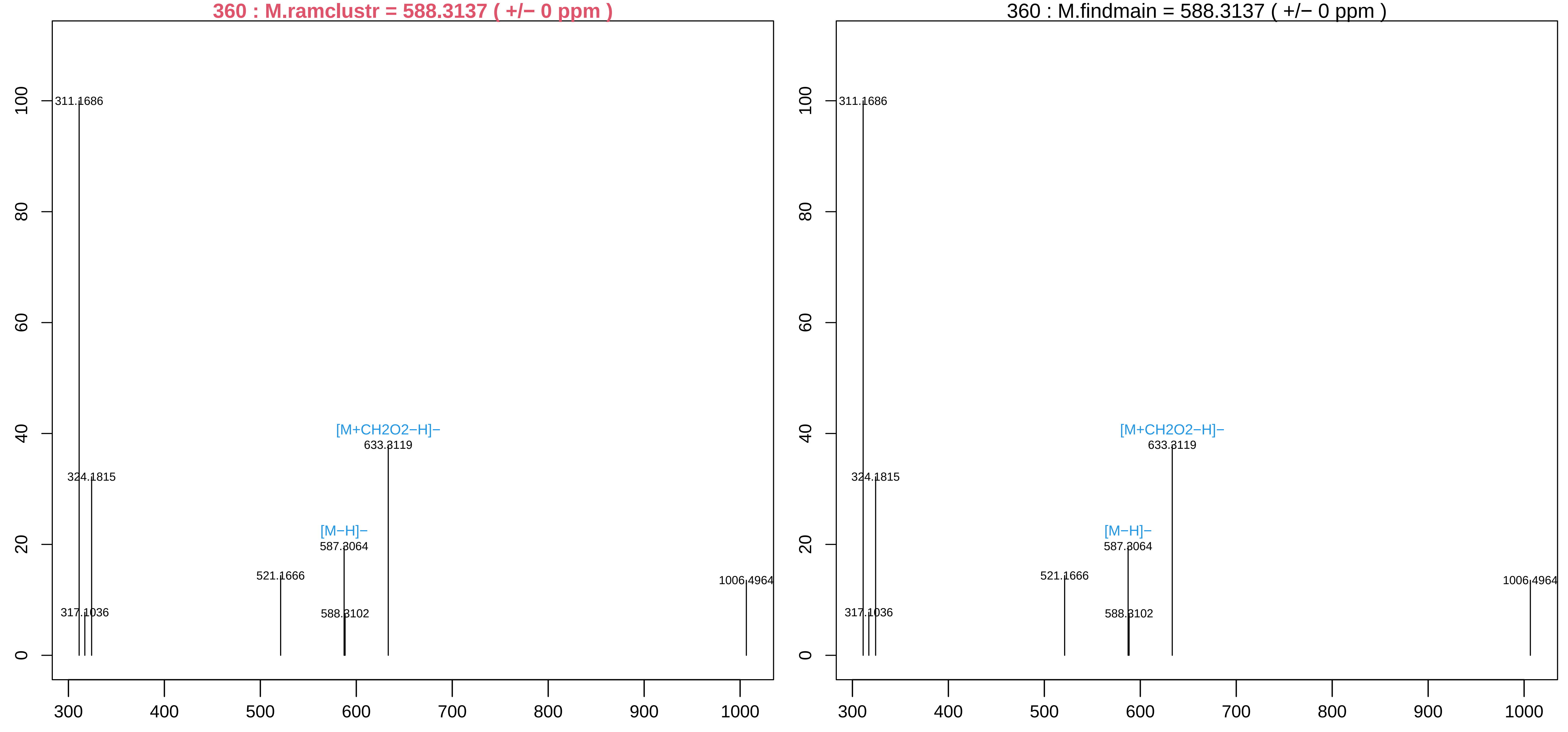
360 : M.ramclustr = 588.3137 (+/− 0 ppm)
311.1686
324.1815
317.1036
521.1666
[M−H]−
587.3064
588.3102
[M+CH2O2−H]−
633.3119
1006.4964
0
20
40
60
80
100
300
400
500
600
700
800
900
1000
360 : M.findmain = 588.3137 (+/− 0 ppm)
311.1686
324.1815
317.1036
521.1666
[M−H]−
587.3064
588.3102
[M+CH2O2−H]−
633.3119
1006.4964
0
20
40
60
80
100
300
400
500
600
700
800
900
1000

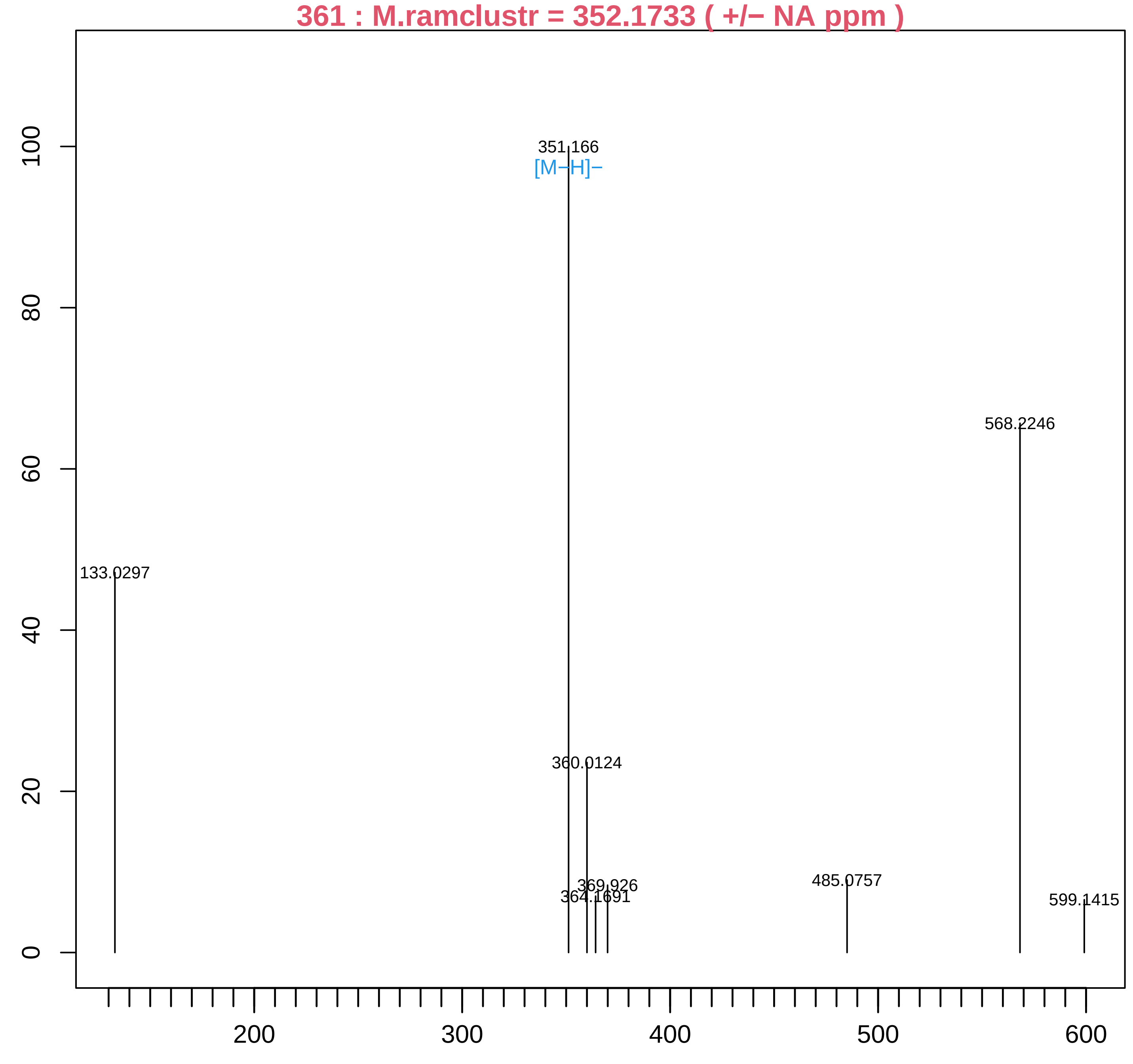
361 : M.ramclustr = 352.1733 (+/− NA ppm)
351.166
[M−H]−
568.2246
133.0297
360.0124
369.926
364.1691
485.0757
599.1415
0
20
40
60
80
100
200
300
400
500
600

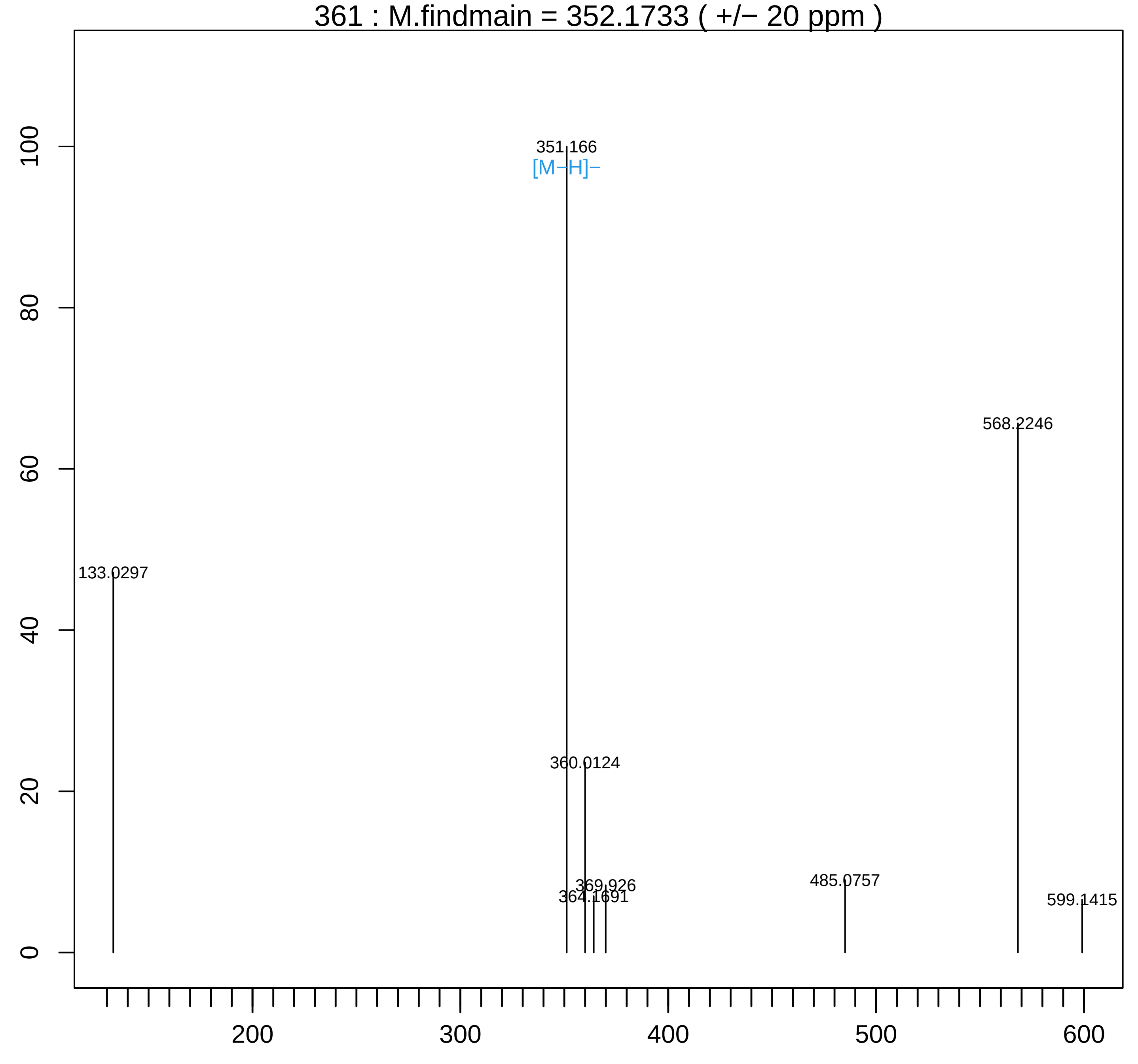
361 : M.findmain = 352.1733 (+/− 20 ppm)
351.166
[M−H]−
568.2246
133.0297
360.0124
369.926
364.1691
485.0757
599.1415
0
20
40
60
80
100
200
300
400
500
600

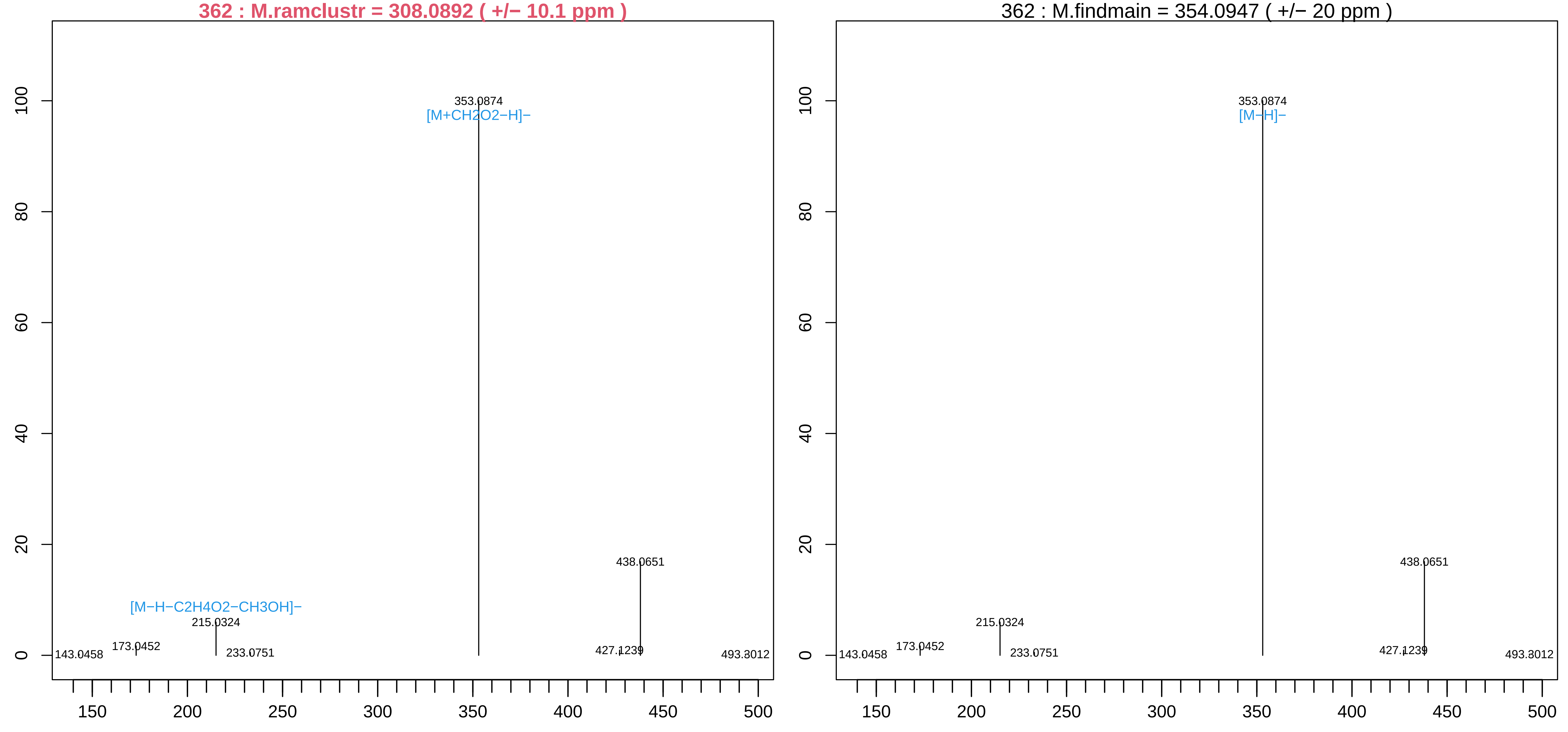
362 : M.ramclustr = 308.0892 (+/− 10.1 ppm)
353.0874
[M+CH2O2−H]−
438.0651
[M−H−C2H4O2−CH3OH]−
215.0324
173.0452
143.0458
233.0751
427.1239
493.3012
362 : M.findmain = 354.0947 (+/− 20 ppm)
353.0874
[M−H]−
438.0651
215.0324
173.0452
143.0458
233.0751
427.1239
493.3012

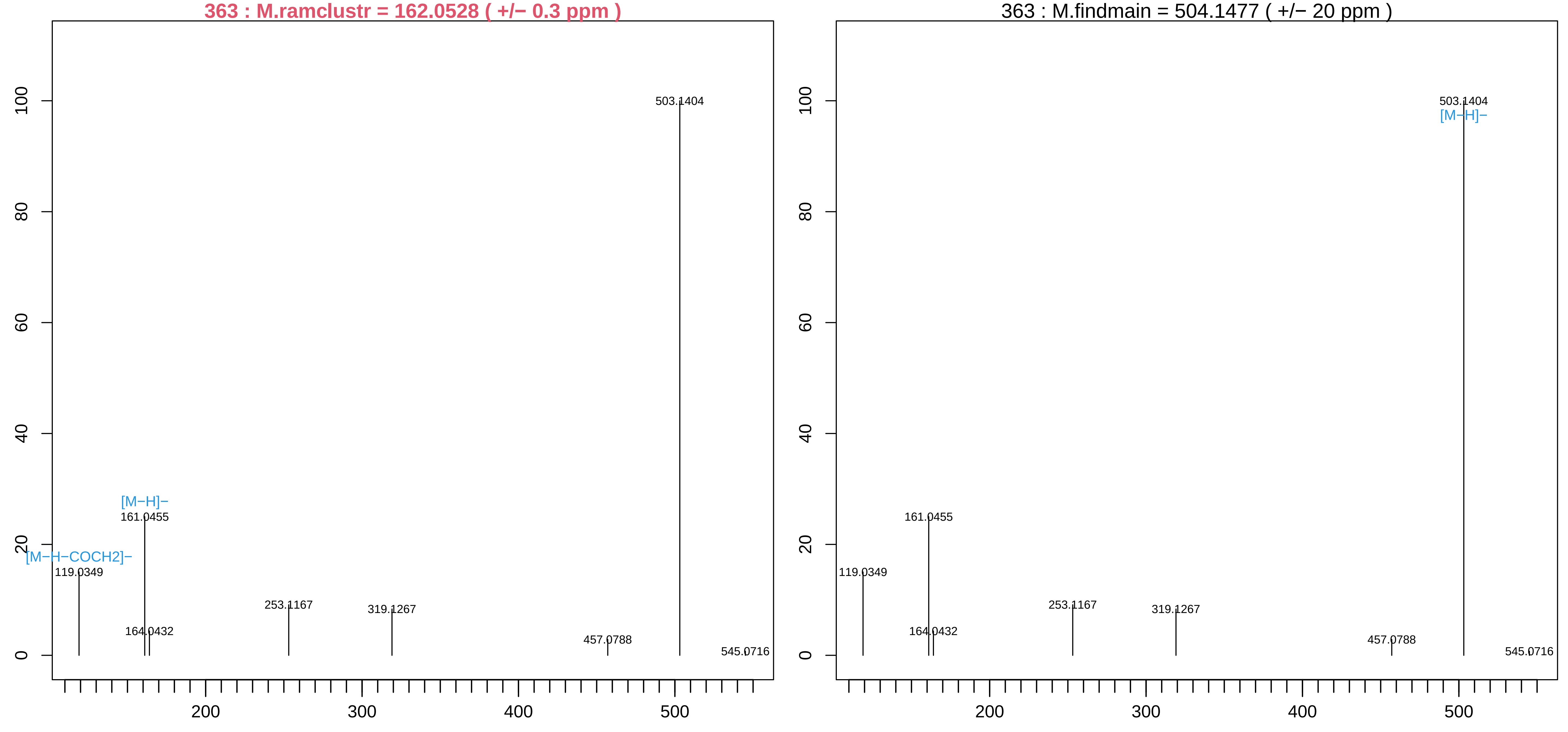
363 : M.ramclustr = 162.0528 (+/− 0.3 ppm)
503.1404
[M−H]−
161.0455
[M−H−COCH2]−
119.0349
164.0432
253.1167
319.1267
457.0788
545.0716
0
20
40
60
80
100
200
300
400
500
363 : M.findmain = 504.1477 (+/− 20 ppm)
503.1404
[M−H]−
161.0455
119.0349
164.0432
253.1167
319.1267
457.0788
545.0716

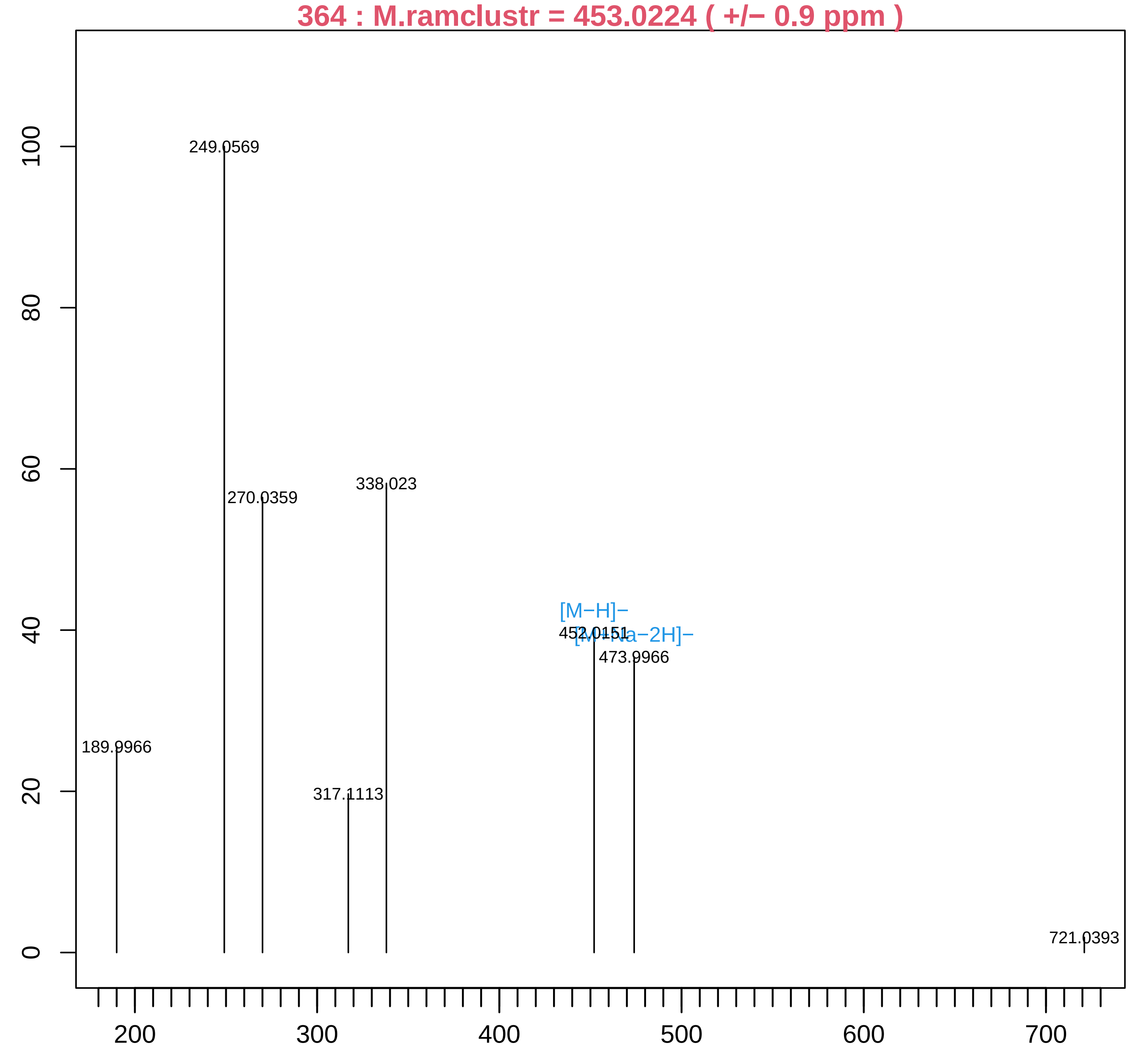
364 : M.ramclustr = 453.0224 (+/− 0.9 ppm)
249.0569
270.0359
338.023
[M−H]−
452.0151
[M+Na−2H]−
473.9966
189.9966
317.1113
721.0393
0
20
40
60
80
100
200
300
400
500
600
700

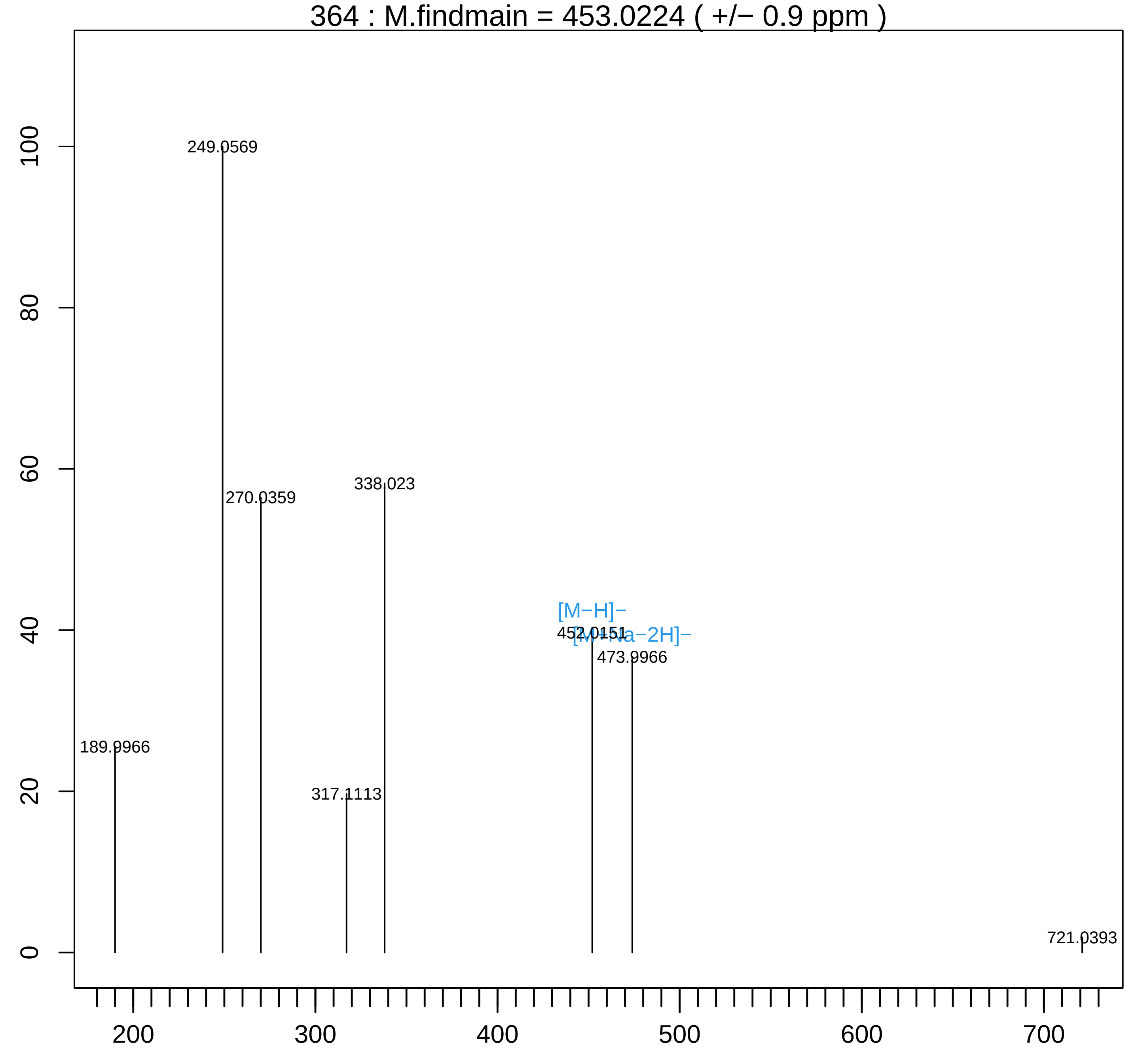
364 : M.findmain = 453.0224 (+/− 0.9 ppm)
249.0569
270.0359
338.023
[M−H]−
452.0151
[M+Na−2H]−
473.9966
189.9966
317.1113
721.0393
0
20
40
60
80
100
200
300
400
500
600
700

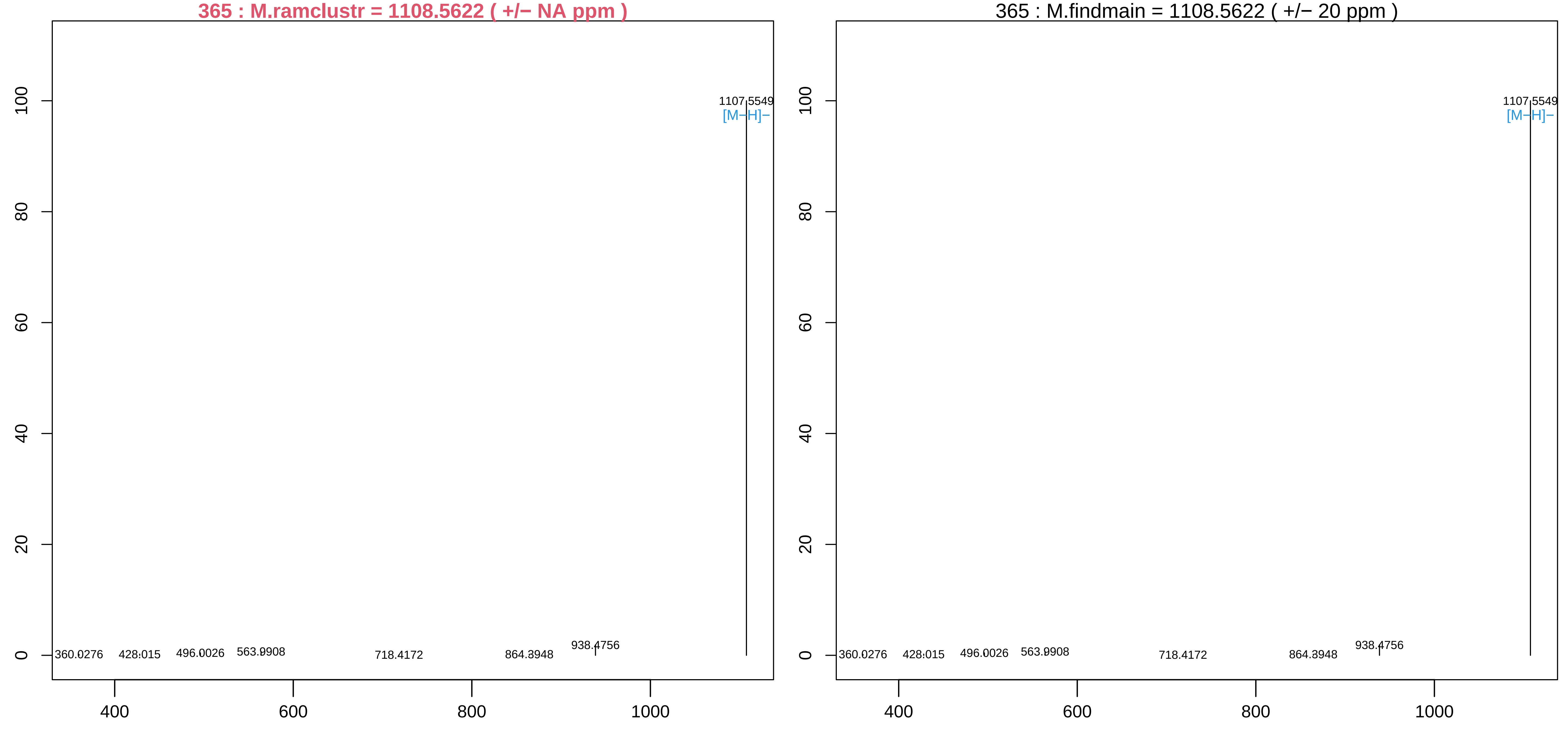

365 : M.ramclustr = 1108.5622 (+/− NA ppm)
1107.5549
[M−H]−
360.0276
428.015
496.0026
563.9908
718.4172
864.3948
938.4756
0
20
40
60
80
100
400
600
800
1000
365 : M.findmain = 1108.5622 (+/− 20 ppm)
1107.5549
[M−H]−
360.0276
428.015
496.0026
563.9908
718.4172
864.3948
938.4756
0
20
40
60
80
100
400
600
800
1000

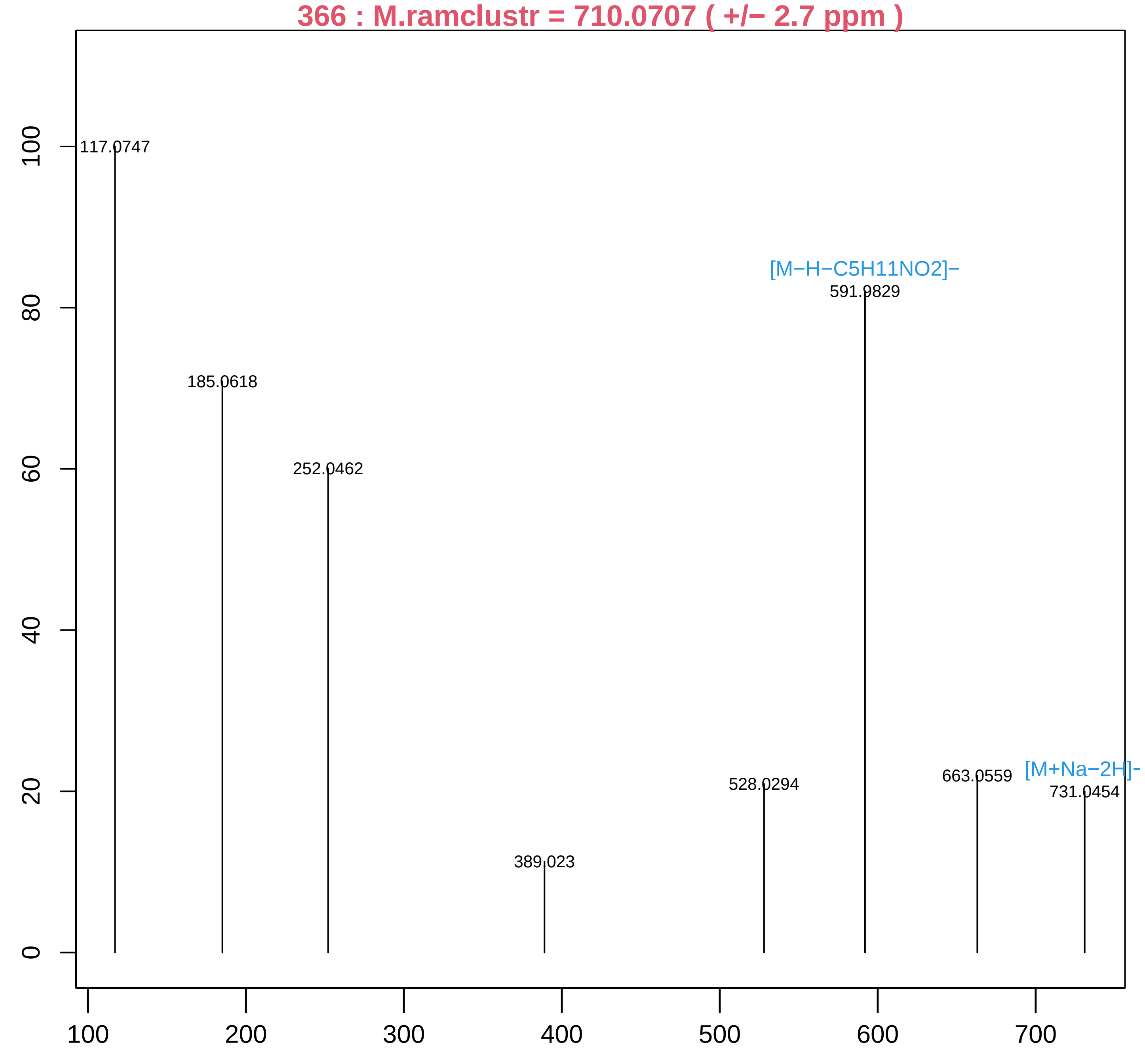
366 : M.ramclustr = 710.0707 (+/− 2.7 ppm)
117.0747
185.0618
252.0462
389.023
528.0294
[M−H−C5H11NO2]−
591.9829
663.0559
[M+Na−2H]-
731.0454
0
20
40
60
80
100
100
200
300
400
500
600
700

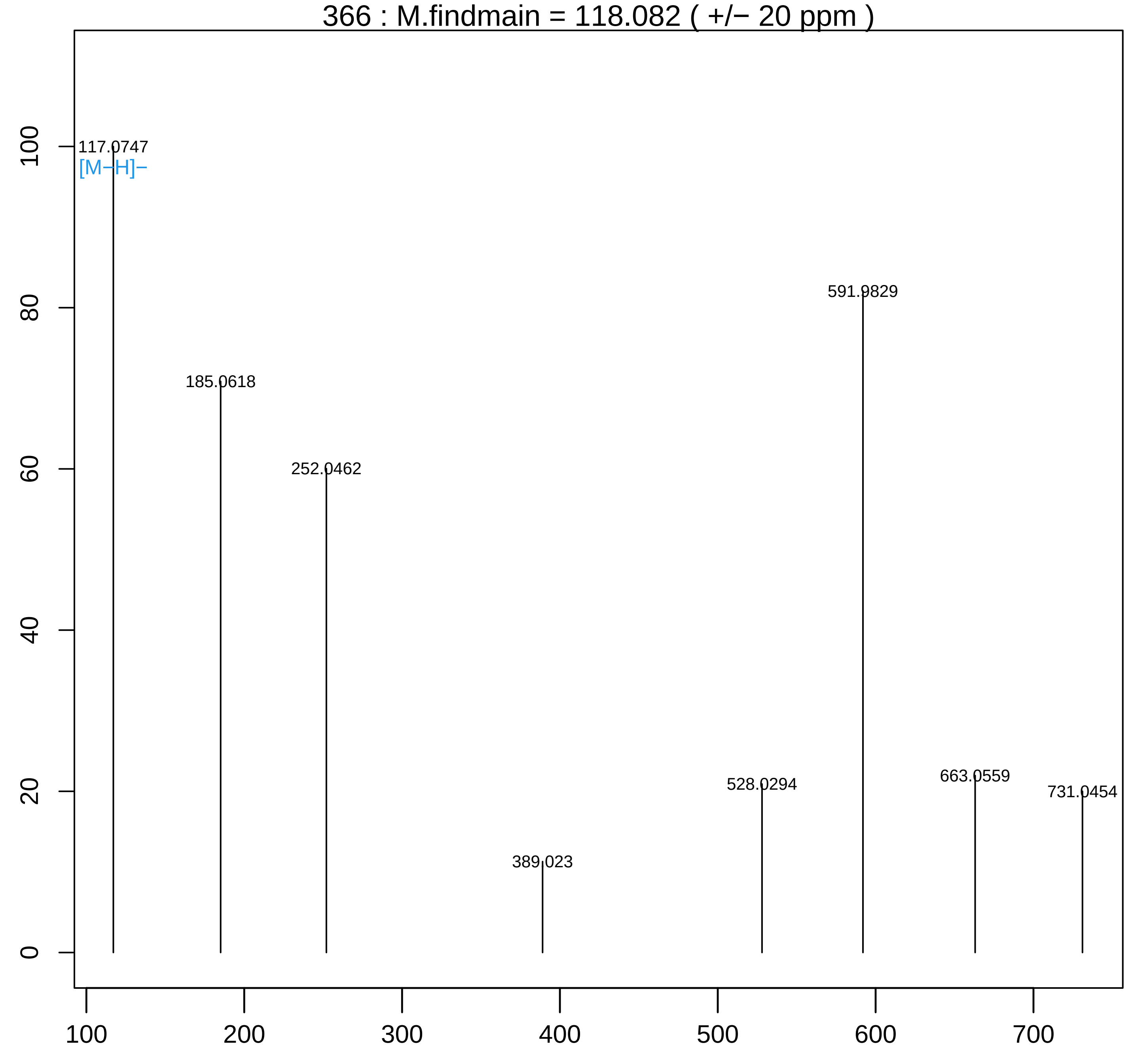
366 : M.findmain = 118.082 (+/− 20 ppm)
117.0747
[M−H]−
185.0618
252.0462
389.023
528.0294
591.9829
663.0559
731.0454
0
20
40
60
80
100
100
200
300
400
500
600
700

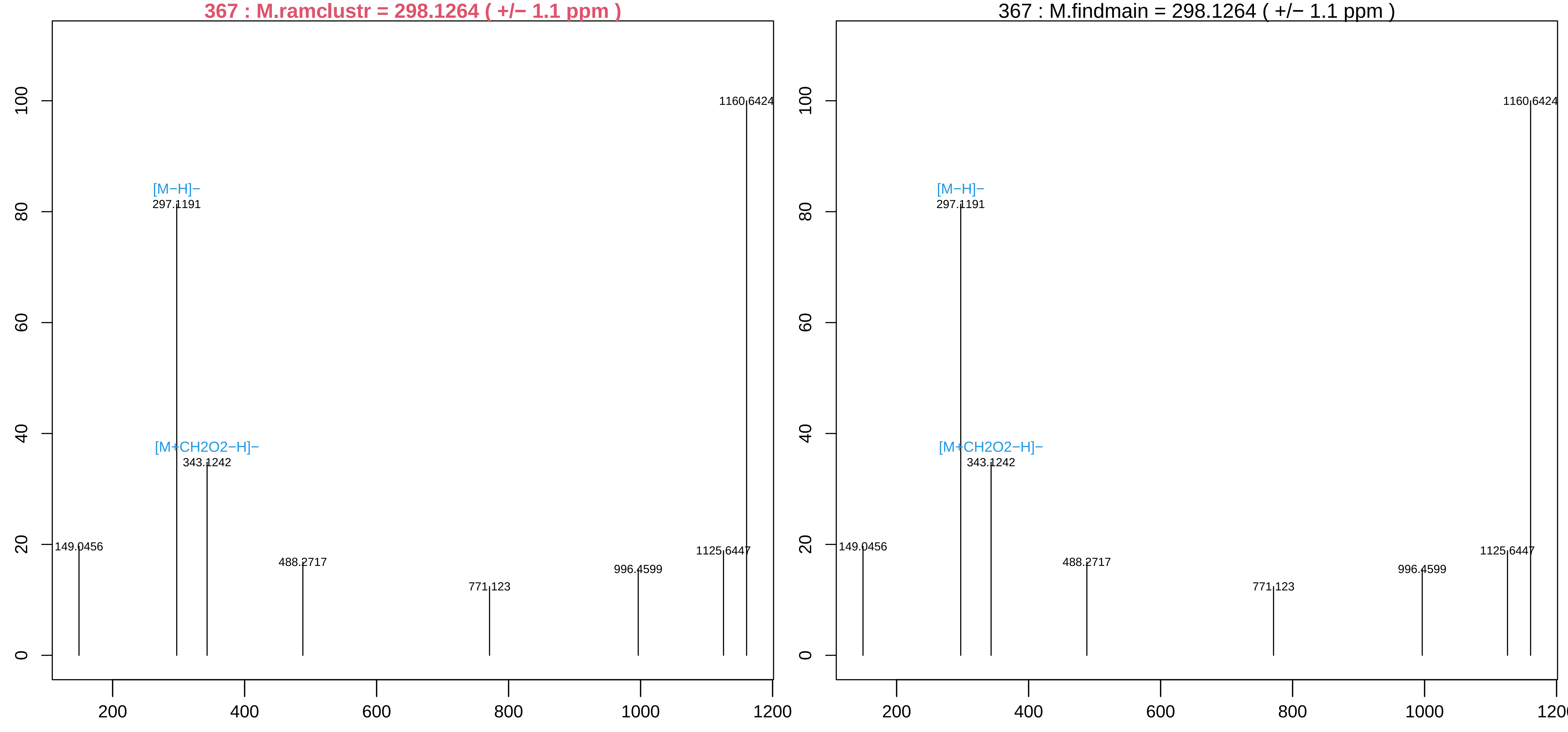

367 : M.ramclustr = 298.1264 (+/− 1.1 ppm)
[M−H]−
297.1191
[M+CH2O2−H]−
343.1242
149.0456
488.2717
771.123
996.4599
1125.6447
1160.6424
367 : M.findmain = 298.1264 (+/− 1.1 ppm)
[M−H]−
297.1191
[M+CH2O2−H]−
343.1242
149.0456
488.2717
771.123
996.4599
1125.6447
1160.6424

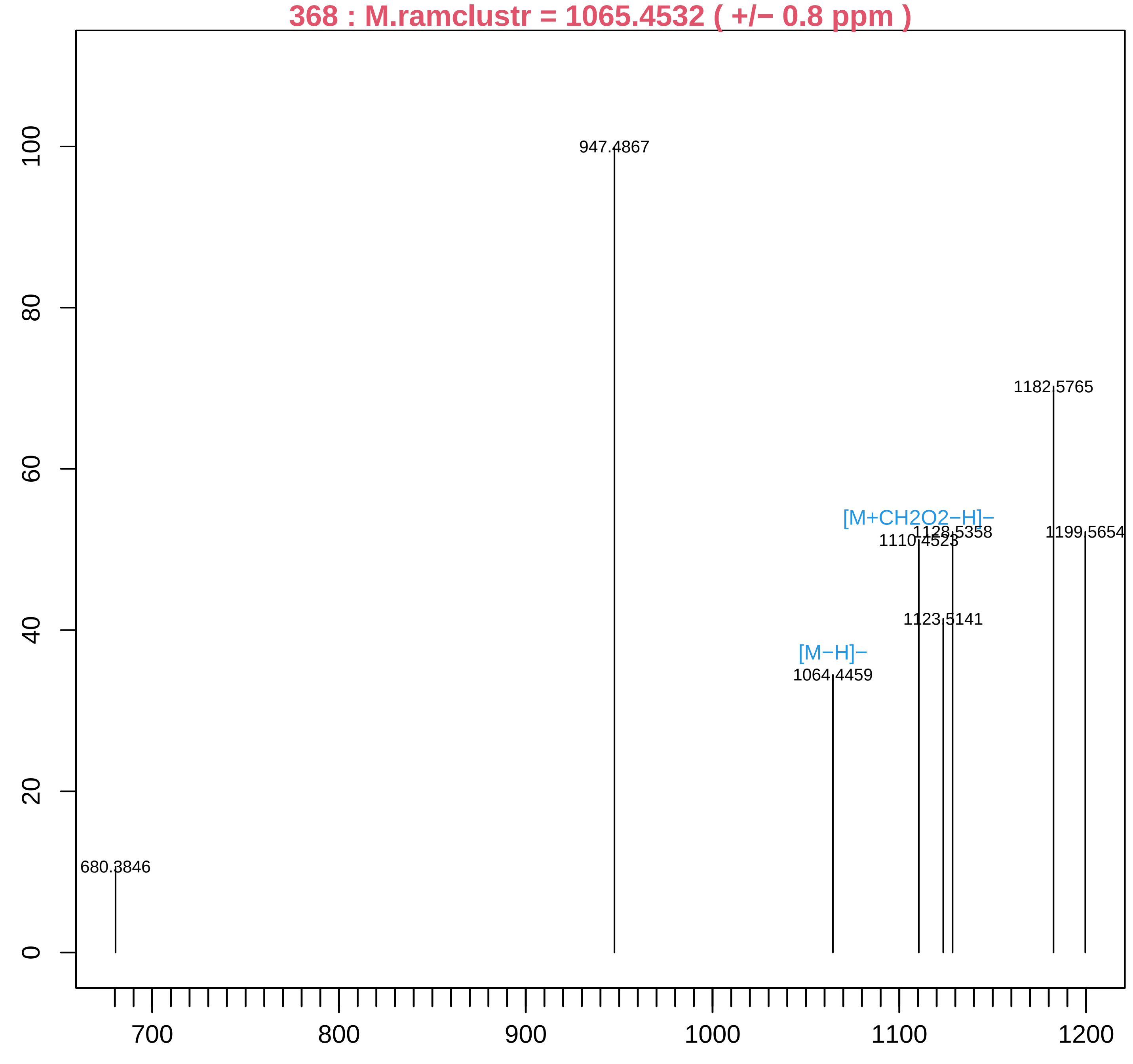

368 : M.ramclustr = 1065.4532 (+/− 0.8 ppm)
947.4867
1182.5765
[M+CH2O2−H]−
1128.5358
1110.4523
1199.5654
1123.5141
[M−H]−
1064.4459
680.3846
0
20
40
60
80
100
700
800
900
1000
1100
1200

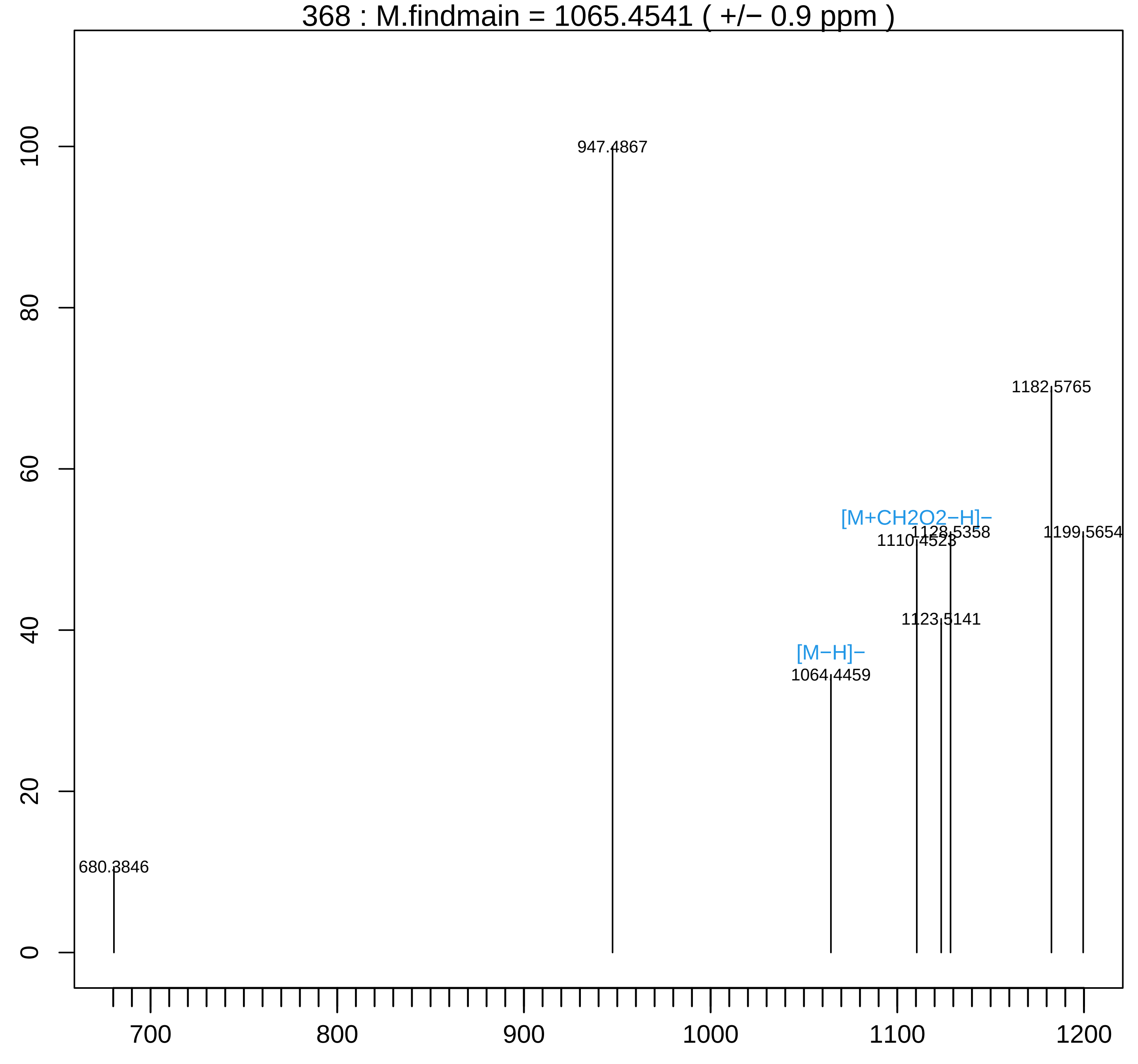

368 : M.findmain = 1065.4541 (+/− 0.9 ppm)
947.4867
1182.5765
[M+CH2O2−H]−
1128.5358
1110.4523
1199.5654
1123.5141
[M−H]−
1064.4459
680.3846
0
20
40
60
80
100
700
800
900
1000
1100
1200

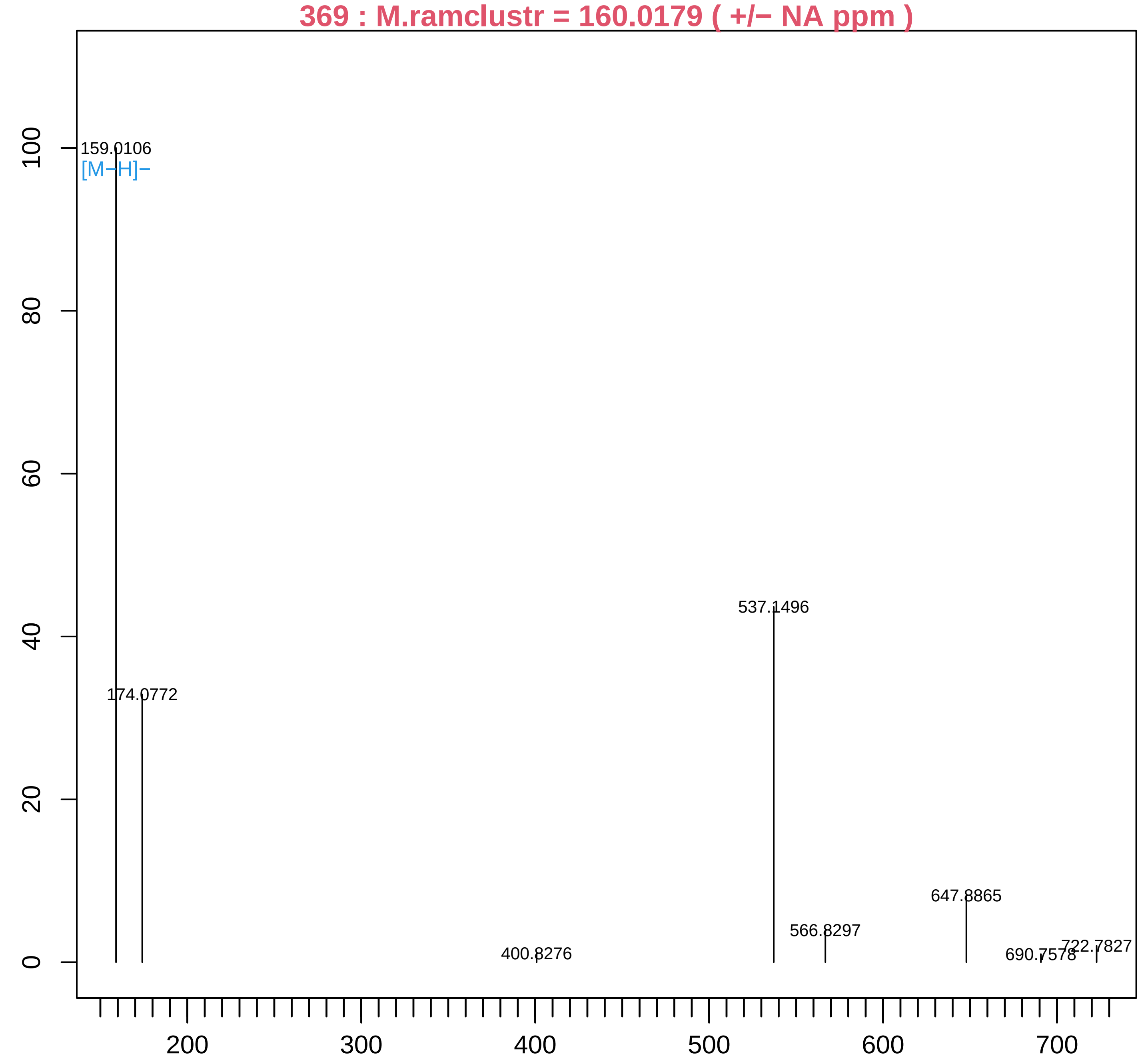

369 : M.ramclustr = 160.0179 (+/− NA ppm)
159.0106
[M−H]−
174.0772
400.8276
537.1496
566.8297
647.8865
690.7578
722.7827
0
20
40
60
80
100
200
300
400
500
600
700

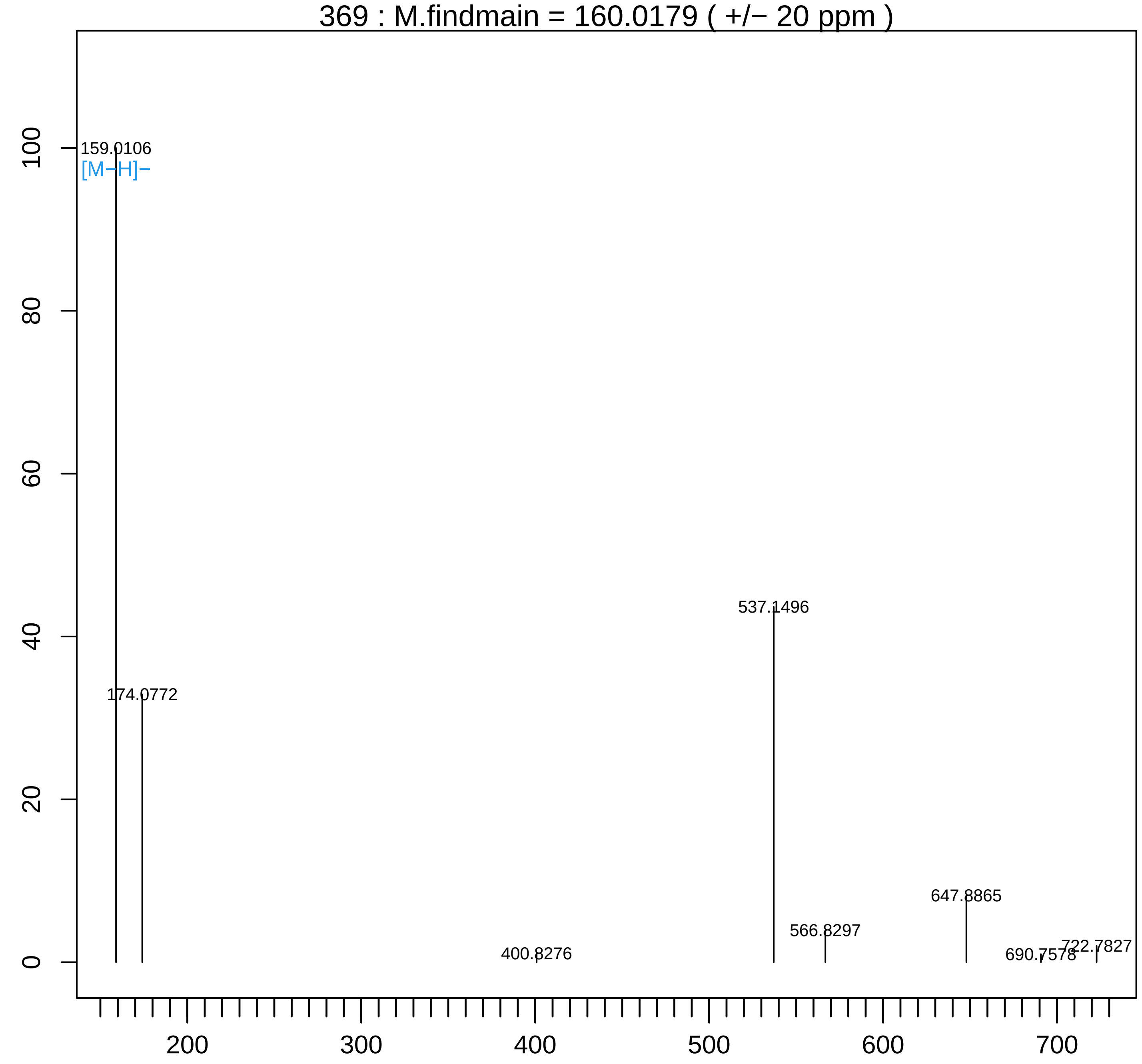

369 : M.findmain = 160.0179 (+/− 20 ppm)
159.0106
[M−H]−
174.0772
400.8276
537.1496
566.8297
647.8865
690.7578
722.7827
0
20
40
60
80
100
200
300
400
500
600
700

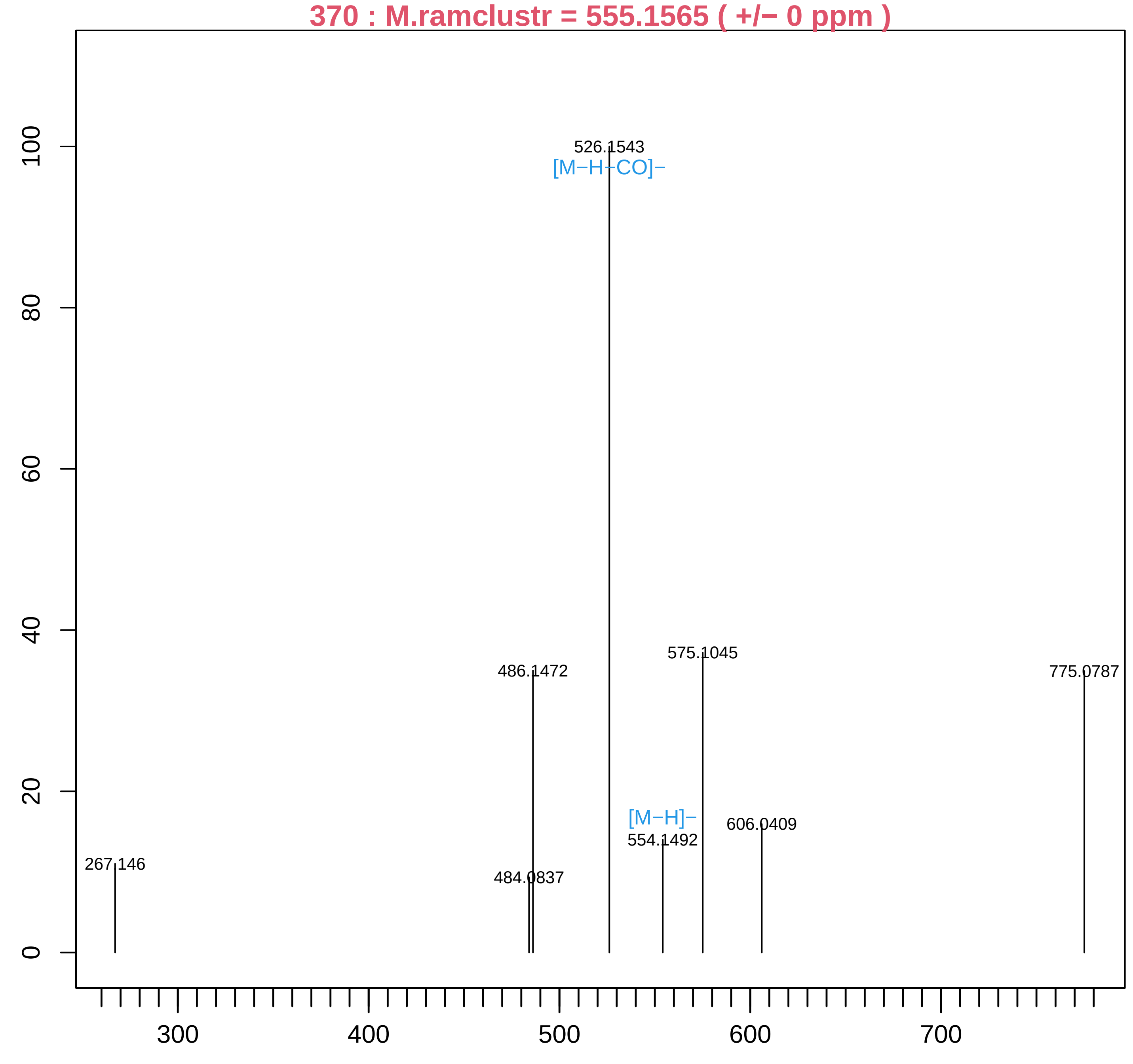
370 : M.ramclustr = 555.1565 (+/− 0 ppm)
526.1543
[M−H−CO]−
486.1472
575.1045
775.0787
[M−H]−
554.1492
606.0409
267.146
484.0837
0
20
40
60
80
100
300
400
500
600
700

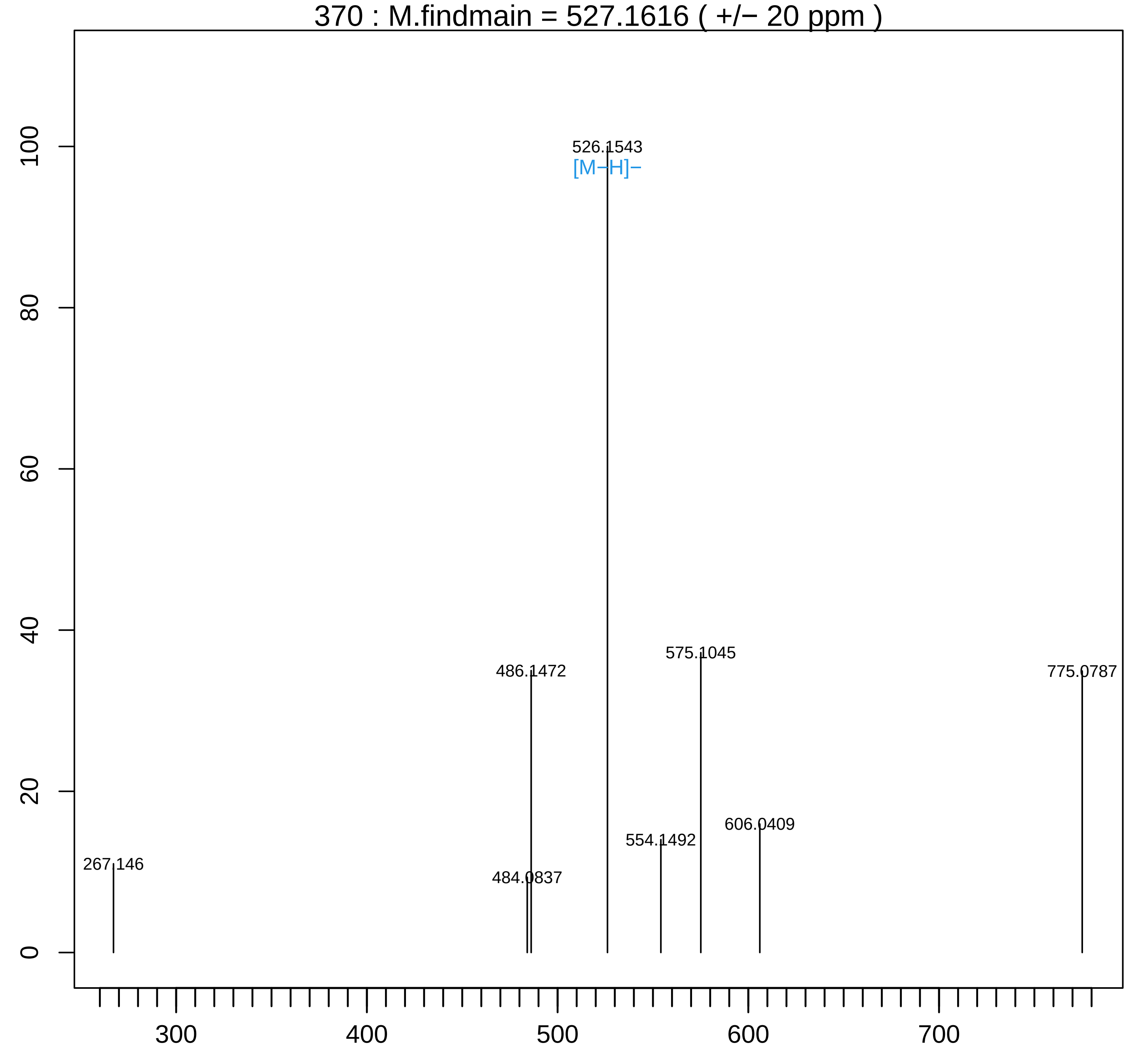
370 : M.findmain = 527.1616 (+/− 20 ppm)
526.1543
[M−H]−
486.1472
575.1045
775.0787
554.1492
606.0409
267.146
484.0837
0
20
40
60
80
100
300
400
500
600
700

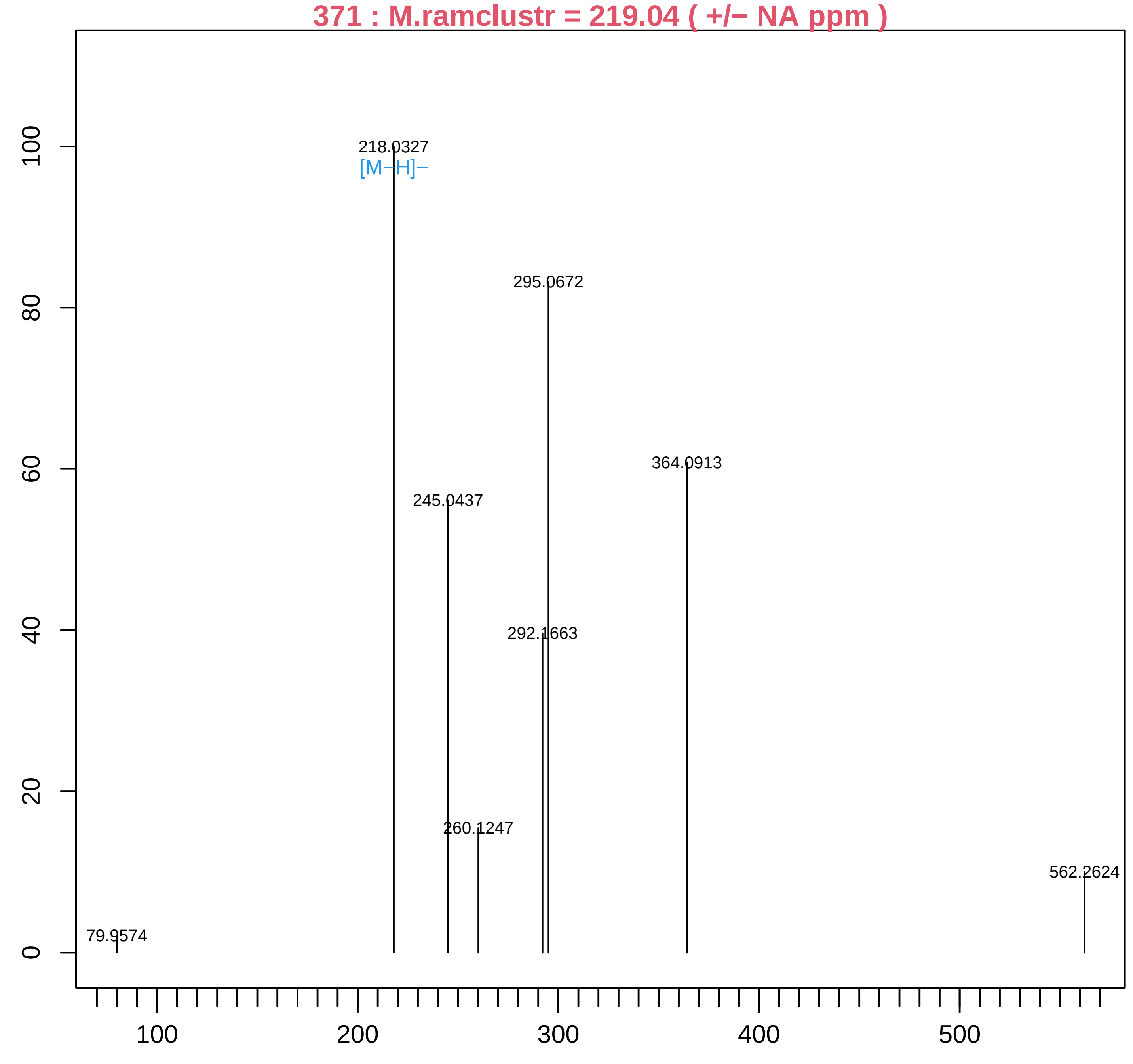

371 : M.ramclustr = 219.04 (+/− NA ppm)
218.0327
[M−H]−
295.0672
364.0913
245.0437
292.1663
260.1247
562.2624
79.9574
0
20
40
60
80
100
100
200
300
400
500

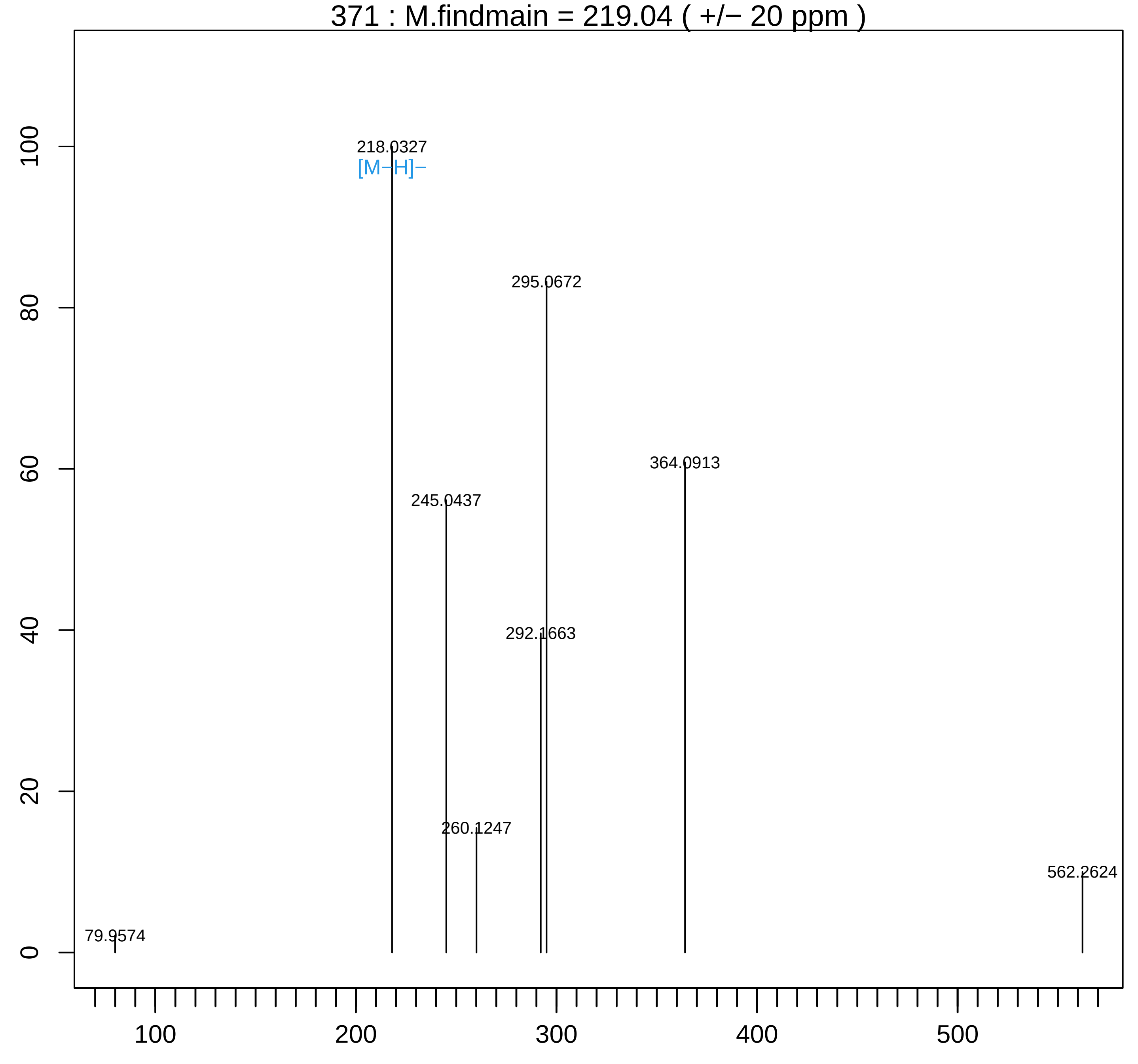

371 : M.findmain = 219.04 (+/− 20 ppm)
218.0327
[M−H]−
295.0672
364.0913
245.0437
292.1663
260.1247
562.2624
79.9574
0
20
40
60
80
100
100
200
300
400
500

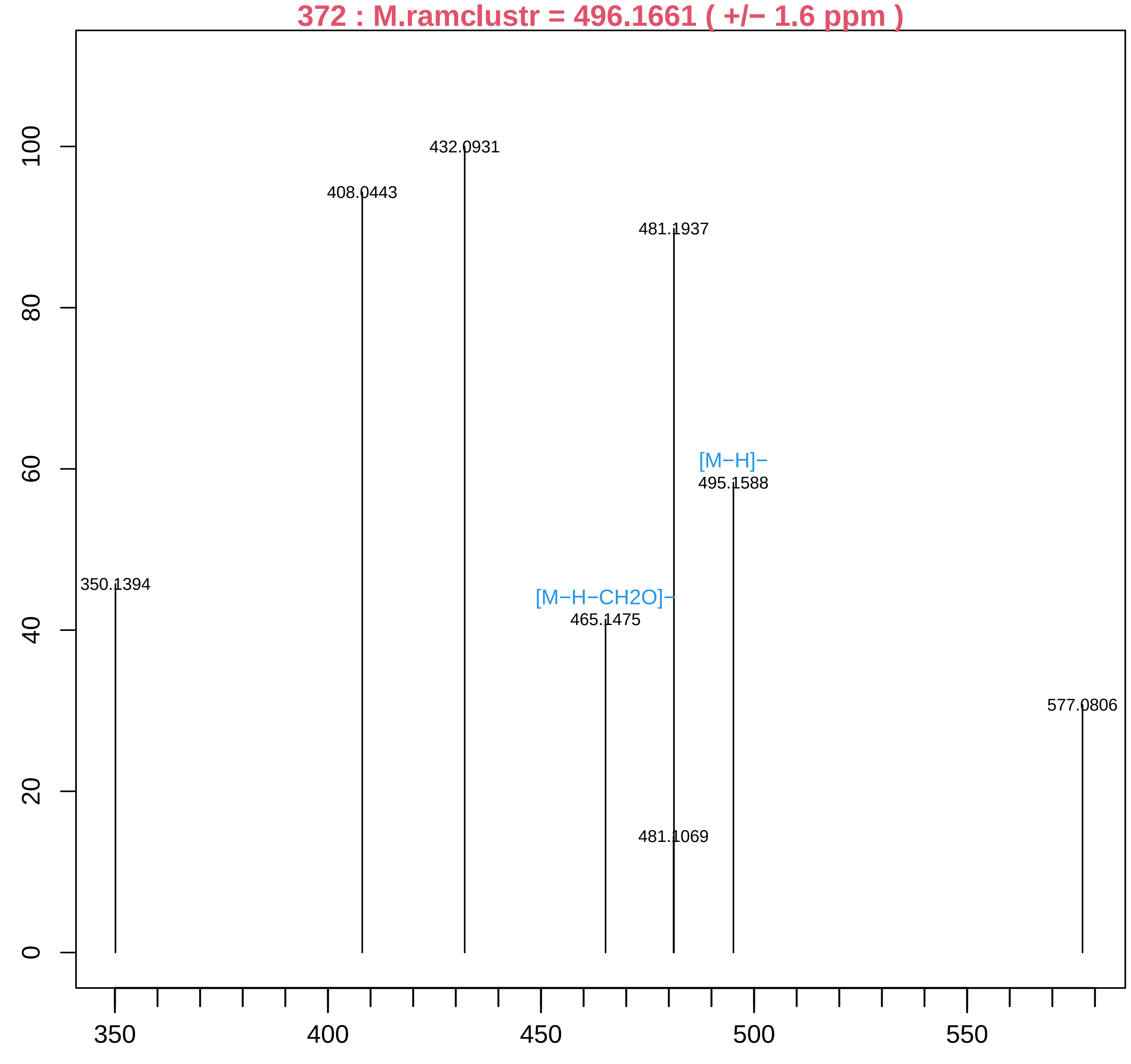
372 : M.ramclustr = 496.1661 (+/− 1.6 ppm)
350.1394
408.0443
432.0931
465.1475
[M−H−CH2O]−
481.1937
481.1069
495.1588
[M−H]−
577.0806
0
20
40
60
80
100
350
400
450
500
550

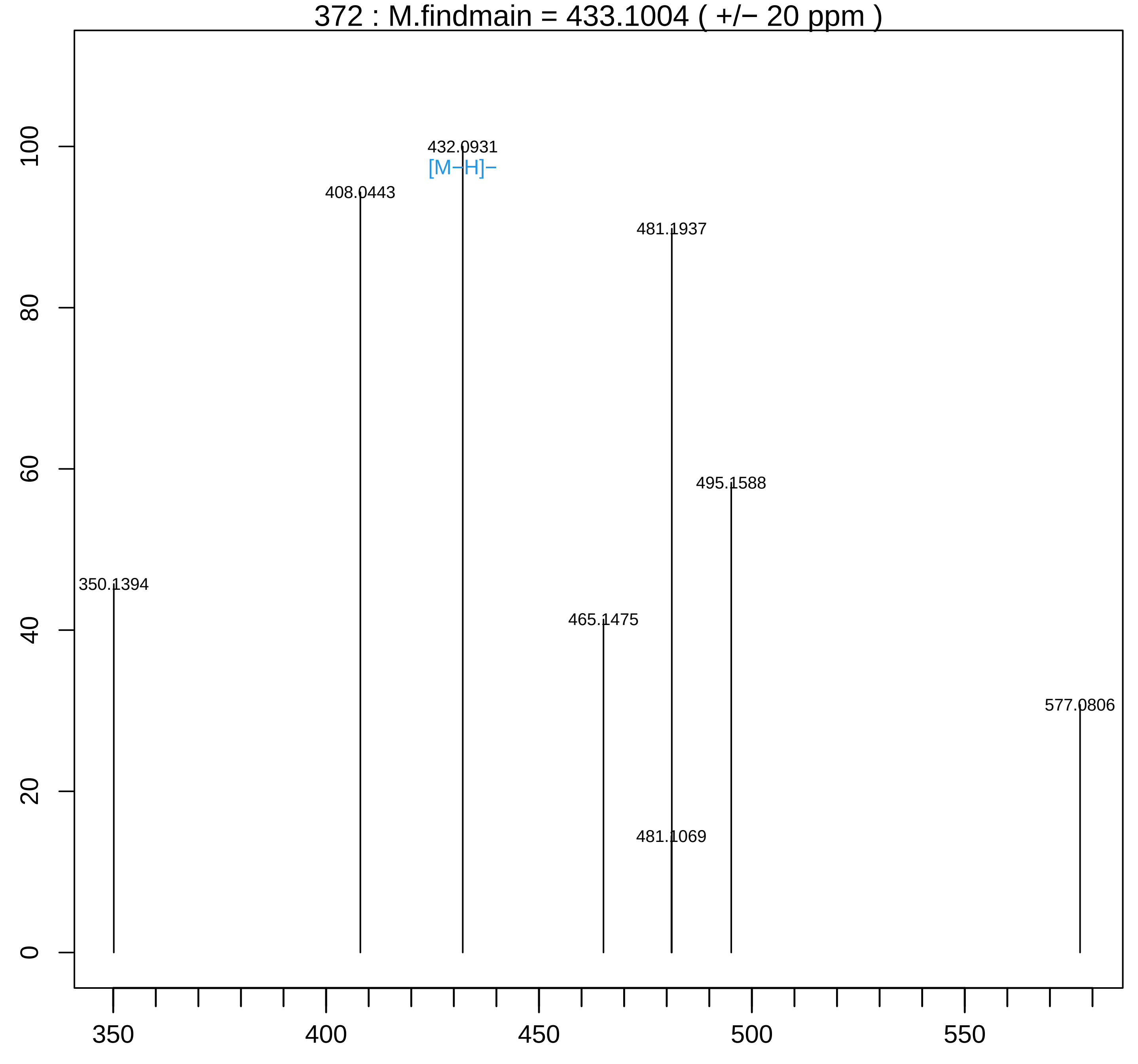
372 : M.findmain = 433.1004 (+/− 20 ppm)
350.1394
408.0443
432.0931
[M−H]−
465.1475
481.1937
481.1069
495.1588
577.0806
0
20
40
60
80
100
350
400
450
500
550

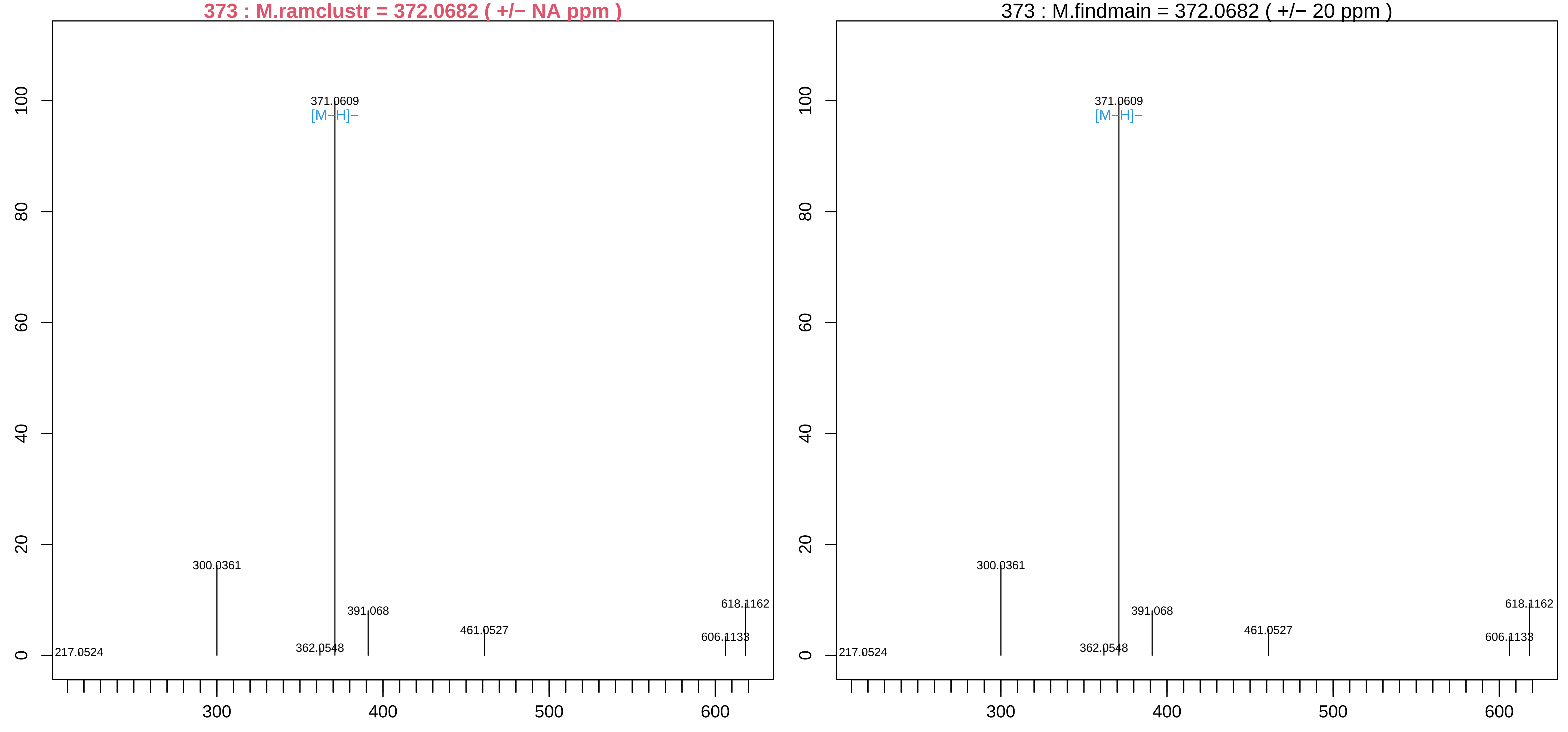

373 : M.ramclustr = 372.0682 (+/− NA ppm)
373 : M.findmain = 372.0682 (+/− 20 ppm)
371.0609
[M−H]−
300.0361
391.068
461.0527
618.1162
606.1133
362.0548
217.0524
0
20
40
60
80
100
300
400
500
600

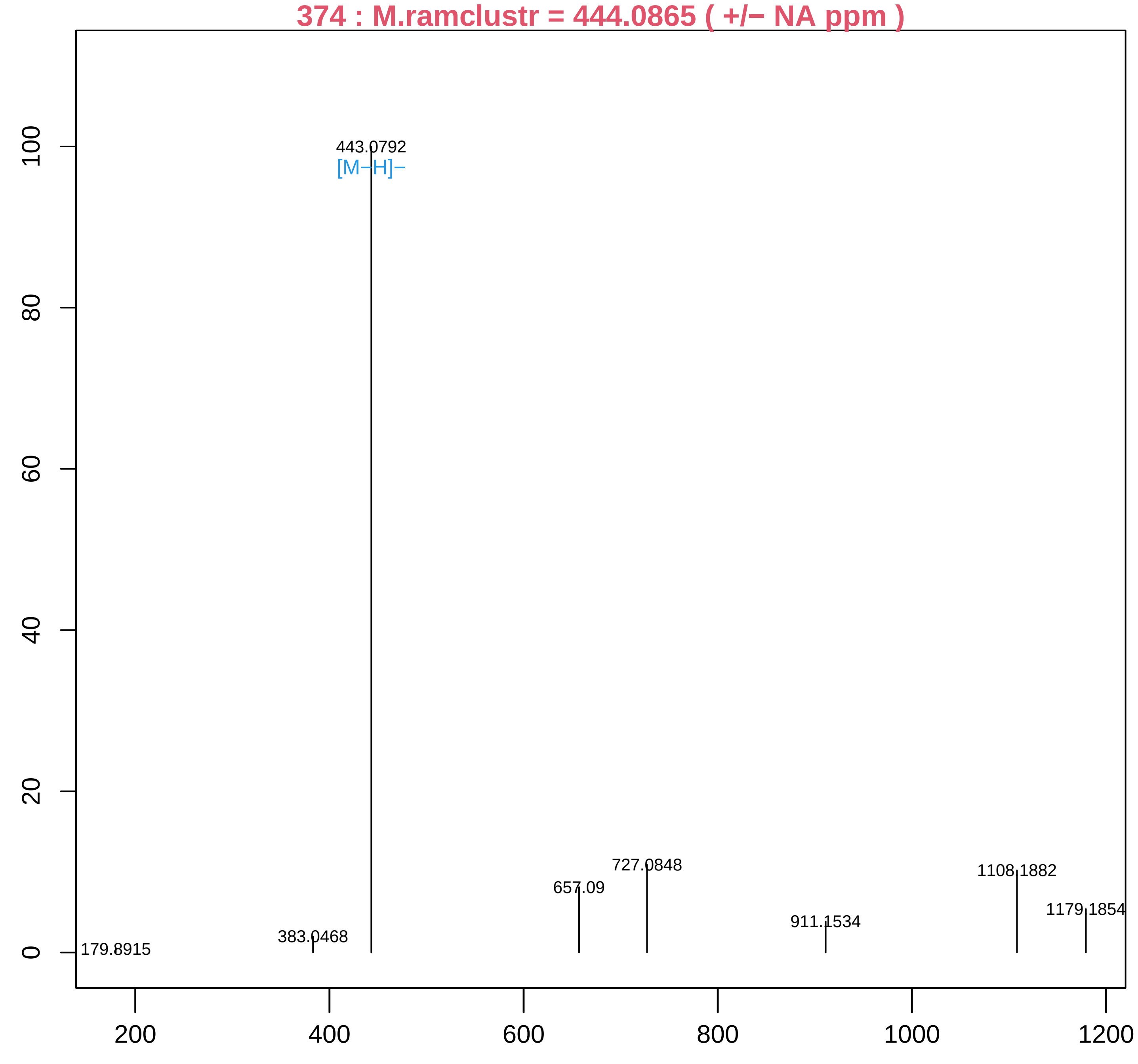
374 : M.ramclustr = 444.0865 (+/− NA ppm)
443.0792
[M−H]−
179.8915
383.0468
657.09
727.0848
911.1534
1108.1882
1179.1854

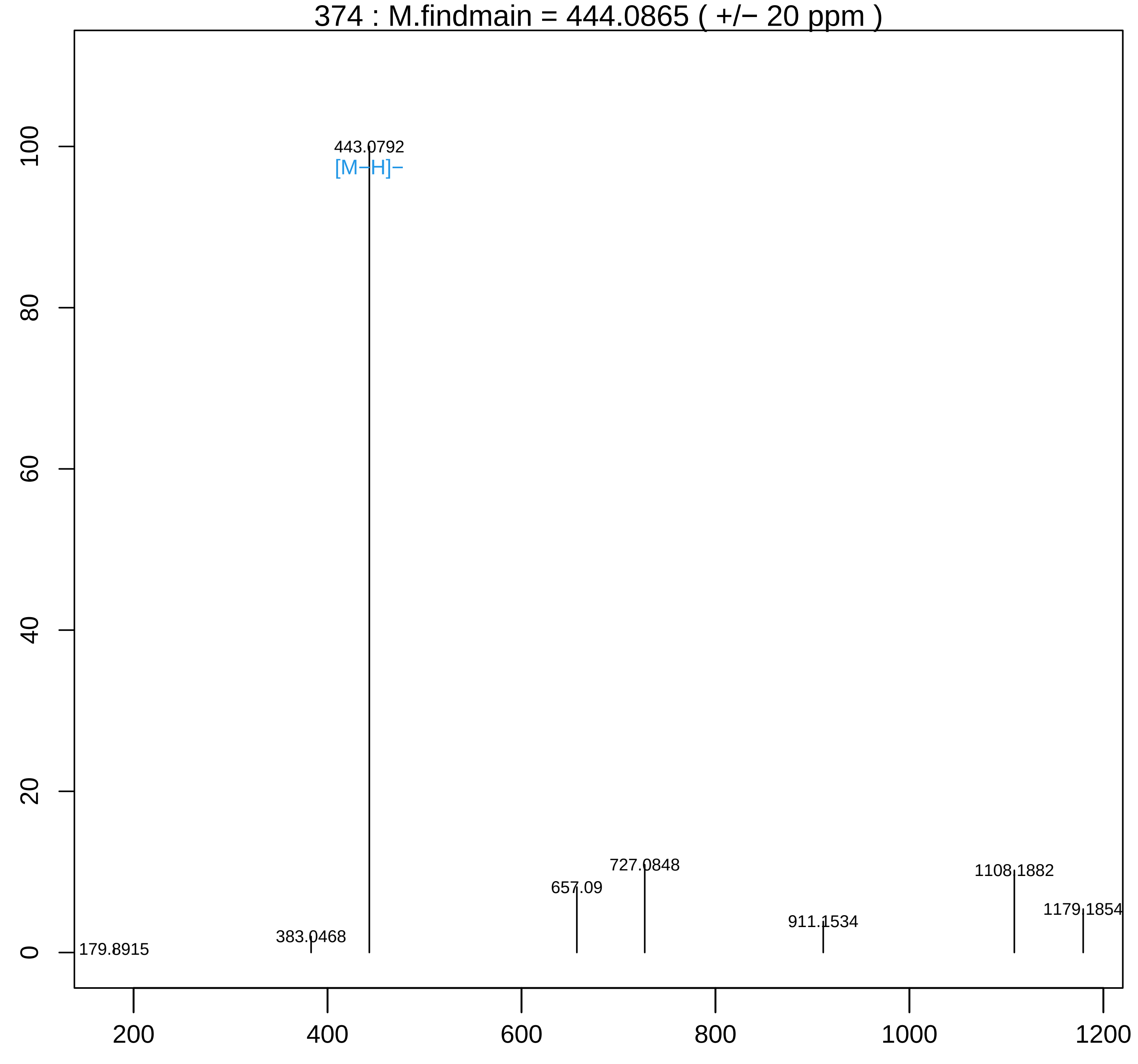
374 : M.findmain = 444.0865 (+/− 20 ppm)
443.0792
[M−H]−
179.8915
383.0468
657.09
727.0848
911.1534
1108.1882
1179.1854

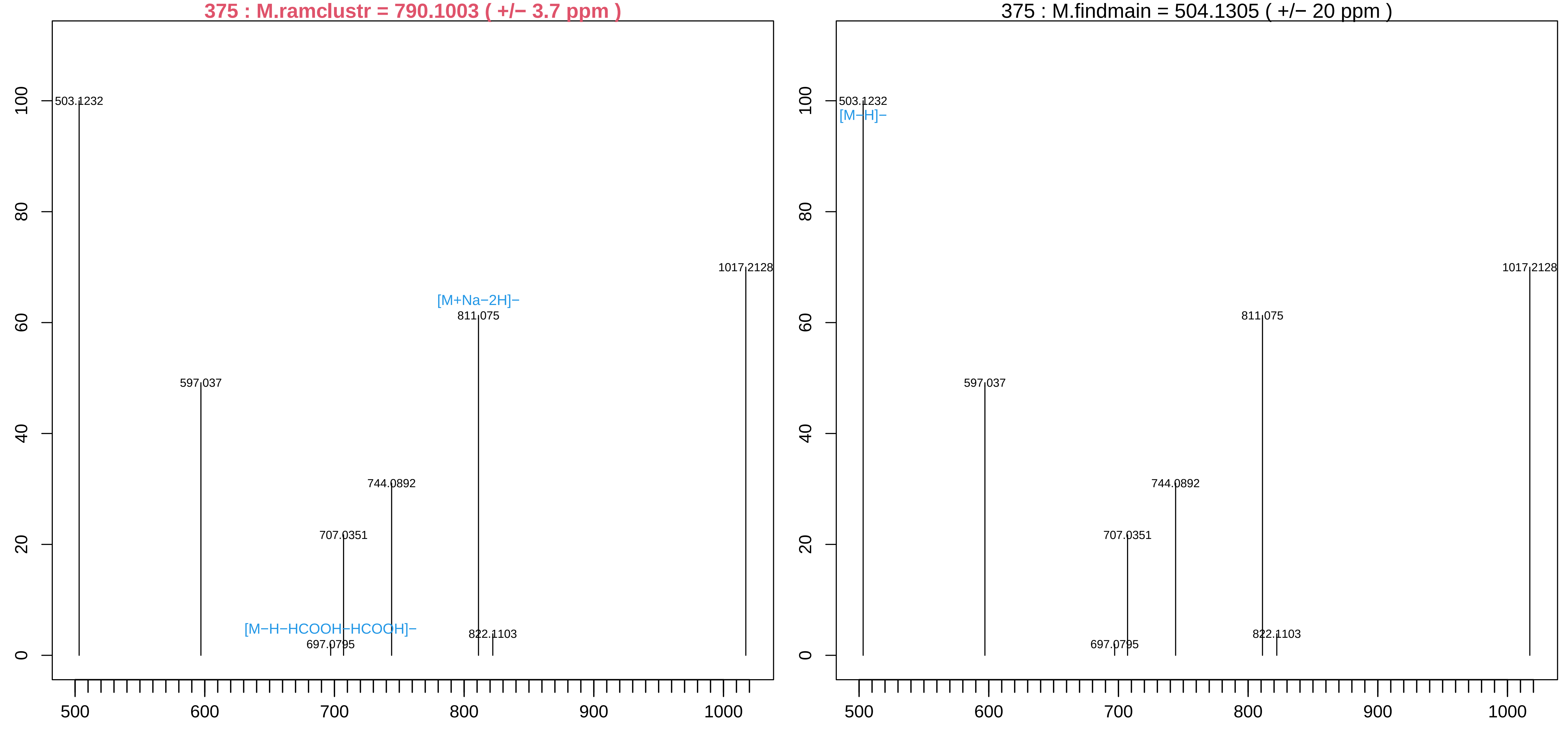

375 : M.ramclustr = 790.1003 (+/− 3.7 ppm)
503.1232
597.037
697.0795
707.0351
744.0892
811.075
822.1103
1017.2128
[M+Na−2H]−
[M−H−HCOOH−HCOOH]−
375 : M.findmain = 504.1305 (+/− 20 ppm)
503.1232
[M−H]−
597.037
697.0795
707.0351
744.0892
811.075
822.1103
1017.2128

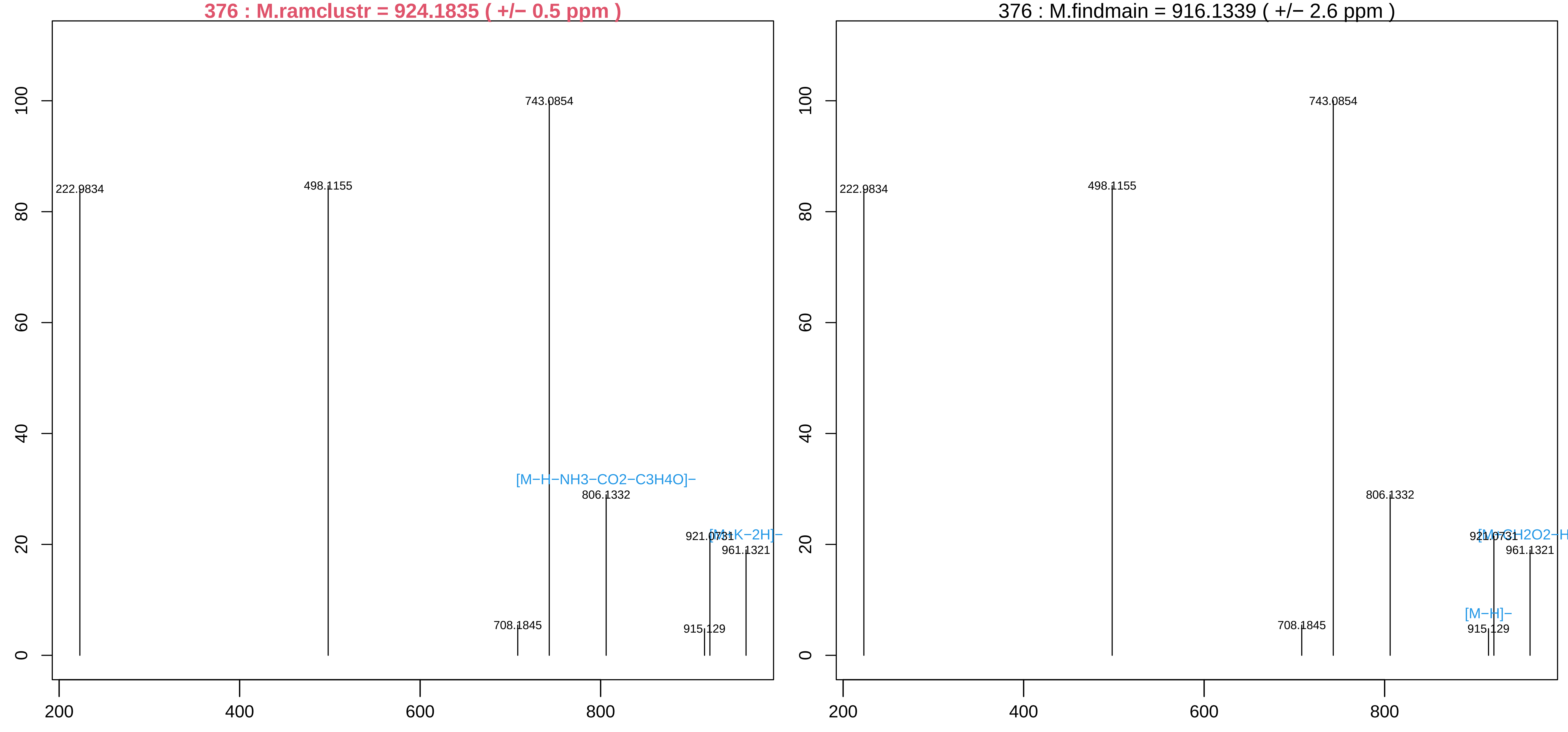
376 : M.ramclustr = 924.1835 (+/− 0.5 ppm)
222.9834
498.1155
743.0854
[M−H−NH3−CO2−C3H4O]−
806.1332
[M+K−2H]−
921.0731
961.1321
708.1845
915.129
376 : M.findmain = 916.1339 (+/− 2.6 ppm)
222.9834
498.1155
743.0854
806.1332
921.0731
961.1321
[M−H]−
708.1845
915.129
0
20
40
60
80
100
200
400
600
800

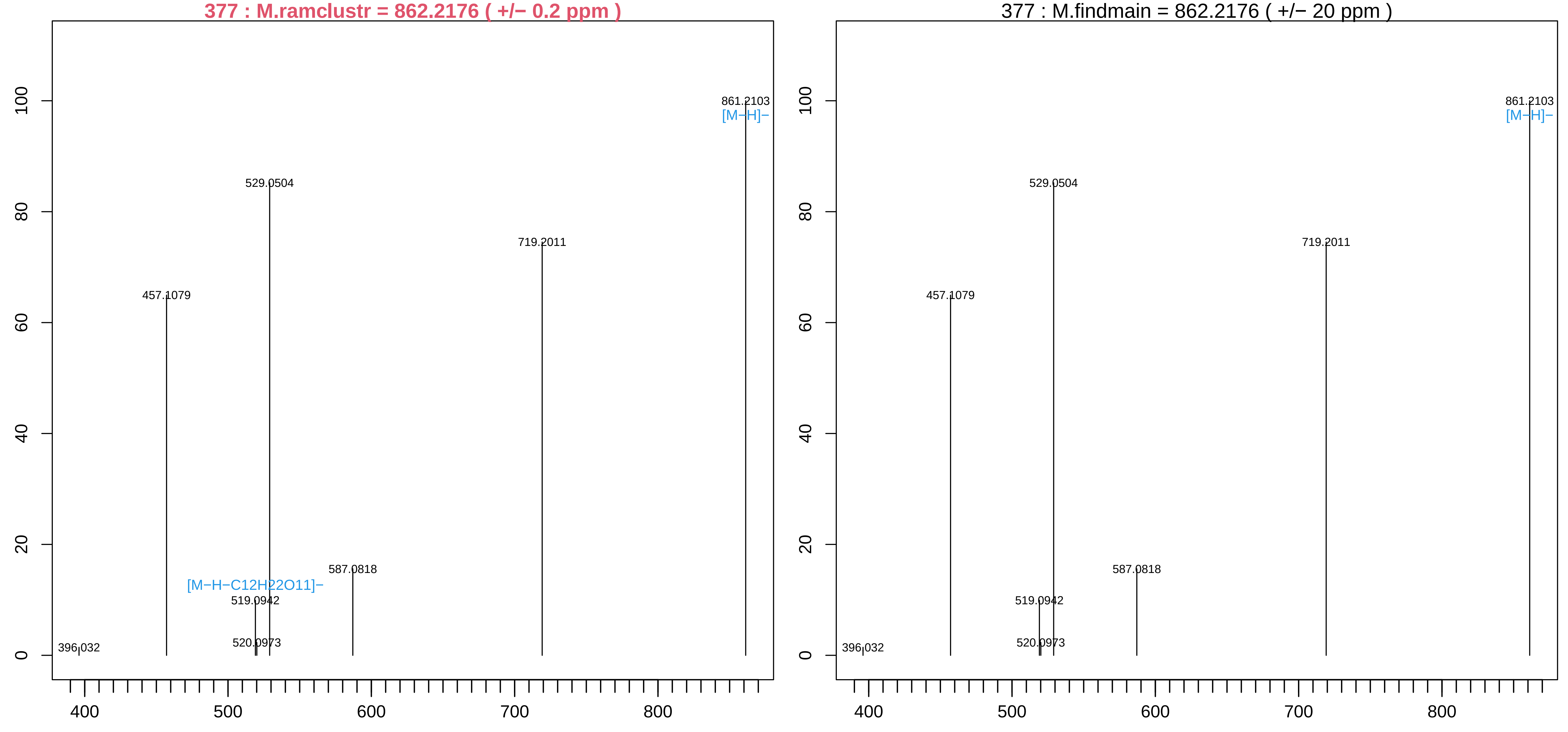
377 : M.ramclustr = 862.2176 (+/− 0.2 ppm)
861.2103
[M−H]−
529.0504
719.2011
457.1079
587.0818
[M−H−C12H22O11]−
519.0942
520.0973
396.032
0
20
40
60
80
100
400
500
600
700
800
377 : M.findmain = 862.2176 (+/− 20 ppm)
861.2103
[M−H]−
529.0504
719.2011
457.1079
587.0818
519.0942
520.0973
396.032
0
20
40
60
80
100
400
500
600
700
800

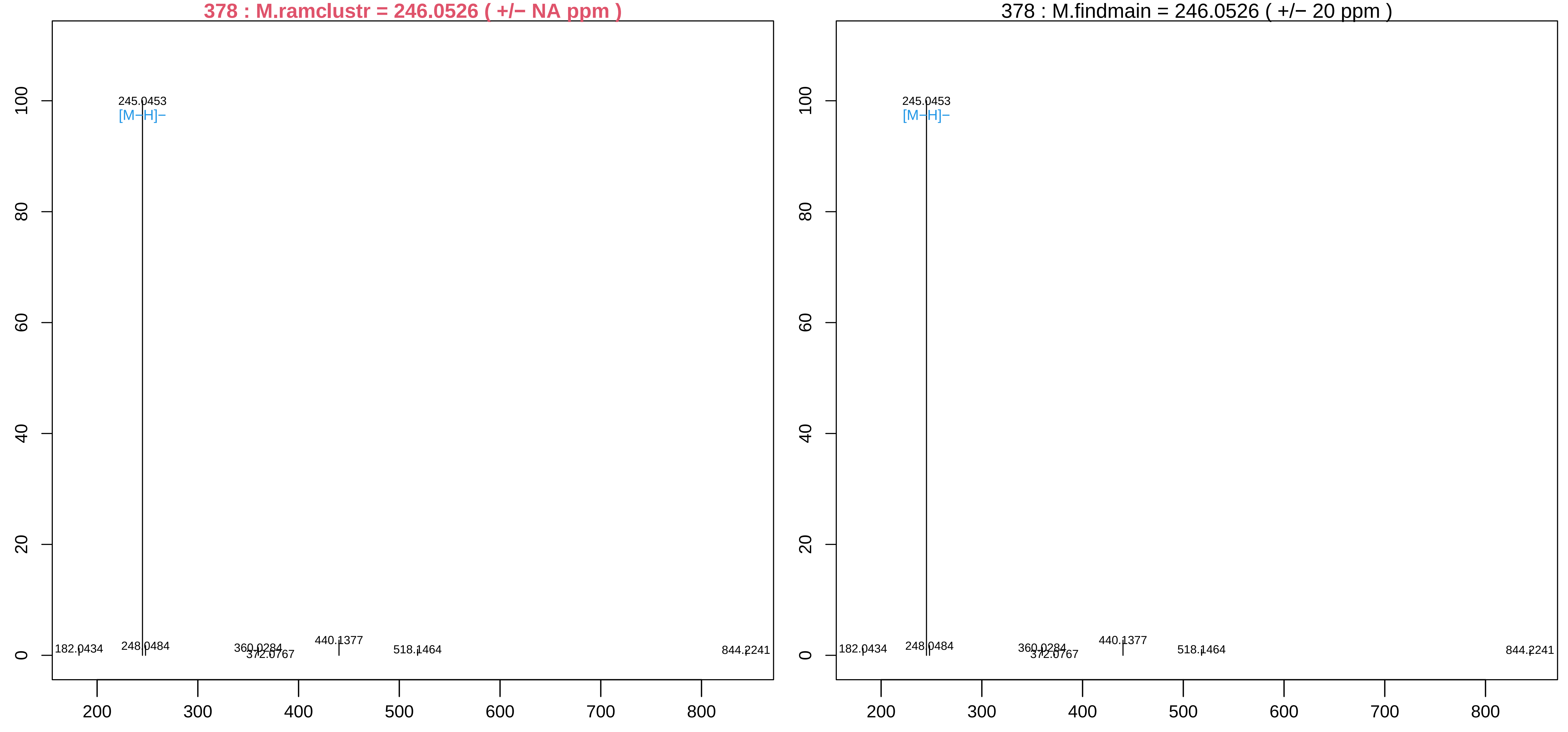
378 : M.ramclustr = 246.0526 (+/− NA ppm)
245.0453
[M−H]−
182.0434
248.0484
360.0284
372.0767
440.1377
518.1464
844.2241
378 : M.findmain = 246.0526 (+/− 20 ppm)
245.0453
[M−H]−
182.0434
248.0484
360.0284
372.0767
440.1377
518.1464
844.2241

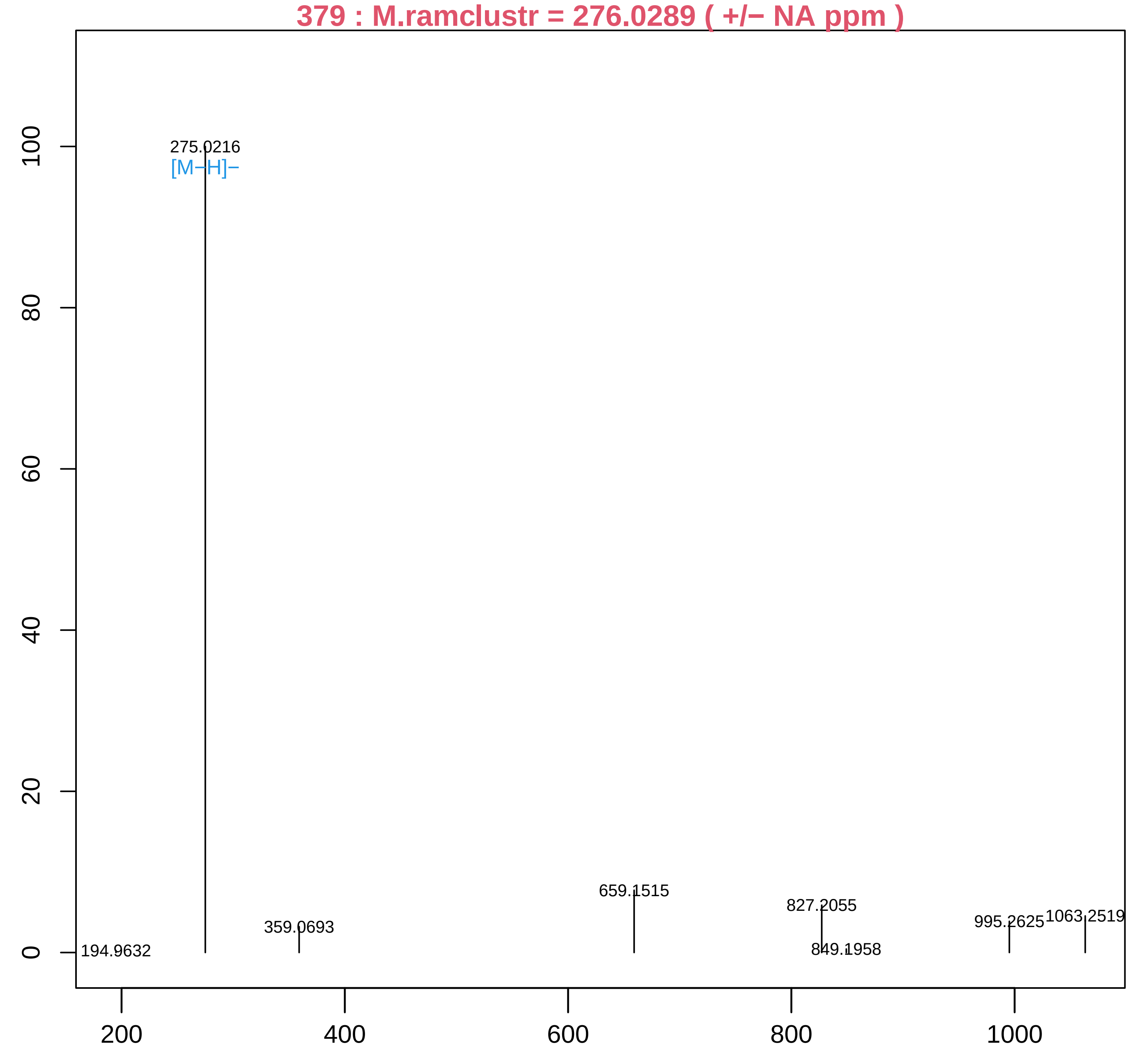
379 : M.ramclustr = 276.0289 (+/− NA ppm)
275.0216
[M−H]−
359.0693
659.1515
827.2055
849.1958
995.2625
1063.2519
194.9632
0
20
40
60
80
100
200
400
600
800
1000

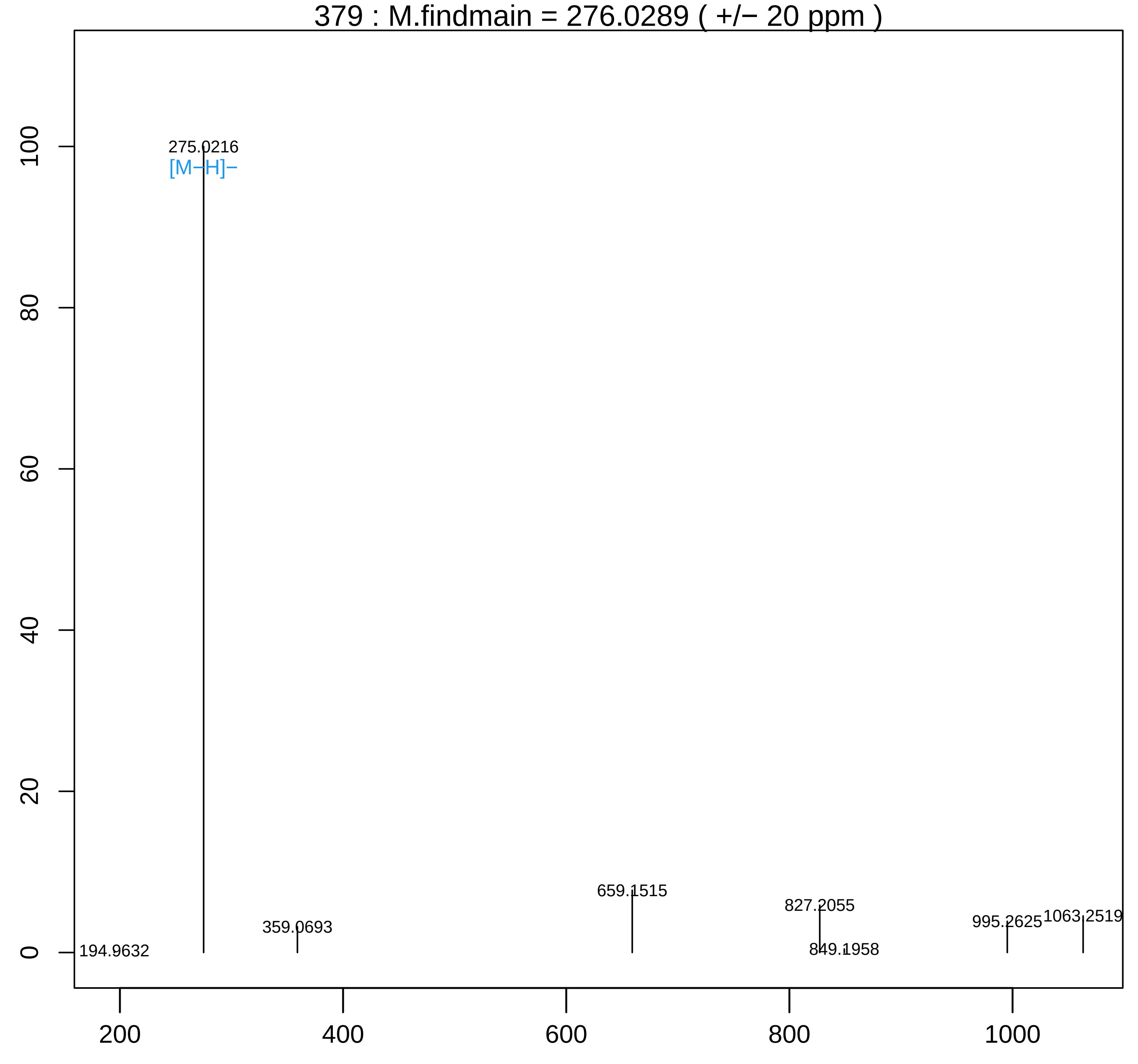
379 : M.findmain = 276.0289 (+/− 20 ppm)
275.0216
[M−H]−
359.0693
659.1515
827.2055
849.1958
995.2625
1063.2519
194.9632
0
20
40
60
80
100
200
400
600
800
1000

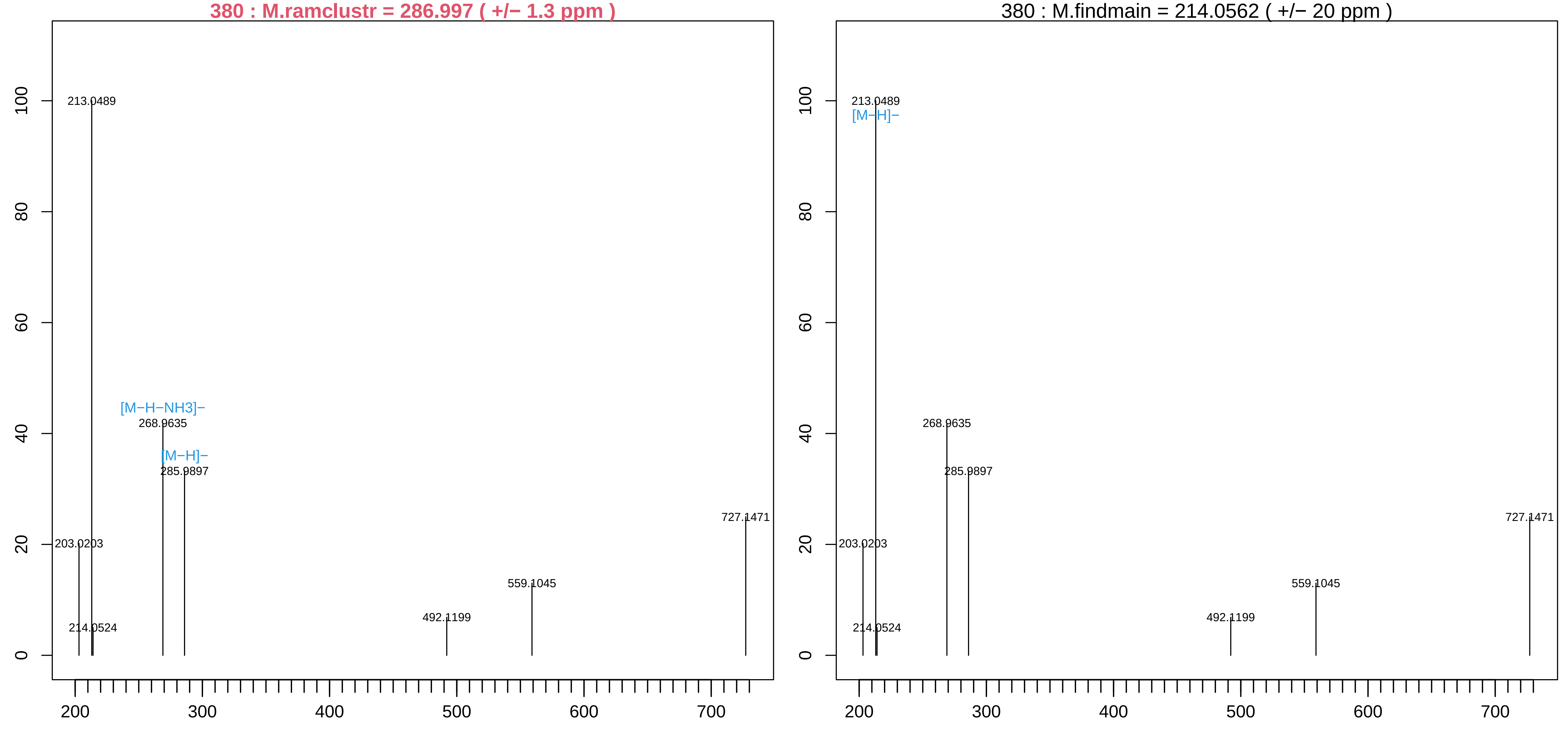

380 : M.ramclustr = 286.997 (+/− 1.3 ppm)
213.0489
[M−H−NH3]−
268.9635
[M−H]−
285.9897
727.1471
203.0203
559.1045
492.1199
214.0524
0
20
40
60
80
100
200
300
400
500
600
700
380 : M.findmain = 214.0562 (+/− 20 ppm)
213.0489
[M−H]−
268.9635
285.9897
727.1471
203.0203
559.1045
492.1199
214.0524

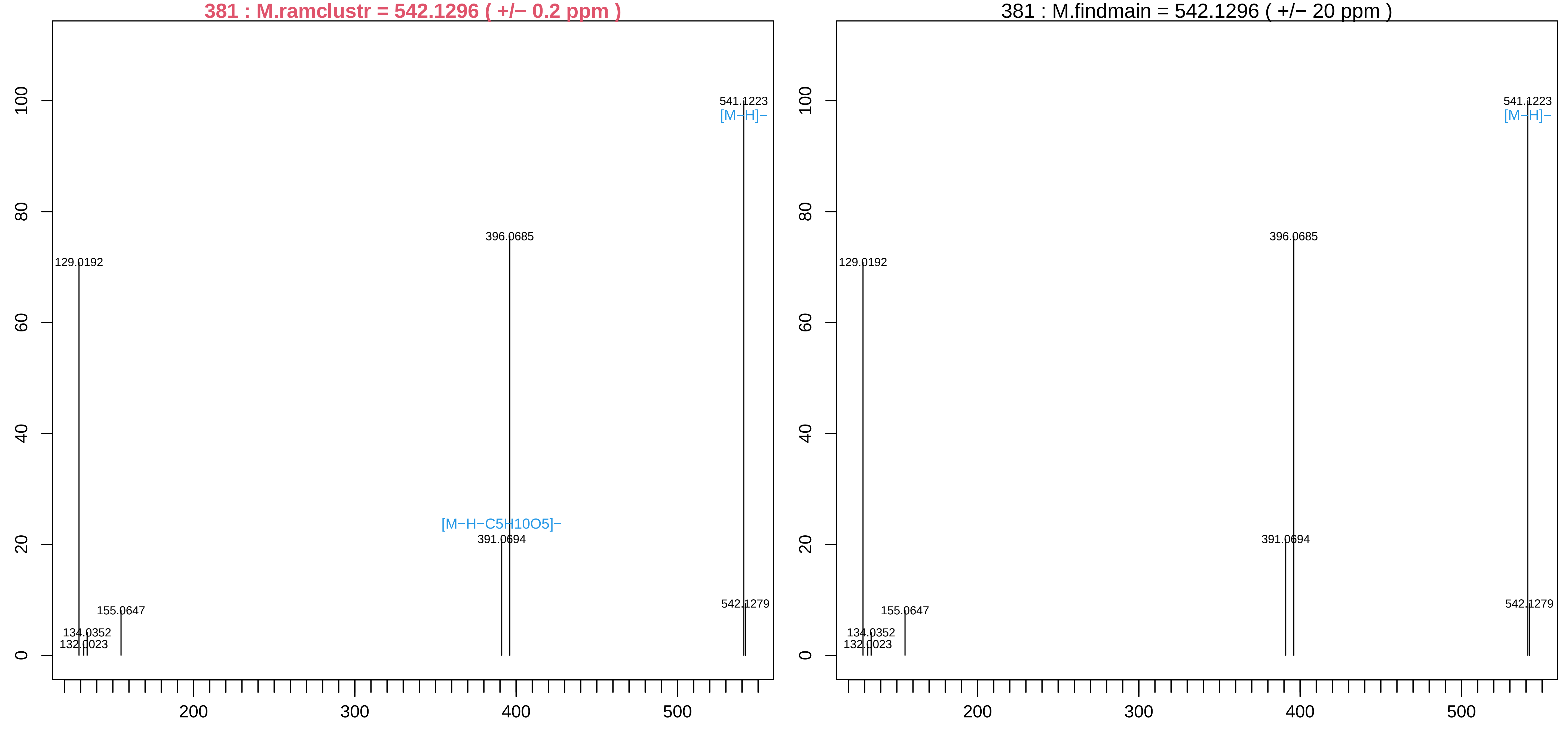
381 : M.ramclustr = 542.1296 (+/− 0.2 ppm)
541.1223
[M−H]−
396.0685
129.0192
[M−H−C5H10O5]−
391.0694
155.0647
134.0352
132.0023
542.1279
381 : M.findmain = 542.1296 (+/− 20 ppm)
541.1223
[M−H]−
396.0685
129.0192
391.0694
155.0647
134.0352
132.0023
542.1279

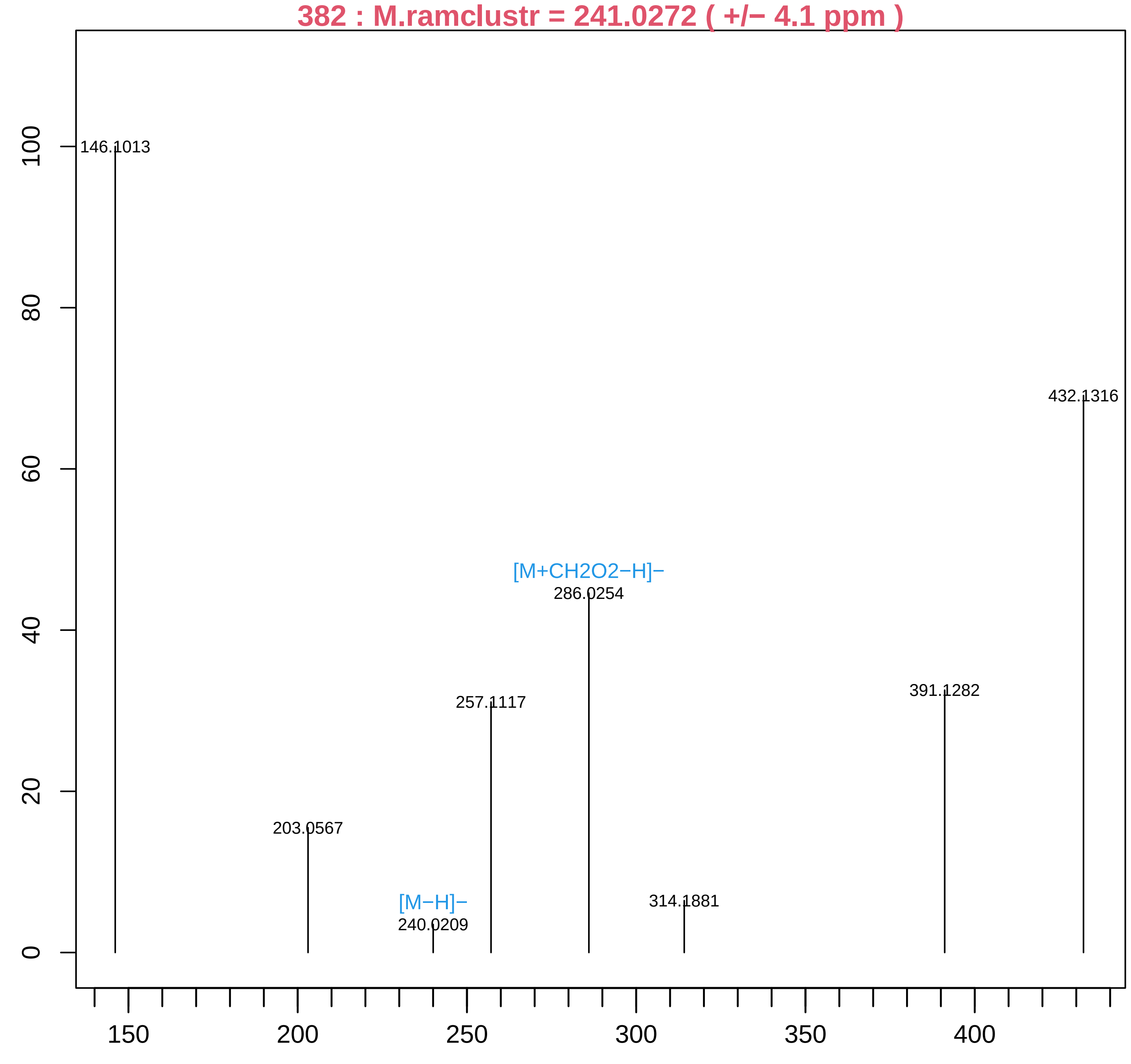
382 : M.ramclustr = 241.0272 (+/− 4.1 ppm)
146.1013
203.0567
[M−H]−
240.0209
257.1117
[M+CH2O2−H]−
286.0254
314.1881
391.1282
432.1316
0
20
40
60
80
100
150
200
250
300
350
400

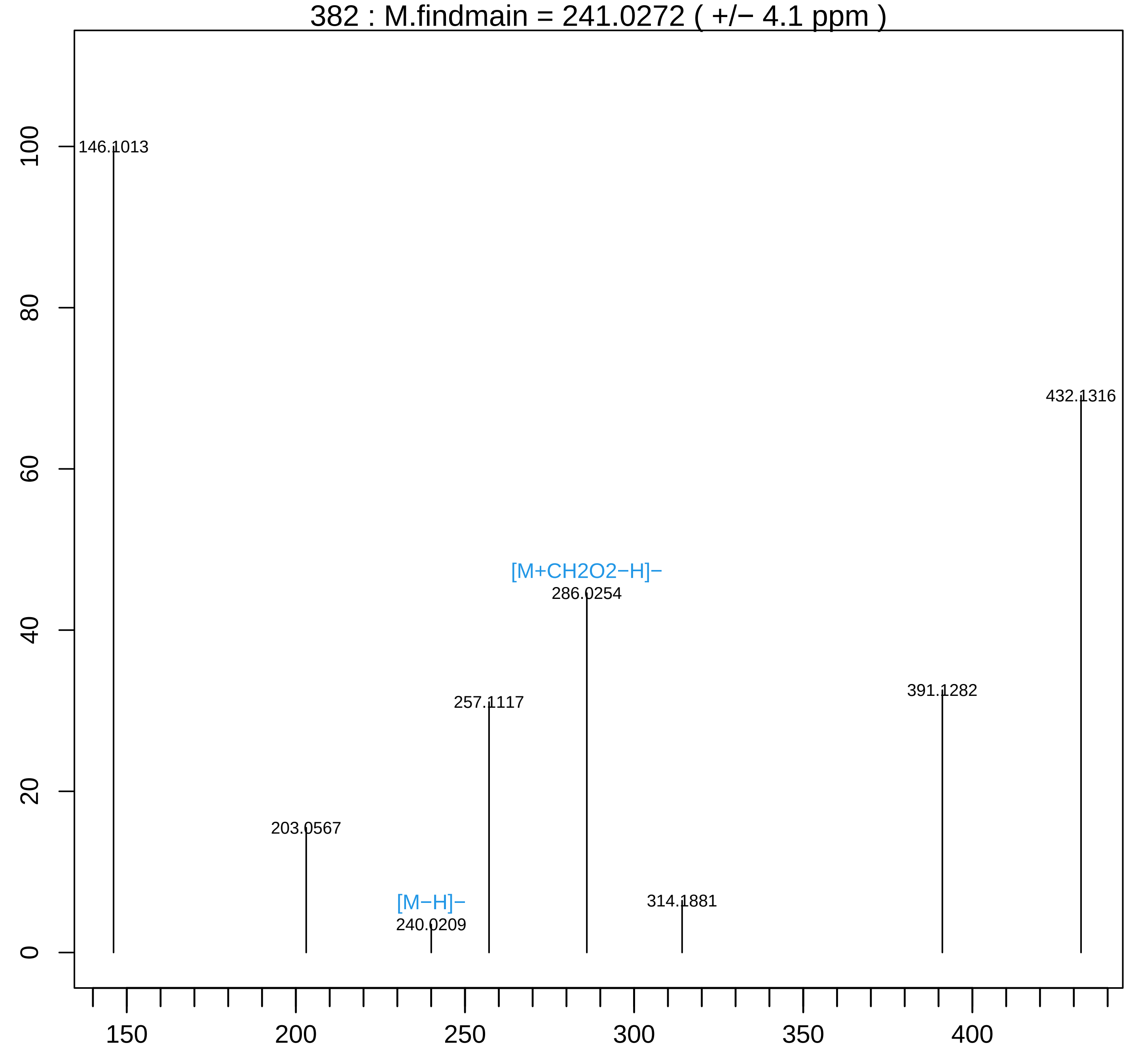
382 : M.findmain = 241.0272 (+/− 4.1 ppm)
146.1013
203.0567
[M−H]−
240.0209
257.1117
[M+CH2O2−H]−
286.0254
314.1881
391.1282
432.1316
0
20
40
60
80
100
150
200
250
300
350
400

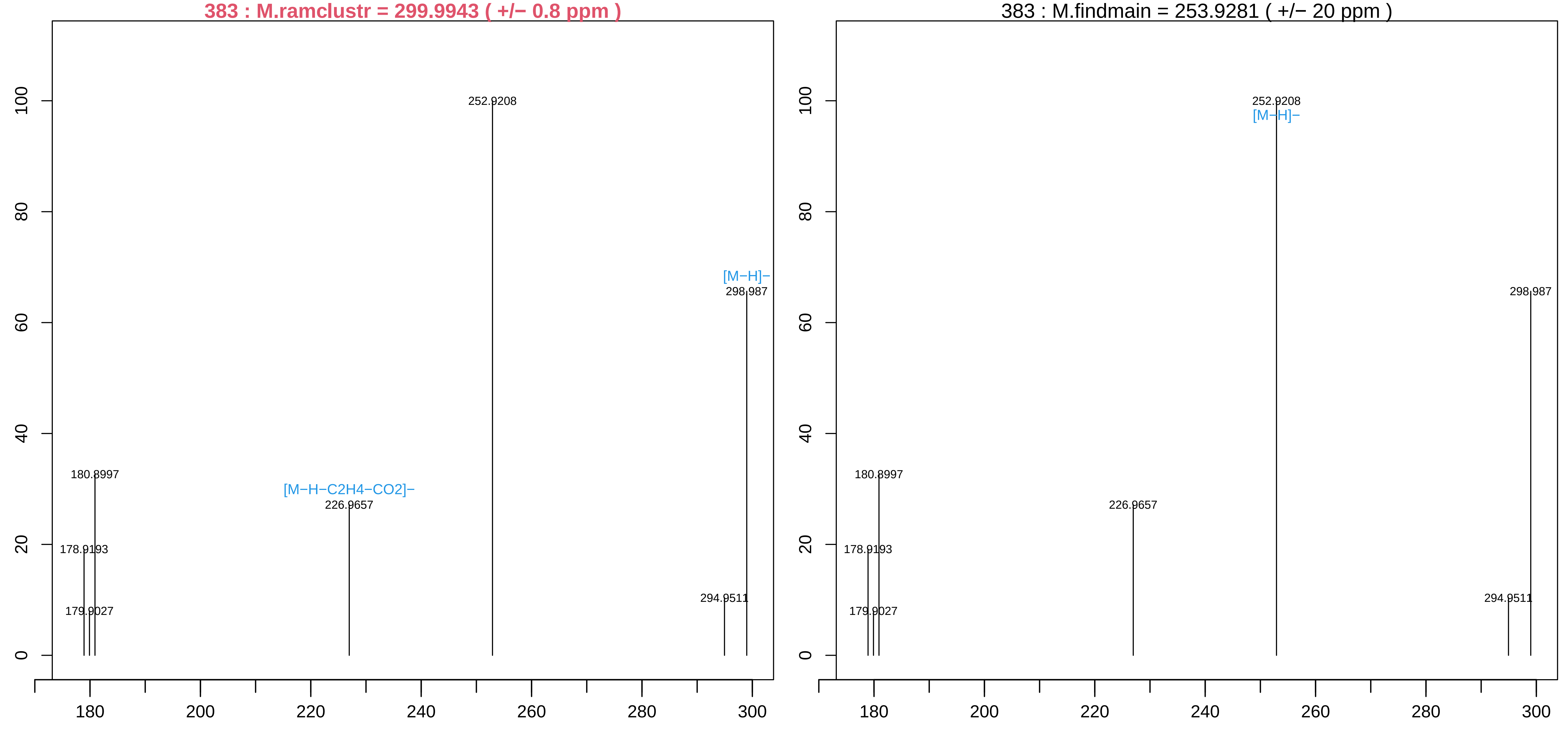
383 : M.ramclustr = 299.9943 (+/− 0.8 ppm)
383 : M.findmain = 253.9281 (+/− 20 ppm)
252.9208
[M−H]−
298.987
180.8997
178.9193
179.9027
[M−H−C2H4−CO2]−
226.9657
294.9511
0
20
40
60
80
100
180
200
220
240
260
280
300

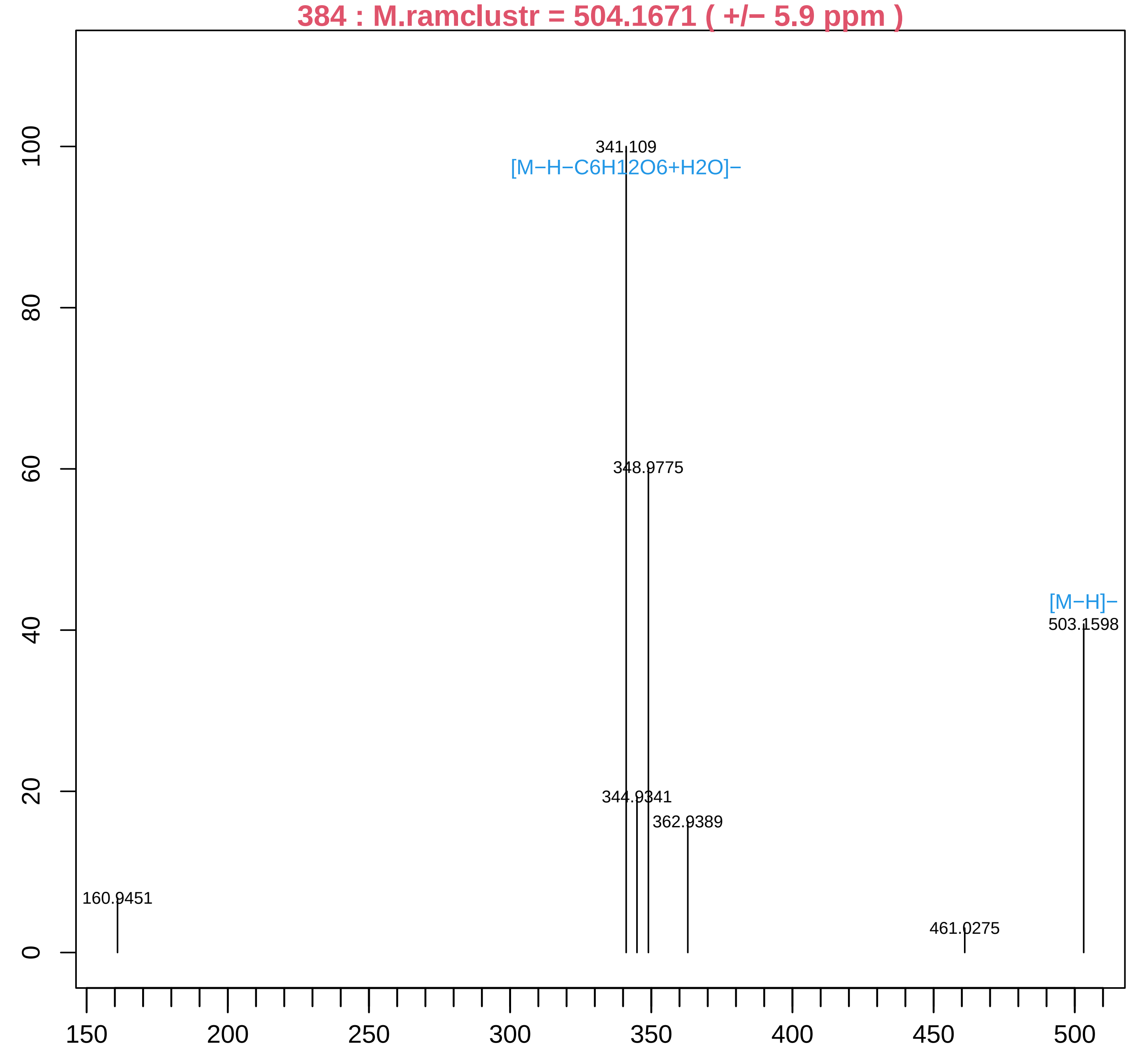
384 : M.ramclustr = 504.1671 (+/− 5.9 ppm)
341.109
[M−H−C6H12O6+H2O]−
348.9775
[M−H]−
503.1598
344.9341
362.9389
160.9451
461.0275
0
20
40
60
80
100
150
200
250
300
350
400
450
500
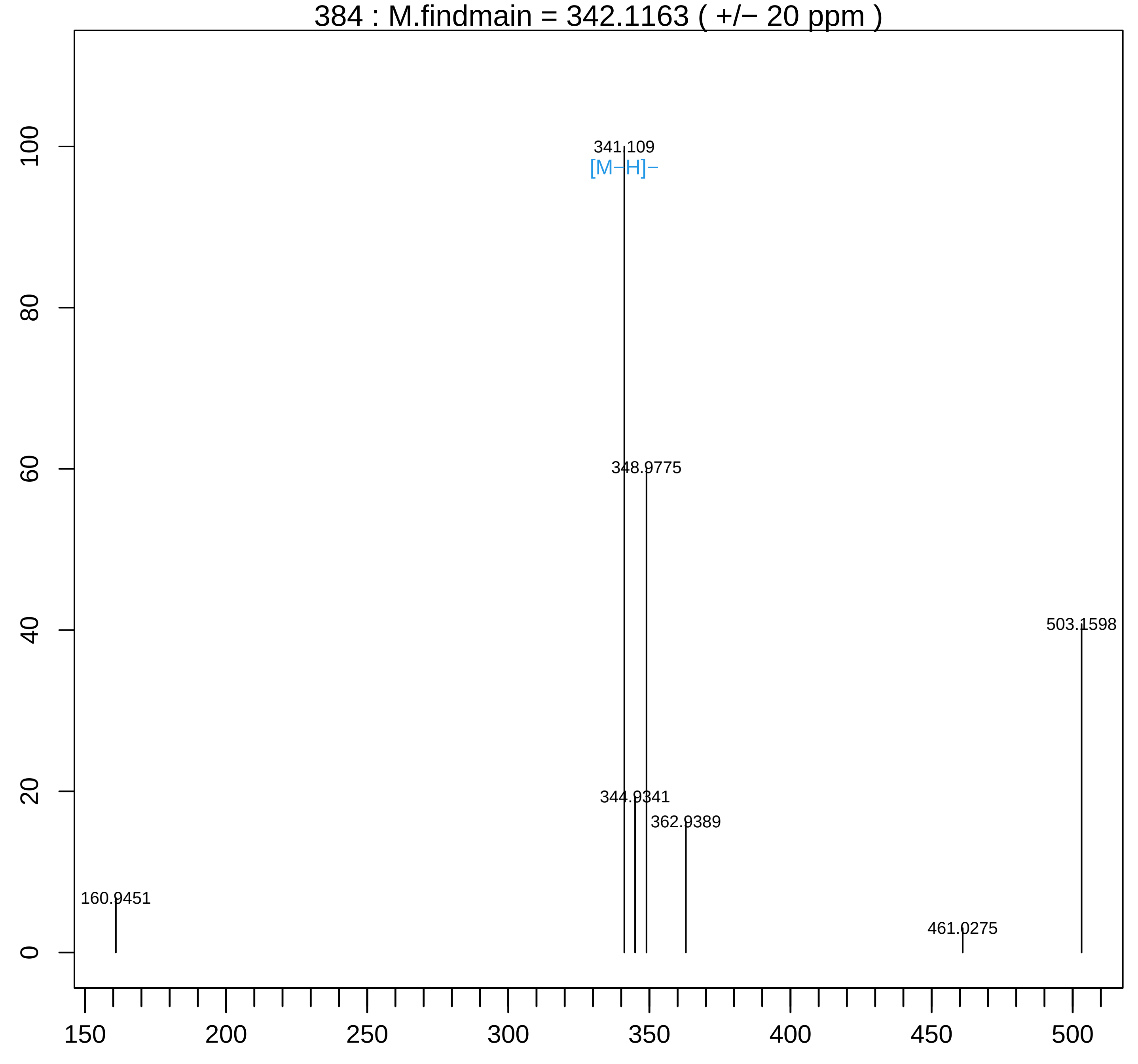
384 : M.findmain = 342.1163 (+/− 20 ppm)
341.109
[M−H]−
348.9775
503.1598
344.9341
362.9389
160.9451
461.0275
0
20
40
60
80
100
150
200
250
300
350
400
450
500

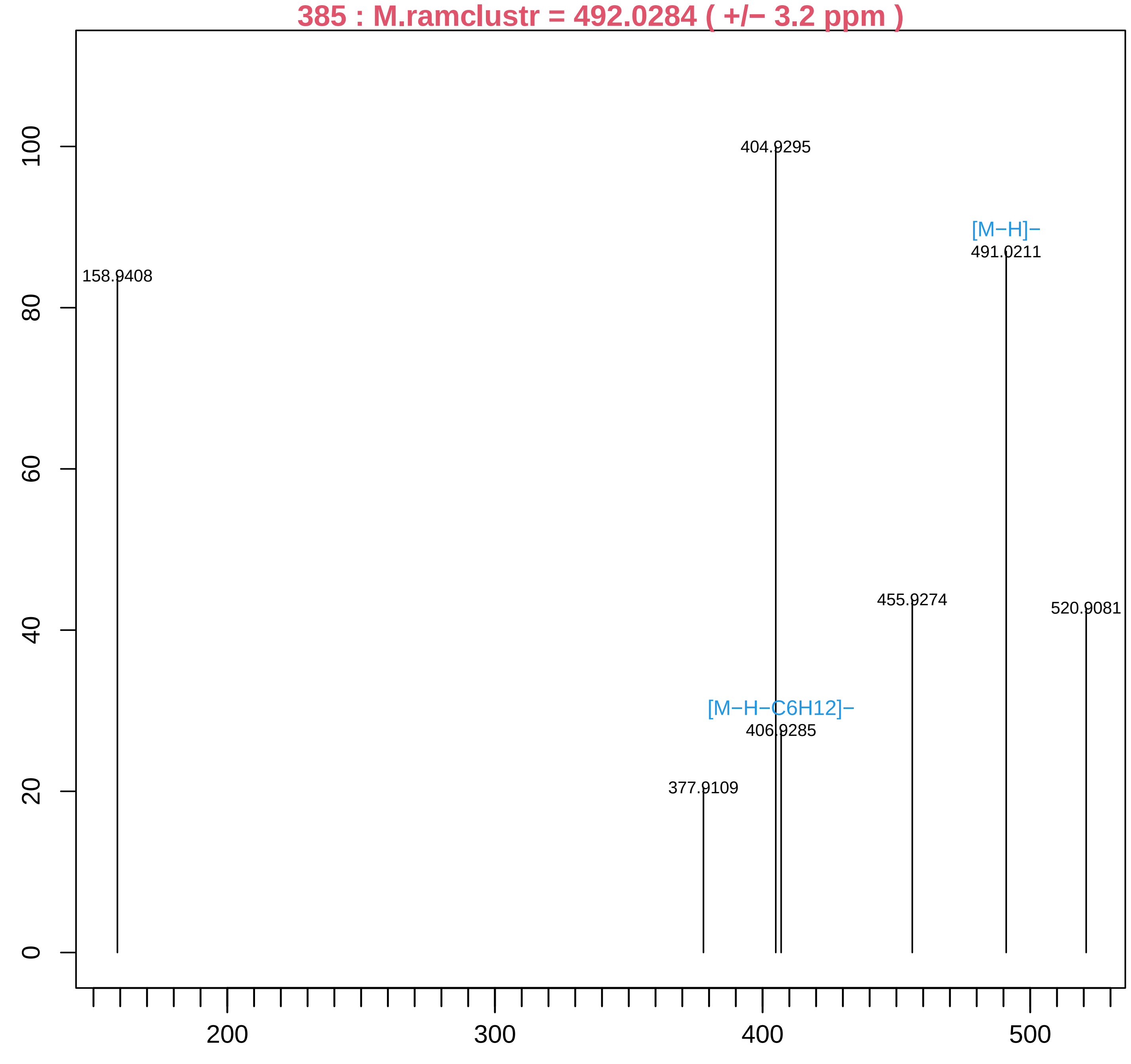
385 : M.ramclustr = 492.0284 (+/− 3.2 ppm)
158.9408
404.9295
[M−H]−
491.0211
455.9274
520.9081
[M−H−C6H12]−
406.9285
377.9109
0
20
40
60
80
100
200
300
400
500

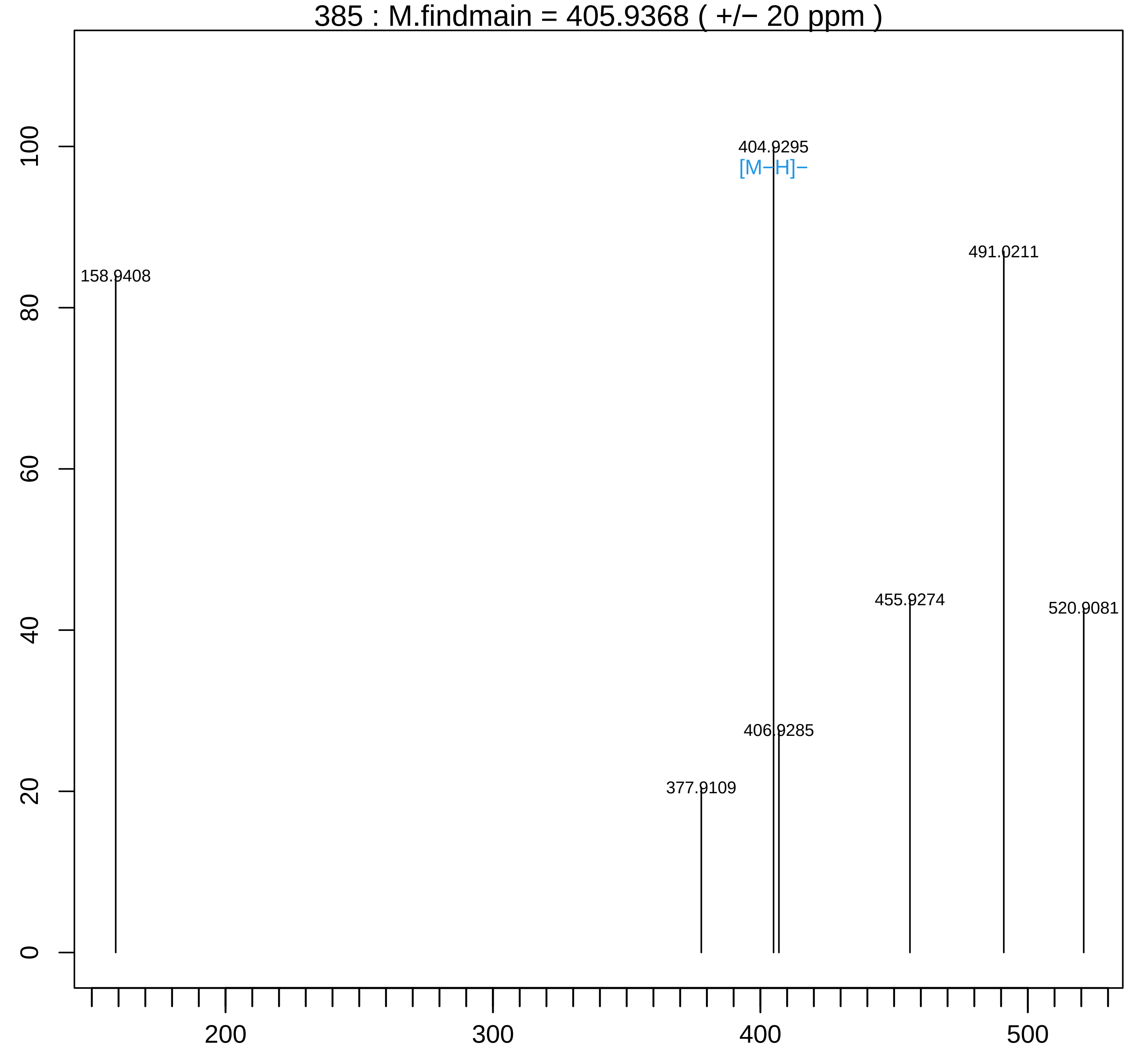
385 : M.findmain = 405.9368 (+/− 20 ppm)
158.9408
404.9295
[M−H]−
491.0211
455.9274
520.9081
406.9285
377.9109
0
20
40
60
80
100
200
300
400
500

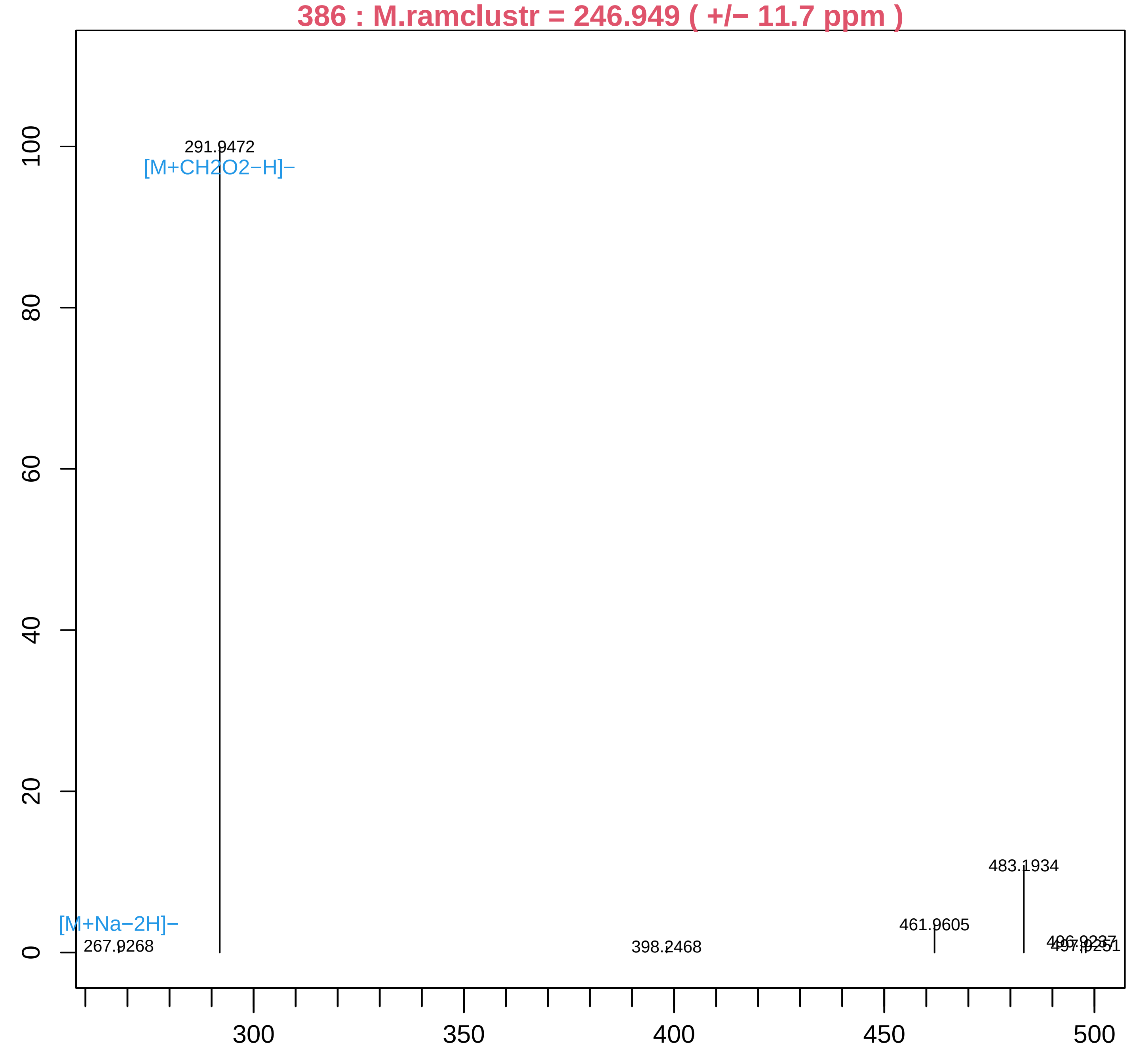

386 : M.ramclustr = 246.949 (+/− 11.7 ppm)
291.9472
[M+CH2O2−H]−
[M+Na−2H]−
267.9268
398.2468
461.9605
483.1934
496.9237
497.9251

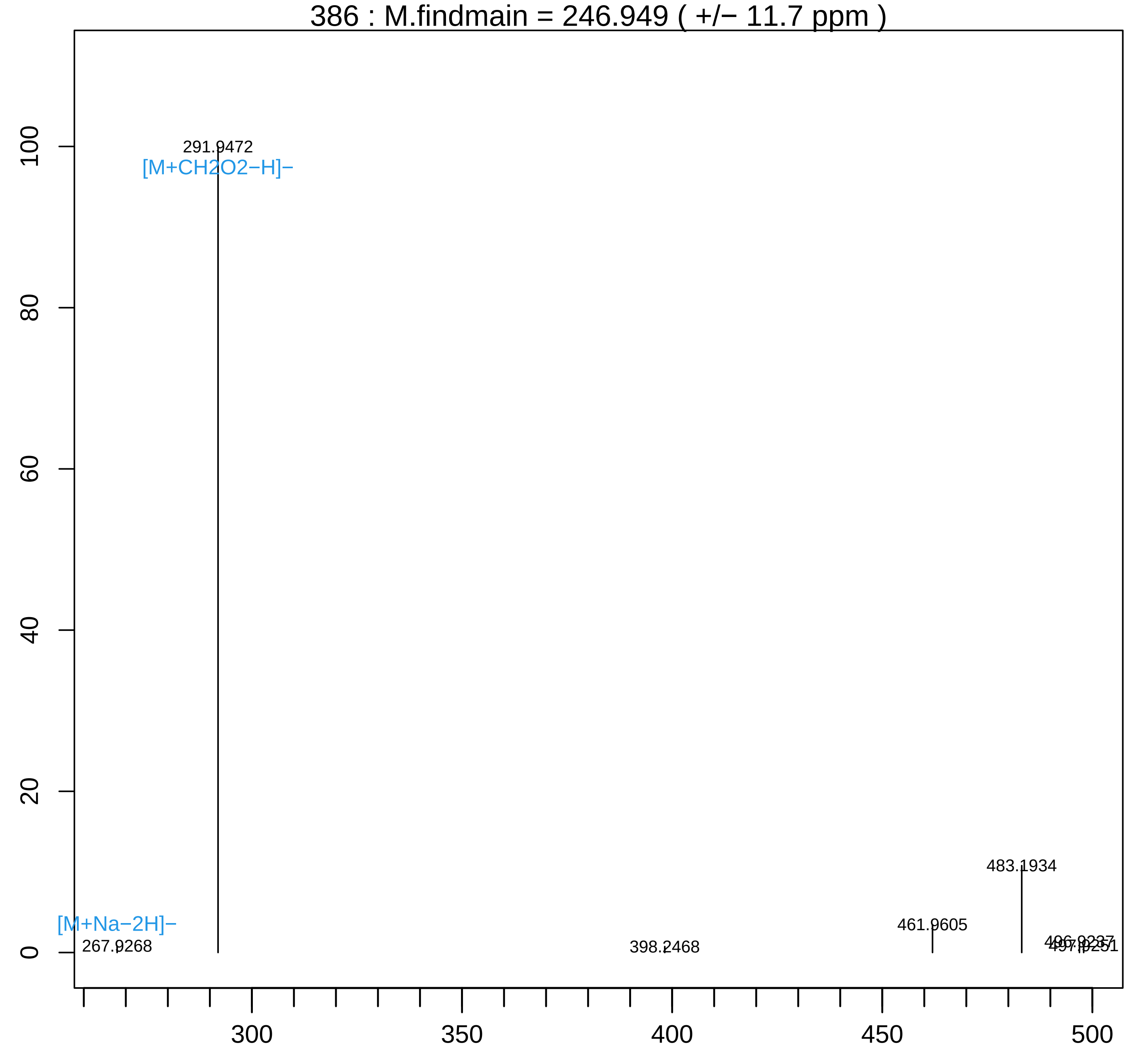

386 : M.findmain = 246.949 (+/− 11.7 ppm)
291.9472
[M+CH2O2−H]−
[M+Na−2H]−
267.9268
398.2468
461.9605
483.1934
496.9237
497.9251

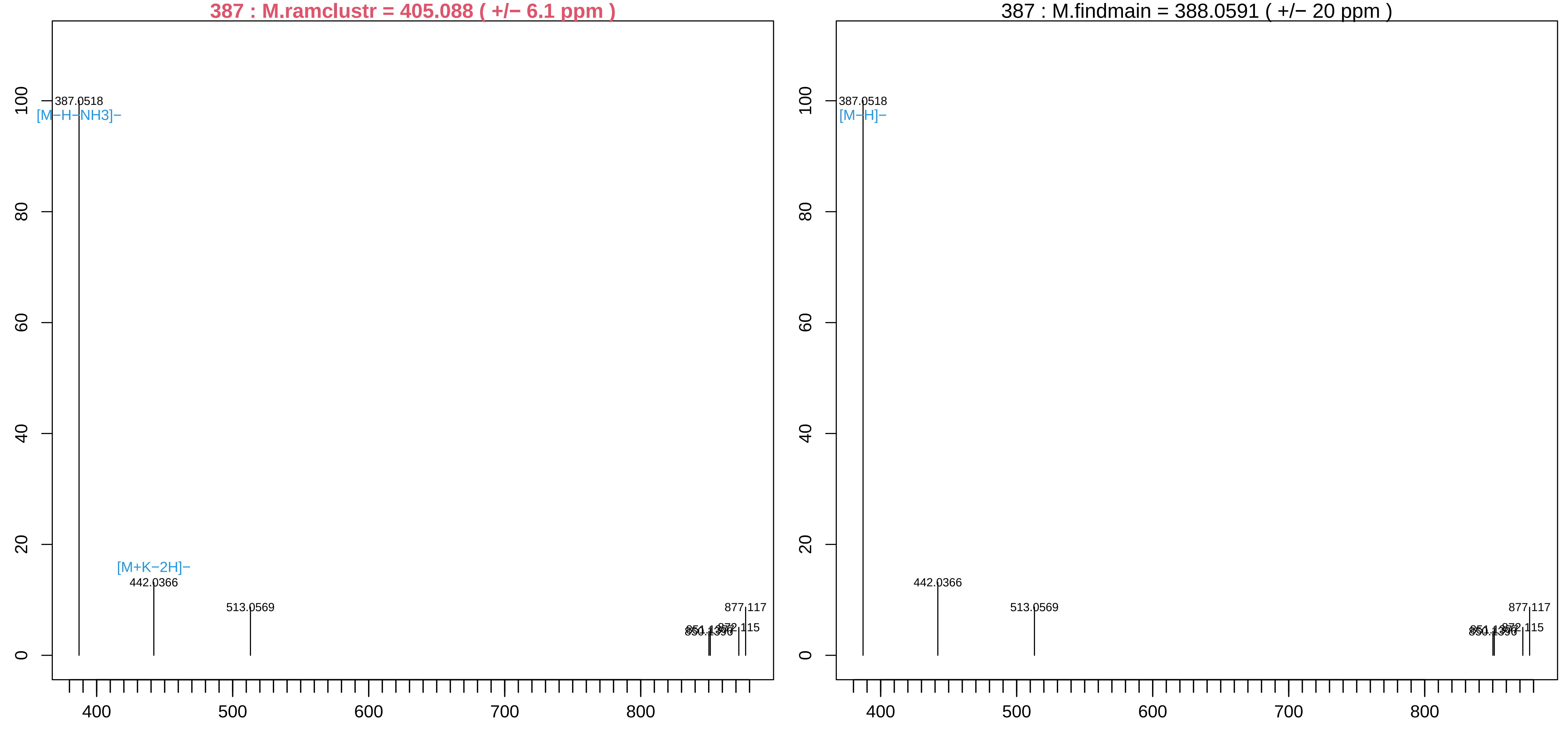

387 : M.ramclustr = 405.088 (+/− 6.1 ppm)
387.0518
[M−H−NH3]−
[M+K−2H]−
442.0366
513.0569
877.117
872.115
850.1396
851.1370
387 : M.findmain = 388.0591 (+/− 20 ppm)
387.0518
[M−H]−
442.0366
513.0569
877.117
872.115
850.1396
851.1370
0
20
40
60
80
100
400
500
600
700
800

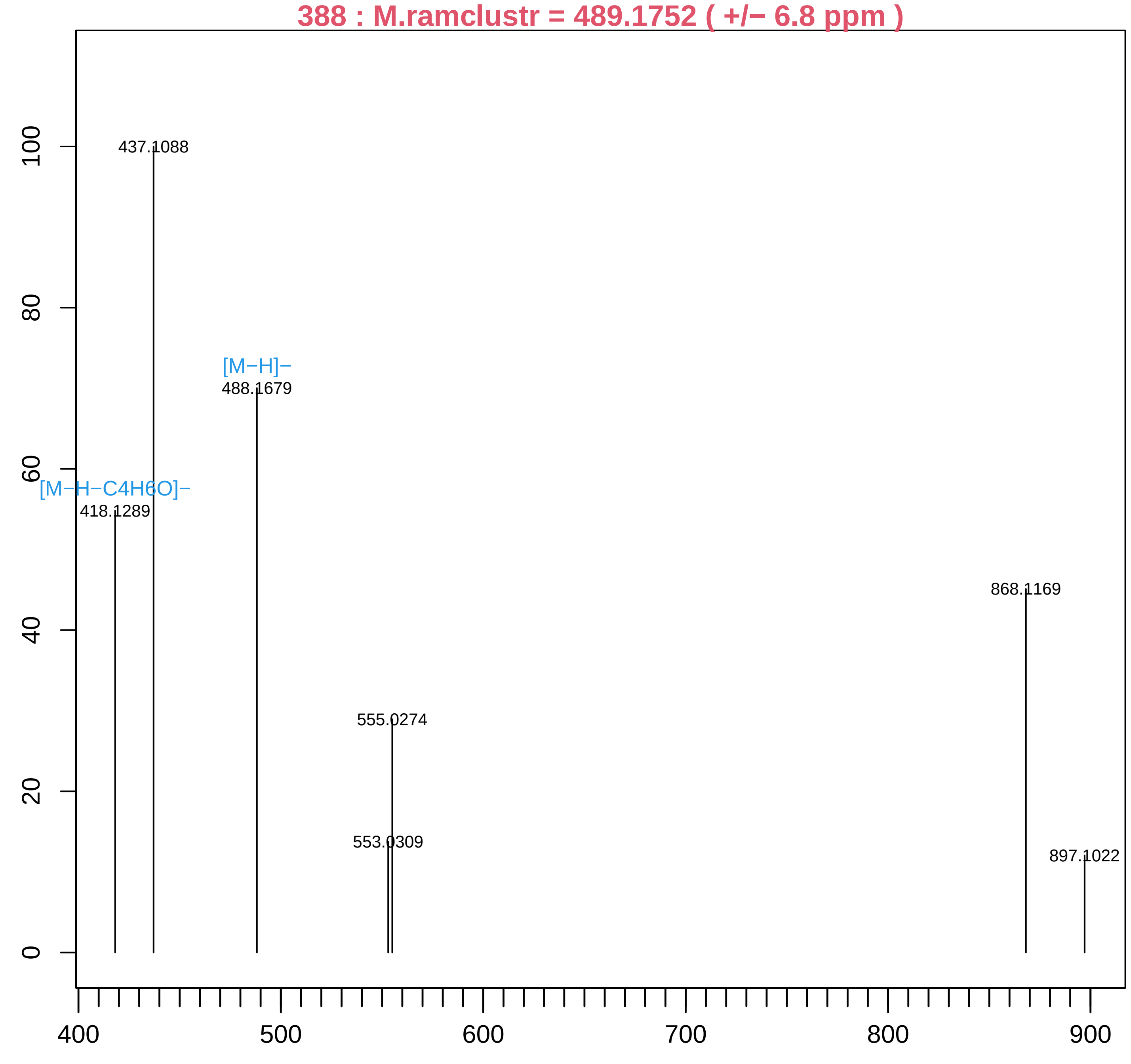
388 : M.ramclustr = 489.1752 (+/− 6.8 ppm)
437.1088
[M−H]−
488.1679
[M−H−C4H6O]−
418.1289
868.1169
555.0274
553.0309
897.1022
0
20
40
60
80
100
400
500
600
700
800
900

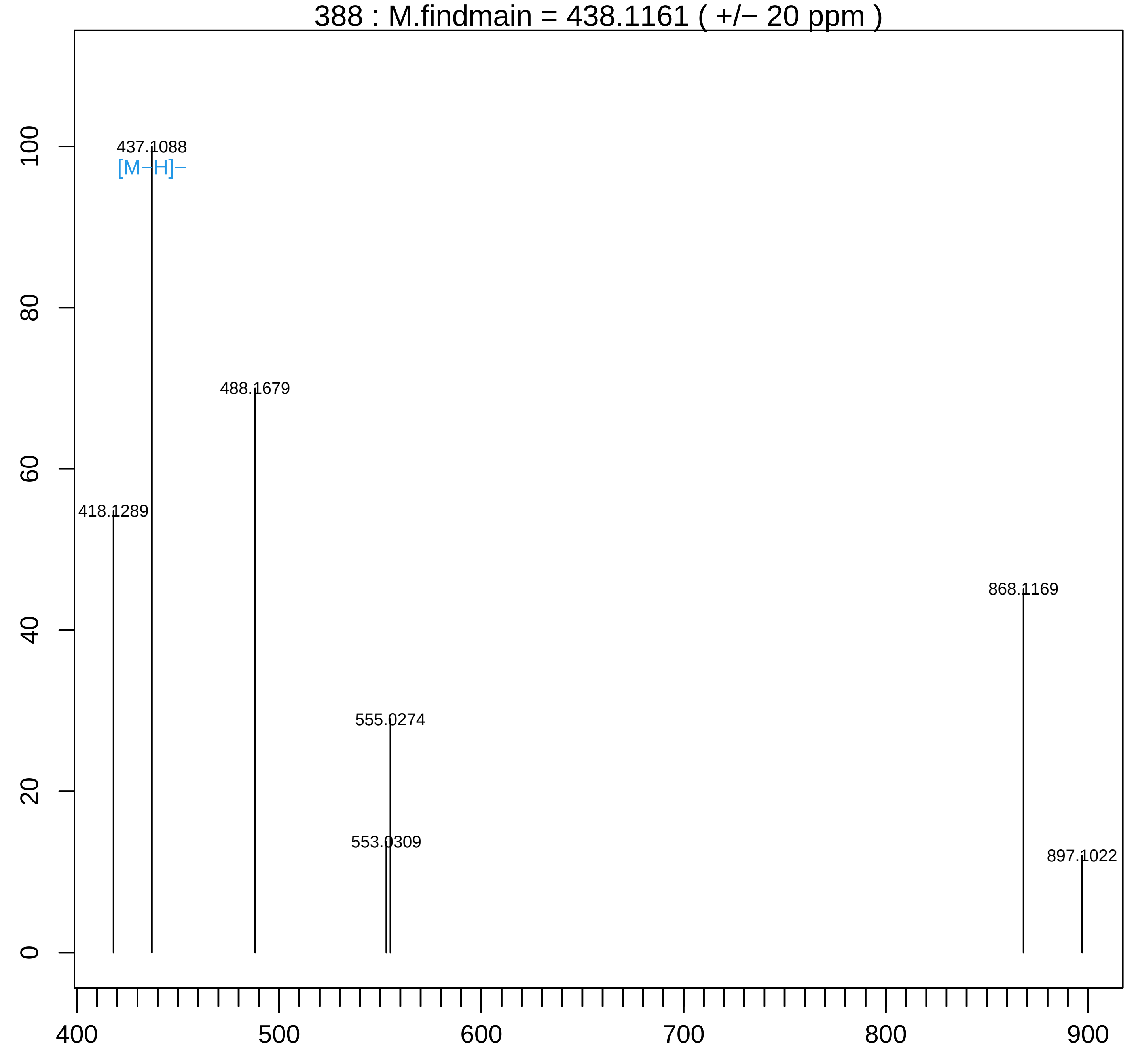
388 : M.findmain = 438.1161 (+/− 20 ppm)
437.1088
[M−H]−
488.1679
418.1289
868.1169
555.0274
553.0309
897.1022
0
20
40
60
80
100
400
500
600
700
800
900

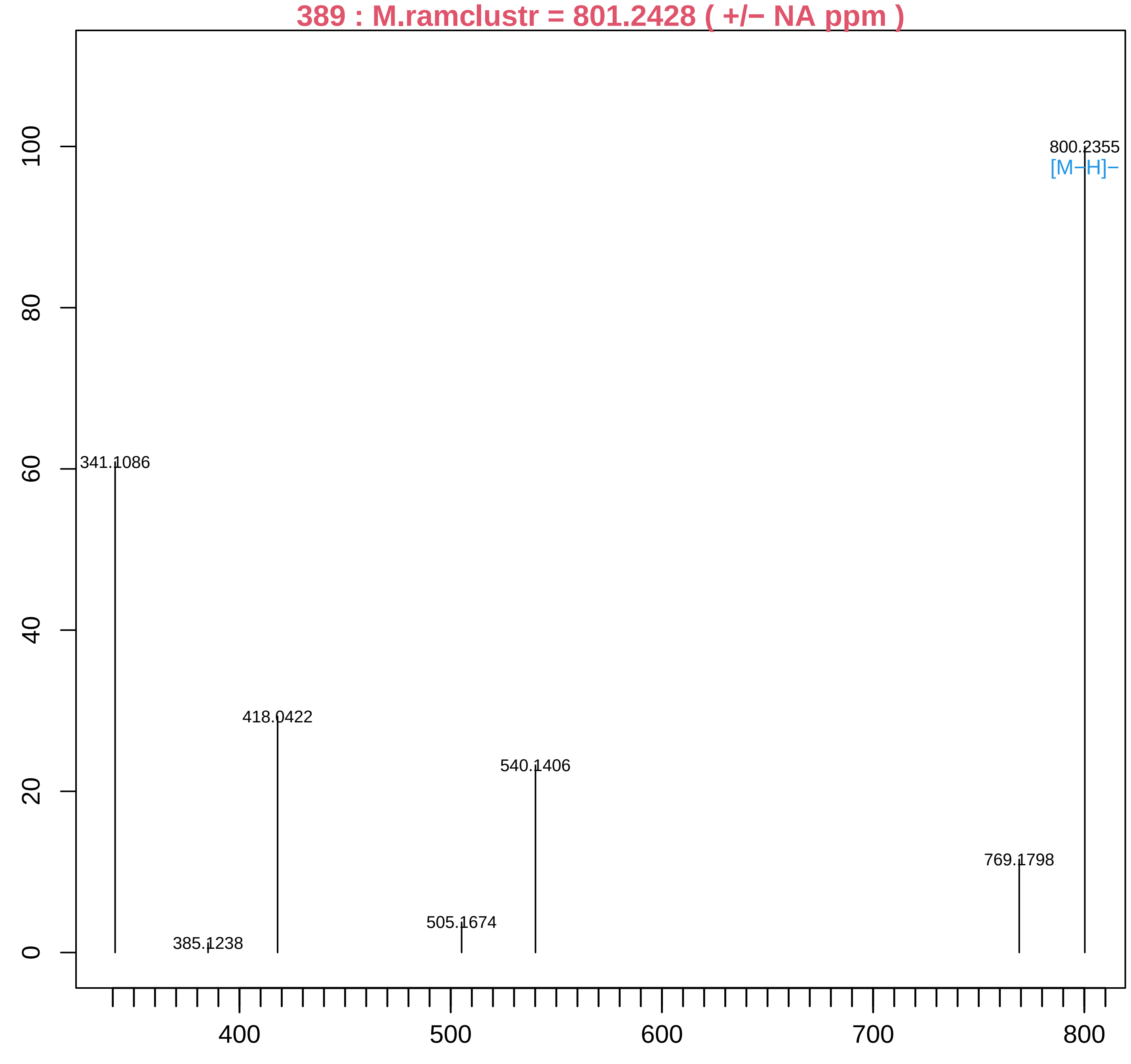
389 : M.ramclustr = 801.2428 (+/− NA ppm)
800.2355
[M−H]−
341.1086
418.0422
540.1406
769.1798
505.1674
385.1238
0
20
40
60
80
100
400
500
600
700
800

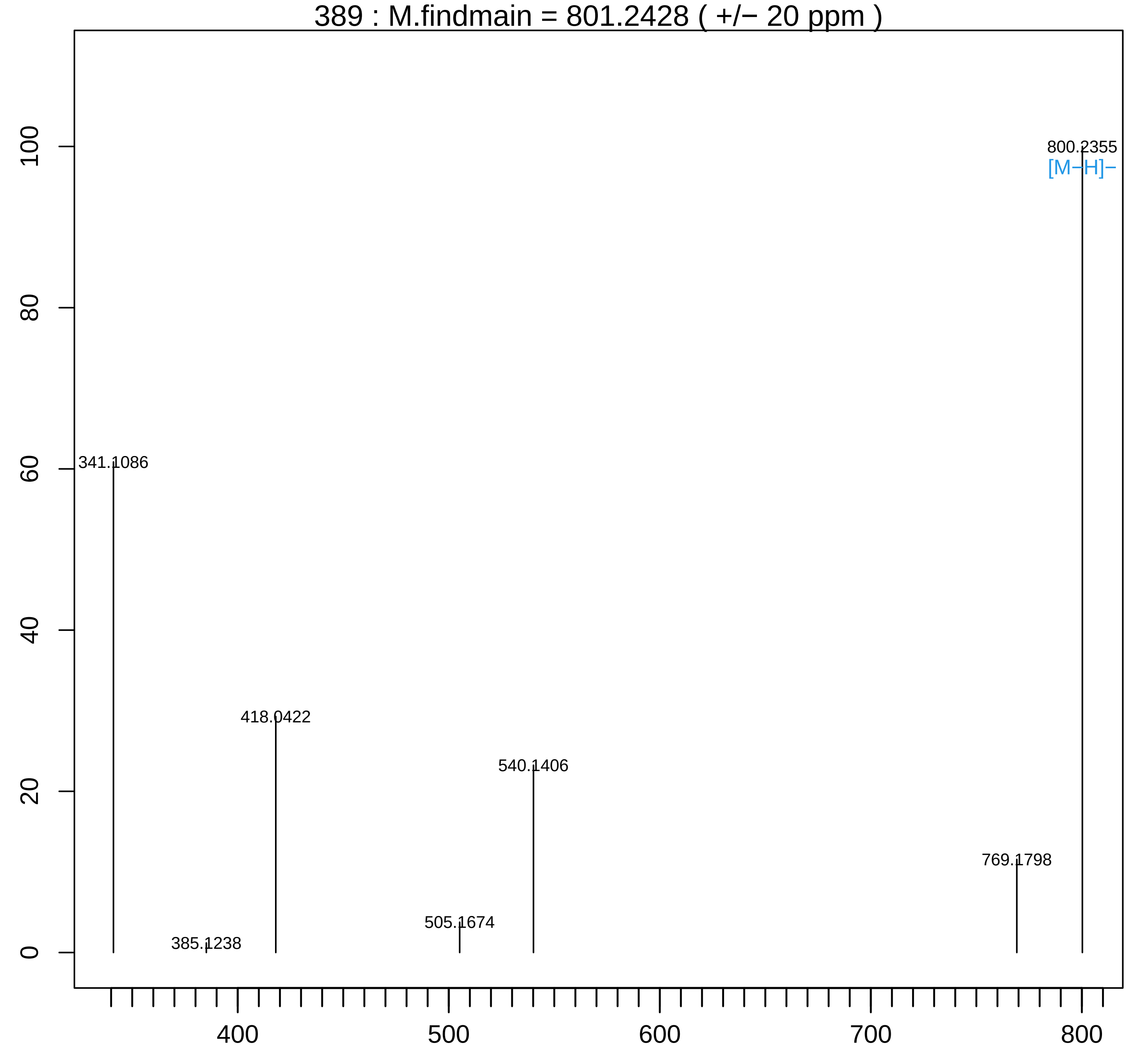
389 : M.findmain = 801.2428 (+/− 20 ppm)
800.2355
[M−H]−
341.1086
418.0422
540.1406
769.1798
505.1674
385.1238
0
20
40
60
80
100
400
500
600
700
800

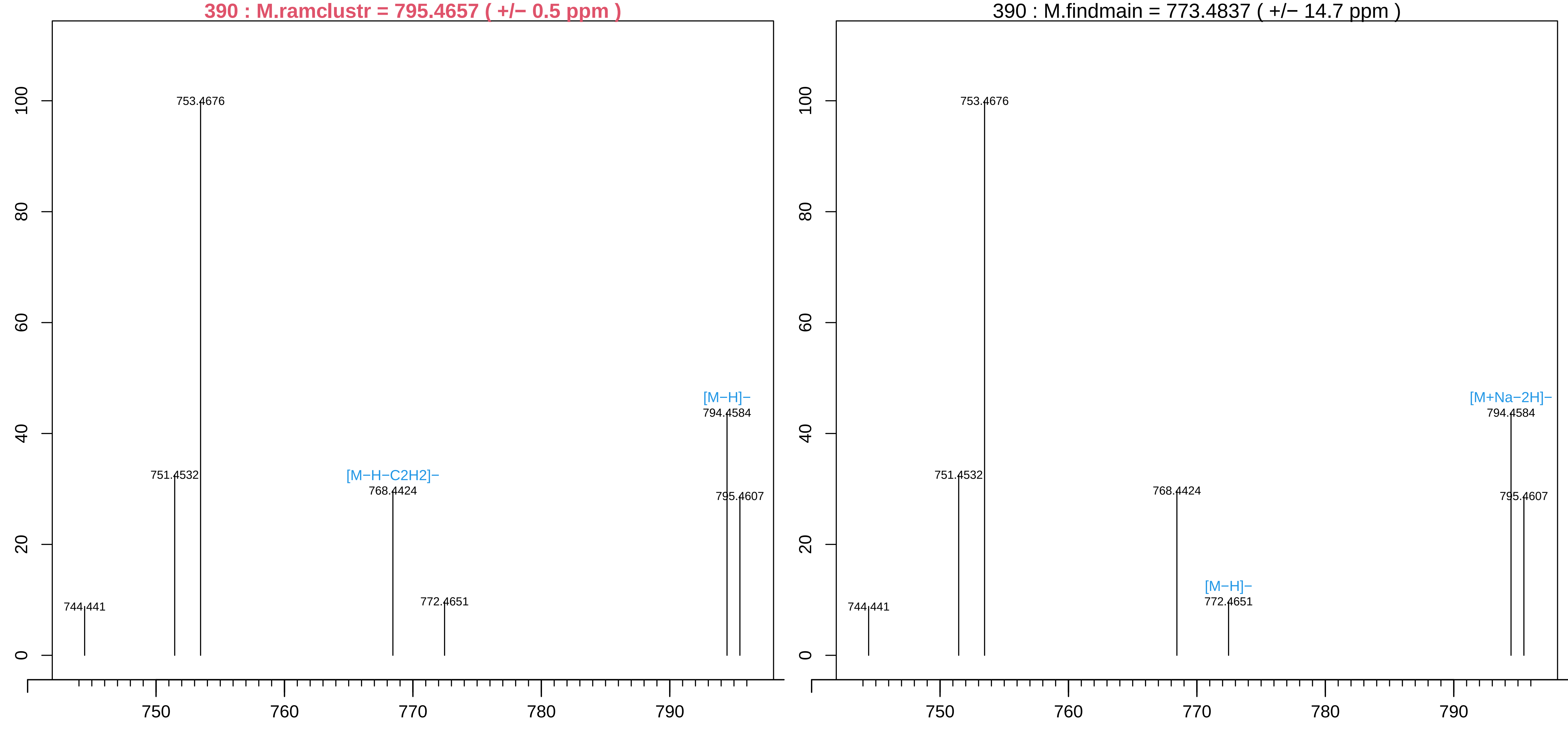

390 : M.ramclustr = 795.4657 (+/− 0.5 ppm)
753.4676
751.4532
744.441
[M−H−C2H2]−
768.4424
772.4651
[M−H]−
794.4584
795.4607
0
20
40
60
80
100
750
760
770
780
790
390 : M.findmain = 773.4837 (+/− 14.7 ppm)
753.4676
751.4532
744.441
768.4424
[M−H]−
772.4651
[M+Na−2H]−
794.4584
795.4607
0
20
40
60
80
100
750
760
770
780
790

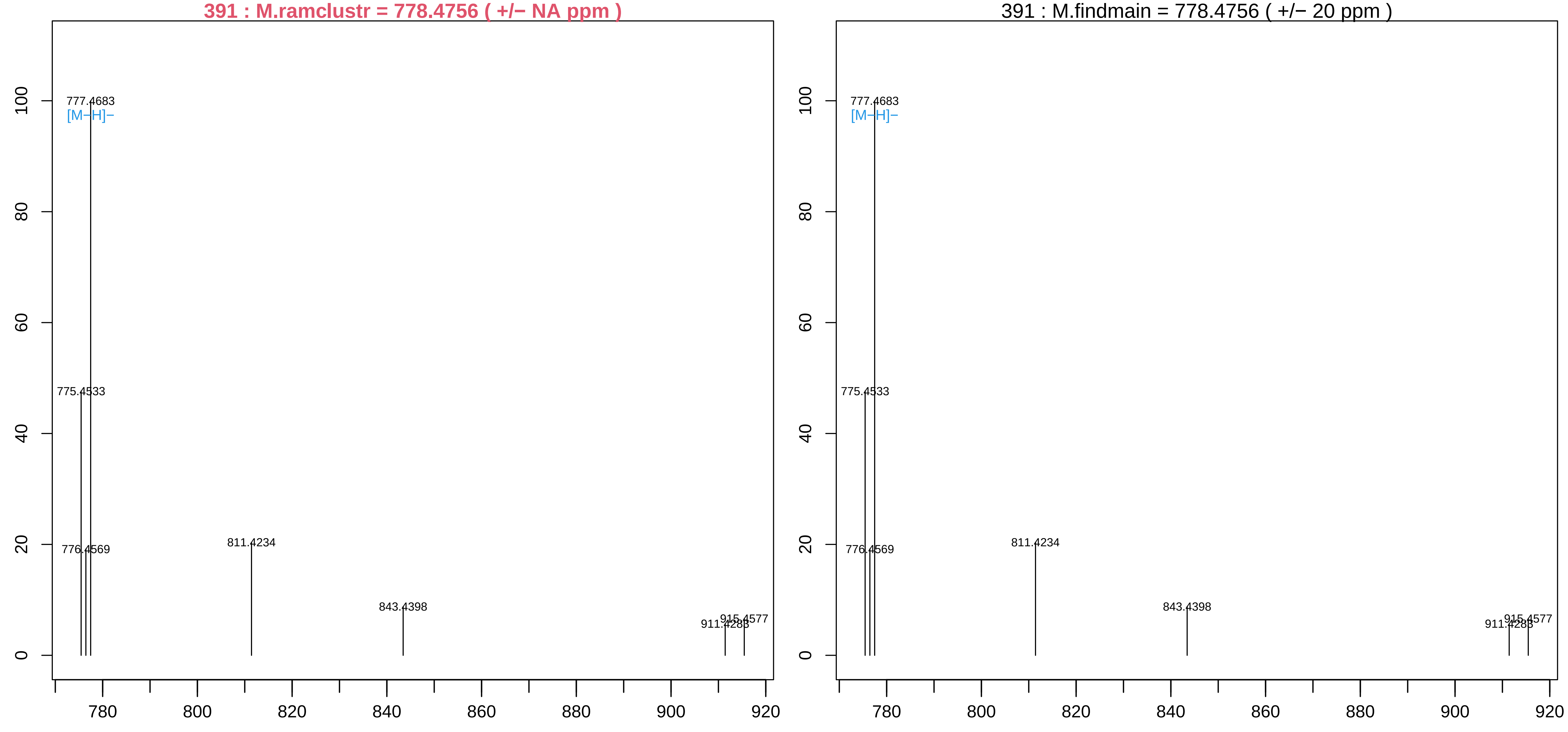

391 : M.ramclustr = 778.4756 (+/− NA ppm)
777.4683
[M−H]−
775.4533
776.4569
811.4234
843.4398
911.4283
915.4577
391 : M.findmain = 778.4756 (+/− 20 ppm)
777.4683
[M−H]−
775.4533
776.4569
811.4234
843.4398
911.4283
915.4577

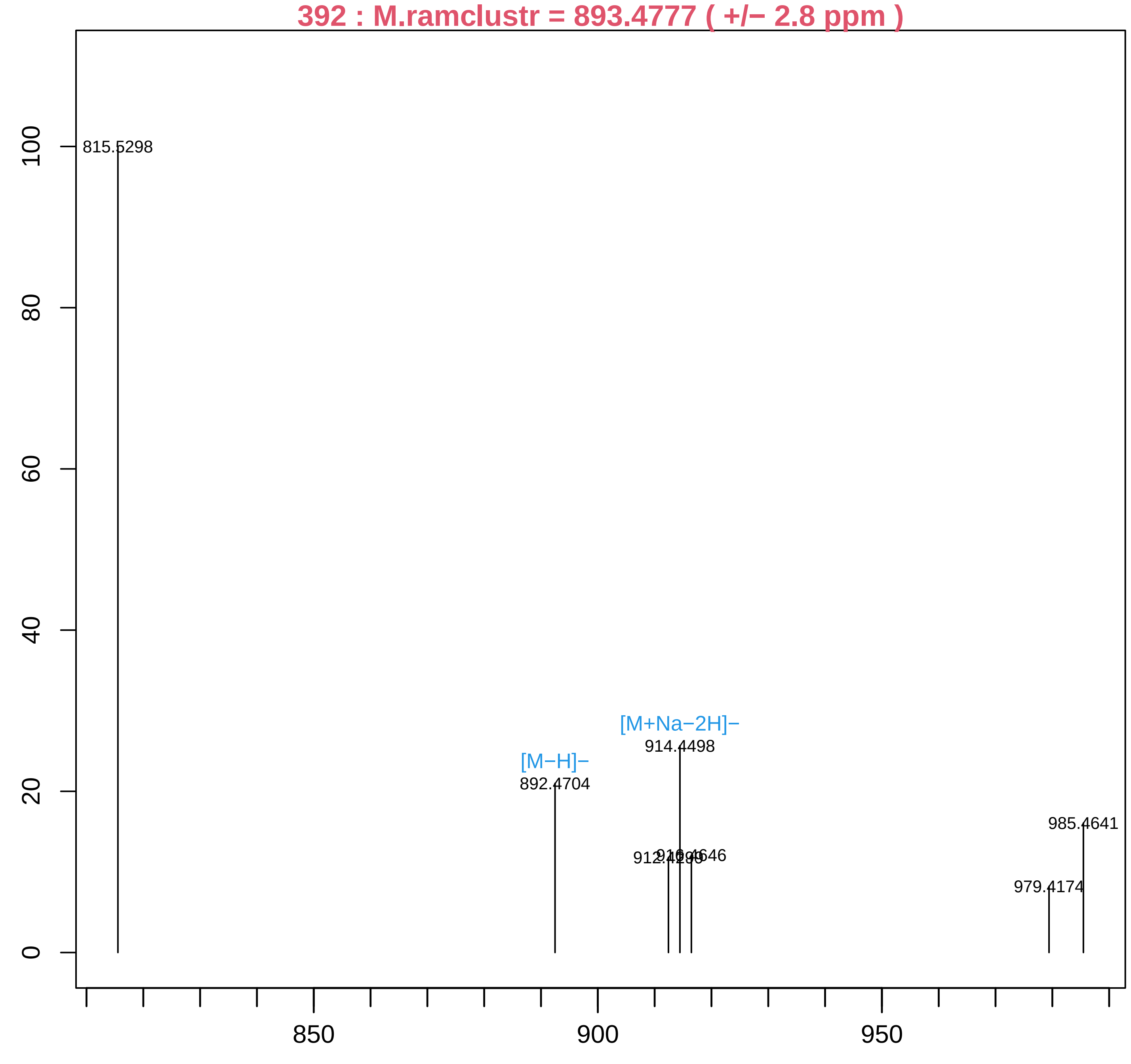
392 : M.ramclustr = 893.4777 (+/− 2.8 ppm)
815.5298
[M−H]−
892.4704
[M+Na−2H]−
914.4498
916.4646
985.4641
979.4174
0
20
40
60
80
100
850
900
950

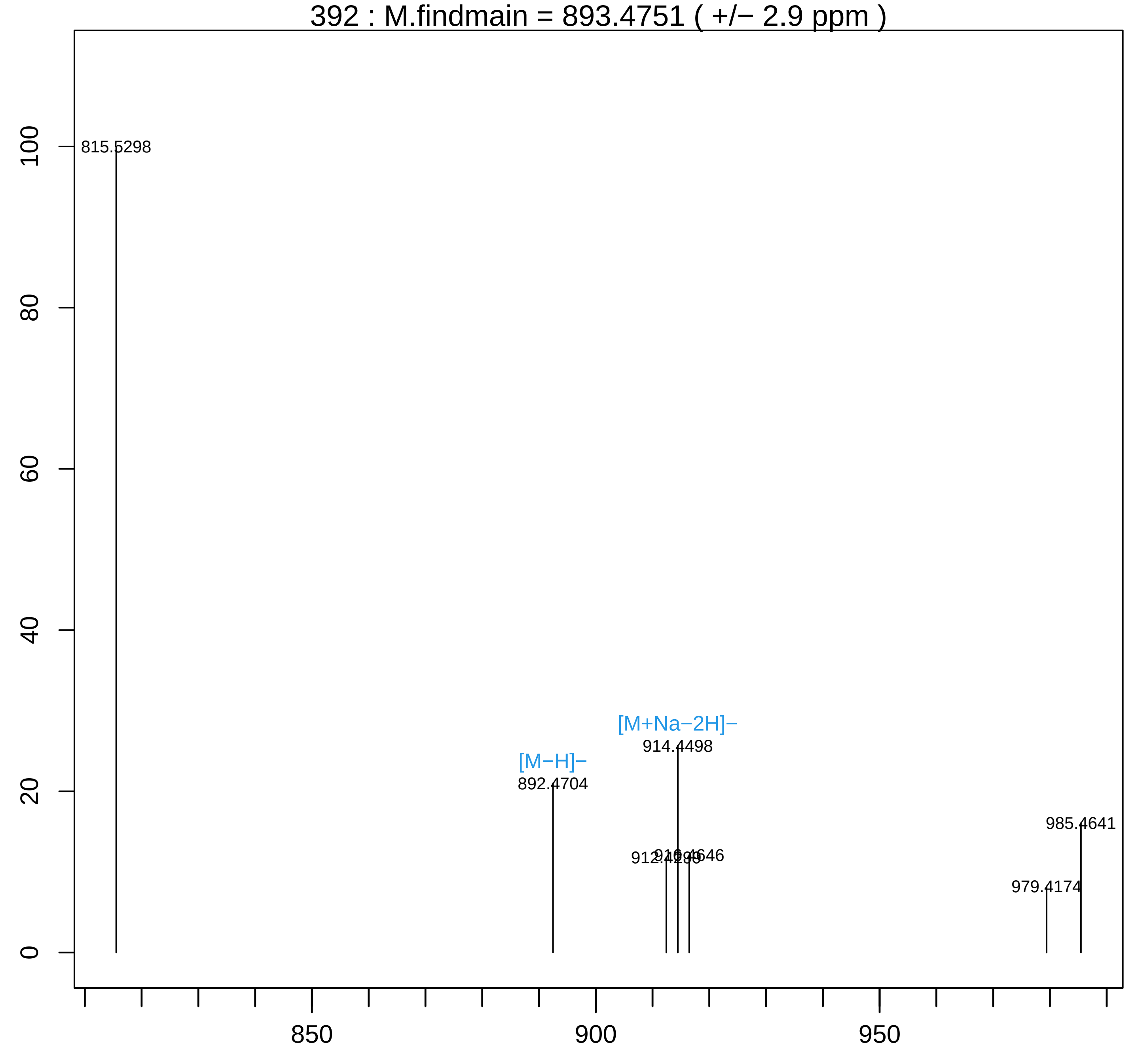
392 : M.findmain = 893.4751 (+/− 2.9 ppm)
815.5298
[M−H]−
892.4704
[M+Na−2H]−
914.4498
916.4646
985.4641
979.4174
0
20
40
60
80
100
850
900
950

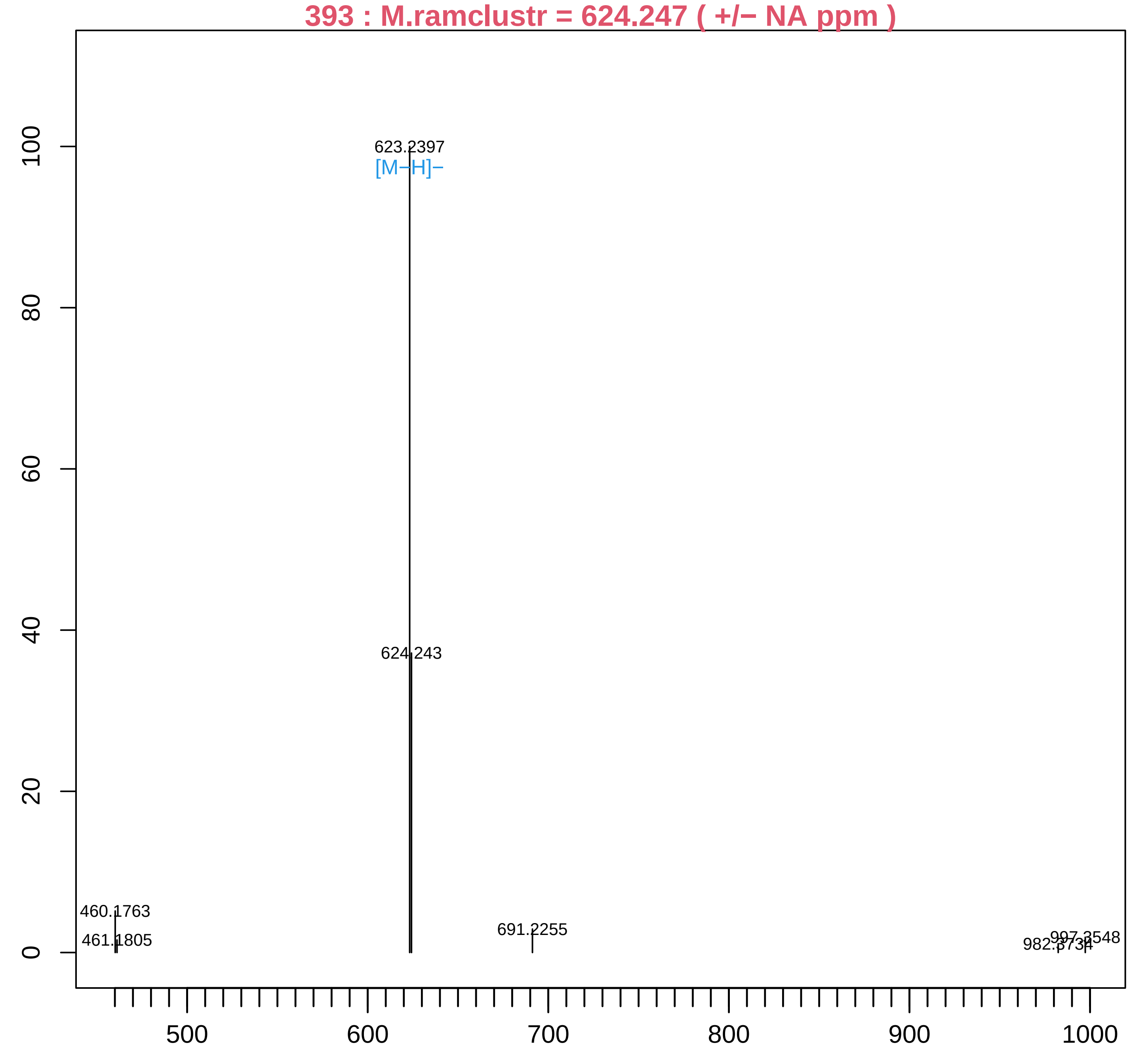
393 : M.ramclustr = 624.247 (+/− NA ppm)
623.2397
[M−H]−
624.243
460.1763
461.1805
691.2255
997.3548
982.3734
0
20
40
60
80
100
500
600
700
800
900
1000

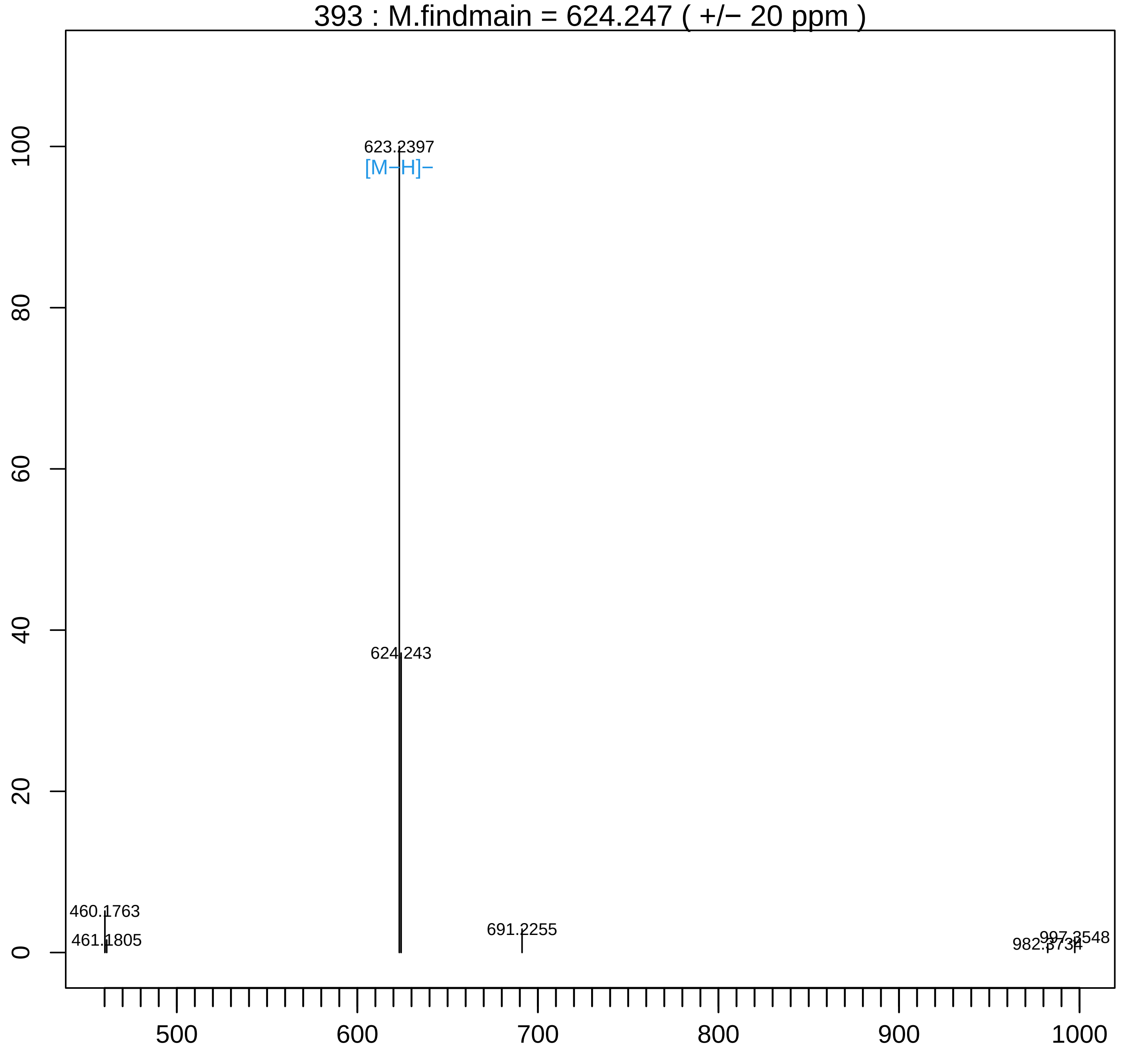
393 : M.findmain = 624.247 (+/− 20 ppm)
623.2397
[M−H]−
624.243
460.1763
461.1805
691.2255
997.3548
982.3734
0
20
40
60
80
100
500
600
700
800
900
1000

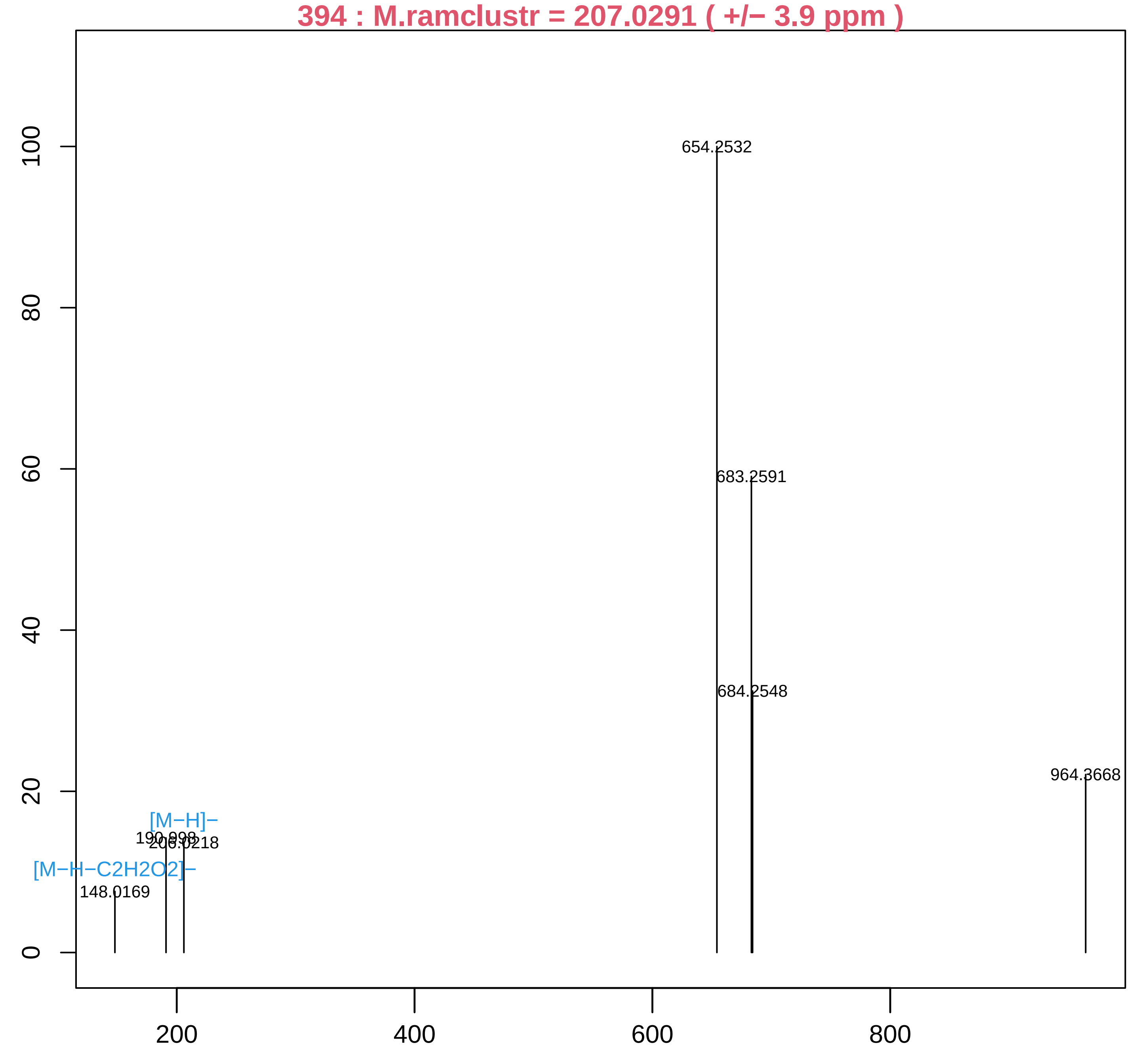
394 : M.ramclustr = 207.0291 (+/− 3.9 ppm)
654.2532
683.2591
684.2548
964.3668
[M−H]−
190.998
206.0218
[M−H−C2H2O2]−
148.0169
0
20
40
60
80
100
200
400
600
800

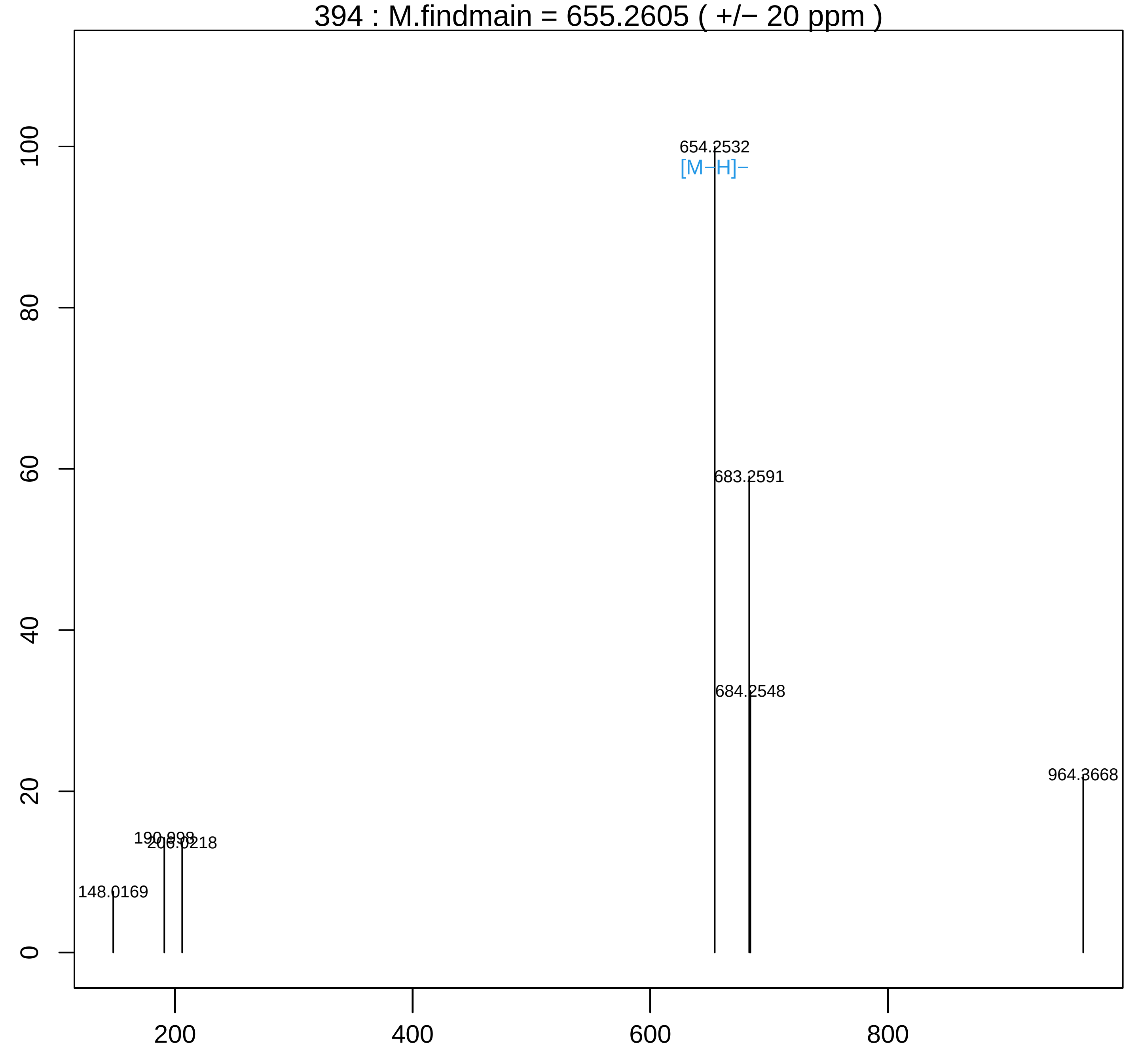
394 : M.findmain = 655.2605 (+/− 20 ppm)
654.2532
[M−H]−
683.2591
684.2548
964.3668
190.998
206.0218
148.0169
0
20
40
60
80
100
200
400
600
800

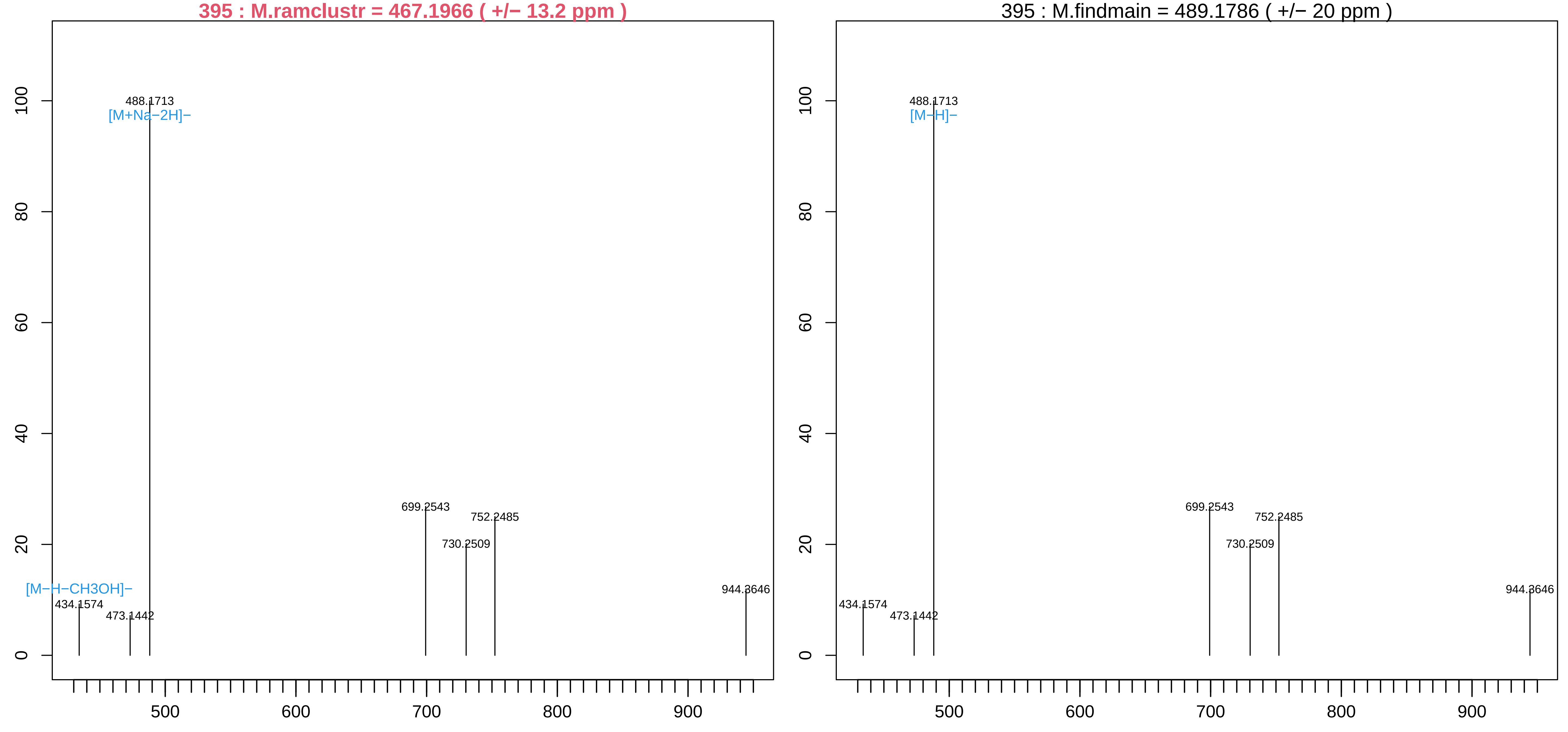
395 : M.ramclustr = 467.1966 (+/− 13.2 ppm)
488.1713
[M+Na−2H]−
[M−H−CH3OH]−
434.1574
473.1442
699.2543
730.2509
752.2485
944.3646
395 : M.findmain = 489.1786 (+/− 20 ppm)
488.1713
[M−H]−
434.1574
473.1442
699.2543
730.2509
752.2485
944.3646
0
20
40
60
80
100
500
600
700
800
900

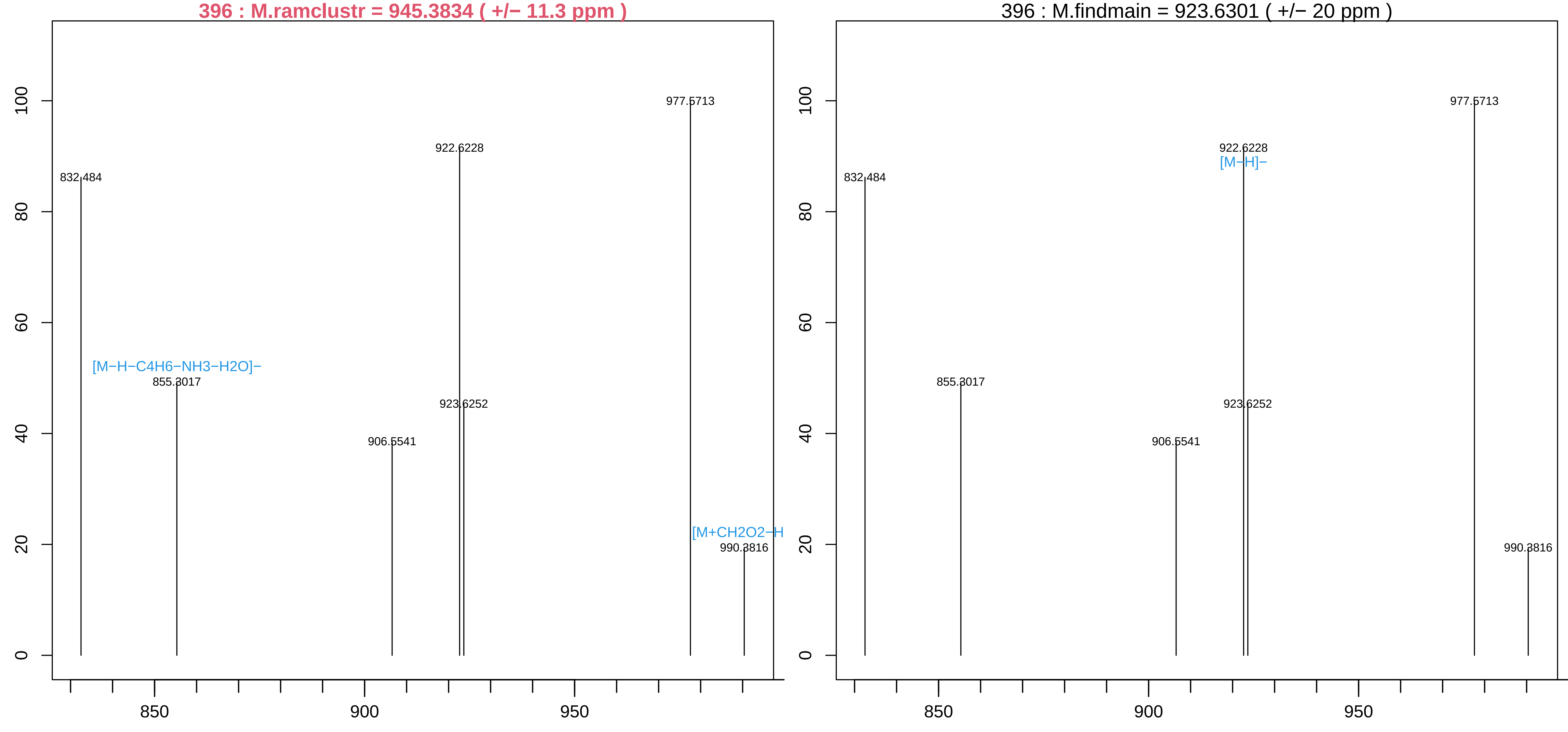

396 : M.ramclustr = 945.3834 (+/− 11.3 ppm)
832.484
[M−H−C4H6−NH3−H2O]−
855.3017
906.5541
922.6228
923.6252
977.5713
[M+CH2O2−H
990.3816
0
20
40
60
80
100
850
900
950
396 : M.findmain = 923.6301 (+/− 20 ppm)
832.484
855.3017
906.5541
922.6228
[M−H]−
923.6252
977.5713
990.3816
0
20
40
60
80
100
850
900
950

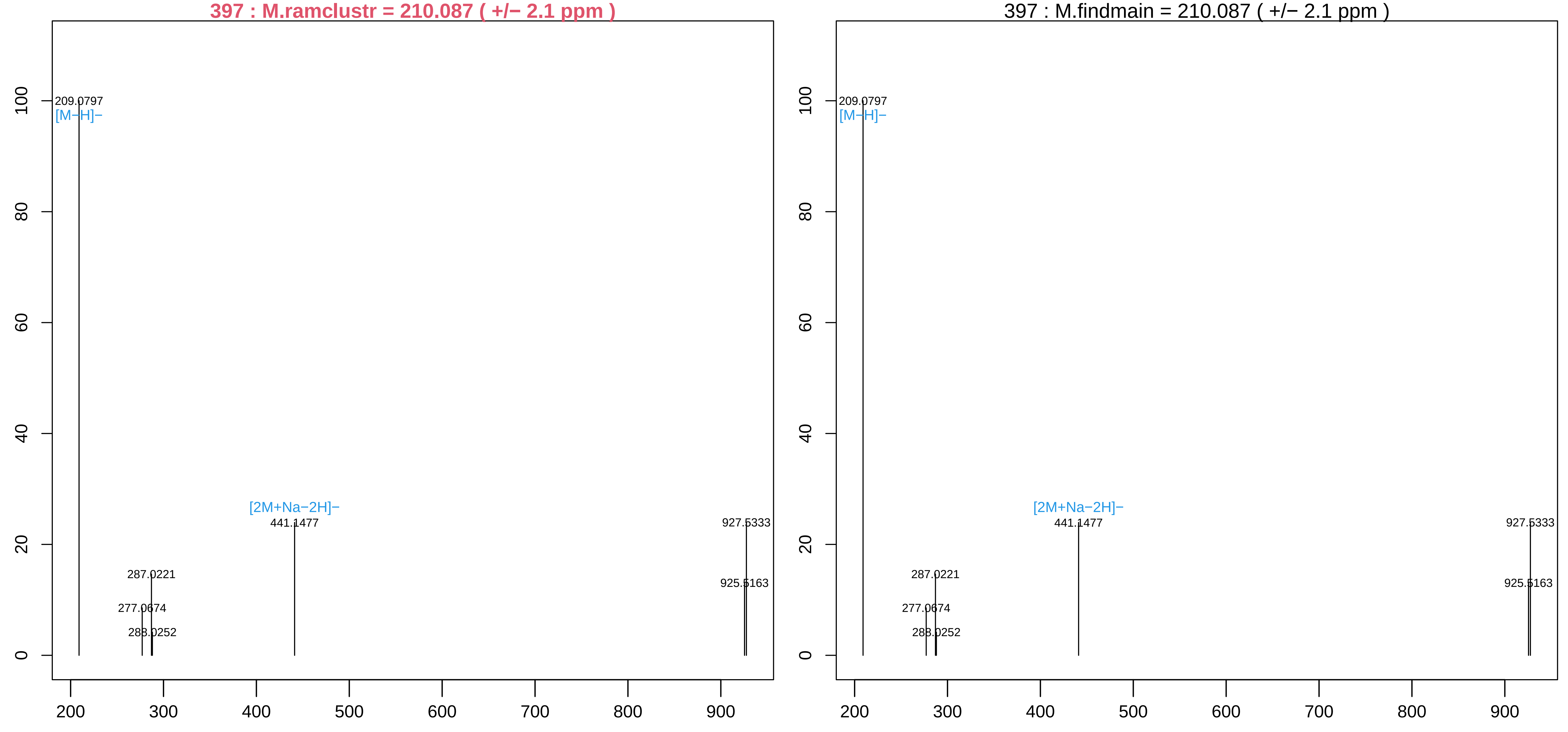
397 : M.ramclustr = 210.087 (+/− 2.1 ppm)
209.0797
[M−H]−
[2M+Na−2H]−
441.1477
927.5333
925.5163
287.0221
277.0674
288.0252
397 : M.findmain = 210.087 (+/− 2.1 ppm)
209.0797
[M−H]−
[2M+Na−2H]−
441.1477
927.5333
925.5163
287.0221
277.0674
288.0252

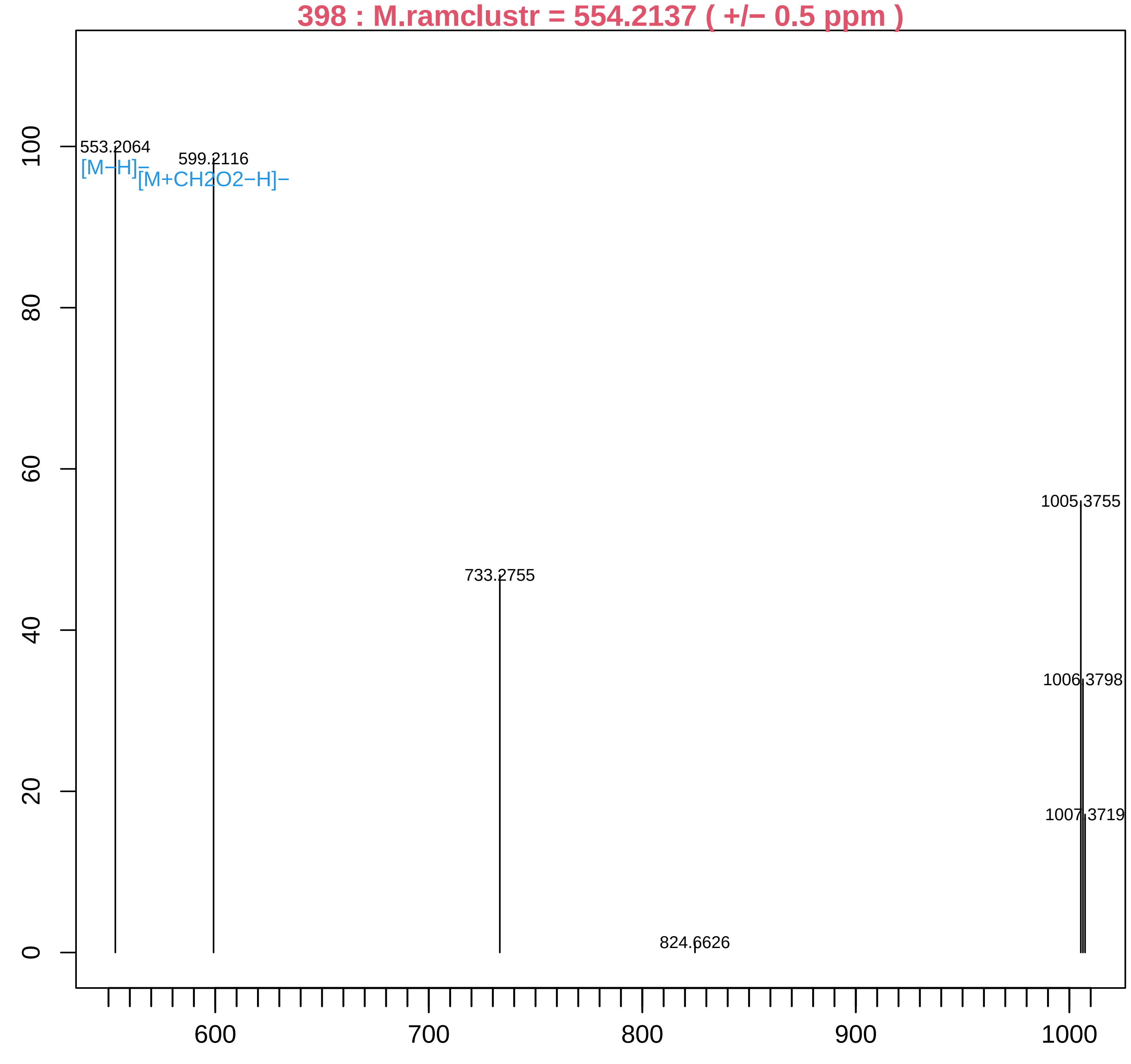
398 : M.ramclustr = 554.2137 (+/− 0.5 ppm)
553.2064
[M−H]−
599.2116
[M+CH2O2−H]−
733.2755
824.6626
1005.3755
1006.3798
1007.3719
0
20
40
60
80
100
600
700
800
900
1000

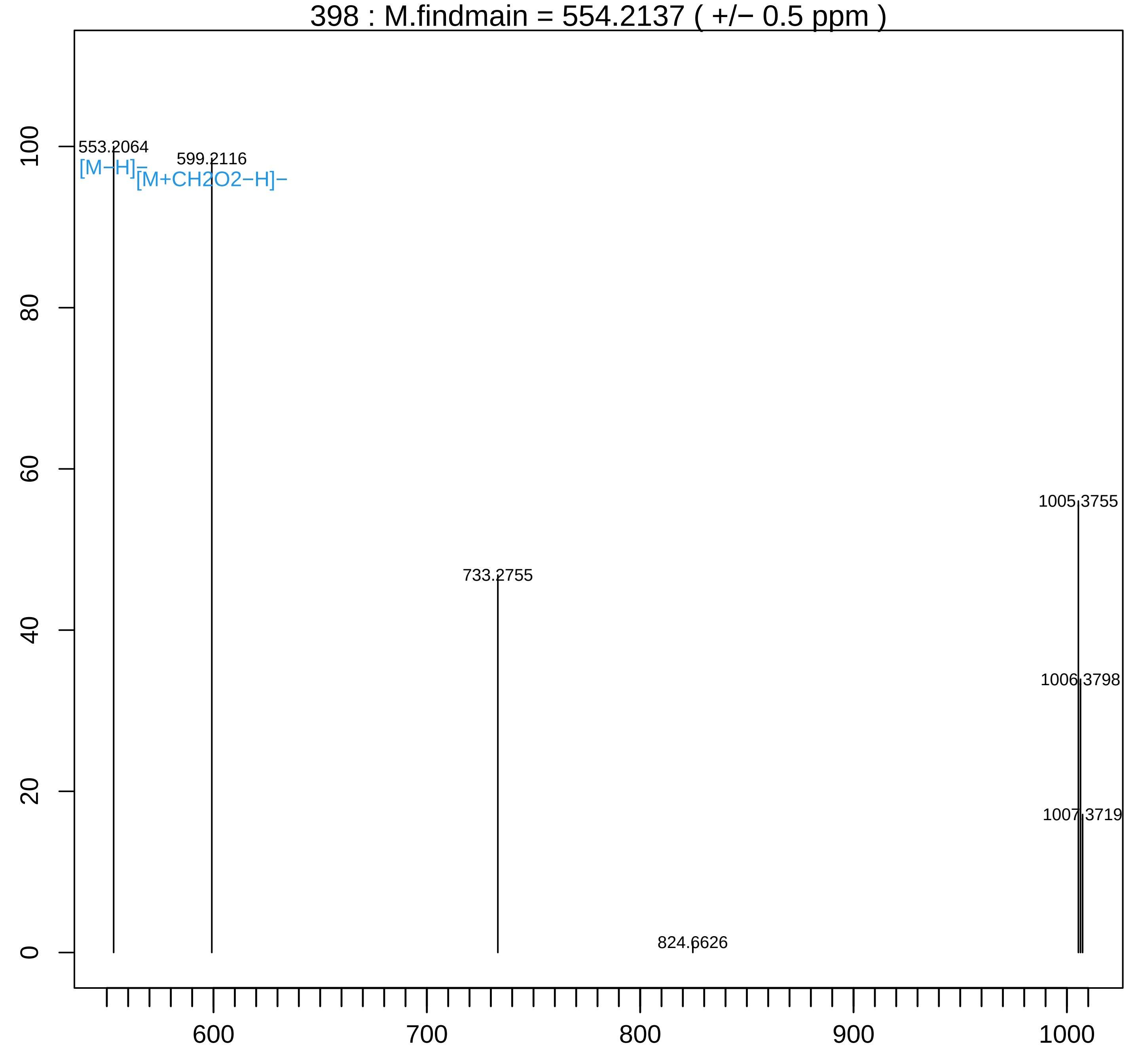
398 : M.findmain = 554.2137 (+/− 0.5 ppm)
553.2064
[M−H]−
599.2116
[M+CH2O2−H]−
733.2755
824.6626
1005.3755
1006.3798
1007.3719
0
20
40
60
80
100
600
700
800
900
1000

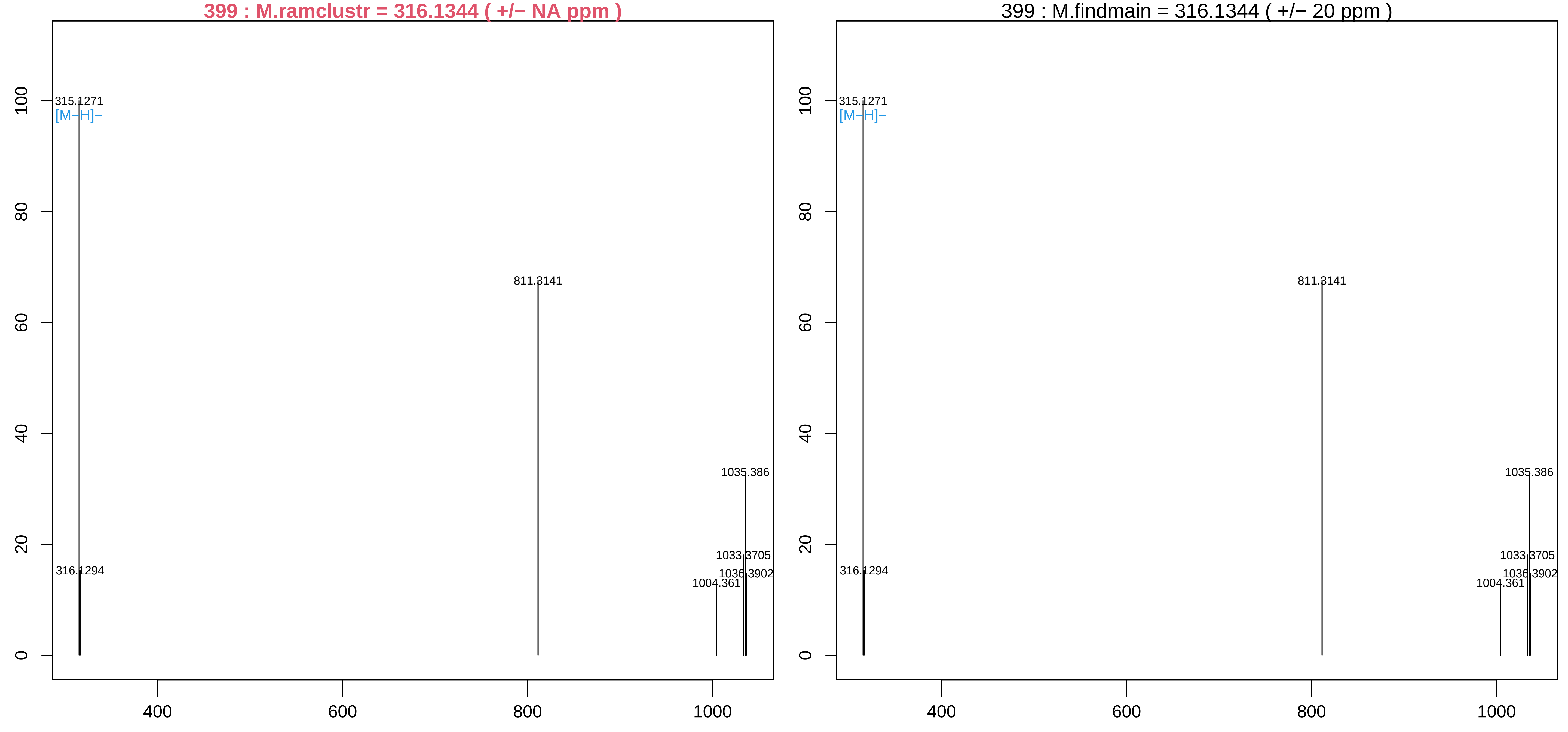
399 : M.ramclustr = 316.1344 (+/− NA ppm)
315.1271
[M−H]−
316.1294
811.3141
1035.386
1033.3705
1036.3902
1004.361
0
20
40
60
80
100
400
600
800
1000
399 : M.findmain = 316.1344 (+/− 20 ppm)
315.1271
[M−H]−
316.1294
811.3141
1035.386
1033.3705
1036.3902
1004.361
0
20
40
60
80
100
400
600
800
1000

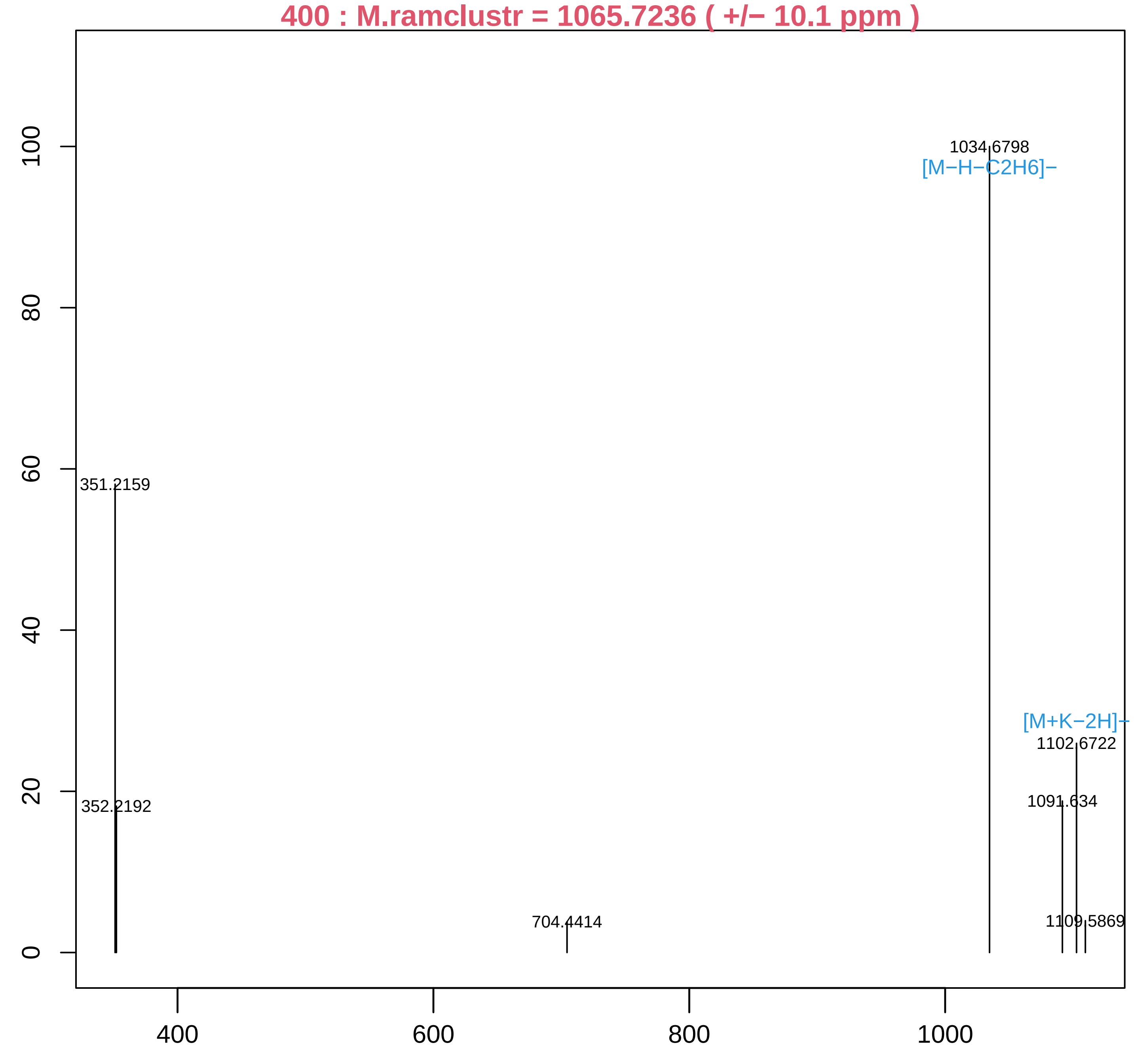

400 : M.ramclustr = 1065.7236 (+/− 10.1 ppm)
1034.6798
[M−H−C2H6]−
351.2159
352.2192
704.4414
[M+K−2H]−
1102.6722
1091.634
1109.5869

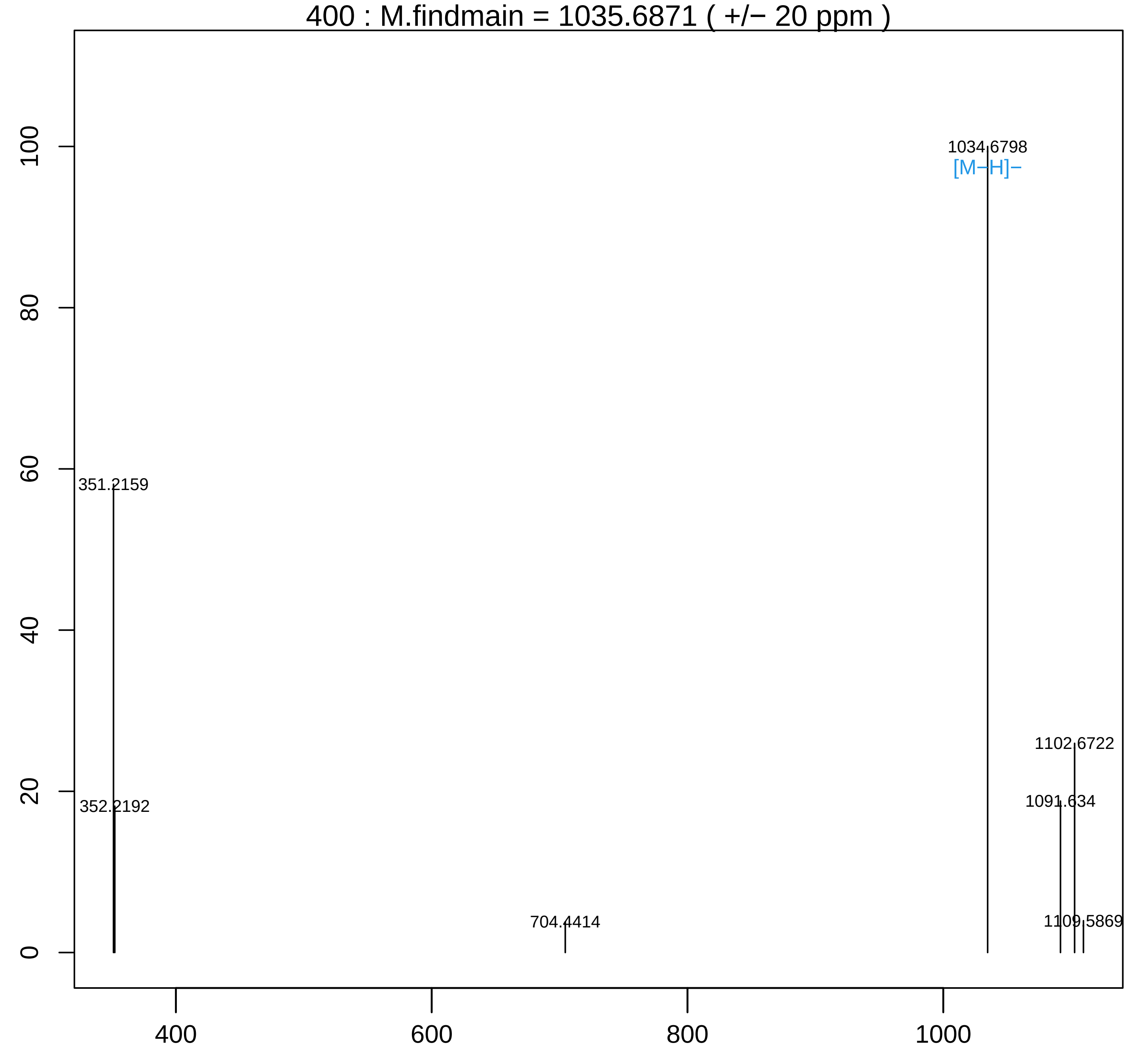

400 : M.findmain = 1035.6871 (+/− 20 ppm)
1034.6798
[M−H]−
351.2159
352.2192
704.4414
1102.6722
1091.634
1109.5869

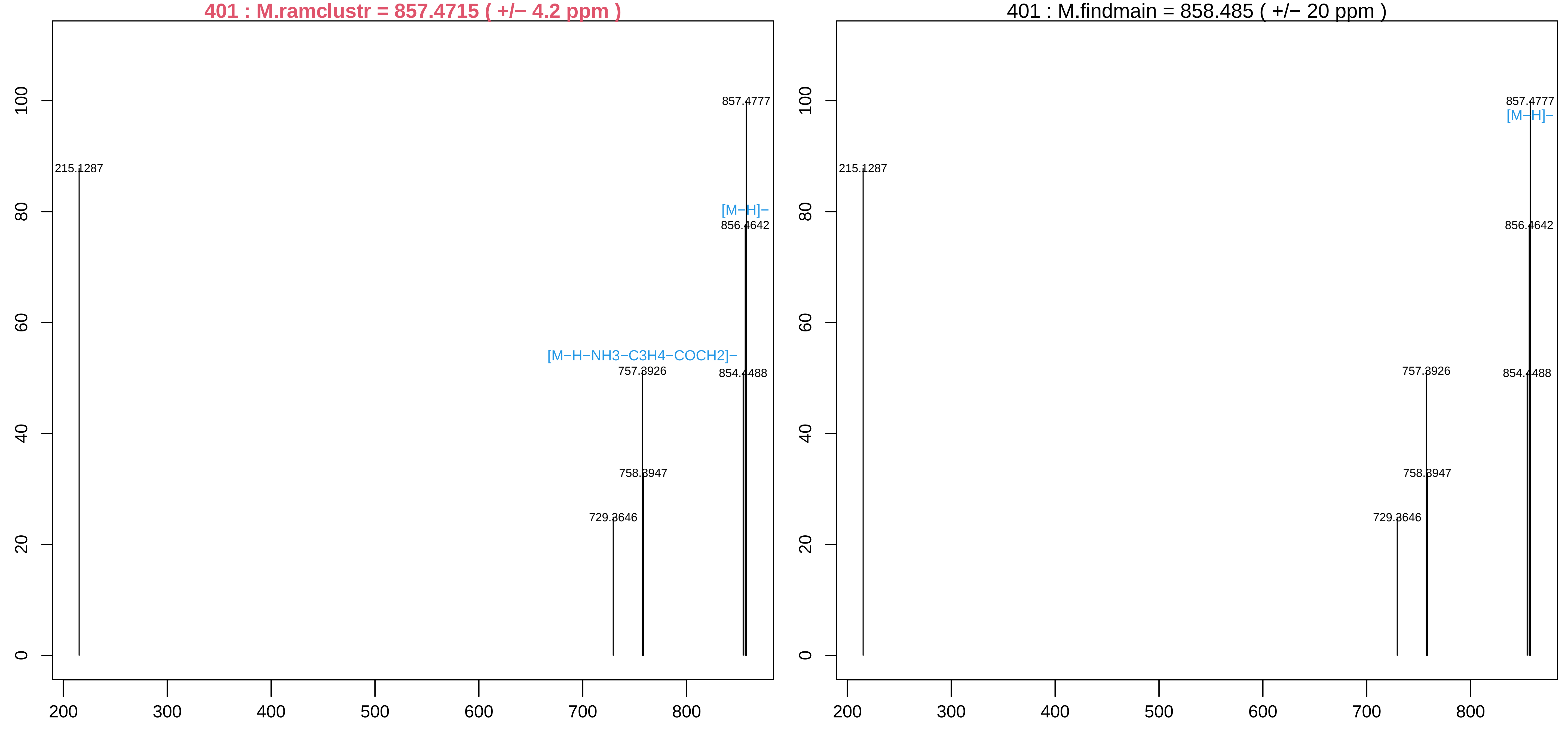
401 : M.ramclustr = 857.4715 (+/− 4.2 ppm)
857.4777
215.1287
[M−H]−
856.4642
[M−H−NH3−C3H4−COCH2]−
757.3926
854.4488
758.3947
729.3646
401 : M.findmain = 858.485 (+/− 20 ppm)
857.4777
[M−H]−
215.1287
856.4642
757.3926
854.4488
758.3947
729.3646
0
20
40
60
80
100
200
300
400
500
600
700
800

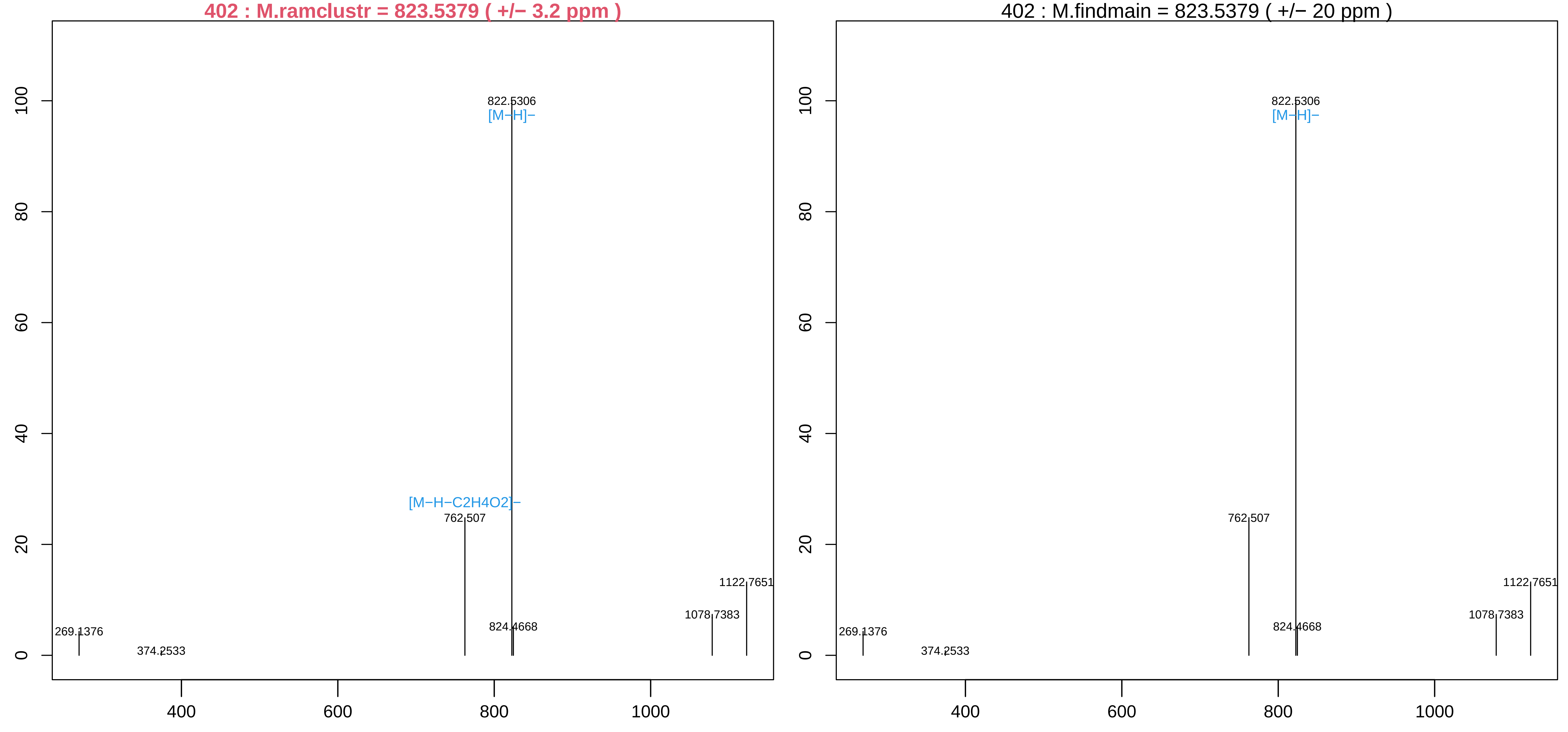
402 : M.ramclustr = 823.5379 (+/− 3.2 ppm)
822.5306
[M−H]−
[M−H−C2H4O2]−
762.507
824.4668
1078.7383
1122.7651
269.1376
374.2533
0
20
40
60
80
100
400
600
800
1000
402 : M.findmain = 823.5379 (+/− 20 ppm)
822.5306
[M−H]−
762.507
824.4668
1078.7383
1122.7651
269.1376
374.2533
0
20
40
60
80
100
400
600
800
1000

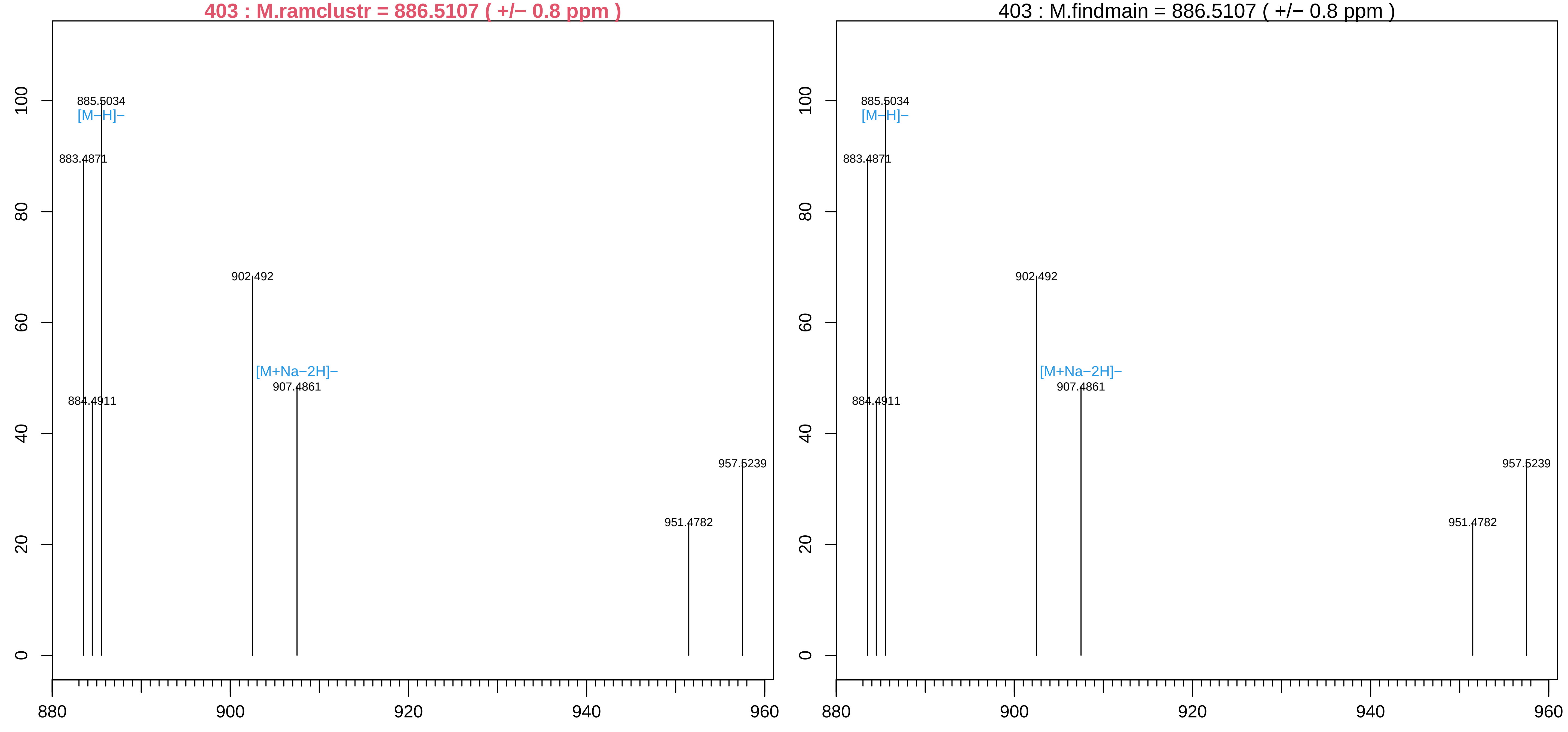
403 : M.ramclustr = 886.5107 (+/− 0.8 ppm)
403 : M.findmain = 886.5107 (+/− 0.8 ppm)
885.5034
[M−H]−
883.4871
884.4911
902.492
[M+Na−2H]−
907.4861
951.4782
957.5239
0
20
40
60
80
100
880
900
920
940
960

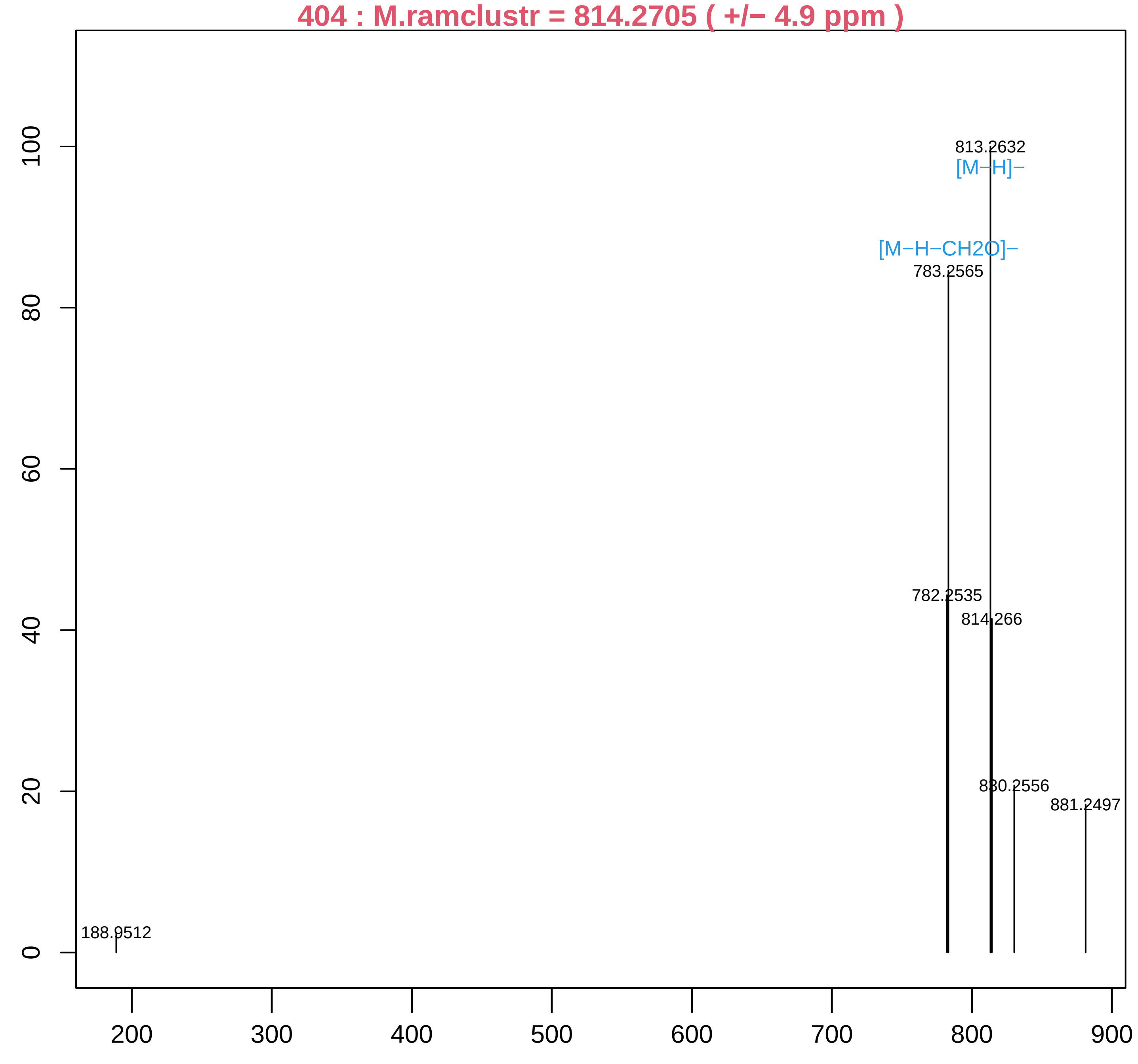
404 : M.ramclustr = 814.2705 (+/− 4.9 ppm)
813.2632
[M−H]−
[M−H−CH2O]−
783.2565
782.2535
814.266
830.2556
881.2497
188.9512
0
20
40
60
80
100
200
300
400
500
600
700
800
900

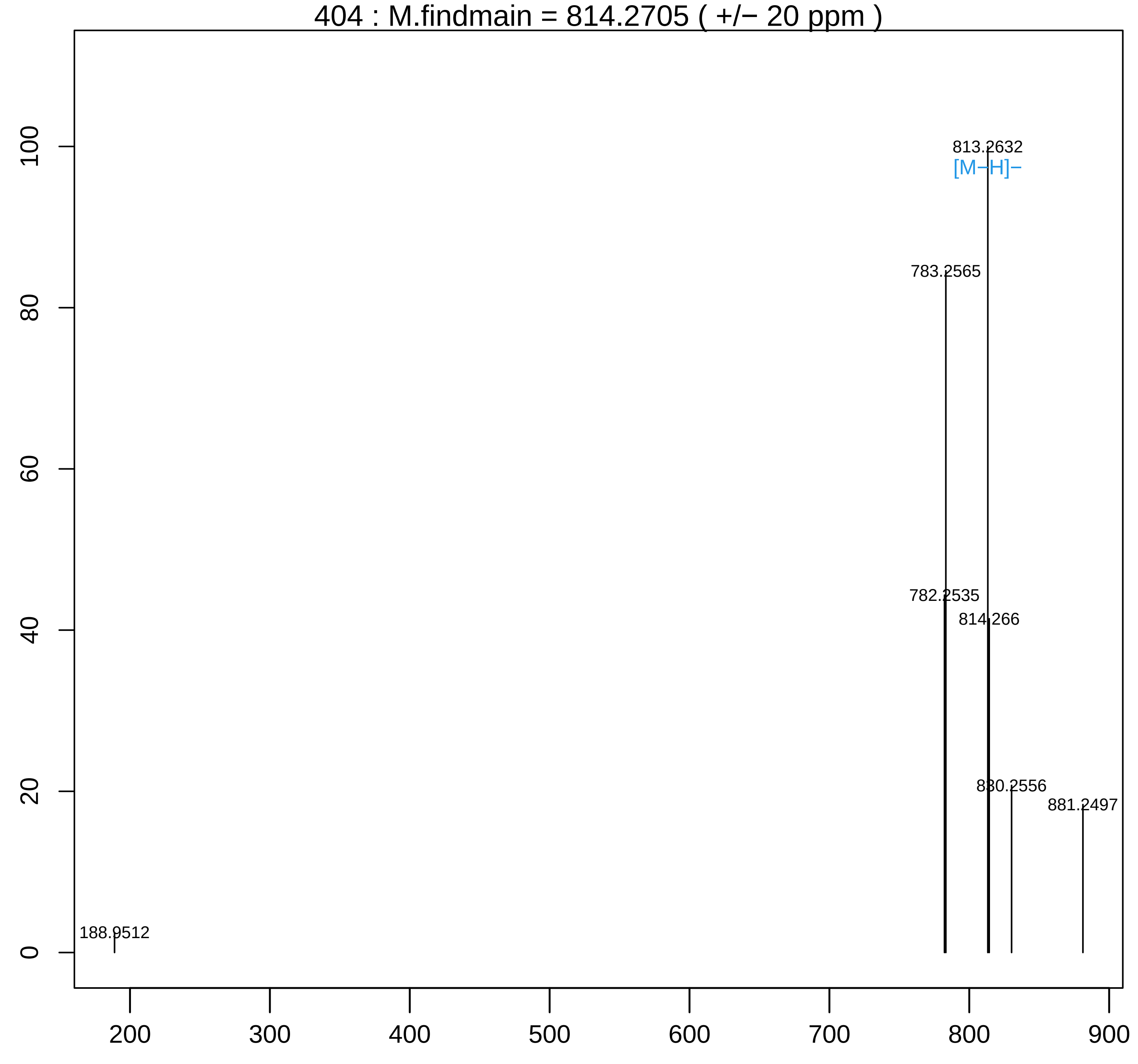
404 : M.findmain = 814.2705 (+/− 20 ppm)
813.2632
[M−H]−
783.2565
782.2535
814.266
830.2556
881.2497
188.9512
0
20
40
60
80
100
200
300
400
500
600
700
800
900

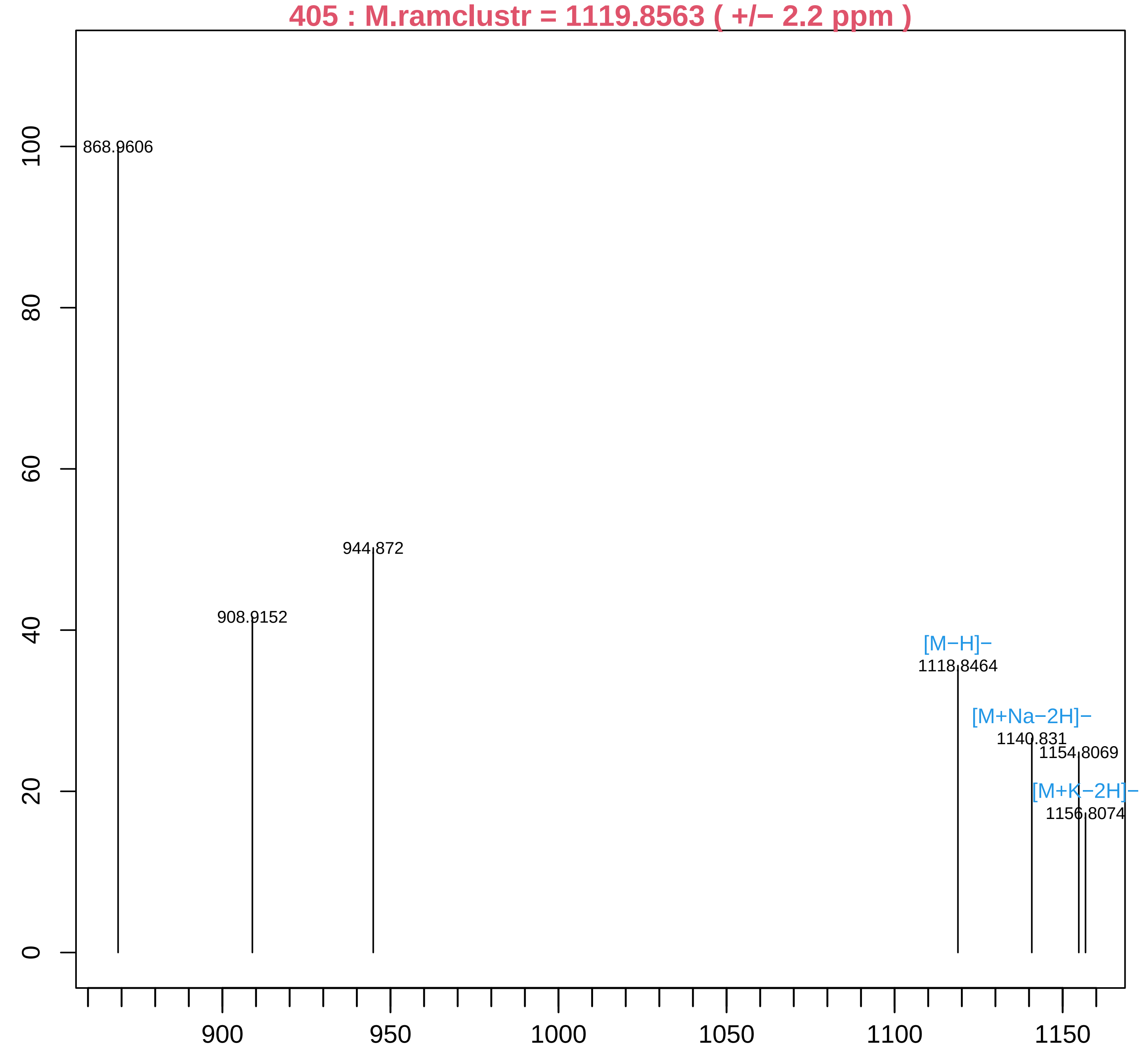
405 : M.ramclustr = 1119.8563 (+/− 2.2 ppm)
868.9606
908.9152
944.872
[M−H]−
1118.8464
[M+Na−2H]−
1140.831
1154.8069
[M+K−2H]−
1156.8074
0
20
40
60
80
100
900
950
1000
1050
1100
1150

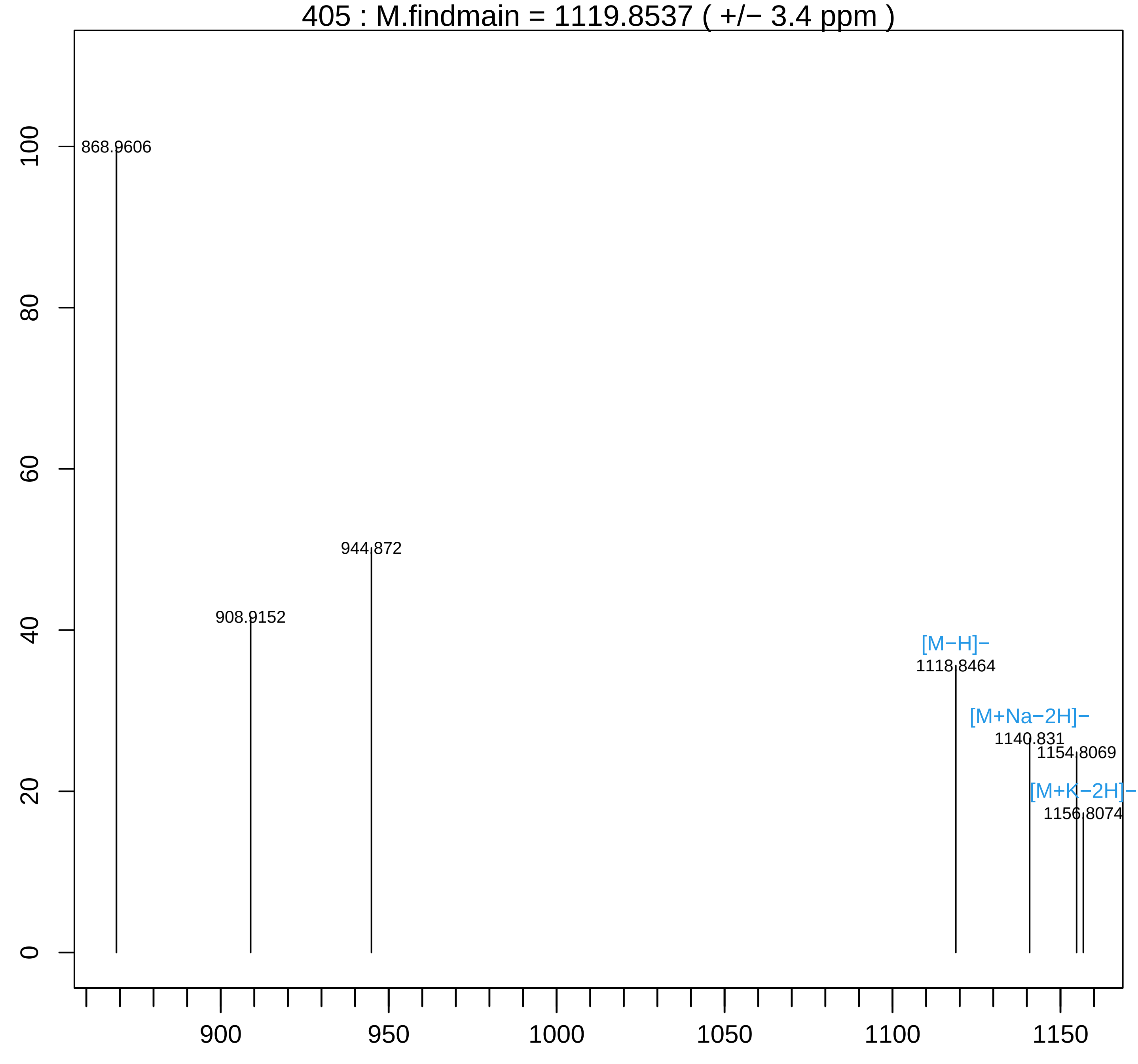
405 : M.findmain = 1119.8537 (+/− 3.4 ppm)
868.9606
908.9152
944.872
[M−H]−
1118.8464
[M+Na−2H]−
1140.831
1154.8069
[M+K−2H]−
1156.8074
0
20
40
60
80
100
900
950
1000
1050
1100
1150

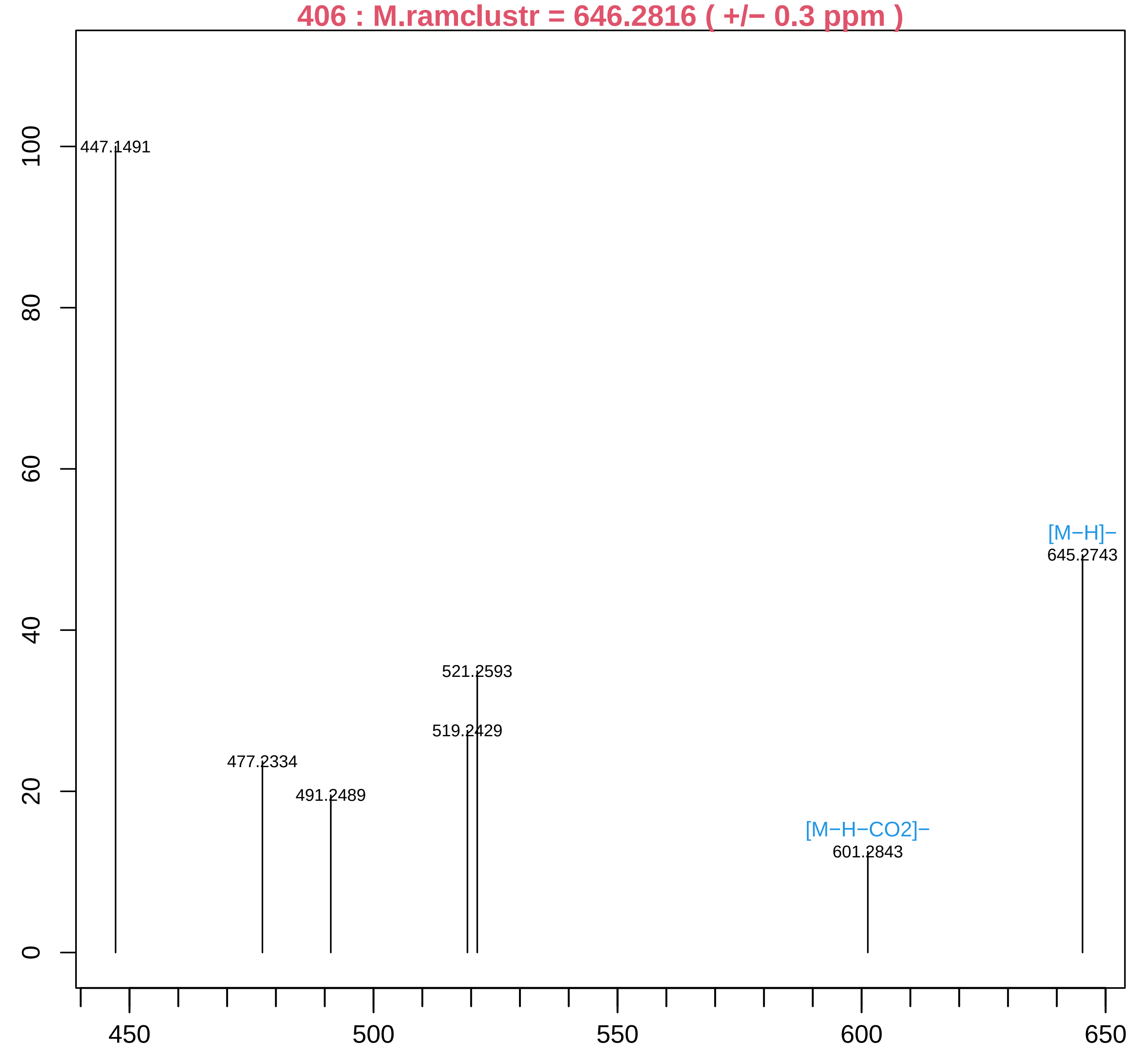
406 : M.ramclustr = 646.2816 (+/− 0.3 ppm)
447.1491
477.2334
491.2489
519.2429
521.2593
[M−H−CO2]−
601.2843
[M−H]−
645.2743
0
20
40
60
80
100
450
500
550
600
650

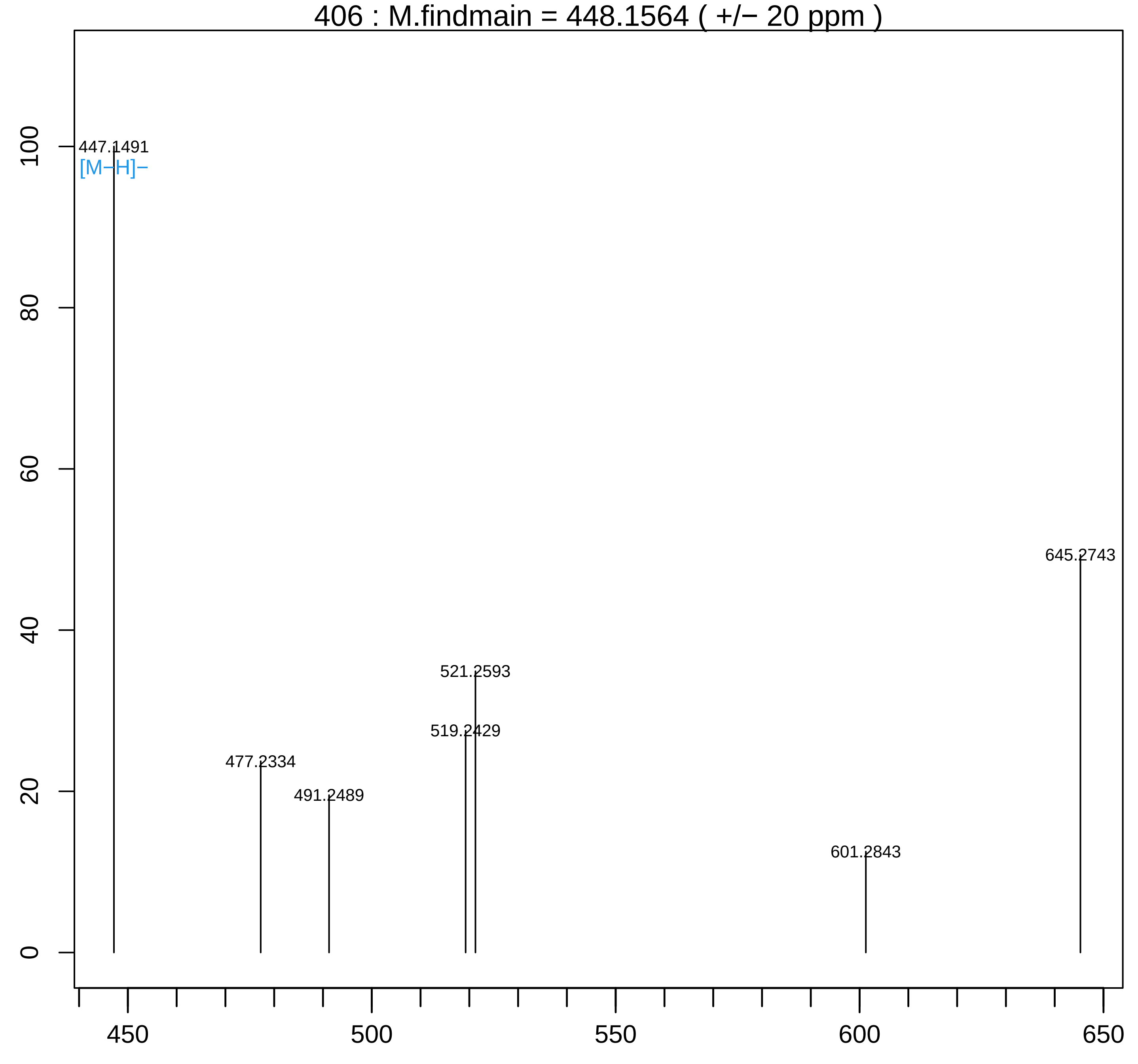
406 : M.findmain = 448.1564 (+/− 20 ppm)
447.1491
[M−H]−
477.2334
491.2489
519.2429
521.2593
601.2843
645.2743
0
20
40
60
80
100
450
500
550
600
650

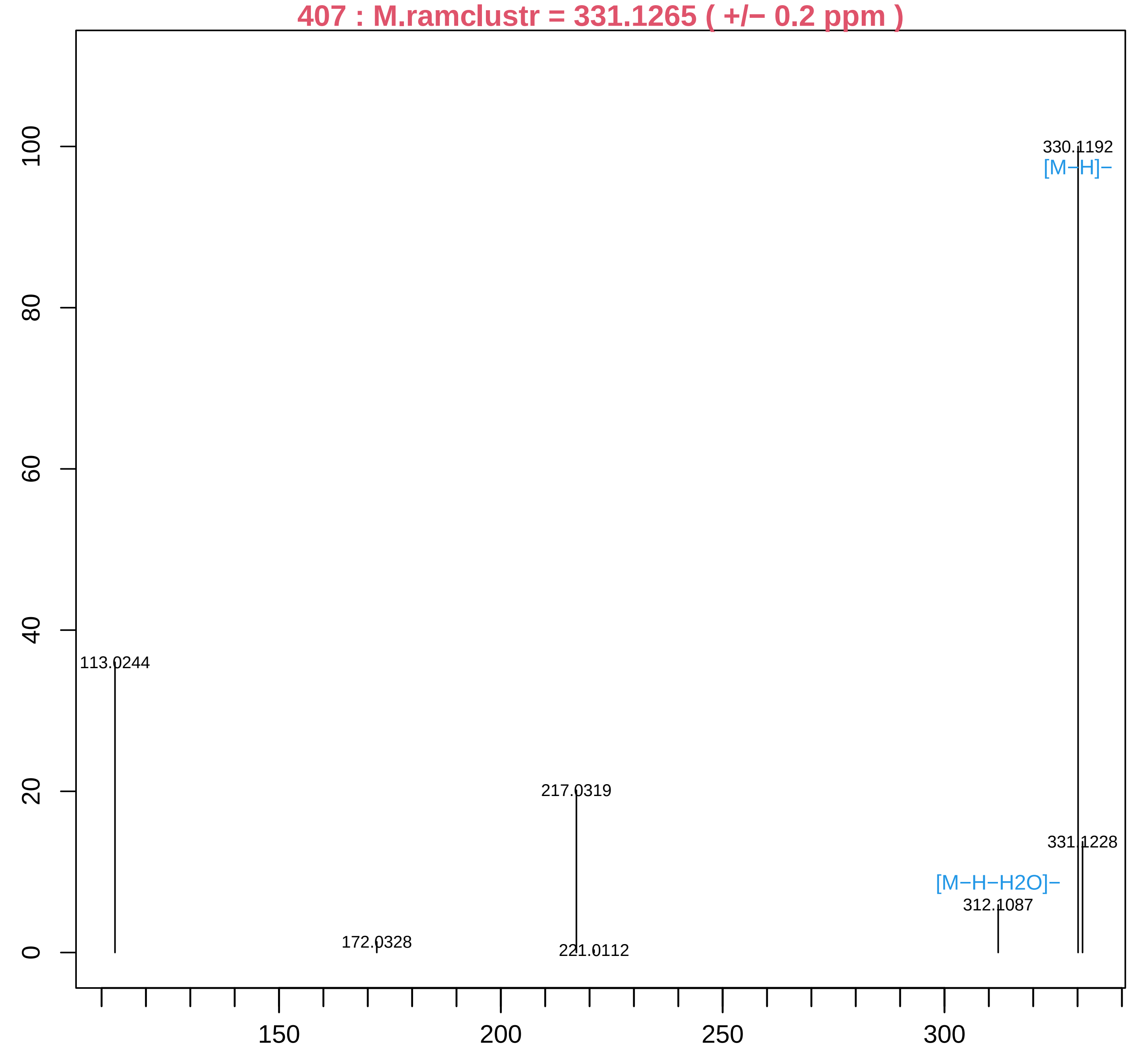

407 : M.ramclustr = 331.1265 (+/− 0.2 ppm)
330.1192
[M−H]−
113.0244
217.0319
331.1228
[M−H−H2O]−
312.1087
172.0328
221.0112
0
20
40
60
80
100
150
200
250
300

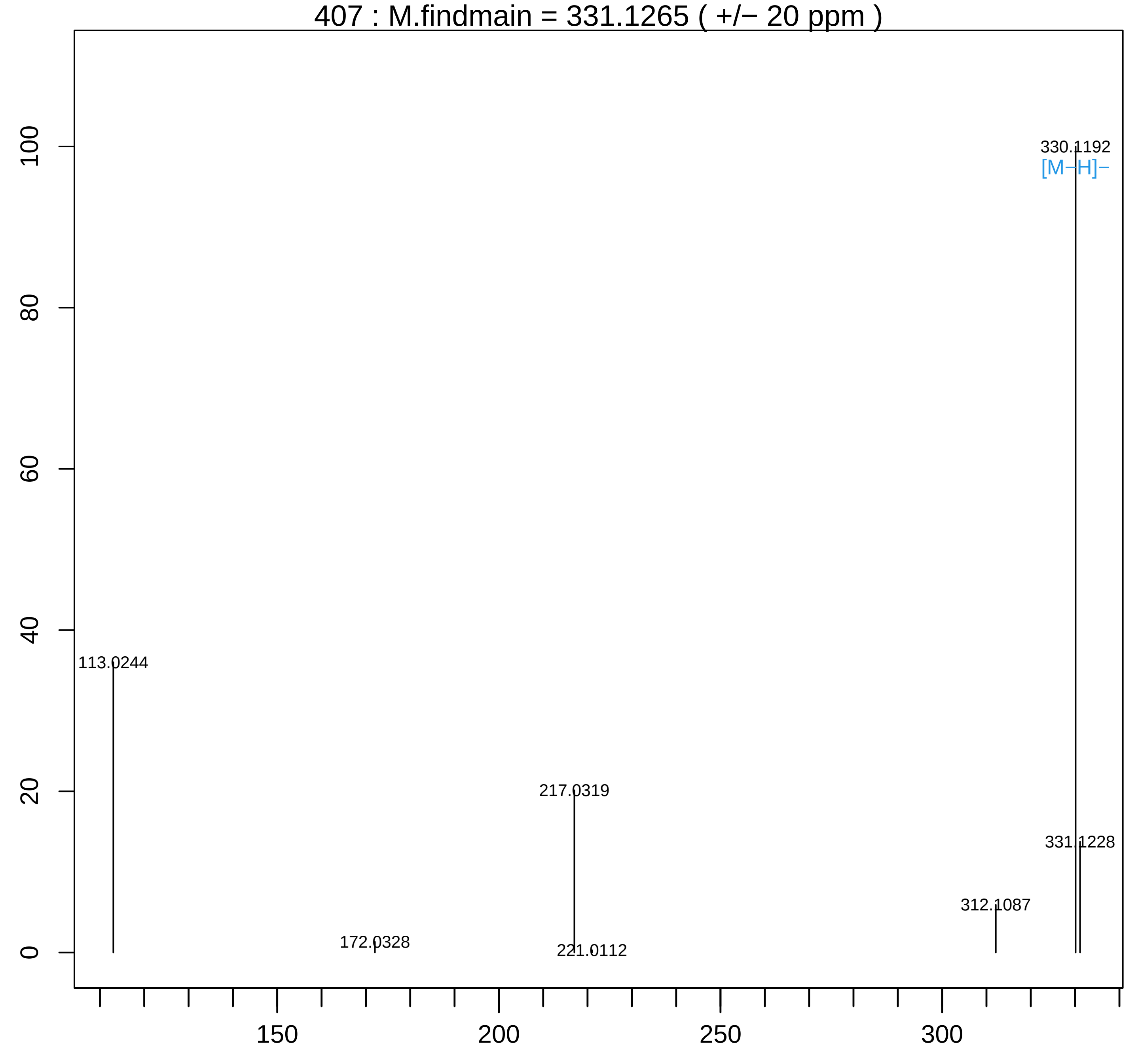

407 : M.findmain = 331.1265 (+/− 20 ppm)
330.1192
[M−H]−
113.0244
217.0319
331.1228
312.1087
172.0328
221.0112
0
20
40
60
80
100
150
200
250
300

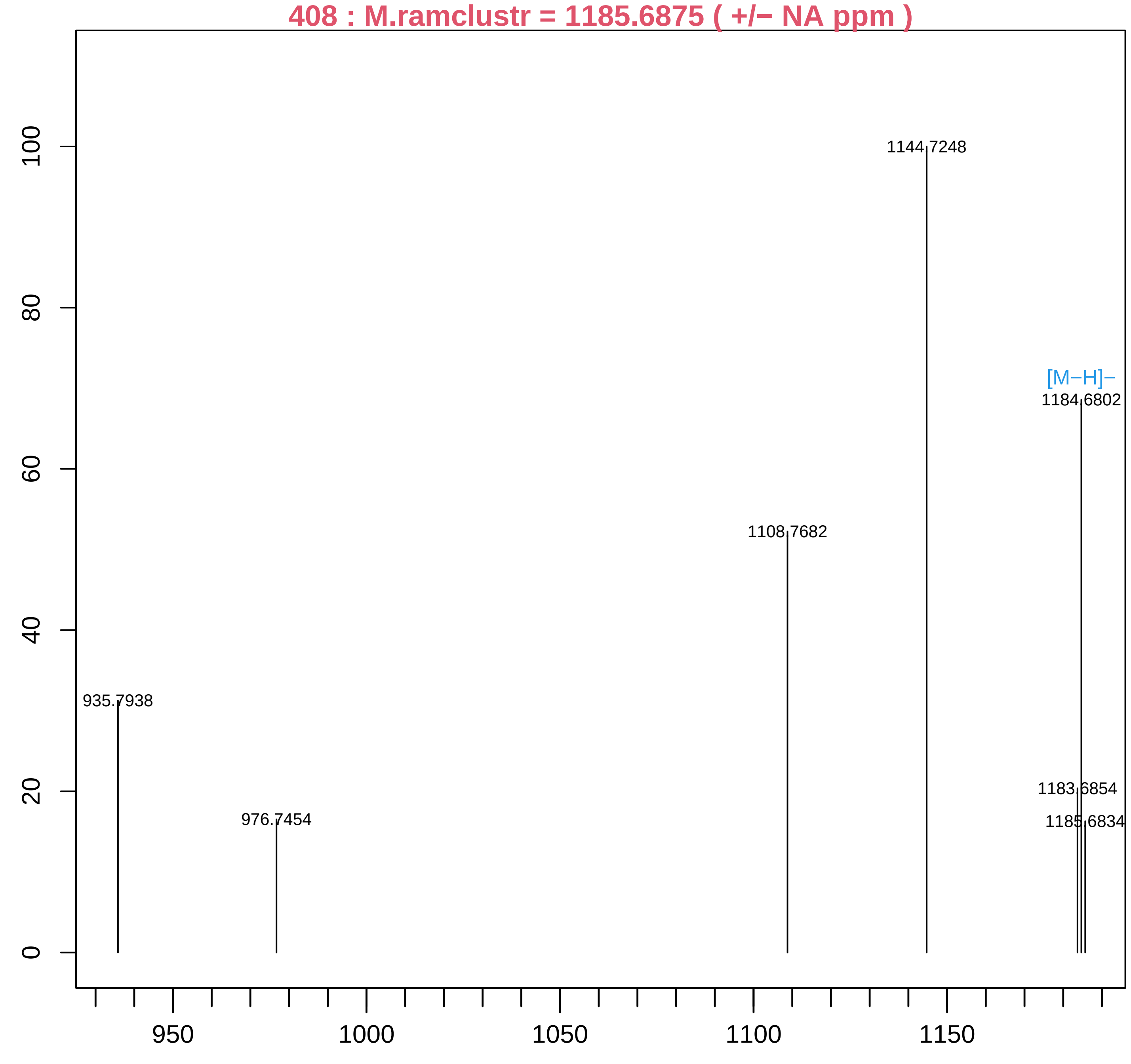

408 : M.ramclustr = 1185.6875 (+/− NA ppm)
[M−H]−
1144.7248
1184.6802
1108.7682
935.7938
976.7454
1183.6854
1185.6834
0
20
40
60
80
100
950
1000
1050
1100
1150

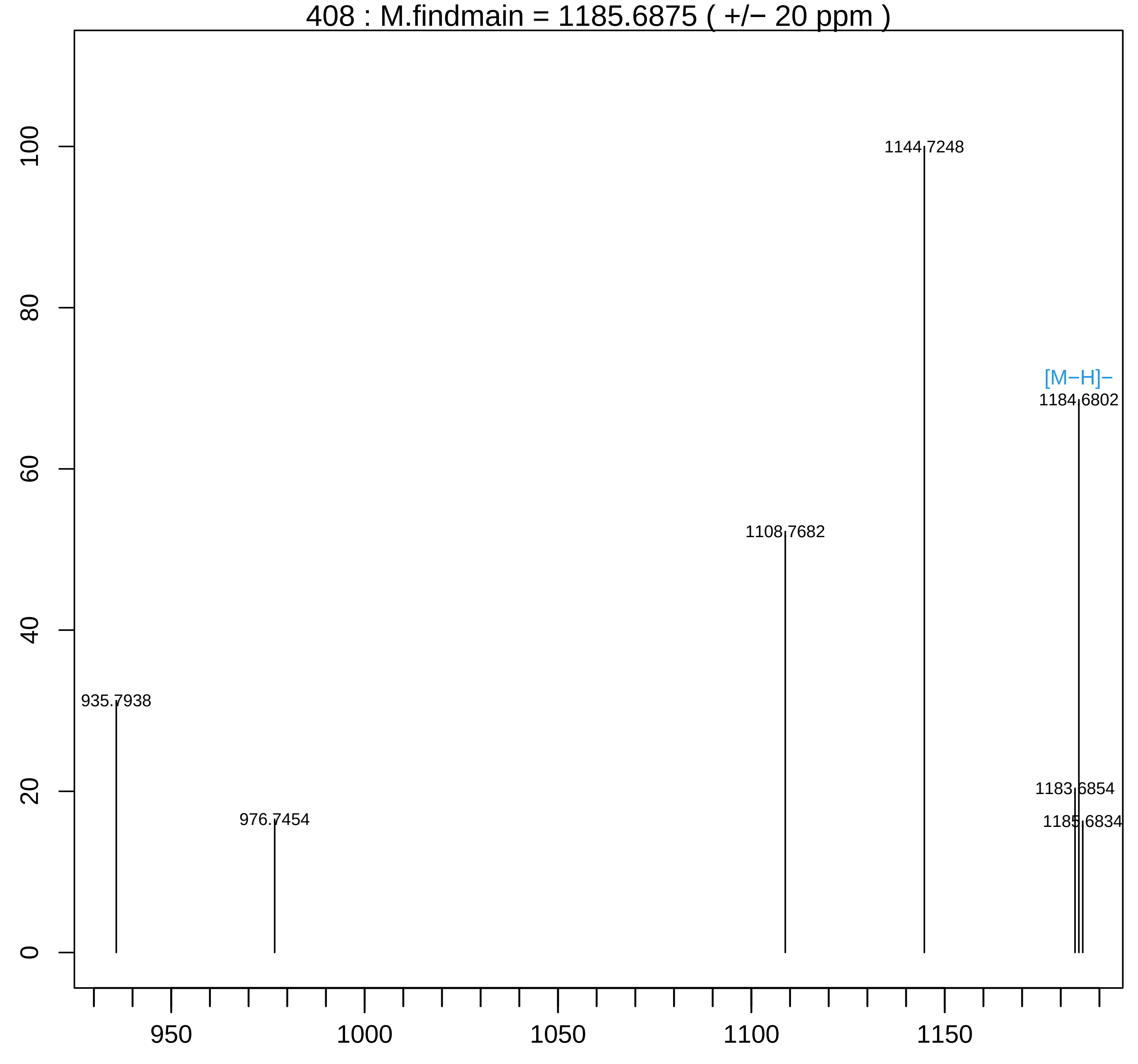

408 : M.findmain = 1185.6875 (+/− 20 ppm)
[M−H]−
1144.7248
1184.6802
1108.7682
935.7938
976.7454
1183.6854
1185.6834
0
20
40
60
80
100
950
1000
1050
1100
1150

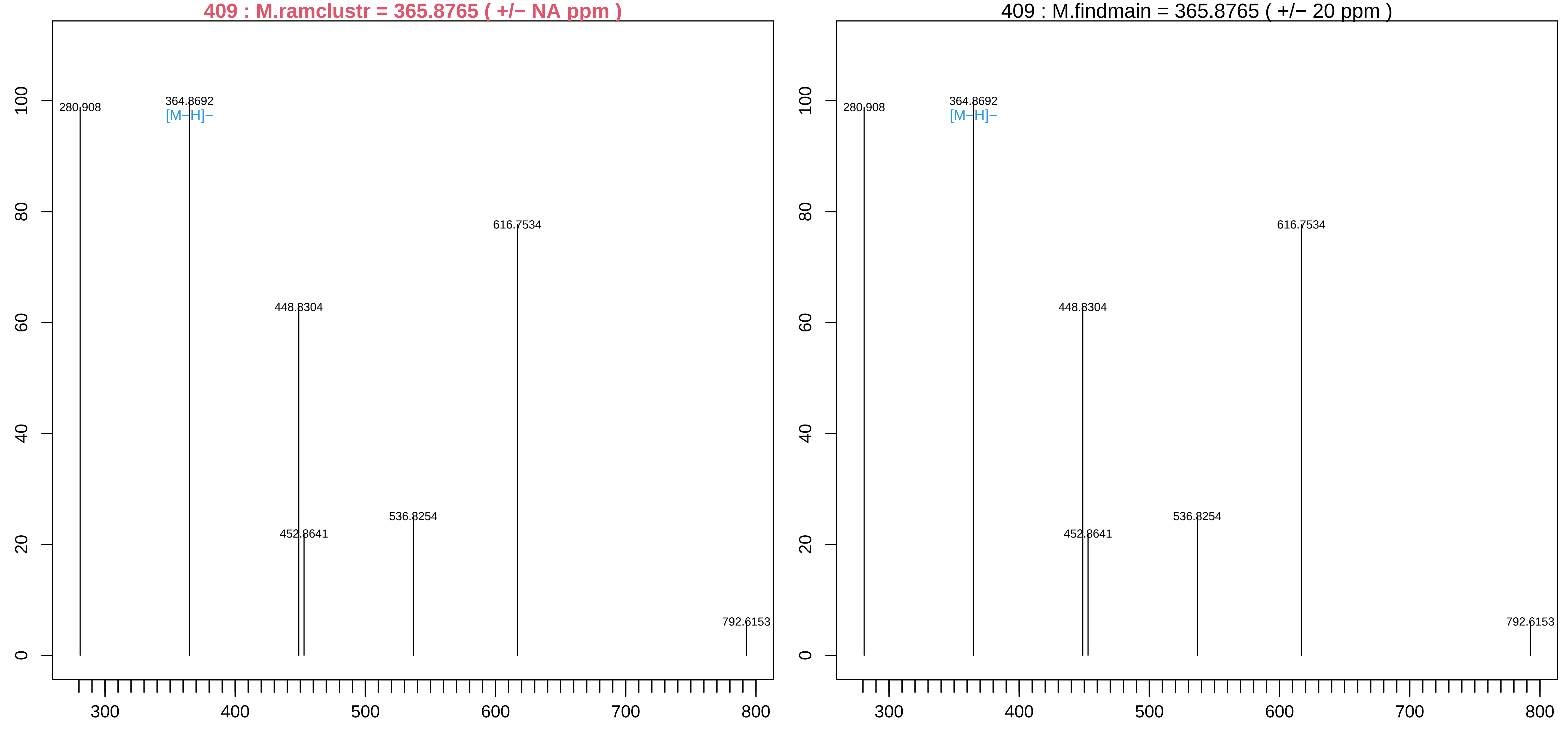
409 : M.ramclustr = 365.8765 (+/− NA ppm)
280.908
364.8692
[M−H]−
448.8304
452.8641
536.8254
616.7534
792.6153
409 : M.findmain = 365.8765 (+/− 20 ppm)
280.908
364.8692
[M−H]−
448.8304
452.8641
536.8254
616.7534
792.6153
0
20
40
60
80
100
300
400
500
600
700
800

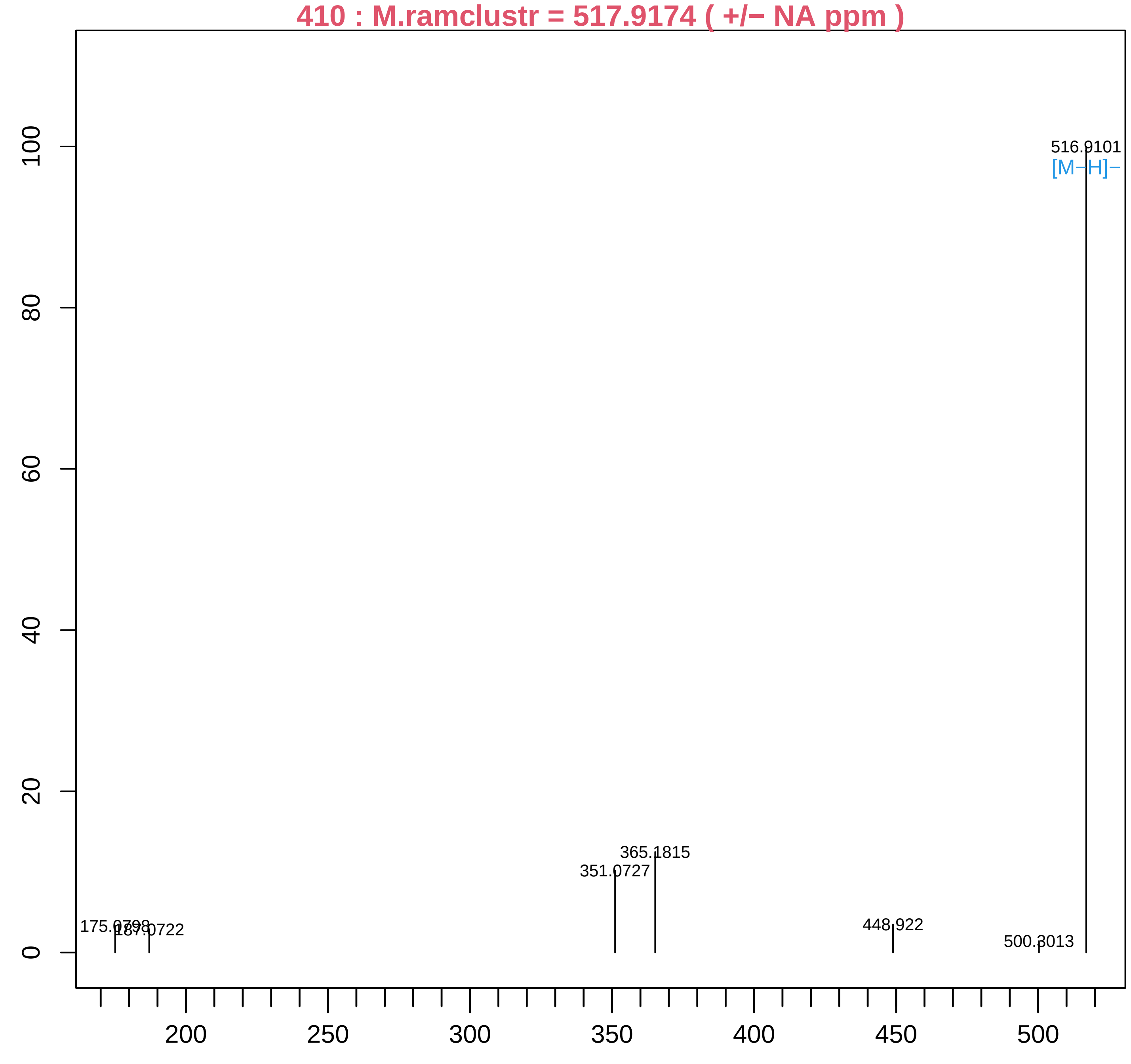
410 : M.ramclustr = 517.9174 (+/− NA ppm)
516.9101
[M−H]−
365.1815
351.0727
175.0798
187.0722
448.922
500.3013
0
20
40
60
80
100
200
250
300
350
400
450
500

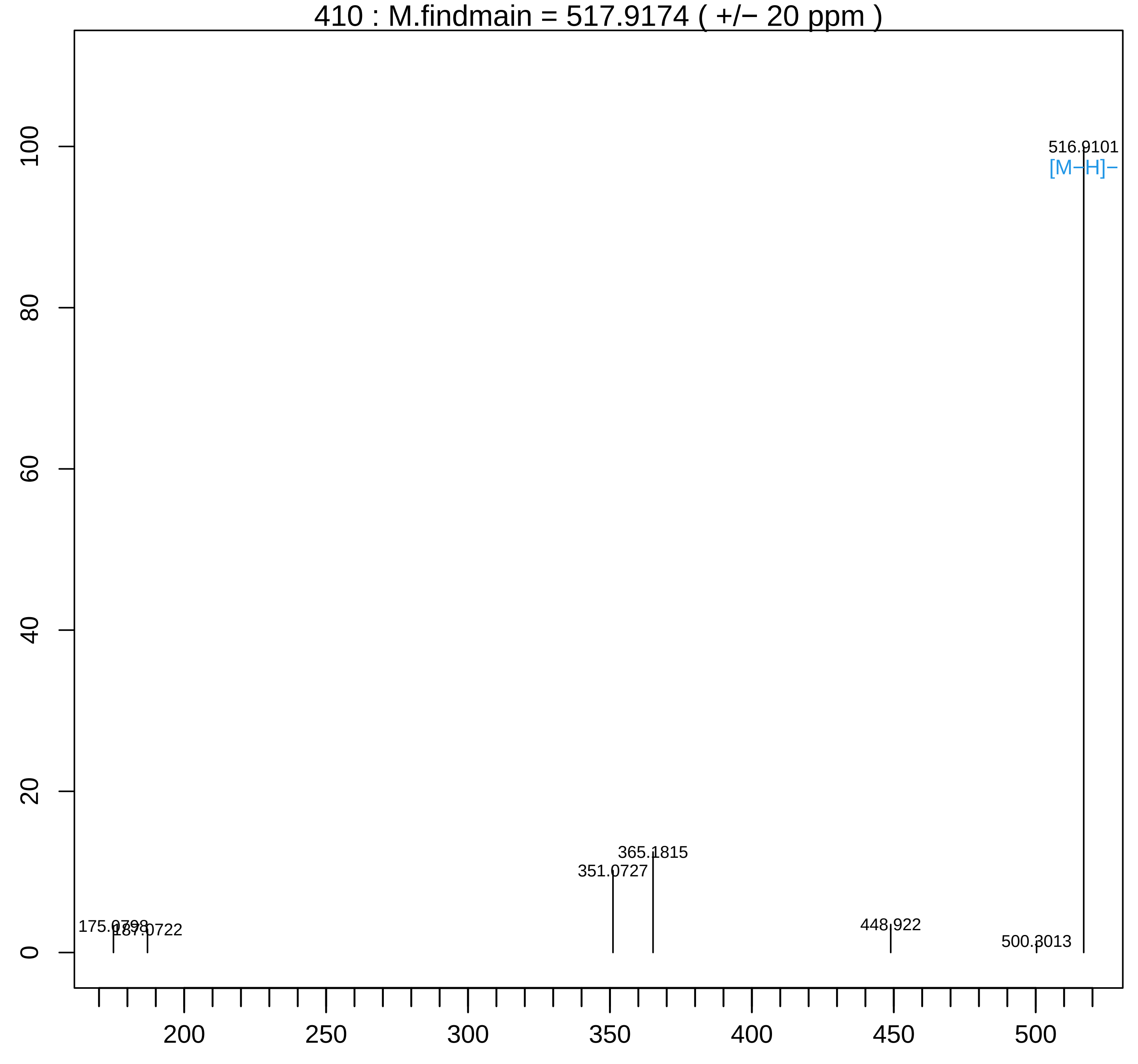
410 : M.findmain = 517.9174 (+/− 20 ppm)
516.9101
[M−H]−
365.1815
351.0727
175.0798
187.0722
448.922
500.3013
0
20
40
60
80
100
200
250
300
350
400
450
500

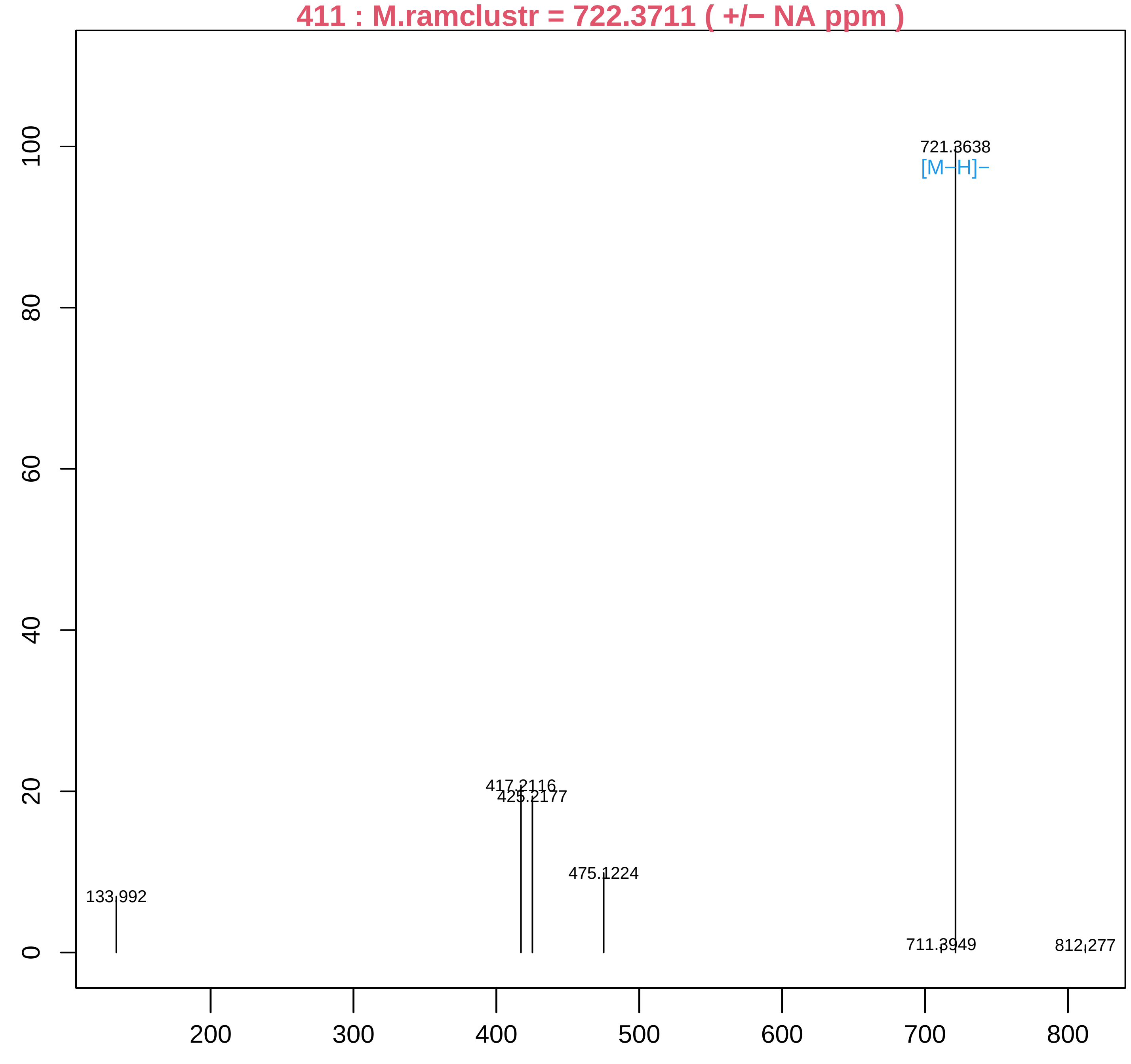

411 : M.ramclustr = 722.3711 (+/− NA ppm)
721.3638
[M−H]−
417.2116
425.2177
475.1224
133.992
711.3949
812.277
0
20
40
60
80
100
200
300
400
500
600
700
800

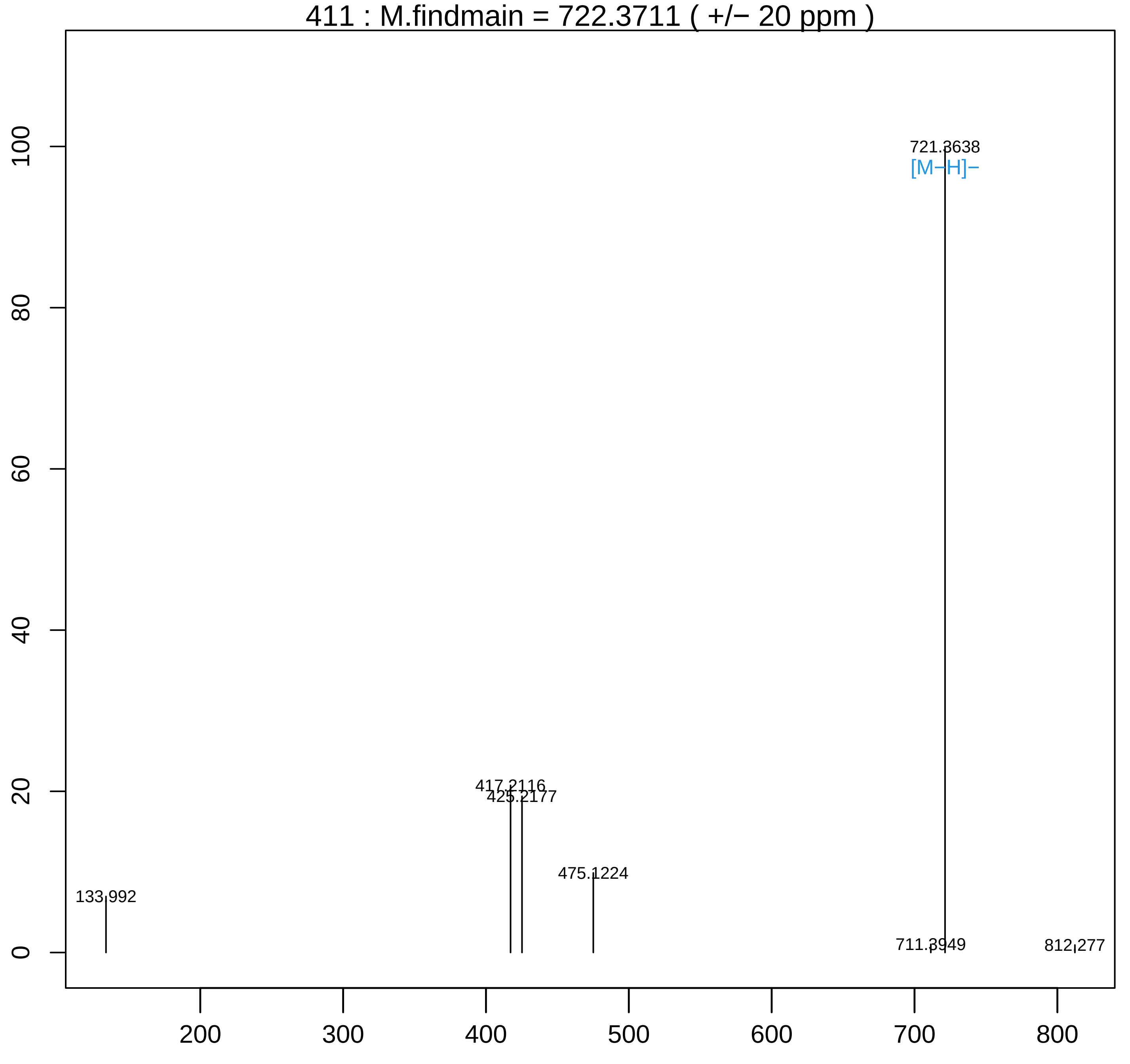

411 : M.findmain = 722.3711 (+/− 20 ppm)
721.3638
[M−H]−
417.2116
425.2177
475.1224
133.992
711.3949
812.277
0
20
40
60
80
100
200
300
400
500
600
700
800

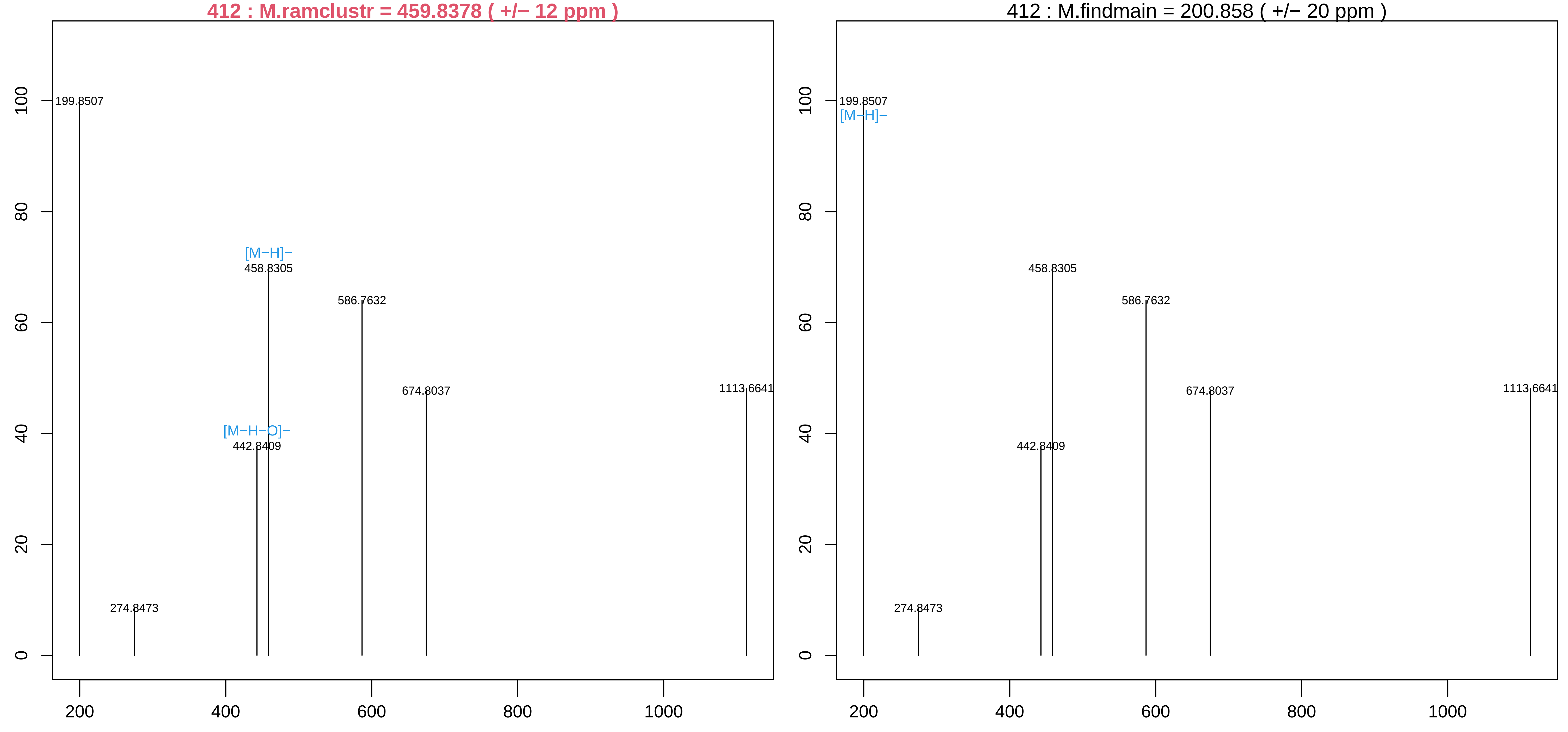
412 : M.ramclustr = 459.8378 (+/− 12 ppm)
199.8507
274.8473
[M−H−O]−
442.8409
[M−H]−
458.8305
586.7632
674.8037
1113.6641
412 : M.findmain = 200.858 (+/− 20 ppm)
199.8507
[M−H]−
274.8473
442.8409
458.8305
586.7632
674.8037
1113.6641

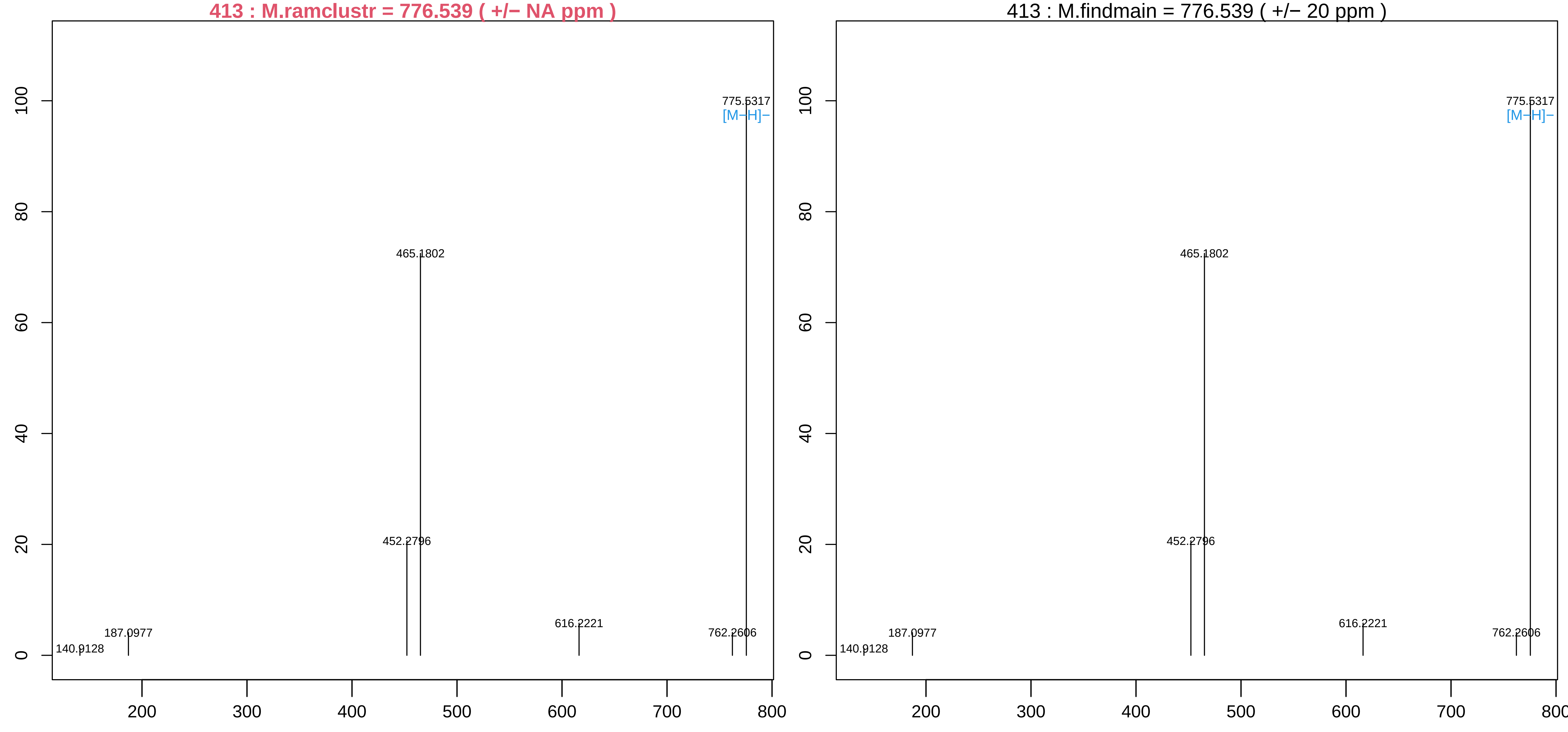

413 : M.ramclustr = 776.539 (+/− NA ppm)
775.5317
[M−H]−
465.1802
452.2796
616.2221
762.2606
187.0977
140.9128
0
20
40
60
80
100
200
300
400
500
600
700
800
413 : M.findmain = 776.539 (+/− 20 ppm)
775.5317
[M−H]−
465.1802
452.2796
616.2221
762.2606
187.0977
140.9128
0
20
40
60
80
100
200
300
400
500
600
700
800

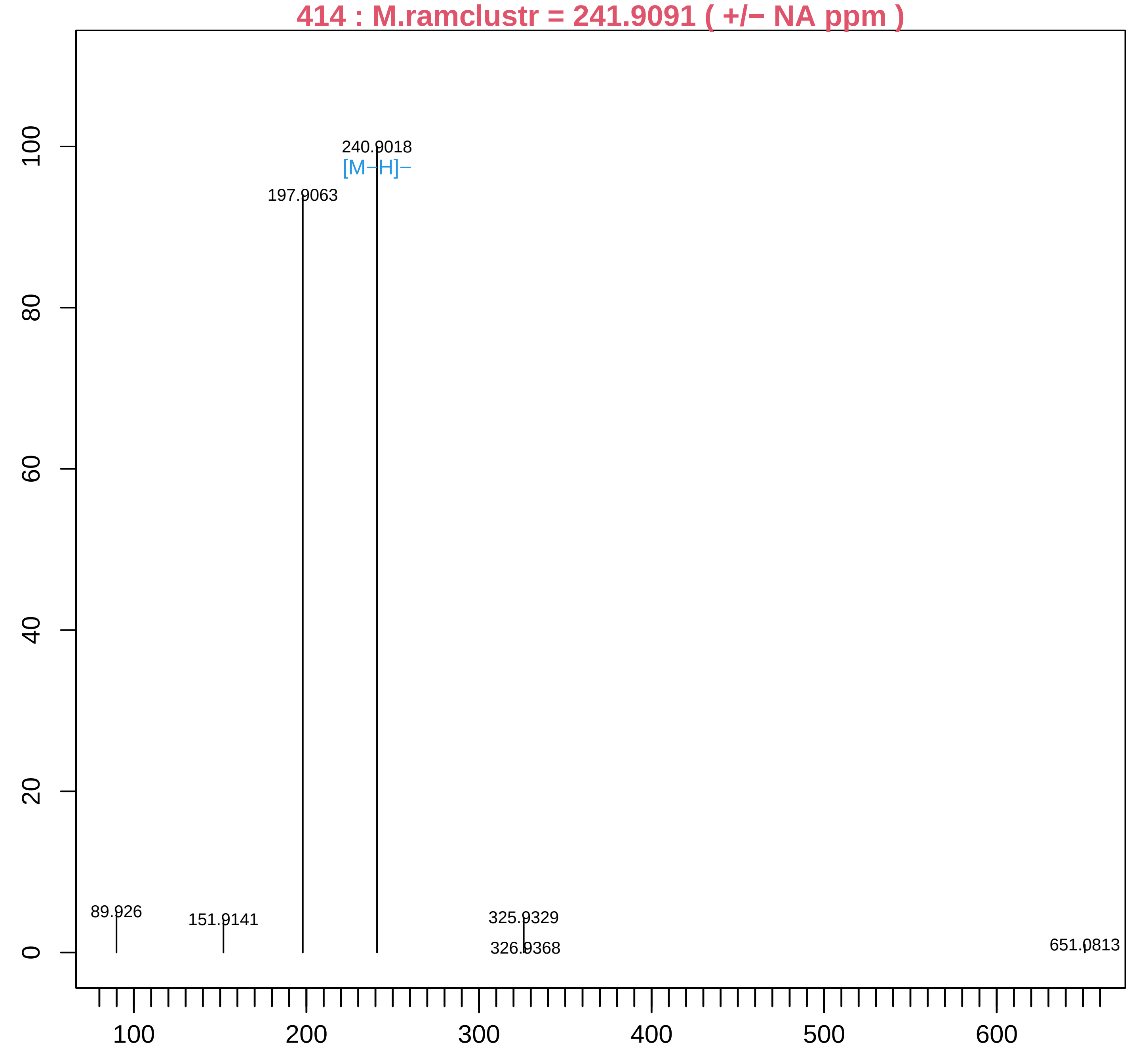

414 : M.ramclustr = 241.9091 (+/− NA ppm)
240.9018
[M−H]−
197.9063
89.926
151.9141
325.9329
326.9368
651.0813
0
20
40
60
80
100
100
200
300
400
500
600

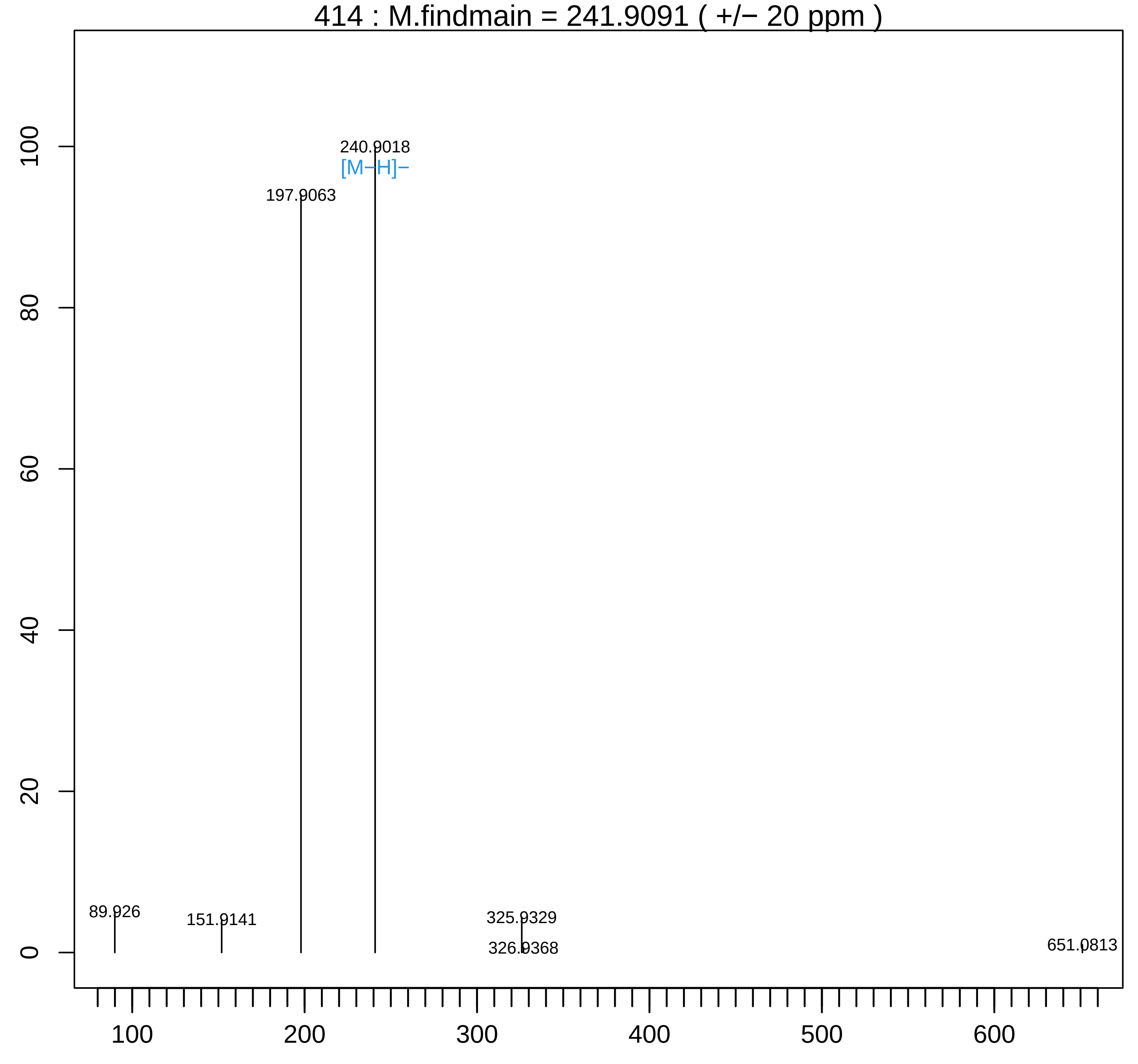

414 : M.findmain = 241.9091 (+/− 20 ppm)
240.9018
[M−H]−
197.9063
89.926
151.9141
325.9329
326.9368
651.0813
0
20
40
60
80
100
100
200
300
400
500
600

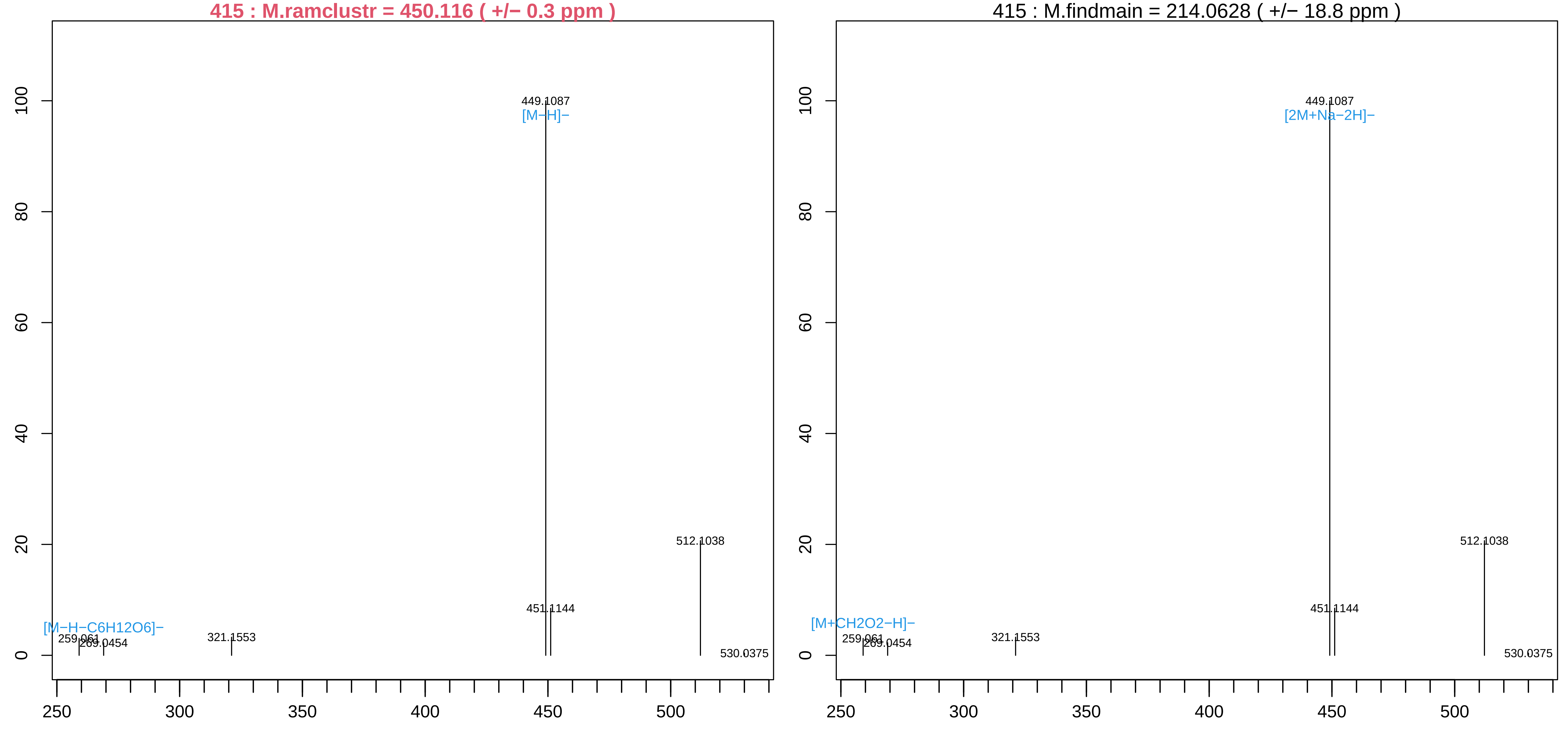
415 : M.ramclustr = 450.116 (+/− 0.3 ppm)
449.1087
[M−H]−
512.1038
451.1144
[M−H−C6H12O6]−
259.061
269.0454
321.1553
530.0375
415 : M.findmain = 214.0628 (+/− 18.8 ppm)
449.1087
[2M+Na−2H]−
512.1038
451.1144
[M+CH2O2−H]−
259.061
269.0454
321.1553
530.0375
0
20
40
60
80
100
250
300
350
400
450
500

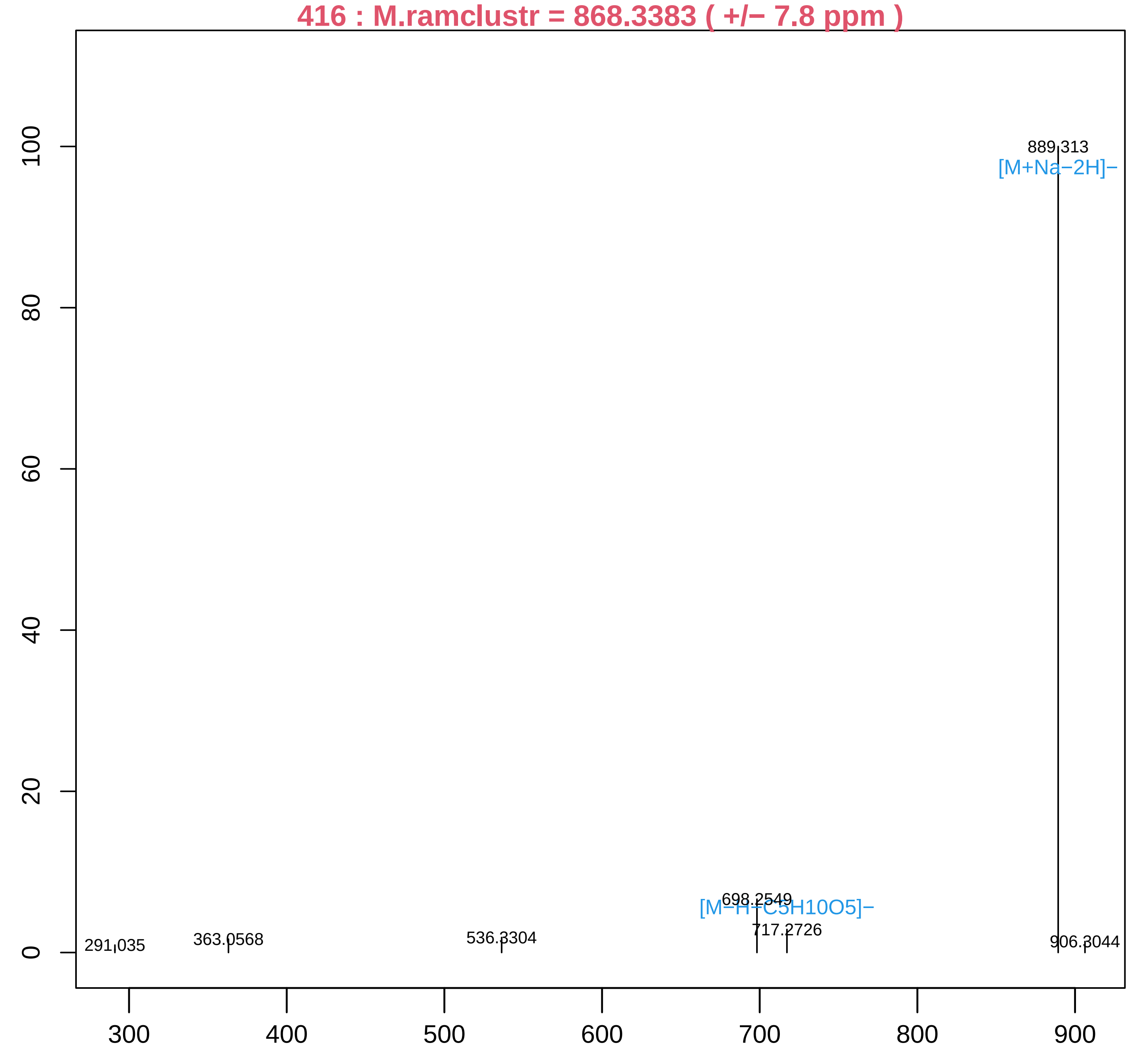

416 : M.ramclustr = 868.3383 (+/− 7.8 ppm)
889.313
[M+Na−2H]−
698.2549
[M−H−C5H10O5]−
717.2726
291.035
363.0568
536.3304
906.3044
0
20
40
60
80
100
300
400
500
600
700
800
900

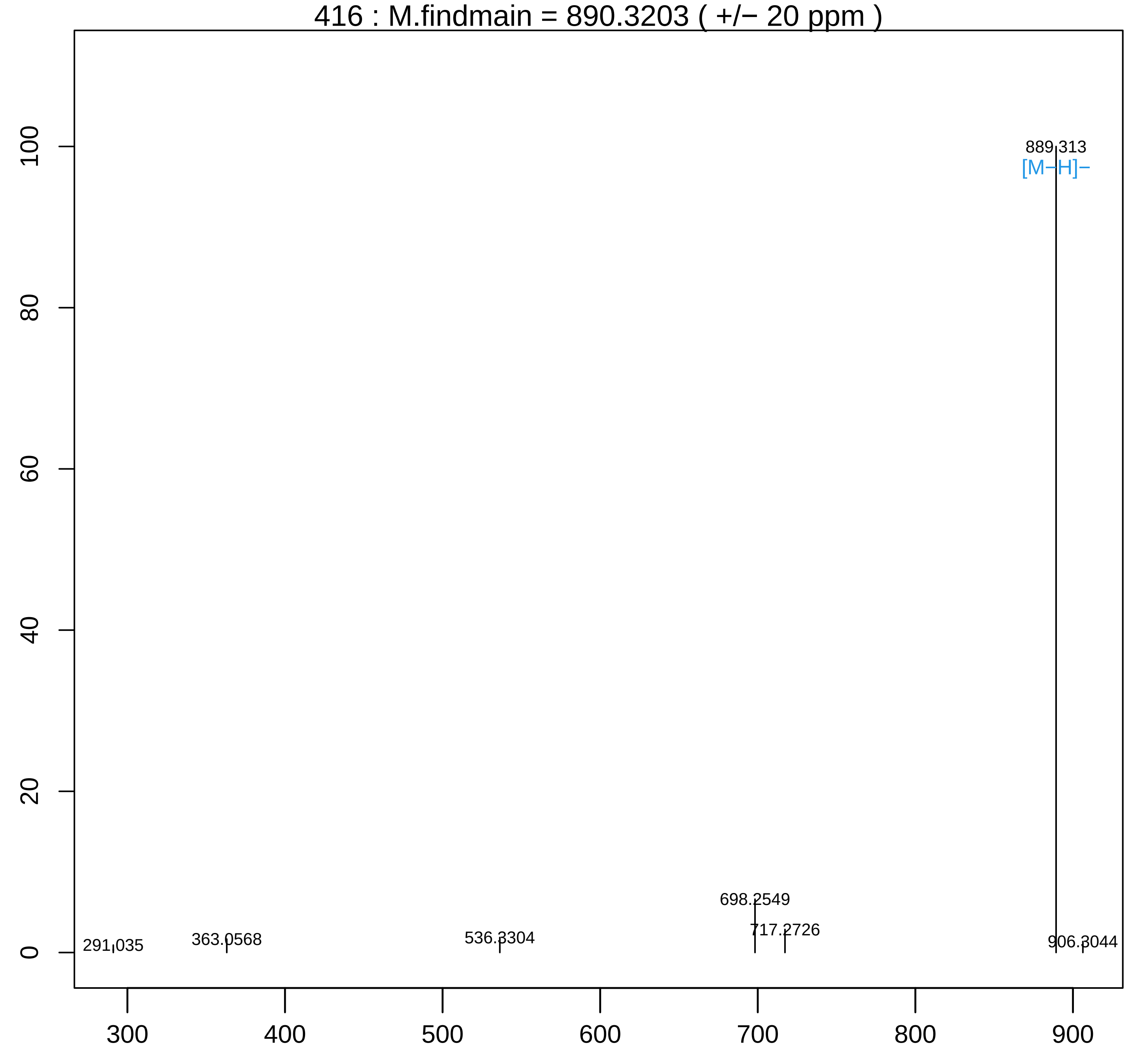

416 : M.findmain = 890.3203 (+/− 20 ppm)
889.313
[M−H]−
698.2549
717.2726
291.035
363.0568
536.3304
906.3044
0
20
40
60
80
100
300
400
500
600
700
800
900

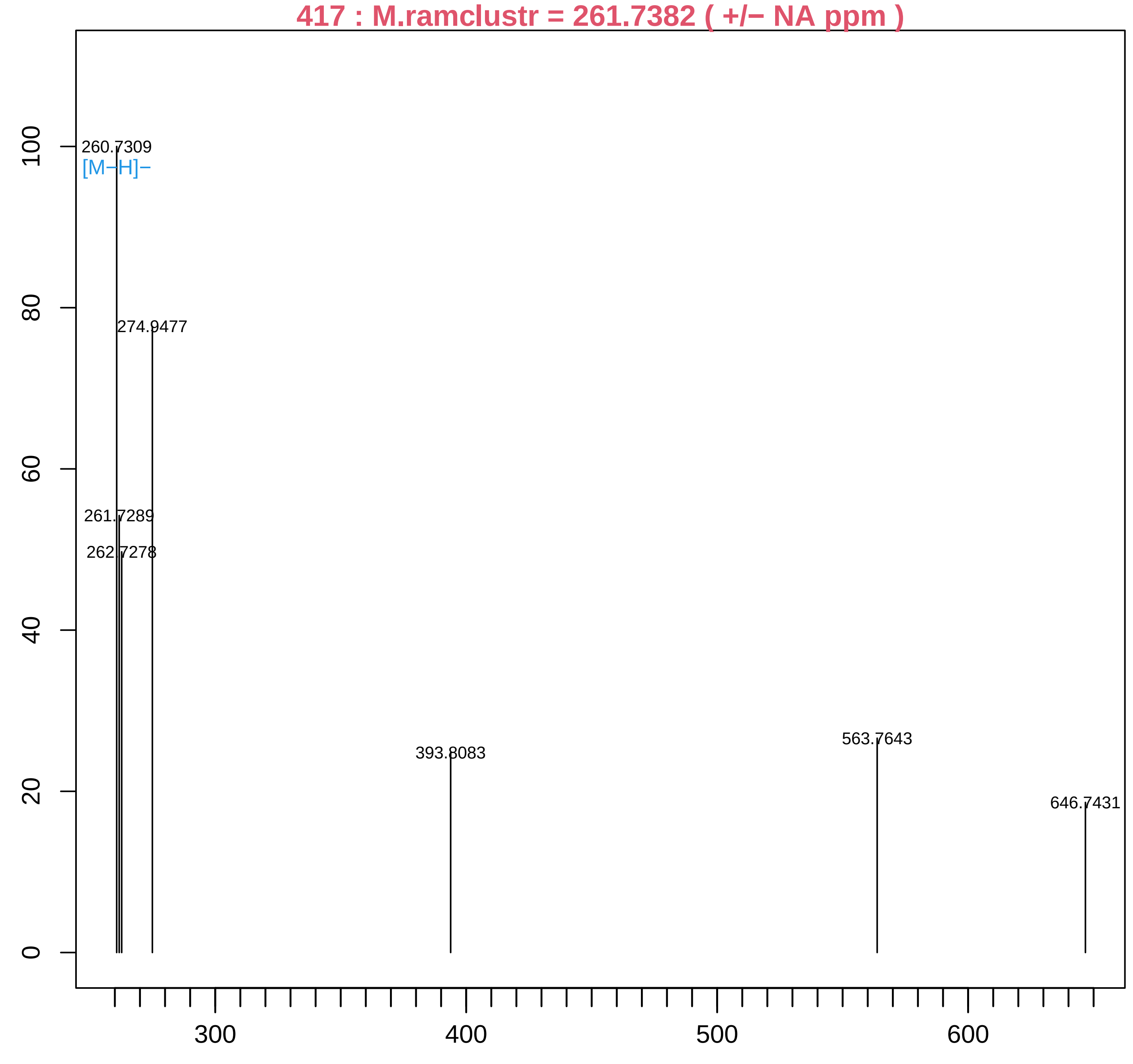
417 : M.ramclustr = 261.7382 (+/− NA ppm)
260.7309
[M−H]−
274.9477
261.7289
262.7278
393.8083
563.7643
646.7431
0
20
40
60
80
100
300
400
500
600

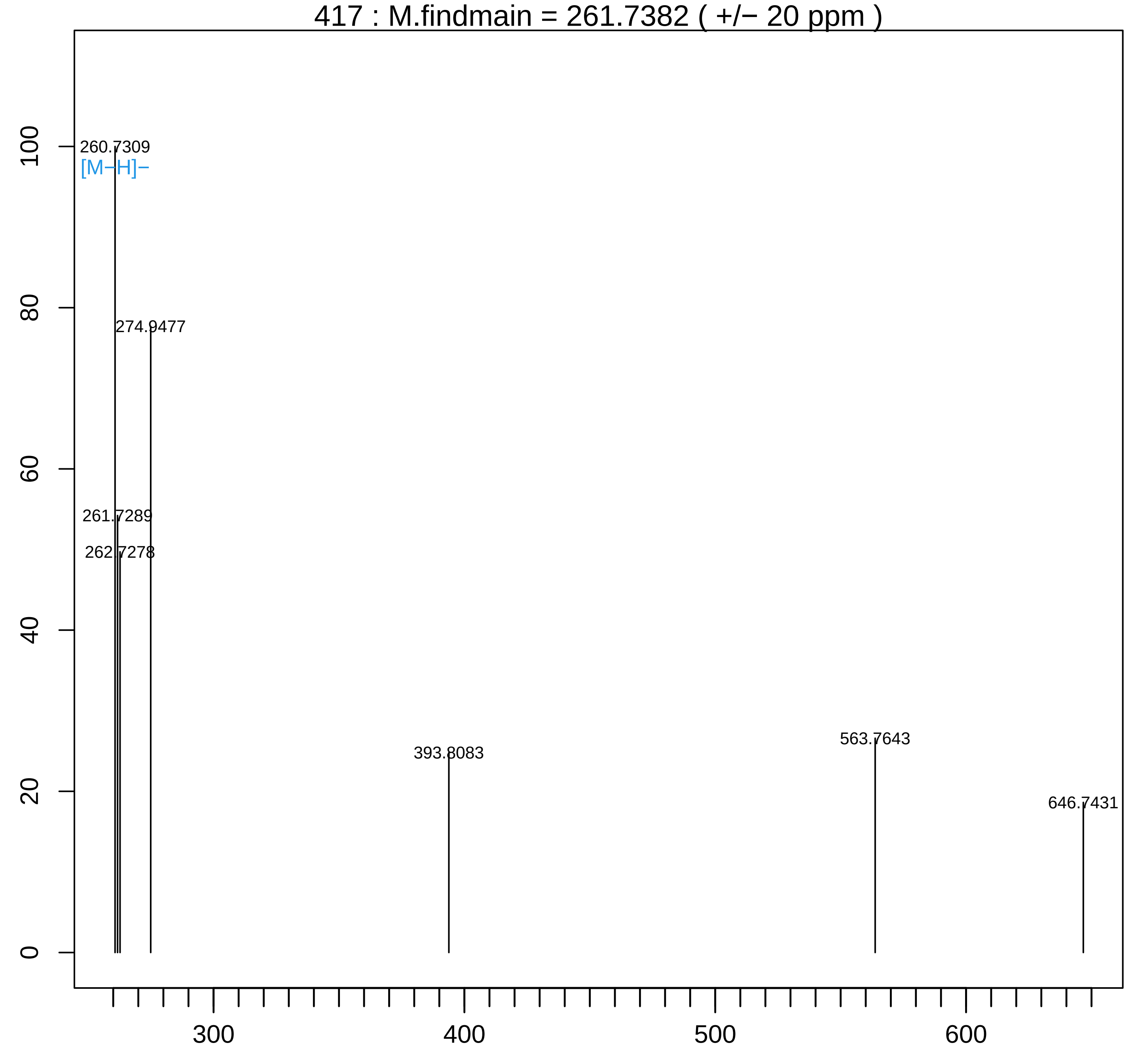
417 : M.findmain = 261.7382 (+/− 20 ppm)
260.7309
[M−H]−
274.9477
261.7289
262.7278
393.8083
563.7643
646.7431
0
20
40
60
80
100
300
400
500
600

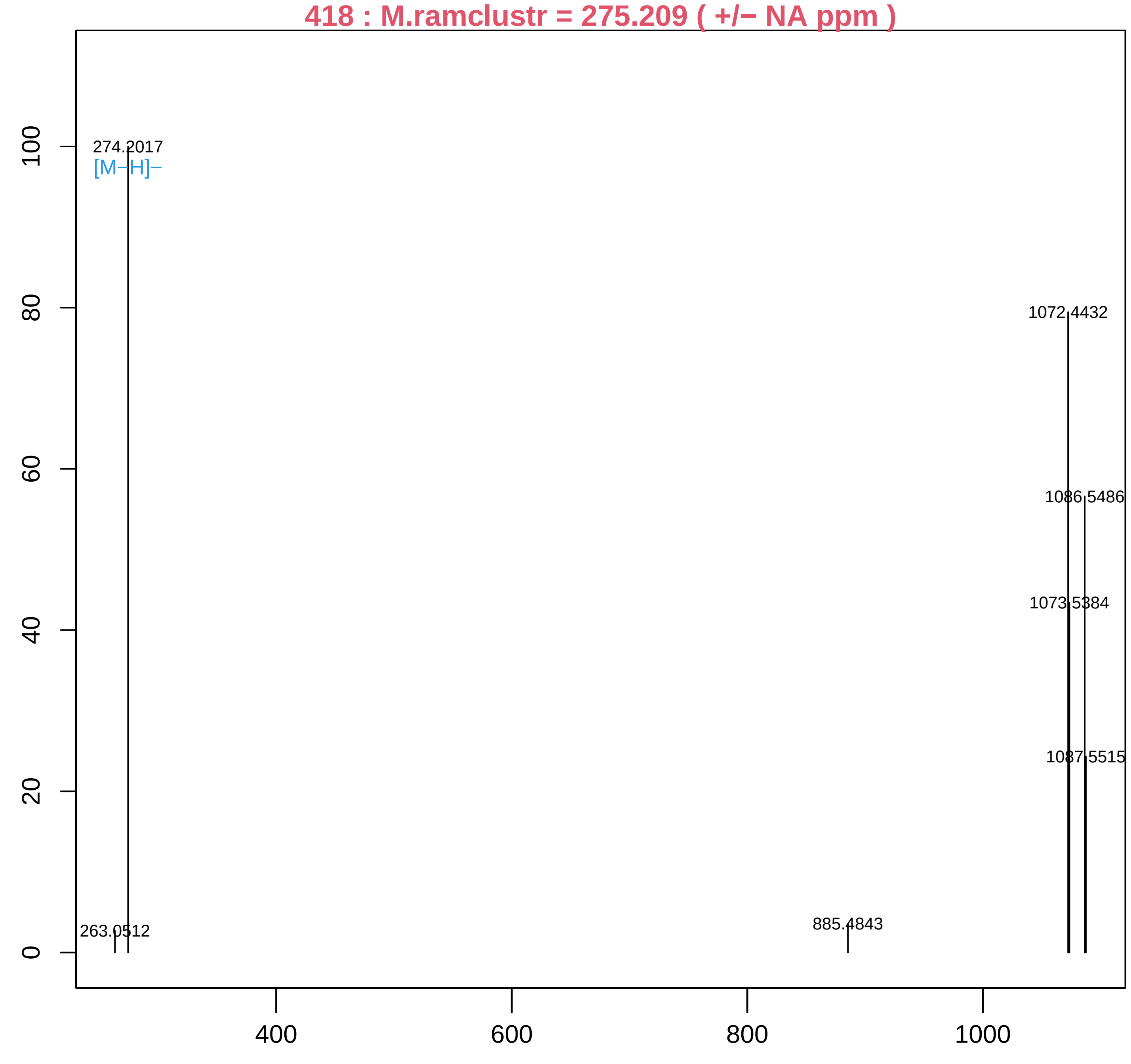

418 : M.ramclustr = 275.209 (+/− NA ppm)
274.2017
[M−H]−
1072.4432
1086.5486
1073.5384
1087.5515
885.4843
263.0512
0
20
40
60
80
100
400
600
800
1000

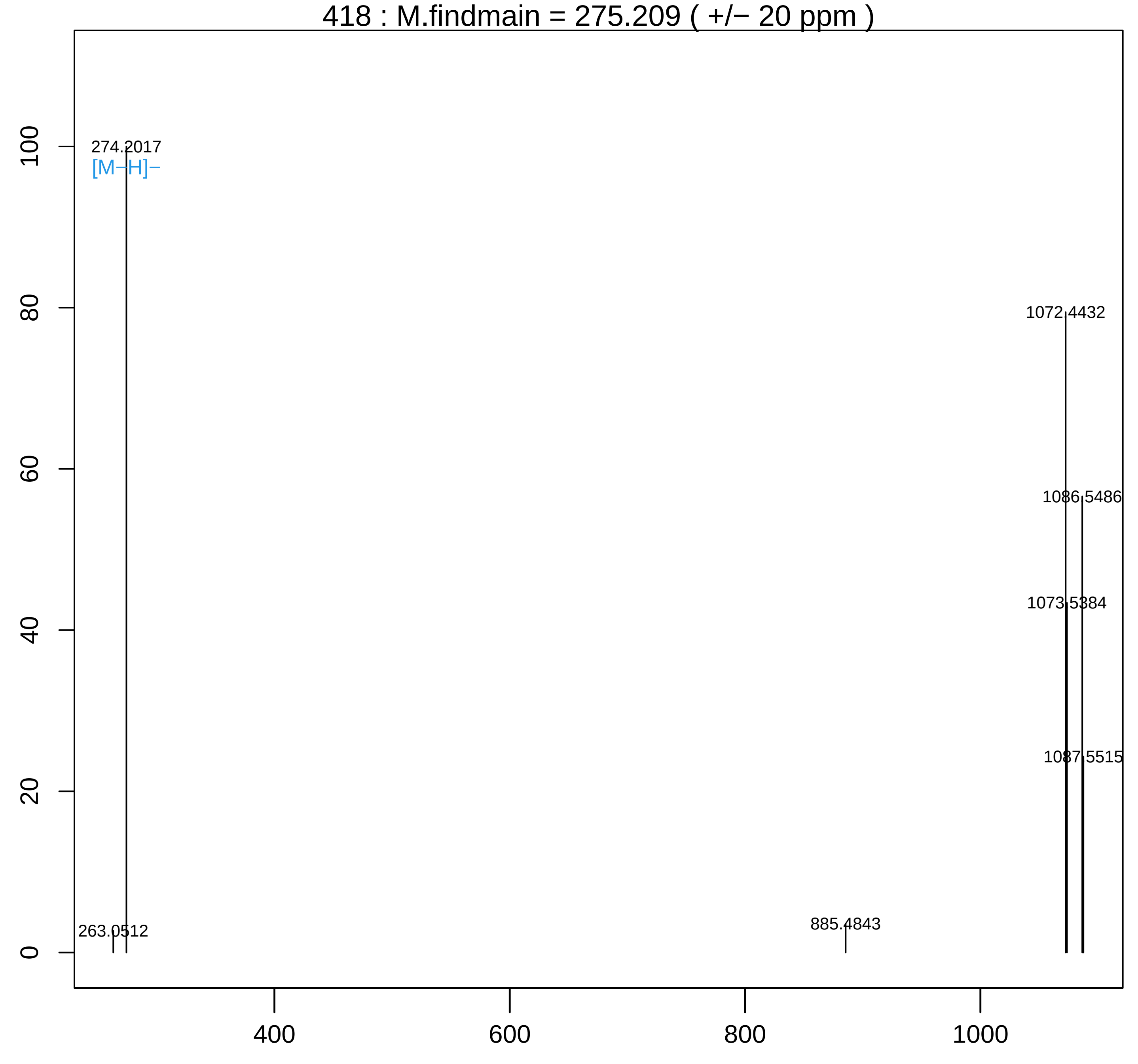

418 : M.findmain = 275.209 (+/− 20 ppm)
274.2017
[M−H]−
1072.4432
1086.5486
1073.5384
1087.5515
885.4843
263.0512
0
20
40
60
80
100
400
600
800
1000

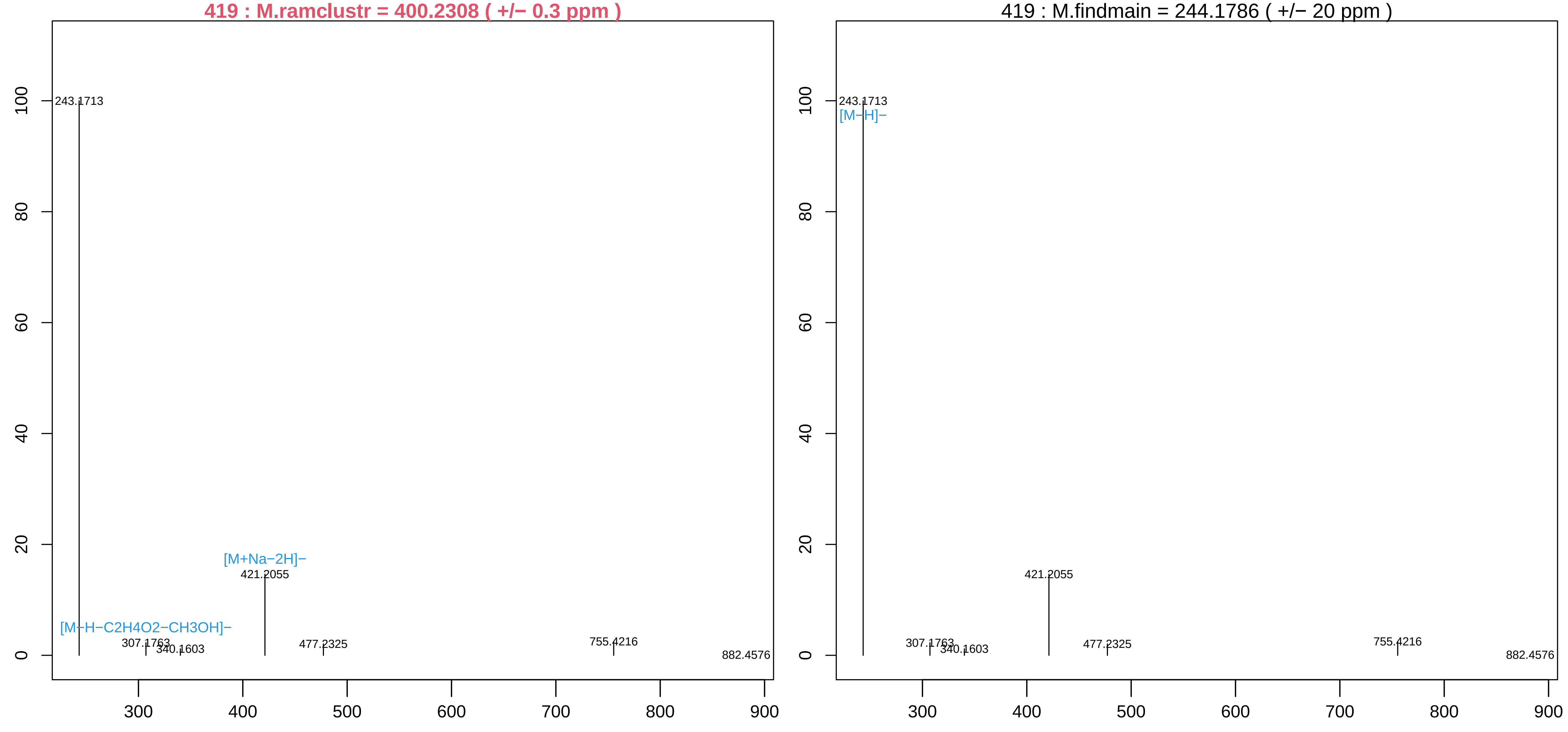
419 : M.ramclustr = 400.2308 (+/− 0.3 ppm)
243.1713
[M−H−C2H4O2−CH3OH]−
307.1763
340.1603
[M+Na−2H]−
421.2055
477.2325
755.4216
882.4576
0
20
40
60
80
100
300
400
500
600
700
800
900
419 : M.findmain = 244.1786 (+/− 20 ppm)
243.1713
[M−H]−
307.1763
340.1603
421.2055
477.2325
755.4216
882.4576
0
20
40
60
80
100
300
400
500
600
700
800
900

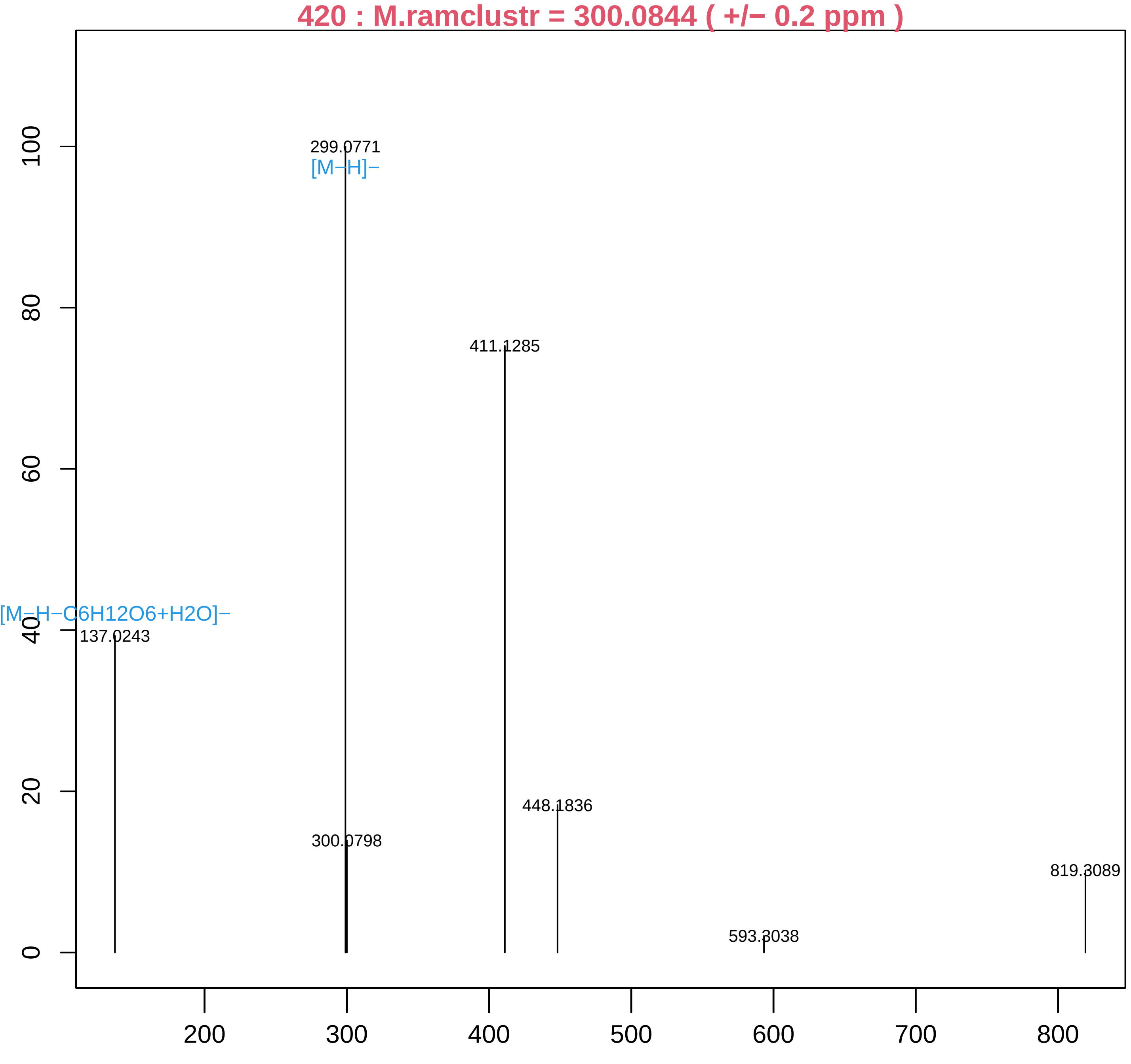

420 : M.ramclustr = 300.0844 (+/− 0.2 ppm)
299.0771
[M−H]−
411.1285
[M−H−C6H12O6+H2O]−
137.0243
448.1836
300.0798
819.3089
593.3038
0
20
40
60
80
100
200
300
400
500
600
700
800

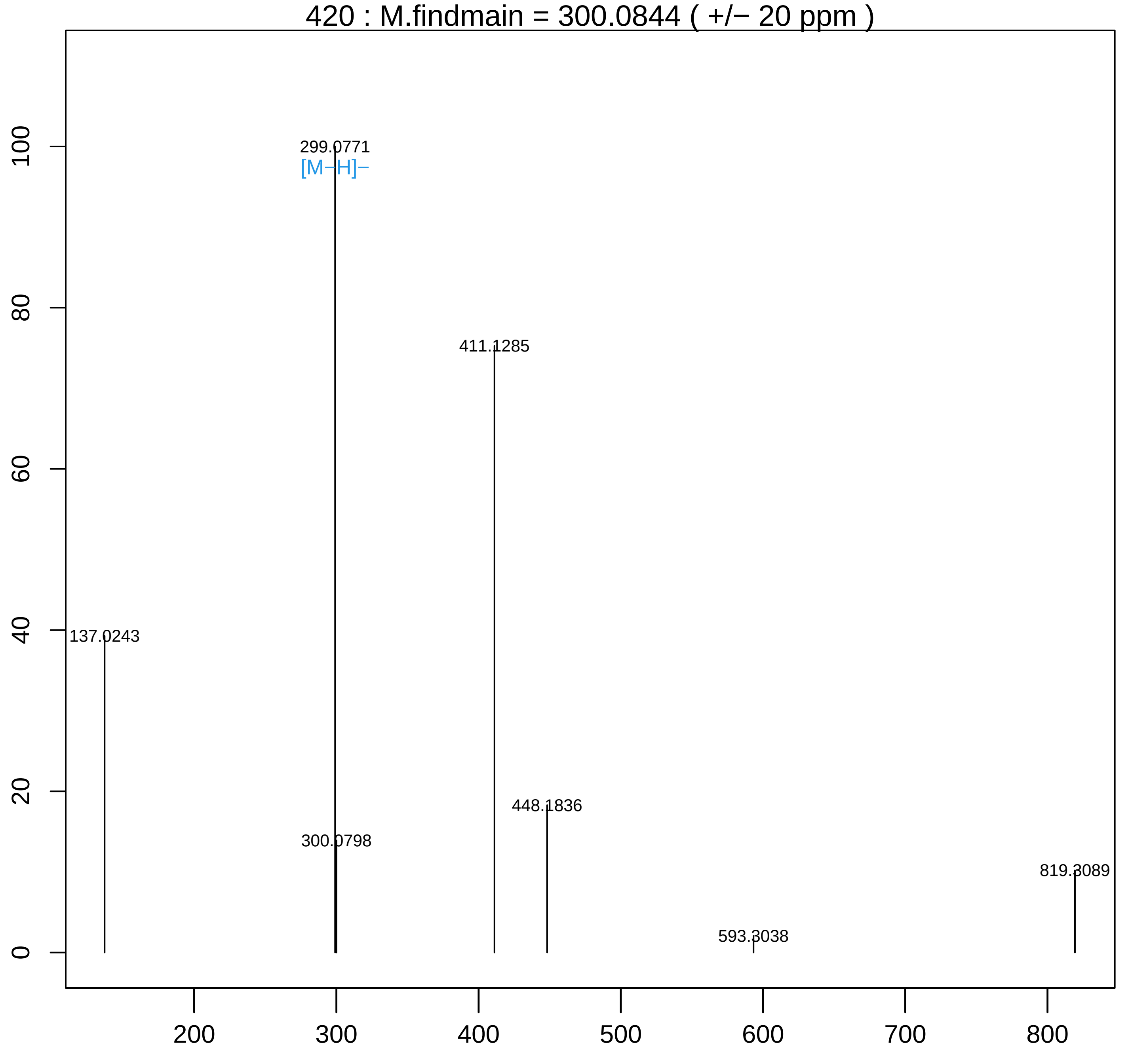

420 : M.findmain = 300.0844 (+/− 20 ppm)
299.0771
[M−H]−
411.1285
137.0243
448.1836
300.0798
819.3089
593.3038
0
20
40
60
80
100
200
300
400
500
600
700
800

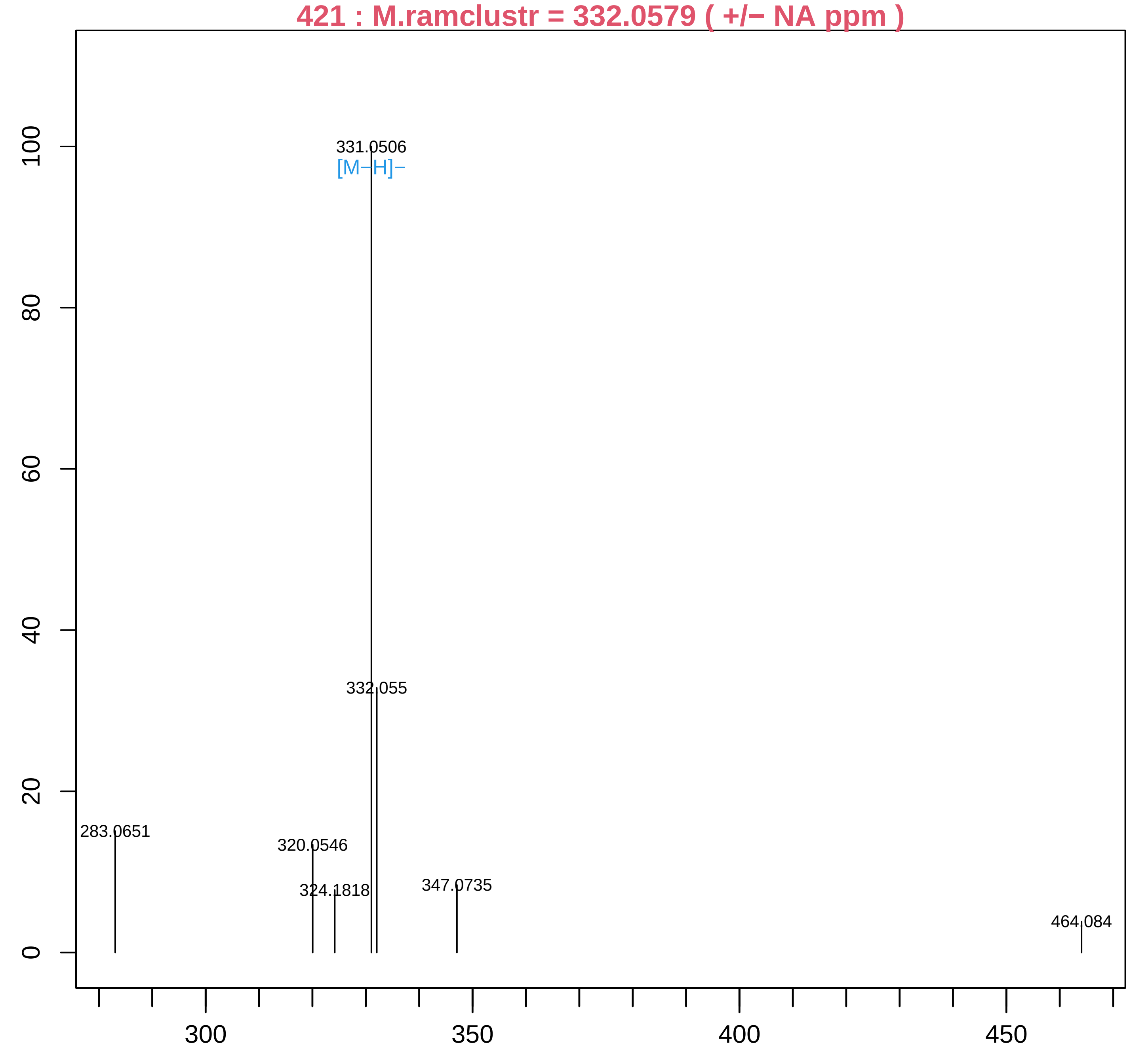
421 : M.ramclustr = 332.0579 (+/− NA ppm)
331.0506
[M−H]−
332.055
283.0651
320.0546
324.1818
347.0735
464.084
0
20
40
60
80
100
300
350
400
450

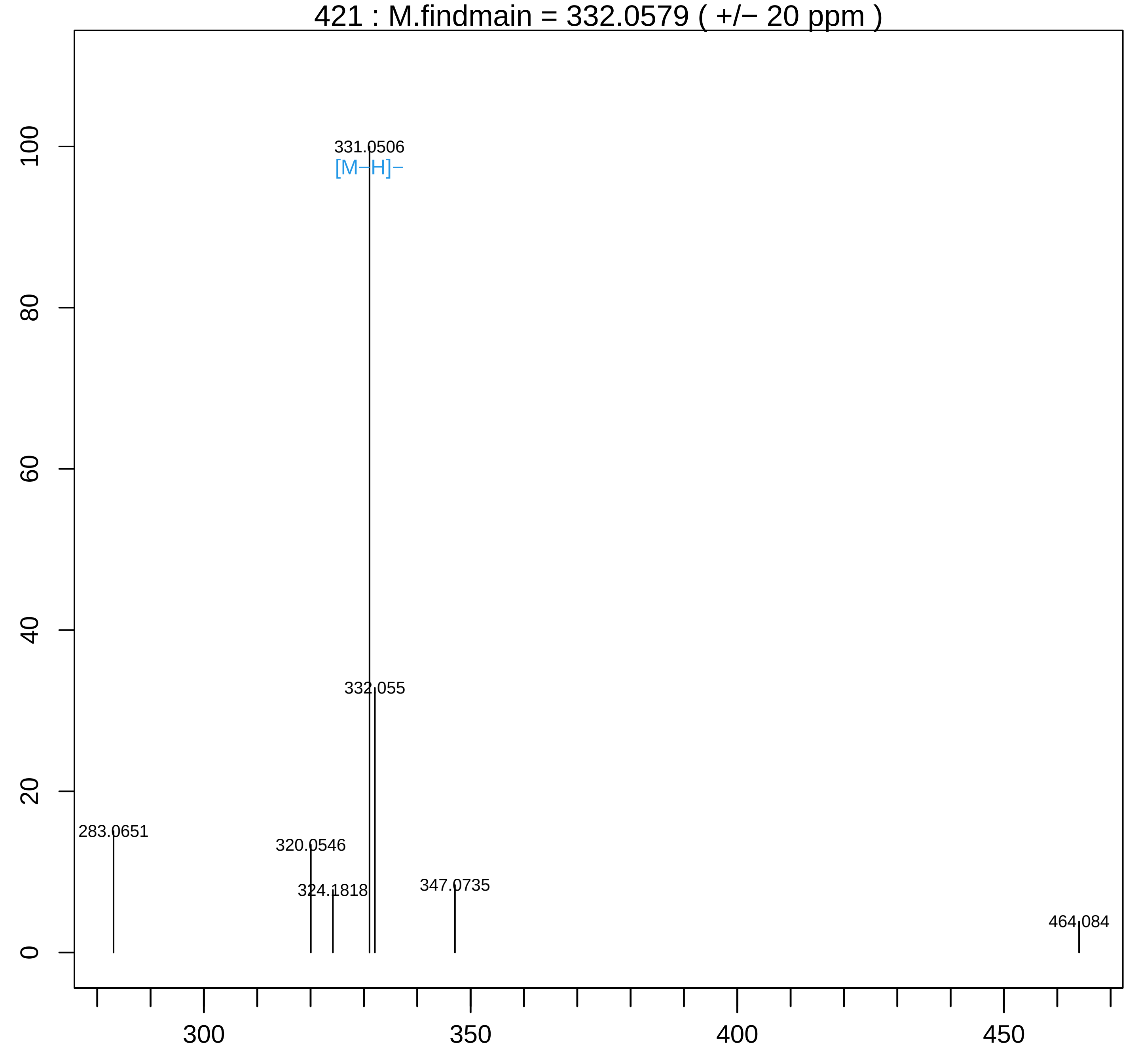
421 : M.findmain = 332.0579 (+/− 20 ppm)
331.0506
[M−H]−
332.055
283.0651
320.0546
324.1818
347.0735
464.084
0
20
40
60
80
100
300
350
400
450

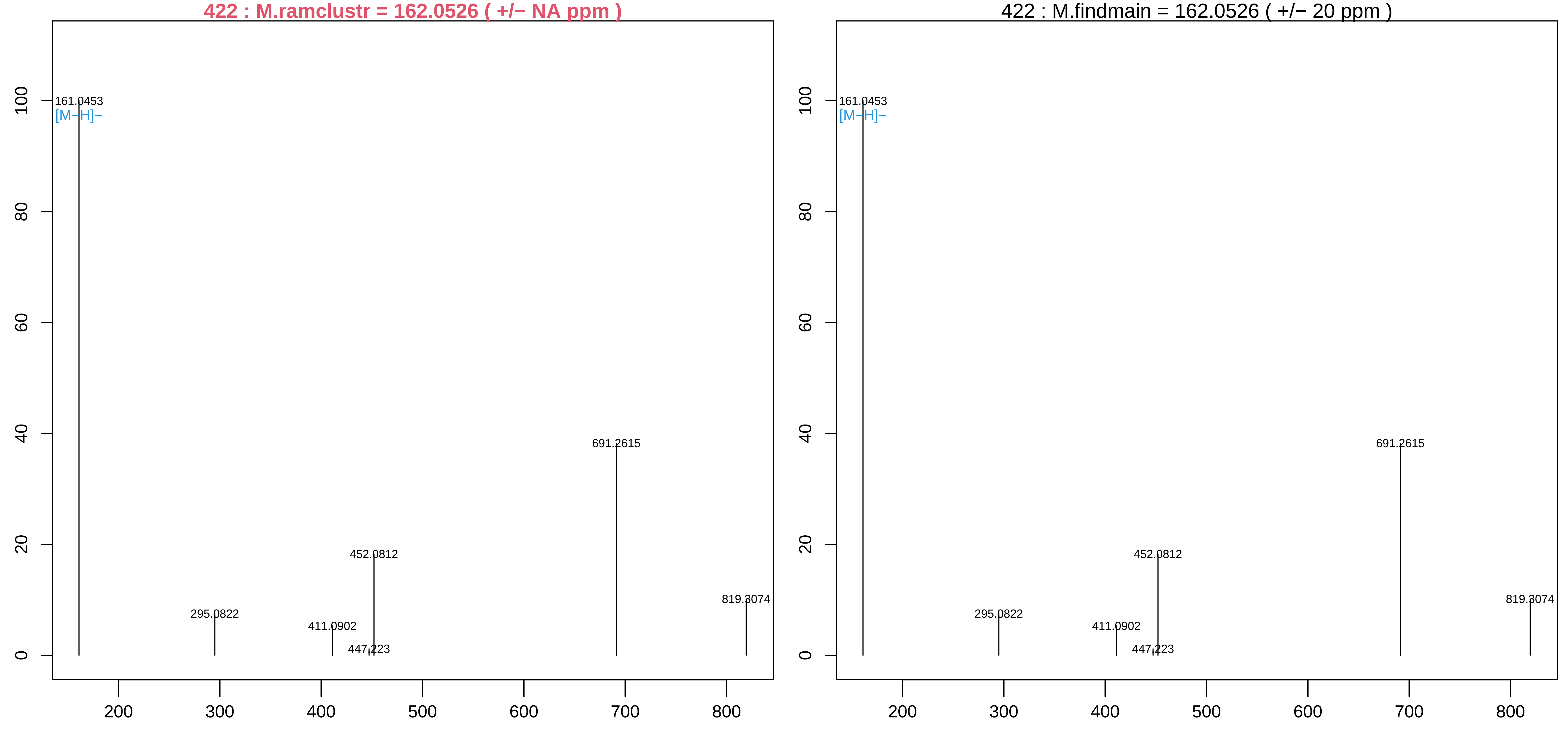
422 : M.ramclustr = 162.0526 (+/− NA ppm)
161.0453
[M−H]−
295.0822
411.0902
447.223
452.0812
691.2615
819.3074
0
20
40
60
80
100
200
300
400
500
600
700
800
422 : M.findmain = 162.0526 (+/− 20 ppm)
161.0453
[M−H]−
295.0822
411.0902
447.223
452.0812
691.2615
819.3074

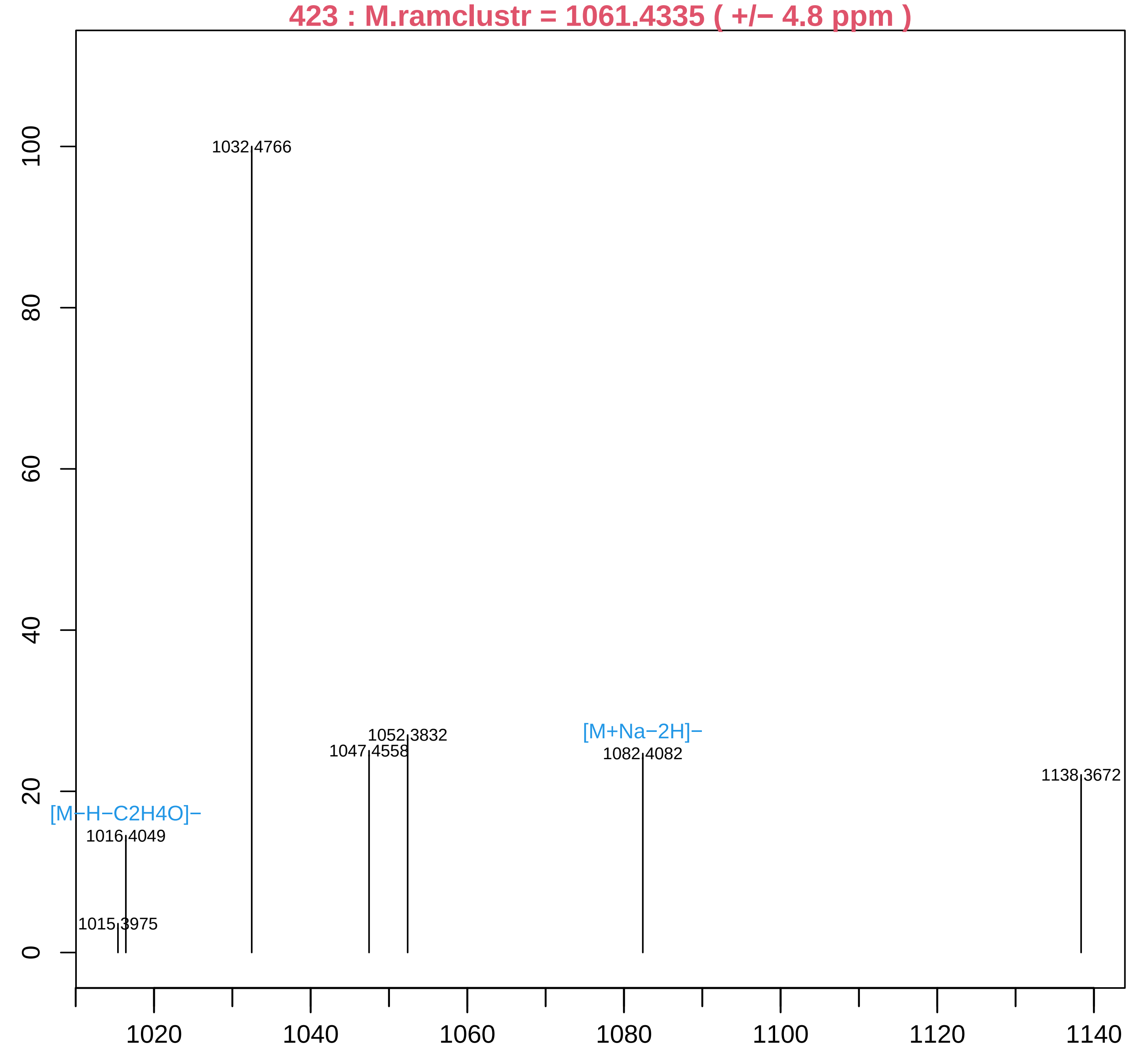

423 : M.ramclustr = 1061.4335 (+/− 4.8 ppm)
1032.4766
1047.4558
1052.3832
[M+Na−2H]−
1082.4082
1138.3672
[M−H−C2H4O]−
1016.4049
1015.3975
0
20
40
60
80
100
1020
1040
1060
1080
1100
1120
1140

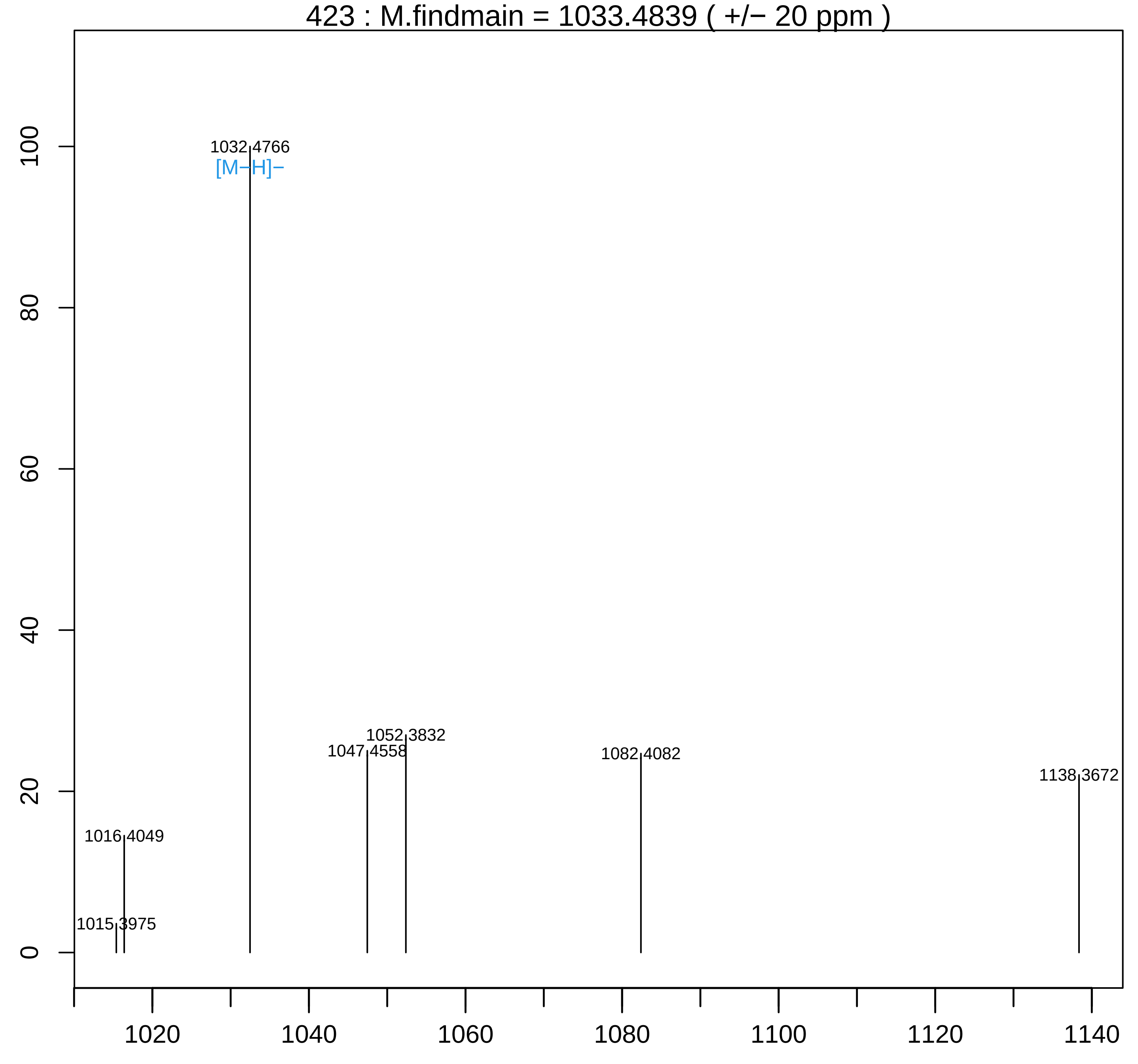

423 : M.findmain = 1033.4839 (+/− 20 ppm)
1032.4766
[M−H]−
1047.4558
1052.3832
1082.4082
1138.3672
1016.4049
1015.3975
0
20
40
60
80
100
1020
1040
1060
1080
1100
1120
1140

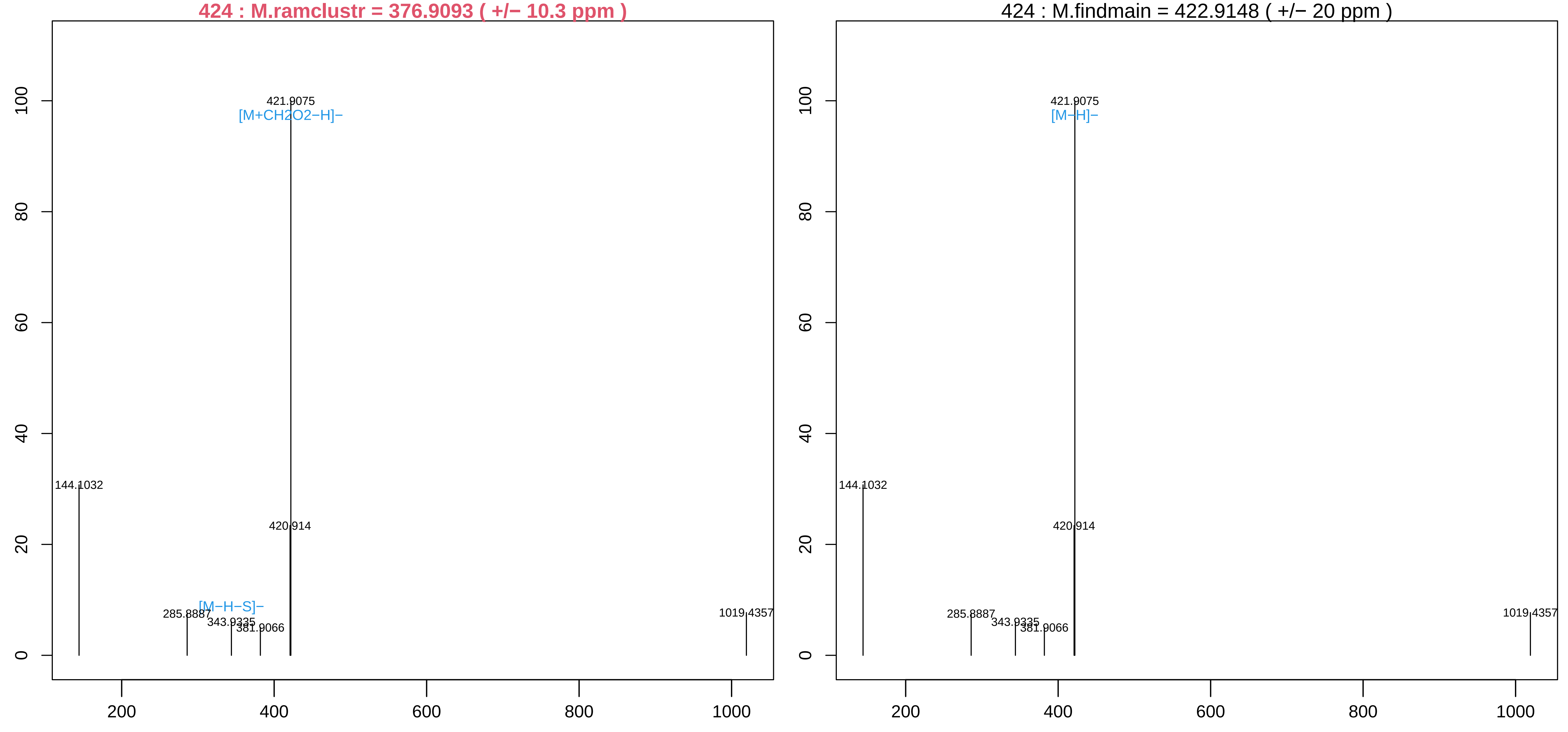

424 : M.ramclustr = 376.9093 (+/− 10.3 ppm)
421.9075
[M+CH2O2−H]−
144.1032
420.914
[M−H−S]−
285.8887
343.9335
381.9066
1019.4357
424 : M.findmain = 422.9148 (+/− 20 ppm)
421.9075
[M−H]−
144.1032
420.914
285.8887
343.9335
381.9066
1019.4357

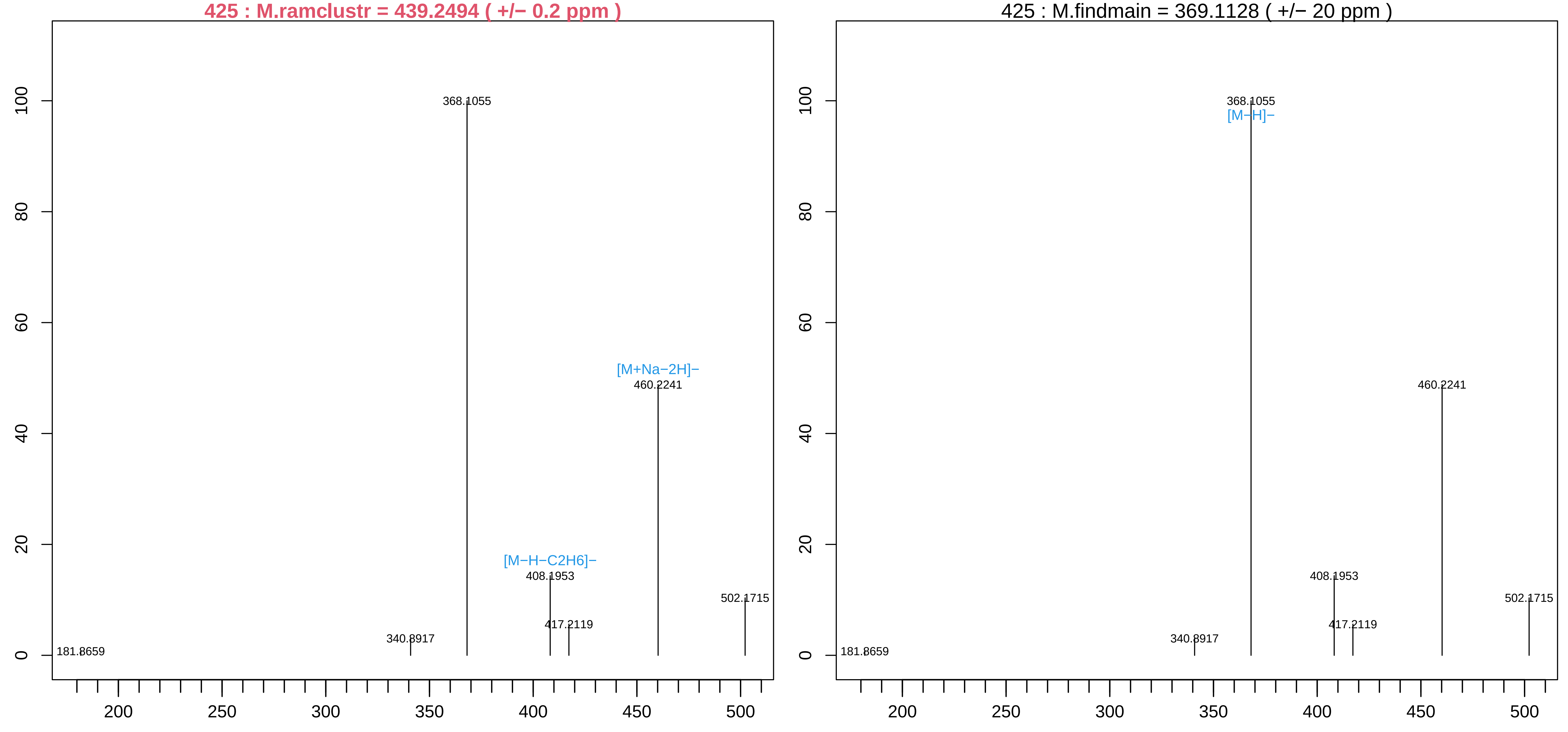
425 : M.ramclustr = 439.2494 (+/− 0.2 ppm)
368.1055
[M+Na−2H]−
460.2241
[M−H−C2H6]−
408.1953
502.1715
417.2119
340.8917
181.8659
425 : M.findmain = 369.1128 (+/− 20 ppm)
368.1055
[M−H]−
460.2241
408.1953
502.1715
417.2119
340.8917
181.8659

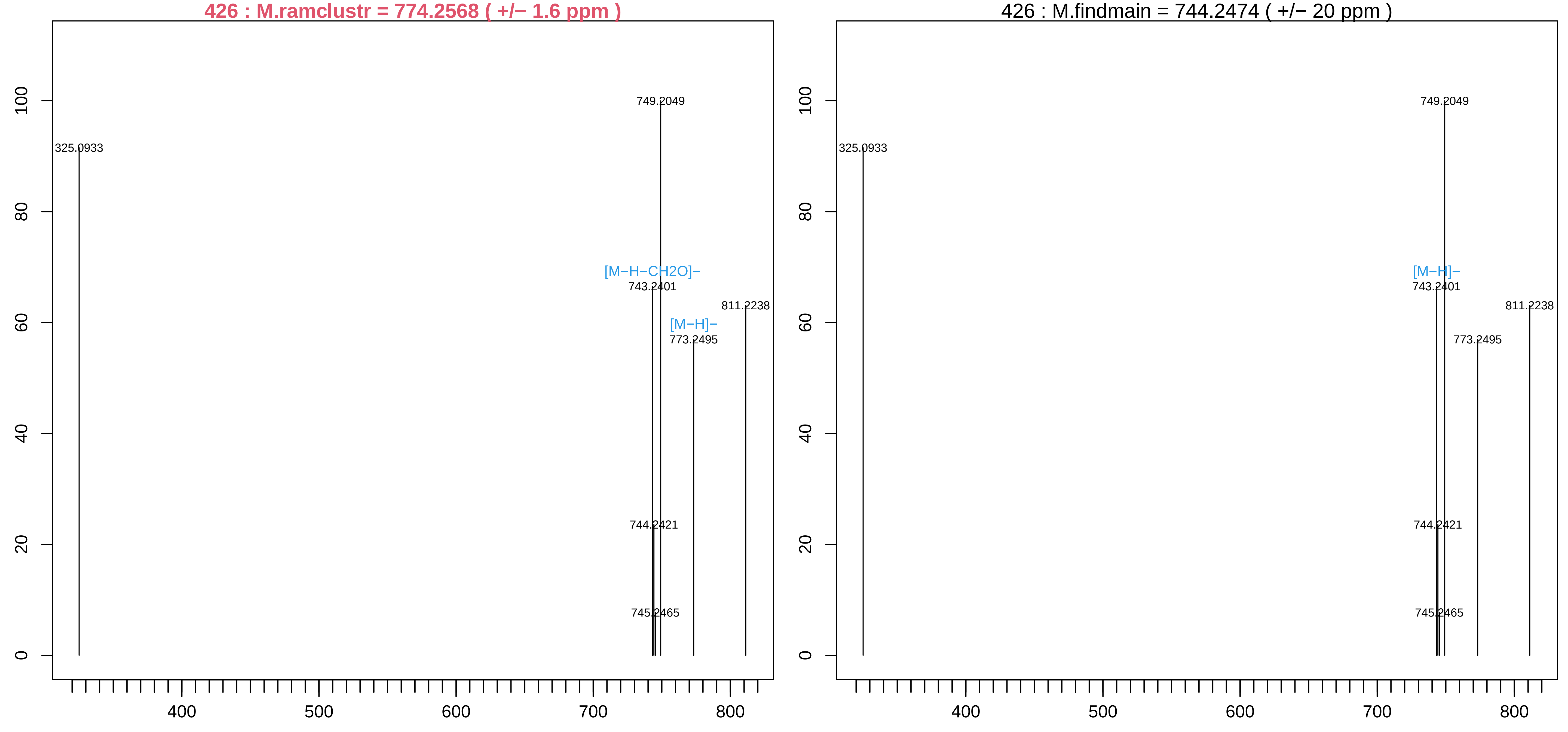
426 : M.ramclustr = 774.2568 (+/− 1.6 ppm)
100
80
60
40
20
0
400
500
600
700
800
325.0933
749.2049
[M−H−CH2O]−
743.2401
811.2238
[M−H]−
773.2495
744.2421
745.2465
426 : M.findmain = 744.2474 (+/− 20 ppm)
100
80
60
40
20
0
400
500
600
700
800
325.0933
749.2049
[M−H]−
743.2401
811.2238
773.2495
744.2421
745.2465

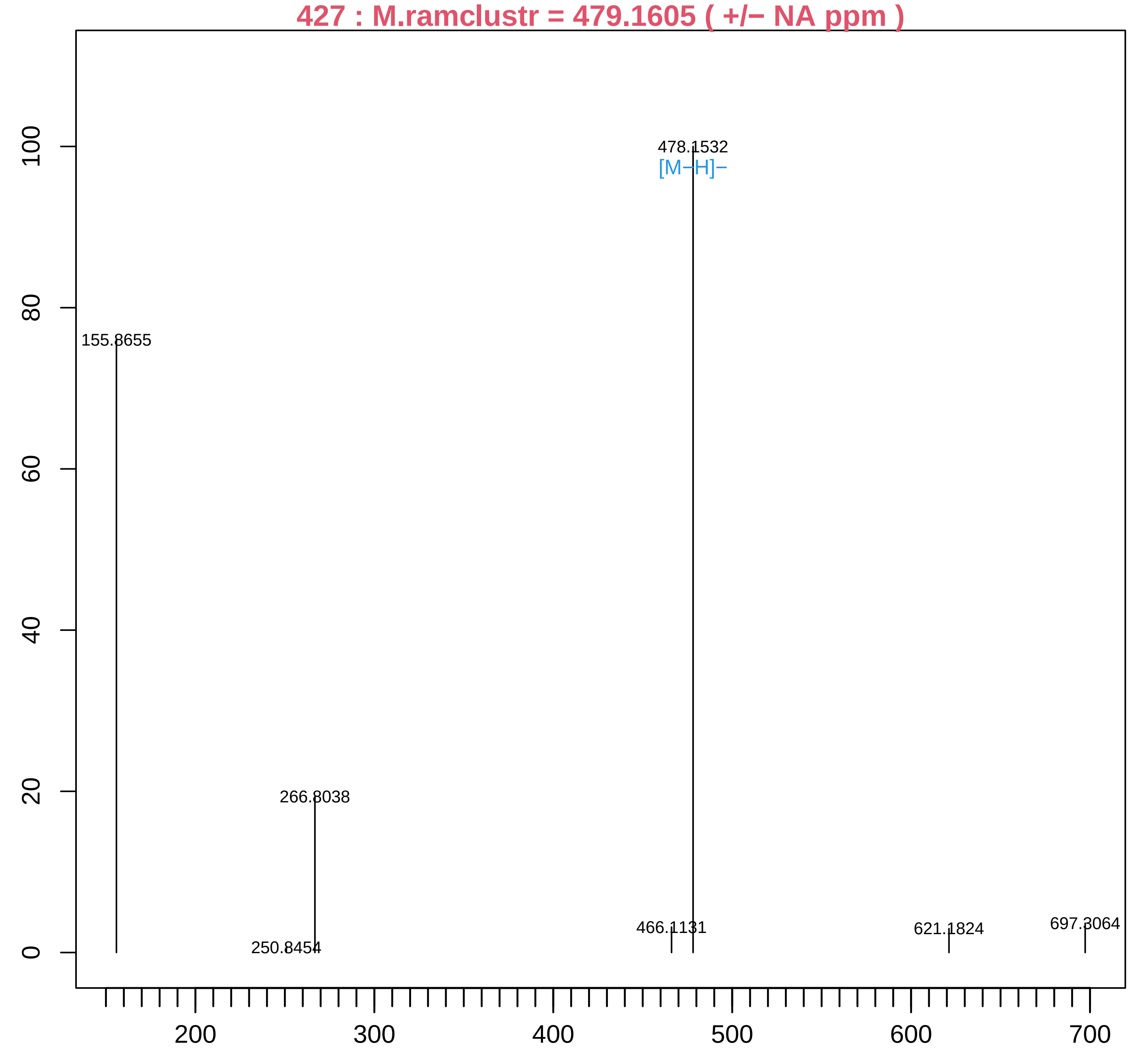

427 : M.ramclustr = 479.1605 (+/− NA ppm)
478.1532
[M−H]−
155.8655
266.8038
250.8454
466.1131
621.1824
697.3064
0
20
40
60
80
100
200
300
400
500
600
700

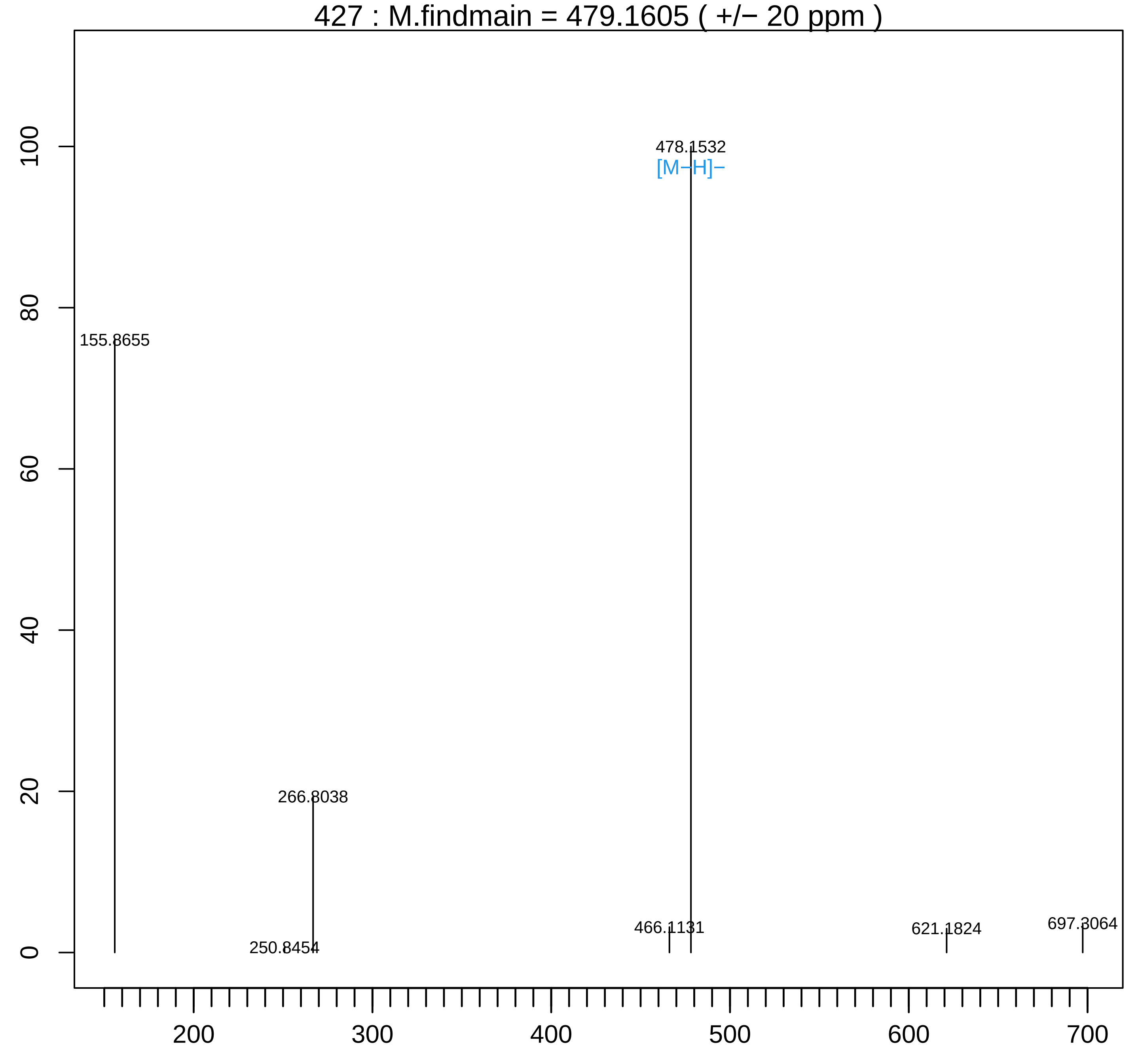

427 : M.findmain = 479.1605 (+/− 20 ppm)
478.1532
[M−H]−
155.8655
266.8038
250.8454
466.1131
621.1824
697.3064
0
20
40
60
80
100
200
300
400
500
600
700

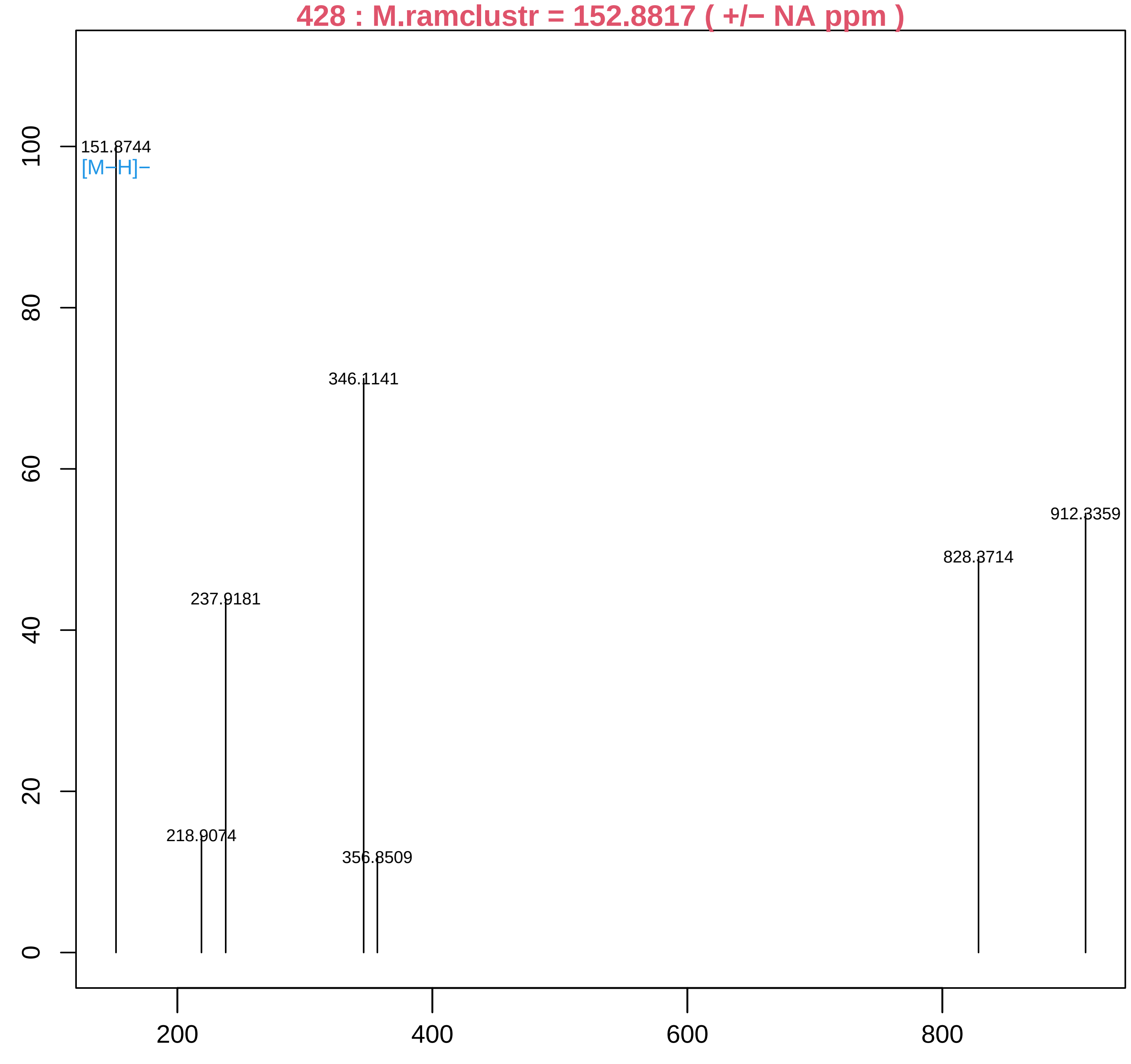
428 : M.ramclustr = 152.8817 (+/− NA ppm)
151.8744
[M−H]−
346.1141
912.3359
828.3714
237.9181
218.9074
356.8509
0
20
40
60
80
100
200
400
600
800

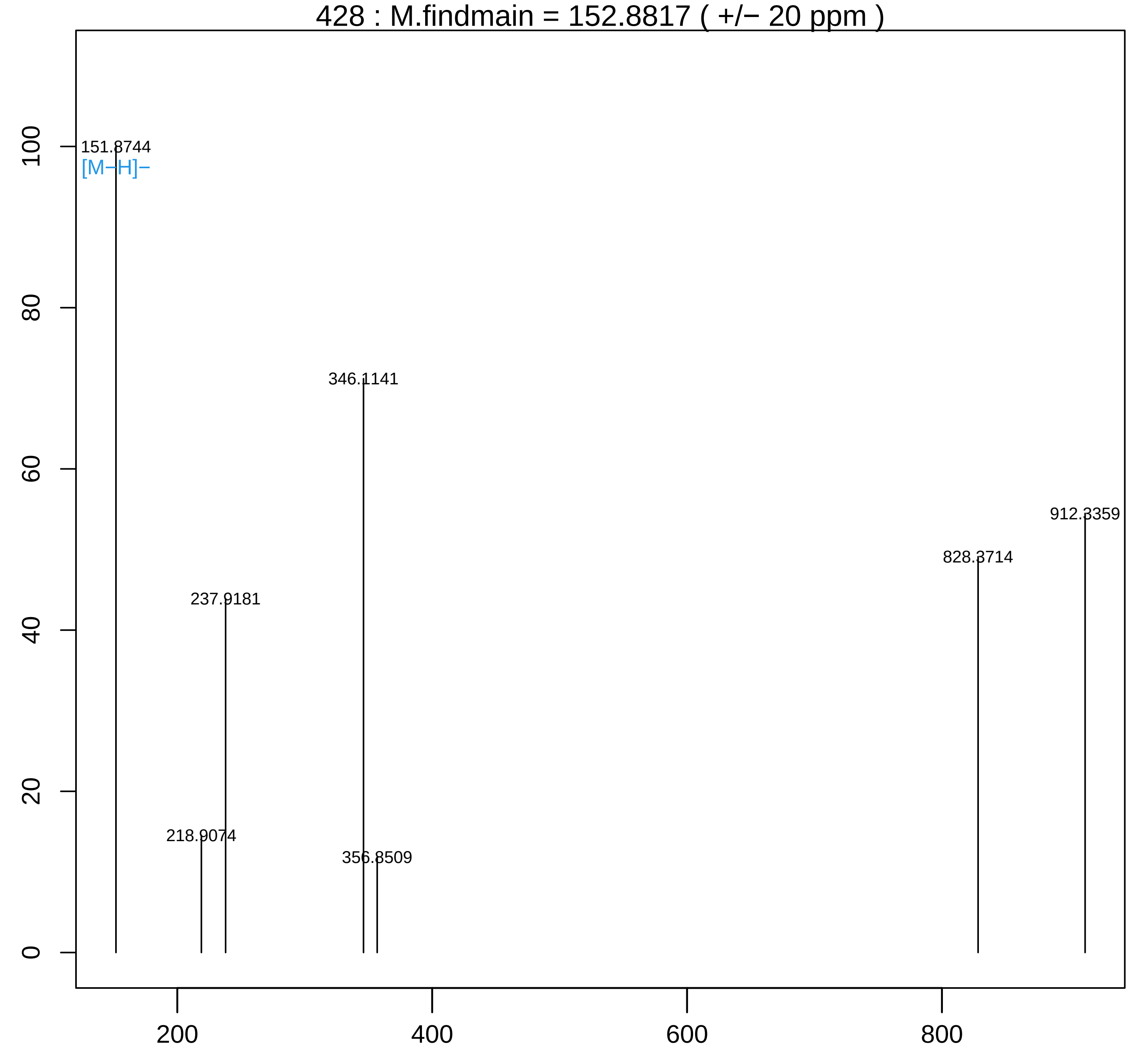
428 : M.findmain = 152.8817 (+/− 20 ppm)
151.8744
[M−H]−
346.1141
912.3359
828.3714
237.9181
218.9074
356.8509
0
20
40
60
80
100
200
400
600
800

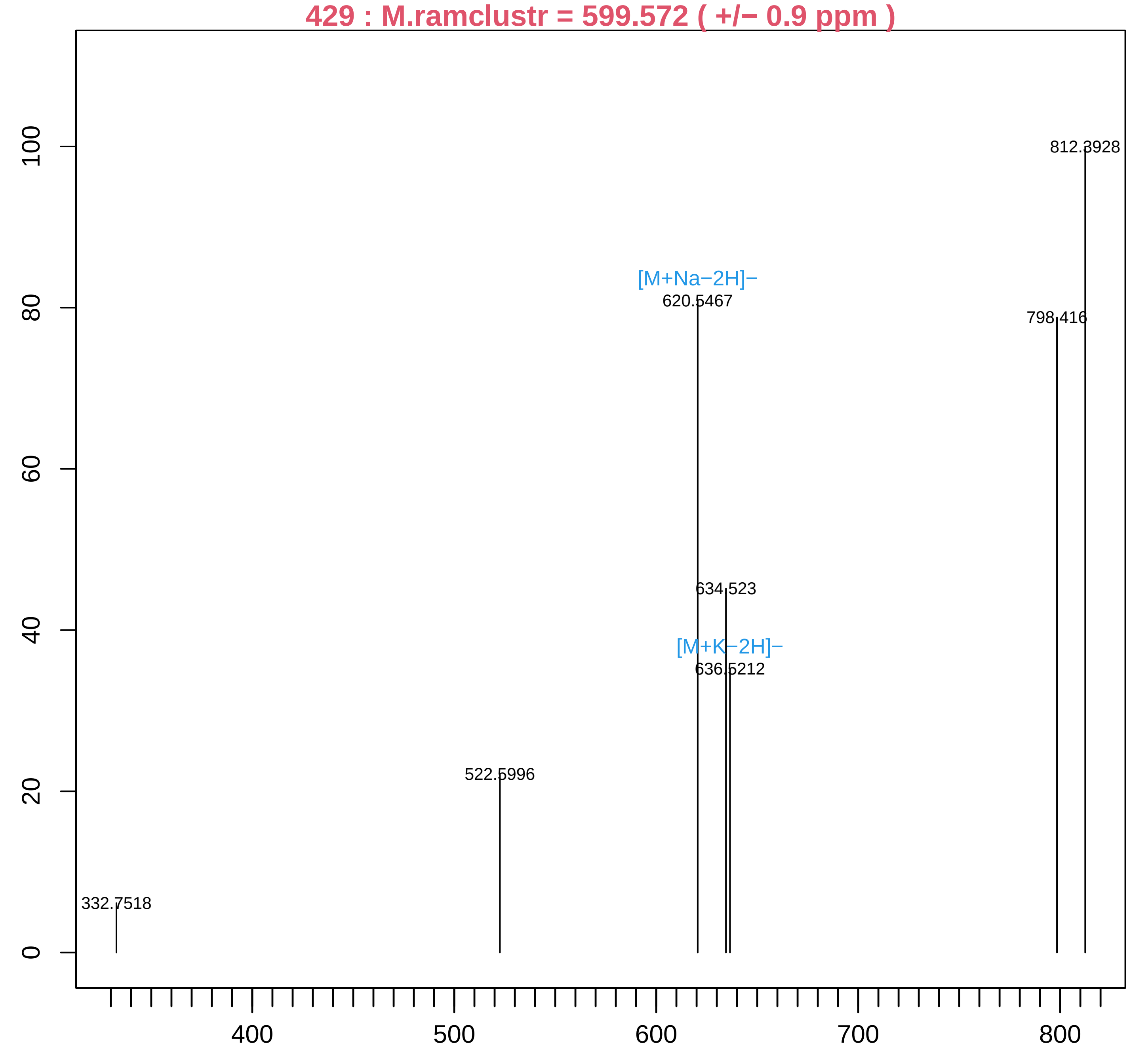
429 : M.ramclustr = 599.572 (+/− 0.9 ppm)
[M+Na−2H]−
620.5467
[M+K−2H]−
636.5212
634.523
812.3928
798.416
522.5996
332.7518
0
20
40
60
80
100
400
500
600
700
800

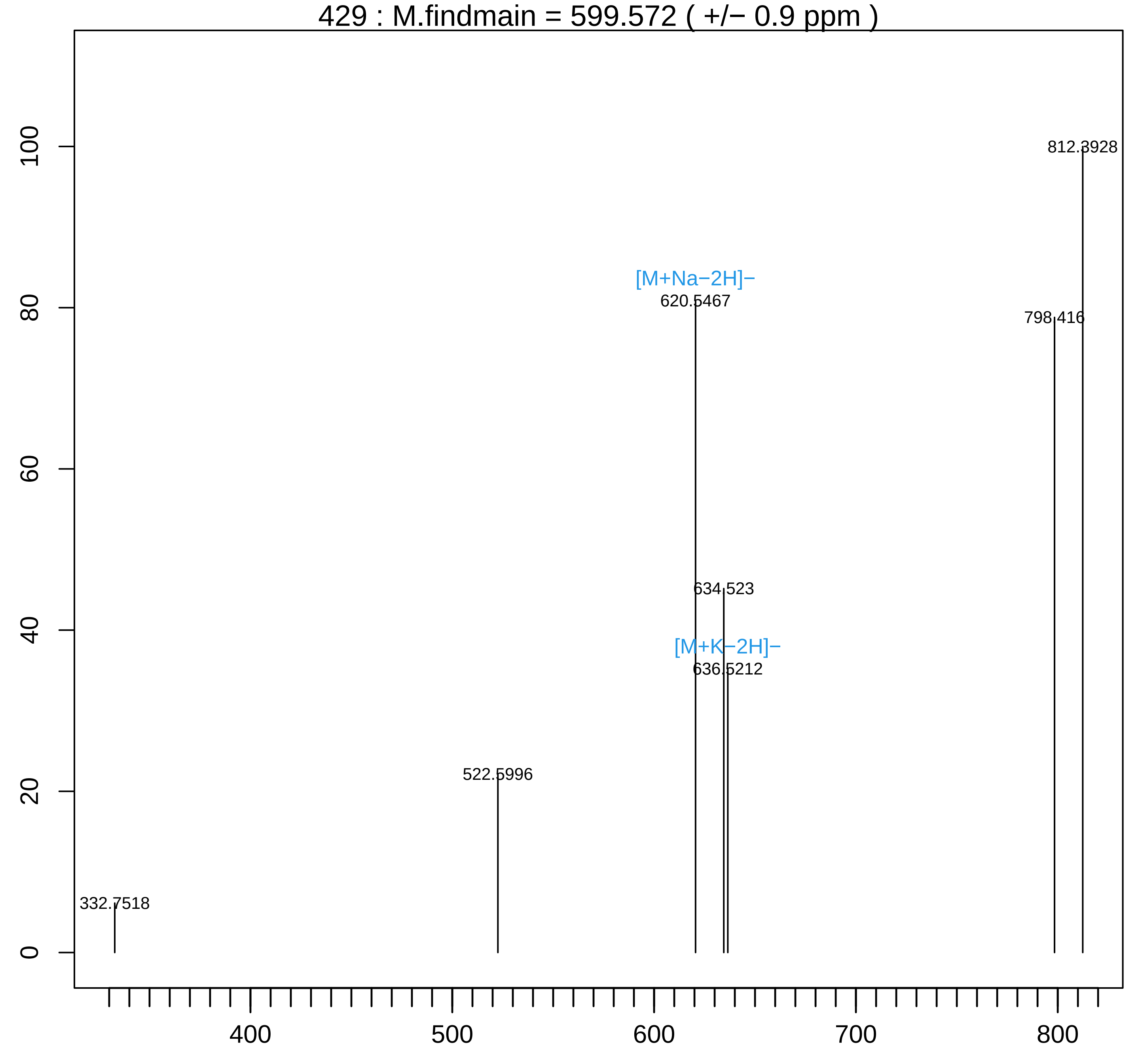
429 : M.findmain = 599.572 (+/− 0.9 ppm)
[M+Na−2H]−
620.5467
[M+K−2H]−
636.5212
634.523
812.3928
798.416
522.5996
332.7518
0
20
40
60
80
100
400
500
600
700
800

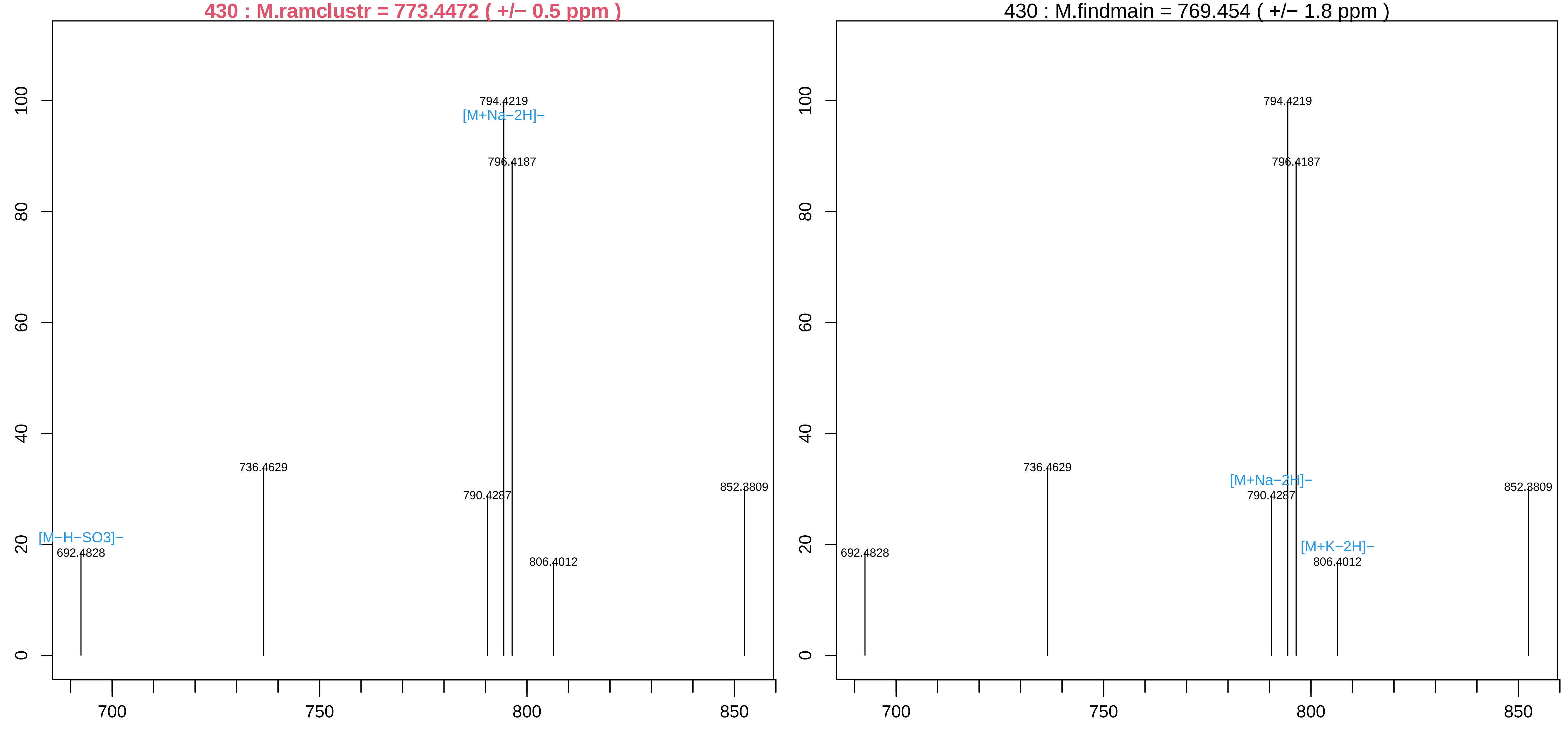
430 : M.ramclustr = 773.4472 (+/− 0.5 ppm)
794.4219
[M+Na−2H]−
796.4187
736.4629
790.4287
852.3809
[M−H−SO3]−
692.4828
806.4012
430 : M.findmain = 769.454 (+/− 1.8 ppm)
794.4219
796.4187
736.4629
[M+Na−2H]−
790.4287
852.3809
692.4828
[M+K−2H]−
806.4012
0
20
40
60
80
100
700
750
800
850

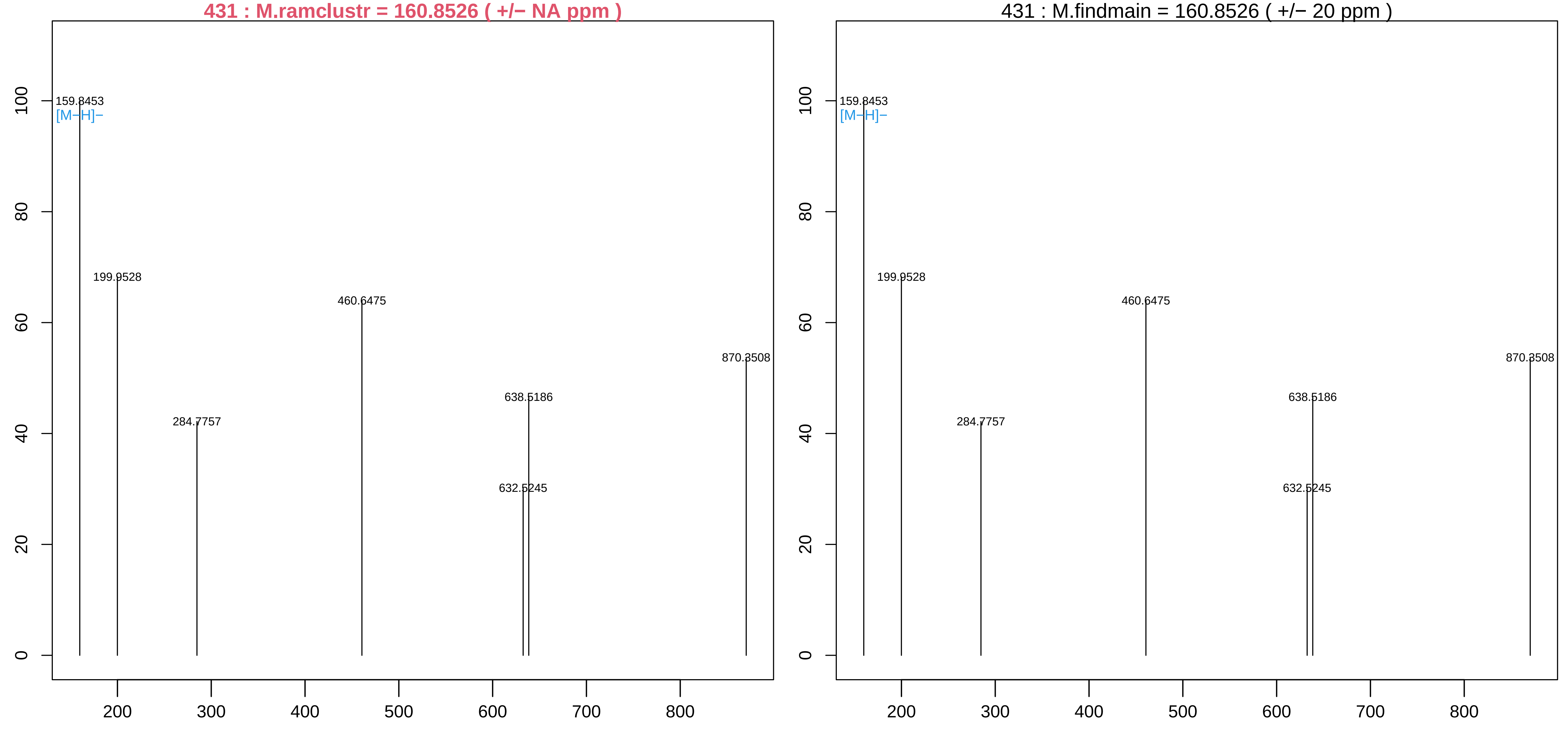
431 : M.ramclustr = 160.8526 (+/− NA ppm)
431 : M.findmain = 160.8526 (+/− 20 ppm)
159.8453
[M−H]−
199.9528
284.7757
460.6475
632.5245
638.5186
870.3508
0
20
40
60
80
100
200
300
400
500
600
700
800

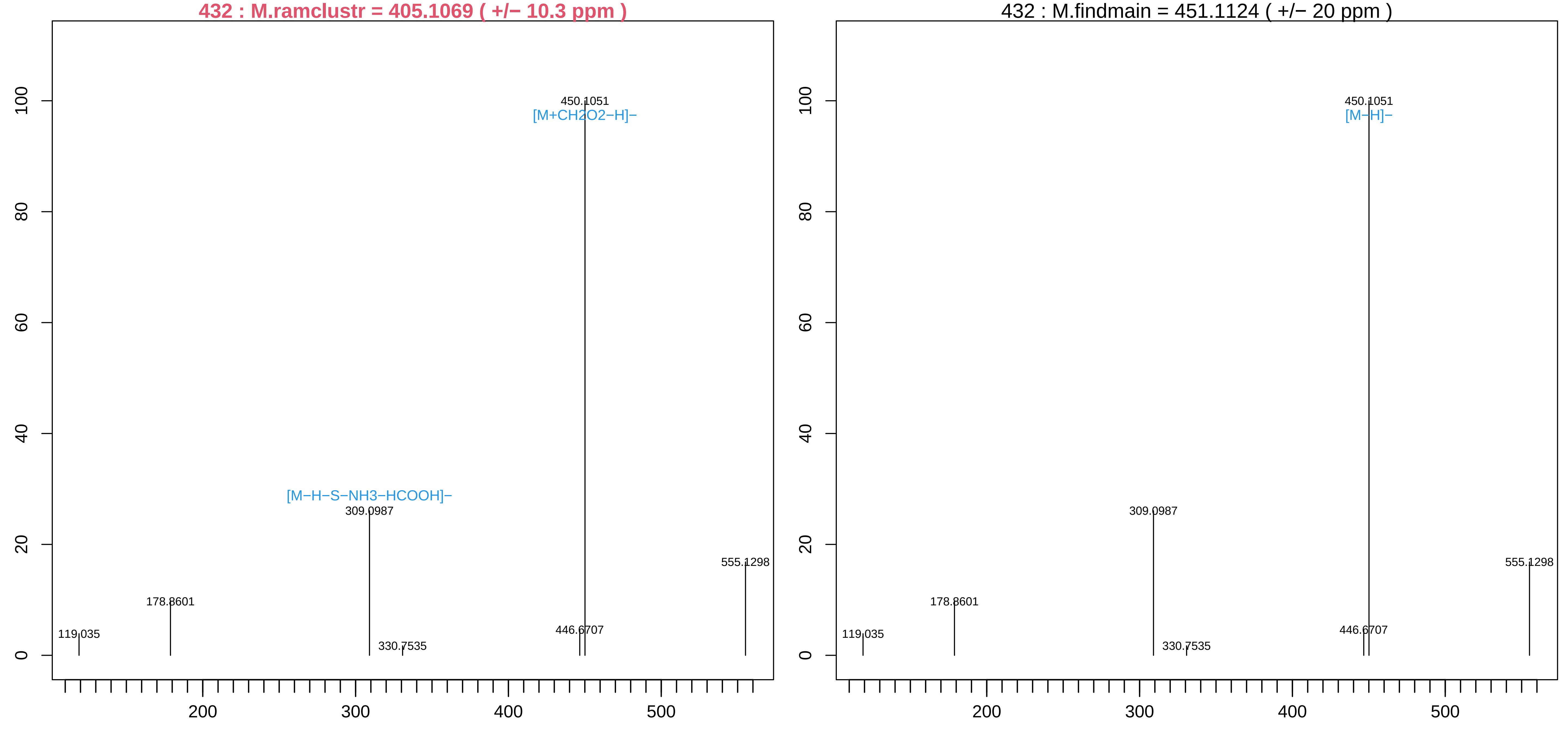

432 : M.ramclustr = 405.1069 (+/− 10.3 ppm)
450.1051
[M+CH2O2−H]−
[M−H−S−NH3−HCOOH]−
309.0987
555.1298
178.8601
119.035
446.6707
330.7535
432 : M.findmain = 451.1124 (+/− 20 ppm)
450.1051
[M−H]−
309.0987
555.1298
178.8601
119.035
446.6707
330.7535

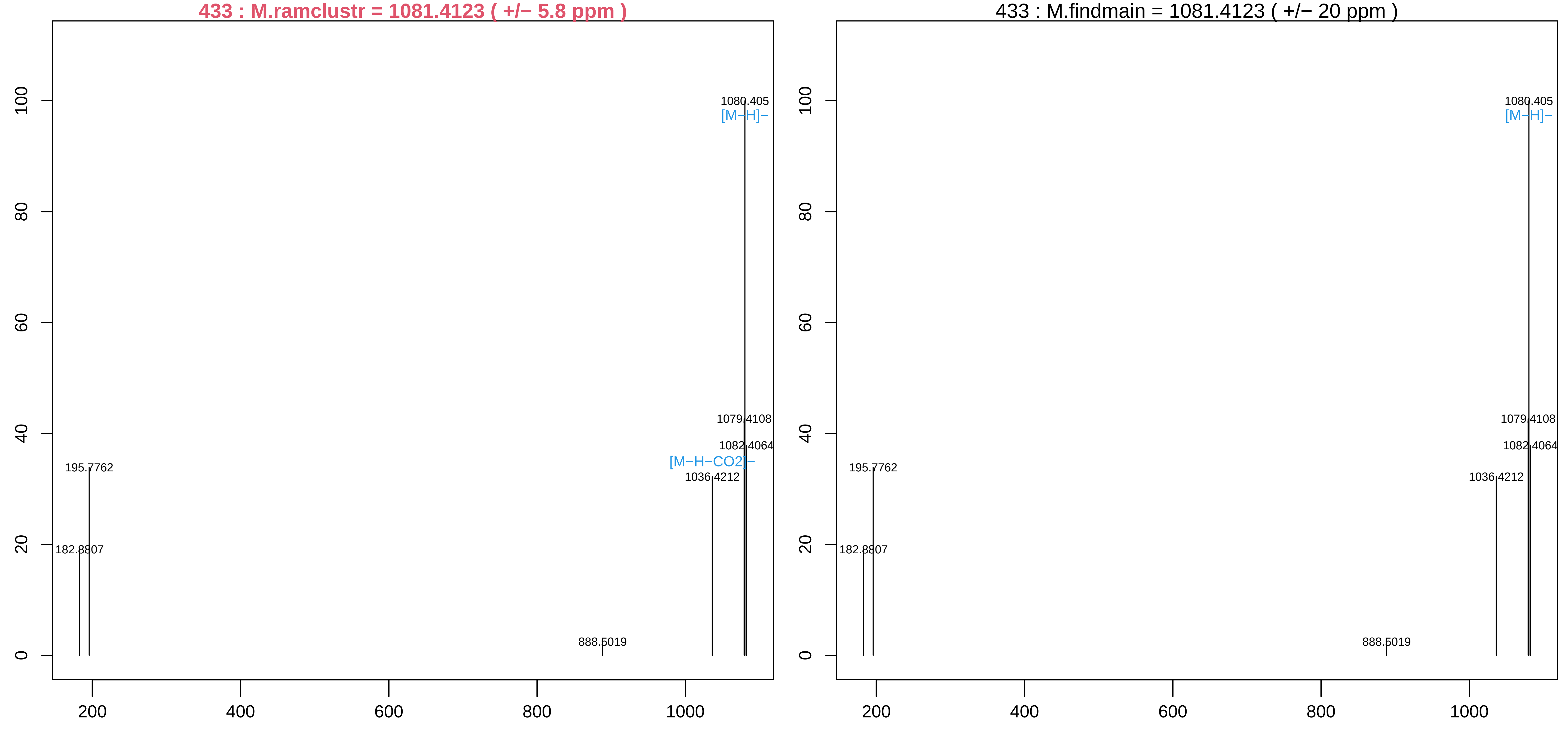

433 : M.ramclustr = 1081.4123 (+/− 5.8 ppm)
1080.405
[M−H]−
1079.4108
1082.4064
[M−H−CO2]−
1036.4212
195.7762
182.8807
888.5019
0
20
40
60
80
100
200
400
600
800
1000
433 : M.findmain = 1081.4123 (+/− 20 ppm)
1080.405
[M−H]−
1079.4108
1082.4064
1036.4212
195.7762
182.8807
888.5019
0
20
40
60
80
100
200
400
600
800
1000

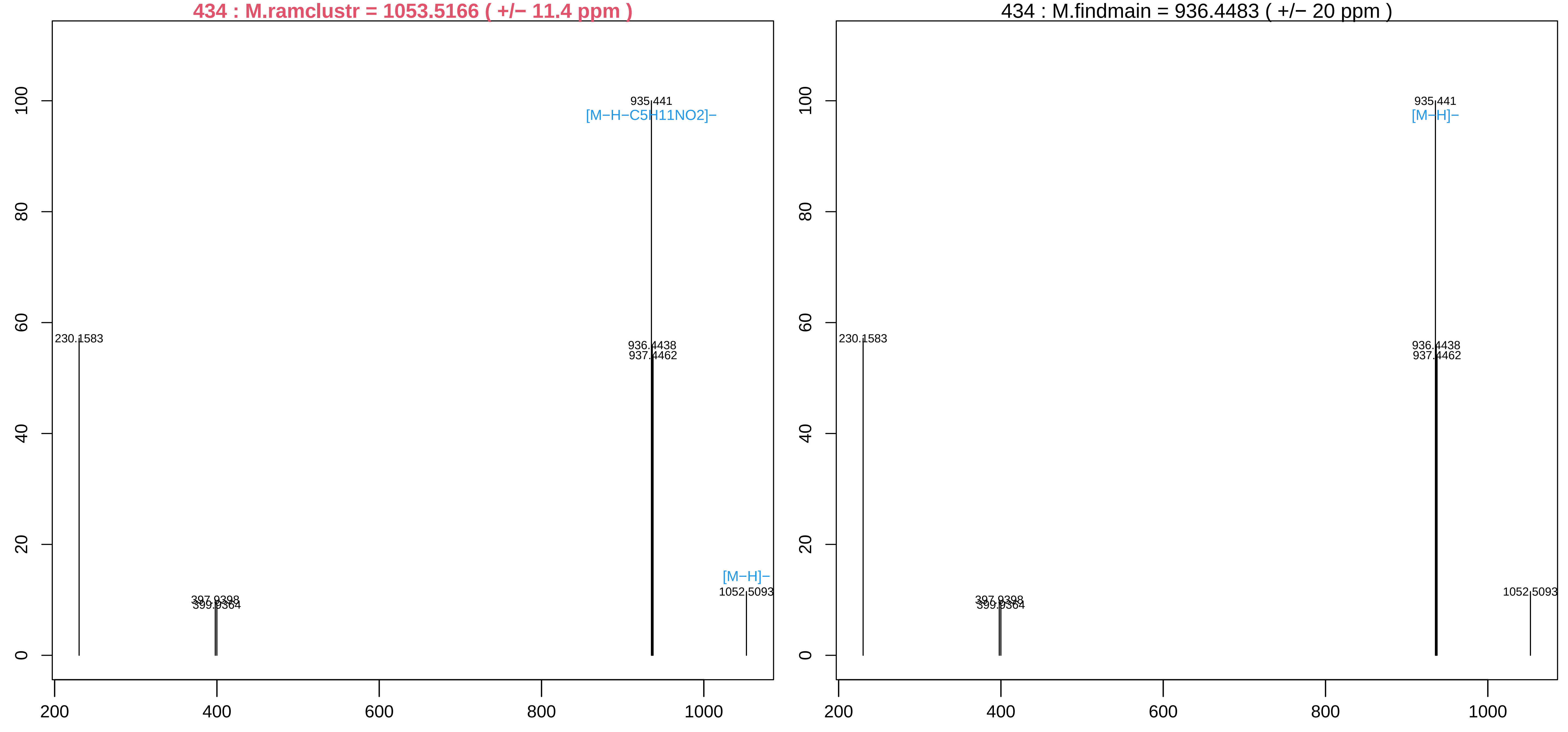
434 : M.ramclustr = 1053.5166 (+/− 11.4 ppm)
935.441
[M−H−C5H11NO2]−
230.1583
936.4438
937.4462
397.9398
399.9364
[M−H]−
1052.5093
434 : M.findmain = 936.4483 (+/− 20 ppm)
935.441
[M−H]−
230.1583
936.4438
937.4462
397.9398
399.9364
1052.5093
0
20
40
60
80
100
200
400
600
800
1000

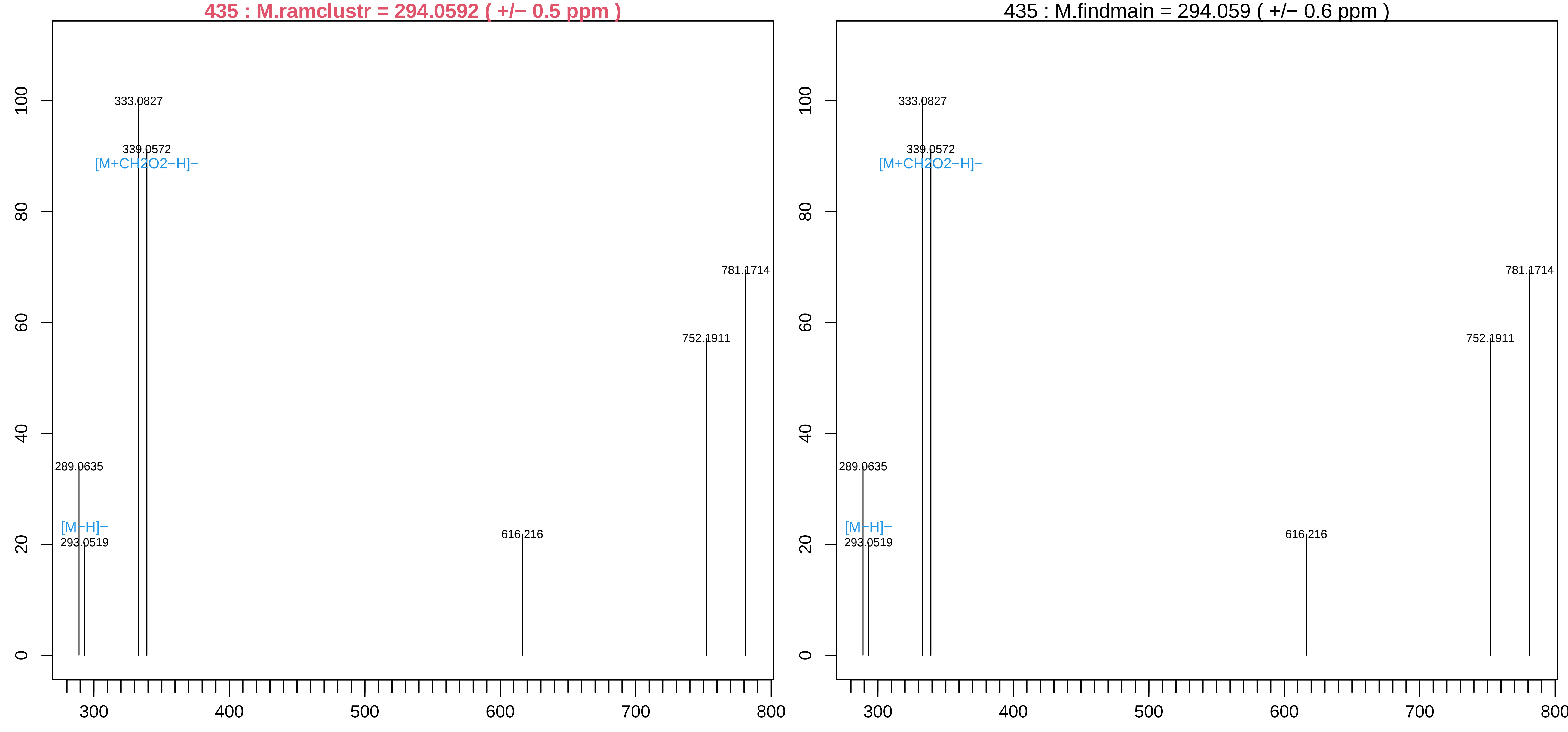

435 : M.ramclustr = 294.0592 (+/− 0.5 ppm)
333.0827
339.0572
[M+CH2O2−H]−
289.0635
[M−H]−
293.0519
616.216
752.1911
781.1714
0
20
40
60
80
100
300
400
500
600
700
800
435 : M.findmain = 294.059 (+/− 0.6 ppm)
333.0827
339.0572
[M+CH2O2−H]−
289.0635
[M−H]−
293.0519
616.216
752.1911
781.1714
0
20
40
60
80
100
300
400
500
600
700
800

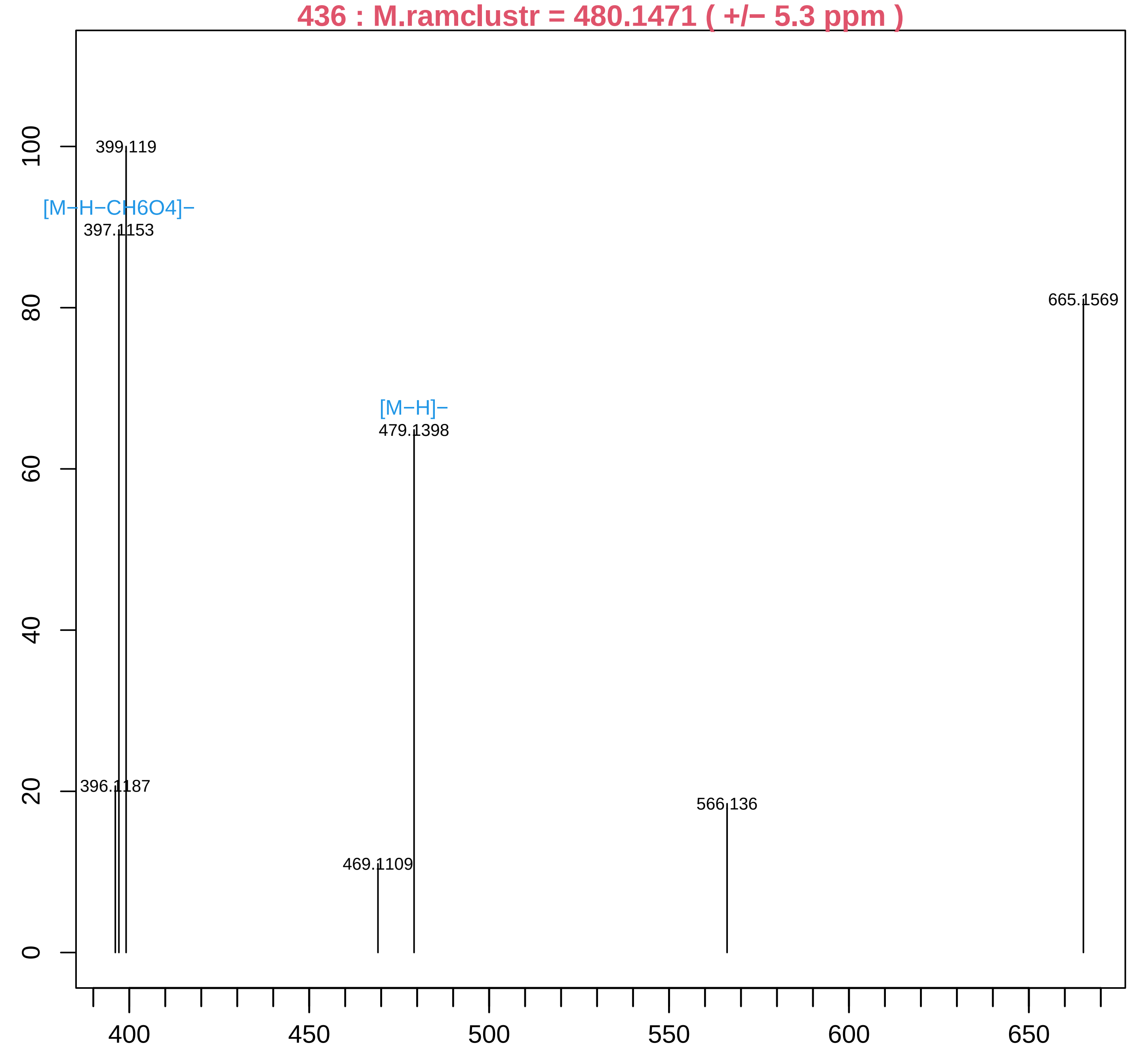

436 : M.ramclustr = 480.1471 (+/− 5.3 ppm)
399.119
[M−H−CH6O4]−
397.1153
665.1569
[M−H]−
479.1398
396.1187
566.136
469.1109
0
20
40
60
80
100
400
450
500
550
600
650

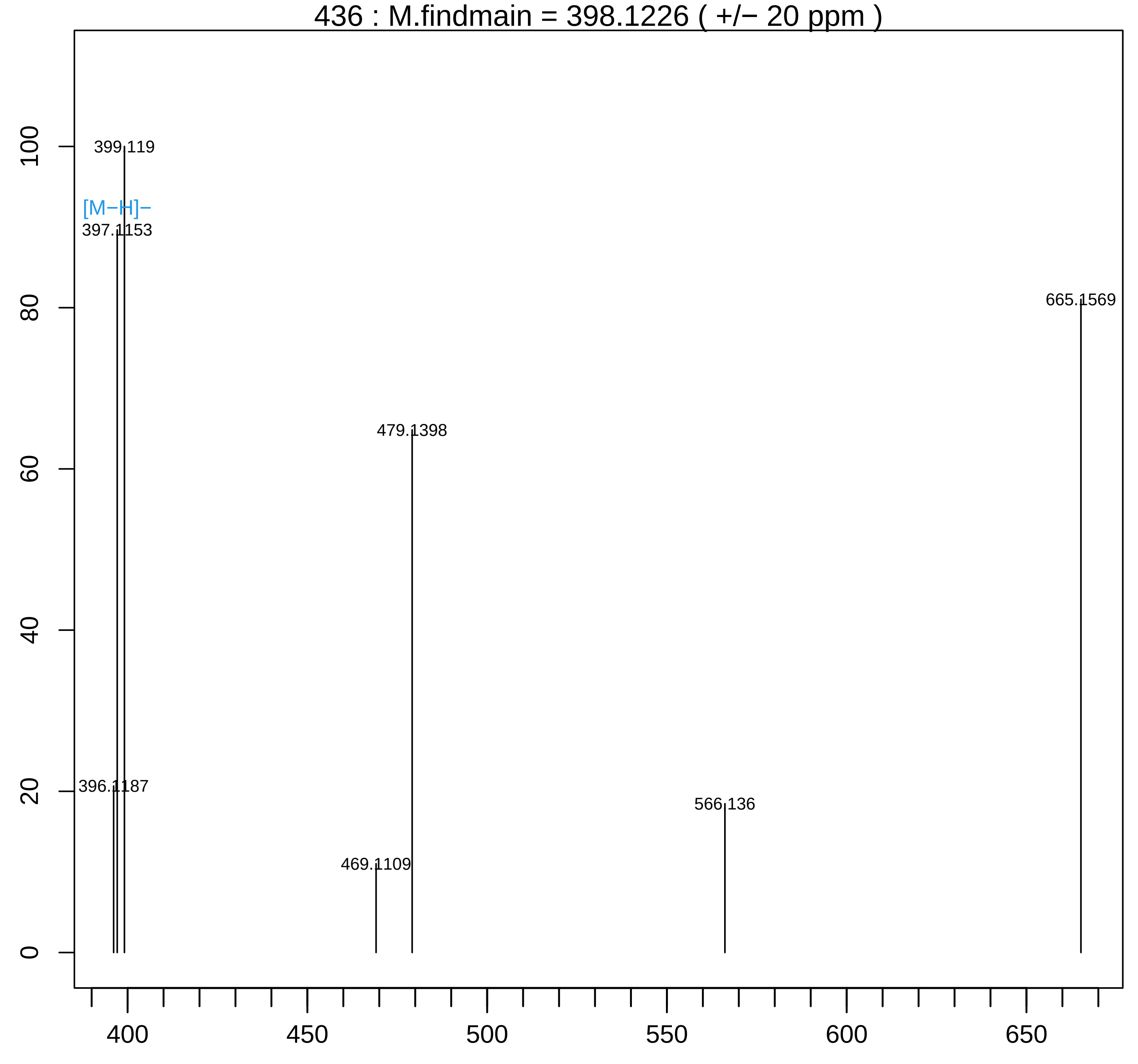

436 : M.findmain = 398.1226 (+/− 20 ppm)
399.119
[M−H]−
397.1153
665.1569
479.1398
396.1187
566.136
469.1109
0
20
40
60
80
100
400
450
500
550
600
650

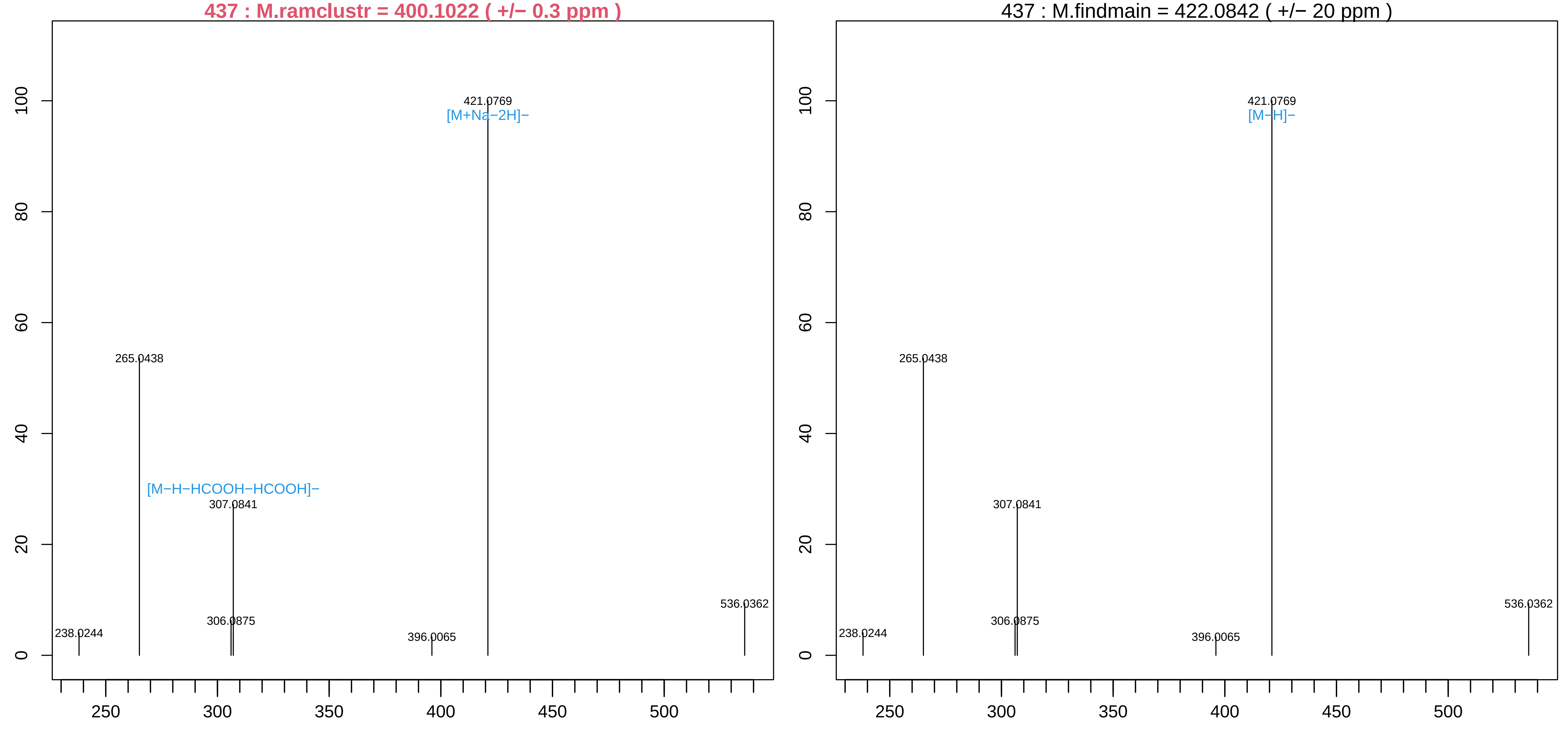

437 : M.ramclustr = 400.1022 (+/− 0.3 ppm)
421.0769
[M+Na−2H]−
265.0438
[M−H−HCOOH−HCOOH]−
307.0841
306.0875
238.0244
396.0065
536.0362
437 : M.findmain = 422.0842 (+/− 20 ppm)
421.0769
[M−H]−
265.0438
307.0841
306.0875
238.0244
396.0065
536.0362
0
20
40
60
80
100
250
300
350
400
450
500

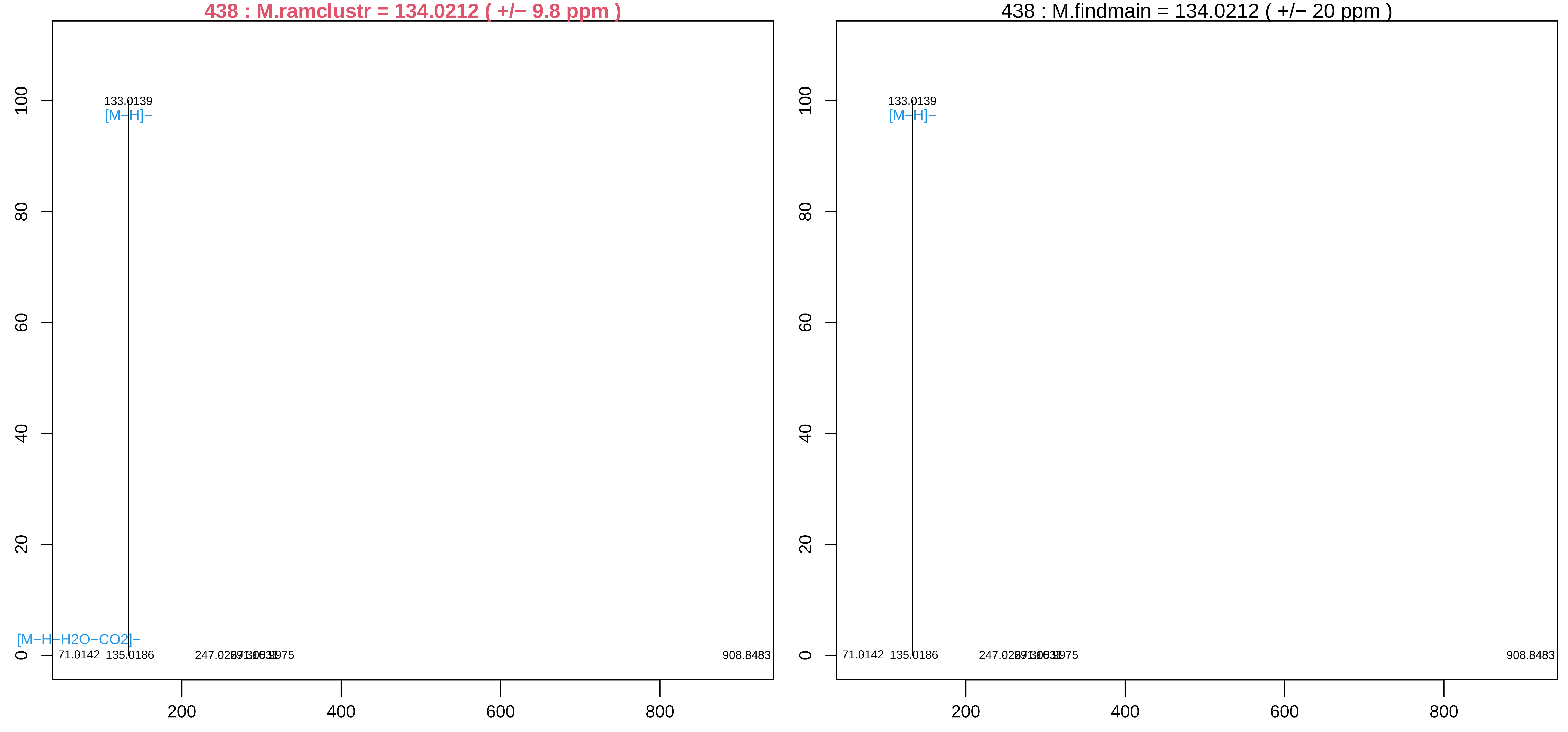

438 : M.ramclustr = 134.0212 (+/− 9.8 ppm)
133.0139
[M−H]−
[M−H−H2O−CO2]−
71.0142
135.0186
908.8483
438 : M.findmain = 134.0212 (+/− 20 ppm)
133.0139
[M−H]−
71.0142
135.0186
908.8483

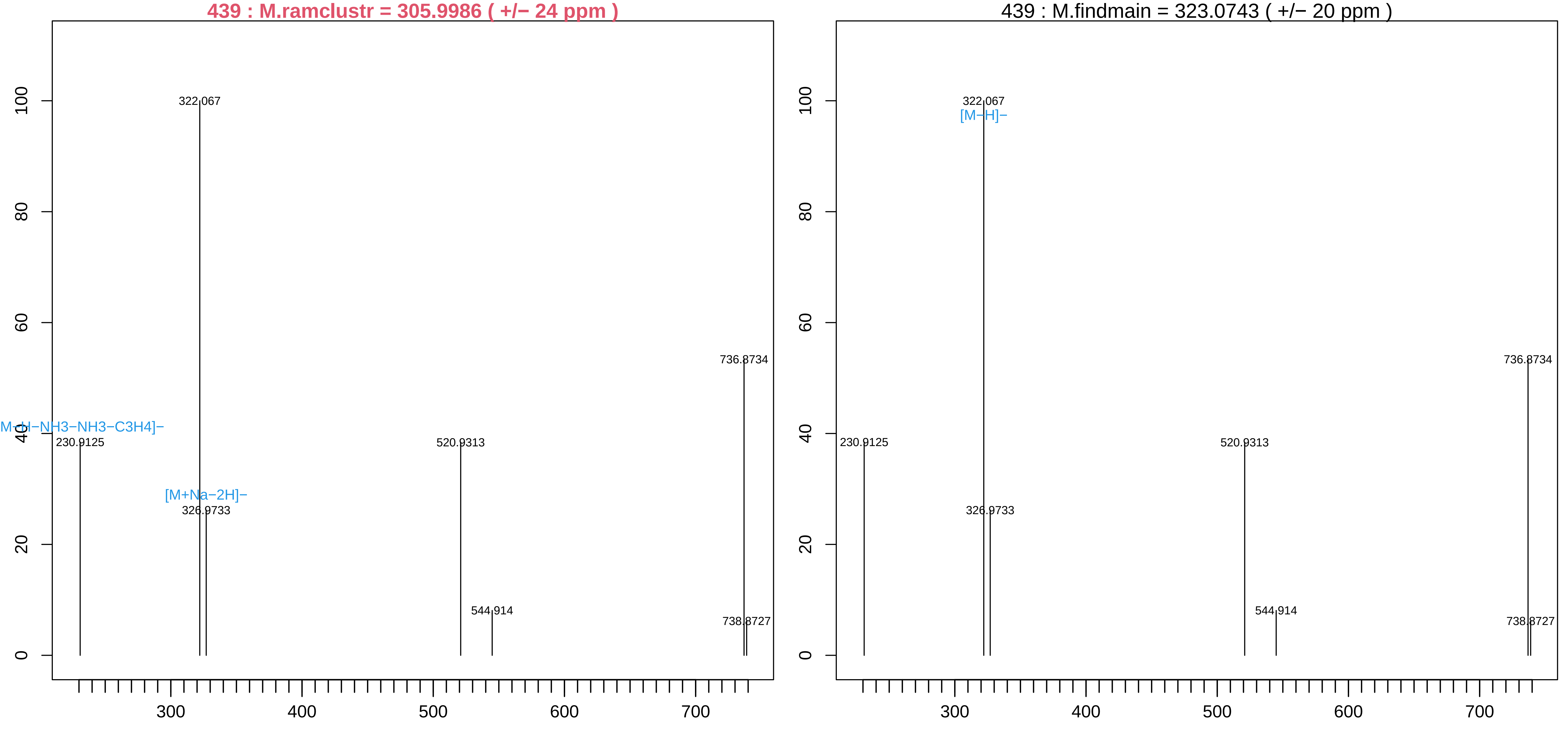

439 : M.ramclustr = 305.9986 (+/− 24 ppm)
322.067
[M−H−NH3−NH3−C3H4]−
230.9125
[M+Na−2H]−
326.9733
520.9313
544.914
736.8734
738.8727
439 : M.findmain = 323.0743 (+/− 20 ppm)
322.067
[M−H]−
230.9125
326.9733
520.9313
544.914
736.8734
738.8727

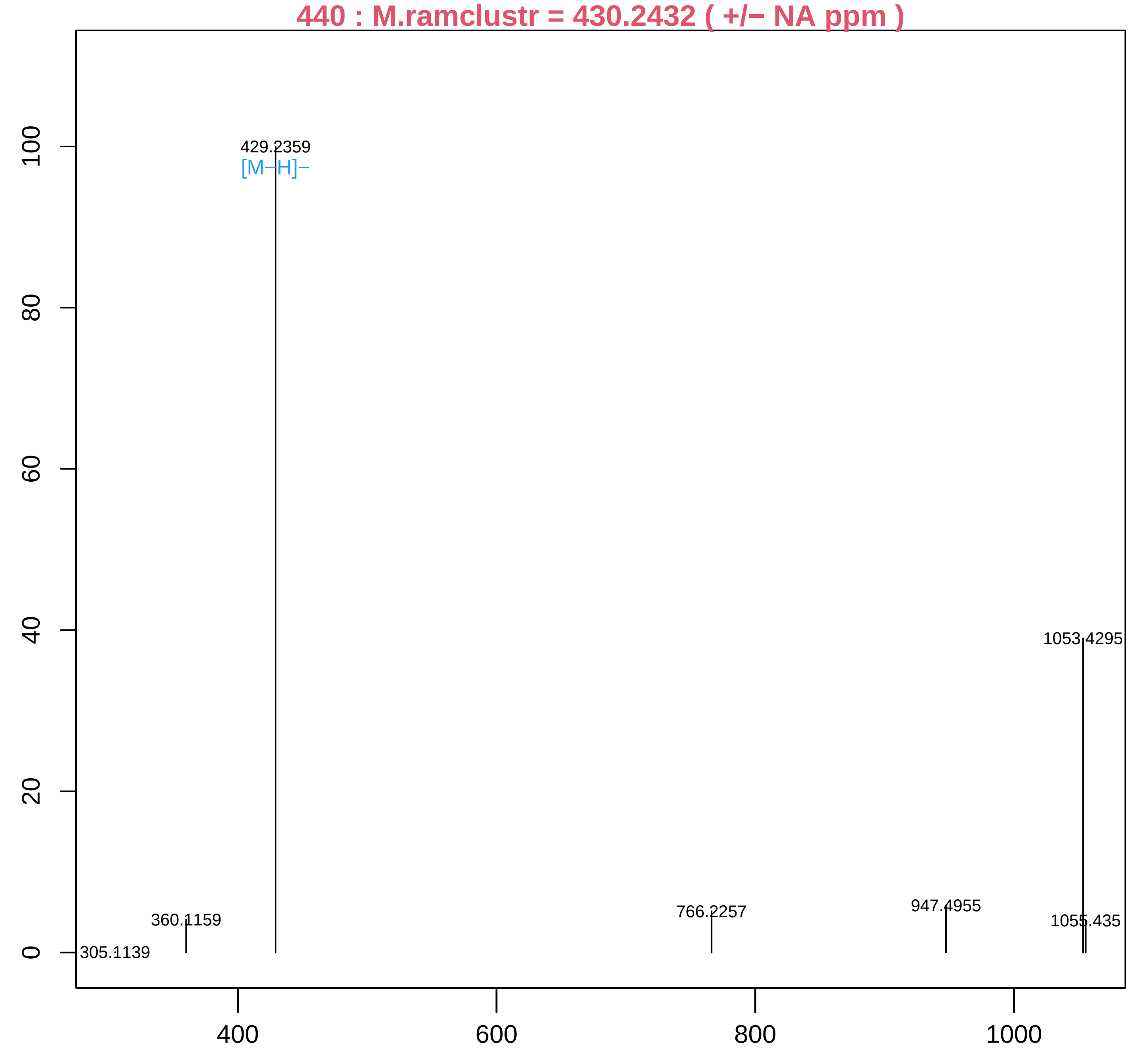
440 : M.ramclustr = 430.2432 (+/− NA ppm)
429.2359
[M−H]−
1053.4295
360.1159
766.2257
947.4955
1055.435
305.1139
0
20
40
60
80
100
400
600
800
1000

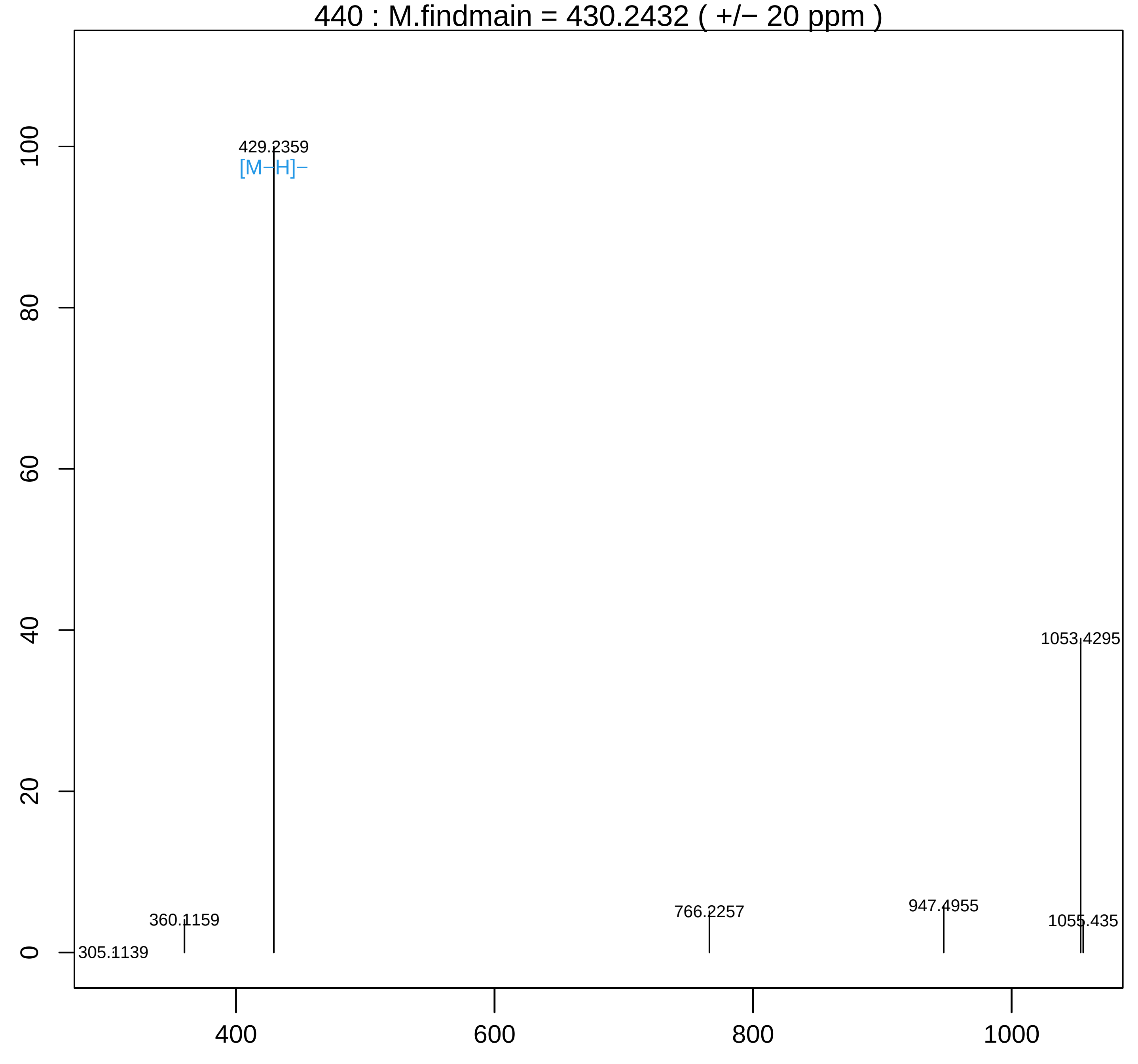
440 : M.findmain = 430.2432 (+/− 20 ppm)
429.2359
[M−H]−
1053.4295
360.1159
766.2257
947.4955
1055.435
305.1139
0
20
40
60
80
100
400
600
800
1000

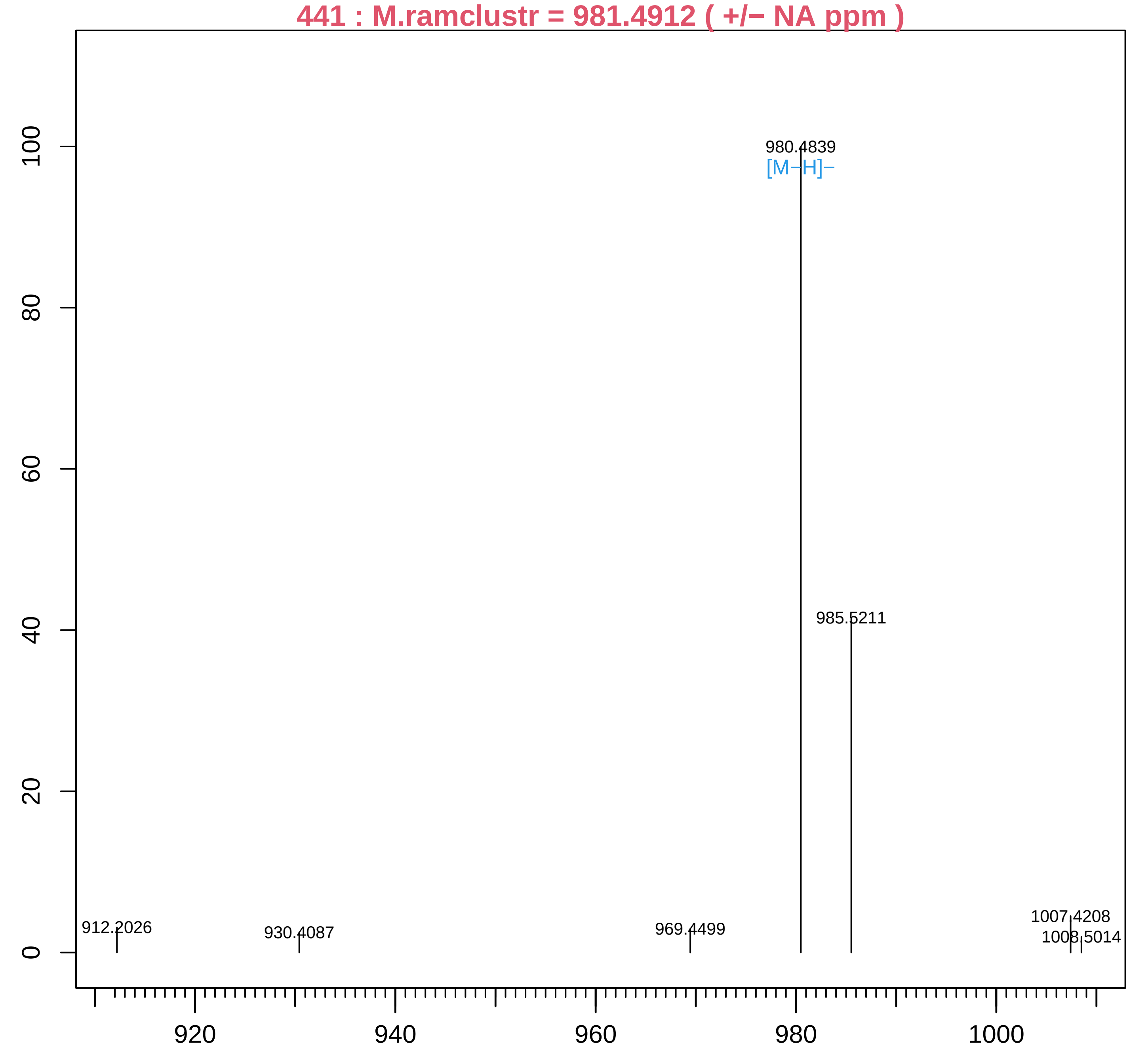
441 : M.ramclustr = 981.4912 (+/− NA ppm)
980.4839
[M−H]−
985.5211
912.2026
930.4087
969.4499
1007.4208
1008.5014
0
20
40
60
80
100
920
940
960
980
1000

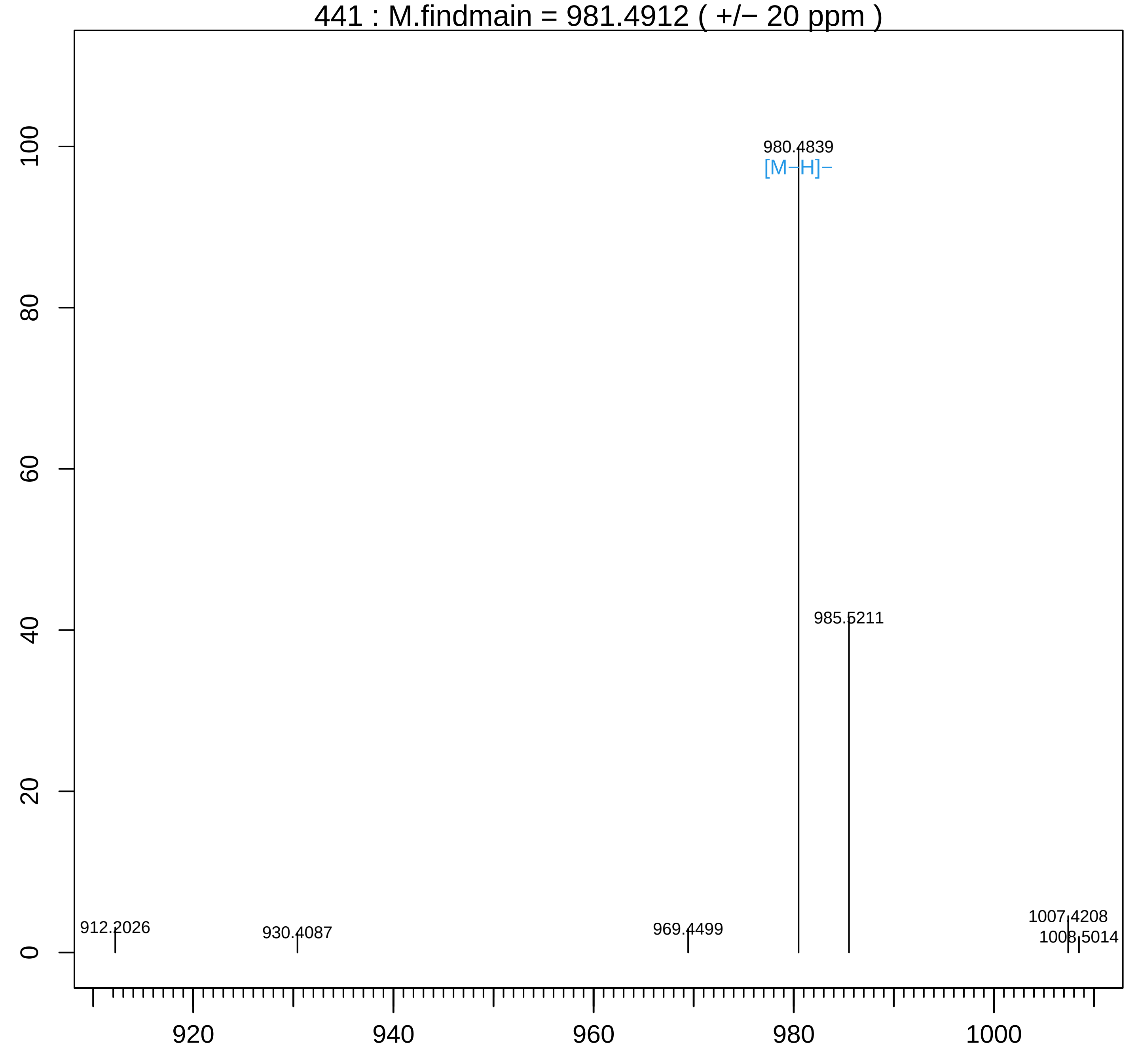
441 : M.findmain = 981.4912 (+/− 20 ppm)
980.4839
[M−H]−
985.5211
912.2026
930.4087
969.4499
1007.4208
1008.5014
0
20
40
60
80
100
920
940
960
980
1000

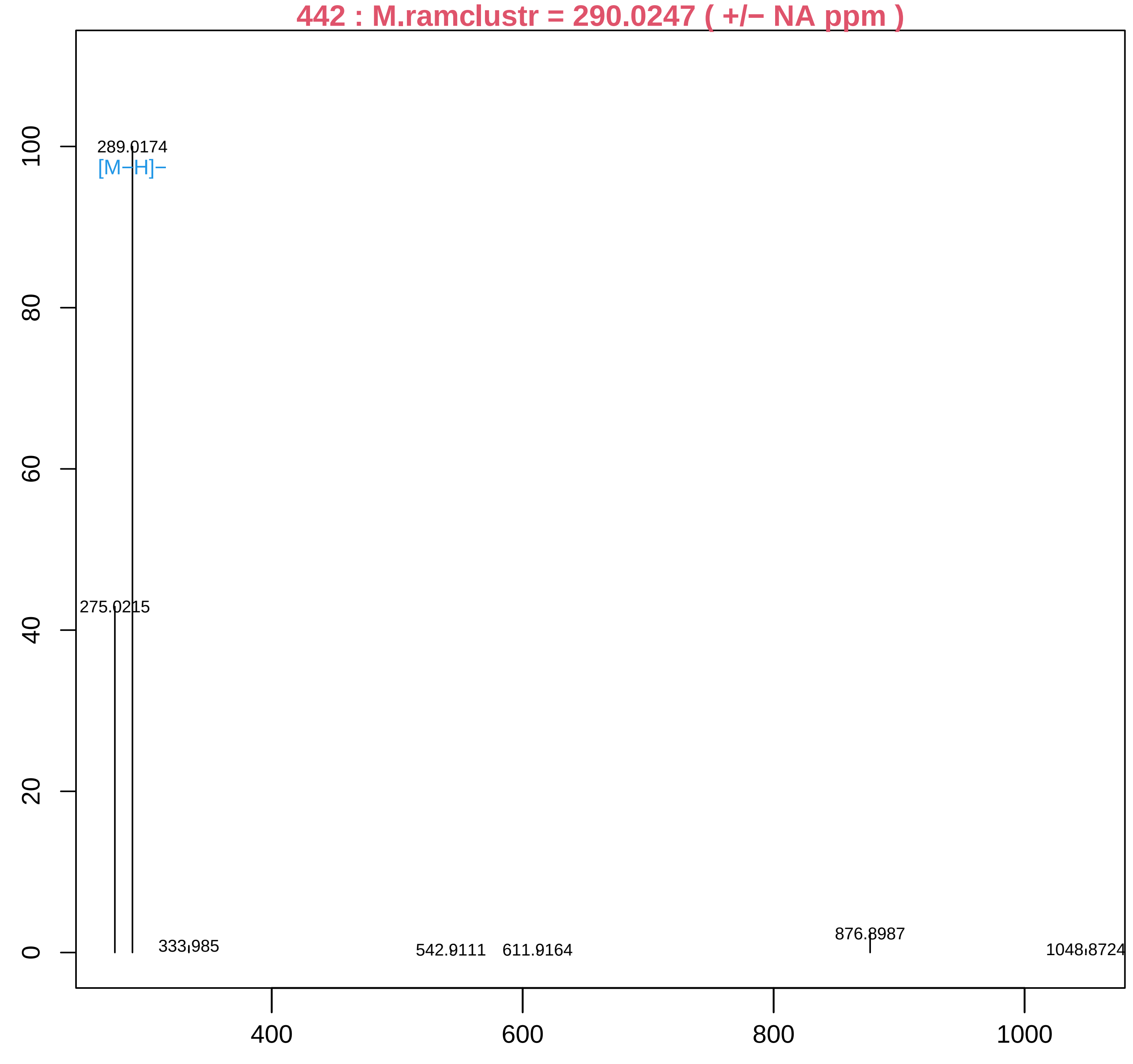
442 : M.ramclustr = 290.0247 (+/− NA ppm)
289.0174
[M−H]−
275.0215
333.985
542.9111
611.9164
876.8987
1048.8724
0
20
40
60
80
100
400
600
800
1000

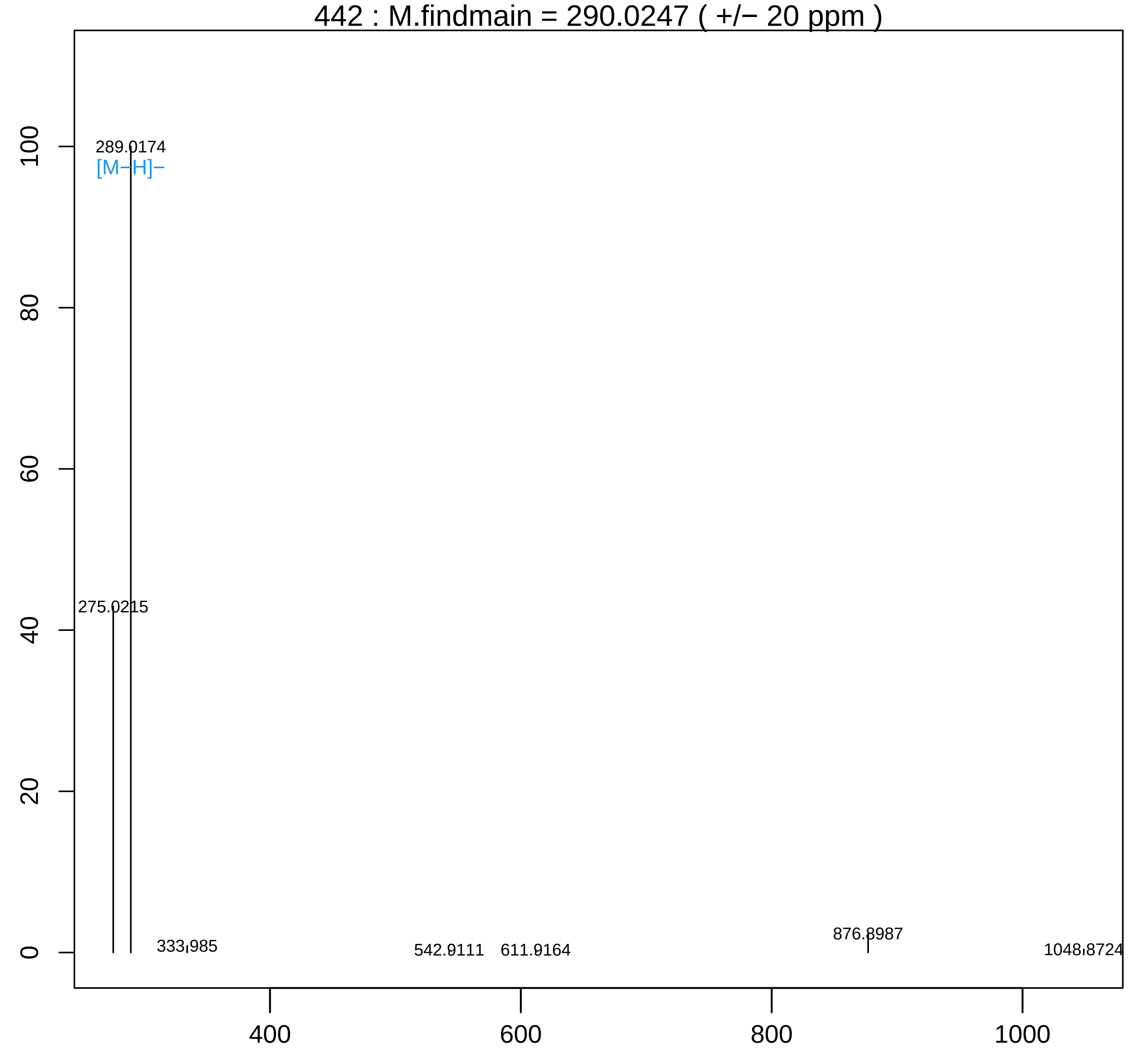
442 : M.findmain = 290.0247 (+/− 20 ppm)
289.0174
[M−H]−
275.0215
333.985
542.9111
611.9164
876.8987
1048.8724
0
20
40
60
80
100
400
600
800
1000

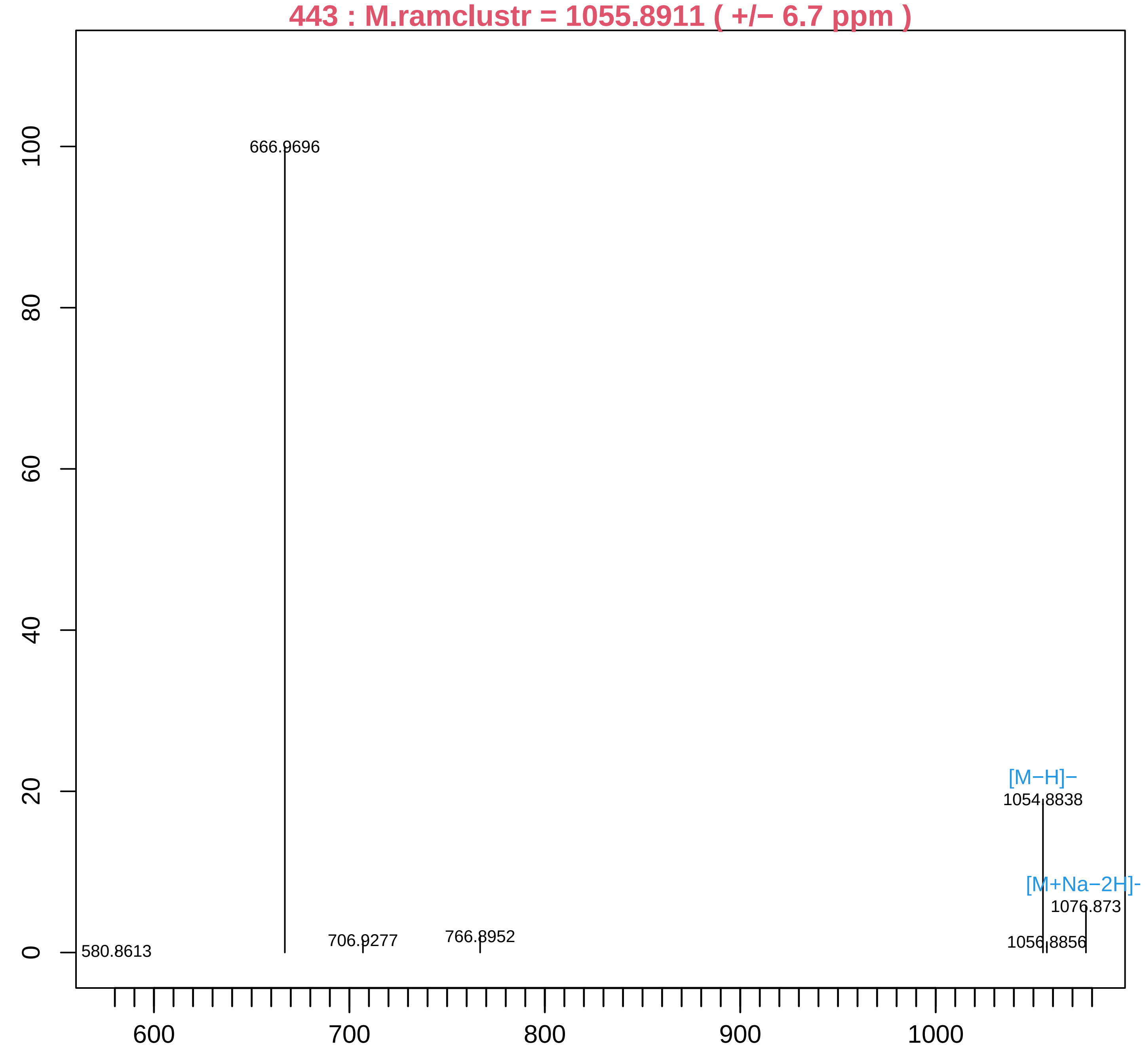
443 : M.ramclustr = 1055.8911 (+/− 6.7 ppm)
666.9696
[M−H]−
1054.8838
[M+Na−2H]-
1076.873
580.8613
706.9277
766.8952
1056.8856
0
20
40
60
80
100
600
700
800
900
1000

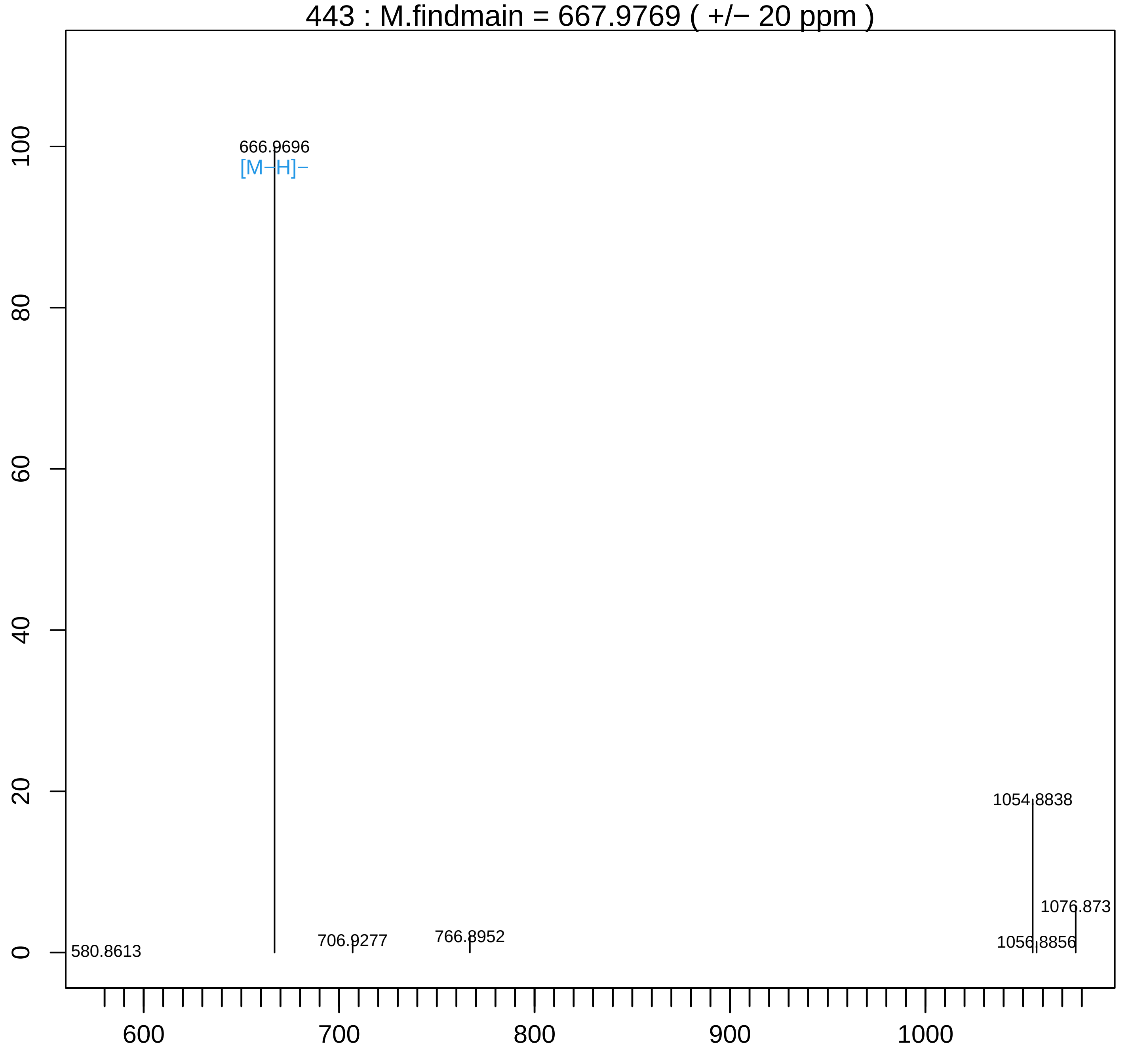
443 : M.findmain = 667.9769 (+/− 20 ppm)
666.9696
[M−H]−
1054.8838
1076.873
580.8613
706.9277
766.8952
1056.8856
0
20
40
60
80
100
600
700
800
900
1000

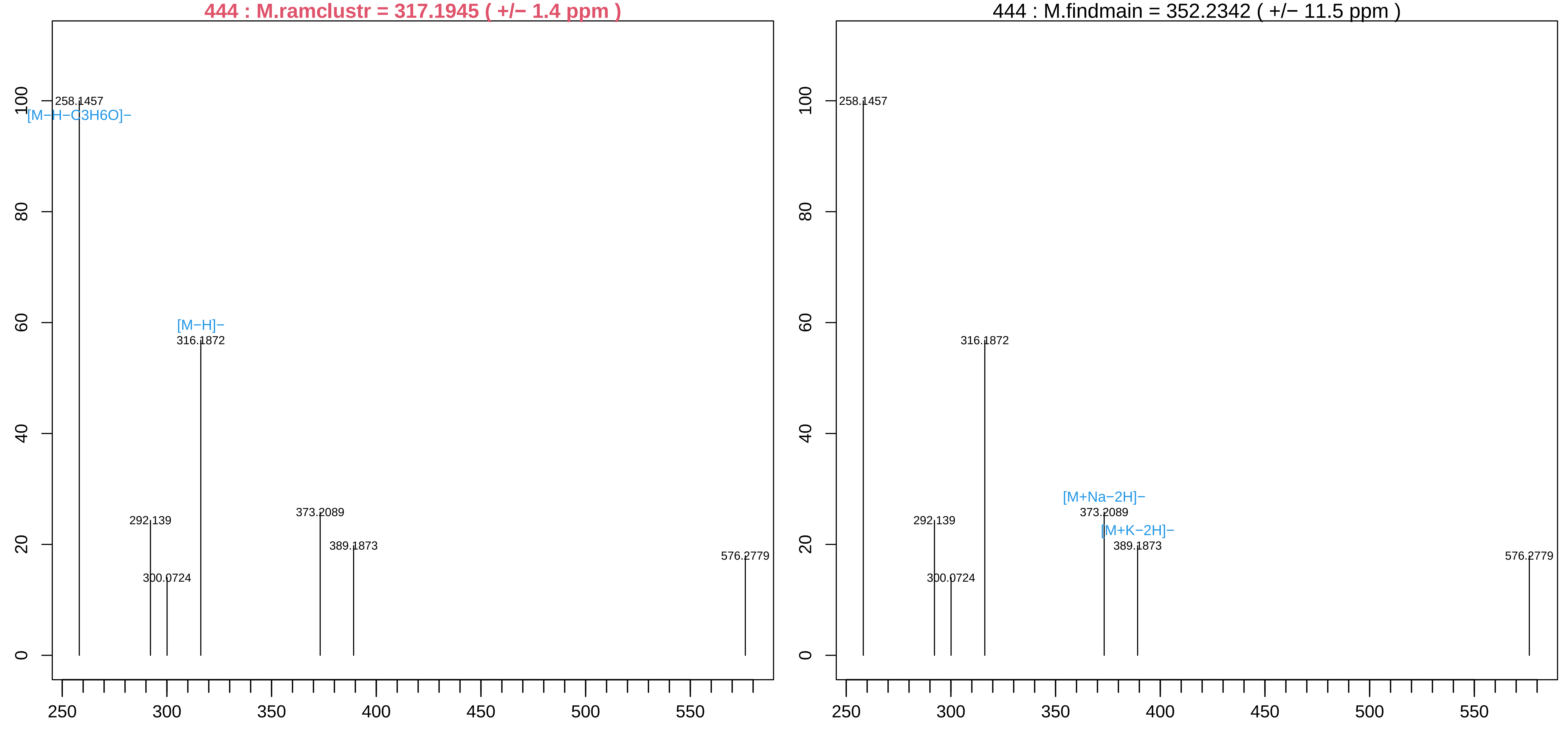
444 : M.ramclustr = 317.1945 (+/− 1.4 ppm)
258.1457
[M−H−C3H6O]−
[M−H]−
316.1872
292.139
300.0724
373.2089
389.1873
576.2779
444 : M.findmain = 352.2342 (+/− 11.5 ppm)
258.1457
316.1872
[M+Na−2H]−
373.2089
[M+K−2H]−
389.1873
292.139
300.0724
576.2779
0
20
40
60
80
100
250
300
350
400
450
500
550

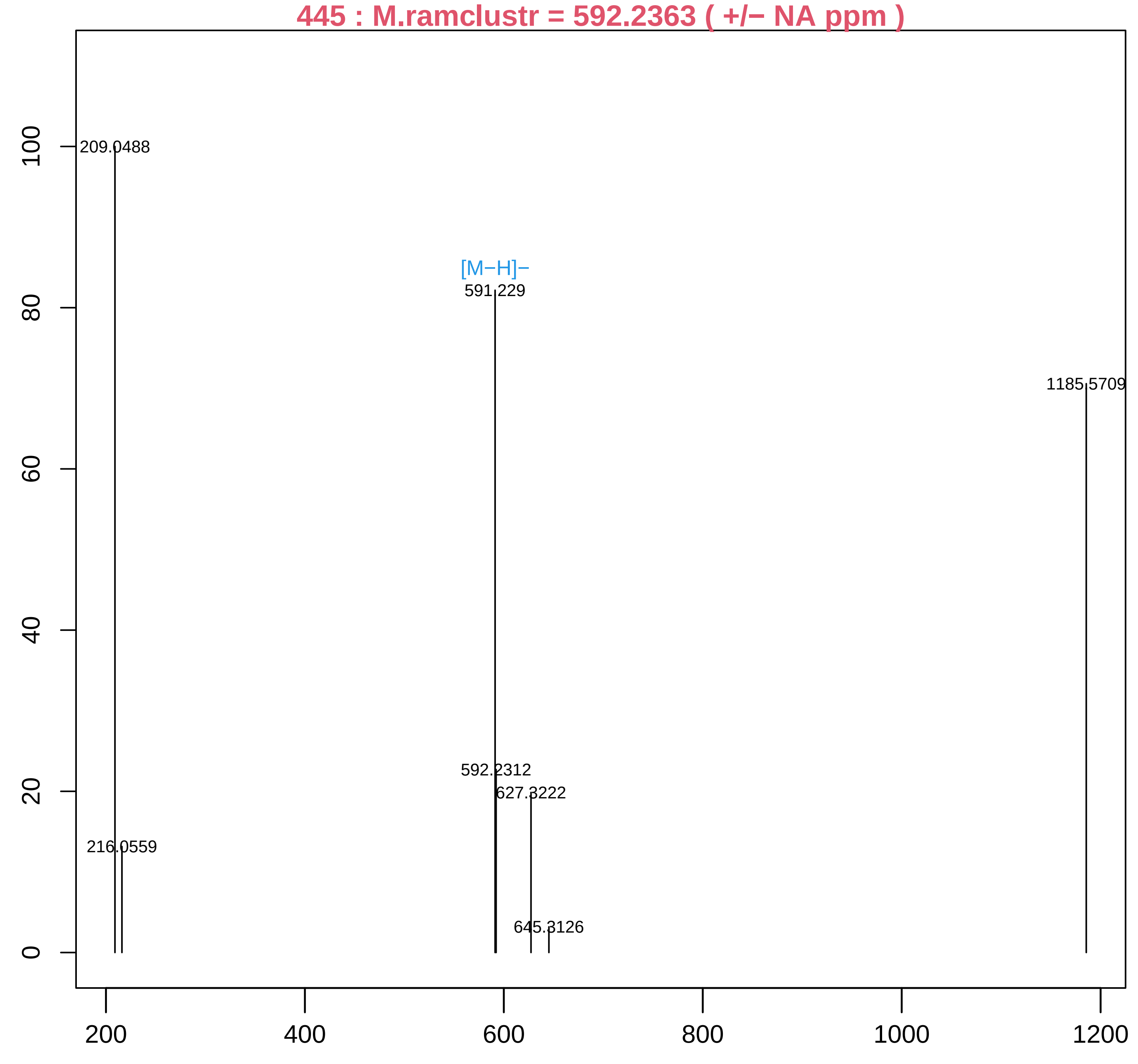
445 : M.ramclustr = 592.2363 (+/− NA ppm)
209.0488
[M−H]−
591.229
1185.5709
592.2312
627.3222
216.0559
645.3126
0
20
40
60
80
100
200
400
600
800
1000
1200

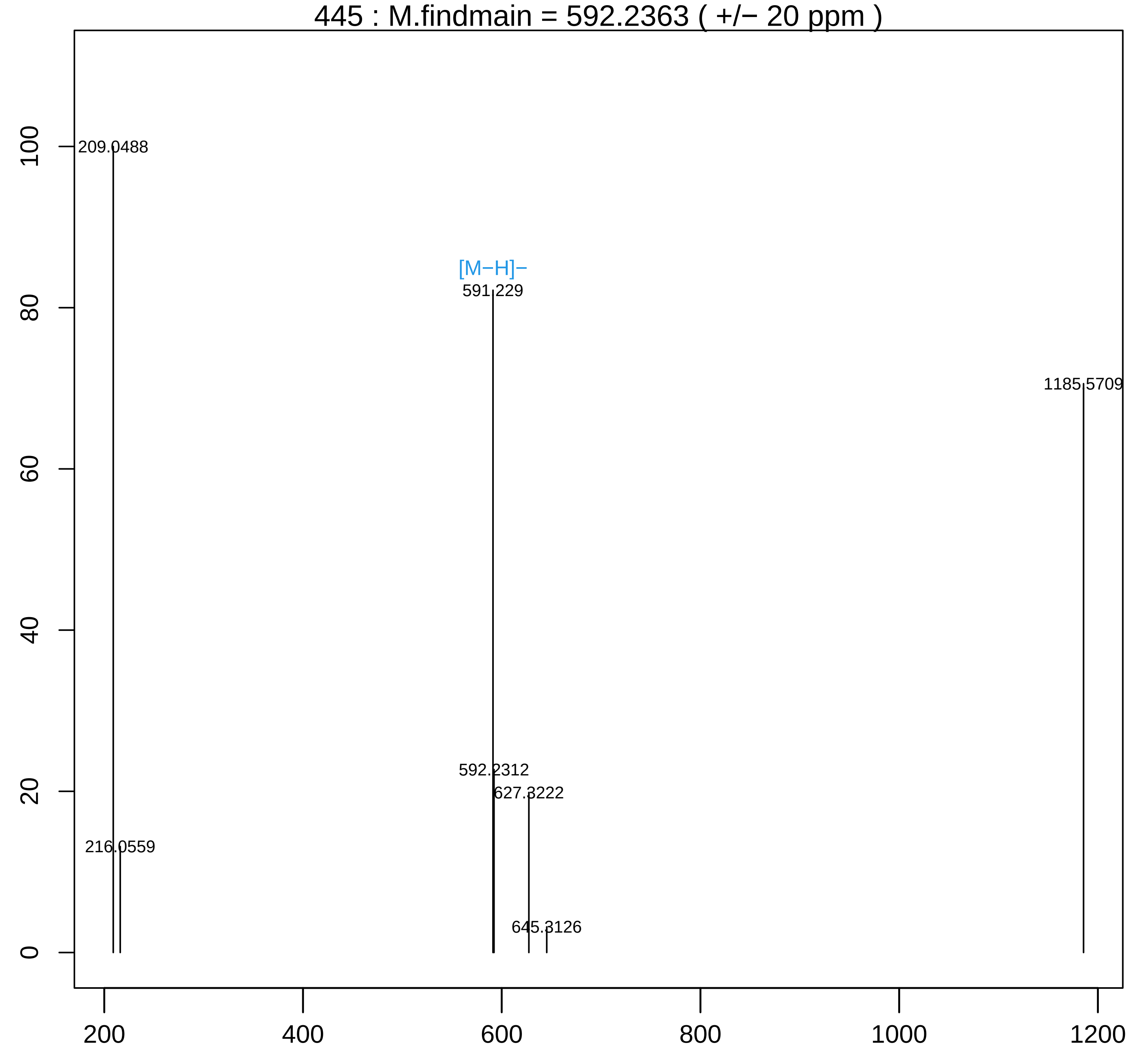
445 : M.findmain = 592.2363 (+/− 20 ppm)
209.0488
[M−H]−
591.229
1185.5709
592.2312
627.3222
216.0559
645.3126
0
20
40
60
80
100
200
400
600
800
1000
1200

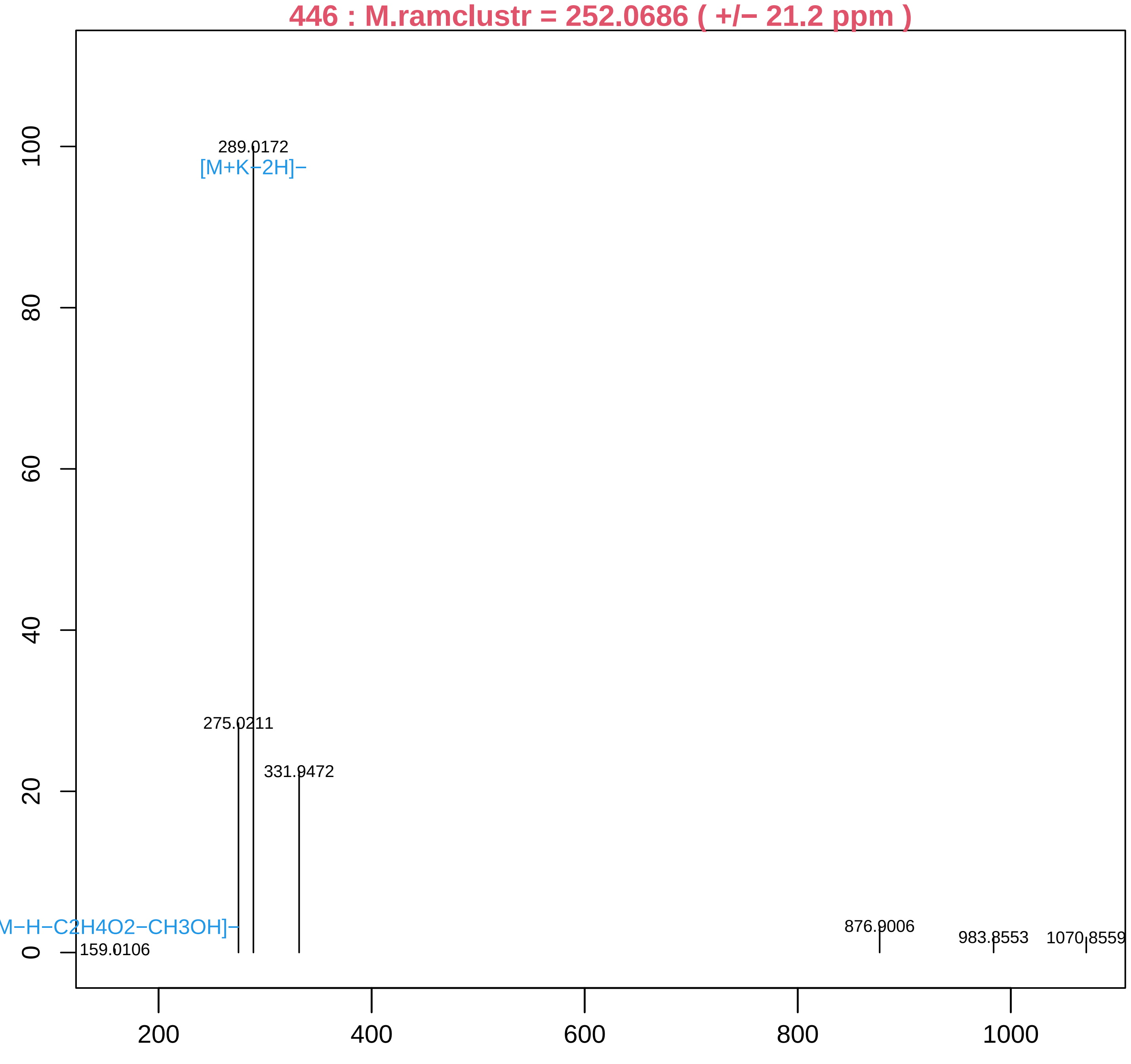

446 : M.ramclustr = 252.0686 (+/− 21.2 ppm)
289.0172
[M+K−2H]−
275.0211
331.9472
M−H−C2H4O2−CH3OH]−
159.0106
876.9006
983.8553
1070.8559

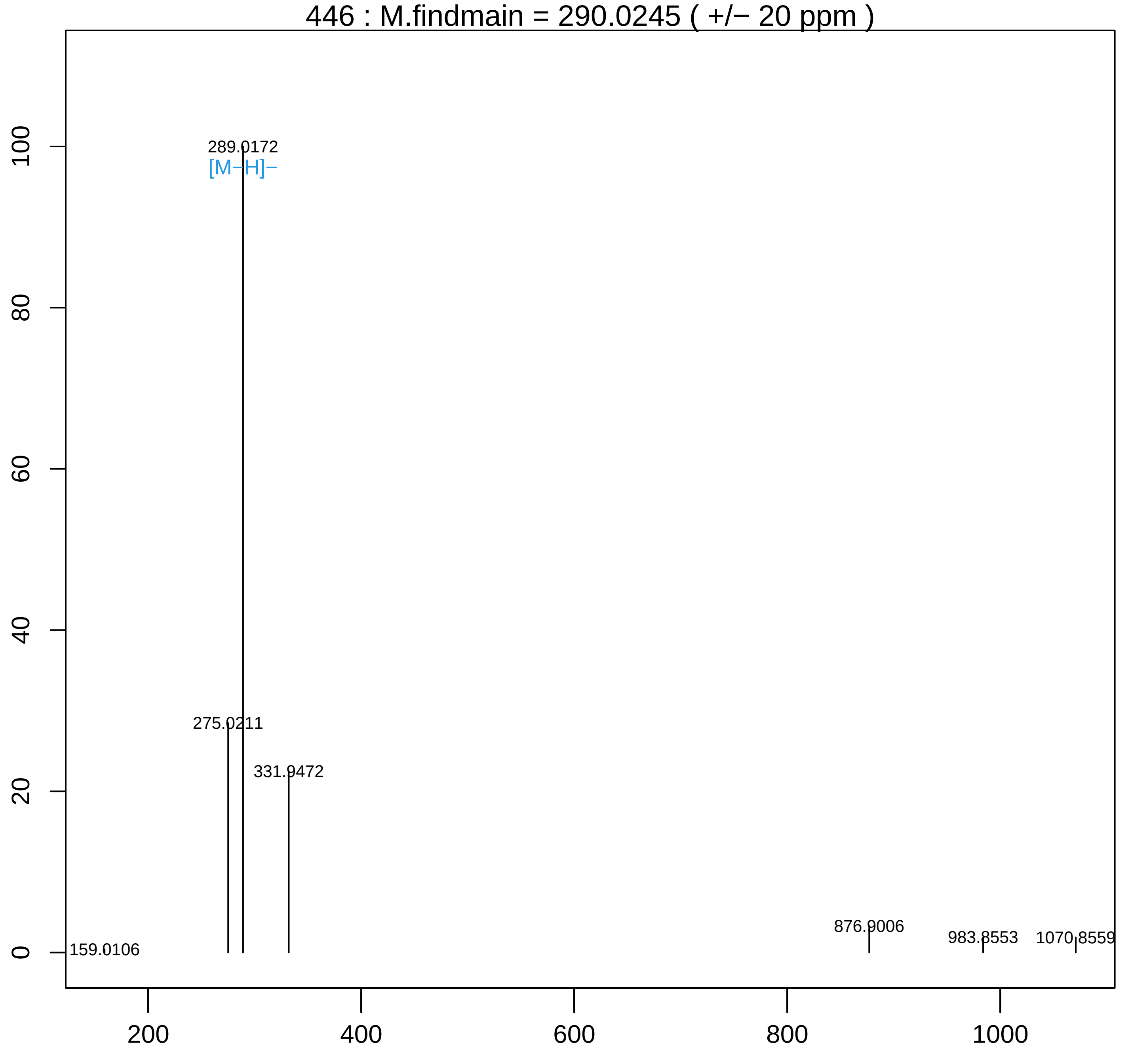

446 : M.findmain = 290.0245 (+/− 20 ppm)
289.0172
[M−H]−
275.0211
331.9472
159.0106
876.9006
983.8553
1070.8559

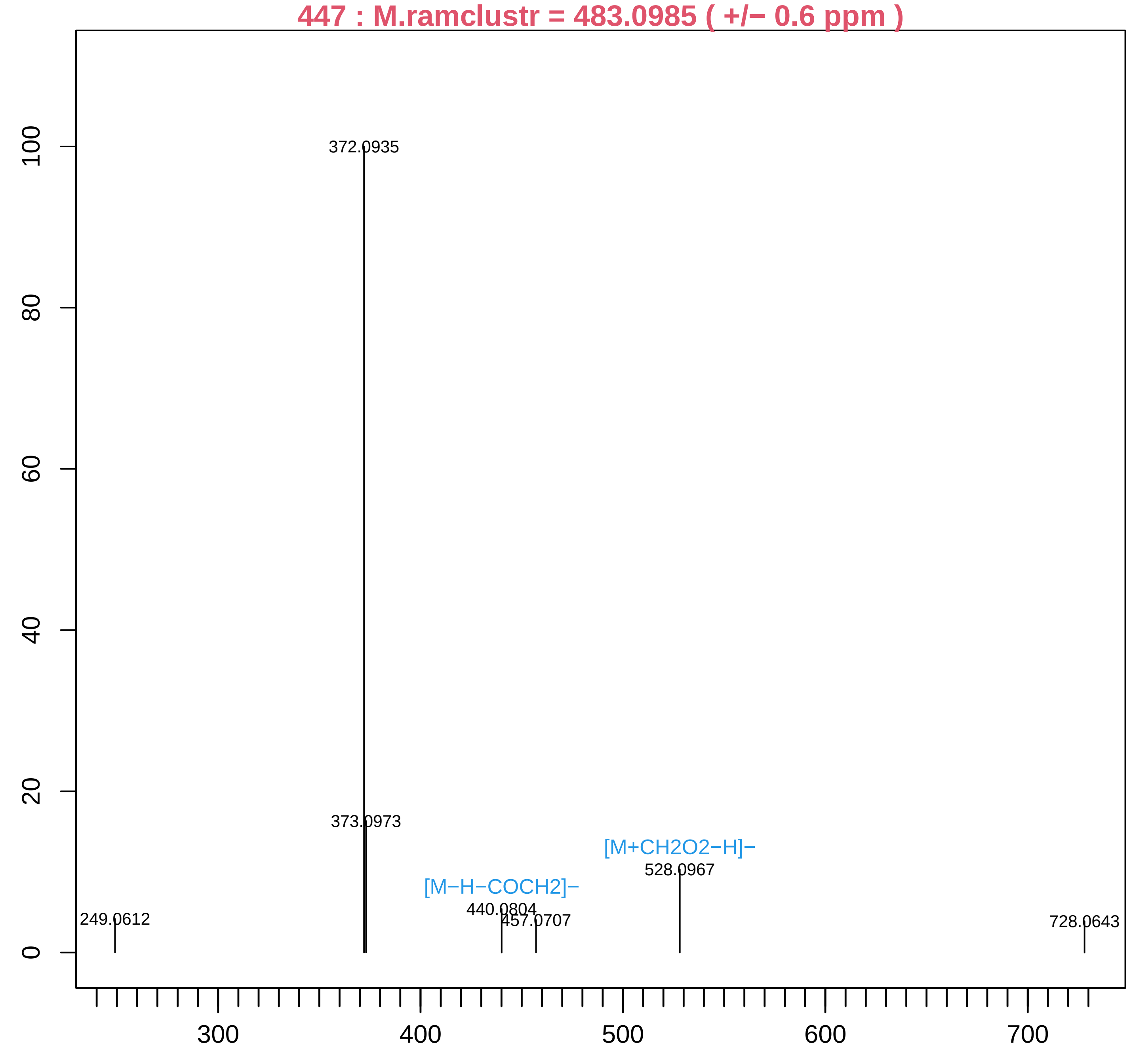
447 : M.ramclustr = 483.0985 (+/− 0.6 ppm)
372.0935
373.0973
[M−H−COCH2]−
440.0804
457.0707
[M+CH2O2−H]−
528.0967
249.0612
728.0643
0
20
40
60
80
100
300
400
500
600
700
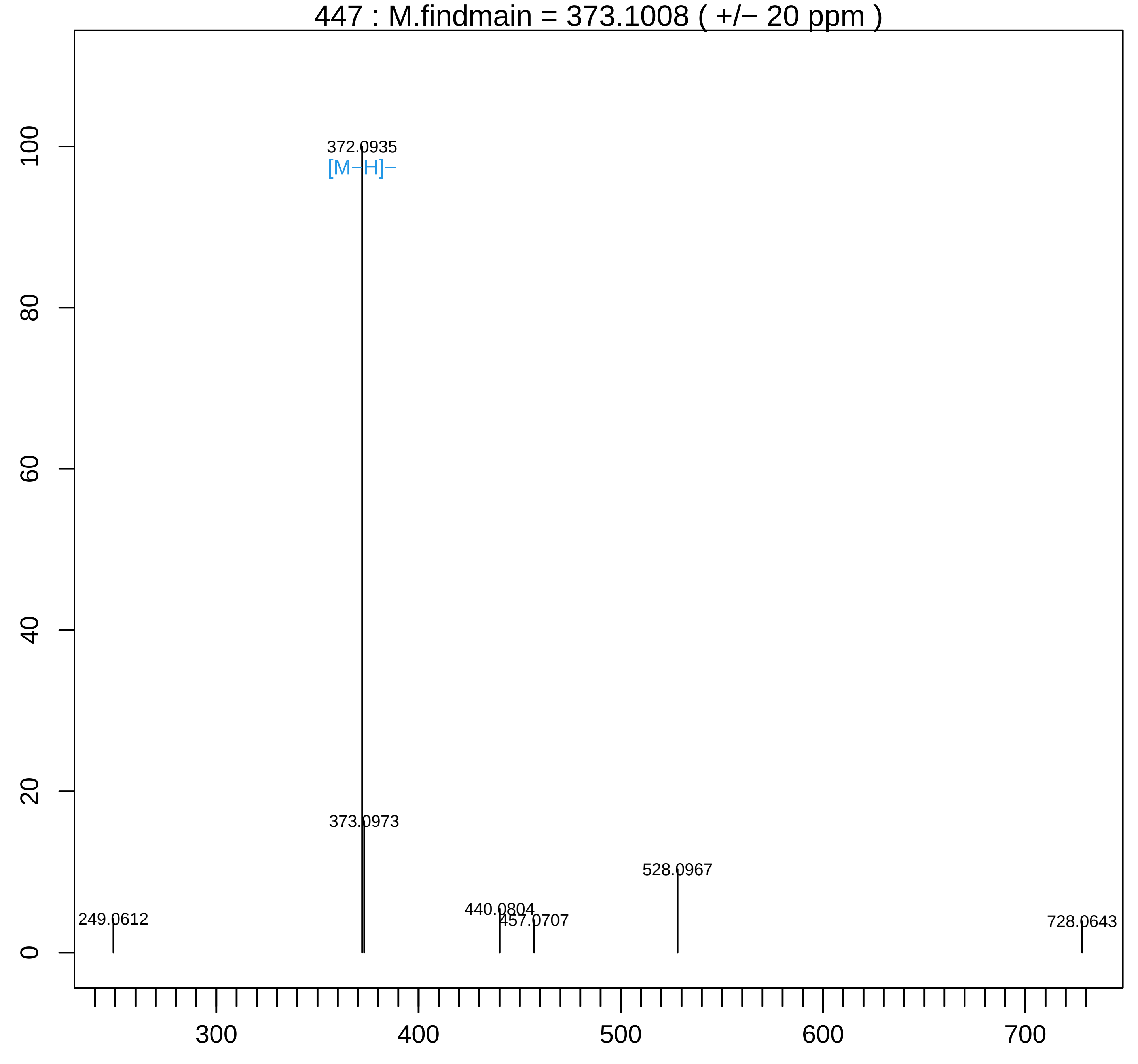
447 : M.findmain = 373.1008 (+/− 20 ppm)
372.0935
[M−H]−
373.0973
440.0804
457.0707
528.0967
249.0612
728.0643
0
20
40
60
80
100
300
400
500
600
700

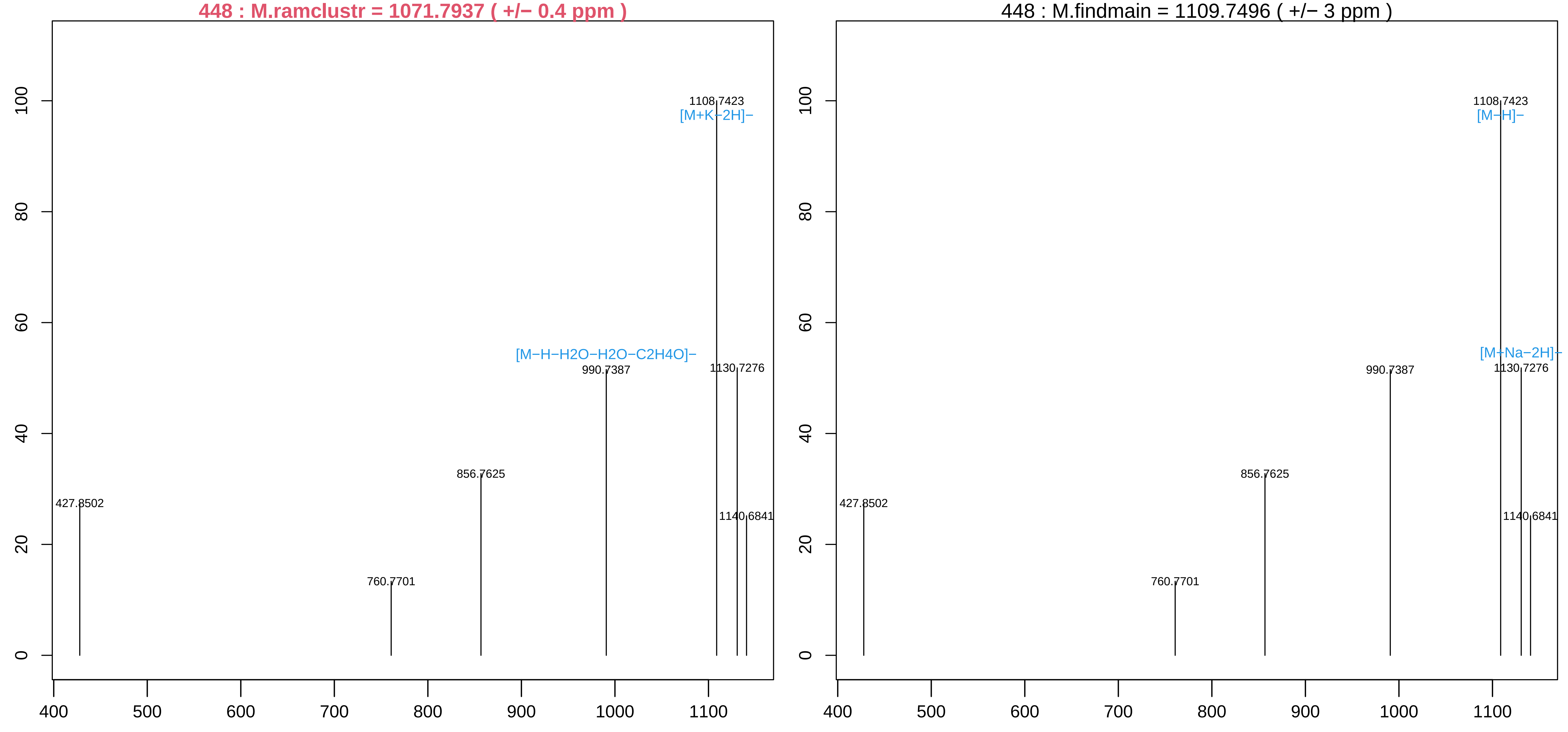

448 : M.ramclustr = 1071.7937 (+/− 0.4 ppm)
1108.7423
[M+K−2H]−
[M−H−H2O−H2O−C2H4O]−
990.7387
1130.7276
856.7625
427.8502
1140.6841
760.7701
0
20
40
60
80
100
400
500
600
700
800
900
1000
1100
448 : M.findmain = 1109.7496 (+/− 3 ppm)
1108.7423
[M−H]−
[M+Na−2H]−
990.7387
1130.7276
856.7625
427.8502
1140.6841
760.7701

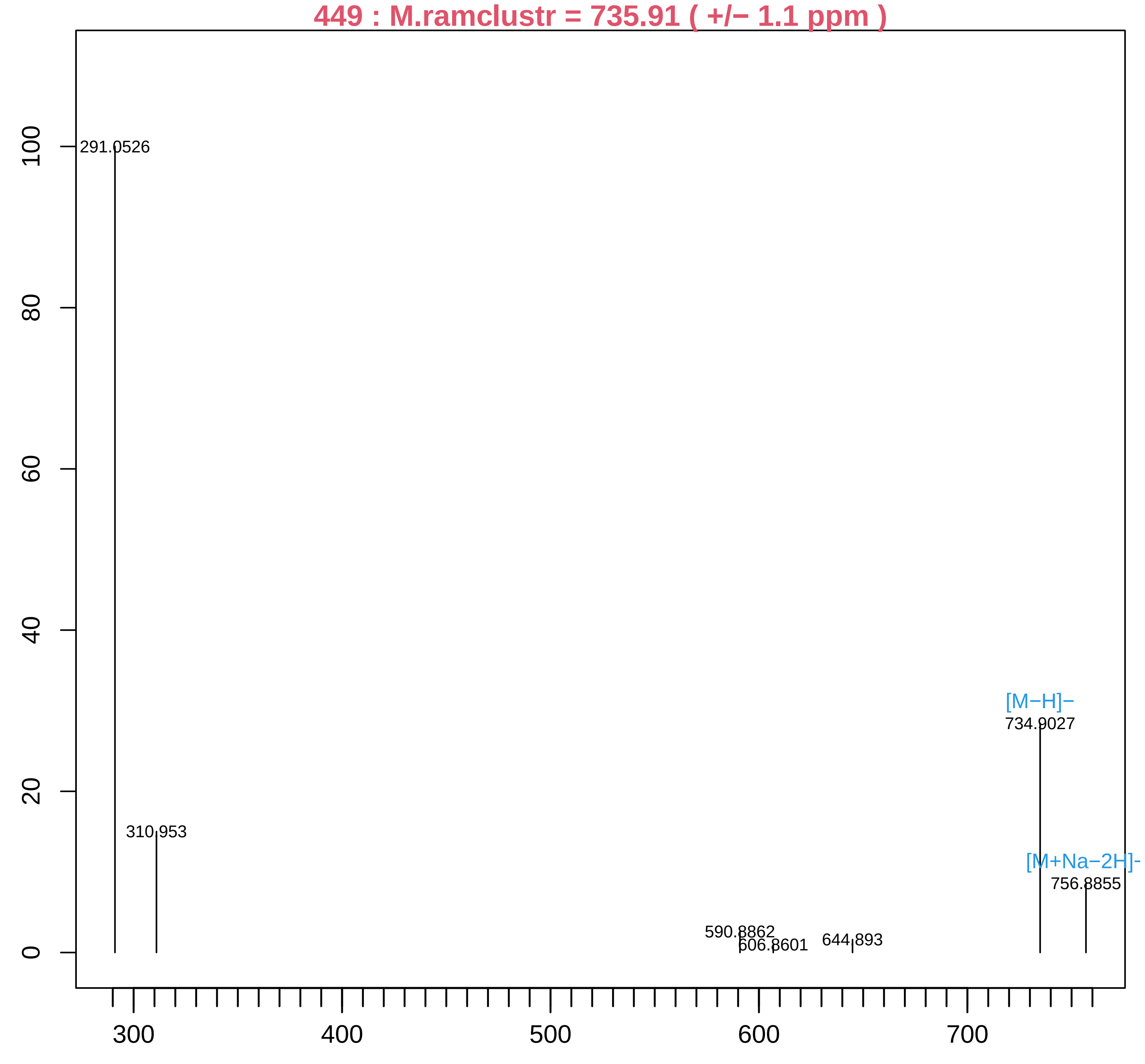

449 : M.ramclustr = 735.91 (+/− 1.1 ppm)
291.0526
310.953
590.8862
606.8601
644.893
[M−H]−
734.9027
[M+Na−2H]-
756.8855
0
20
40
60
80
100
300
400
500
600
700

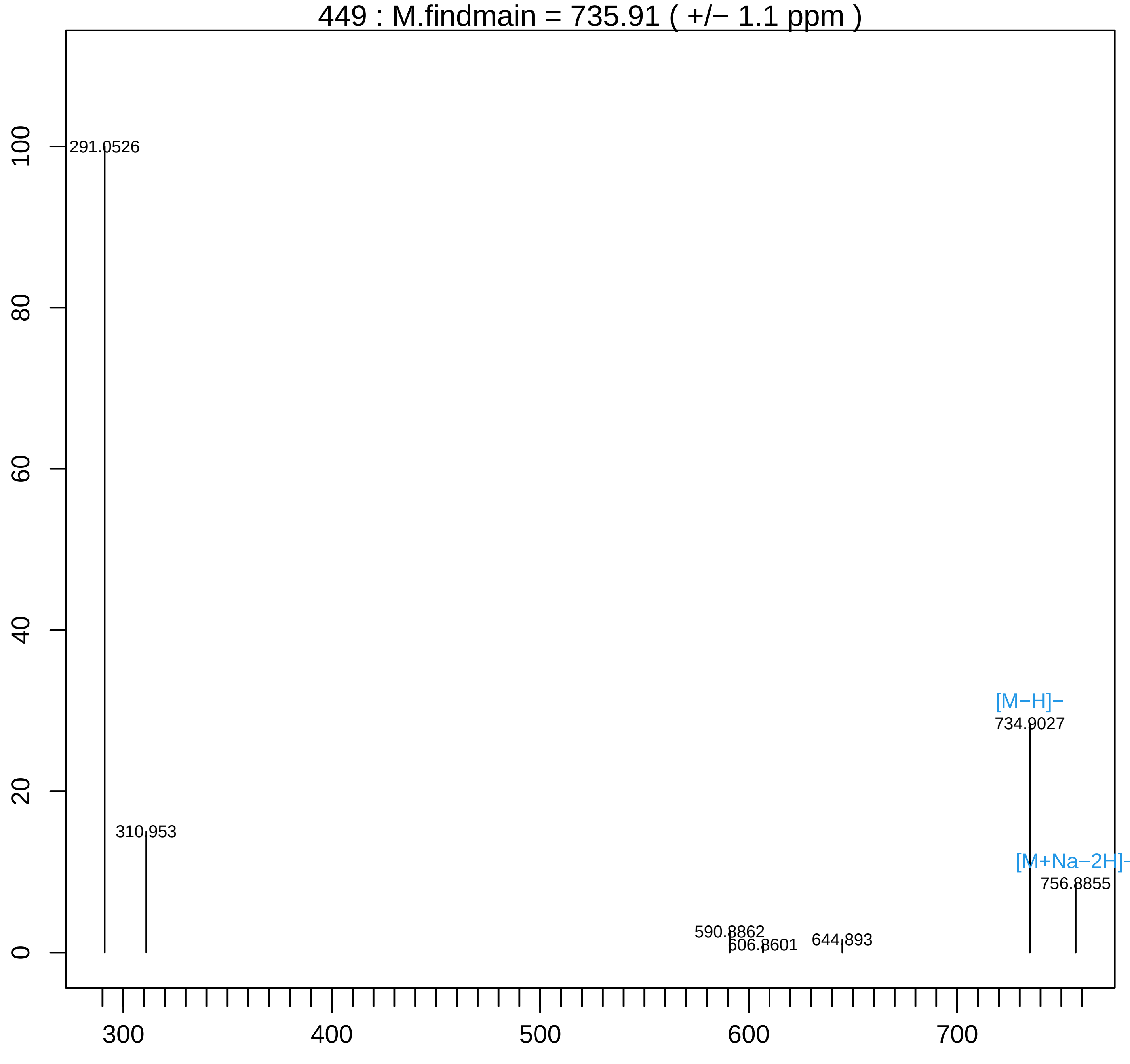

449 : M.findmain = 735.91 (+/− 1.1 ppm)
291.0526
310.953
590.8862
606.8601
644.893
[M−H]−
734.9027
[M+Na−2H]-
756.8855
0
20
40
60
80
100
300
400
500
600
700

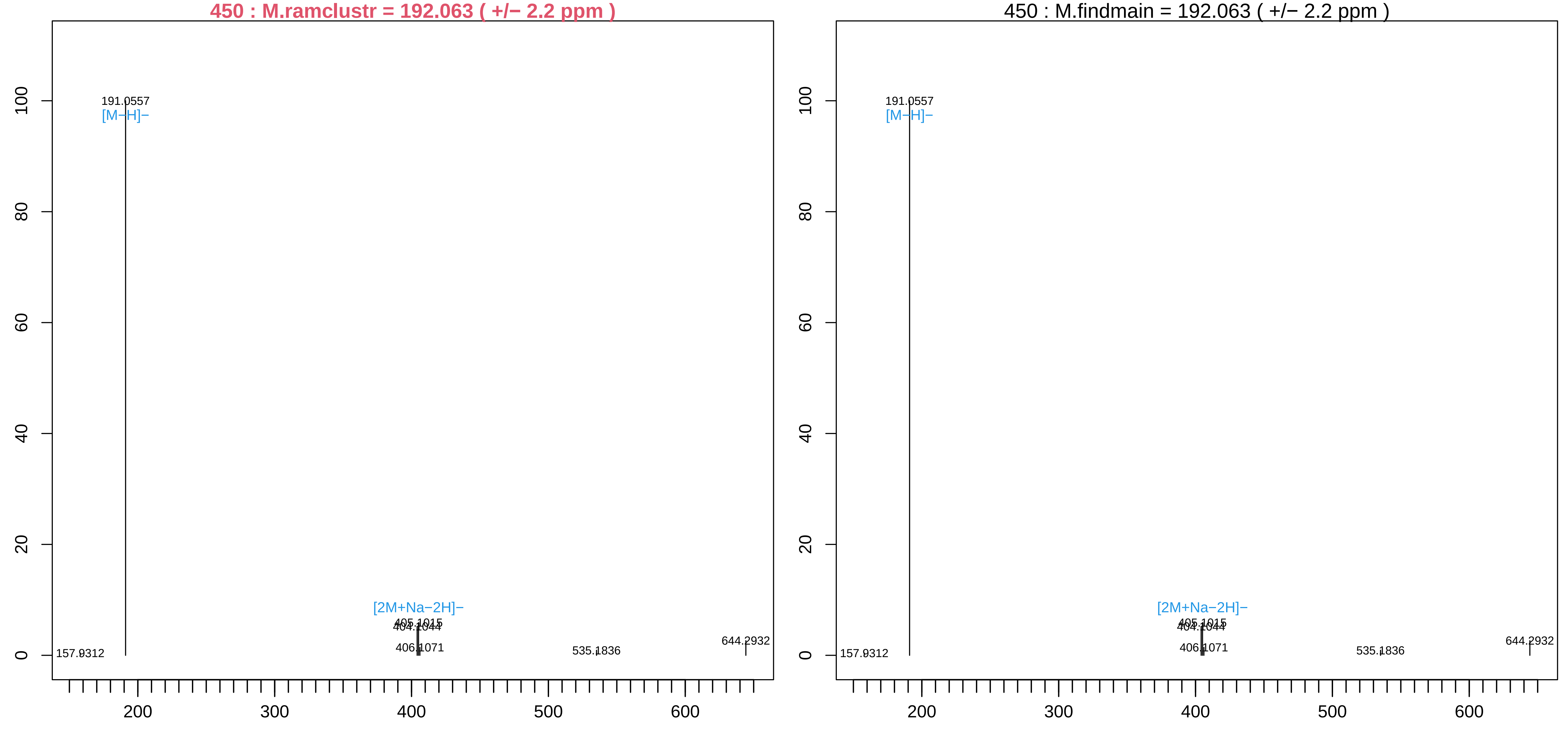
450 : M.ramclustr = 192.063 (+/− 2.2 ppm)
191.0557
[M−H]−
[2M+Na−2H]−
405.1015
404.1044
406.1071
157.9312
535.1836
644.2932
450 : M.findmain = 192.063 (+/− 2.2 ppm)
191.0557
[M−H]−
[2M+Na−2H]−
405.1015
404.1044
406.1071
157.9312
535.1836
644.2932

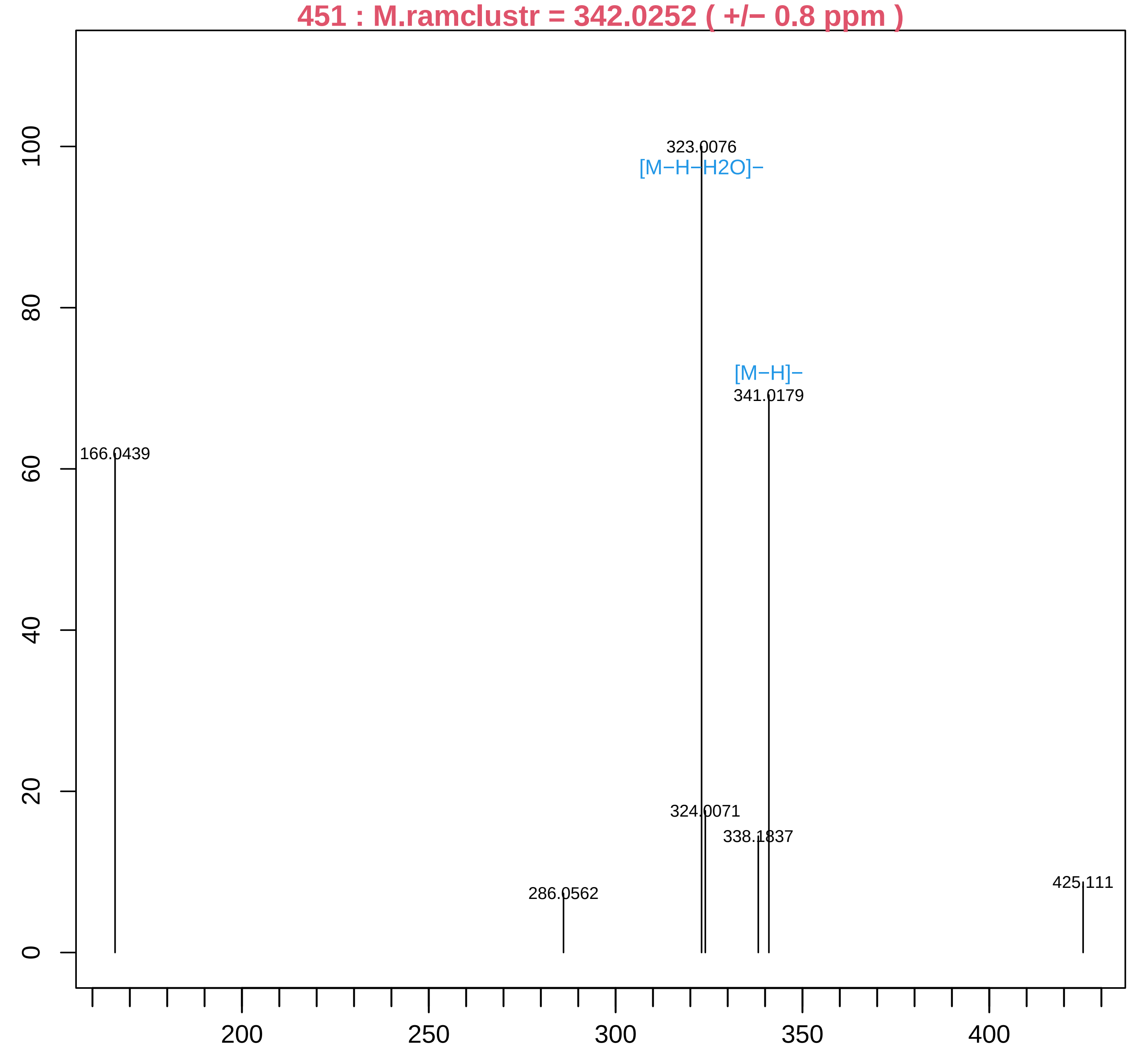
451 : M.ramclustr = 342.0252 (+/− 0.8 ppm)
323.0076
[M−H−H2O]−
[M−H]−
341.0179
166.0439
324.0071
338.1837
286.0562
425.111
0
20
40
60
80
100
200
250
300
350
400

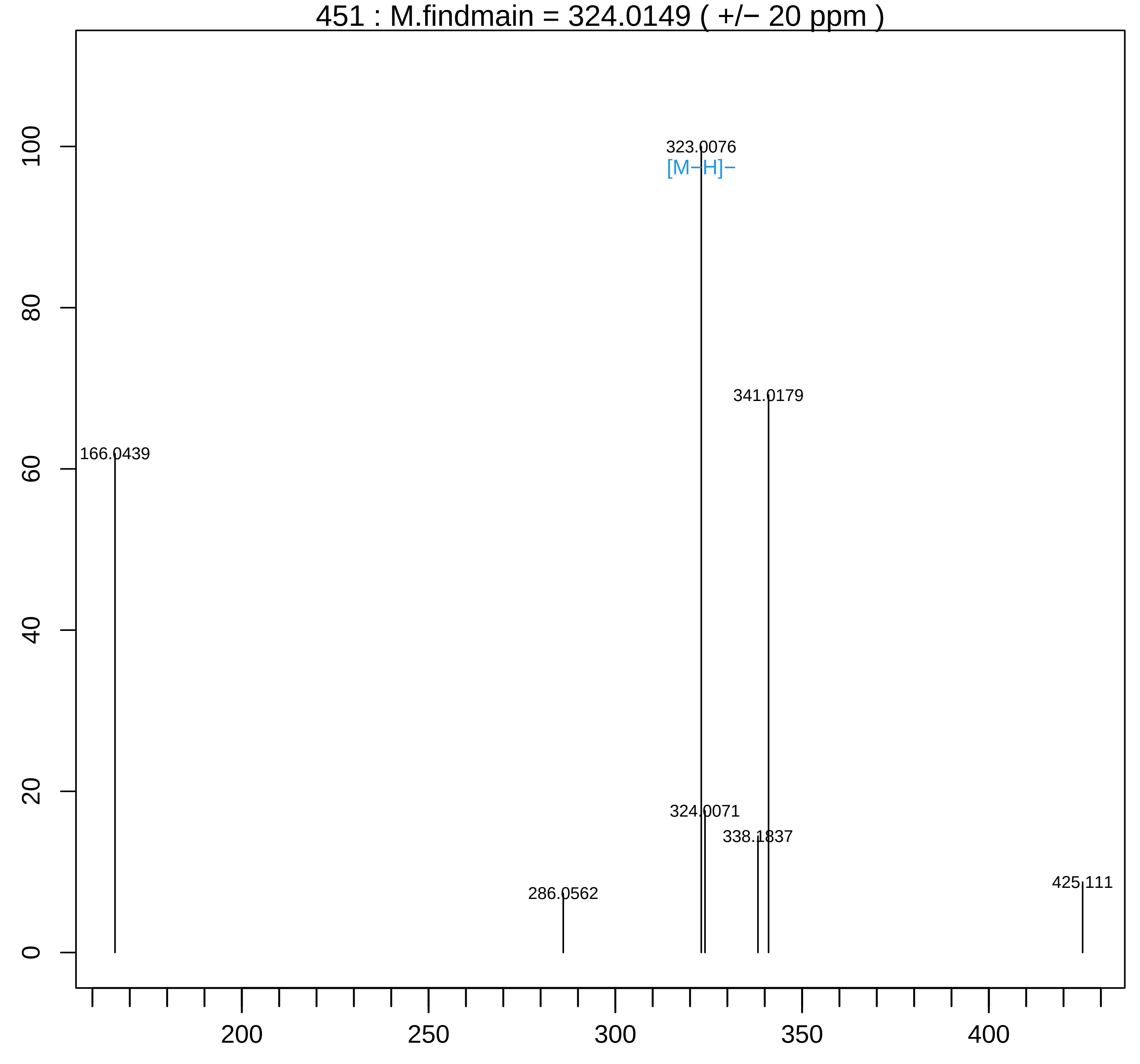
451 : M.findmain = 324.0149 (+/− 20 ppm)
323.0076
[M−H]−
341.0179
166.0439
324.0071
338.1837
286.0562
425.111
0
20
40
60
80
100
200
250
300
350
400

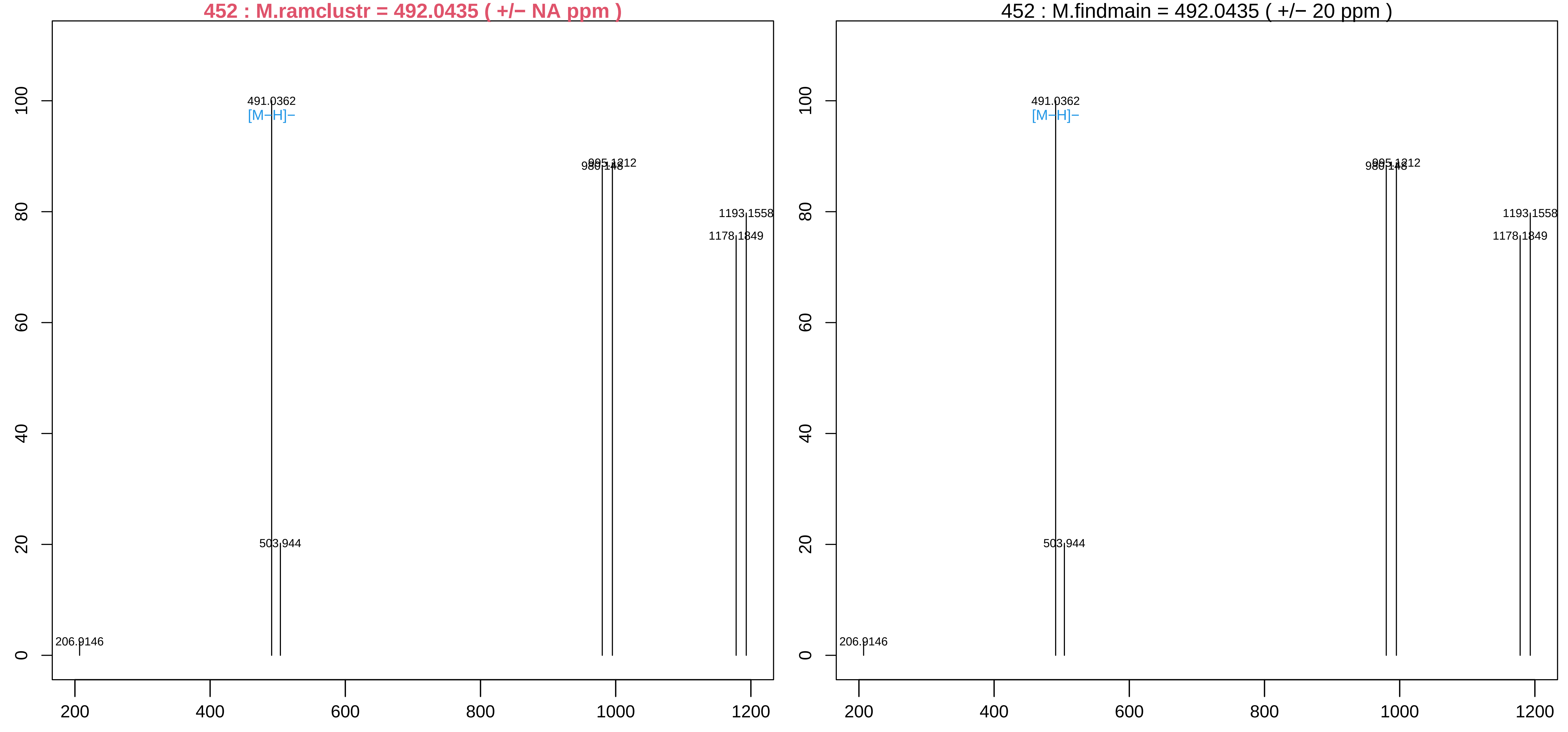
452 : M.ramclustr = 492.0435 (+/− NA ppm)
452 : M.findmain = 492.0435 (+/− 20 ppm)
491.0362
[M−H]−
995.1312
980.148
1193.1558
1178.1849
503.944
206.9146
0
20
40
60
80
100
200
400
600
800
1000
1200

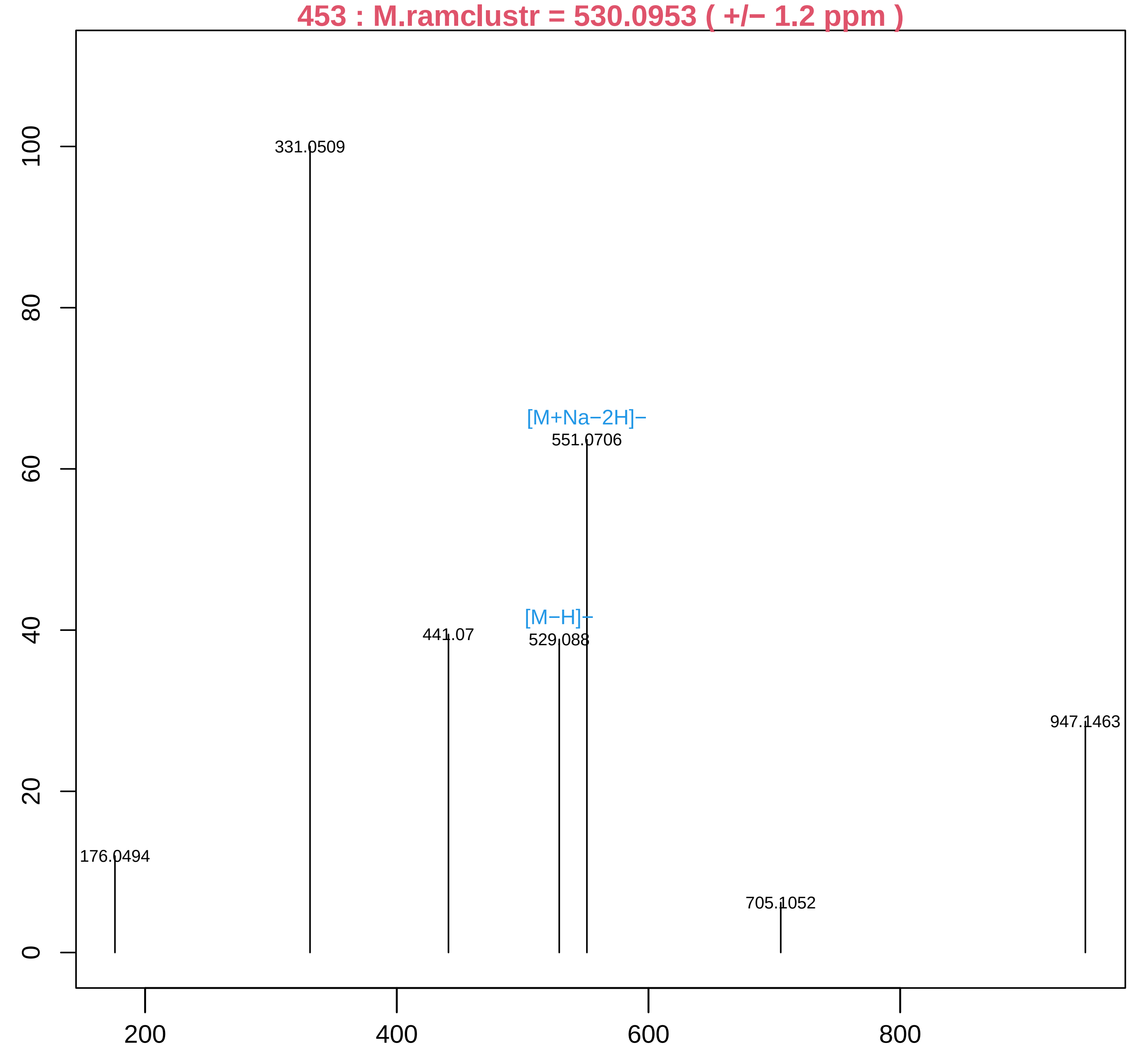

453 : M.ramclustr = 530.0953 (+/− 1.2 ppm)
331.0509
[M+Na−2H]−
551.0706
[M−H]−
529.088
441.07
947.1463
176.0494
705.1052
0
20
40
60
80
100
200
400
600
800

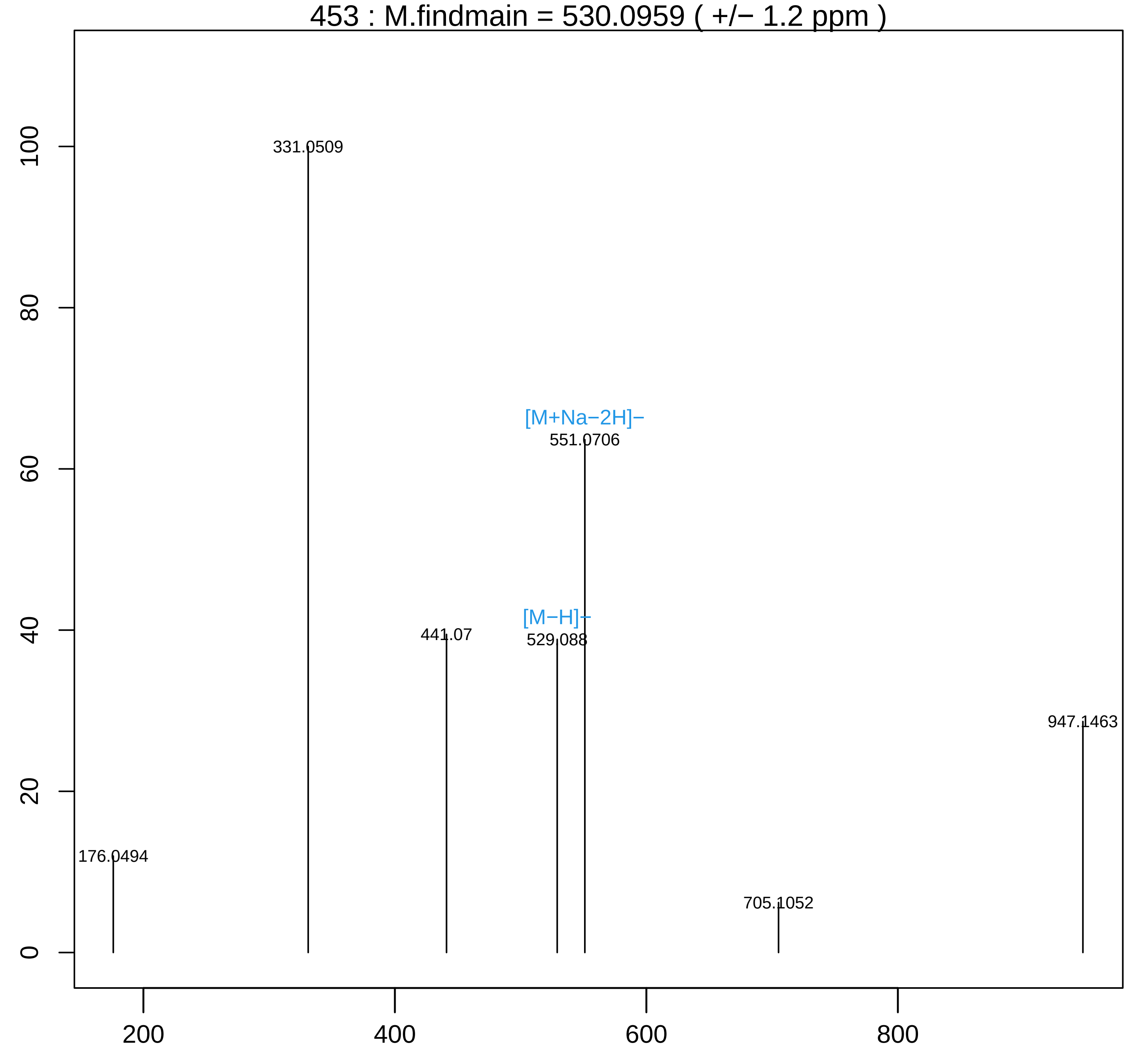

453 : M.findmain = 530.0959 (+/− 1.2 ppm)
331.0509
[M+Na−2H]−
551.0706
[M−H]−
529.088
441.07
947.1463
176.0494
705.1052
0
20
40
60
80
100
200
400
600
800

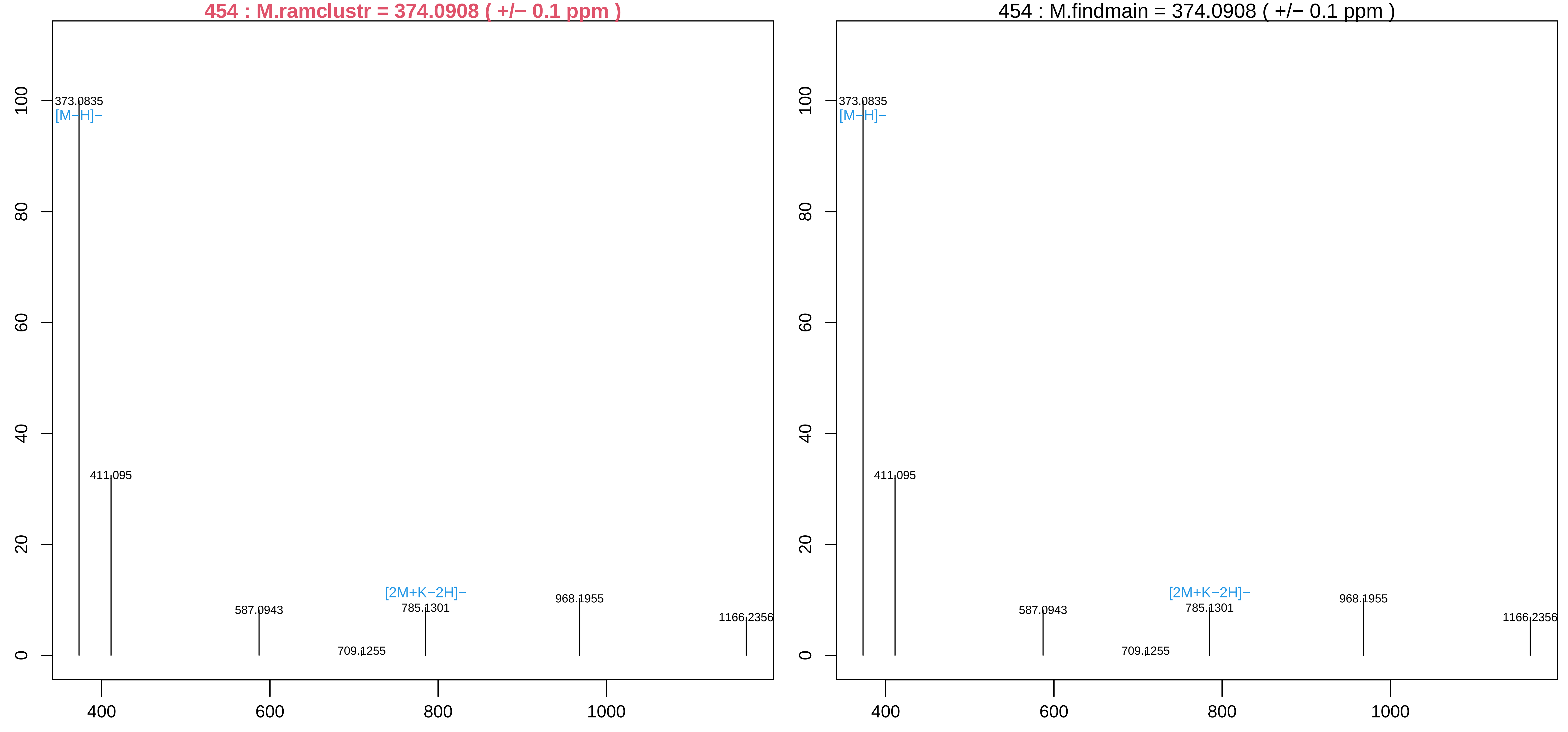
454 : M.ramclustr = 374.0908 (+/− 0.1 ppm)
373.0835
[M−H]−
411.095
587.0943
709.1255
[2M+K−2H]−
785.1301
968.1955
1166.2356
0
20
40
60
80
100
400
600
800
1000
454 : M.findmain = 374.0908 (+/− 0.1 ppm)
373.0835
[M−H]−
411.095
587.0943
709.1255
[2M+K−2H]−
785.1301
968.1955
1166.2356
0
20
40
60
80
100
400
600
800
1000

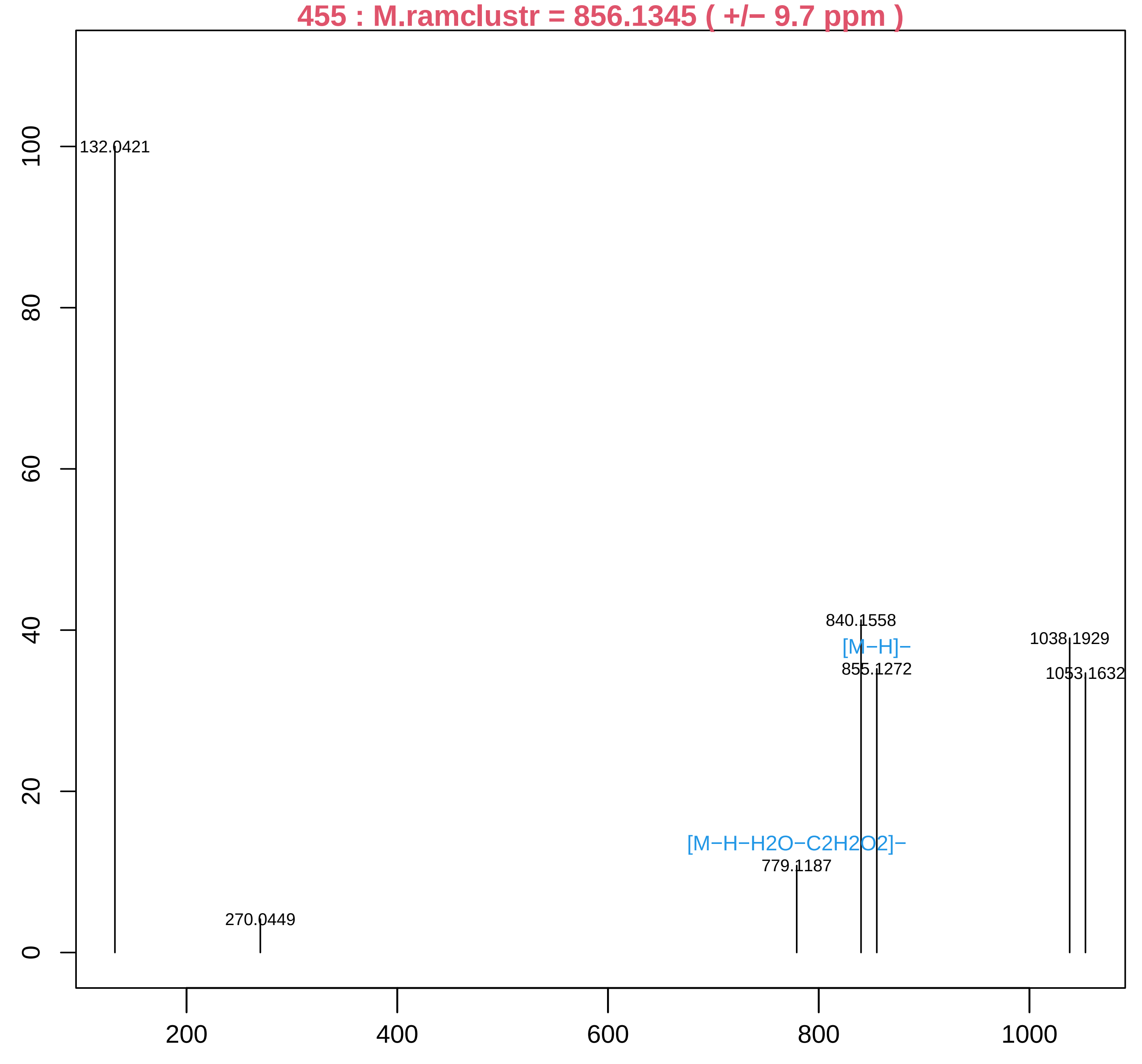
455 : M.ramclustr = 856.1345 (+/− 9.7 ppm)
132.0421
270.0449
779.1187
[M−H−H2O−C2H2O2]−
840.1558
[M−H]−
855.1272
1038.1929
1053.1632
0
20
40
60
80
100
200
400
600
800
1000

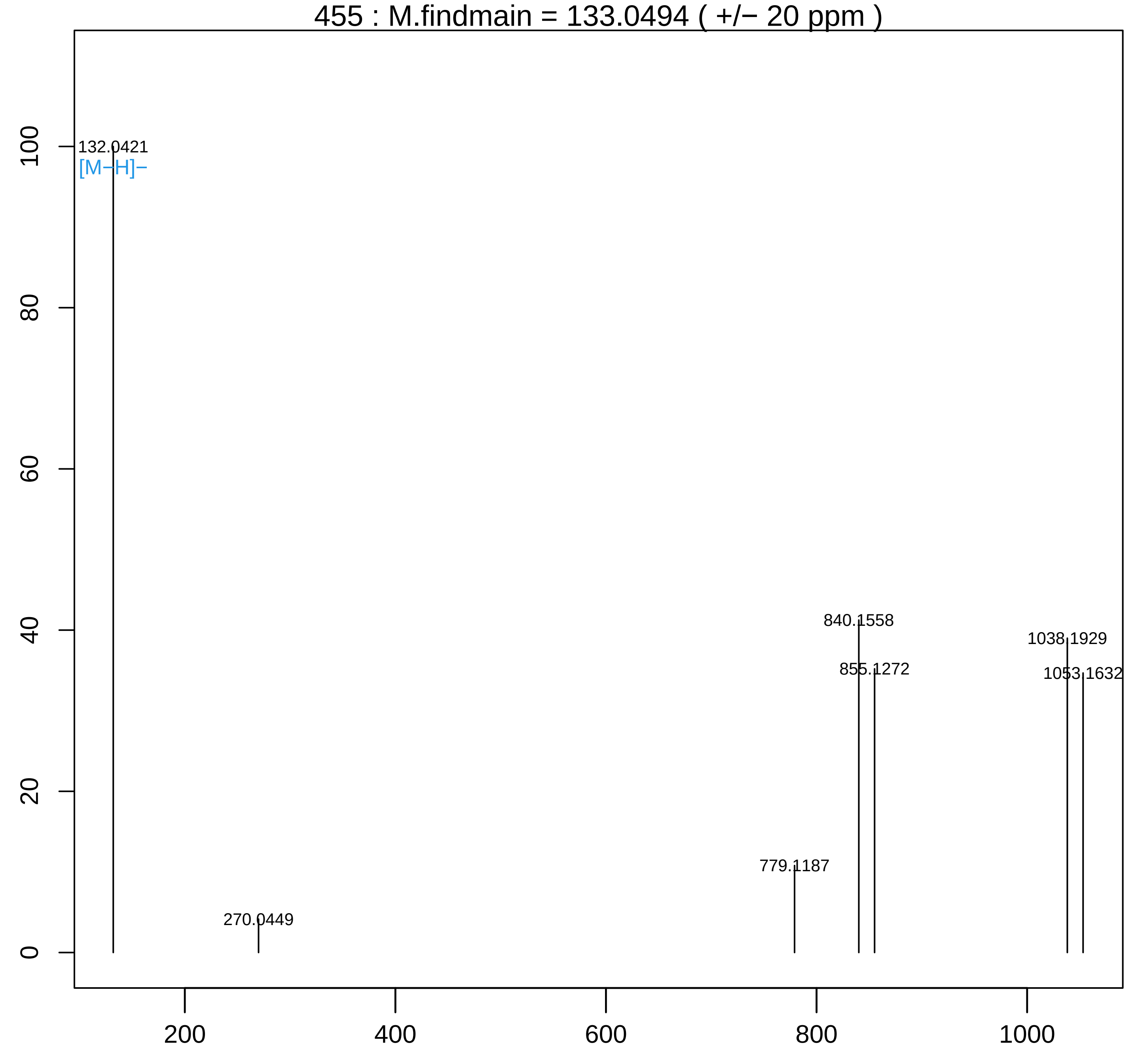
455 : M.findmain = 133.0494 (+/− 20 ppm)
132.0421
[M−H]−
270.0449
779.1187
840.1558
855.1272
1038.1929
1053.1632
0
20
40
60
80
100
200
400
600
800
1000

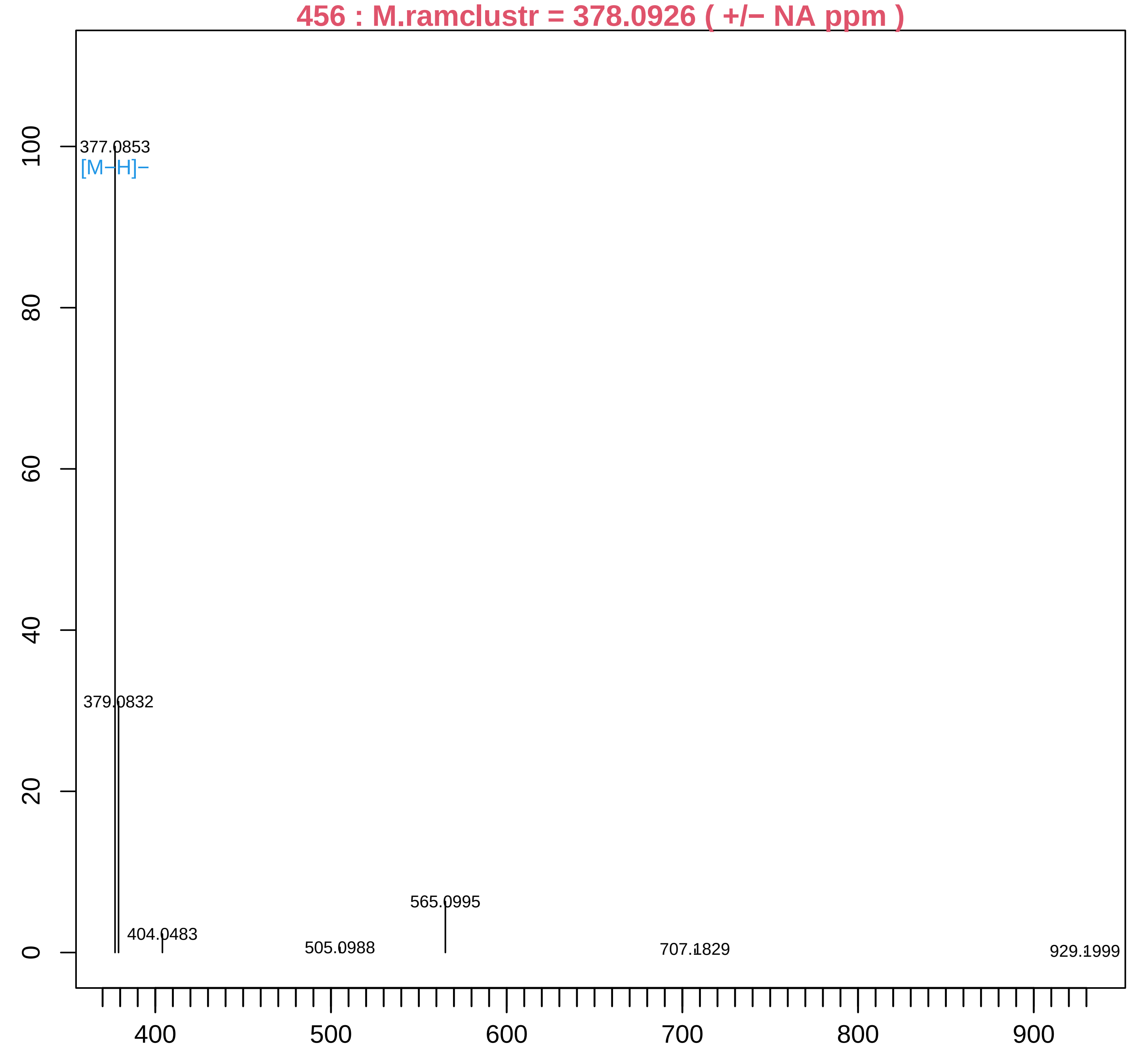
456 : M.ramclustr = 378.0926 (+/− NA ppm)
377.0853
[M−H]−
379.0832
404.0483
505.0988
565.0995
707.1829
929.1999
0
20
40
60
80
100
400
500
600
700
800
900

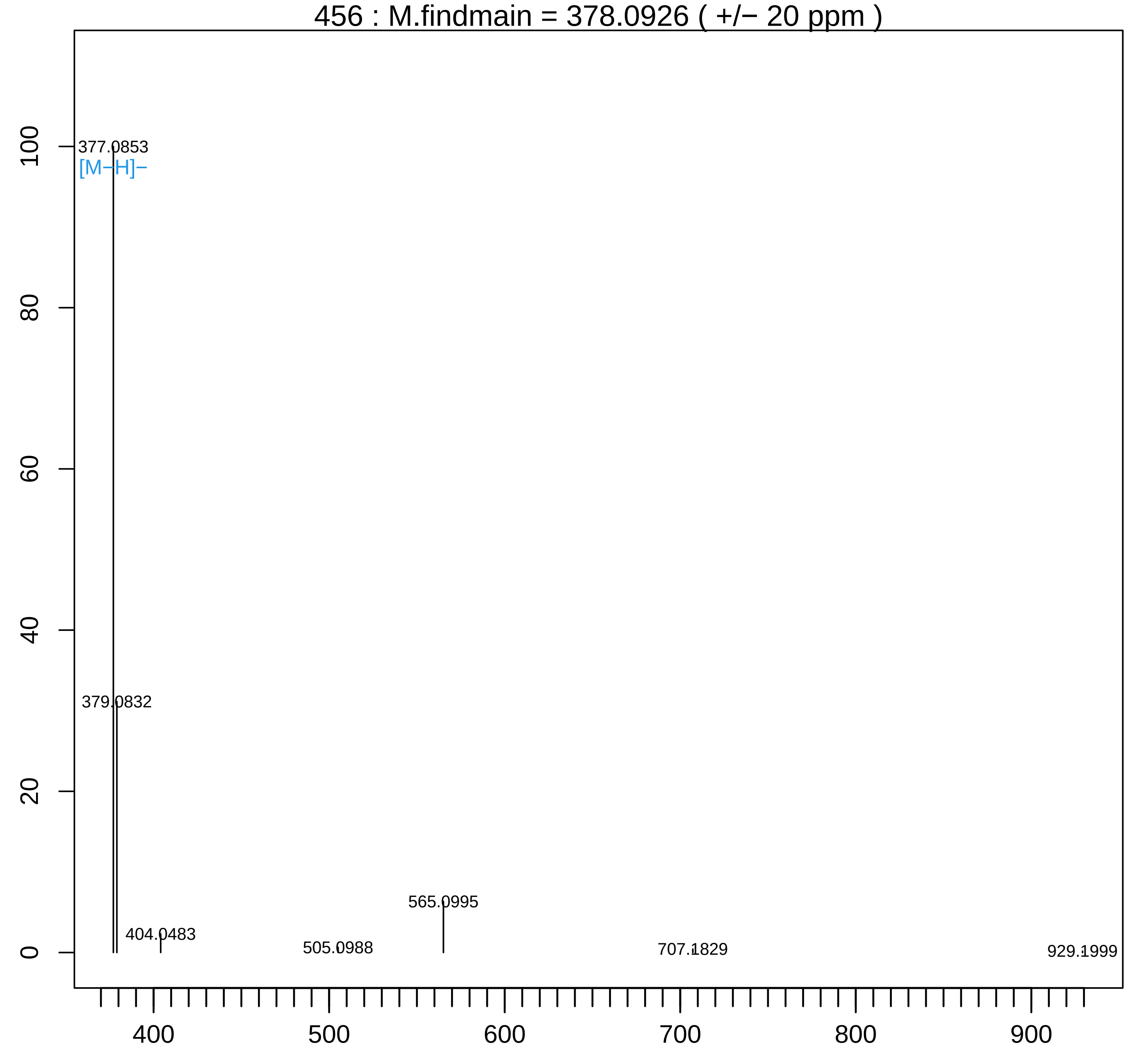
456 : M.findmain = 378.0926 (+/− 20 ppm)
377.0853
[M−H]−
379.0832
404.0483
505.0988
565.0995
707.1829
929.1999
0
20
40
60
80
100
400
500
600
700
800
900

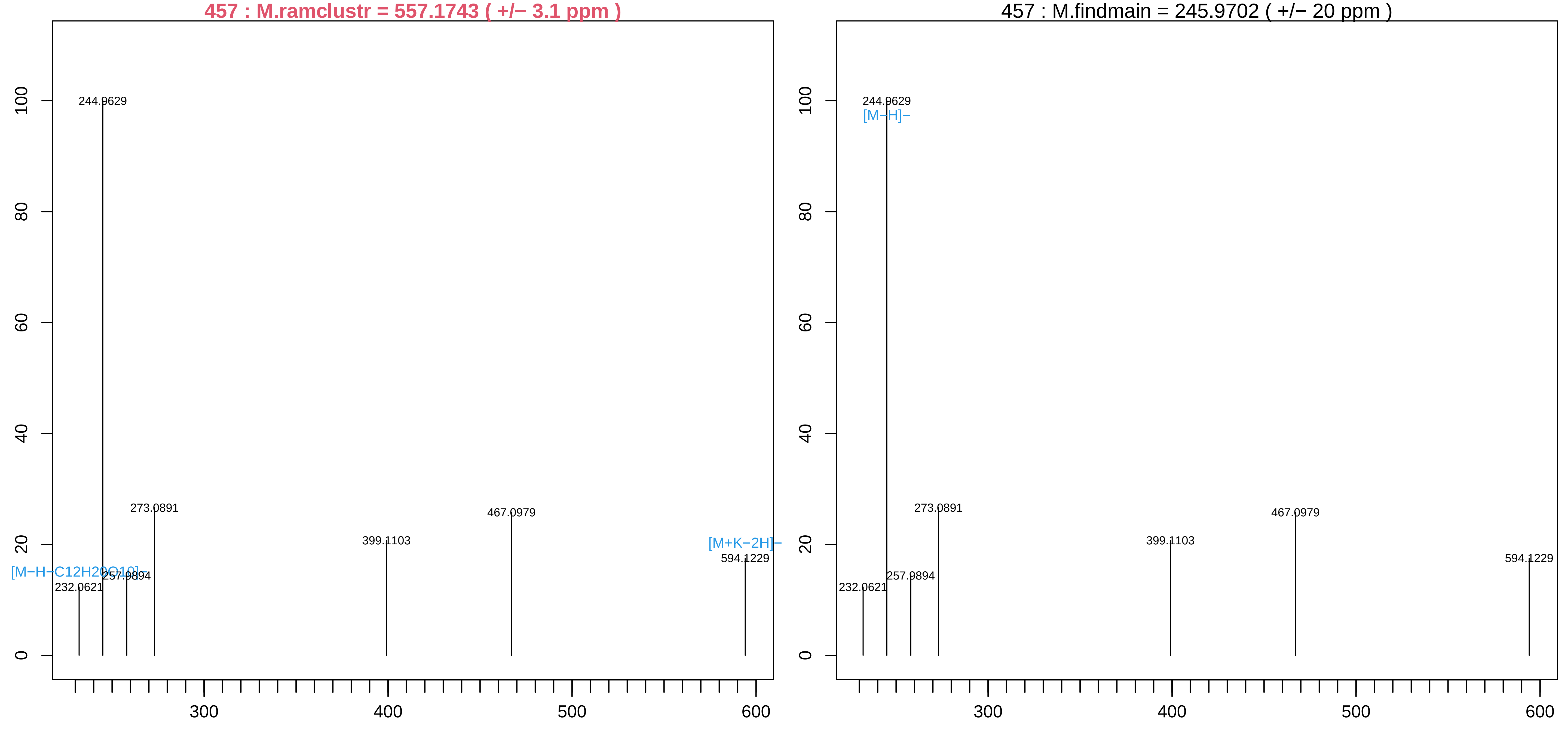
457 : M.ramclustr = 557.1743 (+/− 3.1 ppm)
244.9629
273.0891
467.0979
399.1103
[M+K−2H]−
594.1229
[M−H−C12H20O10]−
257.9894
232.0621
0
20
40
60
80
100
300
400
500
600
457 : M.findmain = 245.9702 (+/− 20 ppm)
244.9629
[M−H]−
273.0891
467.0979
399.1103
594.1229
257.9894
232.0621
0
20
40
60
80
100
300
400
500
600

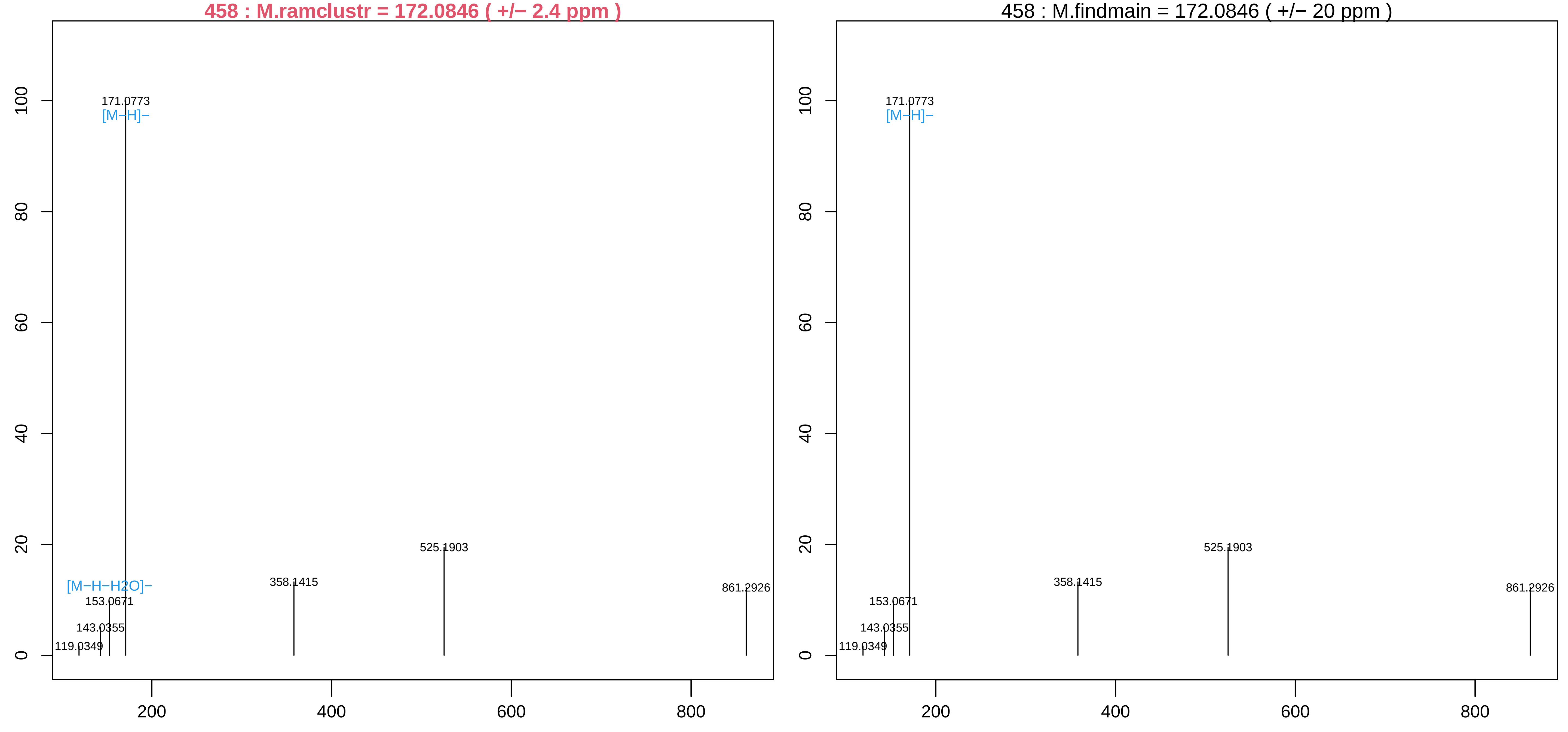

458 : M.ramclustr = 172.0846 (+/− 2.4 ppm)
171.0773
[M−H]−
[M−H−H2O]−
153.0671
143.0355
119.0349
358.1415
525.1903
861.2926
0
20
40
60
80
100
200
400
600
800
458 : M.findmain = 172.0846 (+/− 20 ppm)
171.0773
[M−H]−
153.0671
143.0355
119.0349
358.1415
525.1903
861.2926
0
20
40
60
80
100
200
400
600
800

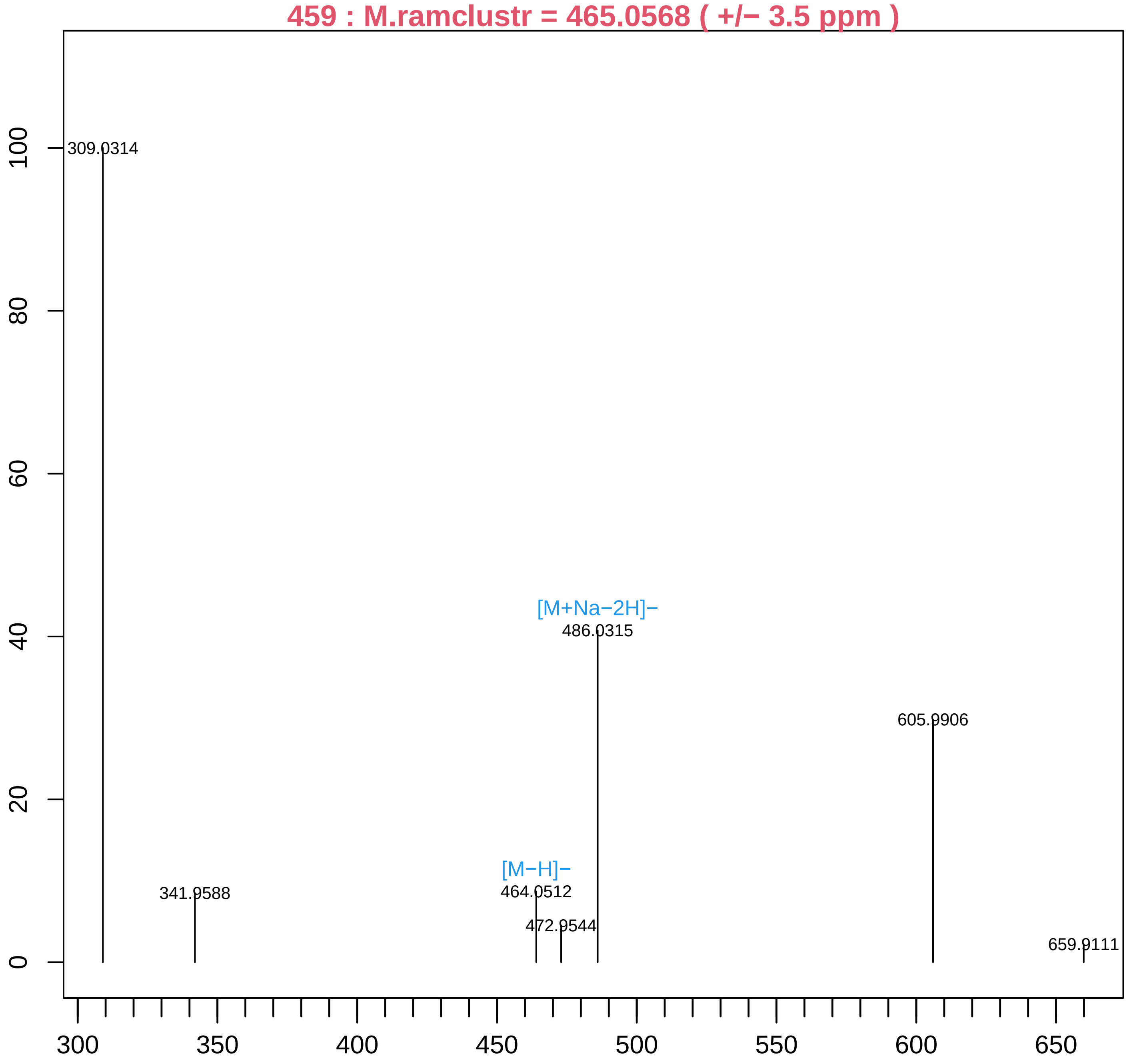
459 : M.ramclustr = 465.0568 (+/− 3.5 ppm)
309.0314
341.9588
[M−H]−
464.0512
472.9544
[M+Na−2H]−
486.0315
605.9906
659.9111
0
20
40
60
80
100
300
350
400
450
500
550
600
650

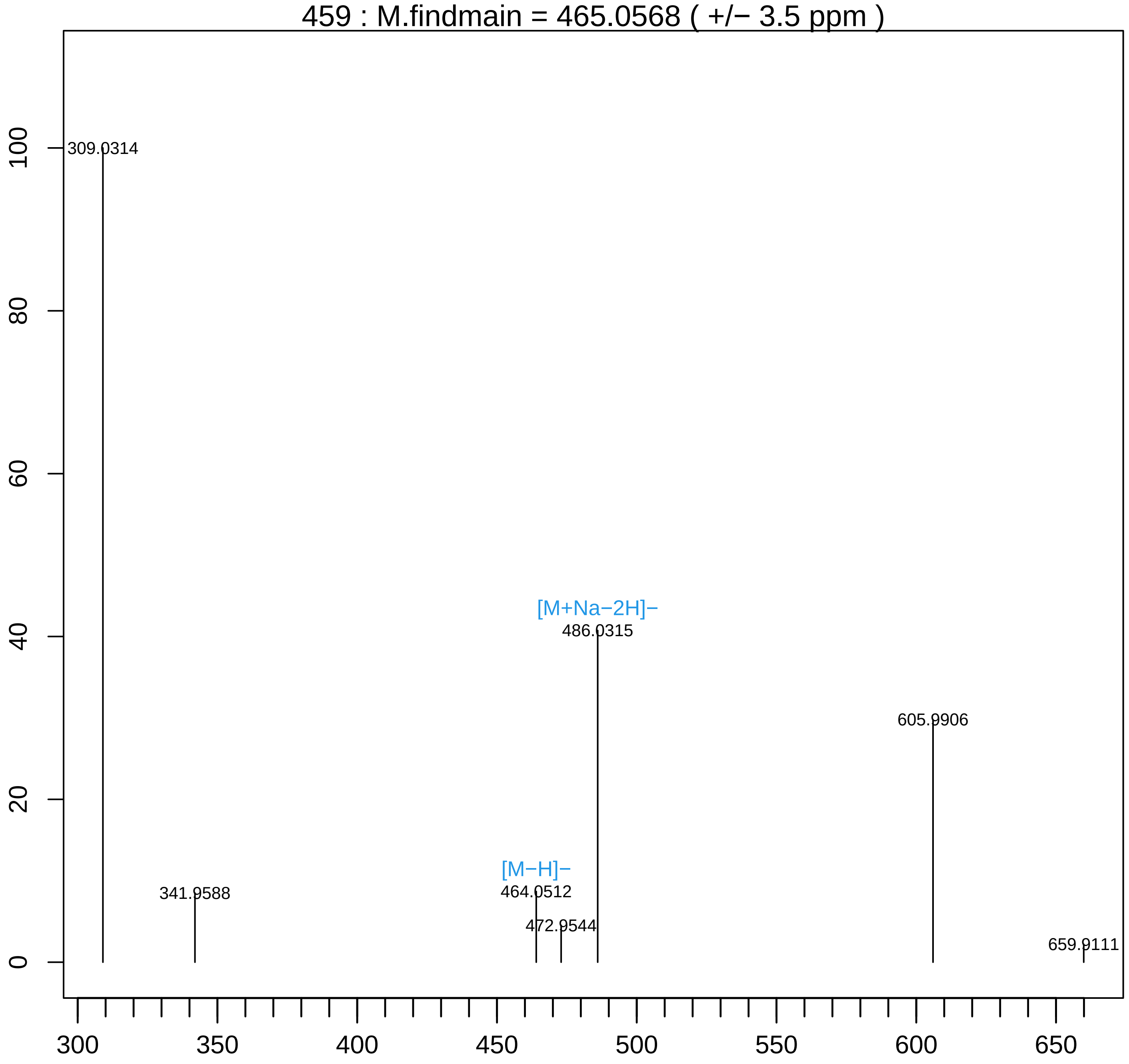
459 : M.findmain = 465.0568 (+/− 3.5 ppm)
309.0314
341.9588
[M−H]−
464.0512
472.9544
[M+Na−2H]−
486.0315
605.9906
659.9111
0
20
40
60
80
100
300
350
400
450
500
550
600
650

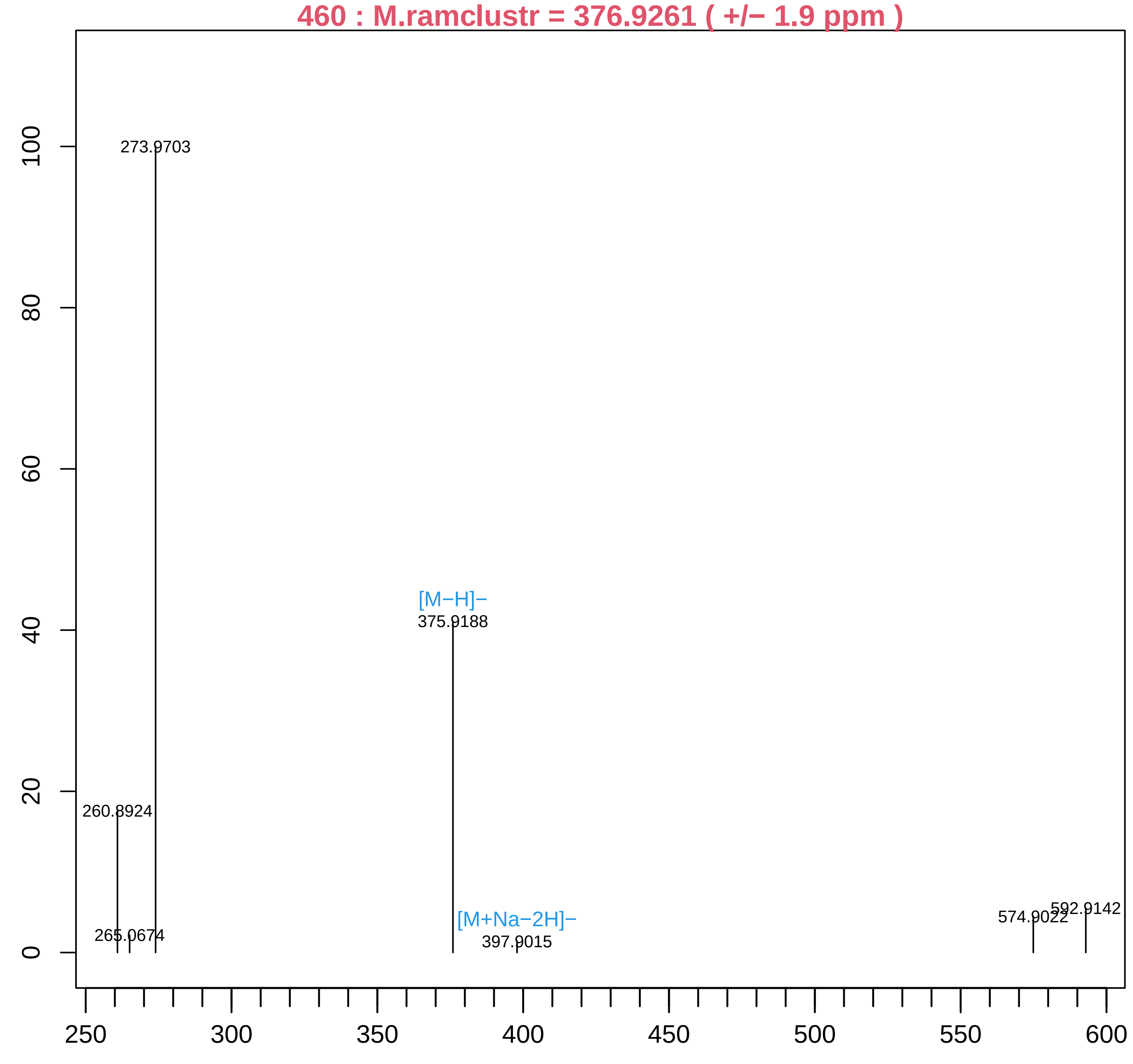

460 : M.ramclustr = 376.9261 (+/− 1.9 ppm)
273.9703
260.8924
265.0674
[M−H]−
375.9188
[M+Na−2H]−
397.9015
574.9022
592.9142

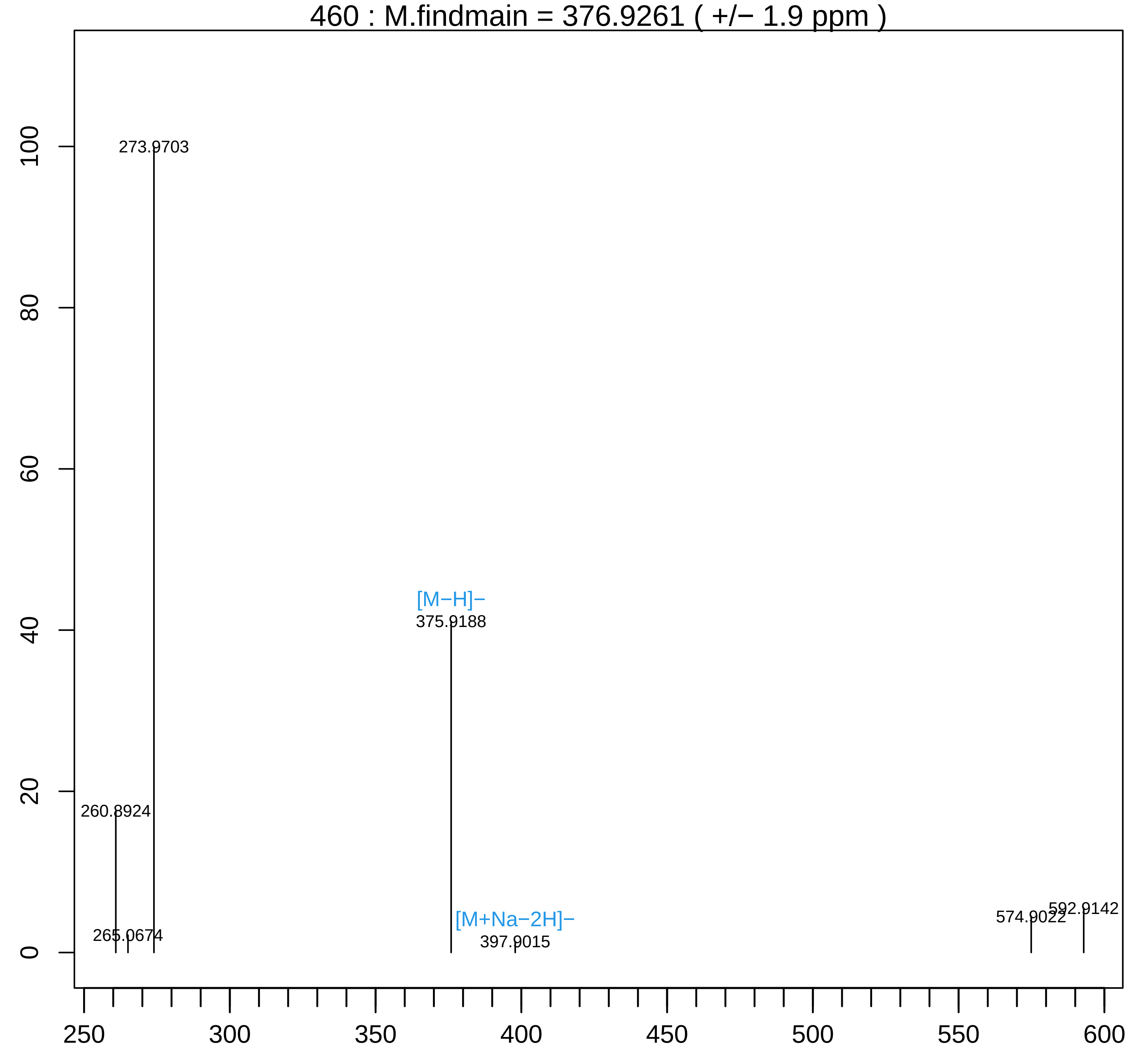

460 : M.findmain = 376.9261 (+/− 1.9 ppm)
273.9703
260.8924
265.0674
[M−H]−
375.9188
[M+Na−2H]−
397.9015
574.9022
592.9142

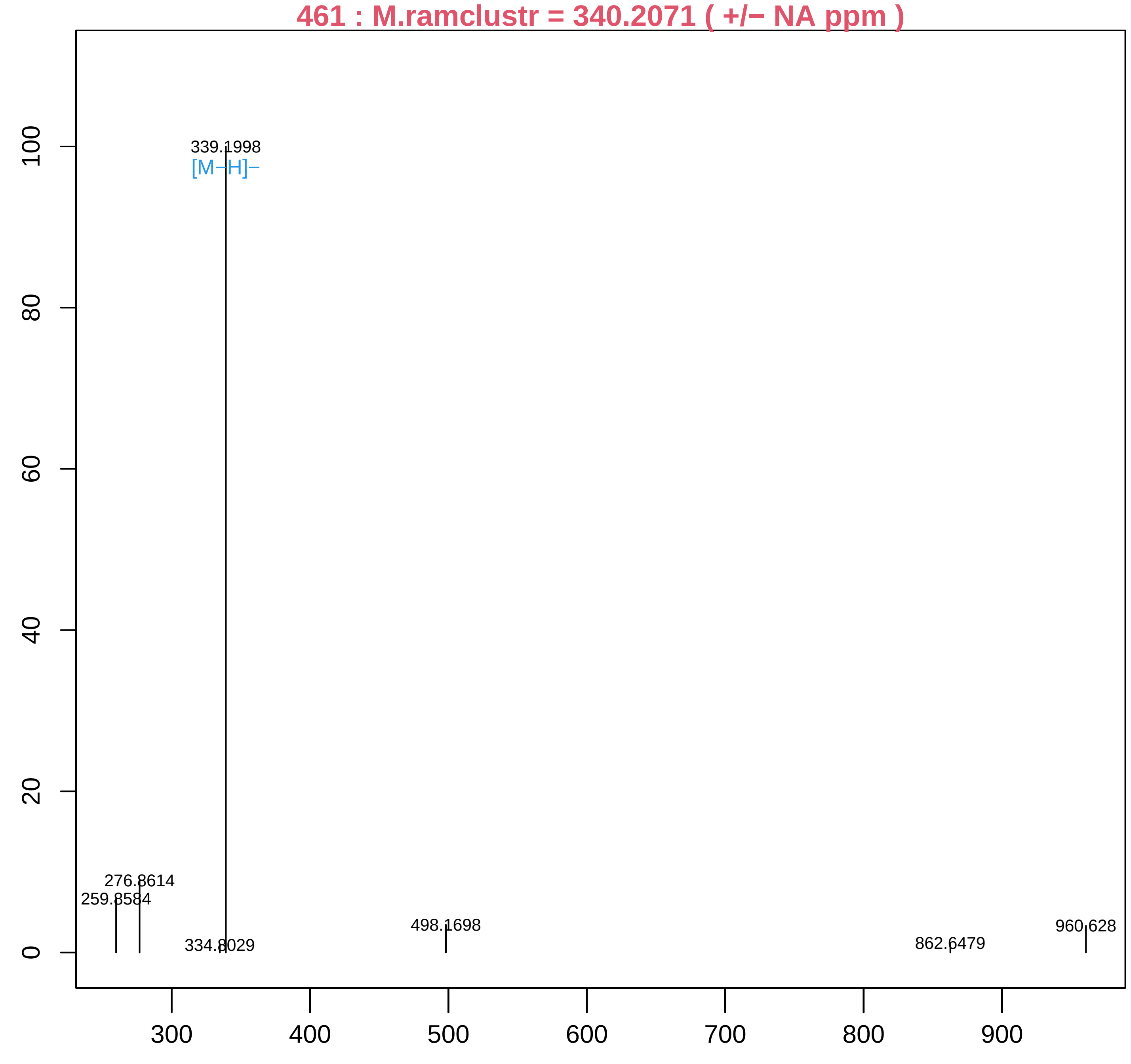
461 : M.ramclustr = 340.2071 (+/− NA ppm)
339.1998
[M−H]−
276.8614
259.8584
334.8029
498.1698
862.6479
960.628
0
20
40
60
80
100
300
400
500
600
700
800
900
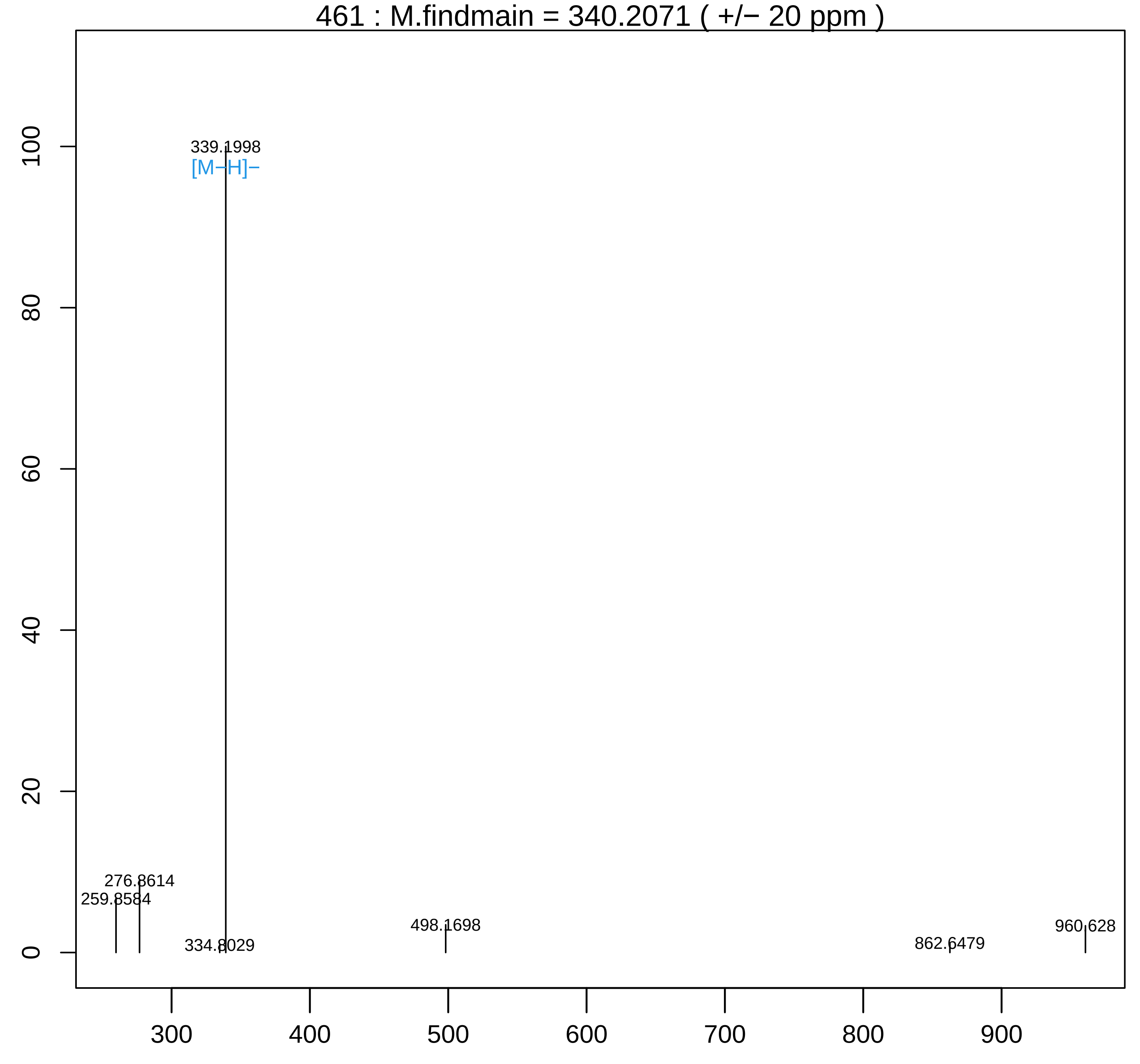
461 : M.findmain = 340.2071 (+/− 20 ppm)
339.1998
[M−H]−
276.8614
259.8584
334.8029
498.1698
862.6479
960.628
0
20
40
60
80
100
300
400
500
600
700
800
900

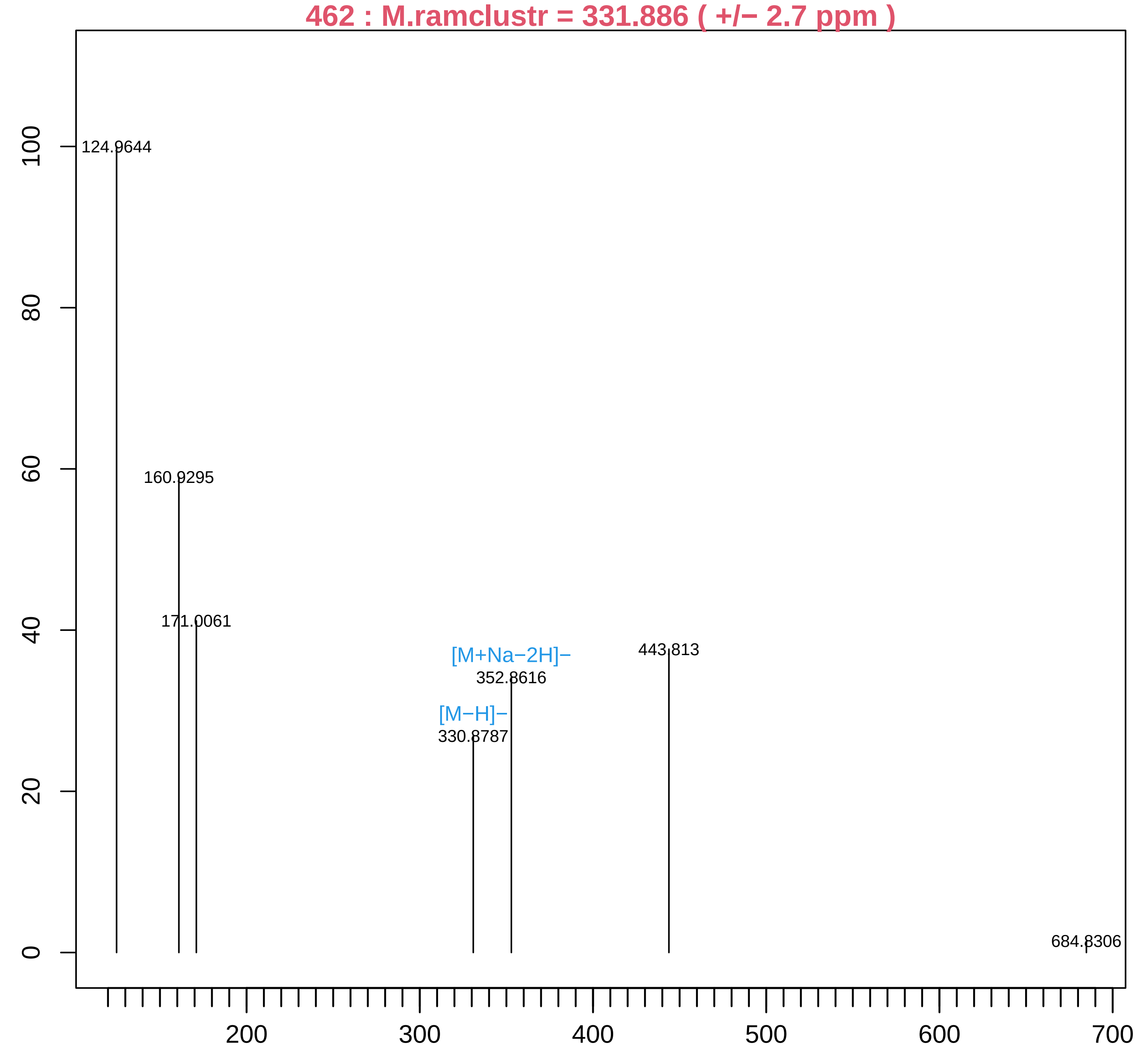
462 : M.ramclustr = 331.886 (+/− 2.7 ppm)
124.9644
160.9295
171.0061
[M+Na−2H]−
352.8616
443.813
[M−H]−
330.8787
684.8306
0
20
40
60
80
100
200
300
400
500
600
700

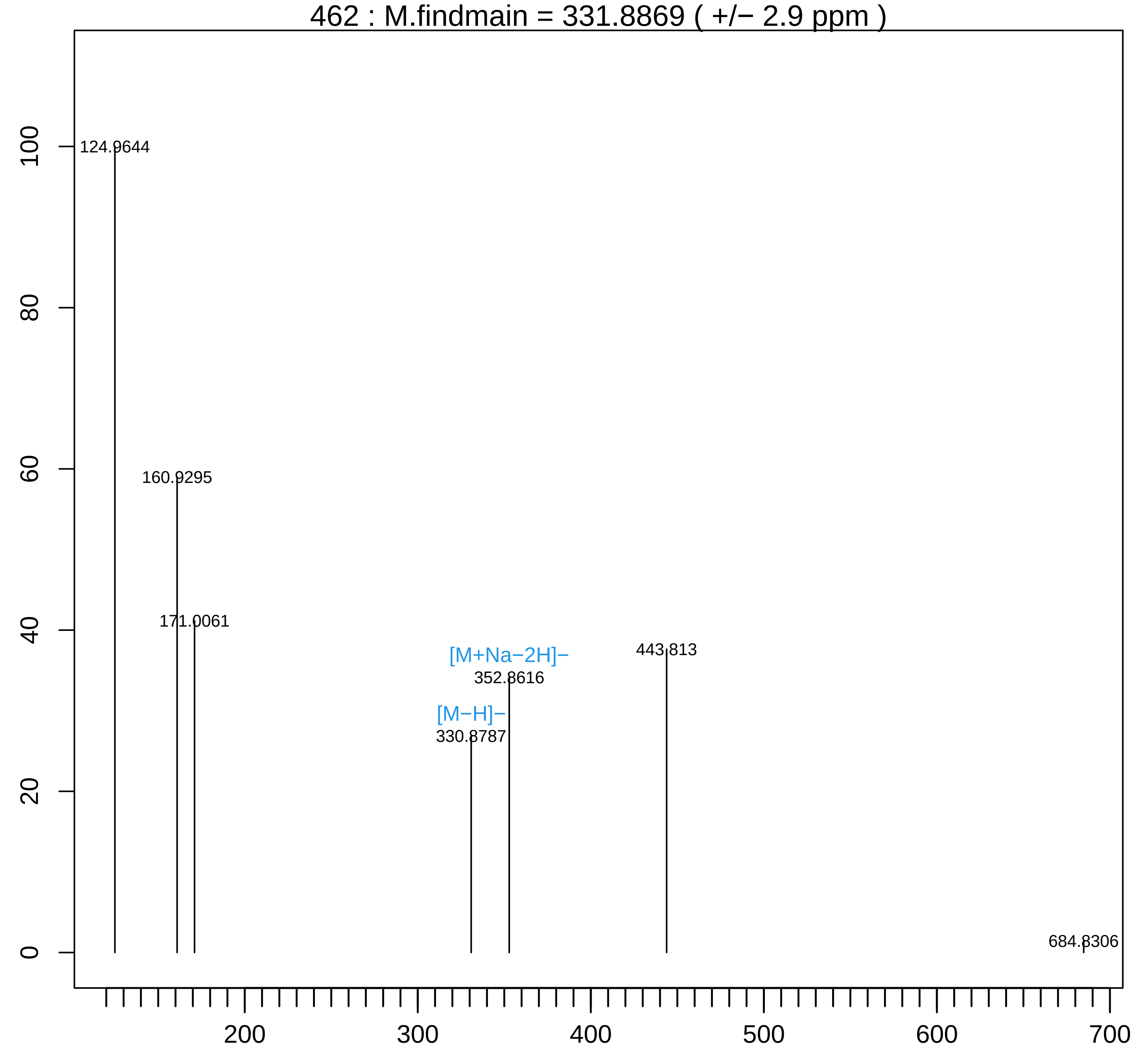
462 : M.findmain = 331.8869 (+/− 2.9 ppm)
124.9644
160.9295
171.0061
[M+Na−2H]−
352.8616
443.813
[M−H]−
330.8787
684.8306
0
20
40
60
80
100
200
300
400
500
600
700

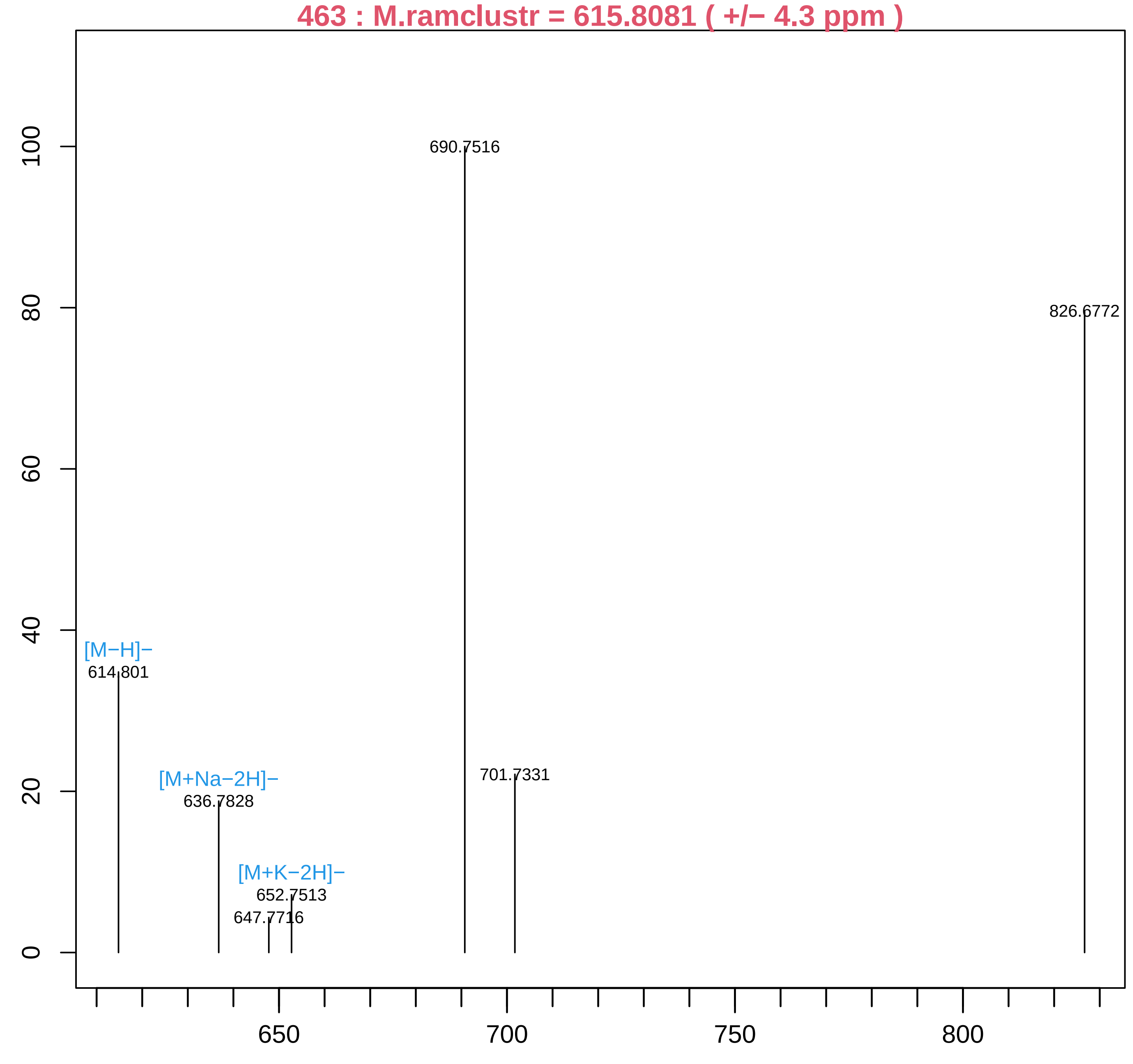
463 : M.ramclustr = 615.8081 (+/− 4.3 ppm)
690.7516
826.6772
[M−H]−
614.801
[M+Na−2H]−
636.7828
701.7331
[M+K−2H]−
652.7513
647.7716
0
20
40
60
80
100
650
700
750
800

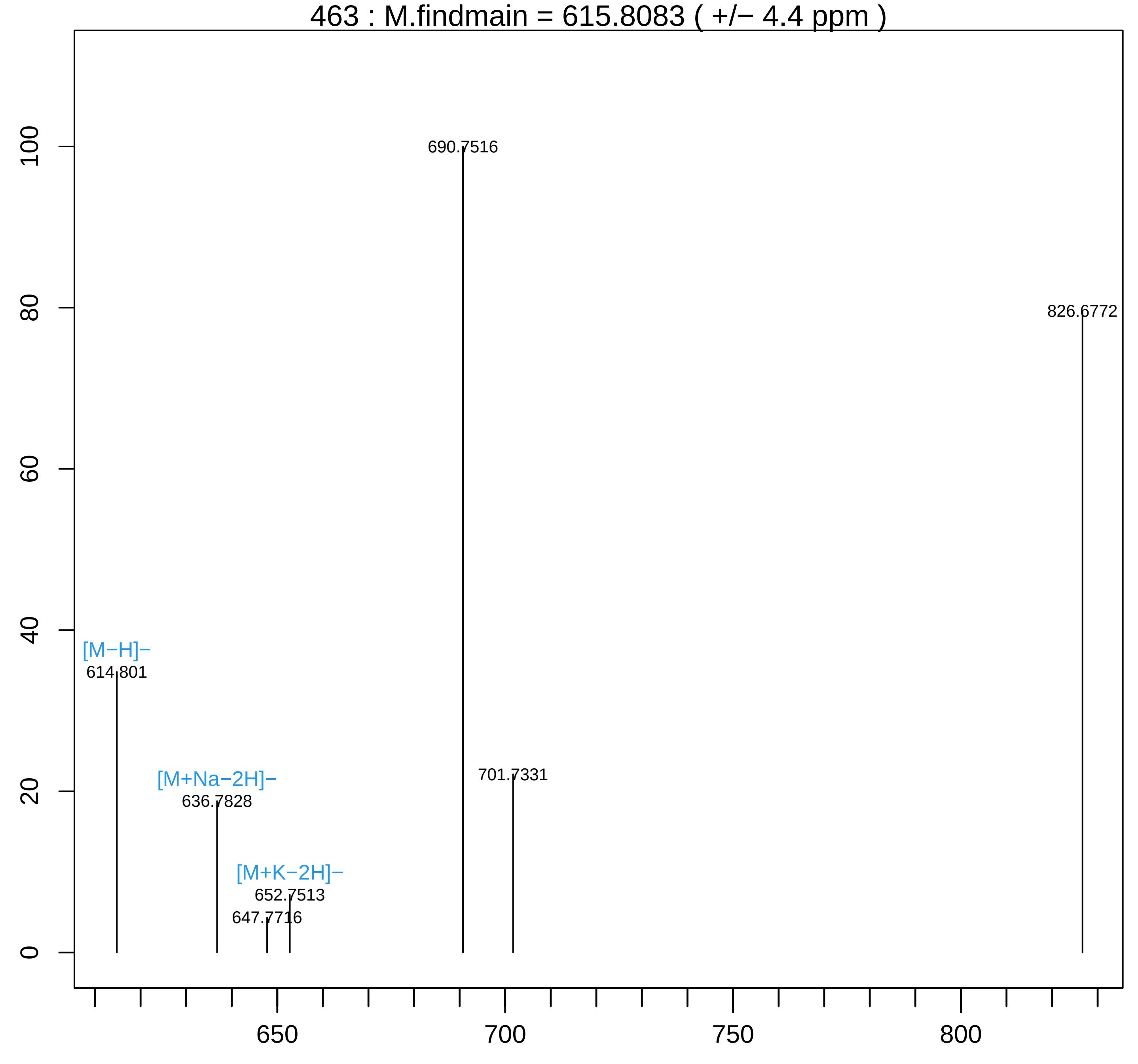
463 : M.findmain = 615.8083 (+/− 4.4 ppm)
690.7516
826.6772
[M−H]−
614.801
[M+Na−2H]−
636.7828
701.7331
[M+K−2H]−
652.7513
647.7716
0
20
40
60
80
100
650
700
750
800

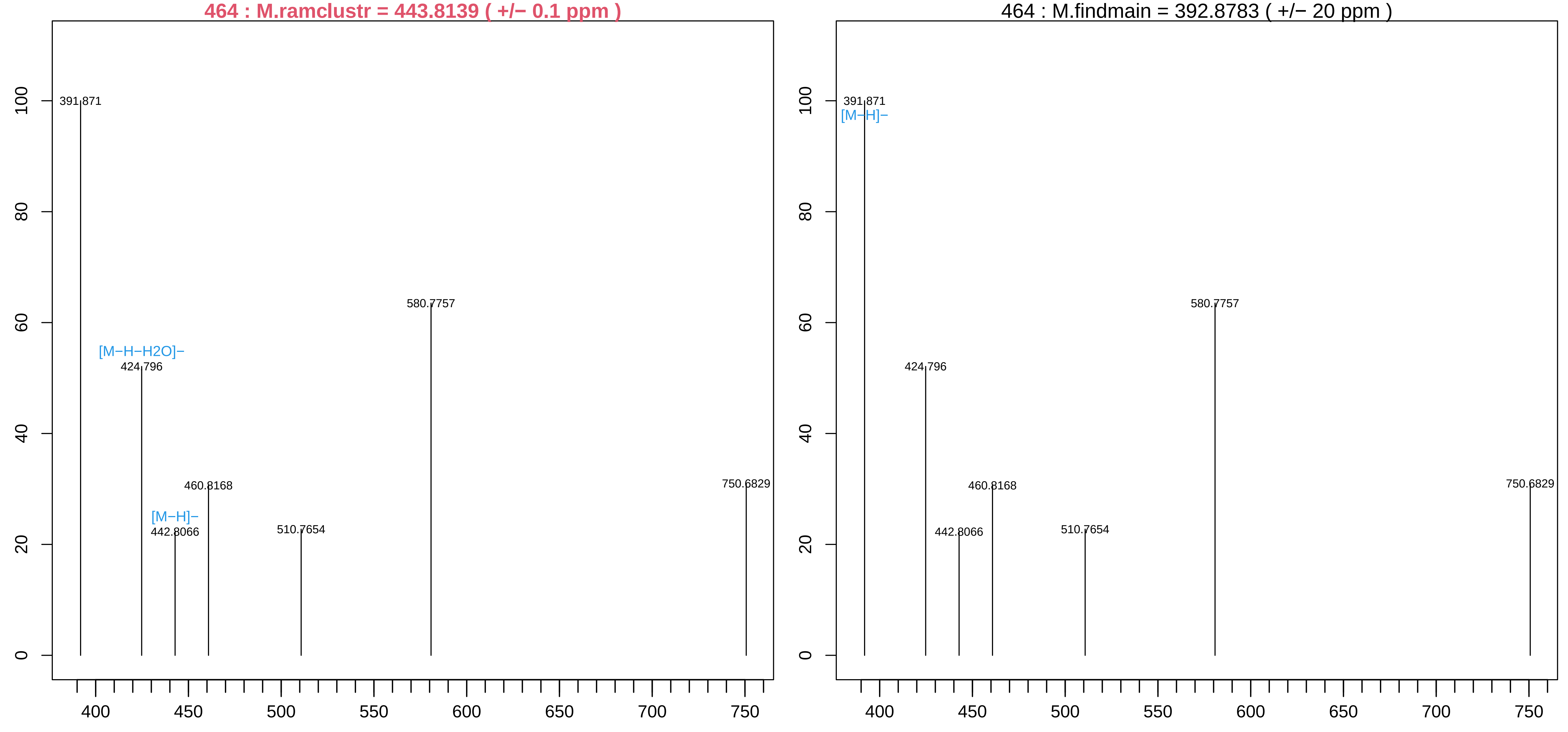
464 : M.ramclustr = 443.8139 (+/− 0.1 ppm)
391.871
[M−H−H2O]−
424.796
[M−H]−
442.8066
460.8168
510.7654
580.7757
750.6829
0
20
40
60
80
100
400
450
500
550
600
650
700
750
464 : M.findmain = 392.8783 (+/− 20 ppm)
391.871
[M−H]−
424.796
442.8066
460.8168
510.7654
580.7757
750.6829

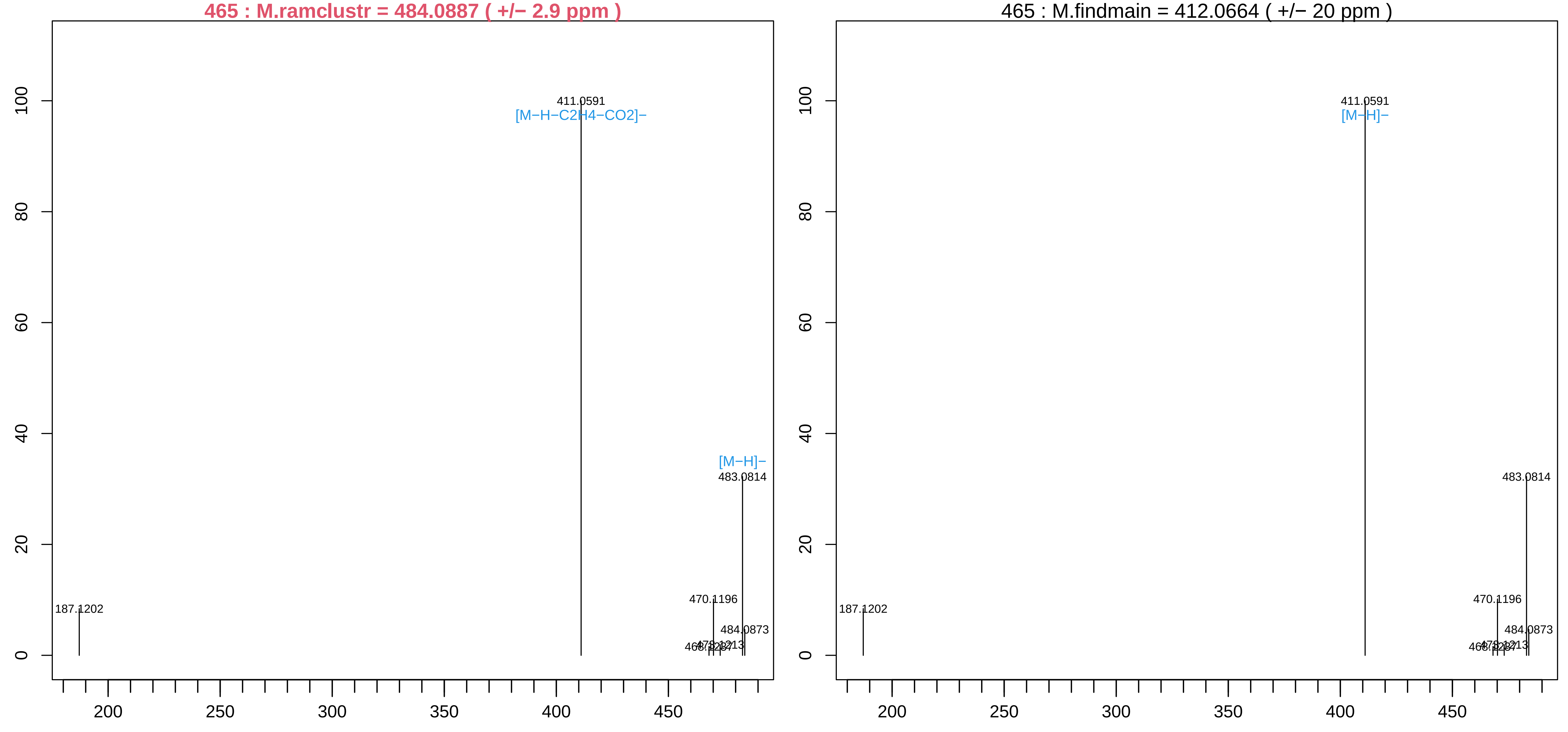
465 : M.ramclustr = 484.0887 (+/− 2.9 ppm)
411.0591
[M−H−C2H4−CO2]−
[M−H]−
483.0814
470.1196
187.1202
484.0873
465 : M.findmain = 412.0664 (+/− 20 ppm)
411.0591
[M−H]−
483.0814
470.1196
187.1202
484.0873
0
20
40
60
80
100
200
250
300
350
400
450

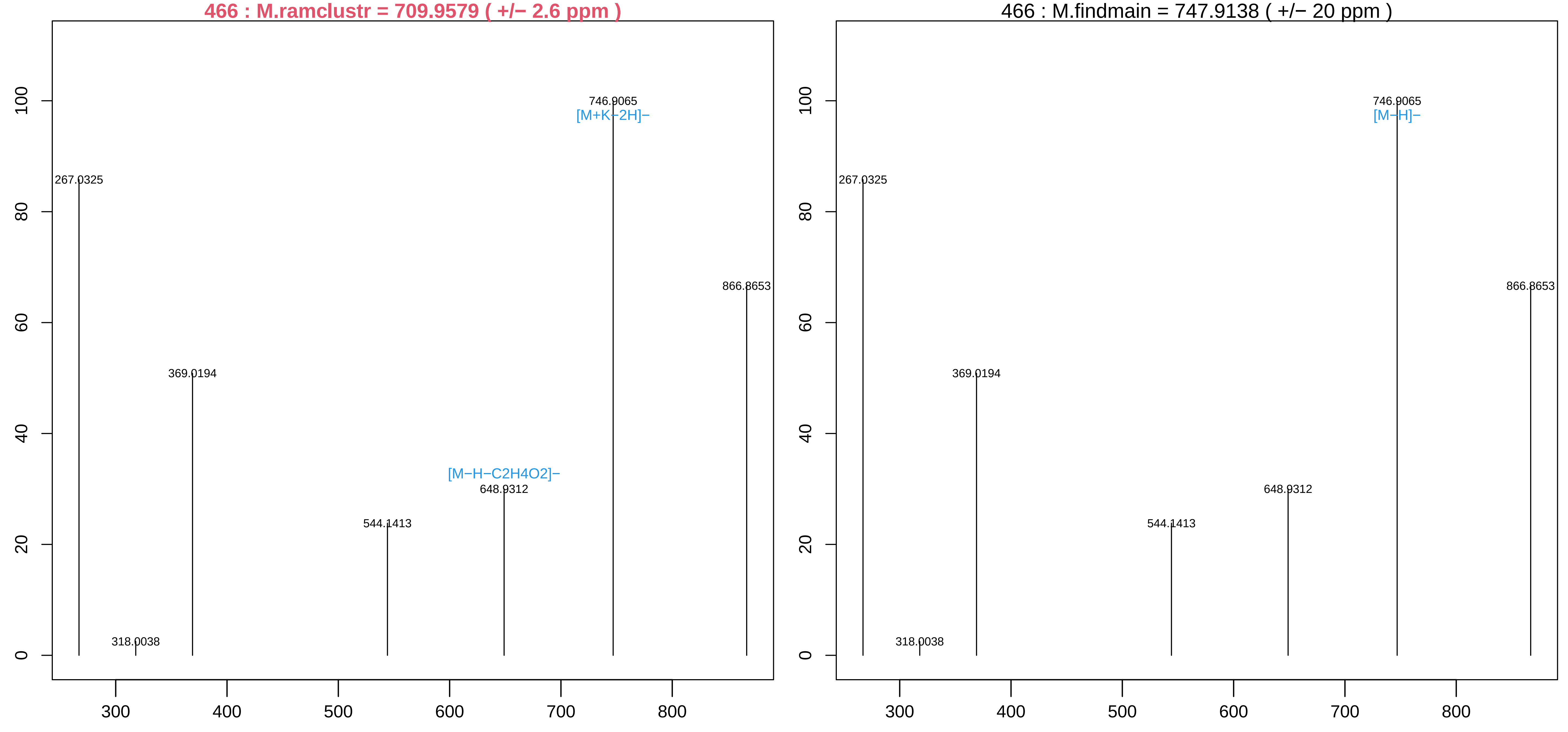
466 : M.ramclustr = 709.9579 (+/− 2.6 ppm)
746.9065
[M+K−2H]−
267.0325
866.8653
369.0194
[M−H−C2H4O2]−
648.9312
544.1413
318.0038
466 : M.findmain = 747.9138 (+/− 20 ppm)
746.9065
[M−H]−
267.0325
866.8653
369.0194
648.9312
544.1413
318.0038
0
20
40
60
80
100
300
400
500
600
700
800

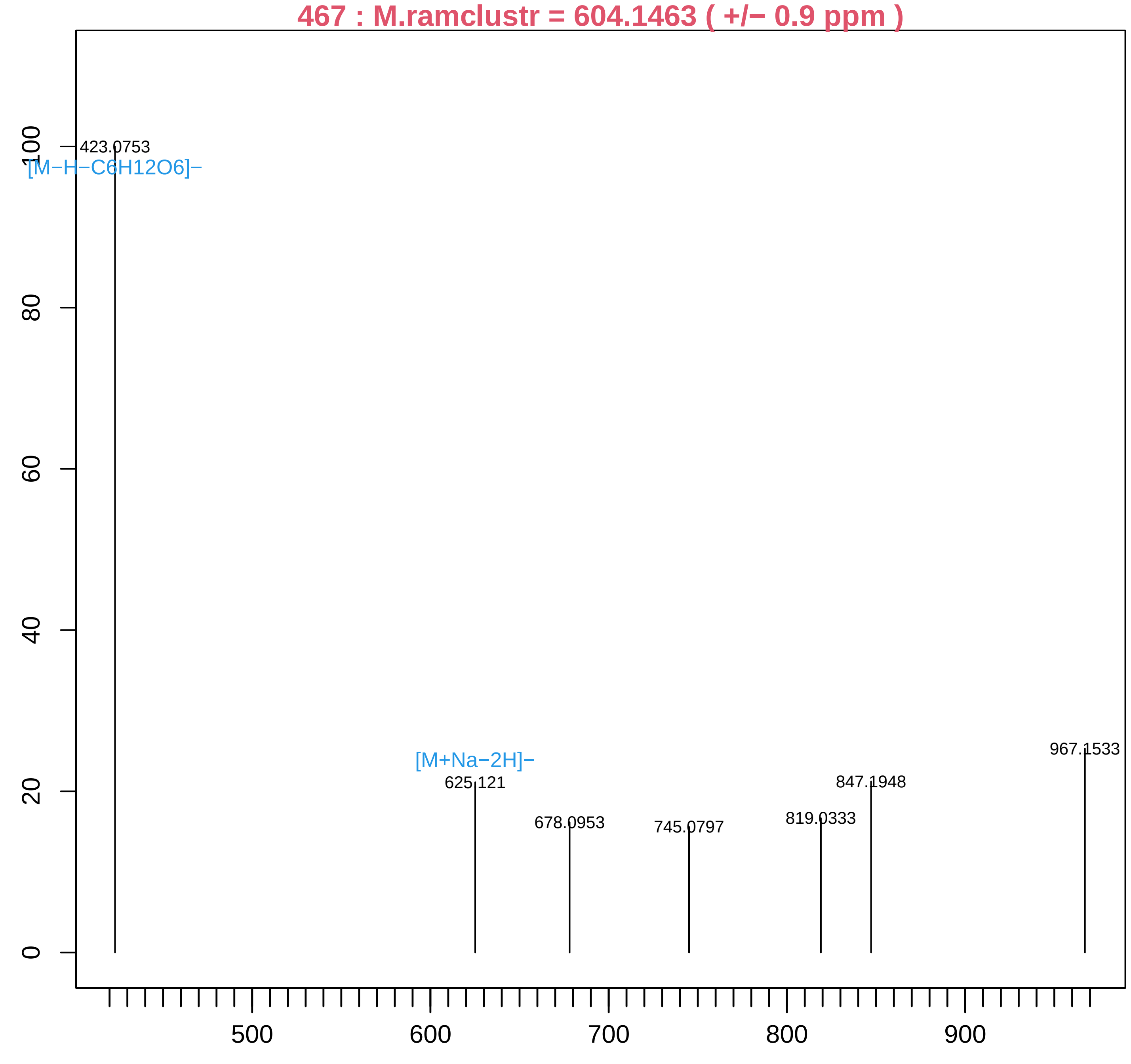

467 : M.ramclustr = 604.1463 (+/− 0.9 ppm)
423.0753
[M−H−C6H12O6]−
[M+Na−2H]−
625.121
678.0953
745.0797
819.0333
847.1948
967.1533
0
20
40
60
80
100
500
600
700
800
900

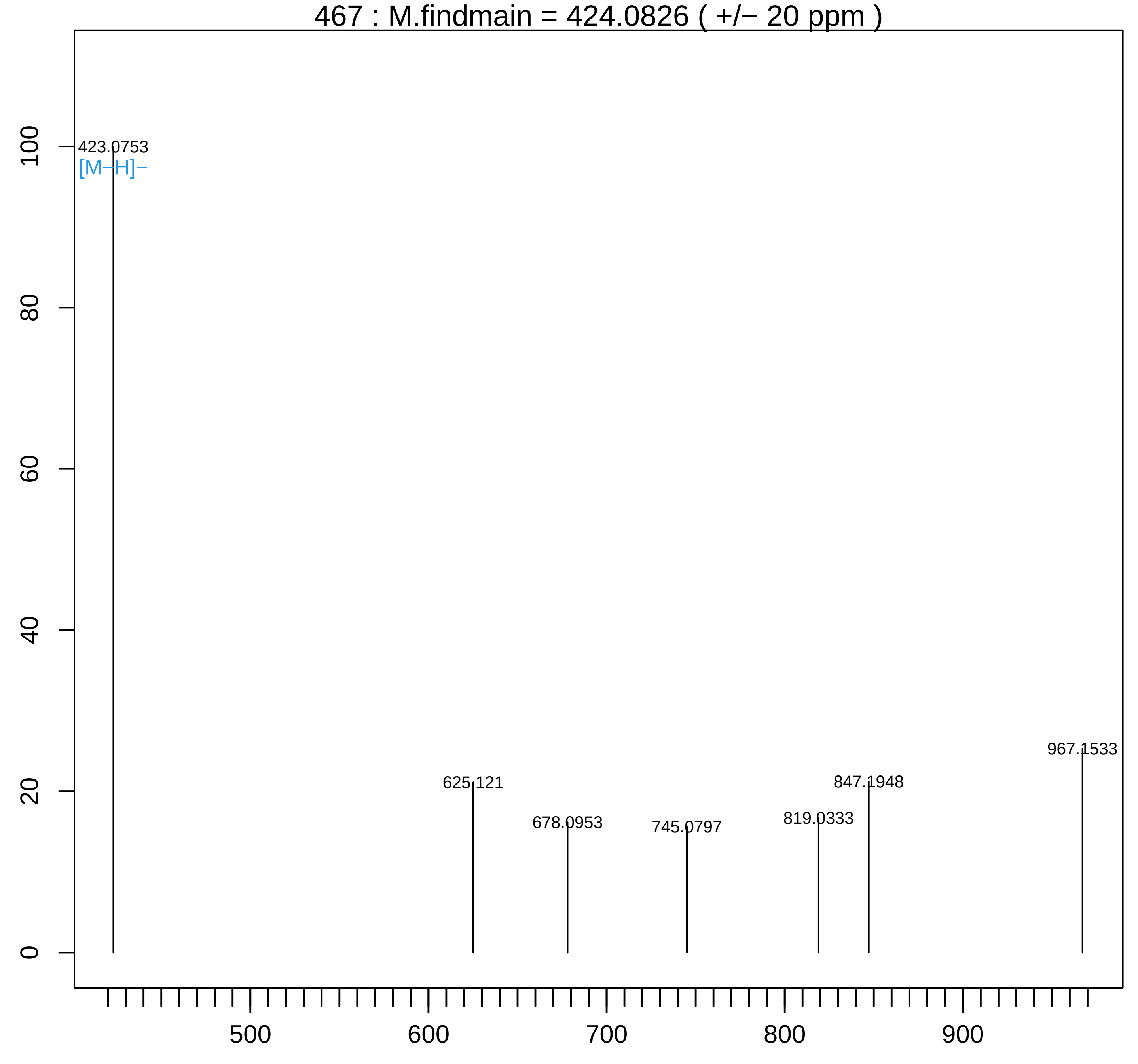

467 : M.findmain = 424.0826 (+/− 20 ppm)
423.0753
[M−H]−
625.121
678.0953
745.0797
819.0333
847.1948
967.1533
0
20
40
60
80
100
500
600
700
800
900

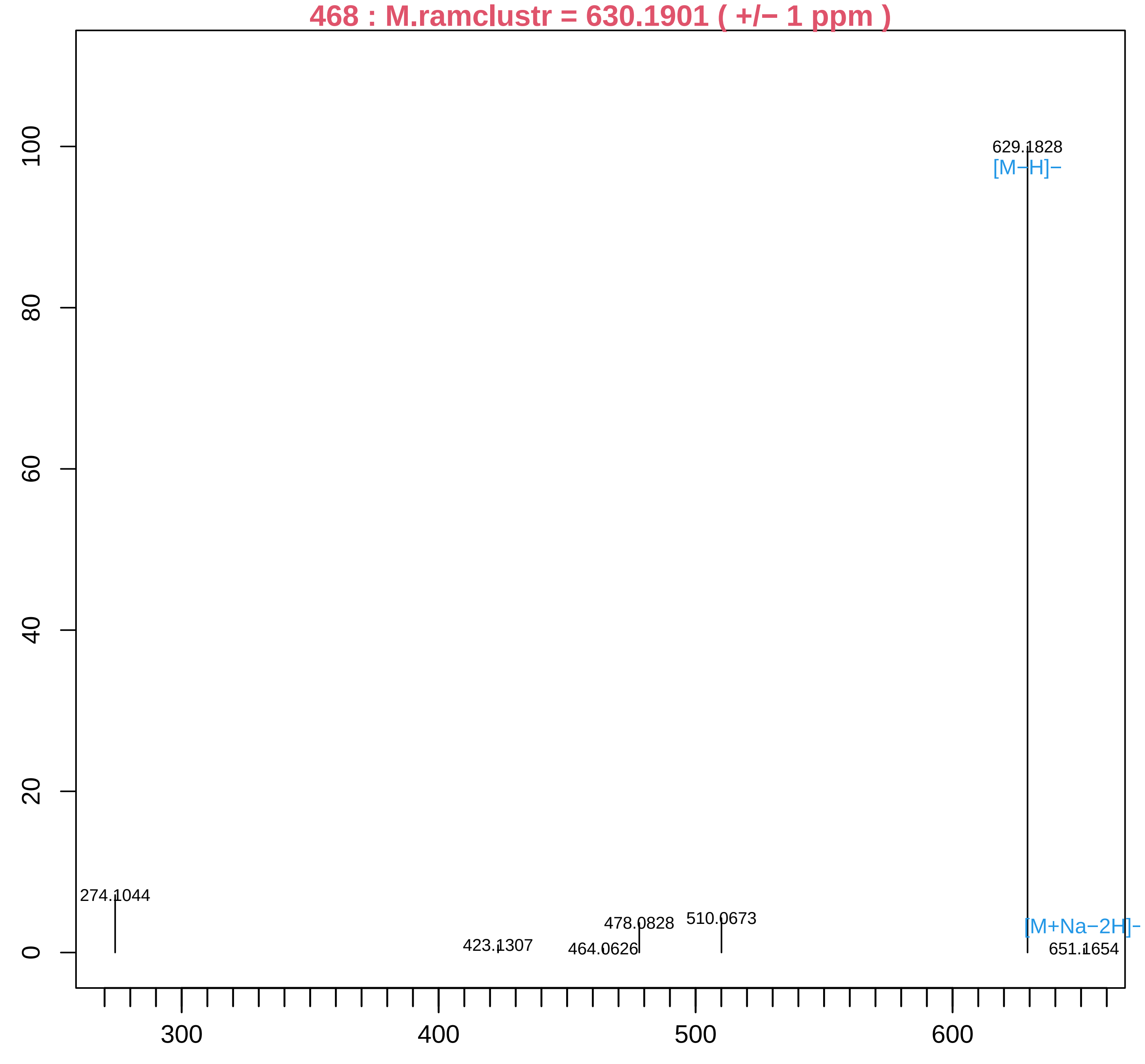
468 : M.ramclustr = 630.1901 (+/− 1 ppm)
629.1828
[M−H]−
274.1044
423.1307
464.0626
478.0828
510.0673
[M+Na−2H]−
651.1654
0
20
40
60
80
100
300
400
500
600

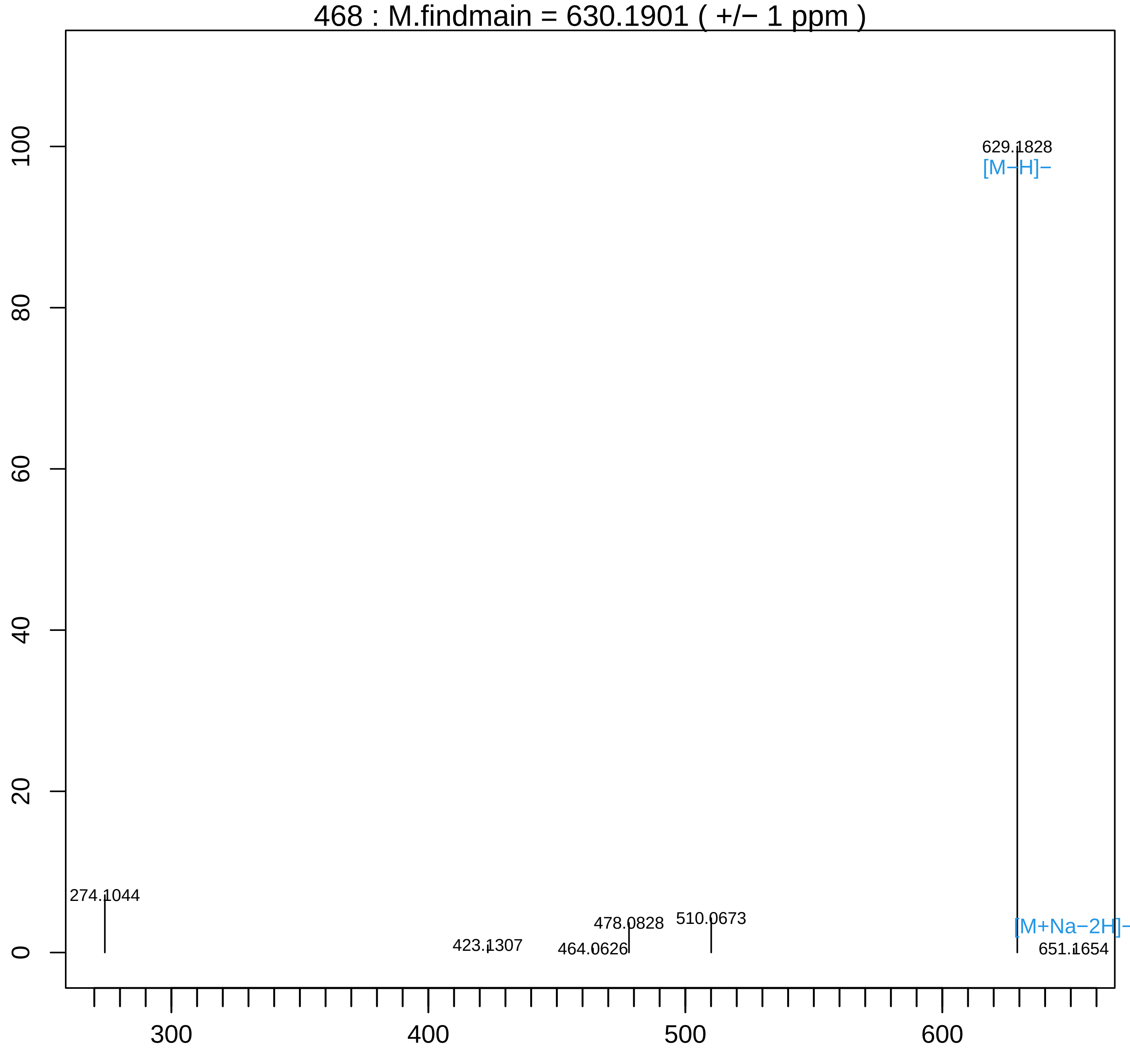
468 : M.findmain = 630.1901 (+/− 1 ppm)
629.1828
[M−H]−
274.1044
423.1307
464.0626
478.0828
510.0673
[M+Na−2H]−
651.1654
0
20
40
60
80
100
300
400
500
600

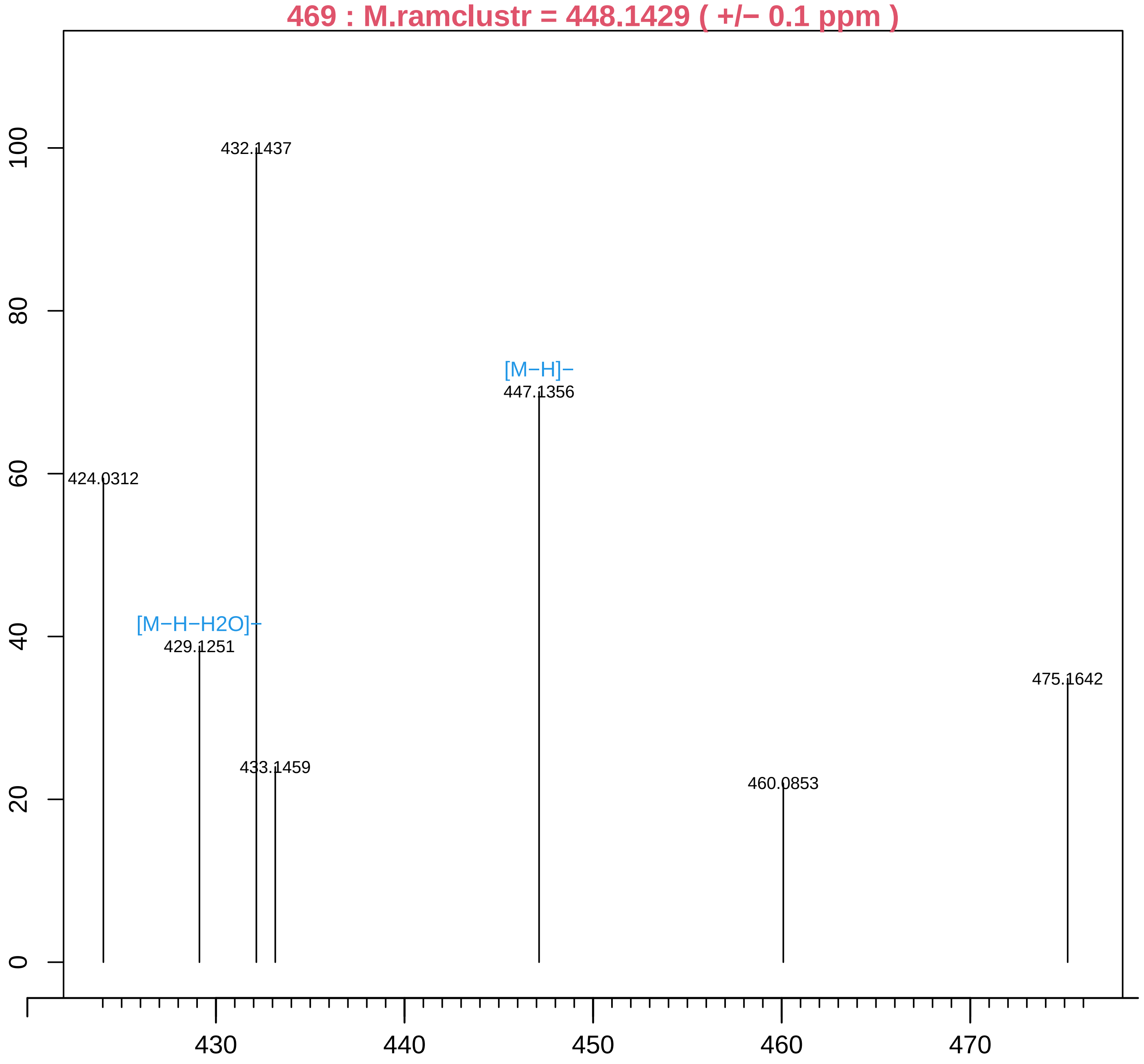
469 : M.ramclustr = 448.1429 (+/− 0.1 ppm)
432.1437
[M−H]−
447.1356
424.0312
[M−H−H2O]−
429.1251
475.1642
433.1459
460.0853
0
20
40
60
80
100
430
440
450
460
470

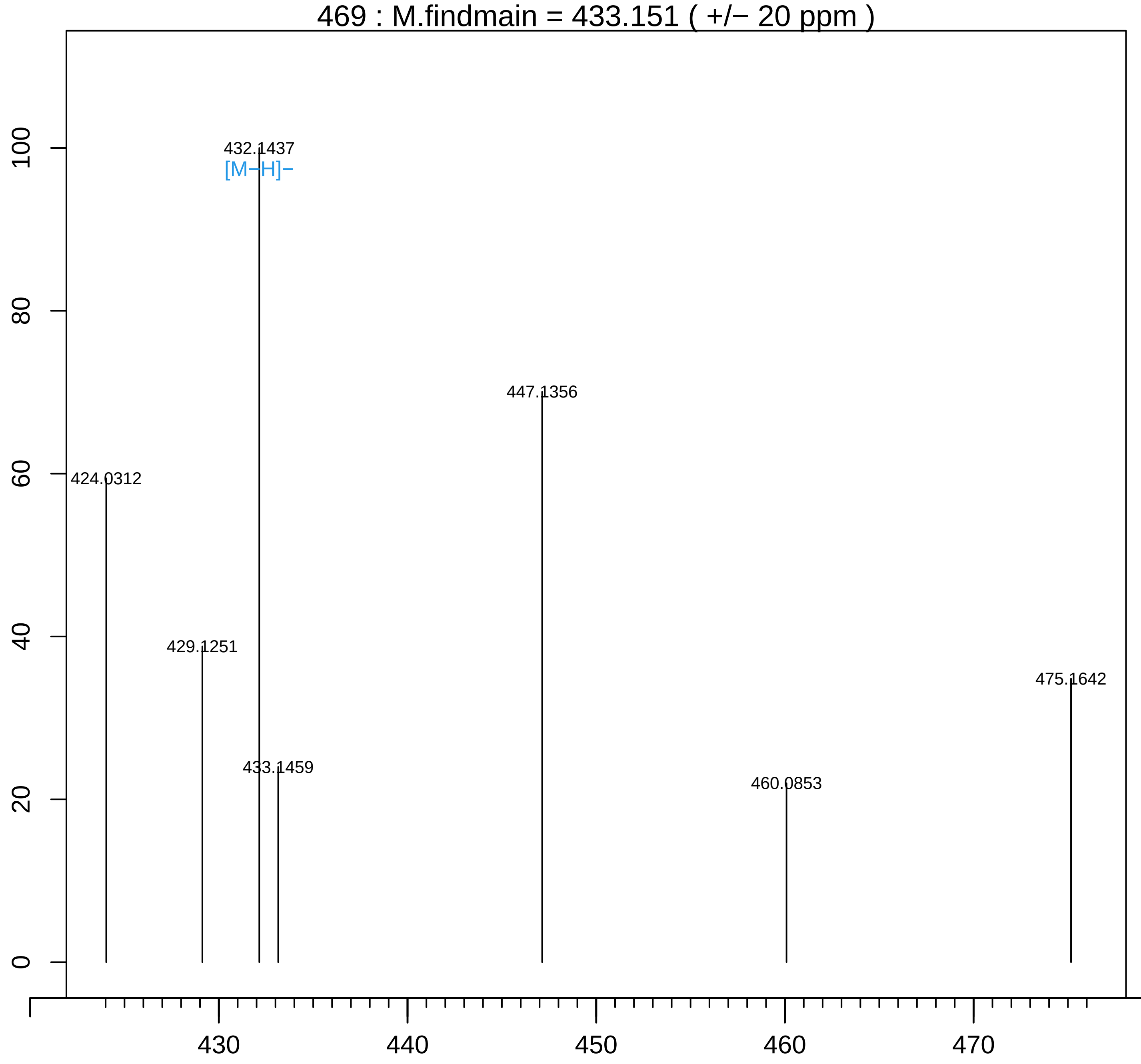
469 : M.findmain = 433.151 (+/− 20 ppm)
432.1437
[M−H]−
447.1356
424.0312
429.1251
475.1642
433.1459
460.0853
0
20
40
60
80
100
430
440
450
460
470

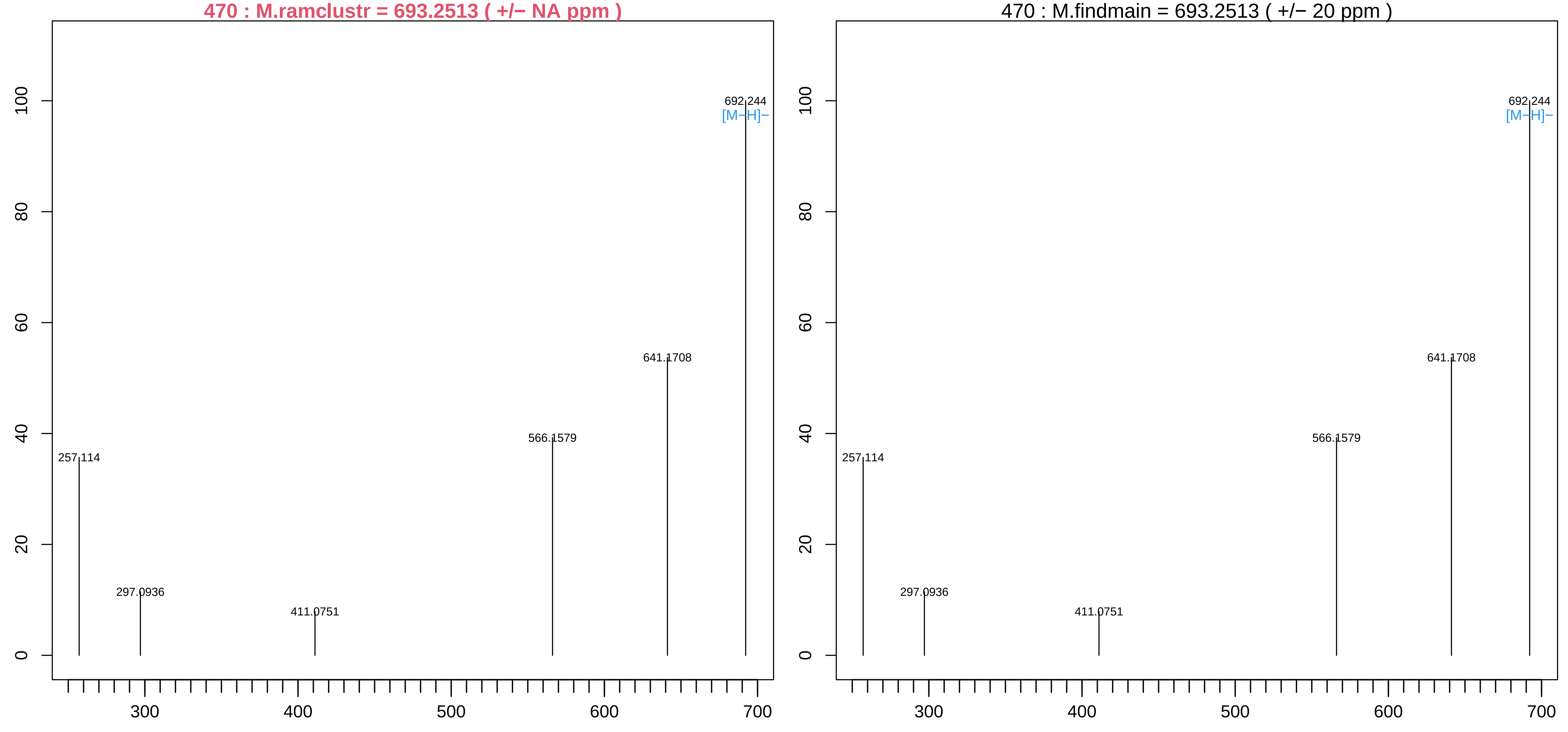

470 : M.ramclustr = 693.2513 (+/− NA ppm)
470 : M.findmain = 693.2513 (+/− 20 ppm)
692.244
[M−H]−
641.1708
566.1579
257.114
297.0936
411.0751
0
20
40
60
80
100
300
400
500
600
700

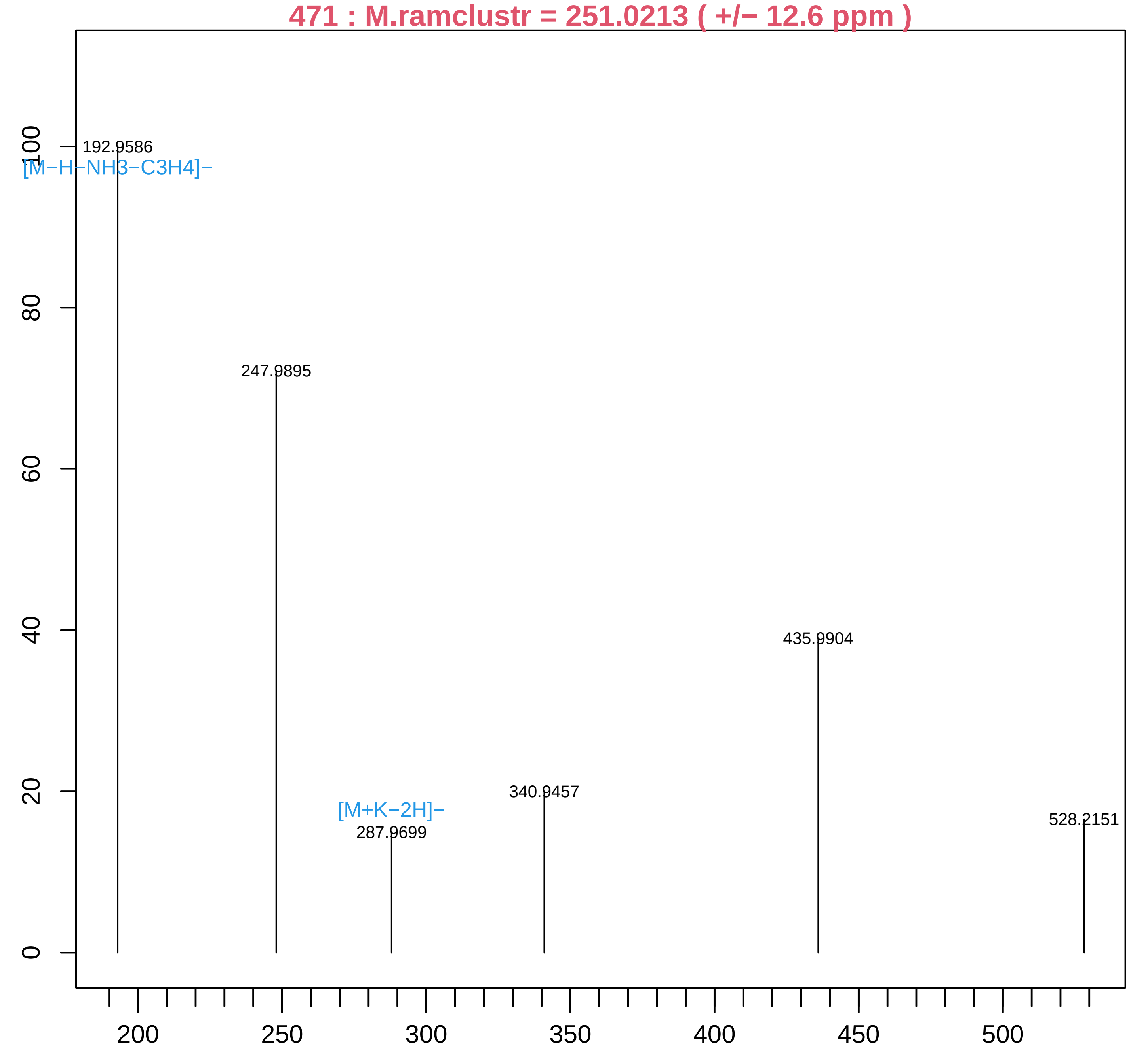

471 : M.ramclustr = 251.0213 (+/− 12.6 ppm)
192.9586
[M−H−NH3−C3H4]−
247.9895
[M+K−2H]−
287.9699
340.9457
435.9904
528.2151
0
20
40
60
80
100
200
250
300
350
400
450
500

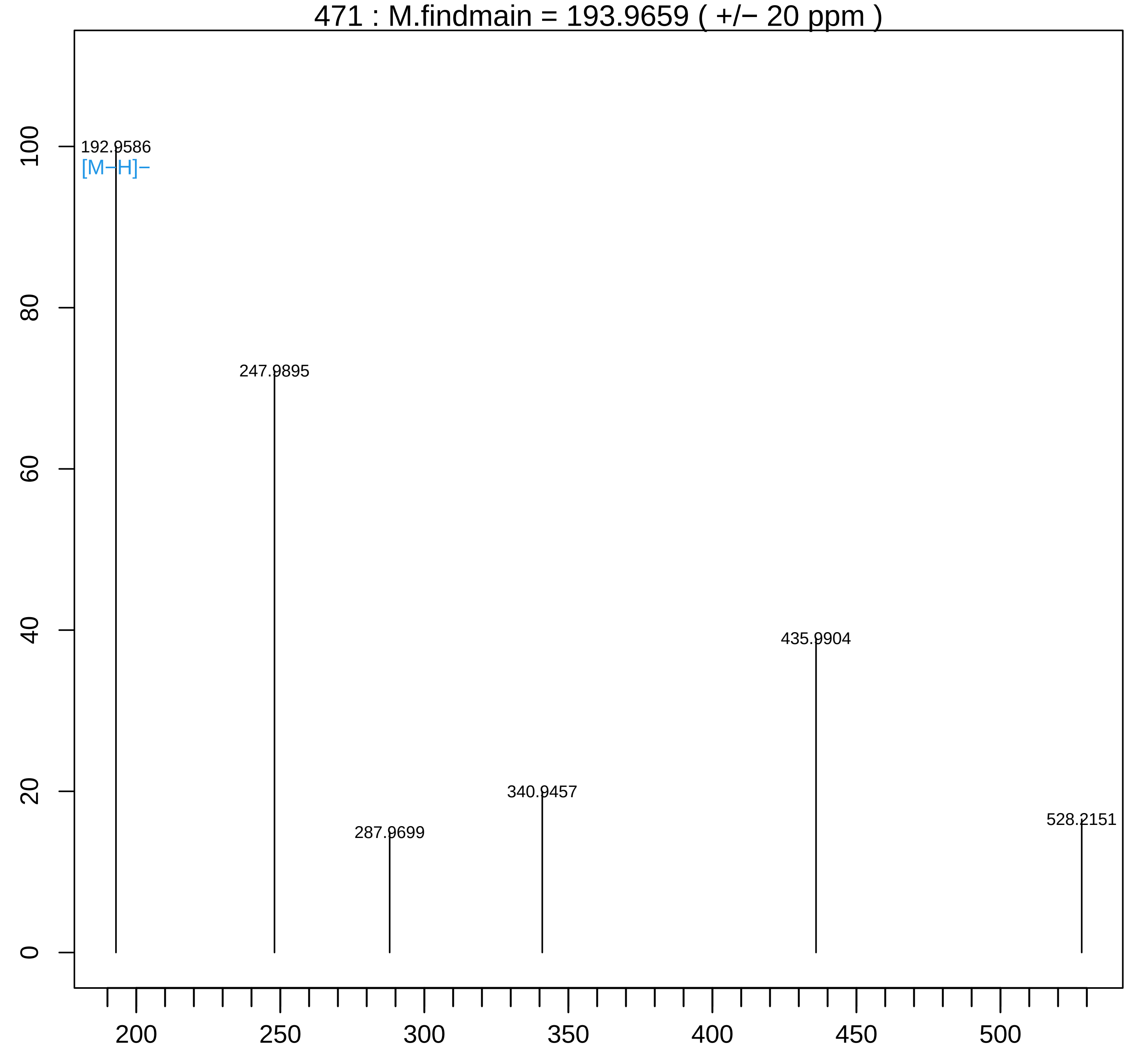

471 : M.findmain = 193.9659 (+/− 20 ppm)
192.9586
[M−H]−
247.9895
287.9699
340.9457
435.9904
528.2151
0
20
40
60
80
100
200
250
300
350
400
450
500

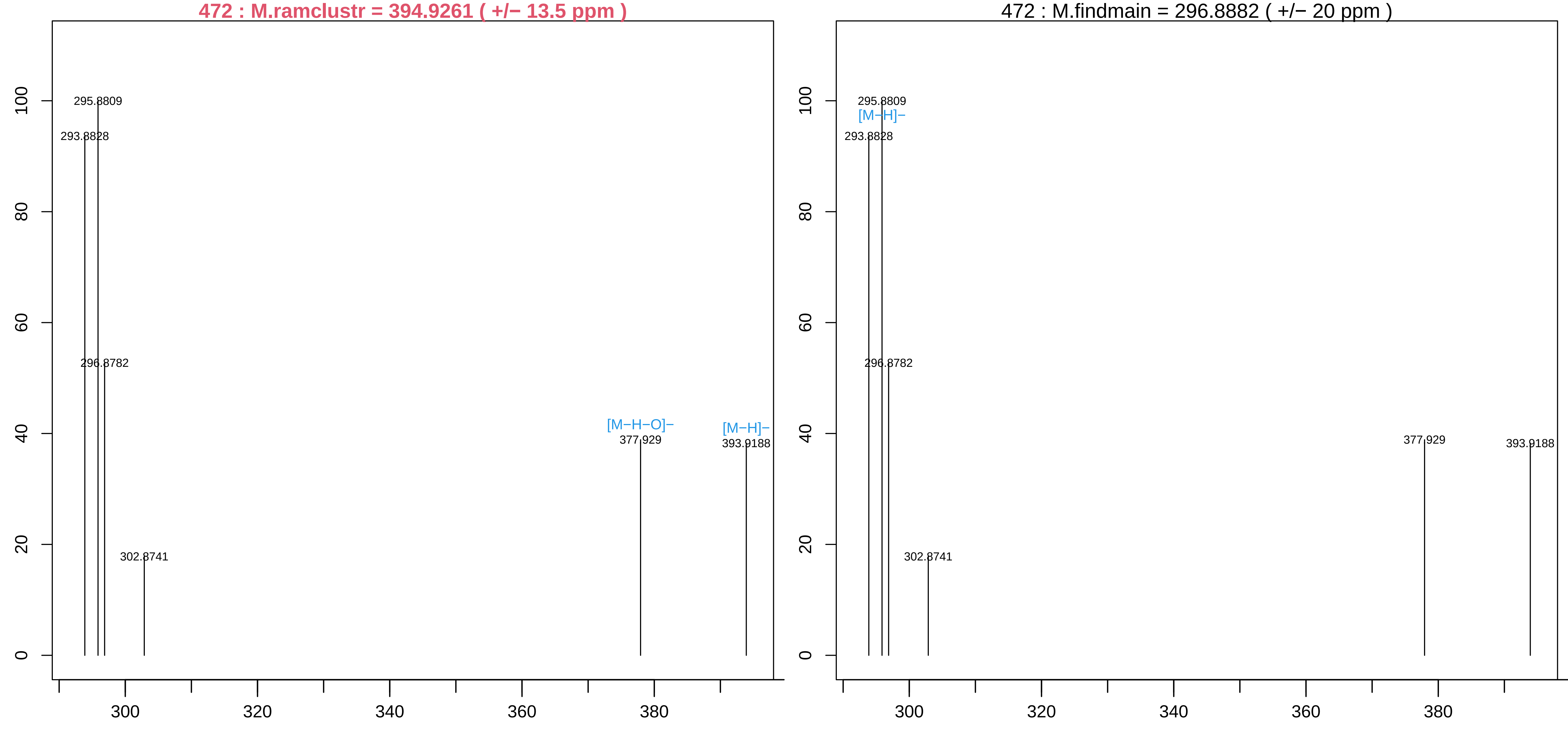
472 : M.ramclustr = 394.9261 (+/− 13.5 ppm)
295.8809
293.8828
296.8782
302.8741
[M−H−O]−
377.929
[M−H]−
393.9188
472 : M.findmain = 296.8882 (+/− 20 ppm)
295.8809
[M−H]−
293.8828
296.8782
302.8741
377.929
393.9188

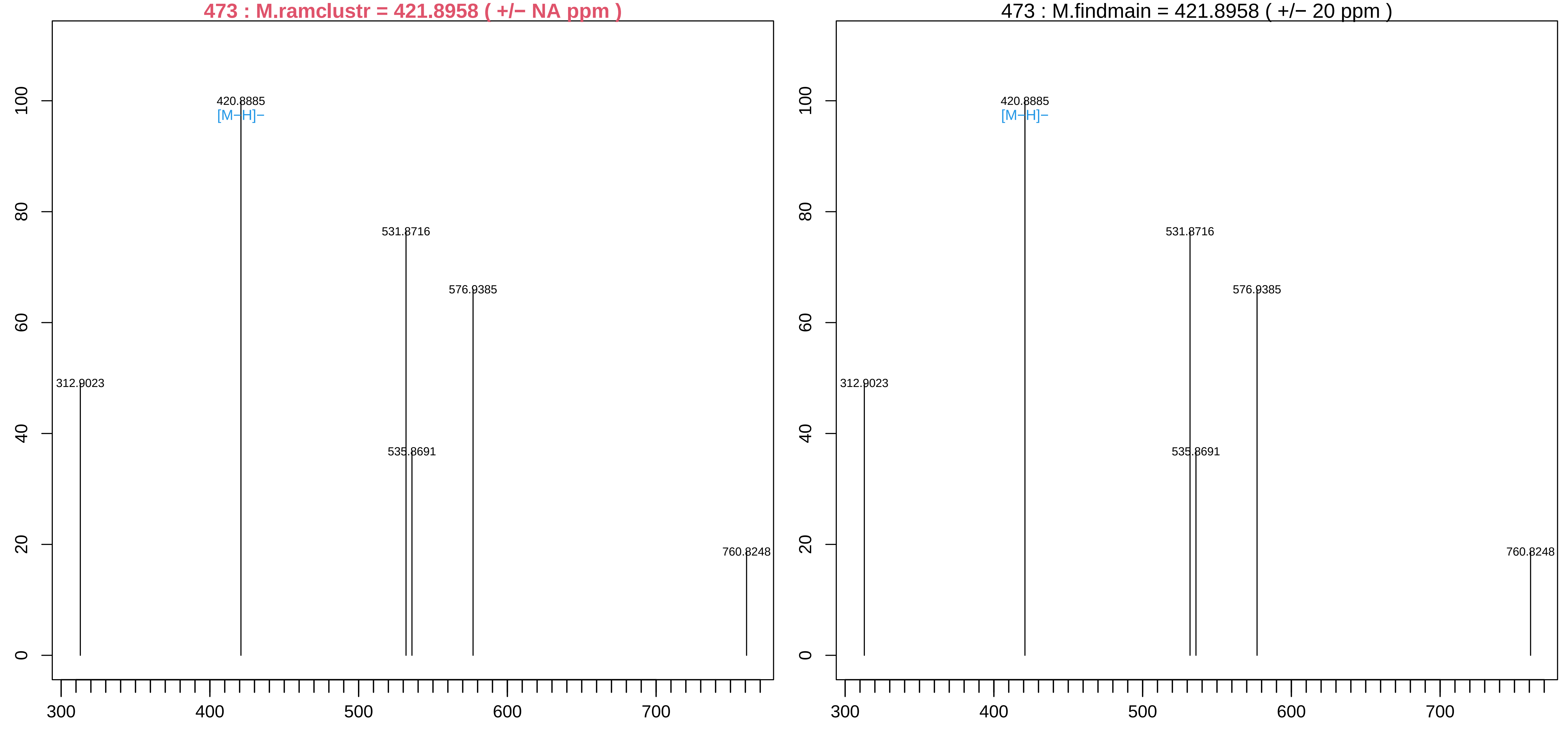
473 : M.ramclustr = 421.8958 (+/− NA ppm)
473 : M.findmain = 421.8958 (+/− 20 ppm)
420.8885
[M−H]−
531.8716
576.9385
312.9023
535.8691
760.8248
0
20
40
60
80
100
300
400
500
600
700

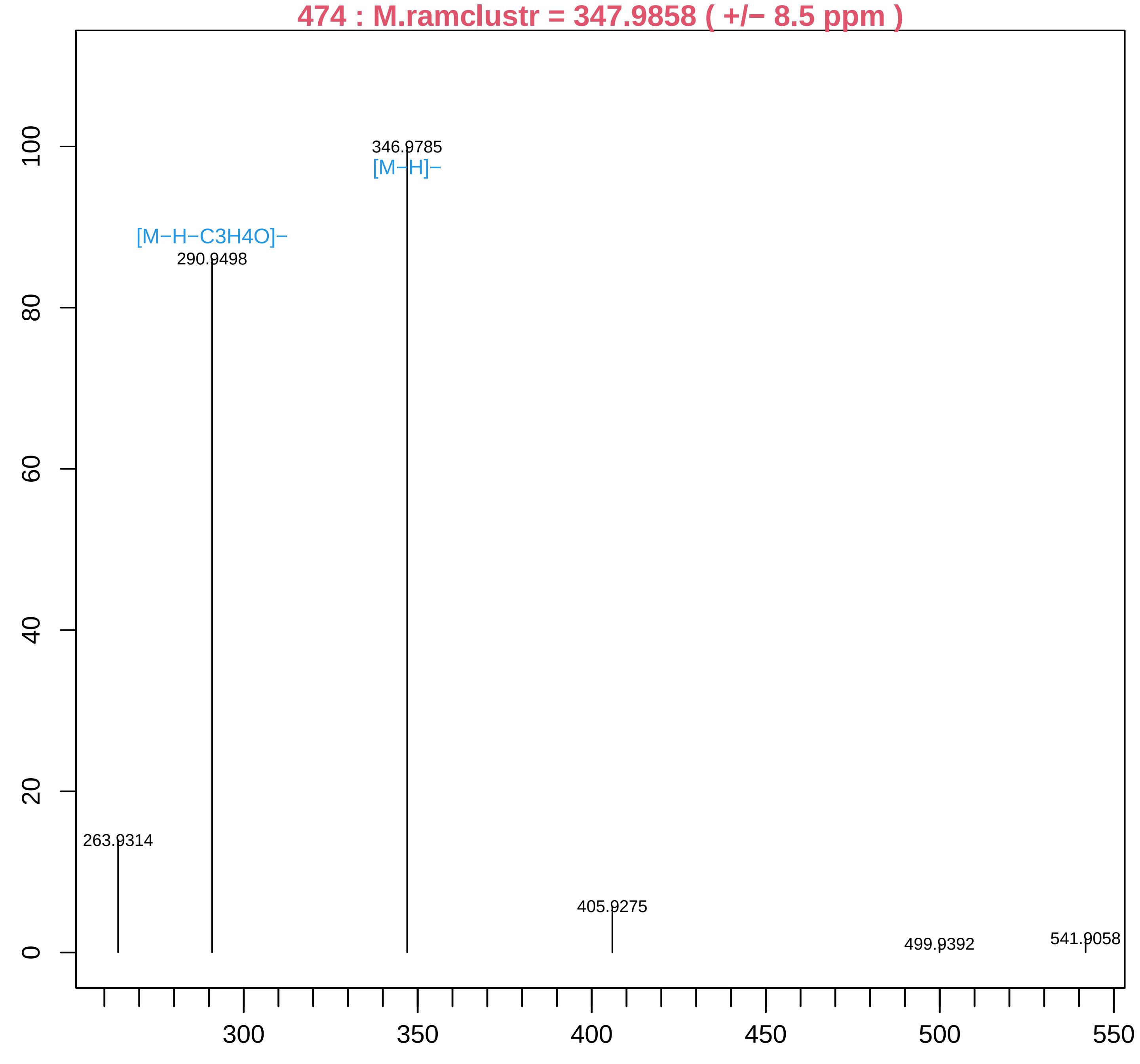
474 : M.ramclustr = 347.9858 (+/− 8.5 ppm)
[M−H−C3H4O]−
290.9498
346.9785
[M−H]−
263.9314
405.9275
499.9392
541.9058
0
20
40
60
80
100
300
350
400
450
500
550

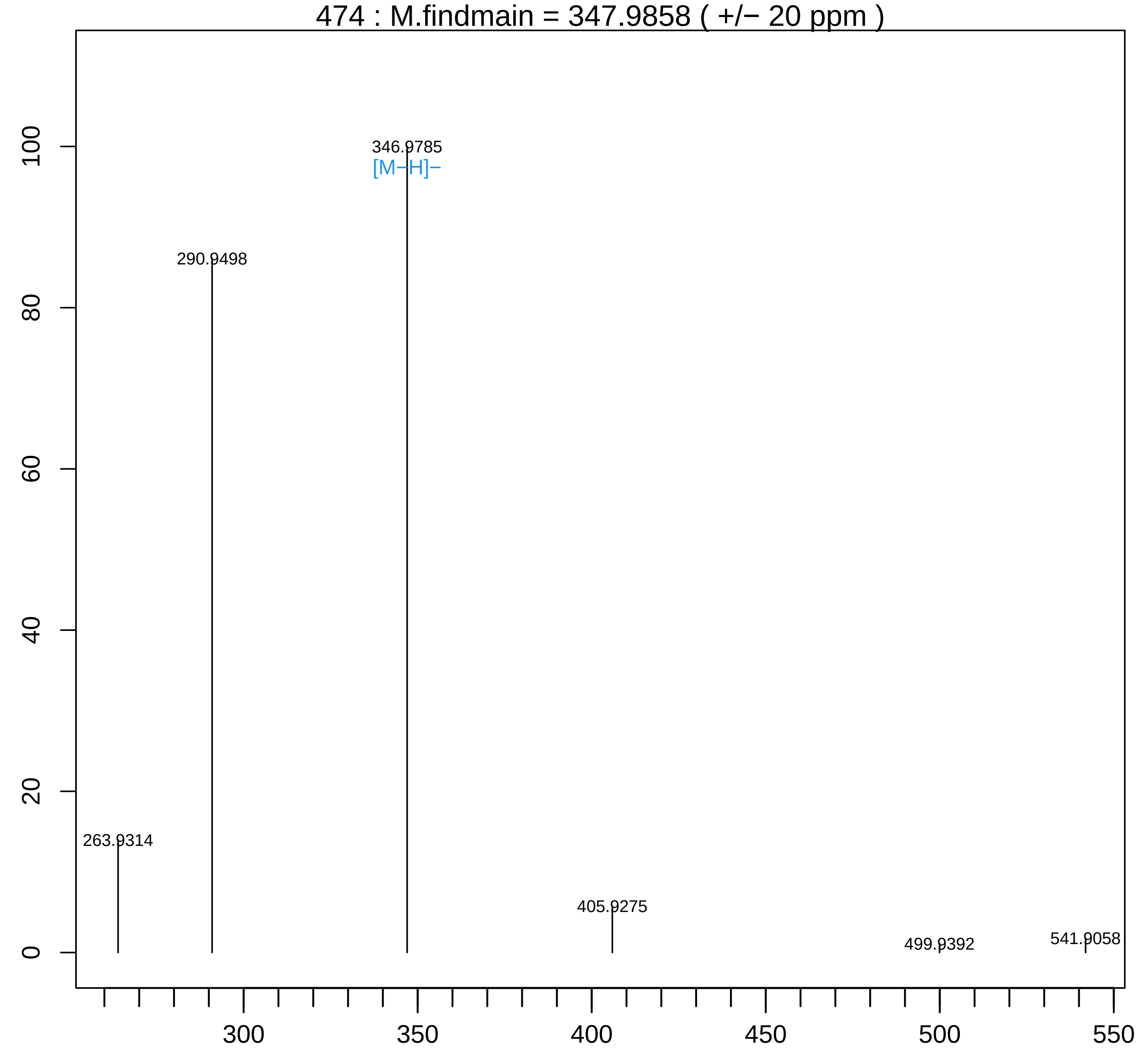
474 : M.findmain = 347.9858 (+/− 20 ppm)
290.9498
346.9785
[M−H]−
263.9314
405.9275
499.9392
541.9058
0
20
40
60
80
100
300
350
400
450
500
550

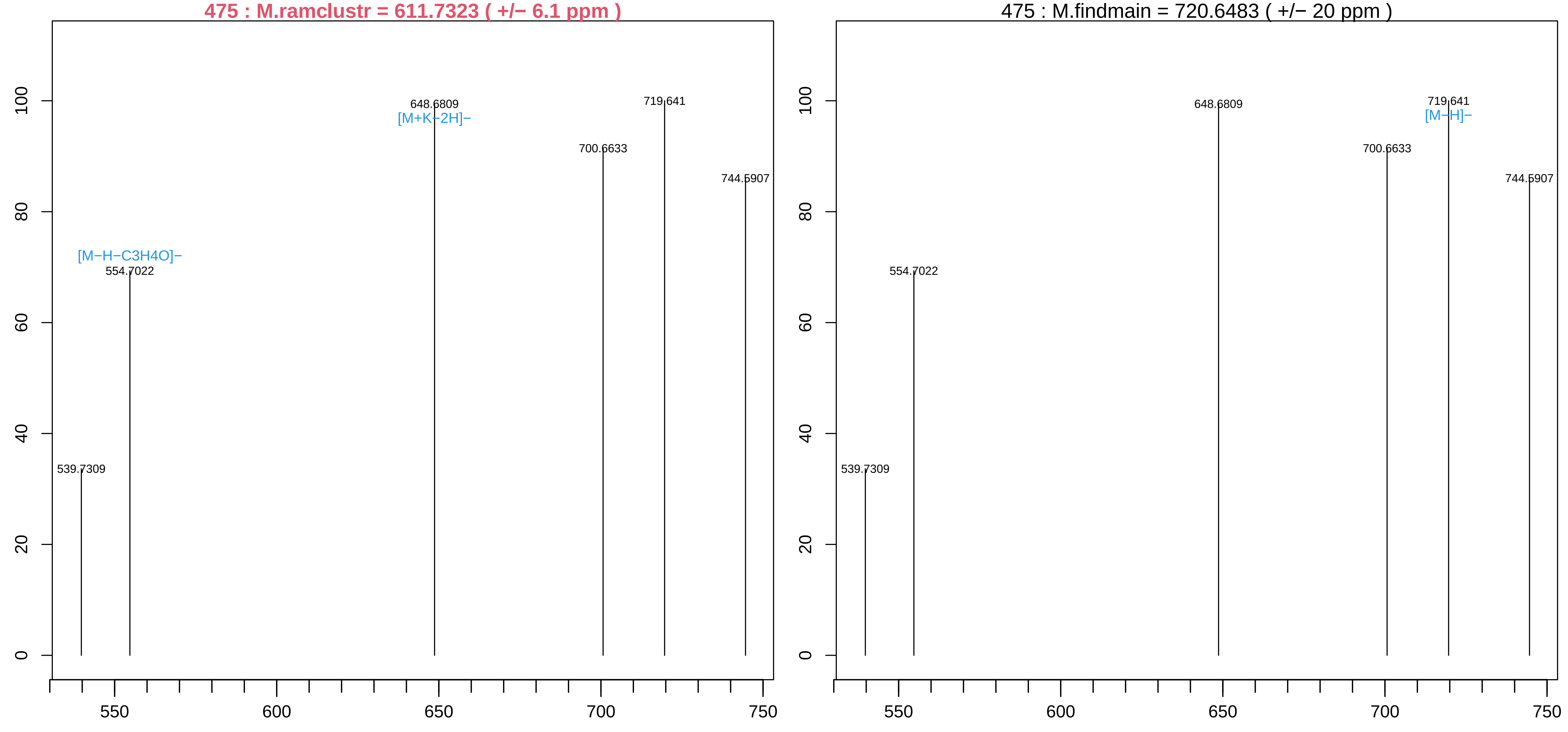

475 : M.ramclustr = 611.7323 (+/− 6.1 ppm)
539.7309
554.7022
[M−H−C3H4O]−
648.6809
[M+K−2H]−
700.6633
719.641
744.5907
475 : M.findmain = 720.6483 (+/− 20 ppm)
539.7309
554.7022
648.6809
700.6633
719.641
[M−H]−
744.5907

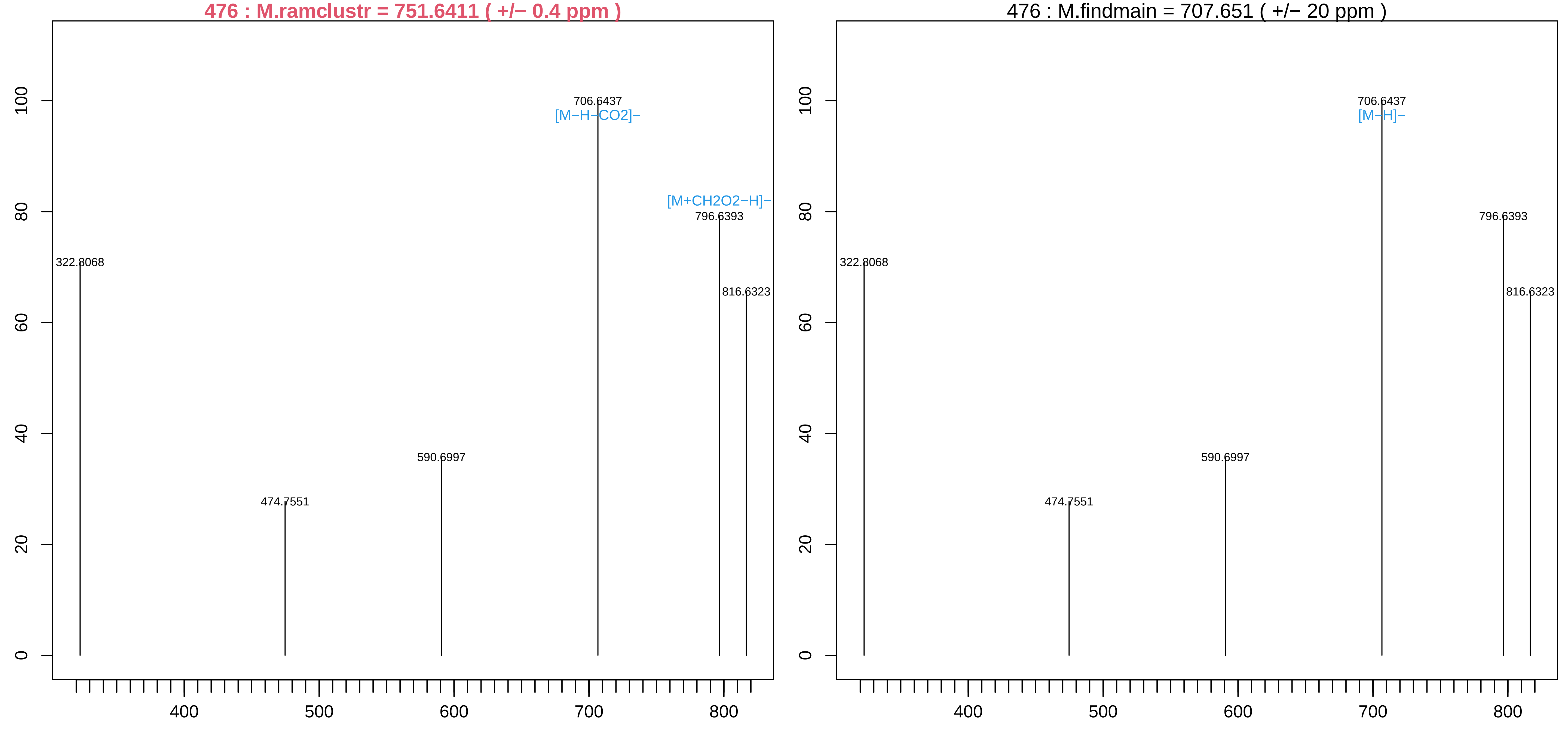
476 : M.ramclustr = 751.6411 (+/− 0.4 ppm)
706.6437
[M−H−CO2]−
[M+CH2O2−H]−
796.6393
322.8068
816.6323
590.6997
474.7551
476 : M.findmain = 707.651 (+/− 20 ppm)
706.6437
[M−H]−
796.6393
322.8068
816.6323
590.6997
474.7551
0
20
40
60
80
100
400
500
600
700
800

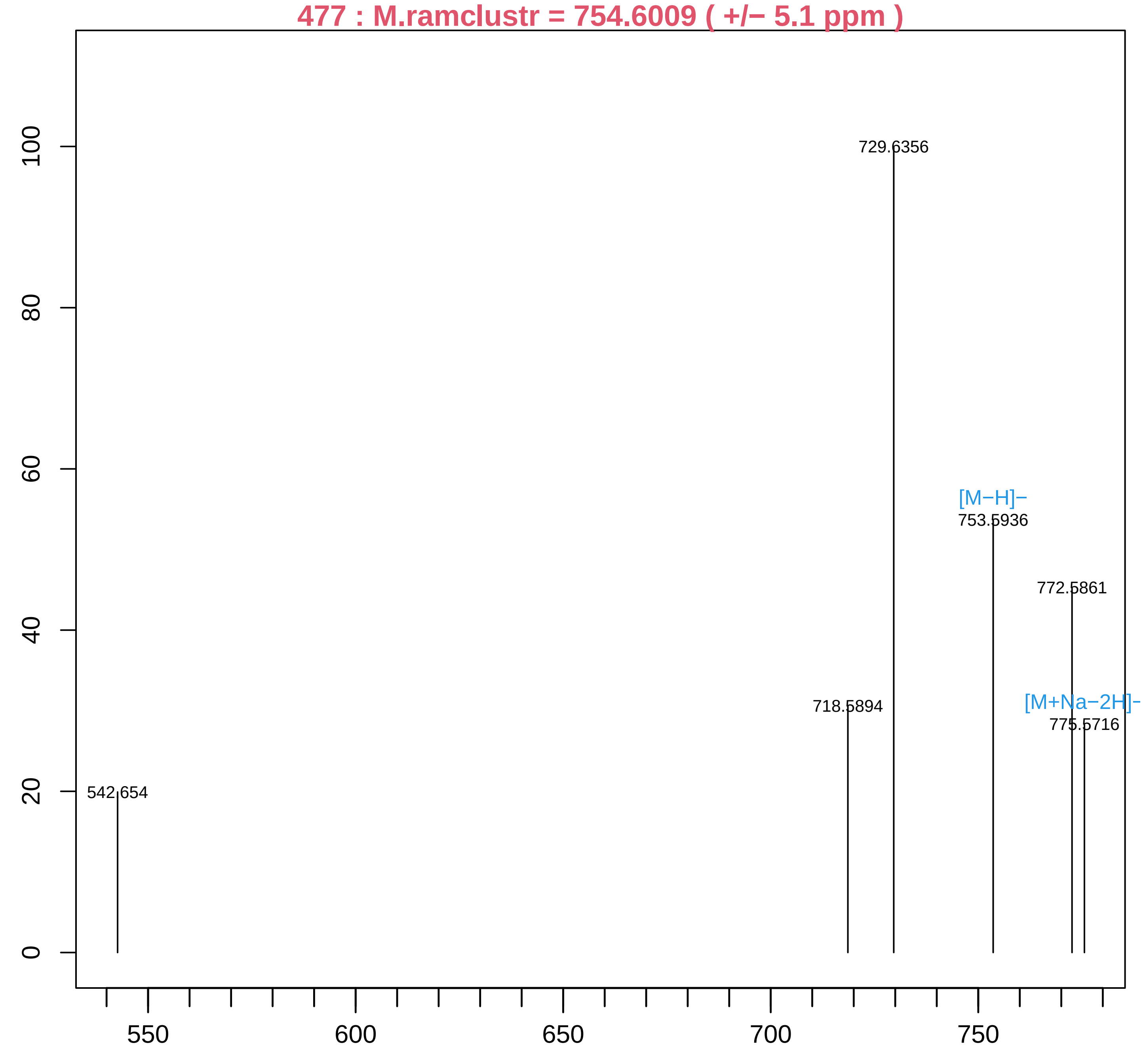
477 : M.ramclustr = 754.6009 (+/− 5.1 ppm)
729.6356
[M−H]−
753.5936
772.5861
718.5894
[M+Na−2H]−
775.5716
542.654
0
20
40
60
80
100
550
600
650
700
750

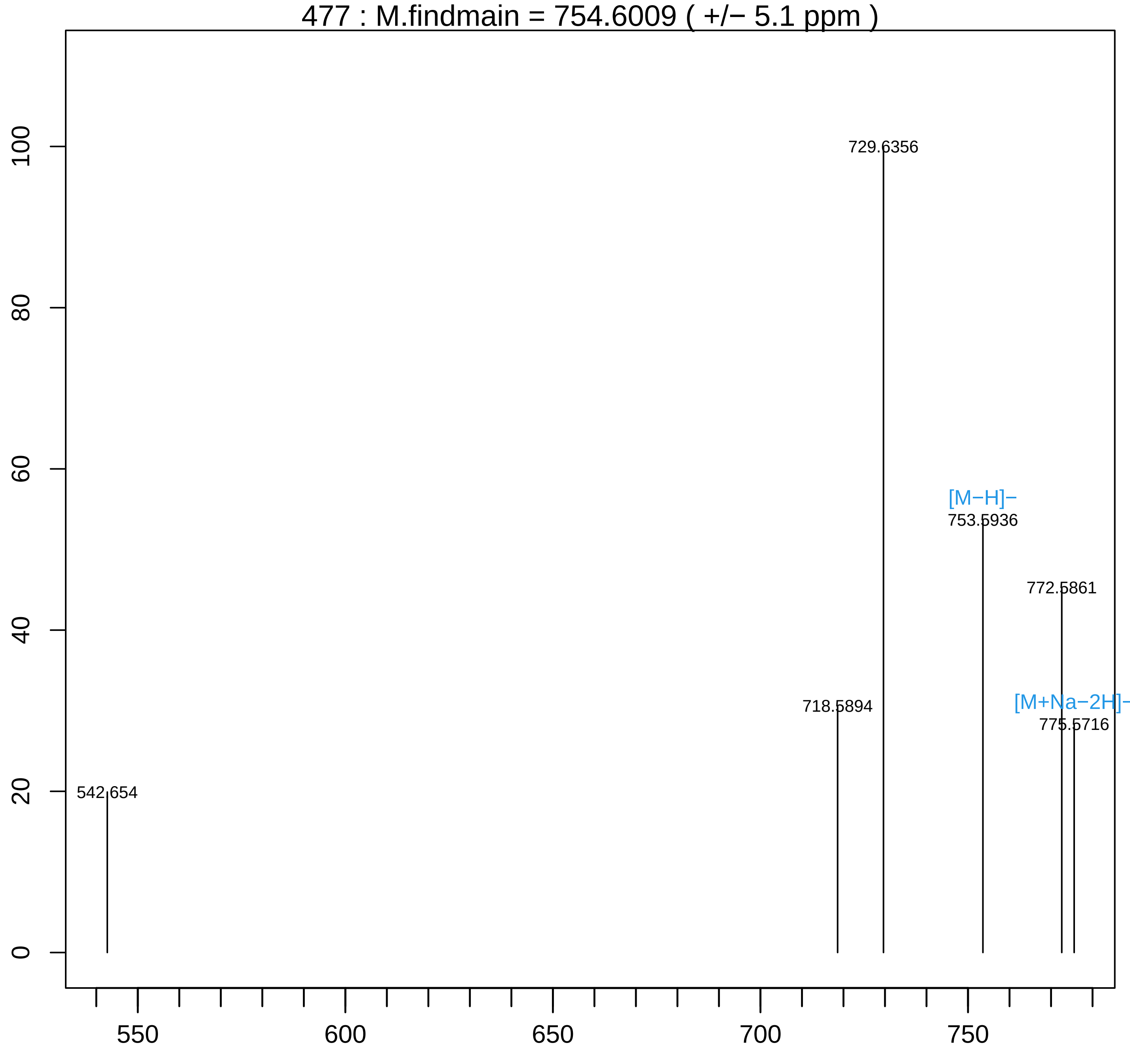
477 : M.findmain = 754.6009 (+/− 5.1 ppm)
729.6356
[M−H]−
753.5936
772.5861
718.5894
[M+Na−2H]−
775.5716
542.654
0
20
40
60
80
100
550
600
650
700
750

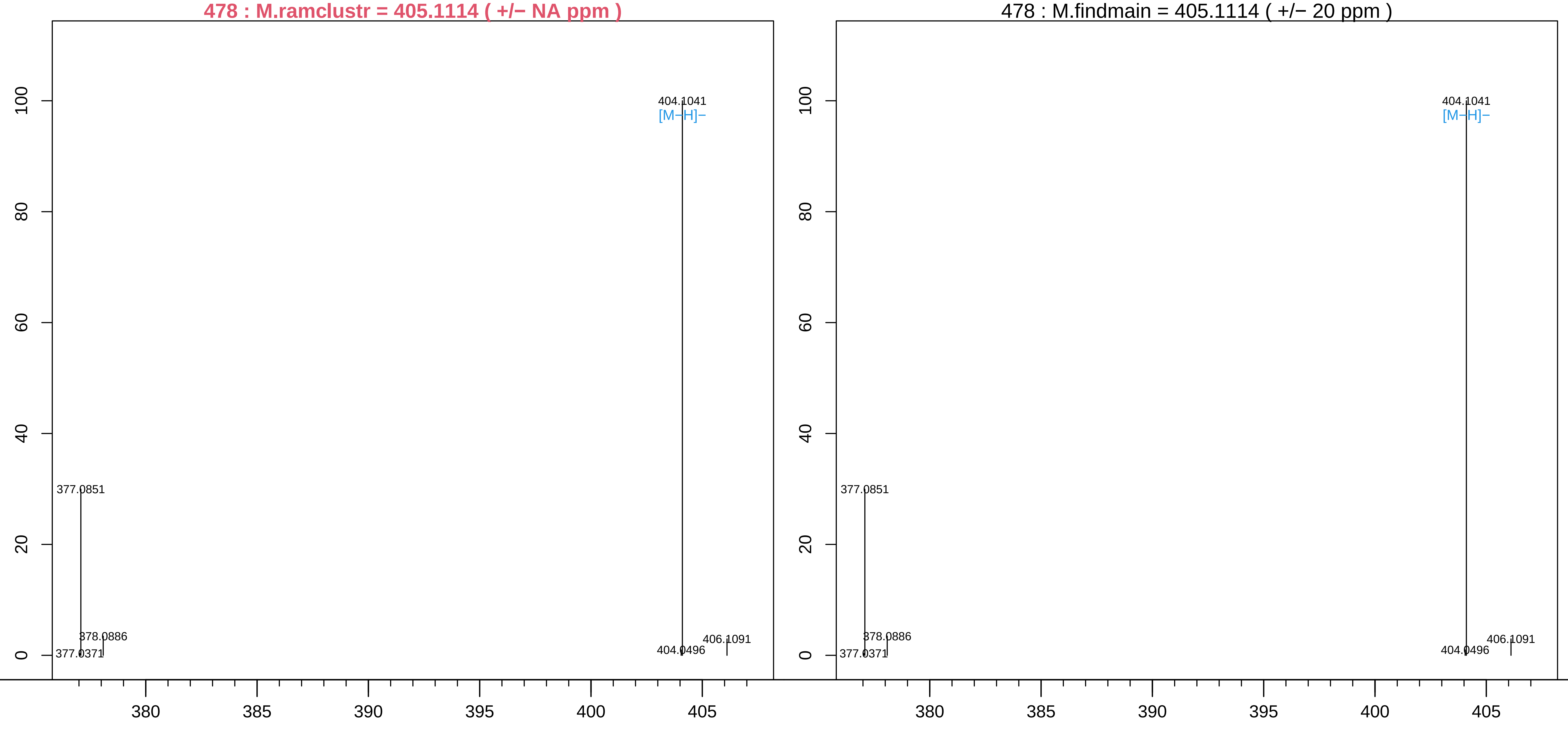

478 : M.ramclustr = 405.1114 (+/− NA ppm)
404.1041
[M−H]−
377.0851
378.0886
377.0371
404.0496
406.1091
0
20
40
60
80
100
380
385
390
395
400
405
478 : M.findmain = 405.1114 (+/− 20 ppm)
404.1041
[M−H]−
377.0851
378.0886
377.0371
404.0496
406.1091

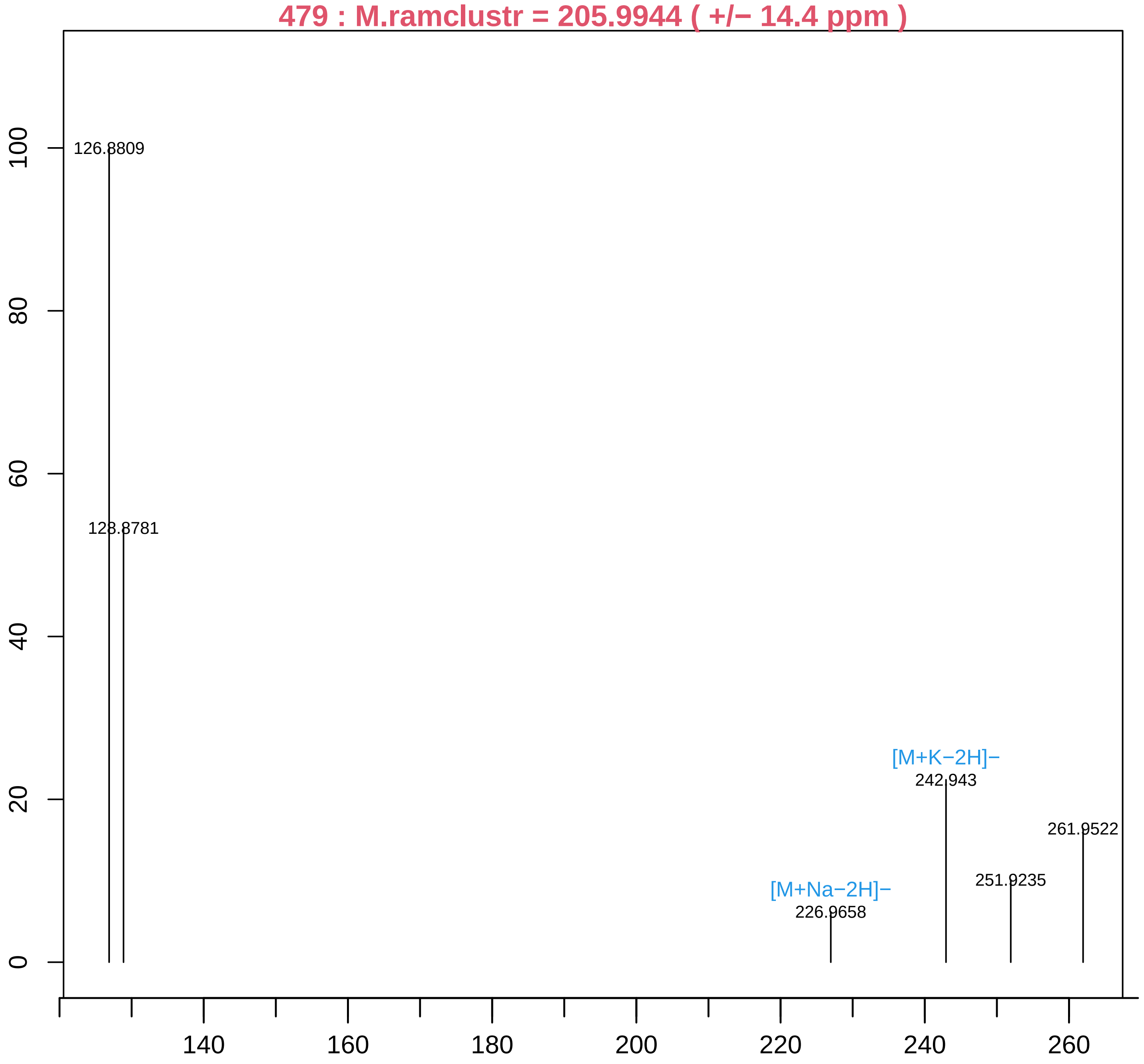
479 : M.ramclustr = 205.9944 (+/− 14.4 ppm)
126.8809
128.8781
[M+Na−2H]−
226.9658
[M+K−2H]−
242.943
251.9235
261.9522
0
20
40
60
80
100
140
160
180
200
220
240
260

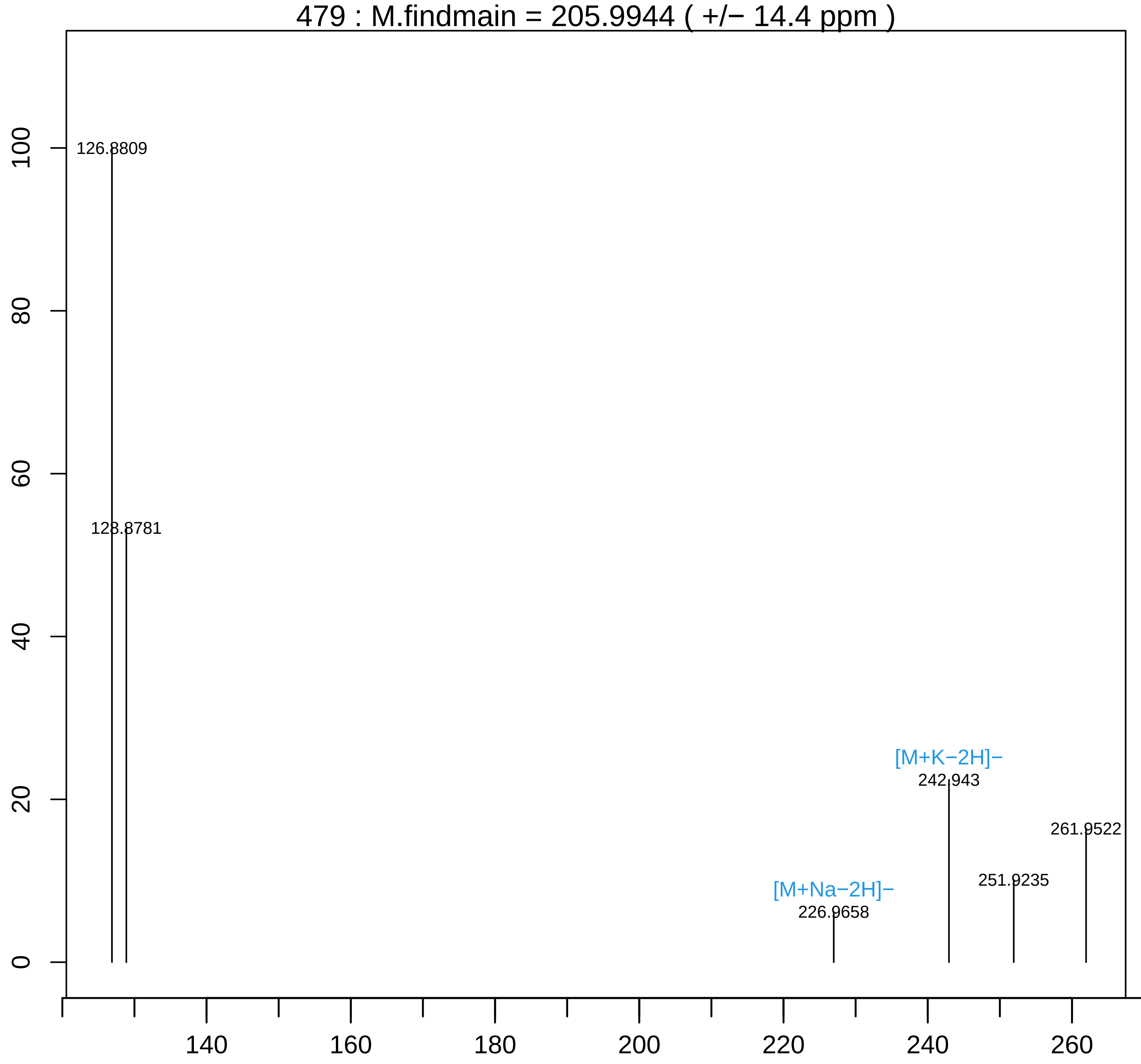
479 : M.findmain = 205.9944 (+/− 14.4 ppm)
126.8809
128.8781
[M+Na−2H]−
226.9658
[M+K−2H]−
242.943
251.9235
261.9522
0
20
40
60
80
100
140
160
180
200
220
240
260

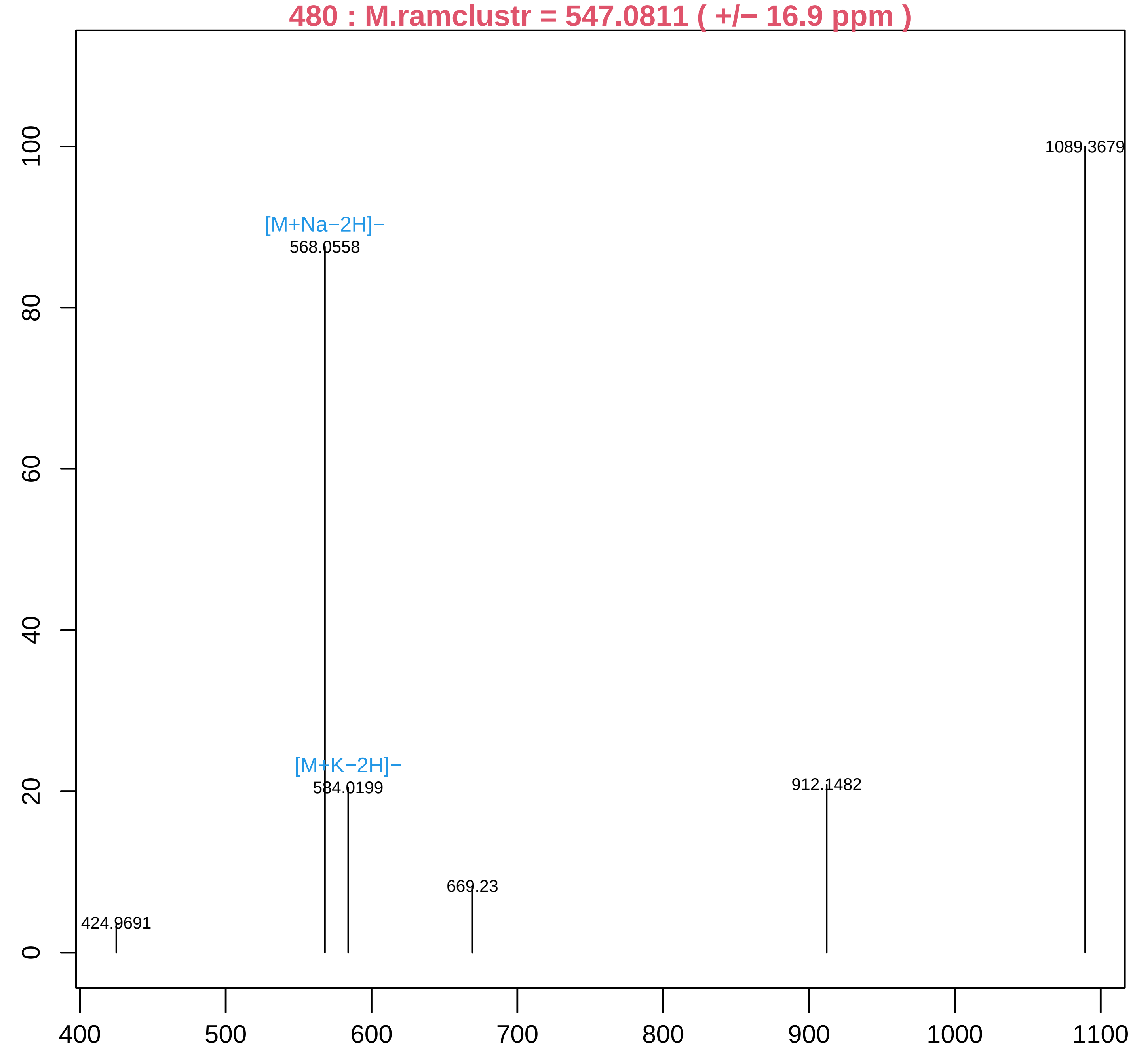
480 : M.ramclustr = 547.0811 (+/− 16.9 ppm)
[M+Na−2H]−
568.0558
[M+K−2H]−
584.0199
424.9691
669.23
912.1482
1089.3679

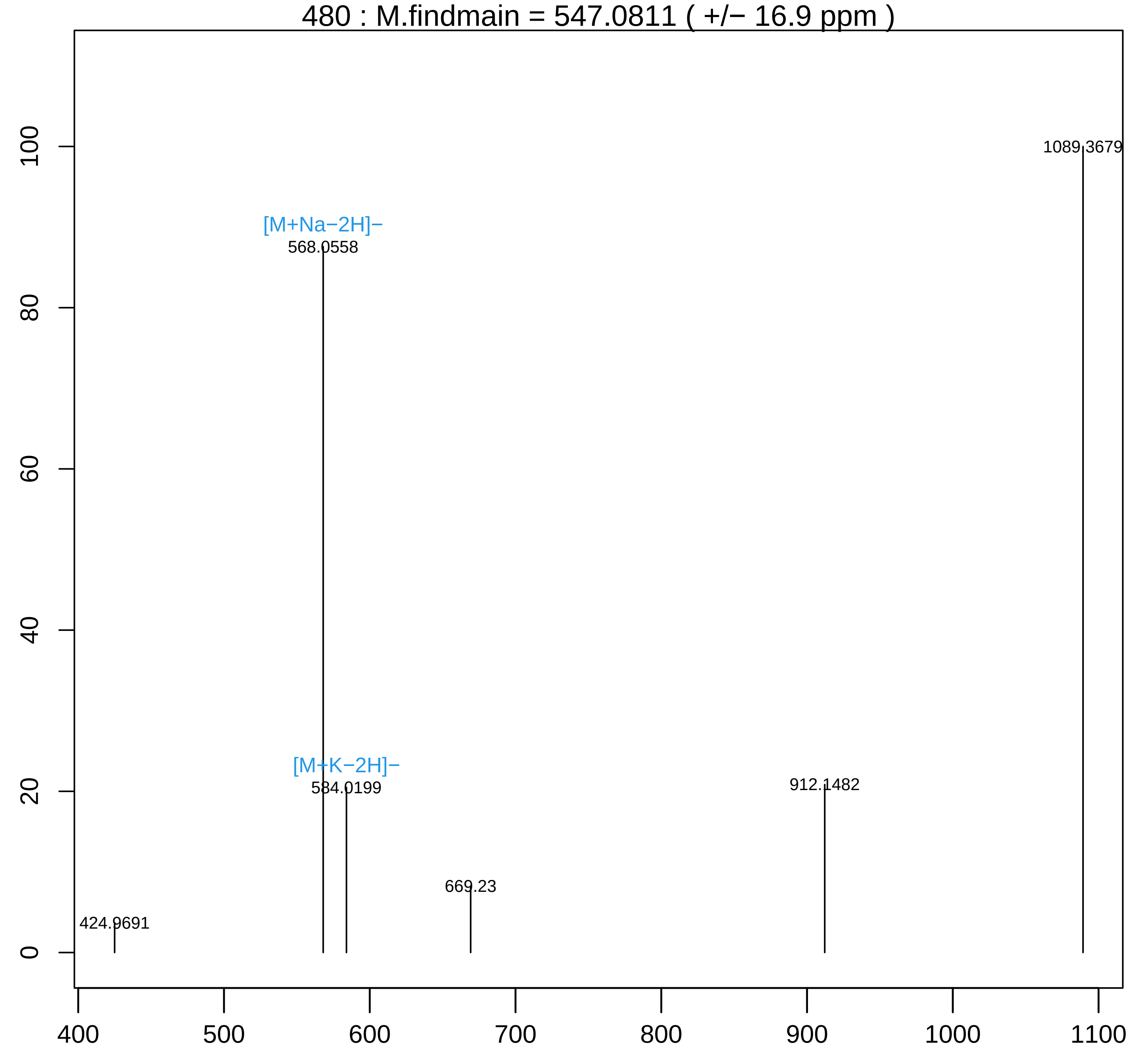
480 : M.findmain = 547.0811 (+/− 16.9 ppm)
[M+Na−2H]−
568.0558
[M+K−2H]−
584.0199
424.9691
669.23
912.1482
1089.3679

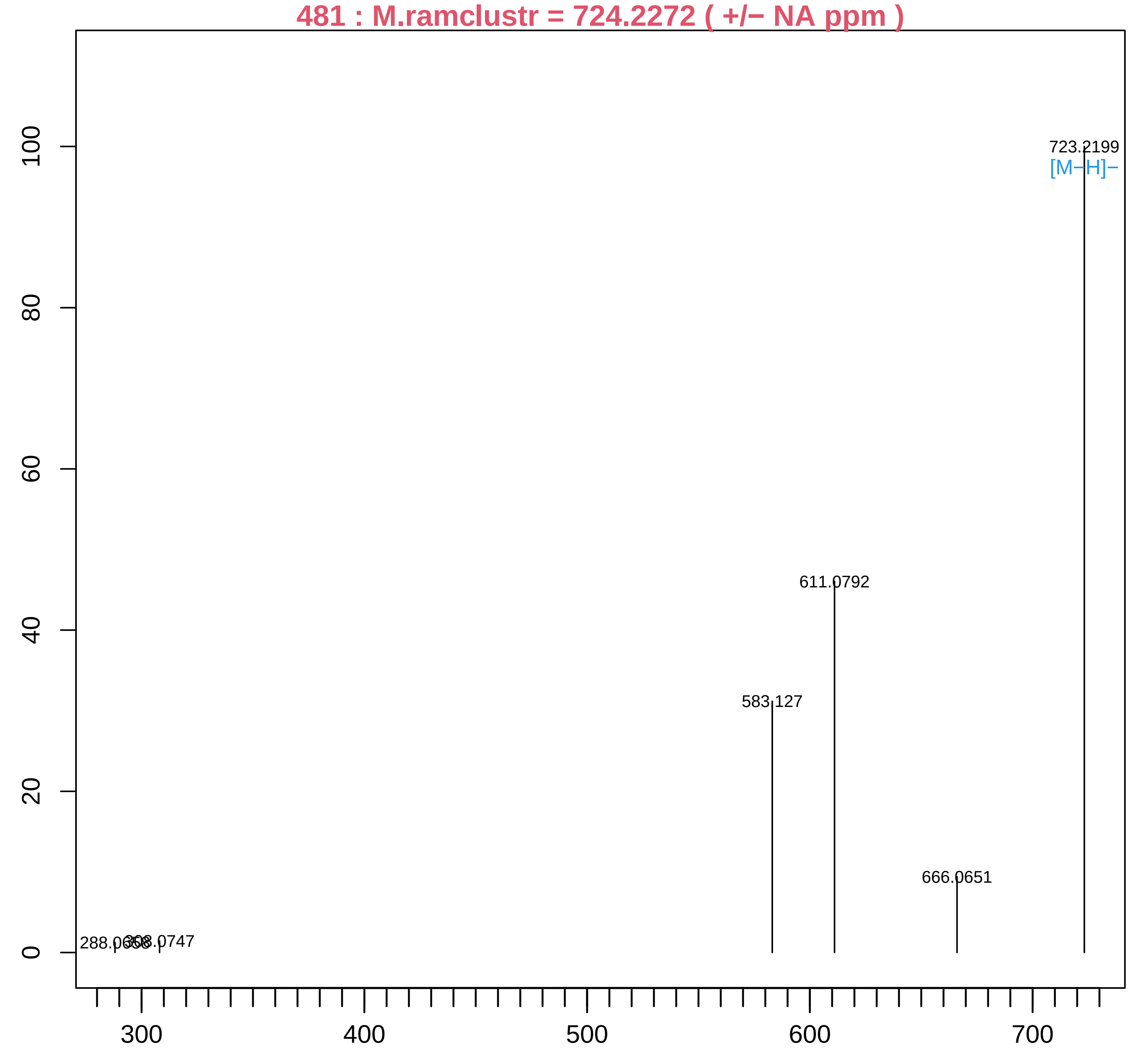
481 : M.ramclustr = 724.2272 (+/− NA ppm)
723.2199
[M−H]−
611.0792
583.127
666.0651
288.0658
308.0747
0
20
40
60
80
100
300
400
500
600
700
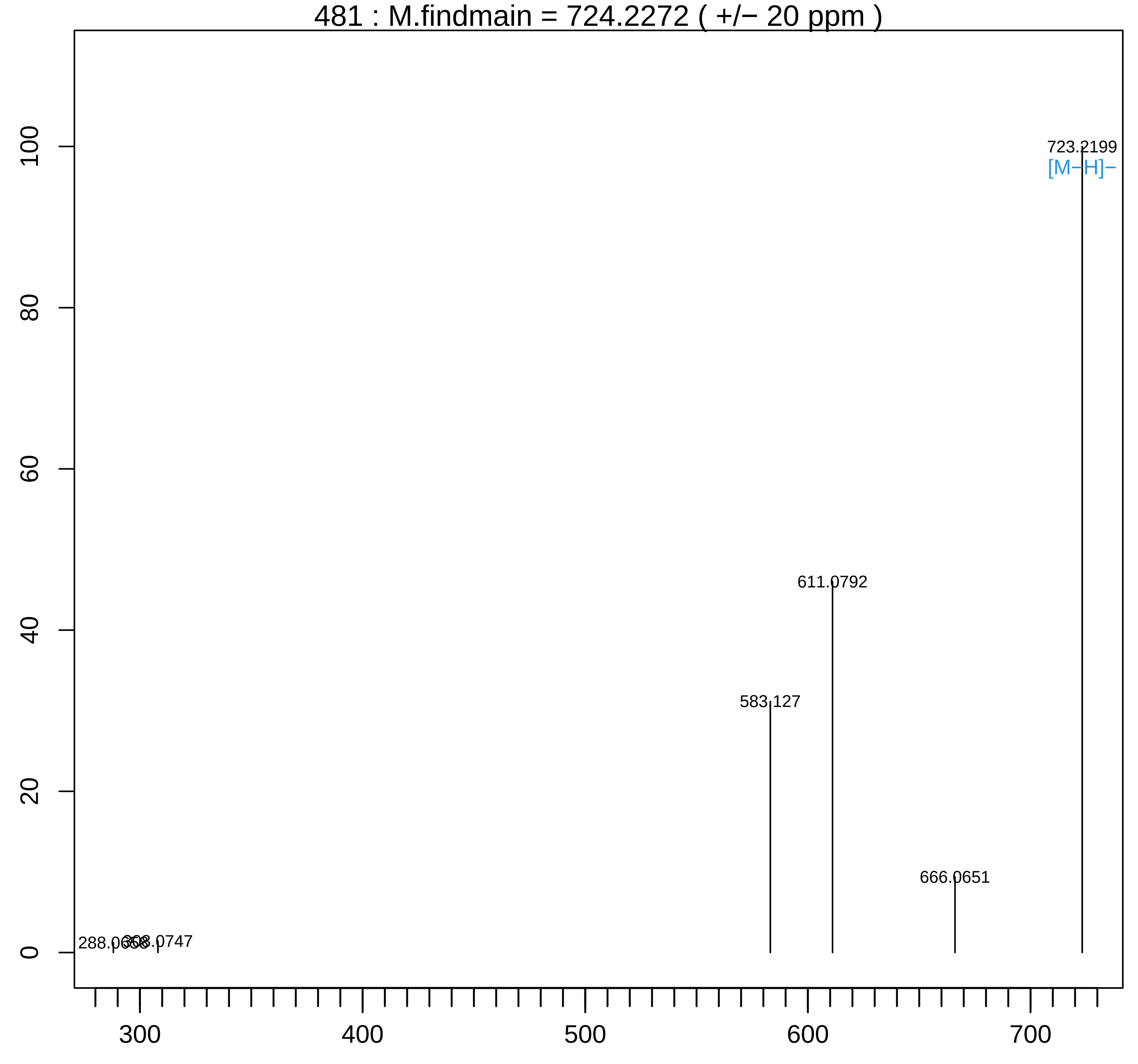
481 : M.findmain = 724.2272 (+/− 20 ppm)
723.2199
[M−H]−
611.0792
583.127
666.0651
288.0658
308.0747
0
20
40
60
80
100
300
400
500
600
700

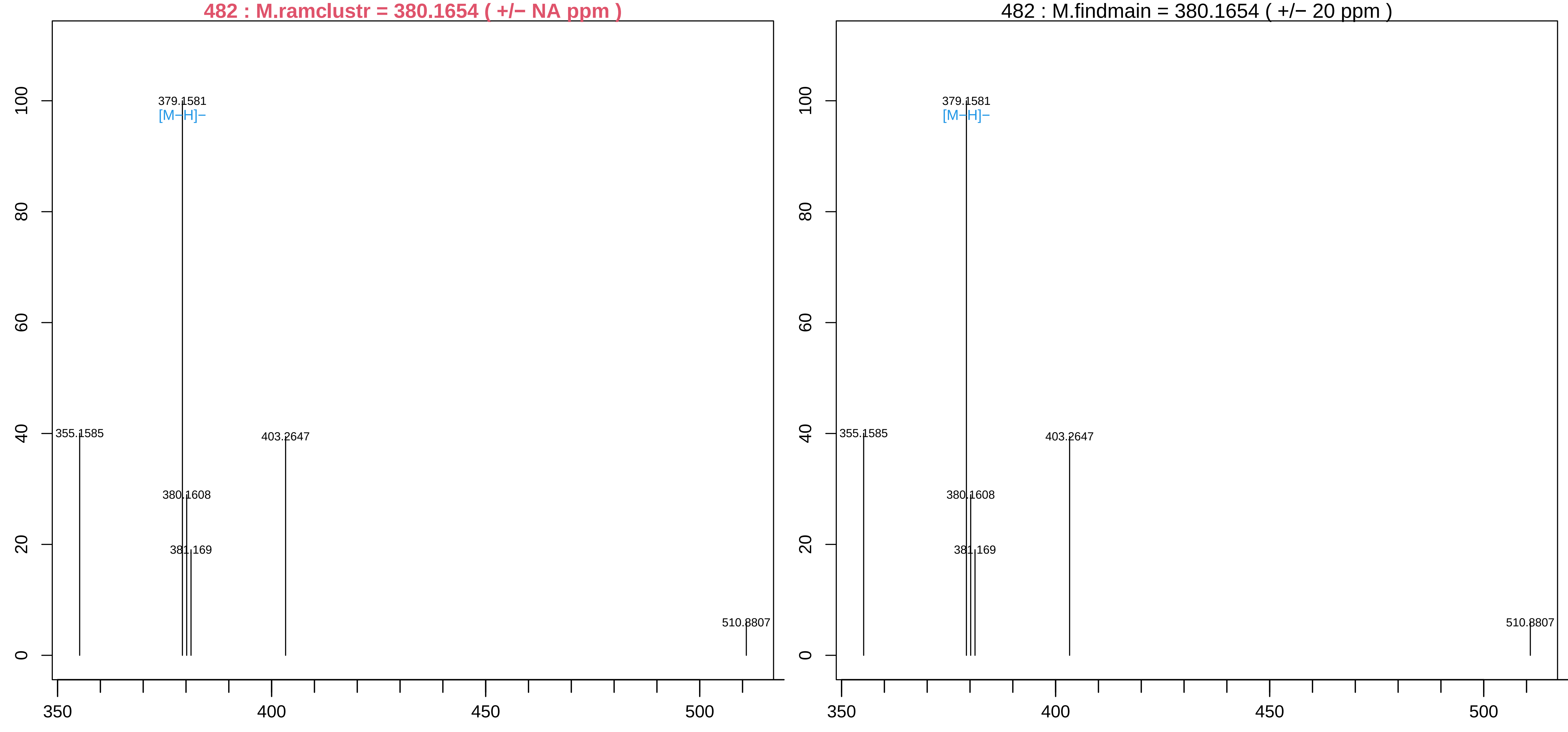
482 : M.ramclustr = 380.1654 (+/− NA ppm)
482 : M.findmain = 380.1654 (+/− 20 ppm)
379.1581
[M−H]−
355.1585
403.2647
380.1608
381.169
510.8807
0
20
40
60
80
100
350
400
450
500

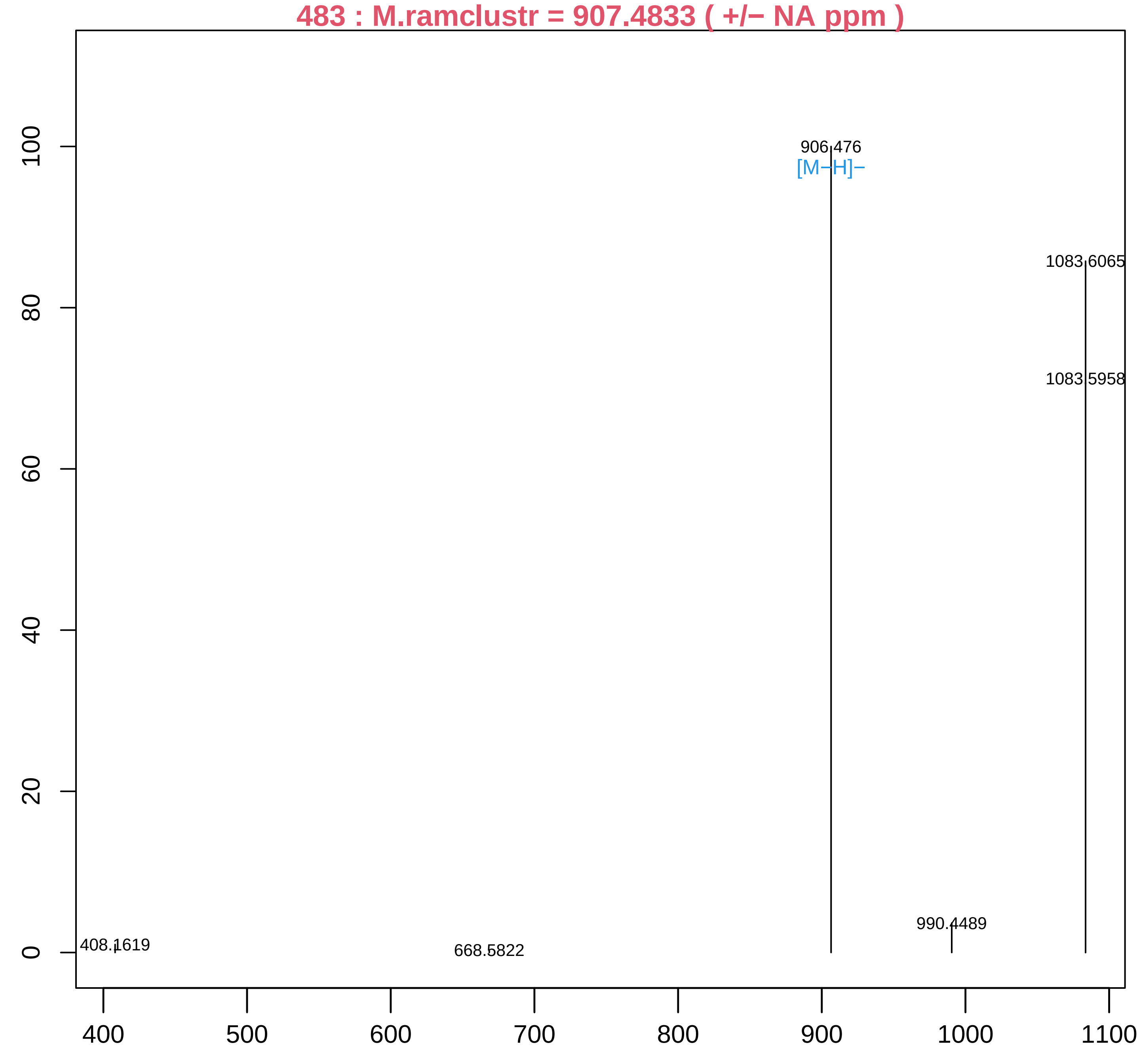
483 : M.ramclustr = 907.4833 (+/− NA ppm)
906.476
[M−H]−
1083.6065
1083.5958
990.4489
408.1619
668.5822
0
20
40
60
80
100
400
500
600
700
800
900
1000
1100

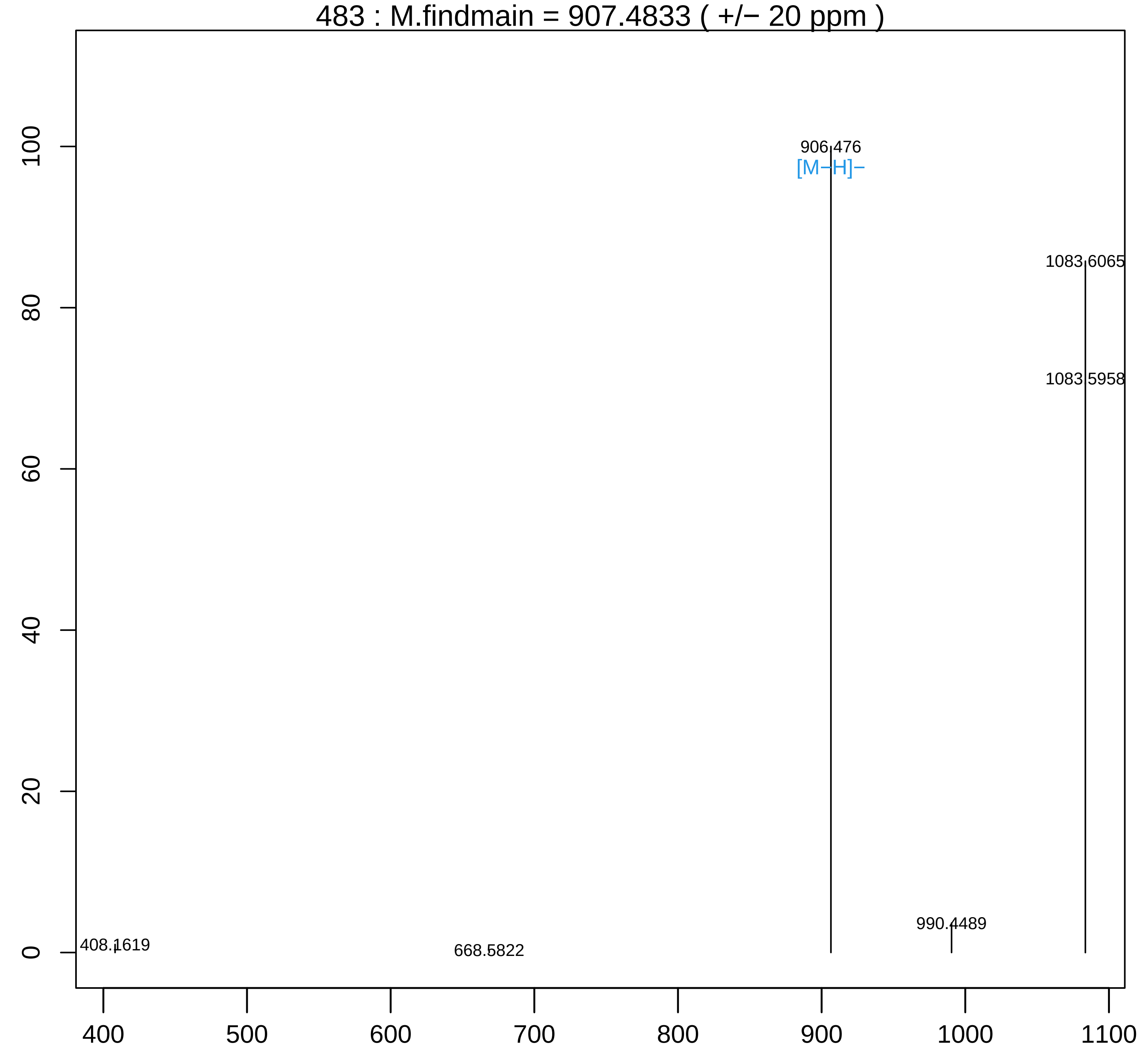
483 : M.findmain = 907.4833 (+/− 20 ppm)
906.476
[M−H]−
1083.6065
1083.5958
990.4489
408.1619
668.5822
0
20
40
60
80
100
400
500
600
700
800
900
1000
1100

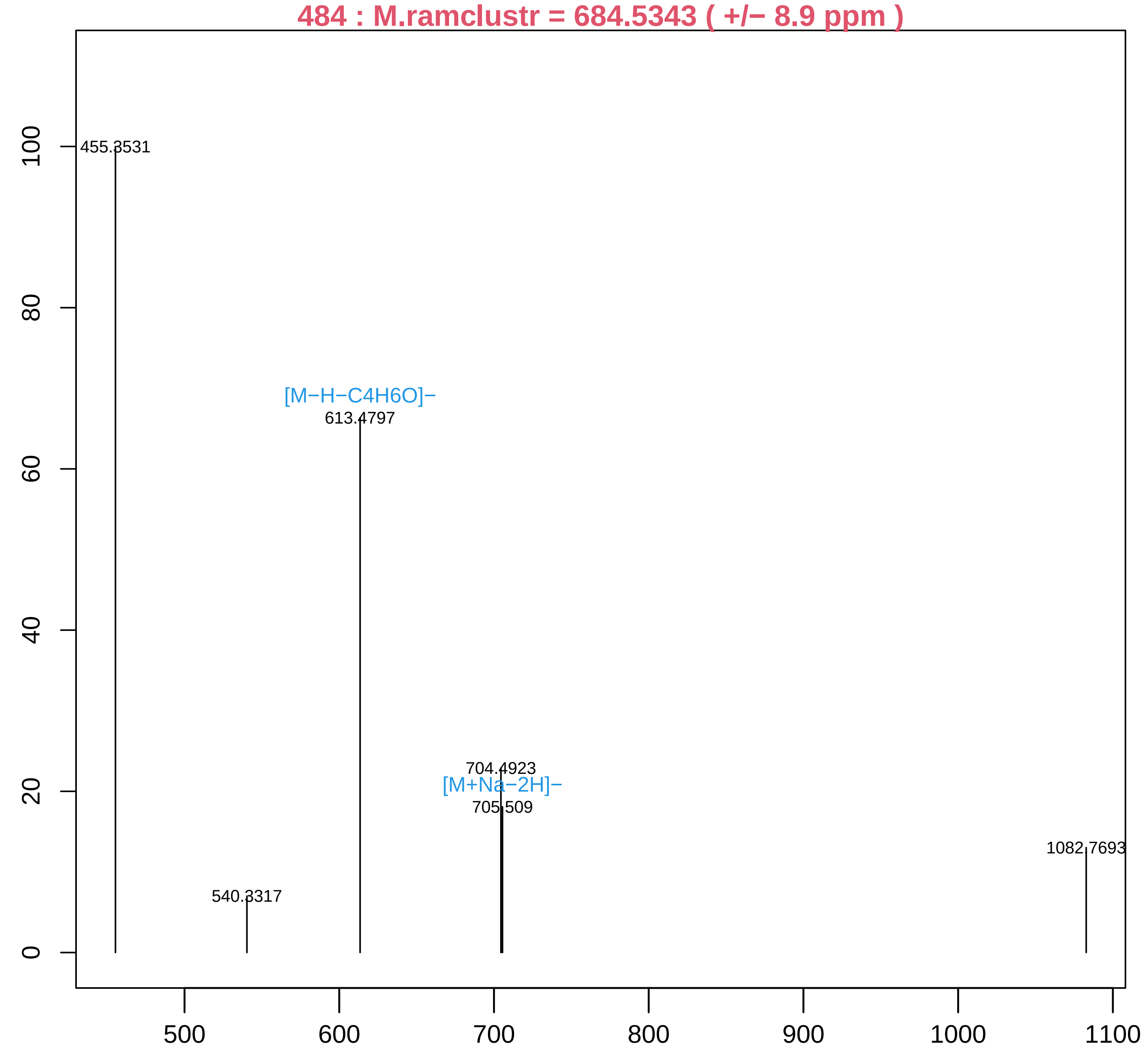
484 : M.ramclustr = 684.5343 (+/− 8.9 ppm)
455.3531
[M−H−C4H6O]−
613.4797
704.4923
[M+Na−2H]−
705.509
540.3317
1082.7693
0
20
40
60
80
100
500
600
700
800
900
1000
1100

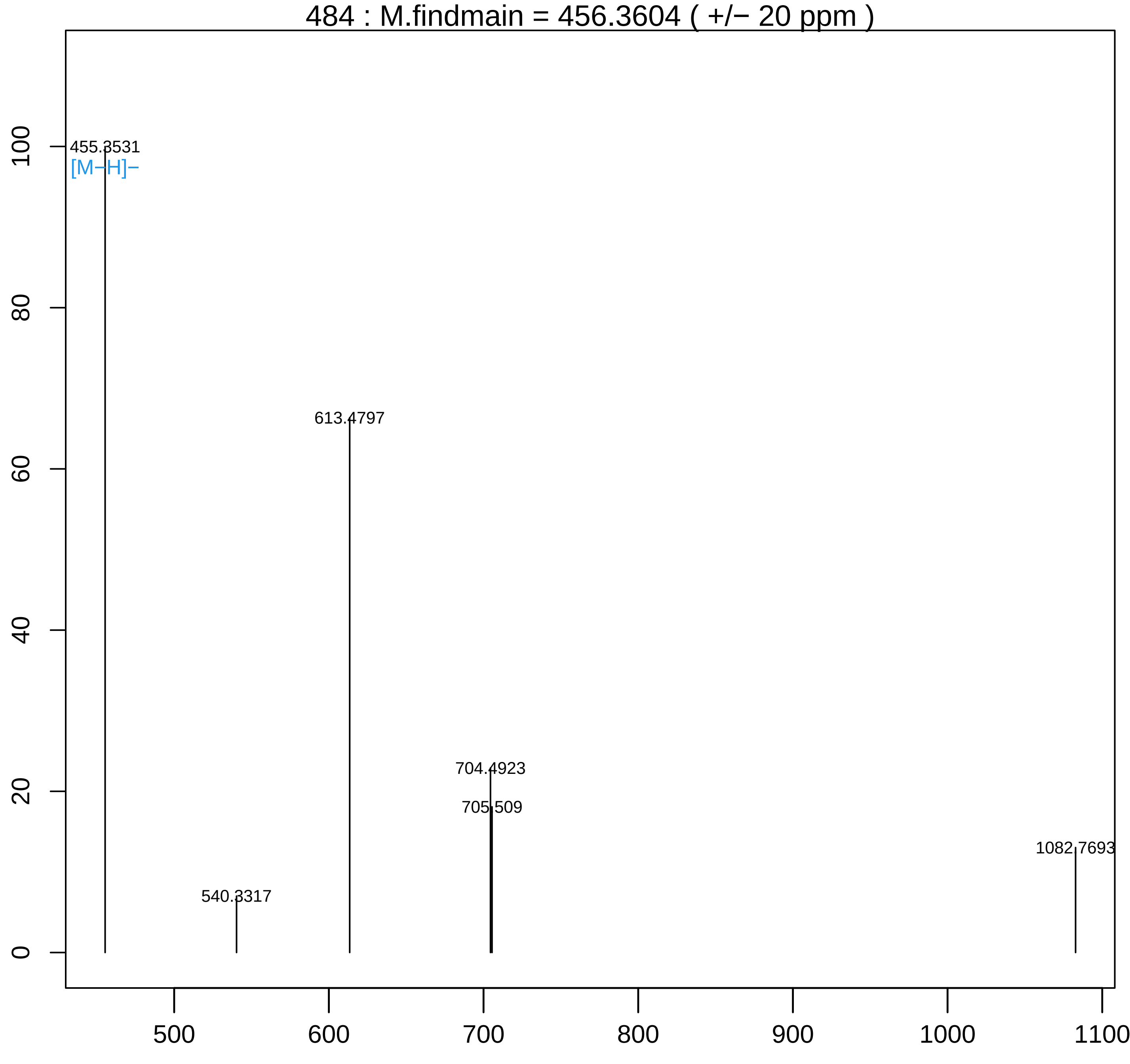
484 : M.findmain = 456.3604 (+/− 20 ppm)
455.3531
[M−H]−
613.4797
704.4923
705.509
540.3317
1082.7693
0
20
40
60
80
100
500
600
700
800
900
1000
1100

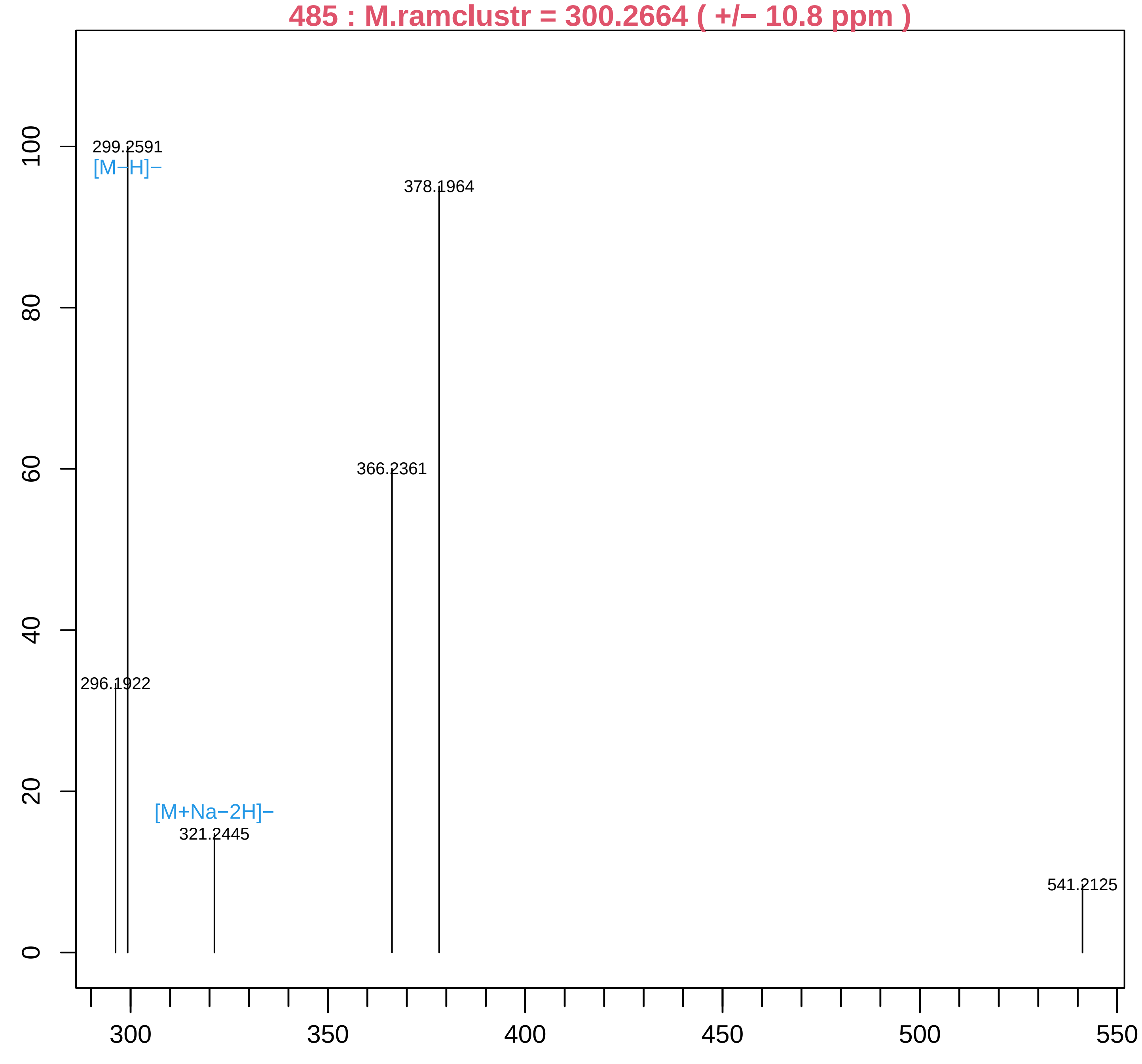
485 : M.ramclustr = 300.2664 (+/− 10.8 ppm)
299.2591
[M−H]−
378.1964
366.2361
296.1922
[M+Na−2H]−
321.2445
541.2125
0
20
40
60
80
100
300
350
400
450
500
550

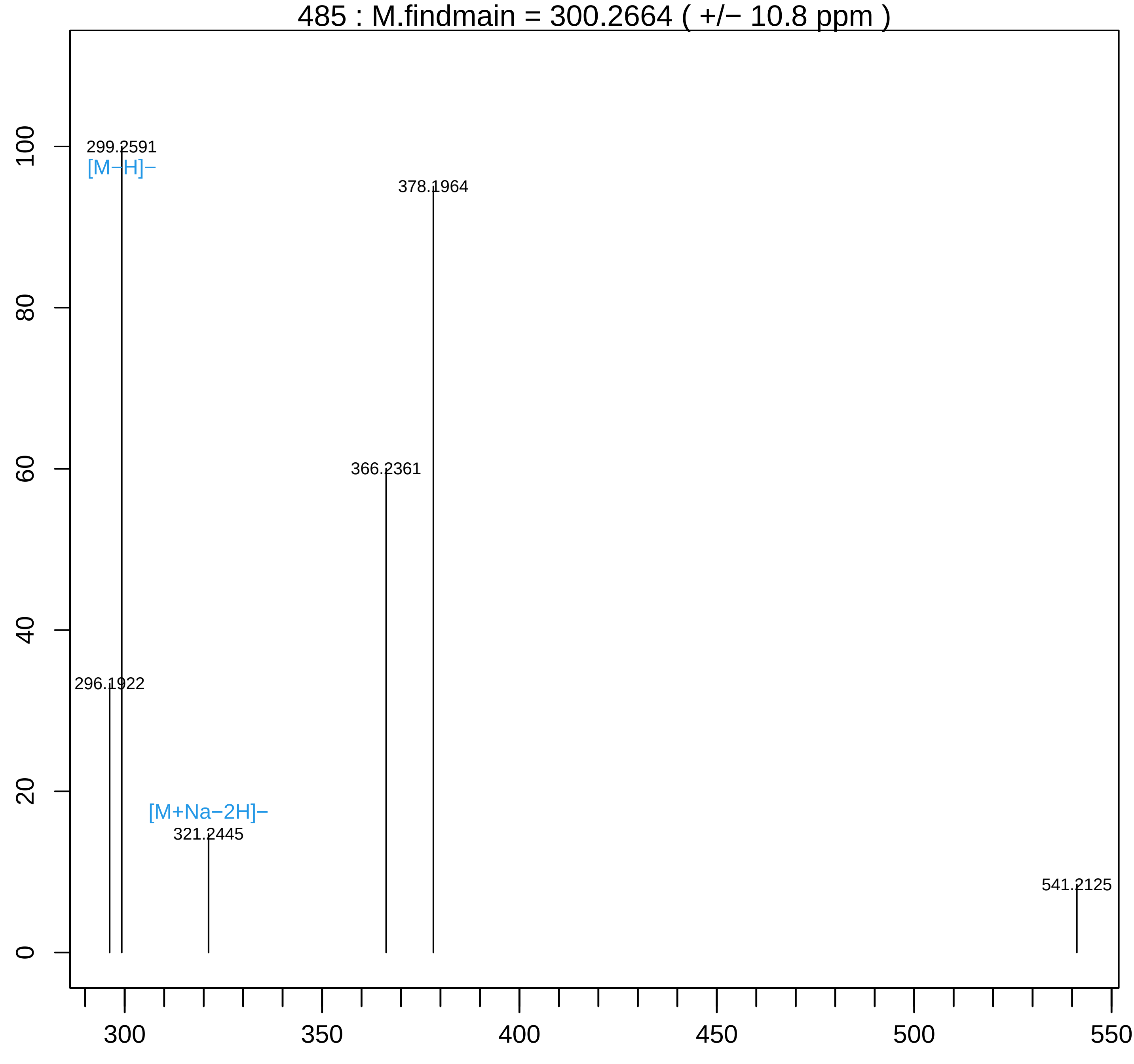
485 : M.findmain = 300.2664 (+/− 10.8 ppm)
299.2591
[M−H]−
378.1964
366.2361
296.1922
[M+Na−2H]−
321.2445
541.2125
0
20
40
60
80
100
300
350
400
450
500
550

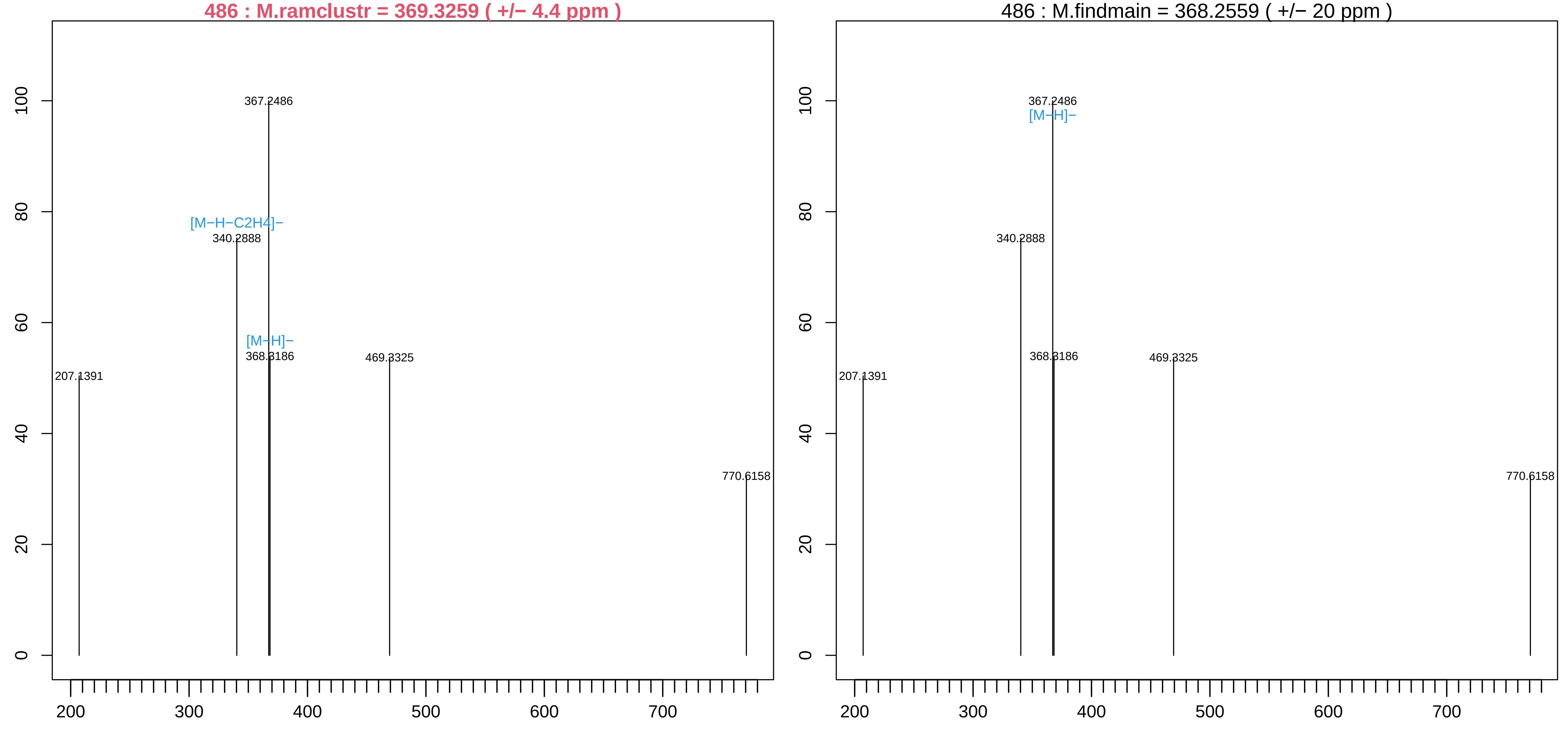
486 : M.ramclustr = 369.3259 (+/− 4.4 ppm)
367.2486
[M−H−C2H4]−
340.2888
[M−H]−
368.3186
469.3325
207.1391
770.6158
486 : M.findmain = 368.2559 (+/− 20 ppm)
367.2486
[M−H]−
340.2888
368.3186
469.3325
207.1391
770.6158

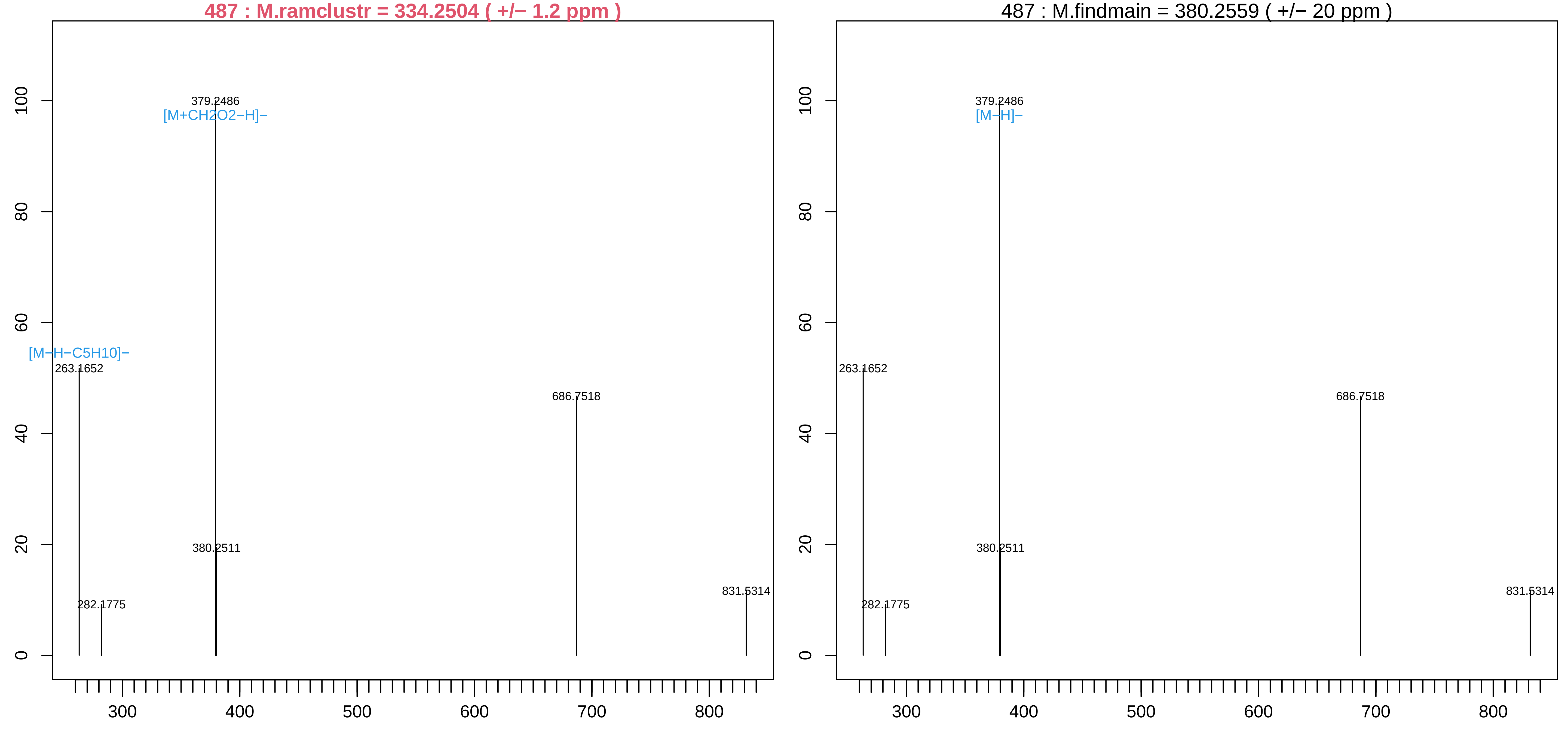

487 : M.ramclustr = 334.2504 (+/− 1.2 ppm)
379.2486
[M+CH2O2−H]−
[M−H−C5H10]−
263.1652
686.7518
380.2511
831.5314
282.1775
487 : M.findmain = 380.2559 (+/− 20 ppm)
379.2486
[M−H]−
263.1652
686.7518
380.2511
831.5314
282.1775

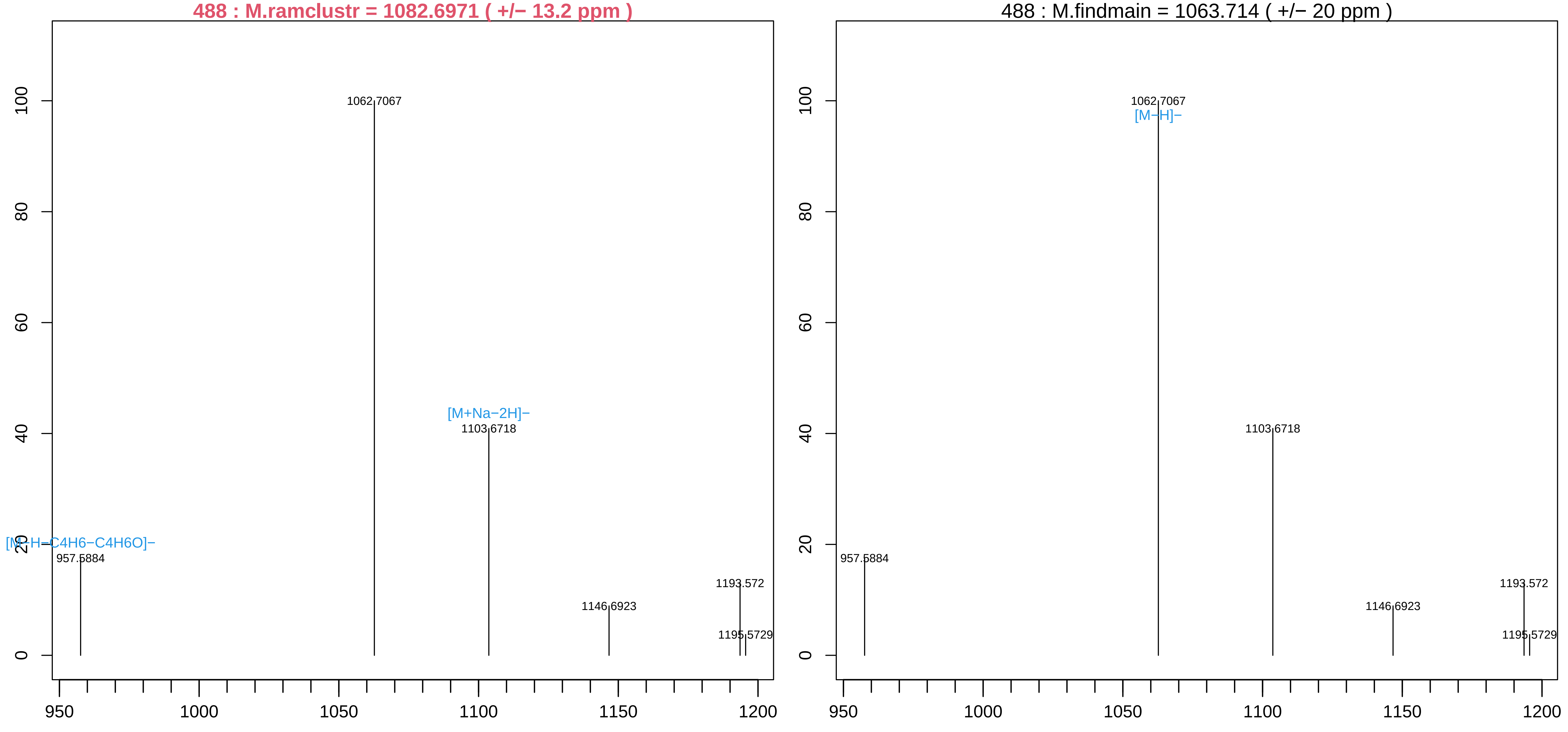
488 : M.ramclustr = 1082.6971 (+/− 13.2 ppm)
1062.7067
[M+Na−2H]−
1103.6718
[M−H−C4H6−C4H6O]−
957.5884
1193.572
1146.6923
1195.5729
488 : M.findmain = 1063.714 (+/− 20 ppm)
1062.7067
[M−H]−
1103.6718
957.5884
1193.572
1146.6923
1195.5729
0
20
40
60
80
100
950
1000
1050
1100
1150
1200

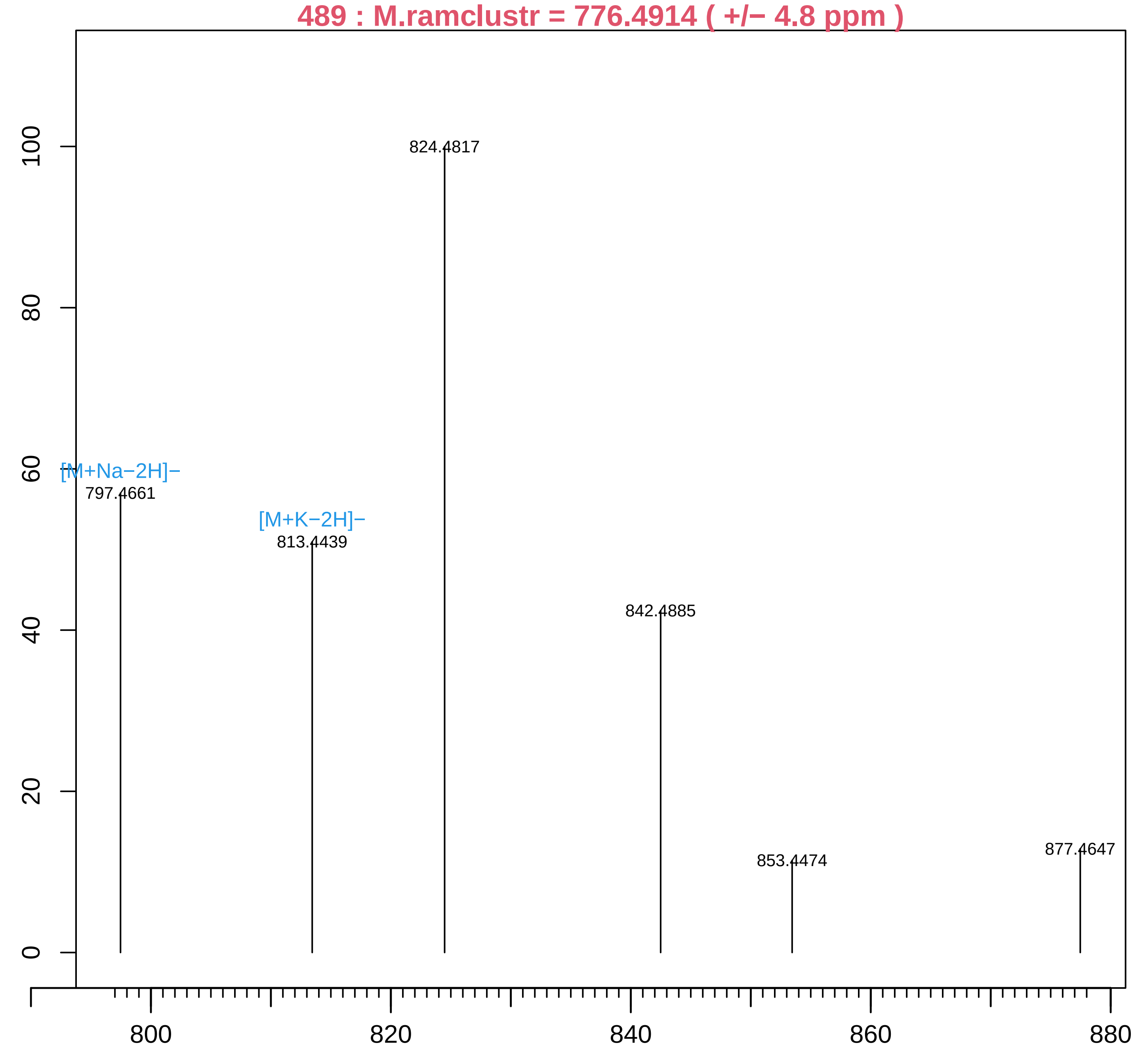
489 : M.ramclustr = 776.4914 (+/− 4.8 ppm)
824.4817
[M+Na−2H]−
797.4661
[M+K−2H]−
813.4439
842.4885
853.4474
877.4647
0
20
40
60
80
100
800
820
840
860
880

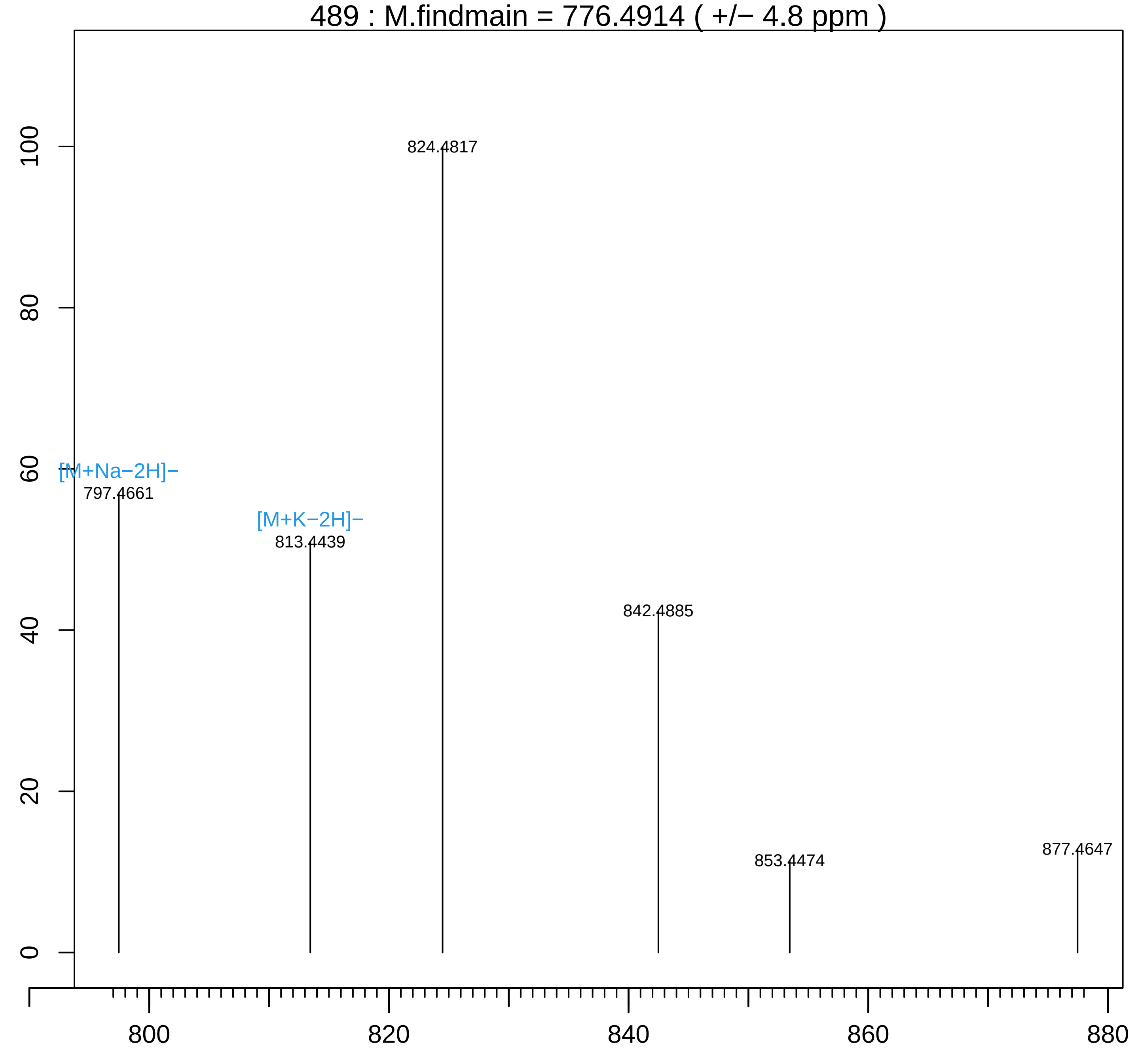
489 : M.findmain = 776.4914 (+/− 4.8 ppm)
824.4817
[M+Na−2H]−
797.4661
[M+K−2H]−
813.4439
842.4885
853.4474
877.4647
0
20
40
60
80
100
800
820
840
860
880

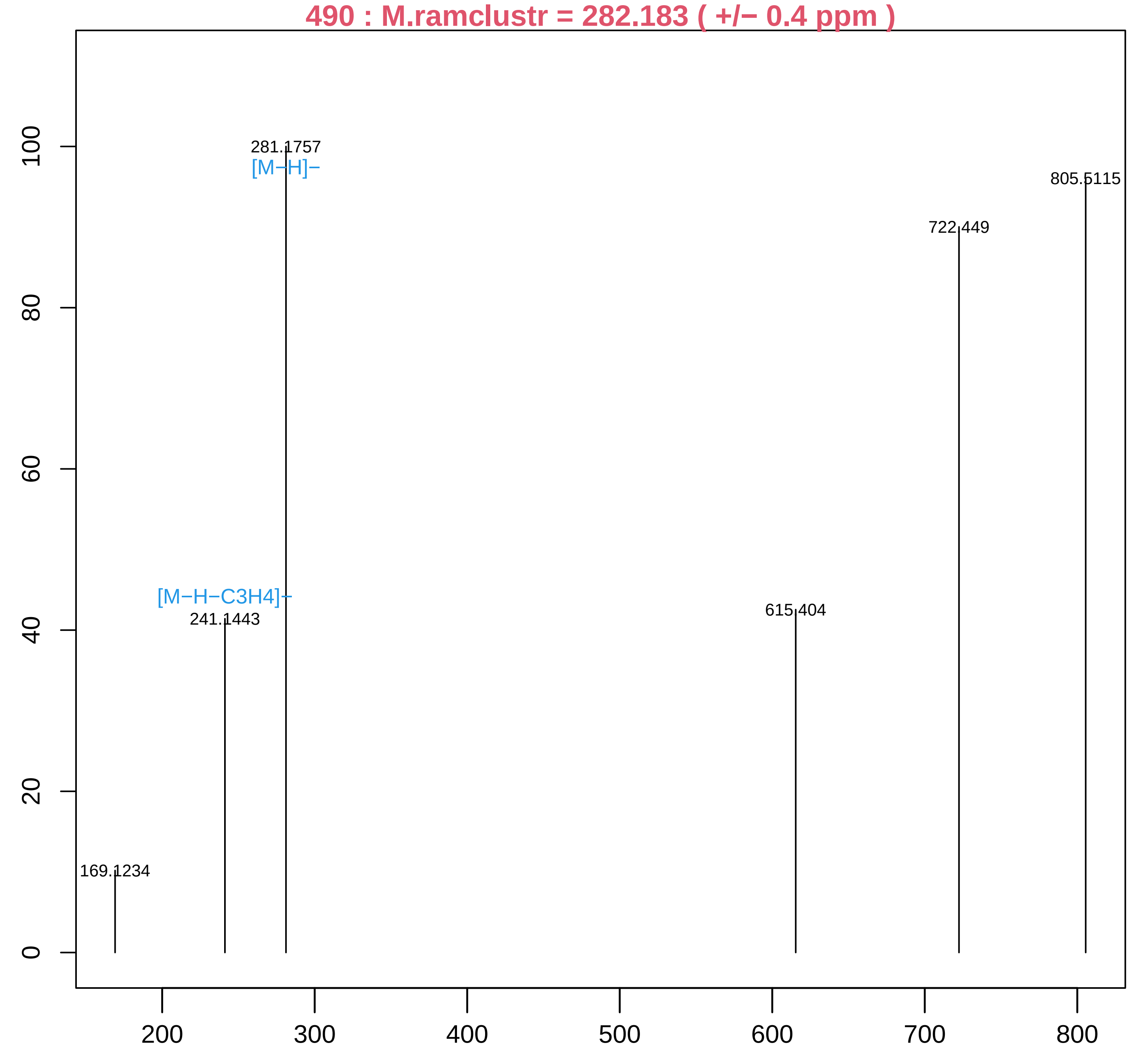
490 : M.ramclustr = 282.183 (+/− 0.4 ppm)
281.1757
[M−H]−
805.5115
722.449
[M−H−C3H4]−
241.1443
615.404
169.1234
0
20
40
60
80
100
200
300
400
500
600
700
800

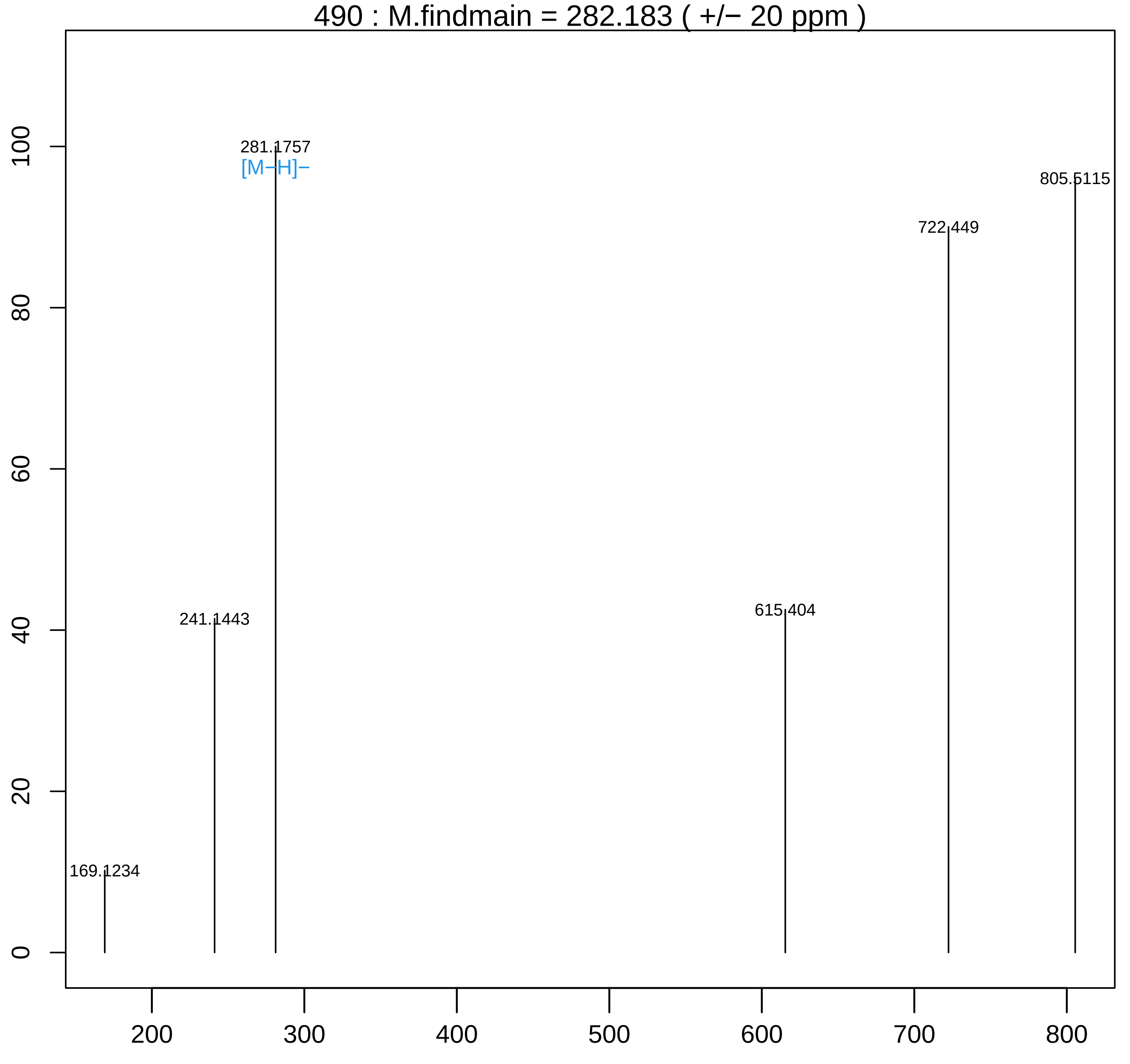
490 : M.findmain = 282.183 (+/− 20 ppm)
281.1757
[M−H]−
805.5115
722.449
241.1443
615.404
169.1234
0
20
40
60
80
100
200
300
400
500
600
700
800

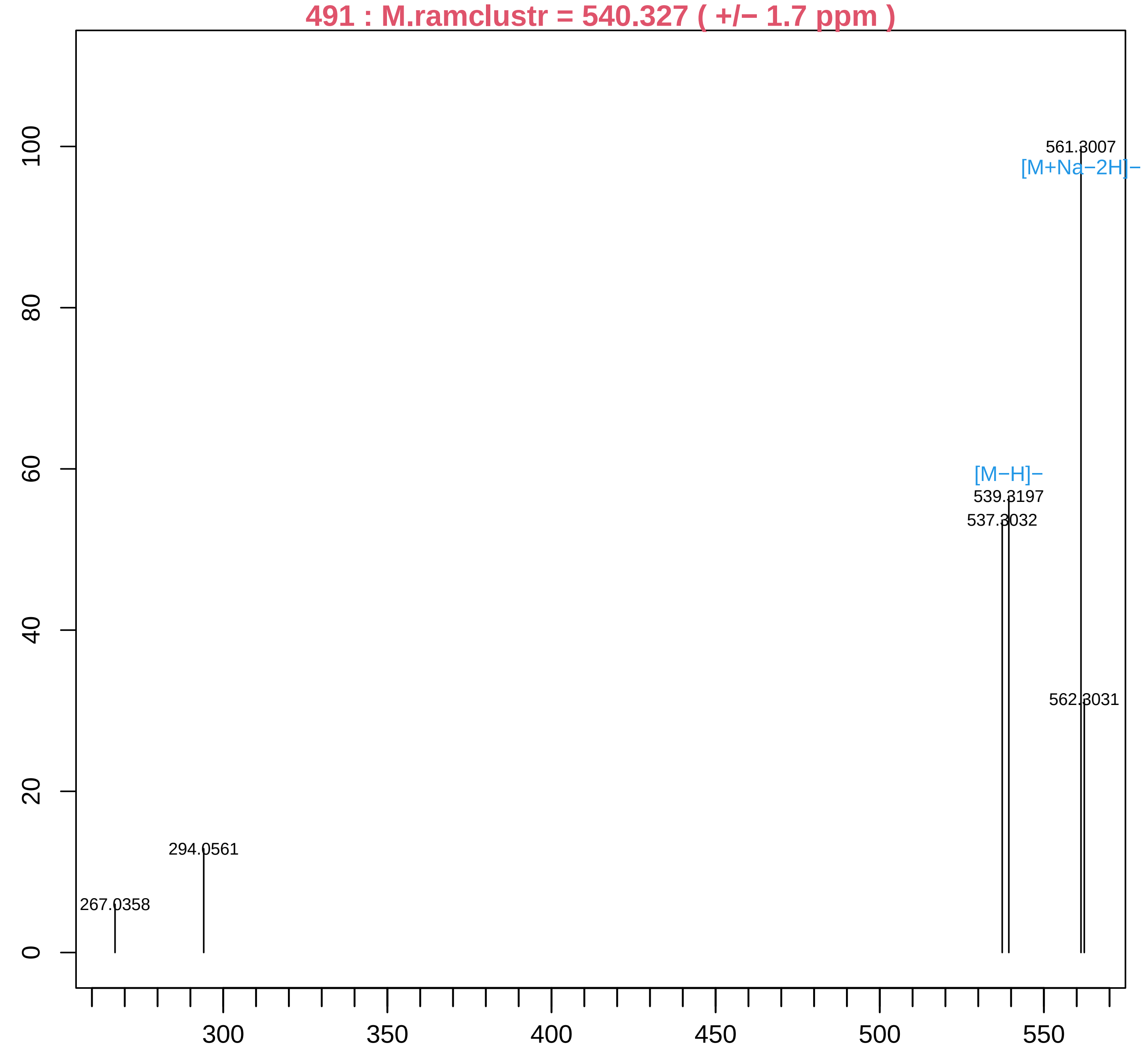
491 : M.ramclustr = 540.327 (+/− 1.7 ppm)
561.3007
[M+Na−2H]−
[M−H]−
539.3197
537.3032
562.3031
294.0561
267.0358
0
20
40
60
80
100
300
350
400
450
500
550

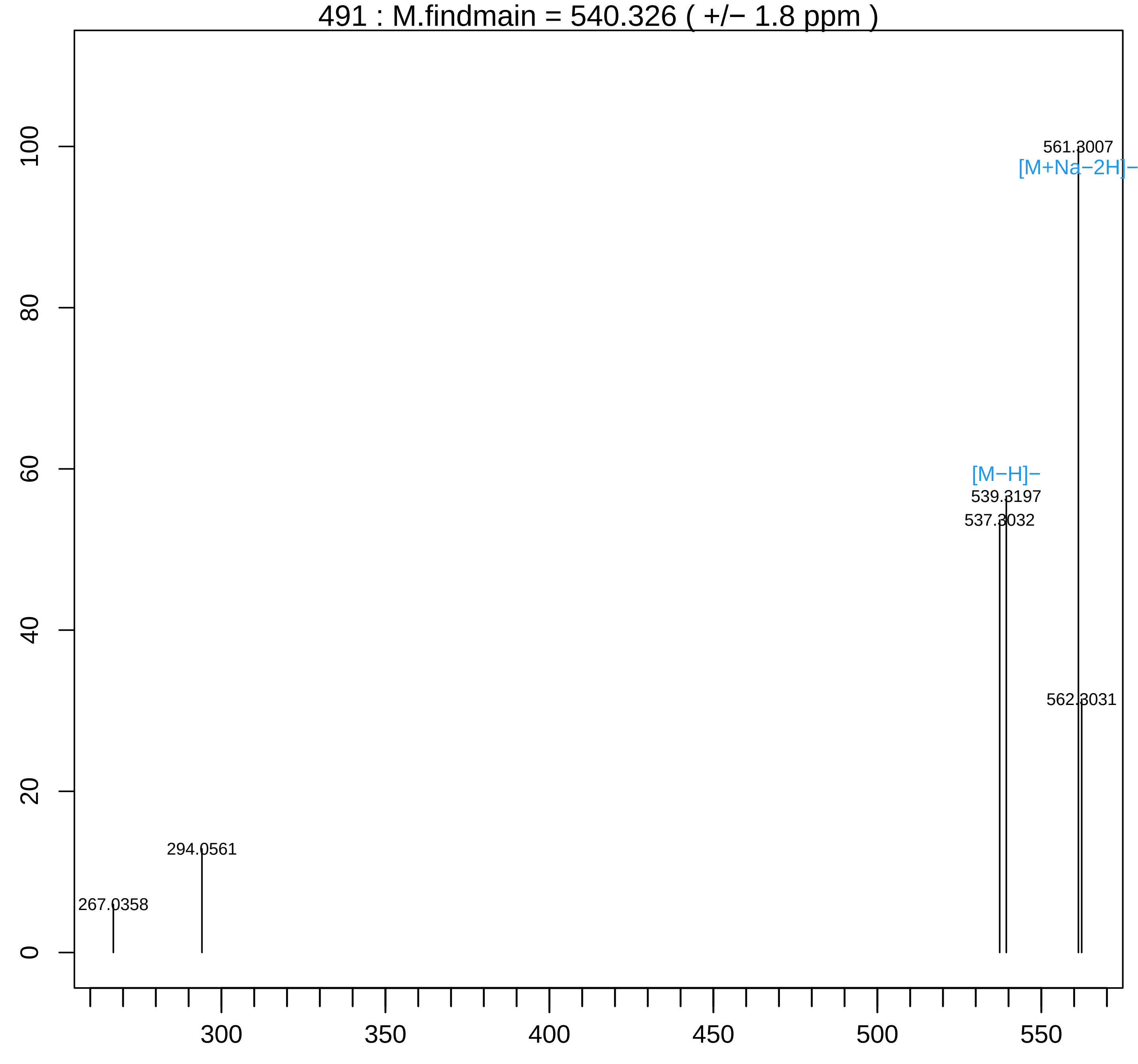
491 : M.findmain = 540.326 (+/− 1.8 ppm)
561.3007
[M+Na−2H]−
[M−H]−
539.3197
537.3032
562.3031
294.0561
267.0358
0
20
40
60
80
100
300
350
400
450
500
550

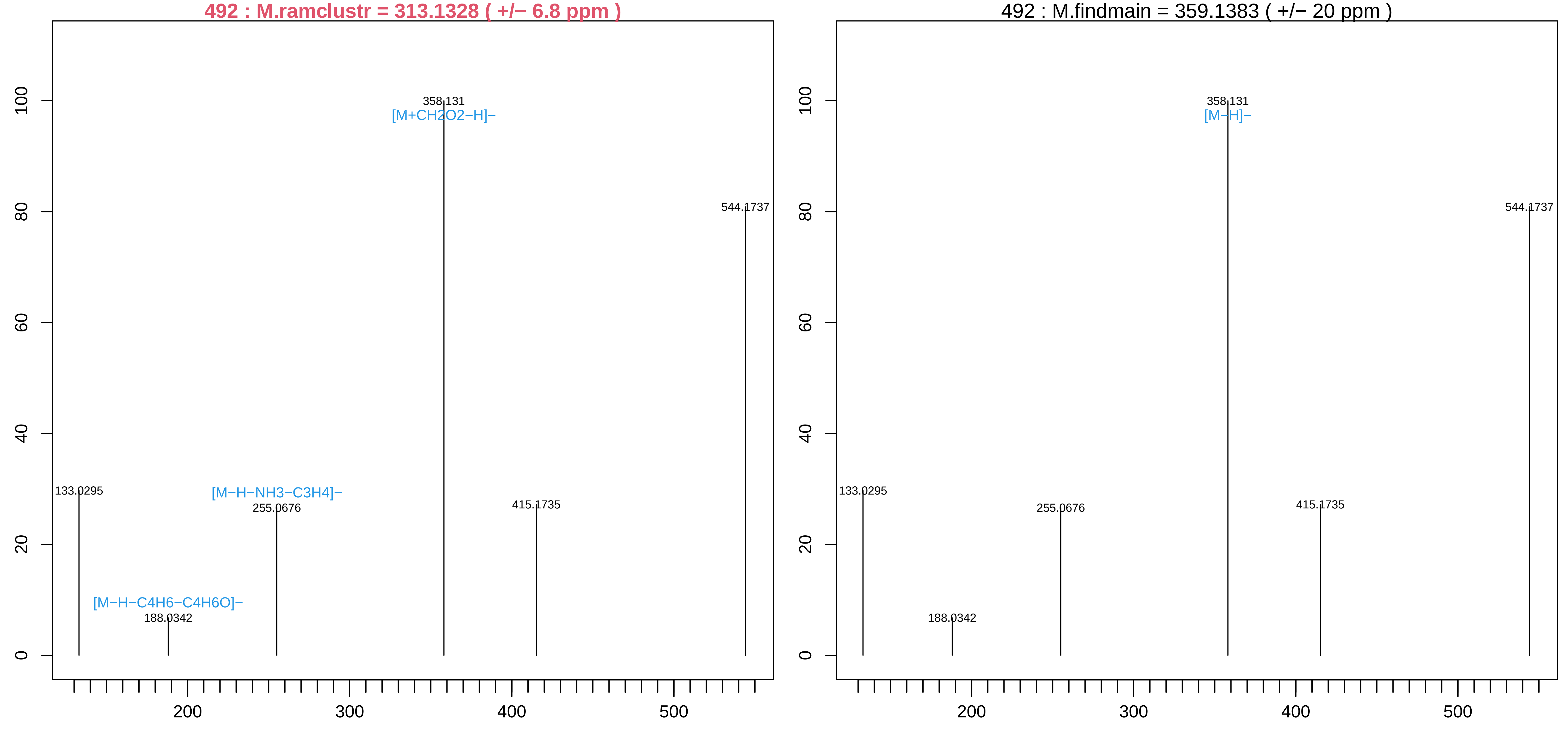
492 : M.ramclustr = 313.1328 (+/− 6.8 ppm)
358.131
[M+CH2O2−H]−
544.1737
133.0295
[M−H−NH3−C3H4]−
255.0676
415.1735
[M−H−C4H6−C4H6O]−
188.0342
492 : M.findmain = 359.1383 (+/− 20 ppm)
358.131
[M−H]−
544.1737
133.0295
255.0676
415.1735
188.0342
0
20
40
60
80
100
200
300
400
500

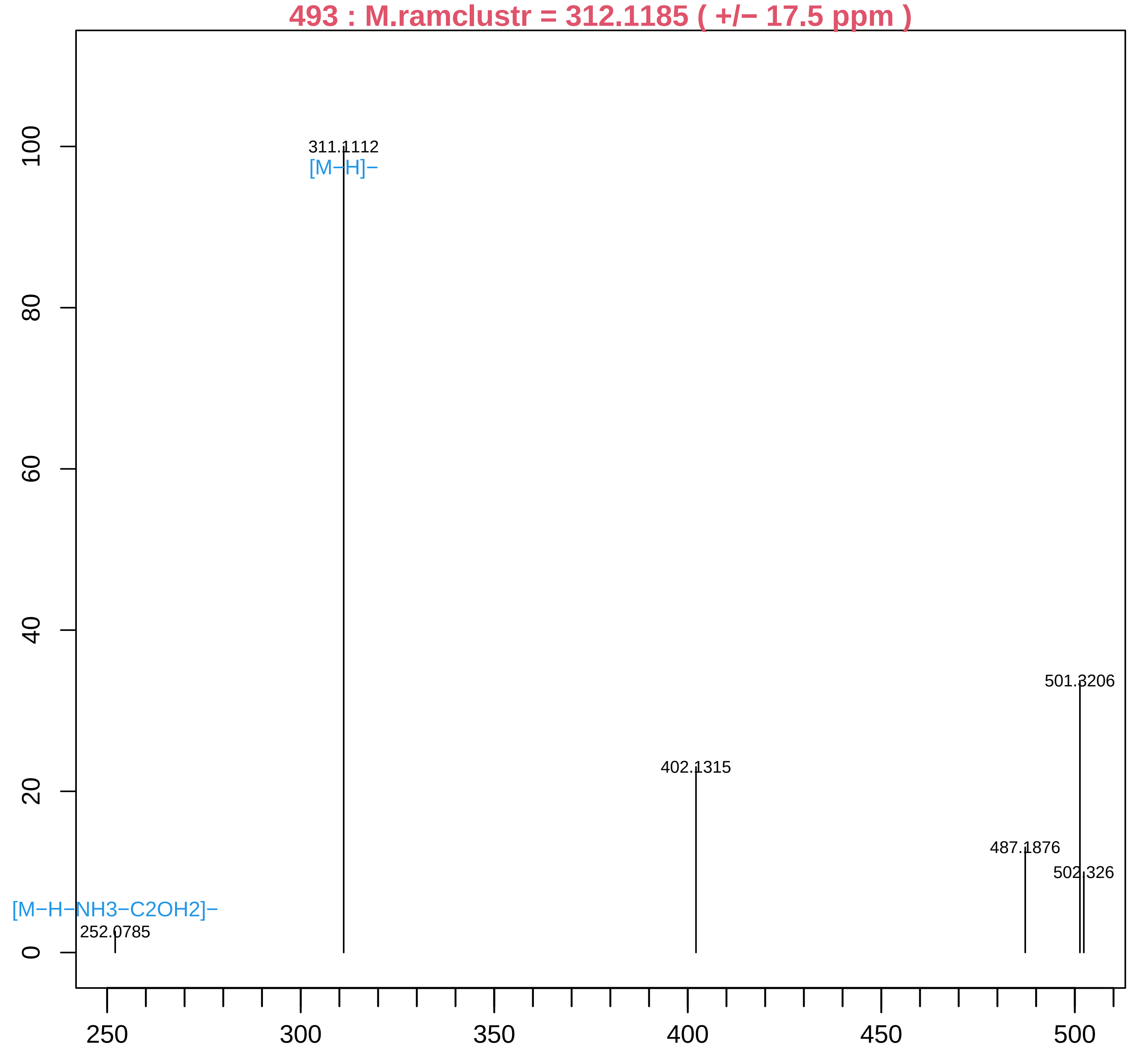
493 : M.ramclustr = 312.1185 (+/− 17.5 ppm)
311.1112
[M−H]−
402.1315
501.3206
487.1876
502.326
[M−H−NH3−C2OH2]−
252.0785
0
20
40
60
80
100
250
300
350
400
450
500

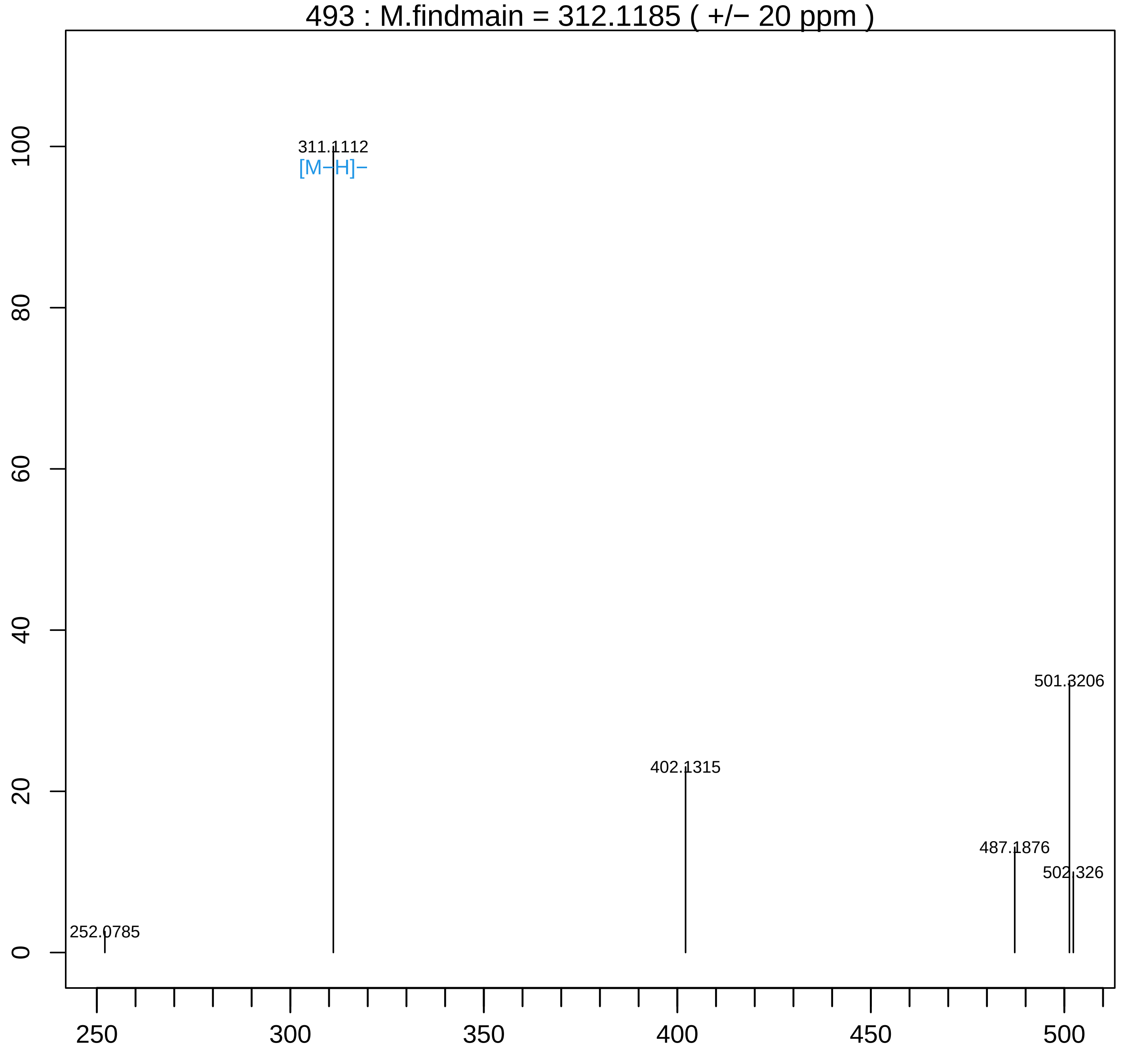
493 : M.findmain = 312.1185 (+/− 20 ppm)
311.1112
[M−H]−
402.1315
501.3206
487.1876
502.326
252.0785
0
20
40
60
80
100
250
300
350
400
450
500

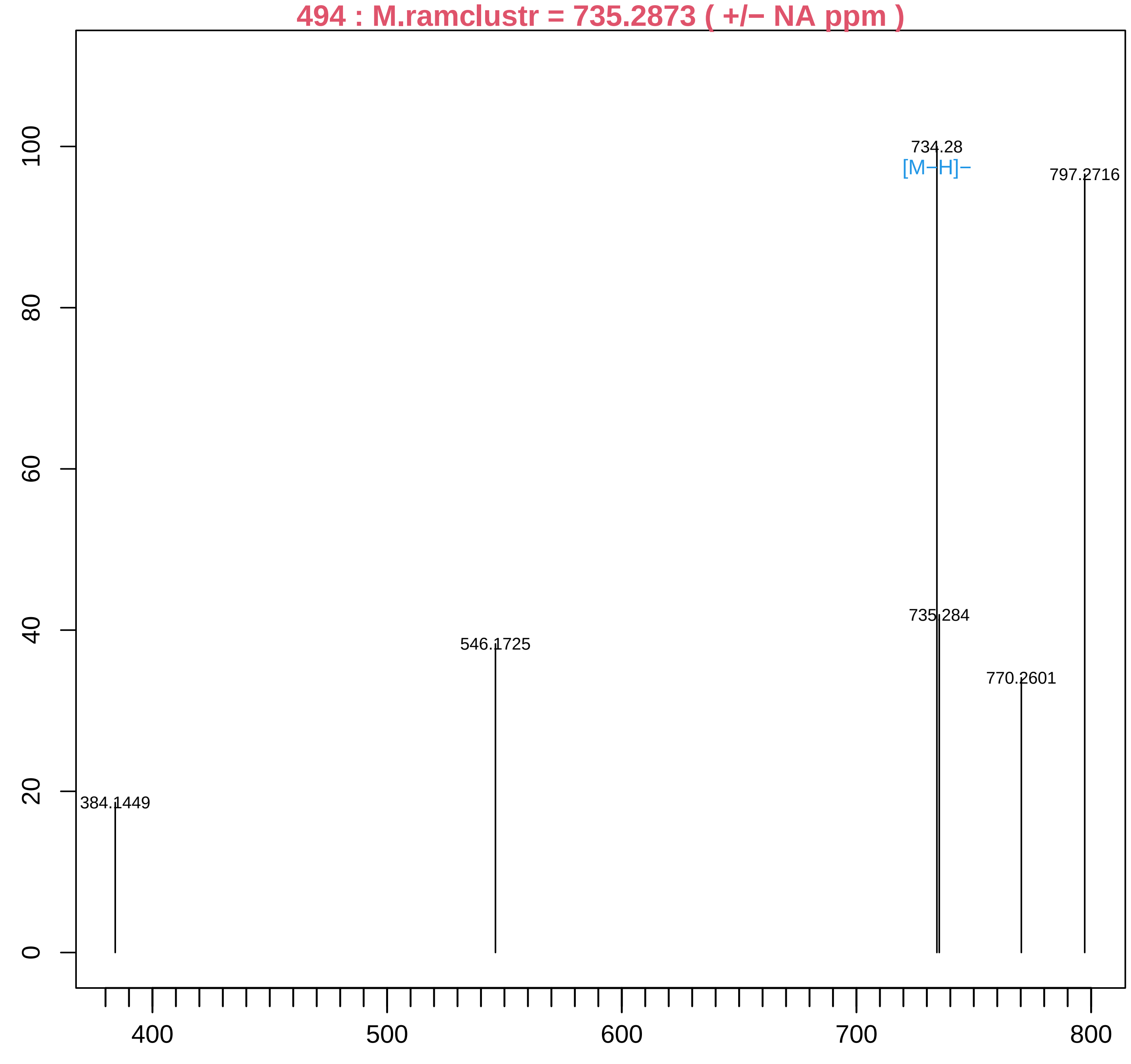
494 : M.ramclustr = 735.2873 (+/− NA ppm)
734.28
[M−H]−
797.2716
735.284
546.1725
770.2601
384.1449
0
20
40
60
80
100
400
500
600
700
800

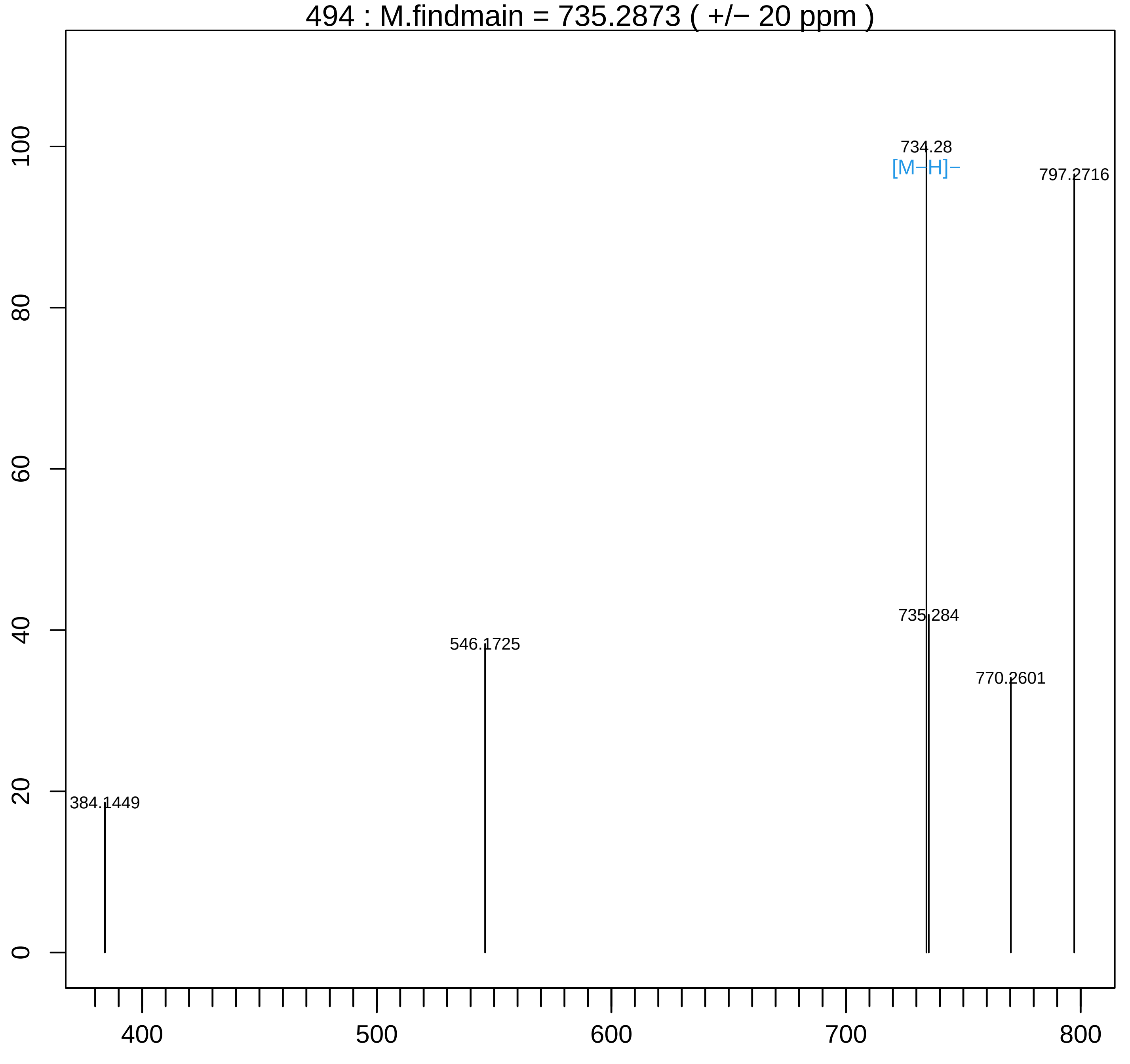
494 : M.findmain = 735.2873 (+/− 20 ppm)
734.28
[M−H]−
797.2716
735.284
546.1725
770.2601
384.1449
0
20
40
60
80
100
400
500
600
700
800

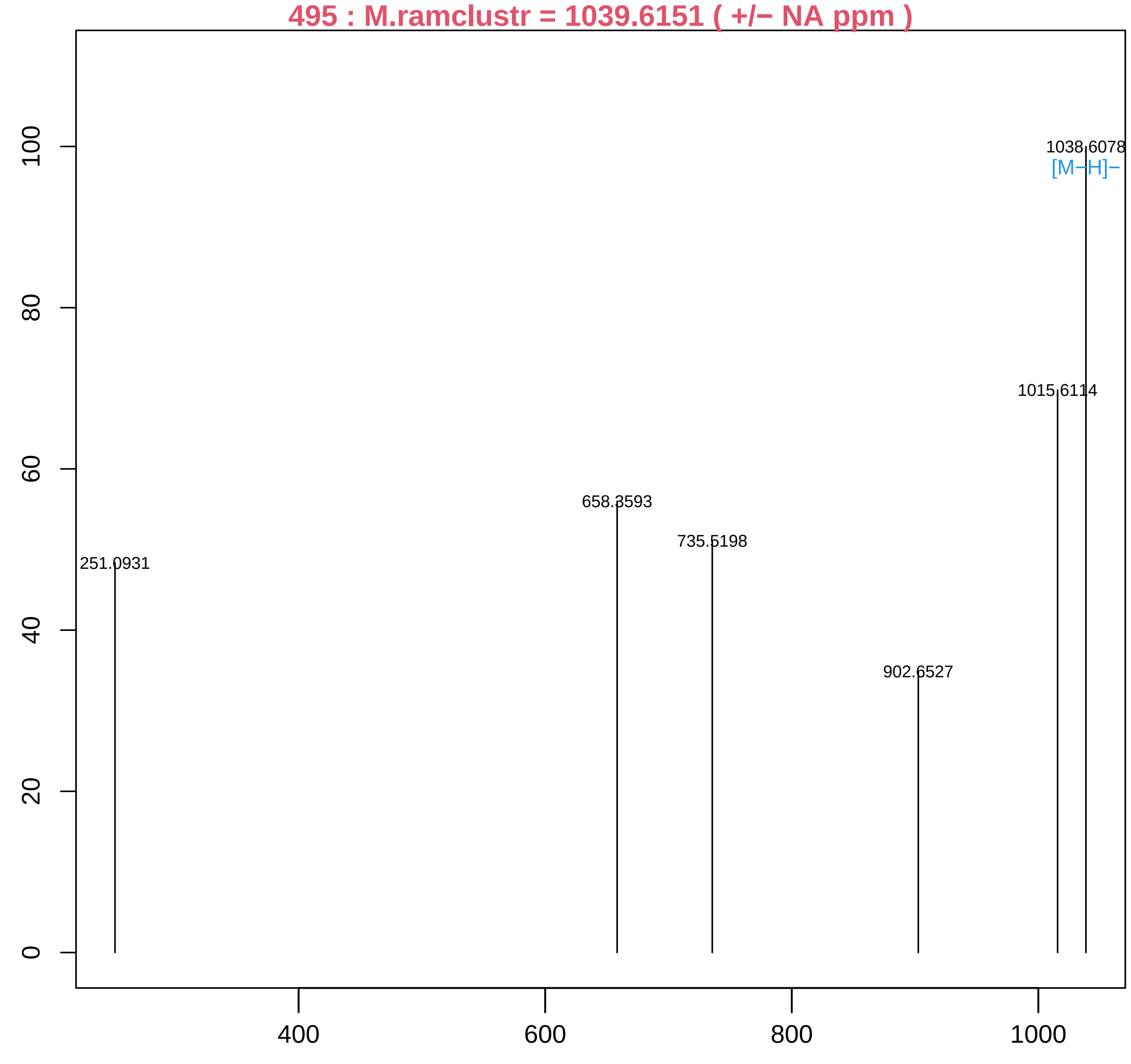
495 : M.ramclustr = 1039.6151 (+/− NA ppm)
1038.6078
[M−H]−
1015.6114
658.3593
735.5198
251.0931
902.6527
0
20
40
60
80
100
400
600
800
1000

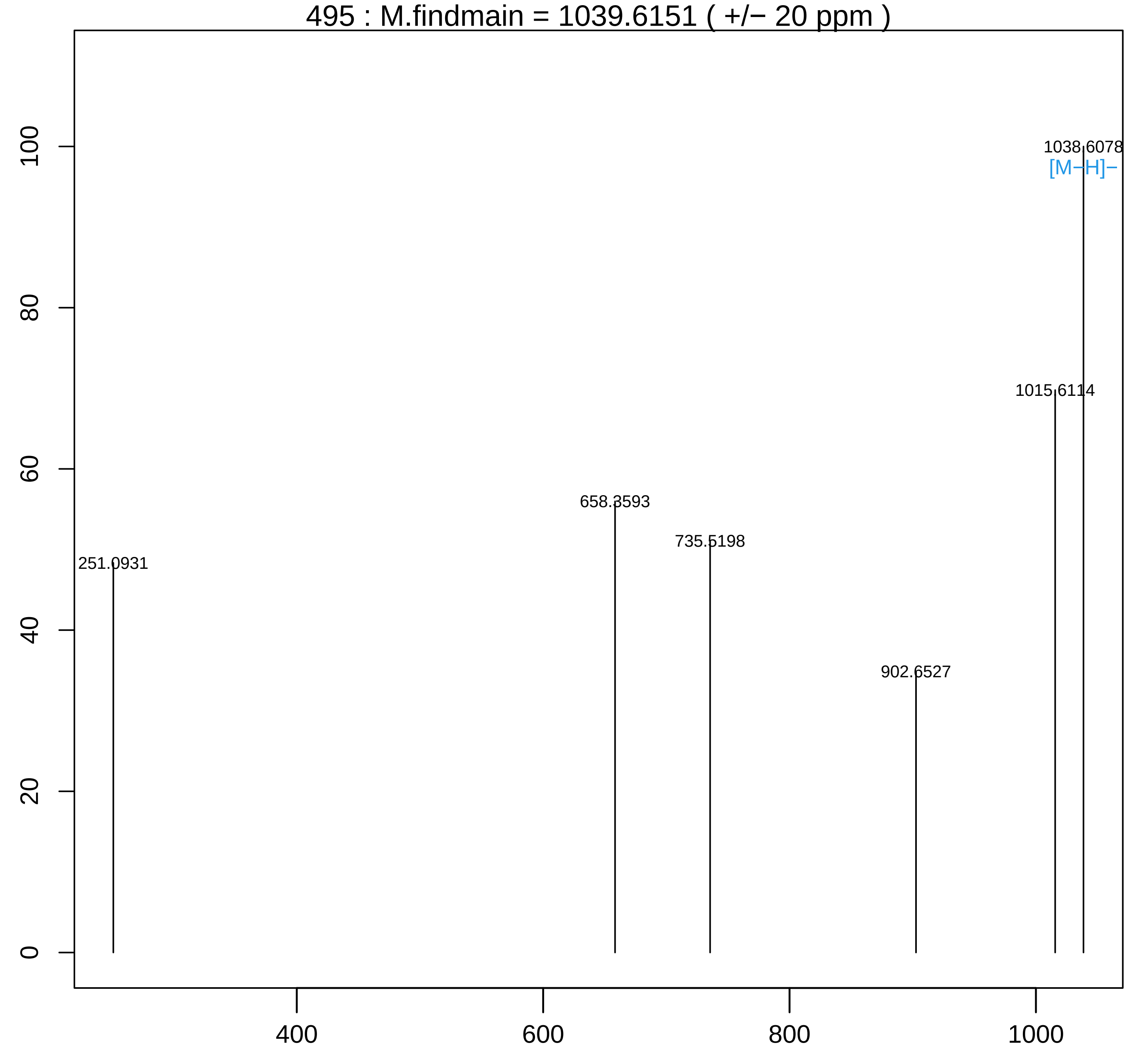
495 : M.findmain = 1039.6151 (+/− 20 ppm)
1038.6078
[M−H]−
1015.6114
658.3593
735.5198
251.0931
902.6527
0
20
40
60
80
100
400
600
800
1000

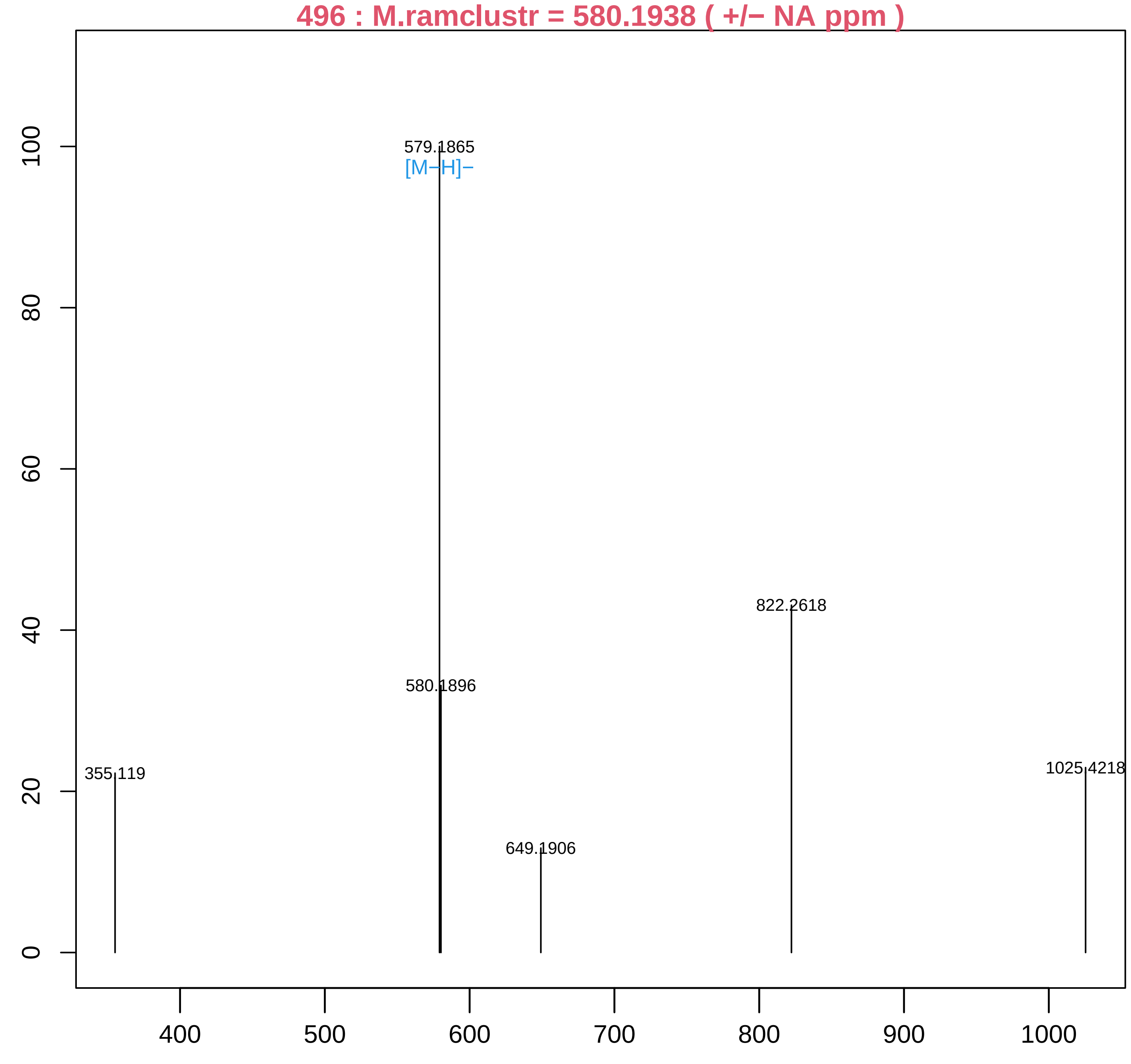
496 : M.ramclustr = 580.1938 (+/− NA ppm)
579.1865
[M−H]−
580.1896
822.2618
649.1906
355.119
1025.4218
0
20
40
60
80
100
400
500
600
700
800
900
1000

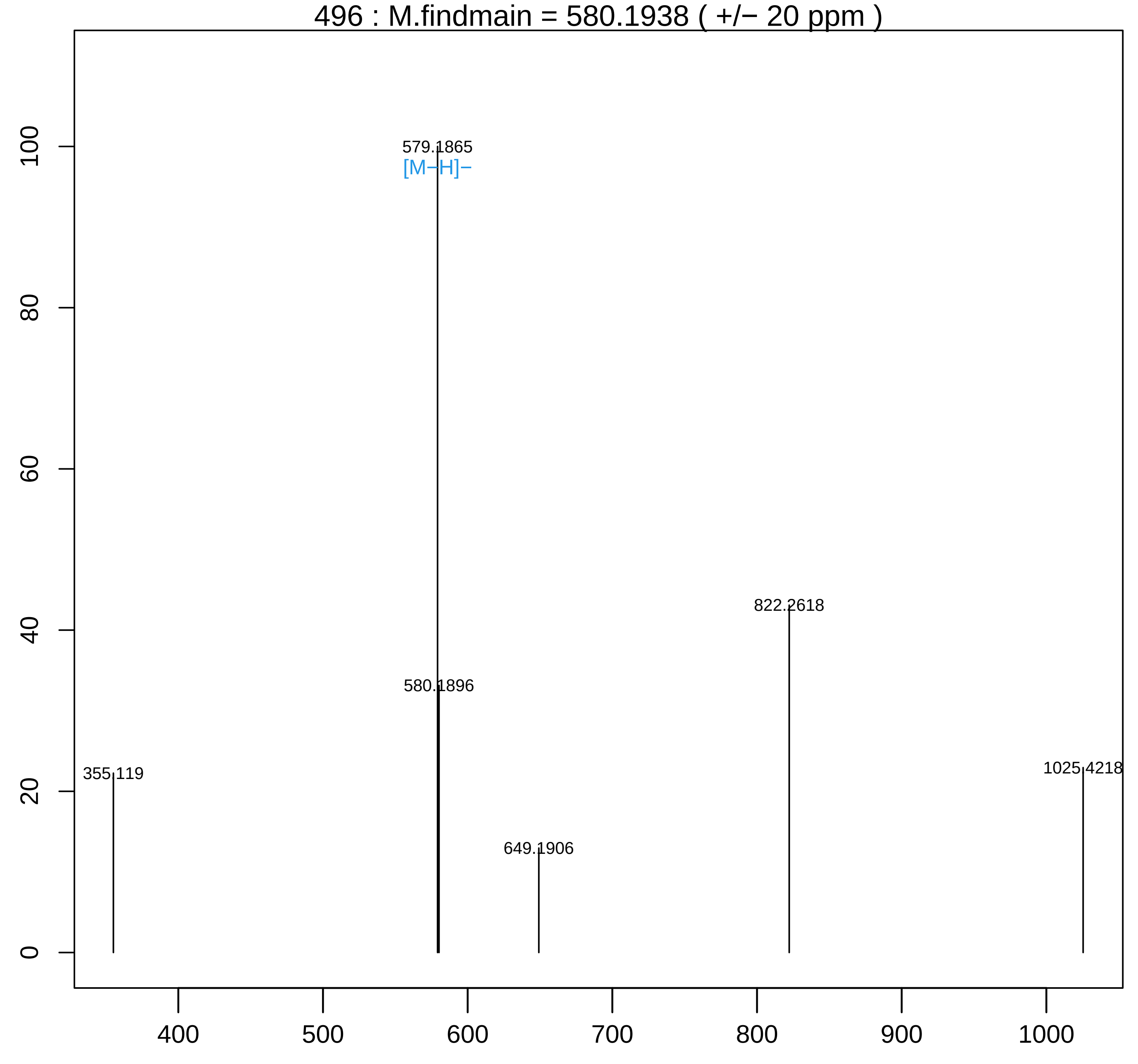
496 : M.findmain = 580.1938 (+/− 20 ppm)
579.1865
[M−H]−
580.1896
822.2618
649.1906
355.119
1025.4218
0
20
40
60
80
100
400
500
600
700
800
900
1000

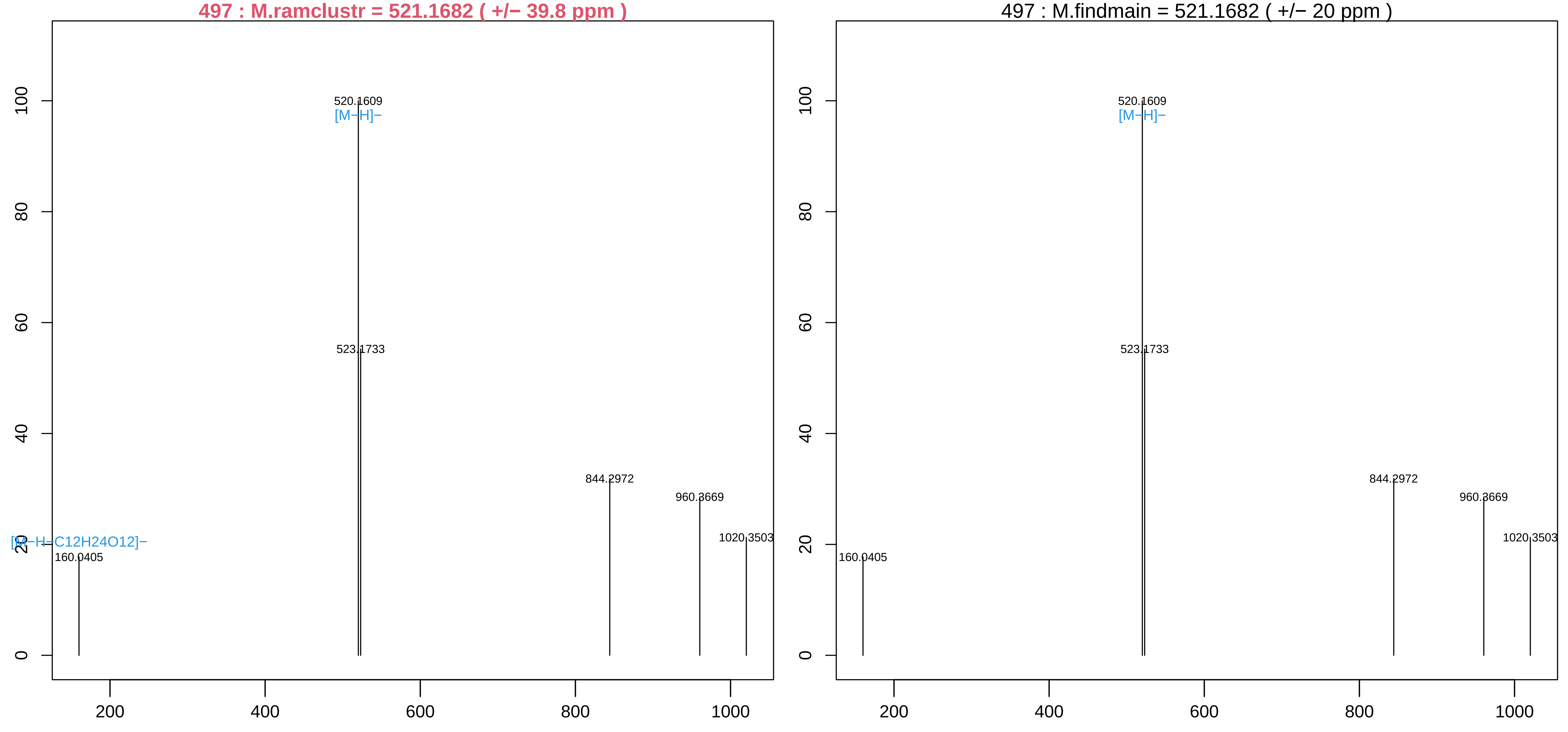
497 : M.ramclustr = 521.1682 (+/− 39.8 ppm)
520.1609
[M−H]−
523.1733
844.2972
960.3669
1020.3503
[M−H−C12H24O12]−
160.0405
497 : M.findmain = 521.1682 (+/− 20 ppm)
520.1609
[M−H]−
523.1733
844.2972
960.3669
1020.3503
160.0405
0
20
40
60
80
100
200
400
600
800
1000

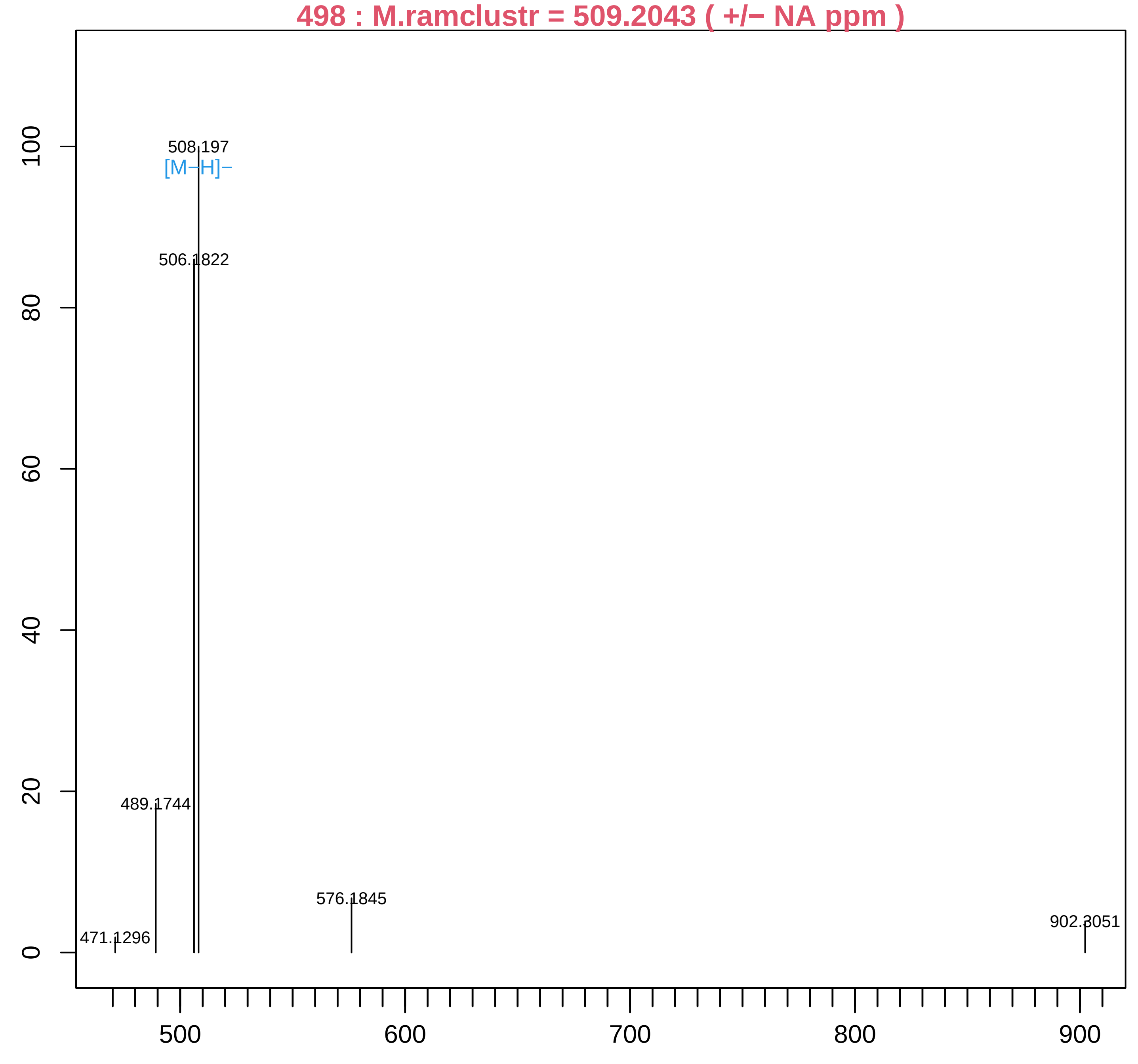
498 : M.ramclustr = 509.2043 (+/− NA ppm)
508.197
[M−H]−
506.1822
489.1744
471.1296
576.1845
902.3051
0
20
40
60
80
100
500
600
700
800
900

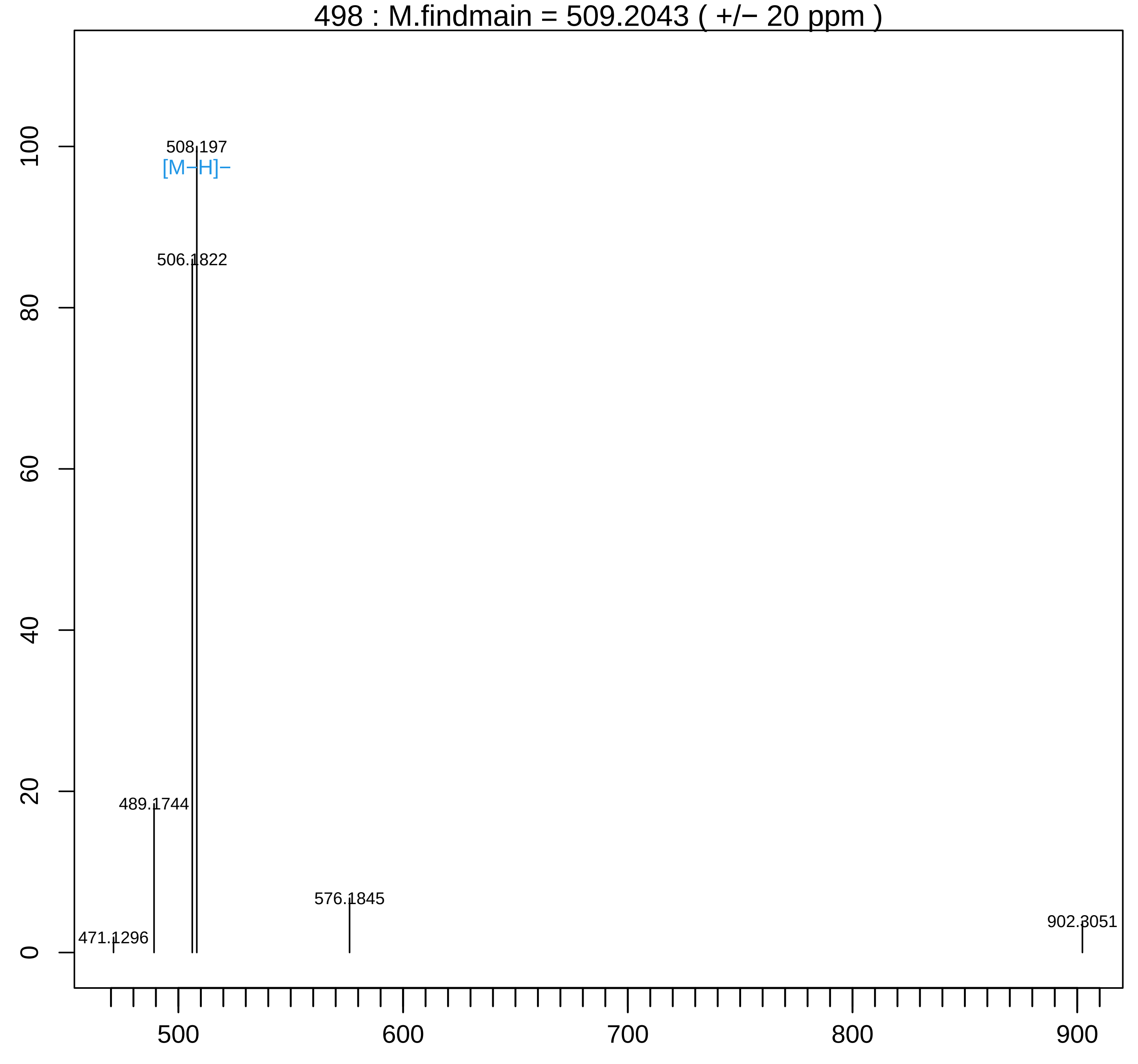
498 : M.findmain = 509.2043 (+/− 20 ppm)
508.197
[M−H]−
506.1822
489.1744
471.1296
576.1845
902.3051
0
20
40
60
80
100
500
600
700
800
900

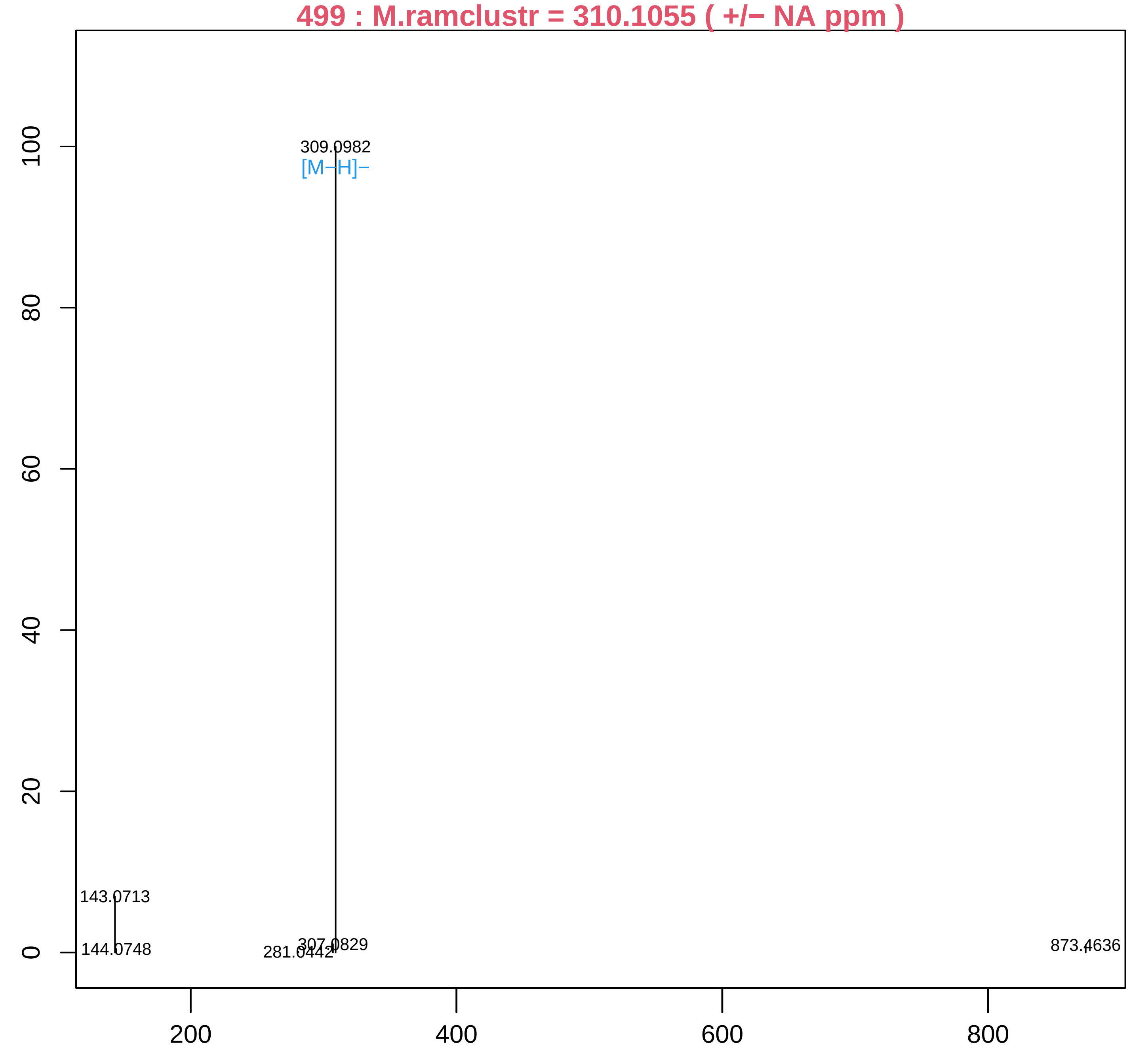

499 : M.ramclustr = 310.1055 (+/− NA ppm)
309.0982
[M−H]−
143.0713
144.0748
281.0442
307.0829
873.4636
0
20
40
60
80
100
200
400
600
800

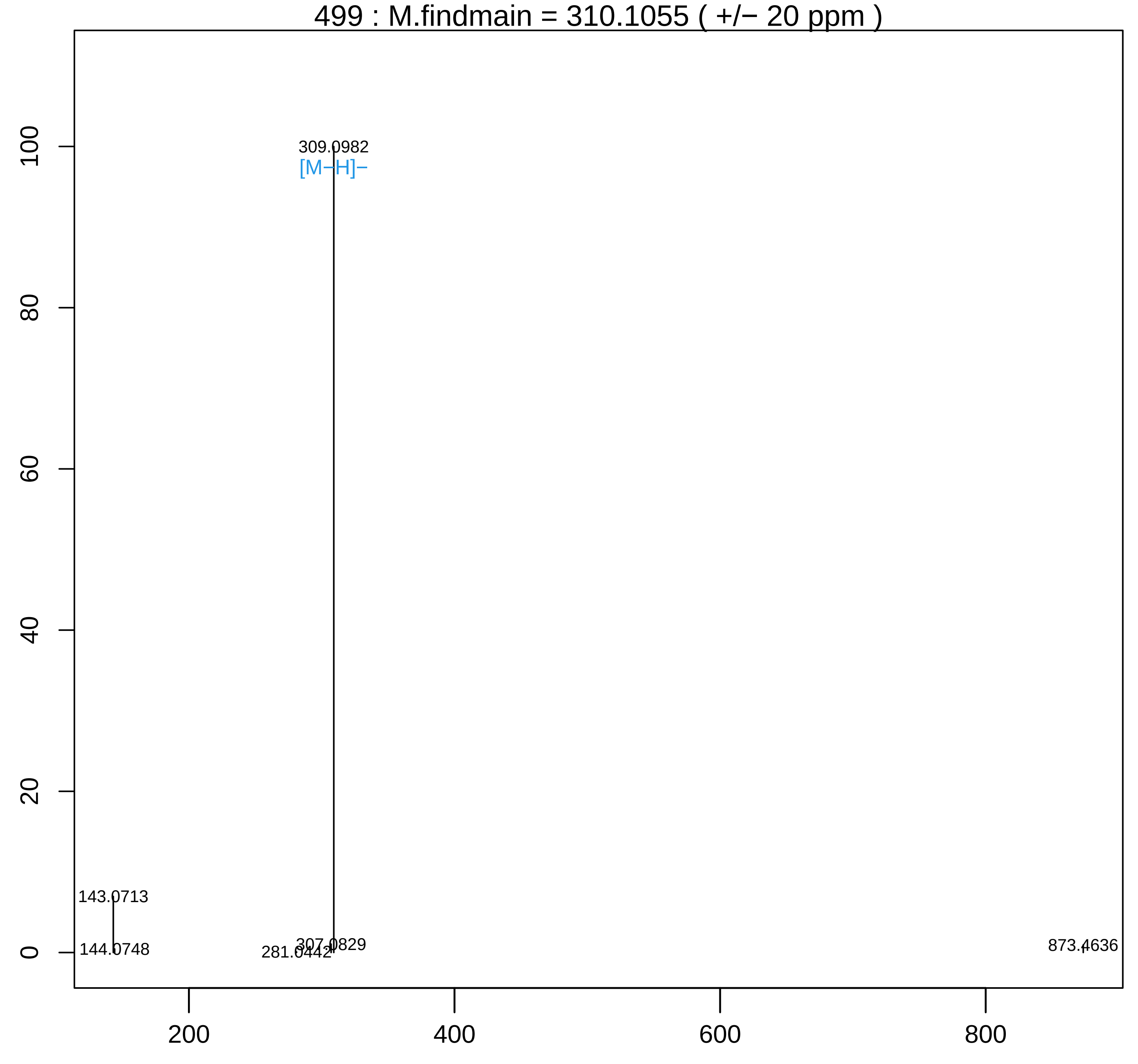

499 : M.findmain = 310.1055 (+/− 20 ppm)
309.0982
[M−H]−
143.0713
144.0748
281.0442
307.0829
873.4636
0
20
40
60
80
100
200
400
600
800

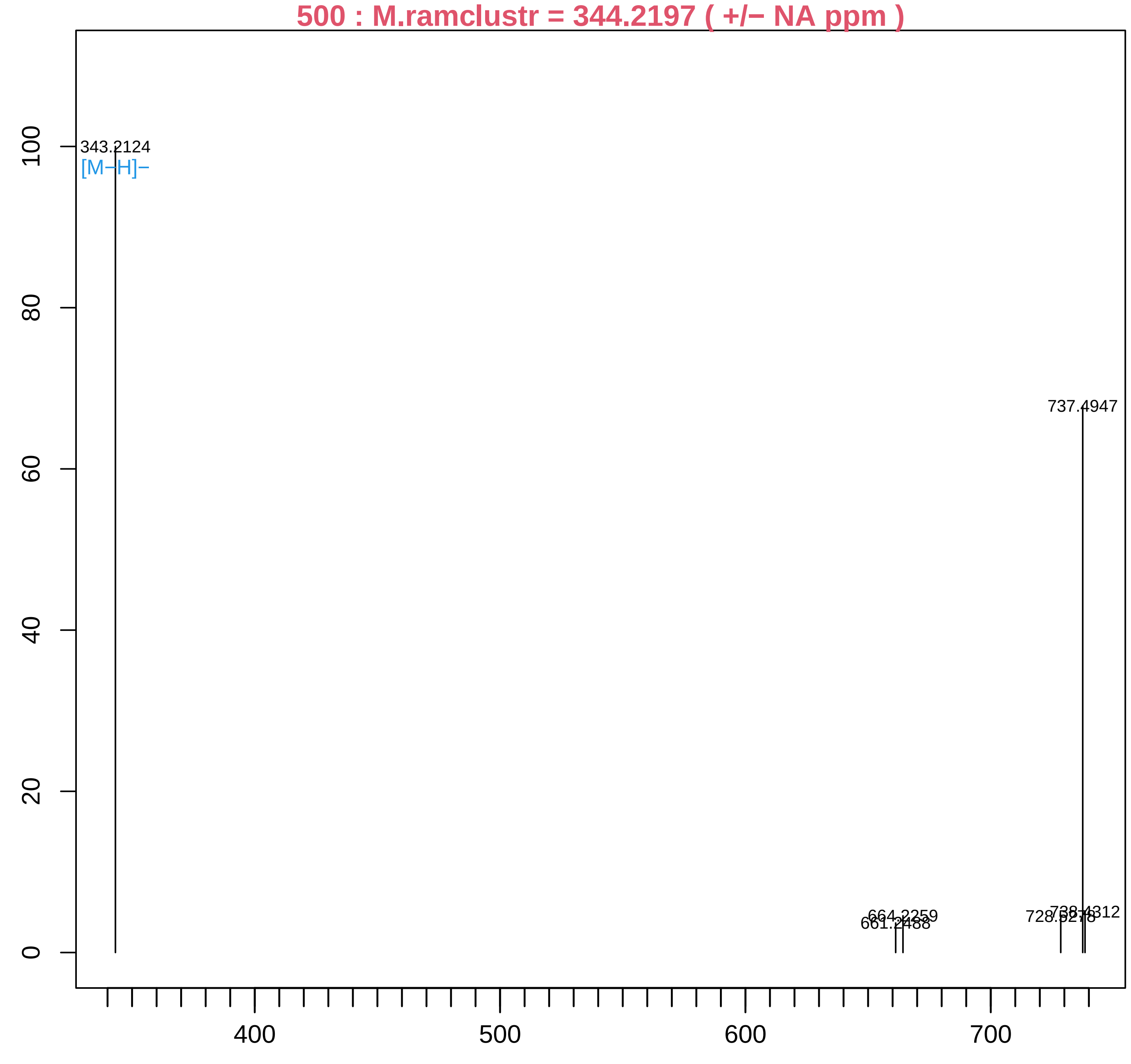
500 : M.ramclustr = 344.2197 (+/− NA ppm)
343.2124
[M−H]−
737.4947
664.2259
661.2488
728.5218
738.4312
0
20
40
60
80
100
400
500
600
700

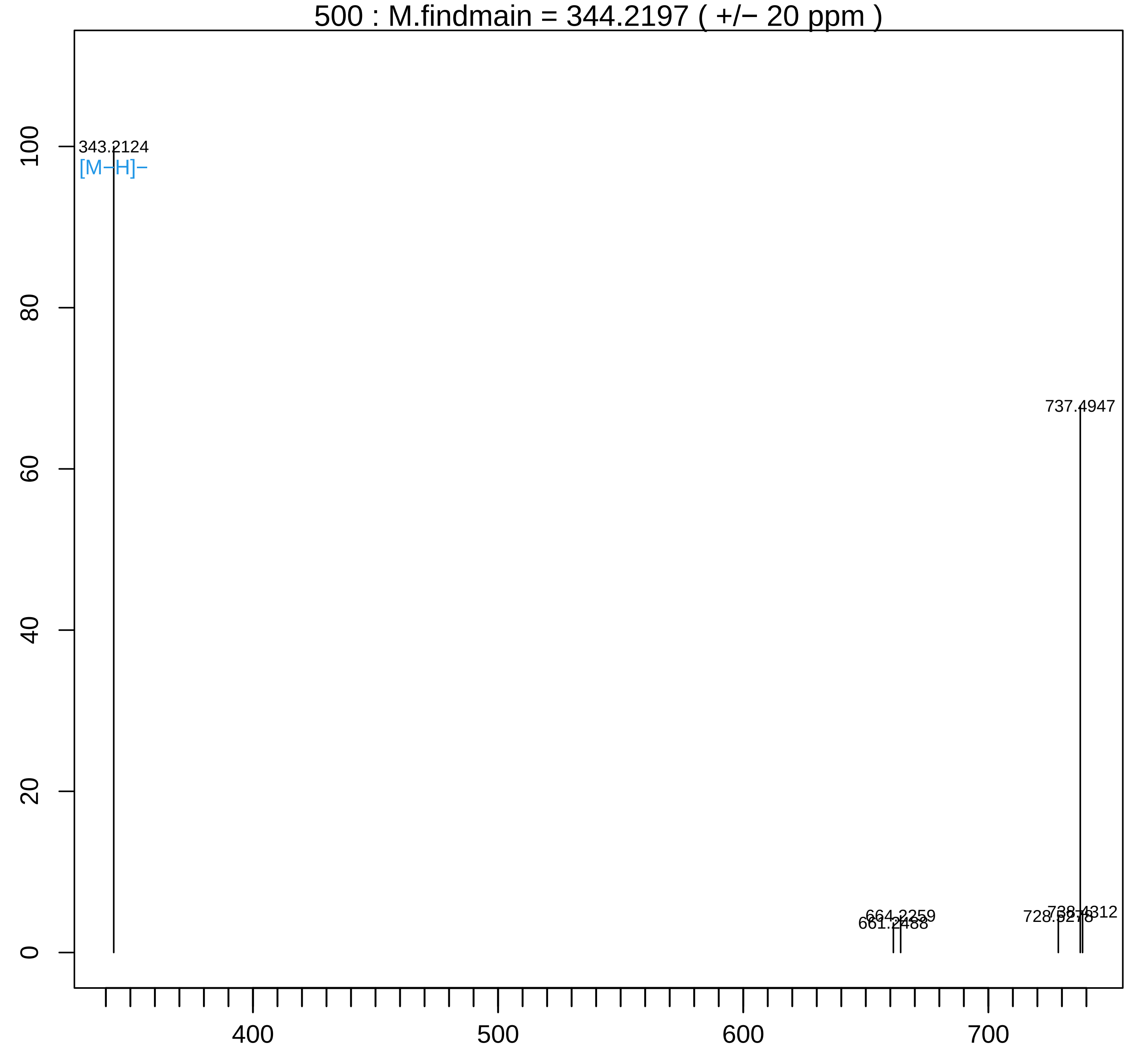
500 : M.findmain = 344.2197 (+/− 20 ppm)
343.2124
[M−H]−
737.4947
664.2259
661.2488
728.5218
738.4312
0
20
40
60
80
100
400
500
600
700

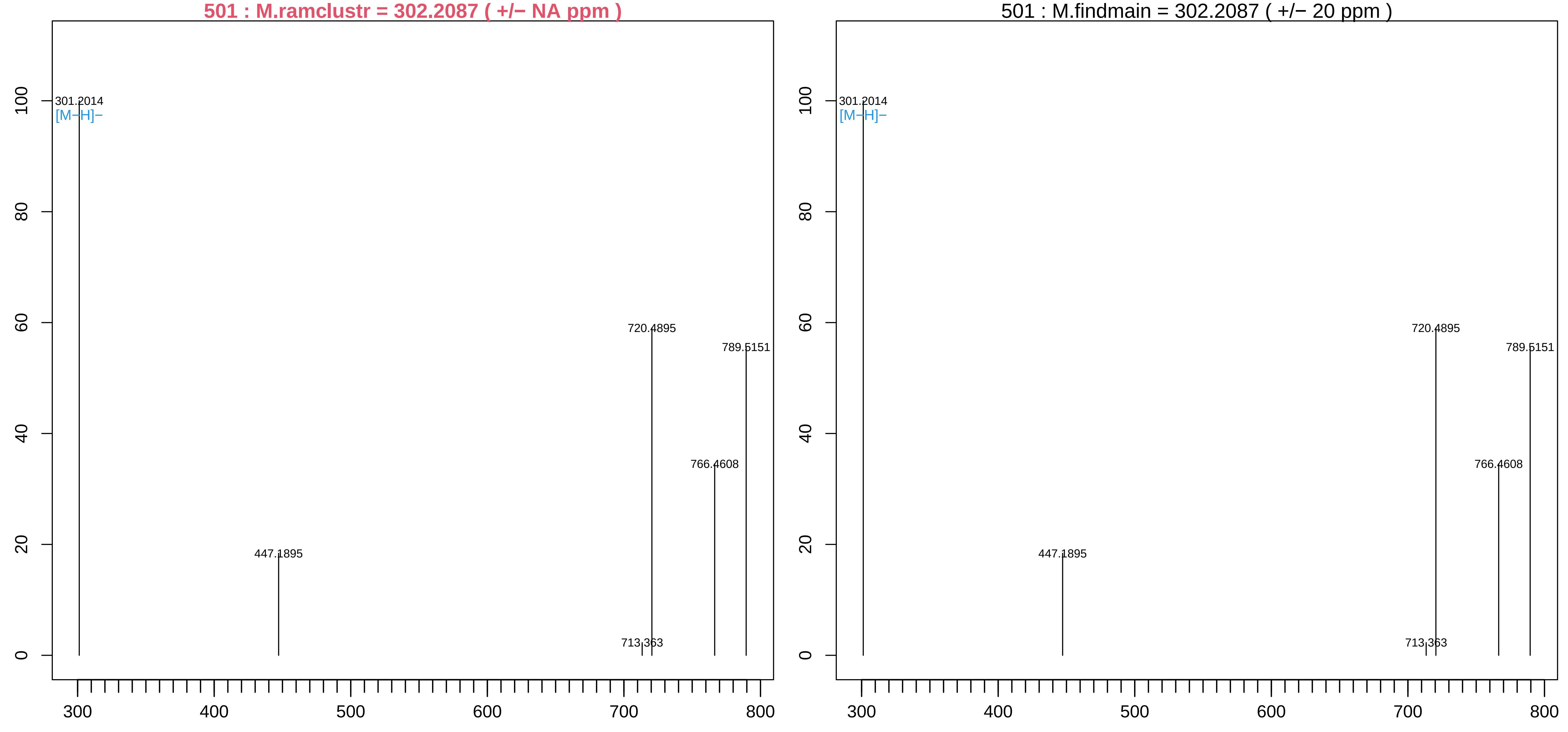

501 : M.ramclustr = 302.2087 (+/− NA ppm)
301.2014
[M−H]−
447.1895
713.363
720.4895
766.4608
789.5151
0
20
40
60
80
100
300
400
500
600
700
800
501 : M.findmain = 302.2087 (+/− 20 ppm)
301.2014
[M−H]−
447.1895
713.363
720.4895
766.4608
789.5151
0
20
40
60
80
100
300
400
500
600
700
800

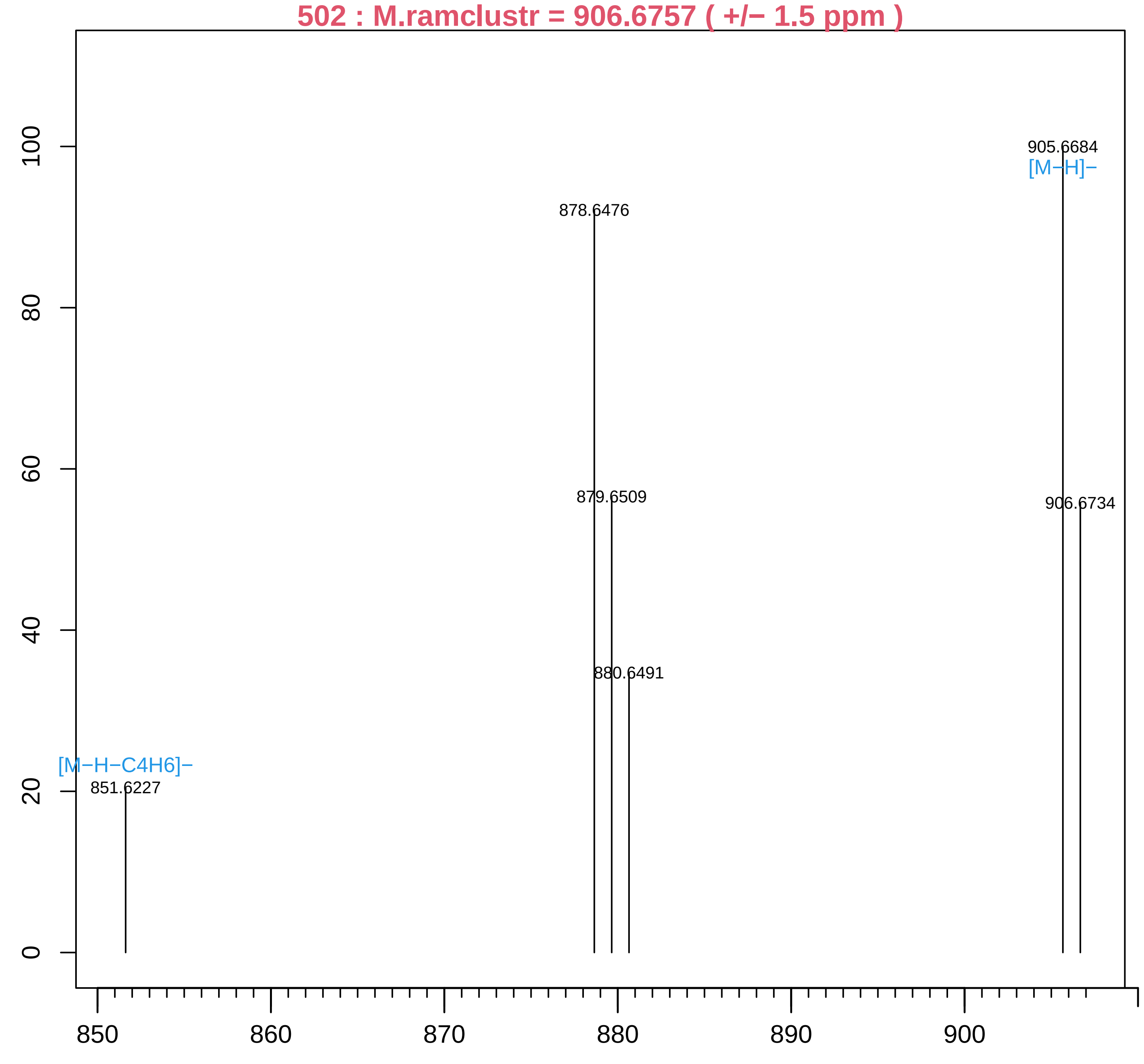

502 : M.ramclustr = 906.6757 (+/− 1.5 ppm)
905.6684
[M−H]−
878.6476
879.6509
906.6734
880.6491
[M−H−C4H6]−
851.6227
0
20
40
60
80
100
850
860
870
880
890
900

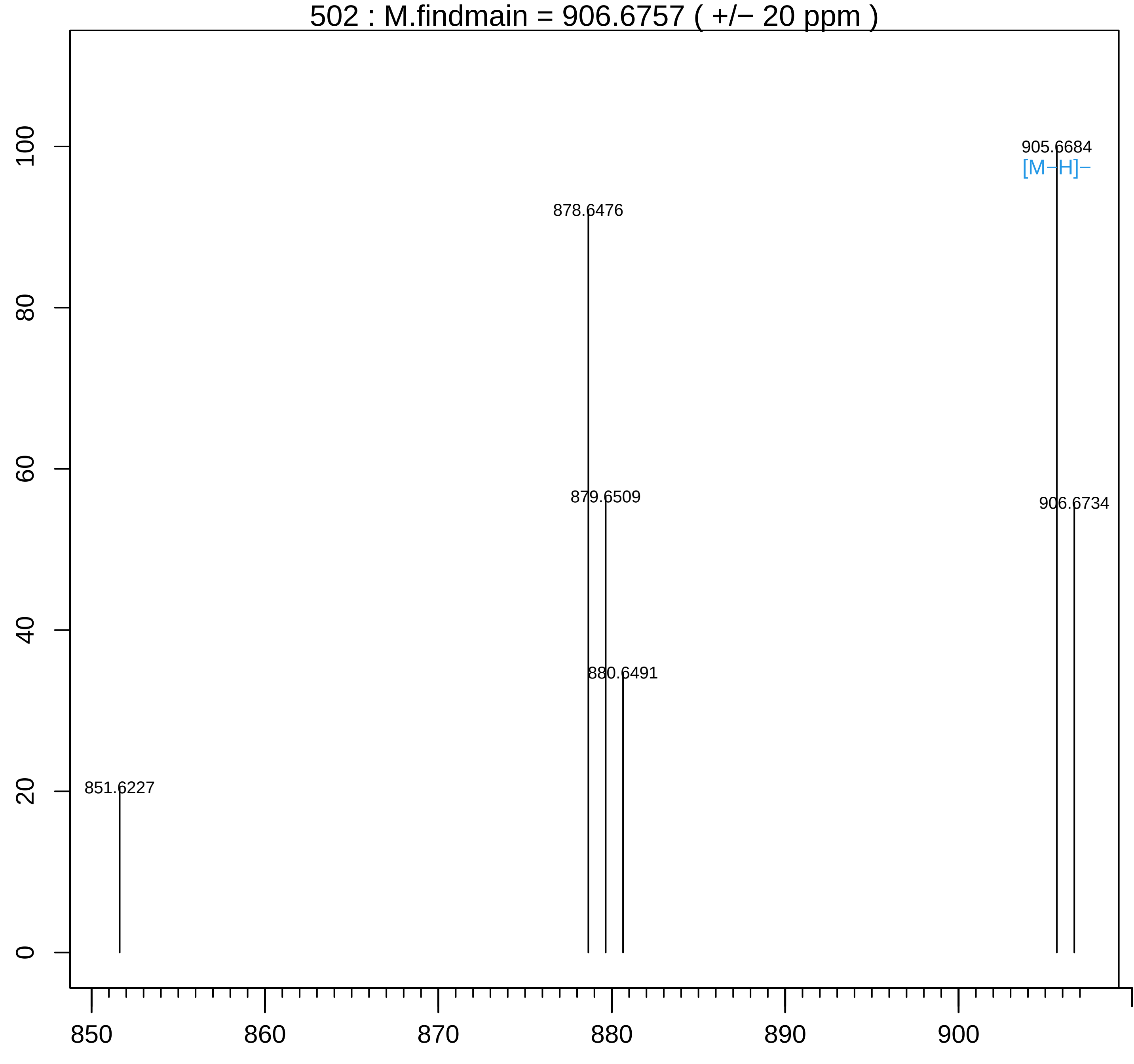

502 : M.findmain = 906.6757 (+/− 20 ppm)
905.6684
[M−H]−
878.6476
879.6509
906.6734
880.6491
851.6227
0
20
40
60
80
100
850
860
870
880
890
900

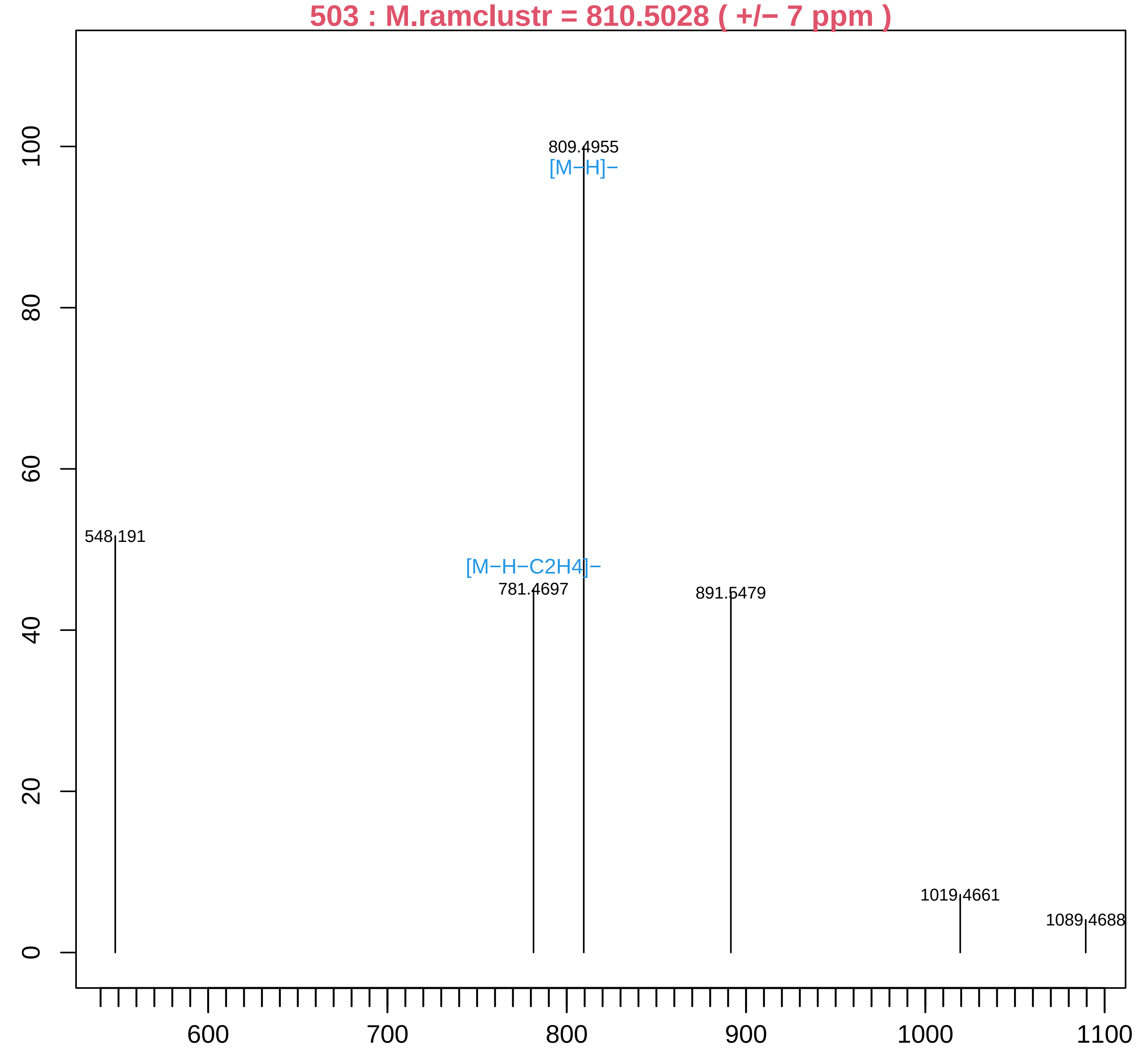

503 : M.ramclustr = 810.5028 (+/− 7 ppm)
809.4955
[M−H]−
548.191
[M−H−C2H4]−
781.4697
891.5479
1019.4661
1089.4688
0
20
40
60
80
100
600
700
800
900
1000
1100

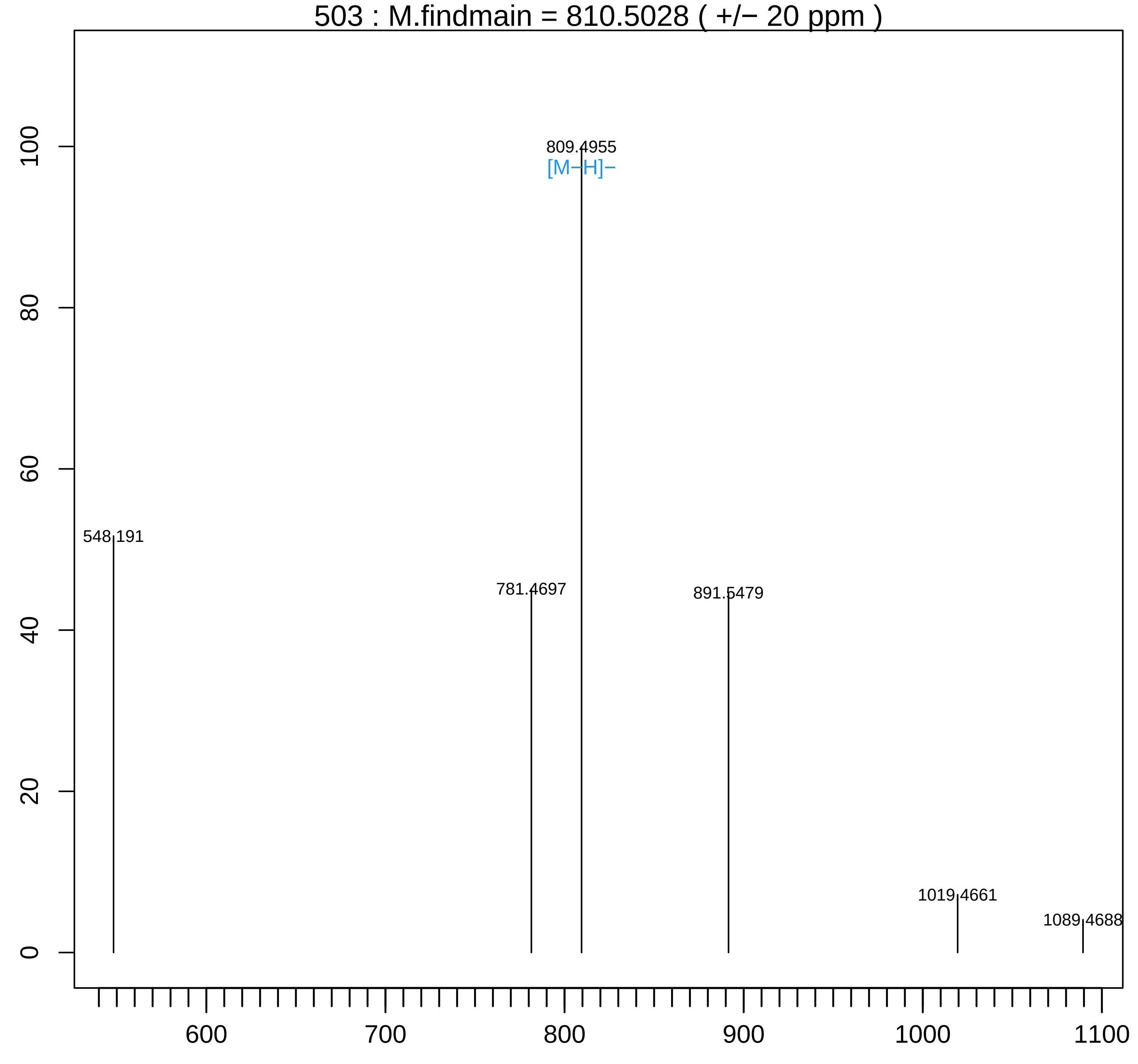

503 : M.findmain = 810.5028 (+/− 20 ppm)
809.4955
[M−H]−
548.191
781.4697
891.5479
1019.4661
1089.4688
0
20
40
60
80
100
600
700
800
900
1000
1100

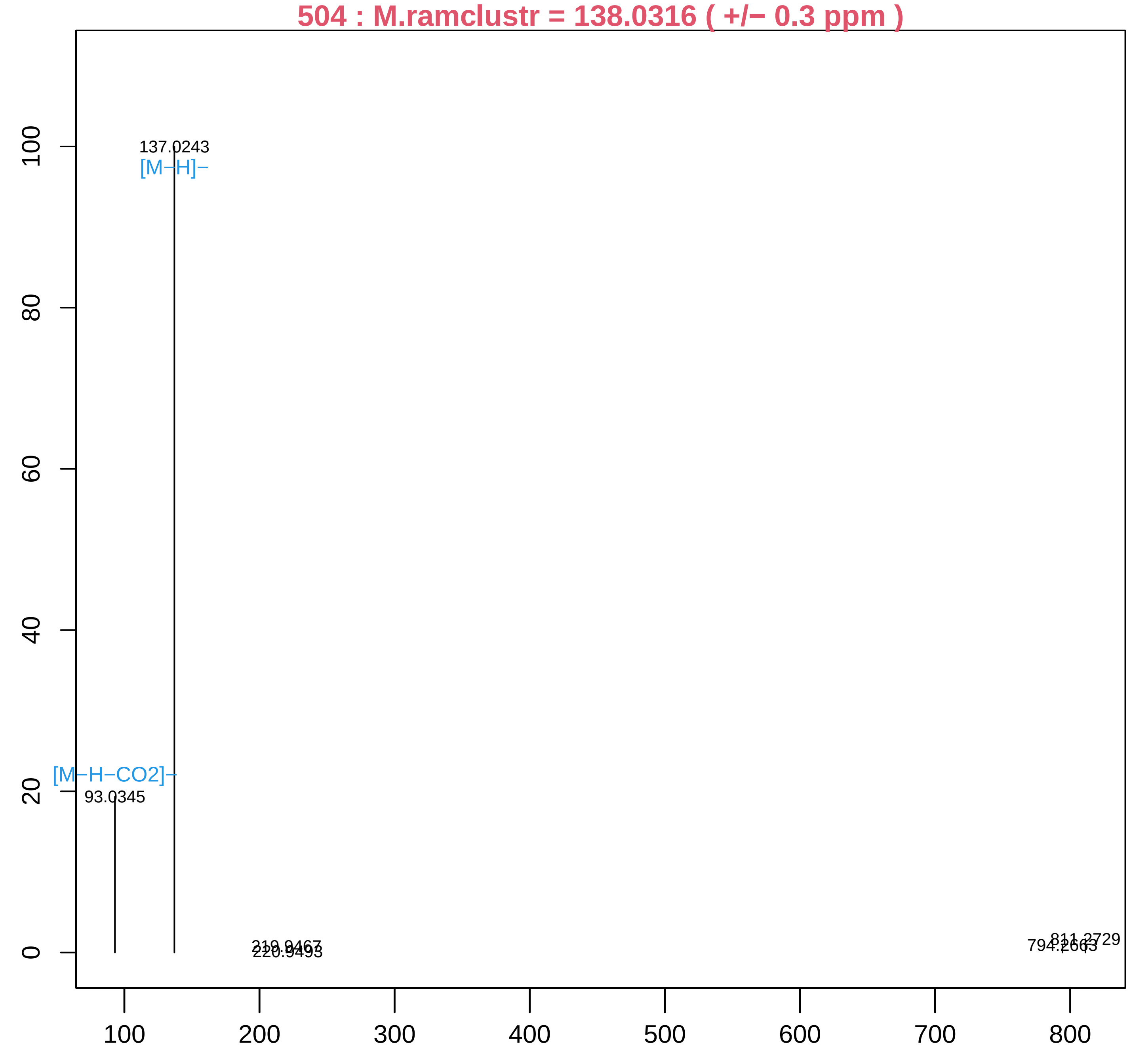

504 : M.ramclustr = 138.0316 (+/− 0.3 ppm)
137.0243
[M−H]−
[M−H−CO2]−
93.0345
219.9467
220.9493
811.2729
794.2663
0
20
40
60
80
100
100
200
300
400
500
600
700
800

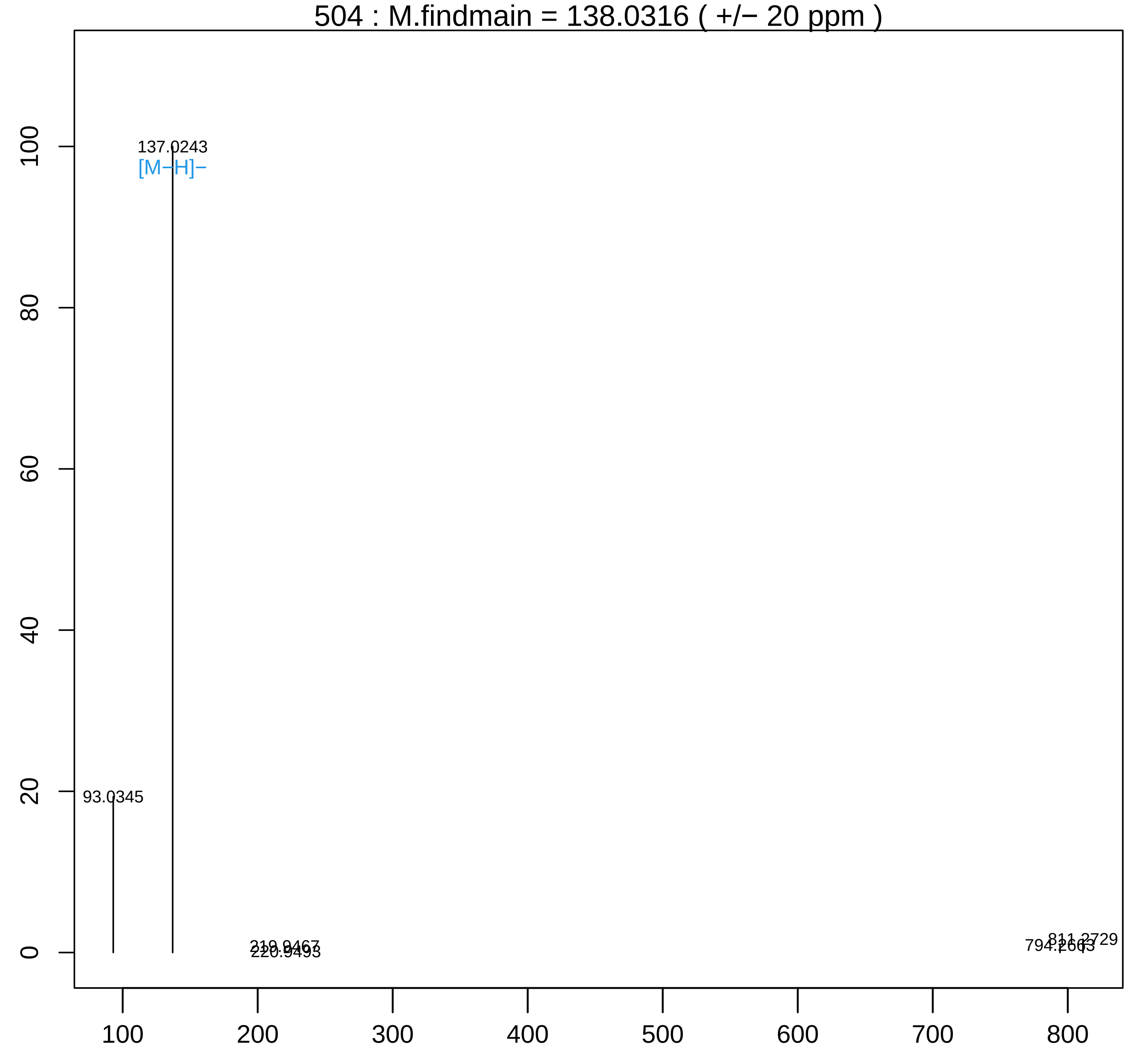

504 : M.findmain = 138.0316 (+/− 20 ppm)
137.0243
[M−H]−
93.0345
219.9467
220.9493
811.2729
794.2663
0
20
40
60
80
100
100
200
300
400
500
600
700
800

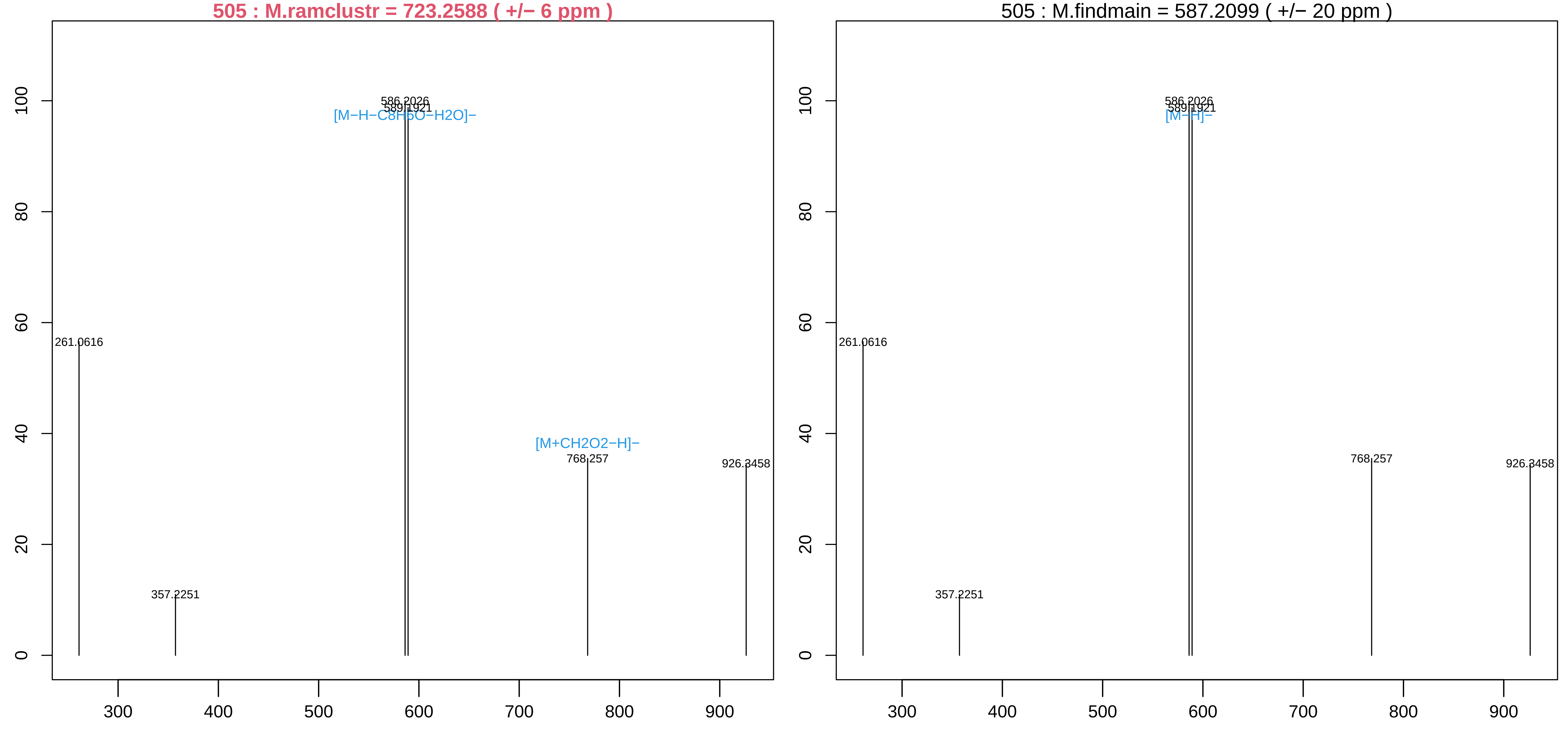
505 : M.ramclustr = 723.2588 (+/− 6 ppm)
586.2026
589.1921
[M−H−C8H6O−H2O]−
261.0616
357.2251
[M+CH2O2−H]−
768.257
926.3458
505 : M.findmain = 587.2099 (+/− 20 ppm)
586.2026
589.1921
[M−H]−
261.0616
357.2251
768.257
926.3458
0
20
40
60
80
100
300
400
500
600
700
800
900

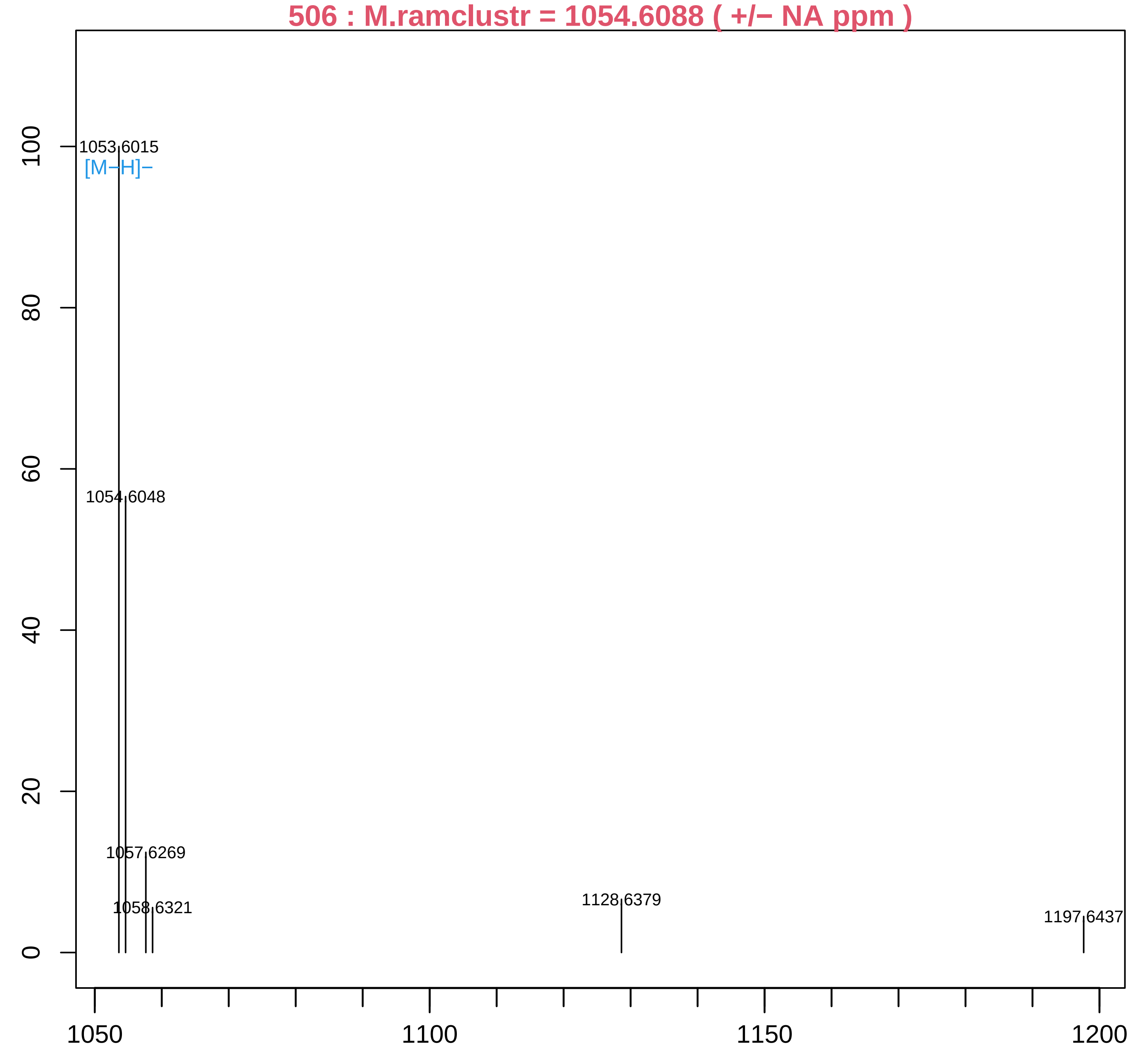

506 : M.ramclustr = 1054.6088 (+/− NA ppm)
1053.6015
[M−H]−
1054.6048
1057.6269
1058.6321
1128.6379
1197.6437
0
20
40
60
80
100
1050
1100
1150
1200

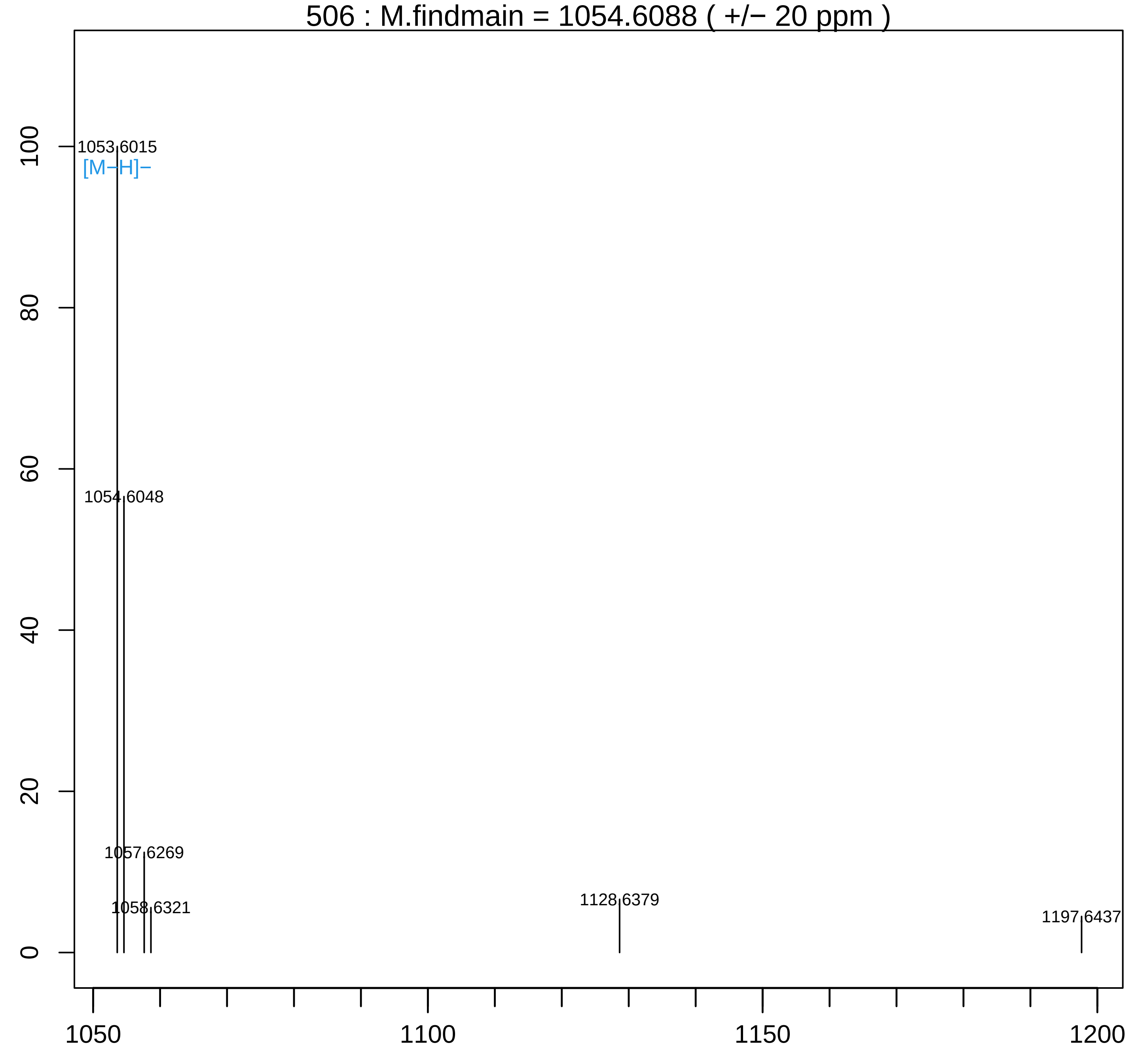

506 : M.findmain = 1054.6088 (+/− 20 ppm)
1053.6015
[M−H]−
1054.6048
1057.6269
1058.6321
1128.6379
1197.6437
0
20
40
60
80
100
1050
1100
1150
1200

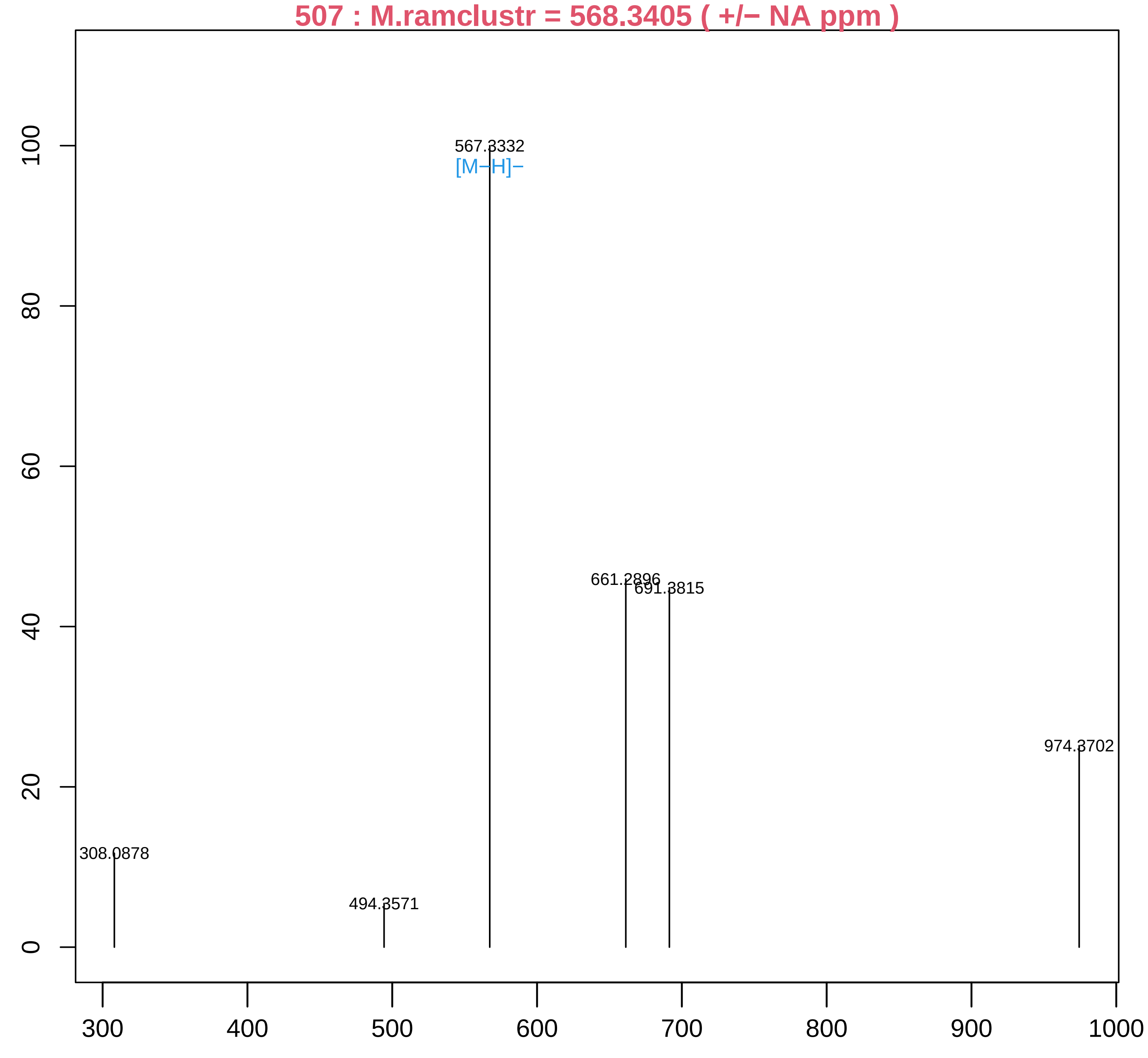

507 : M.ramclustr = 568.3405 (+/− NA ppm)
567.3332
[M−H]−
661.2896
691.3815
974.3702
308.0878
494.3571
0
20
40
60
80
100
300
400
500
600
700
800
900
1000

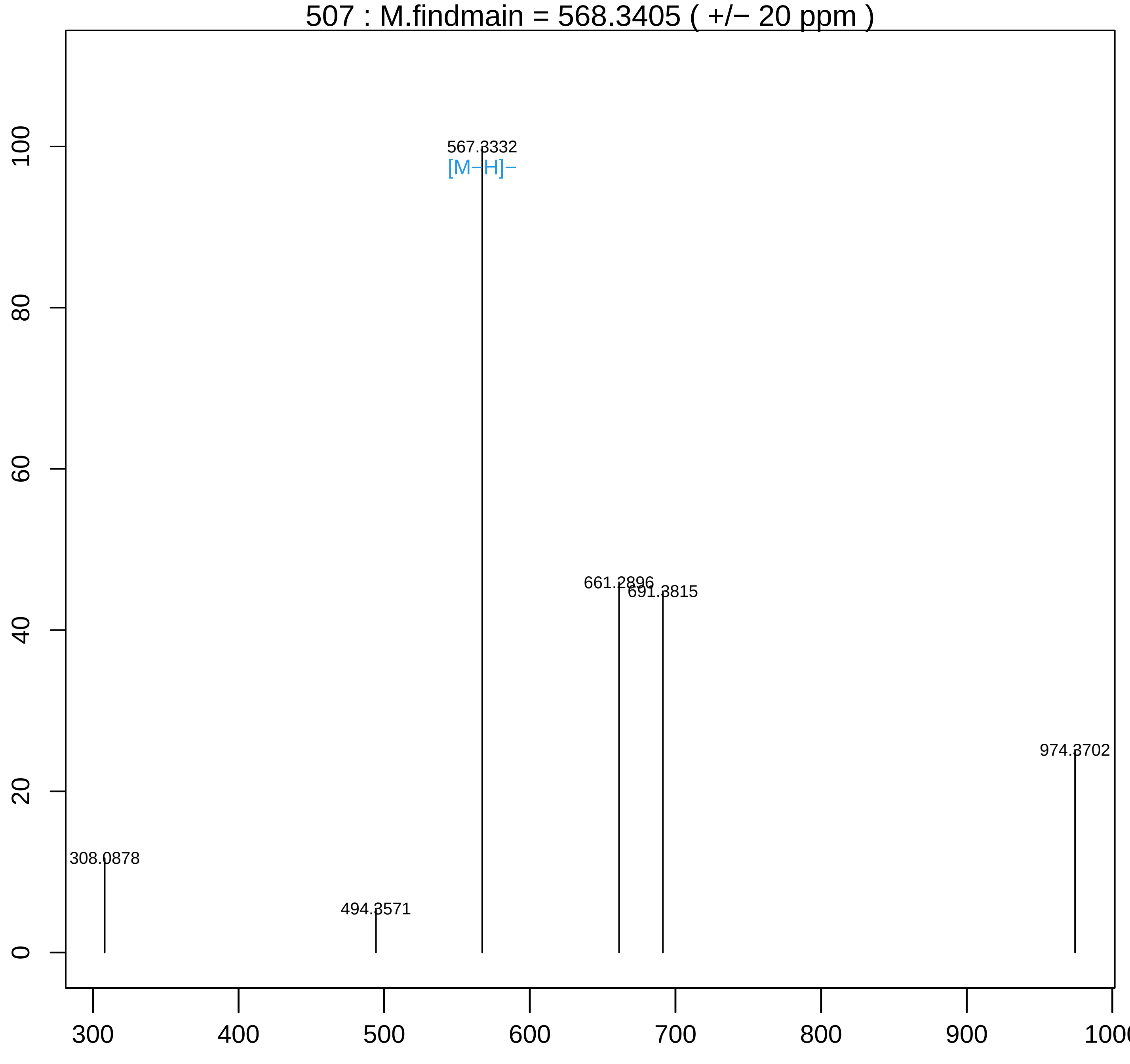

507 : M.findmain = 568.3405 (+/− 20 ppm)
567.3332
[M−H]−
661.2896
691.3815
974.3702
308.0878
494.3571
0
20
40
60
80
100
300
400
500
600
700
800
900

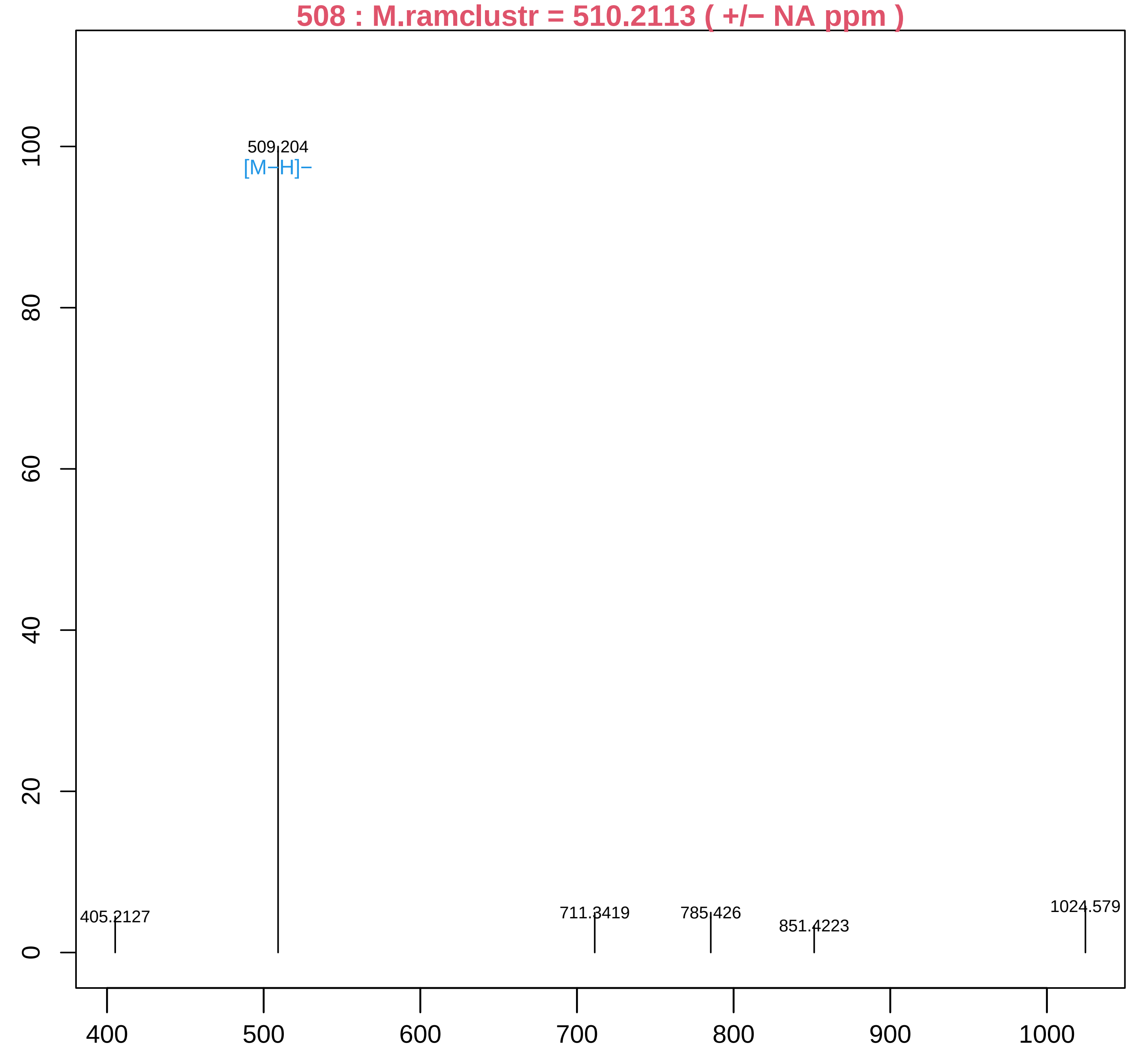
508 : M.ramclustr = 510.2113 (+/− NA ppm)
509.204
[M−H]−
405.2127
711.3419
785.426
851.4223
1024.579
0
20
40
60
80
100
400
500
600
700
800
900
1000

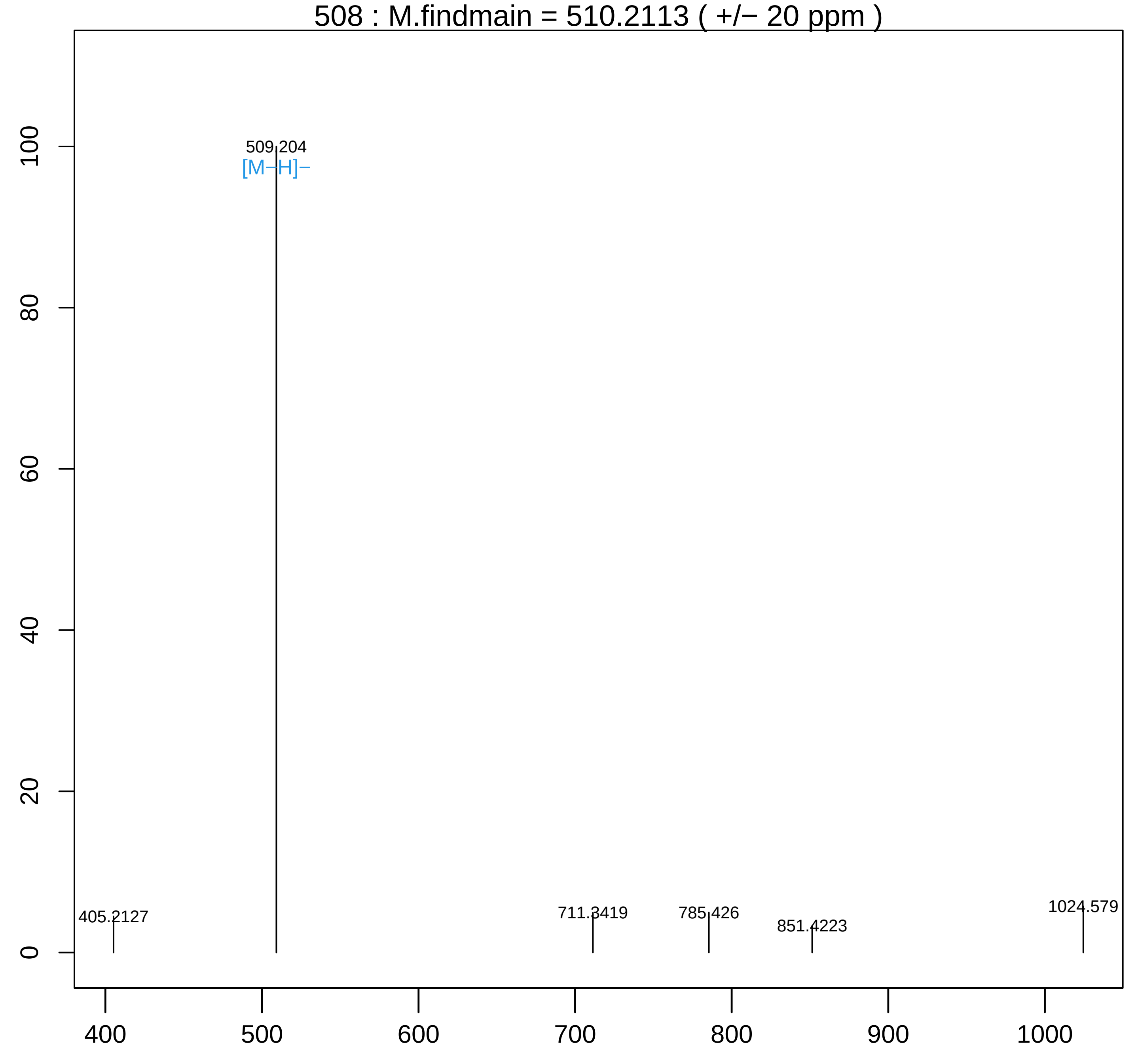
508 : M.findmain = 510.2113 (+/− 20 ppm)
509.204
[M−H]−
405.2127
711.3419
785.426
851.4223
1024.579
0
20
40
60
80
100
400
500
600
700
800
900
1000

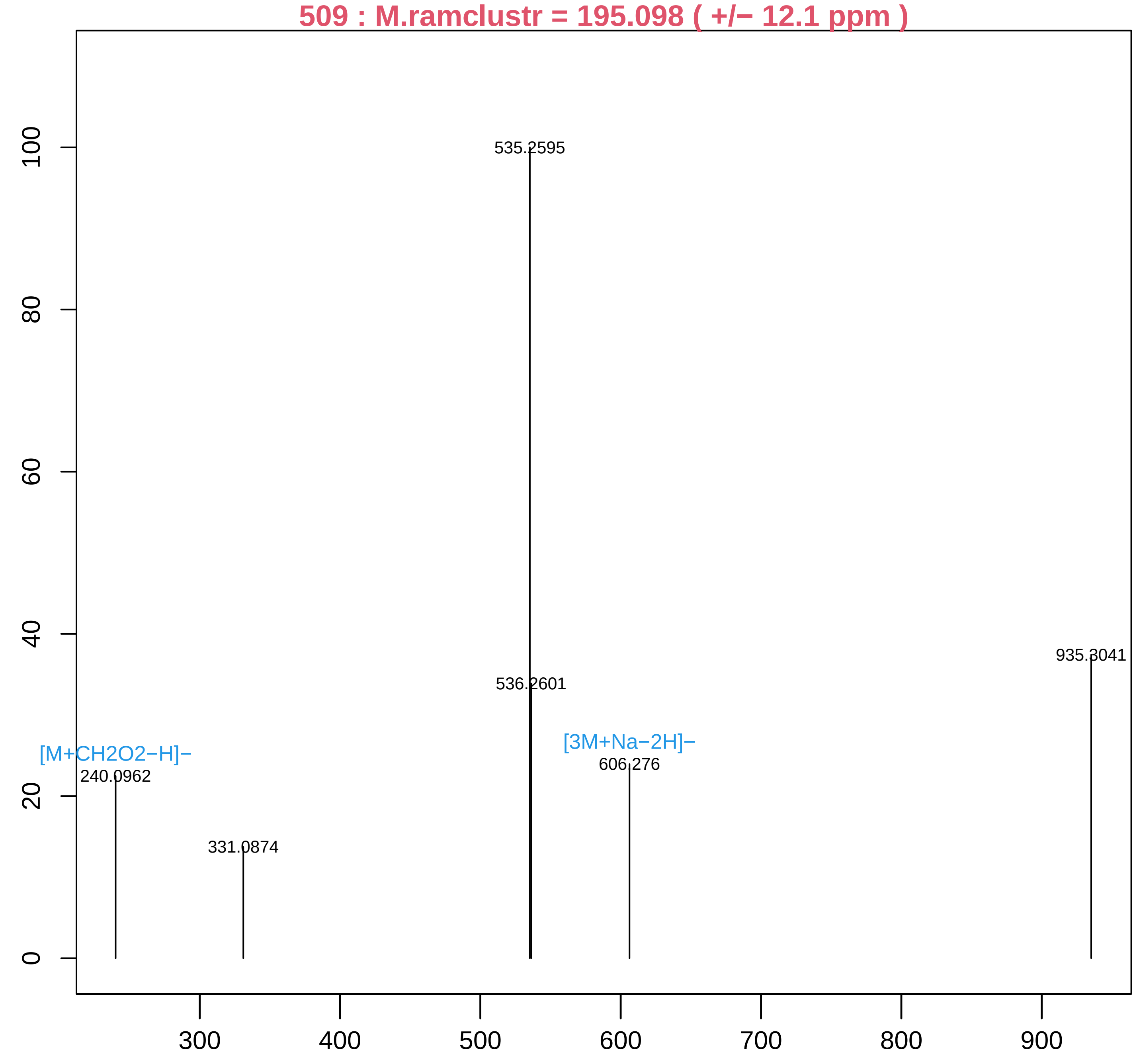

509 : M.ramclustr = 195.098 (+/− 12.1 ppm)
535.2595
536.2601
935.3041
[M+CH2O2−H]−
240.0962
[3M+Na−2H]−
606.276
331.0874

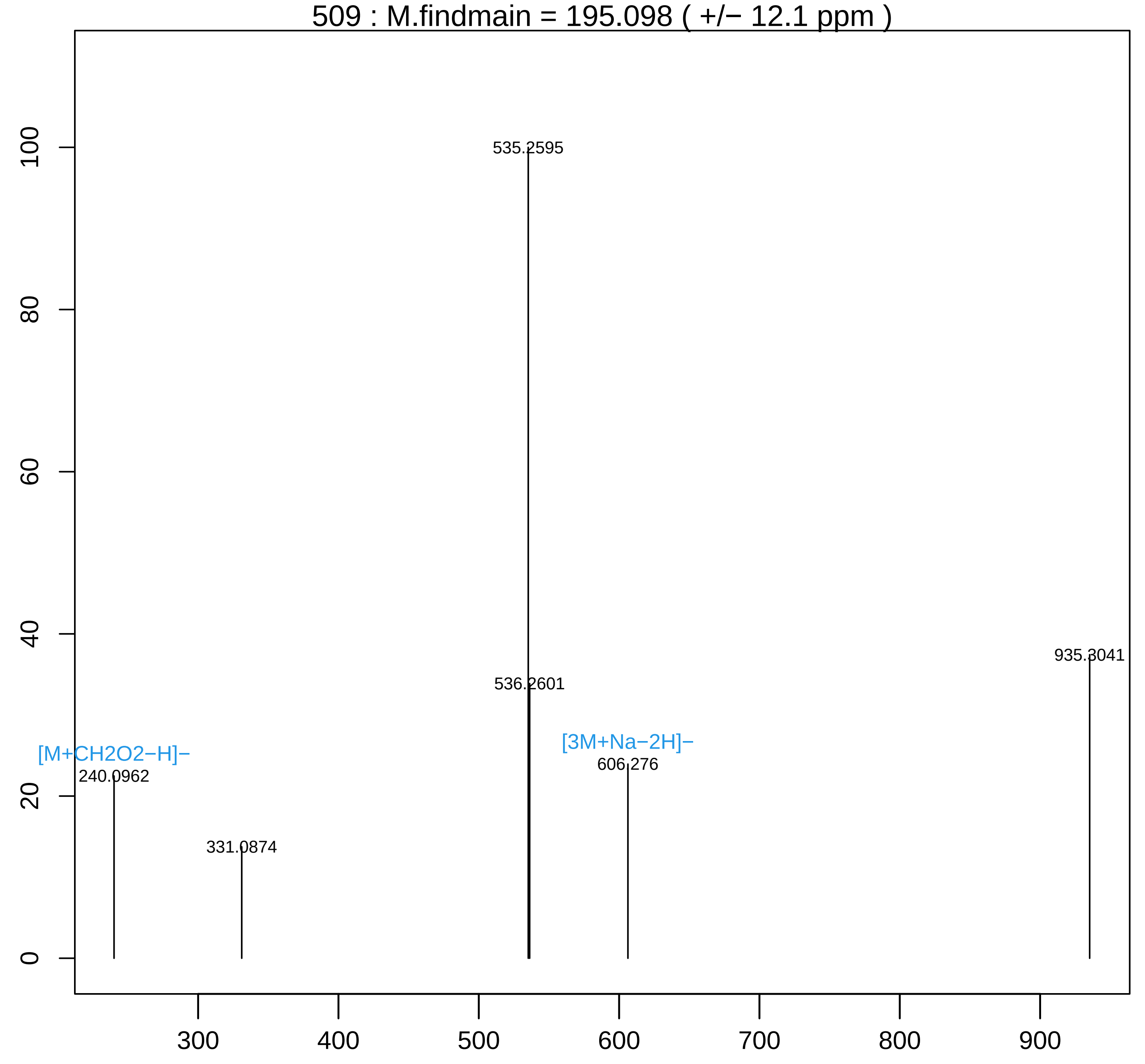

509 : M.findmain = 195.098 (+/− 12.1 ppm)
535.2595
536.2601
935.3041
[M+CH2O2−H]−
240.0962
[3M+Na−2H]−
606.276
331.0874

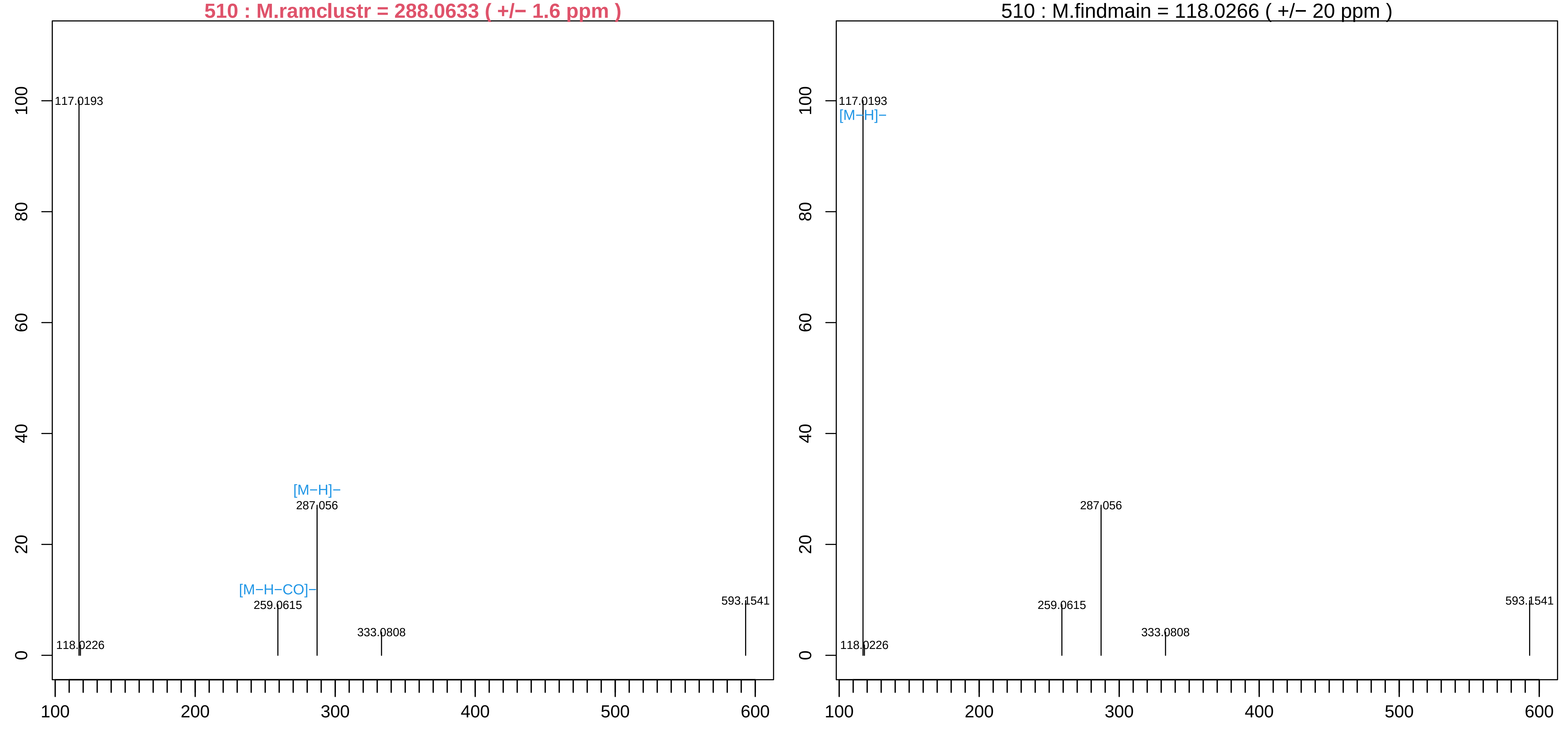
510 : M.ramclustr = 288.0633 (+/− 1.6 ppm)
117.0193
118.0226
[M−H−CO]−
259.0615
[M−H]−
287.056
333.0808
593.1541
510 : M.findmain = 118.0266 (+/− 20 ppm)
117.0193
[M−H]−
118.0226
259.0615
287.056
333.0808
593.1541
0
20
40
60
80
100
100
200
300
400
500
600

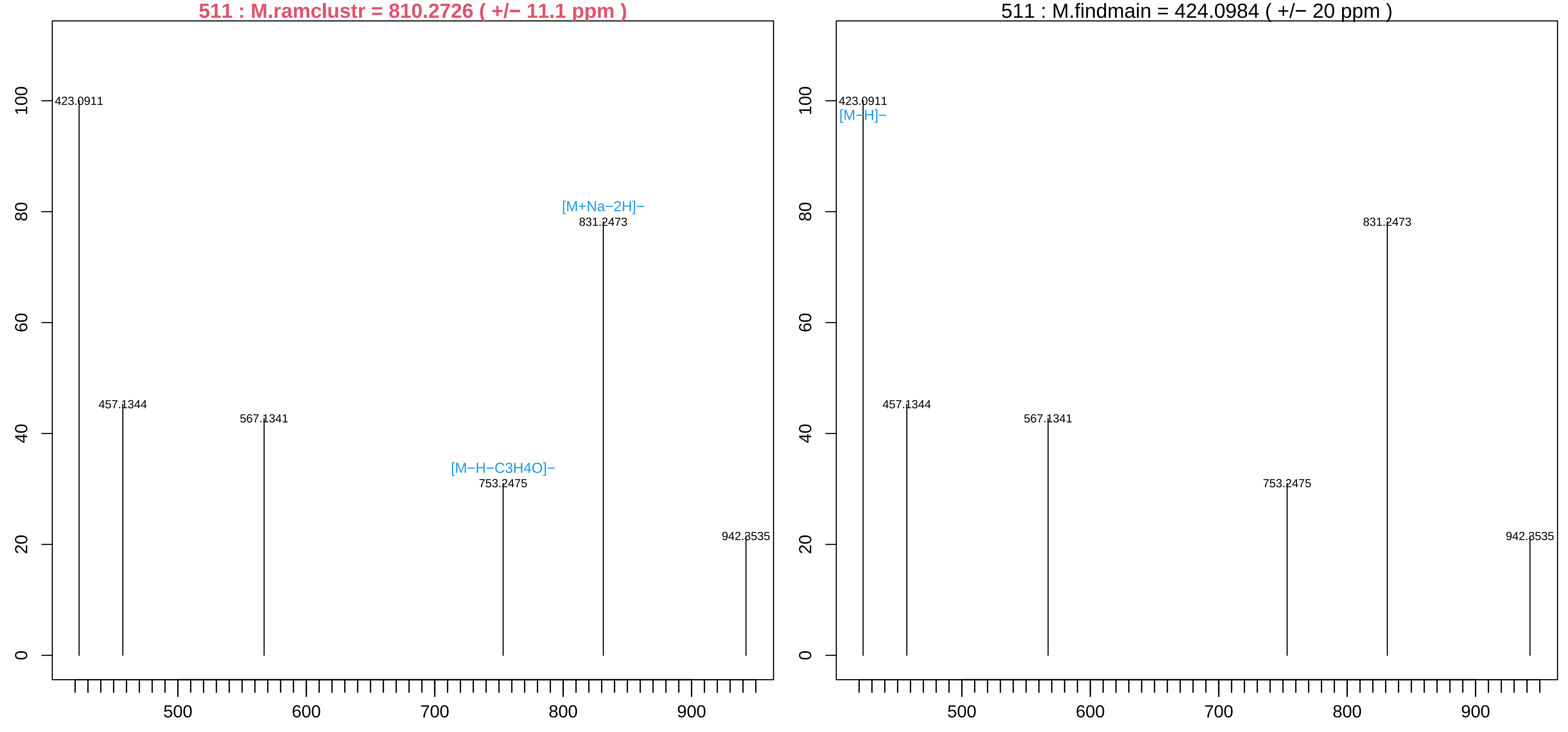
511 : M.ramclustr = 810.2726 (+/− 11.1 ppm)
423.0911
457.1344
567.1341
[M−H−C3H4O]−
753.2475
[M+Na−2H]−
831.2473
942.3535
0
20
40
60
80
100
500
600
700
800
900
511 : M.findmain = 424.0984 (+/− 20 ppm)
423.0911
[M−H]−
457.1344
567.1341
753.2475
831.2473
942.3535
0
20
40
60
80
100
500
600
700
800
900

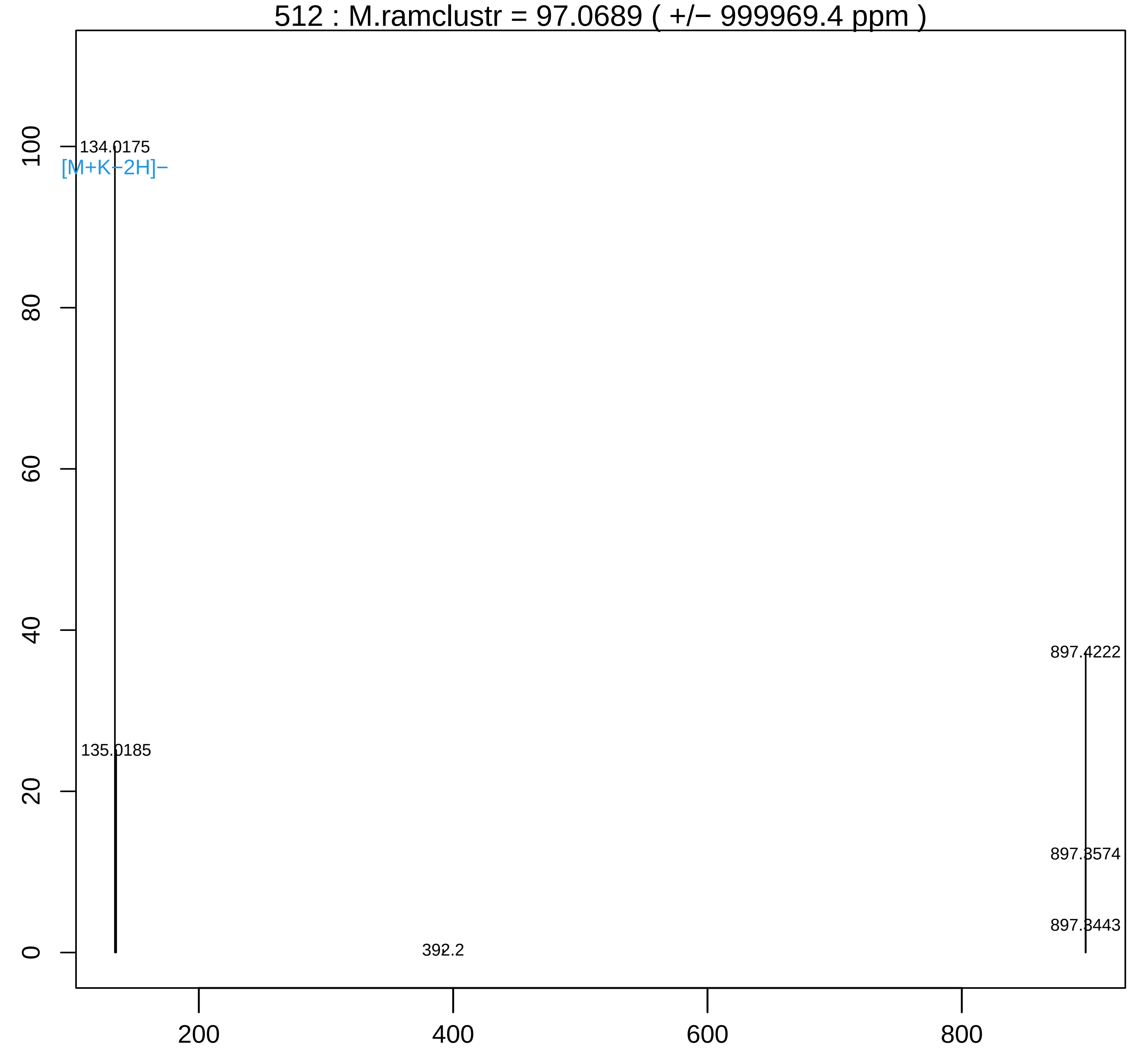

512 : M.ramclustr = 97.0689 (+/− 999969.4 ppm)
134.0175
[M+K−2H]−
135.0185
897.4222
897.3574
897.3443
392.2
0
20
40
60
80
100
200
400
600
800

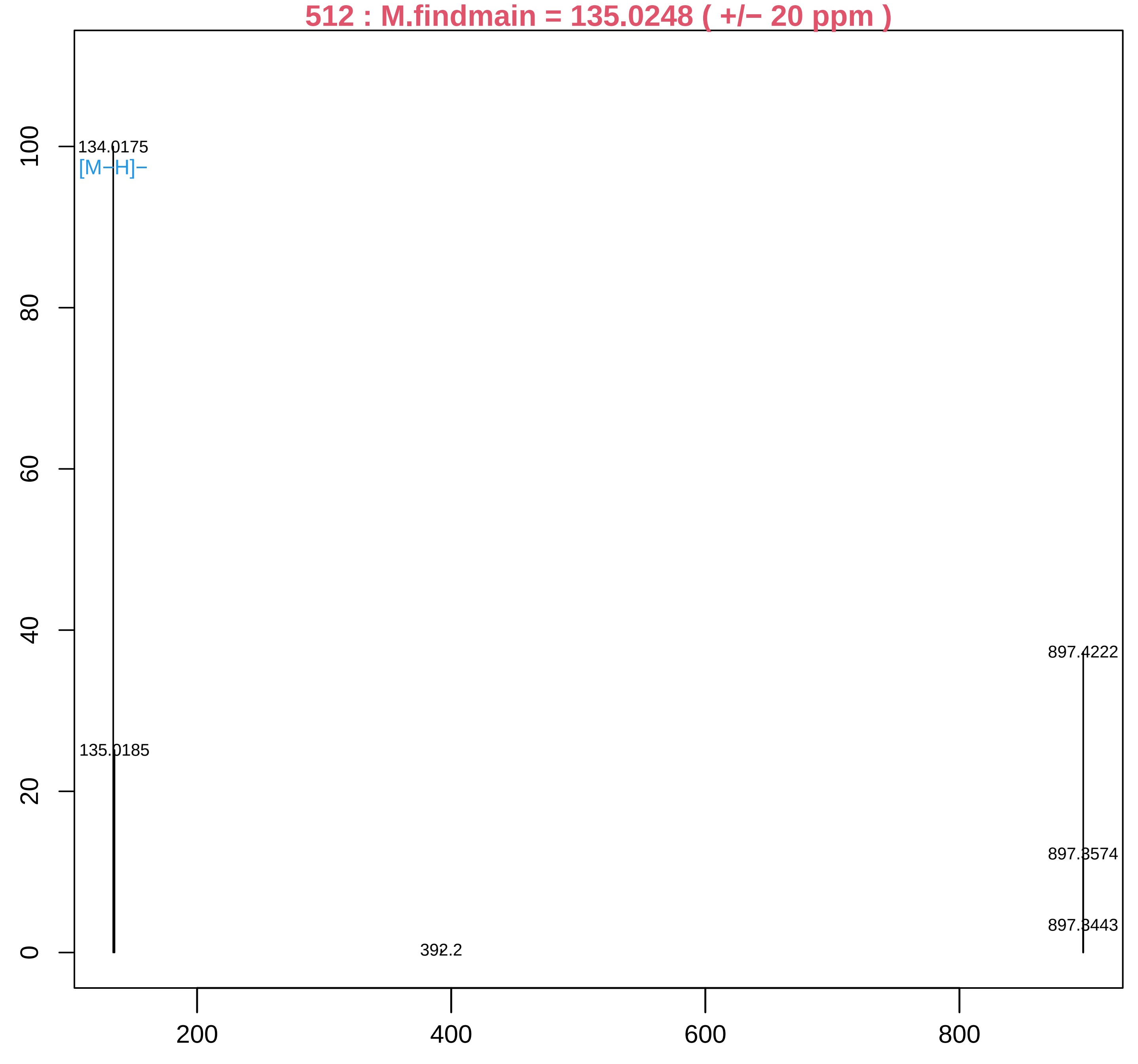

512 : M.findmain = 135.0248 (+/− 20 ppm)
134.0175
[M−H]−
135.0185
897.4222
897.3574
897.3443
392.2
0
20
40
60
80
100
200
400
600
800

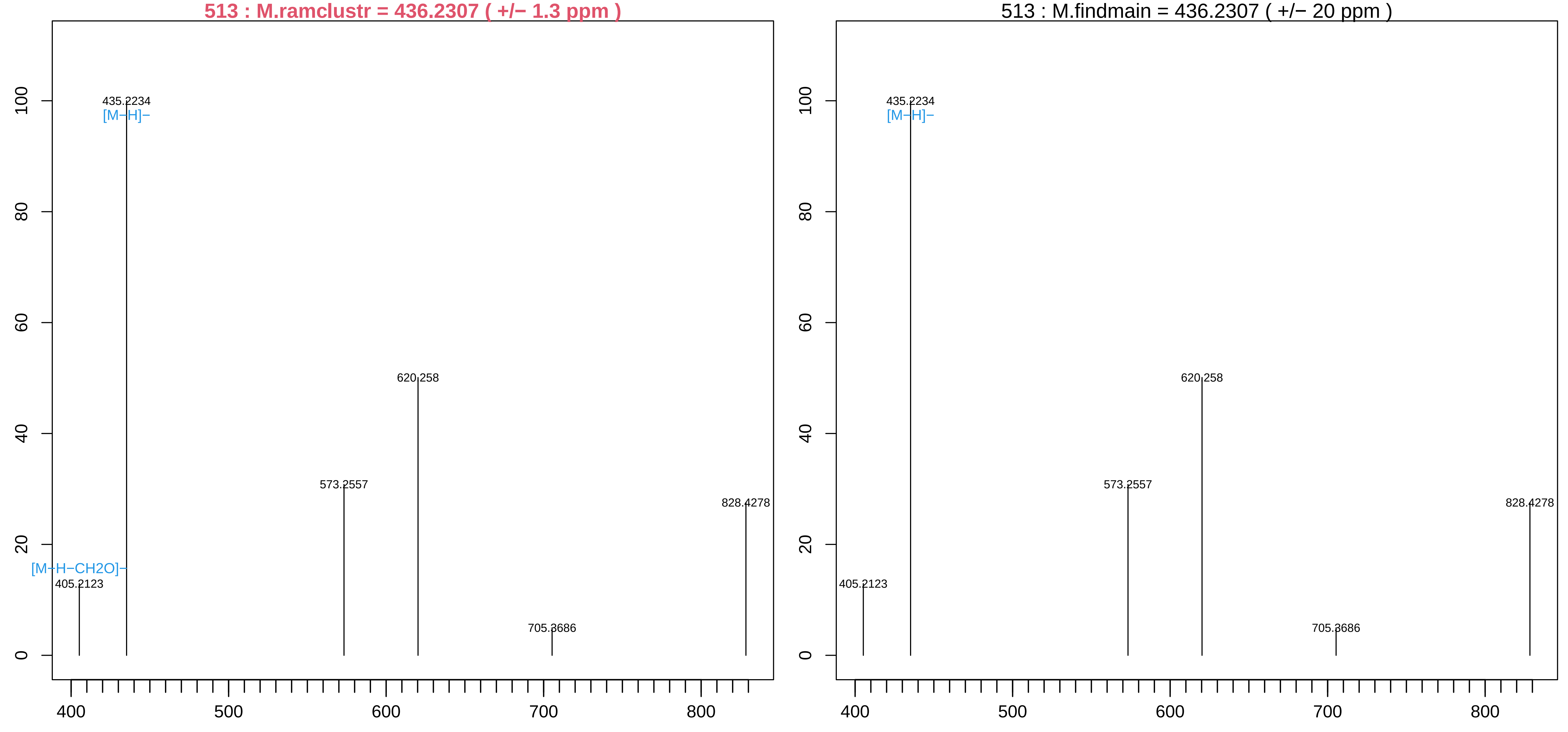
513 : M.ramclustr = 436.2307 (+/− 1.3 ppm)
435.2234
[M−H]−
620.258
573.2557
828.4278
[M−H−CH2O]−
405.2123
705.3686
513 : M.findmain = 436.2307 (+/− 20 ppm)
435.2234
[M−H]−
620.258
573.2557
828.4278
405.2123
705.3686
0
20
40
60
80
100
400
500
600
700
800

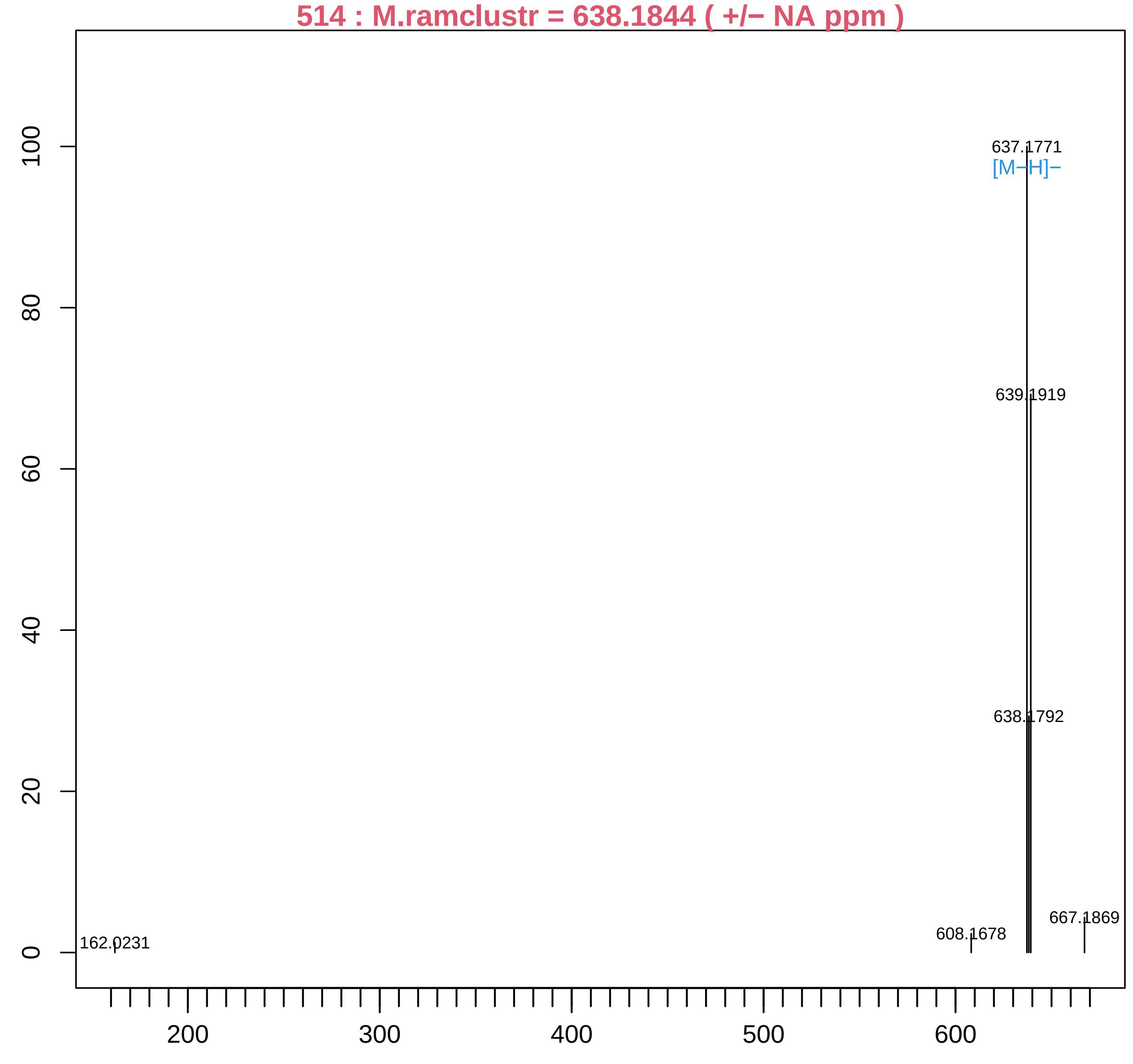
514 : M.ramclustr = 638.1844 (+/− NA ppm)
637.1771
[M−H]−
639.1919
638.1792
608.1678
667.1869
162.0231
0
20
40
60
80
100
200
300
400
500
600

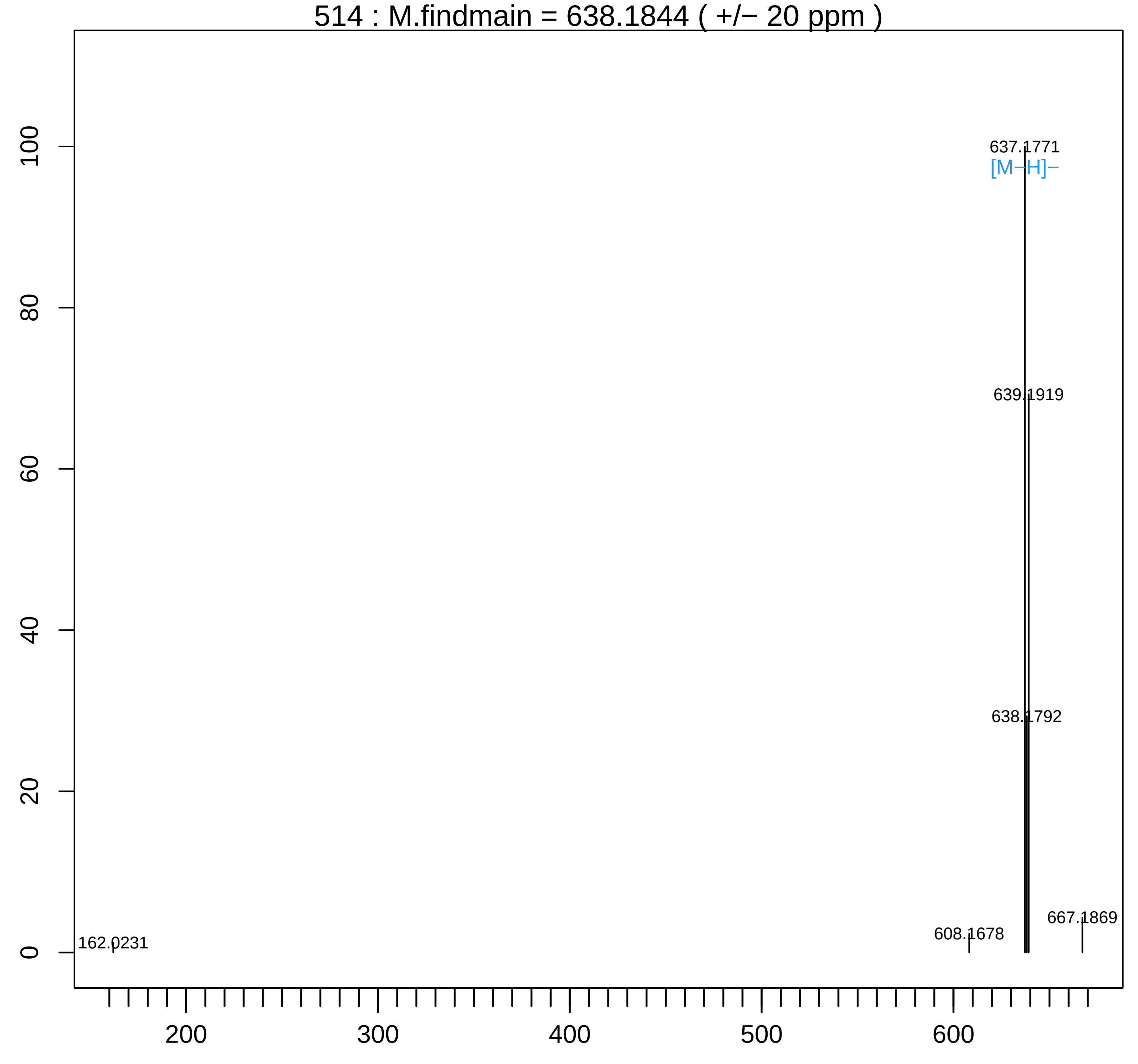
514 : M.findmain = 638.1844 (+/− 20 ppm)
637.1771
[M−H]−
639.1919
638.1792
608.1678
667.1869
162.0231
0
20
40
60
80
100
200
300
400
500
600

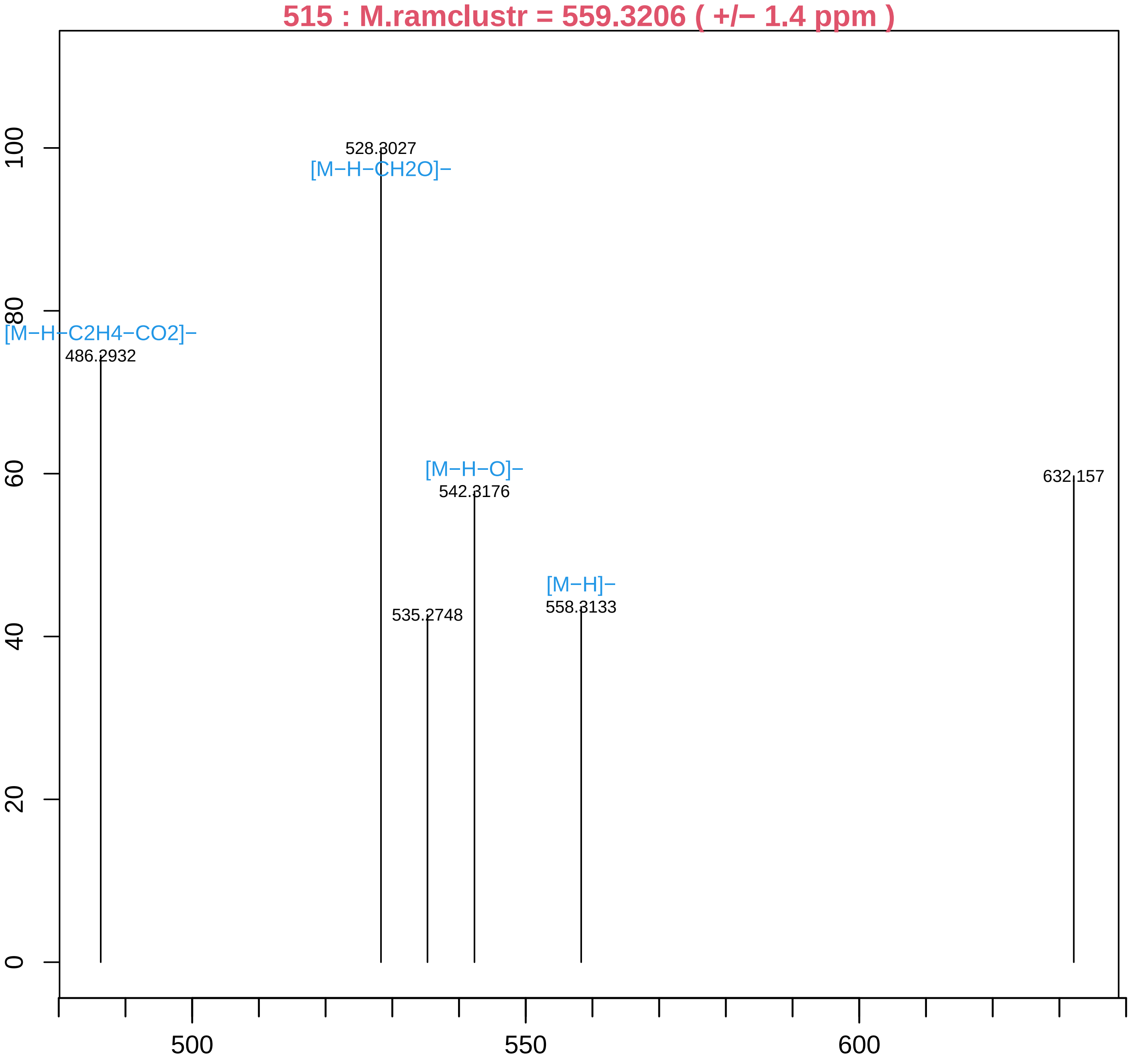

515 : M.ramclustr = 559.3206 (+/− 1.4 ppm)
528.3027
[M−H−CH2O]−
[M−H−C2H4−CO2]−
486.2932
[M−H−O]−
542.3176
535.2748
[M−H]−
558.3133
632.157
0
20
40
60
80
100
500
550
600

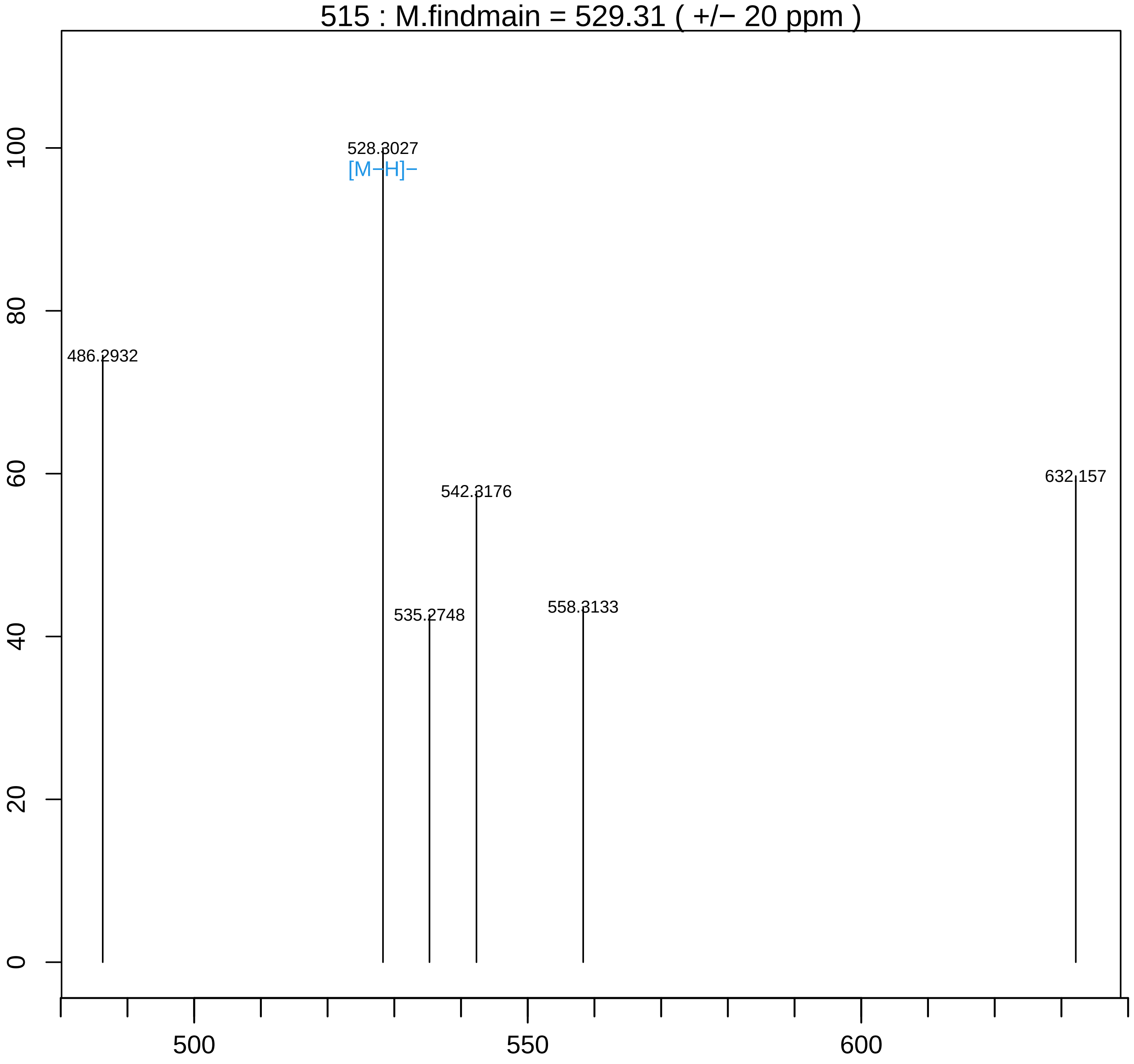

515 : M.findmain = 529.31 (+/− 20 ppm)
528.3027
[M−H]−
486.2932
542.3176
535.2748
558.3133
632.157
0
20
40
60
80
100
500
550
600

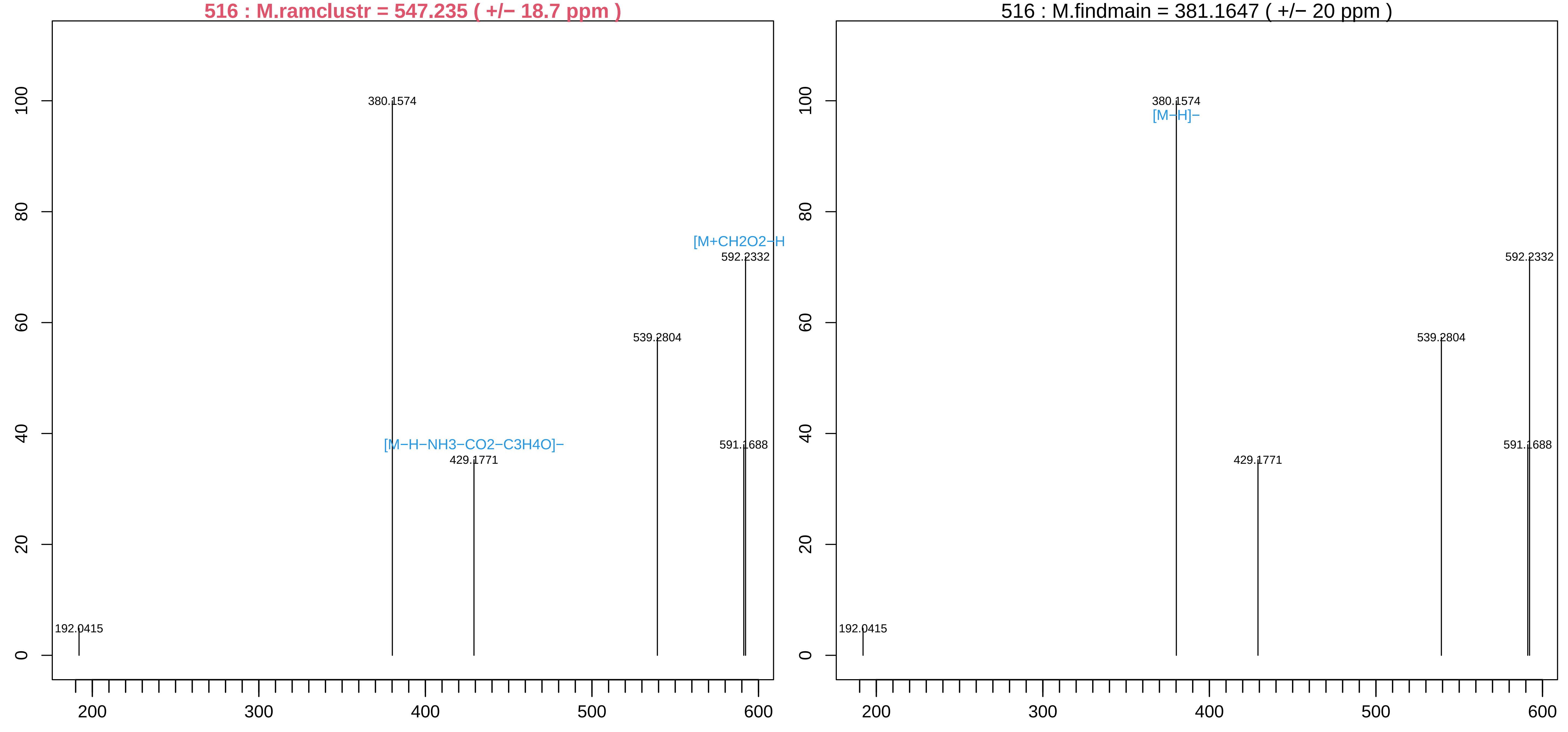
516 : M.ramclustr = 547.235 (+/− 18.7 ppm)
380.1574
[M+CH2O2−H
592.2332
539.2804
[M−H−NH3−CO2−C3H4O]−
429.1771
591.1688
192.0415
516 : M.findmain = 381.1647 (+/− 20 ppm)
380.1574
[M−H]−
592.2332
539.2804
429.1771
591.1688
192.0415
0
20
40
60
80
100
200
300
400
500
600

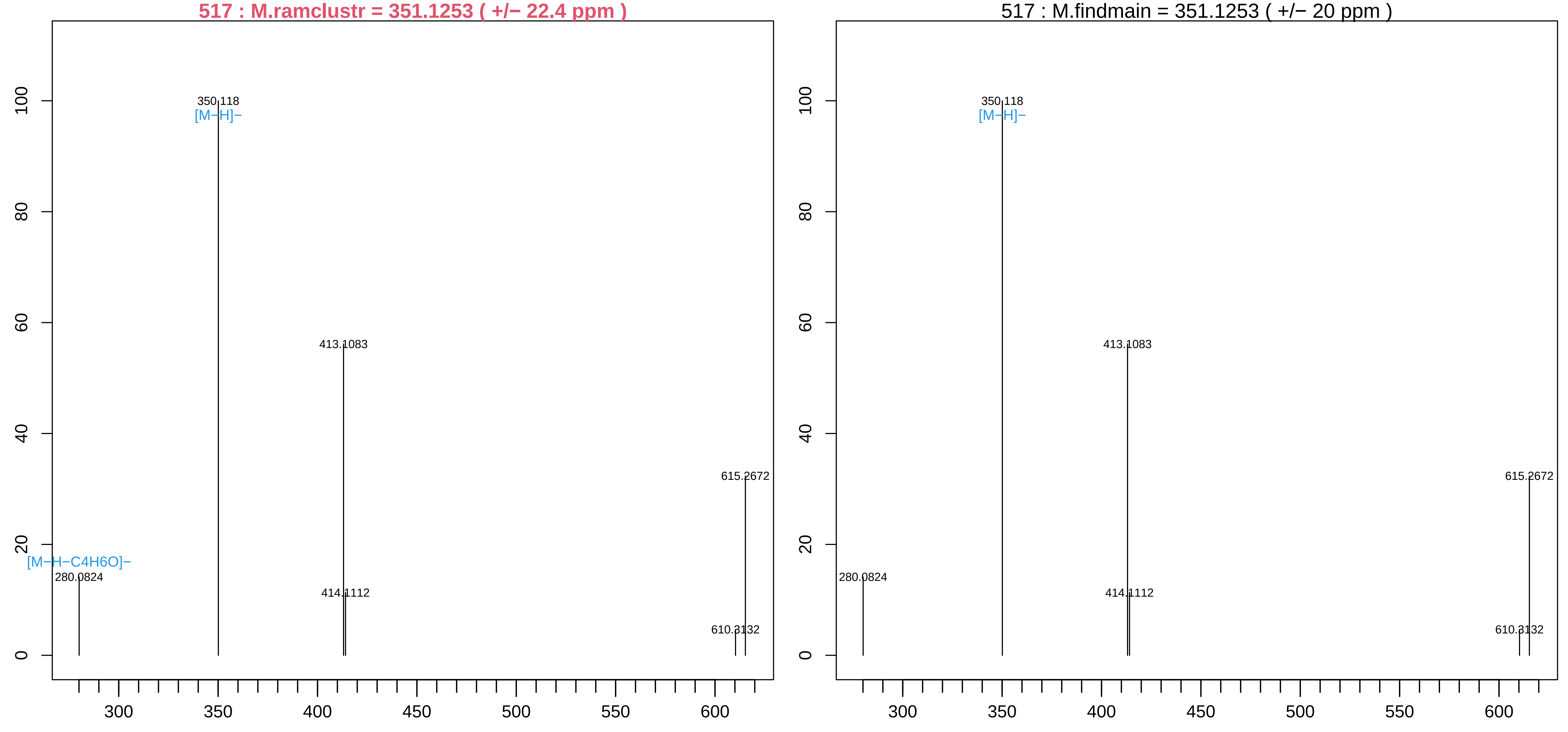

517 : M.ramclustr = 351.1253 (+/− 22.4 ppm)
350.118
[M−H]−
413.1083
615.2672
[M−H−C4H6O]−
280.0824
414.1112
610.3132
517 : M.findmain = 351.1253 (+/− 20 ppm)
350.118
[M−H]−
413.1083
615.2672
280.0824
414.1112
610.3132

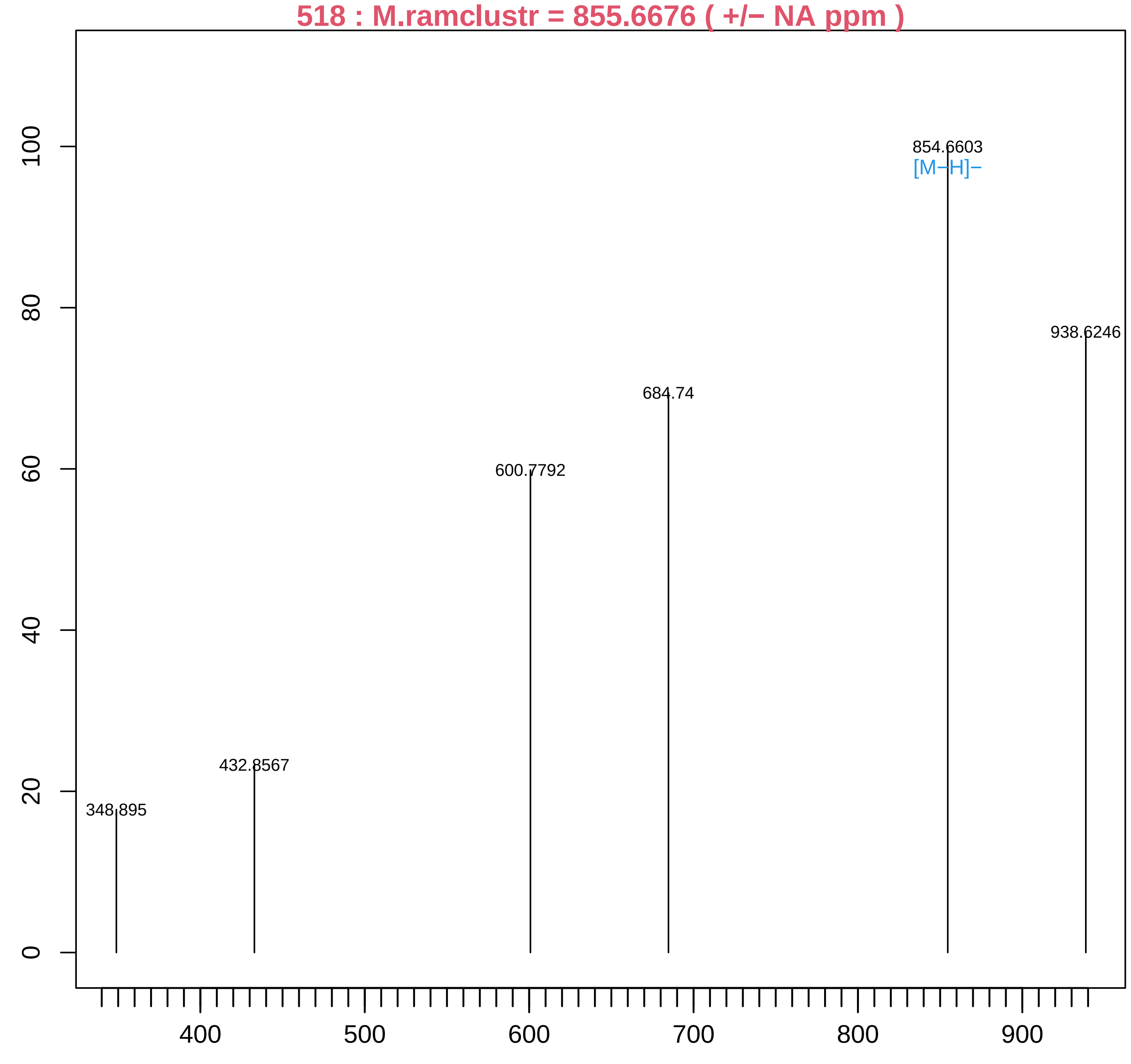

518 : M.ramclustr = 855.6676 (+/− NA ppm)
854.6603
[M−H]−
938.6246
684.74
600.7792
432.8567
348.895

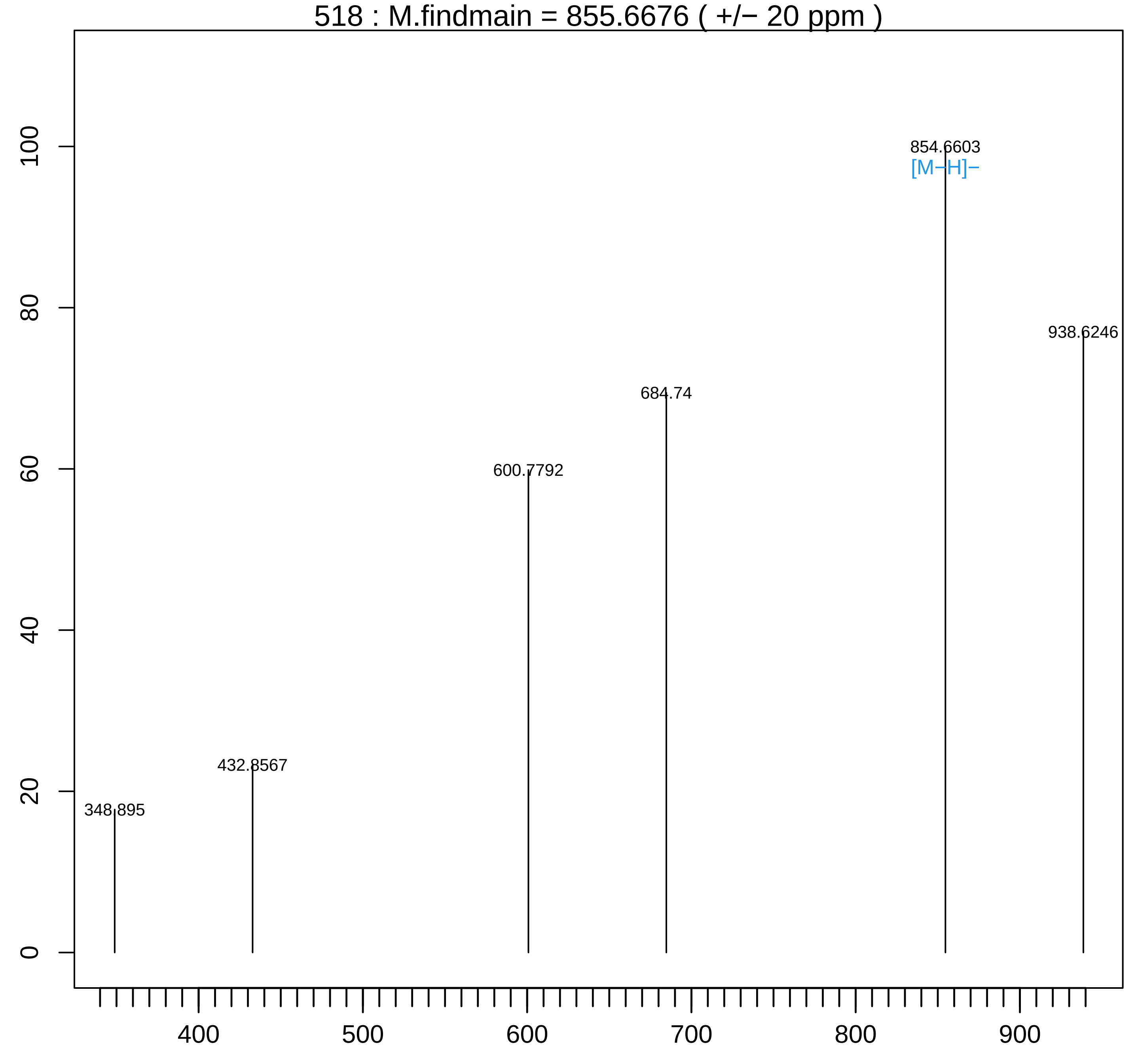

518 : M.findmain = 855.6676 (+/− 20 ppm)
854.6603
[M−H]−
938.6246
684.74
600.7792
432.8567
348.895

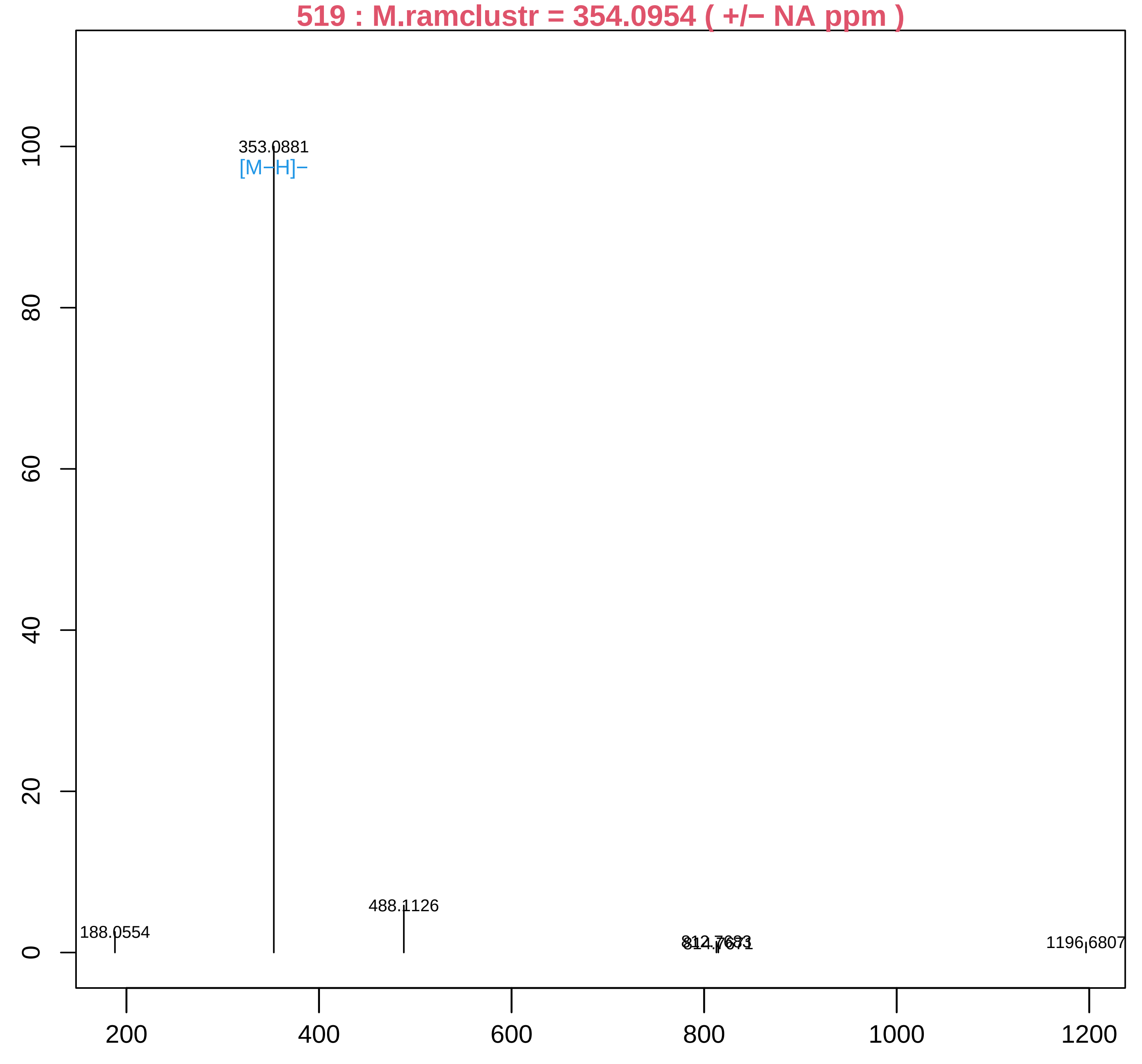
519 : M.ramclustr = 354.0954 (+/− NA ppm)
353.0881
[M−H]−
488.1126
188.0554
1196.6807
0
20
40
60
80
100
200
400
600
800
1000
1200

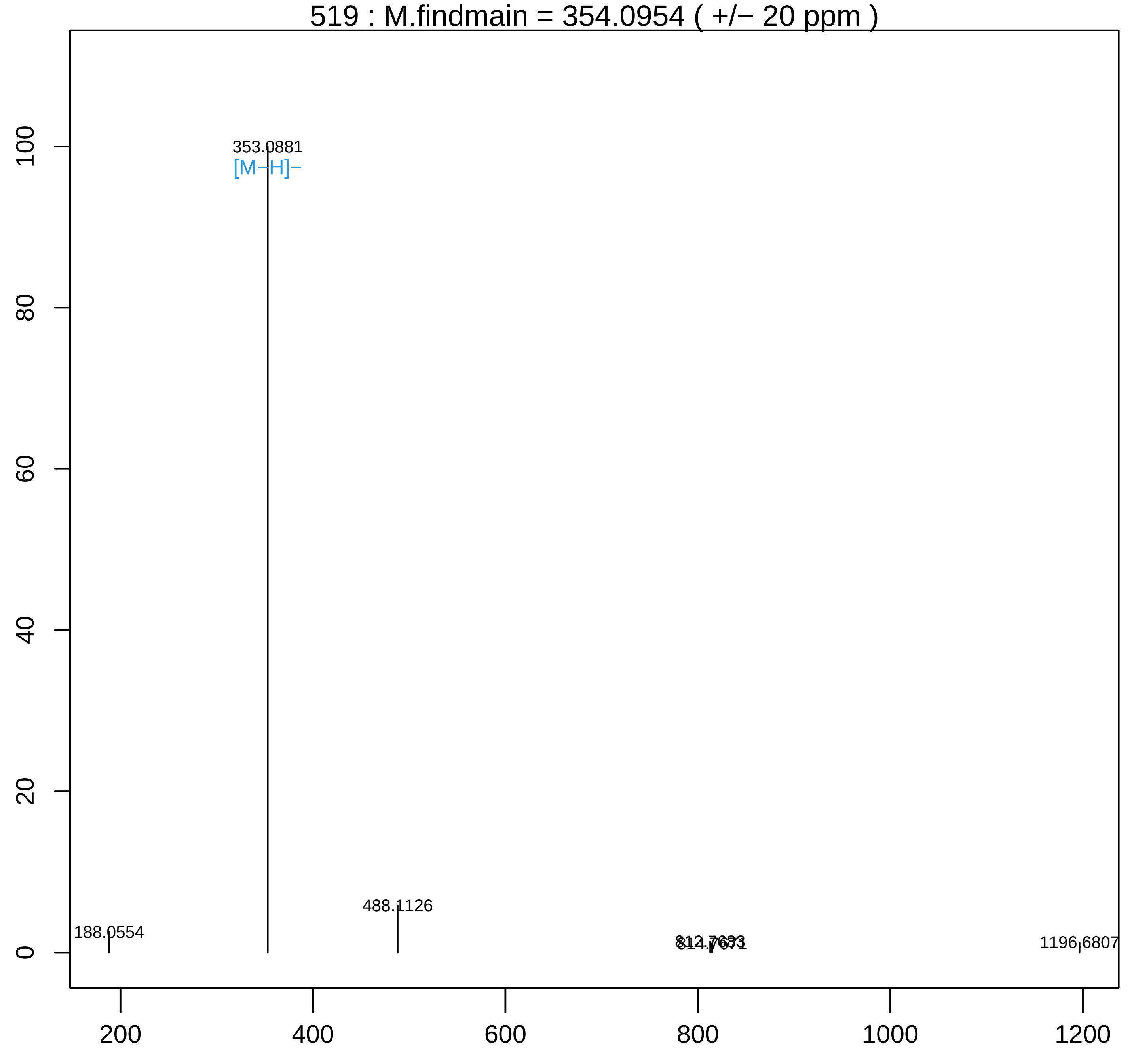
519 : M.findmain = 354.0954 (+/− 20 ppm)
353.0881
[M−H]−
488.1126
188.0554
1196.6807
0
20
40
60
80
100
200
400
600
800
1000
1200

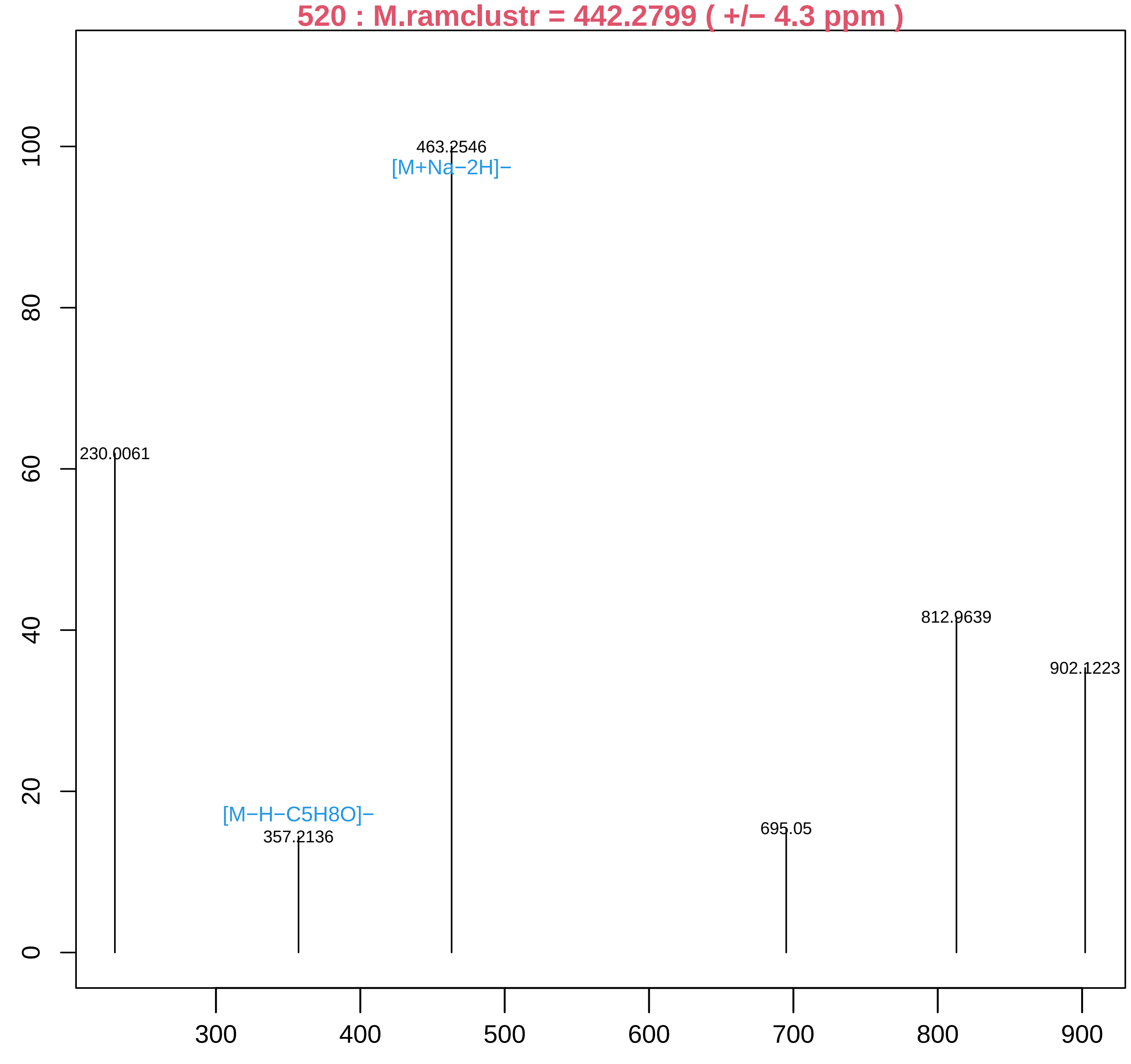
520 : M.ramclustr = 442.2799 (+/− 4.3 ppm)
463.2546
[M+Na−2H]−
230.0061
[M−H−C5H8O]−
357.2136
695.05
812.9639
902.1223
0
20
40
60
80
100
300
400
500
600
700
800
900

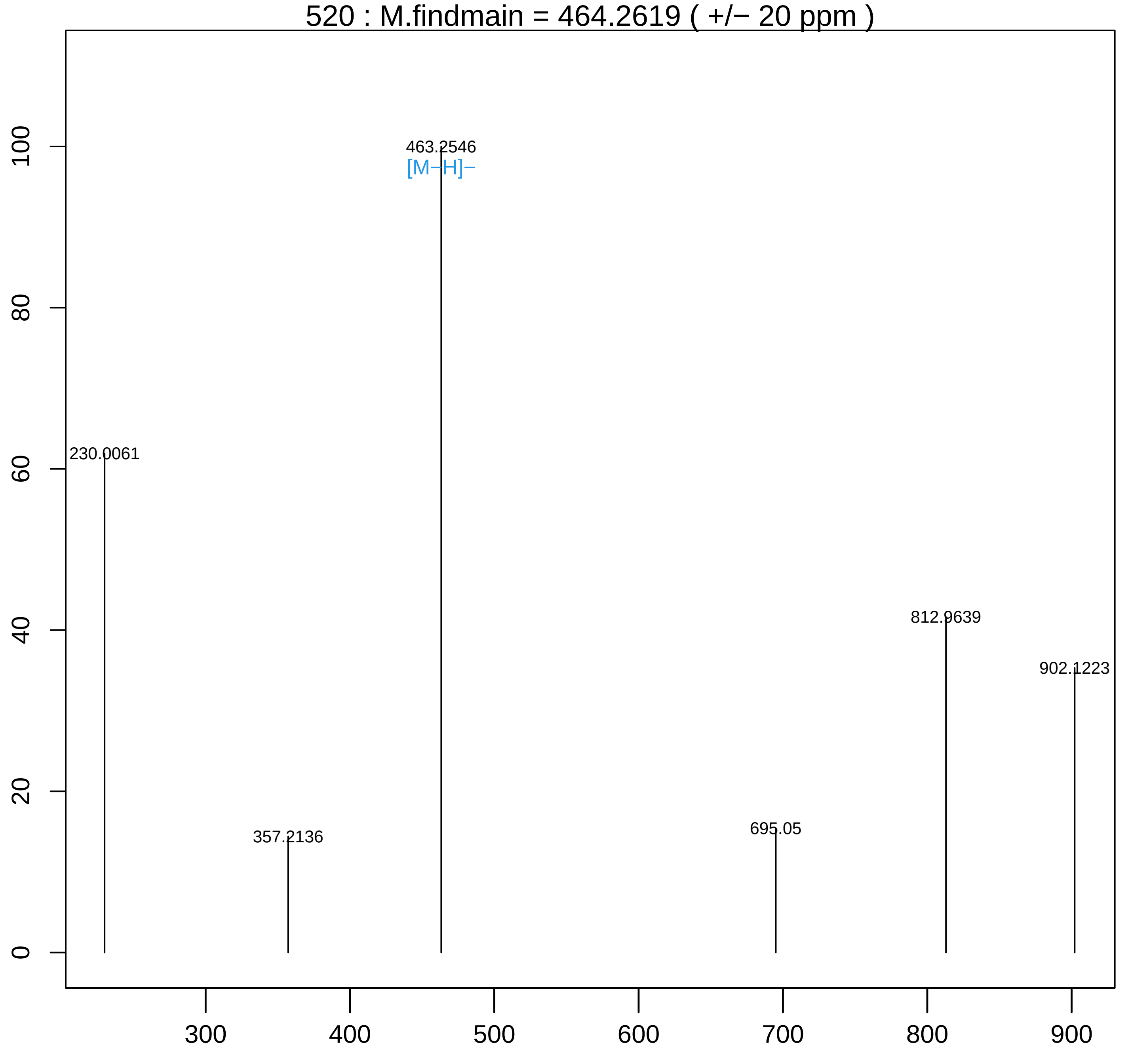
520 : M.findmain = 464.2619 (+/− 20 ppm)
463.2546
[M−H]−
230.0061
357.2136
695.05
812.9639
902.1223
0
20
40
60
80
100
300
400
500
600
700
800
900

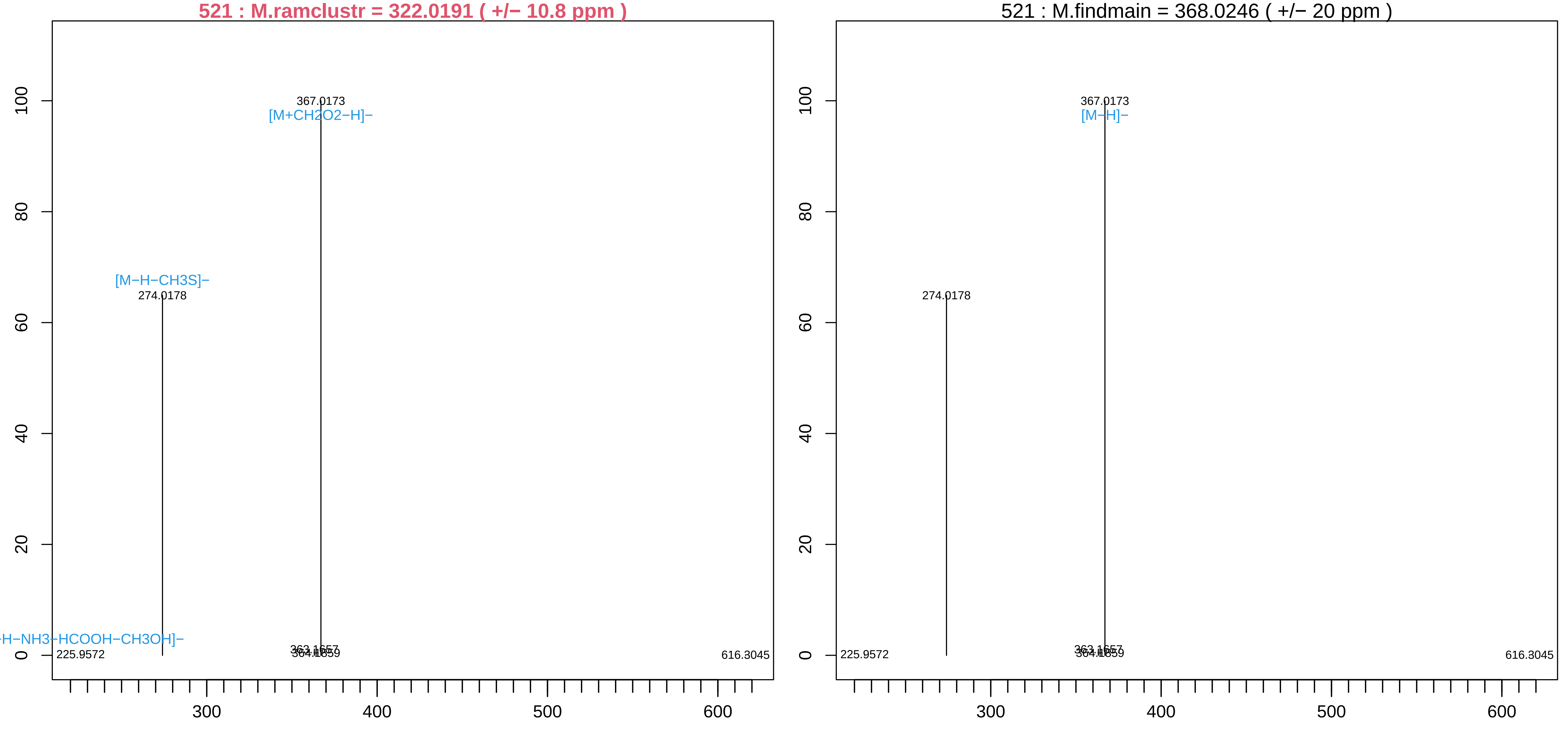

521 : M.ramclustr = 322.0191 (+/− 10.8 ppm)
367.0173
[M+CH2O2−H]−
[M−H−CH3S]−
274.0178
−H−NH3−HCOOH−CH3OH]−
225.9572
363.1657
364.1659
616.3045
521 : M.findmain = 368.0246 (+/− 20 ppm)
367.0173
[M−H]−
274.0178
225.9572
363.1657
364.1659
616.3045
0
20
40
60
80
100
300
400
500
600

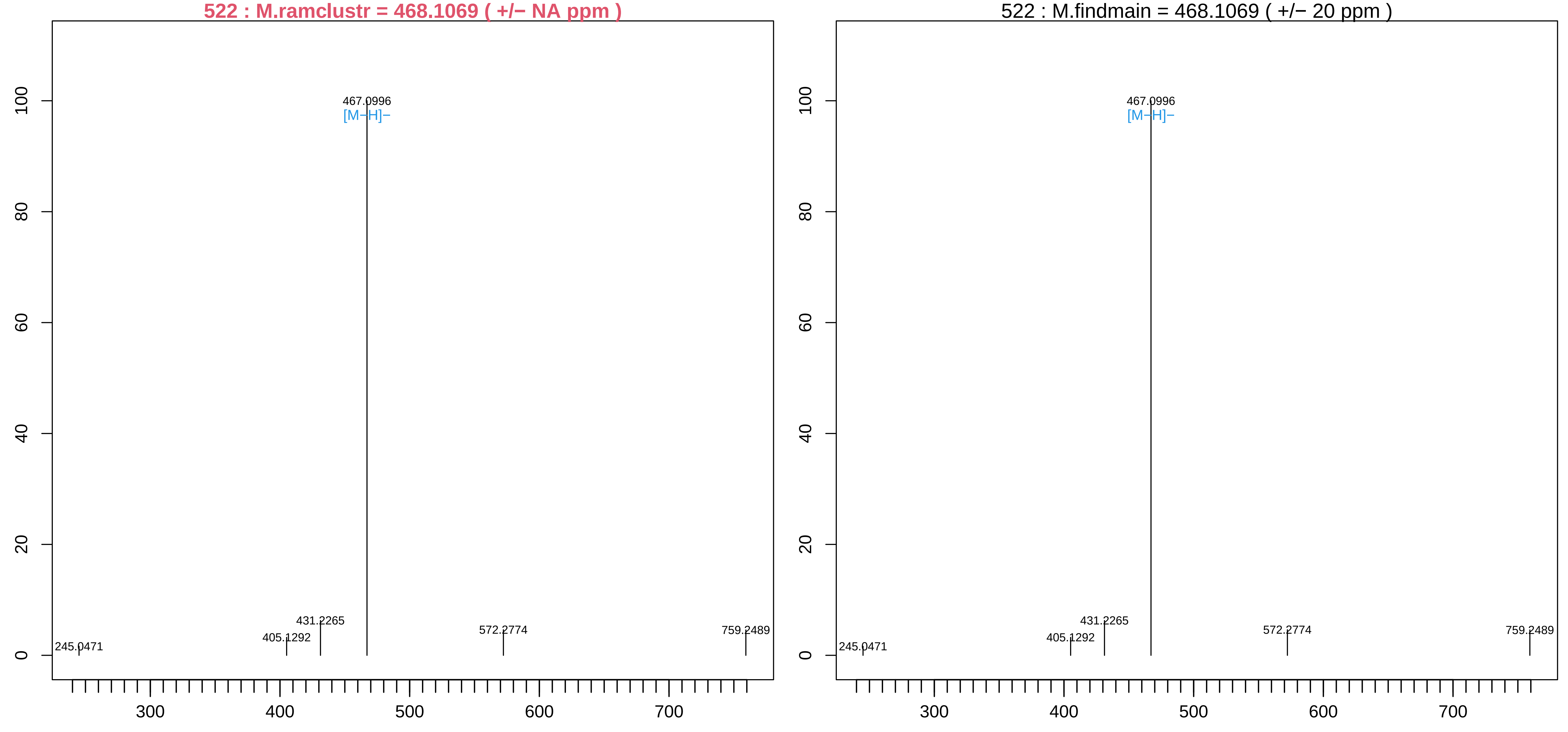

522 : M.ramclustr = 468.1069 (+/− NA ppm)
522 : M.findmain = 468.1069 (+/− 20 ppm)
467.0996
[M−H]−
431.2265
405.1292
245.0471
572.2774
759.2489
0
20
40
60
80
100
300
400
500
600
700

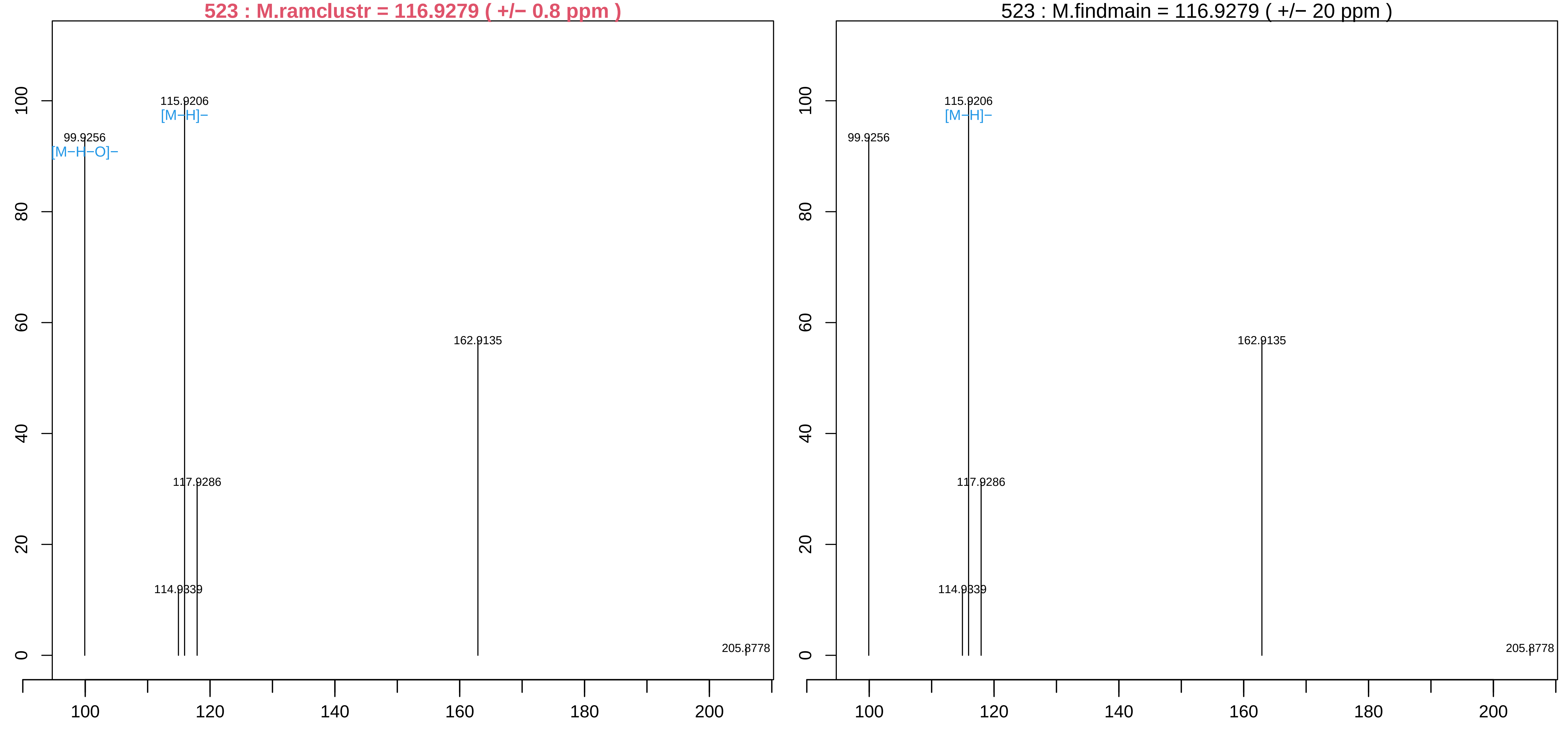
523 : M.ramclustr = 116.9279 (+/− 0.8 ppm)
99.9256
[M−H−O]−
115.9206
[M−H]−
117.9286
114.9339
162.9135
205.8778
523 : M.findmain = 116.9279 (+/− 20 ppm)
99.9256
115.9206
[M−H]−
117.9286
114.9339
162.9135
205.8778
0
20
40
60
80
100
100
120
140
160
180
200

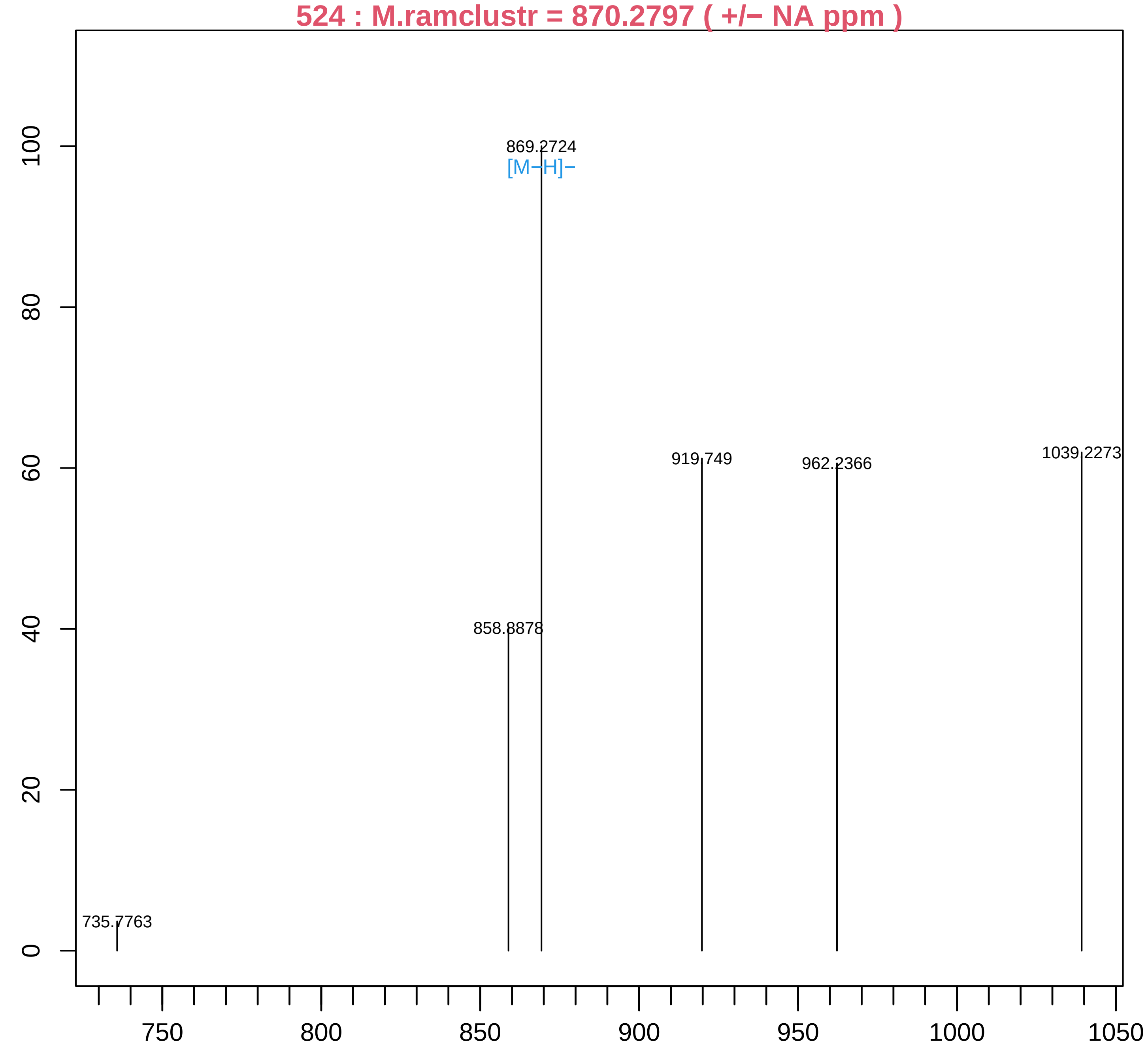
524 : M.ramclustr = 870.2797 (+/− NA ppm)
869.2724
[M−H]−
919.749
962.2366
1039.2273
858.8878
735.7763
0
20
40
60
80
100
750
800
850
900
950
1000
1050

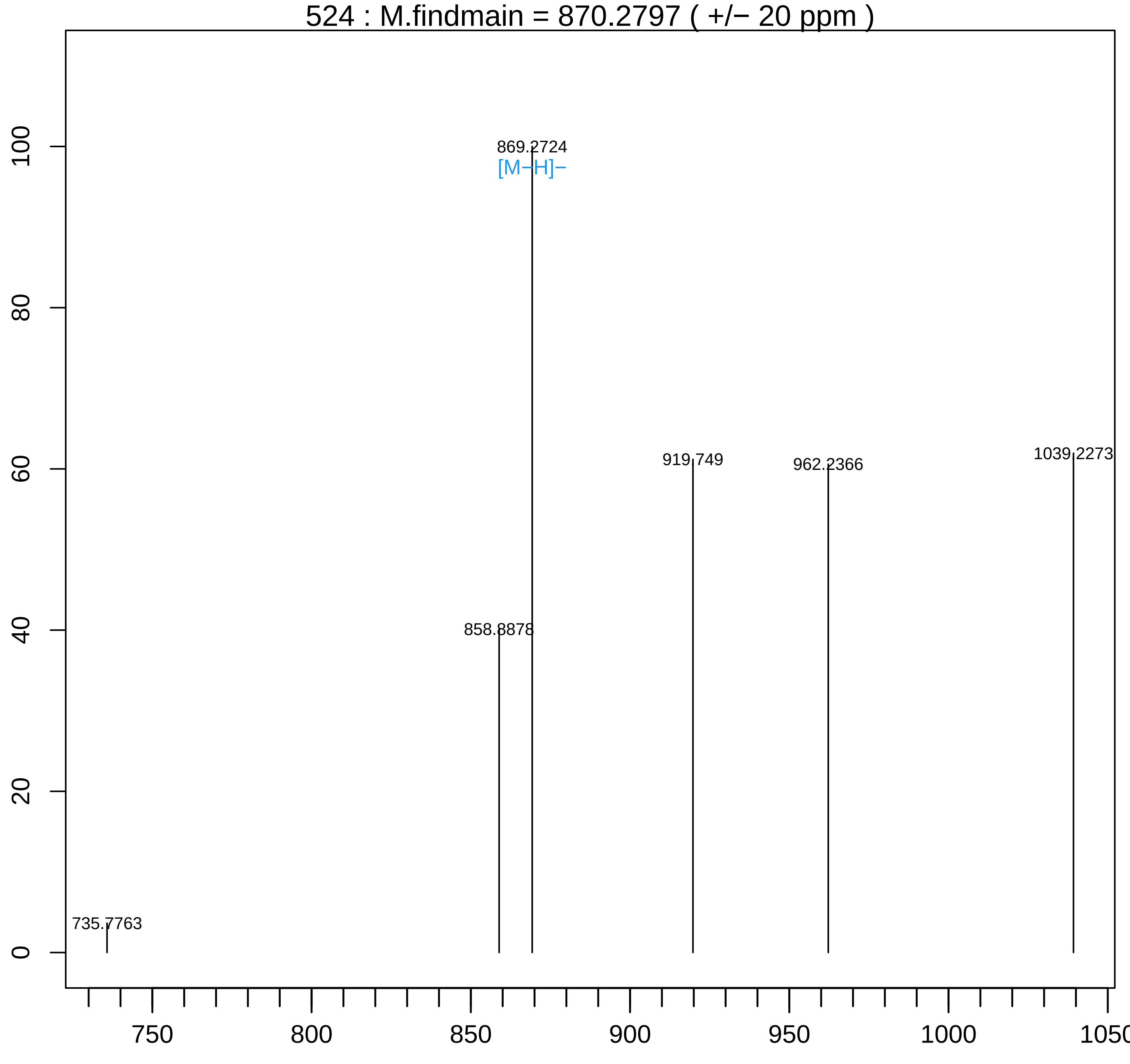
524 : M.findmain = 870.2797 (+/− 20 ppm)
869.2724
[M−H]−
919.749
962.2366
1039.2273
858.8878
735.7763
0
20
40
60
80
100
750
800
850
900
950
1000
1050

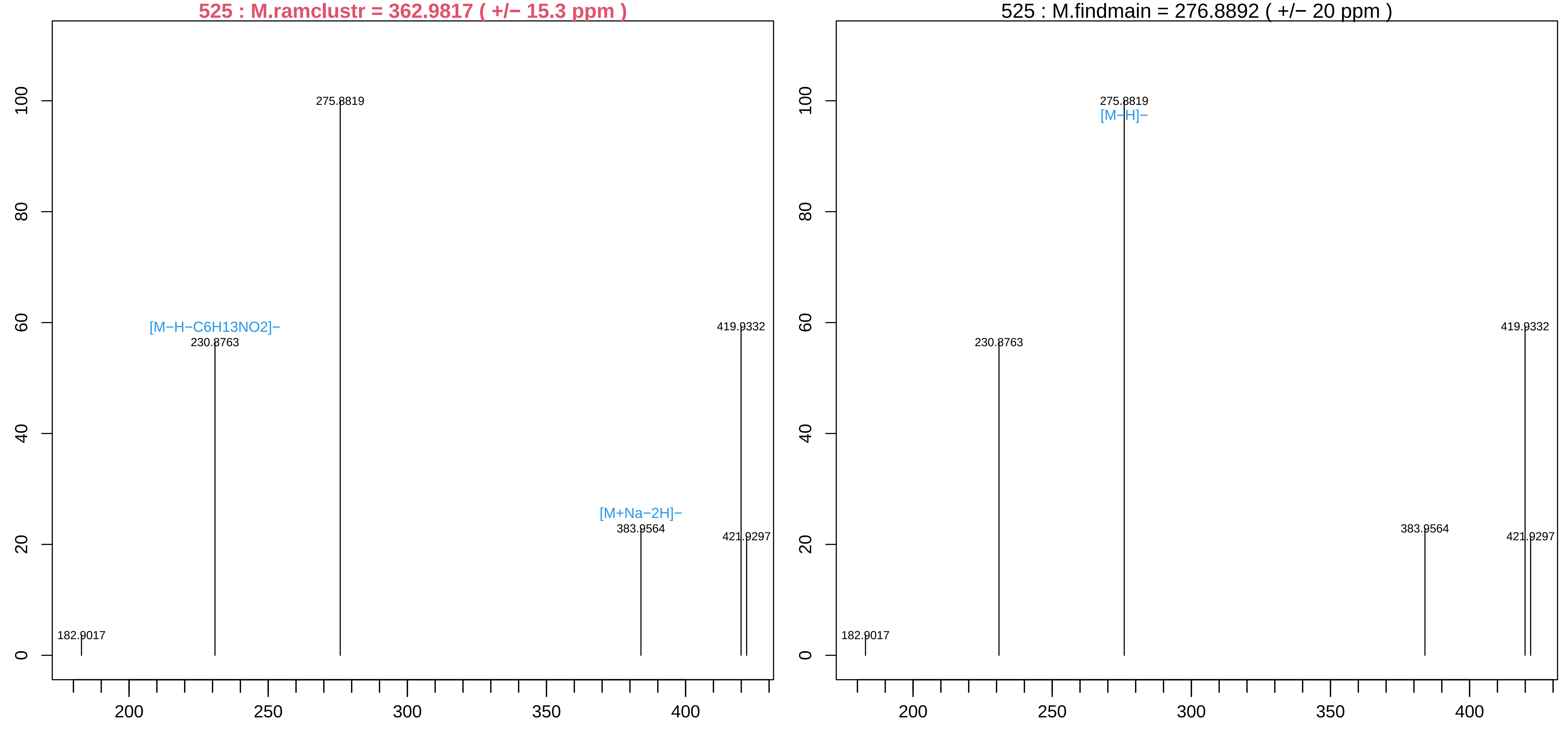

525 : M.ramclustr = 362.9817 (+/− 15.3 ppm)
275.8819
[M−H−C6H13NO2]−
230.8763
419.9332
[M+Na−2H]−
383.9564
421.9297
182.9017
0
20
40
60
80
100
200
250
300
350
400
525 : M.findmain = 276.8892 (+/− 20 ppm)
275.8819
[M−H]−
230.8763
419.9332
383.9564
421.9297
182.9017
0
20
40
60
80
100
200
250
300
350
400

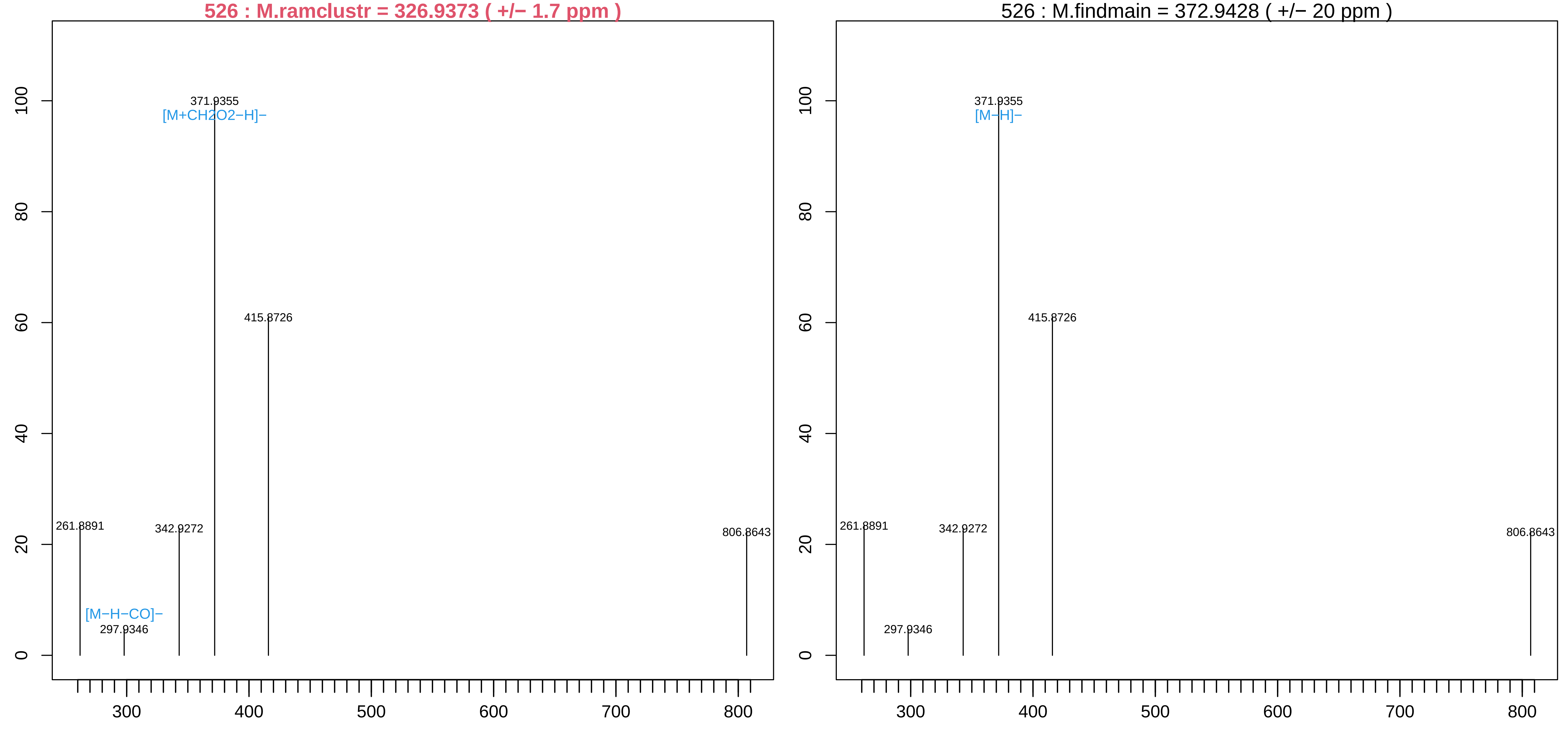
526 : M.ramclustr = 326.9373 (+/− 1.7 ppm)
371.9355
[M+CH2O2−H]−
415.8726
261.8891
342.9272
806.8643
[M−H−CO]−
297.9346
526 : M.findmain = 372.9428 (+/− 20 ppm)
371.9355
[M−H]−
415.8726
261.8891
342.9272
806.8643
297.9346

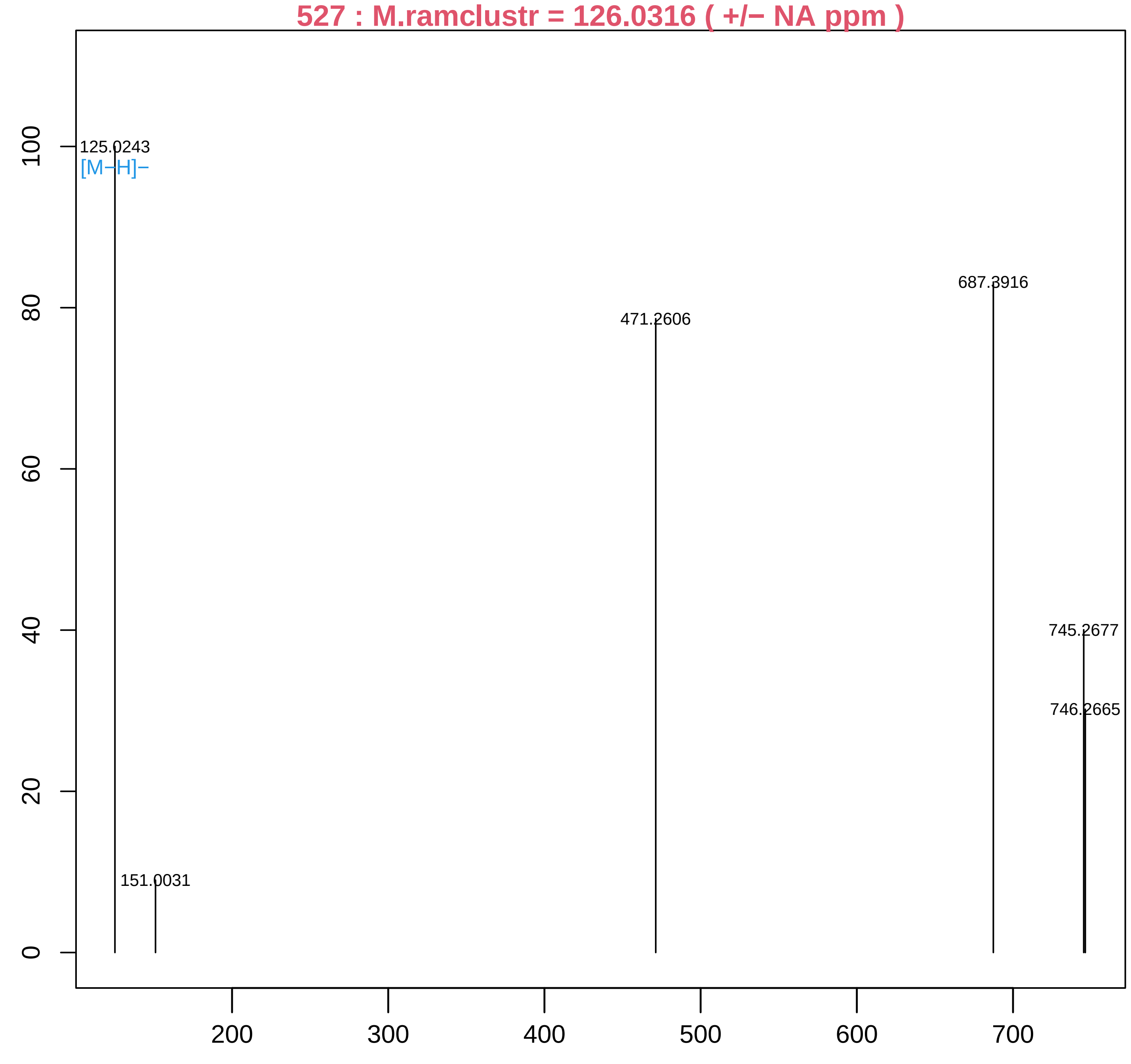
527 : M.ramclustr = 126.0316 (+/− NA ppm)
125.0243
[M−H]−
151.0031
471.2606
687.3916
745.2677
746.2665
0
20
40
60
80
100
200
300
400
500
600
700

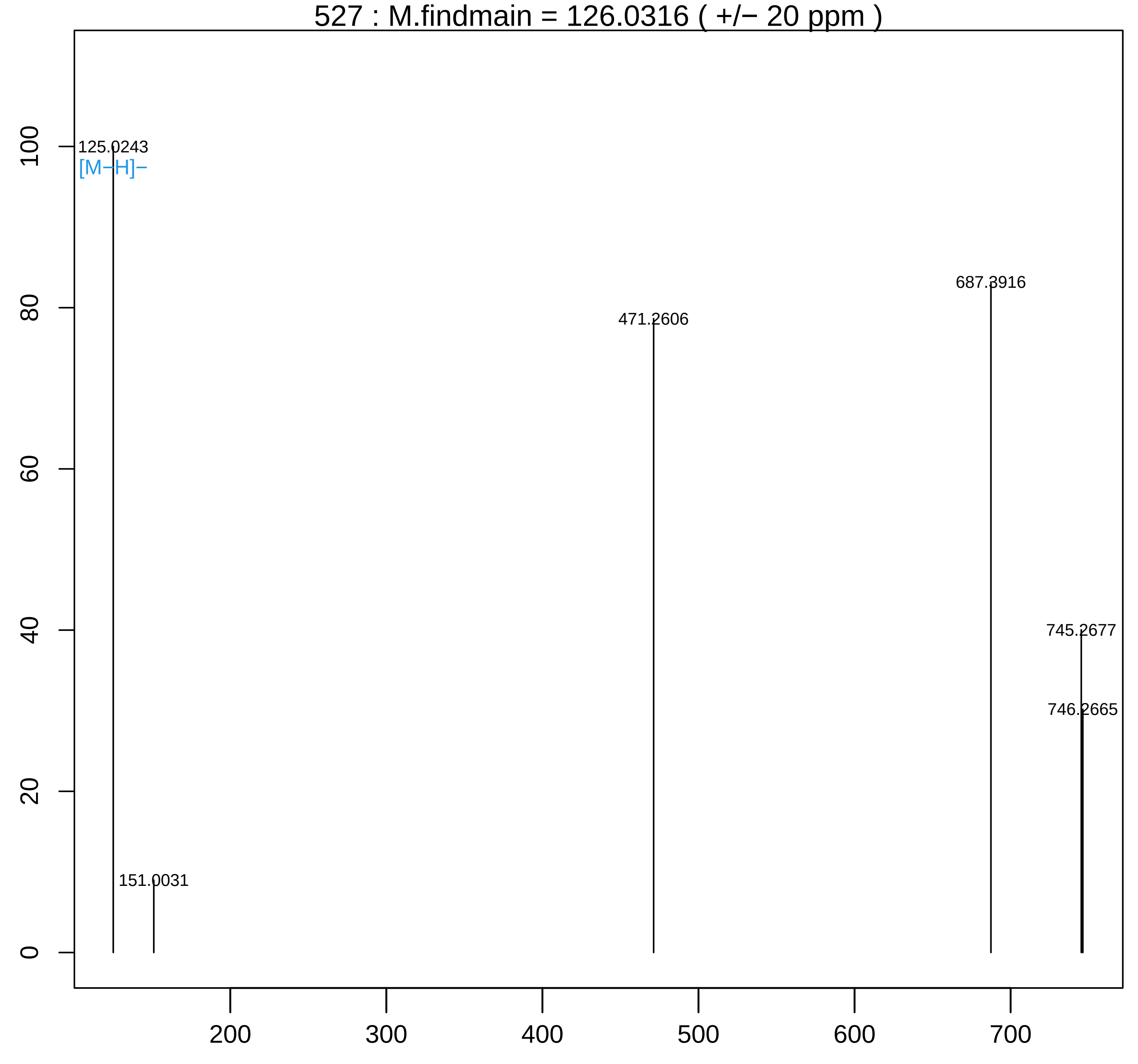
527 : M.findmain = 126.0316 (+/− 20 ppm)
125.0243
[M−H]−
151.0031
471.2606
687.3916
745.2677
746.2665
0
20
40
60
80
100
200
300
400
500
600
700

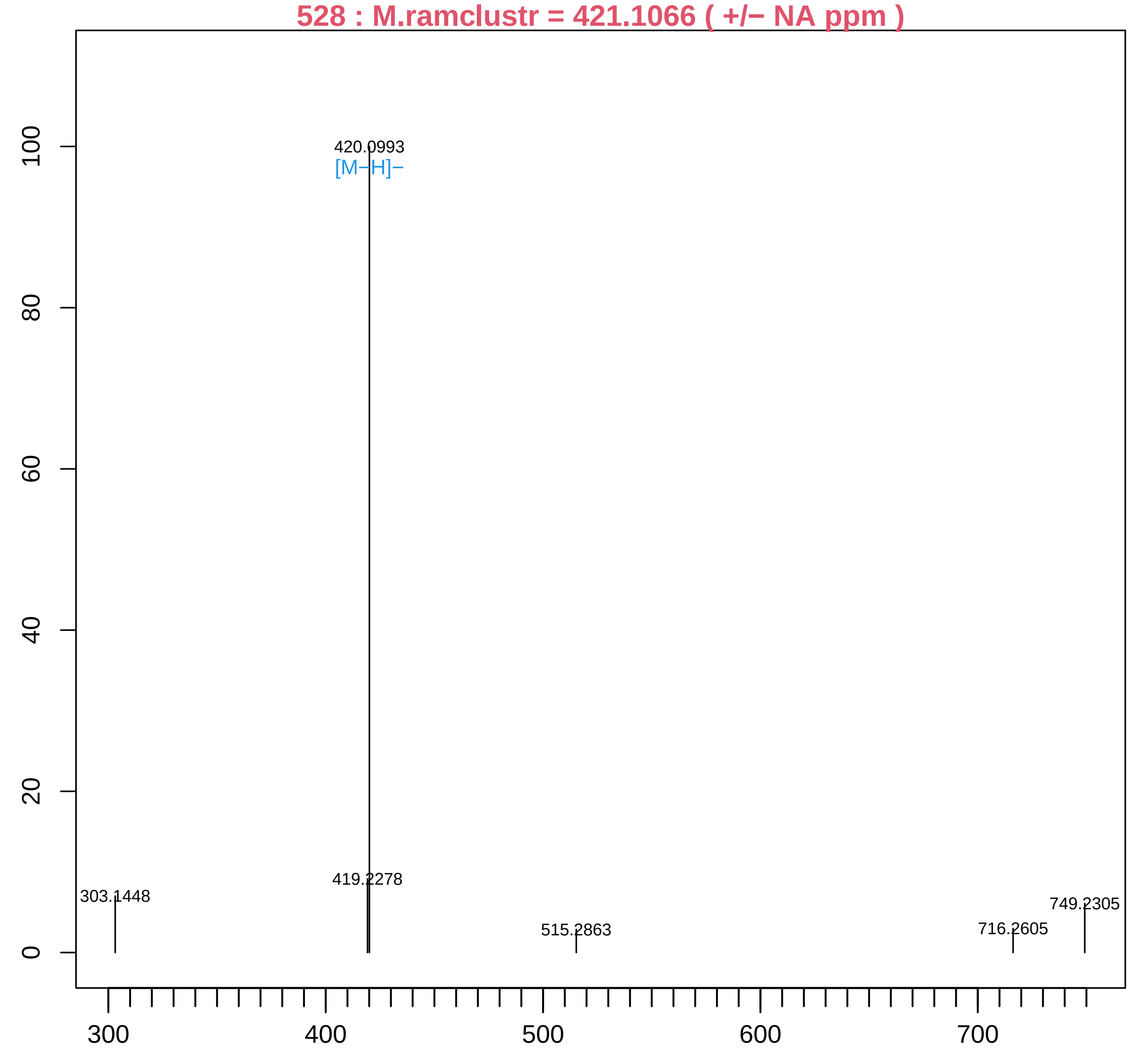
528 : M.ramclustr = 421.1066 (+/− NA ppm)
420.0993
[M−H]−
419.2278
303.1448
515.2863
716.2605
749.2305
0
20
40
60
80
100
300
400
500
600
700

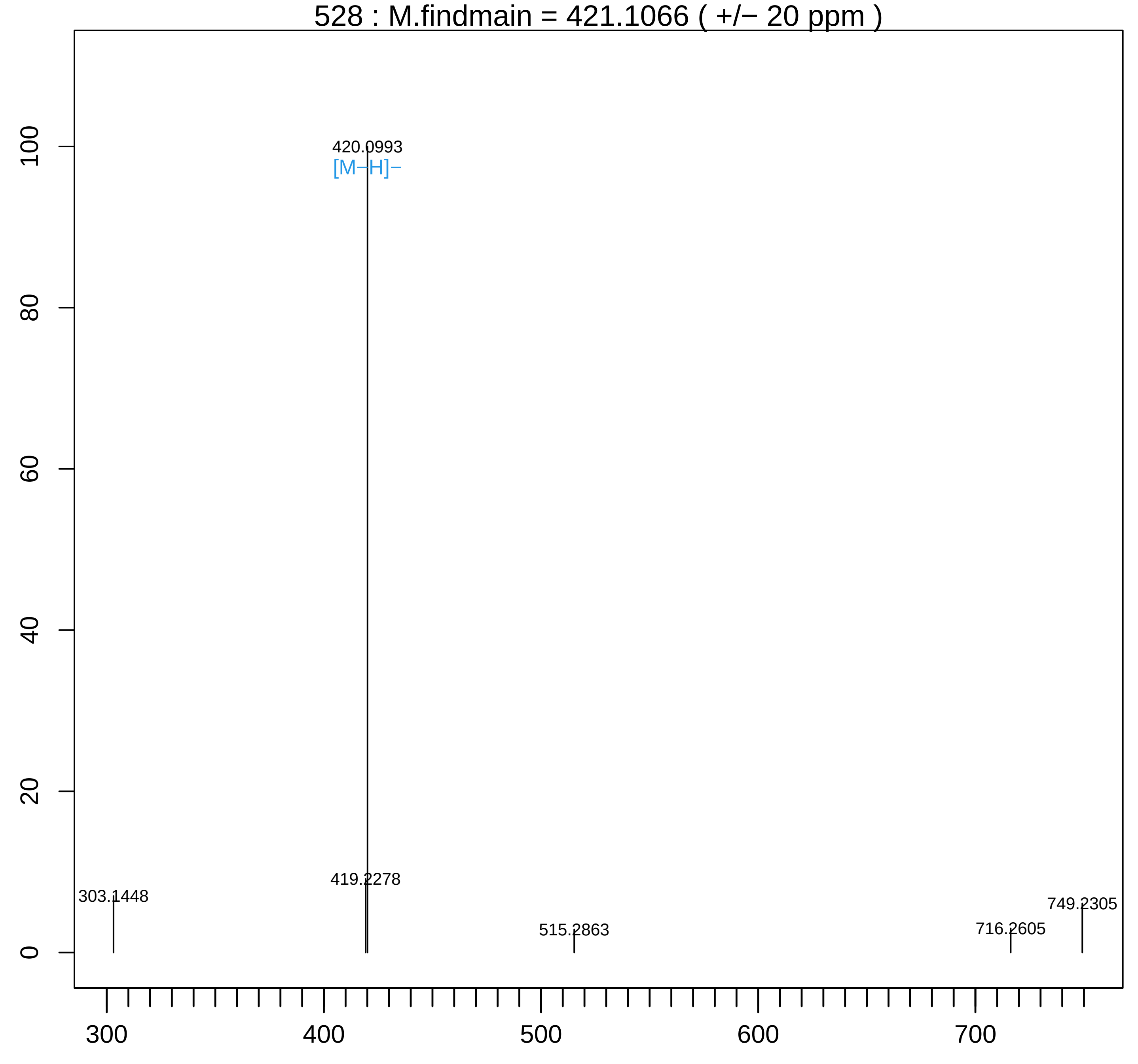
528 : M.findmain = 421.1066 (+/− 20 ppm)
420.0993
[M−H]−
419.2278
303.1448
515.2863
716.2605
749.2305
0
20
40
60
80
100
300
400
500
600
700

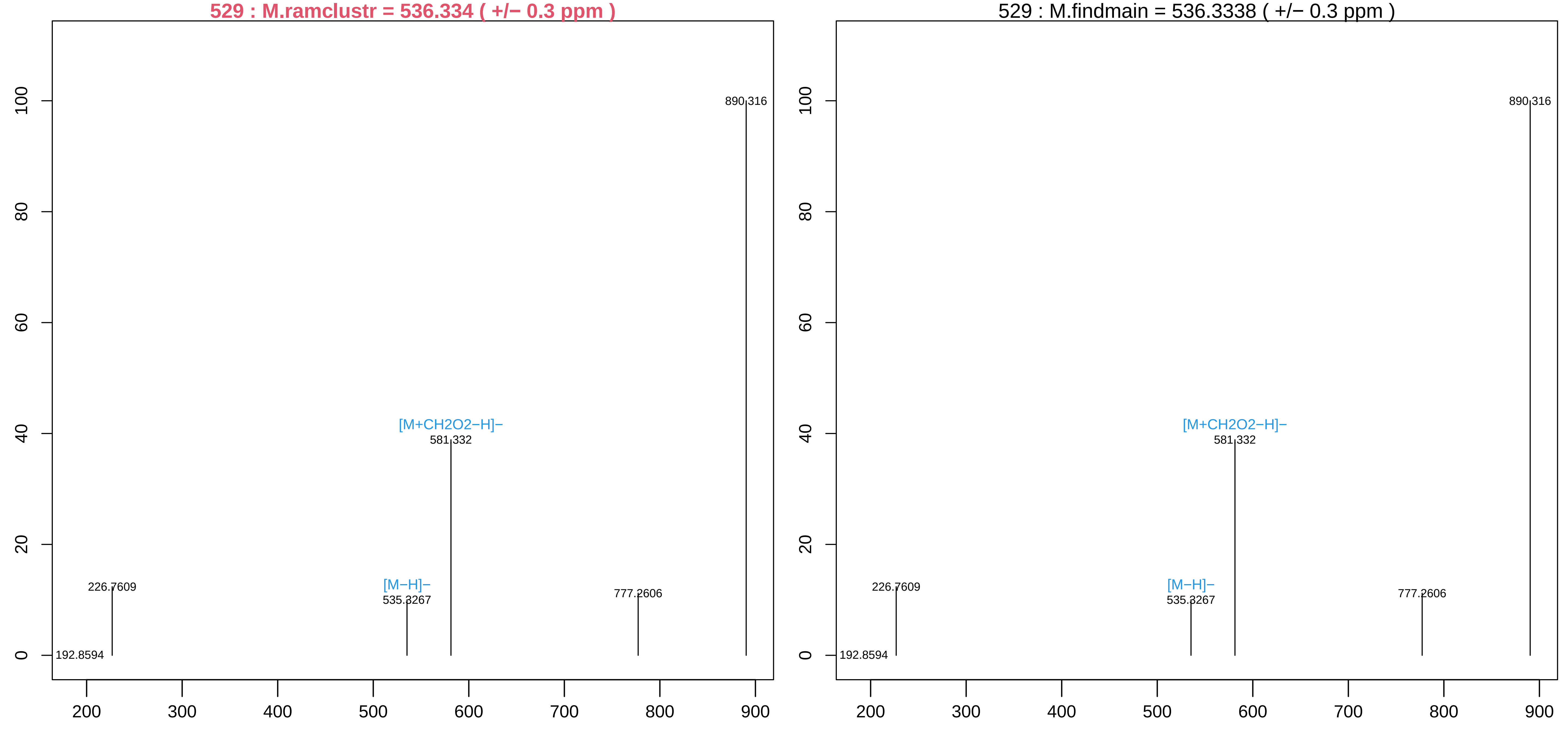
529 : M.ramclustr = 536.334 (+/− 0.3 ppm)
529 : M.findmain = 536.3338 (+/− 0.3 ppm)
0
20
40
60
80
100
200
300
400
500
600
700
800
900
890.316
[M+CH2O2−H]−
581.332
226.7609
[M−H]−
535.3267
777.2606
192.8594

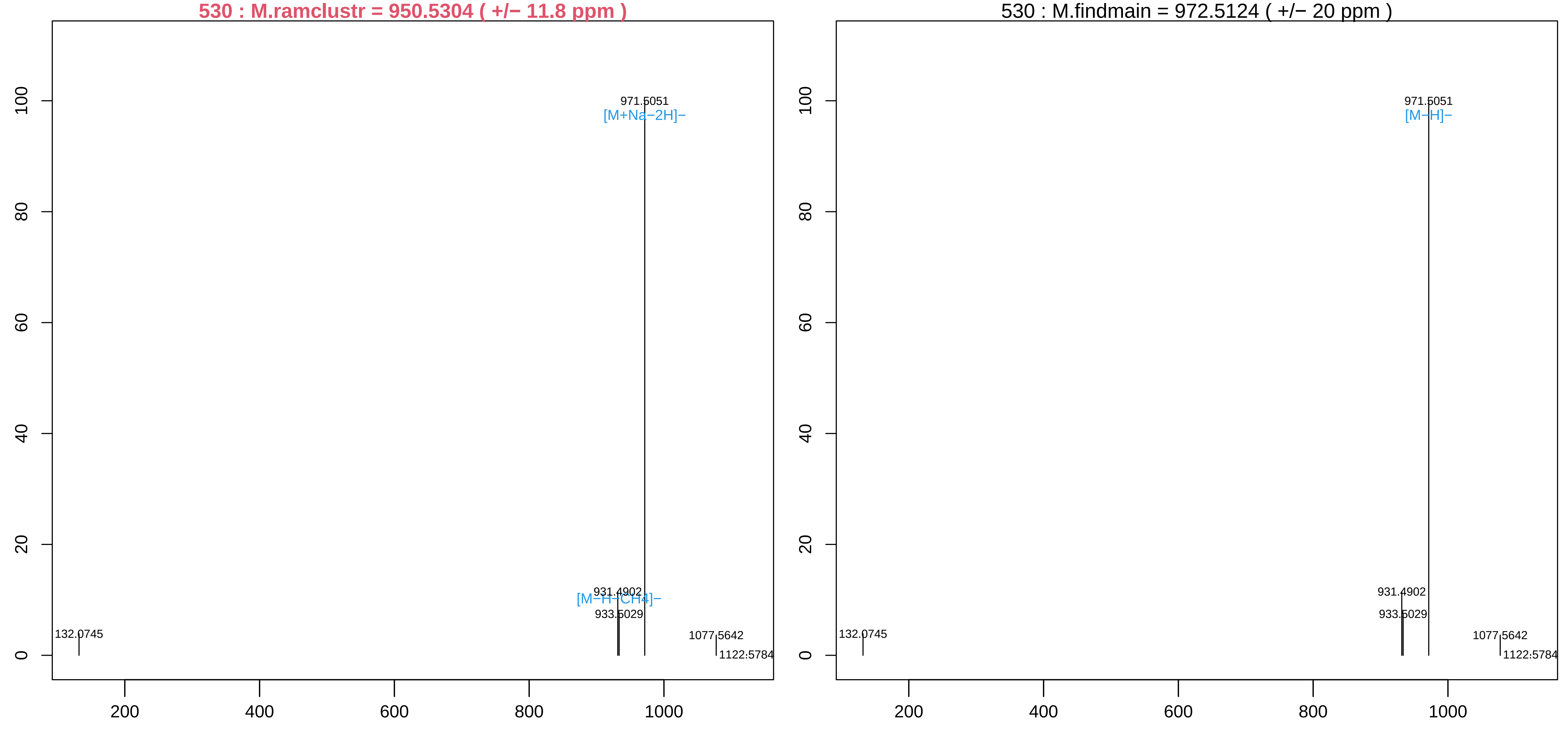
530 : M.ramclustr = 950.5304 (+/− 11.8 ppm)
530 : M.findmain = 972.5124 (+/− 20 ppm)
971.5051
[M+Na−2H]−
[M−H]−
931.4902
[M−H−CH4]−
933.5029
132.0745
1077.5642
1122.5784
0
20
40
60
80
100
200
400
600
800
1000

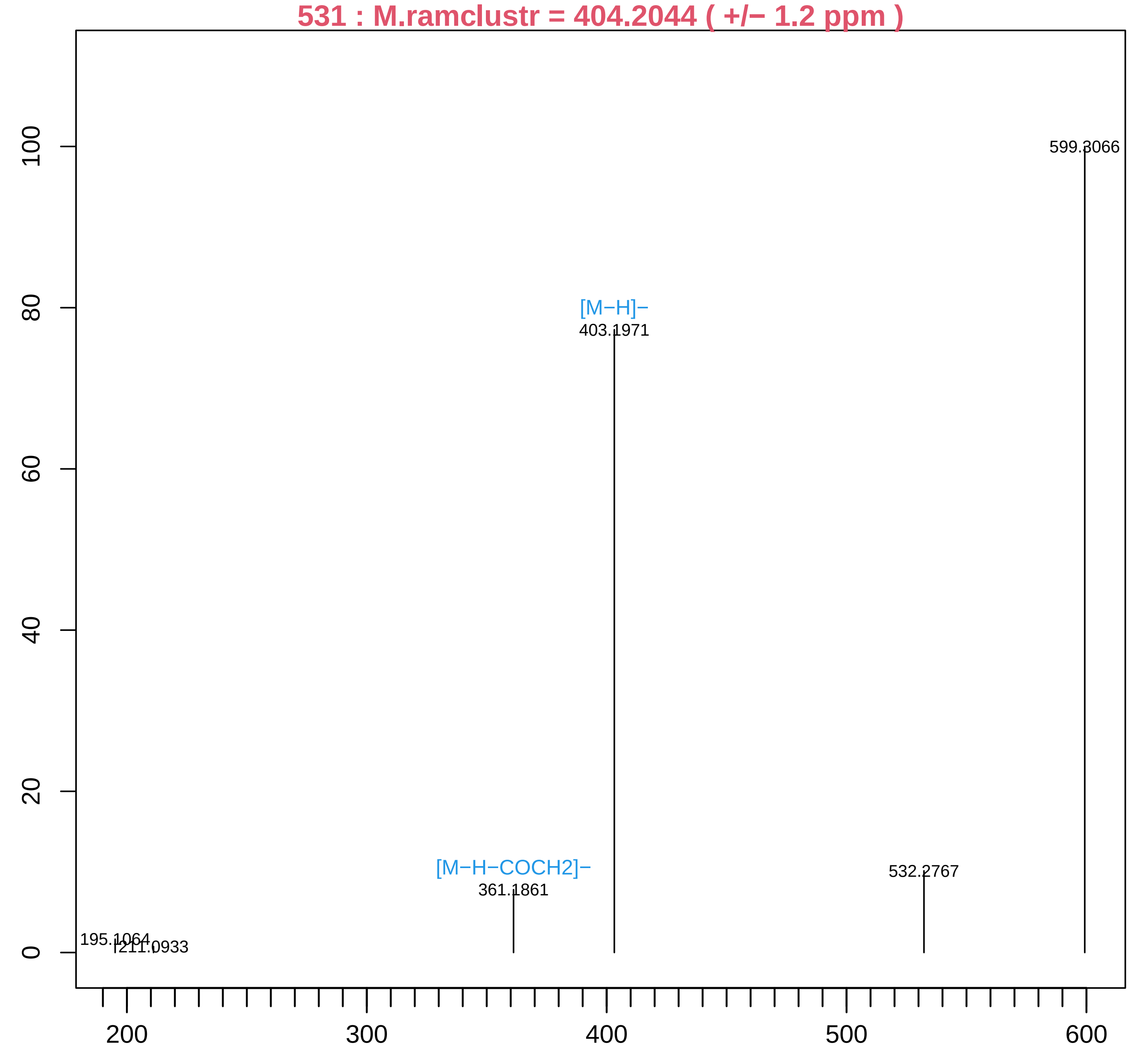

531 : M.ramclustr = 404.2044 (+/− 1.2 ppm)
599.3066
[M−H]−
403.1971
[M−H−COCH2]−
361.1861
532.2767
195.1064
211.0933
0
20
40
60
80
100
200
300
400
500
600

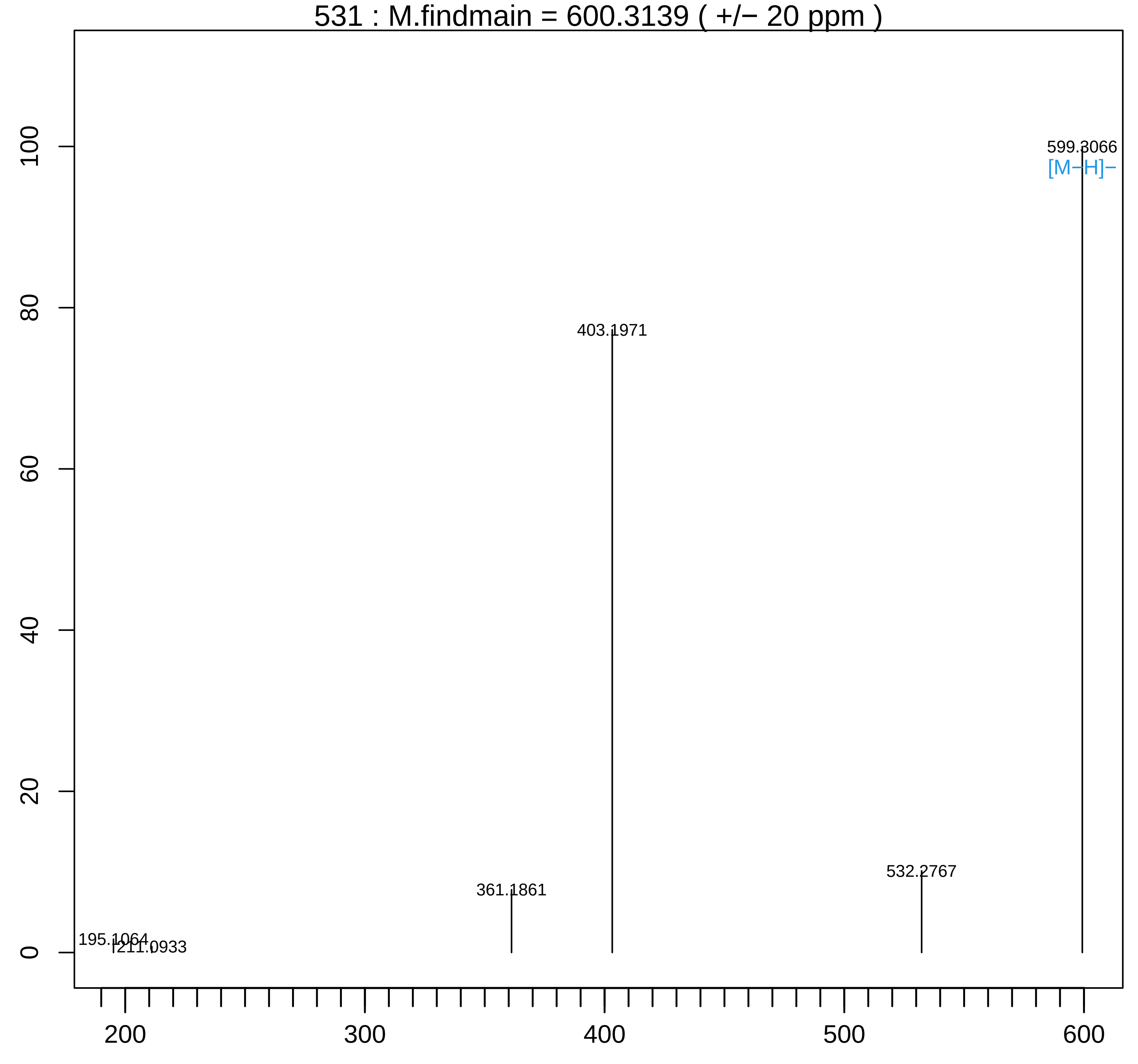

531 : M.findmain = 600.3139 (+/− 20 ppm)
599.3066
[M−H]−
403.1971
361.1861
532.2767
195.1064
211.0933
0
20
40
60
80
100
200
300
400
500
600

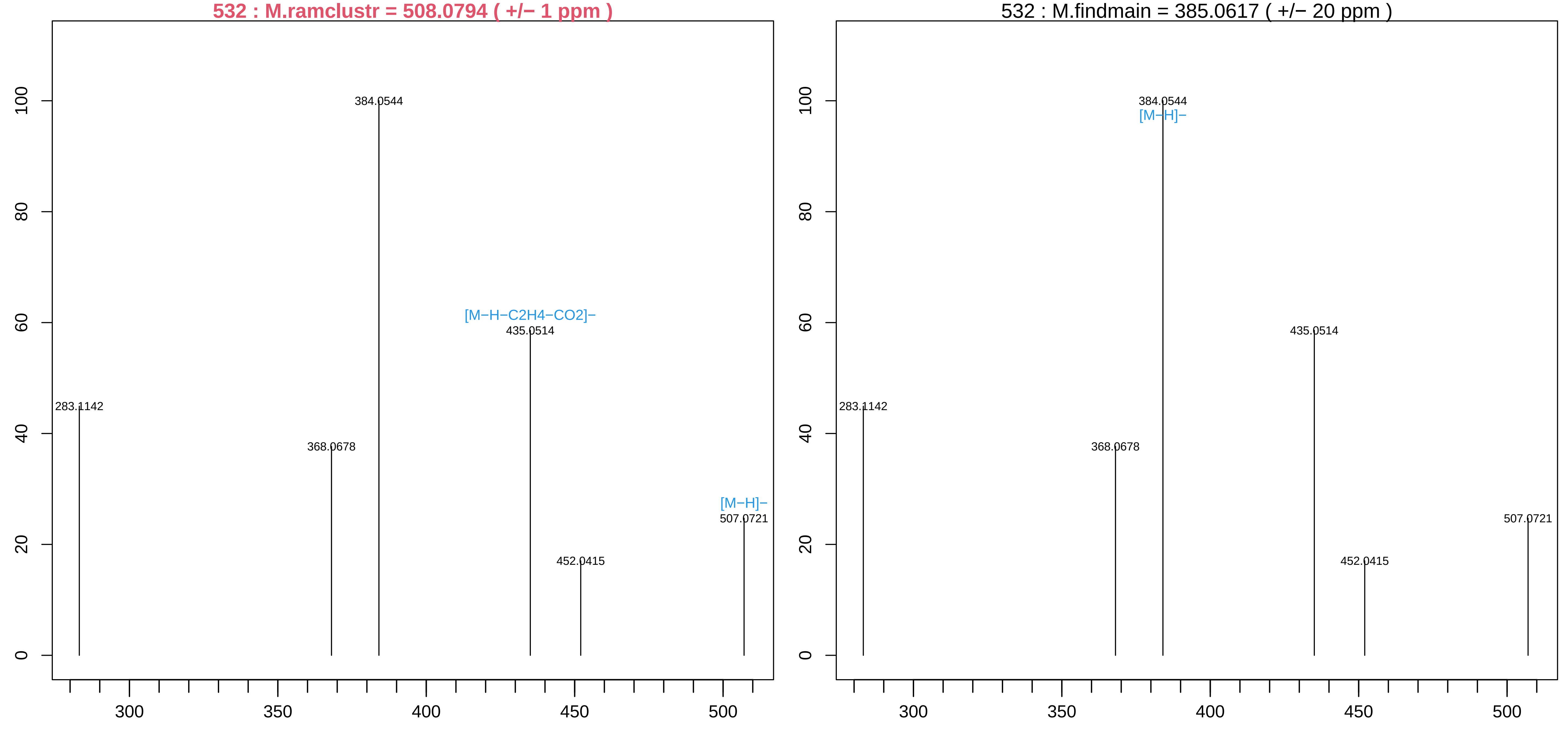

532 : M.ramclustr = 508.0794 (+/− 1 ppm)
384.0544
[M−H−C2H4−CO2]−
435.0514
283.1142
368.0678
[M−H]−
507.0721
452.0415
0
20
40
60
80
100
300
350
400
450
500
532 : M.findmain = 385.0617 (+/− 20 ppm)
384.0544
[M−H]−
435.0514
283.1142
368.0678
507.0721
452.0415
0
20
40
60
80
100
300
350
400
450
500

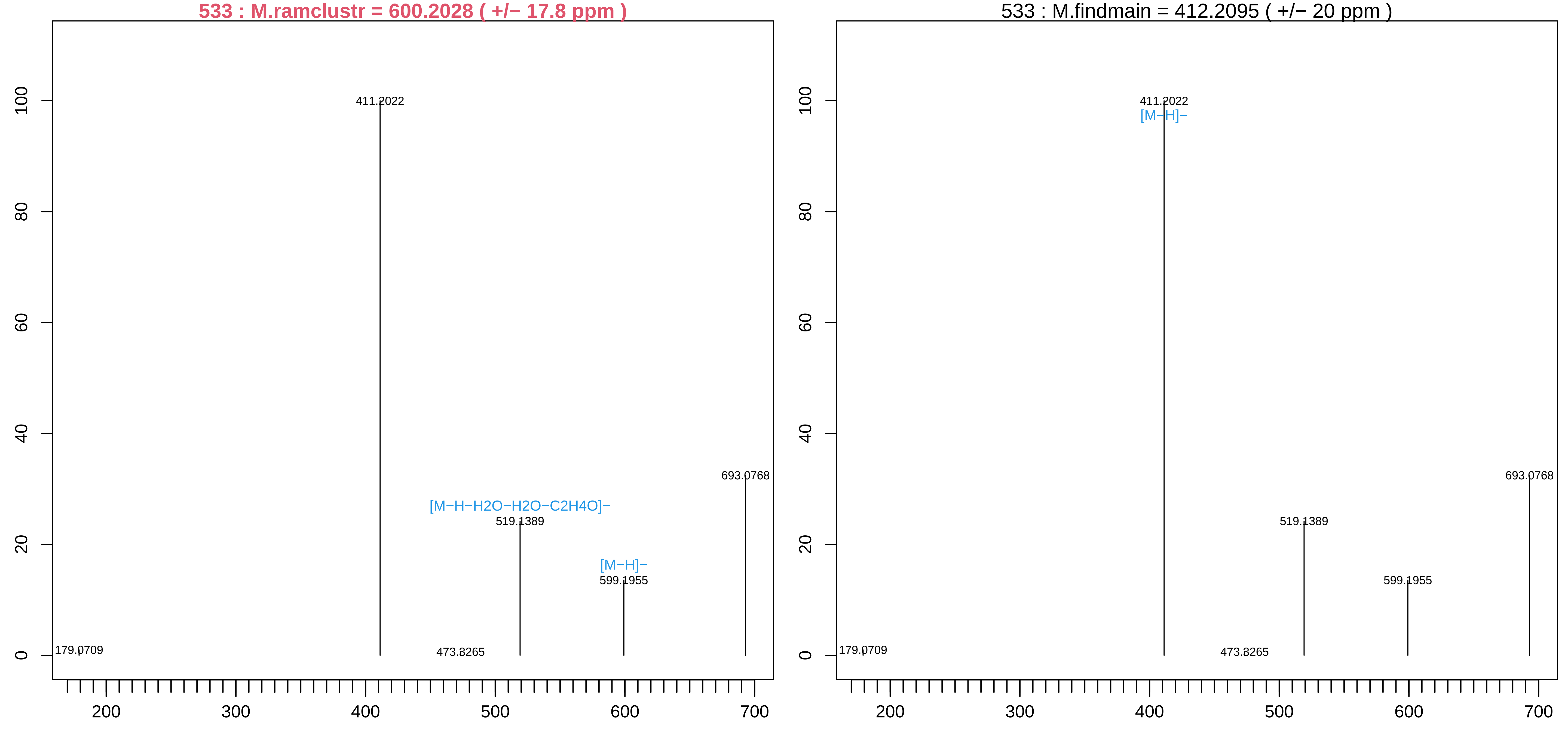

533 : M.ramclustr = 600.2028 (+/− 17.8 ppm)
411.2022
[M−H−H2O−H2O−C2H4O]−
519.1389
[M−H]−
599.1955
693.0768
179.0709
473.8265
533 : M.findmain = 412.2095 (+/− 20 ppm)
411.2022
[M−H]−
519.1389
599.1955
693.0768
179.0709
473.8265

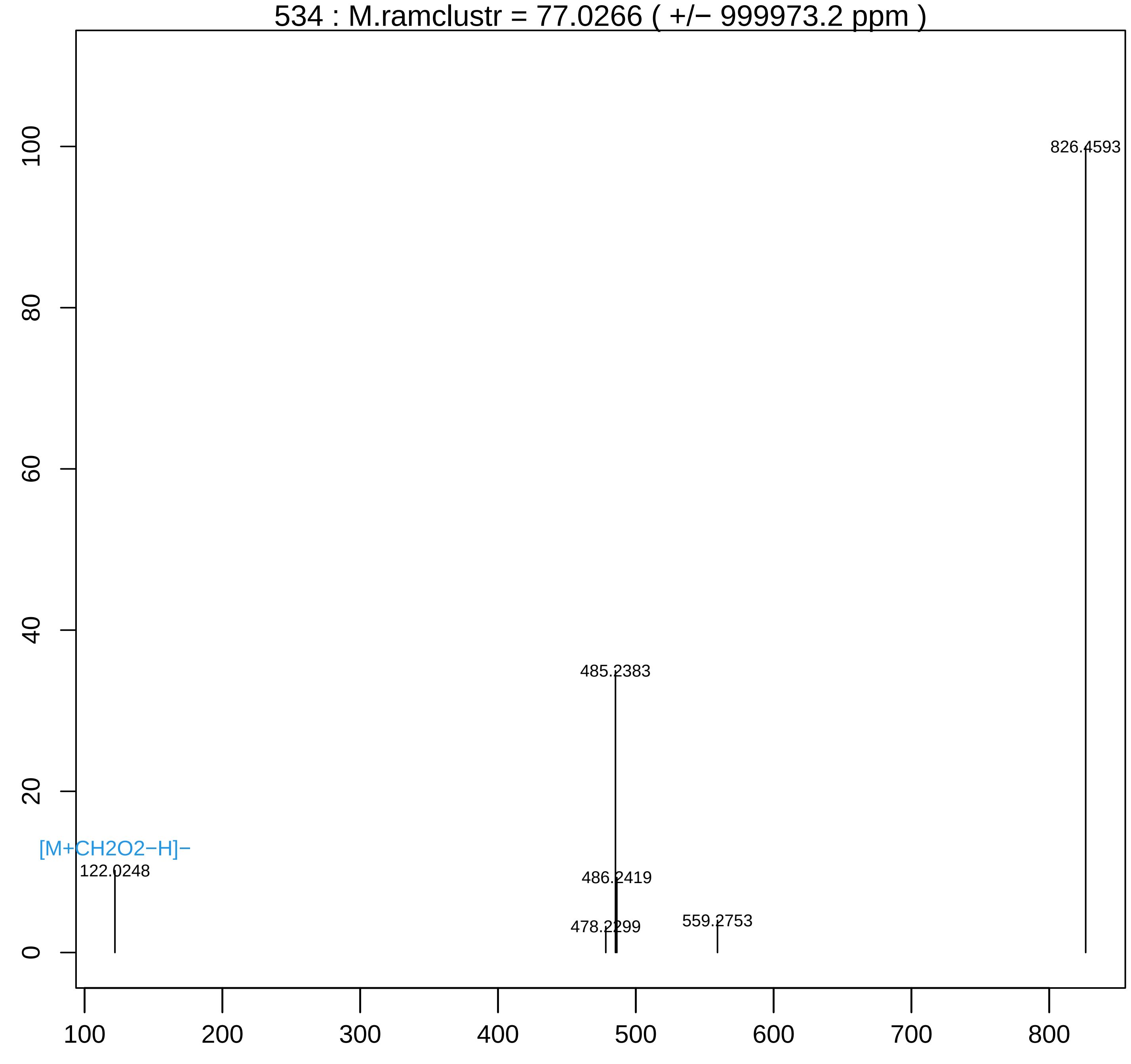
534 : M.ramclustr = 77.0266 (+/− 999973.2 ppm)
826.4593
485.2383
[M+CH2O2−H]−
122.0248
486.2419
478.2299
559.2753
0
20
40
60
80
100
100
200
300
400
500
600
700
800

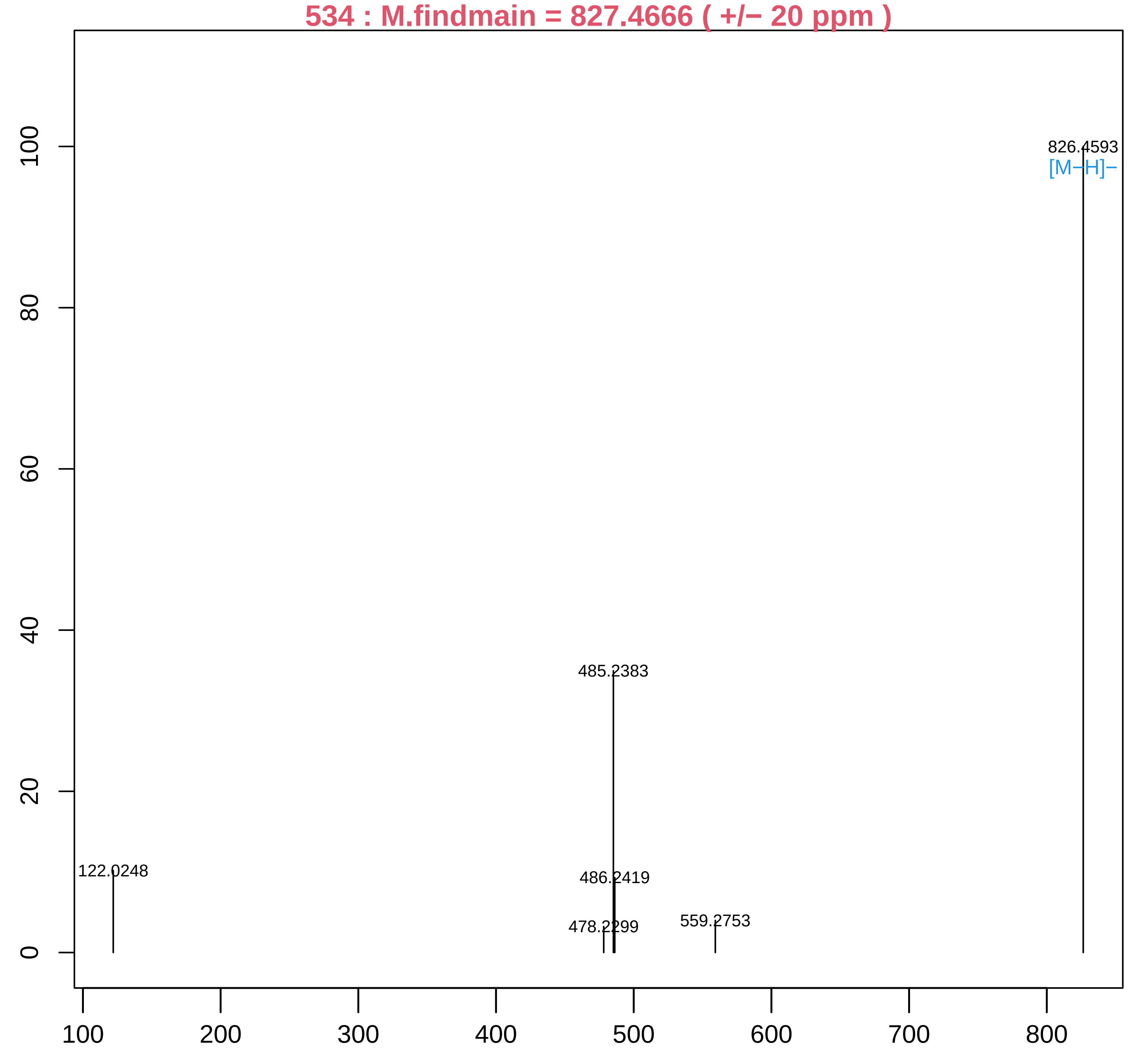
534 : M.findmain = 827.4666 (+/− 20 ppm)
826.4593
[M−H]−
485.2383
122.0248
486.2419
478.2299
559.2753
0
20
40
60
80
100
100
200
300
400
500
600
700
800

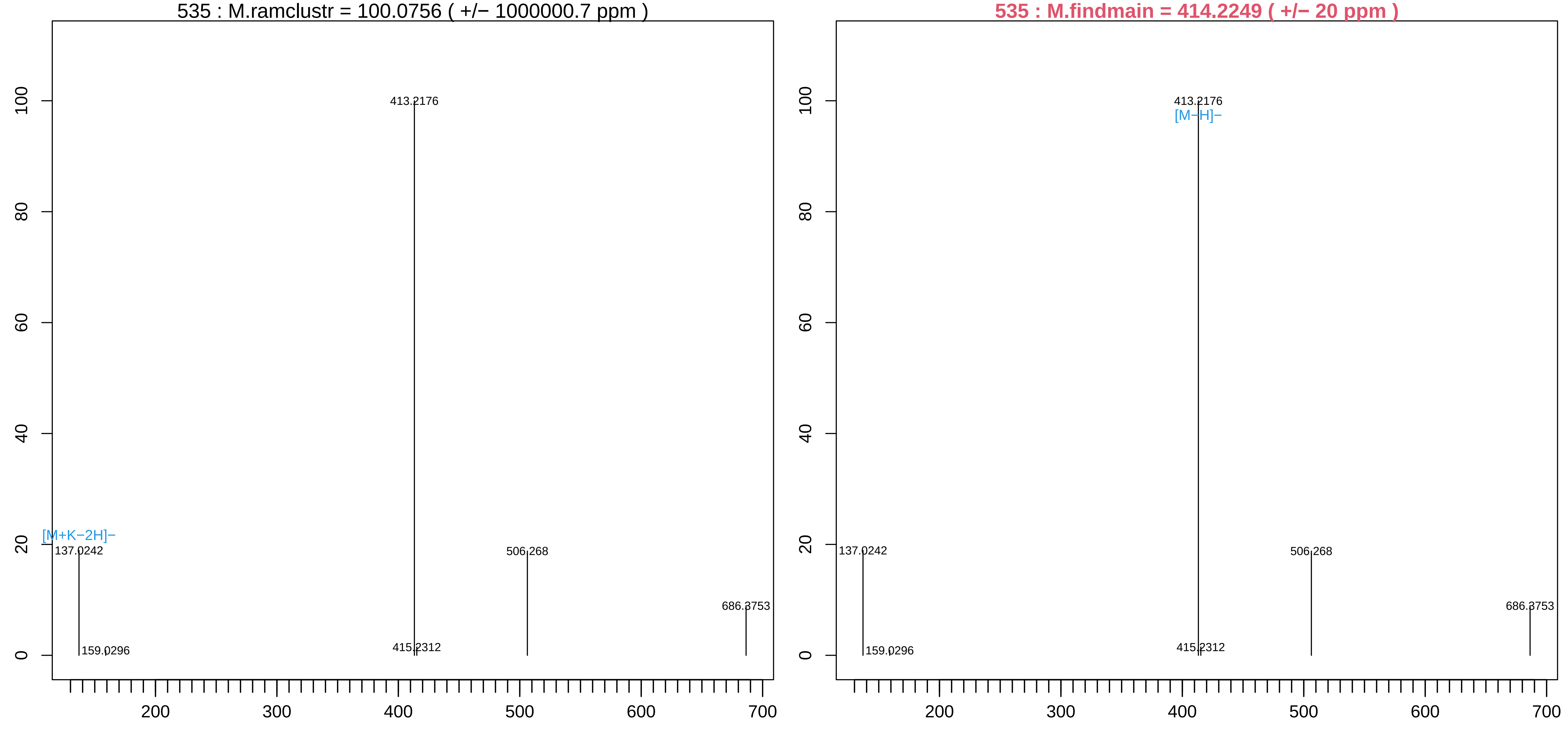

535 : M.ramclustr = 100.0756 (+/− 1000000.7 ppm)
413.2176
[M+K−2H]−
137.0242
506.268
686.3753
159.0296
415.2312
535 : M.findmain = 414.2249 (+/− 20 ppm)
413.2176
[M−H]−
137.0242
506.268
686.3753
159.0296
415.2312
0
20
40
60
80
100
200
300
400
500
600
700

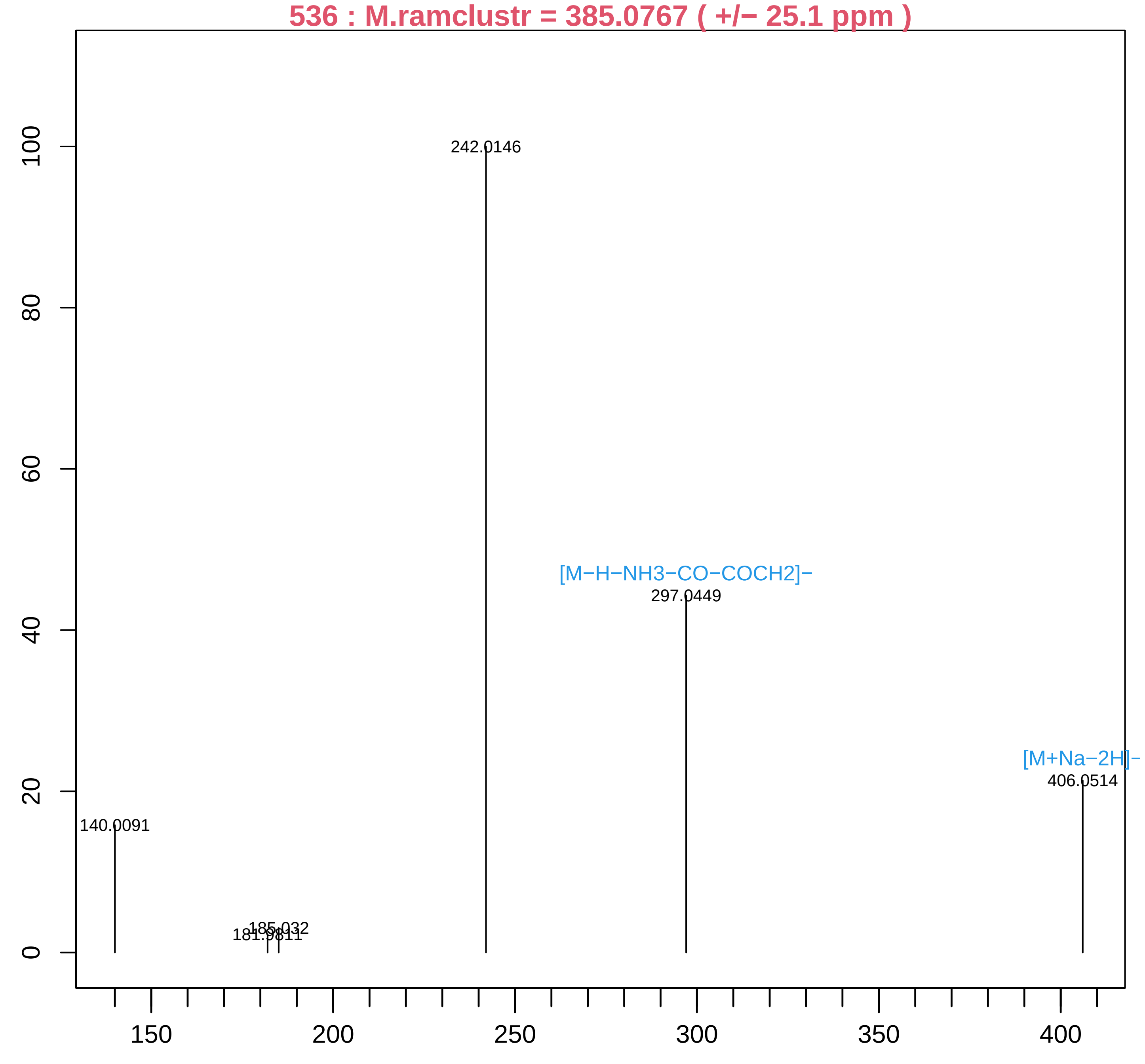
536 : M.ramclustr = 385.0767 (+/− 25.1 ppm)
242.0146
[M−H−NH3−CO−COCH2]−
297.0449
[M+Na−2H]−
406.0514
140.0091
185.032
181.9811
0
20
40
60
80
100
150
200
250
300
350
400

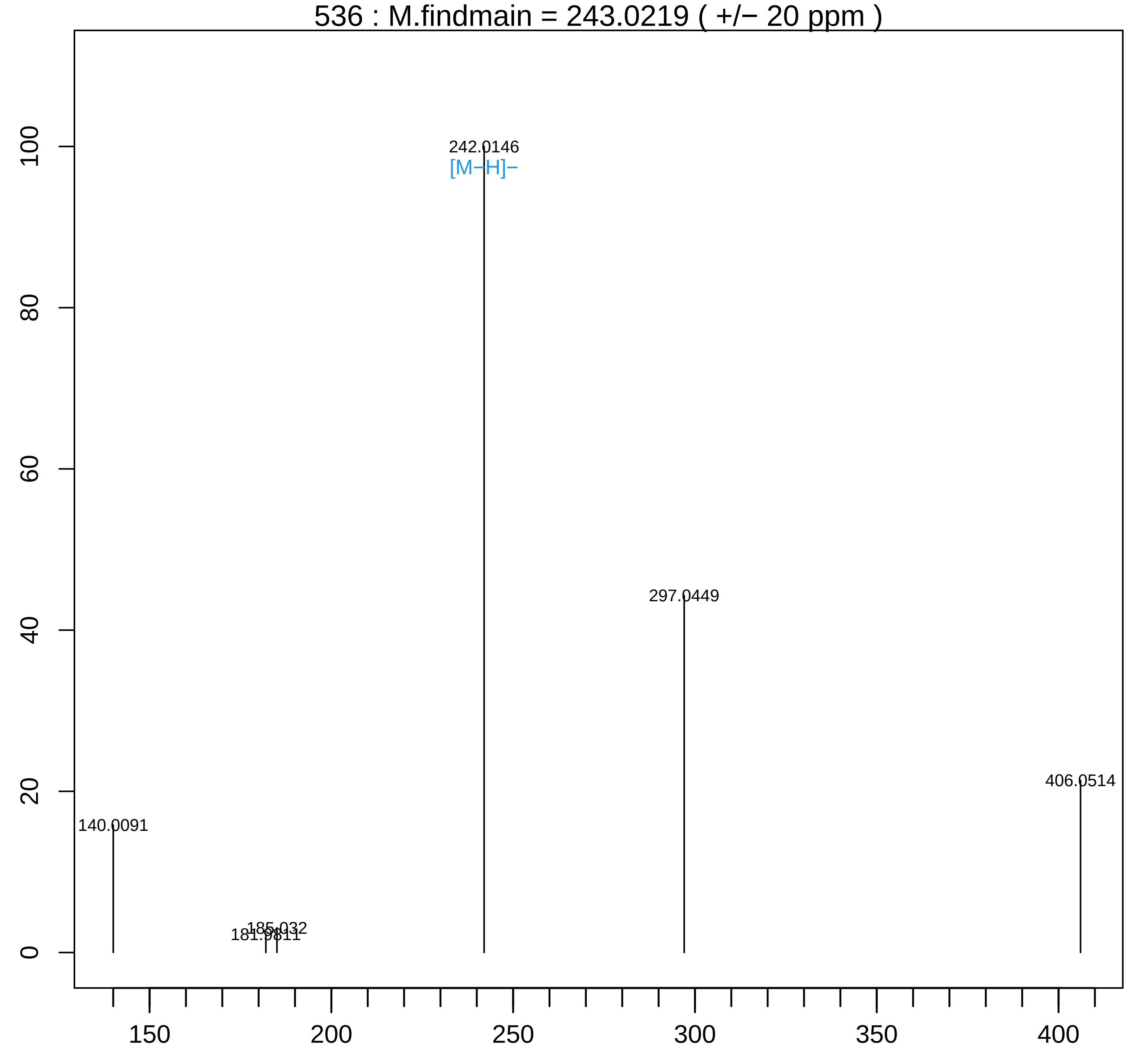
536 : M.findmain = 243.0219 (+/− 20 ppm)
242.0146
[M−H]−
297.0449
406.0514
140.0091
185.032
181.9811
0
20
40
60
80
100
150
200
250
300
350
400

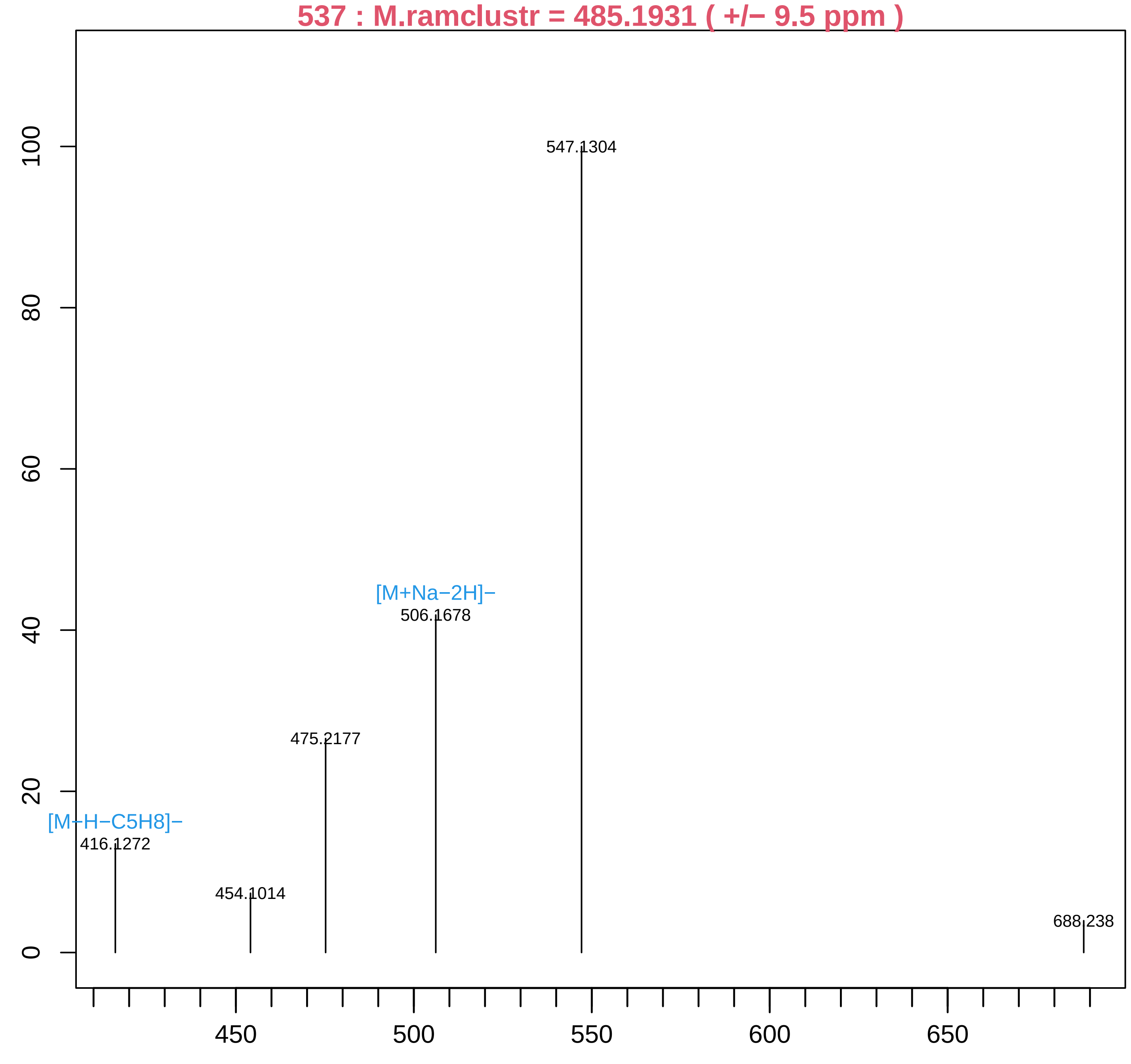
537 : M.ramclustr = 485.1931 (+/− 9.5 ppm)
0
20
40
60
80
100
450
500
550
600
650
547.1304
[M+Na−2H]−
506.1678
475.2177
[M−H−C5H8]−
416.1272
454.1014
688.238

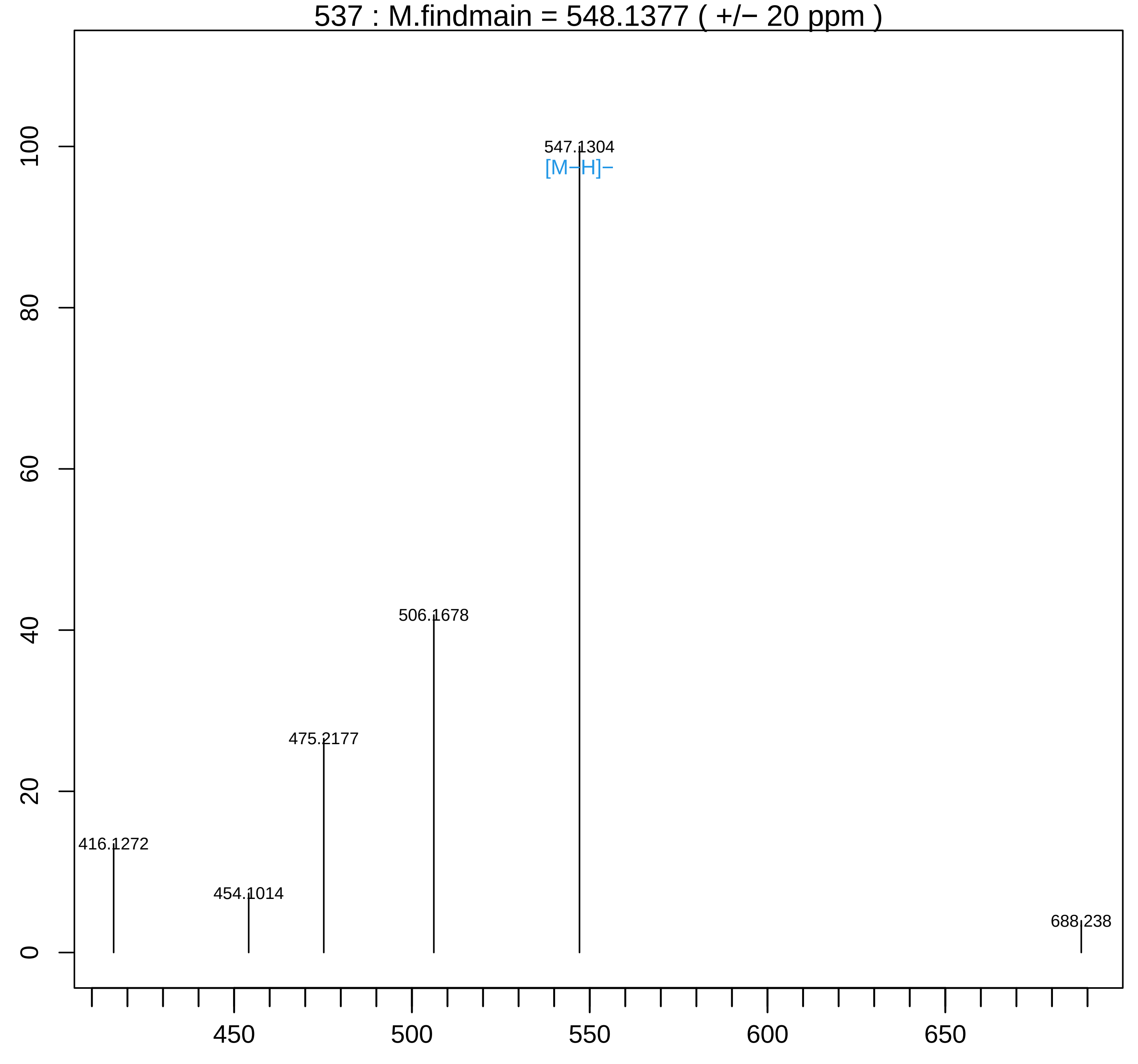
537 : M.findmain = 548.1377 (+/− 20 ppm)
0
20
40
60
80
100
450
500
550
600
650
547.1304
[M−H]−
506.1678
475.2177
416.1272
454.1014
688.238

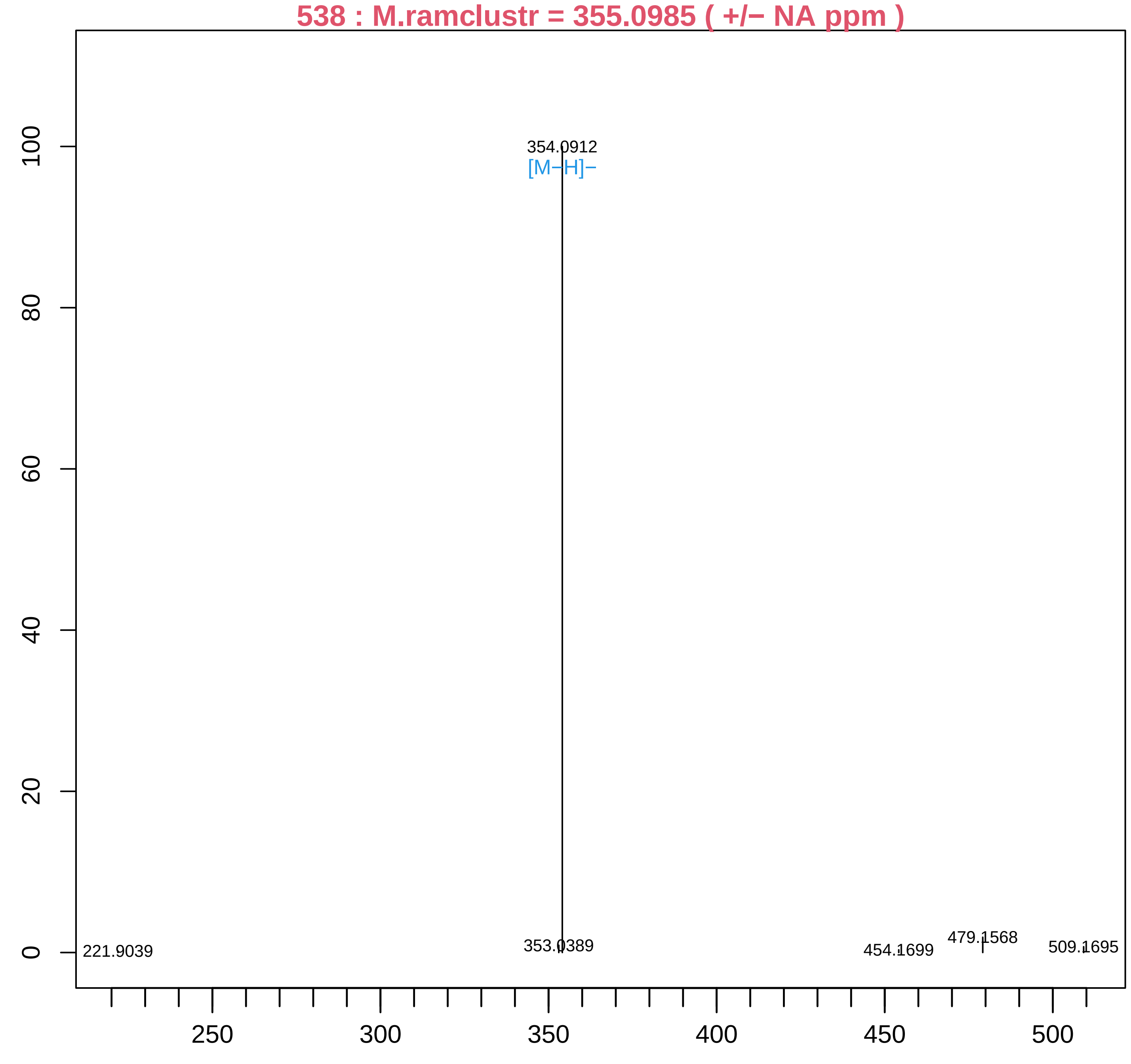
538 : M.ramclustr = 355.0985 (+/− NA ppm)
354.0912
[M−H]−
221.9039
353.0389
454.1699
479.1568
509.1695
0
20
40
60
80
100
250
300
350
400
450
500

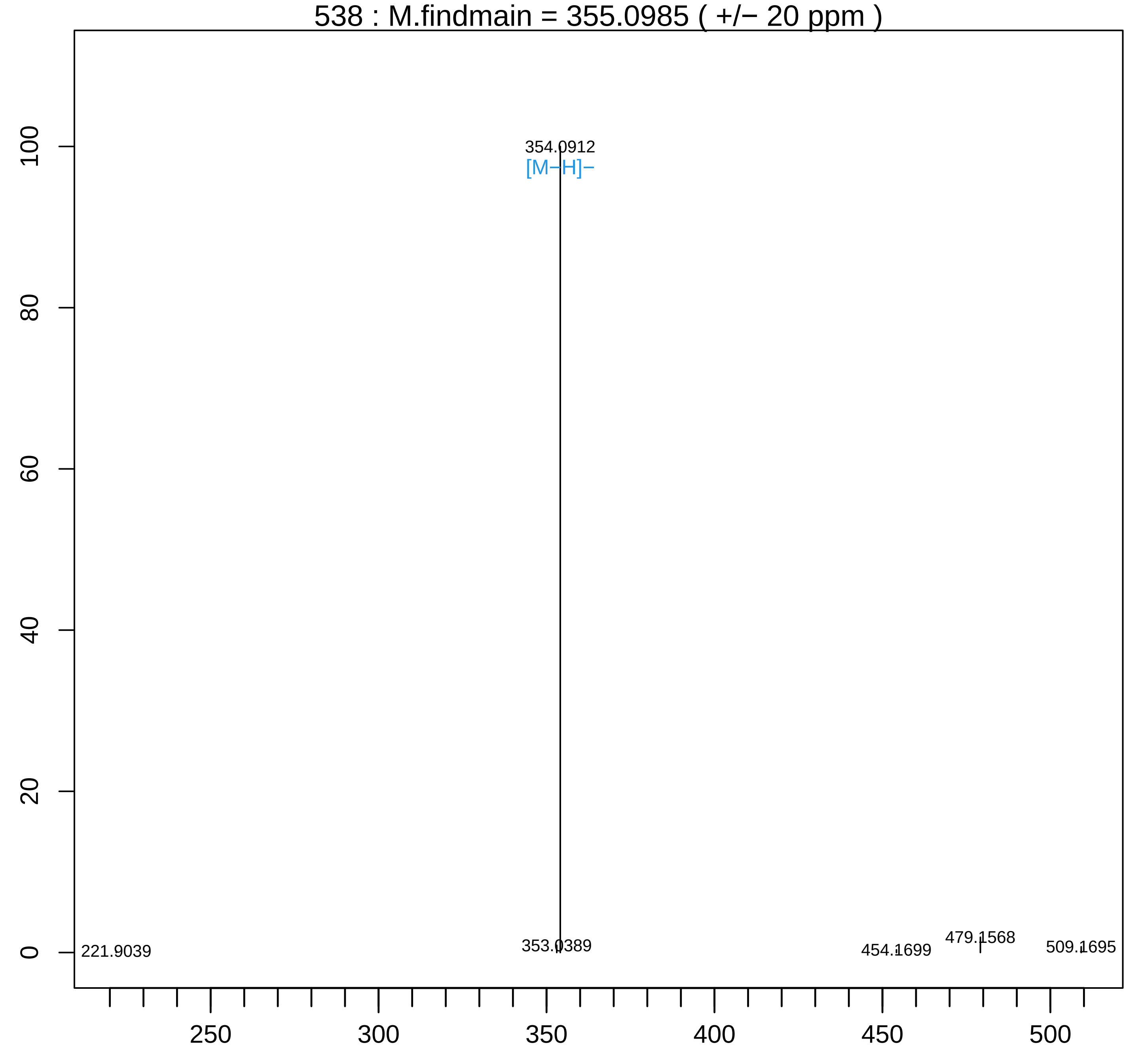
538 : M.findmain = 355.0985 (+/− 20 ppm)
354.0912
[M−H]−
221.9039
353.0389
454.1699
479.1568
509.1695
0
20
40
60
80
100
250
300
350
400
450
500

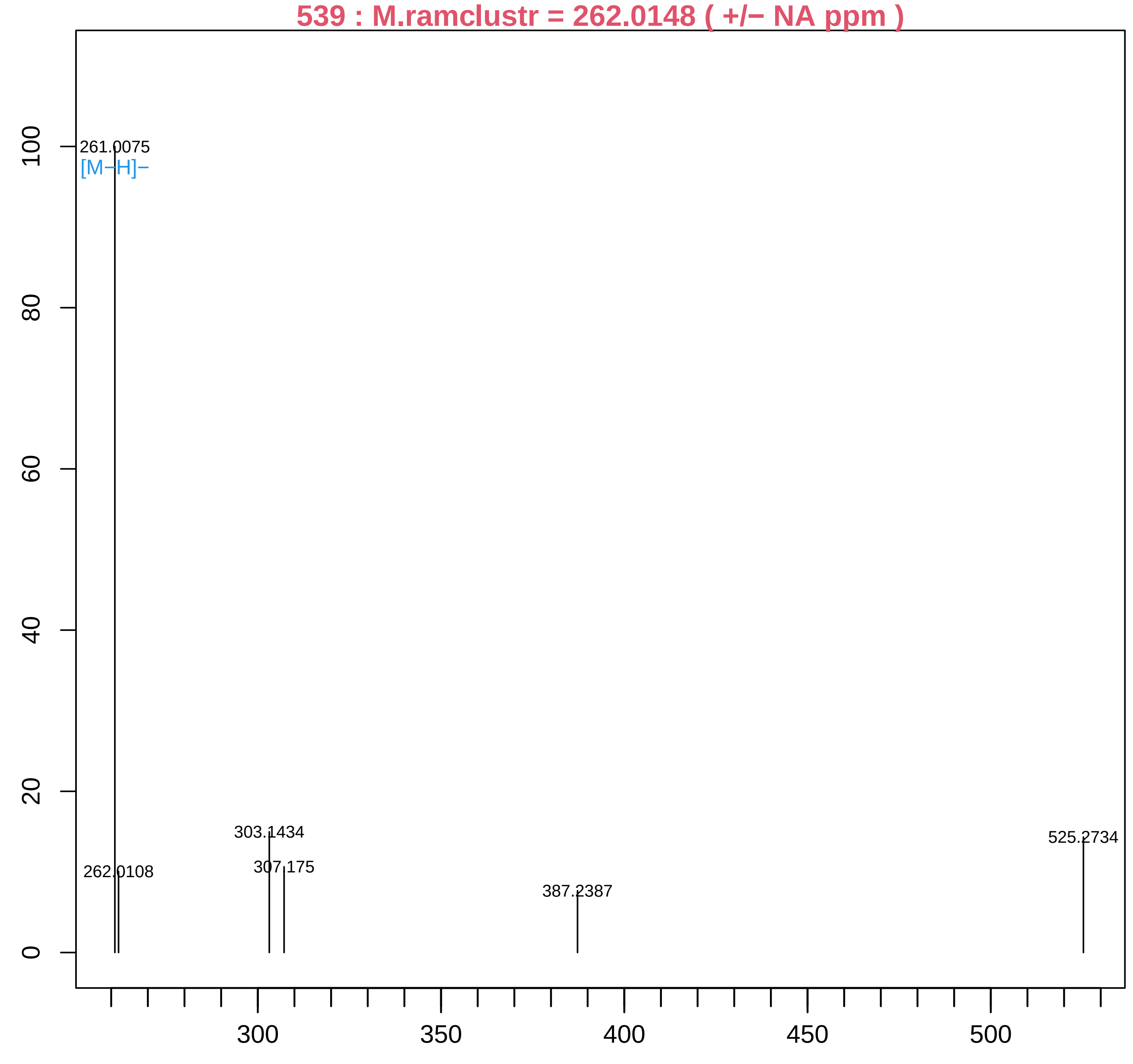
539 : M.ramclustr = 262.0148 (+/− NA ppm)
261.0075
[M−H]−
262.0108
303.1434
307.175
387.2387
525.2734
0
20
40
60
80
100
300
350
400
450
500

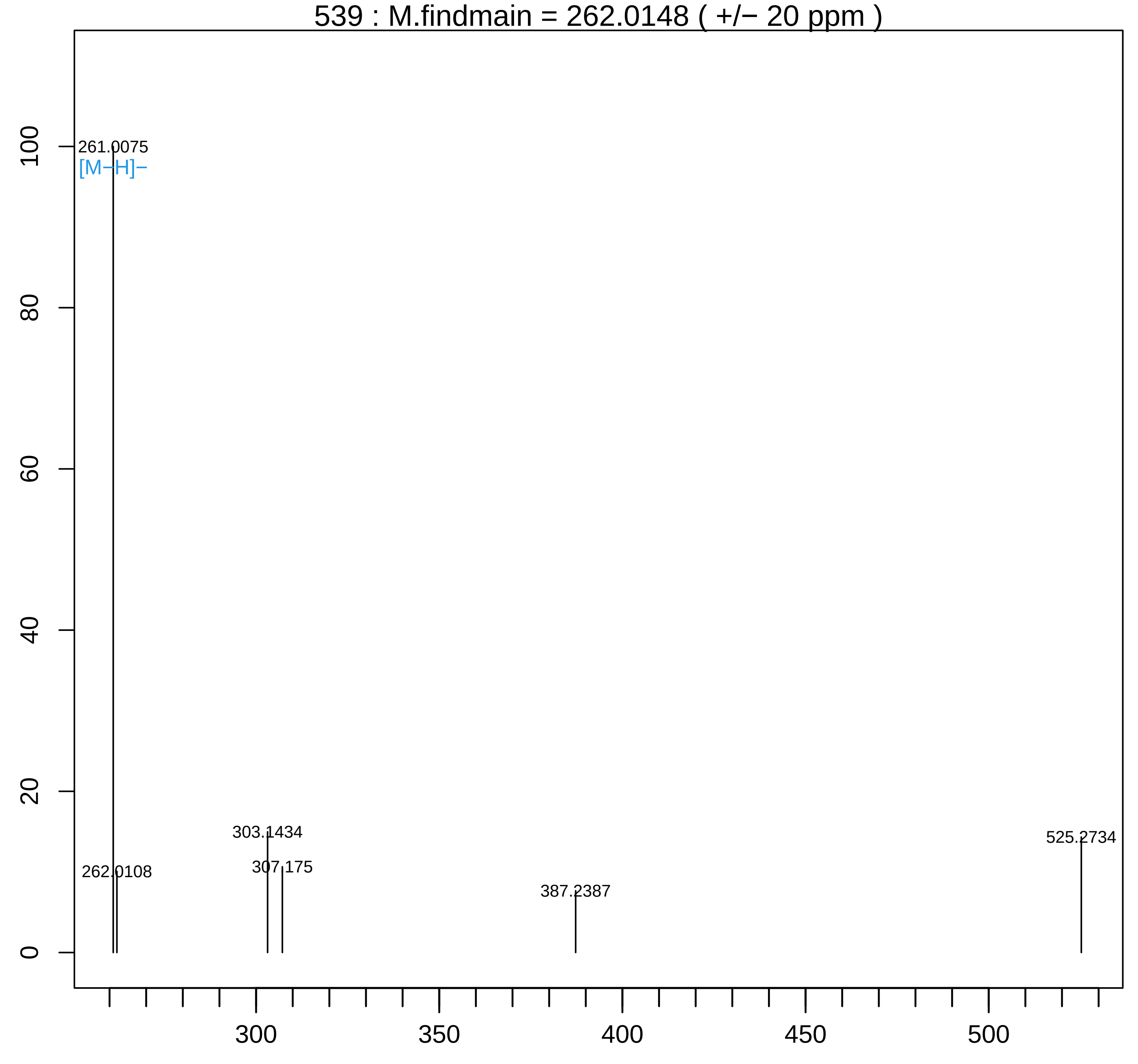
539 : M.findmain = 262.0148 (+/− 20 ppm)
261.0075
[M−H]−
262.0108
303.1434
307.175
387.2387
525.2734
0
20
40
60
80
100
300
350
400
450
500

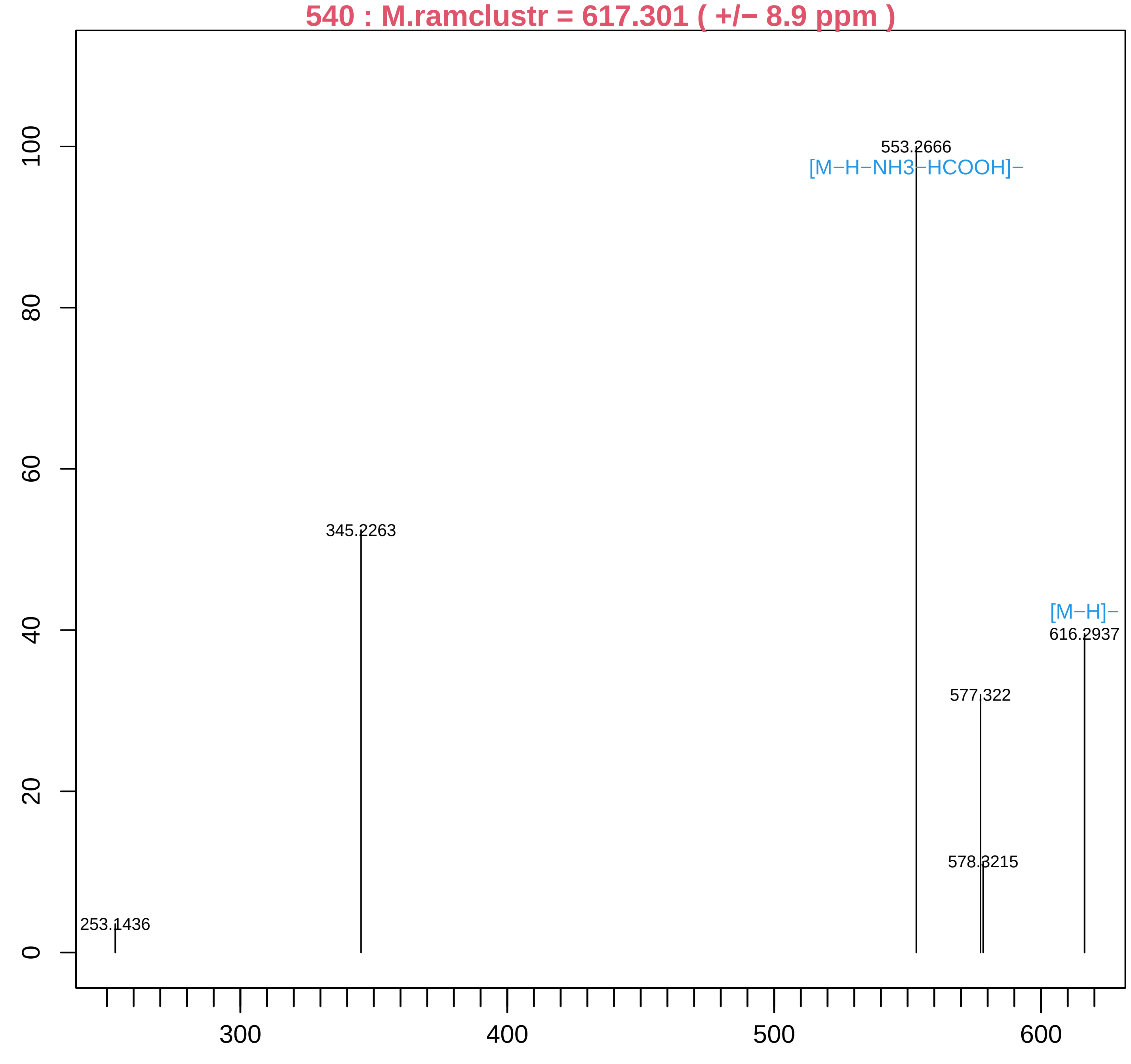
540 : M.ramclustr = 617.301 (+/− 8.9 ppm)
553.2666
[M−H−NH3−HCOOH]−
345.2263
[M−H]−
616.2937
577.322
578.3215
253.1436
0
20
40
60
80
100
300
400
500
600

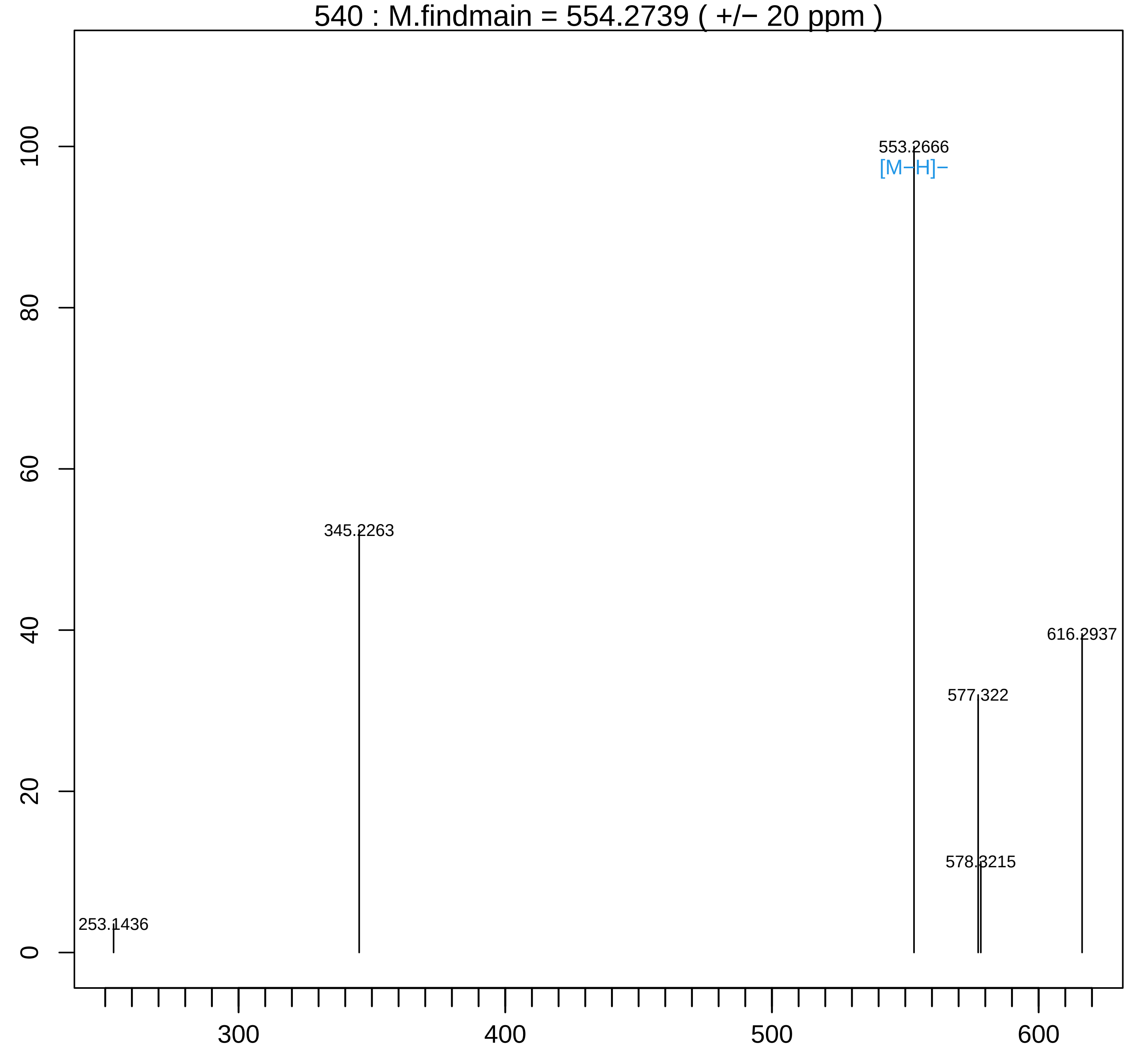
540 : M.findmain = 554.2739 (+/− 20 ppm)
553.2666
[M−H]−
345.2263
616.2937
577.322
578.3215
253.1436
0
20
40
60
80
100
300
400
500
600

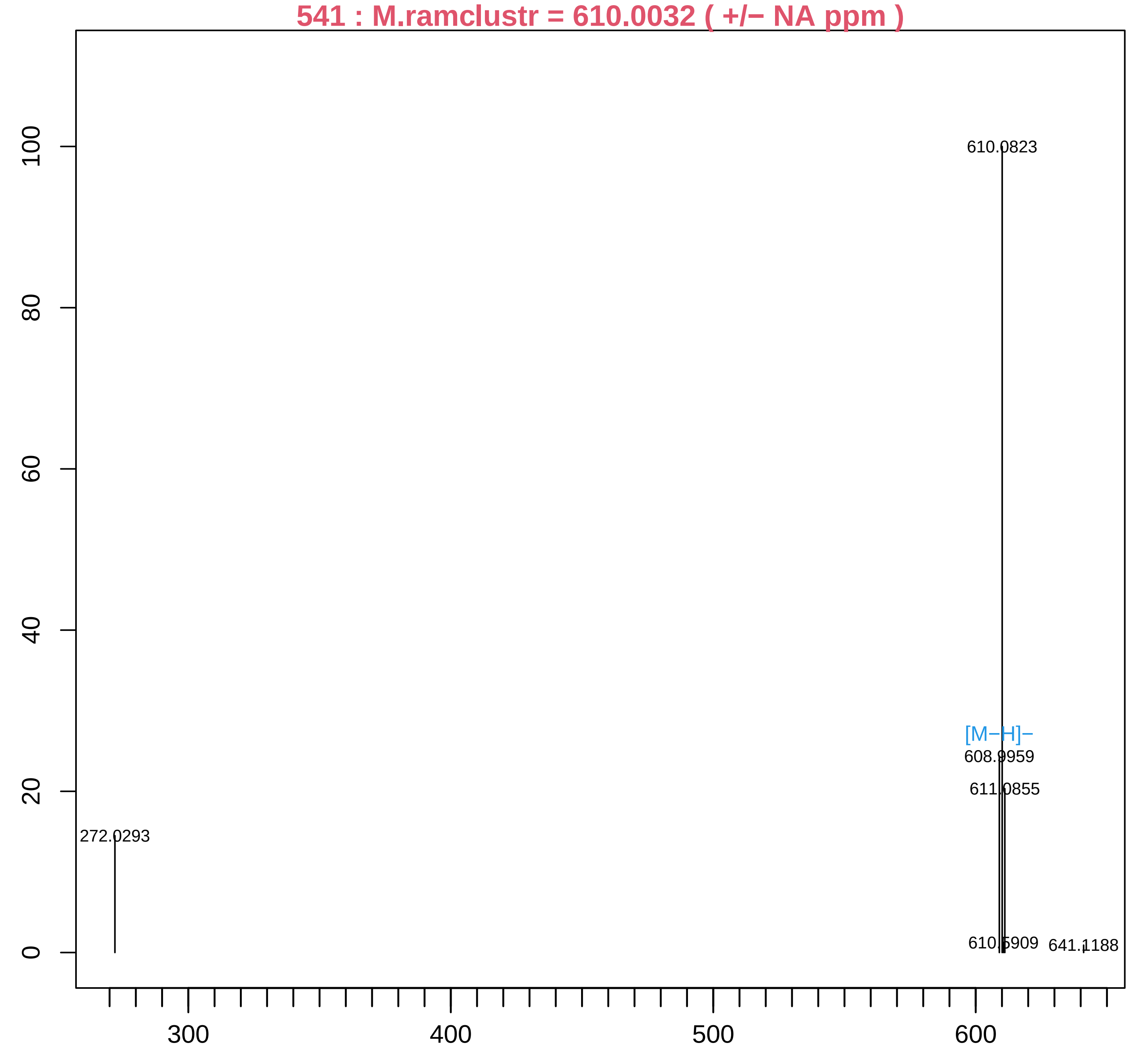
541 : M.ramclustr = 610.0032 (+/− NA ppm)
610.0823
[M−H]−
608.9959
611.0855
272.0293
610.5909
641.1188
0
20
40
60
80
100
300
400
500
600

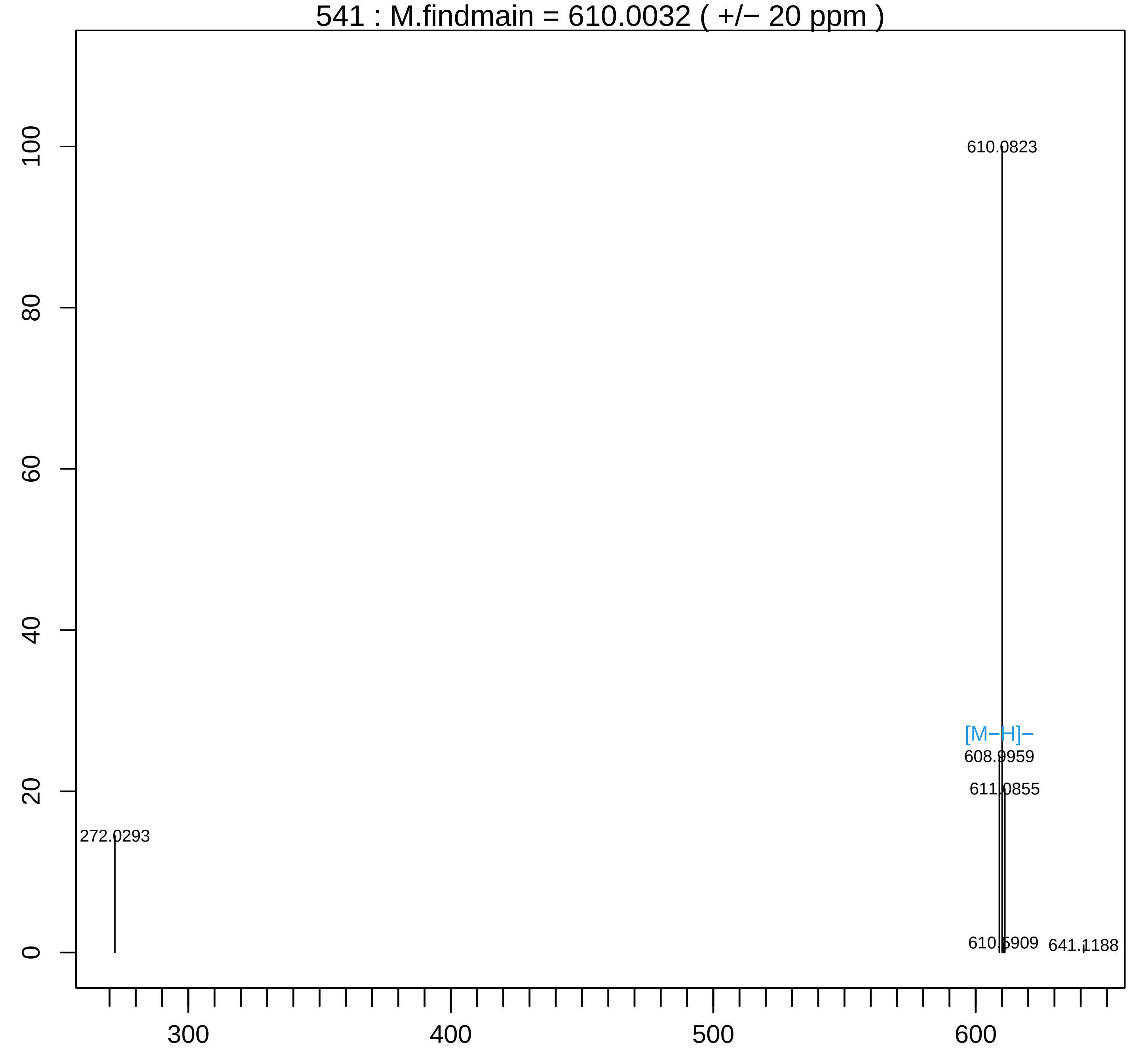
541 : M.findmain = 610.0032 (+/− 20 ppm)
610.0823
[M−H]−
608.9959
611.0855
272.0293
610.5909
641.1188
0
20
40
60
80
100
300
400
500
600

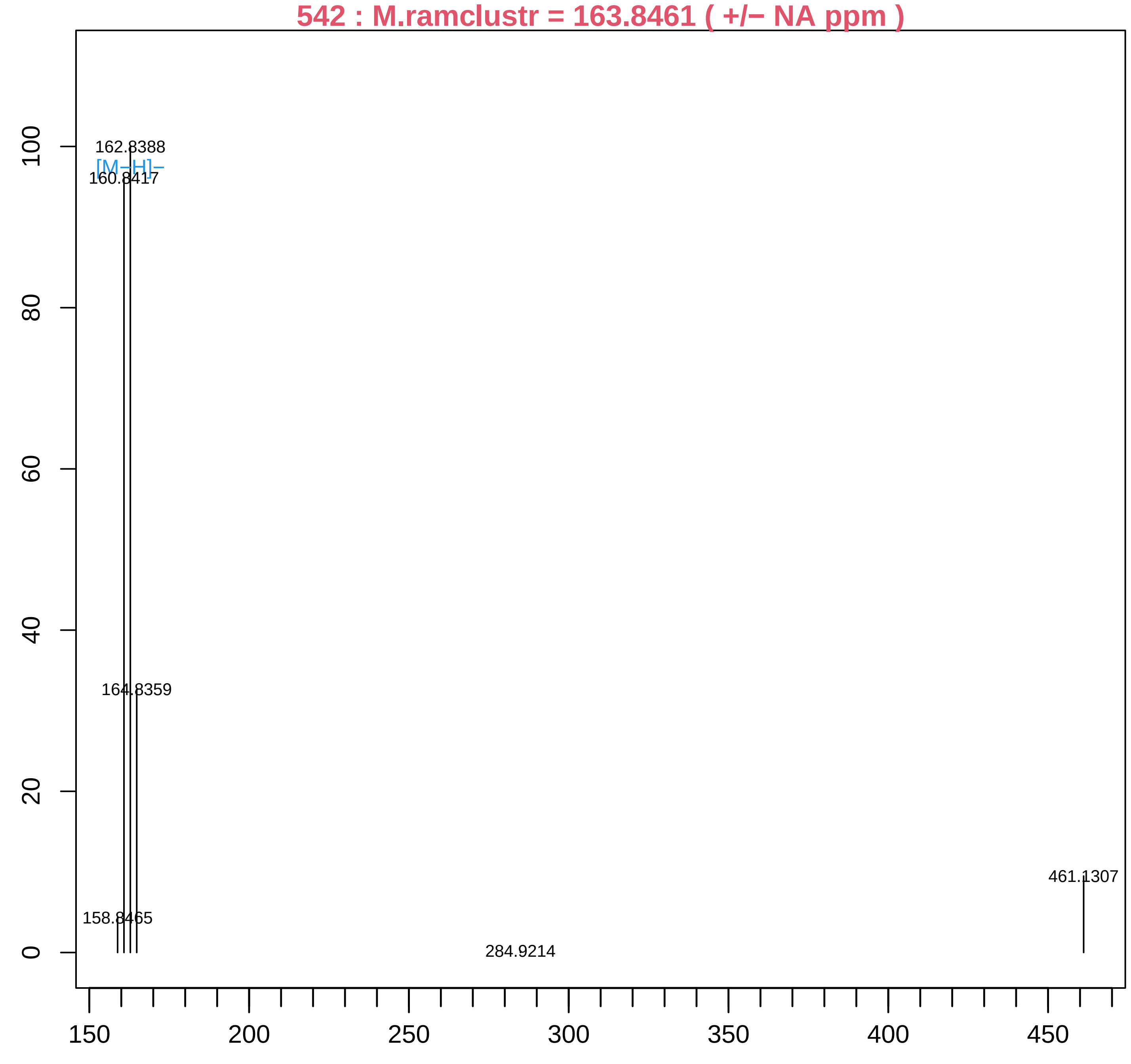

542 : M.ramclustr = 163.8461 (+/− NA ppm)
162.8388
[M−H]−
160.8417
164.8359
461.1307
158.8465
284.9214
0
20
40
60
80
100
150
200
250
300
350
400
450

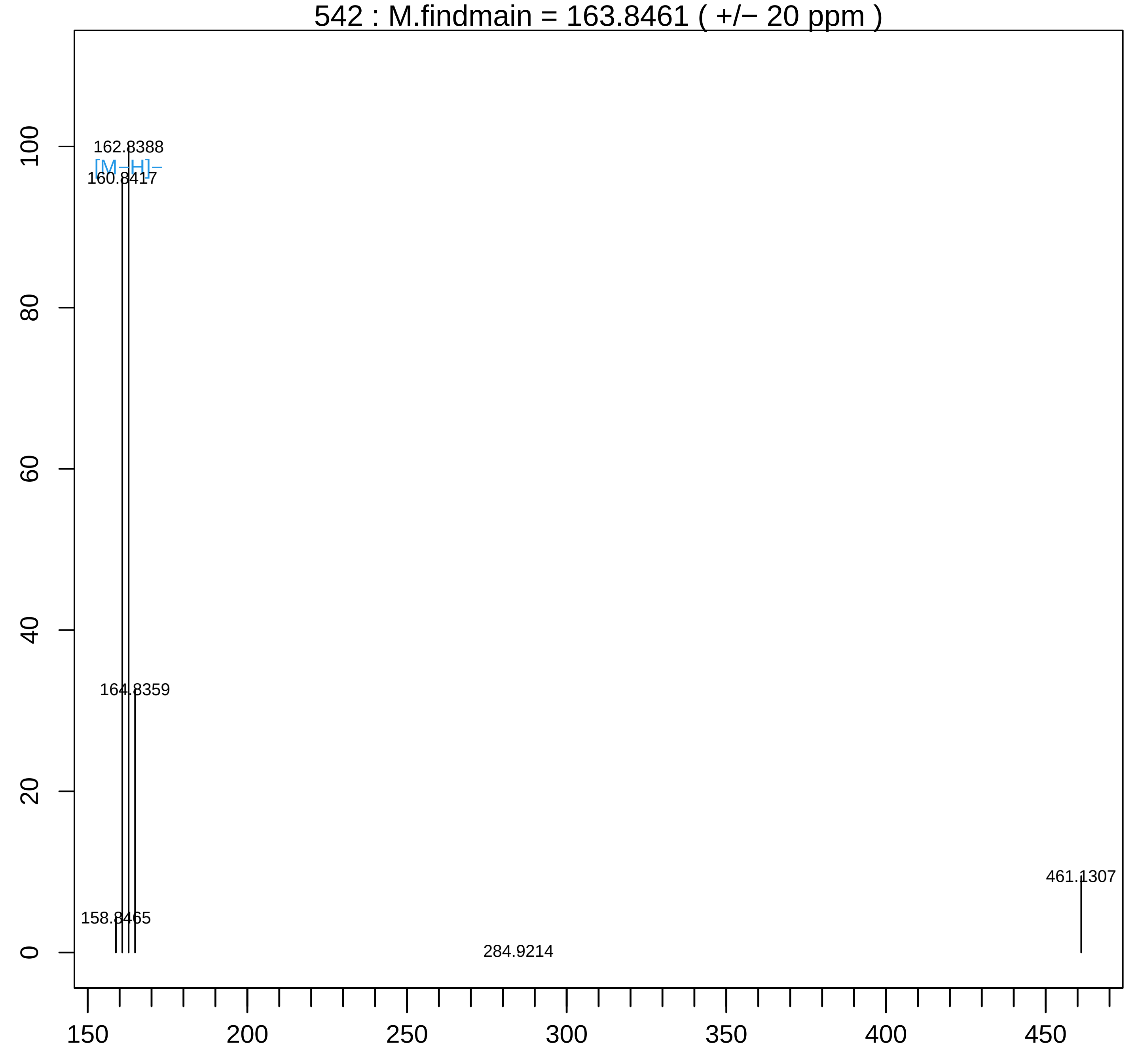

542 : M.findmain = 163.8461 (+/− 20 ppm)
162.8388
[M−H]−
160.8417
164.8359
461.1307
158.8465
284.9214
0
20
40
60
80
100
150
200
250
300
350
400
450

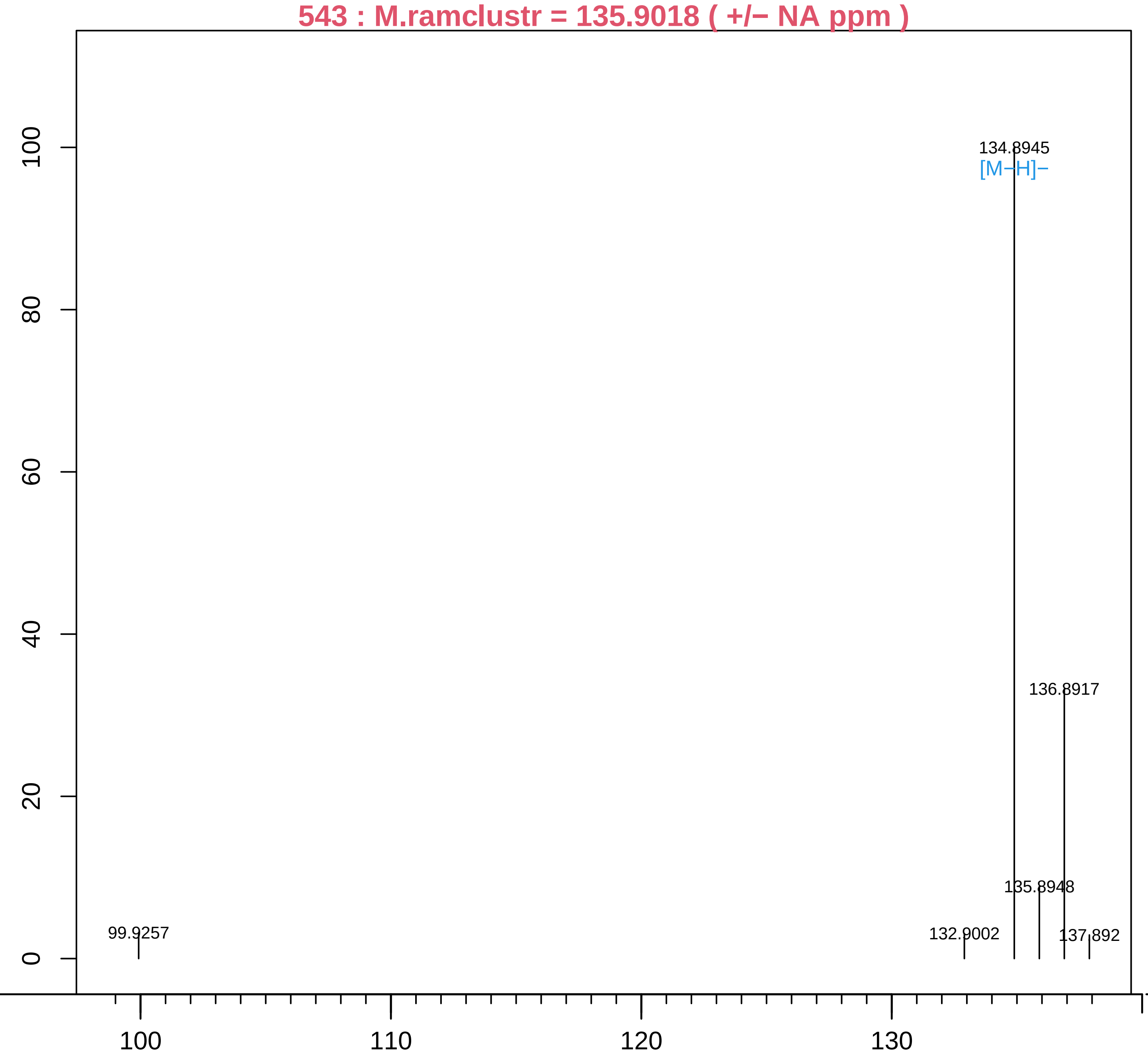
543 : M.ramclustr = 135.9018 (+/− NA ppm)
134.8945
[M−H]−
136.8917
135.8948
99.9257
132.9002
137.892
0
20
40
60
80
100
100
110
120
130

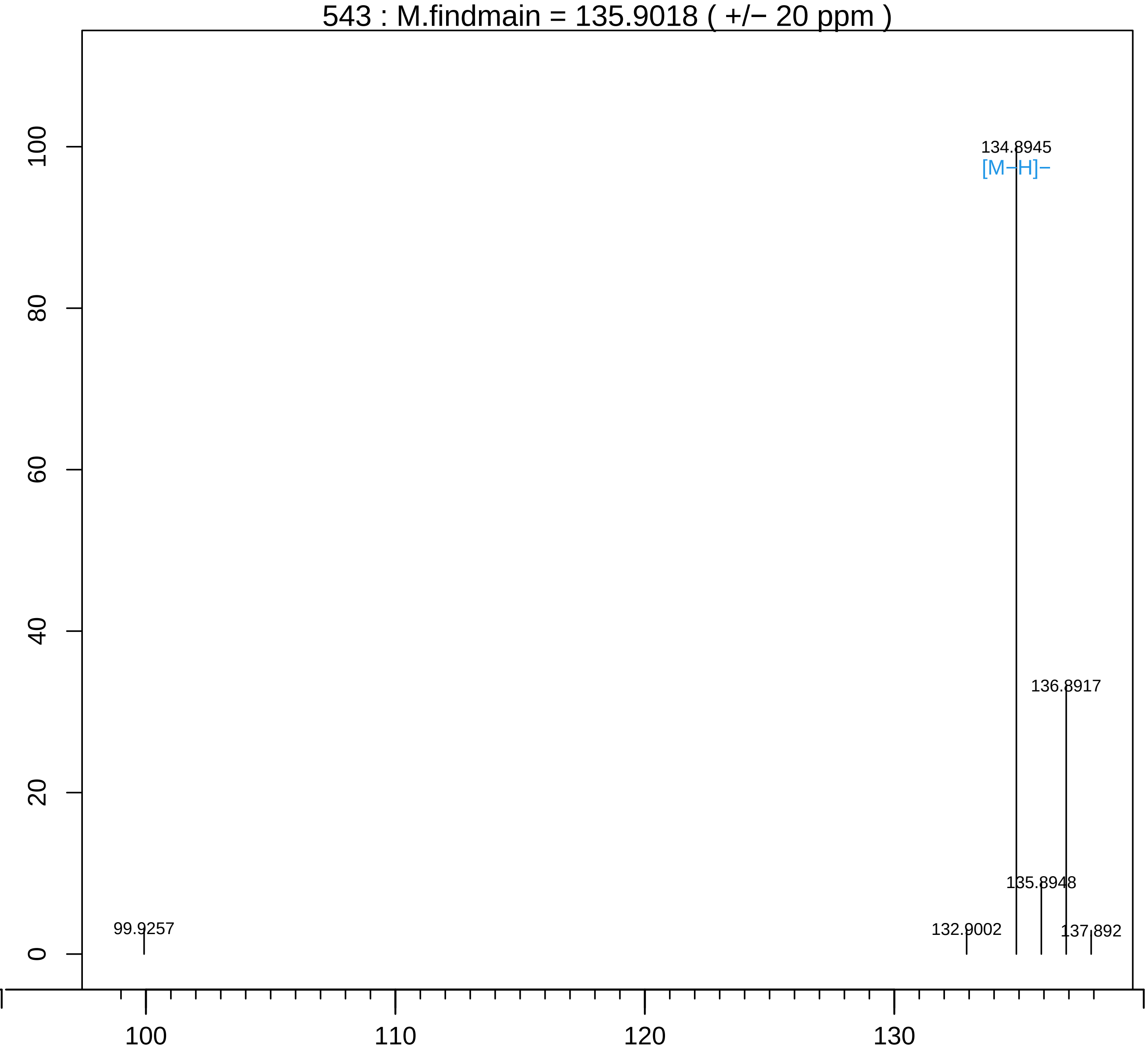
543 : M.findmain = 135.9018 (+/− 20 ppm)
134.8945
[M−H]−
136.8917
135.8948
99.9257
132.9002
137.892
0
20
40
60
80
100
100
110
120
130

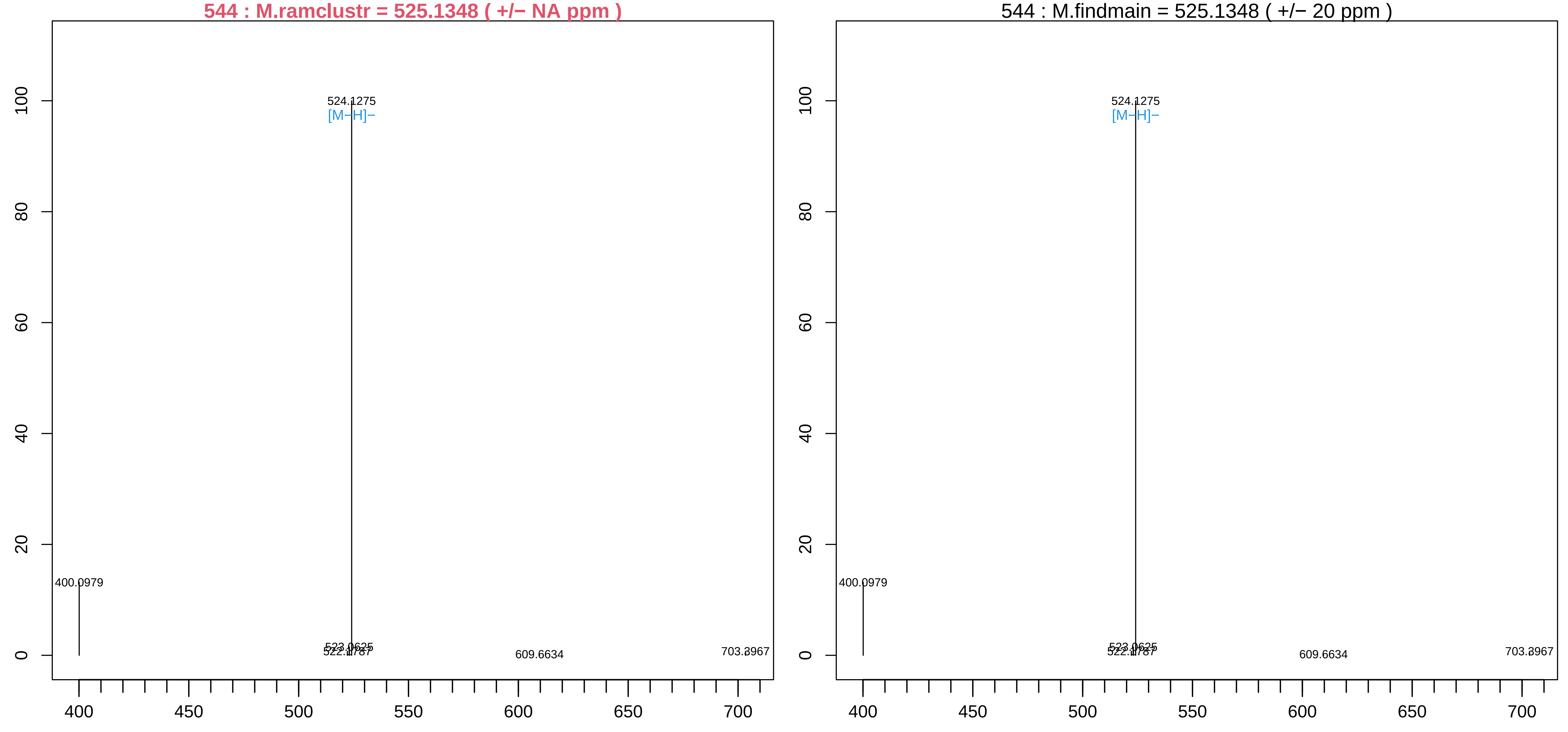

544 : M.ramclustr = 525.1348 (+/− NA ppm)
524.1275
[M−H]−
400.0979
523.0625
522.1787
609.6634
703.3967
0
20
40
60
80
100
400
450
500
550
600
650
700
544 : M.findmain = 525.1348 (+/− 20 ppm)
524.1275
[M−H]−
400.0979
523.0625
522.1787
609.6634
703.3967
0
20
40
60
80
100
400
450
500
550
600
650
700

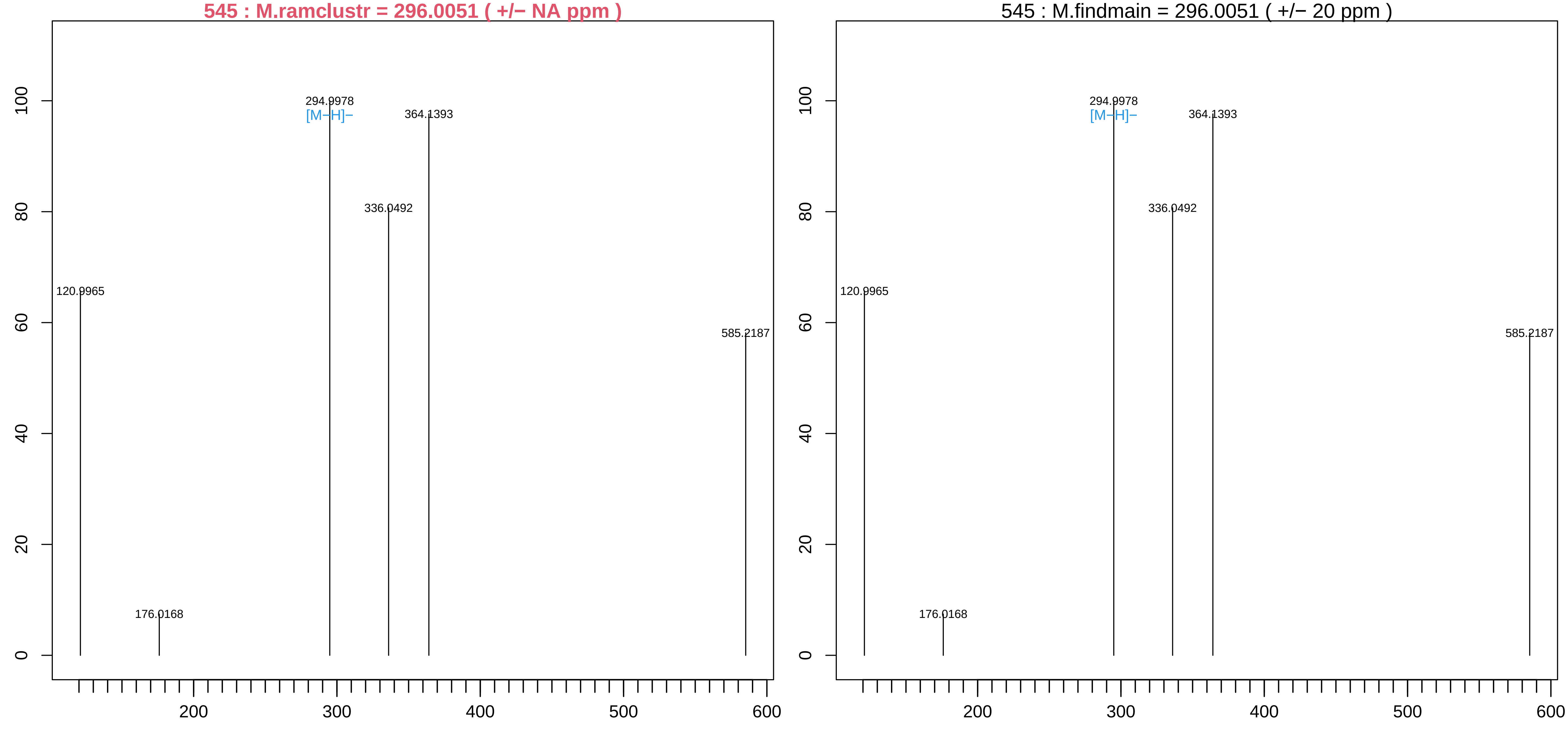

545 : M.ramclustr = 296.0051 (+/− NA ppm)
120.9965
176.0168
294.9978
[M−H]−
336.0492
364.1393
585.2187
545 : M.findmain = 296.0051 (+/− 20 ppm)
120.9965
176.0168
294.9978
[M−H]−
336.0492
364.1393
585.2187
0
20
40
60
80
100
200
300
400
500
600

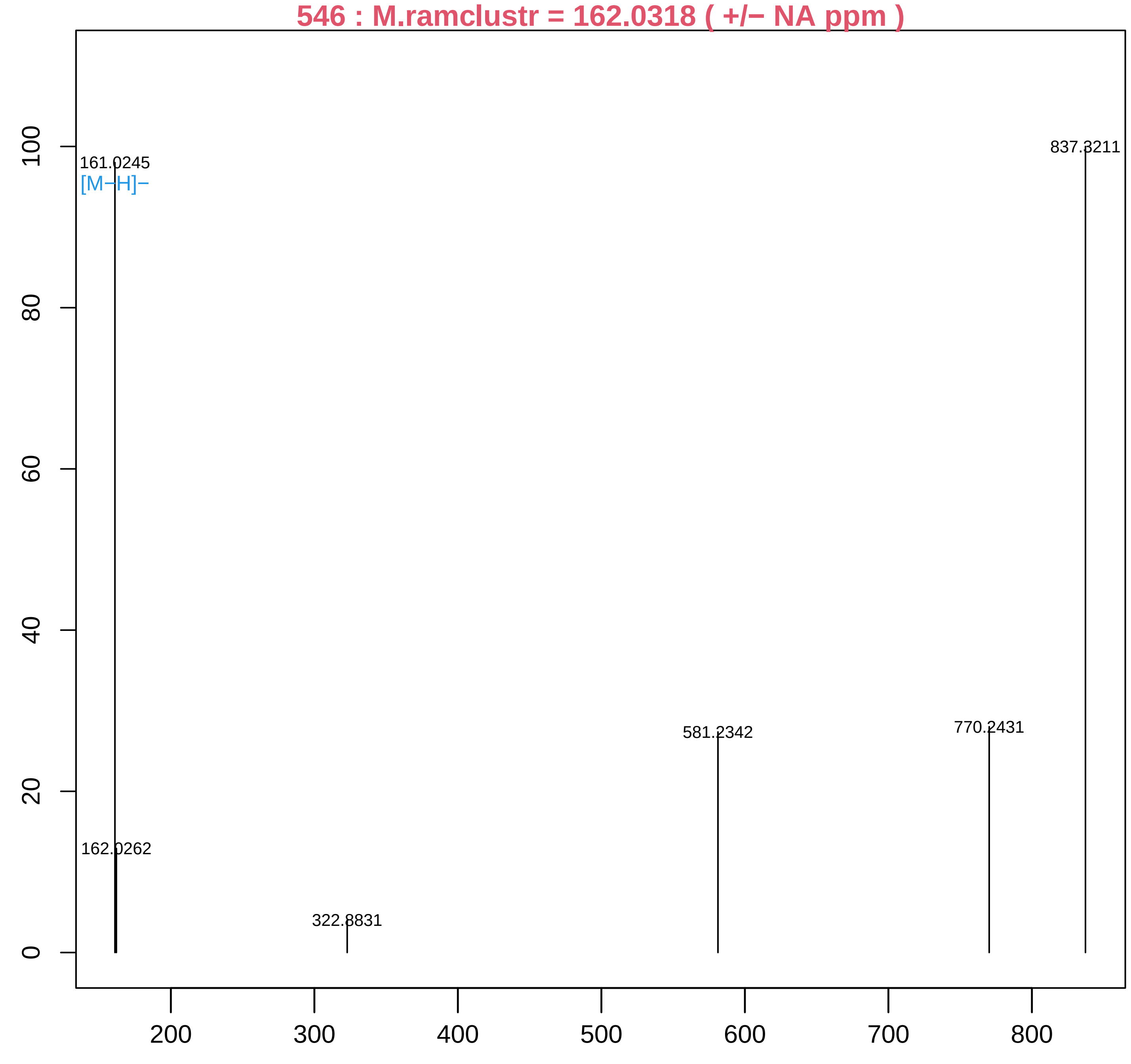

546 : M.ramclustr = 162.0318 (+/− NA ppm)
161.0245
[M−H]−
837.3211
162.0262
322.8831
581.2342
770.2431
0
20
40
60
80
100
200
300
400
500
600
700
800

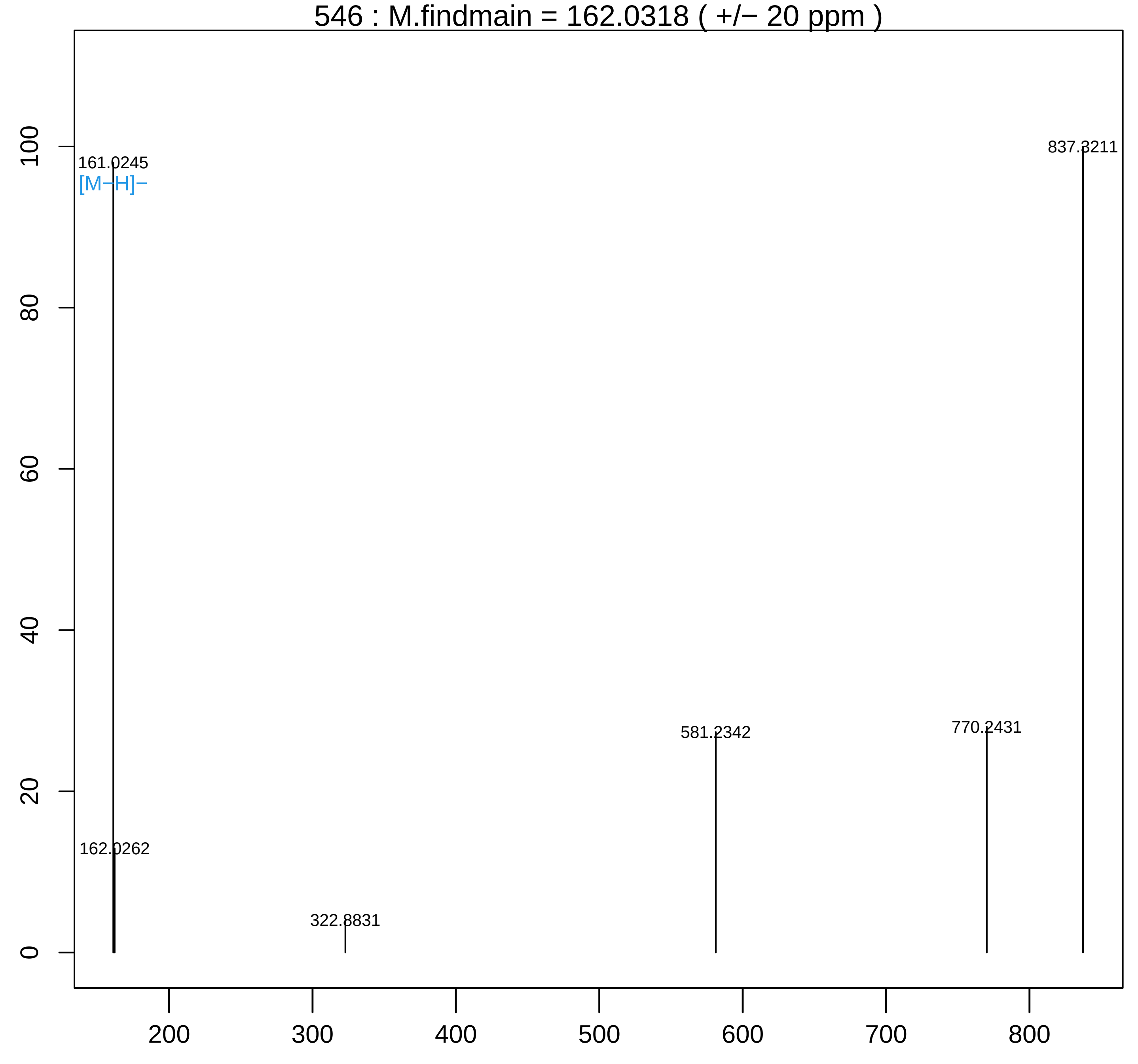

546 : M.findmain = 162.0318 (+/− 20 ppm)
161.0245
[M−H]−
837.3211
162.0262
322.8831
581.2342
770.2431
0
20
40
60
80
100
200
300
400
500
600
700
800

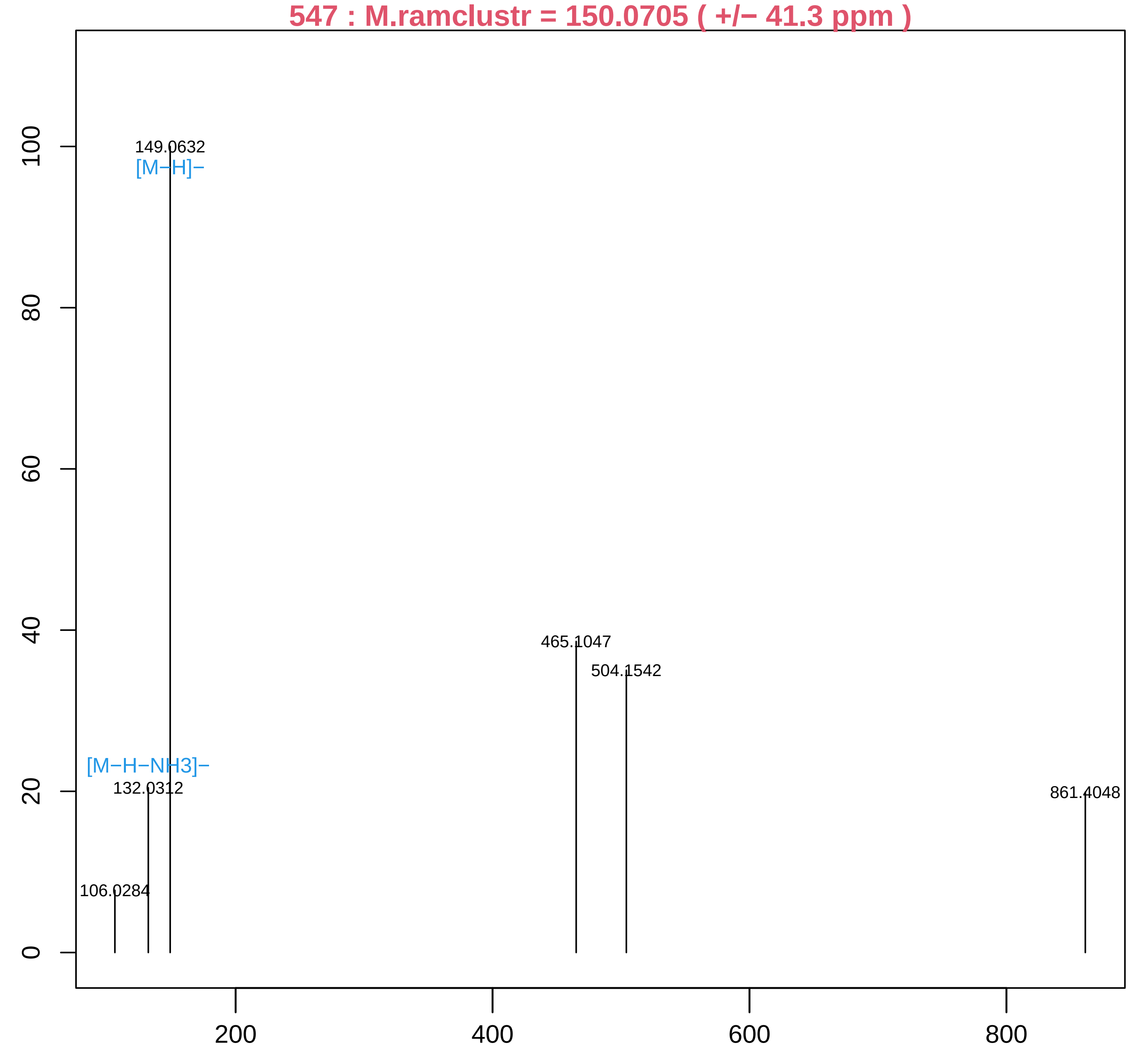
547 : M.ramclustr = 150.0705 (+/− 41.3 ppm)
149.0632
[M−H]−
465.1047
504.1542
[M−H−NH3]−
132.0312
861.4048
106.0284
0
20
40
60
80
100
200
400
600
800

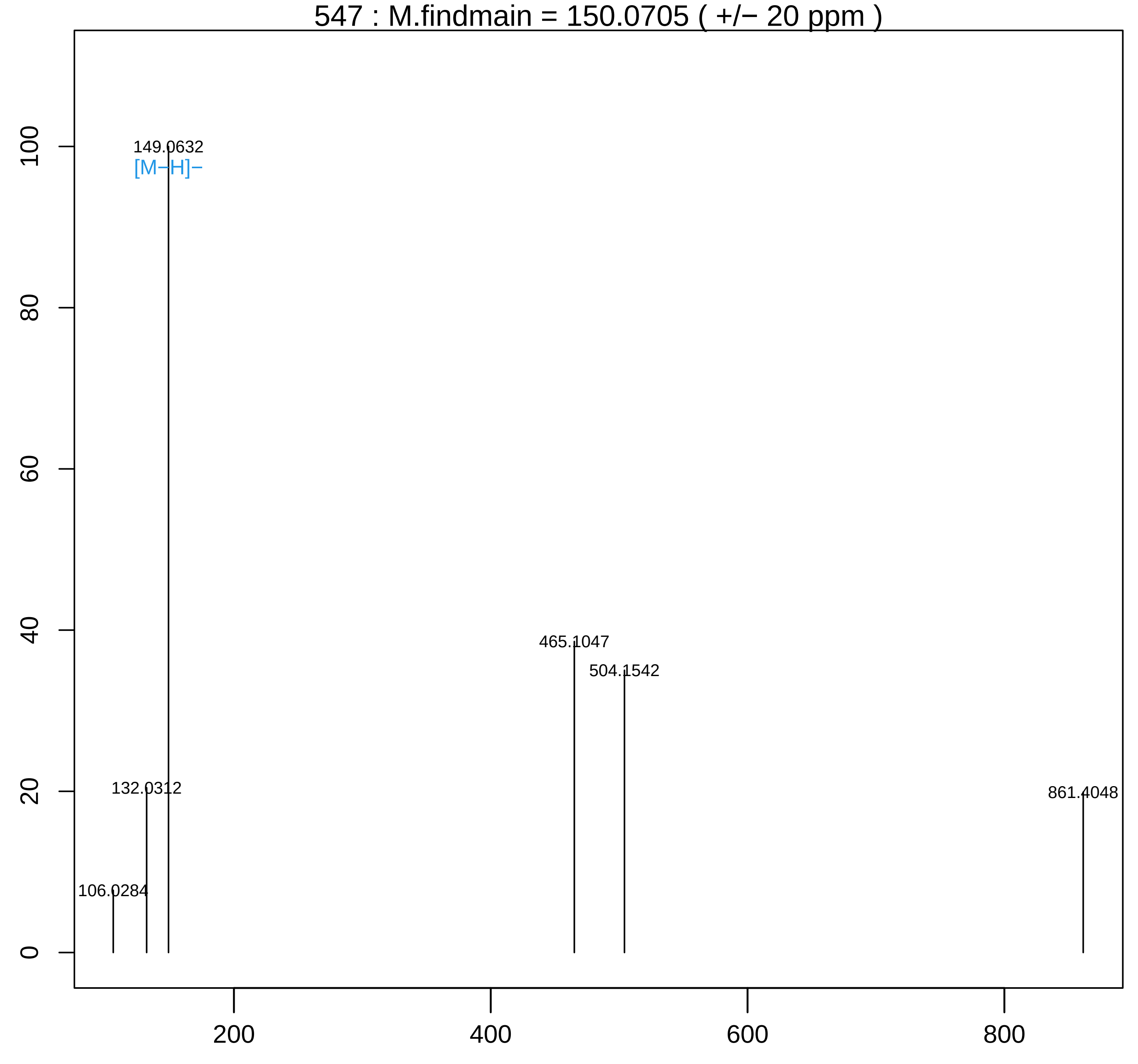
547 : M.findmain = 150.0705 (+/− 20 ppm)
149.0632
[M−H]−
465.1047
504.1542
132.0312
861.4048
106.0284
0
20
40
60
80
100
200
400
600
800

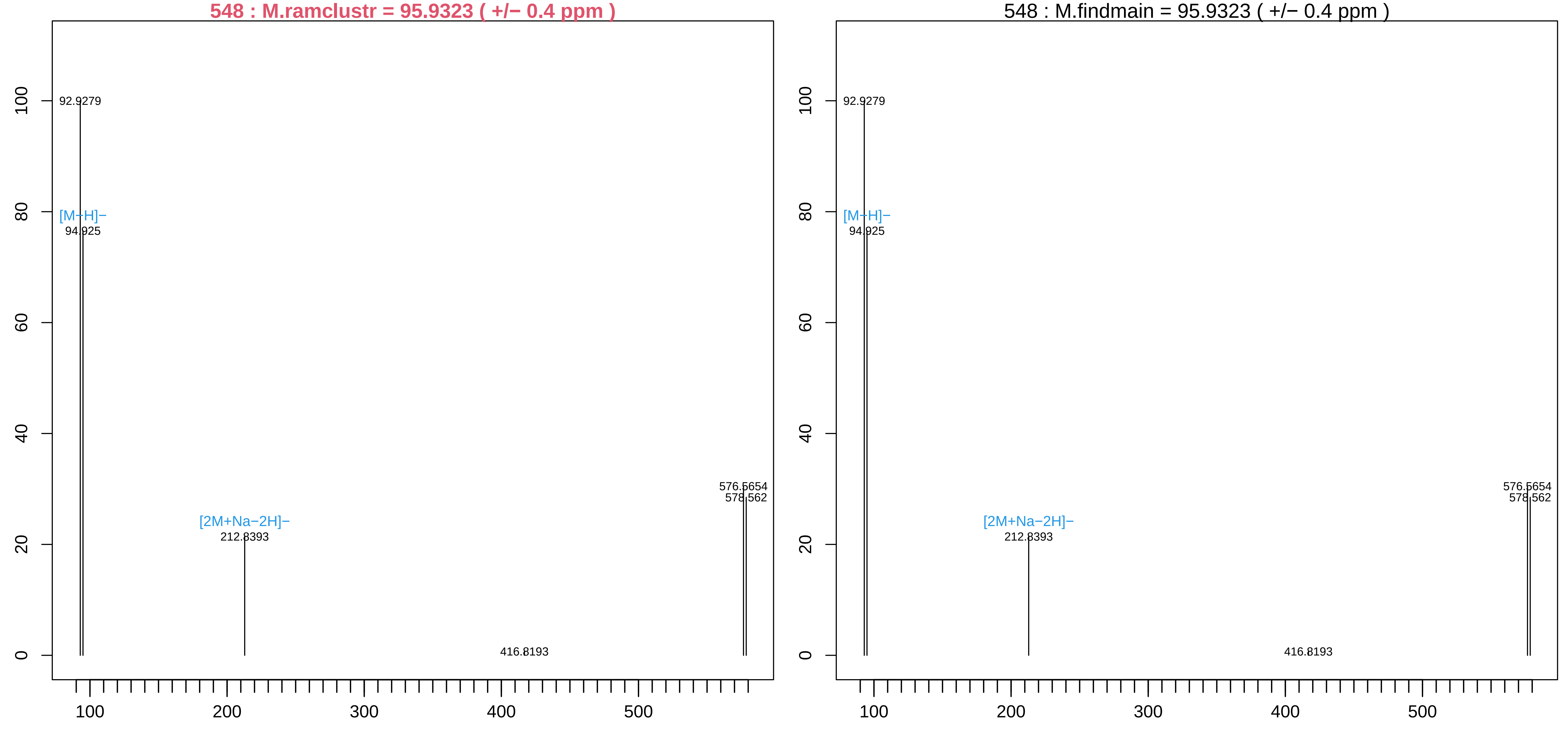
548 : M.ramclustr = 95.9323 (+/− 0.4 ppm)
92.9279
[M−H]−
94.925
[2M+Na−2H]−
212.8393
416.8193
576.5654
578.562
100
80
60
40
20
0
100
200
300
400
500
548 : M.findmain = 95.9323 (+/− 0.4 ppm)
92.9279
[M−H]−
94.925
[2M+Na−2H]−
212.8393
416.8193
576.5654
578.562
100
80
60
40
20
0
100
200
300
400
500

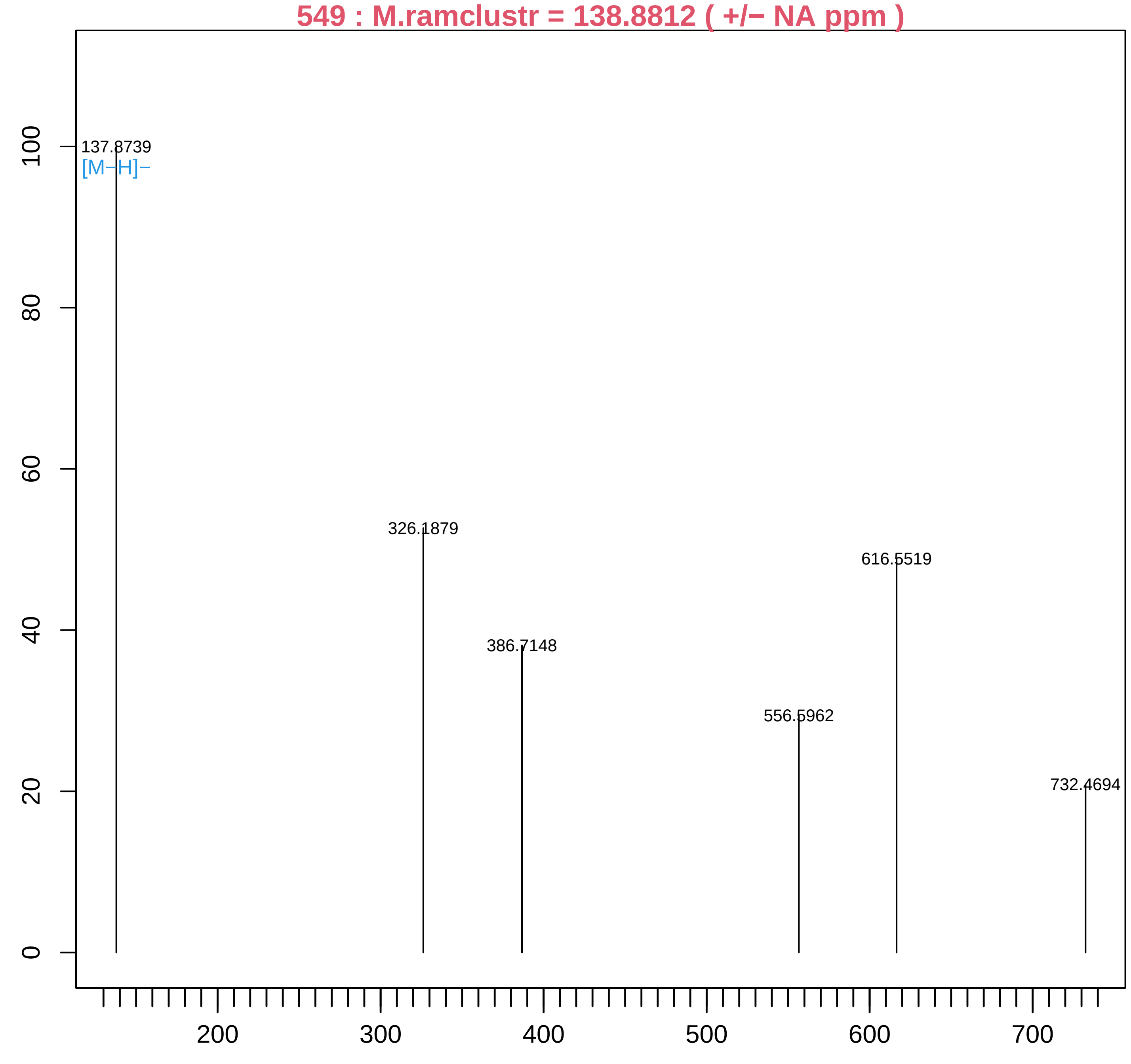
549 : M.ramclustr = 138.8812 (+/− NA ppm)
137.8739
[M−H]−
326.1879
386.7148
556.5962
616.5519
732.4694
0
20
40
60
80
100
200
300
400
500
600
700

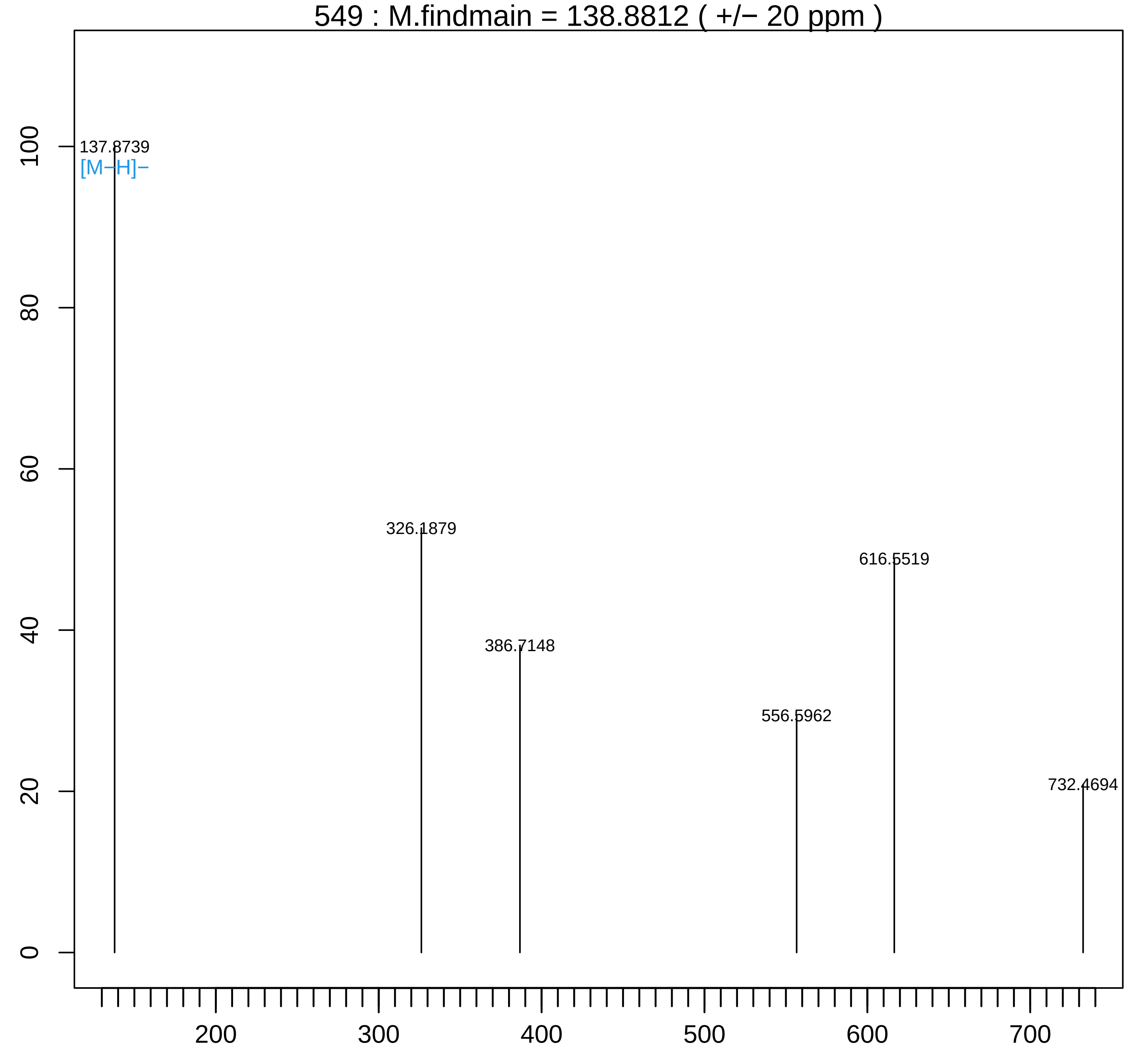
549 : M.findmain = 138.8812 (+/− 20 ppm)
137.8739
[M−H]−
326.1879
386.7148
556.5962
616.5519
732.4694
0
20
40
60
80
100
200
300
400
500
600
700

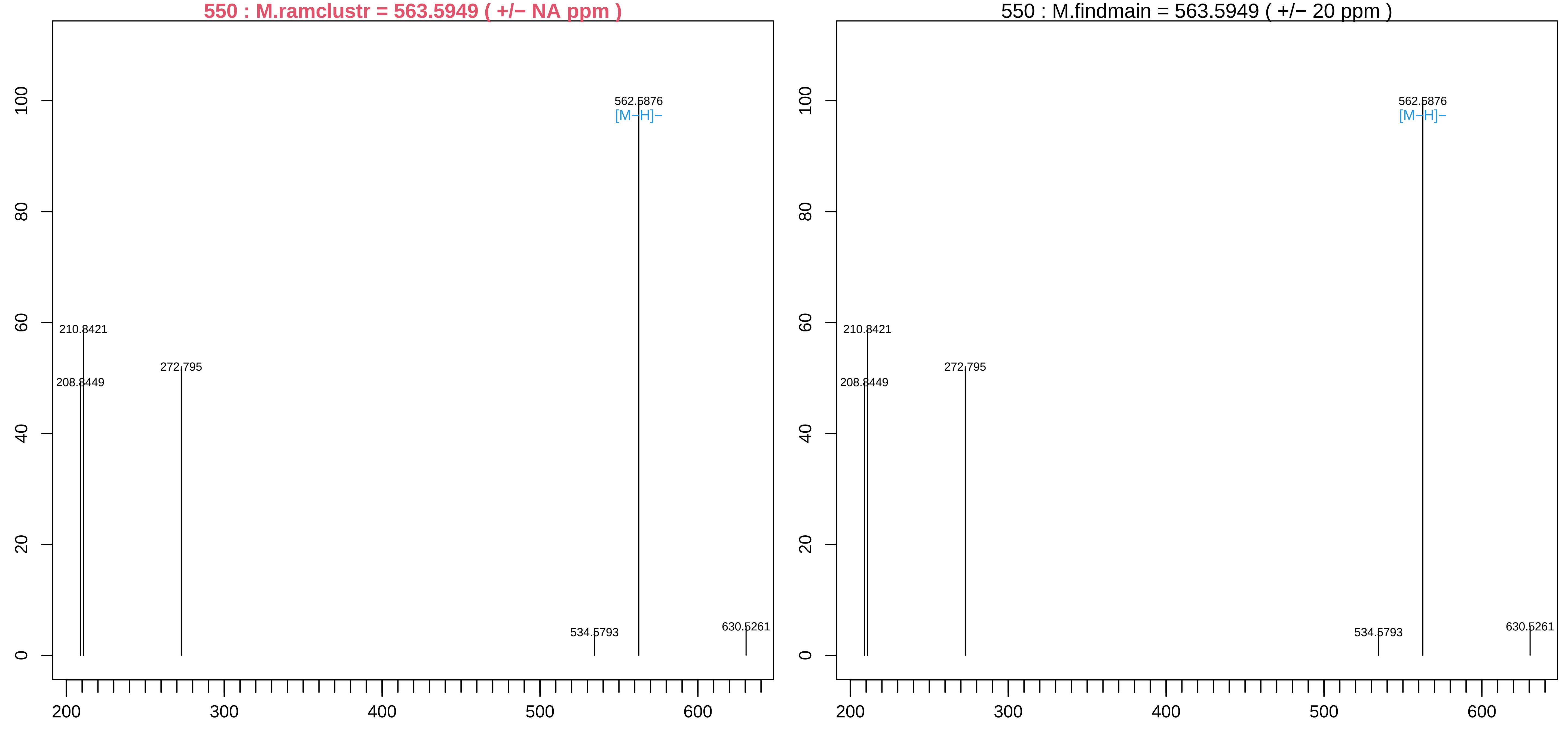
550 : M.ramclustr = 563.5949 (+/− NA ppm)
550 : M.findmain = 563.5949 (+/− 20 ppm)
562.5876
[M−H]−
210.8421
208.8449
272.795
534.5793
630.5261
0
20
40
60
80
100
200
300
400
500
600

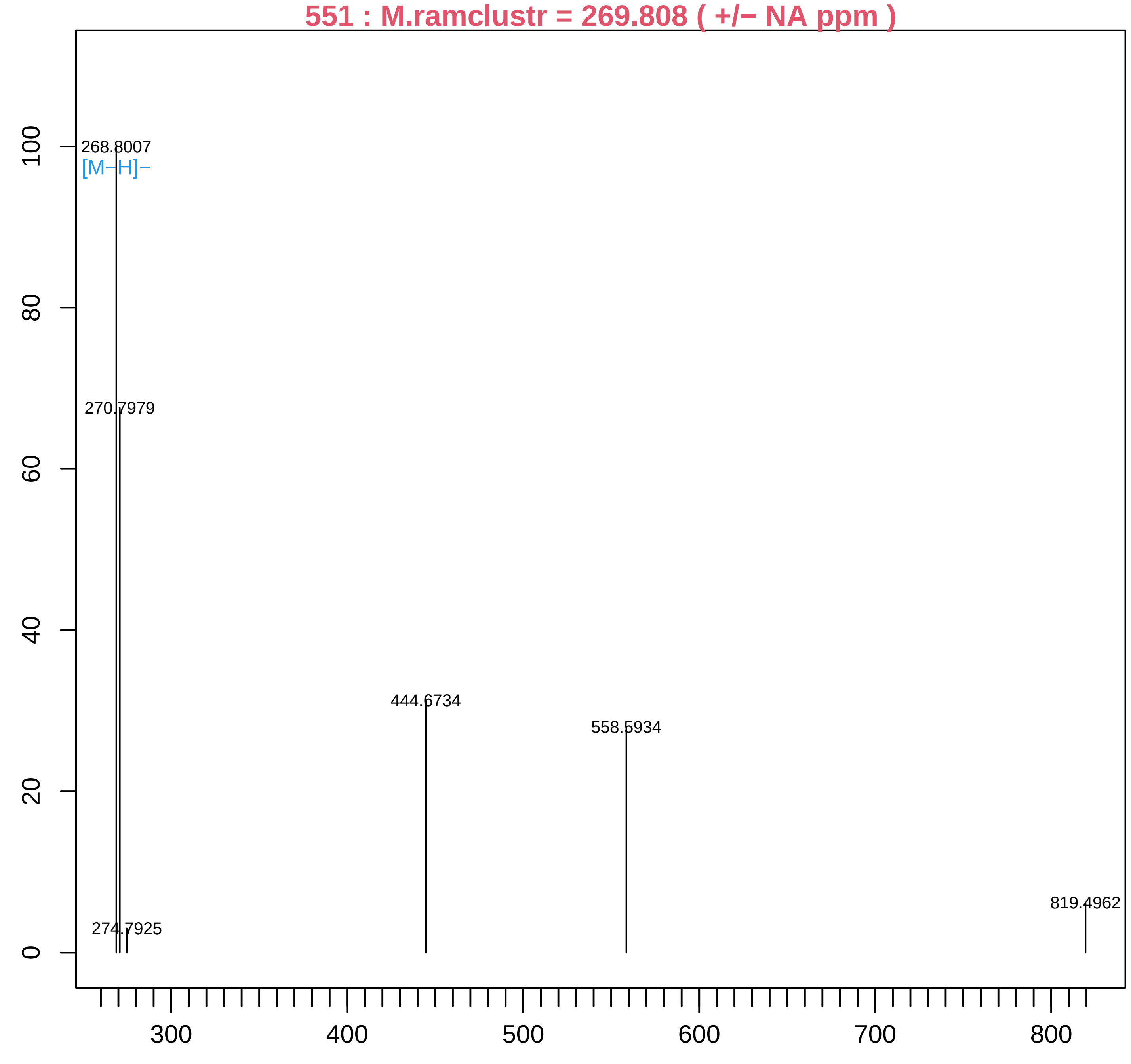
551 : M.ramclustr = 269.808 (+/− NA ppm)
268.8007
[M−H]−
270.7979
444.6734
558.5934
819.4962
274.7925
0
20
40
60
80
100
300
400
500
600
700
800

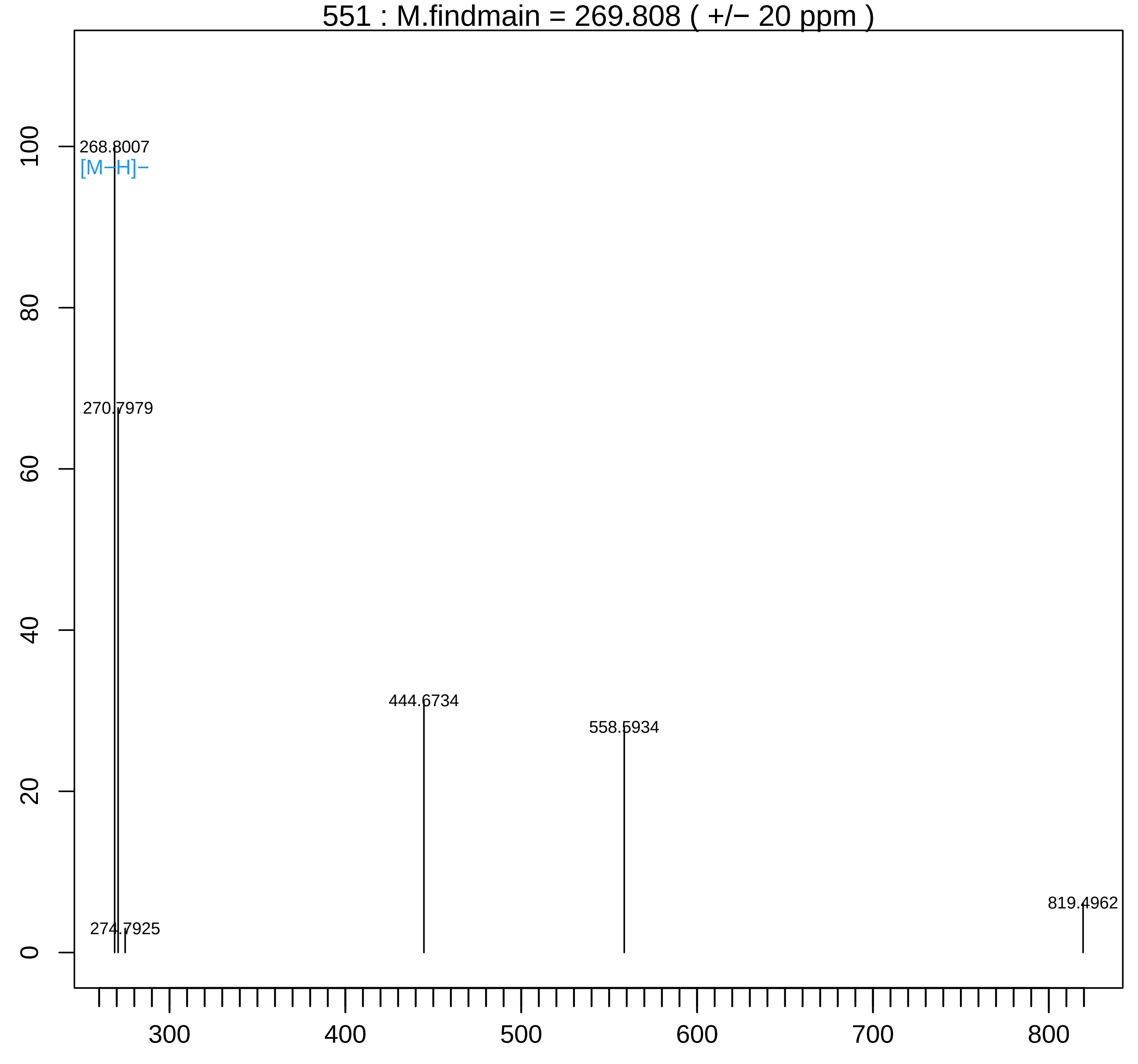
551 : M.findmain = 269.808 (+/− 20 ppm)
268.8007
[M−H]−
270.7979
444.6734
558.5934
819.4962
274.7925
0
20
40
60
80
100
300
400
500
600
700
800

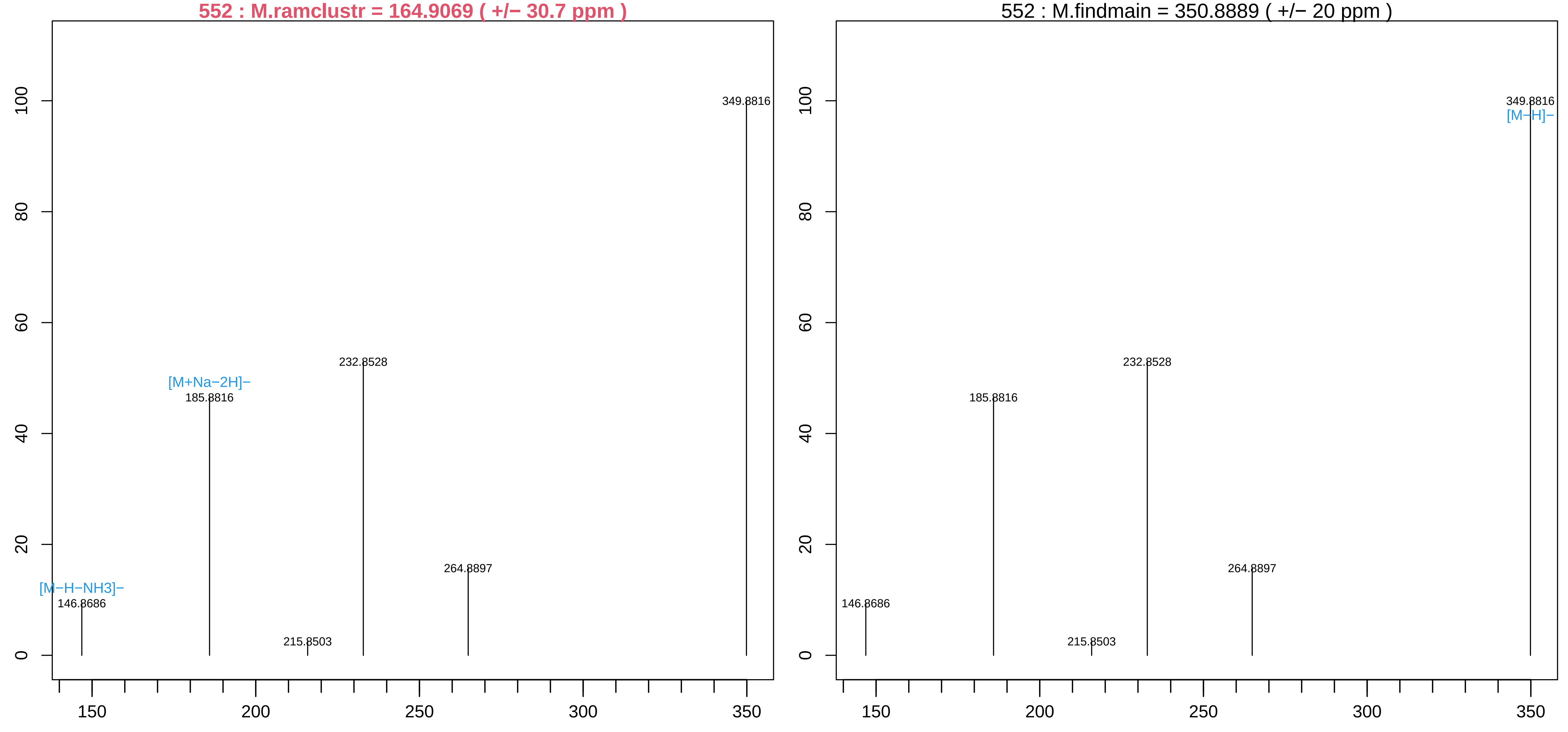
552 : M.ramclustr = 164.9069 (+/− 30.7 ppm)
[M−H−NH3]−
146.8686
[M+Na−2H]−
185.8816
215.8503
232.8528
264.8897
349.8816
552 : M.findmain = 350.8889 (+/− 20 ppm)
146.8686
185.8816
215.8503
232.8528
264.8897
349.8816
[M−H]−

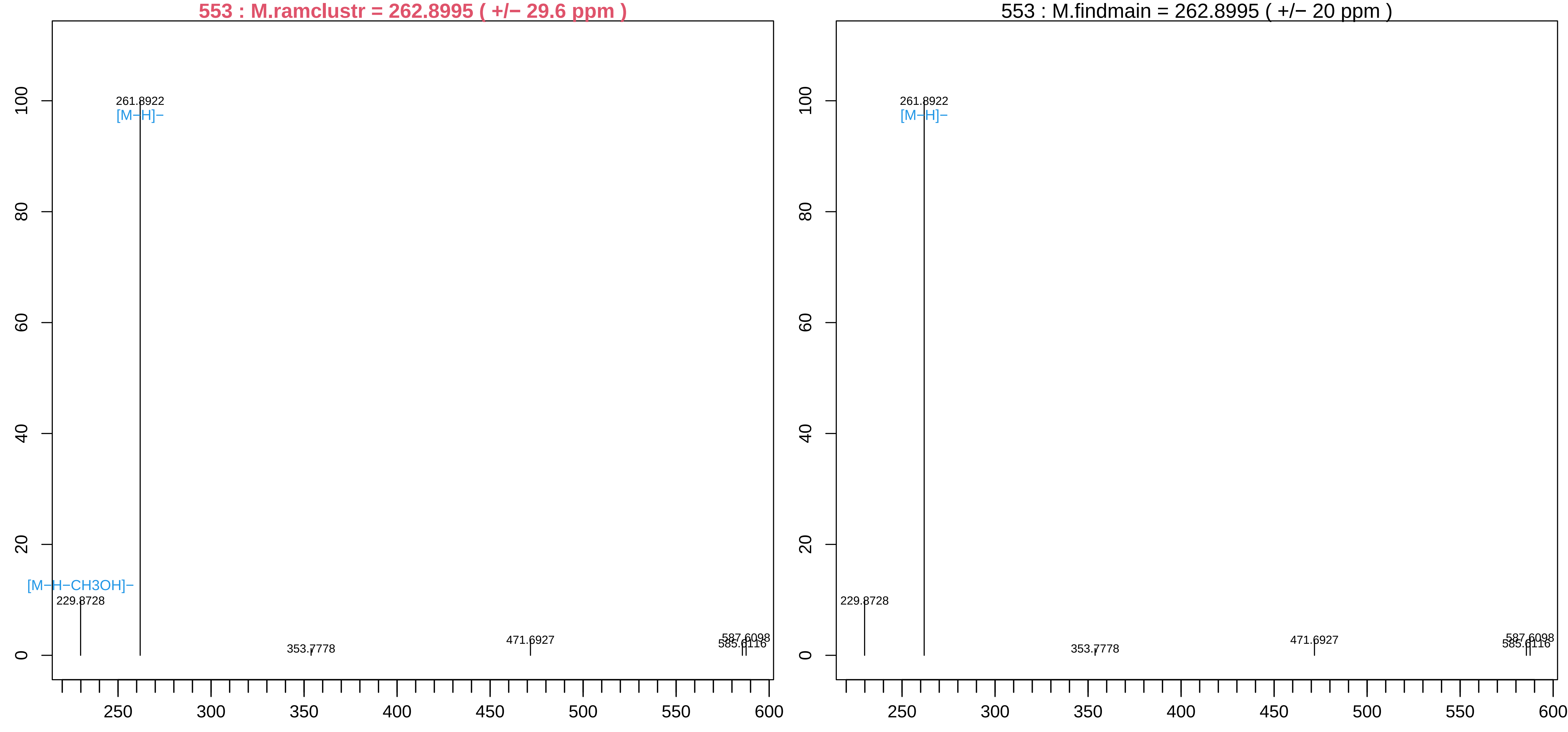
553 : M.ramclustr = 262.8995 (+/− 29.6 ppm)
261.8922
[M−H]−
[M−H−CH3OH]−
229.8728
353.7778
471.6927
587.6098
585.6116
0
20
40
60
80
100
250
300
350
400
450
500
550
600
553 : M.findmain = 262.8995 (+/− 20 ppm)
261.8922
[M−H]−
229.8728
353.7778
471.6927
587.6098
585.6116

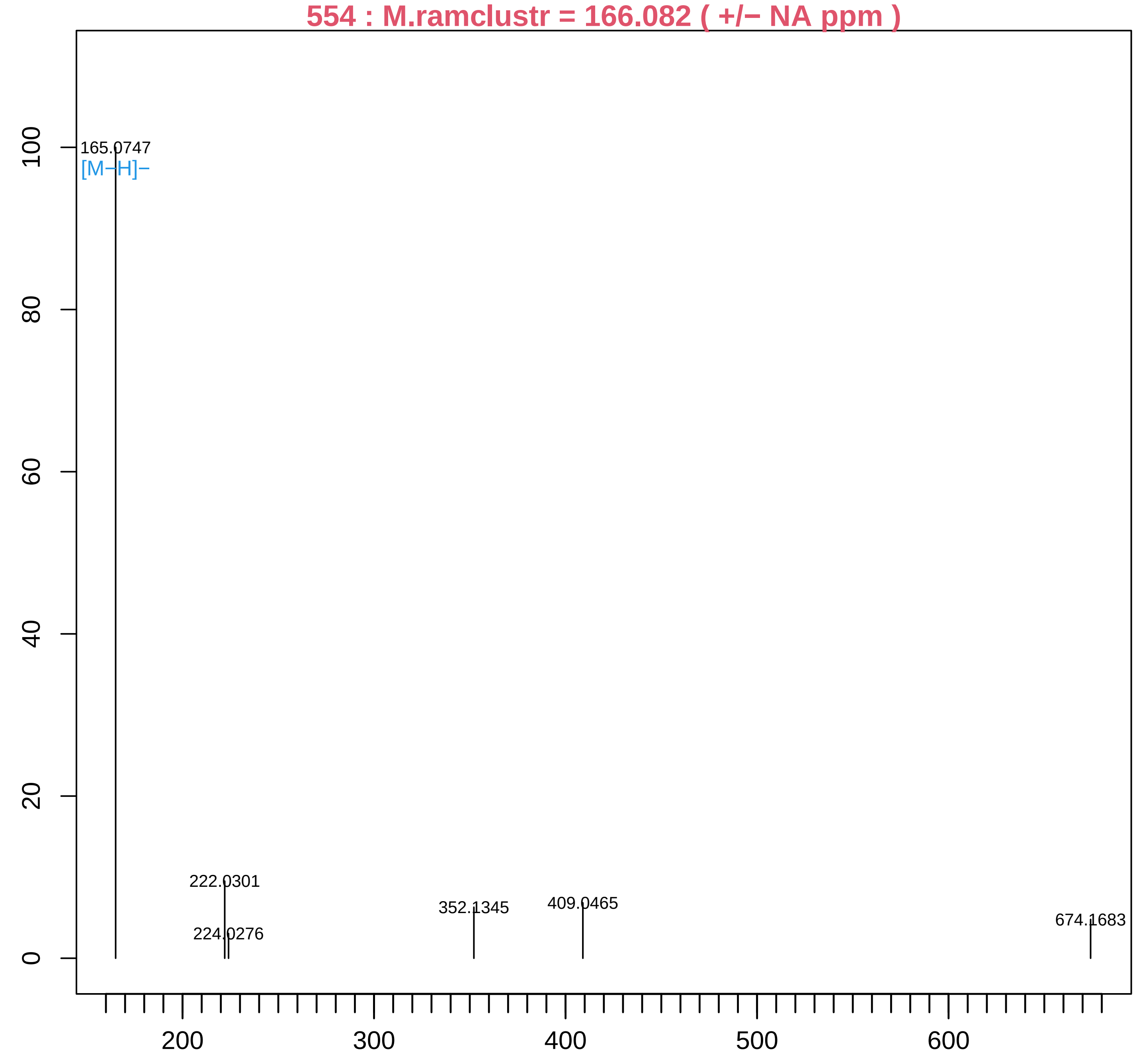
554 : M.ramclustr = 166.082 (+/− NA ppm)
165.0747
[M−H]−
222.0301
224.0276
352.1345
409.0465
674.1683
0
20
40
60
80
100
200
300
400
500
600

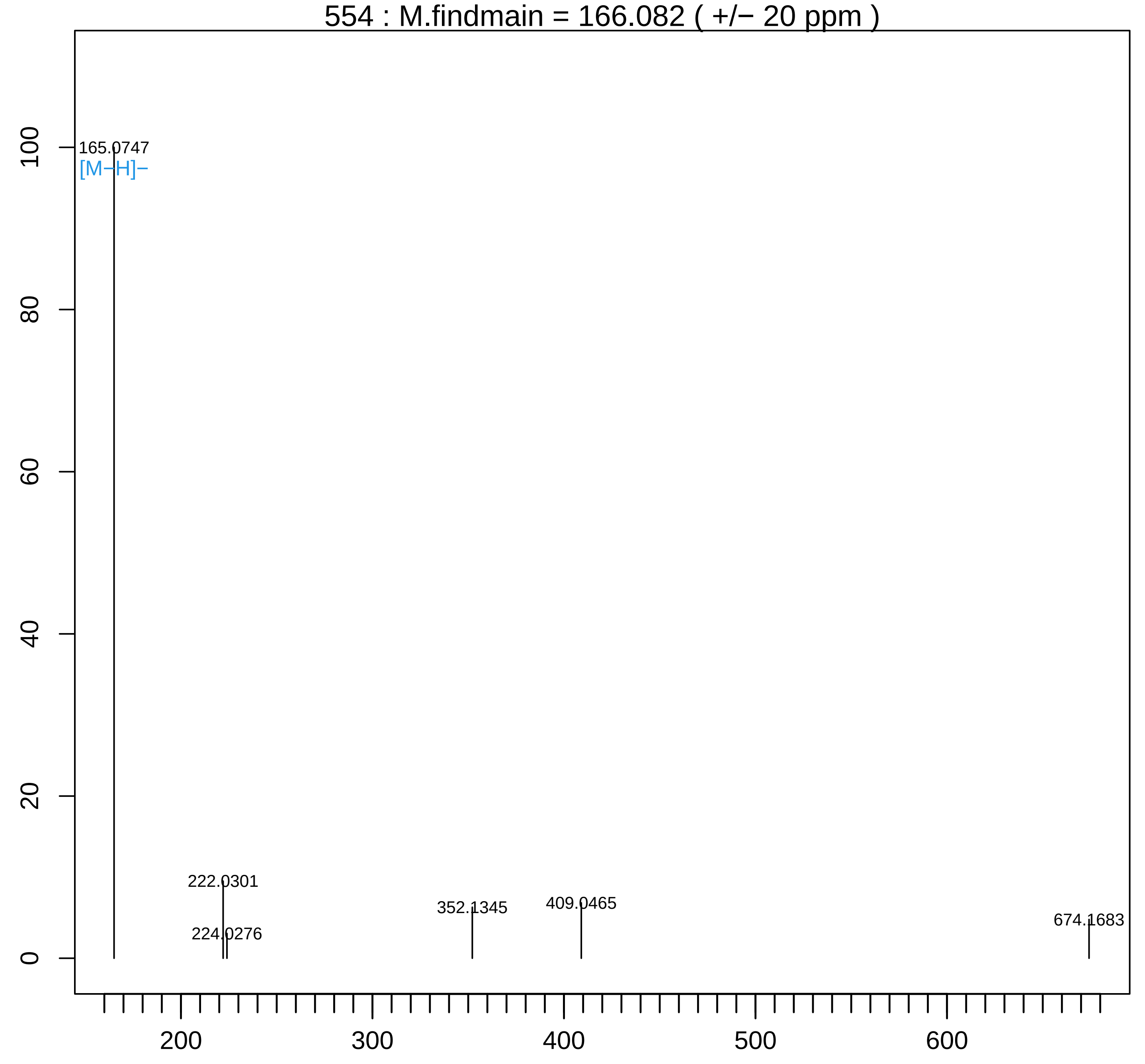
554 : M.findmain = 166.082 (+/− 20 ppm)
165.0747
[M−H]−
222.0301
224.0276
352.1345
409.0465
674.1683
0
20
40
60
80
100
200
300
400
500
600

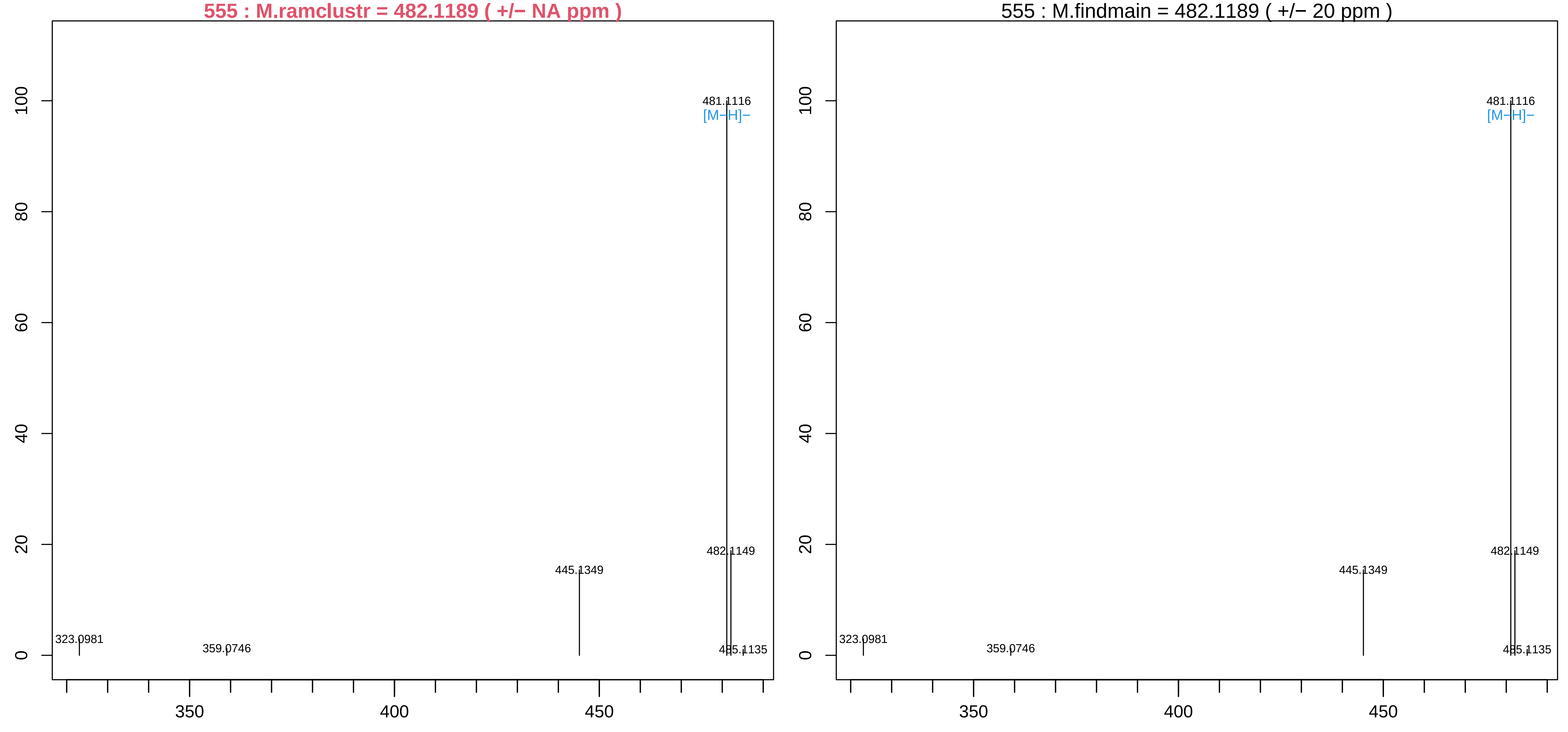
555 : M.ramclustr = 482.1189 (+/− NA ppm)
481.1116
[M−H]−
482.1149
445.1349
323.0981
359.0746
485.1135
555 : M.findmain = 482.1189 (+/− 20 ppm)
481.1116
[M−H]−
482.1149
445.1349
323.0981
359.0746
485.1135
0
20
40
60
80
100
350
400
450

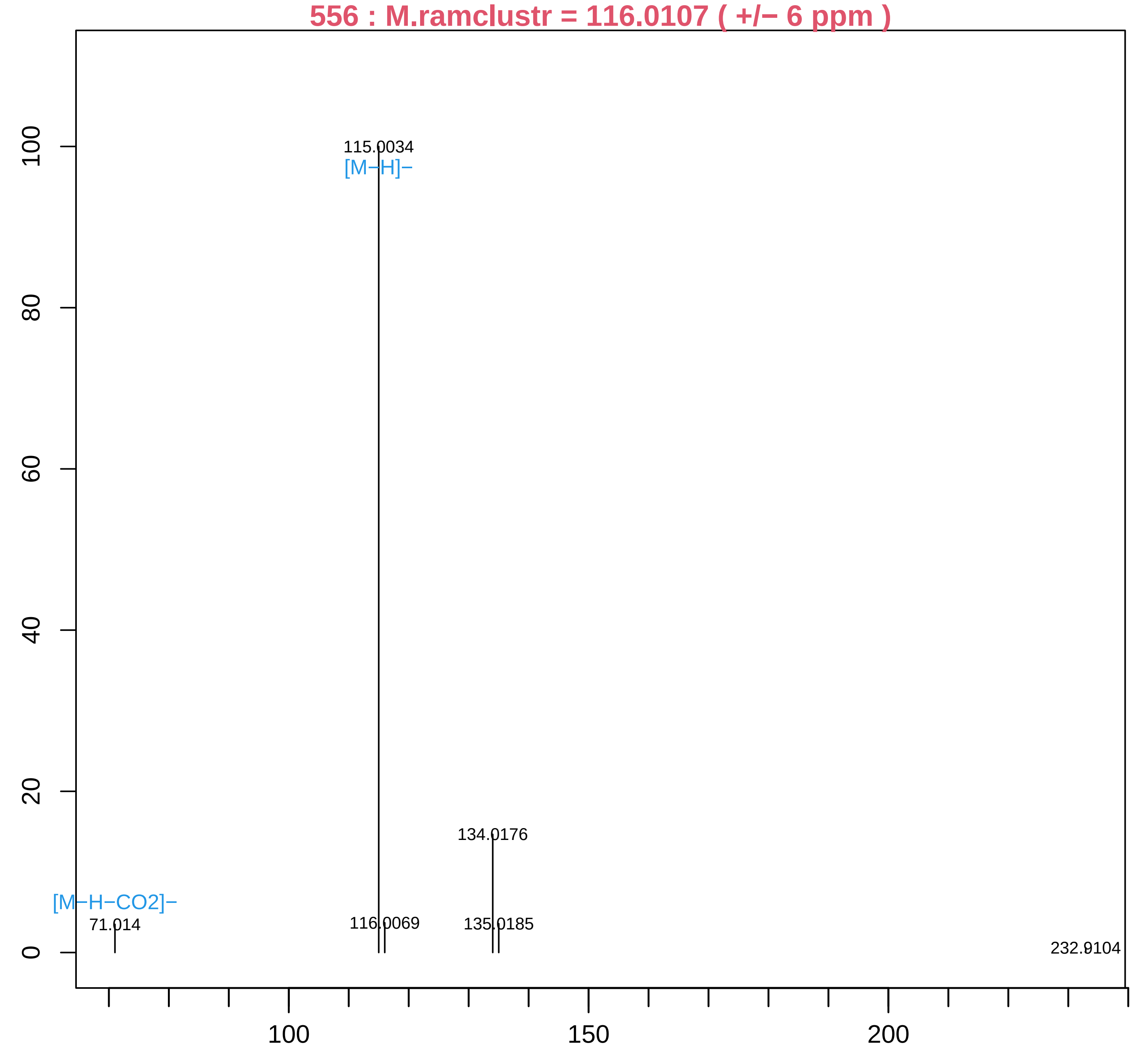
556 : M.ramclustr = 116.0107 (+/− 6 ppm)
115.0034
[M−H]−
134.0176
[M−H−CO2]−
71.014
116.0069
135.0185
232.9104
0
20
40
60
80
100
100
150
200

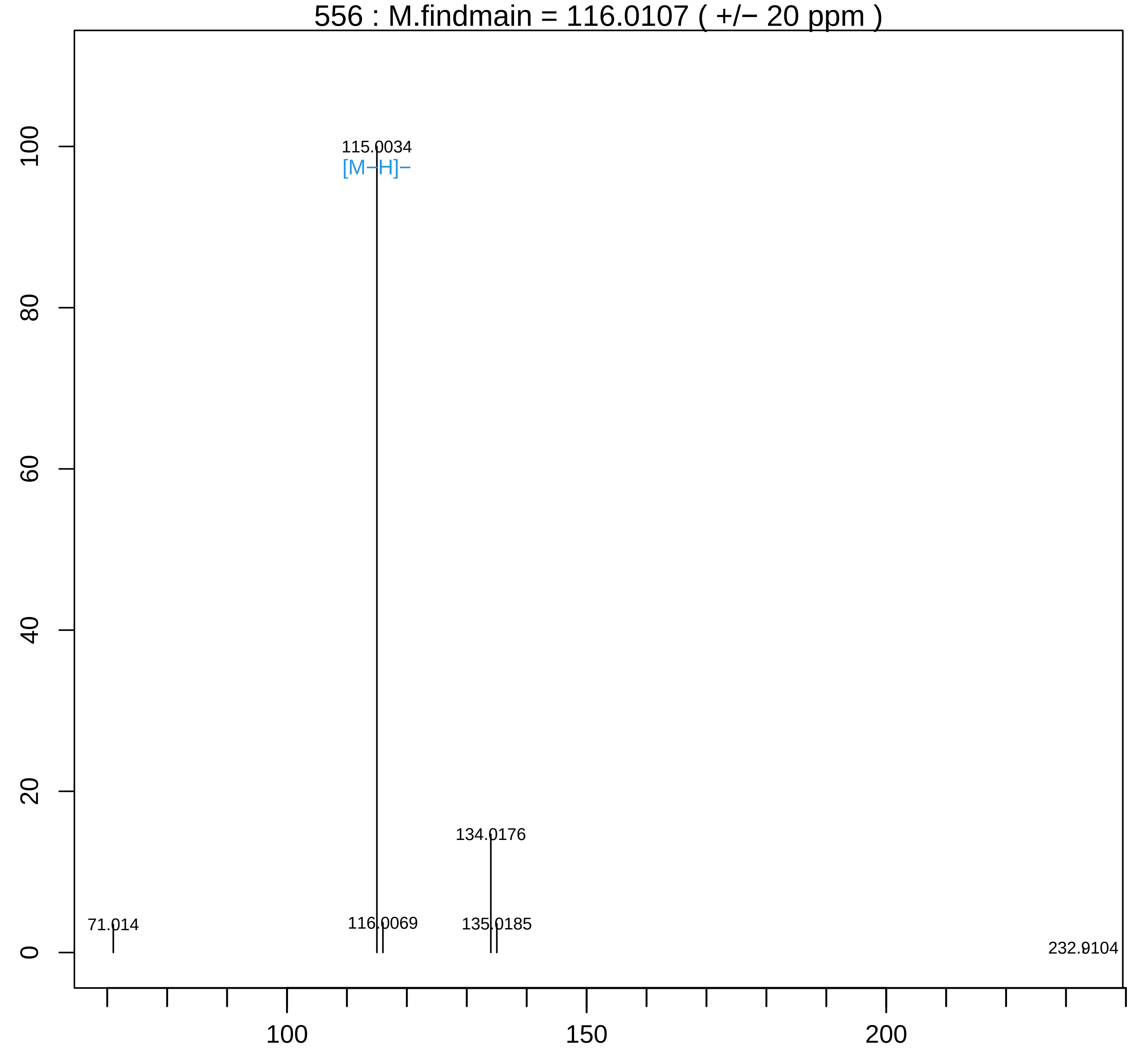
556 : M.findmain = 116.0107 (+/− 20 ppm)
115.0034
[M−H]−
134.0176
71.014
116.0069
135.0185
232.9104
0
20
40
60
80
100
100
150
200

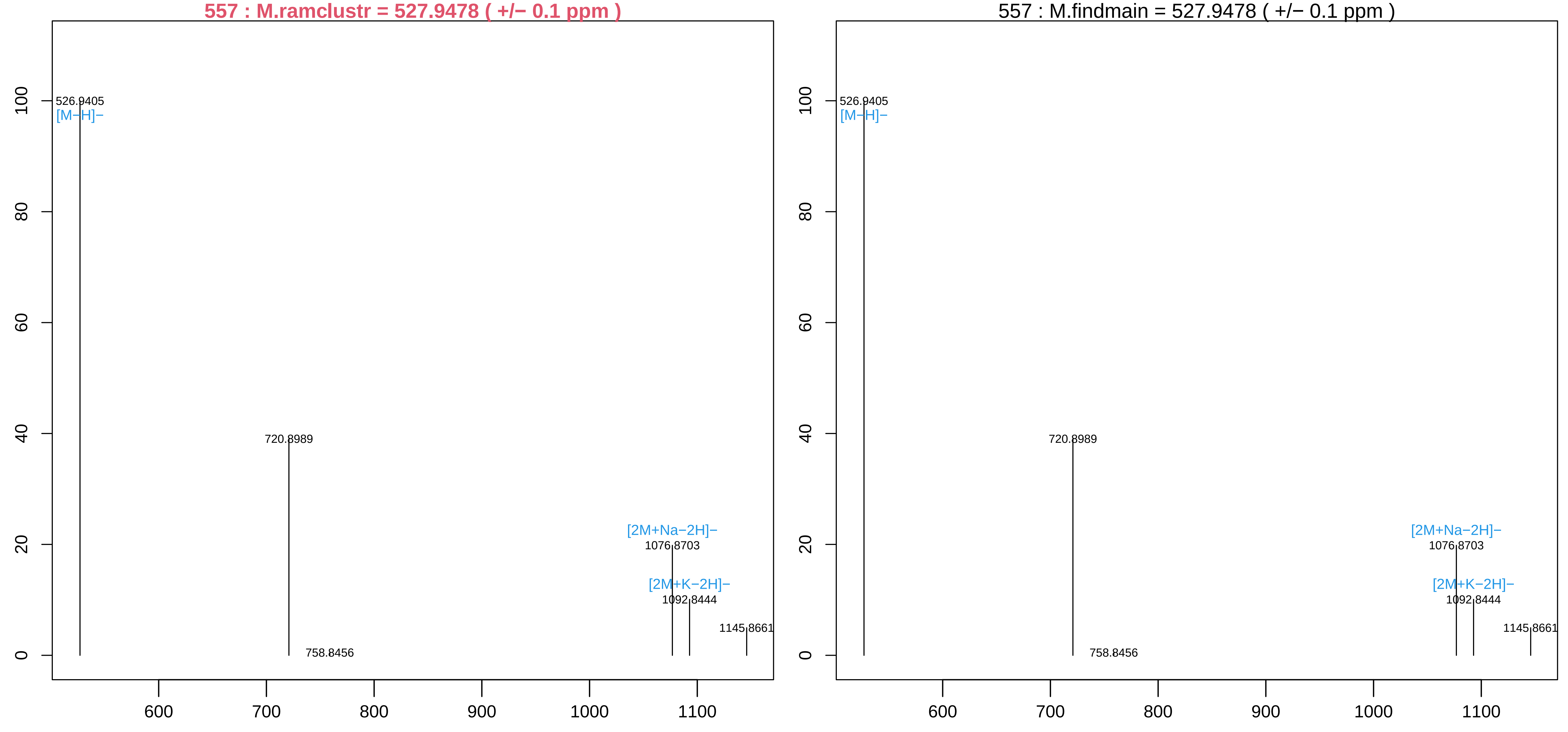

557 : M.ramclustr = 527.9478 (+/− 0.1 ppm)
526.9405
[M−H]−
720.8989
758.8456
[2M+Na−2H]−
1076.8703
[2M+K−2H]−
1092.8444
1145.8661
557 : M.findmain = 527.9478 (+/− 0.1 ppm)
526.9405
[M−H]−
720.8989
758.8456
[2M+Na−2H]−
1076.8703
[2M+K−2H]−
1092.8444
1145.8661

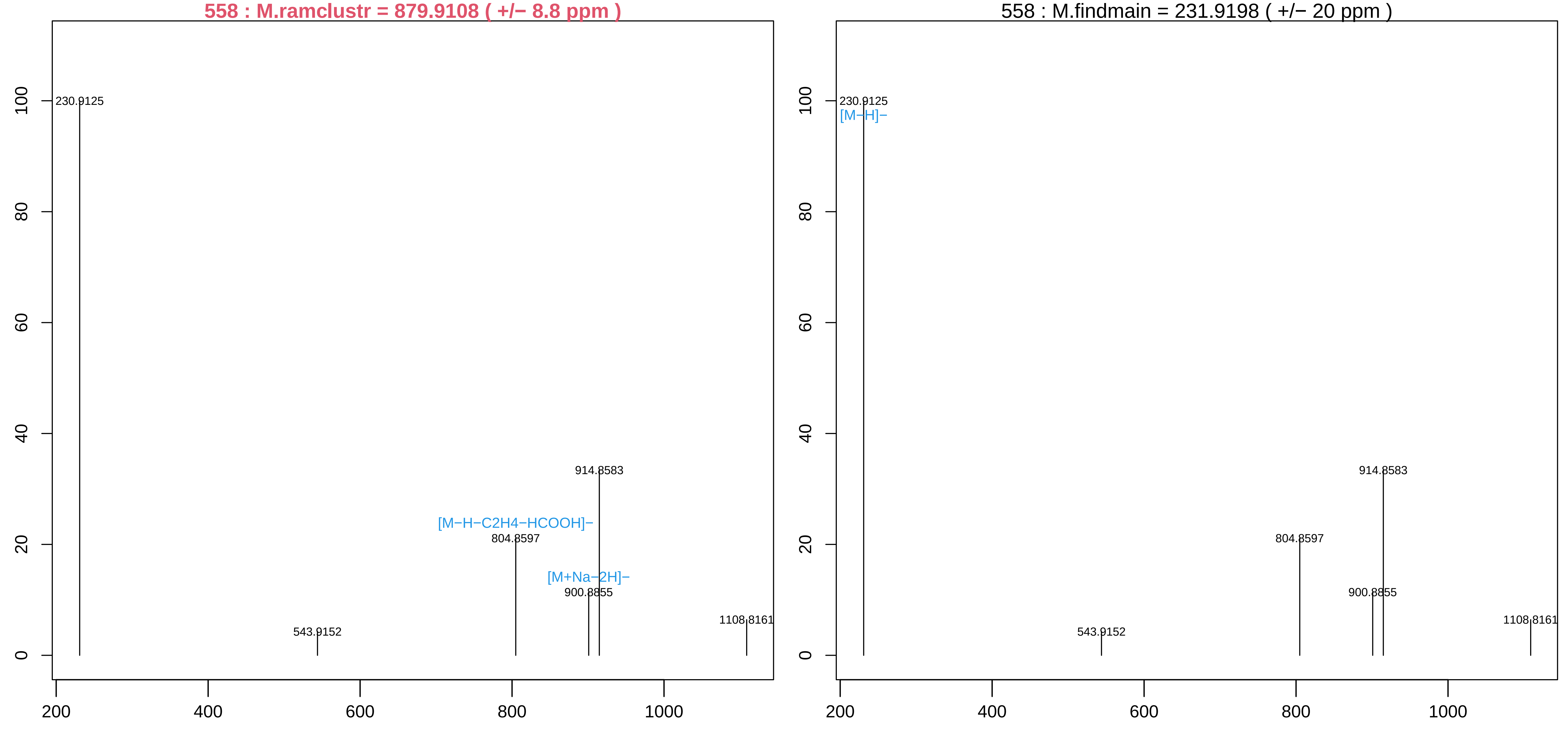
558 : M.ramclustr = 879.9108 (+/− 8.8 ppm)
230.9125
[M−H−C2H4−HCOOH]−
804.8597
914.8583
[M+Na−2H]−
900.8855
543.9152
1108.8161
0
20
40
60
80
100
200
400
600
800
1000
558 : M.findmain = 231.9198 (+/− 20 ppm)
230.9125
[M−H]−
914.8583
804.8597
900.8855
543.9152
1108.8161

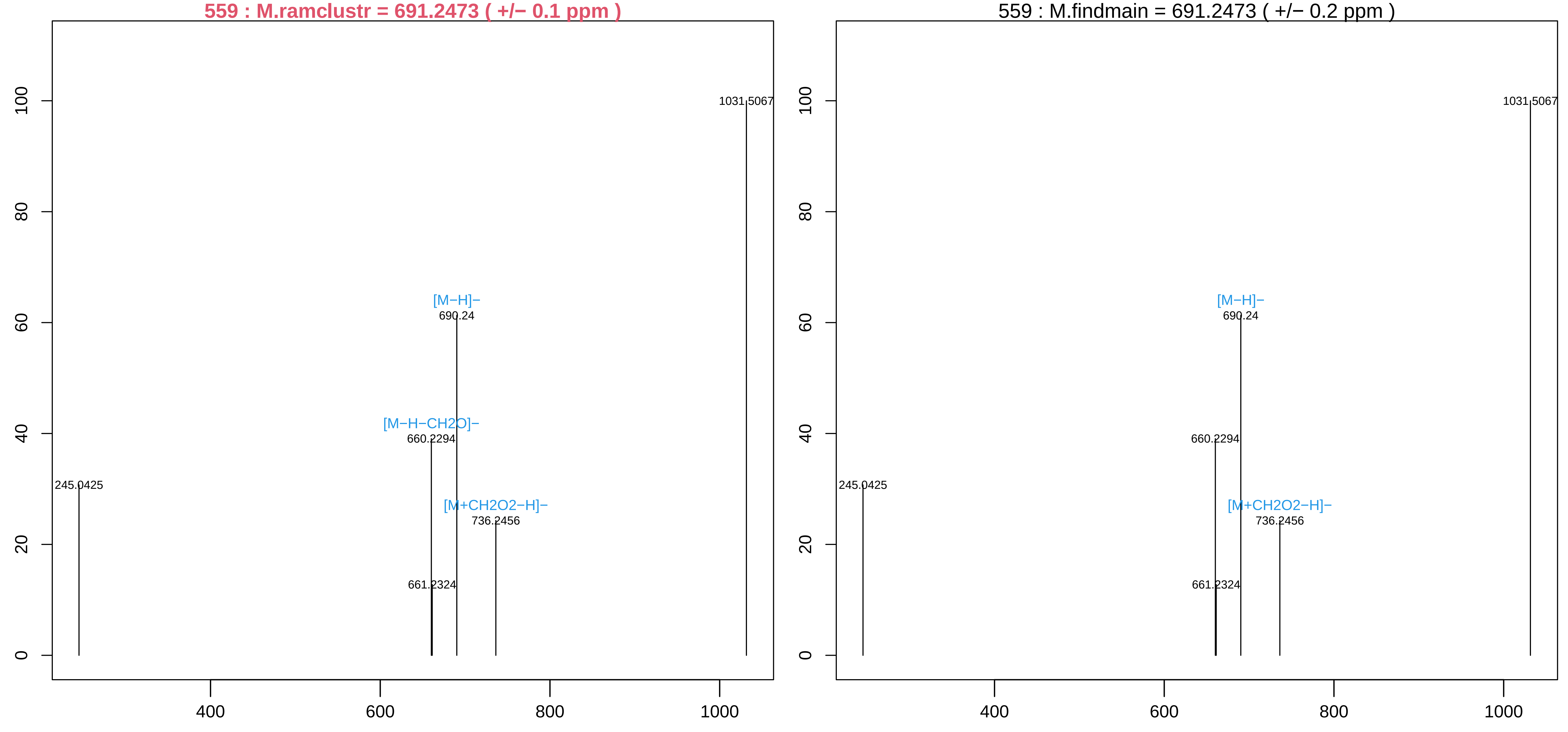
559 : M.ramclustr = 691.2473 (+/− 0.1 ppm)
559 : M.findmain = 691.2473 (+/− 0.2 ppm)
1031.5067
[M−H]−
690.24
[M−H−CH2O]−
660.2294
245.0425
[M+CH2O2−H]−
736.2456
661.2324
0
20
40
60
80
100
400
600
800
1000

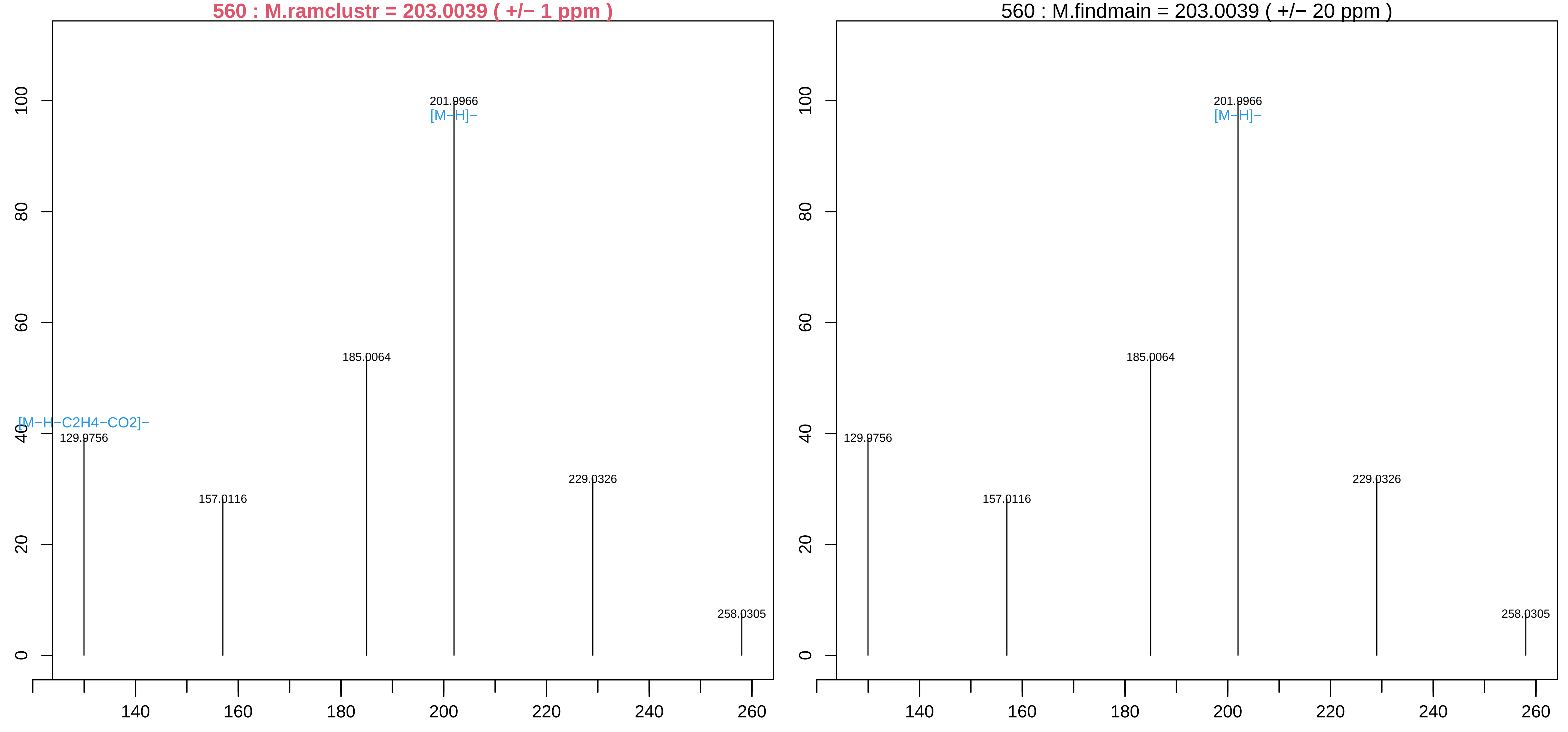

560 : M.ramclustr = 203.0039 (+/− 1 ppm)
201.9966
[M−H]−
185.0064
[M−H−C2H4−CO2]−
129.9756
157.0116
229.0326
258.0305
560 : M.findmain = 203.0039 (+/− 20 ppm)
201.9966
[M−H]−
185.0064
129.9756
157.0116
229.0326
258.0305
0
20
40
60
80
100
140
160
180
200
220
240
260

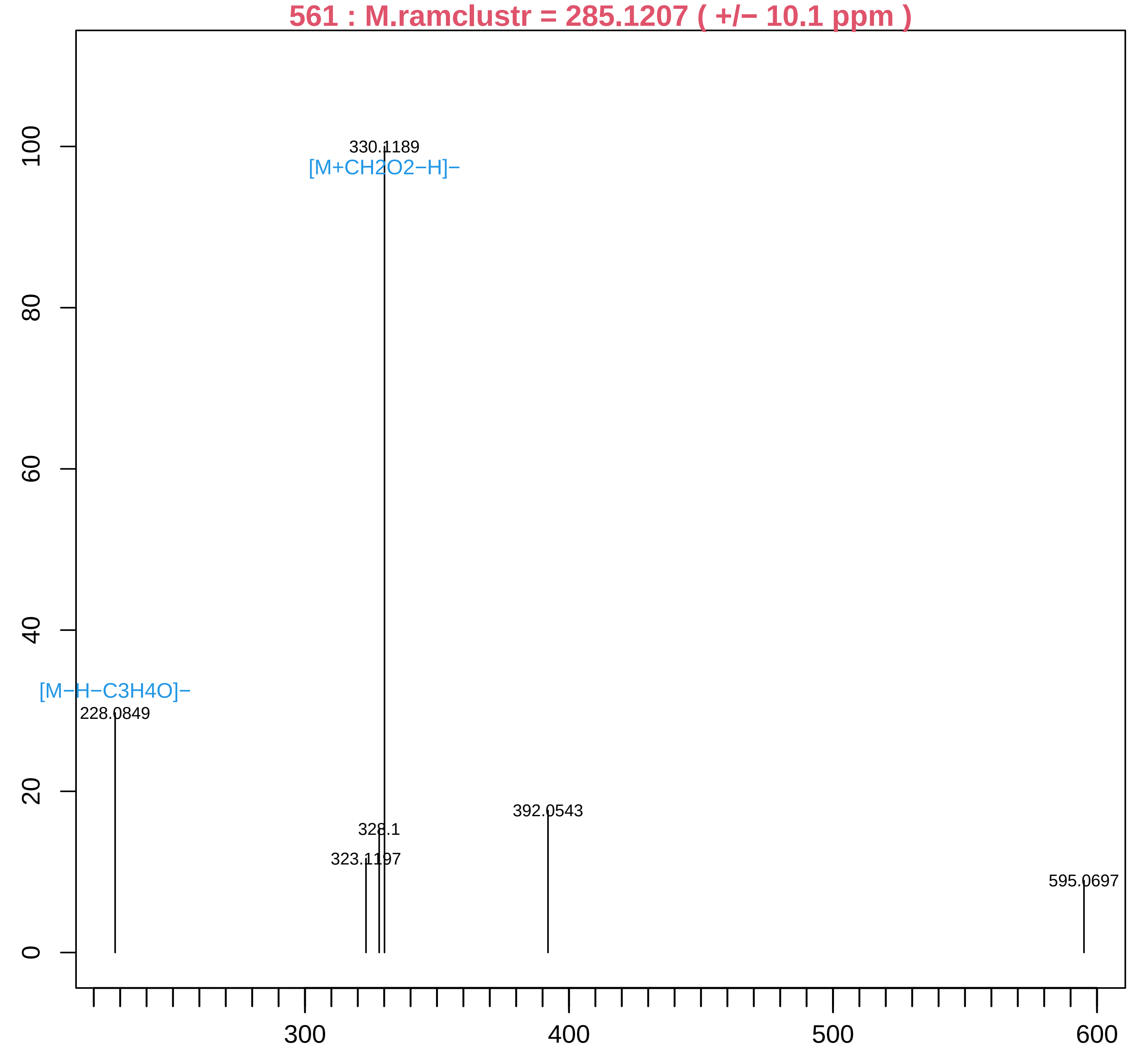

561 : M.ramclustr = 285.1207 (+/− 10.1 ppm)
330.1189
[M+CH2O2−H]−
[M−H−C3H4O]−
228.0849
392.0543
328.1
323.1197
595.0697
0
20
40
60
80
100
300
400
500
600

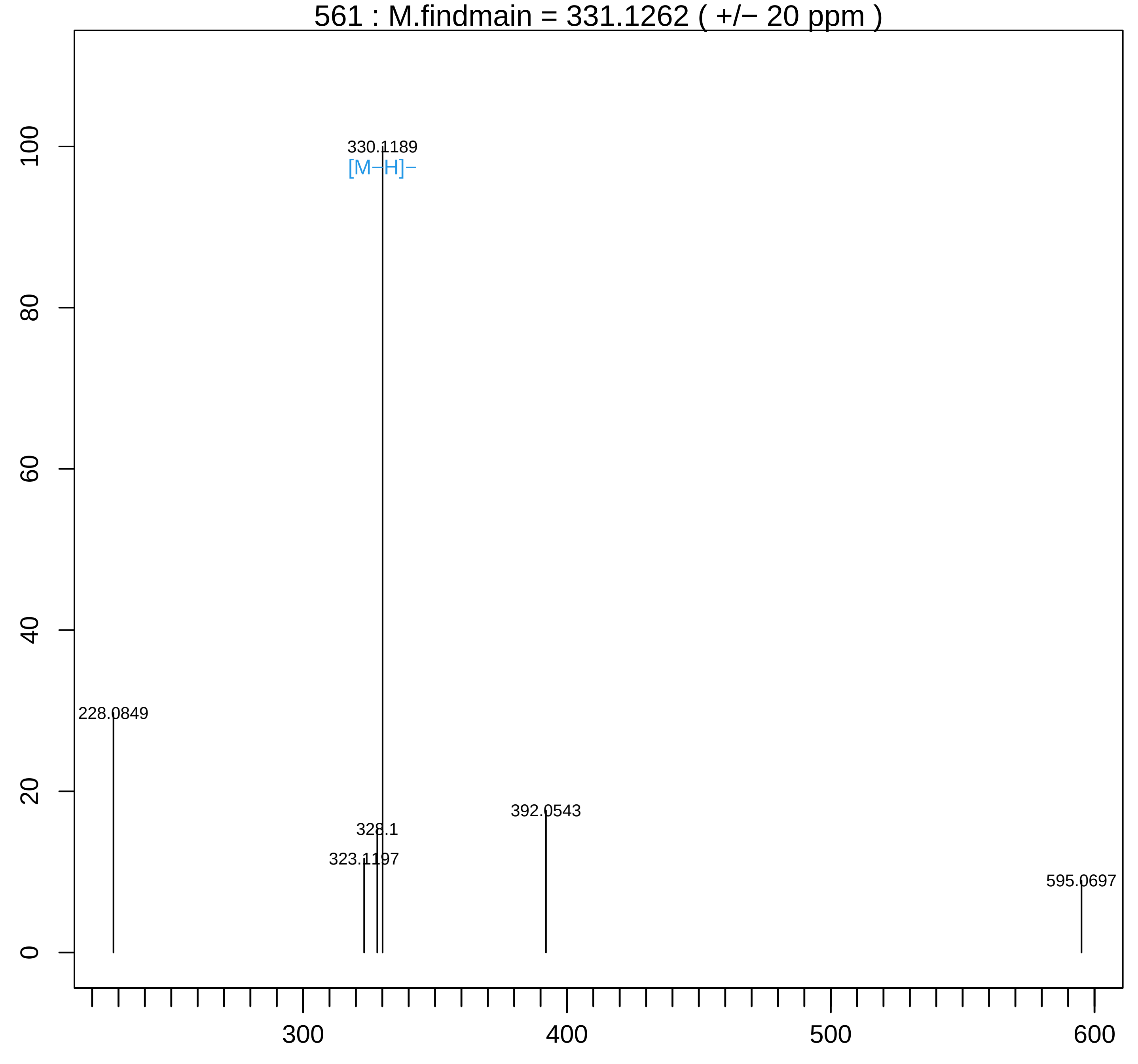

561 : M.findmain = 331.1262 (+/− 20 ppm)
330.1189
[M−H]−
228.0849
392.0543
328.1
323.1197
595.0697
0
20
40
60
80
100
300
400
500
600

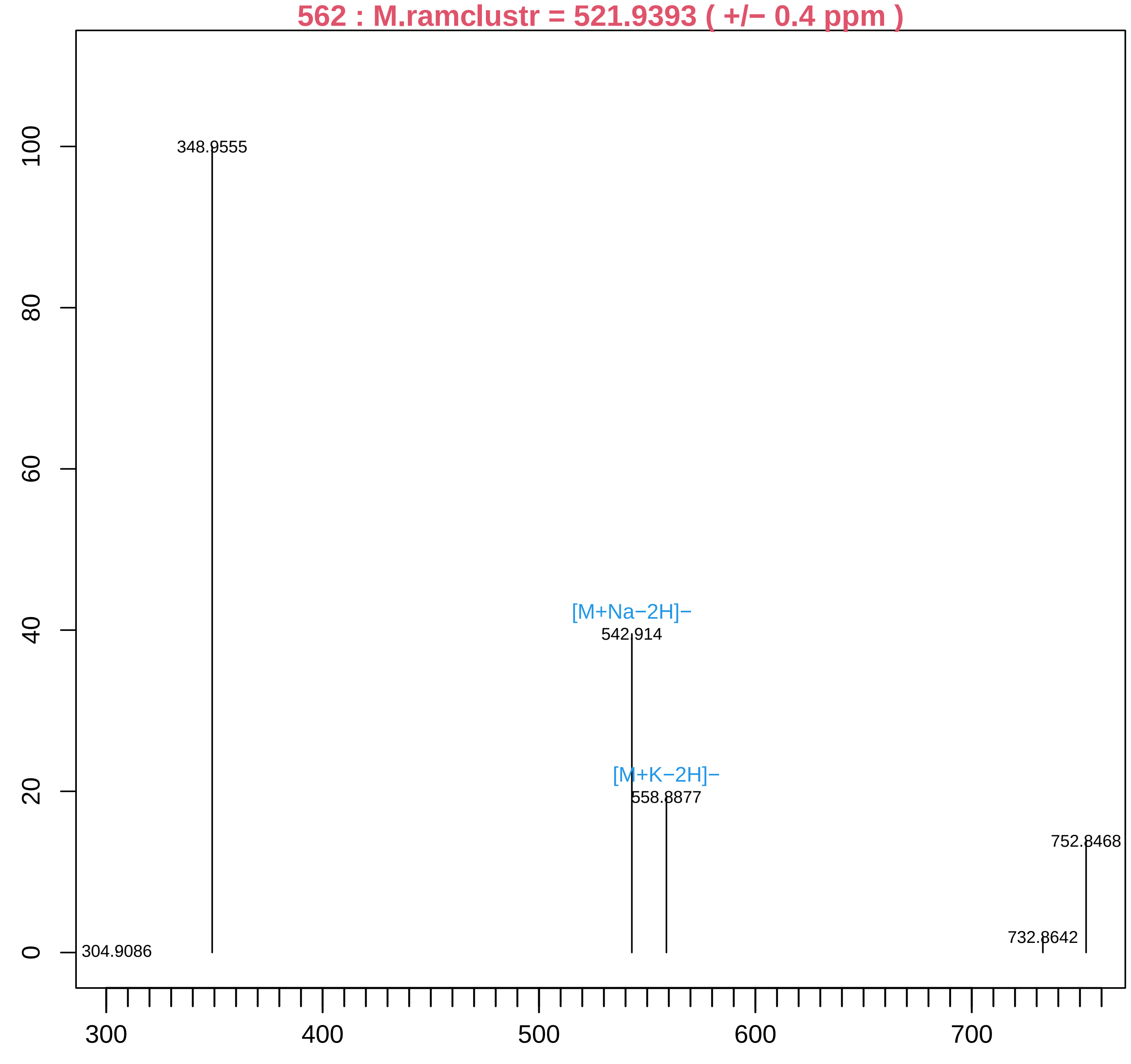
562 : M.ramclustr = 521.9393 (+/− 0.4 ppm)
348.9555
[M+Na−2H]−
542.914
[M+K−2H]−
558.8877
752.8468
732.8642
304.9086
0
20
40
60
80
100
300
400
500
600
700

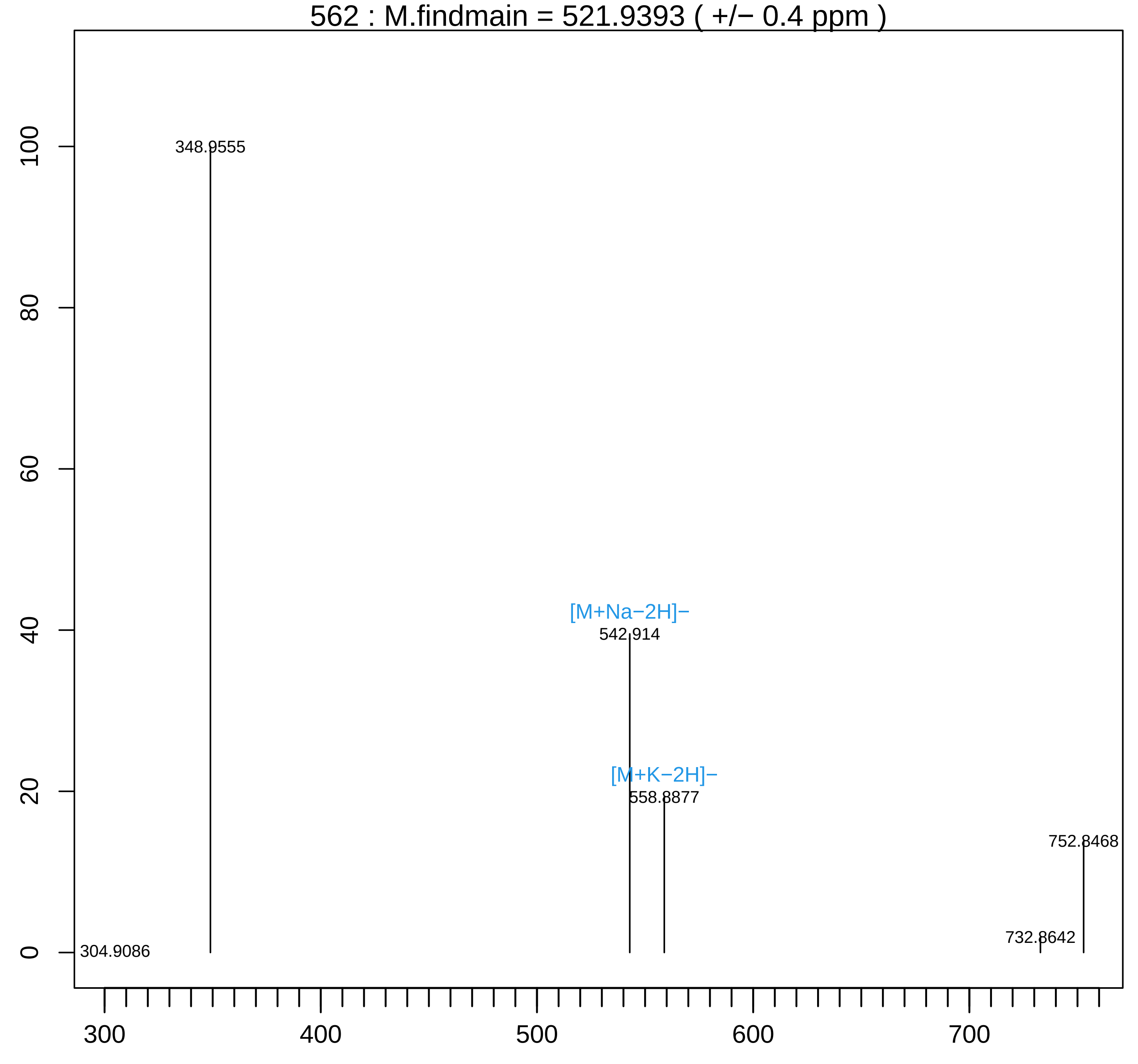
562 : M.findmain = 521.9393 (+/− 0.4 ppm)
348.9555
[M+Na−2H]−
542.914
[M+K−2H]−
558.8877
752.8468
732.8642
304.9086
0
20
40
60
80
100
300
400
500
600
700

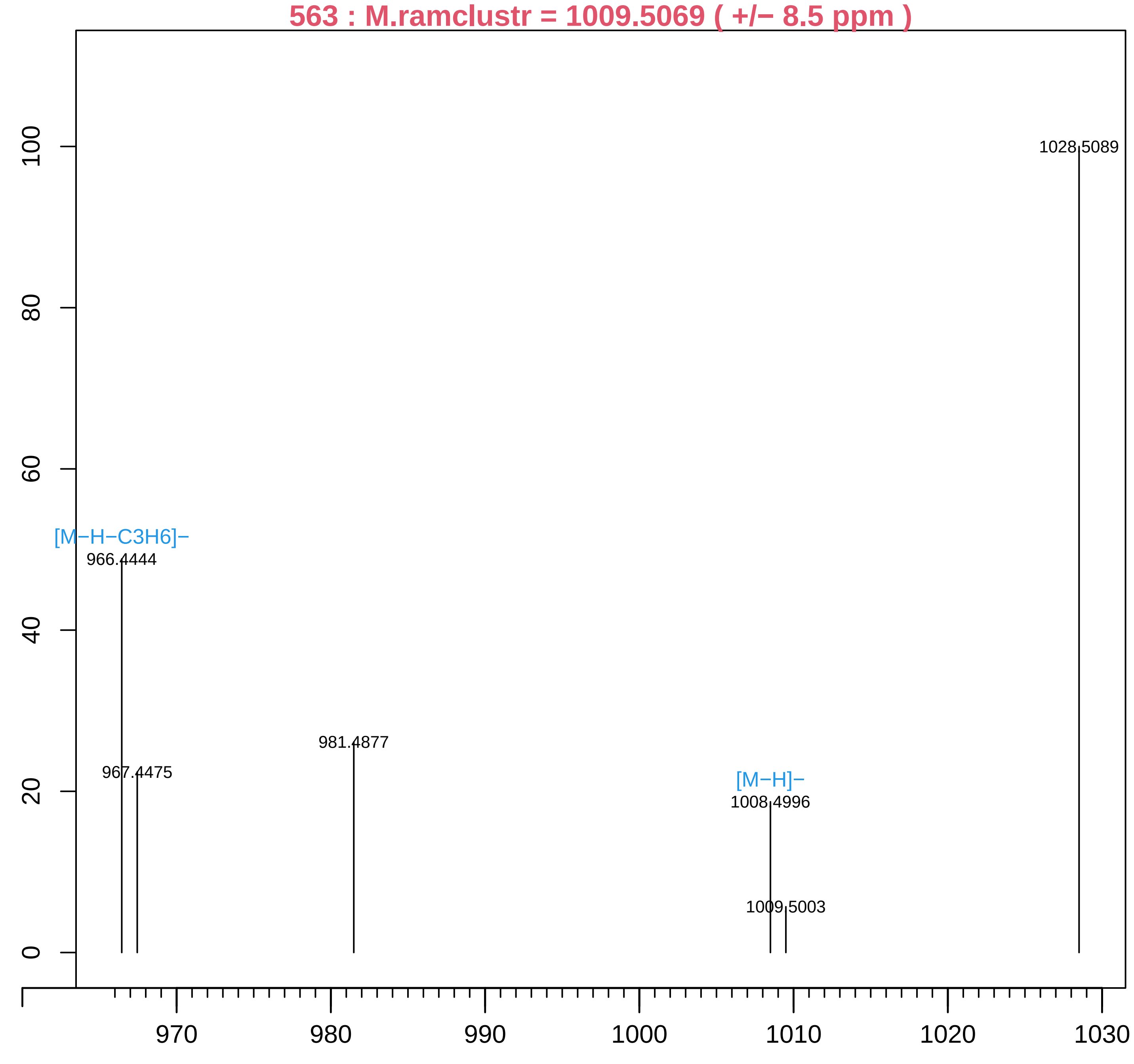
563 : M.ramclustr = 1009.5069 (+/− 8.5 ppm)
[M−H−C3H6]−
966.4444
967.4475
981.4877
[M−H]−
1008.4996
1009.5003
1028.5089
0
20
40
60
80
100
970
980
990
1000
1010
1020
1030

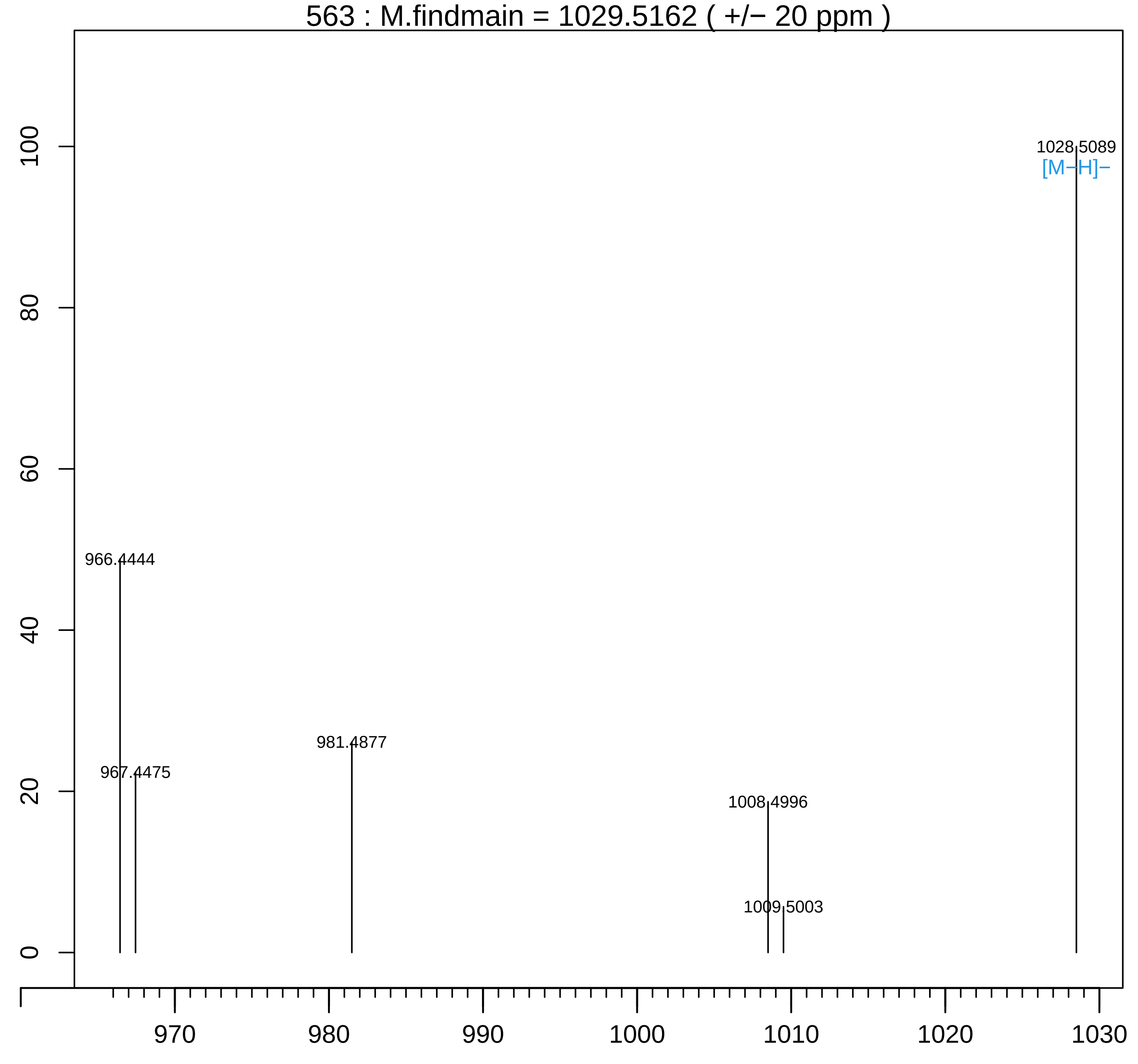
563 : M.findmain = 1029.5162 (+/− 20 ppm)
966.4444
967.4475
981.4877
1008.4996
1009.5003
1028.5089
[M−H]−
0
20
40
60
80
100
970
980
990
1000
1010
1020
1030

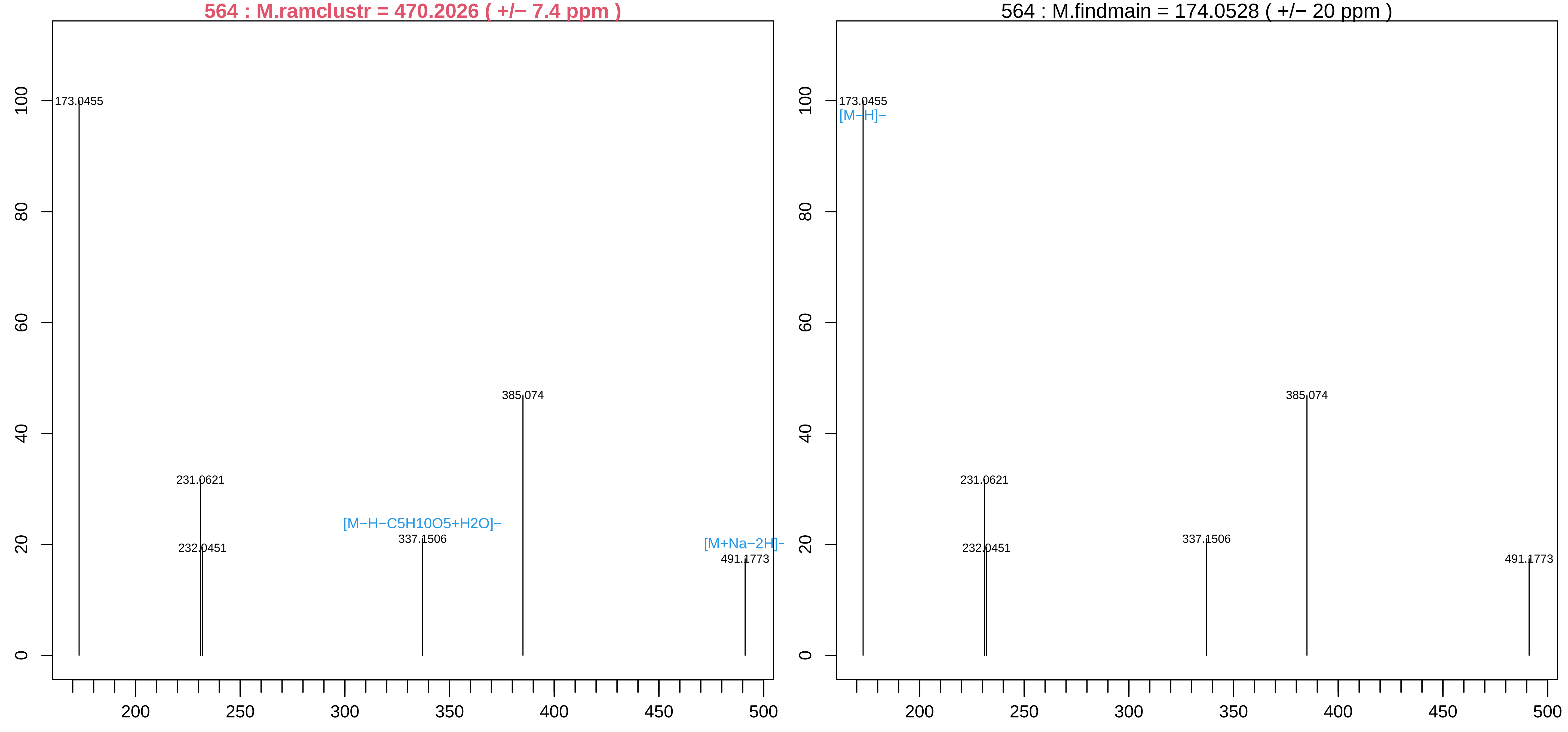

564 : M.ramclustr = 470.2026 (+/− 7.4 ppm)
173.0455
231.0621
232.0451
[M−H−C5H10O5+H2O]−
337.1506
385.074
[M+Na−2H]−
491.1773
0
20
40
60
80
100
200
250
300
350
400
450
500
564 : M.findmain = 174.0528 (+/− 20 ppm)
173.0455
[M−H]−
231.0621
232.0451
337.1506
385.074
491.1773

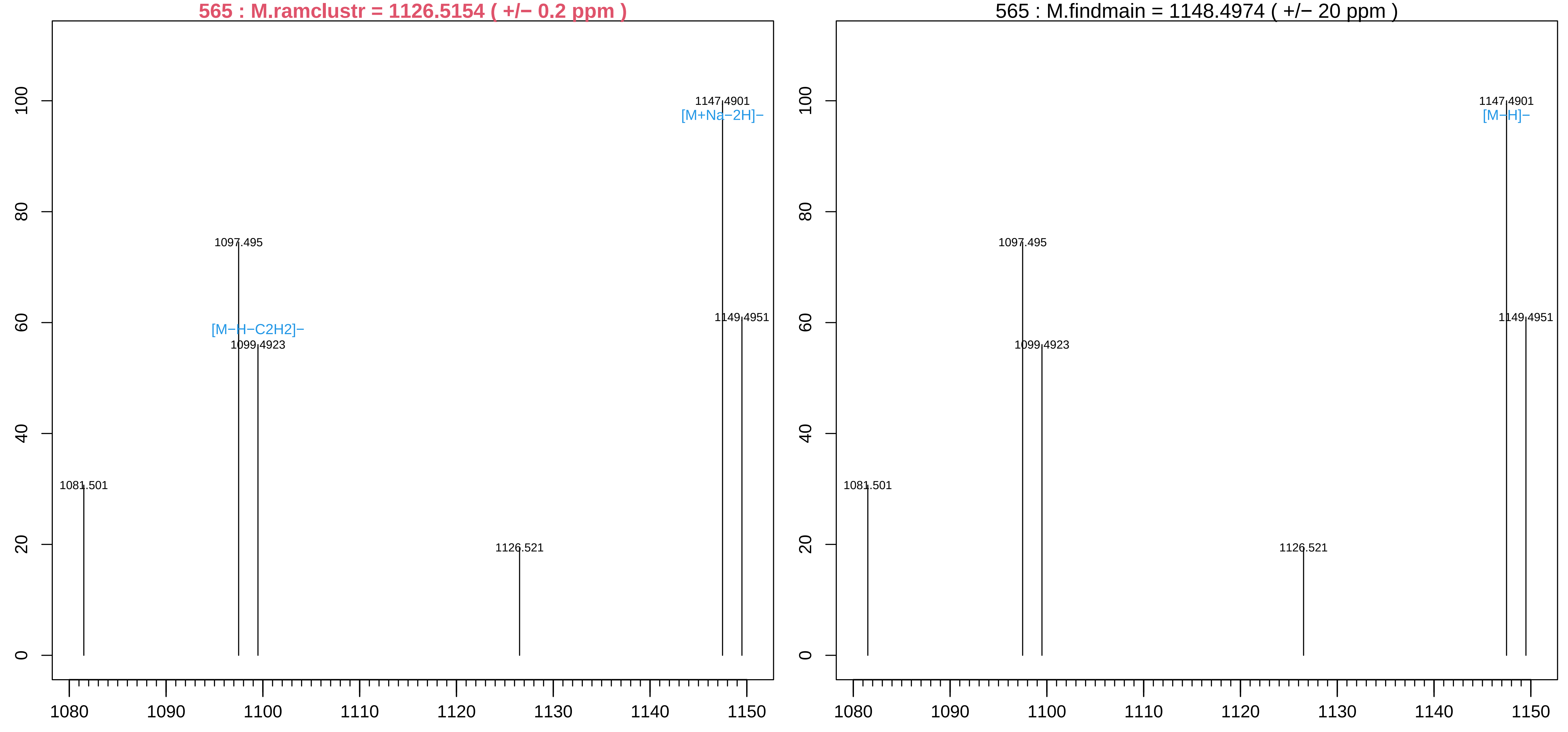
565 : M.ramclustr = 1126.5154 (+/− 0.2 ppm)
565 : M.findmain = 1148.4974 (+/− 20 ppm)
1147.4901
[M+Na−2H]−
1097.495
1149.4951
[M−H−C2H2]−
1099.4923
1081.501
1126.521
[M−H]−
0
20
40
60
80
100
1080
1090
1100
1110
1120
1130
1140
1150

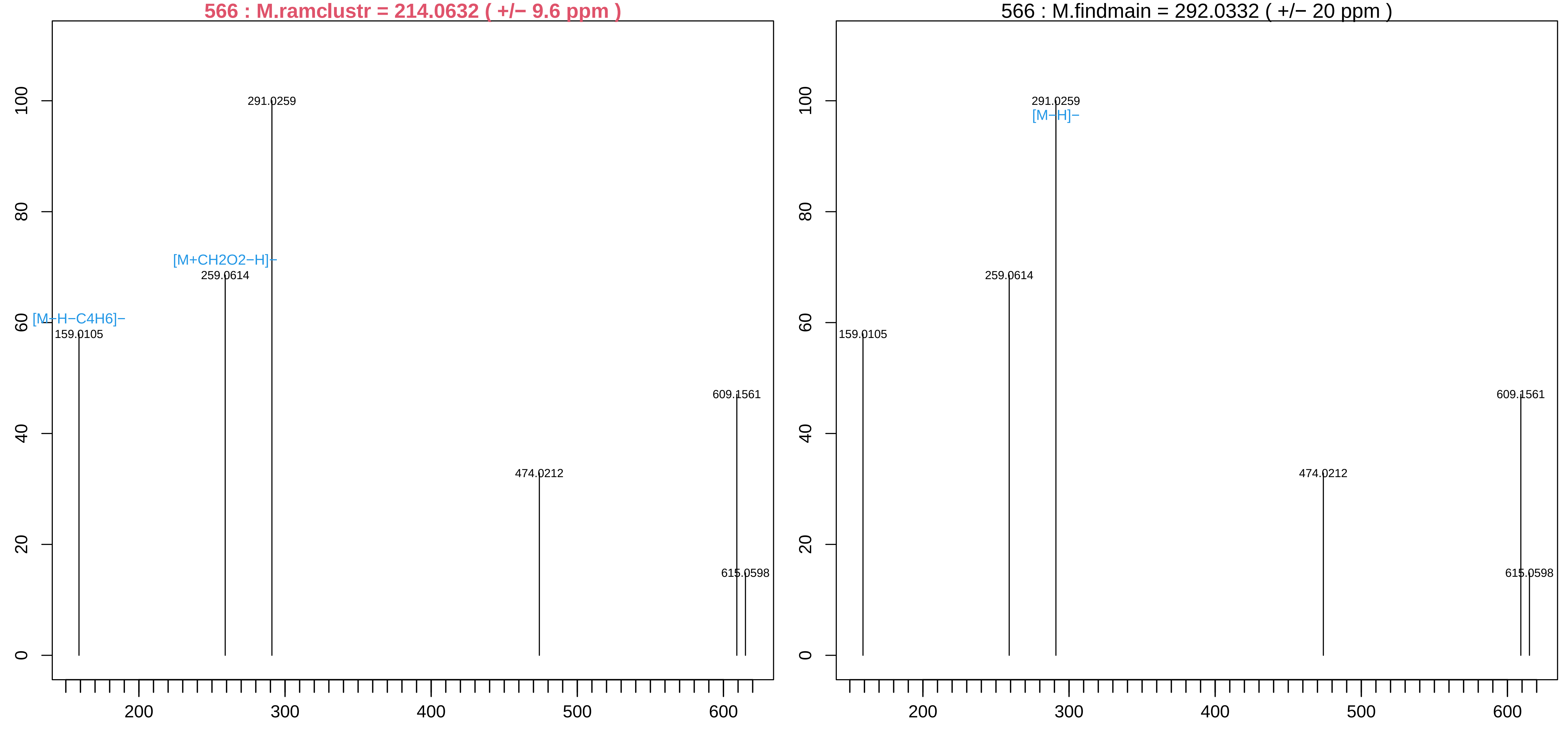
566 : M.ramclustr = 214.0632 (+/− 9.6 ppm)
291.0259
[M+CH2O2−H]−
259.0614
[M−H−C4H6]−
159.0105
609.1561
474.0212
615.0598
566 : M.findmain = 292.0332 (+/− 20 ppm)
291.0259
[M−H]−
259.0614
159.0105
609.1561
474.0212
615.0598
0
20
40
60
80
100
200
300
400
500
600

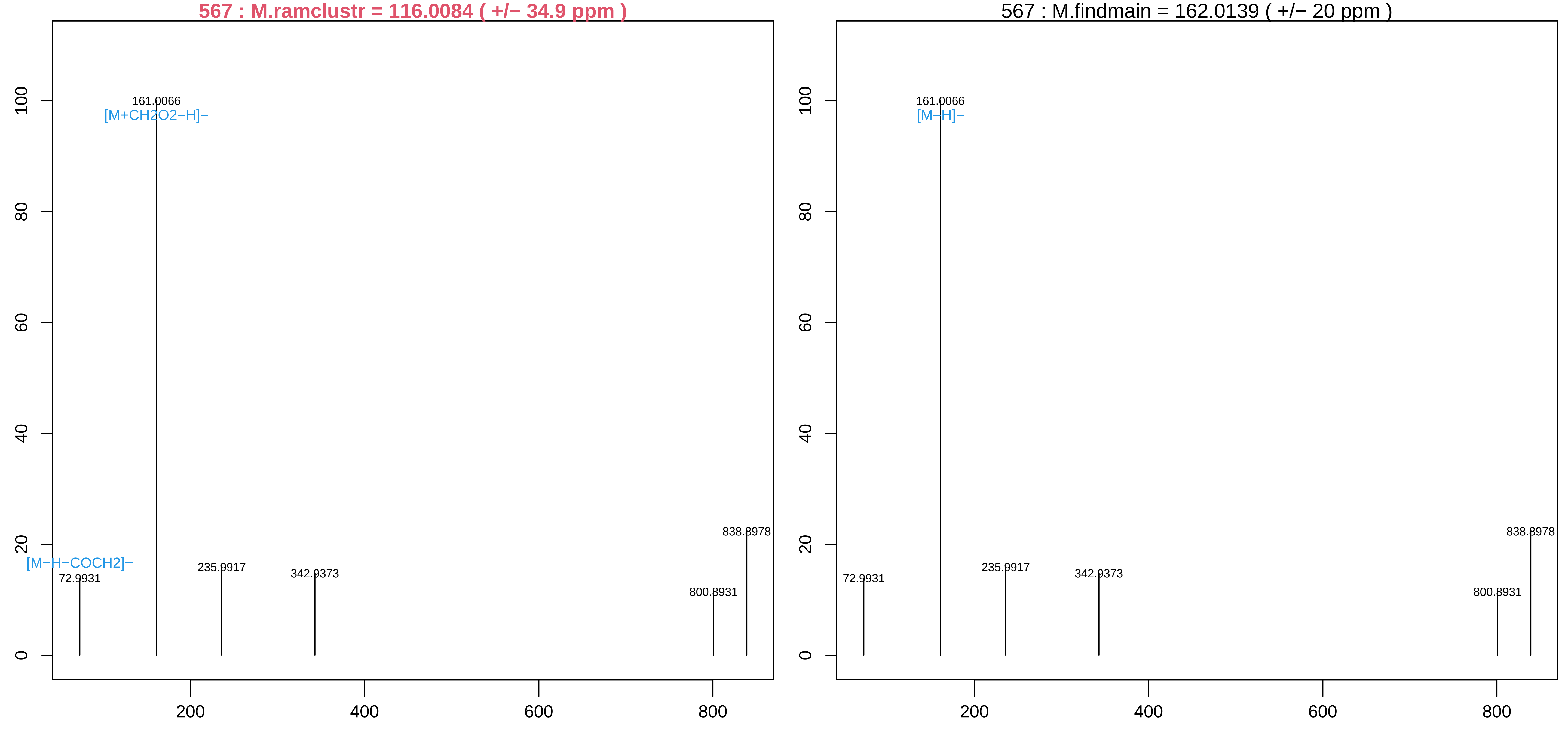
567 : M.ramclustr = 116.0084 (+/− 34.9 ppm)
567 : M.findmain = 162.0139 (+/− 20 ppm)
161.0066
[M+CH2O2−H]−
[M−H]−
[M−H−COCH2]−
72.9931
235.9917
342.9373
800.8931
838.8978
0
20
40
60
80
100
200
400
600
800

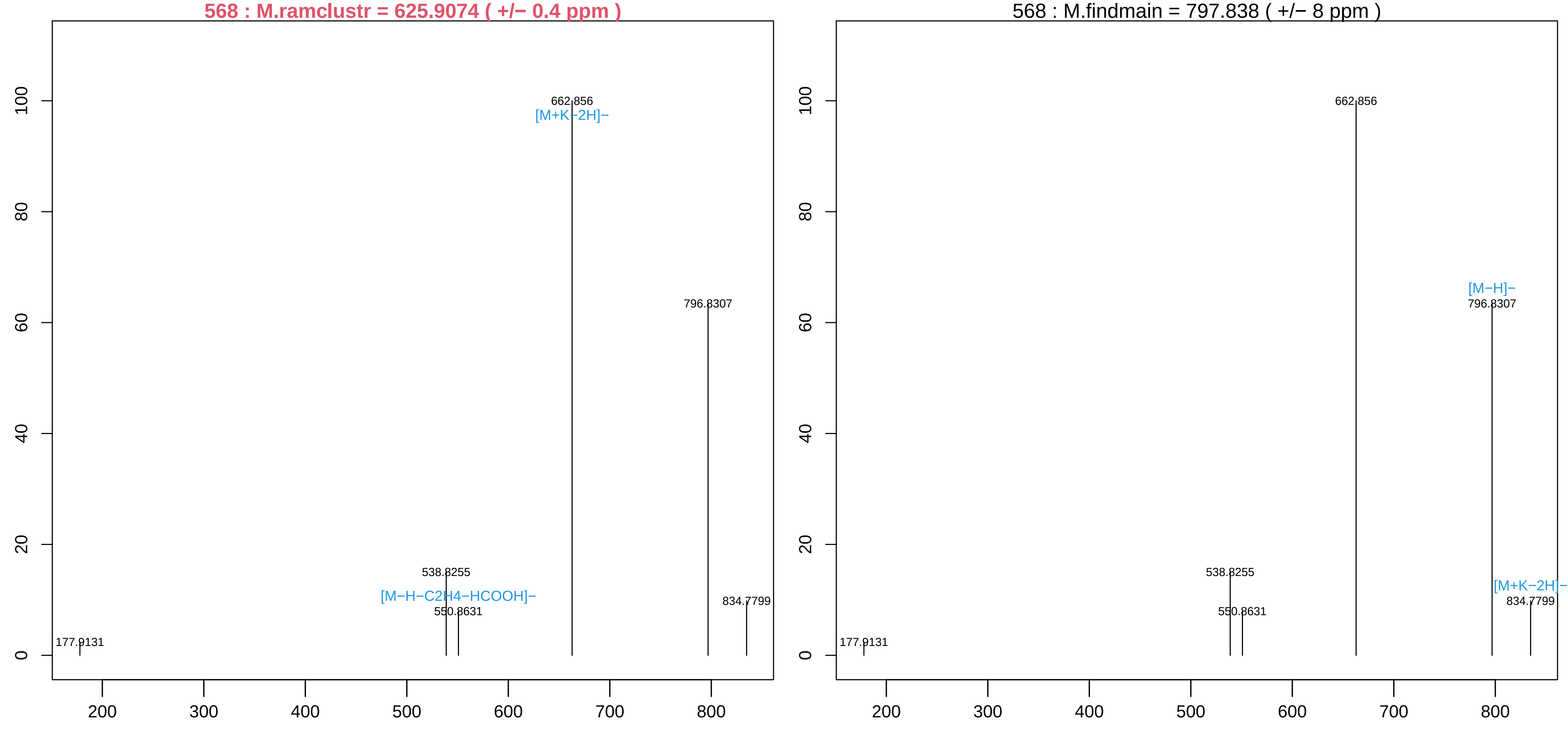
568 : M.ramclustr = 625.9074 (+/− 0.4 ppm)
662.856
[M+K−2H]−
796.8307
538.8255
[M−H−C2H4−HCOOH]−
550.8631
834.7799
177.9131
0
20
40
60
80
100
200
300
400
500
600
700
800
568 : M.findmain = 797.838 (+/− 8 ppm)
662.856
[M−H]−
796.8307
538.8255
[M+K−2H]−
550.8631
834.7799
177.9131

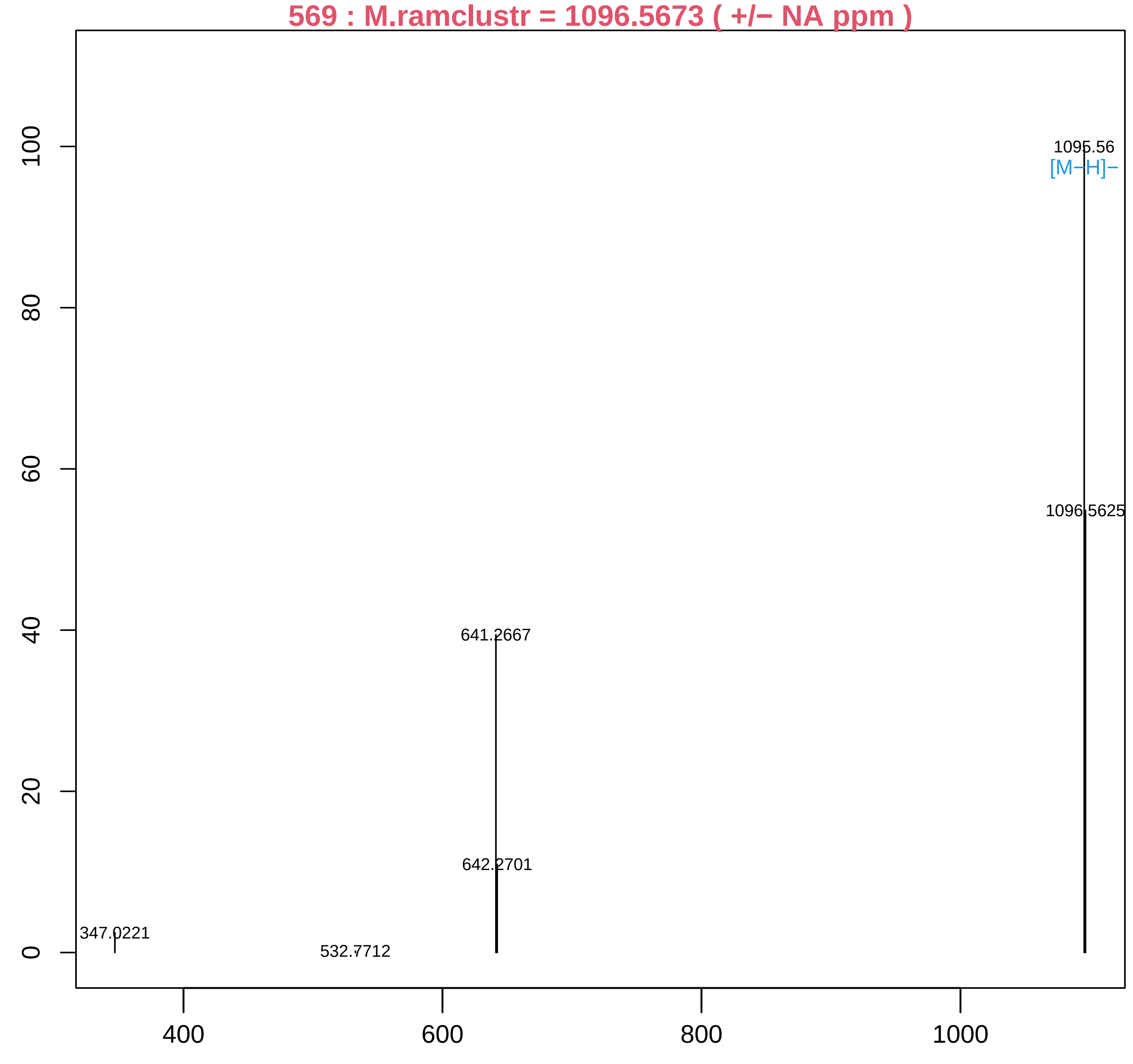
569 : M.ramclustr = 1096.5673 (+/− NA ppm)
1095.56
[M−H]−
1096.5625
641.2667
642.2701
347.0221
532.7712
0
20
40
60
80
100
400
600
800
1000

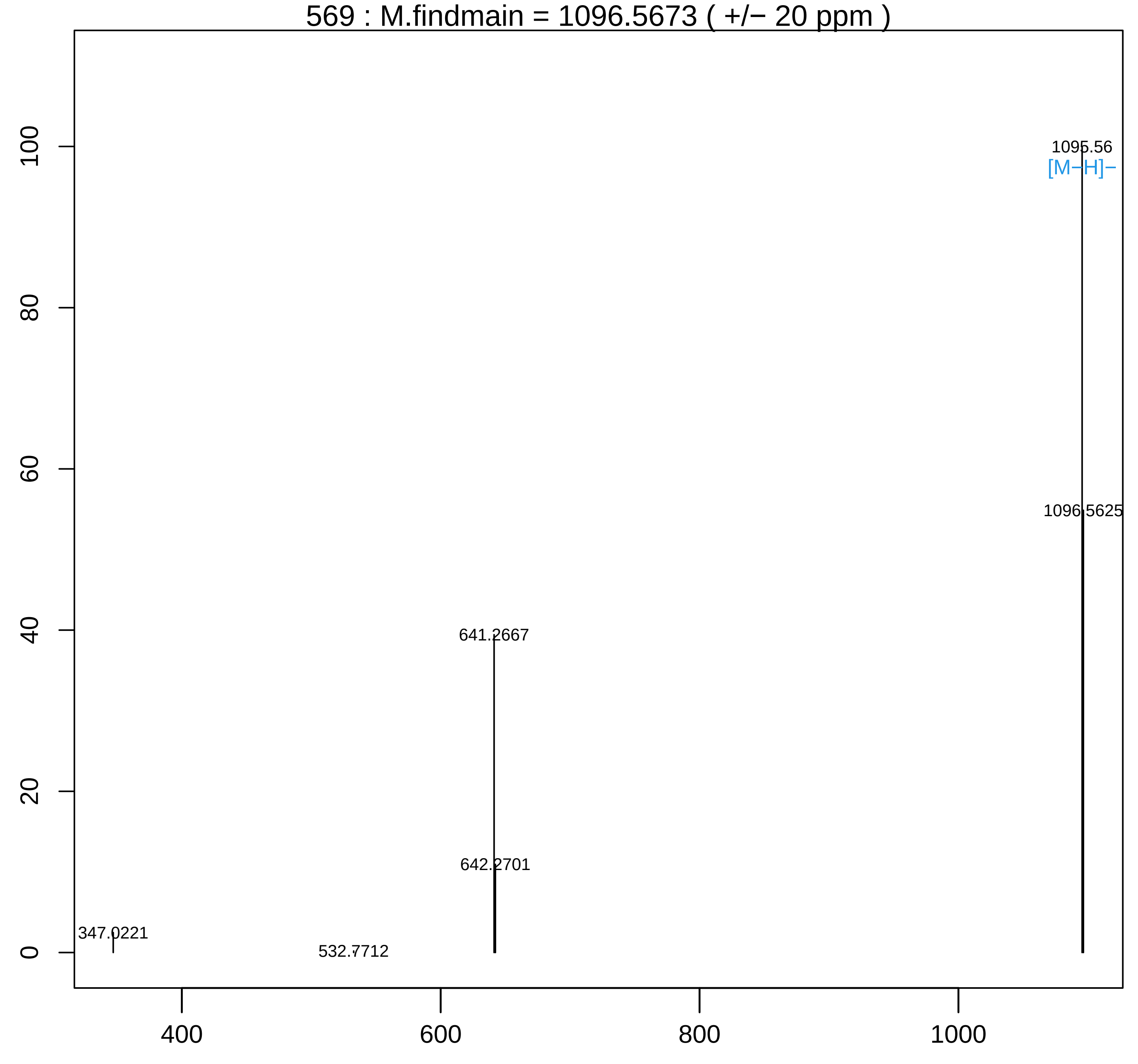
569 : M.findmain = 1096.5673 (+/− 20 ppm)
1095.56
[M−H]−
1096.5625
641.2667
642.2701
347.0221
532.7712
0
20
40
60
80
100
400
600
800
1000

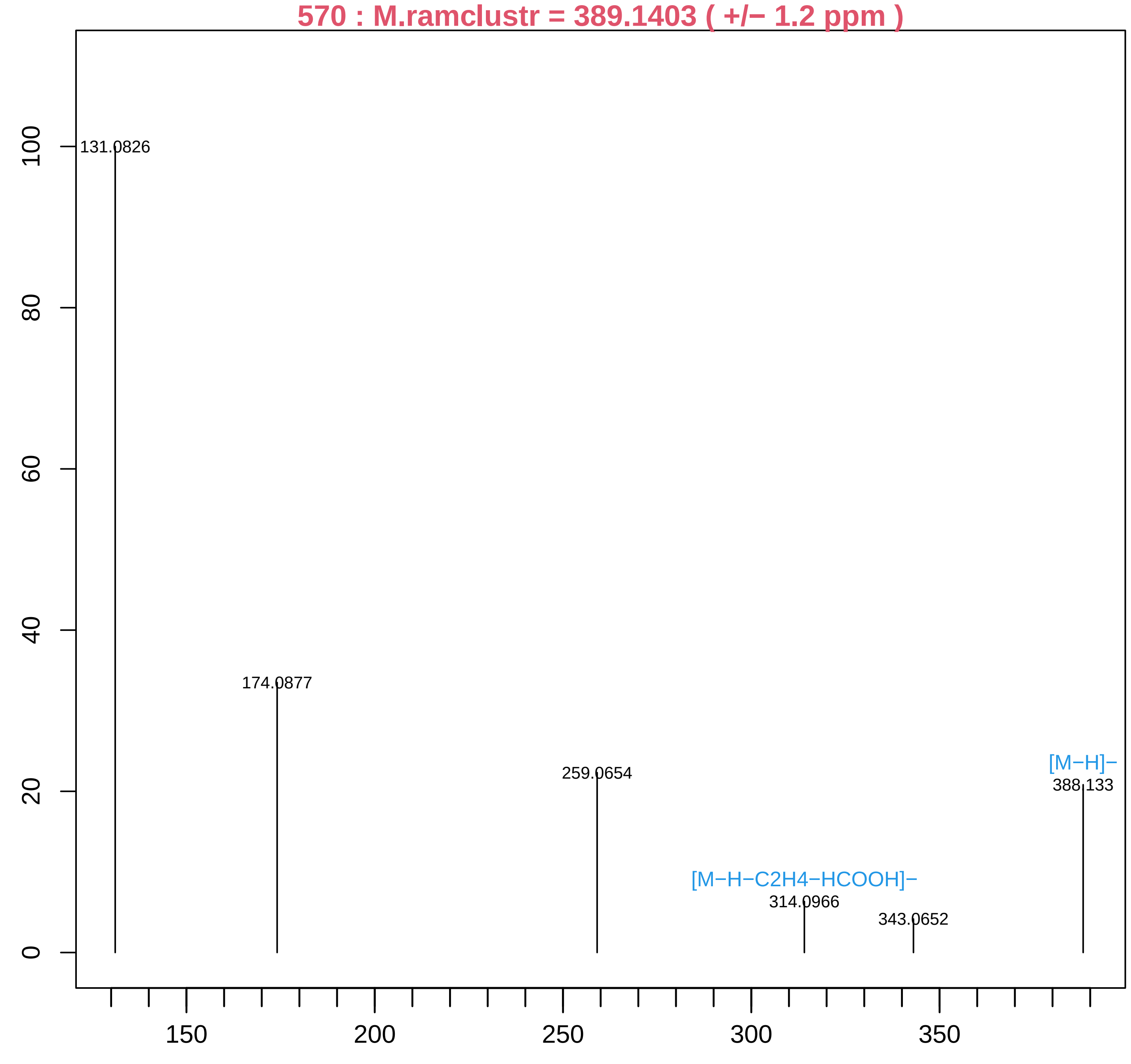

570 : M.ramclustr = 389.1403 (+/− 1.2 ppm)
131.0826
174.0877
259.0654
[M−H−C2H4−HCOOH]−
314.0966
343.0652
[M−H]−
388.133
0
20
40
60
80
100
150
200
250
300
350

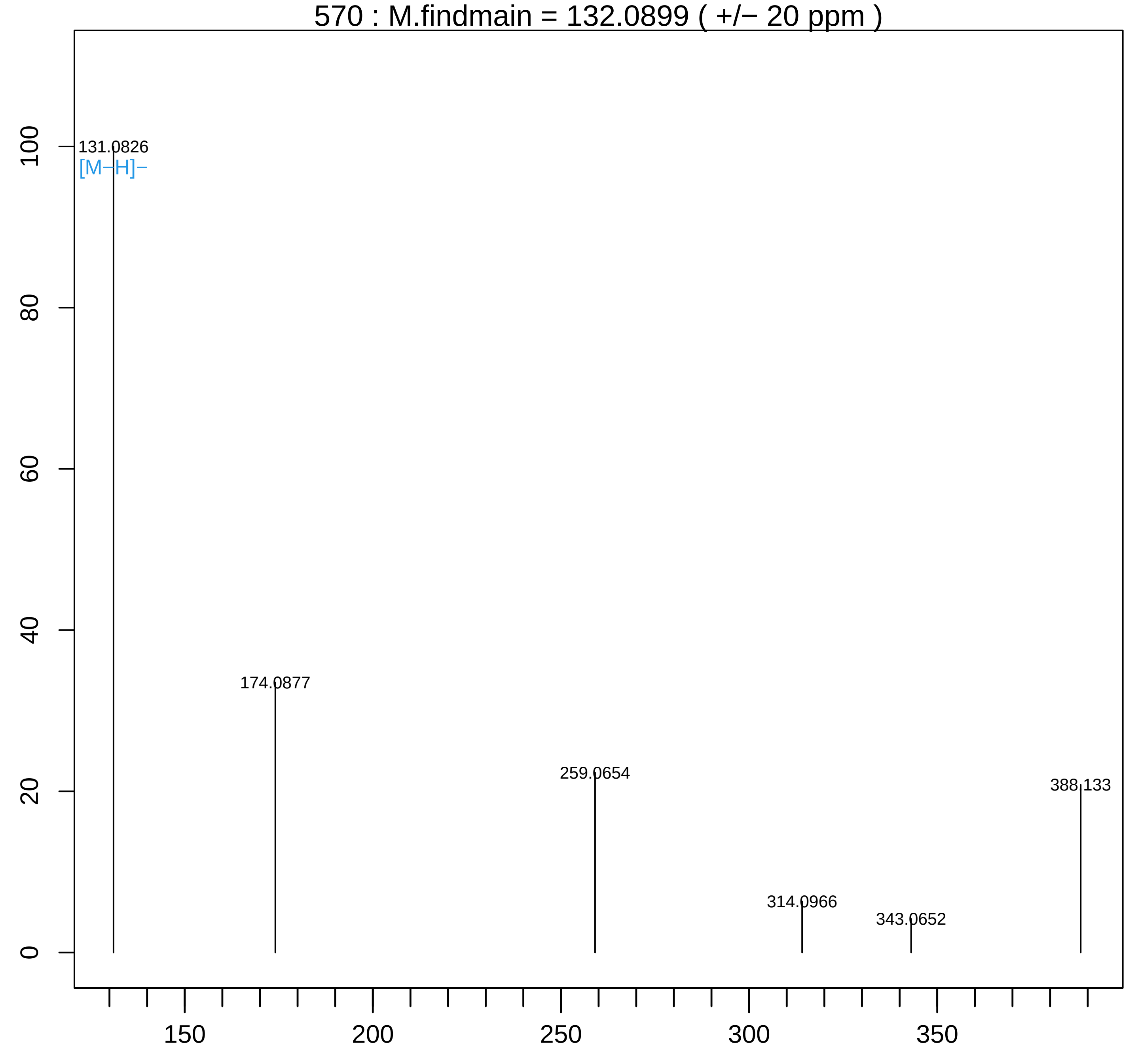

570 : M.findmain = 132.0899 (+/− 20 ppm)
131.0826
[M−H]−
174.0877
259.0654
314.0966
343.0652
388.133
0
20
40
60
80
100
150
200
250
300
350

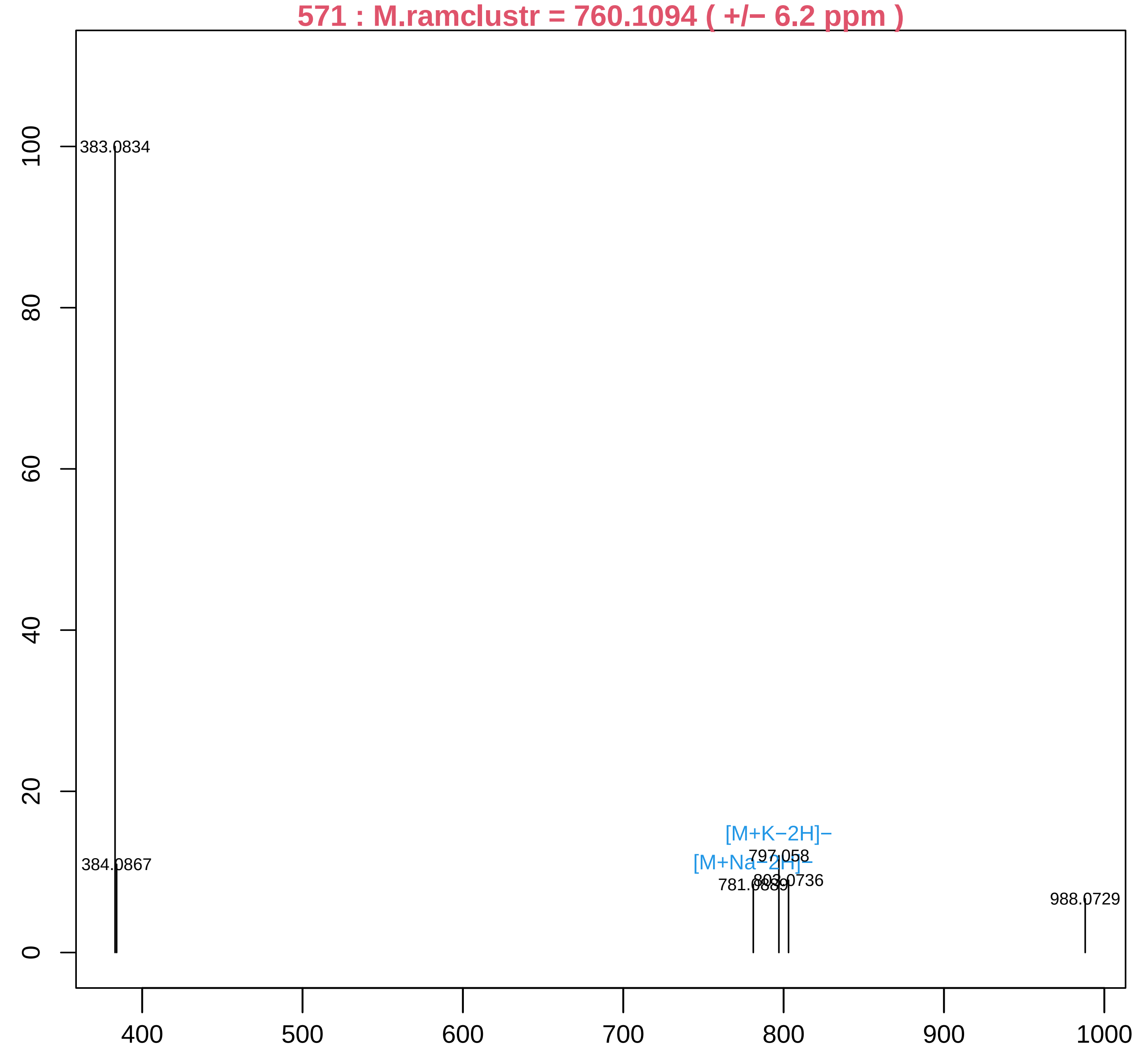
571 : M.ramclustr = 760.1094 (+/− 6.2 ppm)
383.0834
384.0867
[M+K−2H]−
797.058
[M+Na−2H]−
781.0889
803.0736
988.0729
0
20
40
60
80
100
400
500
600
700
800
900
1000

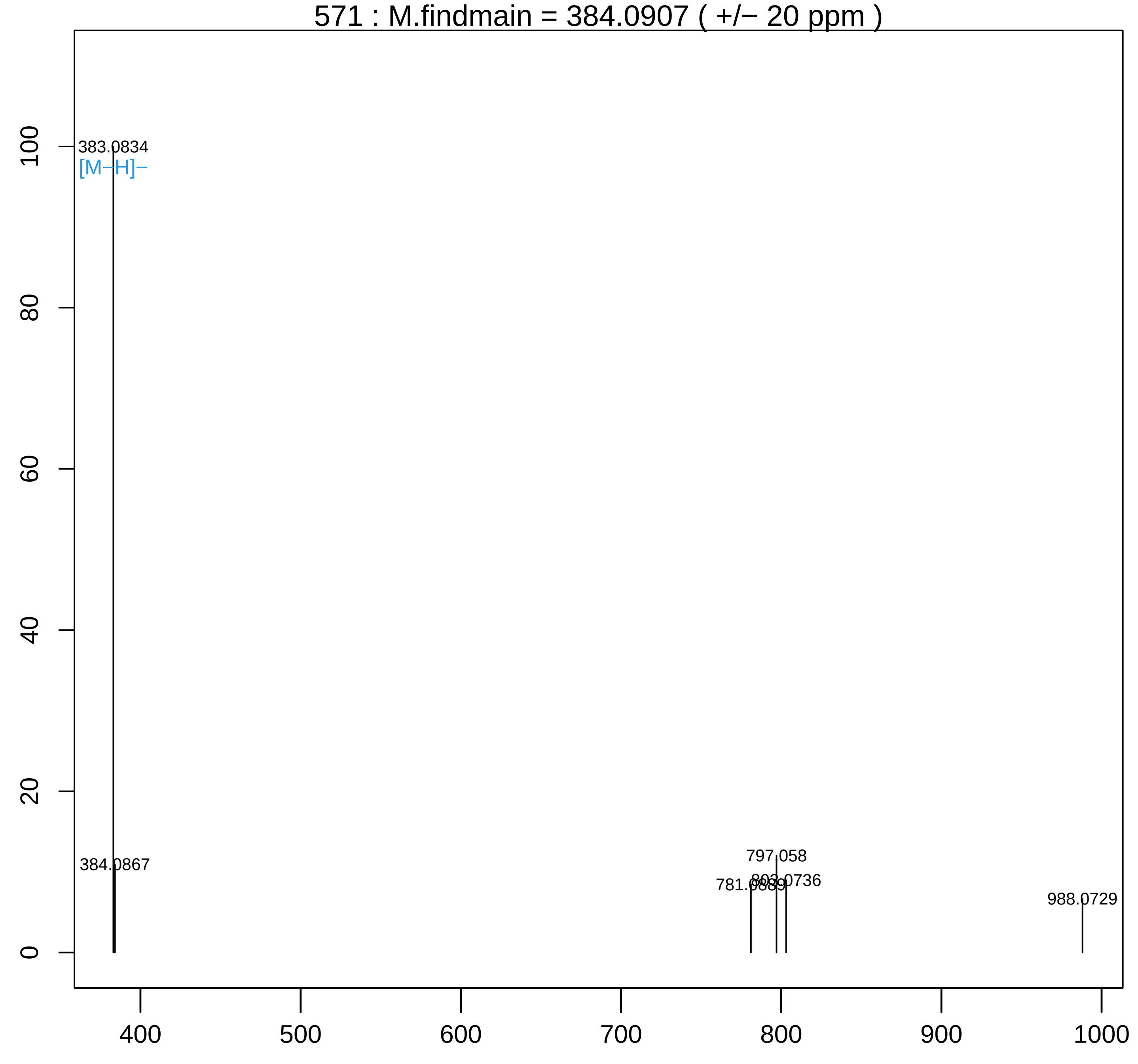
571 : M.findmain = 384.0907 (+/− 20 ppm)
383.0834
[M−H]−
384.0867
797.058
781.0889
803.0736
988.0729
0
20
40
60
80
100
400
500
600
700
800
900
1000

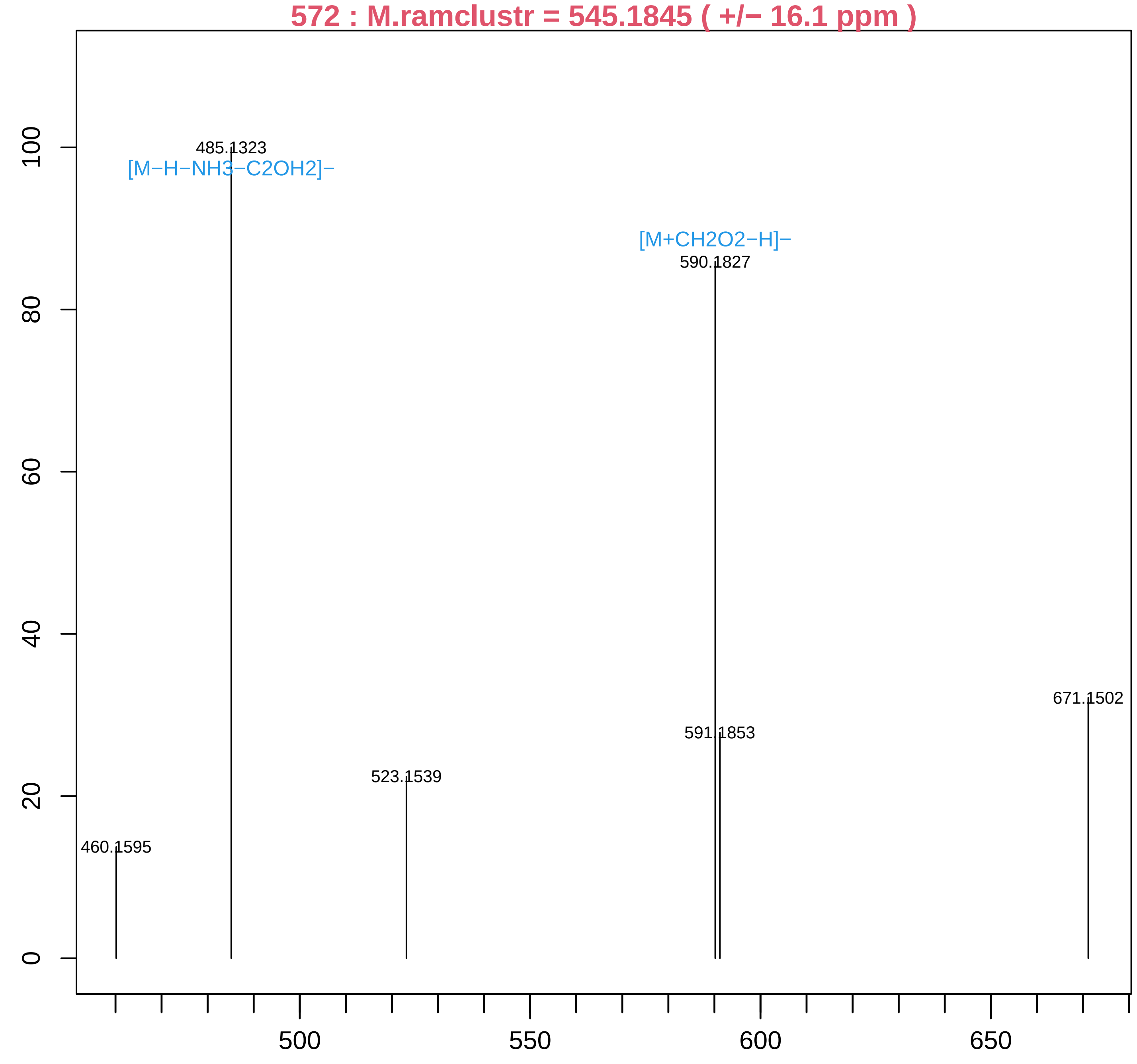

572 : M.ramclustr = 545.1845 (+/− 16.1 ppm)
485.1323
[M−H−NH3−C2OH2]−
[M+CH2O2−H]−
590.1827
591.1853
523.1539
460.1595
671.1502
0
20
40
60
80
100
500
550
600
650

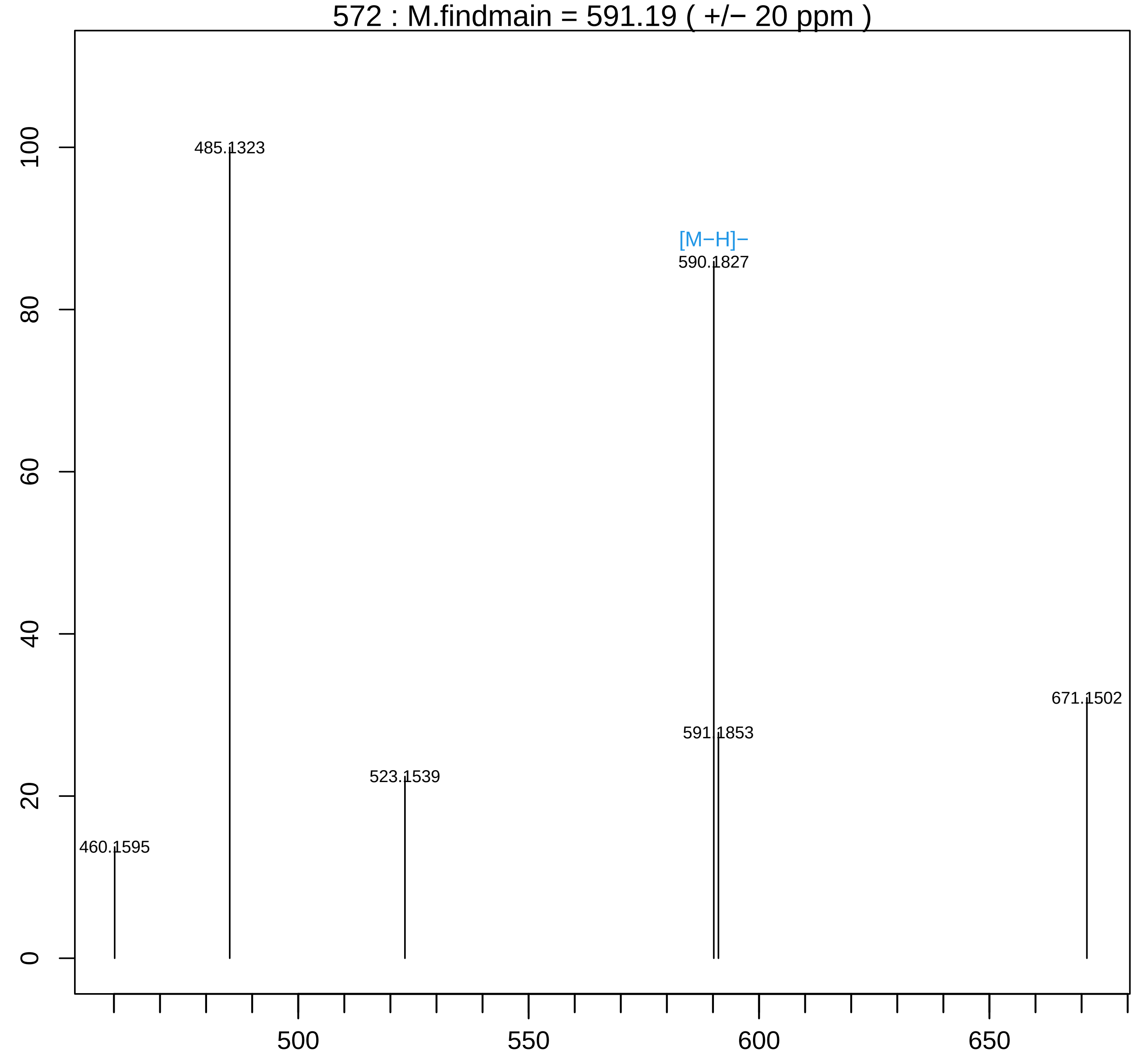

572 : M.findmain = 591.19 (+/− 20 ppm)
485.1323
[M−H]−
590.1827
591.1853
523.1539
460.1595
671.1502
0
20
40
60
80
100
500
550
600
650

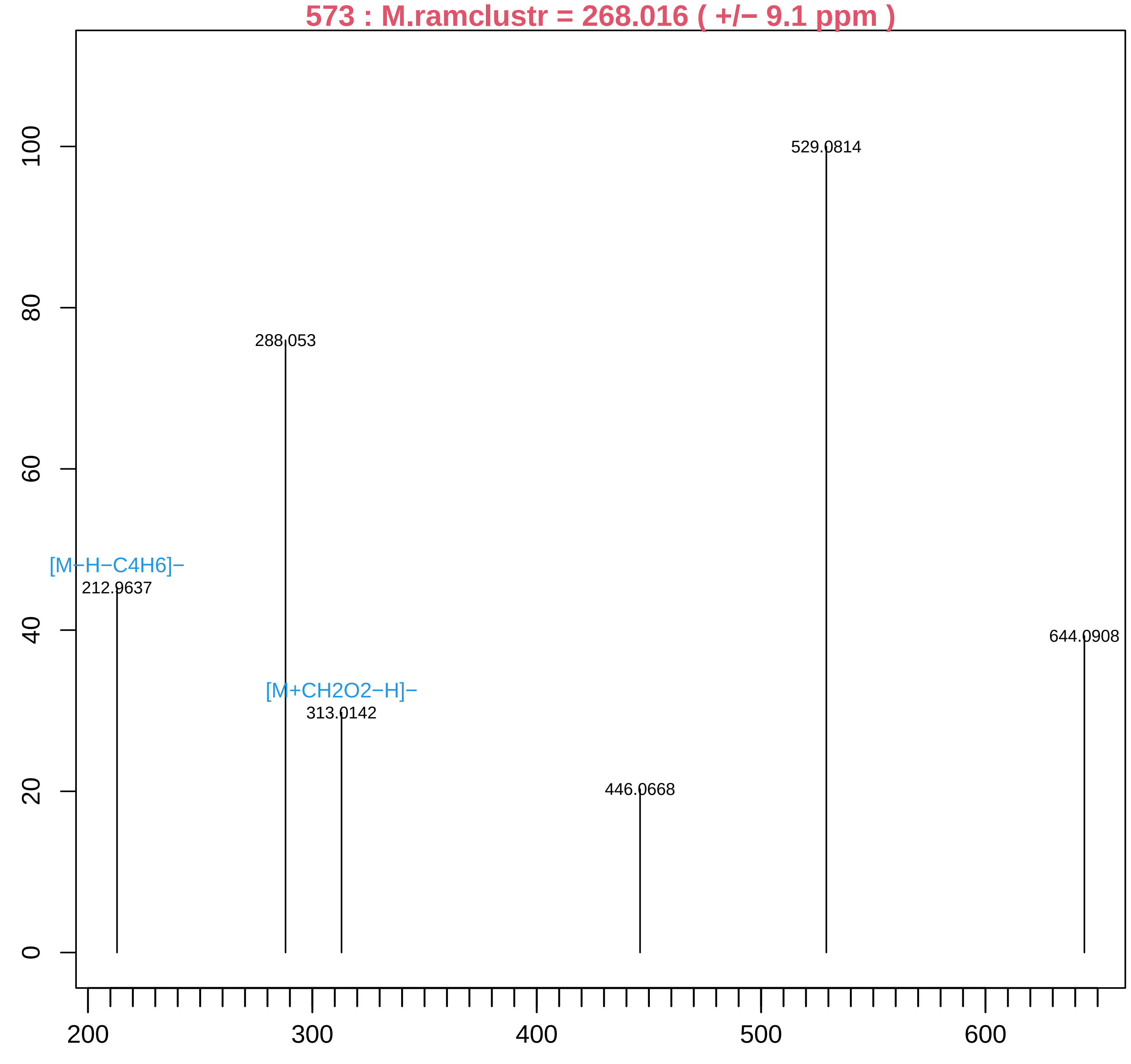
573 : M.ramclustr = 268.016 (+/− 9.1 ppm)
529.0814
288.053
[M−H−C4H6]−
212.9637
644.0908
[M+CH2O2−H]−
313.0142
446.0668
0
20
40
60
80
100
200
300
400
500
600

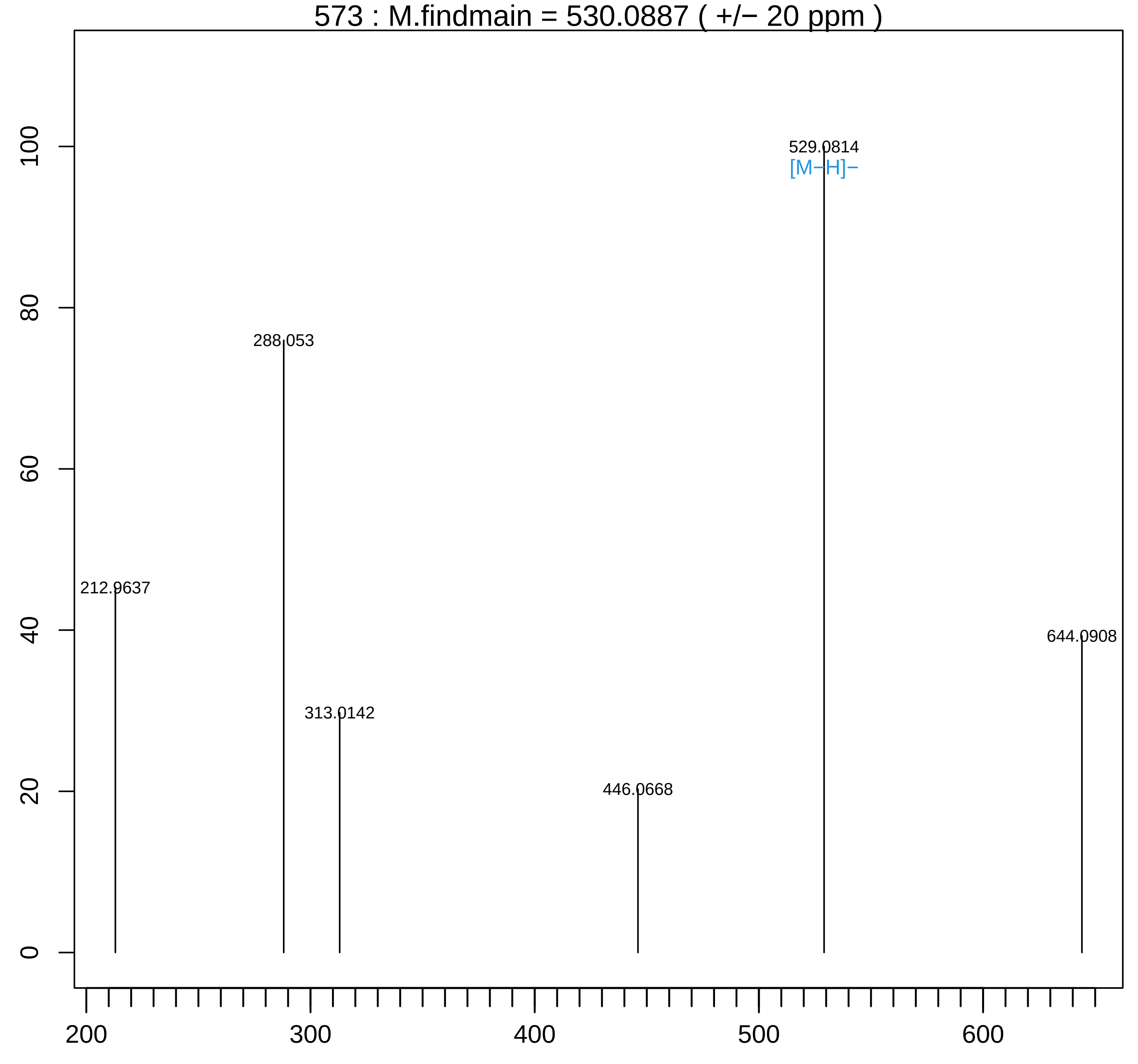
573 : M.findmain = 530.0887 (+/− 20 ppm)
529.0814
[M−H]−
288.053
212.9637
644.0908
313.0142
446.0668
0
20
40
60
80
100
200
300
400
500
600

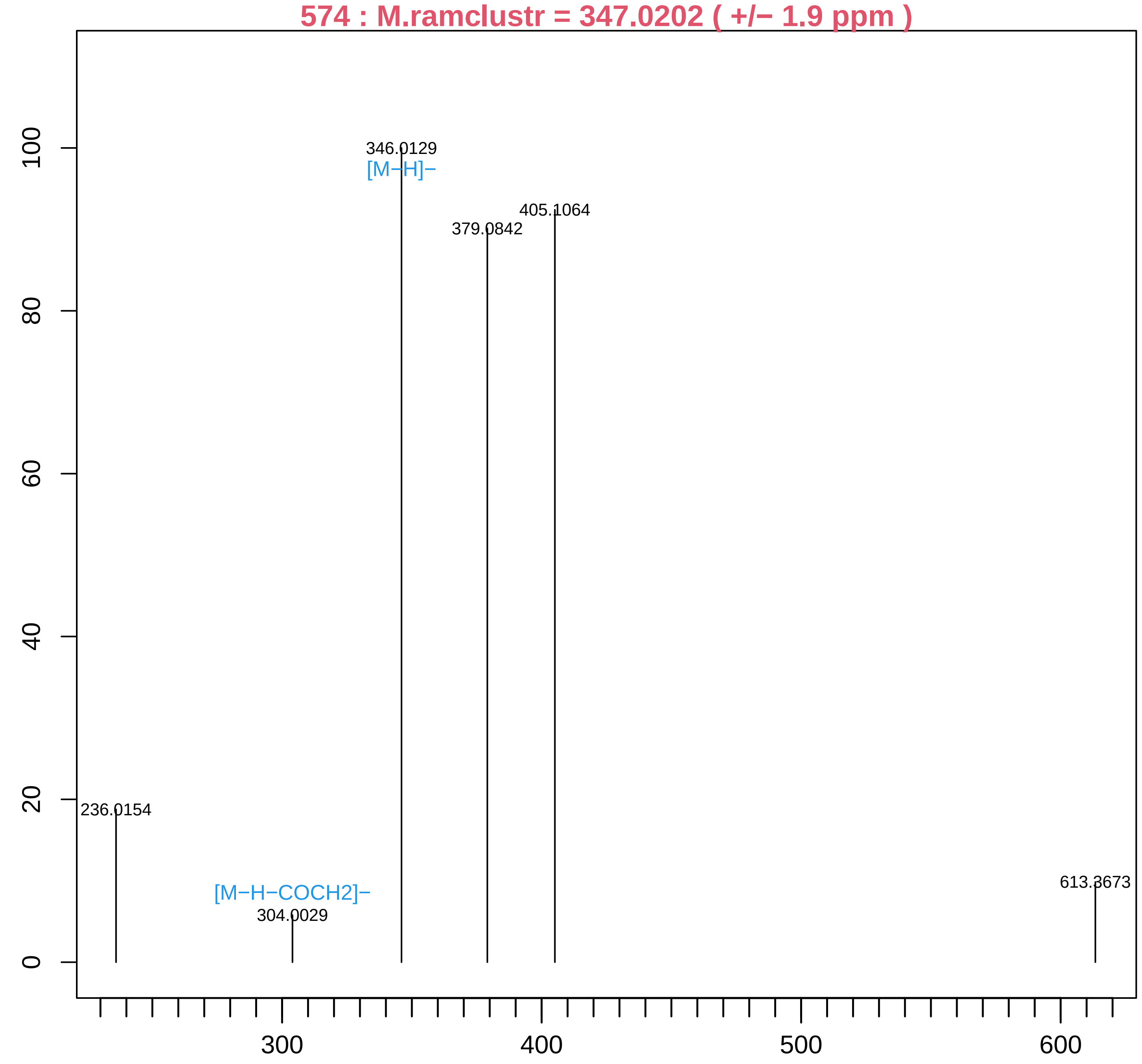
574 : M.ramclustr = 347.0202 (+/− 1.9 ppm)
346.0129
[M−H]−
379.0842
405.1064
236.0154
[M−H−COCH2]−
304.0029
613.3673
0
20
40
60
80
100
300
400
500
600

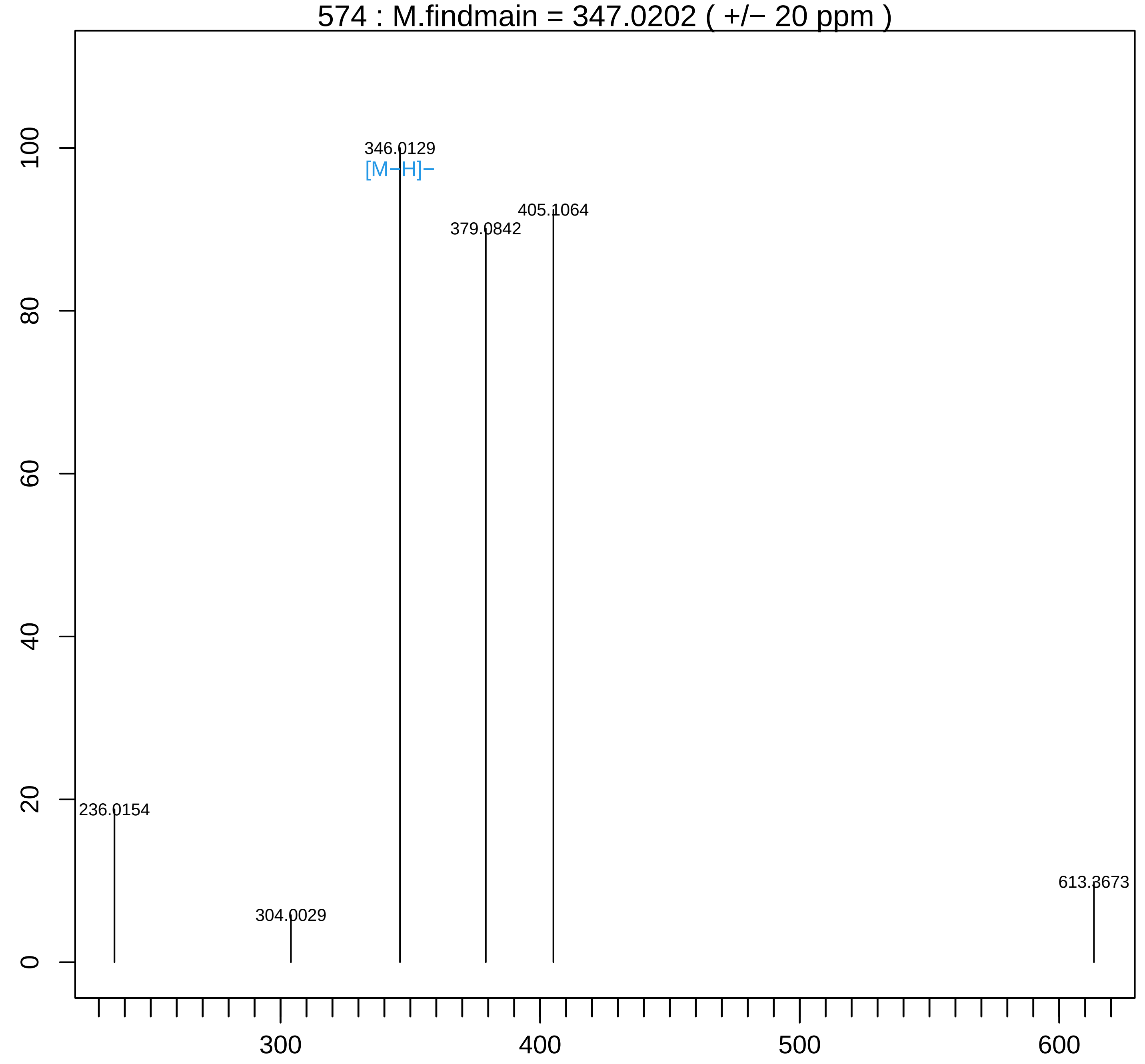
574 : M.findmain = 347.0202 (+/− 20 ppm)
346.0129
[M−H]−
379.0842
405.1064
236.0154
304.0029
613.3673
0
20
40
60
80
100
300
400
500
600

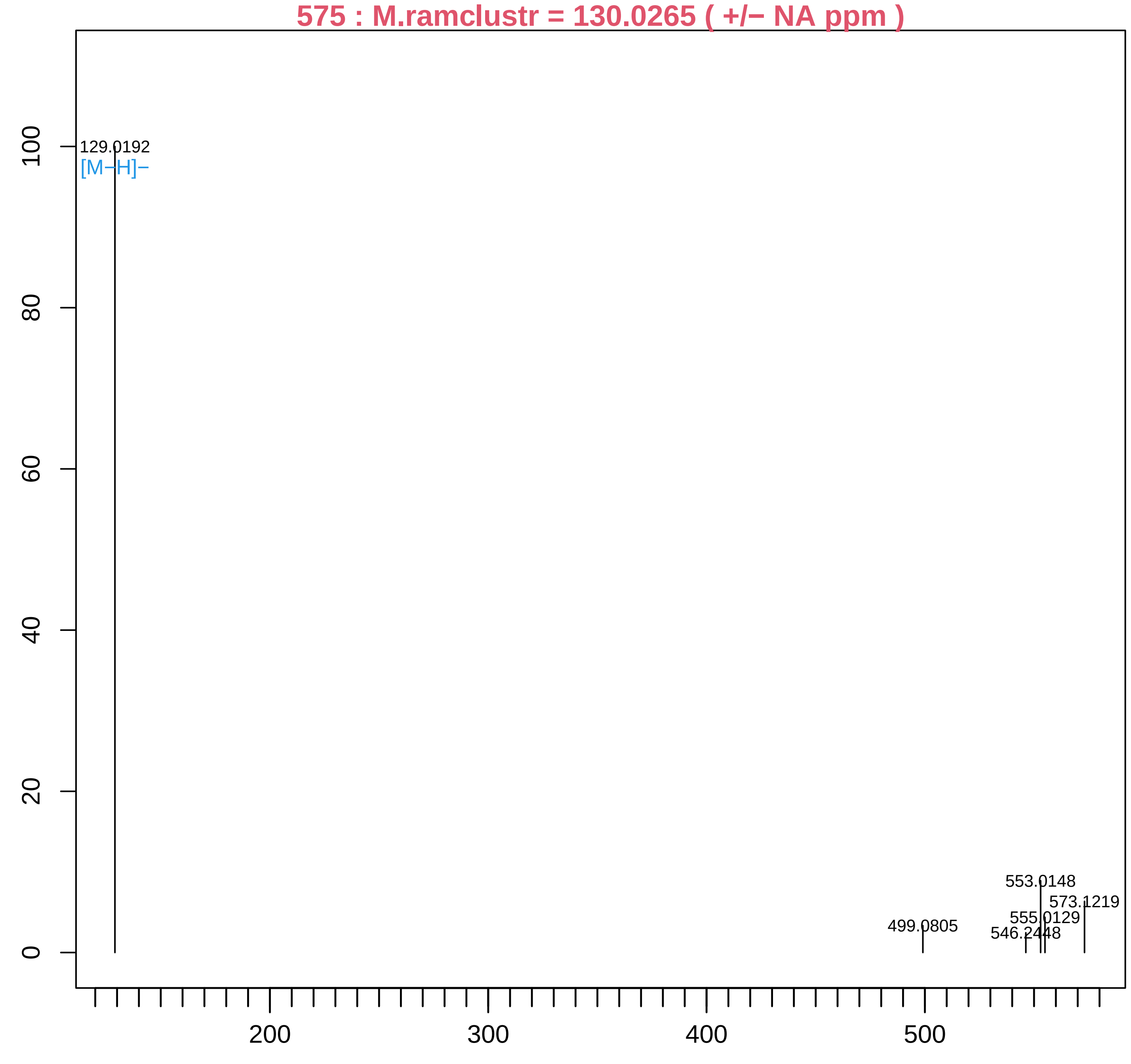
575 : M.ramclustr = 130.0265 (+/− NA ppm)
129.0192
[M−H]−
499.0805
546.2448
553.0148
555.0129
573.1219
0
20
40
60
80
100
200
300
400
500

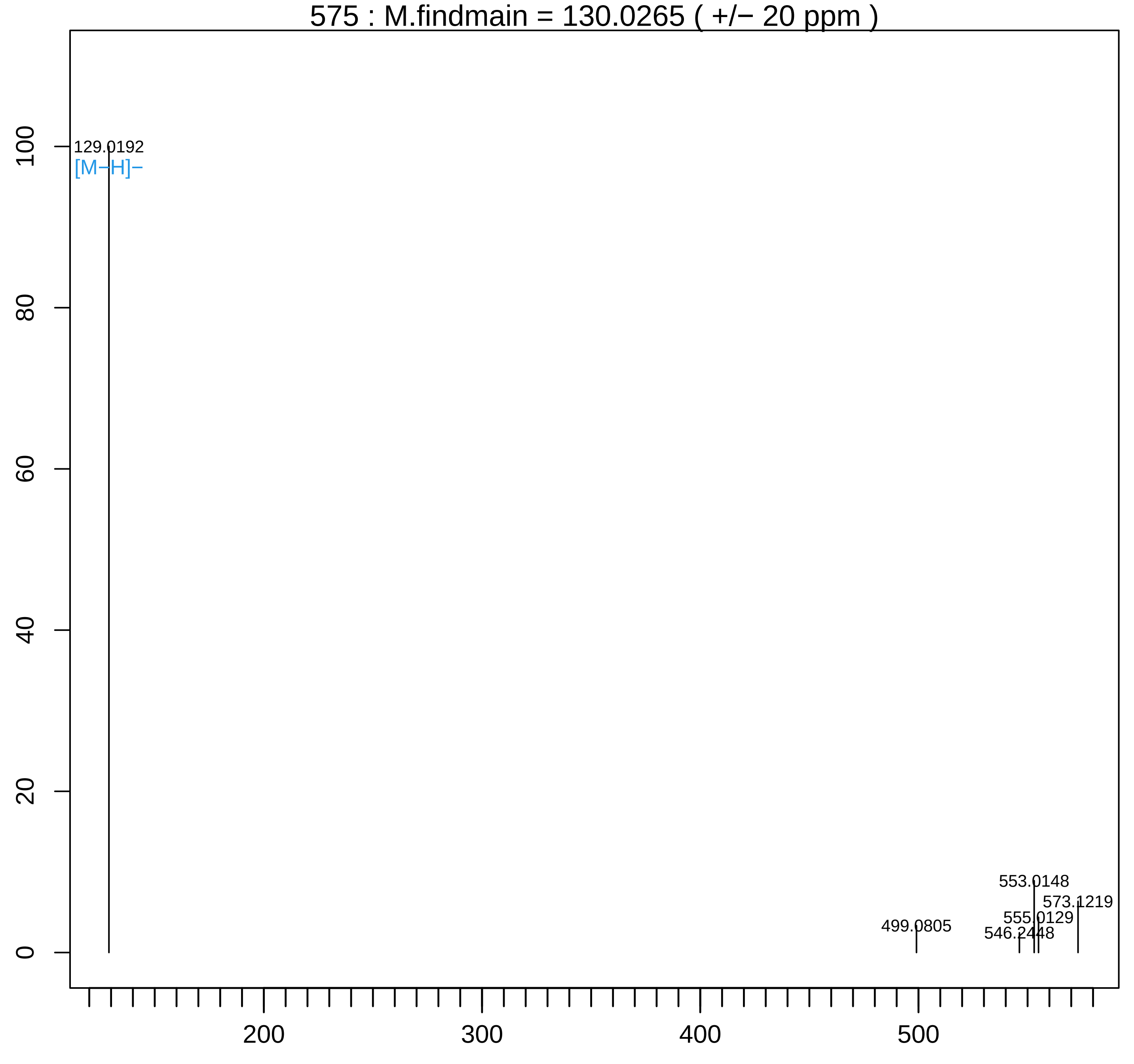
575 : M.findmain = 130.0265 (+/− 20 ppm)
129.0192
[M−H]−
499.0805
546.2448
553.0148
555.0129
573.1219
0
20
40
60
80
100
200
300
400
500

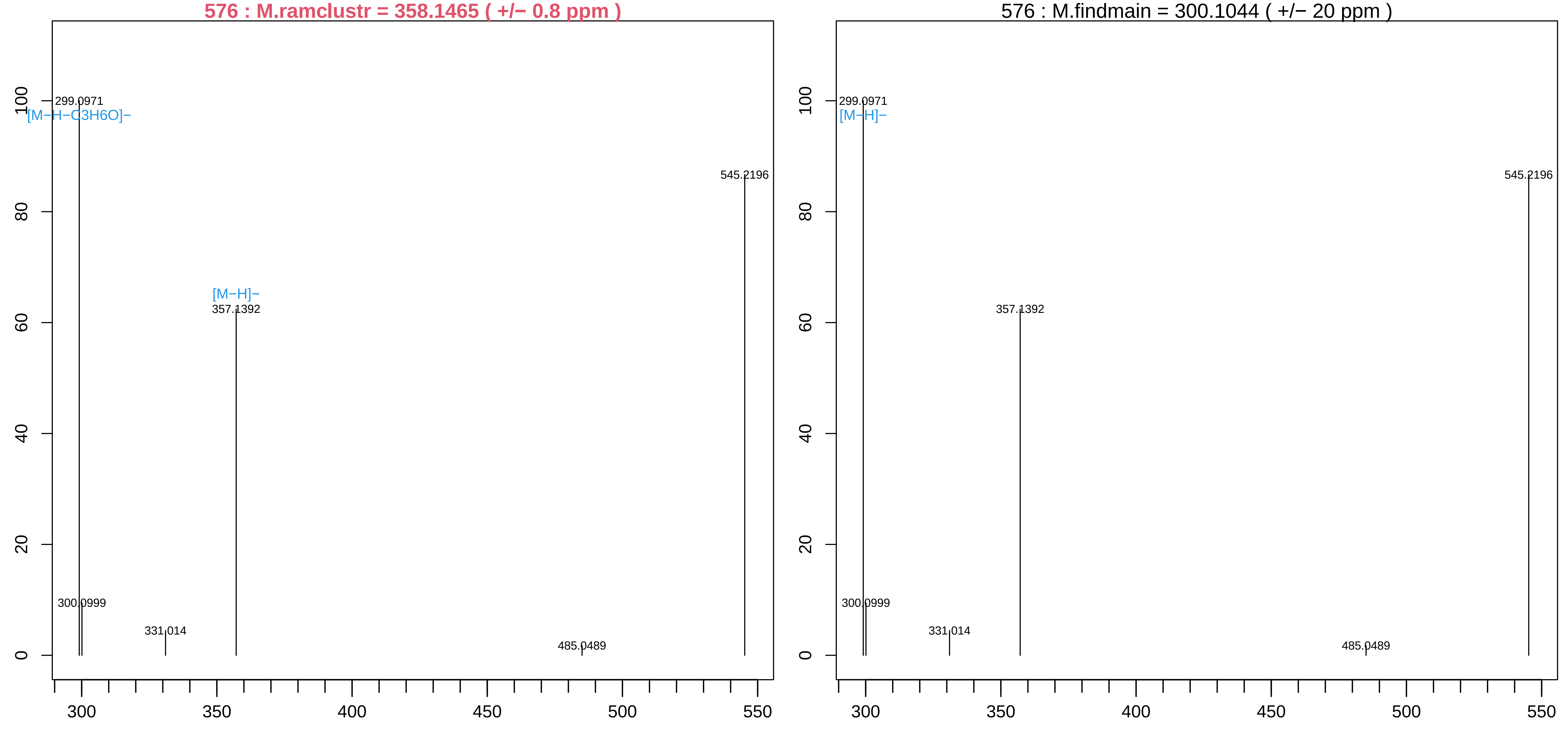
576 : M.ramclustr = 358.1465 (+/− 0.8 ppm)
299.0971
[M−H−C3H6O]−
[M−H]−
357.1392
545.2196
300.0999
331.014
485.0489
576 : M.findmain = 300.1044 (+/− 20 ppm)
299.0971
[M−H]−
357.1392
545.2196
300.0999
331.014
485.0489

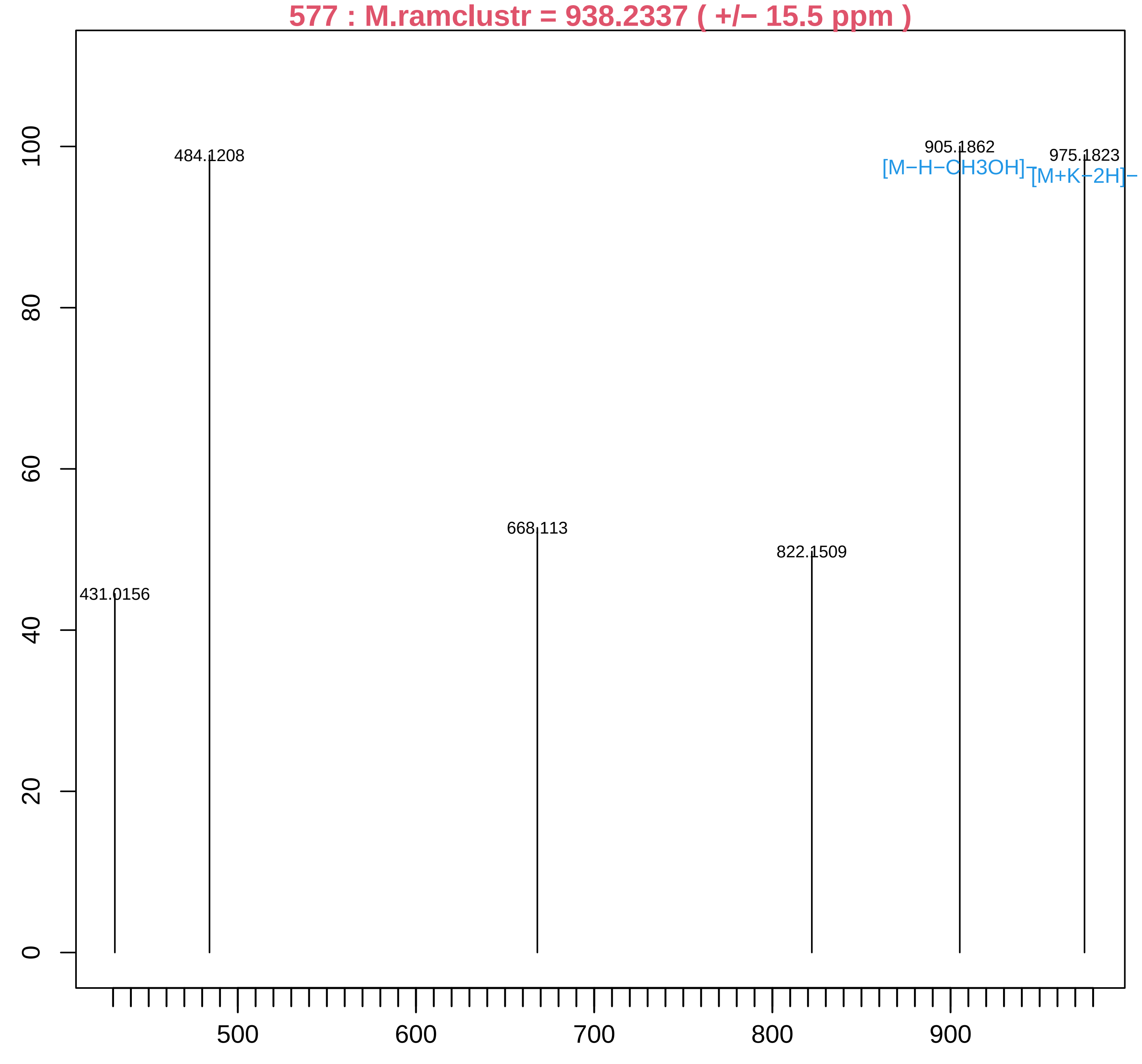

577 : M.ramclustr = 938.2337 (+/− 15.5 ppm)
484.1208
905.1862
[M−H−CH3OH]−
975.1823
[M+K−2H]−
668.113
822.1509
431.0156
0
20
40
60
80
100
500
600
700
800
900

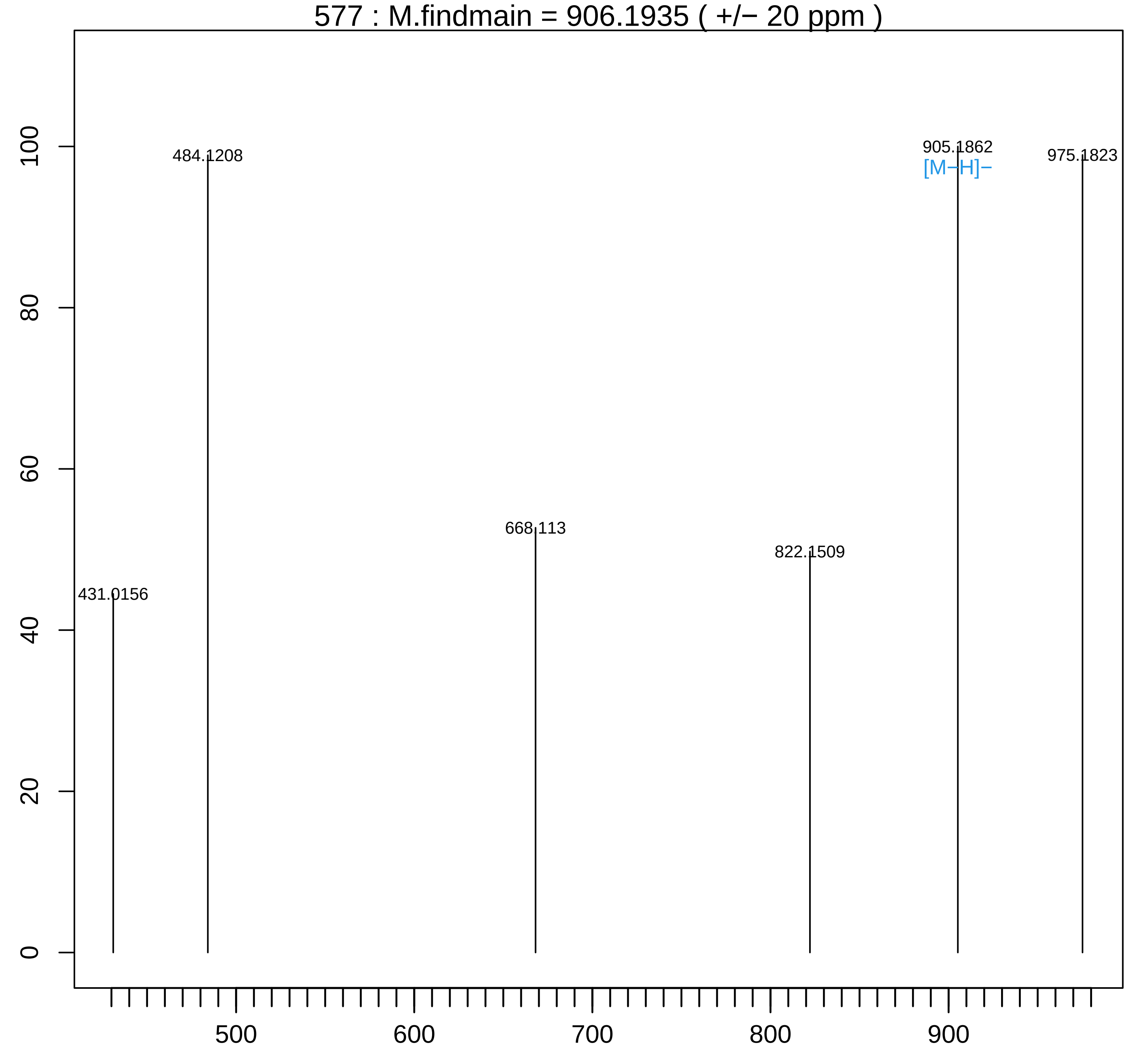

577 : M.findmain = 906.1935 (+/− 20 ppm)
484.1208
905.1862
[M−H]−
975.1823
668.113
822.1509
431.0156
0
20
40
60
80
100
500
600
700
800
900

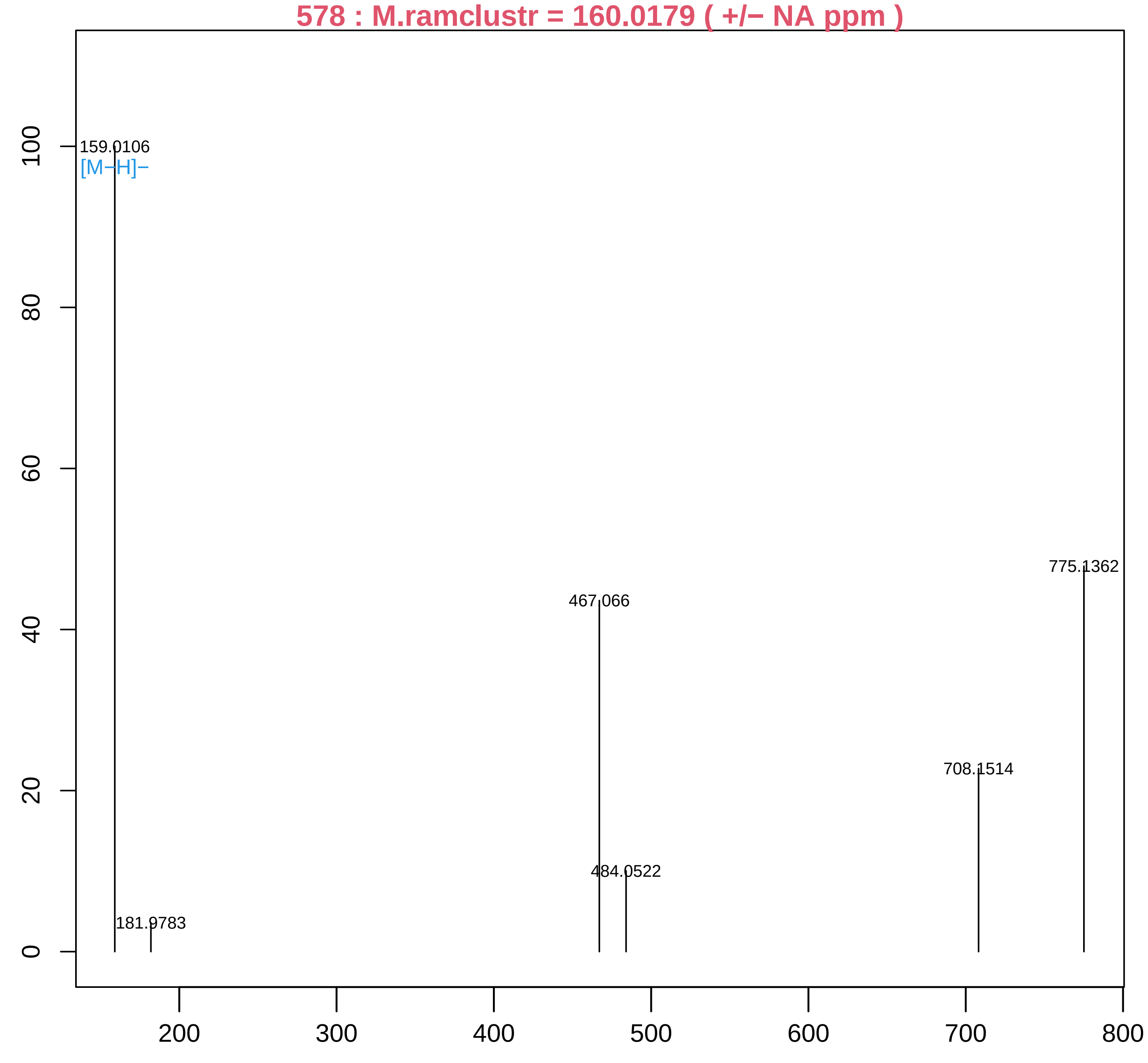
578 : M.ramclustr = 160.0179 (+/− NA ppm)
159.0106
[M−H]−
181.9783
467.066
484.0522
708.1514
775.1362
0
20
40
60
80
100
200
300
400
500
600
700
800

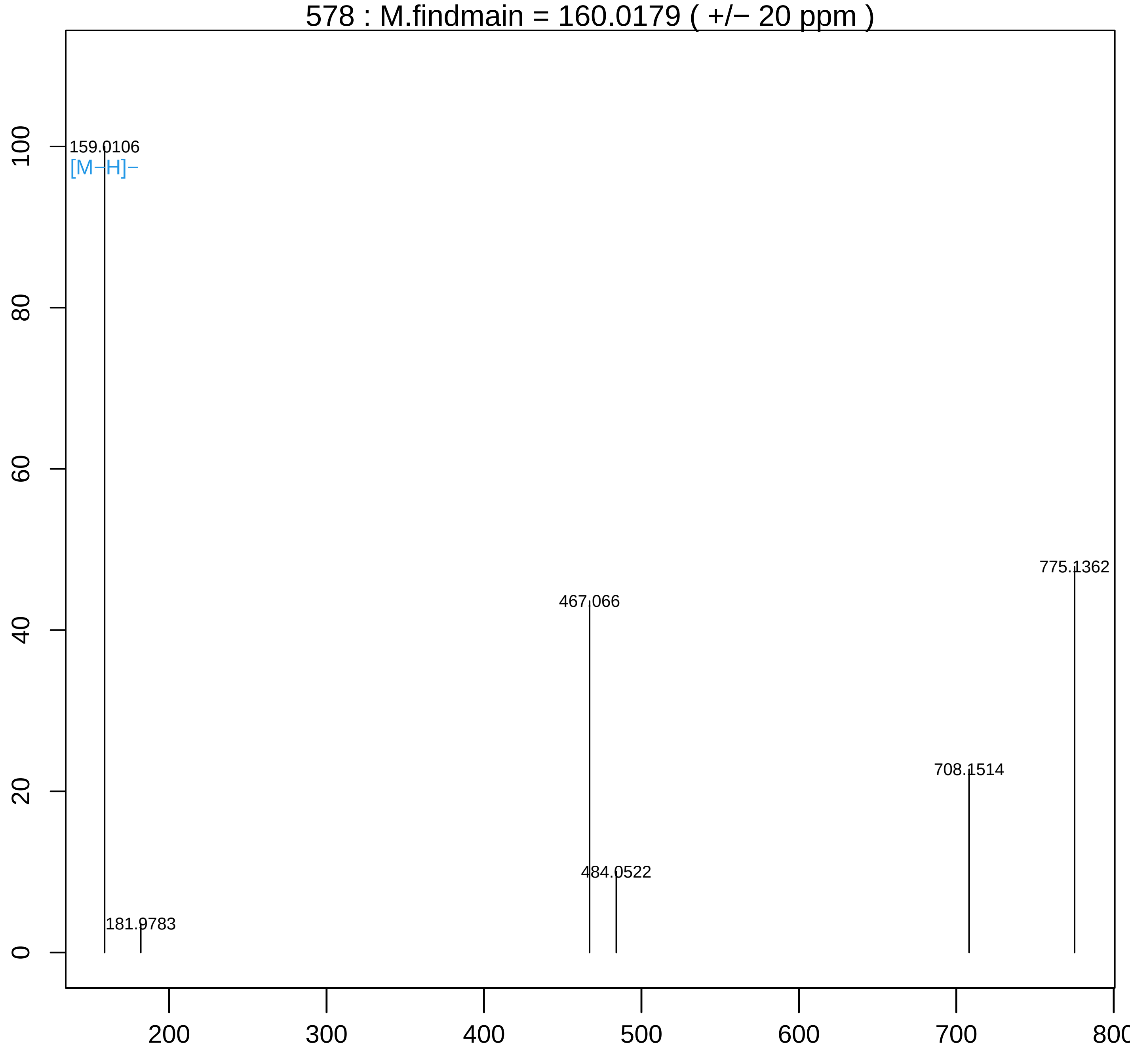
578 : M.findmain = 160.0179 (+/− 20 ppm)
159.0106
[M−H]−
181.9783
467.066
484.0522
708.1514
775.1362
0
20
40
60
80
100
200
300
400
500
600
700
800

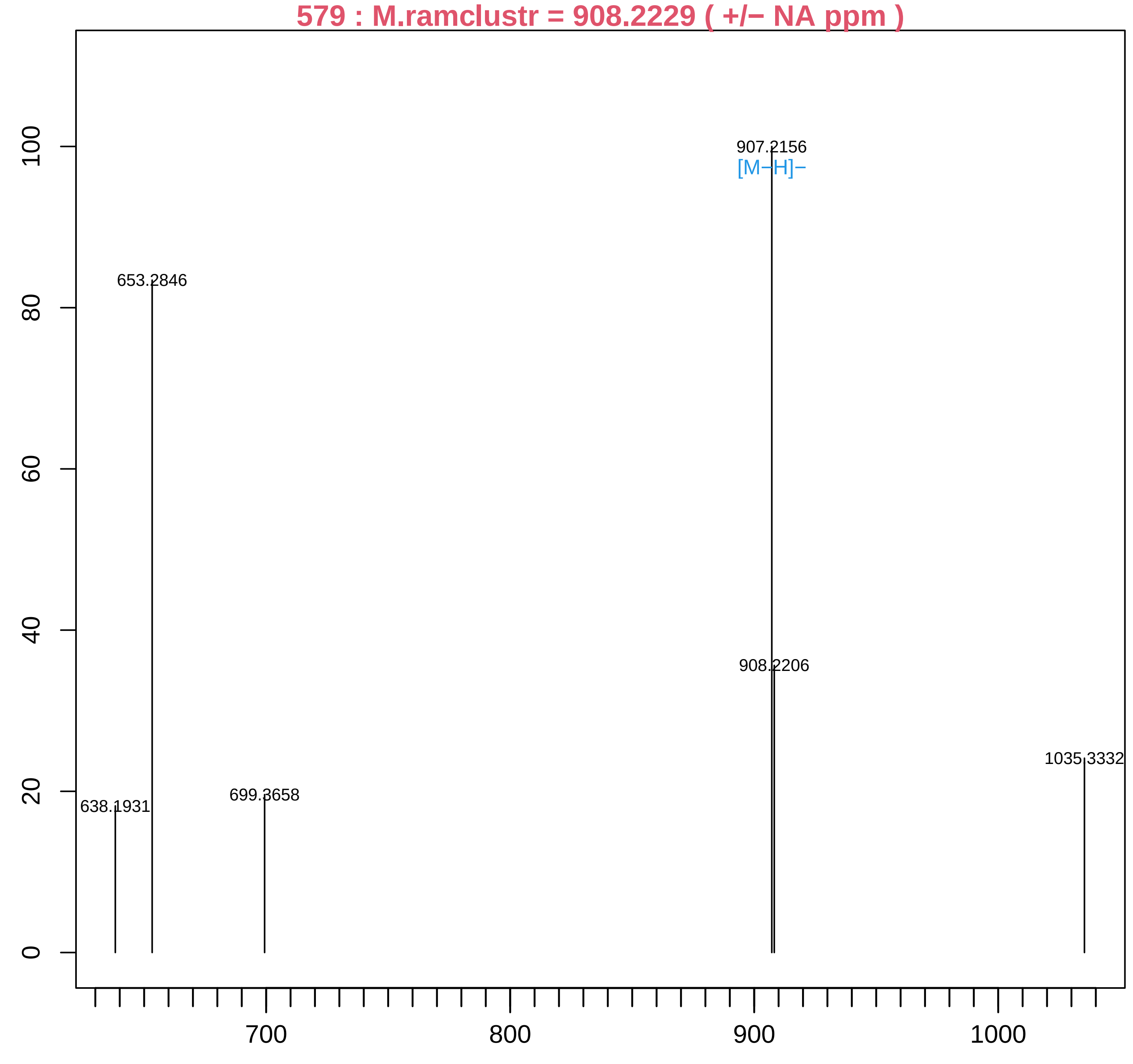
579 : M.ramclustr = 908.2229 (+/− NA ppm)
907.2156
[M−H]−
653.2846
908.2206
1035.3332
699.3658
638.1931
0
20
40
60
80
100
700
800
900
1000

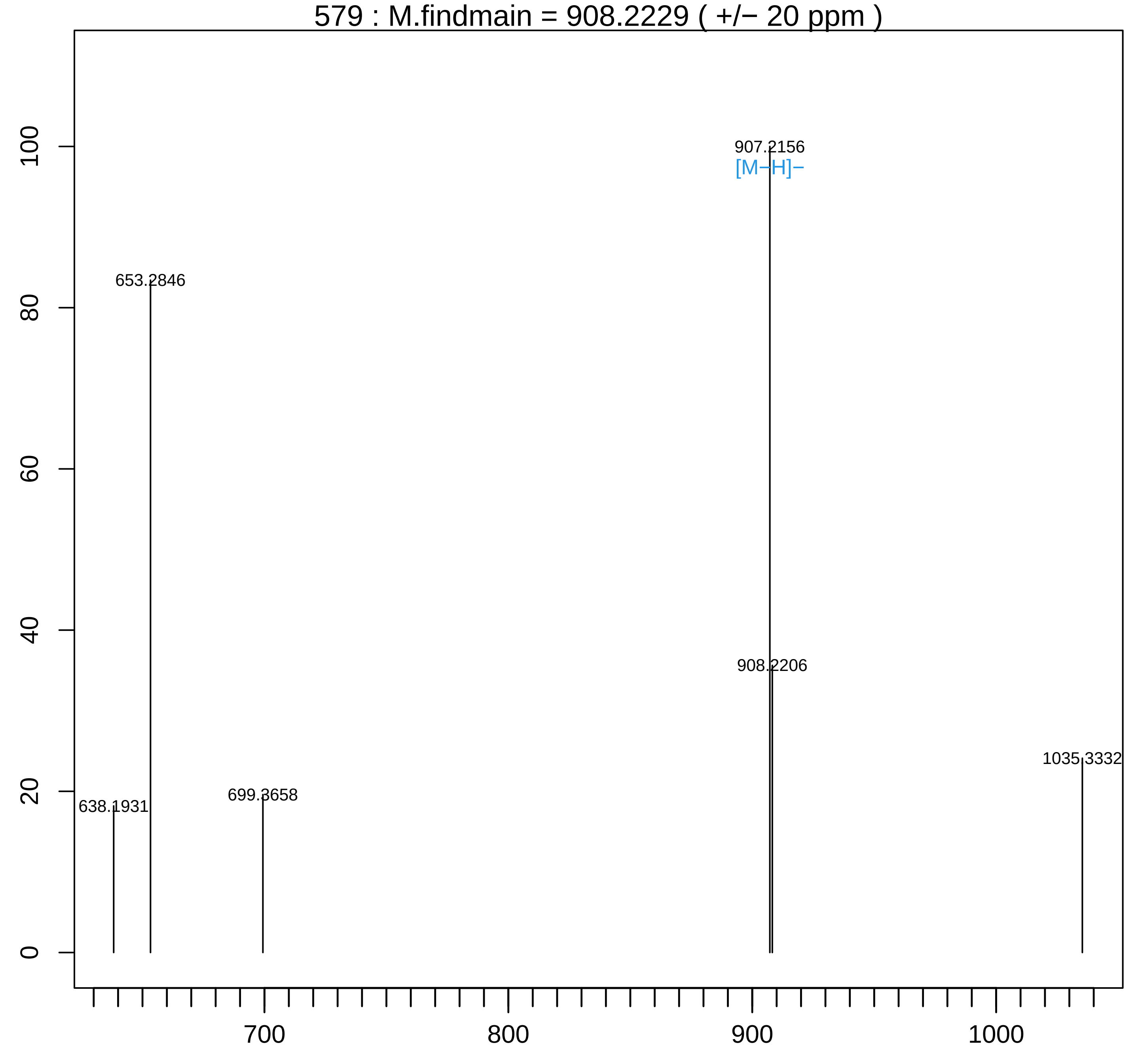
579 : M.findmain = 908.2229 (+/− 20 ppm)
907.2156
[M−H]−
653.2846
908.2206
1035.3332
699.3658
638.1931
0
20
40
60
80
100
700
800
900
1000

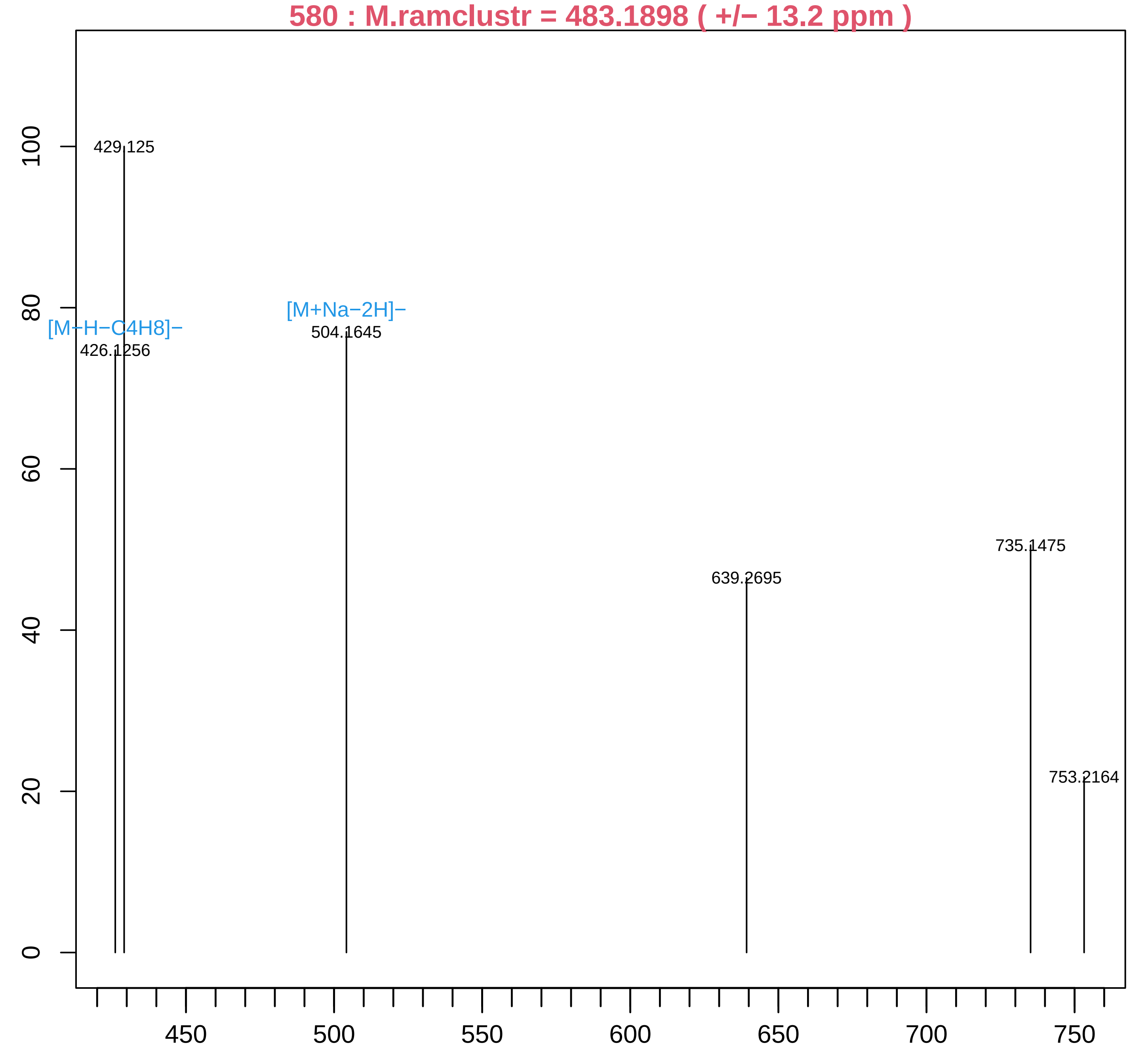

580 : M.ramclustr = 483.1898 (+/− 13.2 ppm)
429.125
[M−H−C4H8]−
426.1256
[M+Na−2H]−
504.1645
639.2695
735.1475
753.2164
0
20
40
60
80
100
450
500
550
600
650
700
750

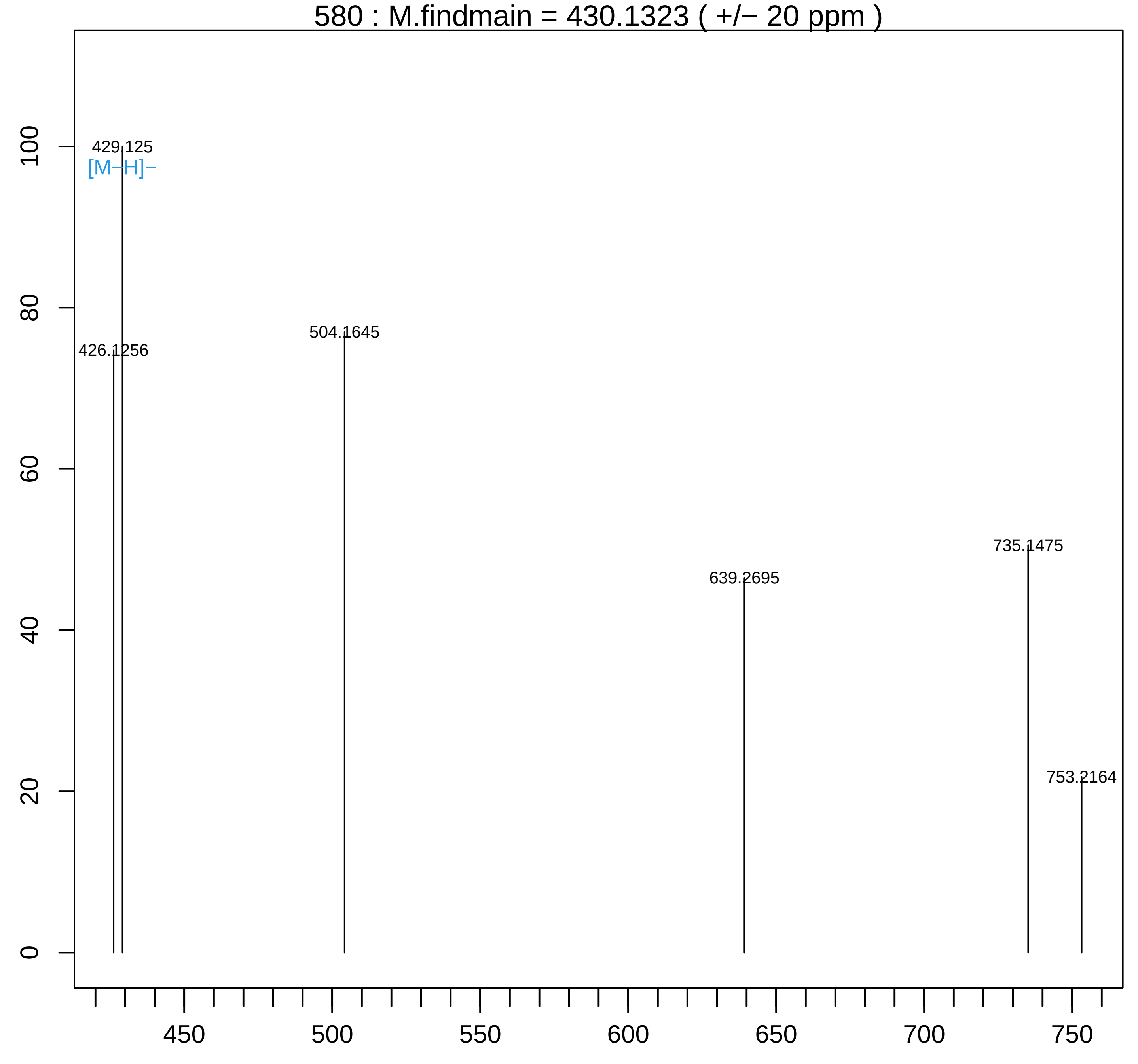

580 : M.findmain = 430.1323 (+/− 20 ppm)
429.125
[M−H]−
426.1256
504.1645
639.2695
735.1475
753.2164
0
20
40
60
80
100
450
500
550
600
650
700
750

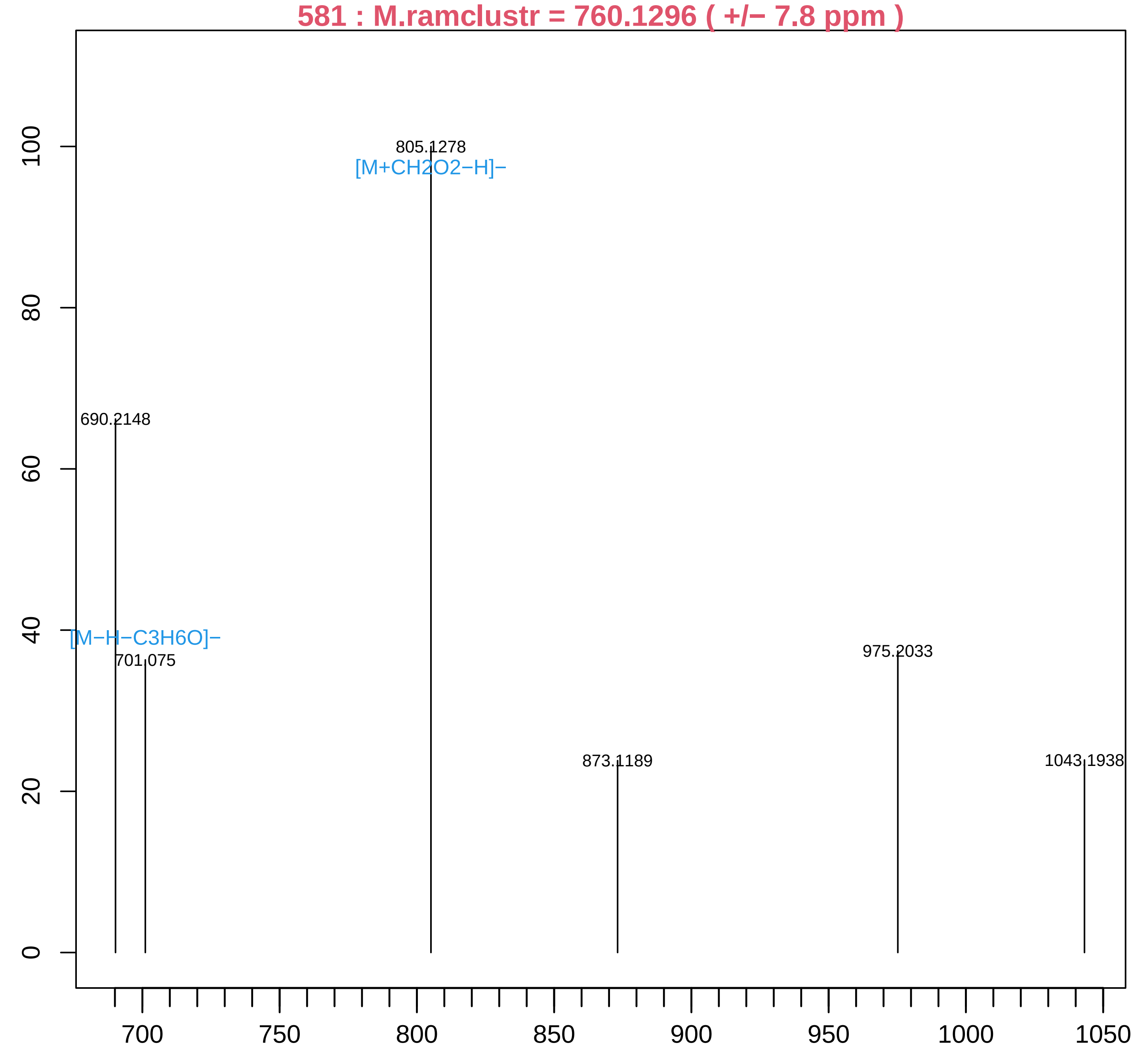
581 : M.ramclustr = 760.1296 (+/− 7.8 ppm)
805.1278
[M+CH2O2−H]−
690.2148
[M−H−C3H6O]−
701.075
975.2033
873.1189
1043.1938
0
20
40
60
80
100
700
750
800
850
900
950
1000
1050

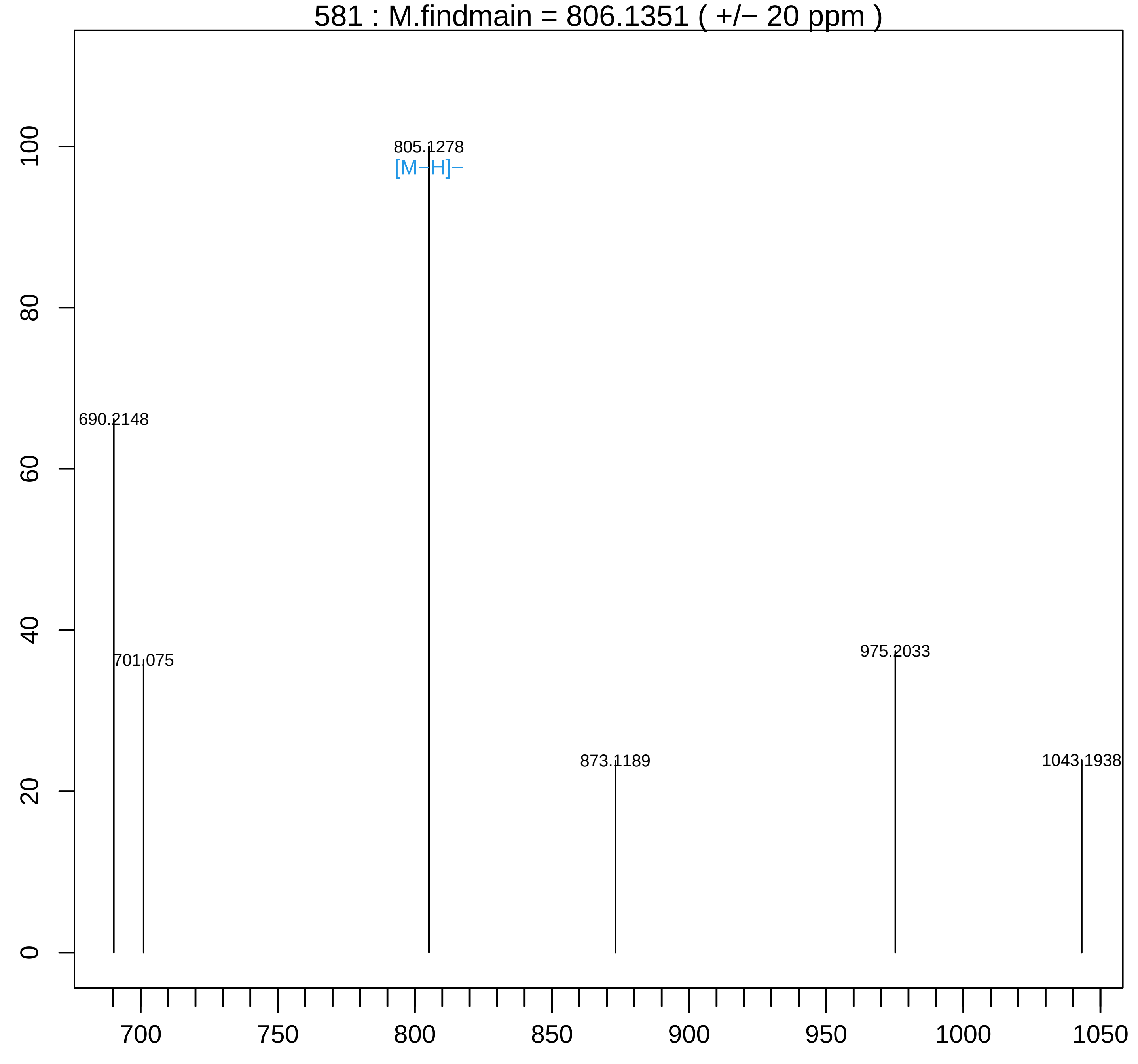
581 : M.findmain = 806.1351 (+/− 20 ppm)
805.1278
[M−H]−
690.2148
701.075
975.2033
873.1189
1043.1938
0
20
40
60
80
100
700
750
800
850
900
950
1000
1050

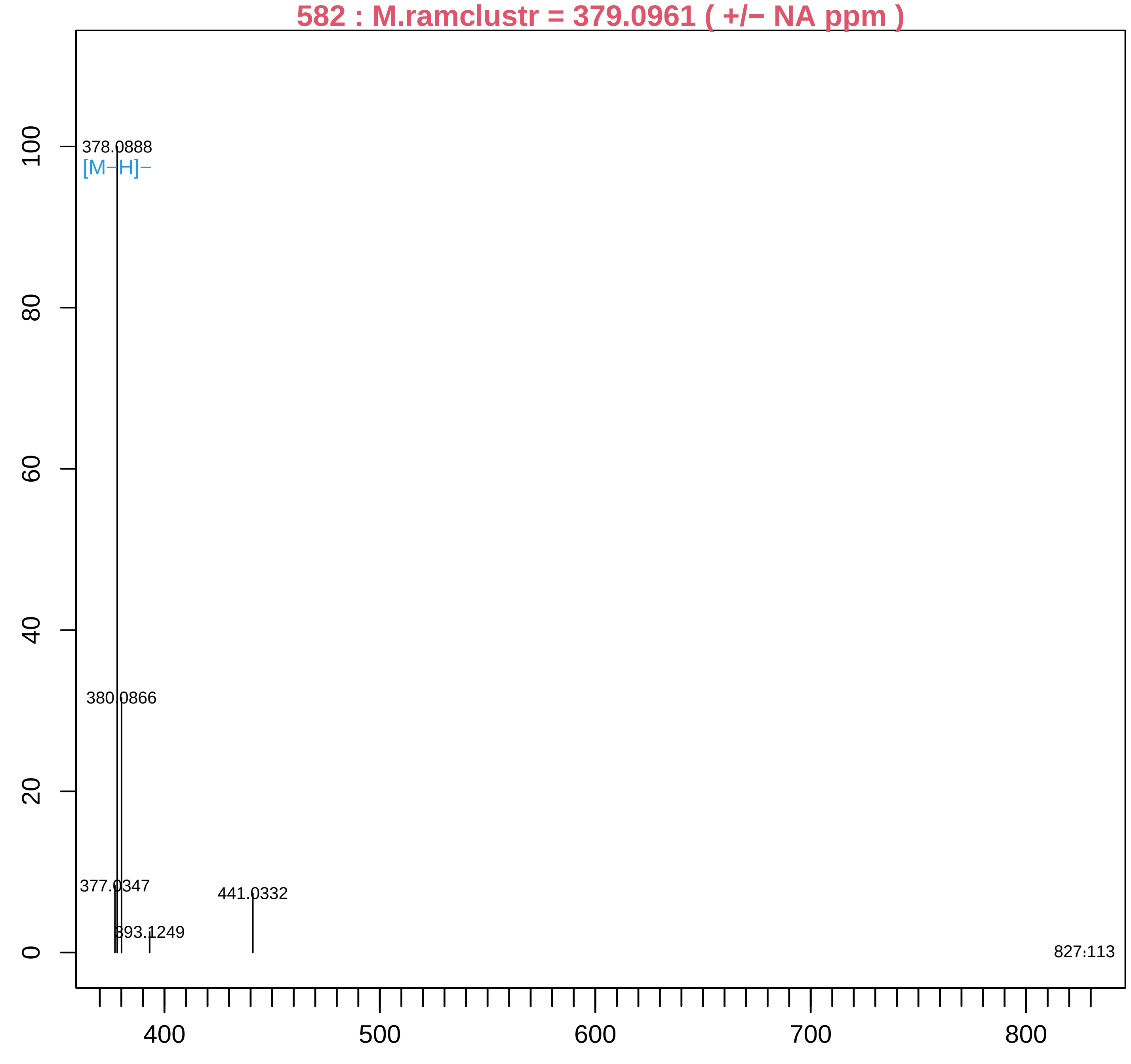

582 : M.ramclustr = 379.0961 (+/− NA ppm)
378.0888
[M−H]−
380.0866
377.0347
441.0332
393.1249
827:113
0
20
40
60
80
100
400
500
600
700
800

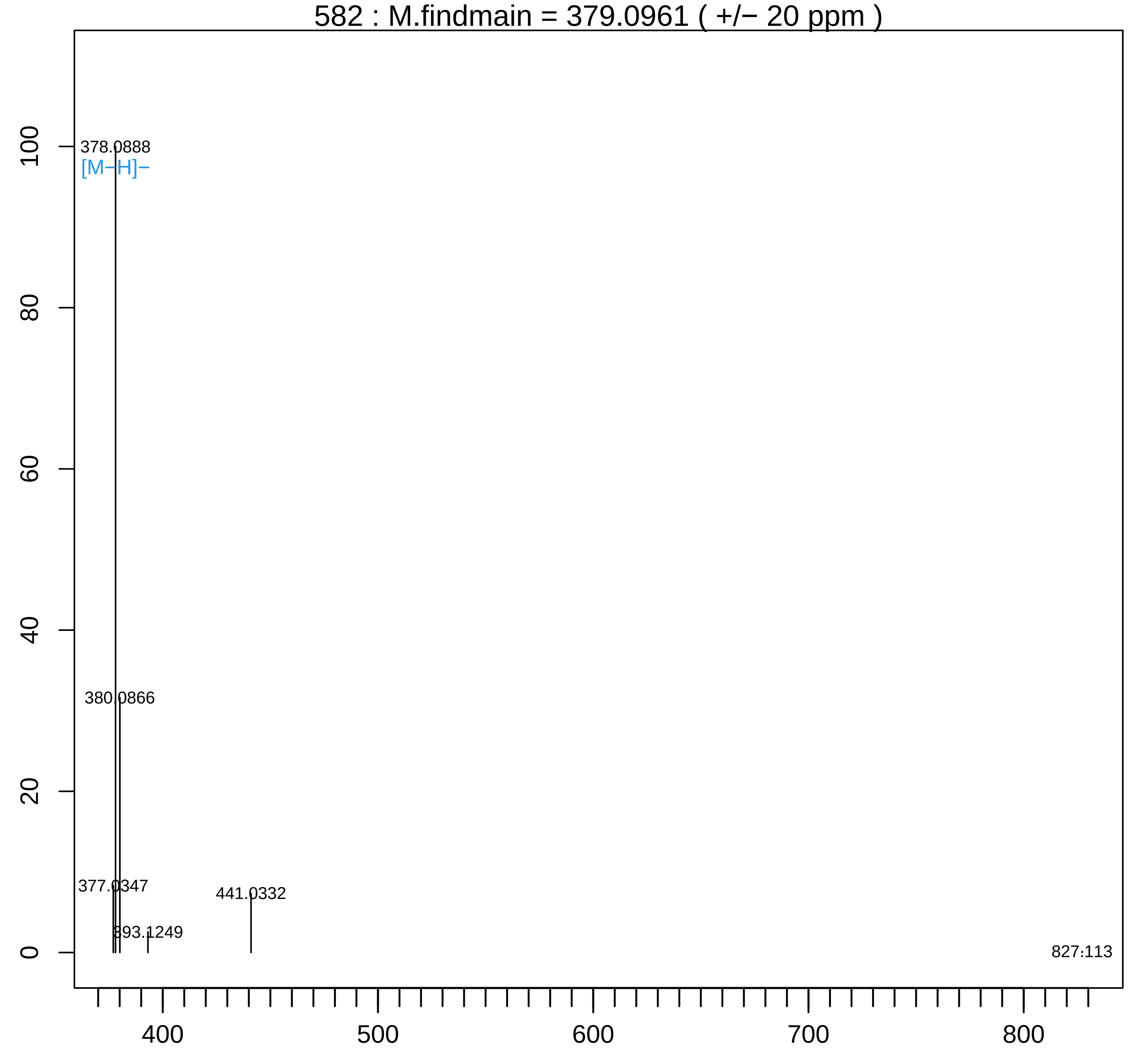

582 : M.findmain = 379.0961 (+/− 20 ppm)
378.0888
[M−H]−
380.0866
377.0347
441.0332
393.1249
827:113
0
20
40
60
80
100
400
500
600
700
800

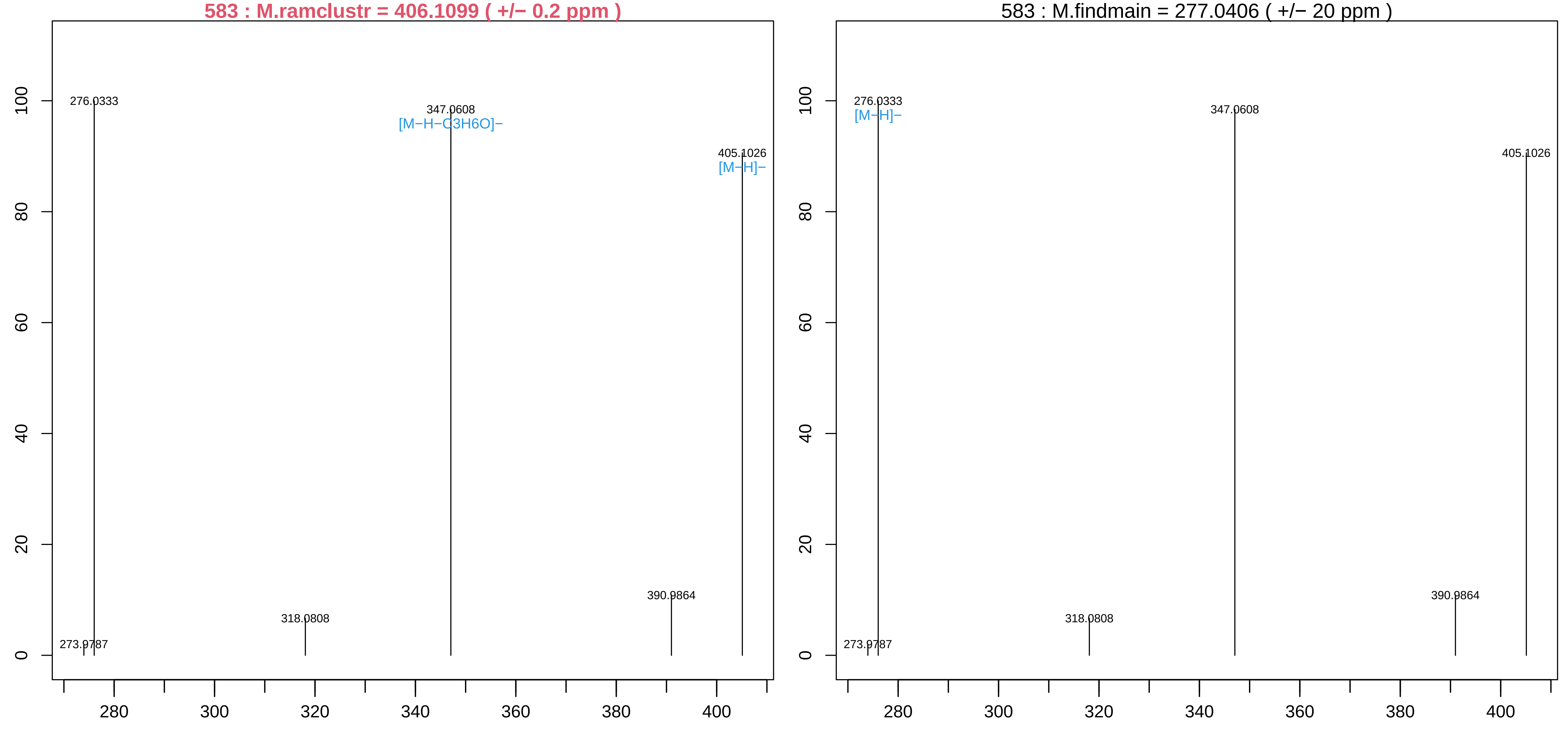

583 : M.ramclustr = 406.1099 (+/− 0.2 ppm)
276.0333
347.0608
[M−H−C3H6O]−
405.1026
[M−H]−
390.9864
318.0808
273.9787
583 : M.findmain = 277.0406 (+/− 20 ppm)
276.0333
[M−H]−
347.0608
405.1026
390.9864
318.0808
273.9787

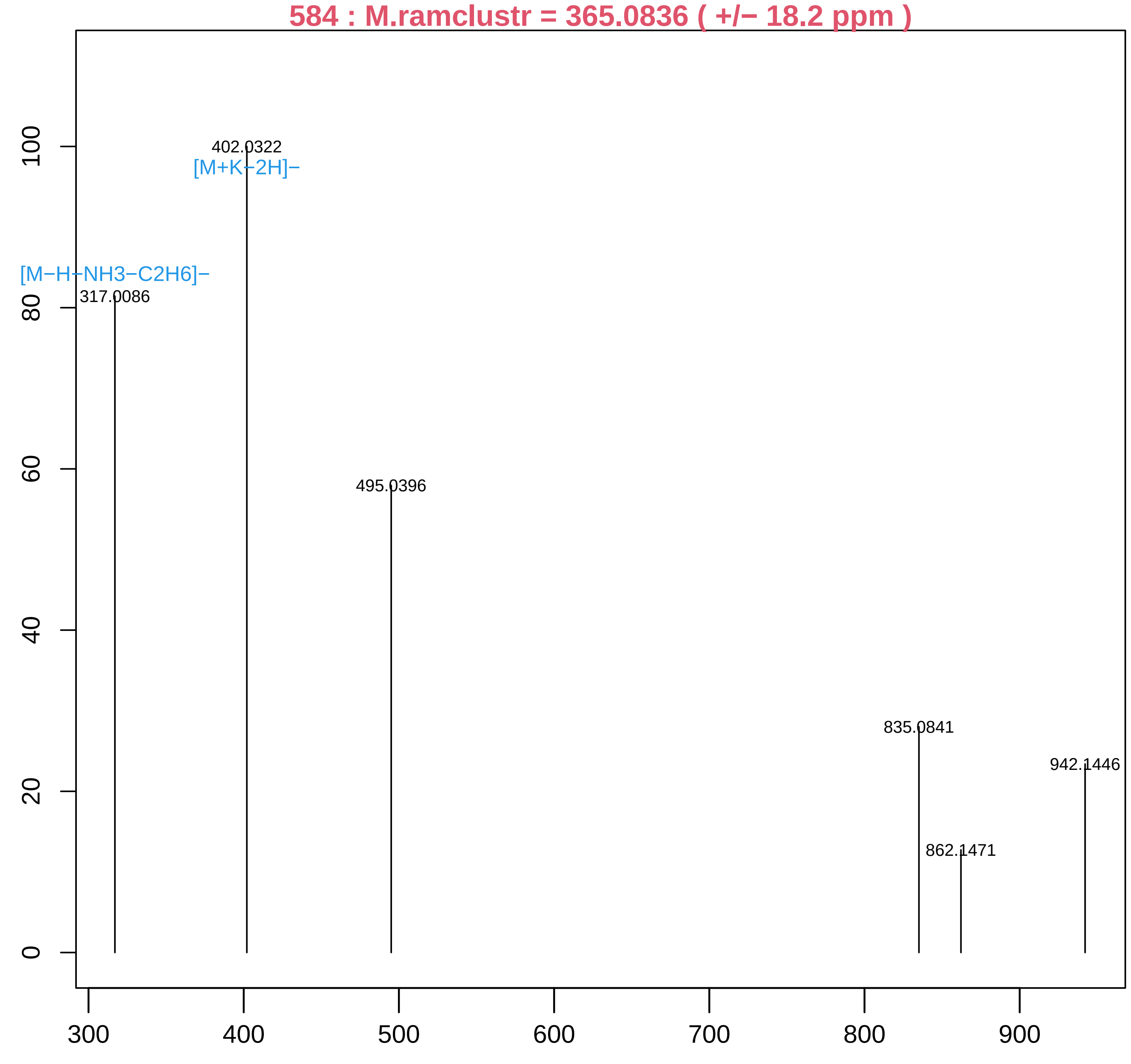

584 : M.ramclustr = 365.0836 (+/− 18.2 ppm)
402.0322
[M+K−2H]−
[M−H−NH3−C2H6]−
317.0086
495.0396
835.0841
942.1446
862.1471
0
20
40
60
80
100
300
400
500
600
700
800
900

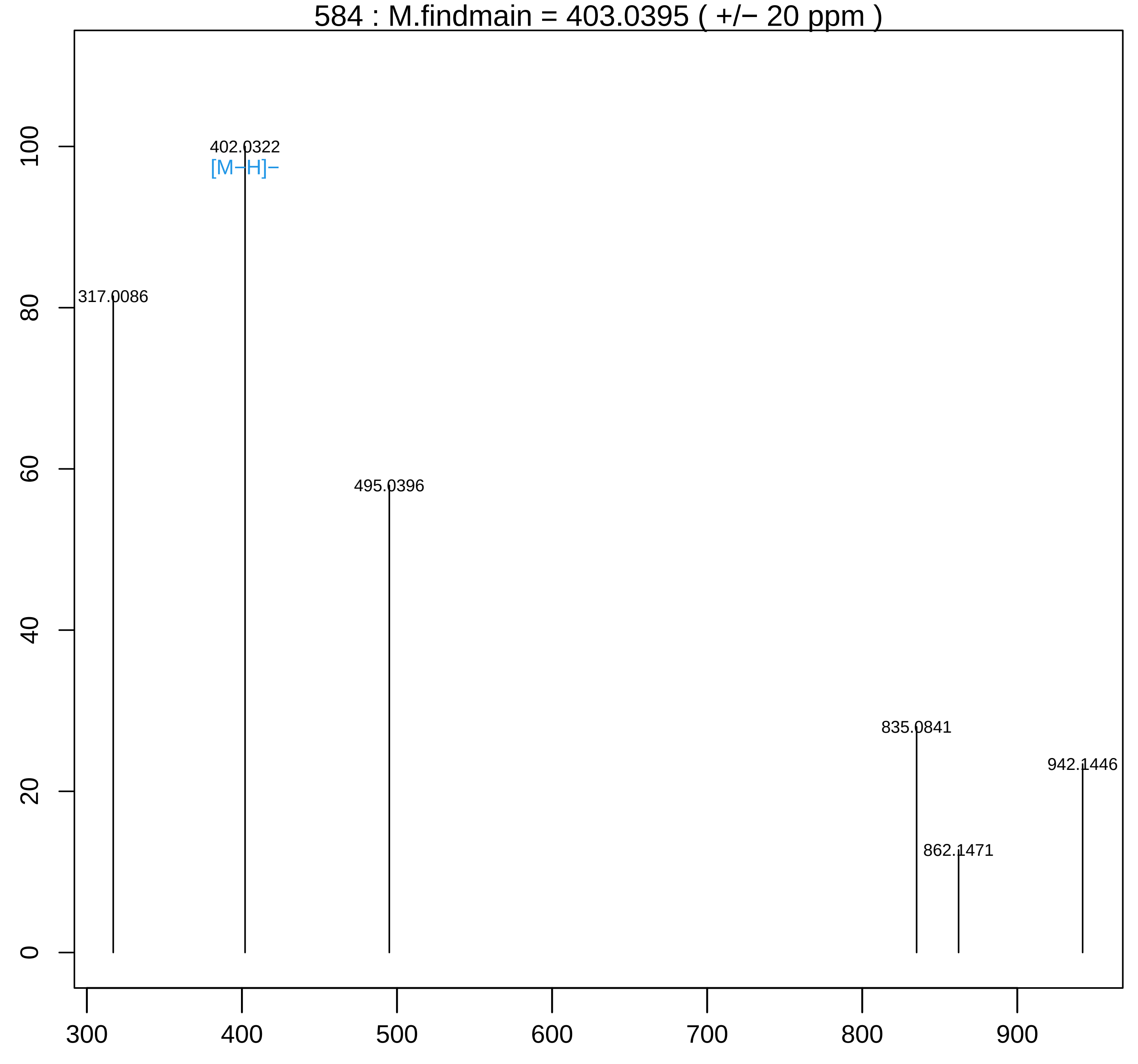

584 : M.findmain = 403.0395 (+/− 20 ppm)
402.0322
[M−H]−
317.0086
495.0396
835.0841
942.1446
862.1471
0
20
40
60
80
100
300
400
500
600
700
800
900

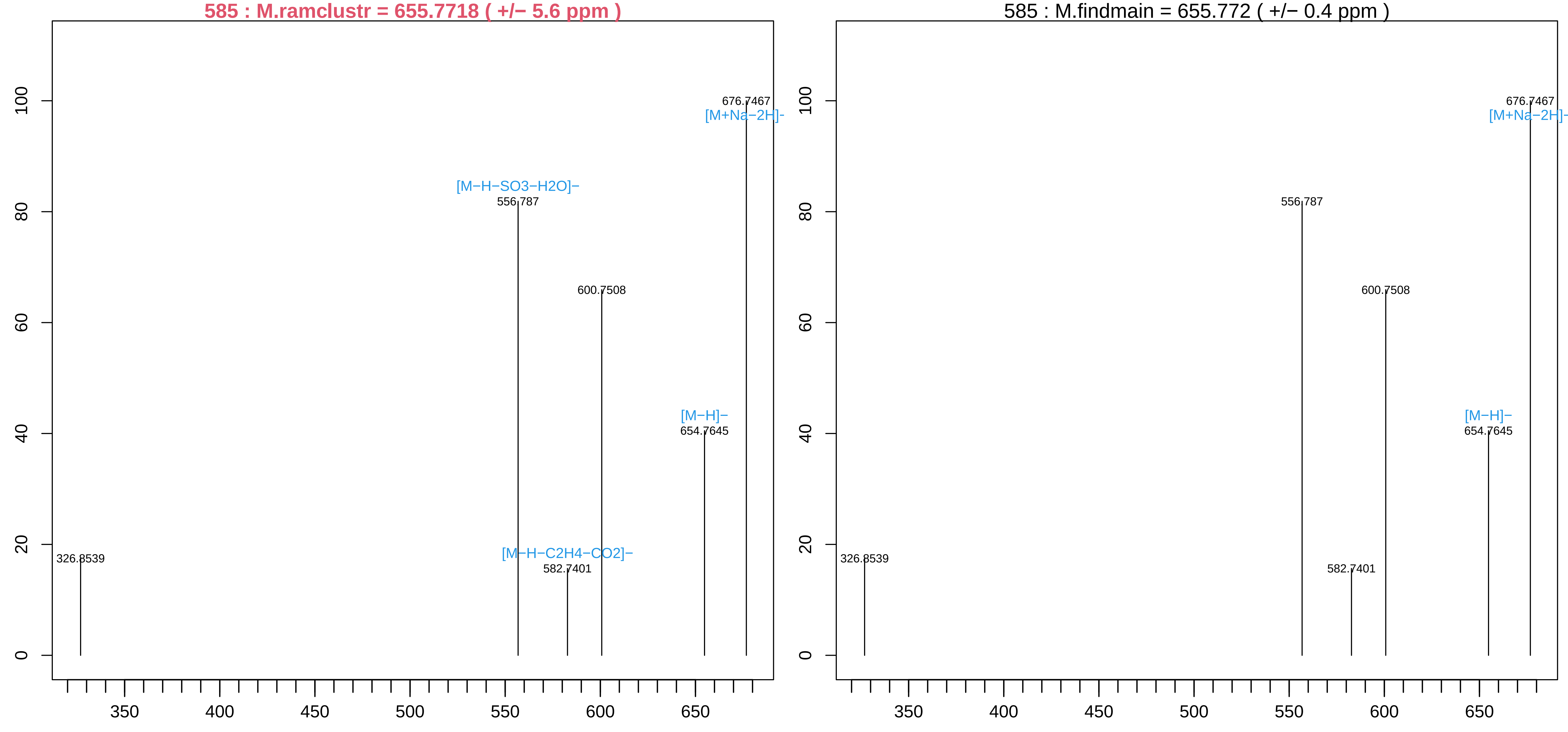
585 : M.ramclustr = 655.7718 (+/− 5.6 ppm)
676.7467
[M+Na−2H]-
[M−H−SO3−H2O]−
556.787
600.7508
[M−H]−
654.7645
[M−H−C2H4−CO2]−
582.7401
326.8539
0
20
40
60
80
100
350
400
450
500
550
600
650
585 : M.findmain = 655.772 (+/− 0.4 ppm)
676.7467
[M+Na−2H]-
556.787
600.7508
[M−H]−
654.7645
582.7401
326.8539
0
20
40
60
80
100
350
400
450
500
550
600
650

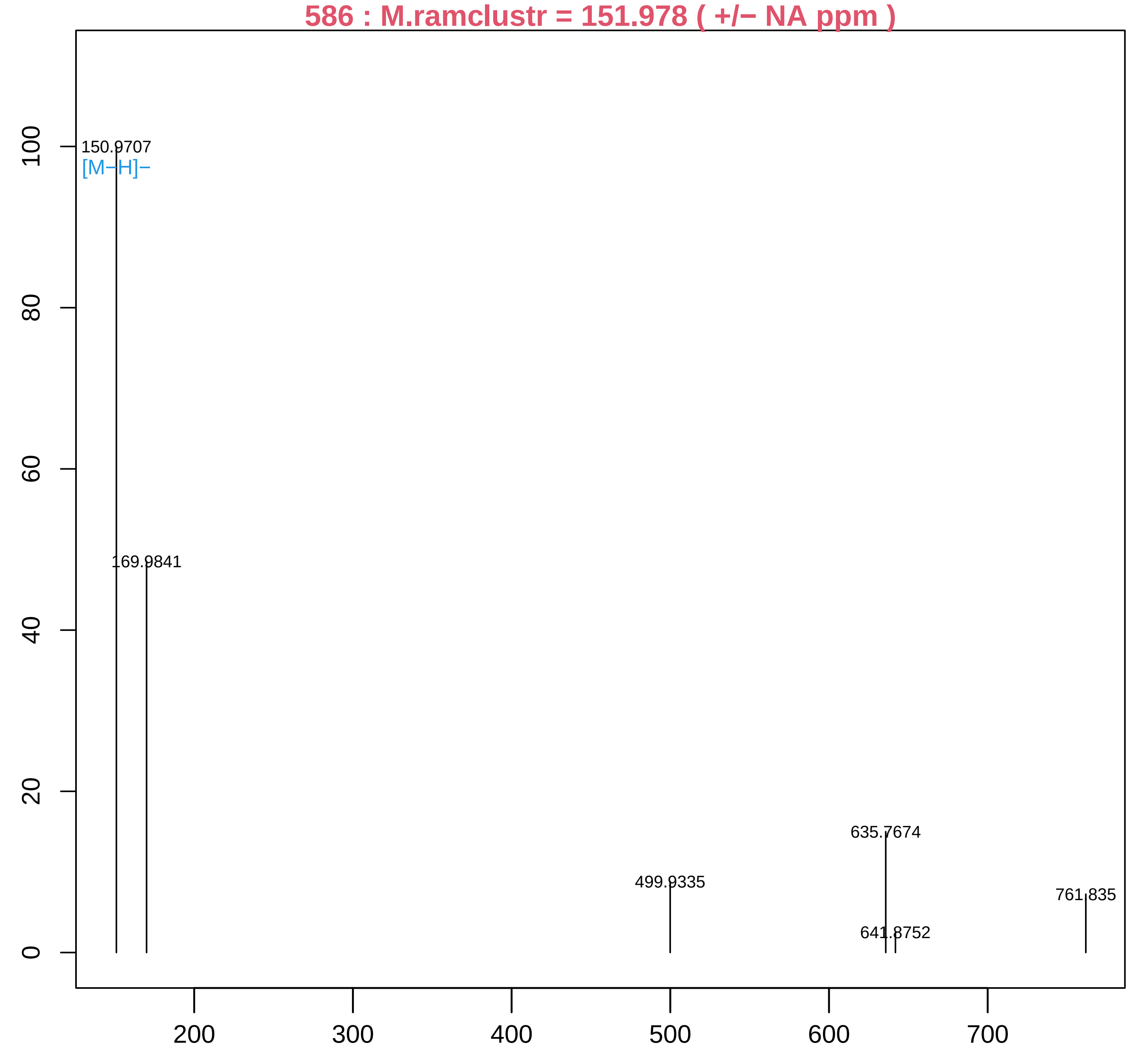
586 : M.ramclustr = 151.978 (+/− NA ppm)
150.9707
[M−H]−
169.9841
499.9335
635.7674
641.8752
761.835
0
20
40
60
80
100
200
300
400
500
600
700

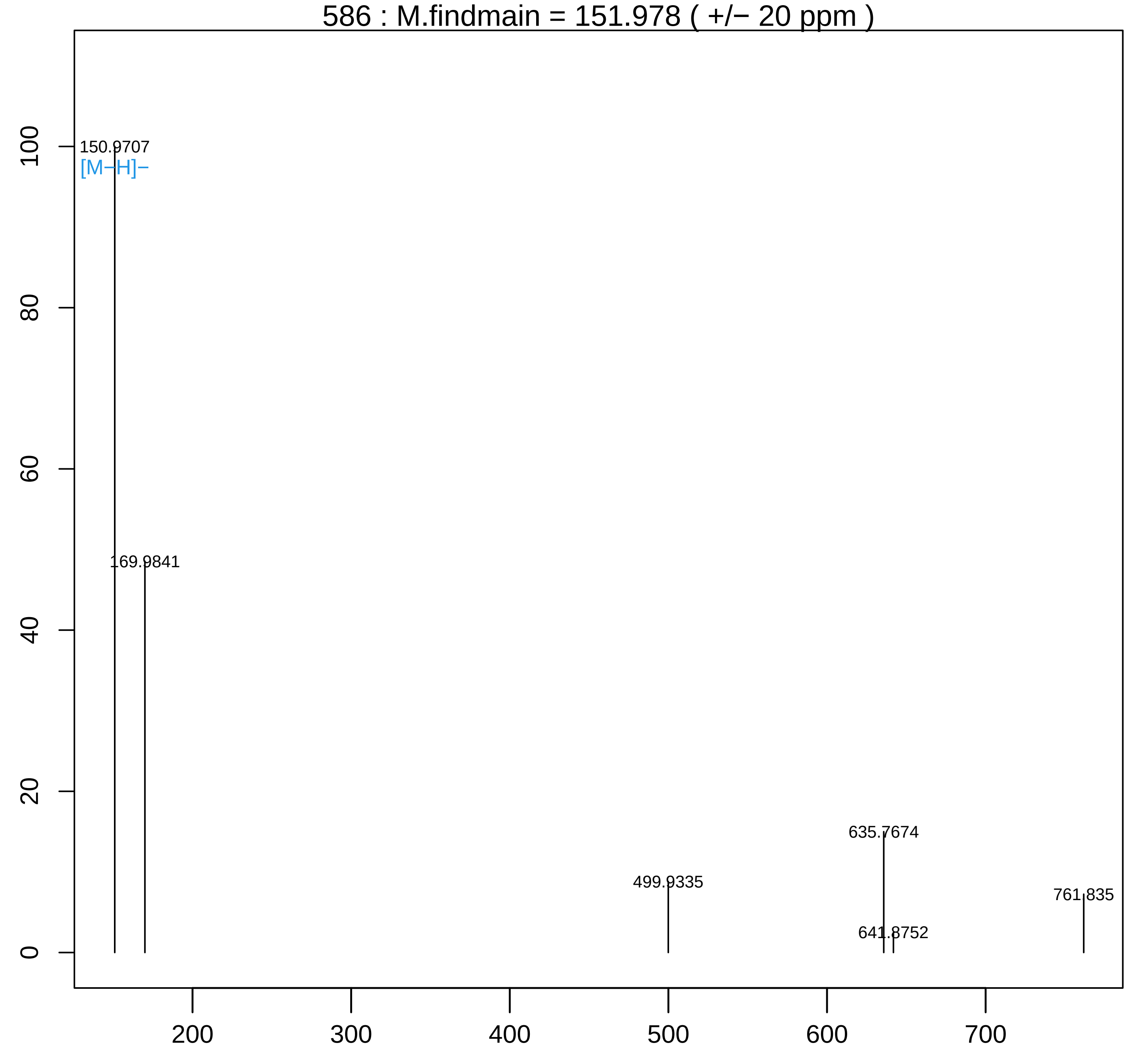
586 : M.findmain = 151.978 (+/− 20 ppm)
150.9707
[M−H]−
169.9841
499.9335
635.7674
641.8752
761.835
0
20
40
60
80
100
200
300
400
500
600
700

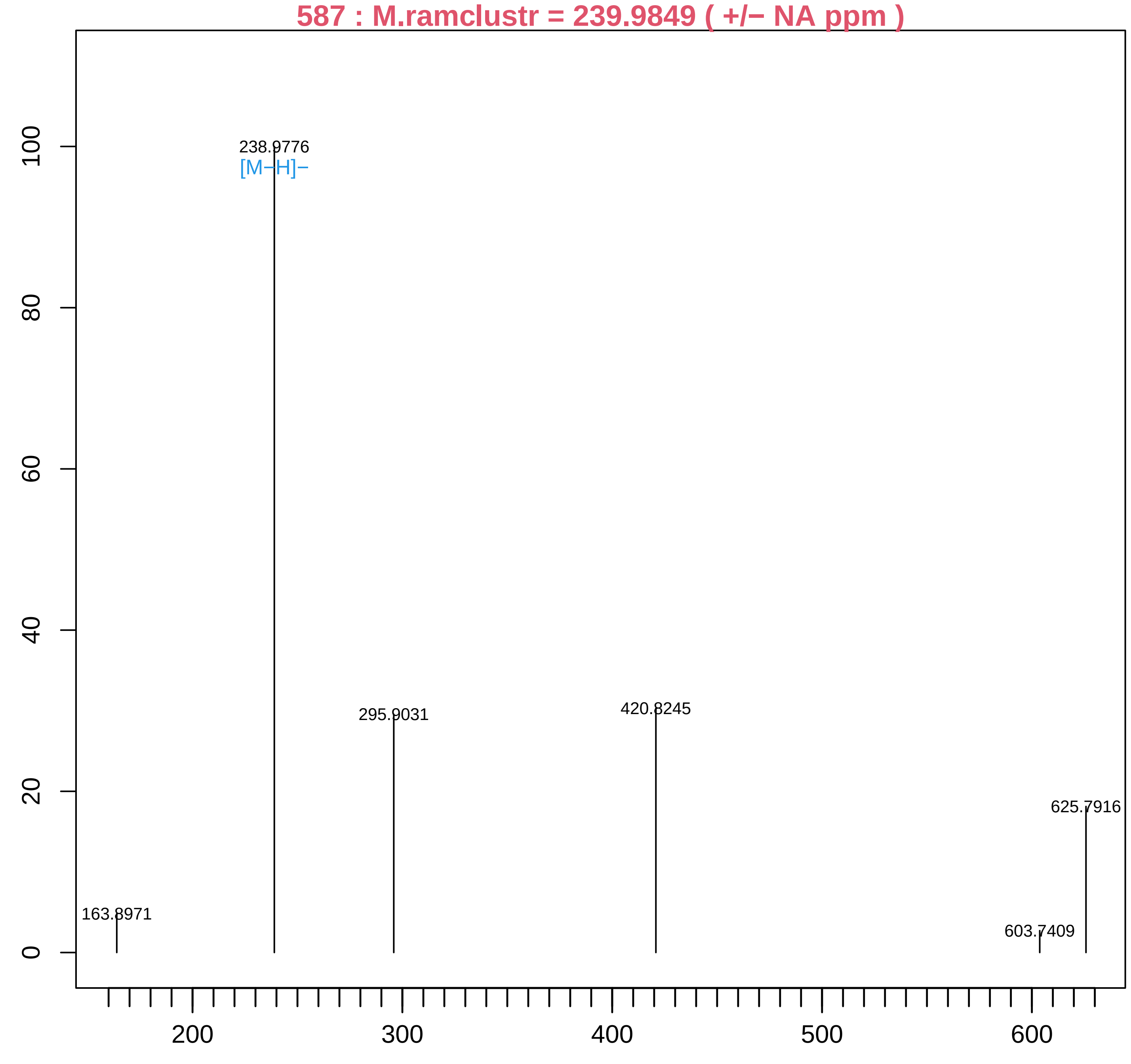
587 : M.ramclustr = 239.9849 (+/− NA ppm)
238.9776
[M−H]−
295.9031
420.8245
625.7916
163.8971
603.7409
0
20
40
60
80
100
200
300
400
500
600

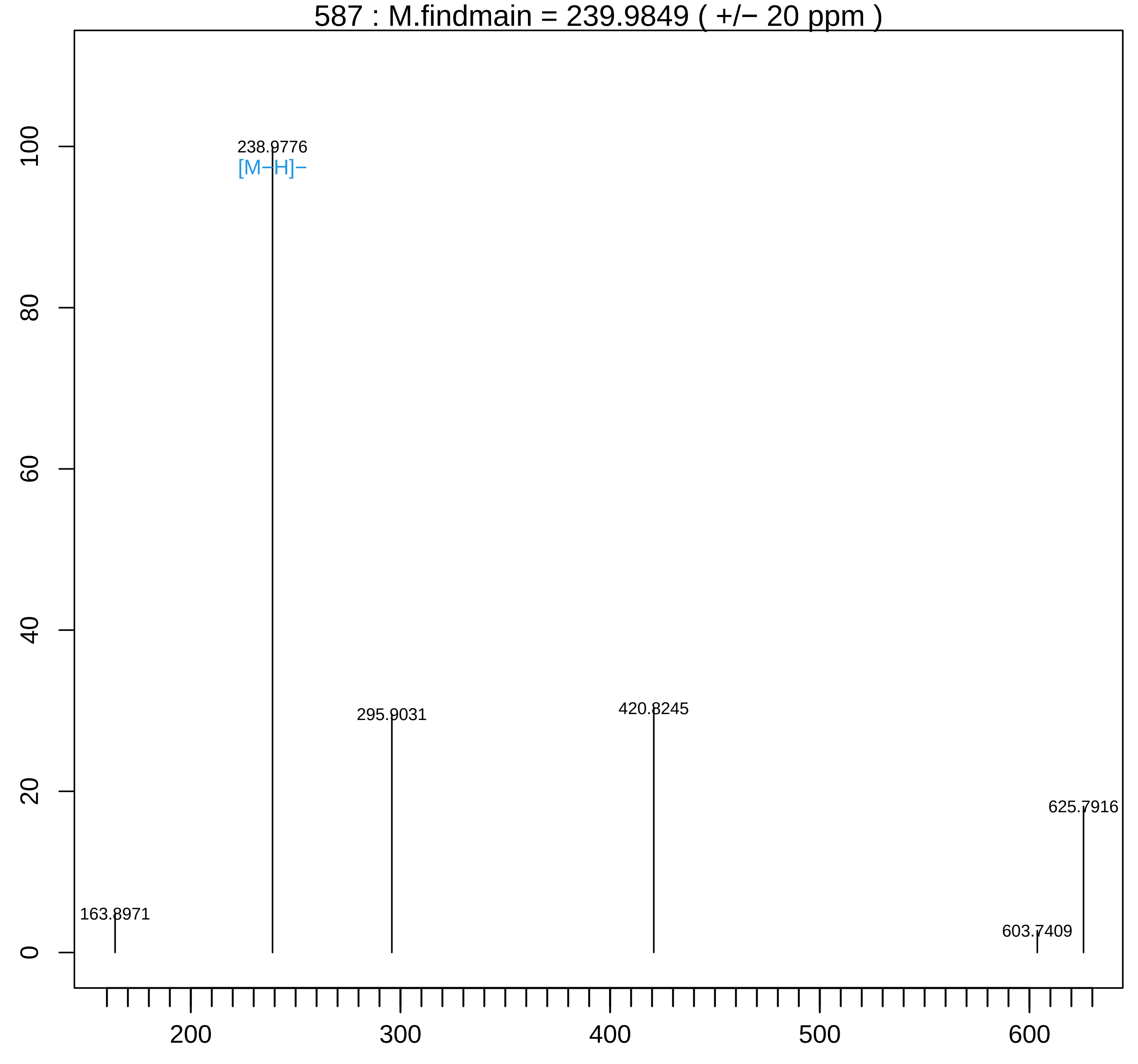
587 : M.findmain = 239.9849 (+/− 20 ppm)
238.9776
[M−H]−
295.9031
420.8245
625.7916
163.8971
603.7409
0
20
40
60
80
100
200
300
400
500
600

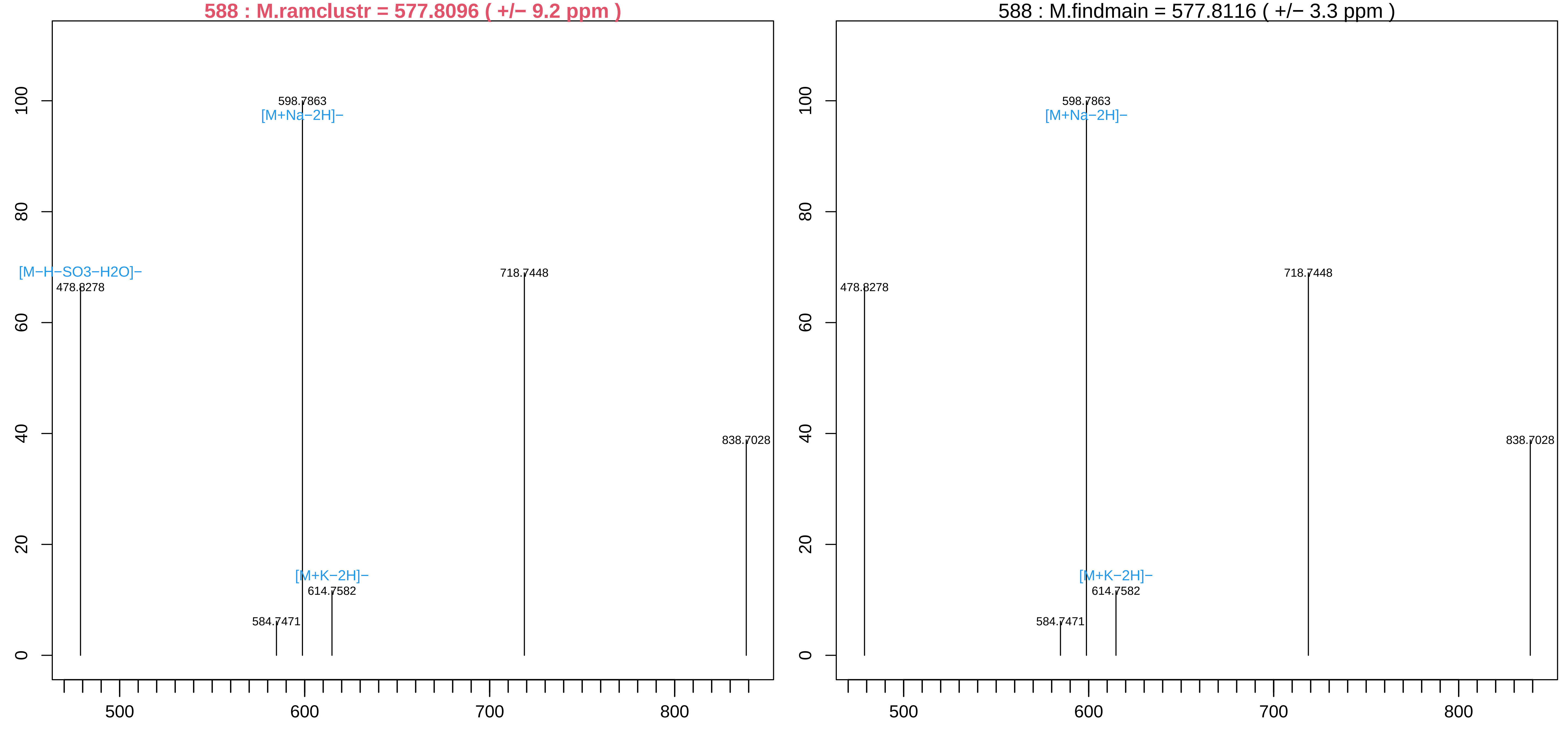

588 : M.ramclustr = 577.8096 (+/− 9.2 ppm)
588 : M.findmain = 577.8116 (+/− 3.3 ppm)
598.7863
[M+Na−2H]−
[M−H−SO3−H2O]−
478.8278
718.7448
838.7028
[M+K−2H]−
614.7582
584.7471
0
20
40
60
80
100
500
600
700
800

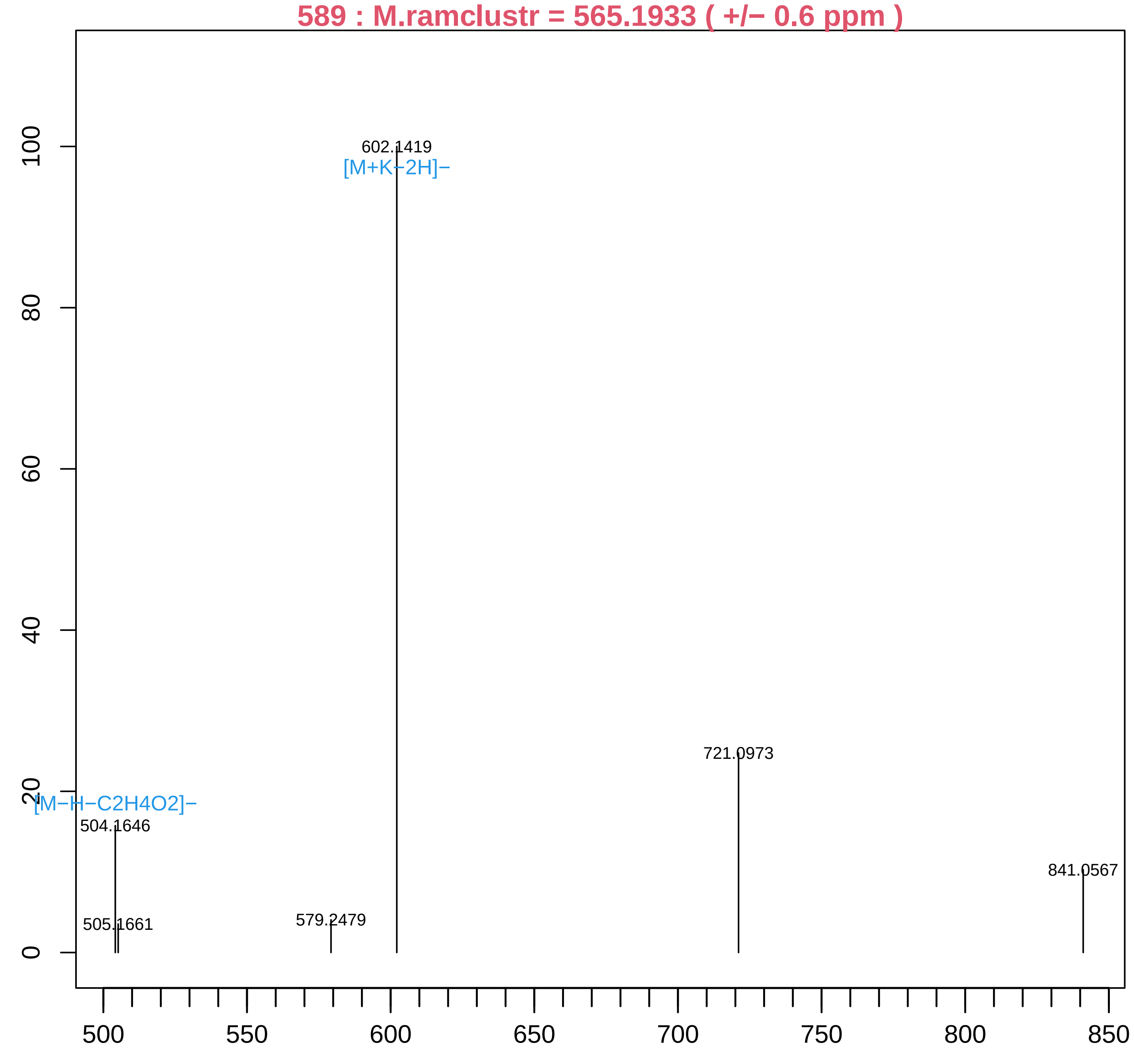
589 : M.ramclustr = 565.1933 (+/− 0.6 ppm)
602.1419
[M+K−2H]−
721.0973
[M−H−C2H4O2]−
504.1646
841.0567
505.1661
579.2479
0
20
40
60
80
100
500
550
600
650
700
750
800
850
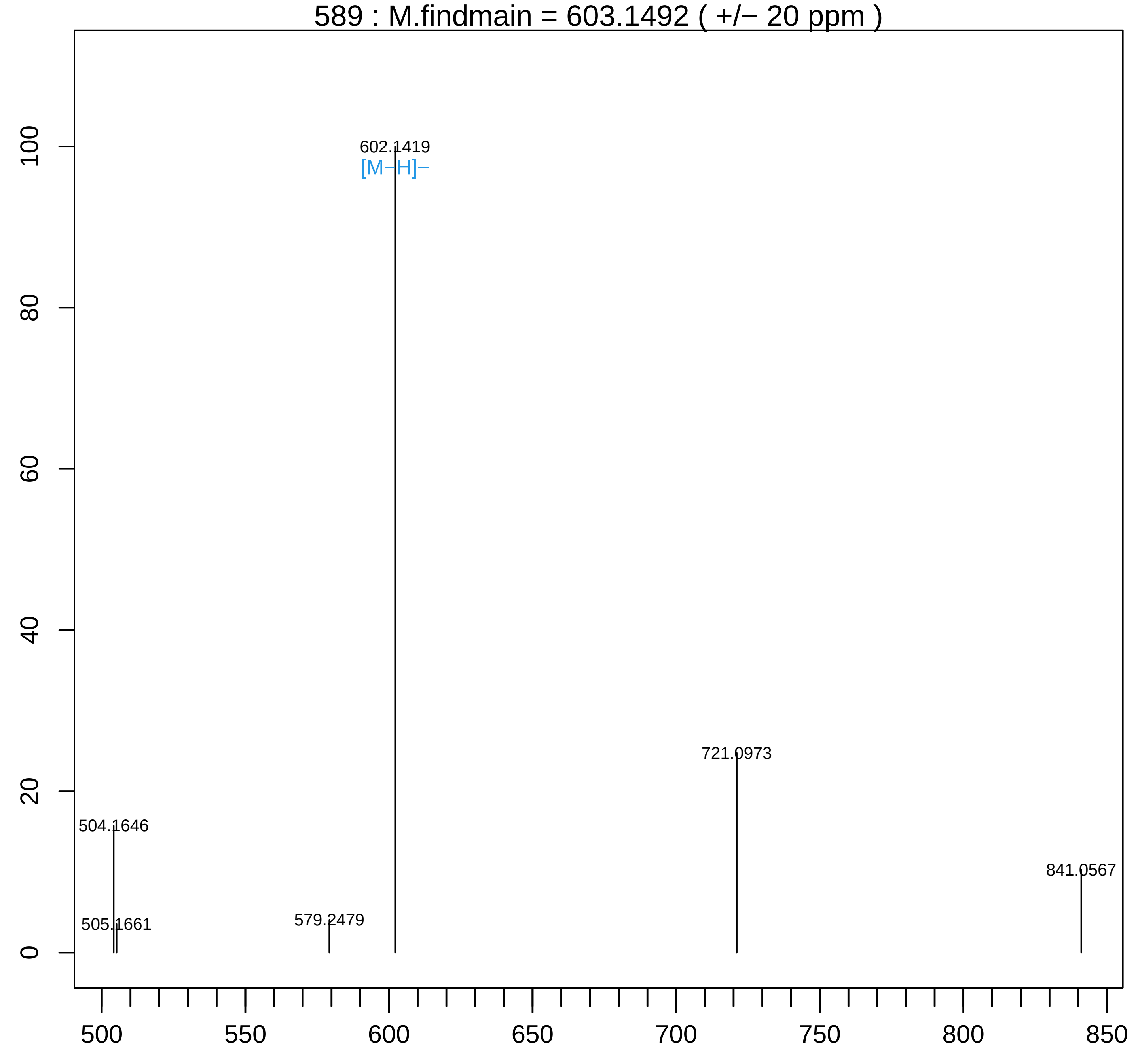
589 : M.findmain = 603.1492 (+/− 20 ppm)
602.1419
[M−H]−
721.0973
504.1646
841.0567
505.1661
579.2479
0
20
40
60
80
100
500
550
600
650
700
750
800
850

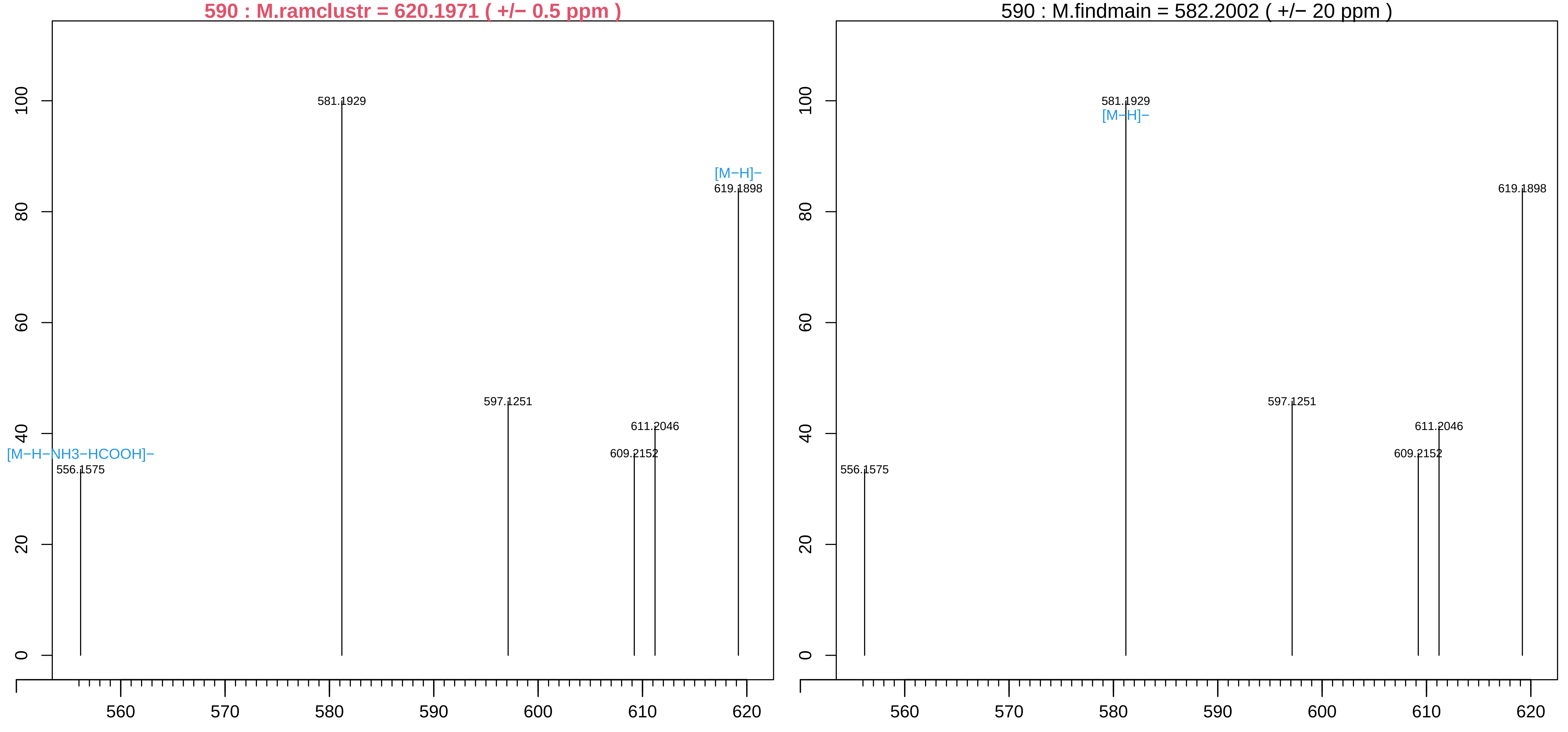

590 : M.ramclustr = 620.1971 (+/− 0.5 ppm)
581.1929
[M−H]−
619.1898
597.1251
611.2046
609.2152
[M−H−NH3−HCOOH]−
556.1575
590 : M.findmain = 582.2002 (+/− 20 ppm)
581.1929
[M−H]−
619.1898
597.1251
611.2046
609.2152
556.1575

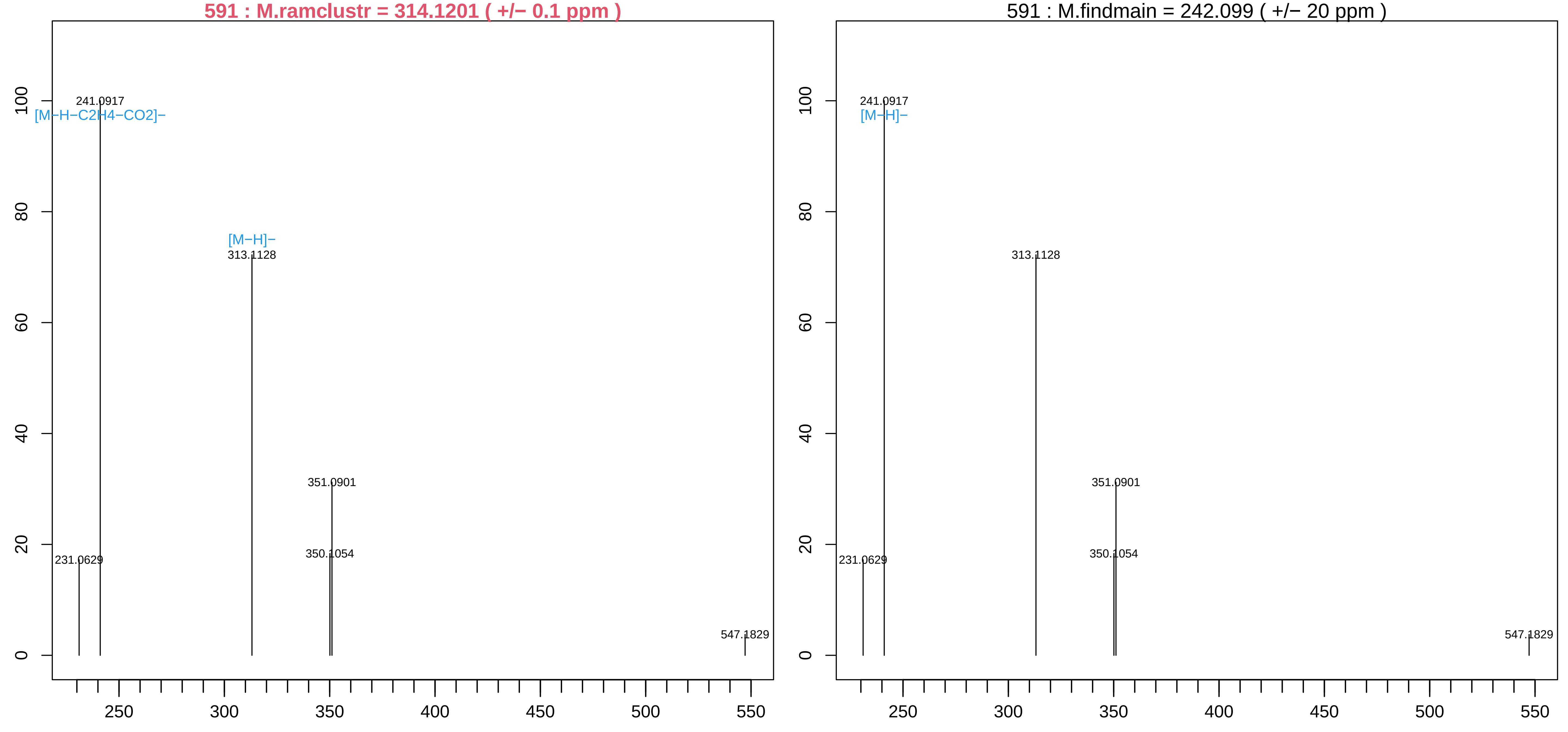
591 : M.ramclustr = 314.1201 (+/− 0.1 ppm)
241.0917
[M−H−C2H4−CO2]−
[M−H]−
313.1128
351.0901
350.1054
231.0629
547.1829
591 : M.findmain = 242.099 (+/− 20 ppm)
241.0917
[M−H]−
313.1128
351.0901
350.1054
231.0629
547.1829
0
20
40
60
80
100
250
300
350
400
450
500
550

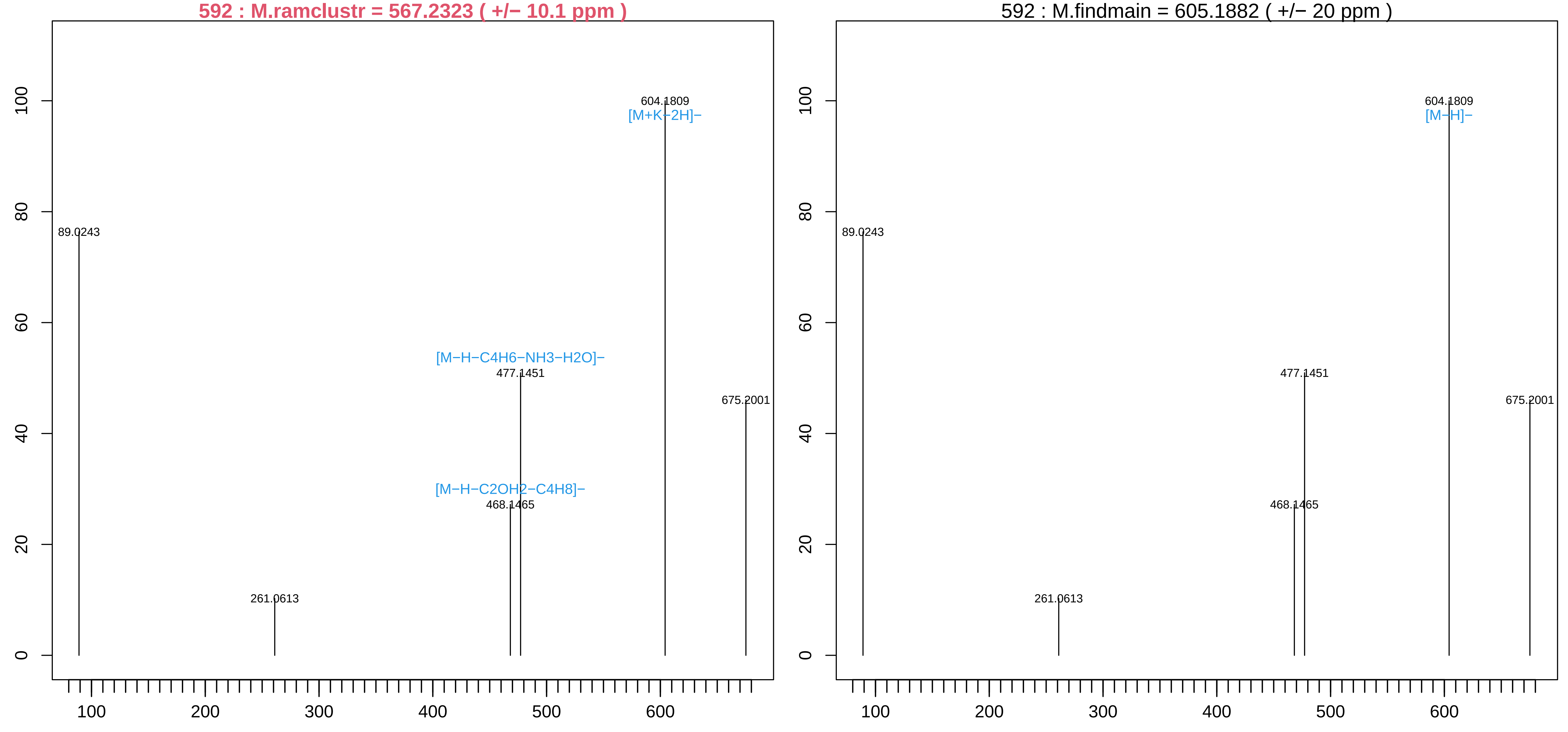
592 : M.ramclustr = 567.2323 (+/− 10.1 ppm)
604.1809
[M+K−2H]−
89.0243
[M−H−C4H6−NH3−H2O]−
477.1451
675.2001
[M−H−C2OH2−C4H8]−
468.1465
261.0613
592 : M.findmain = 605.1882 (+/− 20 ppm)
604.1809
[M−H]−
89.0243
477.1451
675.2001
468.1465
261.0613

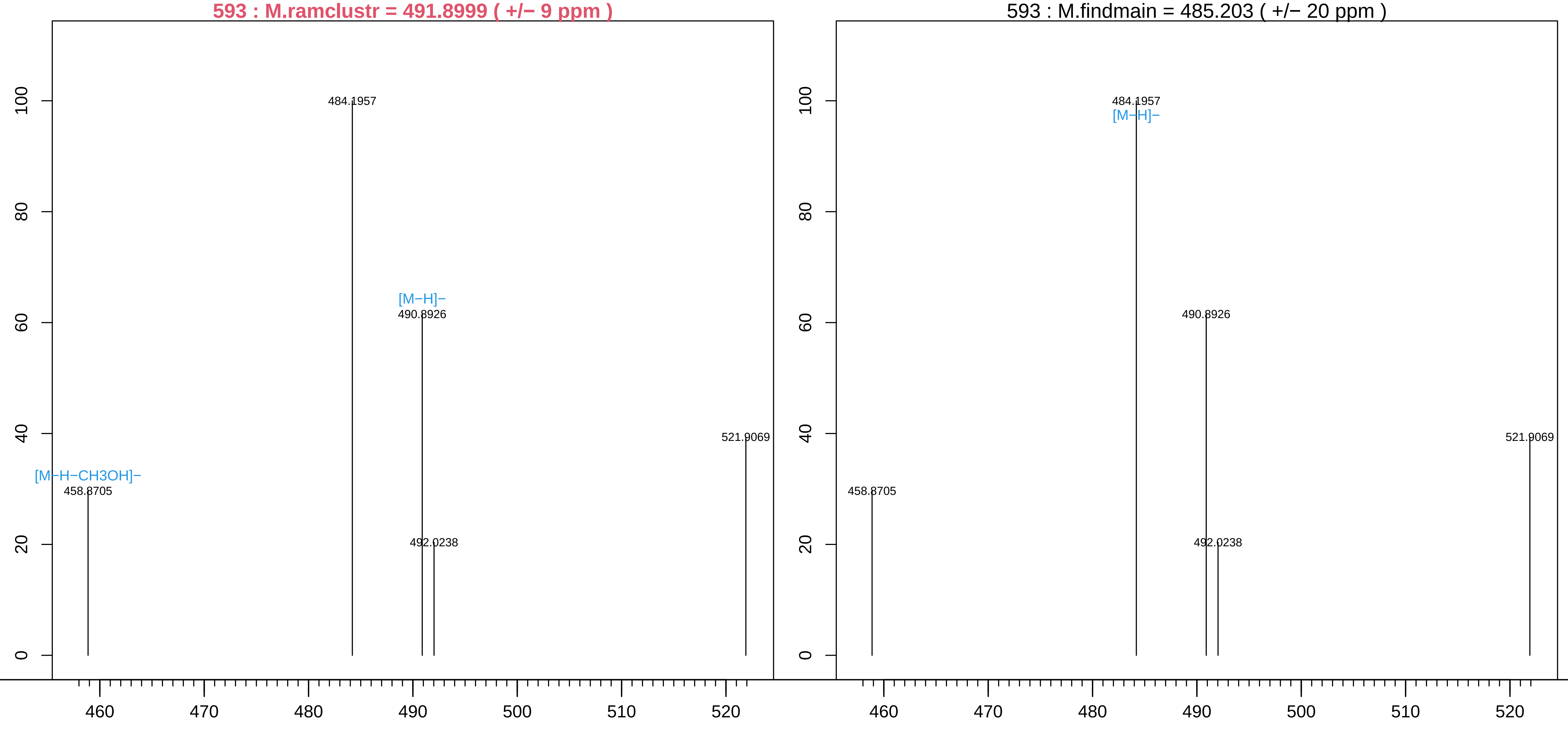
593 : M.ramclustr = 491.8999 (+/− 9 ppm)
593 : M.findmain = 485.203 (+/− 20 ppm)
484.1957
[M−H]−
490.8926
492.0238
521.9069
[M−H−CH3OH]−
458.8705
0
20
40
60
80
100
460
470
480
490
500
510
520

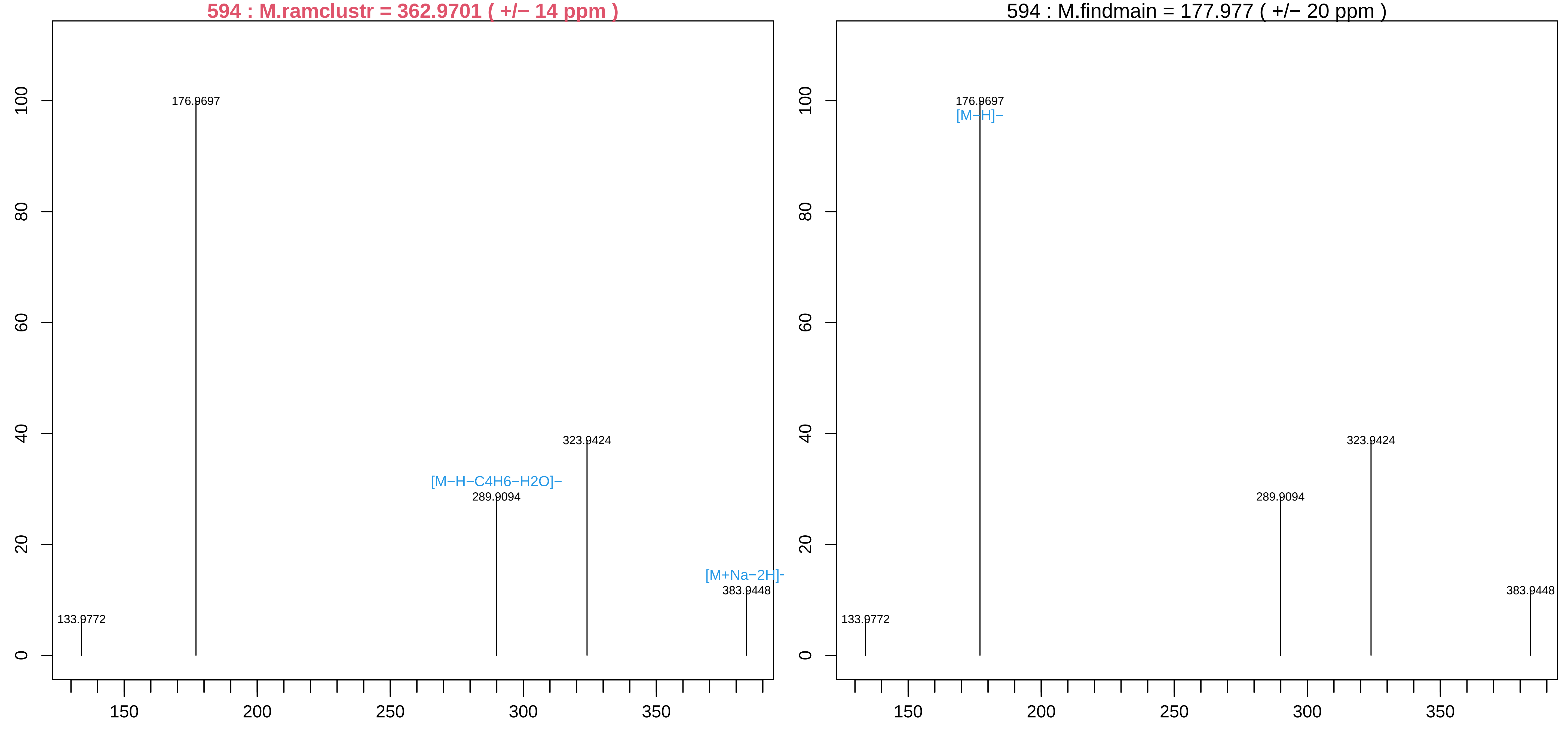
594 : M.ramclustr = 362.9701 (+/− 14 ppm)
176.9697
323.9424
[M−H−C4H6−H2O]−
289.9094
[M+Na−2H]-
383.9448
133.9772
594 : M.findmain = 177.977 (+/− 20 ppm)
176.9697
[M−H]−
323.9424
289.9094
383.9448
133.9772
0
20
40
60
80
100
150
200
250
300
350

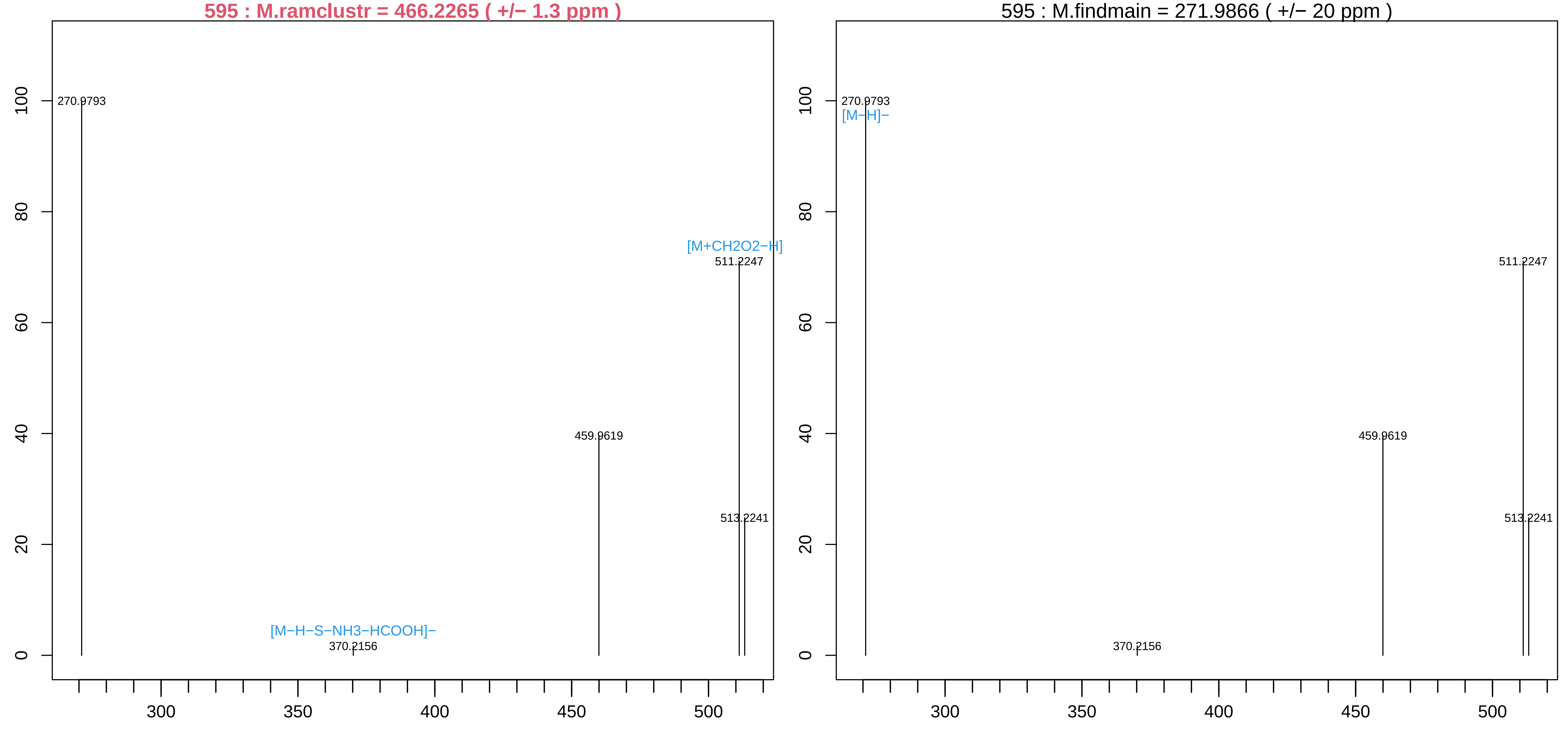

595 : M.ramclustr = 466.2265 (+/− 1.3 ppm)
270.9793
[M+CH2O2−H]
511.2247
459.9619
513.2241
[M−H−S−NH3−HCOOH]−
370.2156
0
20
40
60
80
100
300
350
400
450
500
595 : M.findmain = 271.9866 (+/− 20 ppm)
270.9793
[M−H]−
511.2247
459.9619
513.2241
370.2156
0
20
40
60
80
100
300
350
400
450
500

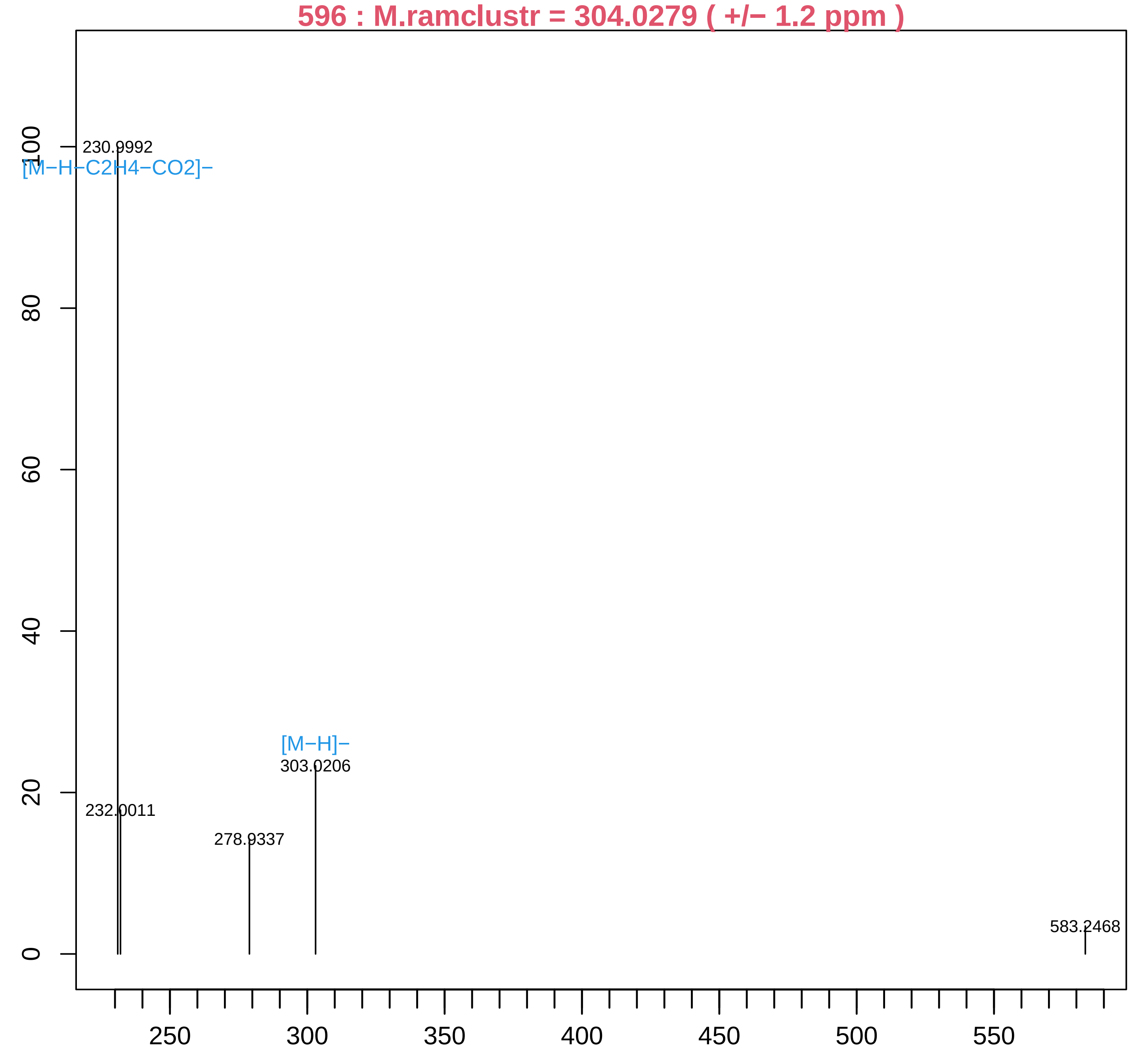

596 : M.ramclustr = 304.0279 (+/− 1.2 ppm)
230.9992
[M−H−C2H4−CO2]−
[M−H]−
303.0206
232.0011
278.9337
583.2468
0
20
40
60
80
100
250
300
350
400
450
500
550

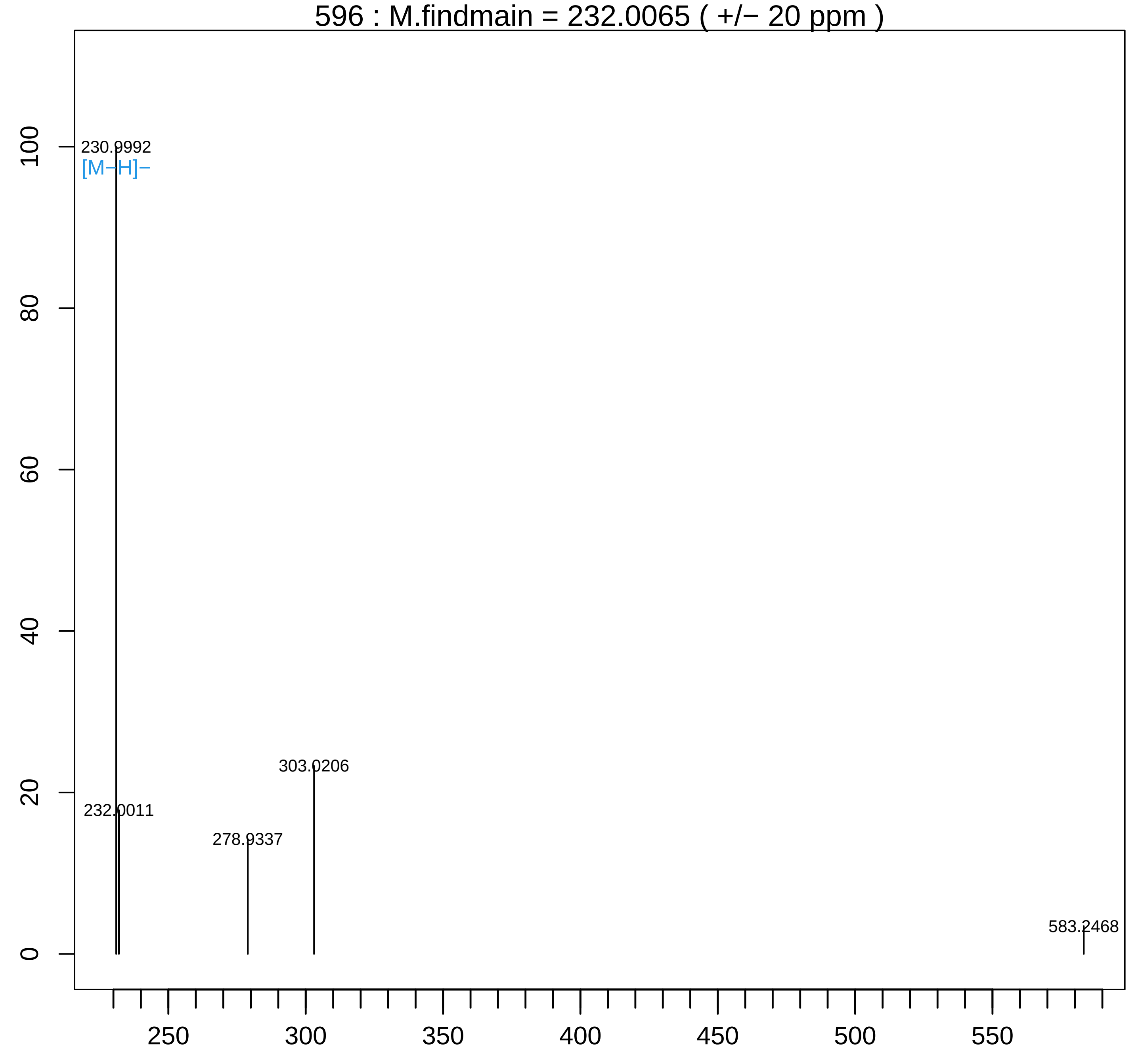

596 : M.findmain = 232.0065 (+/− 20 ppm)
230.9992
[M−H]−
303.0206
232.0011
278.9337
583.2468
0
20
40
60
80
100
250
300
350
400
450
500
550

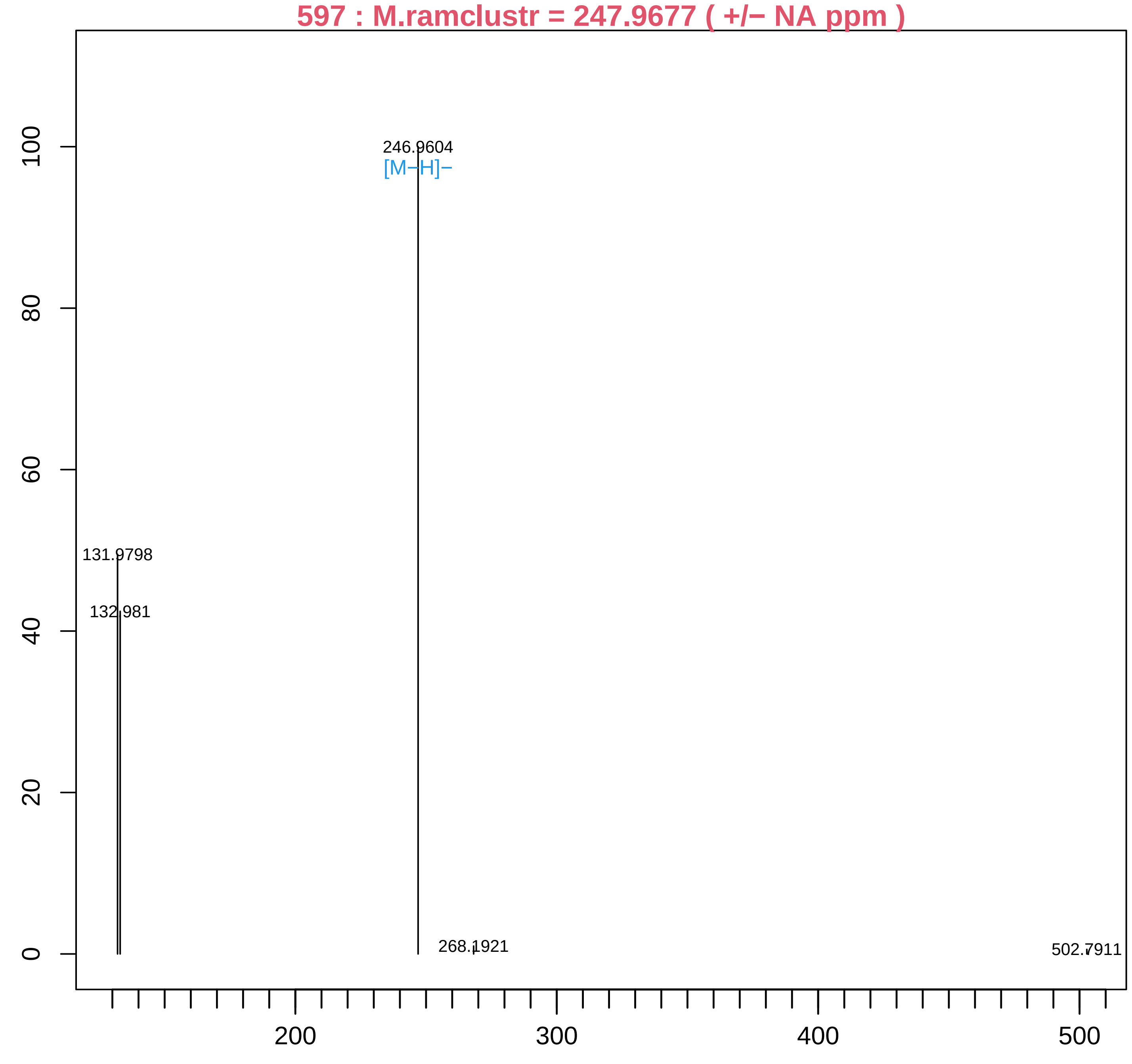
597 : M.ramclustr = 247.9677 (+/− NA ppm)
246.9604
[M−H]−
131.9798
132.981
268.1921
502.7911
0
20
40
60
80
100
200
300
400
500
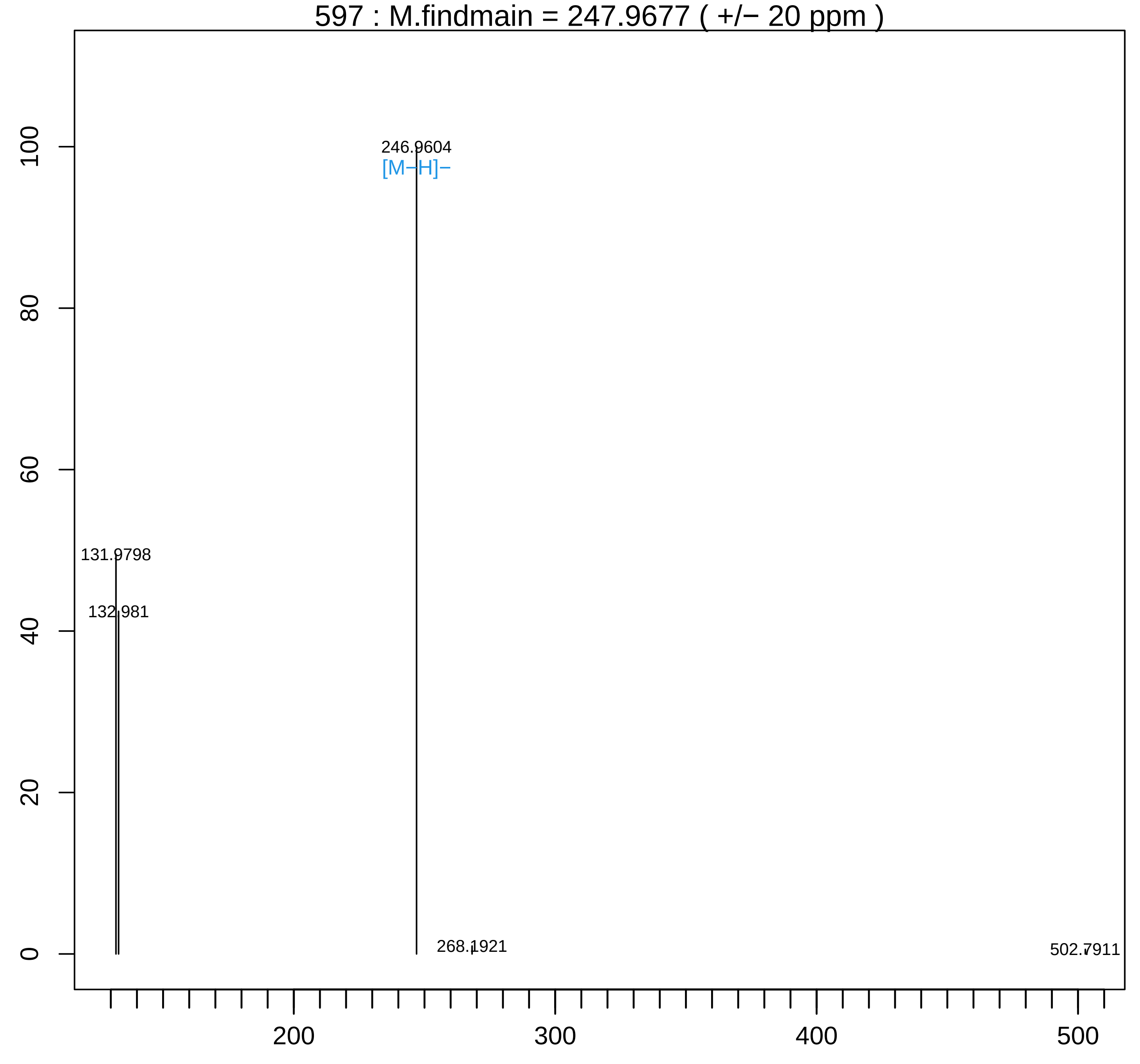
597 : M.findmain = 247.9677 (+/− 20 ppm)
246.9604
[M−H]−
131.9798
132.981
268.1921
502.7911
0
20
40
60
80
100
200
300
400
500

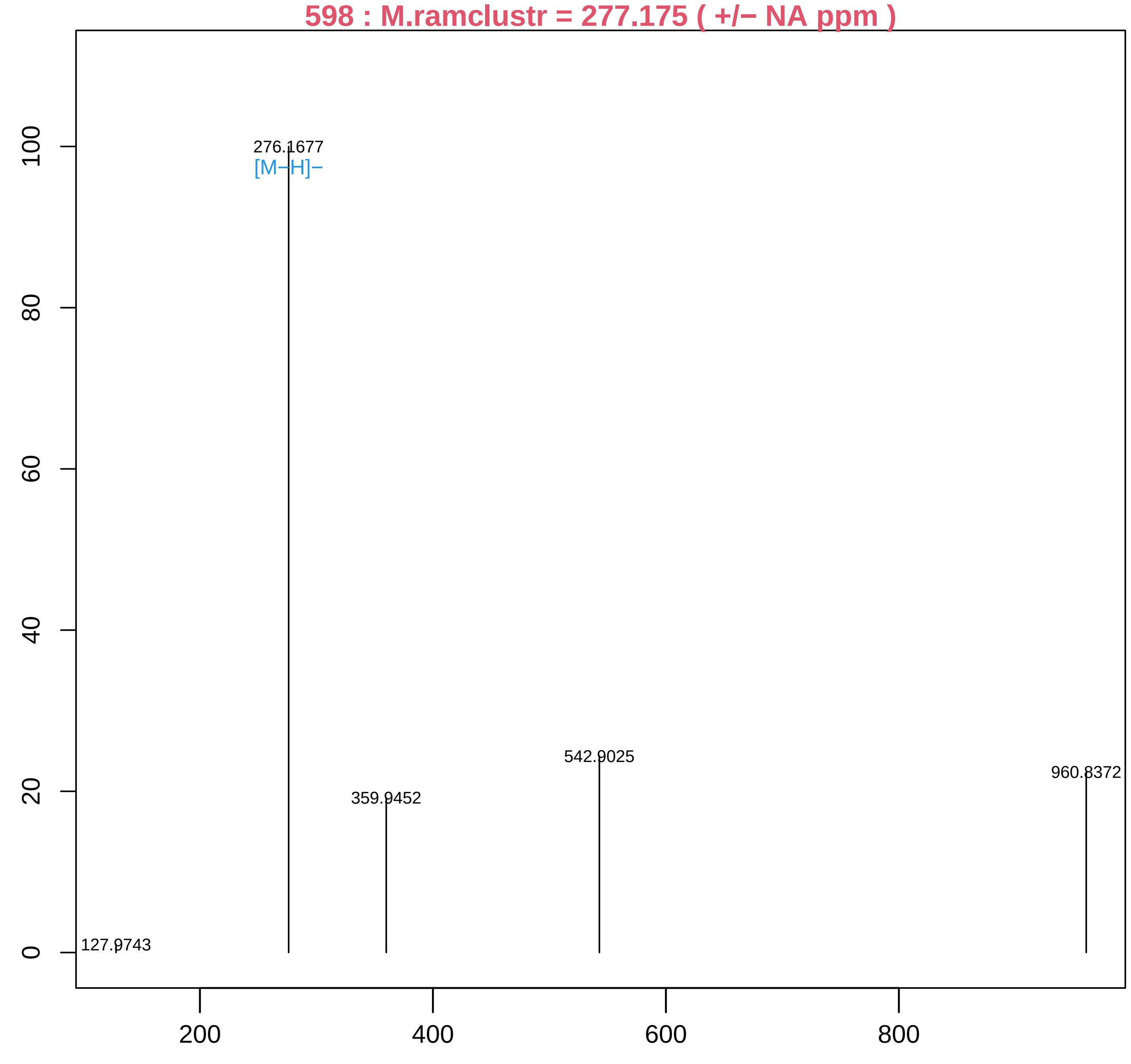

598 : M.ramclustr = 277.175 (+/− NA ppm)
276.1677
[M−H]−
359.9452
542.9025
960.8372
127.9743
0
20
40
60
80
100
200
400
600
800

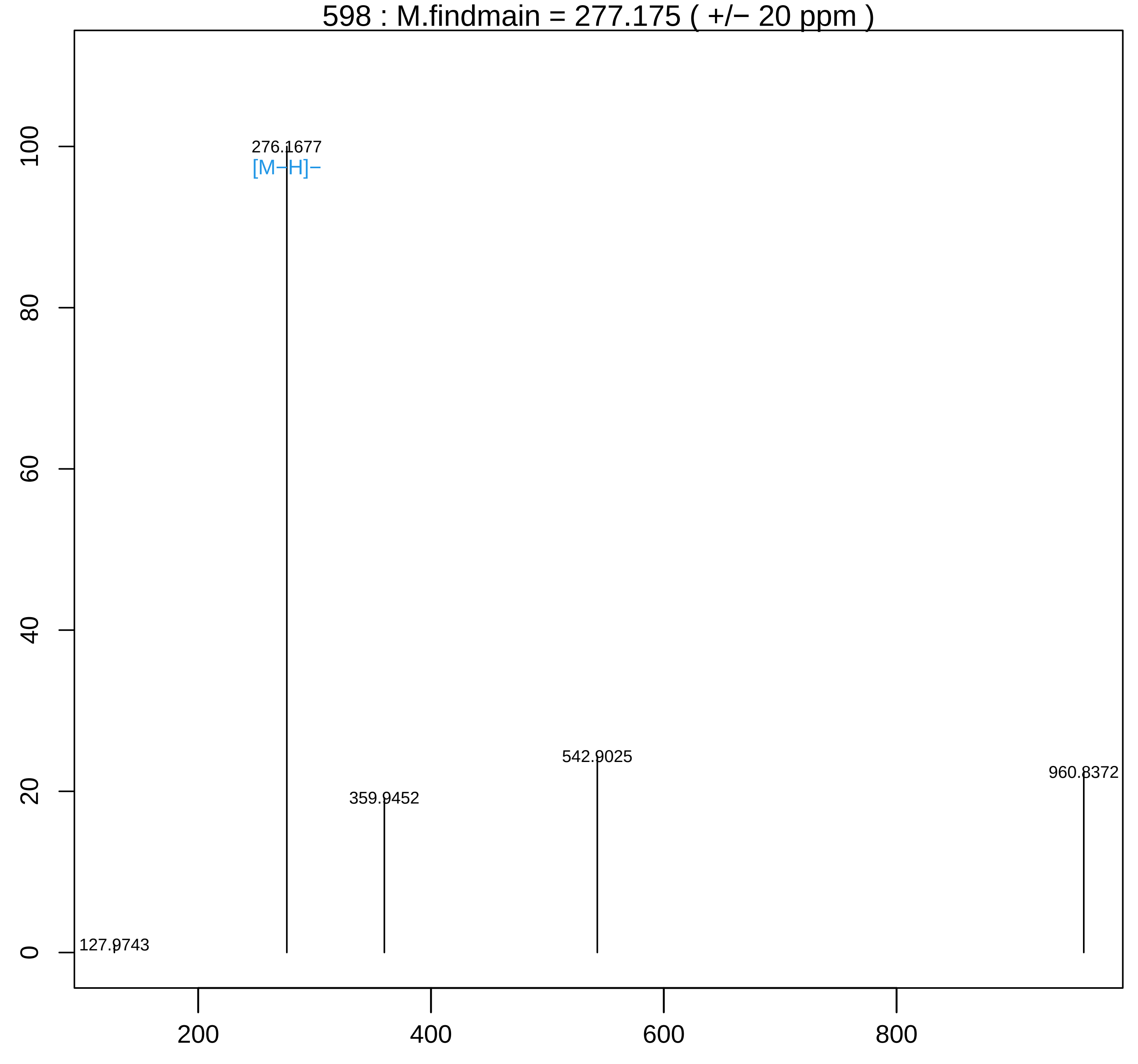

598 : M.findmain = 277.175 (+/− 20 ppm)
276.1677
[M−H]−
359.9452
542.9025
960.8372
127.9743
0
20
40
60
80
100
200
400
600
800

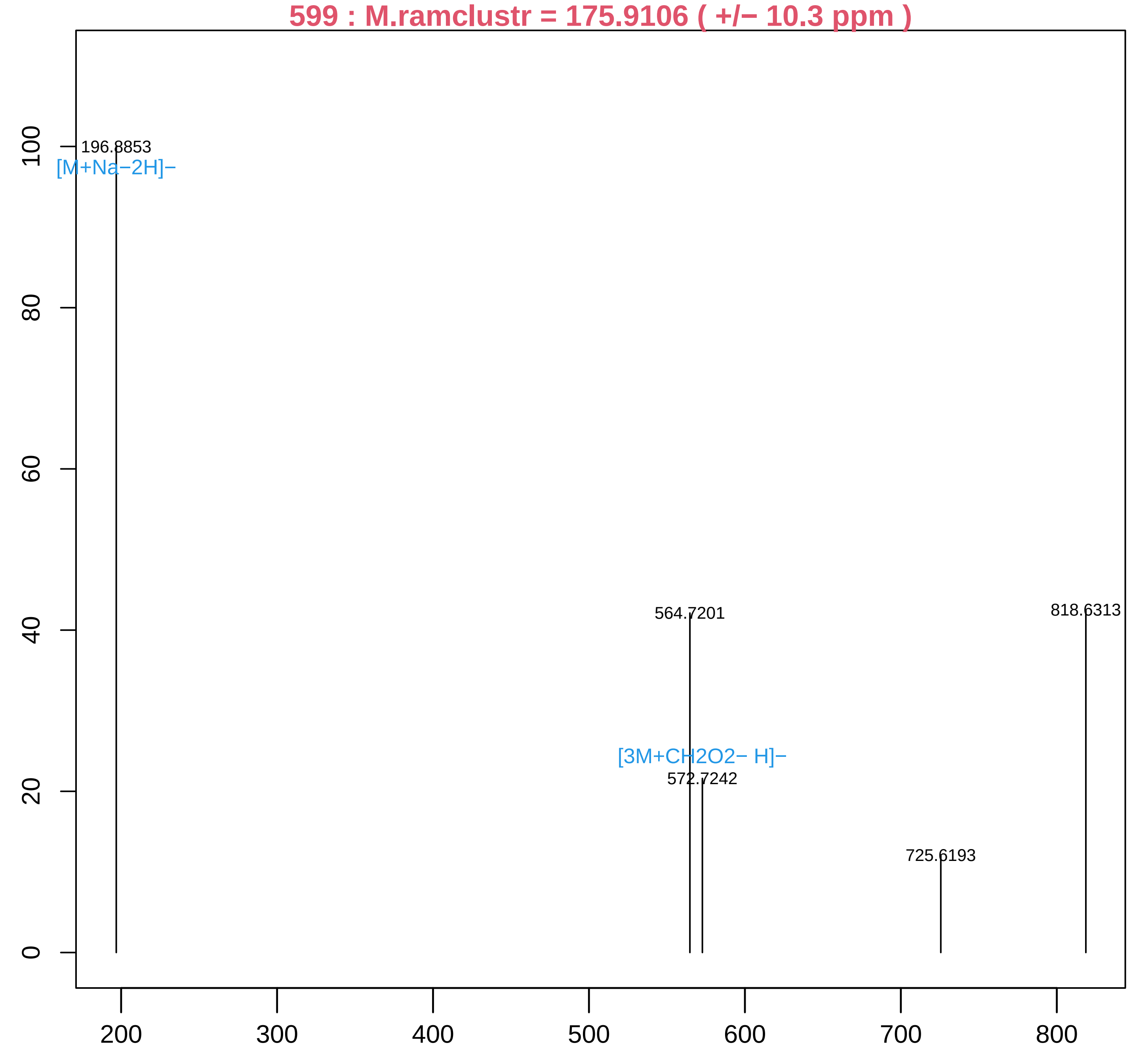
599 : M.ramclustr = 175.9106 (+/− 10.3 ppm)
196.8853
[M+Na−2H]−
564.7201
818.6313
[3M+CH2O2− H]−
572.7242
725.6193
0
20
40
60
80
100
200
300
400
500
600
700
800

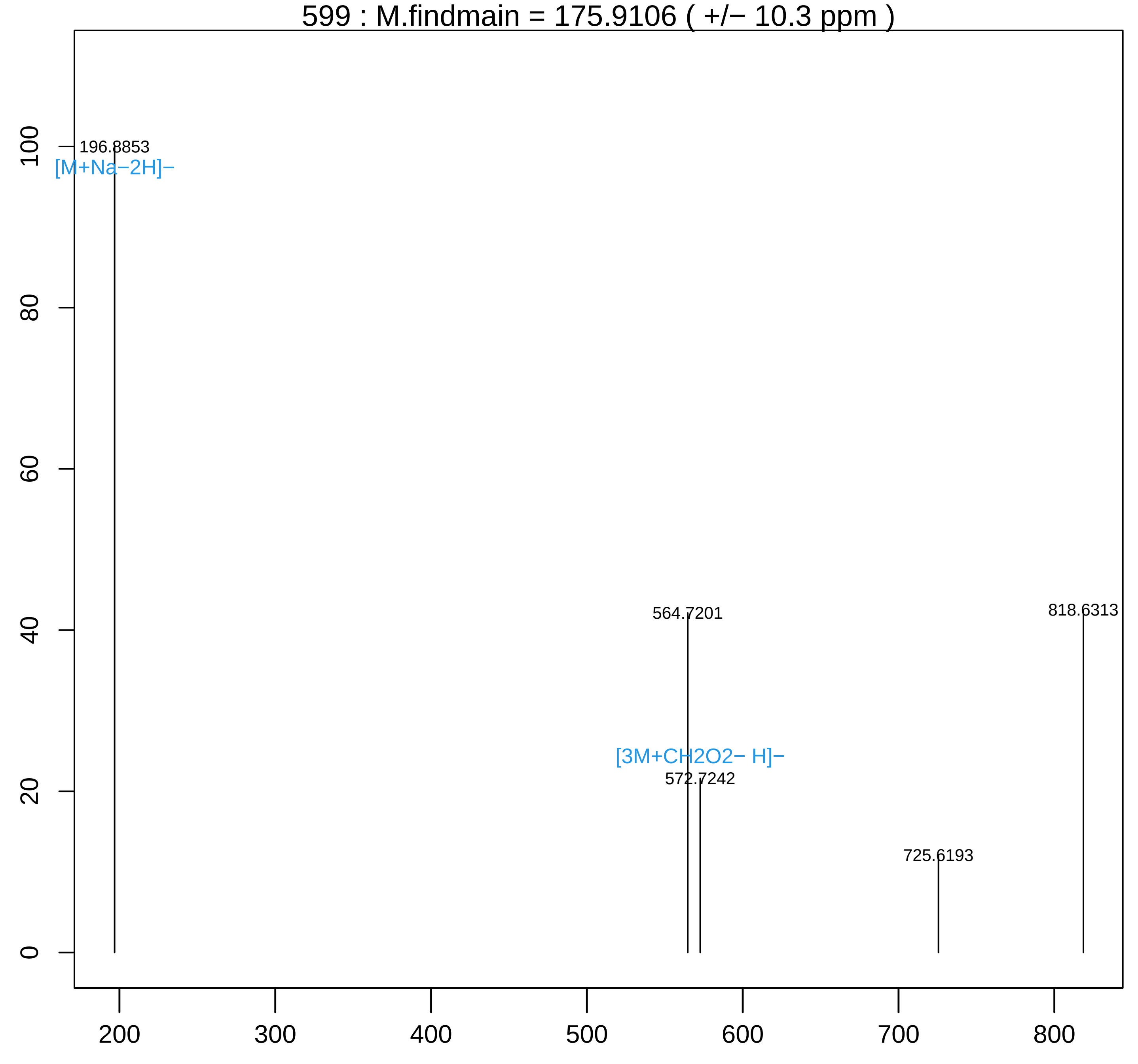
599 : M.findmain = 175.9106 (+/− 10.3 ppm)
196.8853
[M+Na−2H]−
564.7201
818.6313
[3M+CH2O2− H]−
572.7242
725.6193
0
20
40
60
80
100
200
300
400
500
600
700
800

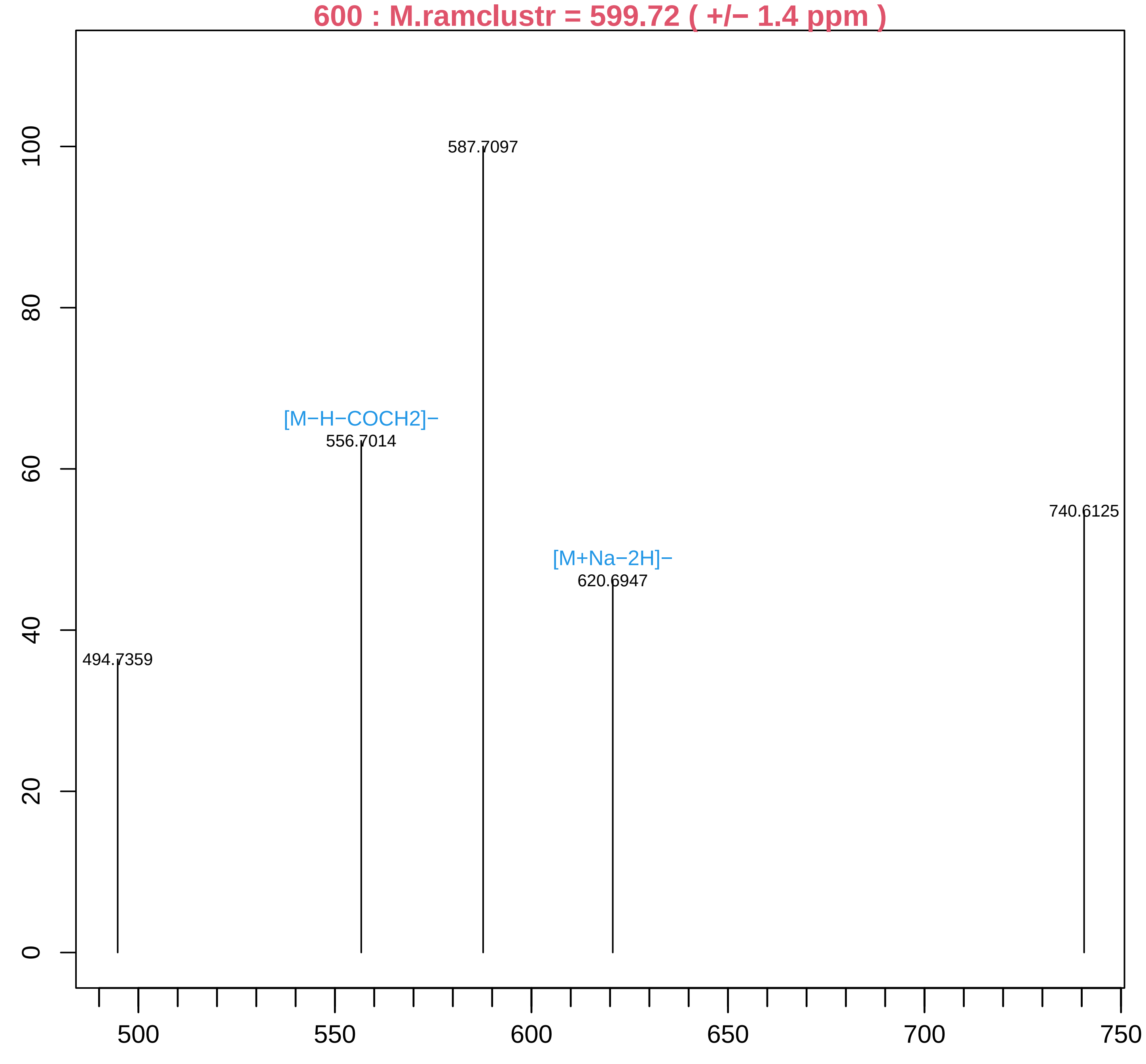
600 : M.ramclustr = 599.72 (+/− 1.4 ppm)
[M−H−COCH2]−
556.7014
587.7097
[M+Na−2H]−
620.6947
740.6125
494.7359
0
20
40
60
80
100
500
550
600
650
700
750

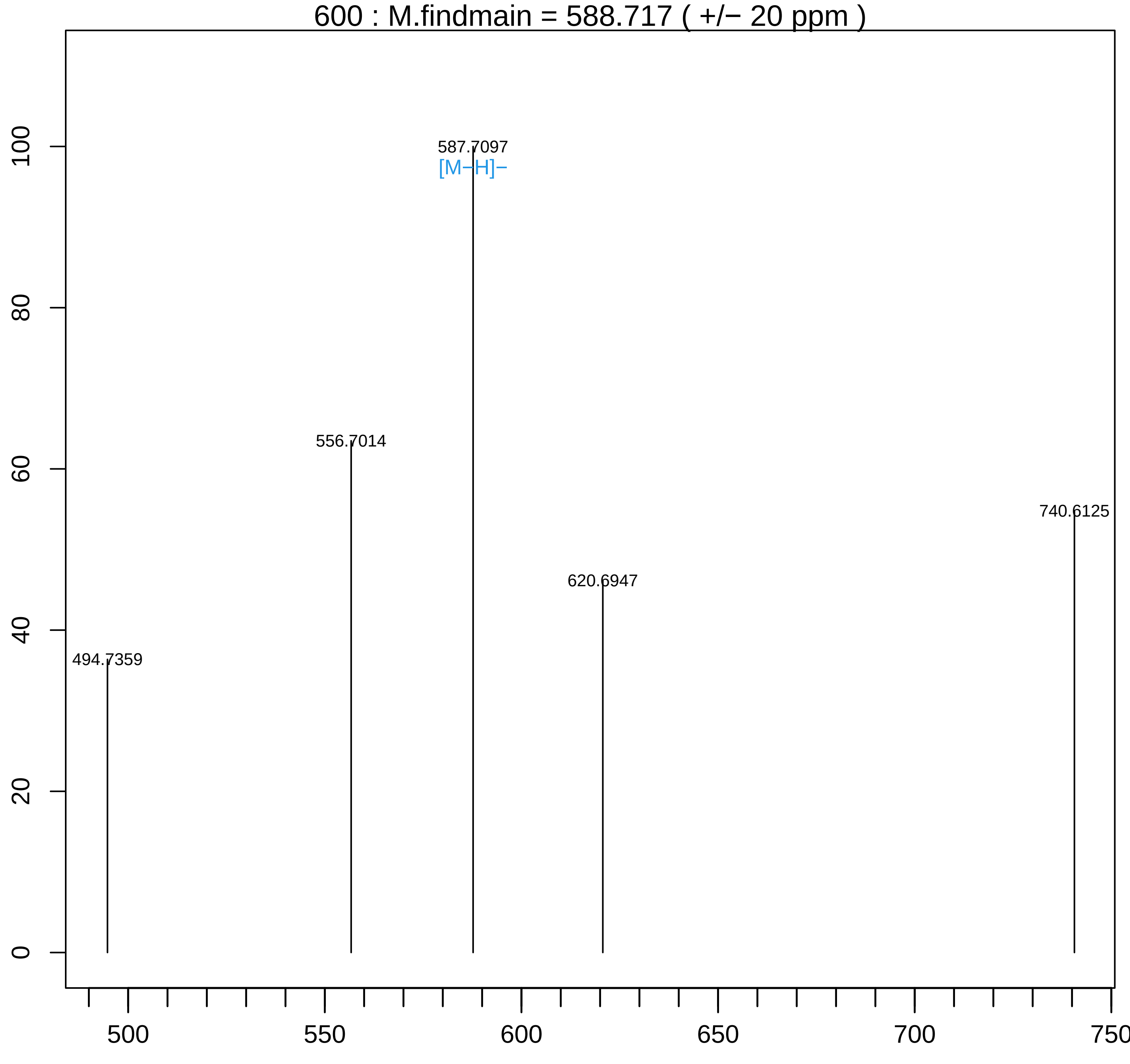
600 : M.findmain = 588.717 (+/− 20 ppm)
587.7097
[M−H]−
556.7014
620.6947
740.6125
494.7359
0
20
40
60
80
100
500
550
600
650
700
750

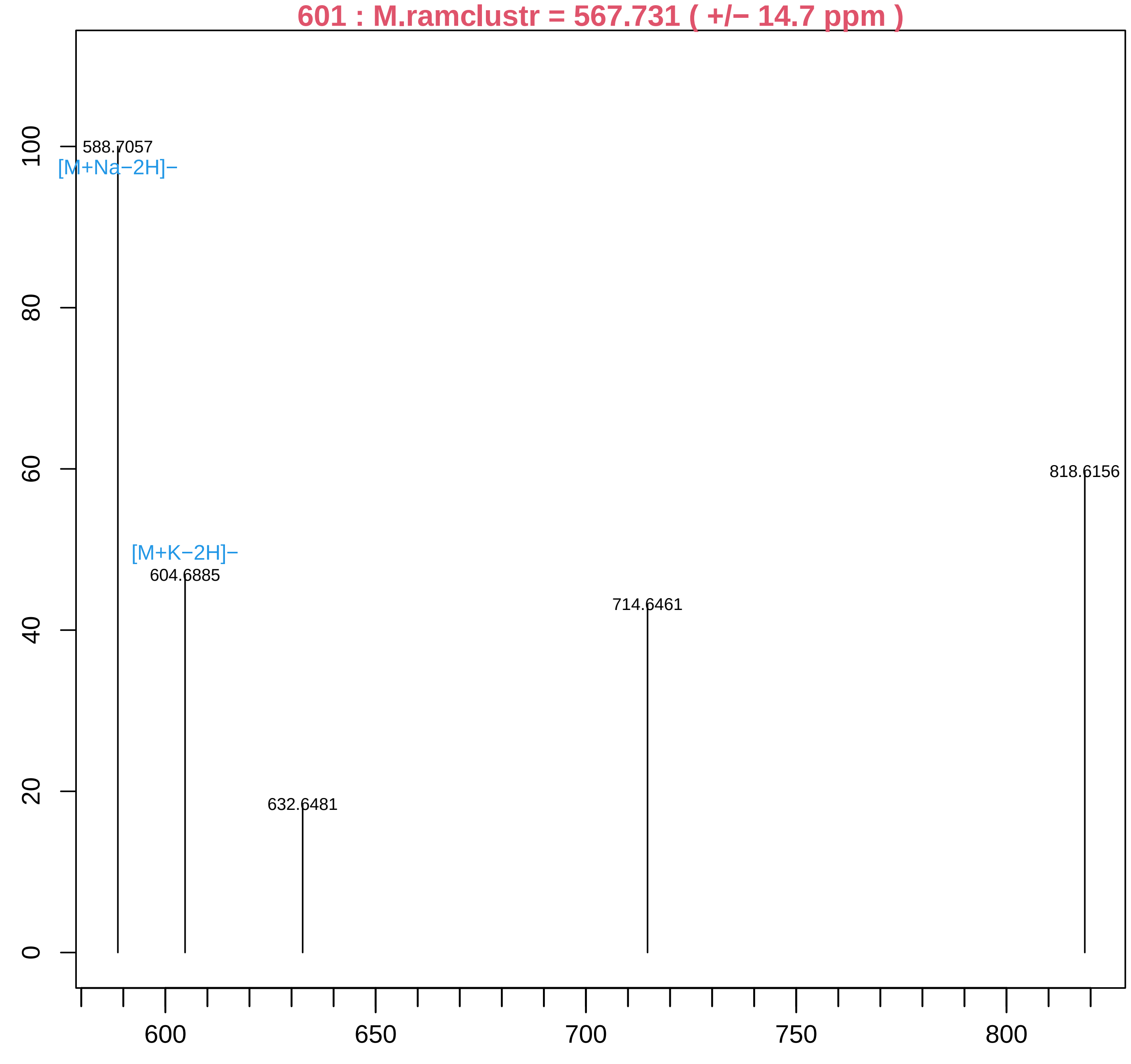
601 : M.ramclustr = 567.731 (+/− 14.7 ppm)
588.7057
[M+Na−2H]−
[M+K−2H]−
604.6885
632.6481
714.6461
818.6156
0
20
40
60
80
100
600
650
700
750
800

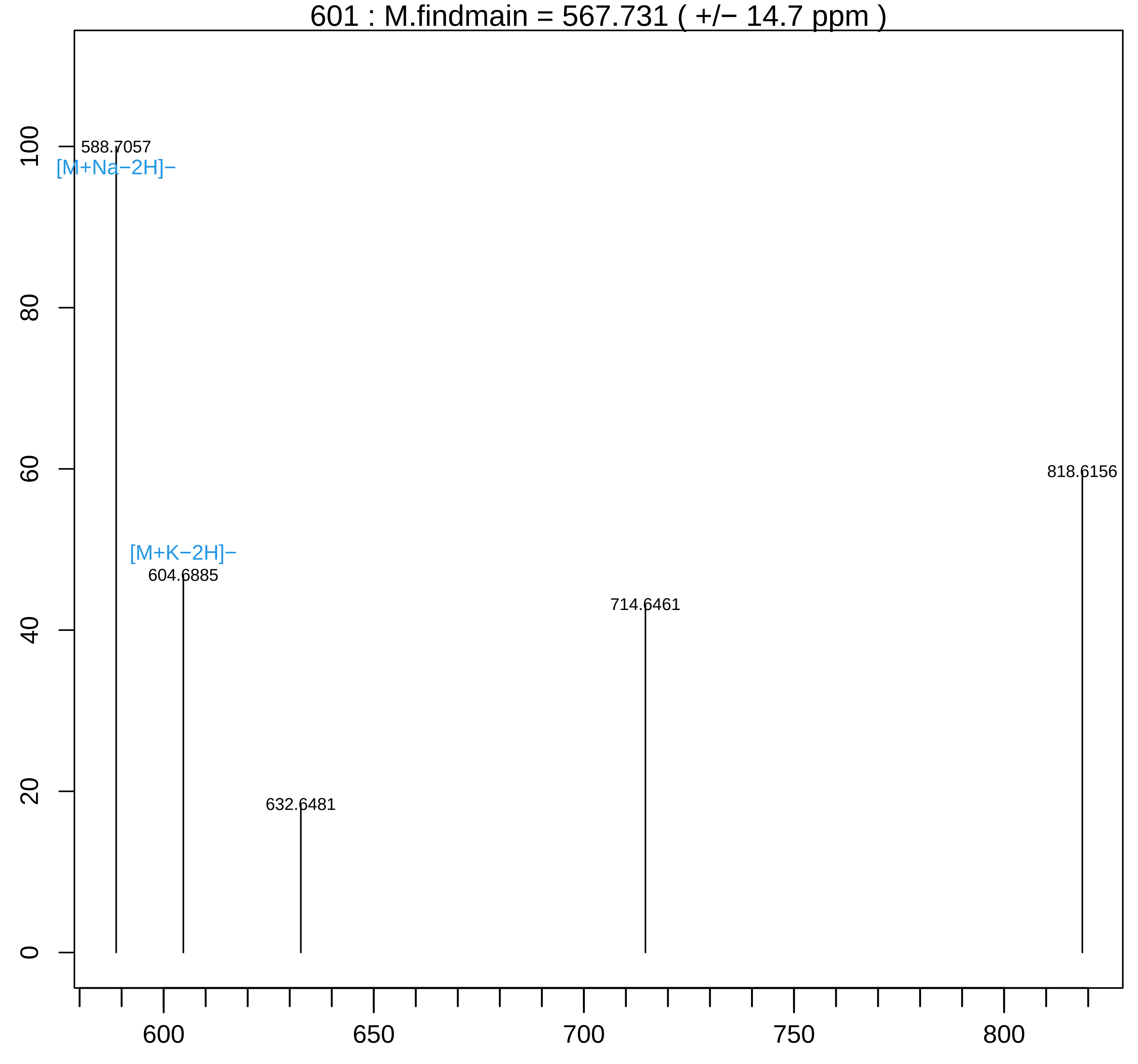
601 : M.findmain = 567.731 (+/− 14.7 ppm)
588.7057
[M+Na−2H]−
[M+K−2H]−
604.6885
632.6481
714.6461
818.6156
0
20
40
60
80
100
600
650
700
750
800

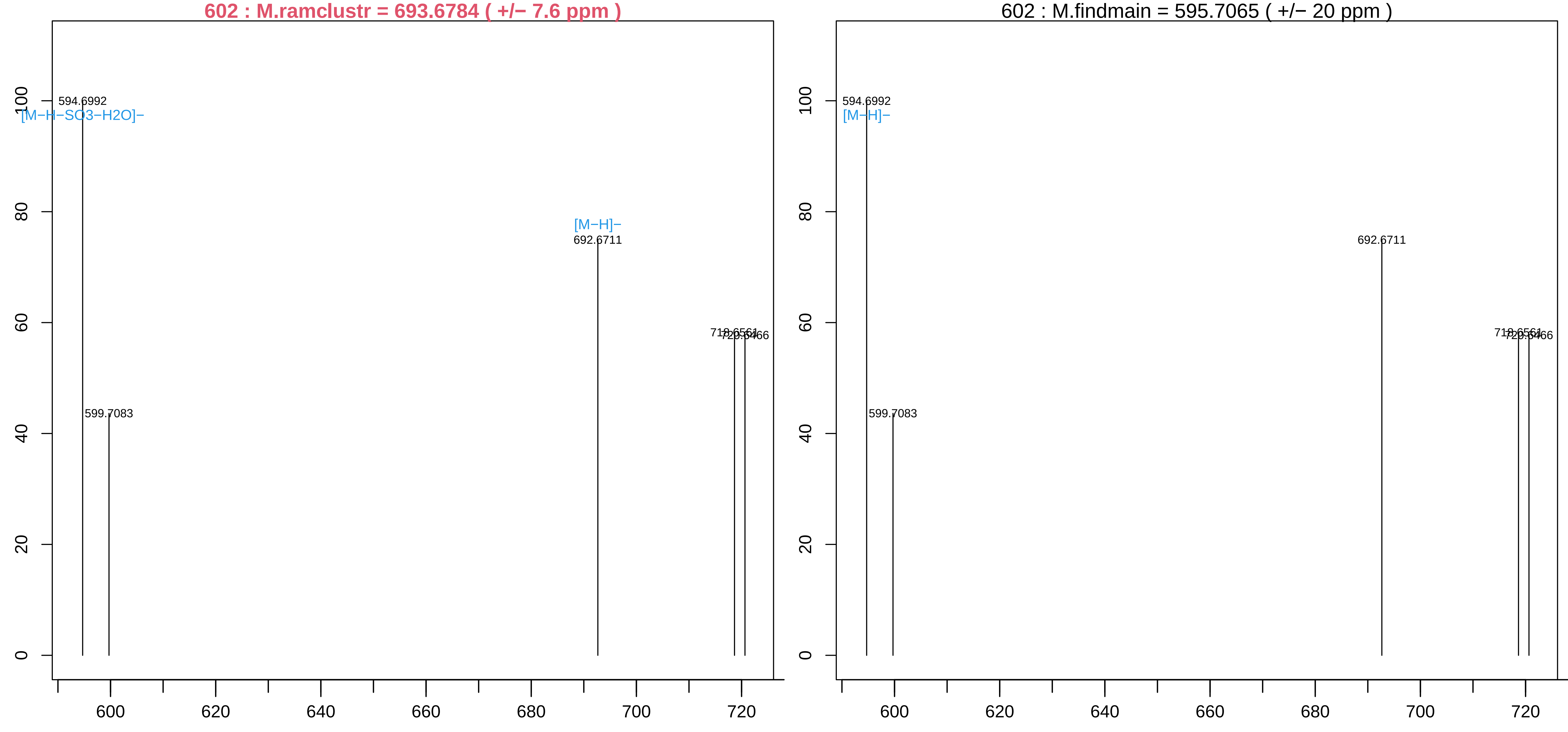
602 : M.ramclustr = 693.6784 (+/− 7.6 ppm)
594.6992
[M−H−SO3−H2O]−
599.7083
[M−H]−
692.6711
718.6561
720.6466
0
20
40
60
80
100
600
620
640
660
680
700
720
602 : M.findmain = 595.7065 (+/− 20 ppm)
594.6992
[M−H]−
599.7083
692.6711
718.6561
720.6466
0
20
40
60
80
100
600
620
640
660
680
700
720

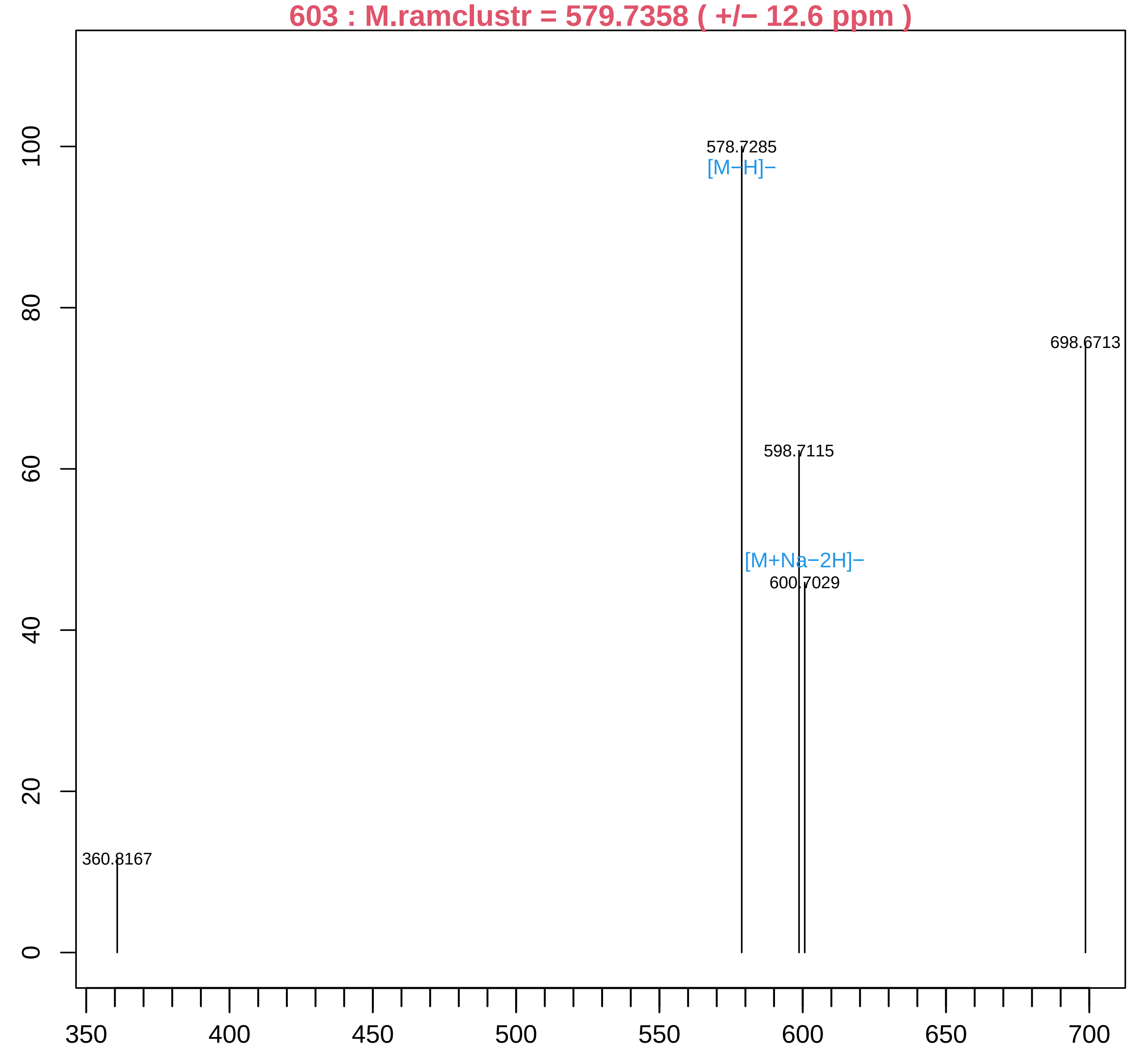

603 : M.ramclustr = 579.7358 (+/− 12.6 ppm)
578.7285
[M−H]−
598.7115
[M+Na−2H]−
600.7029
698.6713
360.8167
0
20
40
60
80
100
350
400
450
500
550
600
650
700

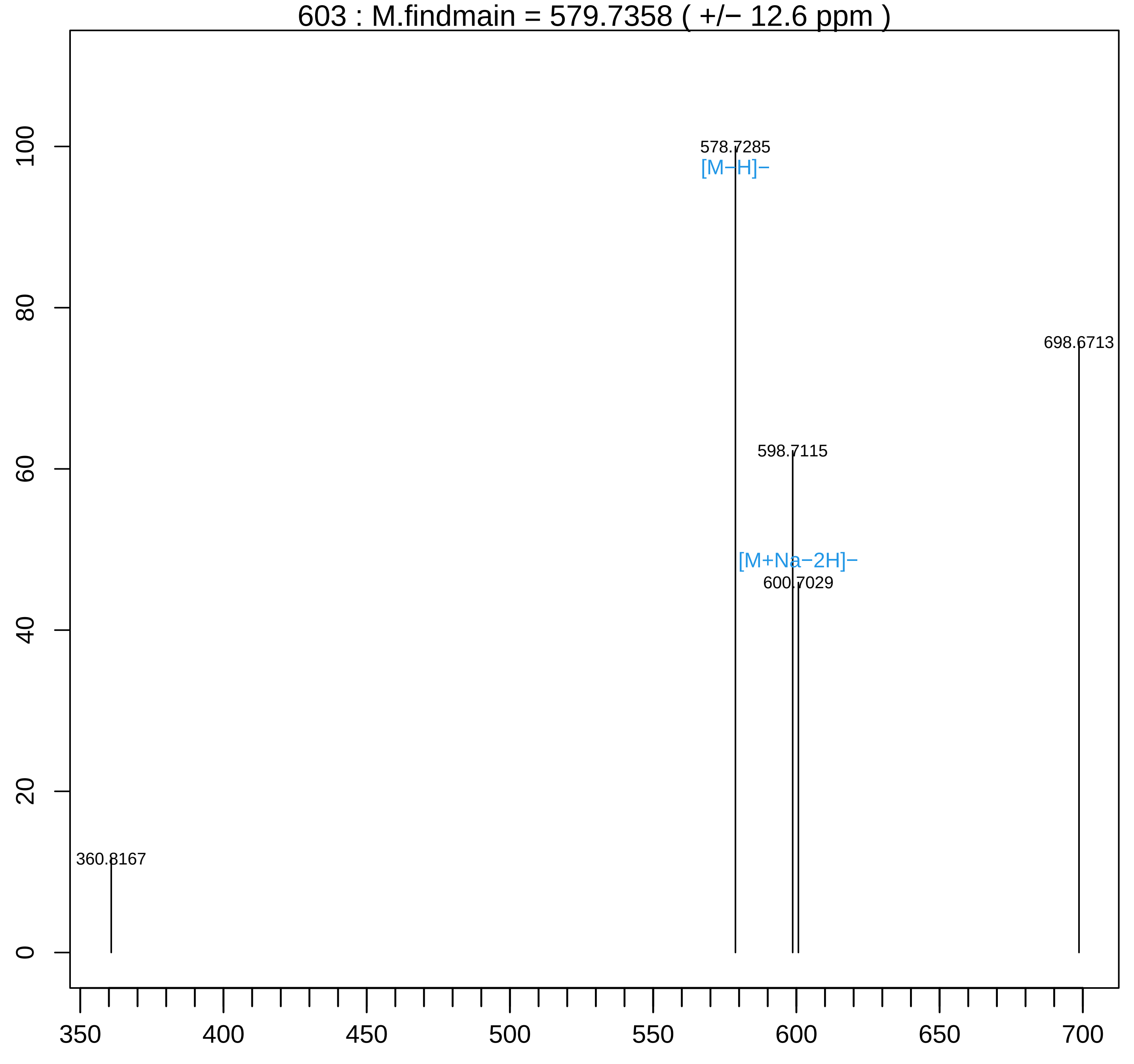

603 : M.findmain = 579.7358 (+/− 12.6 ppm)
578.7285
[M−H]−
598.7115
[M+Na−2H]−
600.7029
698.6713
360.8167
0
20
40
60
80
100
350
400
450
500
550
600
650
700

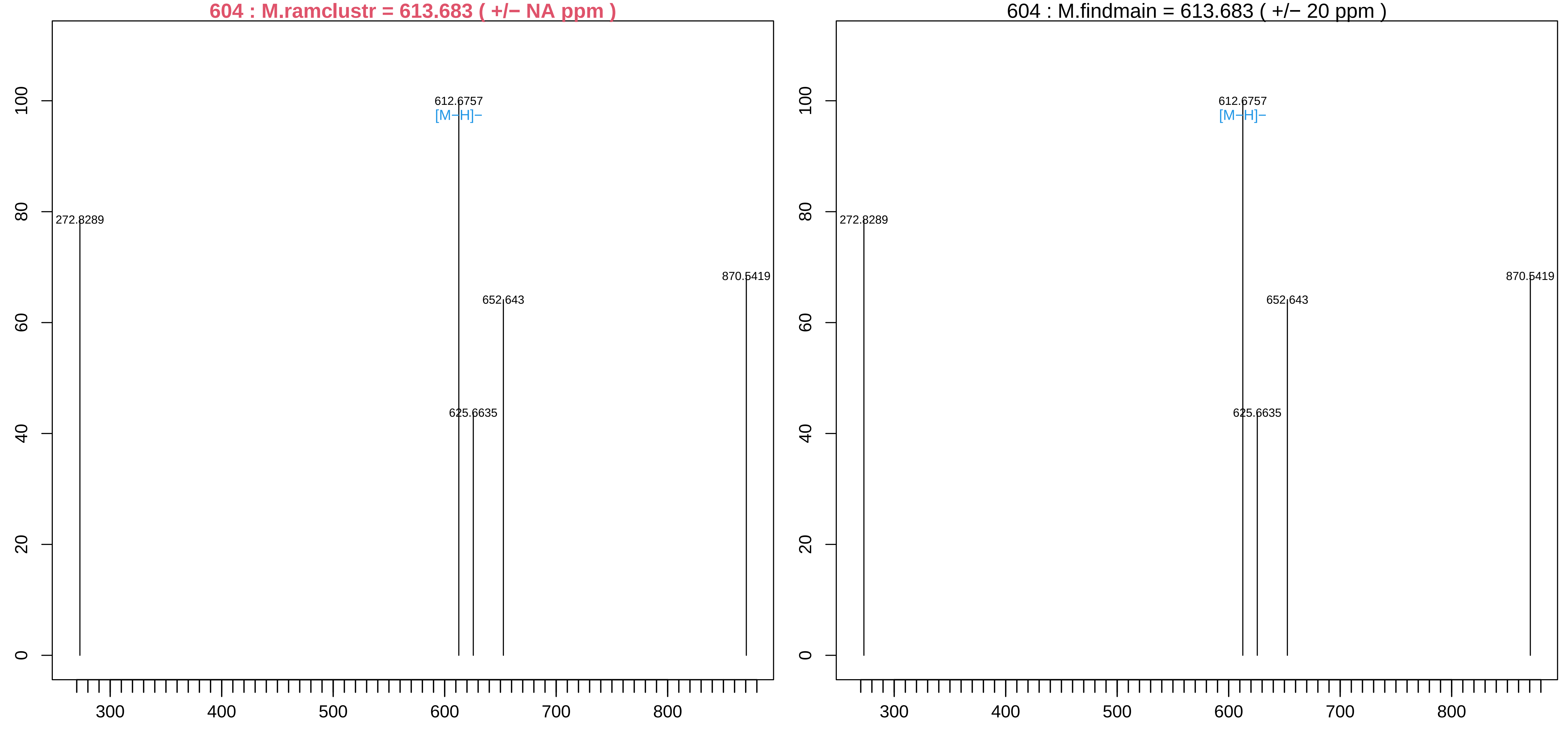
604 : M.ramclustr = 613.683 (+/− NA ppm)
272.8289
612.6757
[M−H]−
625.6635
652.643
870.5419
604 : M.findmain = 613.683 (+/− 20 ppm)
272.8289
612.6757
[M−H]−
625.6635
652.643
870.5419

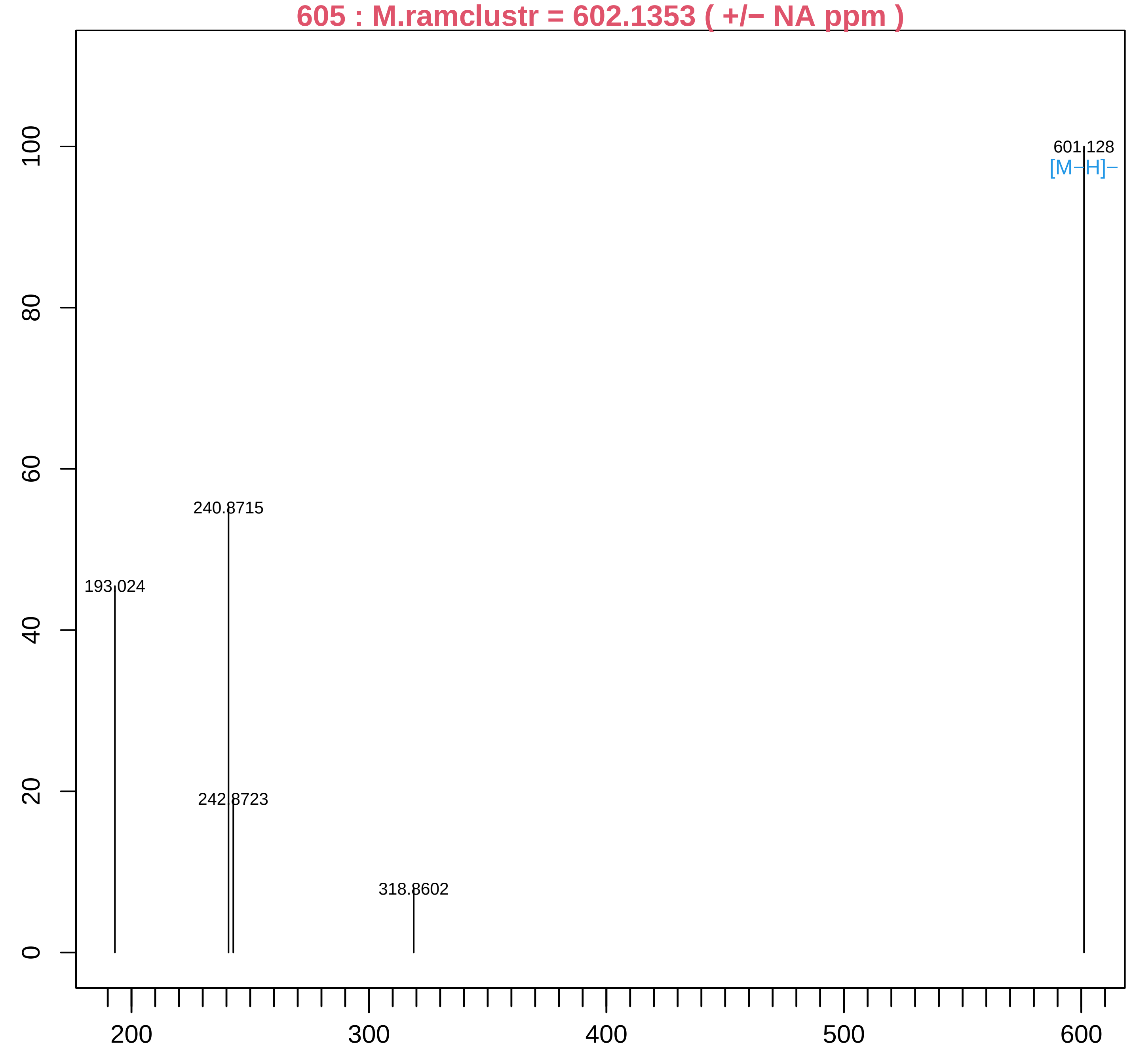
605 : M.ramclustr = 602.1353 (+/− NA ppm)
601.128
[M−H]−
240.8715
193.024
242.8723
318.8602
0
20
40
60
80
100
200
300
400
500
600

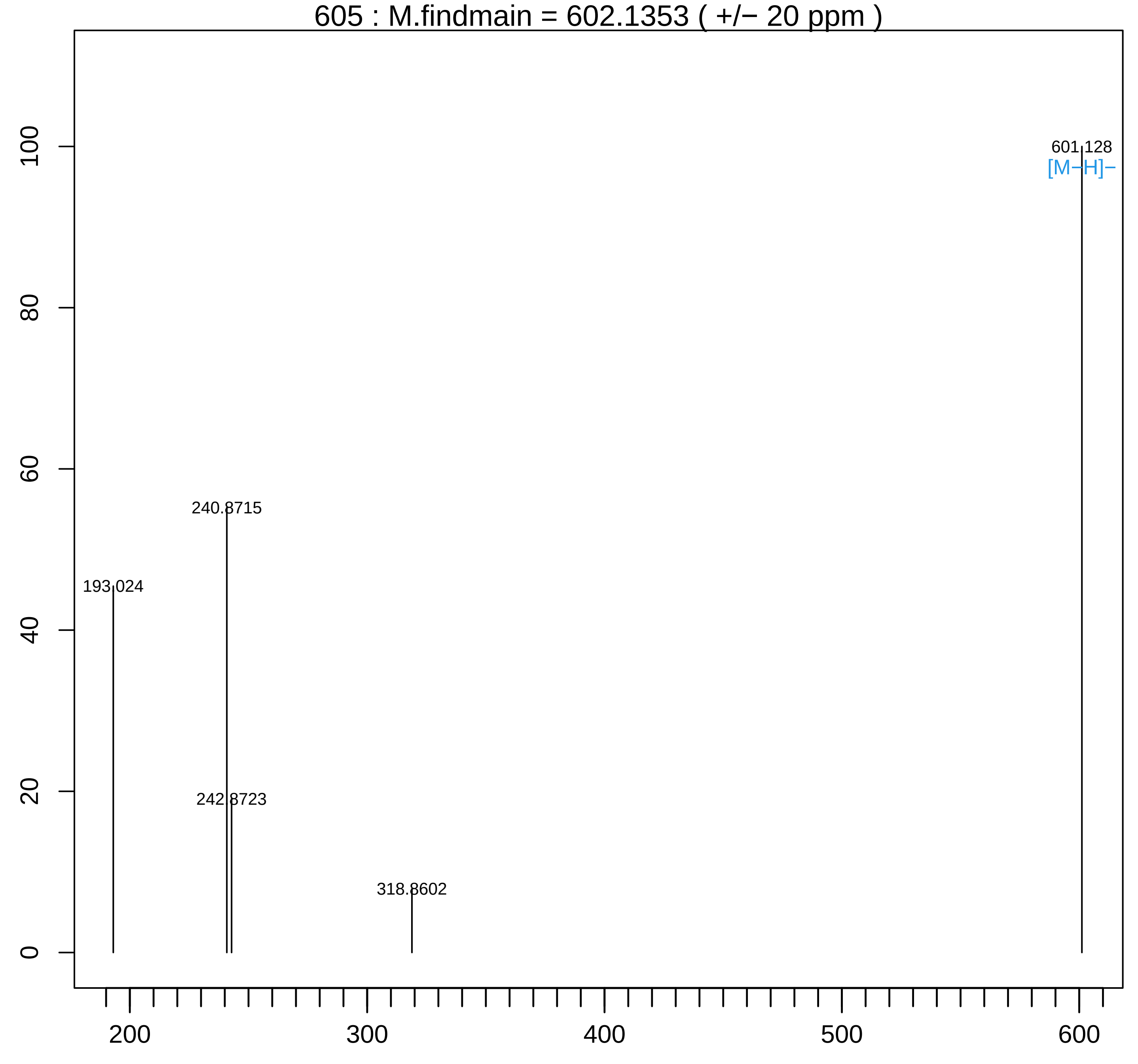
605 : M.findmain = 602.1353 (+/− 20 ppm)
601.128
[M−H]−
240.8715
193.024
242.8723
318.8602
0
20
40
60
80
100
200
300
400
500
600

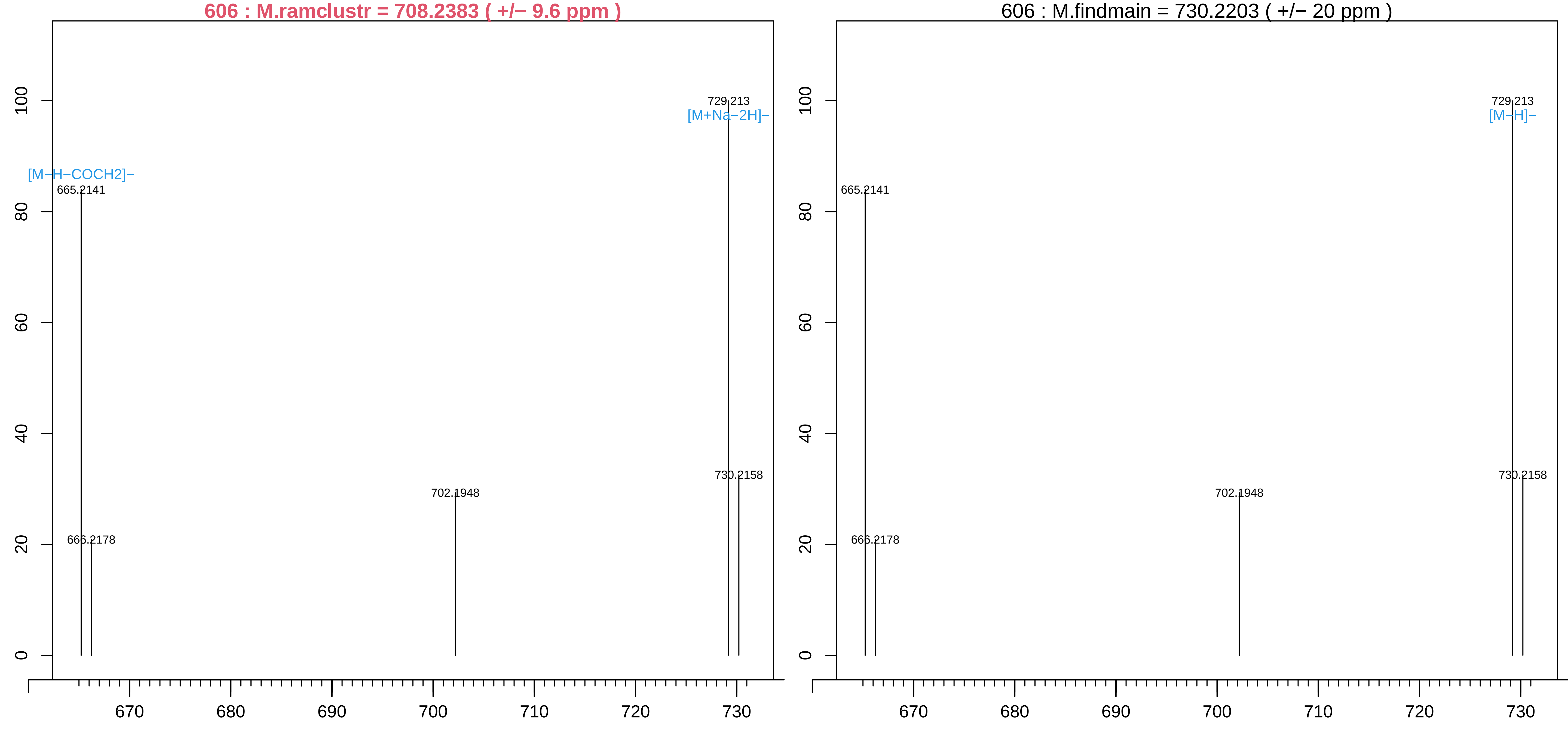
606 : M.ramclustr = 708.2383 (+/− 9.6 ppm)
729.213
[M+Na−2H]−
[M−H−COCH2]−
665.2141
666.2178
702.1948
730.2158
606 : M.findmain = 730.2203 (+/− 20 ppm)
729.213
[M−H]−
665.2141
666.2178
702.1948
730.2158
0
20
40
60
80
100
670
680
690
700
710
720
730

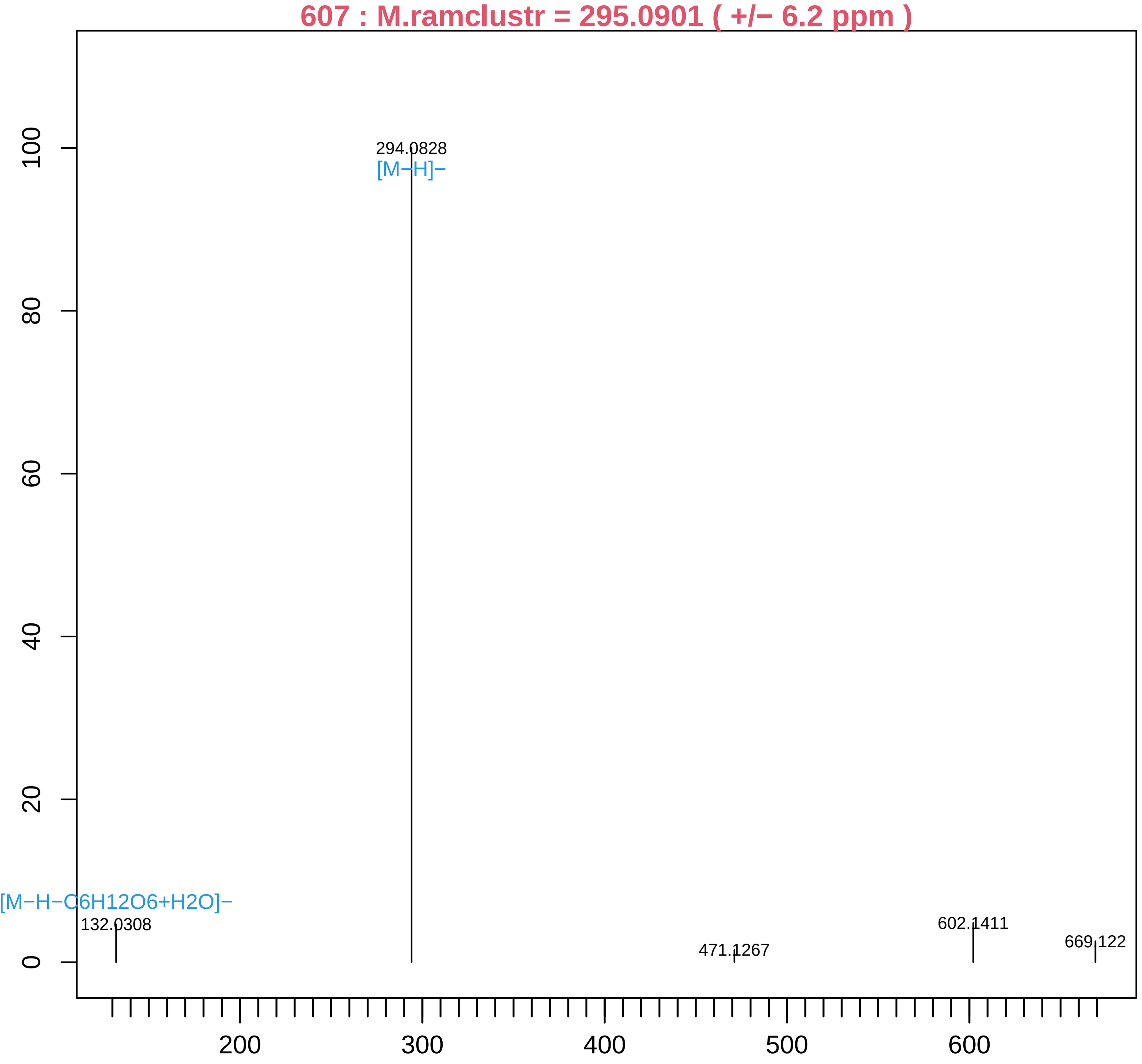

607 : M.ramclustr = 295.0901 (+/− 6.2 ppm)
294.0828
[M−H]−
[M−H−C6H12O6+H2O]−
132.0308
471.1267
602.1411
669.122
0
20
40
60
80
100
200
300
400
500
600

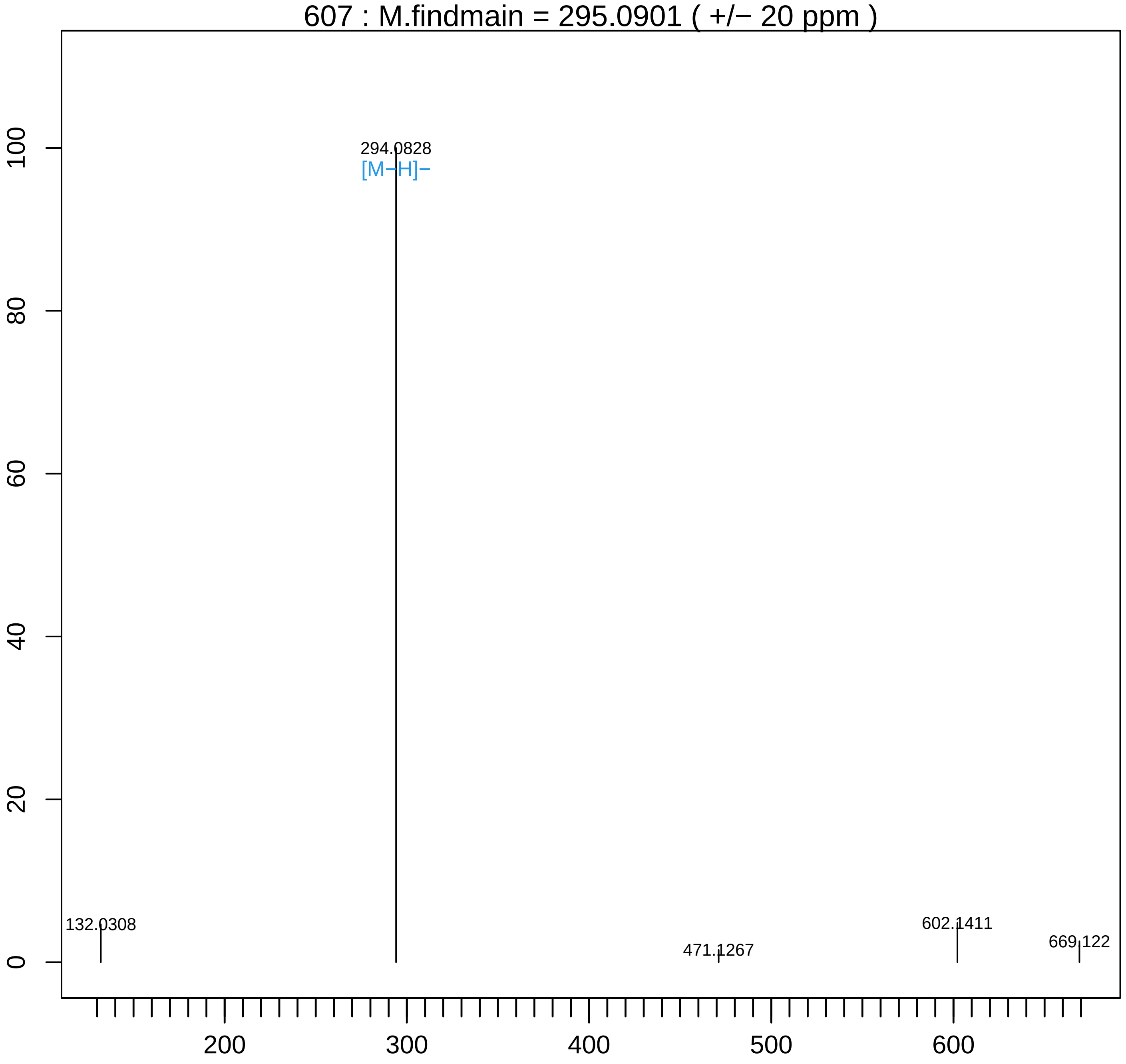

607 : M.findmain = 295.0901 (+/− 20 ppm)
294.0828
[M−H]−
132.0308
471.1267
602.1411
669.122
0
20
40
60
80
100
200
300
400
500
600

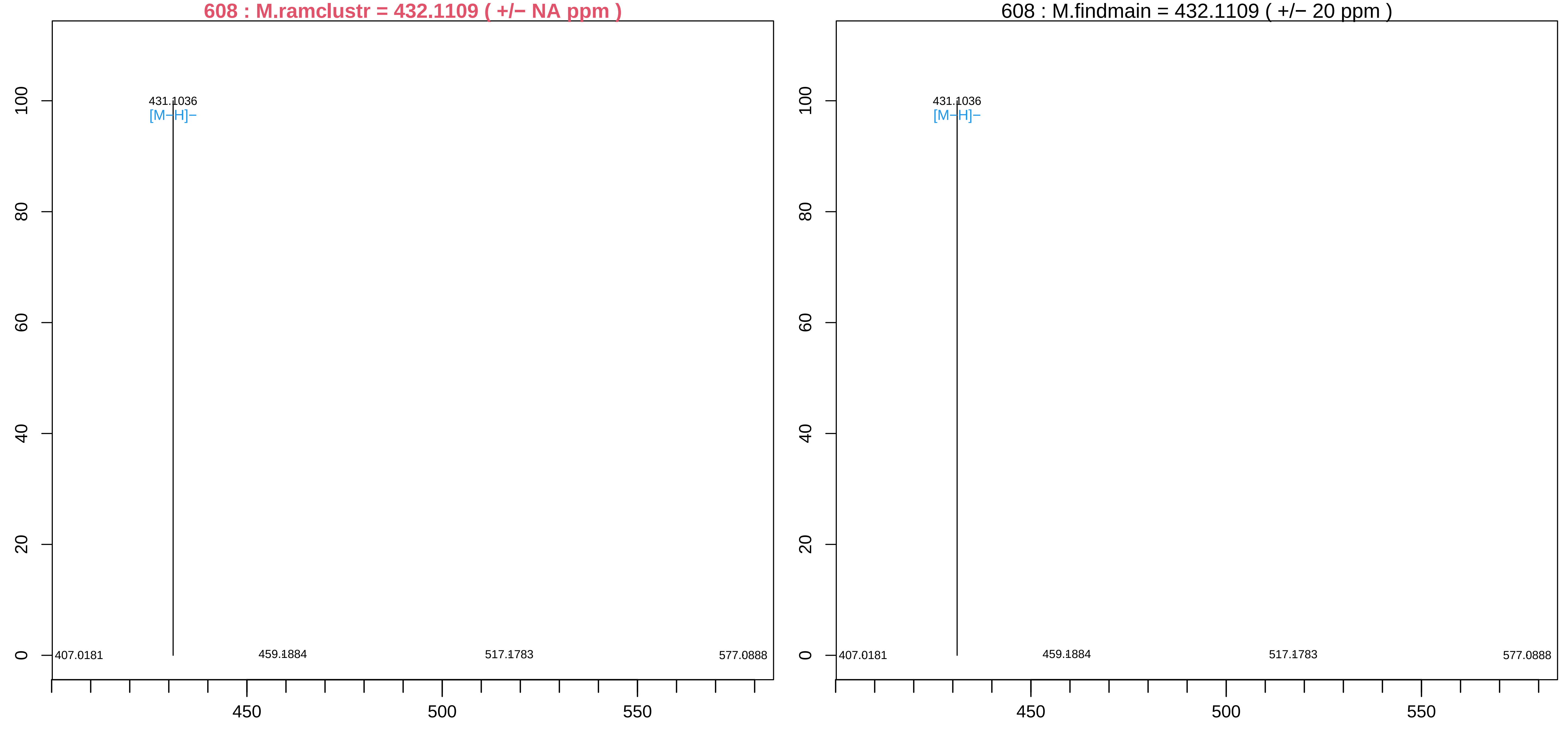
608 : M.ramclustr = 432.1109 (+/− NA ppm)
431.1036
[M−H]−
407.0181
459.1884
517.1783
577.0888
608 : M.findmain = 432.1109 (+/− 20 ppm)
431.1036
[M−H]−
407.0181
459.1884
517.1783
577.0888
0
20
40
60
80
100
450
500
550

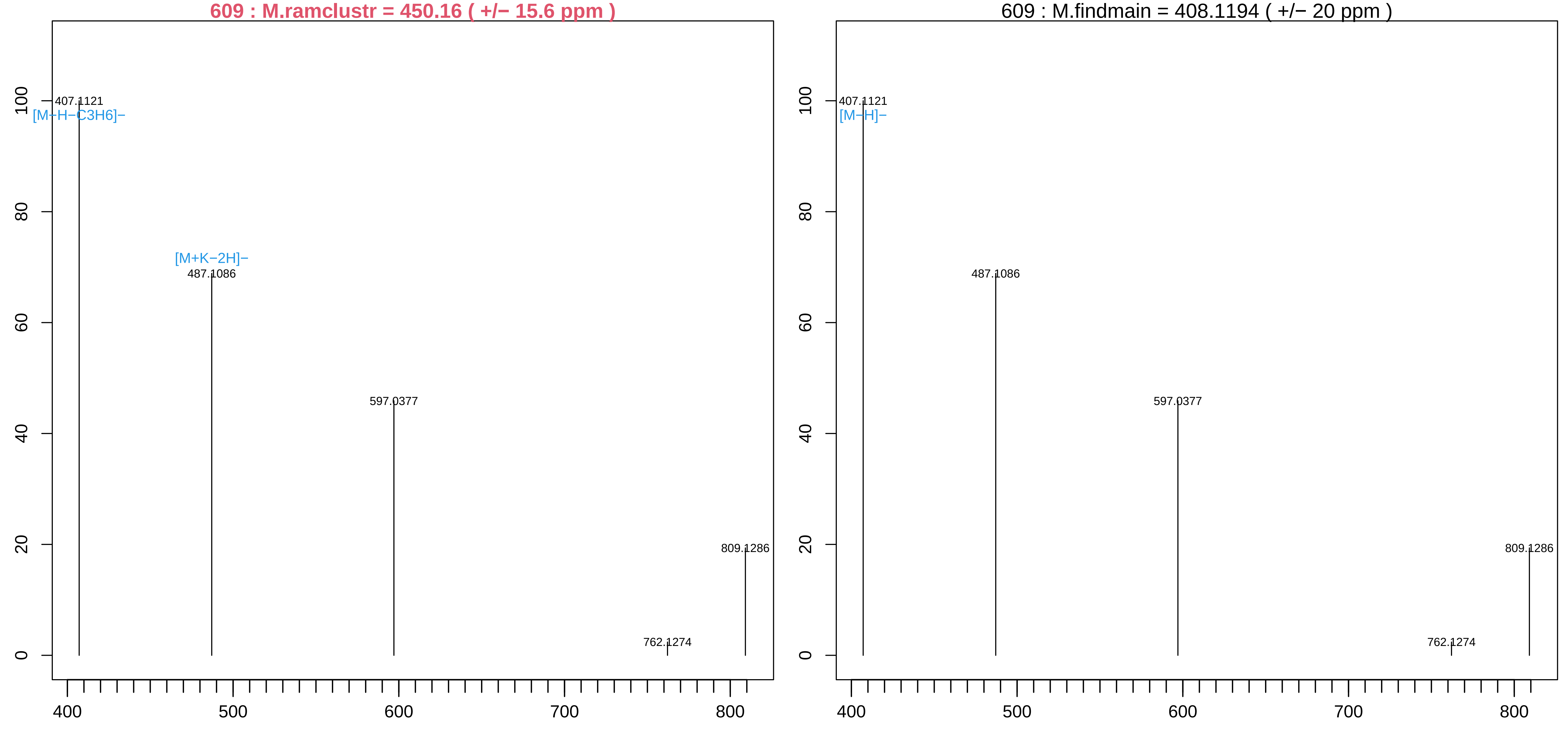
609 : M.ramclustr = 450.16 (+/− 15.6 ppm)
609 : M.findmain = 408.1194 (+/− 20 ppm)
407.1121
[M−H−C3H6]−
[M+K−2H]−
487.1086
597.0377
762.1274
809.1286
407.1121
[M−H]−
487.1086
597.0377
762.1274
809.1286
0
20
40
60
80
100
400
500
600
700
800

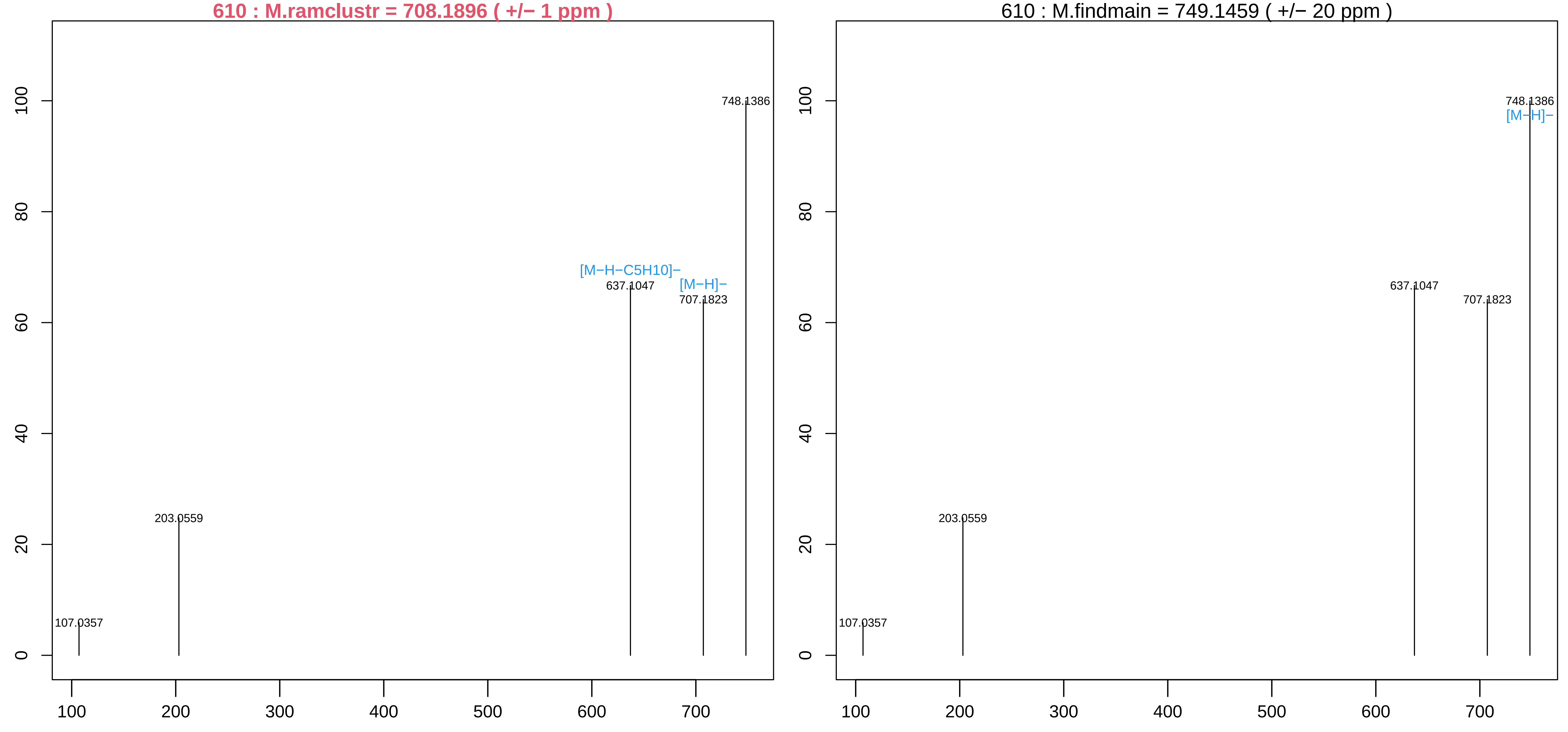

610 : M.ramclustr = 708.1896 (+/− 1 ppm)
748.1386
[M−H−C5H10]−
637.1047
[M−H]−
707.1823
203.0559
107.0357
610 : M.findmain = 749.1459 (+/− 20 ppm)
748.1386
[M−H]−
637.1047
707.1823
203.0559
107.0357

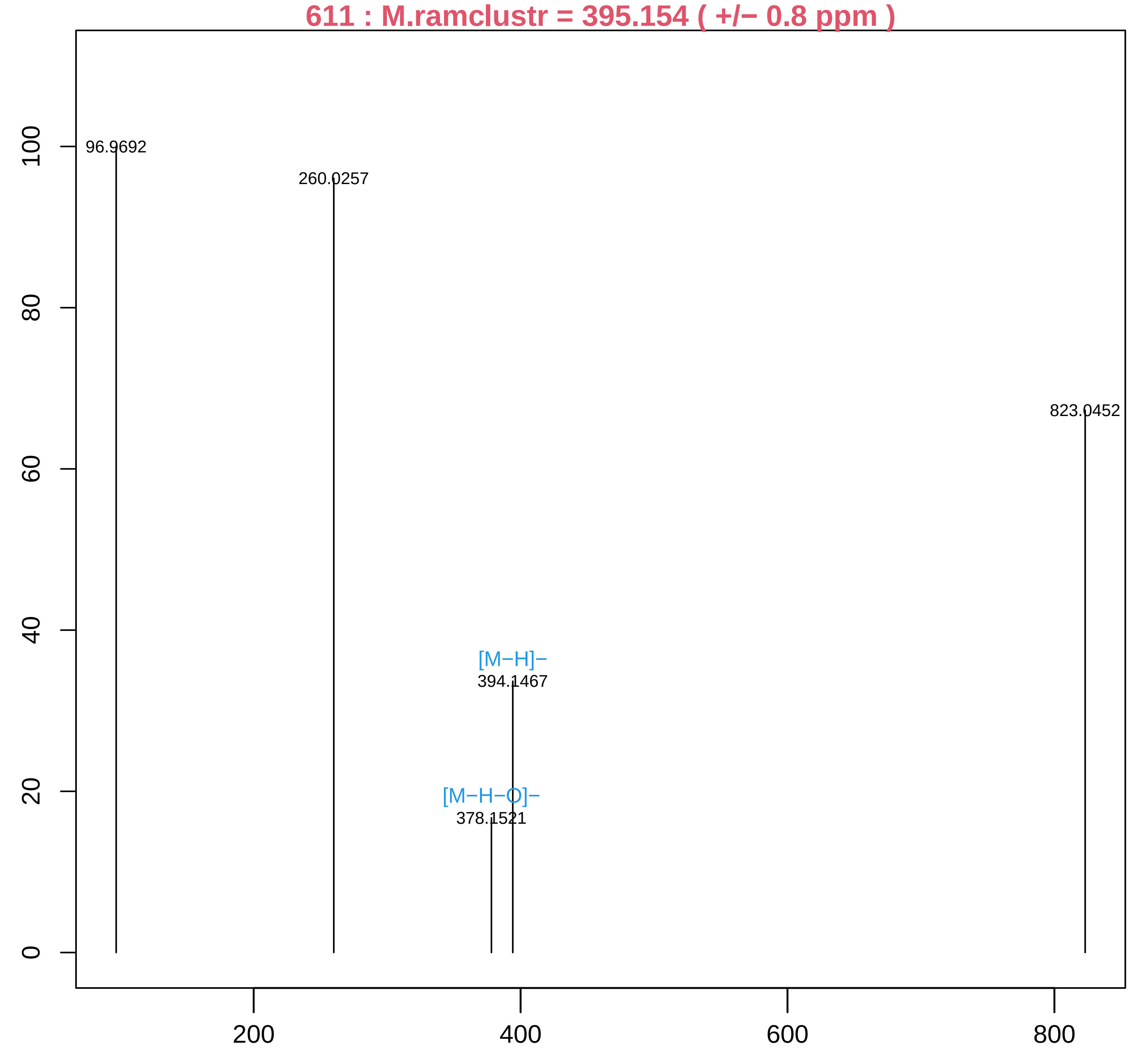
611 : M.ramclustr = 395.154 (+/− 0.8 ppm)
96.9692
260.0257
[M−H]−
394.1467
[M−H−O]−
378.1521
823.0452
0
20
40
60
80
100
200
400
600
800

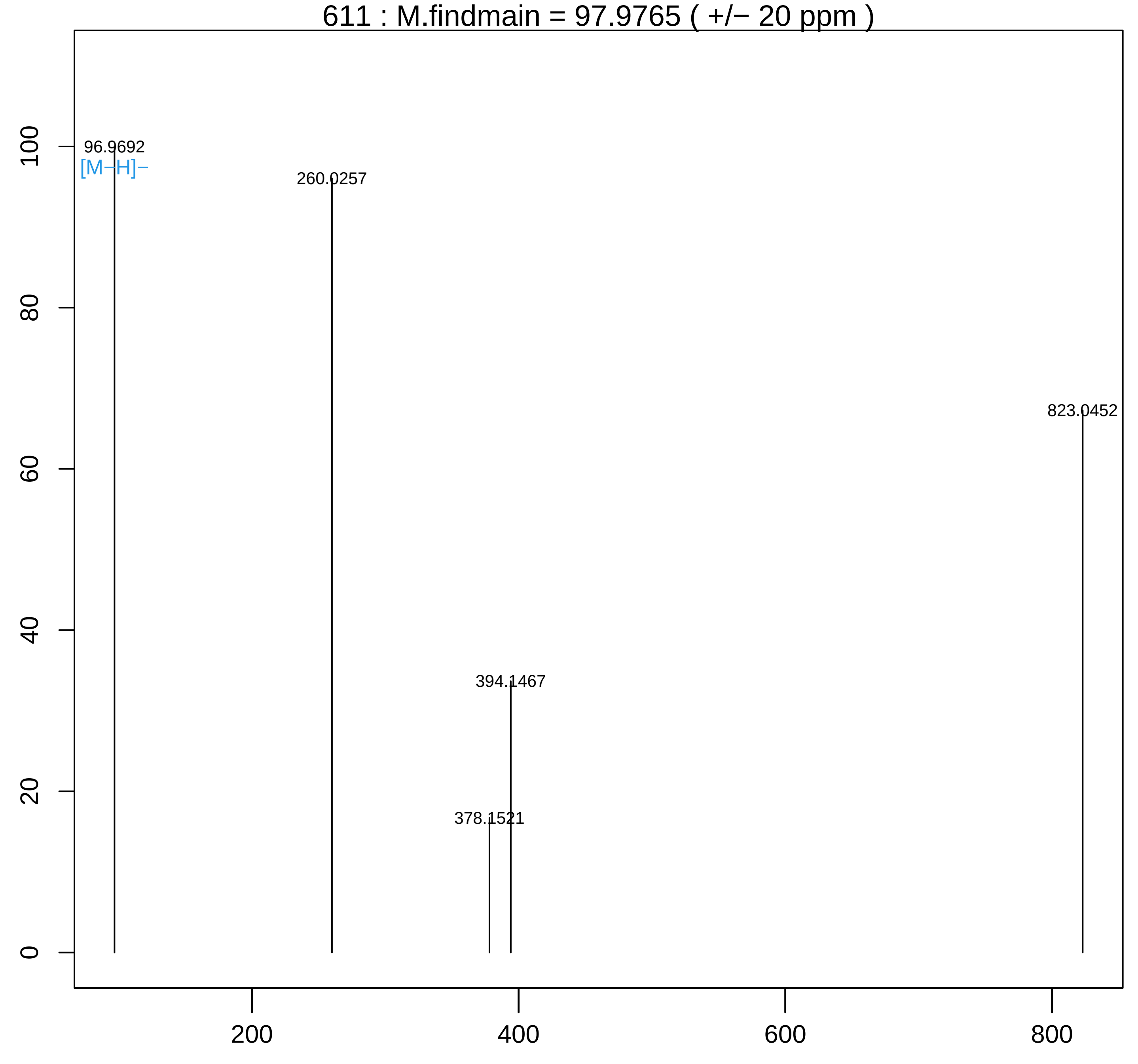
611 : M.findmain = 97.9765 (+/− 20 ppm)
96.9692
[M−H]−
260.0257
394.1467
378.1521
823.0452
0
20
40
60
80
100
200
400
600
800

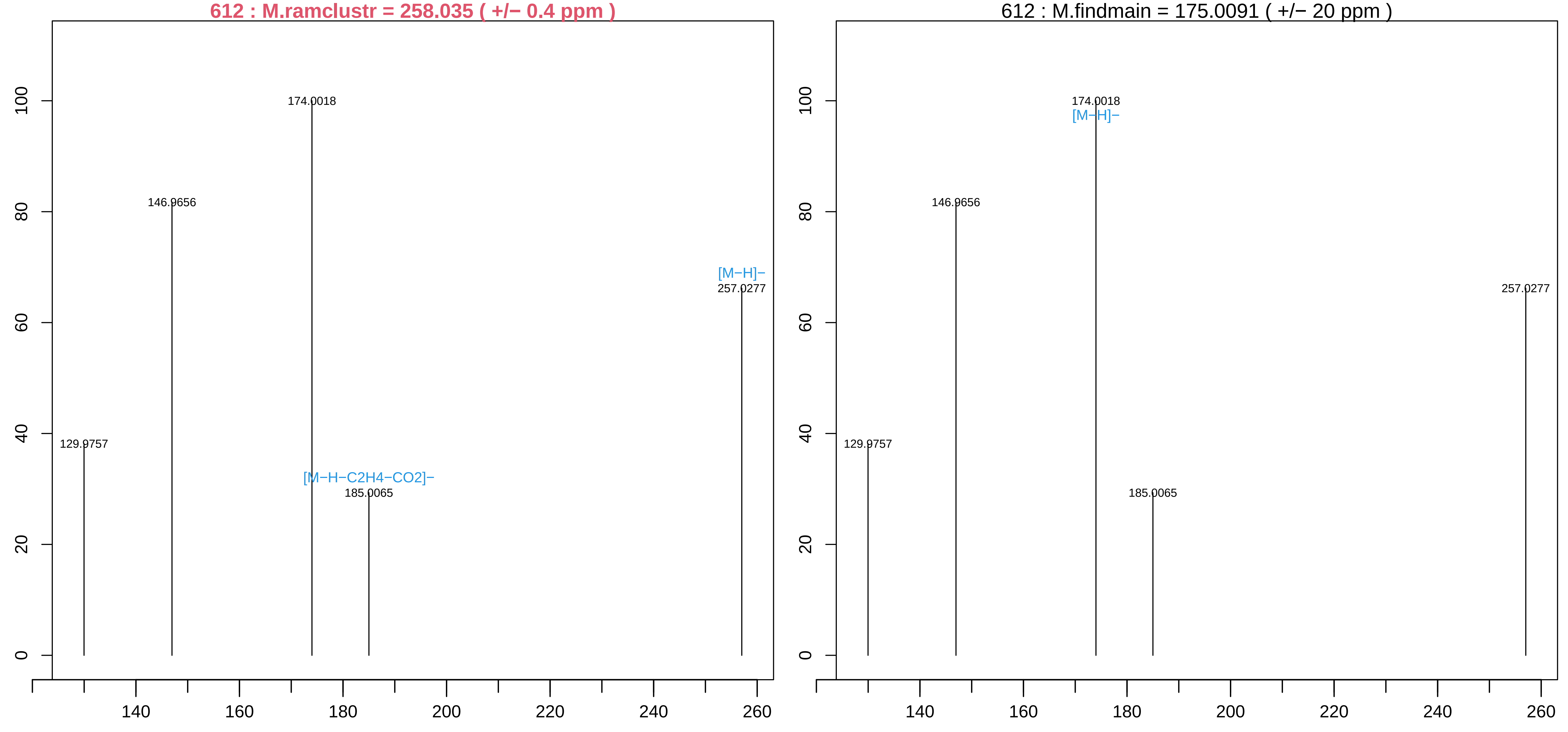
612 : M.ramclustr = 258.035 (+/− 0.4 ppm)
174.0018
146.9656
[M−H]−
257.0277
129.9757
[M−H−C2H4−CO2]−
185.0065
612 : M.findmain = 175.0091 (+/− 20 ppm)
174.0018
[M−H]−
146.9656
257.0277
129.9757
185.0065

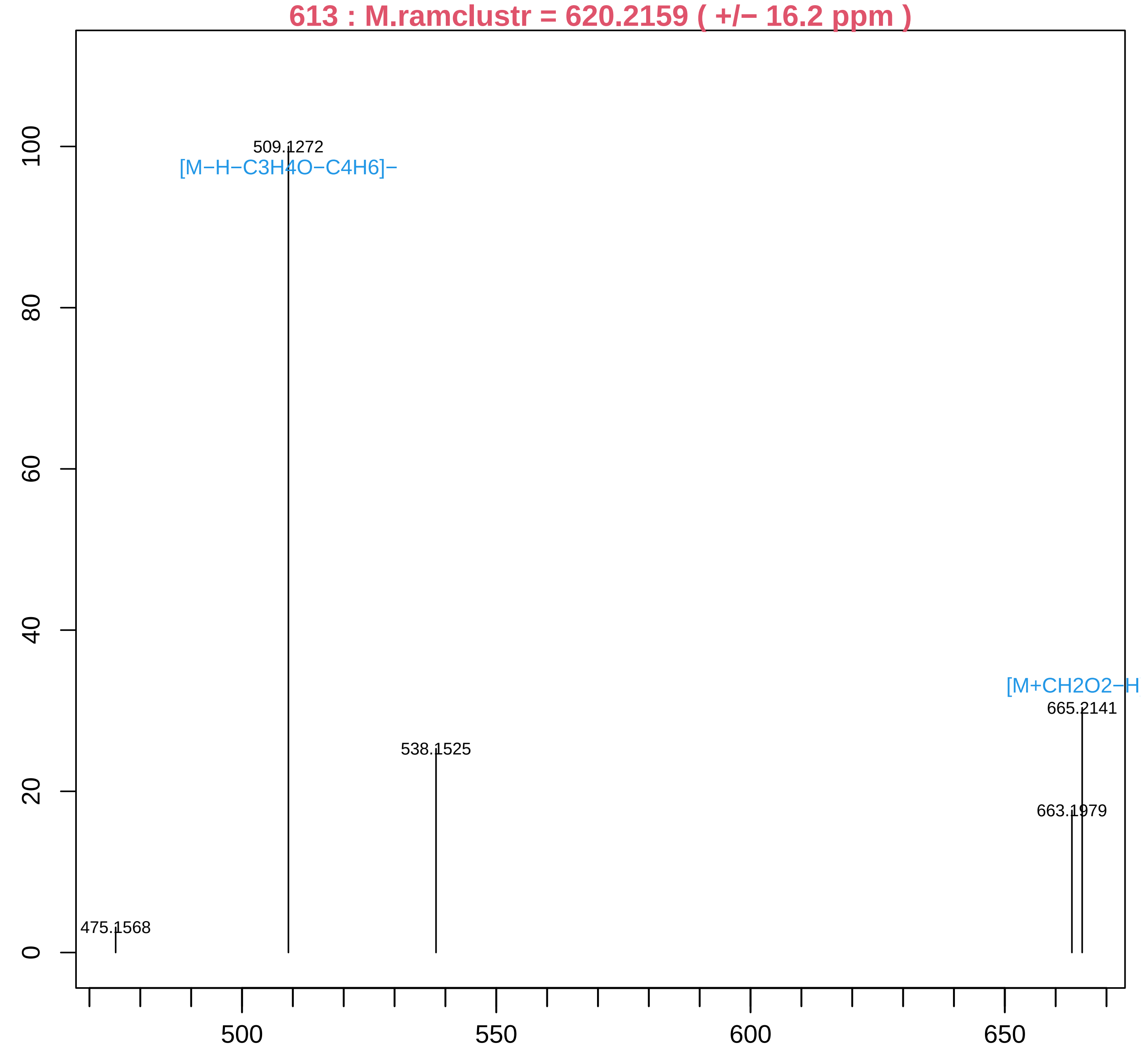

613 : M.ramclustr = 620.2159 (+/− 16.2 ppm)
509.1272
[M−H−C3H4O−C4H6]−
[M+CH2O2−H
665.2141
538.1525
663.1979
475.1568
0
20
40
60
80
100
500
550
600
650

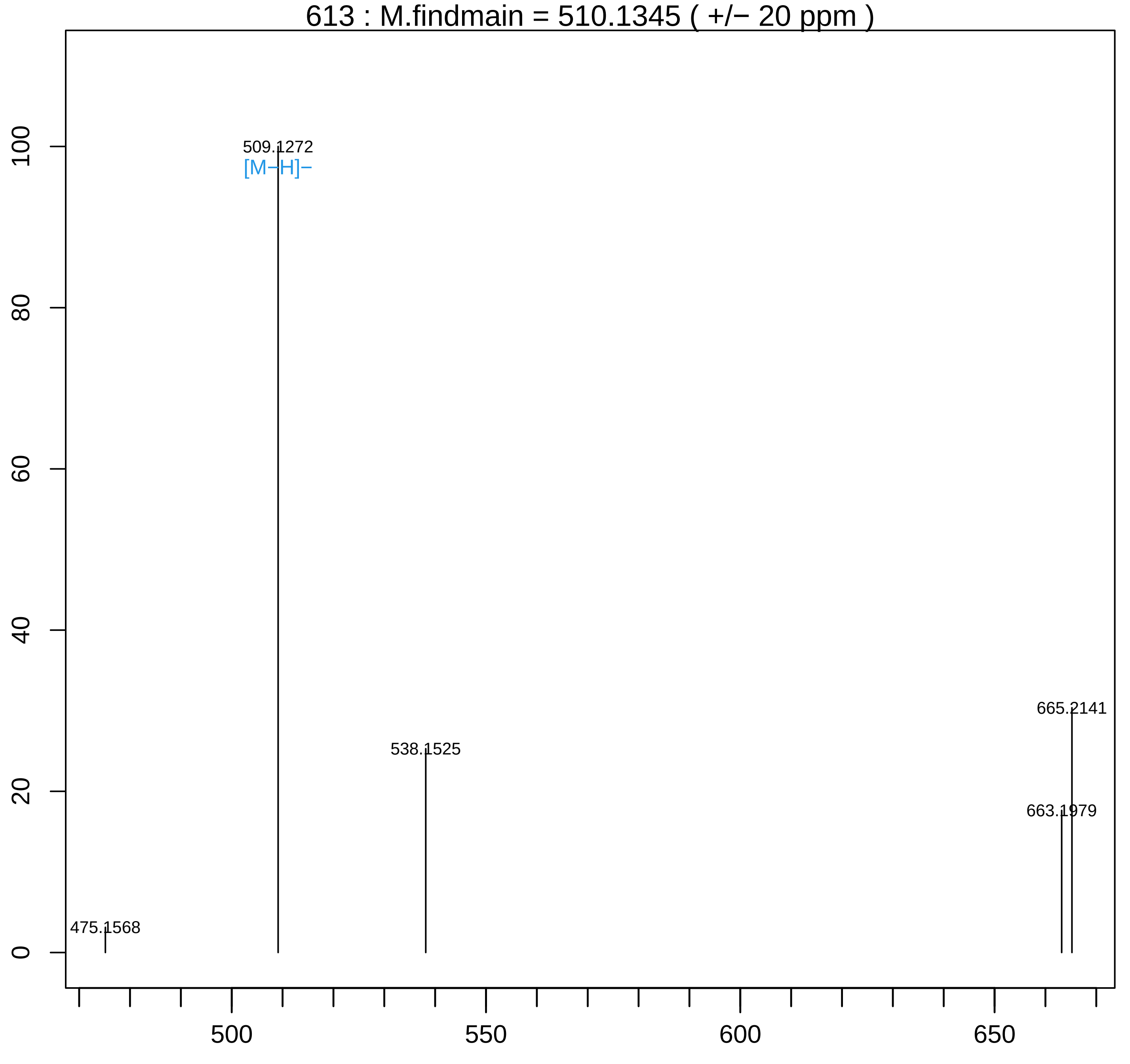

613 : M.findmain = 510.1345 (+/− 20 ppm)
509.1272
[M−H]−
665.2141
538.1525
663.1979
475.1568
0
20
40
60
80
100
500
550
600
650

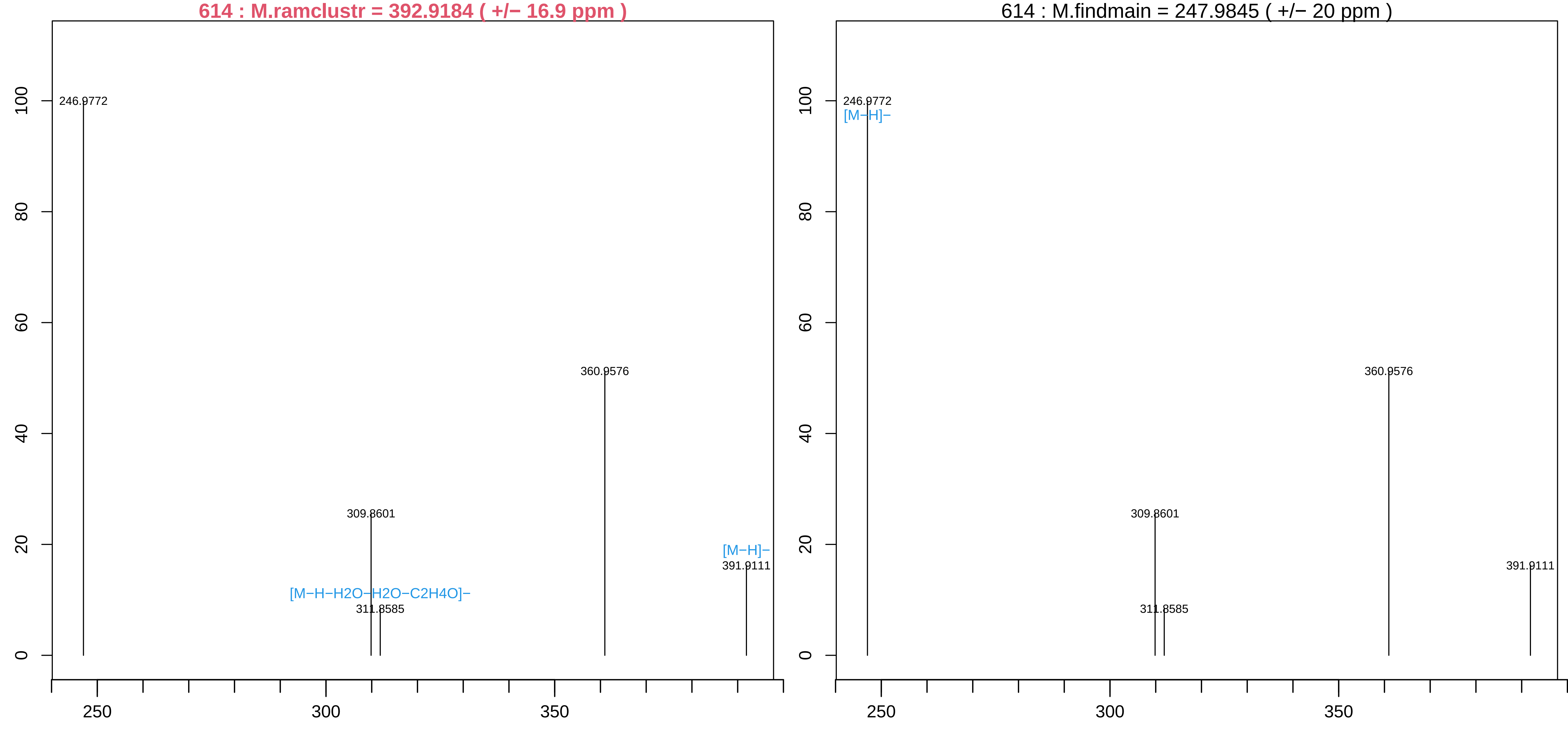

614 : M.ramclustr = 392.9184 (+/− 16.9 ppm)
246.9772
309.8601
[M−H−H2O−H2O−C2H4O]−
311.8585
360.9576
[M−H]−
391.9111
614 : M.findmain = 247.9845 (+/− 20 ppm)
246.9772
[M−H]−
309.8601
311.8585
360.9576
391.9111

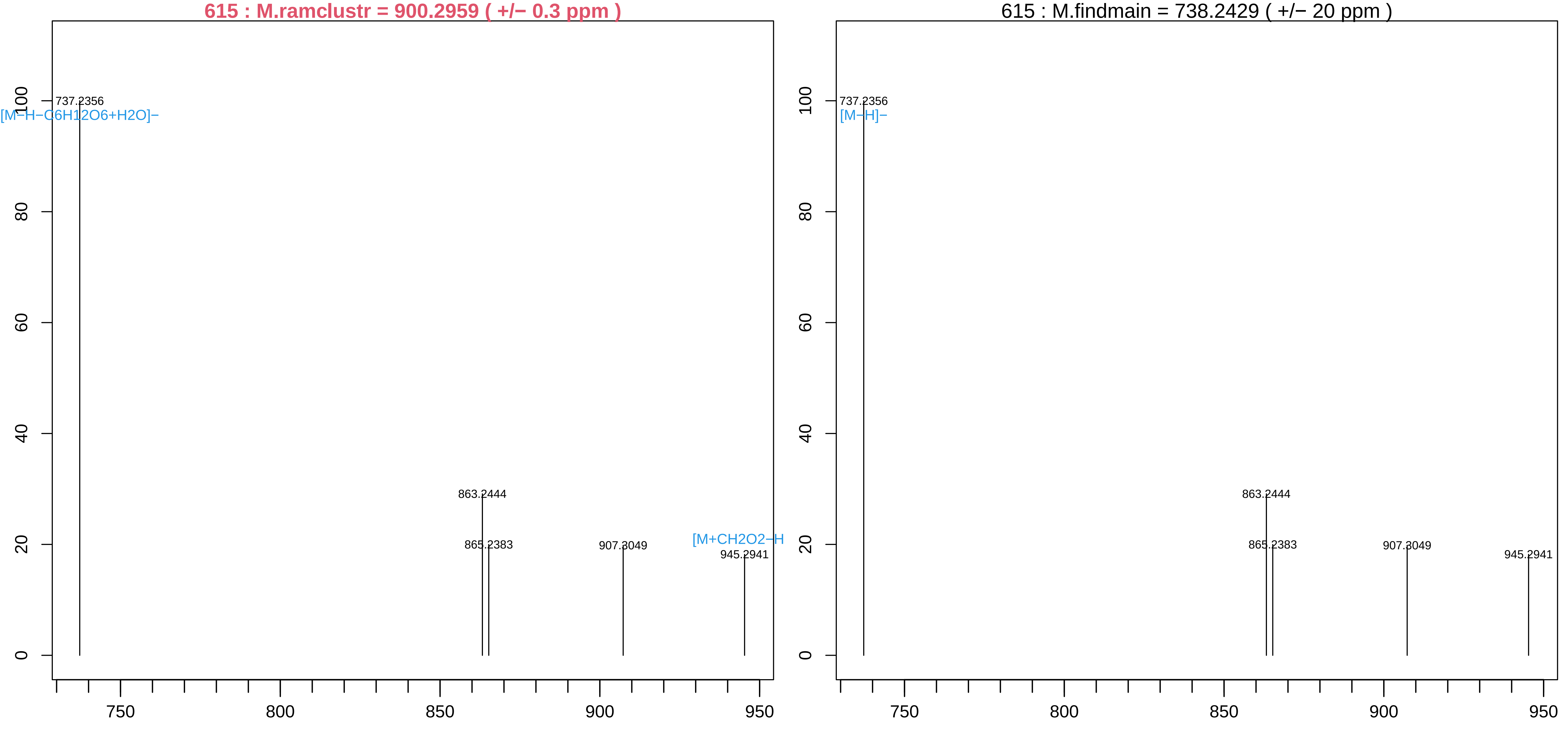

615 : M.ramclustr = 900.2959 (+/− 0.3 ppm)
737.2356
[M−H−C6H12O6+H2O]−
863.2444
865.2383
907.3049
[M+CH2O2−H
945.2941
0
20
40
60
80
100
750
800
850
900
950
615 : M.findmain = 738.2429 (+/− 20 ppm)
737.2356
[M−H]−
863.2444
865.2383
907.3049
945.2941
0
20
40
60
80
100
750
800
850
900
950

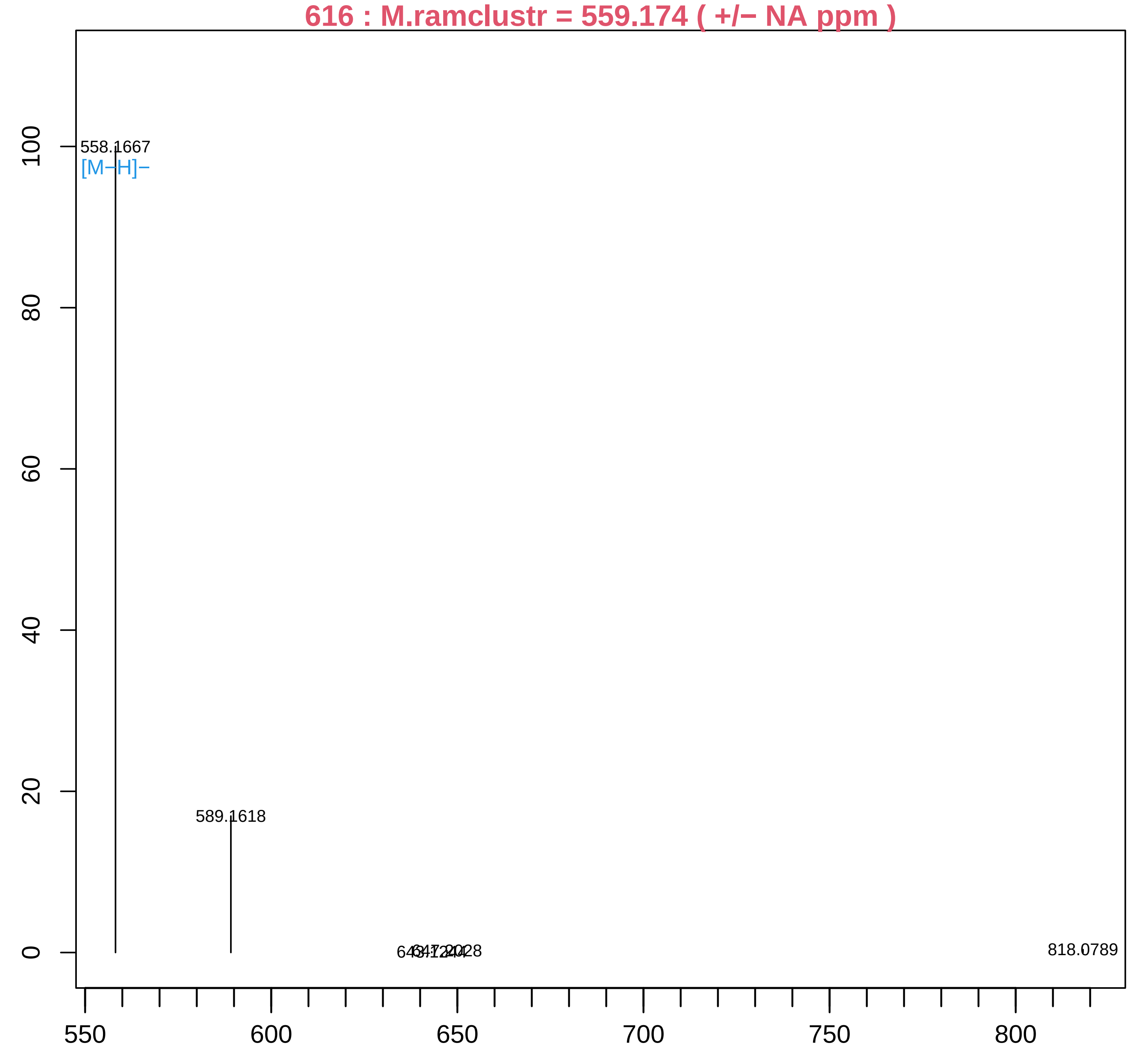
616 : M.ramclustr = 559.174 (+/− NA ppm)
558.1667
[M−H]−
589.1618
818.0789
0
20
40
60
80
100
550
600
650
700
750
800

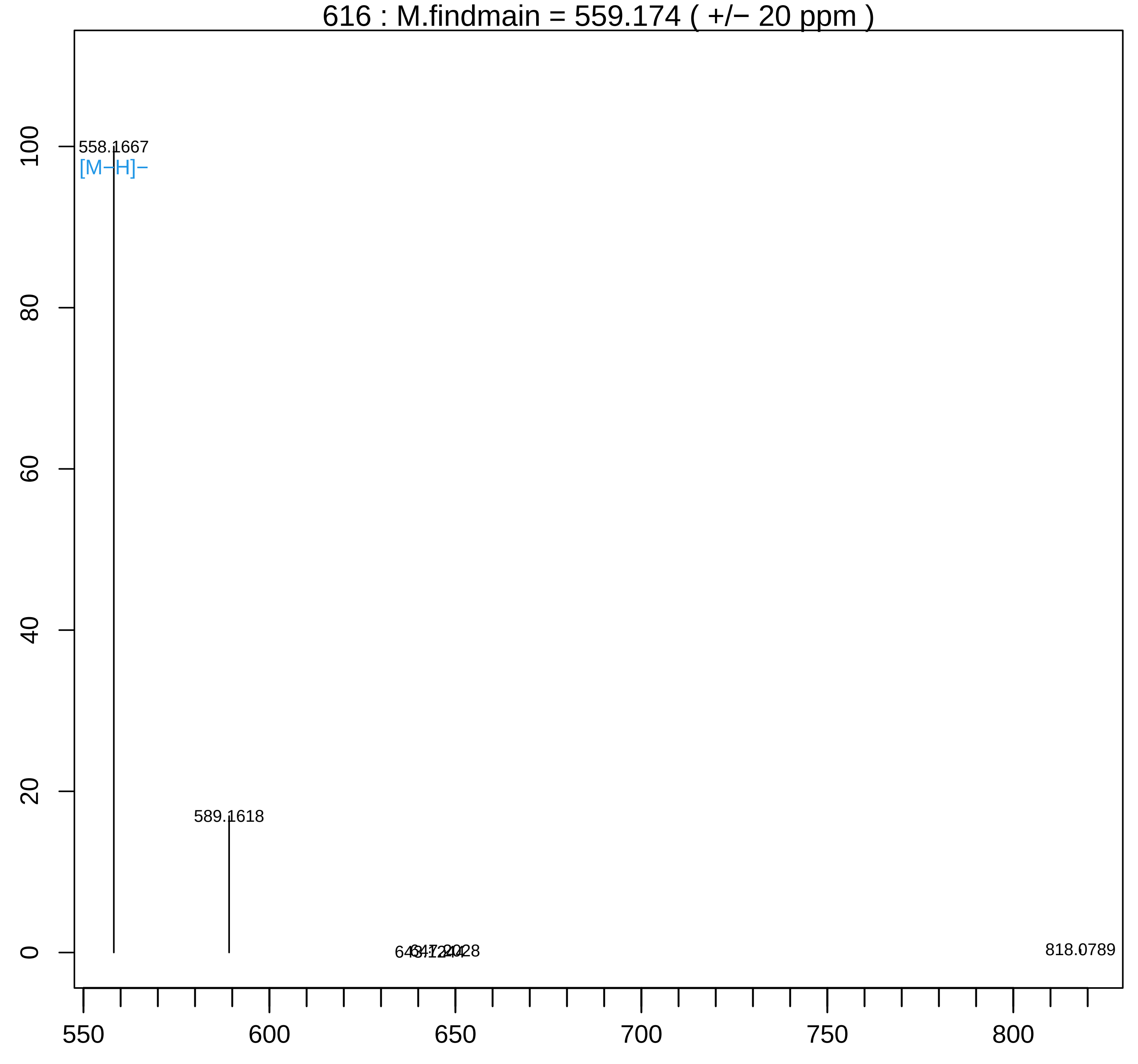
616 : M.findmain = 559.174 (+/− 20 ppm)
558.1667
[M−H]−
589.1618
818.0789
0
20
40
60
80
100
550
600
650
700
750
800

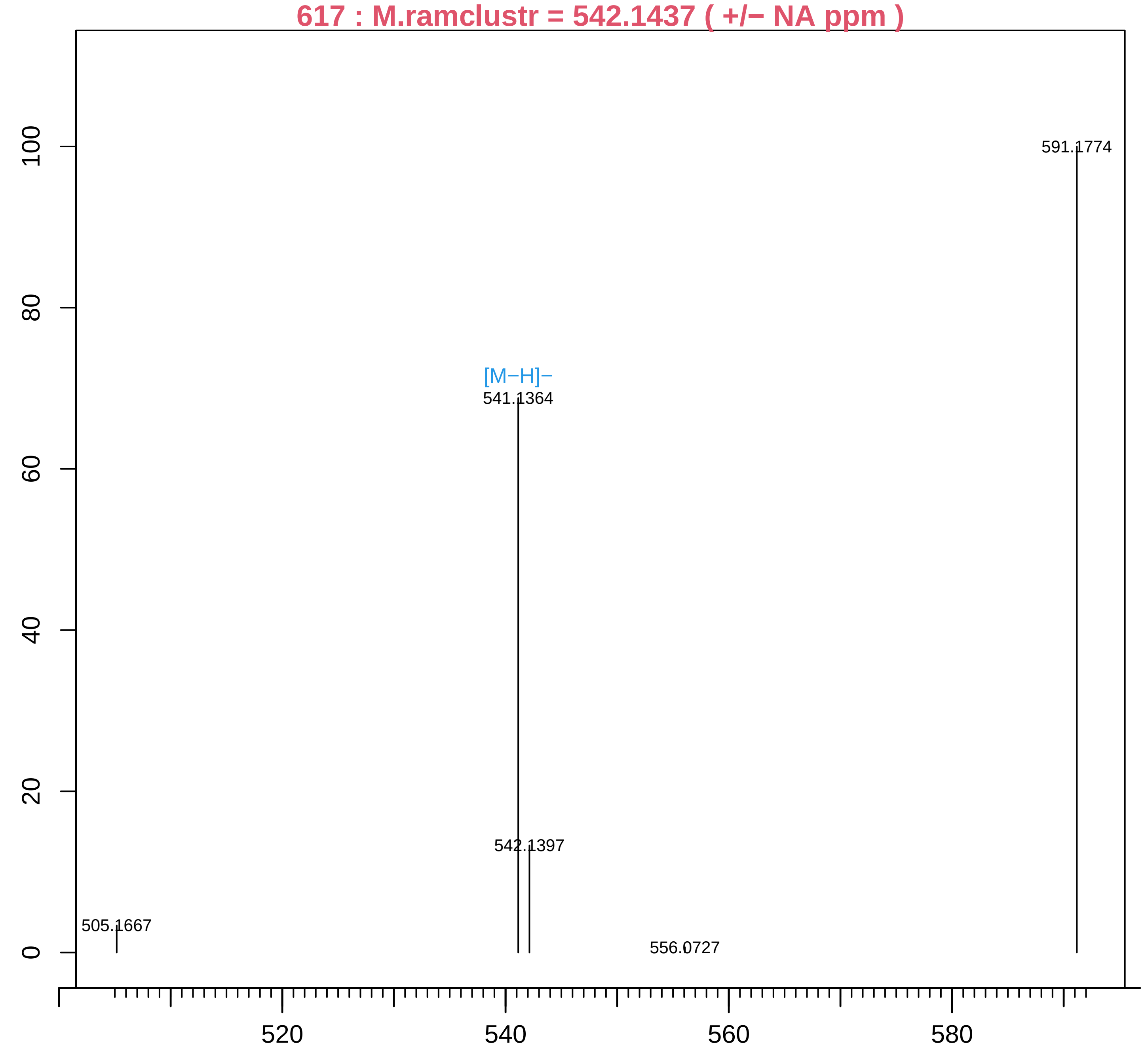
617 : M.ramclustr = 542.1437 (+/− NA ppm)
[M−H]−
541.1364
542.1397
505.1667
556.0727
591.1774
0
20
40
60
80
100
520
540
560
580

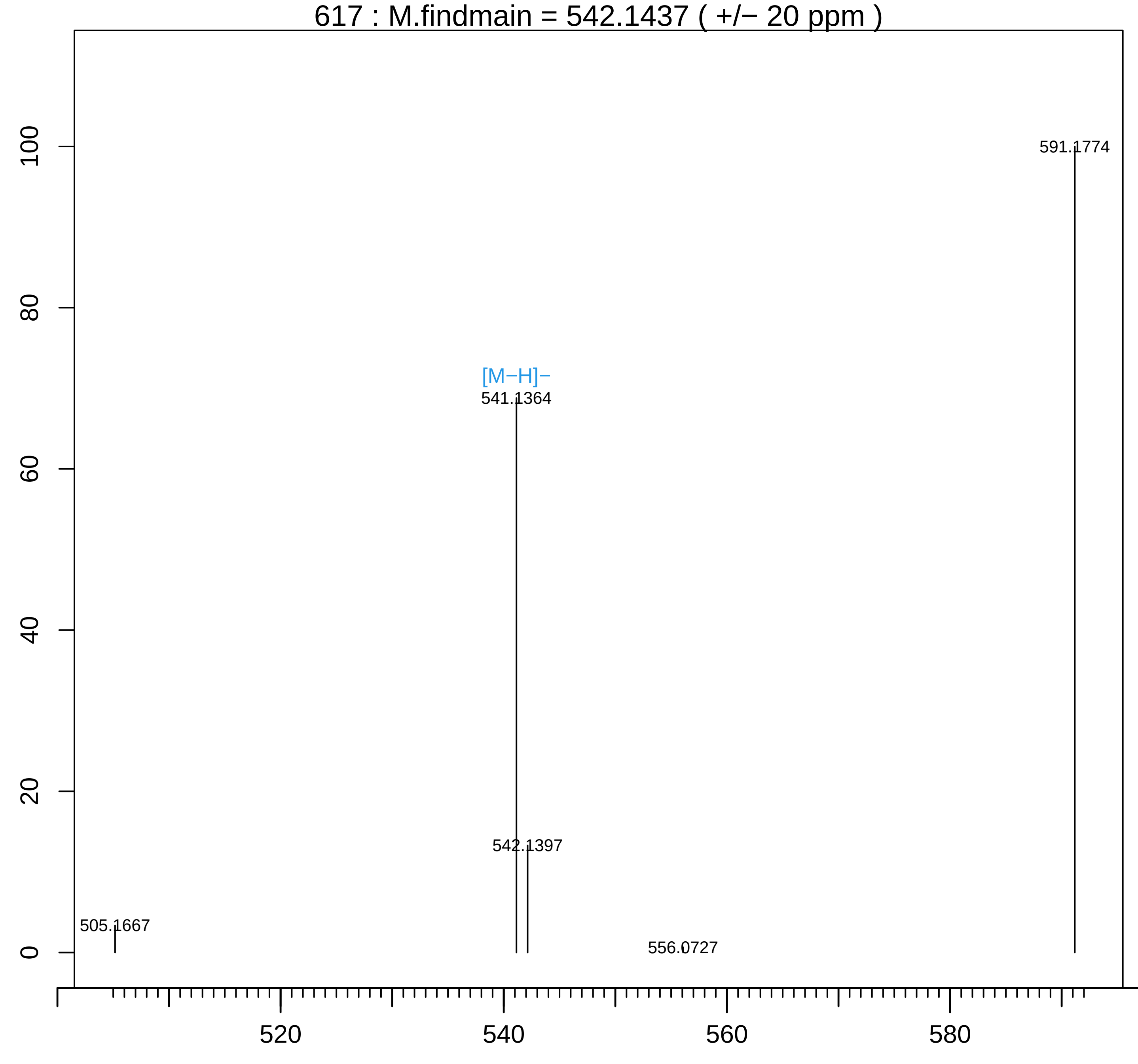
617 : M.findmain = 542.1437 (+/− 20 ppm)
[M−H]−
541.1364
542.1397
505.1667
556.0727
591.1774
0
20
40
60
80
100
520
540
560
580

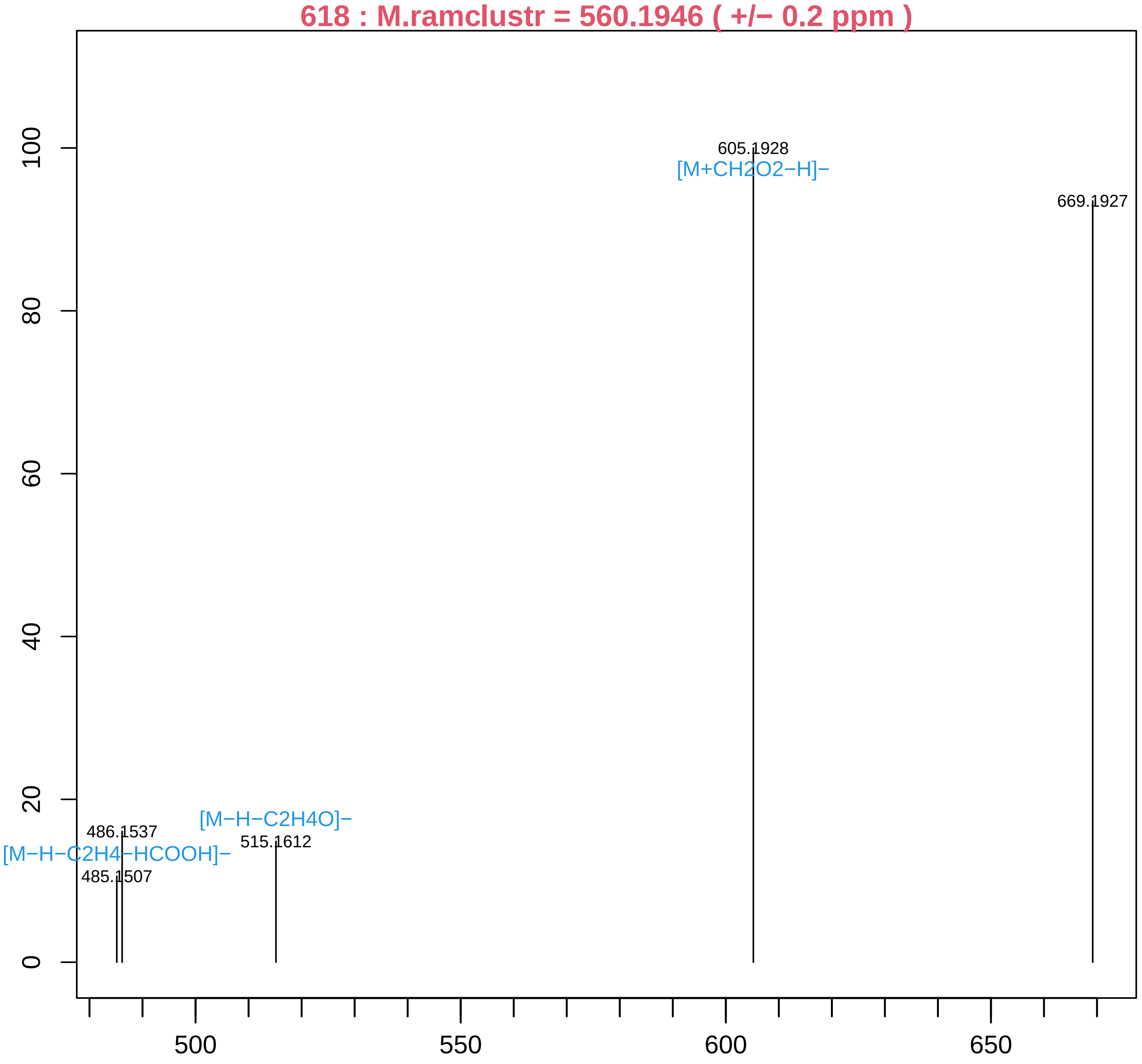
618 : M.ramclustr = 560.1946 (+/− 0.2 ppm)
605.1928
[M+CH2O2−H]−
669.1927
486.1537
[M−H−C2H4O]−
515.1612
[M−H−C2H4−HCOOH]−
485.1507
0
20
40
60
80
100
500
550
600
650

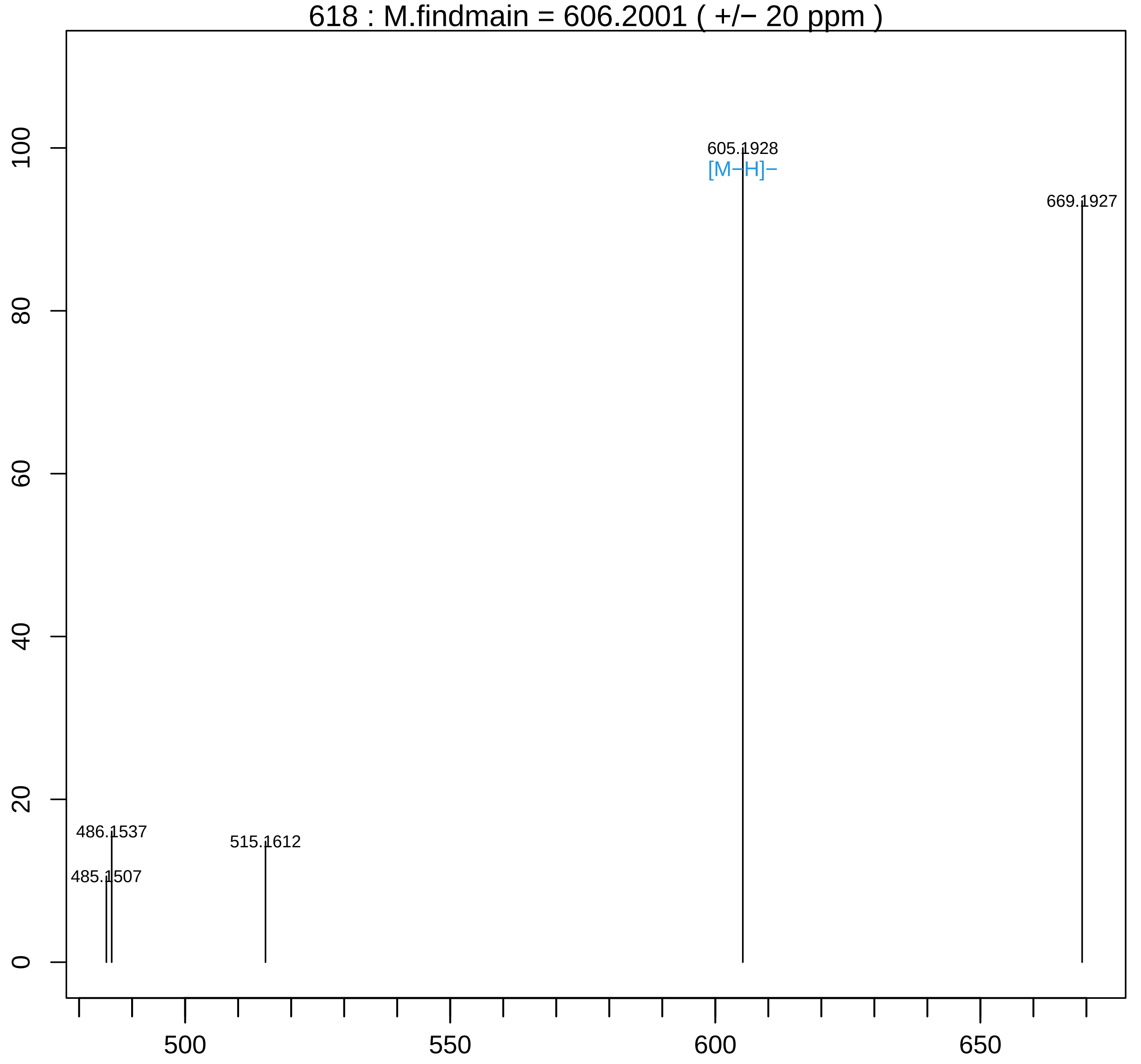
618 : M.findmain = 606.2001 (+/− 20 ppm)
605.1928
[M−H]−
669.1927
486.1537
515.1612
485.1507
0
20
40
60
80
100
500
550
600
650

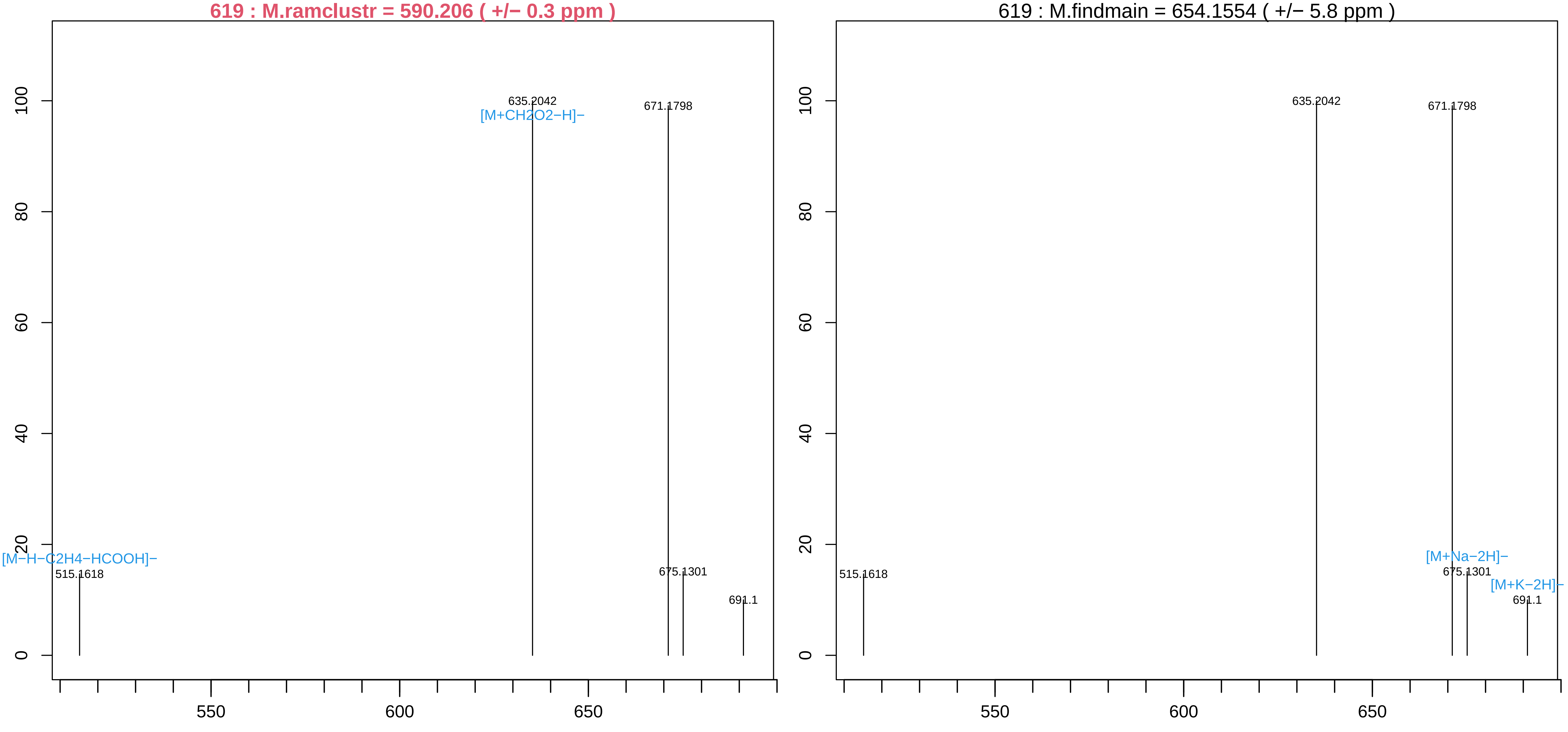

619 : M.ramclustr = 590.206 (+/− 0.3 ppm)
635.2042
[M+CH2O2−H]−
671.1798
[M−H−C2H4−HCOOH]−
515.1618
675.1301
691.1
0
20
40
60
80
100
550
600
650
619 : M.findmain = 654.1554 (+/− 5.8 ppm)
635.2042
671.1798
[M+Na−2H]−
675.1301
[M+K−2H]−
691.1
515.1618

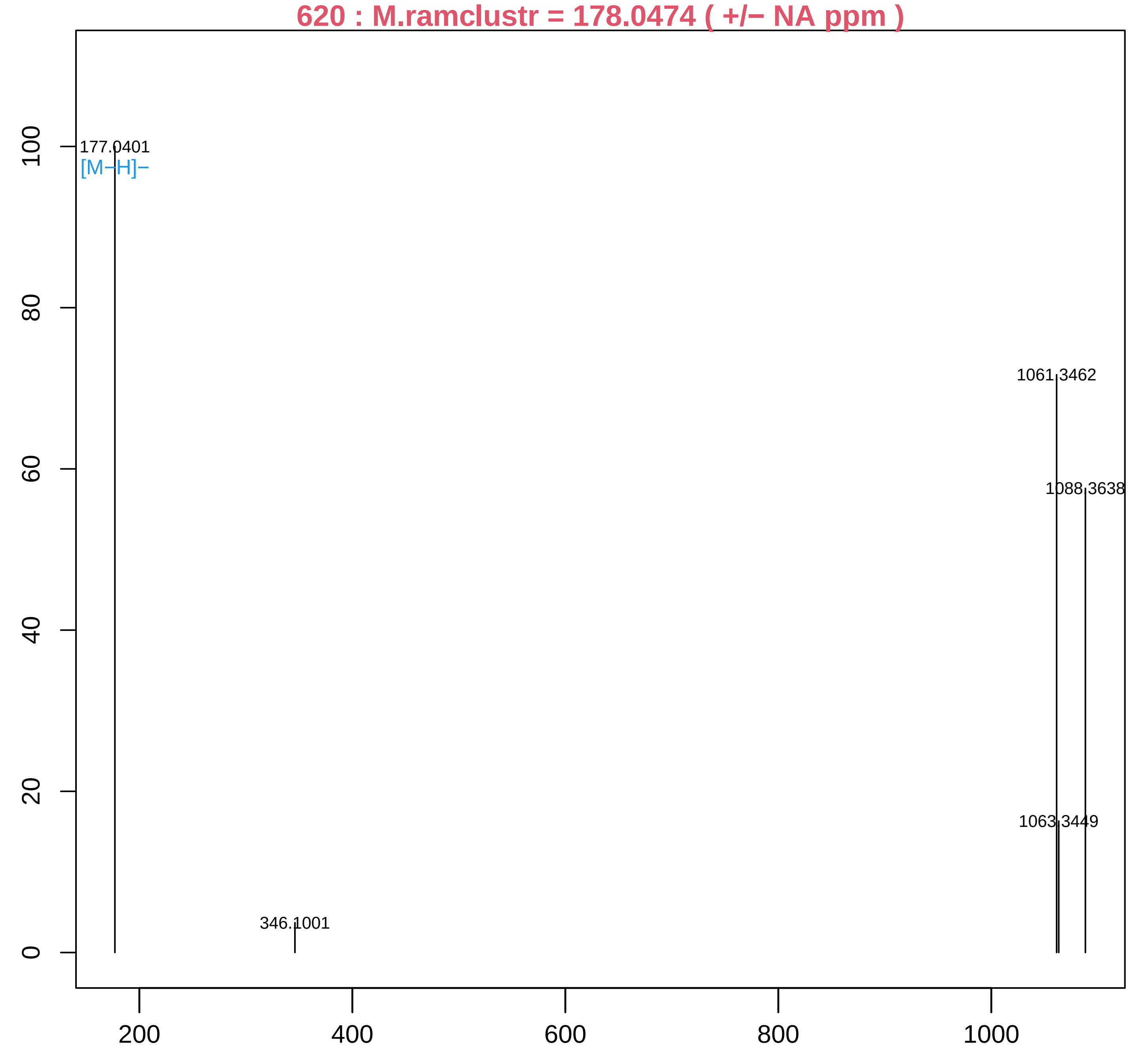

620 : M.ramclustr = 178.0474 (+/− NA ppm)
177.0401
[M−H]−
346.1001
1061.3462
1063.3449
1088.3638
0
20
40
60
80
100
200
400
600
800
1000

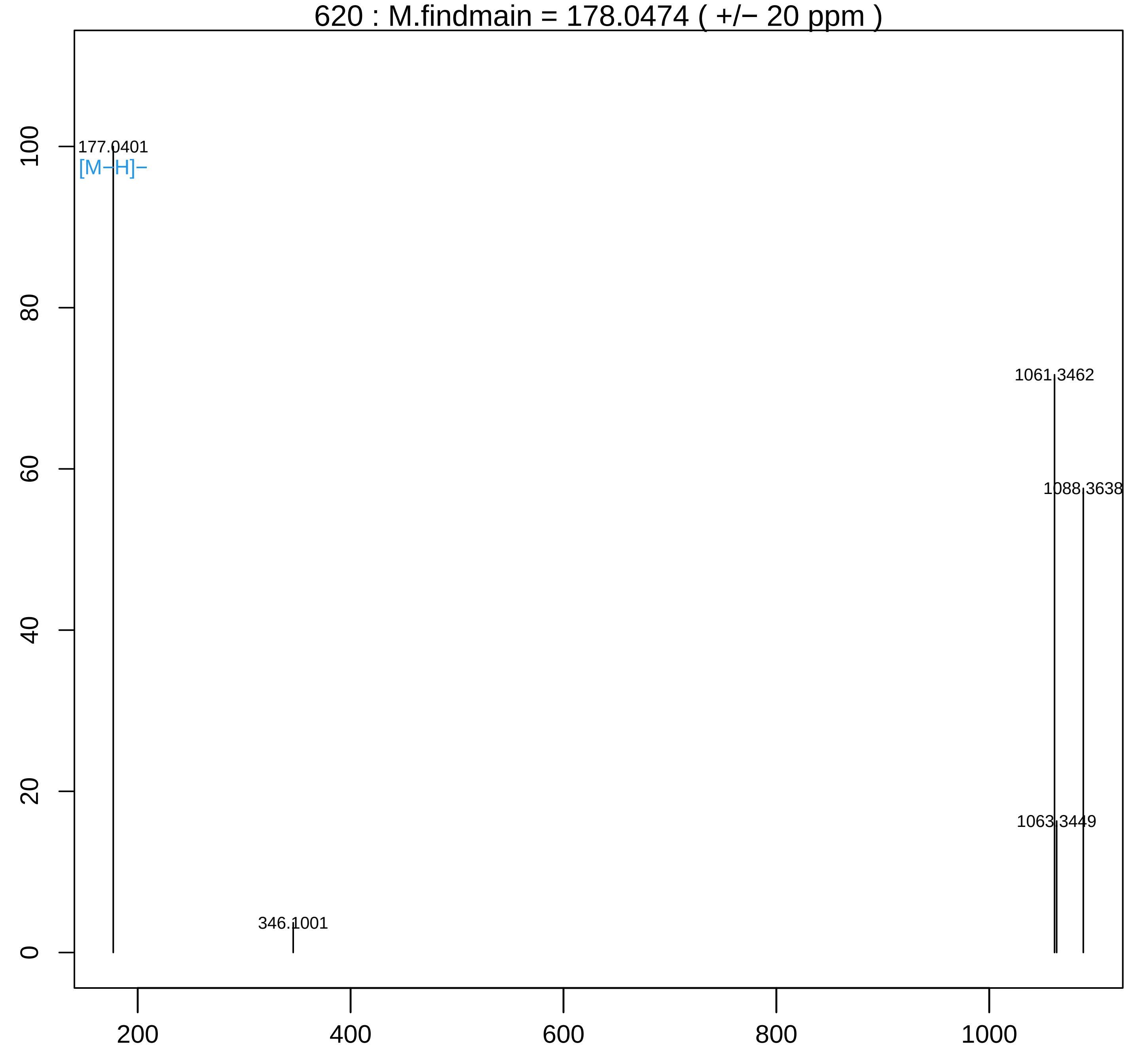

620 : M.findmain = 178.0474 (+/− 20 ppm)
177.0401
[M−H]−
346.1001
1061.3462
1063.3449
1088.3638
0
20
40
60
80
100
200
400
600
800
1000

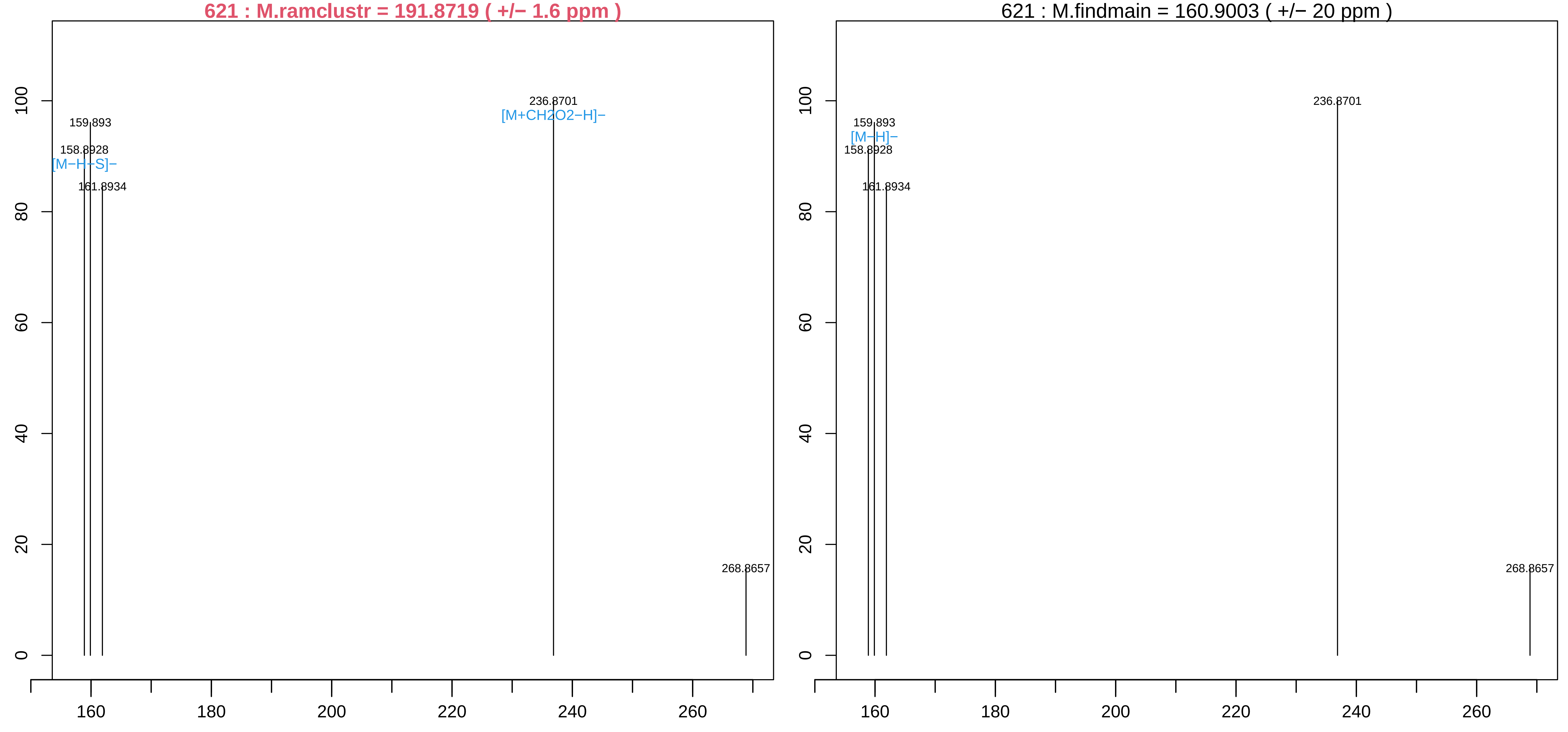
621 : M.ramclustr = 191.8719 (+/− 1.6 ppm)
159.893
158.8928
[M−H−S]−
161.8934
236.8701
[M+CH2O2−H]−
268.8657
621 : M.findmain = 160.9003 (+/− 20 ppm)
159.893
[M−H]−
158.8928
161.8934
236.8701
268.8657
0
20
40
60
80
100
160
180
200
220
240
260

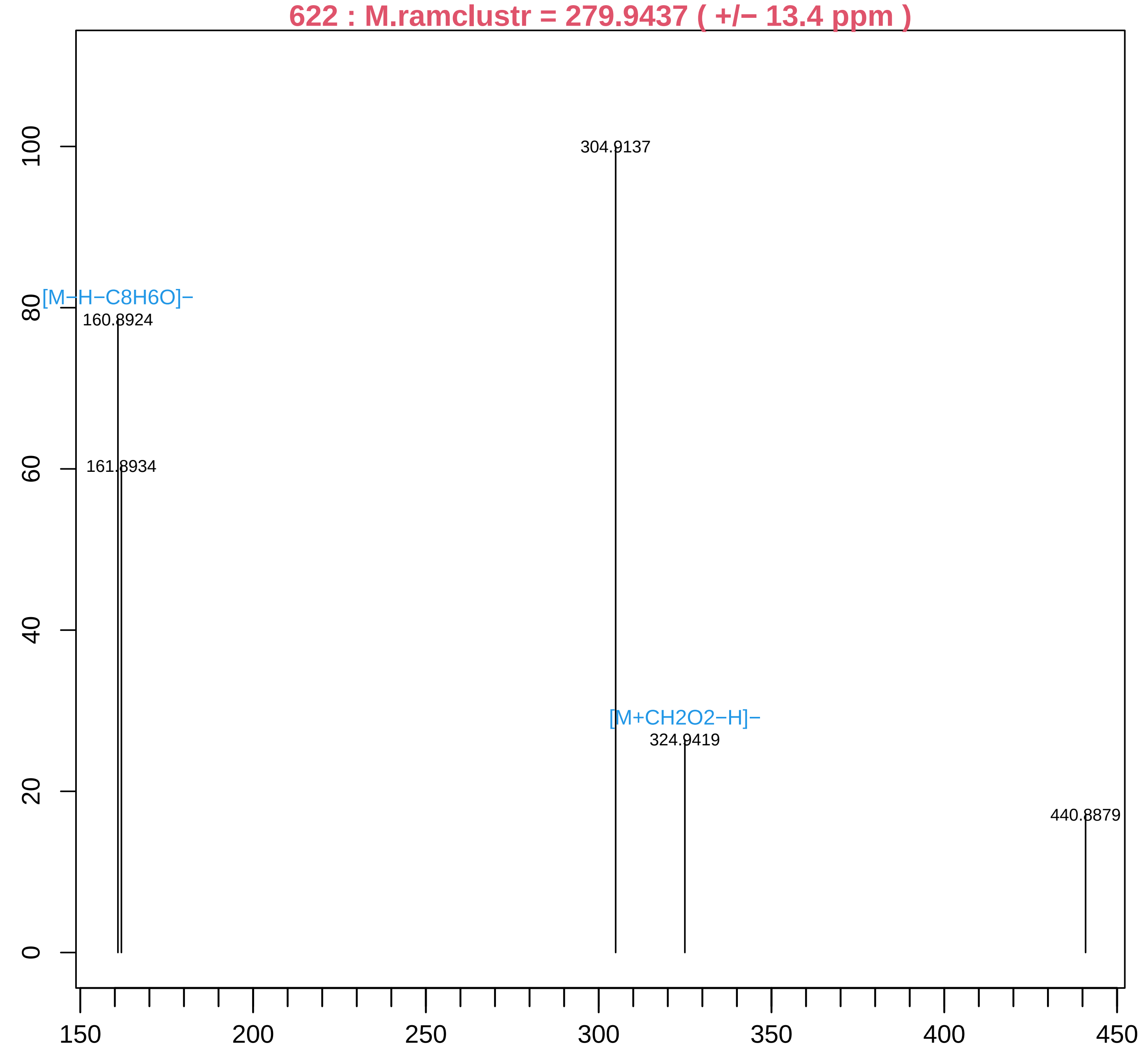

622 : M.ramclustr = 279.9437 (+/− 13.4 ppm)
[M−H−C8H6O]−
160.8924
161.8934
304.9137
[M+CH2O2−H]−
324.9419
440.8879

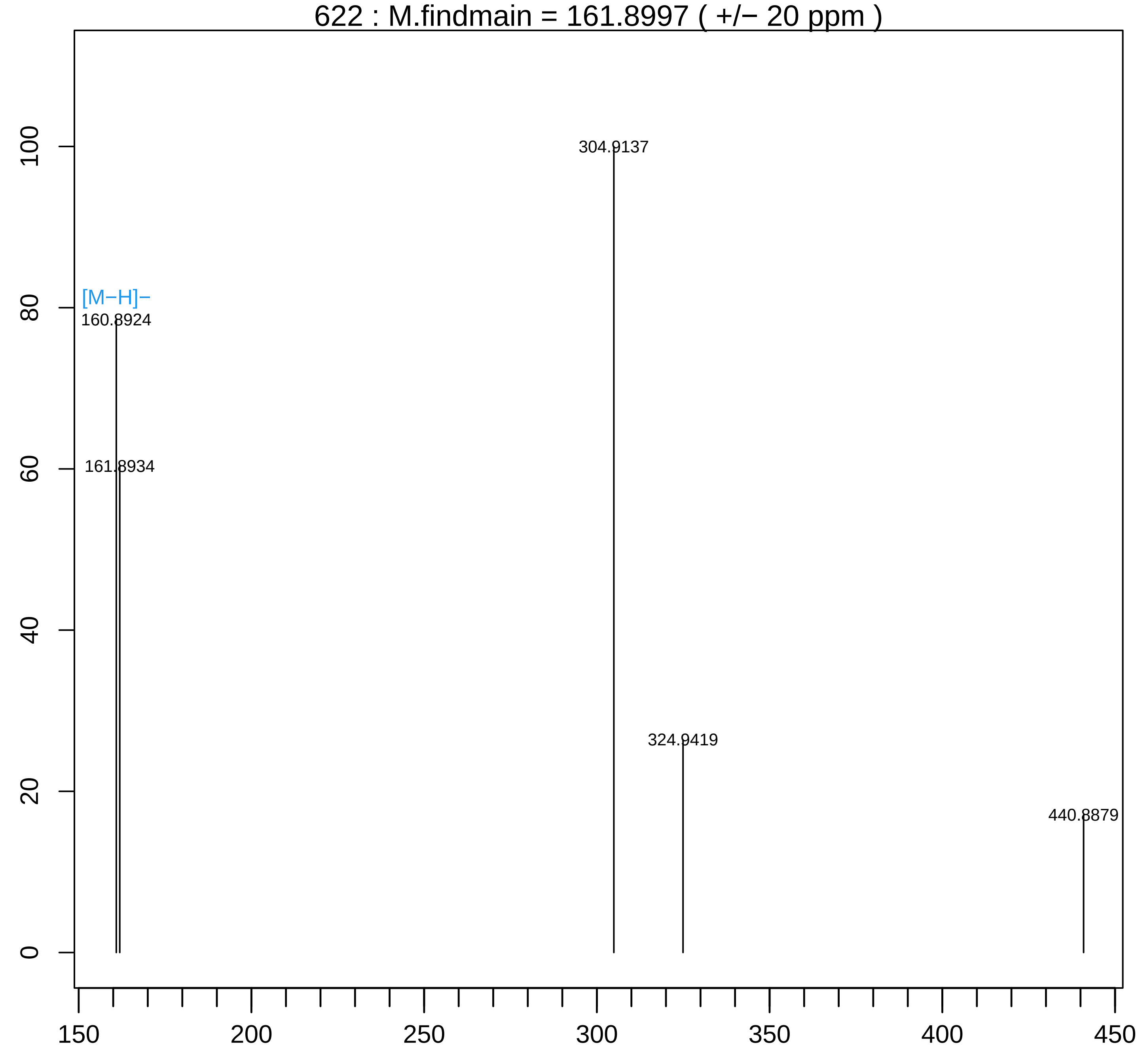

622 : M.findmain = 161.8997 (+/− 20 ppm)
[M−H]−
160.8924
161.8934
304.9137
324.9419
440.8879

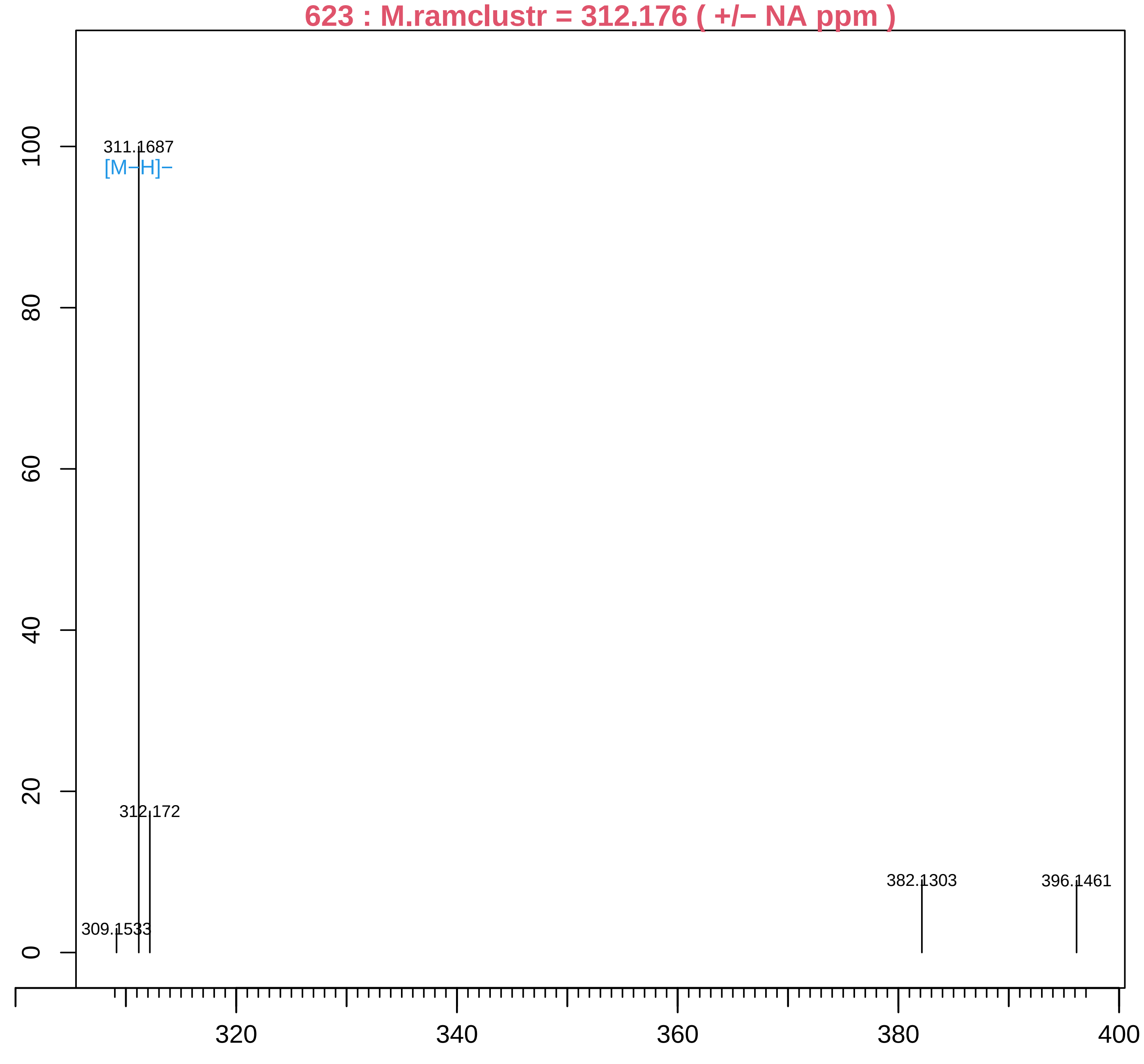

623 : M.ramclustr = 312.176 (+/− NA ppm)
311.1687
[M−H]−
312.172
309.1533
382.1303
396.1461
0
20
40
60
80
100
320
340
360
380
400

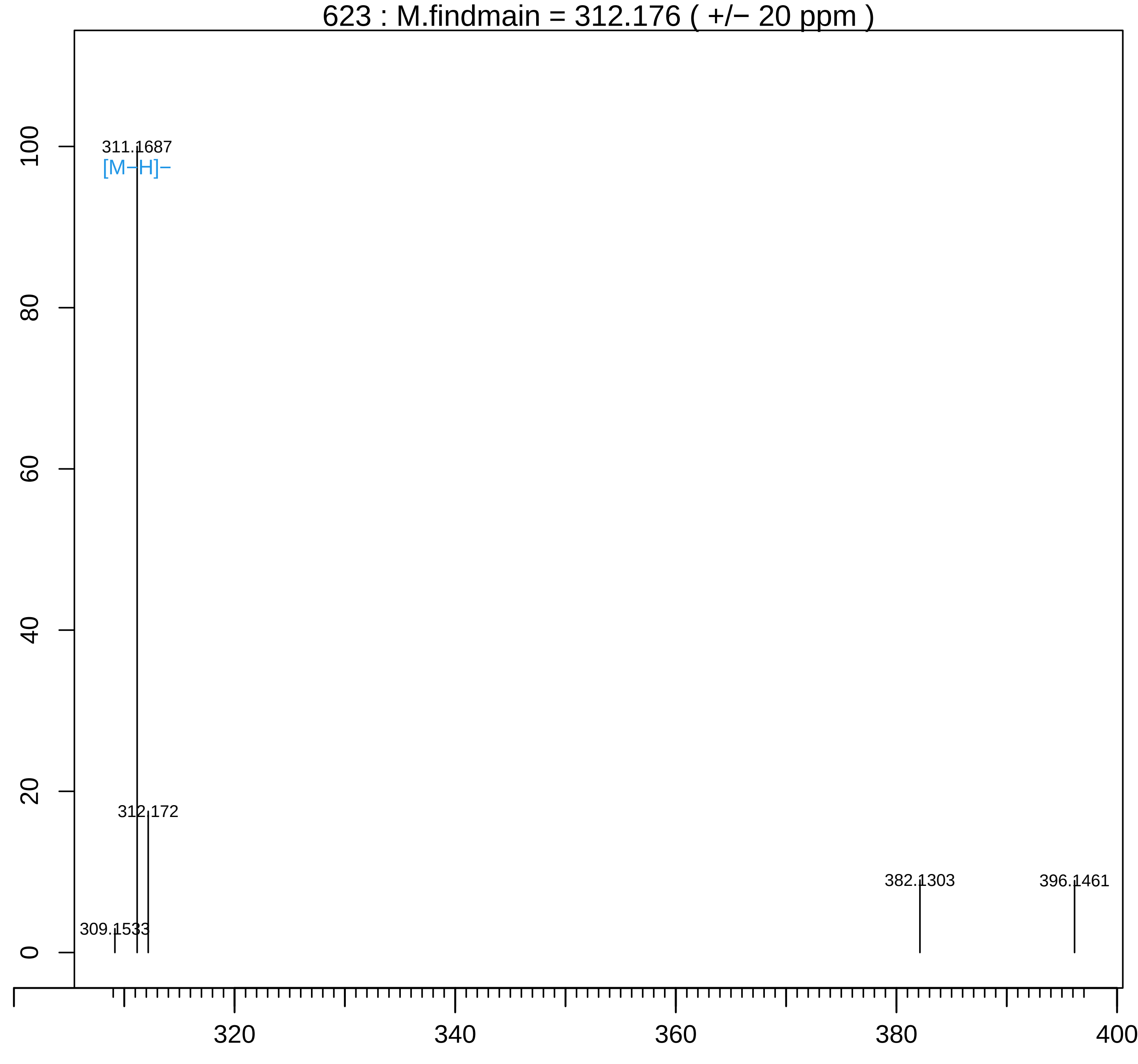

623 : M.findmain = 312.176 (+/− 20 ppm)
311.1687
[M−H]−
312.172
309.1533
382.1303
396.1461
0
20
40
60
80
100
320
340
360
380
400

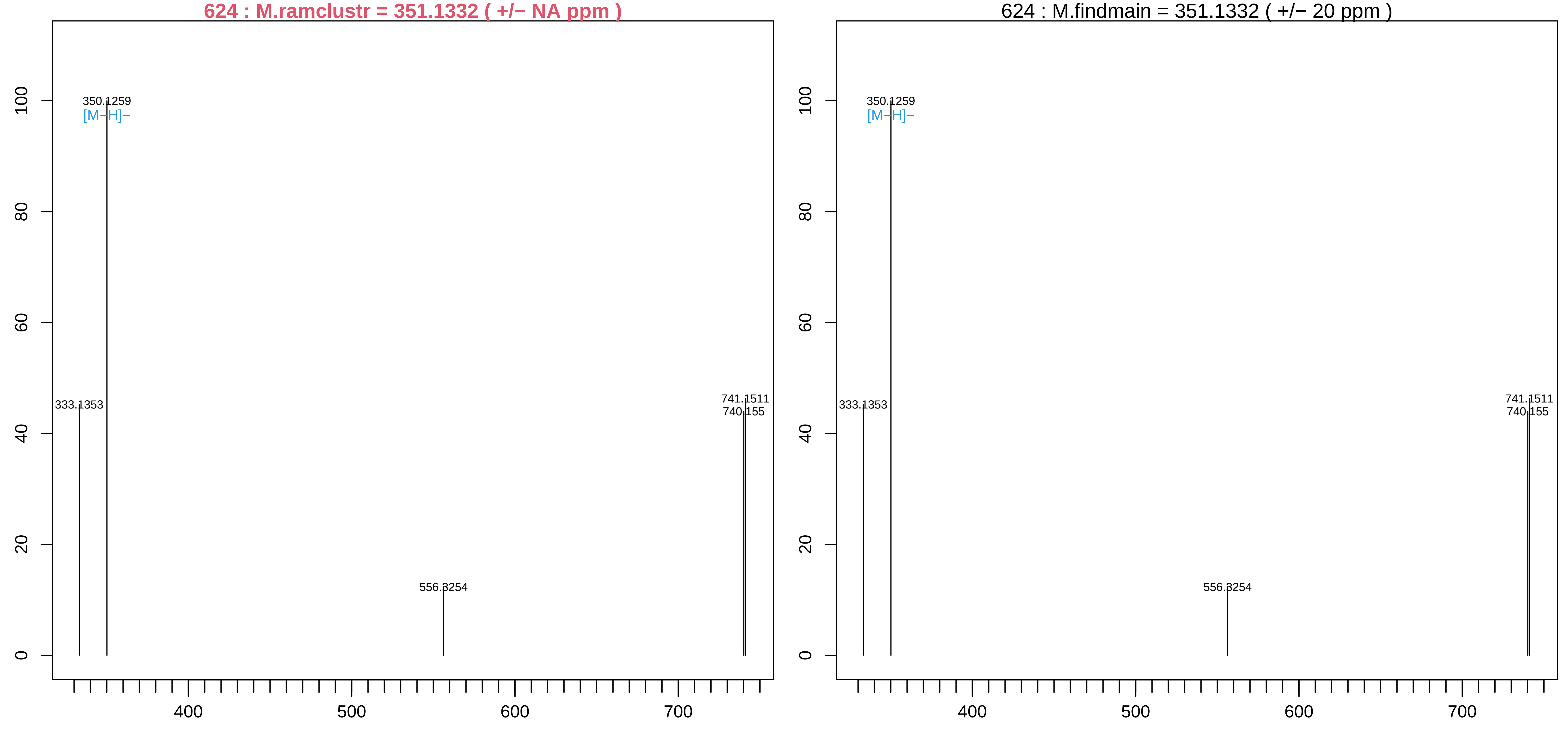
624 : M.ramclustr = 351.1332 (+/− NA ppm)
624 : M.findmain = 351.1332 (+/− 20 ppm)
350.1259
[M−H]−
333.1353
556.3254
741.1511
740.155
0
20
40
60
80
100
400
500
600
700

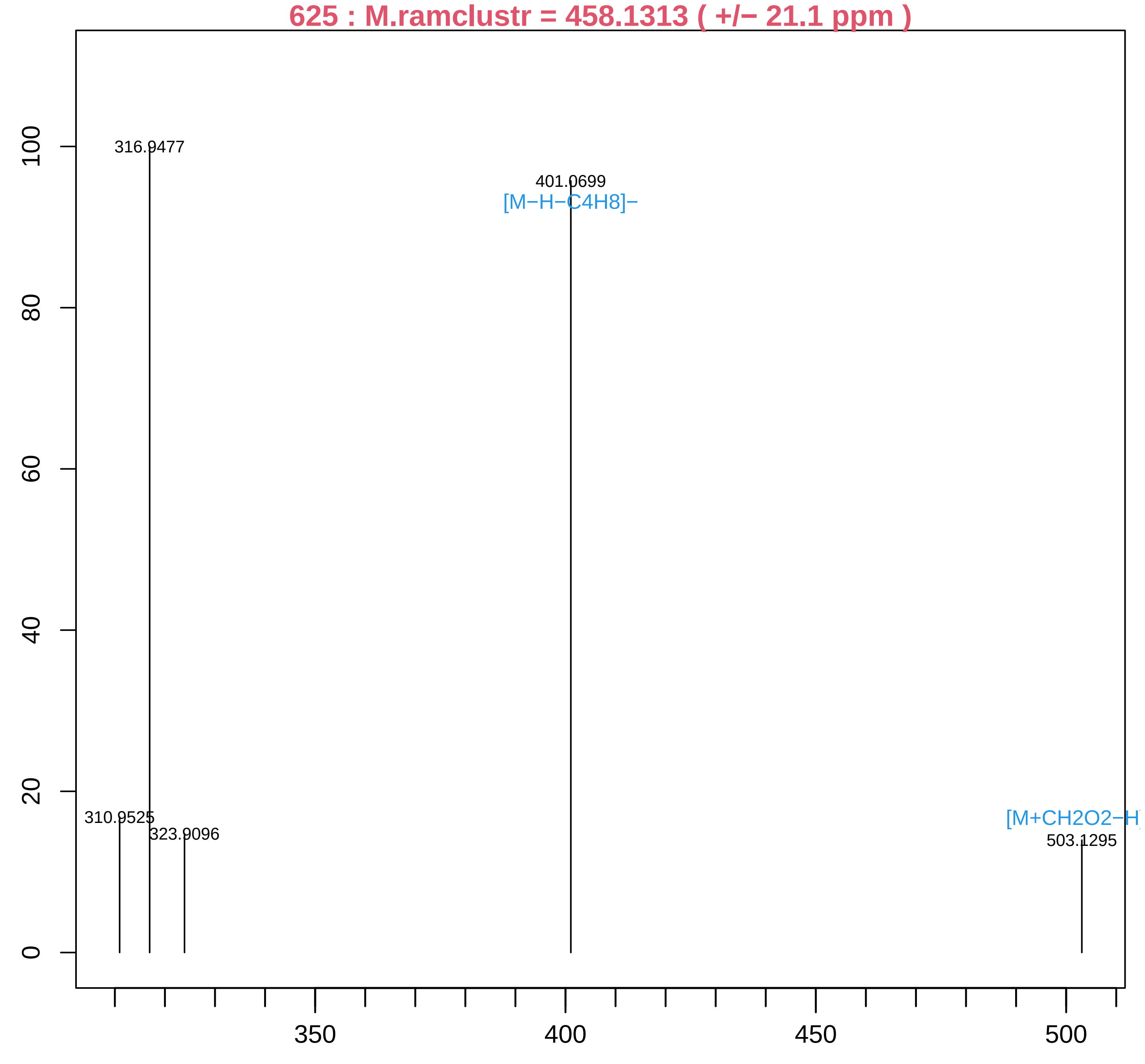
625 : M.ramclustr = 458.1313 (+/− 21.1 ppm)
316.9477
401.0699
[M−H−C4H8]−
310.9525
323.9096
[M+CH2O2−H
503.1295
0
20
40
60
80
100
350
400
450
500

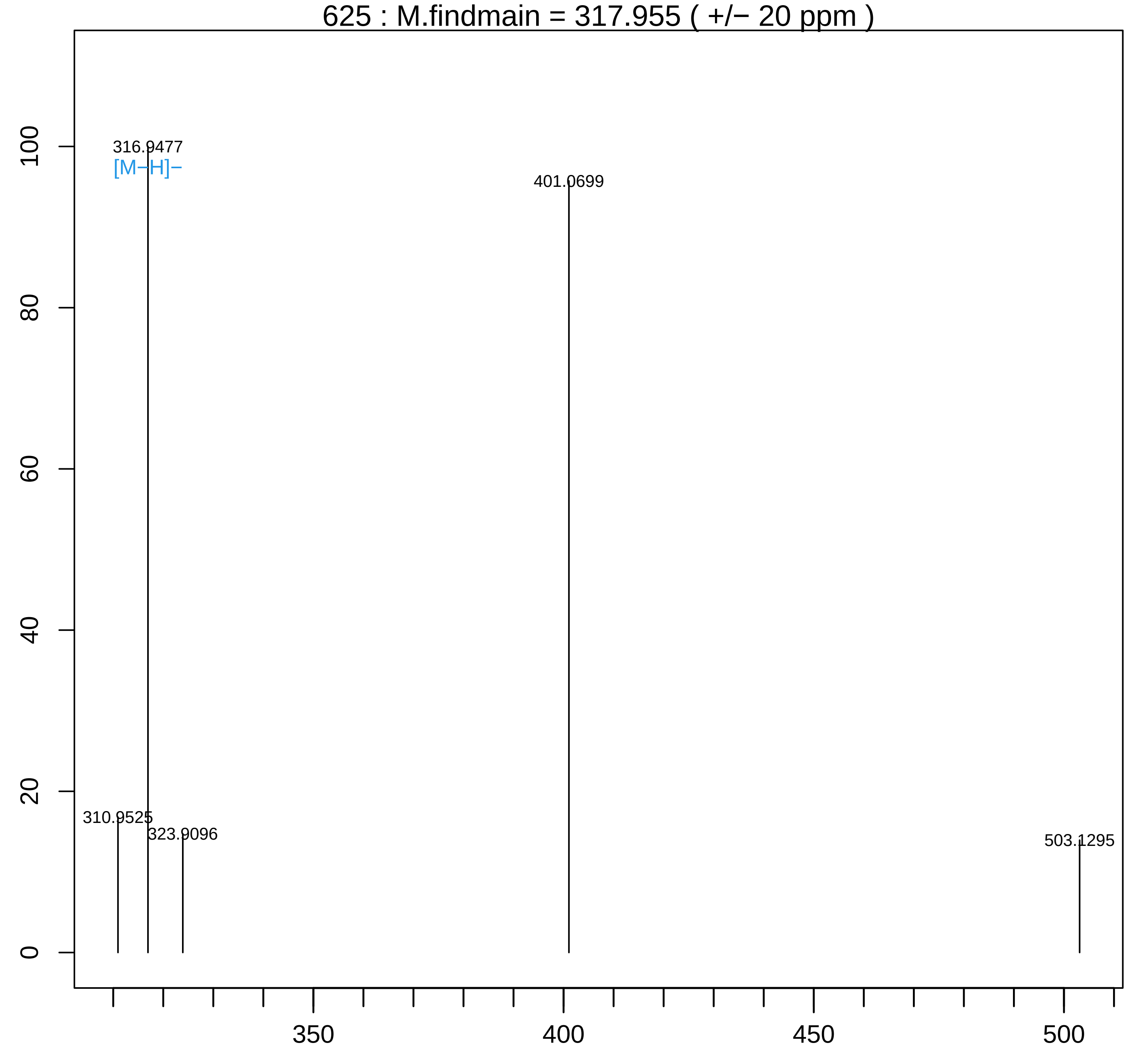
625 : M.findmain = 317.955 (+/− 20 ppm)
316.9477
[M−H]−
401.0699
310.9525
323.9096
503.1295
0
20
40
60
80
100
350
400
450
500

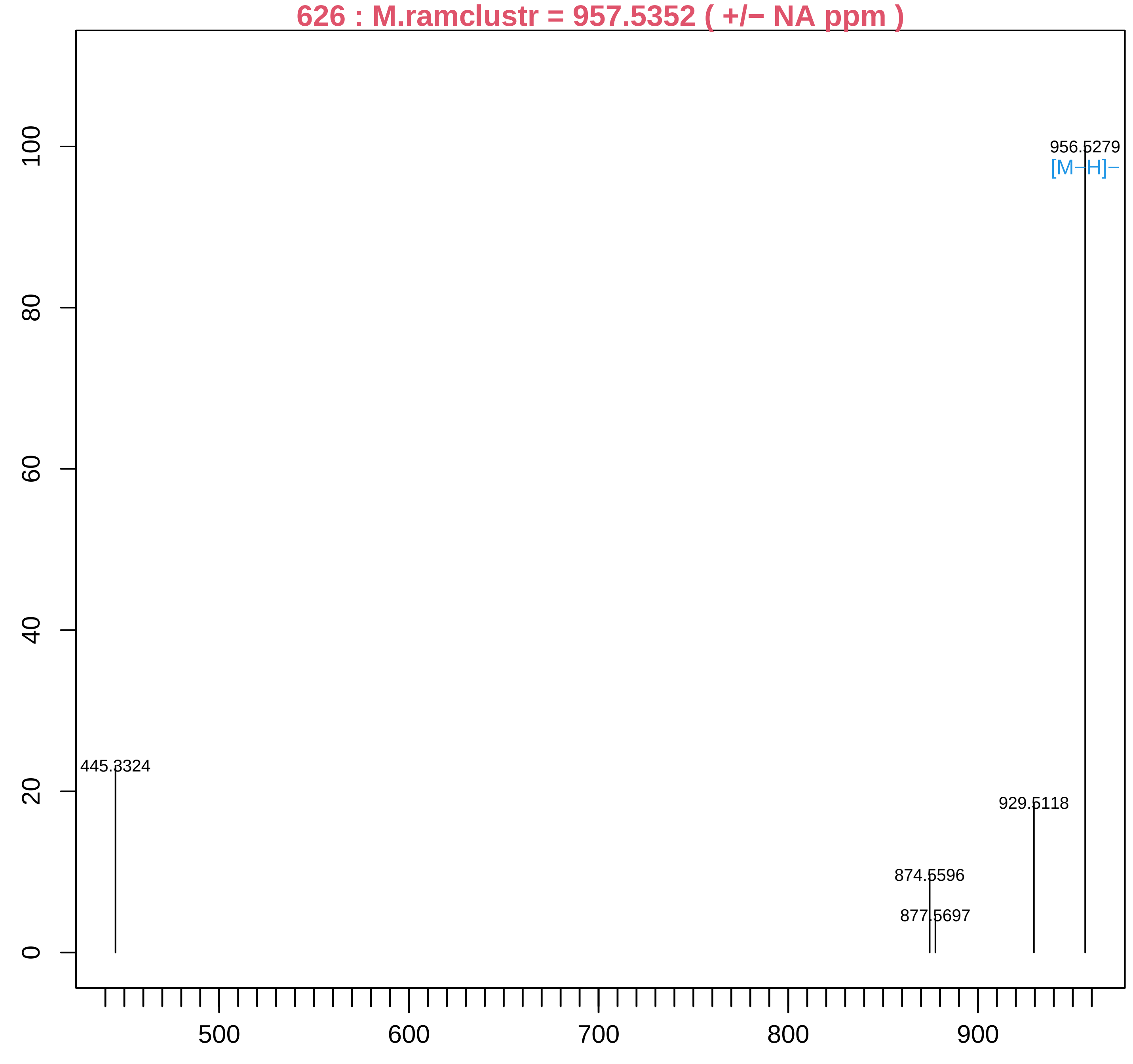
626 : M.ramclustr = 957.5352 (+/− NA ppm)
956.5279
[M−H]−
445.3324
929.5118
874.5596
877.5697
0
20
40
60
80
100
500
600
700
800
900

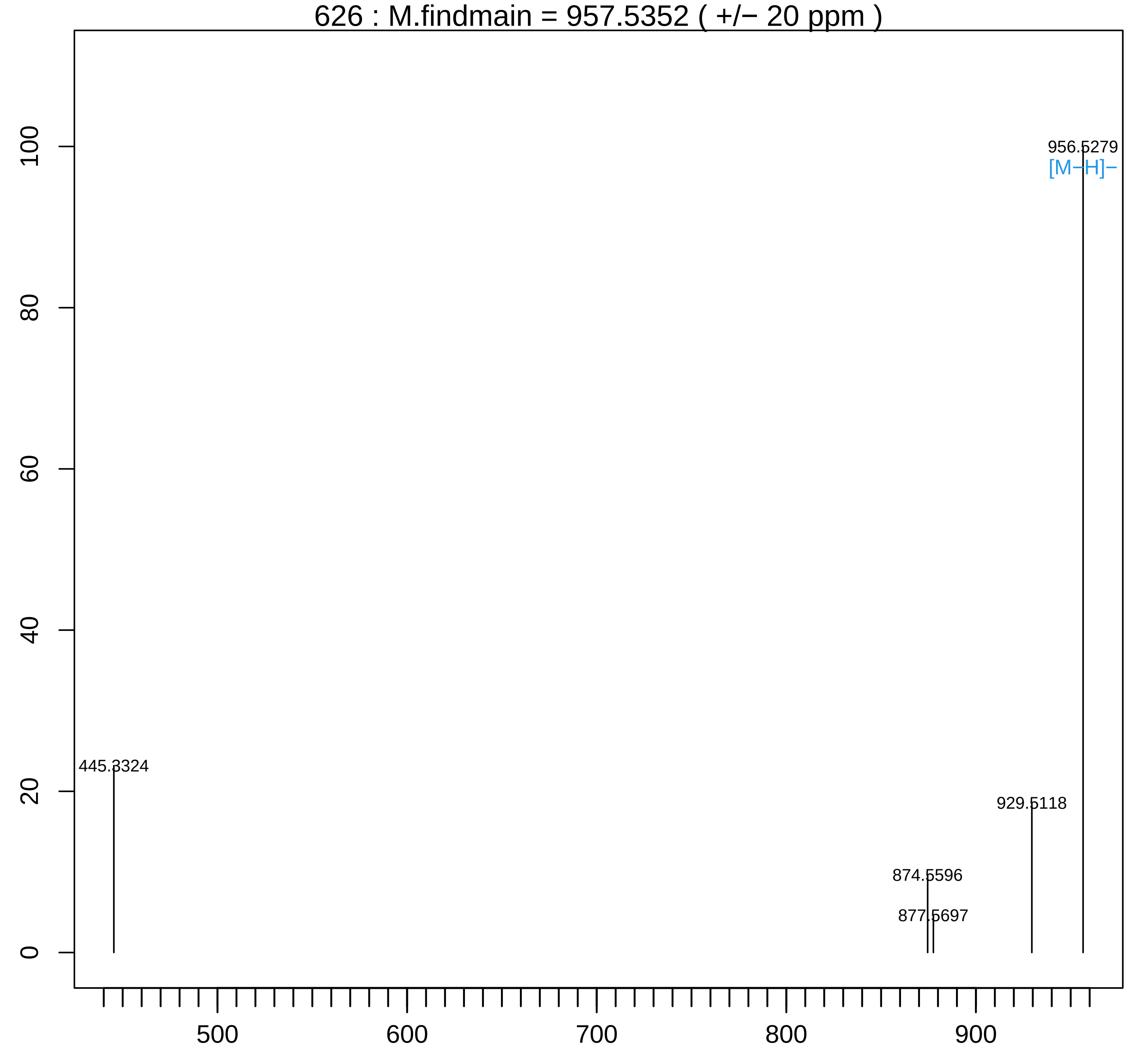
626 : M.findmain = 957.5352 (+/− 20 ppm)
956.5279
[M−H]−
445.3324
929.5118
874.5596
877.5697
0
20
40
60
80
100
500
600
700
800
900

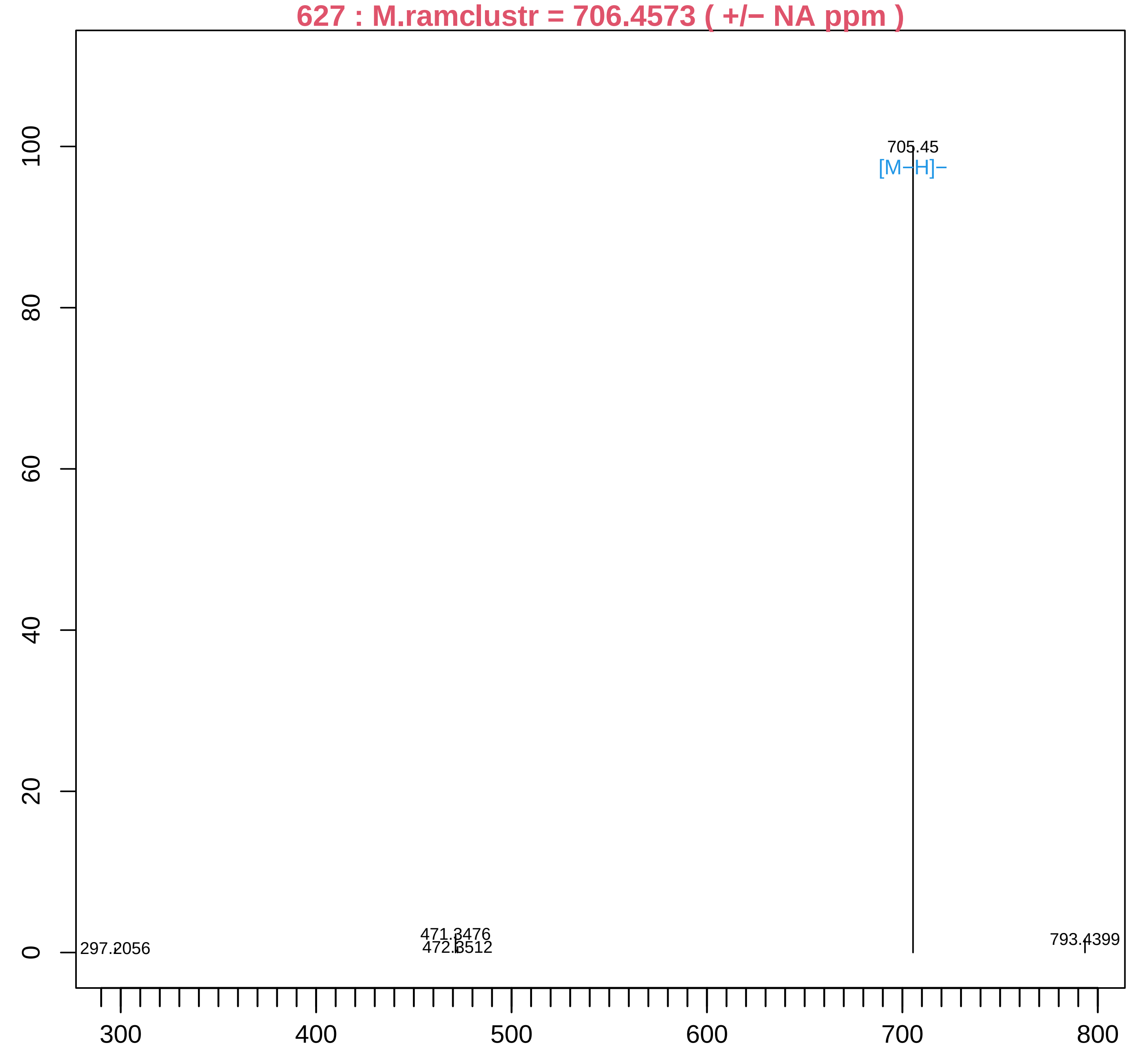

627 : M.ramclustr = 706.4573 (+/− NA ppm)
705.45
[M−H]−
297.2056
471.3476
472.3512
793.4399

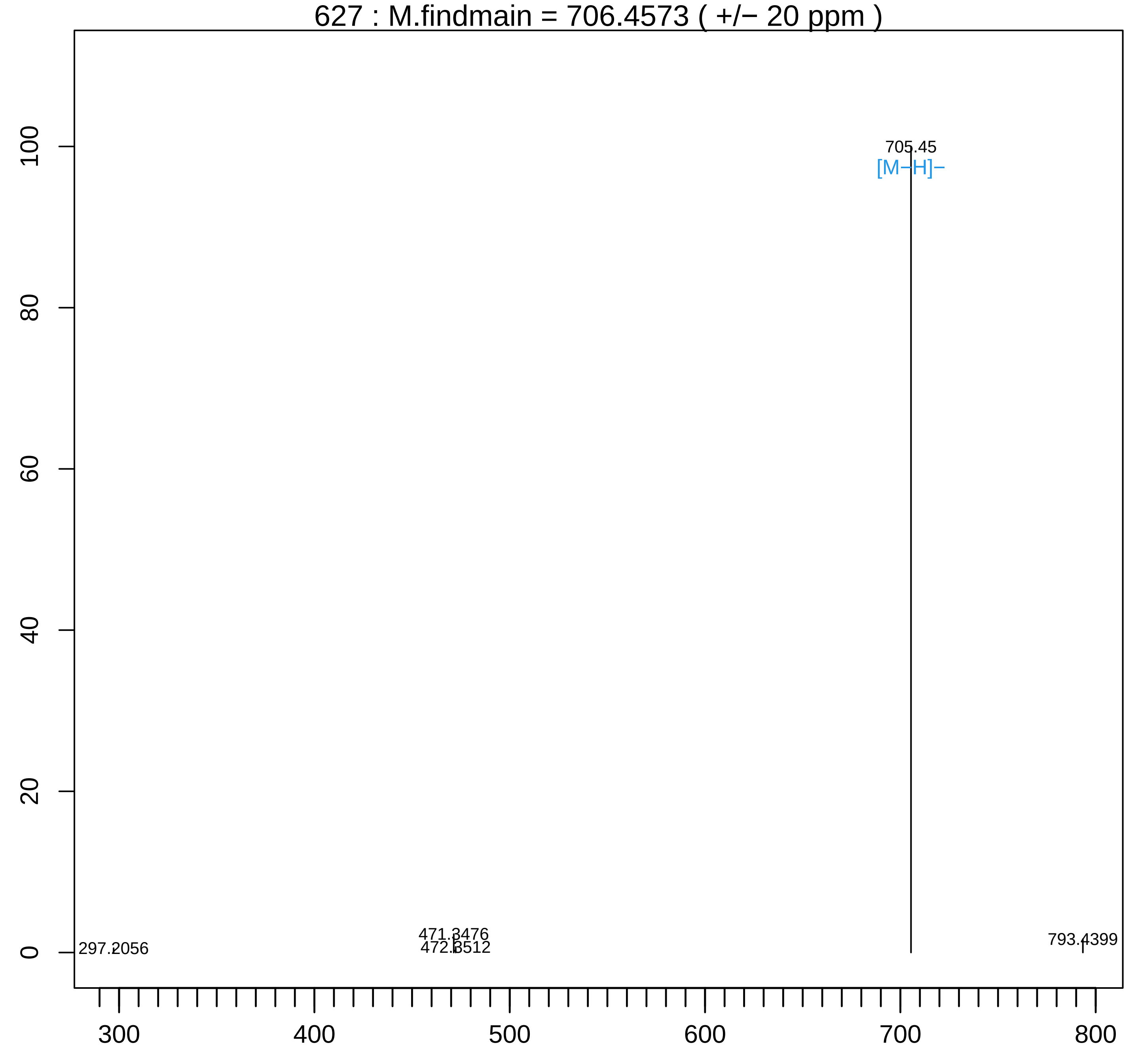

627 : M.findmain = 706.4573 (+/− 20 ppm)
705.45
[M−H]−
297.2056
471.3476
472.3512
793.4399

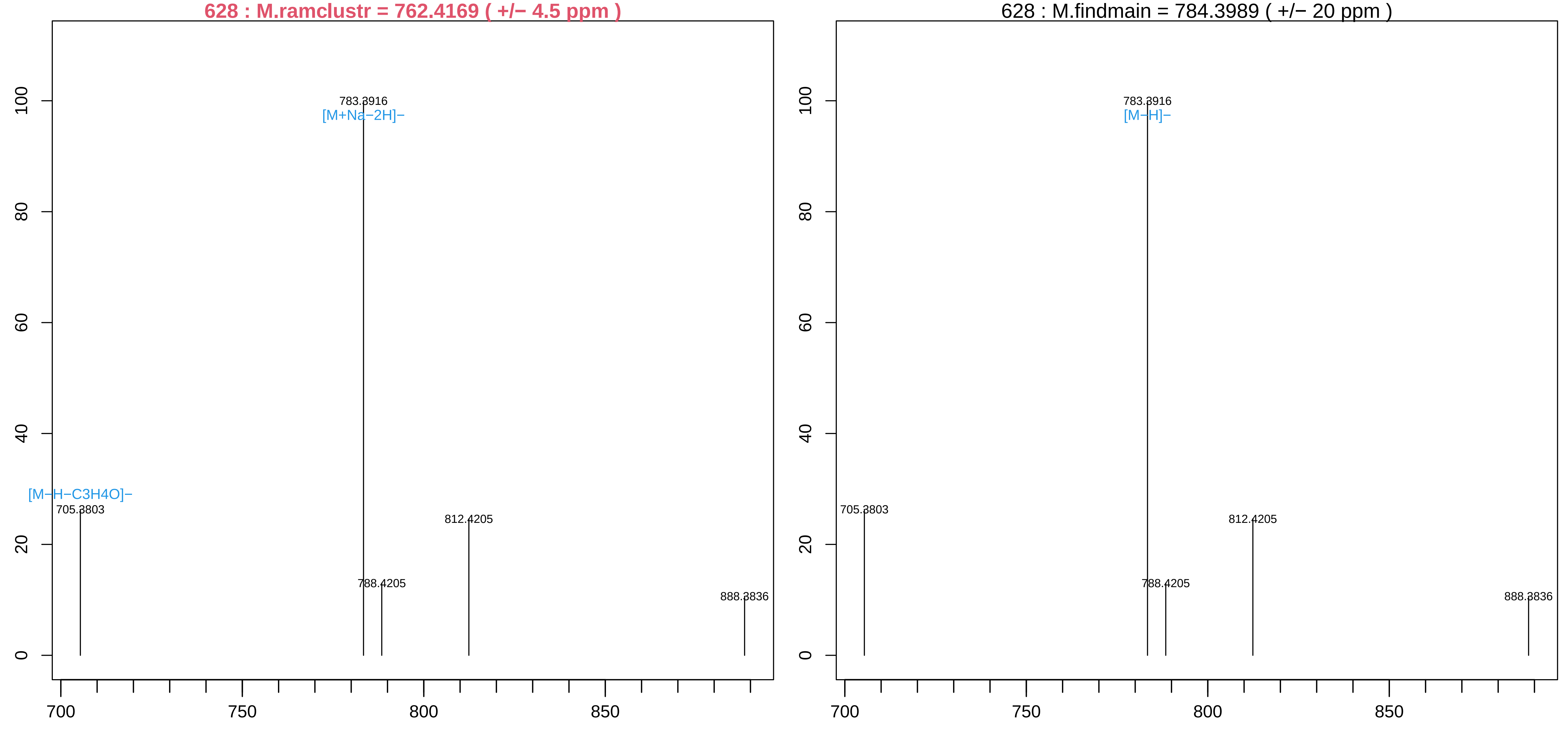
628 : M.ramclustr = 762.4169 (+/− 4.5 ppm)
628 : M.findmain = 784.3989 (+/− 20 ppm)
783.3916
[M+Na−2H]−
[M−H]−
[M−H−C3H4O]−
705.3803
812.4205
788.4205
888.3836
0
20
40
60
80
100
700
750
800
850

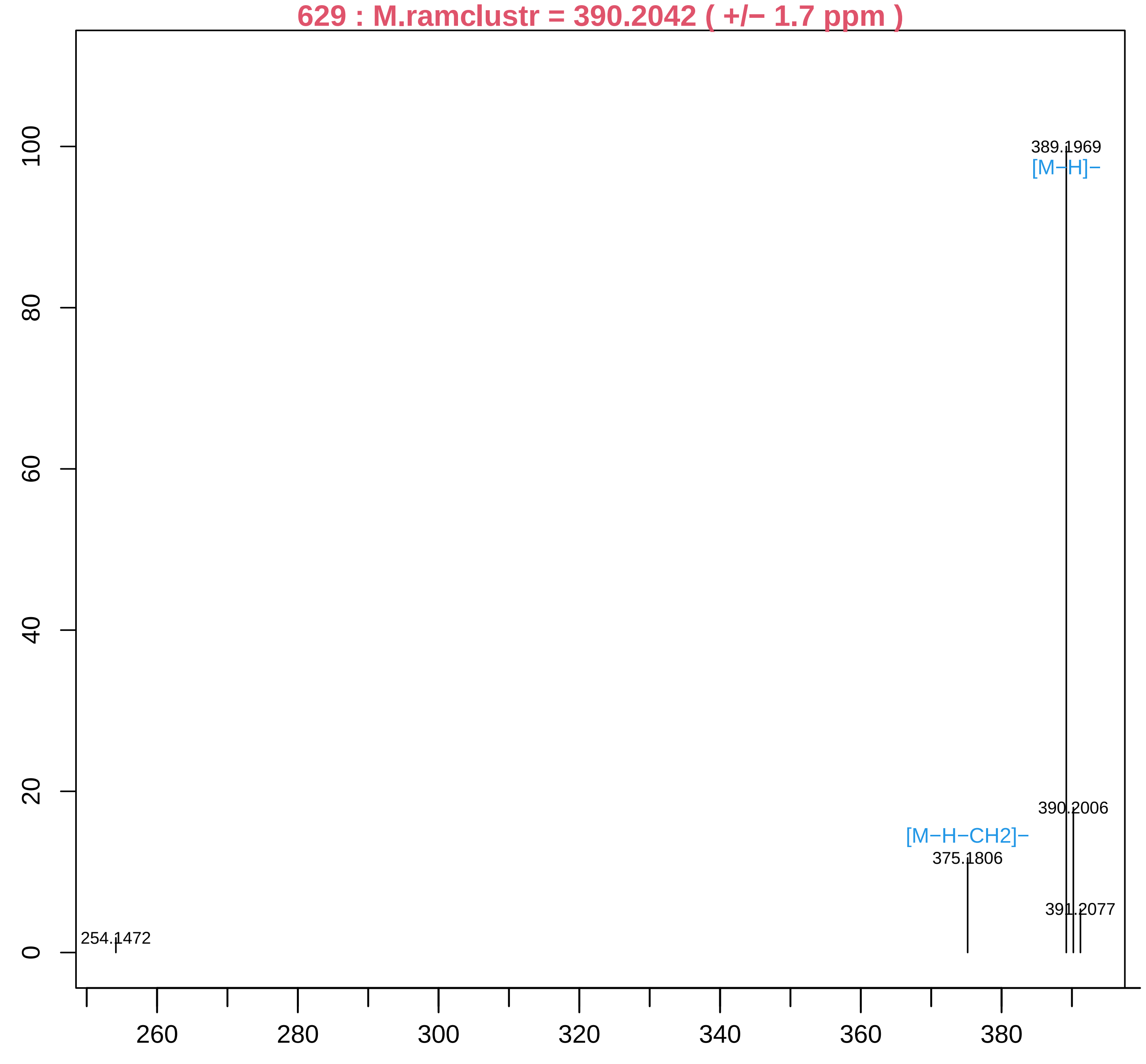
629 : M.ramclustr = 390.2042 (+/− 1.7 ppm)
389.1969
[M−H]−
390.2006
[M−H−CH2]−
375.1806
391.2077
254.1472
0
20
40
60
80
100
260
280
300
320
340
360
380

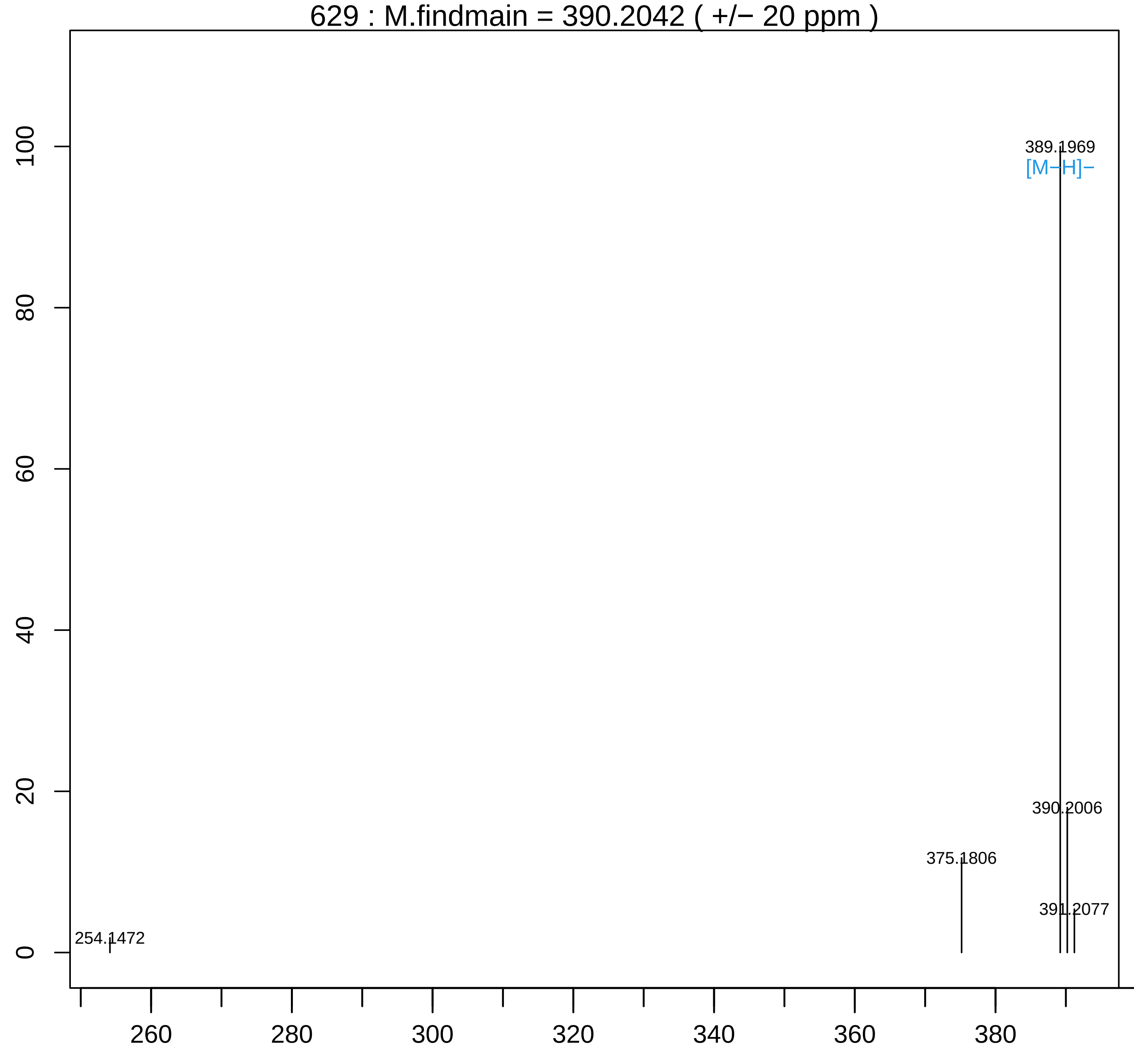
629 : M.findmain = 390.2042 (+/− 20 ppm)
389.1969
[M−H]−
390.2006
375.1806
391.2077
254.1472
0
20
40
60
80
100
260
280
300
320
340
360
380

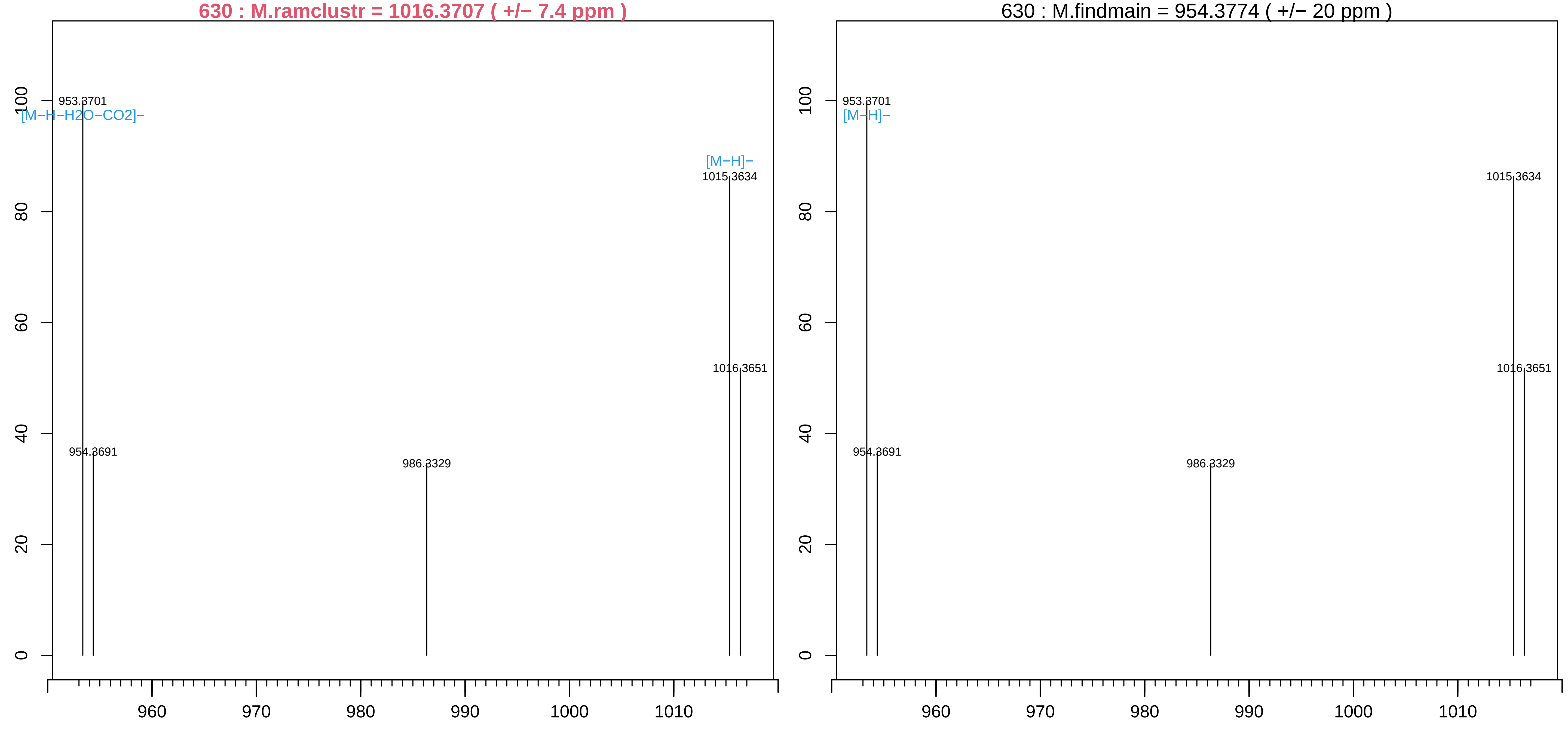
630 : M.ramclustr = 1016.3707 (+/− 7.4 ppm)
953.3701
[M−H−H2O−CO2]−
954.3691
986.3329
[M−H]−
1015.3634
1016.3651
630 : M.findmain = 954.3774 (+/− 20 ppm)
953.3701
[M−H]−
954.3691
986.3329
1015.3634
1016.3651

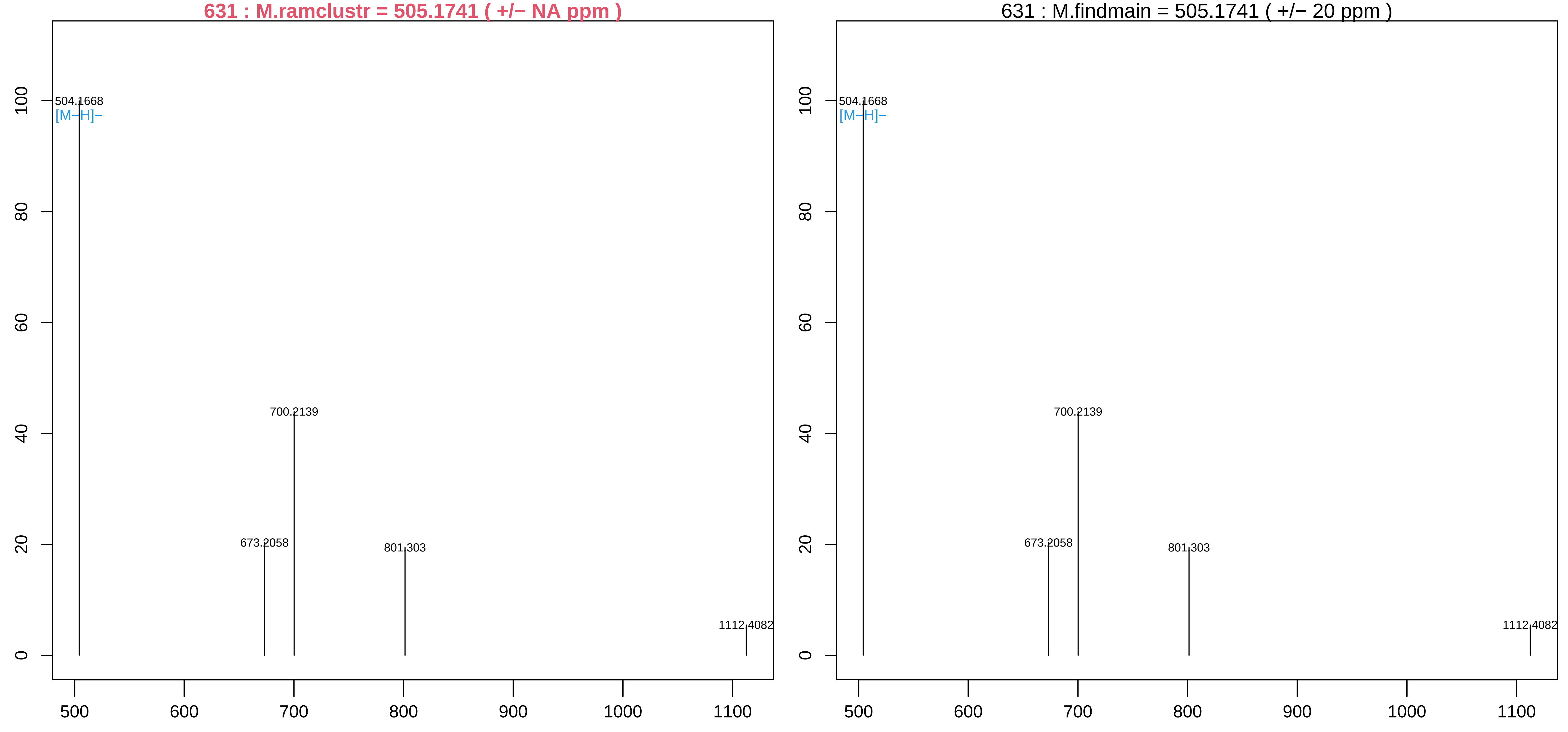

631 : M.ramclustr = 505.1741 (+/− NA ppm)
504.1668
[M−H]−
700.2139
673.2058
801.303
1112.4082
0
20
40
60
80
100
500
600
700
800
900
1000
1100
631 : M.findmain = 505.1741 (+/− 20 ppm)
504.1668
[M−H]−
700.2139
673.2058
801.303
1112.4082
0
20
40
60
80
100
500
600
700
800
900
1000
1100

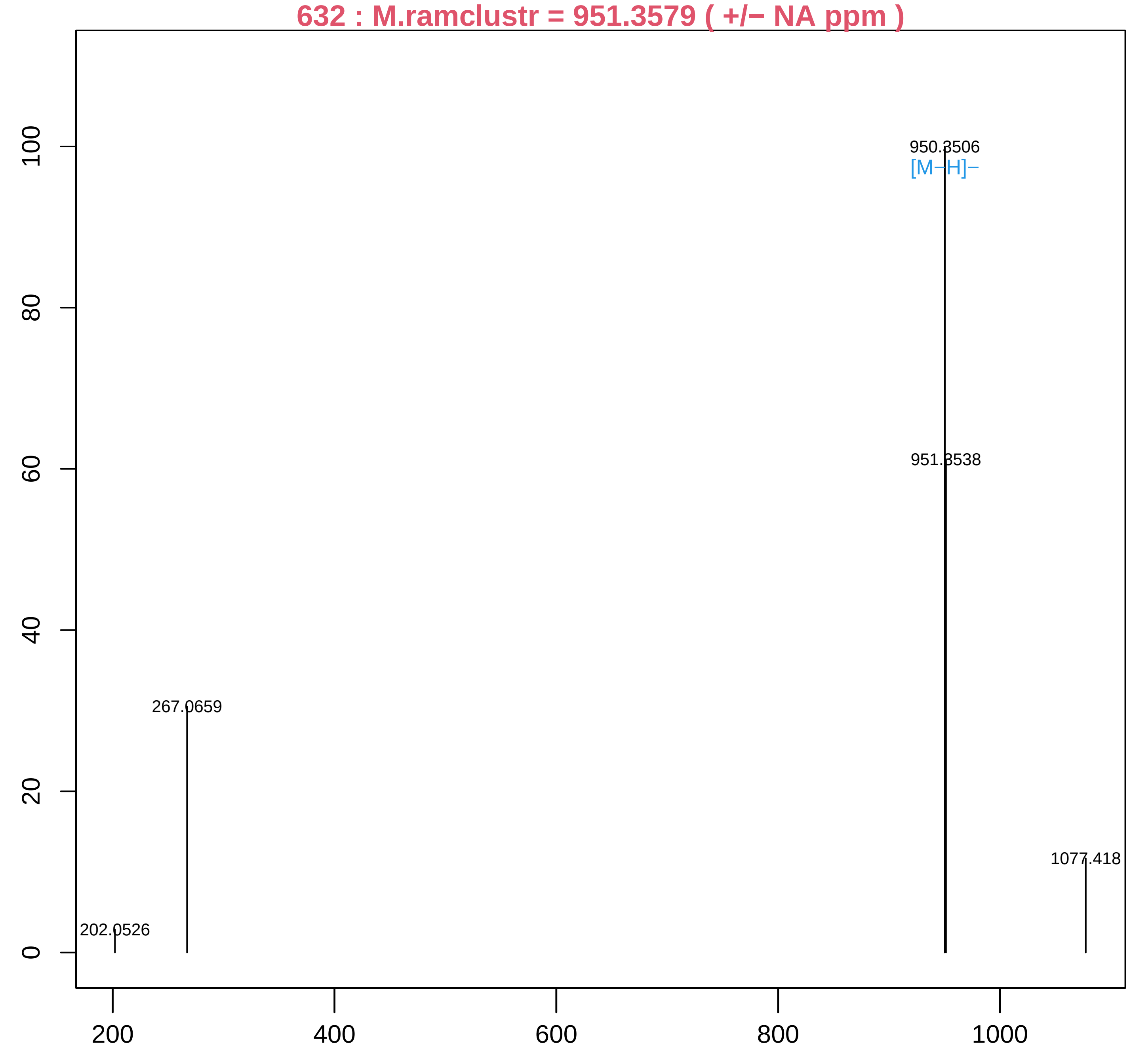
632 : M.ramclustr = 951.3579 (+/− NA ppm)
950.3506
[M−H]−
951.3538
267.0659
1077.418
202.0526
0
20
40
60
80
100
200
400
600
800
1000

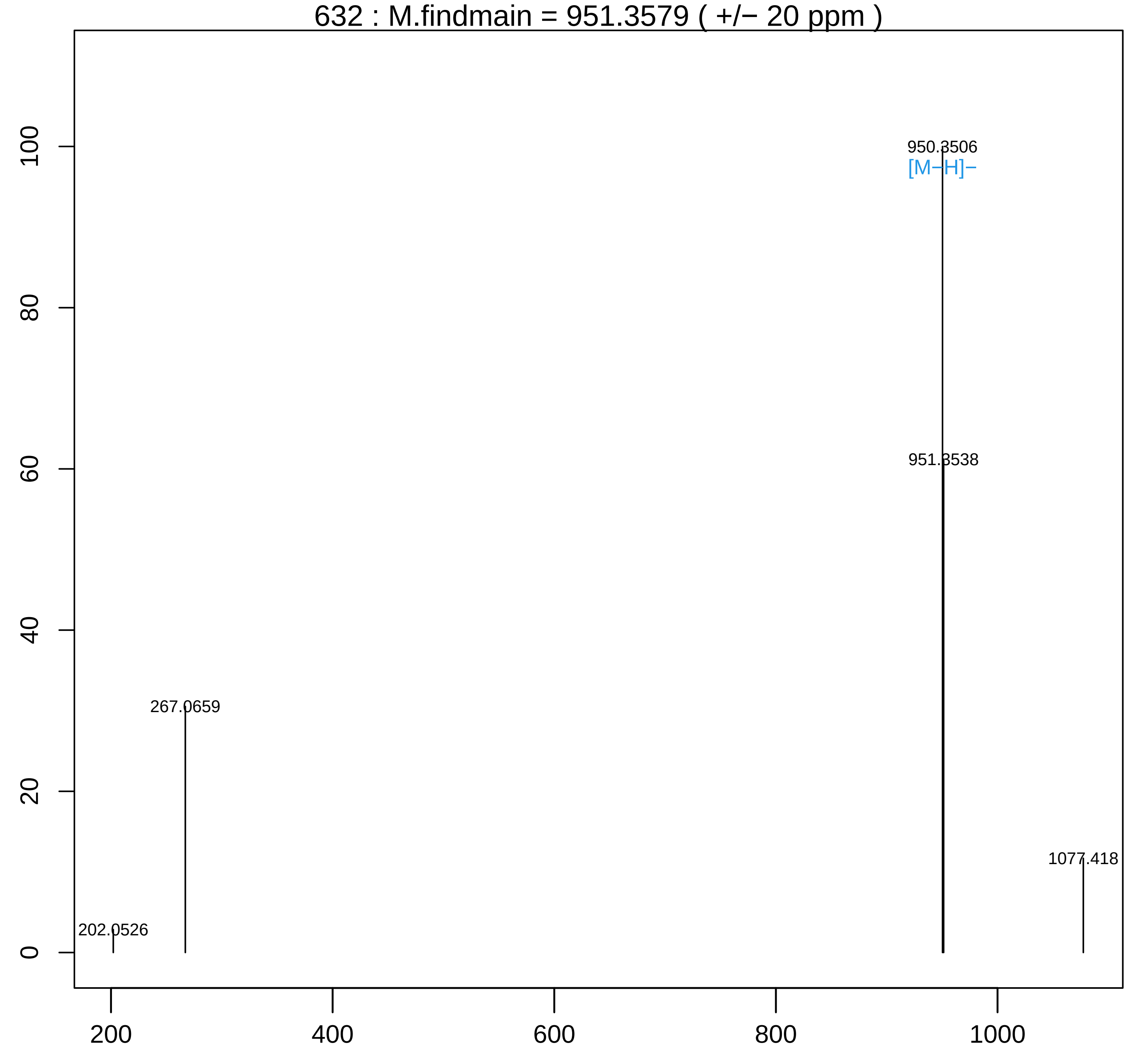
632 : M.findmain = 951.3579 (+/− 20 ppm)
950.3506
[M−H]−
951.3538
267.0659
1077.418
202.0526
0
20
40
60
80
100
200
400
600
800
1000

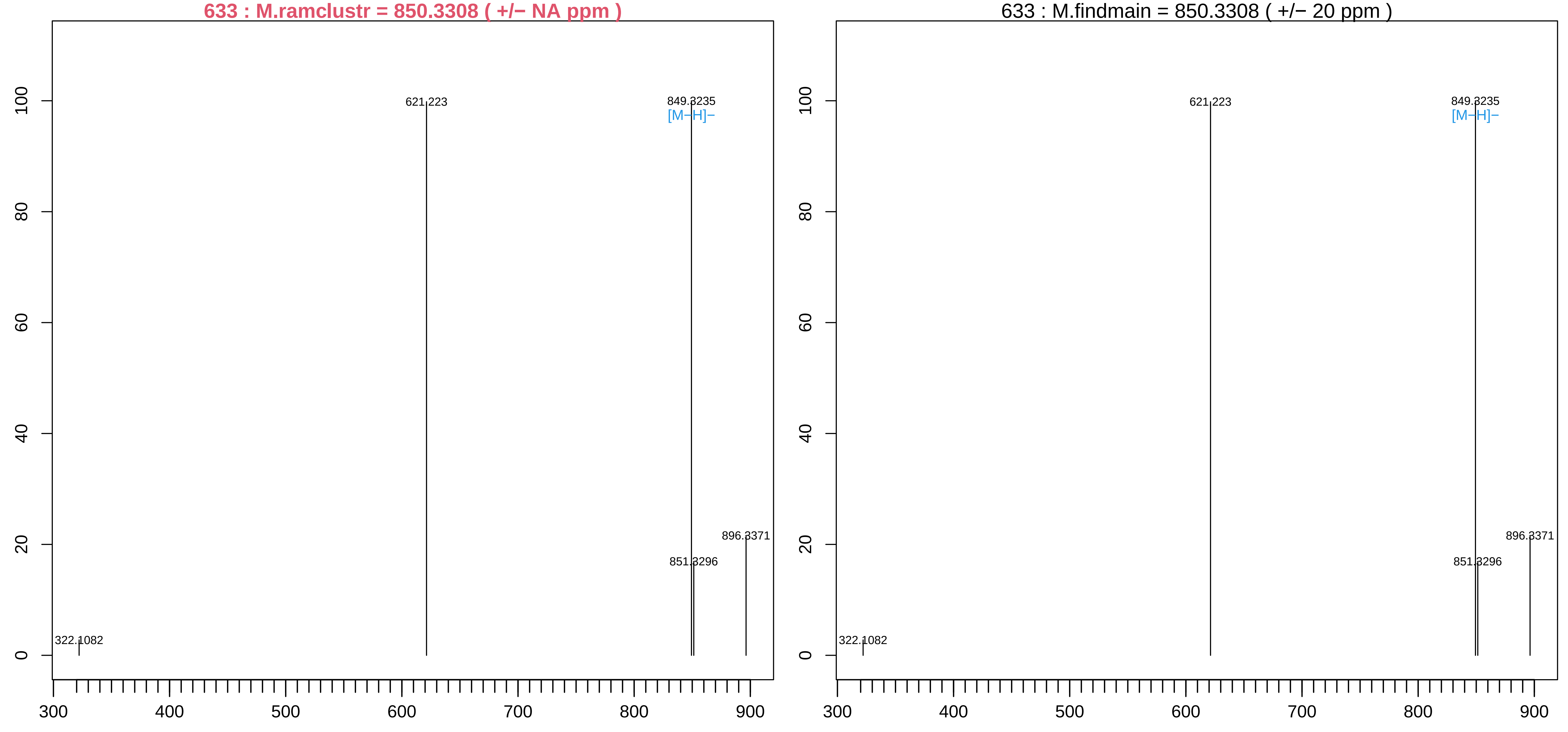
633 : M.ramclustr = 850.3308 (+/− NA ppm)
633 : M.findmain = 850.3308 (+/− 20 ppm)
621.223
849.3235
[M−H]−
851.3296
896.3371
322.1082
0
20
40
60
80
100
300
400
500
600
700
800
900

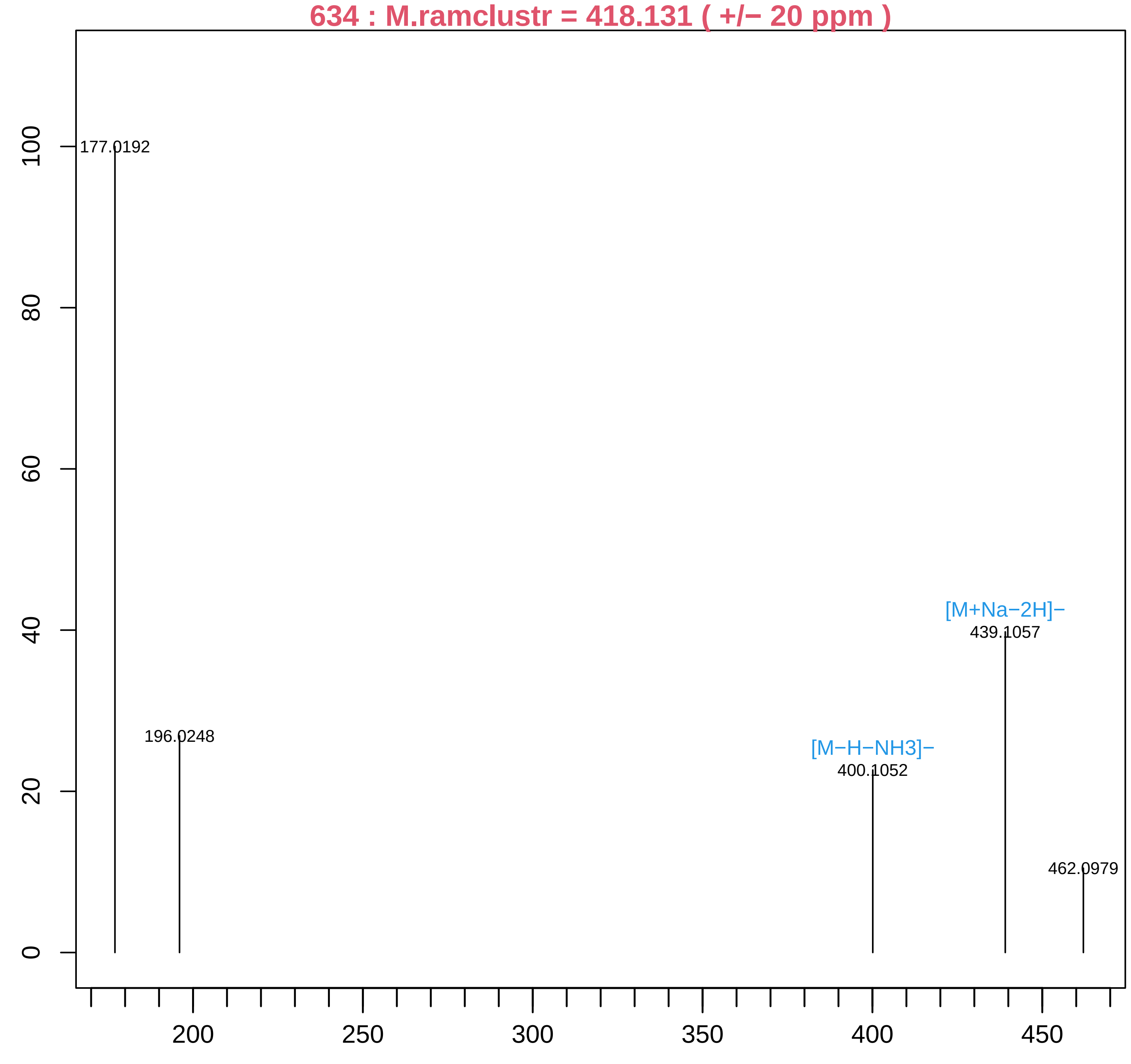
634 : M.ramclustr = 418.131 (+/− 20 ppm)
177.0192
196.0248
[M−H−NH3]−
400.1052
[M+Na−2H]−
439.1057
462.0979
0
20
40
60
80
100
200
250
300
350
400
450

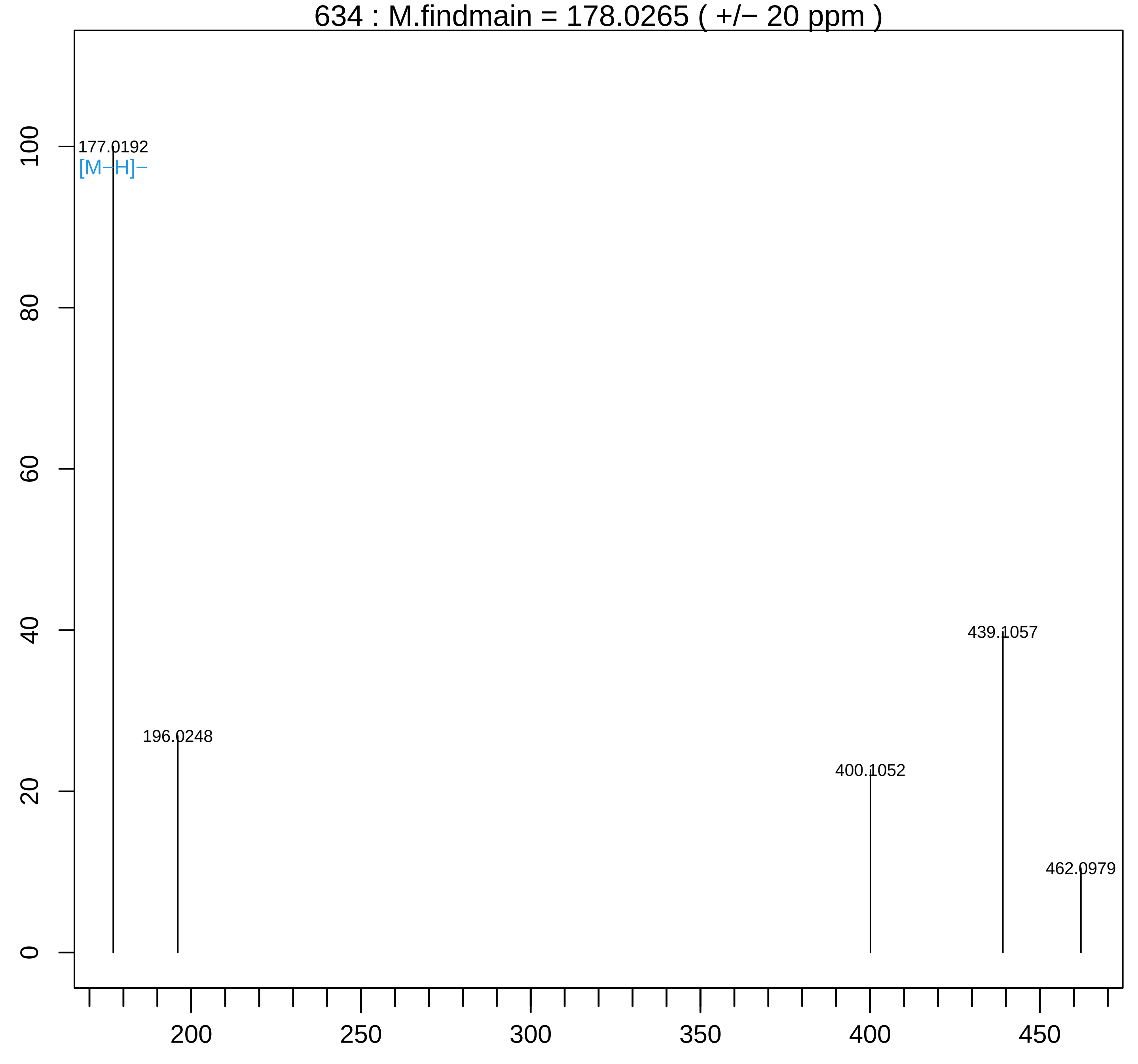
634 : M.findmain = 178.0265 (+/− 20 ppm)
177.0192
[M−H]−
196.0248
400.1052
439.1057
462.0979
0
20
40
60
80
100
200
250
300
350
400
450

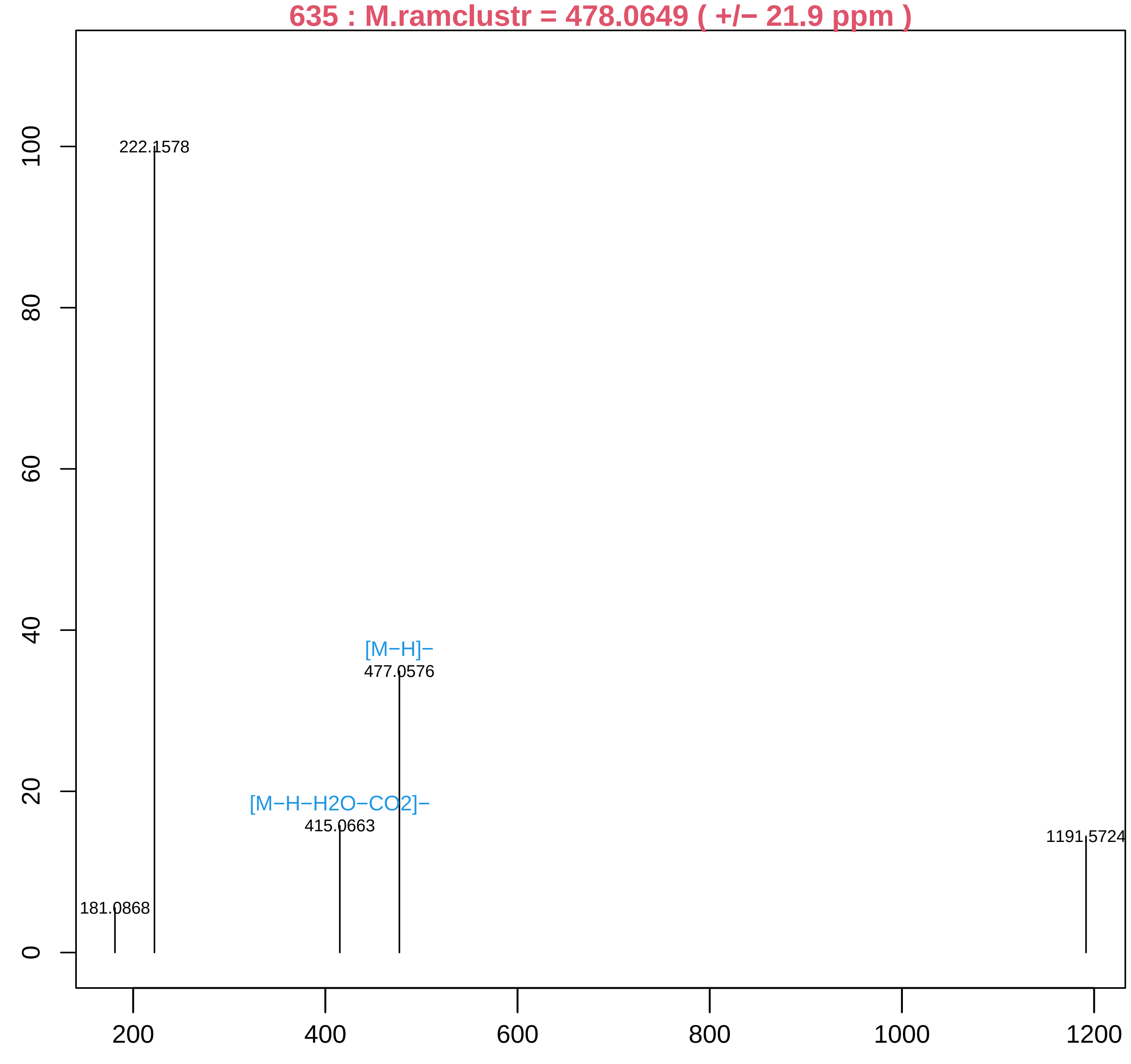
635 : M.ramclustr = 478.0649 (+/− 21.9 ppm)
222.1578
[M−H]−
477.0576
[M−H−H2O−CO2]−
415.0663
1191.5724
181.0868
0
20
40
60
80
100
200
400
600
800
1000
1200

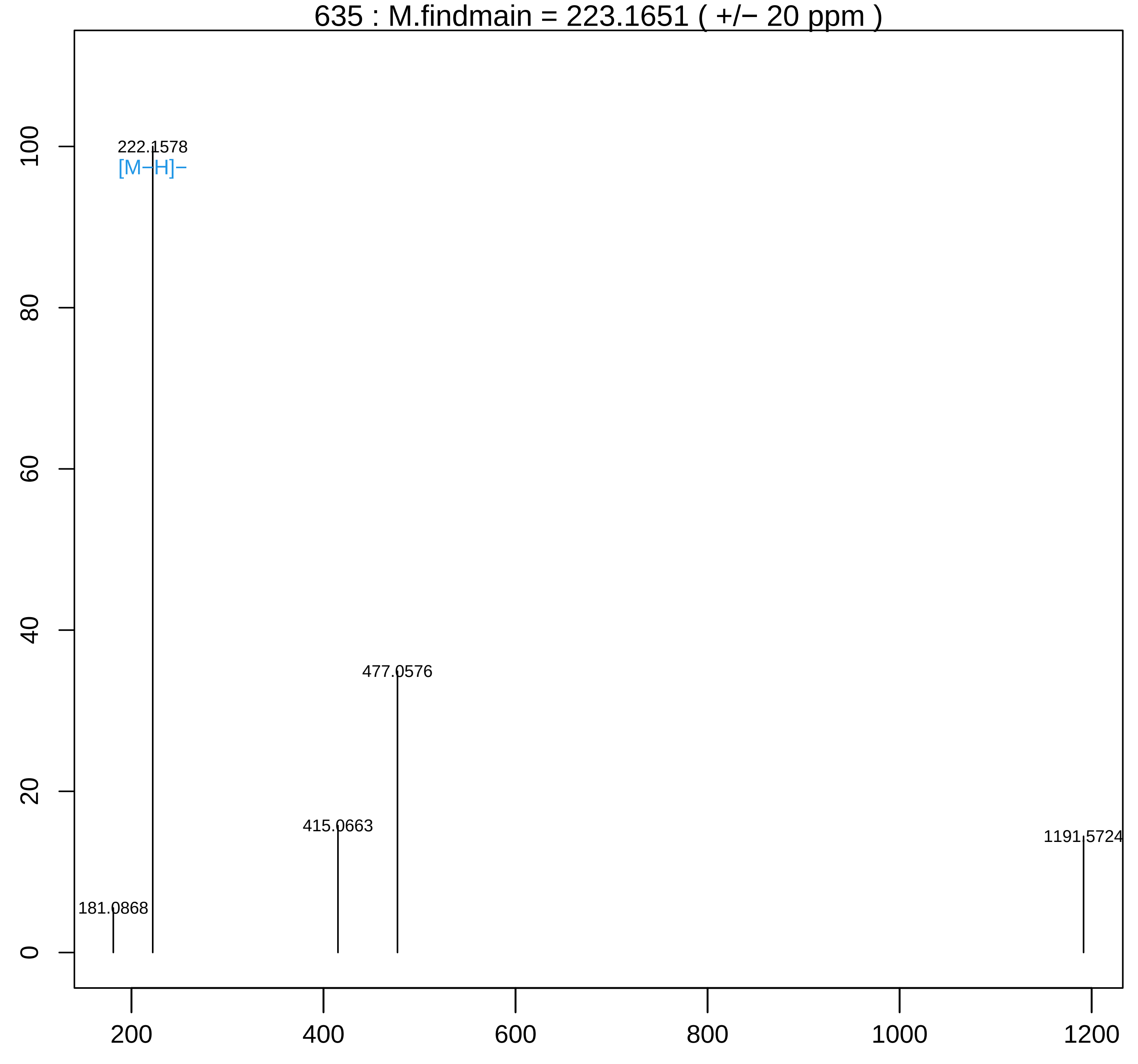
635 : M.findmain = 223.1651 (+/− 20 ppm)
222.1578
[M−H]−
477.0576
415.0663
1191.5724
181.0868
0
20
40
60
80
100
200
400
600
800
1000
1200

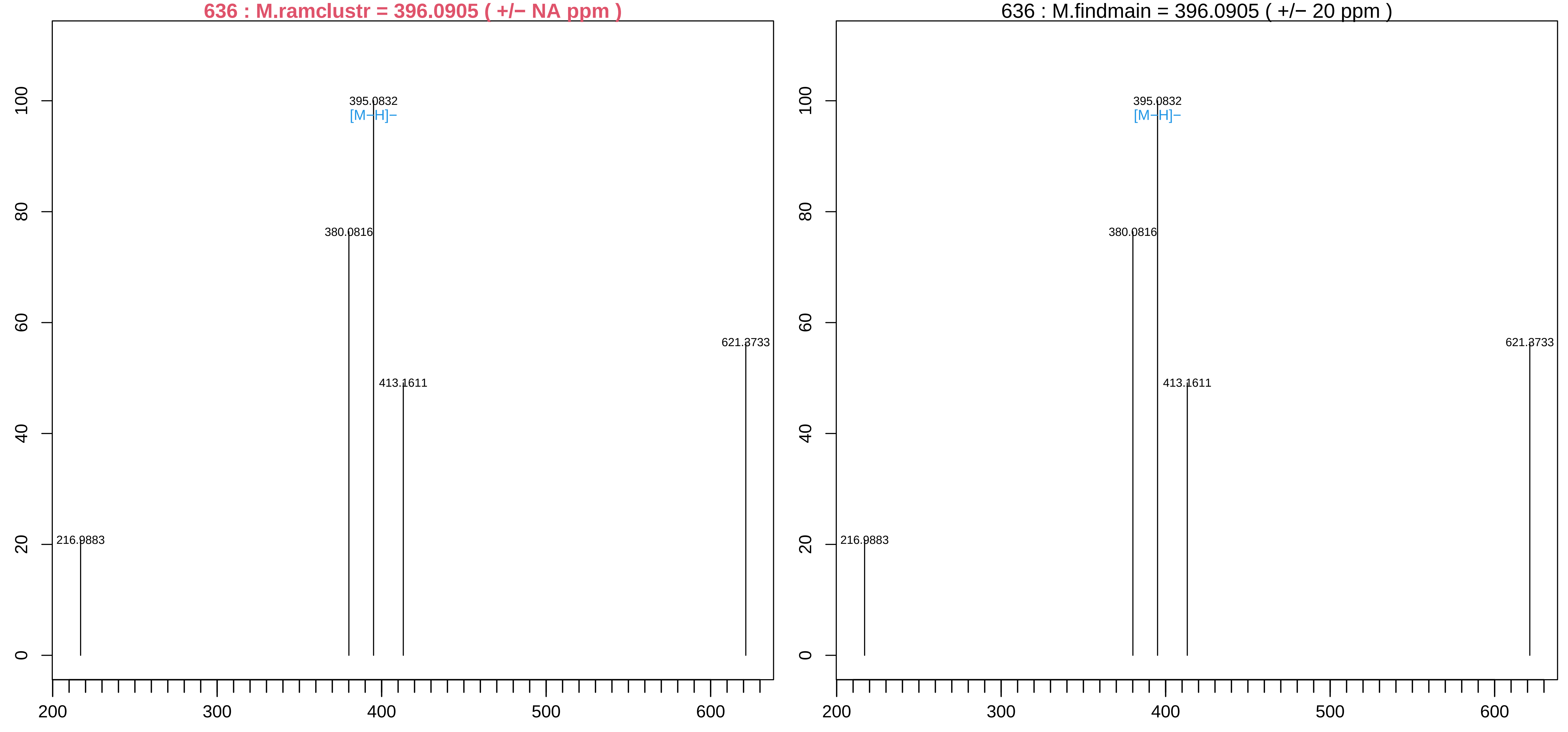

636 : M.ramclustr = 396.0905 (+/− NA ppm)
636 : M.findmain = 396.0905 (+/− 20 ppm)
395.0832
[M−H]−
380.0816
621.3733
413.1611
216.9883
0
20
40
60
80
100
200
300
400
500
600

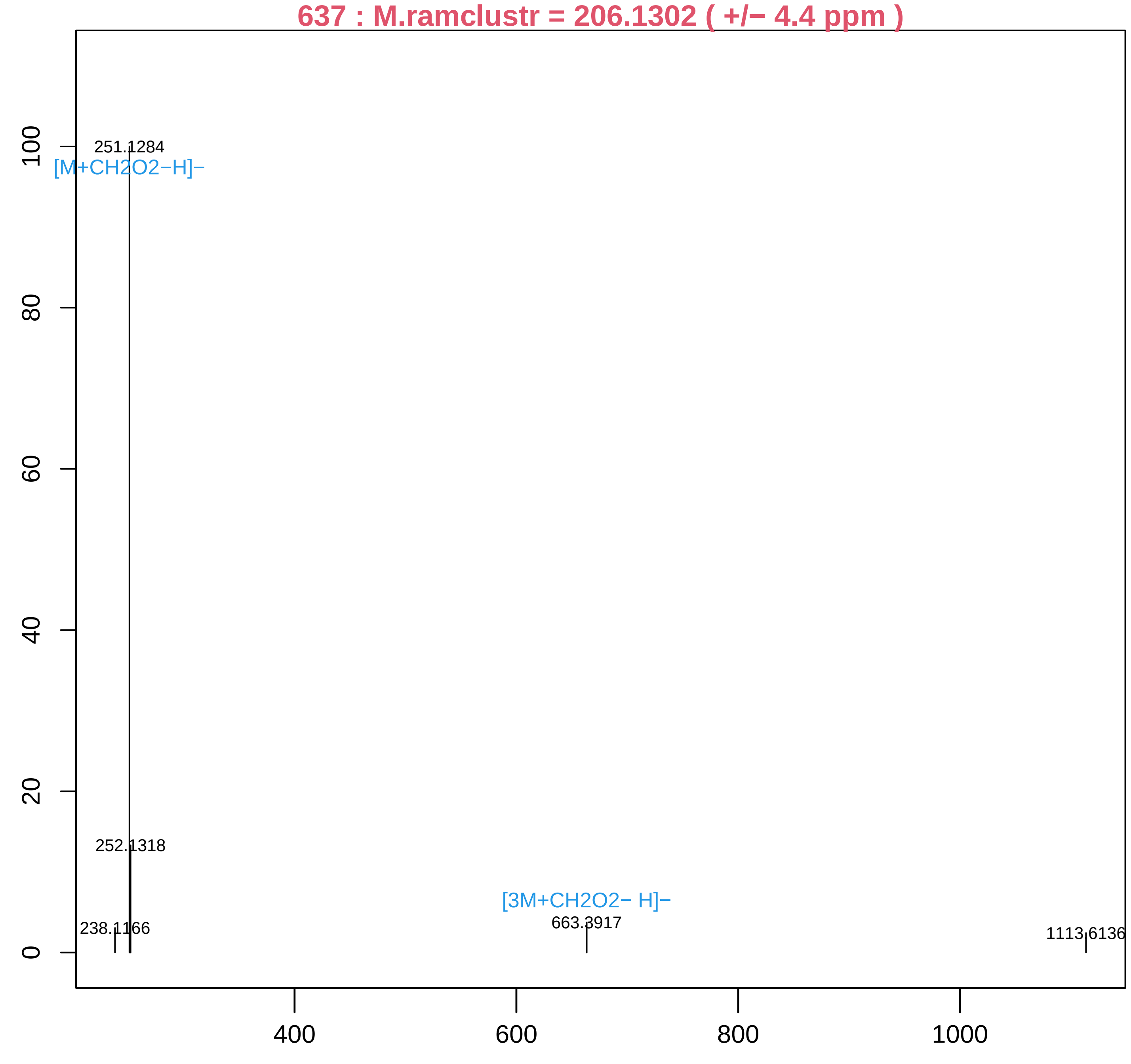

637 : M.ramclustr = 206.1302 (+/− 4.4 ppm)
251.1284
[M+CH2O2−H]−
252.1318
[3M+CH2O2− H]−
238.1166
663.3917
1113.6136
0
20
40
60
80
100
400
600
800
1000

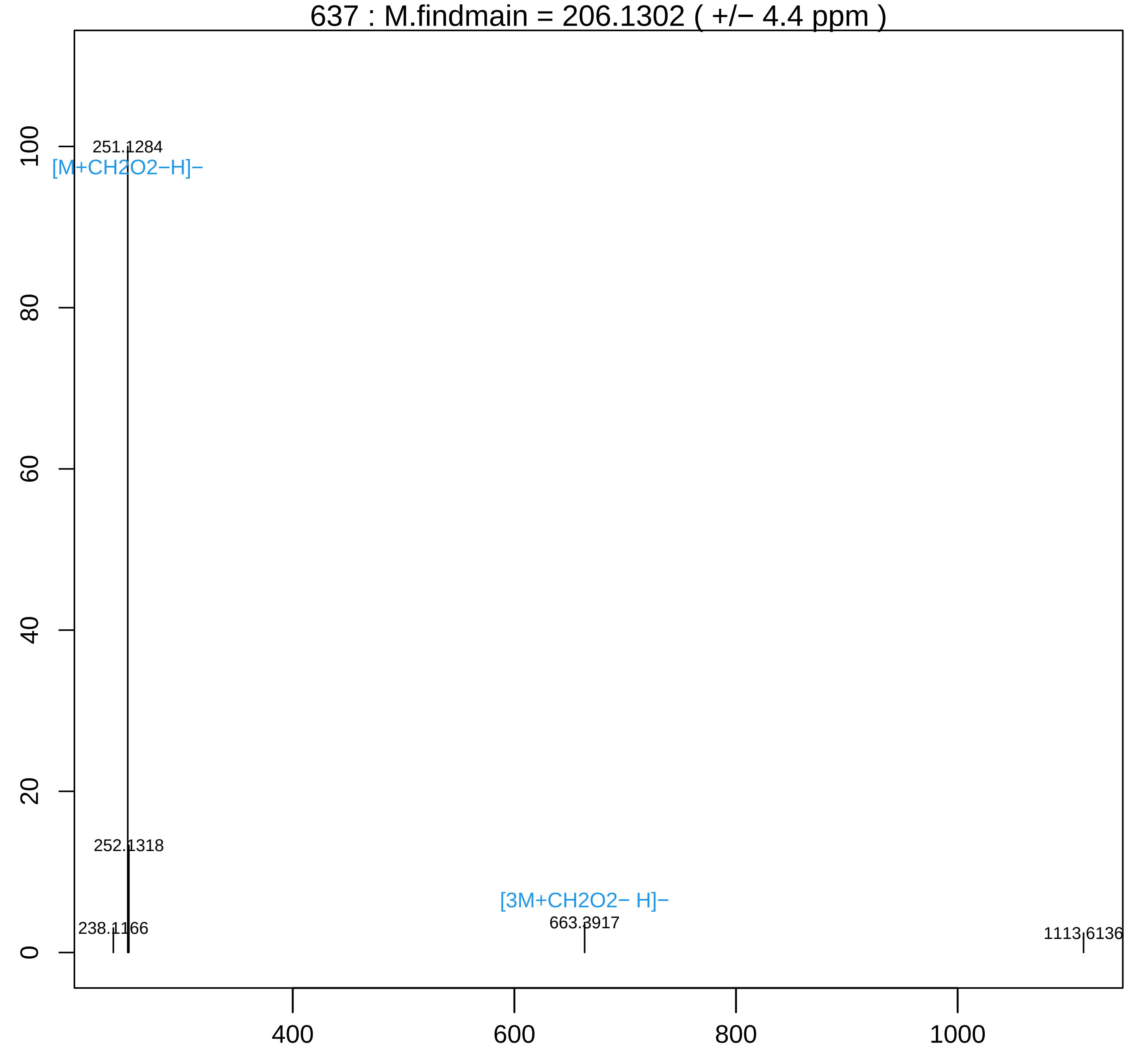

637 : M.findmain = 206.1302 (+/− 4.4 ppm)
251.1284
[M+CH2O2−H]−
252.1318
[3M+CH2O2− H]−
238.1166
663.3917
1113.6136
0
20
40
60
80
100
400
600
800
1000

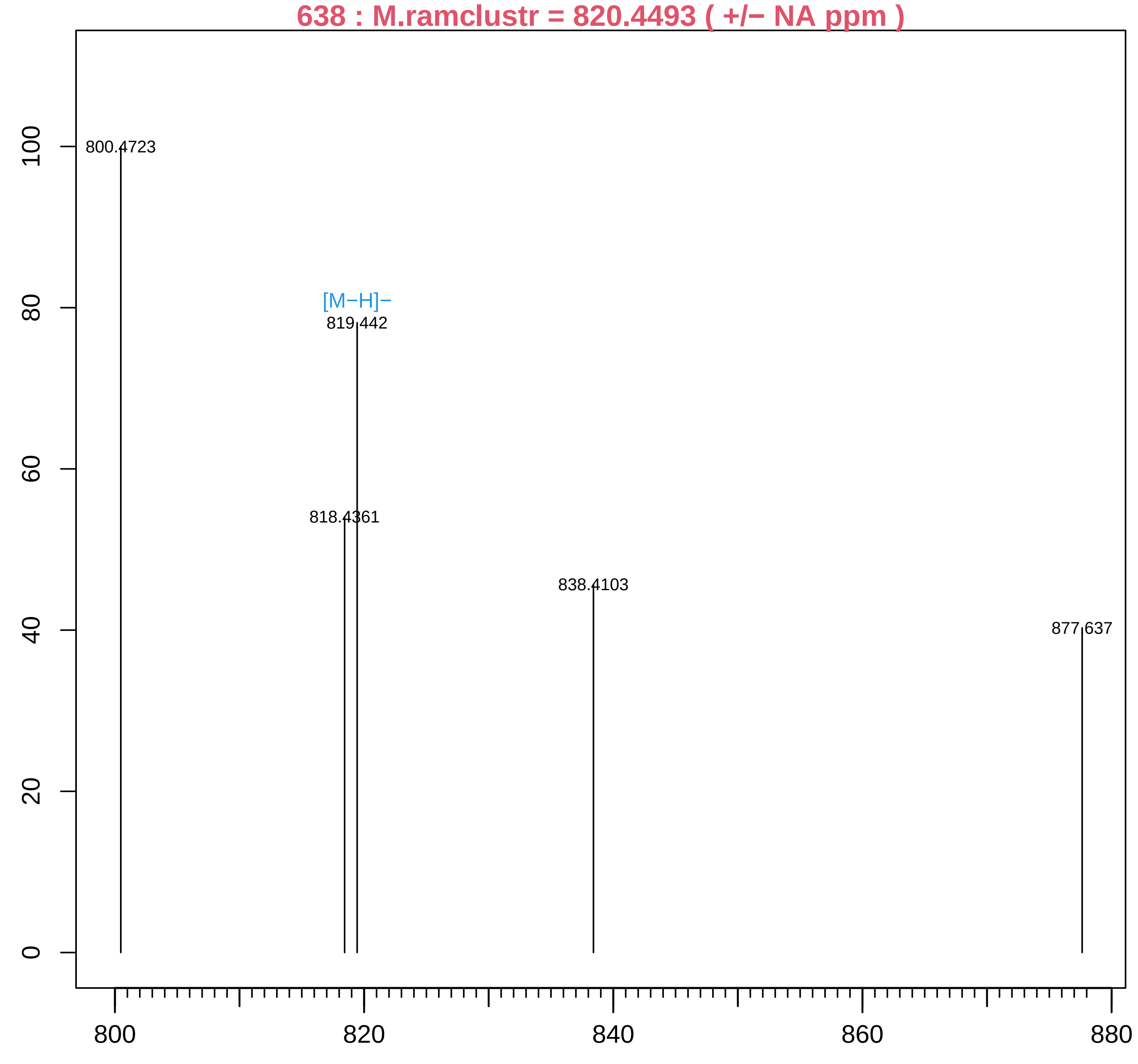

638 : M.ramclustr = 820.4493 (+/− NA ppm)
800.4723
[M−H]−
819.442
818.4361
838.4103
877.637
0
20
40
60
80
100
800
820
840
860
880

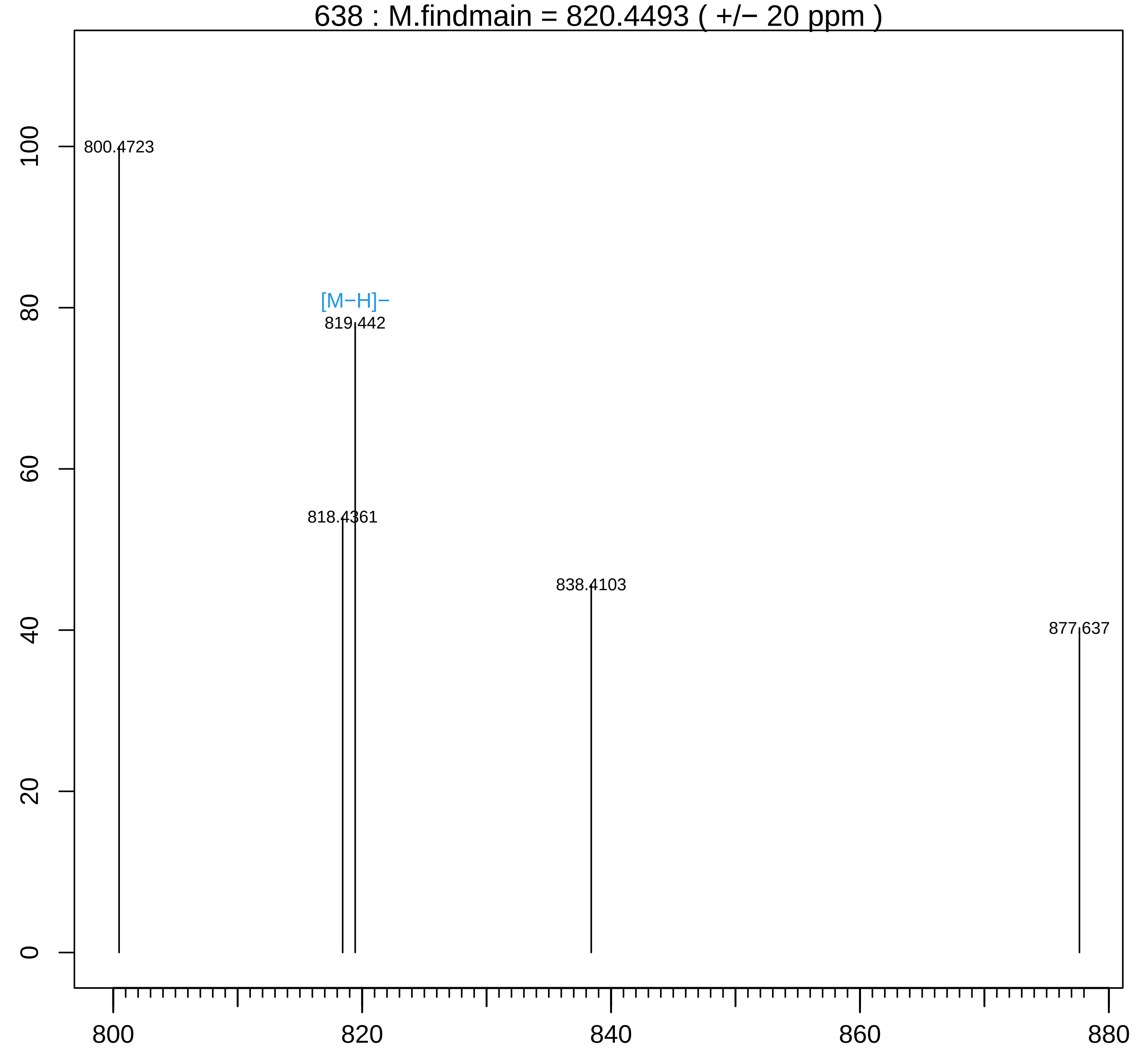

638 : M.findmain = 820.4493 (+/− 20 ppm)
800.4723
[M−H]−
819.442
818.4361
838.4103
877.637
0
20
40
60
80
100
800
820
840
860
880

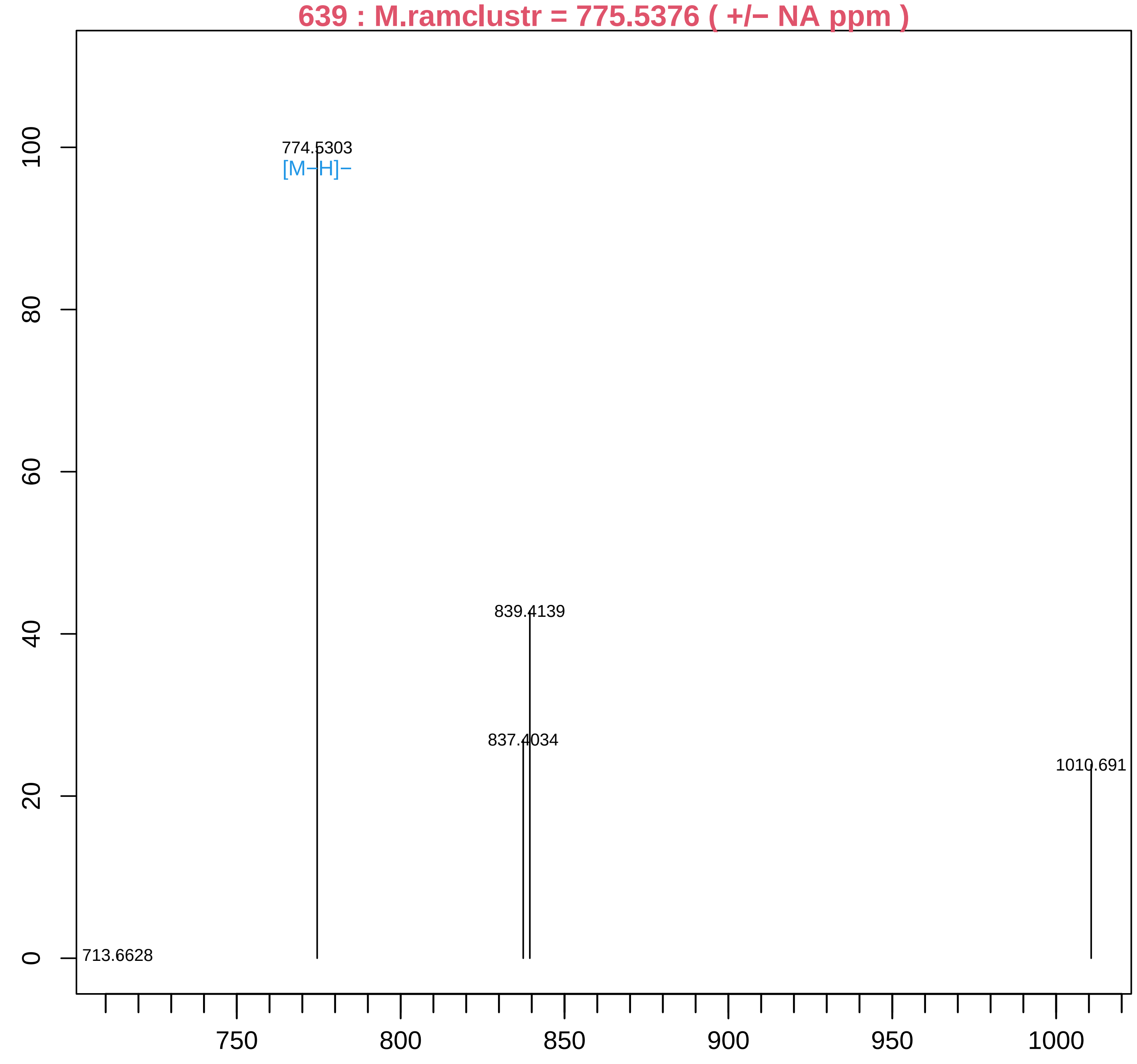

639 : M.ramclustr = 775.5376 (+/− NA ppm)
774.5303
[M−H]−
839.4139
837.4034
1010.691
713.6628
0
20
40
60
80
100
750
800
850
900
950
1000

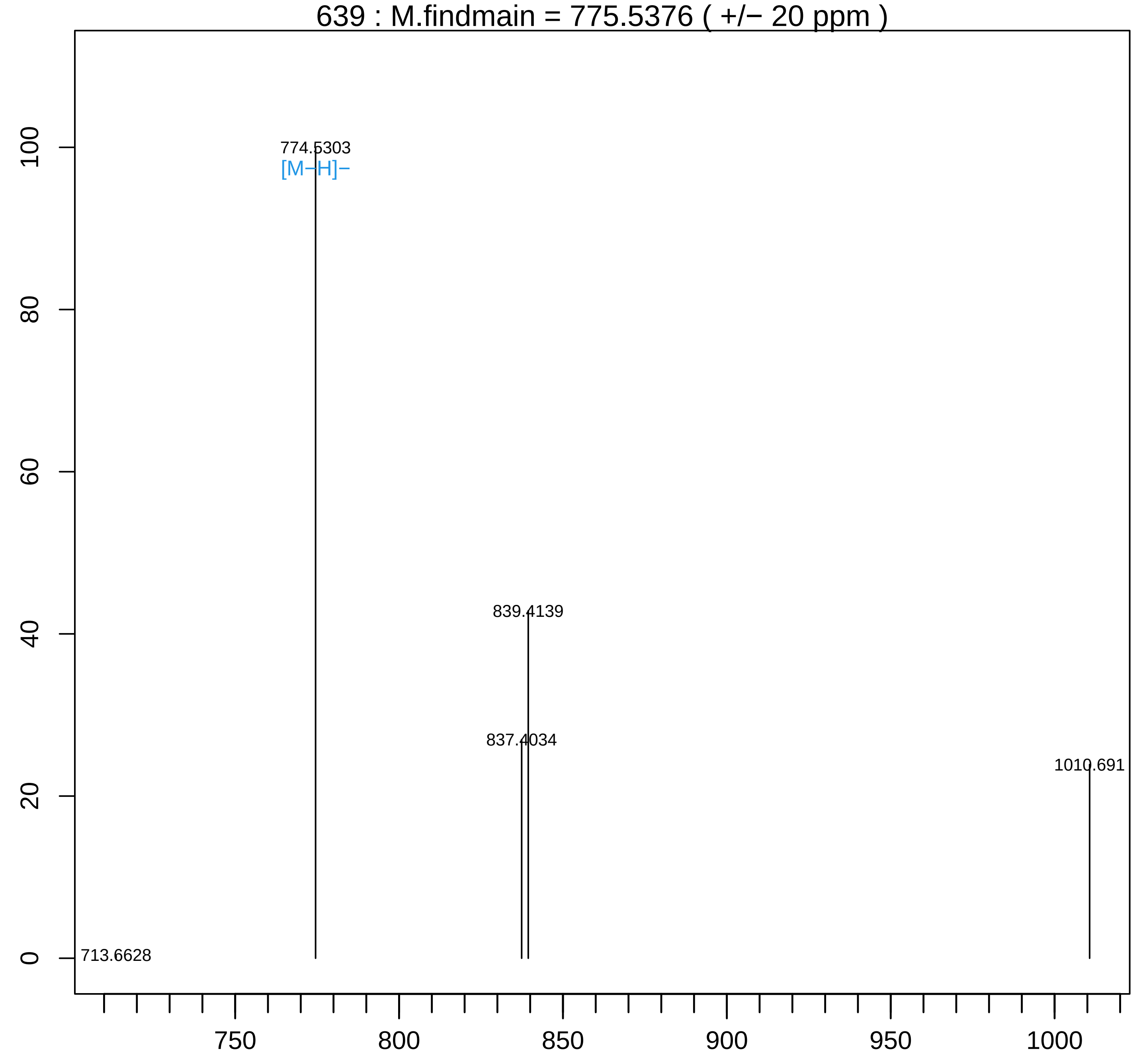

639 : M.findmain = 775.5376 (+/− 20 ppm)
774.5303
[M−H]−
839.4139
837.4034
1010.691
713.6628
0
20
40
60
80
100
750
800
850
900
950
1000

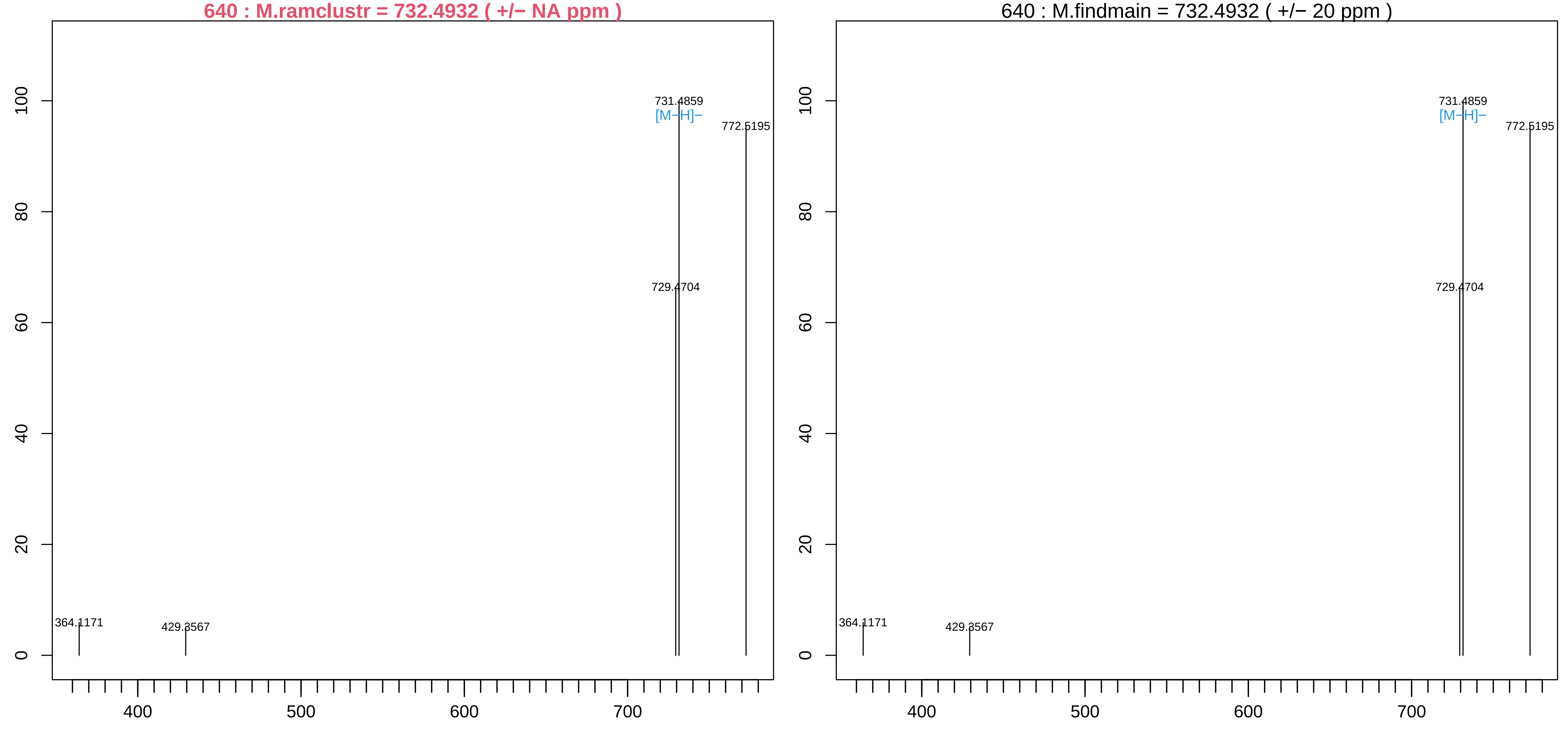
640 : M.ramclustr = 732.4932 (+/− NA ppm)
731.4859
[M−H]−
772.5195
729.4704
364.1171
429.3567
0
20
40
60
80
100
400
500
600
700
640 : M.findmain = 732.4932 (+/− 20 ppm)
731.4859
[M−H]−
772.5195
729.4704
364.1171
429.3567
0
20
40
60
80
100
400
500
600
700

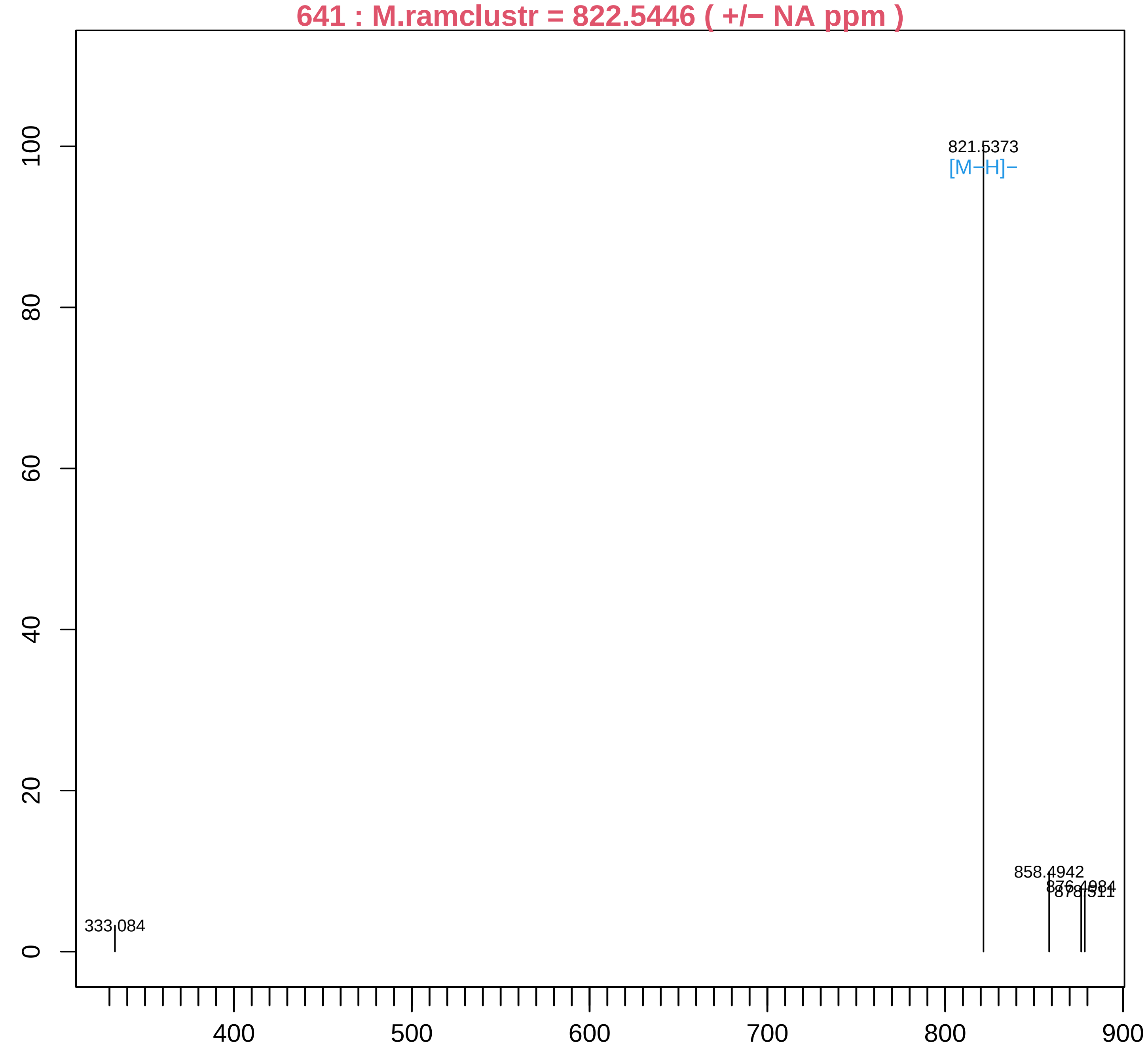
641 : M.ramclustr = 822.5446 (+/− NA ppm)
821.5373
[M−H]−
858.4942
876.4984
878.511
333.084

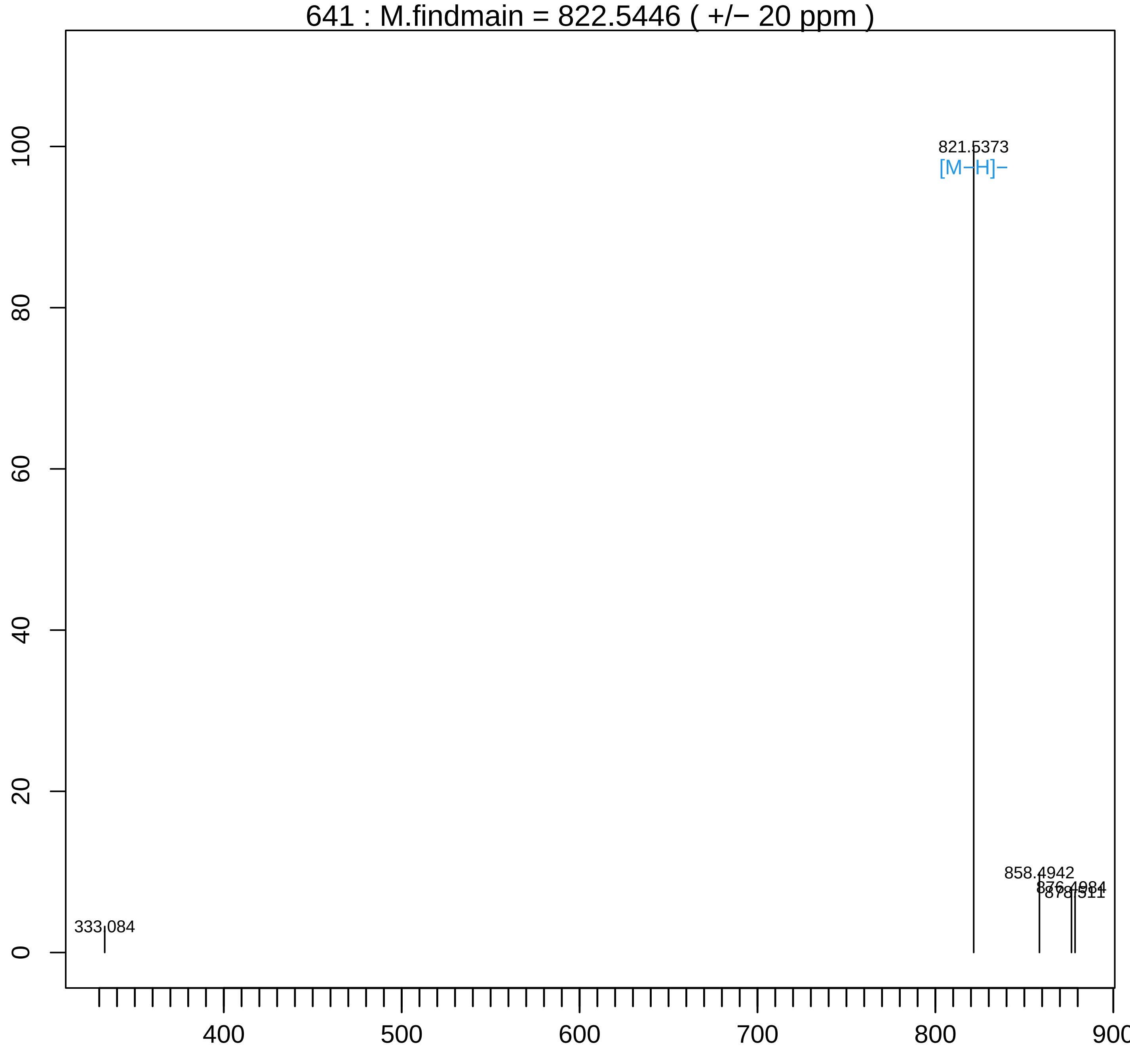
641 : M.findmain = 822.5446 (+/− 20 ppm)
821.5373
[M−H]−
858.4942
876.4984
878.511
333.084

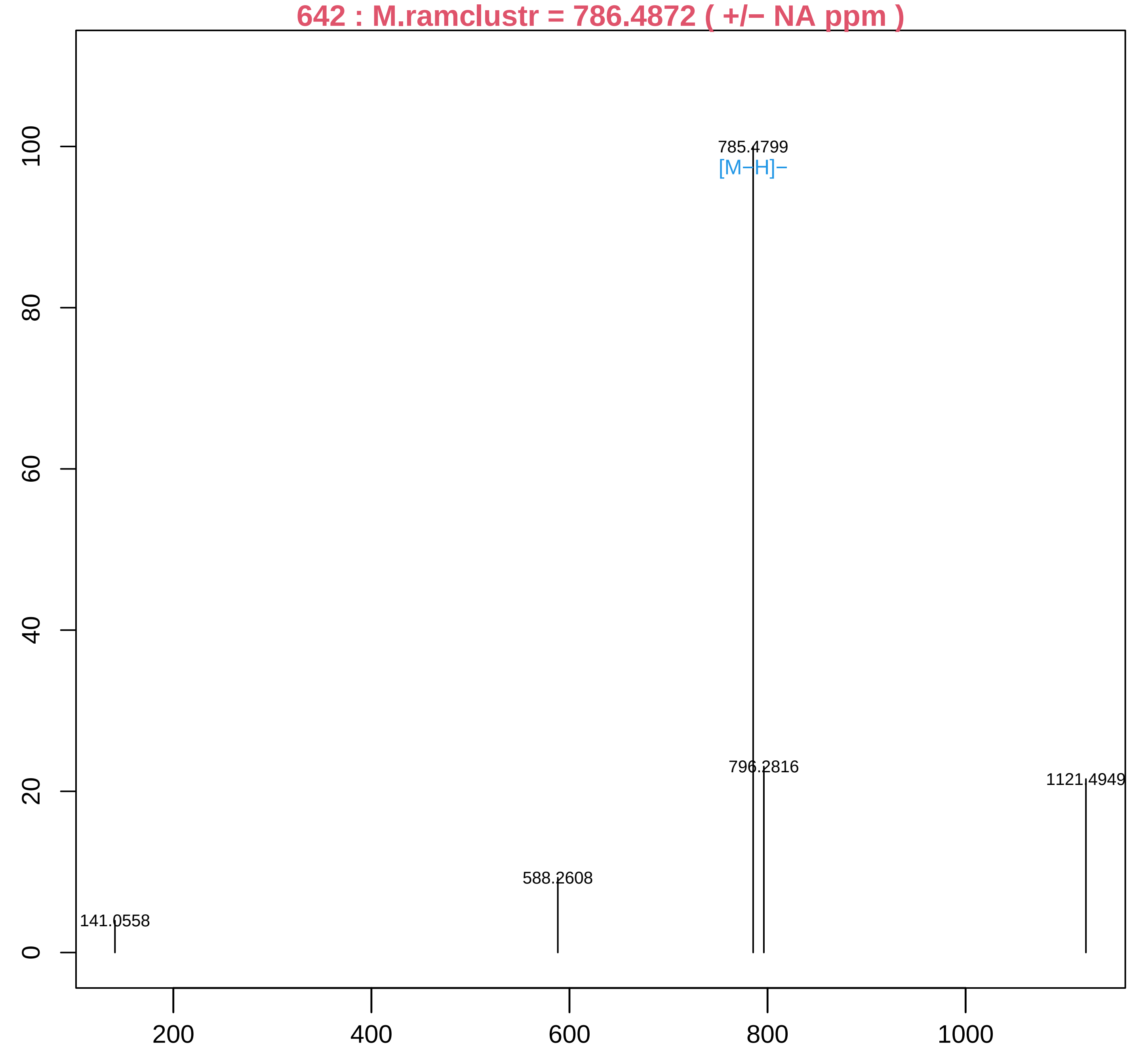

642 : M.ramclustr = 786.4872 (+/− NA ppm)
785.4799
[M−H]−
796.2816
1121.4949
588.2608
141.0558
0
20
40
60
80
100
200
400
600
800
1000

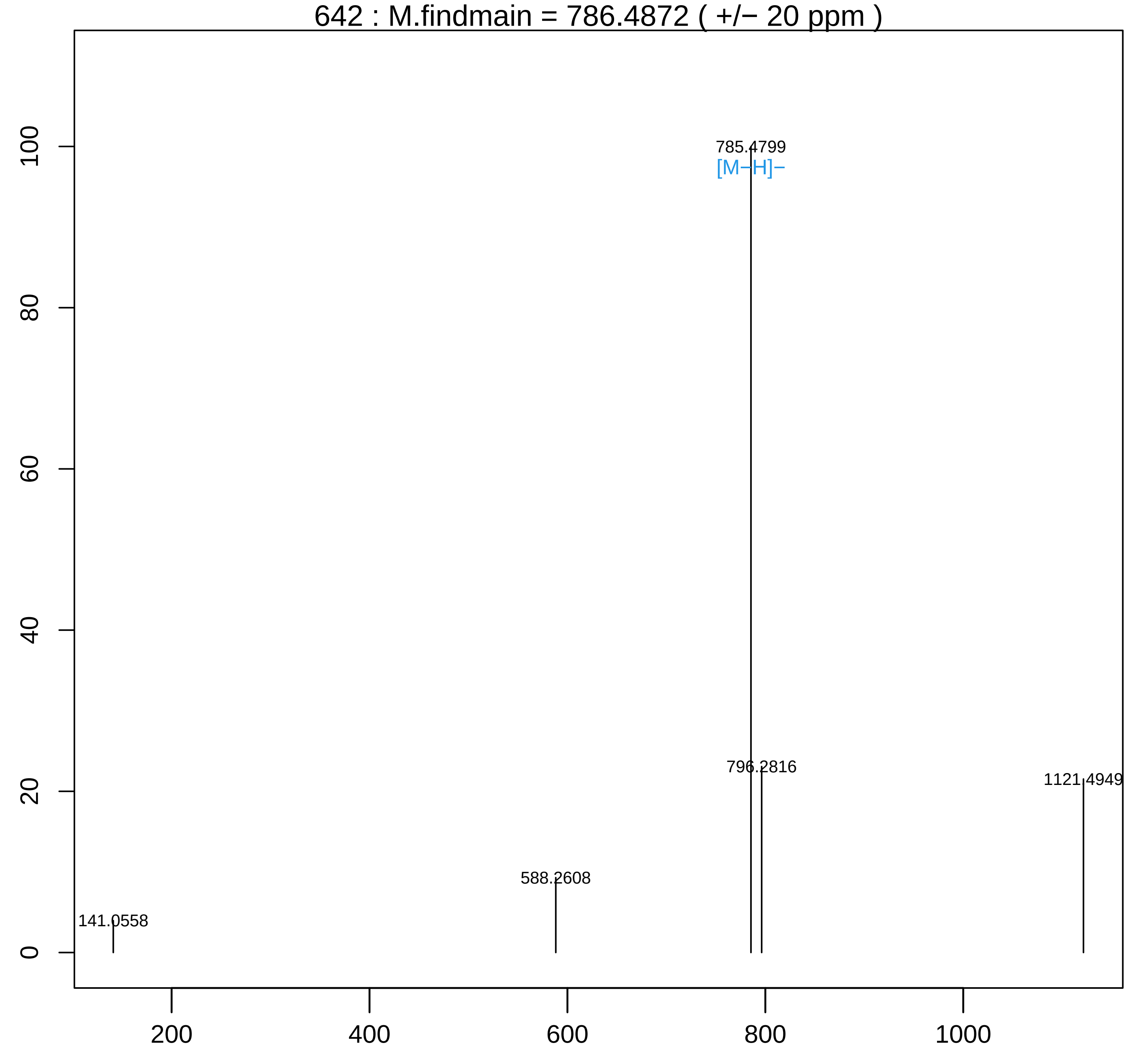

642 : M.findmain = 786.4872 (+/− 20 ppm)
785.4799
[M−H]−
796.2816
1121.4949
588.2608
141.0558
0
20
40
60
80
100
200
400
600
800
1000

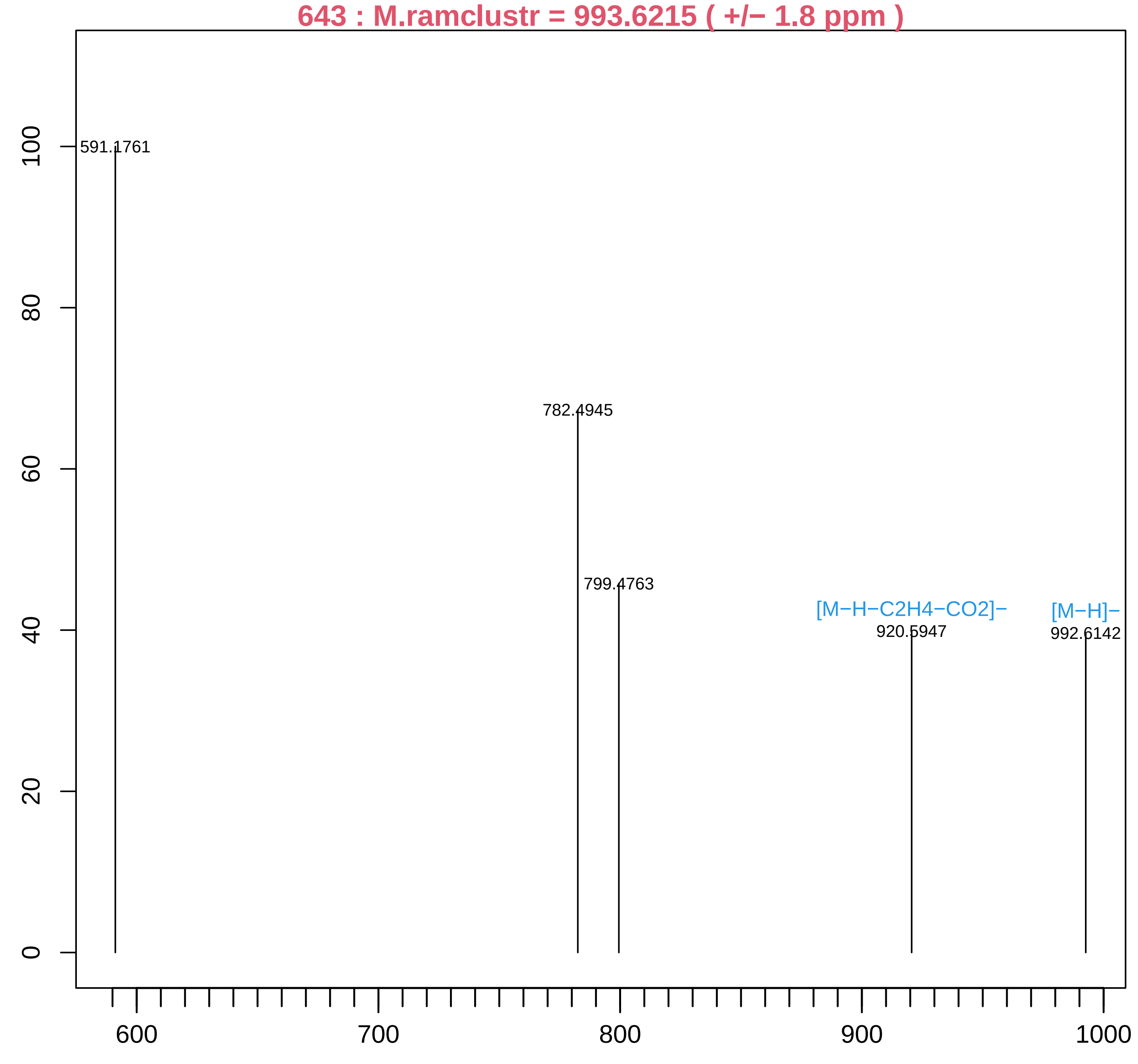

643 : M.ramclustr = 993.6215 (+/− 1.8 ppm)
591.1761
782.4945
799.4763
[M−H−C2H4−CO2]−
920.5947
[M−H]−
992.6142
0
20
40
60
80
100
600
700
800
900
1000

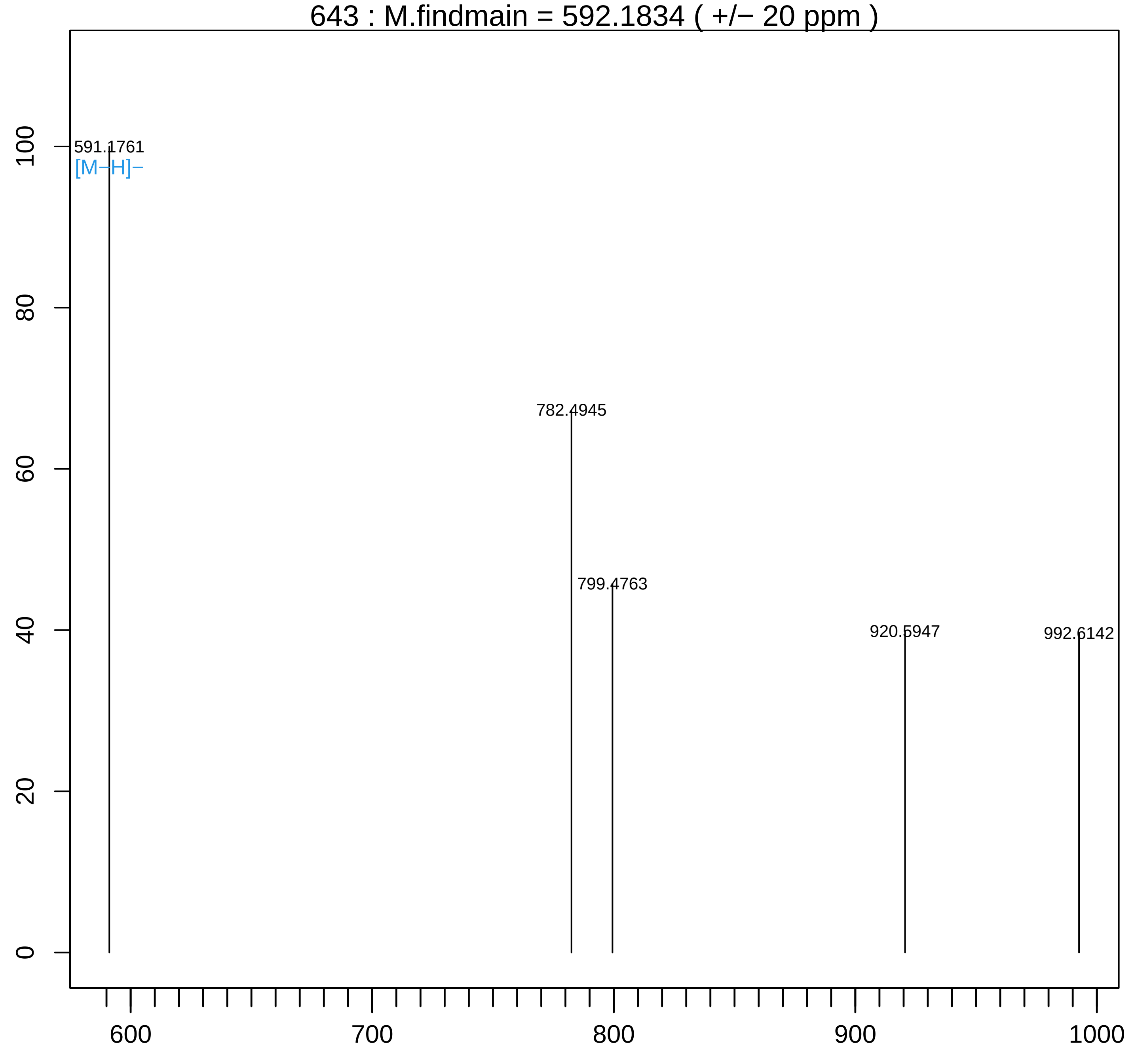

643 : M.findmain = 592.1834 (+/− 20 ppm)
591.1761
[M−H]−
782.4945
799.4763
920.5947
992.6142
0
20
40
60
80
100
600
700
800
900
1000

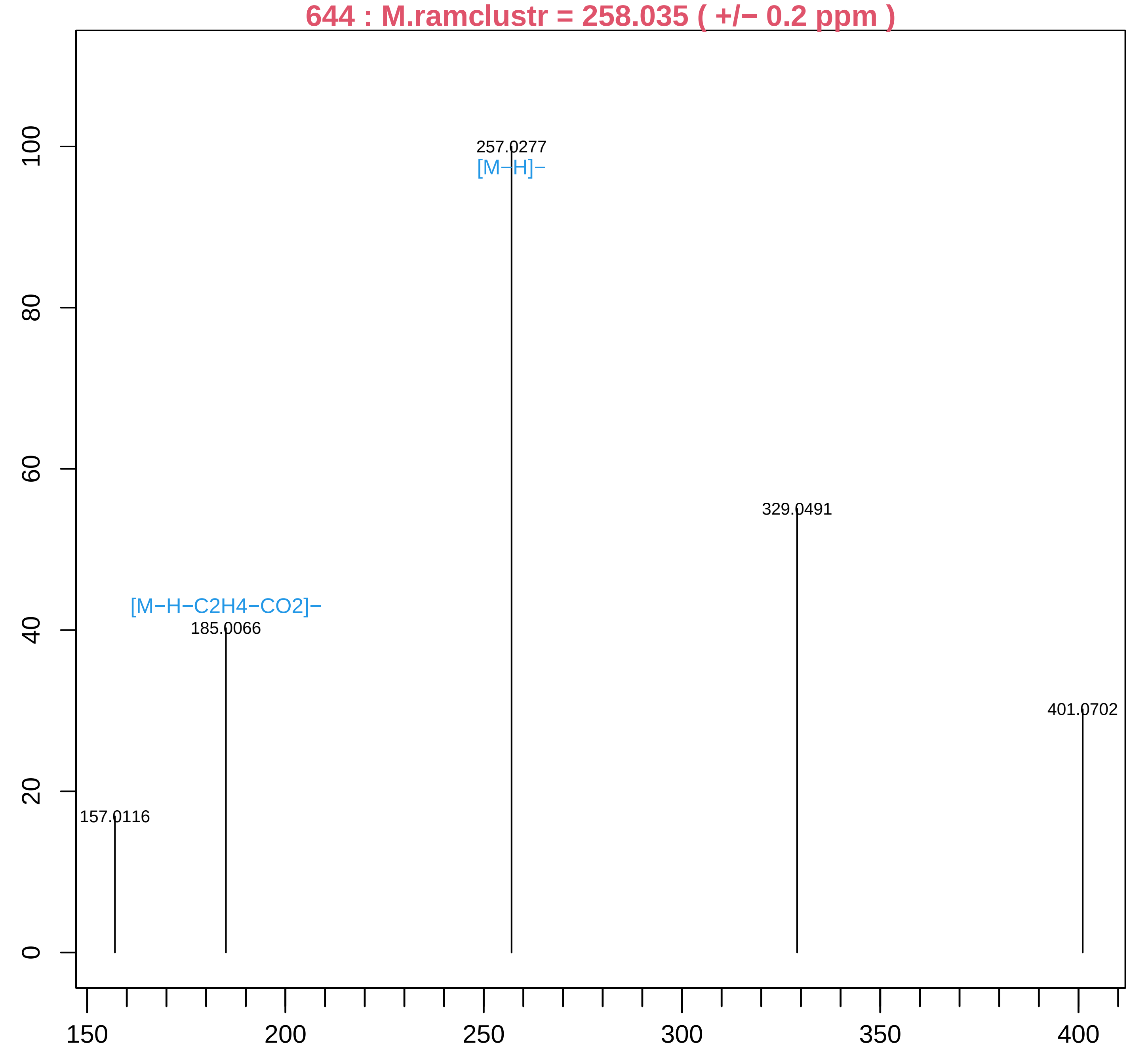
644 : M.ramclustr = 258.035 (+/− 0.2 ppm)
257.0277
[M−H]−
329.0491
[M−H−C2H4−CO2]−
185.0066
401.0702
157.0116
0
20
40
60
80
100
150
200
250
300
350
400

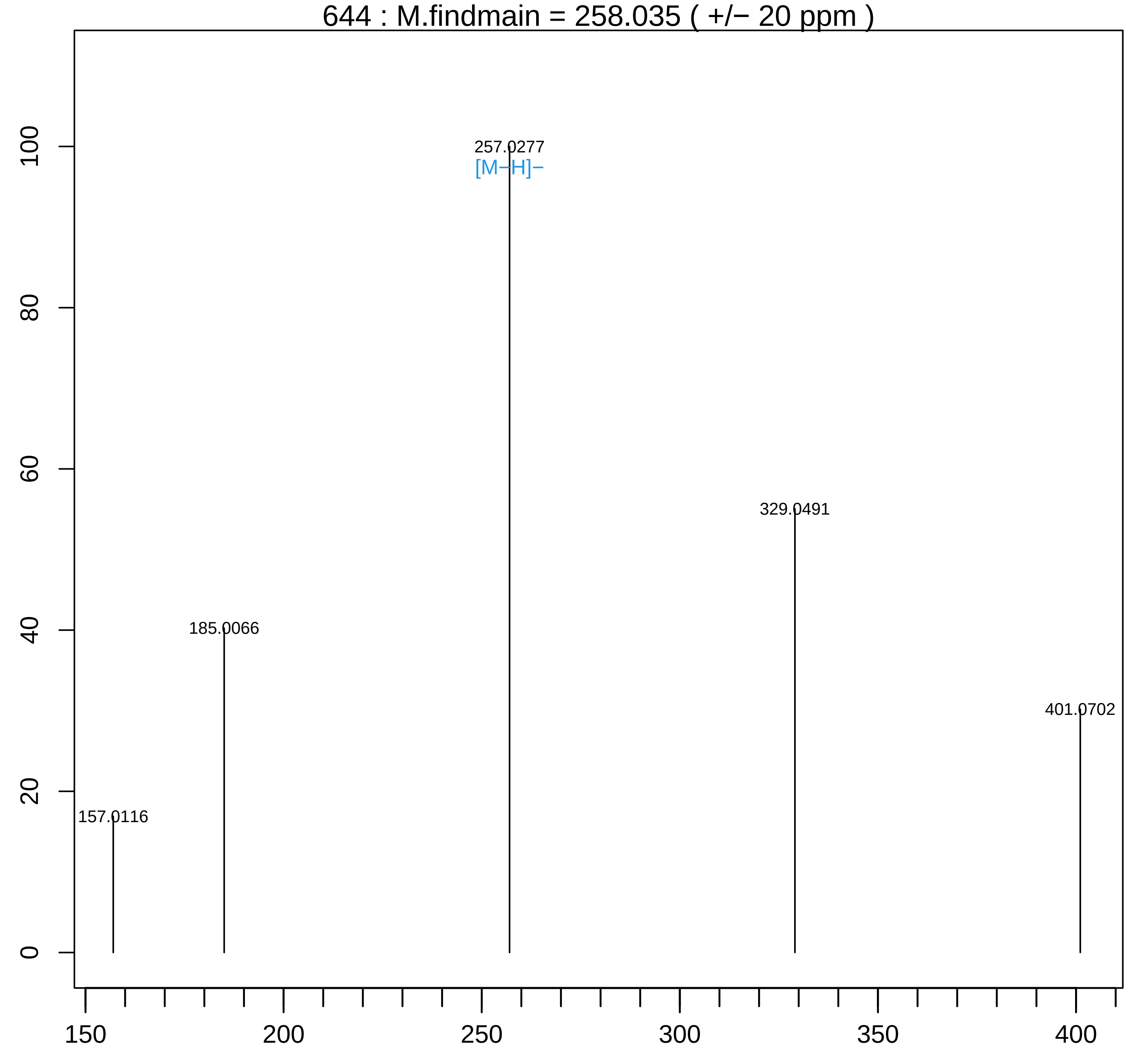
644 : M.findmain = 258.035 (+/− 20 ppm)
257.0277
[M−H]−
329.0491
185.0066
401.0702
157.0116
0
20
40
60
80
100
150
200
250
300
350
400

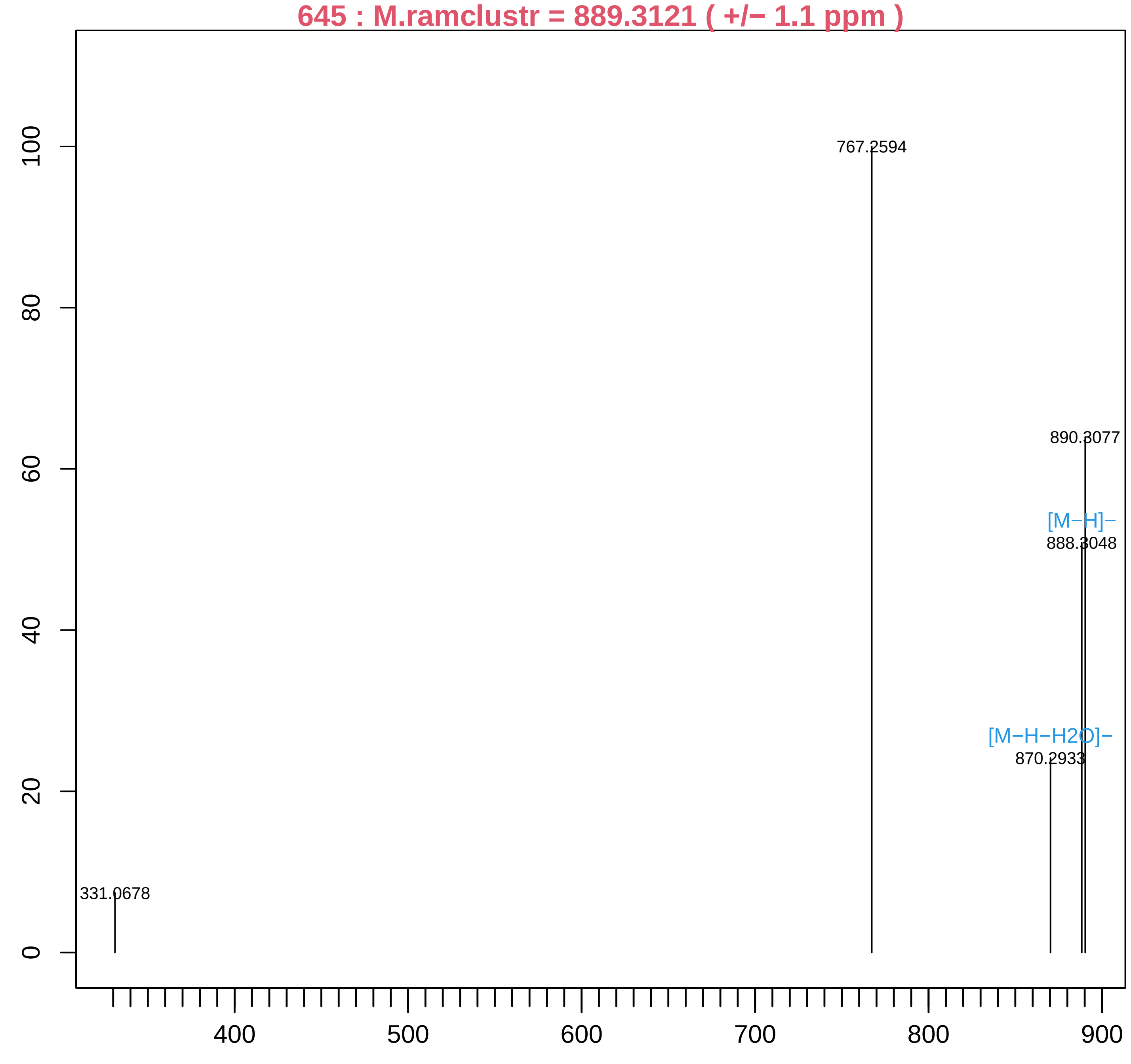

645 : M.ramclustr = 889.3121 (+/− 1.1 ppm)
767.2594
890.3077
[M−H]−
888.3048
[M−H−H2O]−
870.2933
331.0678
0
20
40
60
80
100
400
500
600
700
800
900

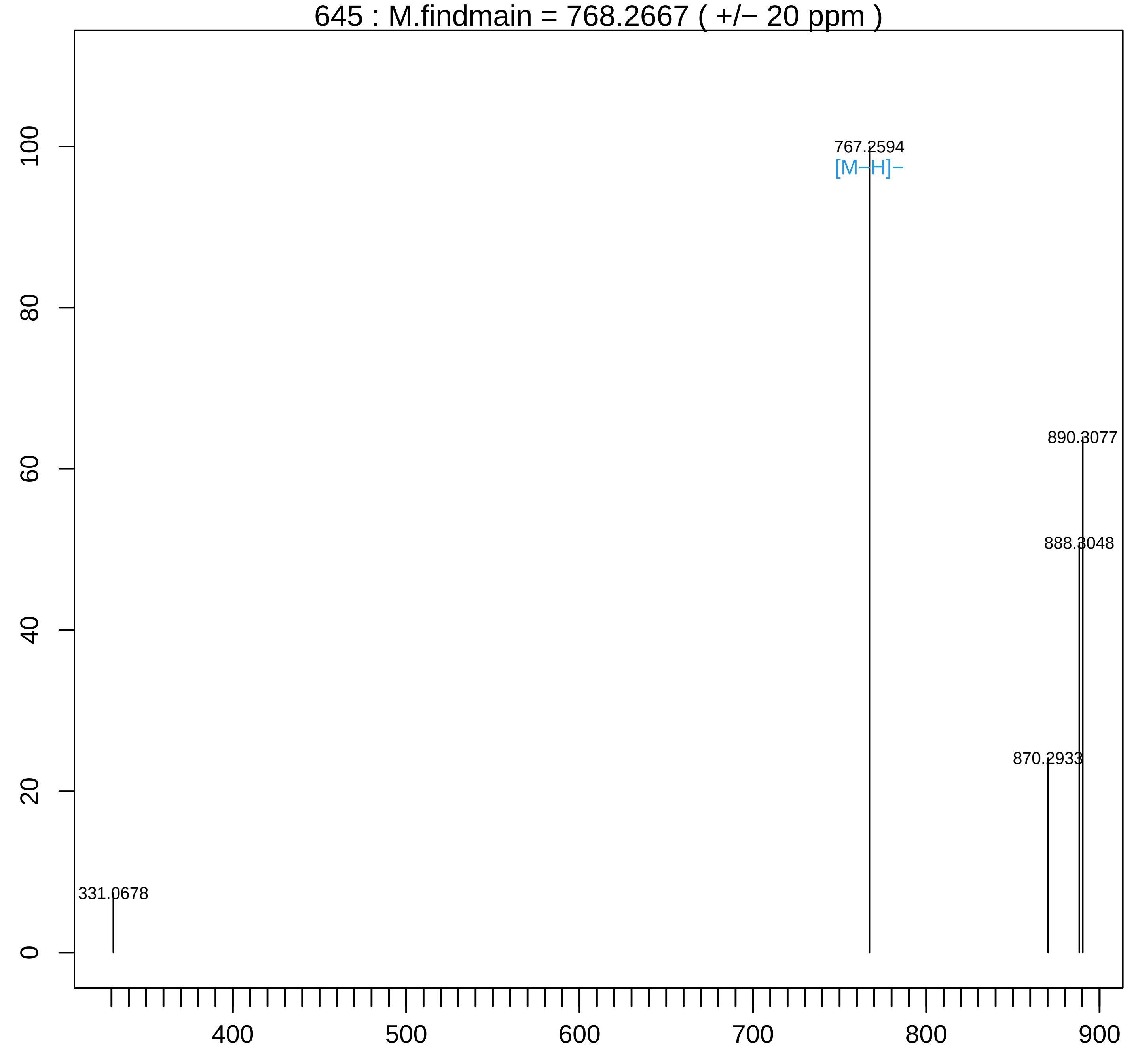

645 : M.findmain = 768.2667 (+/− 20 ppm)
767.2594
[M−H]−
890.3077
888.3048
870.2933
331.0678
0
20
40
60
80
100
400
500
600
700
800
900

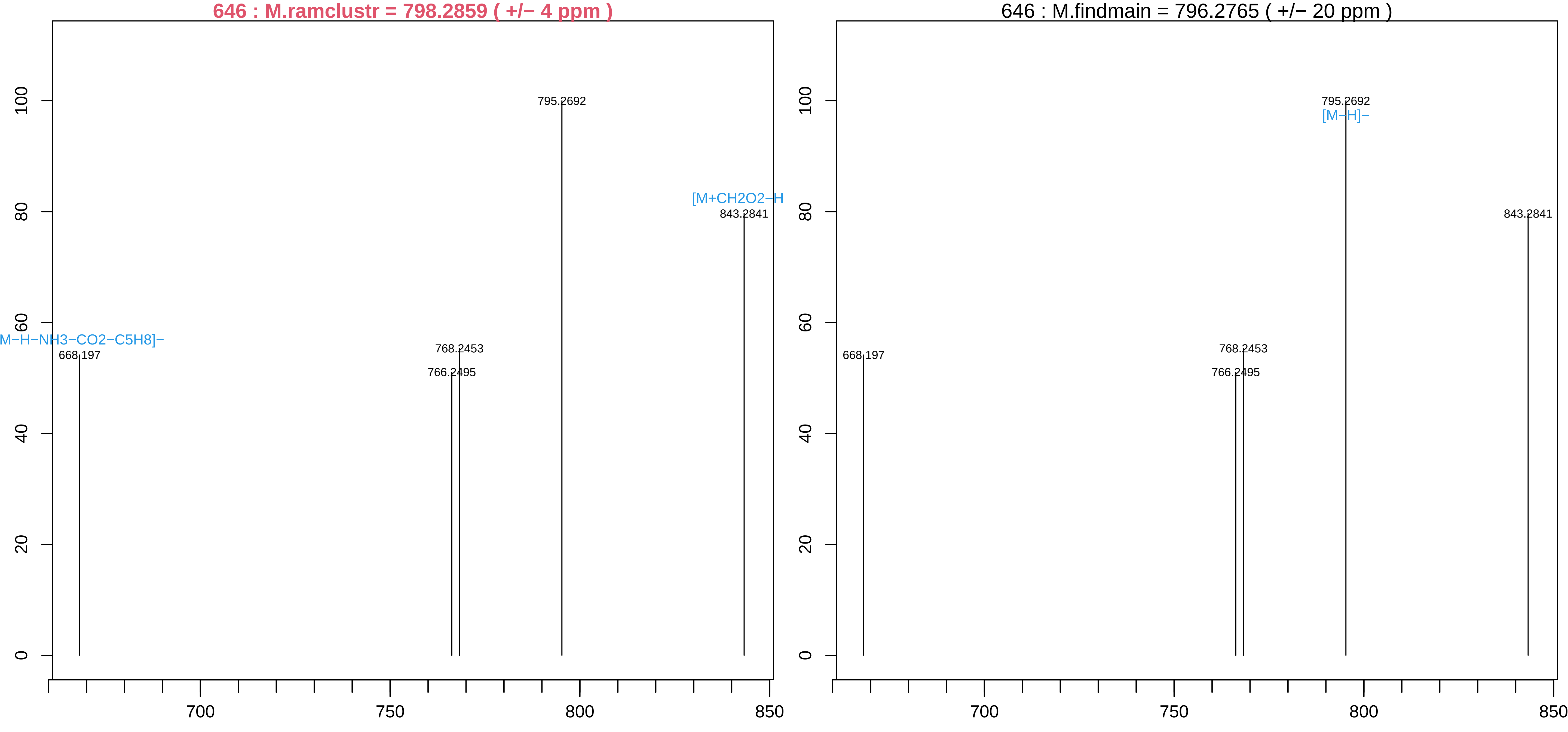
646 : M.ramclustr = 798.2859 (+/− 4 ppm)
795.2692
[M+CH2O2−H
843.2841
M−H−NH3−CO2−C5H8]−
668.197
768.2453
766.2495
646 : M.findmain = 796.2765 (+/− 20 ppm)
795.2692
[M−H]−
843.2841
668.197
768.2453
766.2495
0
20
40
60
80
100
700
750
800
850

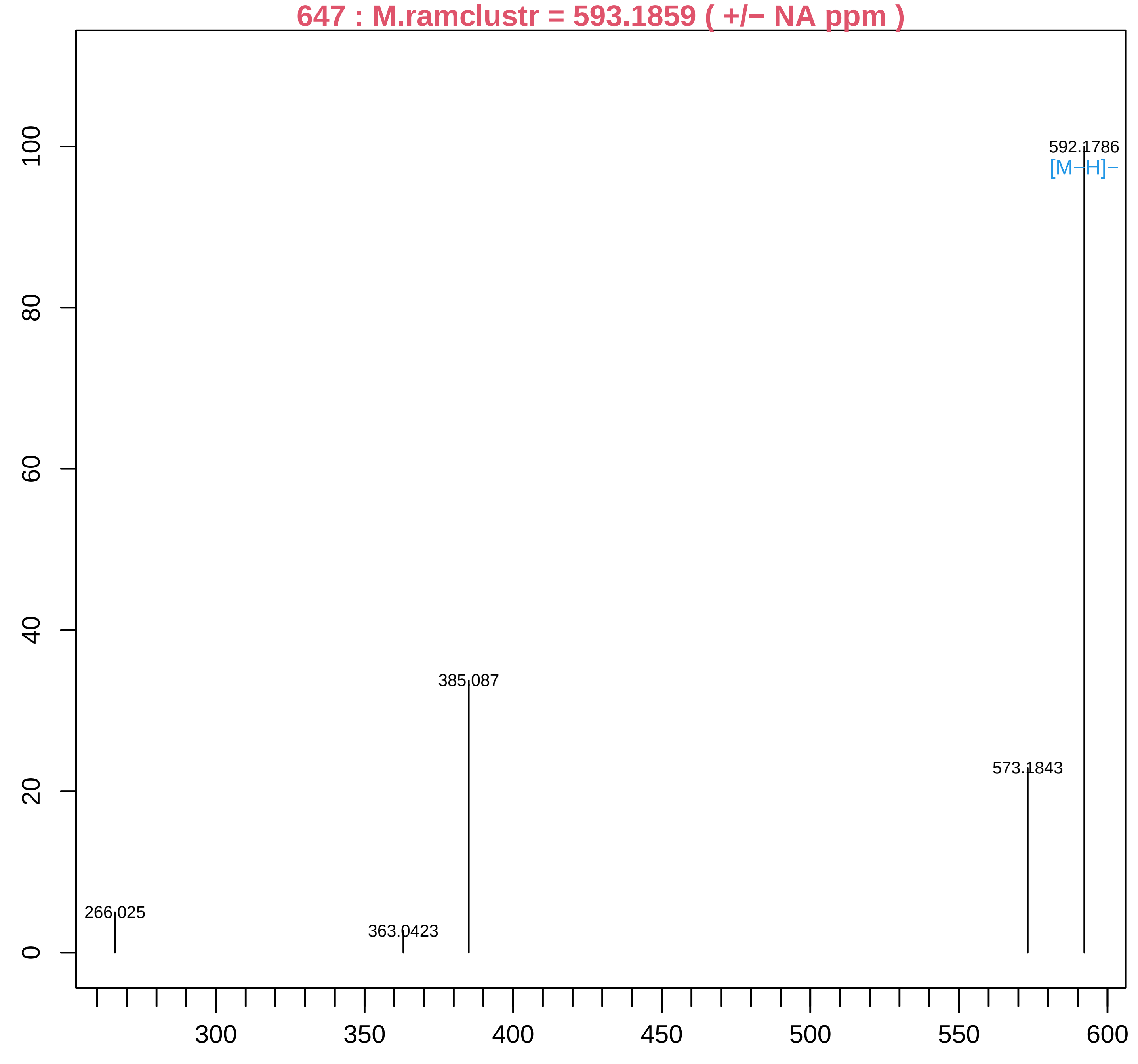
647 : M.ramclustr = 593.1859 (+/− NA ppm)
592.1786
[M−H]−
385.087
573.1843
266.025
363.0423
0
20
40
60
80
100
300
350
400
450
500
550
600

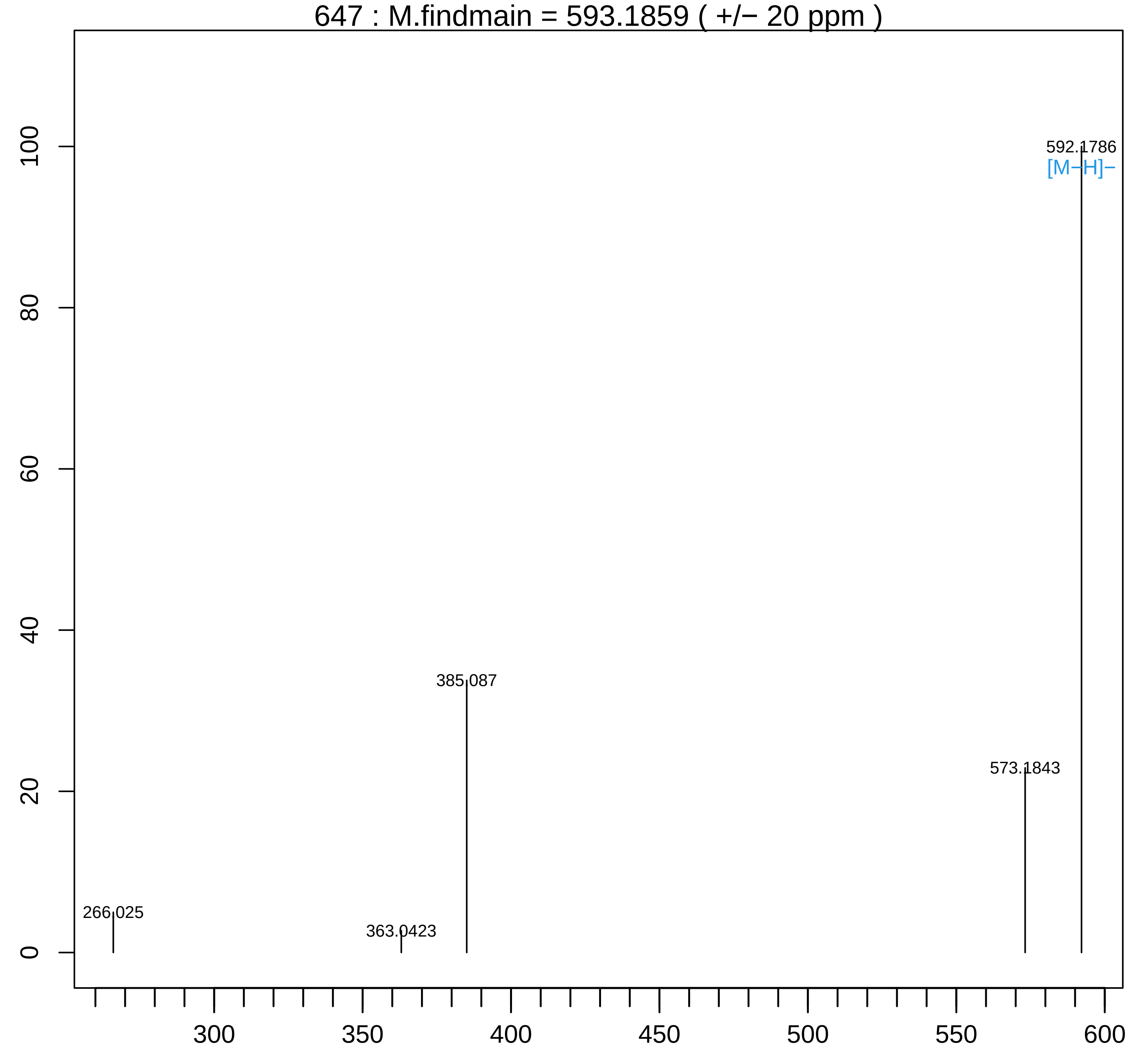
647 : M.findmain = 593.1859 (+/− 20 ppm)
592.1786
[M−H]−
385.087
573.1843
266.025
363.0423
0
20
40
60
80
100
300
350
400
450
500
550
600

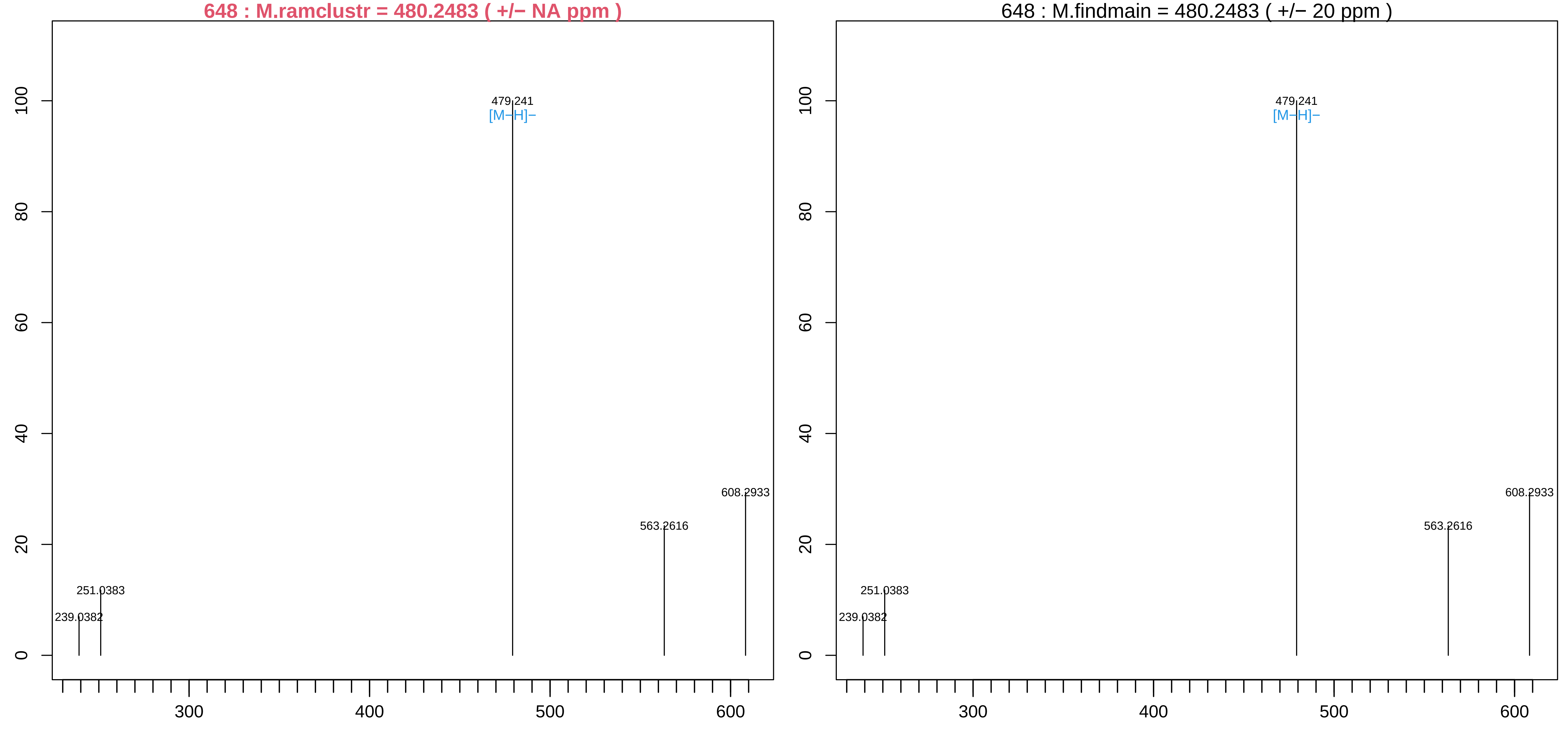

648 : M.ramclustr = 480.2483 (+/− NA ppm)
648 : M.findmain = 480.2483 (+/− 20 ppm)
479.241
[M−H]−
608.2933
563.2616
251.0383
239.0382
0
20
40
60
80
100
300
400
500
600

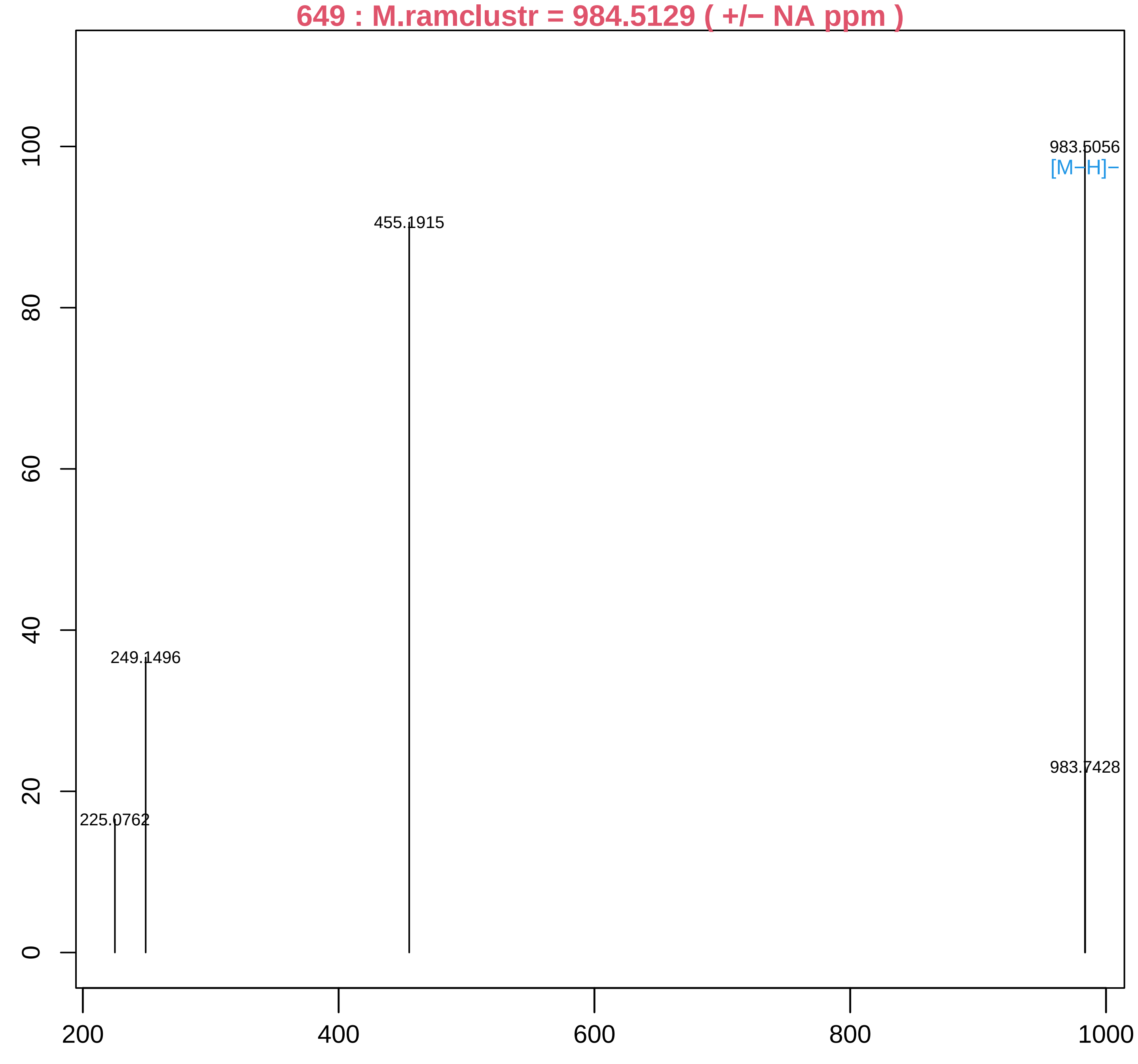

649 : M.ramclustr = 984.5129 (+/− NA ppm)
983.5056
[M−H]−
455.1915
249.1496
983.7428
225.0762
0
20
40
60
80
100
200
400
600
800
1000

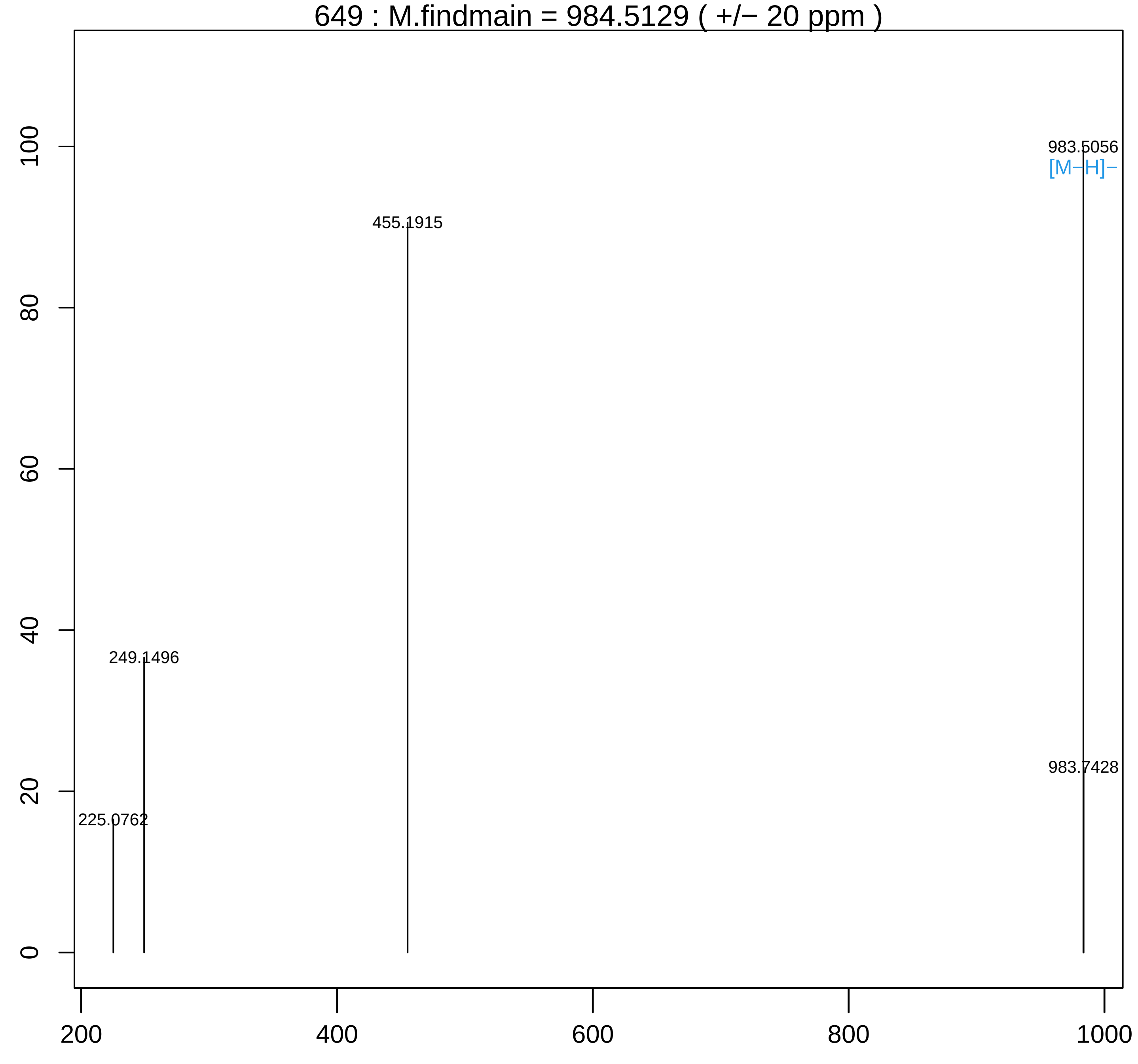

649 : M.findmain = 984.5129 (+/− 20 ppm)
983.5056
[M−H]−
455.1915
249.1496
983.7428
225.0762
0
20
40
60
80
100
200
400
600
800
1000

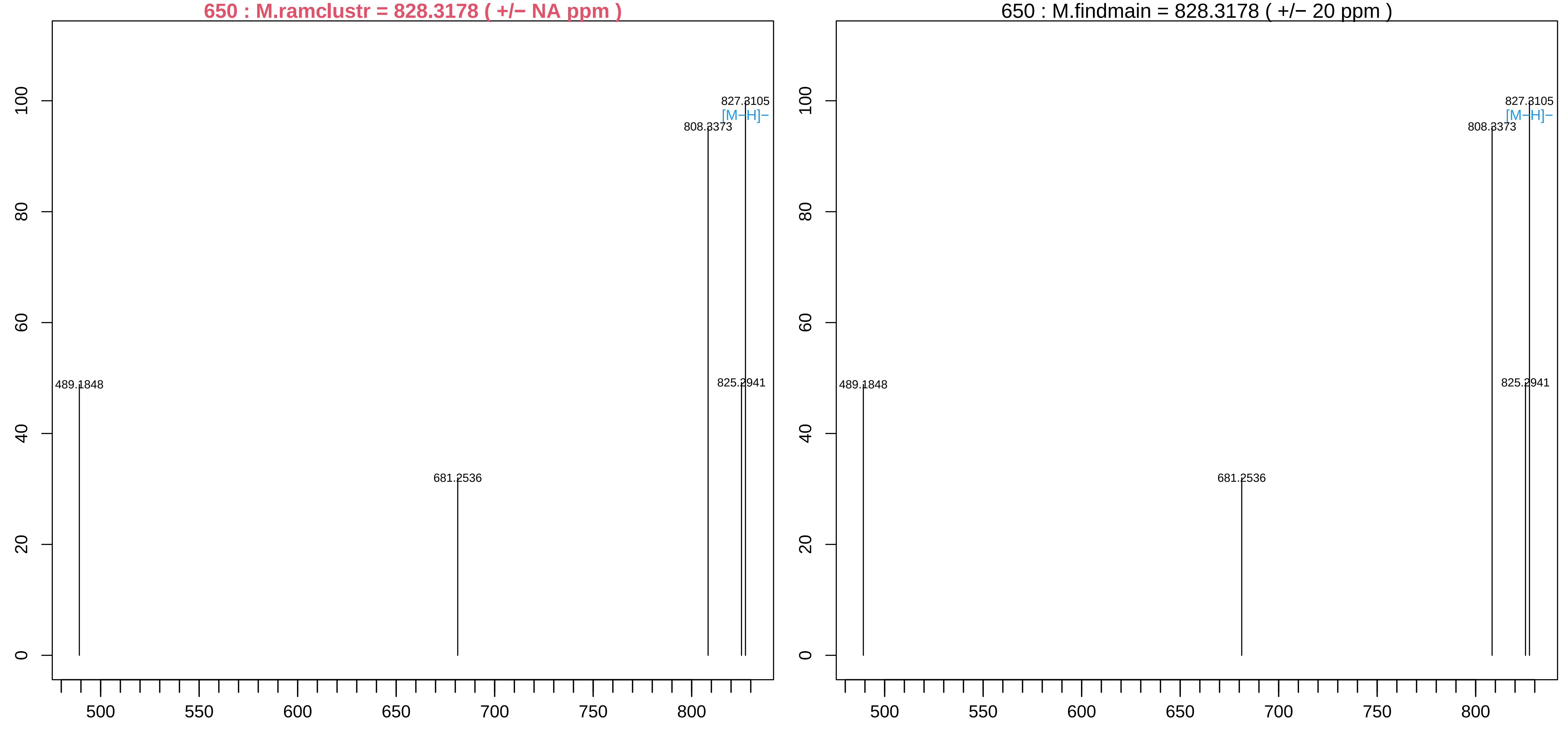
650 : M.ramclustr = 828.3178 (+/− NA ppm)
650 : M.findmain = 828.3178 (+/− 20 ppm)
827.3105
[M−H]−
808.3373
825.2941
489.1848
681.2536
0
20
40
60
80
100
500
550
600
650
700
750
800

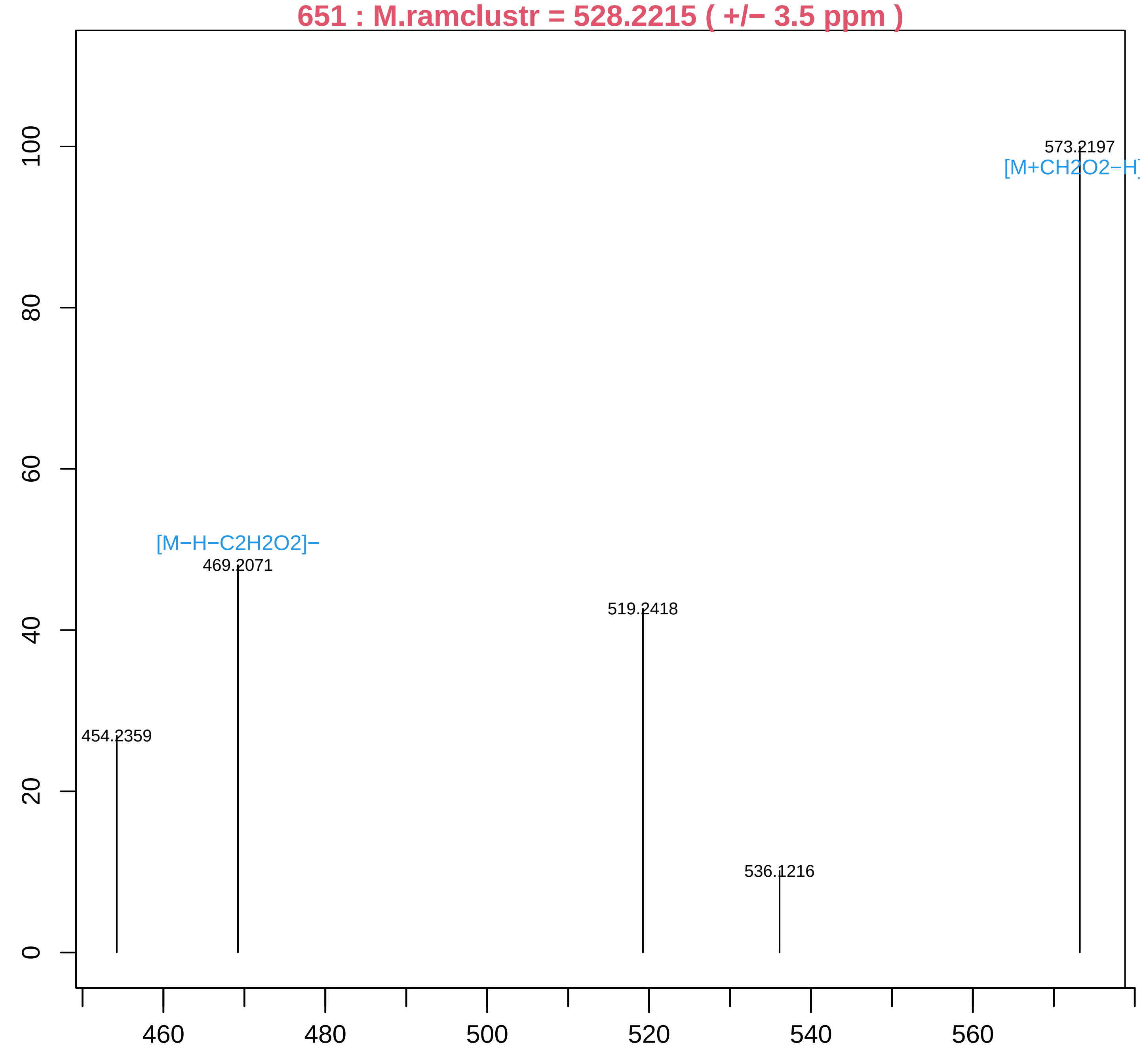
651 : M.ramclustr = 528.2215 (+/− 3.5 ppm)
573.2197
[M+CH2O2−H]
[M−H−C2H2O2]−
469.2071
519.2418
454.2359
536.1216
0
20
40
60
80
100
460
480
500
520
540
560

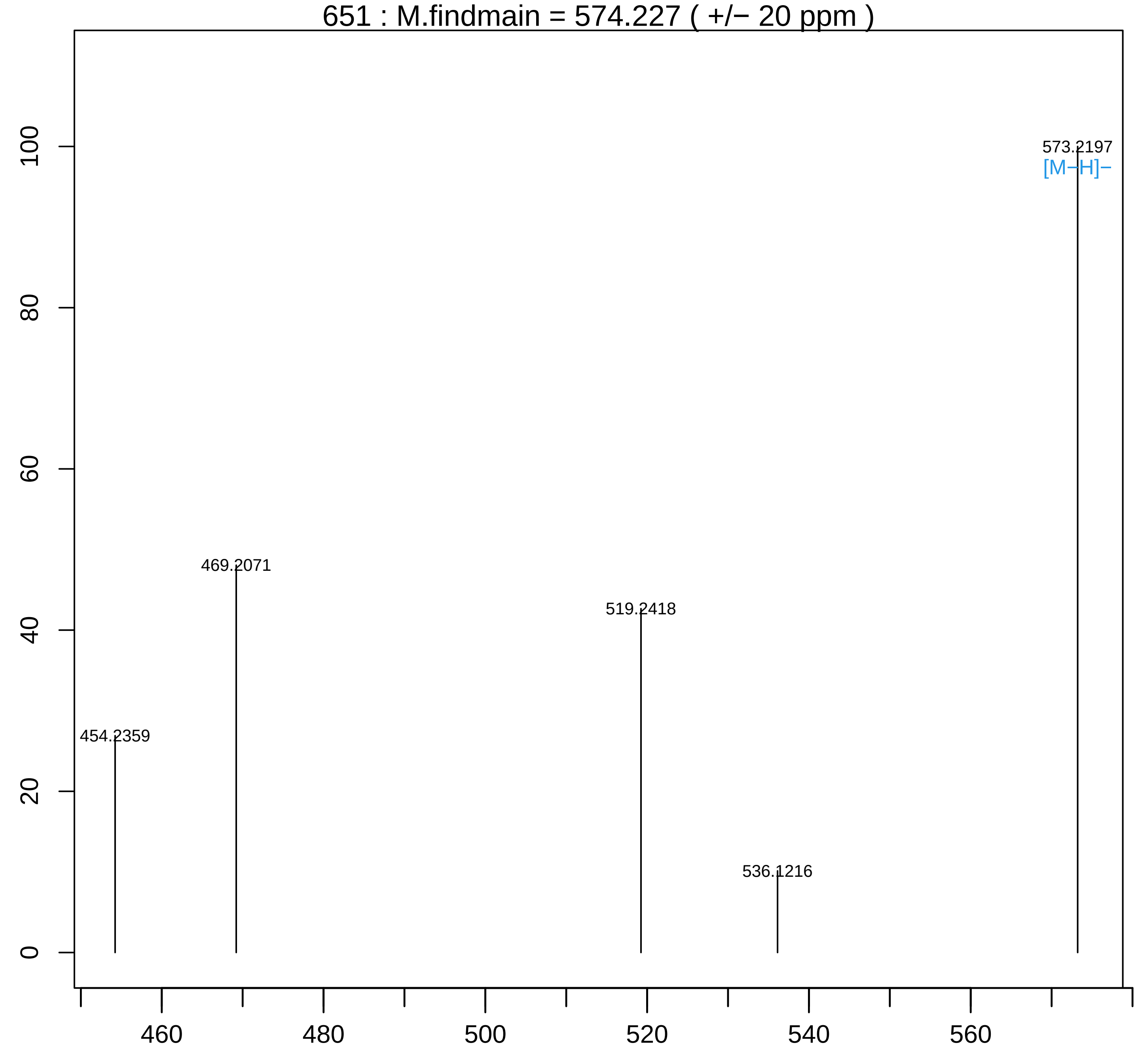
651 : M.findmain = 574.227 (+/− 20 ppm)
573.2197
[M−H]−
469.2071
519.2418
454.2359
536.1216
0
20
40
60
80
100
460
480
500
520
540
560

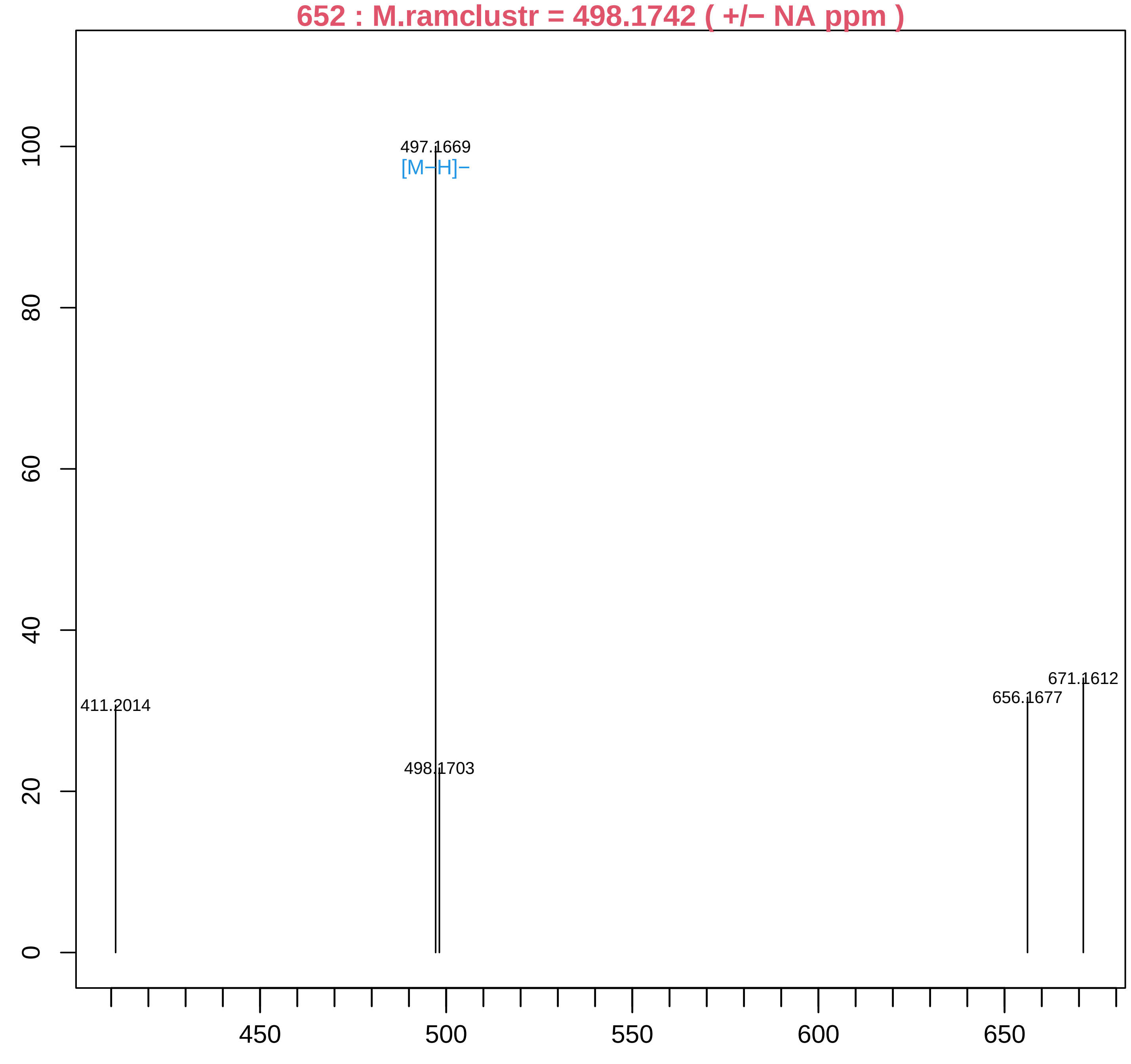
652 : M.ramclustr = 498.1742 (+/− NA ppm)
497.1669
[M−H]−
411.2014
498.1703
656.1677
671.1612
0
20
40
60
80
100
450
500
550
600
650

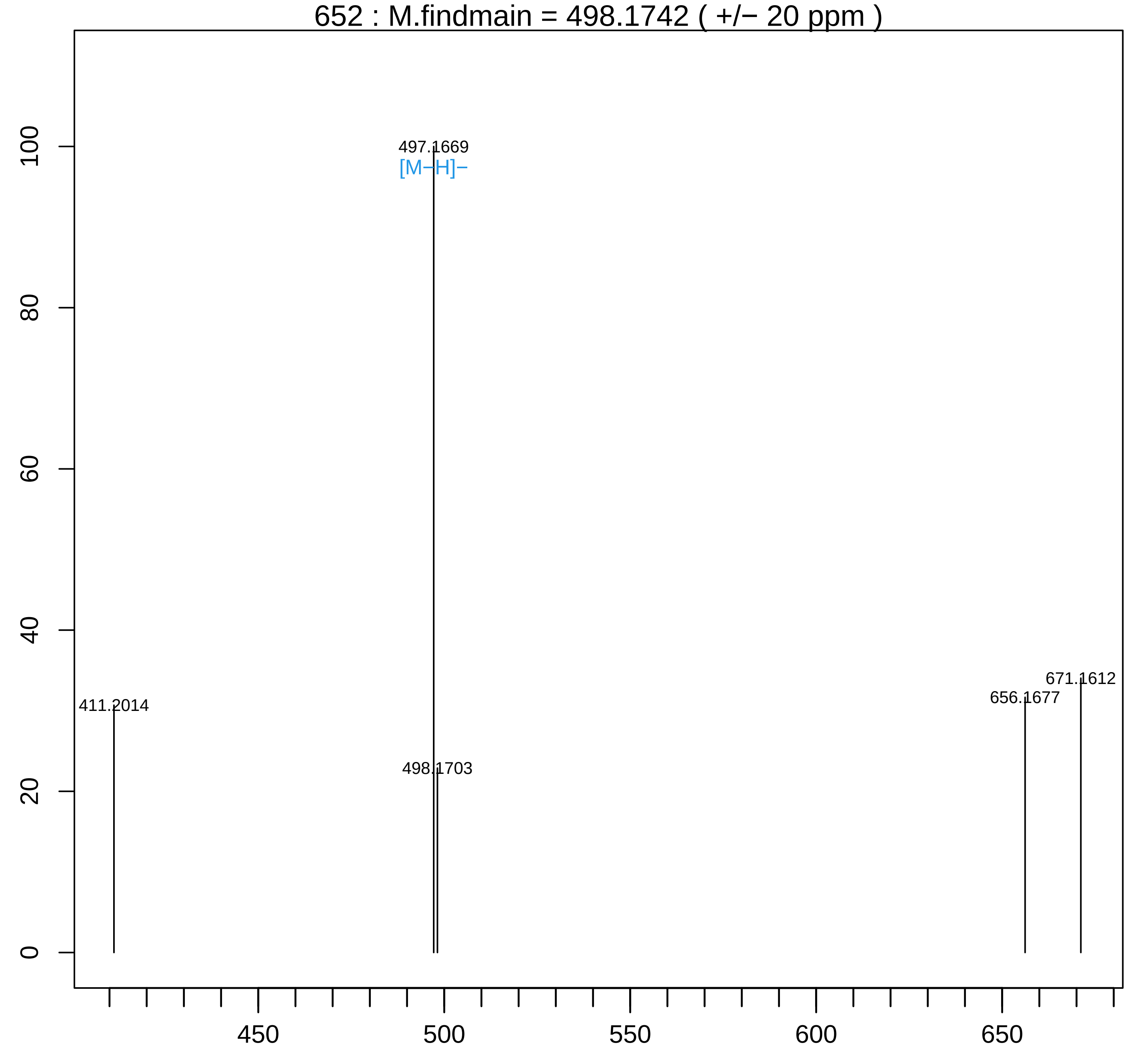
652 : M.findmain = 498.1742 (+/− 20 ppm)
497.1669
[M−H]−
411.2014
498.1703
656.1677
671.1612
0
20
40
60
80
100
450
500
550
600
650

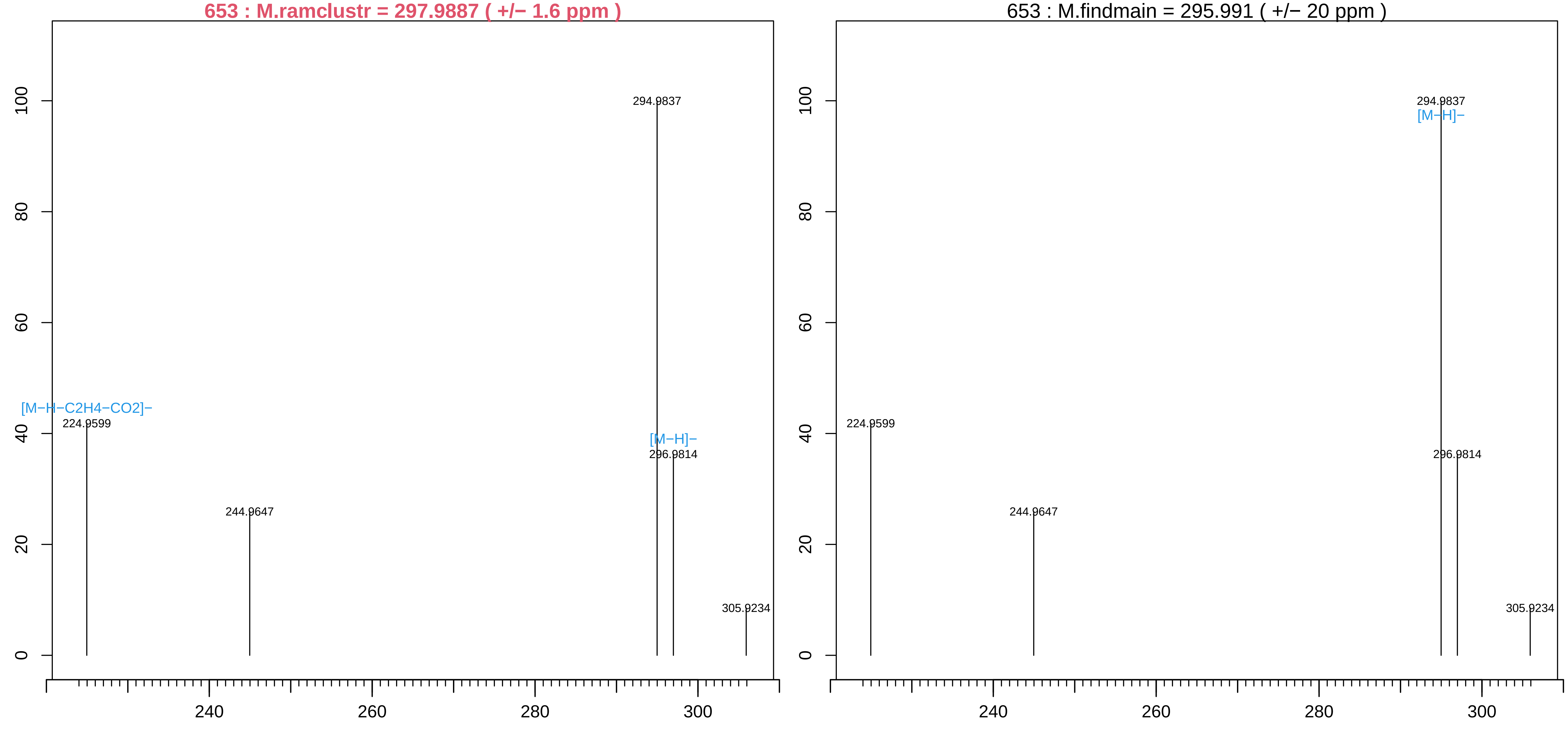
653 : M.ramclustr = 297.9887 (+/− 1.6 ppm)
653 : M.findmain = 295.991 (+/− 20 ppm)
[M−H−C2H4−CO2]−
224.9599
244.9647
294.9837
[M−H]−
296.9814
305.9234
0
20
40
60
80
100
240
260
280
300

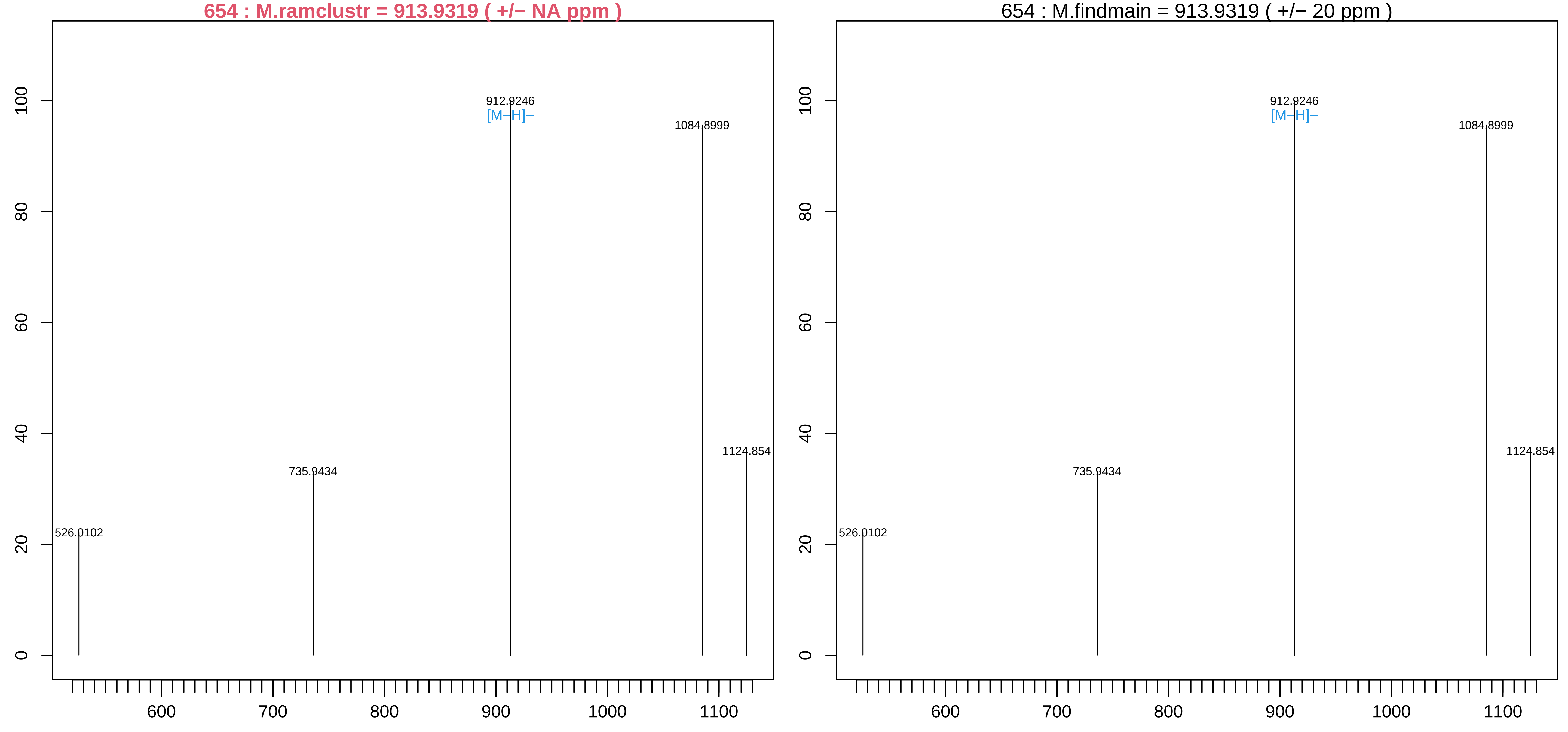

654 : M.ramclustr = 913.9319 (+/− NA ppm)
912.9246
[M−H]−
1084.8999
1124.854
735.9434
526.0102
0
20
40
60
80
100
600
700
800
900
1000
1100
654 : M.findmain = 913.9319 (+/− 20 ppm)
912.9246
[M−H]−
1084.8999
1124.854
735.9434
526.0102
0
20
40
60
80
100
600
700
800
900
1000
1100

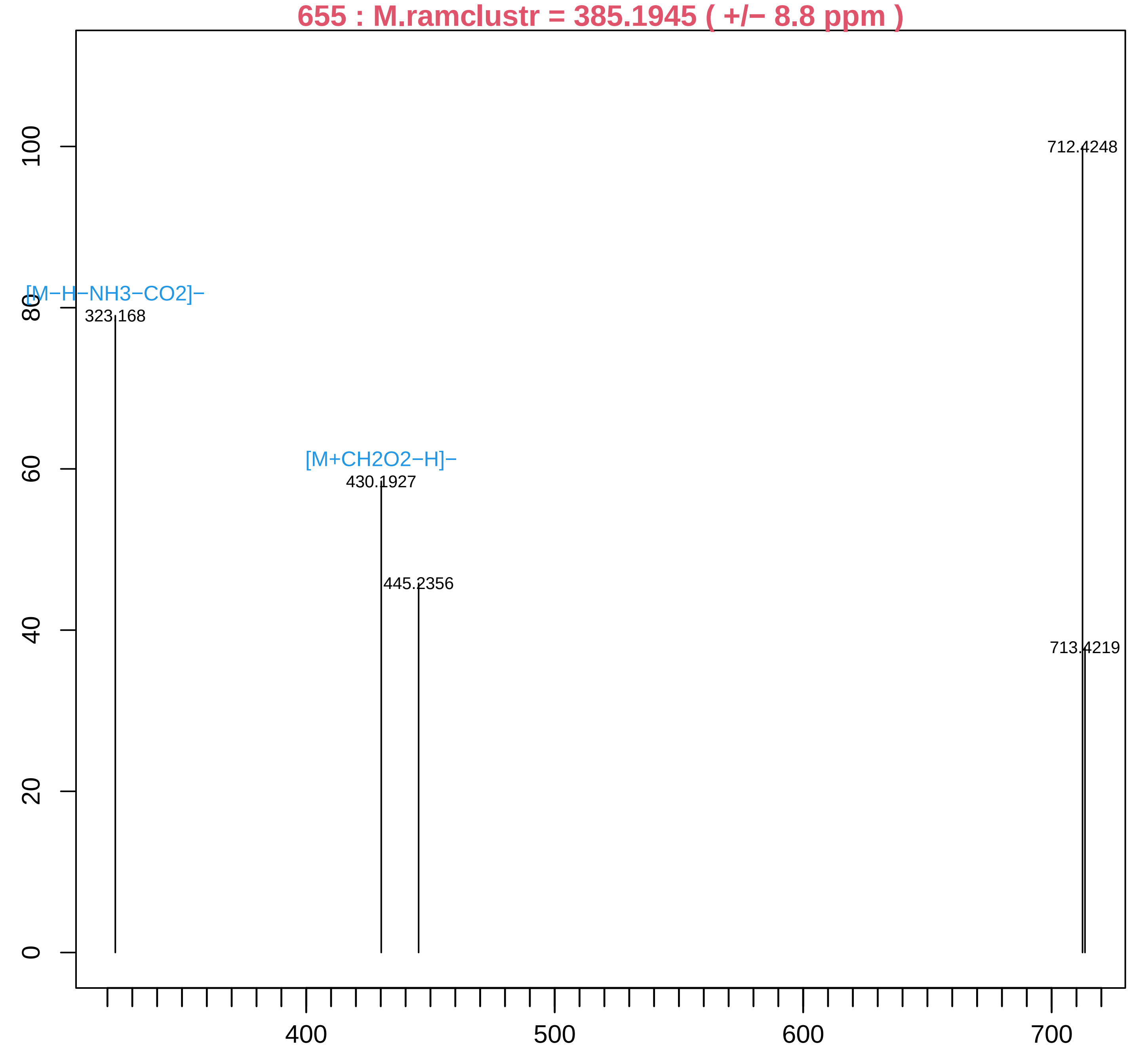
655 : M.ramclustr = 385.1945 (+/− 8.8 ppm)
[M−H−NH3−CO2]−
323.168
[M+CH2O2−H]−
430.1927
445.2356
712.4248
713.4219
0
20
40
60
80
100
400
500
600
700
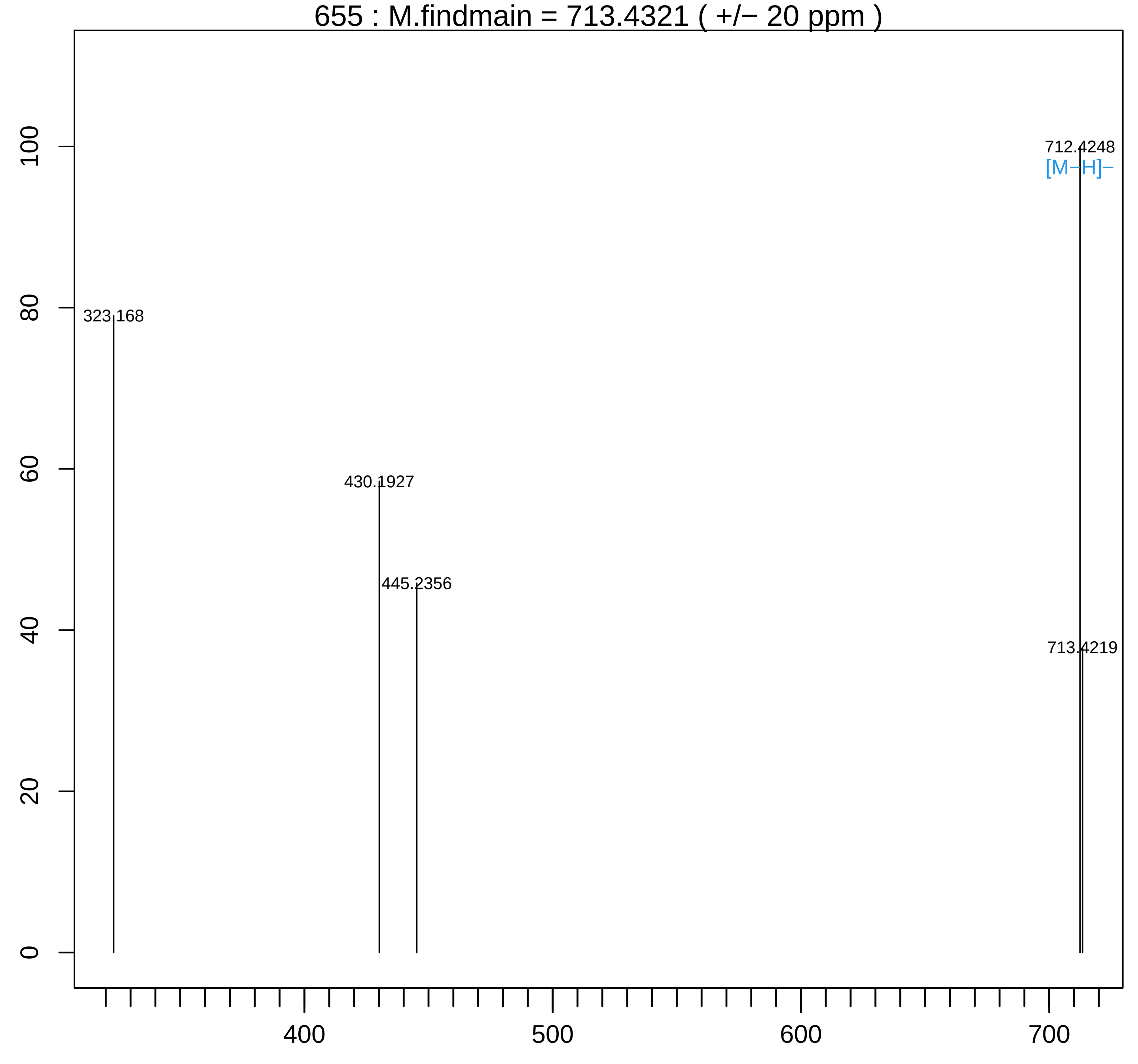
655 : M.findmain = 713.4321 (+/− 20 ppm)
323.168
430.1927
445.2356
712.4248
[M−H]−
713.4219
0
20
40
60
80
100
400
500
600
700

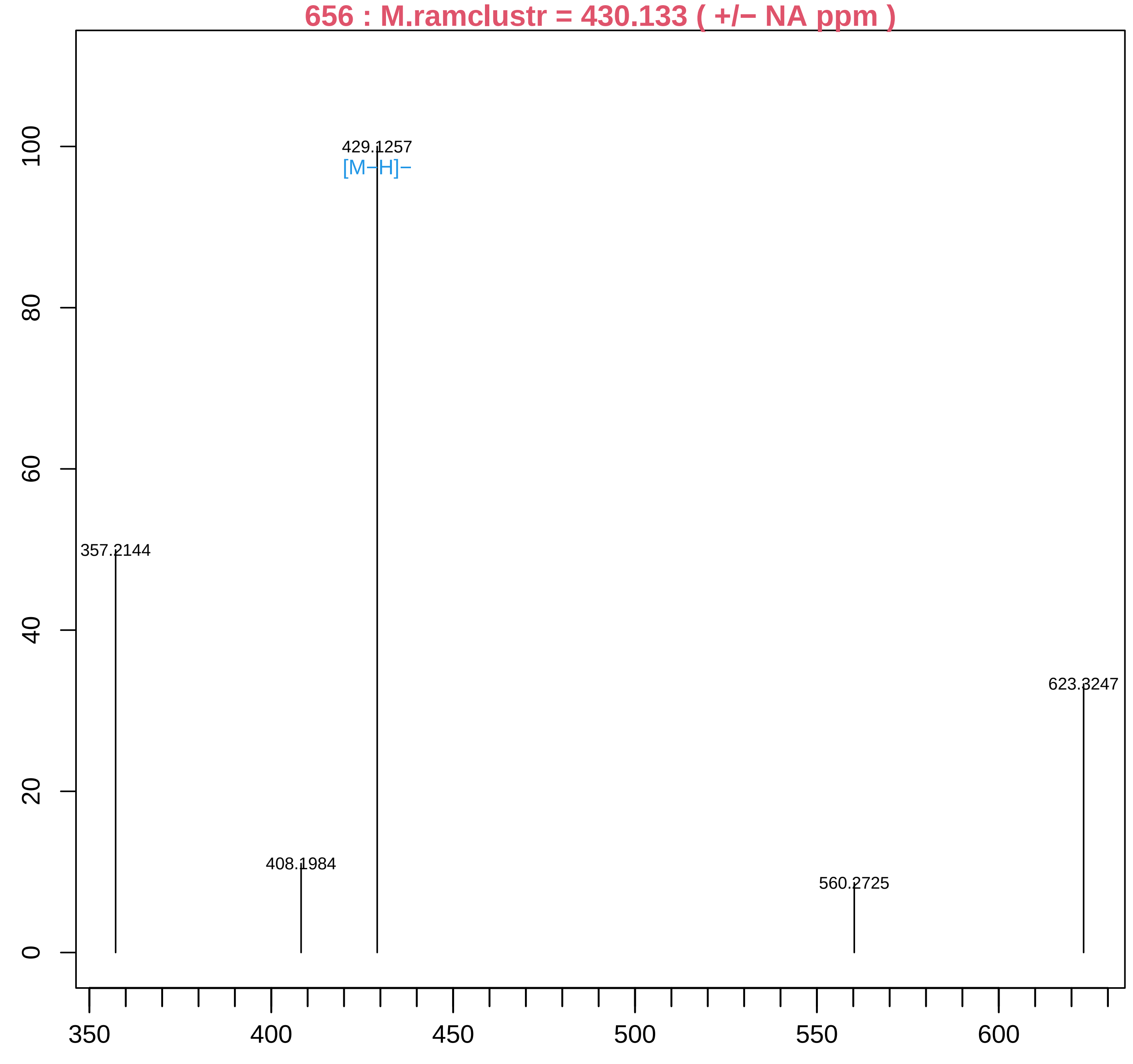
656 : M.ramclustr = 430.133 (+/− NA ppm)
429.1257
[M−H]−
357.2144
408.1984
560.2725
623.3247
0
20
40
60
80
100
350
400
450
500
550
600

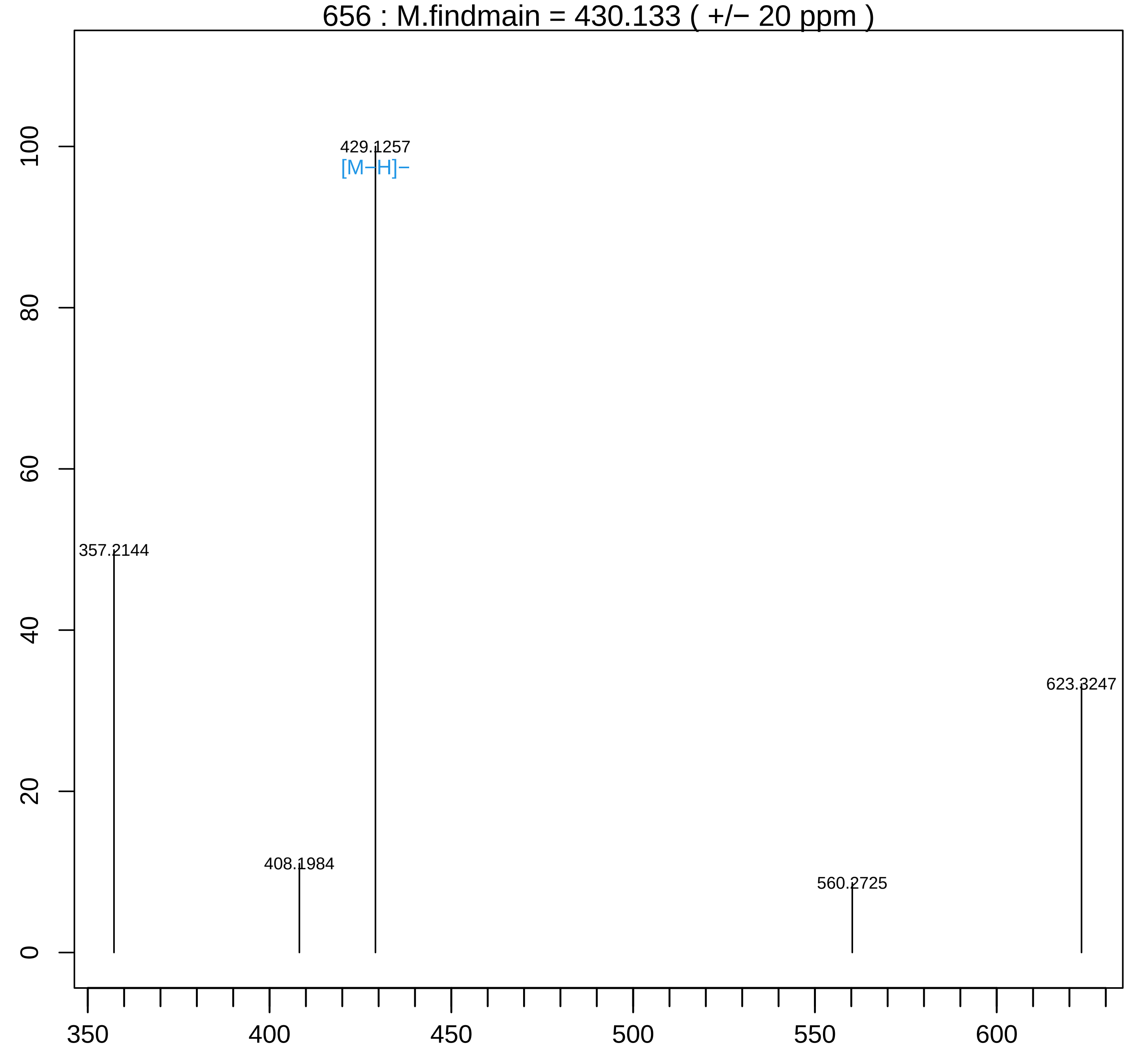
656 : M.findmain = 430.133 (+/− 20 ppm)
429.1257
[M−H]−
357.2144
408.1984
560.2725
623.3247
0
20
40
60
80
100
350
400
450
500
550
600

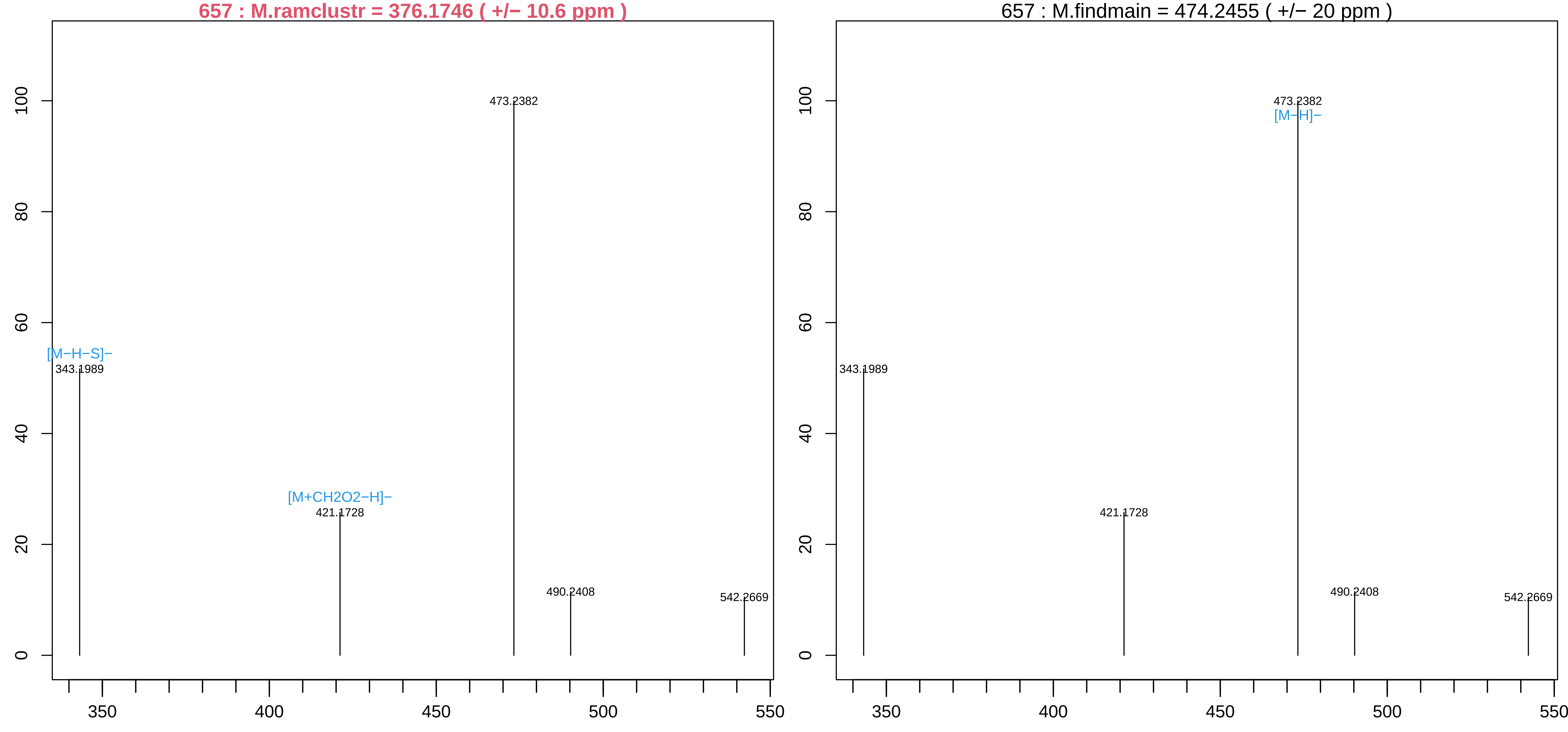
657 : M.ramclustr = 376.1746 (+/− 10.6 ppm)
[M−H−S]−
343.1989
[M+CH2O2−H]−
421.1728
473.2382
490.2408
542.2669
0
20
40
60
80
100
350
400
450
500
550
657 : M.findmain = 474.2455 (+/− 20 ppm)
343.1989
421.1728
473.2382
[M−H]−
490.2408
542.2669
0
20
40
60
80
100
350
400
450
500
550

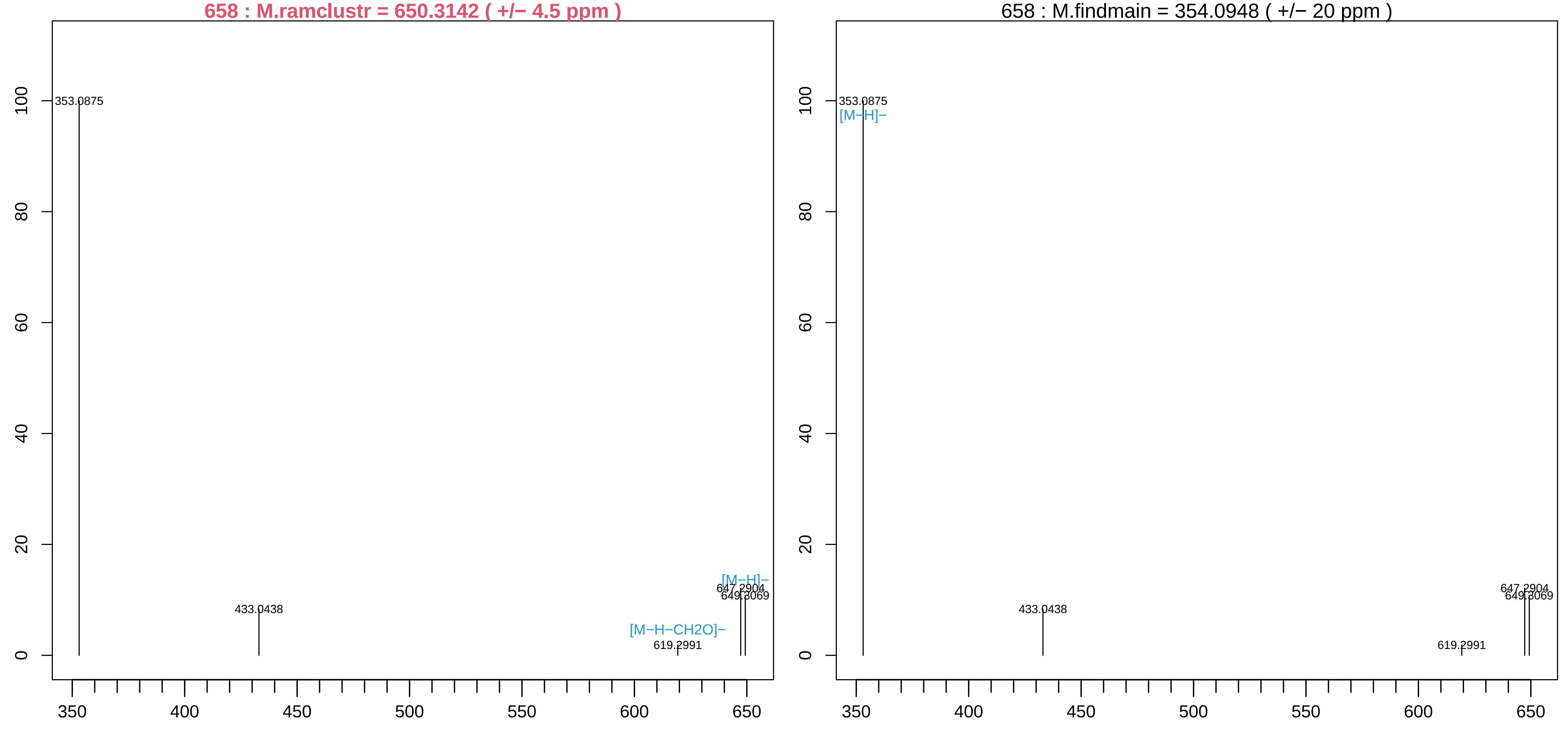

658 : M.ramclustr = 650.3142 (+/− 4.5 ppm)
353.0875
433.0438
[M−H−CH2O]−
619.2991
[M−H]−
647.2904
649.3069
658 : M.findmain = 354.0948 (+/− 20 ppm)
353.0875
[M−H]−
433.0438
619.2991
647.2904
649.3069
0
20
40
60
80
100
350
400
450
500
550
600
650

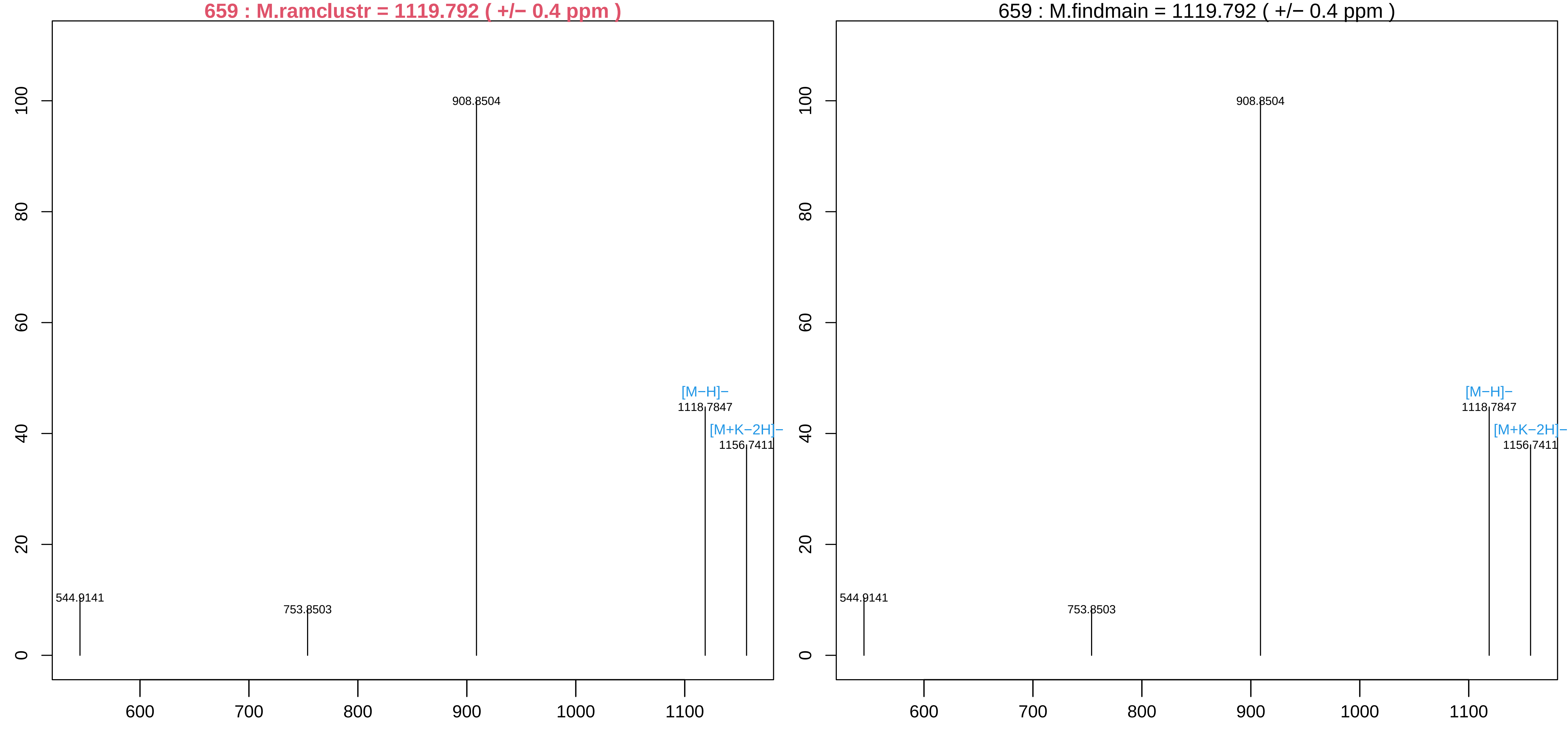
659 : M.ramclustr = 1119.792 (+/− 0.4 ppm)
908.8504
544.9141
753.8503
[M−H]−
1118.7847
[M+K−2H]−
1156.7411
0
20
40
60
80
100
600
700
800
900
1000
1100
659 : M.findmain = 1119.792 (+/− 0.4 ppm)
908.8504
544.9141
753.8503
[M−H]−
1118.7847
[M+K−2H]−
1156.7411
0
20
40
60
80
100
600
700
800
900
1000
1100

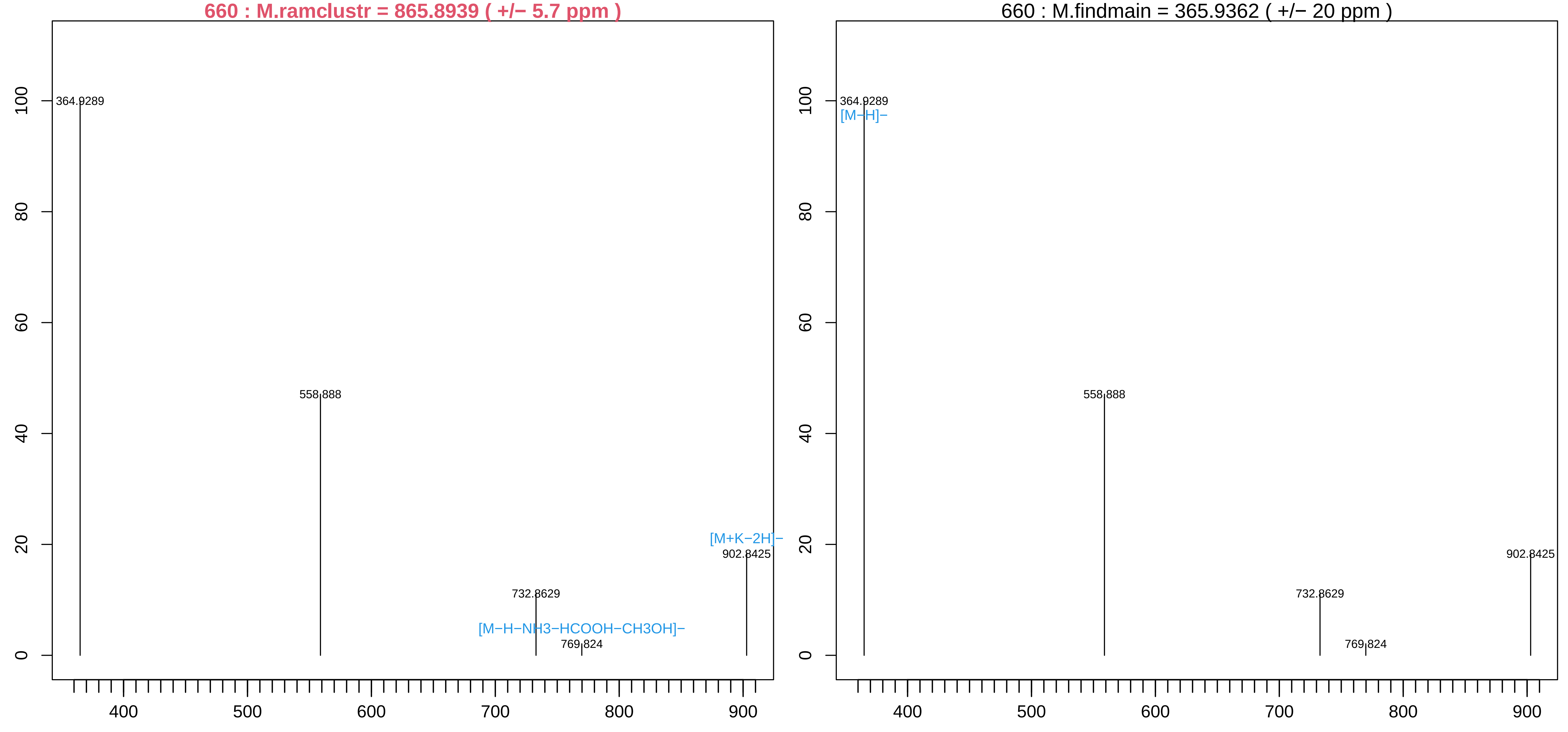
660 : M.ramclustr = 865.8939 (+/− 5.7 ppm)
364.9289
558.888
732.8629
[M−H−NH3−HCOOH−CH3OH]−
769.824
[M+K−2H]−
902.8425
0
20
40
60
80
100
400
500
600
700
800
900
660 : M.findmain = 365.9362 (+/− 20 ppm)
364.9289
[M−H]−
558.888
732.8629
769.824
902.8425

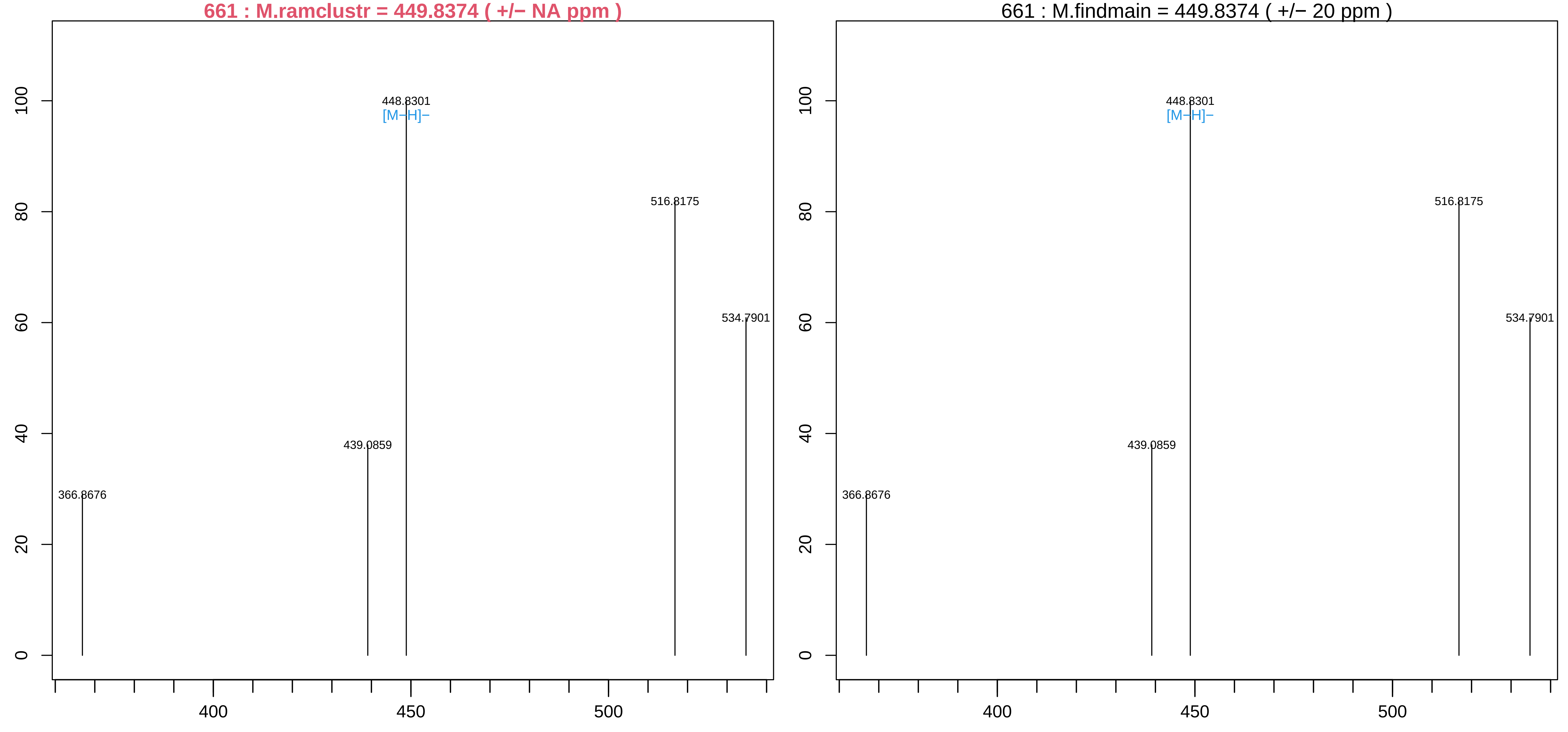
661 : M.ramclustr = 449.8374 (+/− NA ppm)
661 : M.findmain = 449.8374 (+/− 20 ppm)
448.8301
[M−H]−
516.8175
534.7901
439.0859
366.8676
0
20
40
60
80
100
400
450
500

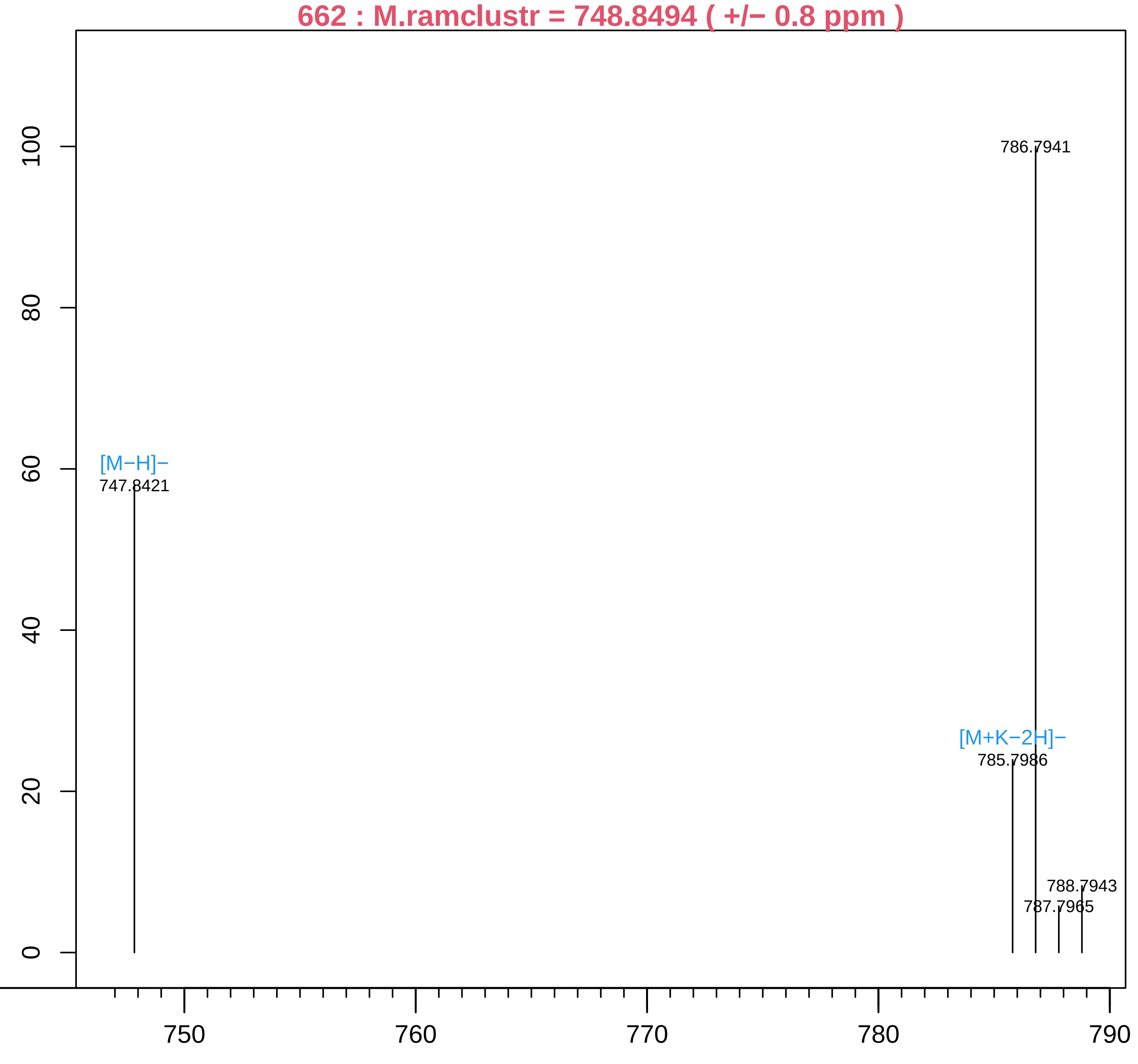

662 : M.ramclustr = 748.8494 (+/− 0.8 ppm)
[M−H]−
747.8421
786.7941
[M+K−2H]−
785.7986
788.7943
787.7965
0
20
40
60
80
100
750
760
770
780
790

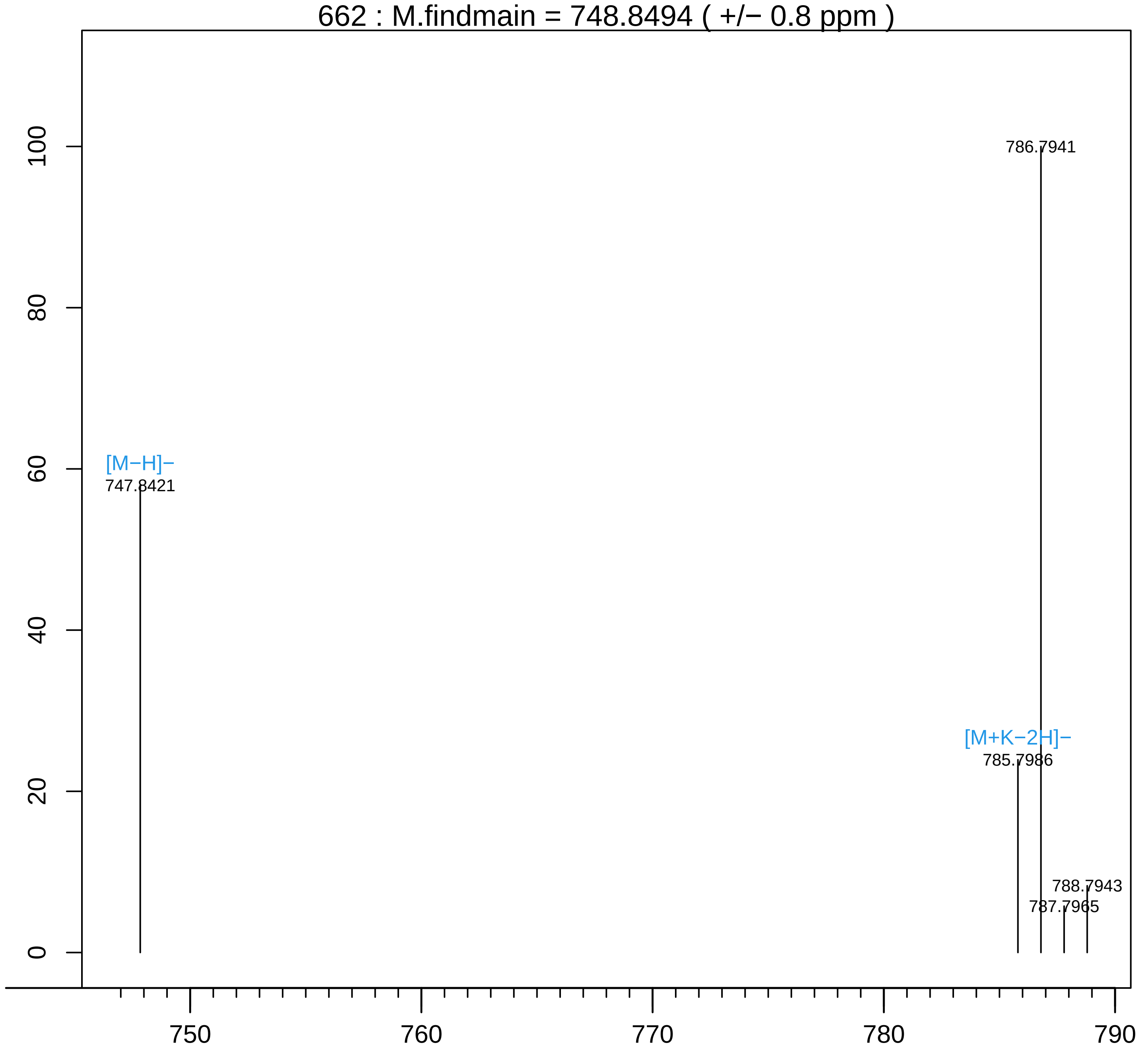

662 : M.findmain = 748.8494 (+/− 0.8 ppm)
[M−H]−
747.8421
786.7941
[M+K−2H]−
785.7986
788.7943
787.7965
0
20
40
60
80
100
750
760
770
780
790

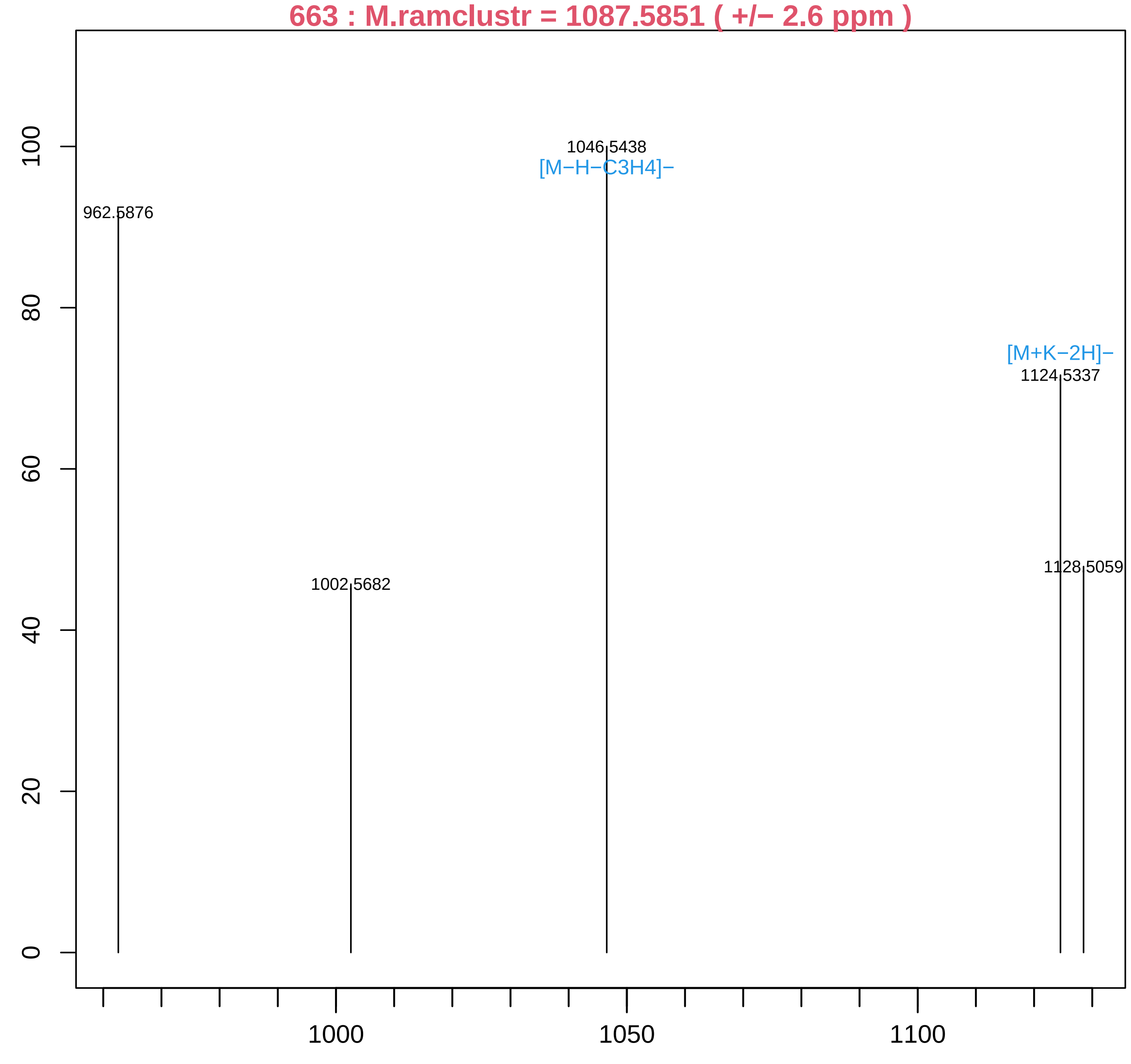
663 : M.ramclustr = 1087.5851 (+/− 2.6 ppm)
962.5876
1002.5682
1046.5438
[M−H−C3H4]−
[M+K−2H]−
1124.5337
1128.5059
0
20
40
60
80
100
1000
1050
1100

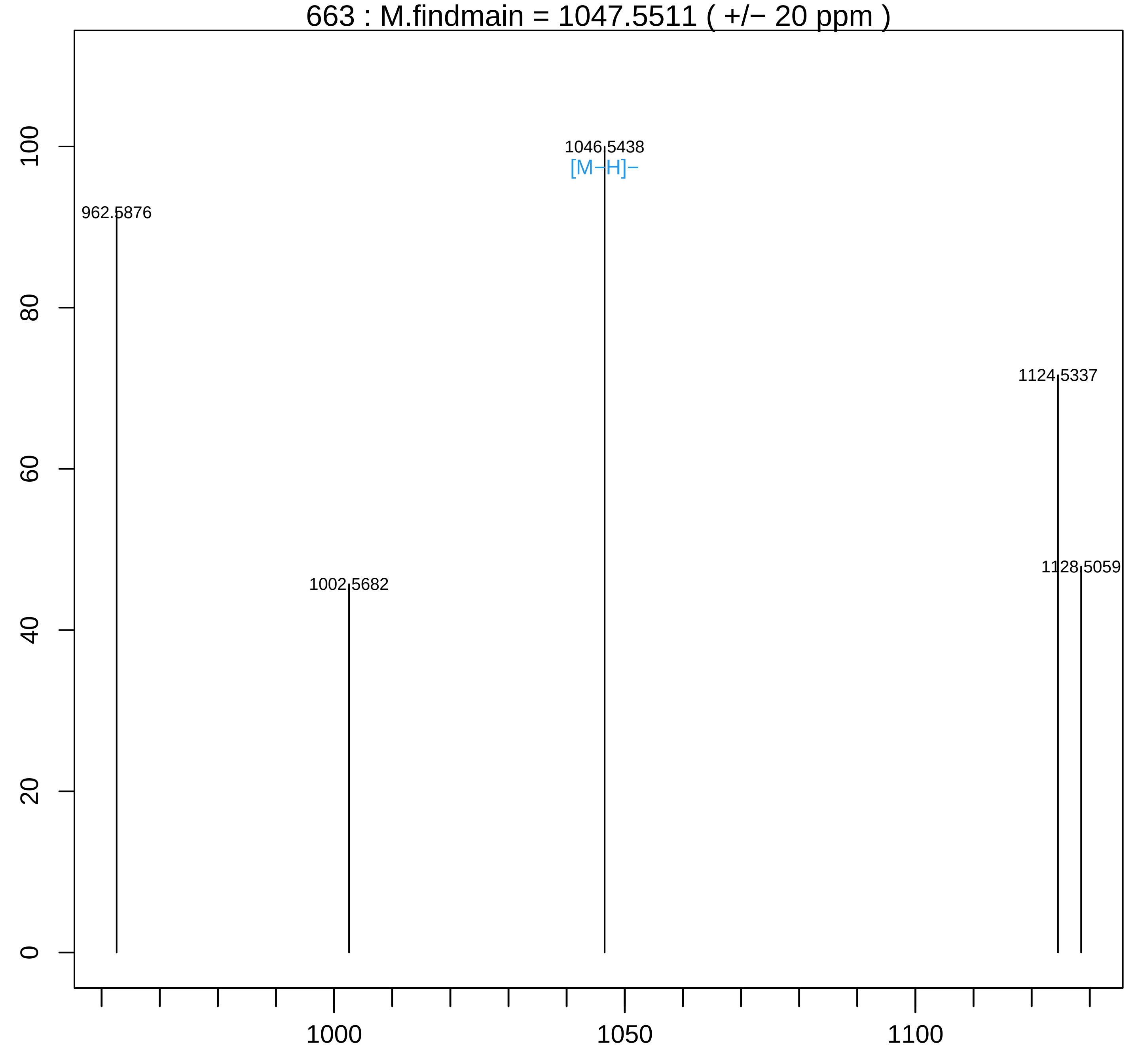
663 : M.findmain = 1047.5511 (+/− 20 ppm)
962.5876
1002.5682
1046.5438
[M−H]−
1124.5337
1128.5059
0
20
40
60
80
100
1000
1050
1100

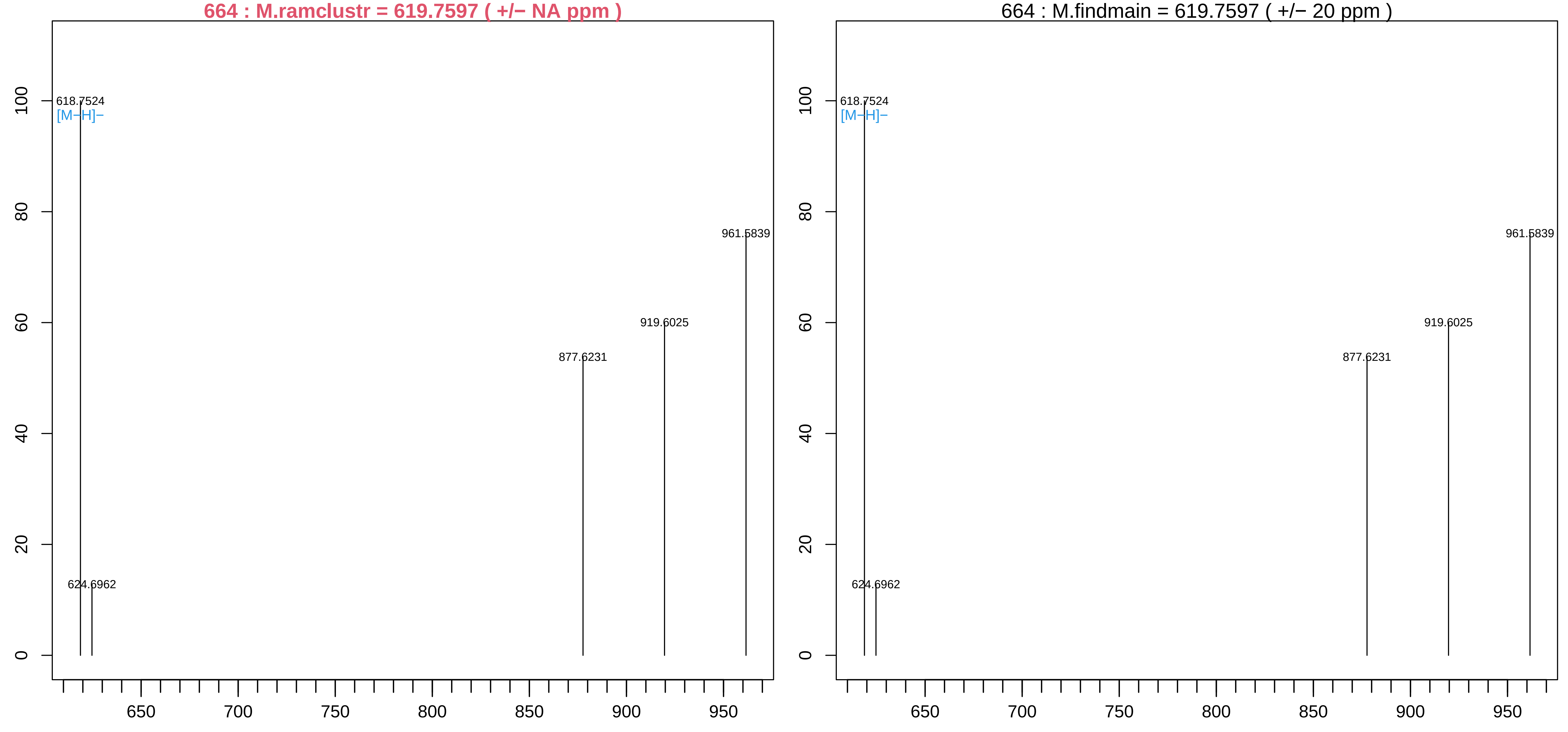

664 : M.ramclustr = 619.7597 (+/− NA ppm)
618.7524
[M−H]−
624.6962
877.6231
919.6025
961.5839
0
20
40
60
80
100
650
700
750
800
850
900
950
664 : M.findmain = 619.7597 (+/− 20 ppm)
618.7524
[M−H]−
624.6962
877.6231
919.6025
961.5839
0
20
40
60
80
100
650
700
750
800
850
900
950

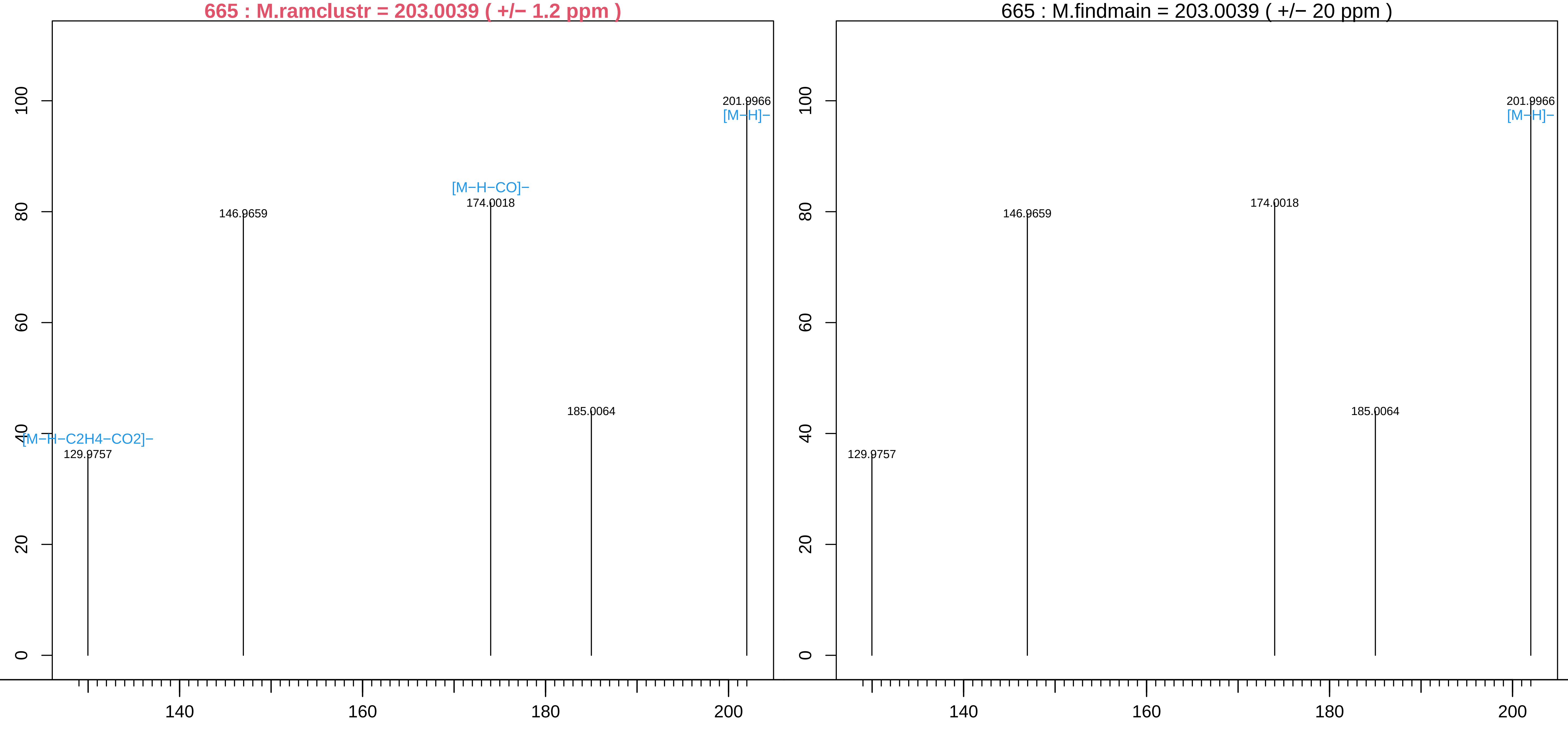
665 : M.ramclustr = 203.0039 (+/− 1.2 ppm)
[M−H]−
201.9966
[M−H−CO]−
174.0018
146.9659
185.0064
[M−H−C2H4−CO2]−
129.9757
0
20
40
60
80
100
140
160
180
200
665 : M.findmain = 203.0039 (+/− 20 ppm)
201.9966
[M−H]−
174.0018
146.9659
185.0064
129.9757
0
20
40
60
80
100
140
160
180
200

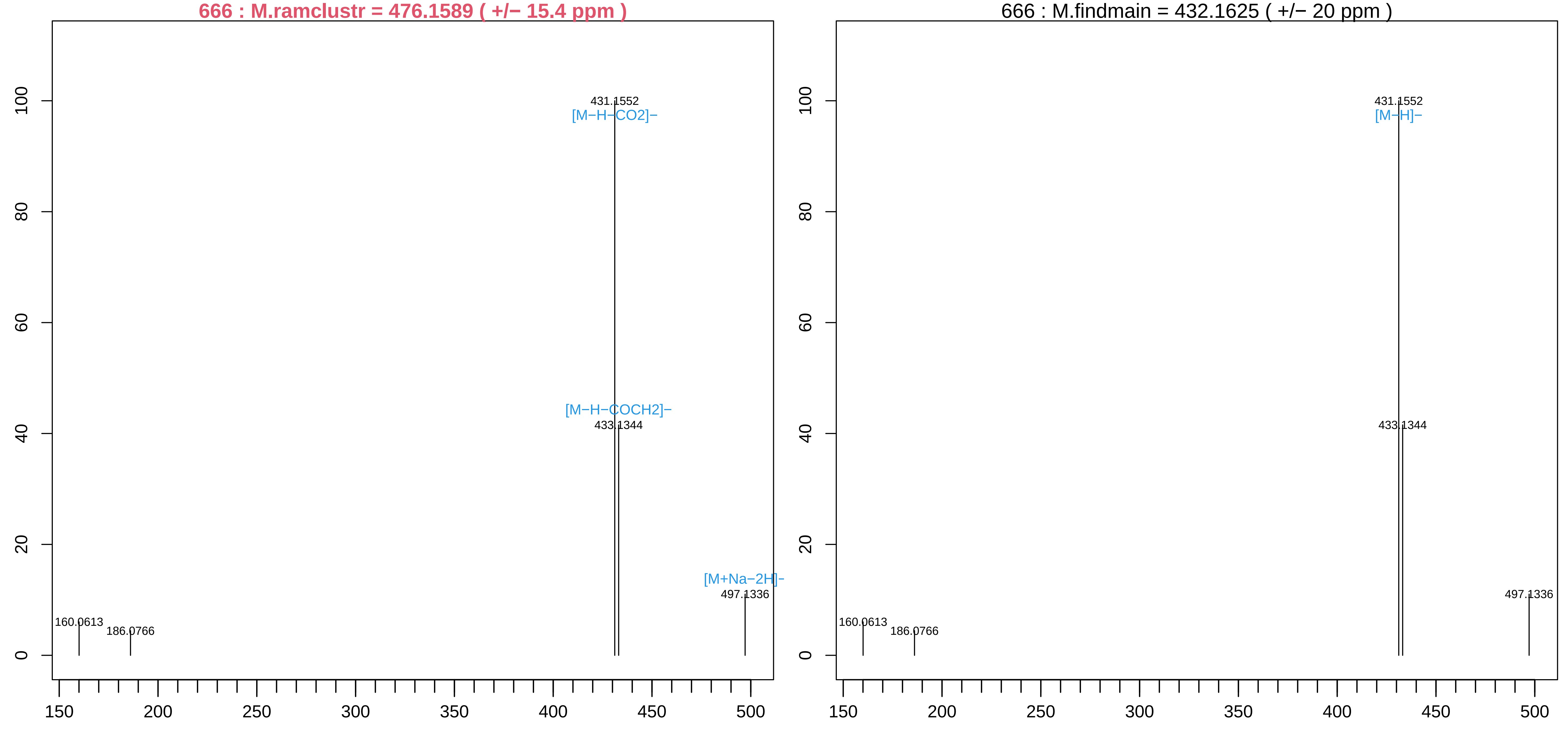

666 : M.ramclustr = 476.1589 (+/− 15.4 ppm)
431.1552
[M−H−CO2]−
[M−H−COCH2]−
433.1344
[M+Na−2H]−
497.1336
160.0613
186.0766
666 : M.findmain = 432.1625 (+/− 20 ppm)
431.1552
[M−H]−
433.1344
497.1336
160.0613
186.0766

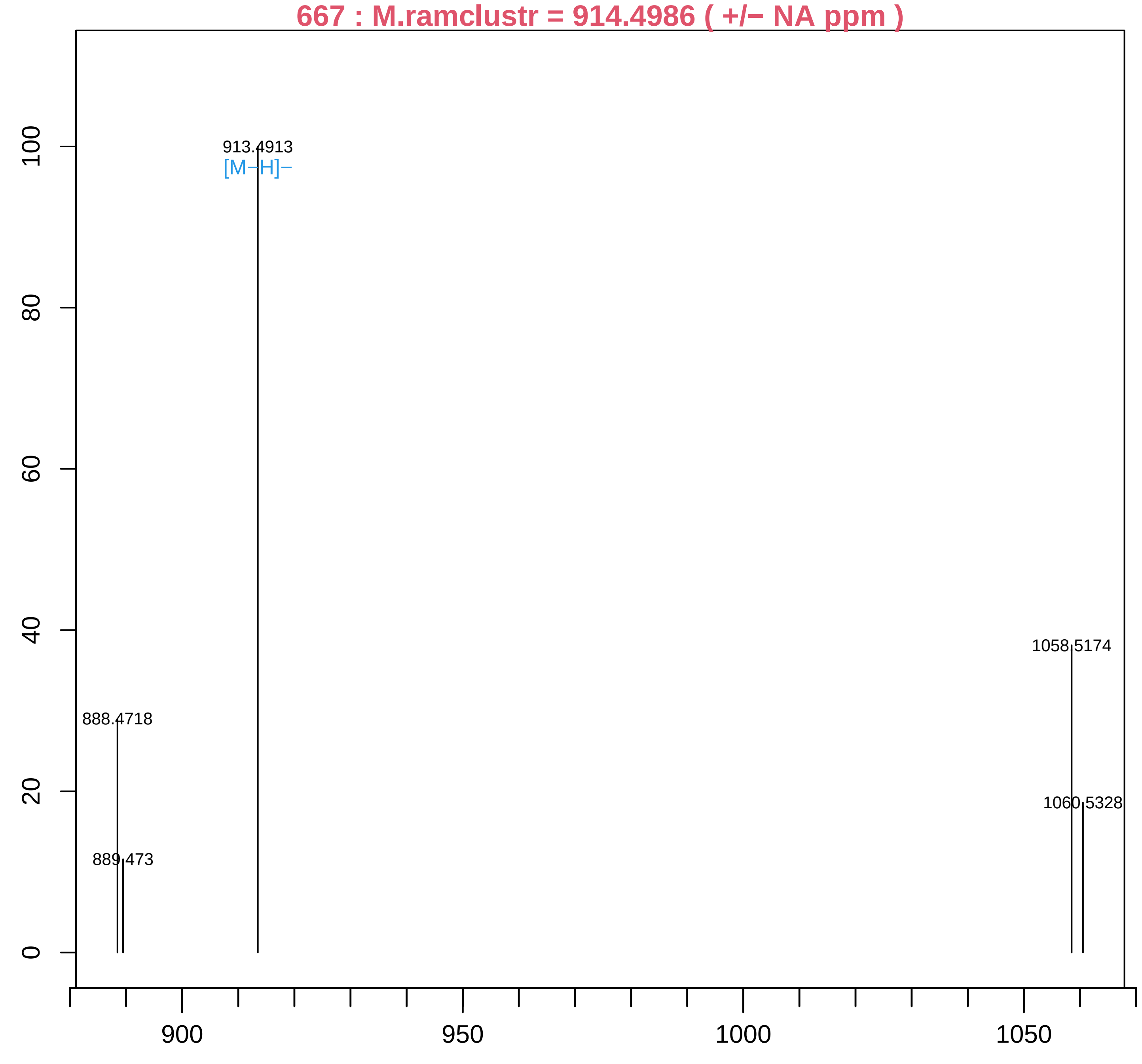

667 : M.ramclustr = 914.4986 (+/− NA ppm)
913.4913
[M−H]−
888.4718
889.473
1058.5174
1060.5328
0
20
40
60
80
100
900
950
1000
1050

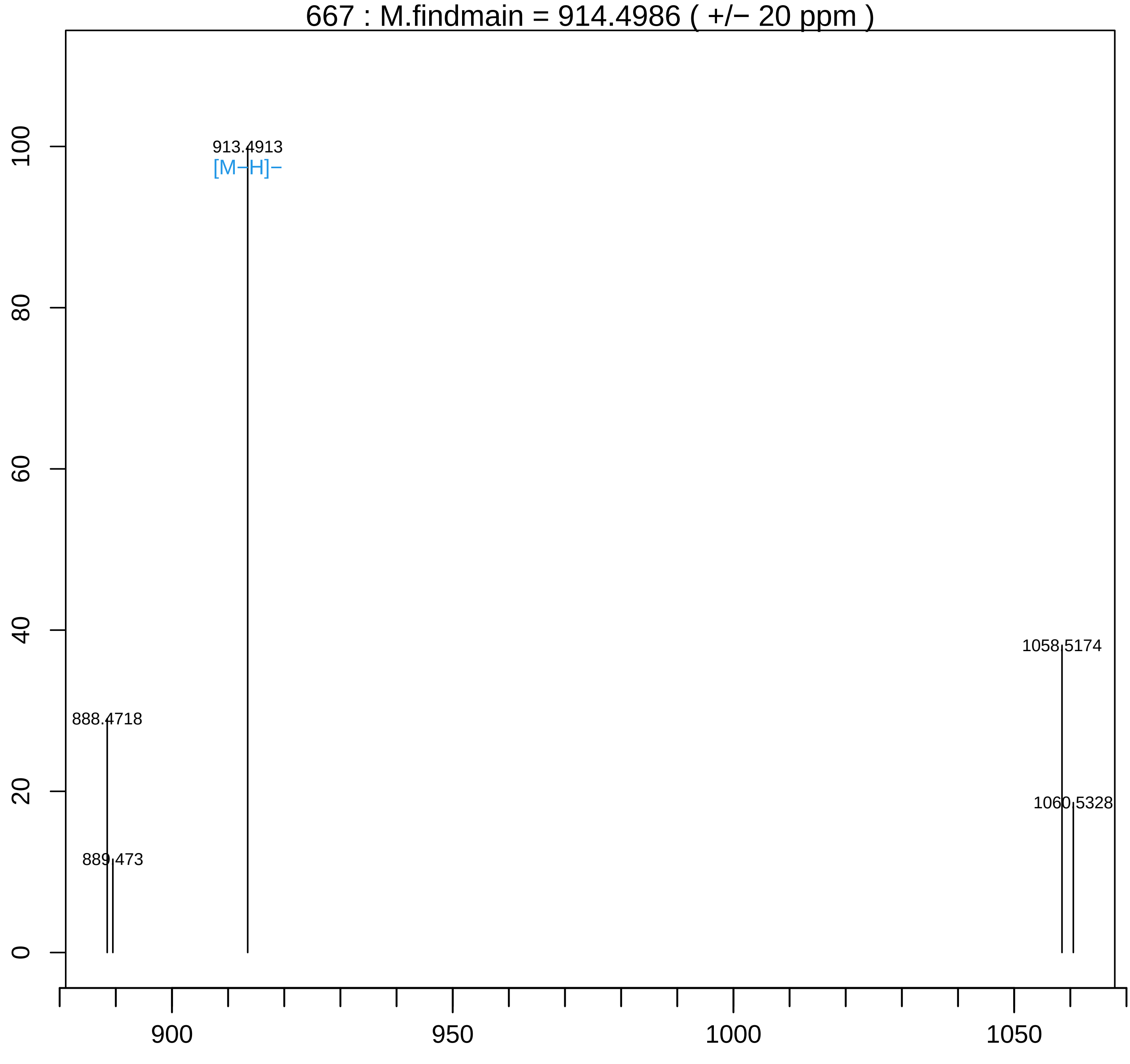

667 : M.findmain = 914.4986 (+/− 20 ppm)
913.4913
[M−H]−
888.4718
889.473
1058.5174
1060.5328
0
20
40
60
80
100
900
950
1000
1050

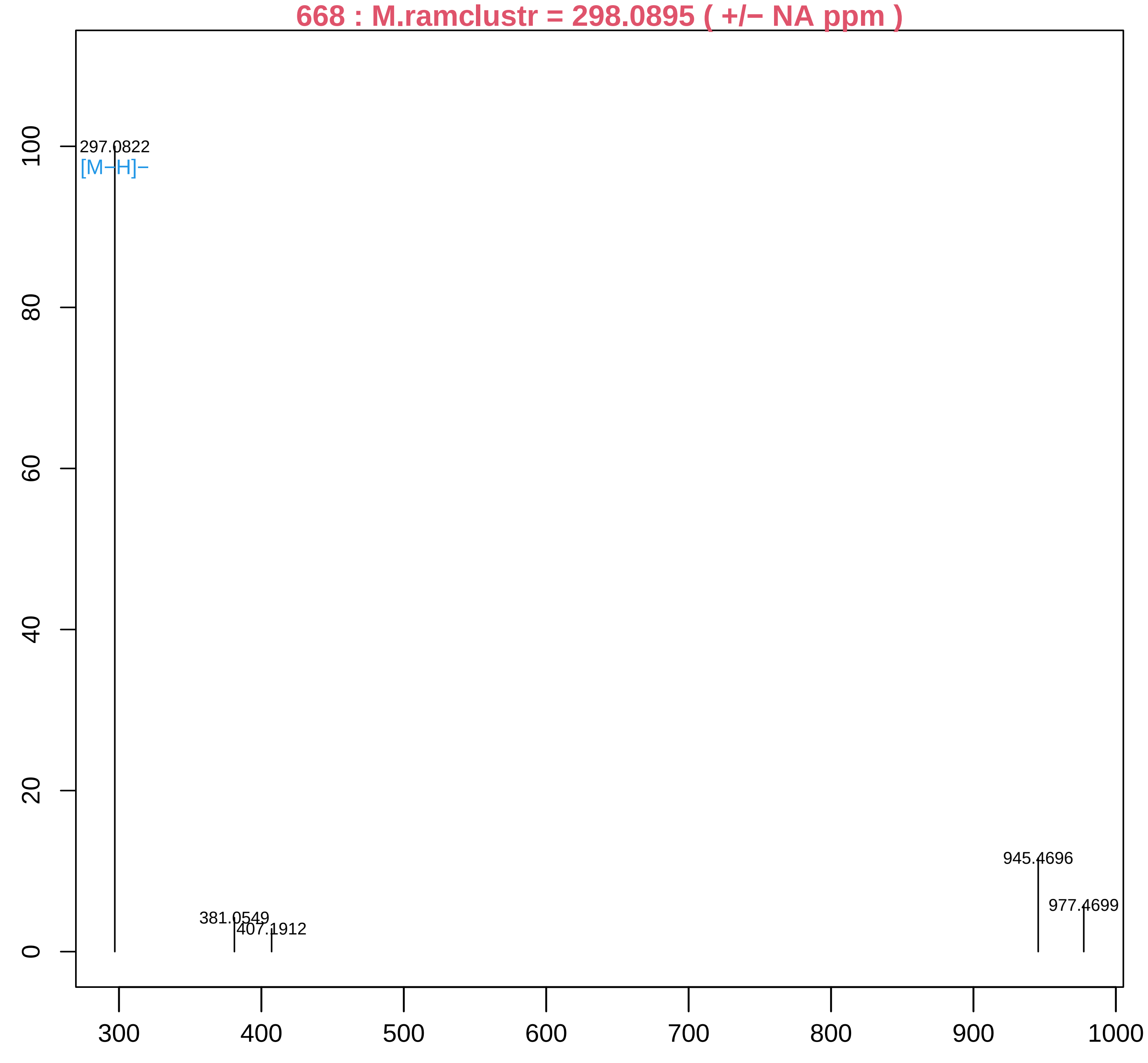

668 : M.ramclustr = 298.0895 (+/− NA ppm)
297.0822
[M−H]−
381.0549
407.1912
945.4696
977.4699
0
20
40
60
80
100
300
400
500
600
700
800
900
1000

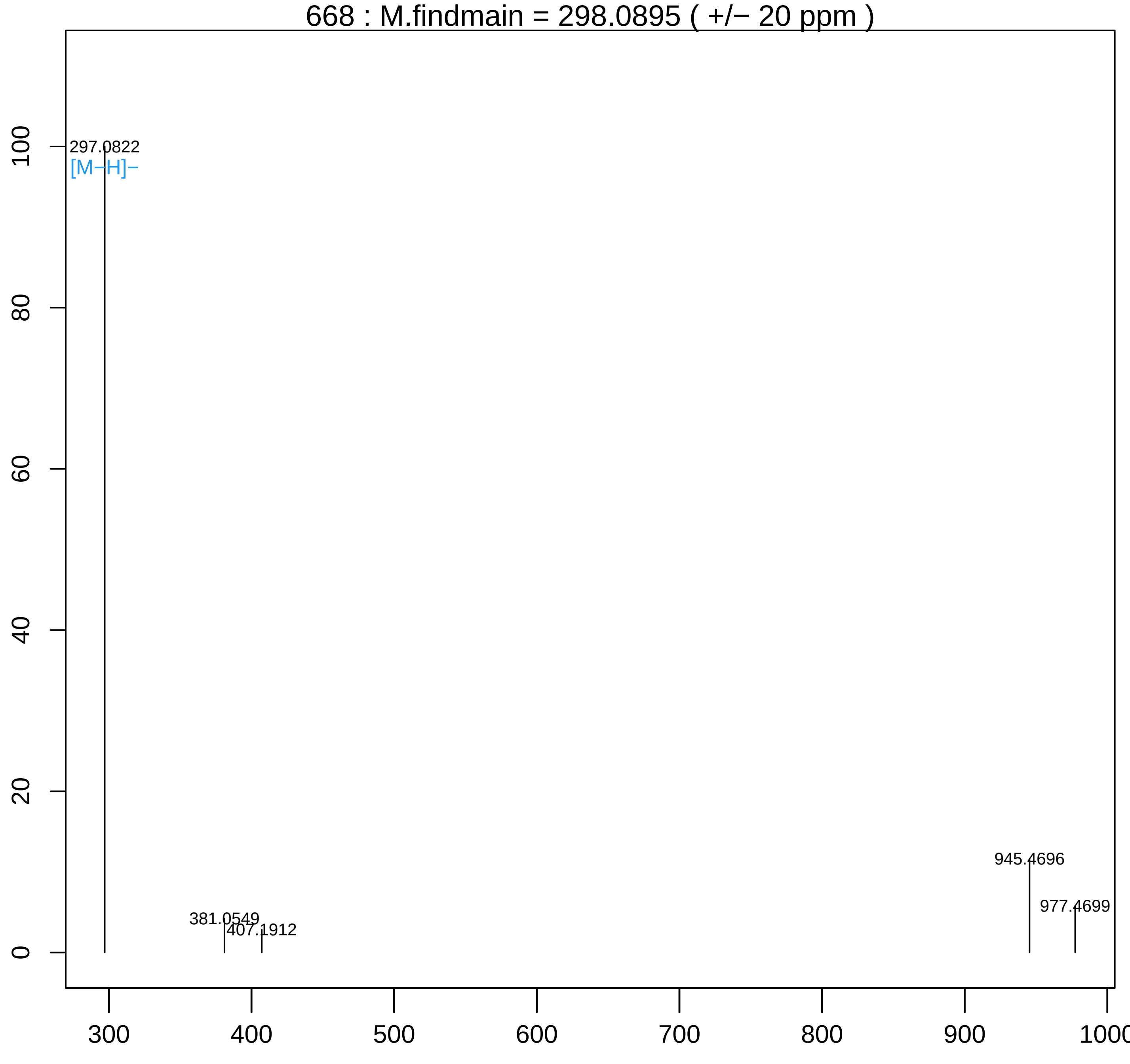

668 : M.findmain = 298.0895 (+/− 20 ppm)
297.0822
[M−H]−
381.0549
407.1912
945.4696
977.4699
0
20
40
60
80
100
300
400
500
600
700
800
900
1000

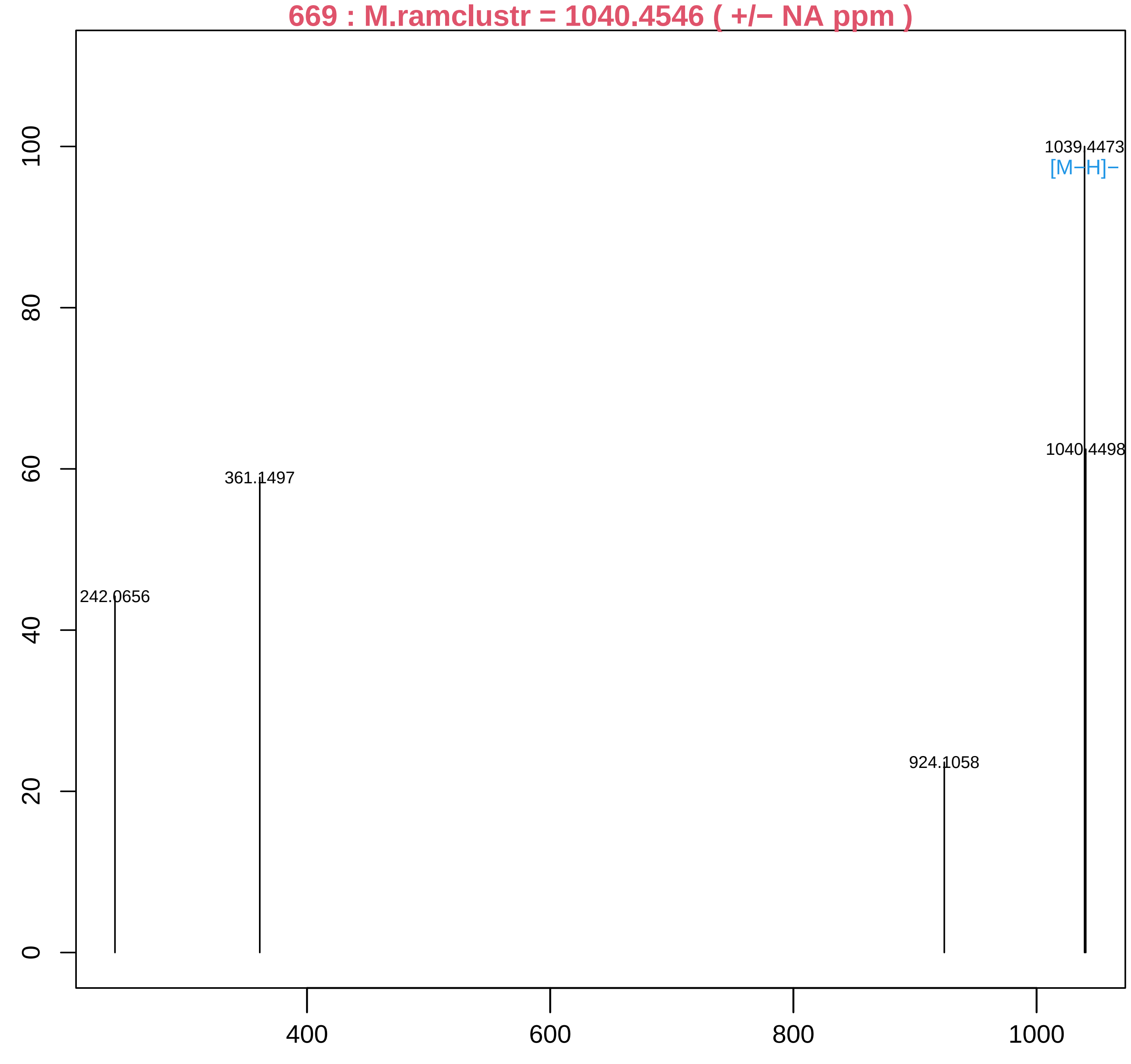

669 : M.ramclustr = 1040.4546 (+/− NA ppm)
1039.4473
[M−H]−
1040.4498
361.1497
242.0656
924.1058
0
20
40
60
80
100
400
600
800
1000

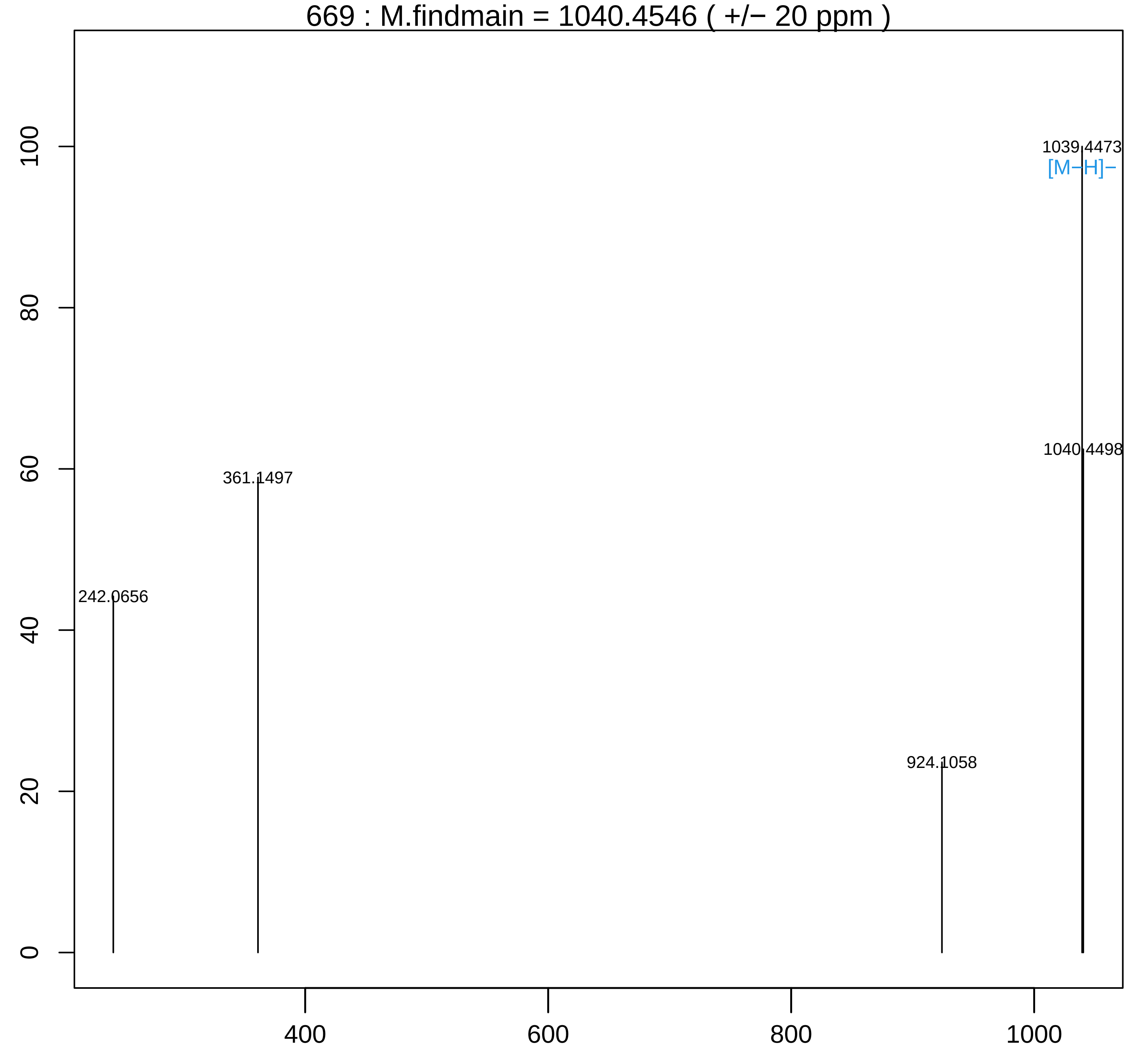

669 : M.findmain = 1040.4546 (+/− 20 ppm)
1039.4473
[M−H]−
1040.4498
361.1497
242.0656
924.1058
0
20
40
60
80
100
400
600
800
1000

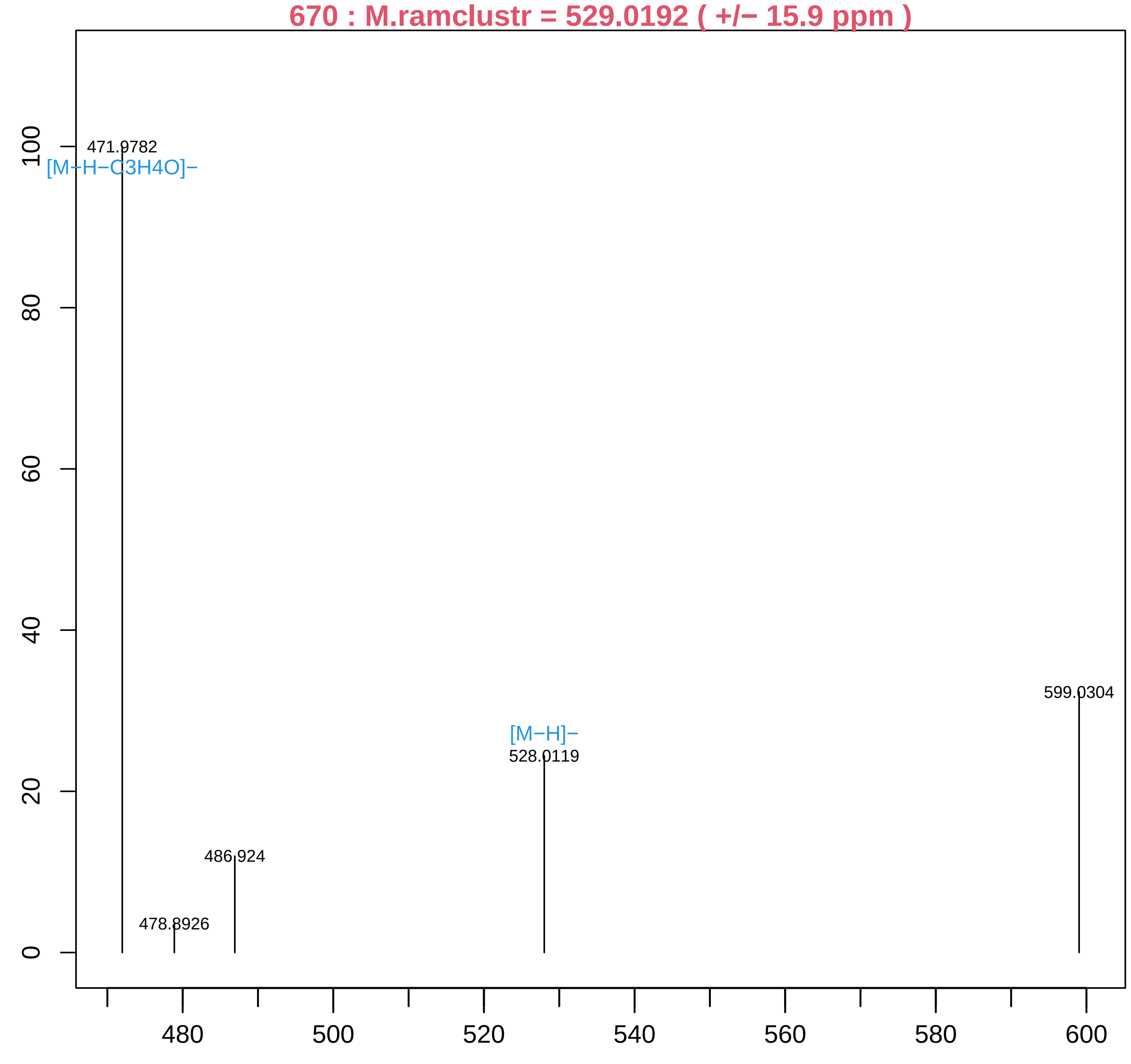
670 : M.ramclustr = 529.0192 (+/− 15.9 ppm)
471.9782
[M−H−C3H4O]−
478.8926
486.924
[M−H]−
528.0119
599.0304
0
20
40
60
80
100
480
500
520
540
560
580
600

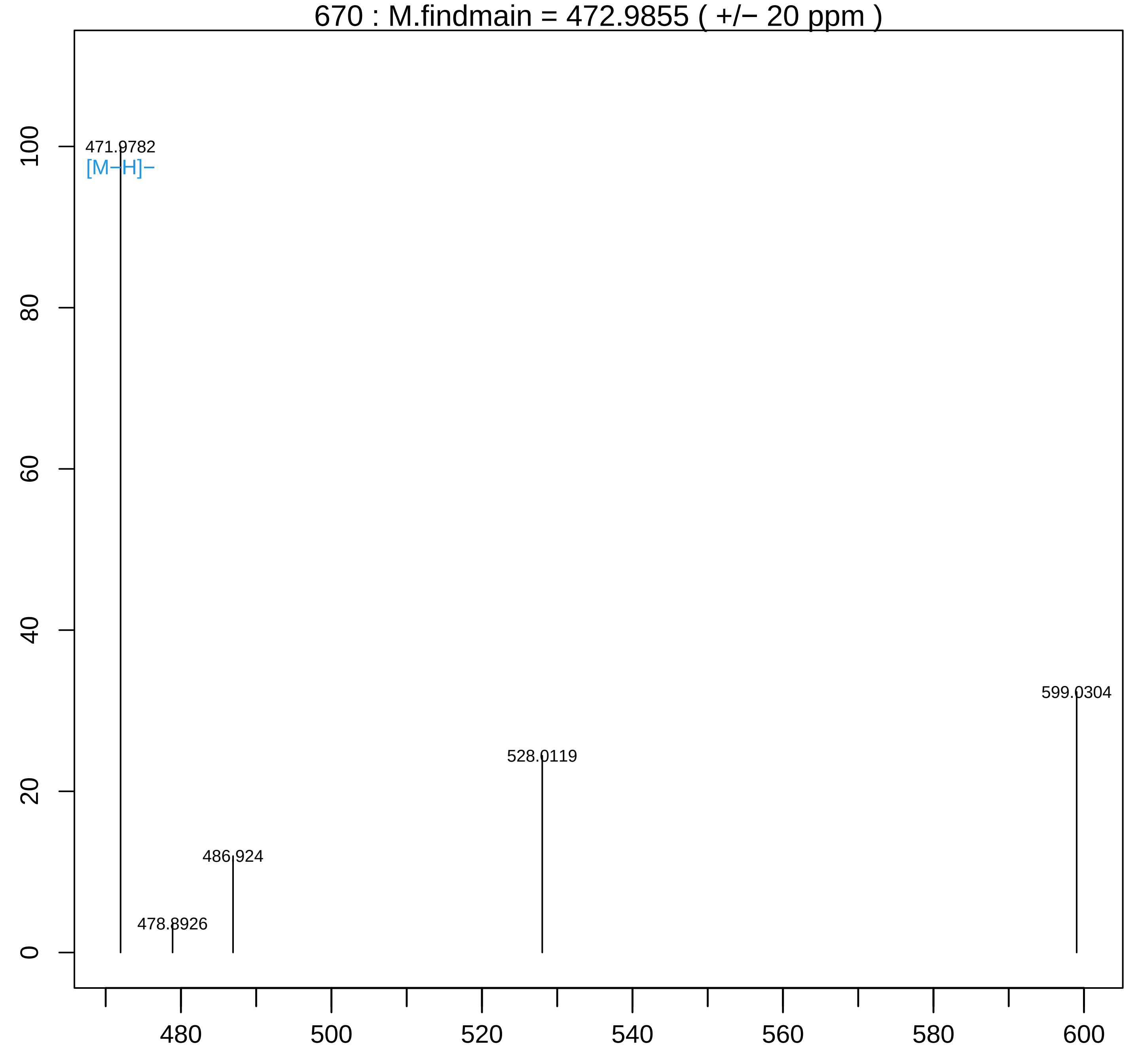
670 : M.findmain = 472.9855 (+/− 20 ppm)
471.9782
[M−H]−
478.8926
486.924
528.0119
599.0304
0
20
40
60
80
100
480
500
520
540
560
580
600

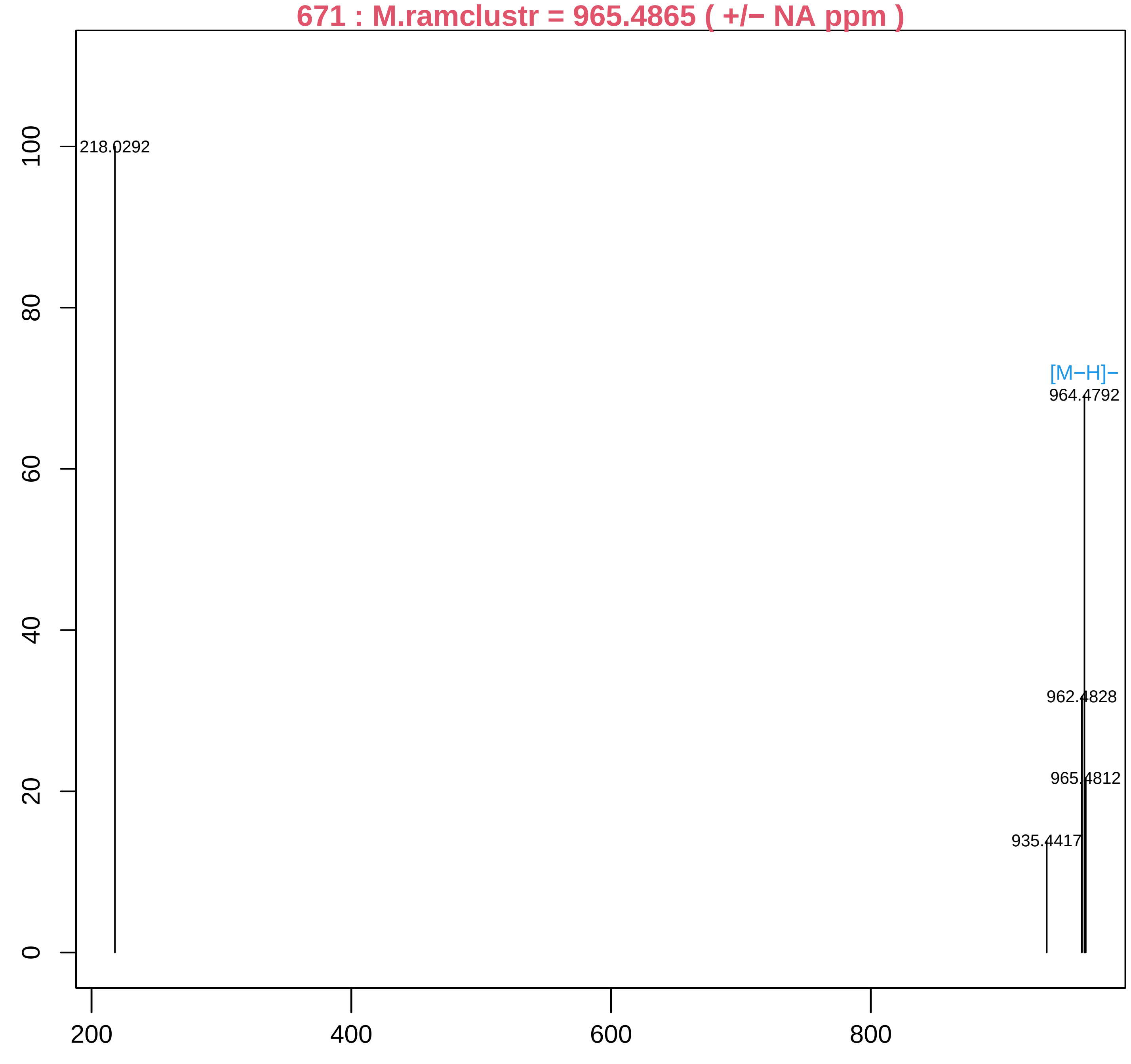
671 : M.ramclustr = 965.4865 (+/− NA ppm)
218.0292
[M−H]−
964.4792
962.4828
965.4812
935.4417
0
20
40
60
80
100
200
400
600
800

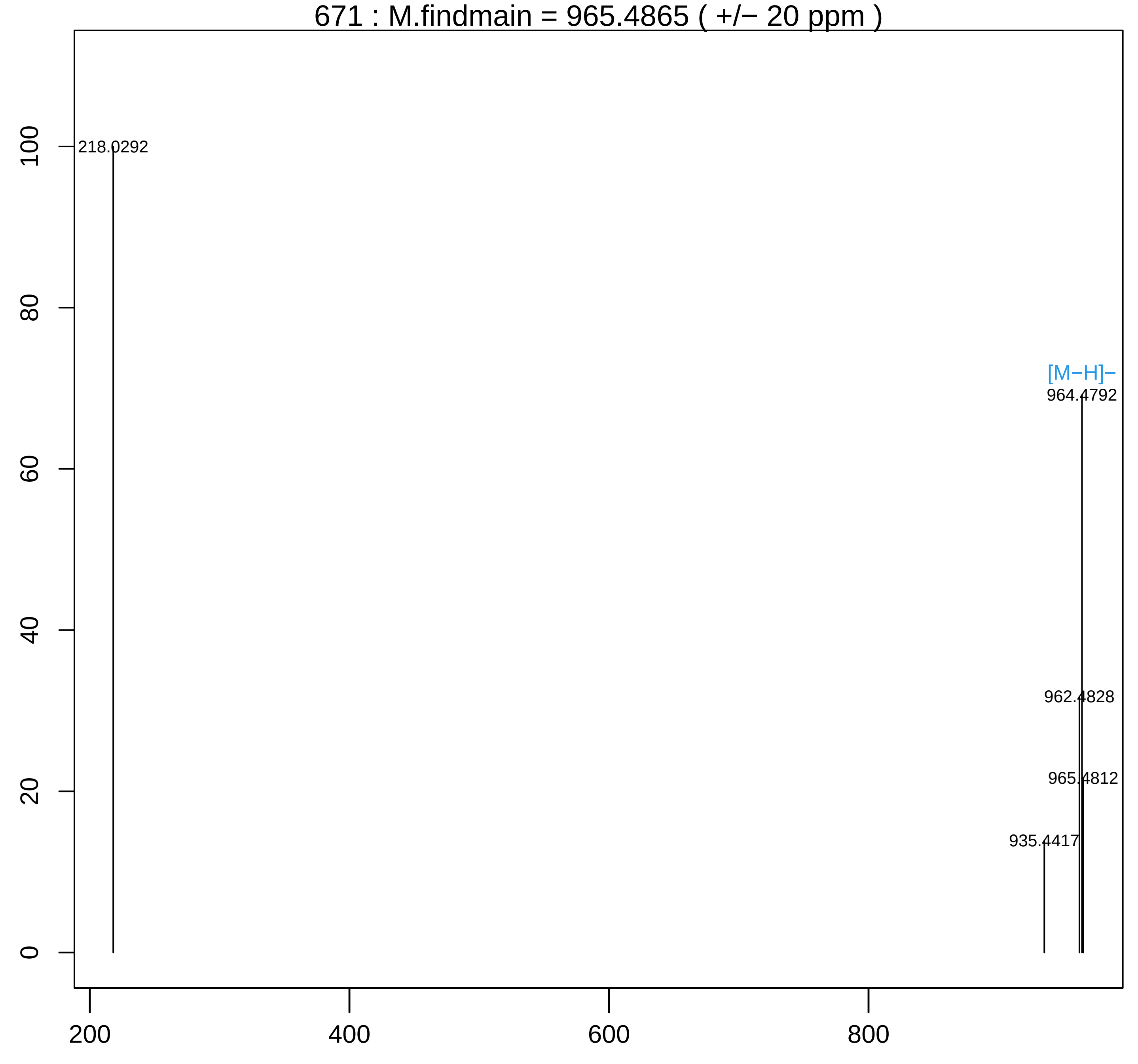
671 : M.findmain = 965.4865 (+/− 20 ppm)
218.0292
[M−H]−
964.4792
962.4828
965.4812
935.4417
0
20
40
60
80
100
200
400
600
800

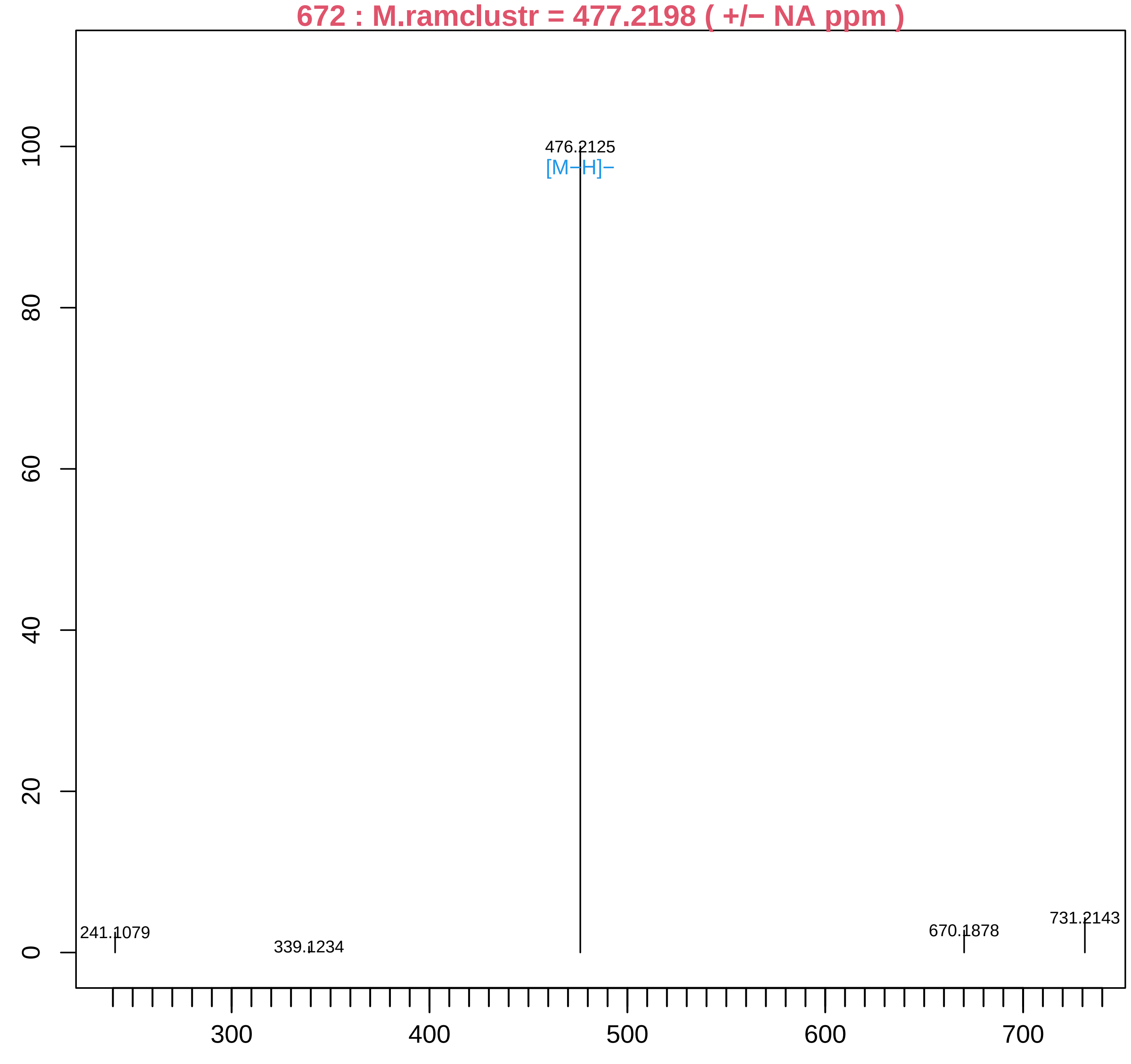
672 : M.ramclustr = 477.2198 (+/− NA ppm)
476.2125
[M−H]−
241.1079
339.1234
670.1878
731.2143
0
20
40
60
80
100
300
400
500
600
700

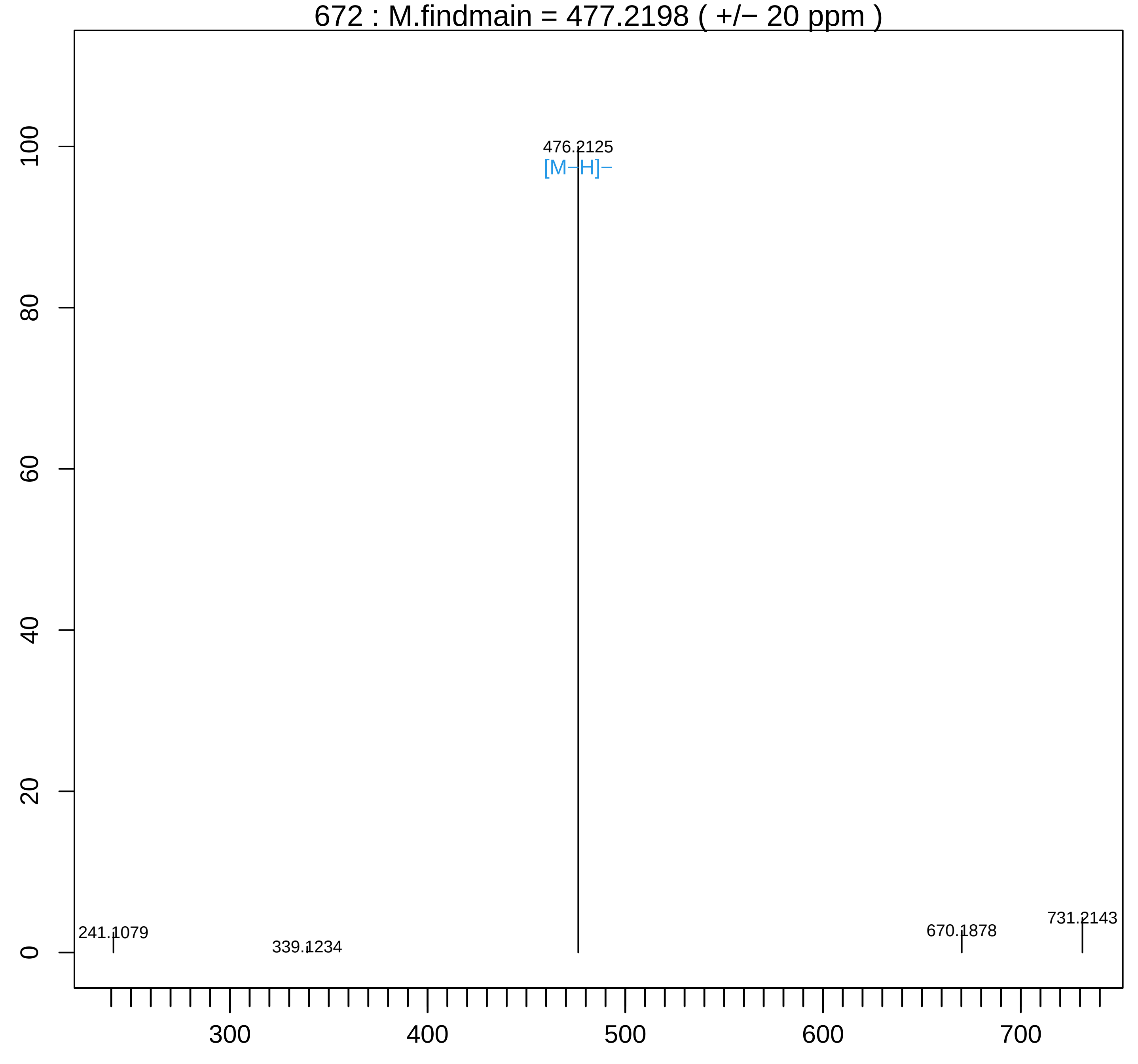
672 : M.findmain = 477.2198 (+/− 20 ppm)
476.2125
[M−H]−
241.1079
339.1234
670.1878
731.2143
0
20
40
60
80
100
300
400
500
600
700

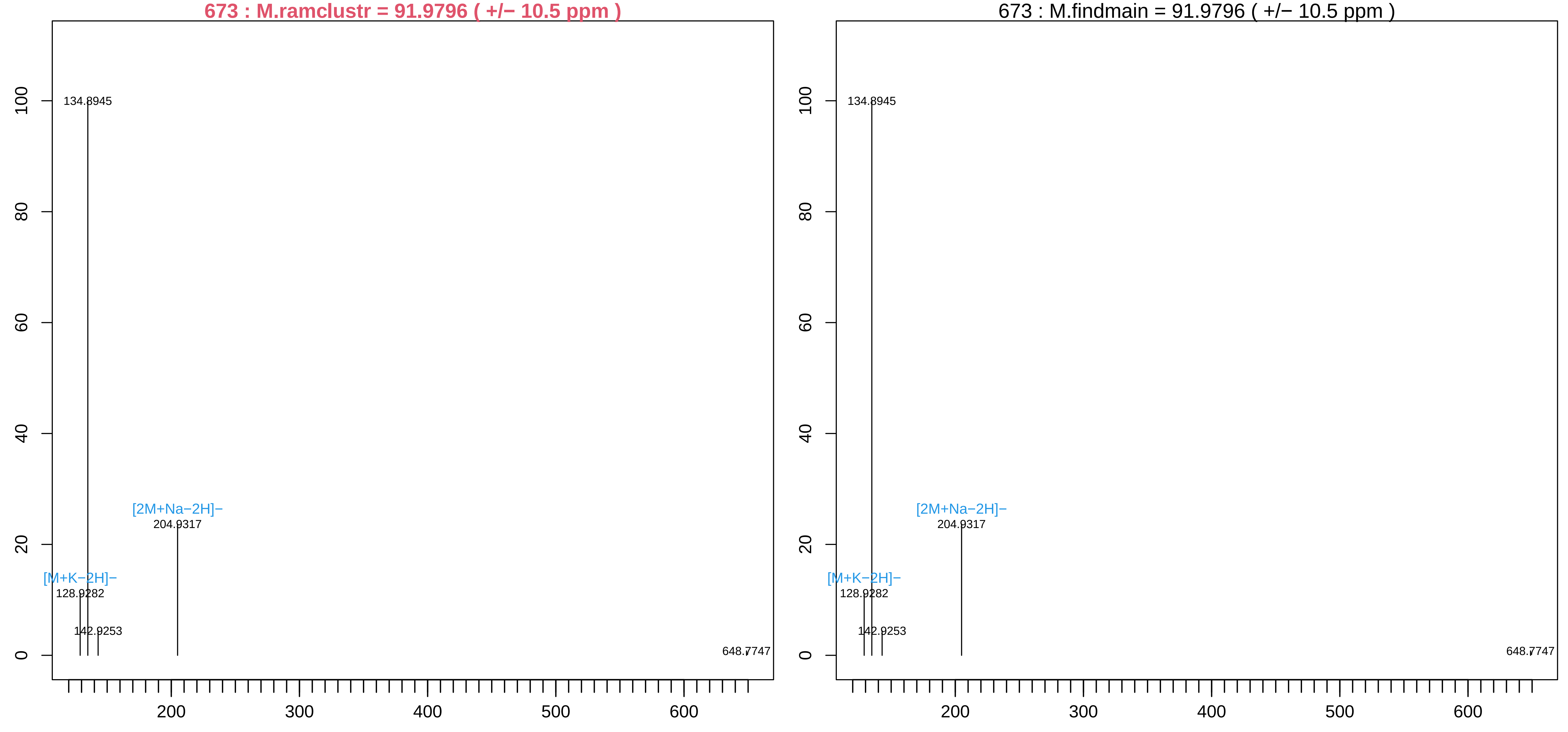
673 : M.ramclustr = 91.9796 (+/− 10.5 ppm)
134.8945
[2M+Na−2H]−
204.9317
[M+K−2H]−
128.9282
142.9253
648.7747
0
20
40
60
80
100
200
300
400
500
600
673 : M.findmain = 91.9796 (+/− 10.5 ppm)
134.8945
[2M+Na−2H]−
204.9317
[M+K−2H]−
128.9282
142.9253
648.7747
0
20
40
60
80
100
200
300
400
500
600

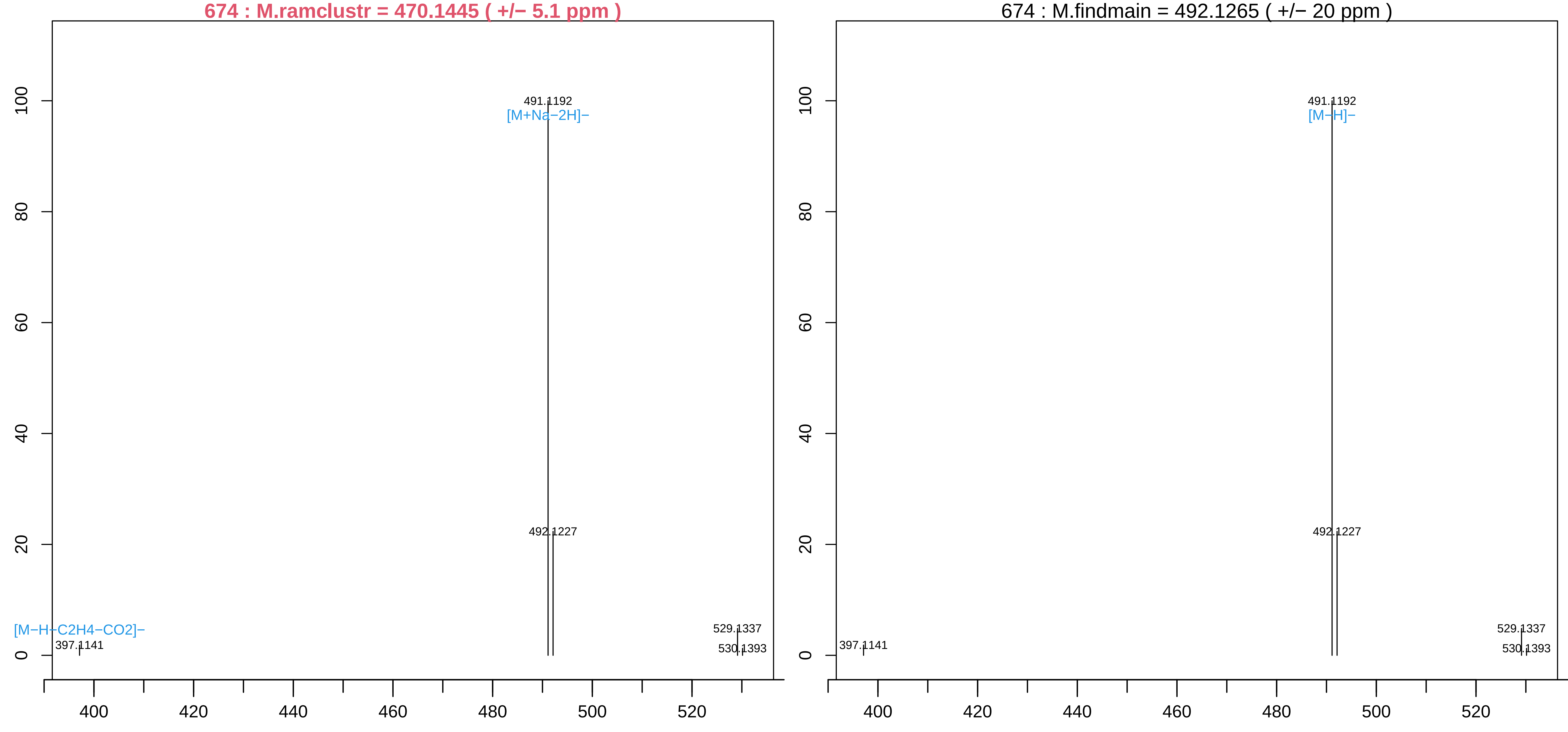

674 : M.ramclustr = 470.1445 (+/− 5.1 ppm)
491.1192
[M+Na−2H]−
492.1227
529.1337
530.1393
[M−H−C2H4−CO2]−
397.1141
674 : M.findmain = 492.1265 (+/− 20 ppm)
491.1192
[M−H]−
492.1227
529.1337
530.1393
397.1141

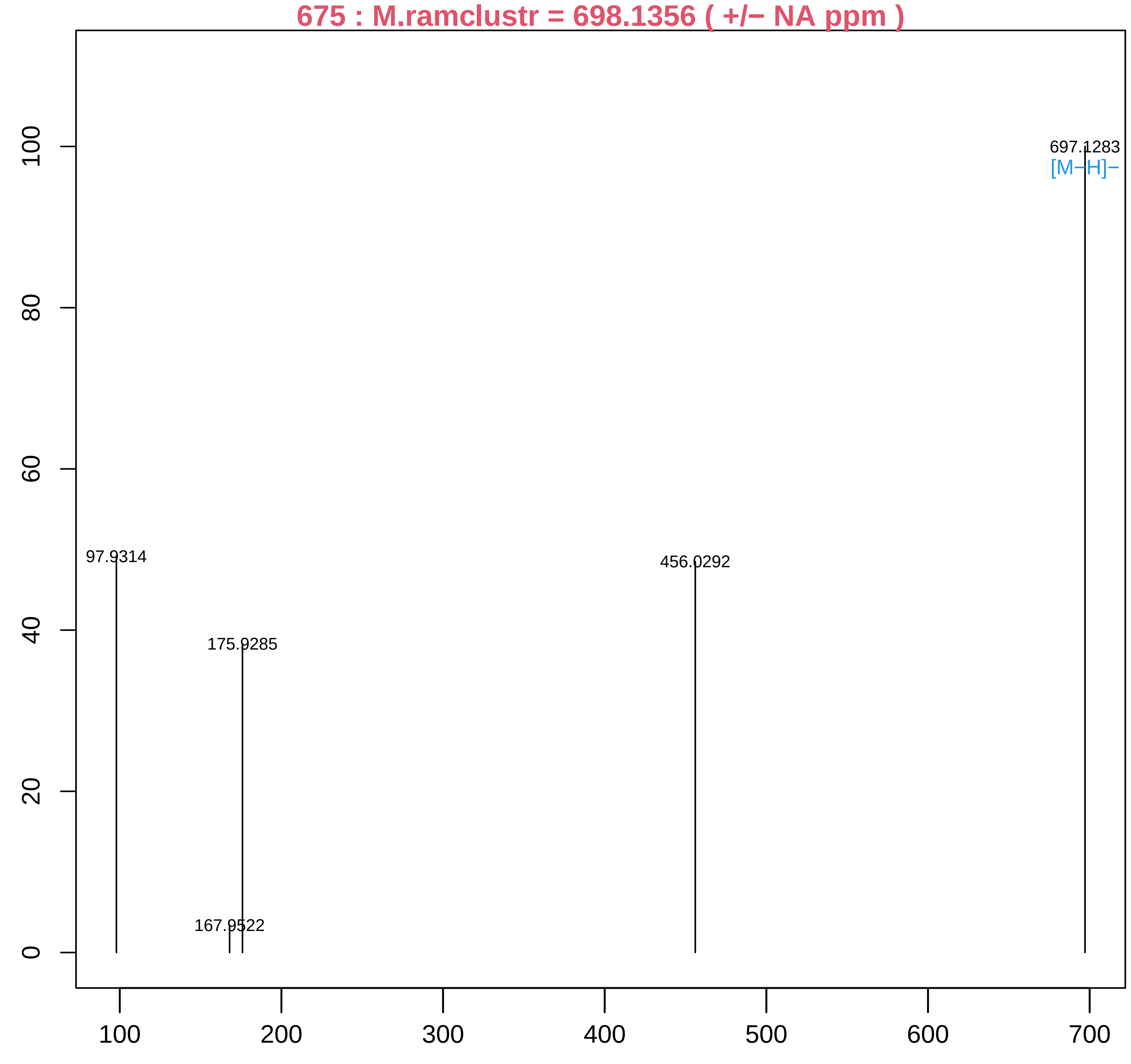
675 : M.ramclustr = 698.1356 (+/− NA ppm)
697.1283
[M−H]−
97.9314
175.9285
167.9522
456.0292
0
20
40
60
80
100
100
200
300
400
500
600
700

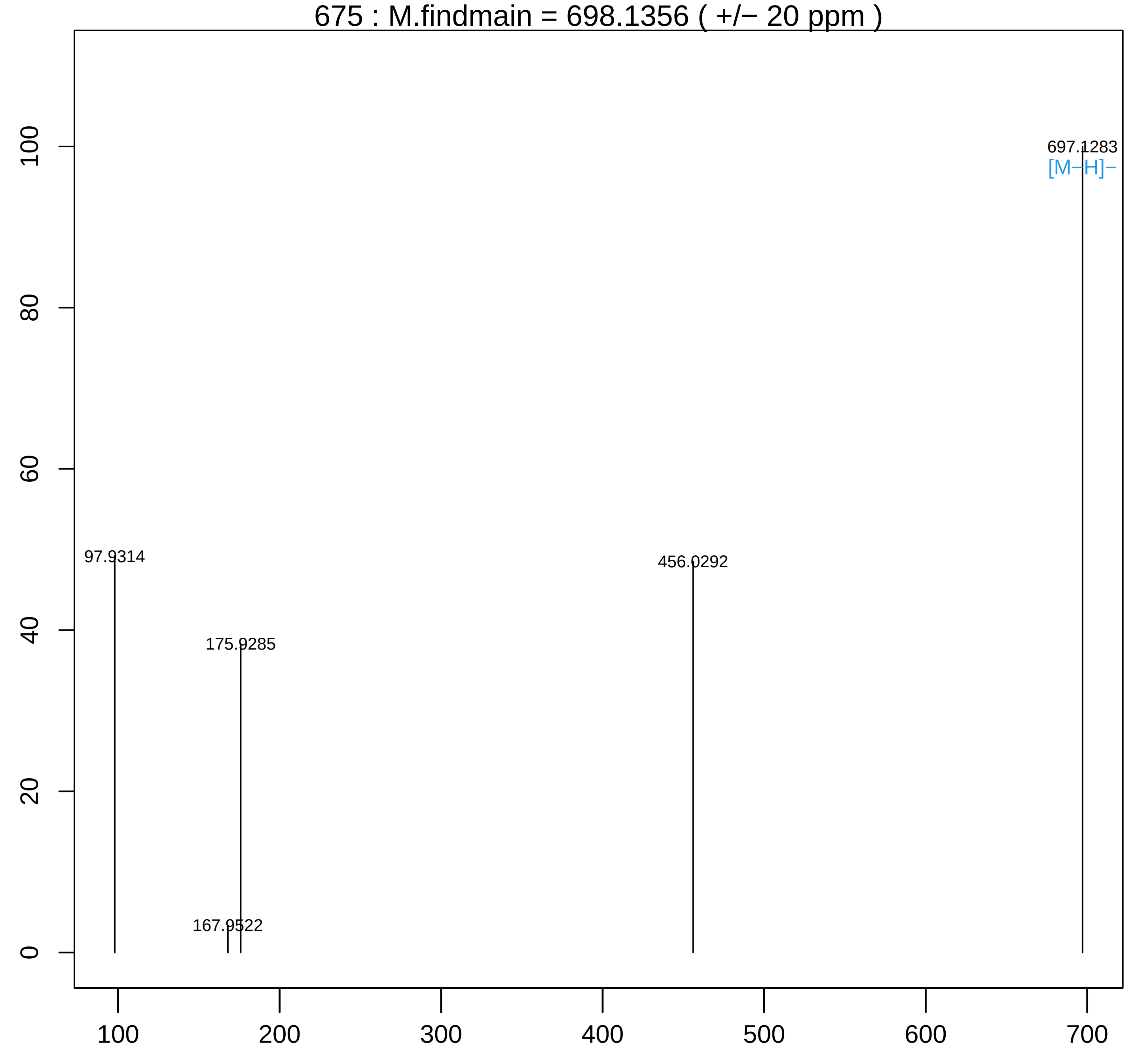
675 : M.findmain = 698.1356 (+/− 20 ppm)
697.1283
[M−H]−
97.9314
175.9285
167.9522
456.0292
0
20
40
60
80
100
100
200
300
400
500
600
700

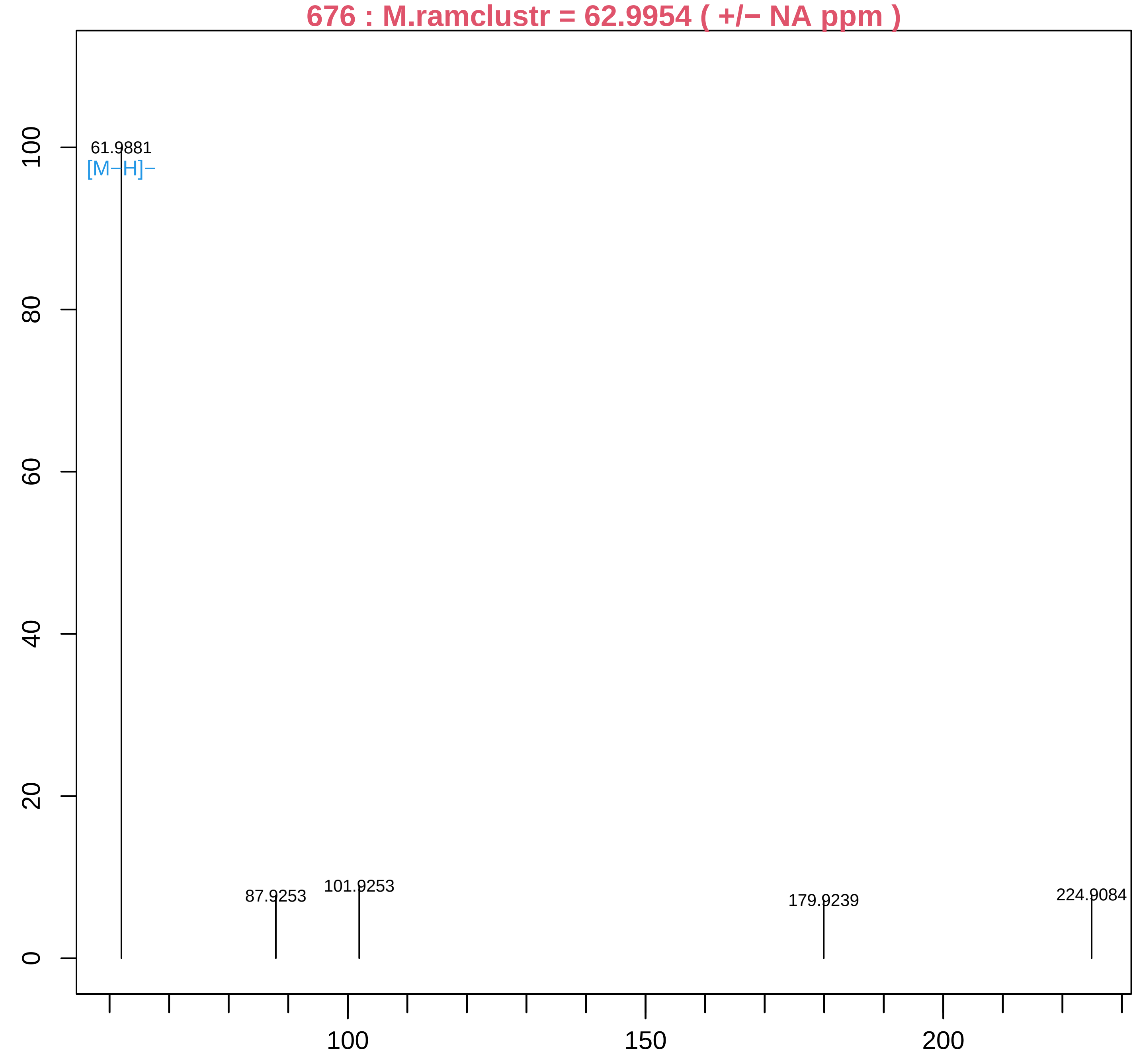
676 : M.ramclustr = 62.9954 (+/− NA ppm)
61.9881
[M−H]−
87.9253
101.9253
179.9239
224.9084
0
20
40
60
80
100
100
150
200

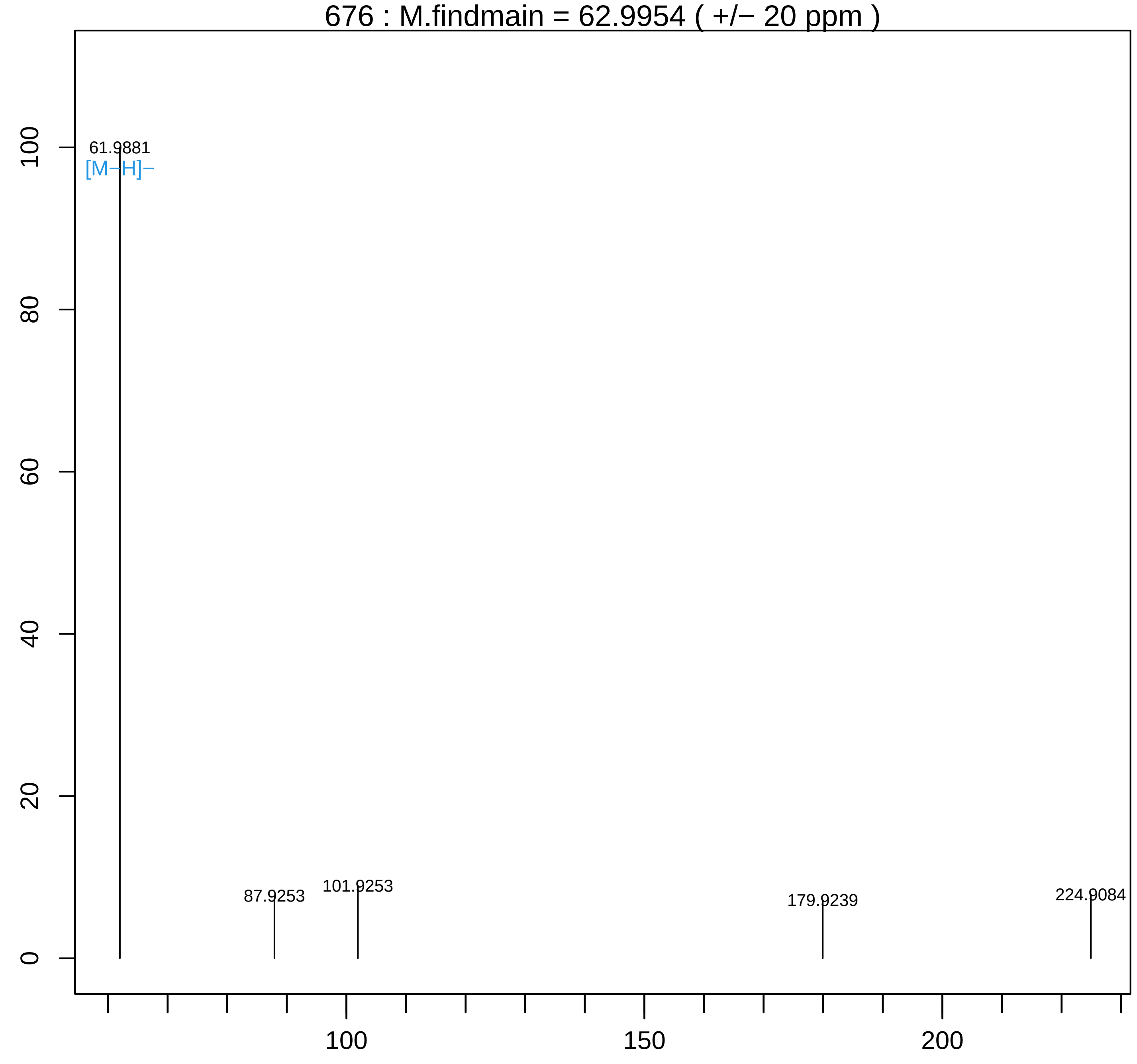
676 : M.findmain = 62.9954 (+/− 20 ppm)
61.9881
[M−H]−
87.9253
101.9253
179.9239
224.9084
0
20
40
60
80
100
100
150
200

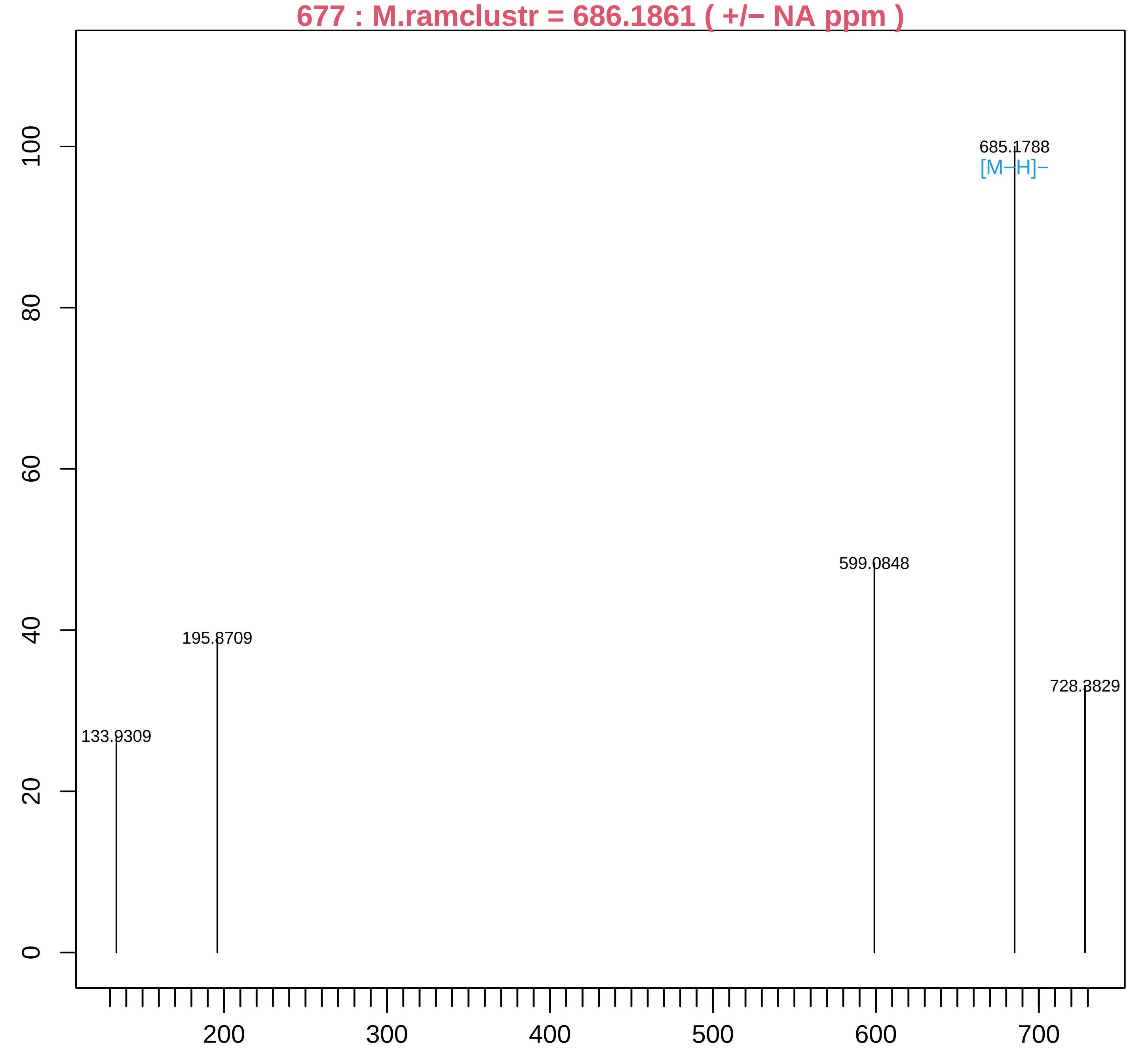
677 : M.ramclustr = 686.1861 (+/− NA ppm)
685.1788
[M−H]−
599.0848
195.8709
728.3829
133.9309

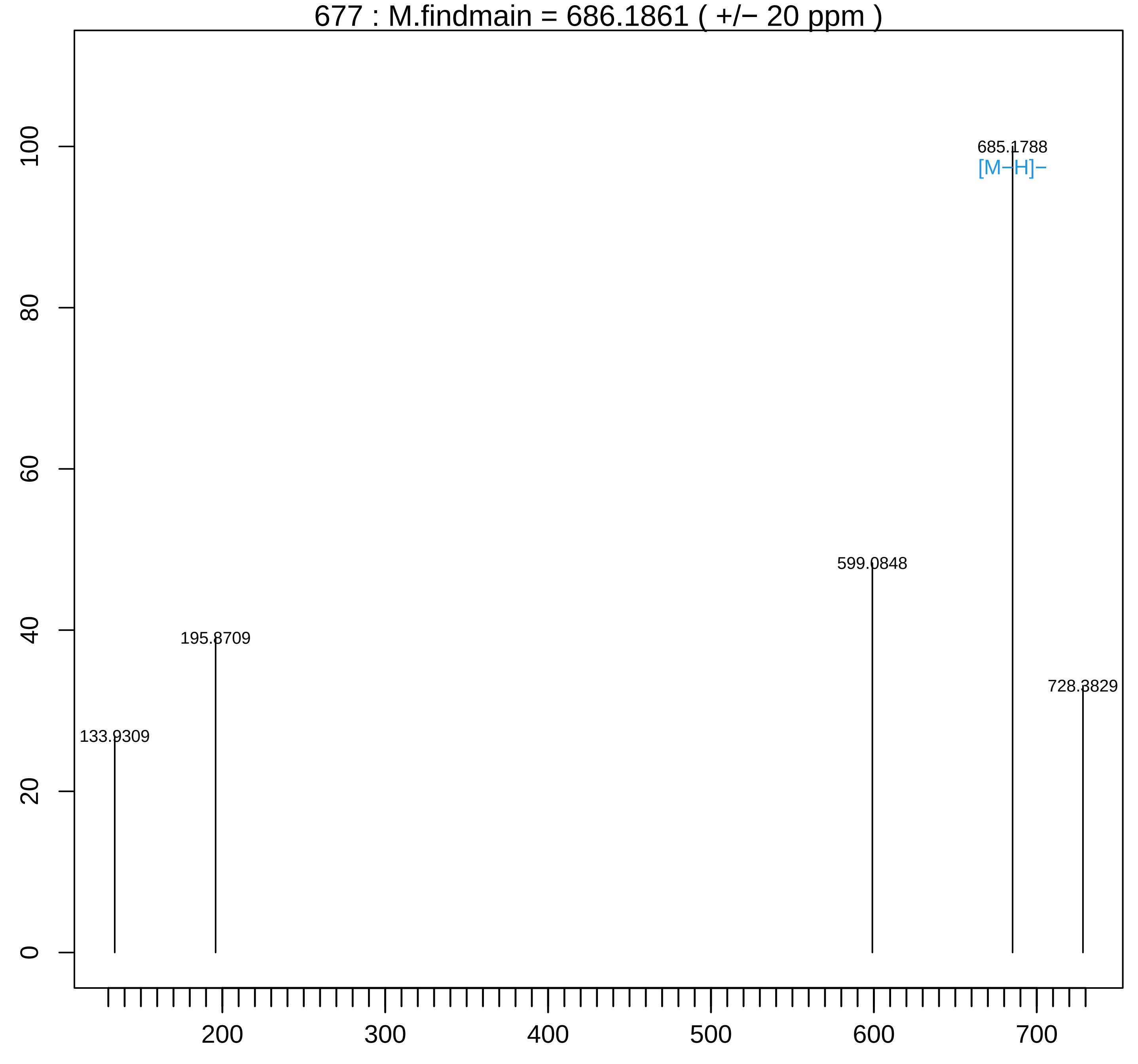
677 : M.findmain = 686.1861 (+/− 20 ppm)
685.1788
[M−H]−
599.0848
195.8709
728.3829
133.9309

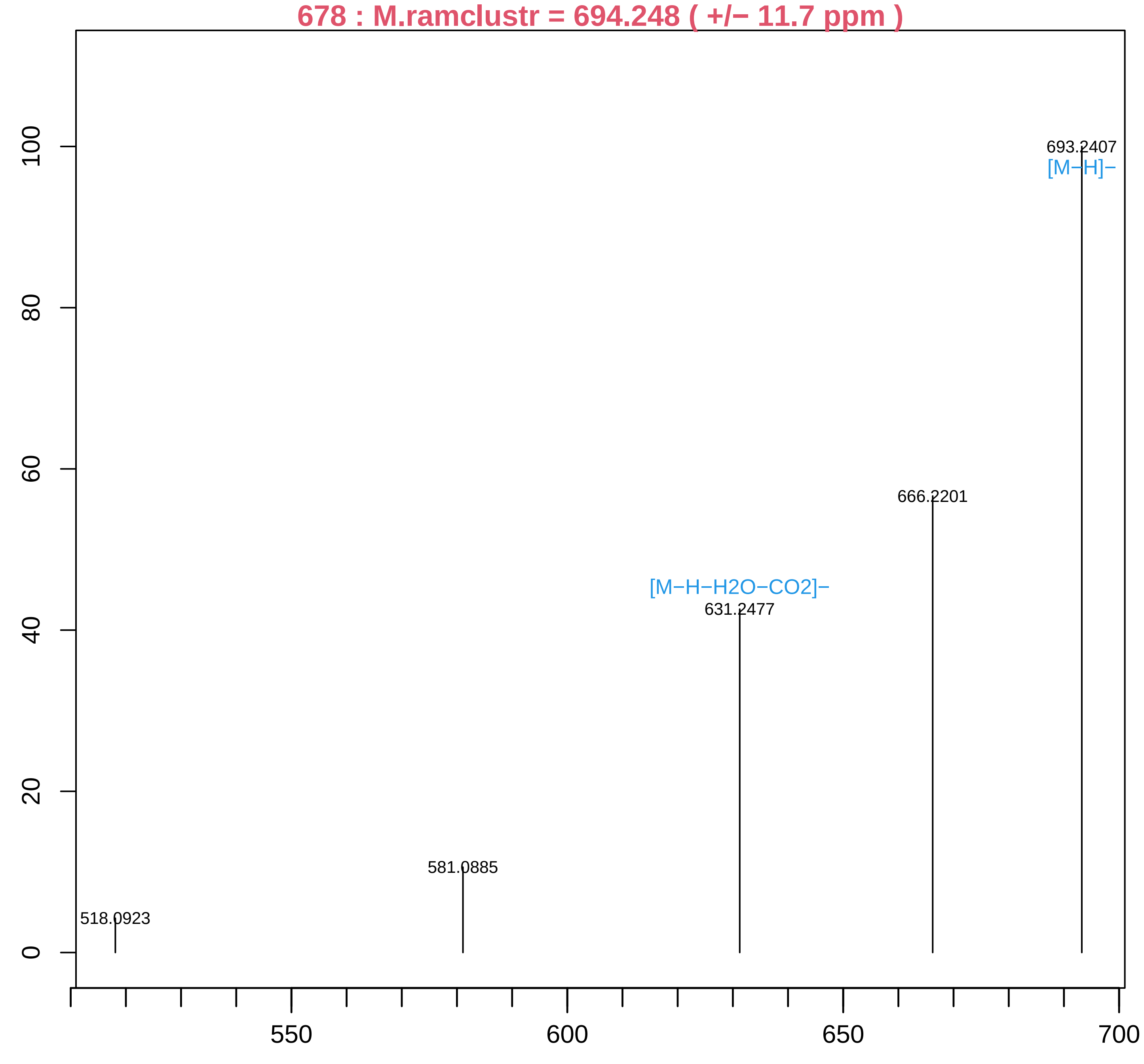
678 : M.ramclustr = 694.248 (+/− 11.7 ppm)
693.2407
[M−H]−
666.2201
[M−H−H2O−CO2]−
631.2477
581.0885
518.0923
0
20
40
60
80
100
550
600
650
700

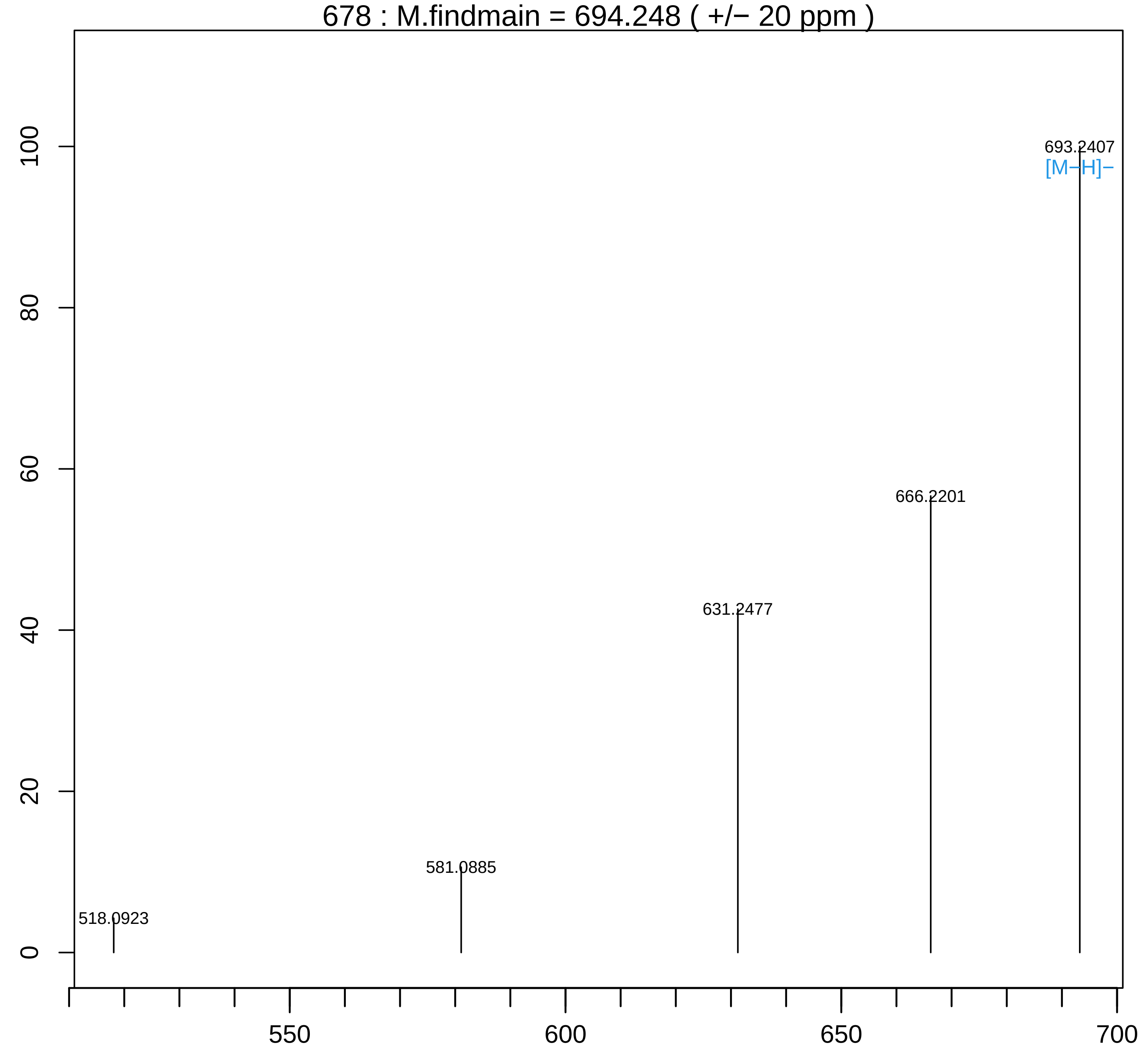
678 : M.findmain = 694.248 (+/− 20 ppm)
693.2407
[M−H]−
666.2201
631.2477
581.0885
518.0923
0
20
40
60
80
100
550
600
650
700

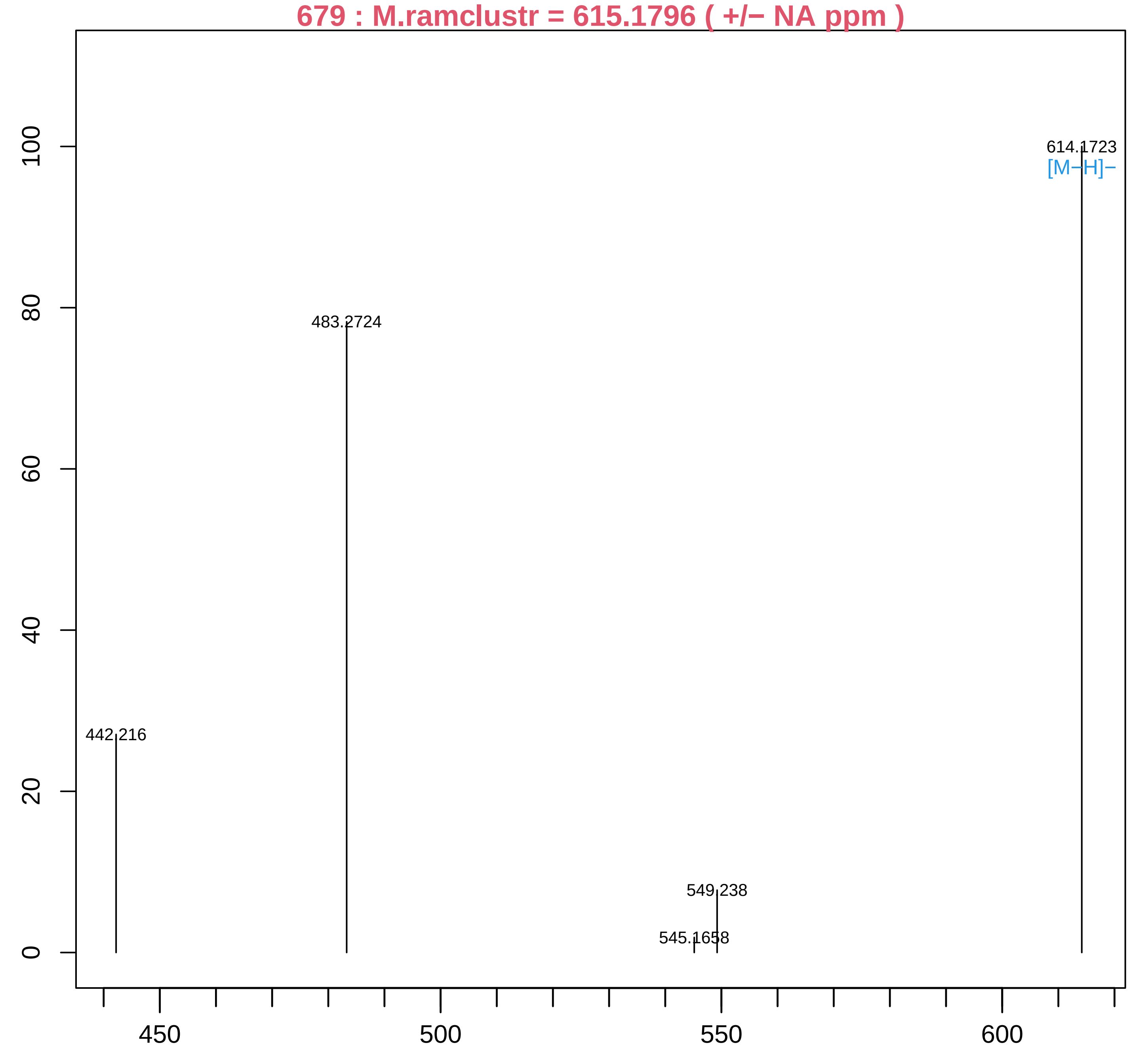
679 : M.ramclustr = 615.1796 (+/− NA ppm)
614.1723
[M−H]−
483.2724
442.216
549.238
545.1658
0
20
40
60
80
100
450
500
550
600

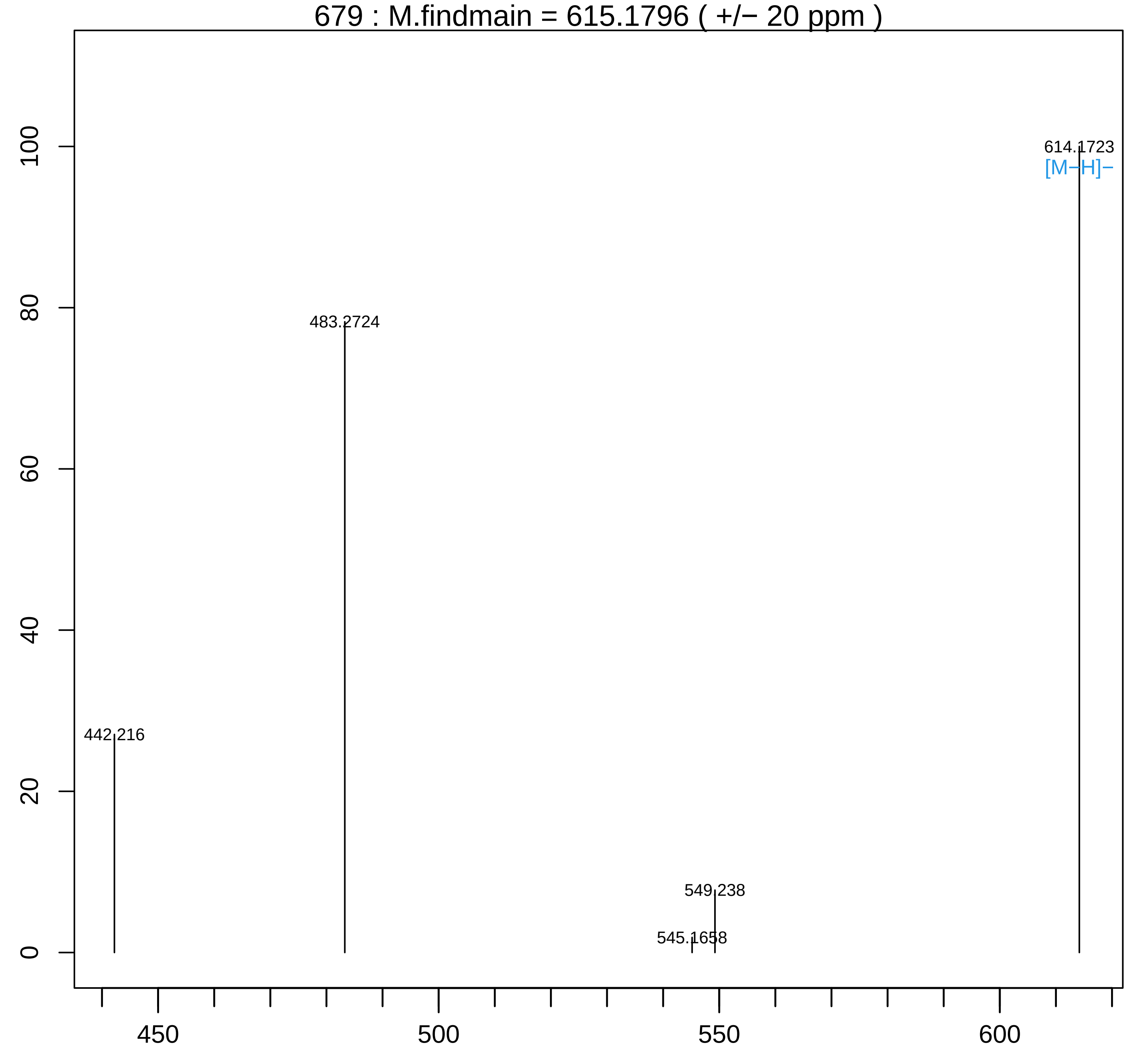
679 : M.findmain = 615.1796 (+/− 20 ppm)
614.1723
[M−H]−
483.2724
442.216
549.238
545.1658
0
20
40
60
80
100
450
500
550
600

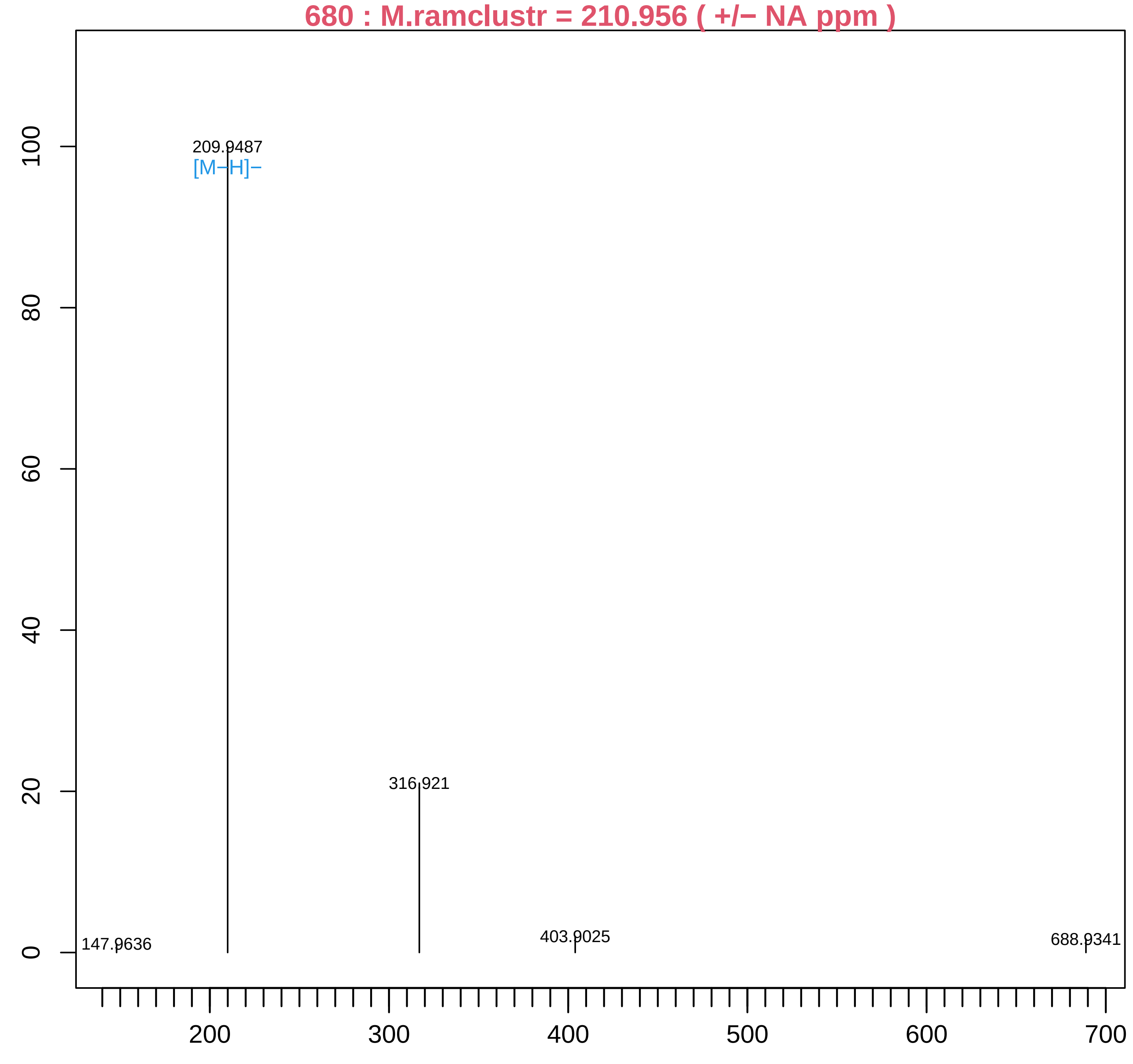
680 : M.ramclustr = 210.956 (+/− NA ppm)
209.9487
[M−H]−
316.921
147.9636
403.9025
688.9341
0
20
40
60
80
100
200
300
400
500
600
700

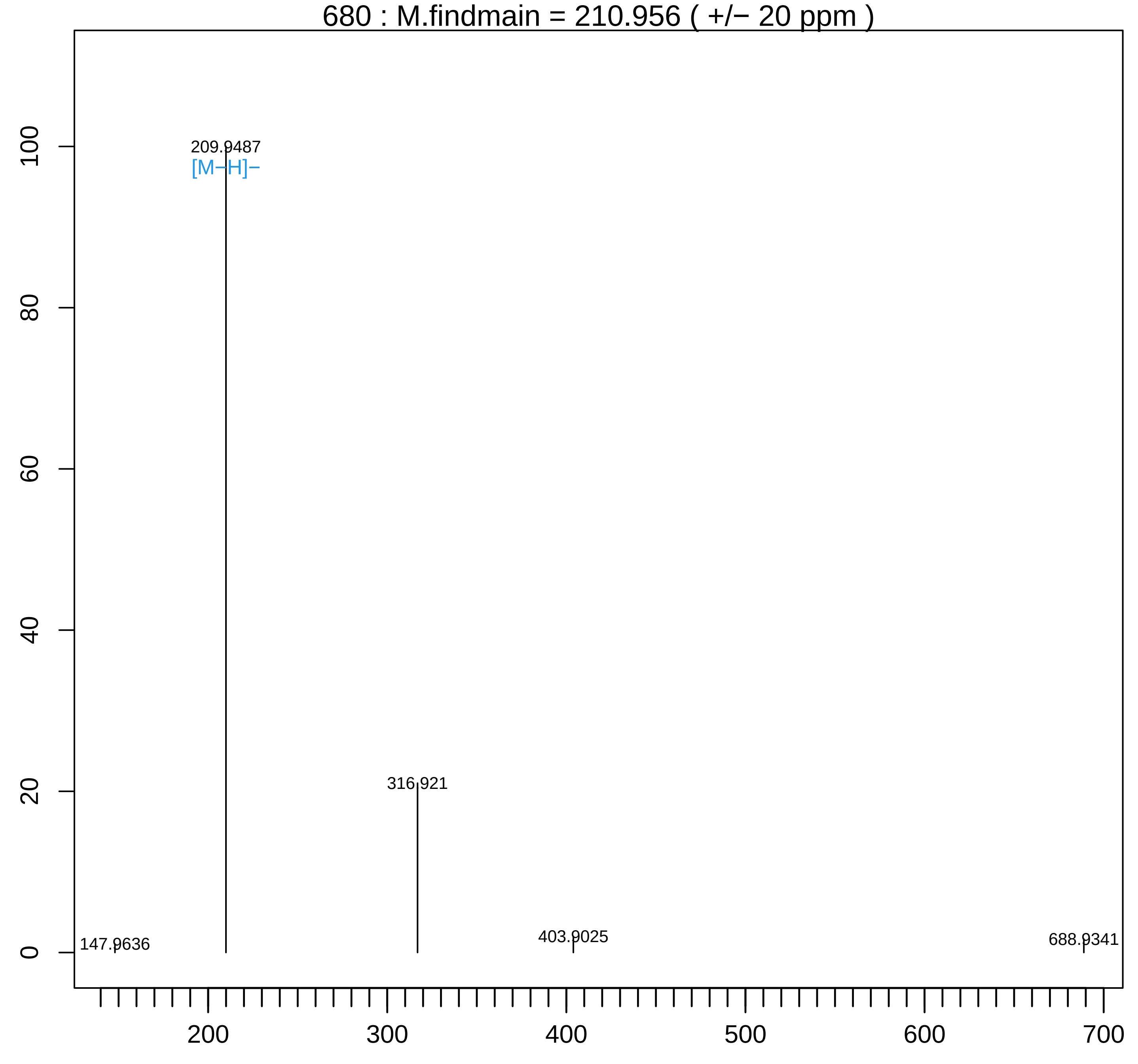
680 : M.findmain = 210.956 (+/− 20 ppm)
209.9487
[M−H]−
316.921
147.9636
403.9025
688.9341
0
20
40
60
80
100
200
300
400
500
600
700

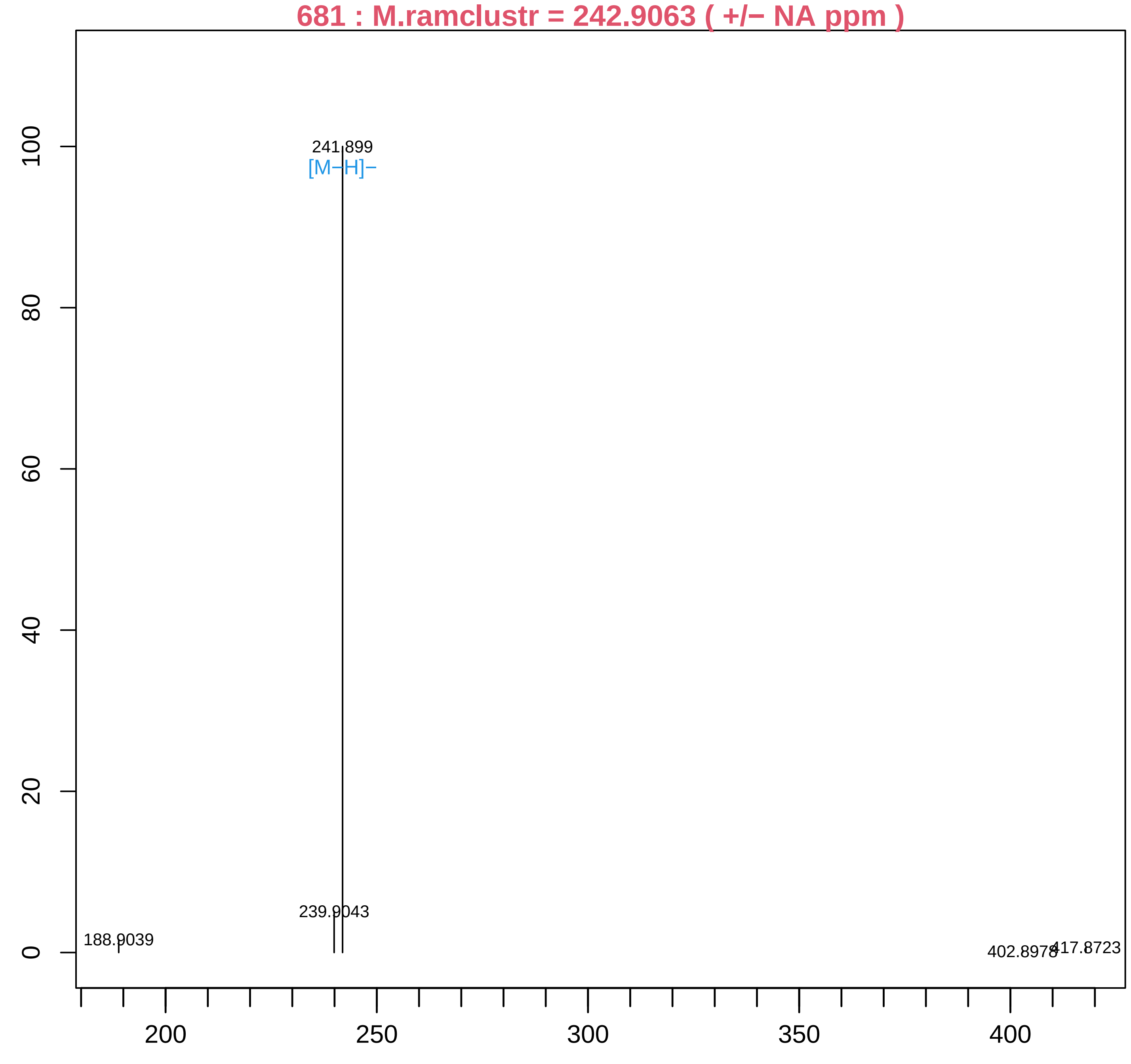
681 : M.ramclustr = 242.9063 (+/− NA ppm)
241.899
[M−H]−
239.9043
188.9039
402.8978
417.8723
0
20
40
60
80
100
200
250
300
350
400

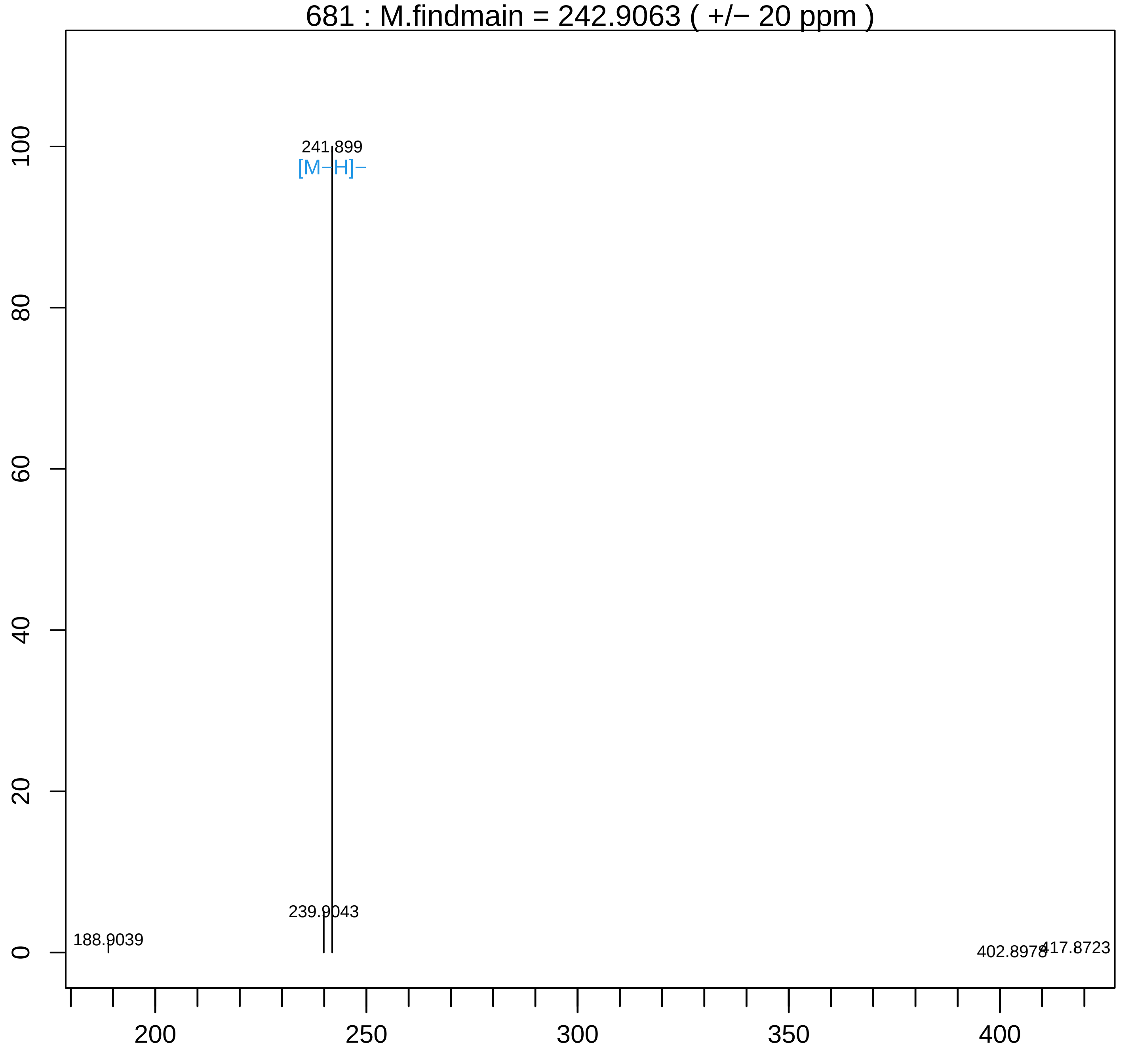
681 : M.findmain = 242.9063 (+/− 20 ppm)
241.899
[M−H]−
239.9043
188.9039
402.8978
417.8723
0
20
40
60
80
100
200
250
300
350
400

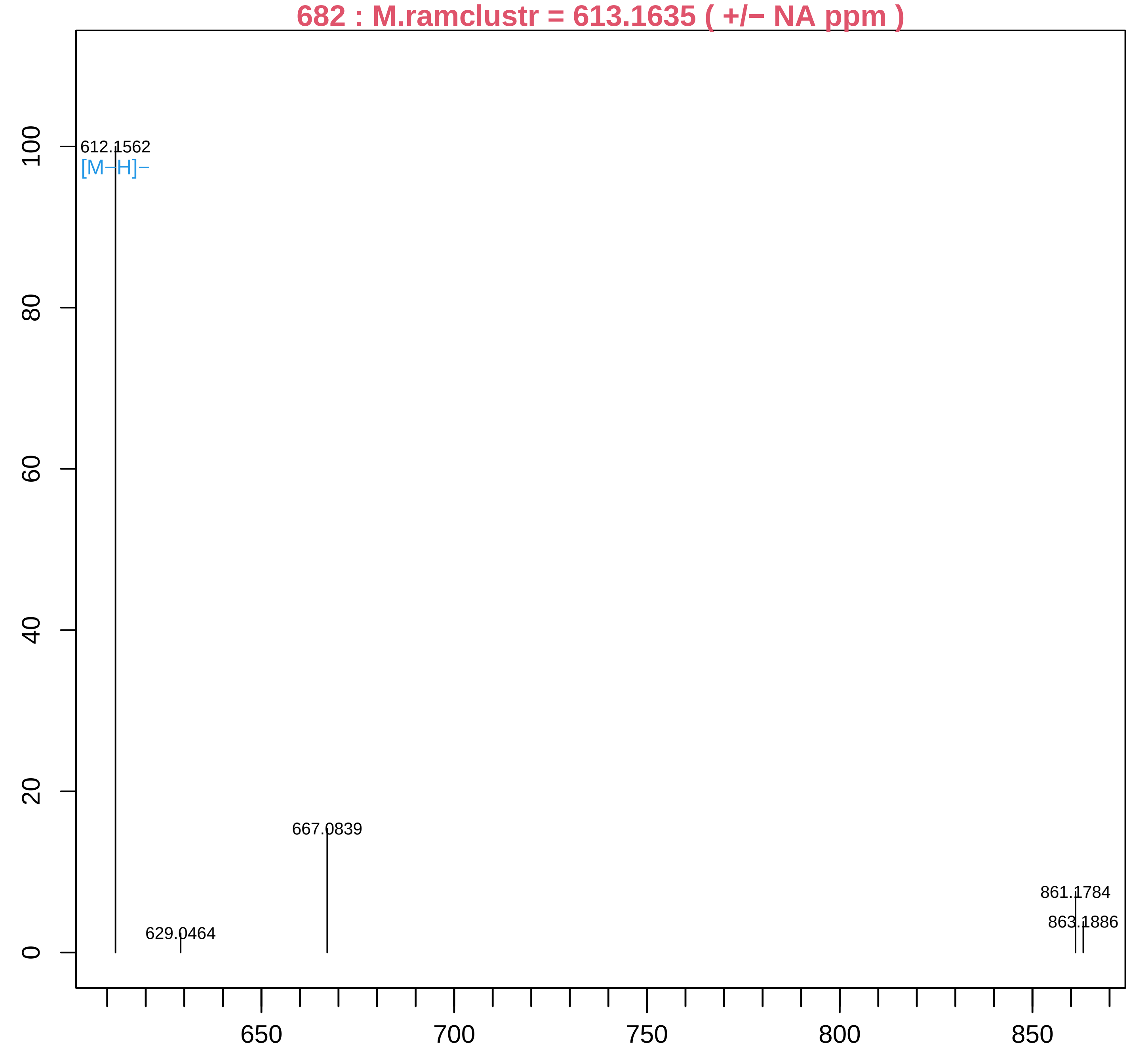
682 : M.ramclustr = 613.1635 (+/− NA ppm)
612.1562
[M−H]−
667.0839
629.0464
861.1784
863.1886
0
20
40
60
80
100
650
700
750
800
850

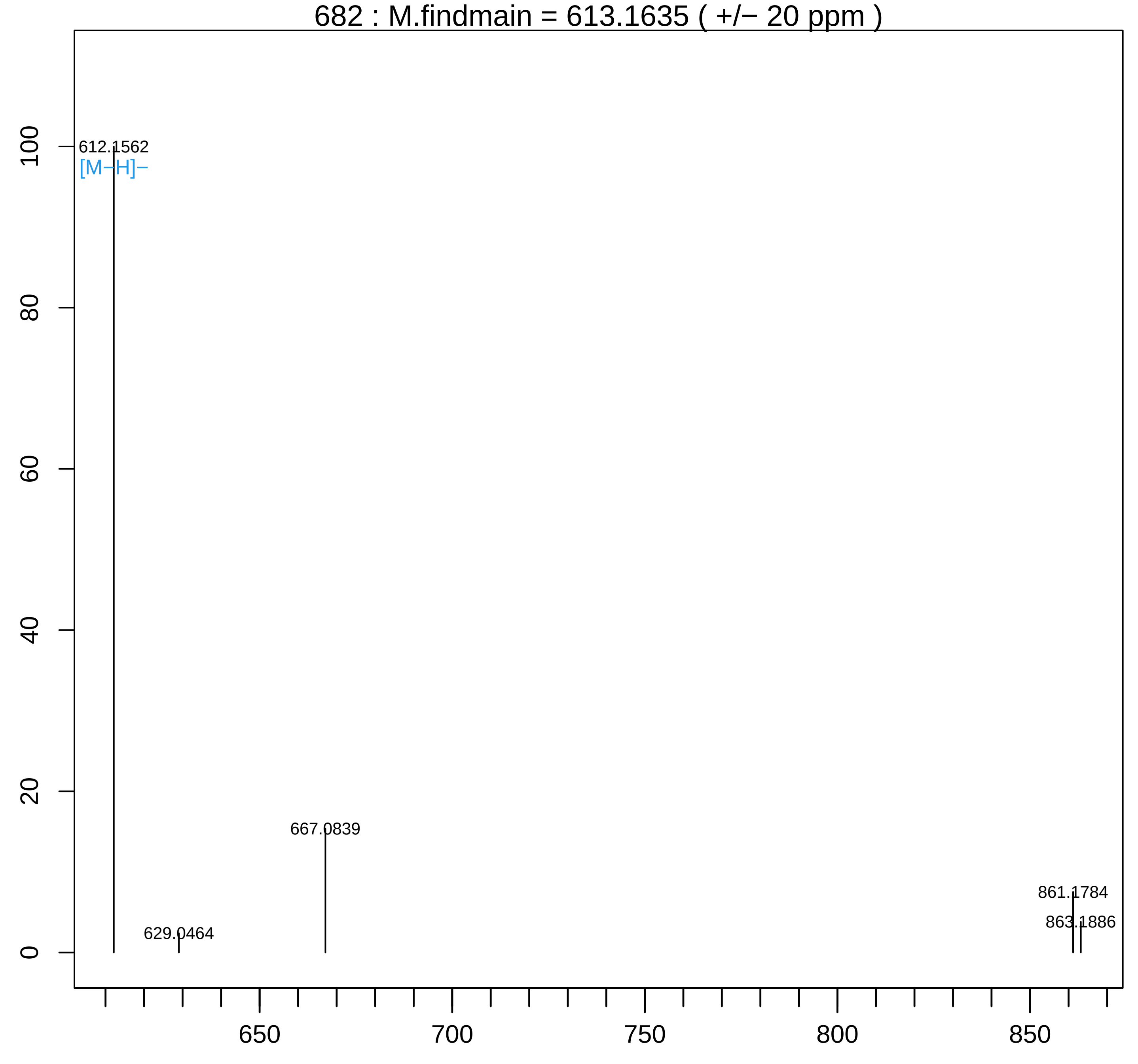
682 : M.findmain = 613.1635 (+/− 20 ppm)
612.1562
[M−H]−
667.0839
629.0464
861.1784
863.1886
0
20
40
60
80
100
650
700
750
800
850

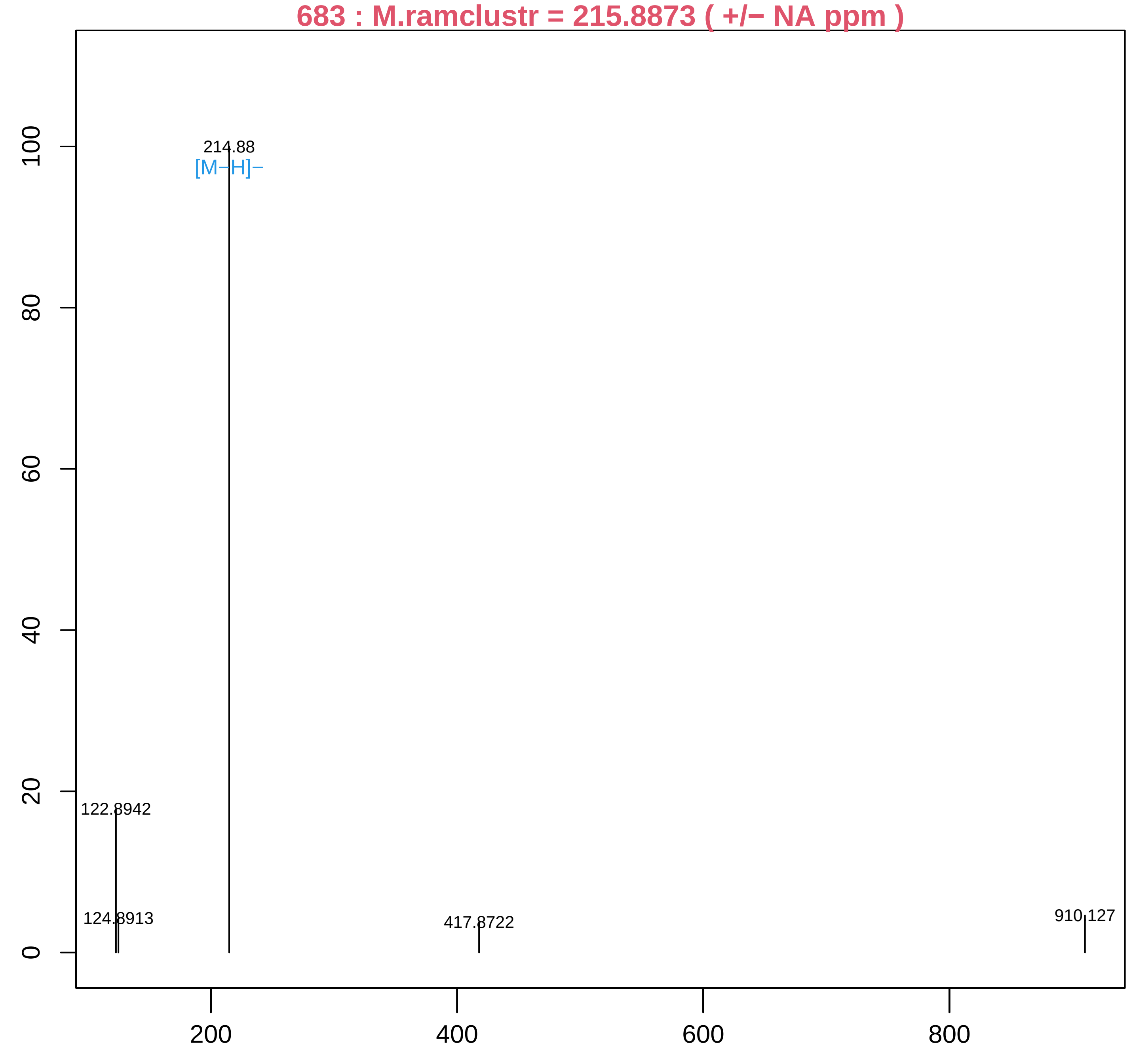
683 : M.ramclustr = 215.8873 (+/− NA ppm)
214.88
[M−H]−
122.8942
124.8913
417.8722
910.127
0
20
40
60
80
100
200
400
600
800

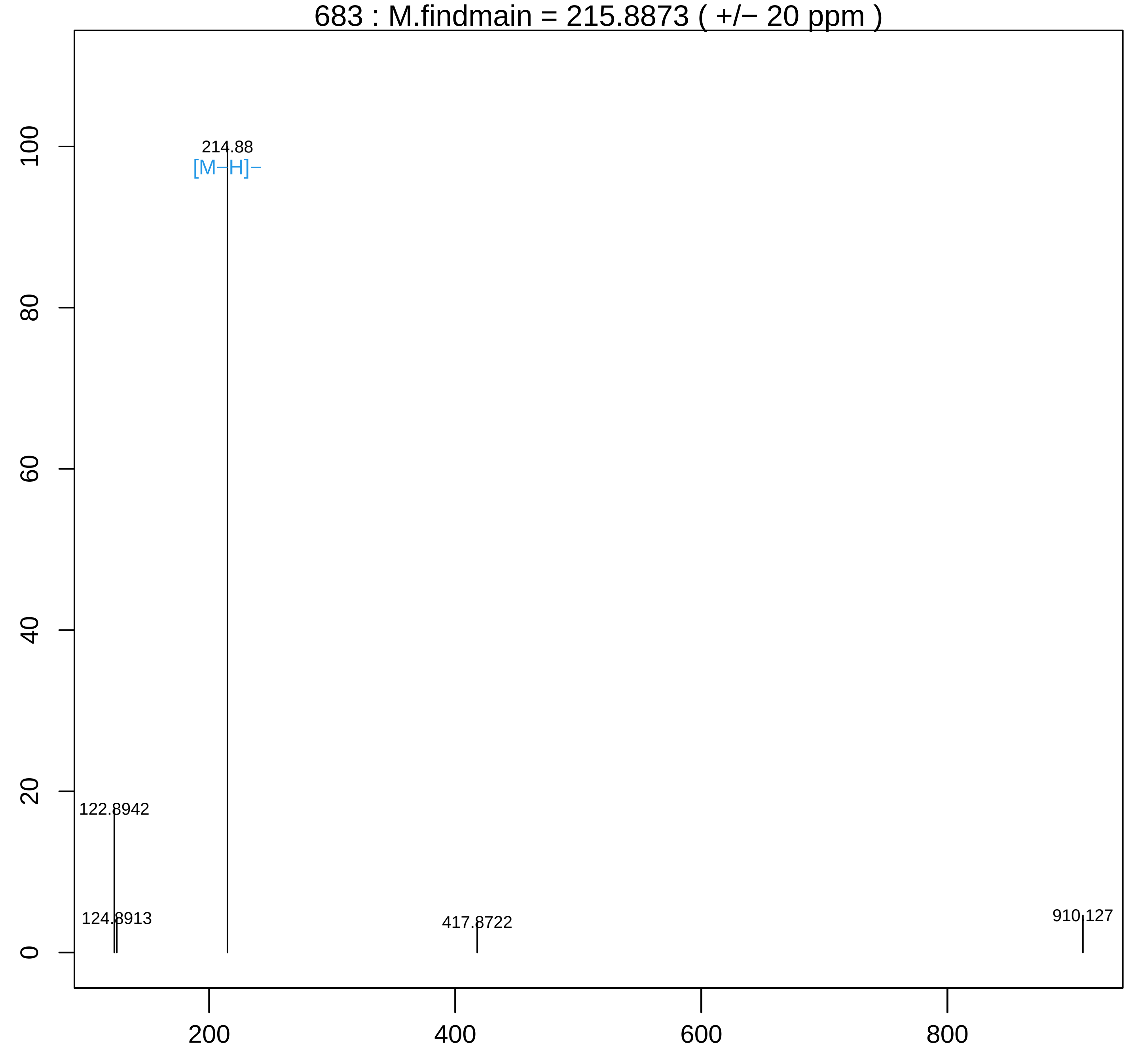
683 : M.findmain = 215.8873 (+/− 20 ppm)
214.88
[M−H]−
122.8942
124.8913
417.8722
910.127
0
20
40
60
80
100
200
400
600
800

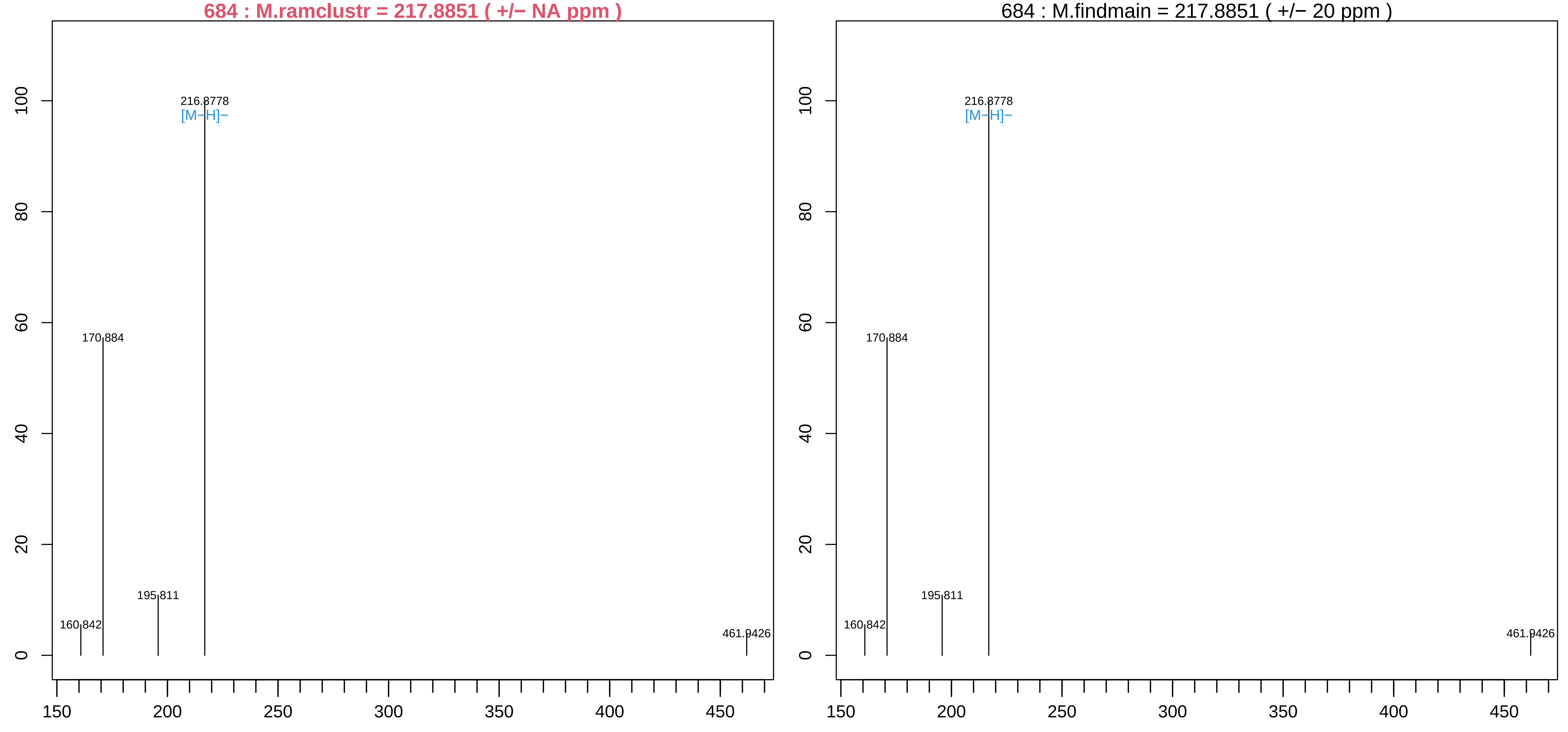
684 : M.ramclustr = 217.8851 (+/− NA ppm)
684 : M.findmain = 217.8851 (+/− 20 ppm)
216.8778
[M−H]−
170.884
195.811
160.842
461.9426
0
20
40
60
80
100
150
200
250
300
350
400
450

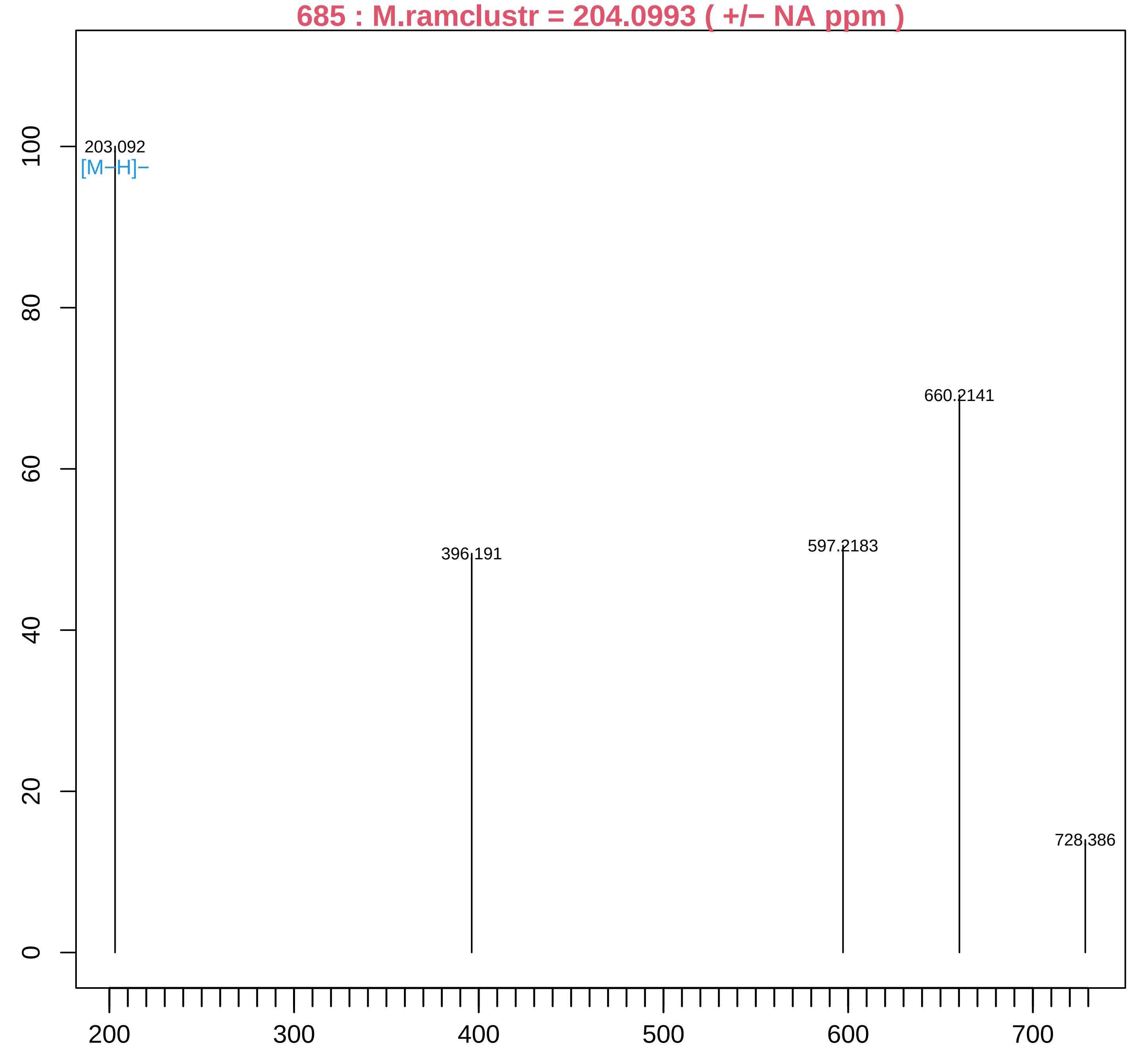
685 : M.ramclustr = 204.0993 (+/− NA ppm)
203.092
[M−H]−
396.191
597.2183
660.2141
728.386
0
20
40
60
80
100
200
300
400
500
600
700

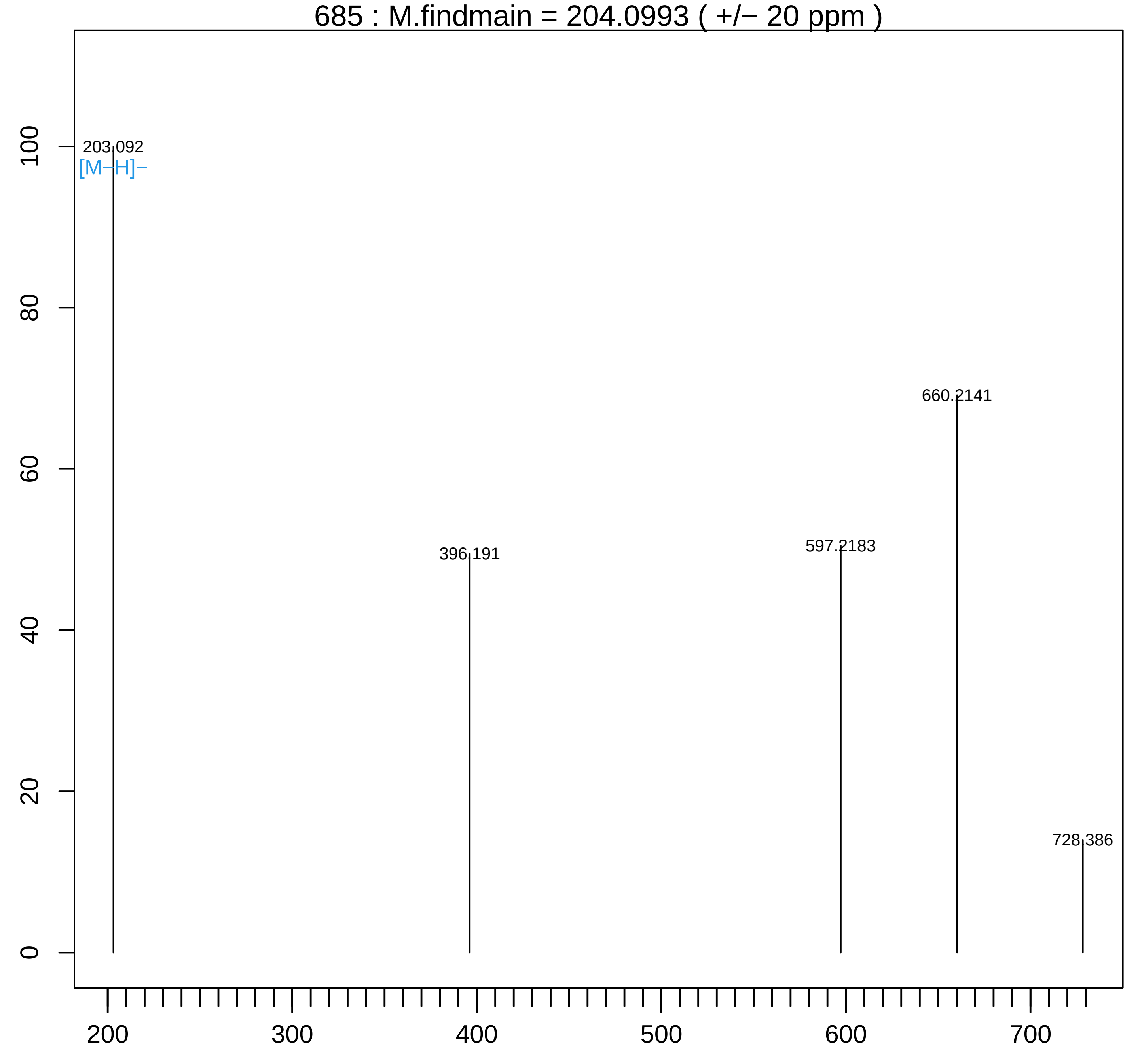
685 : M.findmain = 204.0993 (+/− 20 ppm)
203.092
[M−H]−
396.191
597.2183
660.2141
728.386
0
20
40
60
80
100
200
300
400
500
600
700

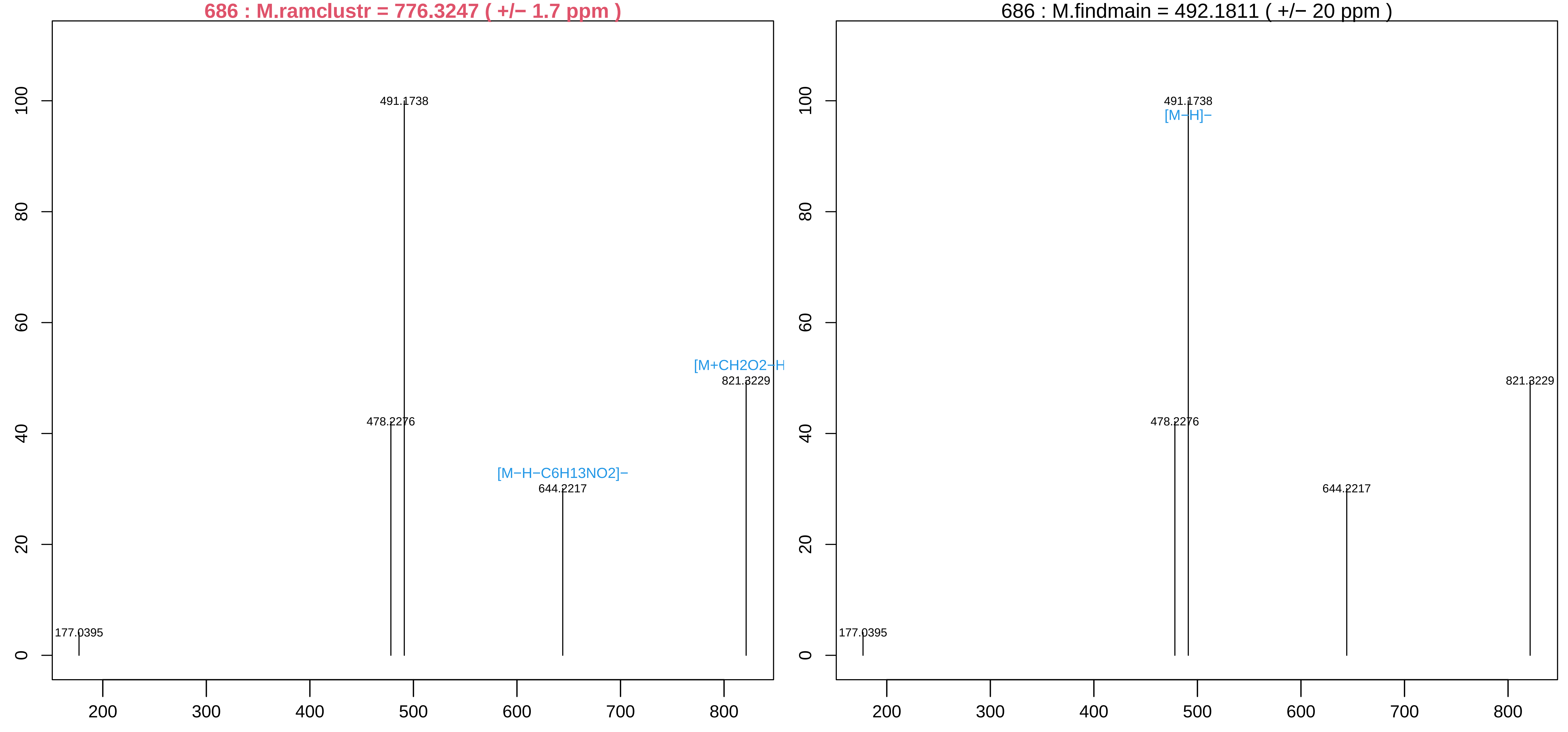

686 : M.ramclustr = 776.3247 (+/− 1.7 ppm)
491.1738
478.2276
644.2217
[M−H−C6H13NO2]−
821.3229
[M+CH2O2−H
177.0395
686 : M.findmain = 492.1811 (+/− 20 ppm)
491.1738
[M−H]−
478.2276
644.2217
821.3229
177.0395
0
20
40
60
80
100
200
300
400
500
600
700
800

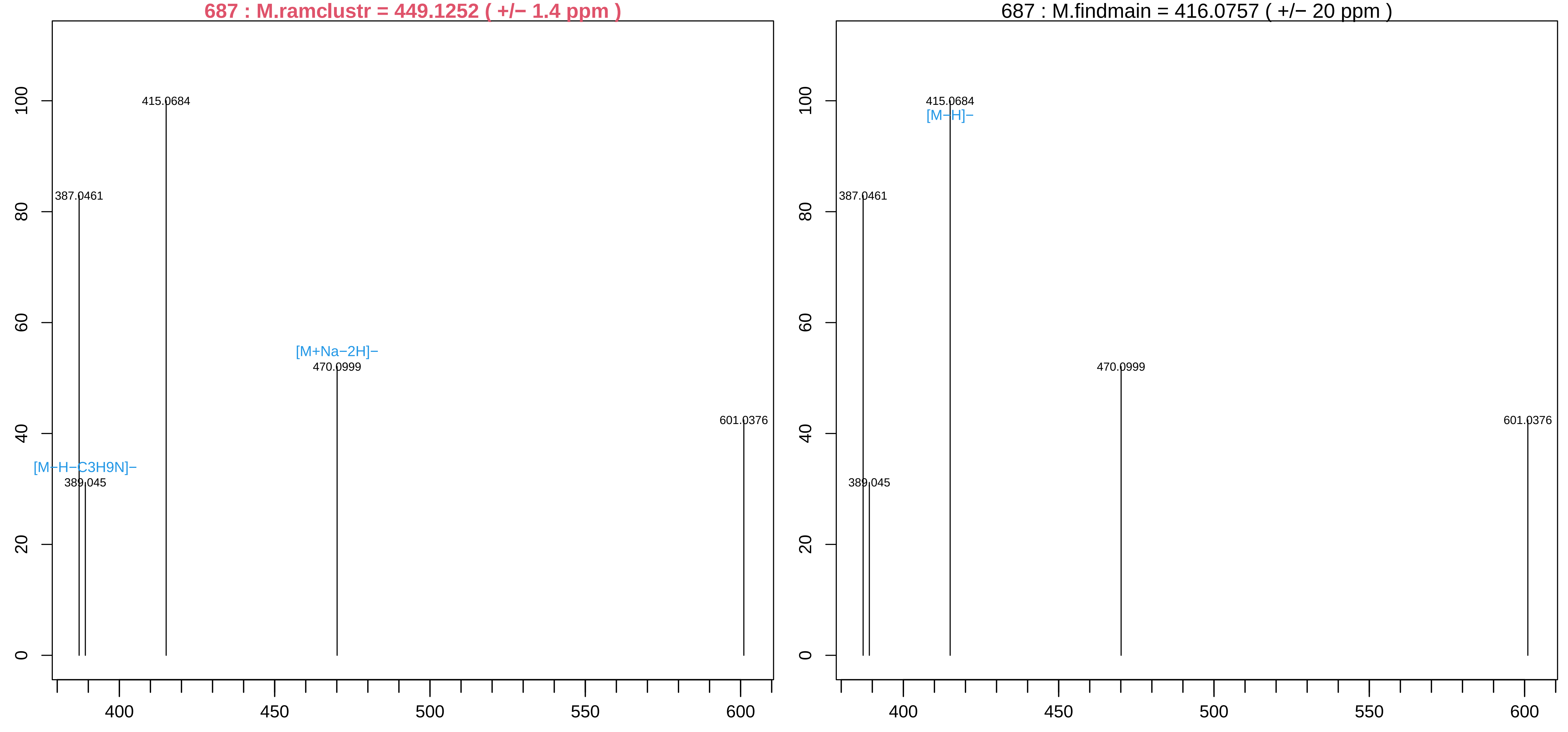
687 : M.ramclustr = 449.1252 (+/− 1.4 ppm)
415.0684
387.0461
[M+Na−2H]−
470.0999
601.0376
[M−H−C3H9N]−
389.045
687 : M.findmain = 416.0757 (+/− 20 ppm)
415.0684
[M−H]−
387.0461
470.0999
601.0376
389.045
0
20
40
60
80
100
400
450
500
550
600

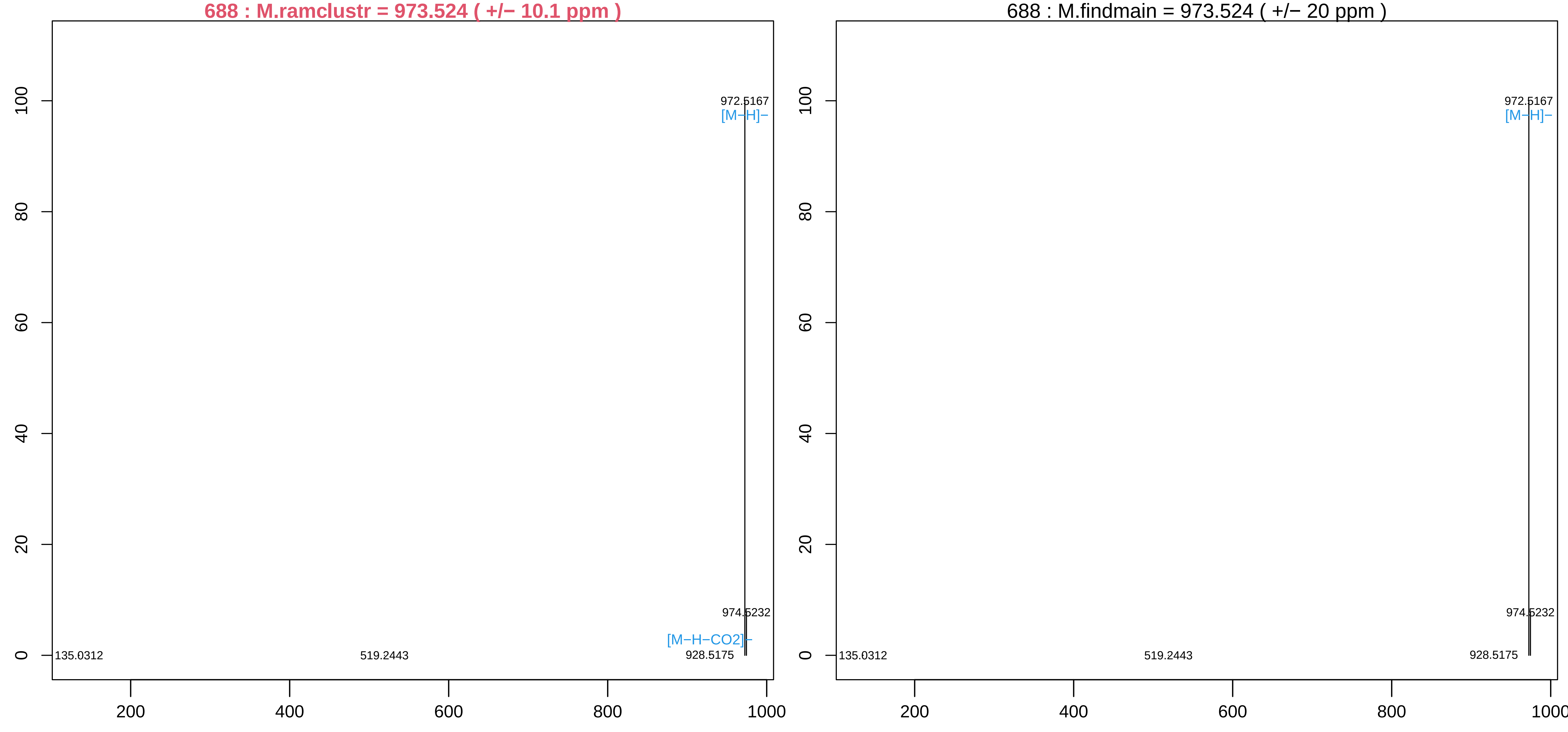
688 : M.ramclustr = 973.524 (+/− 10.1 ppm)
972.5167
[M−H]−
974.5232
[M−H−CO2]−
135.0312
519.2443
928.5175
0
20
40
60
80
100
200
400
600
800
1000
688 : M.findmain = 973.524 (+/− 20 ppm)
972.5167
[M−H]−
974.5232
135.0312
519.2443
928.5175
0
20
40
60
80
100
200
400
600
800
1000

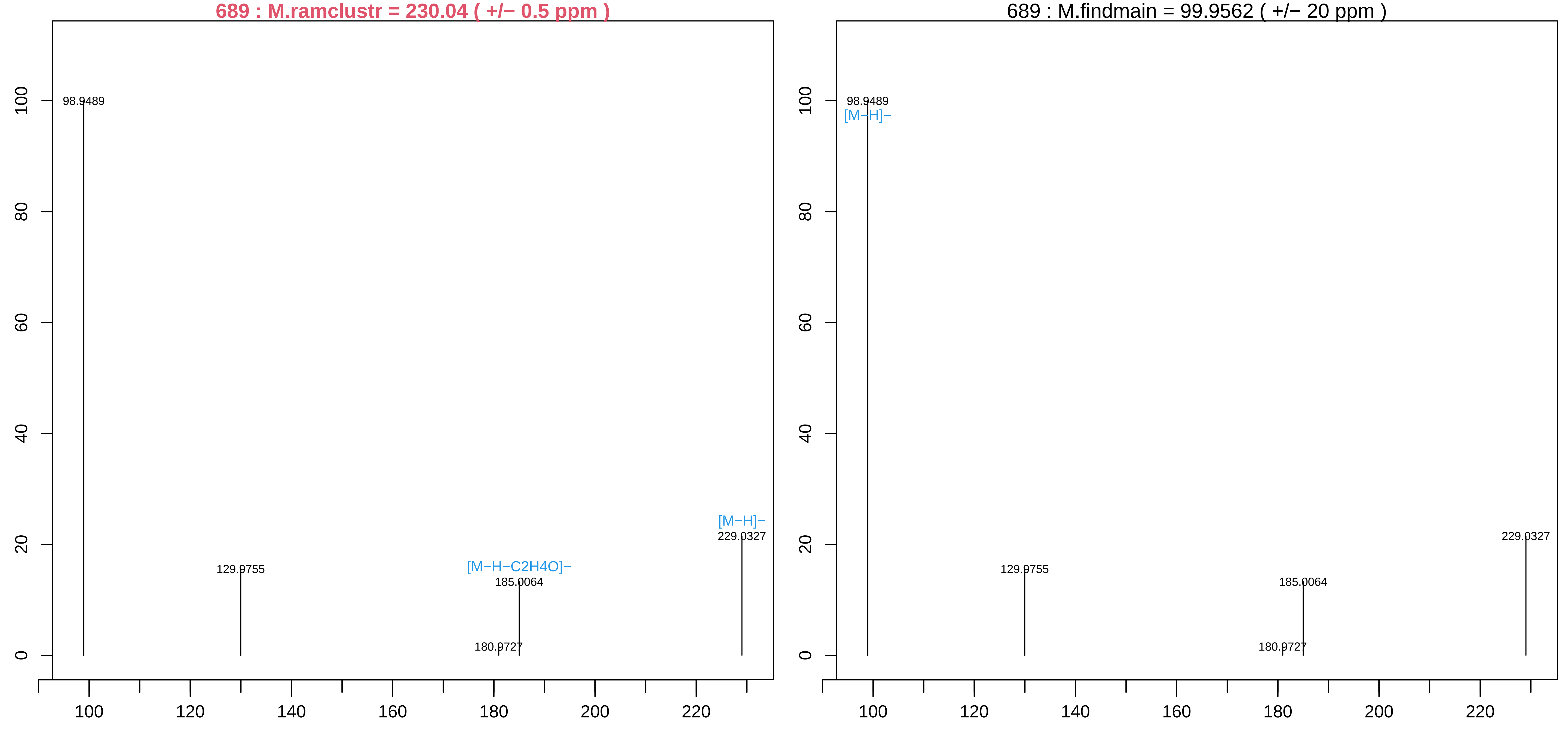

689 : M.ramclustr = 230.04 (+/− 0.5 ppm)
98.9489
129.9755
[M−H−C2H4O]−
185.0064
180.9727
[M−H]−
229.0327
689 : M.findmain = 99.9562 (+/− 20 ppm)
98.9489
[M−H]−
129.9755
185.0064
180.9727
229.0327

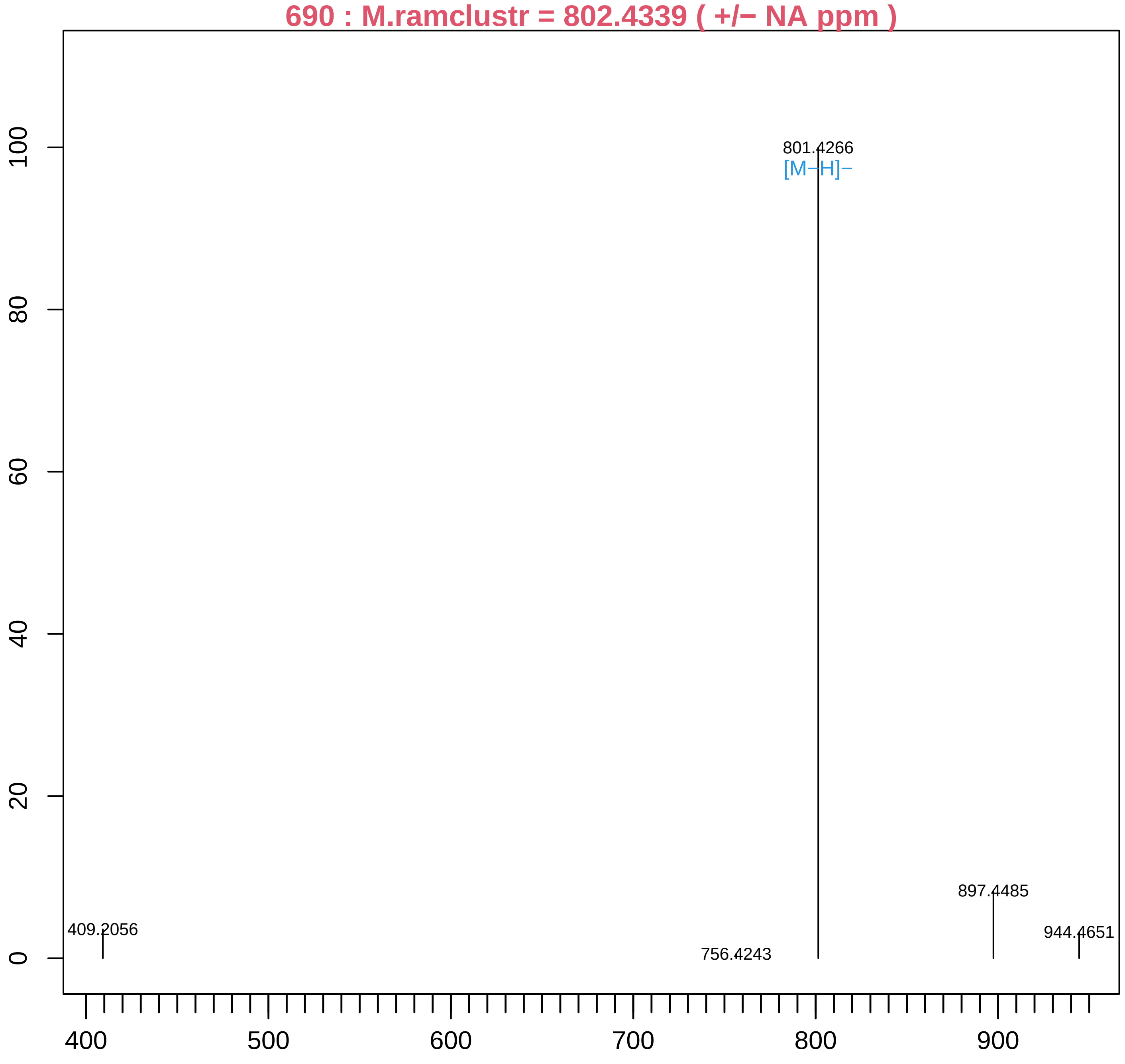
690 : M.ramclustr = 802.4339 (+/− NA ppm)
801.4266
[M−H]−
897.4485
409.2056
756.4243
944.4651
0
20
40
60
80
100
400
500
600
700
800
900

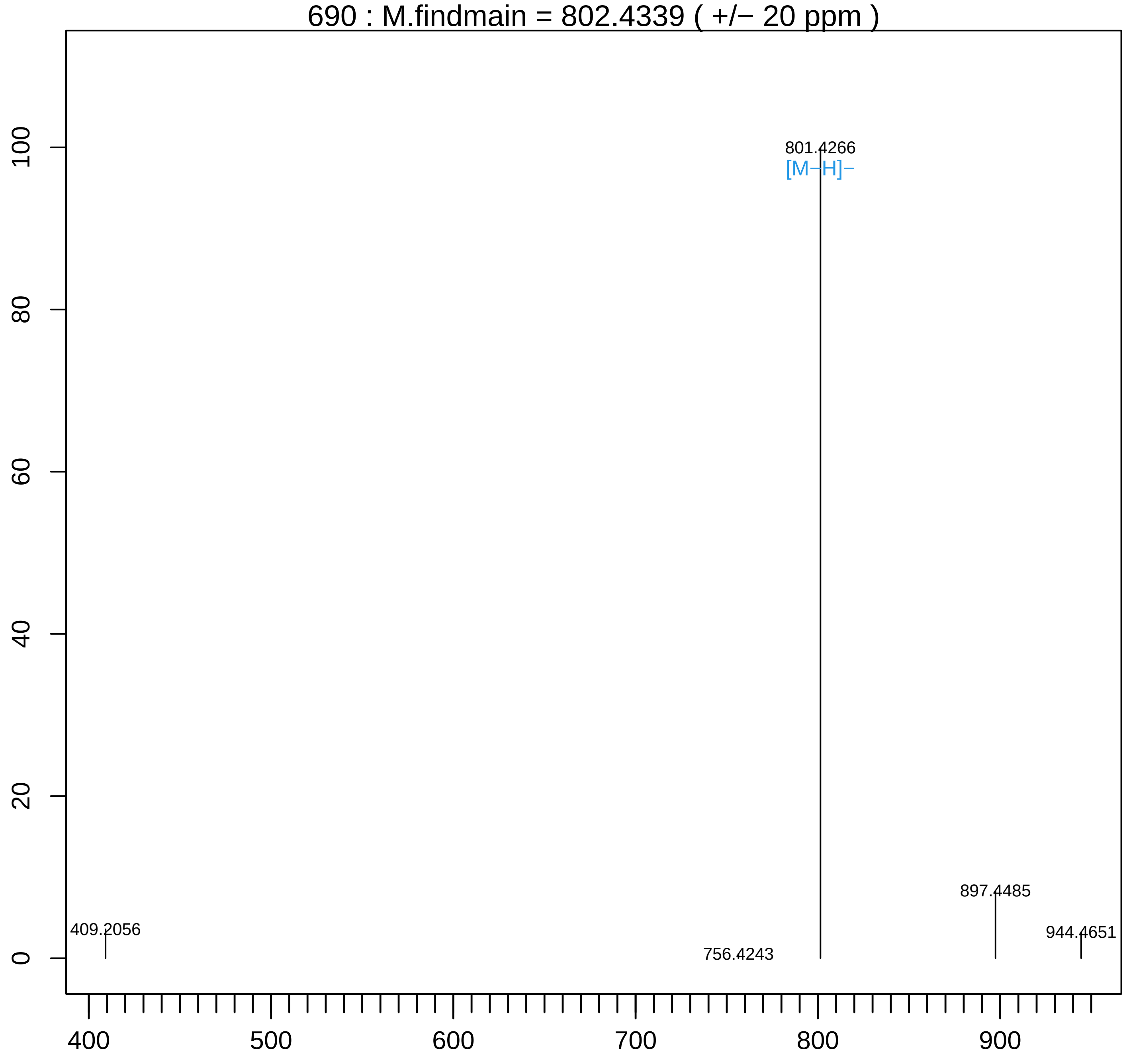
690 : M.findmain = 802.4339 (+/− 20 ppm)
801.4266
[M−H]−
897.4485
409.2056
756.4243
944.4651
0
20
40
60
80
100
400
500
600
700
800
900

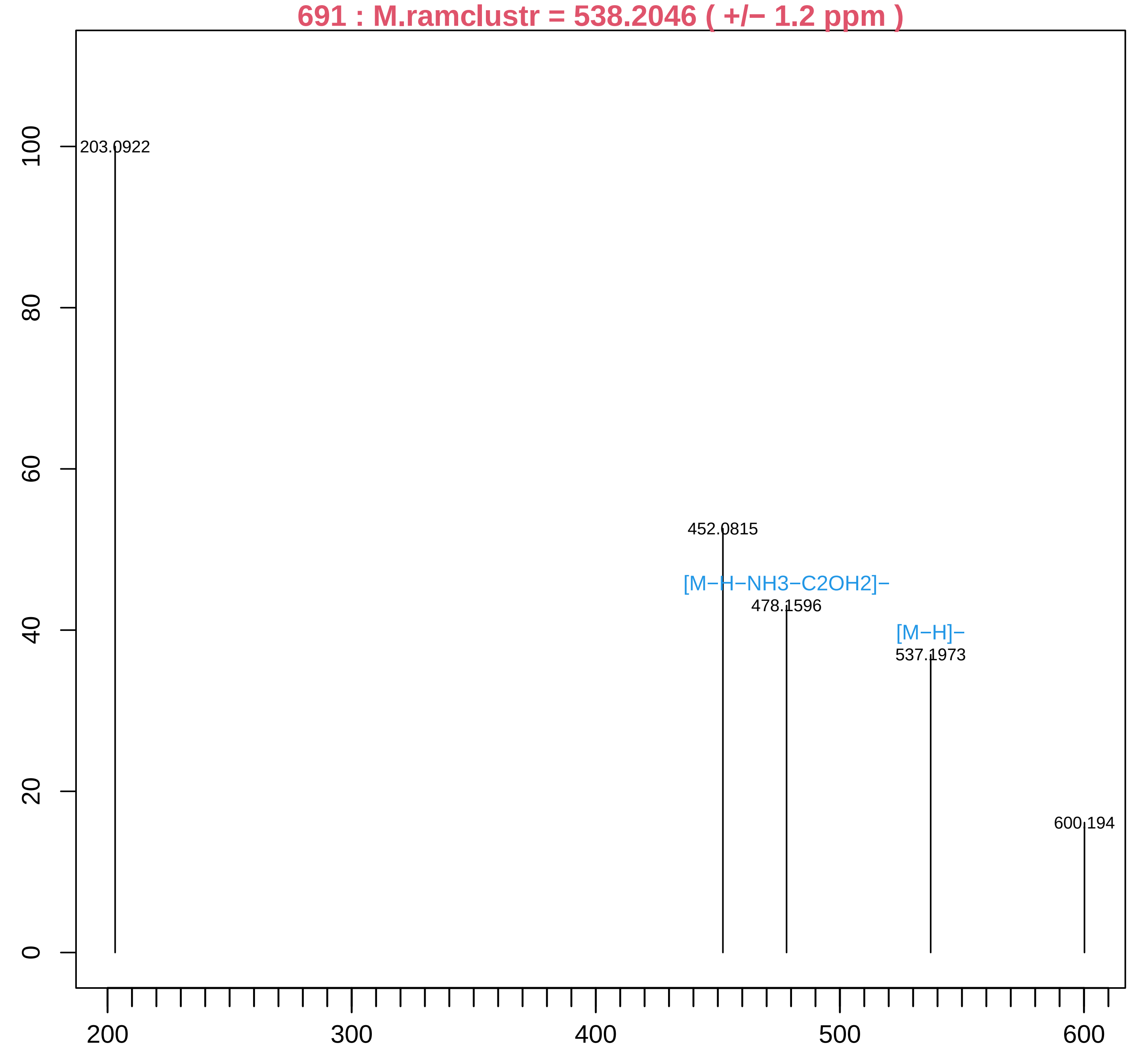
691 : M.ramclustr = 538.2046 (+/− 1.2 ppm)
203.0922
452.0815
[M−H−NH3−C2OH2]−
478.1596
[M−H]−
537.1973
600.194
0
20
40
60
80
100
200
300
400
500
600

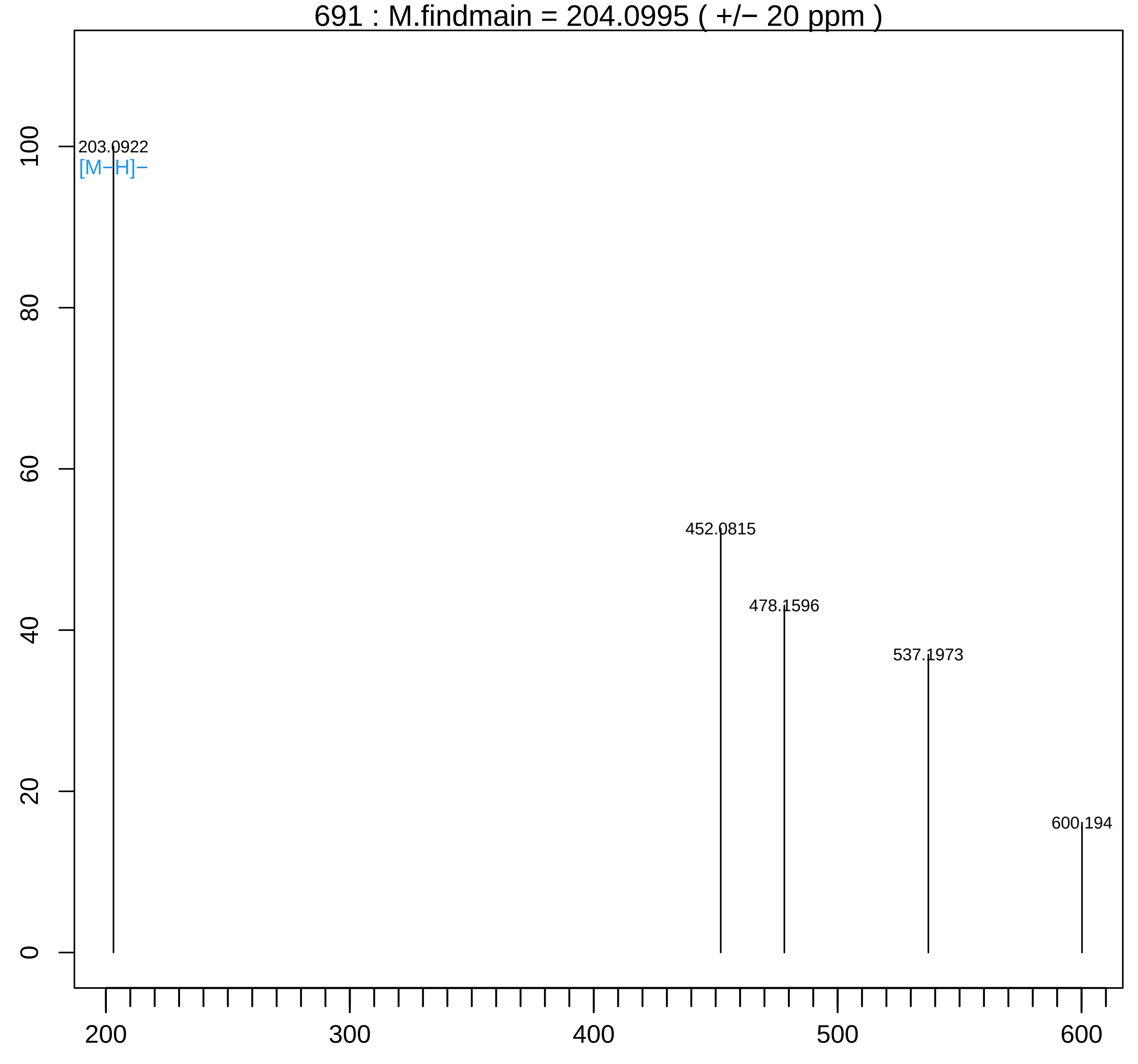
691 : M.findmain = 204.0995 (+/− 20 ppm)
203.0922
[M−H]−
452.0815
478.1596
537.1973
600.194
0
20
40
60
80
100
200
300
400
500
600

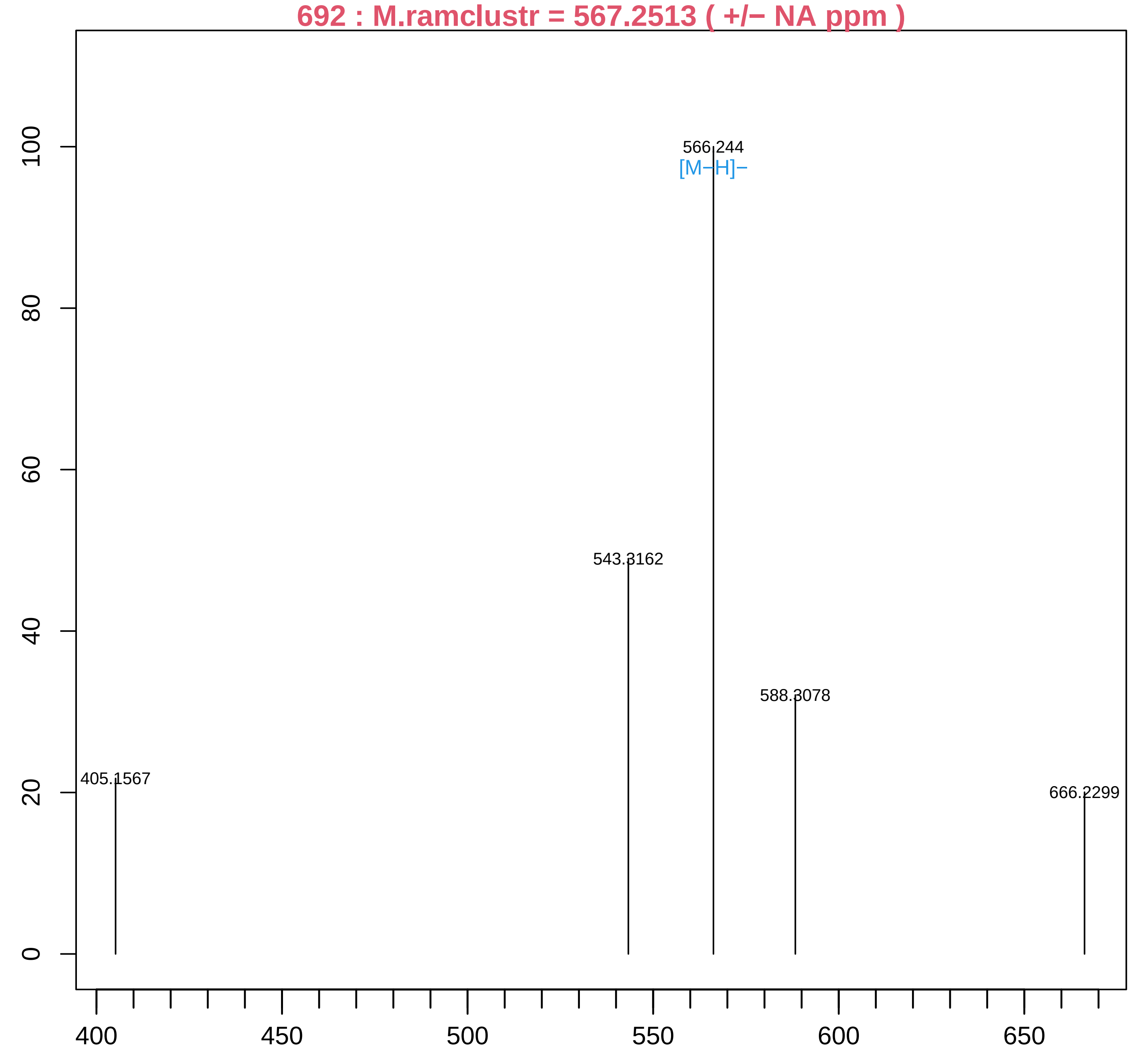
692 : M.ramclustr = 567.2513 (+/− NA ppm)
566.244
[M−H]−
543.3162
588.3078
405.1567
666.2299
0
20
40
60
80
100
400
450
500
550
600
650

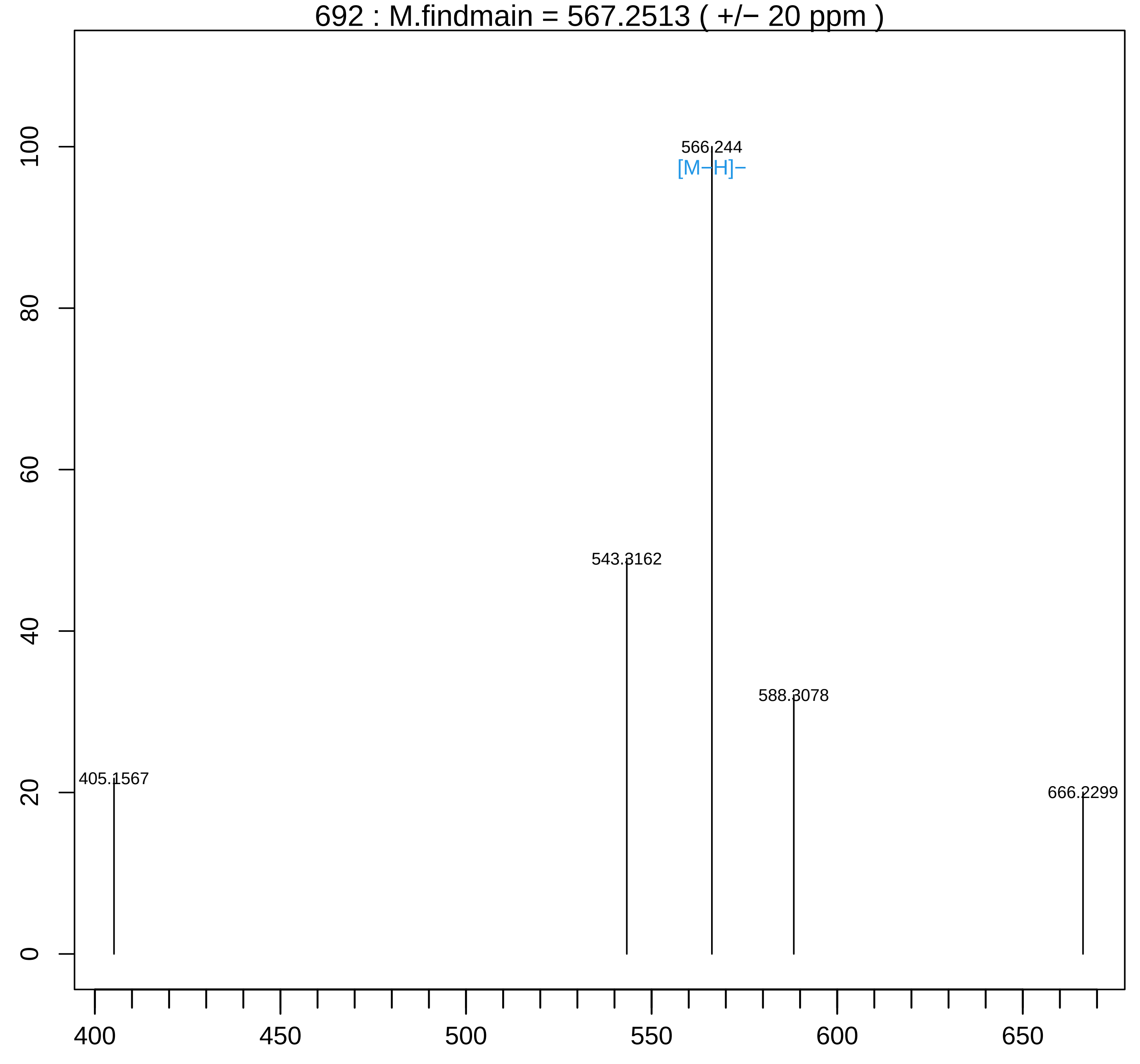
692 : M.findmain = 567.2513 (+/− 20 ppm)
566.244
[M−H]−
543.3162
588.3078
405.1567
666.2299
0
20
40
60
80
100
400
450
500
550
600
650

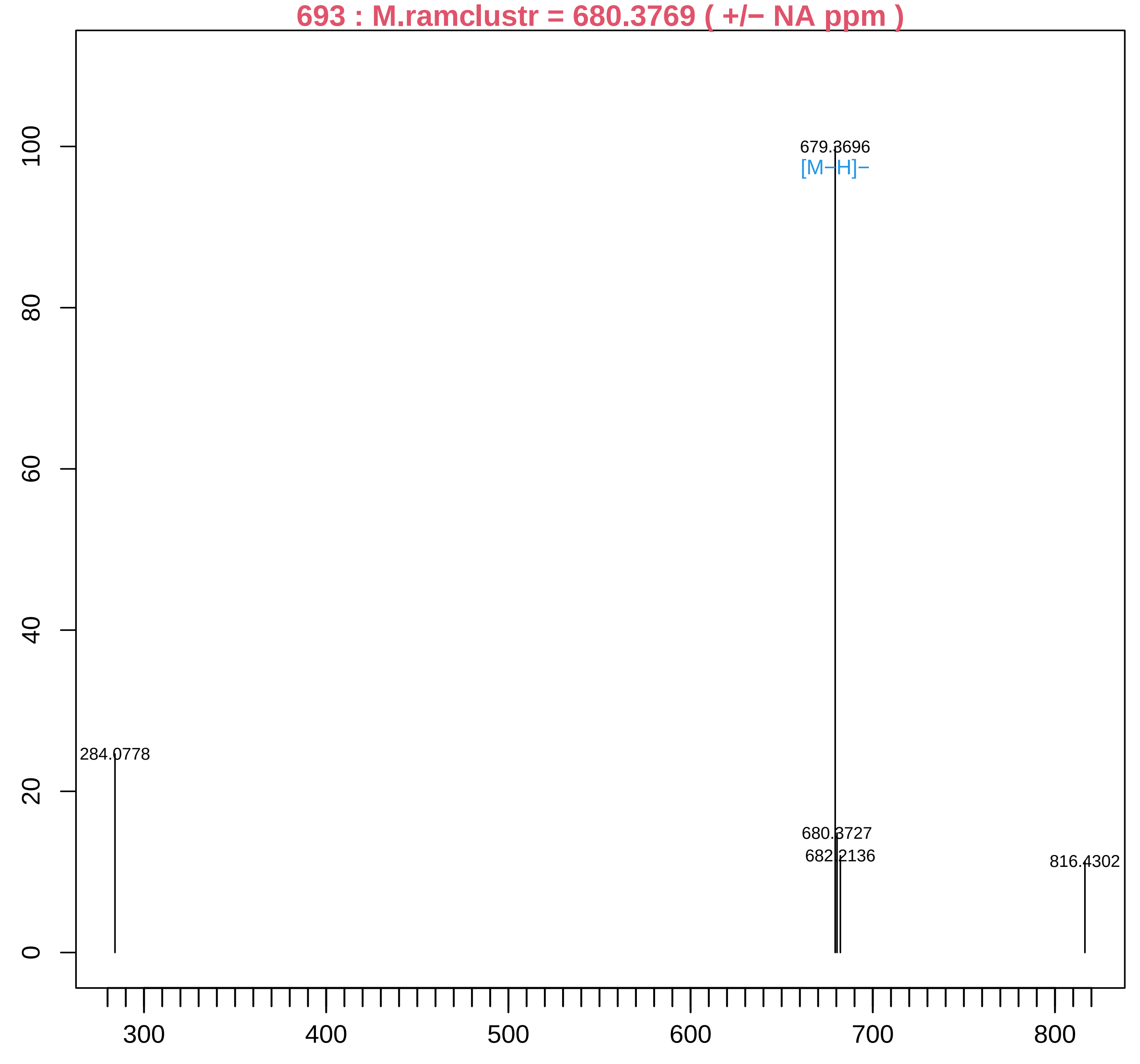
693 : M.ramclustr = 680.3769 (+/− NA ppm)
679.3696
[M−H]−
284.0778
680.3727
682.2136
816.4302
0
20
40
60
80
100
300
400
500
600
700
800
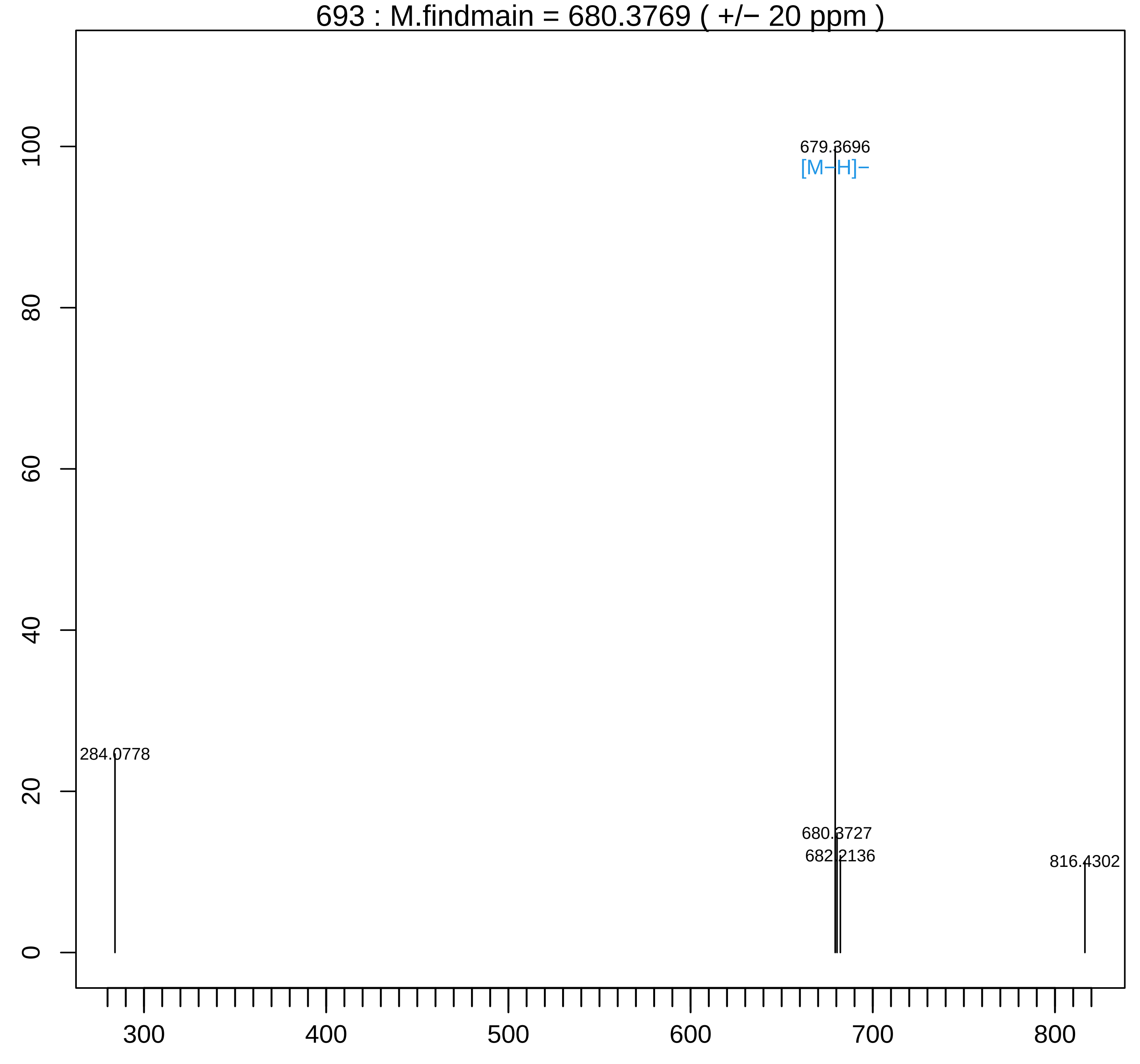
693 : M.findmain = 680.3769 (+/− 20 ppm)
679.3696
[M−H]−
284.0778
680.3727
682.2136
816.4302
0
20
40
60
80
100
300
400
500
600
700
800

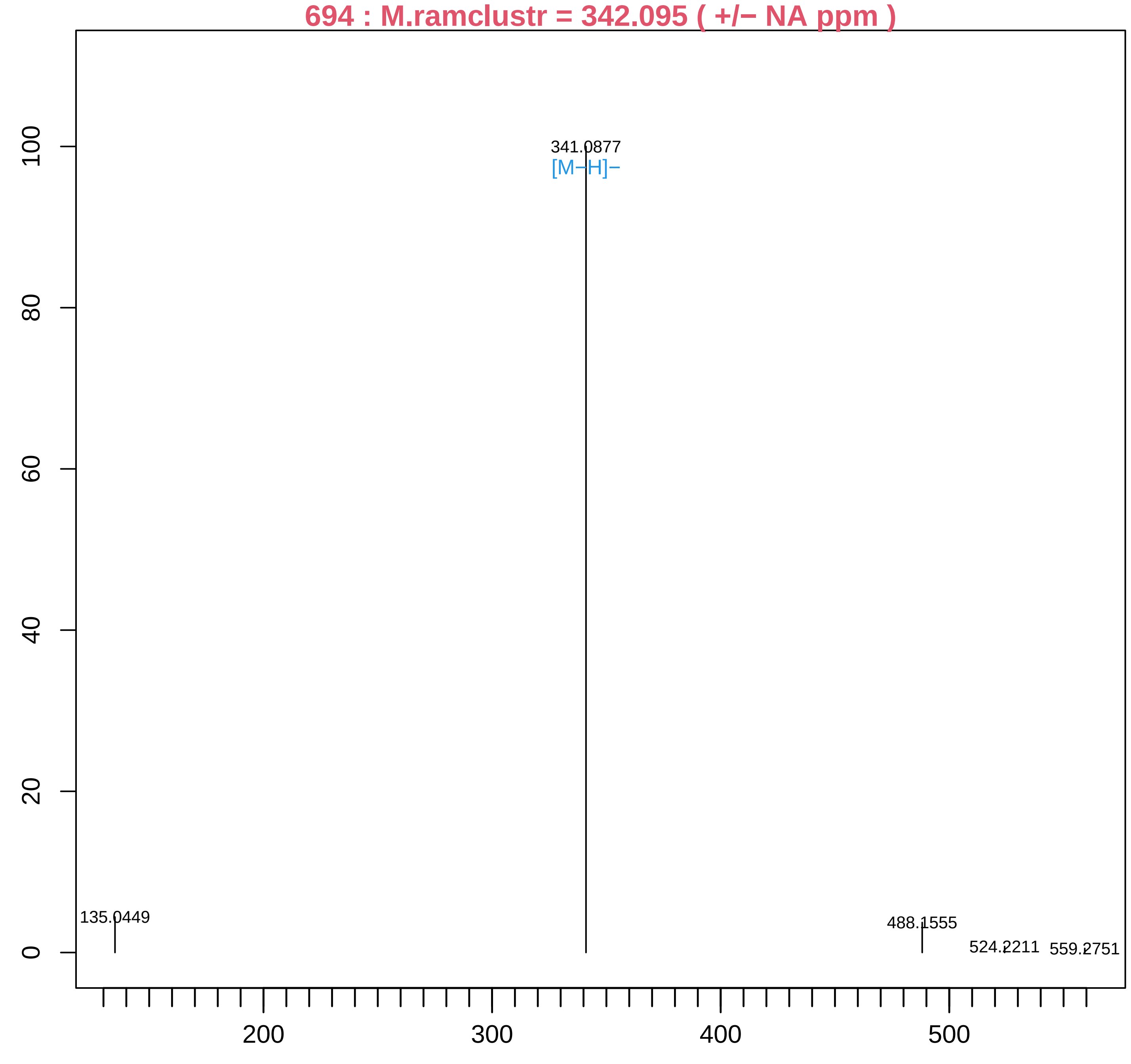

694 : M.ramclustr = 342.095 (+/− NA ppm)
100
80
60
40
20
0
200
300
400
500
341.0877
[M−H]−
135.0449
488.1555
524.2211
559.2751

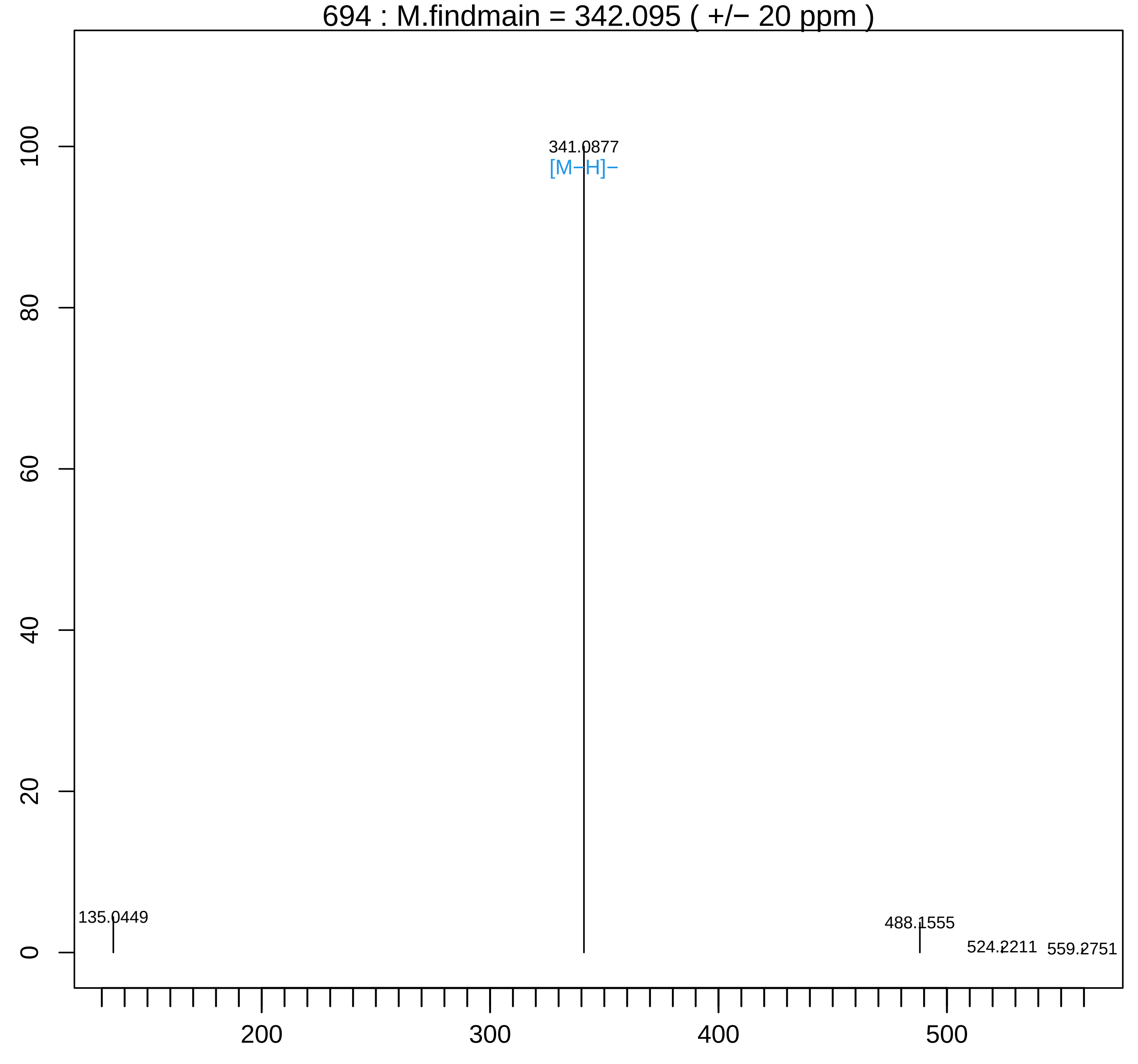

694 : M.findmain = 342.095 (+/− 20 ppm)
100
80
60
40
20
0
200
300
400
500
341.0877
[M−H]−
135.0449
488.1555
524.2211
559.2751

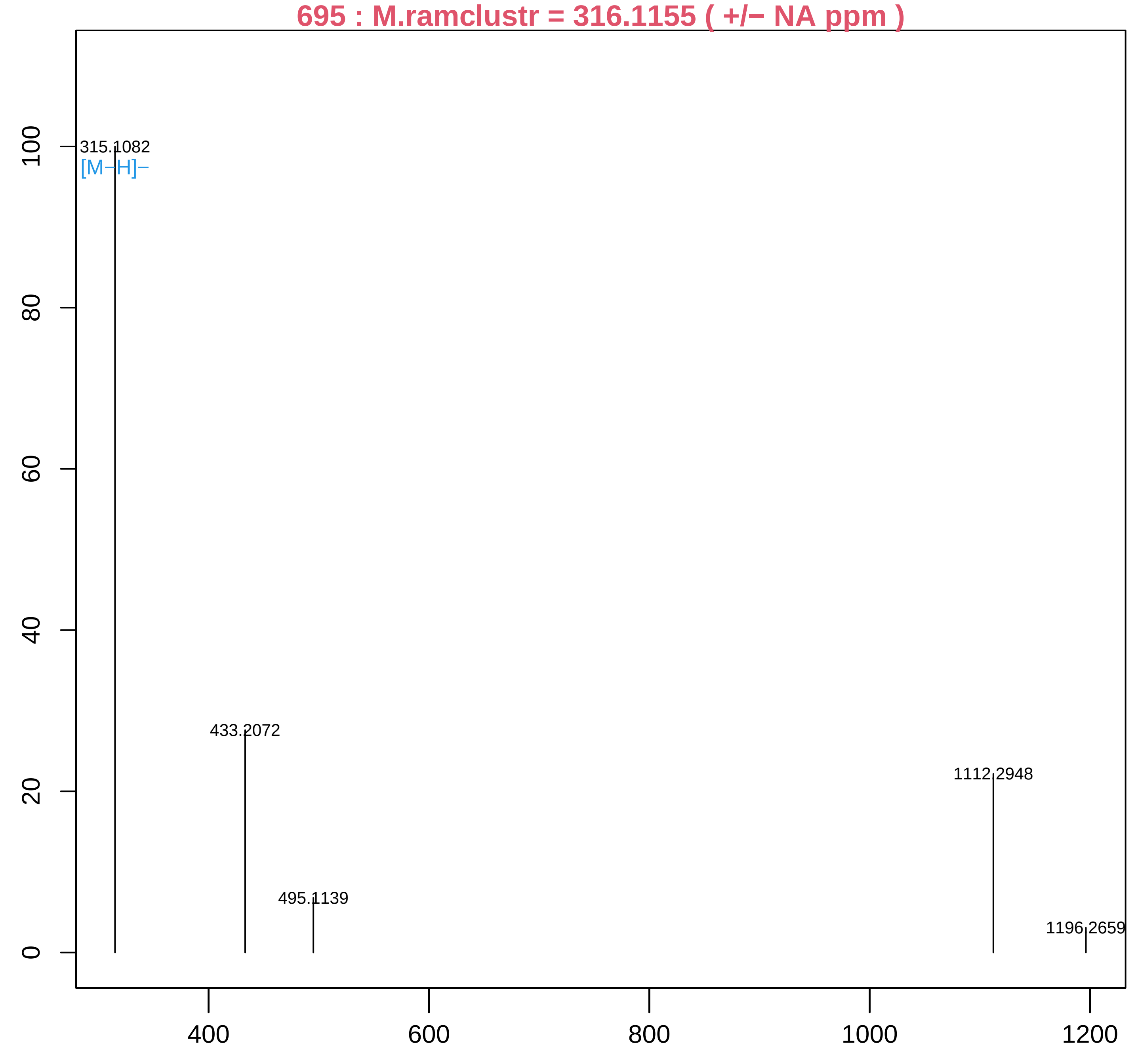
695 : M.ramclustr = 316.1155 (+/− NA ppm)
315.1082
[M−H]−
433.2072
495.1139
1112.2948
1196.2659
0
20
40
60
80
100
400
600
800
1000
1200

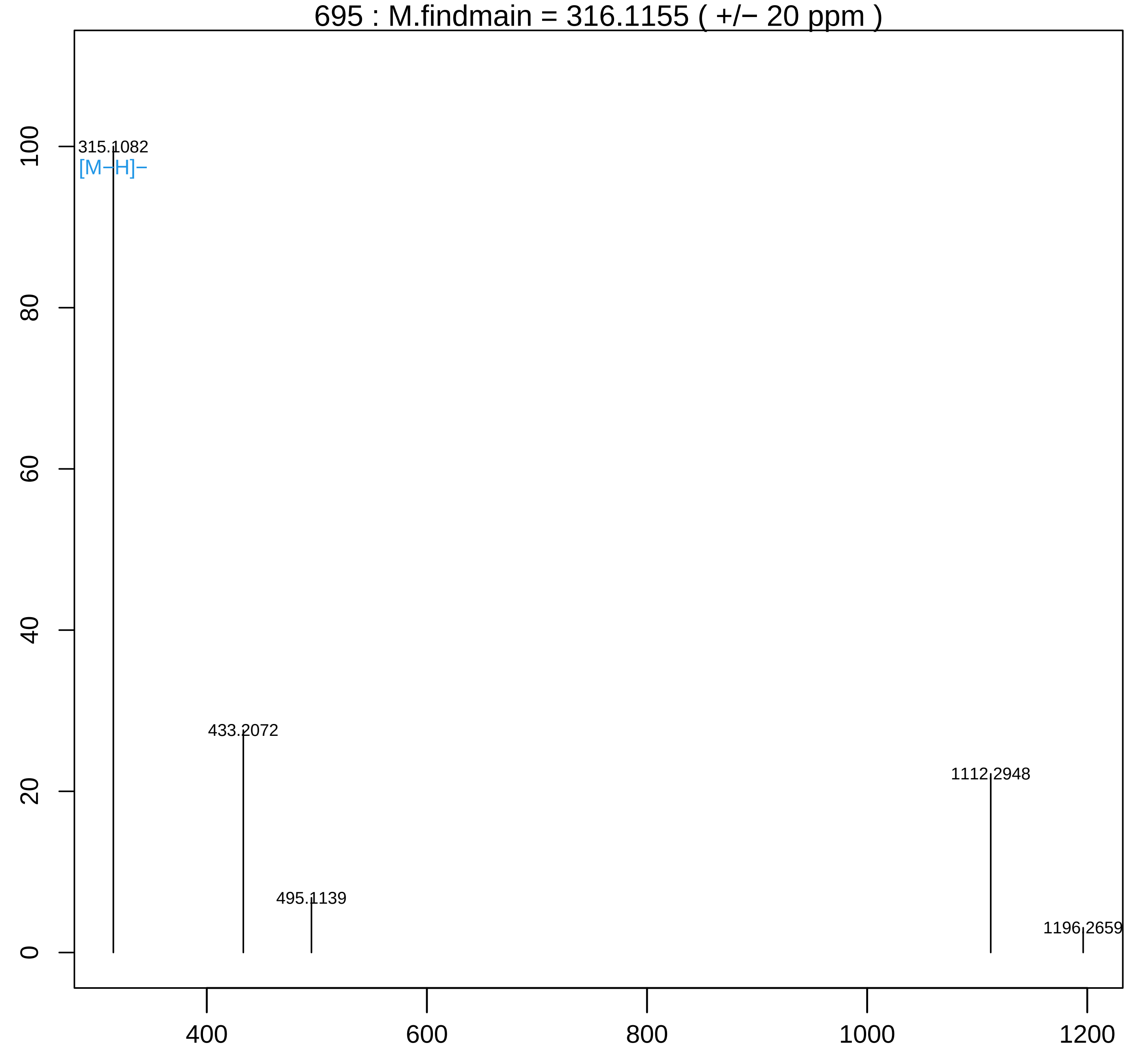
695 : M.findmain = 316.1155 (+/− 20 ppm)
315.1082
[M−H]−
433.2072
495.1139
1112.2948
1196.2659
0
20
40
60
80
100
400
600
800
1000
1200

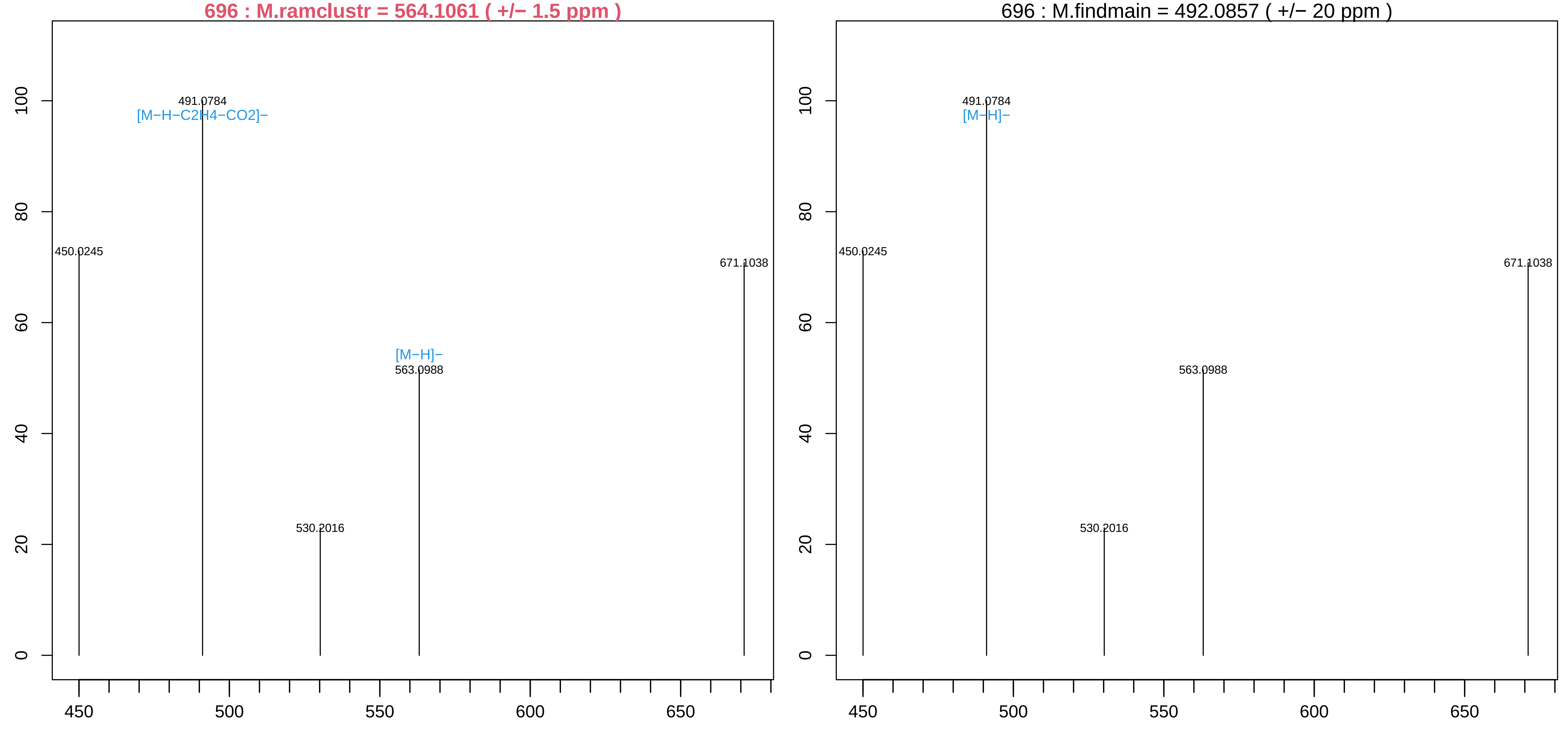
696 : M.ramclustr = 564.1061 (+/− 1.5 ppm)
491.0784
[M−H−C2H4−CO2]−
450.0245
671.1038
[M−H]−
563.0988
530.2016
696 : M.findmain = 492.0857 (+/− 20 ppm)
491.0784
[M−H]−
450.0245
671.1038
563.0988
530.2016
0
20
40
60
80
100
450
500
550
600
650

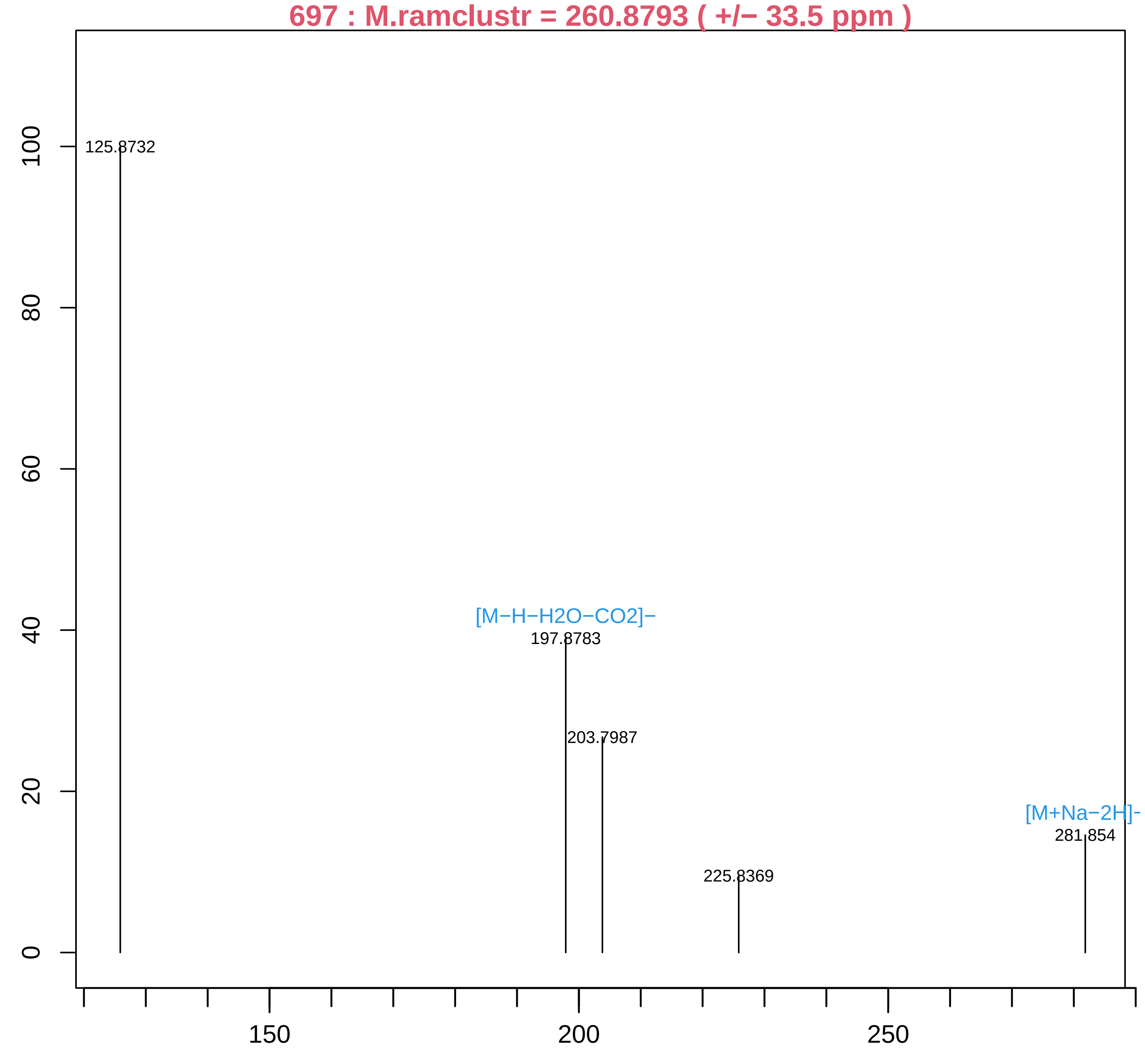
697 : M.ramclustr = 260.8793 (+/− 33.5 ppm)
125.8732
[M−H−H2O−CO2]−
197.8783
203.7987
225.8369
[M+Na−2H]-
281.854
0
20
40
60
80
100
150
200
250

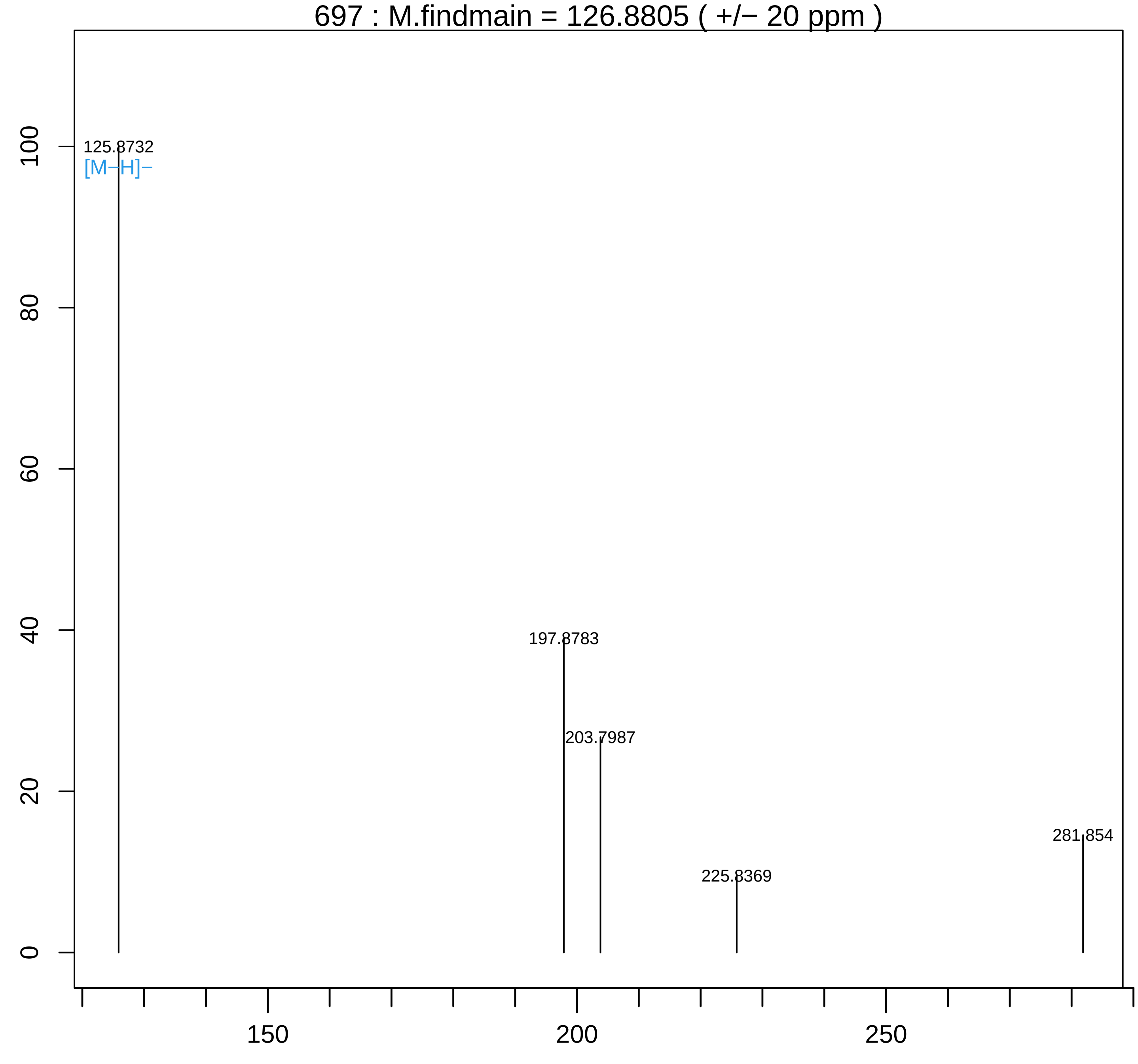
697 : M.findmain = 126.8805 (+/− 20 ppm)
125.8732
[M−H]−
197.8783
203.7987
225.8369
281.854
0
20
40
60
80
100
150
200
250

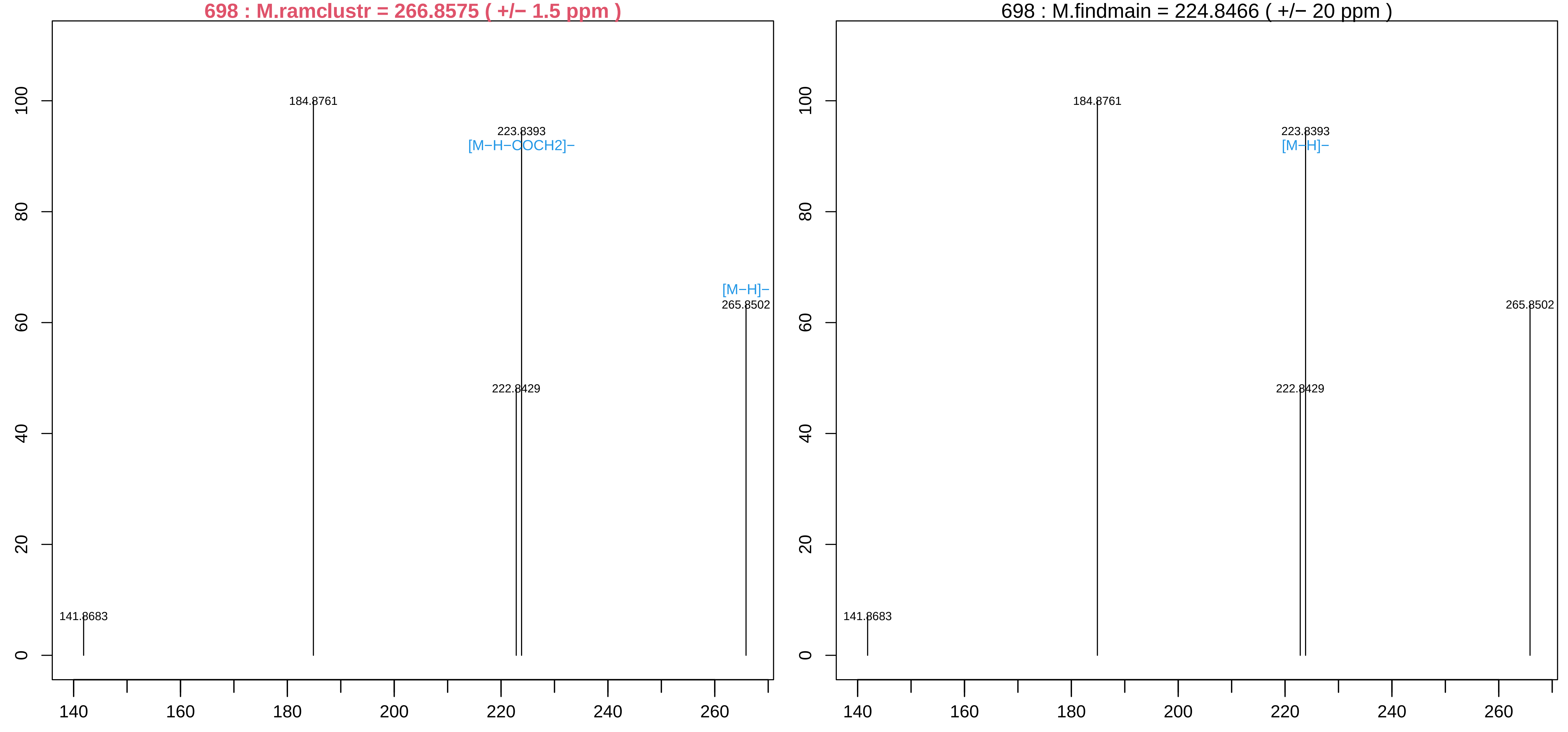
698 : M.ramclustr = 266.8575 (+/− 1.5 ppm)
184.8761
223.8393
[M−H−COCH2]−
[M−H]−
265.8502
222.8429
141.8683
698 : M.findmain = 224.8466 (+/− 20 ppm)
184.8761
223.8393
[M−H]−
265.8502
222.8429
141.8683

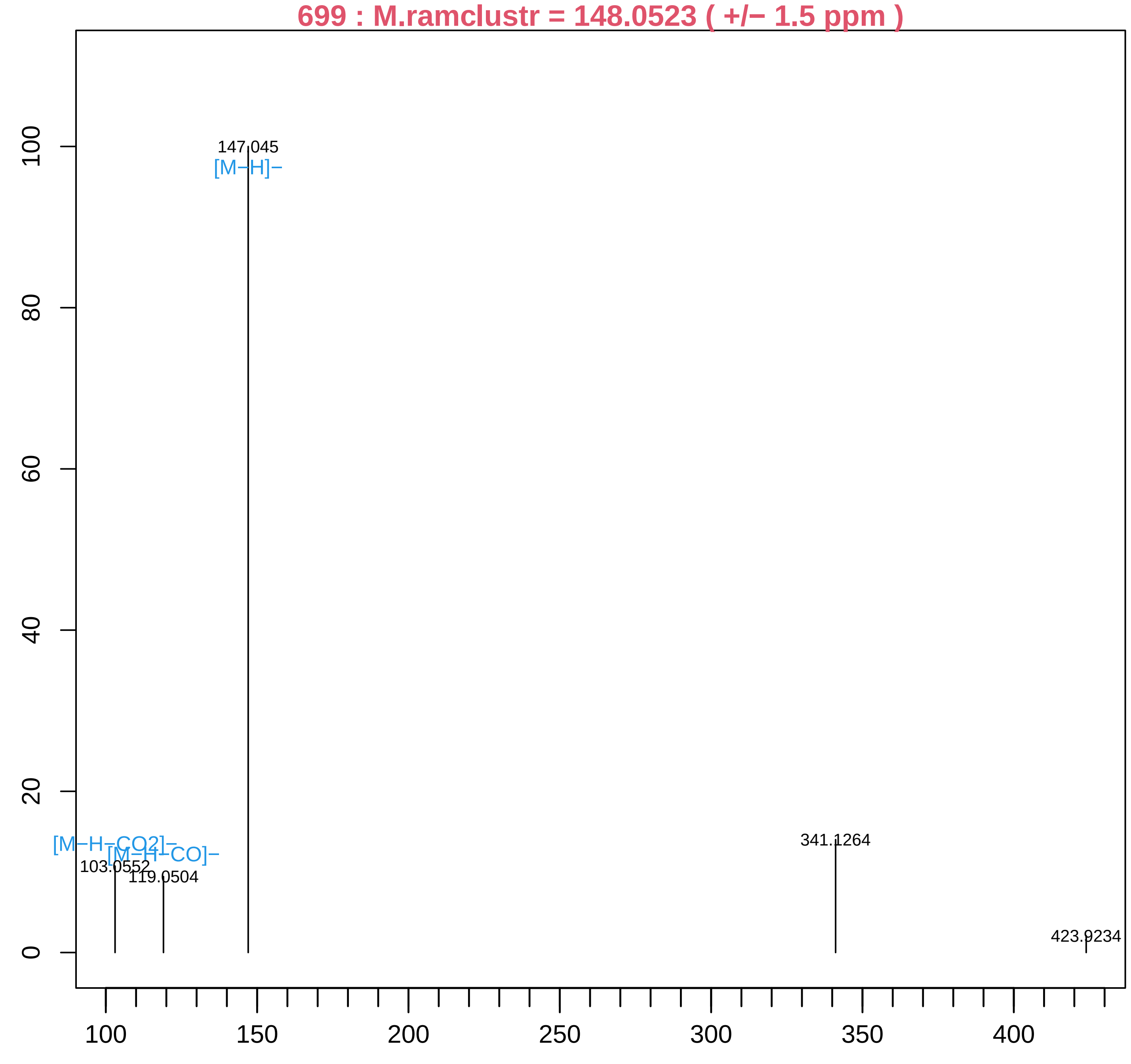
699 : M.ramclustr = 148.0523 (+/− 1.5 ppm)
147.045
[M−H]−
[M−H−CO2]−
[M−H−CO]−
103.0552
119.0504
341.1264
423.9234
0
20
40
60
80
100
100
150
200
250
300
350
400
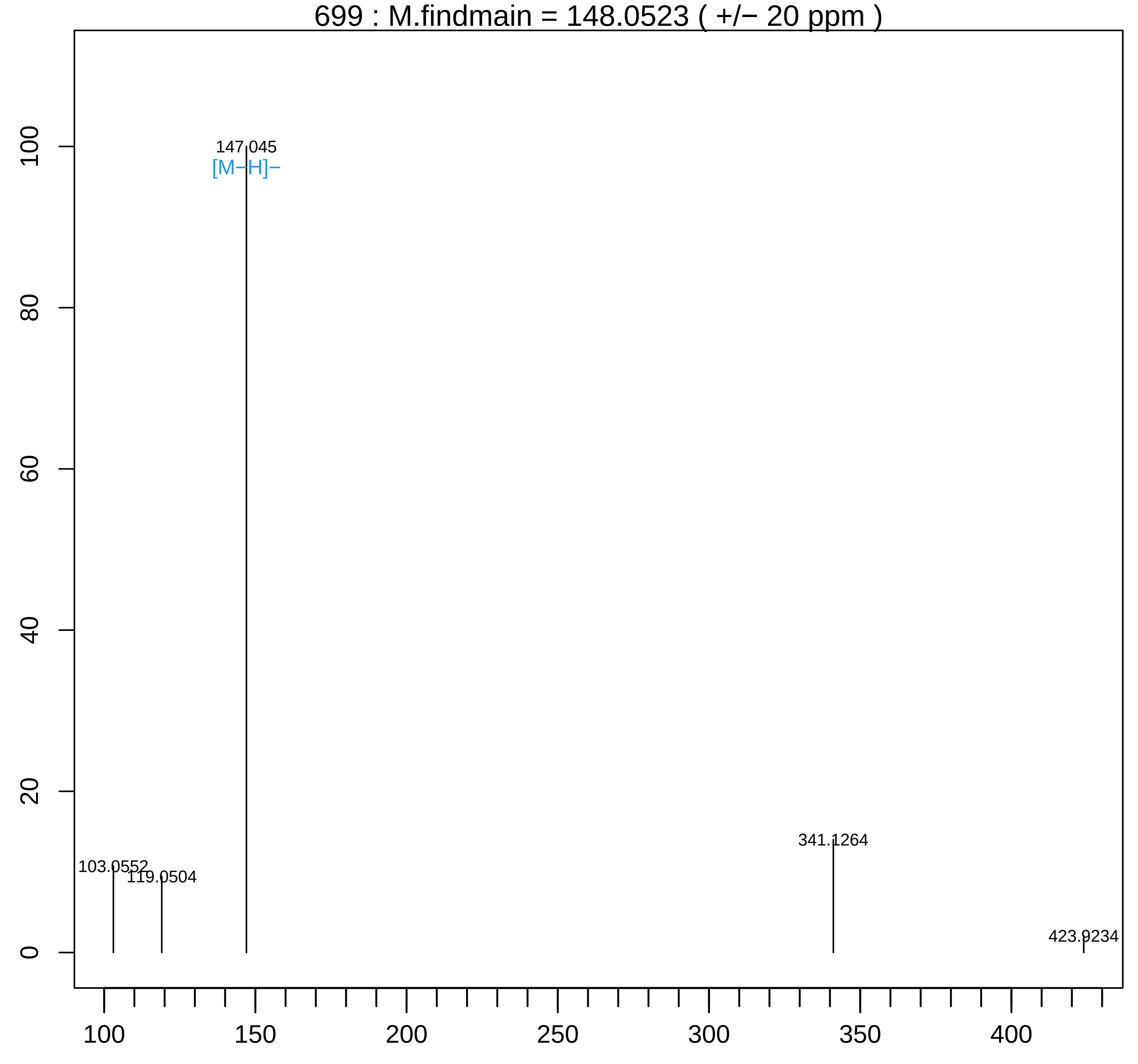
699 : M.findmain = 148.0523 (+/− 20 ppm)
147.045
[M−H]−
103.0552
119.0504
341.1264
423.9234
0
20
40
60
80
100
100
150
200
250
300
350
400

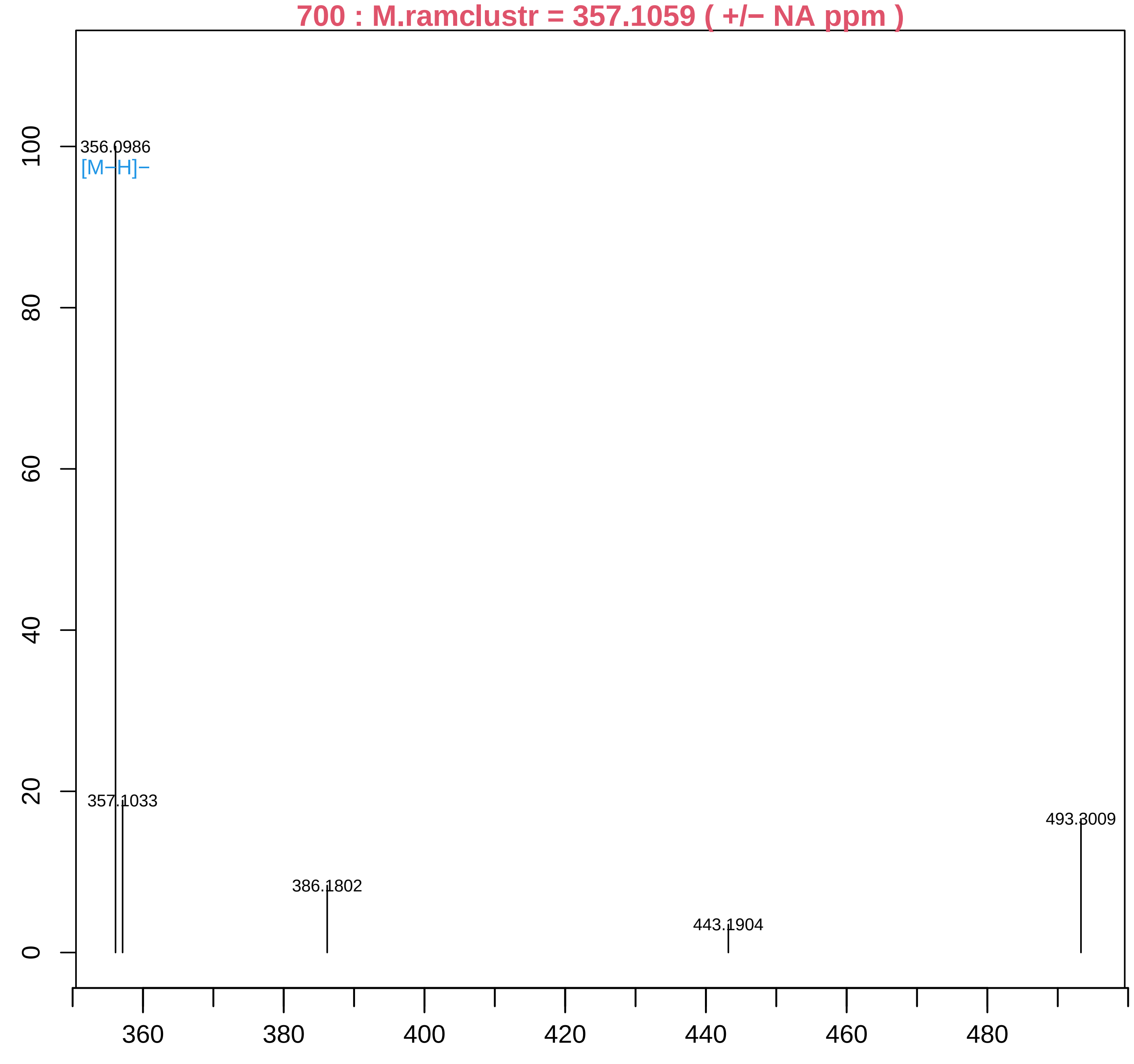

700 : M.ramclustr = 357.1059 (+/− NA ppm)
356.0986
[M−H]−
357.1033
386.1802
443.1904
493.3009
0
20
40
60
80
100
360
380
400
420
440
460
480

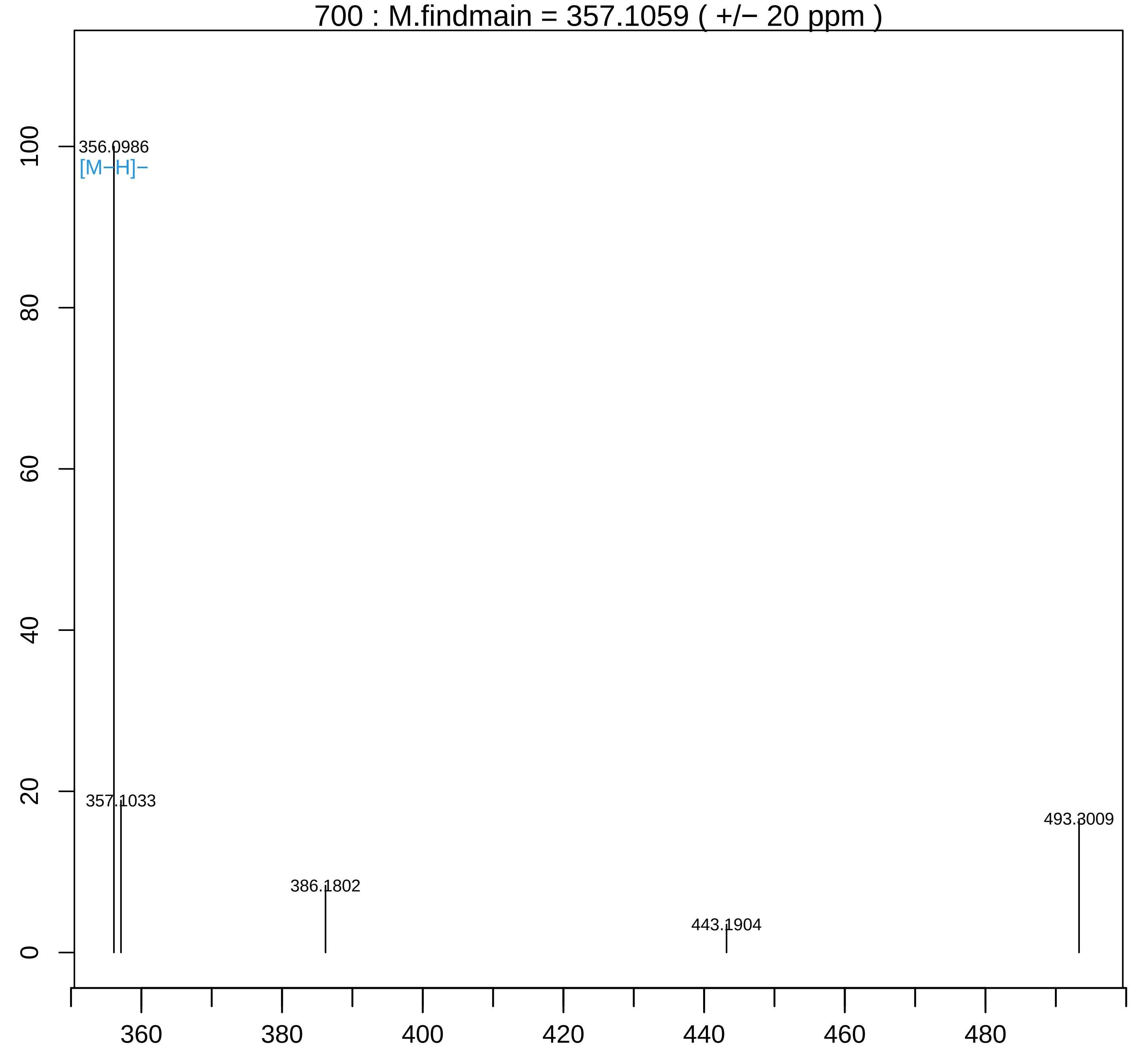

700 : M.findmain = 357.1059 (+/− 20 ppm)
356.0986
[M−H]−
357.1033
386.1802
443.1904
493.3009
0
20
40
60
80
100
360
380
400
420
440
460
480

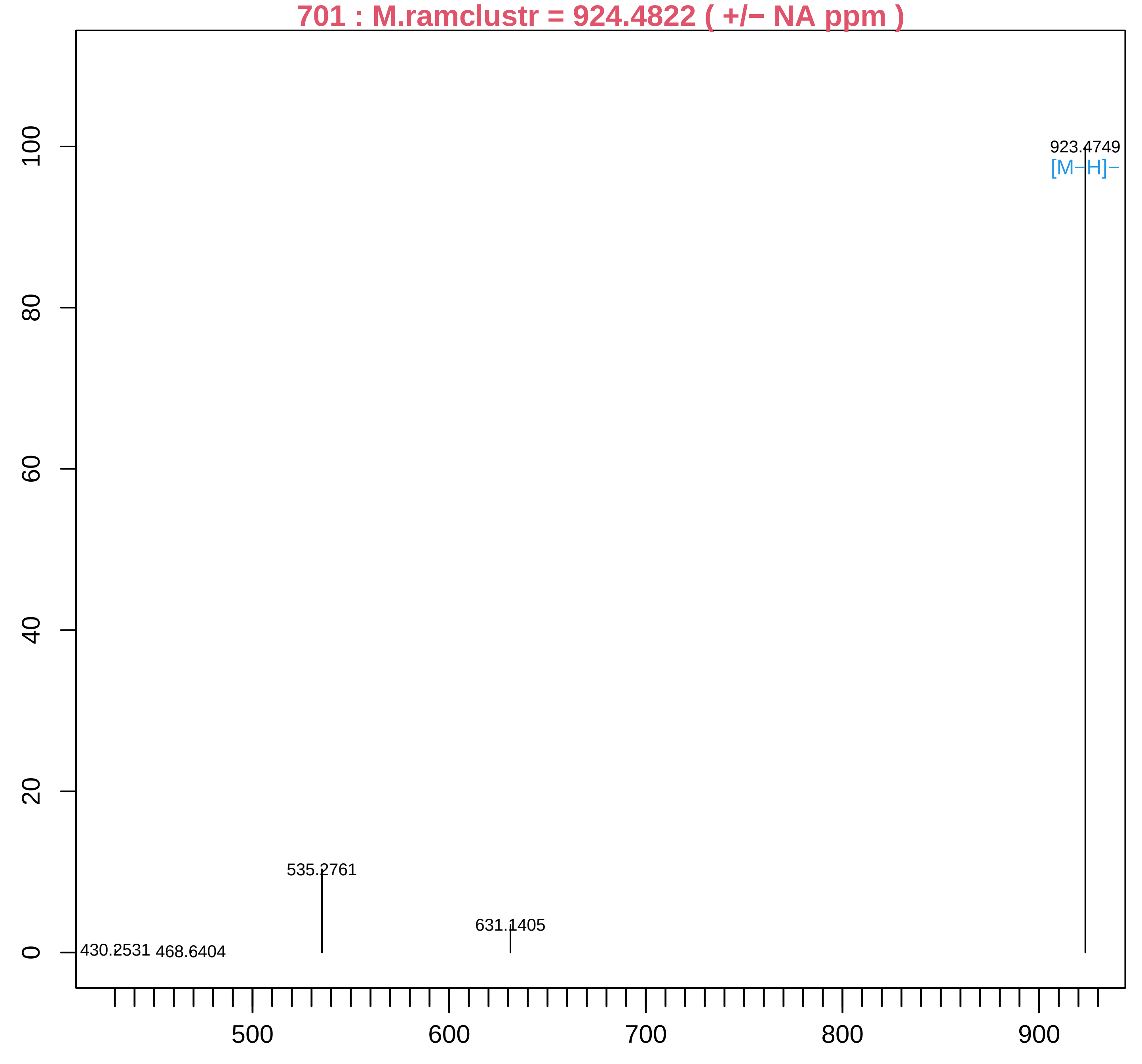

701 : M.ramclustr = 924.4822 (+/− NA ppm)
923.4749
[M−H]−
535.2761
631.1405
430.2531
468.6404
0
20
40
60
80
100
500
600
700
800
900

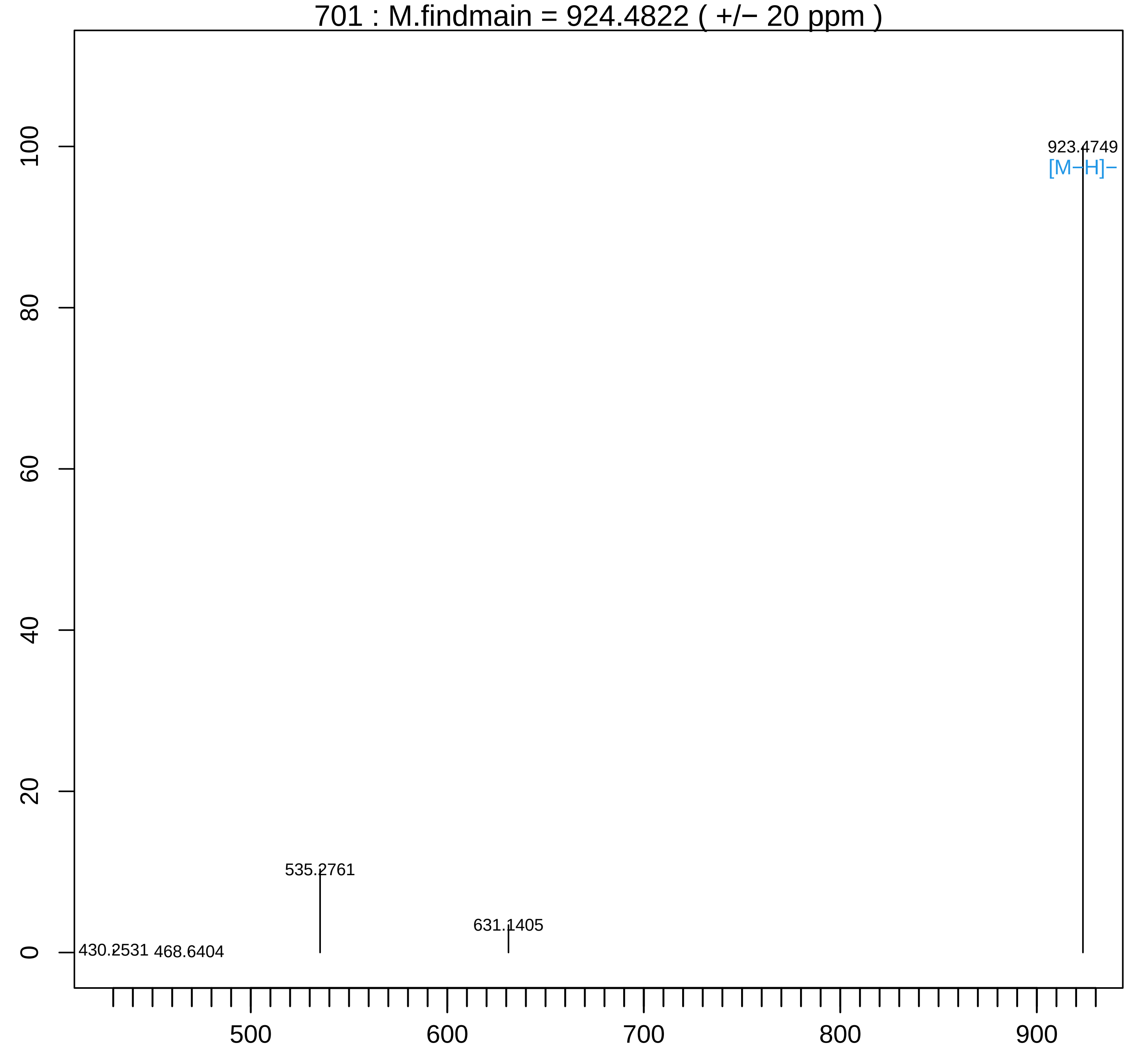

701 : M.findmain = 924.4822 (+/− 20 ppm)
923.4749
[M−H]−
535.2761
631.1405
430.2531
468.6404
0
20
40
60
80
100
500
600
700
800
900

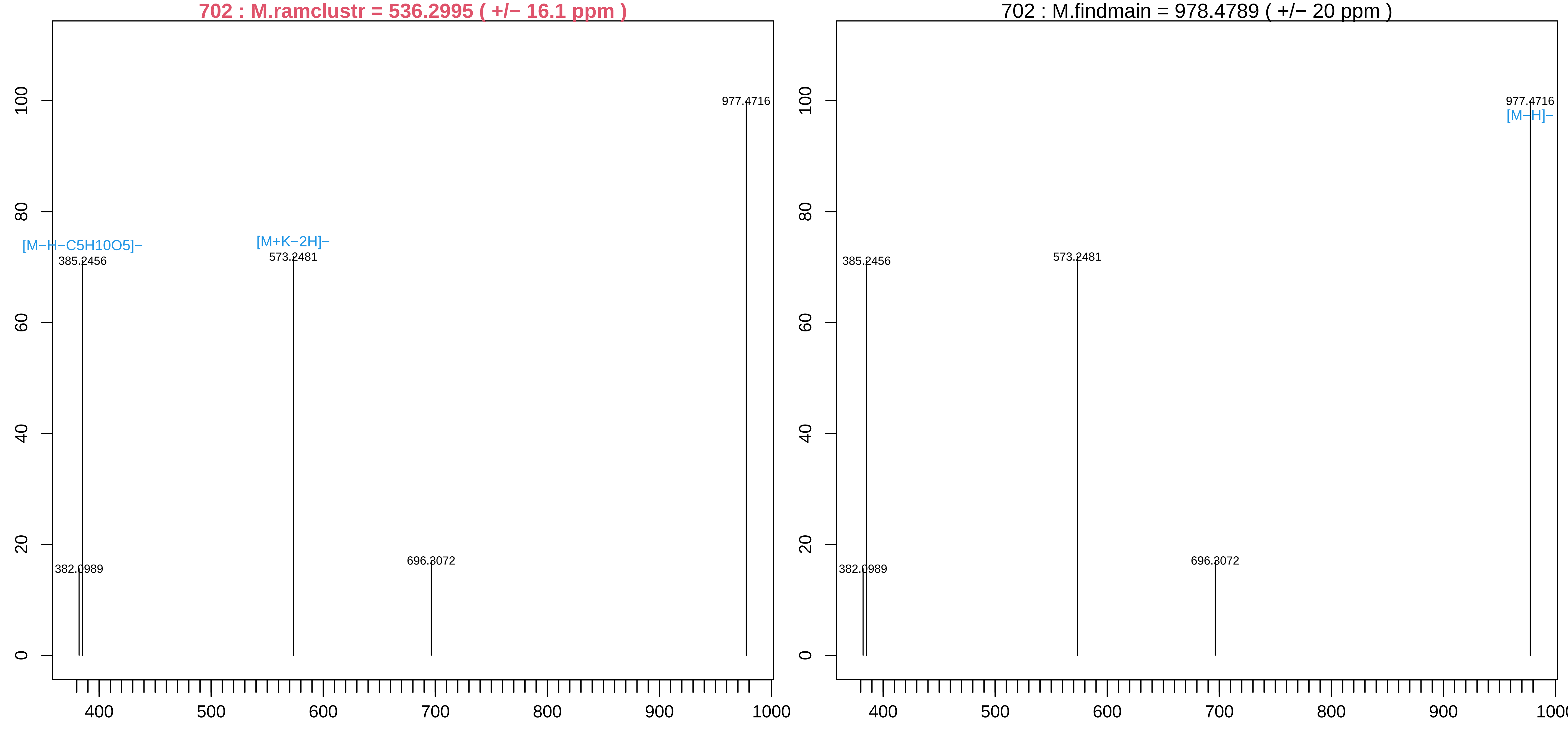
702 : M.ramclustr = 536.2995 (+/− 16.1 ppm)
[M−H−C5H10O5]−
385.2456
382.0989
[M+K−2H]−
573.2481
696.3072
977.4716
702 : M.findmain = 978.4789 (+/− 20 ppm)
385.2456
382.0989
573.2481
696.3072
977.4716
[M−H]−

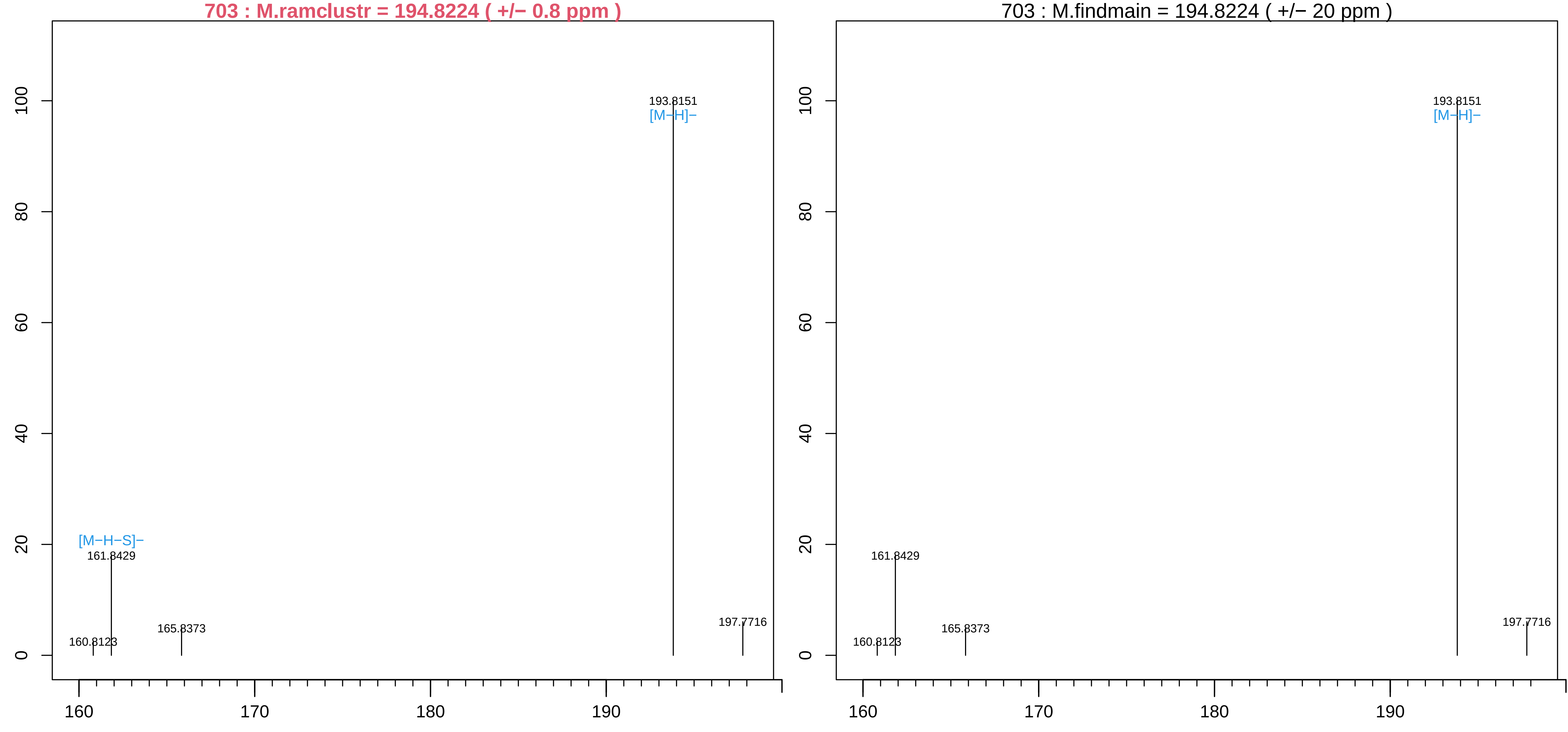

703 : M.ramclustr = 194.8224 (+/− 0.8 ppm)
193.8151
[M−H]−
[M−H−S]−
161.8429
160.8123
165.8373
197.7716
0
20
40
60
80
100
160
170
180
190
703 : M.findmain = 194.8224 (+/− 20 ppm)
193.8151
[M−H]−
161.8429
160.8123
165.8373
197.7716
0
20
40
60
80
100
160
170
180
190

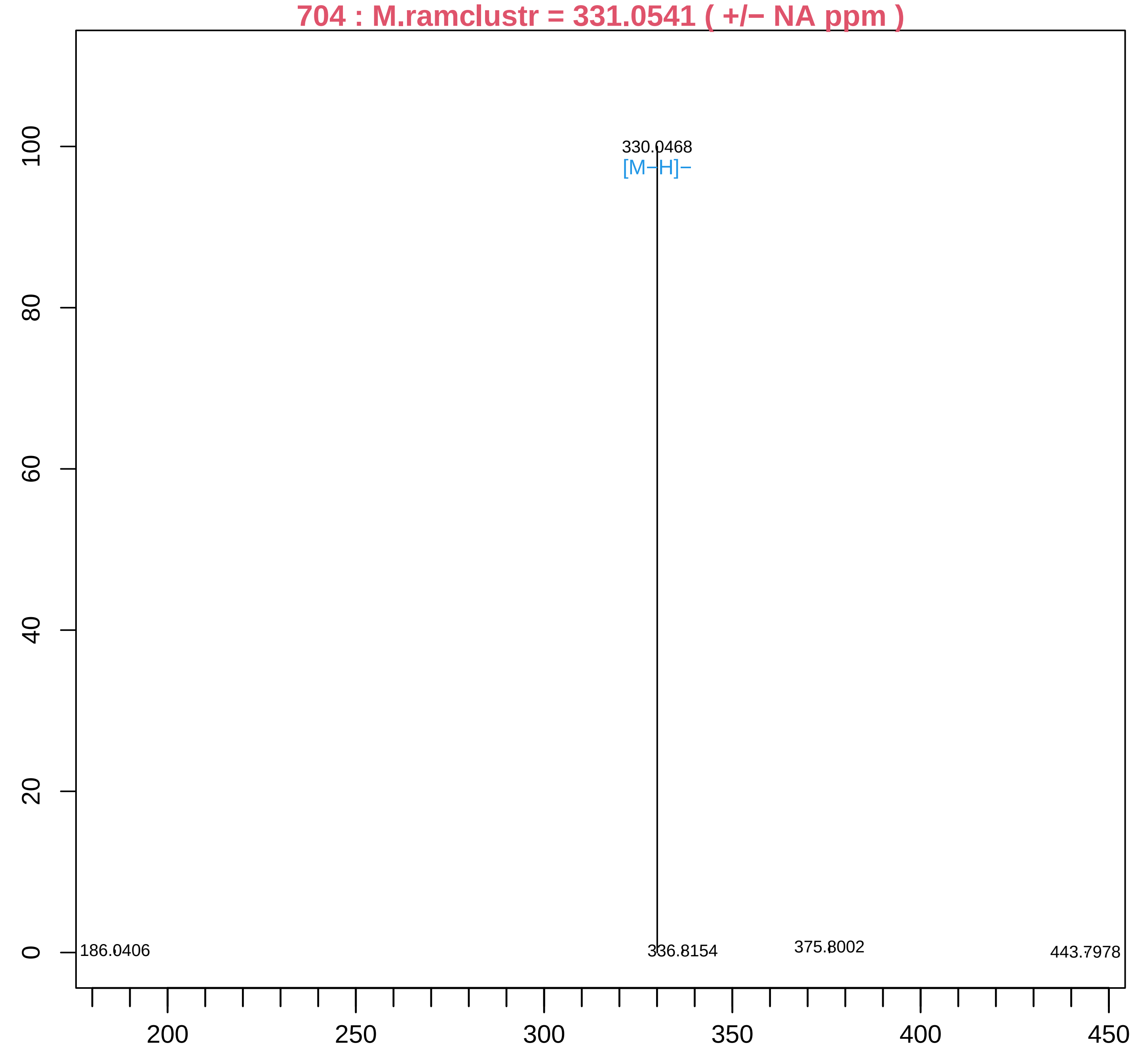
704 : M.ramclustr = 331.0541 (+/− NA ppm)
330.0468
[M−H]−
186.0406
336.8154
375.8002
443.7978
0
20
40
60
80
100
200
250
300
350
400
450

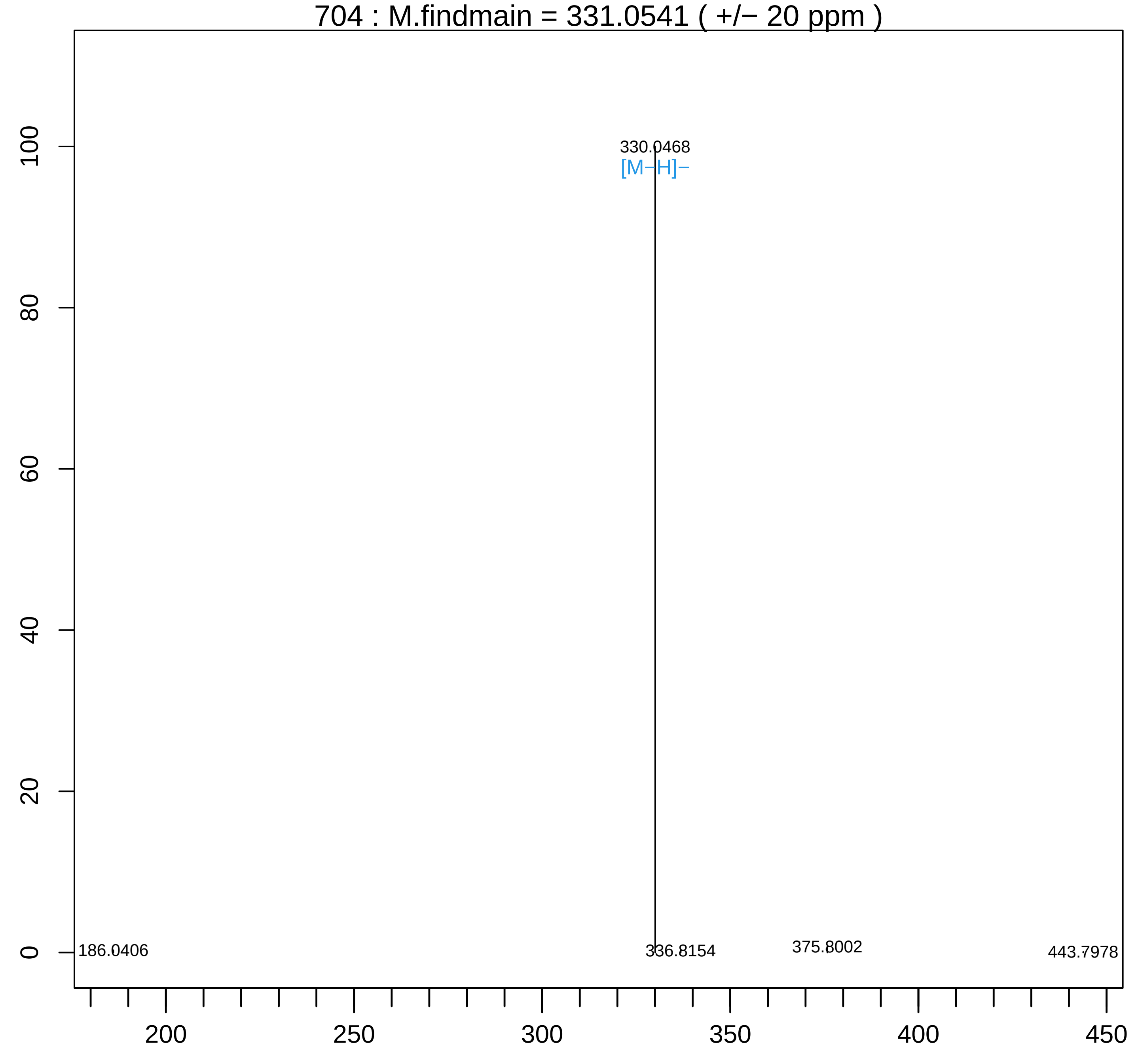
704 : M.findmain = 331.0541 (+/− 20 ppm)
330.0468
[M−H]−
186.0406
336.8154
375.8002
443.7978
0
20
40
60
80
100
200
250
300
350
400
450

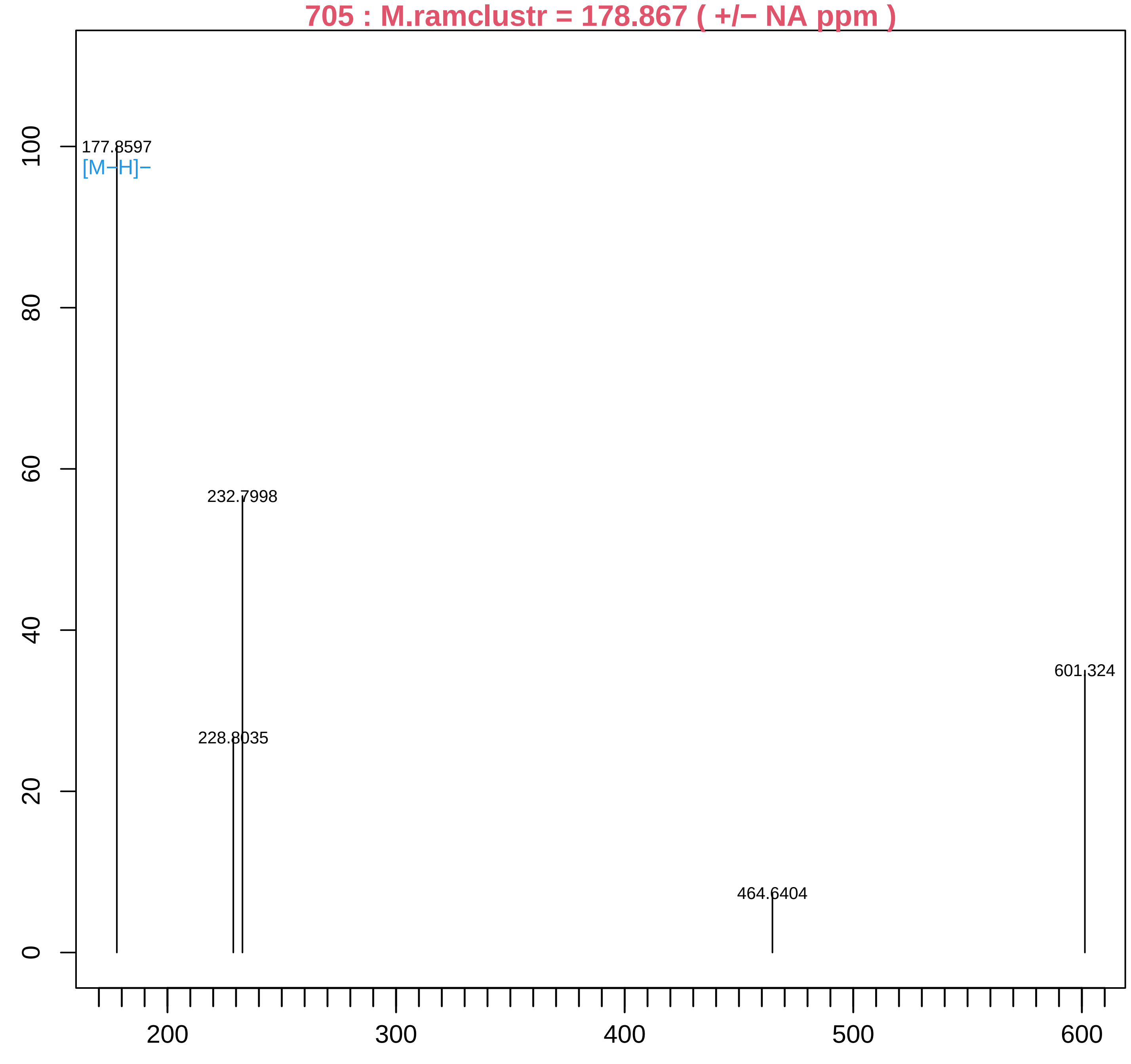

705 : M.ramclustr = 178.867 (+/− NA ppm)
177.8597
[M−H]−
232.7998
228.8035
464.6404
601.324
0
20
40
60
80
100
200
300
400
500
600

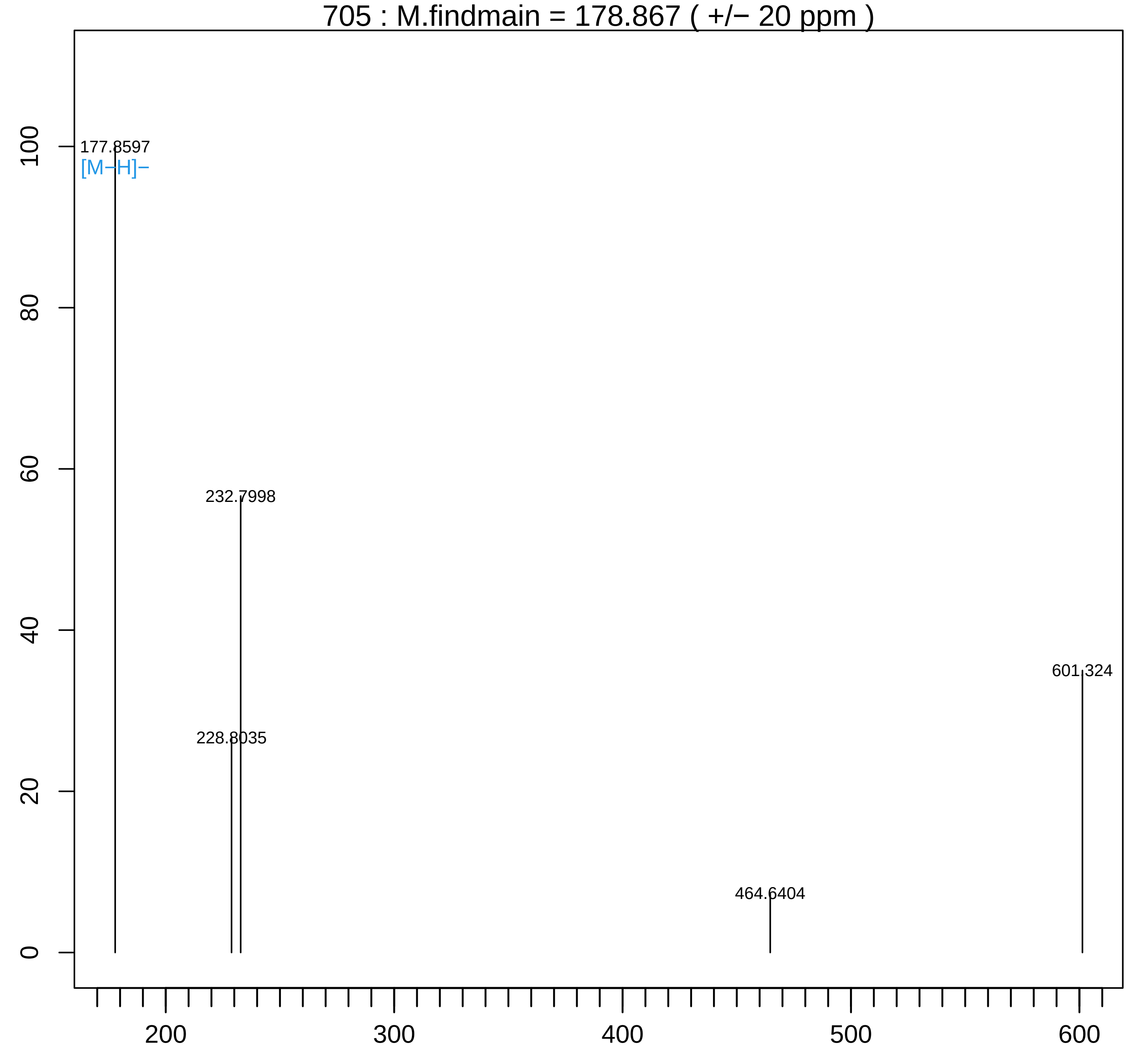

705 : M.findmain = 178.867 (+/− 20 ppm)
177.8597
[M−H]−
232.7998
228.8035
464.6404
601.324
0
20
40
60
80
100
200
300
400
500
600

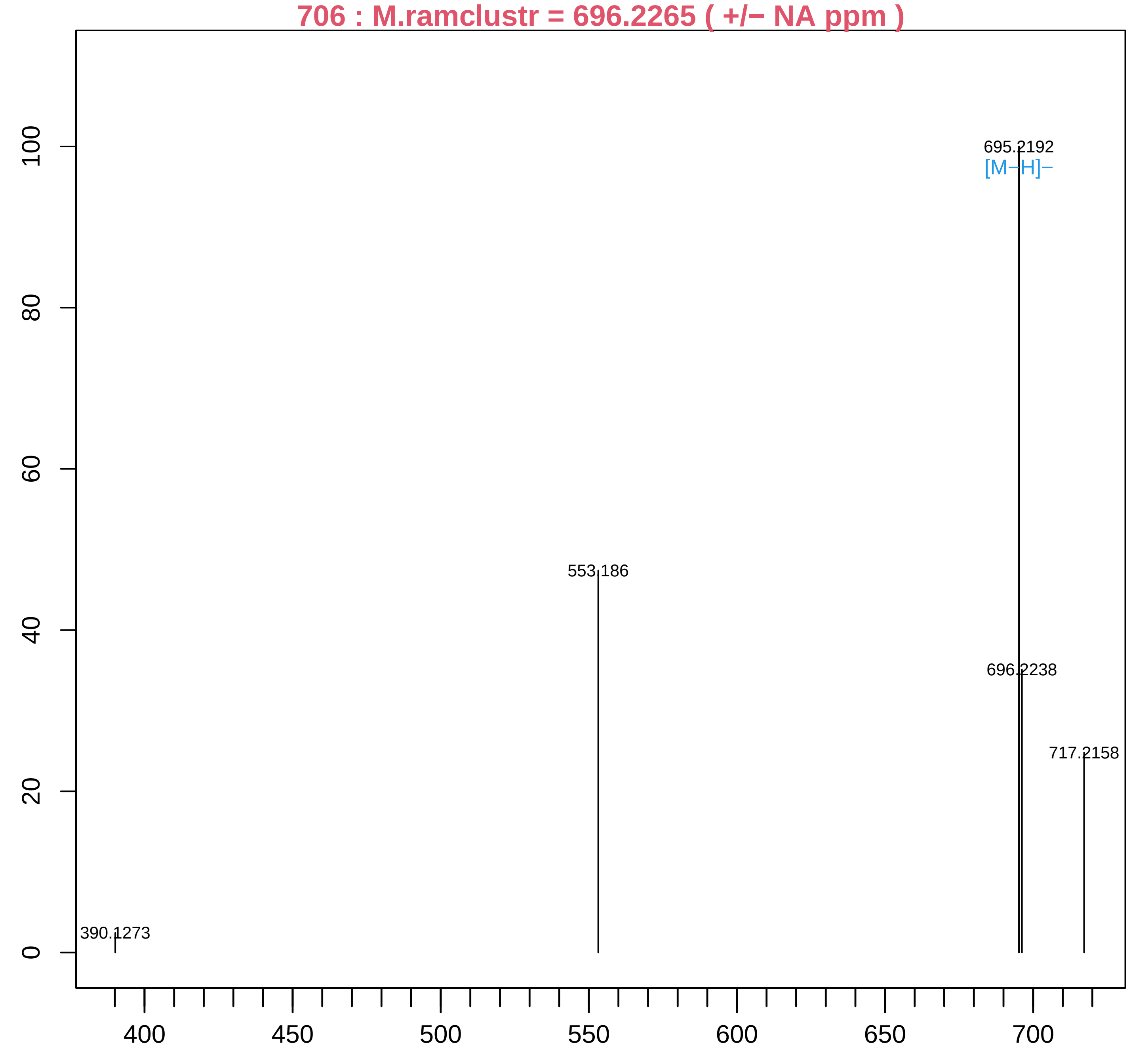
706 : M.ramclustr = 696.2265 (+/− NA ppm)
695.2192
[M−H]−
553.186
696.2238
717.2158
390.1273
0
20
40
60
80
100
400
450
500
550
600
650
700

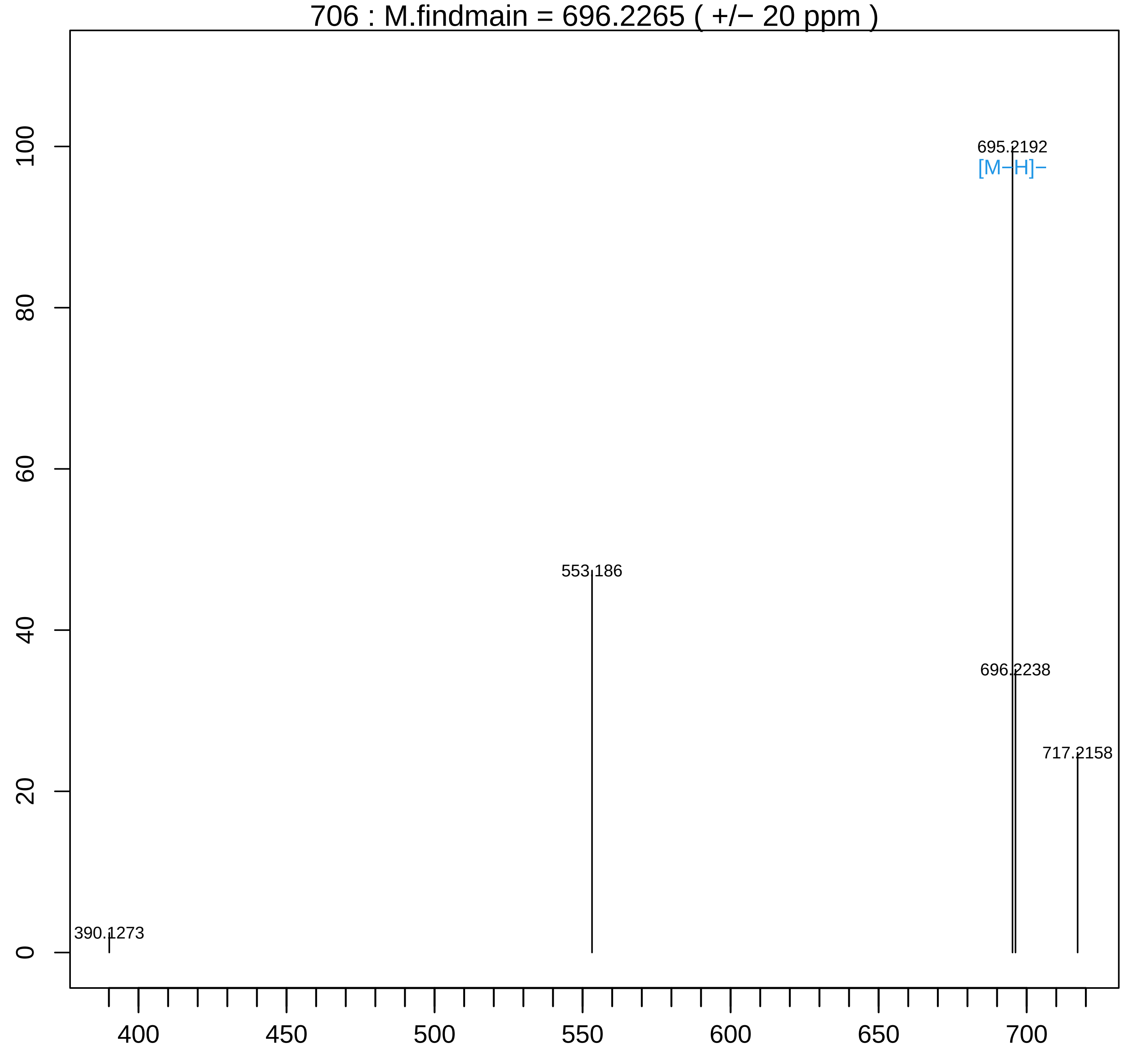
706 : M.findmain = 696.2265 (+/− 20 ppm)
695.2192
[M−H]−
553.186
696.2238
717.2158
390.1273
0
20
40
60
80
100
400
450
500
550
600
650
700

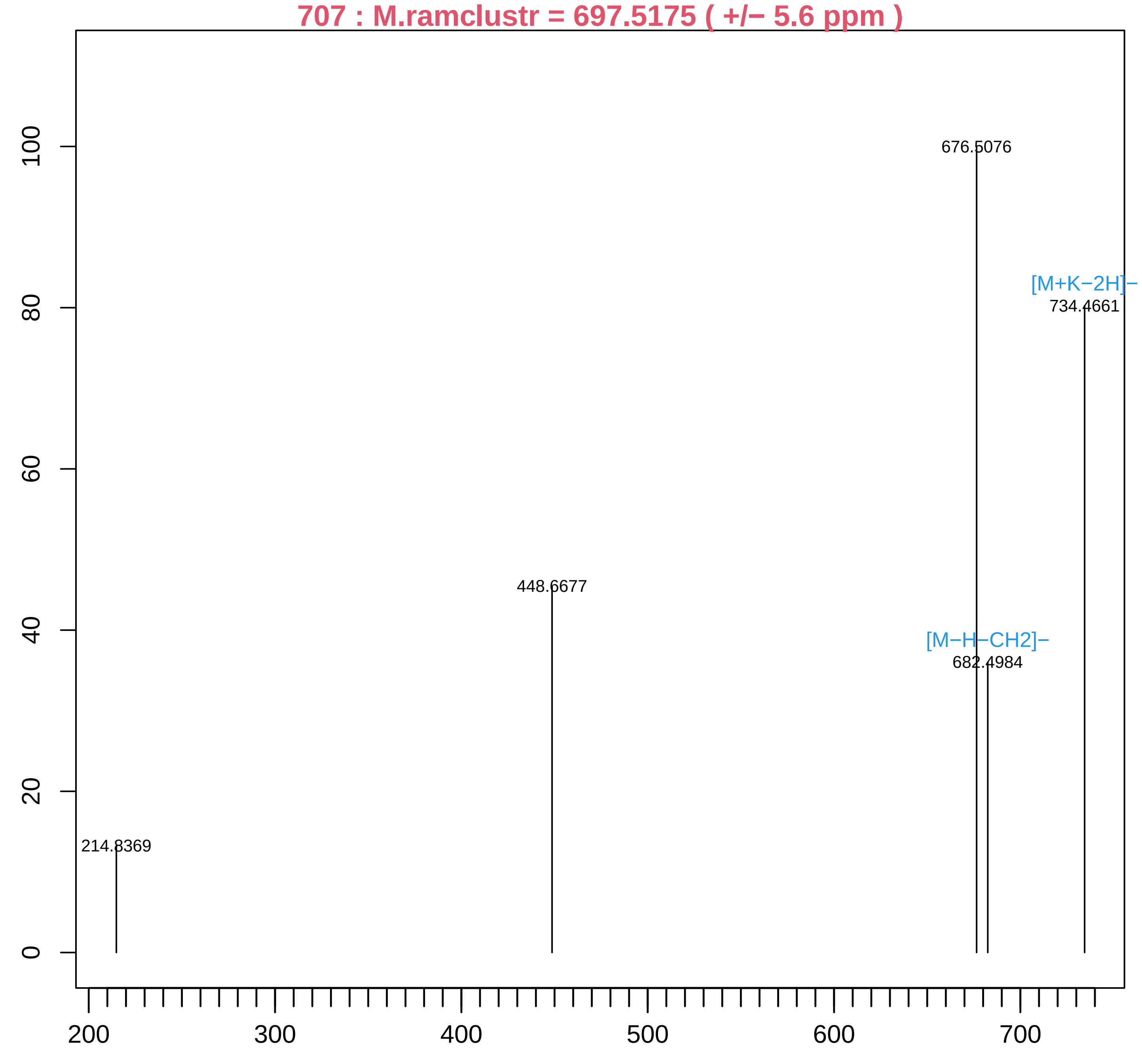

707 : M.ramclustr = 697.5175 (+/− 5.6 ppm)
676.5076
[M+K−2H]−
734.4661
448.6677
[M−H−CH2]−
682.4984
214.8369
0
20
40
60
80
100
200
300
400
500
600
700

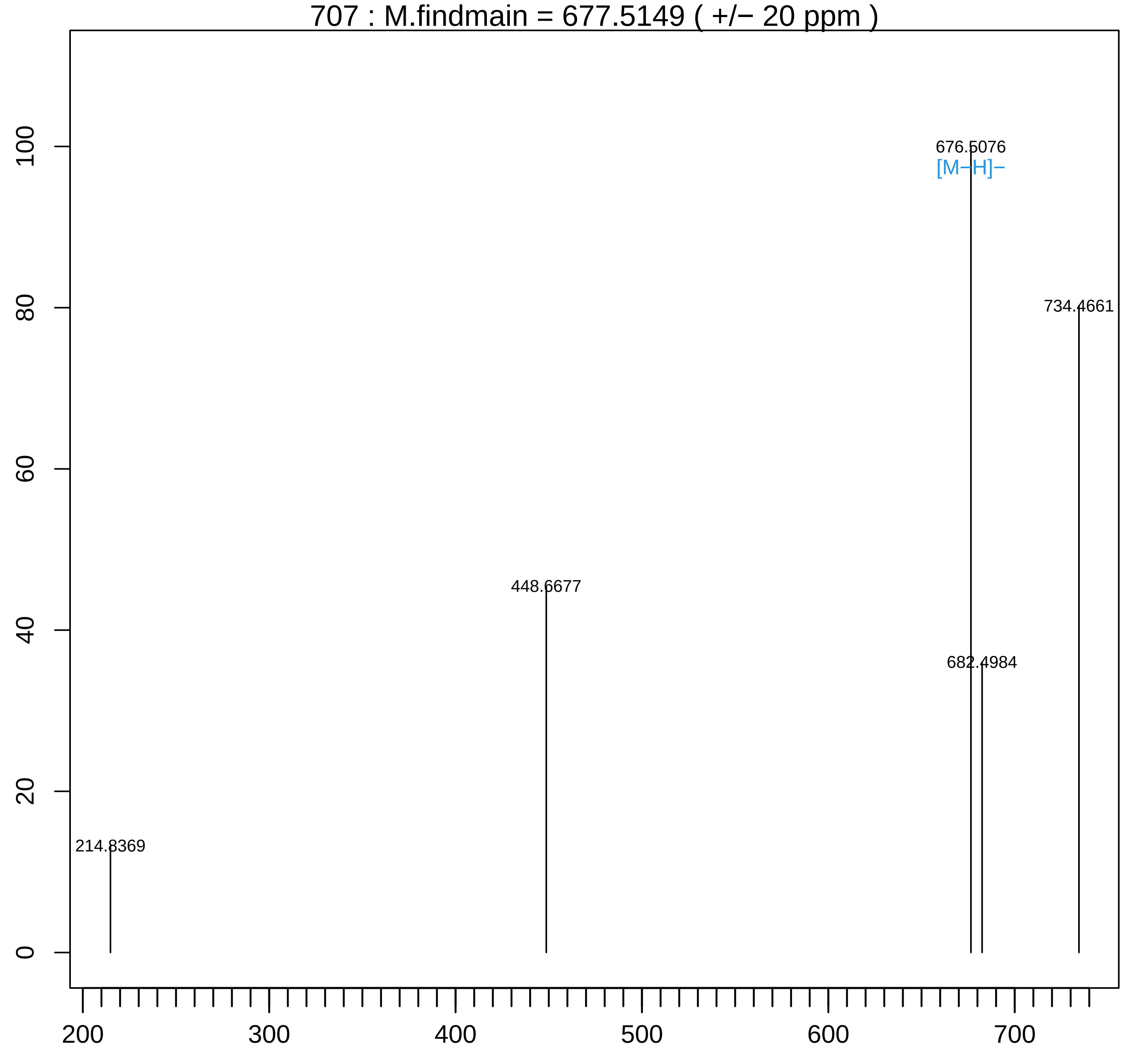

707 : M.findmain = 677.5149 (+/− 20 ppm)
676.5076
[M−H]−
734.4661
448.6677
682.4984
214.8369
0
20
40
60
80
100
200
300
400
500
600
700

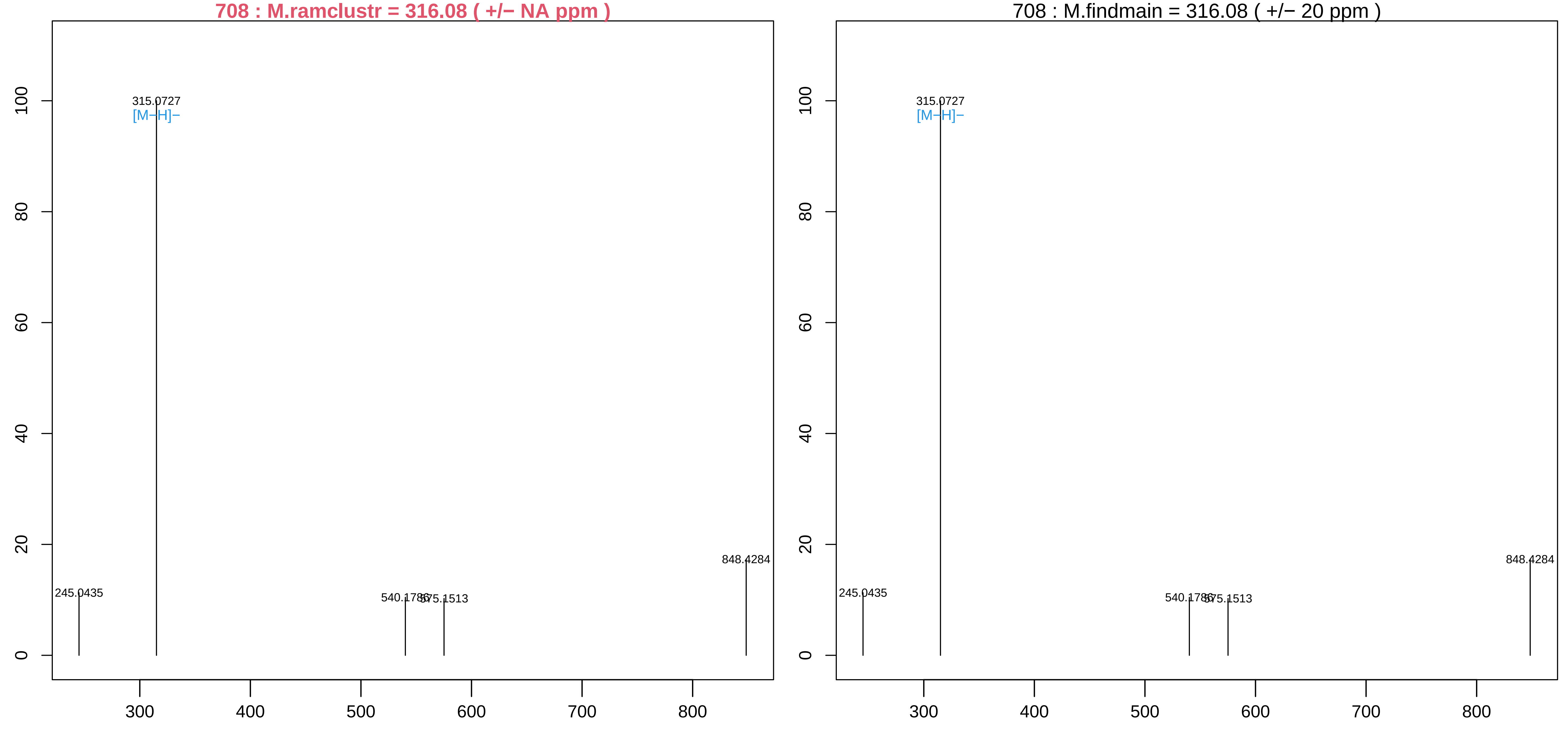
708 : M.ramclustr = 316.08 (+/− NA ppm)
315.0727
[M−H]−
245.0435
540.1786
575.1513
848.4284
708 : M.findmain = 316.08 (+/− 20 ppm)
315.0727
[M−H]−
245.0435
540.1786
575.1513
848.4284

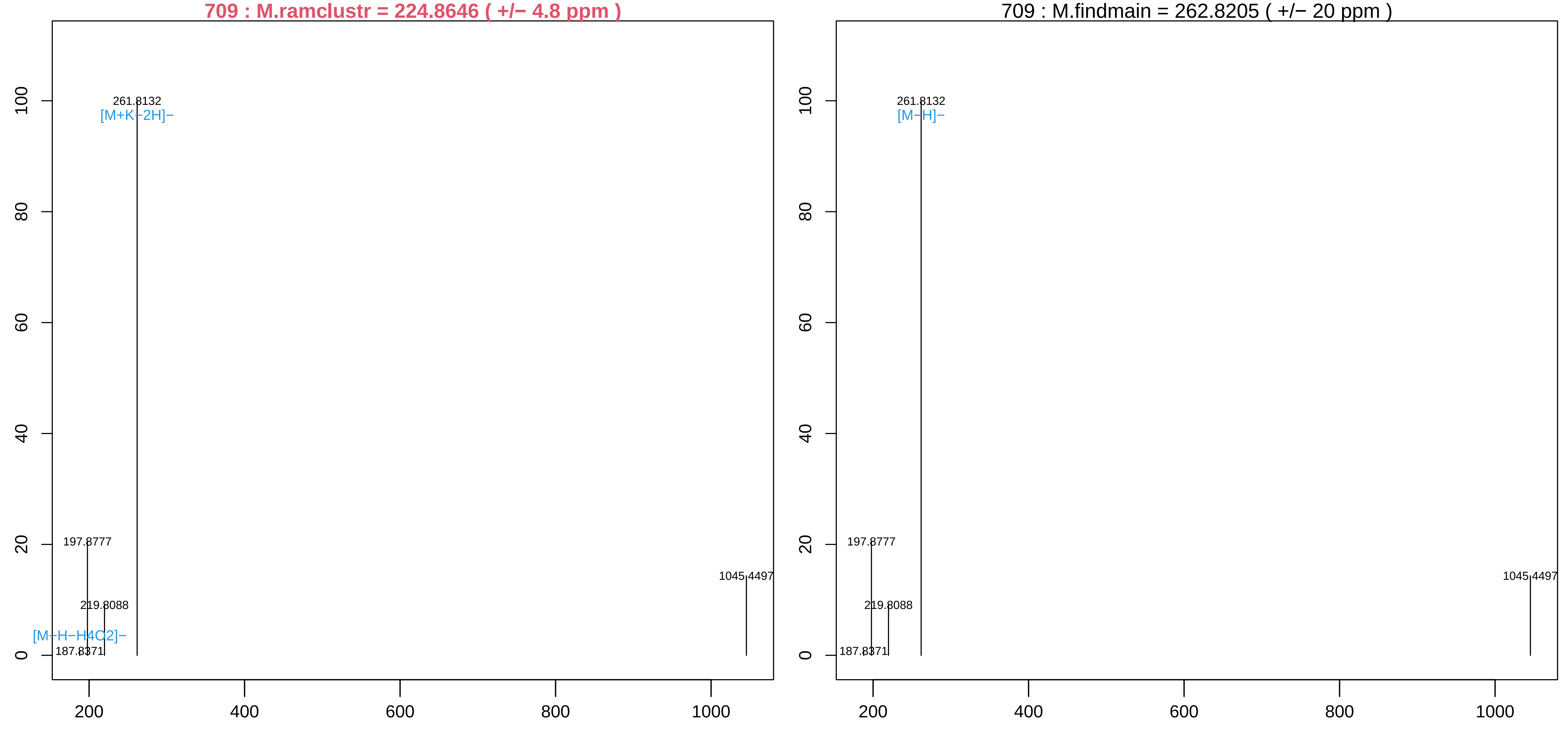
709 : M.ramclustr = 224.8646 (+/− 4.8 ppm)
261.8132
[M+K−2H]−
197.8777
219.8088
[M−H−H4C2]−
187.8371
1045.4497
0
20
40
60
80
100
200
400
600
800
1000
709 : M.findmain = 262.8205 (+/− 20 ppm)
261.8132
[M−H]−
197.8777
219.8088
187.8371
1045.4497
0
20
40
60
80
100
200
400
600
800
1000

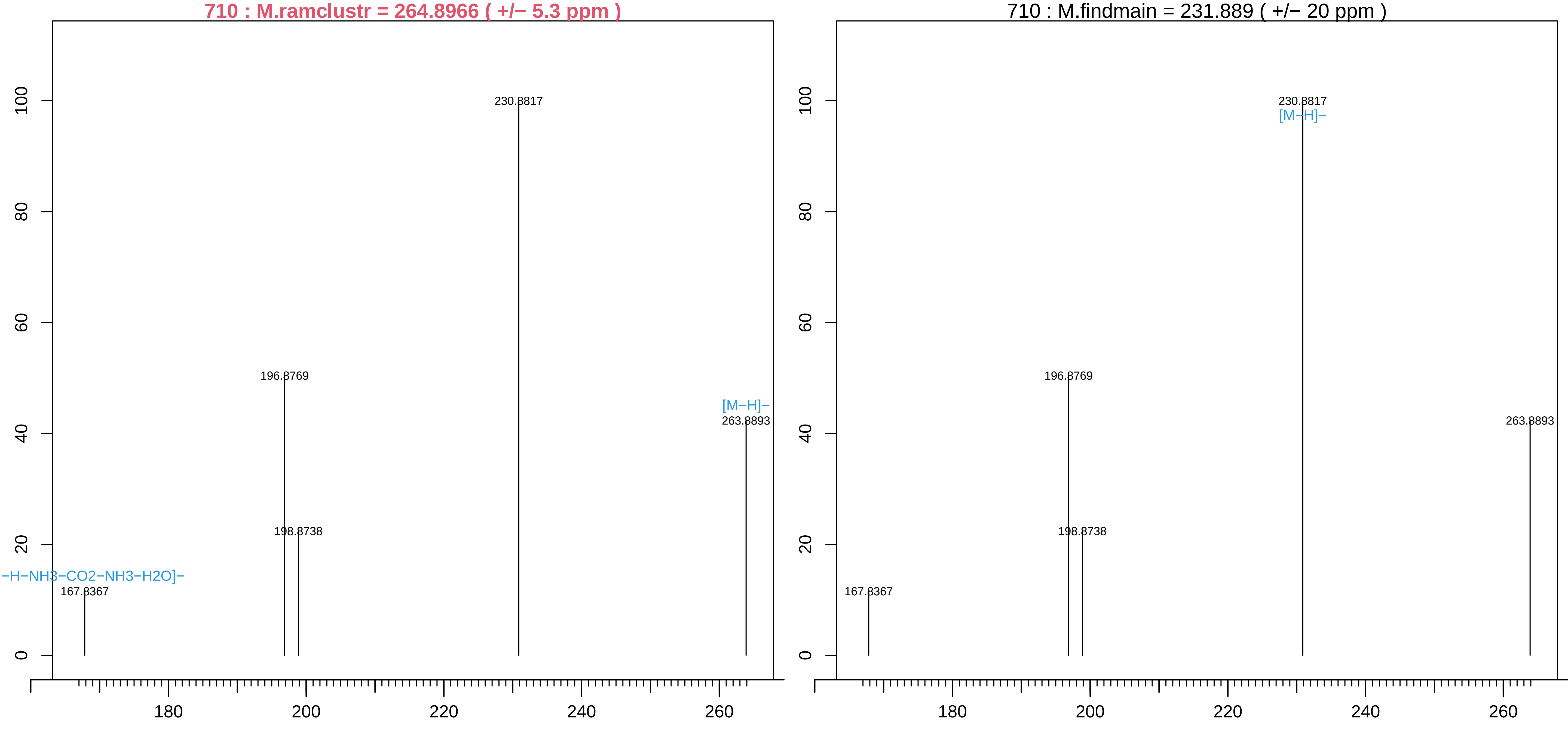

710 : M.ramclustr = 264.8966 (+/− 5.3 ppm)
230.8817
196.8769
[M−H]−
263.8893
198.8738
−H−NH3−CO2−NH3−H2O]−
167.8367
710 : M.findmain = 231.889 (+/− 20 ppm)
230.8817
[M−H]−
196.8769
263.8893
198.8738
167.8367
0
20
40
60
80
100
180
200
220
240
260

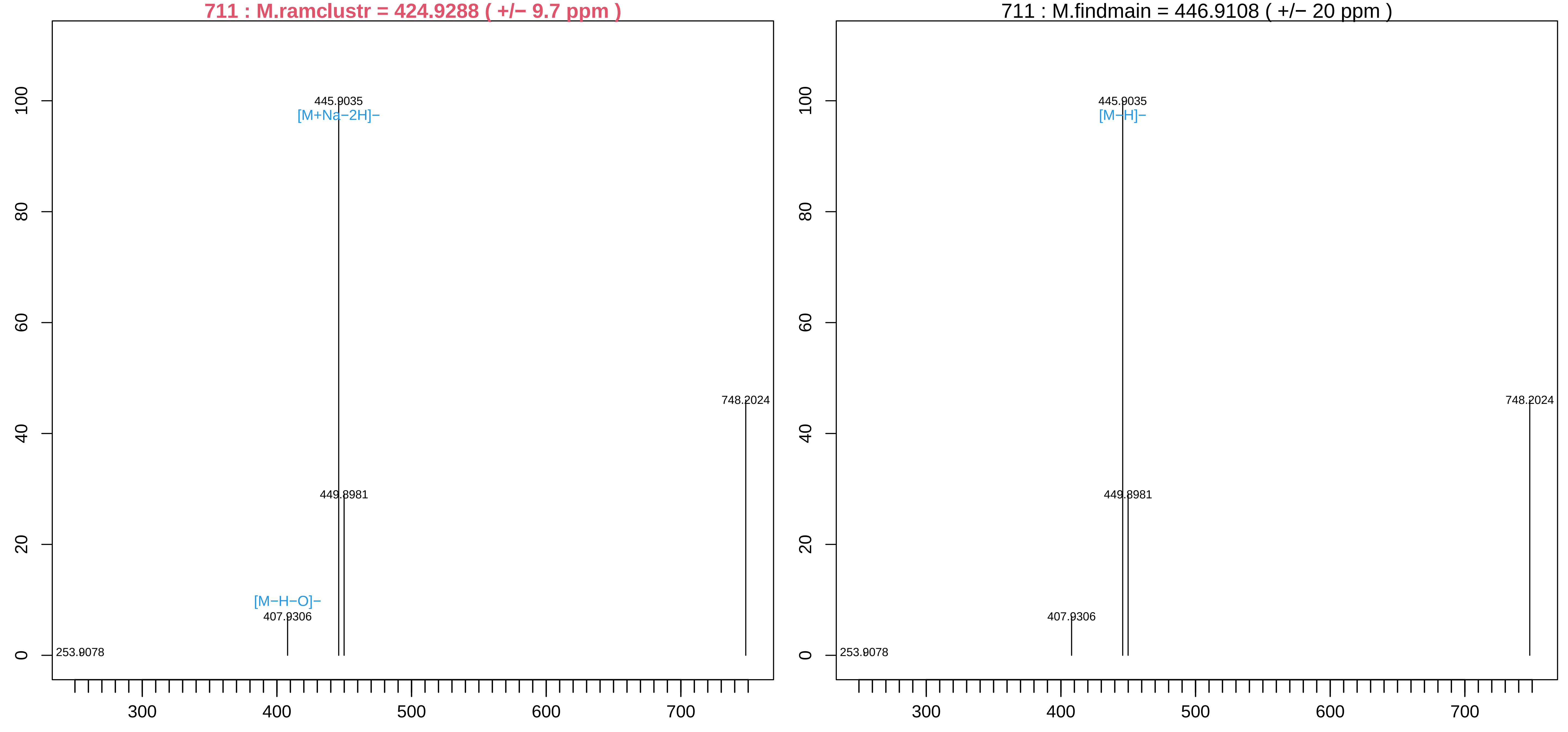
711 : M.ramclustr = 424.9288 (+/− 9.7 ppm)
445.9035
[M+Na−2H]−
748.2024
449.8981
[M−H−O]−
407.9306
253.9078
711 : M.findmain = 446.9108 (+/− 20 ppm)
445.9035
[M−H]−
748.2024
449.8981
407.9306
253.9078
0
20
40
60
80
100
300
400
500
600
700

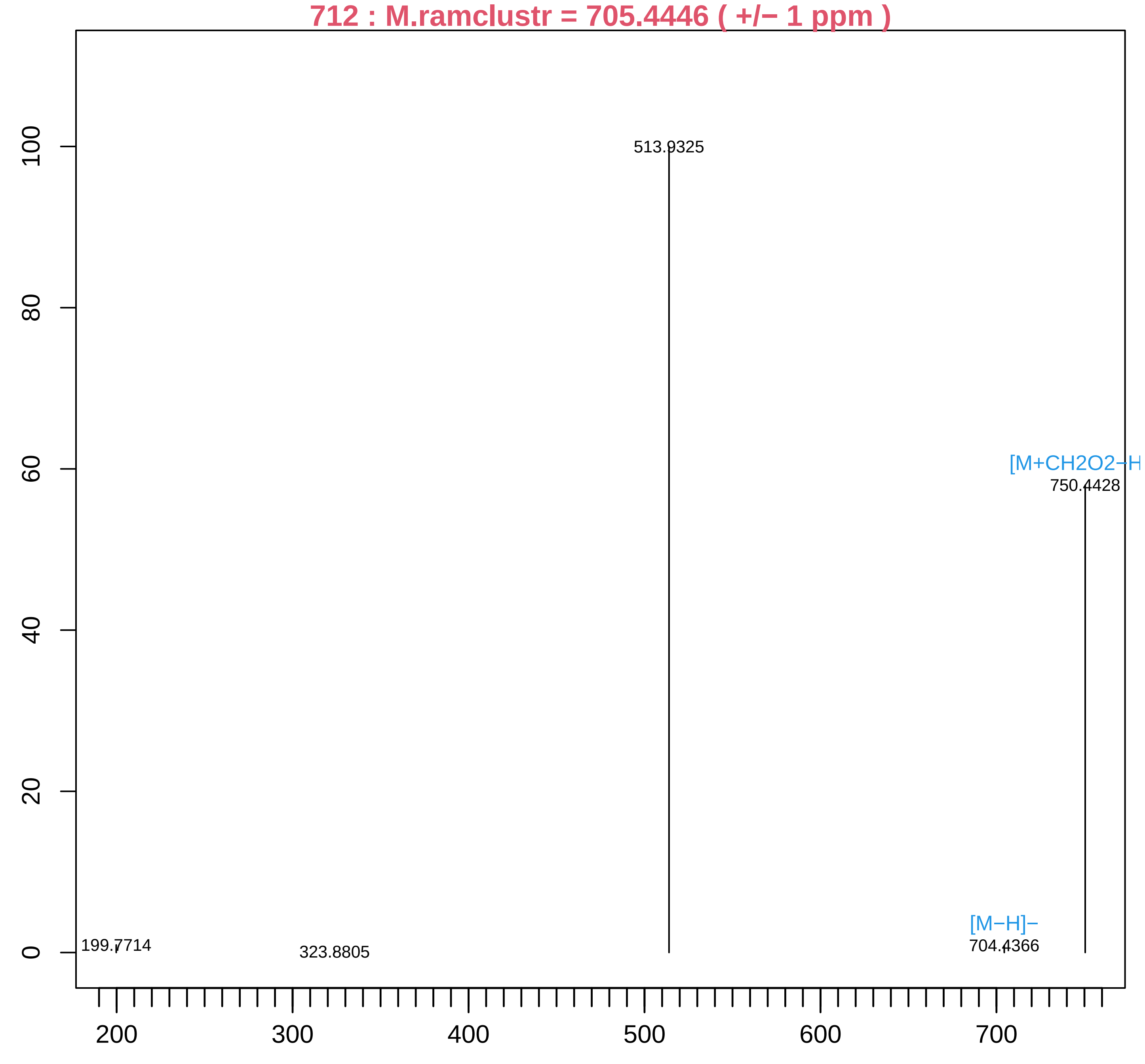
712 : M.ramclustr = 705.4446 (+/− 1 ppm)
513.9325
[M+CH2O2−H
750.4428
[M−H]−
704.4366
199.7714
323.8805
0
20
40
60
80
100
200
300
400
500
600
700

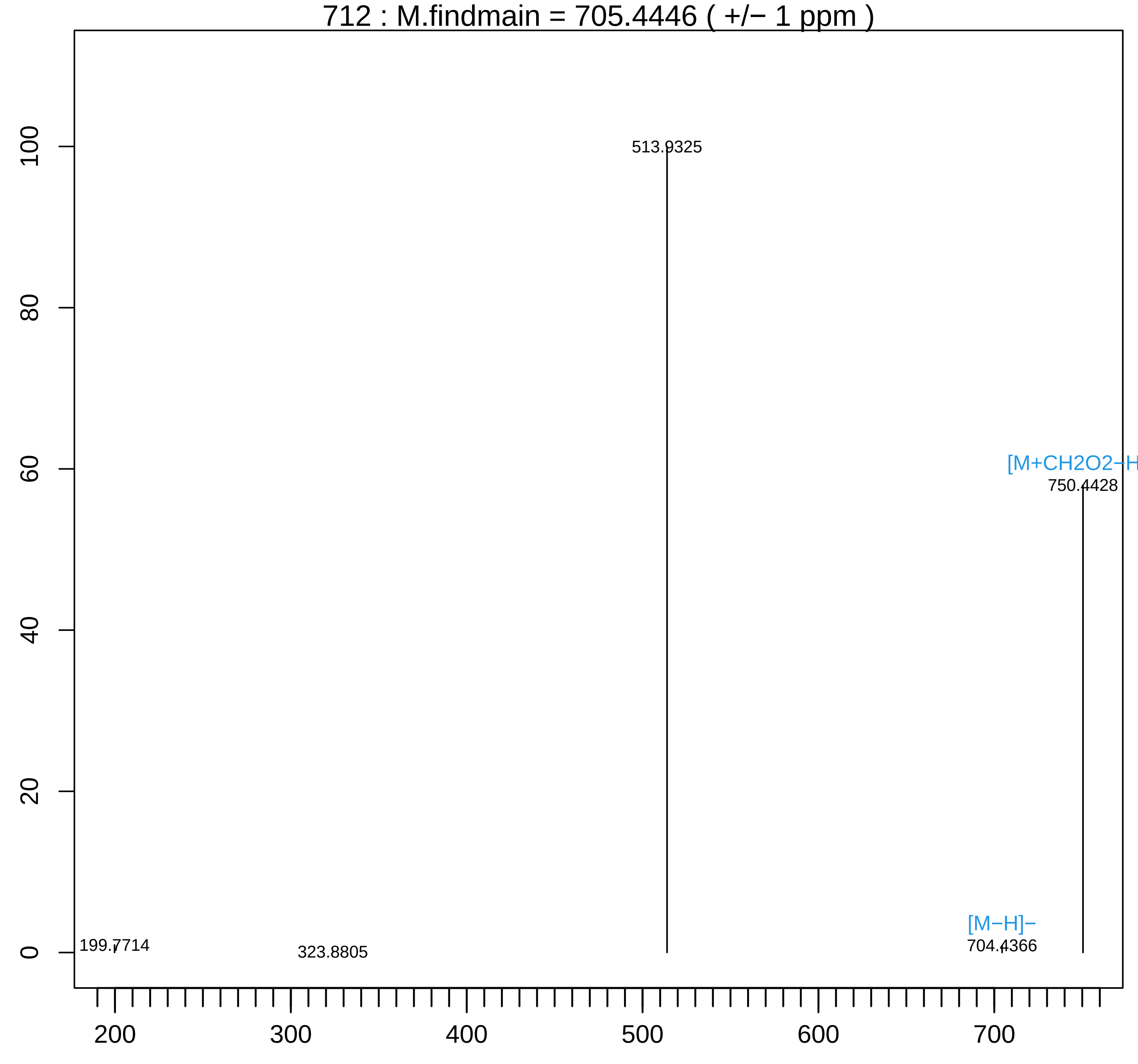
712 : M.findmain = 705.4446 (+/− 1 ppm)
513.9325
[M+CH2O2−H
750.4428
[M−H]−
704.4366
199.7714
323.8805
0
20
40
60
80
100
200
300
400
500
600
700

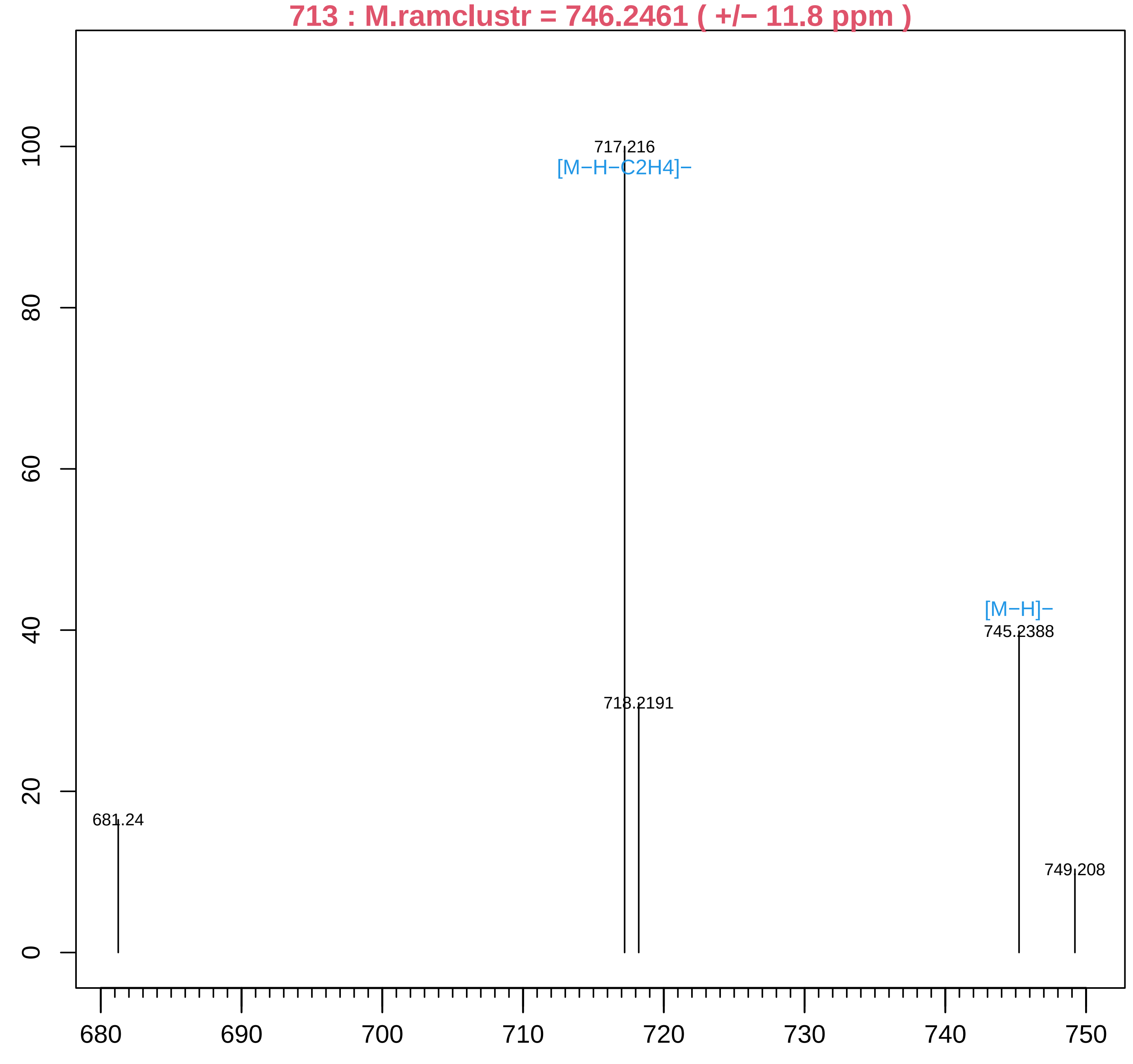
713 : M.ramclustr = 746.2461 (+/− 11.8 ppm)
717.216
[M−H−C2H4]−
718.2191
[M−H]−
745.2388
681.24
749.208
0
20
40
60
80
100
680
690
700
710
720
730
740
750

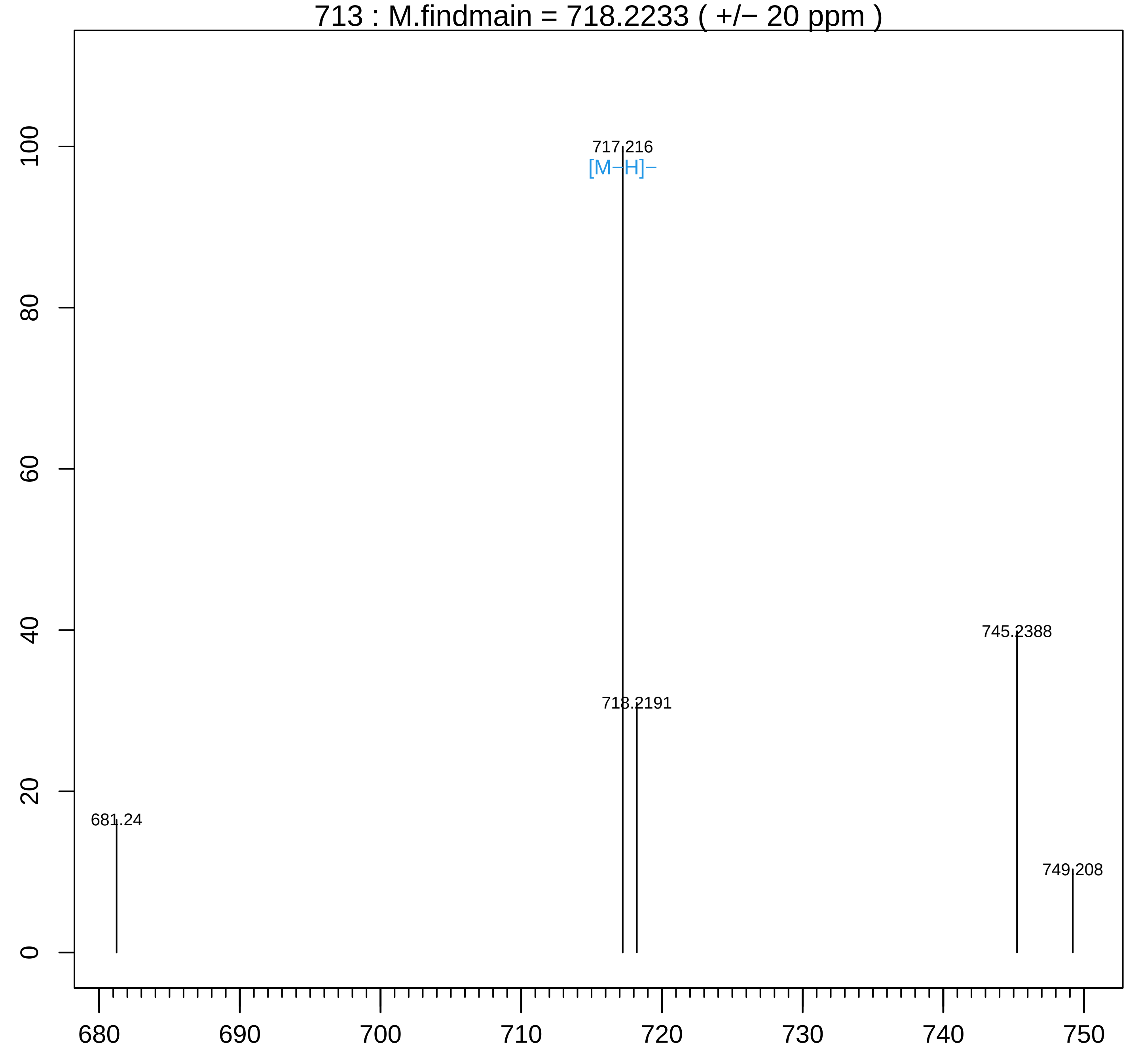
713 : M.findmain = 718.2233 (+/− 20 ppm)
717.216
[M−H]−
718.2191
745.2388
681.24
749.208
0
20
40
60
80
100
680
690
700
710
720
730
740
750

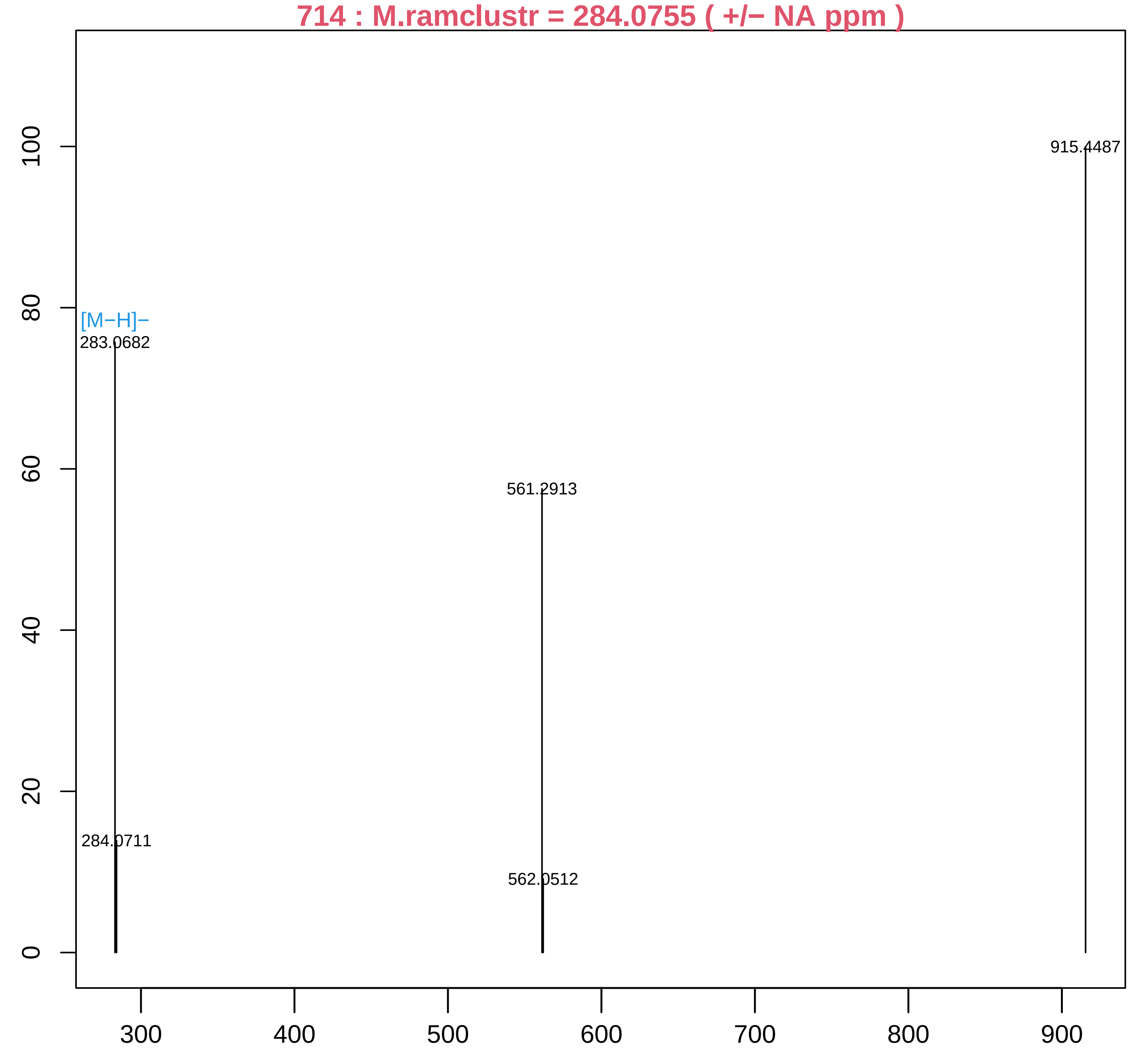
714 : M.ramclustr = 284.0755 (+/− NA ppm)
[M−H]−
283.0682
284.0711
561.2913
562.0512
915.4487
0
20
40
60
80
100
300
400
500
600
700
800
900

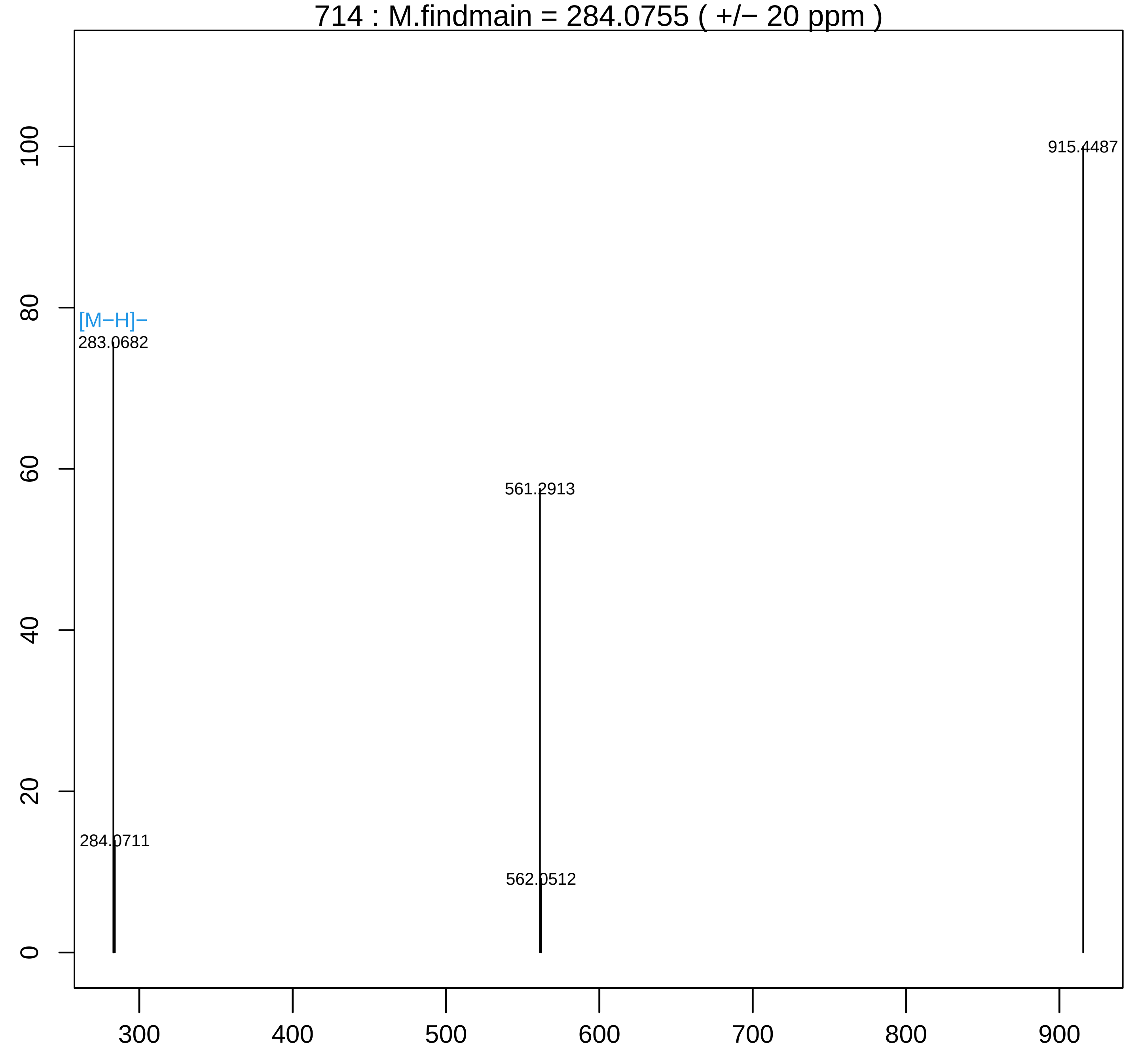
714 : M.findmain = 284.0755 (+/− 20 ppm)
[M−H]−
283.0682
284.0711
561.2913
562.0512
915.4487
0
20
40
60
80
100
300
400
500
600
700
800
900

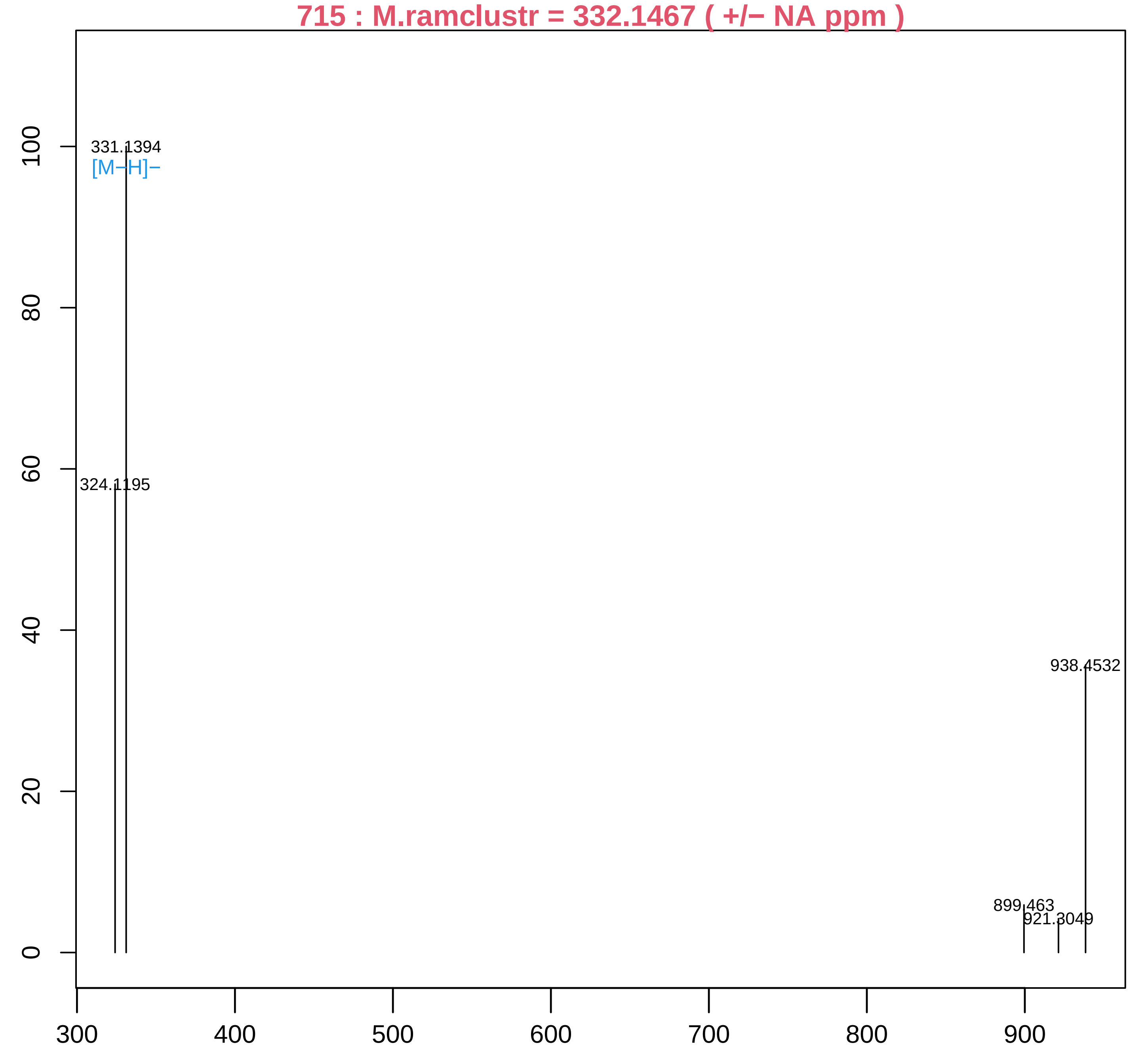

715 : M.ramclustr = 332.1467 (+/− NA ppm)
331.1394
[M−H]−
324.1195
938.4532
899.463
921.3049
0
20
40
60
80
100
300
400
500
600
700
800
900

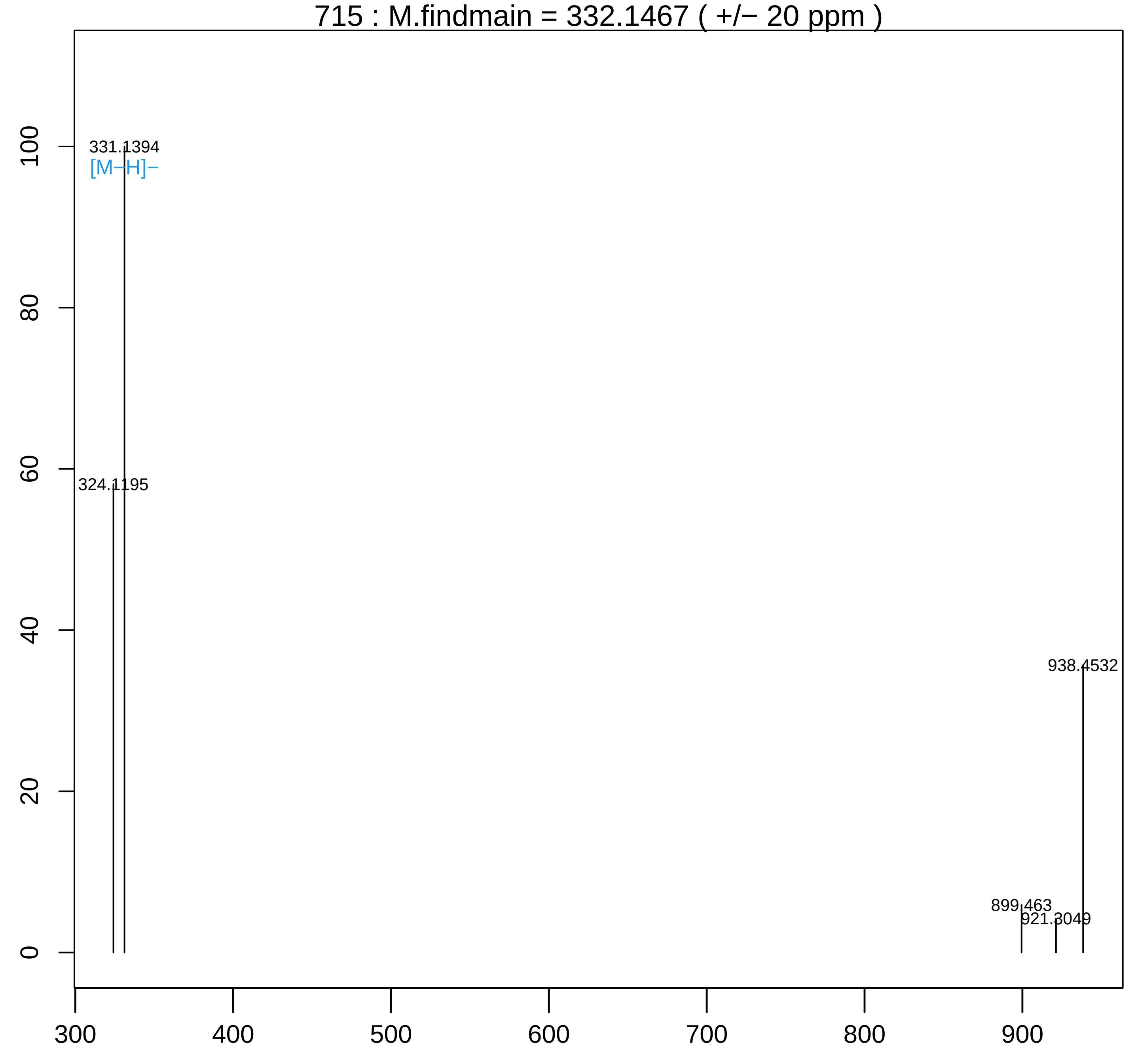

715 : M.findmain = 332.1467 (+/− 20 ppm)
331.1394
[M−H]−
324.1195
938.4532
899.463
921.3049
0
20
40
60
80
100
300
400
500
600
700
800
900

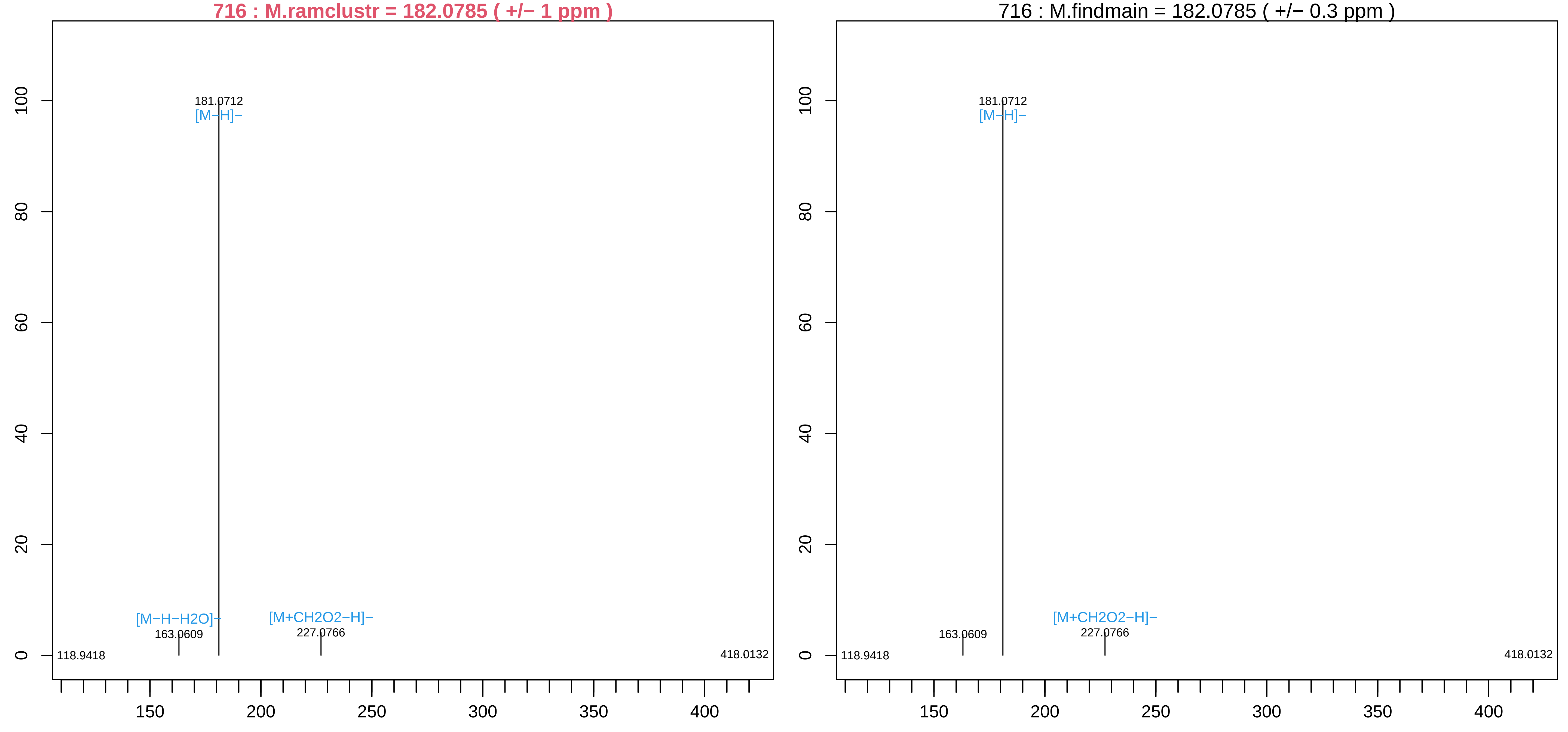

716 : M.ramclustr = 182.0785 (+/− 1 ppm)
181.0712
[M−H]−
[M−H−H2O]−
163.0609
[M+CH2O2−H]−
227.0766
118.9418
418.0132
716 : M.findmain = 182.0785 (+/− 0.3 ppm)
181.0712
[M−H]−
163.0609
[M+CH2O2−H]−
227.0766
118.9418
418.0132

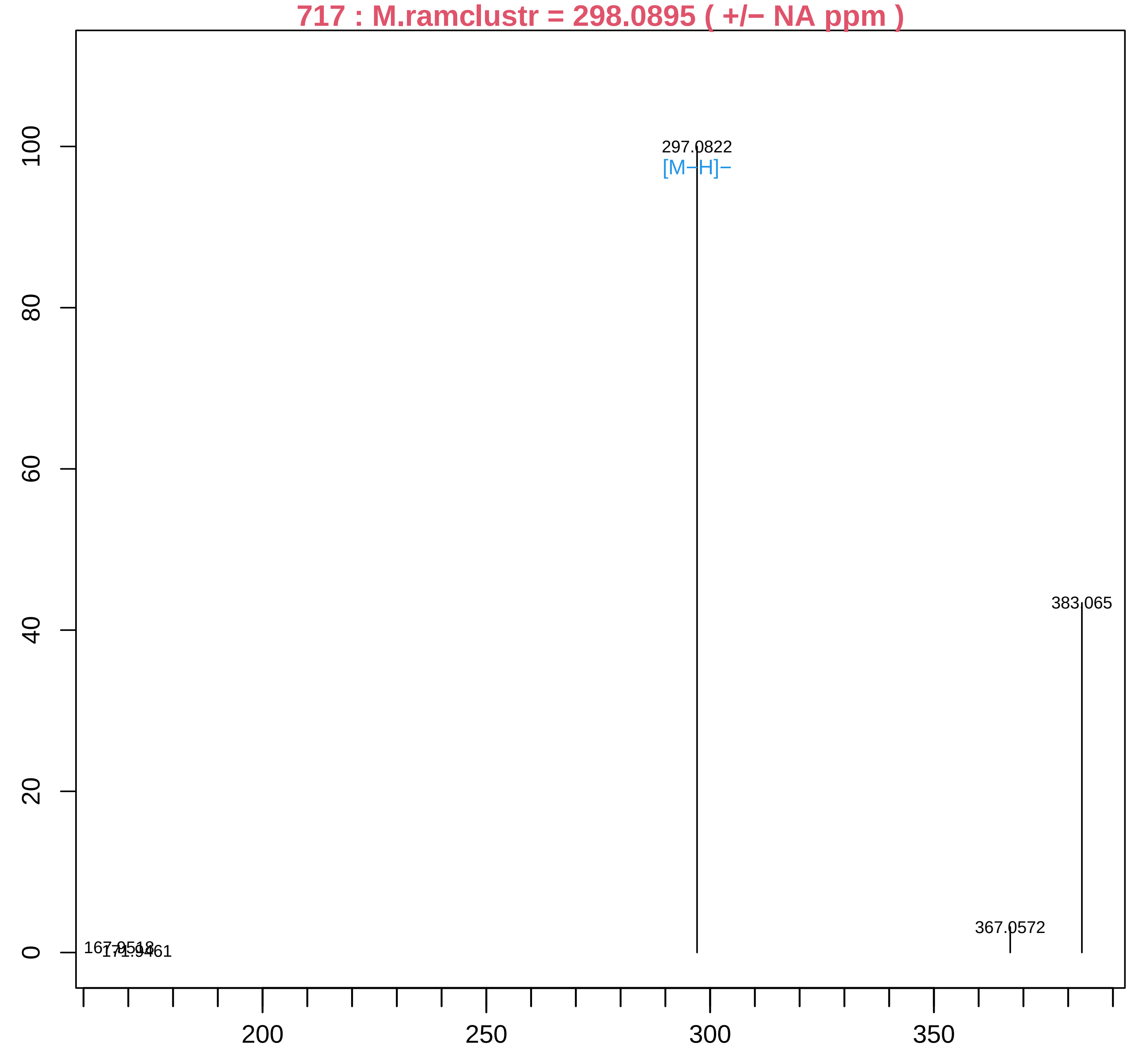

717 : M.ramclustr = 298.0895 (+/− NA ppm)
297.0822
[M−H]−
383.065
367.0572
167.9518
171.9461
0
20
40
60
80
100
200
250
300
350

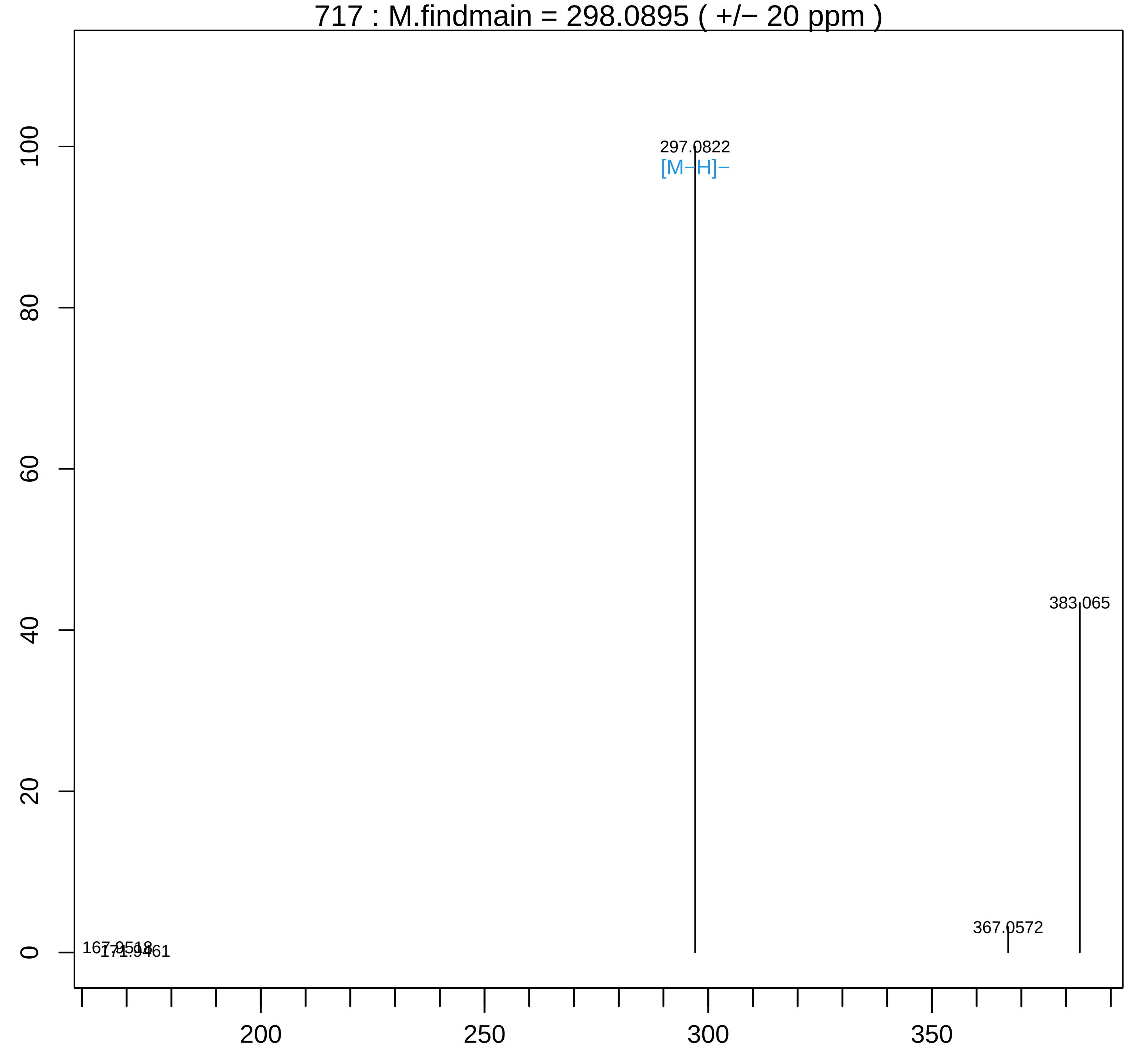

717 : M.findmain = 298.0895 (+/− 20 ppm)
297.0822
[M−H]−
383.065
367.0572
167.9518
171.9461
0
20
40
60
80
100
200
250
300
350

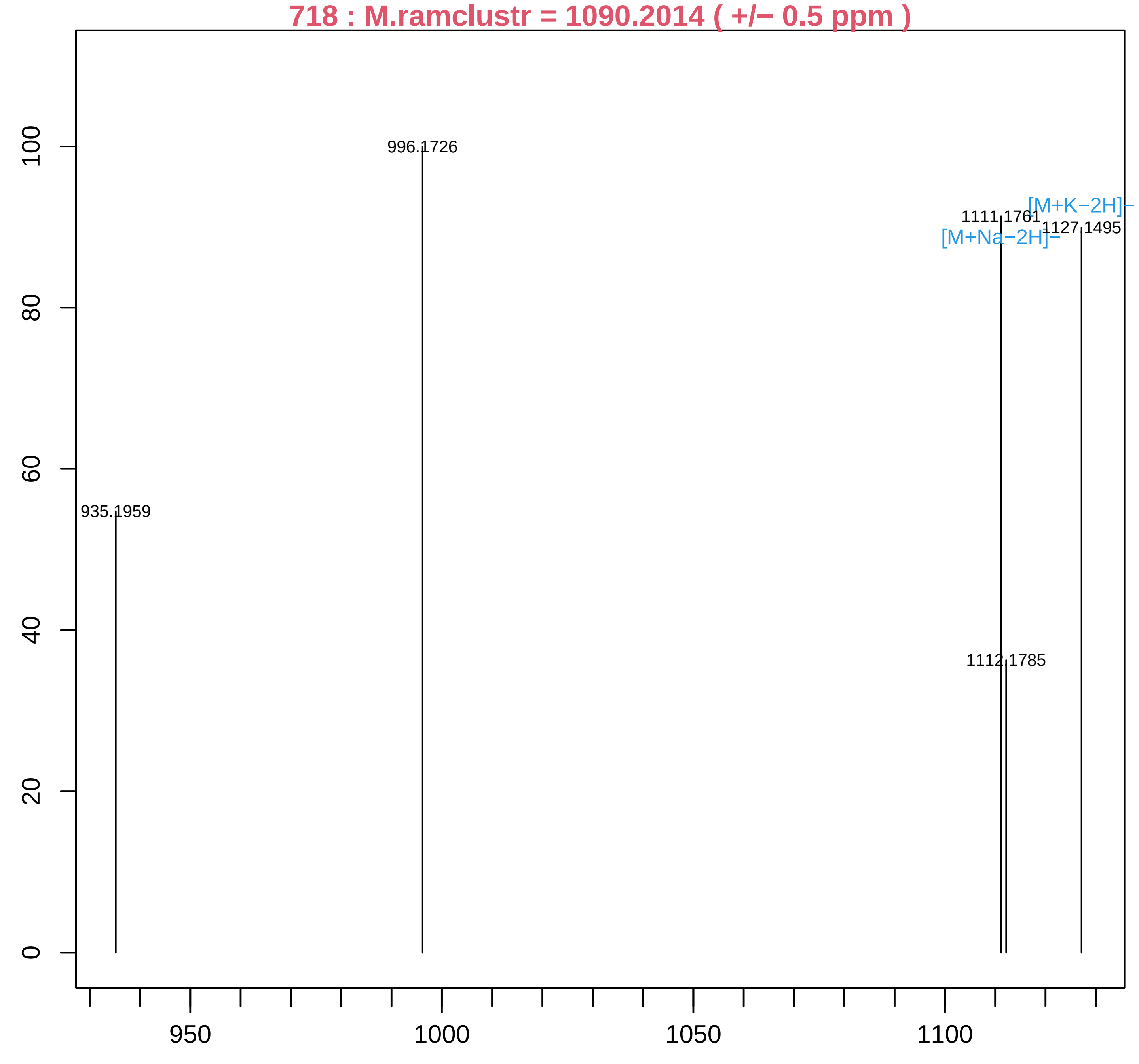
718 : M.ramclustr = 1090.2014 (+/− 0.5 ppm)
996.1726
935.1959
1111.1761
[M+Na−2H]−
[M+K−2H]−
1127.1495
1112.1785
0
20
40
60
80
100
950
1000
1050
1100

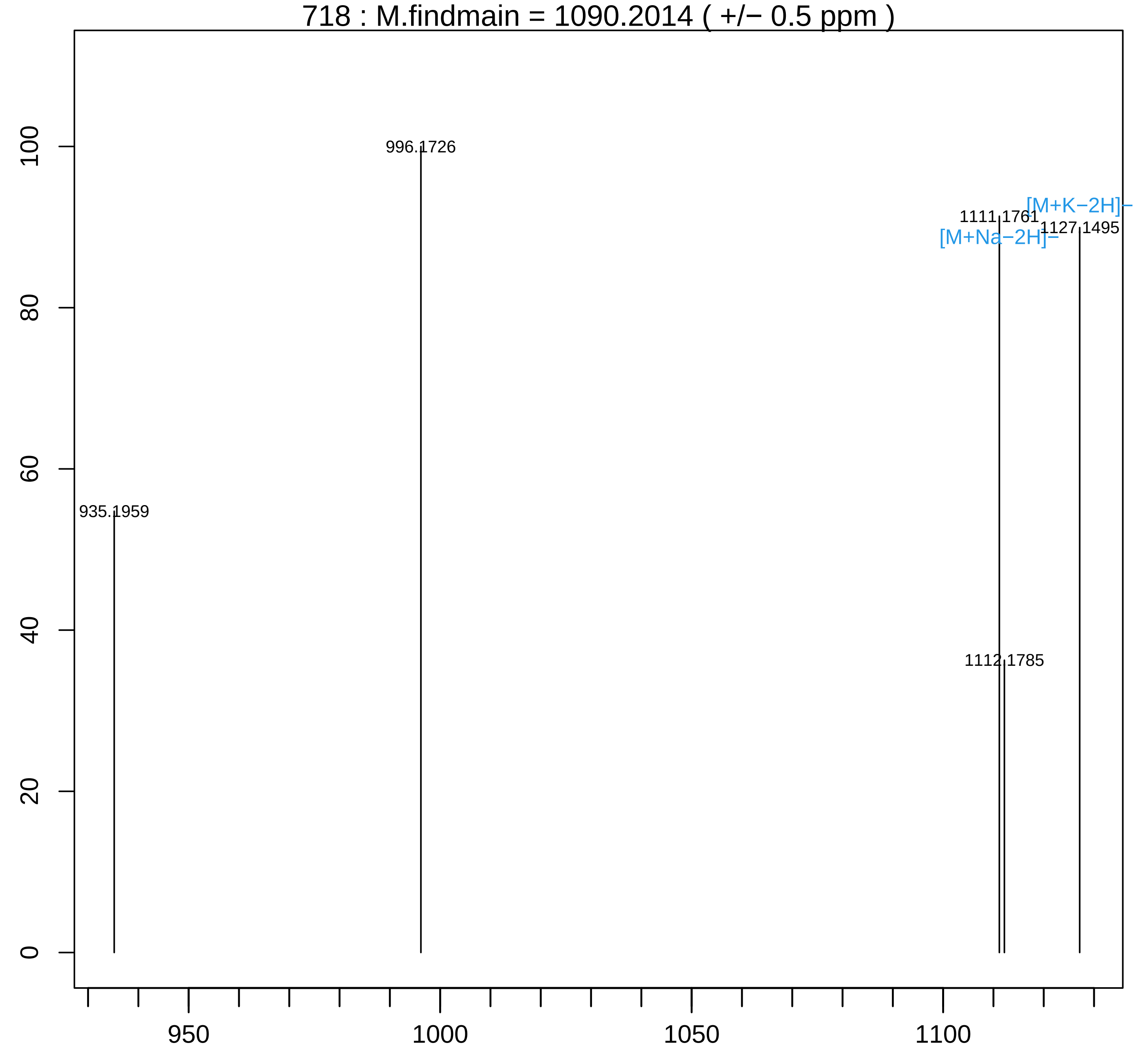
718 : M.findmain = 1090.2014 (+/− 0.5 ppm)
996.1726
935.1959
1111.1761
[M+Na−2H]−
[M+K−2H]−
1127.1495
1112.1785
0
20
40
60
80
100
950
1000
1050
1100

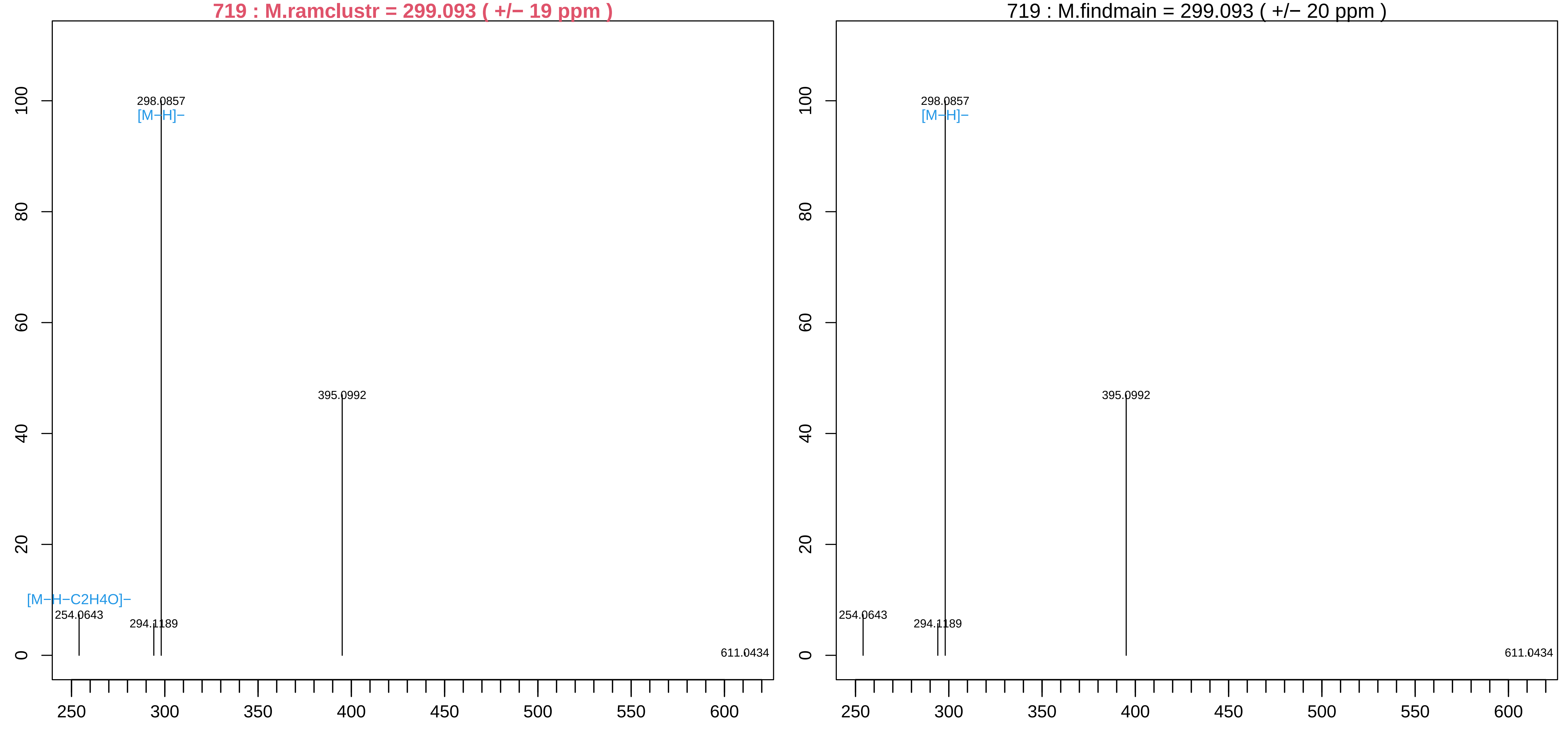
719 : M.ramclustr = 299.093 (+/− 19 ppm)
298.0857
[M−H]−
395.0992
[M−H−C2H4O]−
254.0643
294.1189
611.0434
719 : M.findmain = 299.093 (+/− 20 ppm)
298.0857
[M−H]−
395.0992
254.0643
294.1189
611.0434

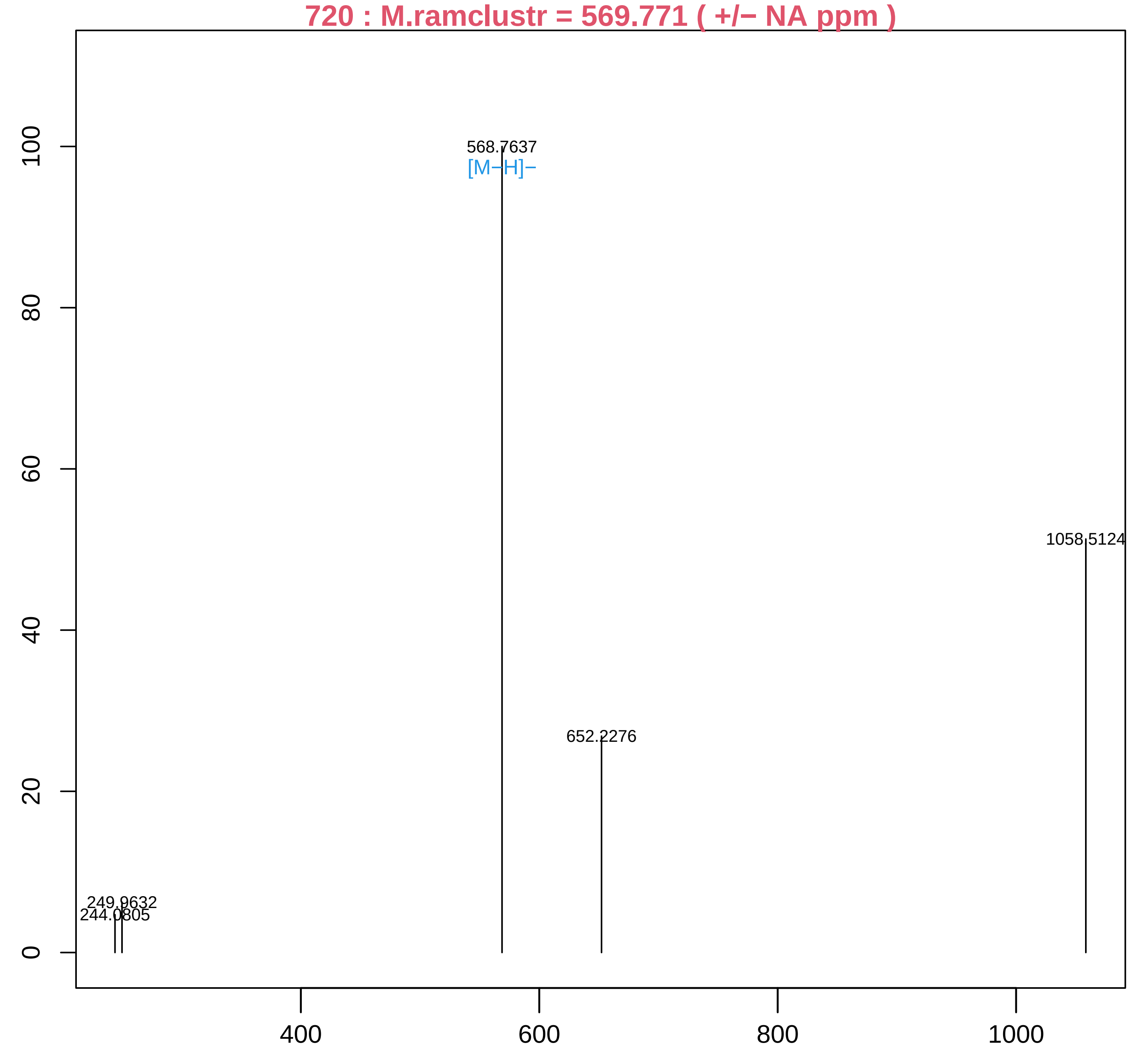
720 : M.ramclustr = 569.771 (+/− NA ppm)
568.7637
[M−H]−
1058.5124
652.2276
249.9632
244.0805
0
20
40
60
80
100
400
600
800
1000
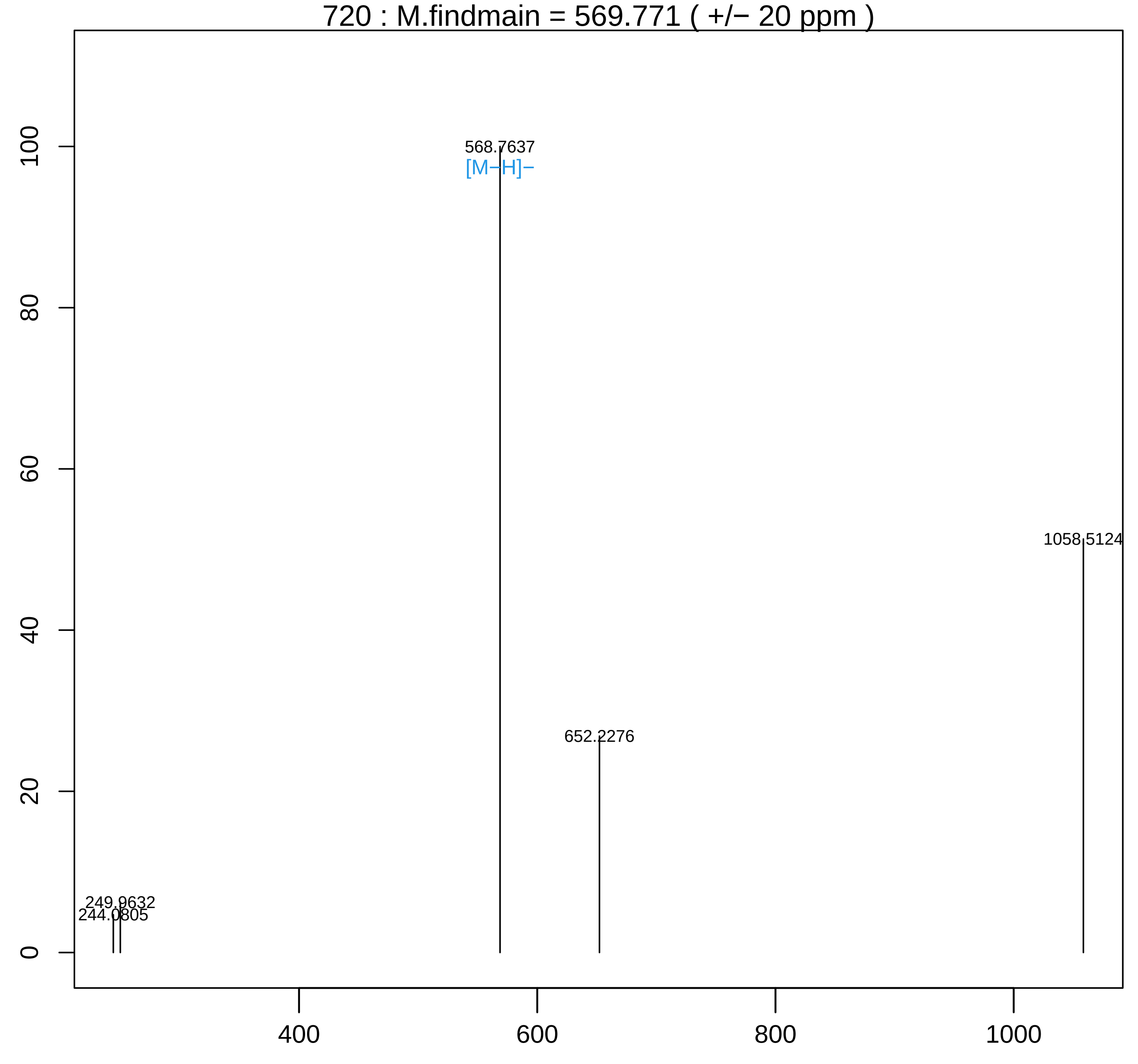
720 : M.findmain = 569.771 (+/− 20 ppm)
568.7637
[M−H]−
1058.5124
652.2276
249.9632
244.0805
0
20
40
60
80
100
400
600
800
1000

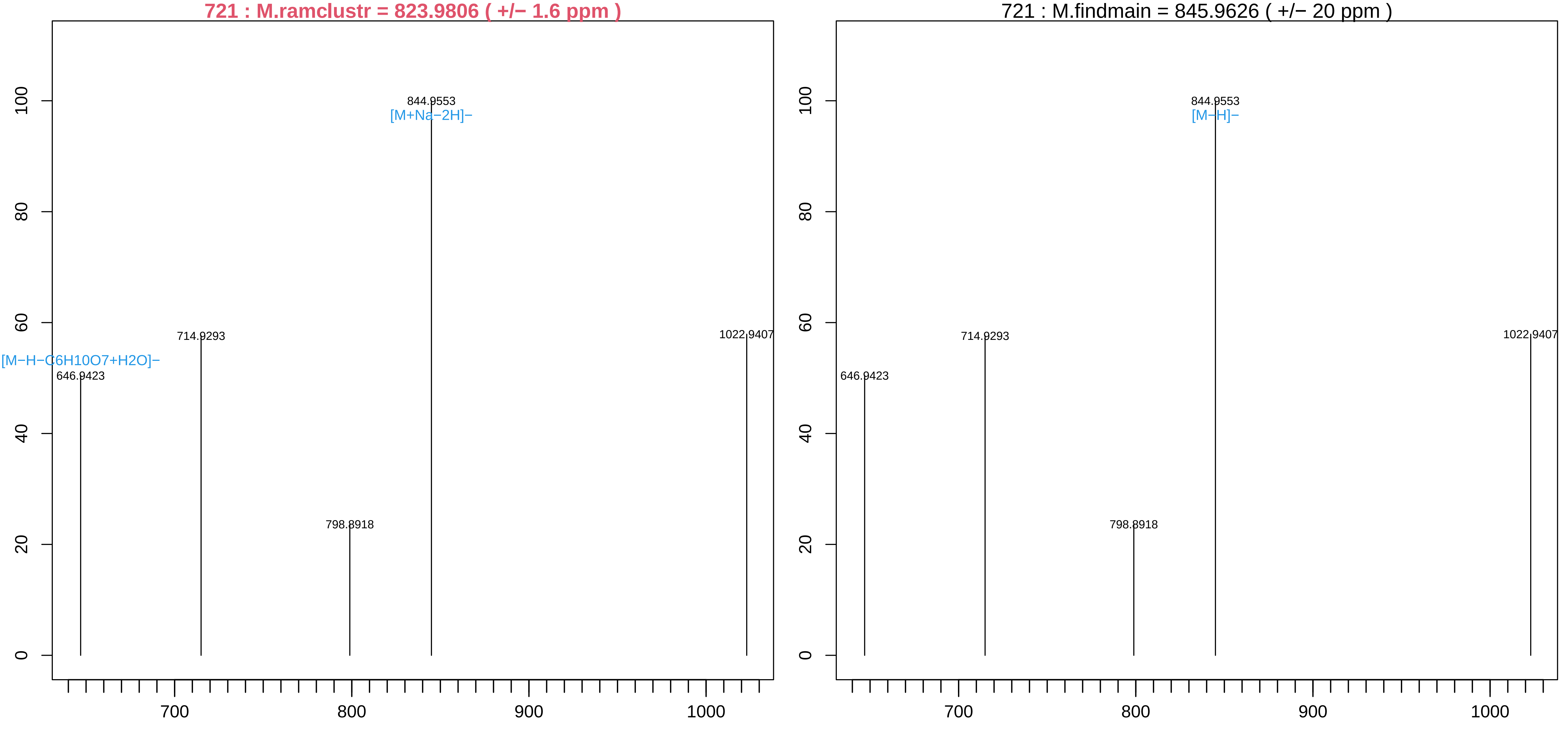

721 : M.ramclustr = 823.9806 (+/− 1.6 ppm)
844.9553
[M+Na−2H]−
714.9293
1022.9407
[M−H−C6H10O7+H2O]−
646.9423
798.8918
0
20
40
60
80
100
700
800
900
1000
721 : M.findmain = 845.9626 (+/− 20 ppm)
844.9553
[M−H]−
714.9293
1022.9407
646.9423
798.8918
0
20
40
60
80
100
700
800
900
1000

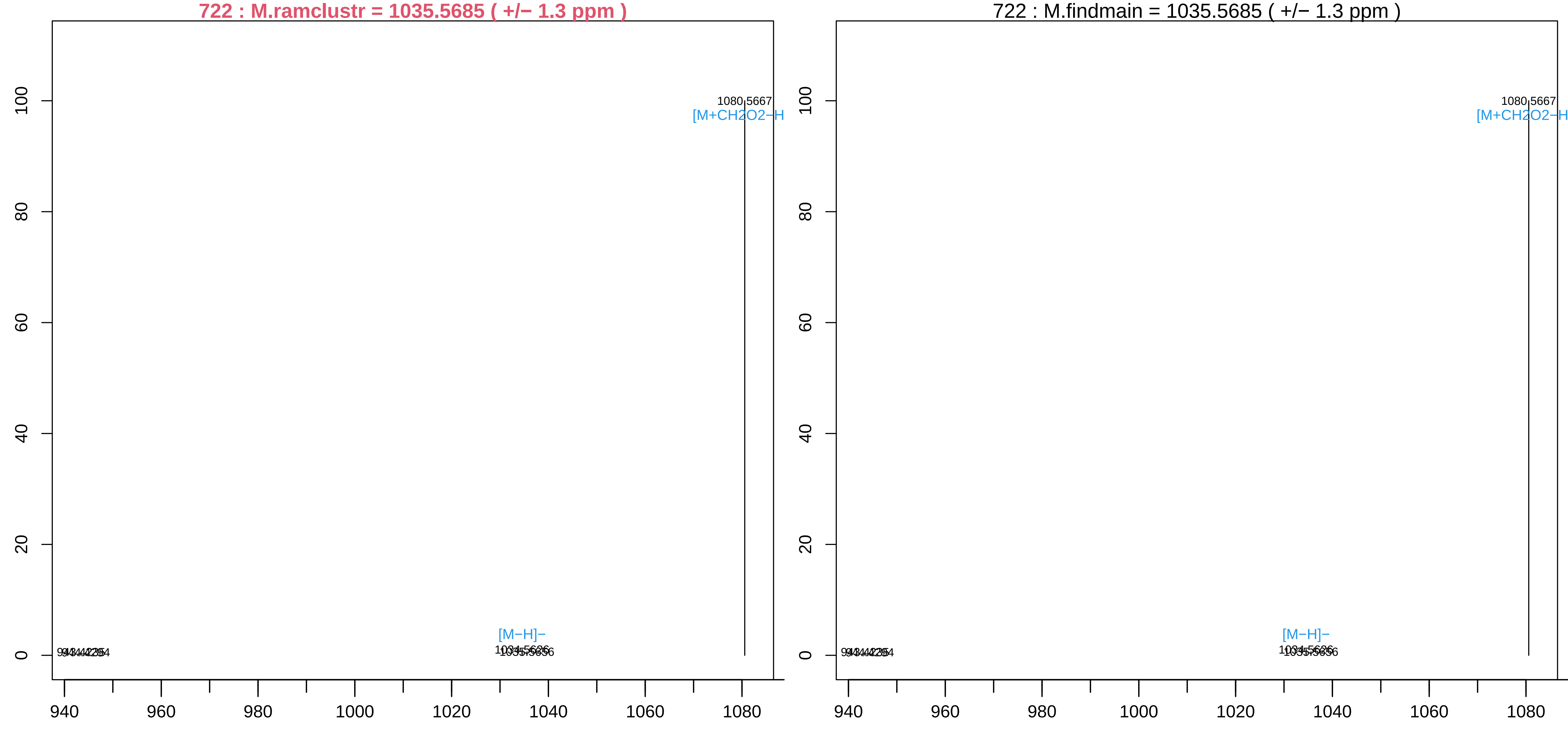
722 : M.ramclustr = 1035.5685 (+/− 1.3 ppm)
722 : M.findmain = 1035.5685 (+/− 1.3 ppm)
1080.5667
[M+CH2O2−H
[M−H]−
0
20
40
60
80
100
940
960
980
1000
1020
1040
1060
1080

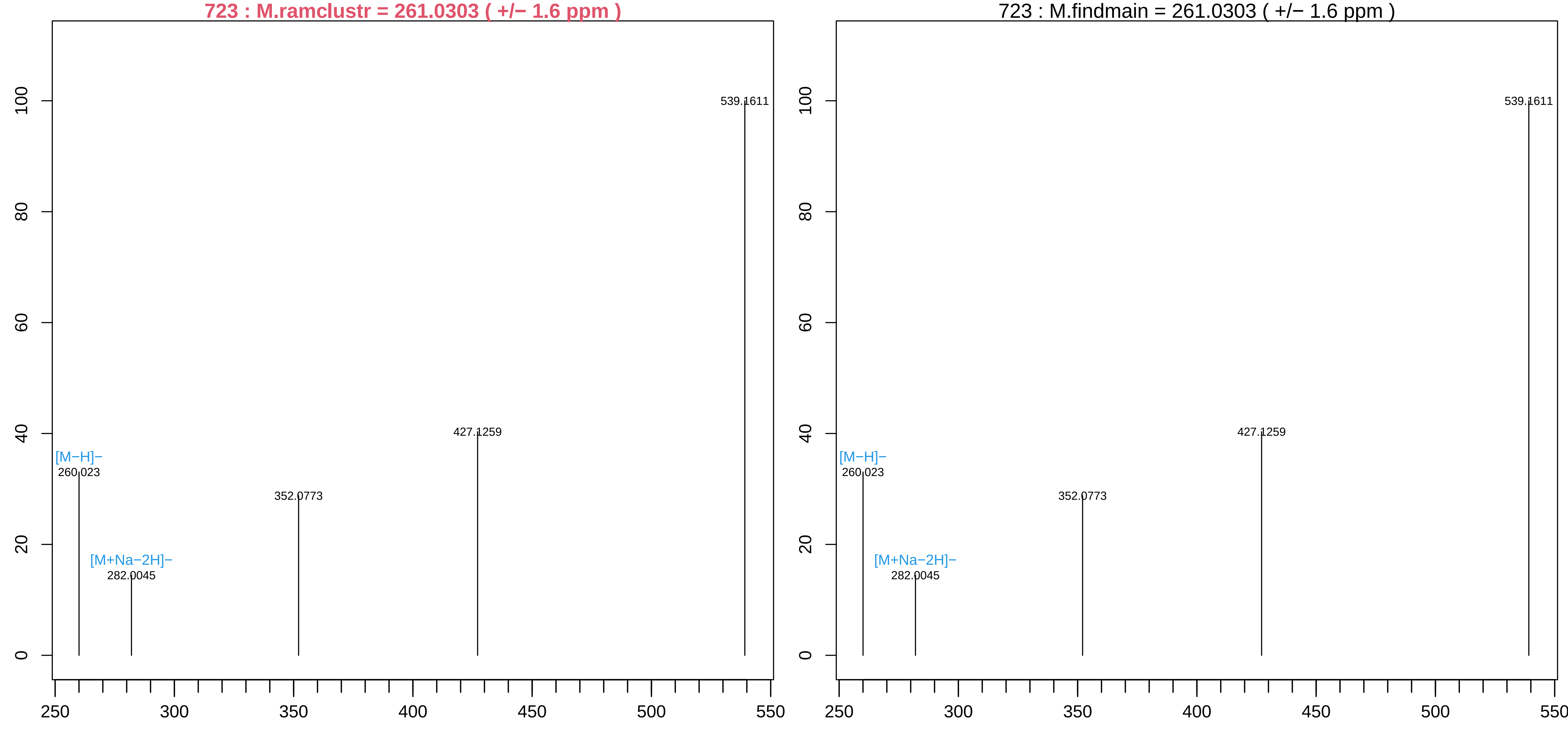
723 : M.ramclustr = 261.0303 (+/− 1.6 ppm)
723 : M.findmain = 261.0303 (+/− 1.6 ppm)
539.1611
427.1259
[M−H]−
260.023
352.0773
[M+Na−2H]−
282.0045
0
20
40
60
80
100
250
300
350
400
450
500
550

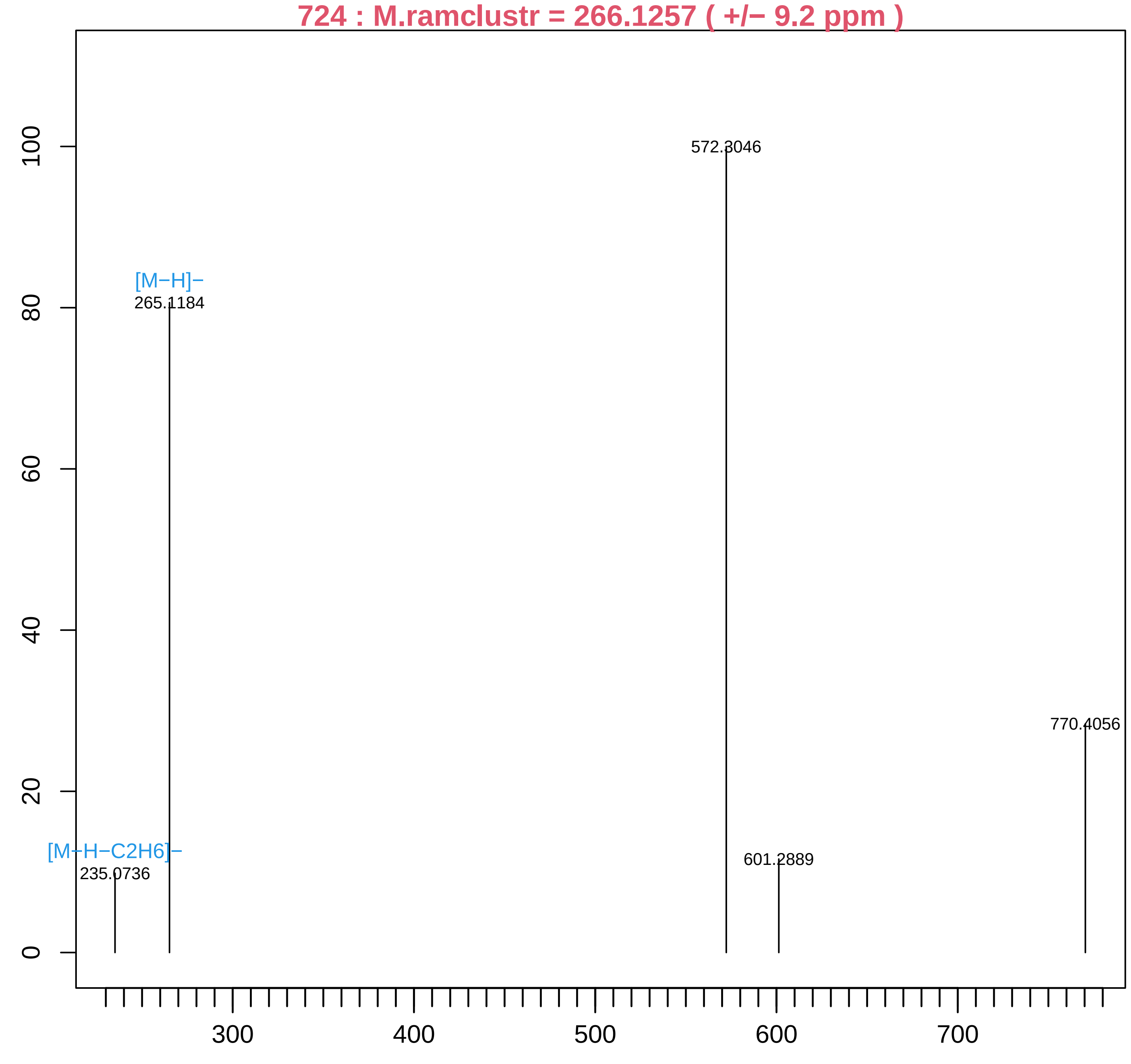
724 : M.ramclustr = 266.1257 (+/− 9.2 ppm)
[M−H]−
265.1184
572.3046
770.4056
601.2889
[M−H−C2H6]−
235.0736
0
20
40
60
80
100
300
400
500
600
700

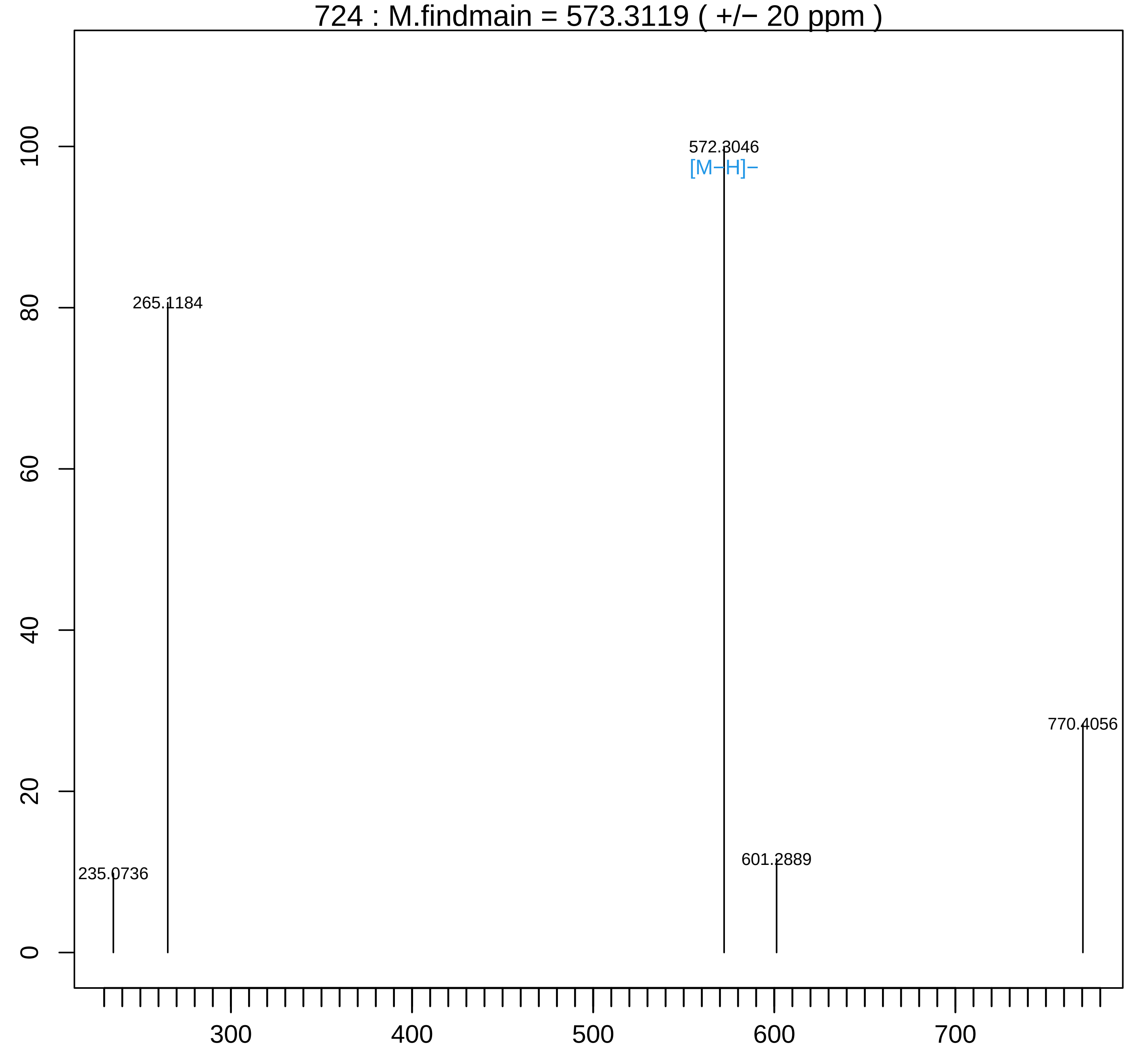
724 : M.findmain = 573.3119 (+/− 20 ppm)
572.3046
[M−H]−
265.1184
770.4056
601.2889
235.0736
0
20
40
60
80
100
300
400
500
600
700

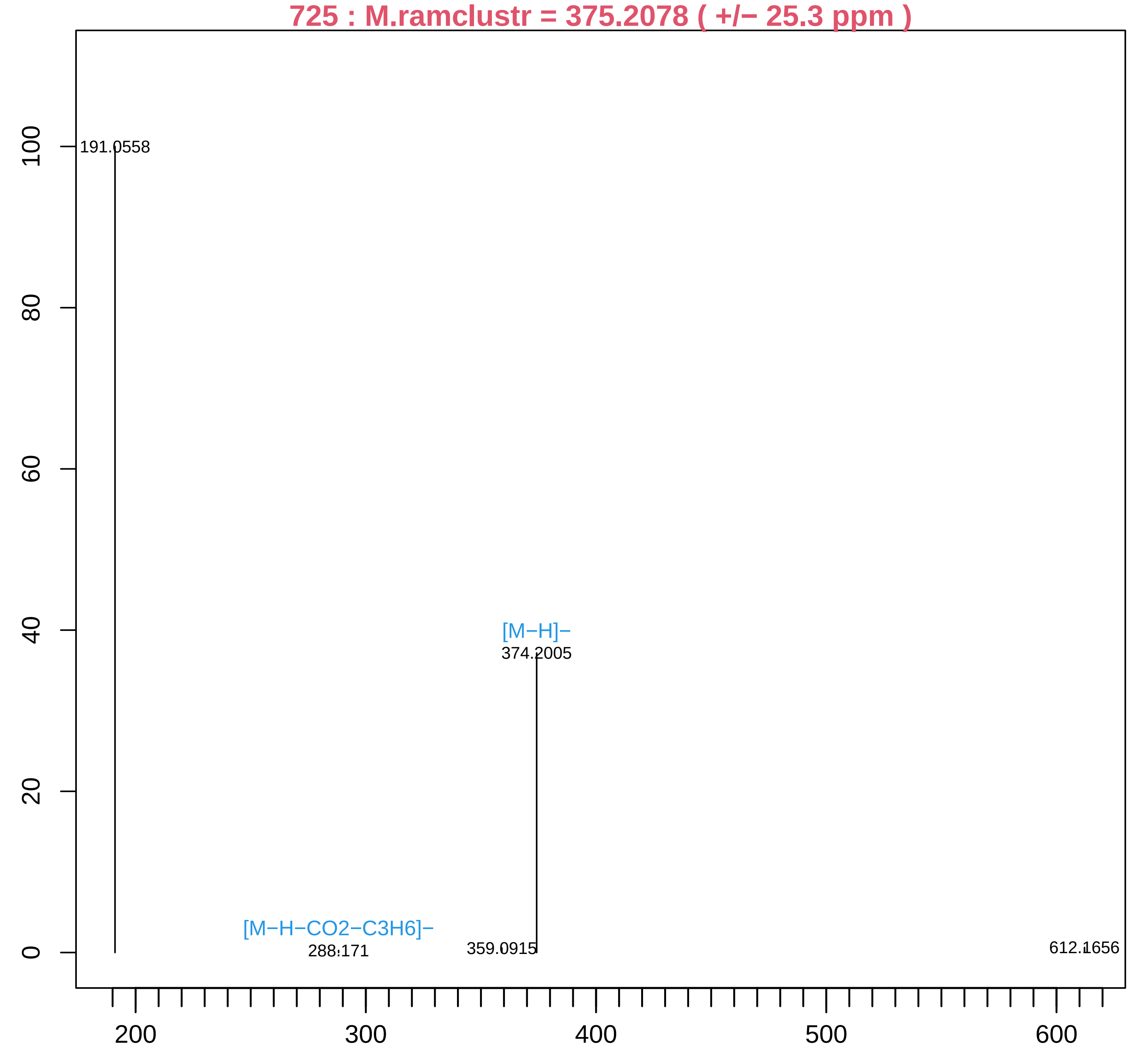

725 : M.ramclustr = 375.2078 (+/− 25.3 ppm)
191.0558
[M−H]−
374.2005
[M−H−CO2−C3H6]−
288.171
359.0915
612.1656
0
20
40
60
80
100
200
300
400
500
600

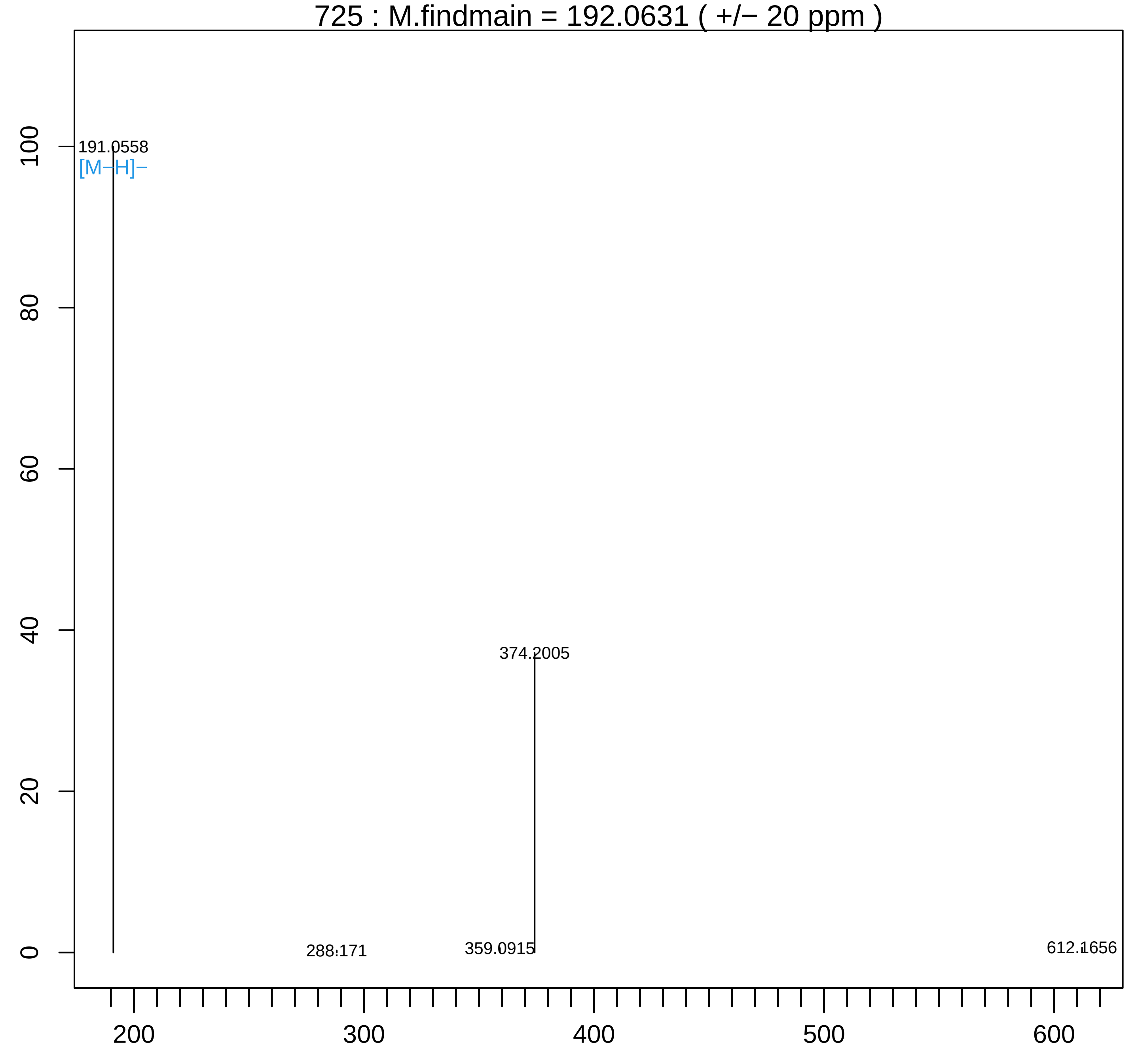

725 : M.findmain = 192.0631 (+/− 20 ppm)
191.0558
[M−H]−
374.2005
288.171
359.0915
612.1656
0
20
40
60
80
100
200
300
400
500
600

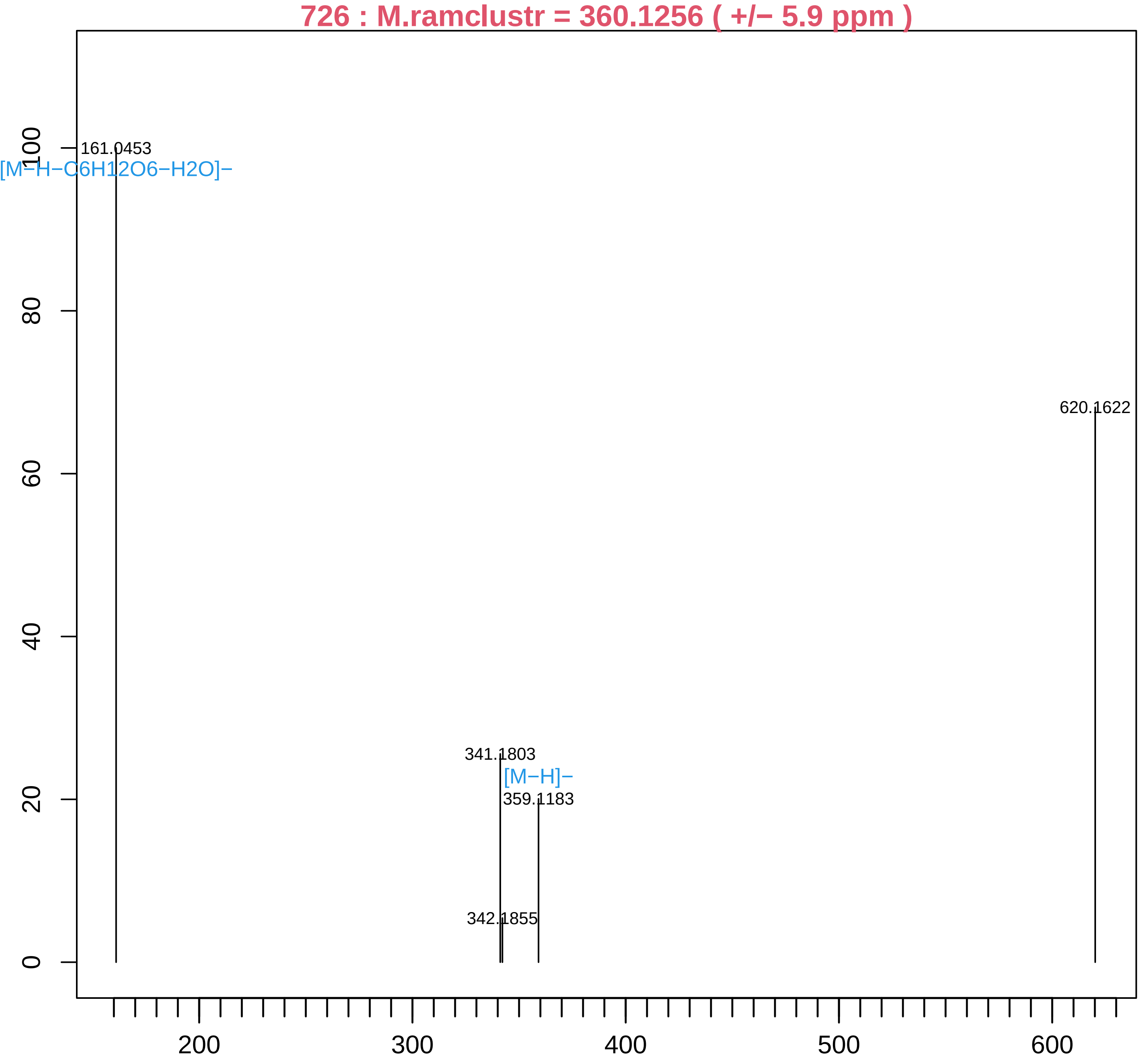
726 : M.ramclustr = 360.1256 (+/− 5.9 ppm)
161.0453
[M−H−C6H12O6−H2O]−
620.1622
341.1803
[M−H]−
359.1183
342.1855
0
20
40
60
80
100
200
300
400
500
600

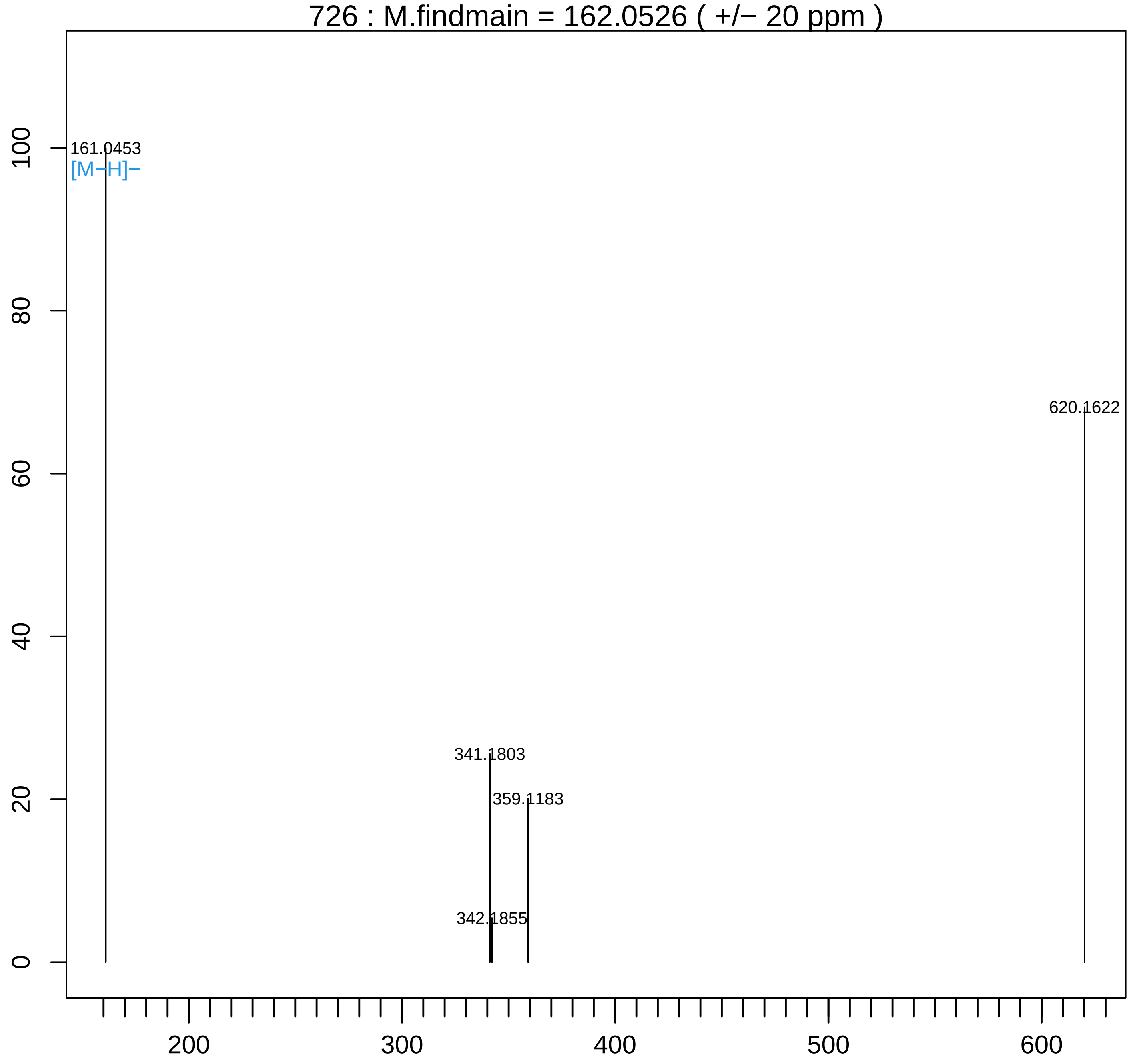
726 : M.findmain = 162.0526 (+/− 20 ppm)
161.0453
[M−H]−
620.1622
341.1803
359.1183
342.1855
0
20
40
60
80
100
200
300
400
500
600

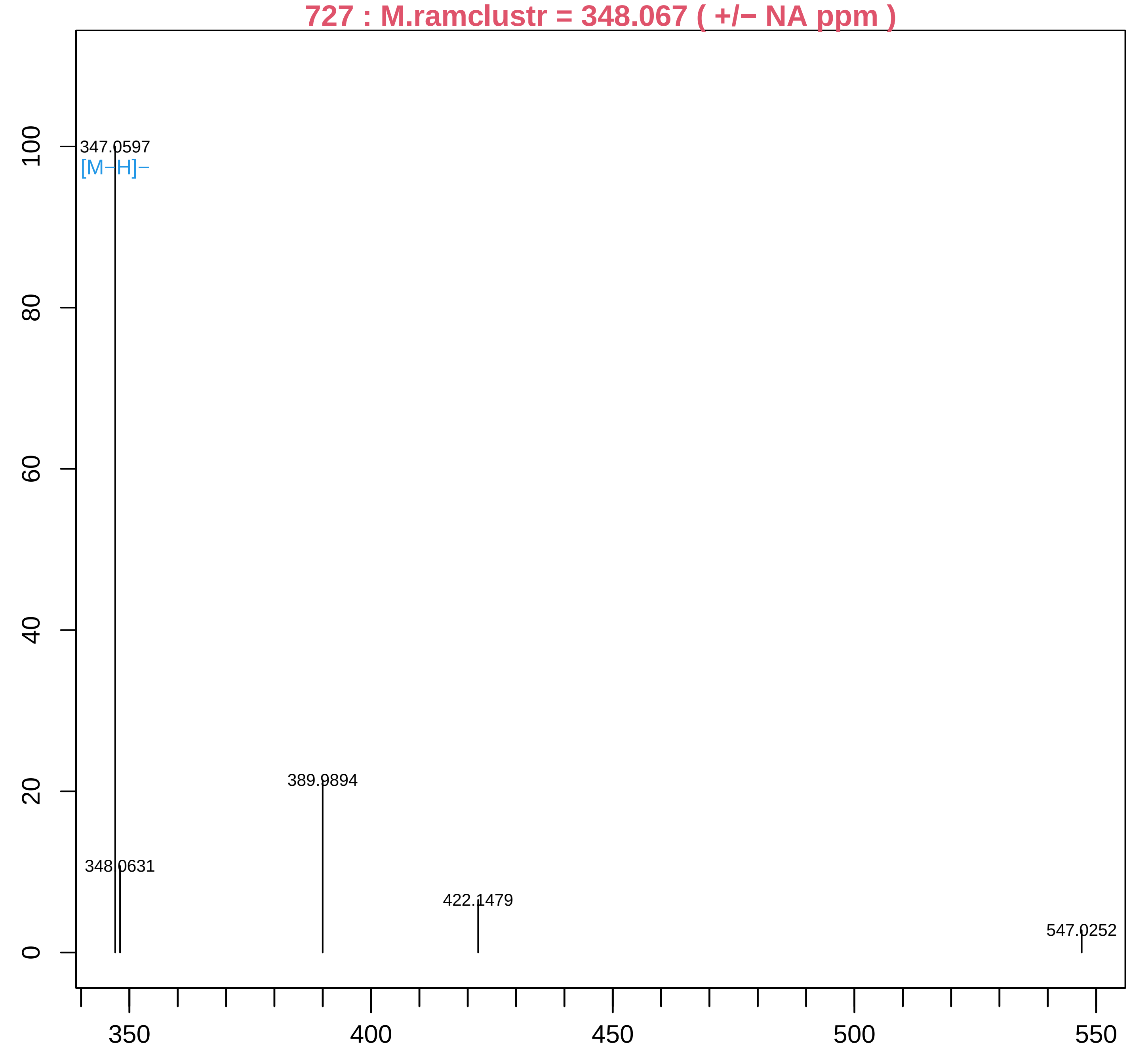
727 : M.ramclustr = 348.067 (+/− NA ppm)
347.0597
[M−H]−
348.0631
389.9894
422.1479
547.0252
0
20
40
60
80
100
350
400
450
500
550

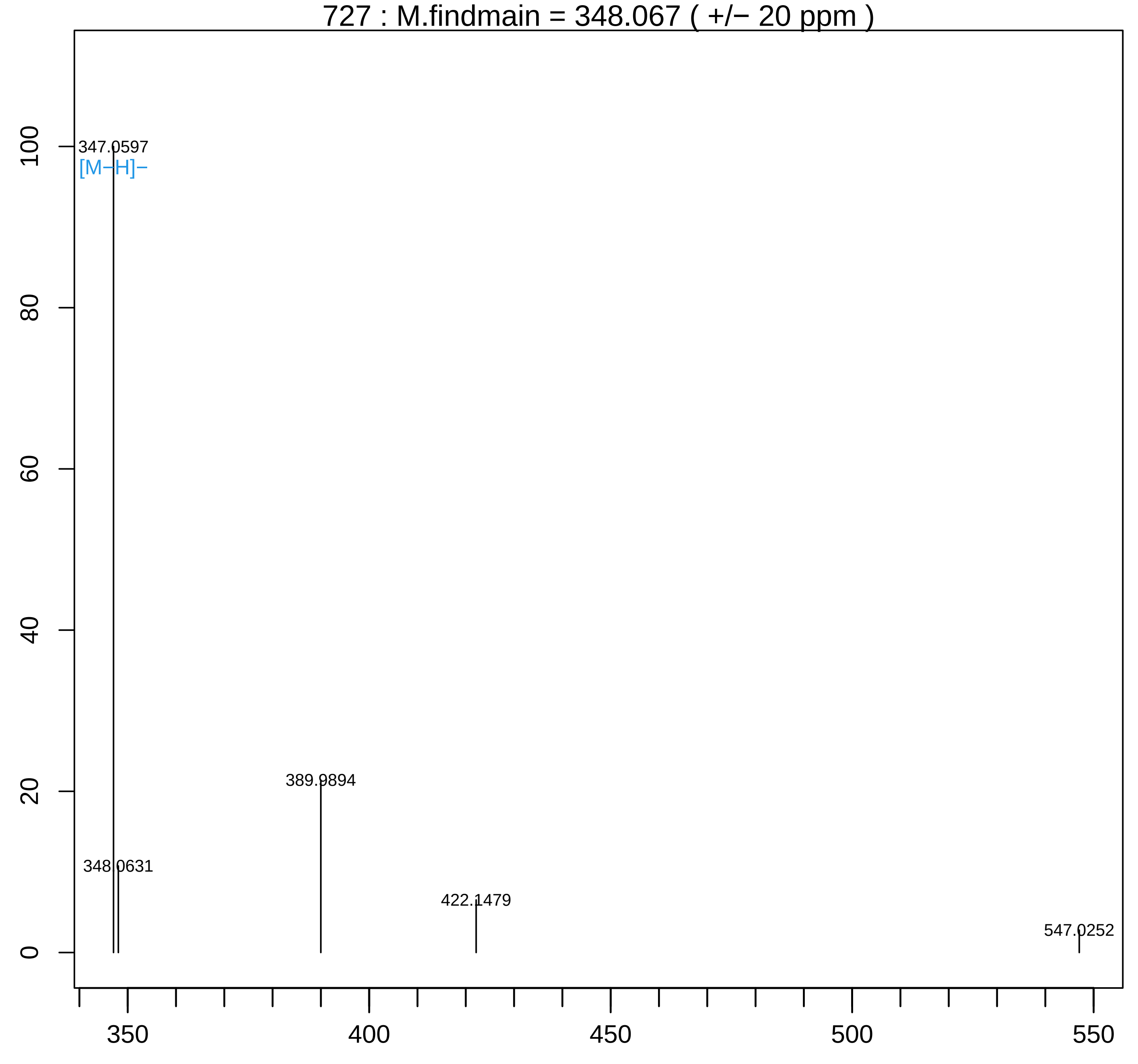
727 : M.findmain = 348.067 (+/− 20 ppm)
347.0597
[M−H]−
348.0631
389.9894
422.1479
547.0252
0
20
40
60
80
100
350
400
450
500
550

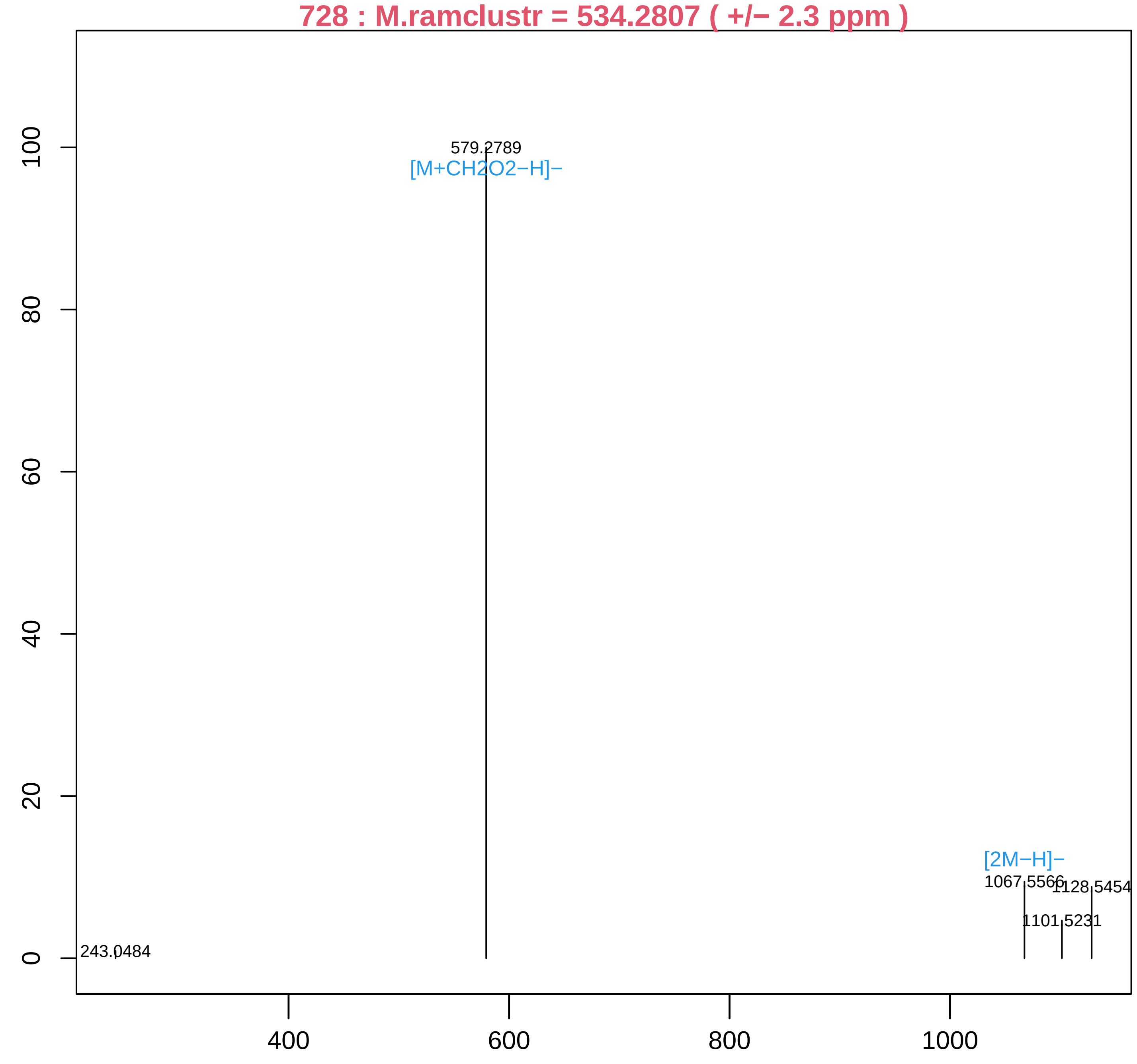
728 : M.ramclustr = 534.2807 (+/− 2.3 ppm)
579.2789
[M+CH2O2−H]−
[2M−H]−
1067.5566
1128.5454
1101.5231
243.0484
0
20
40
60
80
100
400
600
800
1000

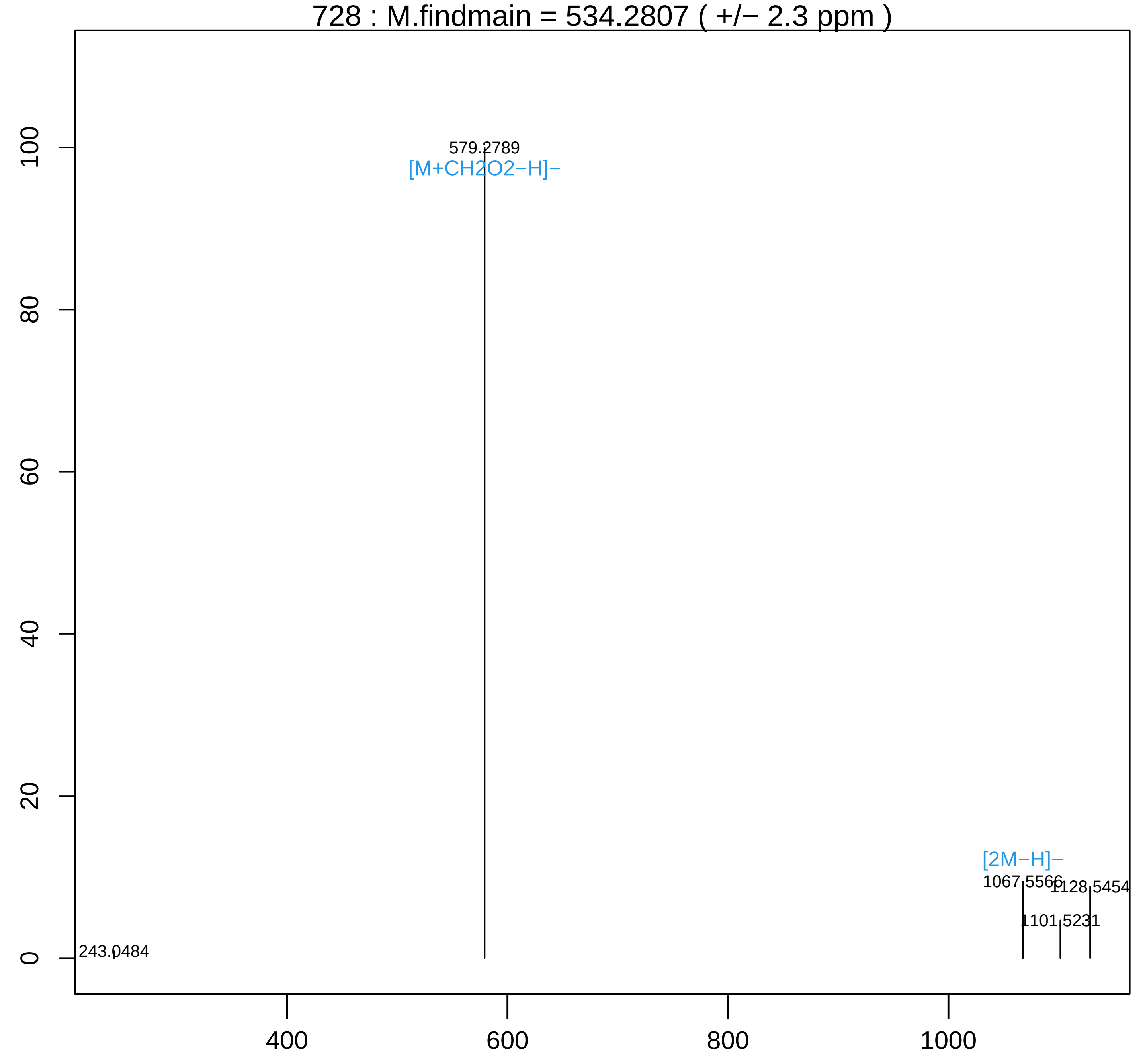
728 : M.findmain = 534.2807 (+/− 2.3 ppm)
579.2789
[M+CH2O2−H]−
[2M−H]−
1067.5566
1128.5454
1101.5231
243.0484
0
20
40
60
80
100
400
600
800
1000

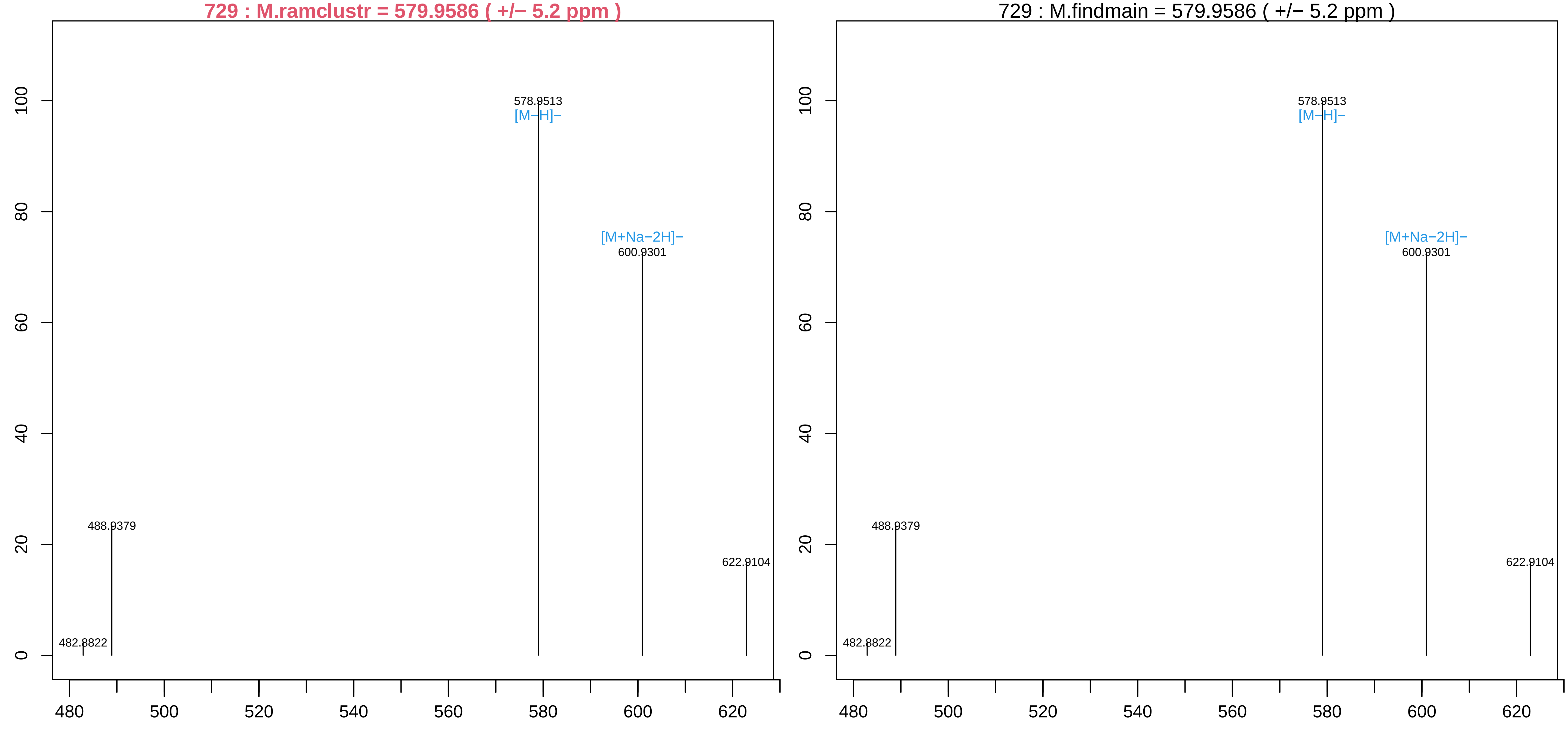

729 : M.ramclustr = 579.9586 (+/− 5.2 ppm)
578.9513
[M−H]−
[M+Na−2H]−
600.9301
488.9379
622.9104
482.8822
0
20
40
60
80
100
480
500
520
540
560
580
600
620
729 : M.findmain = 579.9586 (+/− 5.2 ppm)
578.9513
[M−H]−
[M+Na−2H]−
600.9301
488.9379
622.9104
482.8822
0
20
40
60
80
100
480
500
520
540
560
580
600
620

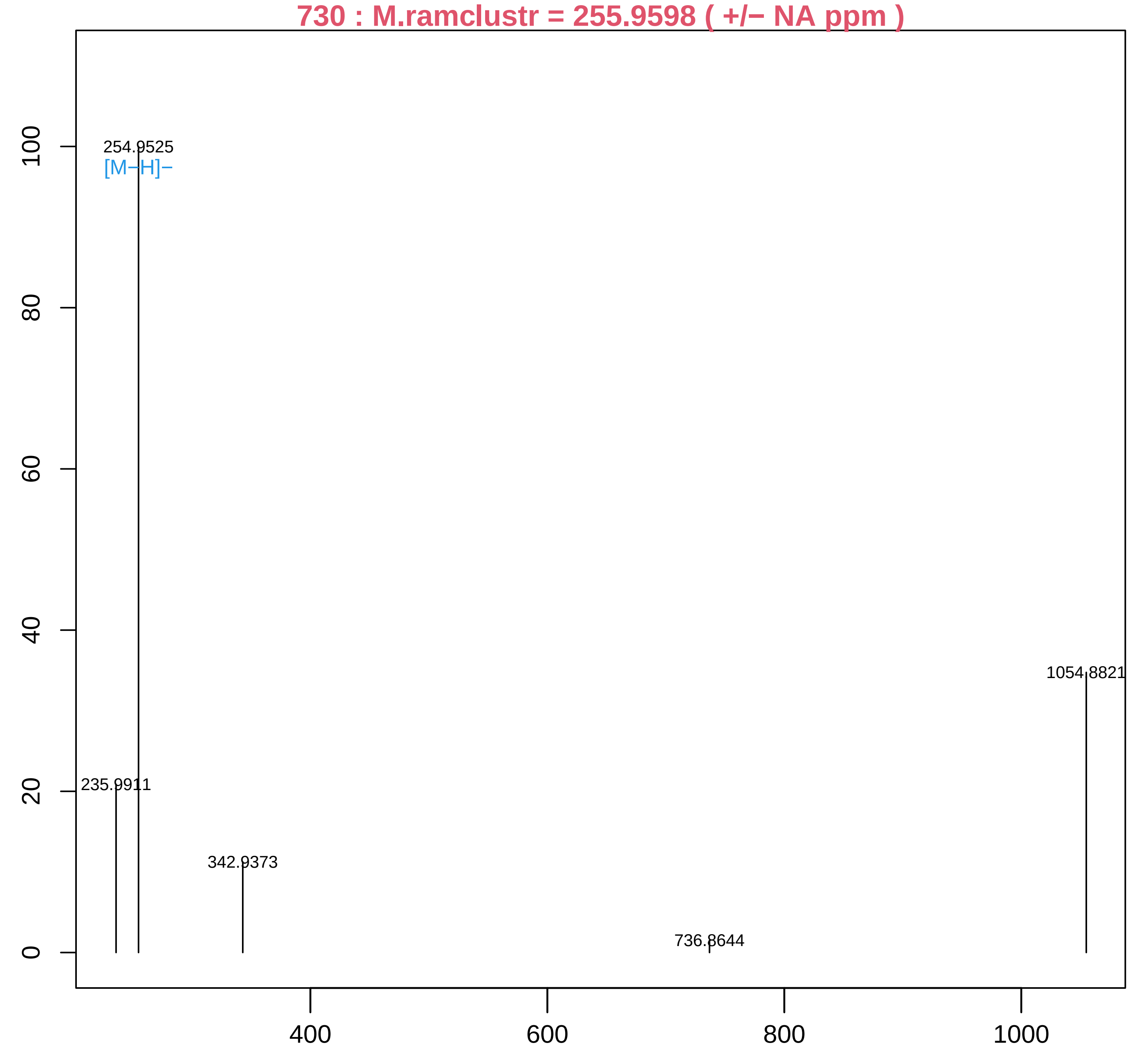

730 : M.ramclustr = 255.9598 (+/− NA ppm)
254.9525
[M−H]−
235.9911
342.9373
736.8644
1054.8821
0
20
40
60
80
100
400
600
800
1000

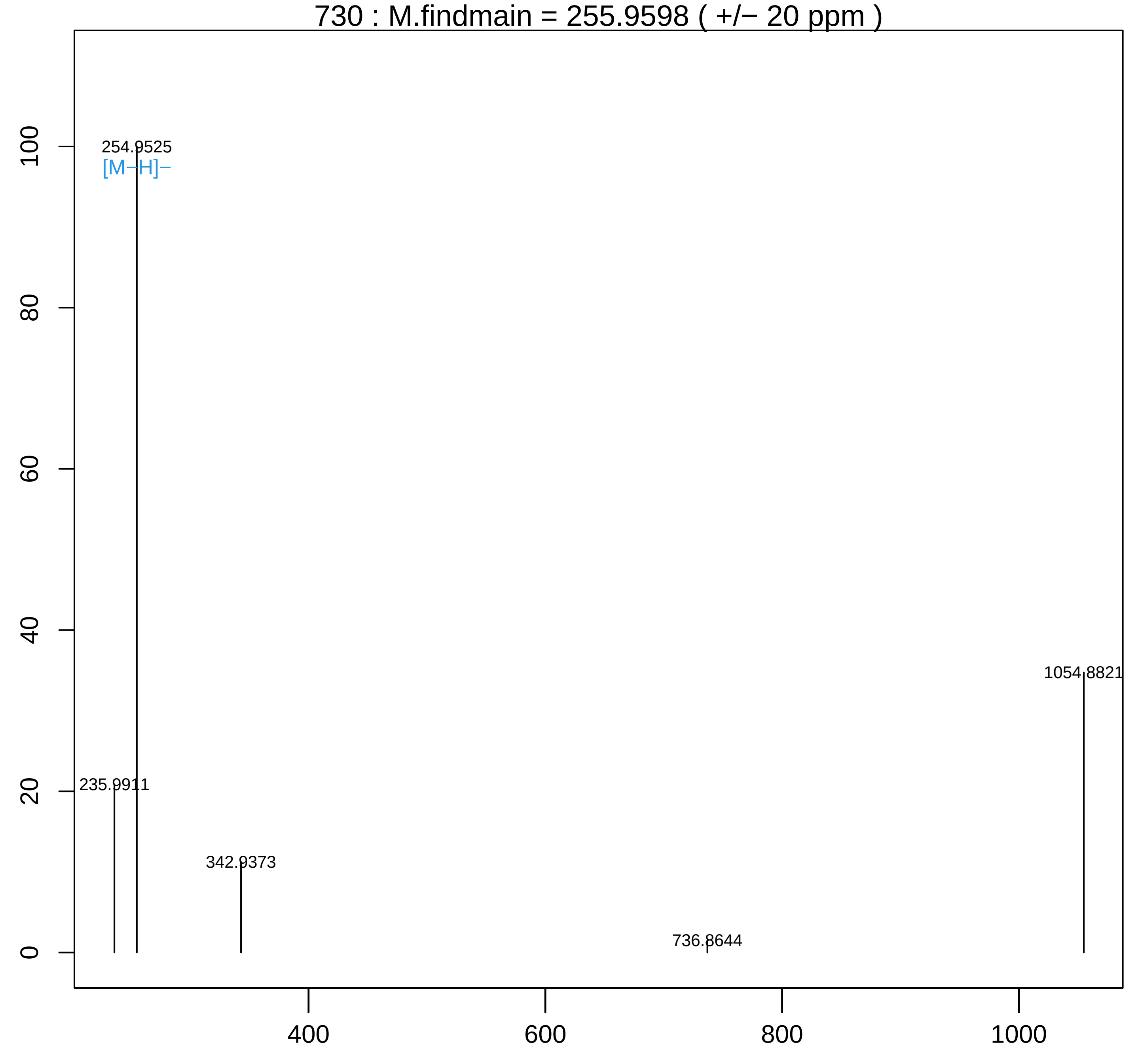

730 : M.findmain = 255.9598 (+/− 20 ppm)
254.9525
[M−H]−
235.9911
342.9373
736.8644
1054.8821
0
20
40
60
80
100
400
600
800
1000

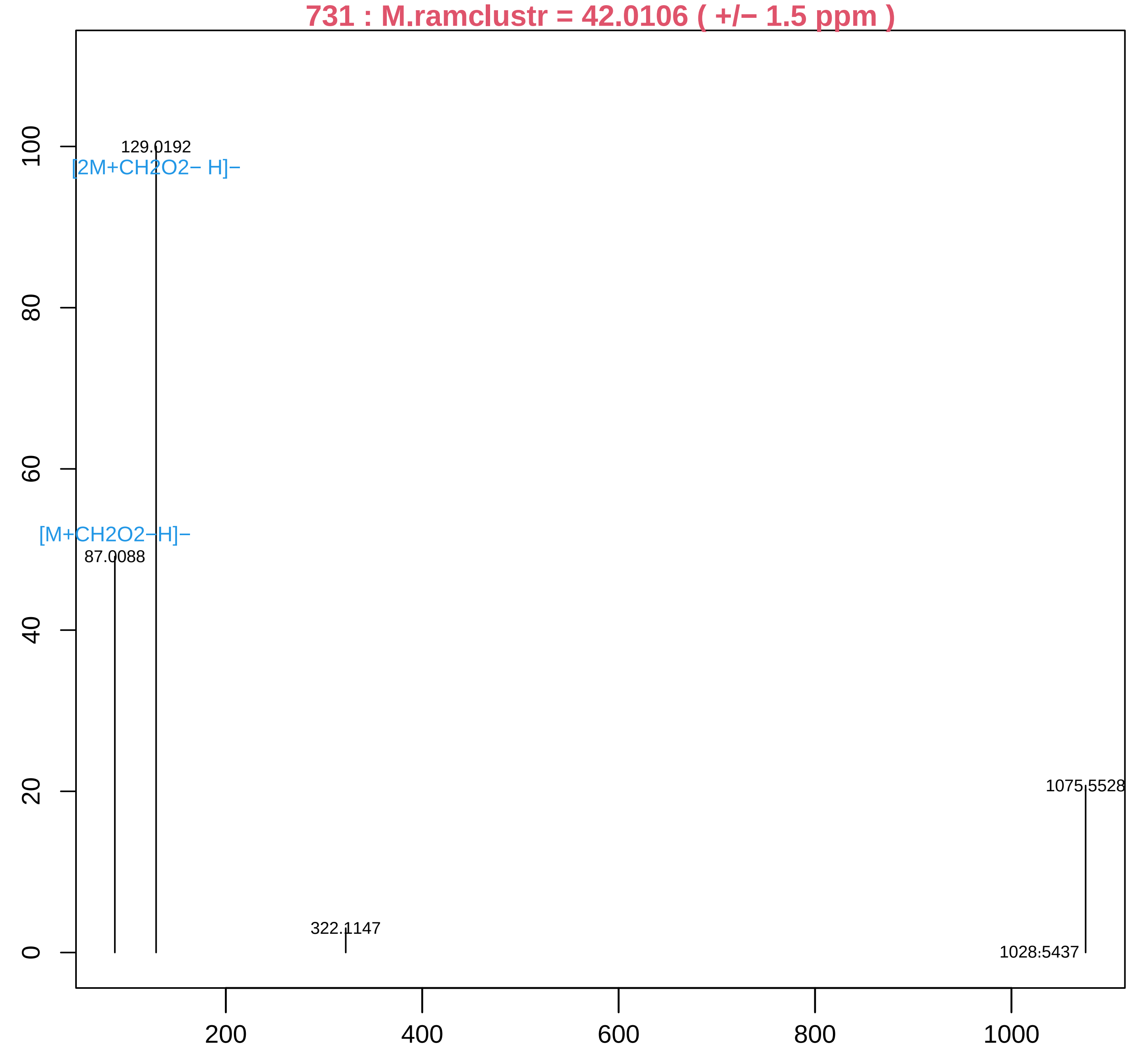
731 : M.ramclustr = 42.0106 (+/− 1.5 ppm)
129.0192
[2M+CH2O2− H]−
[M+CH2O2−H]−
87.0088
1075.5528
322.1147
1028.5437
0
20
40
60
80
100
200
400
600
800
1000

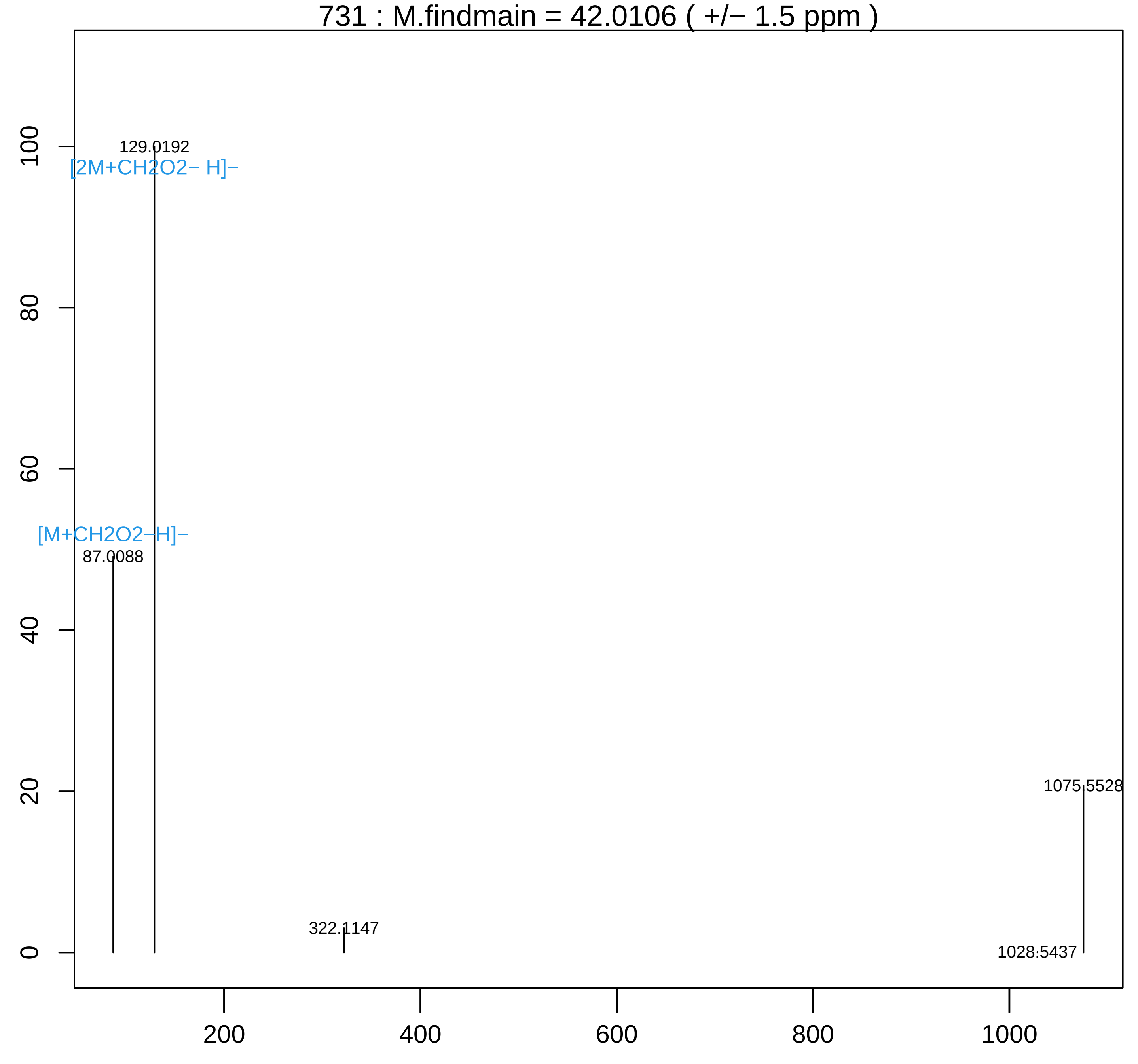
731 : M.findmain = 42.0106 (+/− 1.5 ppm)
129.0192
[2M+CH2O2− H]−
[M+CH2O2−H]−
87.0088
1075.5528
322.1147
1028.5437
0
20
40
60
80
100
200
400
600
800
1000

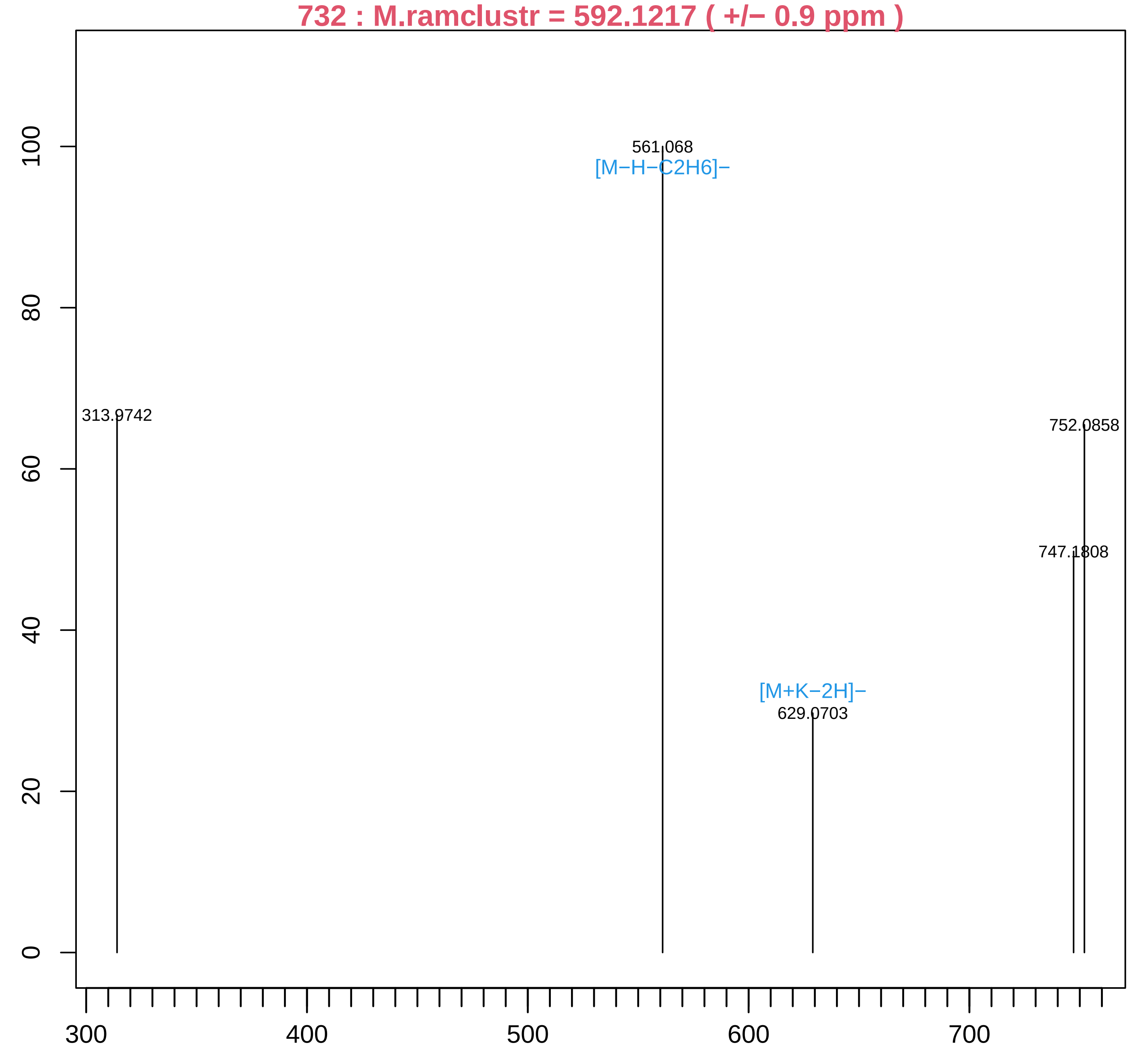

732 : M.ramclustr = 592.1217 (+/− 0.9 ppm)
561.068
[M−H−C2H6]−
313.9742
752.0858
747.1808
[M+K−2H]−
629.0703
0
20
40
60
80
100
300
400
500
600
700

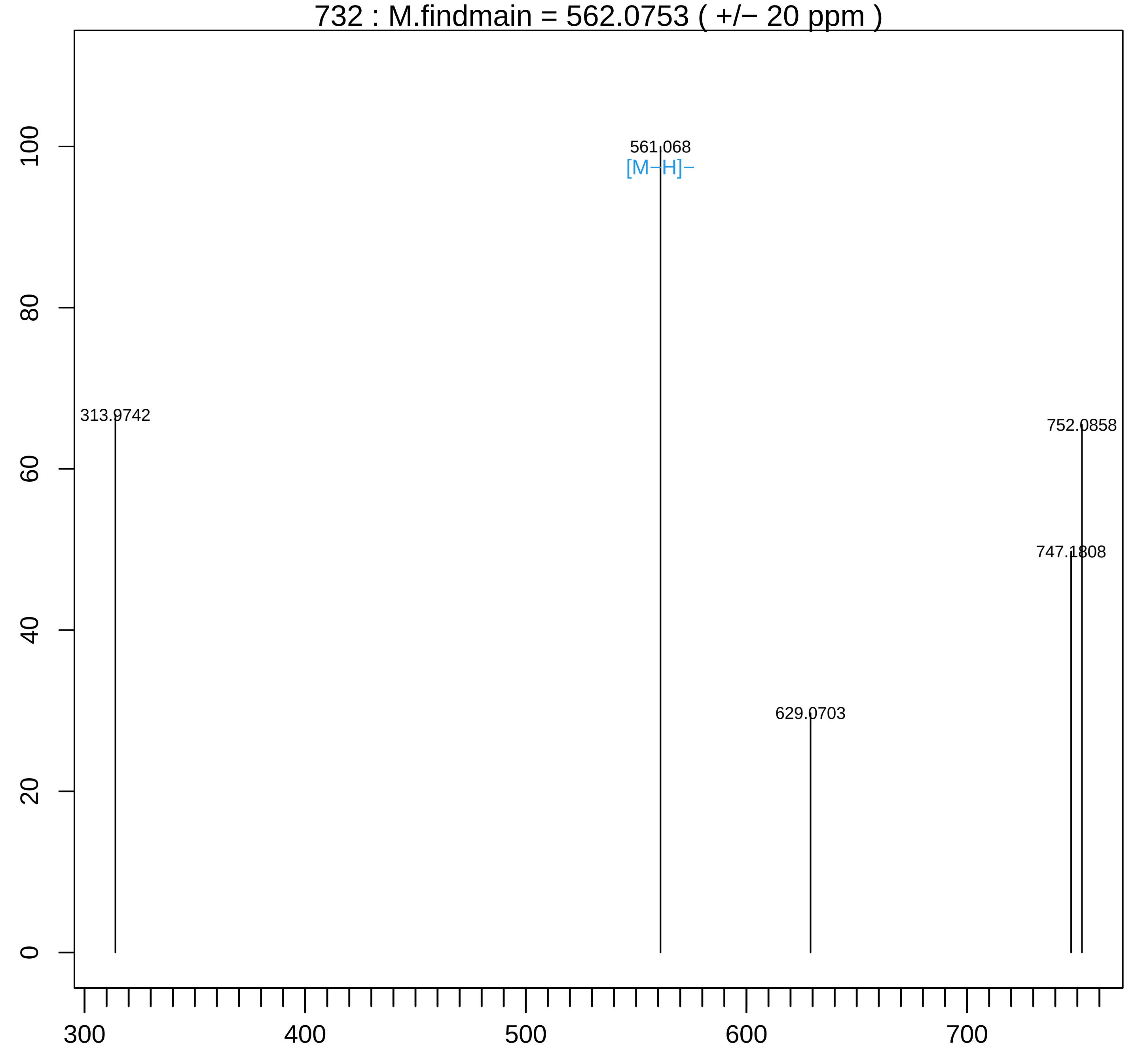

732 : M.findmain = 562.0753 (+/− 20 ppm)
561.068
[M−H]−
313.9742
752.0858
747.1808
629.0703
0
20
40
60
80
100
300
400
500
600
700

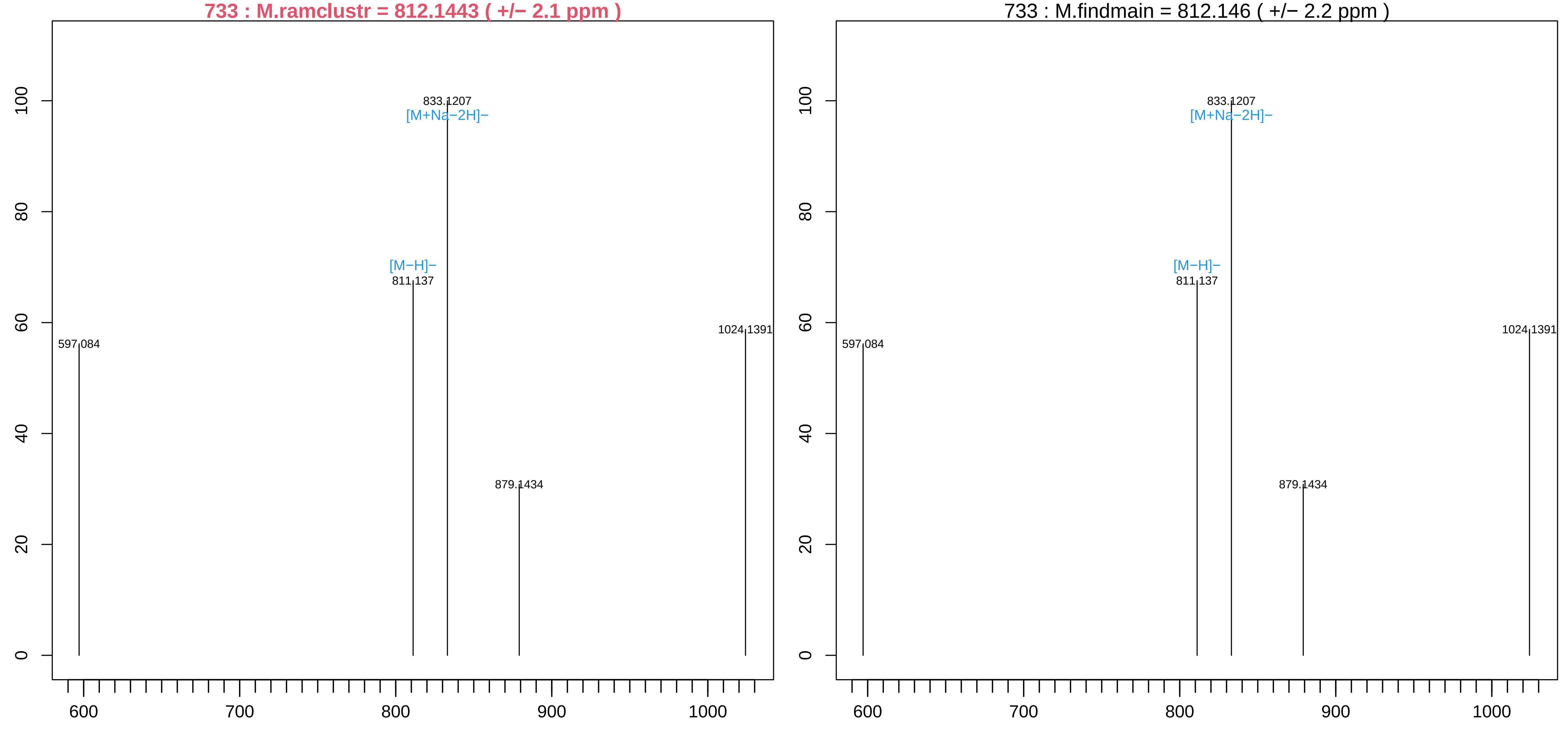
733 : M.ramclustr = 812.1443 (+/− 2.1 ppm)
833.1207
[M+Na−2H]−
[M−H]−
811.137
597.084
1024.1391
879.1434
733 : M.findmain = 812.146 (+/− 2.2 ppm)
833.1207
[M+Na−2H]−
[M−H]−
811.137
597.084
1024.1391
879.1434

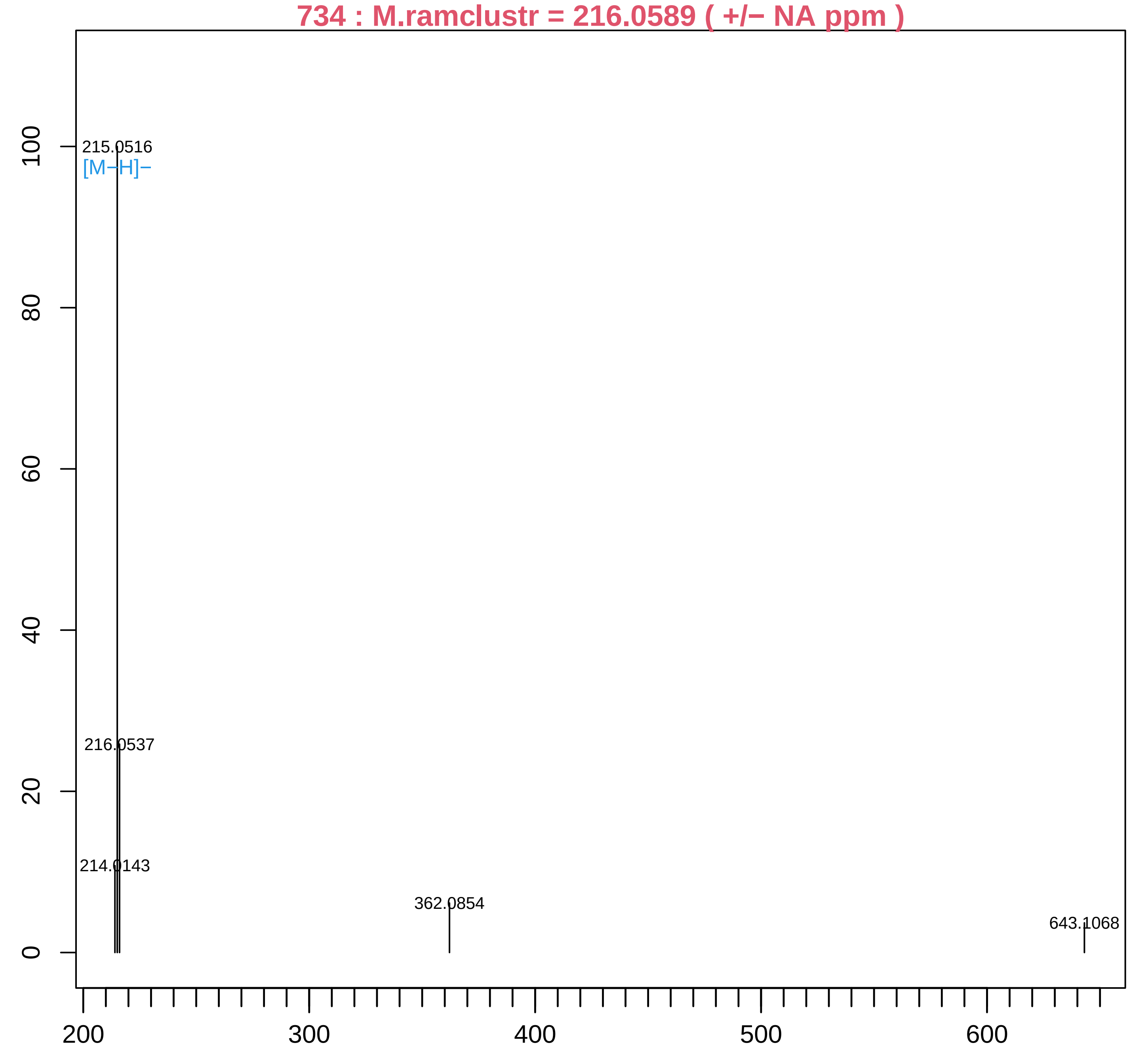
734 : M.ramclustr = 216.0589 (+/− NA ppm)
215.0516
[M−H]−
216.0537
214.0143
362.0854
643.1068
0
20
40
60
80
100
200
300
400
500
600

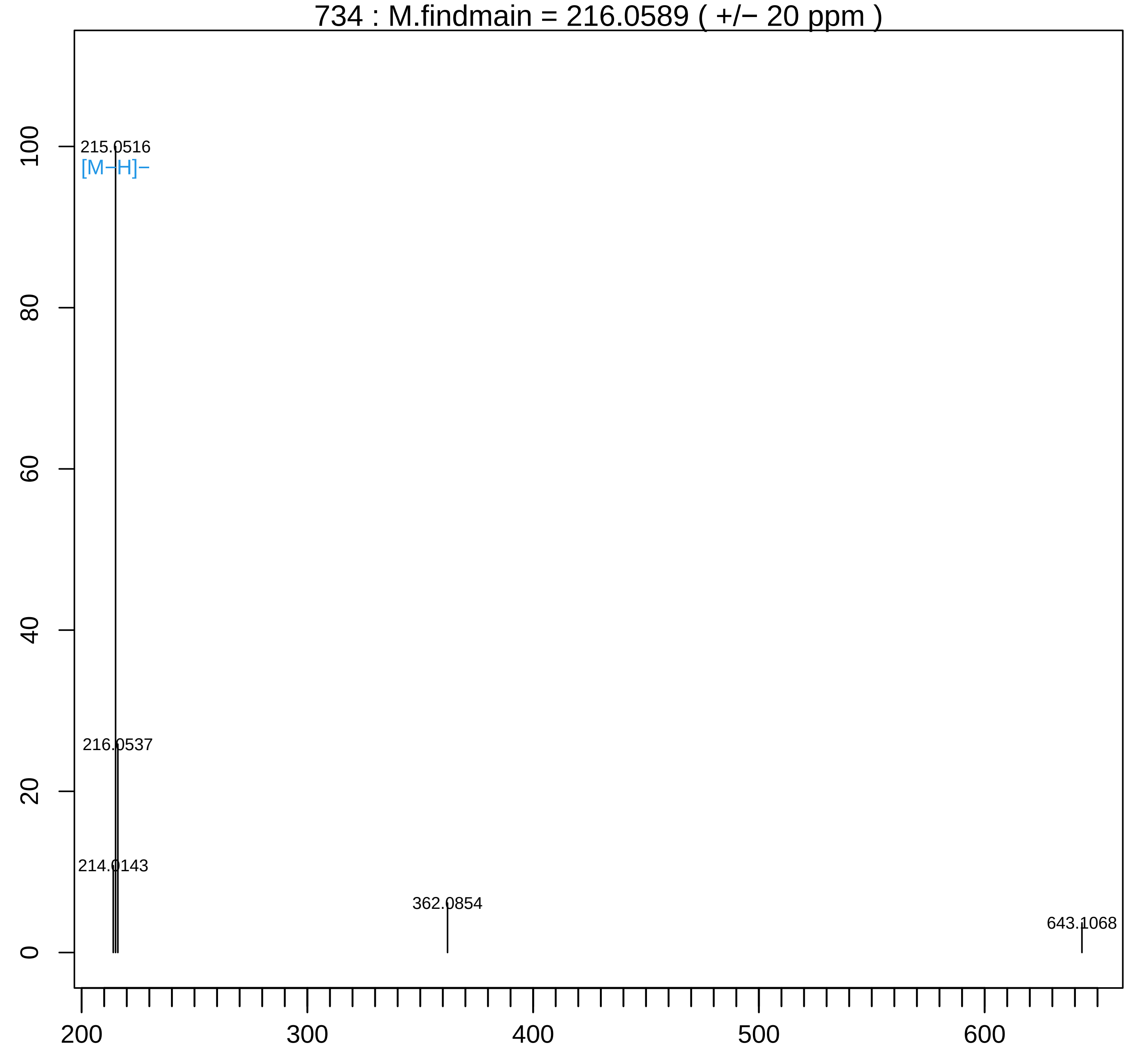
734 : M.findmain = 216.0589 (+/− 20 ppm)
215.0516
[M−H]−
216.0537
214.0143
362.0854
643.1068
0
20
40
60
80
100
200
300
400
500
600

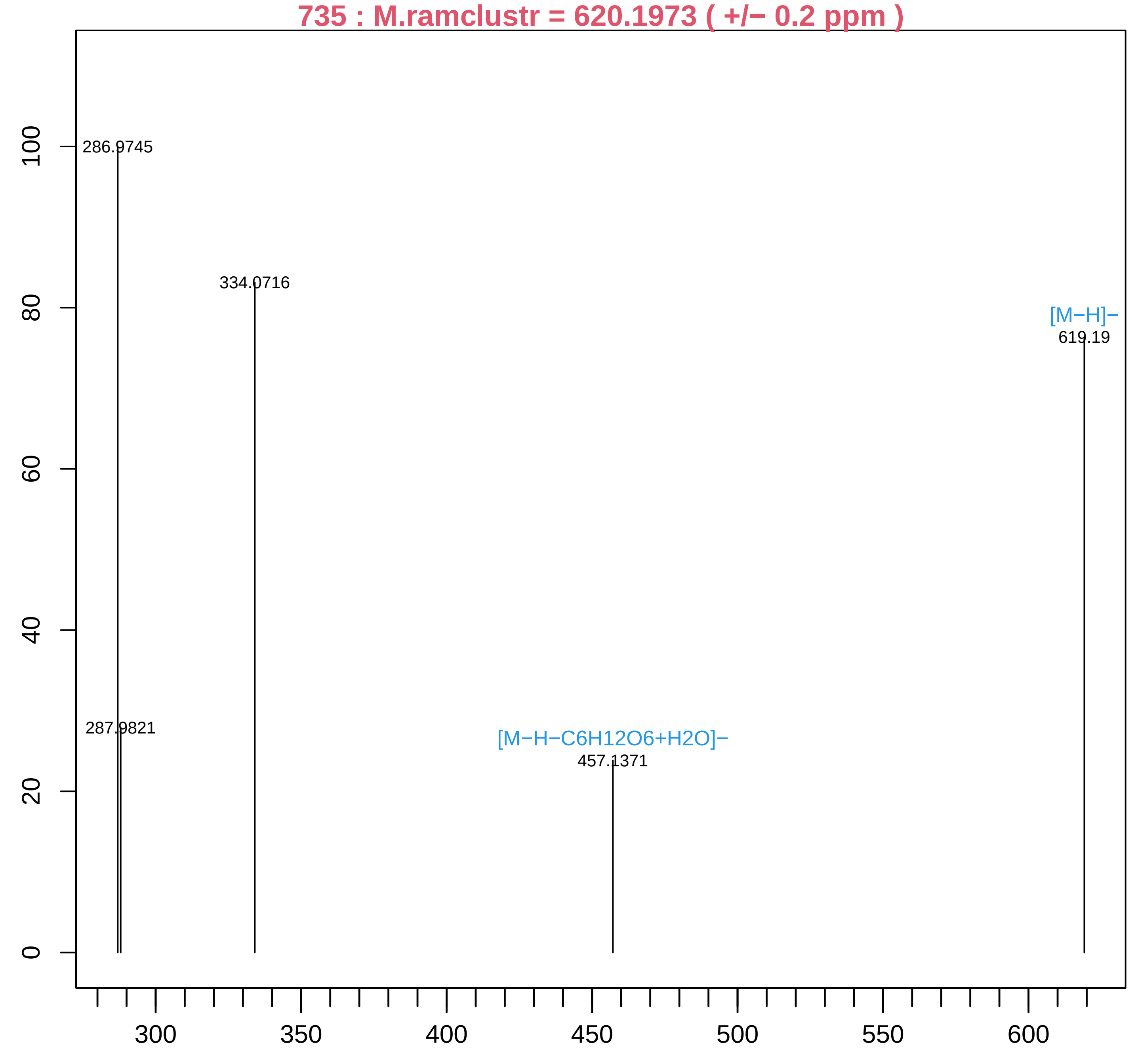

735 : M.ramclustr = 620.1973 (+/− 0.2 ppm)
286.9745
334.0716
[M−H]−
619.19
287.9821
[M−H−C6H12O6+H2O]−
457.1371
0
20
40
60
80
100
300
350
400
450
500
550
600

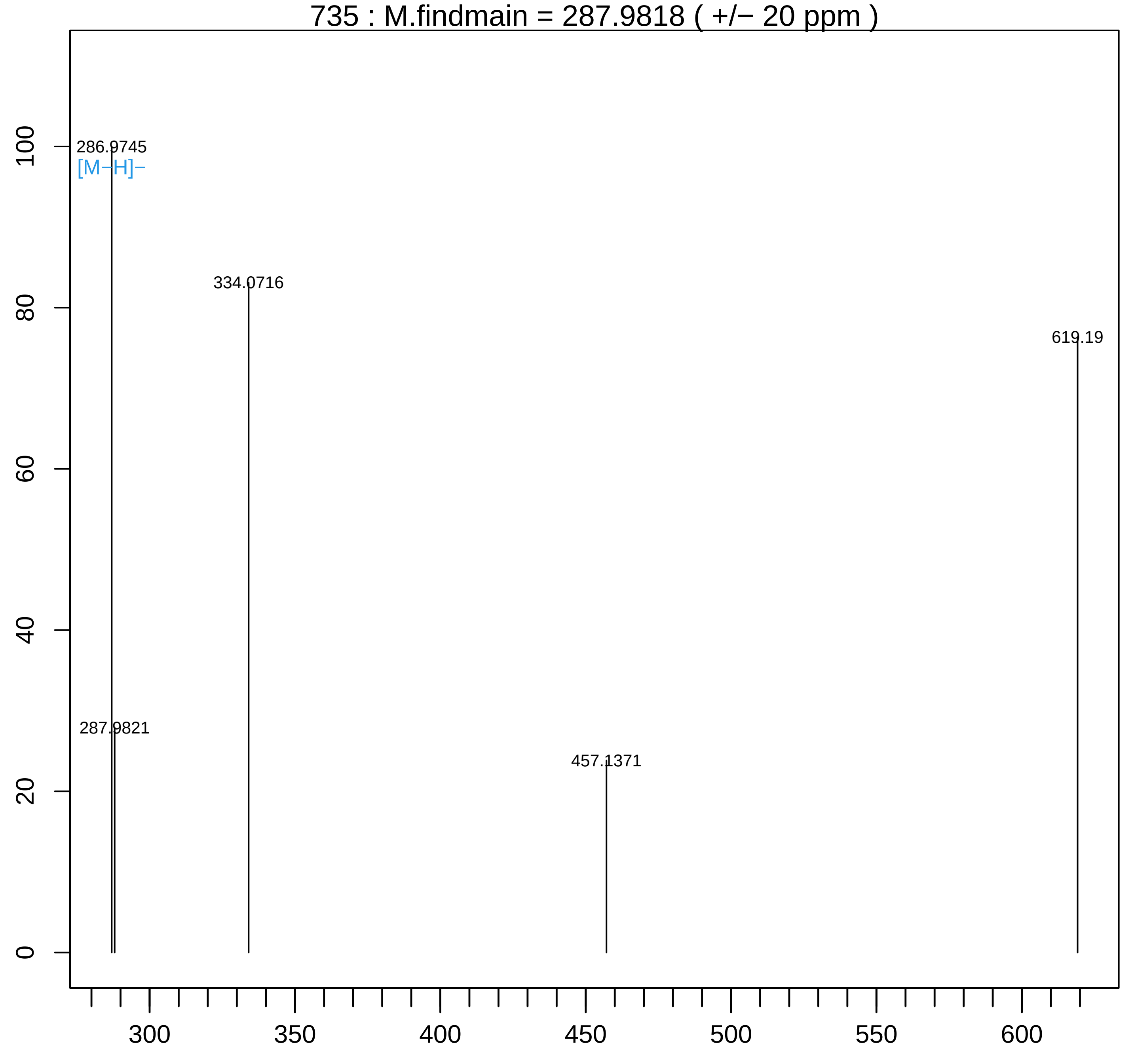

735 : M.findmain = 287.9818 (+/− 20 ppm)
286.9745
[M−H]−
334.0716
619.19
287.9821
457.1371
0
20
40
60
80
100
300
350
400
450
500
550
600

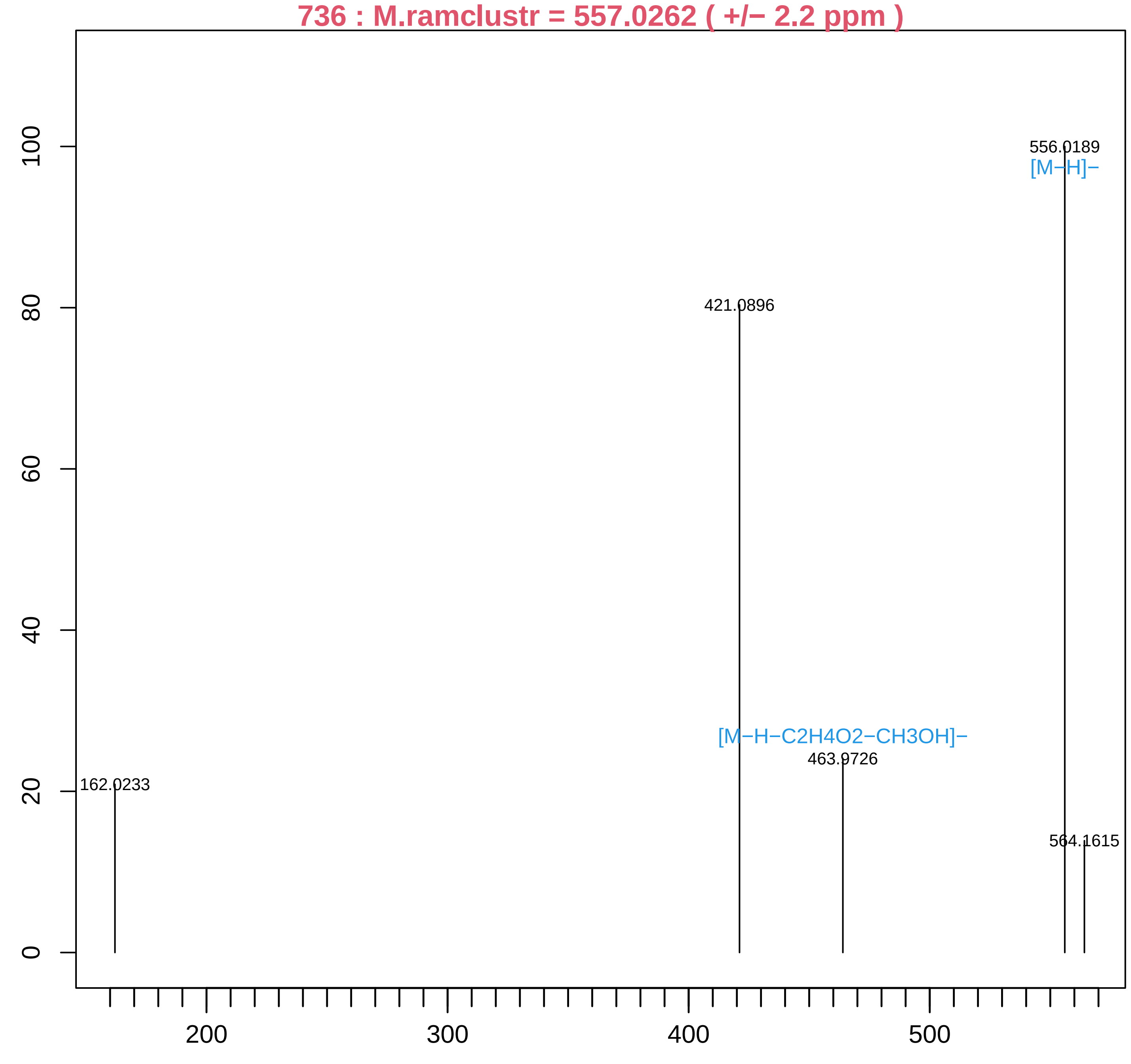
736 : M.ramclustr = 557.0262 (+/− 2.2 ppm)
556.0189
[M−H]−
421.0896
[M−H−C2H4O2−CH3OH]−
463.9726
162.0233
564.1615
0
20
40
60
80
100
200
300
400
500

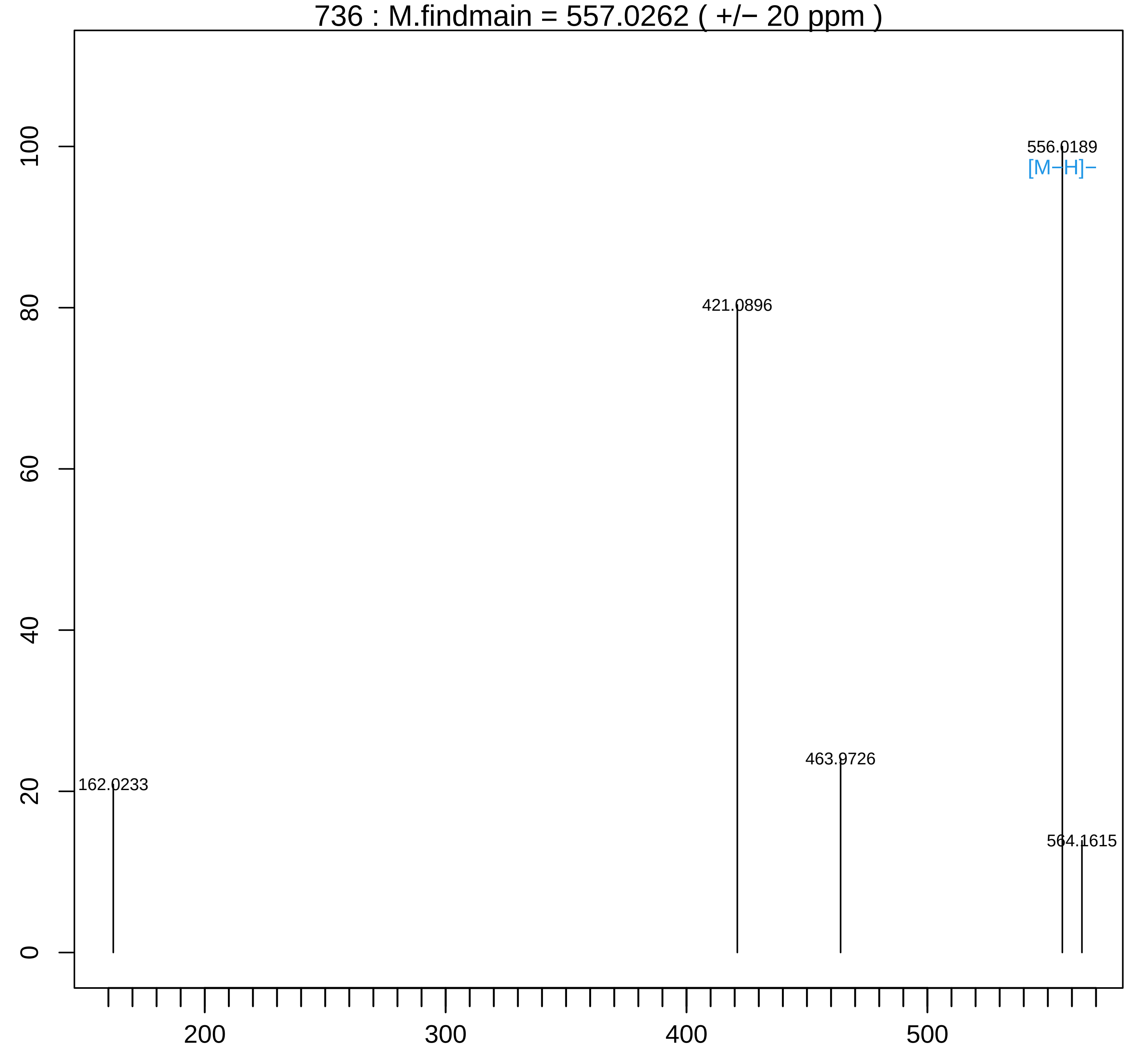
736 : M.findmain = 557.0262 (+/− 20 ppm)
556.0189
[M−H]−
421.0896
463.9726
162.0233
564.1615
0
20
40
60
80
100
200
300
400
500

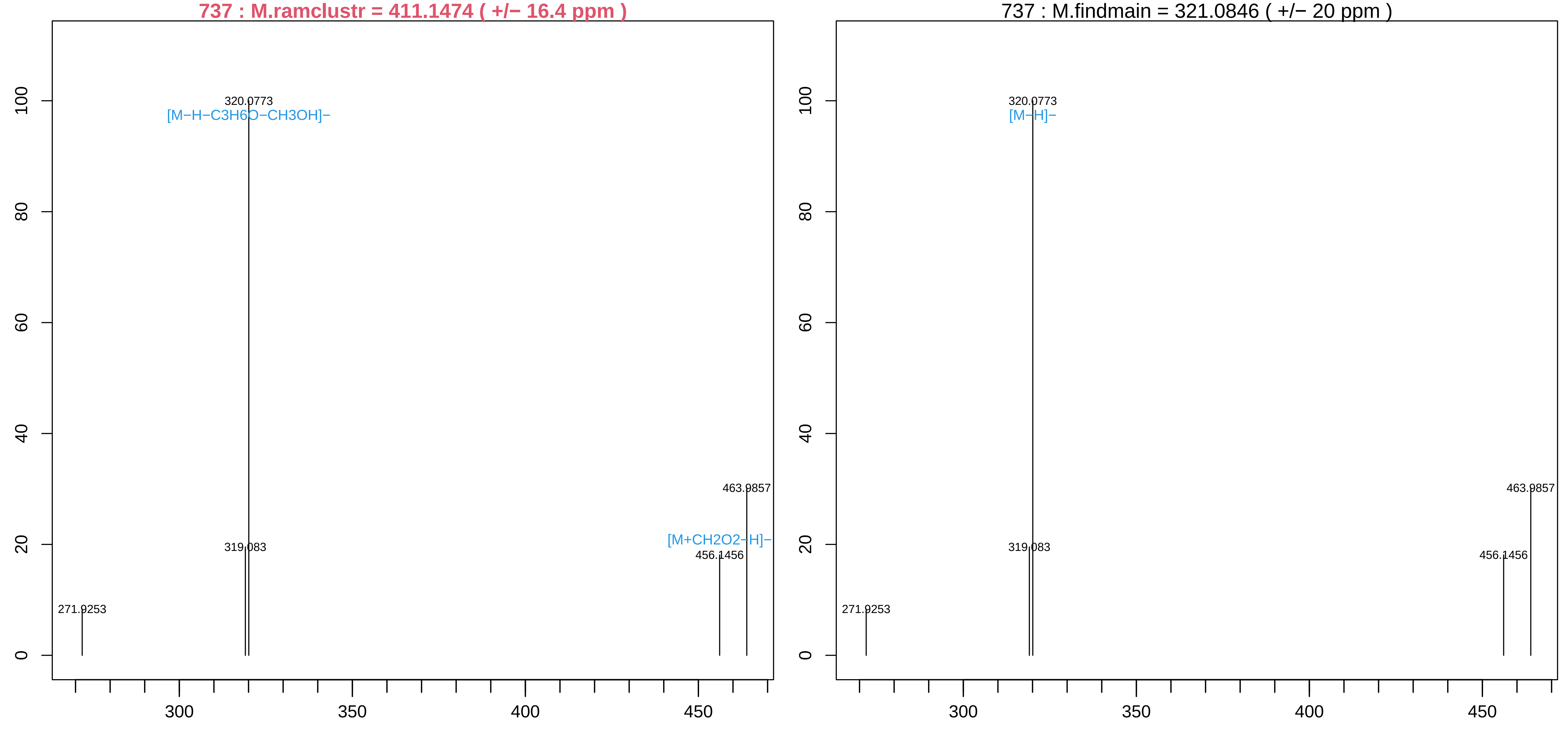
737 : M.ramclustr = 411.1474 (+/− 16.4 ppm)
320.0773
[M−H−C3H6O−CH3OH]−
319.083
271.9253
463.9857
[M+CH2O2−H]−
456.1456
737 : M.findmain = 321.0846 (+/− 20 ppm)
320.0773
[M−H]−
319.083
271.9253
463.9857
456.1456
0
20
40
60
80
100
300
350
400
450

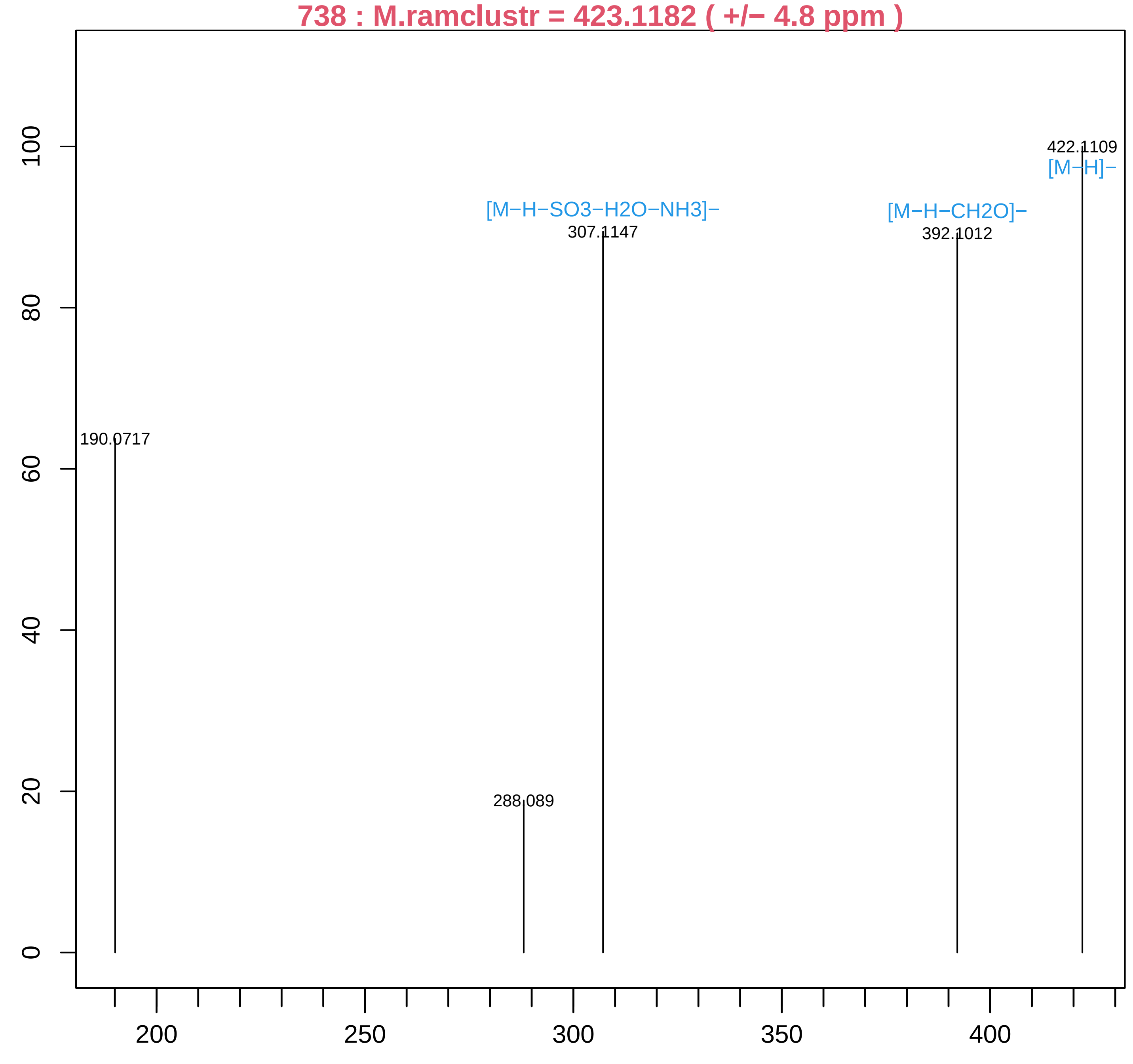
738 : M.ramclustr = 423.1182 (+/− 4.8 ppm)
422.1109
[M−H]−
[M−H−SO3−H2O−NH3]−
307.1147
[M−H−CH2O]−
392.1012
190.0717
288.089
0
20
40
60
80
100
200
250
300
350
400

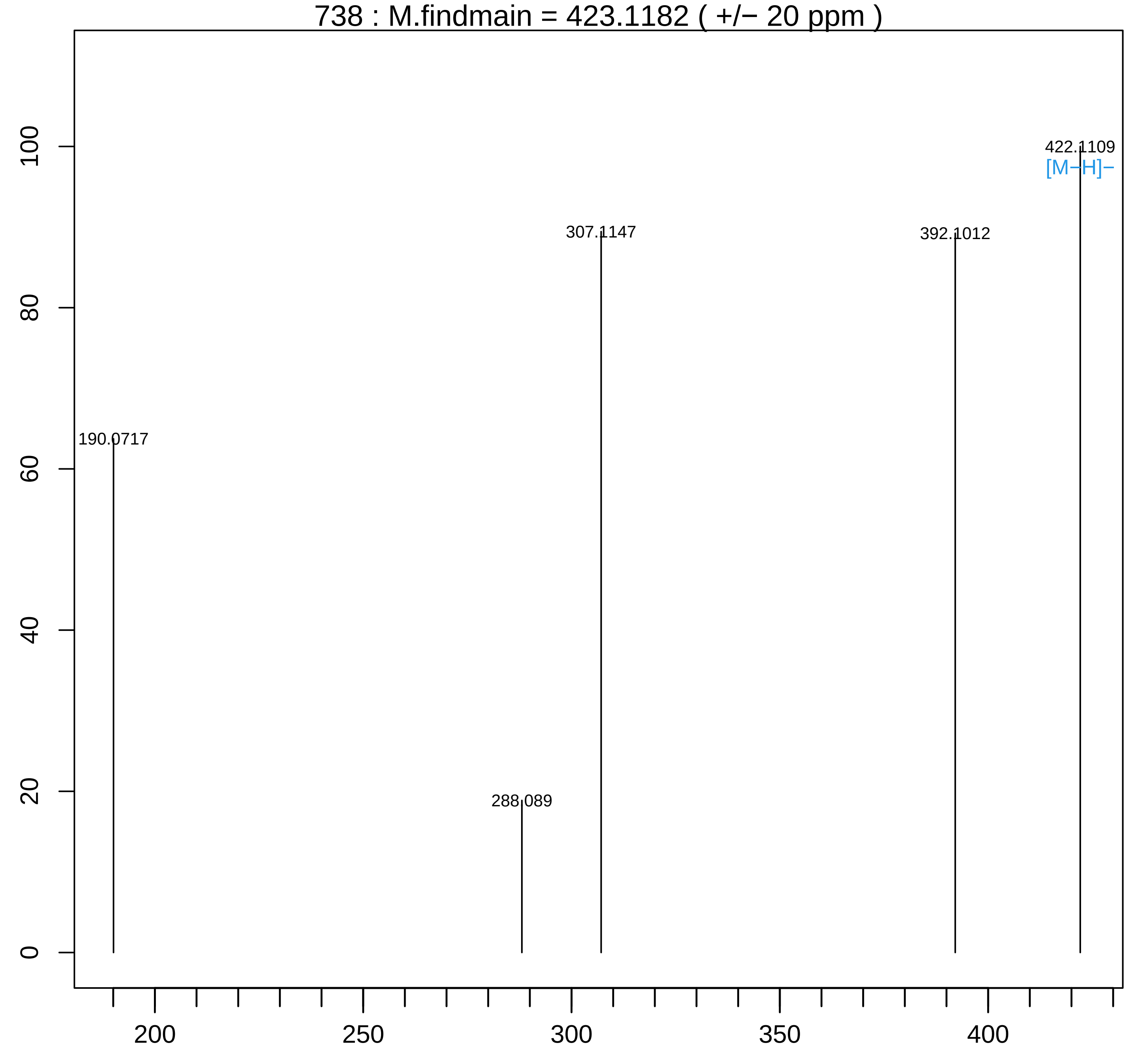
738 : M.findmain = 423.1182 (+/− 20 ppm)
422.1109
[M−H]−
307.1147
392.1012
190.0717
288.089
0
20
40
60
80
100
200
250
300
350
400

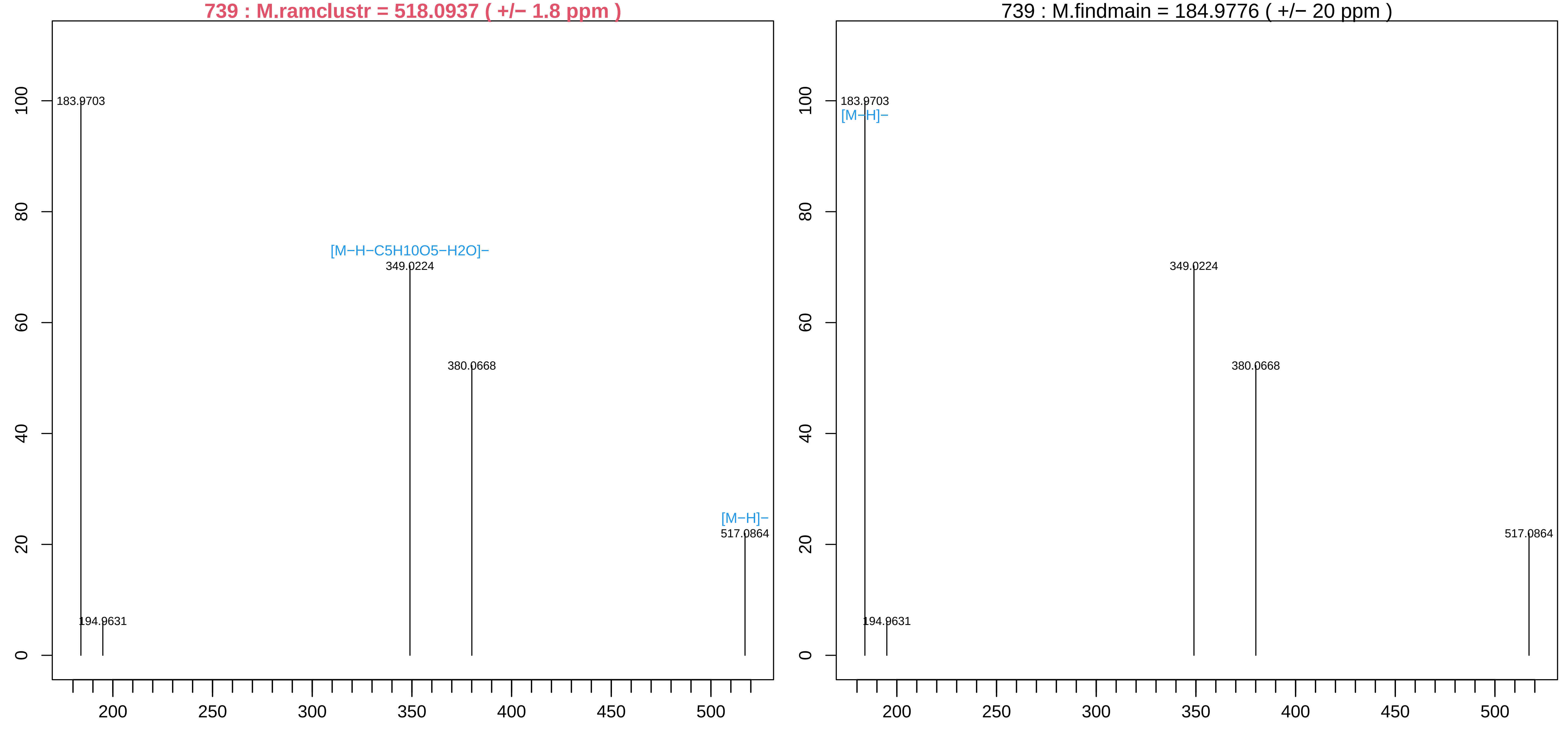
739 : M.ramclustr = 518.0937 (+/− 1.8 ppm)
183.9703
194.9631
[M−H−C5H10O5−H2O]−
349.0224
380.0668
[M−H]−
517.0864
739 : M.findmain = 184.9776 (+/− 20 ppm)
183.9703
[M−H]−
194.9631
349.0224
380.0668
517.0864

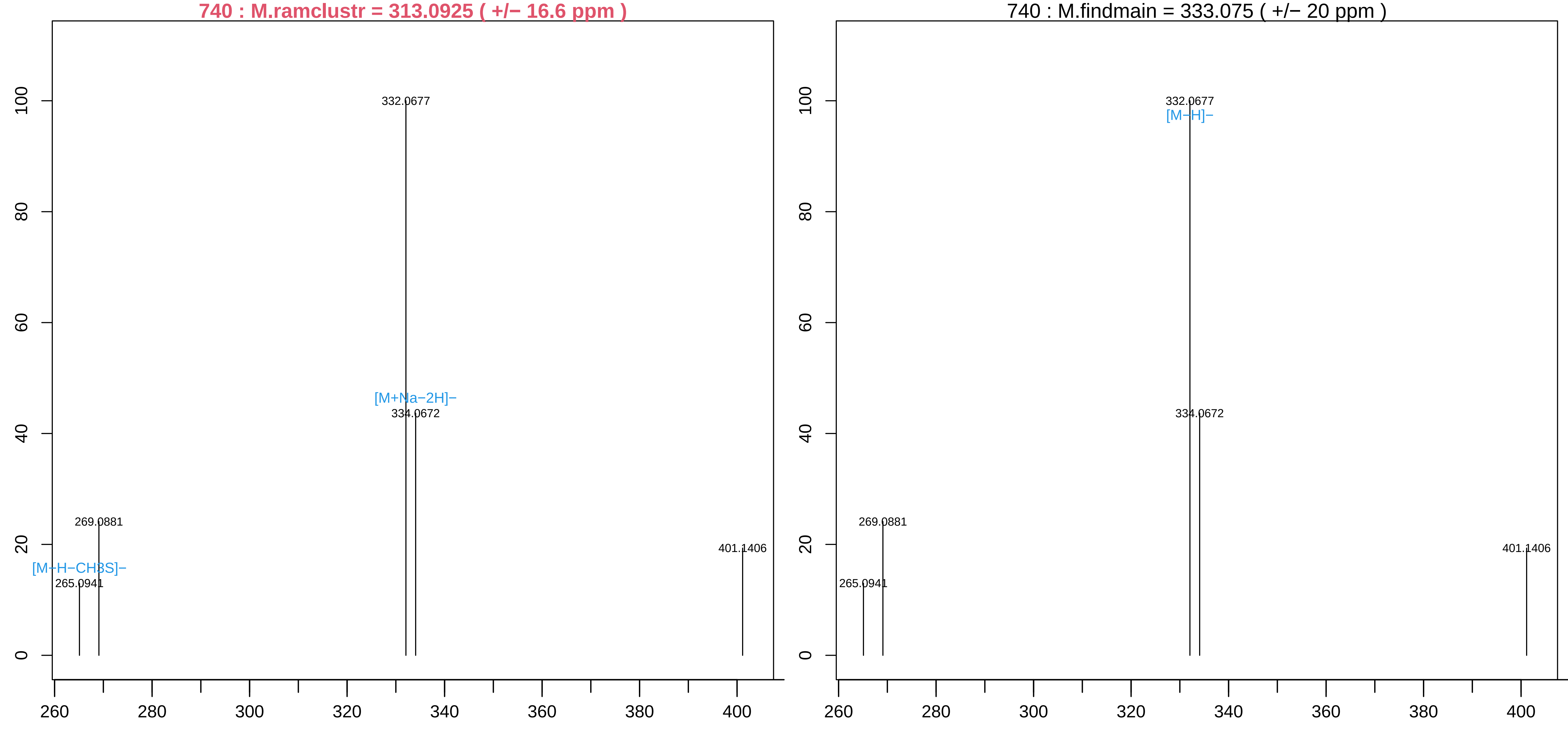
740 : M.ramclustr = 313.0925 (+/− 16.6 ppm)
332.0677
[M+Na−2H]−
334.0672
269.0881
[M−H−CH3S]−
265.0941
401.1406
740 : M.findmain = 333.075 (+/− 20 ppm)
332.0677
[M−H]−
334.0672
269.0881
265.0941
401.1406

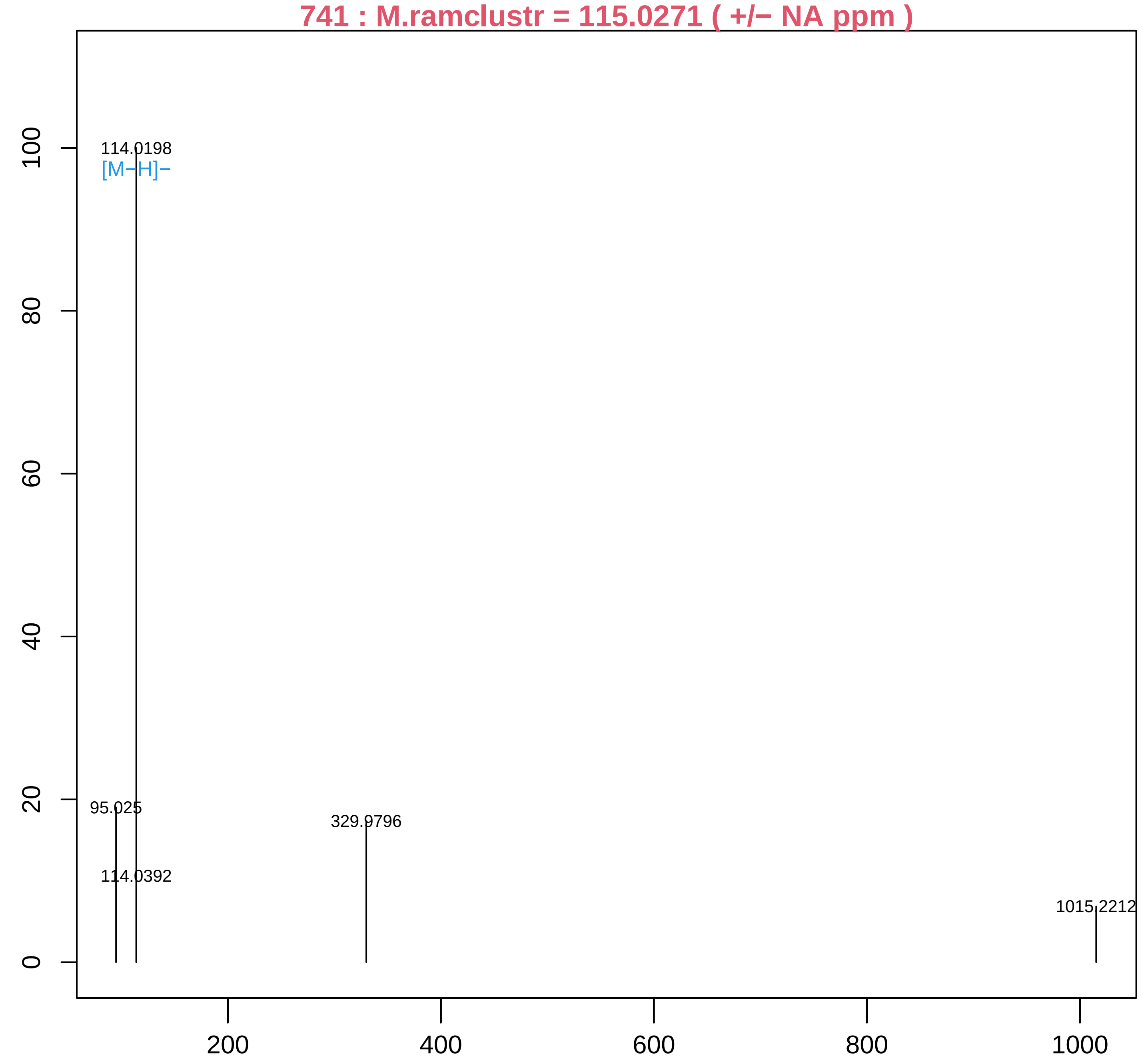
741 : M.ramclustr = 115.0271 (+/− NA ppm)
114.0198
[M−H]−
95.025
114.0392
329.9796
1015.2212
0
20
40
60
80
100
200
400
600
800
1000

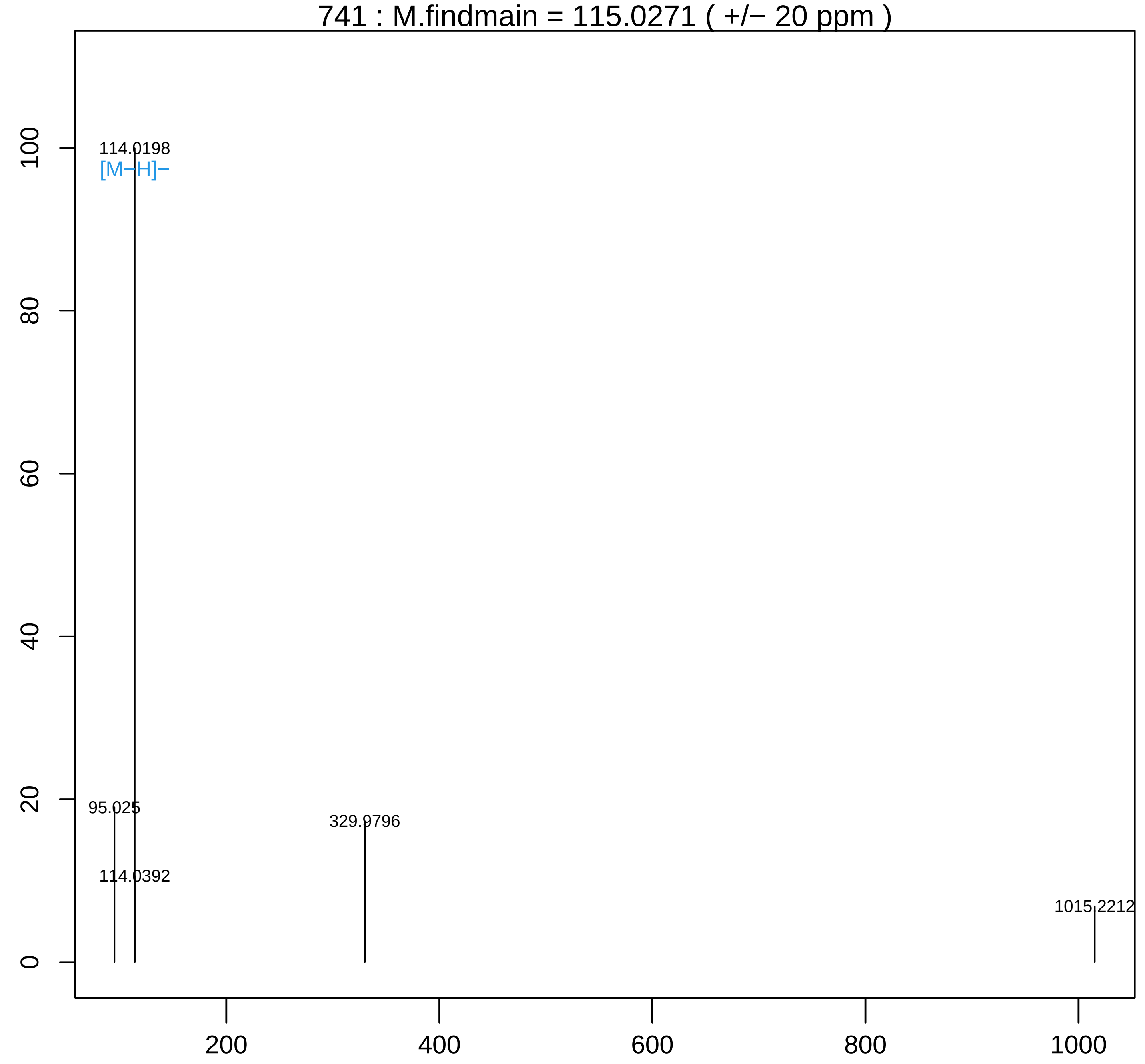
741 : M.findmain = 115.0271 (+/− 20 ppm)
114.0198
[M−H]−
95.025
114.0392
329.9796
1015.2212
0
20
40
60
80
100
200
400
600
800
1000

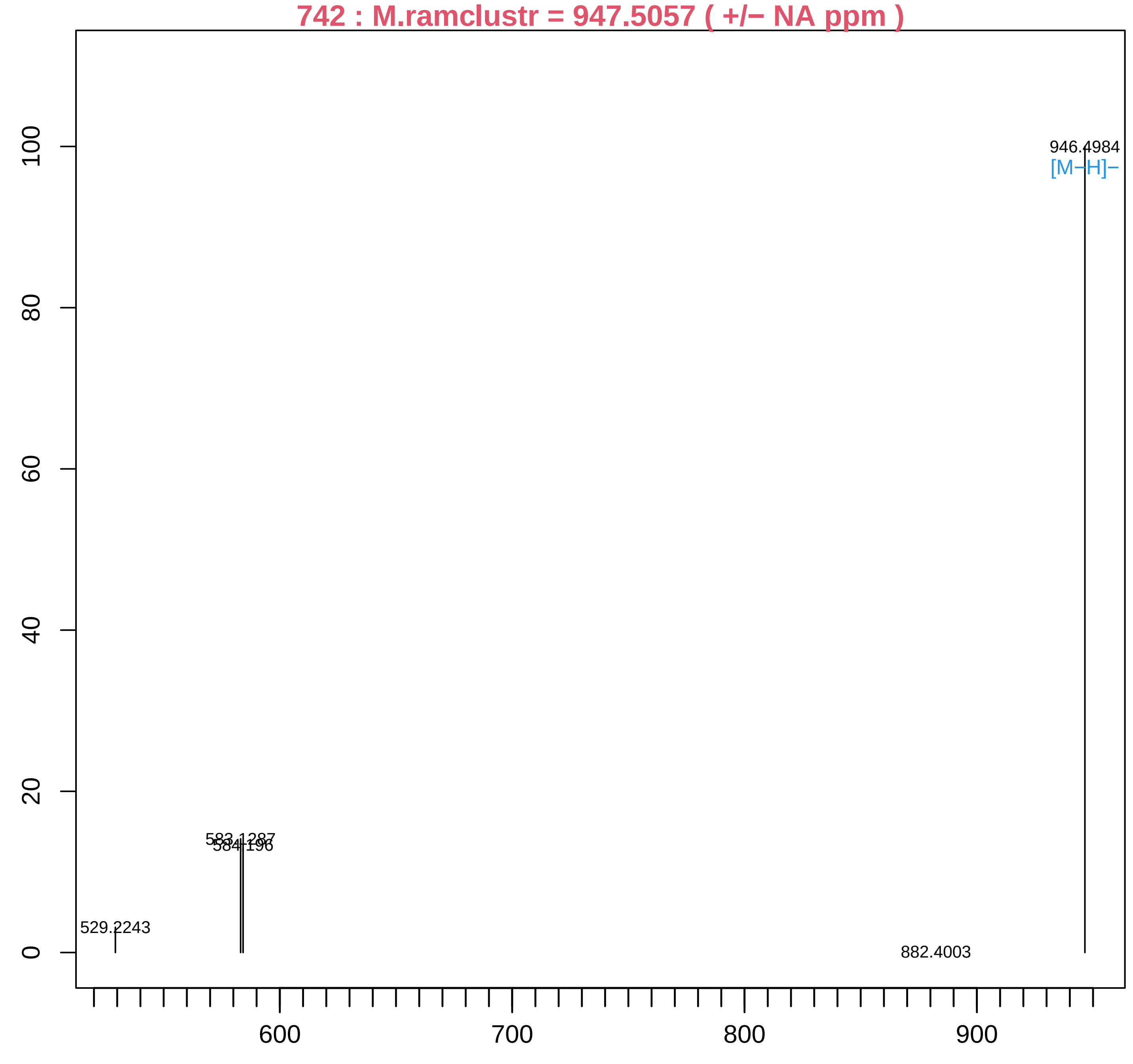
742 : M.ramclustr = 947.5057 (+/− NA ppm)
946.4984
[M−H]−
583.1287
584.196
529.2243
882.4003
0
20
40
60
80
100
600
700
800
900

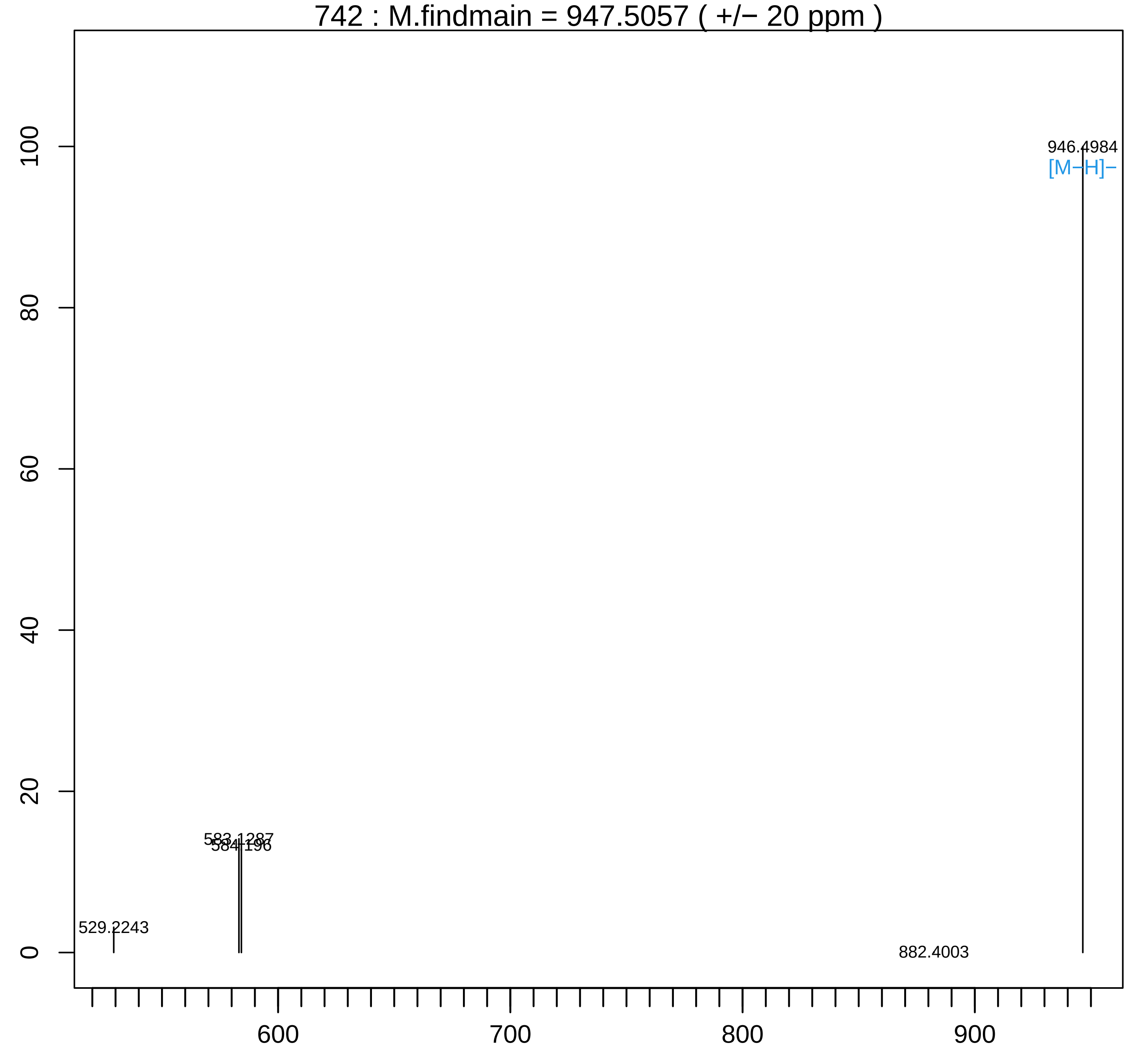
742 : M.findmain = 947.5057 (+/− 20 ppm)
946.4984
[M−H]−
583.1287
584.196
529.2243
882.4003
0
20
40
60
80
100
600
700
800
900

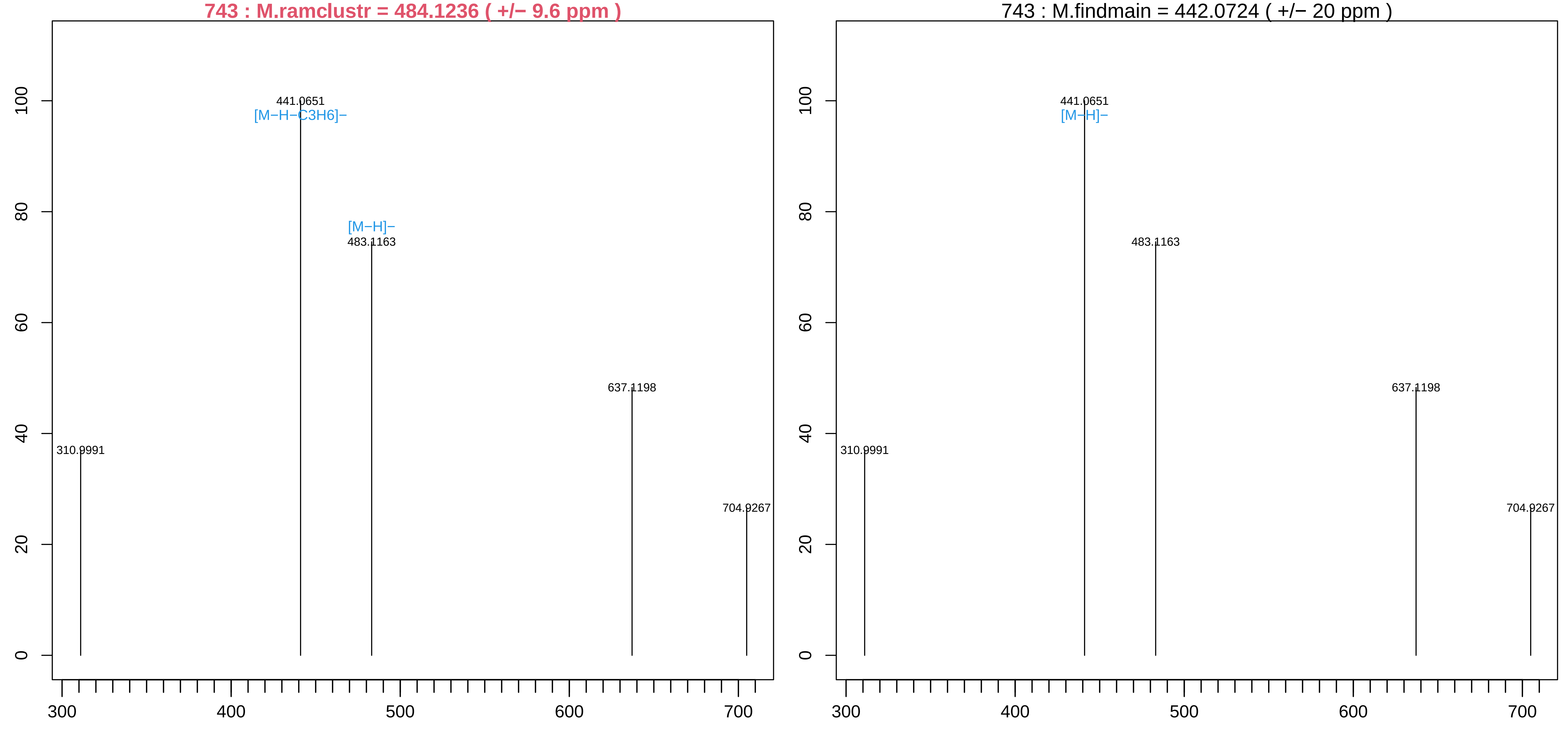
743 : M.ramclustr = 484.1236 (+/− 9.6 ppm)
441.0651
[M−H−C3H6]−
[M−H]−
483.1163
637.1198
310.9991
704.9267
743 : M.findmain = 442.0724 (+/− 20 ppm)
441.0651
[M−H]−
483.1163
637.1198
310.9991
704.9267
0
20
40
60
80
100
300
400
500
600
700

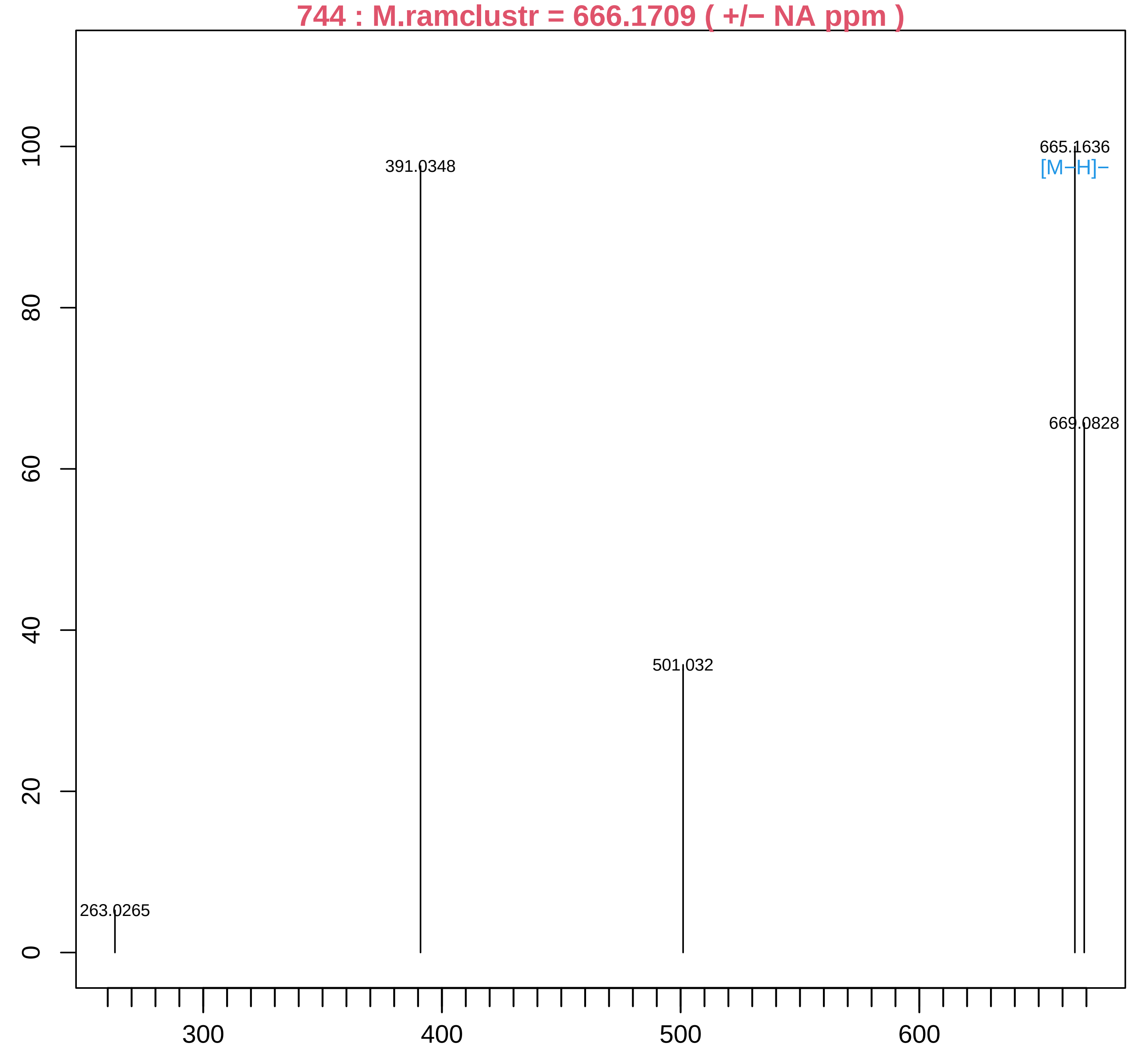

744 : M.ramclustr = 666.1709 (+/− NA ppm)
391.0348
501.032
665.1636
[M−H]−
669.0828
263.0265
0
20
40
60
80
100
300
400
500
600

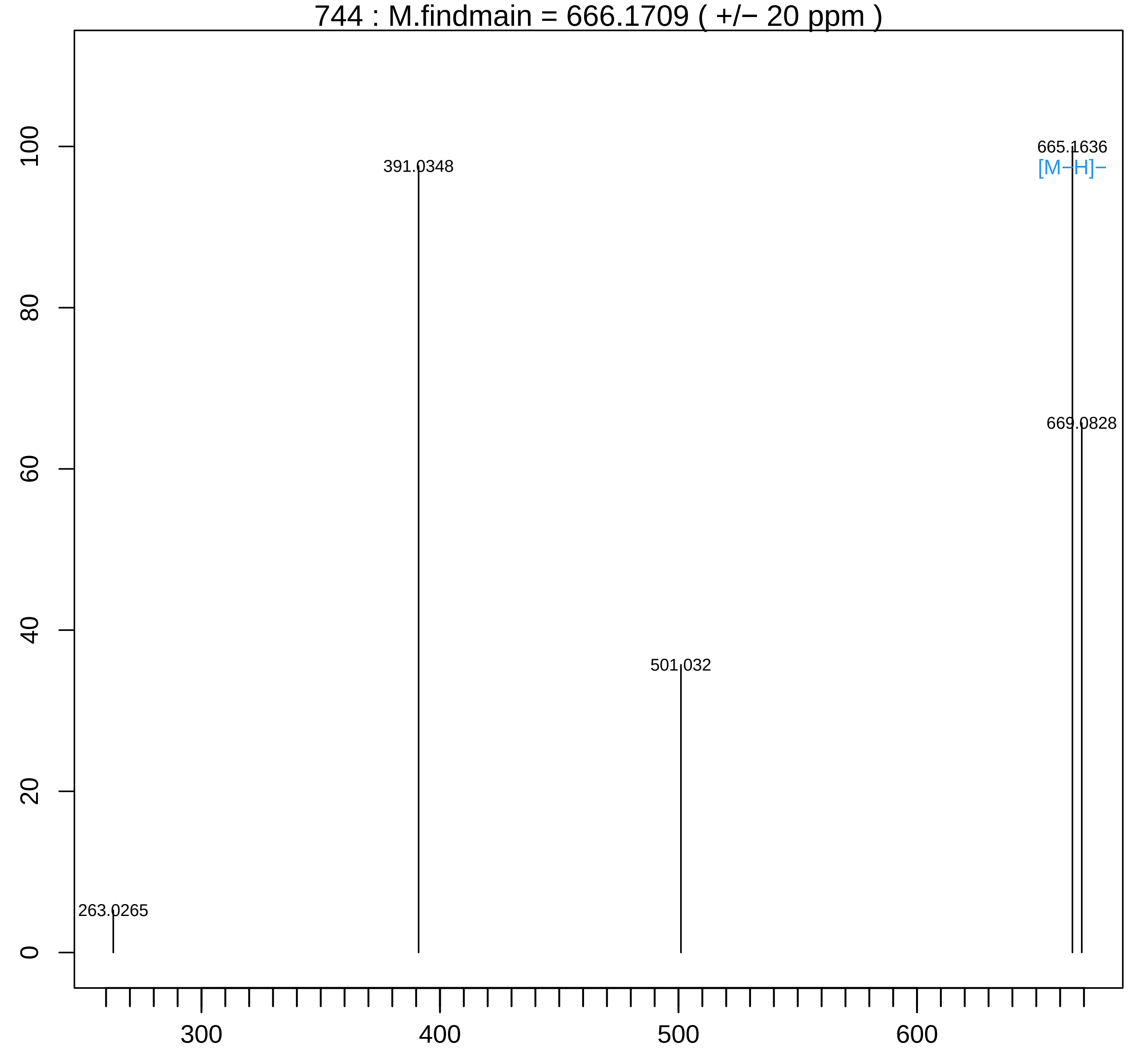

744 : M.findmain = 666.1709 (+/− 20 ppm)
391.0348
501.032
665.1636
[M−H]−
669.0828
263.0265
0
20
40
60
80
100
300
400
500
600

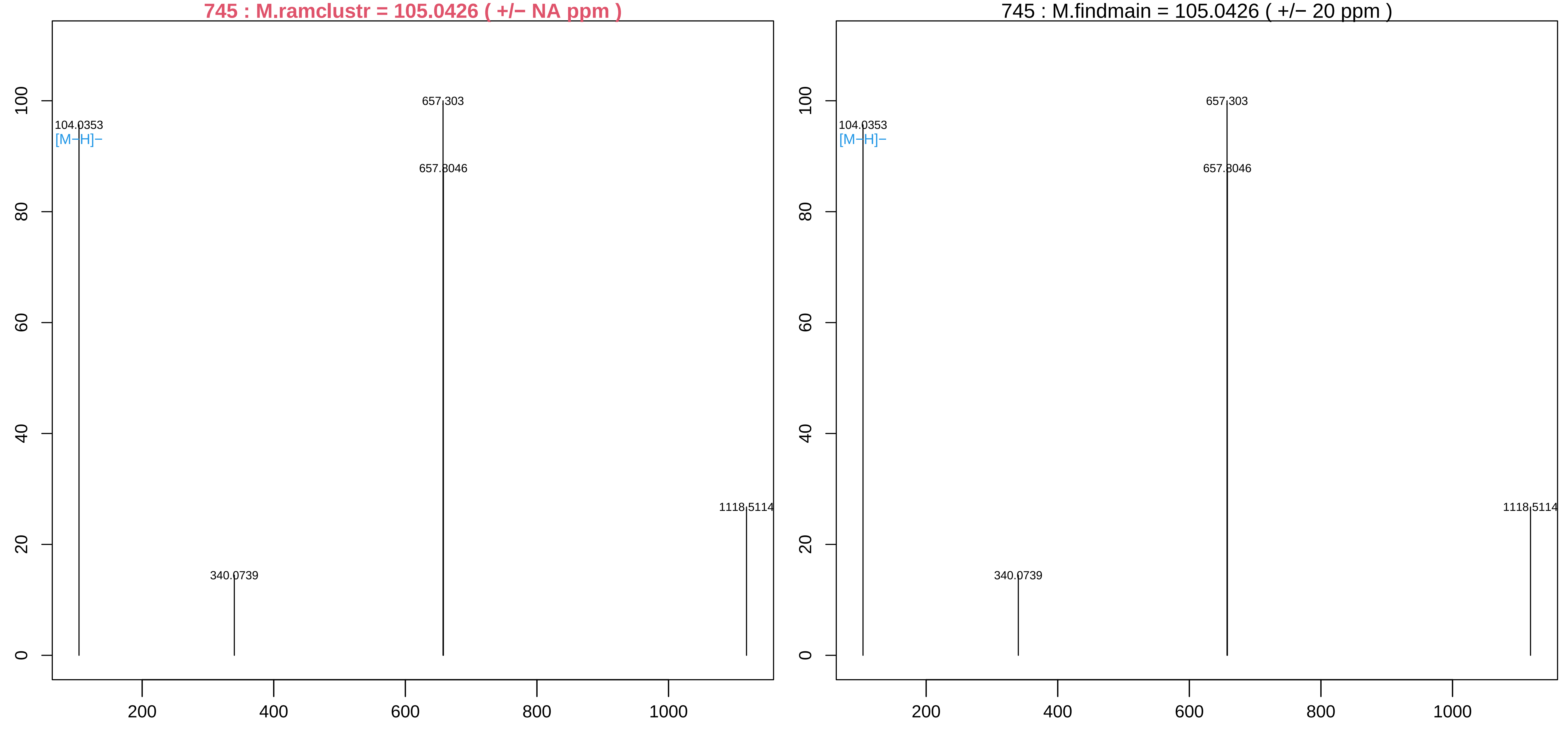
745 : M.ramclustr = 105.0426 (+/− NA ppm)
104.0353
[M−H]−
657.303
657.3046
340.0739
1118.5114
0
20
40
60
80
100
200
400
600
800
1000
745 : M.findmain = 105.0426 (+/− 20 ppm)
104.0353
[M−H]−
657.303
657.3046
340.0739
1118.5114
0
20
40
60
80
100
200
400
600
800
1000

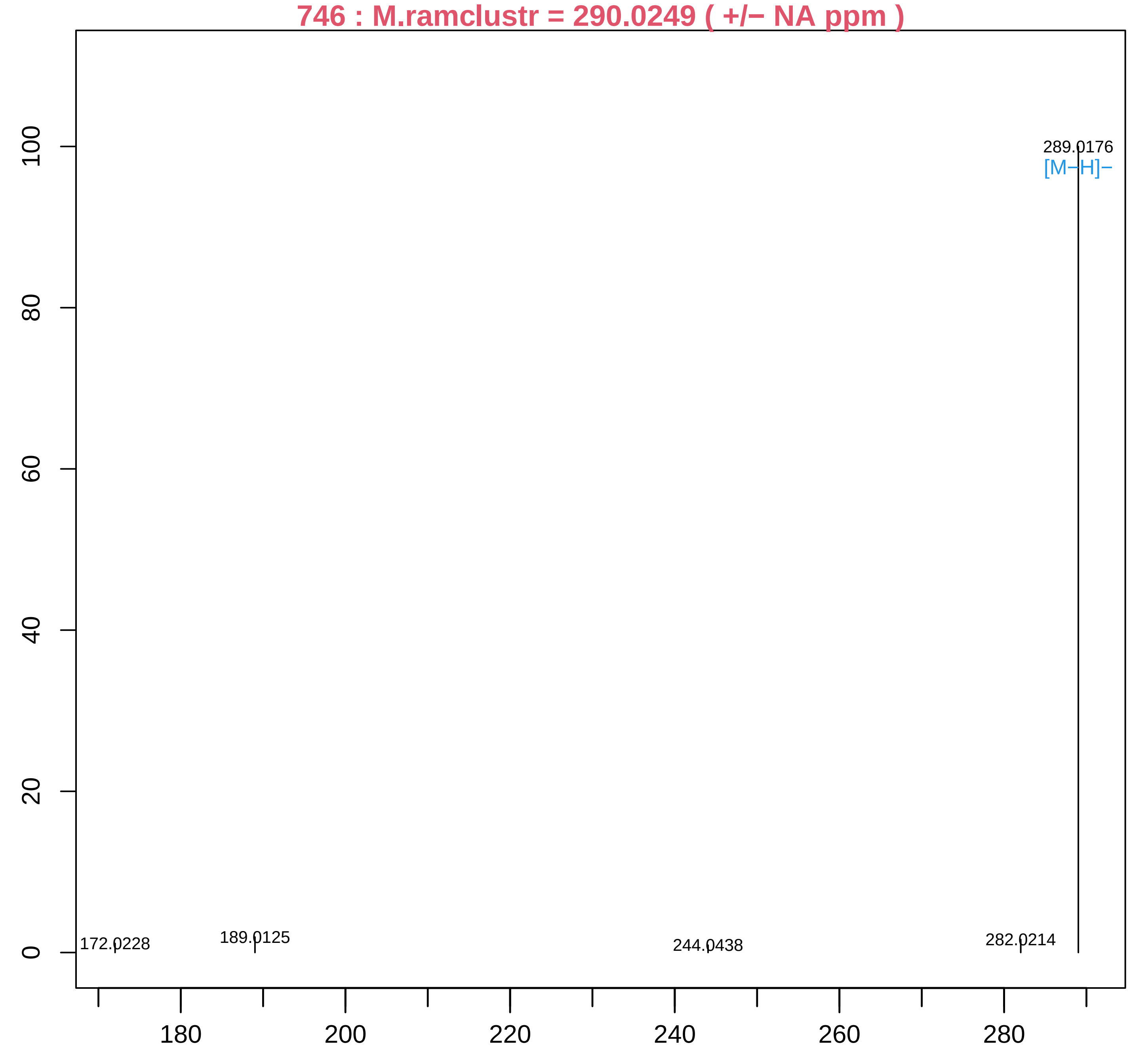

746 : M.ramclustr = 290.0249 (+/− NA ppm)
289.0176
[M−H]−
172.0228
189.0125
244.0438
282.0214
0
20
40
60
80
100
180
200
220
240
260
280

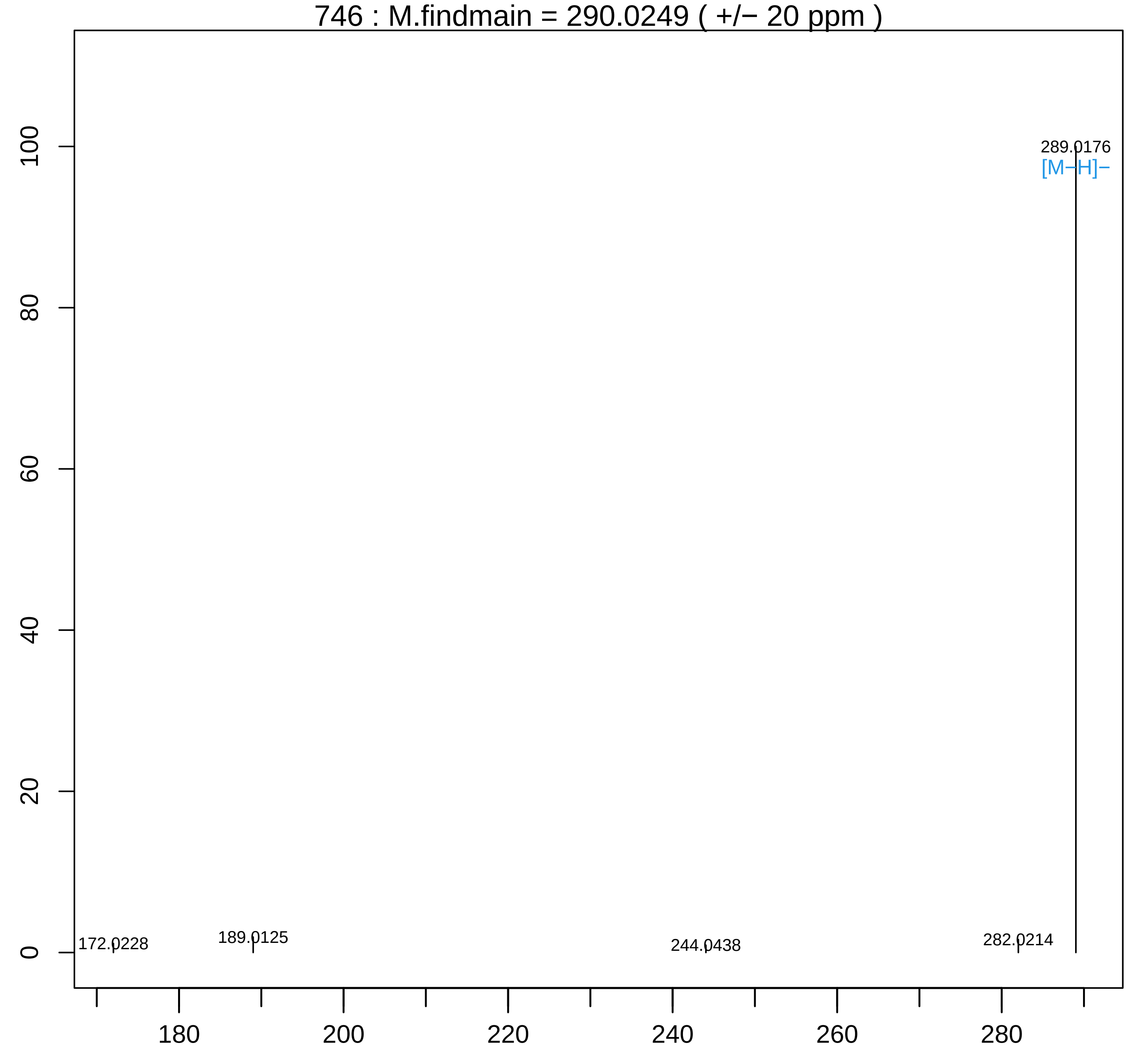

746 : M.findmain = 290.0249 (+/− 20 ppm)
289.0176
[M−H]−
172.0228
189.0125
244.0438
282.0214
0
20
40
60
80
100
180
200
220
240
260
280

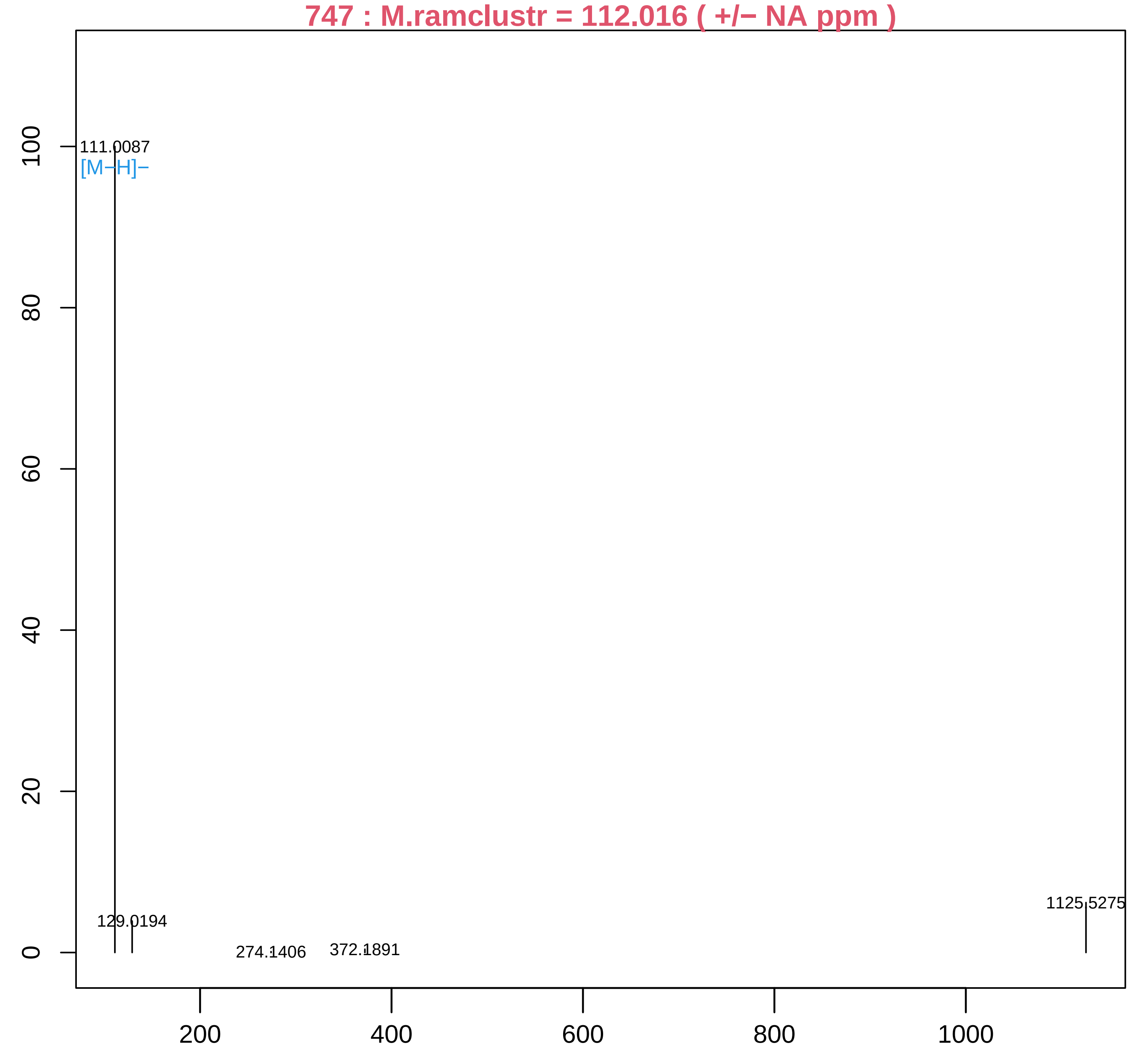
747 : M.ramclustr = 112.016 (+/− NA ppm)
111.0087
[M−H]−
129.0194
274.1406
372.1891
1125.5275
0
20
40
60
80
100
200
400
600
800
1000

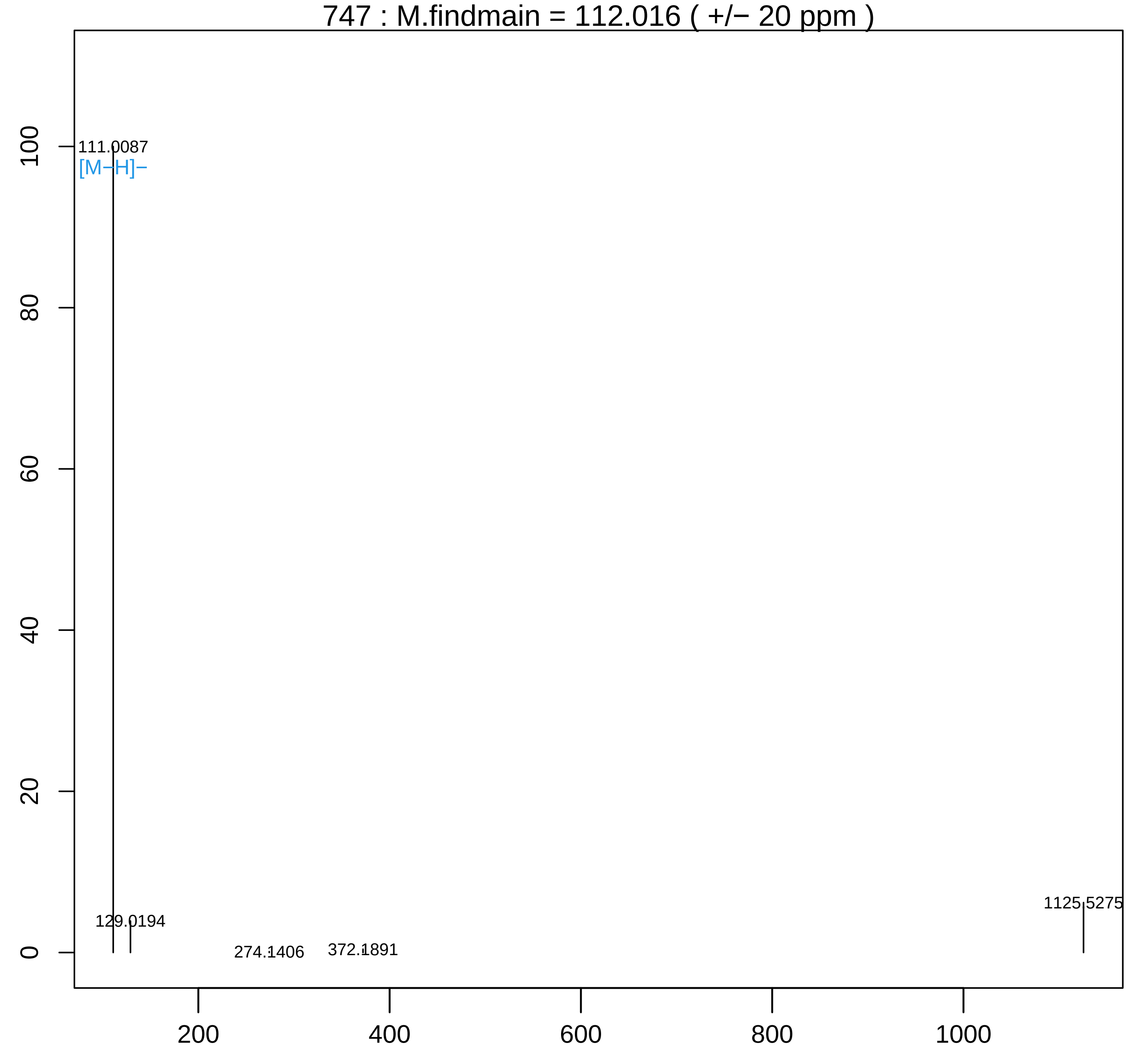
747 : M.findmain = 112.016 (+/− 20 ppm)
111.0087
[M−H]−
129.0194
274.1406
372.1891
1125.5275
0
20
40
60
80
100
200
400
600
800
1000

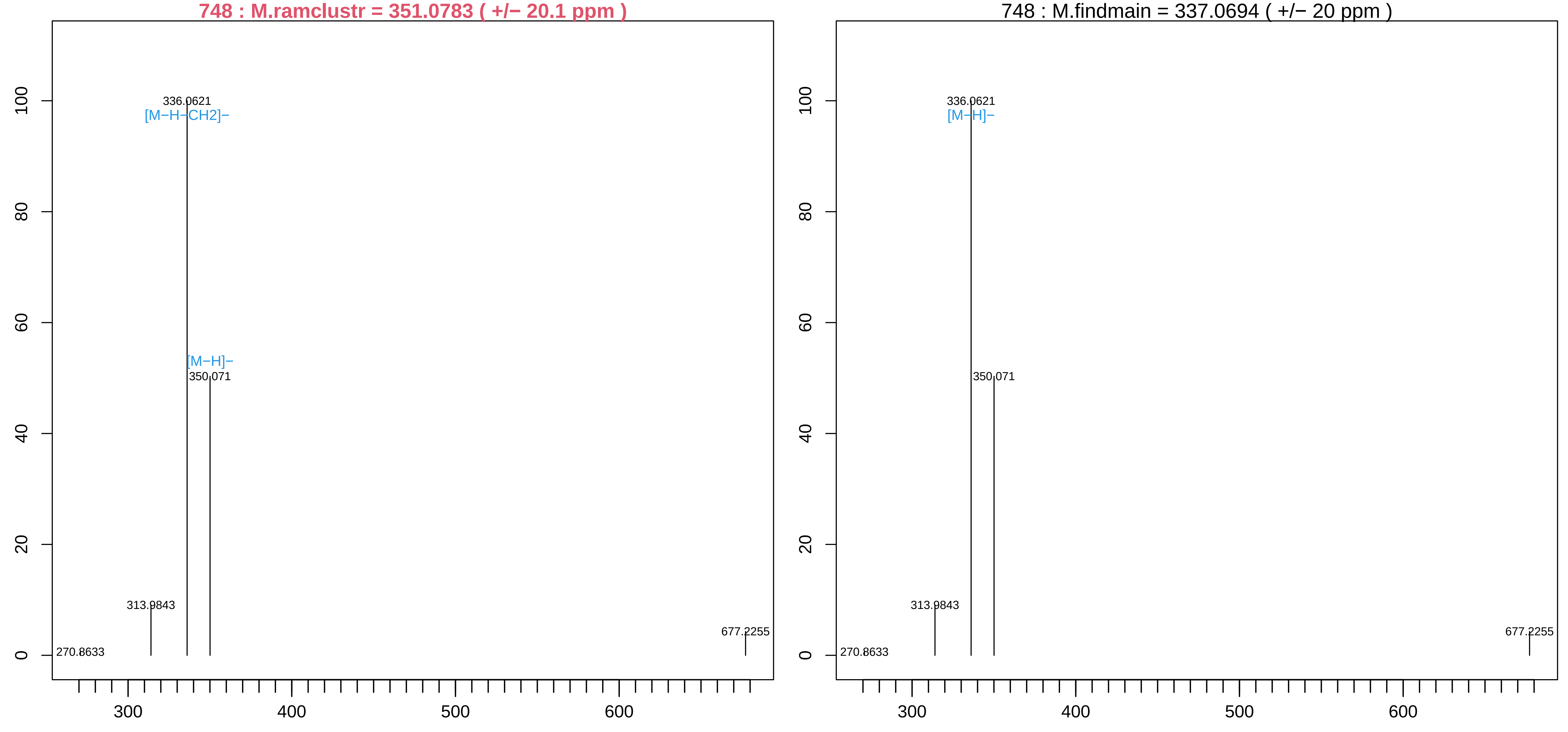
748 : M.ramclustr = 351.0783 (+/− 20.1 ppm)
336.0621
[M−H−CH2]−
[M−H]−
350.071
313.9843
270.8633
677.2255
748 : M.findmain = 337.0694 (+/− 20 ppm)
336.0621
[M−H]−
350.071
313.9843
270.8633
677.2255
0
20
40
60
80
100
300
400
500
600

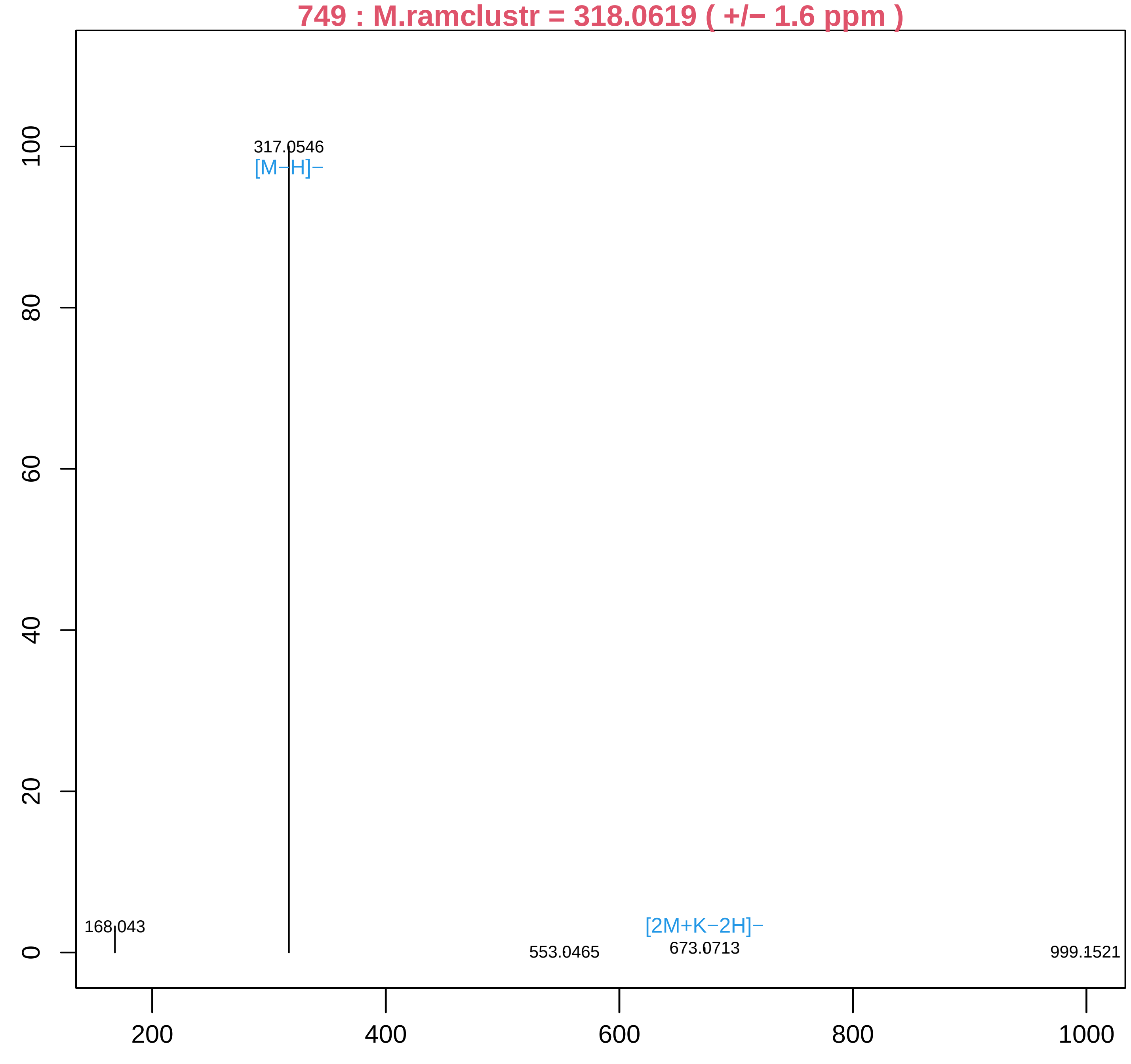
749 : M.ramclustr = 318.0619 (+/− 1.6 ppm)
317.0546
[M−H]−
168.043
553.0465
[2M+K−2H]−
673.0713
999.1521
0
20
40
60
80
100
200
400
600
800
1000

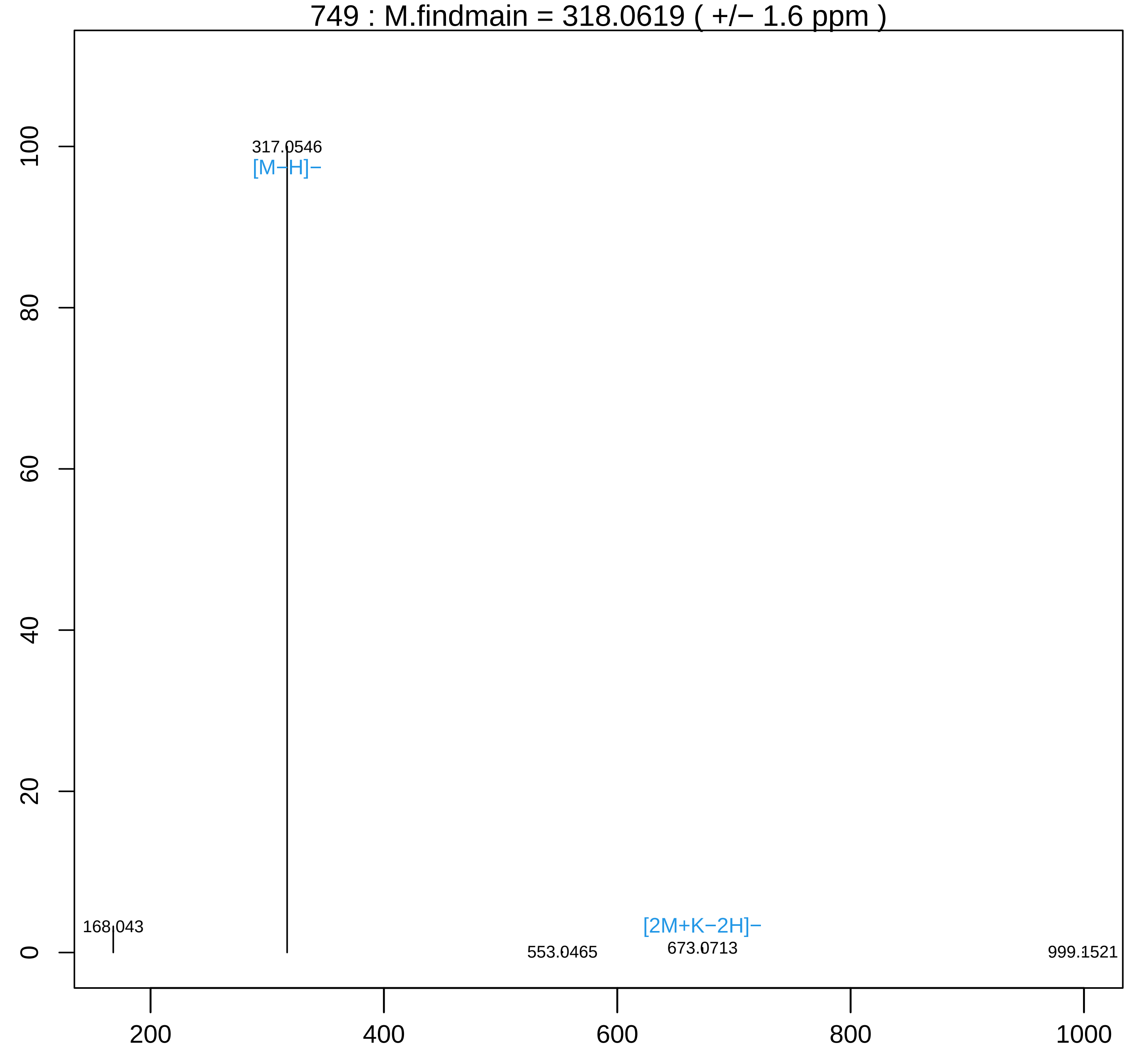
749 : M.findmain = 318.0619 (+/− 1.6 ppm)
317.0546
[M−H]−
168.043
553.0465
[2M+K−2H]−
673.0713
999.1521
0
20
40
60
80
100
200
400
600
800
1000

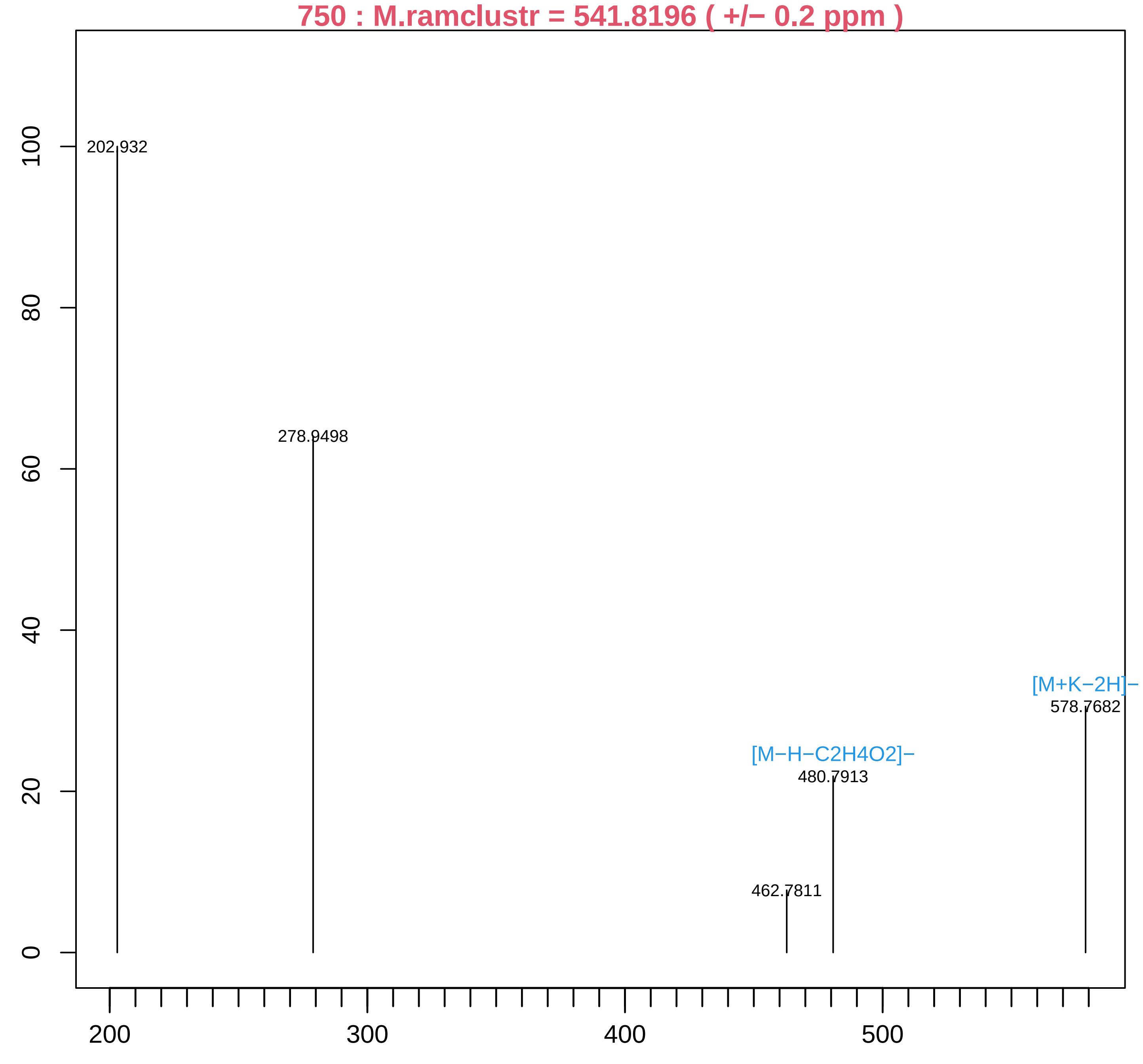

750 : M.ramclustr = 541.8196 (+/− 0.2 ppm)
202.932
278.9498
[M−H−C2H4O2]−
480.7913
462.7811
[M+K−2H]−
578.7682
0
20
40
60
80
100
200
300
400
500

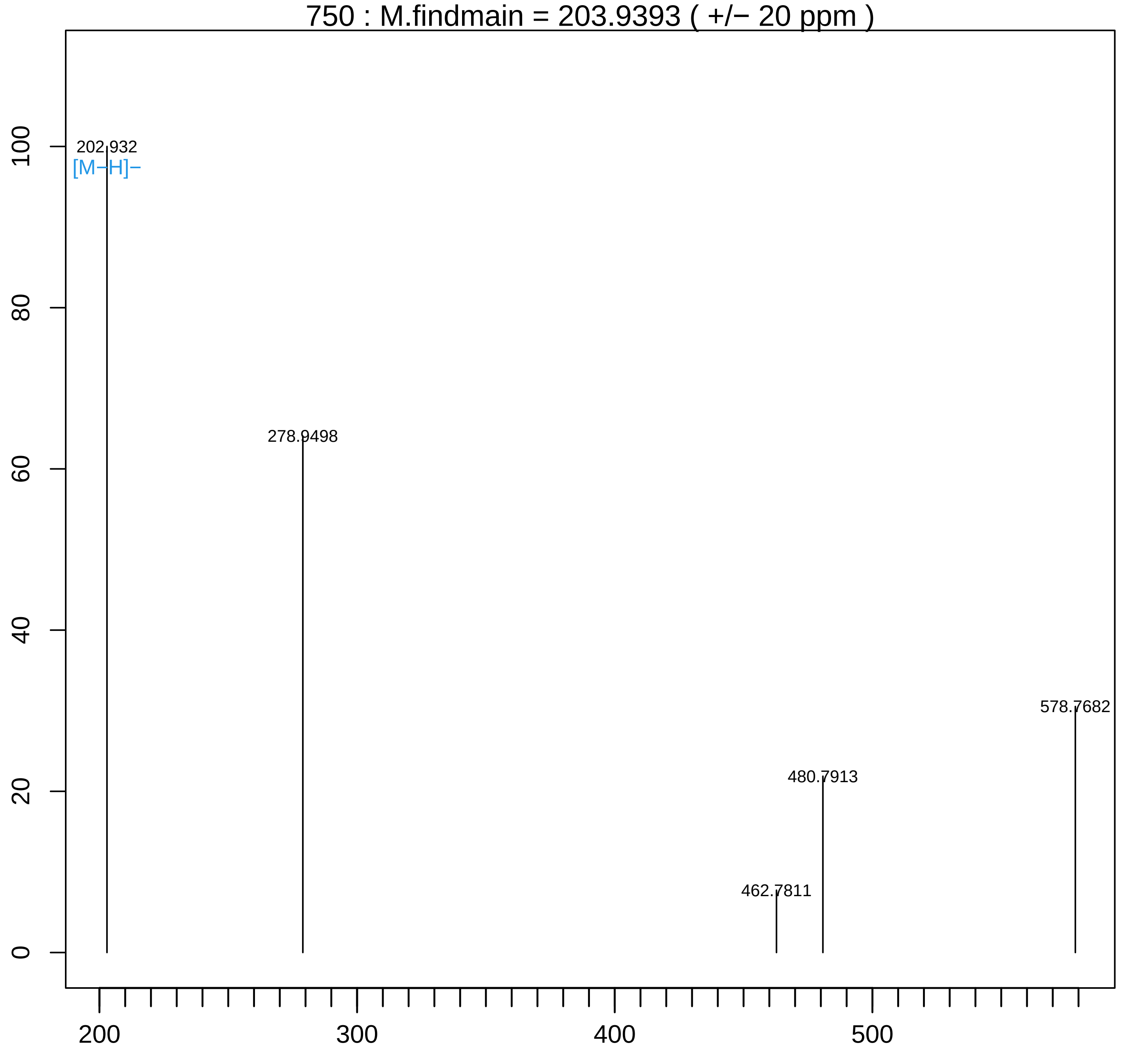

750 : M.findmain = 203.9393 (+/− 20 ppm)
202.932
[M−H]−
278.9498
480.7913
462.7811
578.7682
0
20
40
60
80
100
200
300
400
500

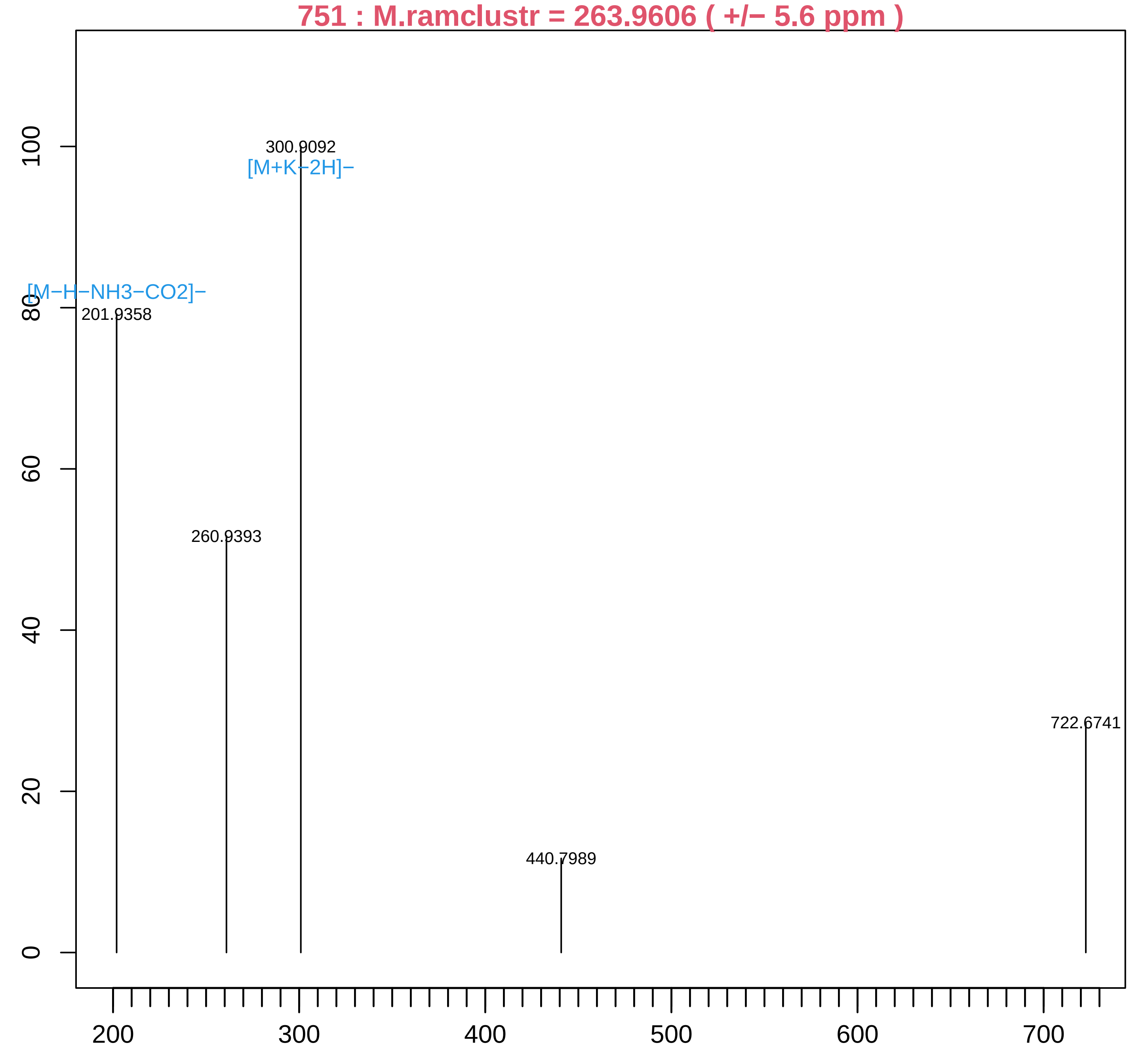
751 : M.ramclustr = 263.9606 (+/− 5.6 ppm)
300.9092
[M+K−2H]−
[M−H−NH3−CO2]−
201.9358
260.9393
440.7989
722.6741
0
20
40
60
80
100
200
300
400
500
600
700

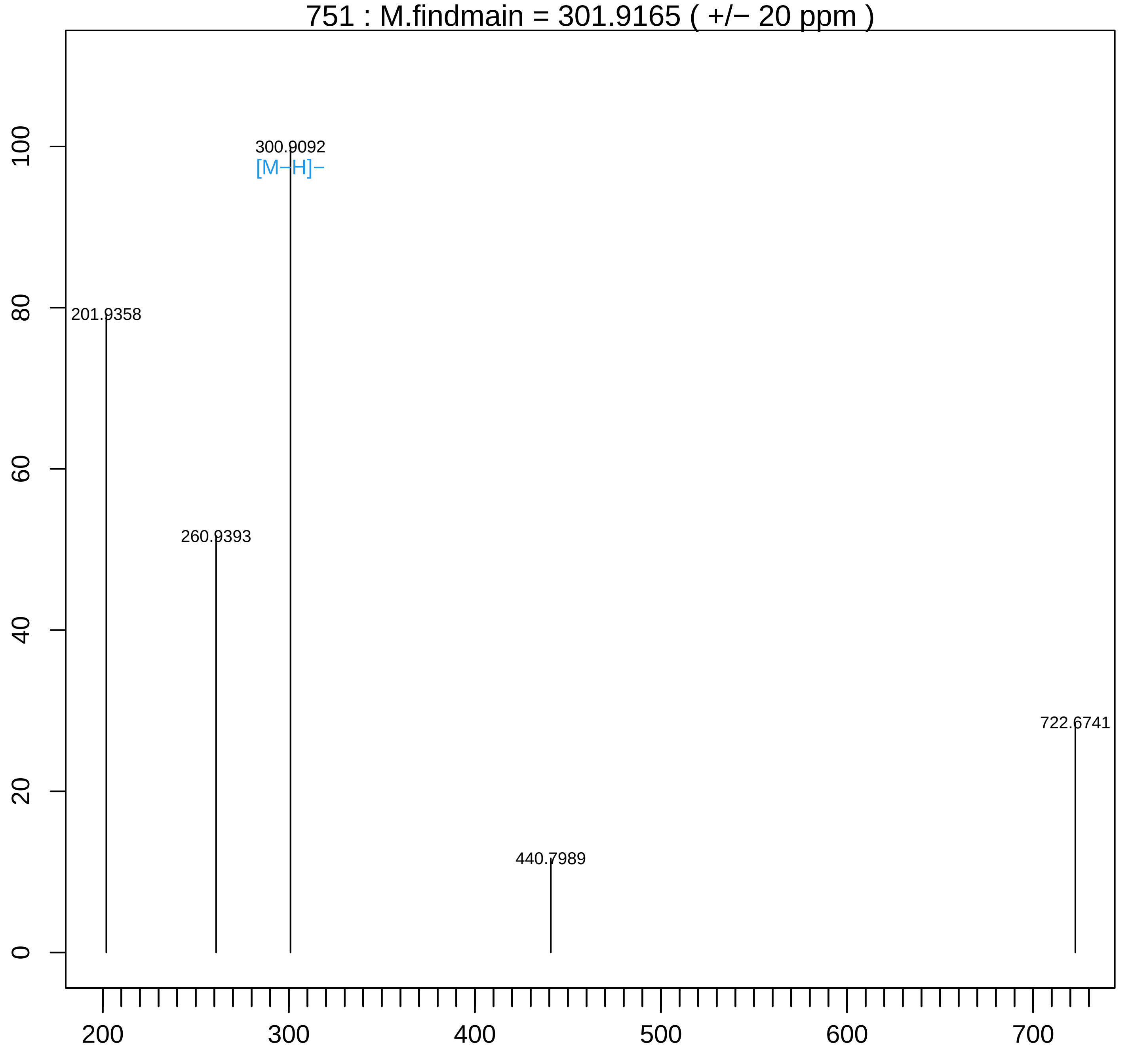
751 : M.findmain = 301.9165 (+/− 20 ppm)
300.9092
[M−H]−
201.9358
260.9393
440.7989
722.6741
0
20
40
60
80
100
200
300
400
500
600
700

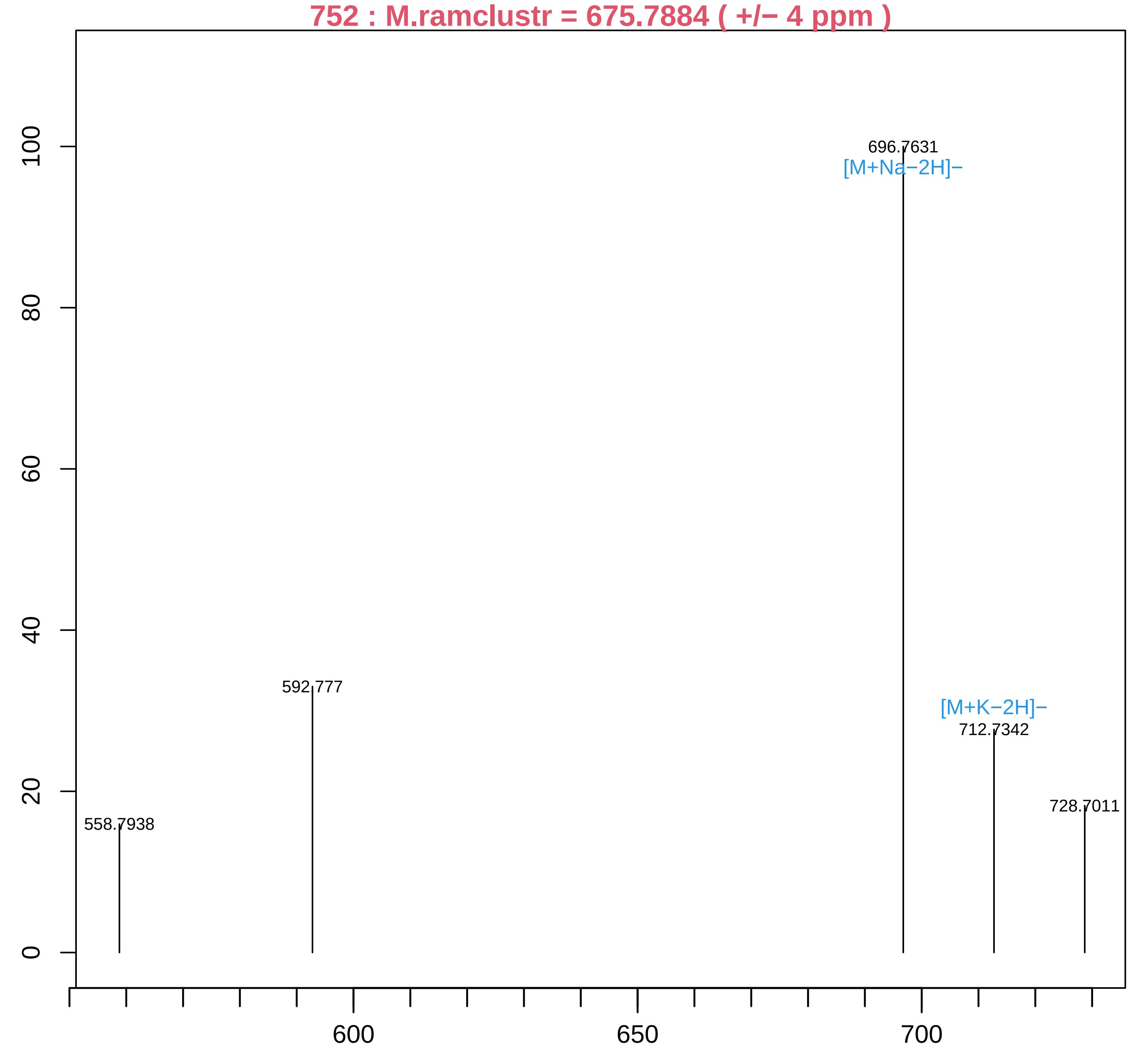
752 : M.ramclustr = 675.7884 (+/− 4 ppm)
696.7631
[M+Na−2H]−
592.777
[M+K−2H]−
712.7342
728.7011
558.7938
0
20
40
60
80
100
600
650
700

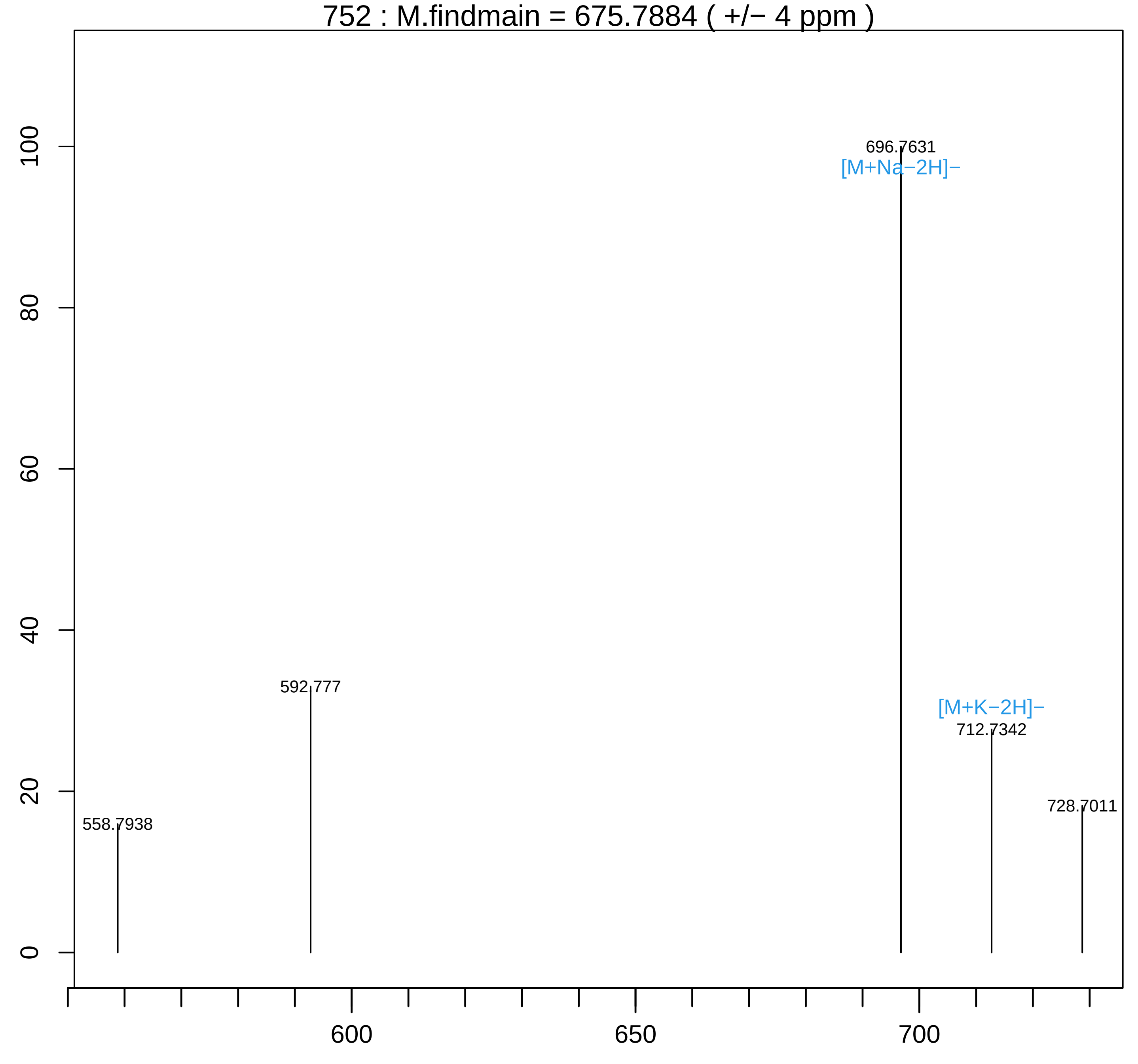
752 : M.findmain = 675.7884 (+/− 4 ppm)
696.7631
[M+Na−2H]−
592.777
[M+K−2H]−
712.7342
728.7011
558.7938
0
20
40
60
80
100
600
650
700

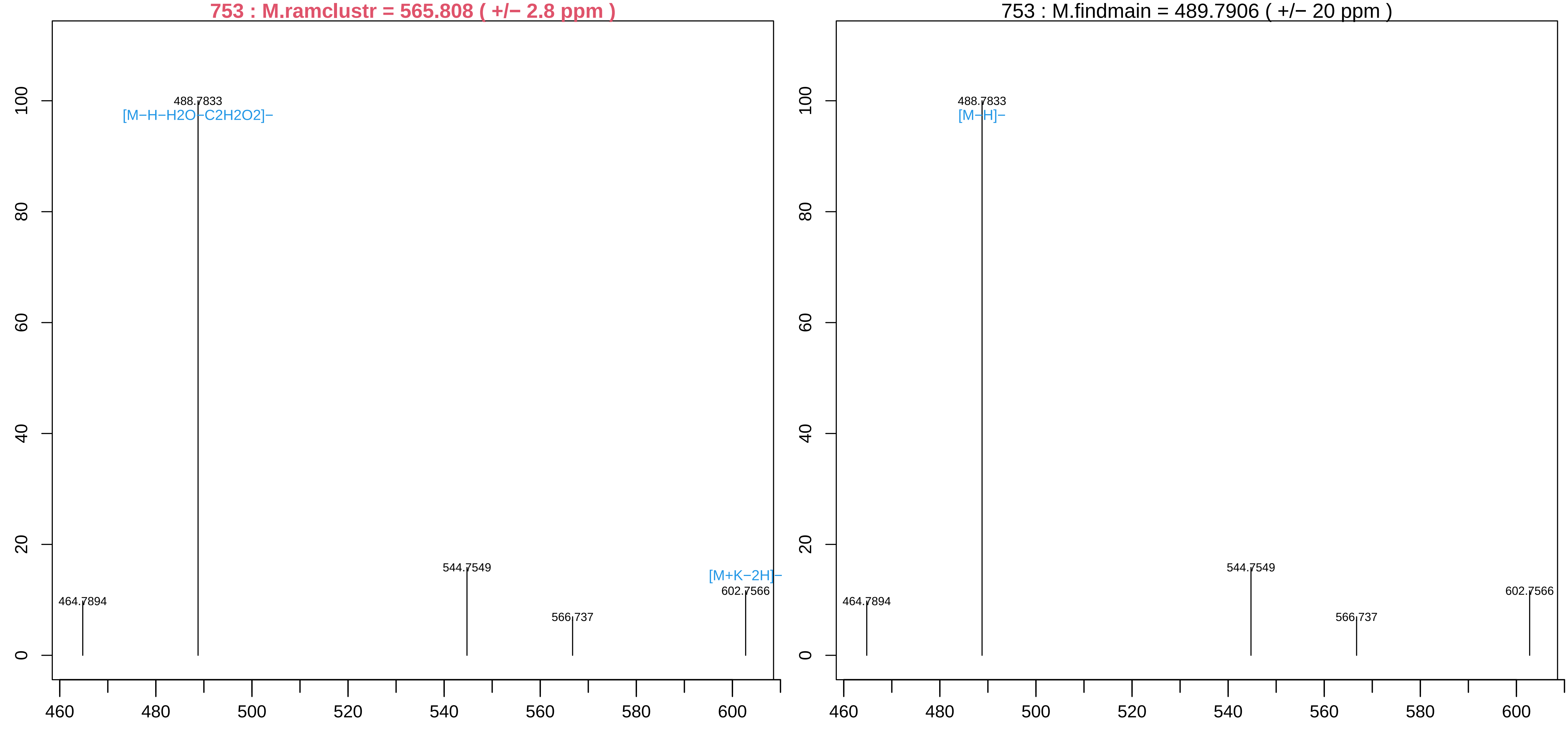
753 : M.ramclustr = 565.808 (+/− 2.8 ppm)
488.7833
[M−H−H2O−C2H2O2]−
464.7894
544.7549
566.737
[M+K−2H]−
602.7566
753 : M.findmain = 489.7906 (+/− 20 ppm)
488.7833
[M−H]−
464.7894
544.7549
566.737
602.7566

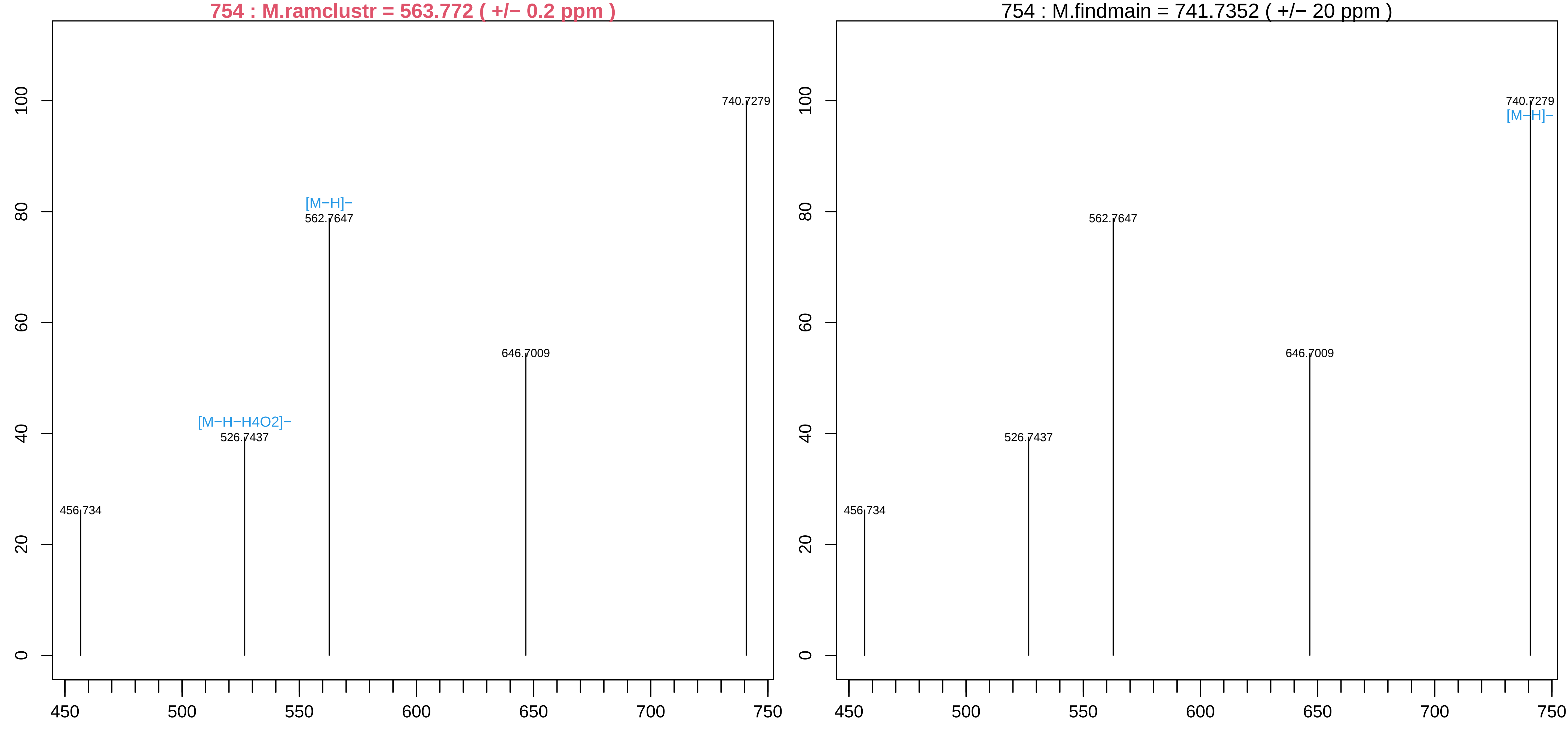
754 : M.ramclustr = 563.772 (+/− 0.2 ppm)
[M−H]−
562.7647
[M−H−H4O2]−
526.7437
456.734
646.7009
740.7279
754 : M.findmain = 741.7352 (+/− 20 ppm)
740.7279
[M−H]−
562.7647
526.7437
456.734
646.7009
0
20
40
60
80
100
450
500
550
600
650
700
750

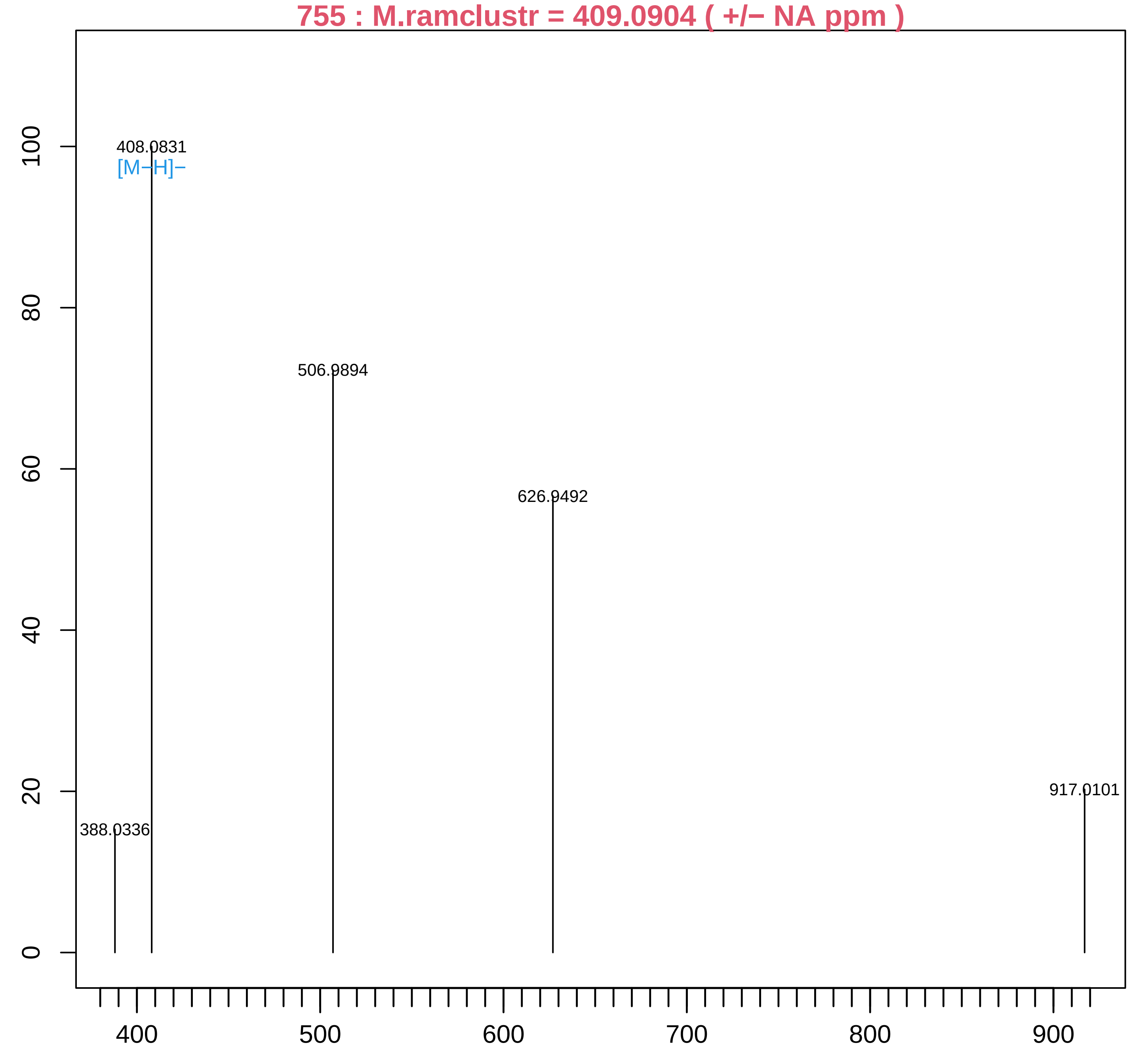
755 : M.ramclustr = 409.0904 (+/− NA ppm)
408.0831
[M−H]−
506.9894
626.9492
917.0101
388.0336
0
20
40
60
80
100
400
500
600
700
800
900

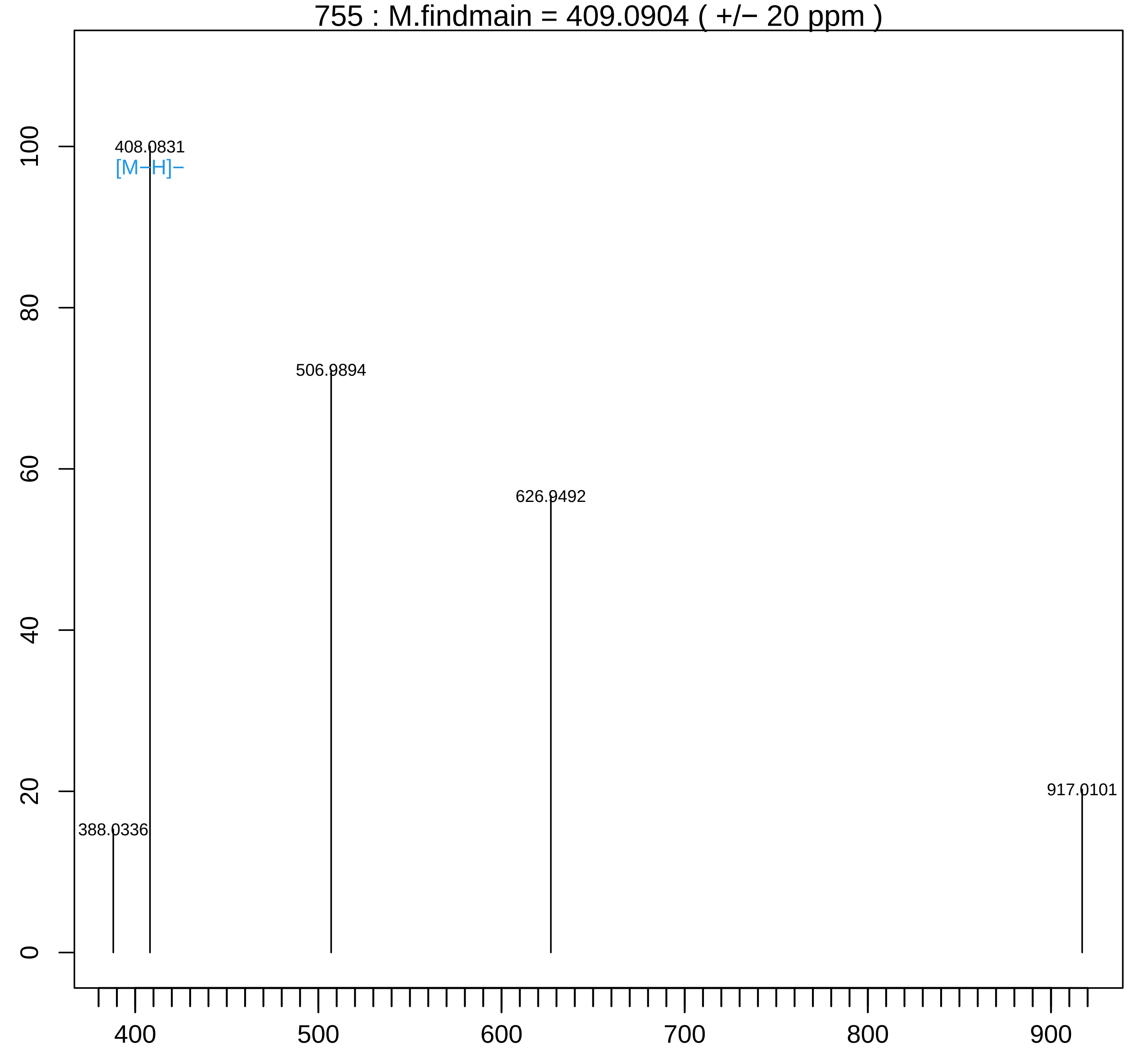
755 : M.findmain = 409.0904 (+/− 20 ppm)
408.0831
[M−H]−
506.9894
626.9492
917.0101
388.0336
0
20
40
60
80
100
400
500
600
700
800
900

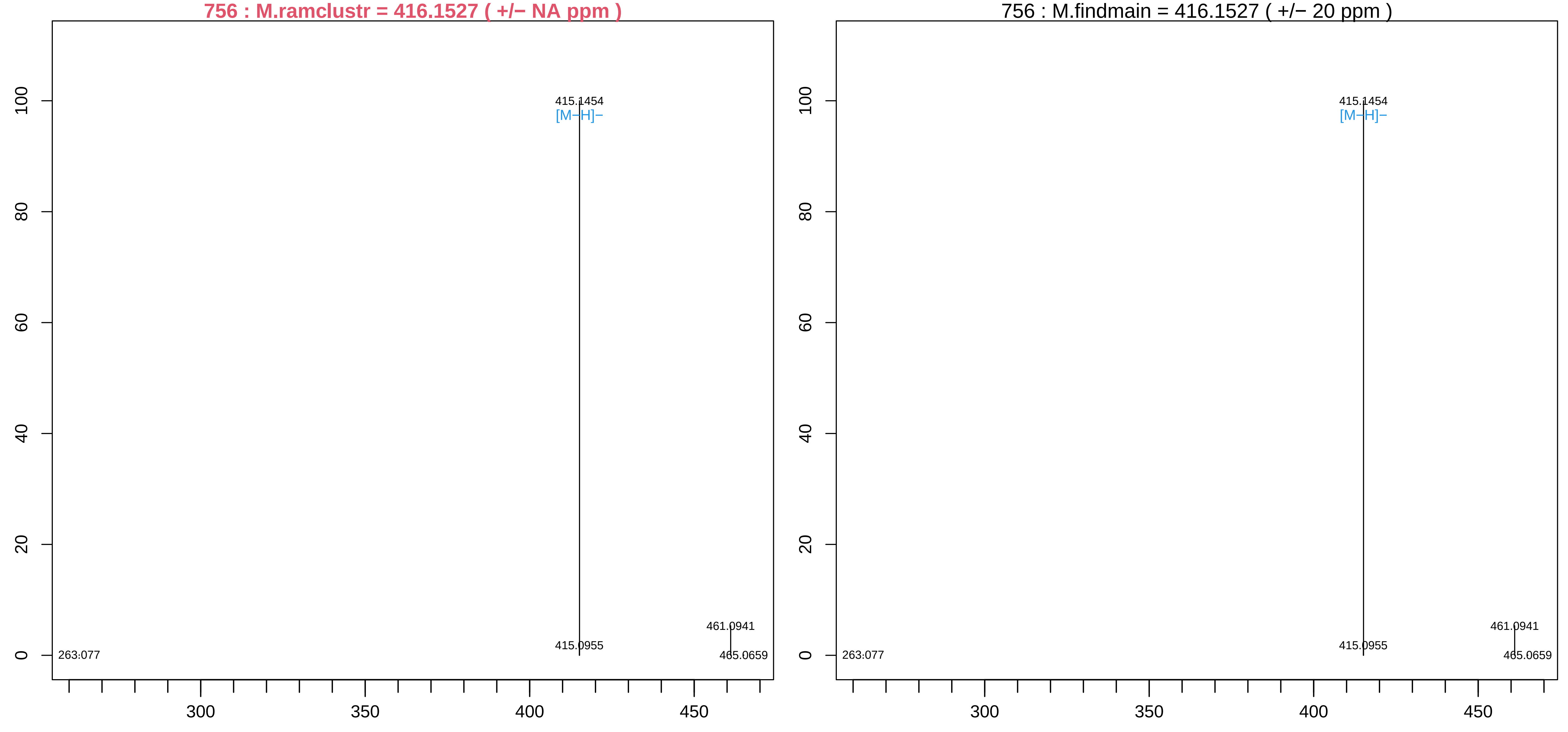
756 : M.ramclustr = 416.1527 (+/− NA ppm)
756 : M.findmain = 416.1527 (+/− 20 ppm)
415.1454
[M−H]−
415.0955
461.0941
465.0659
263:077
0
20
40
60
80
100
300
350
400
450

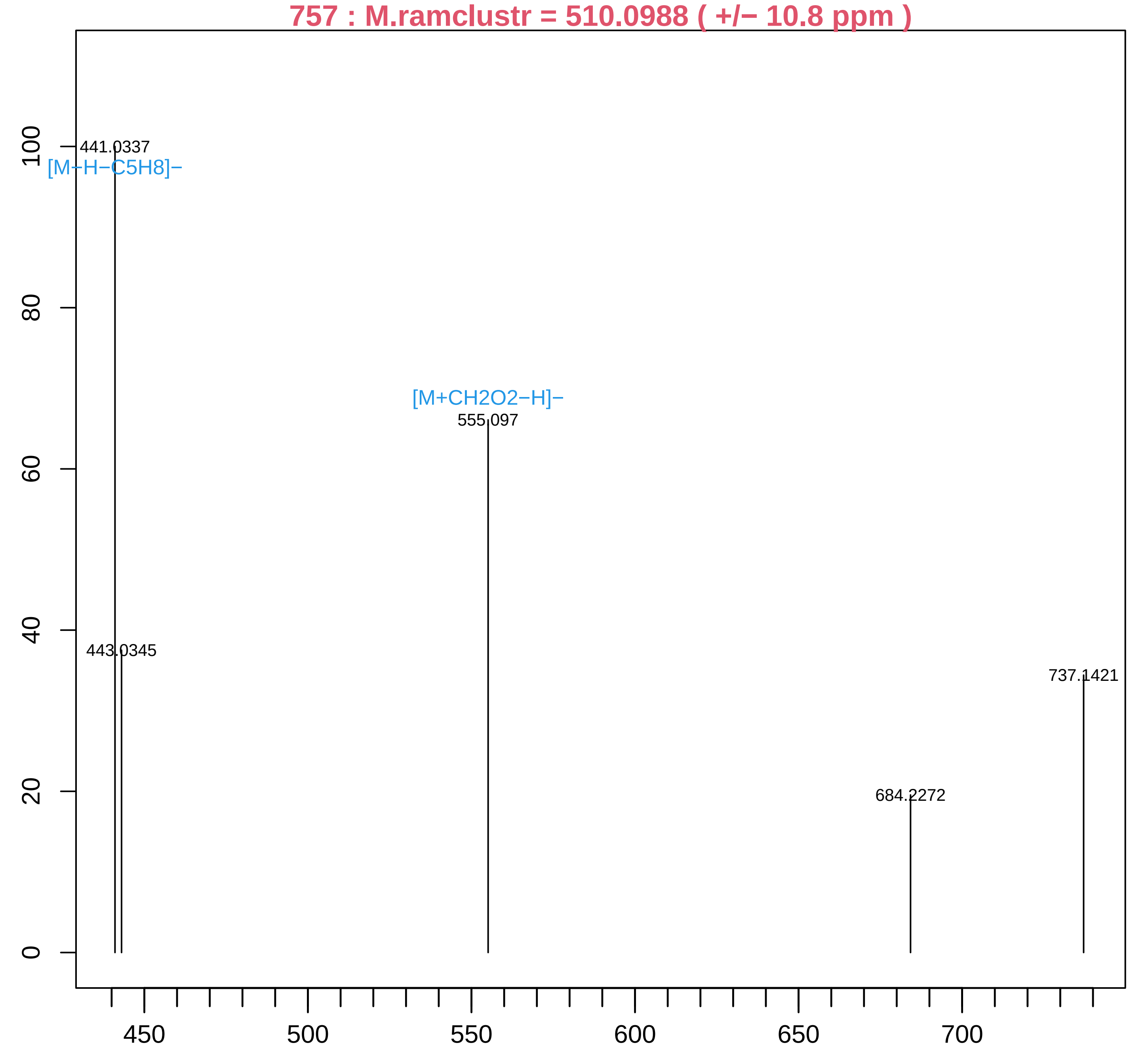
757 : M.ramclustr = 510.0988 (+/− 10.8 ppm)
441.0337
[M−H−C5H8]−
[M+CH2O2−H]−
555.097
443.0345
737.1421
684.2272
0
20
40
60
80
100
450
500
550
600
650
700
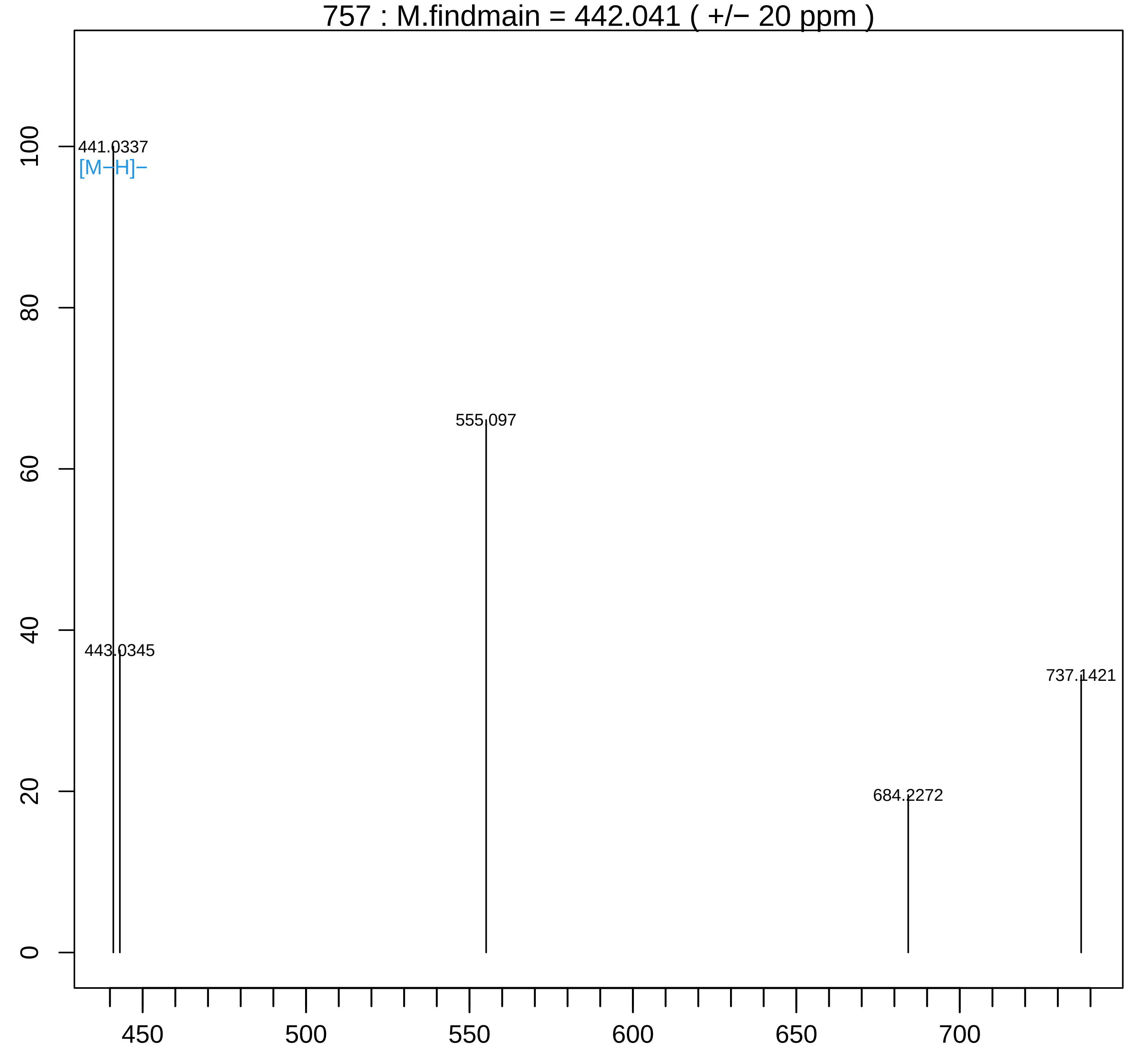
757 : M.findmain = 442.041 (+/− 20 ppm)
441.0337
[M−H]−
555.097
443.0345
737.1421
684.2272
0
20
40
60
80
100
450
500
550
600
650
700

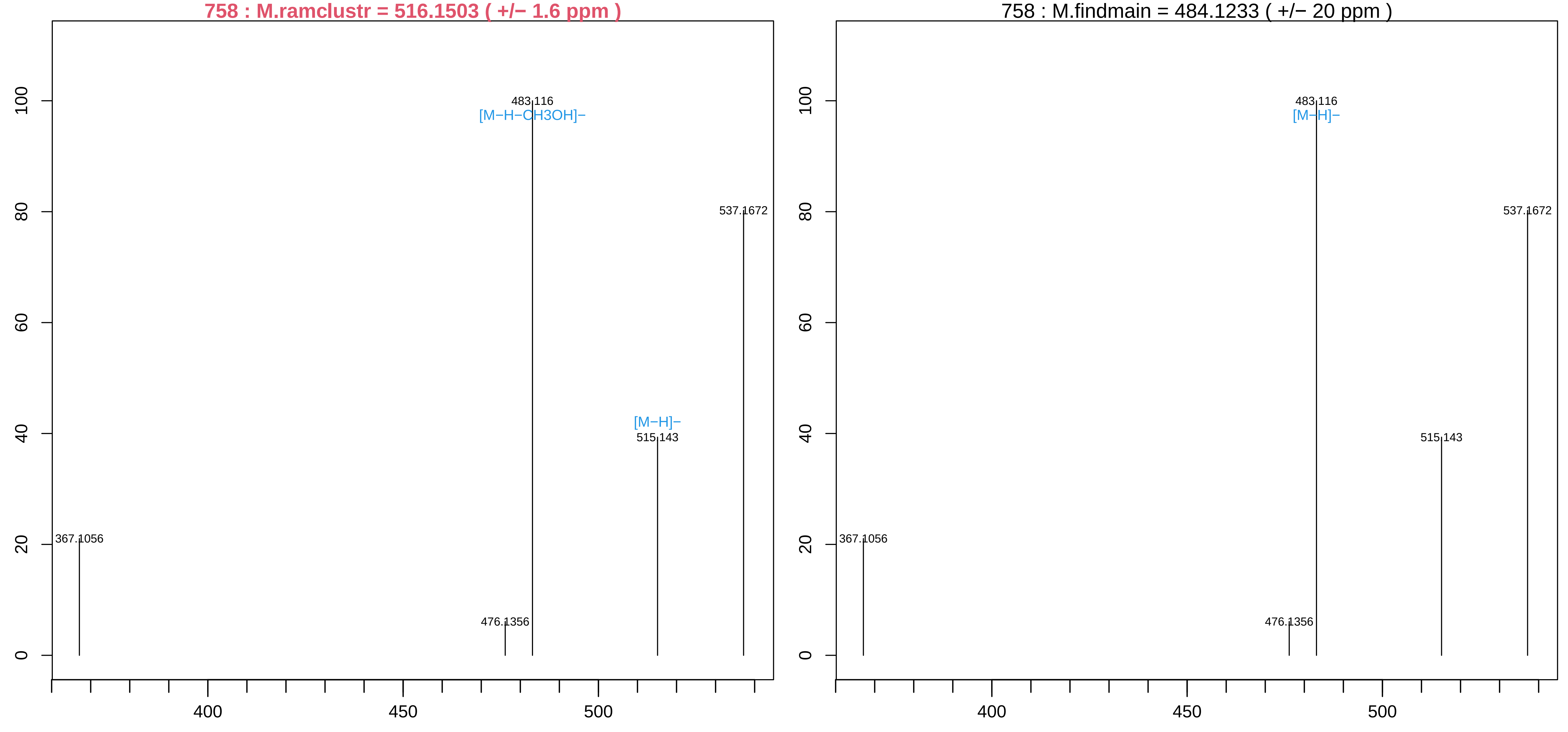
758 : M.ramclustr = 516.1503 (+/− 1.6 ppm)
483.116
[M−H−CH3OH]−
537.1672
[M−H]−
515.143
367.1056
476.1356
0
20
40
60
80
100
400
450
500
758 : M.findmain = 484.1233 (+/− 20 ppm)
483.116
[M−H]−
537.1672
515.143
367.1056
476.1356
0
20
40
60
80
100
400
450
500

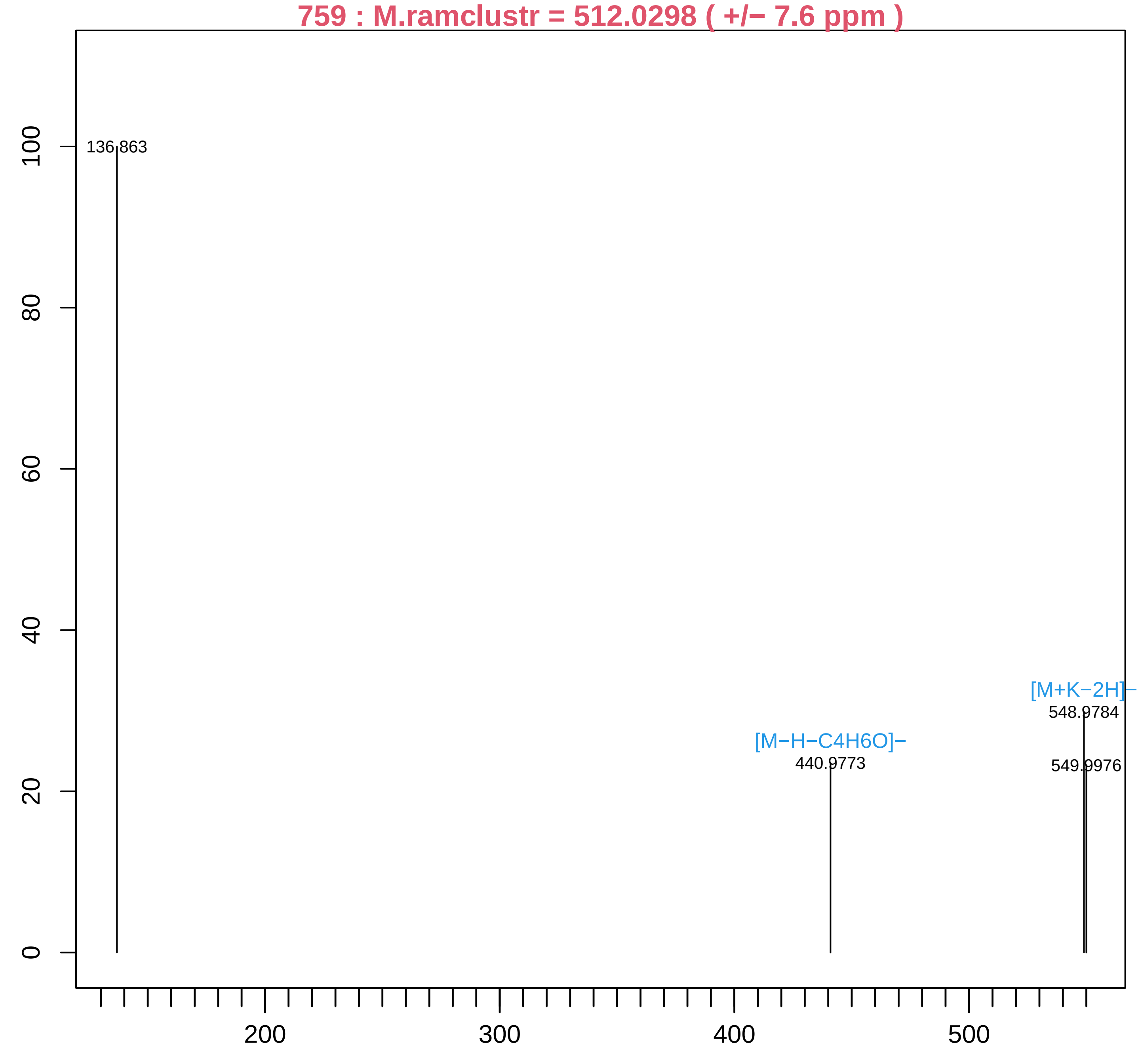
759 : M.ramclustr = 512.0298 (+/− 7.6 ppm)
136.863
[M−H−C4H6O]−
440.9773
[M+K−2H]−
548.9784
549.9976
0
20
40
60
80
100
200
300
400
500

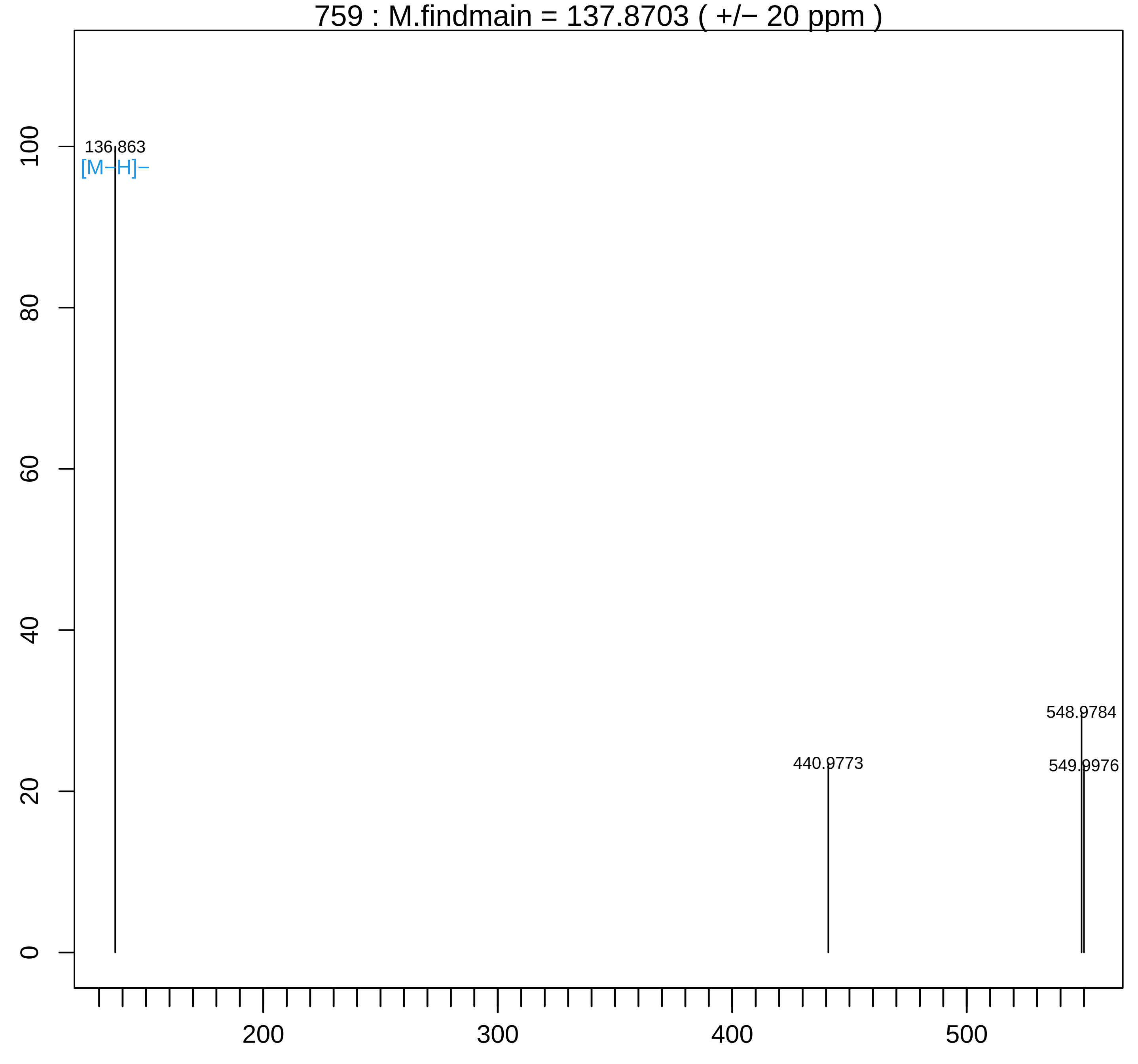
759 : M.findmain = 137.8703 (+/− 20 ppm)
136.863
[M−H]−
440.9773
548.9784
549.9976
0
20
40
60
80
100
200
300
400
500

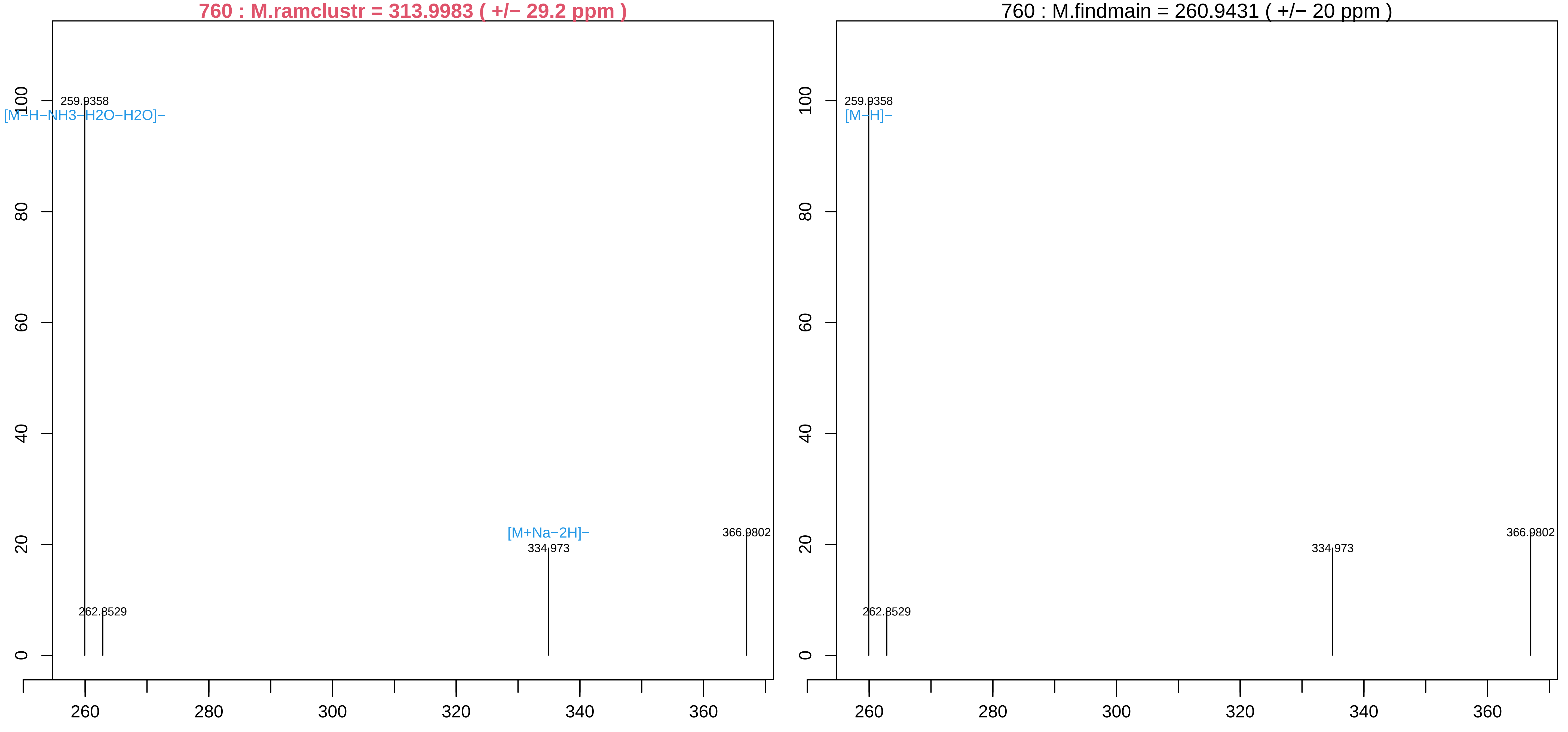
760 : M.ramclustr = 313.9983 (+/− 29.2 ppm)
259.9358
[M−H−NH3−H2O−H2O]−
262.8529
[M+Na−2H]−
334.973
366.9802
0
20
40
60
80
100
260
280
300
320
340
360
760 : M.findmain = 260.9431 (+/− 20 ppm)
259.9358
[M−H]−
262.8529
334.973
366.9802
0
20
40
60
80
100
260
280
300
320
340
360

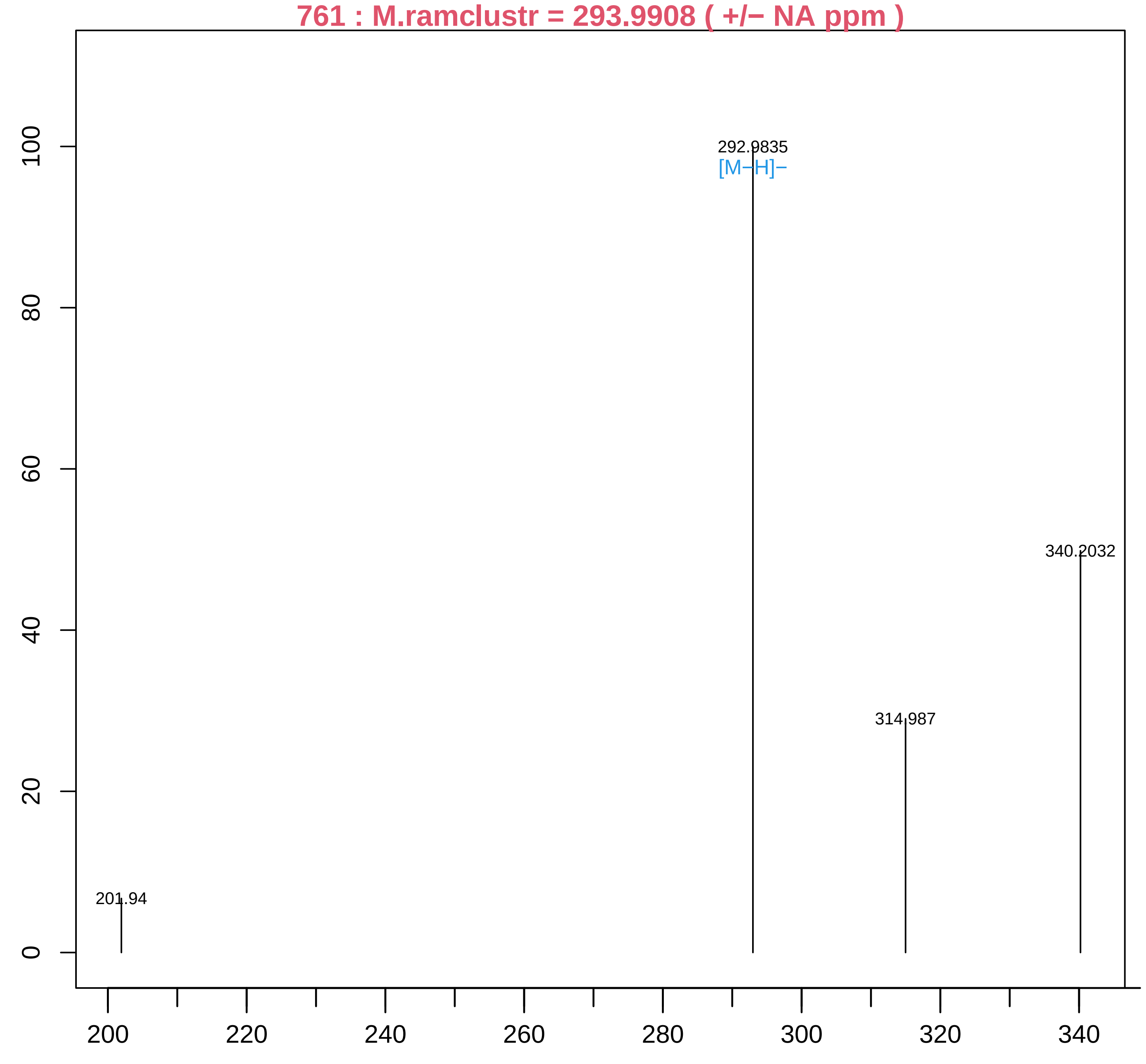
761 : M.ramclustr = 293.9908 (+/− NA ppm)
292.9835
[M−H]−
340.2032
314.987
201.94
0
20
40
60
80
100
200
220
240
260
280
300
320
340

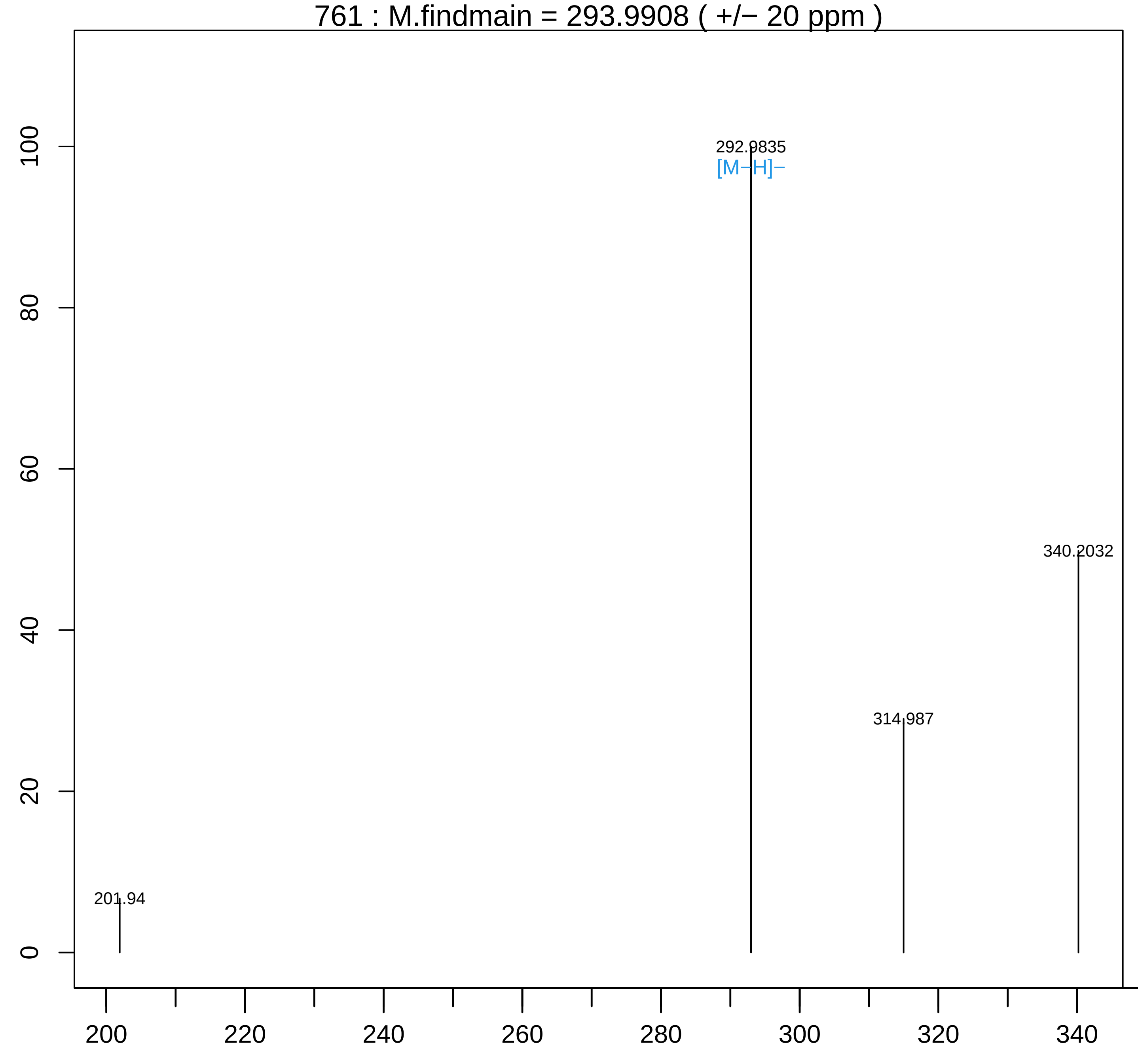
761 : M.findmain = 293.9908 (+/− 20 ppm)
292.9835
[M−H]−
340.2032
314.987
201.94
0
20
40
60
80
100
200
220
240
260
280
300
320
340

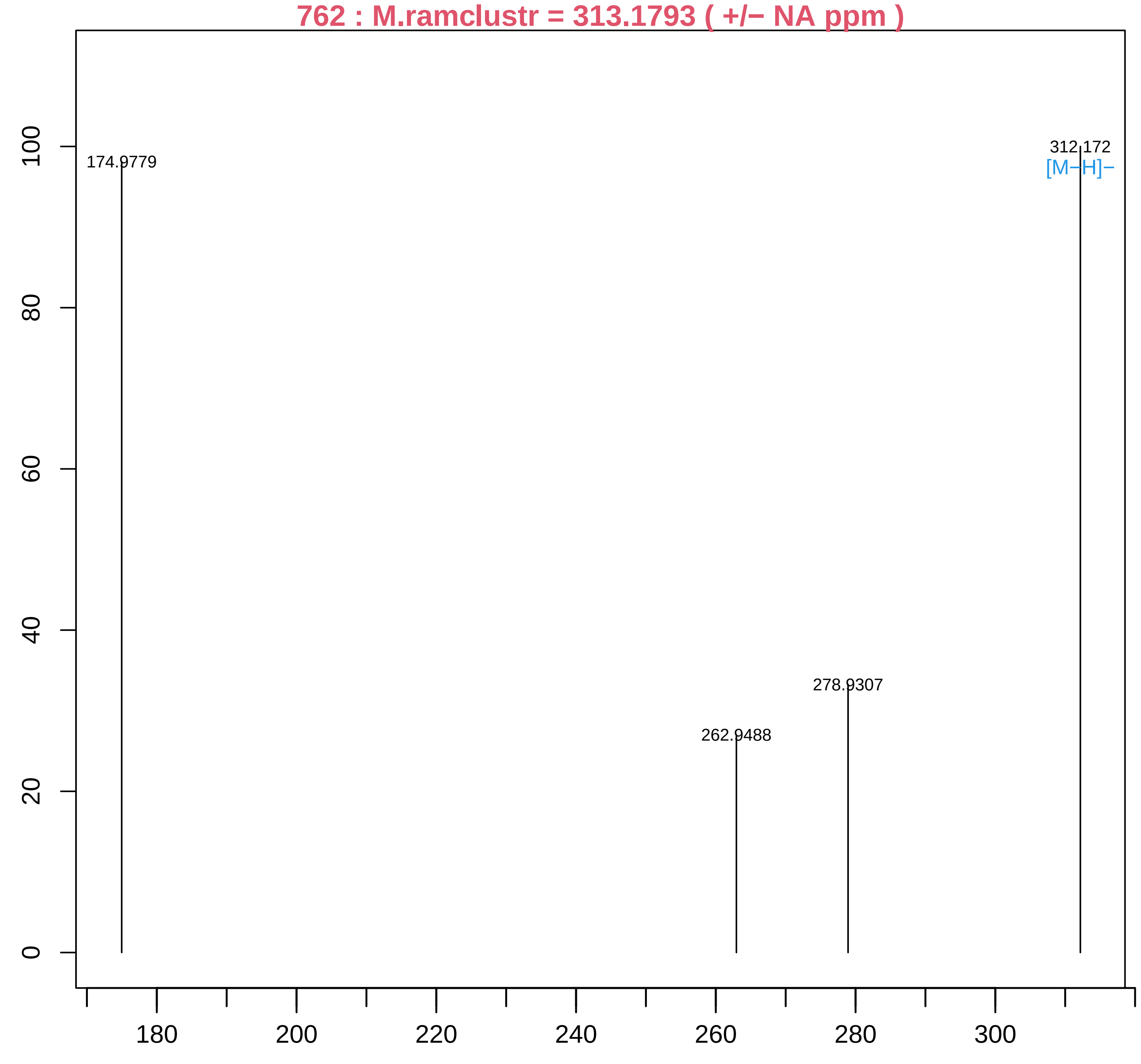
762 : M.ramclustr = 313.1793 (+/− NA ppm)
174.9779
262.9488
278.9307
312.172
[M−H]−
0
20
40
60
80
100
180
200
220
240
260
280
300

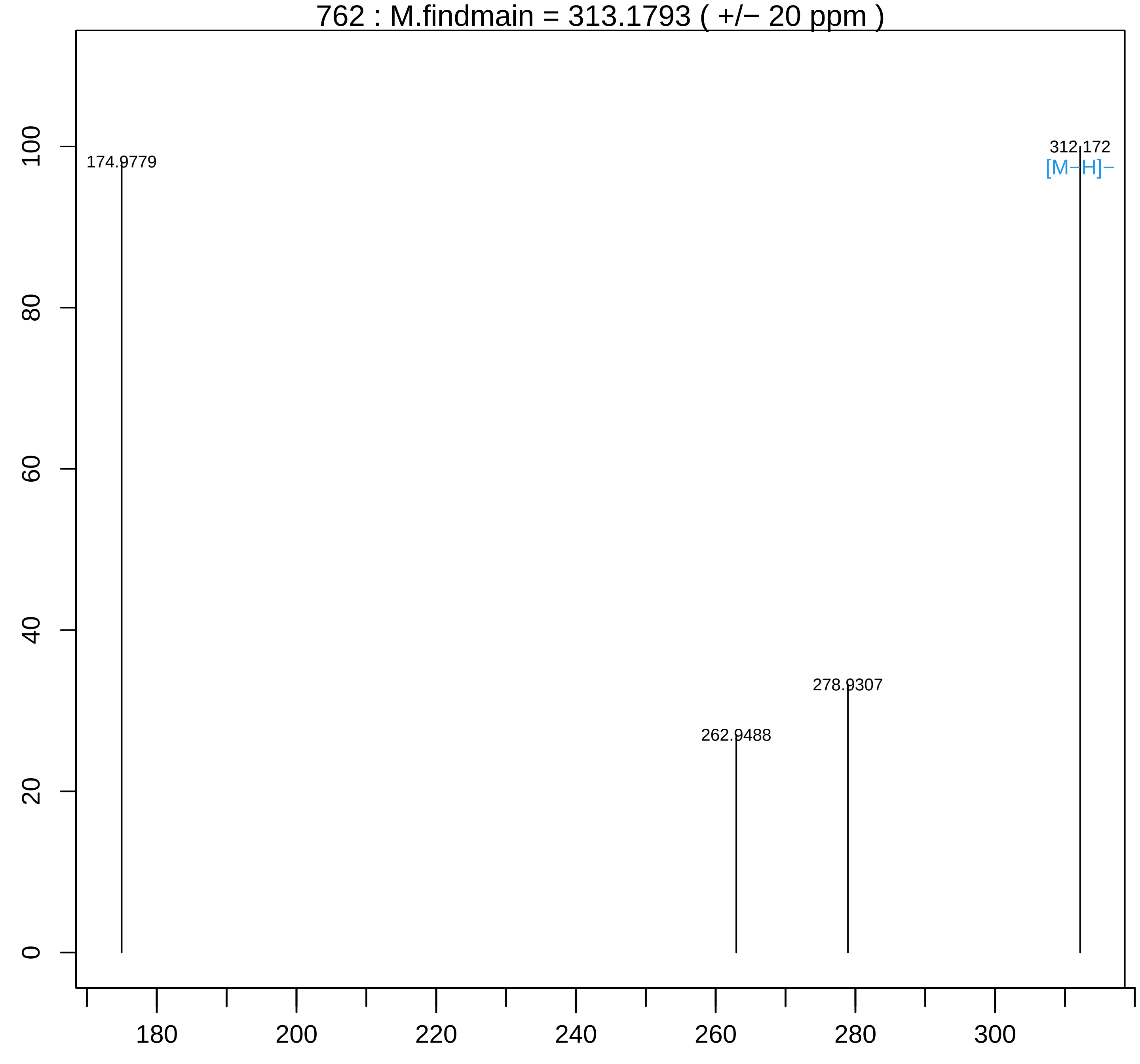
762 : M.findmain = 313.1793 (+/− 20 ppm)
174.9779
262.9488
278.9307
312.172
[M−H]−
0
20
40
60
80
100
180
200
220
240
260
280
300

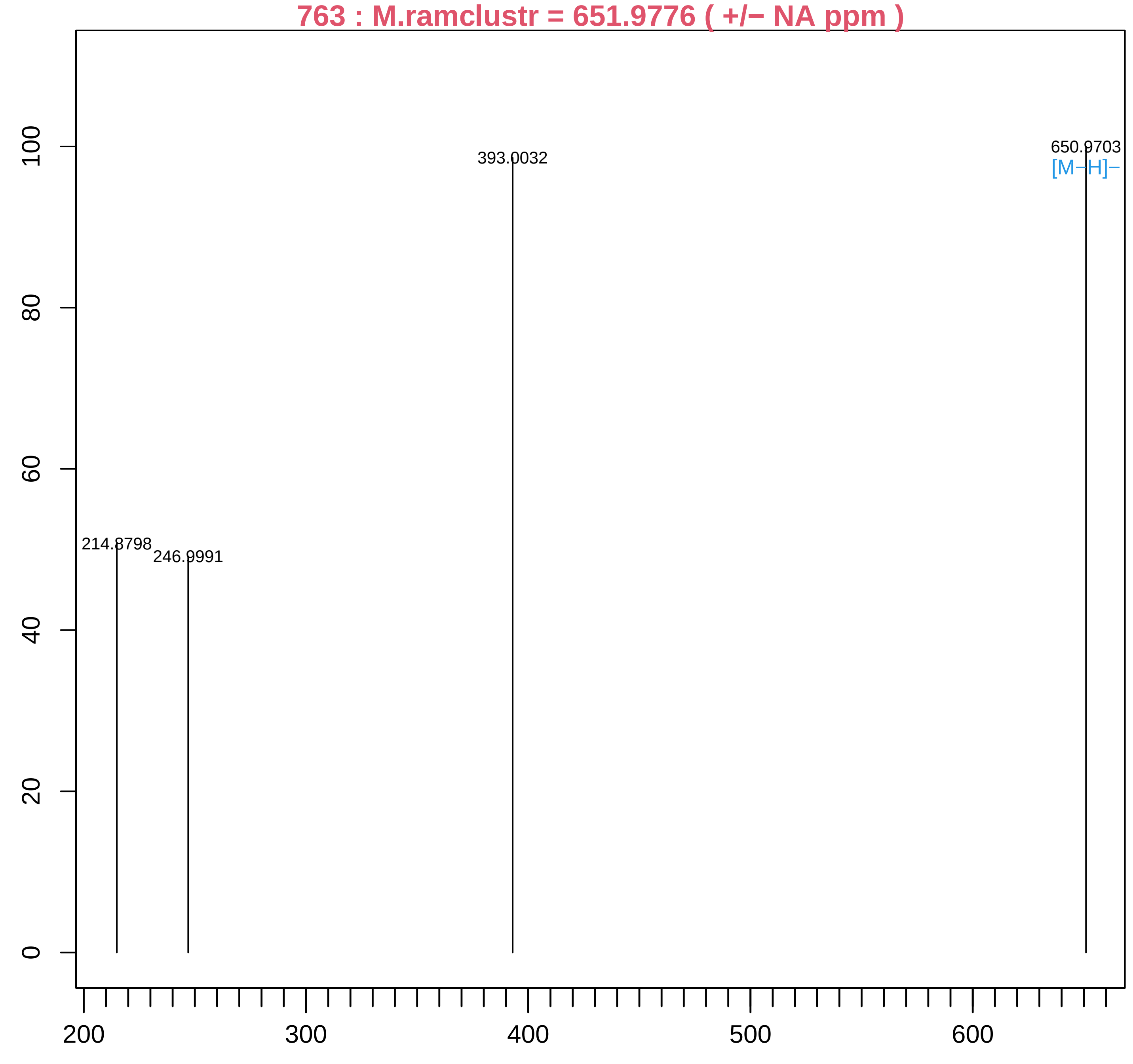

763 : M.ramclustr = 651.9776 (+/− NA ppm)
650.9703
[M−H]−
393.0032
214.8798
246.9991
0
20
40
60
80
100
200
300
400
500
600

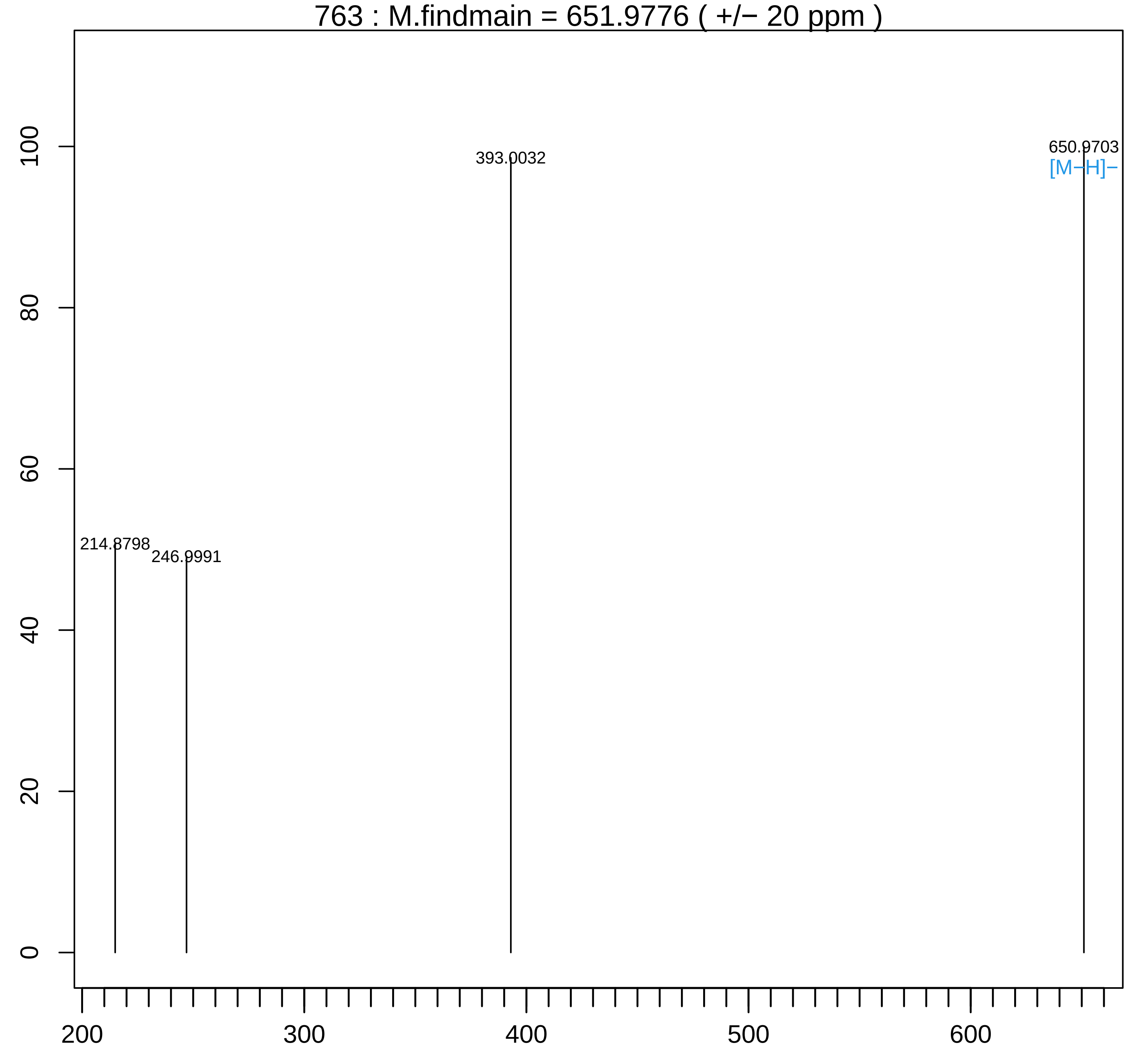

763 : M.findmain = 651.9776 (+/− 20 ppm)
650.9703
[M−H]−
393.0032
214.8798
246.9991
0
20
40
60
80
100
200
300
400
500
600

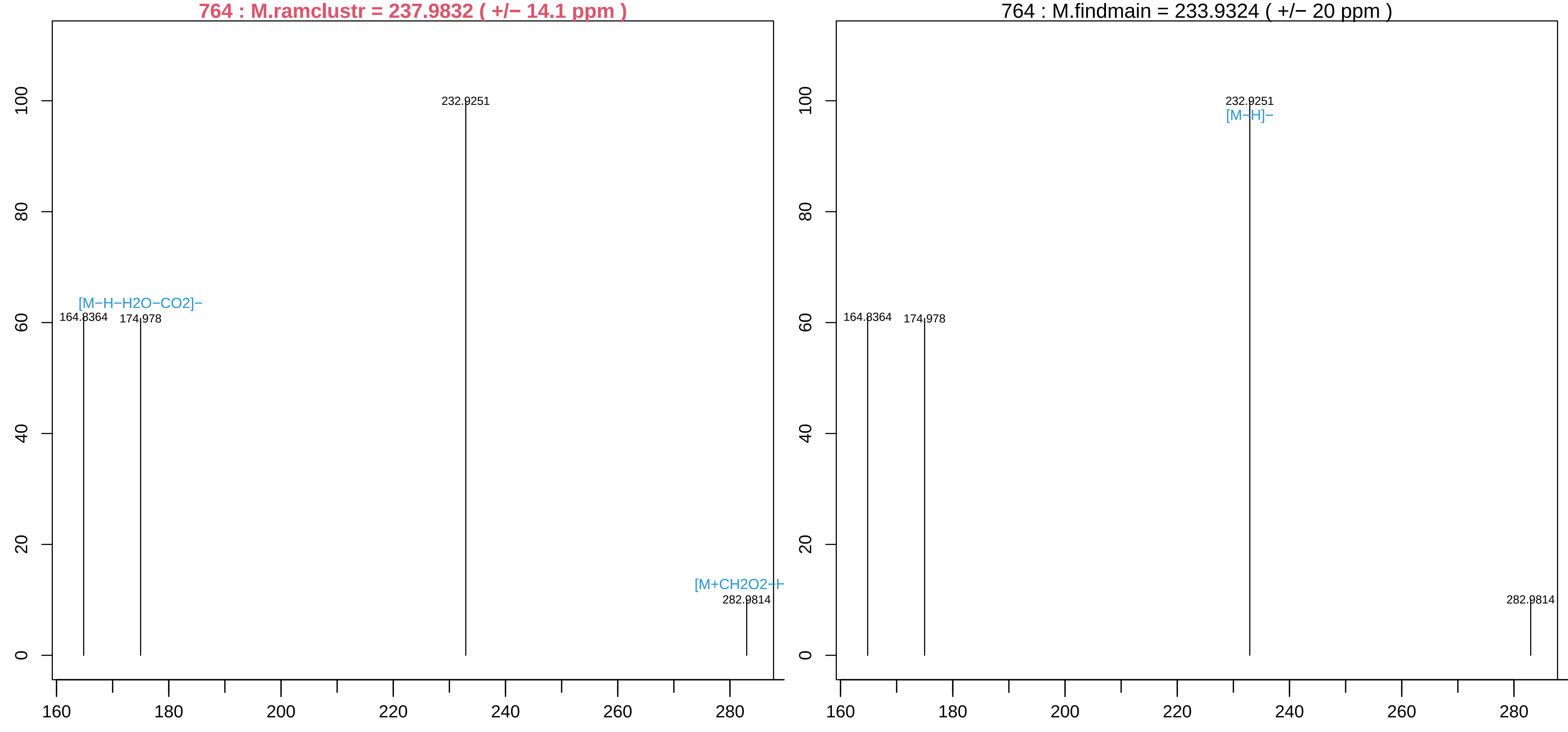
764 : M.ramclustr = 237.9832 (+/− 14.1 ppm)
[M−H−H2O−CO2]−
164.8364
174.978
232.9251
[M+CH2O2−H
282.9814
764 : M.findmain = 233.9324 (+/− 20 ppm)
164.8364
174.978
232.9251
[M−H]−
282.9814
0
20
40
60
80
100
160
180
200
220
240
260
280

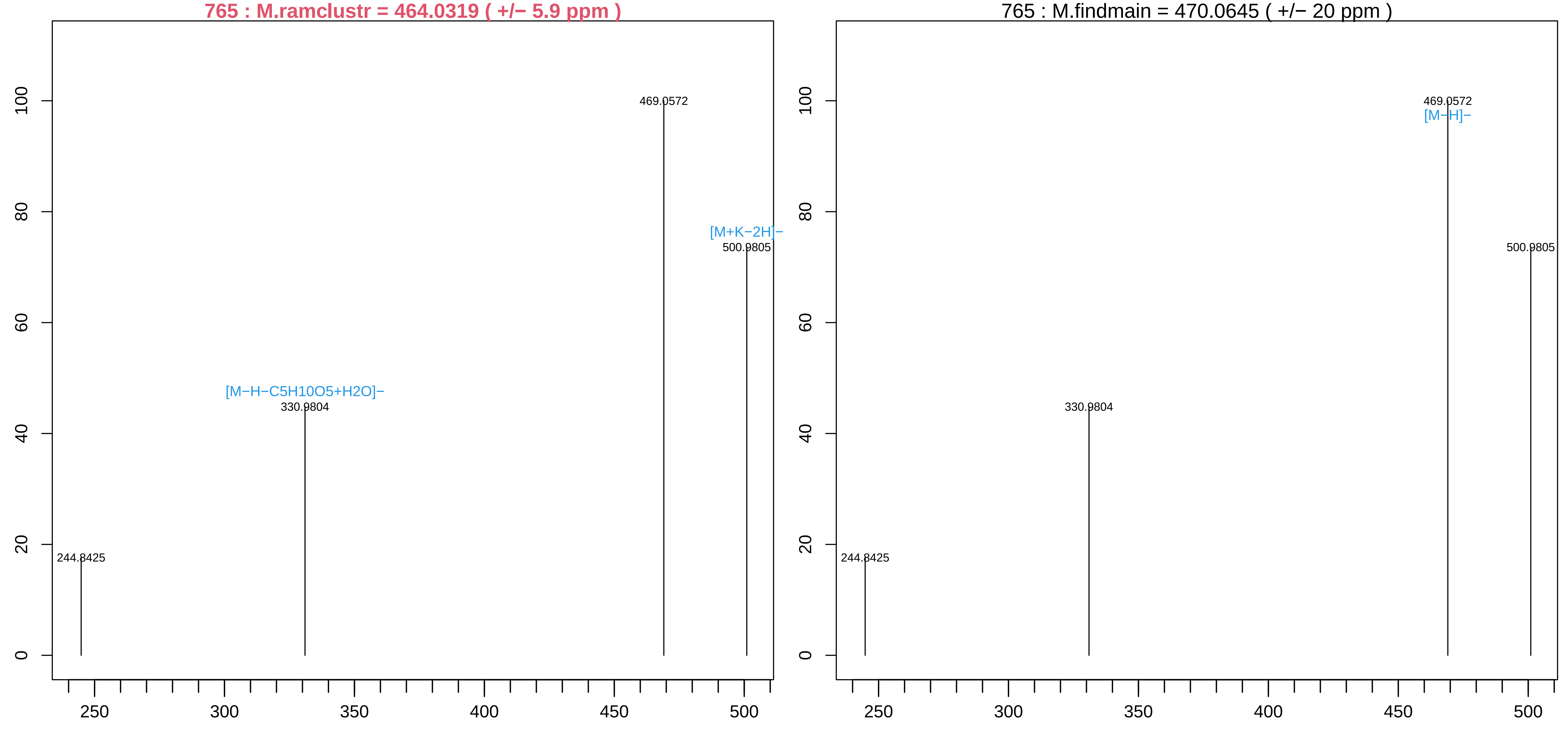
765 : M.ramclustr = 464.0319 (+/− 5.9 ppm)
469.0572
[M+K−2H]−
500.9805
[M−H−C5H10O5+H2O]−
330.9804
244.8425
765 : M.findmain = 470.0645 (+/− 20 ppm)
469.0572
[M−H]−
500.9805
330.9804
244.8425
0
20
40
60
80
100
250
300
350
400
450
500

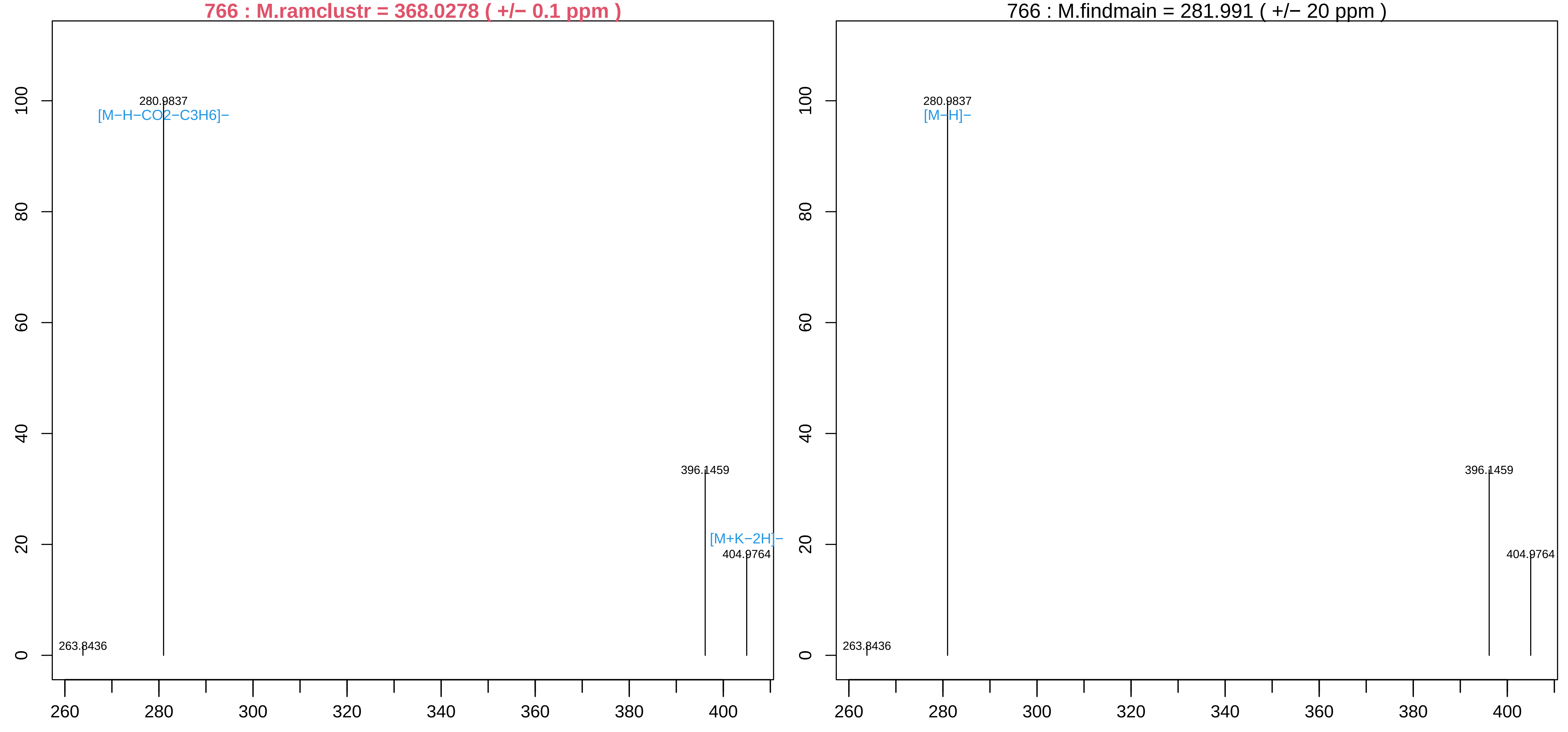
766 : M.ramclustr = 368.0278 (+/− 0.1 ppm)
280.9837
[M−H−CO2−C3H6]−
396.1459
[M+K−2H]−
404.9764
263.8436
0
20
40
60
80
100
260
280
300
320
340
360
380
400
766 : M.findmain = 281.991 (+/− 20 ppm)
280.9837
[M−H]−
396.1459
404.9764
263.8436

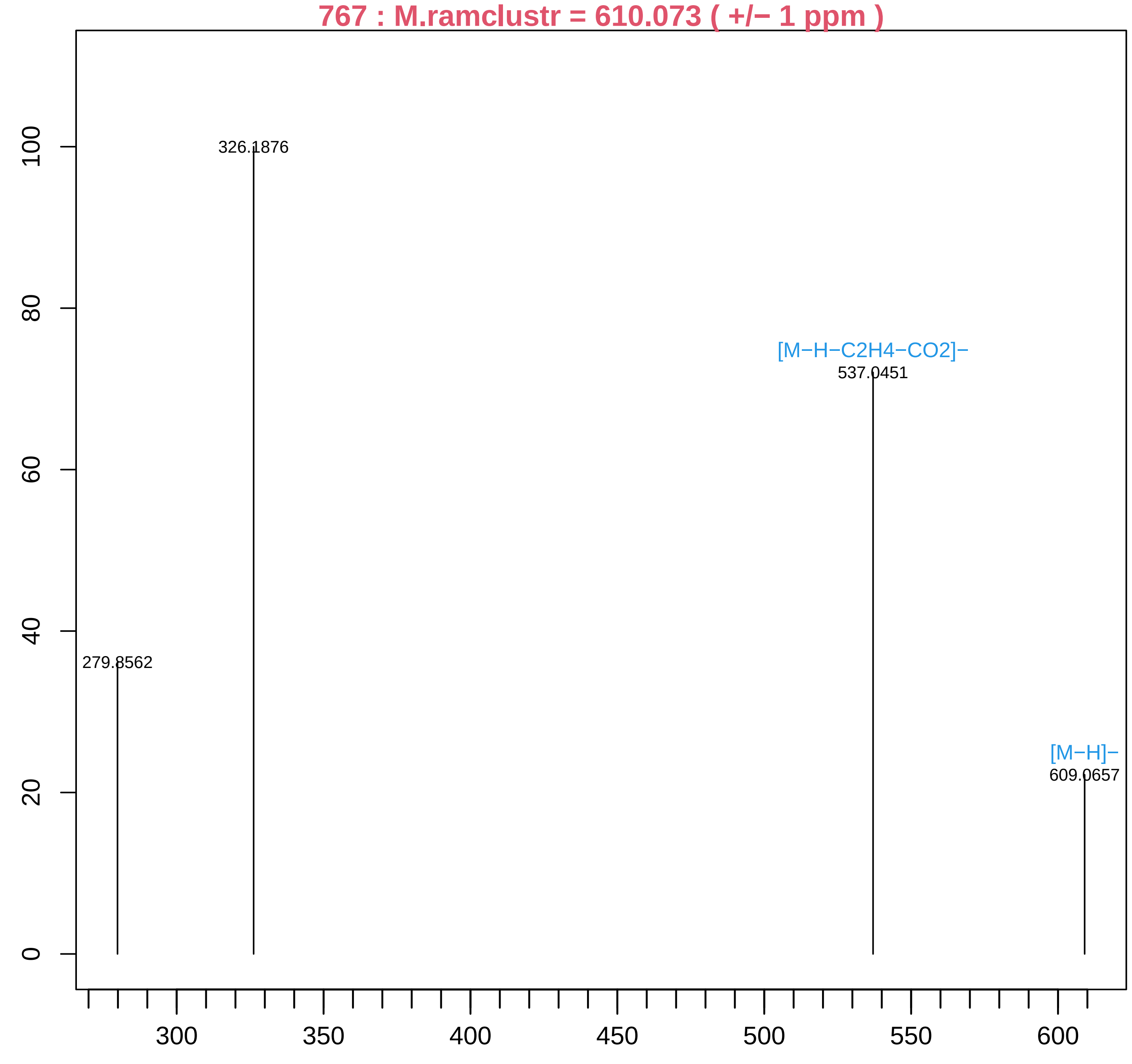

767 : M.ramclustr = 610.073 (+/− 1 ppm)
326.1876
[M−H−C2H4−CO2]−
537.0451
279.8562
[M−H]−
609.0657
0
20
40
60
80
100
300
350
400
450
500
550
600

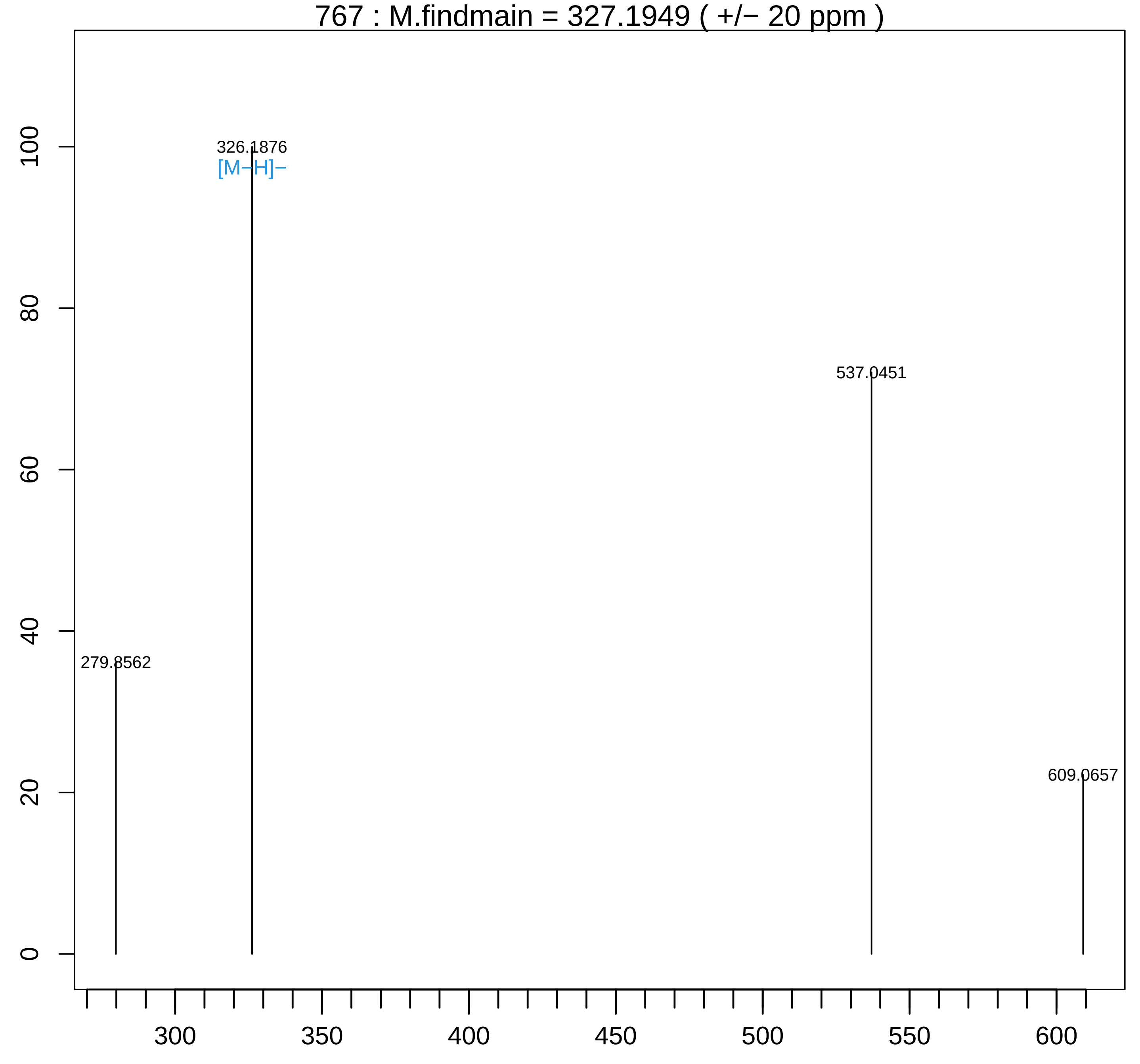

767 : M.findmain = 327.1949 (+/− 20 ppm)
326.1876
[M−H]−
537.0451
279.8562
609.0657
0
20
40
60
80
100
300
350
400
450
500
550
600

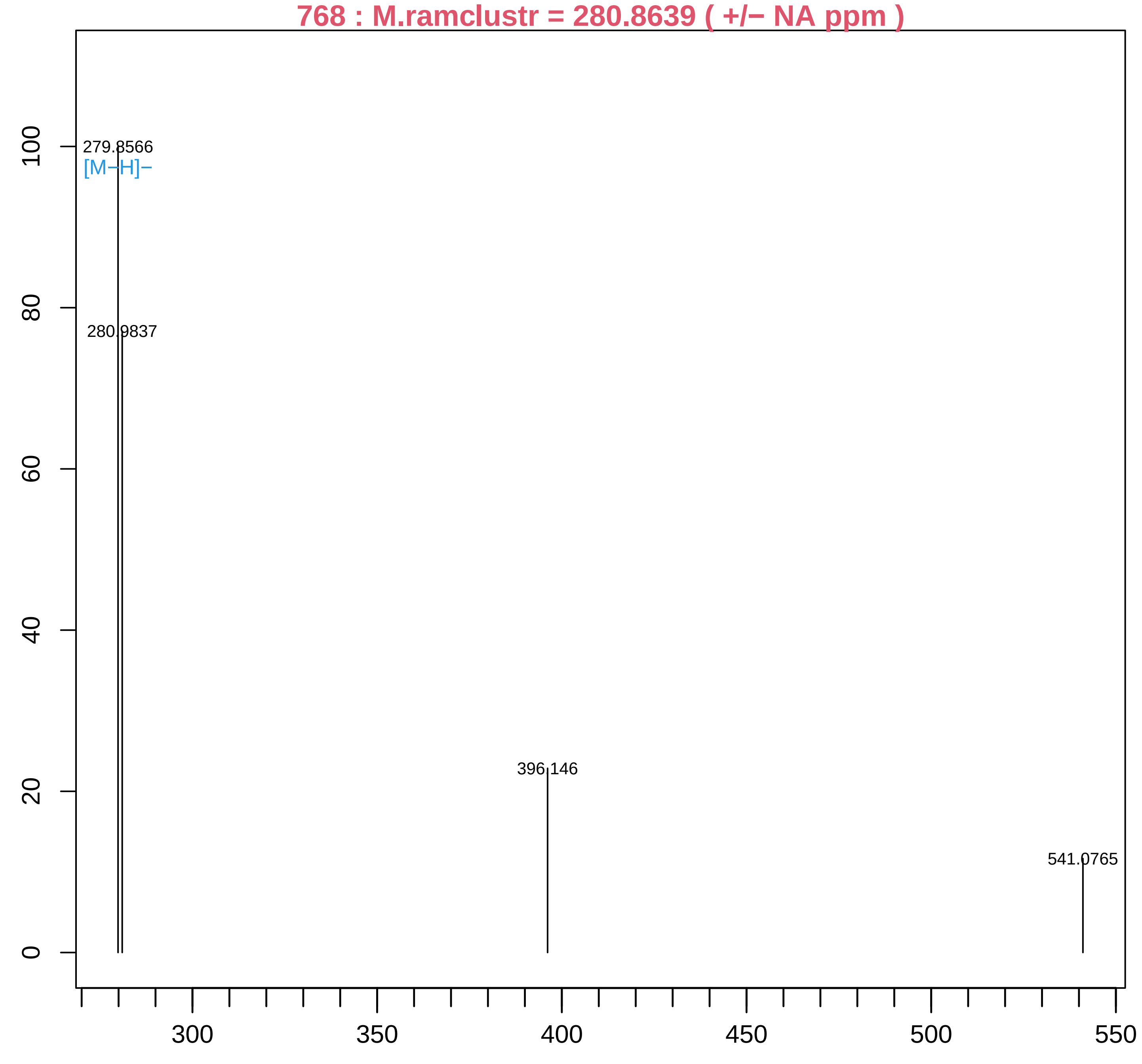

768 : M.ramclustr = 280.8639 (+/− NA ppm)
279.8566
[M−H]−
280.9837
396.146
541.0765
0
20
40
60
80
100
300
350
400
450
500
550

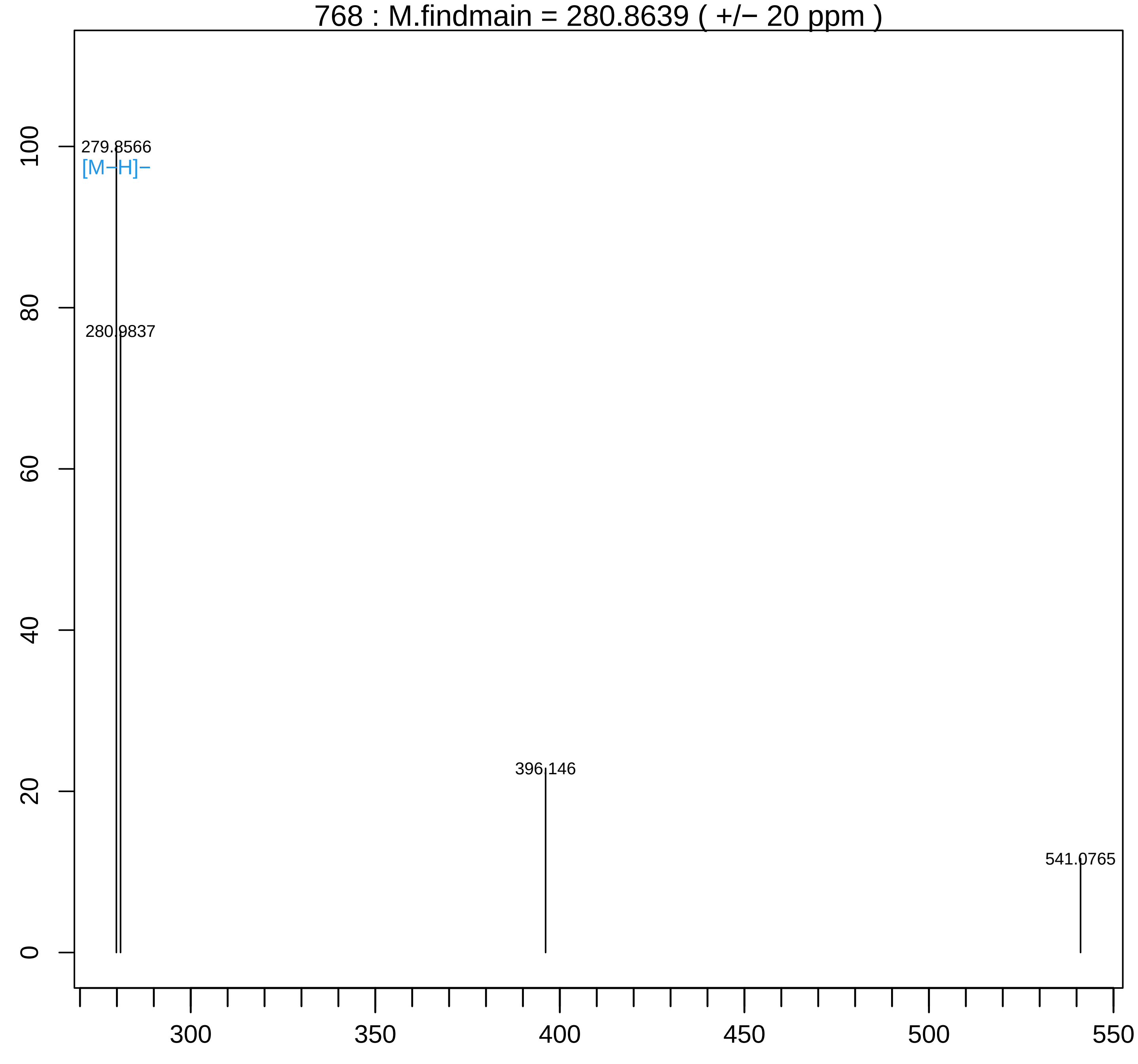

768 : M.findmain = 280.8639 (+/− 20 ppm)
279.8566
[M−H]−
280.9837
396.146
541.0765
0
20
40
60
80
100
300
350
400
450
500
550

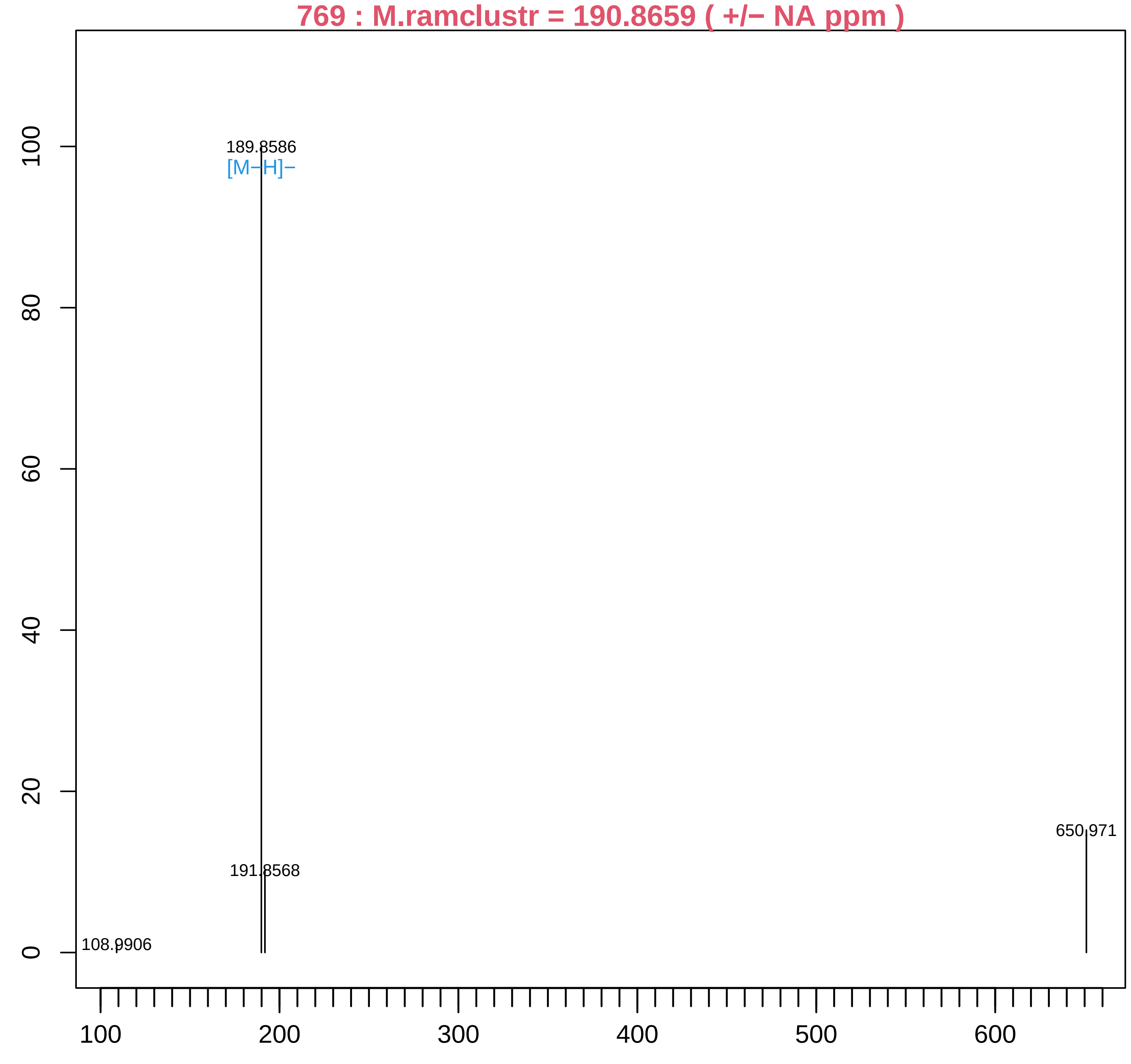
769 : M.ramclustr = 190.8659 (+/− NA ppm)
189.8586
[M−H]−
191.8568
108.0906
650.971
0
20
40
60
80
100
100
200
300
400
500
600

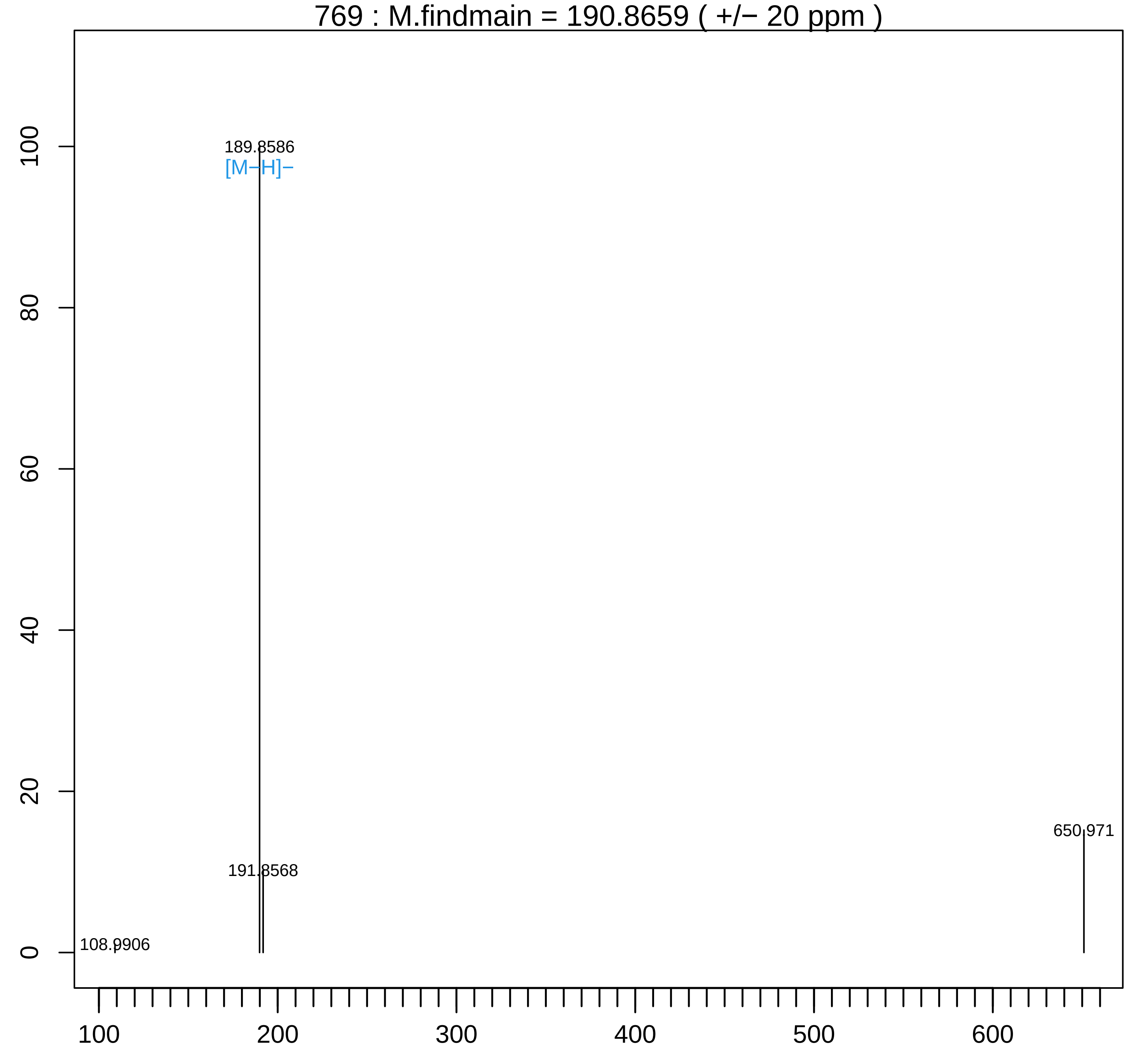
769 : M.findmain = 190.8659 (+/− 20 ppm)
189.8586
[M−H]−
191.8568
108.0906
650.971
0
20
40
60
80
100
100
200
300
400
500
600

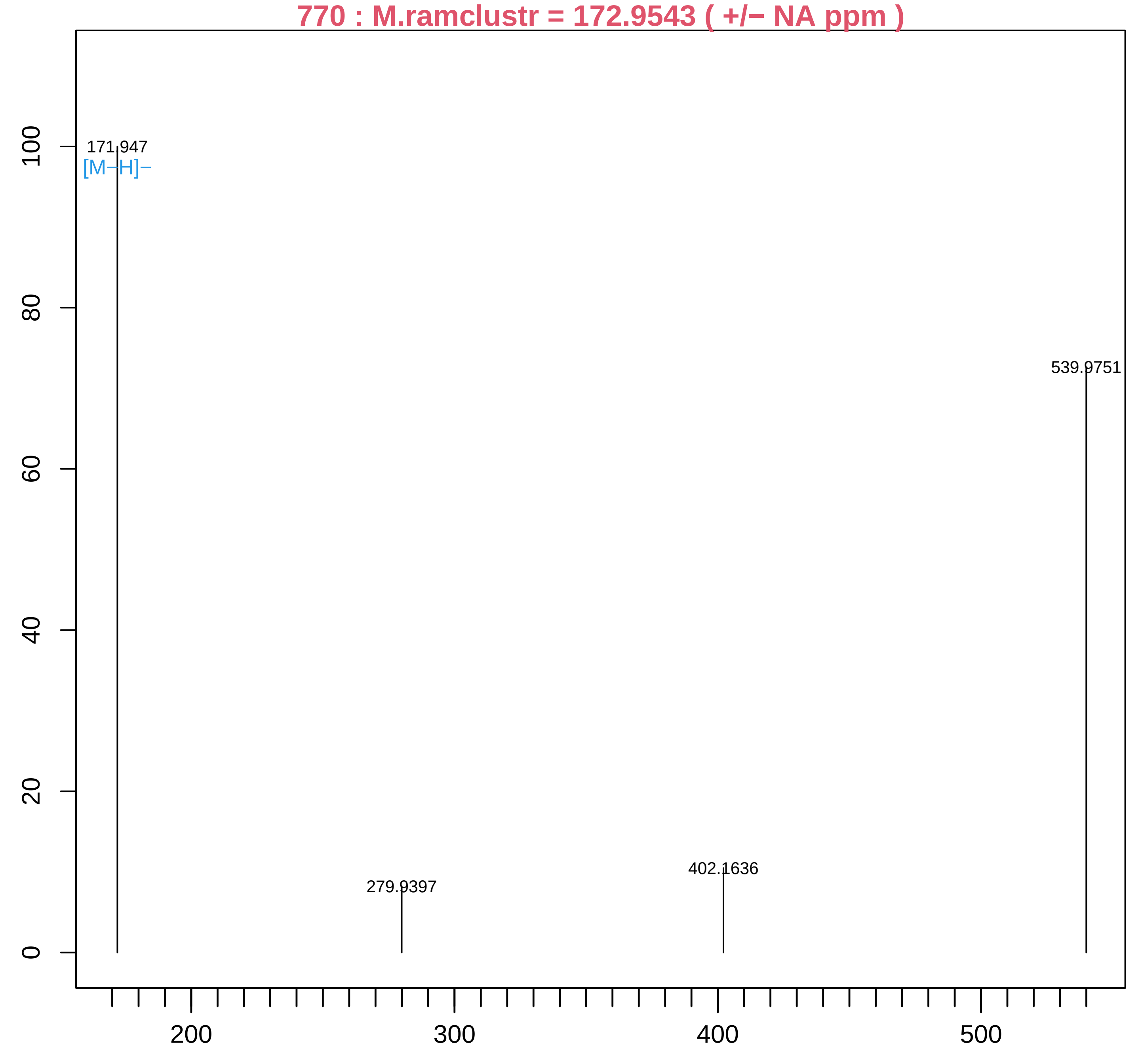

770 : M.ramclustr = 172.9543 (+/− NA ppm)
171.947
[M−H]−
539.9751
279.9397
402.1636
0
20
40
60
80
100
200
300
400
500

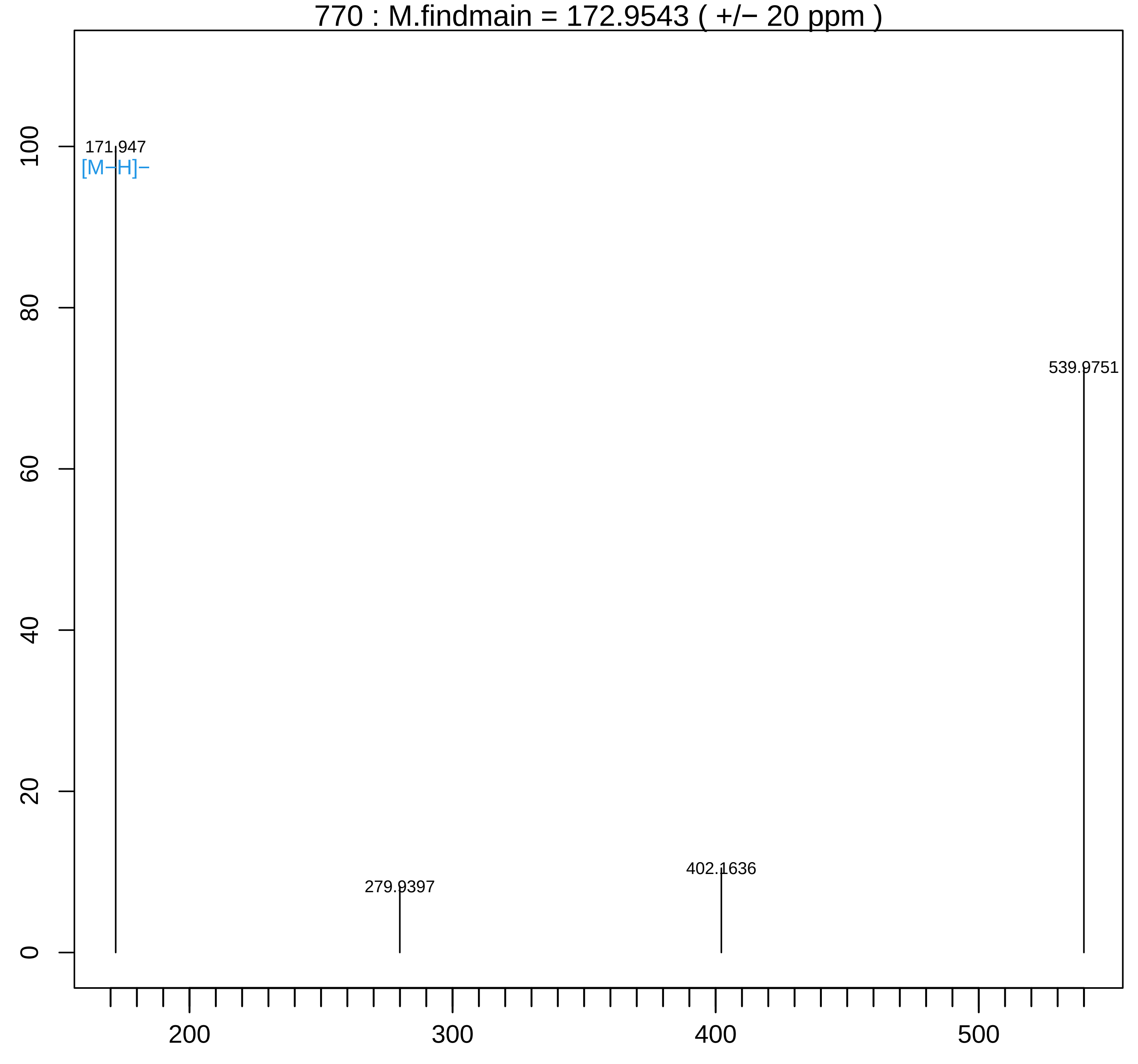

770 : M.findmain = 172.9543 (+/− 20 ppm)
171.947
[M−H]−
539.9751
279.9397
402.1636
0
20
40
60
80
100
200
300
400
500

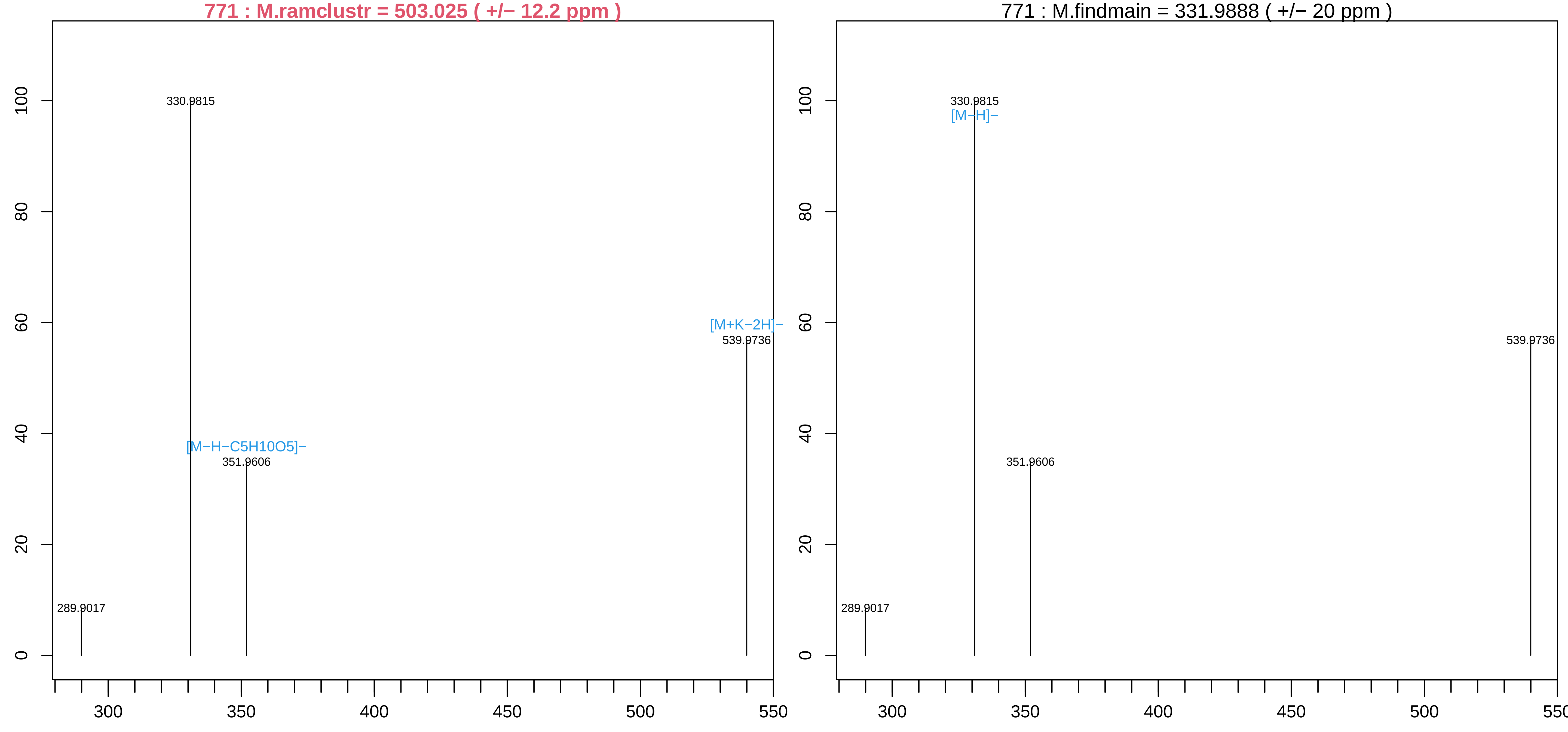
771 : M.ramclustr = 503.025 (+/− 12.2 ppm)
330.9815
[M−H−C5H10O5]−
351.9606
[M+K−2H]−
539.9736
289.9017
771 : M.findmain = 331.9888 (+/− 20 ppm)
330.9815
[M−H]−
351.9606
539.9736
289.9017

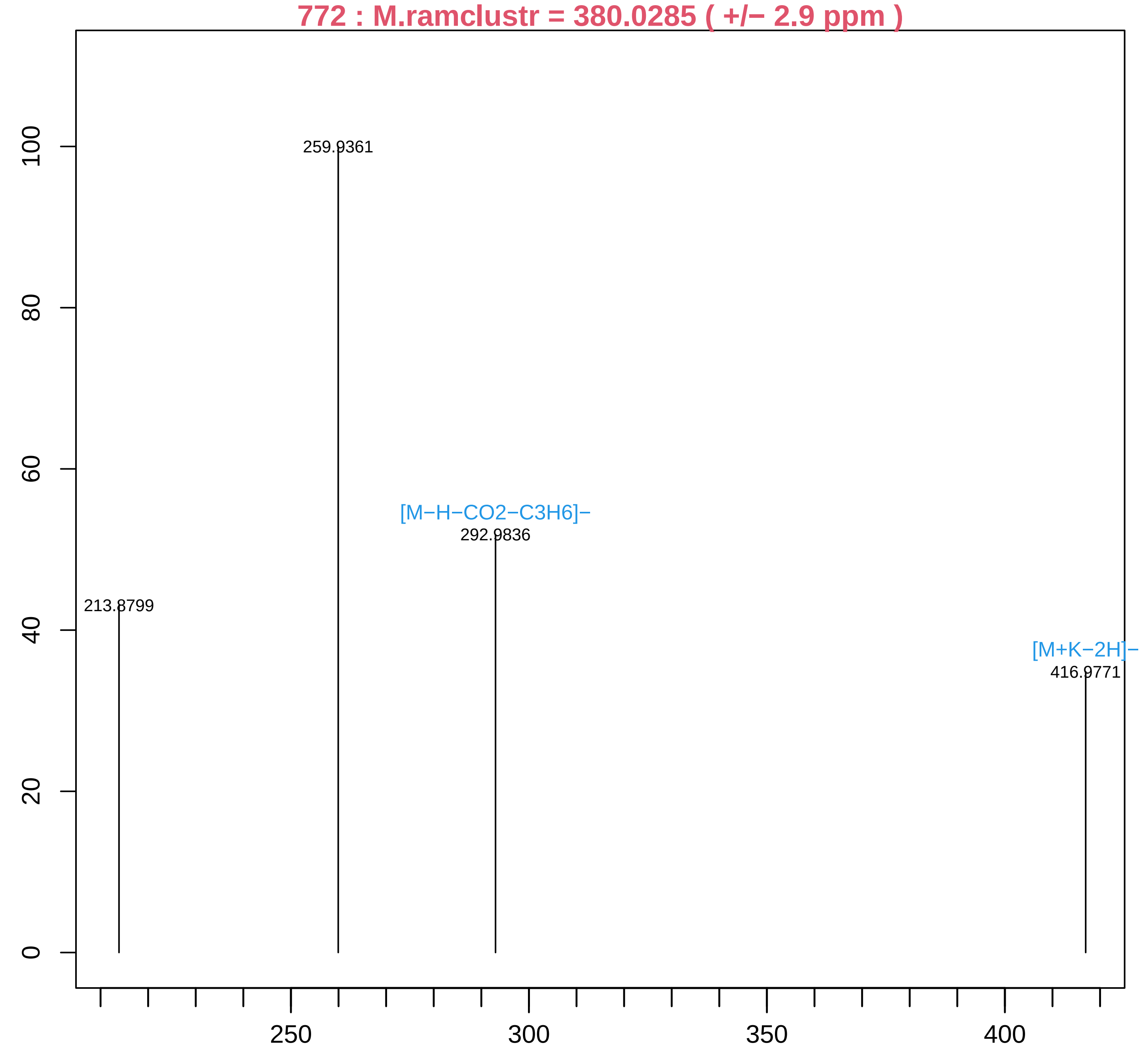

772 : M.ramclustr = 380.0285 (+/− 2.9 ppm)
259.9361
[M−H−CO2−C3H6]−
292.9836
213.8799
[M+K−2H]−
416.9771
0
20
40
60
80
100
250
300
350
400

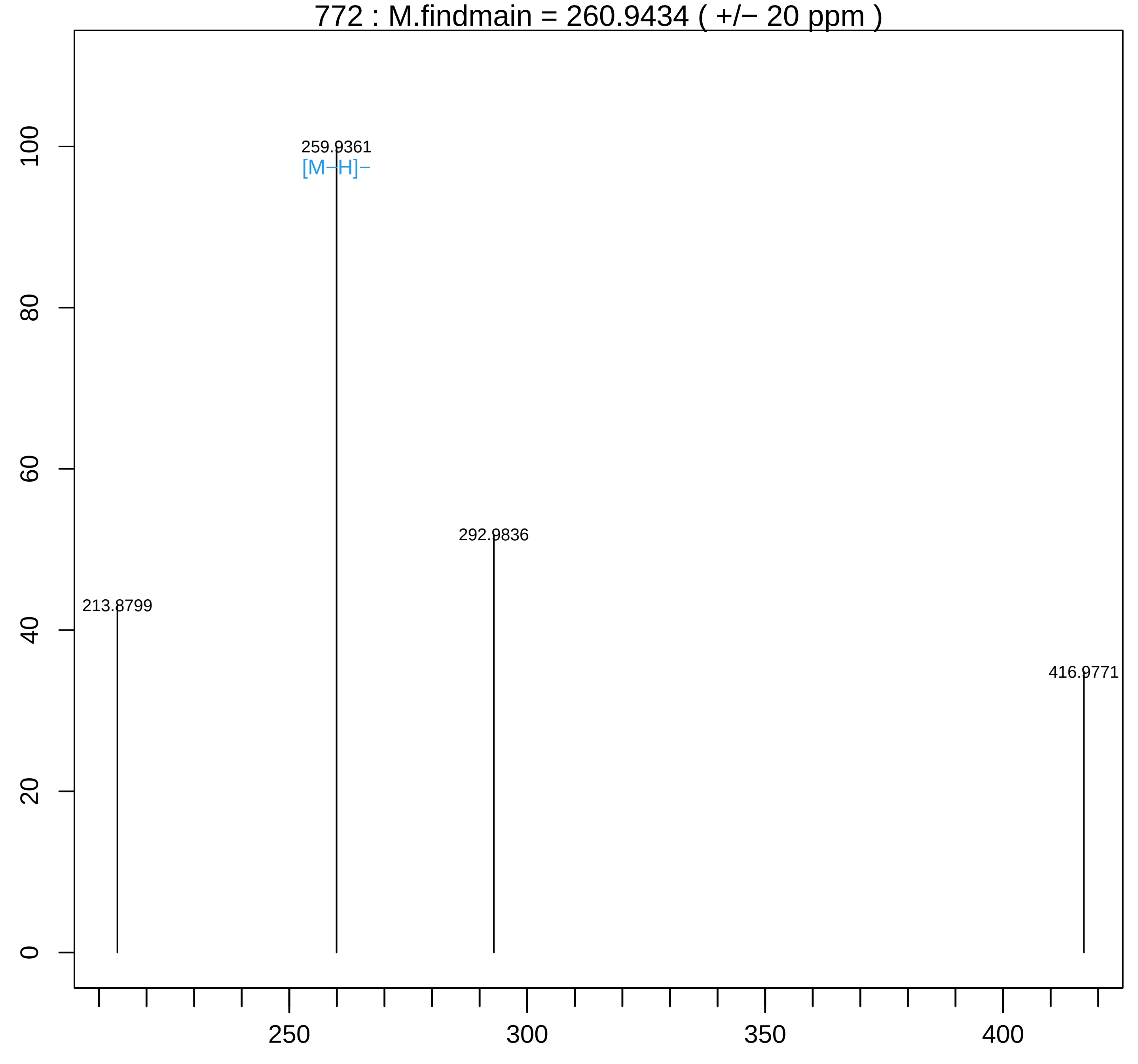

772 : M.findmain = 260.9434 (+/− 20 ppm)
259.9361
[M−H]−
292.9836
213.8799
416.9771
0
20
40
60
80
100
250
300
350
400

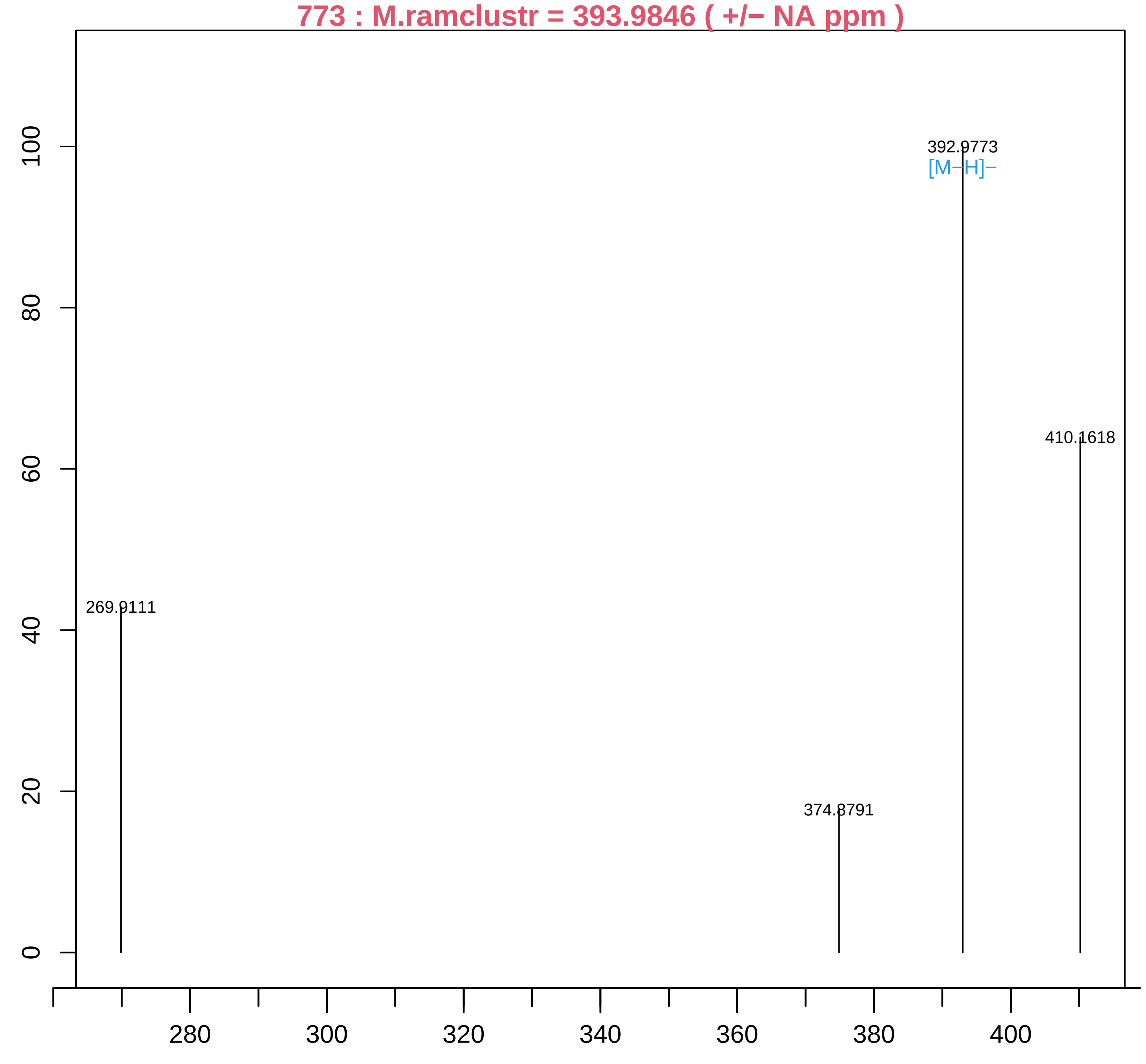
773 : M.ramclustr = 393.9846 (+/− NA ppm)
392.9773
[M−H]−
410.1618
269.9111
374.8791
0
20
40
60
80
100
280
300
320
340
360
380
400

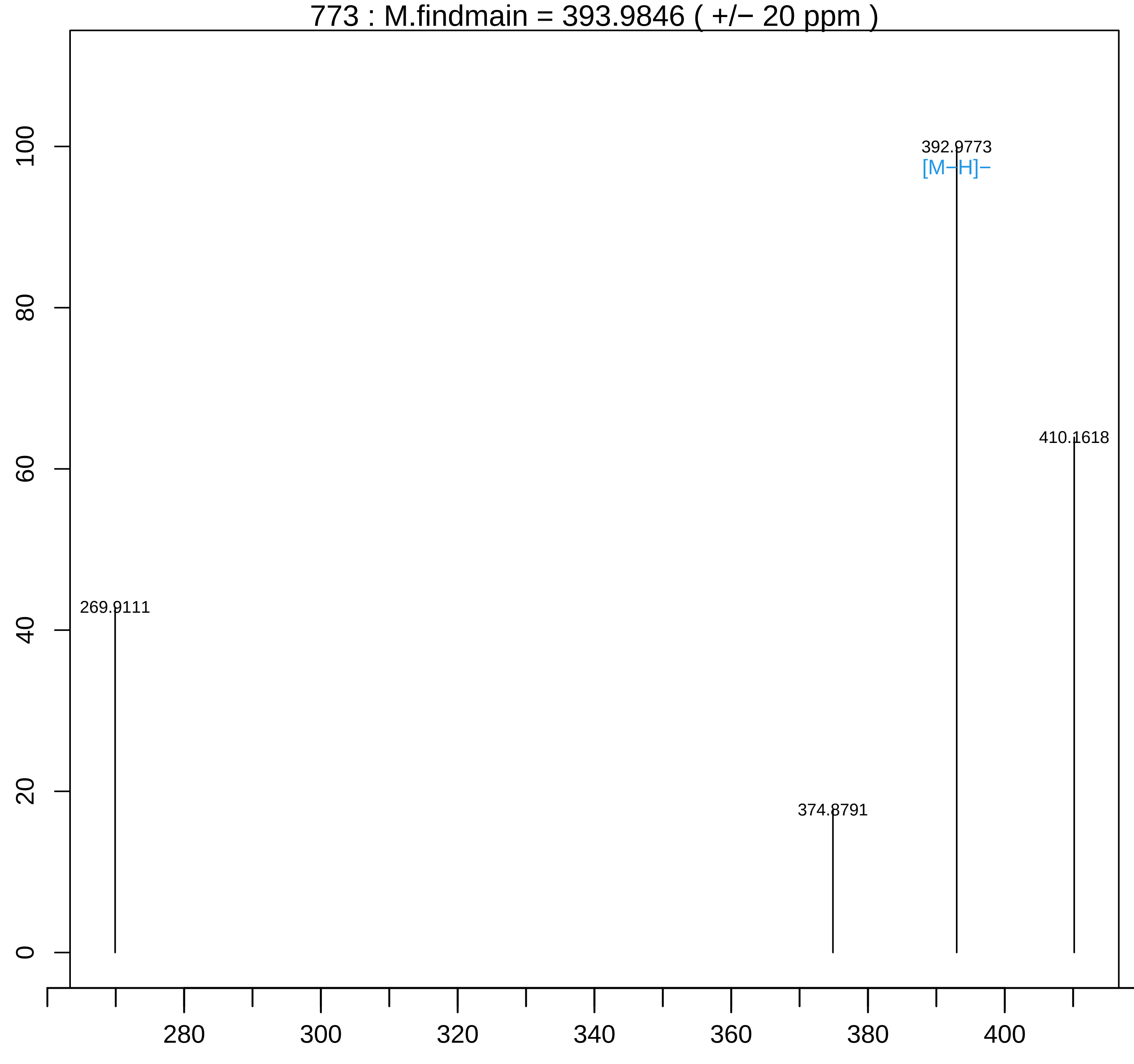
773 : M.findmain = 393.9846 (+/− 20 ppm)
392.9773
[M−H]−
410.1618
269.9111
374.8791
0
20
40
60
80
100
280
300
320
340
360
380
400

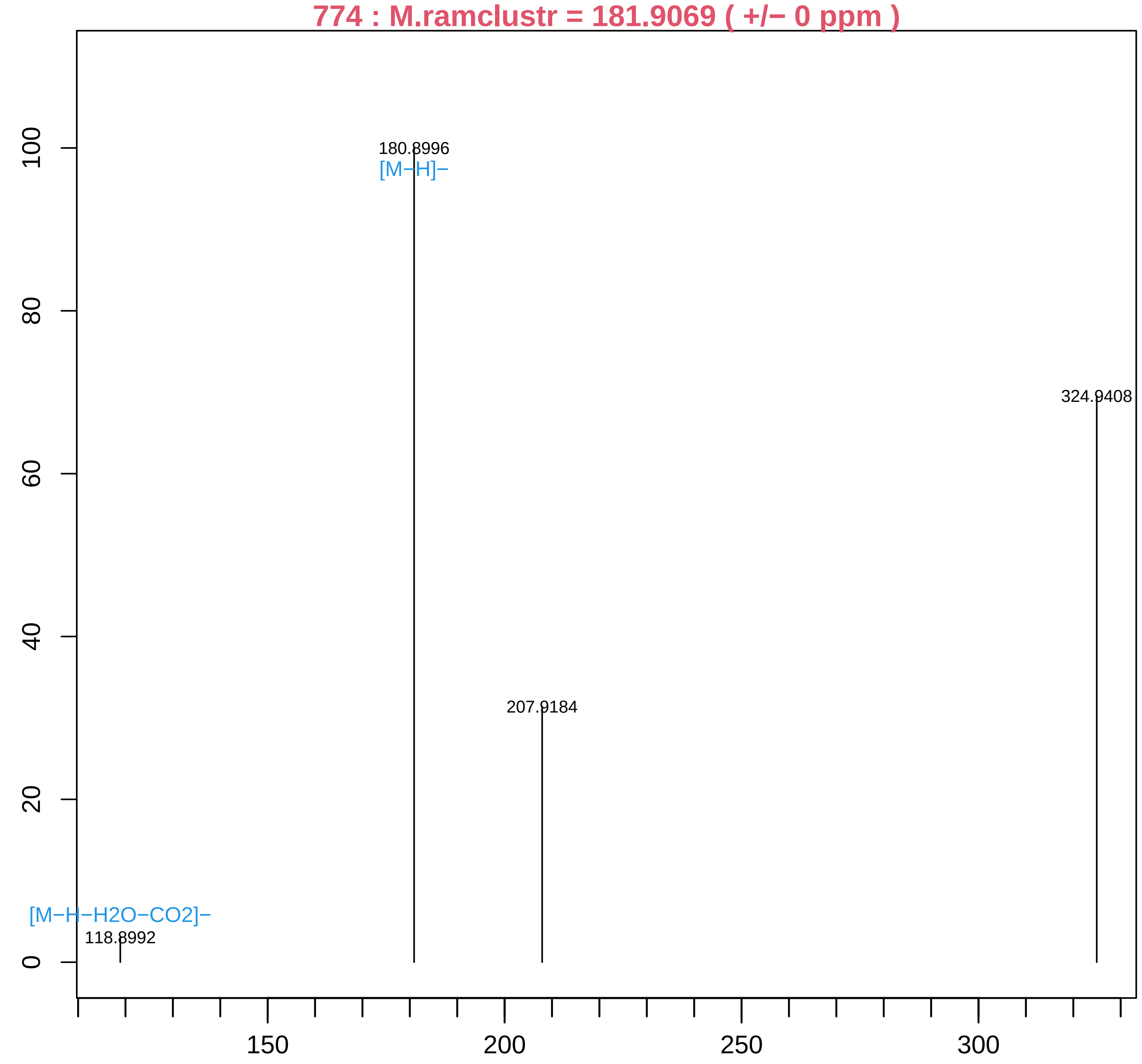
774 : M.ramclustr = 181.9069 (+/− 0 ppm)
180.8996
[M−H]−
324.9408
207.9184
[M−H−H2O−CO2]−
118.8992
0
20
40
60
80
100
150
200
250
300

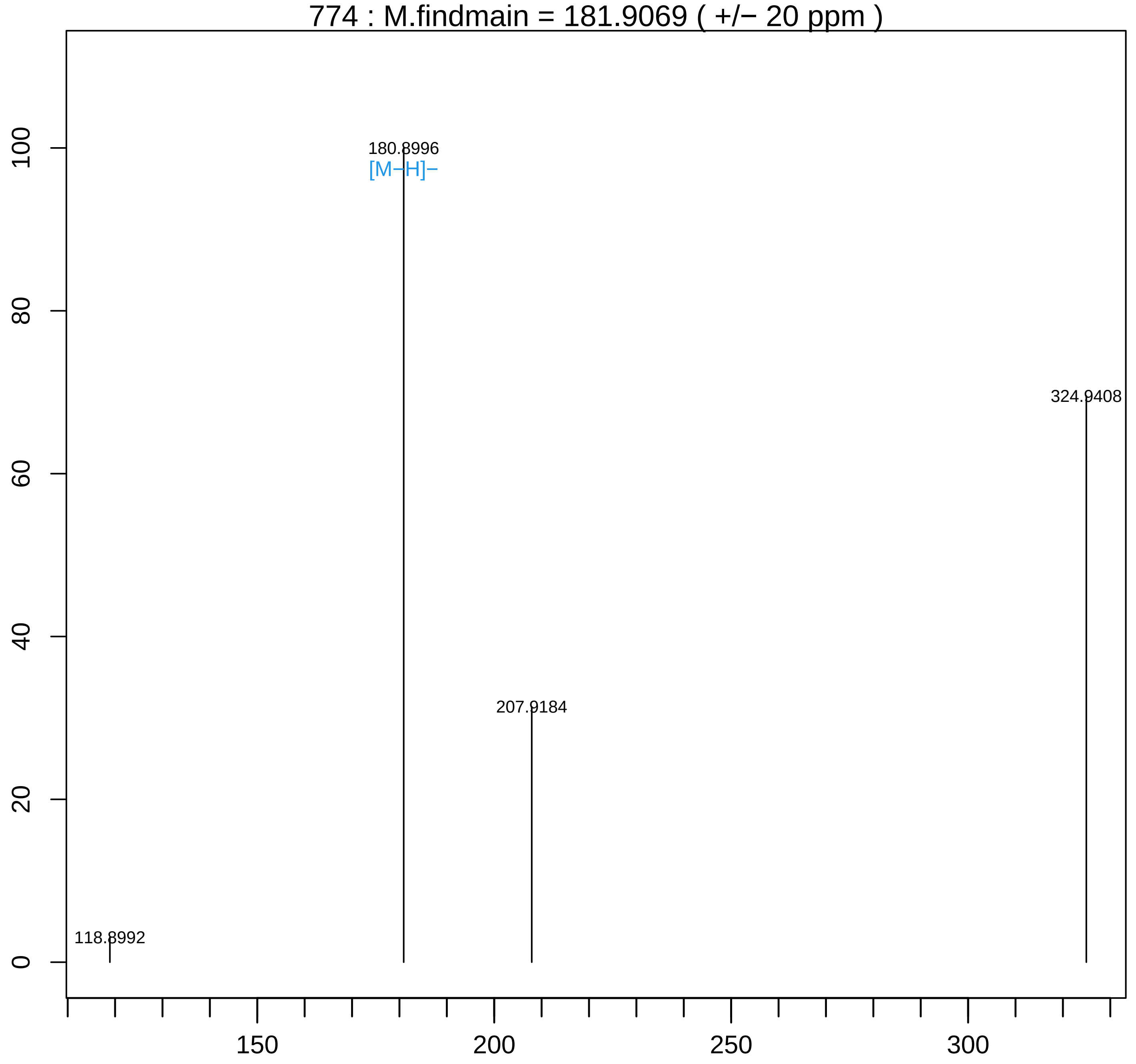
774 : M.findmain = 181.9069 (+/− 20 ppm)
180.8996
[M−H]−
324.9408
207.9184
118.8992
0
20
40
60
80
100
150
200
250
300

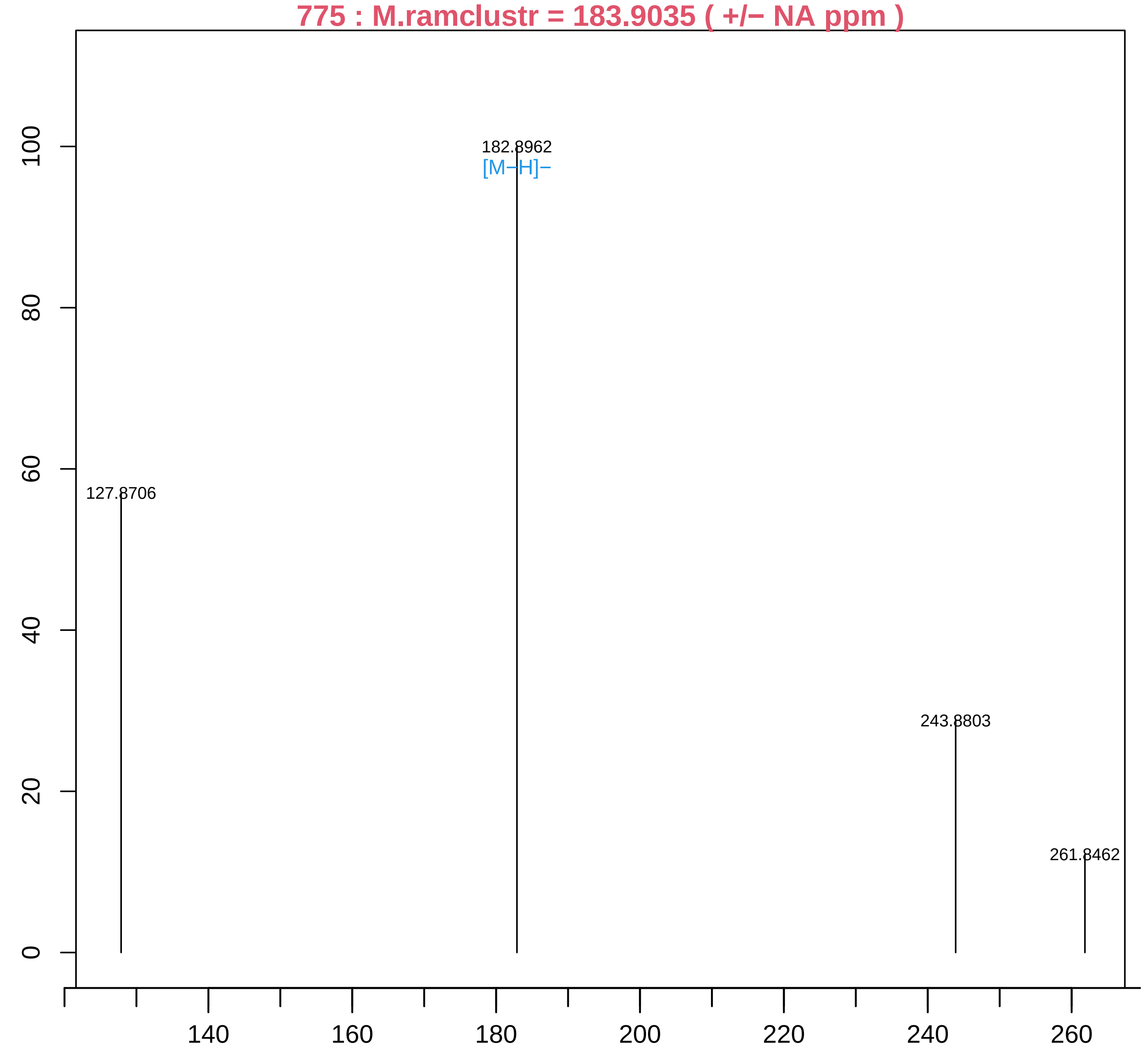
775 : M.ramclustr = 183.9035 (+/− NA ppm)
182.8962
[M−H]−
127.8706
243.8803
261.8462
0
20
40
60
80
100
140
160
180
200
220
240
260

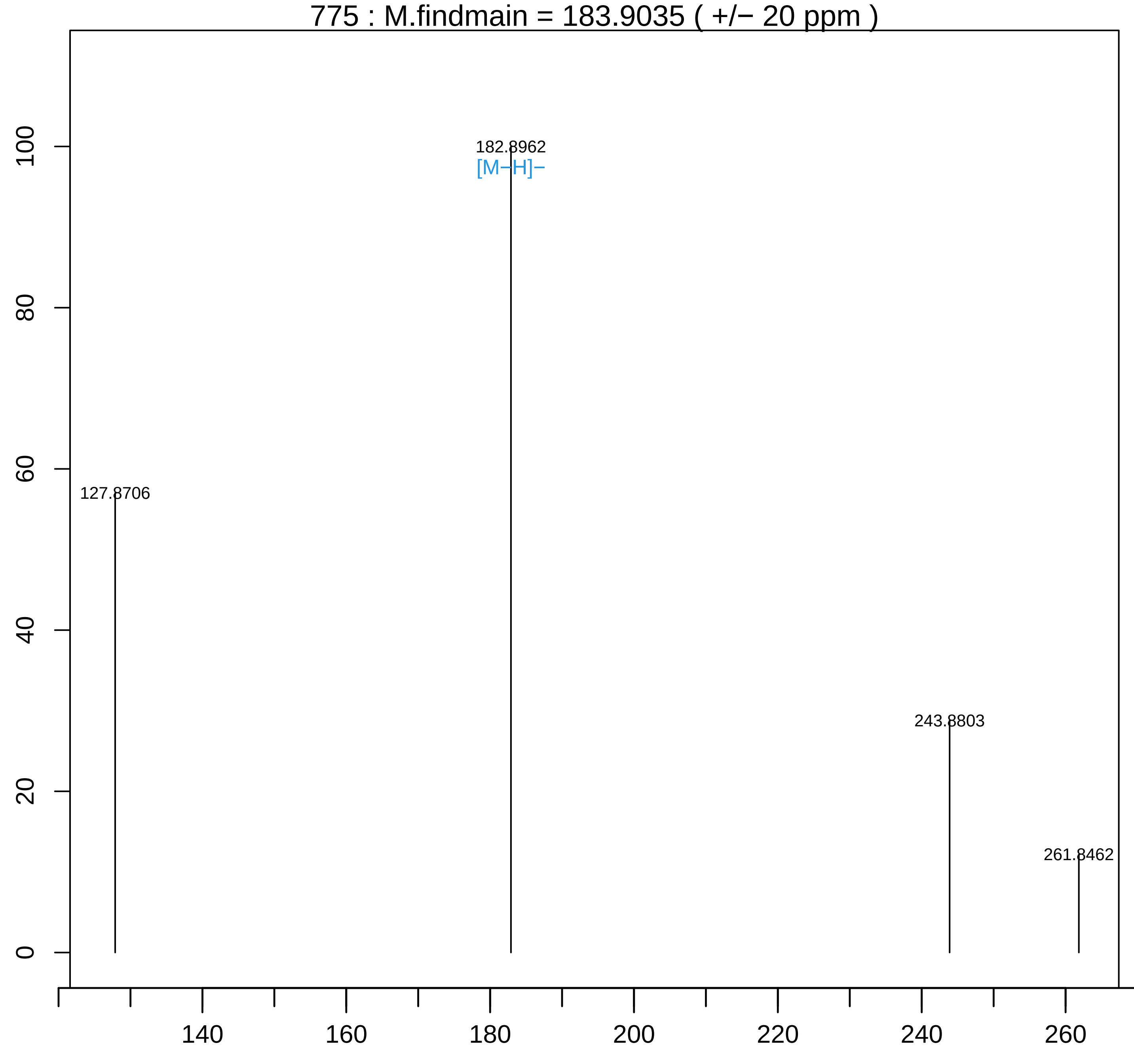
775 : M.findmain = 183.9035 (+/− 20 ppm)
182.8962
[M−H]−
127.8706
243.8803
261.8462
0
20
40
60
80
100
140
160
180
200
220
240
260

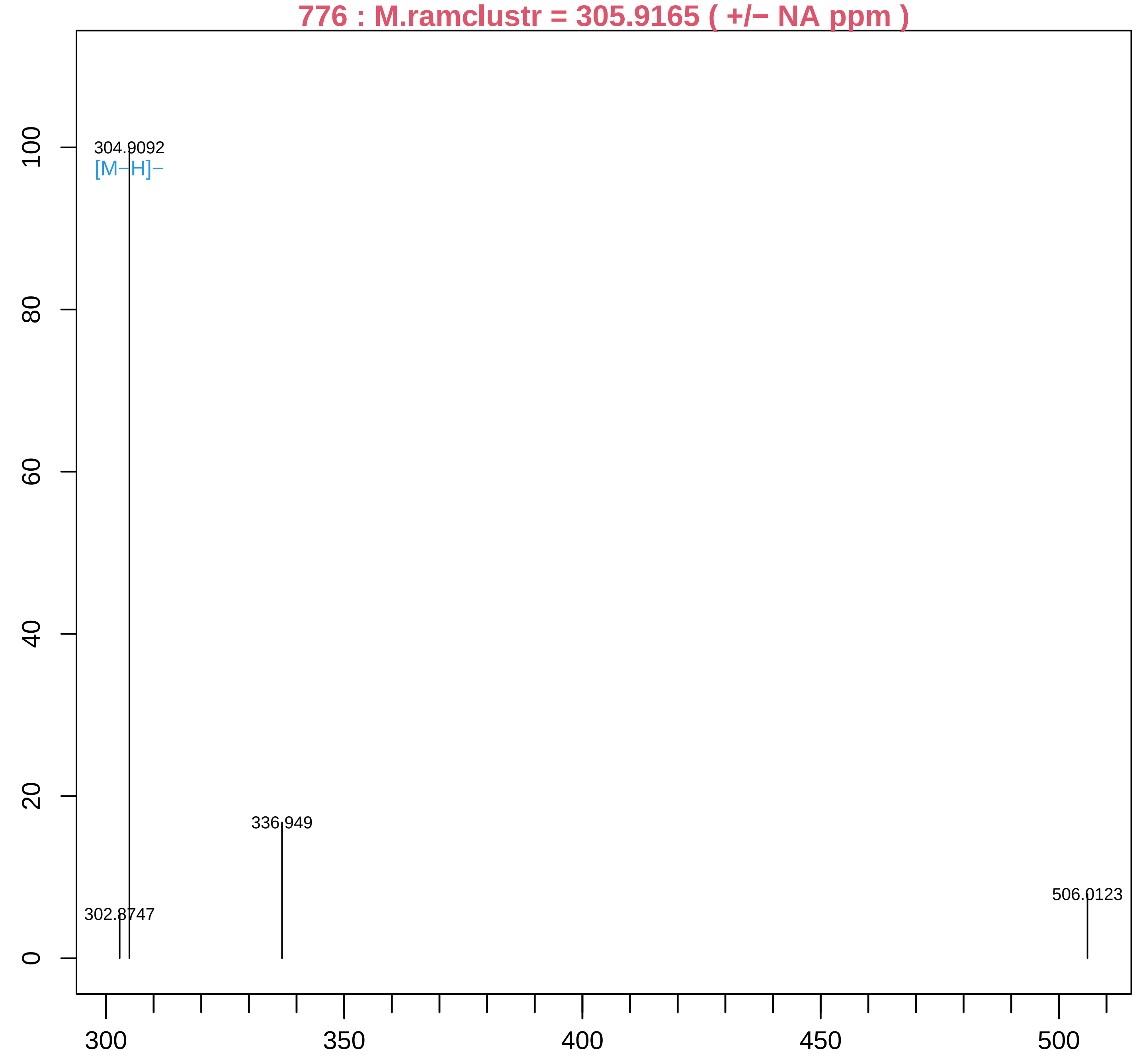

776 : M.ramclustr = 305.9165 (+/− NA ppm)
304.9092
[M−H]−
336.949
302.8747
506.0123
0
20
40
60
80
100
300
350
400
450
500

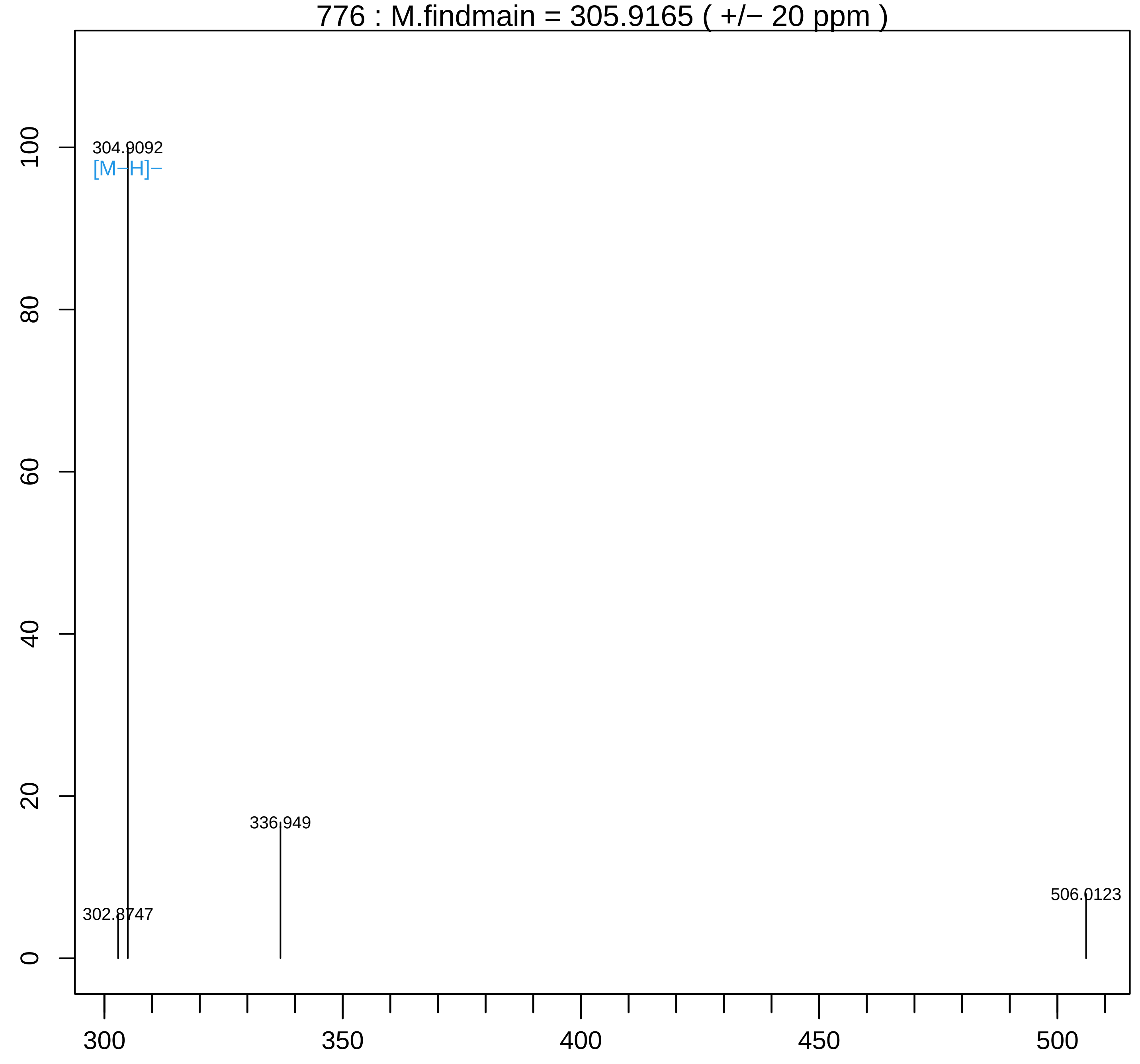

776 : M.findmain = 305.9165 (+/− 20 ppm)
304.9092
[M−H]−
336.949
302.8747
506.0123
0
20
40
60
80
100
300
350
400
450
500

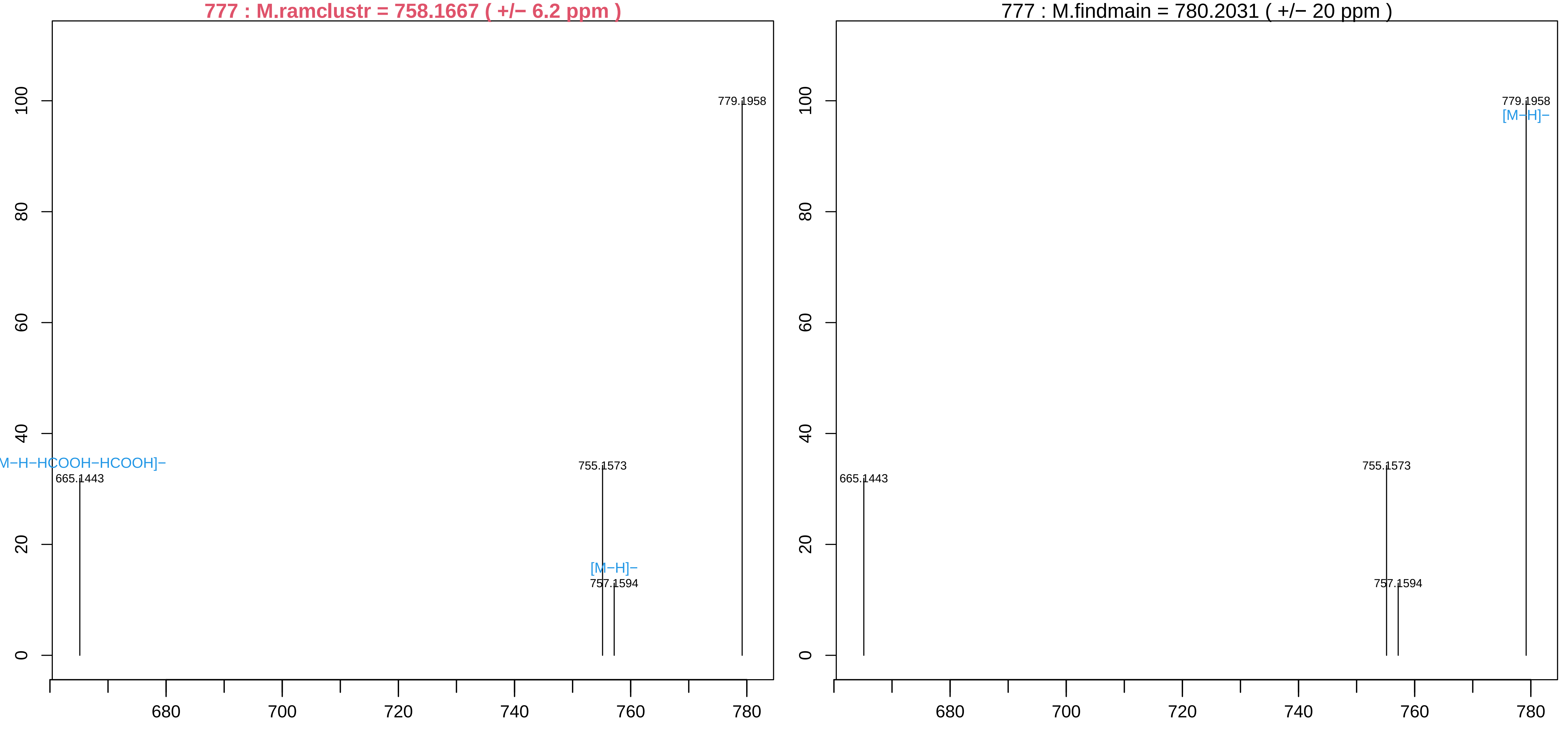
777 : M.ramclustr = 758.1667 (+/− 6.2 ppm)
[M−H−HCOOH−HCOOH]−
665.1443
755.1573
[M−H]−
757.1594
779.1958
777 : M.findmain = 780.2031 (+/− 20 ppm)
779.1958
[M−H]−
665.1443
755.1573
757.1594
0
20
40
60
80
100
680
700
720
740
760
780

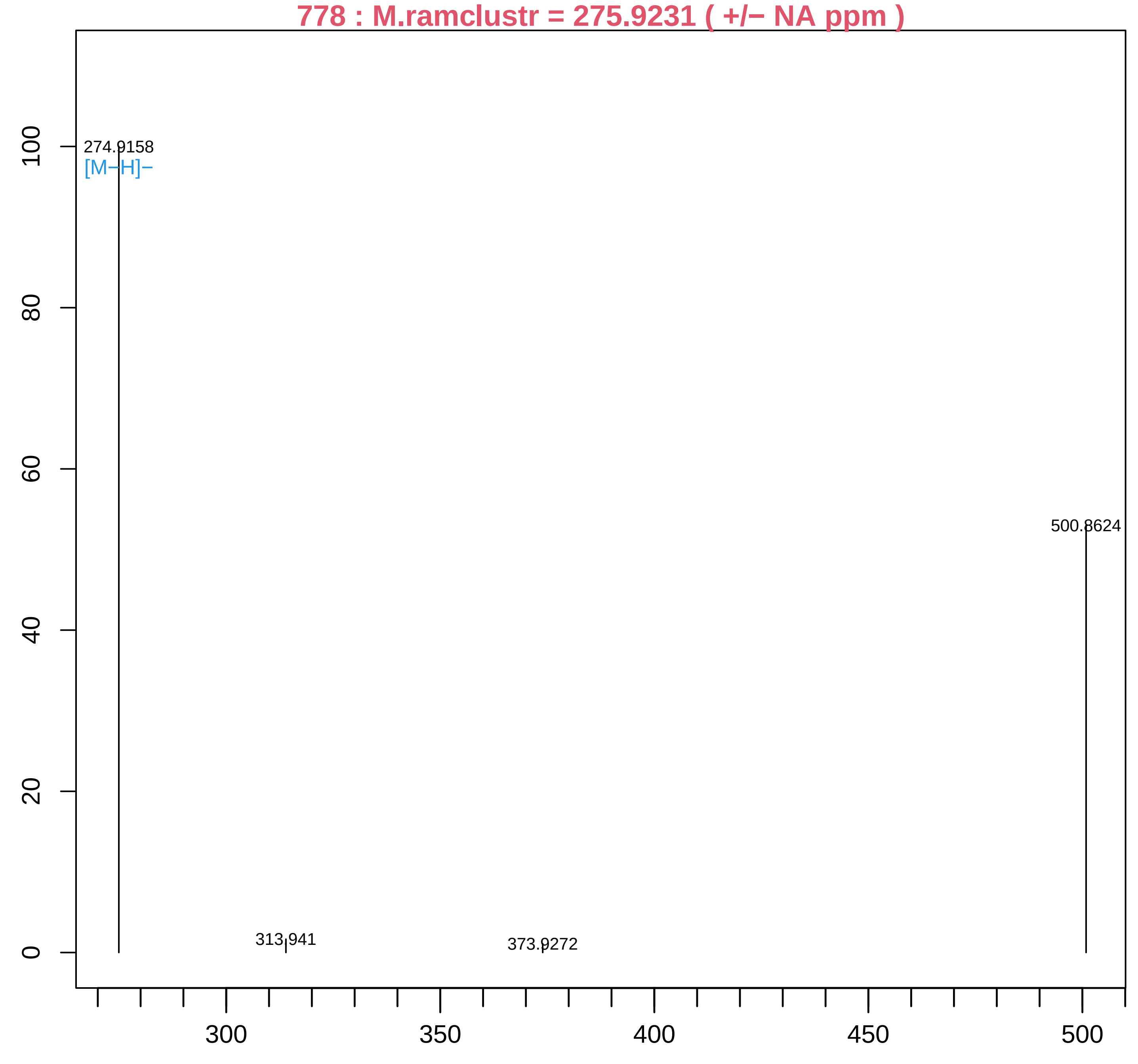
778 : M.ramclustr = 275.9231 (+/− NA ppm)
274.9158
[M−H]−
313.941
373.9272
500.8624
0
20
40
60
80
100
300
350
400
450
500

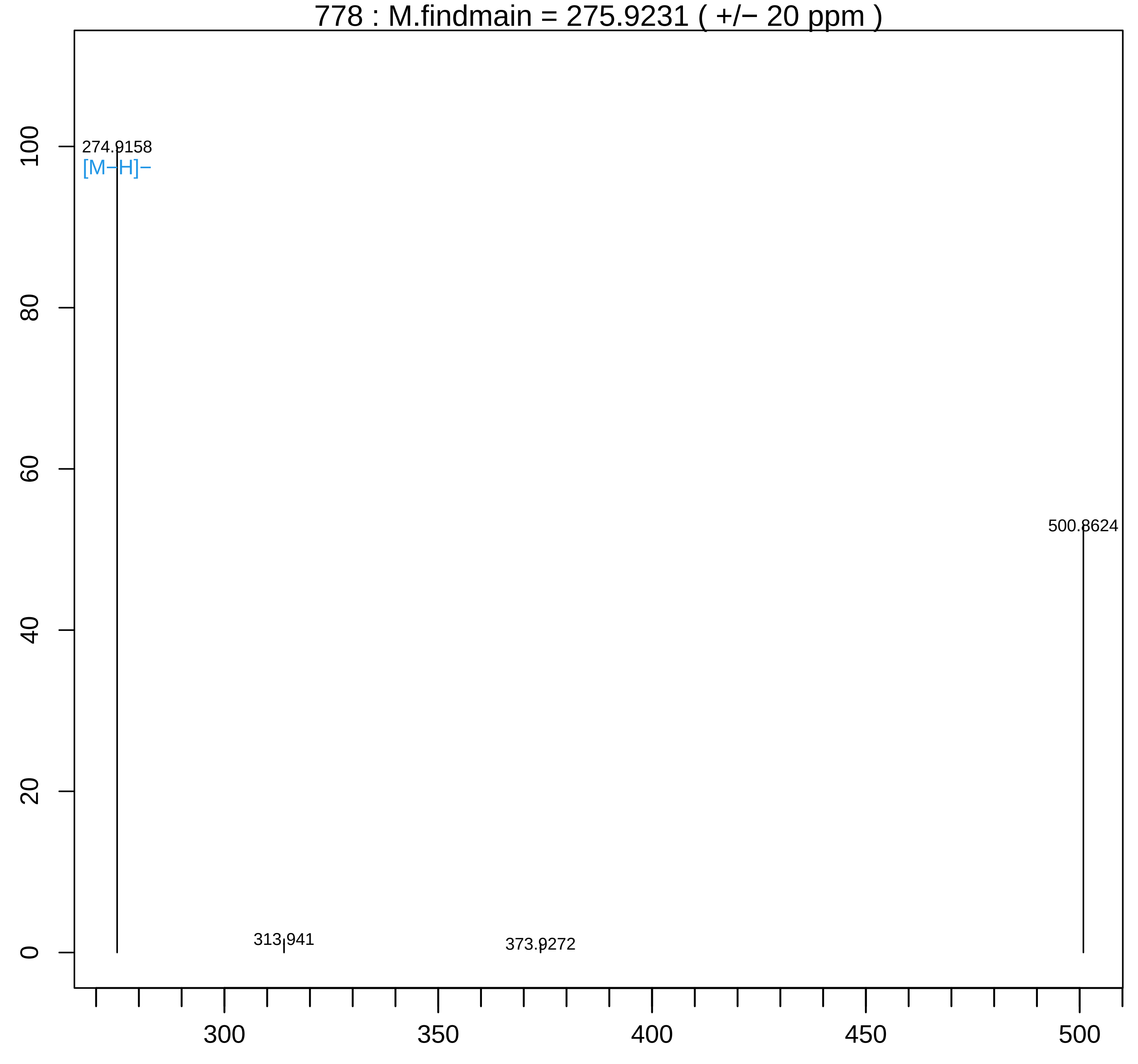
778 : M.findmain = 275.9231 (+/− 20 ppm)
274.9158
[M−H]−
313.941
373.9272
500.8624
0
20
40
60
80
100
300
350
400
450
500

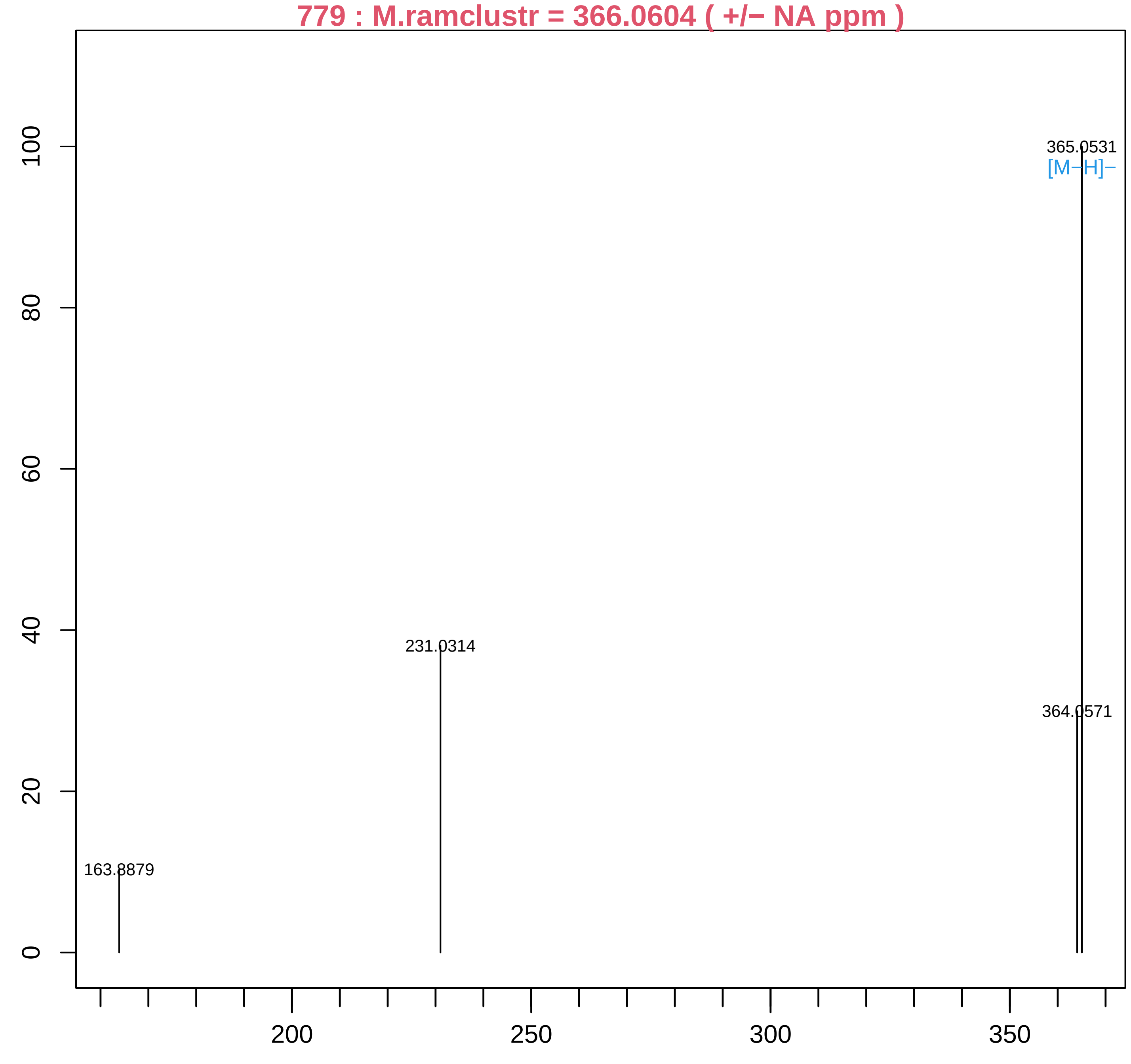
779 : M.ramclustr = 366.0604 (+/− NA ppm)
365.0531
[M−H]−
231.0314
364.0571
163.8879
0
20
40
60
80
100
200
250
300
350

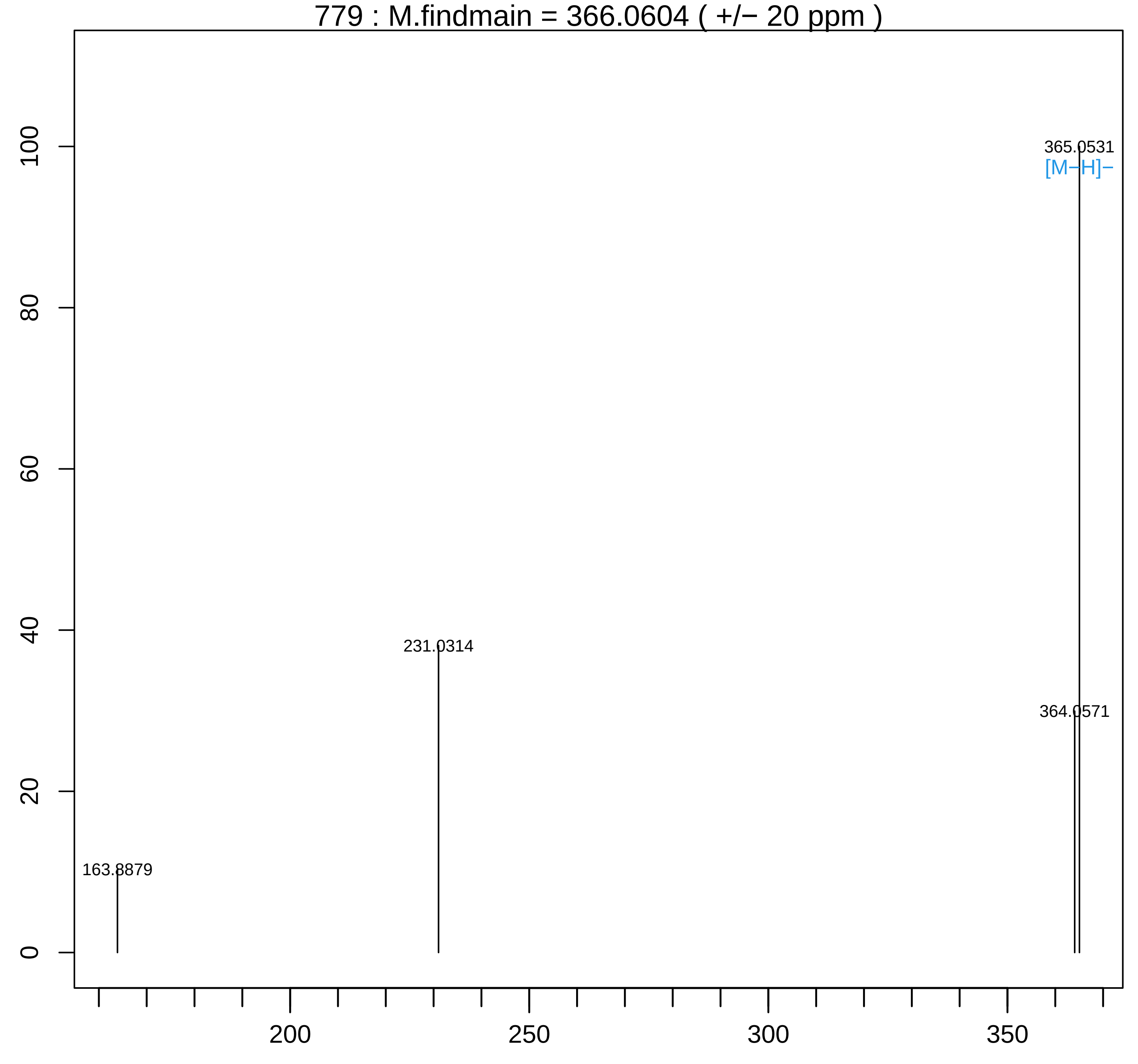
779 : M.findmain = 366.0604 (+/− 20 ppm)
365.0531
[M−H]−
231.0314
364.0571
163.8879
0
20
40
60
80
100
200
250
300
350

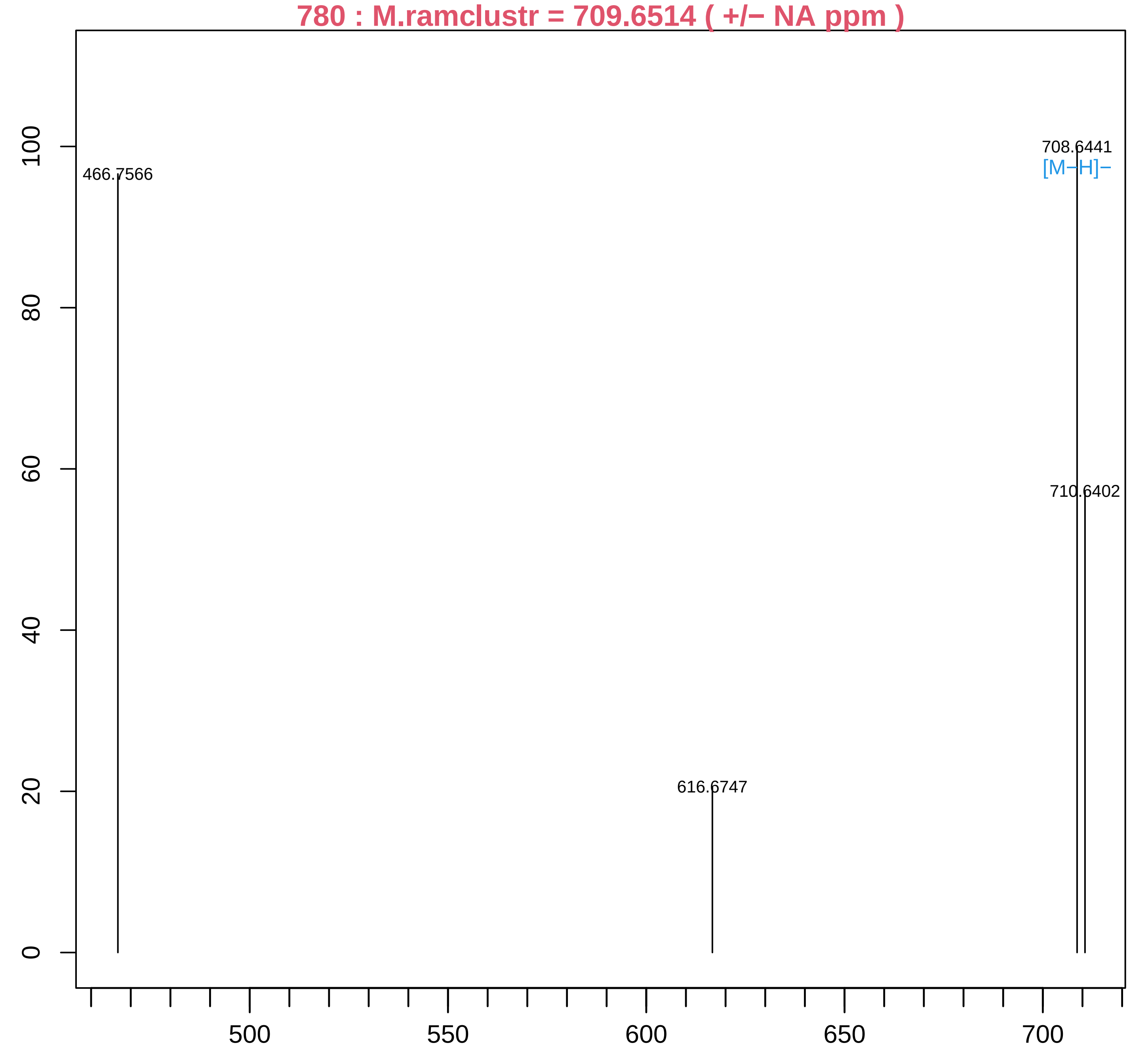
780 : M.ramclustr = 709.6514 (+/− NA ppm)
466.7566
616.6747
708.6441
[M−H]−
710.6402
0
20
40
60
80
100
500
550
600
650
700

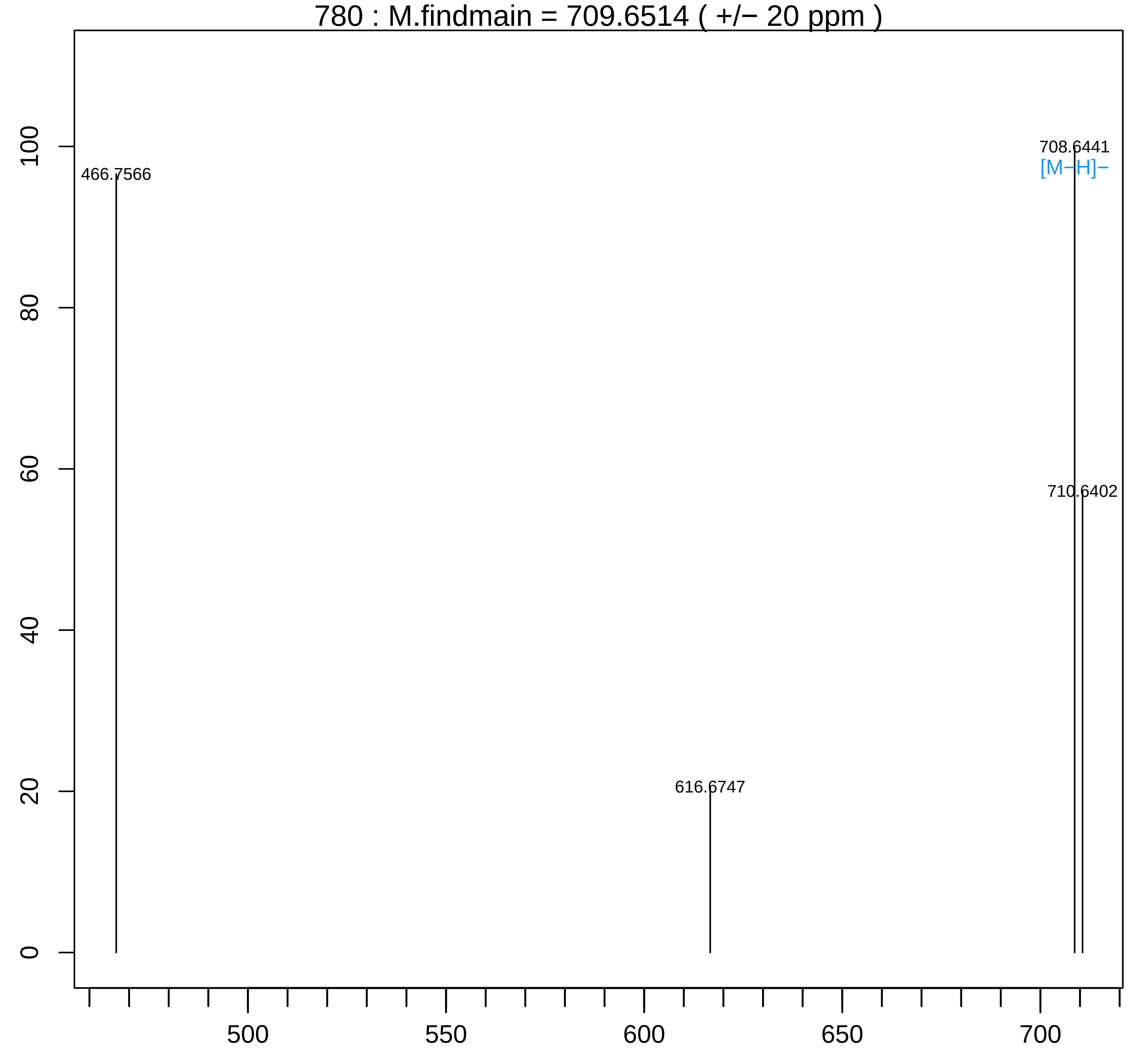
780 : M.findmain = 709.6514 (+/− 20 ppm)
466.7566
616.6747
708.6441
[M−H]−
710.6402
0
20
40
60
80
100
500
550
600
650
700

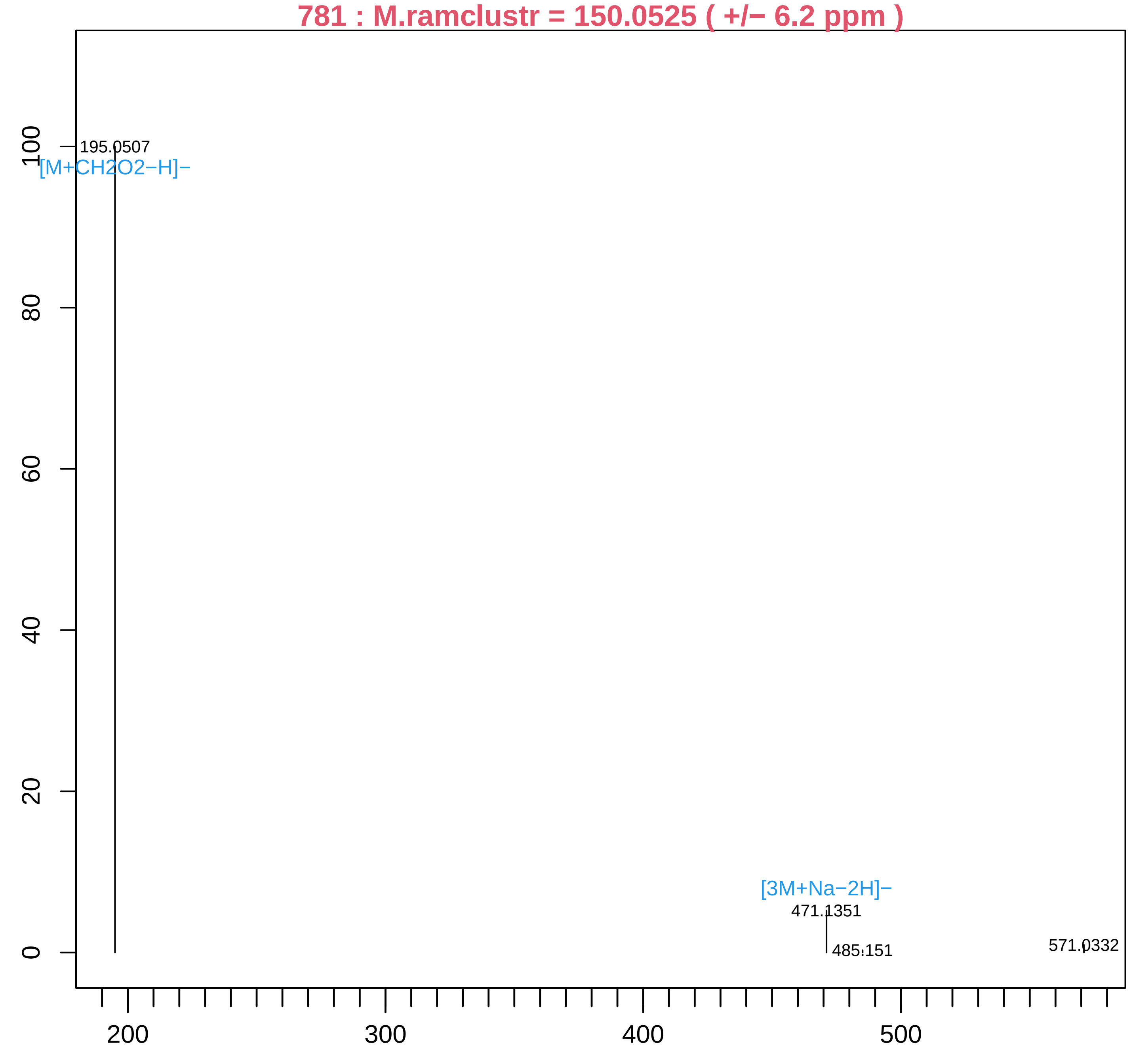
781 : M.ramclustr = 150.0525 (+/− 6.2 ppm)
195.0507
[M+CH2O2−H]−
[3M+Na−2H]−
471.1351
485.151
571.0332
0
20
40
60
80
100
200
300
400
500

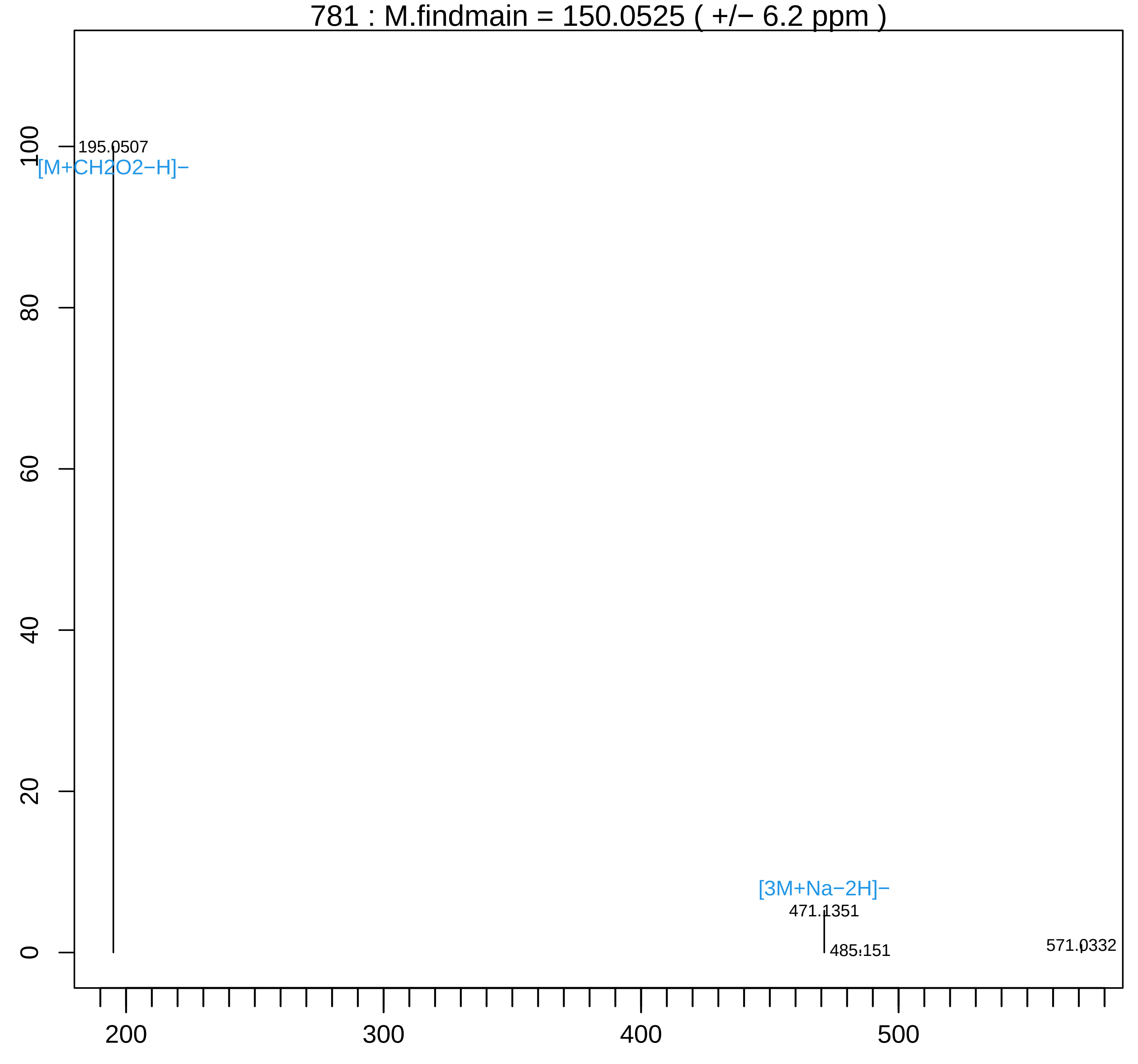
781 : M.findmain = 150.0525 (+/− 6.2 ppm)
195.0507
[M+CH2O2−H]−
[3M+Na−2H]−
471.1351
485.151
571.0332
0
20
40
60
80
100
200
300
400
500

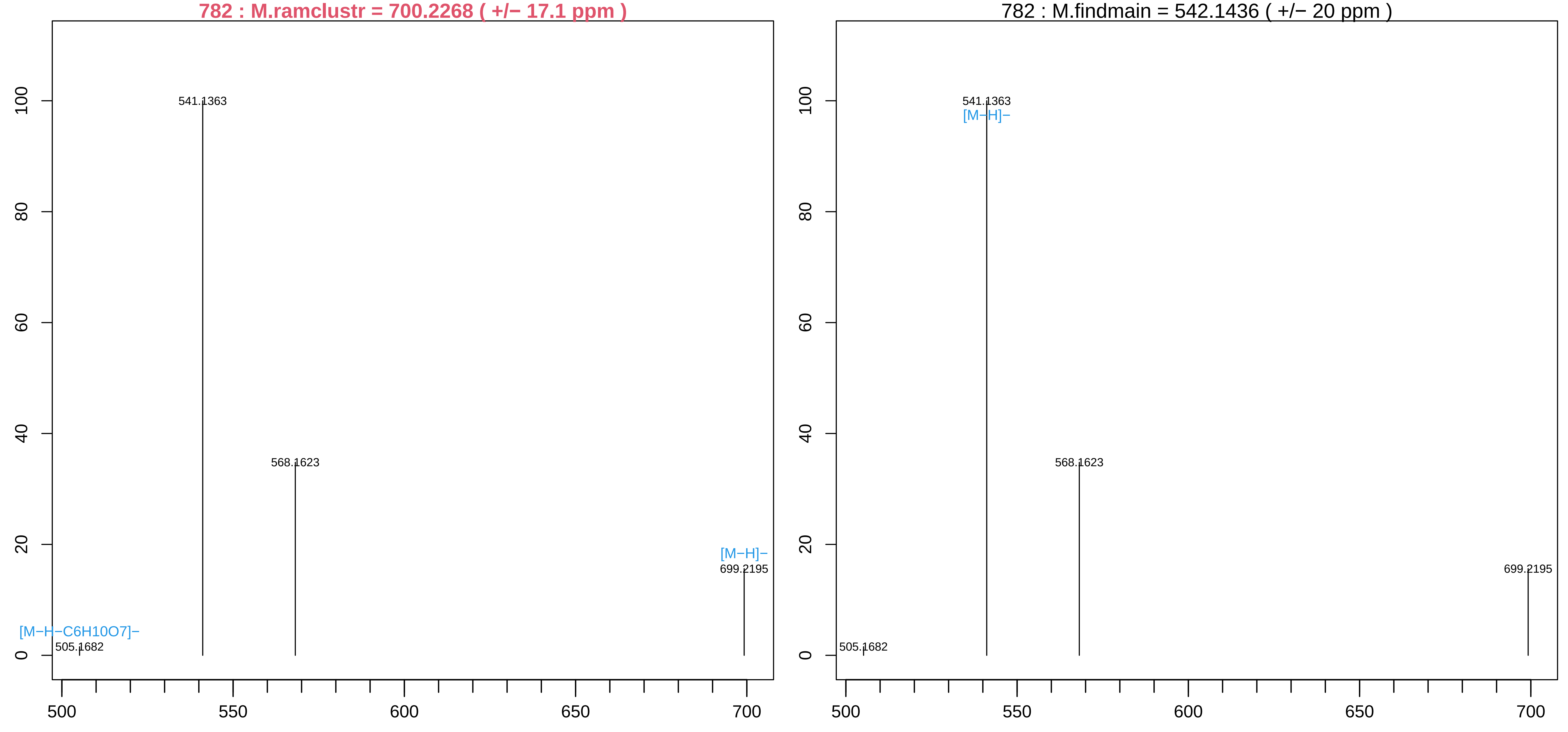
782 : M.ramclustr = 700.2268 (+/− 17.1 ppm)
541.1363
568.1623
[M−H]−
699.2195
[M−H−C6H10O7]−
505.1682
782 : M.findmain = 542.1436 (+/− 20 ppm)
541.1363
[M−H]−
568.1623
699.2195
505.1682

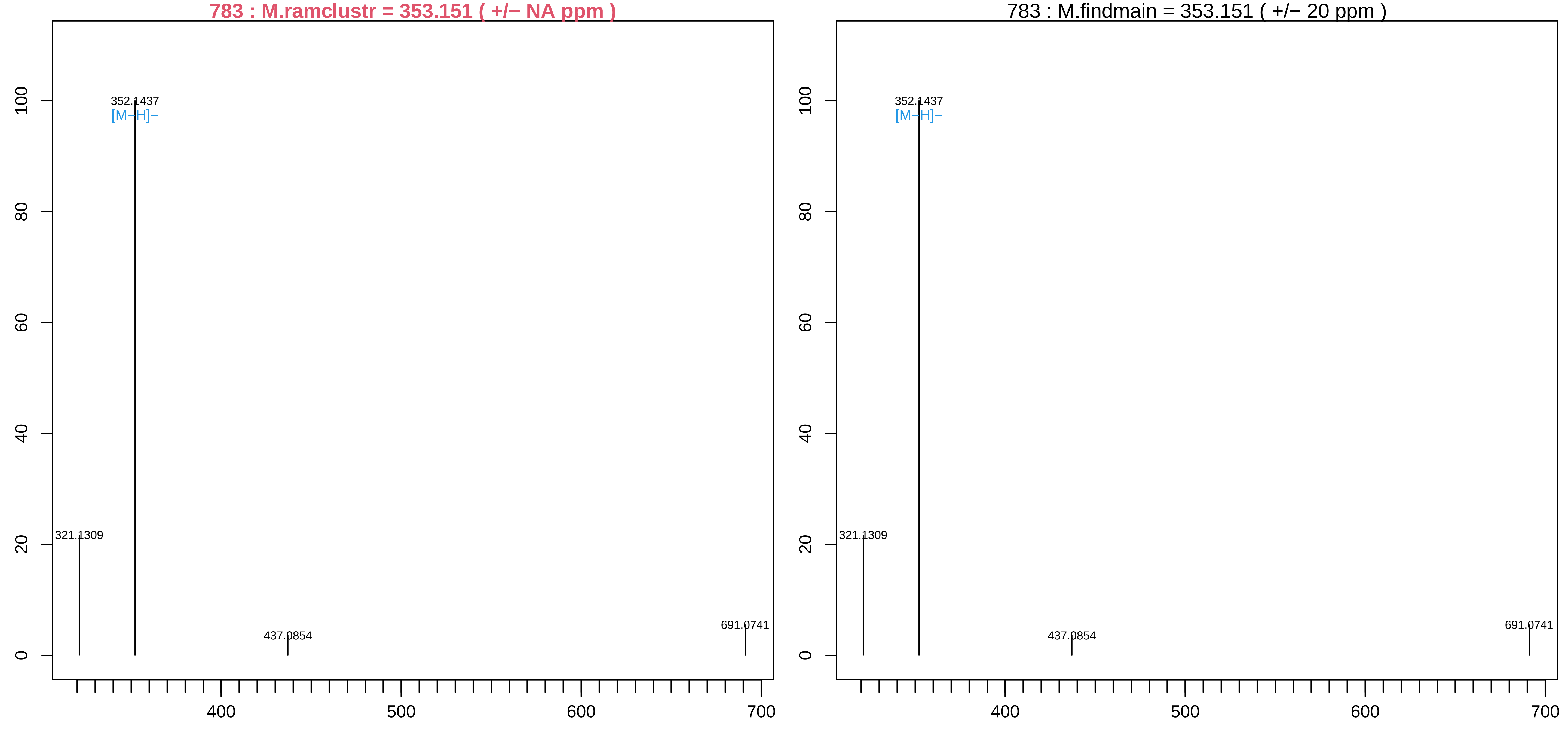
783 : M.ramclustr = 353.151 (+/− NA ppm)
352.1437
[M−H]−
321.1309
437.0854
691.0741
0
20
40
60
80
100
400
500
600
700
783 : M.findmain = 353.151 (+/− 20 ppm)
352.1437
[M−H]−
321.1309
437.0854
691.0741
0
20
40
60
80
100
400
500
600
700

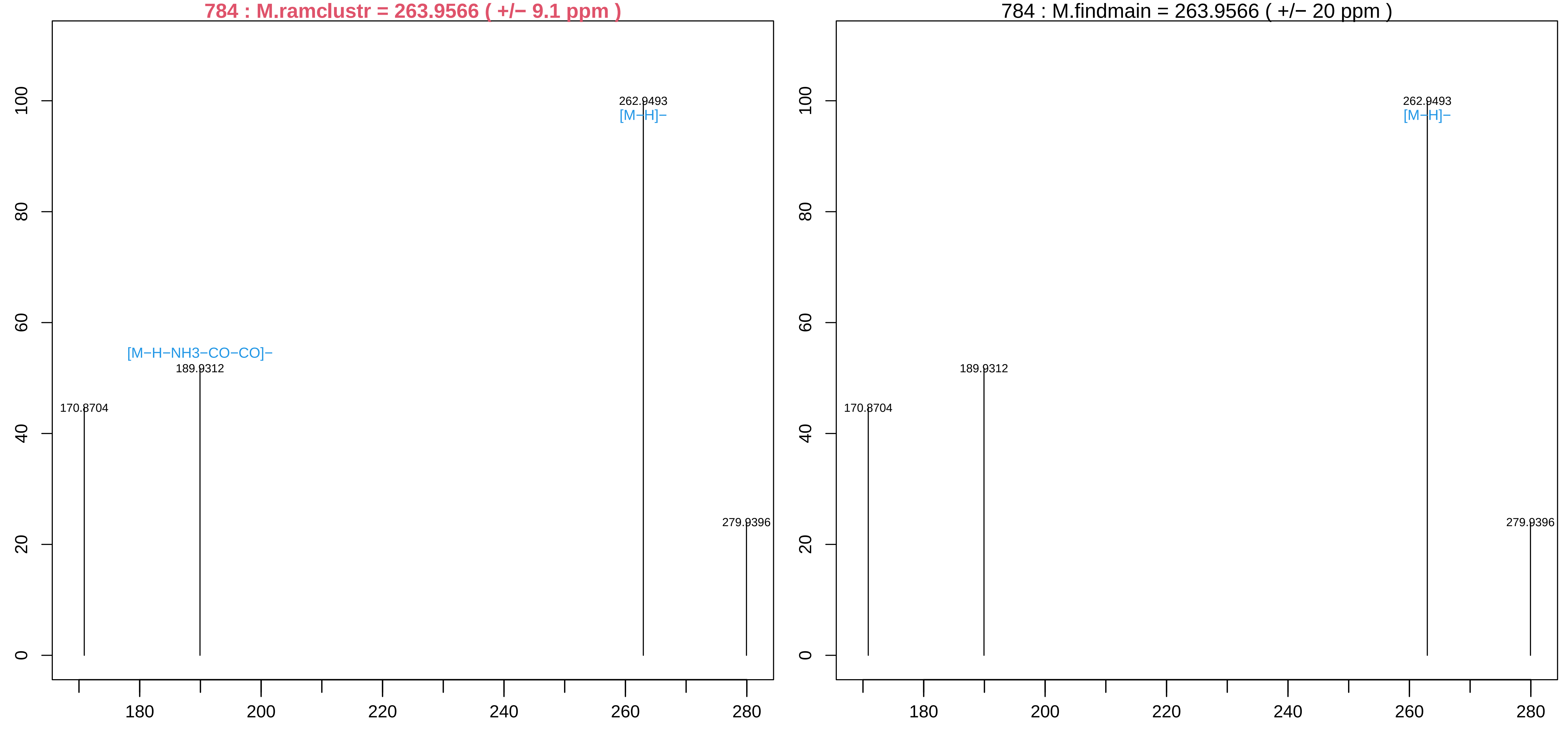

784 : M.ramclustr = 263.9566 (+/− 9.1 ppm)
262.9493
[M−H]−
[M−H−NH3−CO−CO]−
189.9312
170.8704
279.9396
0
20
40
60
80
100
180
200
220
240
260
280
784 : M.findmain = 263.9566 (+/− 20 ppm)
262.9493
[M−H]−
189.9312
170.8704
279.9396

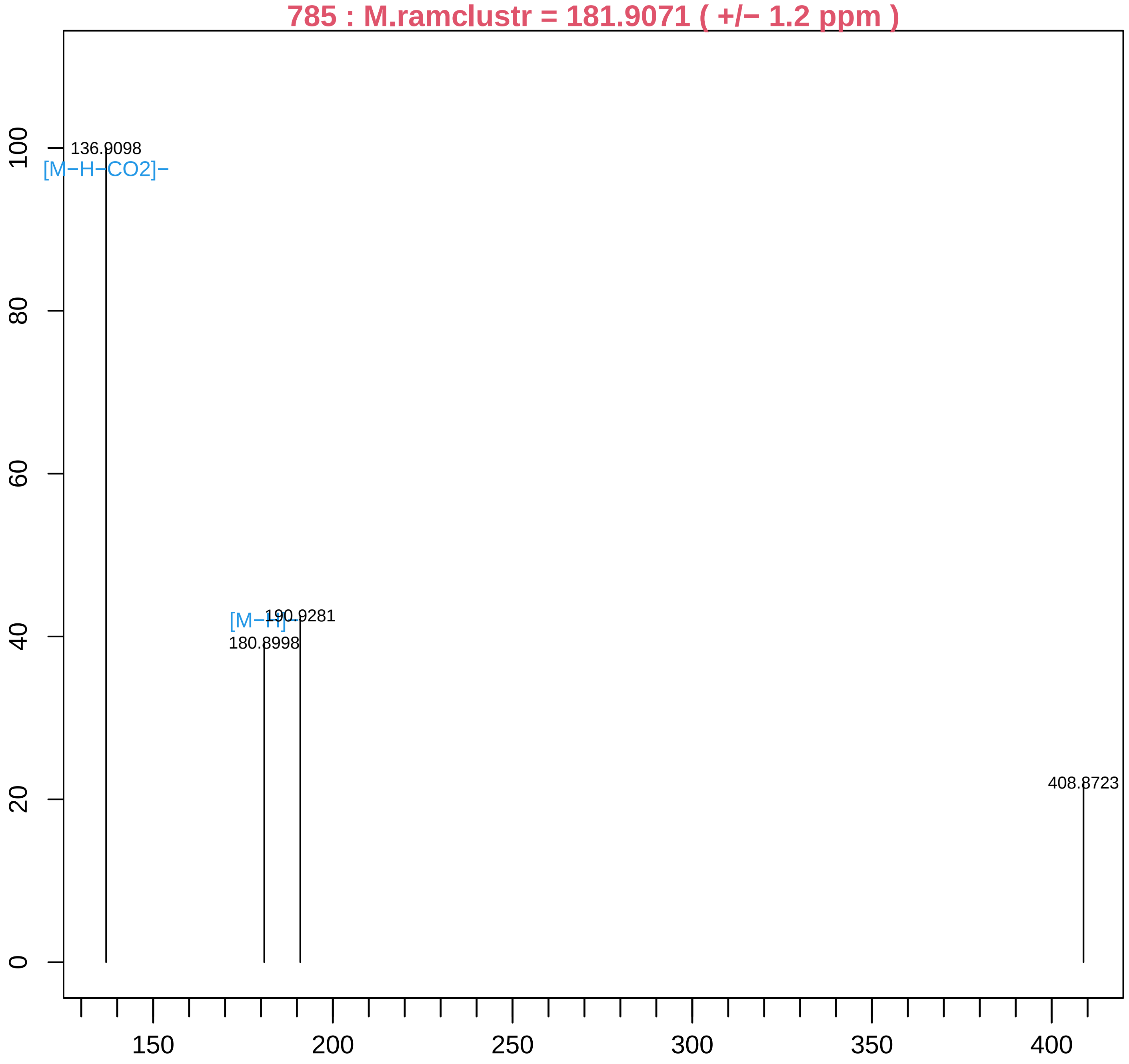
785 : M.ramclustr = 181.9071 (+/− 1.2 ppm)
136.9098
[M−H−CO2]−
[M−H]−
190.9281
180.8998
408.8723
0
20
40
60
80
100
150
200
250
300
350
400

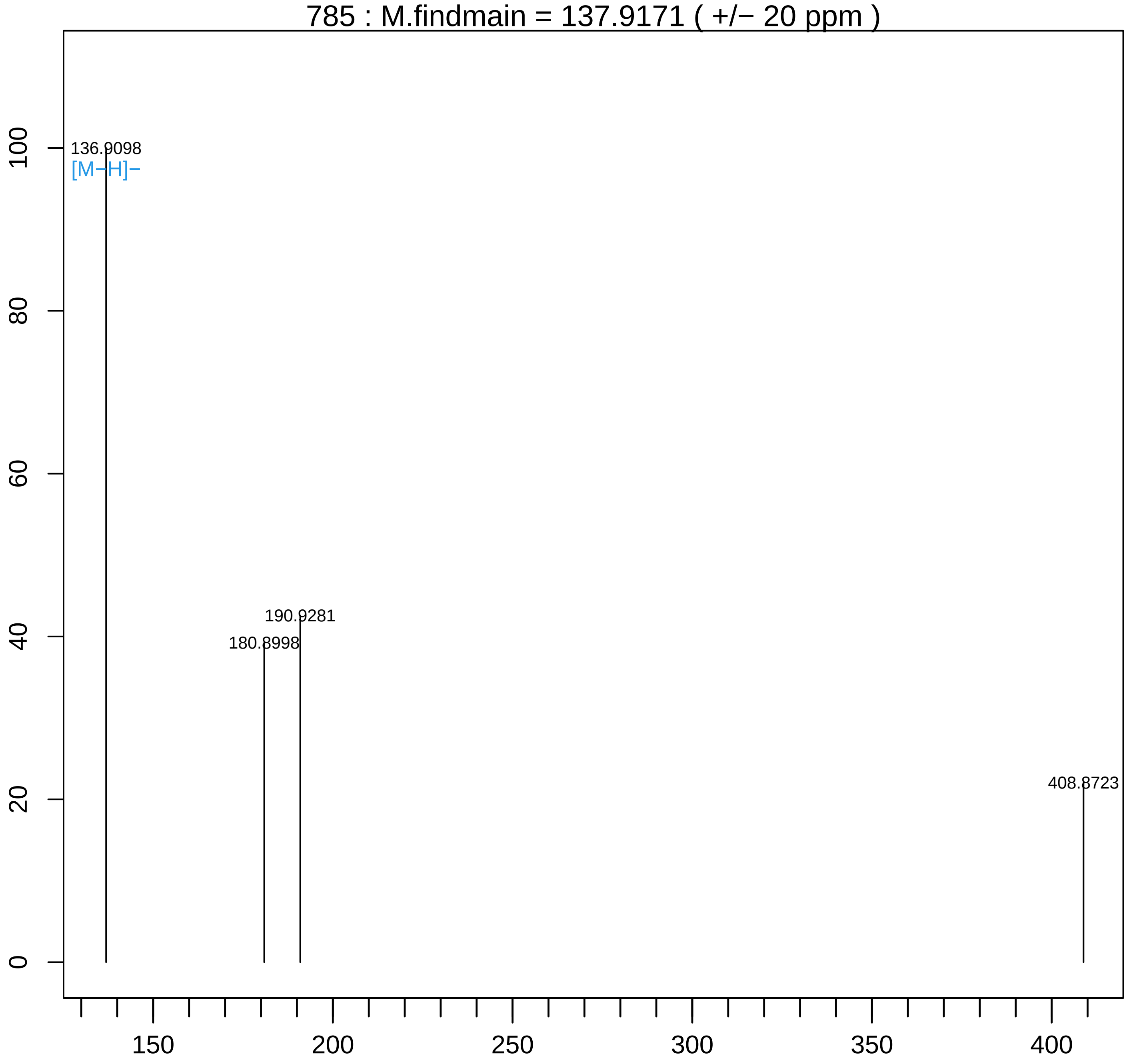
785 : M.findmain = 137.9171 (+/− 20 ppm)
136.9098
[M−H]−
190.9281
180.8998
408.8723
0
20
40
60
80
100
150
200
250
300
350
400

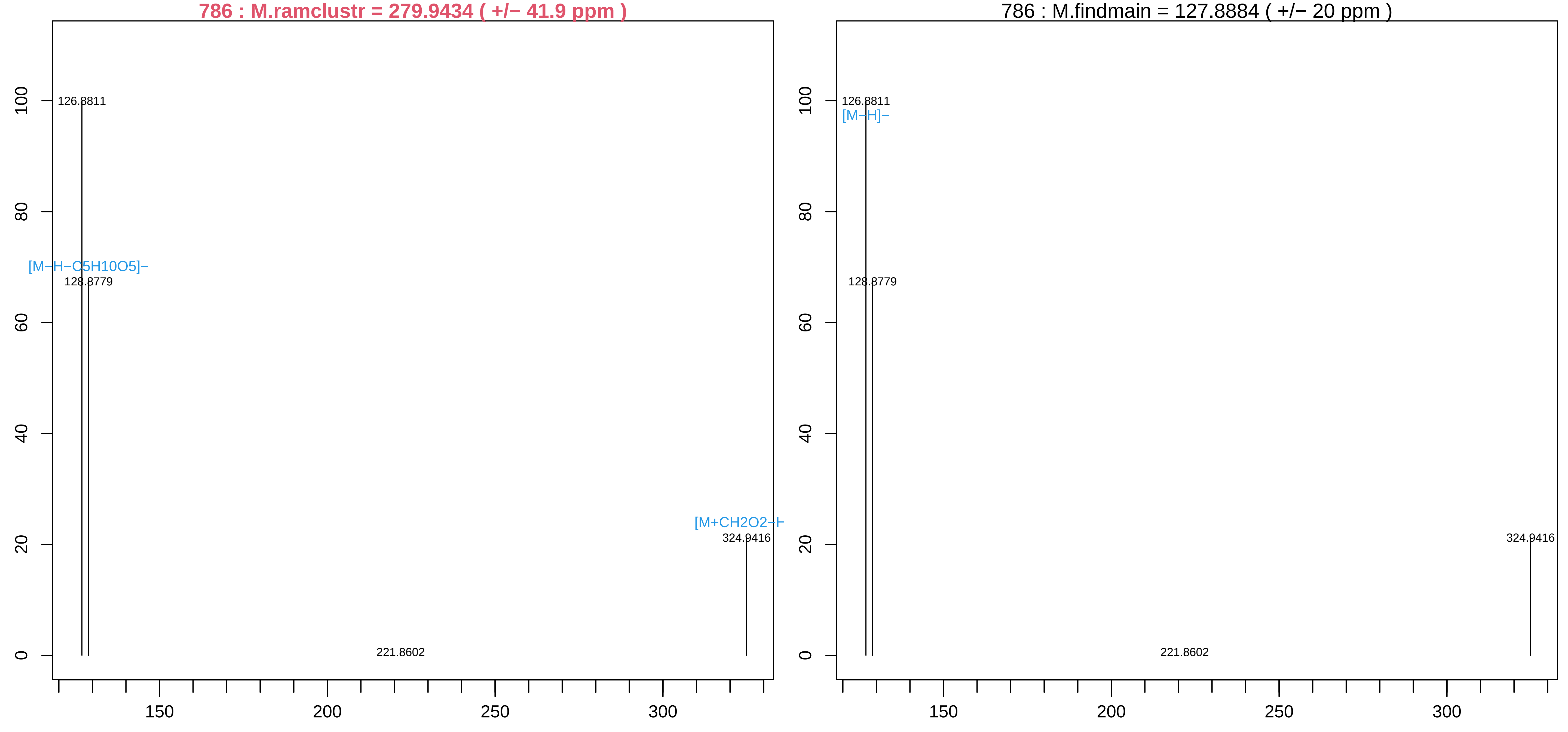

786 : M.ramclustr = 279.9434 (+/− 41.9 ppm)
126.8811
[M−H−C5H10O5]−
128.8779
[M+CH2O2−H
324.9416
221.8602
786 : M.findmain = 127.8884 (+/− 20 ppm)
126.8811
[M−H]−
128.8779
324.9416
221.8602

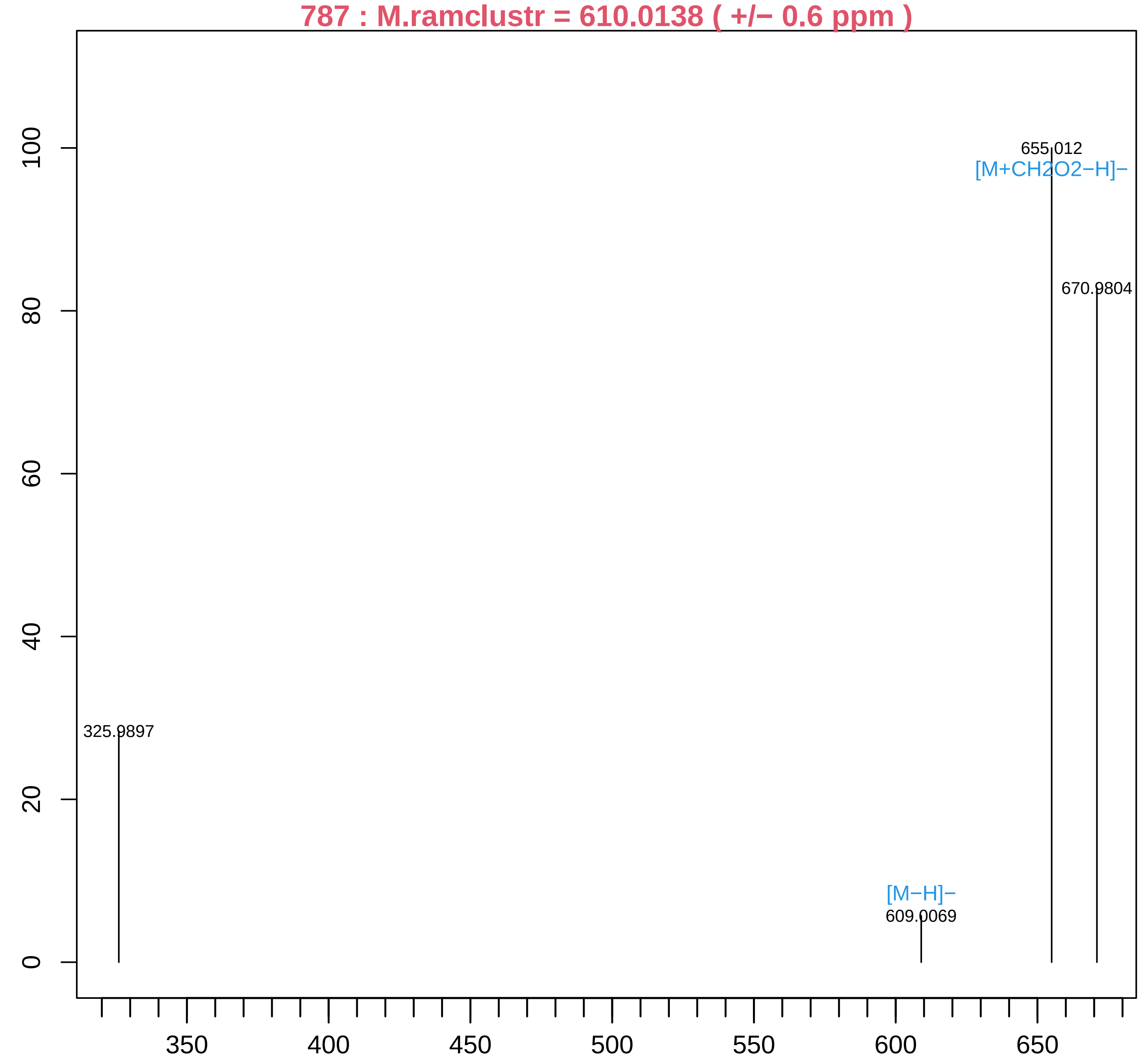
787 : M.ramclustr = 610.0138 (+/− 0.6 ppm)
655.012
[M+CH2O2−H]−
670.9804
325.9897
[M−H]−
609.0069
0
20
40
60
80
100
350
400
450
500
550
600
650

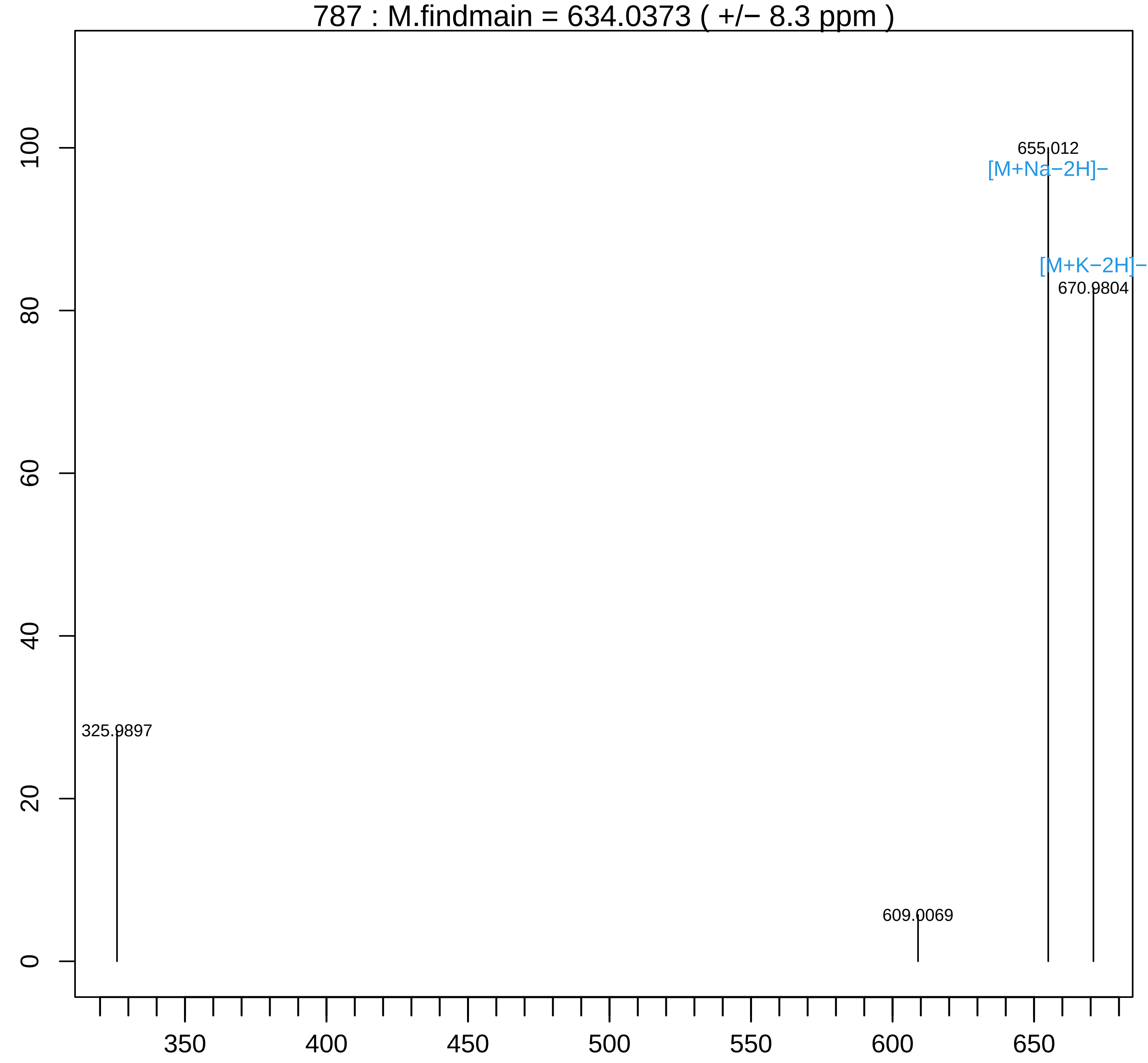
787 : M.findmain = 634.0373 (+/− 8.3 ppm)
655.012
[M+Na−2H]−
[M+K−2H]−
670.9804
325.9897
609.0069
0
20
40
60
80
100
350
400
450
500
550
600
650

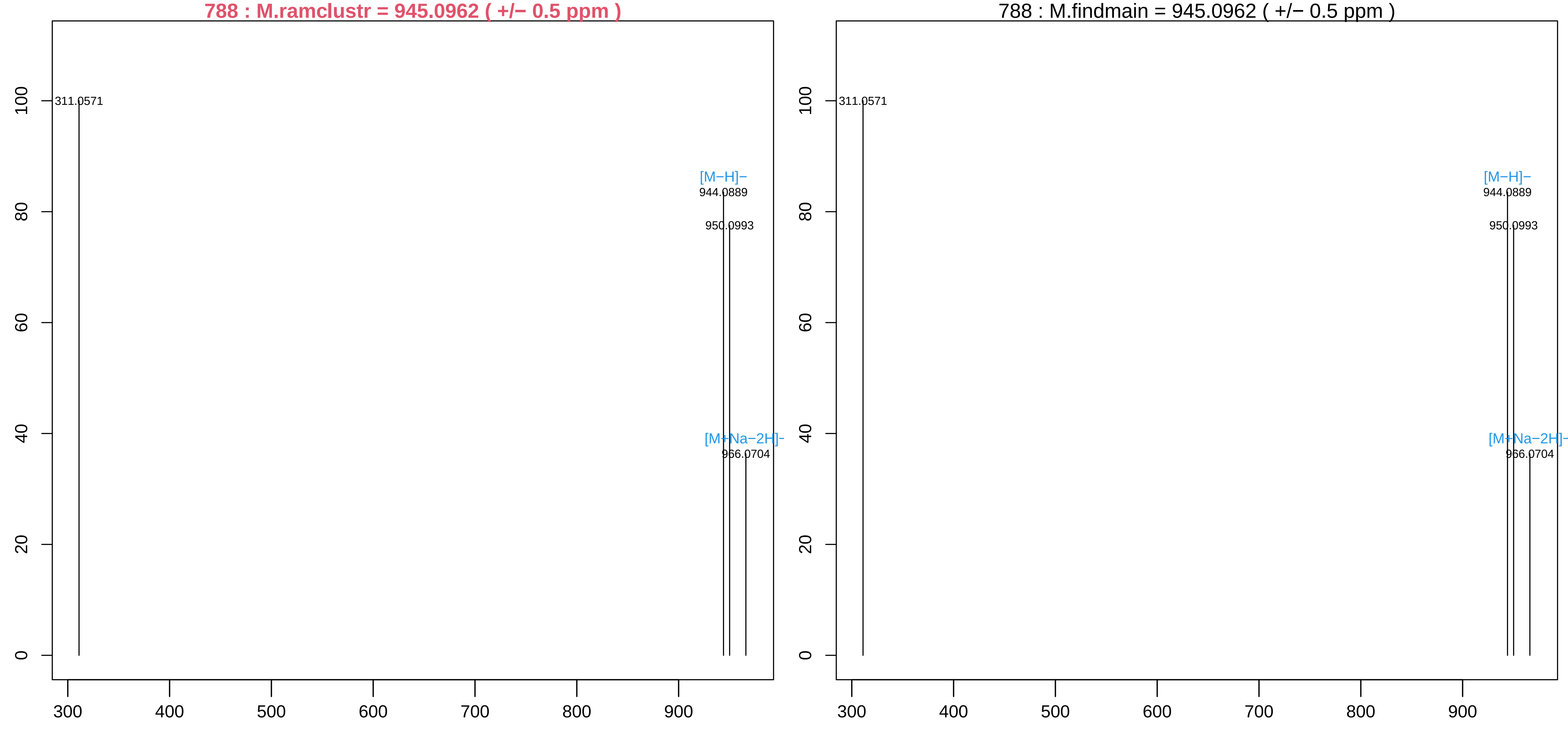
788 : M.ramclustr = 945.0962 (+/− 0.5 ppm)
311.0571
[M−H]−
944.0889
950.0993
[M+Na−2H]−
966.0704
0
20
40
60
80
100
300
400
500
600
700
800
900
788 : M.findmain = 945.0962 (+/− 0.5 ppm)
311.0571
[M−H]−
944.0889
950.0993
[M+Na−2H]−
966.0704

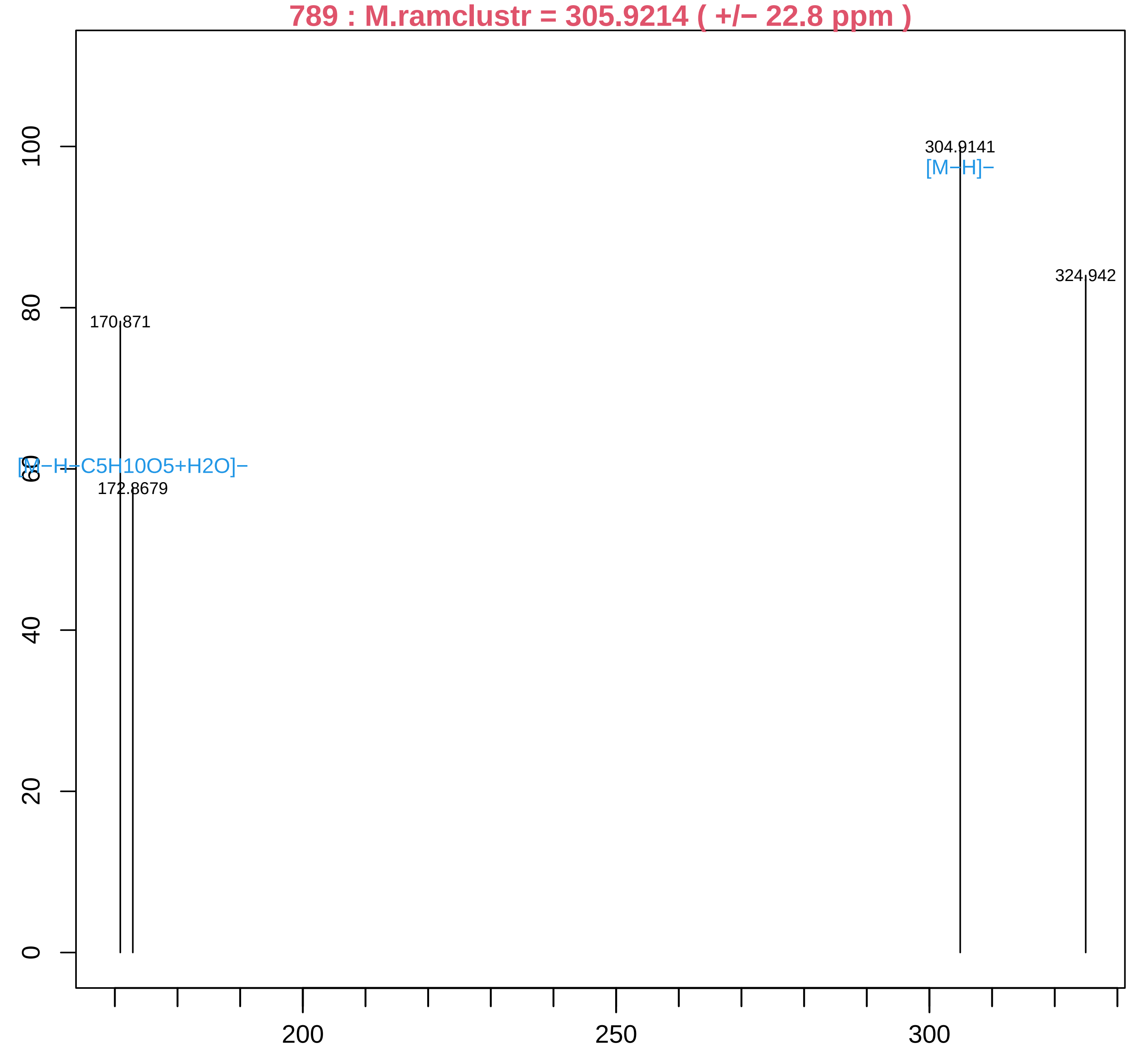

789 : M.ramclustr = 305.9214 (+/− 22.8 ppm)
304.9141
[M−H]−
324.942
170.871
[M−H−C5H10O5+H2O]−
172.8679
0
20
40
60
80
100
200
250
300

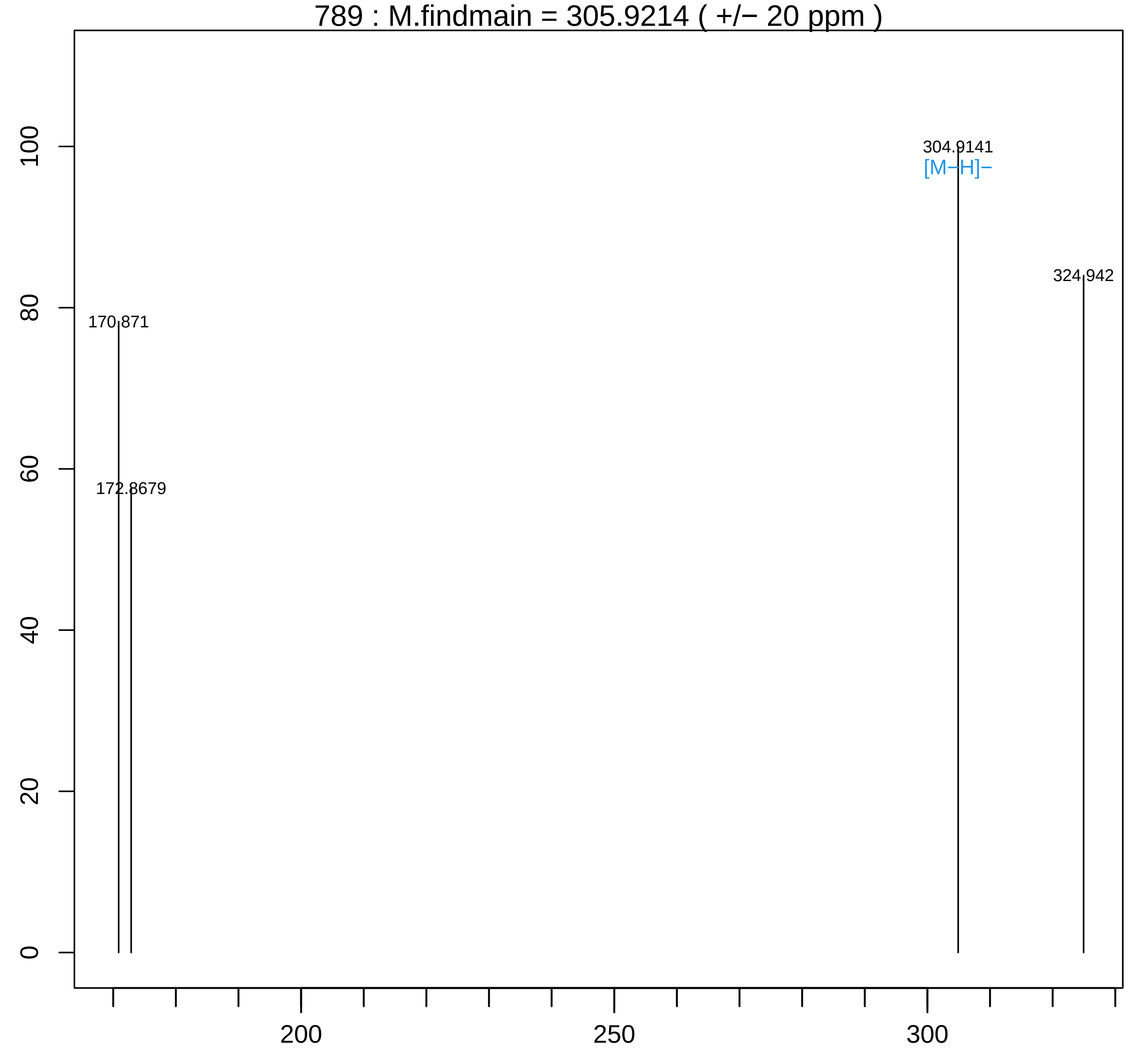

789 : M.findmain = 305.9214 (+/− 20 ppm)
304.9141
[M−H]−
324.942
170.871
172.8679
0
20
40
60
80
100
200
250
300

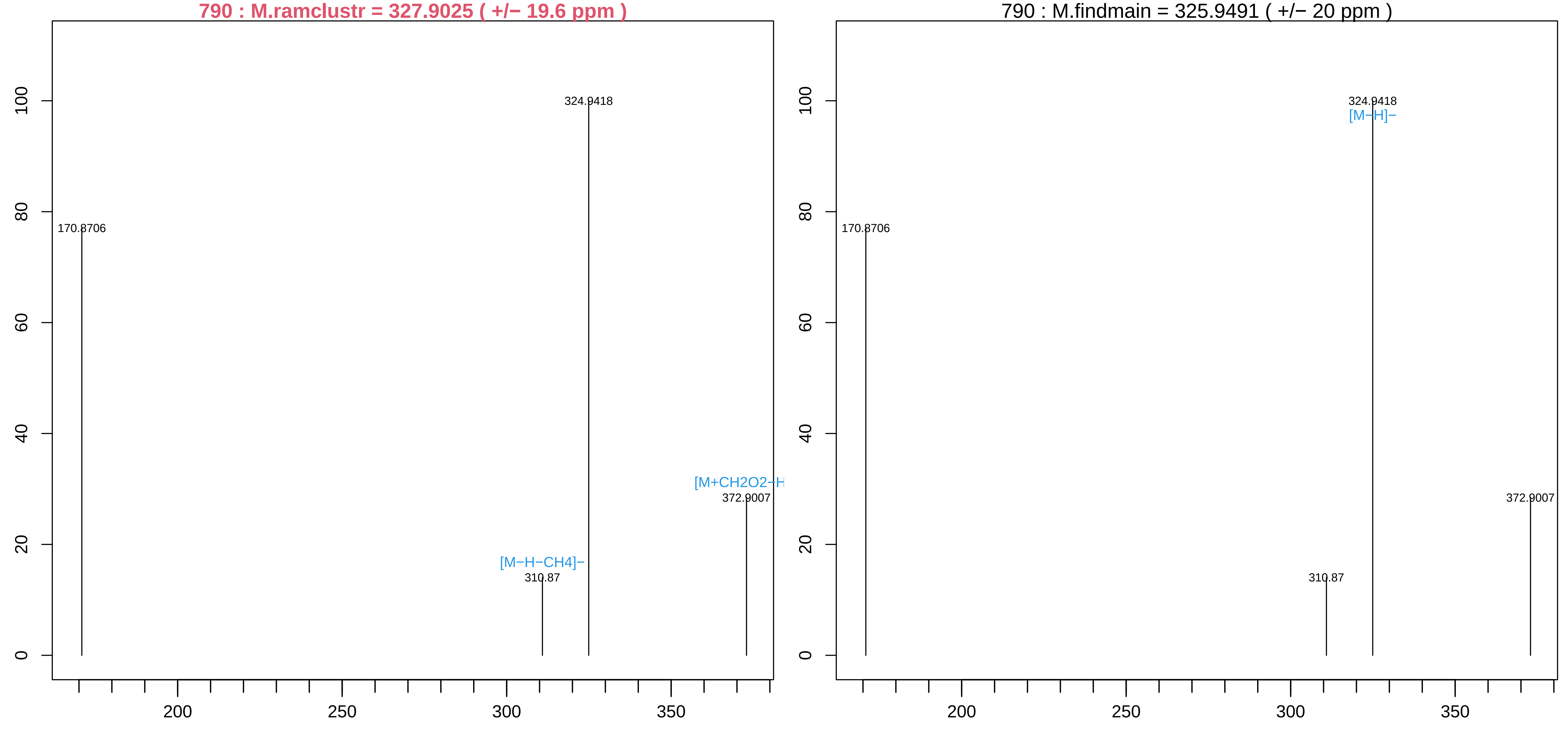
790 : M.ramclustr = 327.9025 (+/− 19.6 ppm)
324.9418
170.8706
[M+CH2O2−H
372.9007
[M−H−CH4]−
310.87
0
20
40
60
80
100
200
250
300
350
790 : M.findmain = 325.9491 (+/− 20 ppm)
324.9418
[M−H]−
170.8706
372.9007
310.87
0
20
40
60
80
100
200
250
300
350

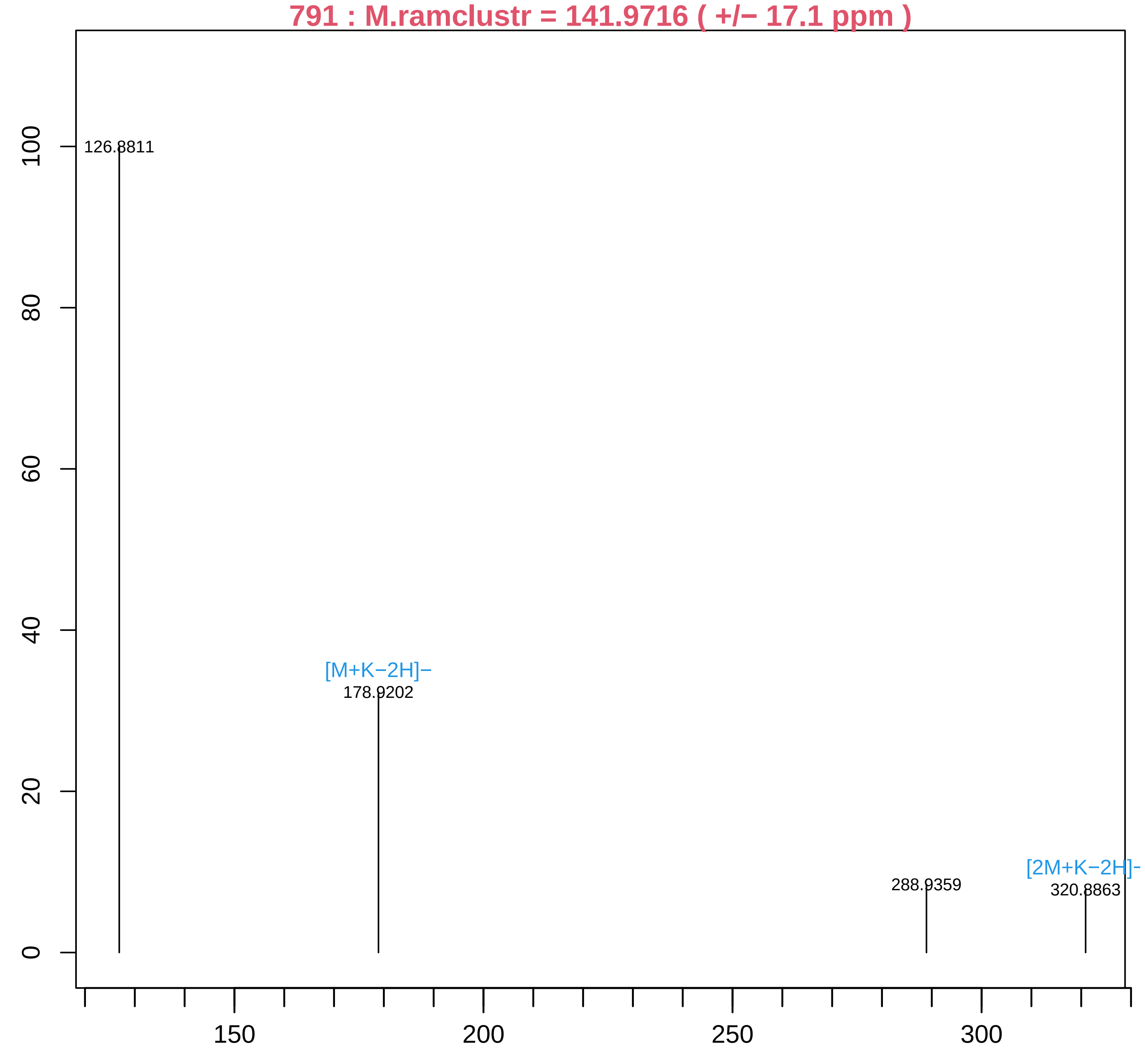
791 : M.ramclustr = 141.9716 (+/− 17.1 ppm)
126.8811
[M+K−2H]−
178.9202
288.9359
[2M+K−2H]-
320.8863
0
20
40
60
80
100
150
200
250
300

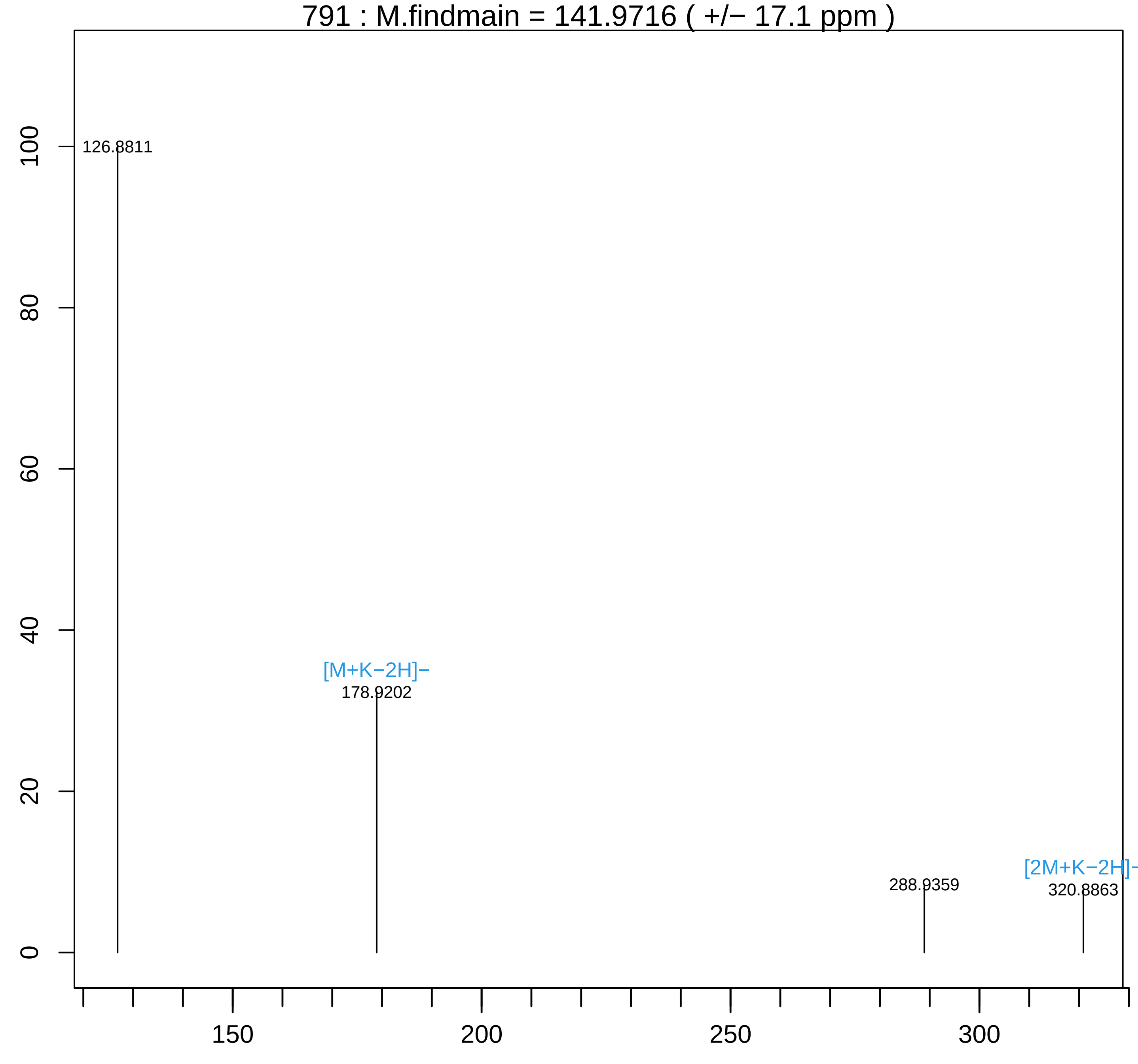
791 : M.findmain = 141.9716 (+/− 17.1 ppm)
126.8811
[M+K−2H]−
178.9202
288.9359
[2M+K−2H]-
320.8863
0
20
40
60
80
100
150
200
250
300

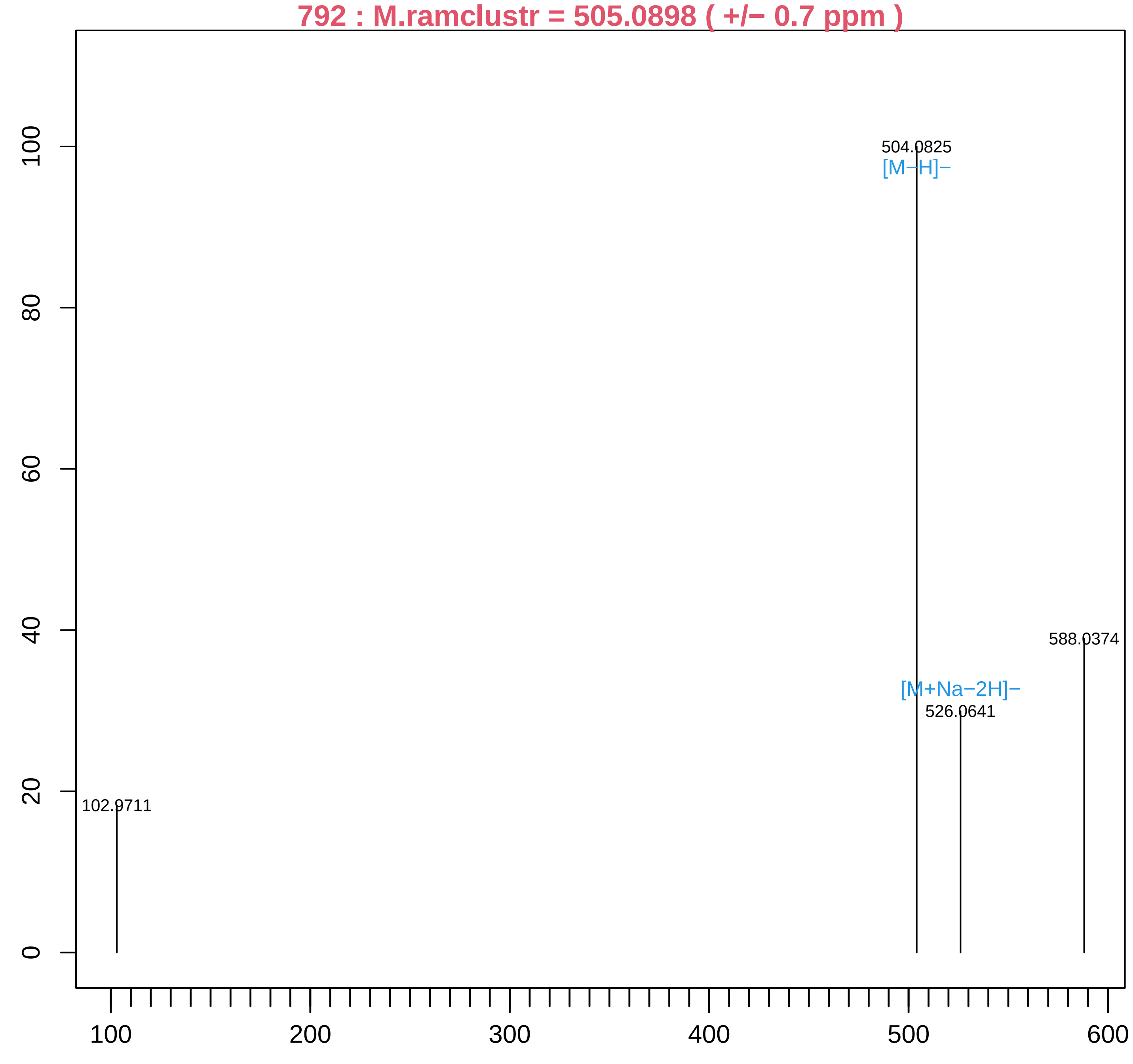
792 : M.ramclustr = 505.0898 (+/− 0.7 ppm)
504.0825
[M−H]−
588.0374
[M+Na−2H]−
526.0641
102.9711
0
20
40
60
80
100
100
200
300
400
500
600
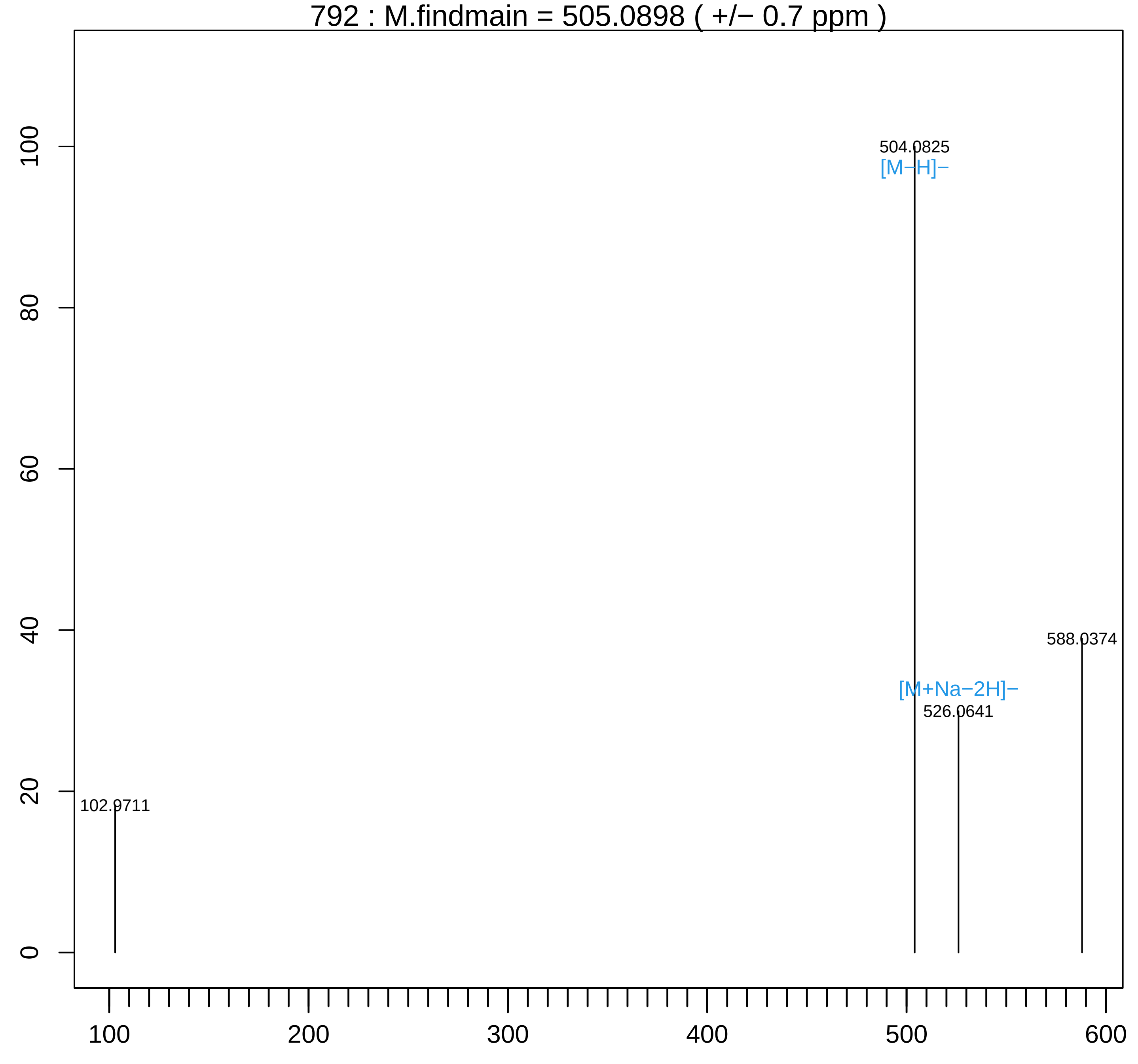
792 : M.findmain = 505.0898 (+/− 0.7 ppm)
504.0825
[M−H]−
588.0374
[M+Na−2H]−
526.0641
102.9711
0
20
40
60
80
100
100
200
300
400
500
600

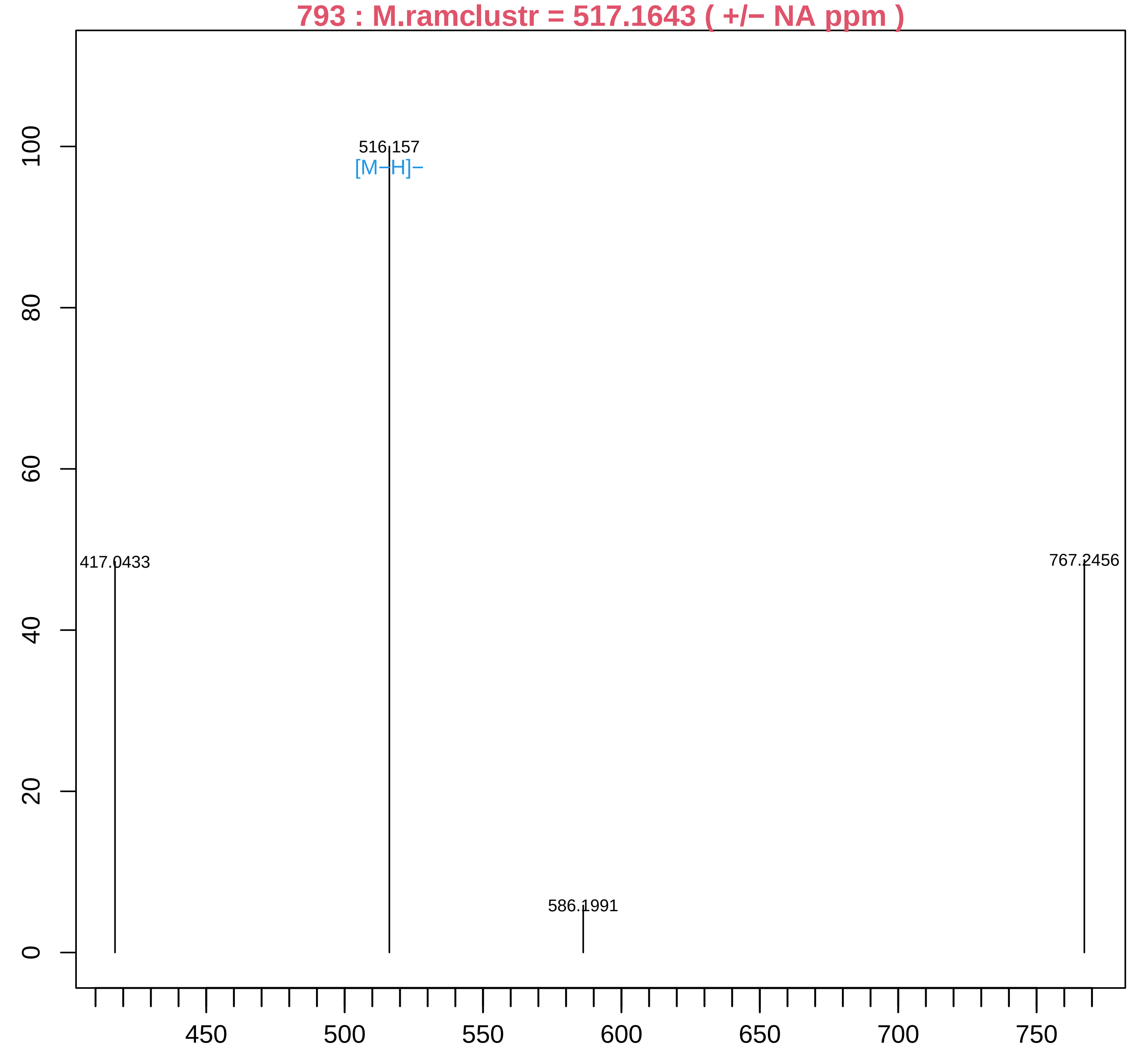
793 : M.ramclustr = 517.1643 (+/− NA ppm)
516.157
[M−H]−
417.0433
767.2456
586.1991

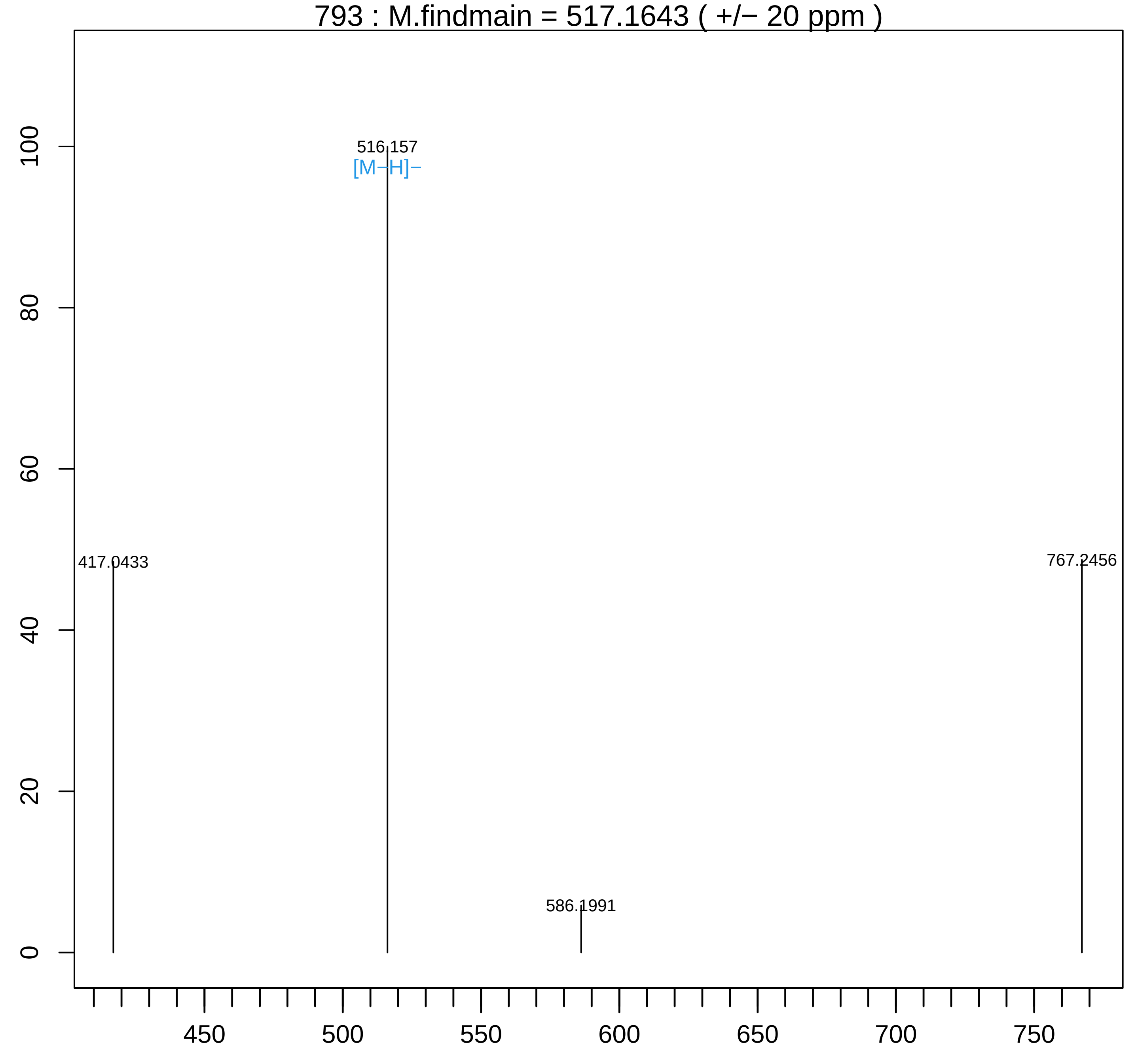
793 : M.findmain = 517.1643 (+/− 20 ppm)
516.157
[M−H]−
417.0433
767.2456
586.1991

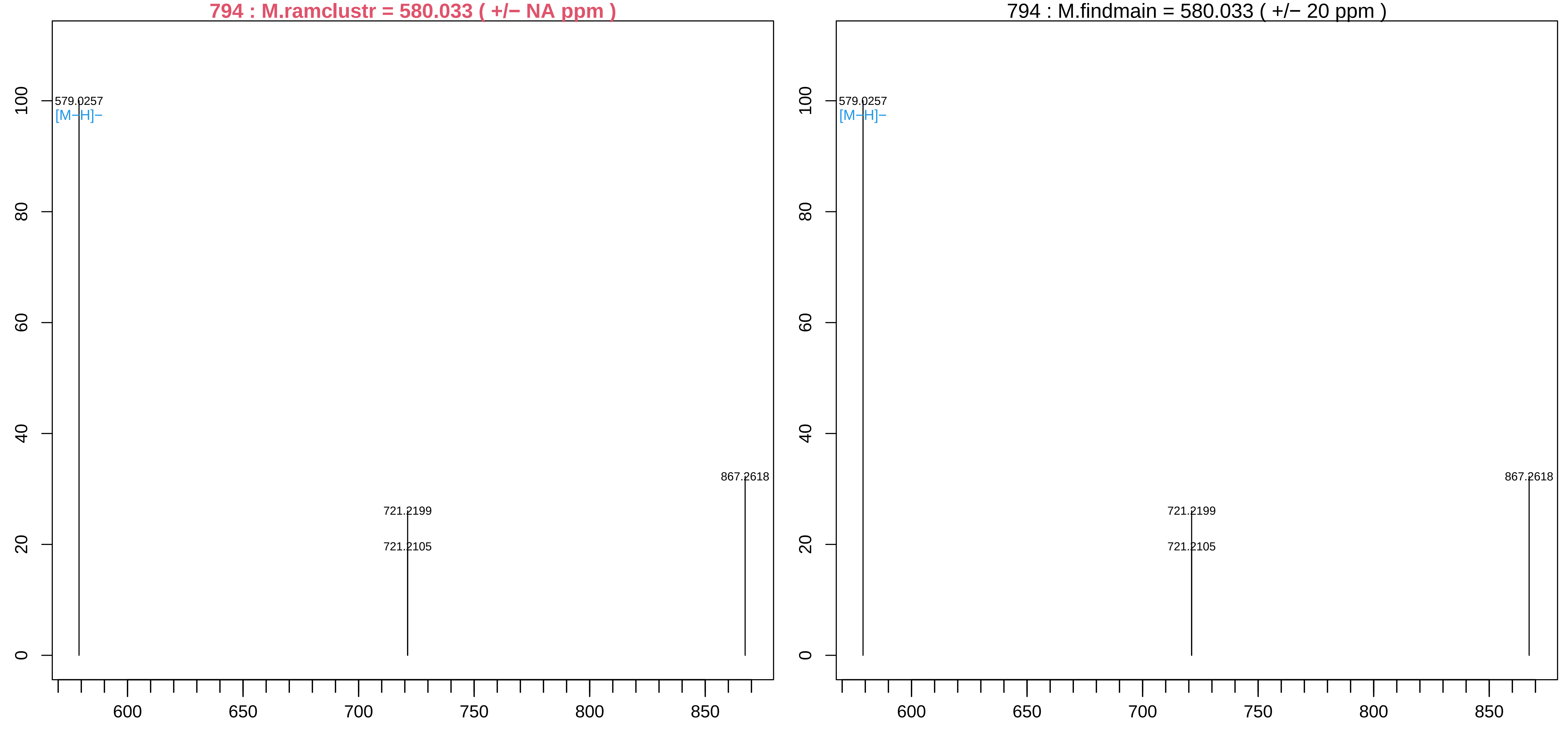
794 : M.ramclustr = 580.033 (+/− NA ppm)
579.0257
[M−H]−
721.2199
721.2105
867.2618
794 : M.findmain = 580.033 (+/− 20 ppm)
579.0257
[M−H]−
721.2199
721.2105
867.2618
0
20
40
60
80
100
600
650
700
750
800
850

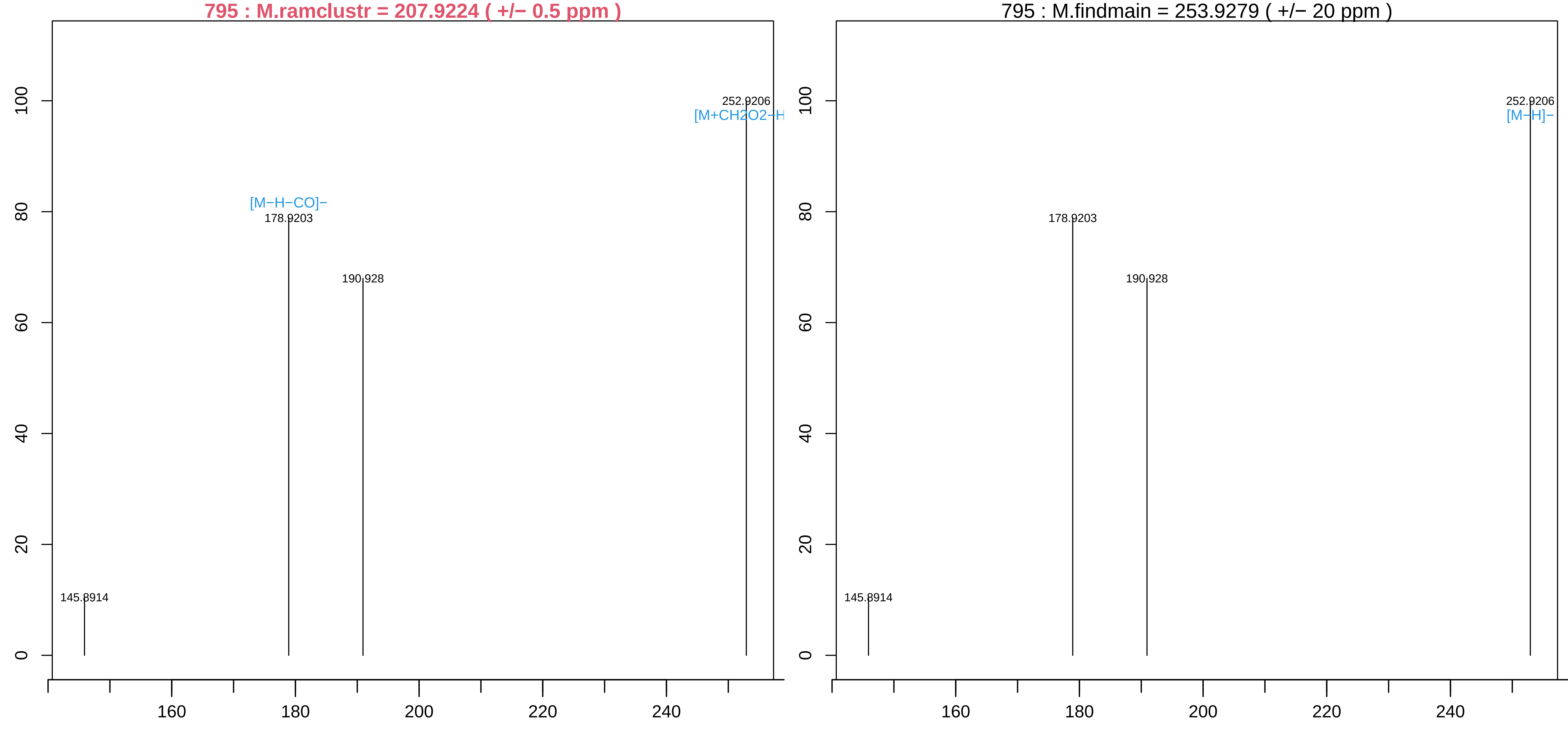

795 : M.ramclustr = 207.9224 (+/− 0.5 ppm)
252.9206
[M+CH2O2−H
[M−H−CO]−
178.9203
190.928
145.8914
0
20
40
60
80
100
160
180
200
220
240
795 : M.findmain = 253.9279 (+/− 20 ppm)
252.9206
[M−H]−
178.9203
190.928
145.8914
0
20
40
60
80
100
160
180
200
220
240

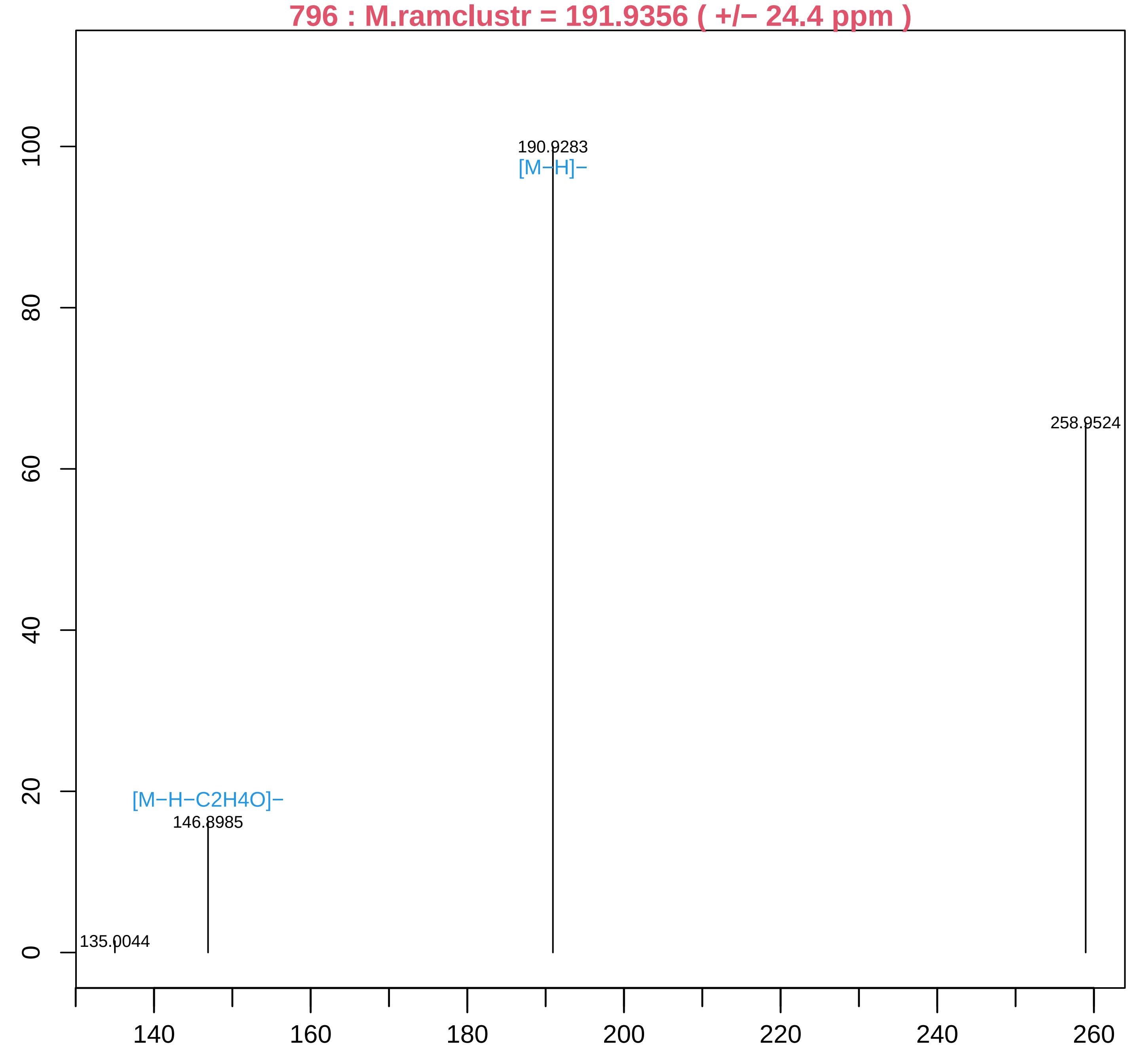

796 : M.ramclustr = 191.9356 (+/− 24.4 ppm)
190.9283
[M−H]−
258.9524
[M−H−C2H4O]−
146.8985
135.0044
0
20
40
60
80
100
140
160
180
200
220
240
260

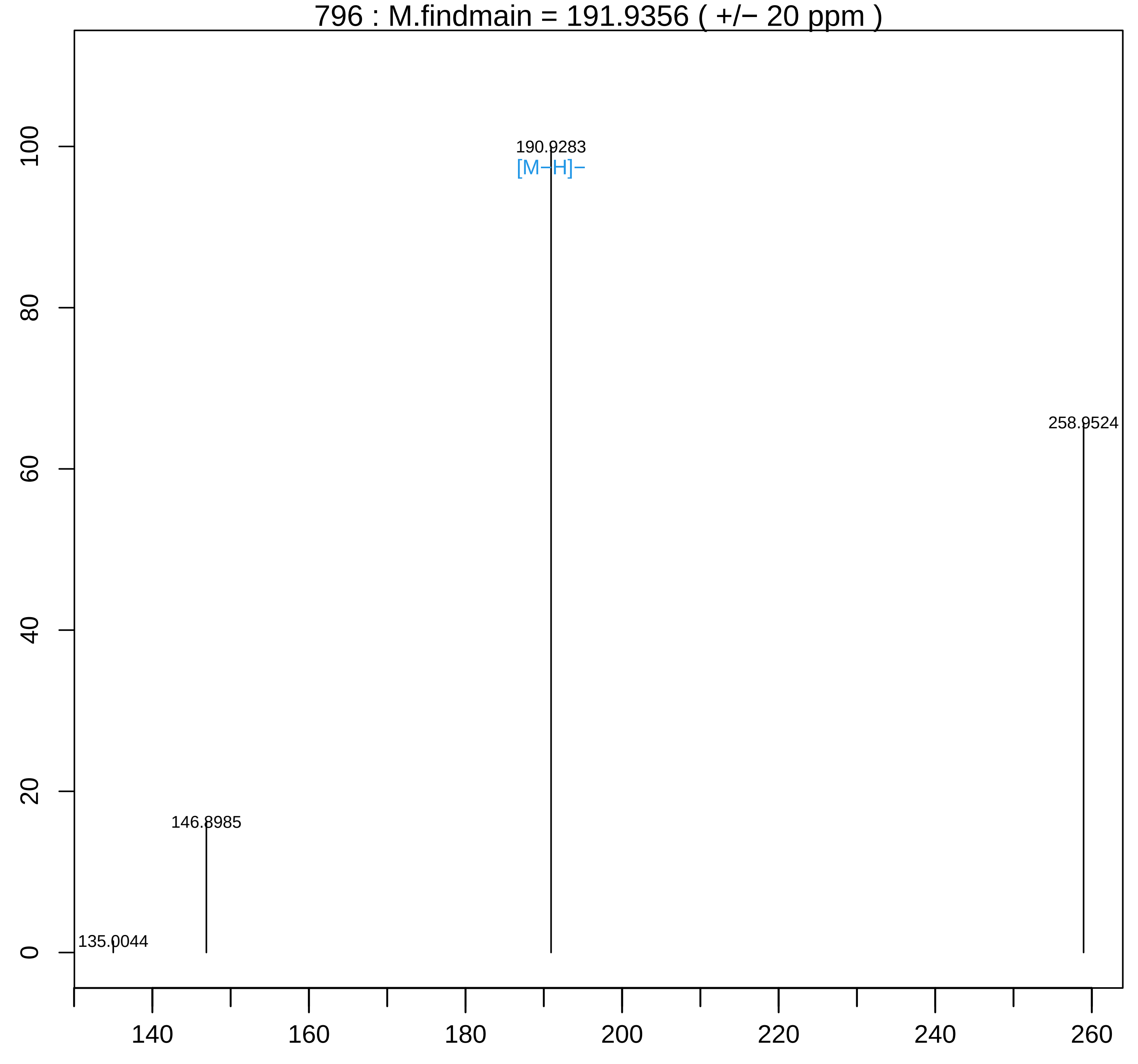

796 : M.findmain = 191.9356 (+/− 20 ppm)
190.9283
[M−H]−
258.9524
146.8985
135.0044
0
20
40
60
80
100
140
160
180
200
220
240
260

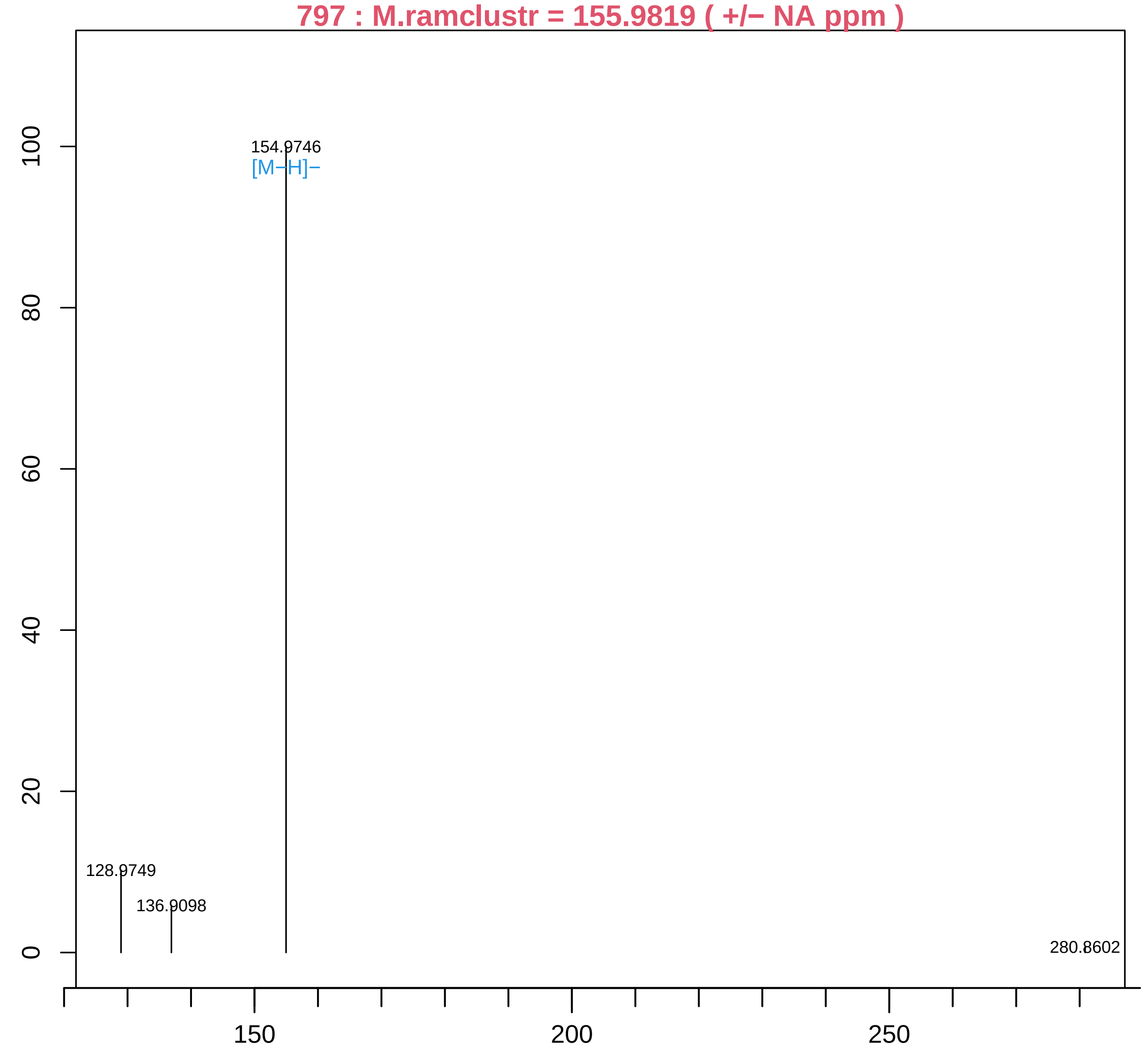
797 : M.ramclustr = 155.9819 (+/− NA ppm)
154.9746
[M−H]−
128.9749
136.9098
280.8602
0
20
40
60
80
100
150
200
250

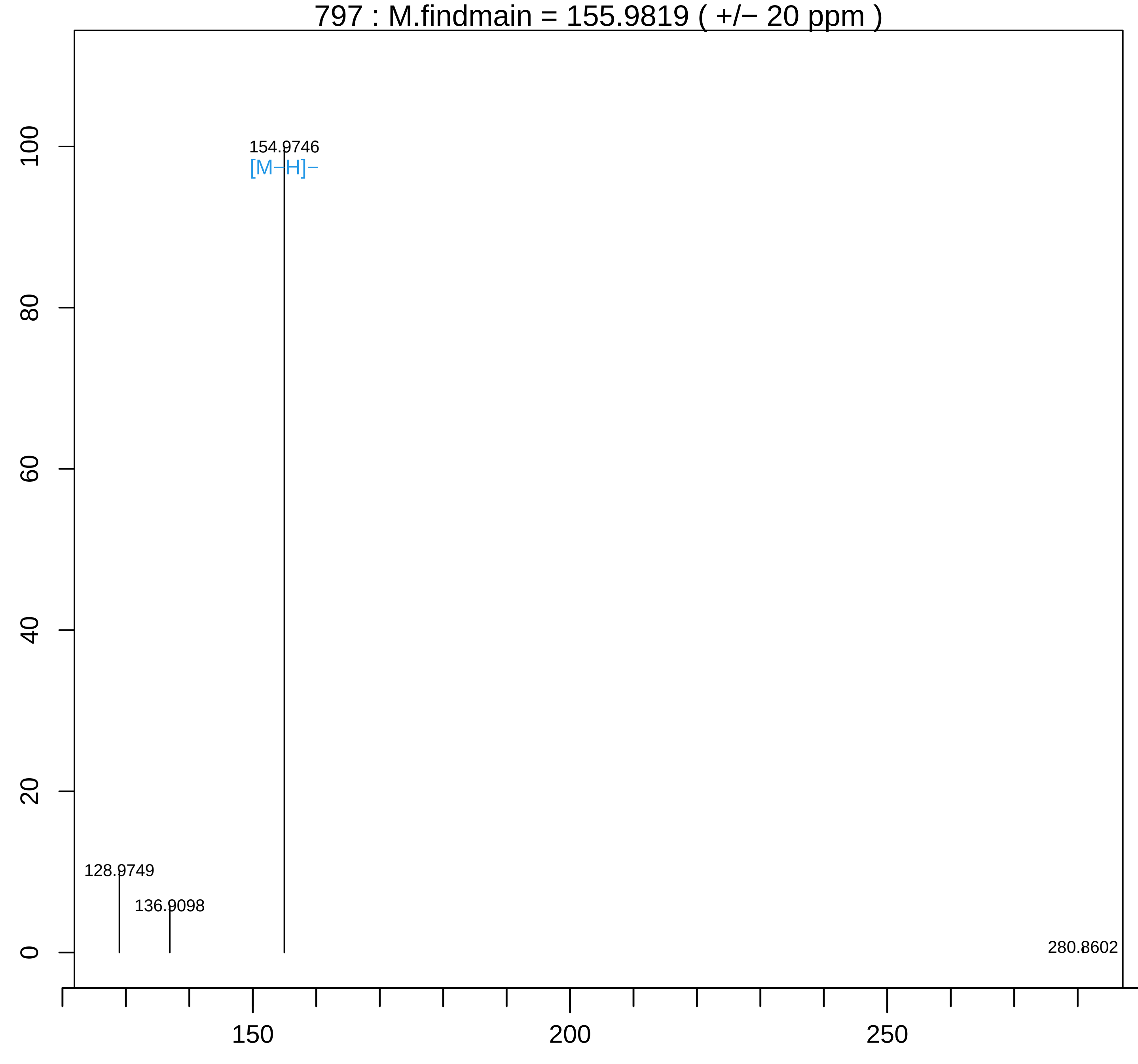
797 : M.findmain = 155.9819 (+/− 20 ppm)
154.9746
[M−H]−
128.9749
136.9098
280.8602
0
20
40
60
80
100
150
200
250

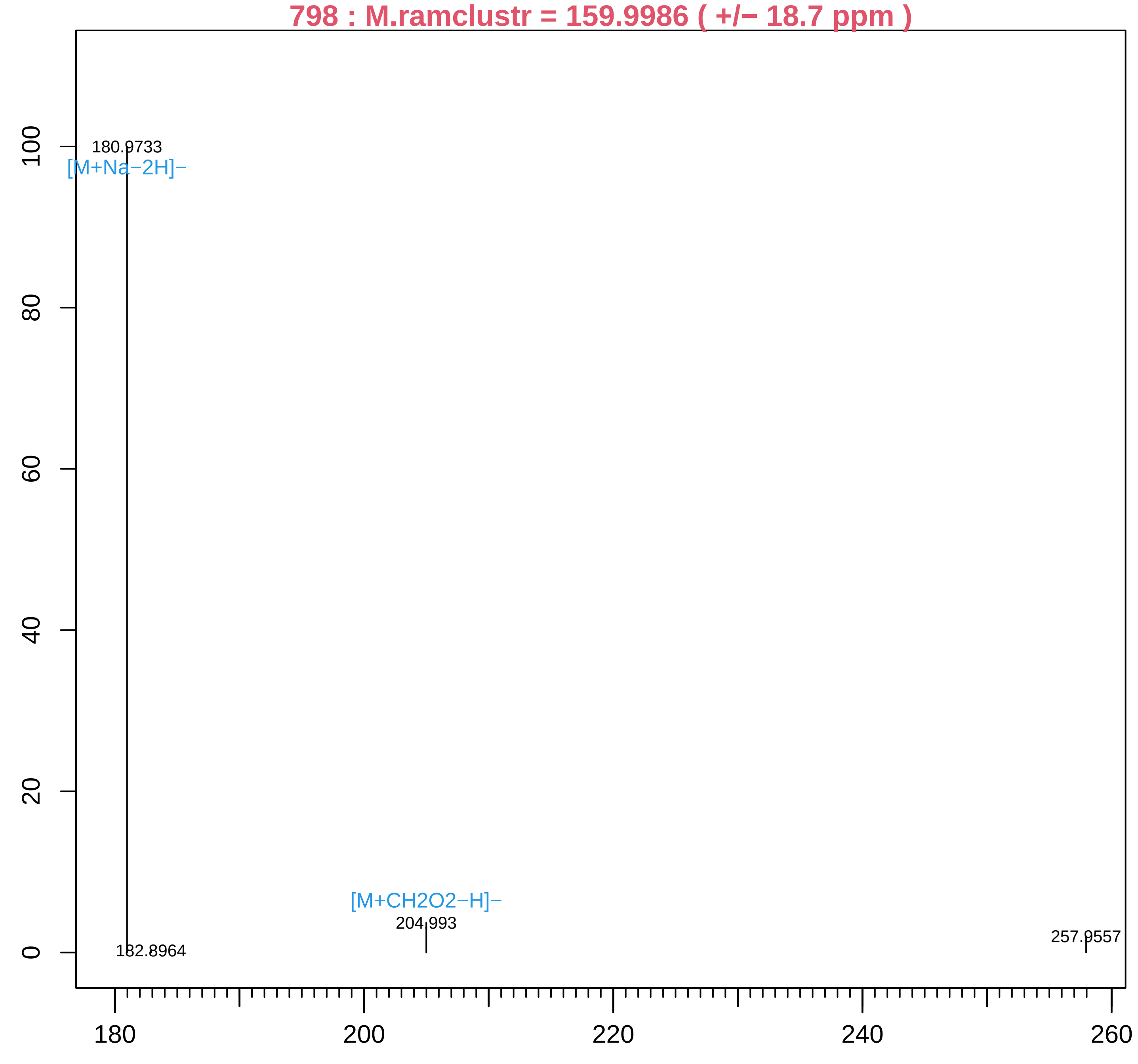
798 : M.ramclustr = 159.9986 (+/− 18.7 ppm)
180.9733
[M+Na−2H]−
[M+CH2O2−H]−
204.993
257.9557
182.8964
0
20
40
60
80
100
180
200
220
240
260

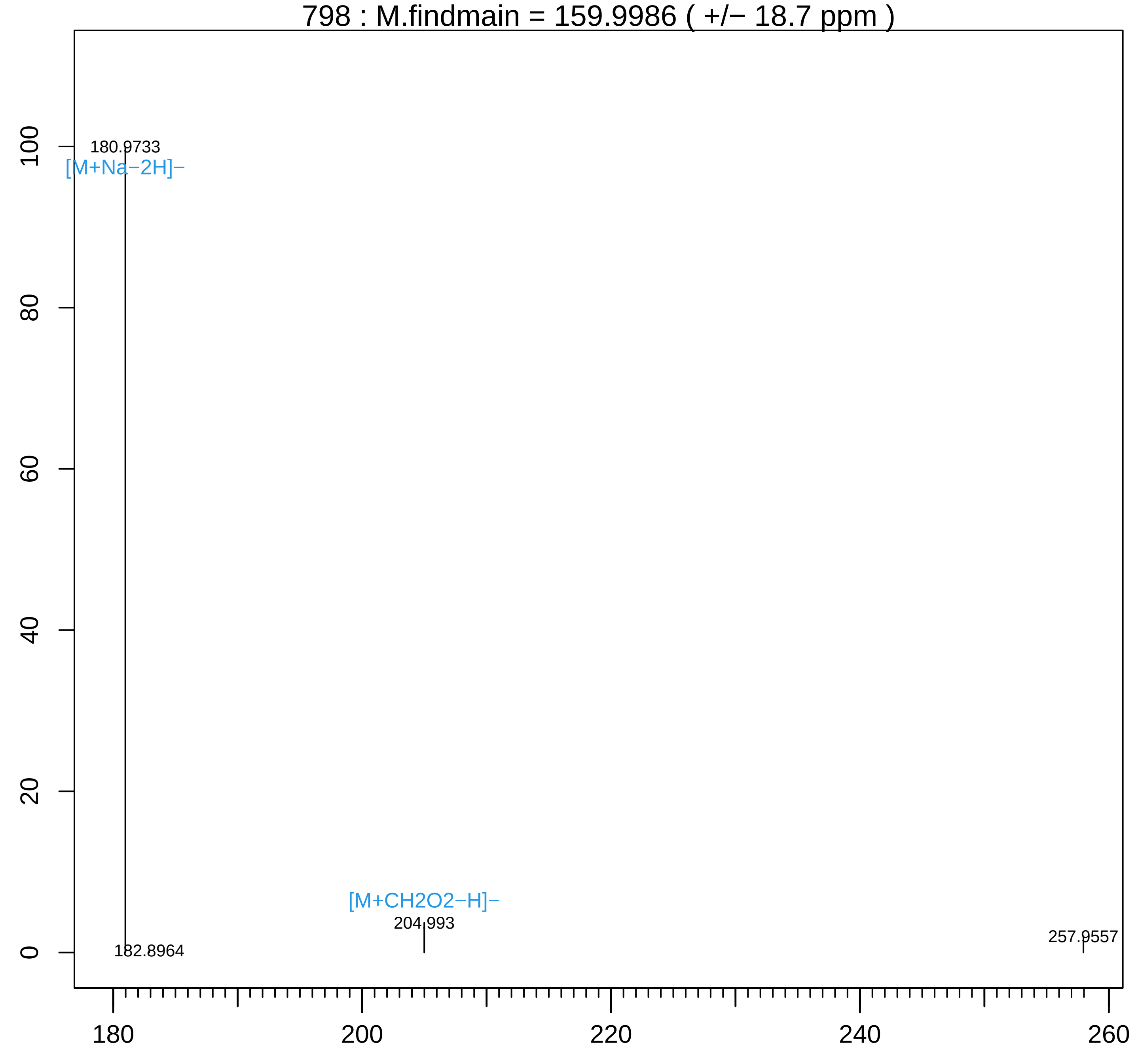
798 : M.findmain = 159.9986 (+/− 18.7 ppm)
180.9733
[M+Na−2H]−
[M+CH2O2−H]−
204.993
257.9557
182.8964
0
20
40
60
80
100
180
200
220
240
260

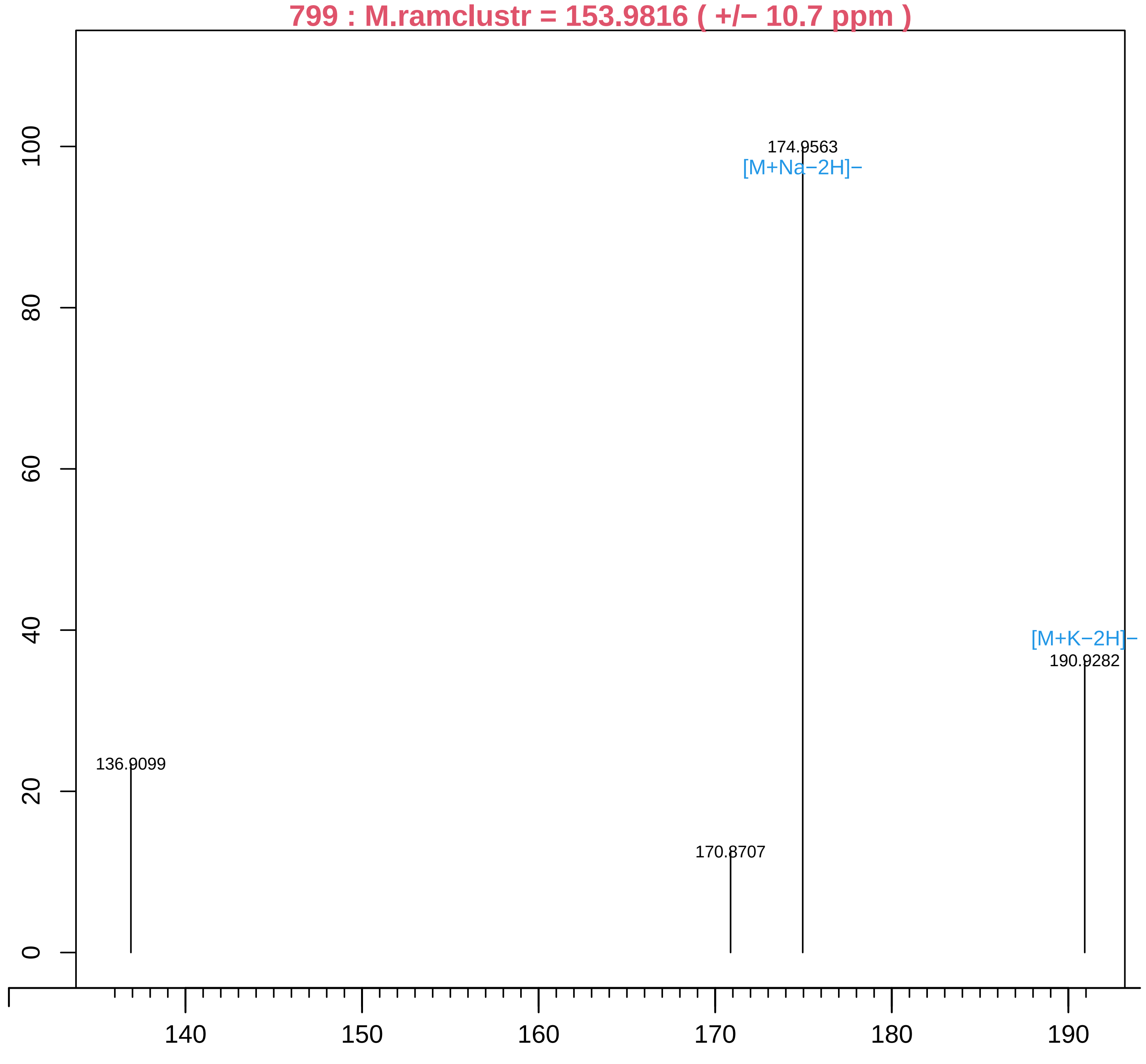
799 : M.ramclustr = 153.9816 (+/− 10.7 ppm)
174.9563
[M+Na−2H]−
[M+K−2H]−
190.9282
136.9099
170.8707
0
20
40
60
80
100
140
150
160
170
180
190

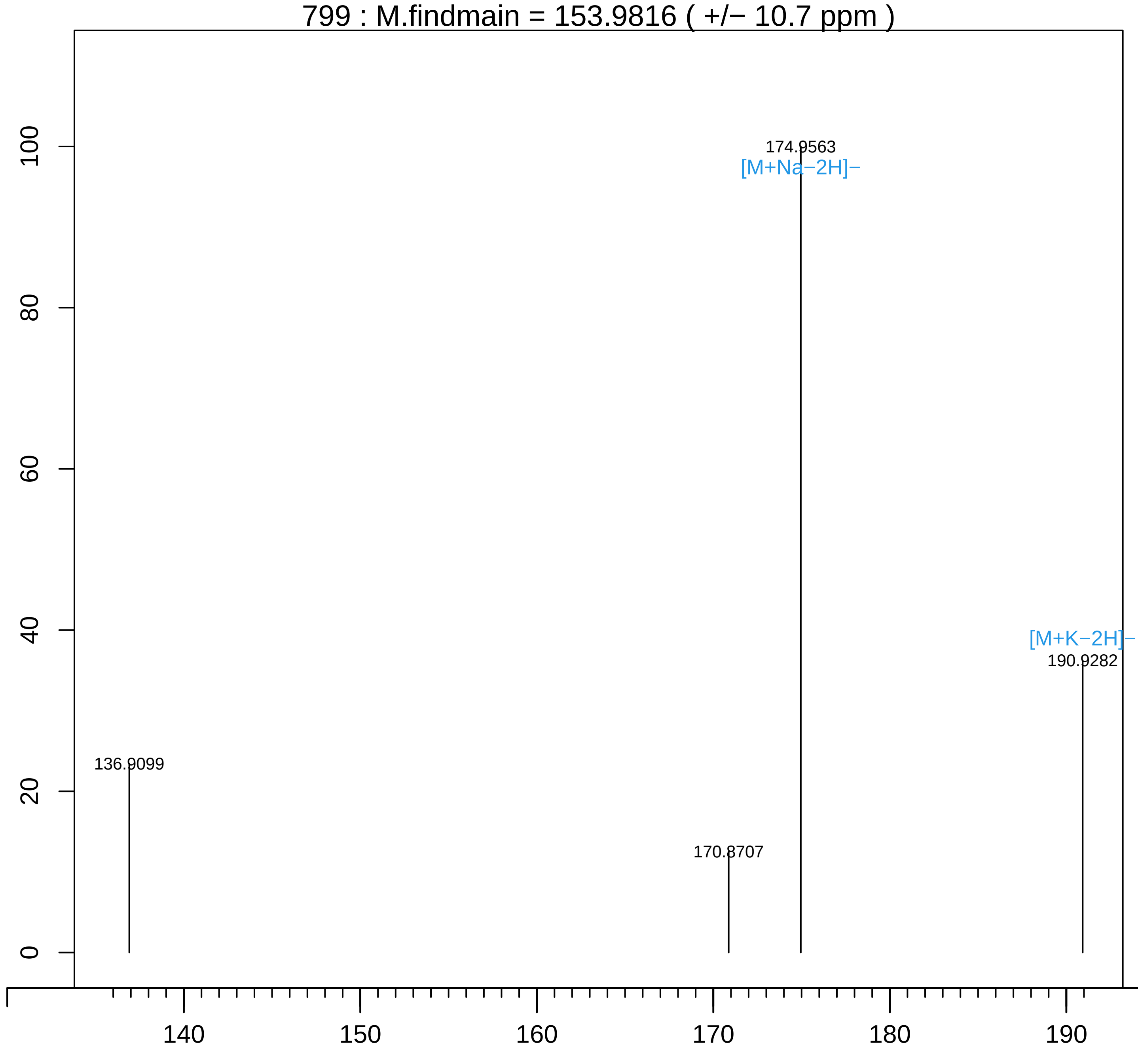
799 : M.findmain = 153.9816 (+/− 10.7 ppm)
174.9563
[M+Na−2H]−
[M+K−2H]−
190.9282
136.9099
170.8707
0
20
40
60
80
100
140
150
160
170
180
190

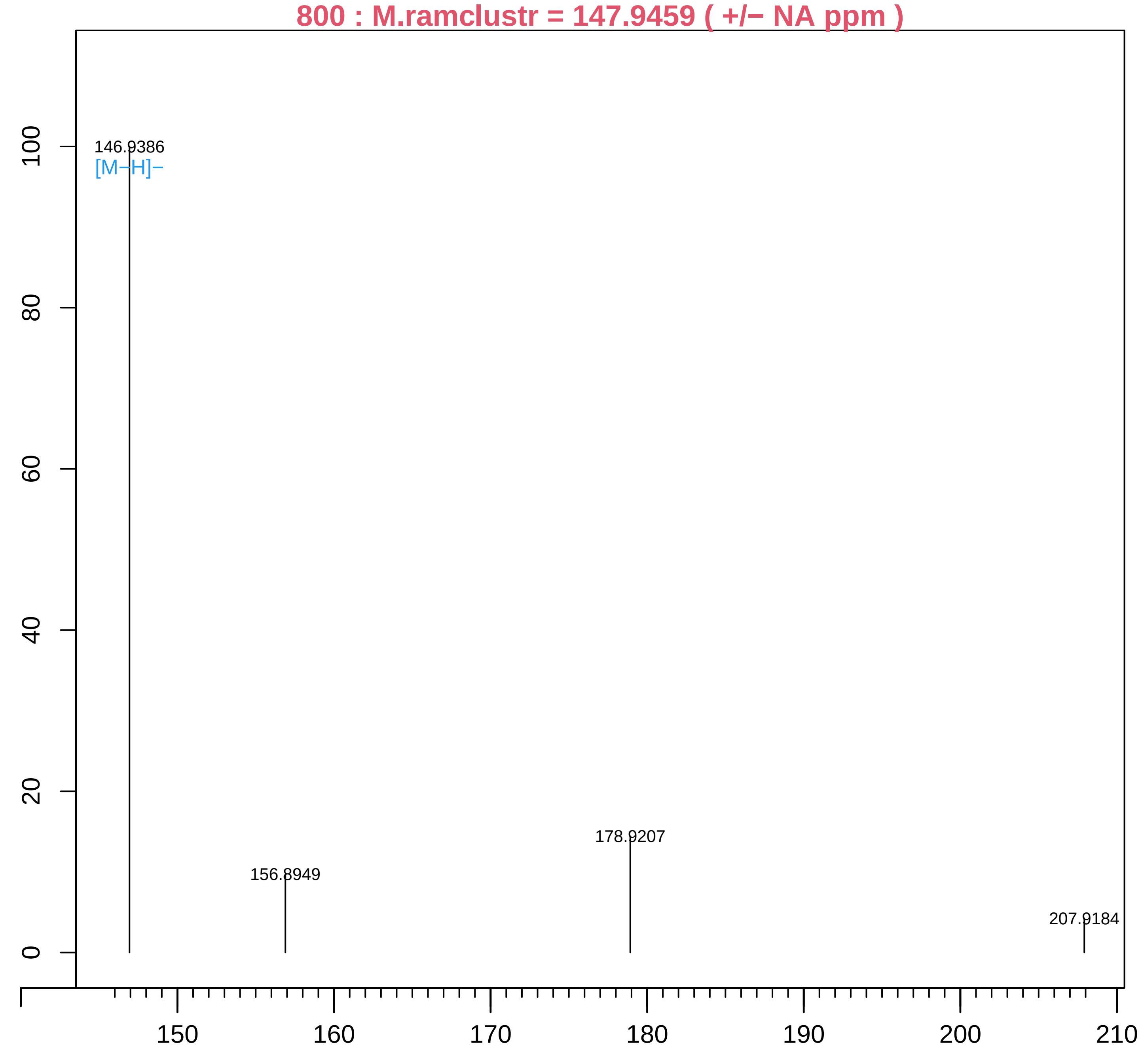
800 : M.ramclustr = 147.9459 (+/− NA ppm)
146.9386
[M−H]−
156.8949
178.9207
207.9184
0
20
40
60
80
100
150
160
170
180
190
200
210

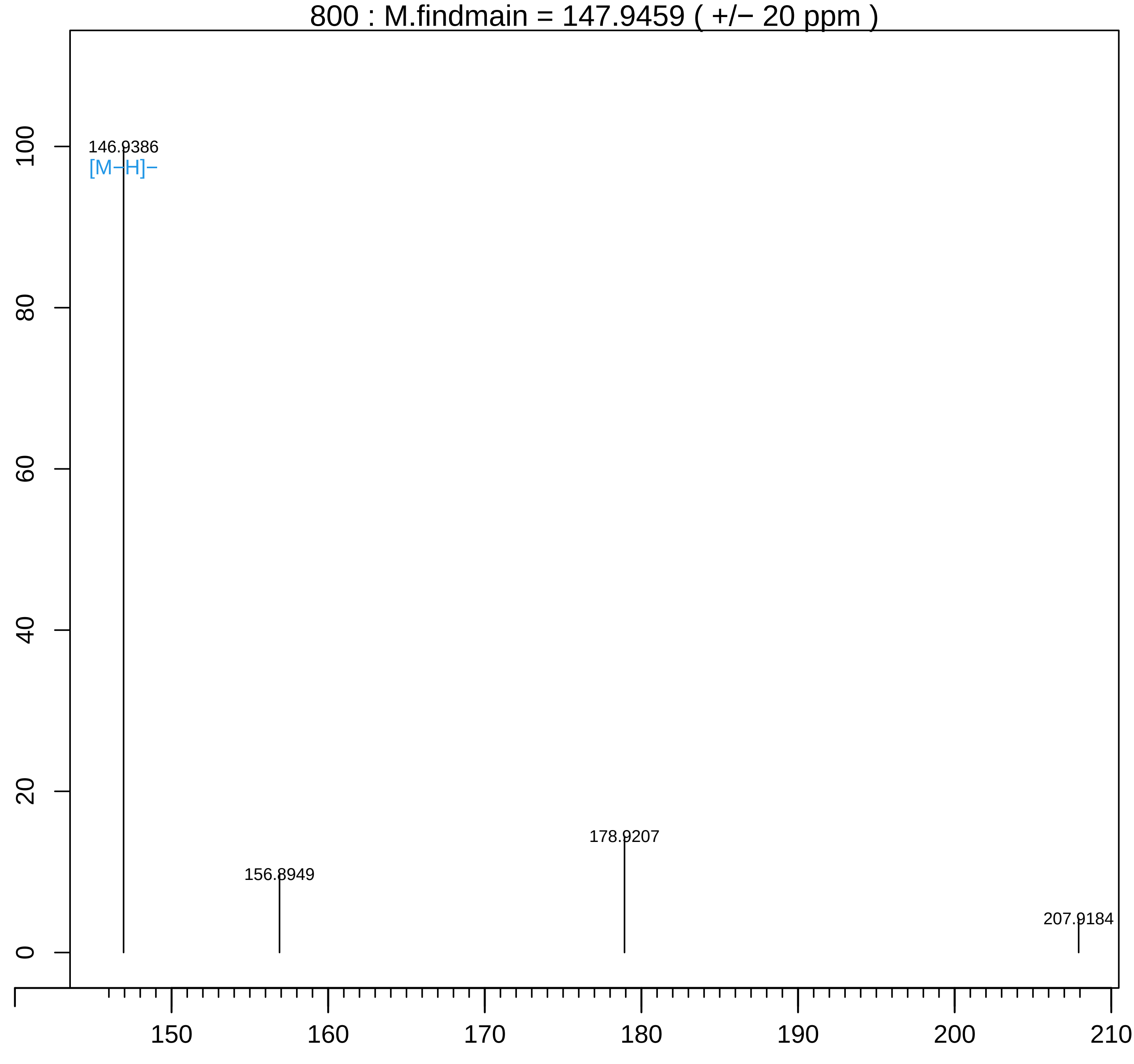
800 : M.findmain = 147.9459 (+/− 20 ppm)
146.9386
[M−H]−
156.8949
178.9207
207.9184
0
20
40
60
80
100
150
160
170
180
190
200
210

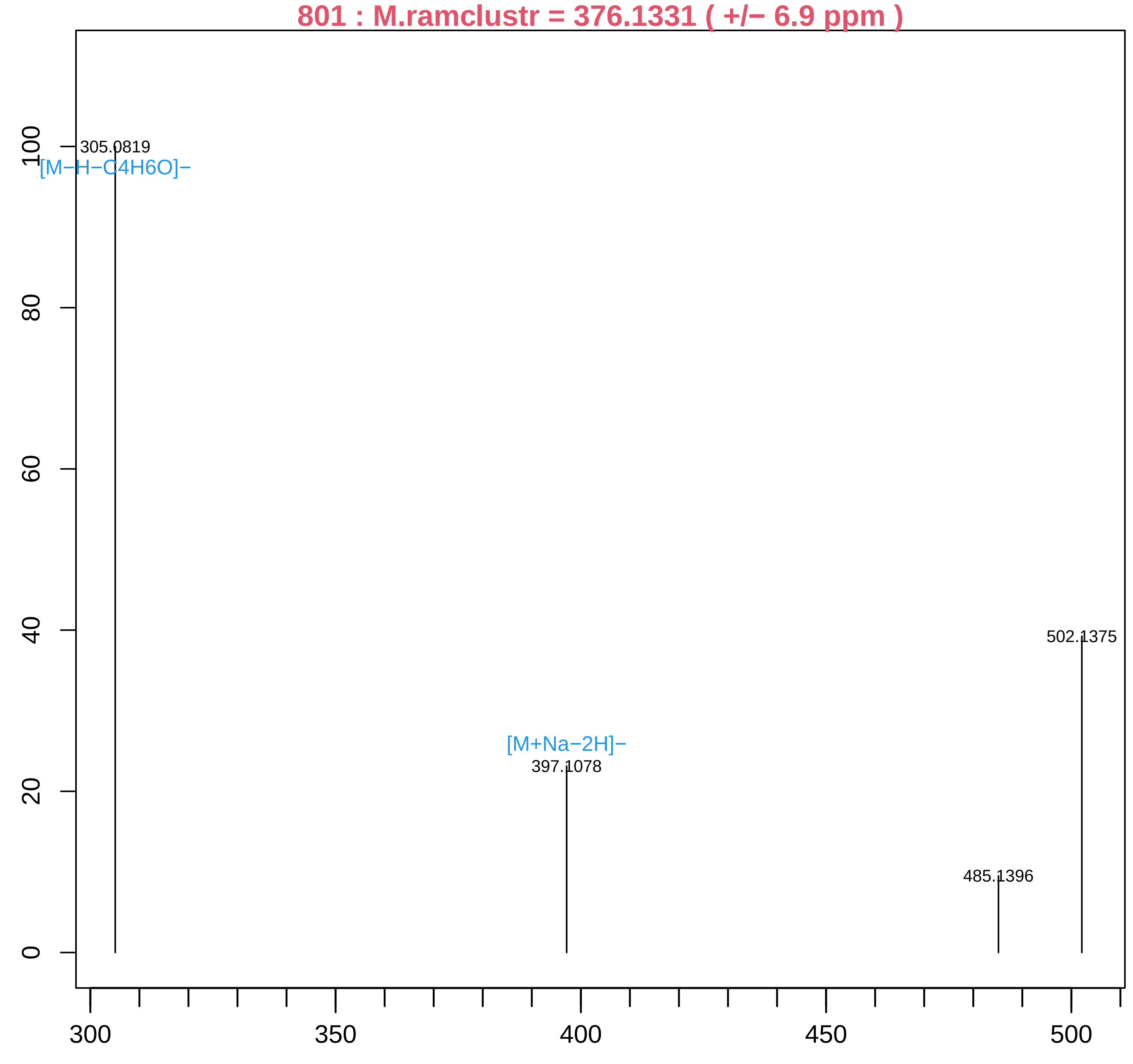

801 : M.ramclustr = 376.1331 (+/− 6.9 ppm)
305.0819
[M−H−C4H6O]−
[M+Na−2H]−
397.1078
485.1396
502.1375
0
20
40
60
80
100
300
350
400
450
500

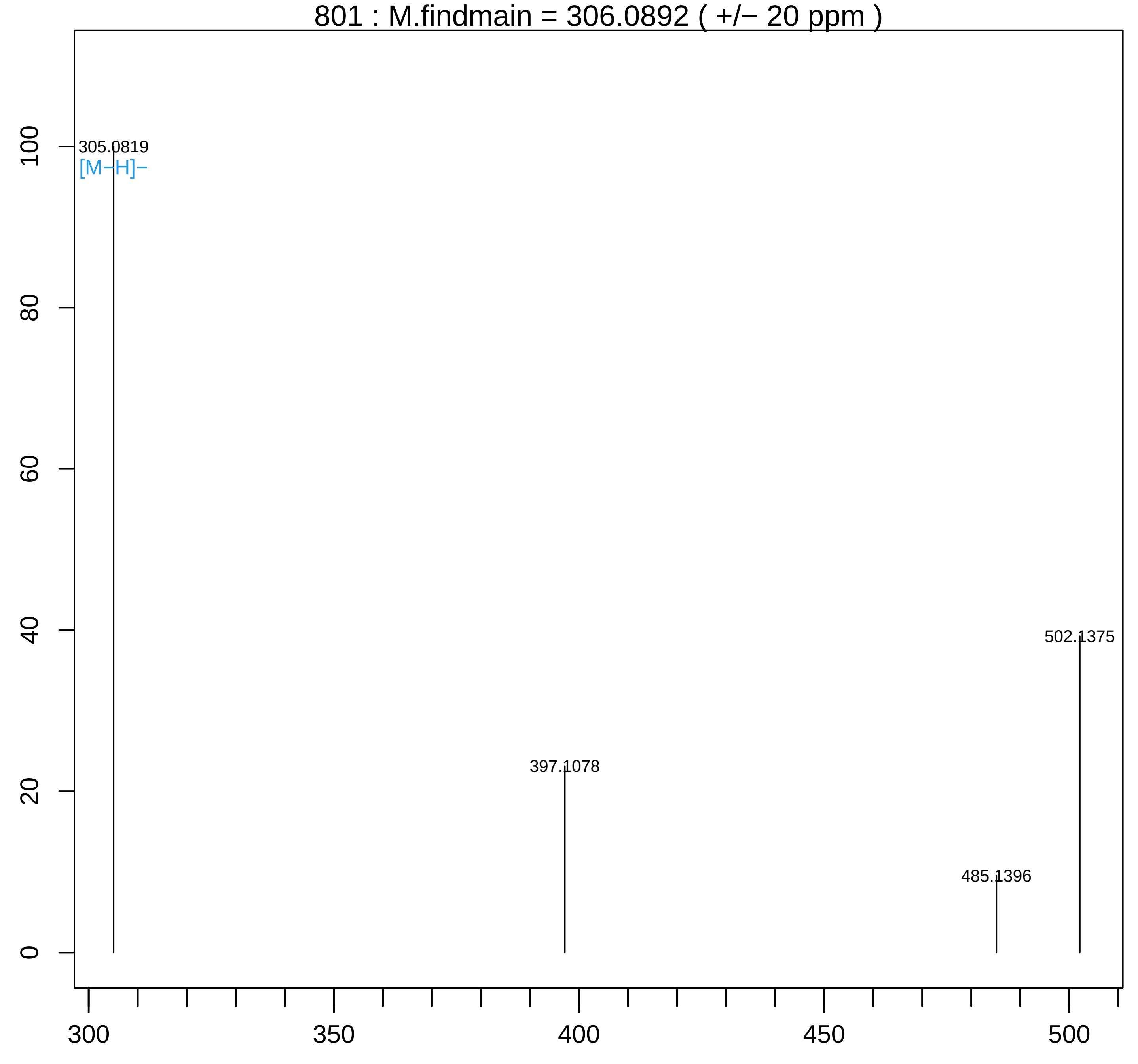

801 : M.findmain = 306.0892 (+/− 20 ppm)
305.0819
[M−H]−
397.1078
485.1396
502.1375
0
20
40
60
80
100
300
350
400
450
500

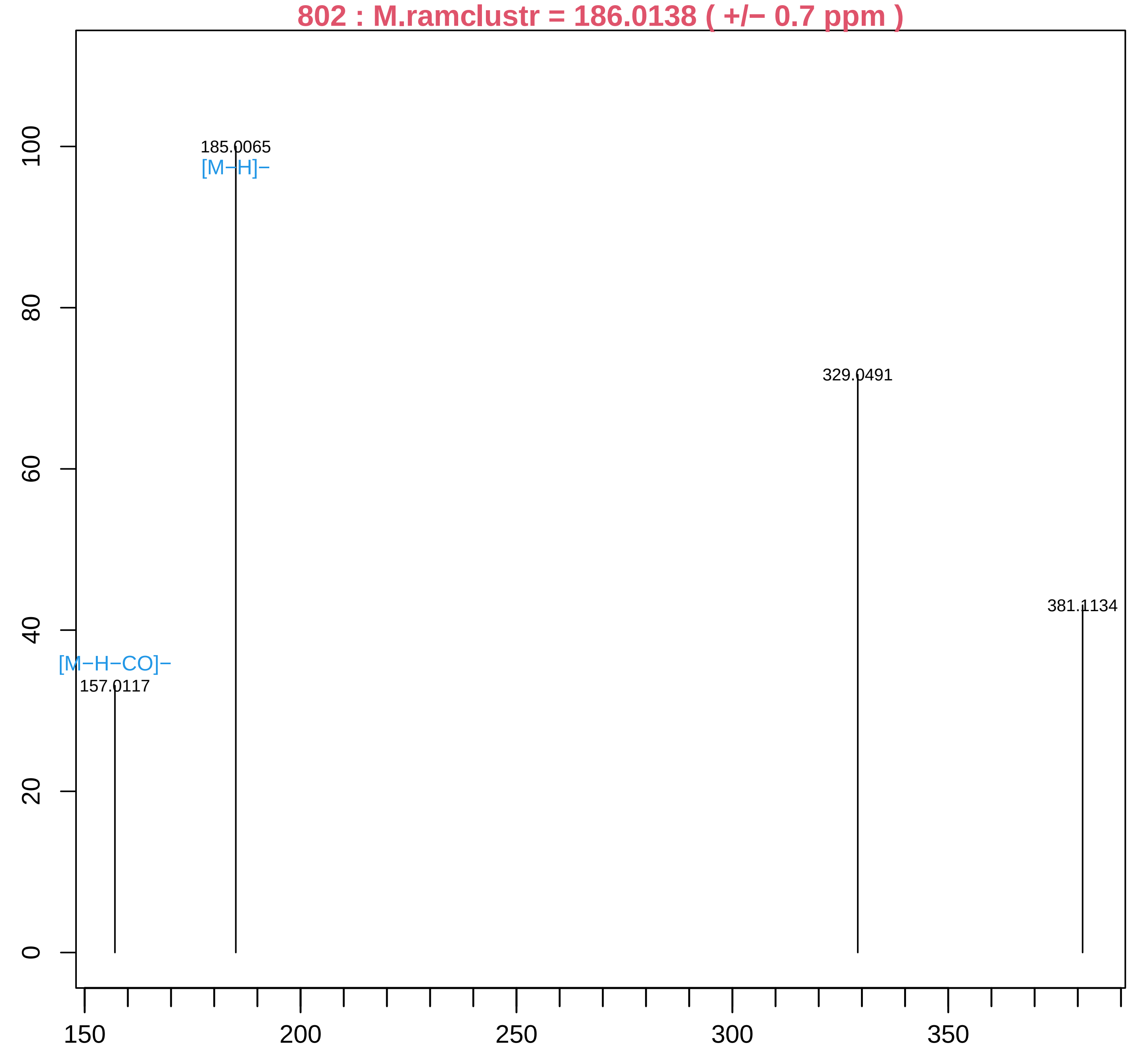
802 : M.ramclustr = 186.0138 (+/− 0.7 ppm)
185.0065
[M−H]−
329.0491
381.1134
[M−H−CO]−
157.0117
0
20
40
60
80
100
150
200
250
300
350

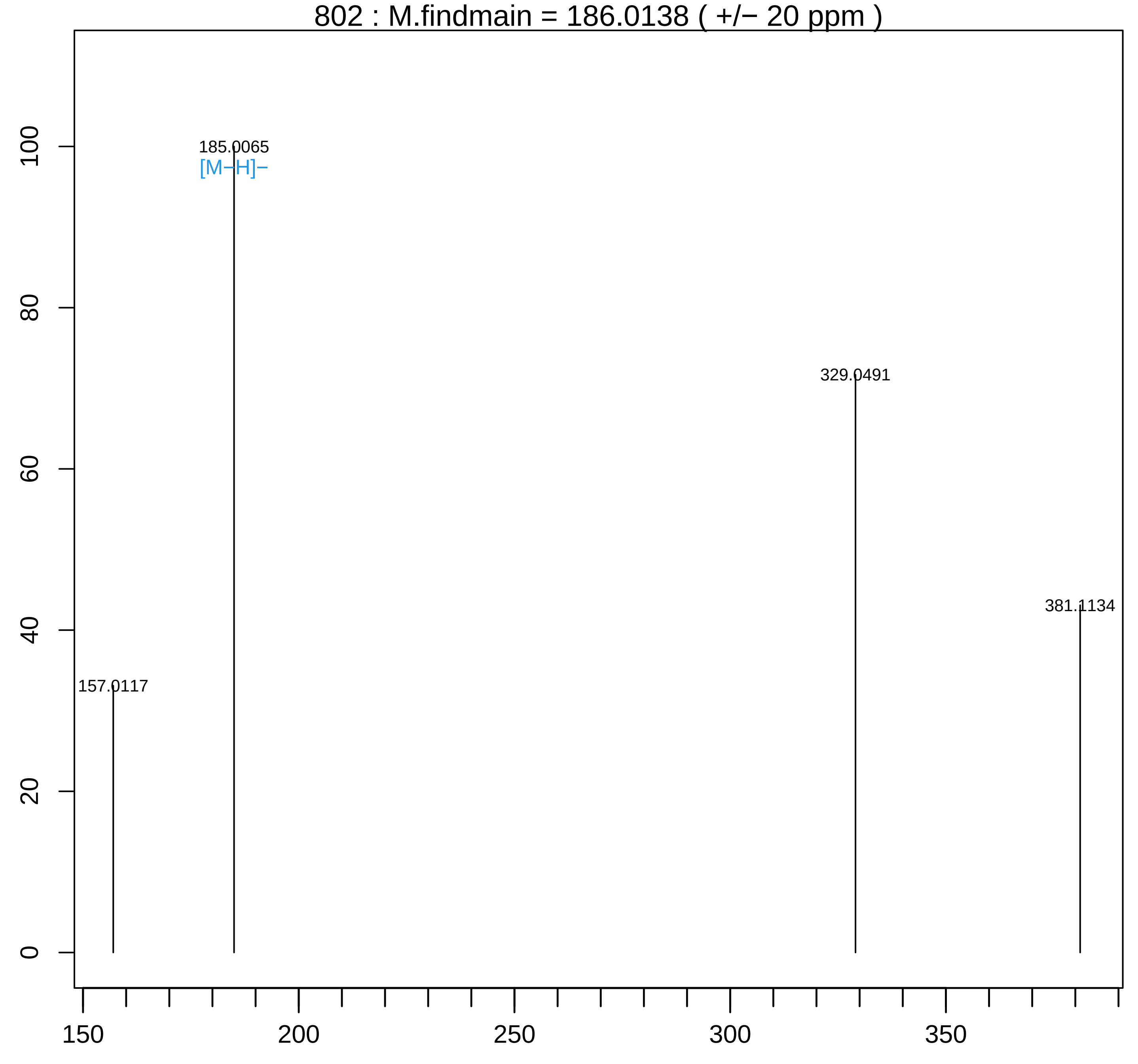
802 : M.findmain = 186.0138 (+/− 20 ppm)
185.0065
[M−H]−
329.0491
381.1134
157.0117
0
20
40
60
80
100
150
200
250
300
350

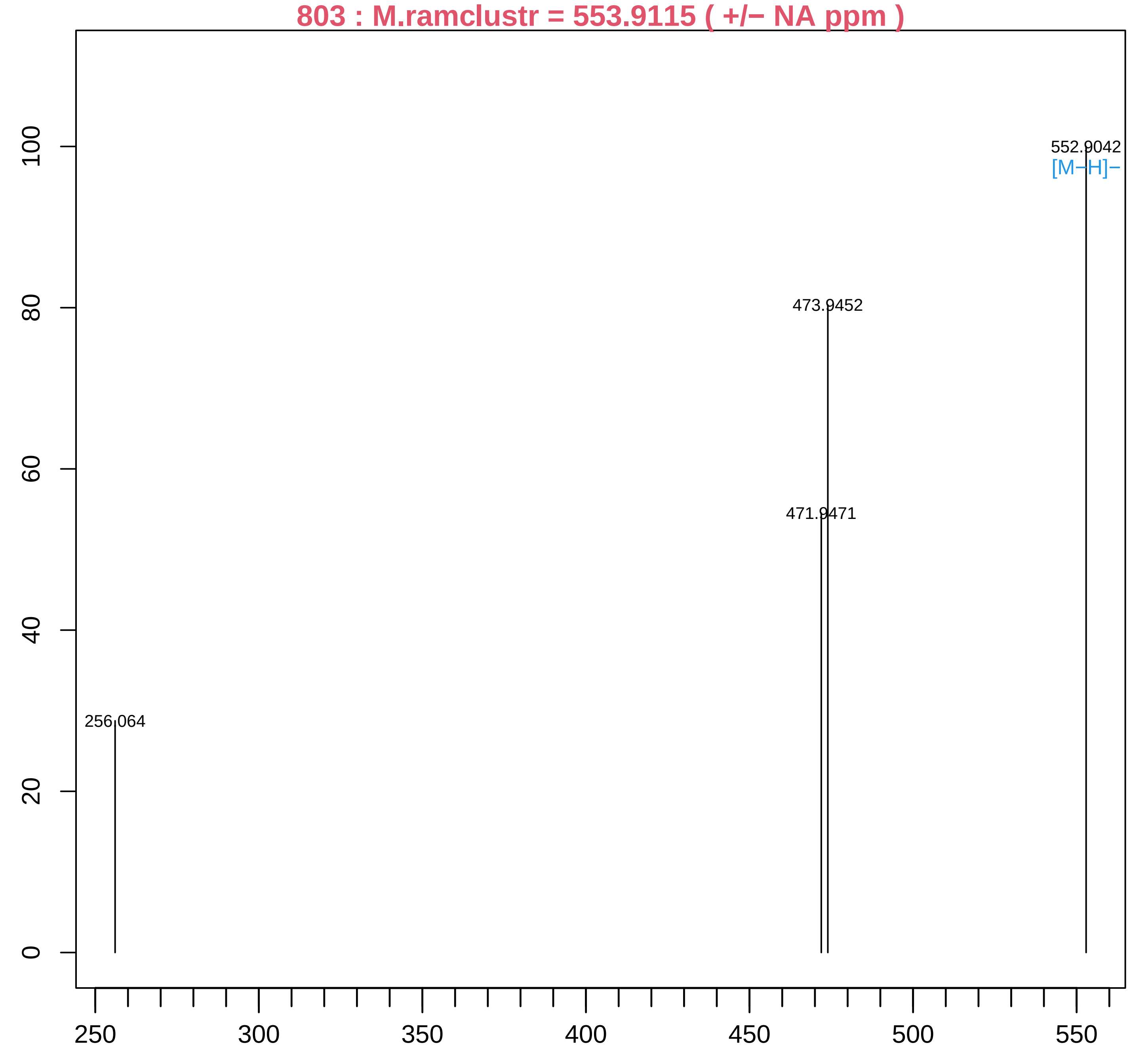

803 : M.ramclustr = 553.9115 (+/− NA ppm)
552.9042
[M−H]−
473.9452
471.9471
256.064
0
20
40
60
80
100
250
300
350
400
450
500
550

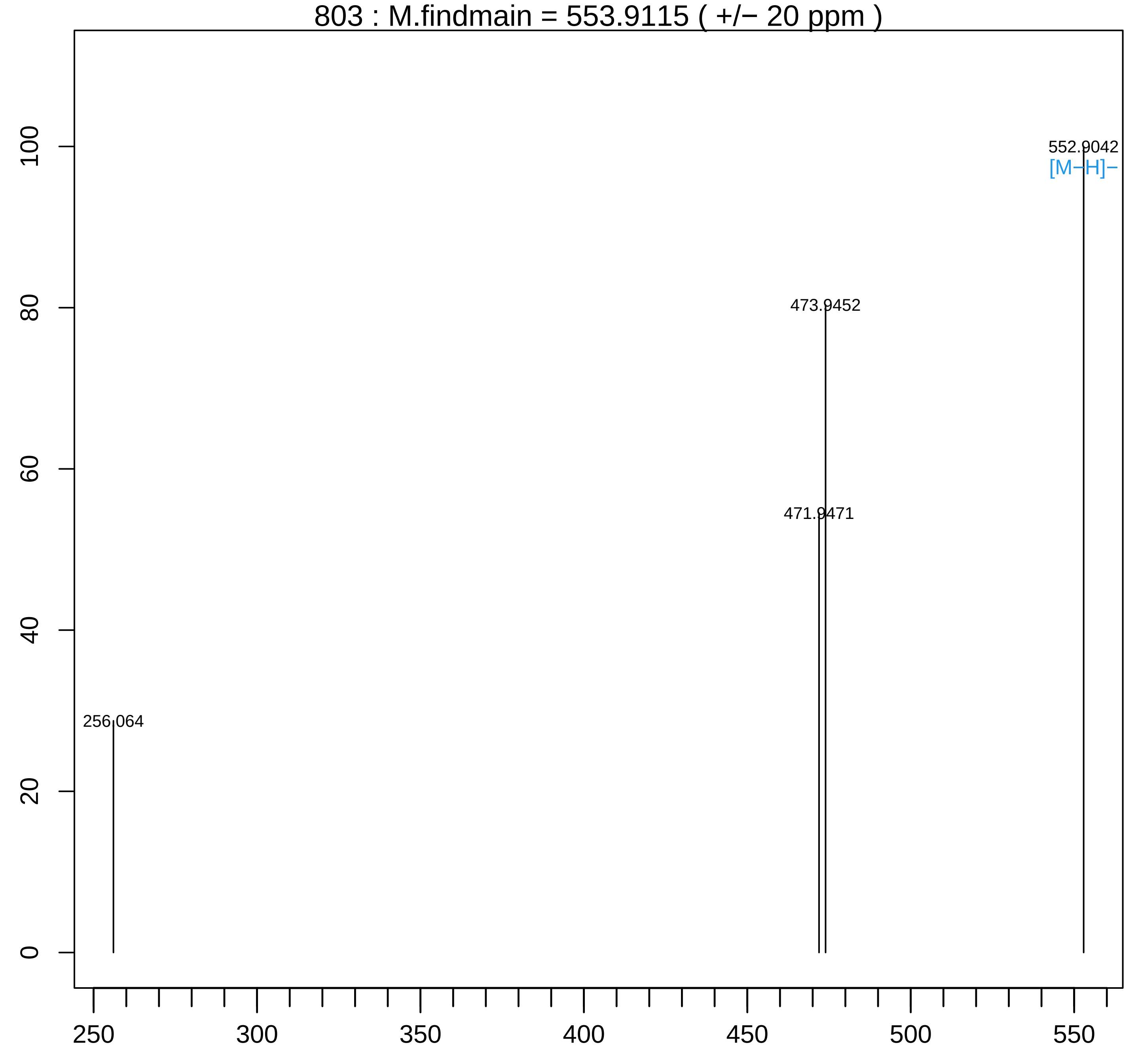

803 : M.findmain = 553.9115 (+/− 20 ppm)
552.9042
[M−H]−
473.9452
471.9471
256.064
0
20
40
60
80
100
250
300
350
400
450
500
550

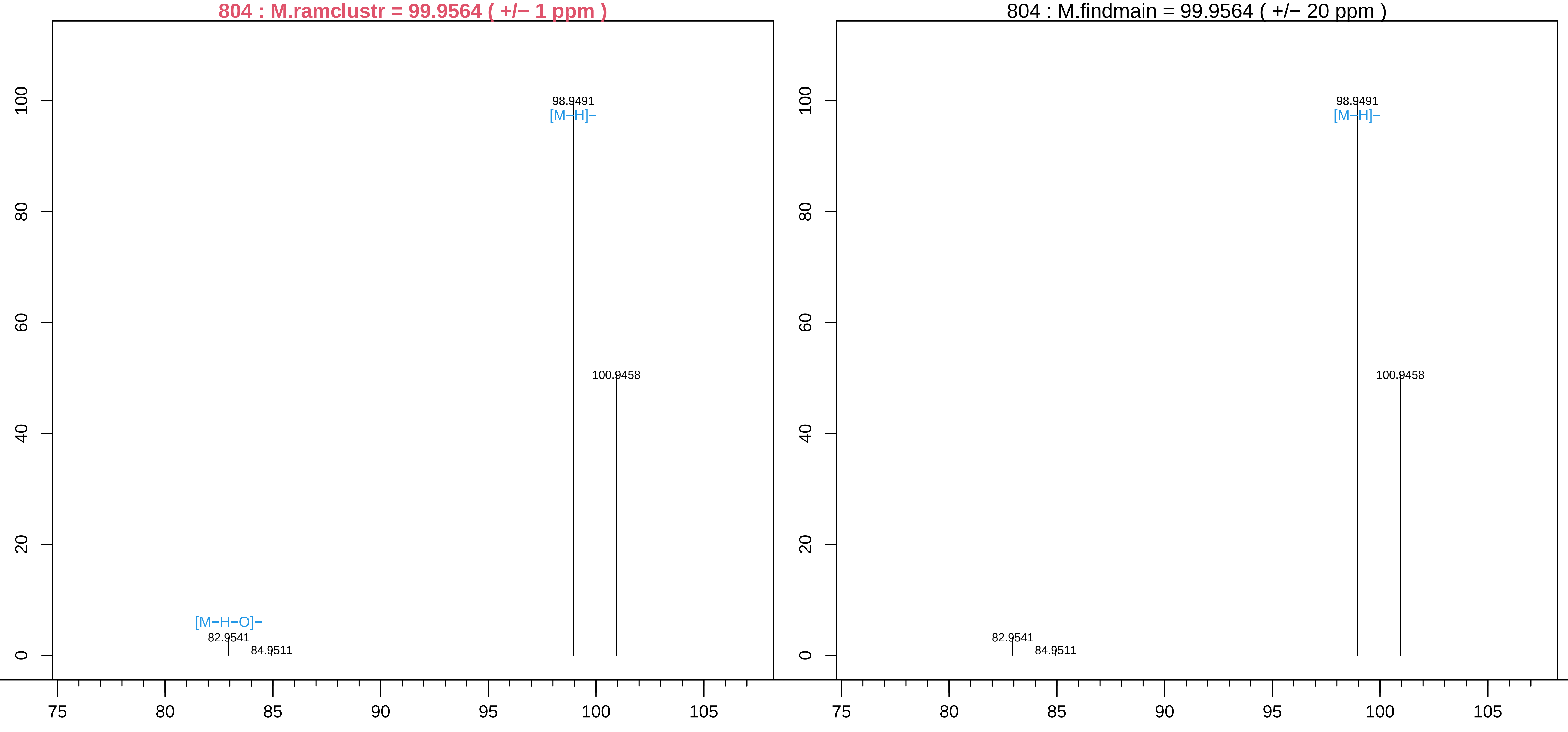

804 : M.ramclustr = 99.9564 (+/− 1 ppm)
98.9491
[M−H]−
100.9458
[M−H−O]−
82.9541
84.9511
804 : M.findmain = 99.9564 (+/− 20 ppm)
98.9491
[M−H]−
100.9458
82.9541
84.9511

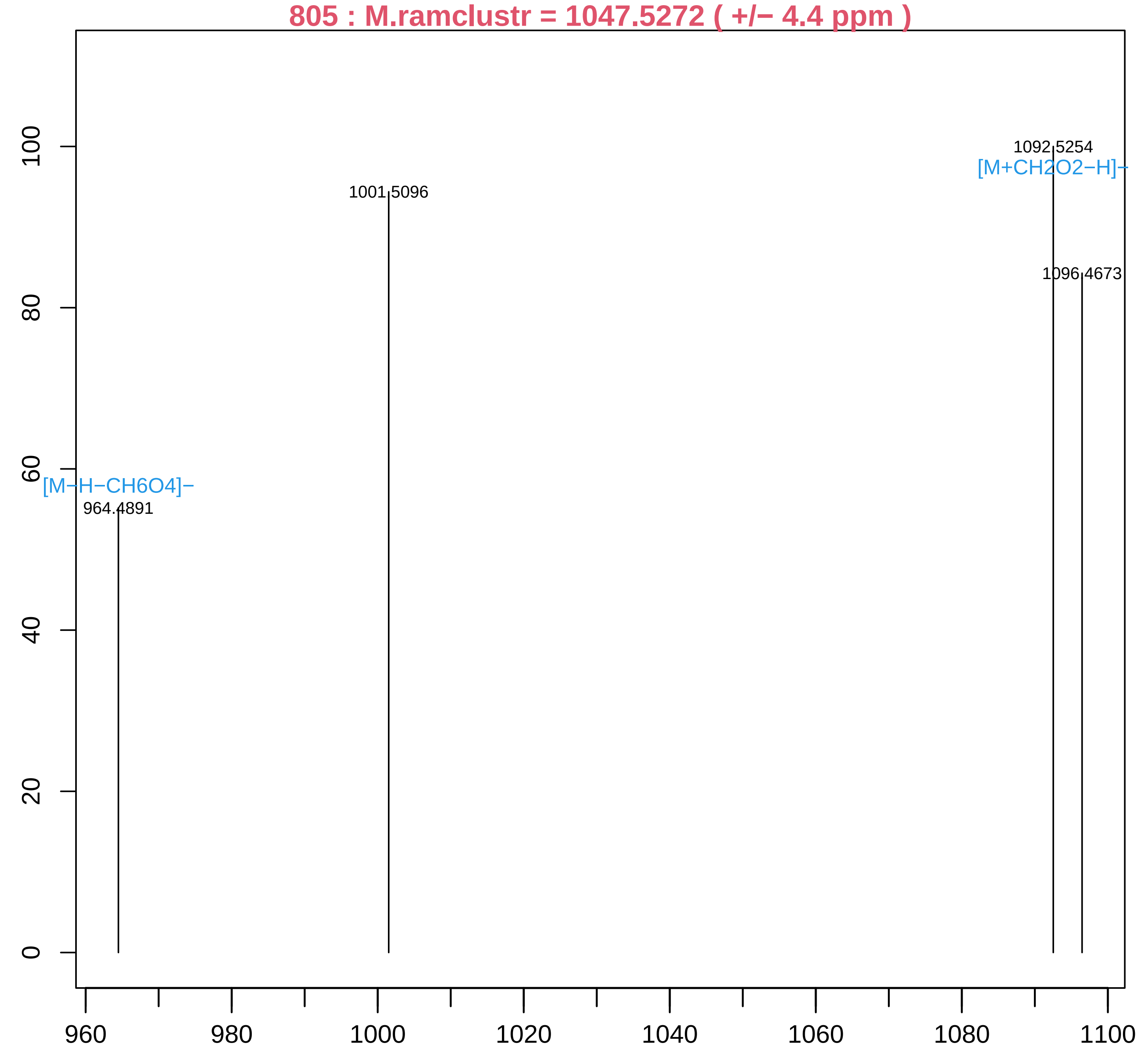
805 : M.ramclustr = 1047.5272 (+/− 4.4 ppm)
1092.5254
[M+CH2O2−H]−
1001.5096
1096.4673
[M−H−CH6O4]−
964.4891
0
20
40
60
80
100
960
980
1000
1020
1040
1060
1080
1100

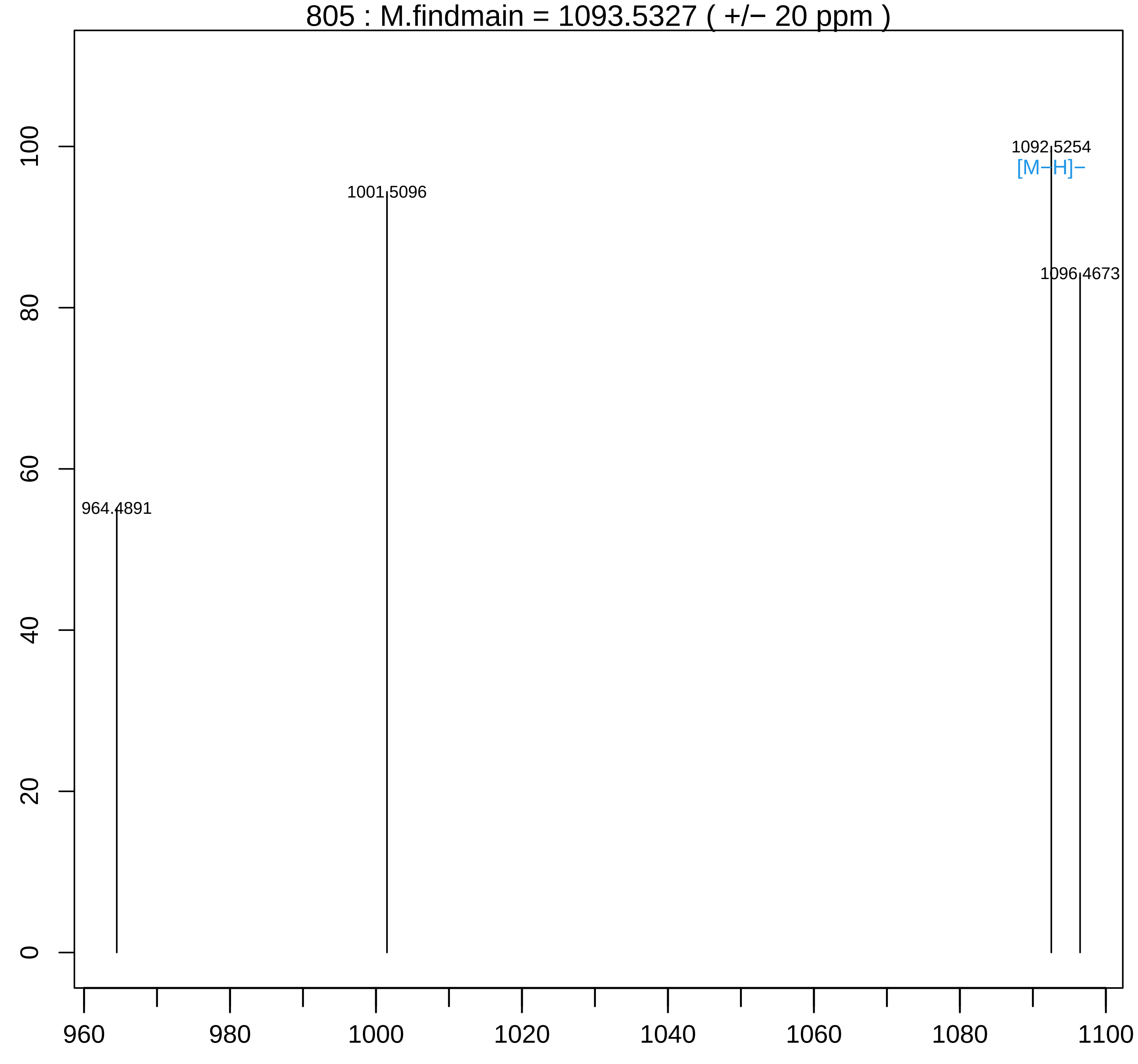
805 : M.findmain = 1093.5327 (+/− 20 ppm)
1092.5254
[M−H]−
1001.5096
1096.4673
964.4891
0
20
40
60
80
100
960
980
1000
1020
1040
1060
1080
1100

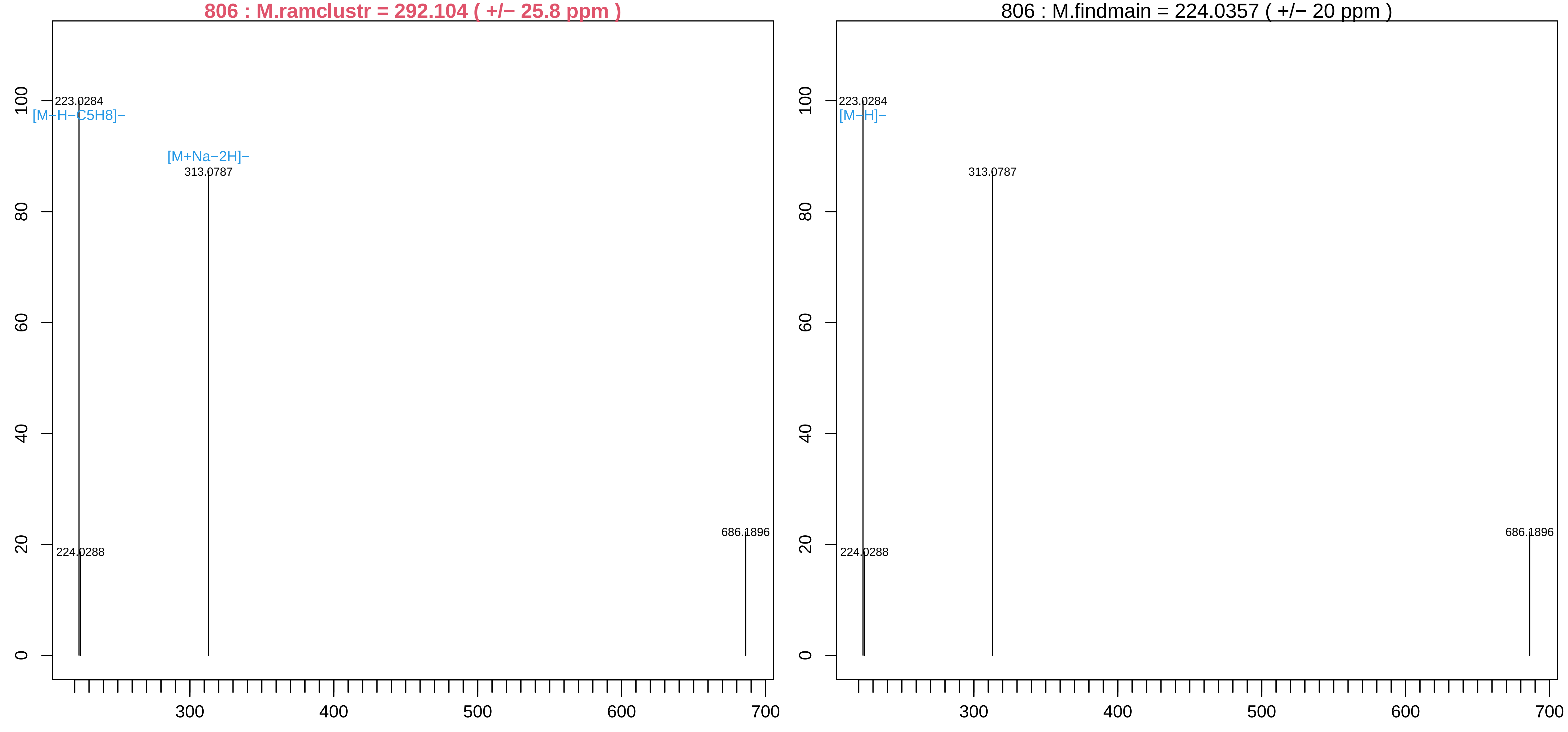
806 : M.ramclustr = 292.104 (+/− 25.8 ppm)
223.0284
[M−H−C5H8]−
[M+Na−2H]−
313.0787
224.0288
686.1896
806 : M.findmain = 224.0357 (+/− 20 ppm)
223.0284
[M−H]−
313.0787
224.0288
686.1896

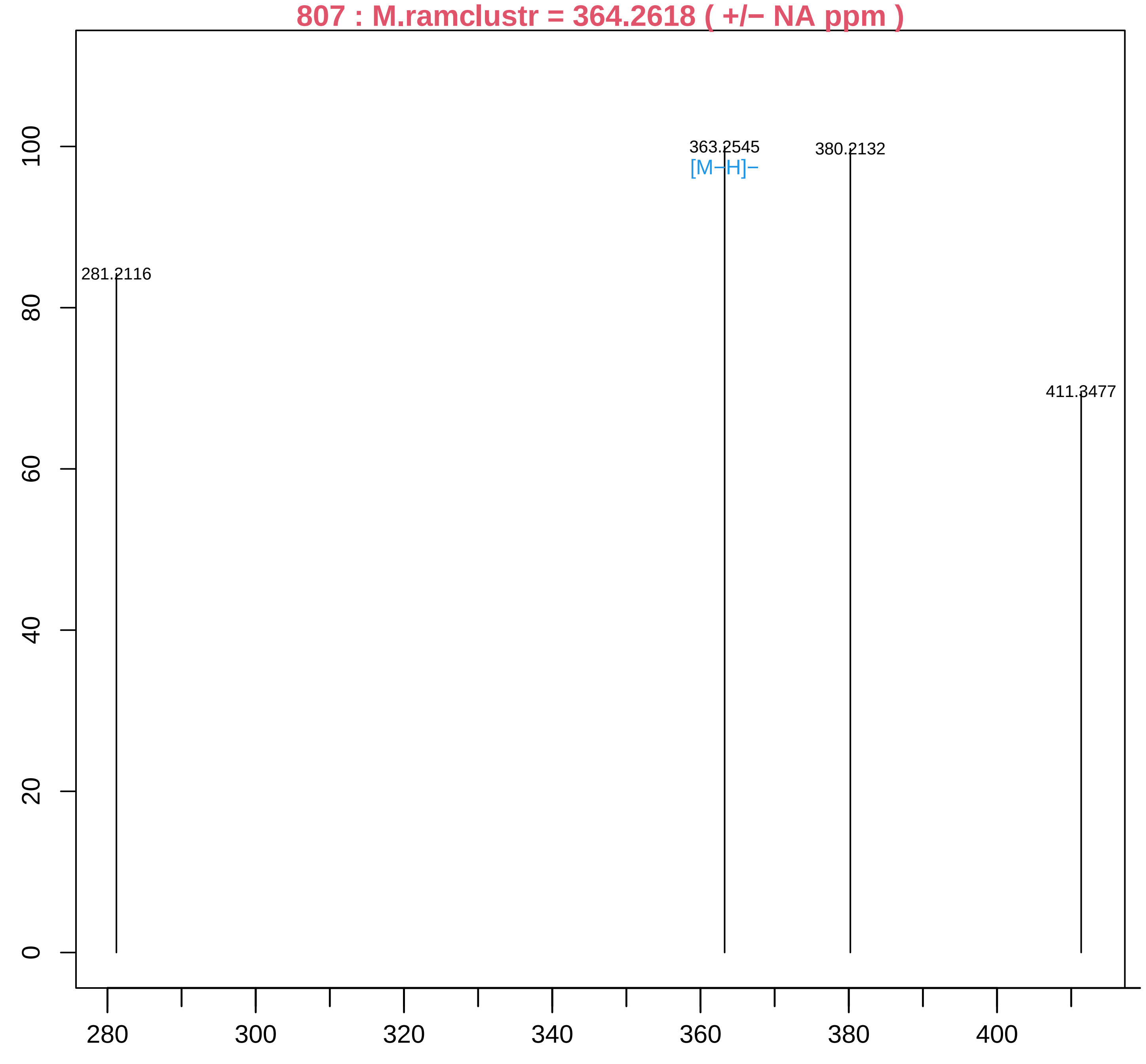
807 : M.ramclustr = 364.2618 (+/− NA ppm)
281.2116
363.2545
[M−H]−
380.2132
411.3477
0
20
40
60
80
100
280
300
320
340
360
380
400

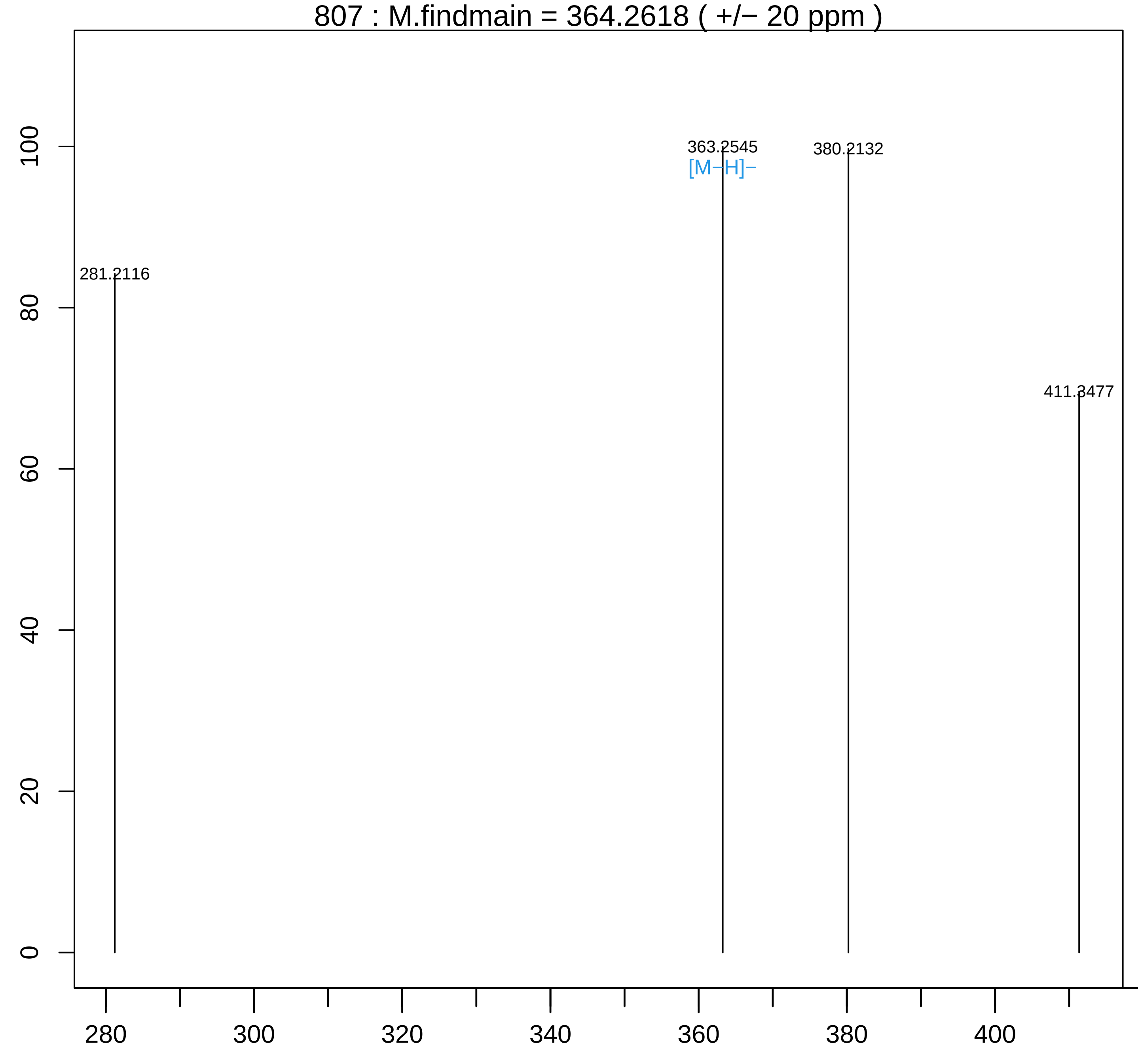
807 : M.findmain = 364.2618 (+/− 20 ppm)
281.2116
363.2545
[M−H]−
380.2132
411.3477
0
20
40
60
80
100
280
300
320
340
360
380
400

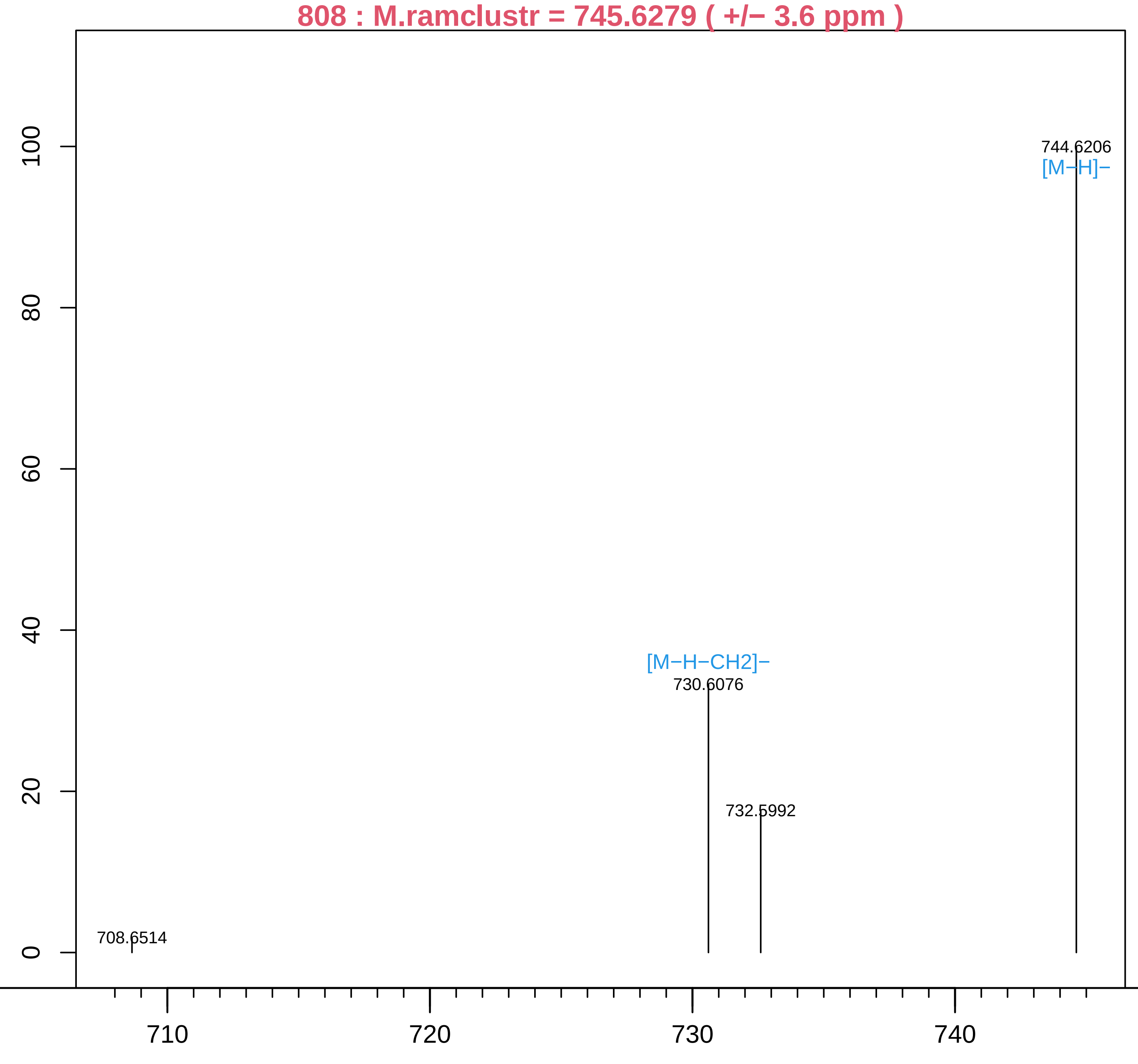

808 : M.ramclustr = 745.6279 (+/− 3.6 ppm)
744.6206
[M−H]−
[M−H−CH2]−
730.6076
732.5992
708.6514
0
20
40
60
80
100
710
720
730
740

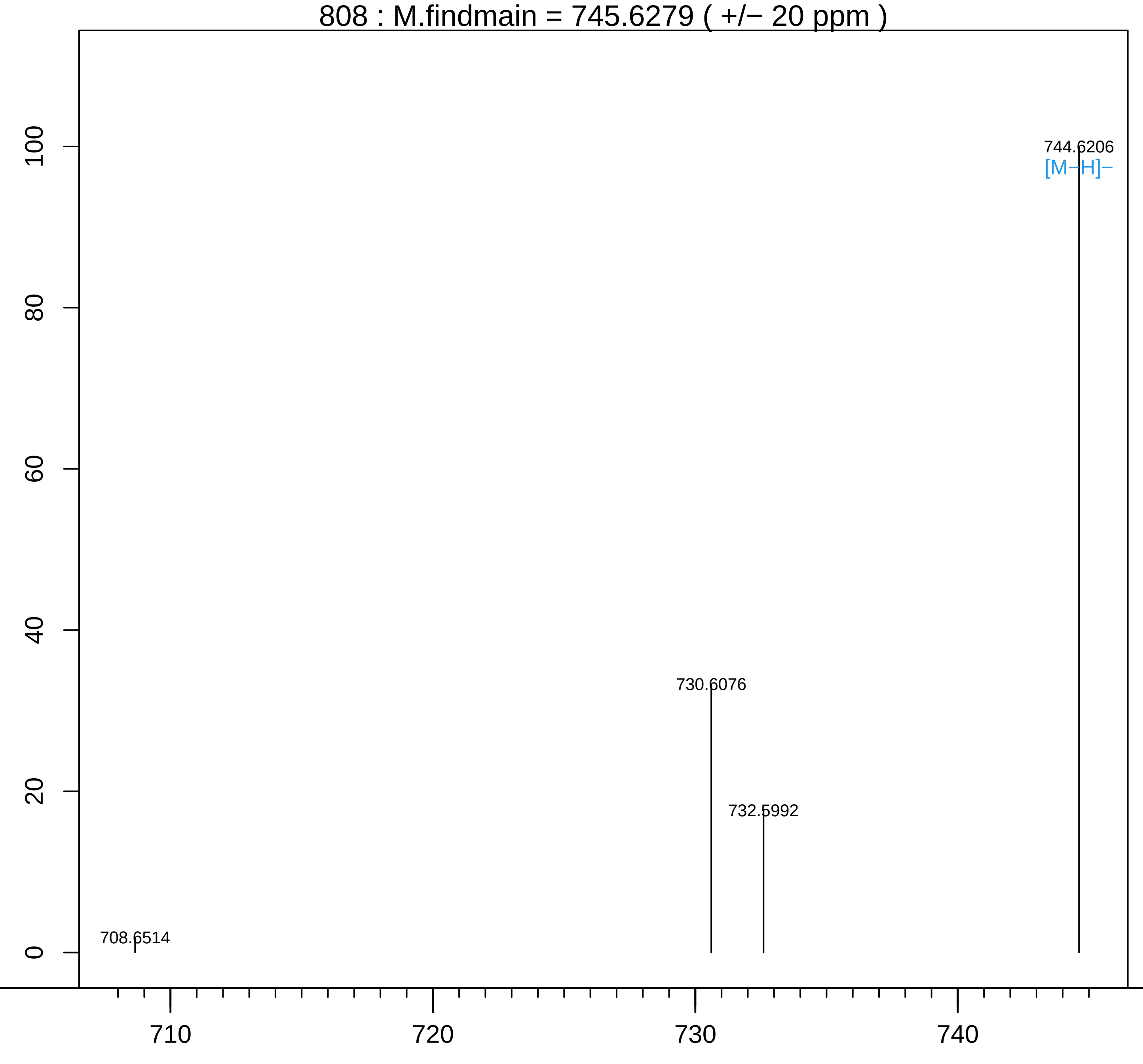

808 : M.findmain = 745.6279 (+/− 20 ppm)
744.6206
[M−H]−
730.6076
732.5992
708.6514
0
20
40
60
80
100
710
720
730
740

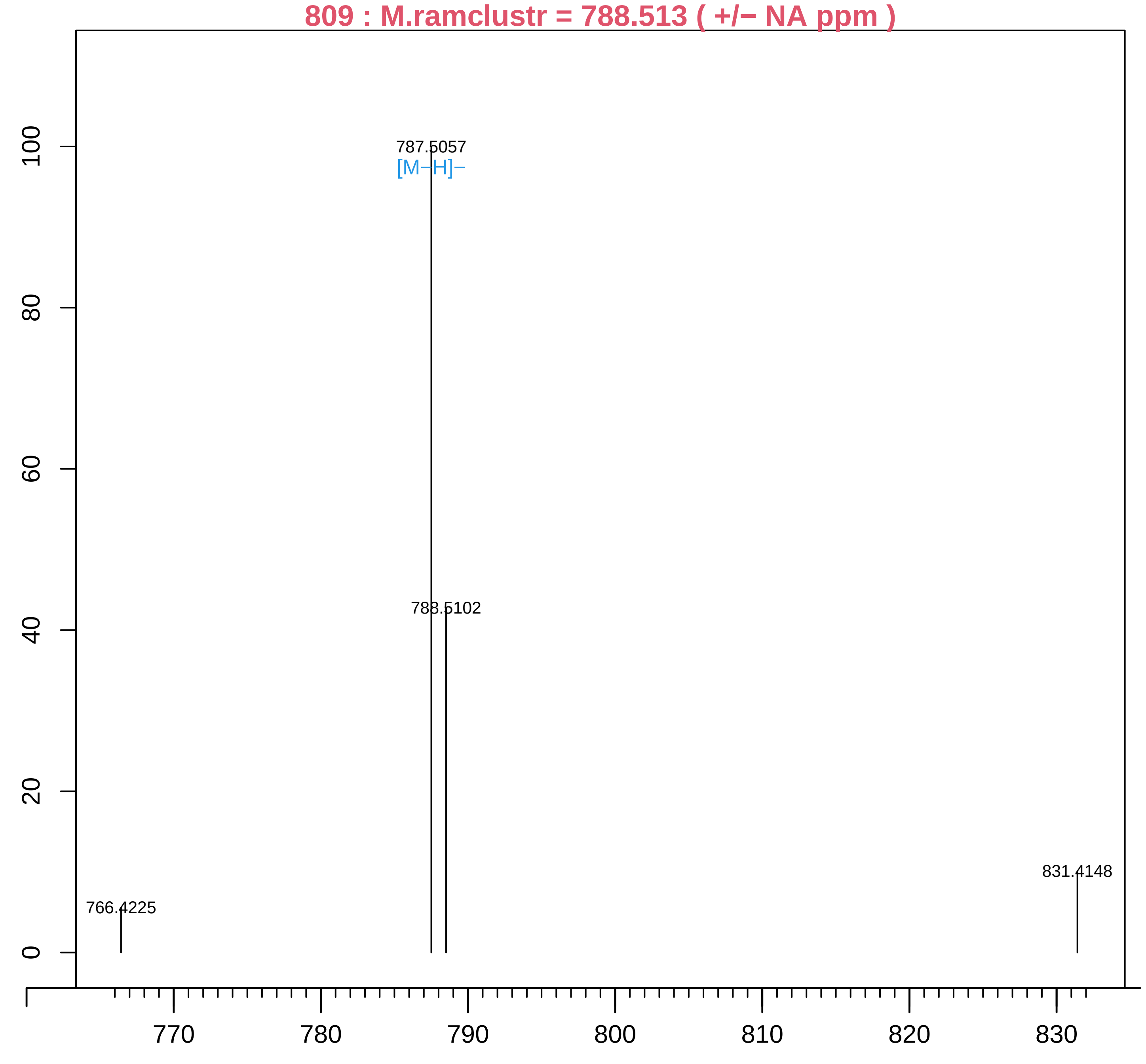
809 : M.ramclustr = 788.513 (+/− NA ppm)
787.5057
[M−H]−
788.5102
766.4225
831.4148
0
20
40
60
80
100
770
780
790
800
810
820
830

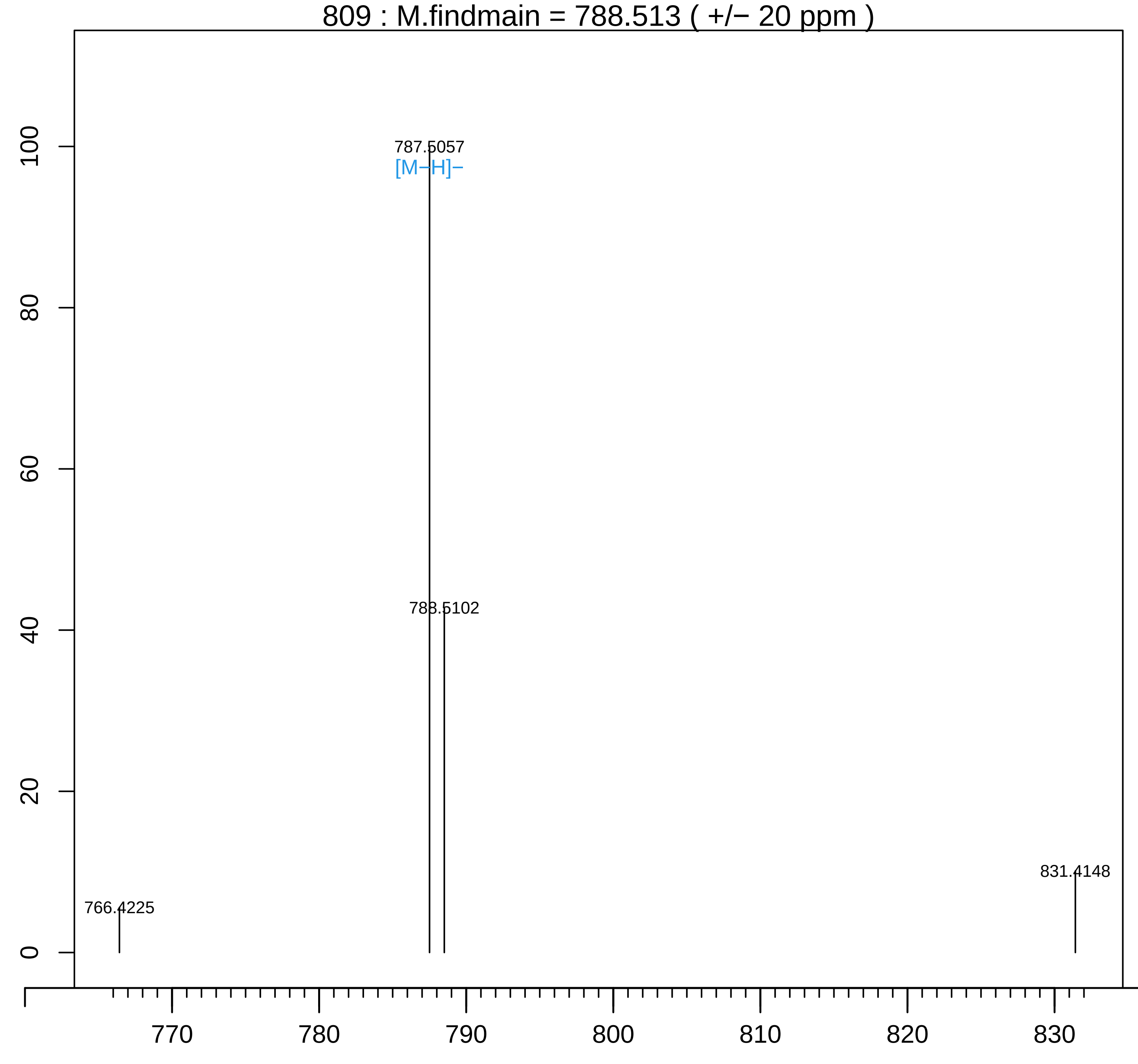
809 : M.findmain = 788.513 (+/− 20 ppm)
787.5057
[M−H]−
788.5102
766.4225
831.4148
0
20
40
60
80
100
770
780
790
800
810
820
830

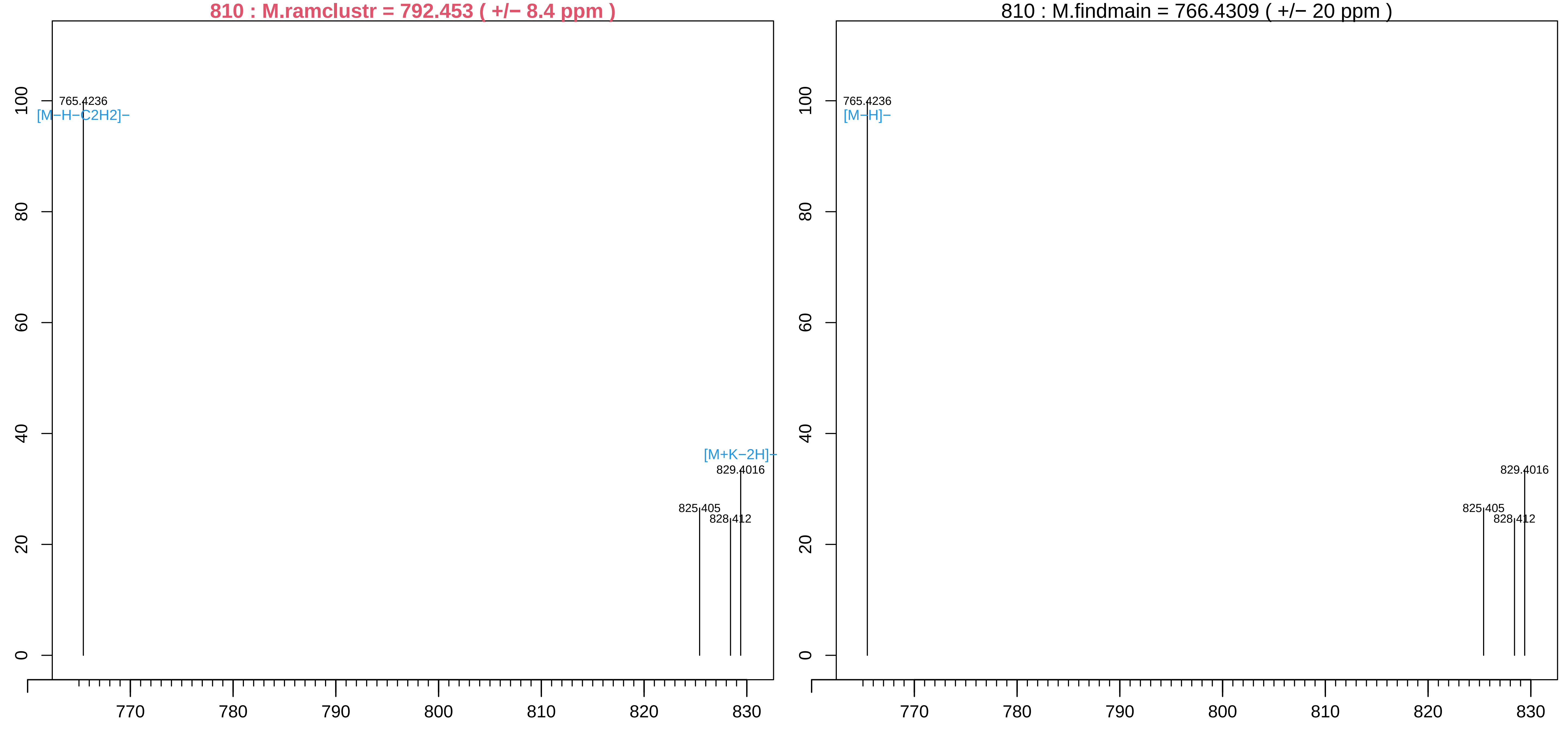
810 : M.ramclustr = 792.453 (+/− 8.4 ppm)
765.4236
[M−H−C2H2]−
[M+K−2H]−
829.4016
825.405
828.412
810 : M.findmain = 766.4309 (+/− 20 ppm)
765.4236
[M−H]−
829.4016
825.405
828.412

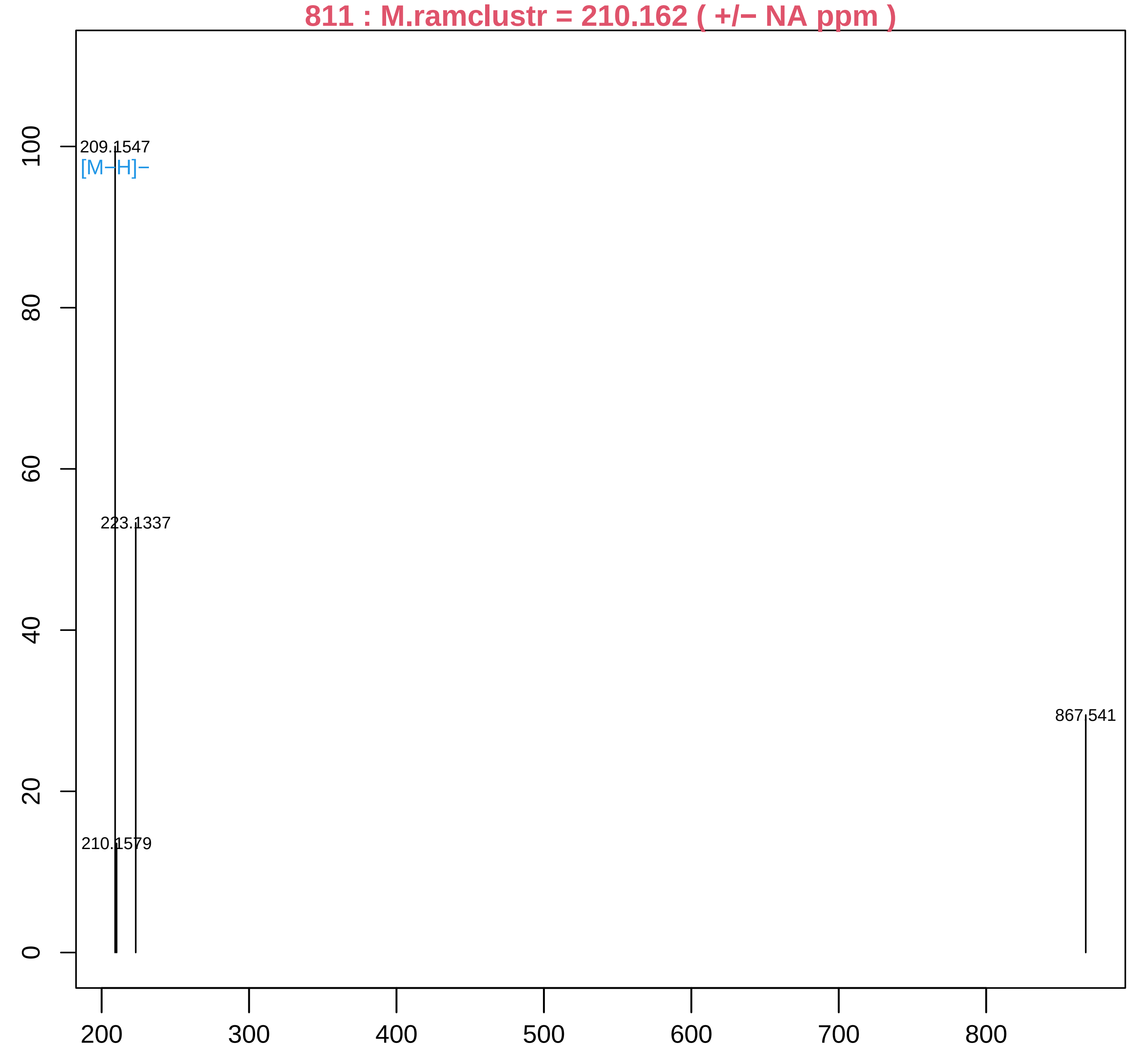
811 : M.ramclustr = 210.162 (+/− NA ppm)
209.1547
[M−H]−
223.1337
867.541
210.1579
0
20
40
60
80
100
200
300
400
500
600
700
800
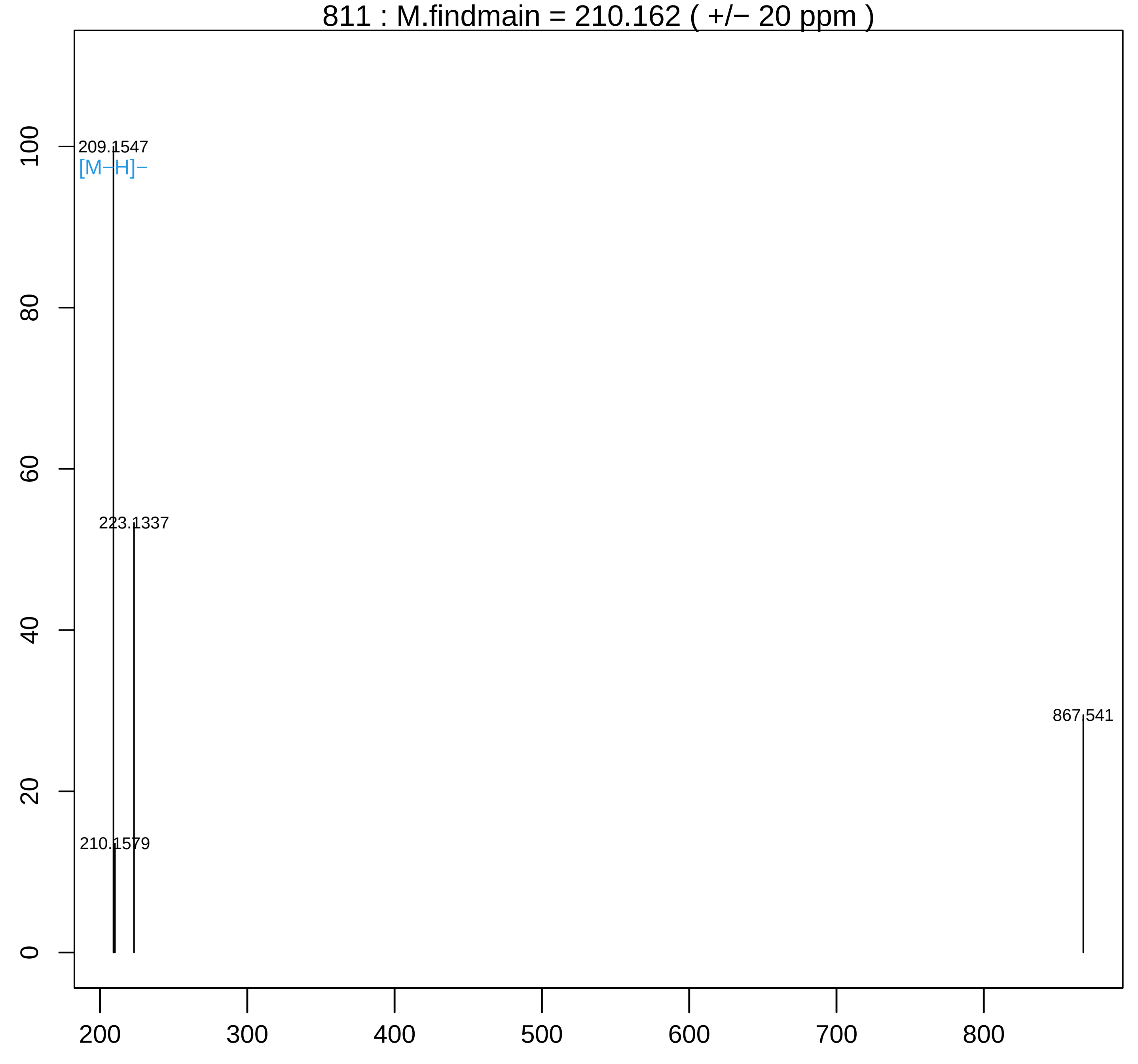
811 : M.findmain = 210.162 (+/− 20 ppm)
209.1547
[M−H]−
223.1337
867.541
210.1579
0
20
40
60
80
100
200
300
400
500
600
700
800

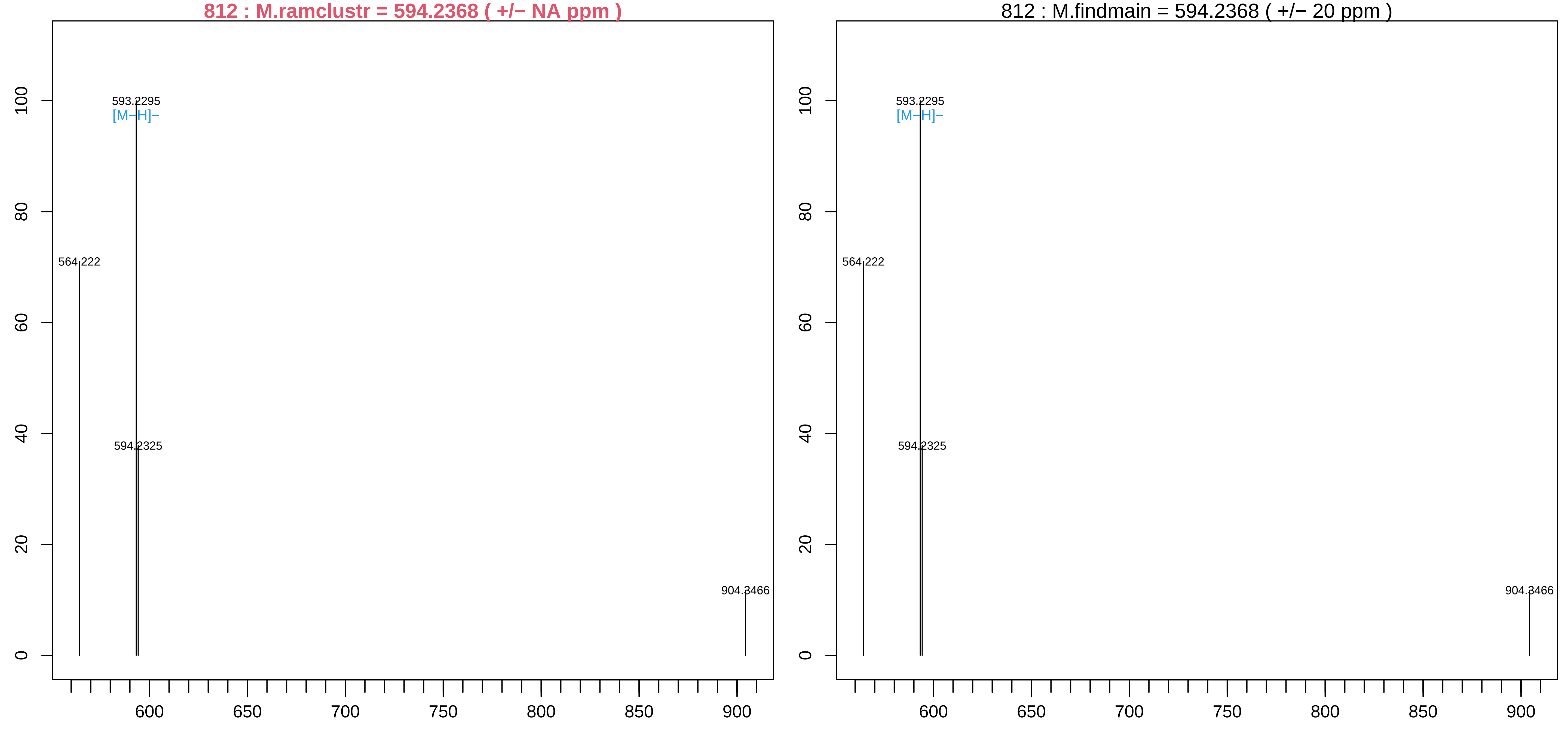
812 : M.ramclustr = 594.2368 (+/− NA ppm)
812 : M.findmain = 594.2368 (+/− 20 ppm)
593.2295
[M−H]−
564.222
594.2325
904.3466
0
20
40
60
80
100
600
650
700
750
800
850
900

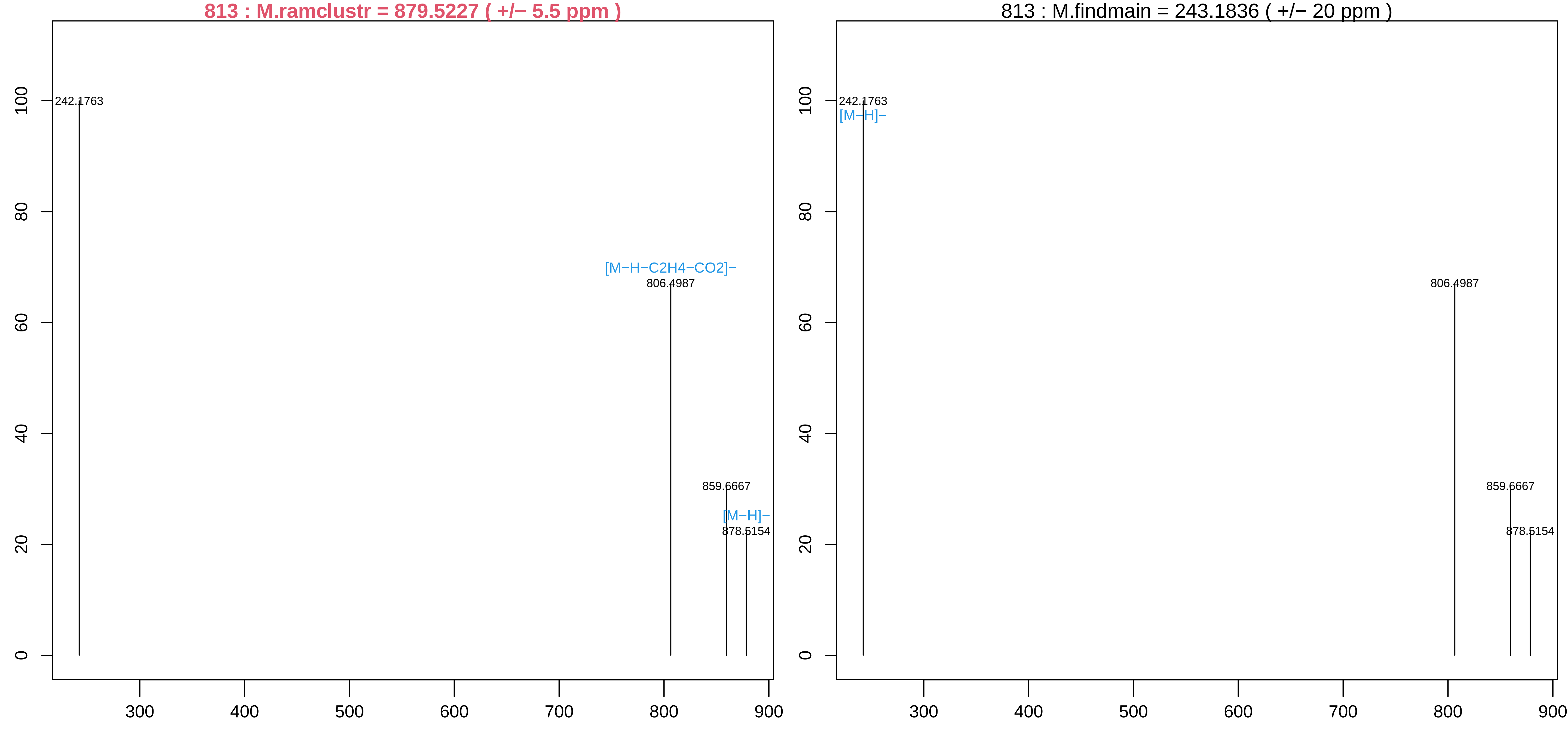

813 : M.ramclustr = 879.5227 (+/− 5.5 ppm)
242.1763
[M−H−C2H4−CO2]−
806.4987
859.6667
[M−H]−
878.5154
813 : M.findmain = 243.1836 (+/− 20 ppm)
242.1763
[M−H]−
806.4987
859.6667
878.5154
0
20
40
60
80
100
300
400
500
600
700
800
900

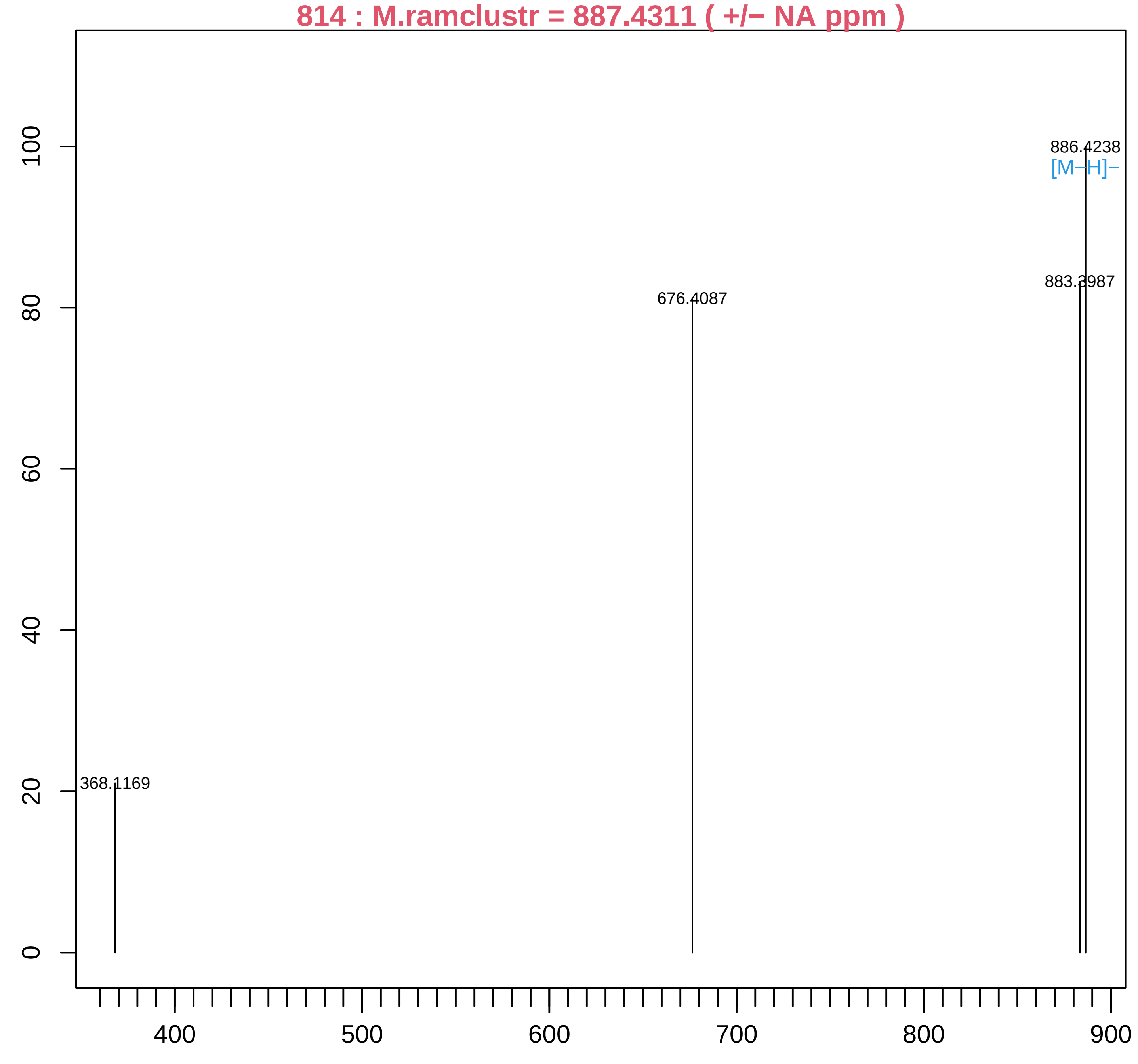
814 : M.ramclustr = 887.4311 (+/− NA ppm)
886.4238
[M−H]−
883.3987
676.4087
368.1169
0
20
40
60
80
100
400
500
600
700
800
900

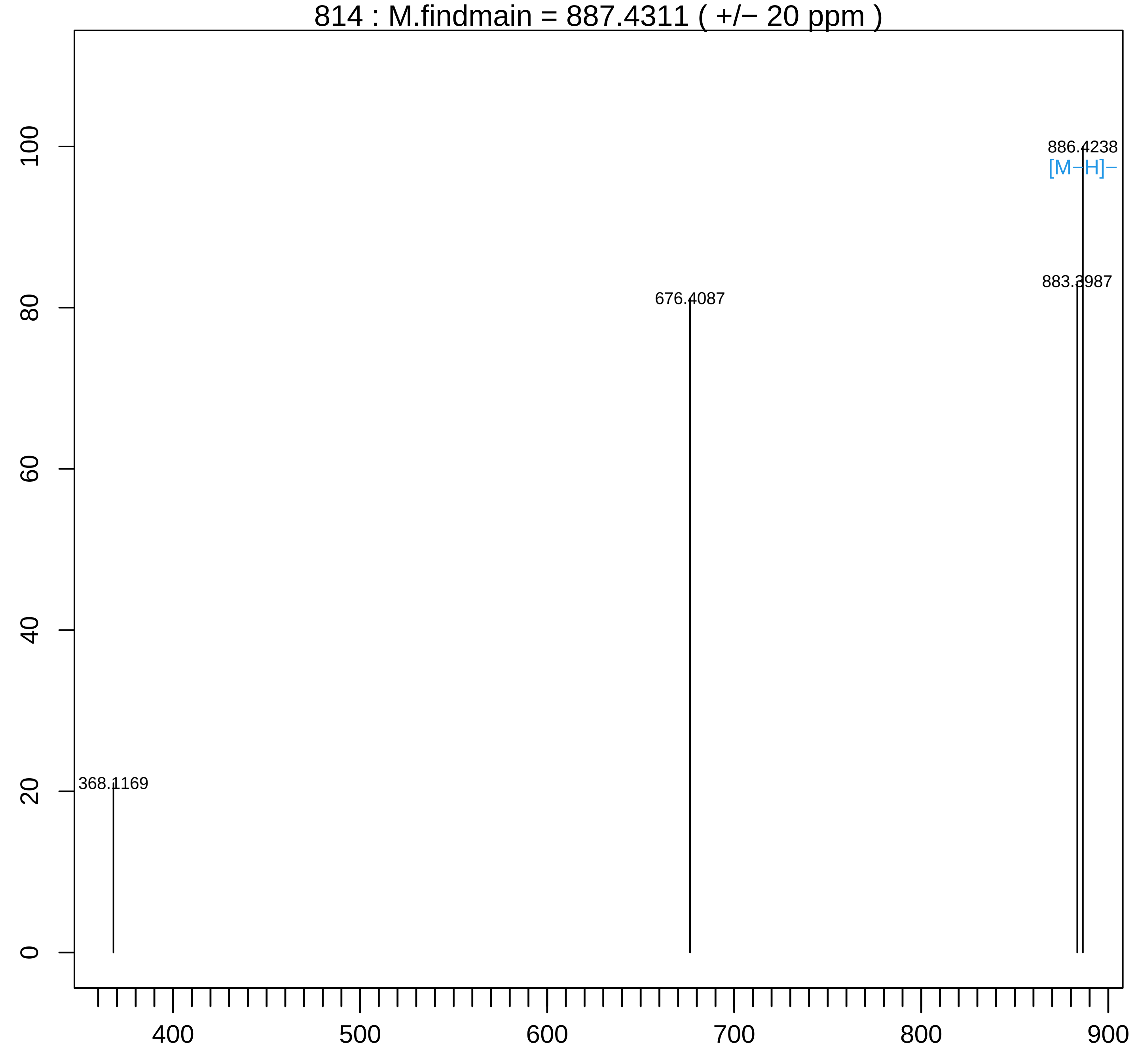
814 : M.findmain = 887.4311 (+/− 20 ppm)
886.4238
[M−H]−
883.3987
676.4087
368.1169
0
20
40
60
80
100
400
500
600
700
800
900

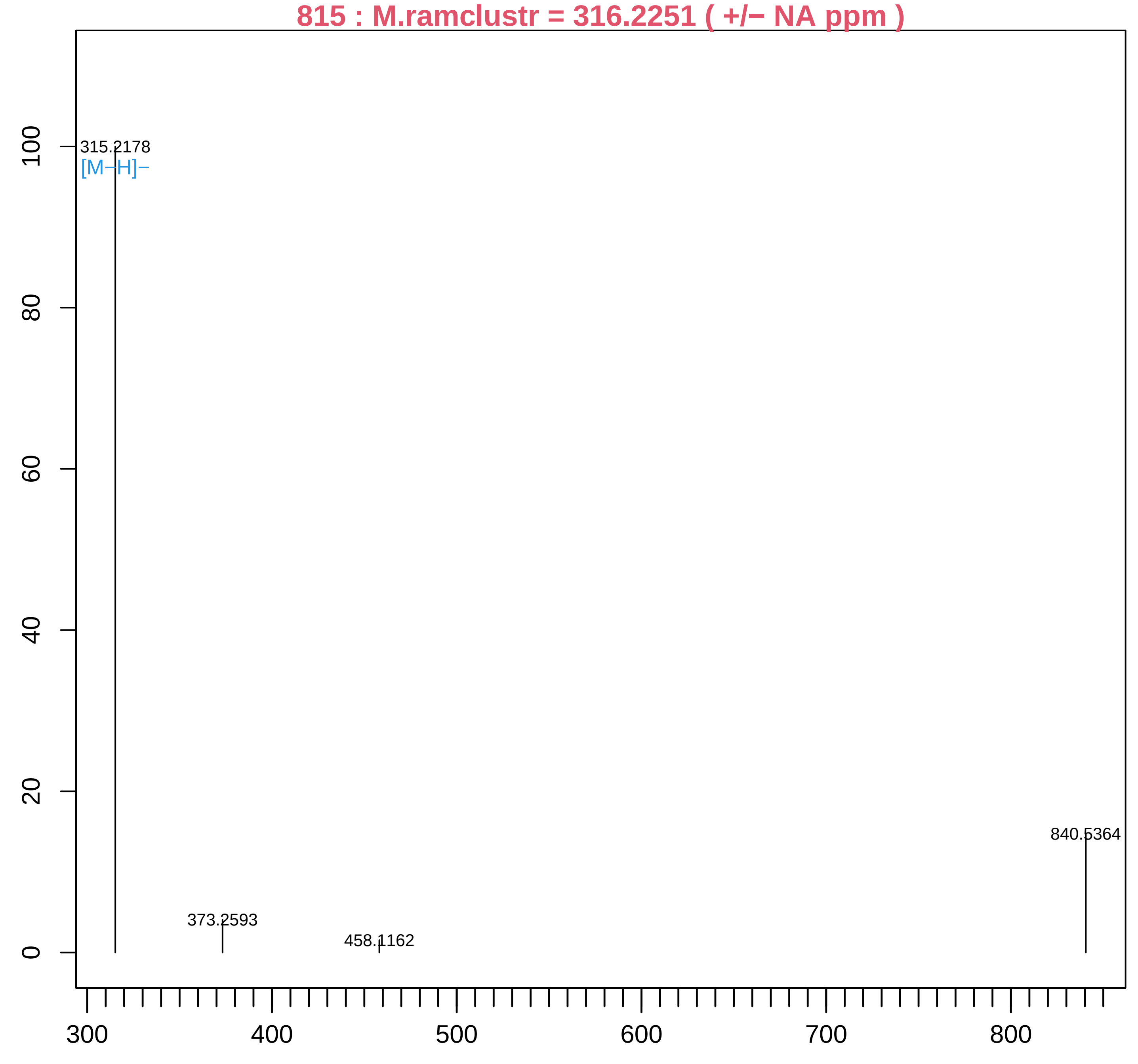
815 : M.ramclustr = 316.2251 (+/− NA ppm)
315.2178
[M−H]−
373.2593
458.1162
840.5364
0
20
40
60
80
100
300
400
500
600
700
800

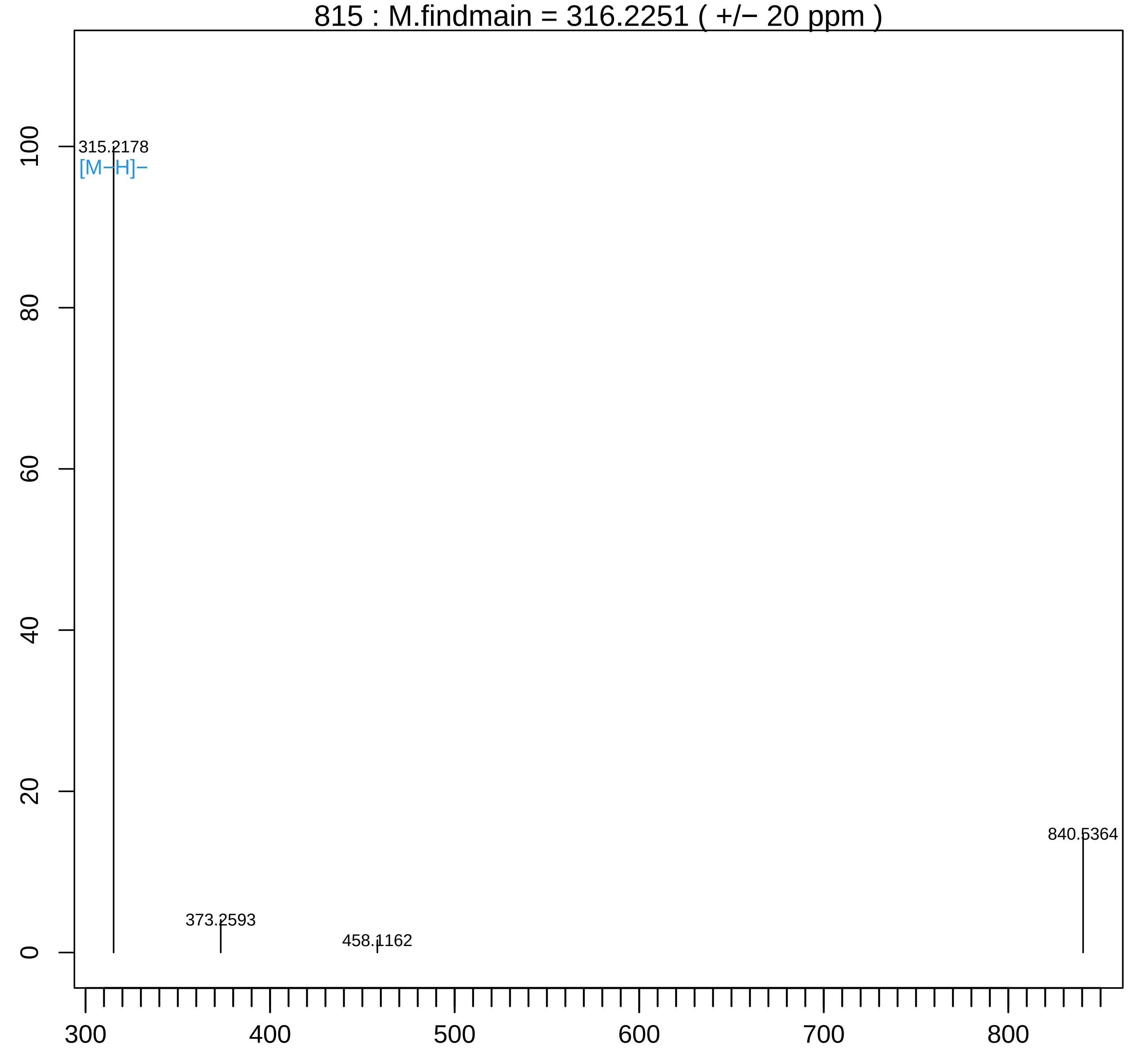
815 : M.findmain = 316.2251 (+/− 20 ppm)
315.2178
[M−H]−
373.2593
458.1162
840.5364
0
20
40
60
80
100
300
400
500
600
700
800

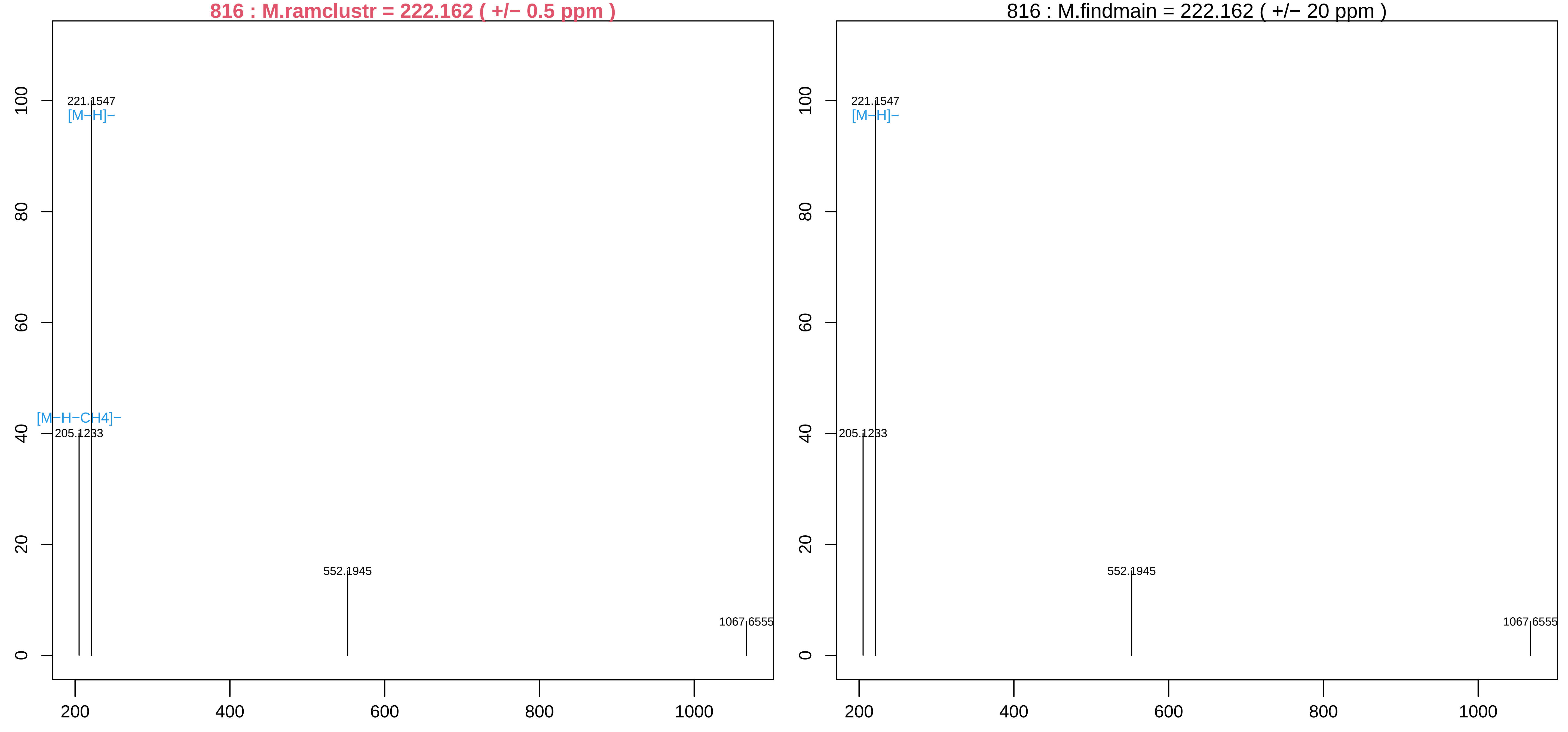
816 : M.ramclustr = 222.162 (+/− 0.5 ppm)
221.1547
[M−H]−
[M−H−CH4]−
205.1233
552.1945
1067.6555
0
20
40
60
80
100
200
400
600
800
1000
816 : M.findmain = 222.162 (+/− 20 ppm)
221.1547
[M−H]−
205.1233
552.1945
1067.6555
0
20
40
60
80
100
200
400
600
800
1000

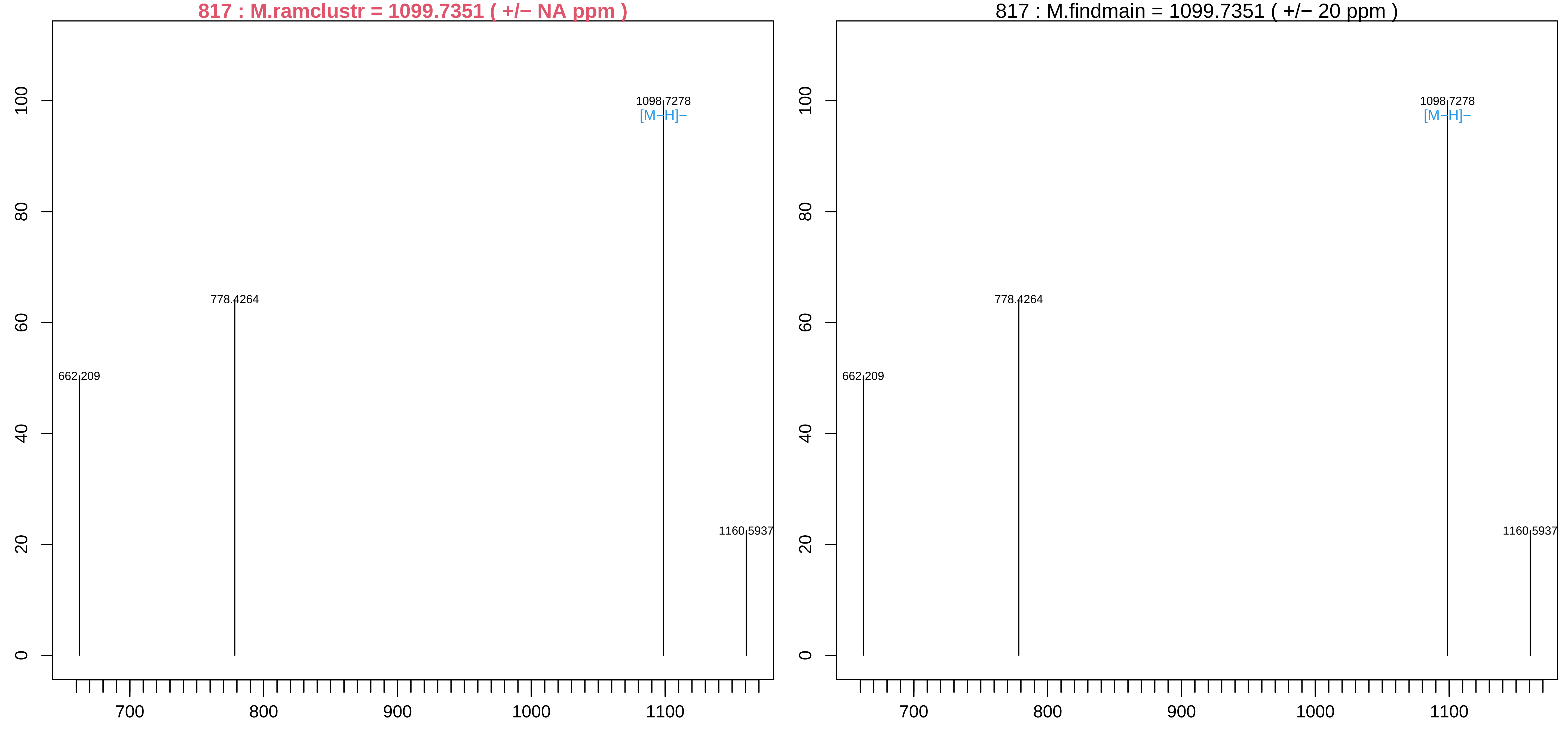

817 : M.ramclustr = 1099.7351 (+/− NA ppm)
1098.7278
[M−H]−
778.4264
662.209
1160.5937
817 : M.findmain = 1099.7351 (+/− 20 ppm)
1098.7278
[M−H]−
778.4264
662.209
1160.5937

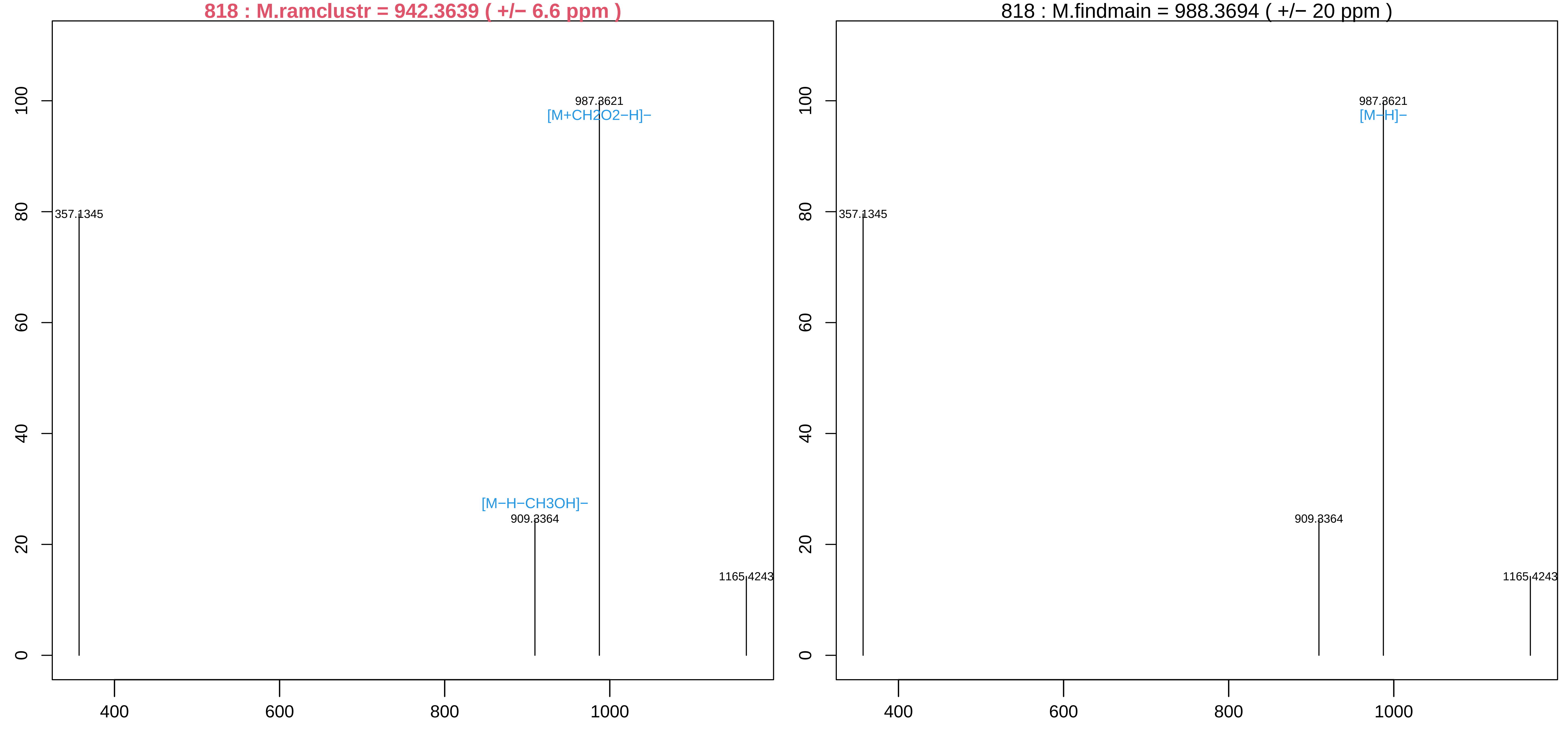

818 : M.ramclustr = 942.3639 (+/− 6.6 ppm)
987.3621
[M+CH2O2−H]−
357.1345
[M−H−CH3OH]−
909.3364
1165.4243
0
20
40
60
80
100
400
600
800
1000
818 : M.findmain = 988.3694 (+/− 20 ppm)
987.3621
[M−H]−
357.1345
909.3364
1165.4243

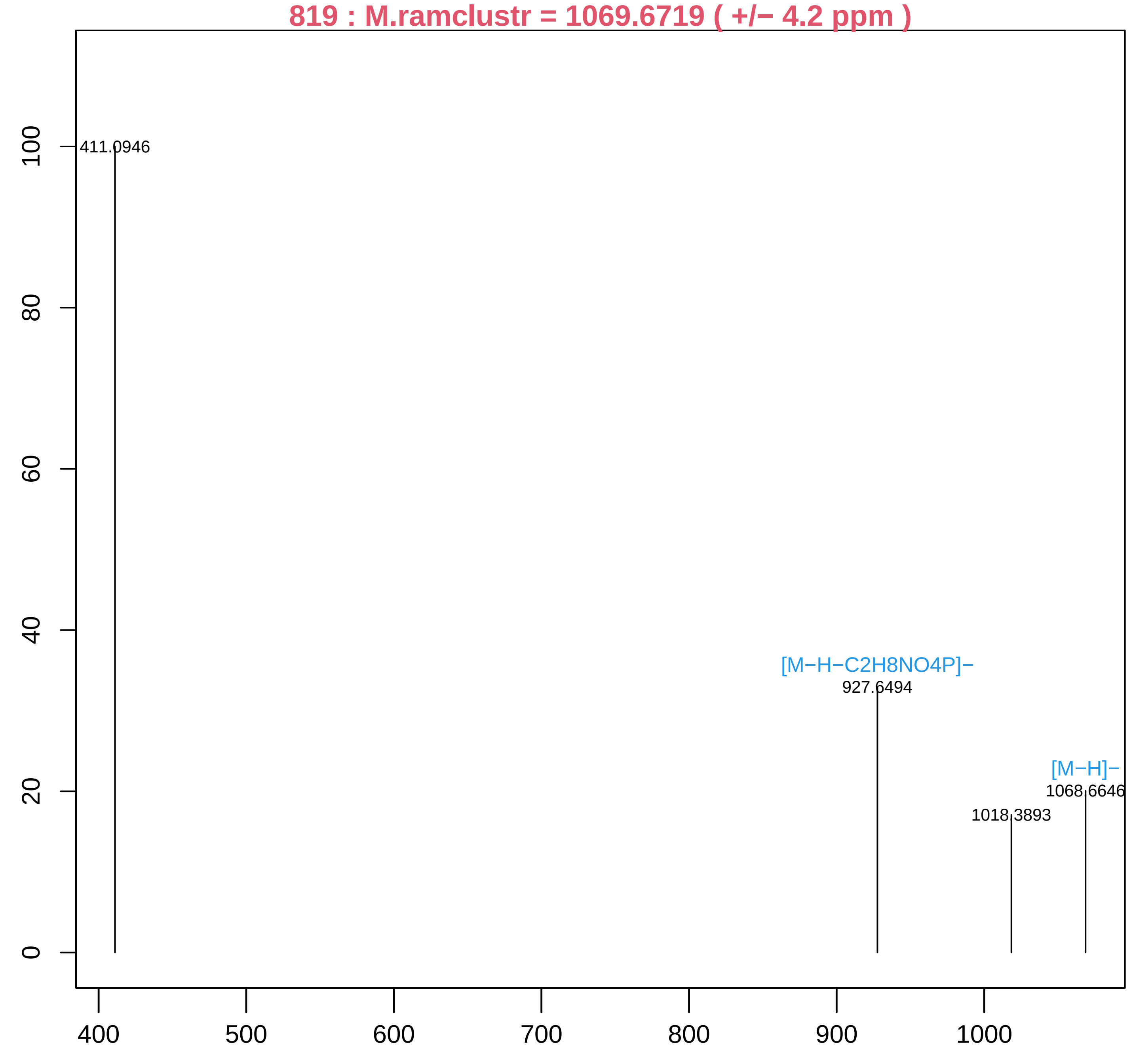
819 : M.ramclustr = 1069.6719 (+/− 4.2 ppm)
411.0946
[M−H−C2H8NO4P]−
927.6494
1018.3893
[M−H]−
1068.6646
0
20
40
60
80
100
400
500
600
700
800
900
1000

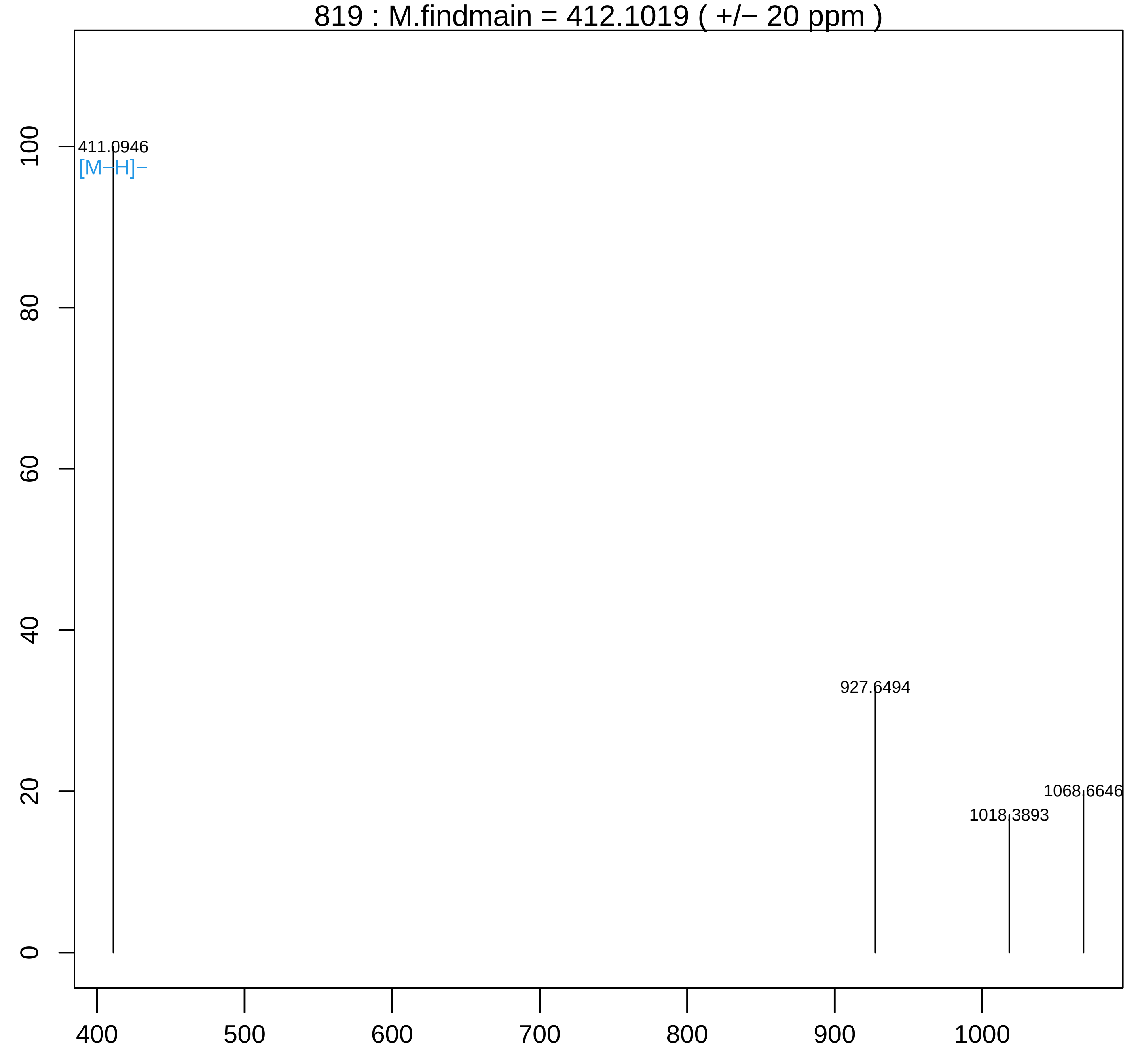
819 : M.findmain = 412.1019 (+/− 20 ppm)
411.0946
[M−H]−
927.6494
1018.3893
1068.6646
0
20
40
60
80
100
400
500
600
700
800
900
1000

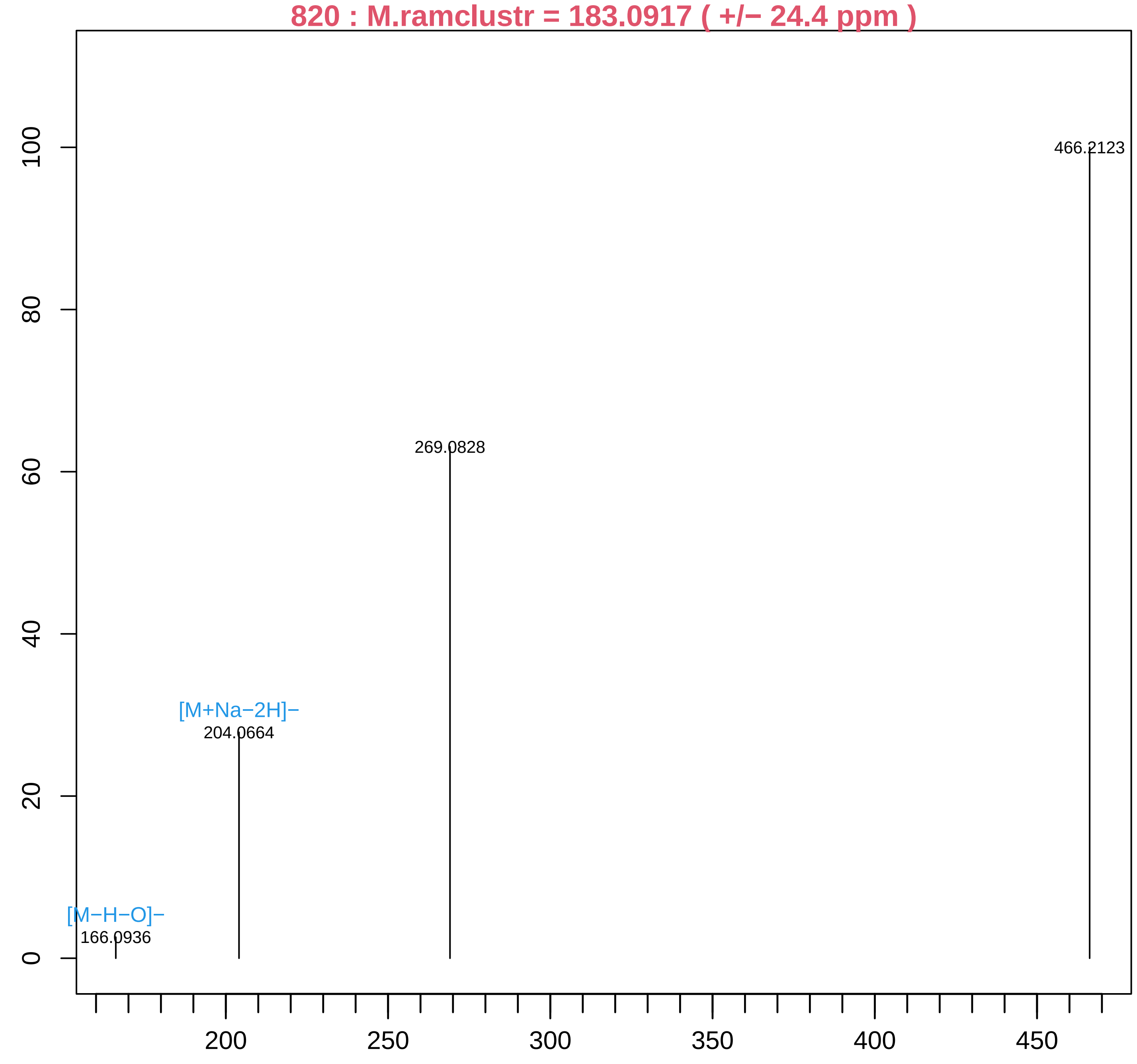

820 : M.ramclustr = 183.0917 (+/− 24.4 ppm)
466.2123
269.0828
[M+Na−2H]−
204.0664
[M−H−O]−
166.0936
0
20
40
60
80
100
200
250
300
350
400
450

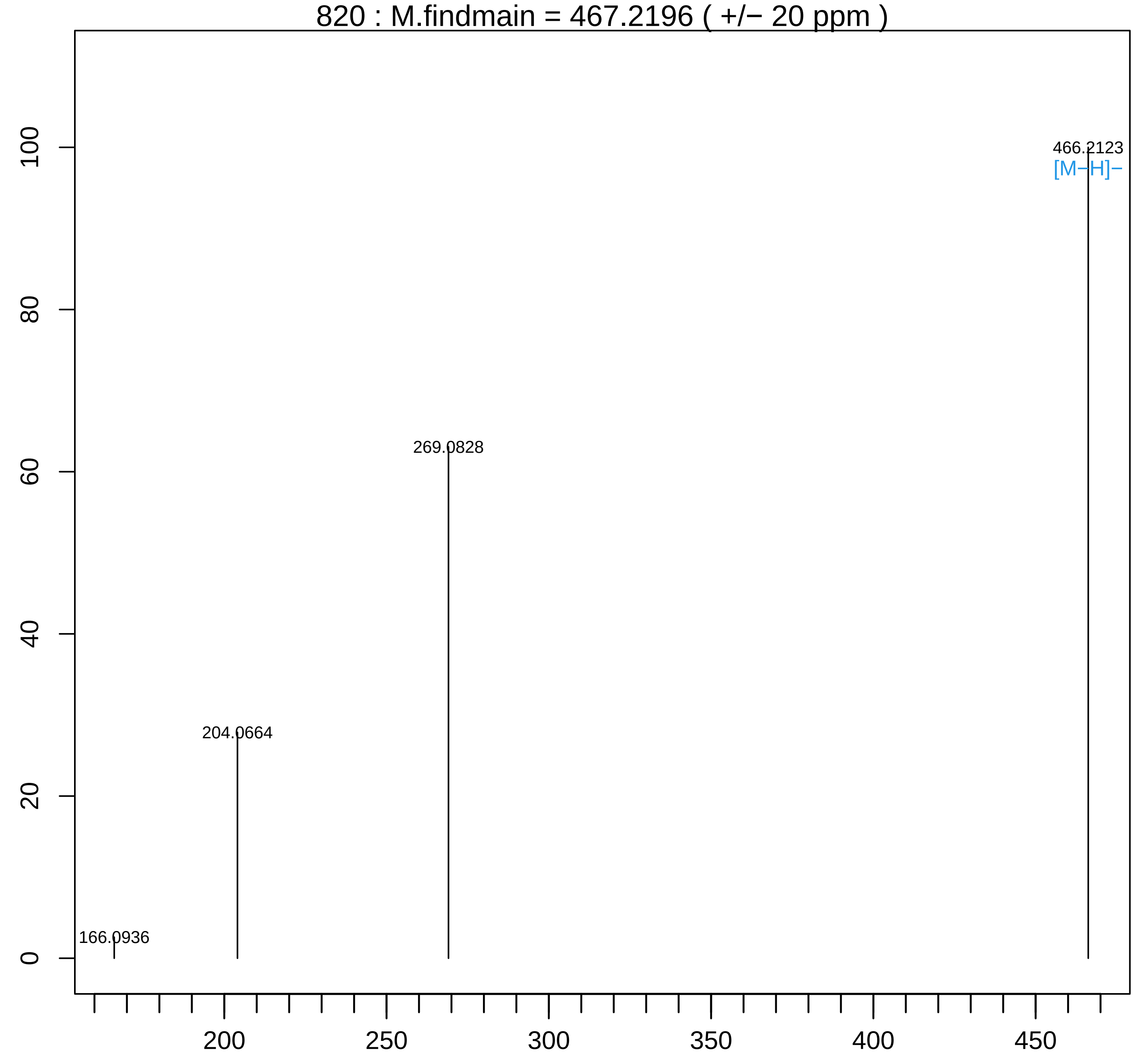

820 : M.findmain = 467.2196 (+/− 20 ppm)
466.2123
[M−H]−
269.0828
204.0664
166.0936
0
20
40
60
80
100
200
250
300
350
400
450

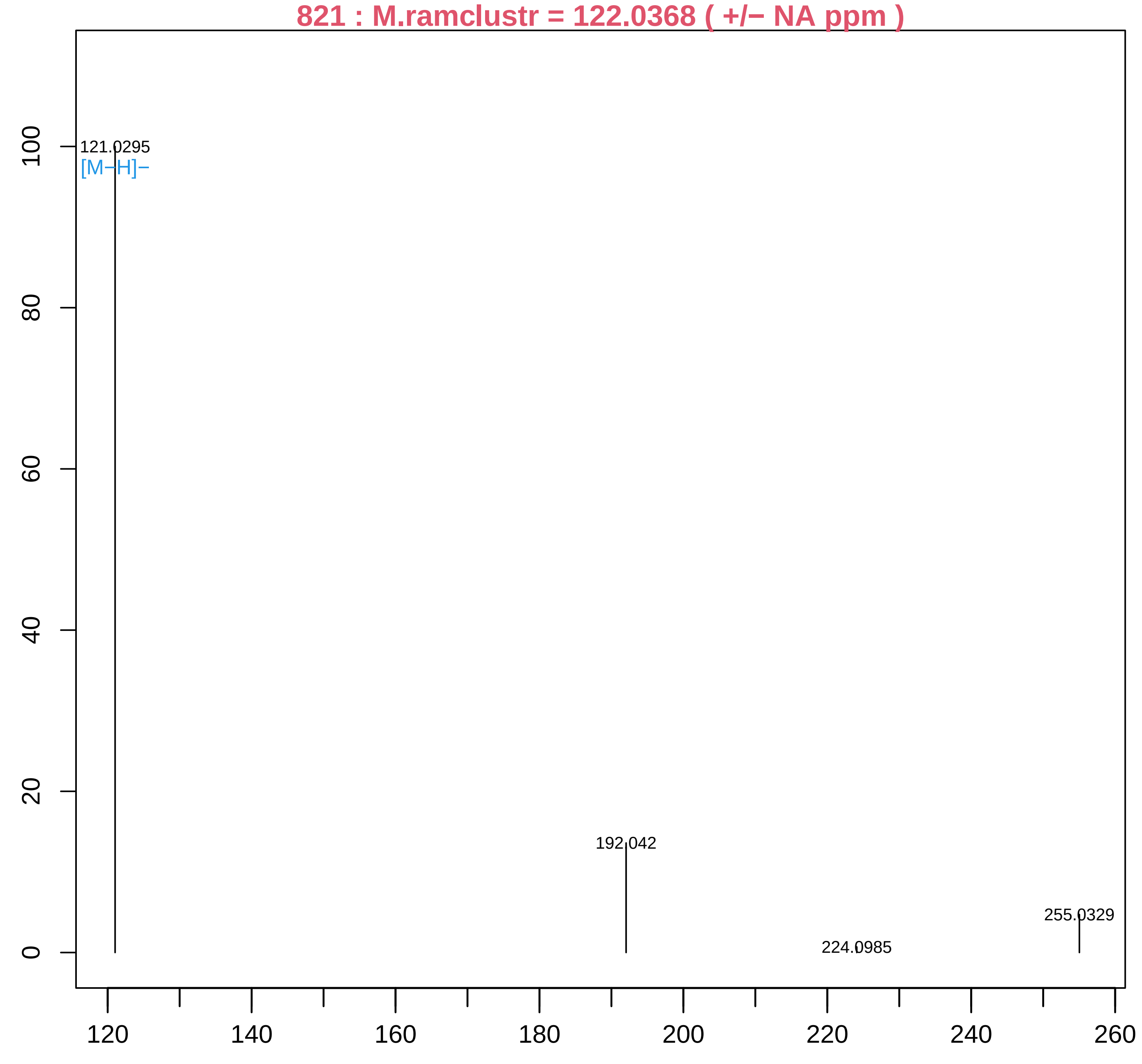

821 : M.ramclustr = 122.0368 (+/− NA ppm)
121.0295
[M−H]−
192.042
224.0985
255.0329
0
20
40
60
80
100
120
140
160
180
200
220
240
260

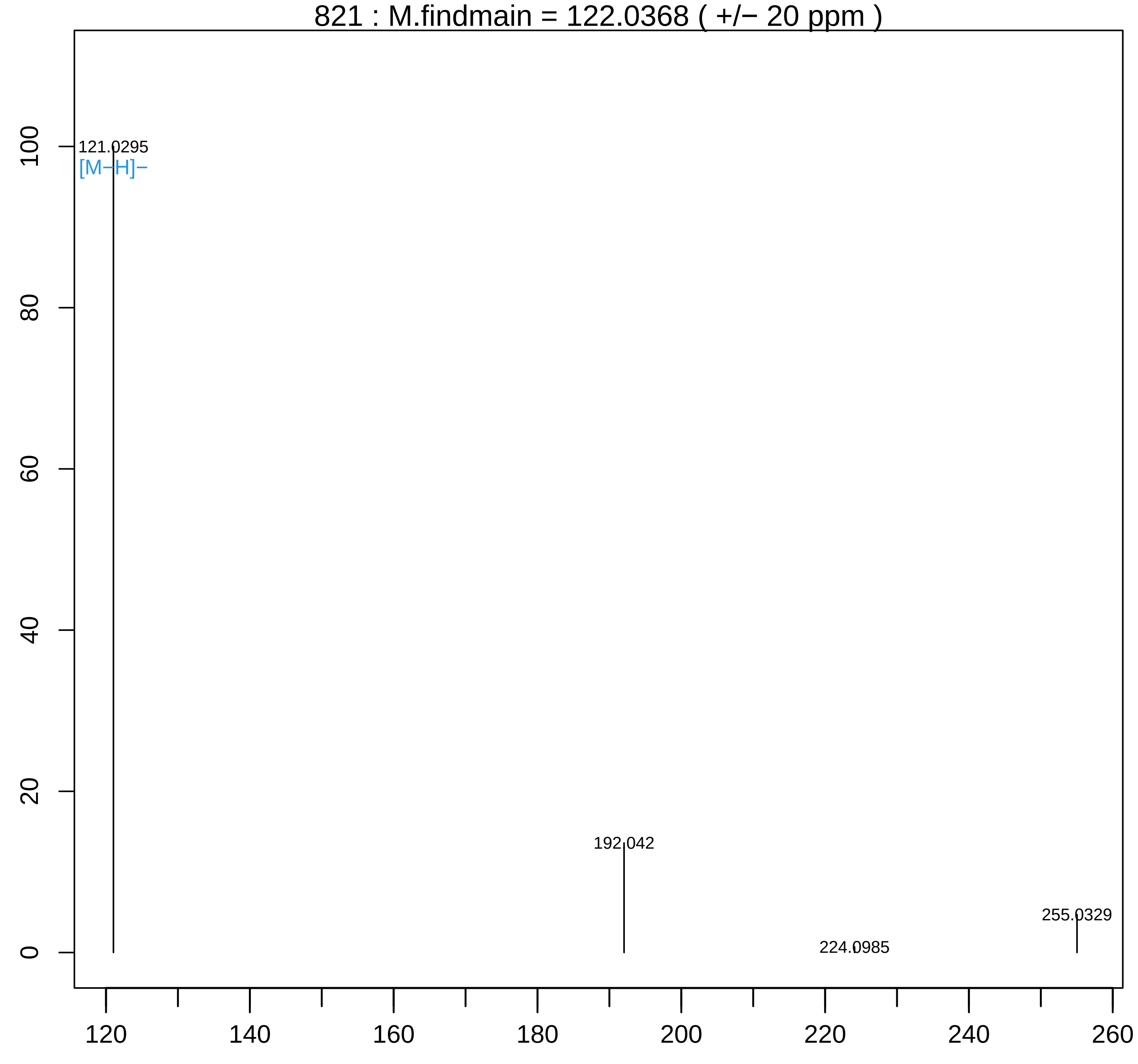

821 : M.findmain = 122.0368 (+/− 20 ppm)
121.0295
[M−H]−
192.042
224.0985
255.0329
0
20
40
60
80
100
120
140
160
180
200
220
240
260

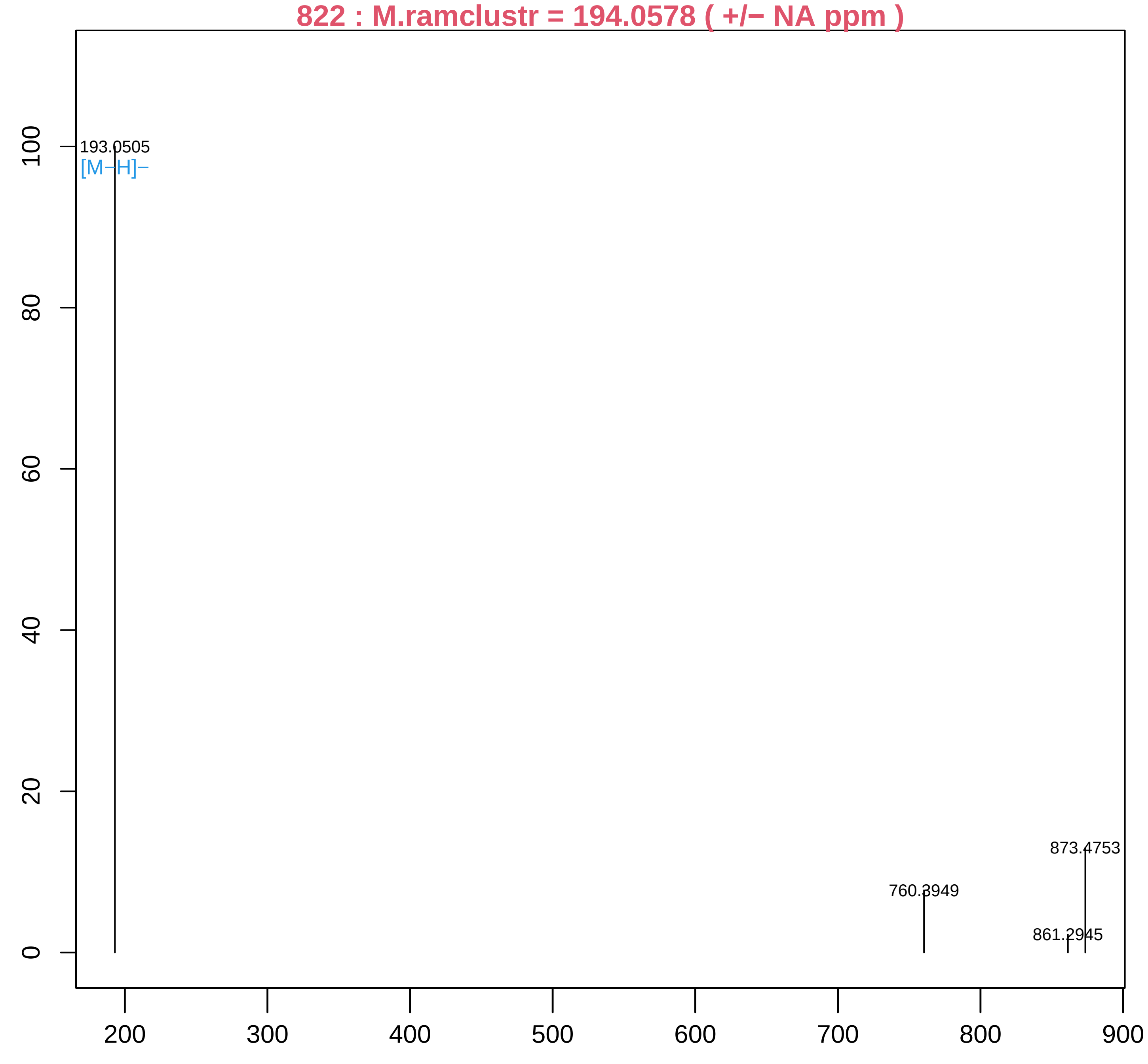

822 : M.ramclustr = 194.0578 (+/− NA ppm)
193.0505
[M−H]−
873.4753
760.3949
861.2945
0
20
40
60
80
100
200
300
400
500
600
700
800
900

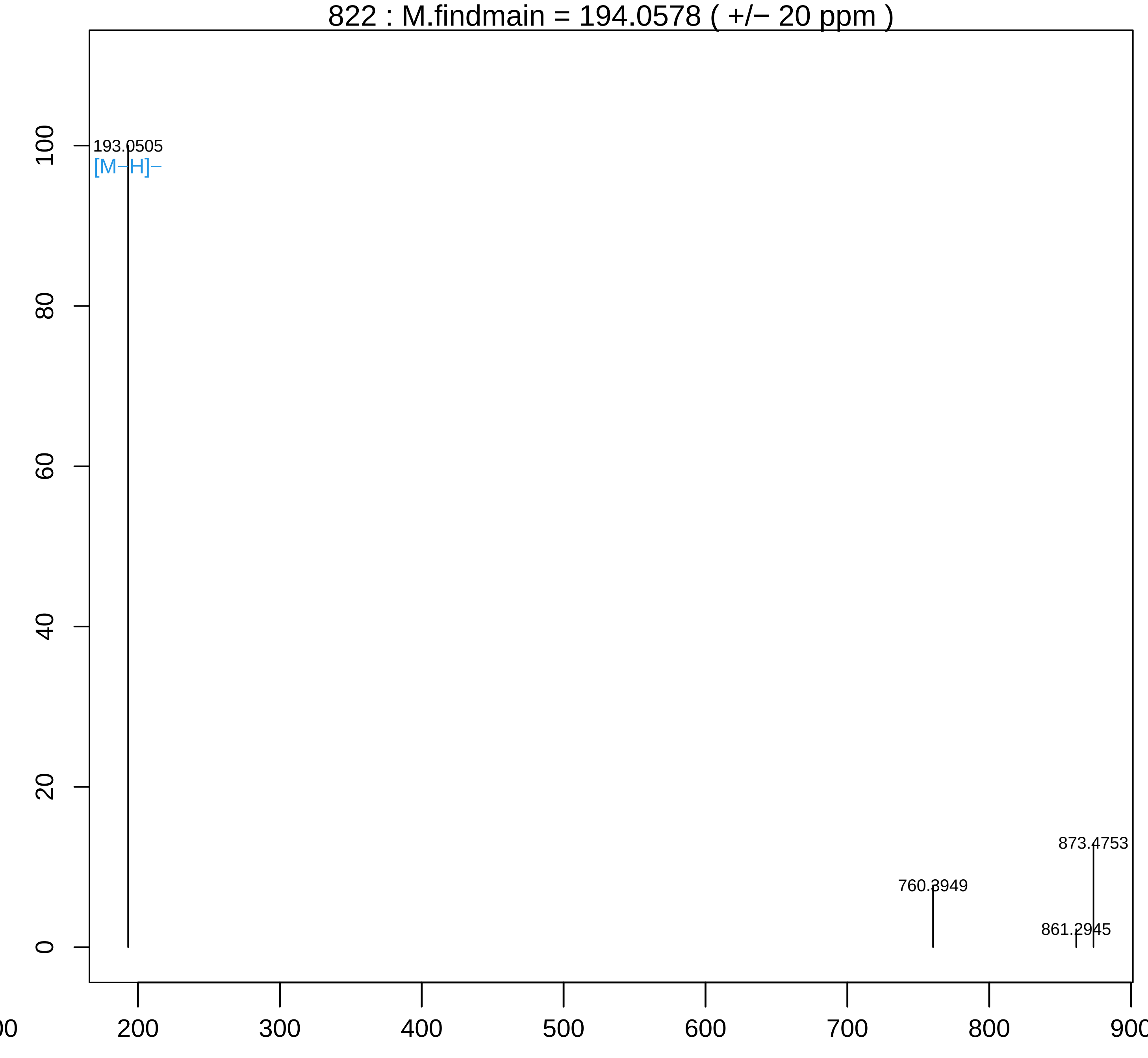

822 : M.findmain = 194.0578 (+/− 20 ppm)
193.0505
[M−H]−
873.4753
760.3949
861.2945
0
20
40
60
80
100
200
300
400
500
600
700
800
900

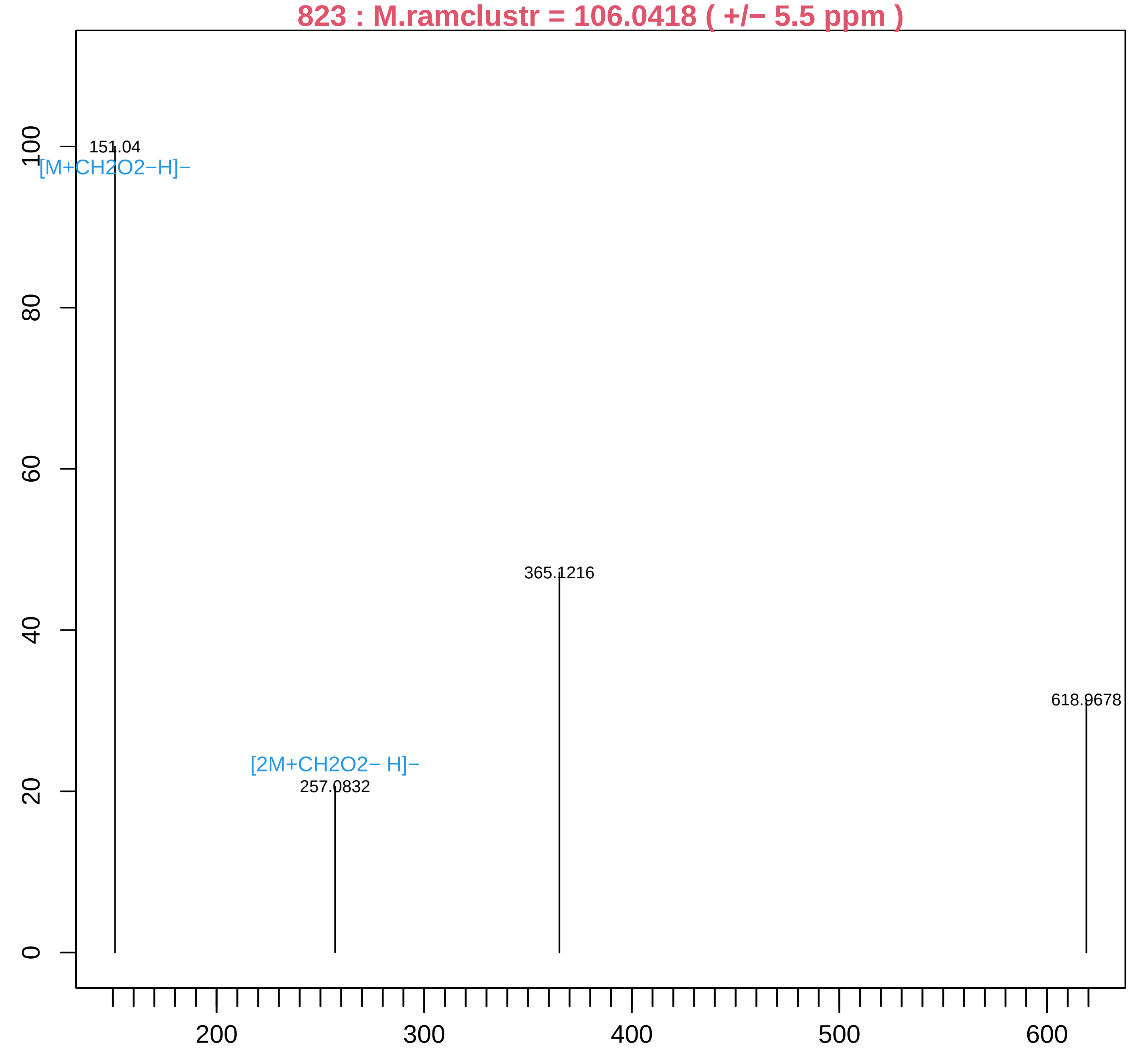
823 : M.ramclustr = 106.0418 (+/− 5.5 ppm)
151.04
[M+CH2O2−H]−
365.1216
618.9678
[2M+CH2O2− H]−
257.0832
0
20
40
60
80
100
200
300
400
500
600

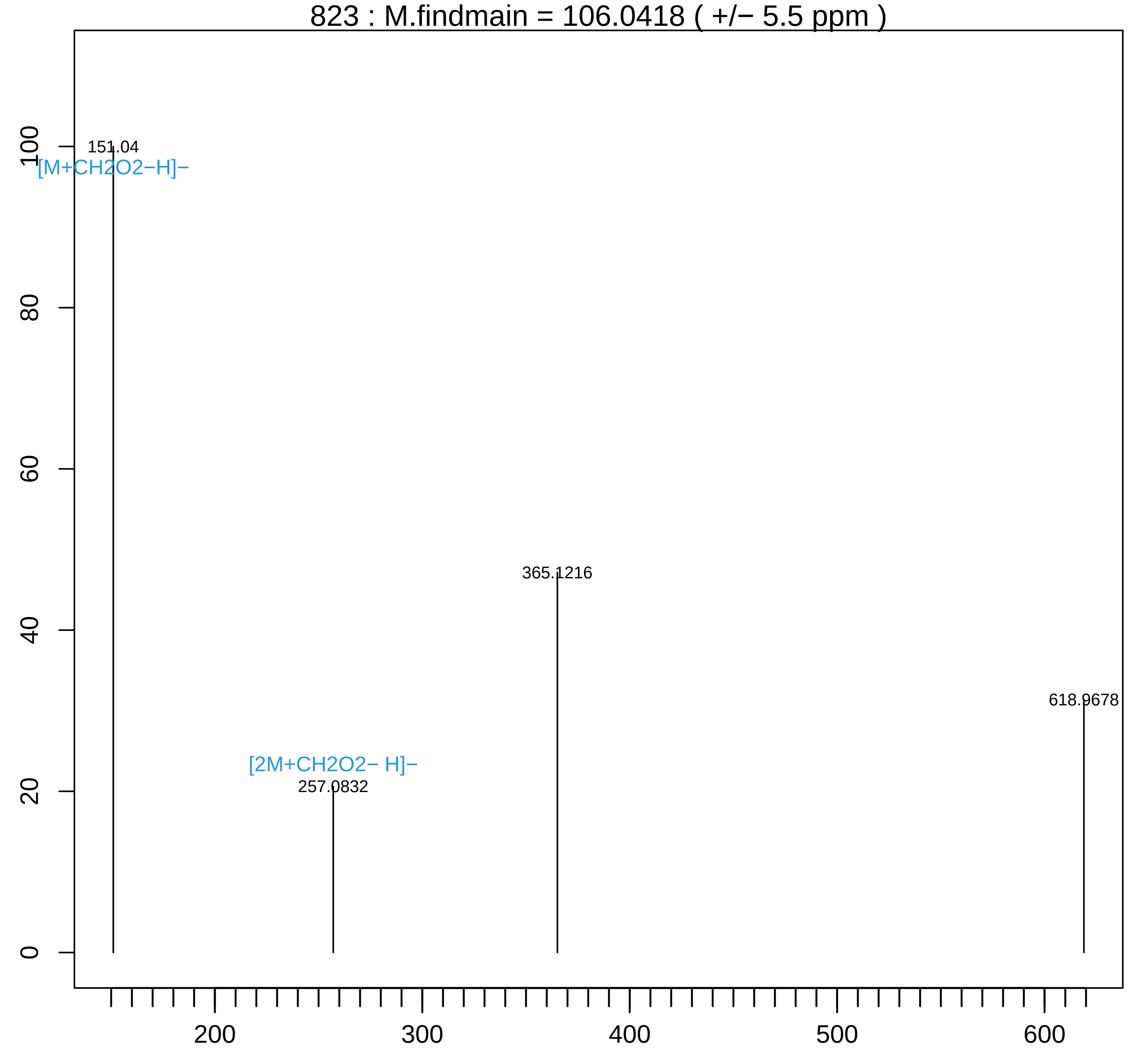
823 : M.findmain = 106.0418 (+/− 5.5 ppm)
151.04
[M+CH2O2−H]−
365.1216
618.9678
[2M+CH2O2− H]−
257.0832
0
20
40
60
80
100
200
300
400
500
600

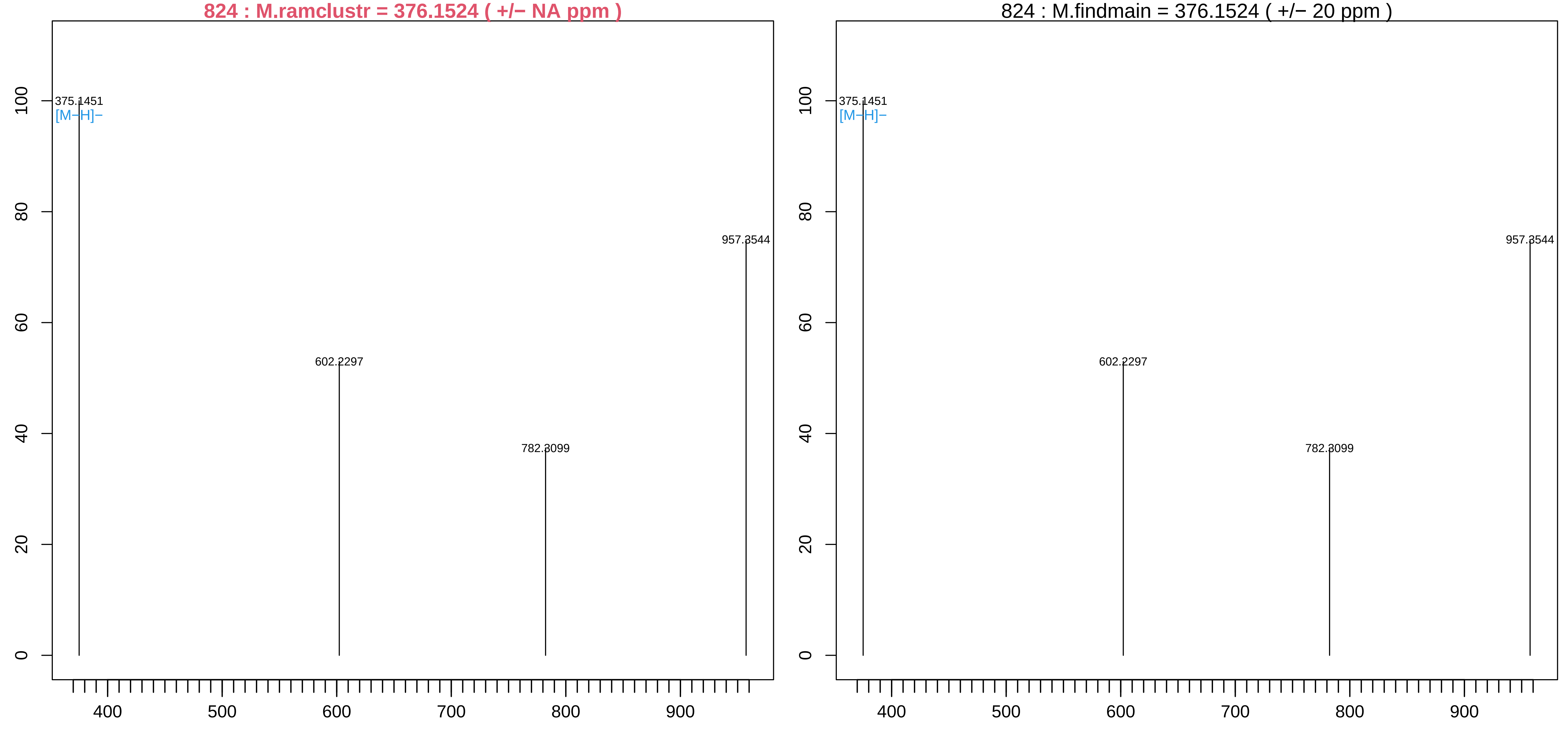

824 : M.ramclustr = 376.1524 (+/− NA ppm)
375.1451
[M−H]−
602.2297
782.3099
957.3544
824 : M.findmain = 376.1524 (+/− 20 ppm)
375.1451
[M−H]−
602.2297
782.3099
957.3544

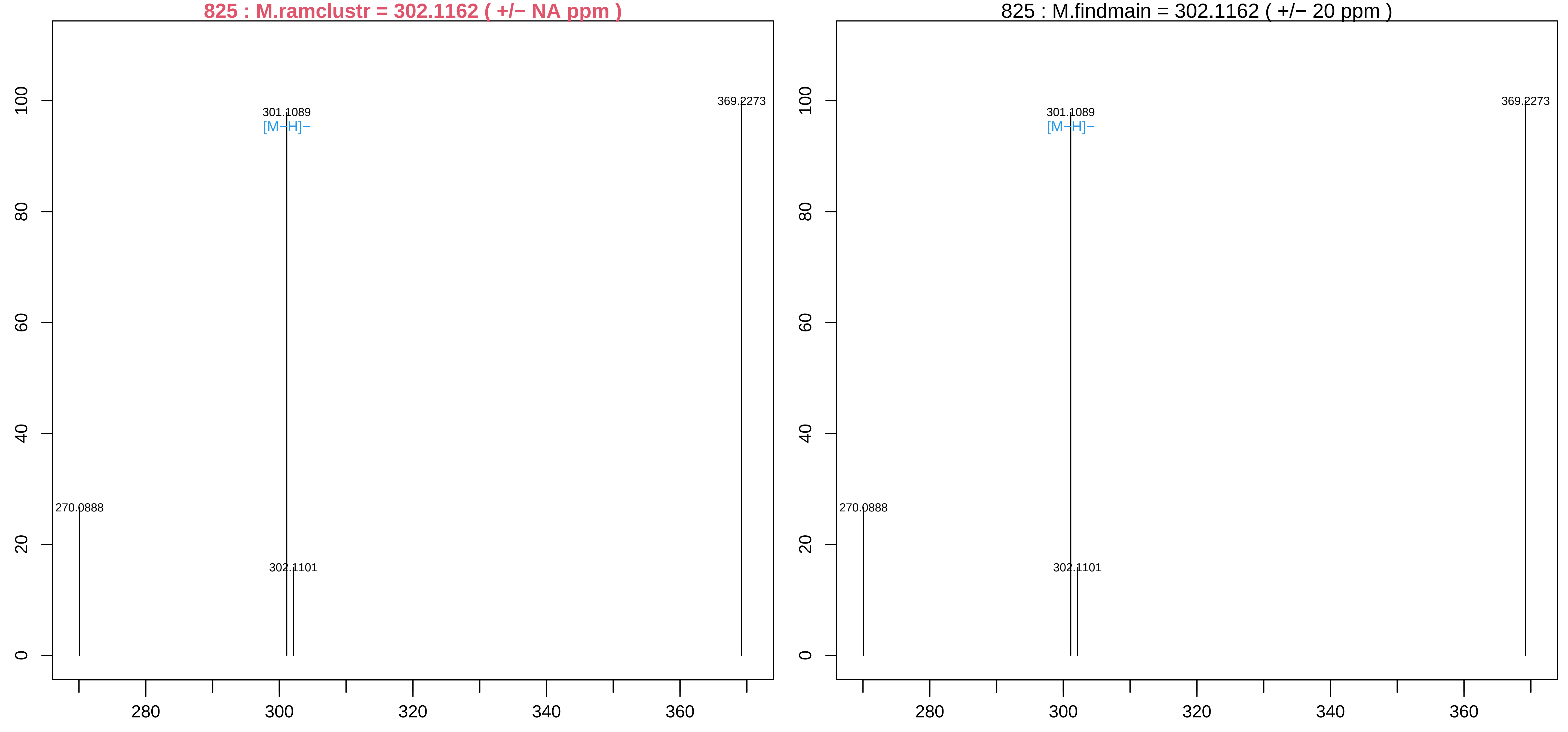
825 : M.ramclustr = 302.1162 (+/− NA ppm)
825 : M.findmain = 302.1162 (+/− 20 ppm)
301.1089
[M−H]−
369.2273
270.0888
302.1101
0
20
40
60
80
100
280
300
320
340
360

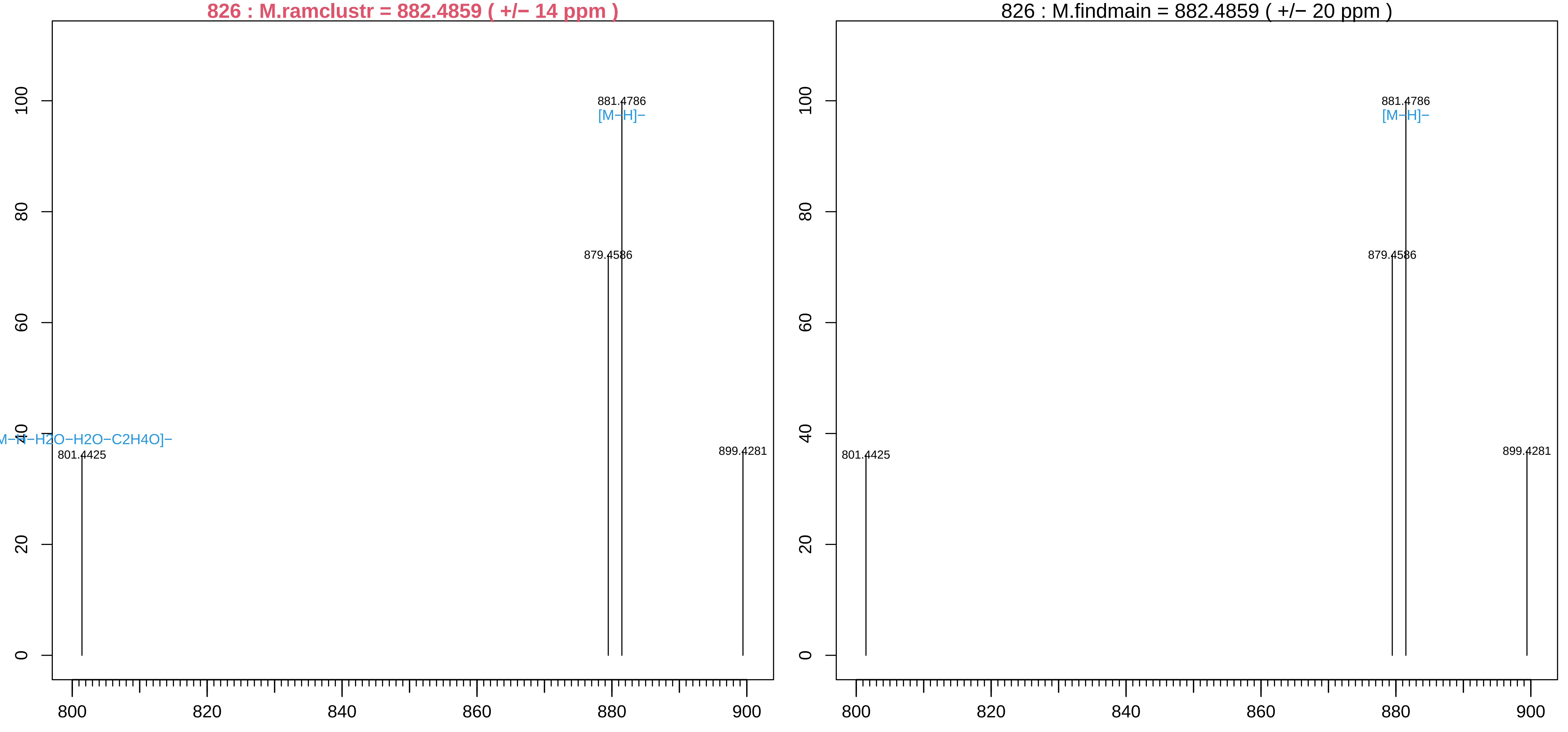

826 : M.ramclustr = 882.4859 (+/− 14 ppm)
881.4786
[M−H]−
879.4586
[M−H−H2O−H2O−C2H4O]−
801.4425
899.4281
826 : M.findmain = 882.4859 (+/− 20 ppm)
881.4786
[M−H]−
879.4586
801.4425
899.4281

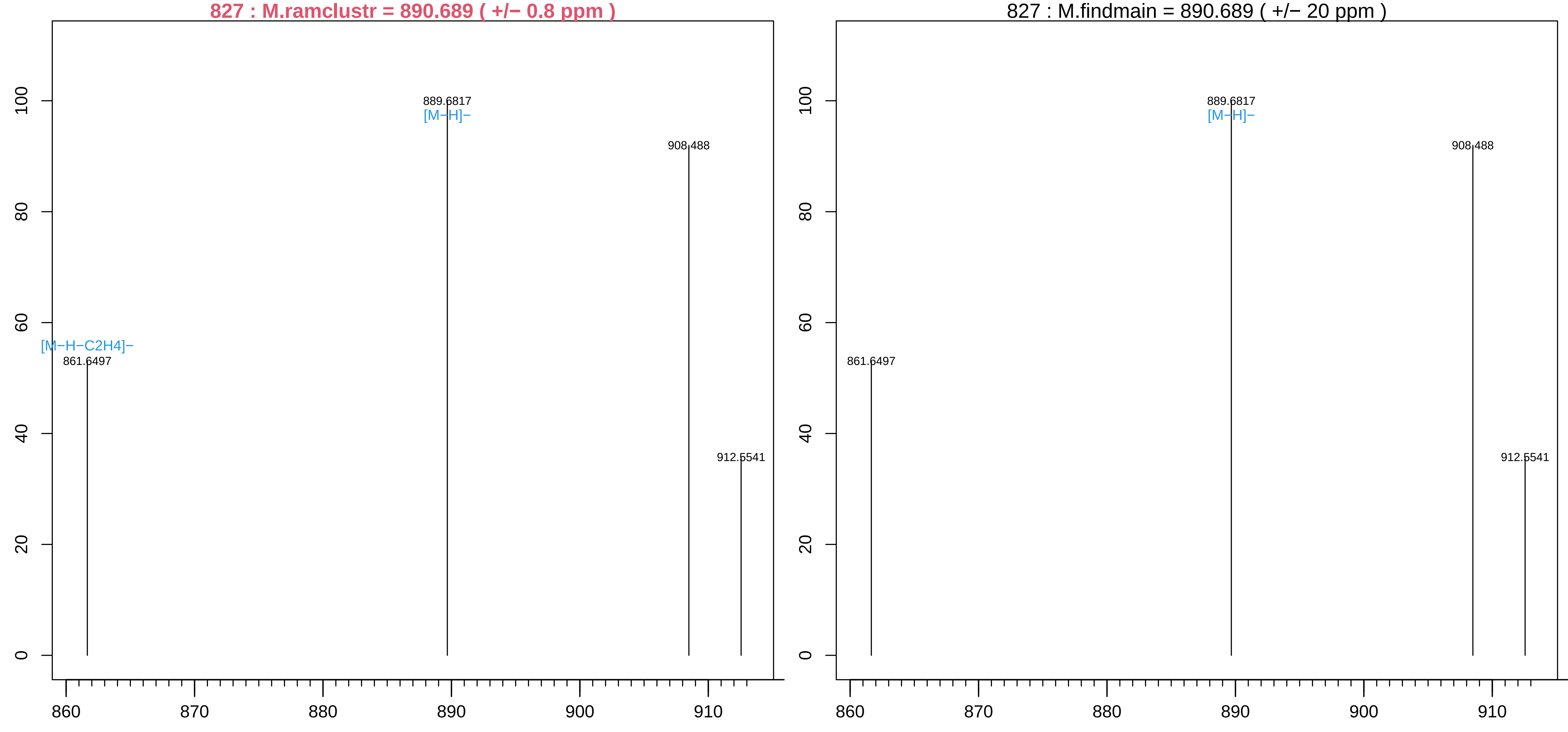

827 : M.ramclustr = 890.689 (+/− 0.8 ppm)
889.6817
[M−H]−
908.488
912.5541
[M−H−C2H4]−
861.6497
0
20
40
60
80
100
860
870
880
890
900
910
827 : M.findmain = 890.689 (+/− 20 ppm)
889.6817
[M−H]−
908.488
912.5541
861.6497
0
20
40
60
80
100
860
870
880
890
900
910

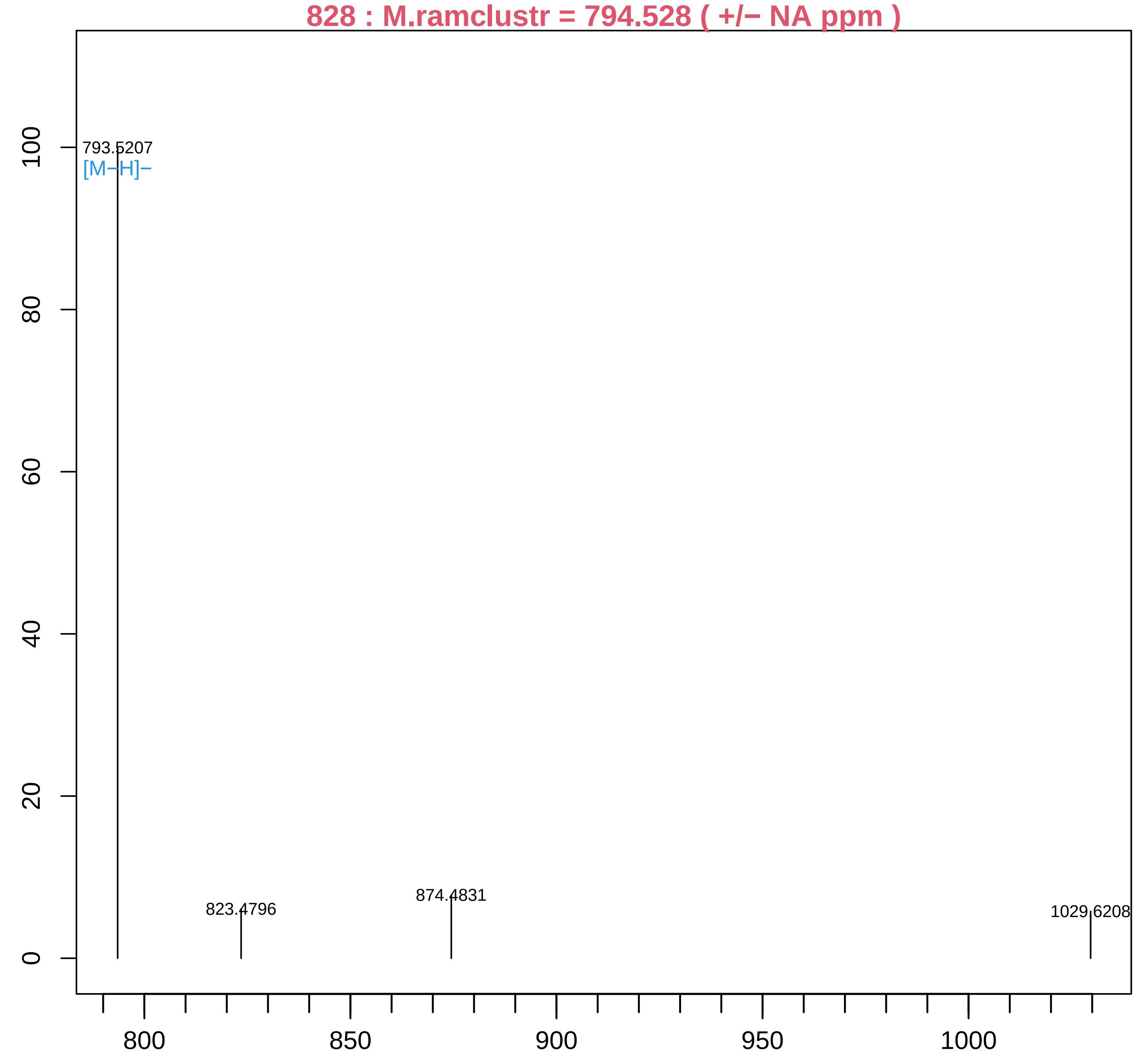
828 : M.ramclustr = 794.528 (+/− NA ppm)
793.5207
[M−H]−
823.4796
874.4831
1029.6208
0
20
40
60
80
100
800
850
900
950
1000

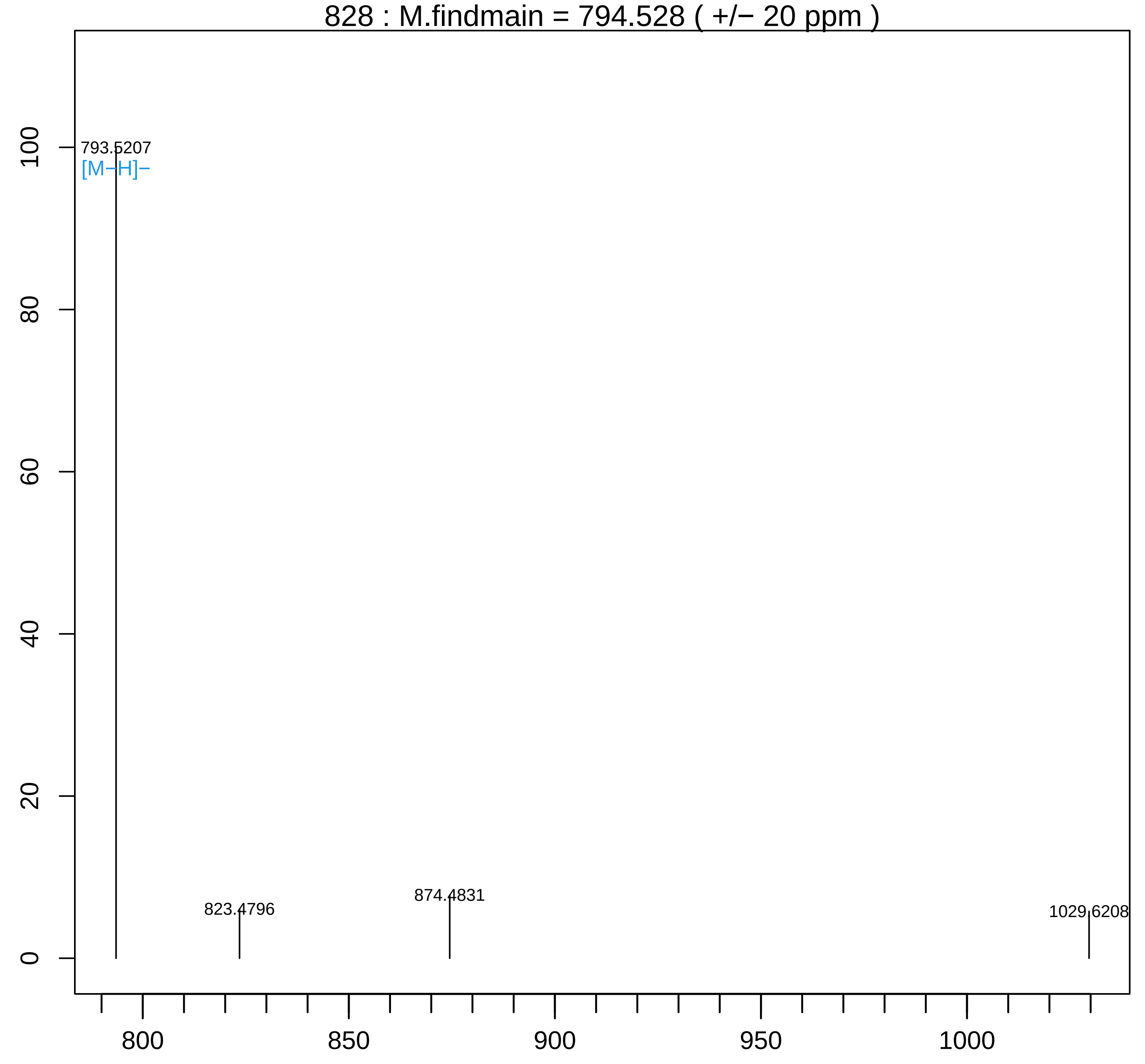
828 : M.findmain = 794.528 (+/− 20 ppm)
793.5207
[M−H]−
823.4796
874.4831
1029.6208
0
20
40
60
80
100
800
850
900
950
1000

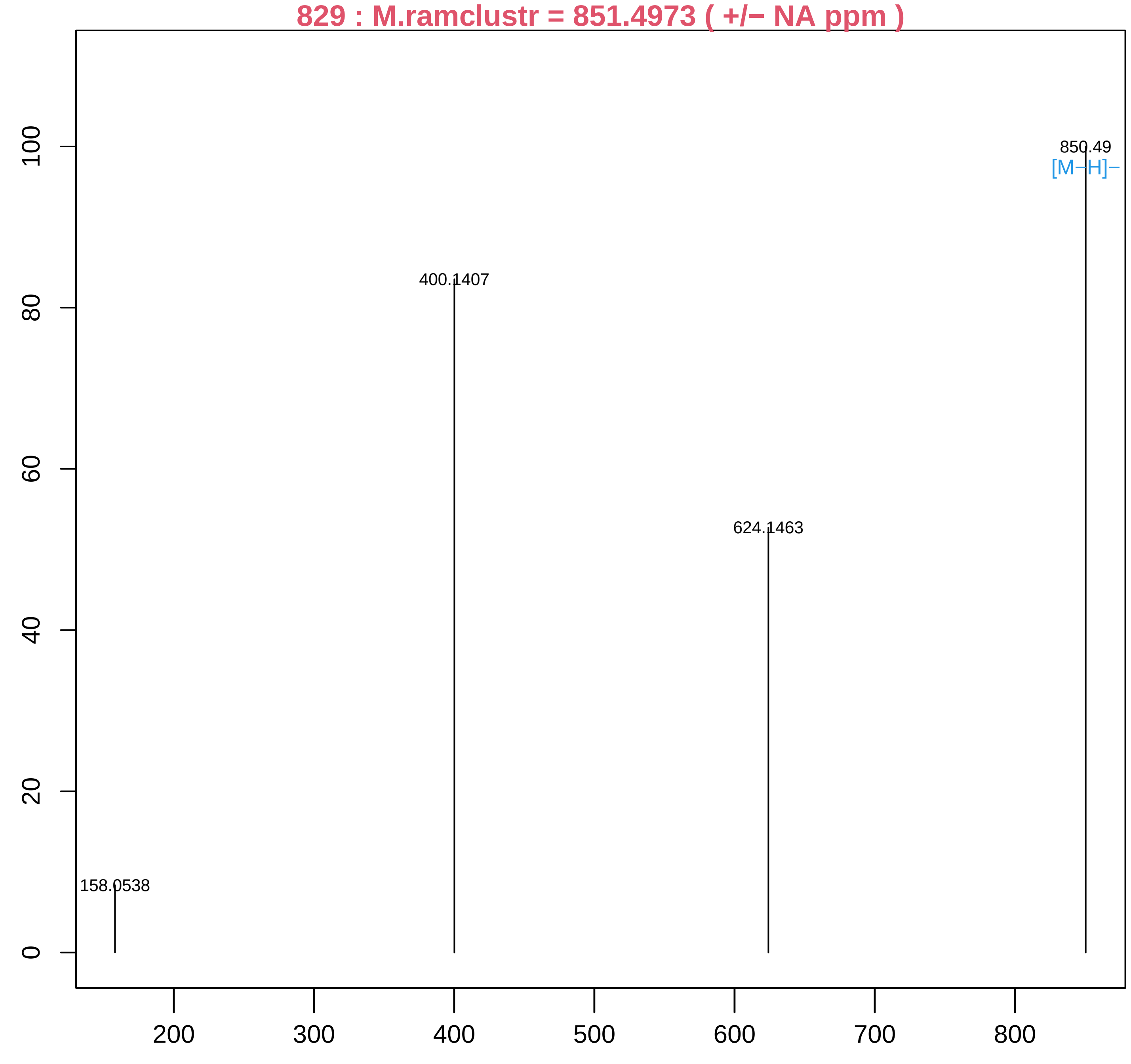
829 : M.ramclustr = 851.4973 (+/− NA ppm)
850.49
[M−H]−
400.1407
624.1463
158.0538
0
20
40
60
80
100
200
300
400
500
600
700
800

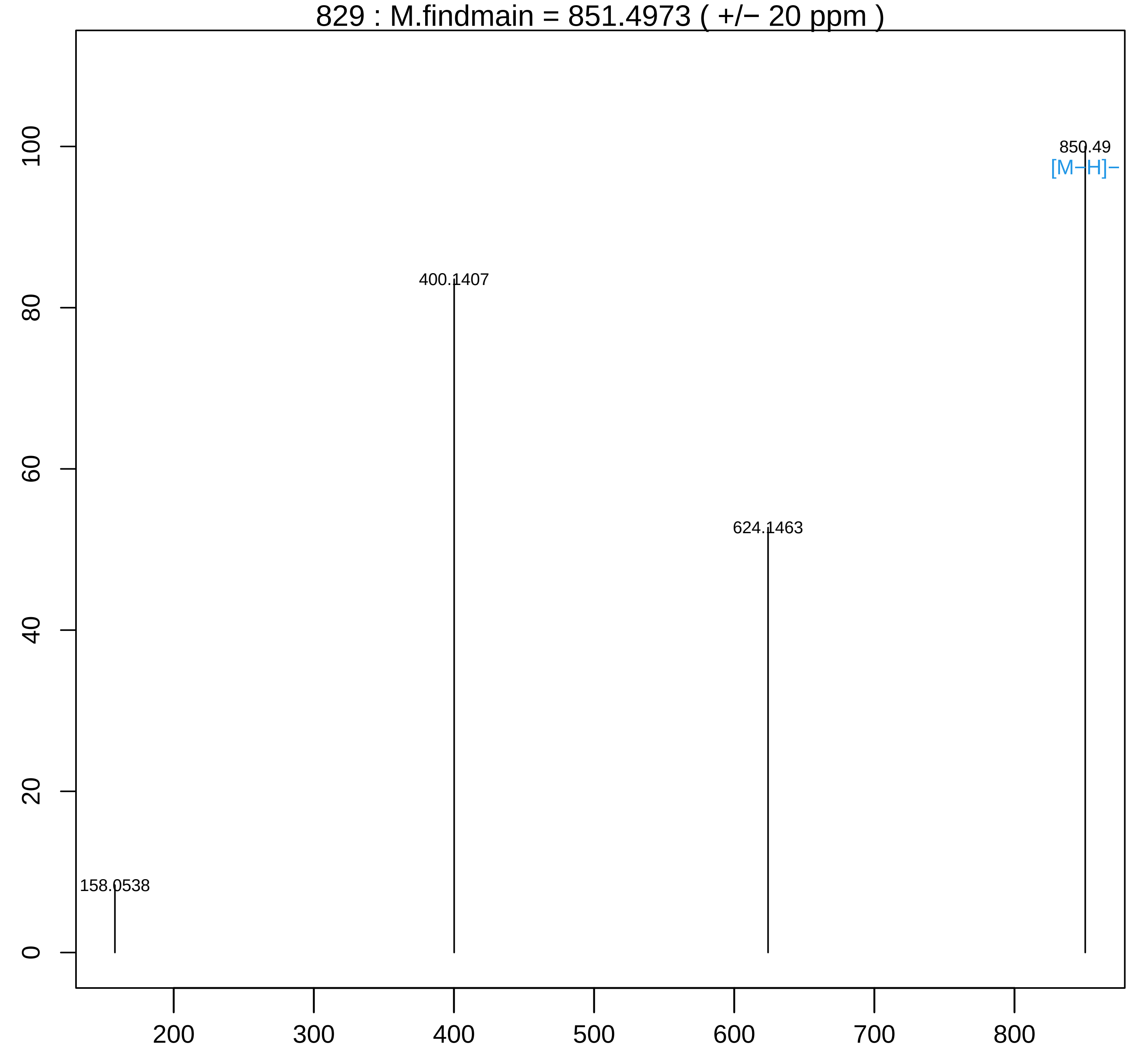
829 : M.findmain = 851.4973 (+/− 20 ppm)
850.49
[M−H]−
400.1407
624.1463
158.0538
0
20
40
60
80
100
200
300
400
500
600
700
800

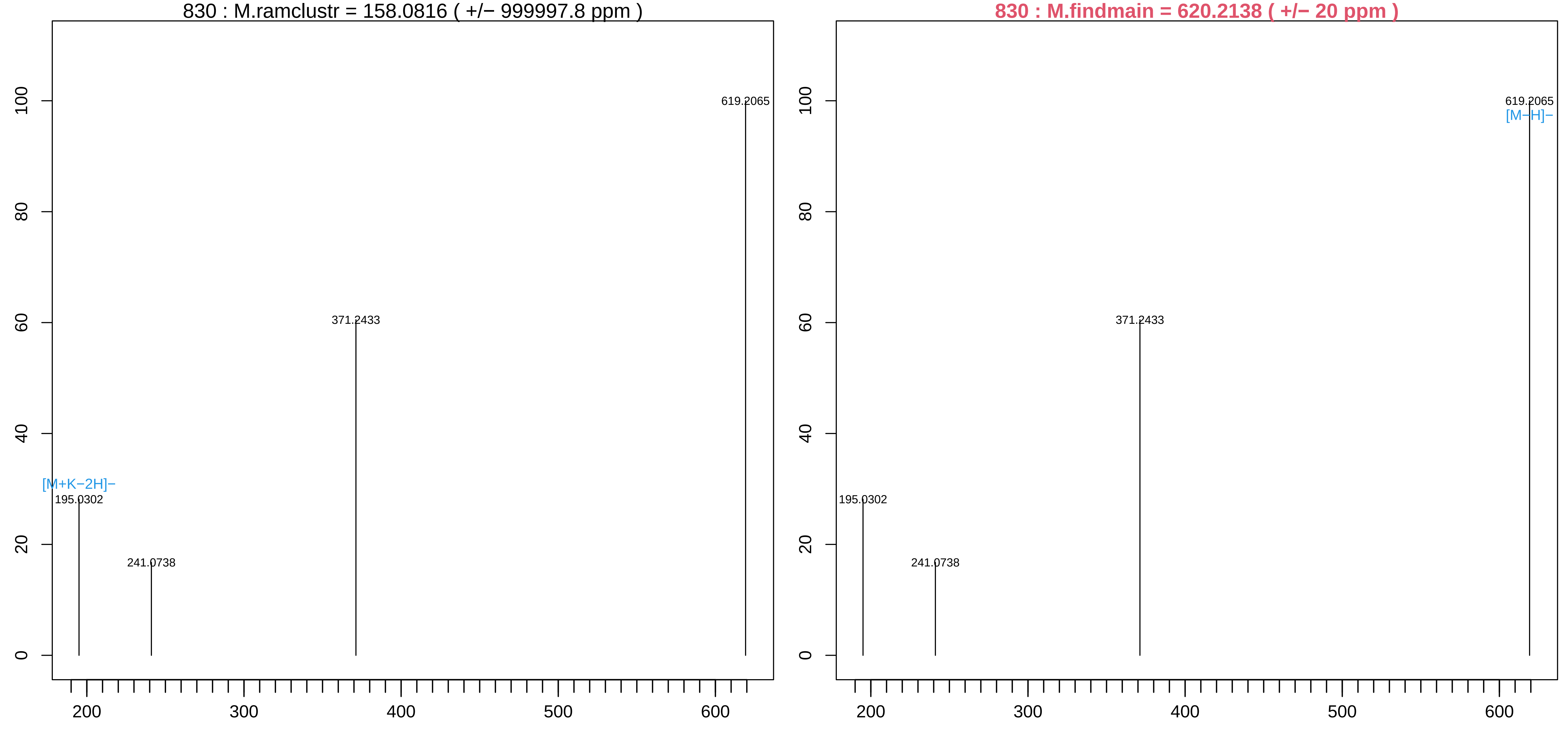
830 : M.ramclustr = 158.0816 (+/− 999997.8 ppm)
619.2065
371.2433
[M+K−2H]−
195.0302
241.0738
0
20
40
60
80
100
200
300
400
500
600
830 : M.findmain = 620.2138 (+/− 20 ppm)
619.2065
[M−H]−
371.2433
195.0302
241.0738
0
20
40
60
80
100
200
300
400
500
600

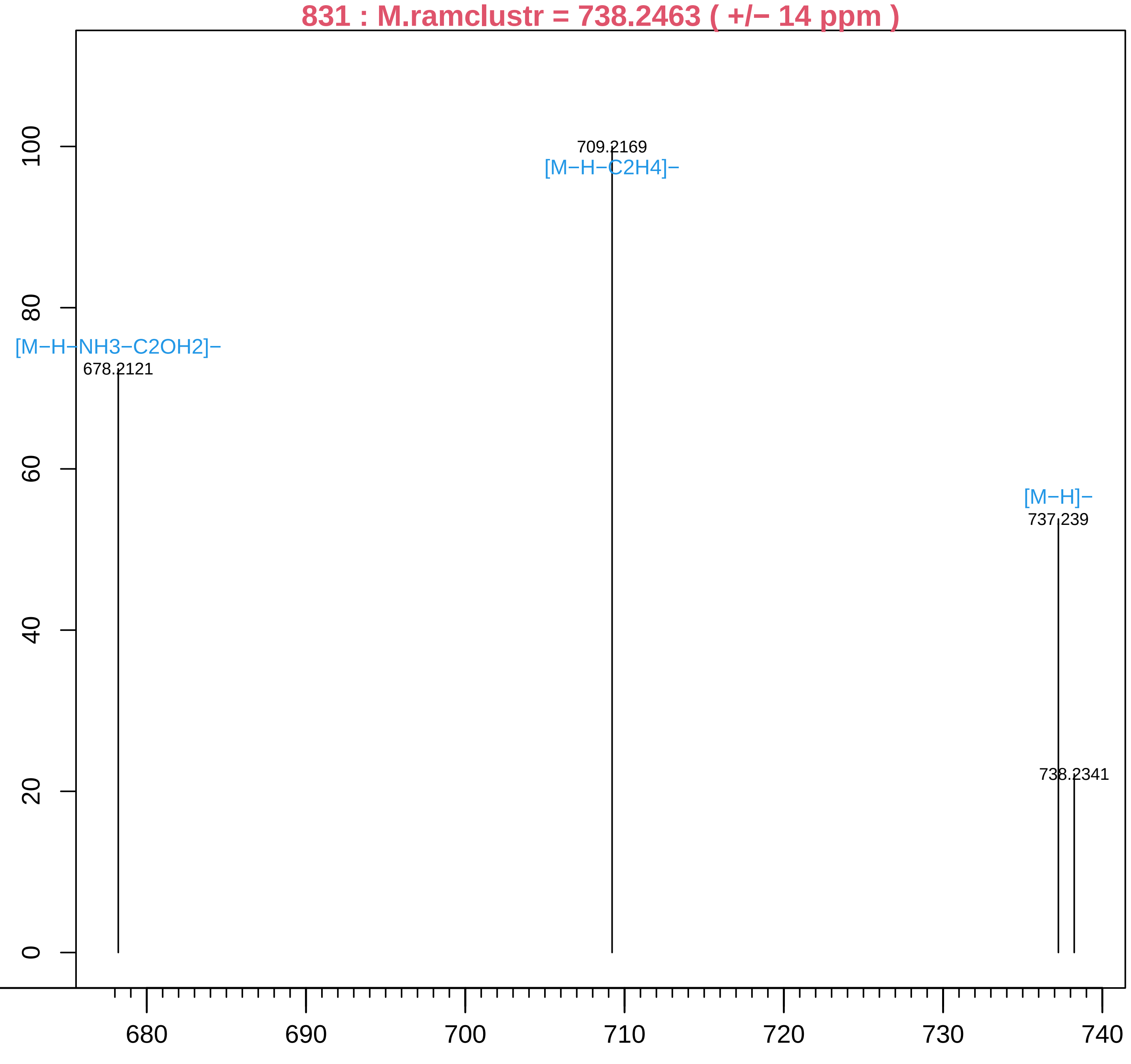

831 : M.ramclustr = 738.2463 (+/− 14 ppm)
709.2169
[M−H−C2H4]−
[M−H−NH3−C2OH2]−
678.2121
[M−H]−
737.239
738.2341
0
20
40
60
80
100
680
690
700
710
720
730
740

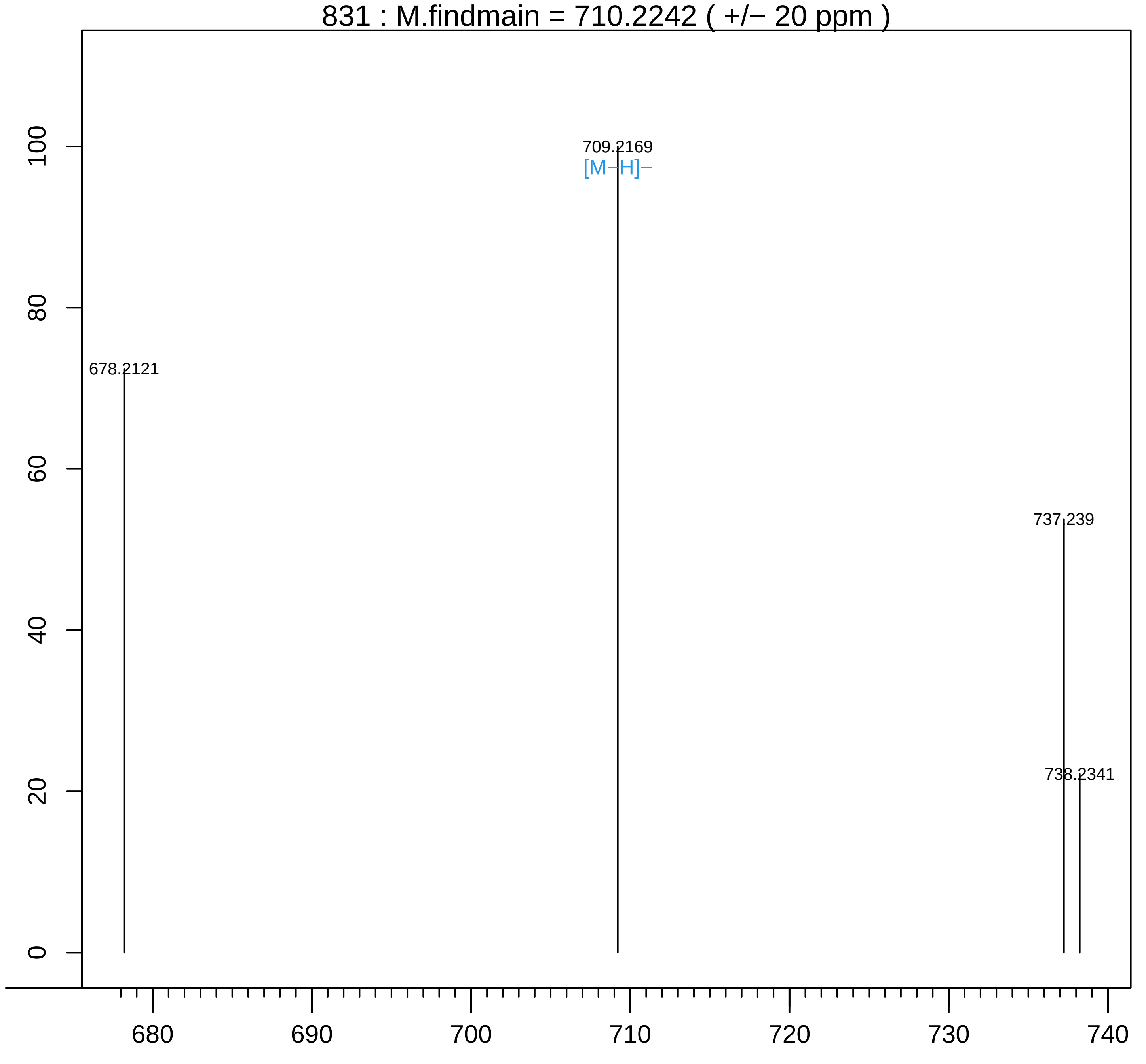

831 : M.findmain = 710.2242 (+/− 20 ppm)
709.2169
[M−H]−
678.2121
737.239
738.2341
0
20
40
60
80
100
680
690
700
710
720
730
740

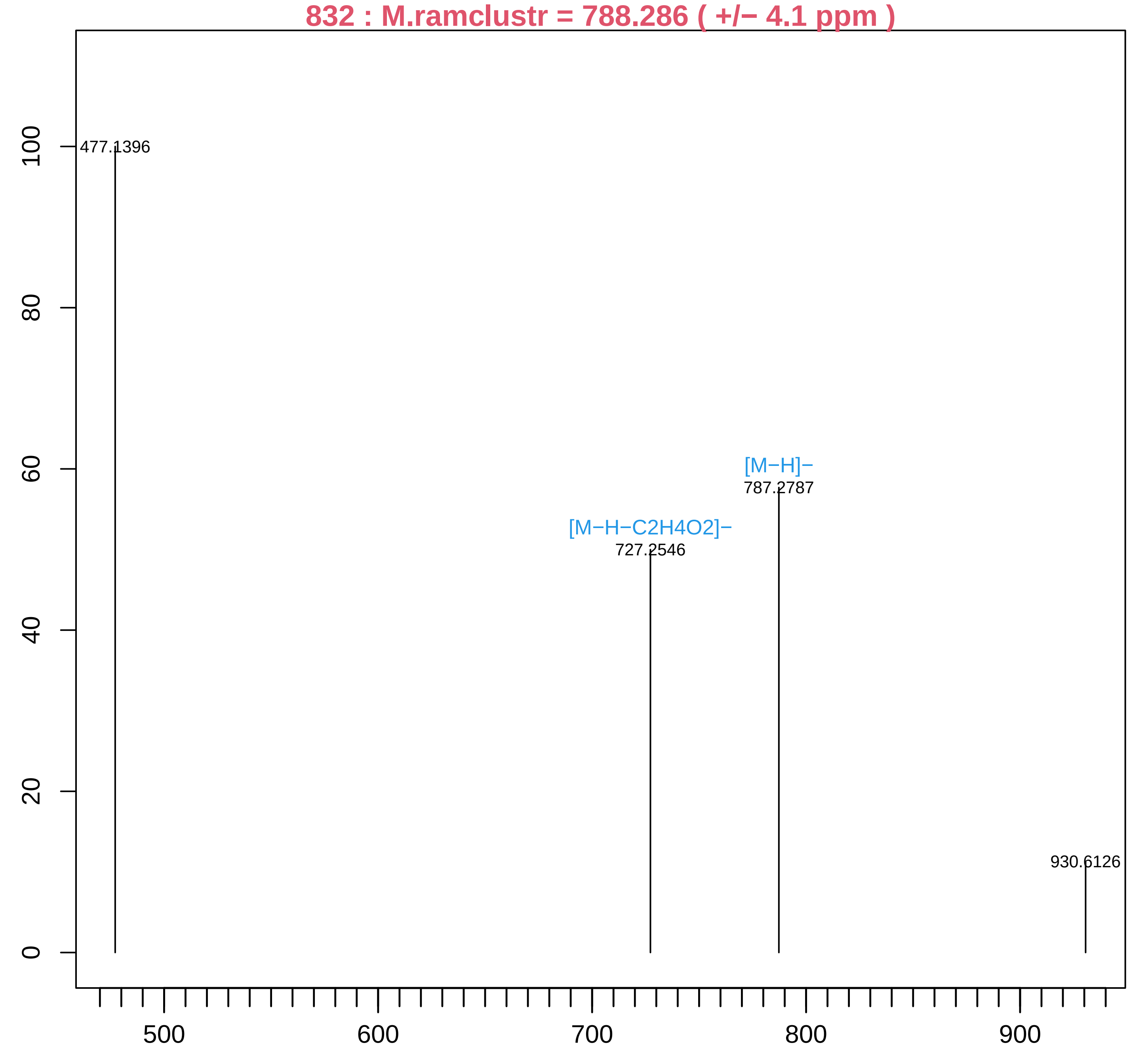
832 : M.ramclustr = 788.286 (+/− 4.1 ppm)
477.1396
[M−H−C2H4O2]−
727.2546
[M−H]−
787.2787
930.6126
0
20
40
60
80
100
500
600
700
800
900

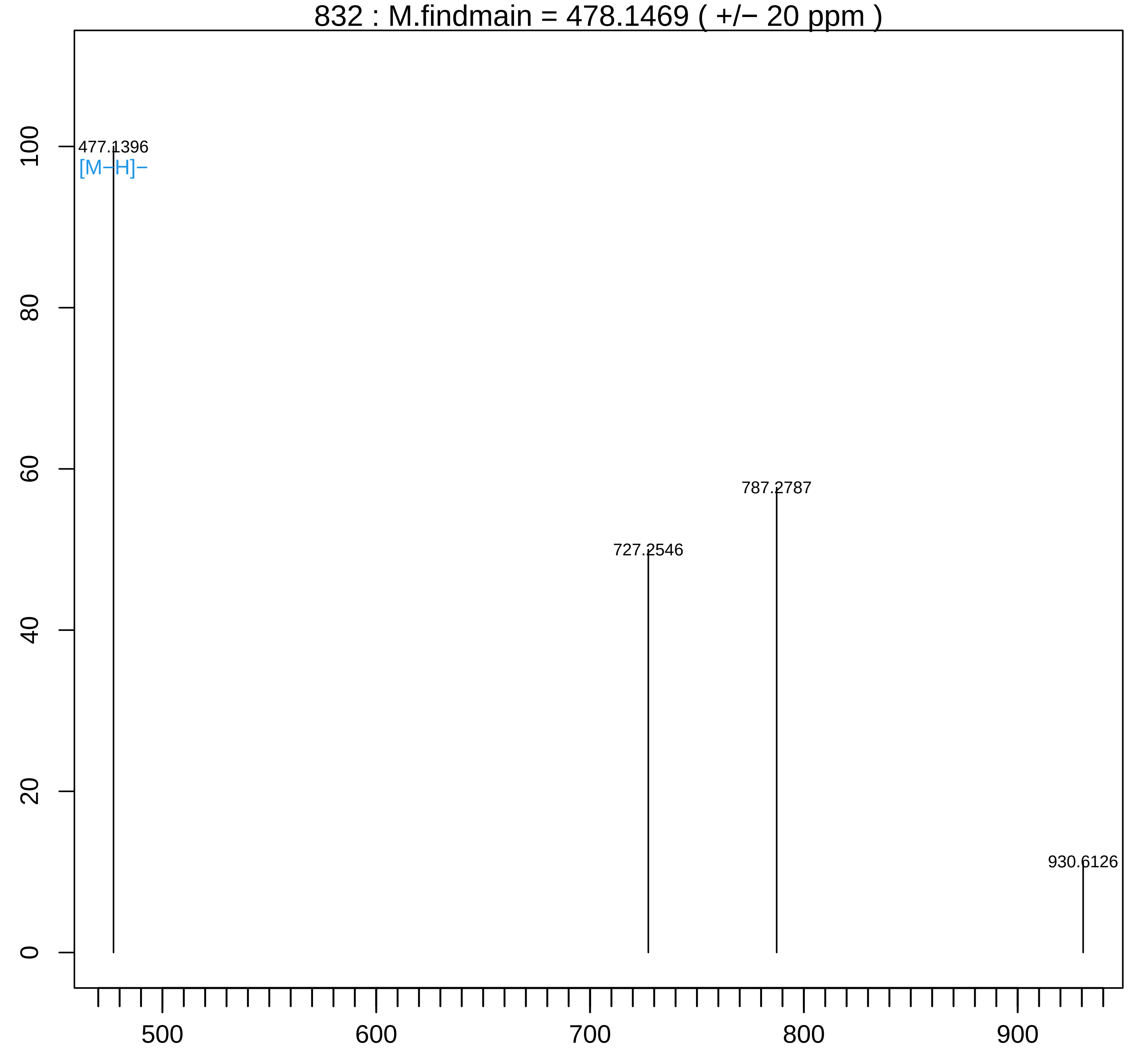
832 : M.findmain = 478.1469 (+/− 20 ppm)
477.1396
[M−H]−
727.2546
787.2787
930.6126
0
20
40
60
80
100
500
600
700
800
900

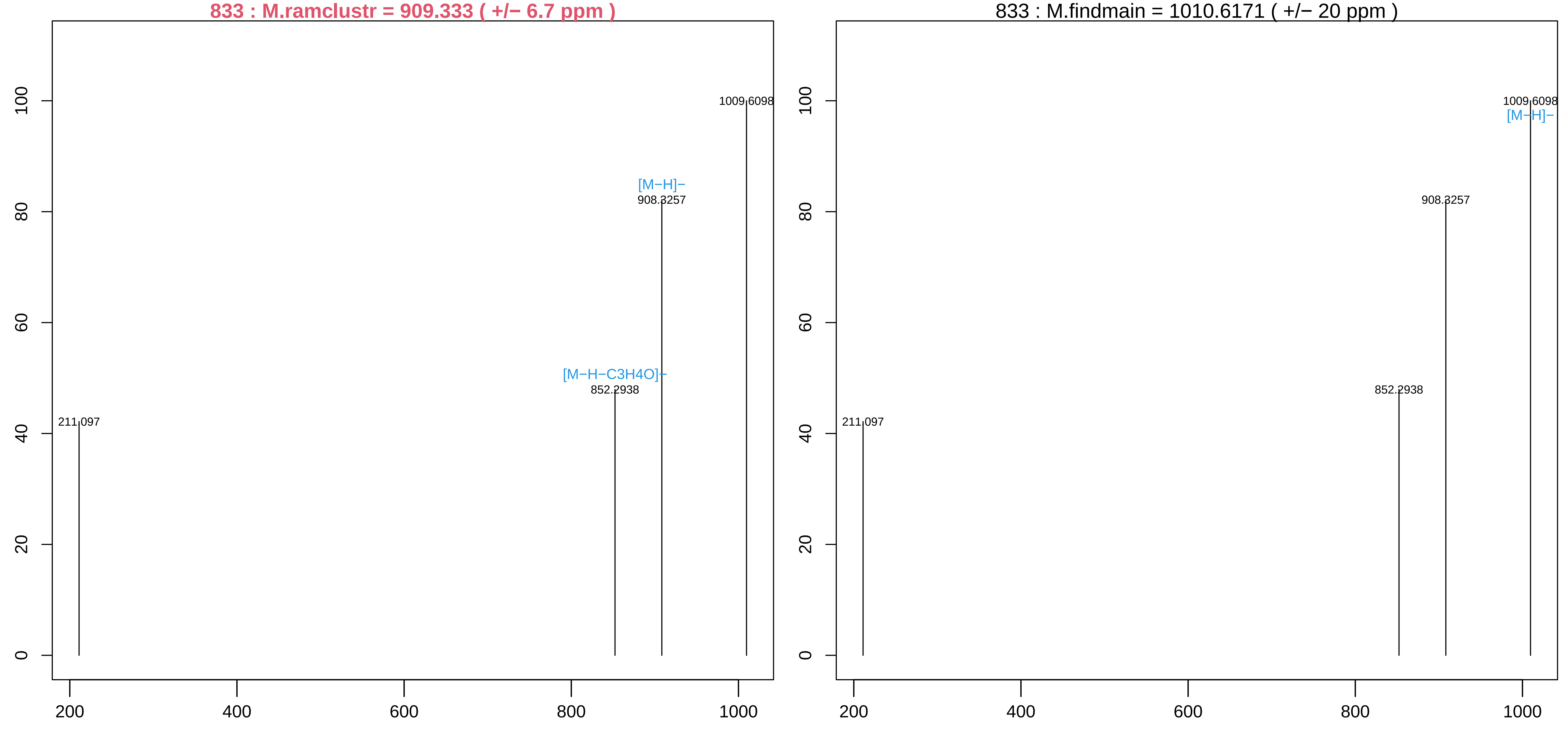
833 : M.ramclustr = 909.333 (+/− 6.7 ppm)
1009.6098
[M−H]−
908.3257
[M−H−C3H4O]−
852.2938
211.097
833 : M.findmain = 1010.6171 (+/− 20 ppm)
1009.6098
[M−H]−
908.3257
852.2938
211.097

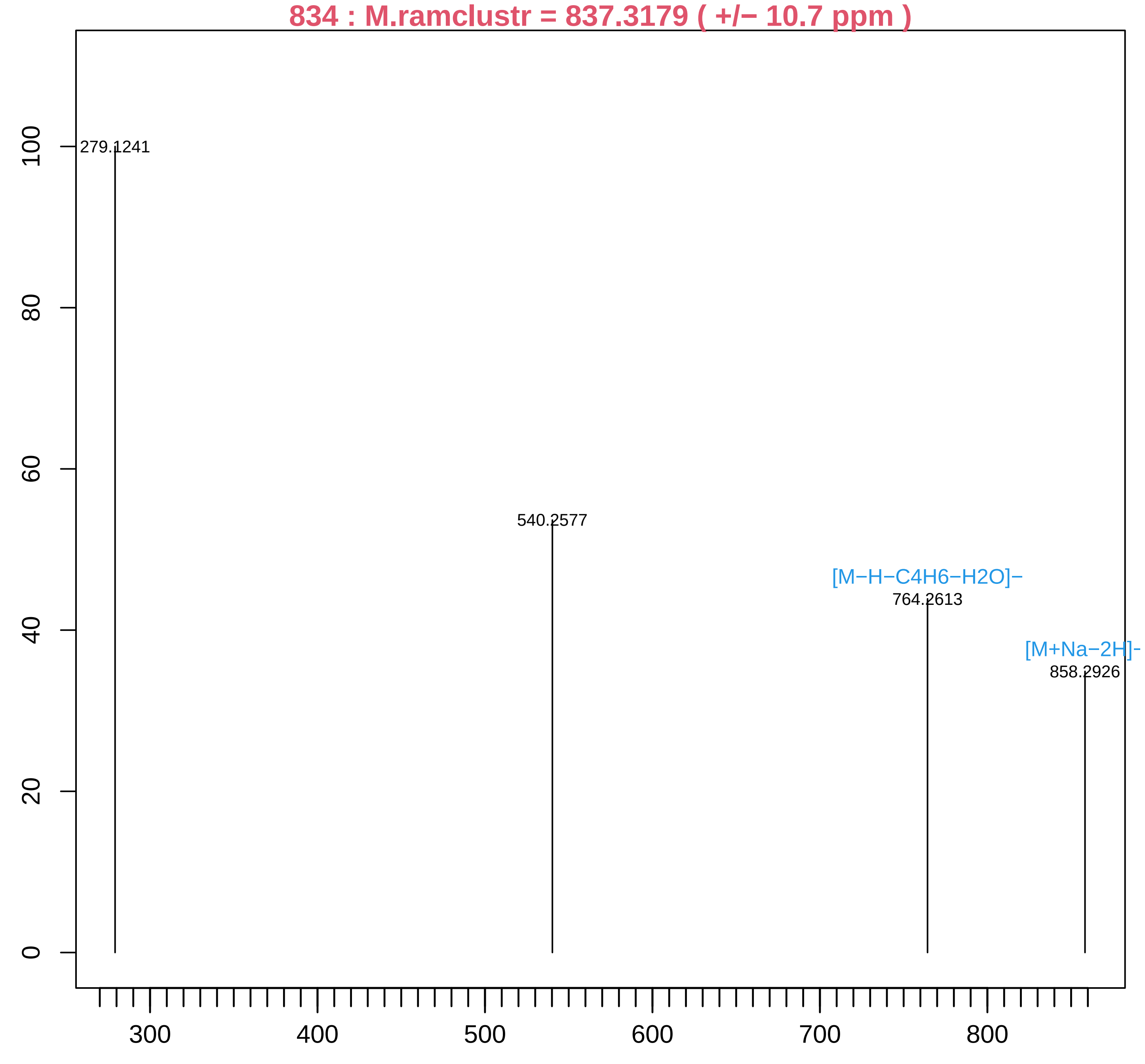
834 : M.ramclustr = 837.3179 (+/− 10.7 ppm)
279.1241
540.2577
[M−H−C4H6−H2O]−
764.2613
[M+Na−2H]-
858.2926
0
20
40
60
80
100
300
400
500
600
700
800

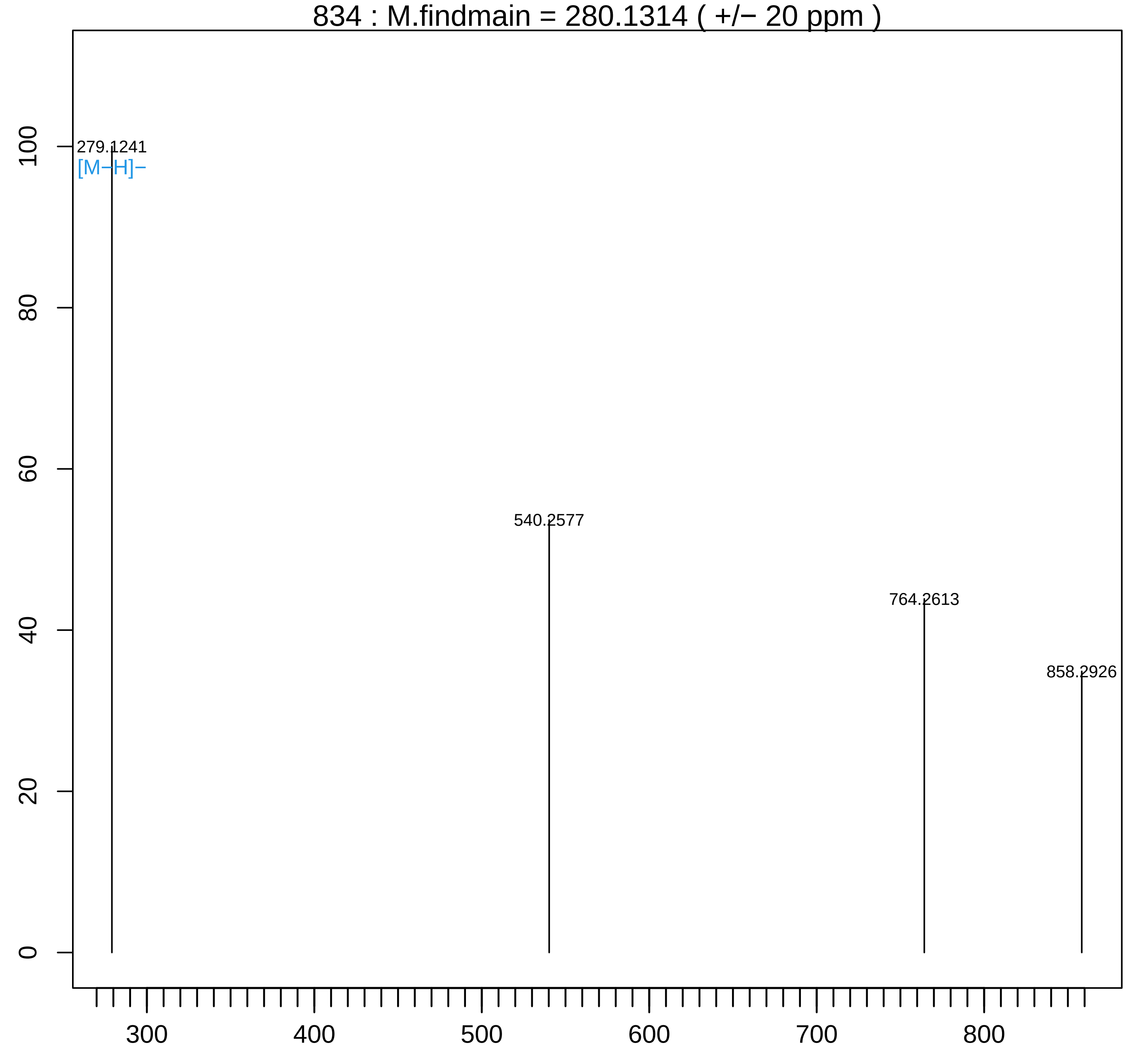
834 : M.findmain = 280.1314 (+/− 20 ppm)
279.1241
[M−H]−
540.2577
764.2613
858.2926
0
20
40
60
80
100
300
400
500
600
700
800

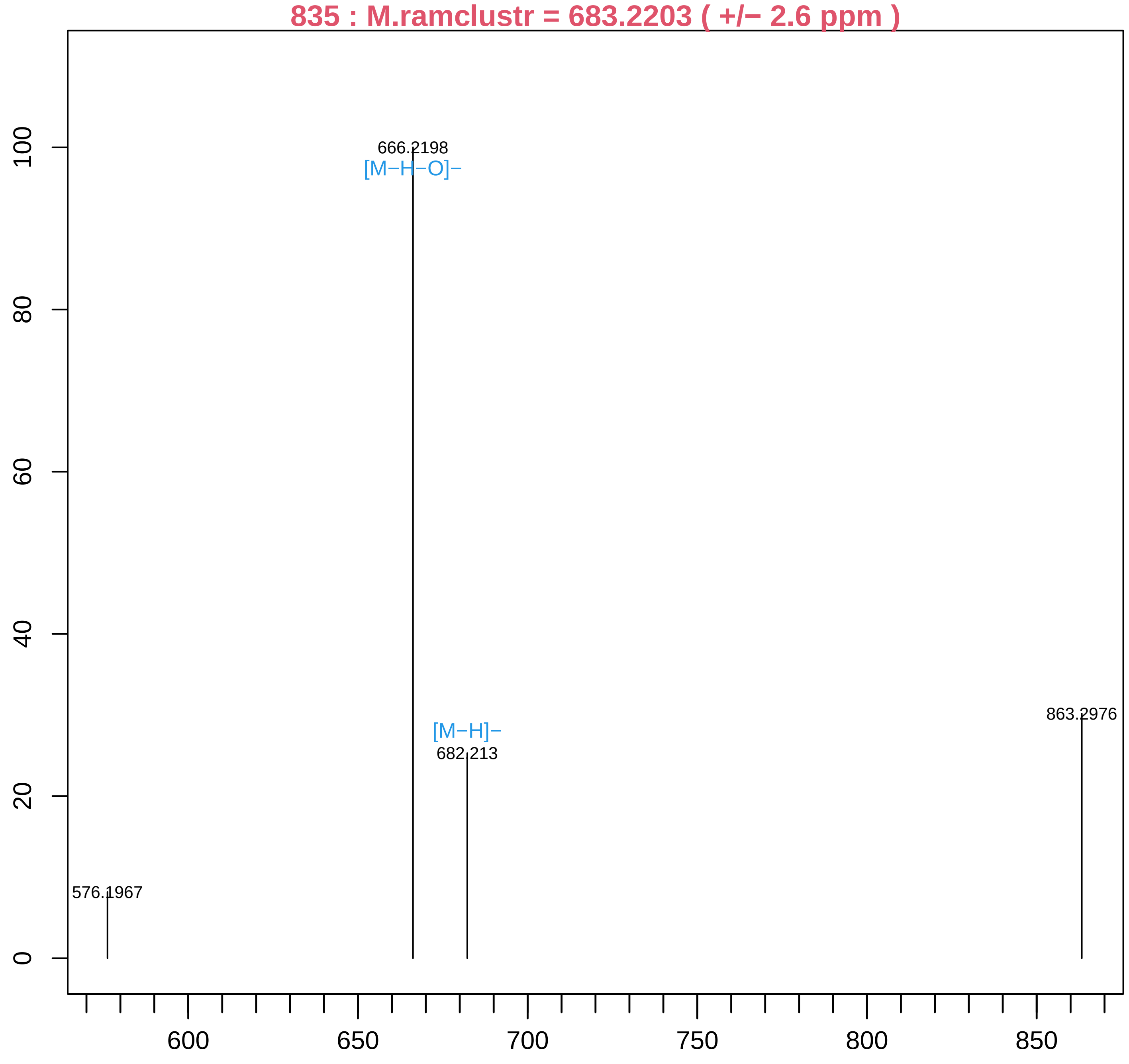

835 : M.ramclustr = 683.2203 (+/− 2.6 ppm)
666.2198
[M−H−O]−
[M−H]−
682.213
863.2976
576.1967
0
20
40
60
80
100
600
650
700
750
800
850

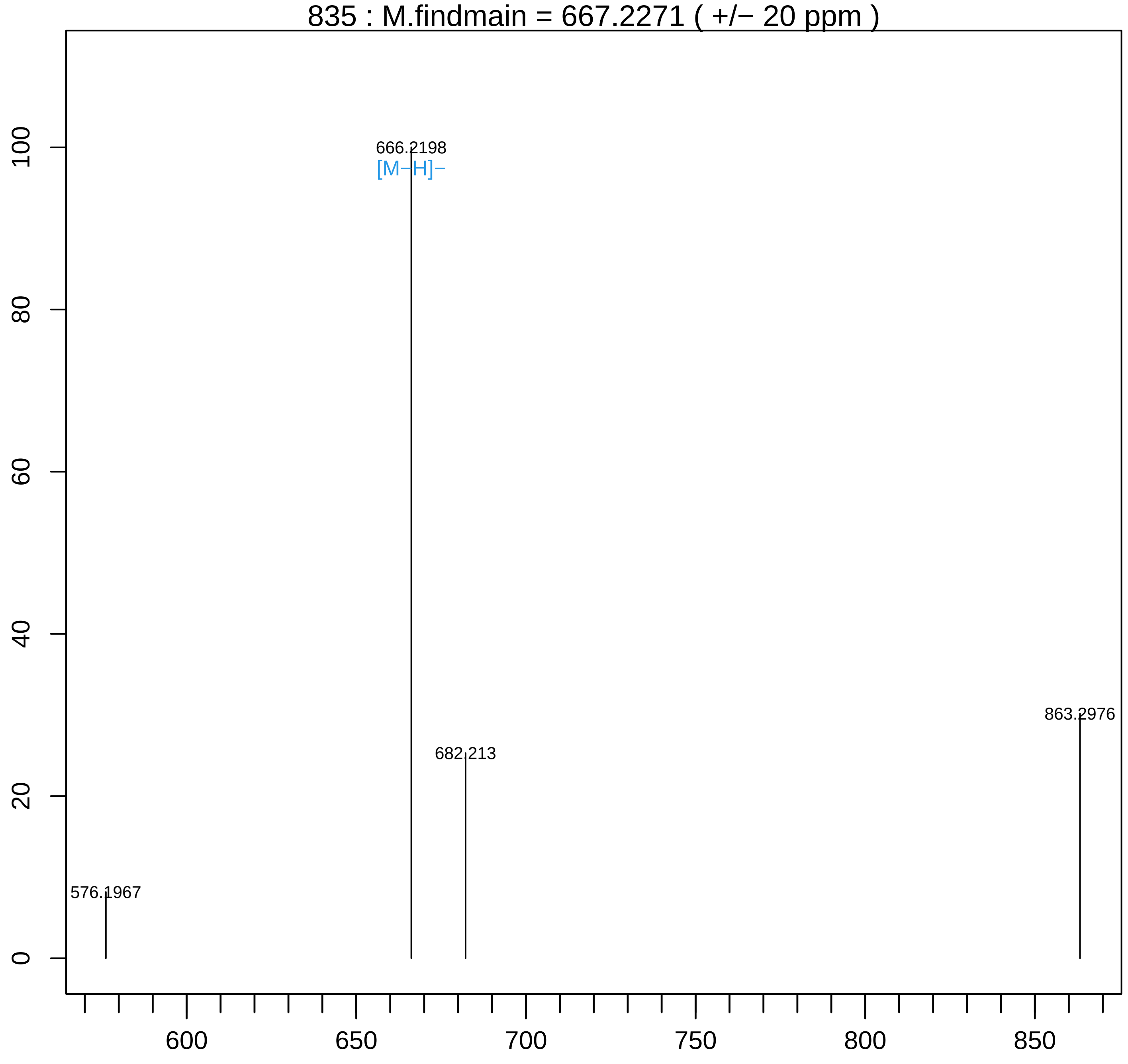

835 : M.findmain = 667.2271 (+/− 20 ppm)
666.2198
[M−H]−
682.213
863.2976
576.1967
0
20
40
60
80
100
600
650
700
750
800
850

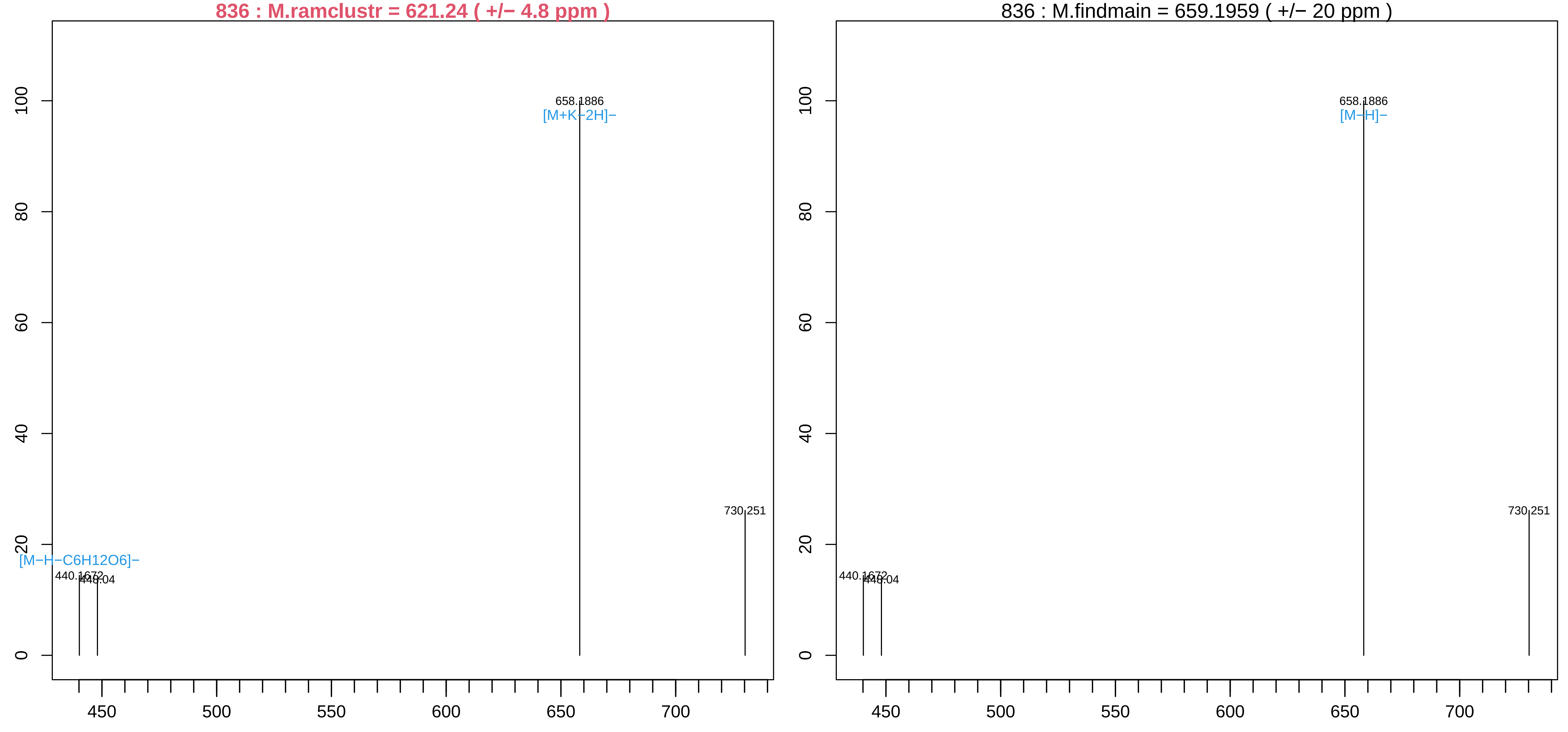
836 : M.ramclustr = 621.24 (+/− 4.8 ppm)
658.1886
[M+K−2H]−
730.251
[M−H−C6H12O6]−
440.1672
446.04
836 : M.findmain = 659.1959 (+/− 20 ppm)
658.1886
[M−H]−
730.251
440.1672
446.04
0
20
40
60
80
100
450
500
550
600
650
700

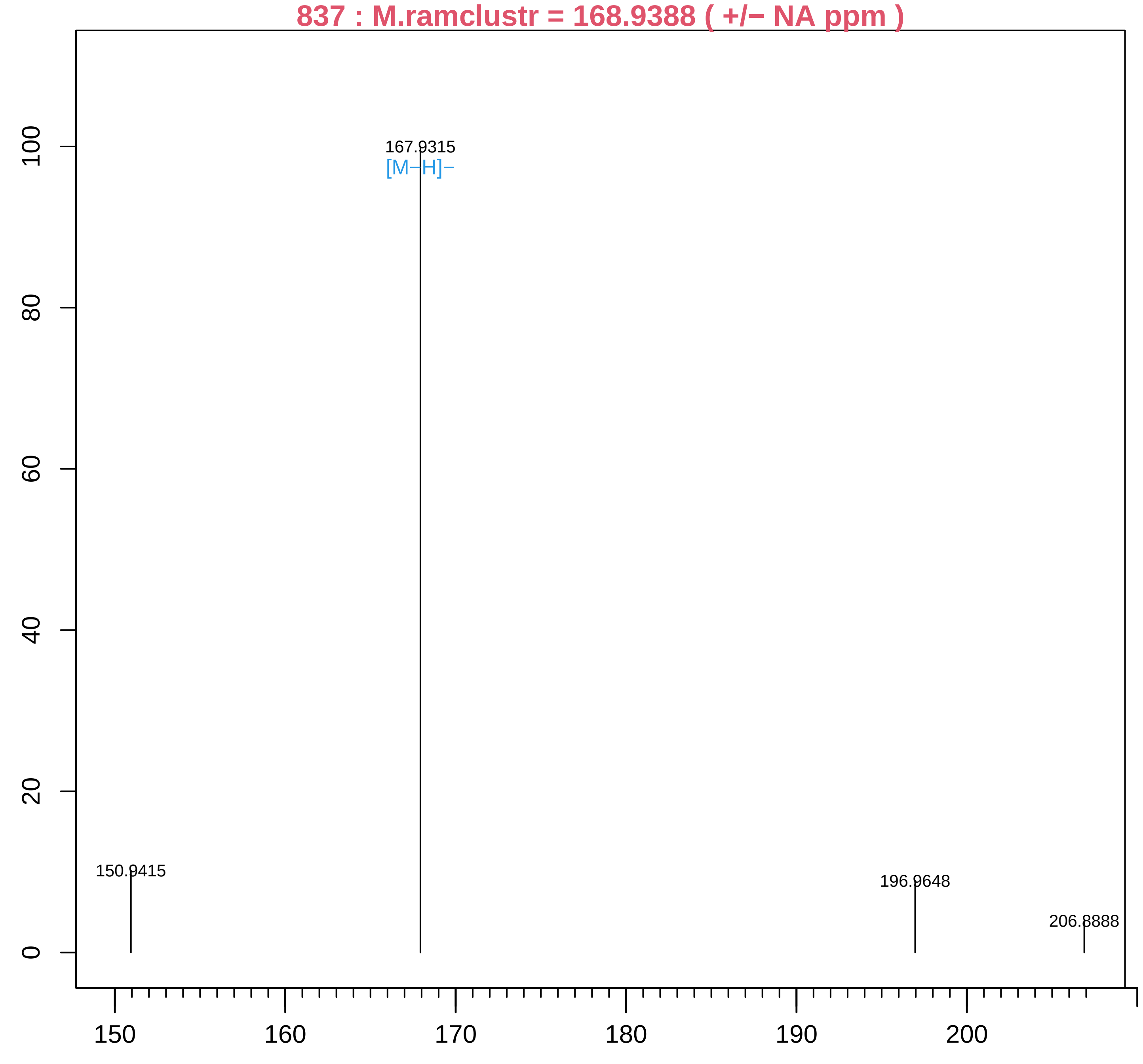
837 : M.ramclustr = 168.9388 (+/− NA ppm)
167.9315
[M−H]−
150.9415
196.9648
206.8888
0
20
40
60
80
100
150
160
170
180
190
200

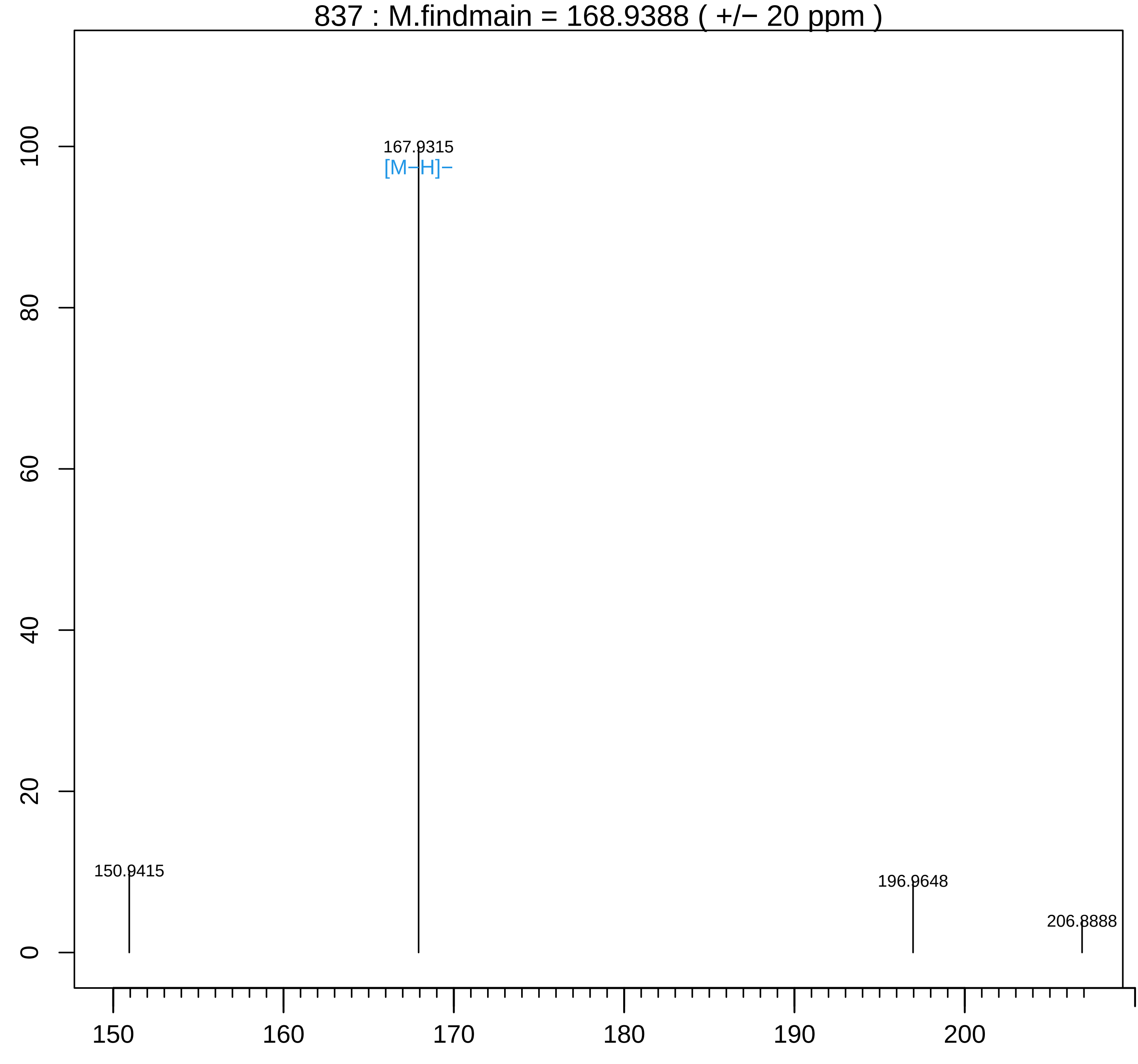
837 : M.findmain = 168.9388 (+/− 20 ppm)
167.9315
[M−H]−
150.9415
196.9648
206.8888
0
20
40
60
80
100
150
160
170
180
190
200

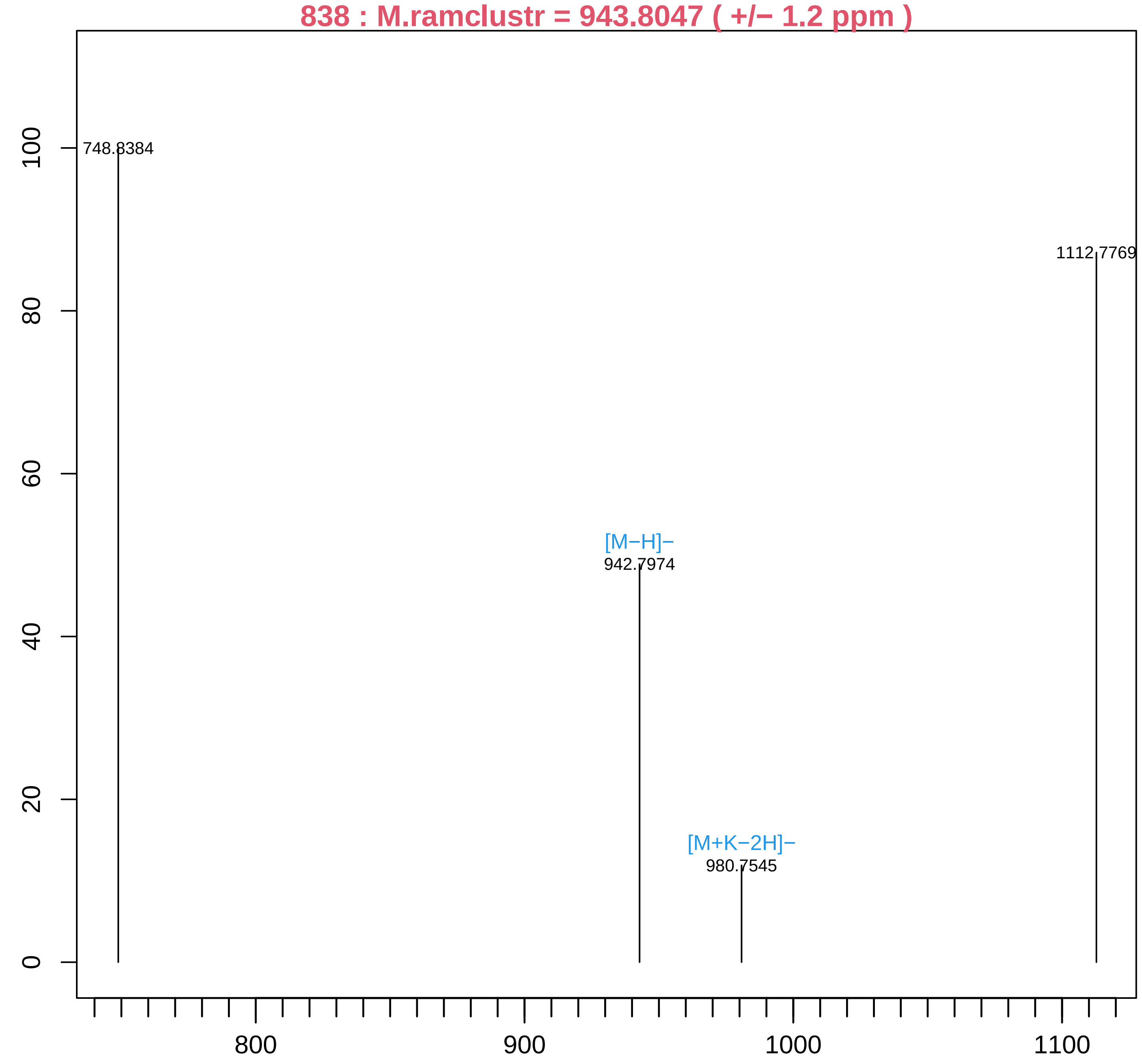
838 : M.ramclustr = 943.8047 (+/− 1.2 ppm)
748.8384
1112.7769
[M−H]−
942.7974
[M+K−2H]−
980.7545
0
20
40
60
80
100
800
900
1000
1100
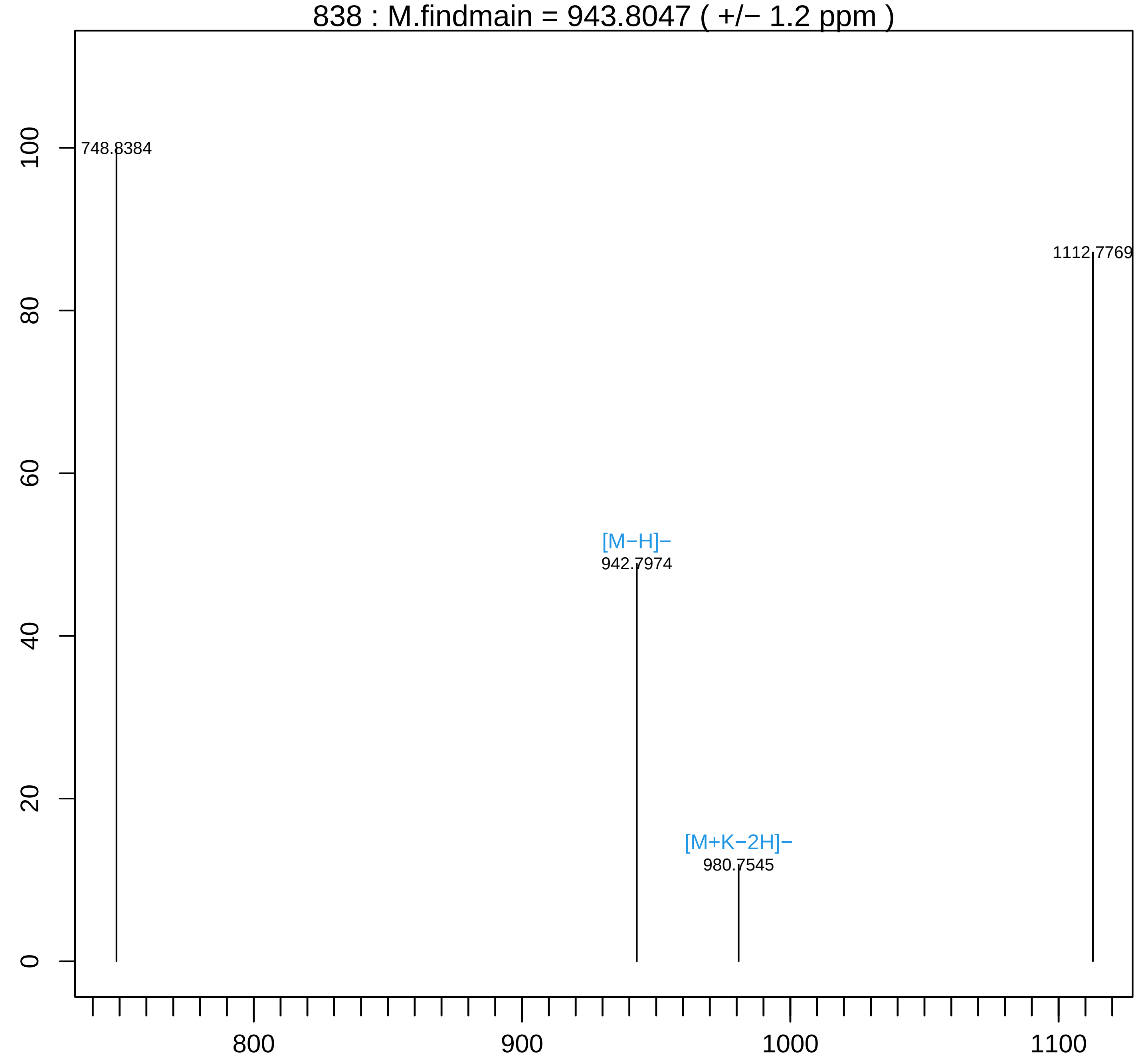
838 : M.findmain = 943.8047 (+/− 1.2 ppm)
748.8384
1112.7769
[M−H]−
942.7974
[M+K−2H]−
980.7545
0
20
40
60
80
100
800
900
1000
1100

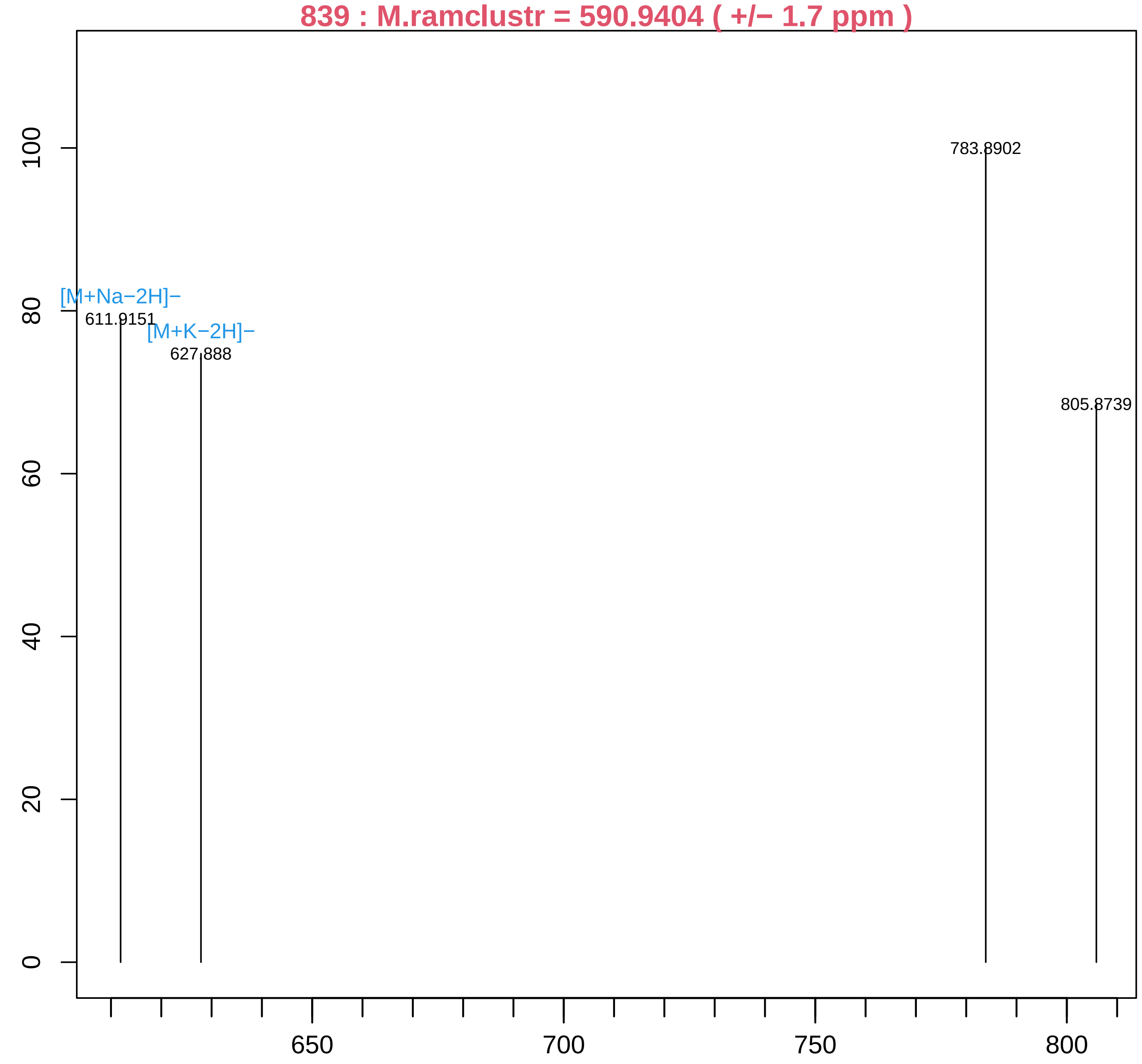

839 : M.ramclustr = 590.9404 (+/− 1.7 ppm)
[M+Na−2H]−
611.9151
[M+K−2H]−
627.888
783.8902
805.8739
0
20
40
60
80
100
650
700
750
800

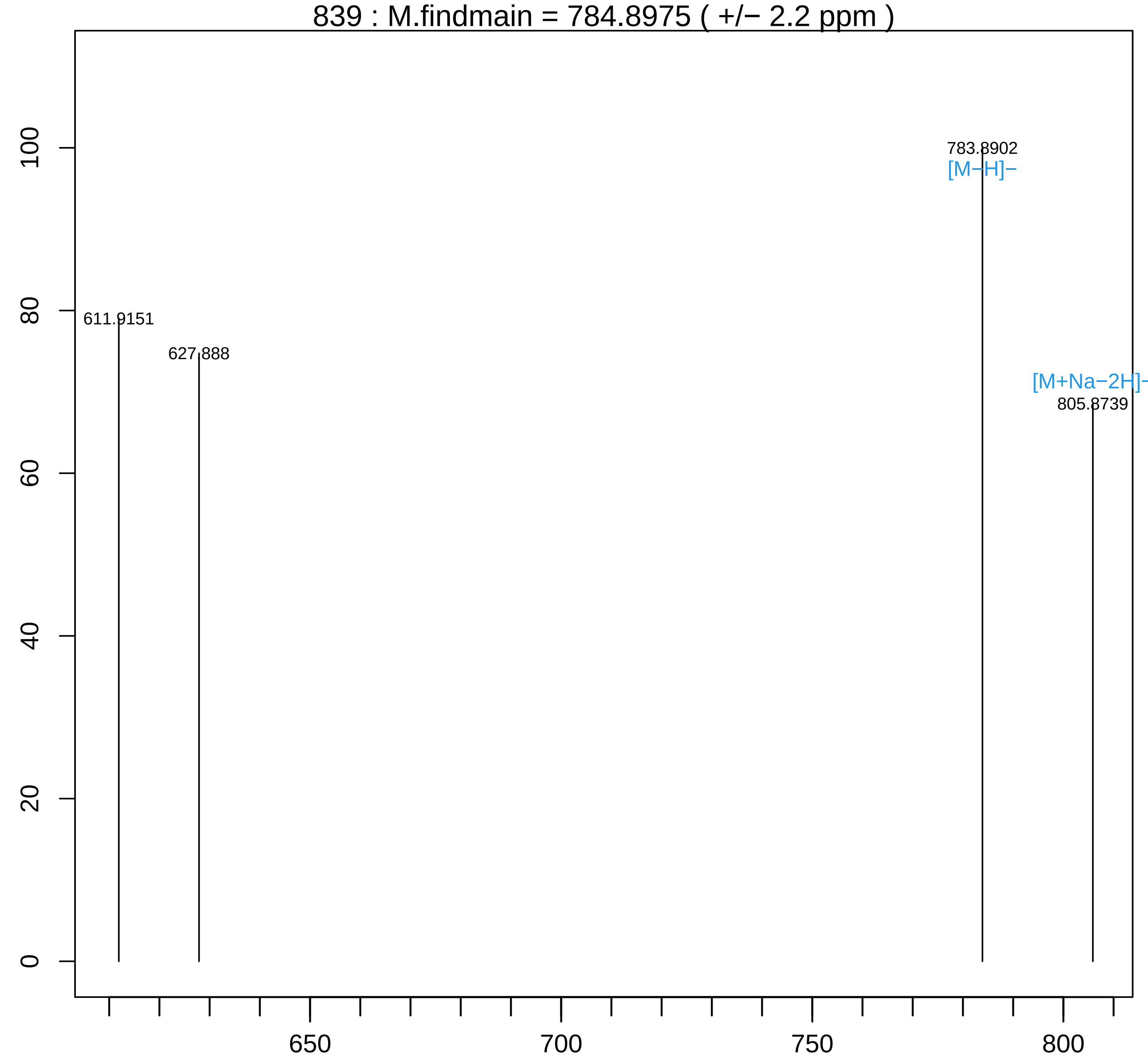

839 : M.findmain = 784.8975 (+/− 2.2 ppm)
611.9151
627.888
783.8902
[M−H]−
[M+Na−2H]−
805.8739
0
20
40
60
80
100
650
700
750
800

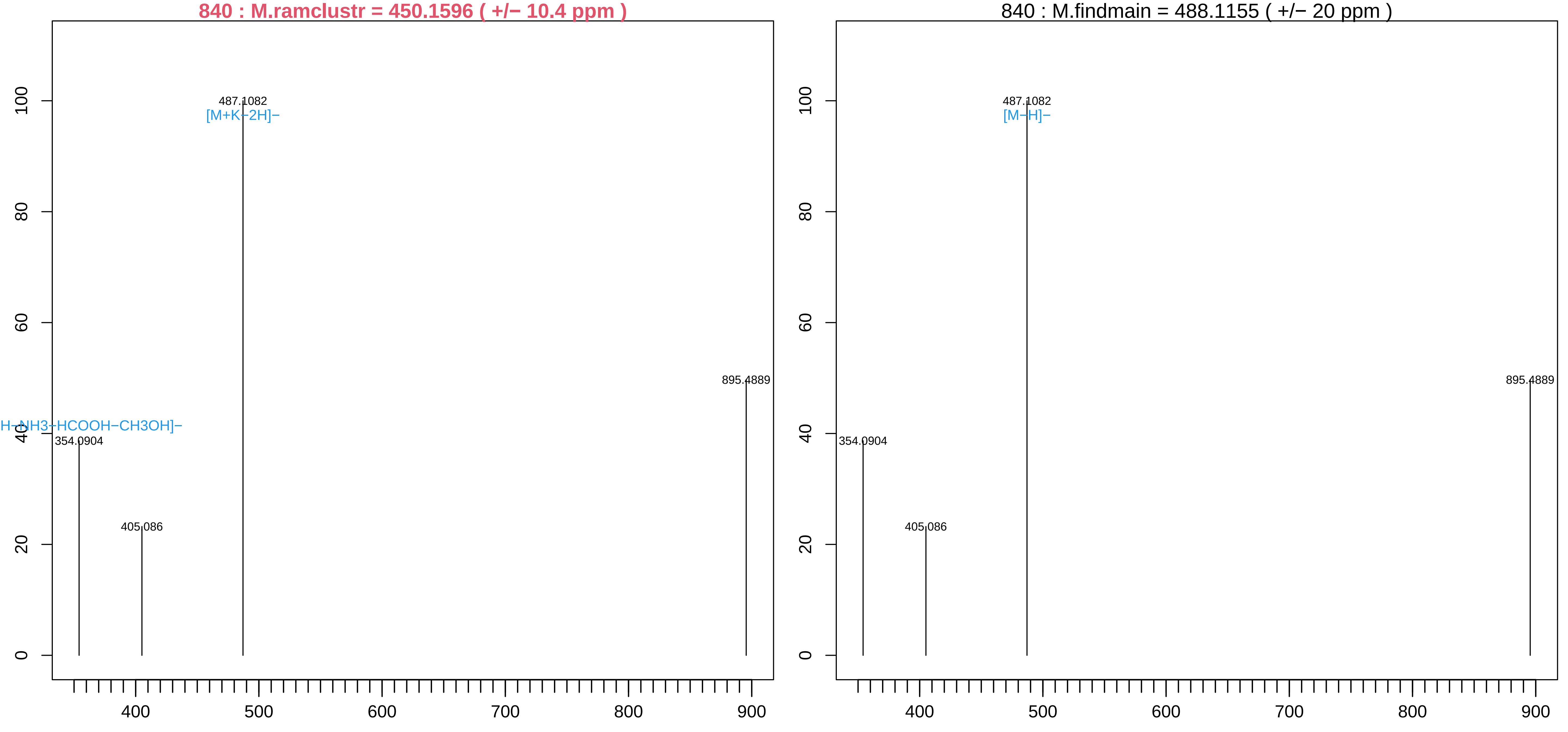

840 : M.ramclustr = 450.1596 (+/− 10.4 ppm)
487.1082
[M+K−2H]−
H−NH3−HCOOH−CH3OH]−
354.0904
405.086
895.4889
840 : M.findmain = 488.1155 (+/− 20 ppm)
487.1082
[M−H]−
354.0904
405.086
895.4889
0
20
40
60
80
100
400
500
600
700
800
900

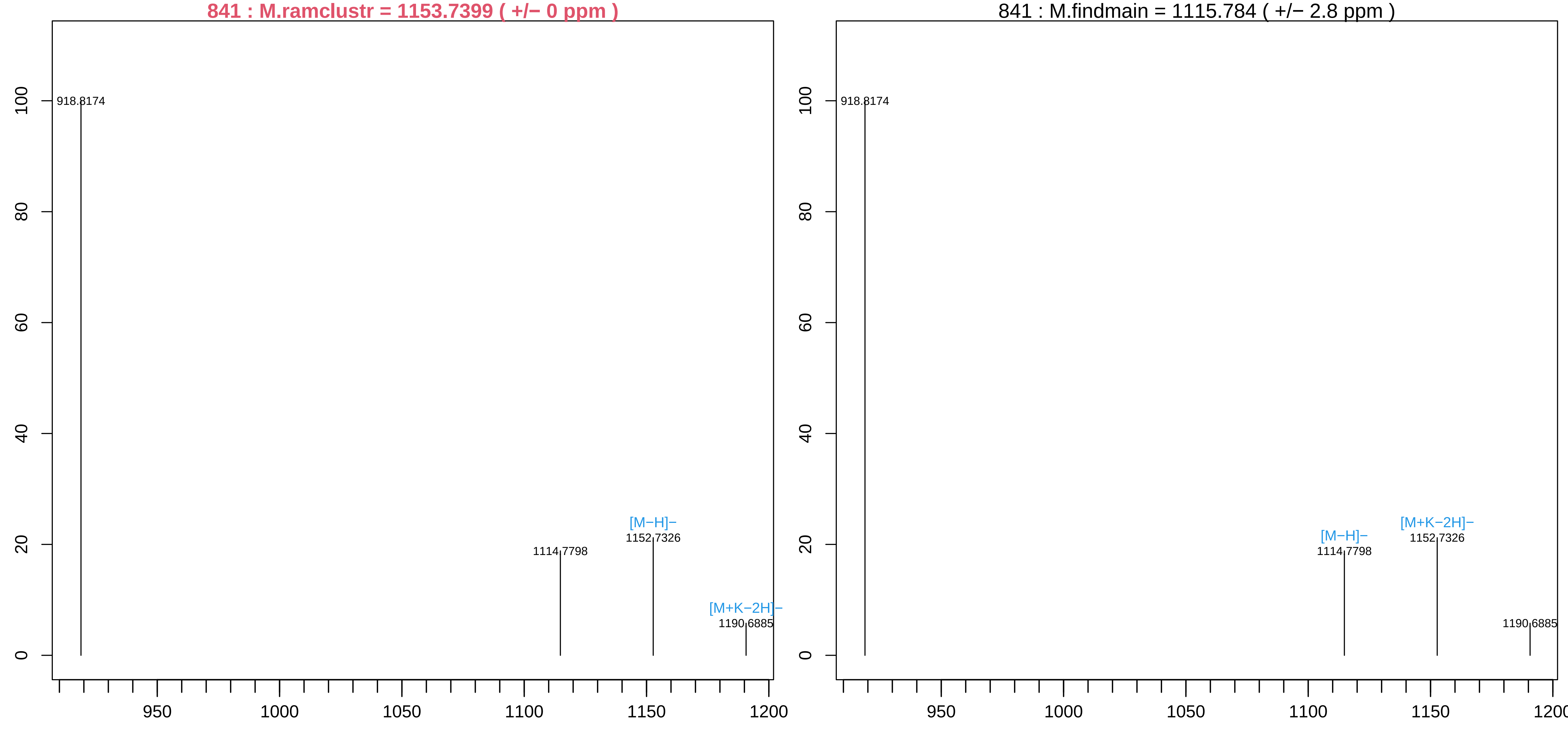

841 : M.ramclustr = 1153.7399 (+/− 0 ppm)
918.8174
1114.7798
[M−H]−
1152.7326
[M+K−2H]−
1190.6885
0
20
40
60
80
100
950
1000
1050
1100
1150
1200
841 : M.findmain = 1115.784 (+/− 2.8 ppm)
918.8174
[M−H]−
1114.7798
[M+K−2H]−
1152.7326
1190.6885

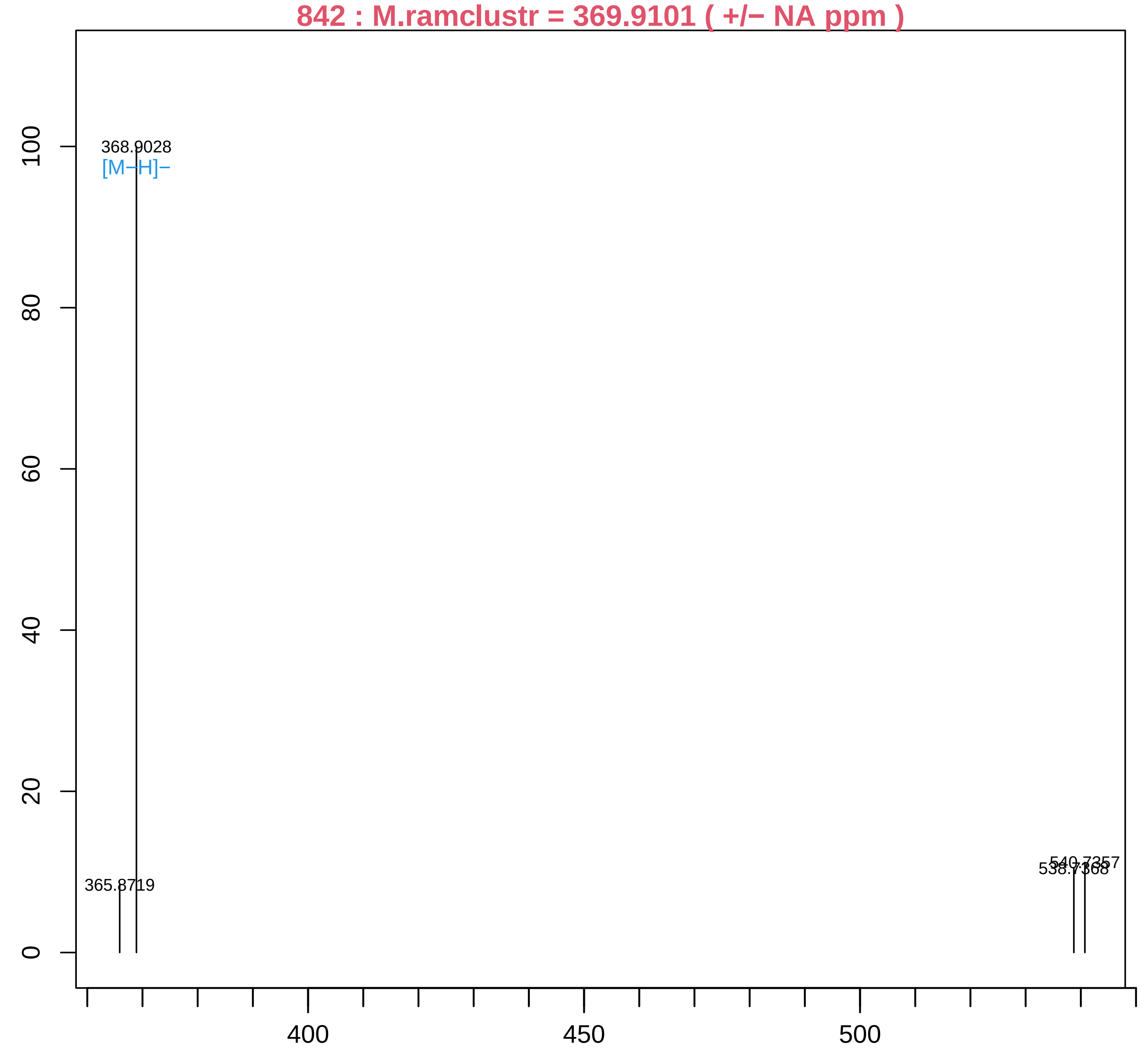

842 : M.ramclustr = 369.9101 (+/− NA ppm)
368.9028
[M−H]−
365.8719
540.7357
538.7368
0
20
40
60
80
100
400
450
500

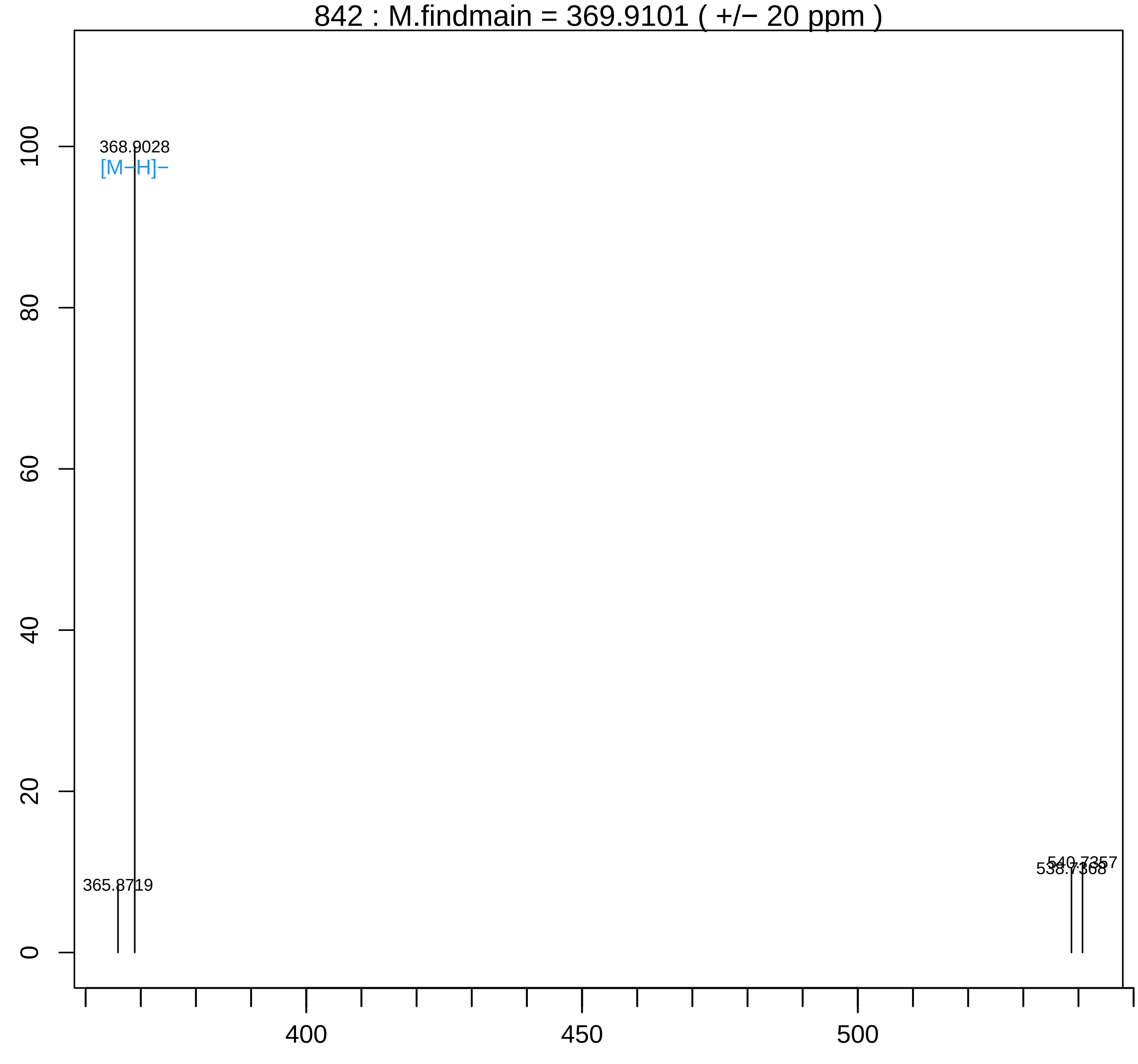

842 : M.findmain = 369.9101 (+/− 20 ppm)
368.9028
[M−H]−
365.8719
540.7357
538.7368
0
20
40
60
80
100
400
450
500

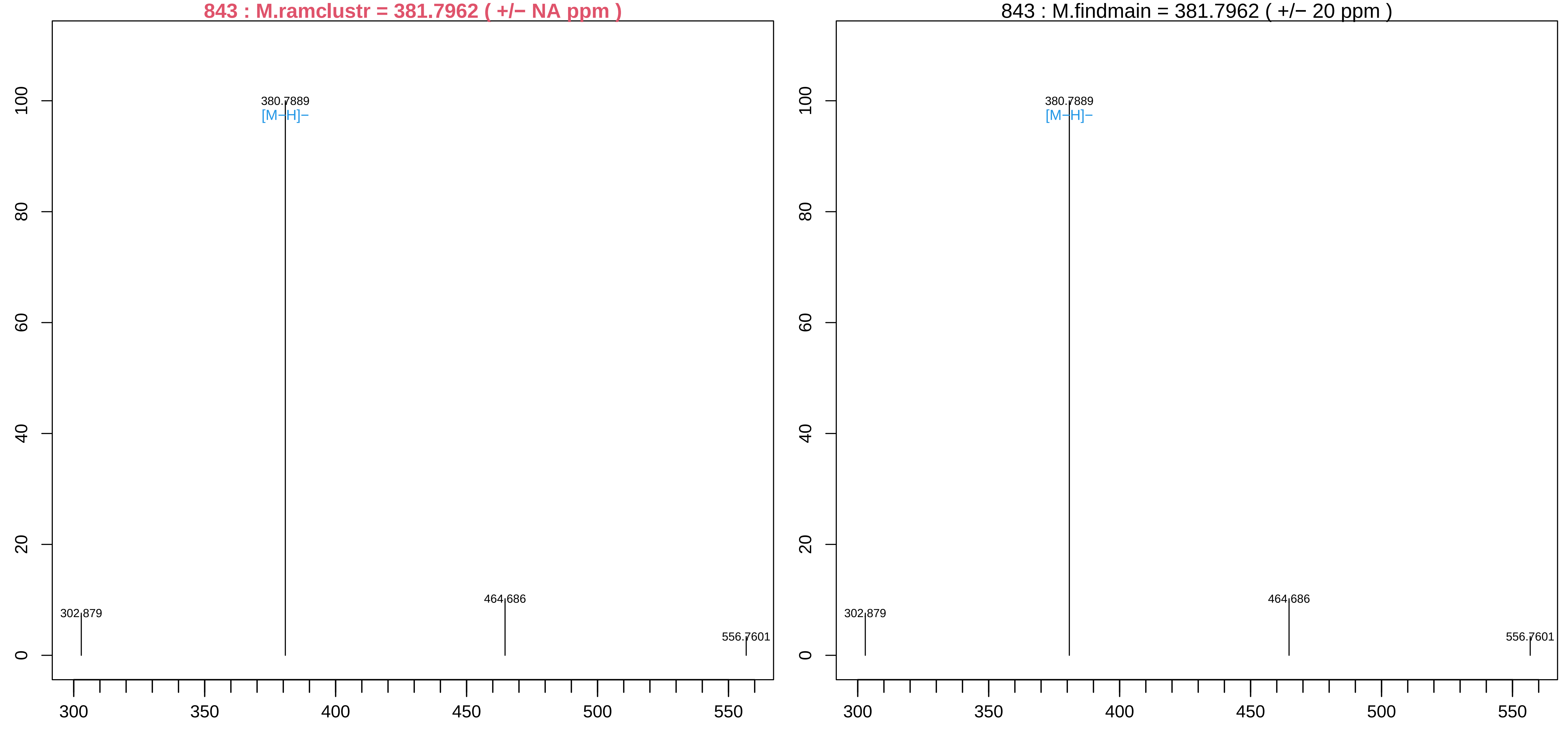

843 : M.ramclustr = 381.7962 (+/− NA ppm)
380.7889
[M−H]−
302.879
464.686
556.7601
843 : M.findmain = 381.7962 (+/− 20 ppm)
380.7889
[M−H]−
302.879
464.686
556.7601

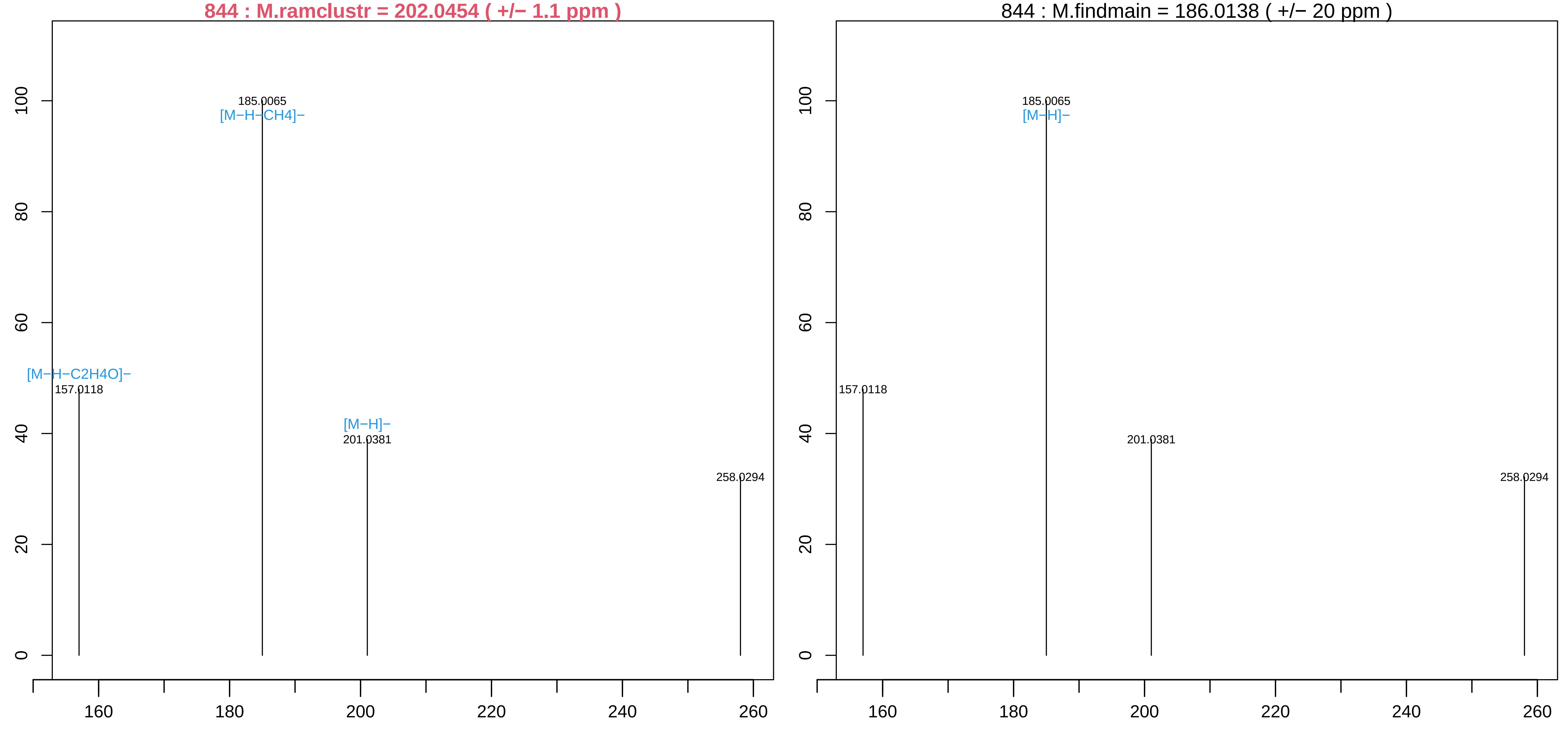
844 : M.ramclustr = 202.0454 (+/− 1.1 ppm)
185.0065
[M−H−CH4]−
[M−H−C2H4O]−
157.0118
[M−H]−
201.0381
258.0294
844 : M.findmain = 186.0138 (+/− 20 ppm)
185.0065
[M−H]−
157.0118
201.0381
258.0294
0
20
40
60
80
100
160
180
200
220
240
260

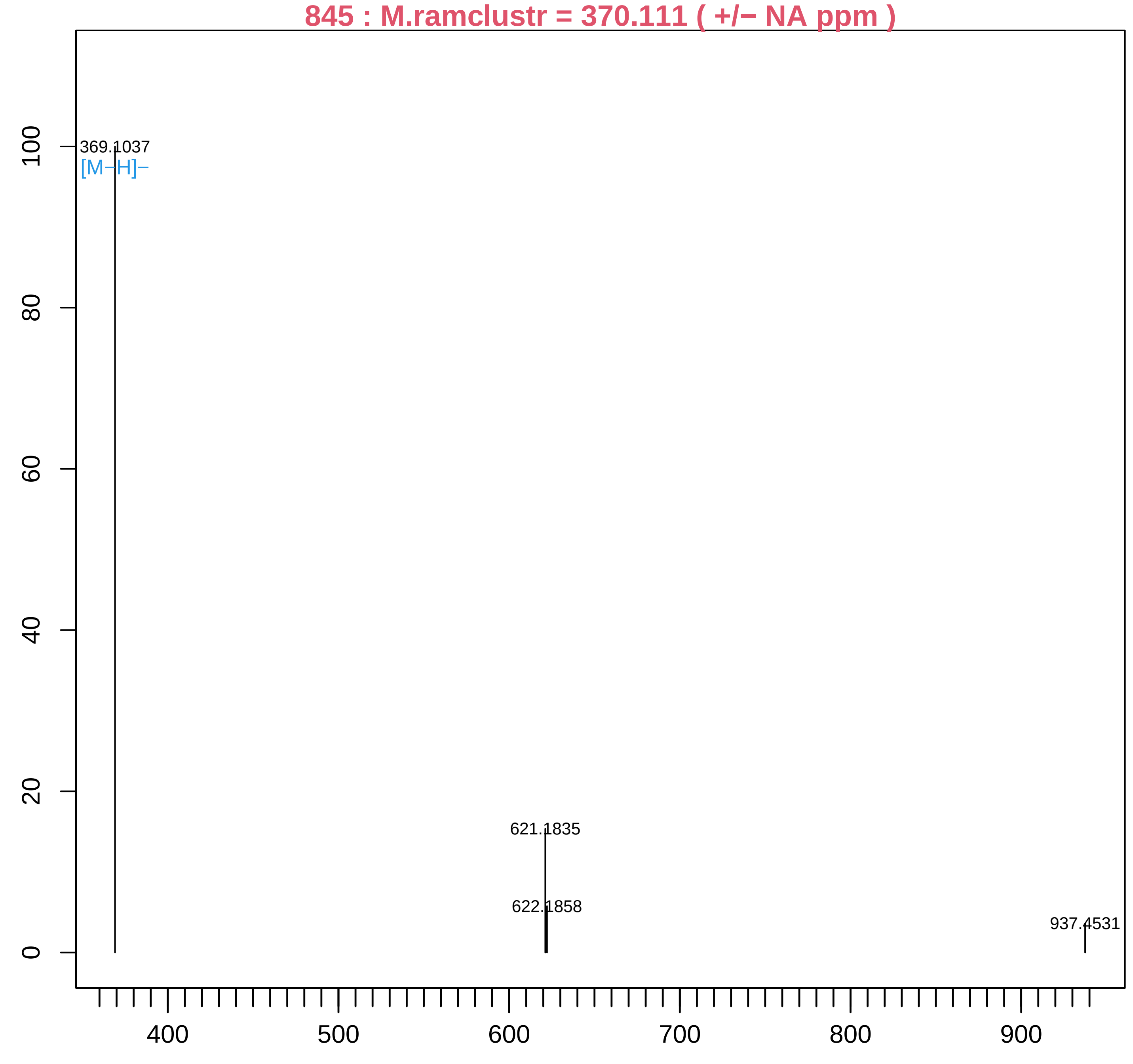
845 : M.ramclustr = 370.111 (+/− NA ppm)
369.1037
[M−H]−
621.1835
622.1858
937.4531
0
20
40
60
80
100
400
500
600
700
800
900

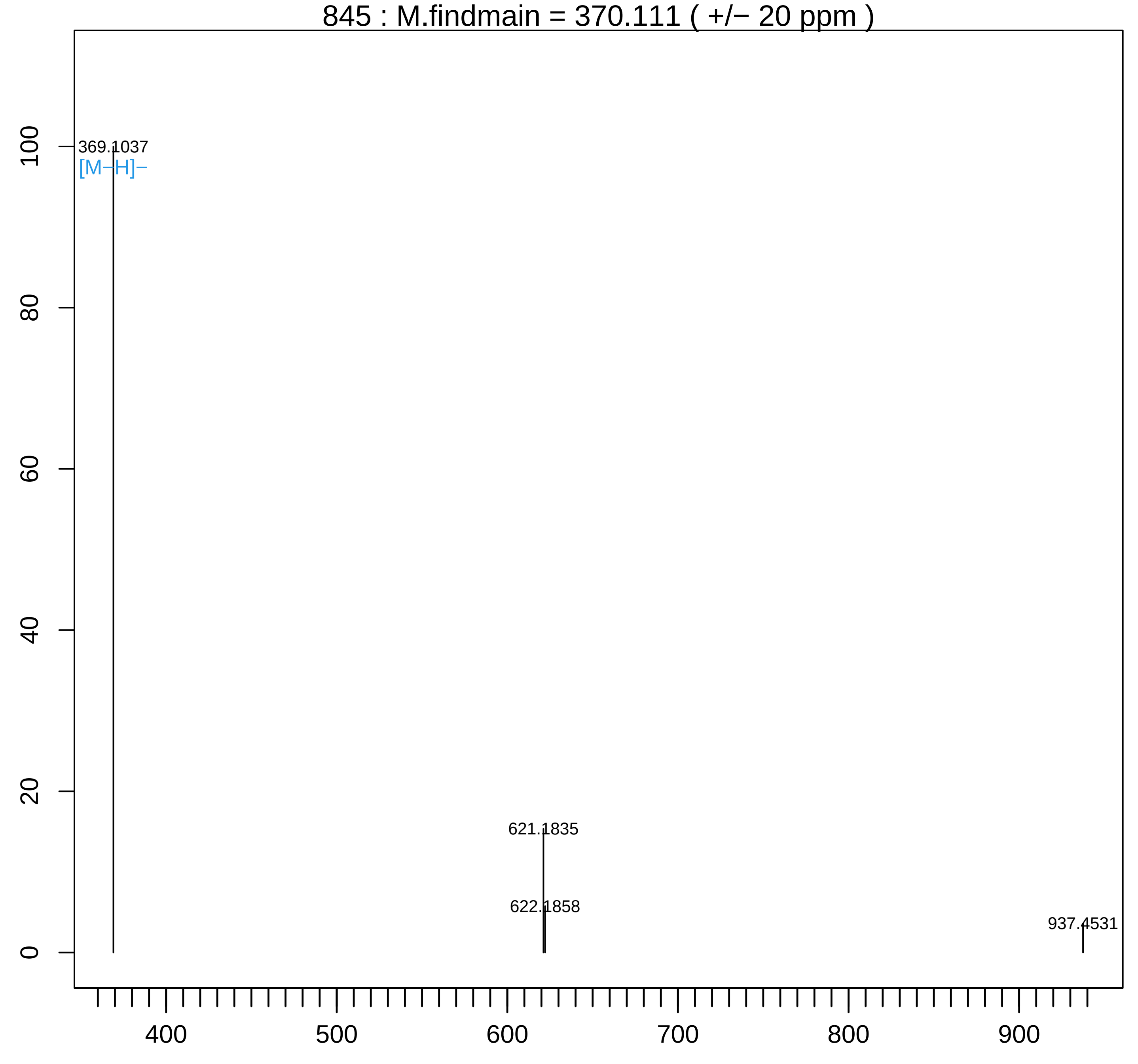
845 : M.findmain = 370.111 (+/− 20 ppm)
369.1037
[M−H]−
621.1835
622.1858
937.4531
0
20
40
60
80
100
400
500
600
700
800
900

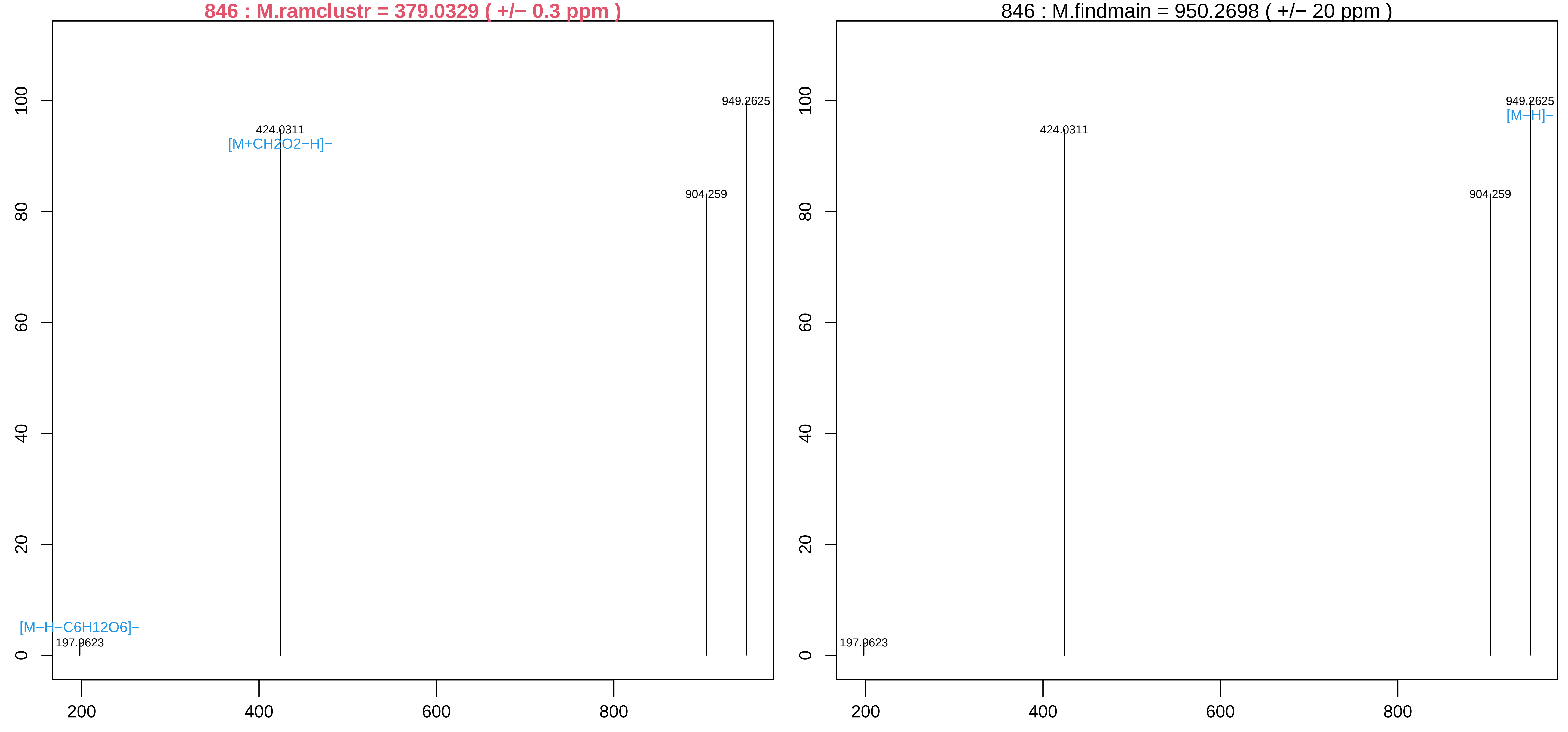
846 : M.ramclustr = 379.0329 (+/− 0.3 ppm)
949.2625
424.0311
[M+CH2O2−H]−
904.259
[M−H−C6H12O6]−
197.9623
846 : M.findmain = 950.2698 (+/− 20 ppm)
949.2625
[M−H]−
424.0311
904.259
197.9623
0
20
40
60
80
100
200
400
600
800

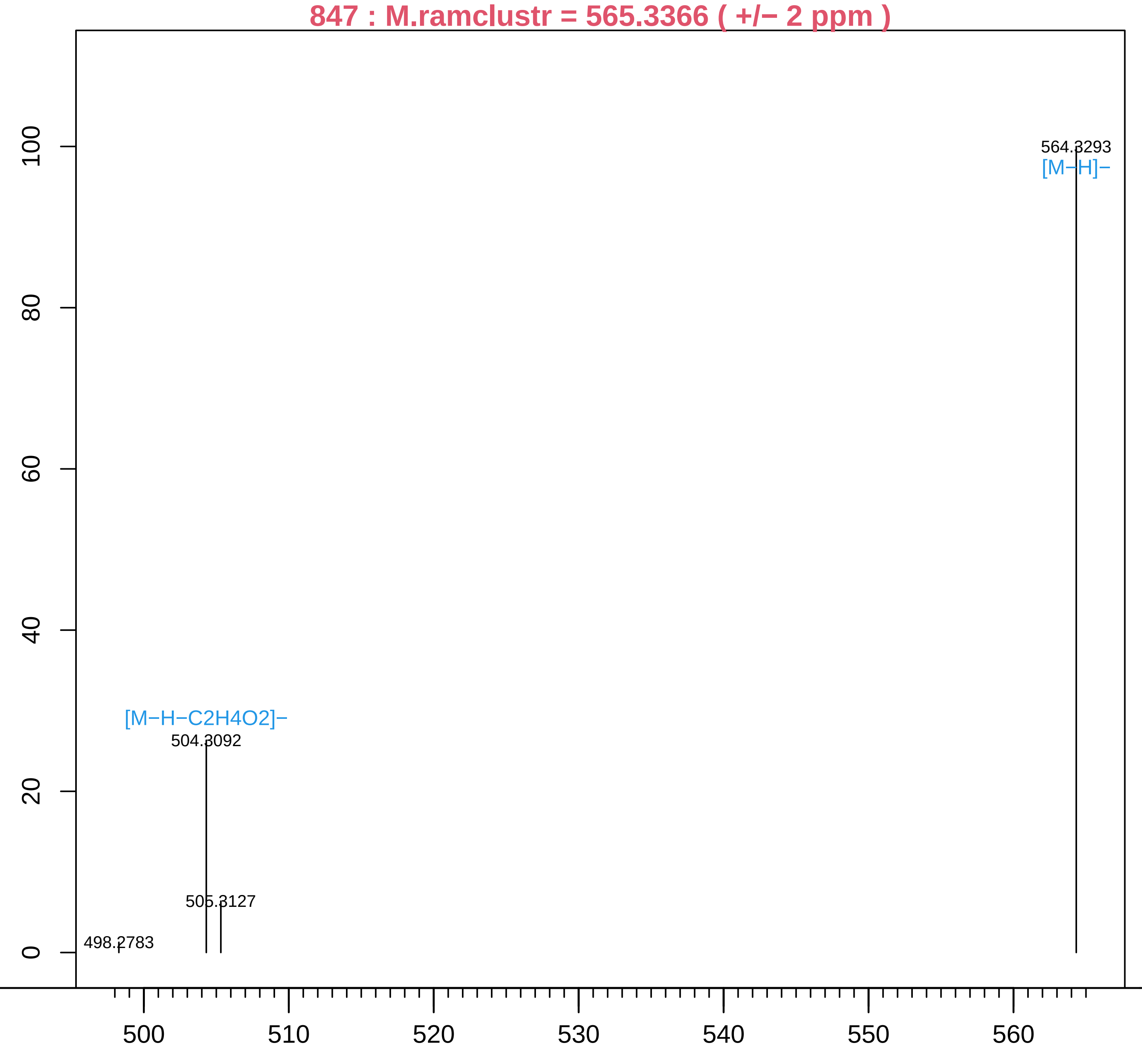

847 : M.ramclustr = 565.3366 (+/− 2 ppm)
564.3293
[M−H]−
[M−H−C2H4O2]−
504.3092
505.3127
498.2783
0
20
40
60
80
100
500
510
520
530
540
550
560

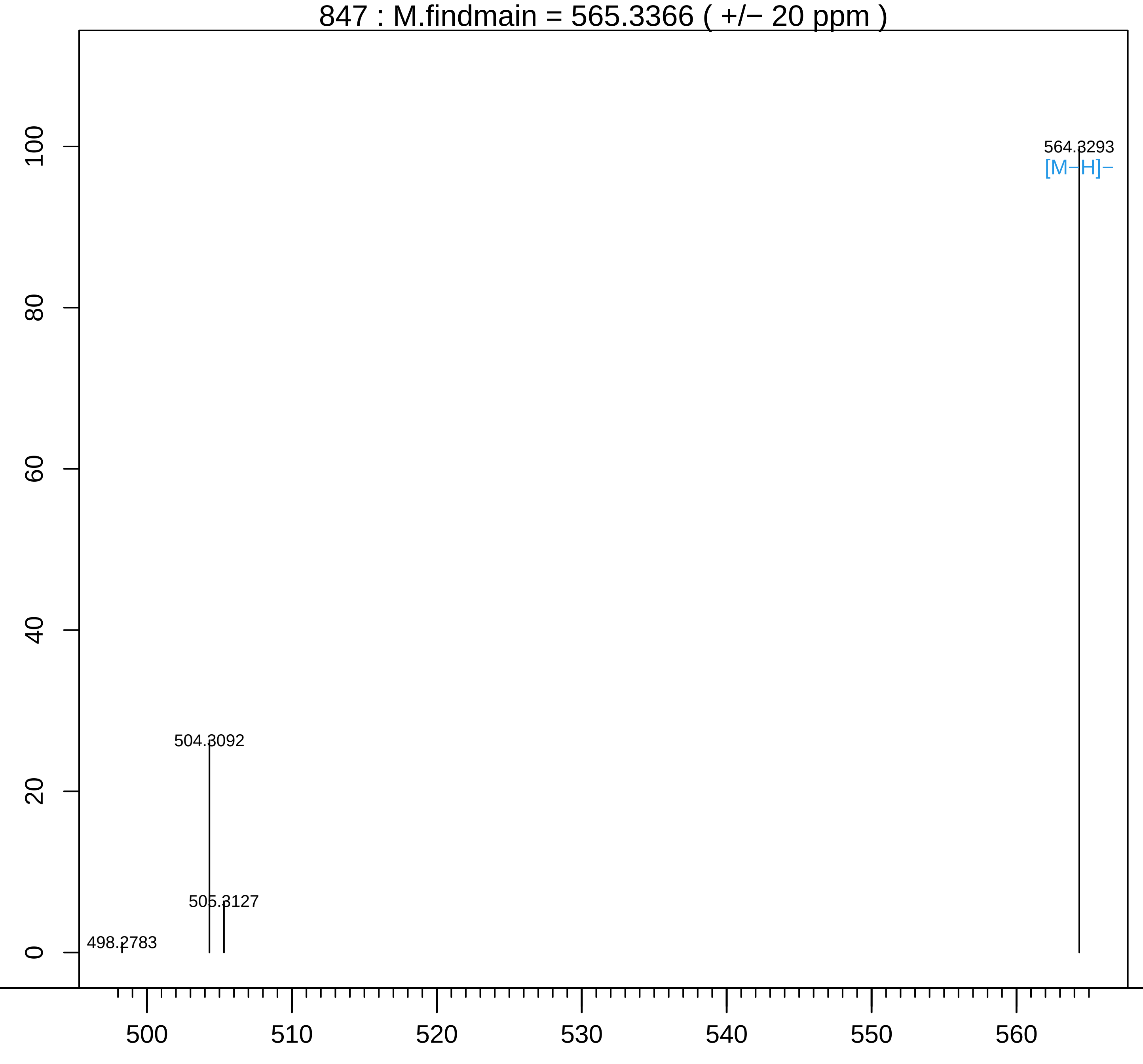

847 : M.findmain = 565.3366 (+/− 20 ppm)
564.3293
[M−H]−
504.3092
505.3127
498.2783
0
20
40
60
80
100
500
510
520
530
540
550
560

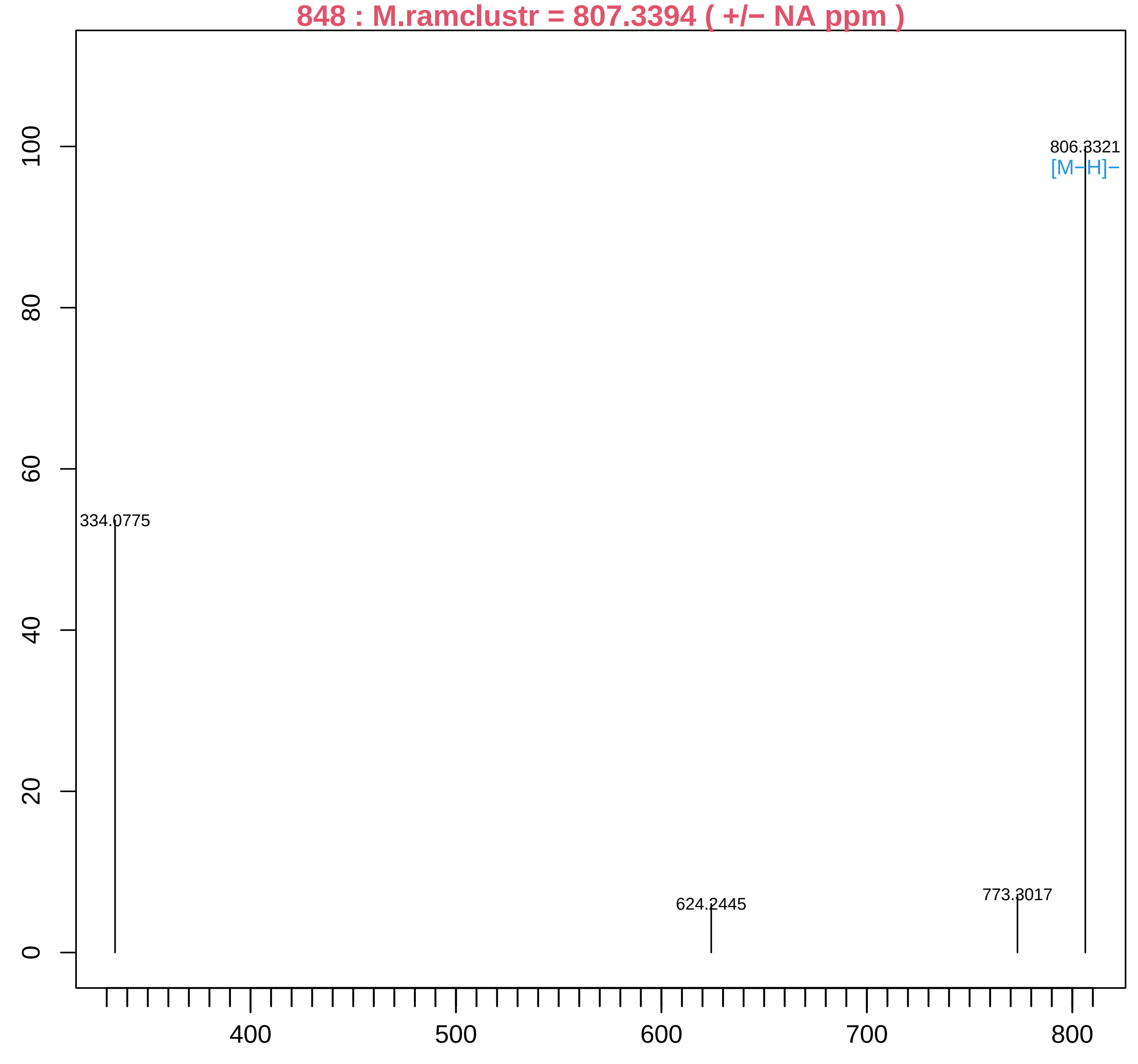
848 : M.ramclustr = 807.3394 (+/− NA ppm)
806.3321
[M−H]−
334.0775
624.2445
773.3017
0
20
40
60
80
100
400
500
600
700
800

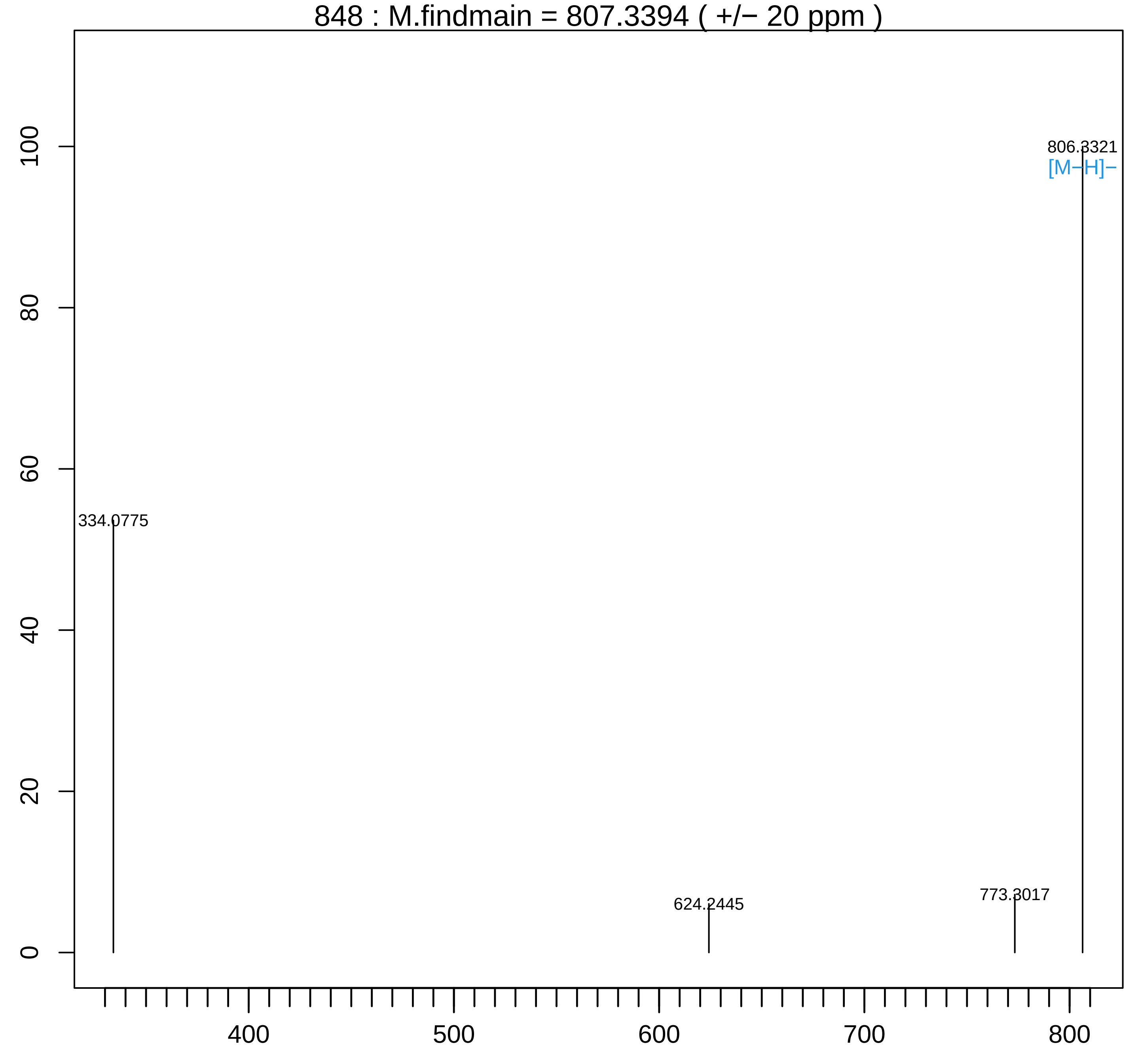
848 : M.findmain = 807.3394 (+/− 20 ppm)
806.3321
[M−H]−
334.0775
624.2445
773.3017
0
20
40
60
80
100
400
500
600
700
800

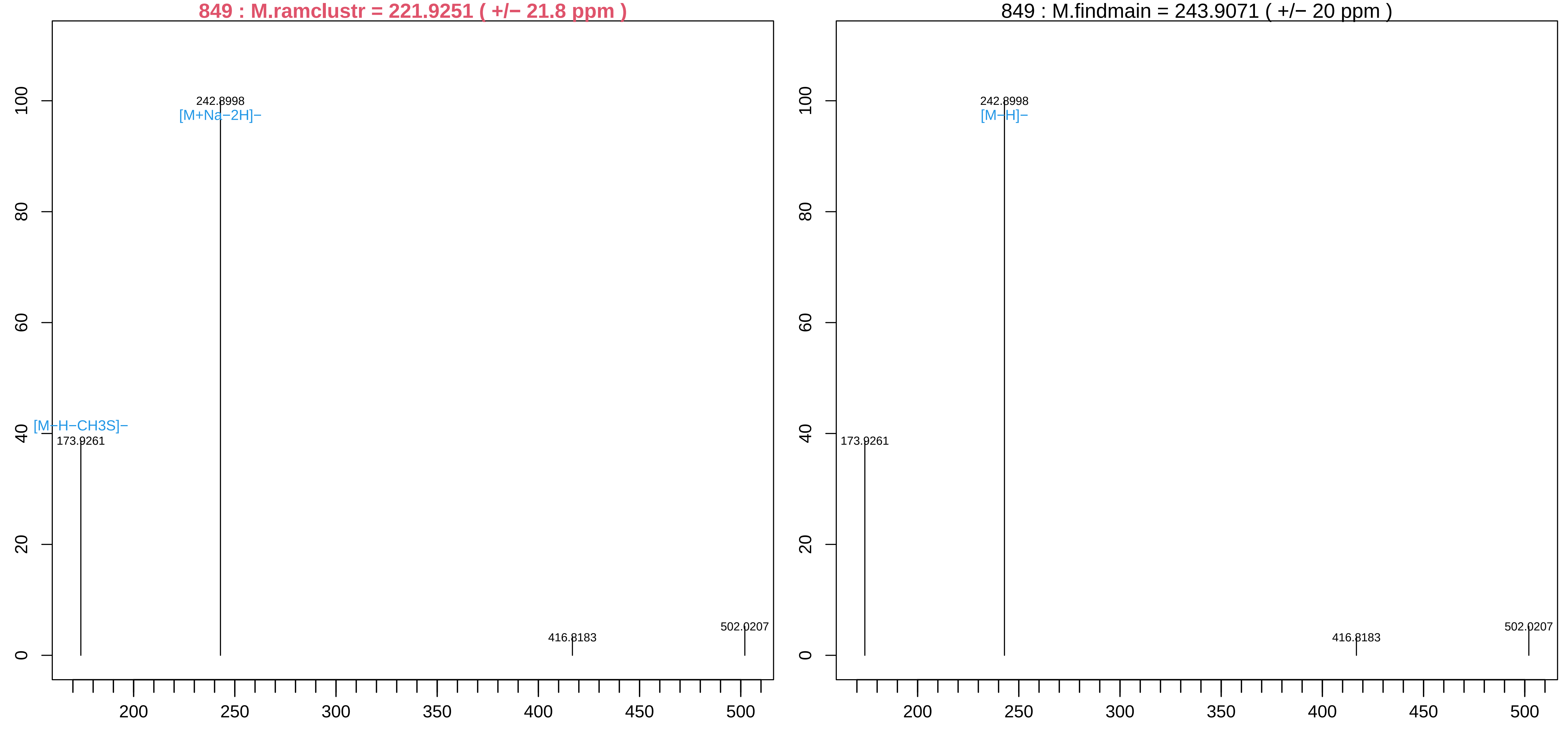

849 : M.ramclustr = 221.9251 (+/− 21.8 ppm)
242.8998
[M+Na−2H]−
[M−H−CH3S]−
173.9261
416.8183
502.0207
849 : M.findmain = 243.9071 (+/− 20 ppm)
242.8998
[M−H]−
173.9261
416.8183
502.0207
0
20
40
60
80
100
200
250
300
350
400
450
500

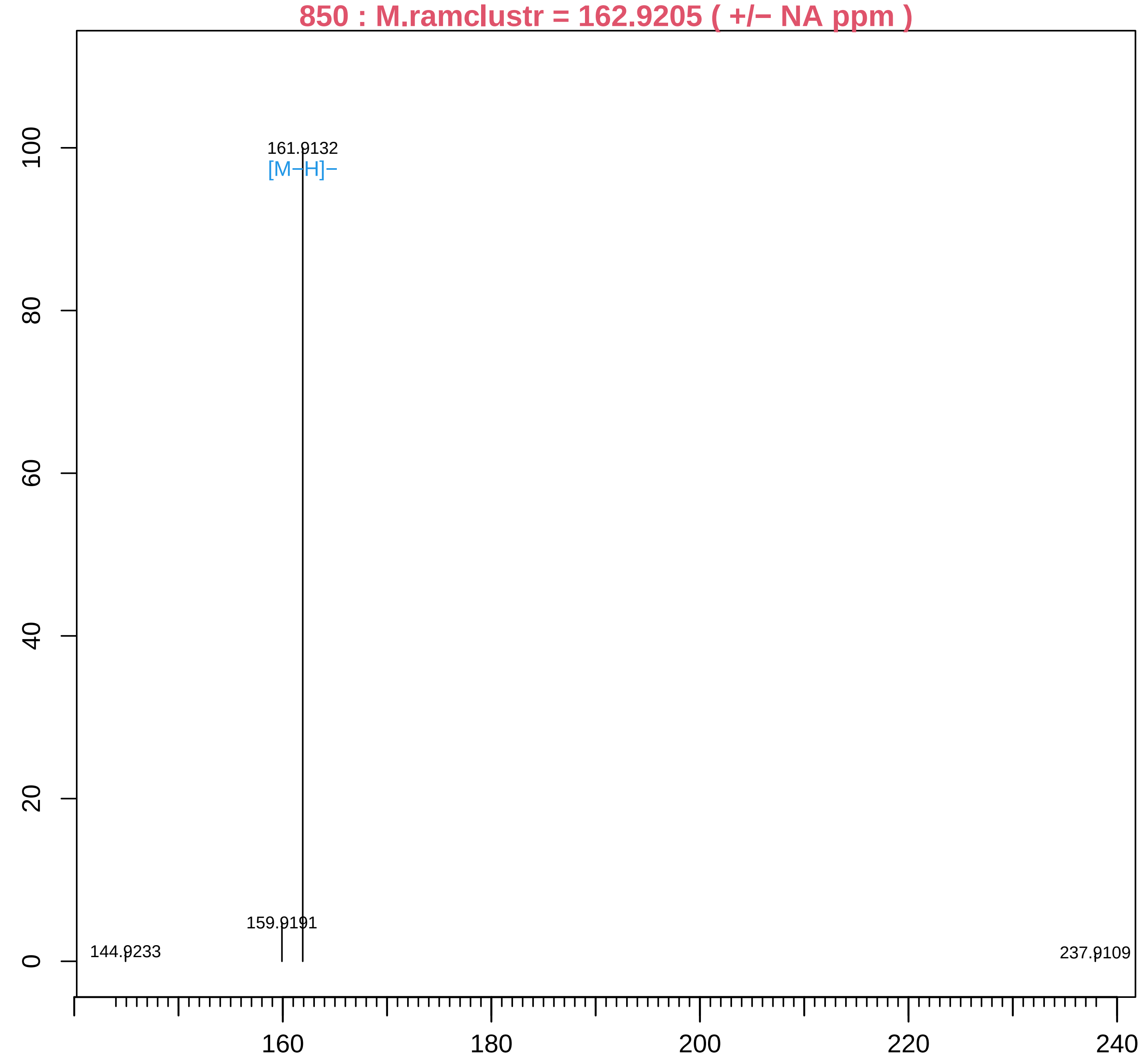
850 : M.ramclustr = 162.9205 (+/− NA ppm)
161.9132
[M−H]−
159.9191
144.9233
237.9109
0
20
40
60
80
100
160
180
200
220
240

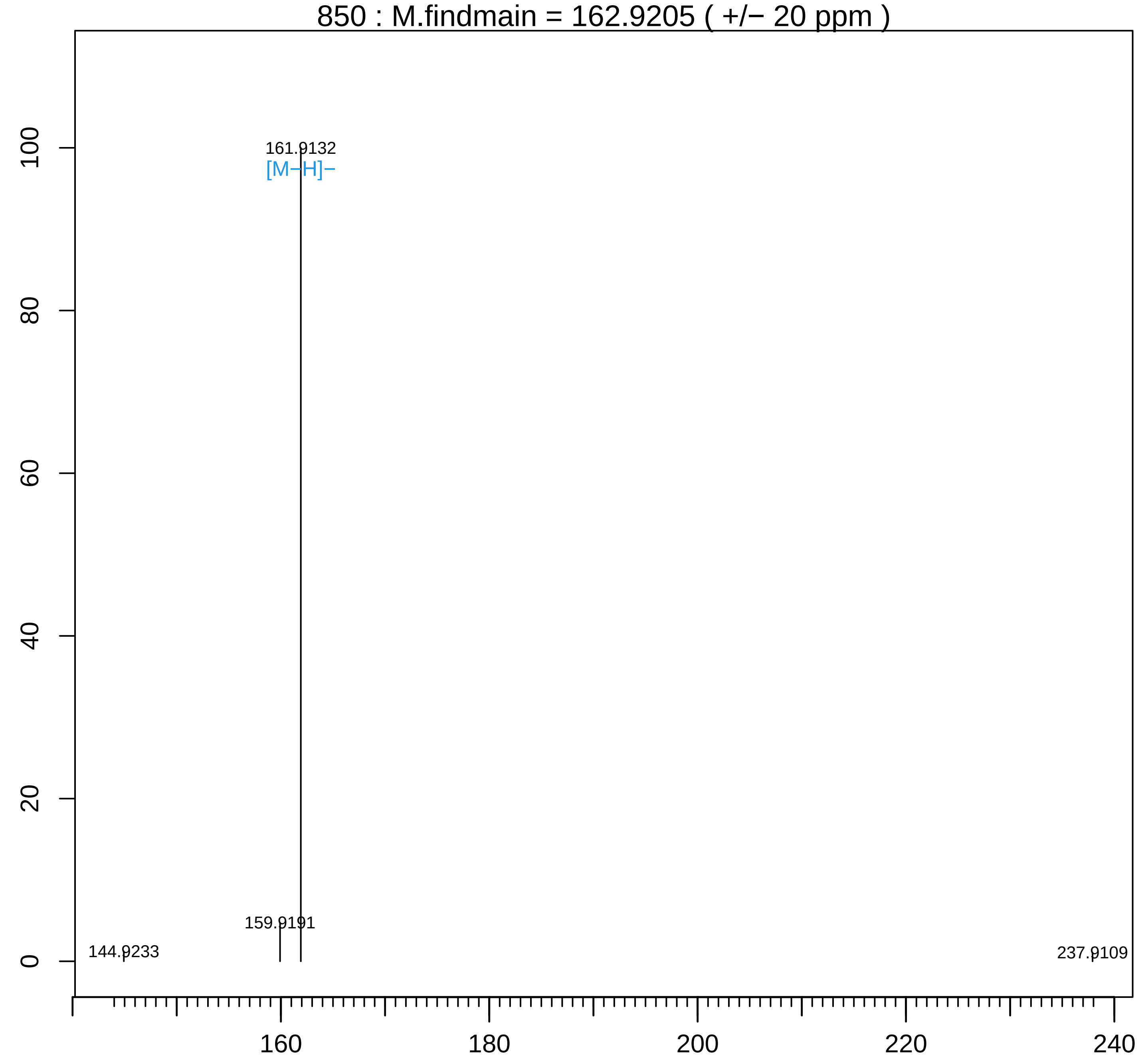
850 : M.findmain = 162.9205 (+/− 20 ppm)
161.9132
[M−H]−
159.9191
144.9233
237.9109
0
20
40
60
80
100
160
180
200
220
240

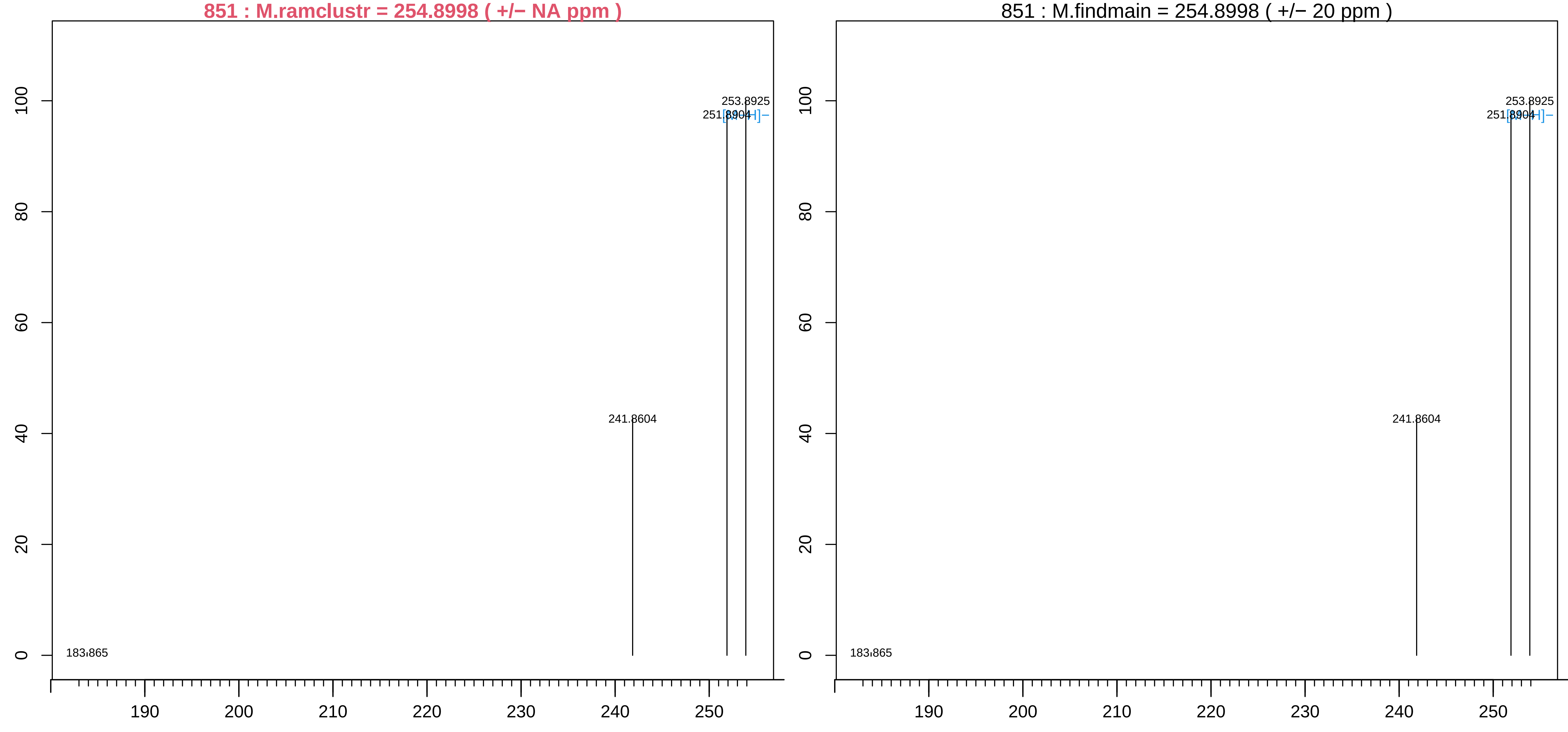

851 : M.ramclustr = 254.8998 (+/− NA ppm)
253.8925
251.8904
[M−H]−
241.8604
183.865
100
80
60
40
20
0
190
200
210
220
230
240
250
851 : M.findmain = 254.8998 (+/− 20 ppm)
253.8925
251.8904
[M−H]−
241.8604
183.865
100
80
60
40
20
0
190
200
210
220
230
240
250

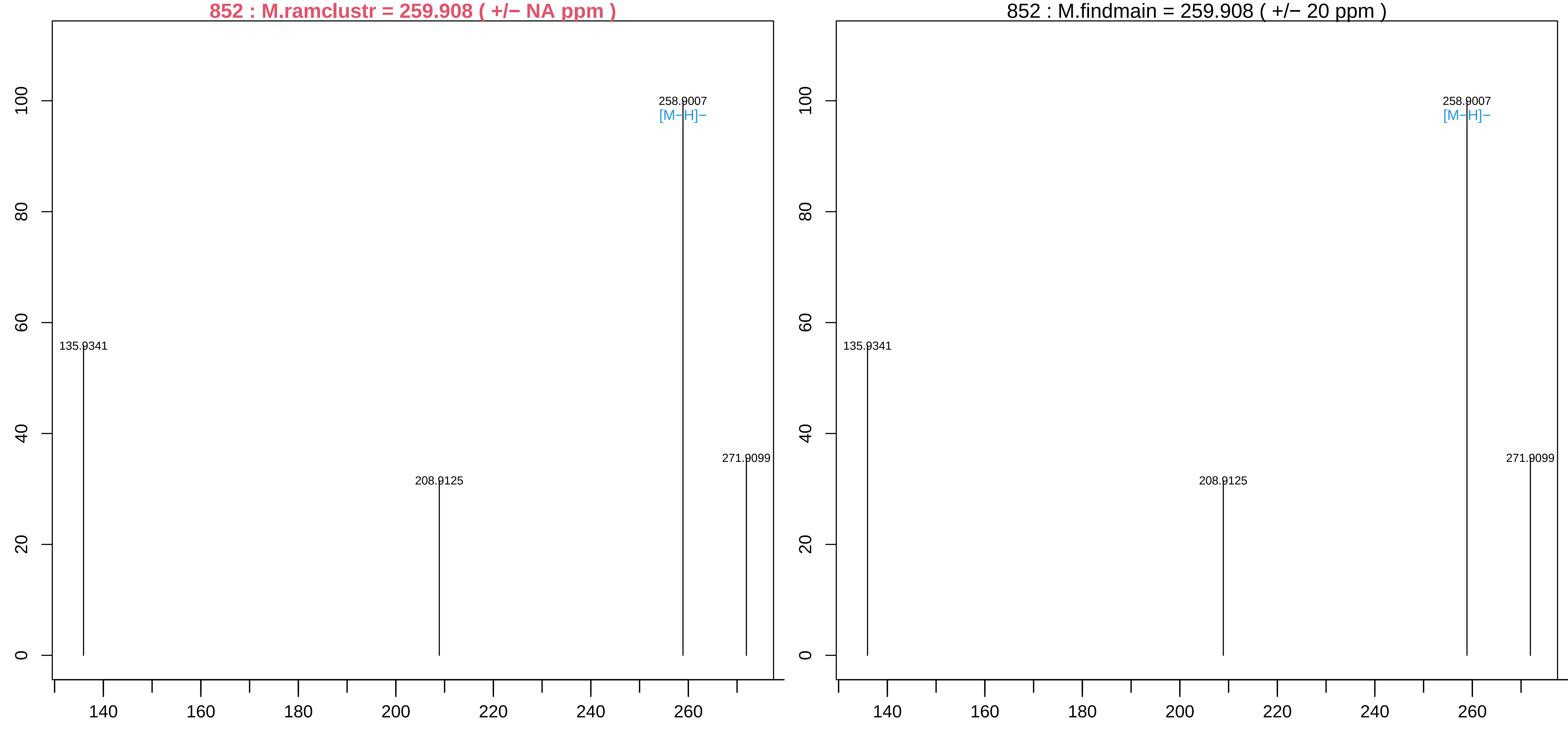
852 : M.ramclustr = 259.908 (+/− NA ppm)
852 : M.findmain = 259.908 (+/− 20 ppm)
258.9007
[M−H]−
135.9341
208.9125
271.9099
0
20
40
60
80
100
140
160
180
200
220
240
260

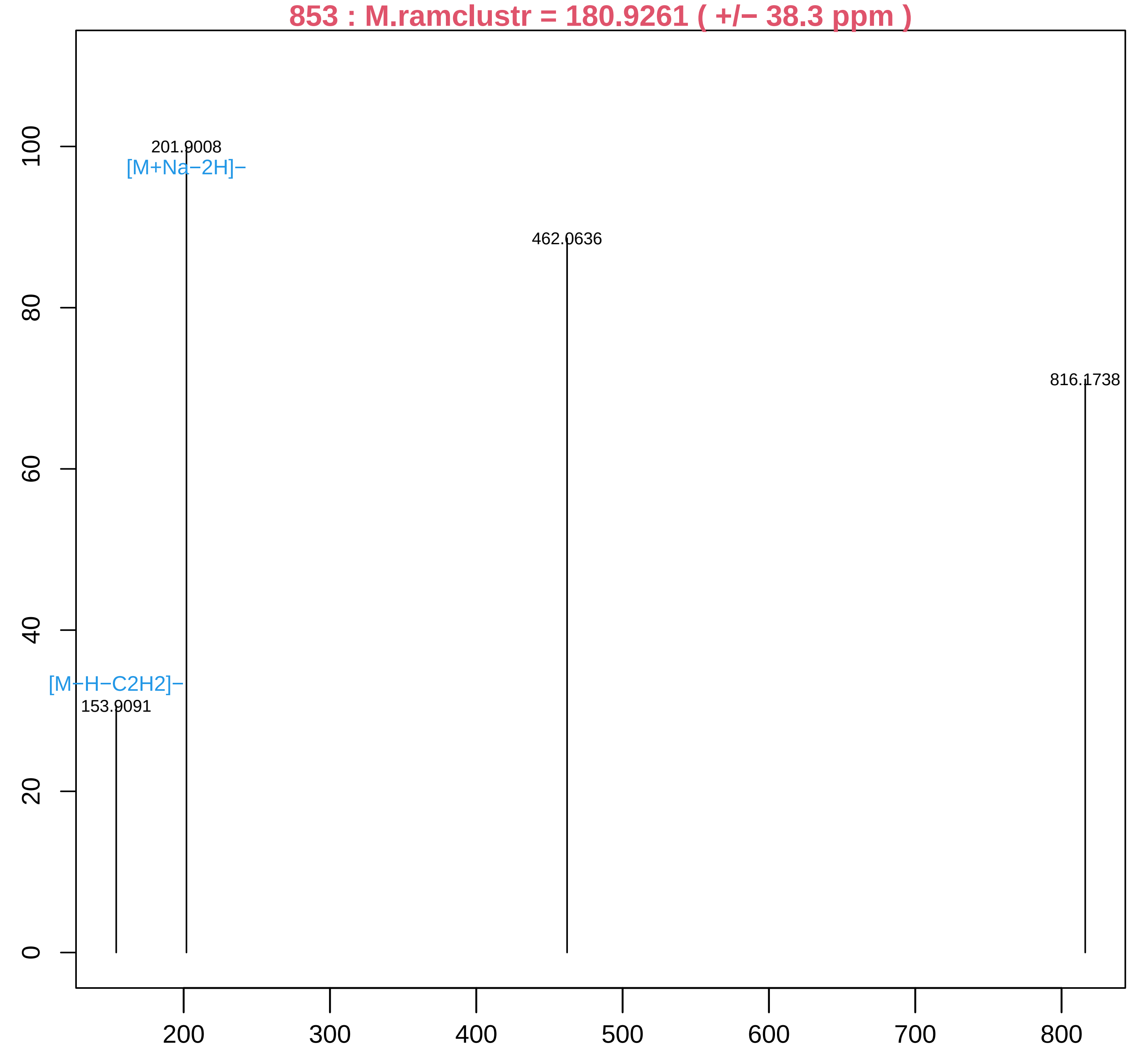
853 : M.ramclustr = 180.9261 (+/− 38.3 ppm)
201.9008
[M+Na−2H]−
462.0636
816.1738
[M−H−C2H2]−
153.9091
0
20
40
60
80
100
200
300
400
500
600
700
800

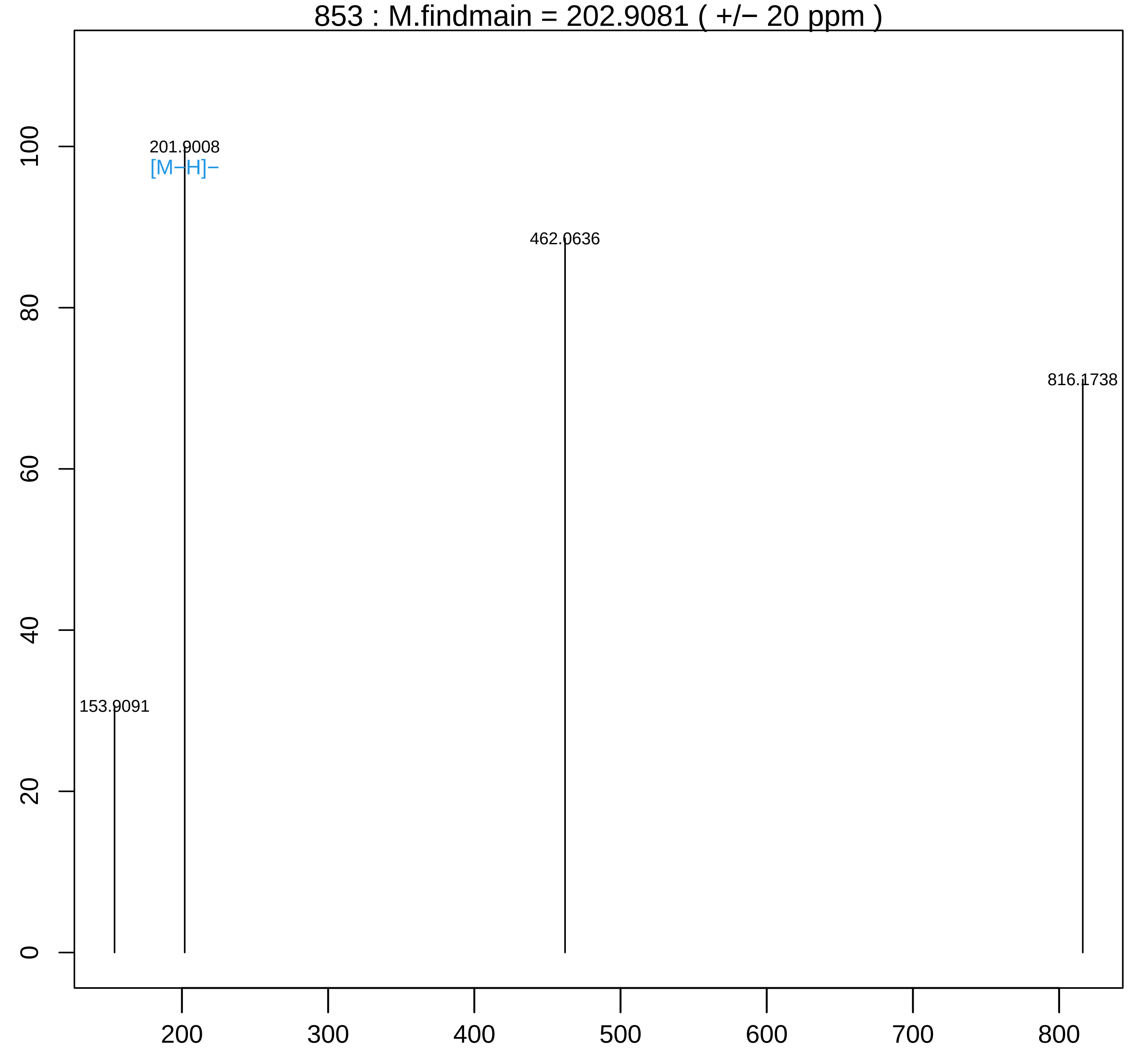
853 : M.findmain = 202.9081 (+/− 20 ppm)
201.9008
[M−H]−
462.0636
816.1738
153.9091
0
20
40
60
80
100
200
300
400
500
600
700
800

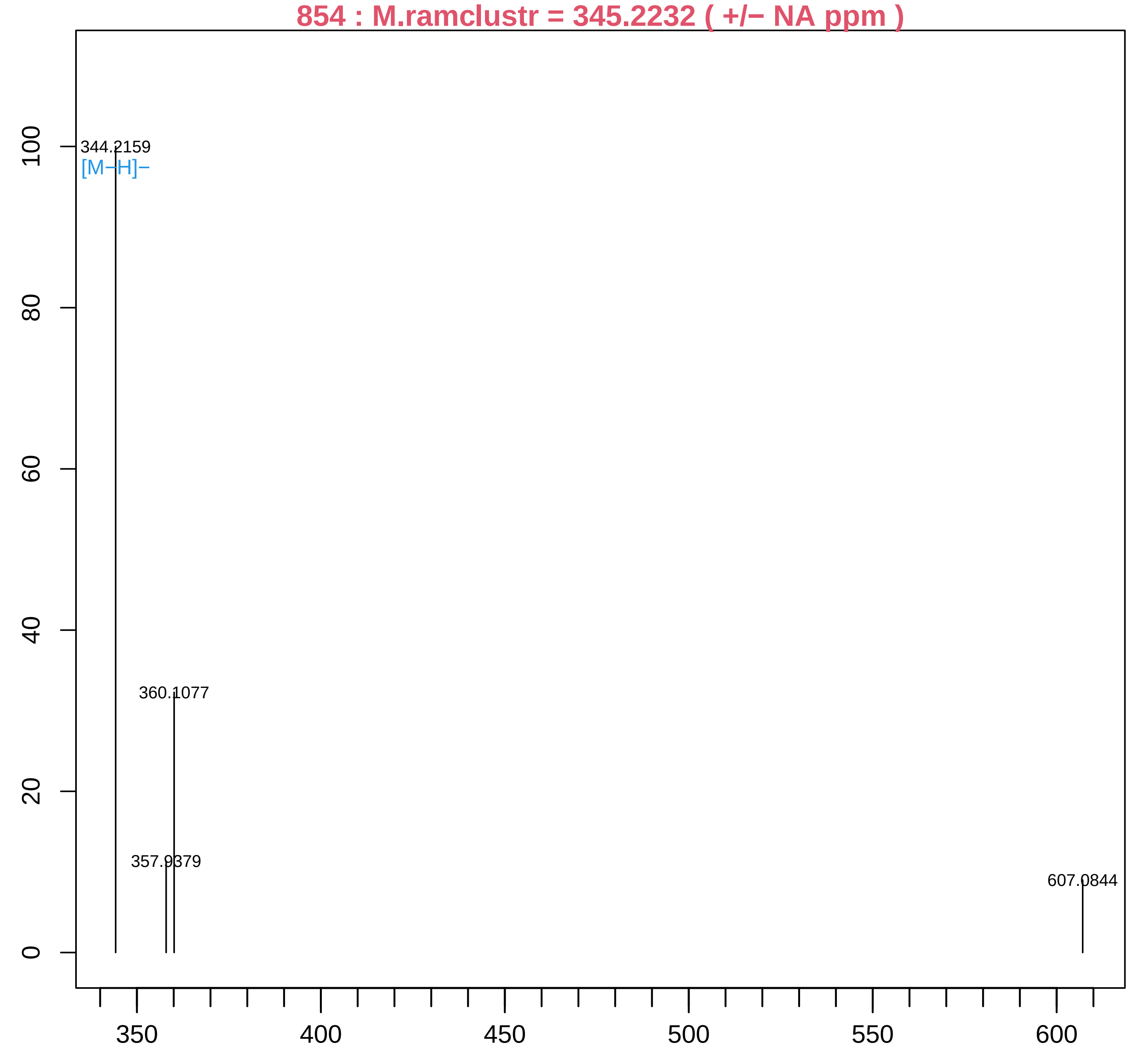

854 : M.ramclustr = 345.2232 (+/− NA ppm)
344.2159
[M−H]−
360.1077
357.9379
607.0844
0
20
40
60
80
100
350
400
450
500
550
600

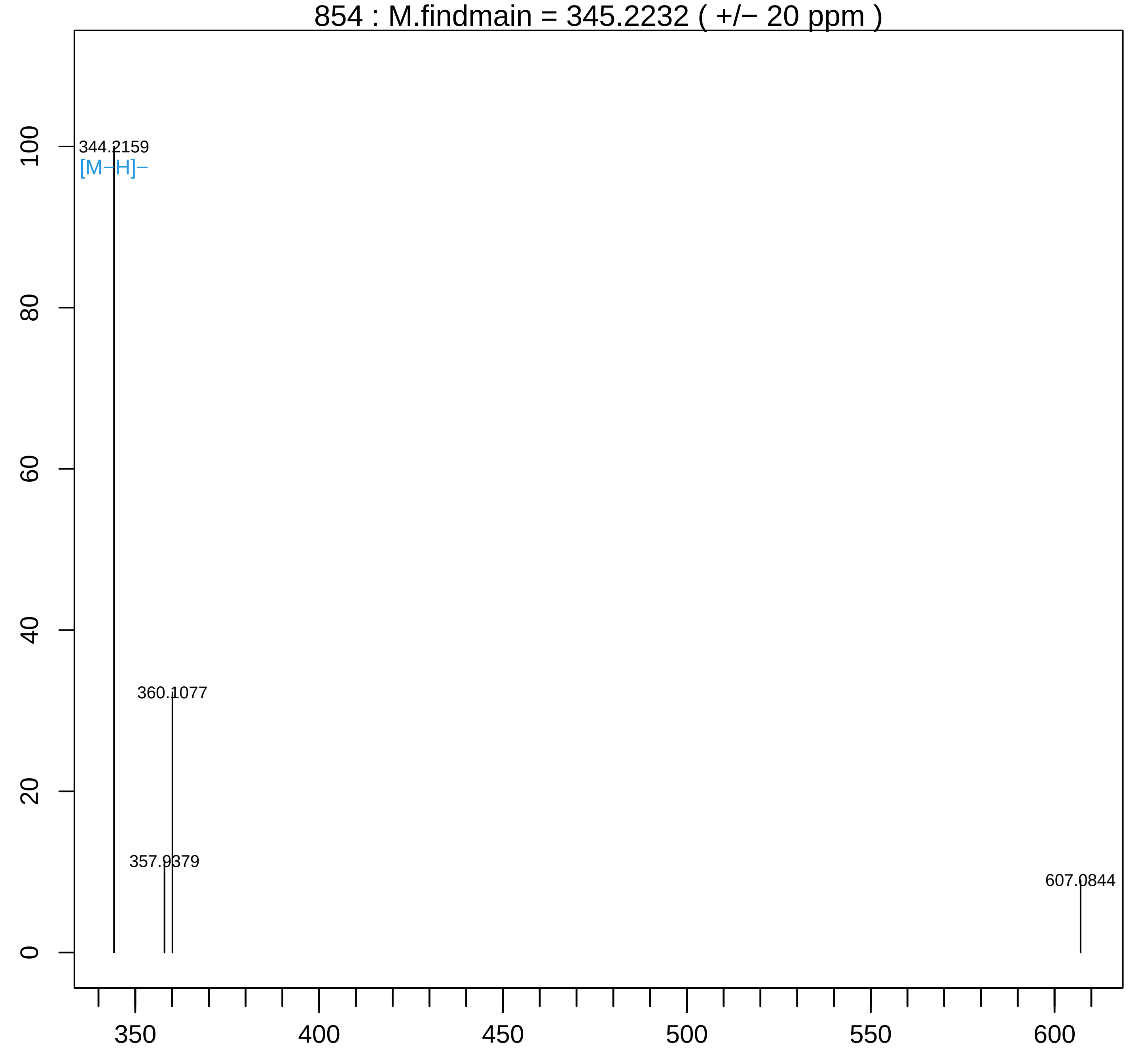

854 : M.findmain = 345.2232 (+/− 20 ppm)
344.2159
[M−H]−
360.1077
357.9379
607.0844
0
20
40
60
80
100
350
400
450
500
550
600

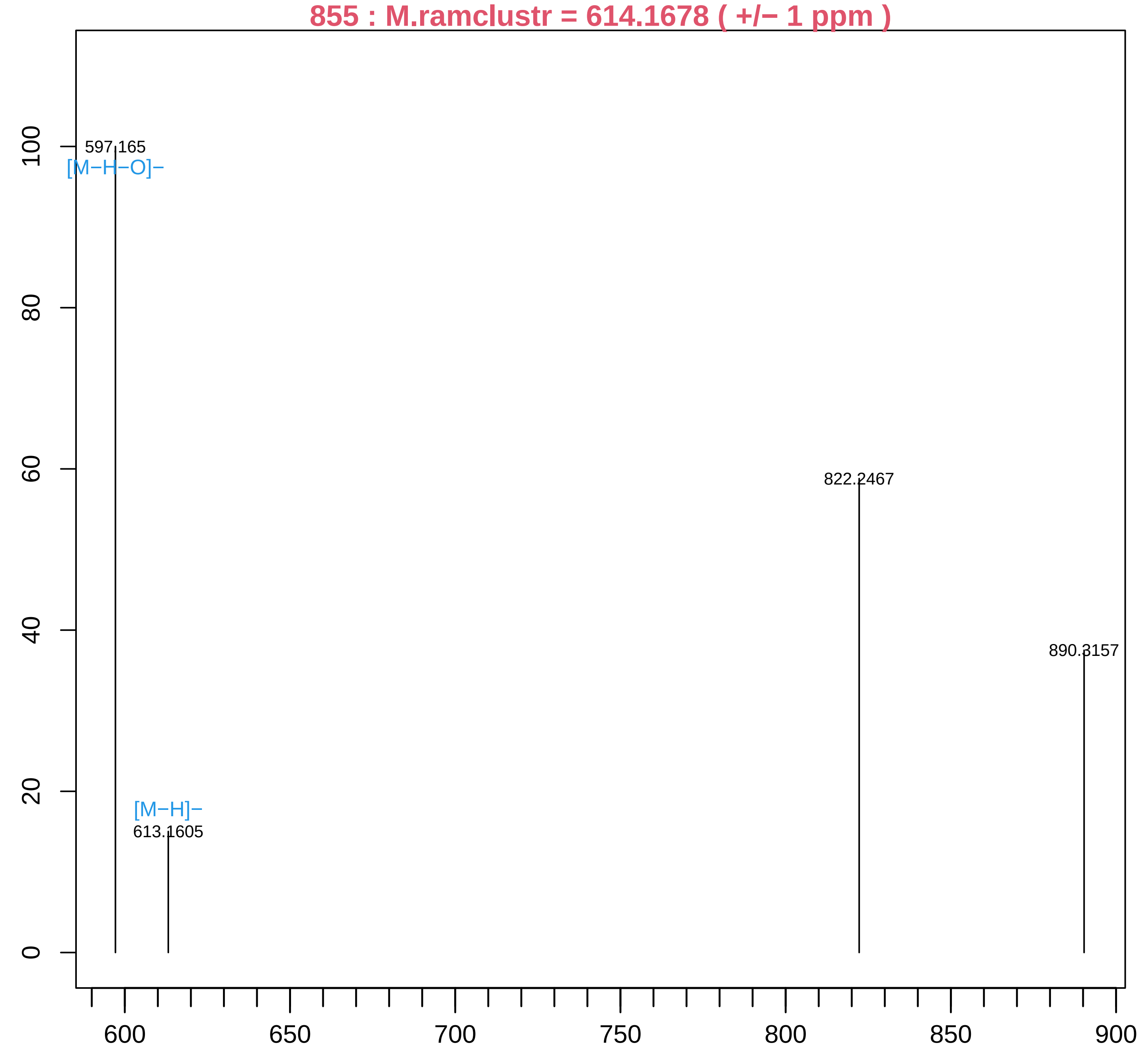
855 : M.ramclustr = 614.1678 (+/− 1 ppm)
597.165
[M−H−O]−
[M−H]−
613.1605
822.2467
890.3157
0
20
40
60
80
100
600
650
700
750
800
850
900

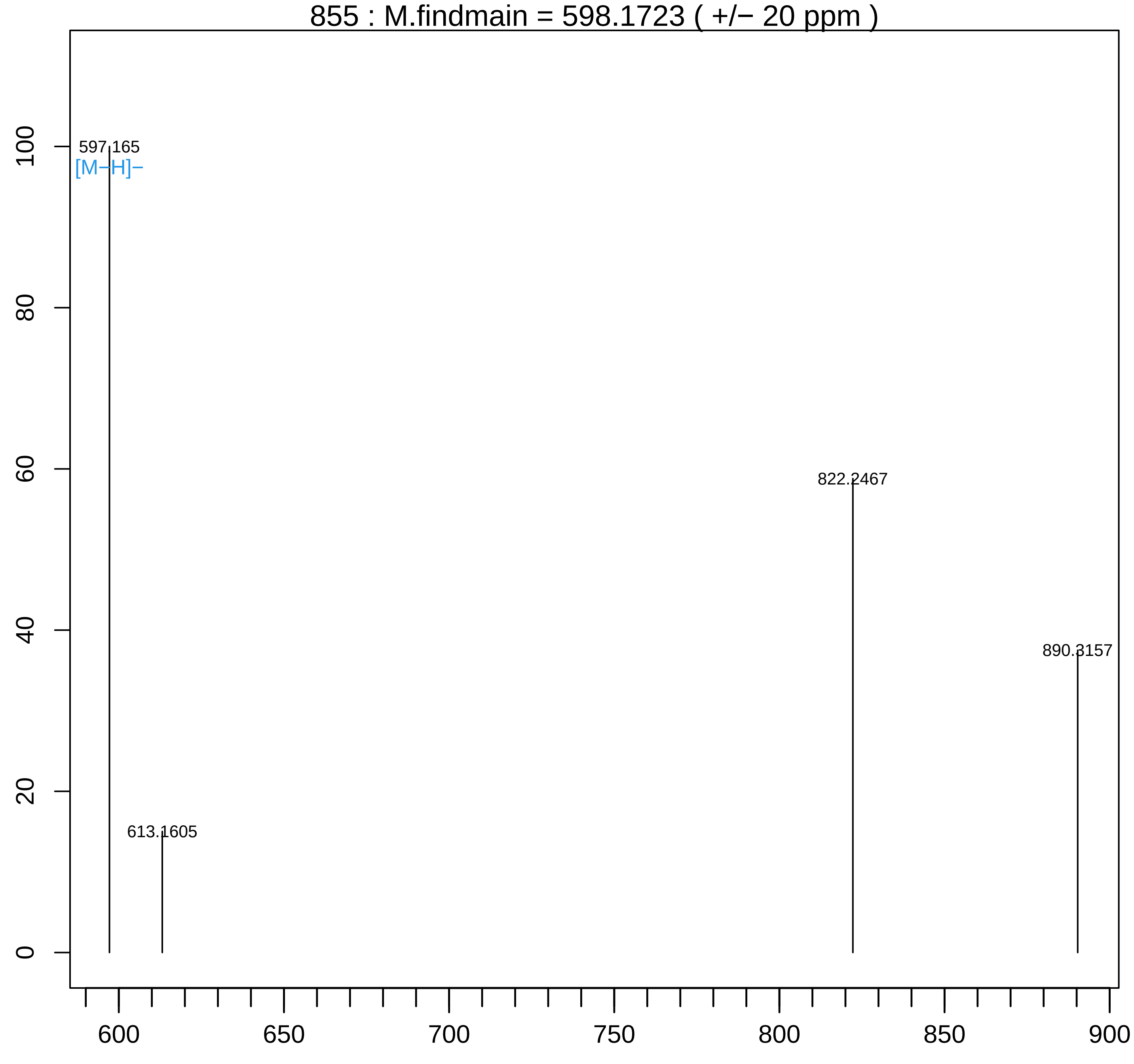
855 : M.findmain = 598.1723 (+/− 20 ppm)
597.165
[M−H]−
613.1605
822.2467
890.3157
0
20
40
60
80
100
600
650
700
750
800
850
900

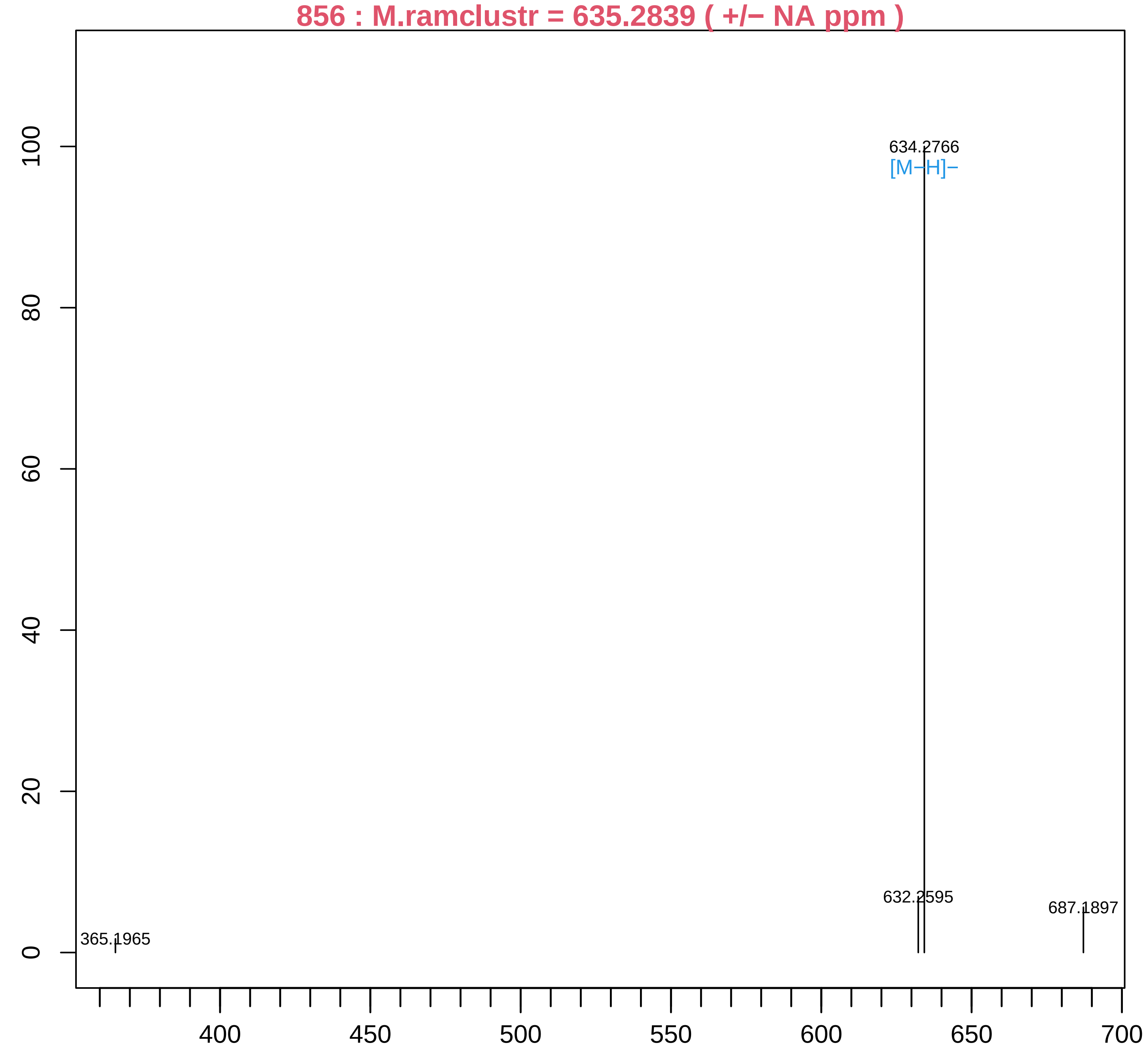
856 : M.ramclustr = 635.2839 (+/− NA ppm)
634.2766
[M−H]−
632.2595
687.1897
365.1965
0
20
40
60
80
100
400
450
500
550
600
650
700

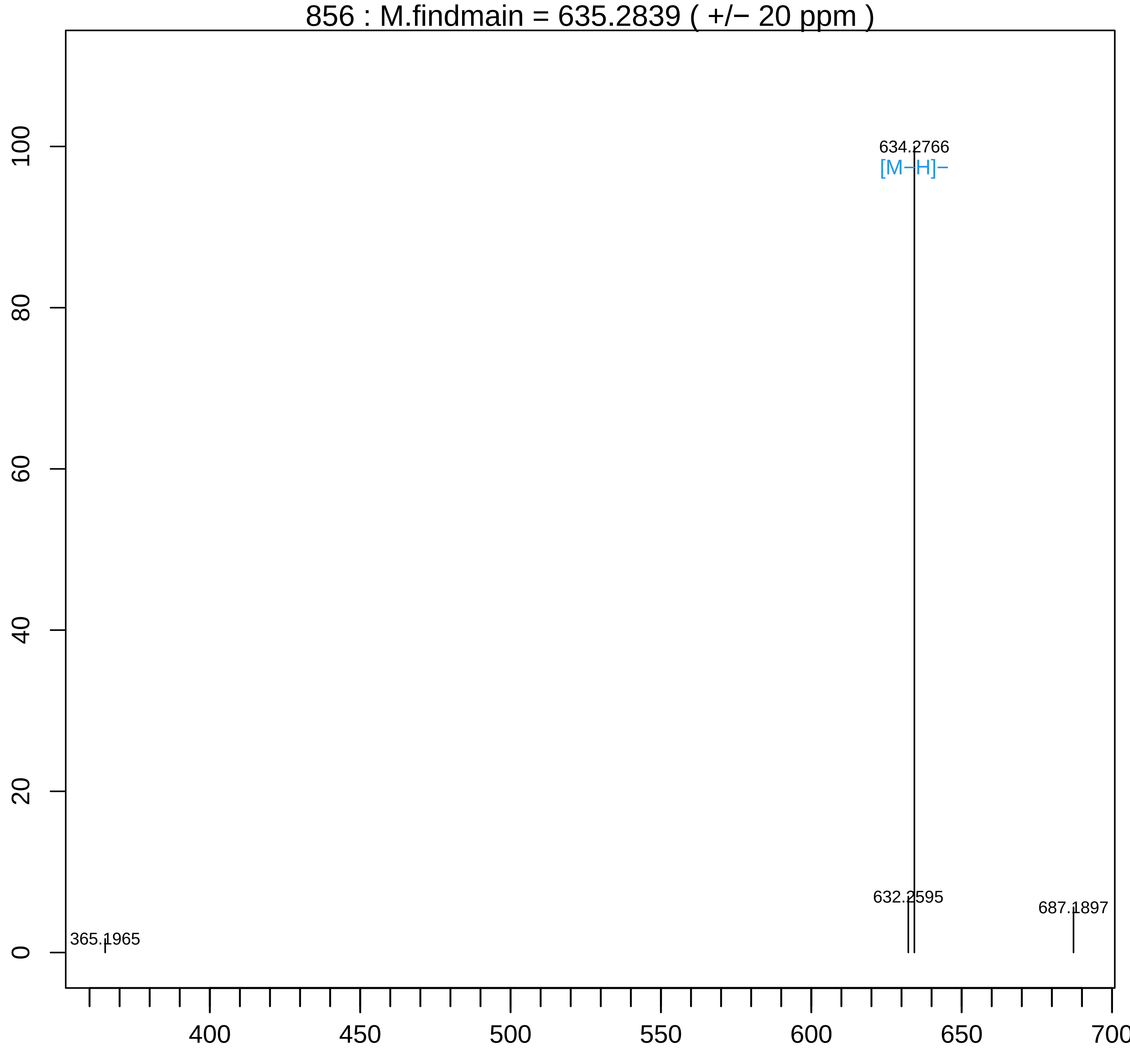
856 : M.findmain = 635.2839 (+/− 20 ppm)
634.2766
[M−H]−
632.2595
687.1897
365.1965
0
20
40
60
80
100
400
450
500
550
600
650
700

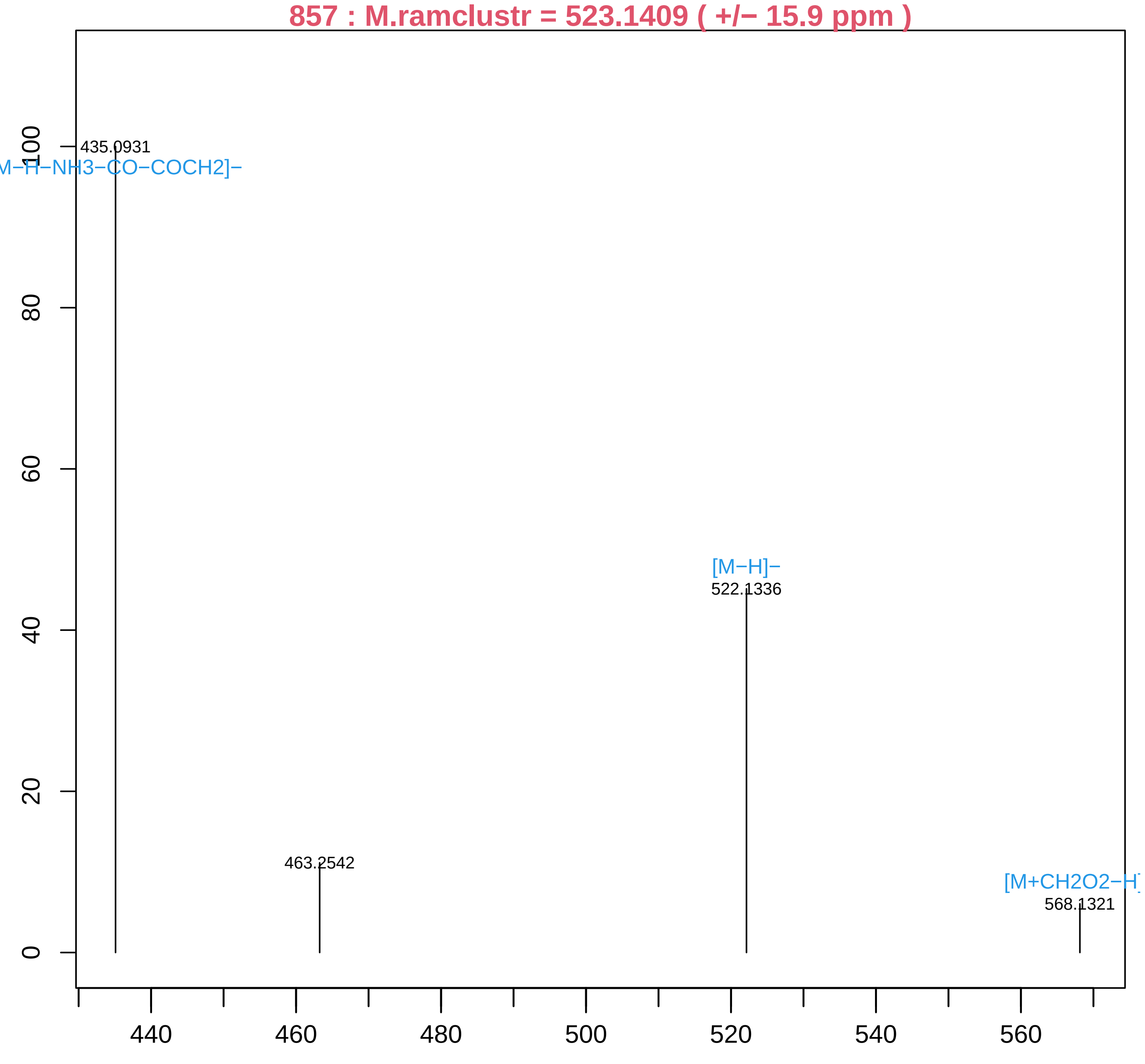

857 : M.ramclustr = 523.1409 (+/− 15.9 ppm)
435.0931
M−H−NH3−CO−COCH2]−
463.2542
[M−H]−
522.1336
[M+CH2O2−H
568.1321
0
20
40
60
80
100
440
460
480
500
520
540
560

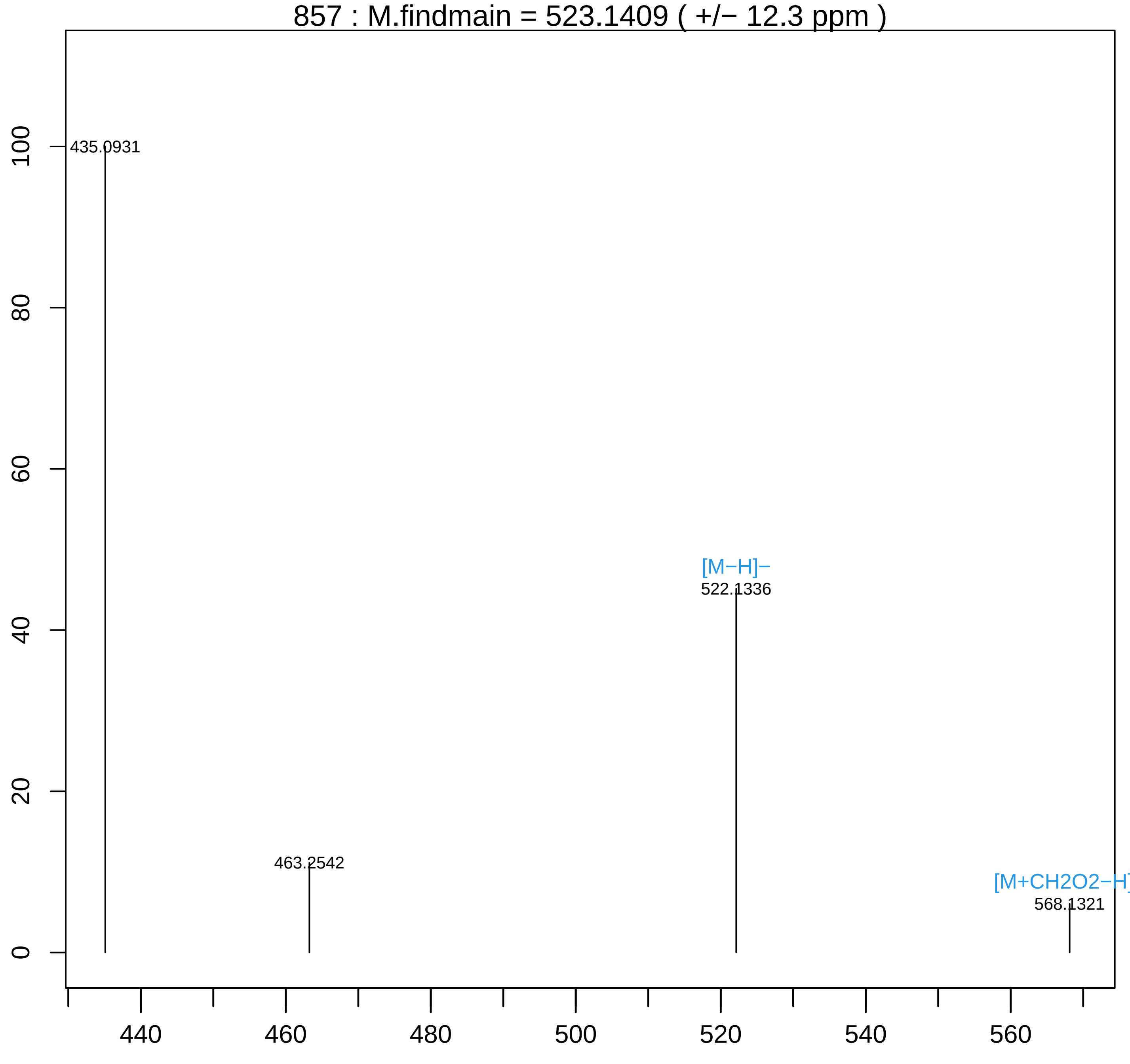

857 : M.findmain = 523.1409 (+/− 12.3 ppm)
435.0931
463.2542
[M−H]−
522.1336
[M+CH2O2−H
568.1321
0
20
40
60
80
100
440
460
480
500
520
540
560

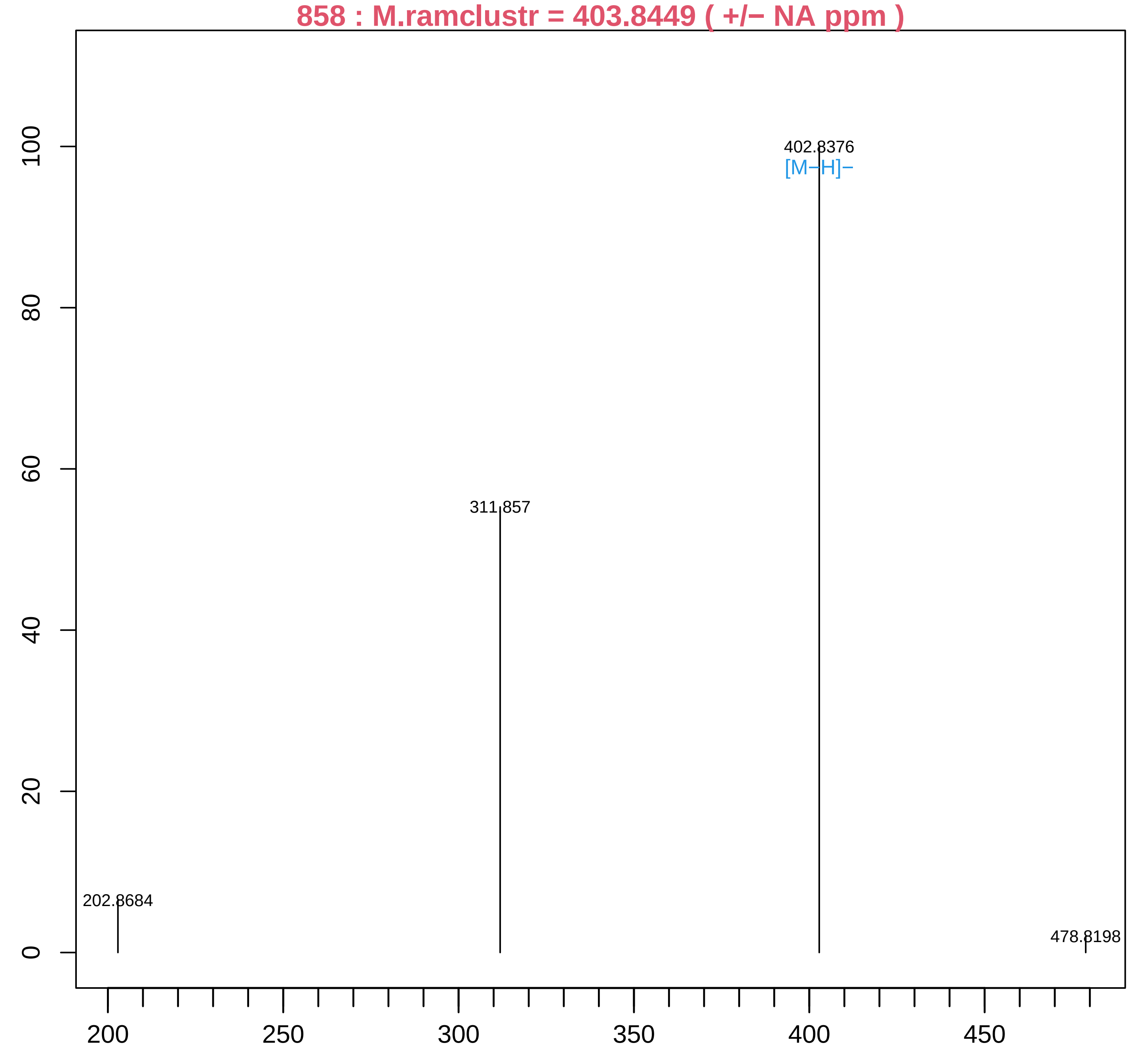
858 : M.ramclustr = 403.8449 (+/− NA ppm)
402.8376
[M−H]−
311.857
202.8684
478.8198
0
20
40
60
80
100
200
250
300
350
400
450

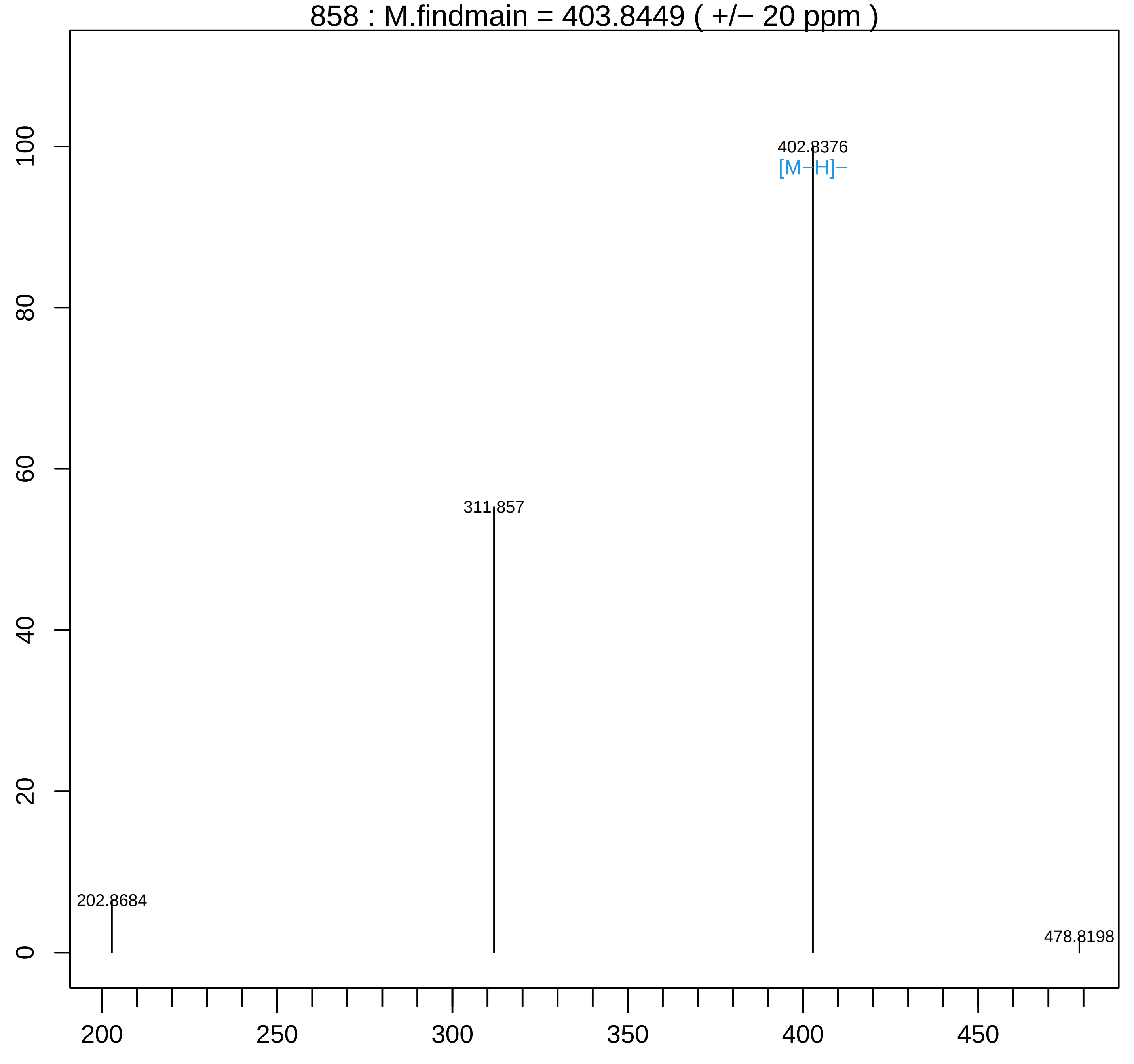
858 : M.findmain = 403.8449 (+/− 20 ppm)
402.8376
[M−H]−
311.857
202.8684
478.8198
0
20
40
60
80
100
200
250
300
350
400
450

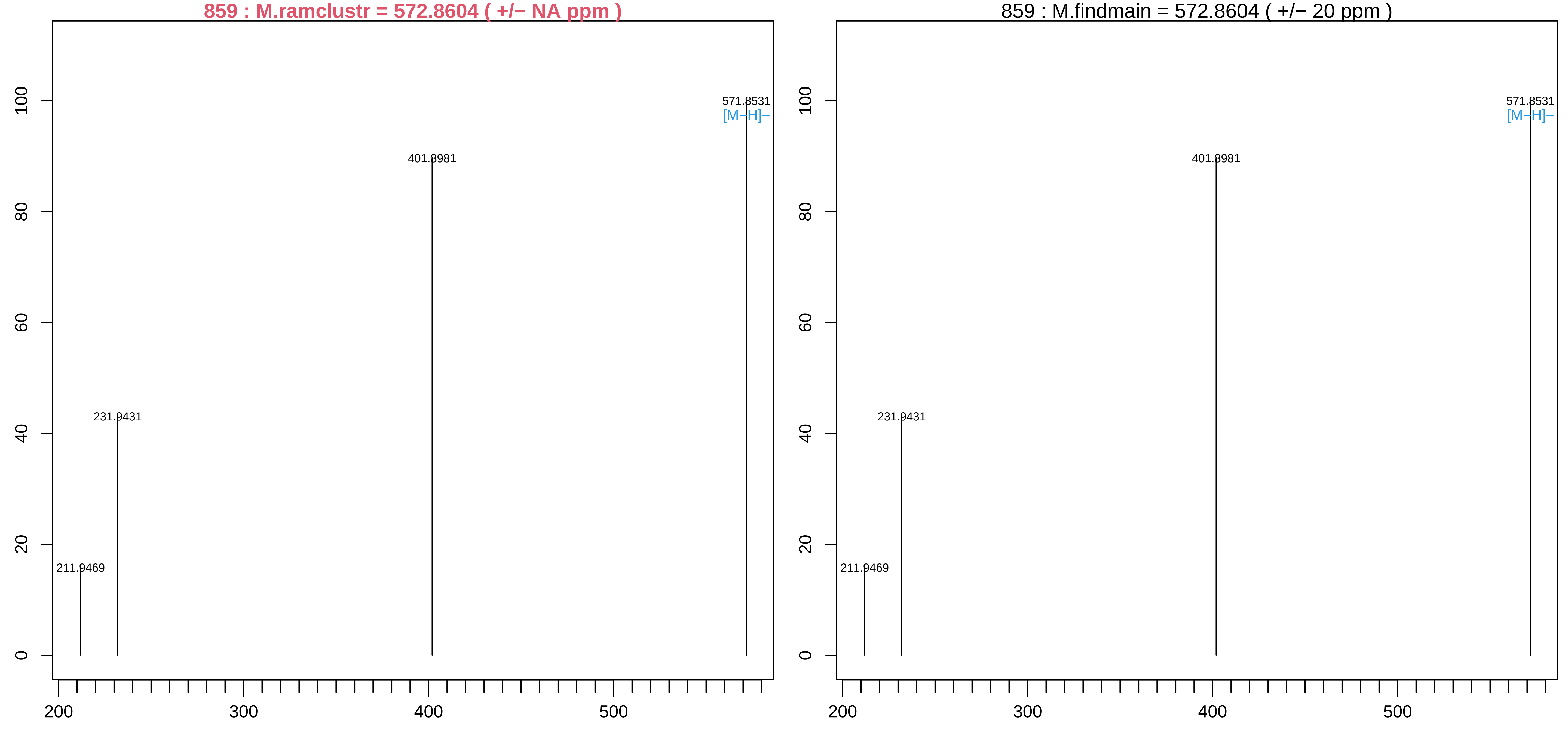
859 : M.ramclustr = 572.8604 (+/− NA ppm)
571.8531
[M−H]−
401.8981
231.9431
211.9469
859 : M.findmain = 572.8604 (+/− 20 ppm)
571.8531
[M−H]−
401.8981
231.9431
211.9469

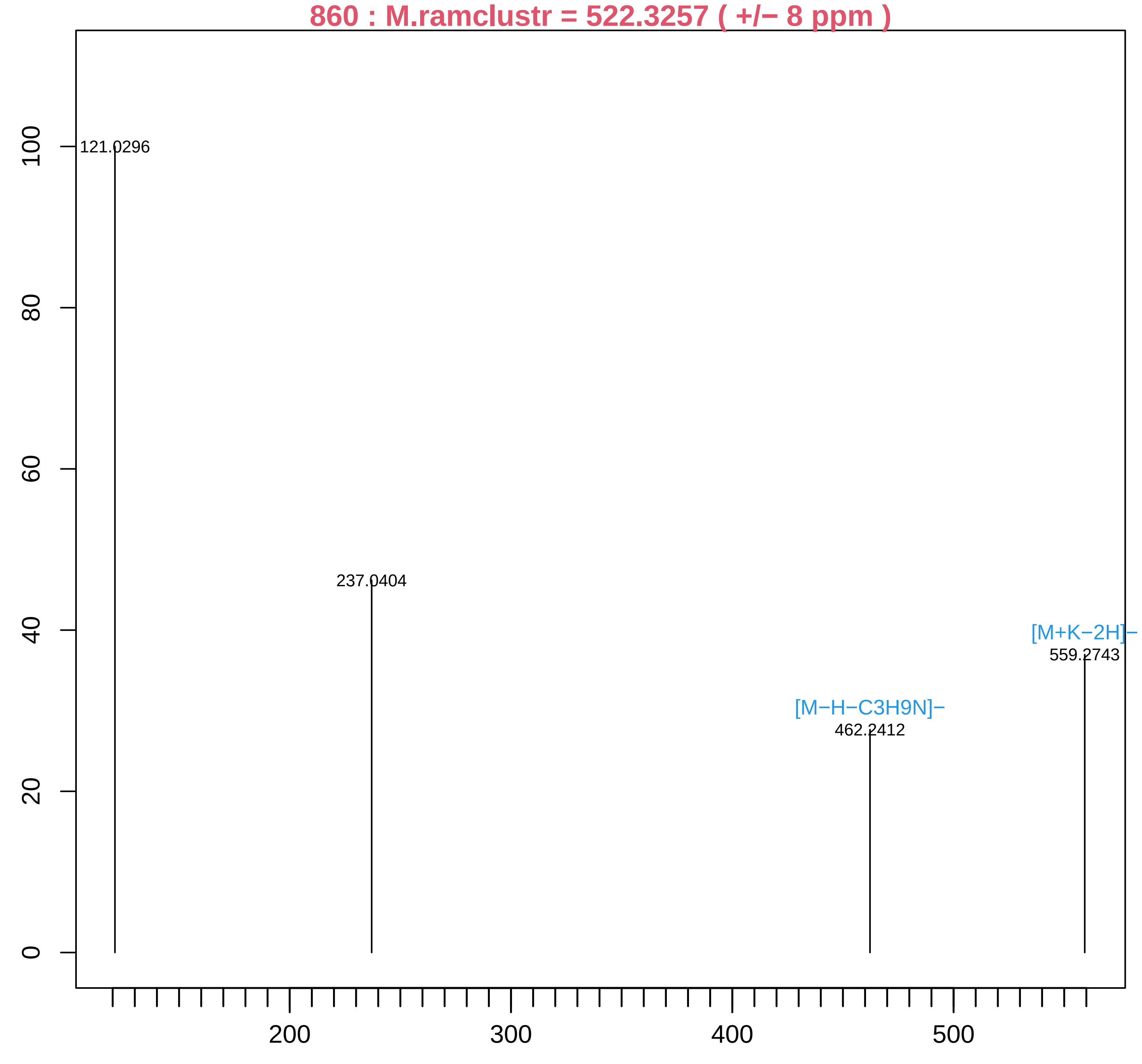

860 : M.ramclustr = 522.3257 (+/− 8 ppm)
121.0296
237.0404
[M−H−C3H9N]−
462.2412
[M+K−2H]−
559.2743
0
20
40
60
80
100
200
300
400
500

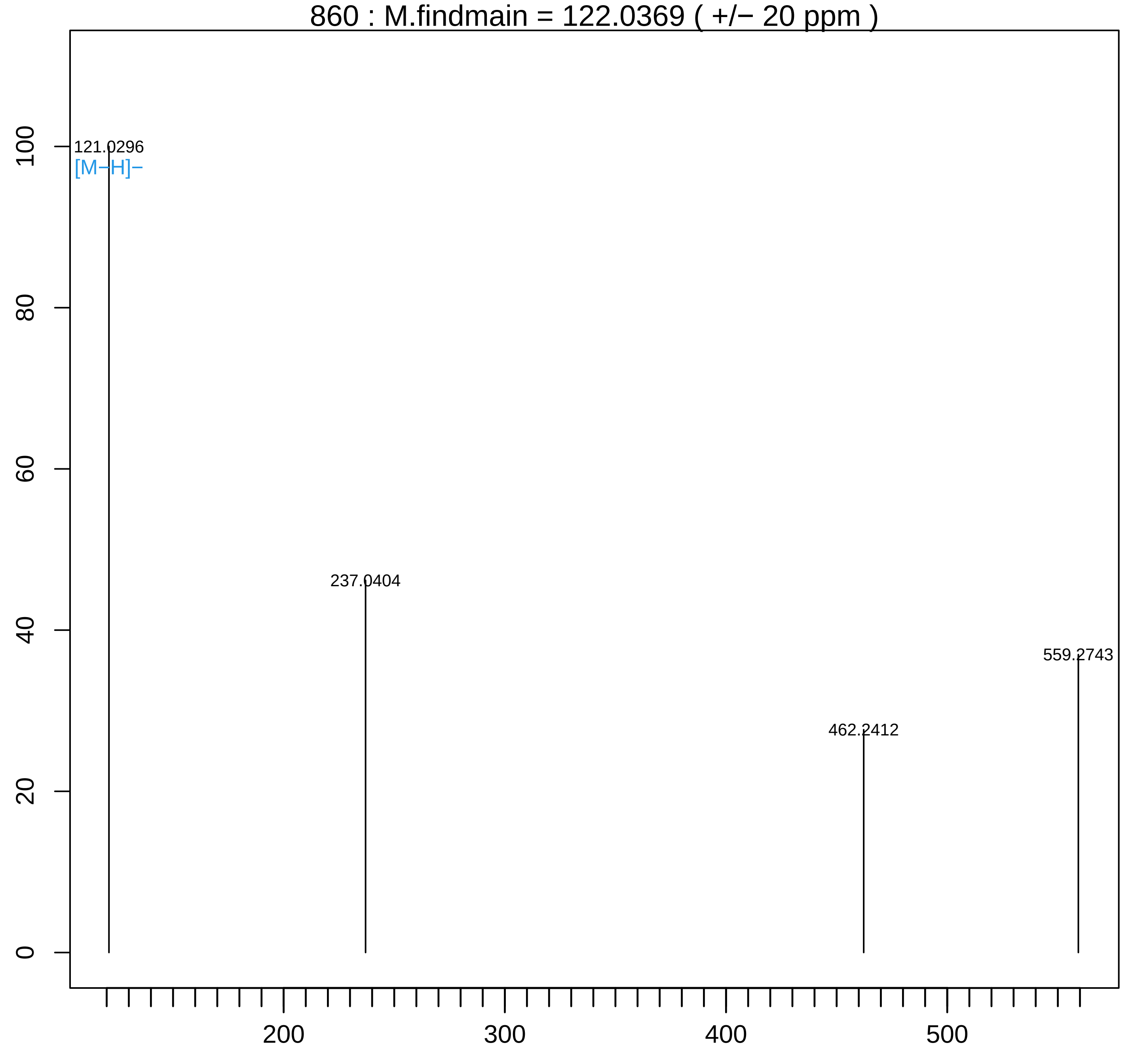

860 : M.findmain = 122.0369 (+/− 20 ppm)
121.0296
[M−H]−
237.0404
462.2412
559.2743
0
20
40
60
80
100
200
300
400
500

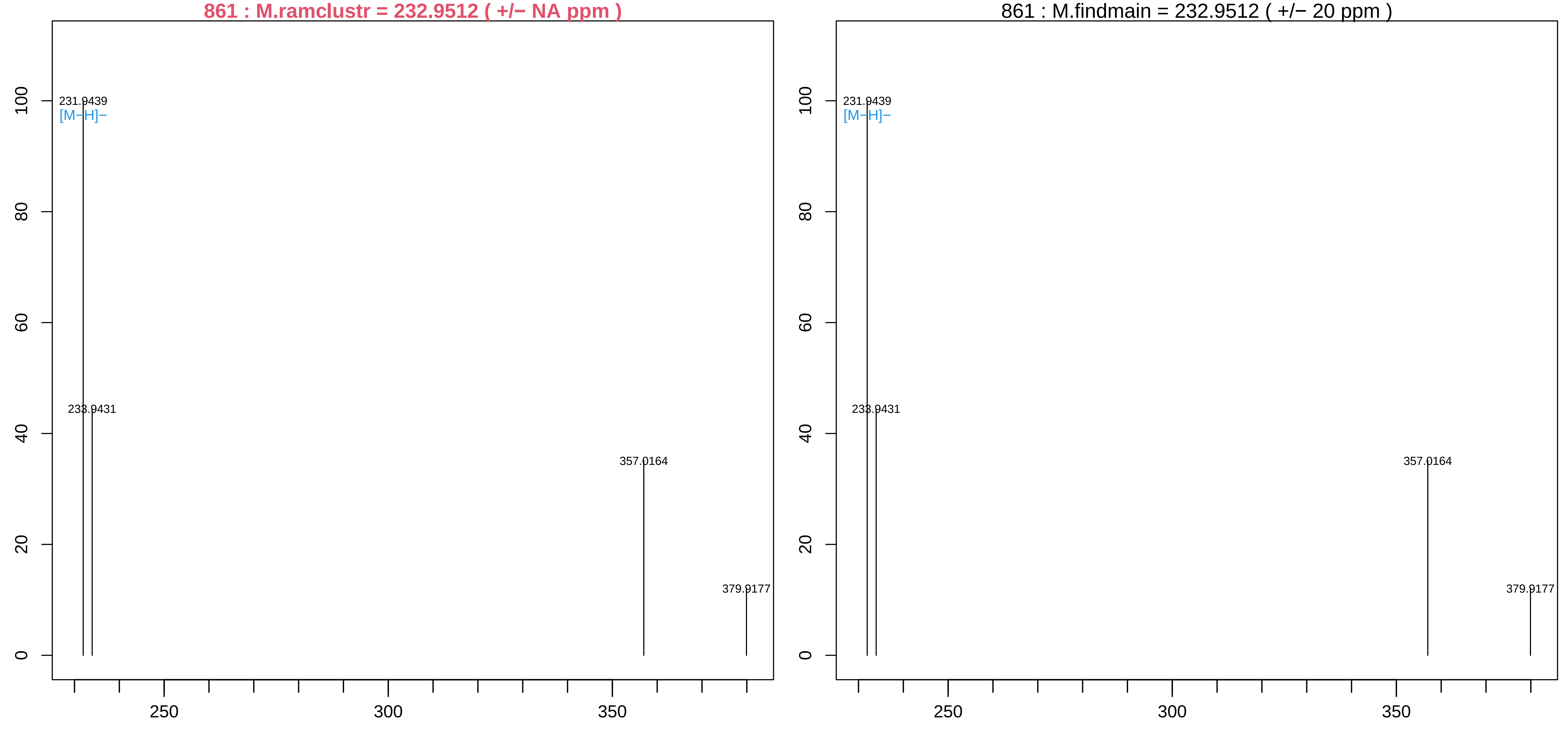

861 : M.ramclustr = 232.9512 (+/− NA ppm)
231.9439
[M−H]−
233.9431
357.0164
379.9177
861 : M.findmain = 232.9512 (+/− 20 ppm)
231.9439
[M−H]−
233.9431
357.0164
379.9177
0
20
40
60
80
100
250
300
350

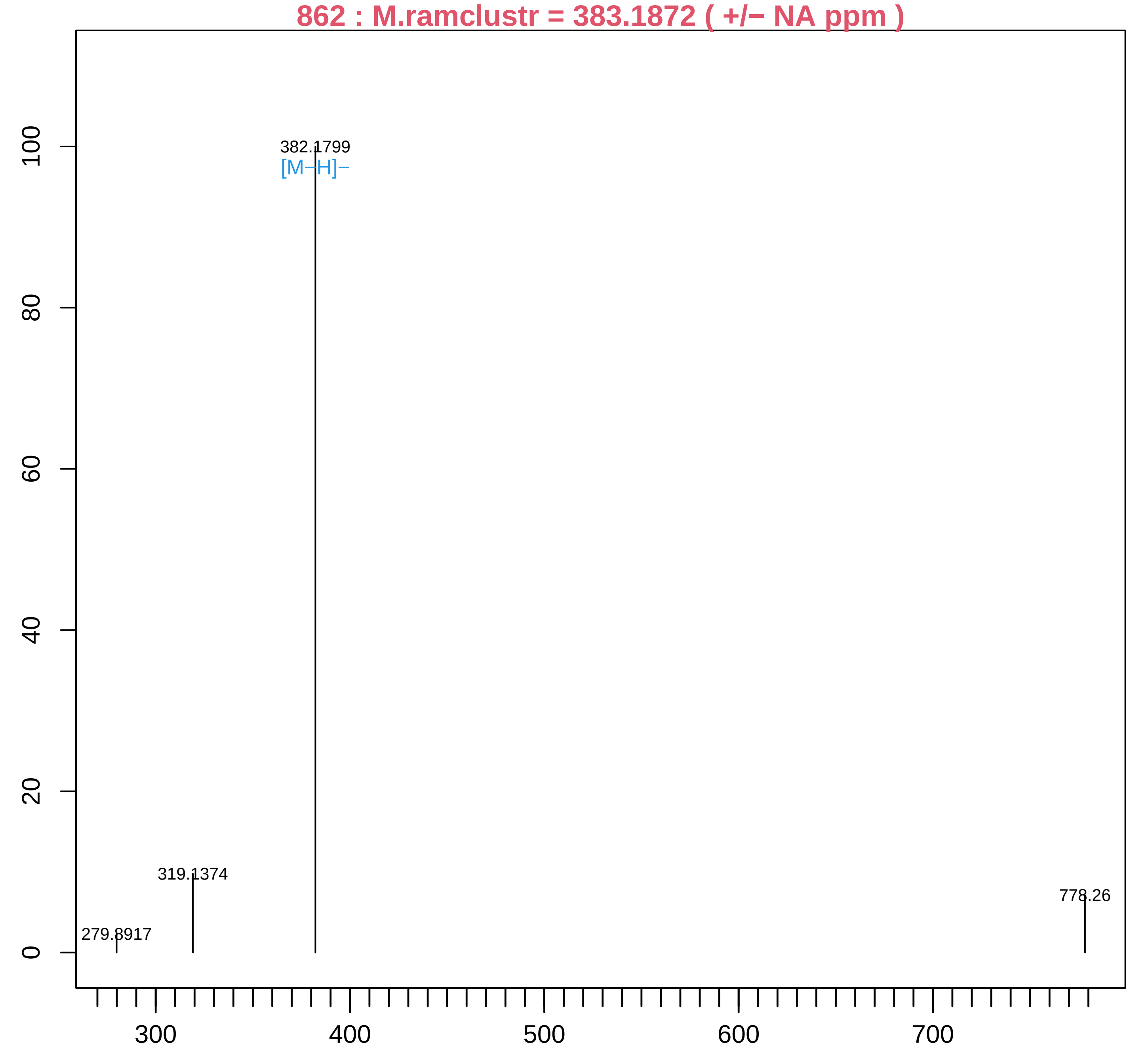
862 : M.ramclustr = 383.1872 (+/− NA ppm)
382.1799
[M−H]−
319.1374
279.8917
778.26
0
20
40
60
80
100
300
400
500
600
700

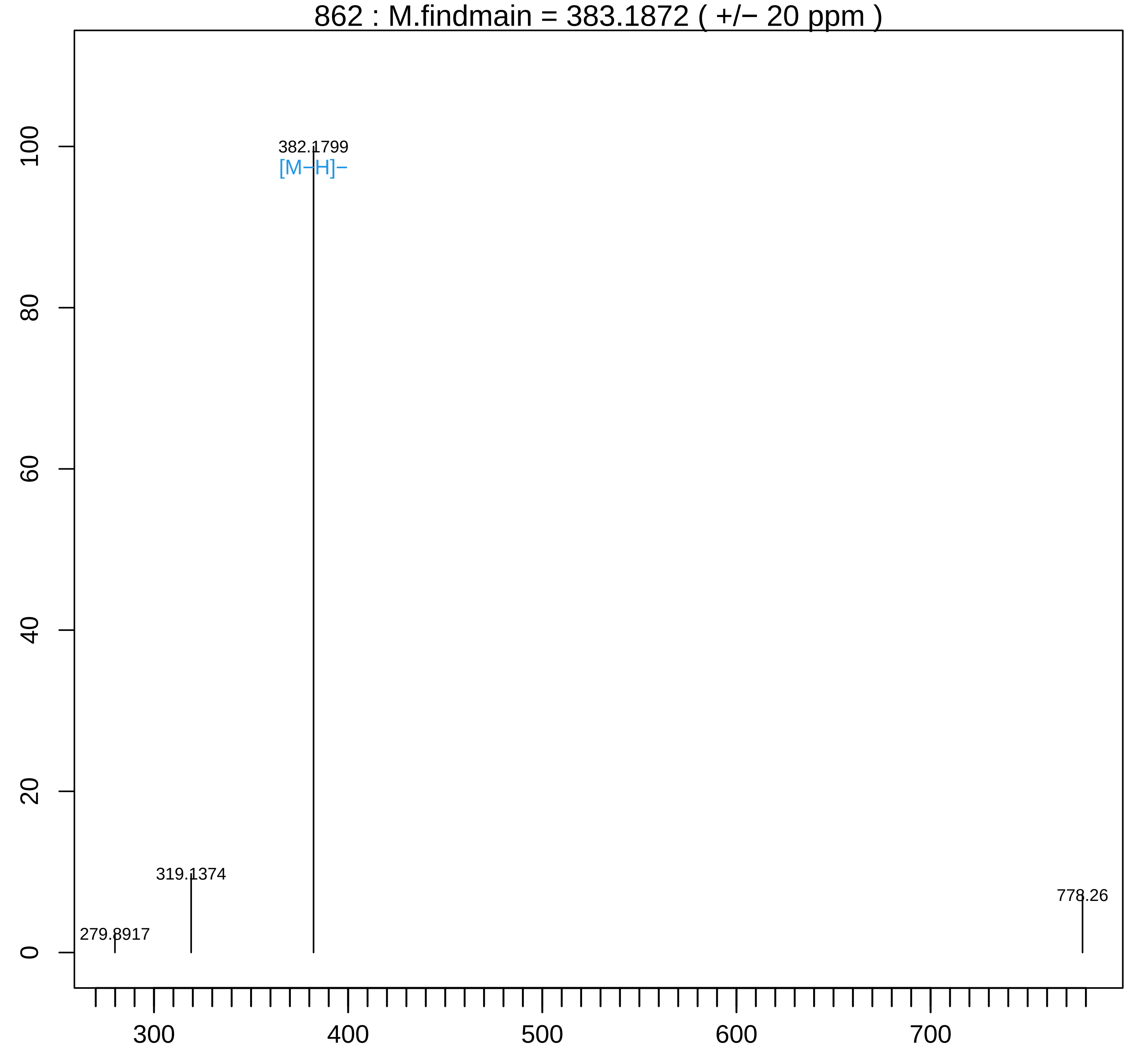
862 : M.findmain = 383.1872 (+/− 20 ppm)
382.1799
[M−H]−
319.1374
279.8917
778.26
0
20
40
60
80
100
300
400
500
600
700

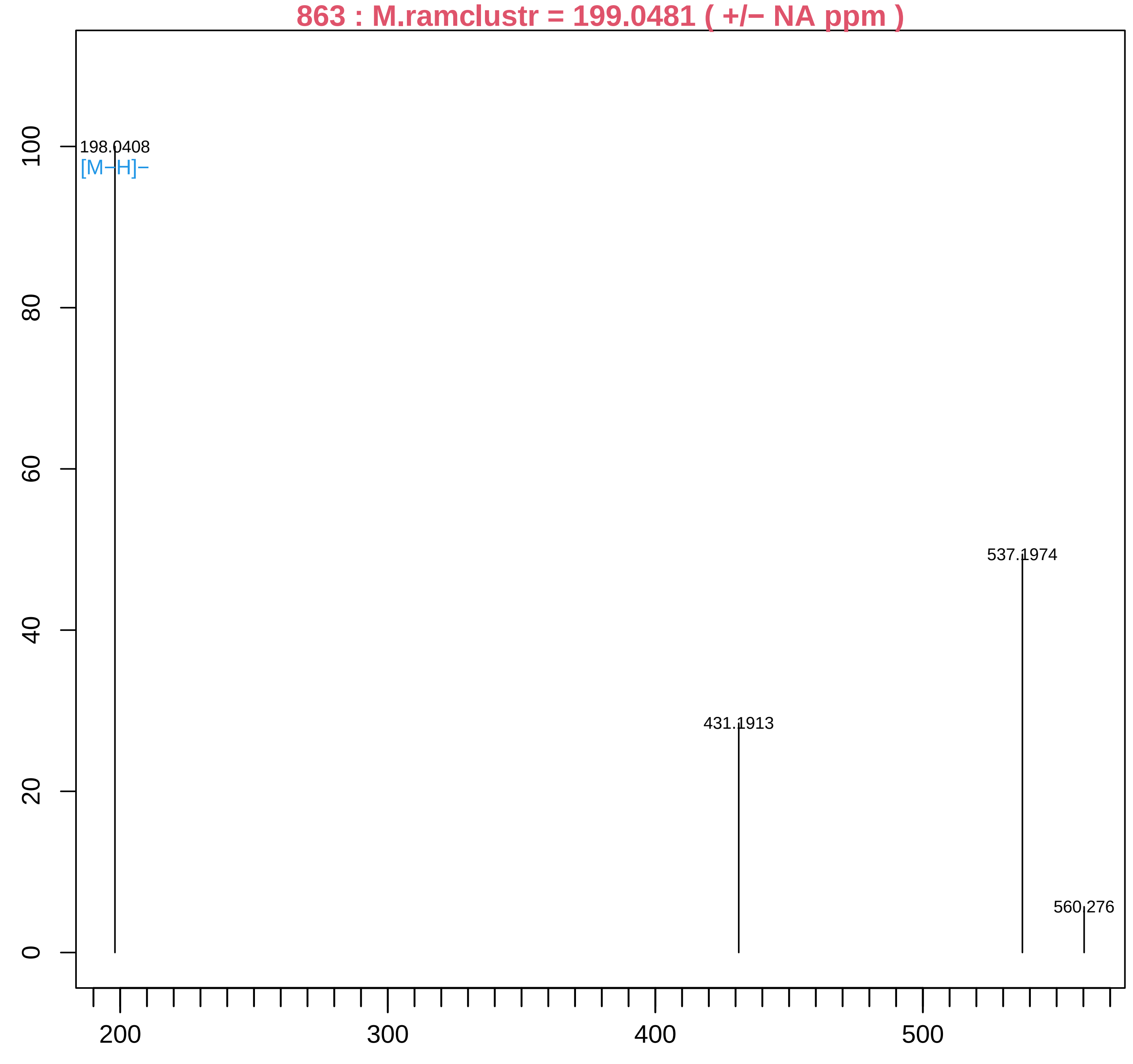

863 : M.ramclustr = 199.0481 (+/− NA ppm)
198.0408
[M−H]−
431.1913
537.1974
560.276
0
20
40
60
80
100
200
300
400
500

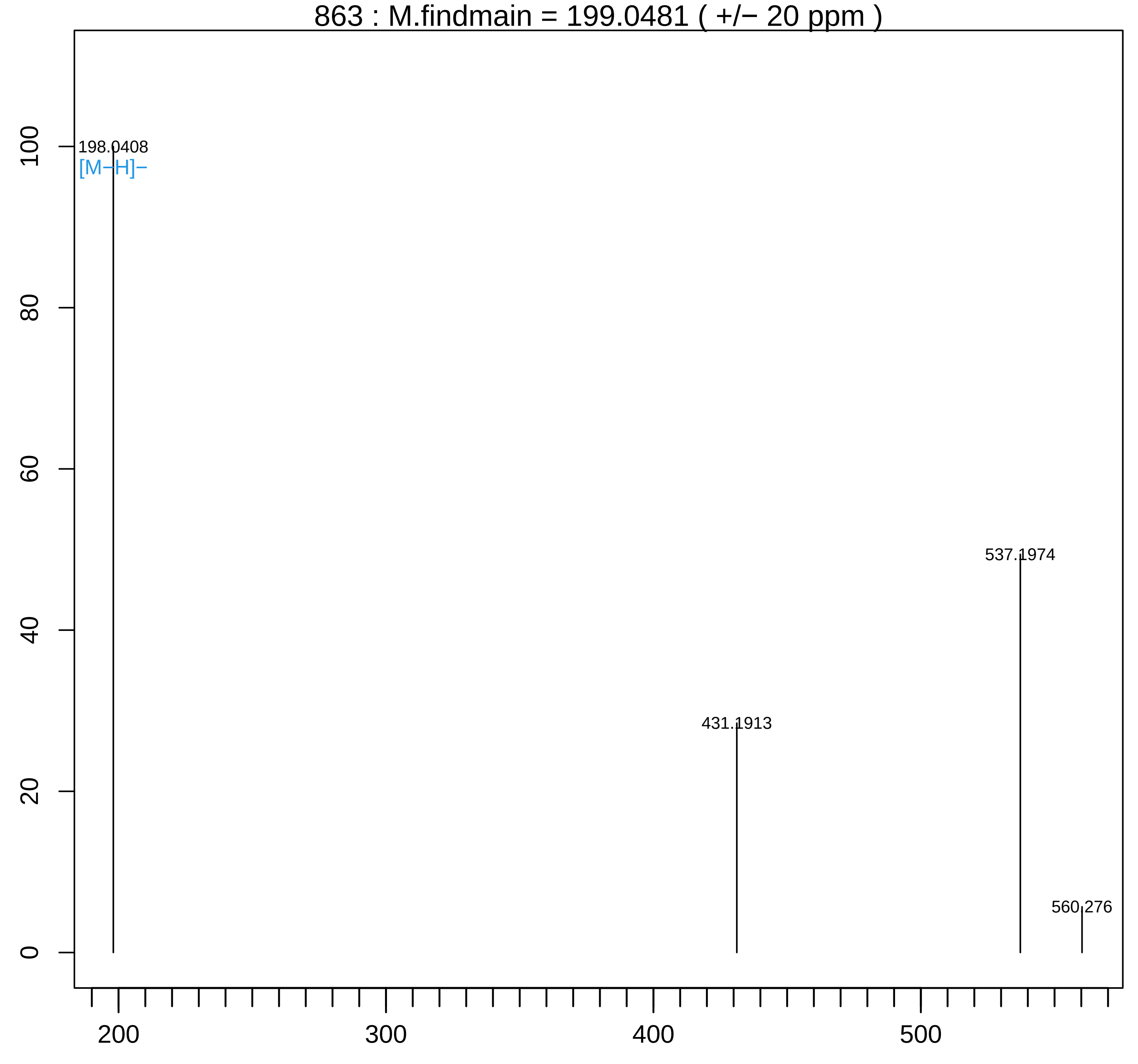

863 : M.findmain = 199.0481 (+/− 20 ppm)
198.0408
[M−H]−
431.1913
537.1974
560.276
0
20
40
60
80
100
200
300
400
500

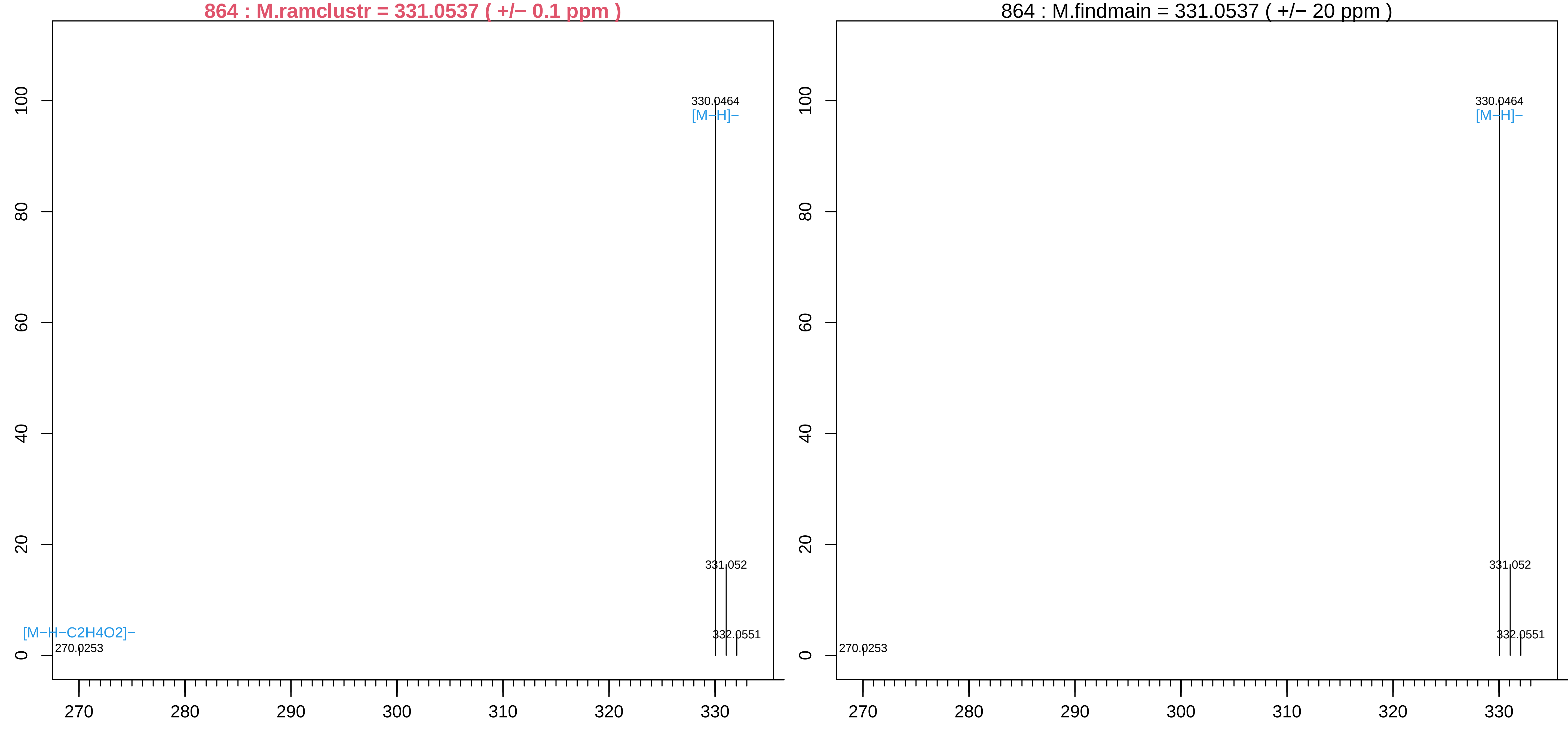

864 : M.ramclustr = 331.0537 (+/− 0.1 ppm)
330.0464
[M−H]−
331.052
332.0551
[M−H−C2H4O2]−
270.0253
0
20
40
60
80
100
270
280
290
300
310
320
330
864 : M.findmain = 331.0537 (+/− 20 ppm)
330.0464
[M−H]−
331.052
332.0551
270.0253

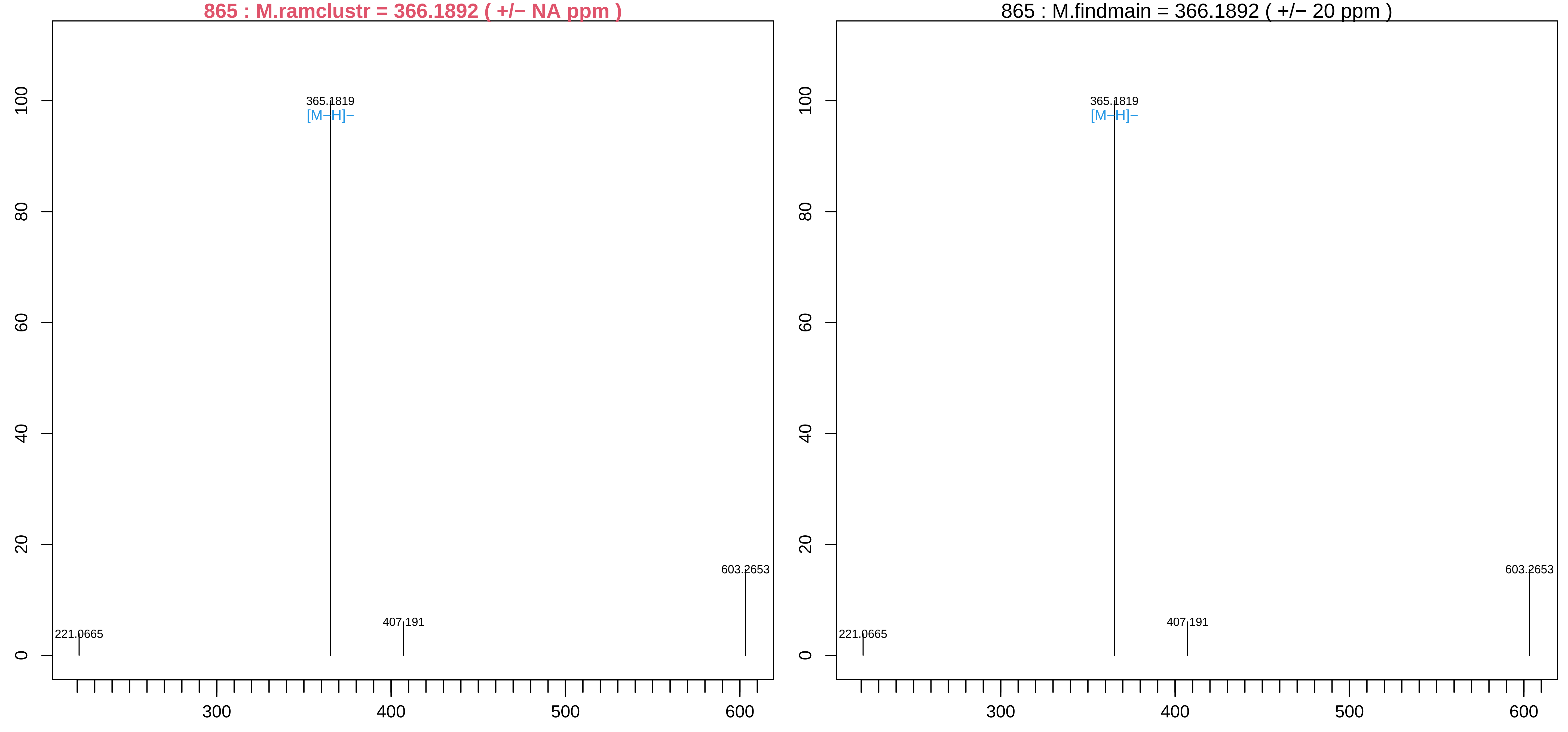

865 : M.ramclustr = 366.1892 (+/− NA ppm)
365.1819
[M−H]−
221.0665
407.191
603.2653
0
20
40
60
80
100
300
400
500
600
865 : M.findmain = 366.1892 (+/− 20 ppm)
365.1819
[M−H]−
221.0665
407.191
603.2653
0
20
40
60
80
100
300
400
500
600

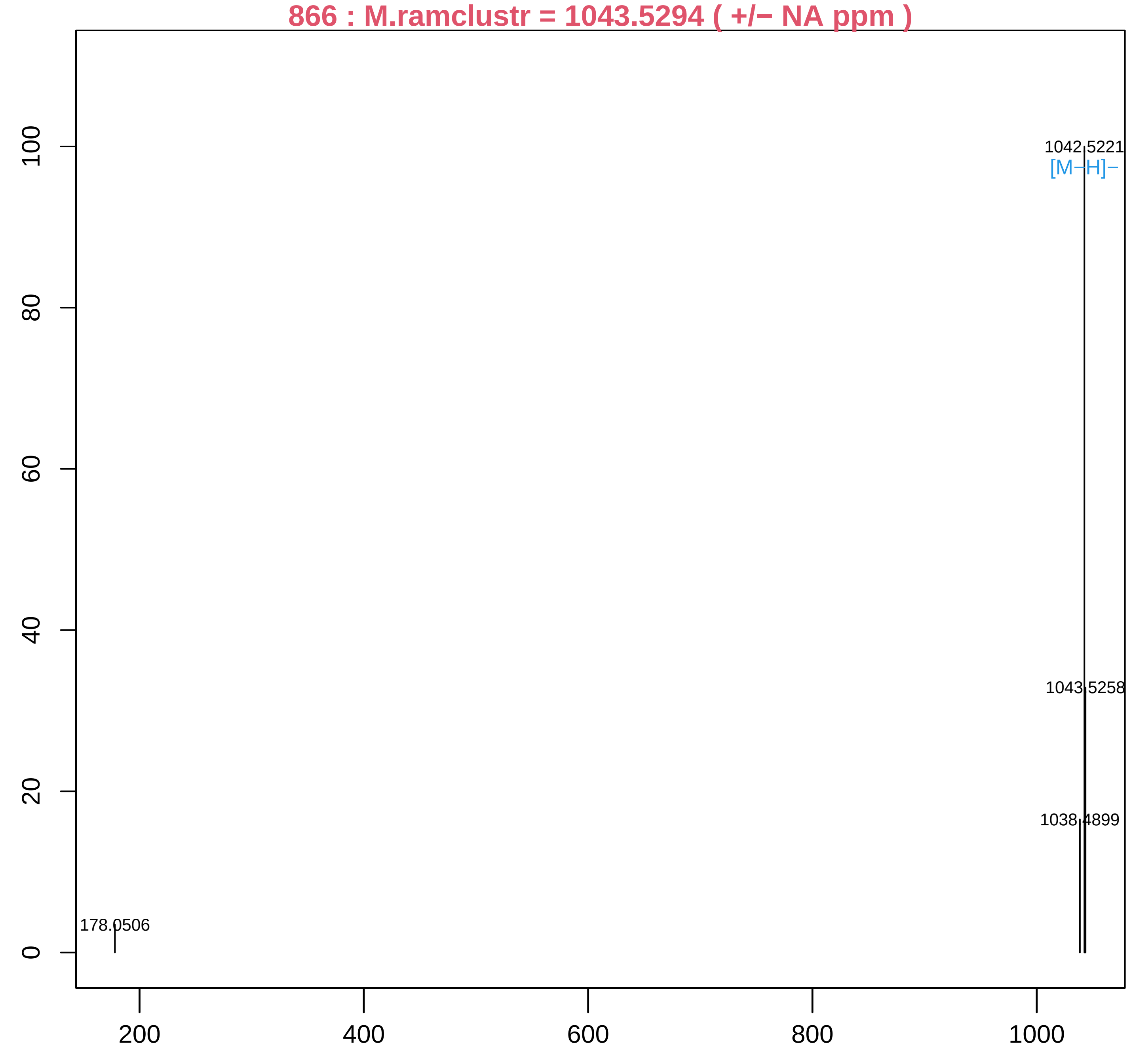
866 : M.ramclustr = 1043.5294 (+/− NA ppm)
1042.5221
[M−H]−
1043.5258
1038.4899
178.0506
0
20
40
60
80
100
200
400
600
800
1000

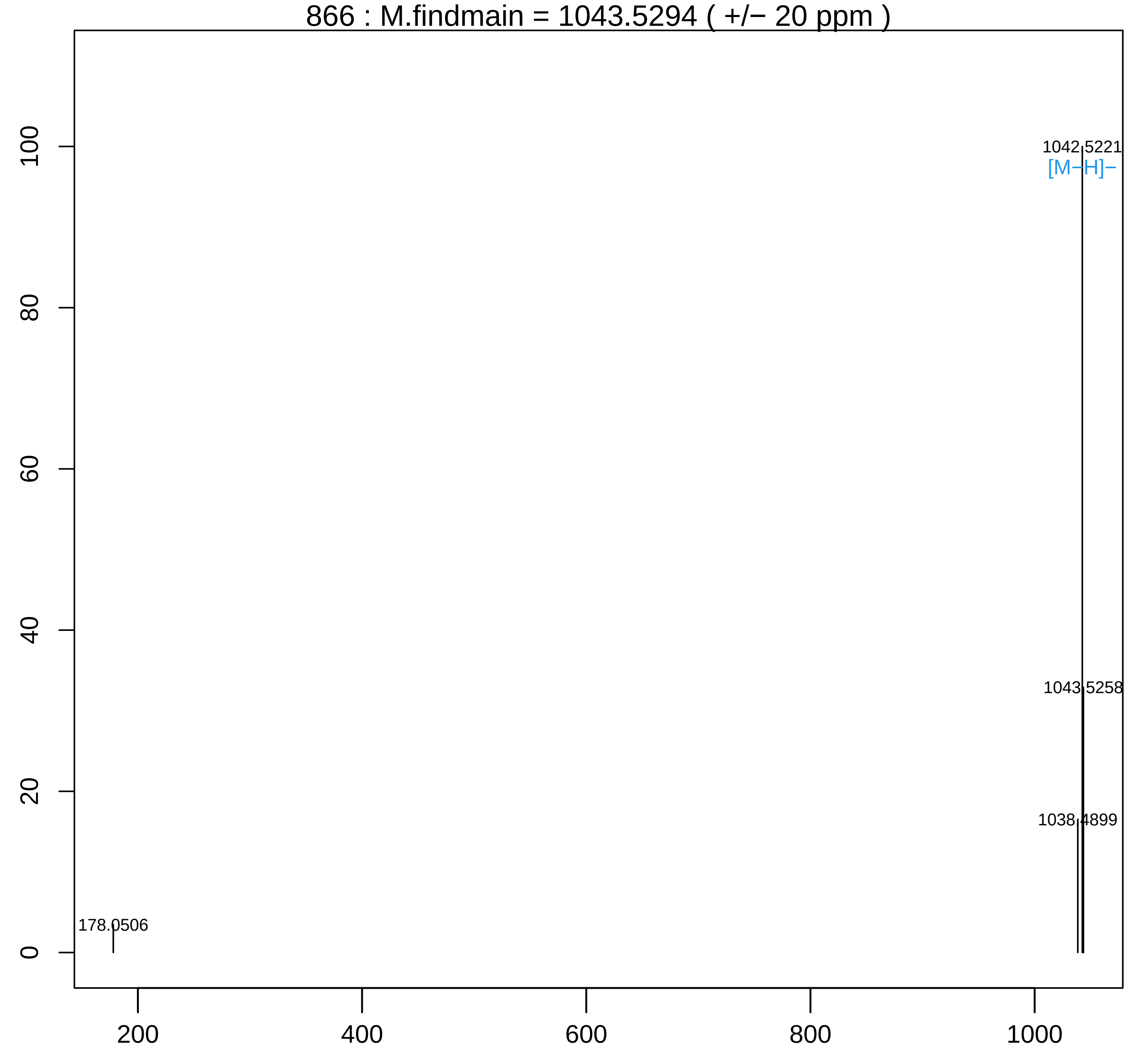
866 : M.findmain = 1043.5294 (+/− 20 ppm)
1042.5221
[M−H]−
1043.5258
1038.4899
178.0506
0
20
40
60
80
100
200
400
600
800
1000

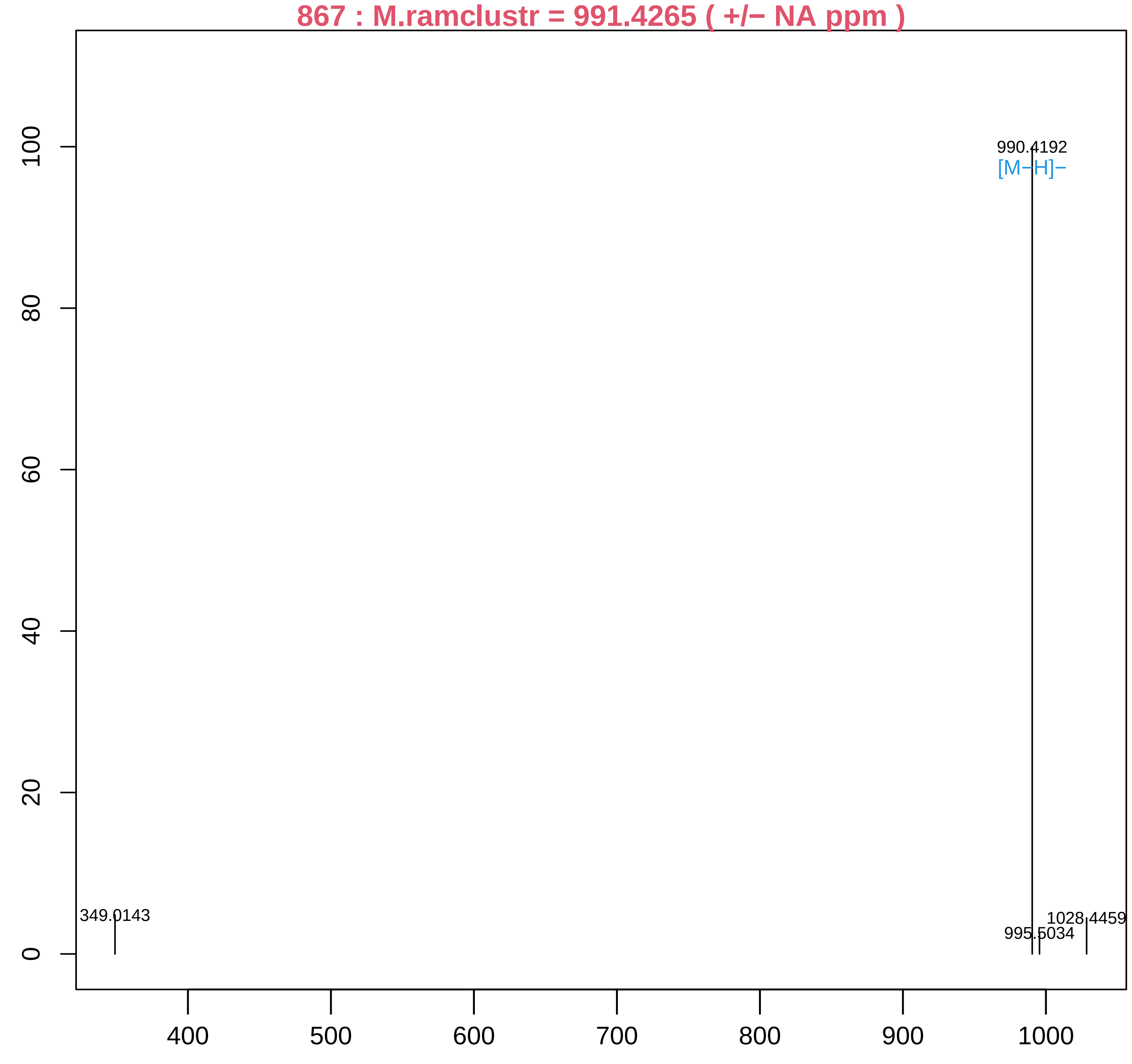

867 : M.ramclustr = 991.4265 (+/− NA ppm)
990.4192
[M−H]−
349.0143
995.5034
1028.4459
0
20
40
60
80
100
400
500
600
700
800
900
1000

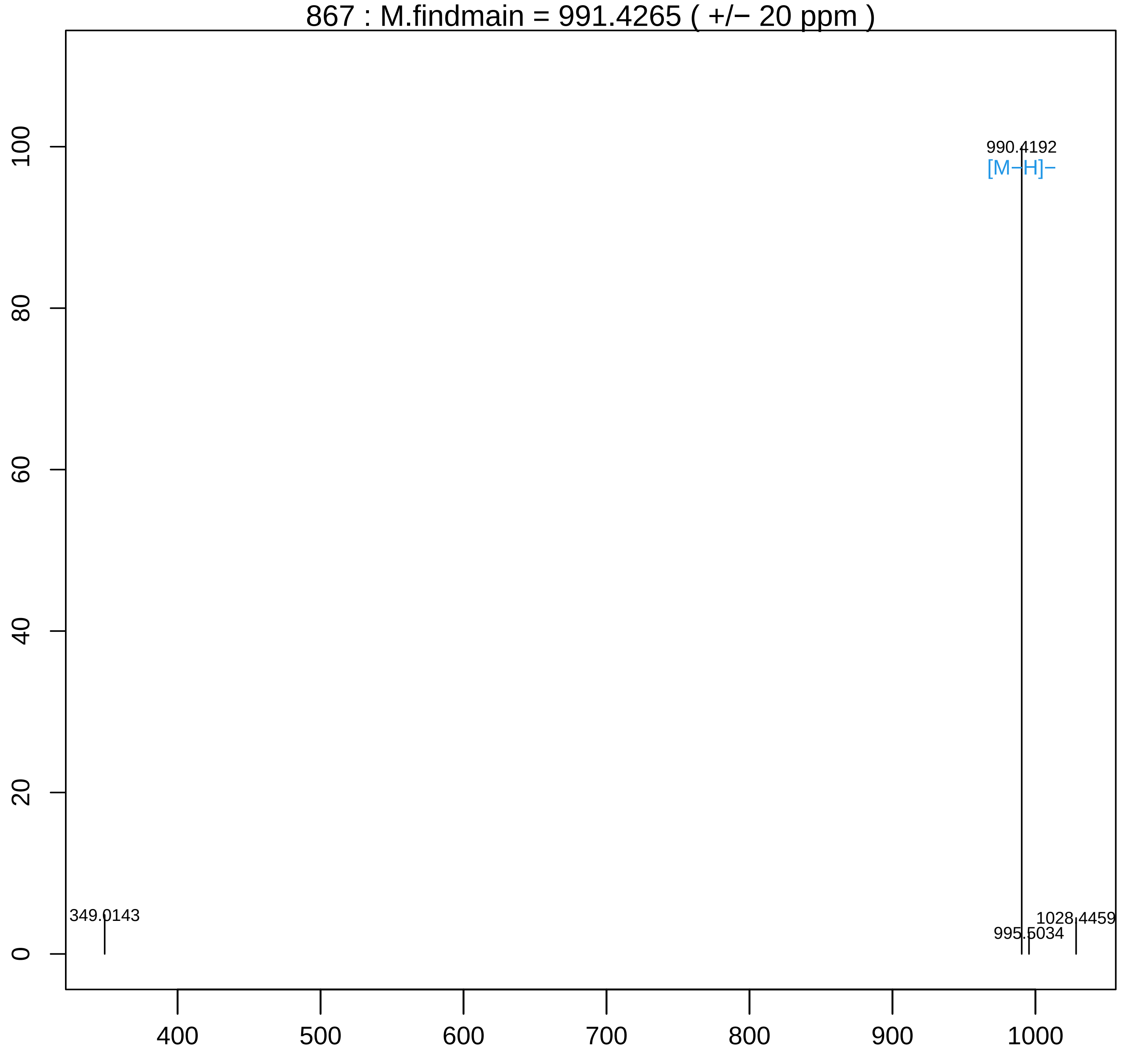

867 : M.findmain = 991.4265 (+/− 20 ppm)
990.4192
[M−H]−
349.0143
995.5034
1028.4459
0
20
40
60
80
100
400
500
600
700
800
900
1000

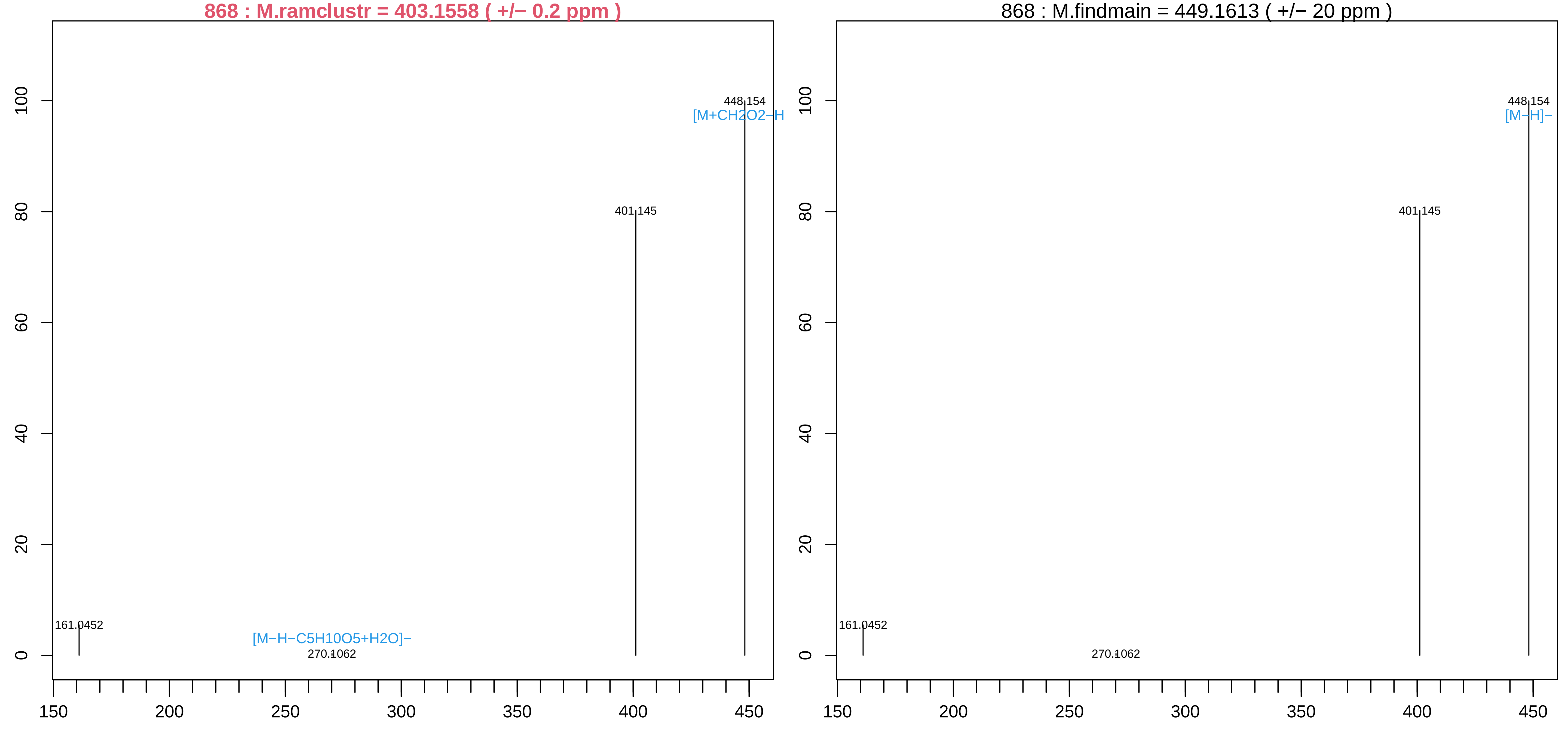

868 : M.ramclustr = 403.1558 (+/− 0.2 ppm)
448.154
[M+CH2O2−H
401.145
161.0452
[M−H−C5H10O5+H2O]−
270.1062
868 : M.findmain = 449.1613 (+/− 20 ppm)
448.154
[M−H]−
401.145
161.0452
270.1062

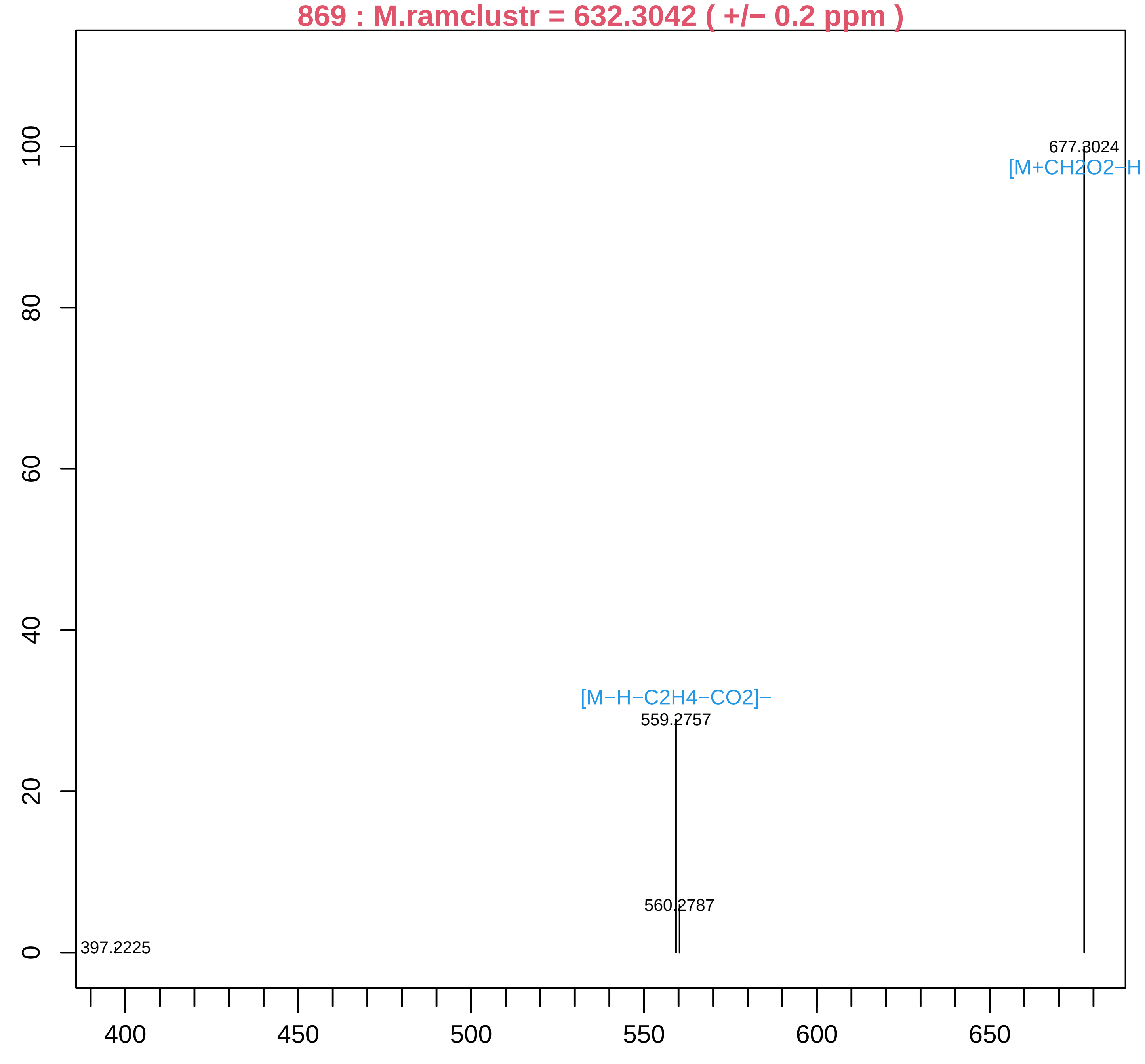

869 : M.ramclustr = 632.3042 (+/− 0.2 ppm)
677.3024
[M+CH2O2−H
[M−H−C2H4−CO2]−
559.2757
560.2787
397.2225
0
20
40
60
80
100
400
450
500
550
600
650

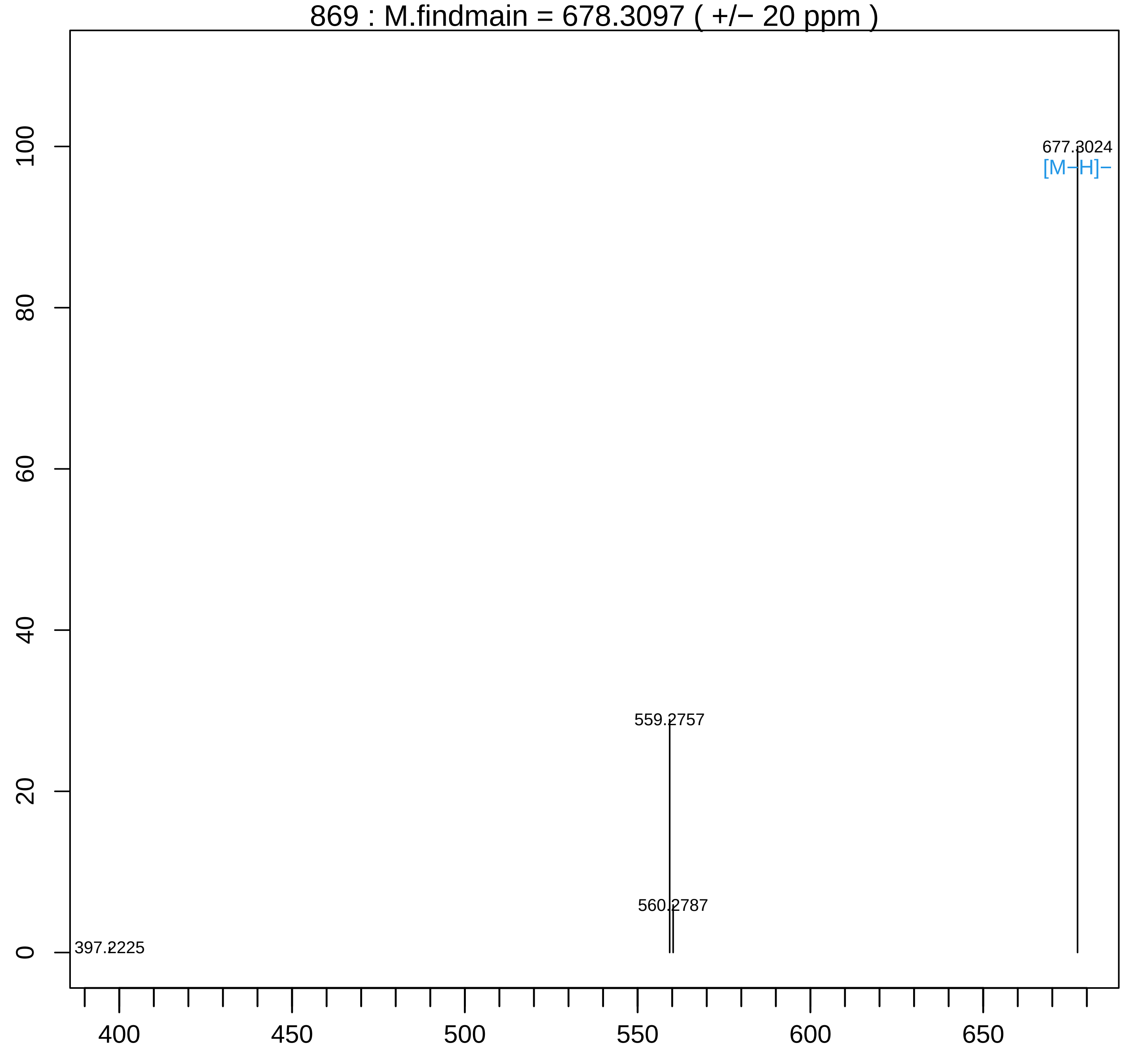

869 : M.findmain = 678.3097 (+/− 20 ppm)
677.3024
[M−H]−
559.2757
560.2787
397.2225
0
20
40
60
80
100
400
450
500
550
600
650

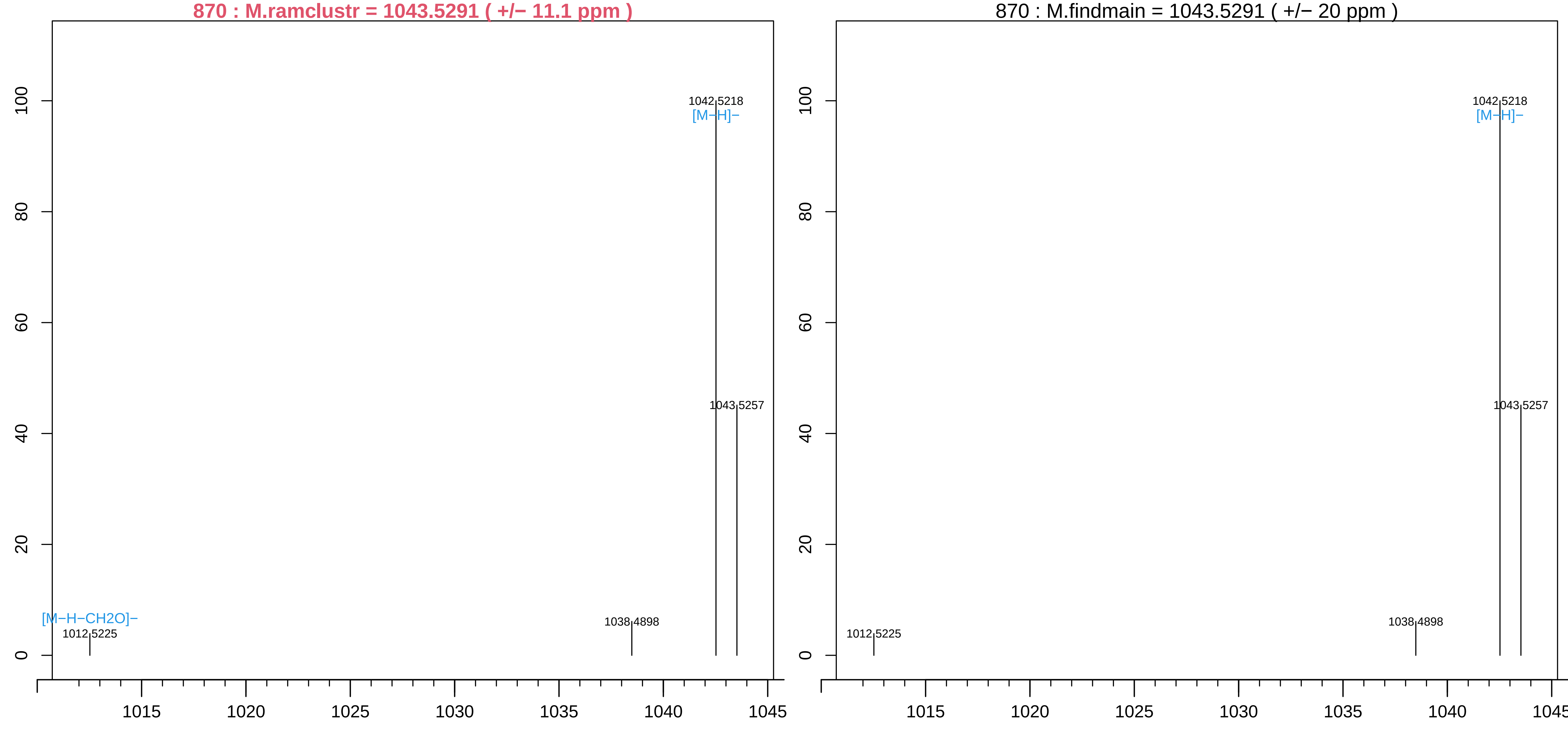

870 : M.ramclustr = 1043.5291 (+/− 11.1 ppm)
870 : M.findmain = 1043.5291 (+/− 20 ppm)
1042.5218
[M−H]−
1043.5257
1038.4898
1012.5225
[M−H−CH2O]−
0
20
40
60
80
100
1015
1020
1025
1030
1035
1040
1045

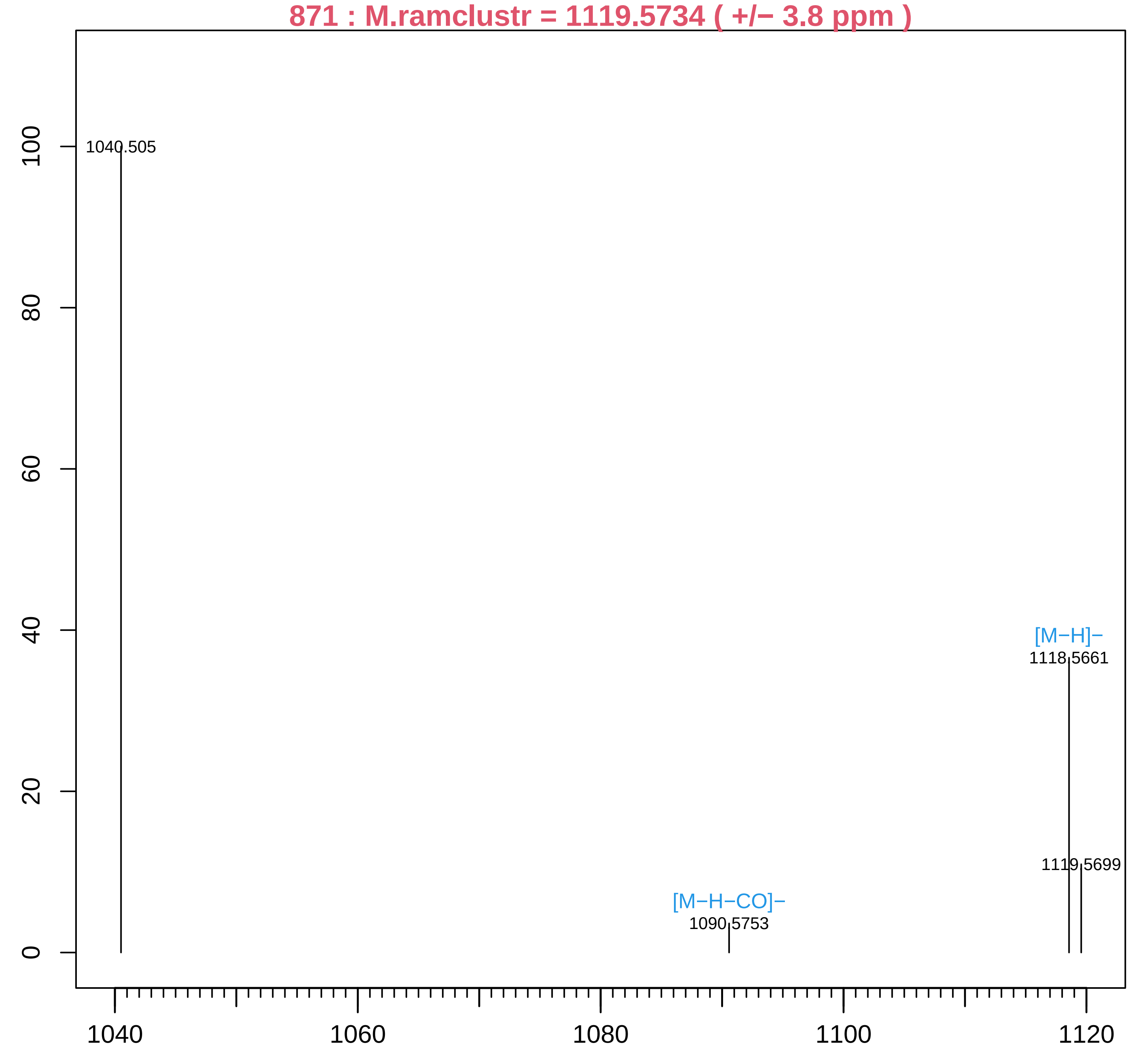
871 : M.ramclustr = 1119.5734 (+/− 3.8 ppm)
1040.505
[M−H]−
1118.5661
1119.5699
[M−H−CO]−
1090.5753
0
20
40
60
80
100
1040
1060
1080
1100
1120

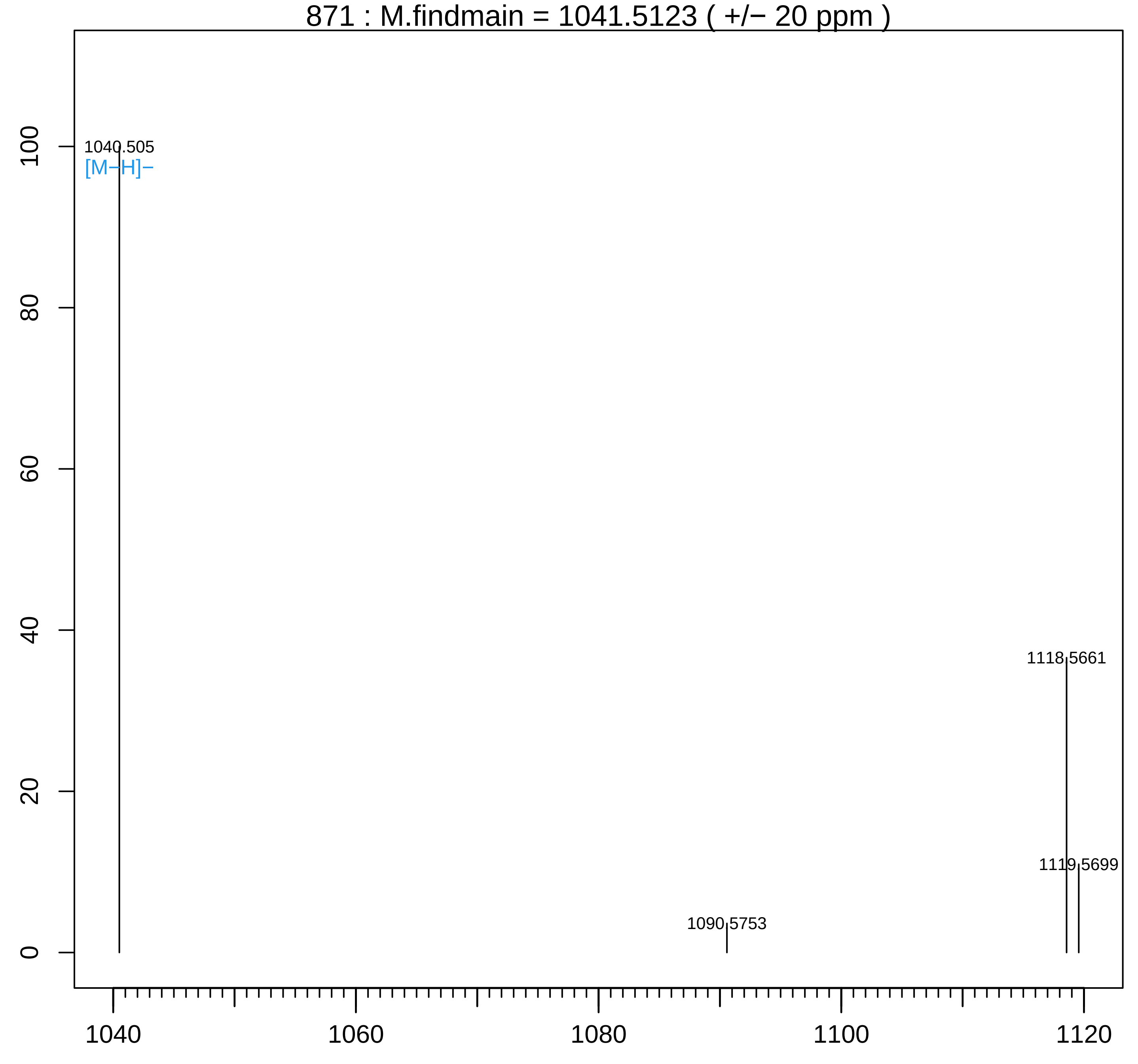
871 : M.findmain = 1041.5123 (+/− 20 ppm)
1040.505
[M−H]−
1118.5661
1119.5699
1090.5753
0
20
40
60
80
100
1040
1060
1080
1100
1120

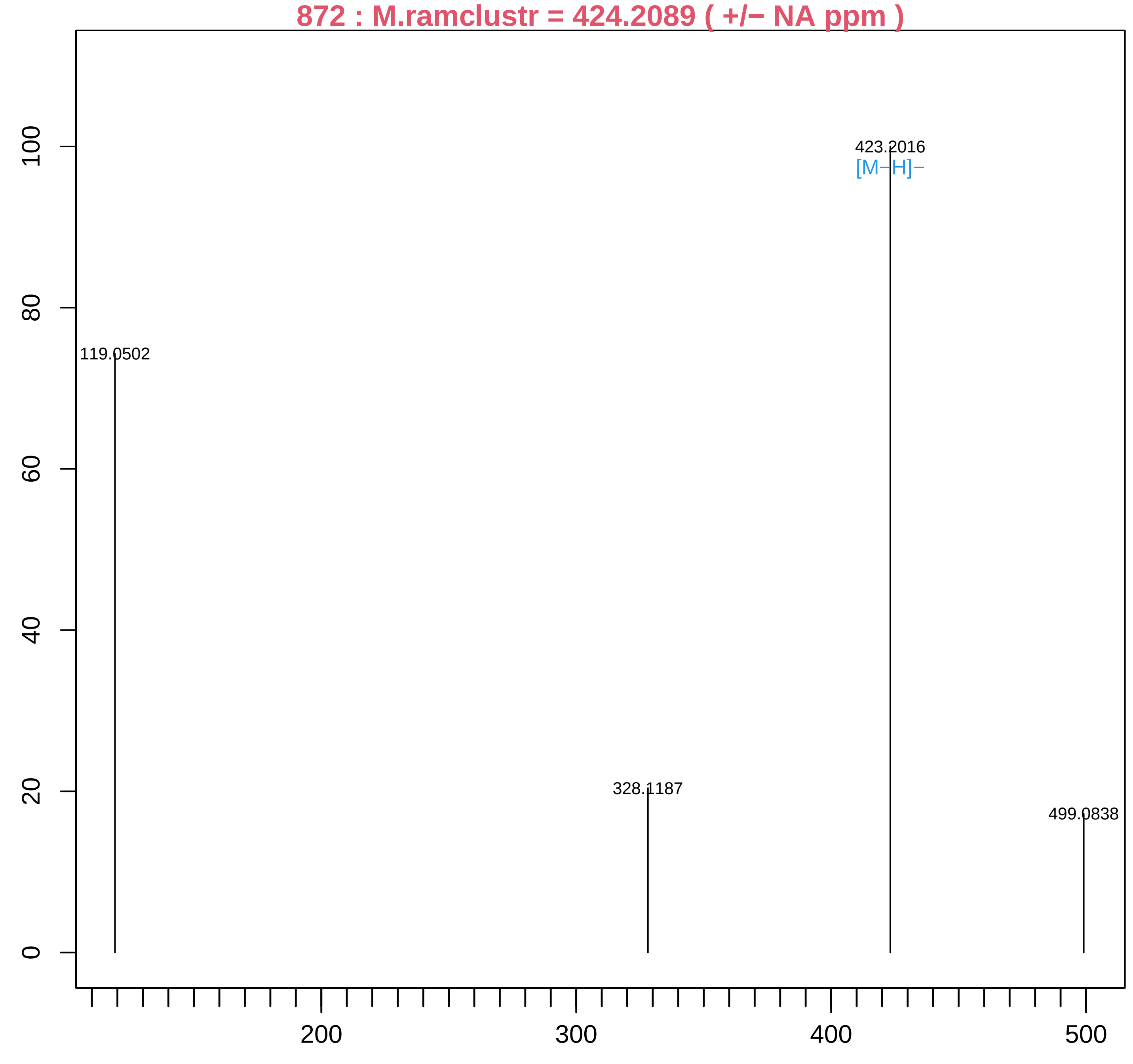
872 : M.ramclustr = 424.2089 (+/− NA ppm)
423.2016
[M−H]−
119.0502
328.1187
499.0838
0
20
40
60
80
100
200
300
400
500

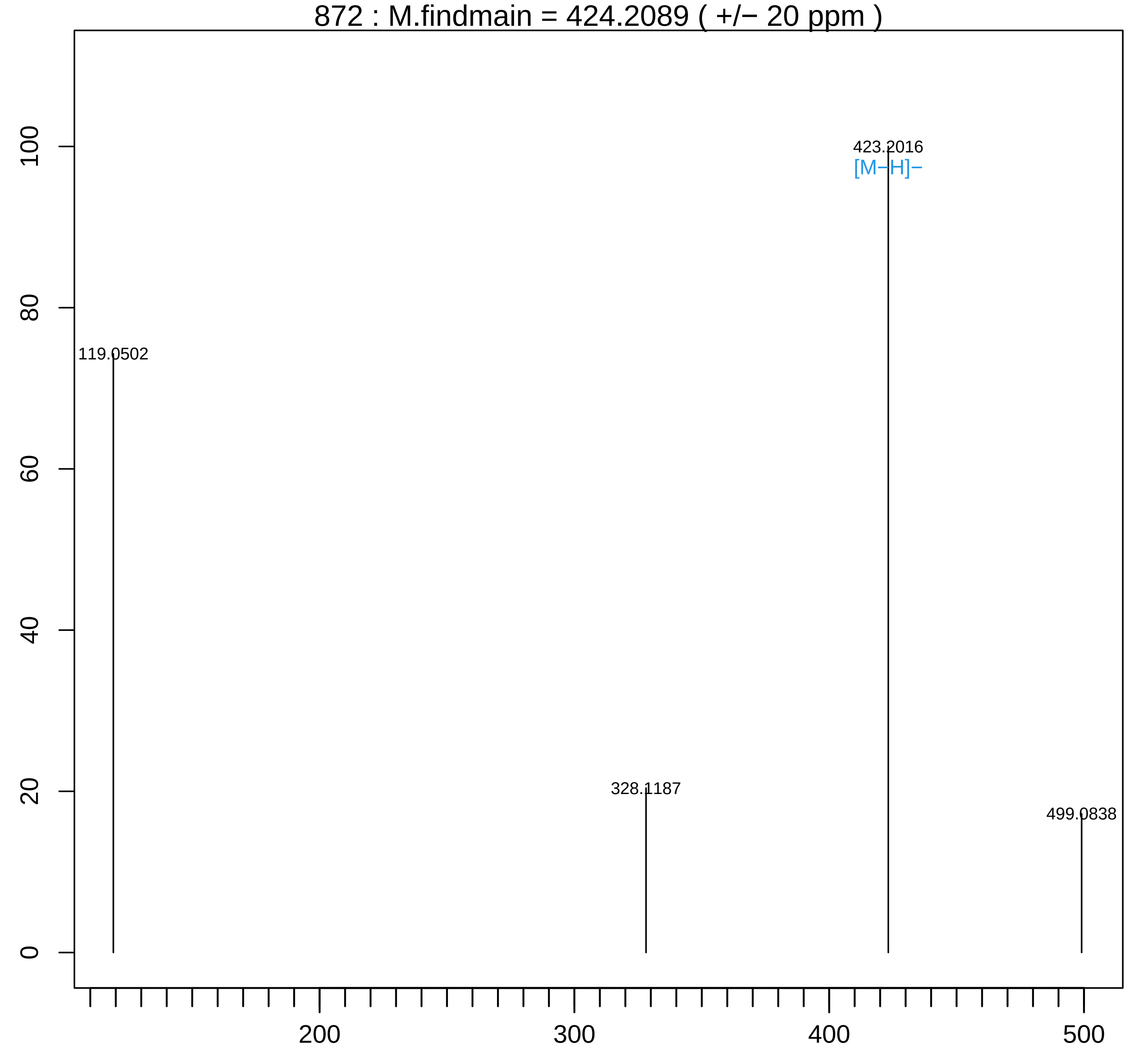
872 : M.findmain = 424.2089 (+/− 20 ppm)
423.2016
[M−H]−
119.0502
328.1187
499.0838
0
20
40
60
80
100
200
300
400
500

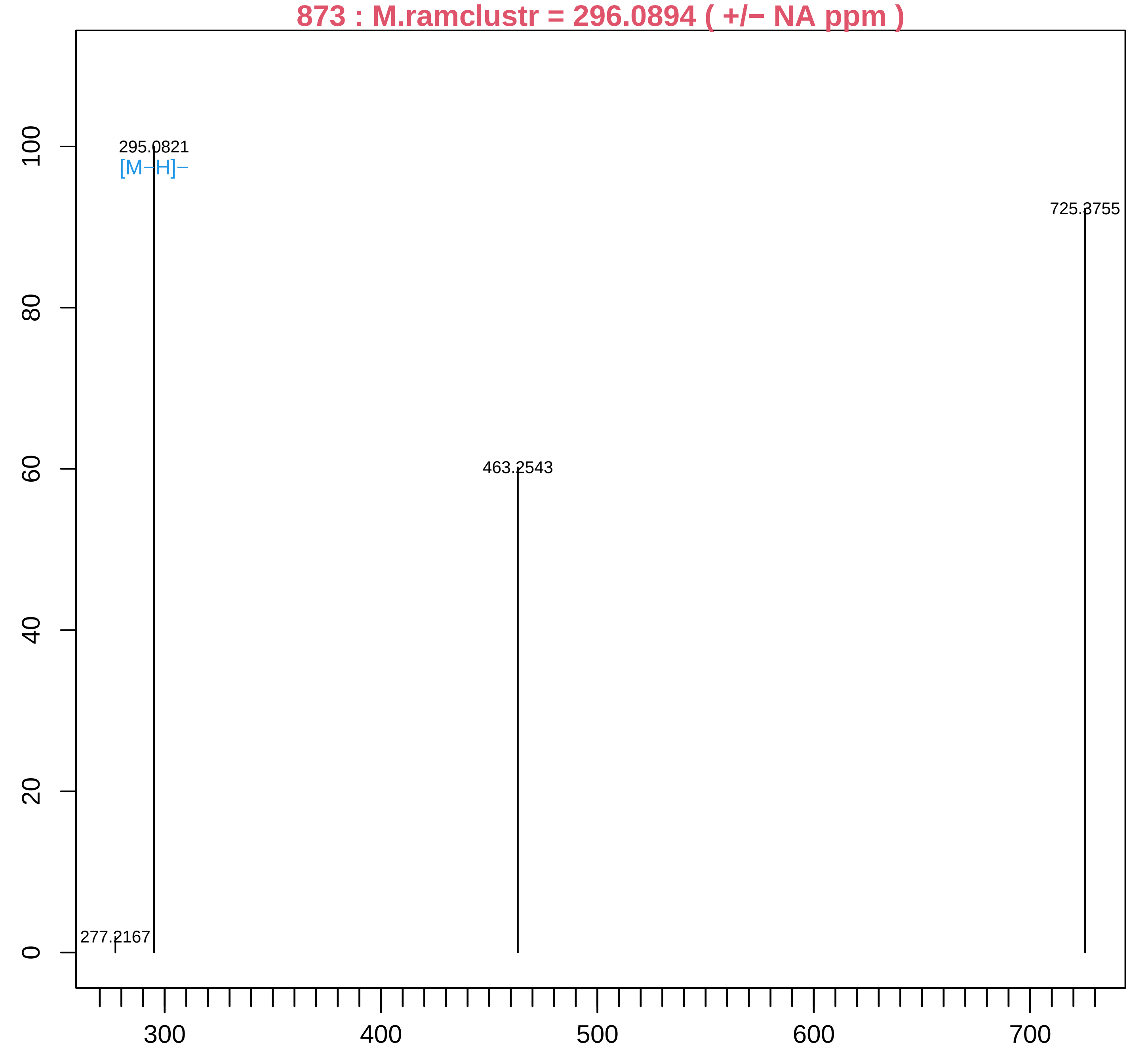

873 : M.ramclustr = 296.0894 (+/− NA ppm)
295.0821
[M−H]−
725.3755
463.2543
277.2167
0
20
40
60
80
100
300
400
500
600
700

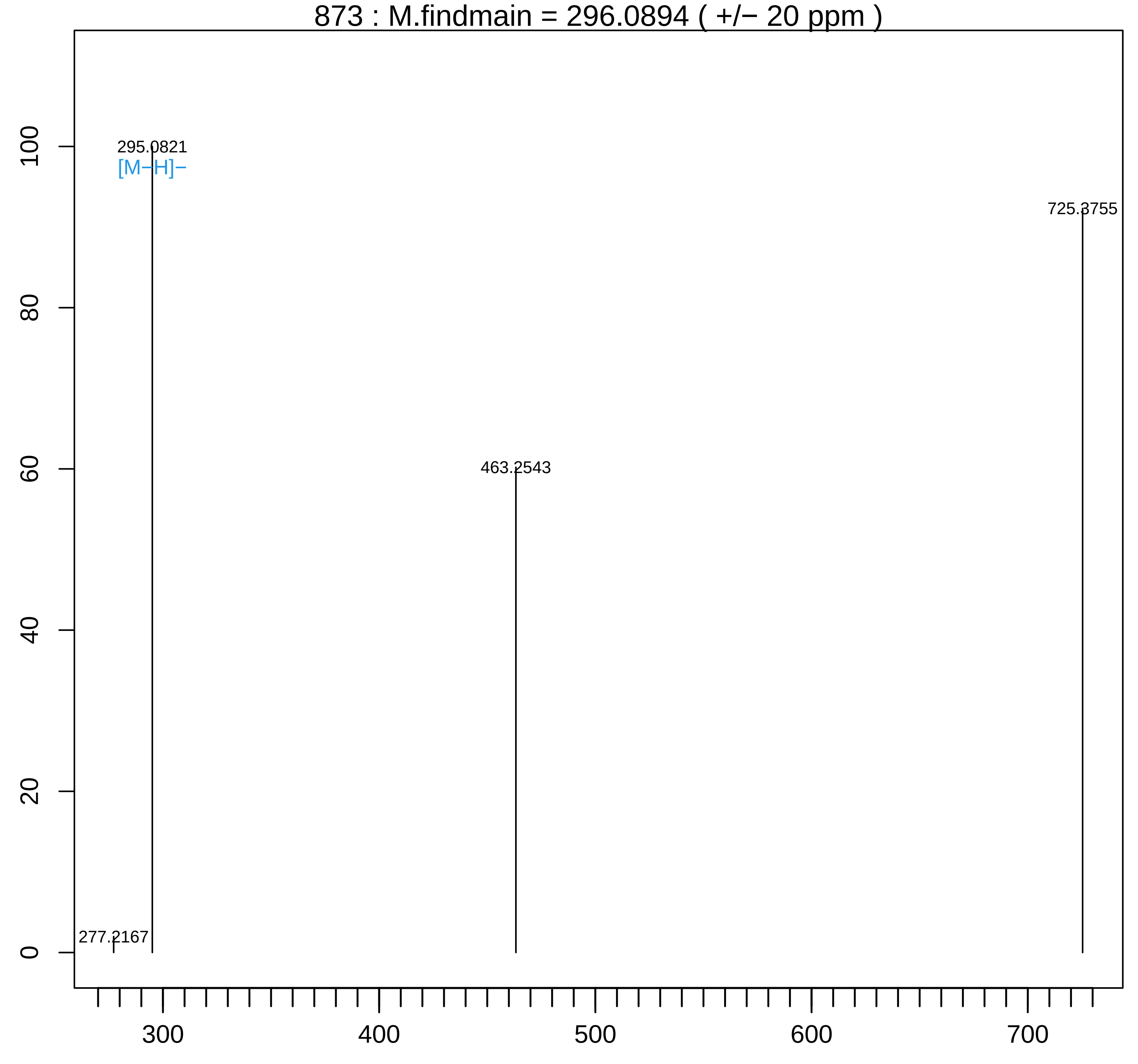

873 : M.findmain = 296.0894 (+/− 20 ppm)
295.0821
[M−H]−
725.3755
463.2543
277.2167
0
20
40
60
80
100
300
400
500
600
700

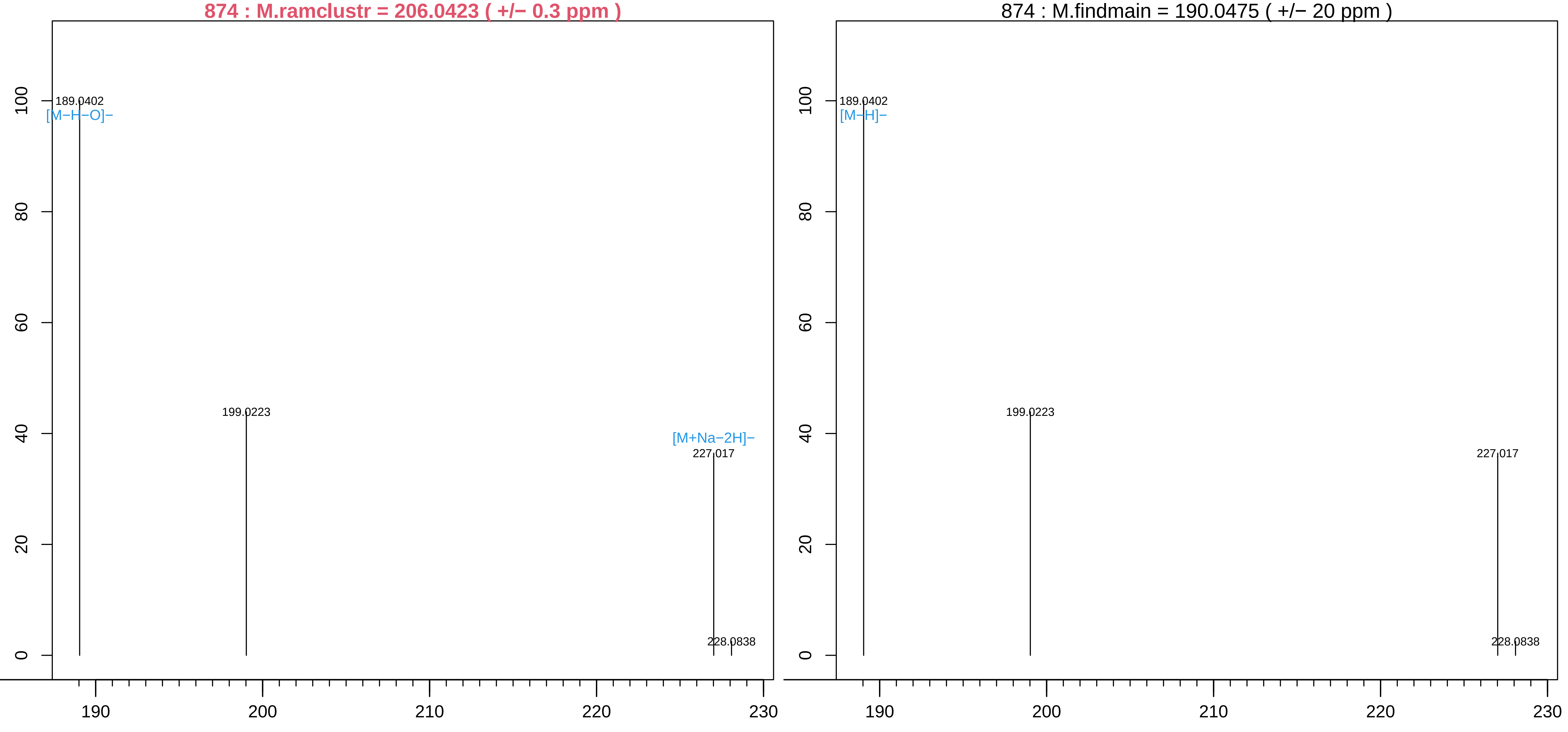

874 : M.ramclustr = 206.0423 (+/− 0.3 ppm)
189.0402
[M−H−O]−
199.0223
[M+Na−2H]−
227.017
228.0838
874 : M.findmain = 190.0475 (+/− 20 ppm)
189.0402
[M−H]−
199.0223
227.017
228.0838
0
20
40
60
80
100
190
200
210
220
230

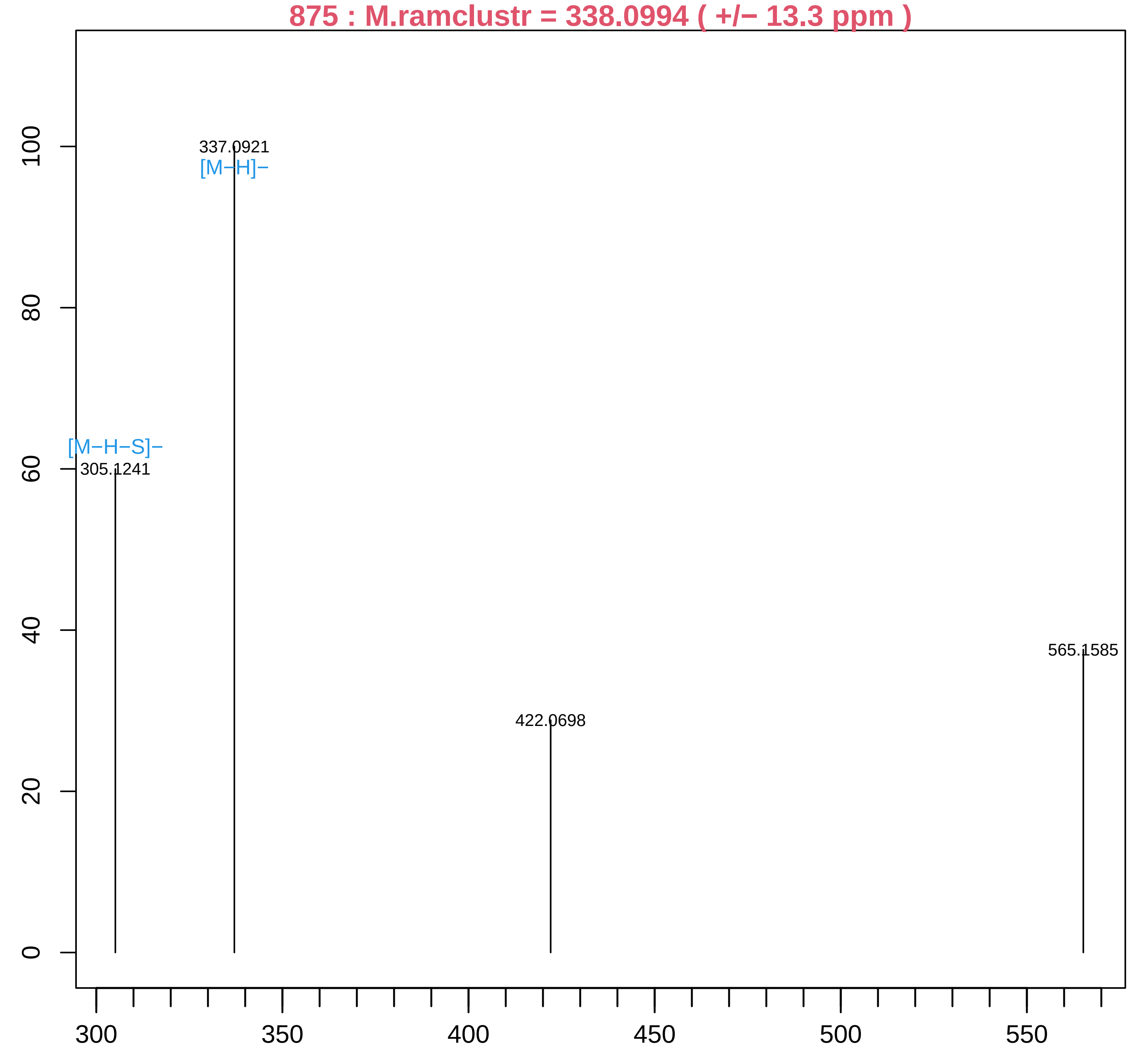

875 : M.ramclustr = 338.0994 (+/− 13.3 ppm)
337.0921
[M−H]−
[M−H−S]−
305.1241
422.0698
565.1585
0
20
40
60
80
100
300
350
400
450
500
550

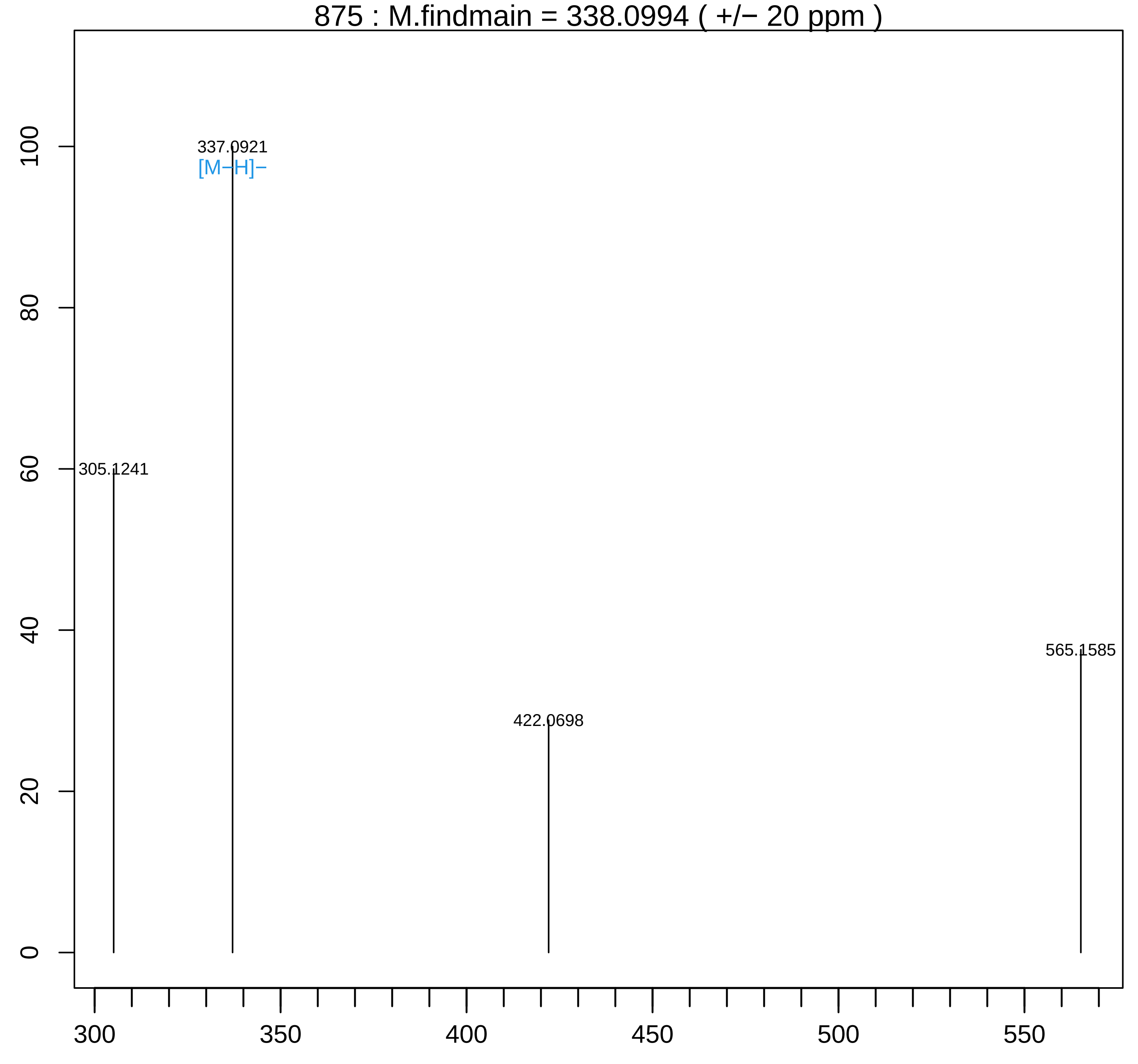

875 : M.findmain = 338.0994 (+/− 20 ppm)
337.0921
[M−H]−
305.1241
422.0698
565.1585
0
20
40
60
80
100
300
350
400
450
500
550

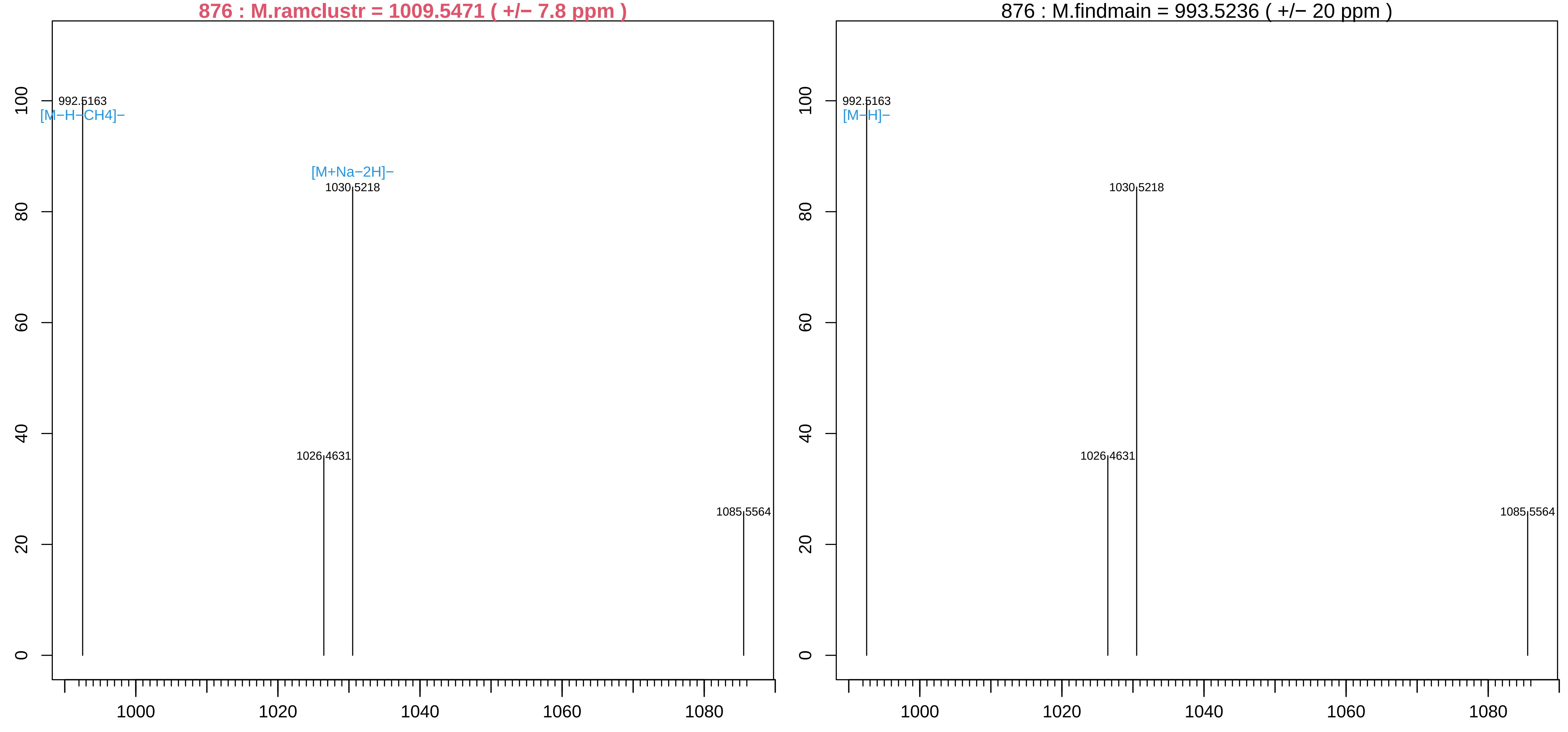

876 : M.ramclustr = 1009.5471 (+/− 7.8 ppm)
992.5163
[M−H−CH4]−
[M+Na−2H]−
1030.5218
1026.4631
1085.5564
876 : M.findmain = 993.5236 (+/− 20 ppm)
992.5163
[M−H]−
1030.5218
1026.4631
1085.5564
0
20
40
60
80
100
1000
1020
1040
1060
1080

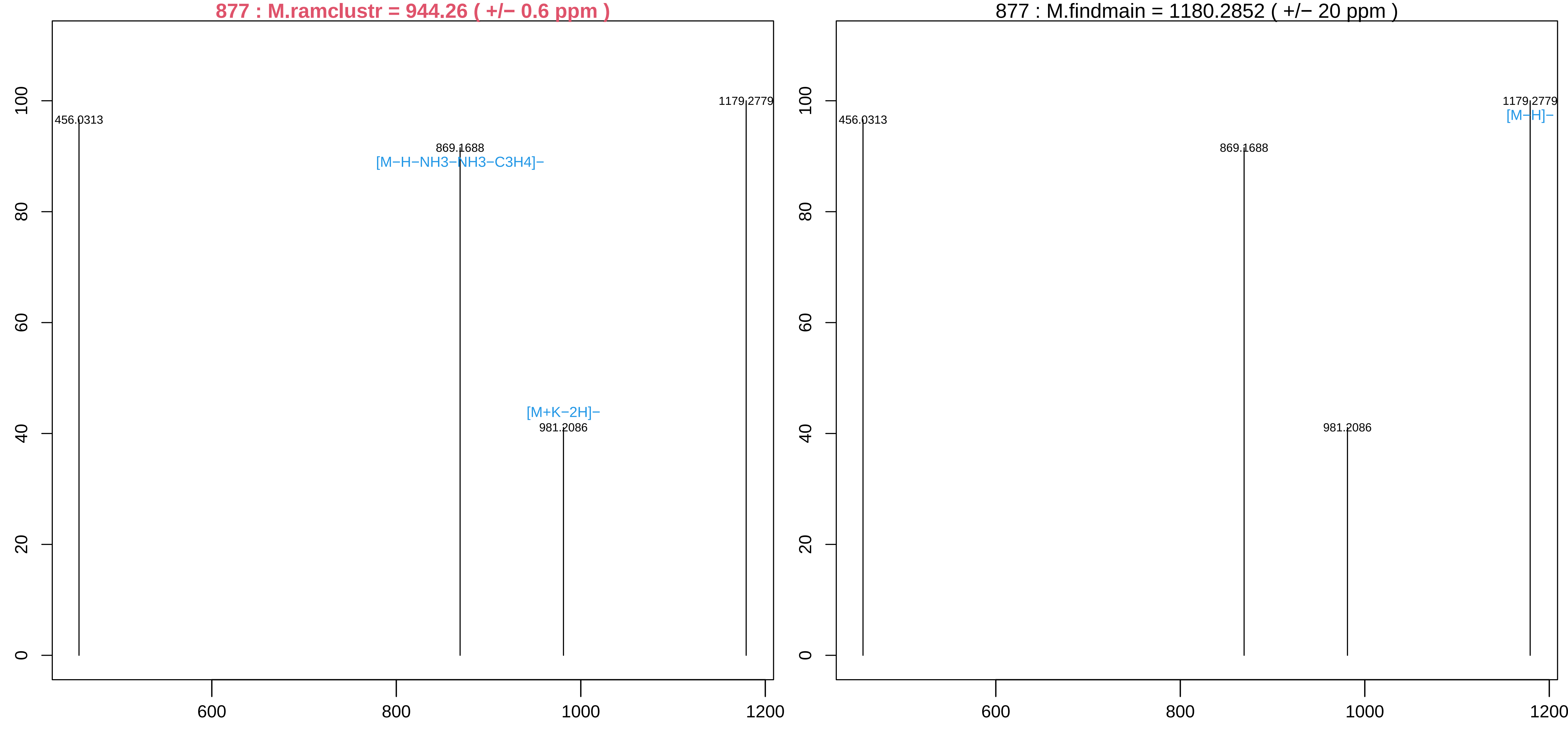

877 : M.ramclustr = 944.26 (+/− 0.6 ppm)
456.0313
869.1688
[M−H−NH3−NH3−C3H4]−
[M+K−2H]−
981.2086
1179.2779
0
20
40
60
80
100
600
800
1000
1200
877 : M.findmain = 1180.2852 (+/− 20 ppm)
456.0313
869.1688
981.2086
1179.2779
[M−H]−

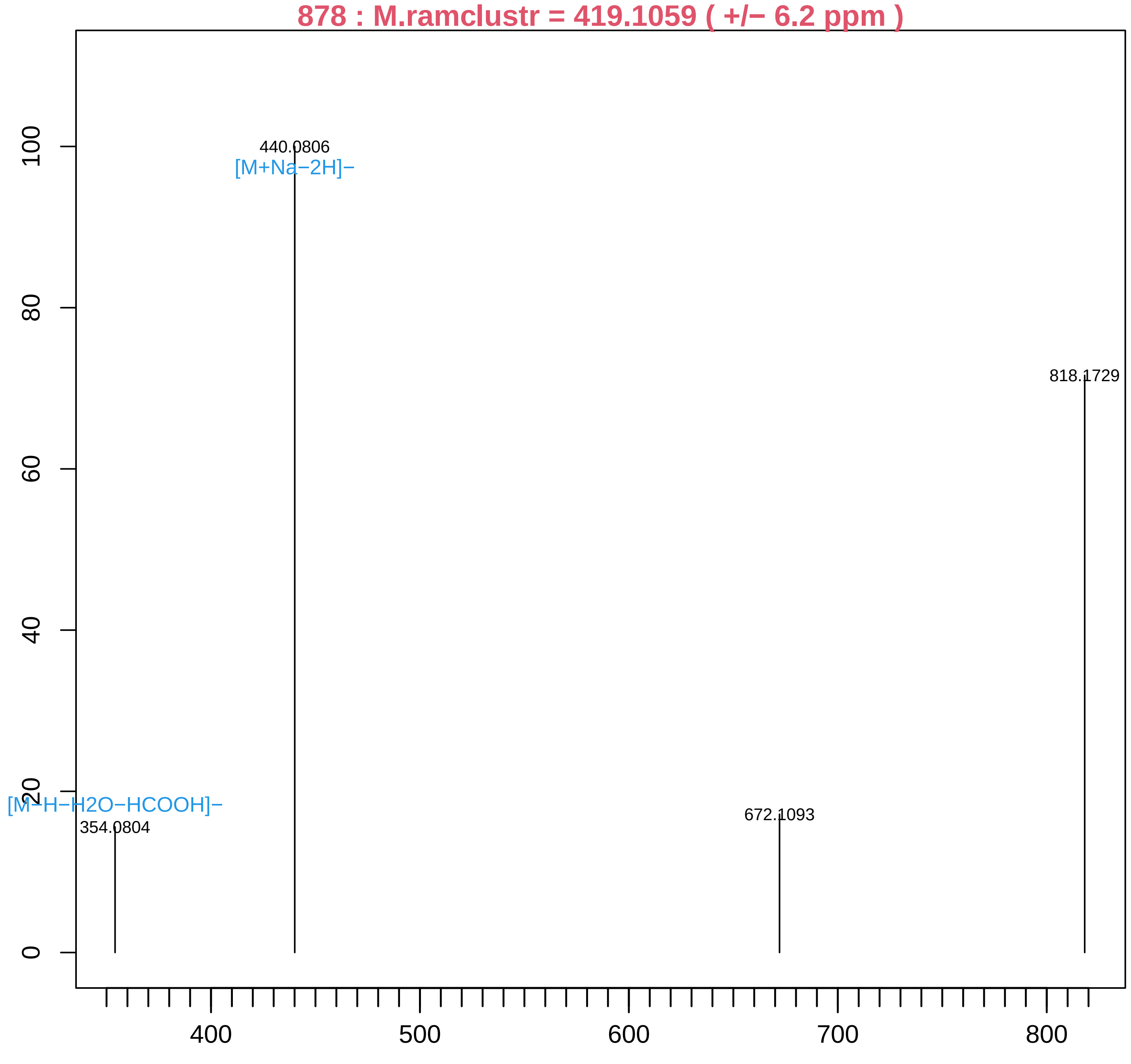
878 : M.ramclustr = 419.1059 (+/− 6.2 ppm)
440.0806
[M+Na−2H]−
818.1729
[M−H−H2O−HCOOH]−
354.0804
672.1093
0
20
40
60
80
100
400
500
600
700
800

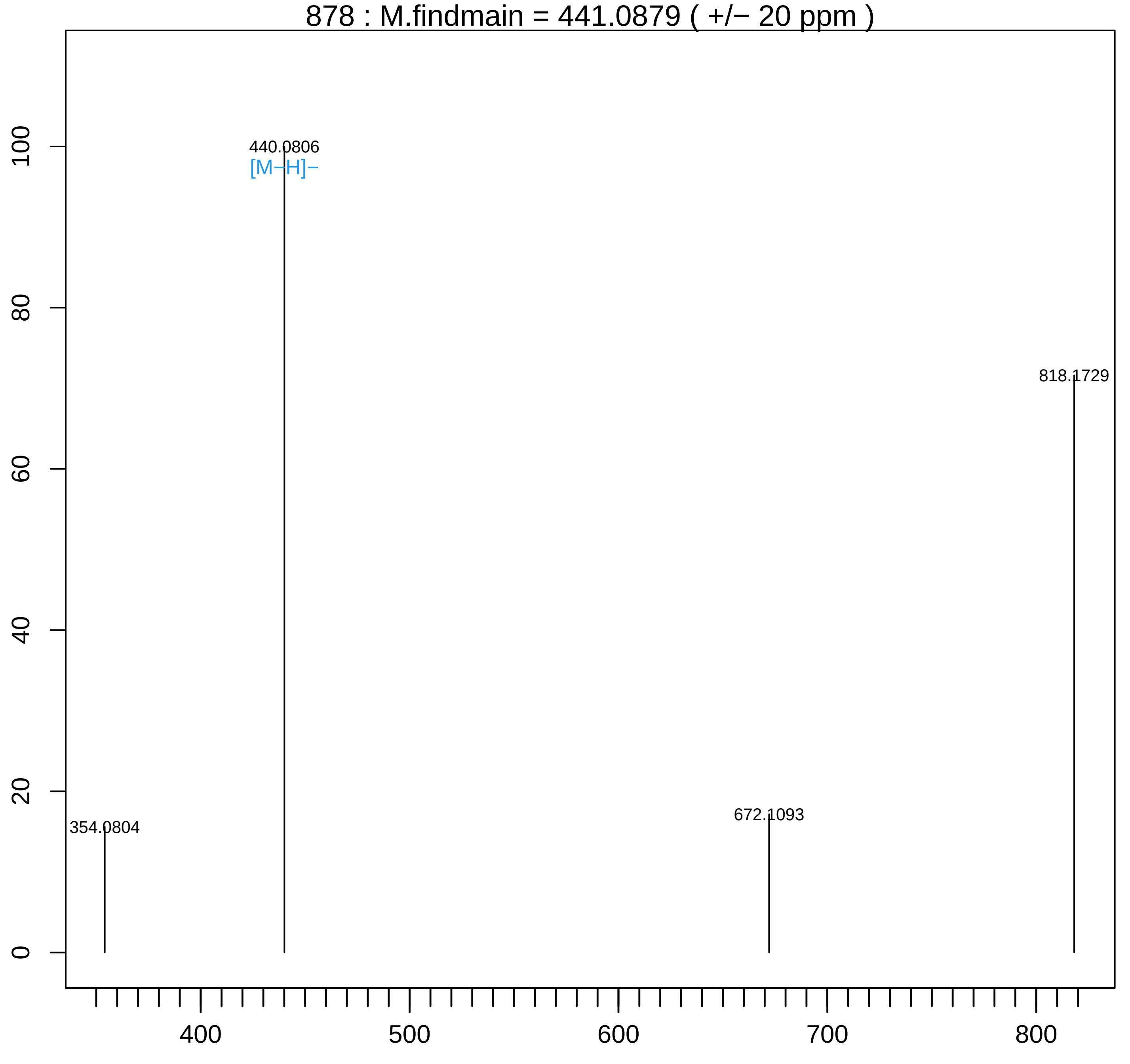
878 : M.findmain = 441.0879 (+/− 20 ppm)
440.0806
[M−H]−
818.1729
354.0804
672.1093
0
20
40
60
80
100
400
500
600
700
800

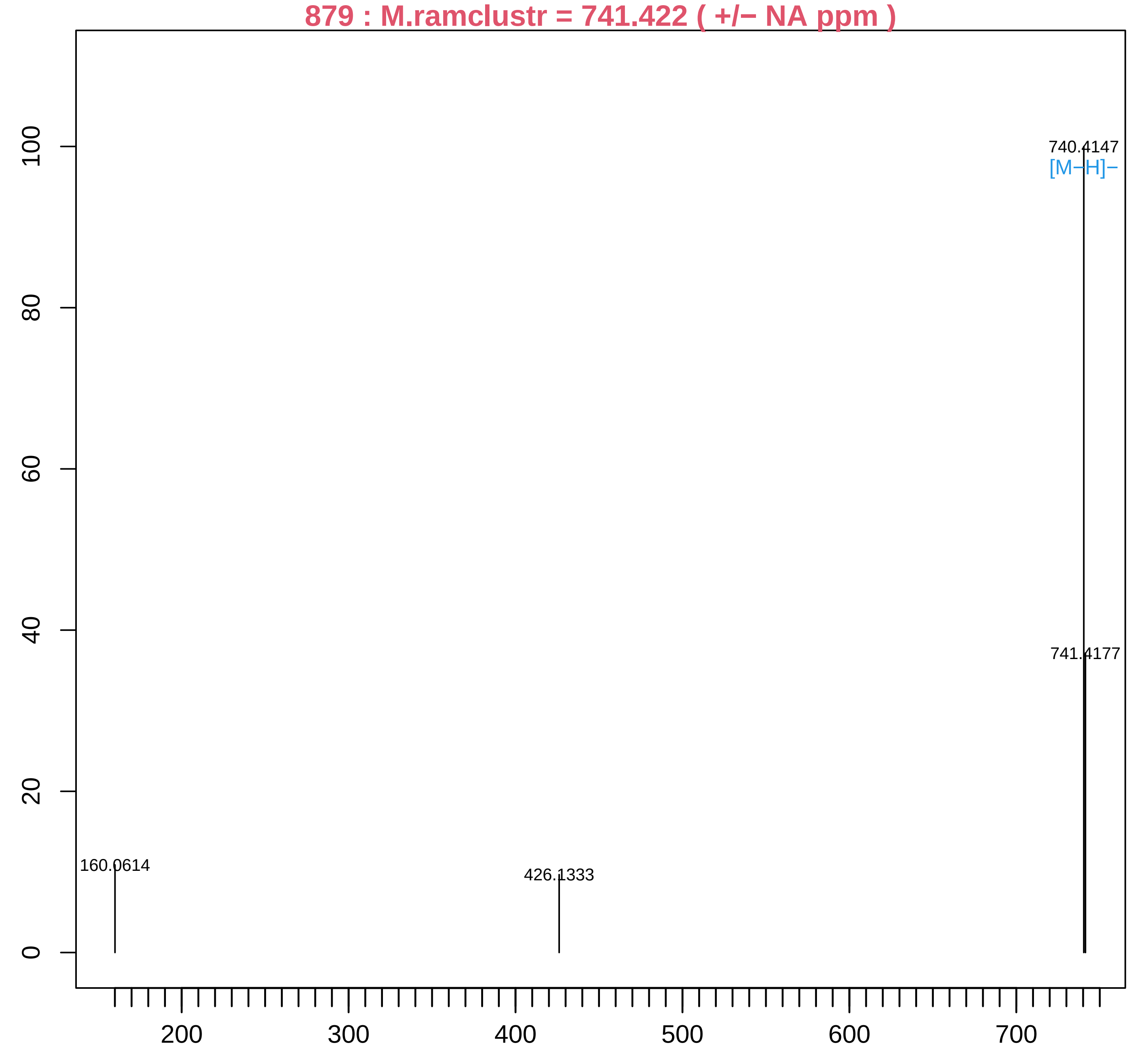
879 : M.ramclustr = 741.422 (+/− NA ppm)
740.4147
[M−H]−
741.4177
160.0614
426.1333
0
20
40
60
80
100
200
300
400
500
600
700

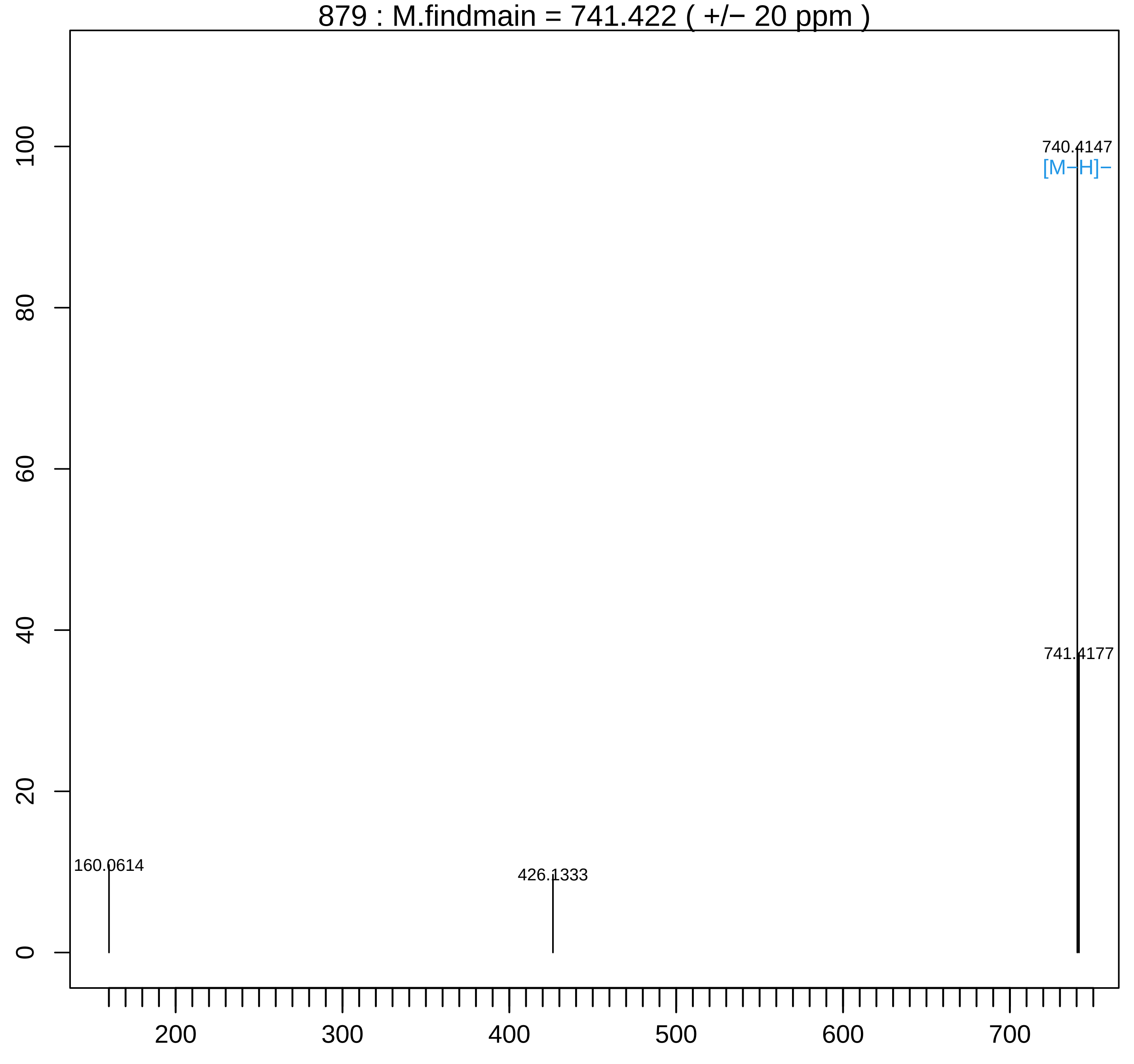
879 : M.findmain = 741.422 (+/− 20 ppm)
740.4147
[M−H]−
741.4177
160.0614
426.1333
0
20
40
60
80
100
200
300
400
500
600
700

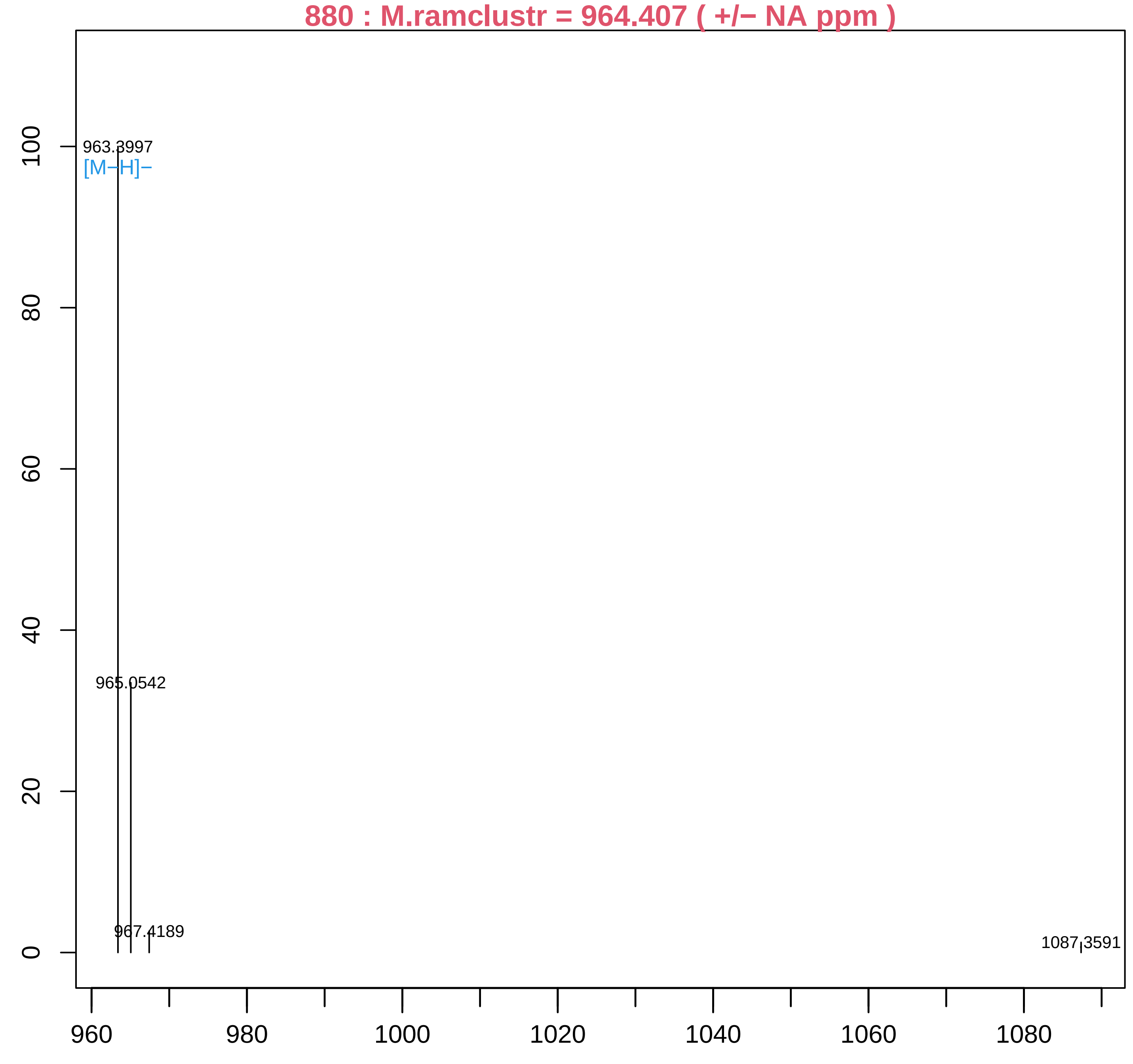
880 : M.ramclustr = 964.407 (+/− NA ppm)
963.3997
[M−H]−
965.0542
967.4189
1087.3591
0
20
40
60
80
100
960
980
1000
1020
1040
1060
1080

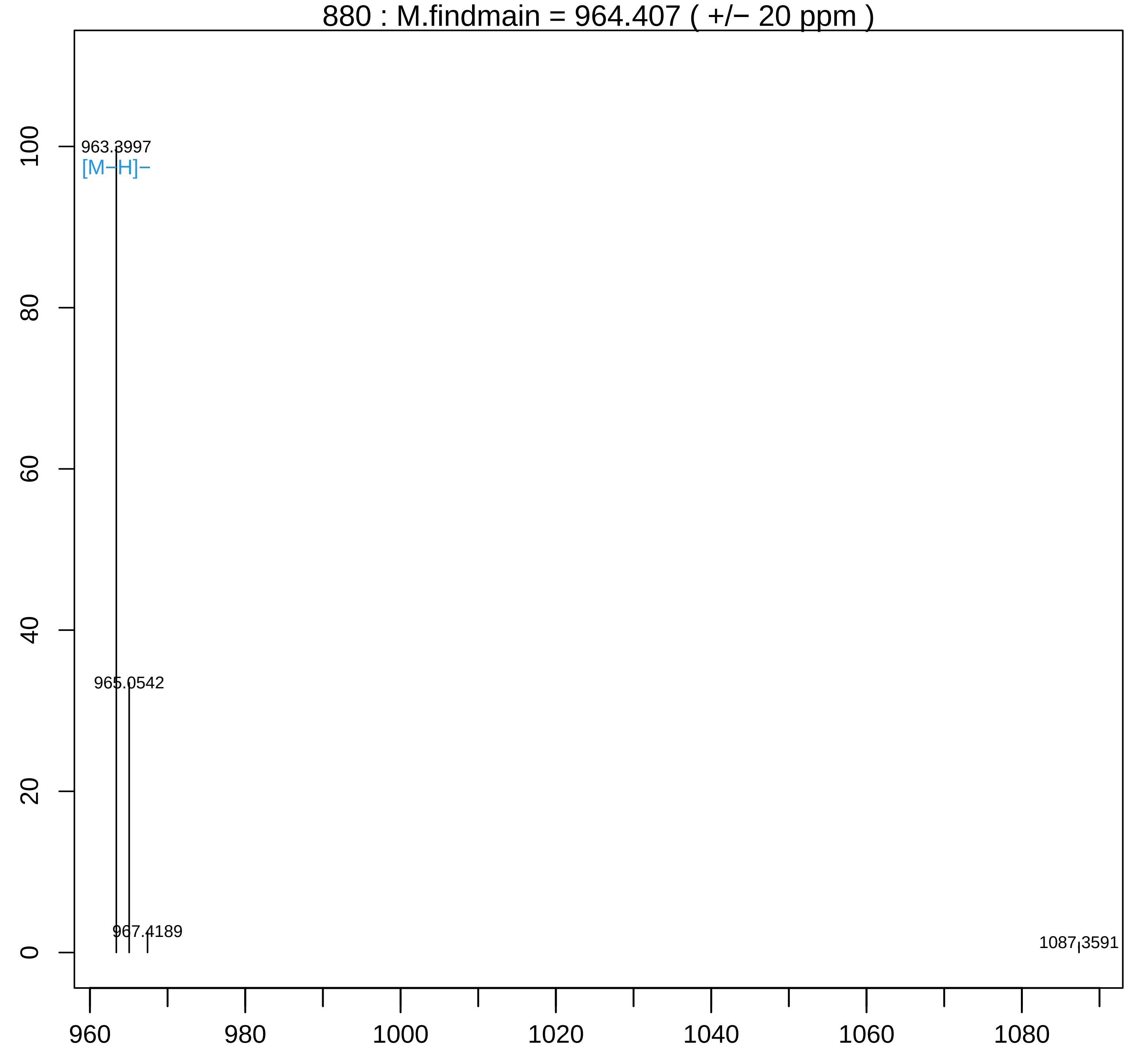
880 : M.findmain = 964.407 (+/− 20 ppm)
963.3997
[M−H]−
965.0542
967.4189
1087.3591
0
20
40
60
80
100
960
980
1000
1020
1040
1060
1080

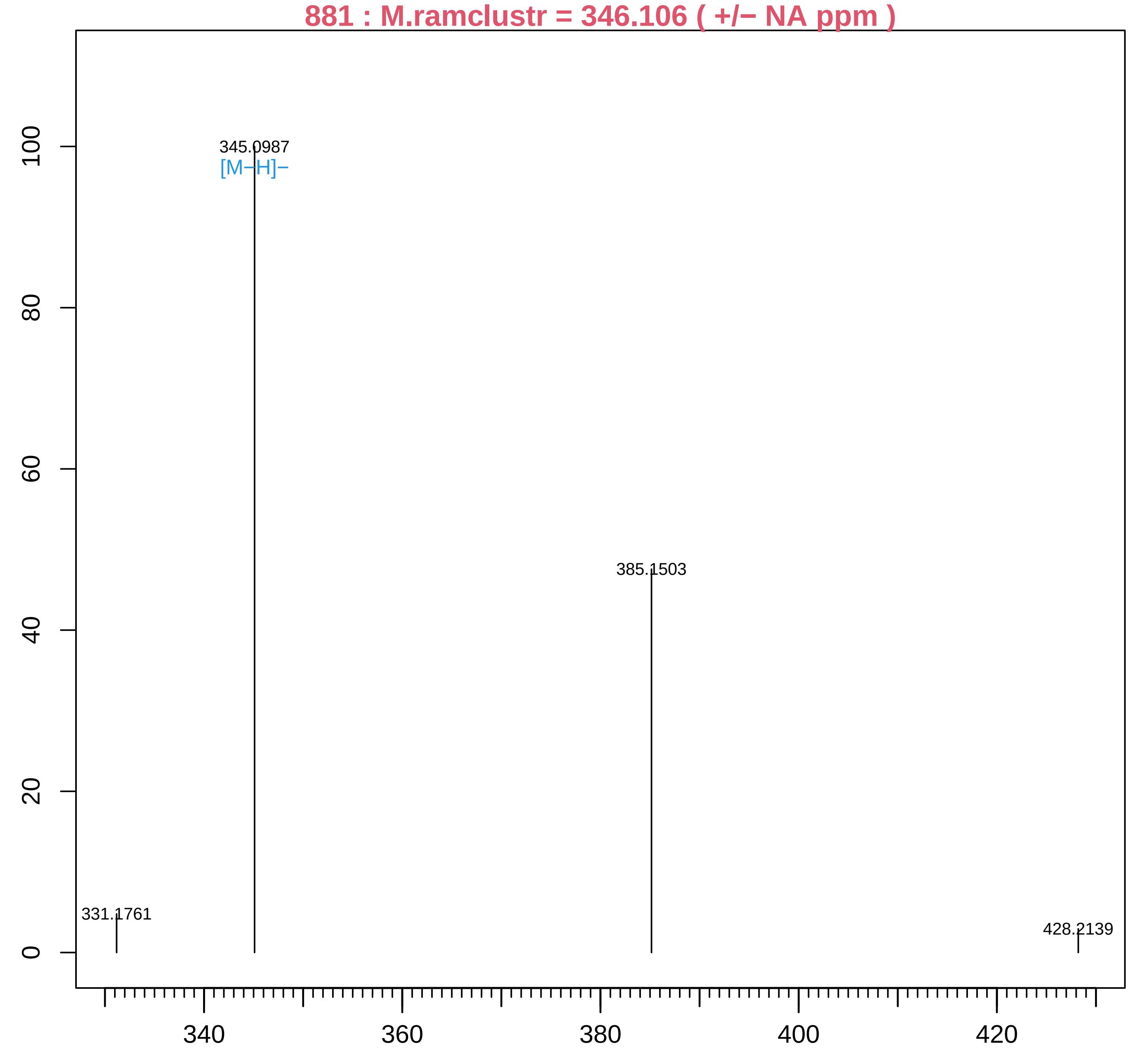
881 : M.ramclustr = 346.106 (+/− NA ppm)
345.0987
[M−H]−
385.1503
331.1761
428.2139
0
20
40
60
80
100
340
360
380
400
420

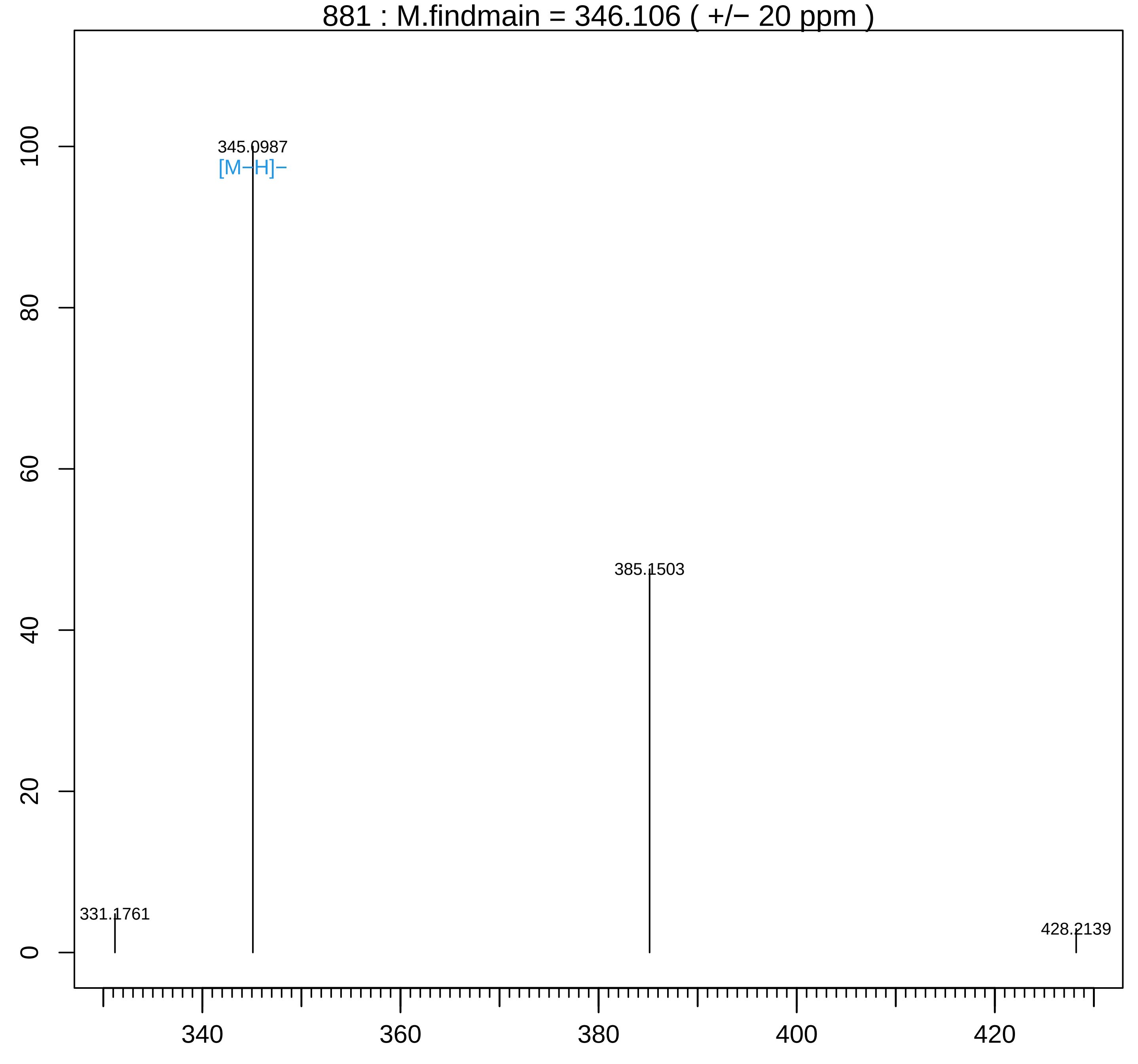
881 : M.findmain = 346.106 (+/− 20 ppm)
345.0987
[M−H]−
385.1503
331.1761
428.2139
0
20
40
60
80
100
340
360
380
400
420

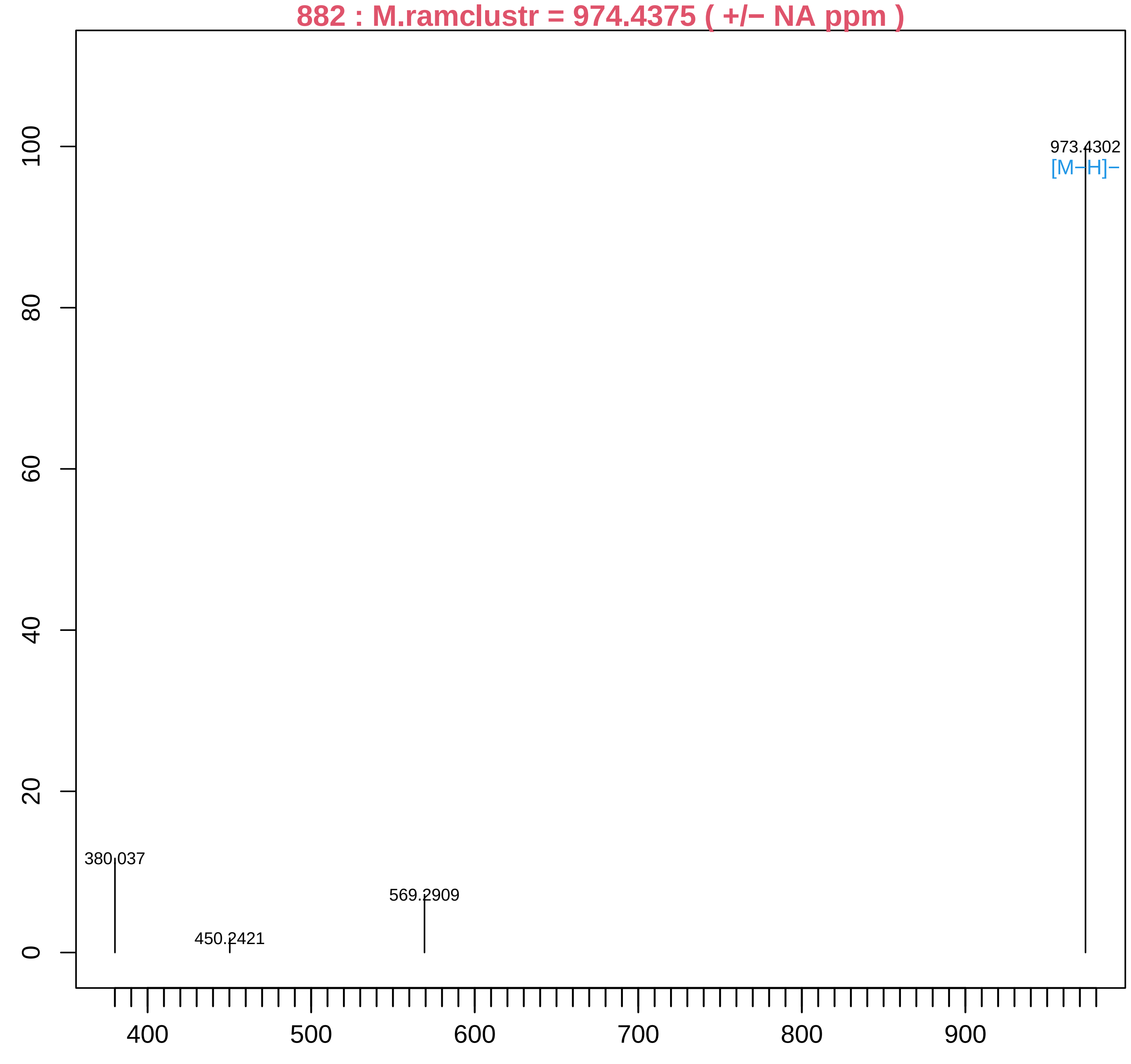
882 : M.ramclustr = 974.4375 (+/− NA ppm)
973.4302
[M−H]−
380.037
450.2421
569.2909
0
20
40
60
80
100
400
500
600
700
800
900

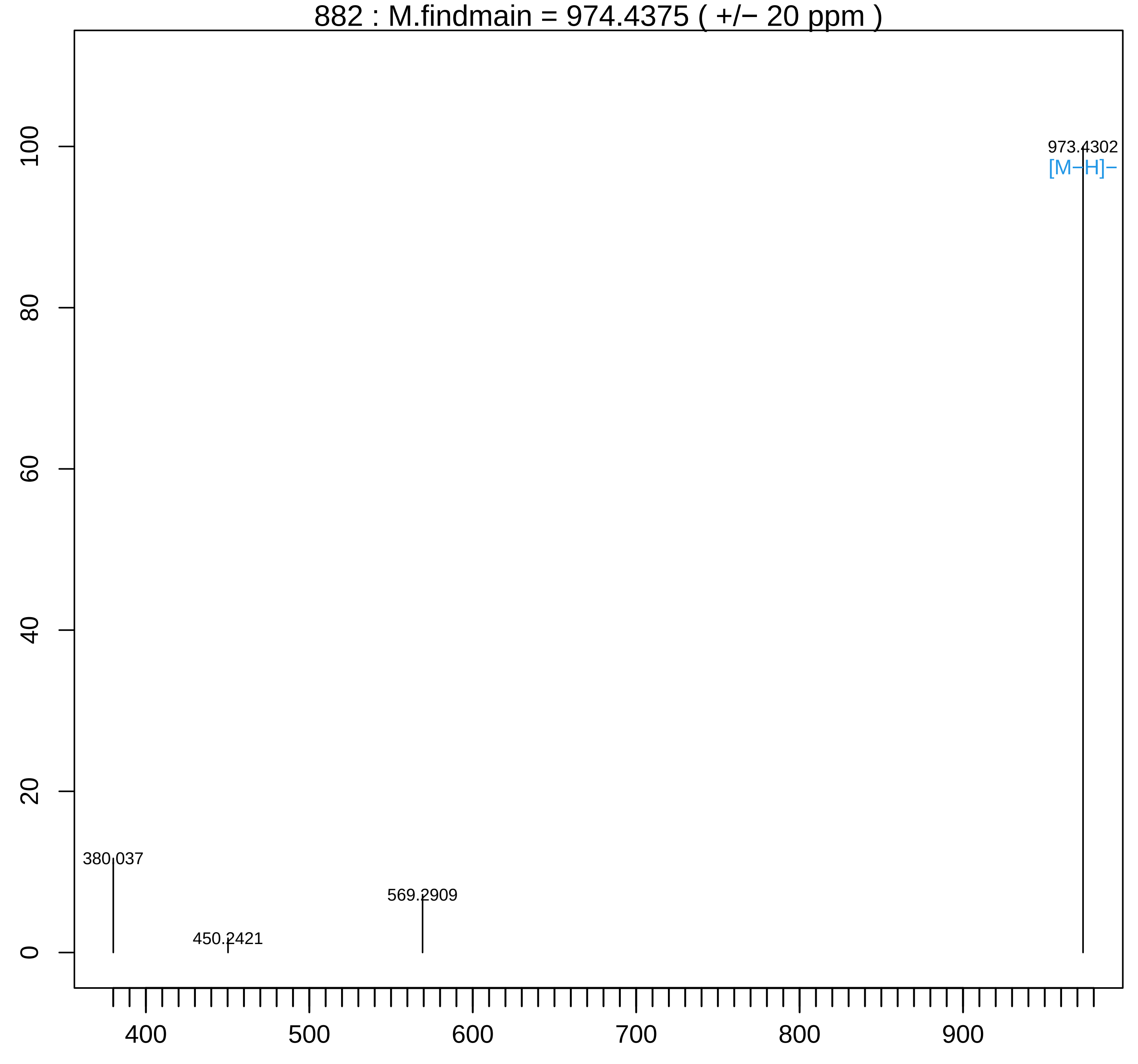
882 : M.findmain = 974.4375 (+/− 20 ppm)
973.4302
[M−H]−
380.037
450.2421
569.2909
0
20
40
60
80
100
400
500
600
700
800
900

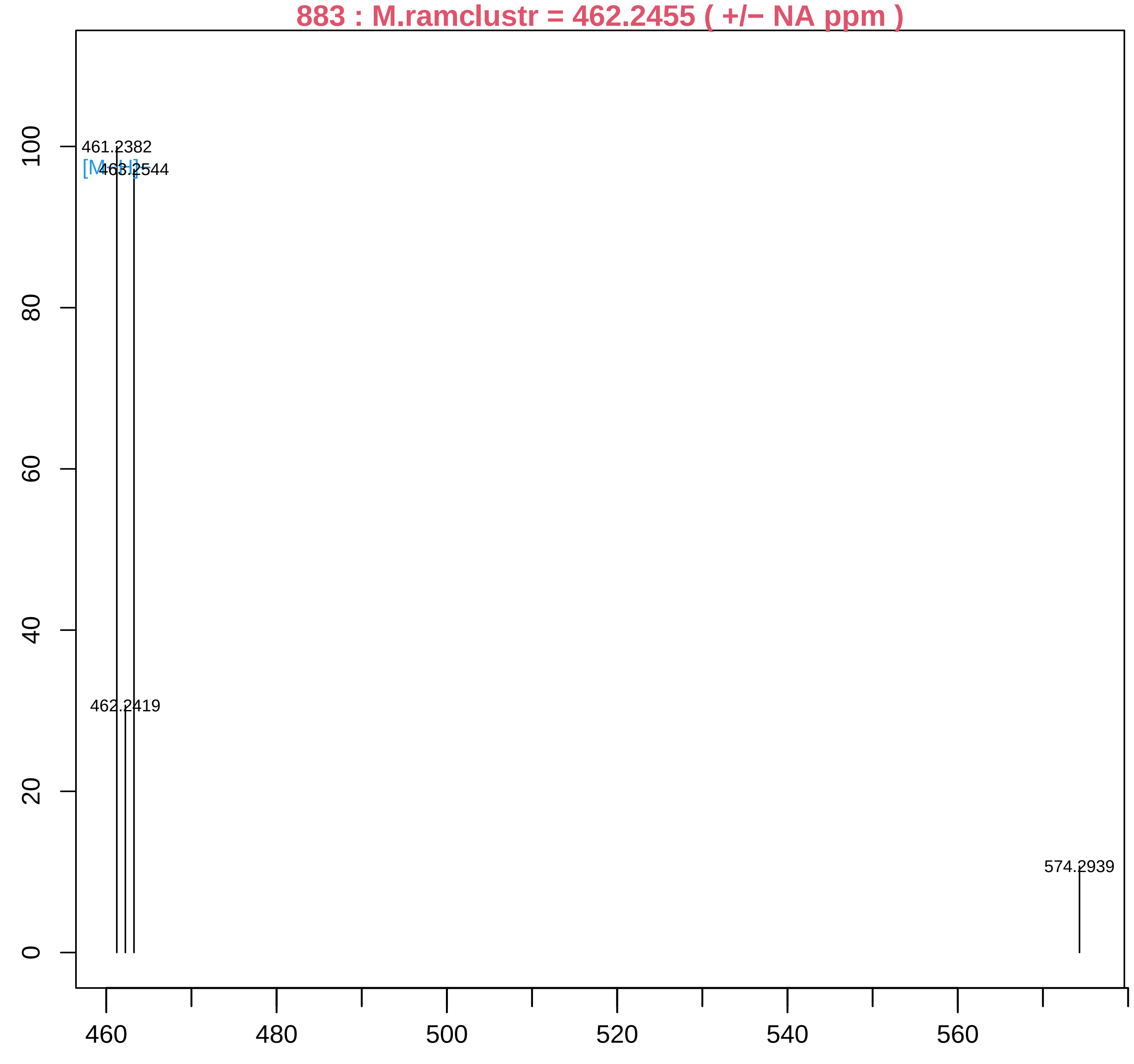
883 : M.ramclustr = 462.2455 (+/− NA ppm)
461.2382
[M+H]+
463.2544
462.2419
574.2939
0
20
40
60
80
100
460
480
500
520
540
560

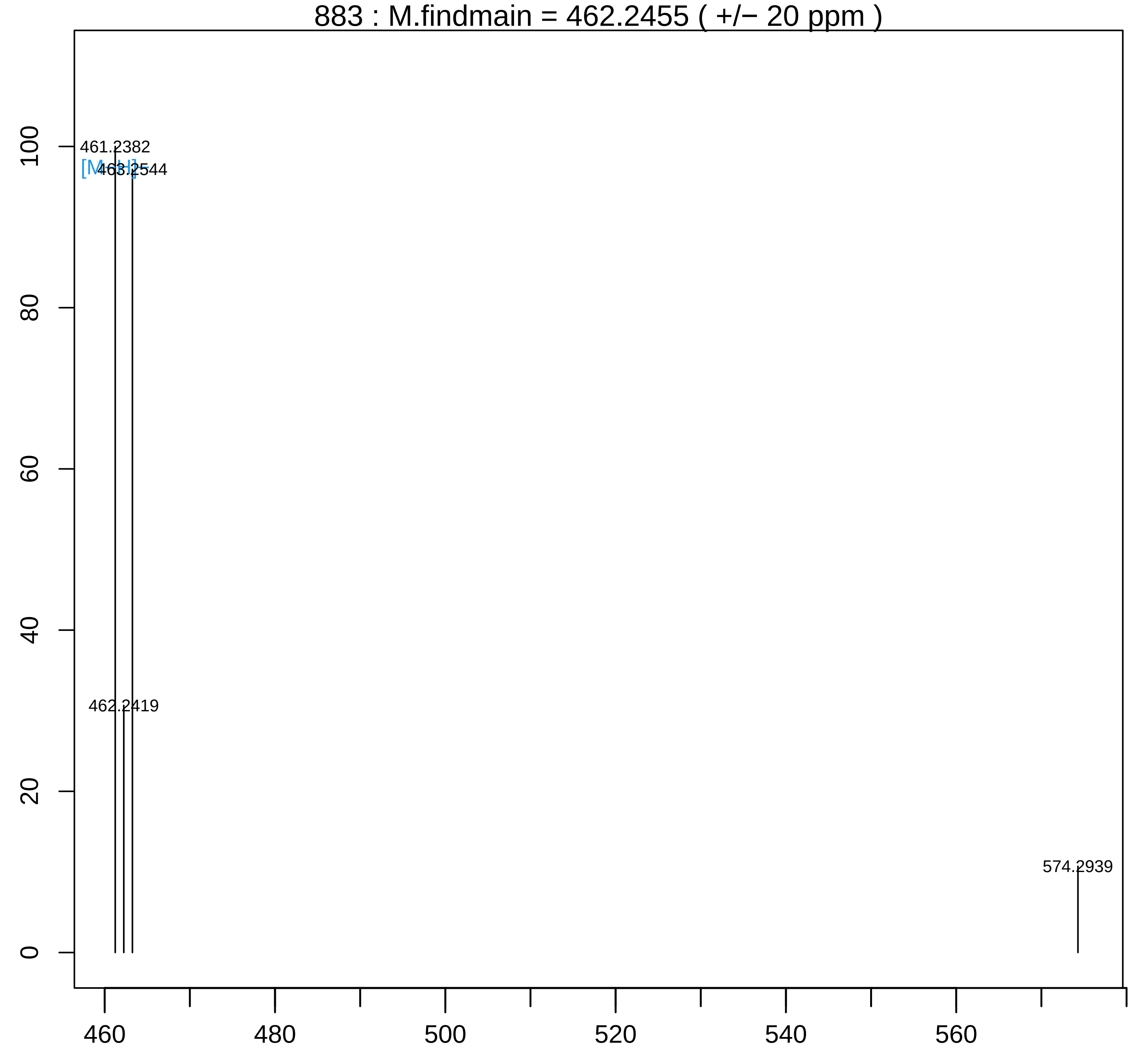
883 : M.findmain = 462.2455 (+/− 20 ppm)
461.2382
[M+H]+
463.2544
462.2419
574.2939
0
20
40
60
80
100
460
480
500
520
540
560

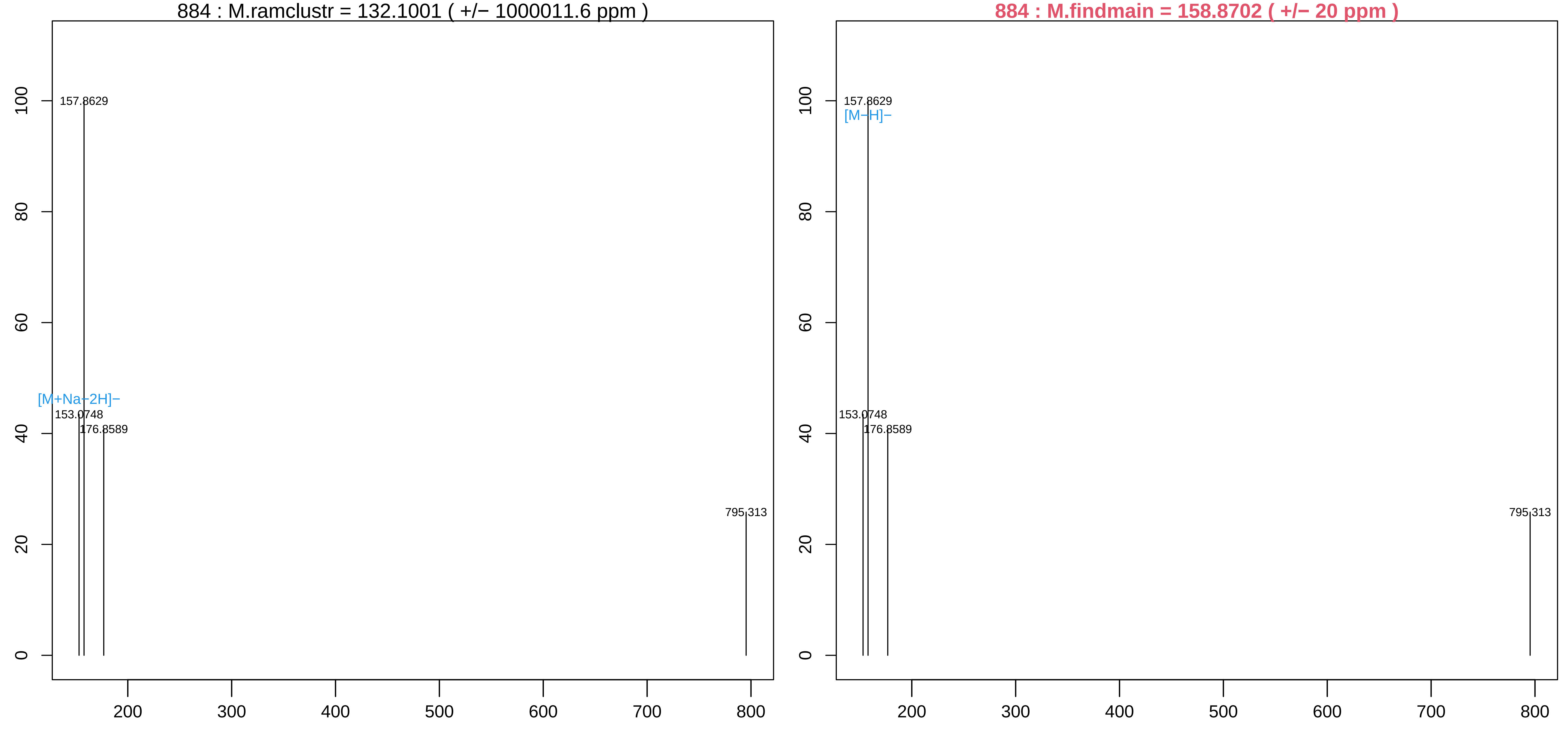
884 : M.ramclustr = 132.1001 (+/− 1000011.6 ppm)
157.8629
[M+Na−2H]−
153.0748
176.8589
795.313
884 : M.findmain = 158.8702 (+/− 20 ppm)
157.8629
[M−H]−
153.0748
176.8589
795.313
0
20
40
60
80
100
200
300
400
500
600
700
800

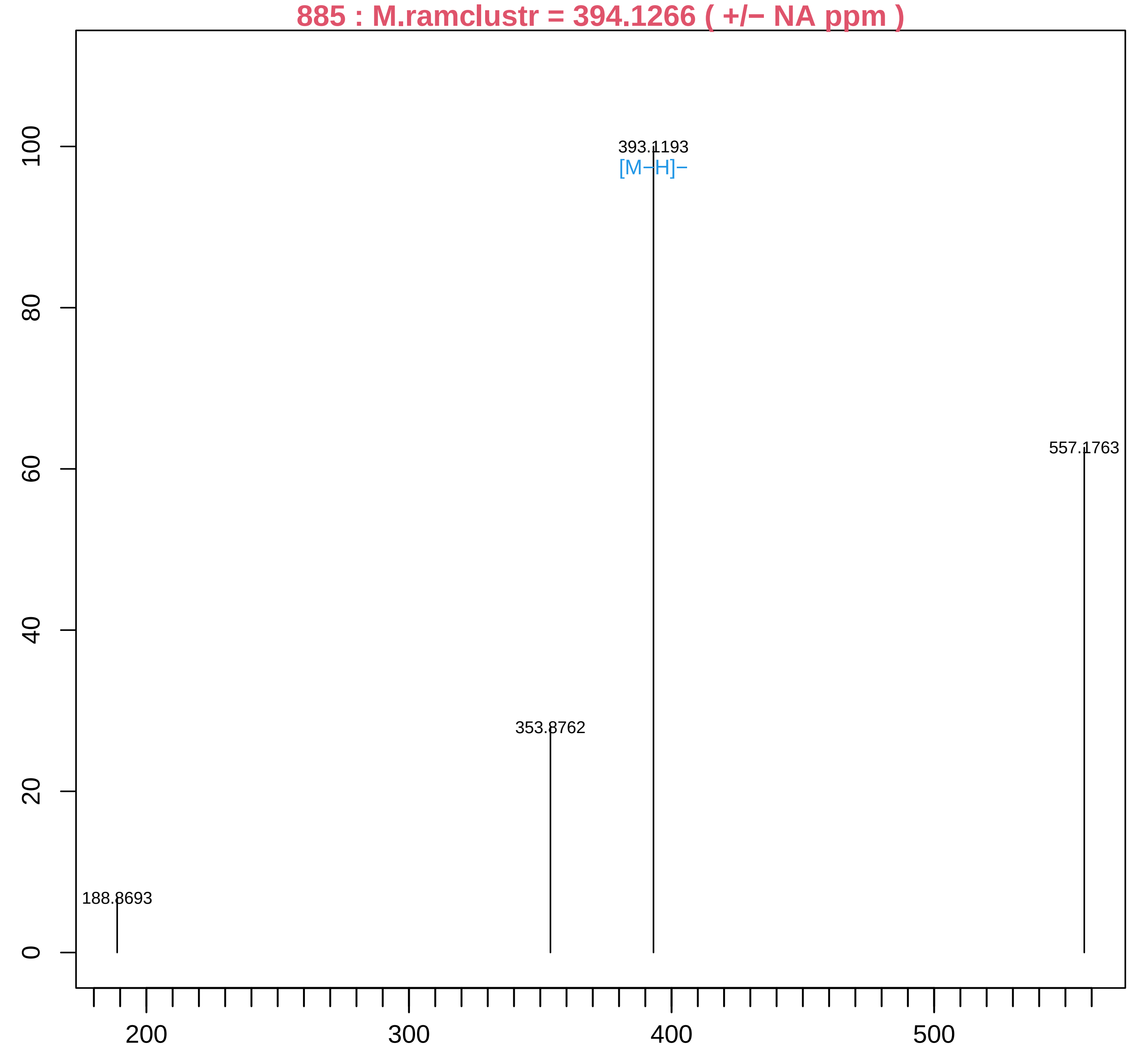
885 : M.ramclustr = 394.1266 (+/− NA ppm)
393.1193
[M−H]−
557.1763
353.8762
188.8693
0
20
40
60
80
100
200
300
400
500

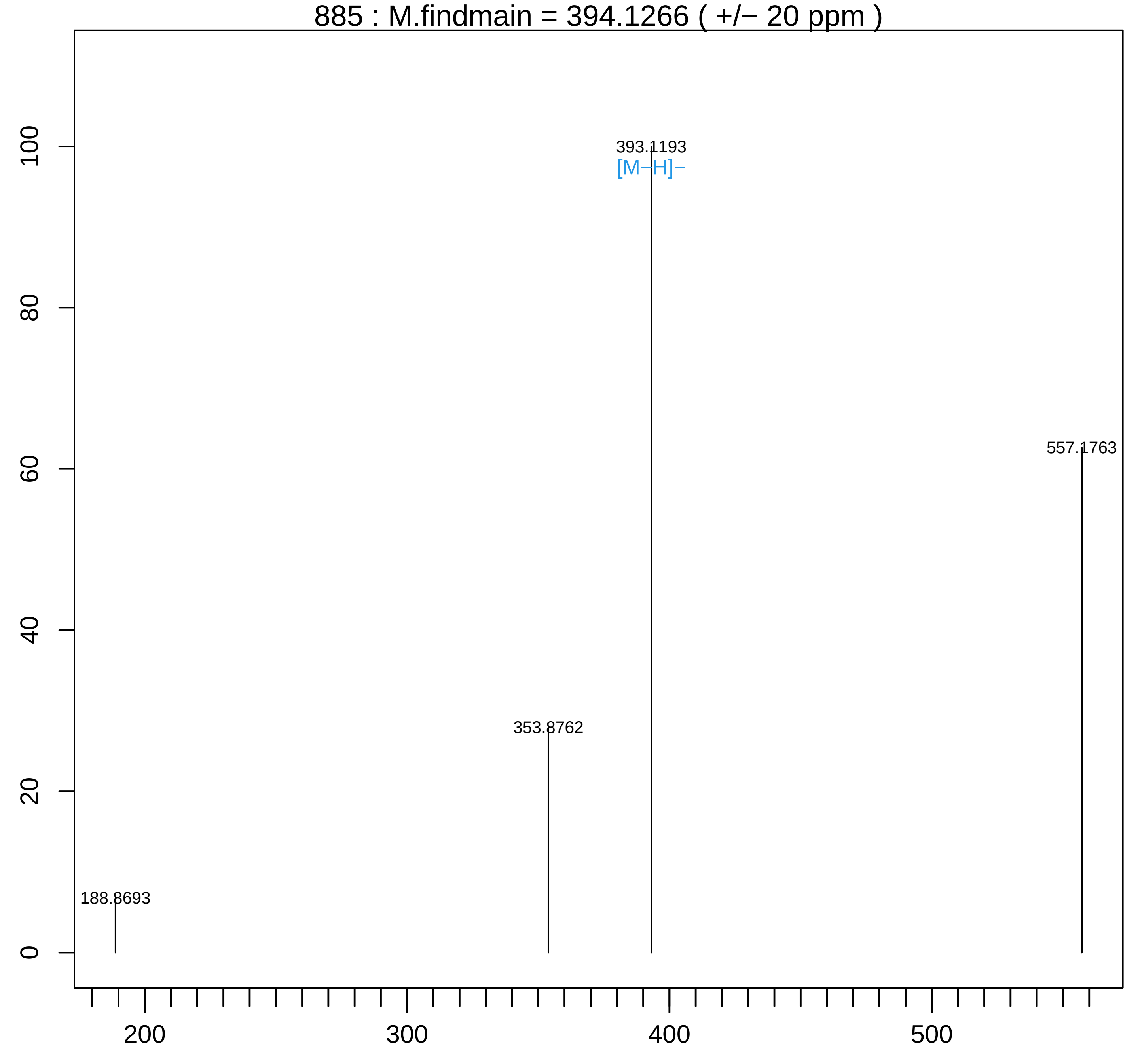
885 : M.findmain = 394.1266 (+/− 20 ppm)
393.1193
[M−H]−
557.1763
353.8762
188.8693
0
20
40
60
80
100
200
300
400
500

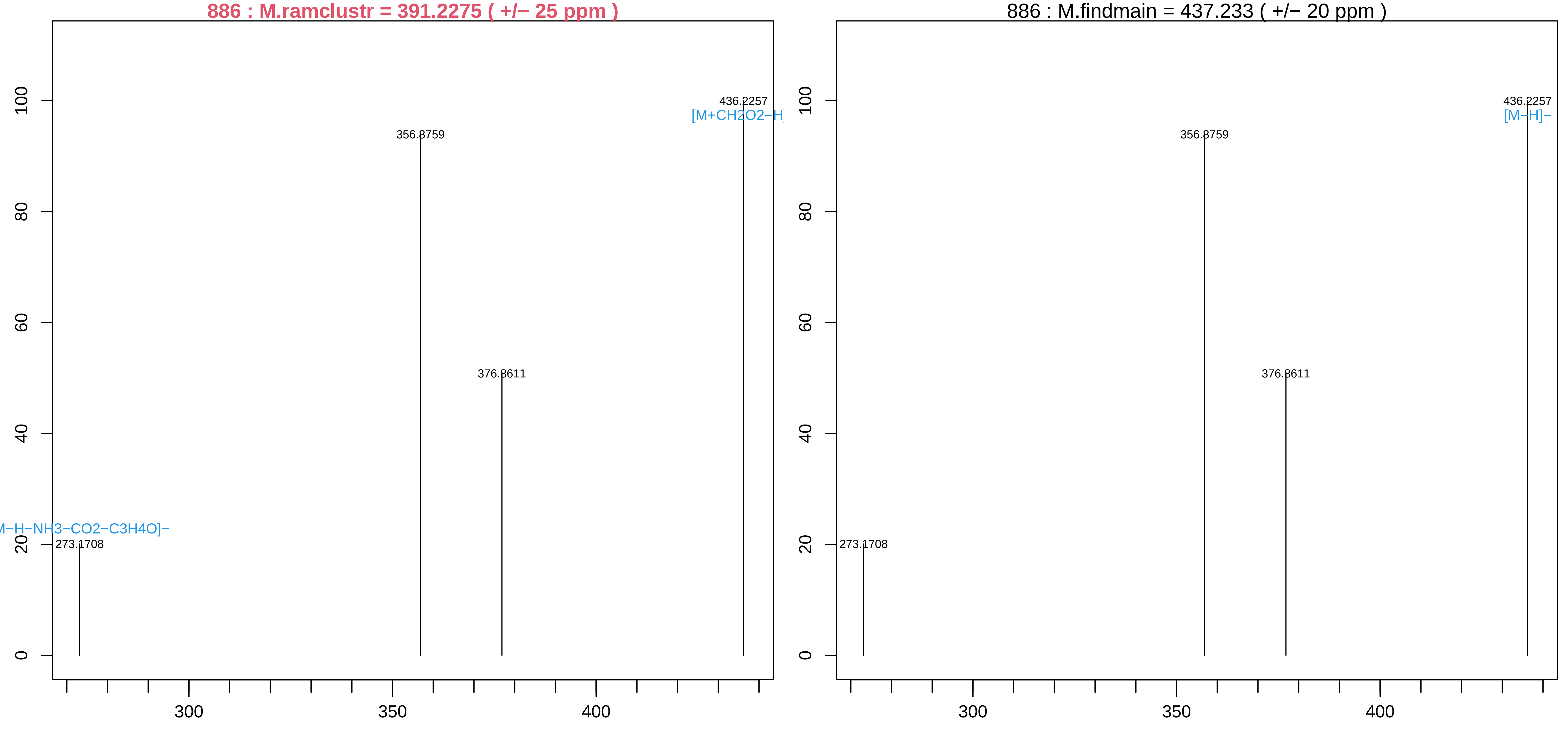

886 : M.ramclustr = 391.2275 (+/− 25 ppm)
886 : M.findmain = 437.233 (+/− 20 ppm)
436.2257
[M+CH2O2−H]
356.8759
376.8611
273.1708
M−H−NH3−CO2−C3H4O]−
[M−H]−
0
20
40
60
80
100
300
350
400

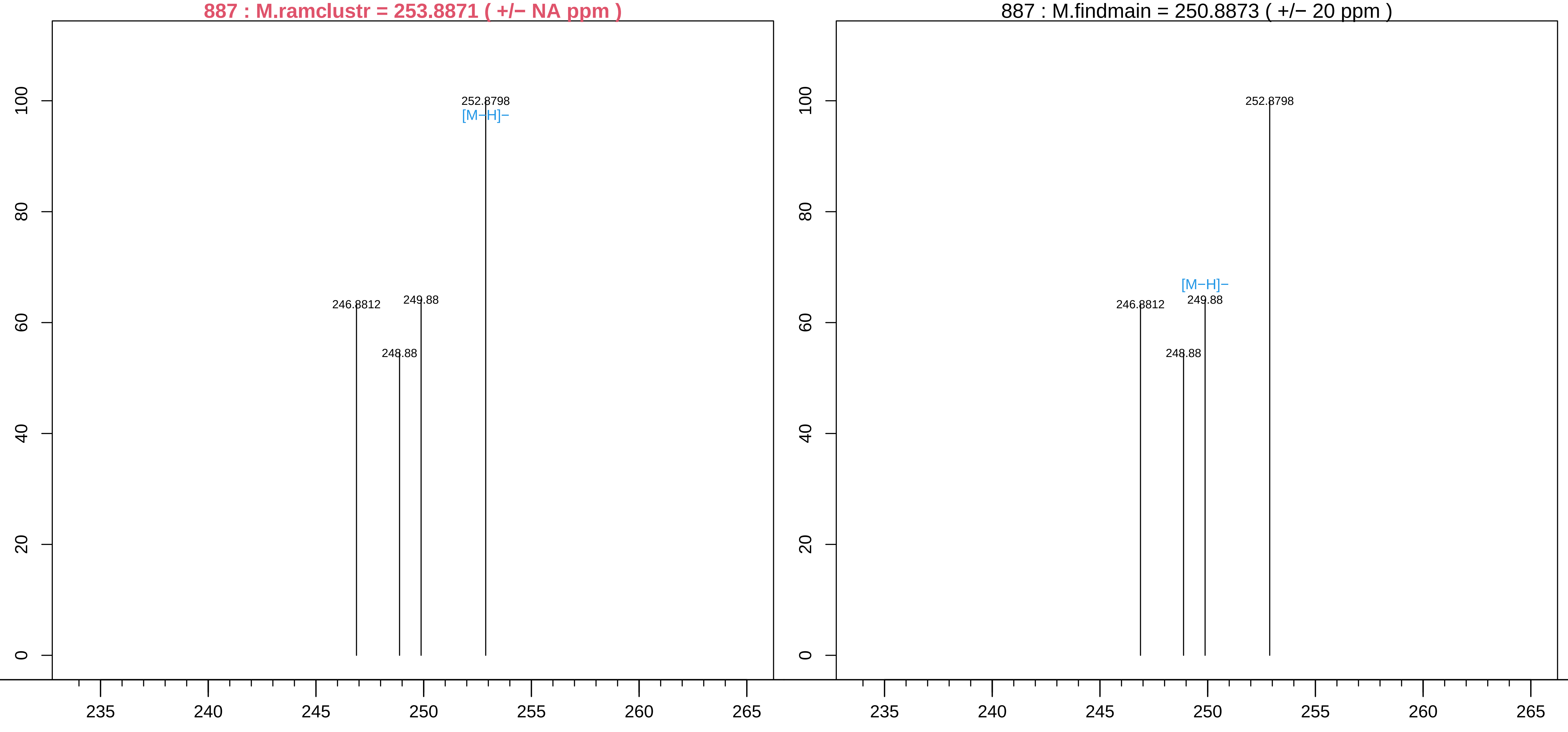
887 : M.ramclustr = 253.8871 (+/− NA ppm)
252.8798
[M−H]−
246.8812
249.88
248.88
887 : M.findmain = 250.8873 (+/− 20 ppm)
252.8798
[M−H]−
246.8812
249.88
248.88
0
20
40
60
80
100
235
240
245
250
255
260
265

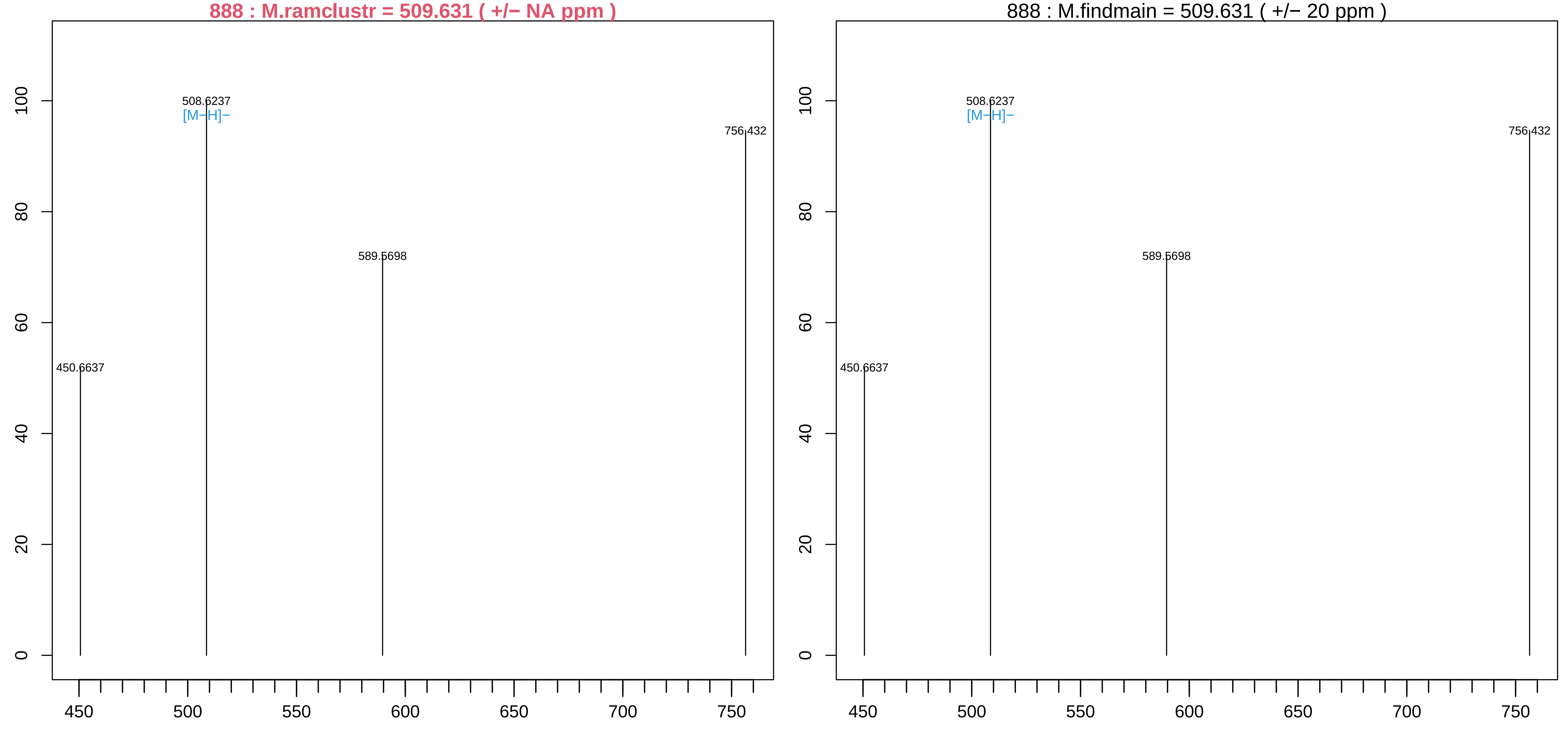
888 : M.ramclustr = 509.631 (+/− NA ppm)
508.6237
[M−H]−
756.432
589.5698
450.6637
888 : M.findmain = 509.631 (+/− 20 ppm)
508.6237
[M−H]−
756.432
589.5698
450.6637
0
20
40
60
80
100
450
500
550
600
650
700
750

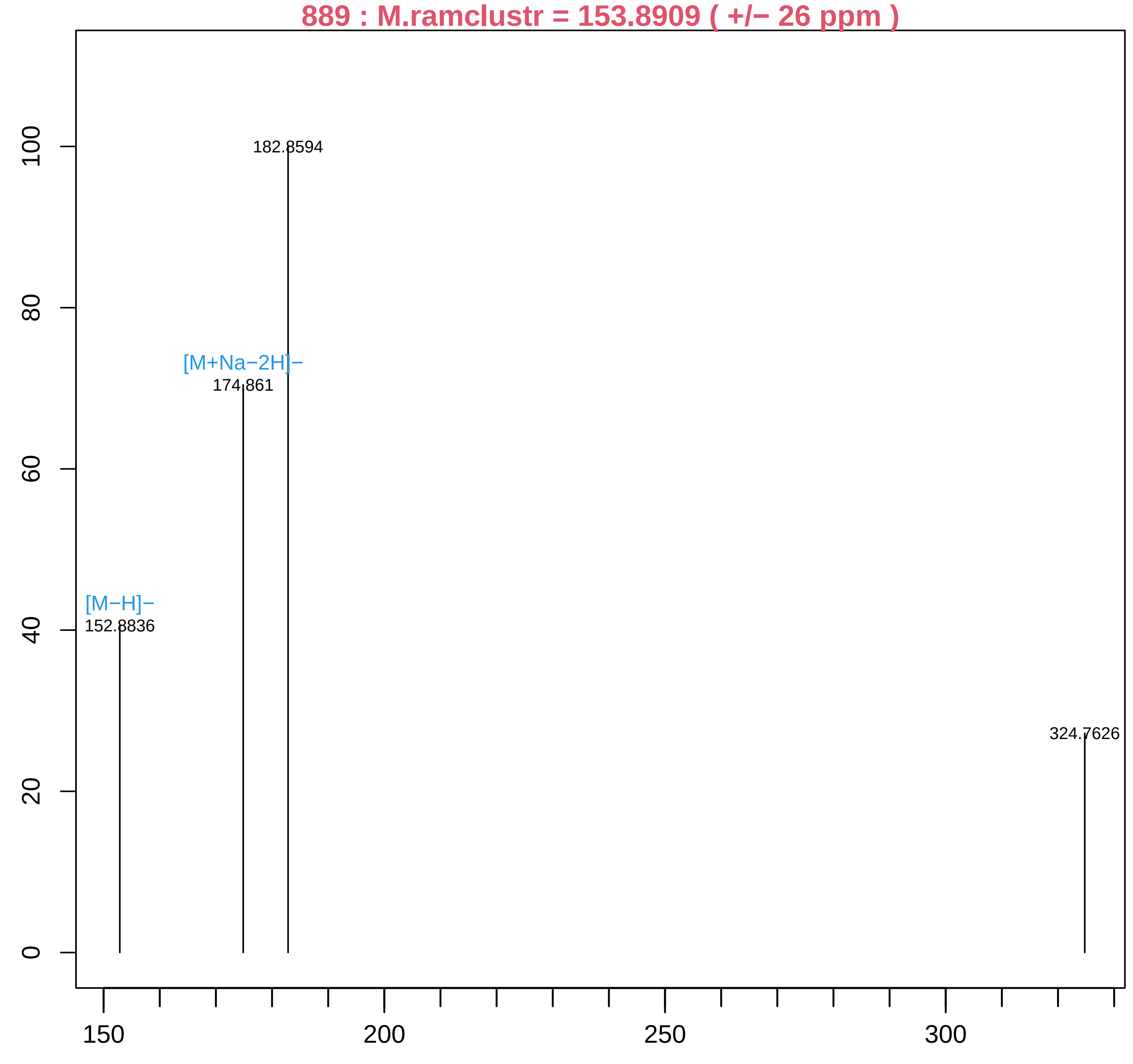

889 : M.ramclustr = 153.8909 (+/− 26 ppm)
0
20
40
60
80
100
150
200
250
300
[M−H]−
152.8836
[M+Na−2H]−
174.861
182.8594
324.7626

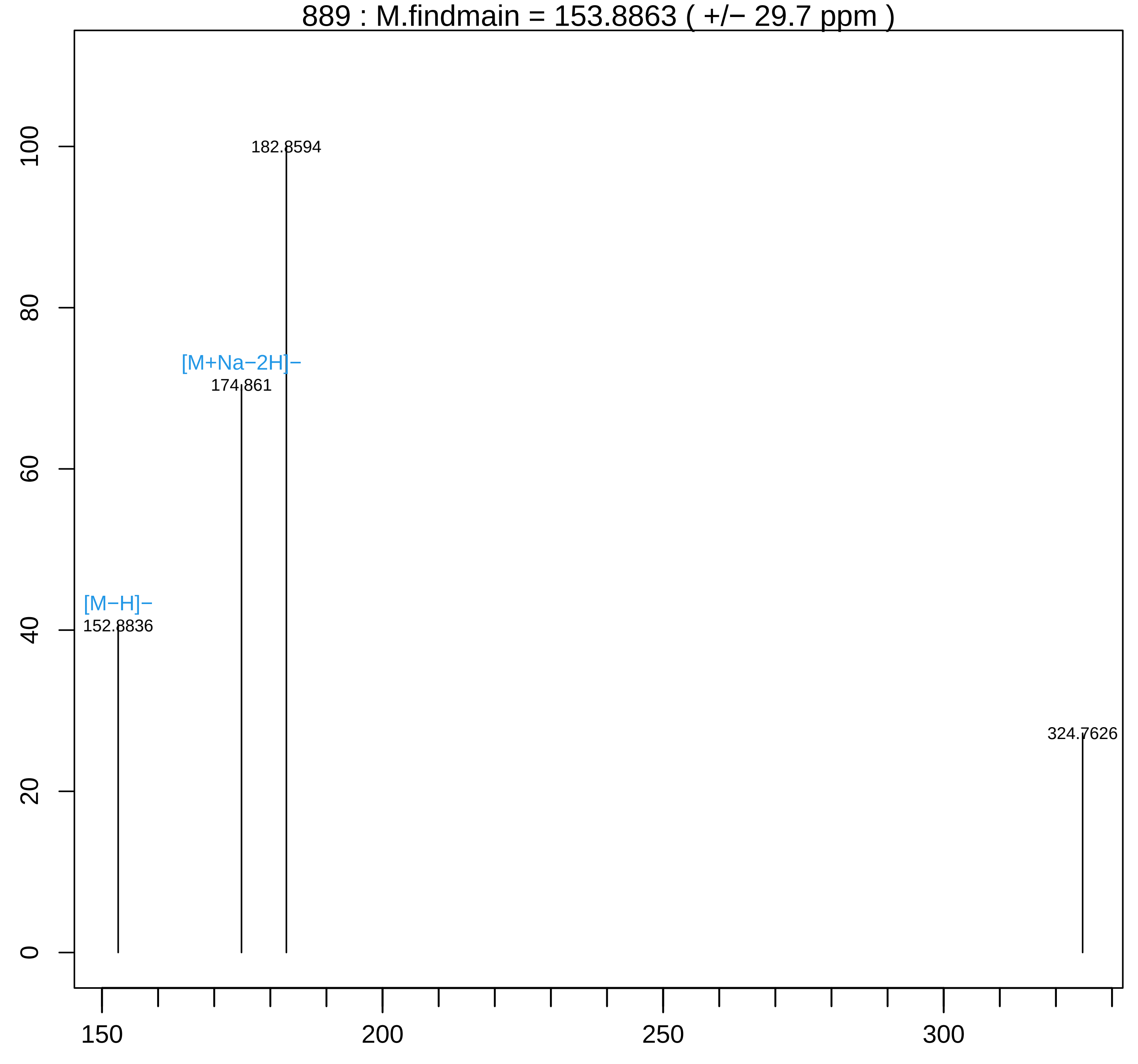

889 : M.findmain = 153.8863 (+/− 29.7 ppm)
0
20
40
60
80
100
150
200
250
300
[M−H]−
152.8836
[M+Na−2H]−
174.861
182.8594
324.7626

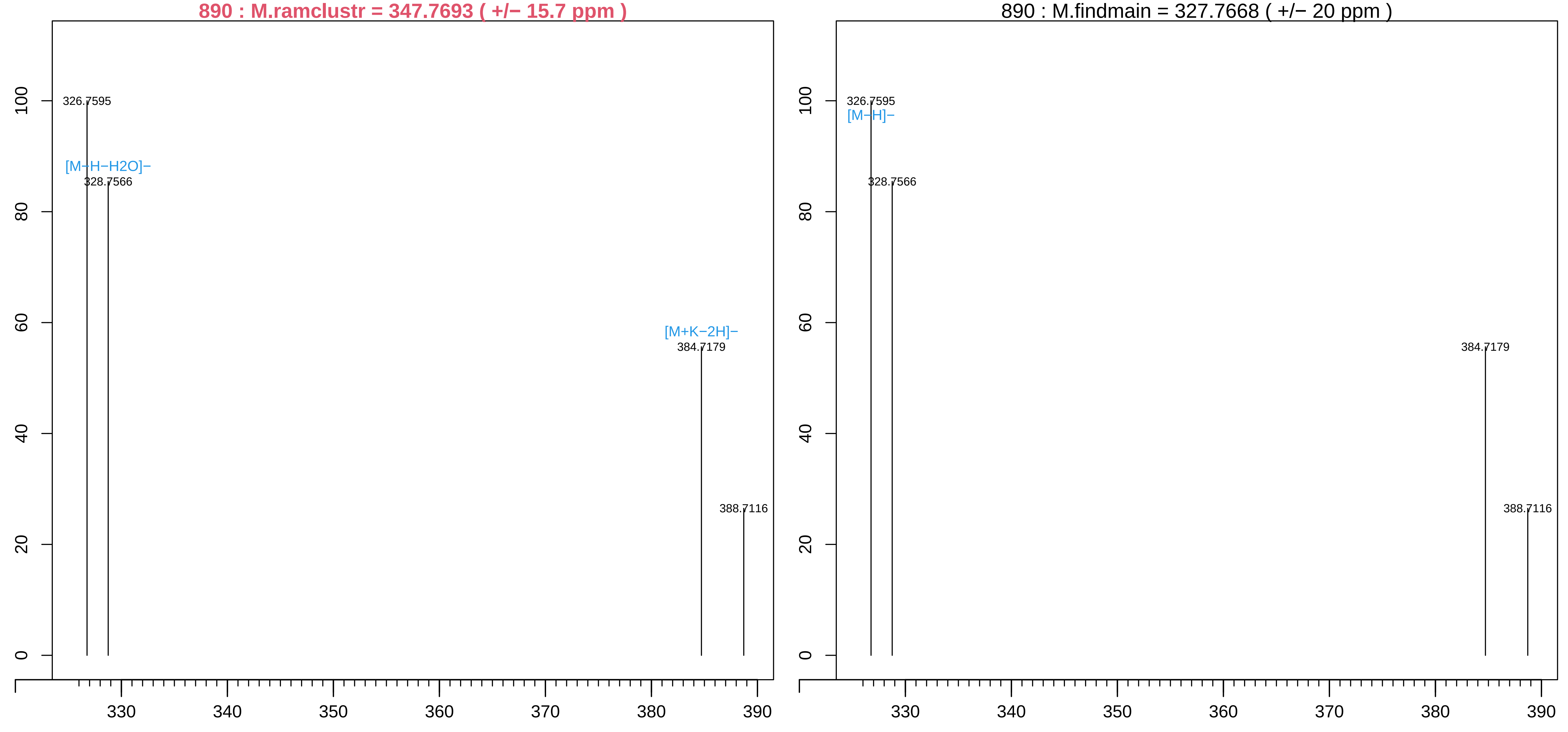
890 : M.ramclustr = 347.7693 (+/− 15.7 ppm)
326.7595
[M−H−H2O]−
328.7566
[M+K−2H]−
384.7179
388.7116
890 : M.findmain = 327.7668 (+/− 20 ppm)
326.7595
[M−H]−
328.7566
384.7179
388.7116
0
20
40
60
80
100
330
340
350
360
370
380
390

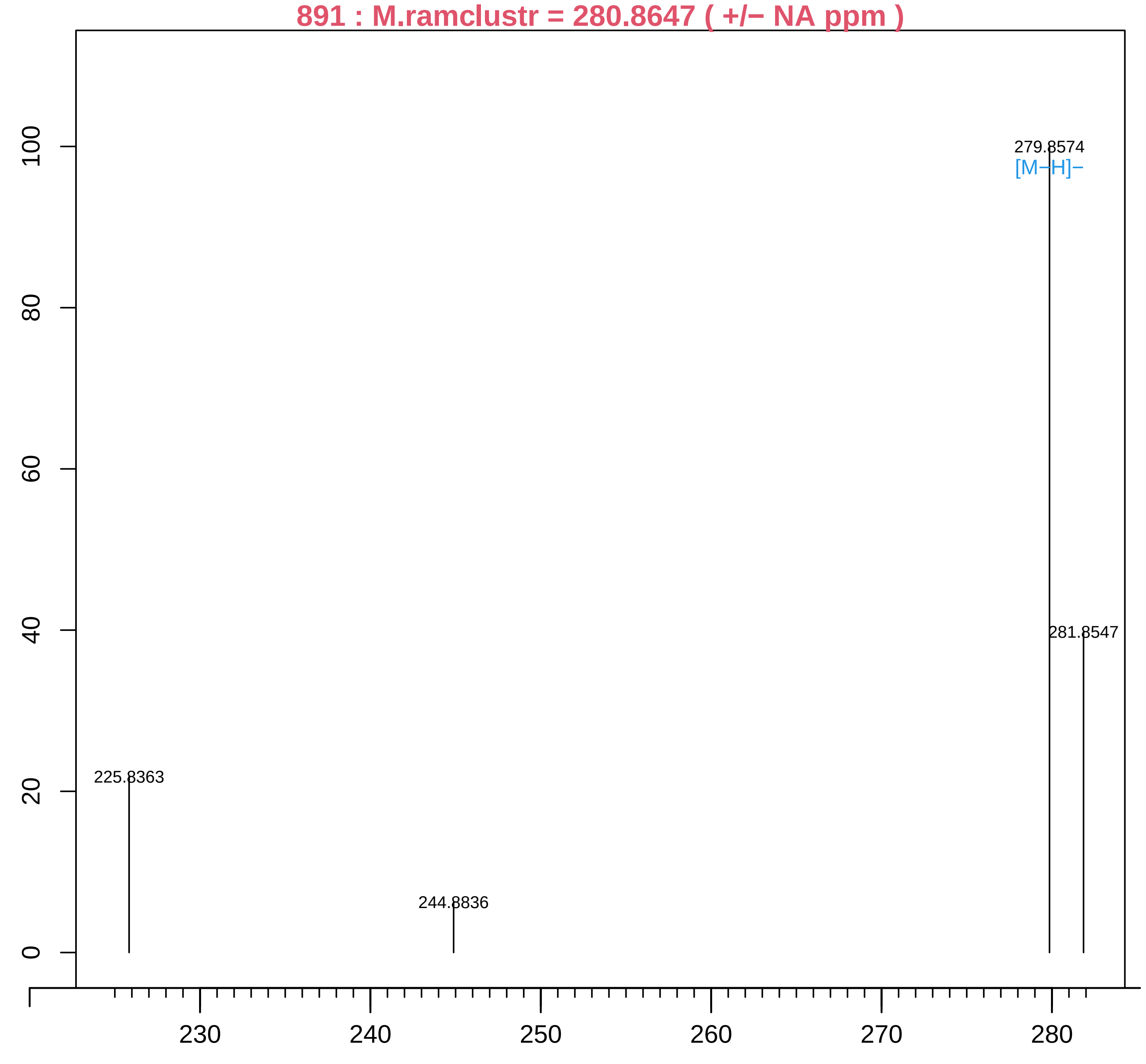
891 : M.ramclustr = 280.8647 (+/− NA ppm)
279.8574
[M−H]−
281.8547
225.8363
244.8836
0
20
40
60
80
100
230
240
250
260
270
280

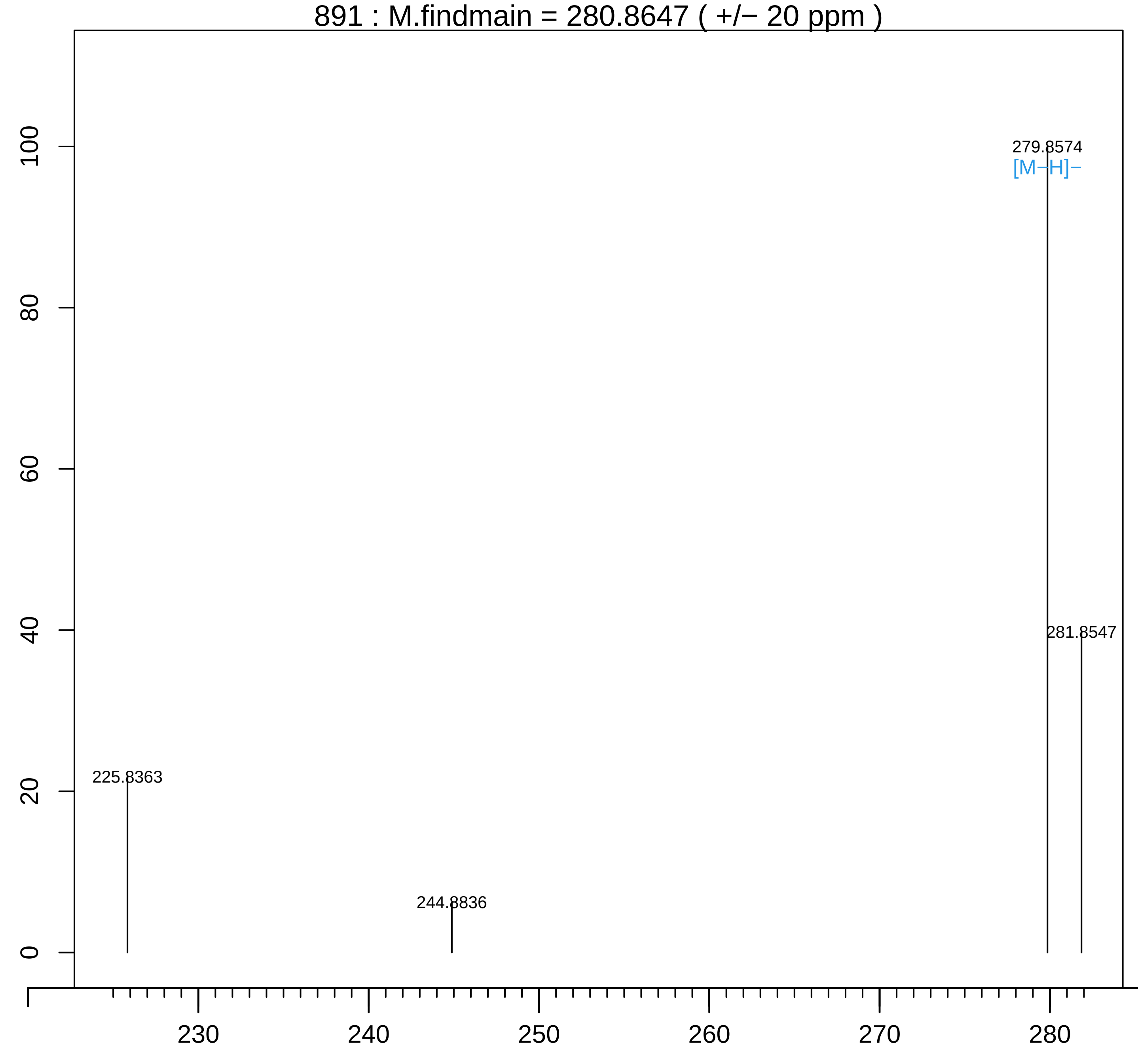
891 : M.findmain = 280.8647 (+/− 20 ppm)
279.8574
[M−H]−
281.8547
225.8363
244.8836
0
20
40
60
80
100
230
240
250
260
270
280

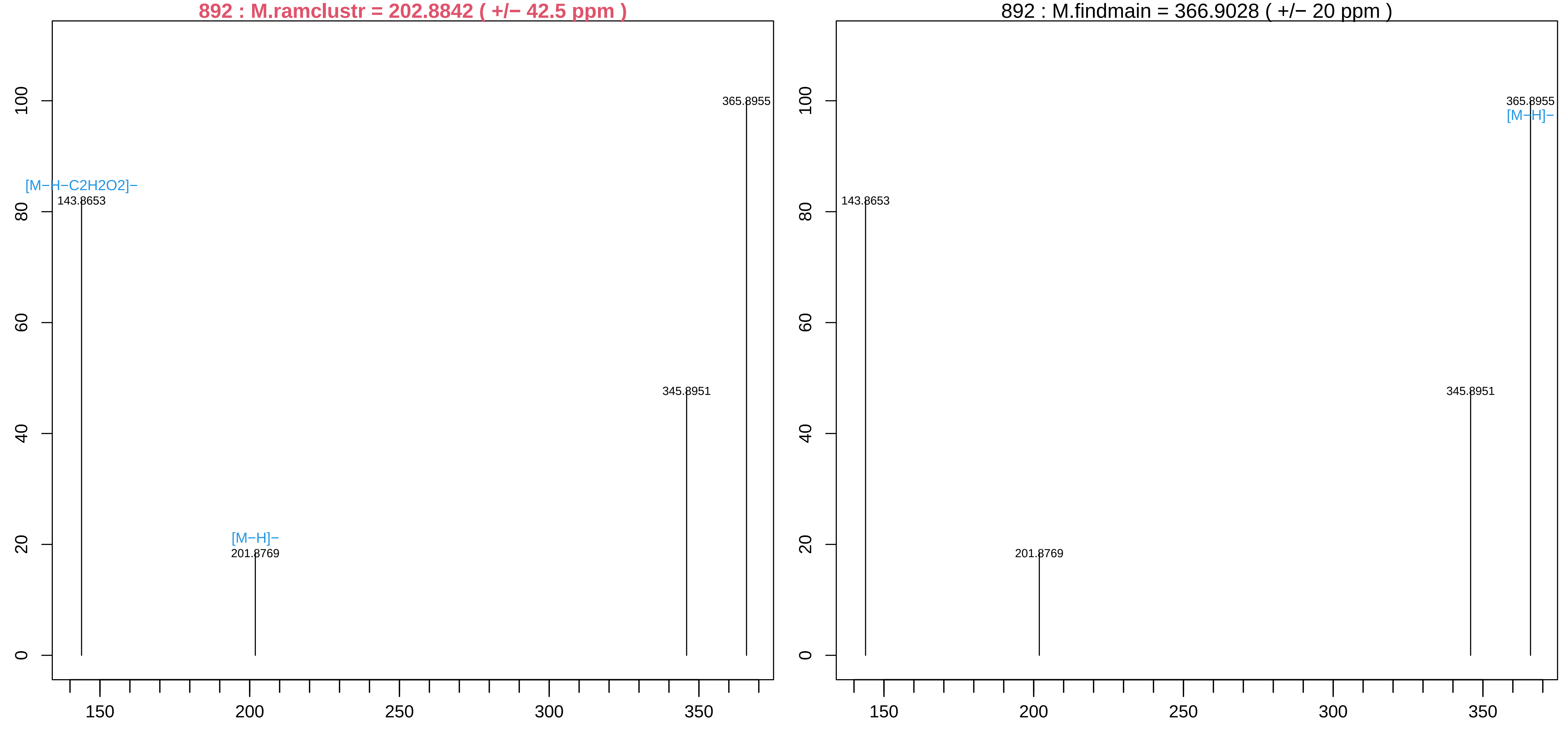
892 : M.ramclustr = 202.8842 (+/− 42.5 ppm)
[M−H−C2H2O2]−
143.8653
[M−H]−
201.8769
345.8951
365.8955
892 : M.findmain = 366.9028 (+/− 20 ppm)
143.8653
201.8769
345.8951
365.8955
[M−H]−
0
20
40
60
80
100
150
200
250
300
350

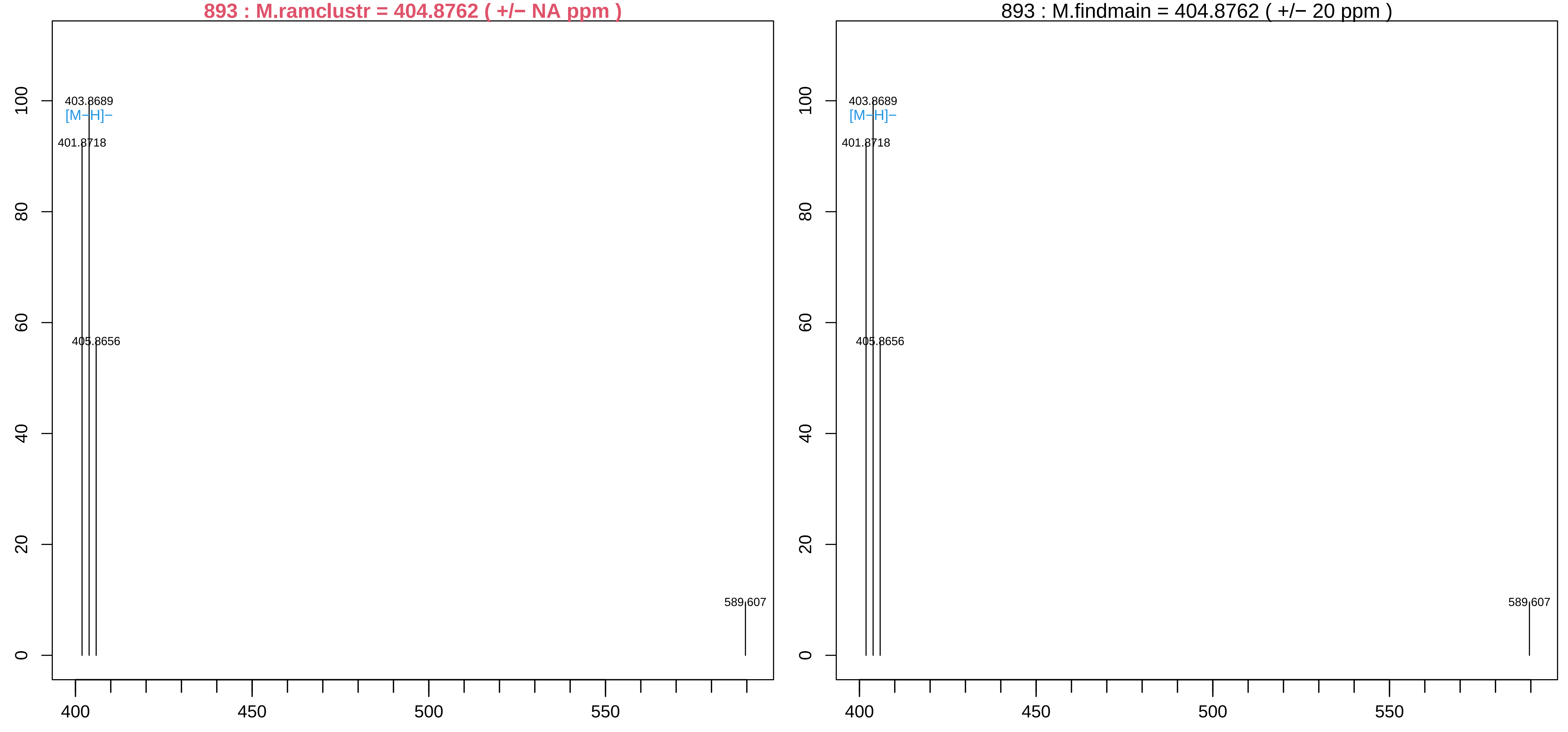
893 : M.ramclustr = 404.8762 (+/− NA ppm)
403.8689
[M−H]−
401.8718
405.8656
589.607
0
20
40
60
80
100
400
450
500
550
893 : M.findmain = 404.8762 (+/− 20 ppm)
403.8689
[M−H]−
401.8718
405.8656
589.607
0
20
40
60
80
100
400
450
500
550

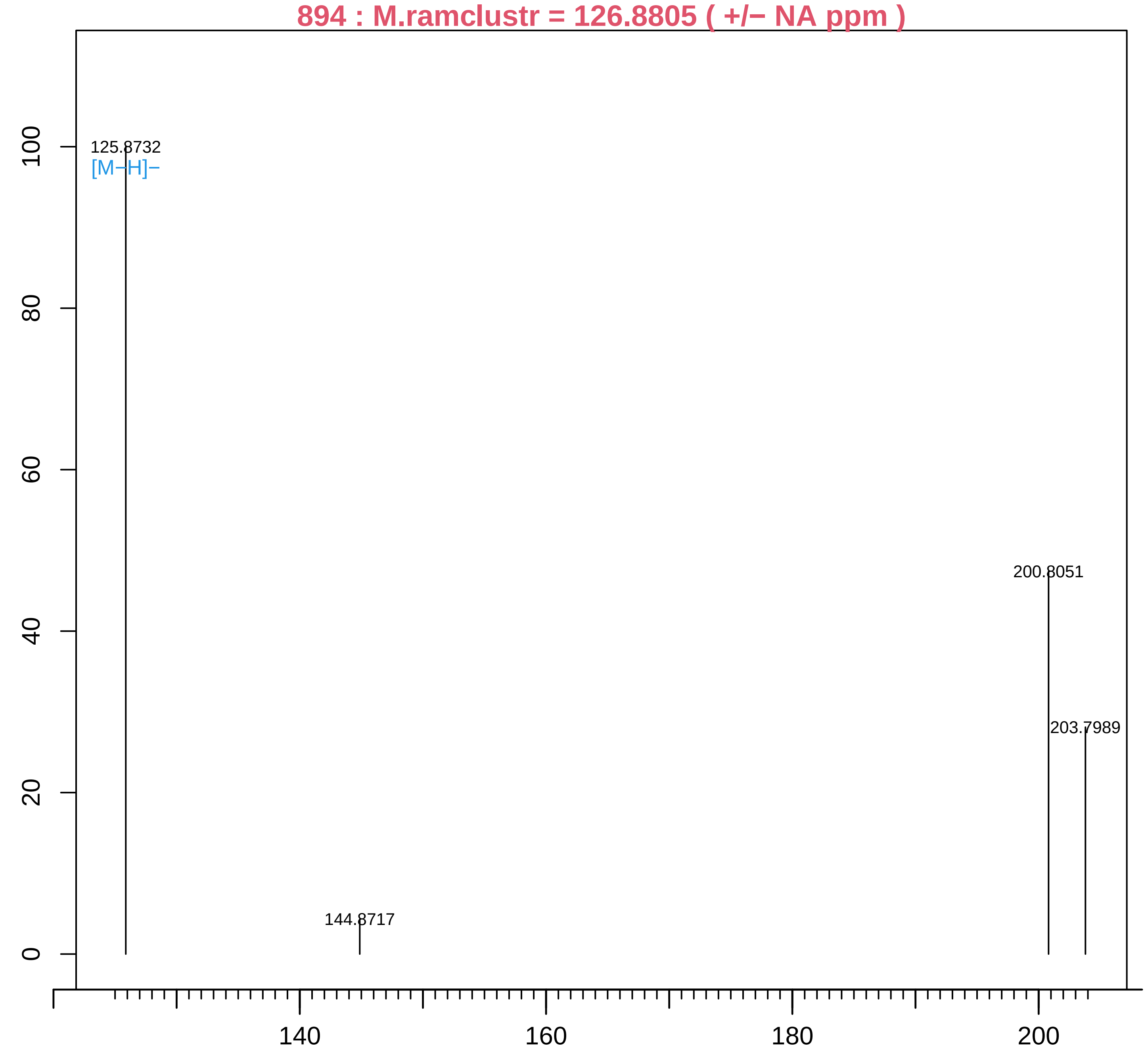
894 : M.ramclustr = 126.8805 (+/− NA ppm)
125.8732
[M−H]−
144.8717
200.8051
203.7989
0
20
40
60
80
100
140
160
180
200

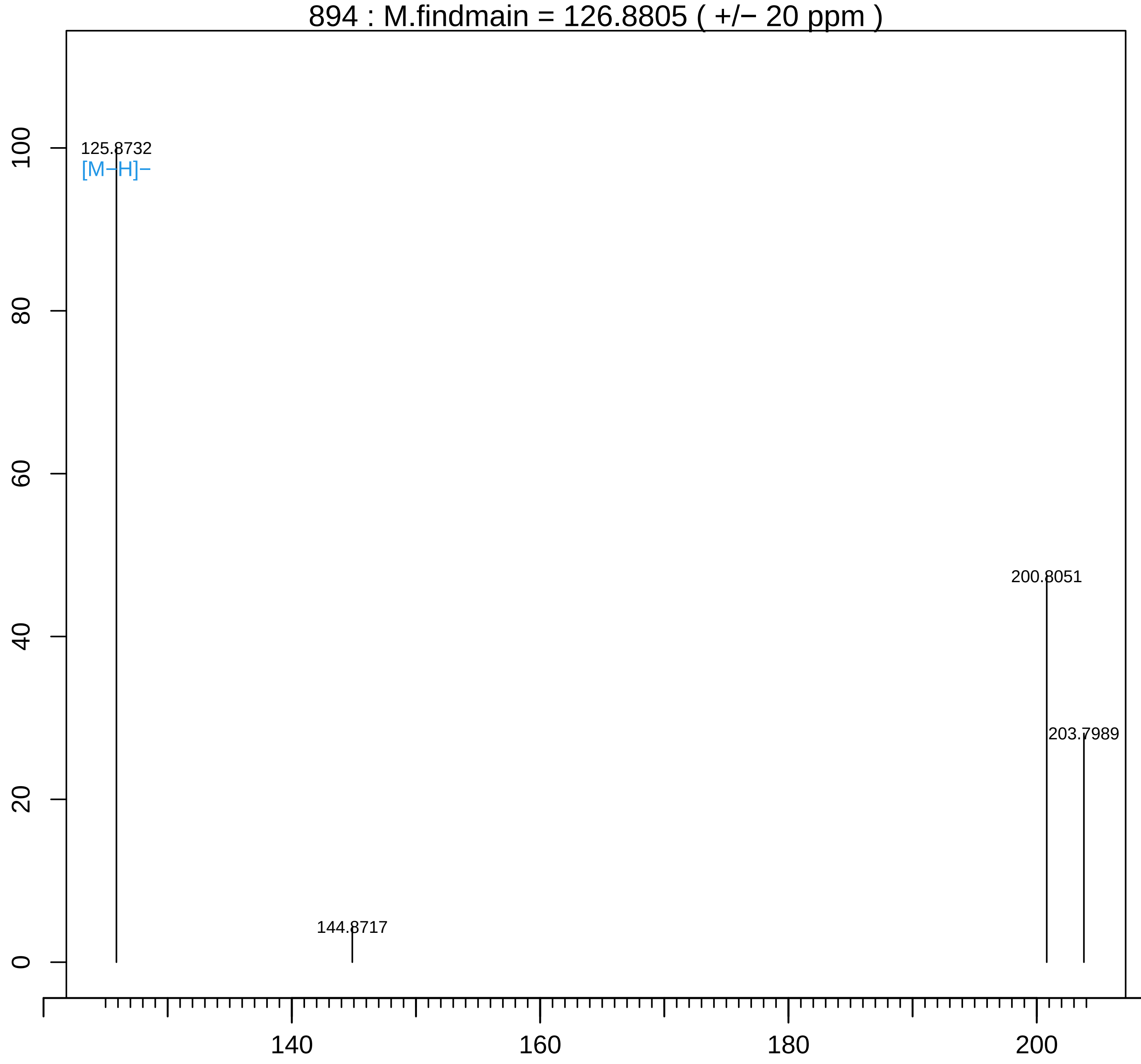
894 : M.findmain = 126.8805 (+/− 20 ppm)
125.8732
[M−H]−
144.8717
200.8051
203.7989
0
20
40
60
80
100
140
160
180
200

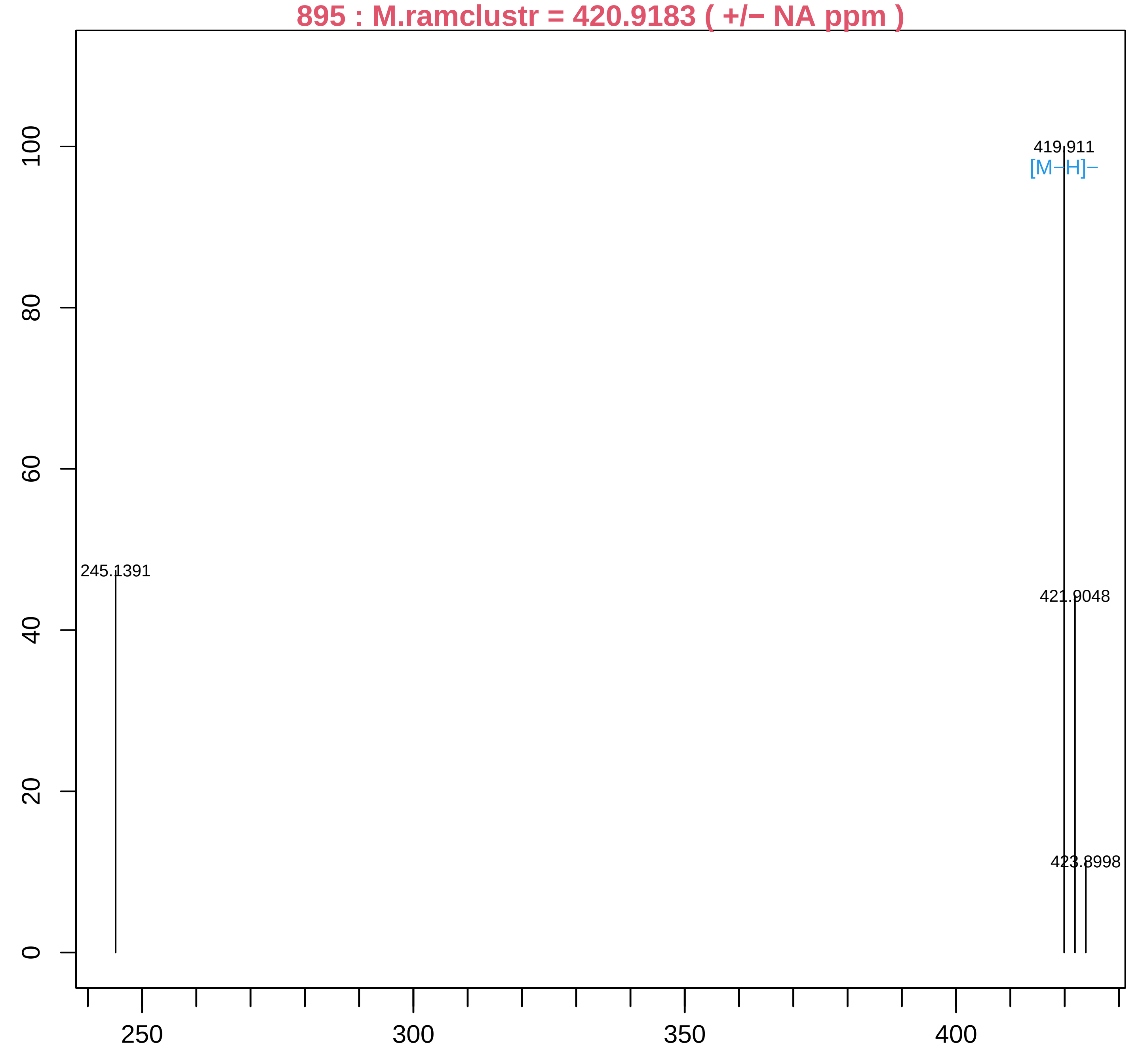
895 : M.ramclustr = 420.9183 (+/− NA ppm)
419.911
[M−H]−
245.1391
421.9048
423.8998
0
20
40
60
80
100
250
300
350
400

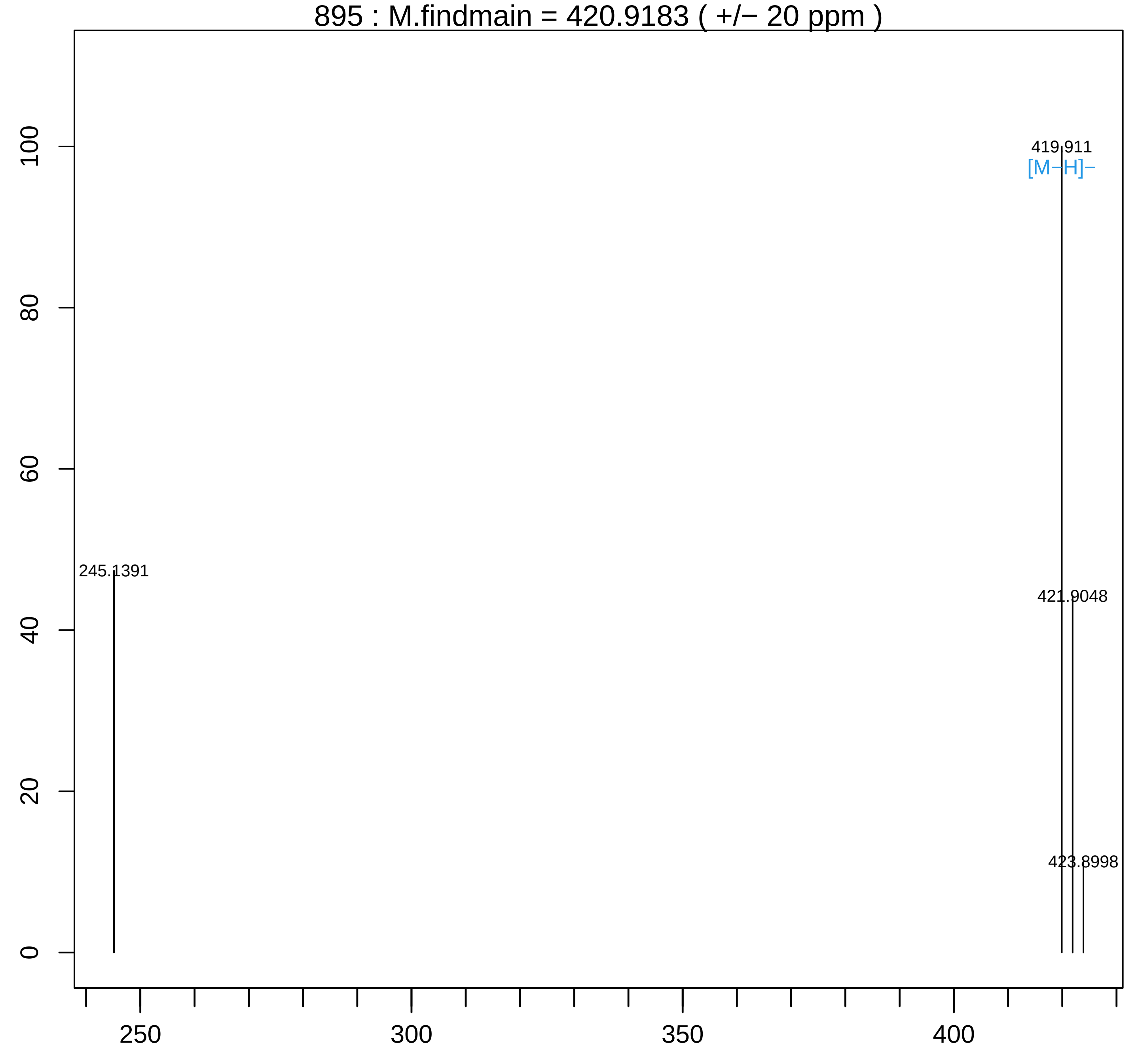
895 : M.findmain = 420.9183 (+/− 20 ppm)
419.911
[M−H]−
245.1391
421.9048
423.8998
0
20
40
60
80
100
250
300
350
400

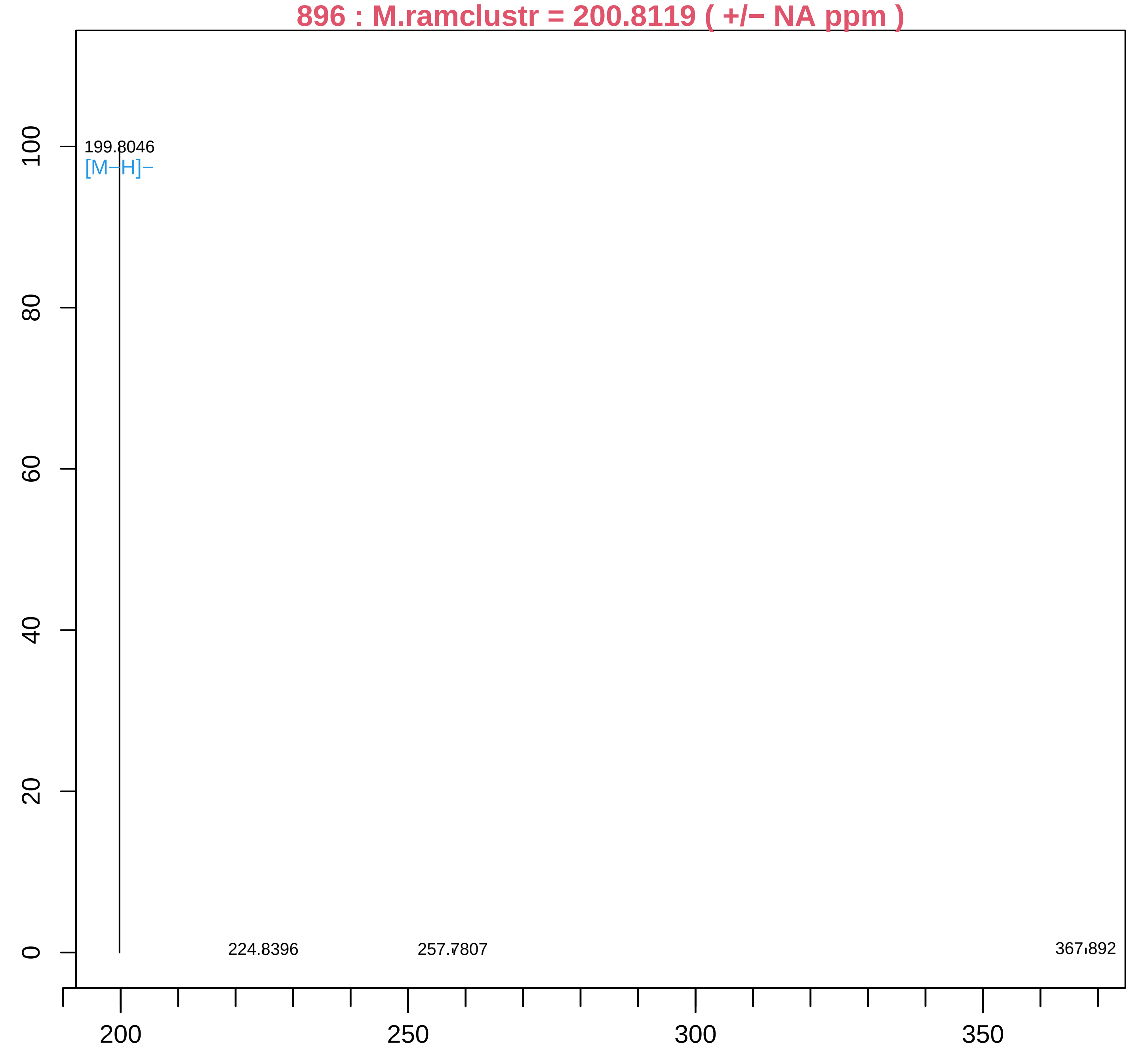
896 : M.ramclustr = 200.8119 (+/− NA ppm)
199.8046
[M−H]−
224.8396
257.7807
367.892
0
20
40
60
80
100
200
250
300
350

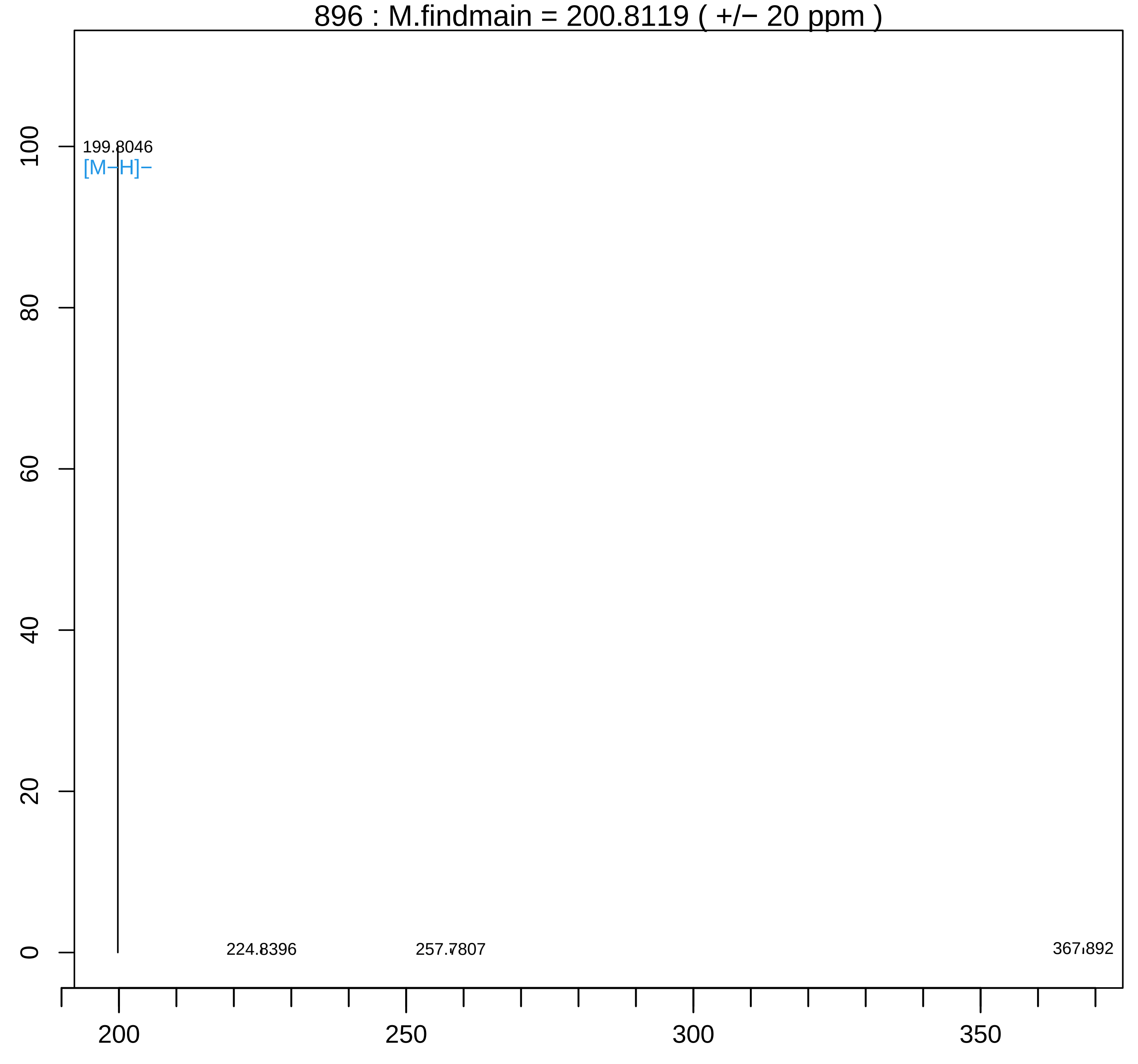
896 : M.findmain = 200.8119 (+/− 20 ppm)
199.8046
[M−H]−
224.8396
257.7807
367.892
0
20
40
60
80
100
200
250
300
350

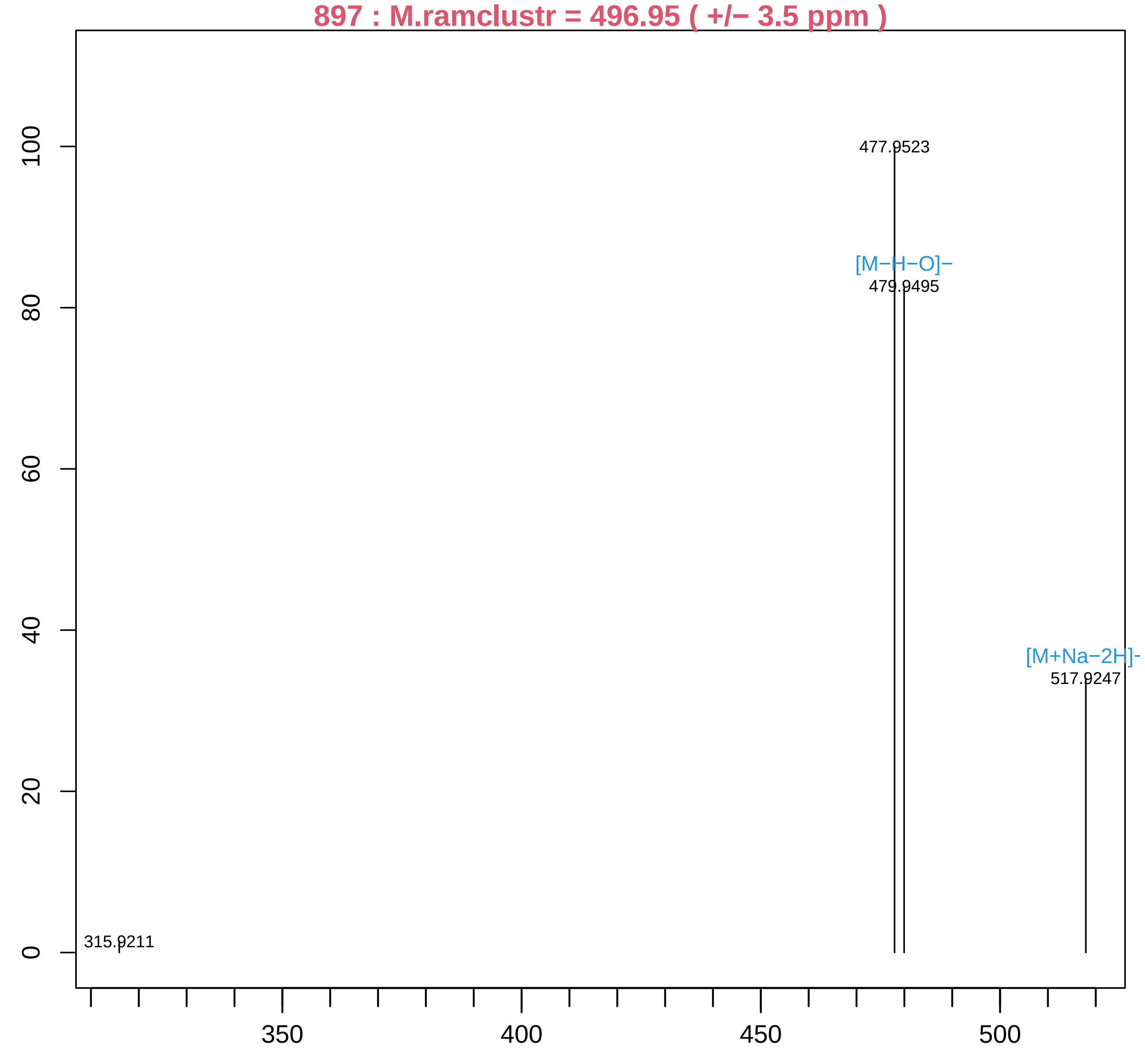

897 : M.ramclustr = 496.95 (+/− 3.5 ppm)
477.9523
[M−H−O]−
479.9495
[M+Na−2H]-
517.9247
315.9211
0
20
40
60
80
100
350
400
450
500

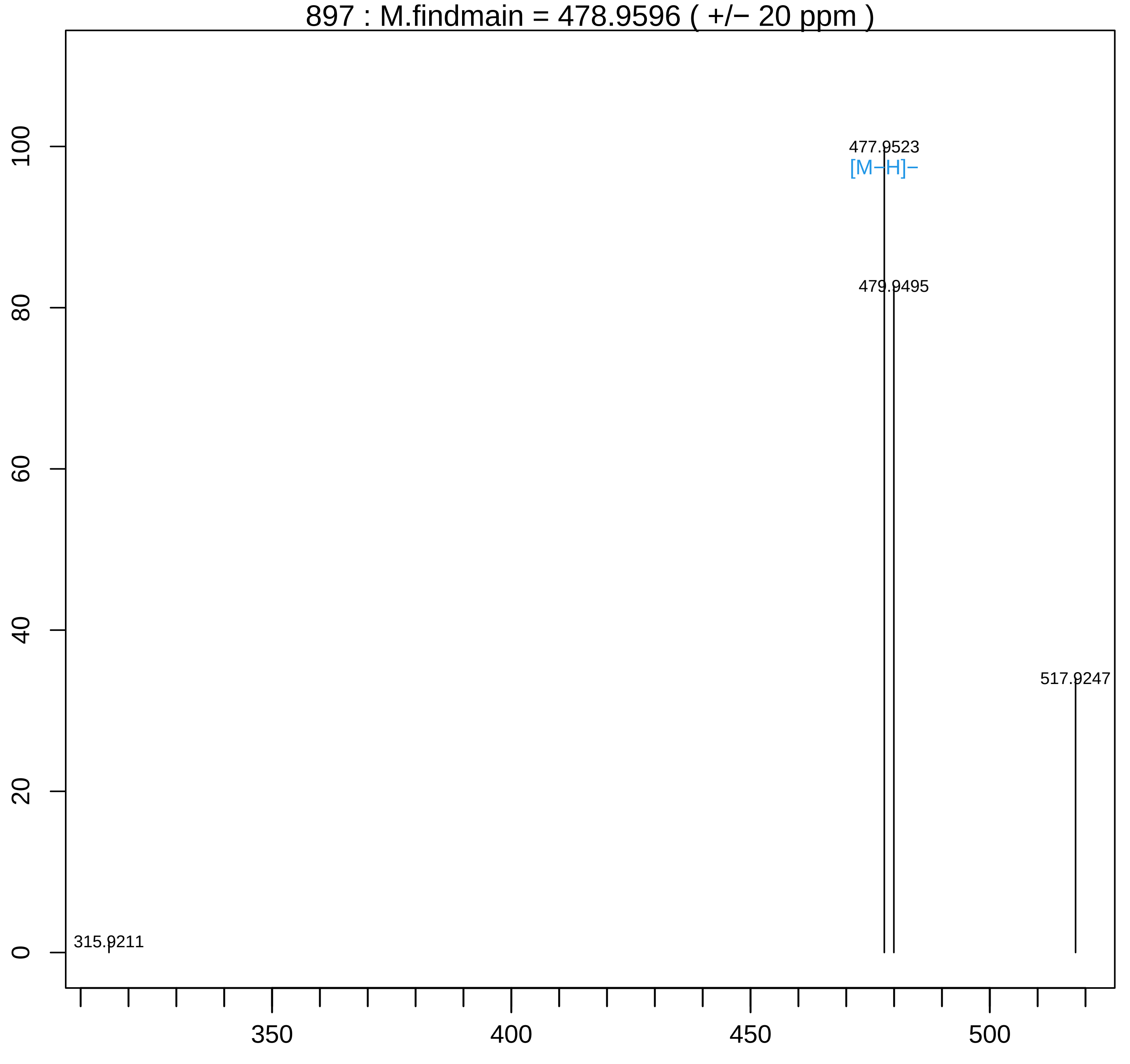

897 : M.findmain = 478.9596 (+/− 20 ppm)
477.9523
[M−H]−
479.9495
517.9247
315.9211
0
20
40
60
80
100
350
400
450
500

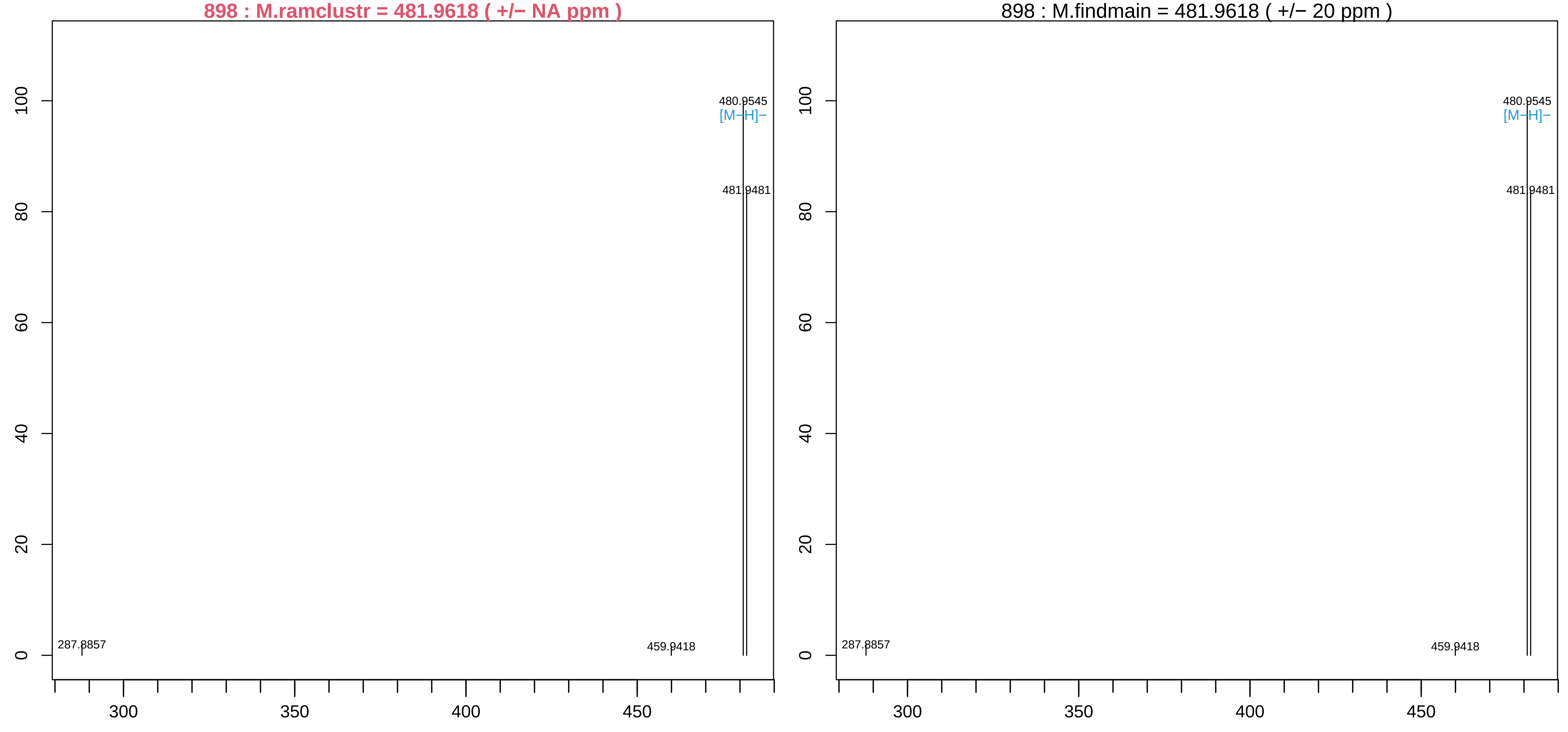
898 : M.ramclustr = 481.9618 (+/− NA ppm)
898 : M.findmain = 481.9618 (+/− 20 ppm)
480.9545
[M−H]−
481.9481
287.8857
459.9418
0
20
40
60
80
100
300
350
400
450

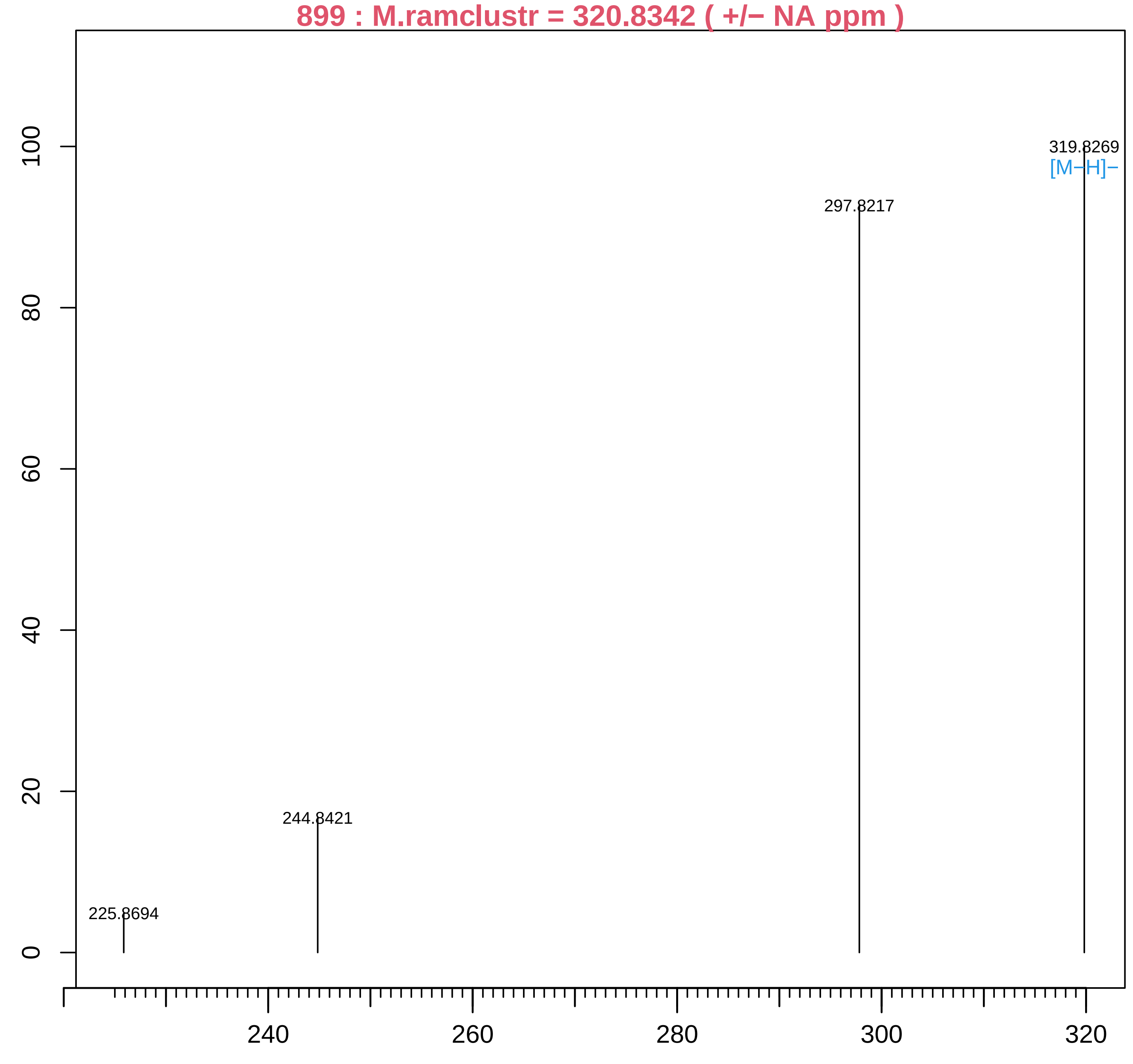
899 : M.ramclustr = 320.8342 (+/− NA ppm)
319.8269
[M−H]−
297.8217
244.8421
225.8694
0
20
40
60
80
100
240
260
280
300
320

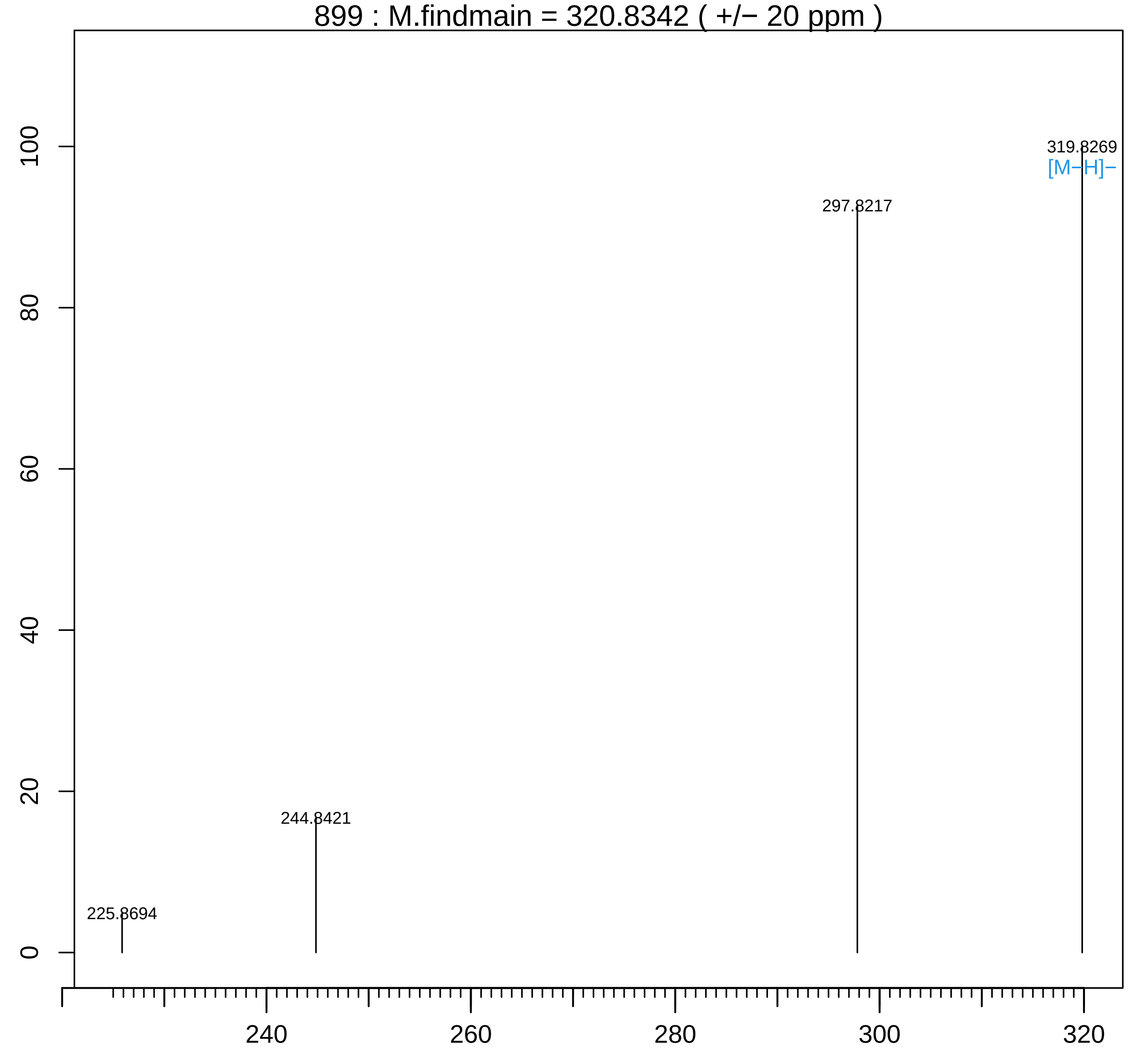
899 : M.findmain = 320.8342 (+/− 20 ppm)
319.8269
[M−H]−
297.8217
244.8421
225.8694
0
20
40
60
80
100
240
260
280
300
320

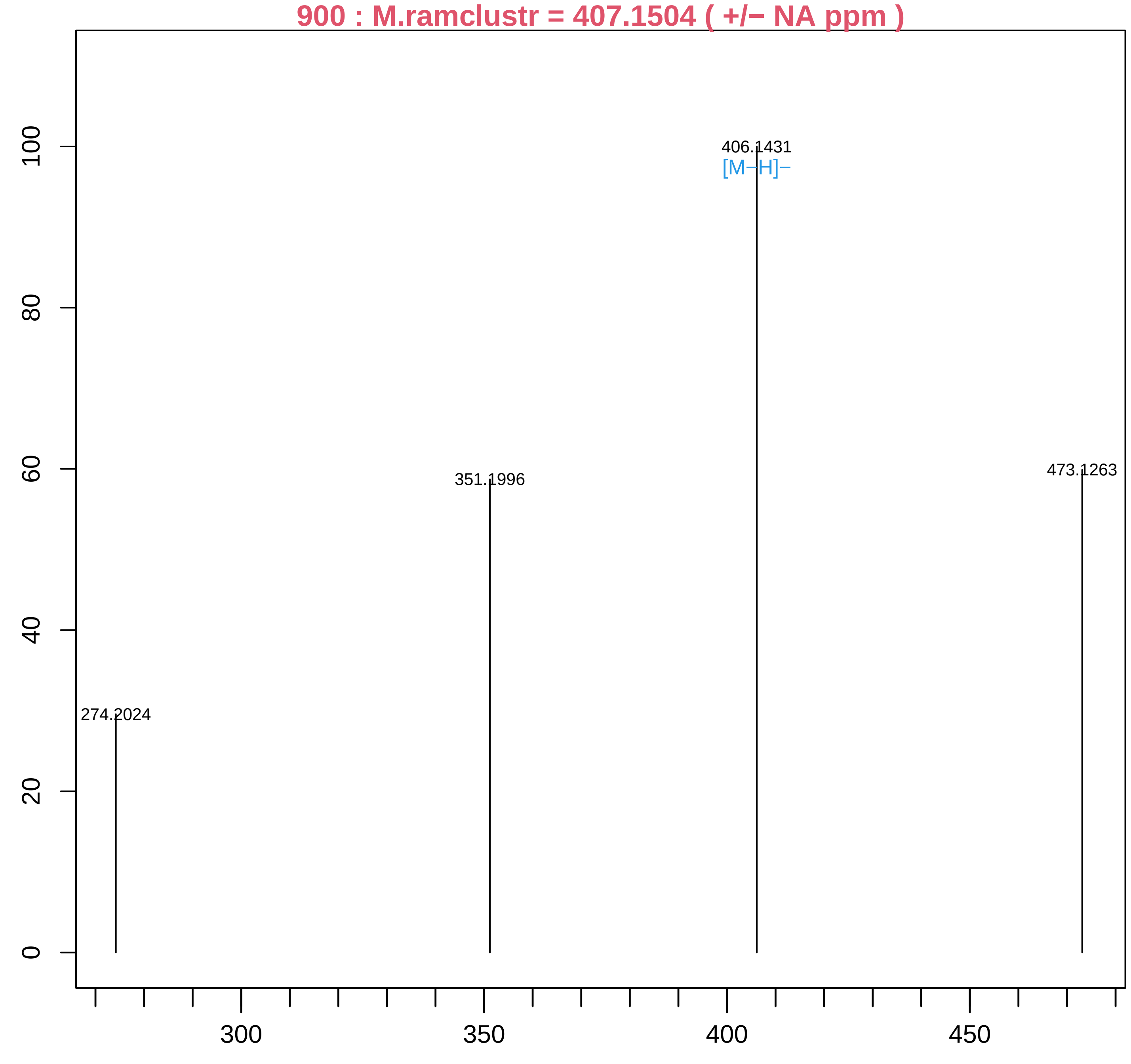
900 : M.ramclustr = 407.1504 (+/− NA ppm)
406.1431
[M−H]−
351.1996
473.1263
274.2024
0
20
40
60
80
100
300
350
400
450

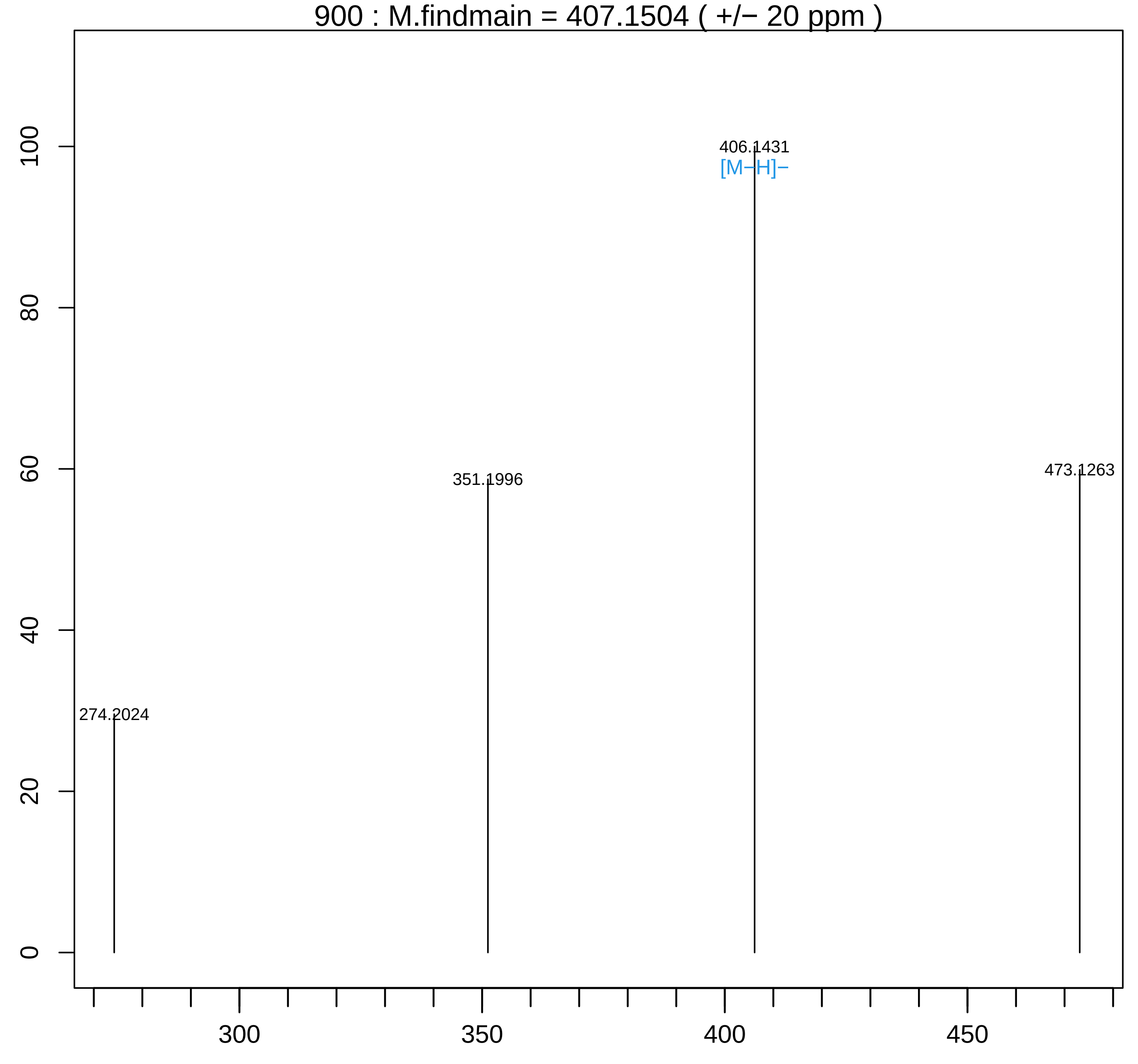
900 : M.findmain = 407.1504 (+/− 20 ppm)
406.1431
[M−H]−
351.1996
473.1263
274.2024
0
20
40
60
80
100
300
350
400
450

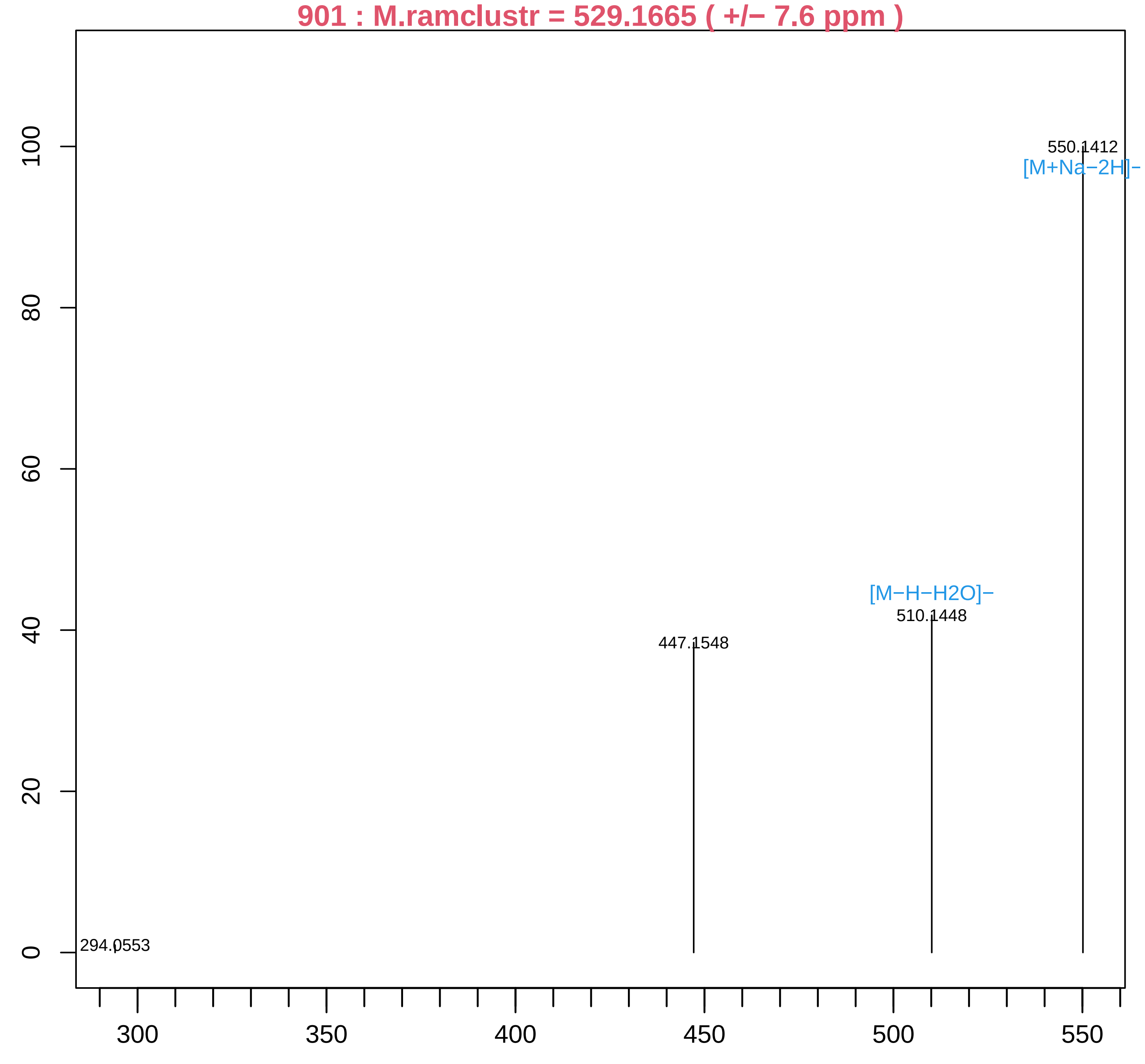

901 : M.ramclustr = 529.1665 (+/− 7.6 ppm)
550.1412
[M+Na−2H]−
[M−H−H2O]−
510.1448
447.1548
294.0553
0
20
40
60
80
100
300
350
400
450
500
550

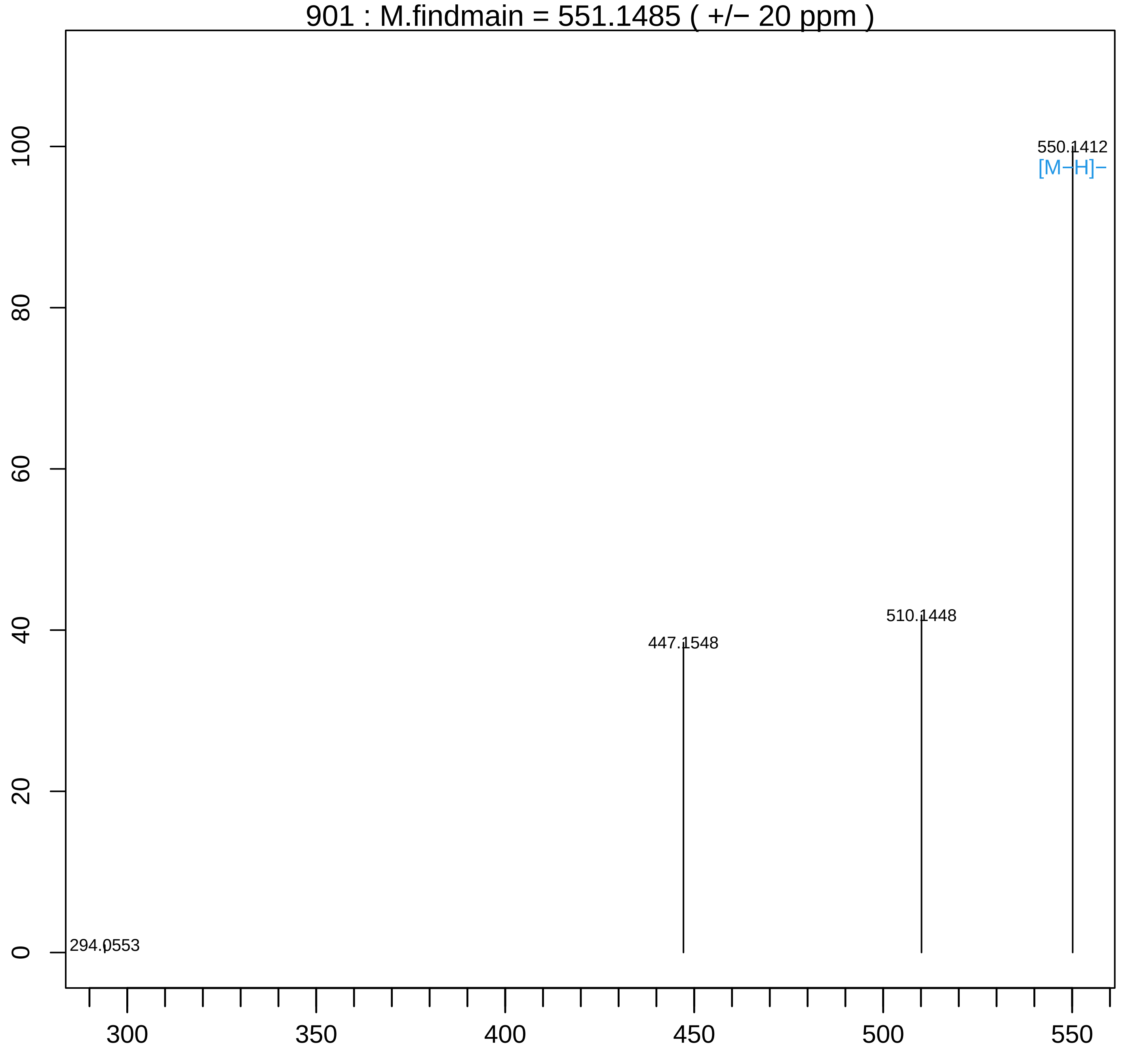

901 : M.findmain = 551.1485 (+/− 20 ppm)
550.1412
[M−H]−
510.1448
447.1548
294.0553
0
20
40
60
80
100
300
350
400
450
500
550

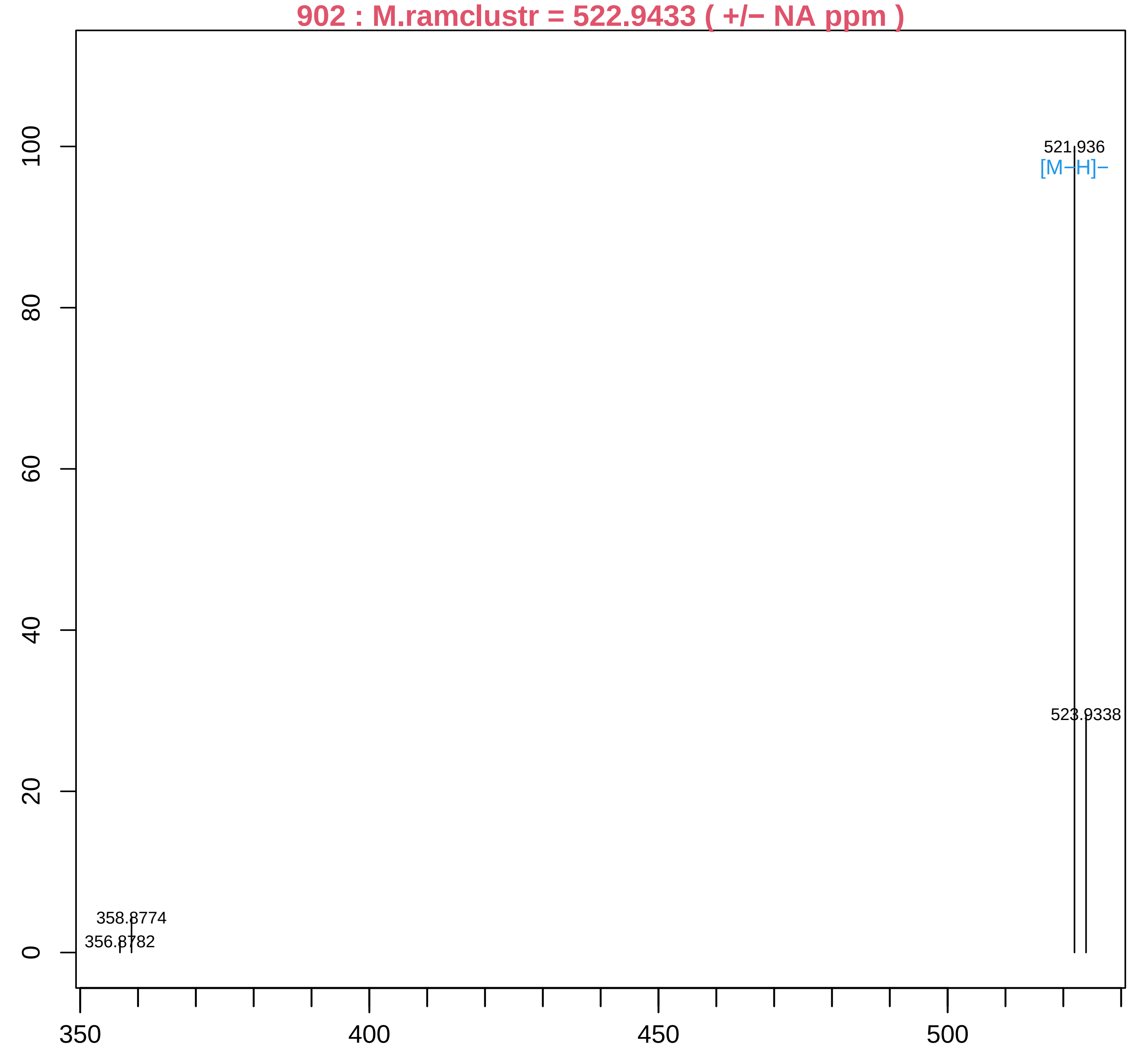
902 : M.ramclustr = 522.9433 (+/− NA ppm)
521.936
[M−H]−
523.9338
358.8774
356.8782
0
20
40
60
80
100
350
400
450
500

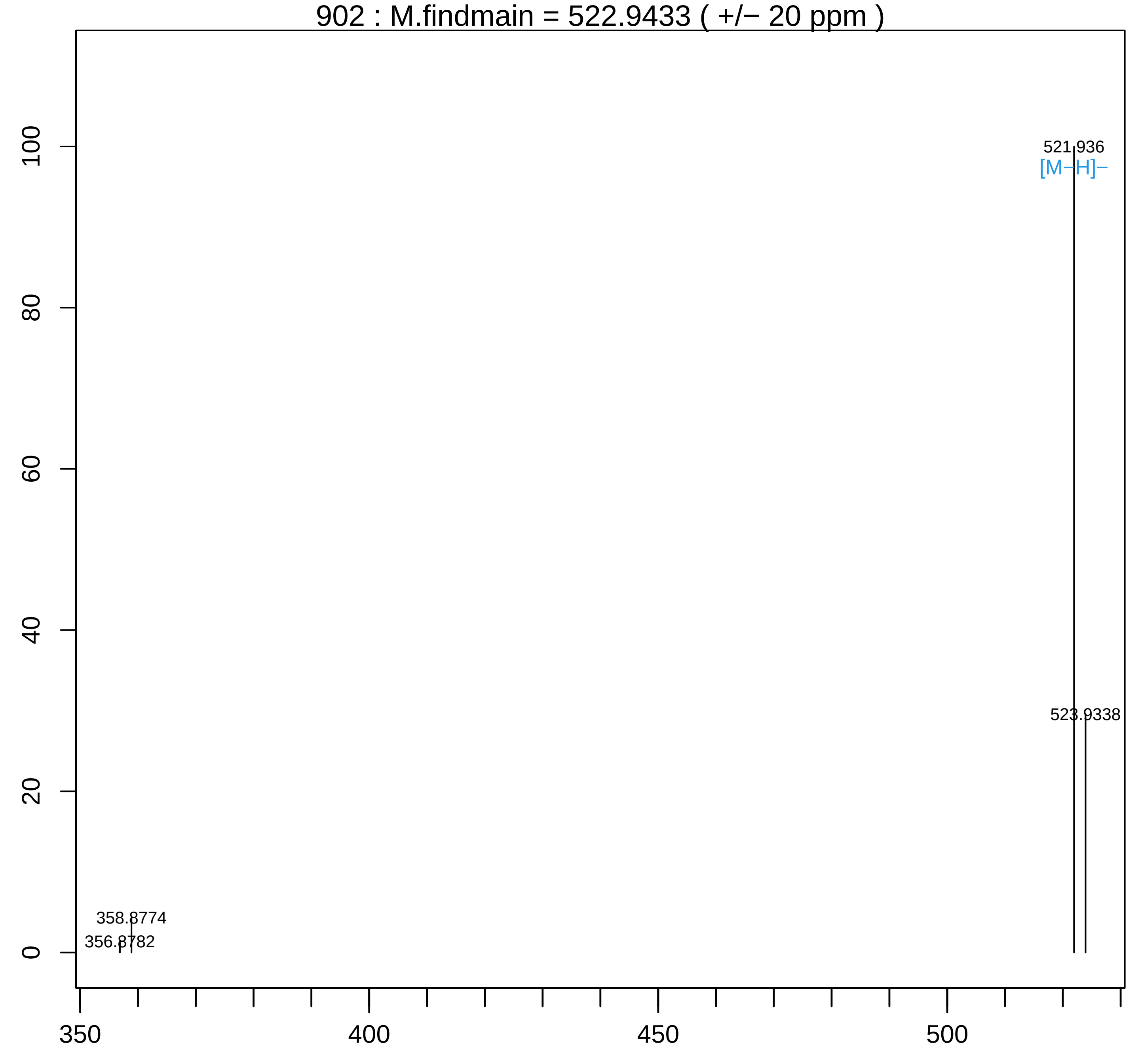
902 : M.findmain = 522.9433 (+/− 20 ppm)
521.936
[M−H]−
523.9338
358.8774
356.8782
0
20
40
60
80
100
350
400
450
500

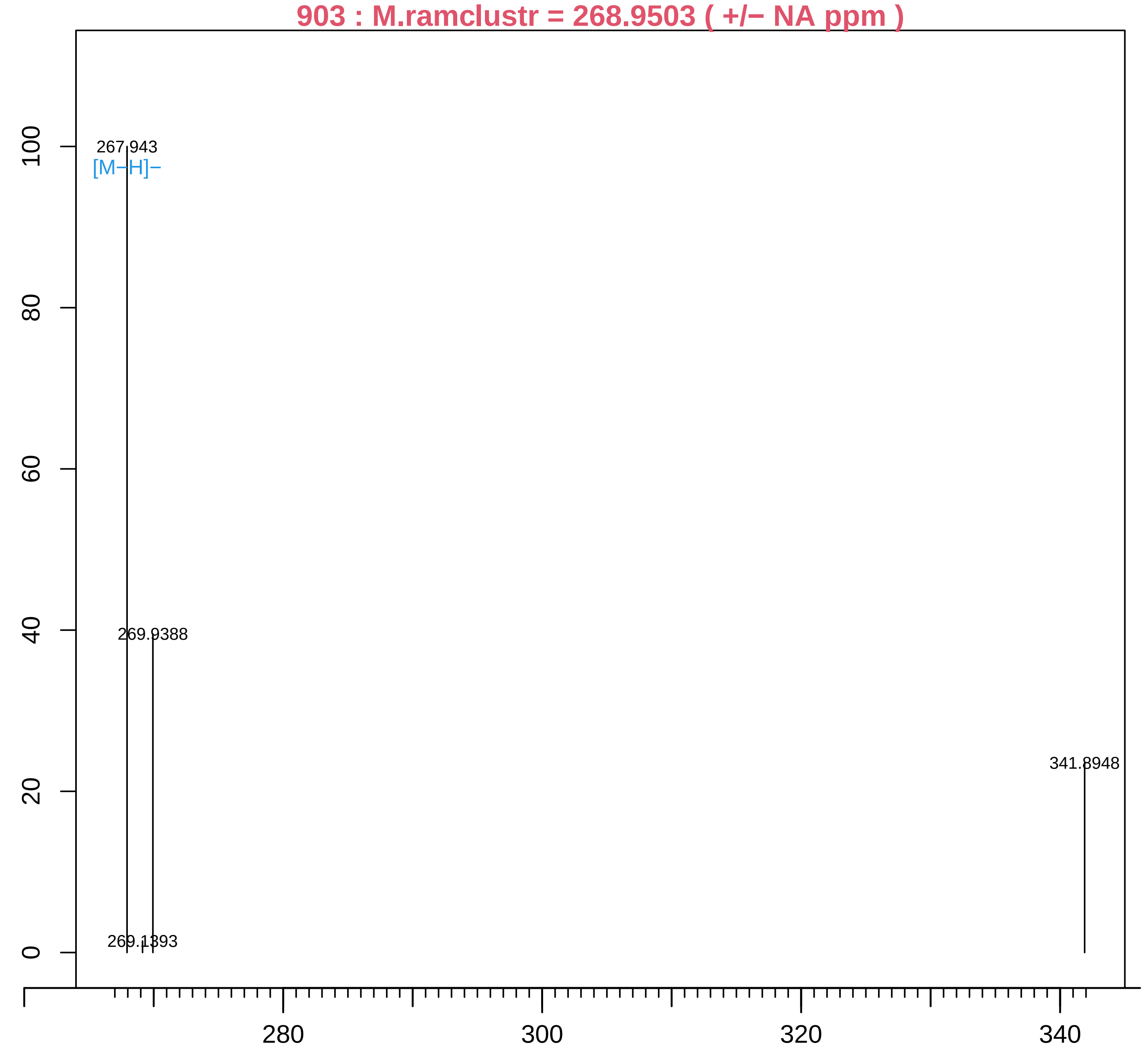

903 : M.ramclustr = 268.9503 (+/− NA ppm)
267.943
[M−H]−
269.9388
341.8948
269.1393
0
20
40
60
80
100
280
300
320
340

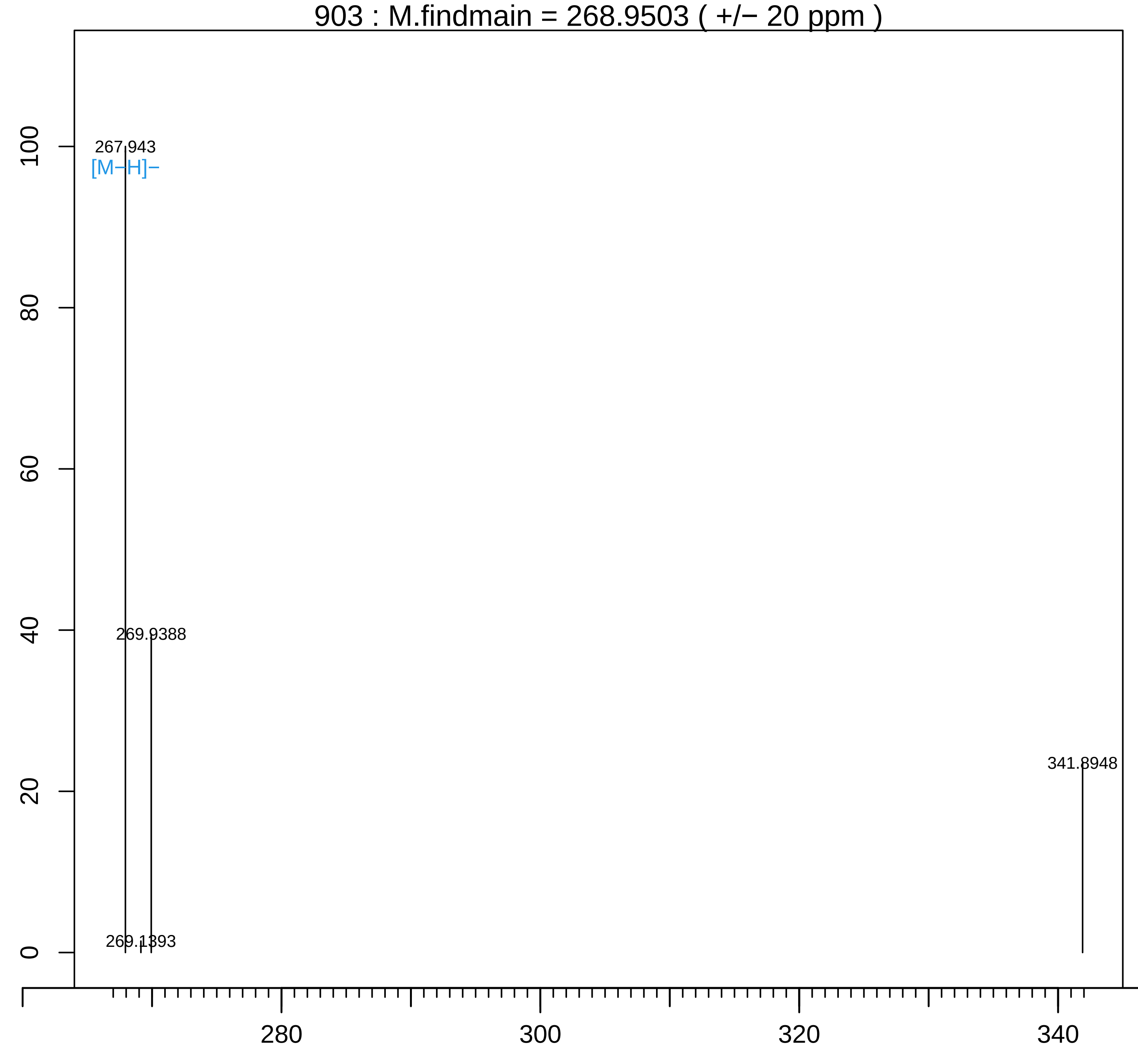

903 : M.findmain = 268.9503 (+/− 20 ppm)
267.943
[M−H]−
269.9388
341.8948
269.1393
0
20
40
60
80
100
280
300
320
340

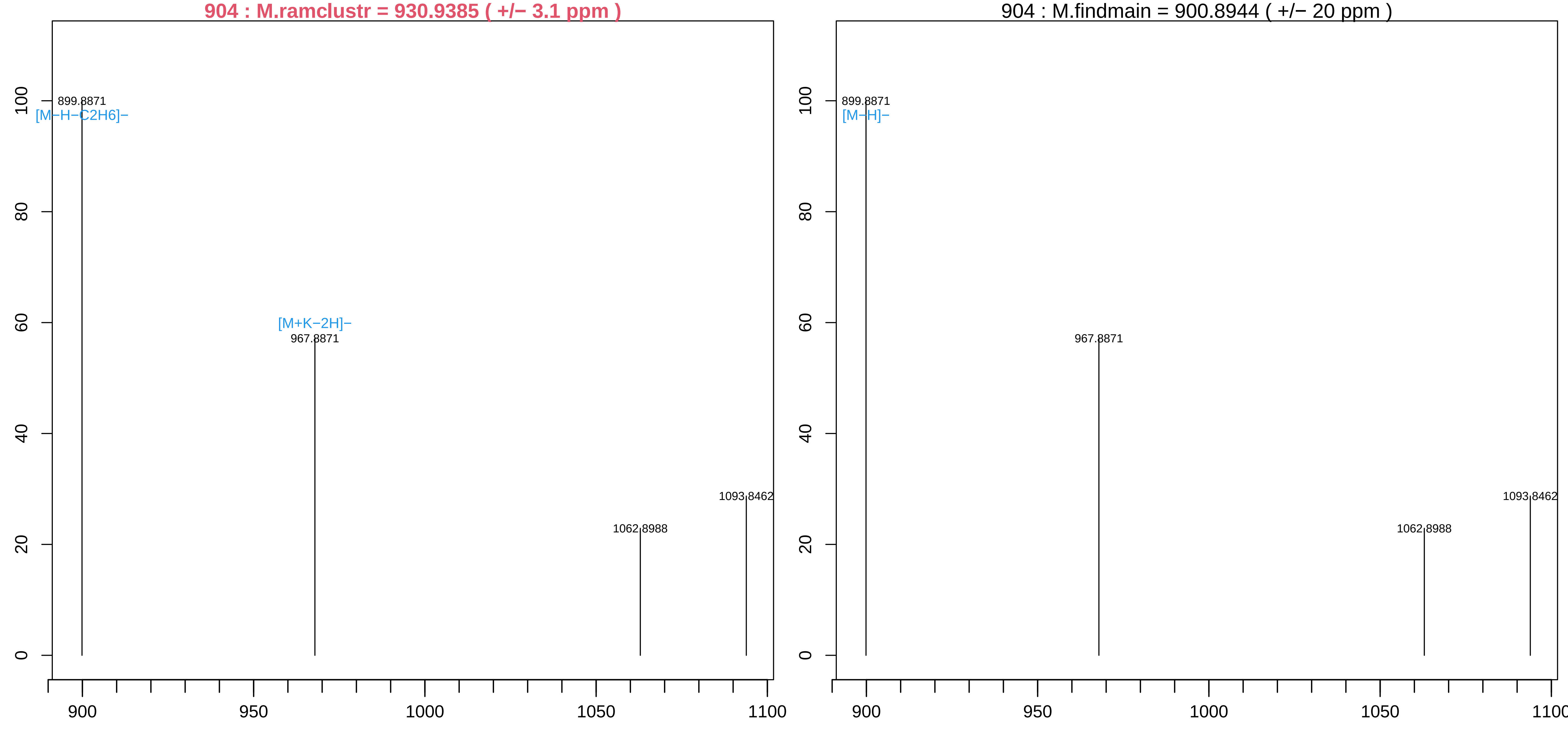
904 : M.ramclustr = 930.9385 (+/− 3.1 ppm)
899.8871
[M−H−C2H6]−
[M+K−2H]−
967.8871
1062.8988
1093.8462
904 : M.findmain = 900.8944 (+/− 20 ppm)
899.8871
[M−H]−
967.8871
1062.8988
1093.8462

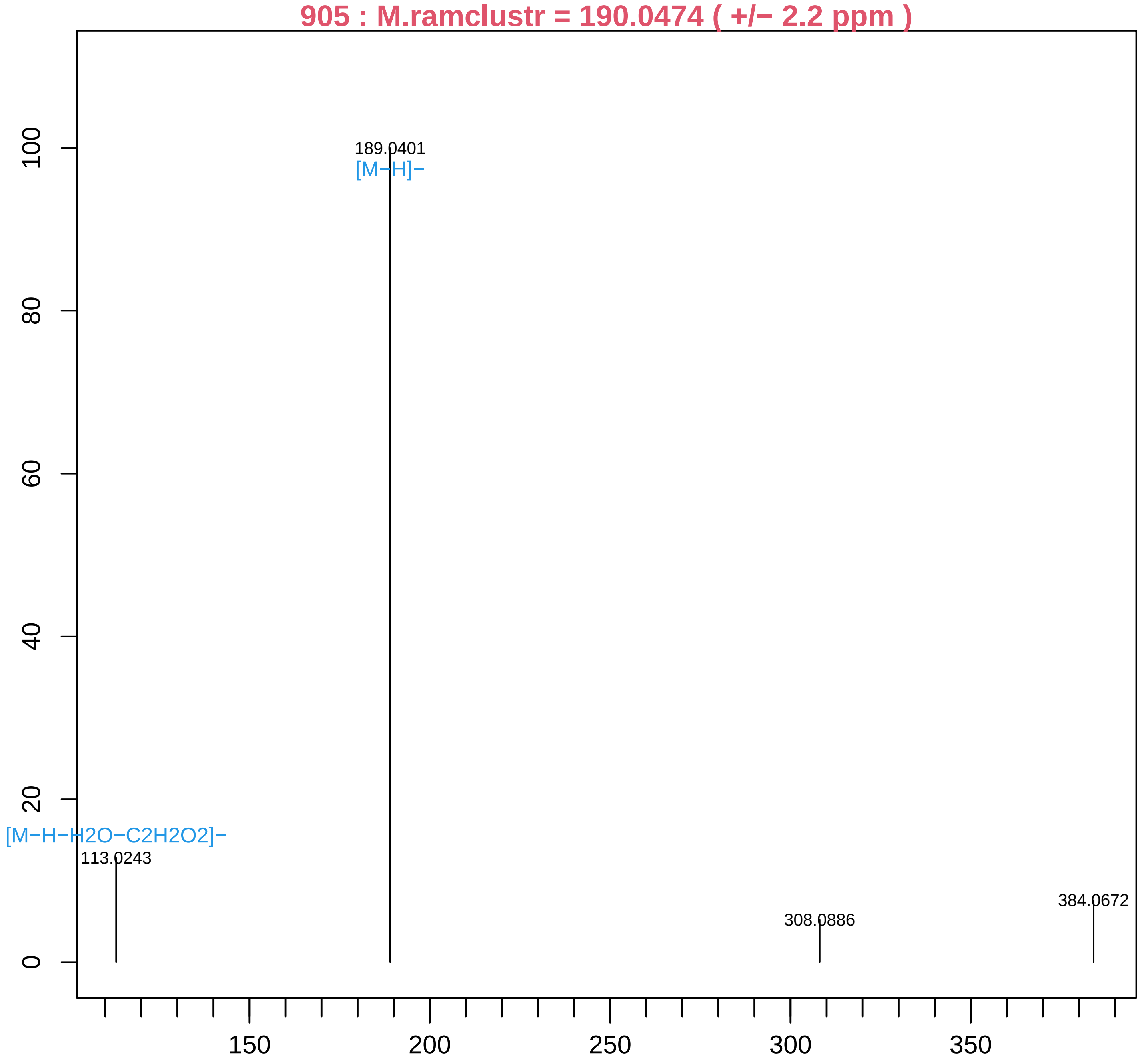
905 : M.ramclustr = 190.0474 (+/− 2.2 ppm)
189.0401
[M−H]−
[M−H−H2O−C2H2O2]−
113.0243
308.0886
384.0672
0
20
40
60
80
100
150
200
250
300
350

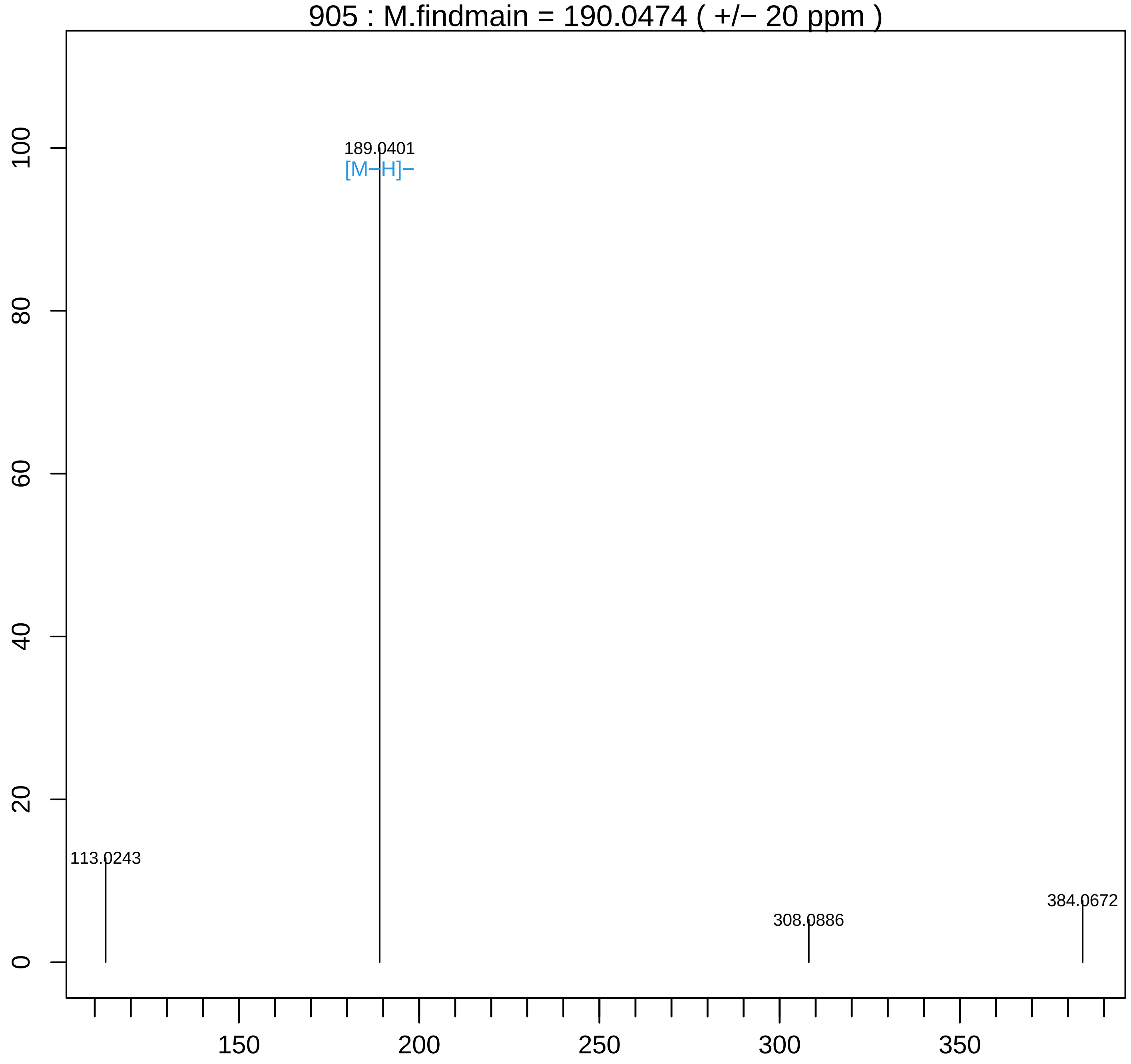
905 : M.findmain = 190.0474 (+/− 20 ppm)
189.0401
[M−H]−
113.0243
308.0886
384.0672
0
20
40
60
80
100
150
200
250
300
350

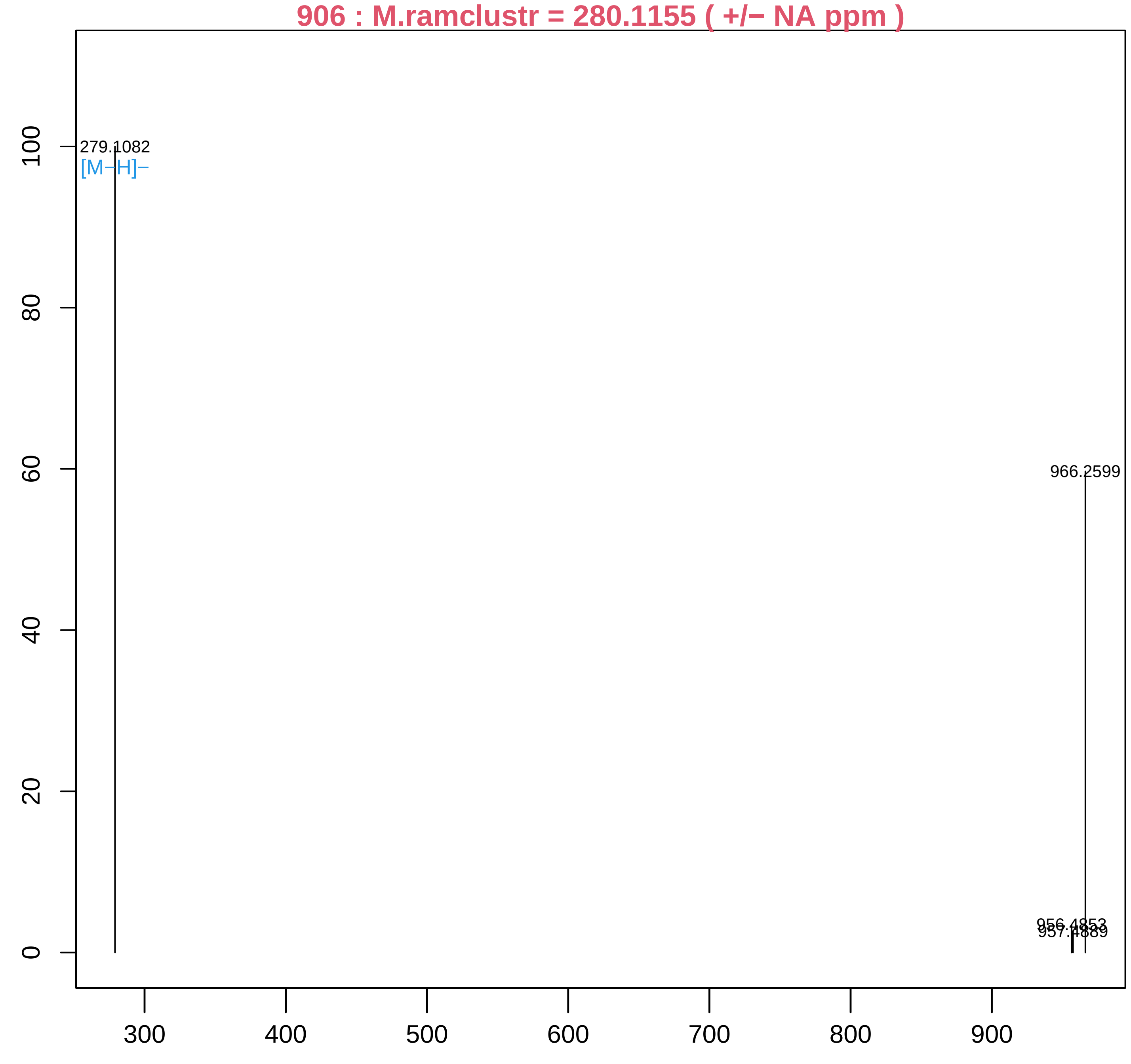
906 : M.ramclustr = 280.1155 (+/− NA ppm)
279.1082
[M−H]−
966.2599
956.4853
957.4889
0
20
40
60
80
100
300
400
500
600
700
800
900

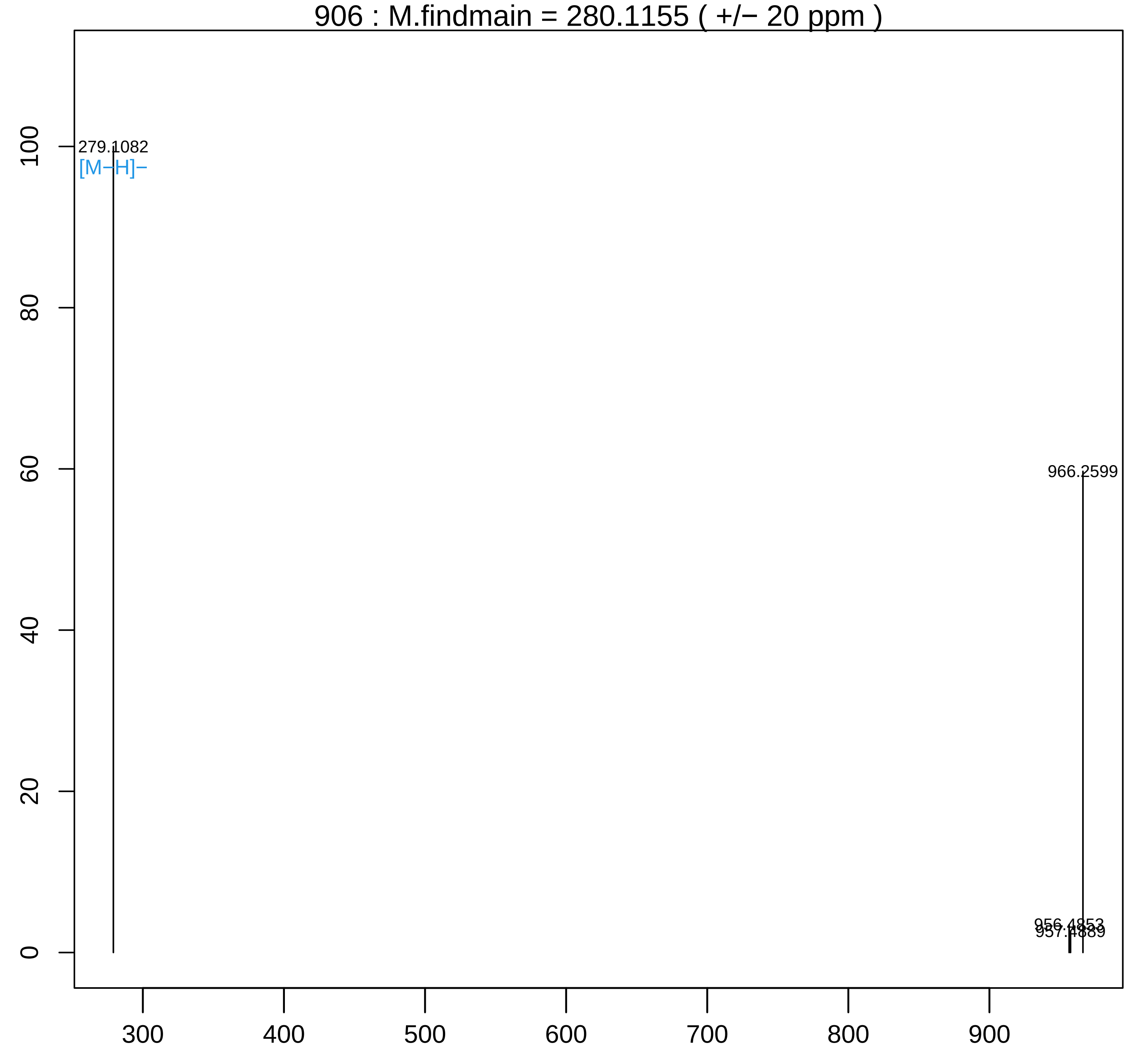
906 : M.findmain = 280.1155 (+/− 20 ppm)
279.1082
[M−H]−
966.2599
956.4853
957.4889
0
20
40
60
80
100
300
400
500
600
700
800
900

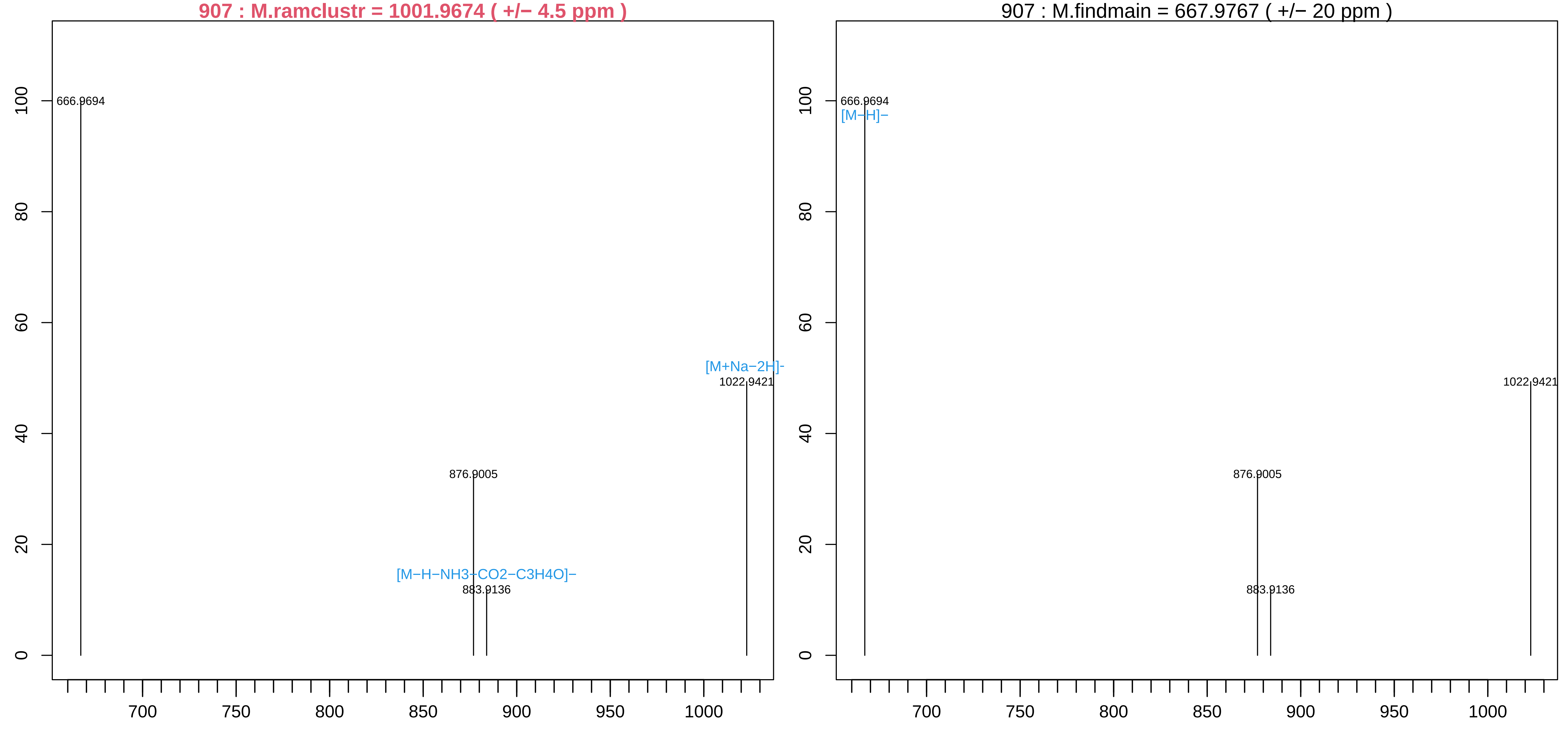
907 : M.ramclustr = 1001.9674 (+/− 4.5 ppm)
666.9694
[M−H−NH3−CO2−C3H4O]−
883.9136
876.9005
[M+Na−2H]-
1022.9421
907 : M.findmain = 667.9767 (+/− 20 ppm)
666.9694
[M−H]−
876.9005
883.9136
1022.9421
0
20
40
60
80
100
700
750
800
850
900
950
1000

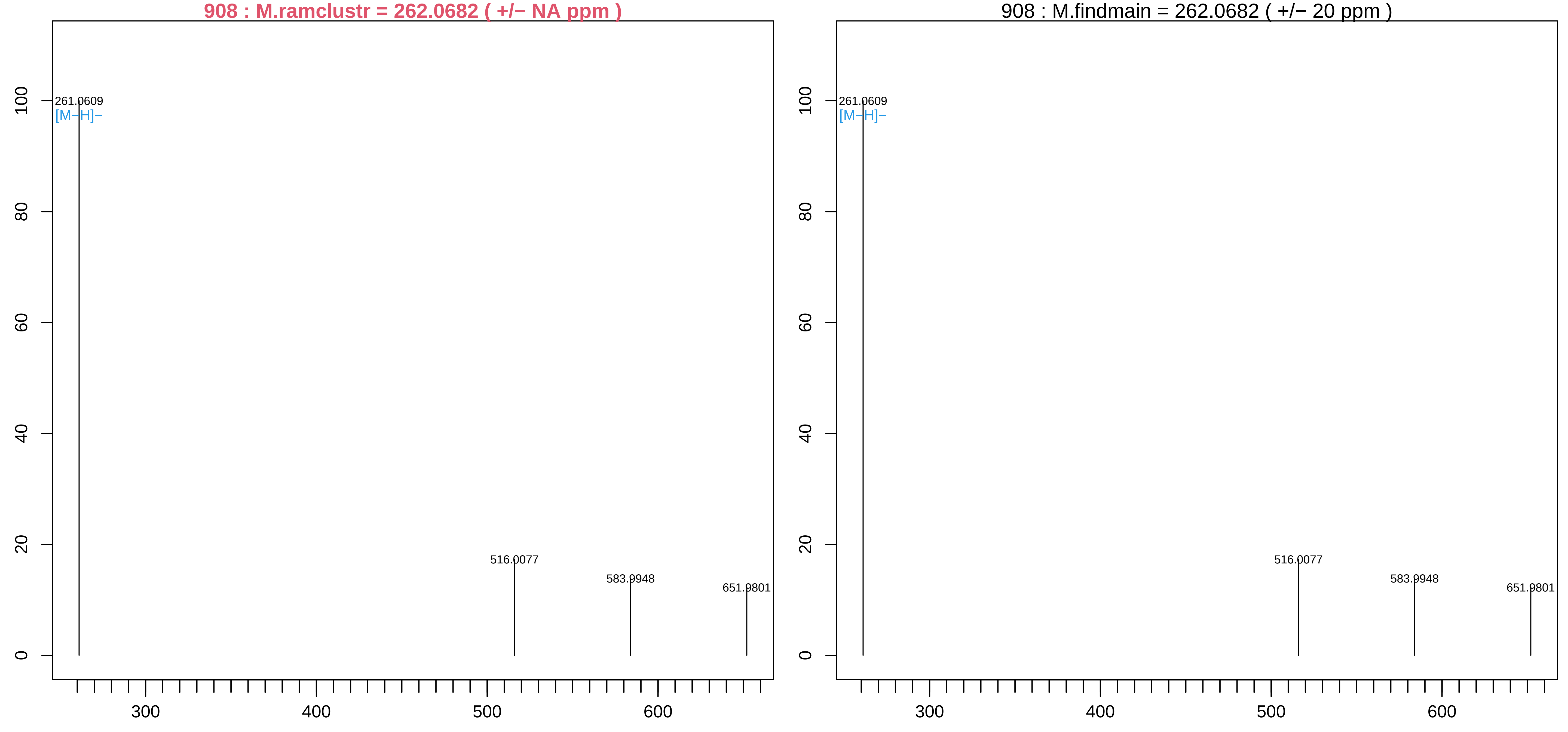

908 : M.ramclustr = 262.0682 (+/− NA ppm)
261.0609
[M−H]−
516.0077
583.9948
651.9801
0
20
40
60
80
100
300
400
500
600
908 : M.findmain = 262.0682 (+/− 20 ppm)
261.0609
[M−H]−
516.0077
583.9948
651.9801
0
20
40
60
80
100
300
400
500
600

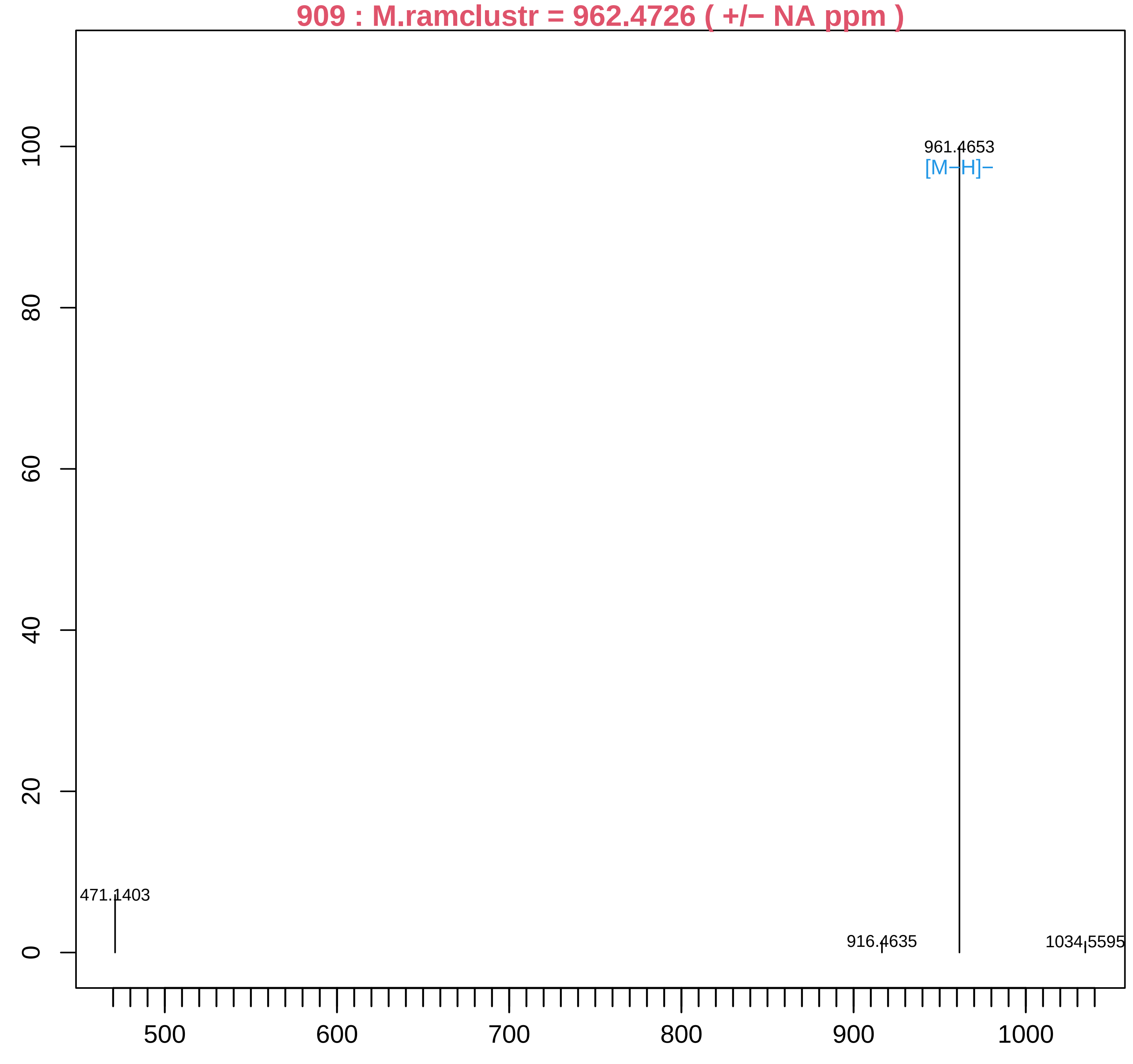
909 : M.ramclustr = 962.4726 (+/− NA ppm)
961.4653
[M−H]−
471.1403
916.4635
1034.5595
0
20
40
60
80
100
500
600
700
800
900
1000

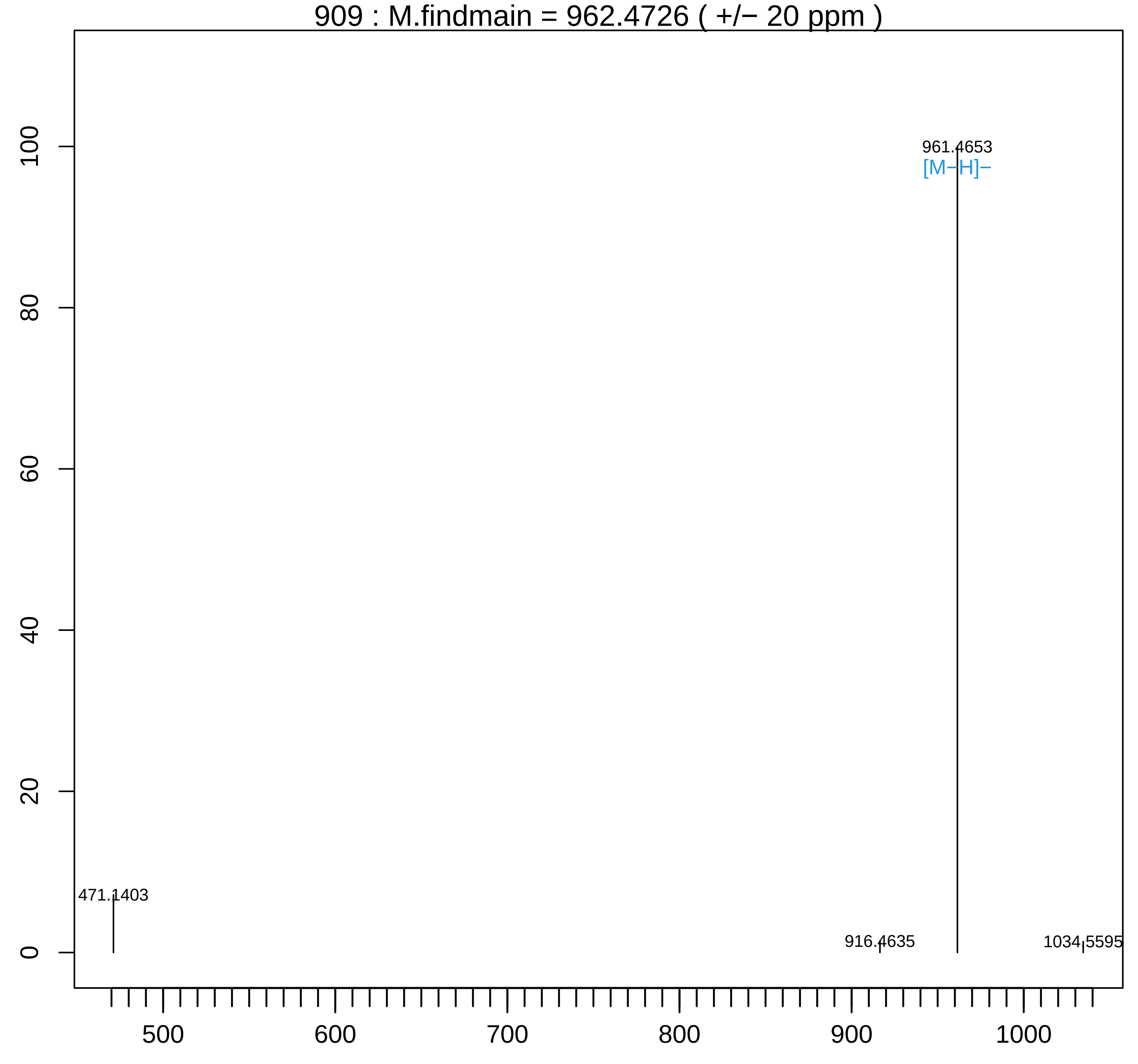
909 : M.findmain = 962.4726 (+/− 20 ppm)
961.4653
[M−H]−
471.1403
916.4635
1034.5595
0
20
40
60
80
100
500
600
700
800
900
1000

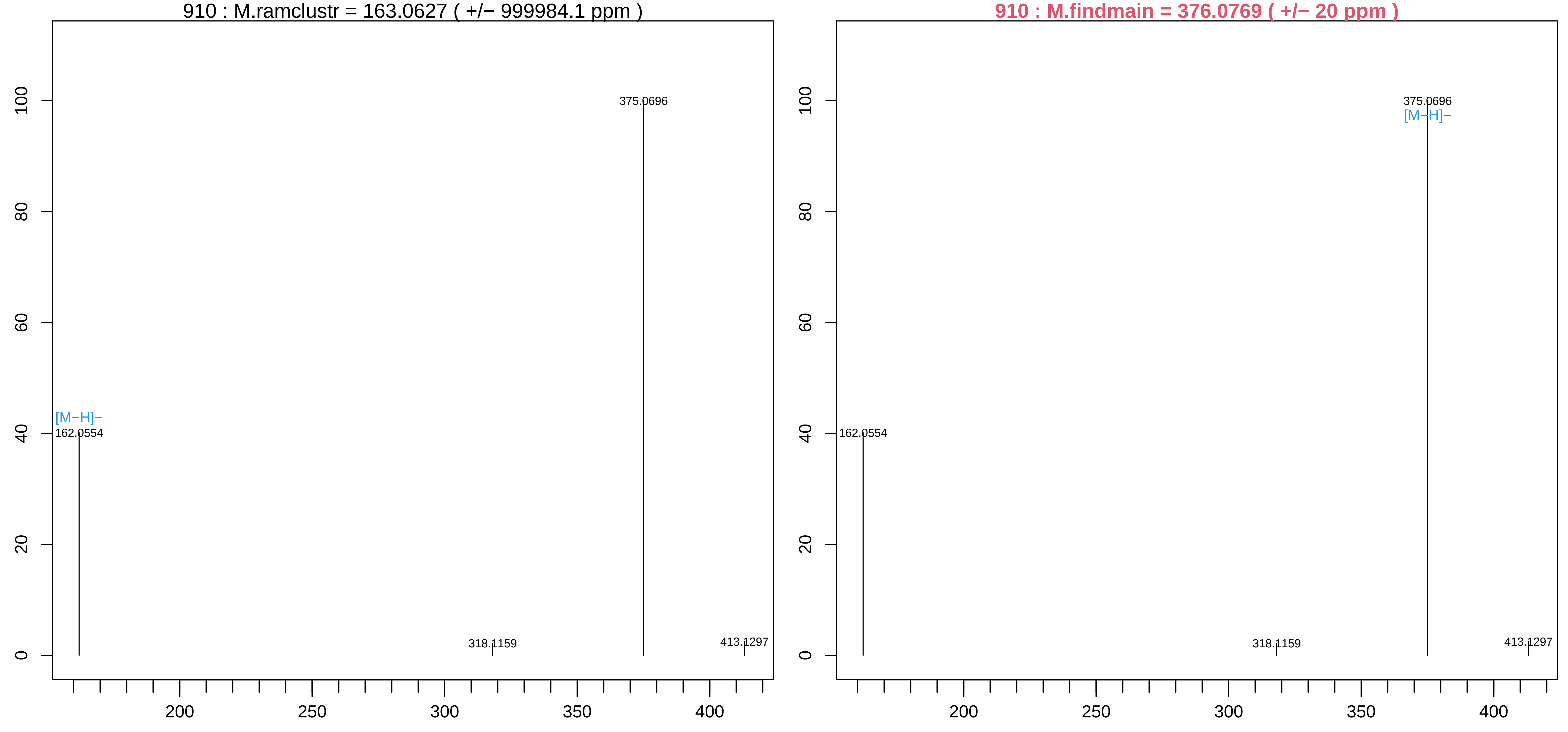
910 : M.ramclustr = 163.0627 (+/− 999984.1 ppm)
375.0696
[M−H]−
162.0554
318.1159
413.1297
0
20
40
60
80
100
200
250
300
350
400
910 : M.findmain = 376.0769 (+/− 20 ppm)
375.0696
[M−H]−
162.0554
318.1159
413.1297
0
20
40
60
80
100
200
250
300
350
400

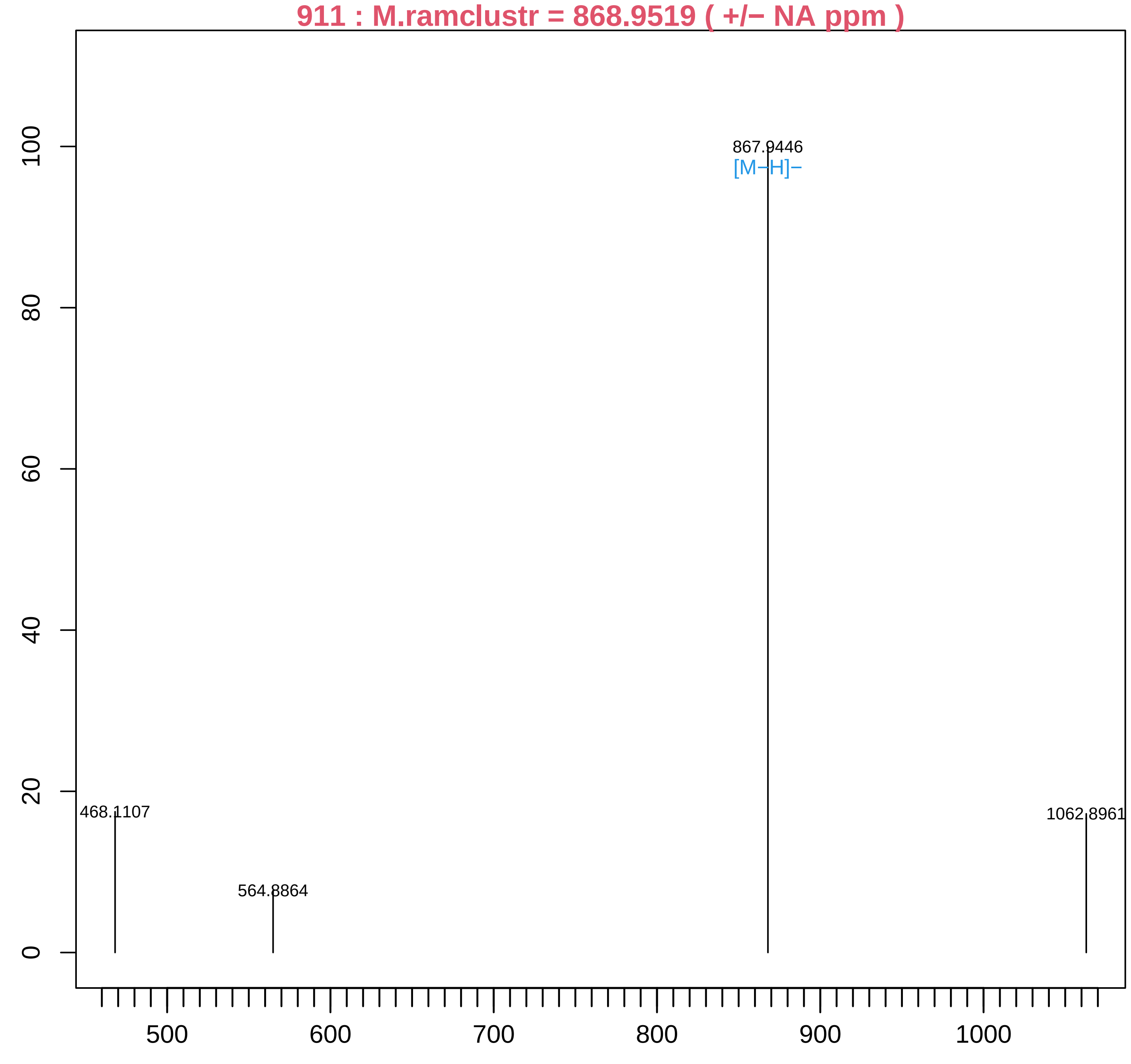
911 : M.ramclustr = 868.9519 (+/− NA ppm)
867.9446
[M−H]−
468.1107
564.8864
1062.8961
0
20
40
60
80
100
500
600
700
800
900
1000

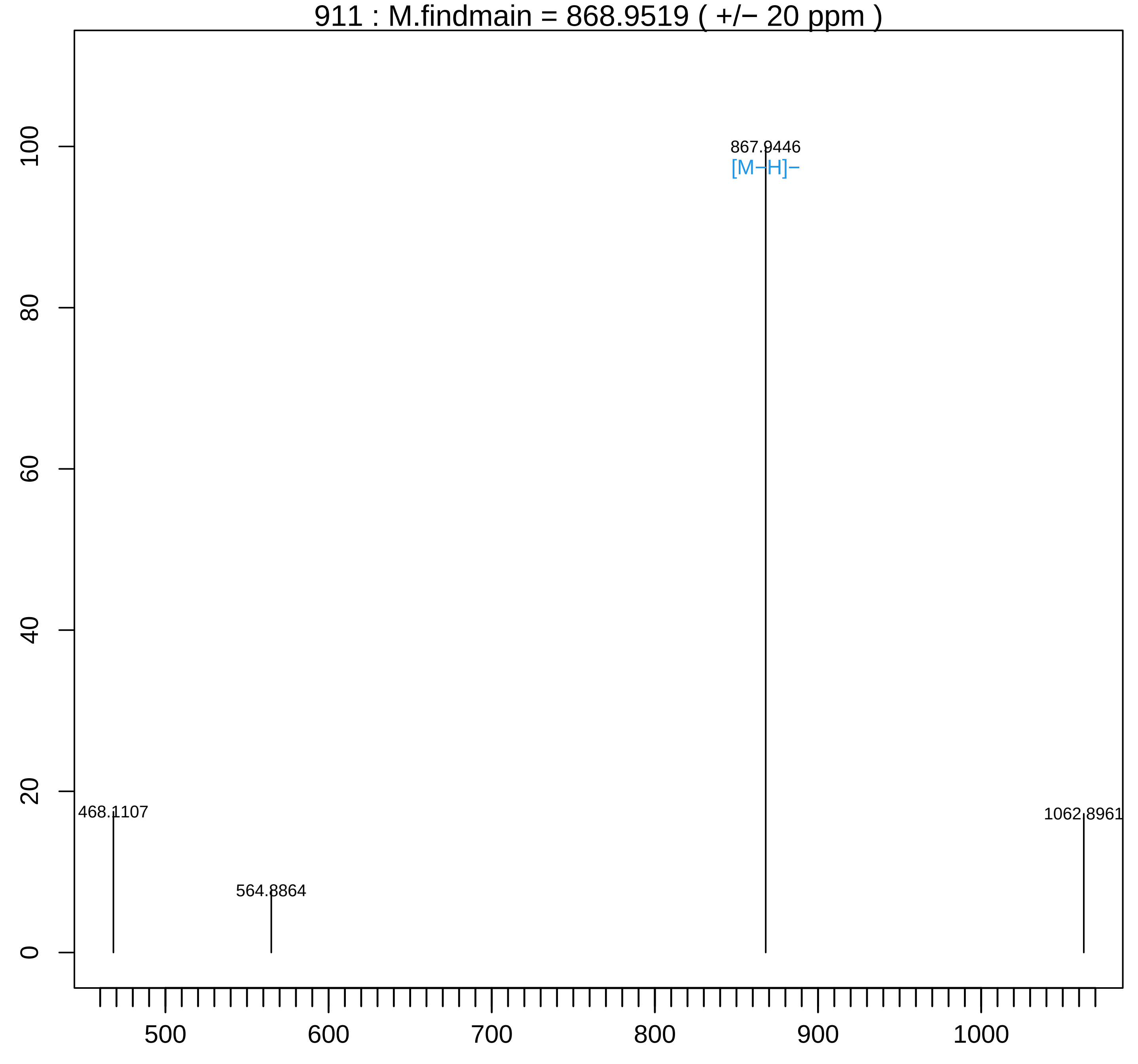
911 : M.findmain = 868.9519 (+/− 20 ppm)
867.9446
[M−H]−
468.1107
564.8864
1062.8961
0
20
40
60
80
100
500
600
700
800
900
1000

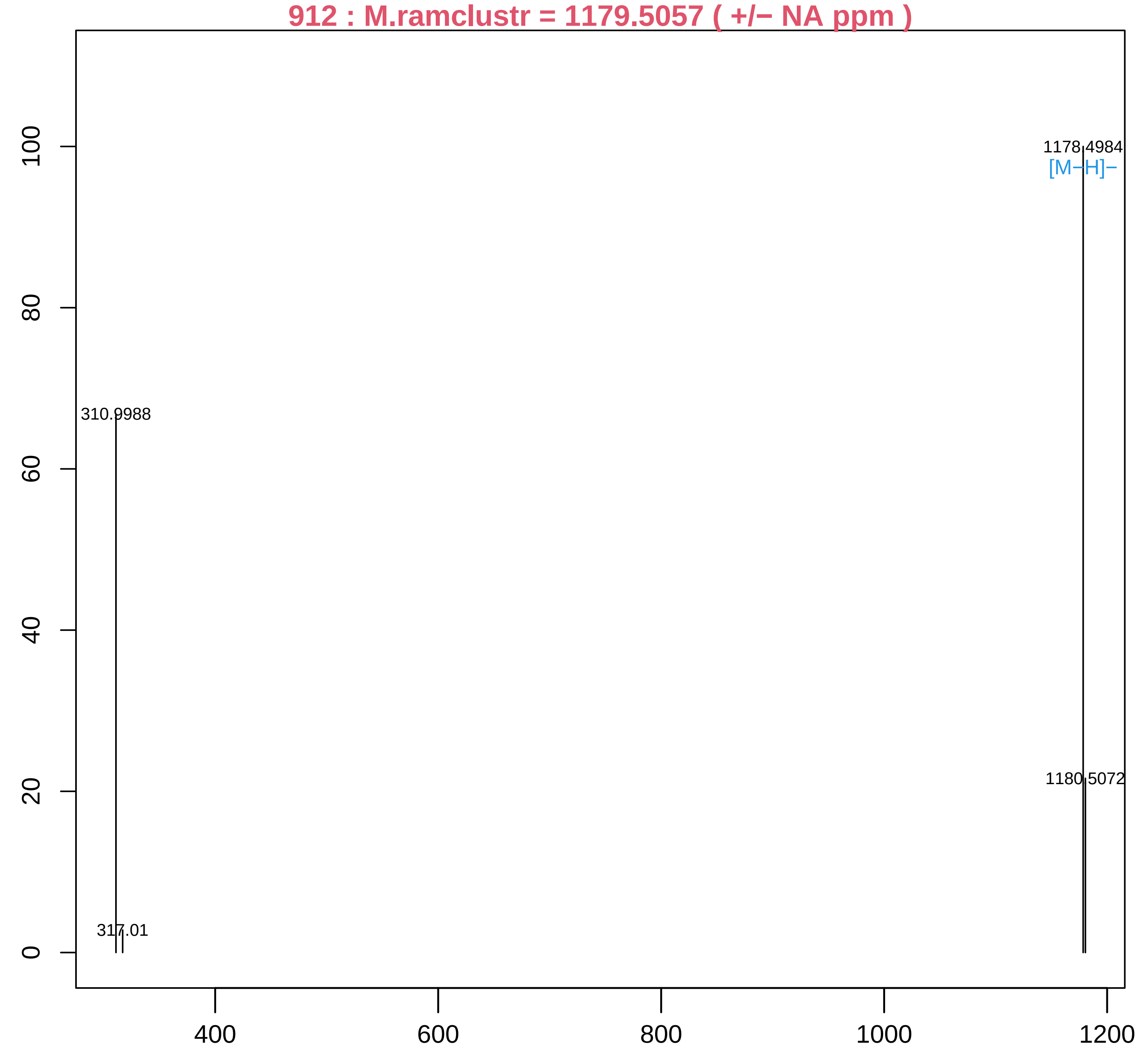

912 : M.ramclustr = 1179.5057 (+/− NA ppm)
1178.4984
[M−H]−
310.9988
1180.5072
317.01
0
20
40
60
80
100
400
600
800
1000
1200

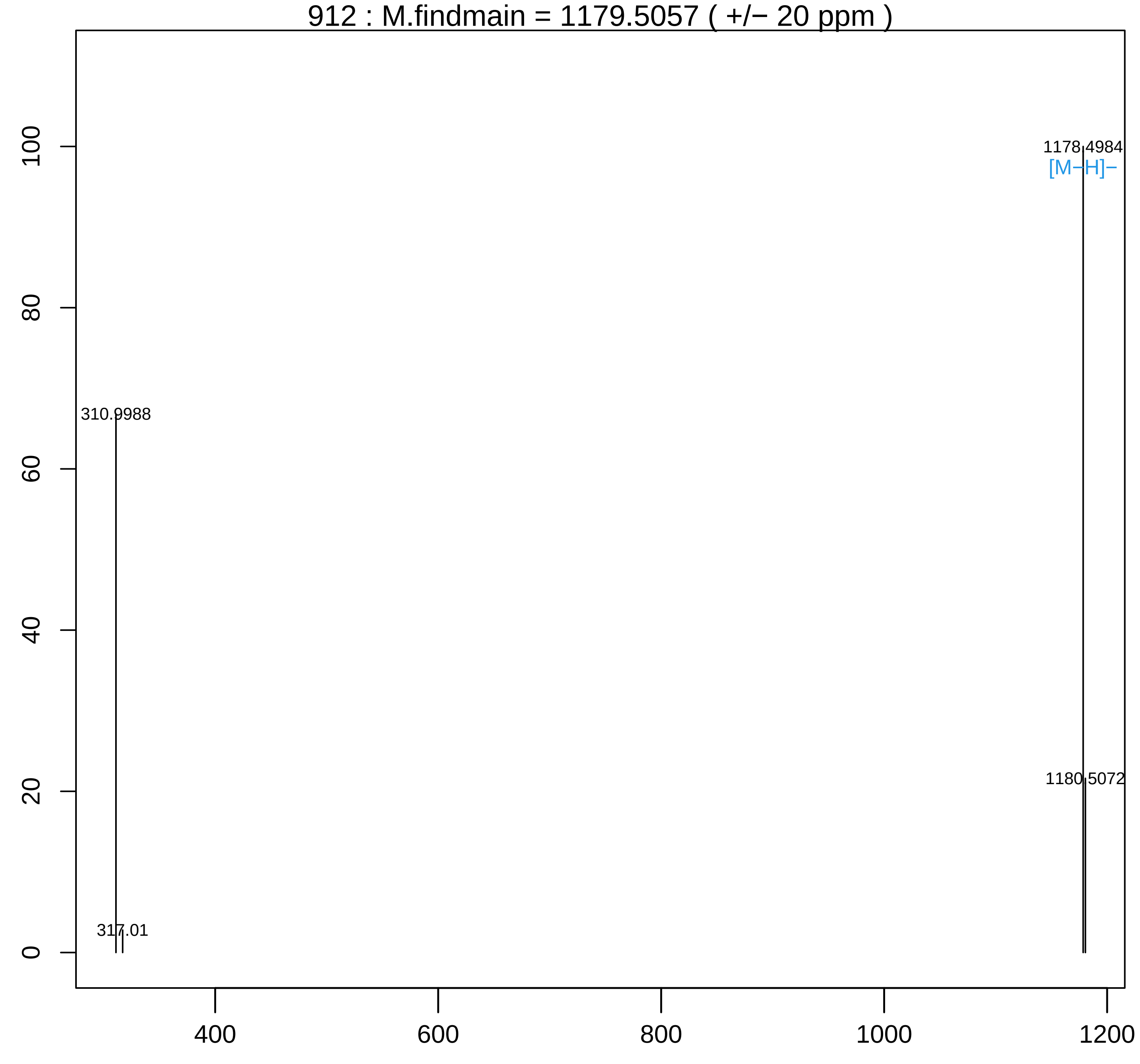

912 : M.findmain = 1179.5057 (+/− 20 ppm)
1178.4984
[M−H]−
310.9988
1180.5072
317.01
0
20
40
60
80
100
400
600
800
1000
1200

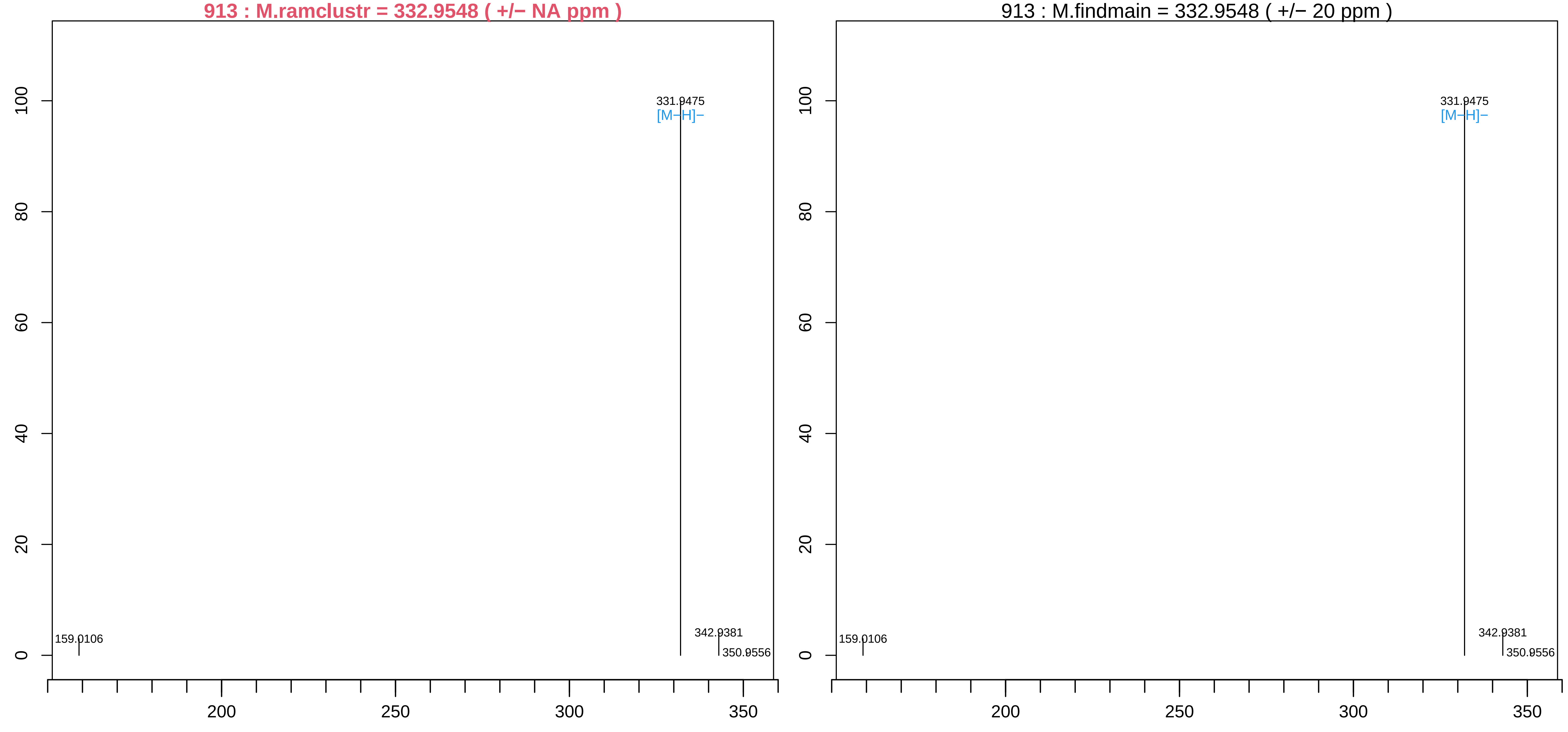

913 : M.ramclustr = 332.9548 (+/− NA ppm)
331.9475
[M−H]−
159.0106
342.9381
350.9556
913 : M.findmain = 332.9548 (+/− 20 ppm)
331.9475
[M−H]−
159.0106
342.9381
350.9556
0
20
40
60
80
100
200
250
300
350

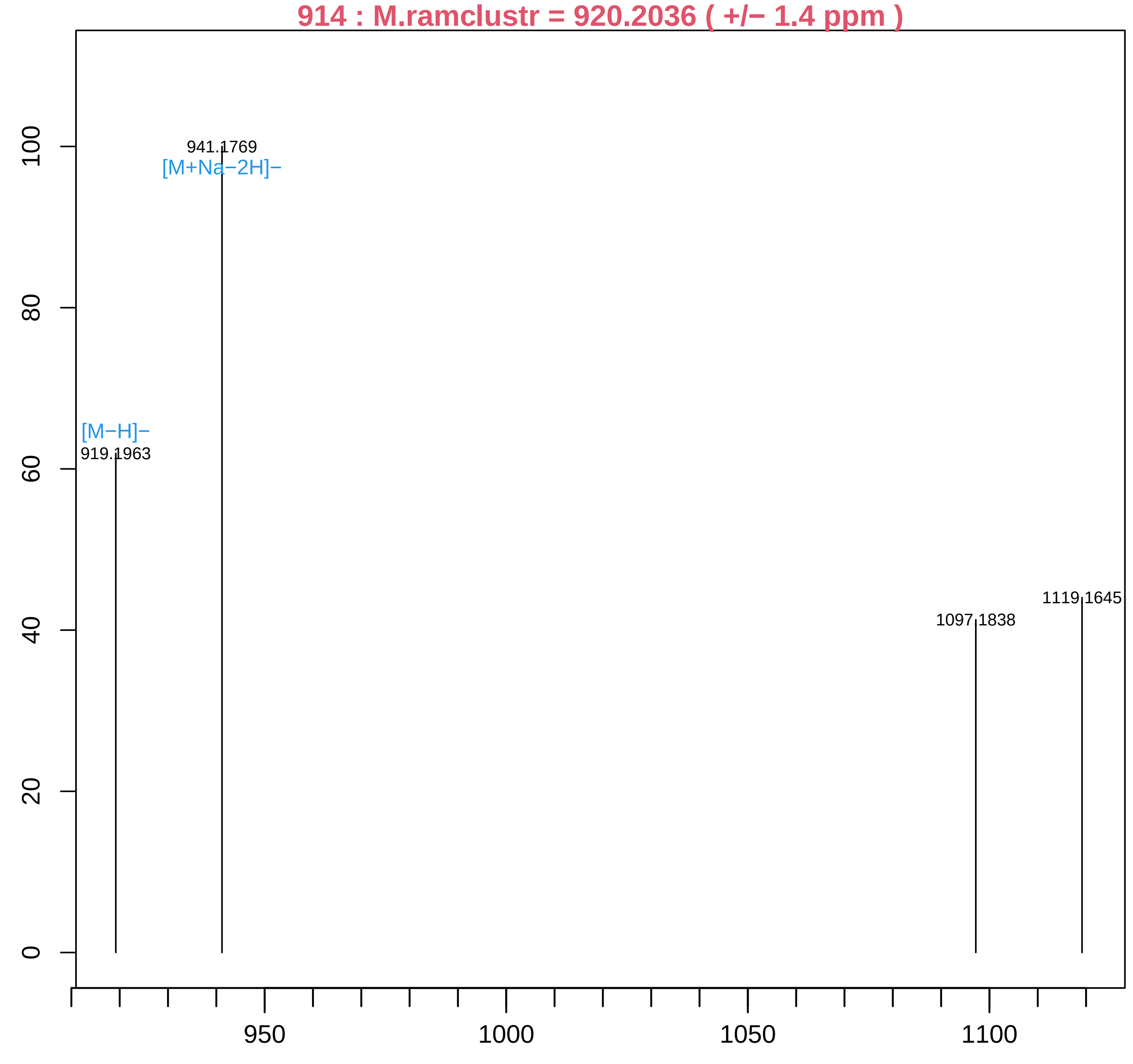
914 : M.ramclustr = 920.2036 (+/− 1.4 ppm)
941.1769
[M+Na−2H]−
[M−H]−
919.1963
1097.1838
1119.1645
0
20
40
60
80
100
950
1000
1050
1100

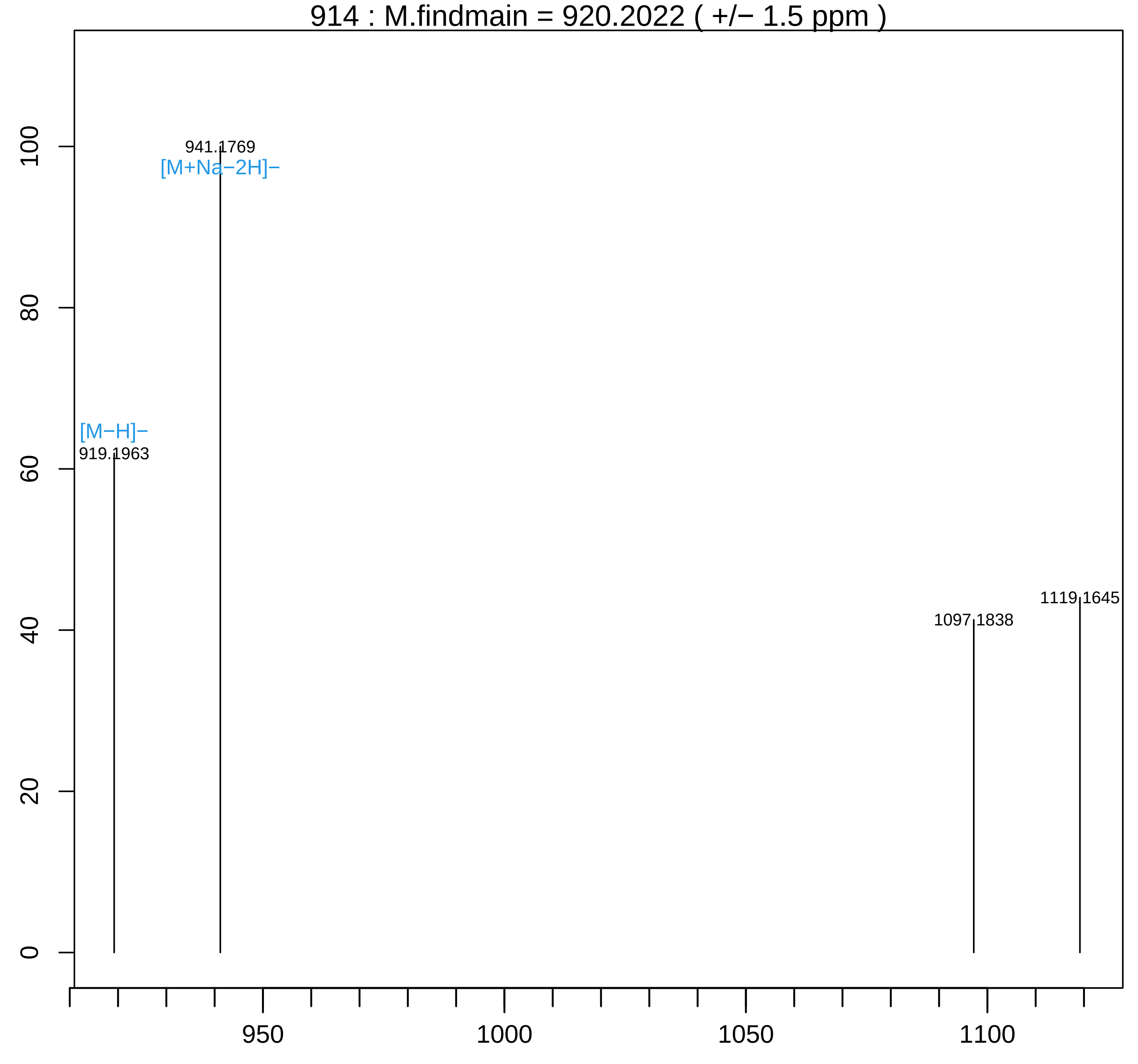
914 : M.findmain = 920.2022 (+/− 1.5 ppm)
941.1769
[M+Na−2H]−
[M−H]−
919.1963
1097.1838
1119.1645
0
20
40
60
80
100
950
1000
1050
1100

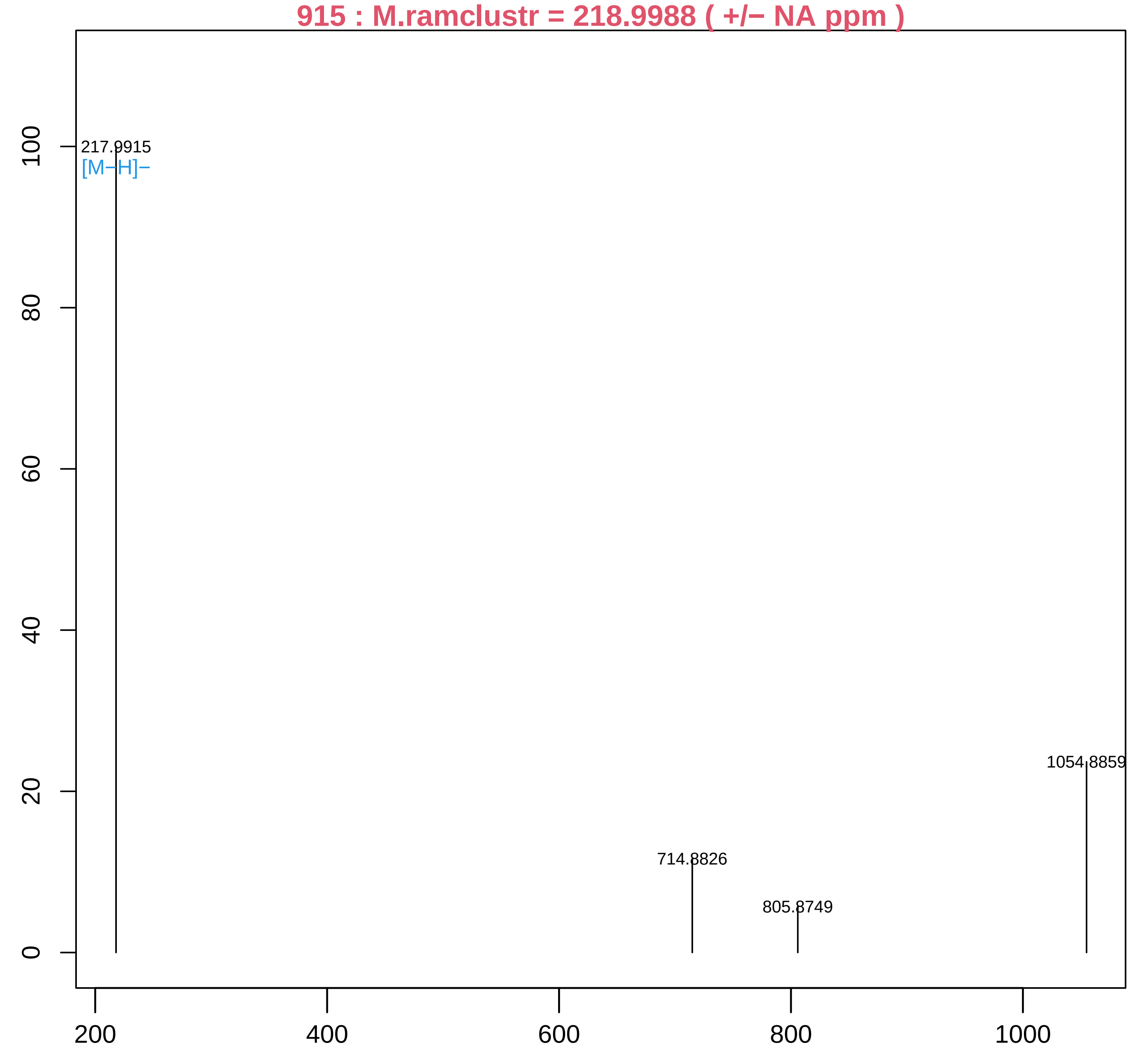

915 : M.ramclustr = 218.9988 (+/− NA ppm)
217.9915
[M−H]−
714.8826
805.8749
1054.8859
0
20
40
60
80
100
200
400
600
800
1000

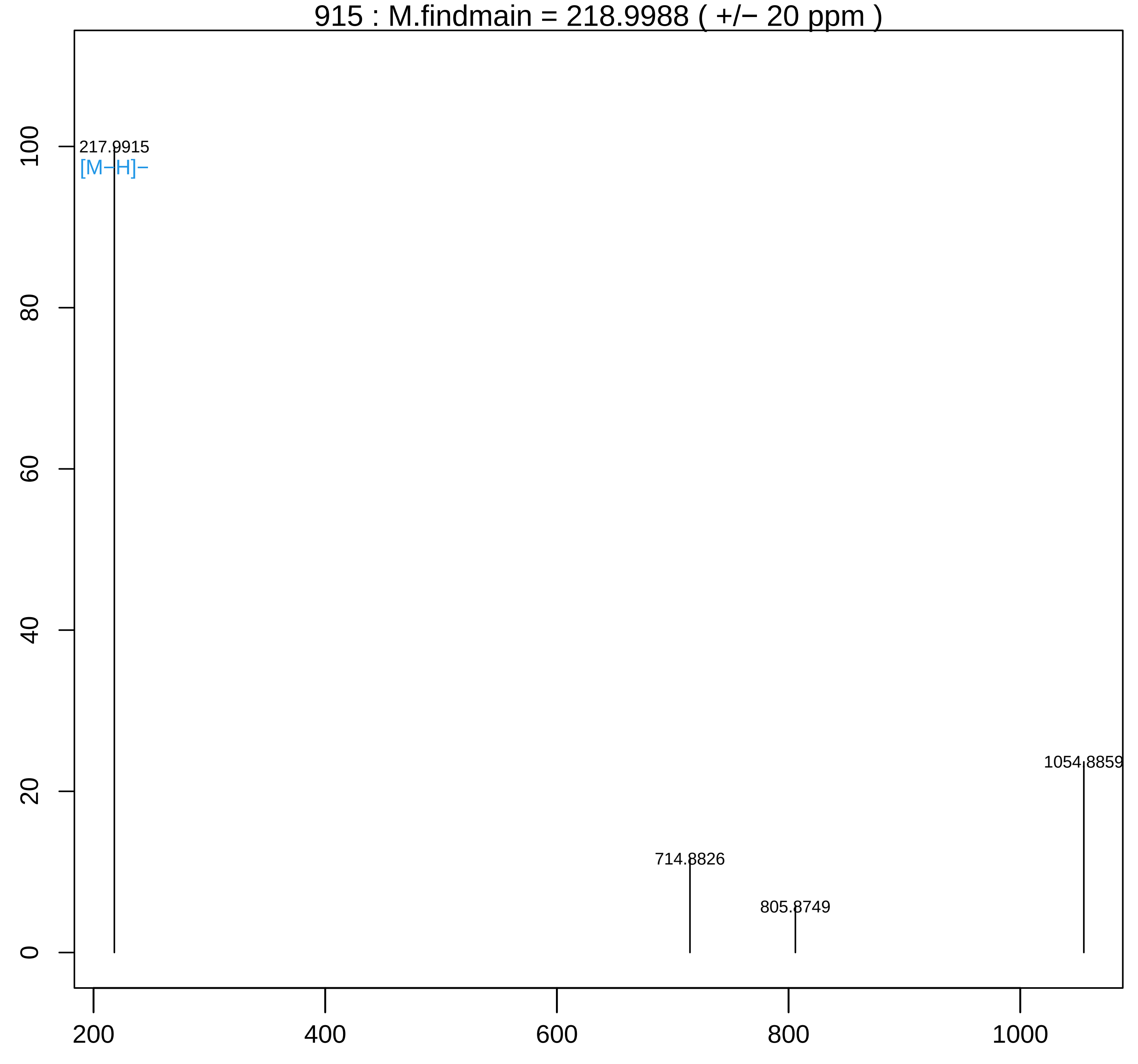

915 : M.findmain = 218.9988 (+/− 20 ppm)
217.9915
[M−H]−
714.8826
805.8749
1054.8859
0
20
40
60
80
100
200
400
600
800
1000

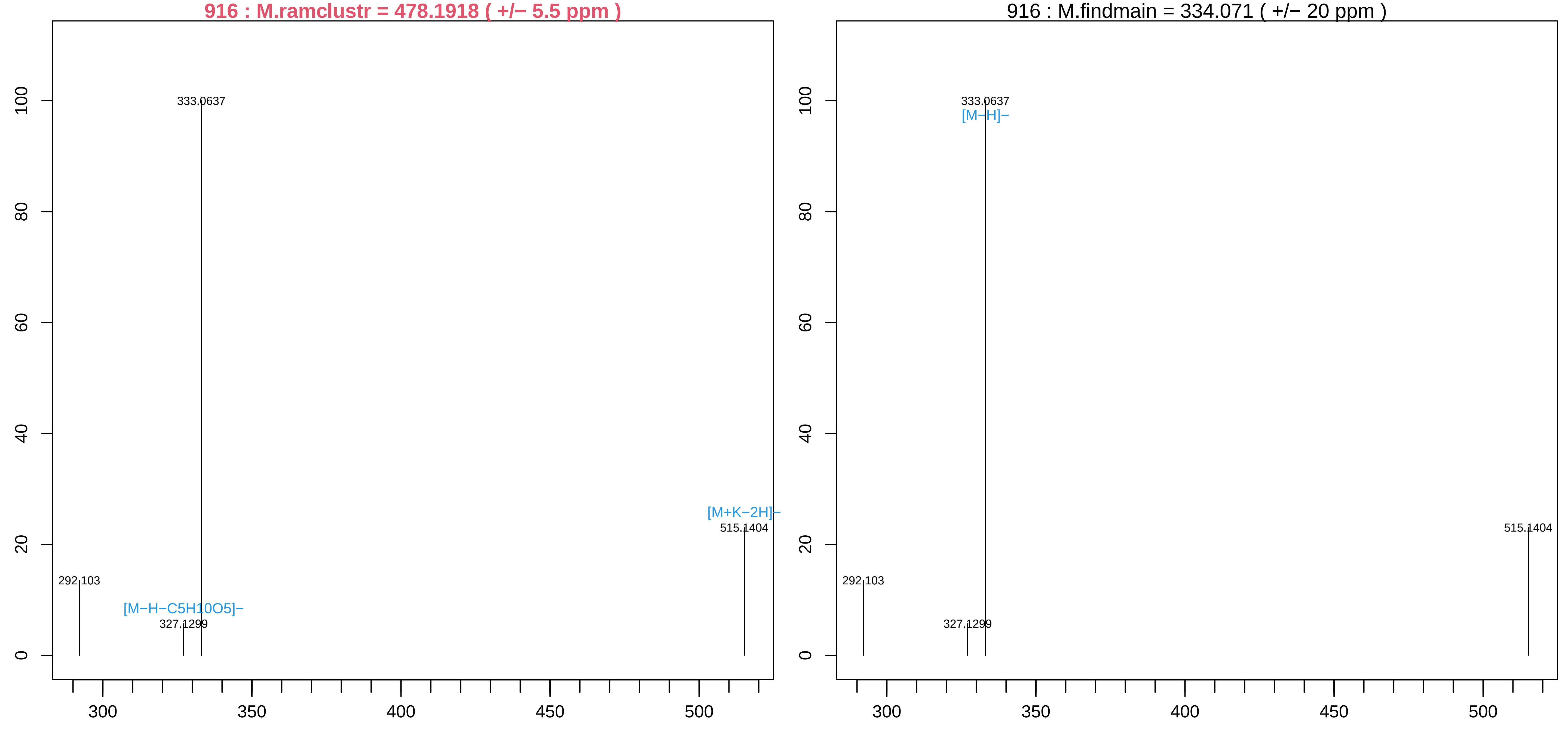

916 : M.ramclustr = 478.1918 (+/− 5.5 ppm)
333.0637
[M+K−2H]−
515.1404
292.103
[M−H−C5H10O5]−
327.1299
916 : M.findmain = 334.071 (+/− 20 ppm)
333.0637
[M−H]−
515.1404
292.103
327.1299
0
20
40
60
80
100
300
350
400
450
500

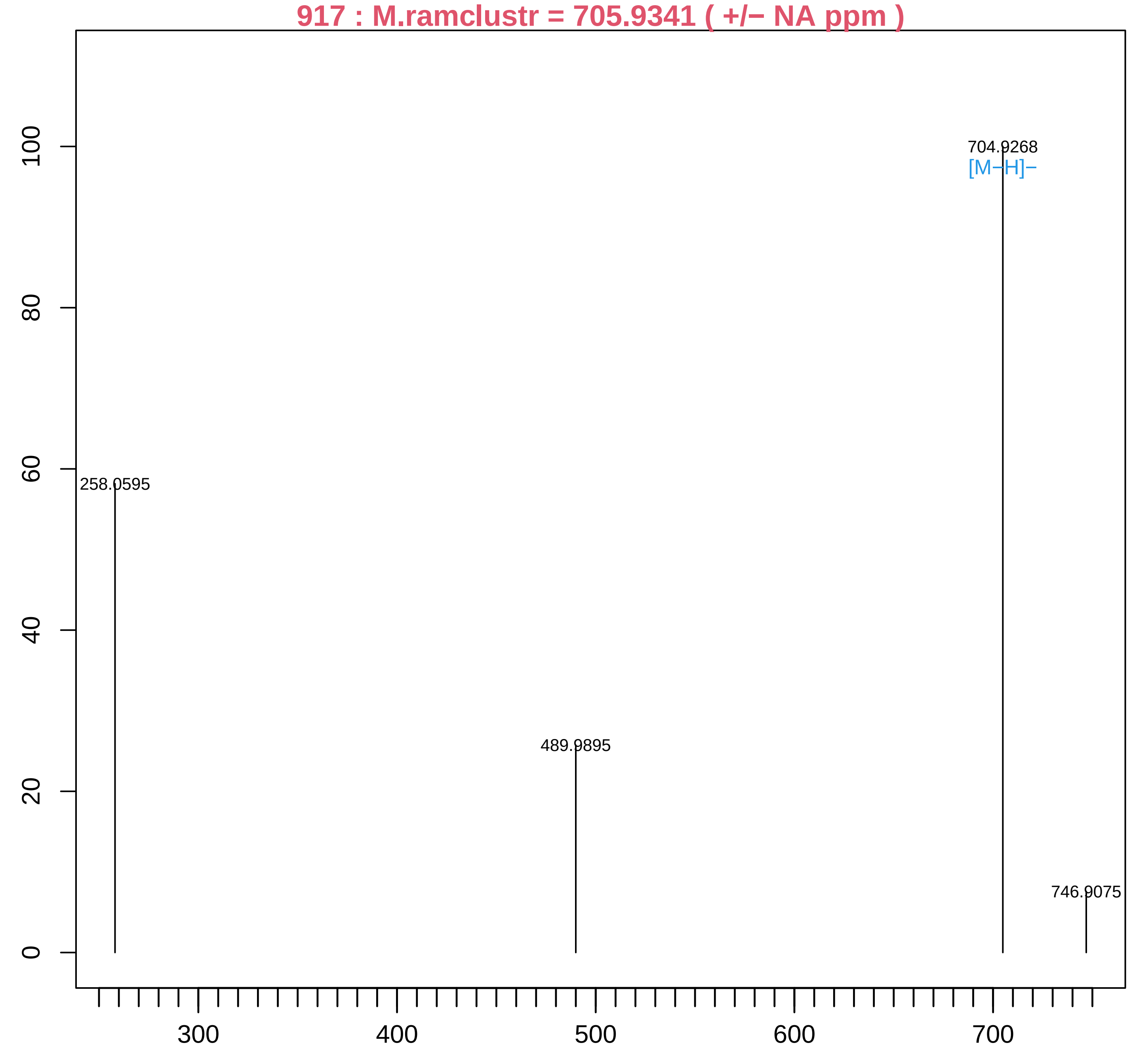
917 : M.ramclustr = 705.9341 (+/− NA ppm)
704.9268
[M−H]−
258.0595
489.9895
746.9075
0
20
40
60
80
100
300
400
500
600
700

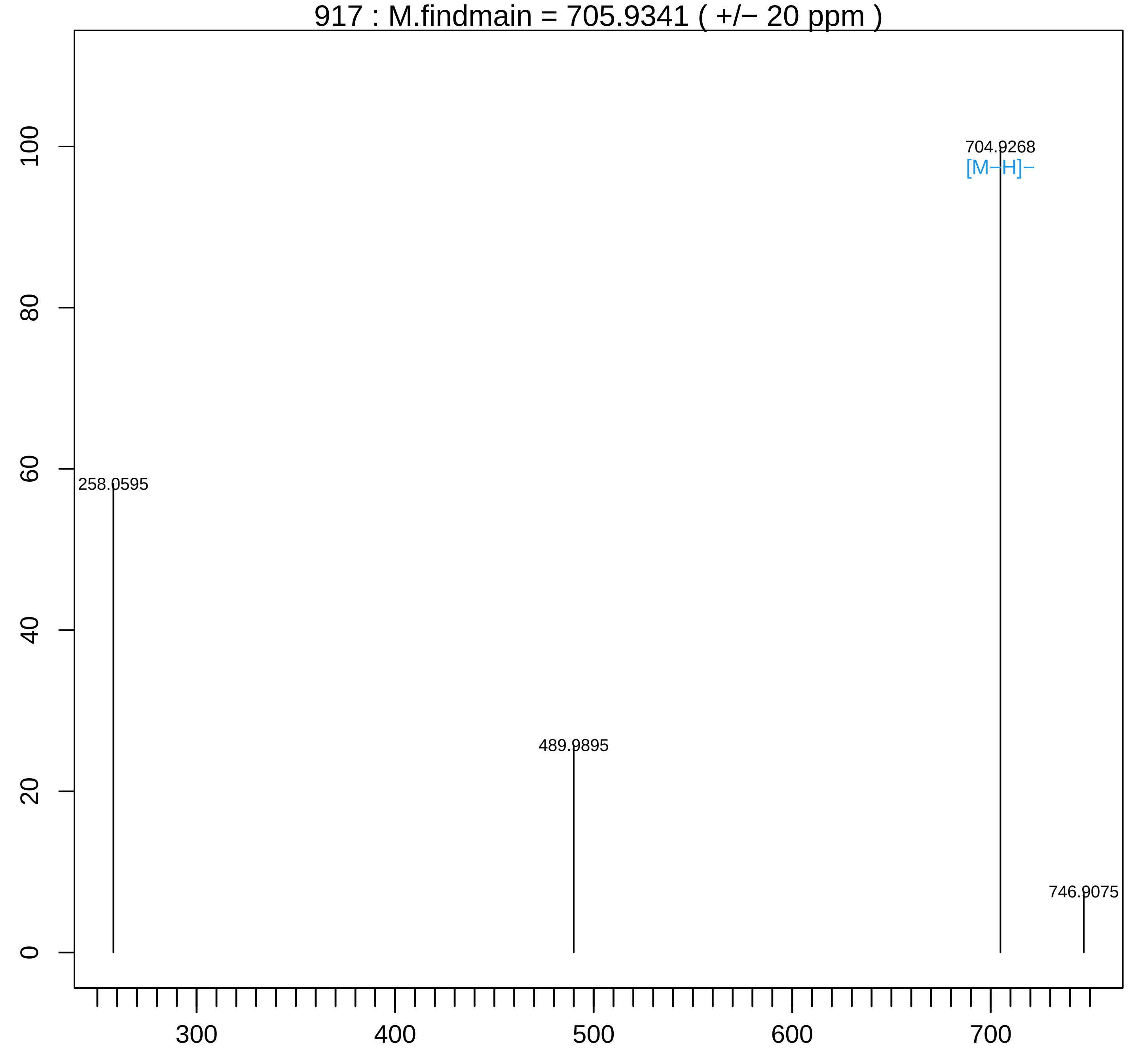
917 : M.findmain = 705.9341 (+/− 20 ppm)
704.9268
[M−H]−
258.0595
489.9895
746.9075
0
20
40
60
80
100
300
400
500
600
700

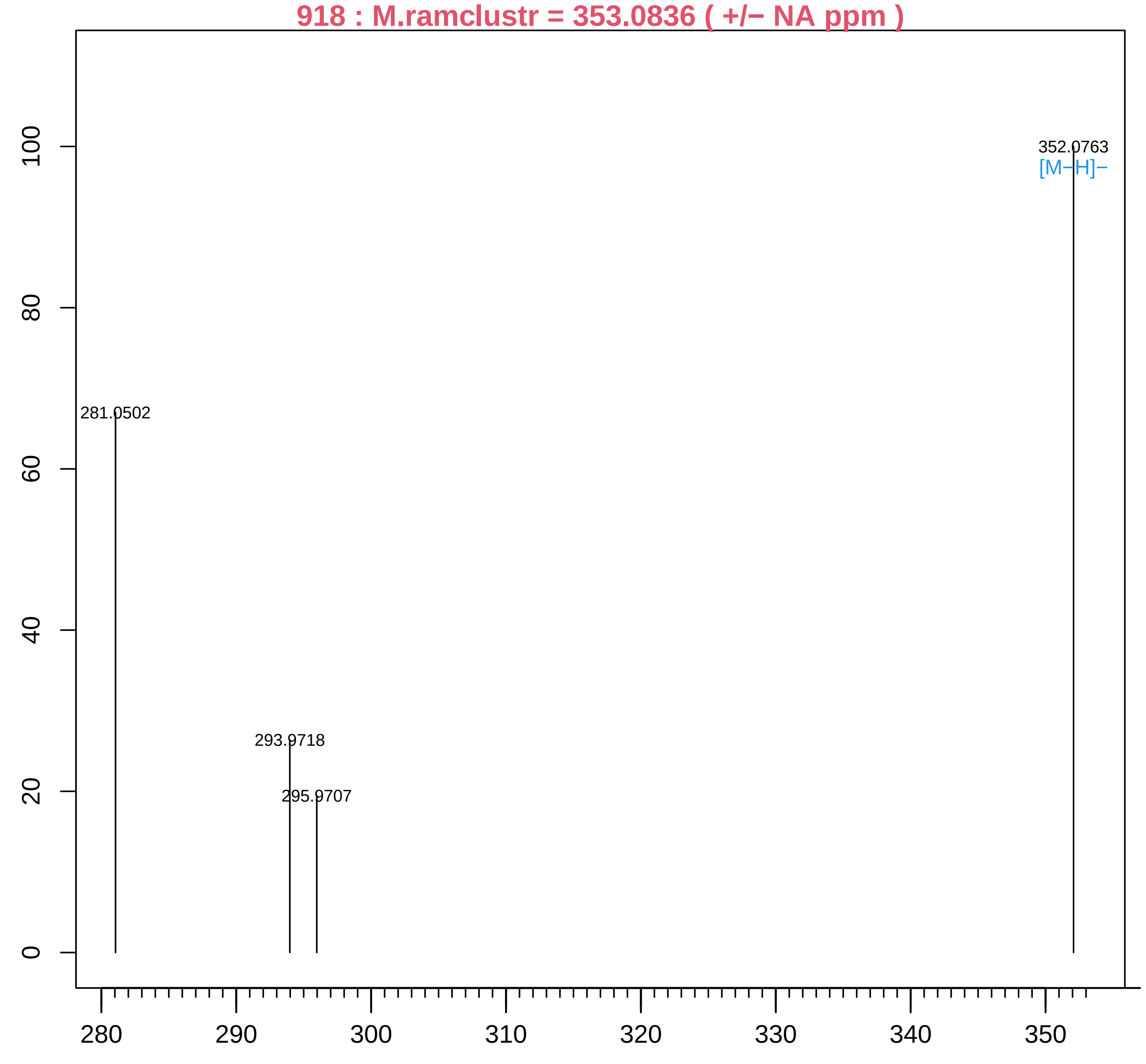
918 : M.ramclustr = 353.0836 (+/− NA ppm)
281.0502
293.9718
295.9707
352.0763
[M−H]−
0
20
40
60
80
100
280
290
300
310
320
330
340
350
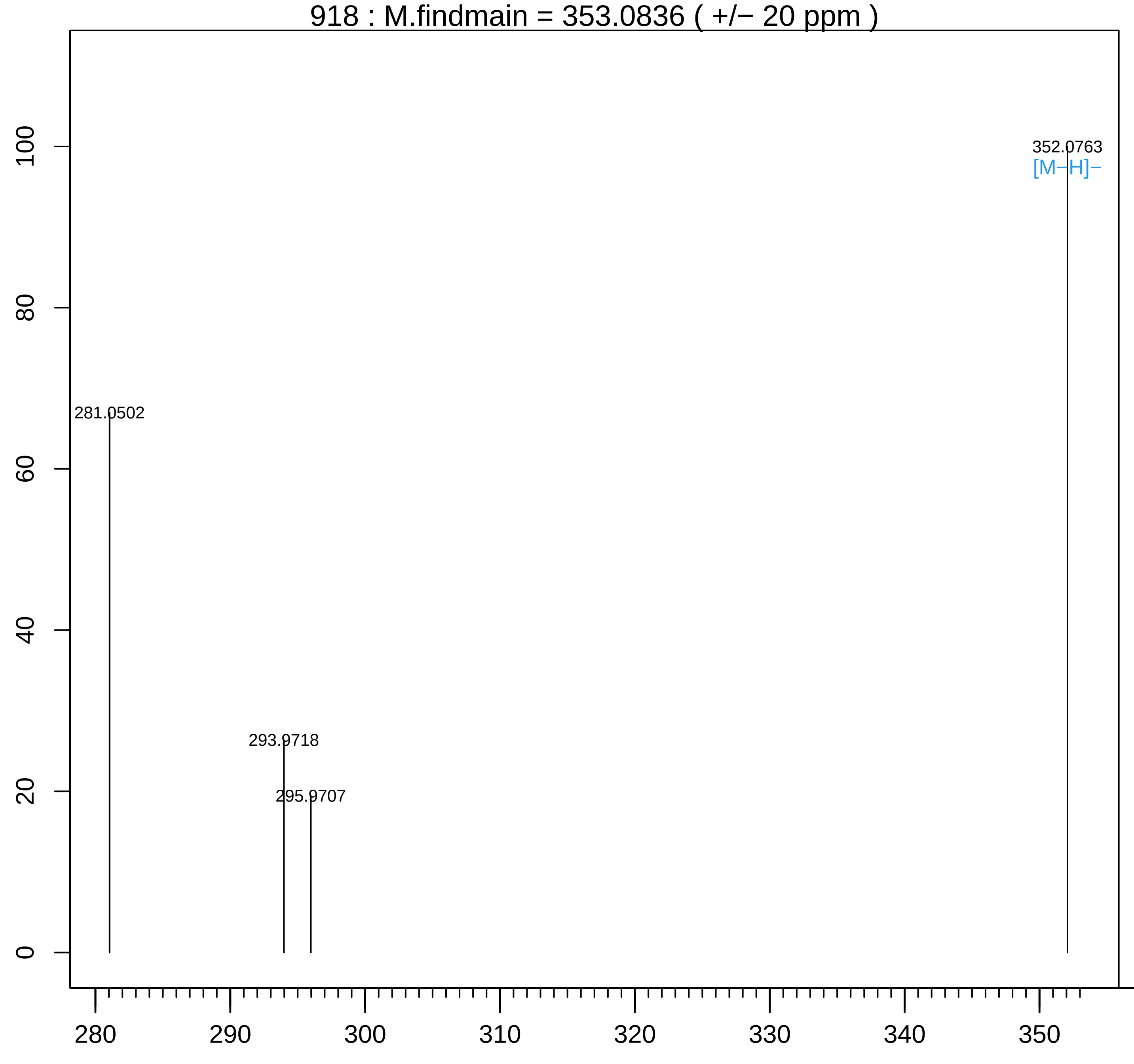
918 : M.findmain = 353.0836 (+/− 20 ppm)
281.0502
293.9718
295.9707
352.0763
[M−H]−
0
20
40
60
80
100
280
290
300
310
320
330
340
350

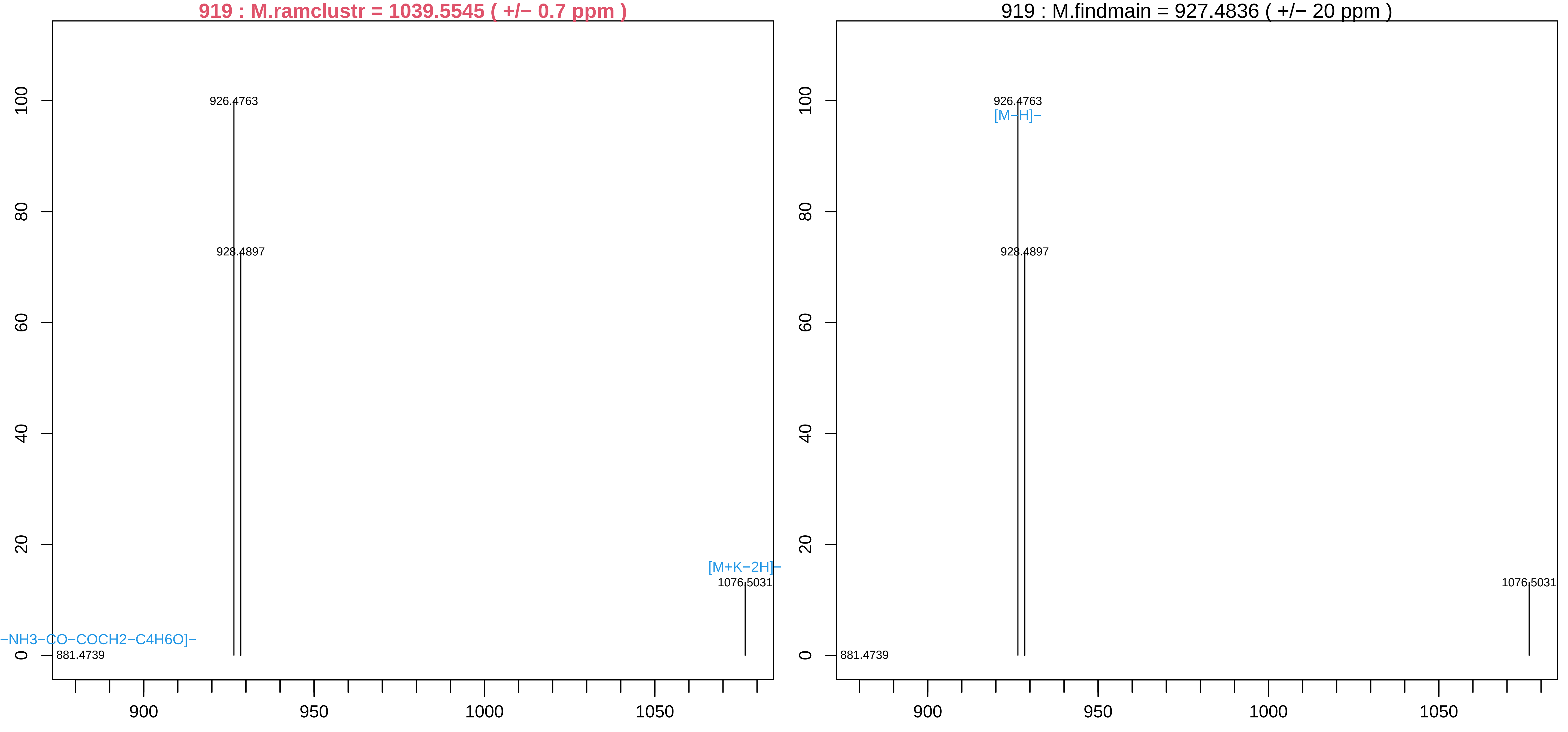
919 : M.ramclustr = 1039.5545 (+/− 0.7 ppm)
926.4763
928.4897
[M+K−2H]−
1076.5031
−NH3−CO−COCH2−C4H6O]−
881.4739
919 : M.findmain = 927.4836 (+/− 20 ppm)
926.4763
[M−H]−
928.4897
1076.5031
881.4739
0
20
40
60
80
100
900
950
1000
1050

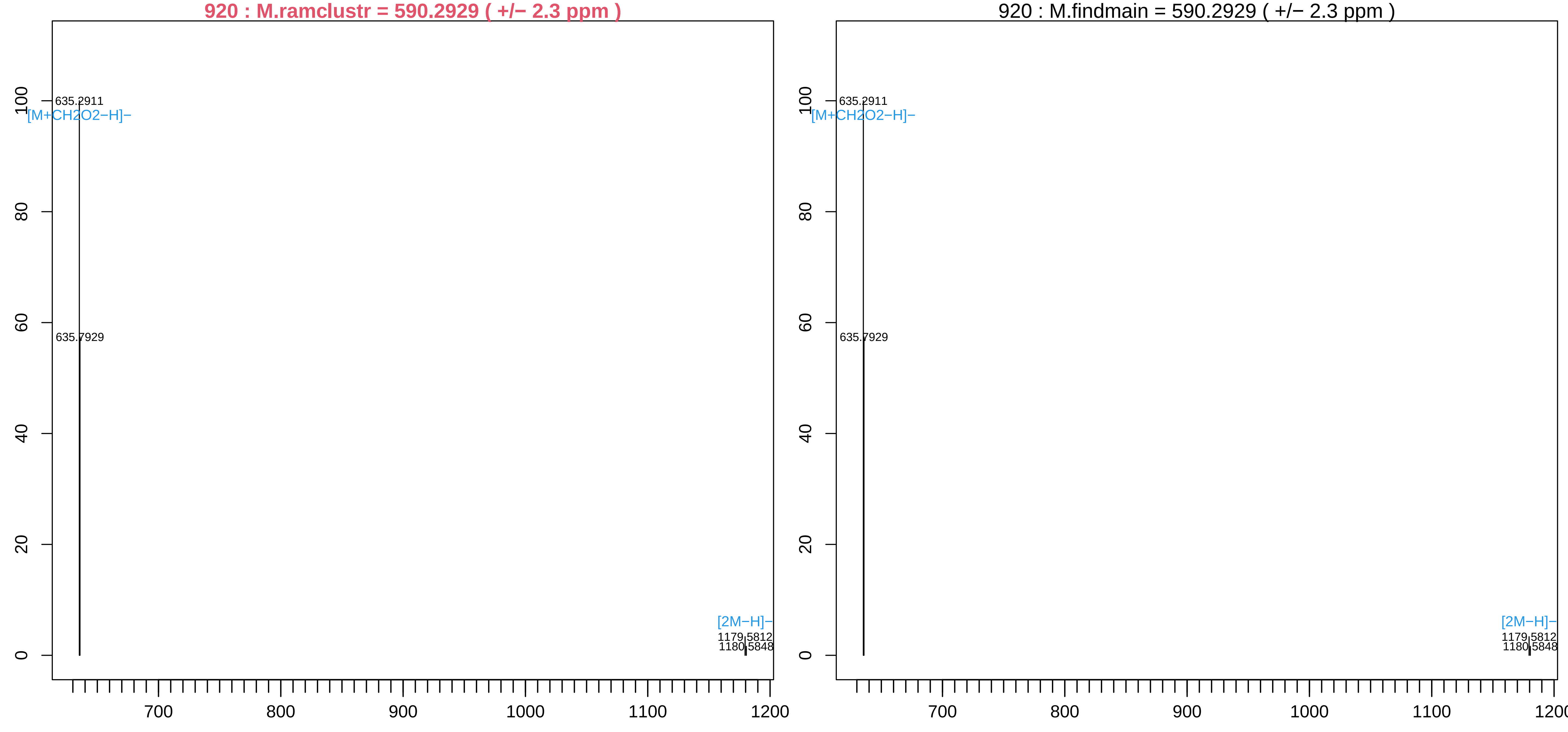
920 : M.ramclustr = 590.2929 (+/− 2.3 ppm)
635.2911
[M+CH2O2−H]−
635.7929
[2M−H]−
1179.5812
1180.5848
0
20
40
60
80
100
700
800
900
1000
1100
1200
920 : M.findmain = 590.2929 (+/− 2.3 ppm)
635.2911
[M+CH2O2−H]−
635.7929
[2M−H]−
1179.5812
1180.5848

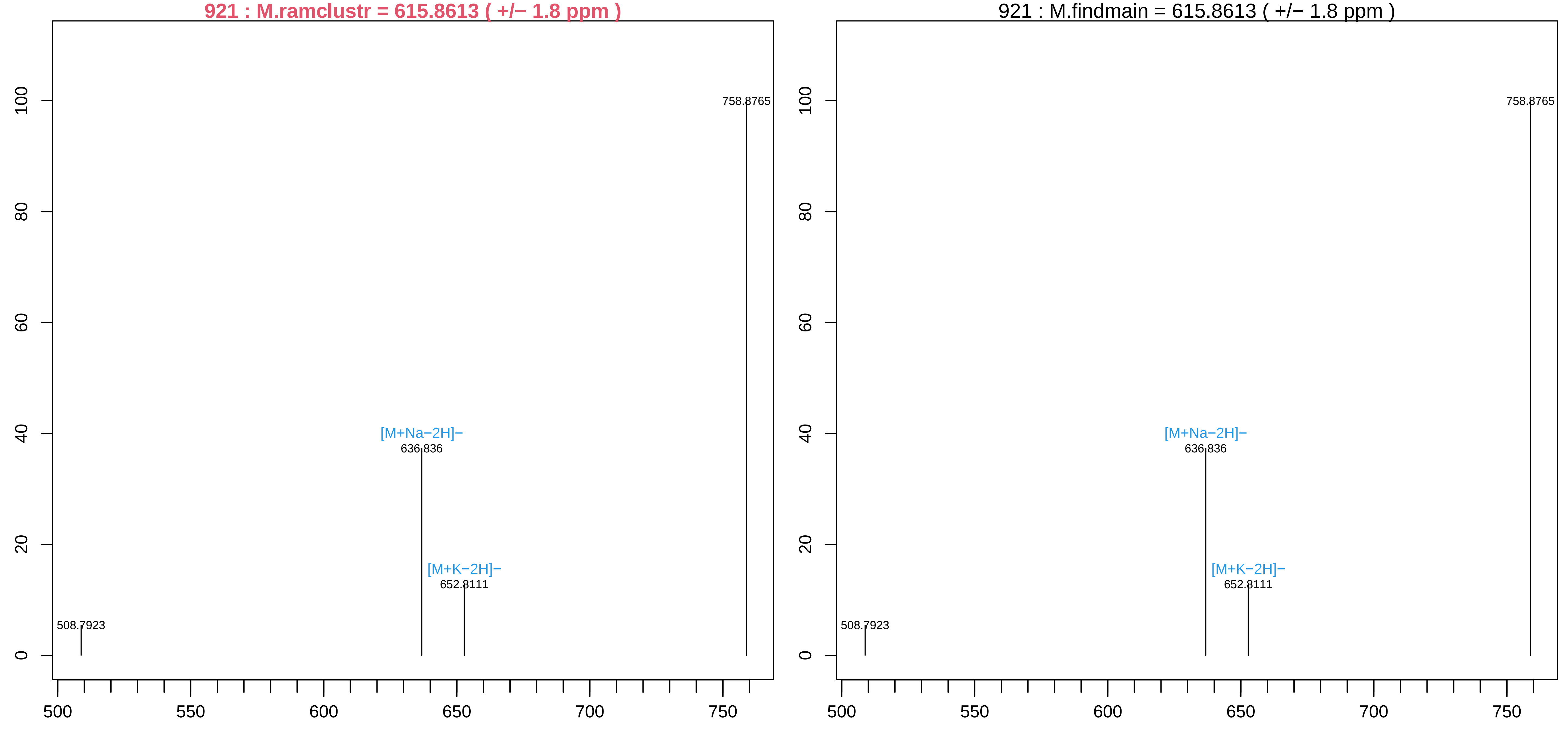

921 : M.ramclustr = 615.8613 (+/− 1.8 ppm)
758.8765
[M+Na−2H]−
636.836
[M+K−2H]−
652.8111
508.7923
921 : M.findmain = 615.8613 (+/− 1.8 ppm)
758.8765
[M+Na−2H]−
636.836
[M+K−2H]−
652.8111
508.7923

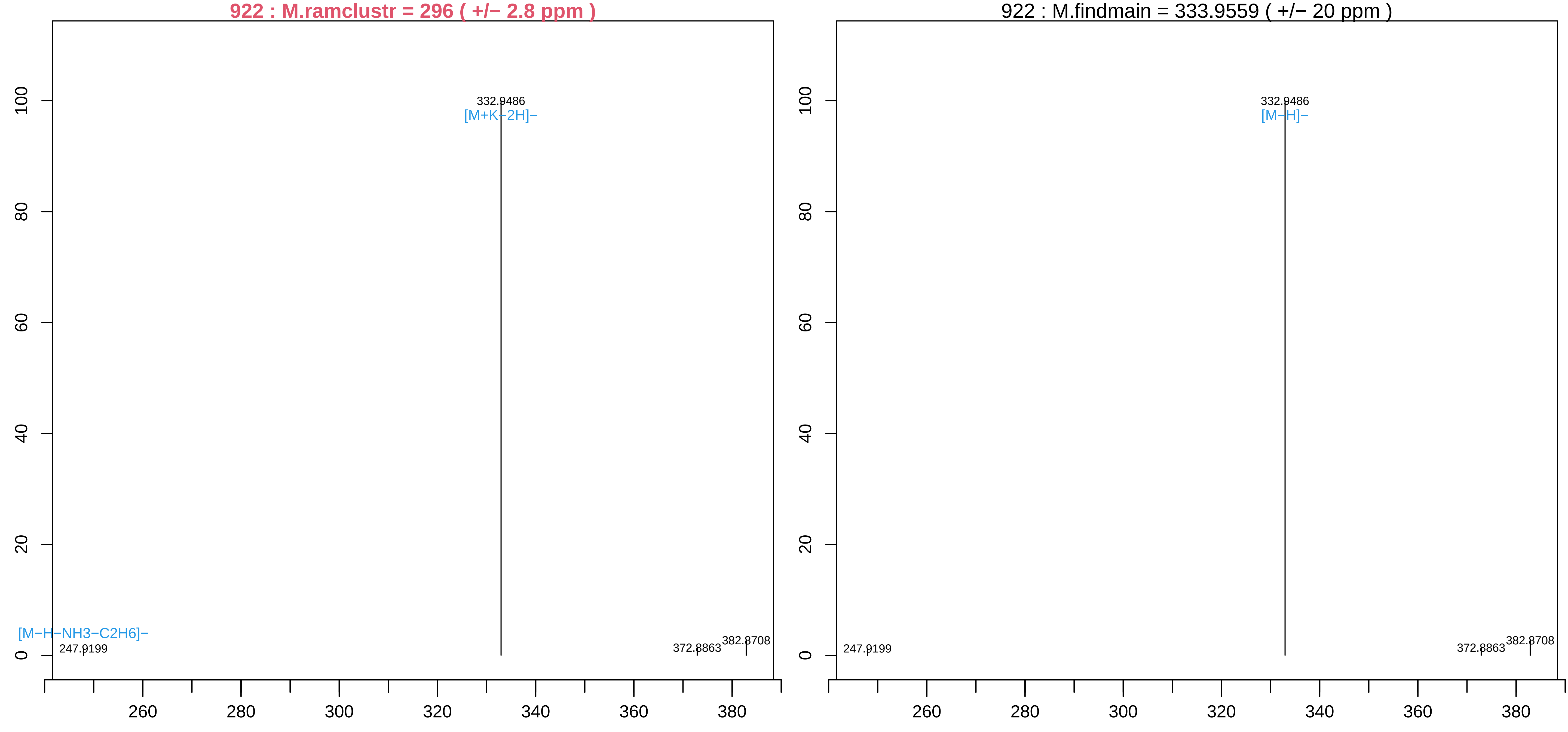

922 : M.ramclustr = 296 (+/− 2.8 ppm)
332.9486
[M+K−2H]−
[M−H−NH3−C2H6]−
247.9199
372.8863
382.8708
922 : M.findmain = 333.9559 (+/− 20 ppm)
332.9486
[M−H]−
247.9199
372.8863
382.8708
0
20
40
60
80
100
260
280
300
320
340
360
380

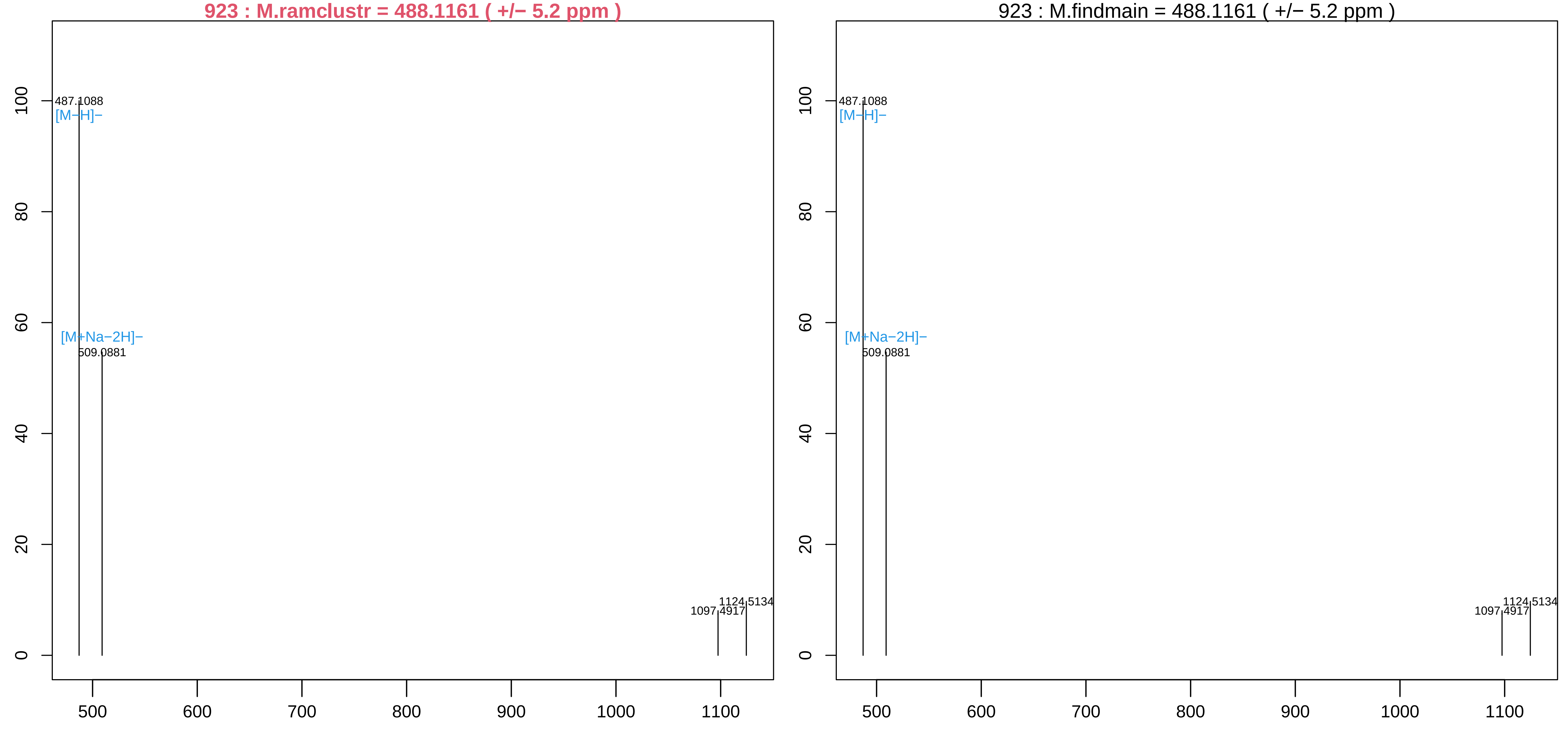

923 : M.ramclustr = 488.1161 (+/− 5.2 ppm)
487.1088
[M−H]−
[M+Na−2H]−
509.0881
1124.5134
1097.4917
0
20
40
60
80
100
500
600
700
800
900
1000
1100
923 : M.findmain = 488.1161 (+/− 5.2 ppm)
487.1088
[M−H]−
[M+Na−2H]−
509.0881
1124.5134
1097.4917
0
20
40
60
80
100
500
600
700
800
900
1000
1100

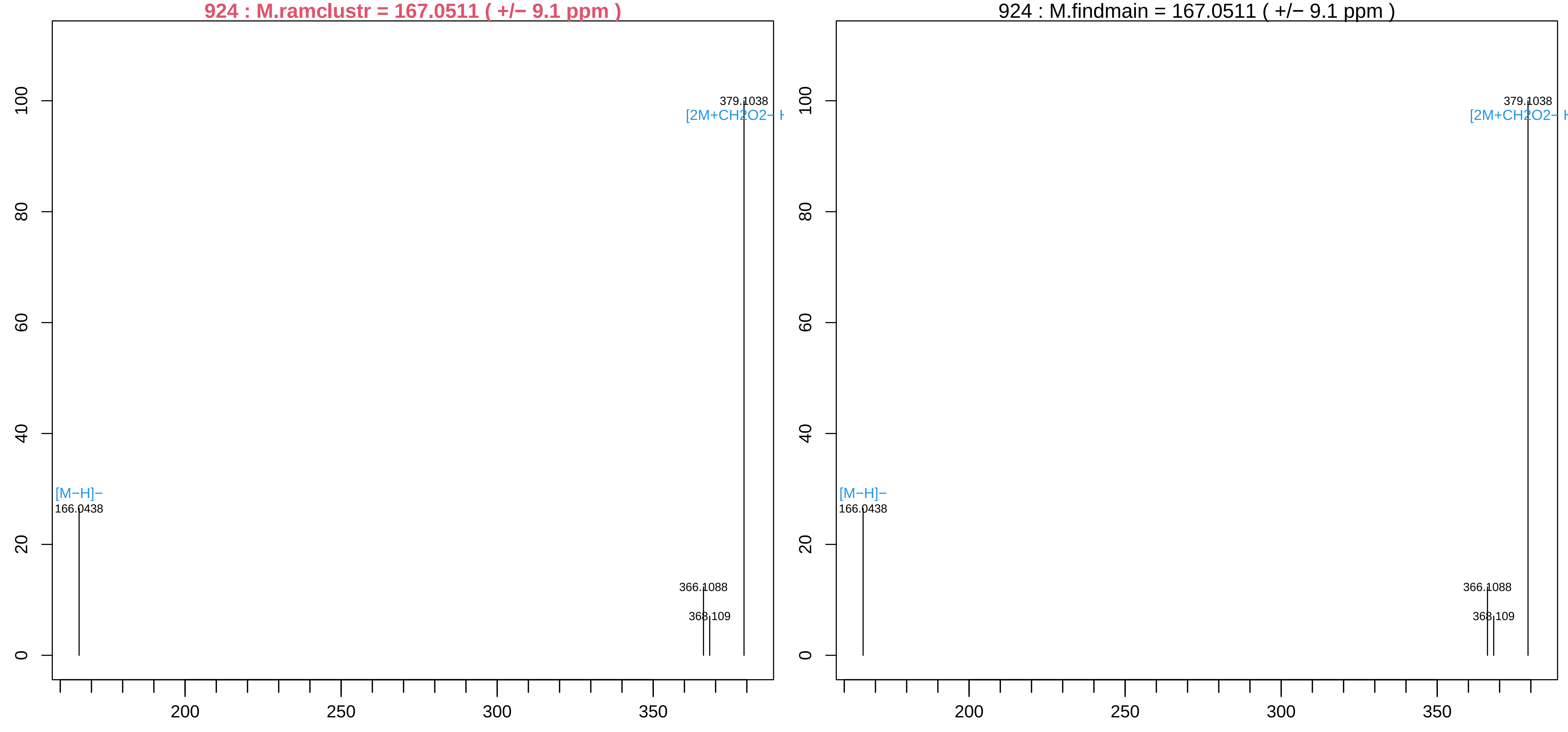
924 : M.ramclustr = 167.0511 (+/− 9.1 ppm)
924 : M.findmain = 167.0511 (+/− 9.1 ppm)
379.1038
[2M+CH2O2−H
[M−H]−
166.0438
366.1088
368.109
0
20
40
60
80
100
200
250
300
350

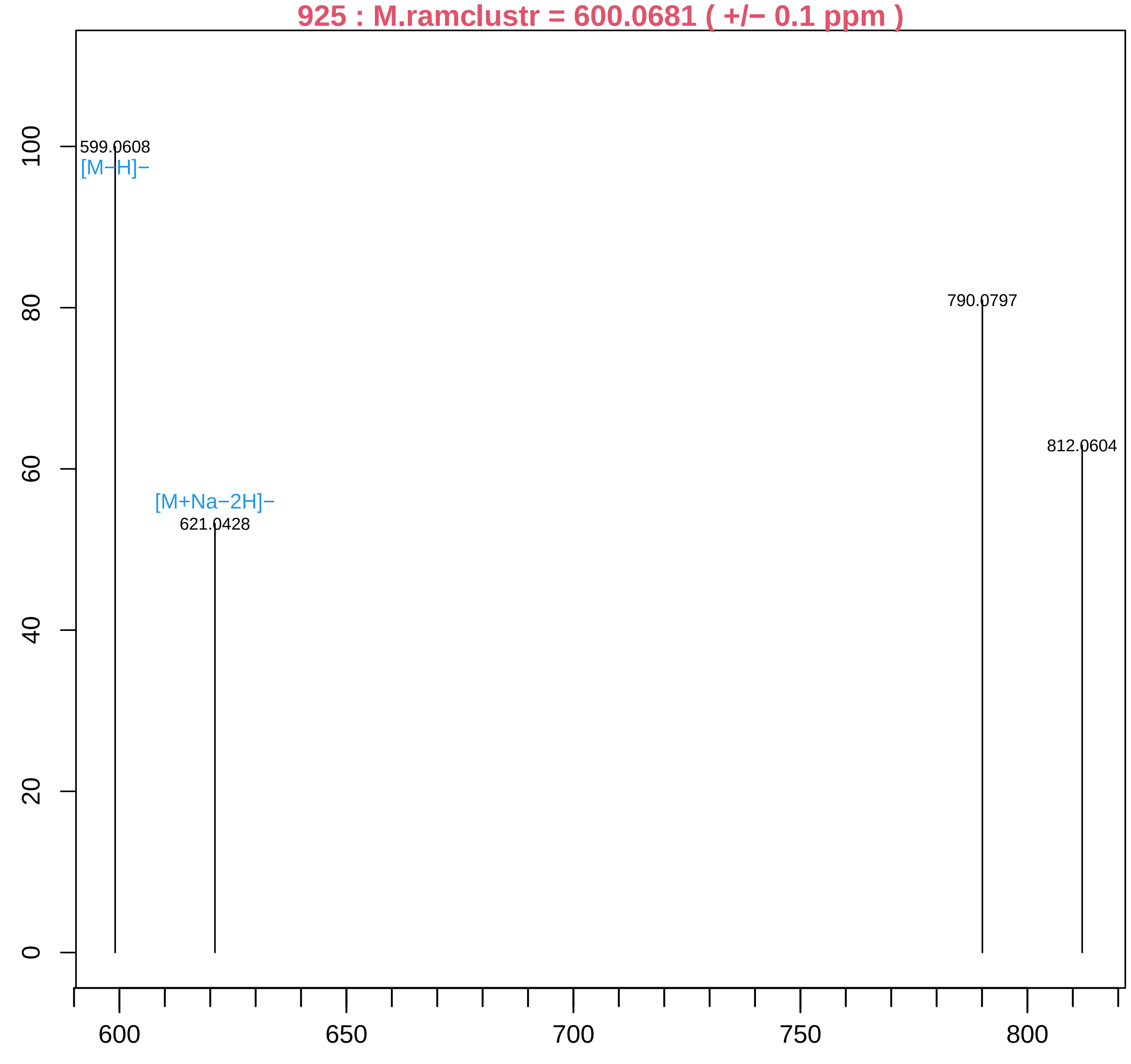
925 : M.ramclustr = 600.0681 (+/− 0.1 ppm)
599.0608
[M−H]−
[M+Na−2H]−
621.0428
790.0797
812.0604
0
20
40
60
80
100
600
650
700
750
800

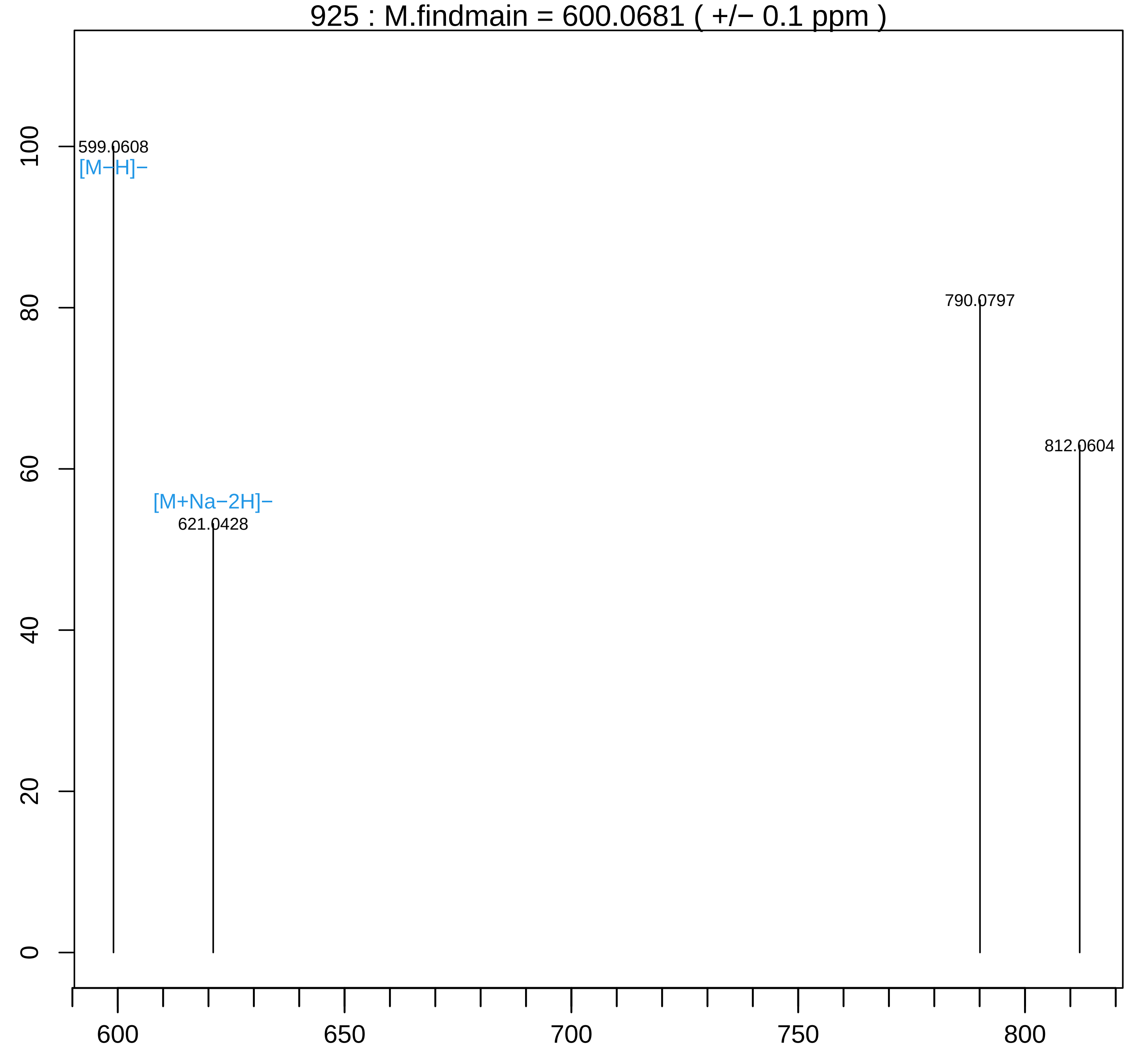
925 : M.findmain = 600.0681 (+/− 0.1 ppm)
599.0608
[M−H]−
[M+Na−2H]−
621.0428
790.0797
812.0604
0
20
40
60
80
100
600
650
700
750
800

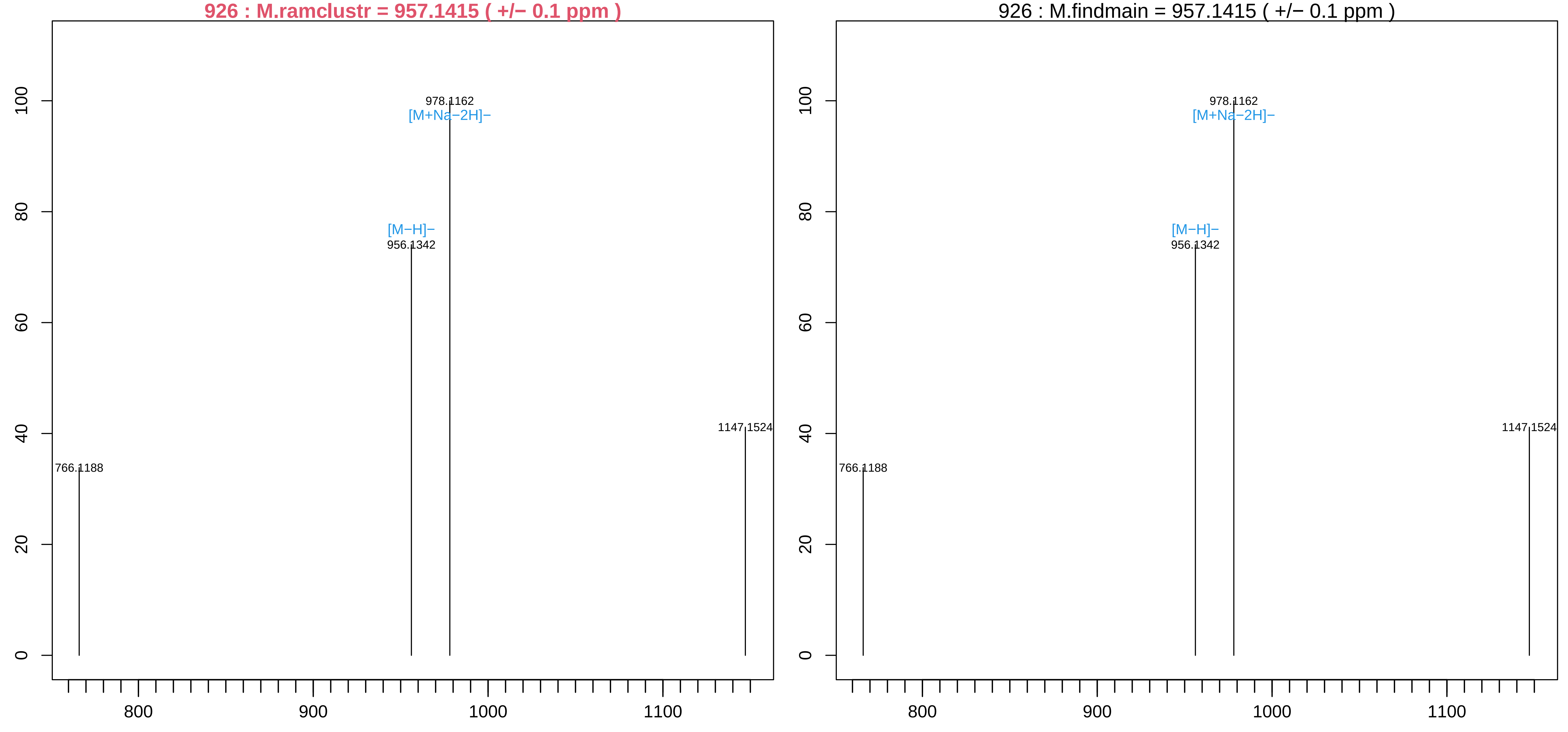
926 : M.ramclustr = 957.1415 (+/− 0.1 ppm)
978.1162
[M+Na−2H]−
[M−H]−
956.1342
1147.1524
766.1188
0
20
40
60
80
100
800
900
1000
1100
926 : M.findmain = 957.1415 (+/− 0.1 ppm)
978.1162
[M+Na−2H]−
[M−H]−
956.1342
1147.1524
766.1188
0
20
40
60
80
100
800
900
1000
1100

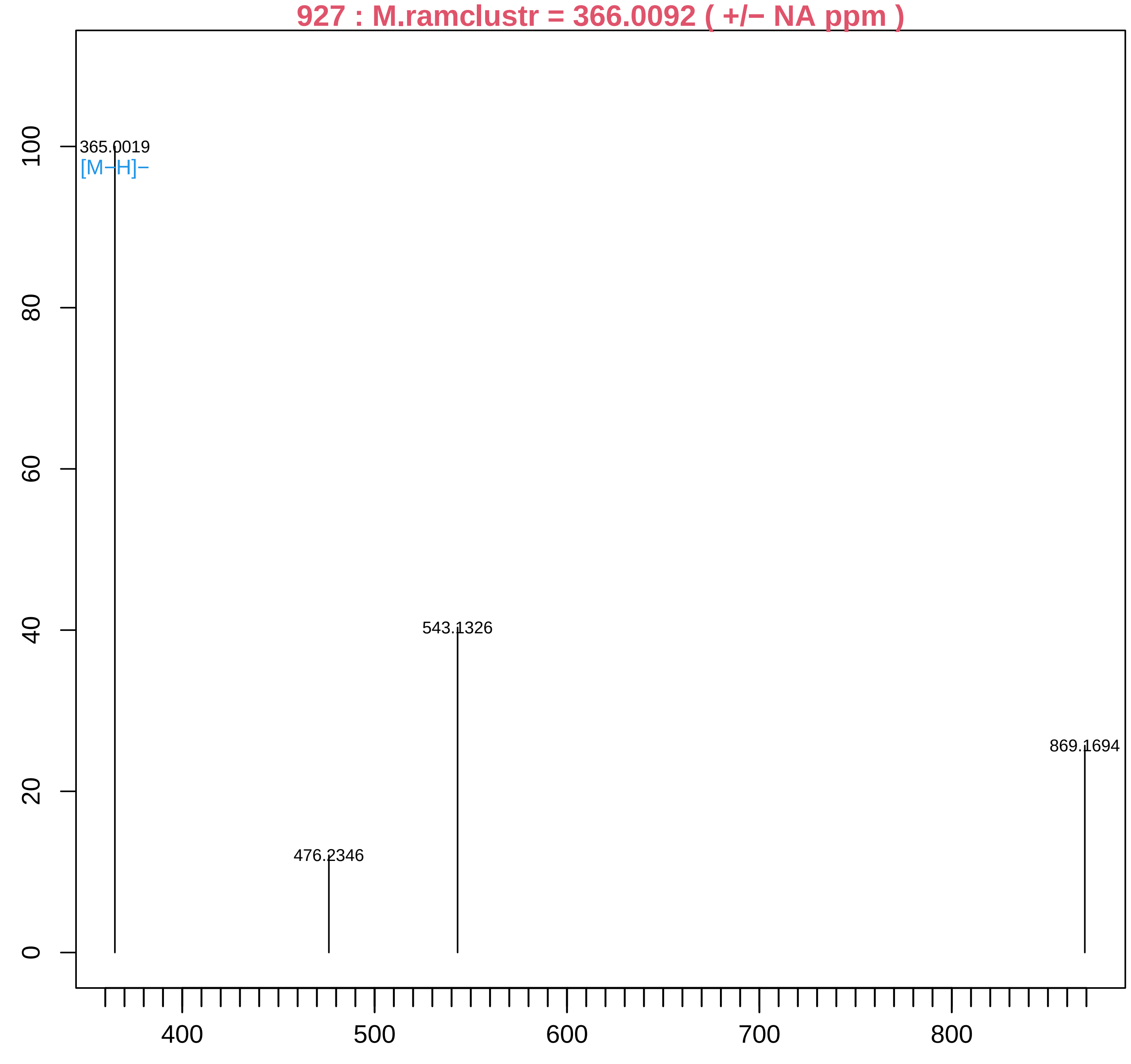
927 : M.ramclustr = 366.0092 (+/− NA ppm)
365.0019
[M−H]−
476.2346
543.1326
869.1694
0
20
40
60
80
100
400
500
600
700
800

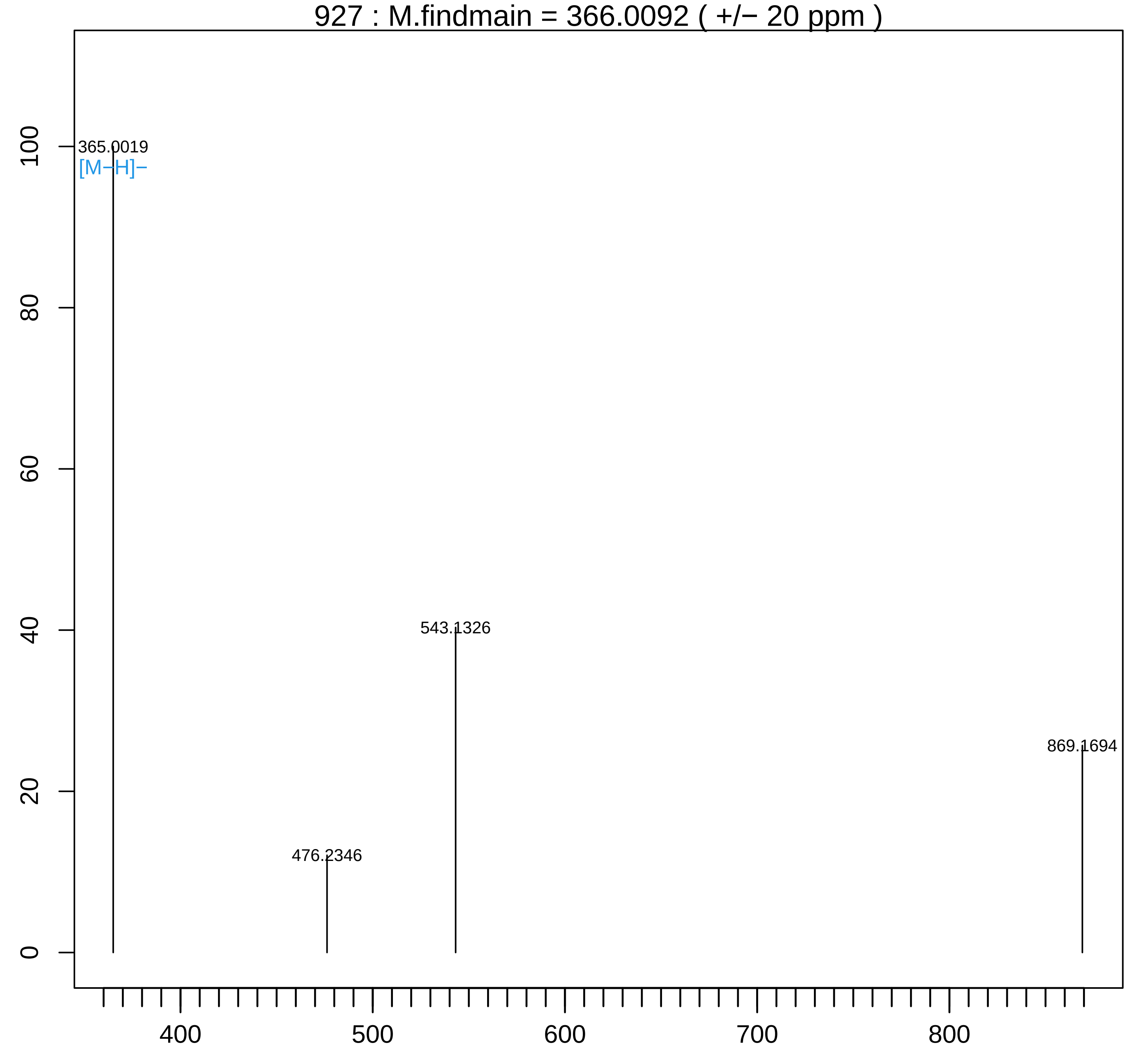
927 : M.findmain = 366.0092 (+/− 20 ppm)
365.0019
[M−H]−
476.2346
543.1326
869.1694
0
20
40
60
80
100
400
500
600
700
800

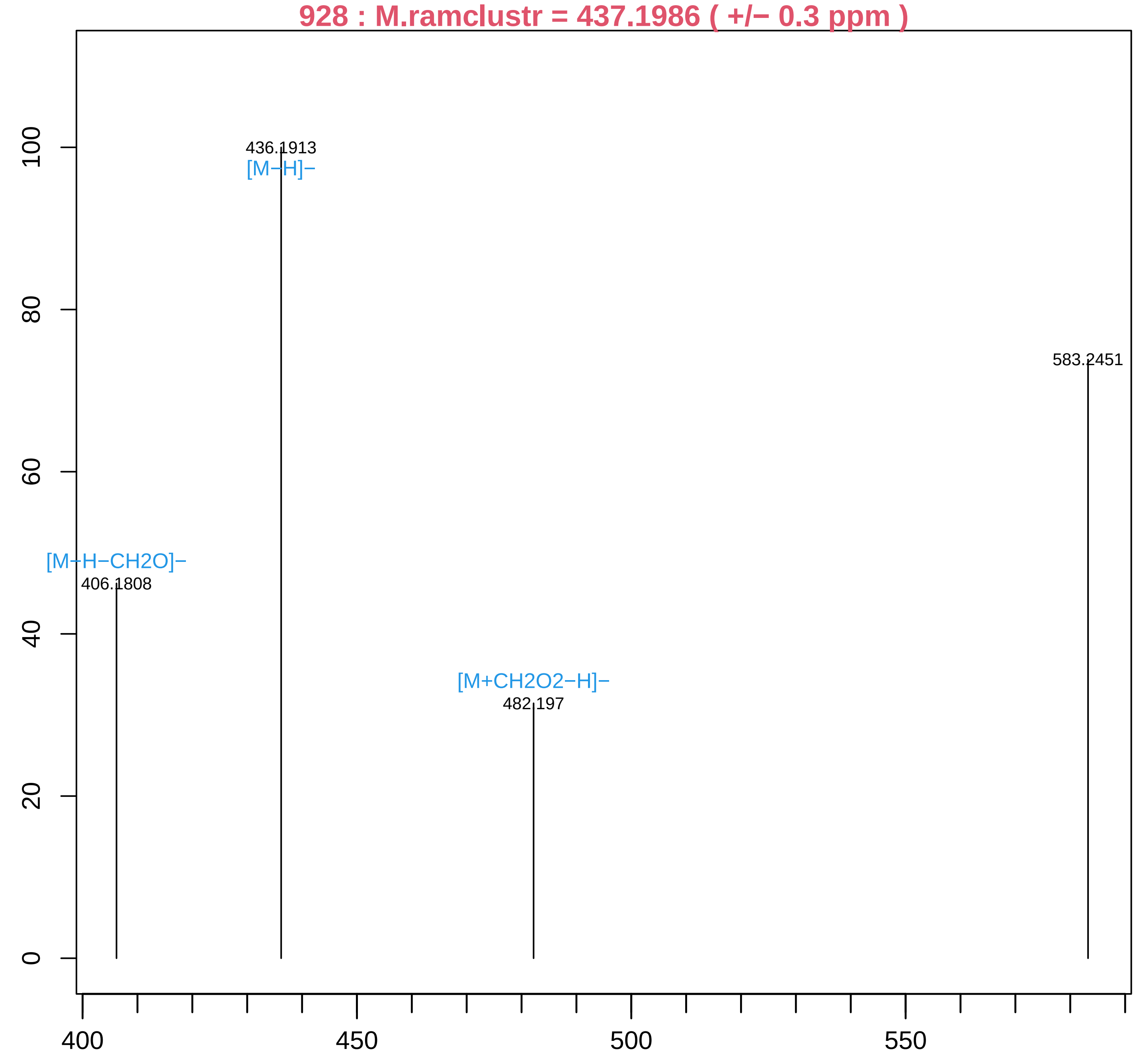

928 : M.ramclustr = 437.1986 (+/− 0.3 ppm)
436.1913
[M−H]−
583.2451
[M−H−CH2O]−
406.1808
[M+CH2O2−H]−
482.197
0
20
40
60
80
100
400
450
500
550

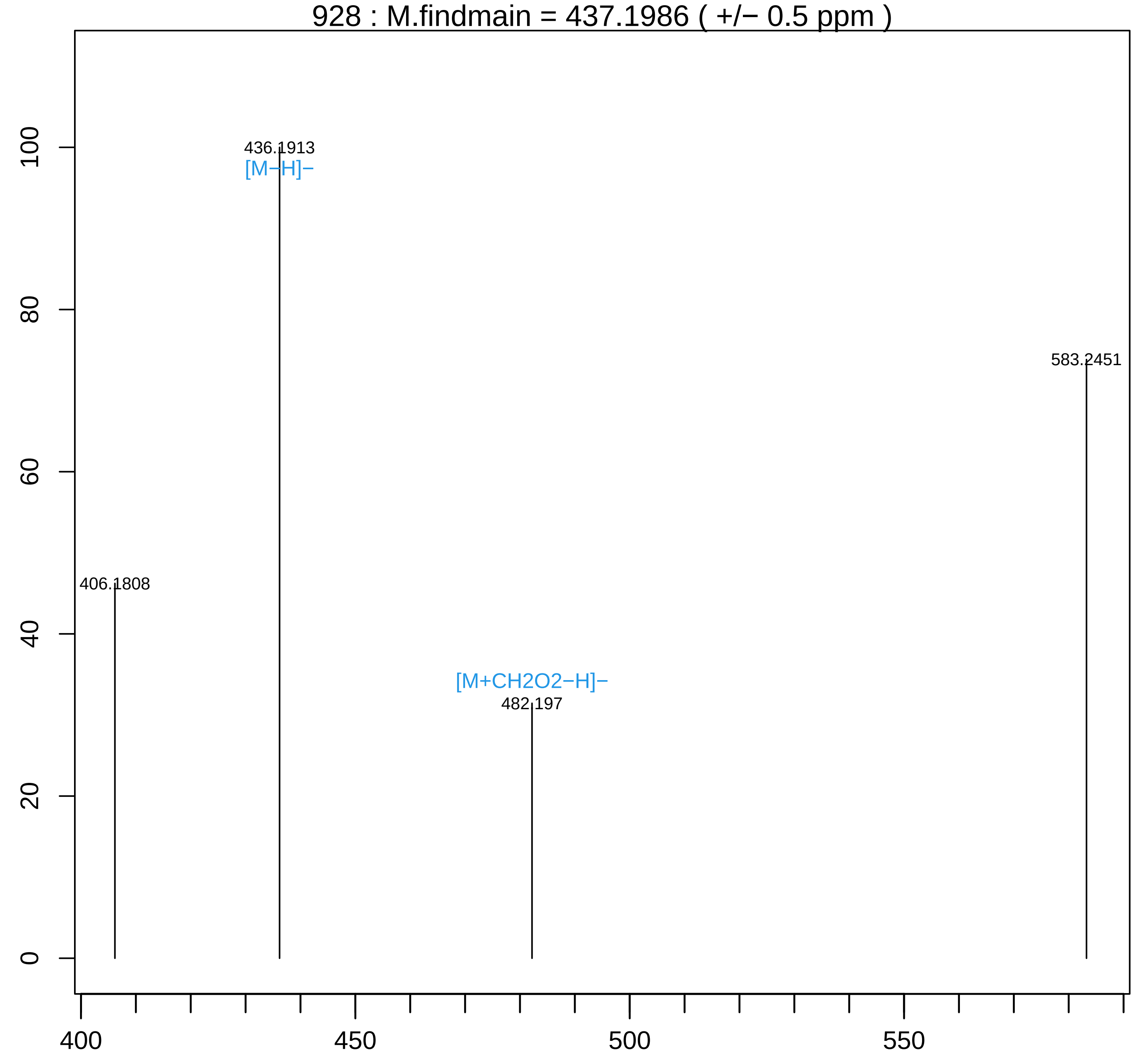

928 : M.findmain = 437.1986 (+/− 0.5 ppm)
436.1913
[M−H]−
583.2451
406.1808
[M+CH2O2−H]−
482.197
0
20
40
60
80
100
400
450
500
550

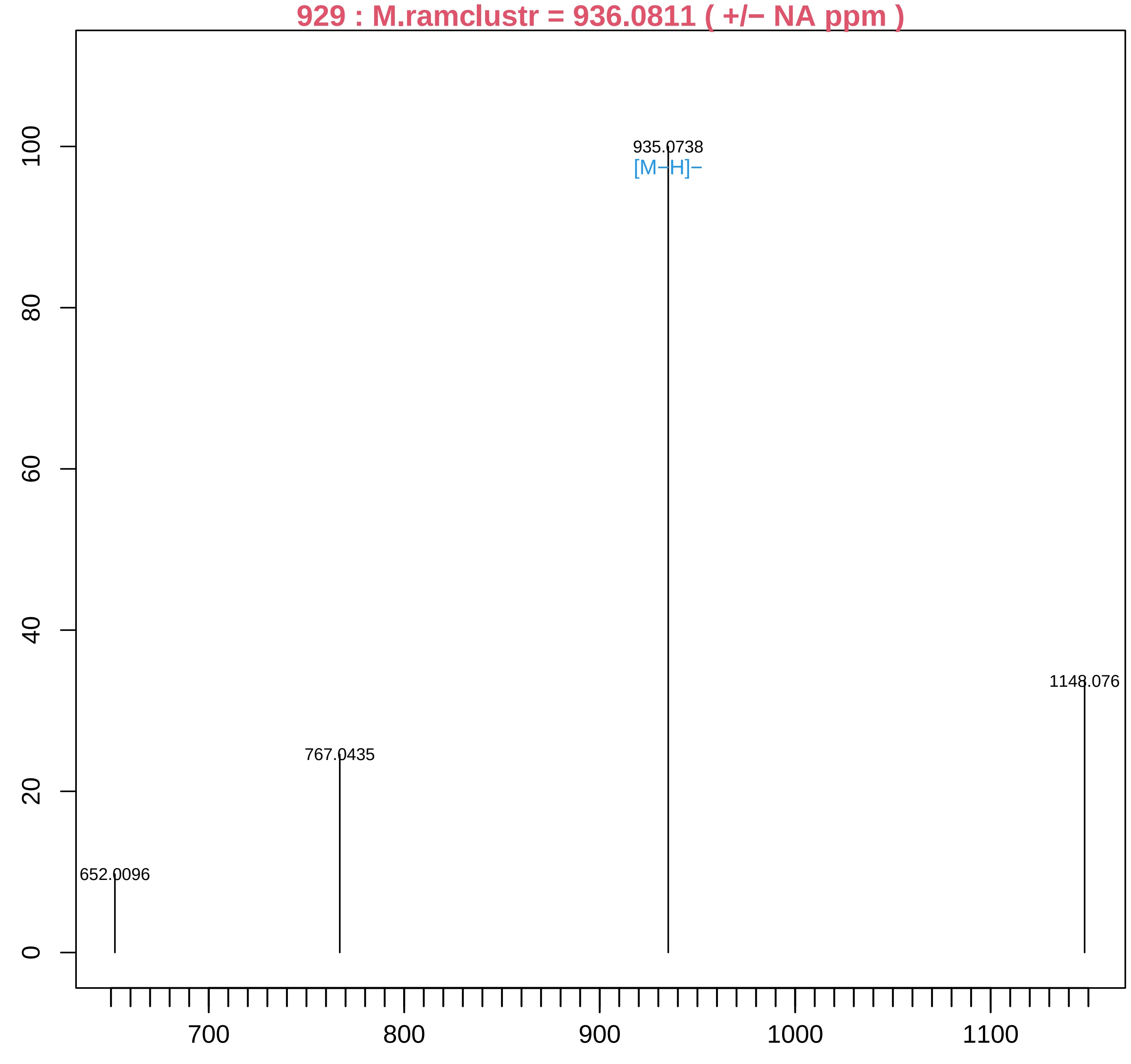
929 : M.ramclustr = 936.0811 (+/− NA ppm)
935.0738
[M−H]−
767.0435
652.0096
1148.076
0
20
40
60
80
100
700
800
900
1000
1100

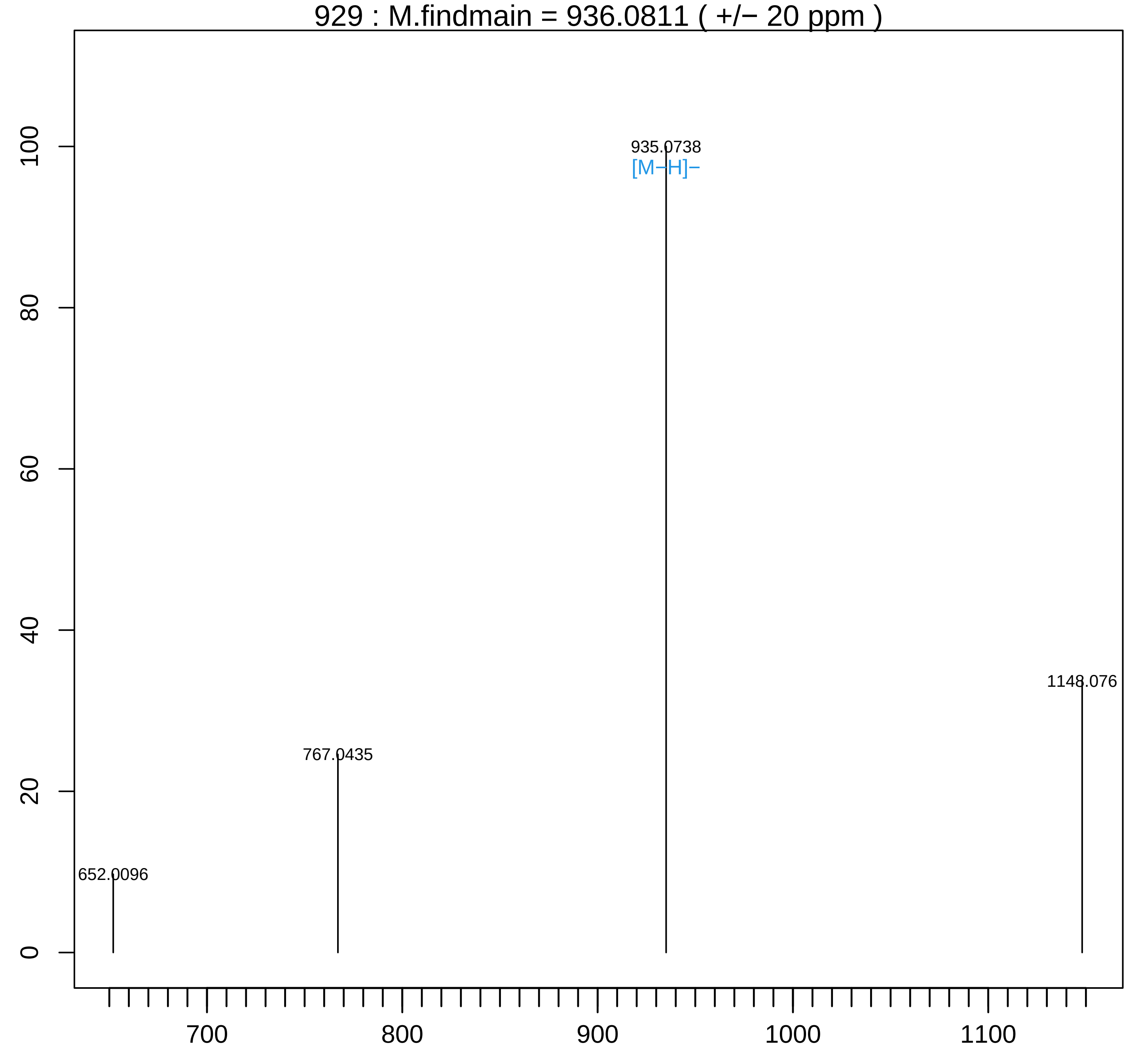
929 : M.findmain = 936.0811 (+/− 20 ppm)
935.0738
[M−H]−
767.0435
652.0096
1148.076
0
20
40
60
80
100
700
800
900
1000
1100

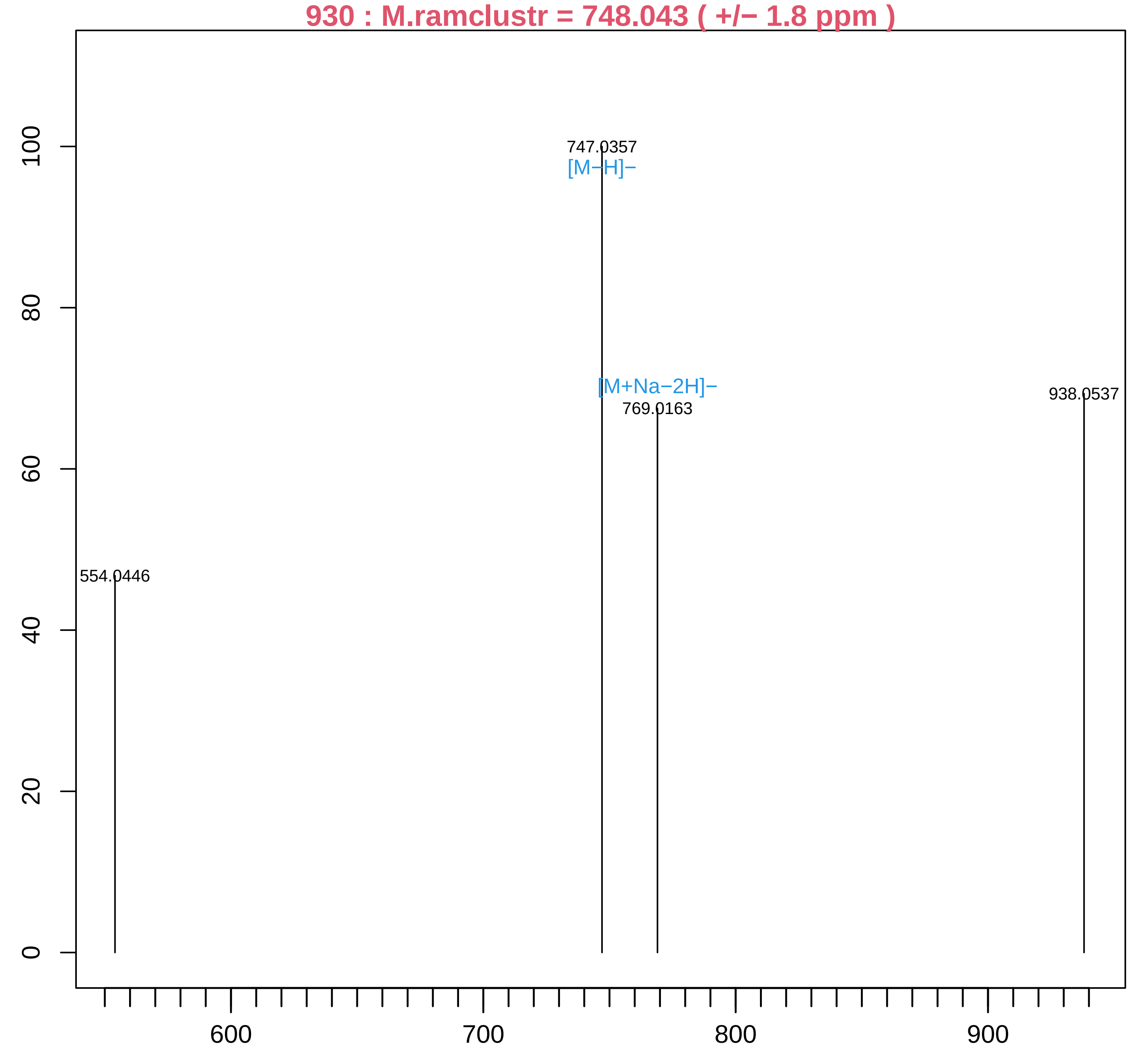
930 : M.ramclustr = 748.043 (+/− 1.8 ppm)
747.0357
[M−H]−
[M+Na−2H]−
769.0163
938.0537
554.0446
0
20
40
60
80
100
600
700
800
900

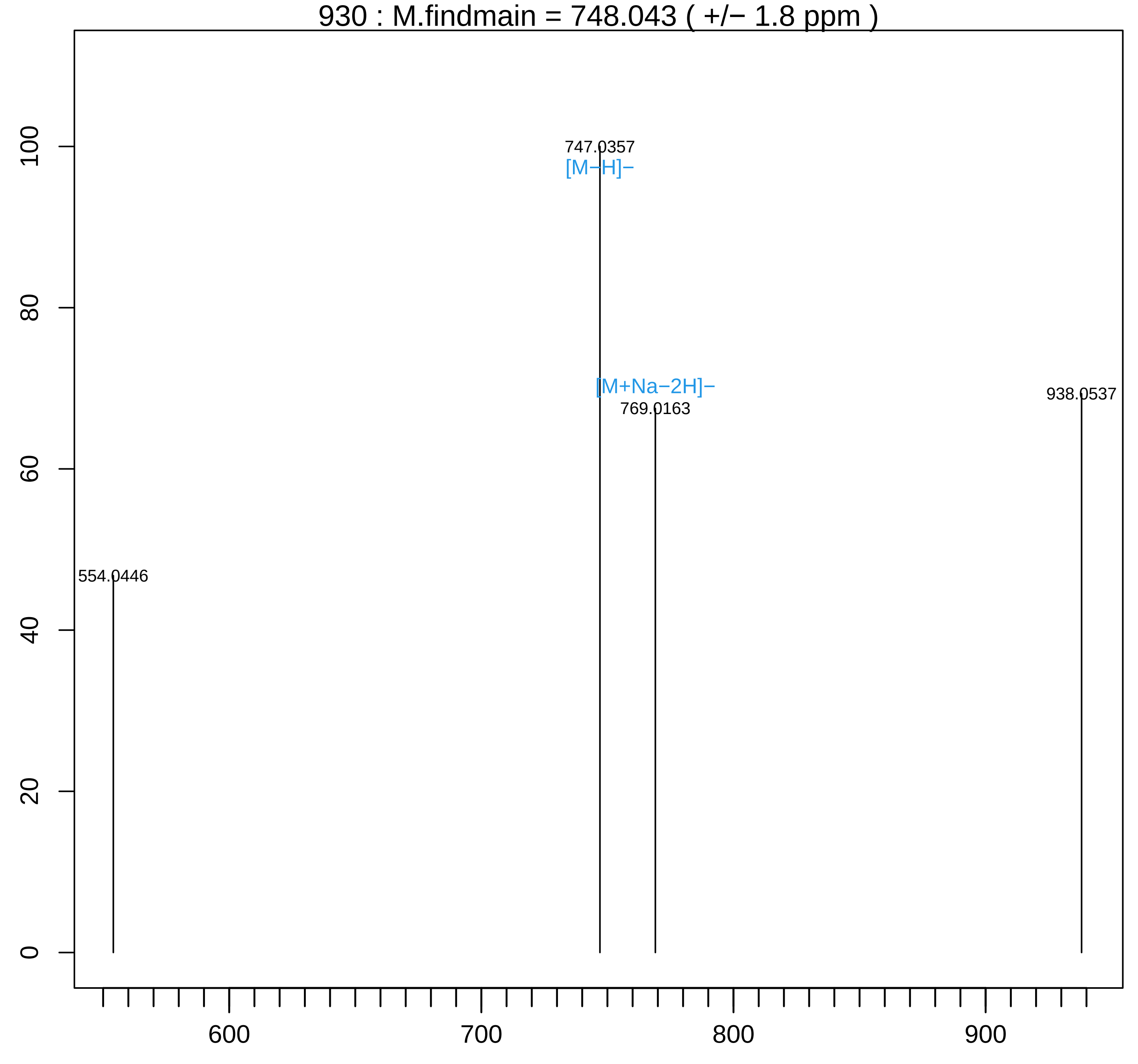
930 : M.findmain = 748.043 (+/− 1.8 ppm)
747.0357
[M−H]−
[M+Na−2H]−
769.0163
938.0537
554.0446
0
20
40
60
80
100
600
700
800
900

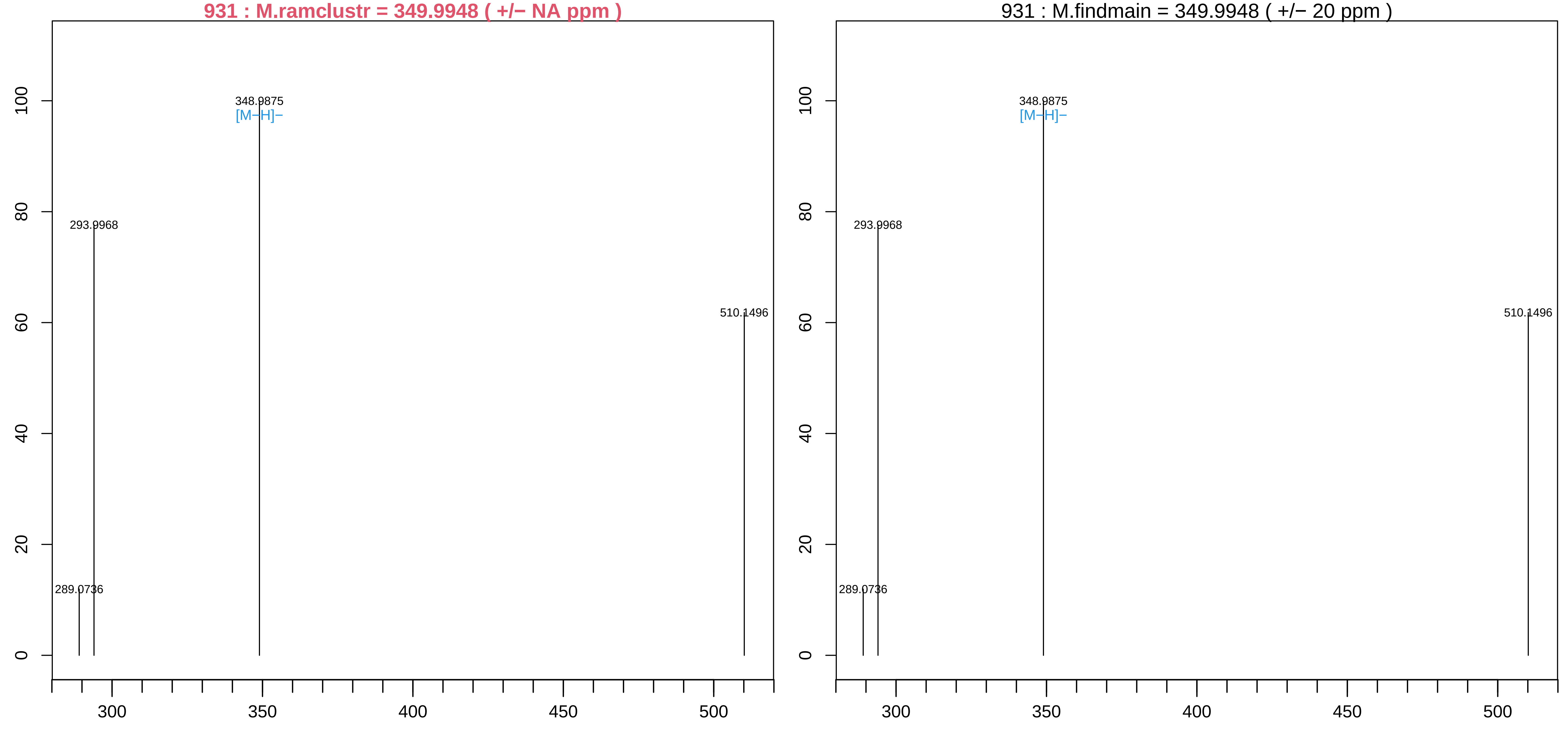
931 : M.ramclustr = 349.9948 (+/− NA ppm)
348.9875
[M−H]−
293.9968
510.1496
289.0736
0
20
40
60
80
100
300
350
400
450
500
931 : M.findmain = 349.9948 (+/− 20 ppm)
348.9875
[M−H]−
293.9968
510.1496
289.0736
0
20
40
60
80
100
300
350
400
450
500

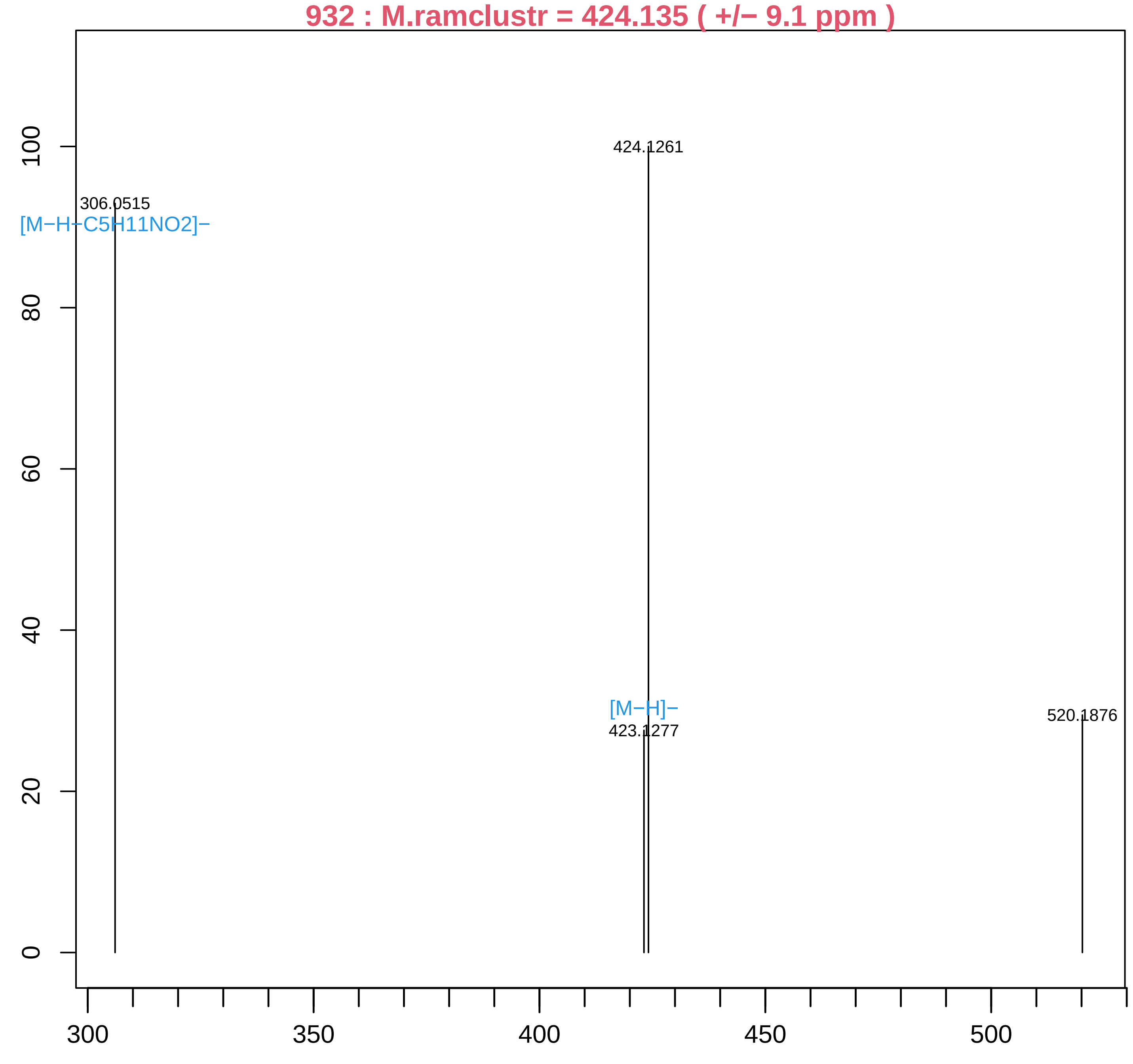

932 : M.ramclustr = 424.135 (+/− 9.1 ppm)
424.1261
306.0515
[M−H−C5H11NO2]−
[M−H]−
423.1277
520.1876
0
20
40
60
80
100
300
350
400
450
500

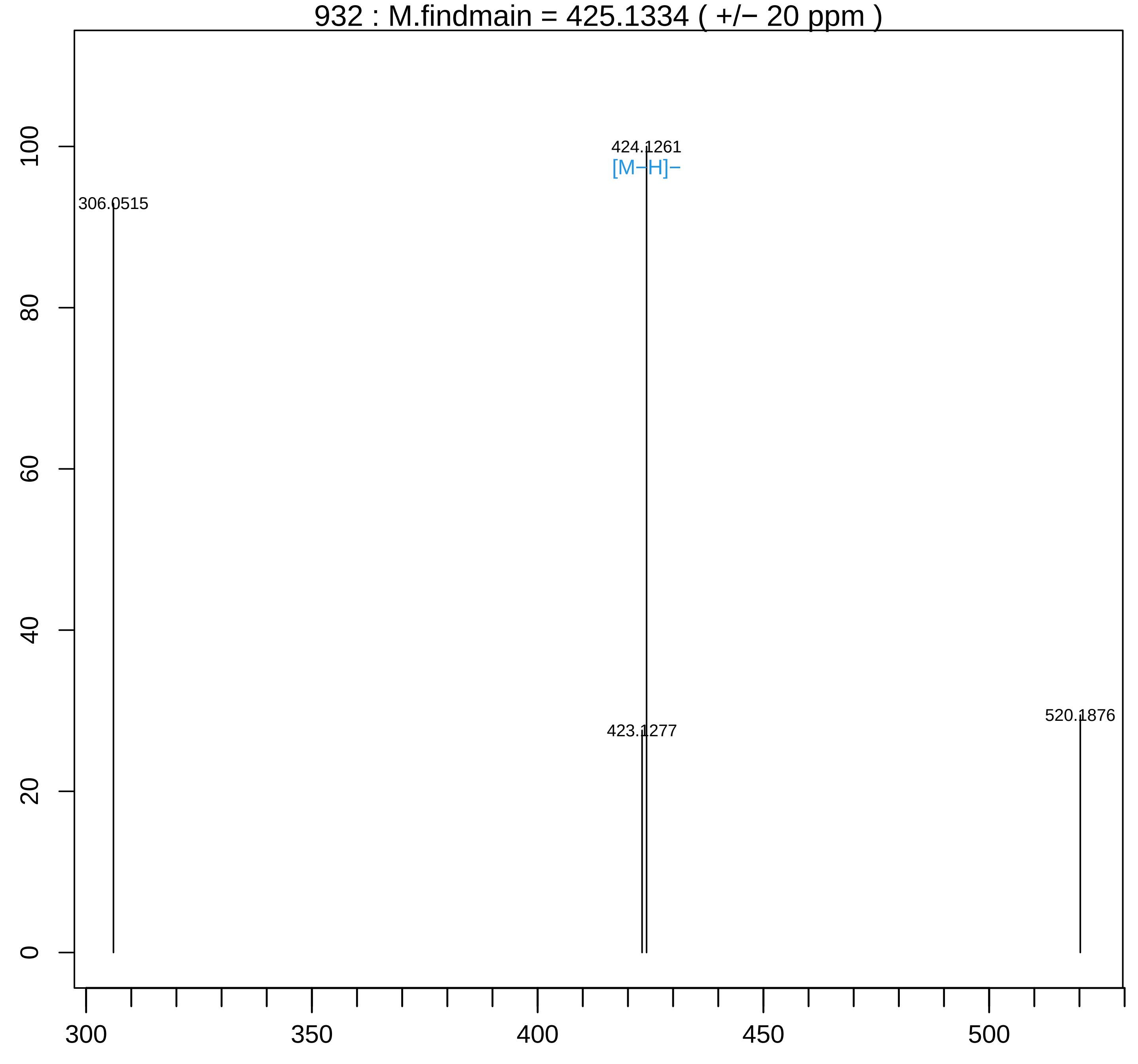

932 : M.findmain = 425.1334 (+/− 20 ppm)
424.1261
[M−H]−
306.0515
423.1277
520.1876
0
20
40
60
80
100
300
350
400
450
500

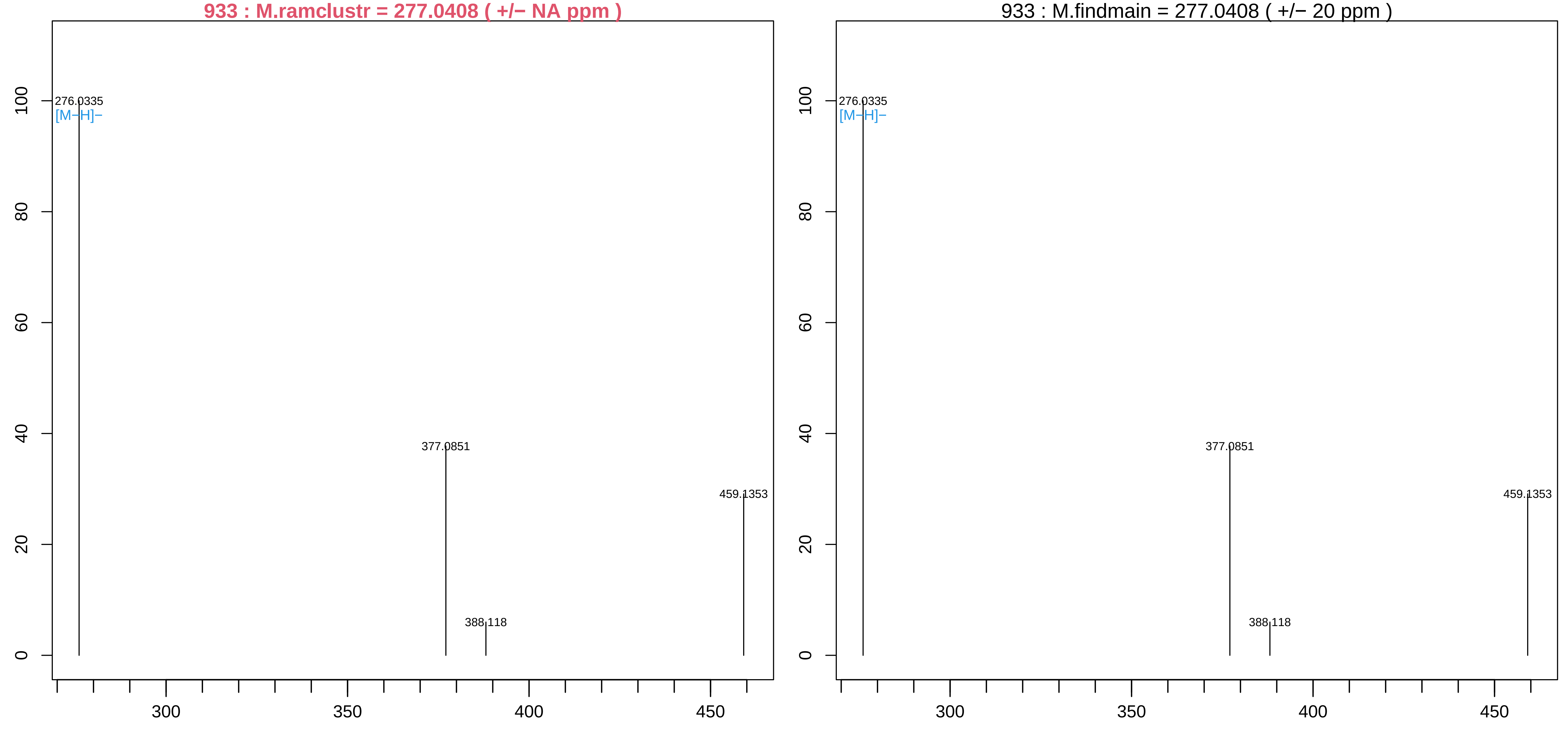
933 : M.ramclustr = 277.0408 (+/− NA ppm)
276.0335
[M−H]−
377.0851
388.118
459.1353
0
20
40
60
80
100
300
350
400
450
933 : M.findmain = 277.0408 (+/− 20 ppm)
276.0335
[M−H]−
377.0851
388.118
459.1353
0
20
40
60
80
100
300
350
400
450

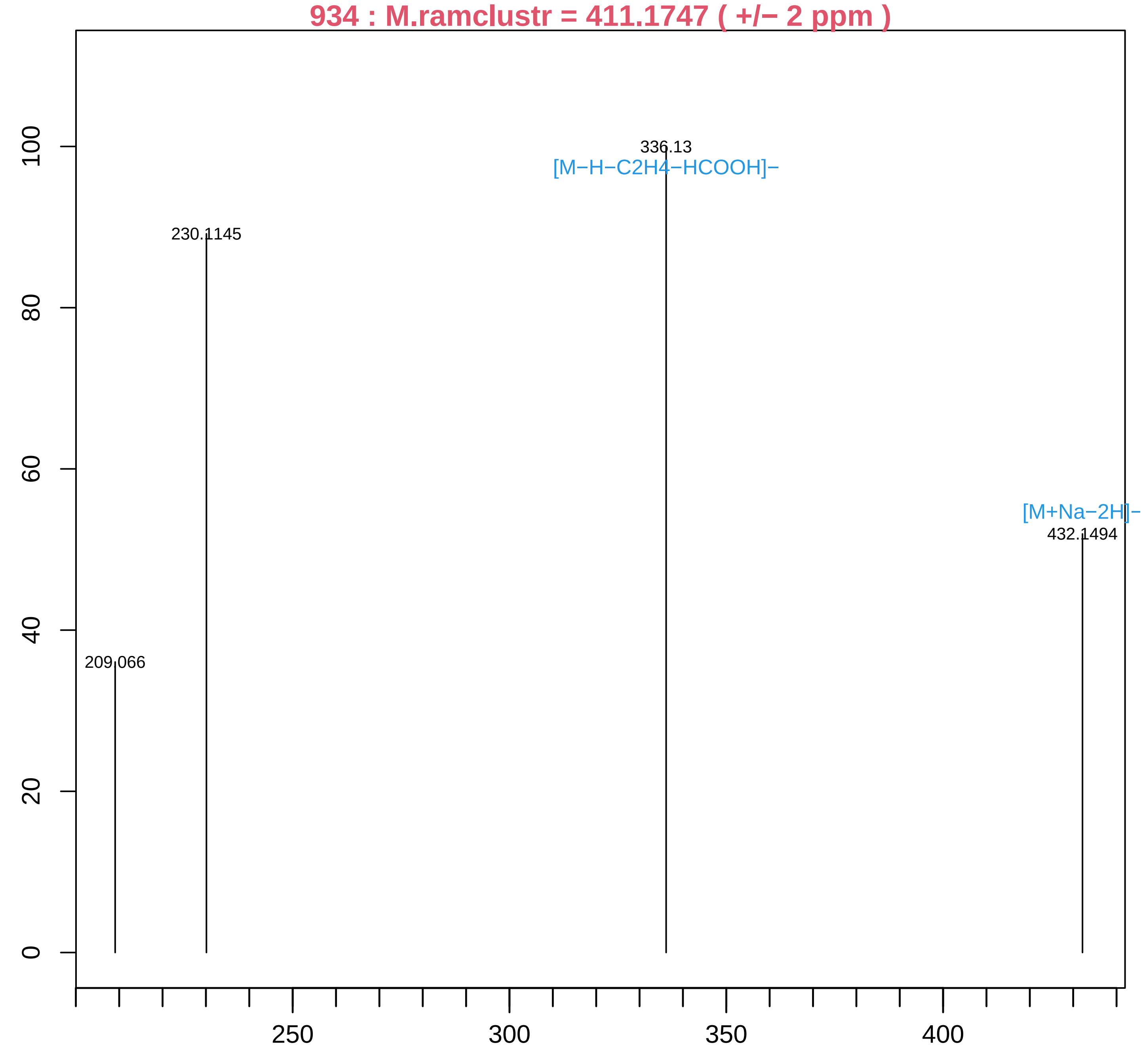
934 : M.ramclustr = 411.1747 (+/− 2 ppm)
336.13
[M−H−C2H4−HCOOH]−
230.1145
209.066
[M+Na−2H]−
432.1494
0
20
40
60
80
100
250
300
350
400

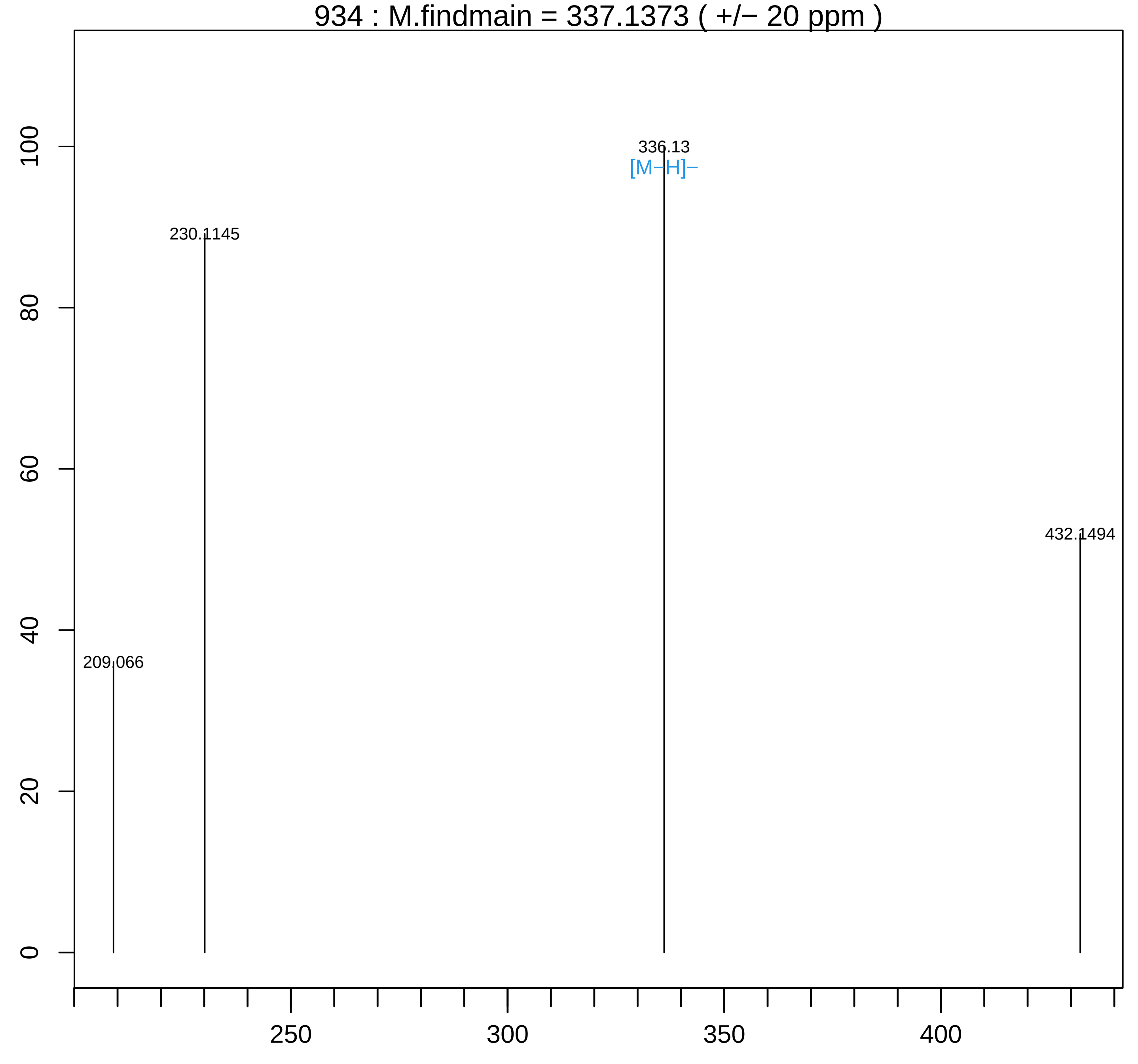
934 : M.findmain = 337.1373 (+/− 20 ppm)
336.13
[M−H]−
230.1145
209.066
432.1494
0
20
40
60
80
100
250
300
350
400

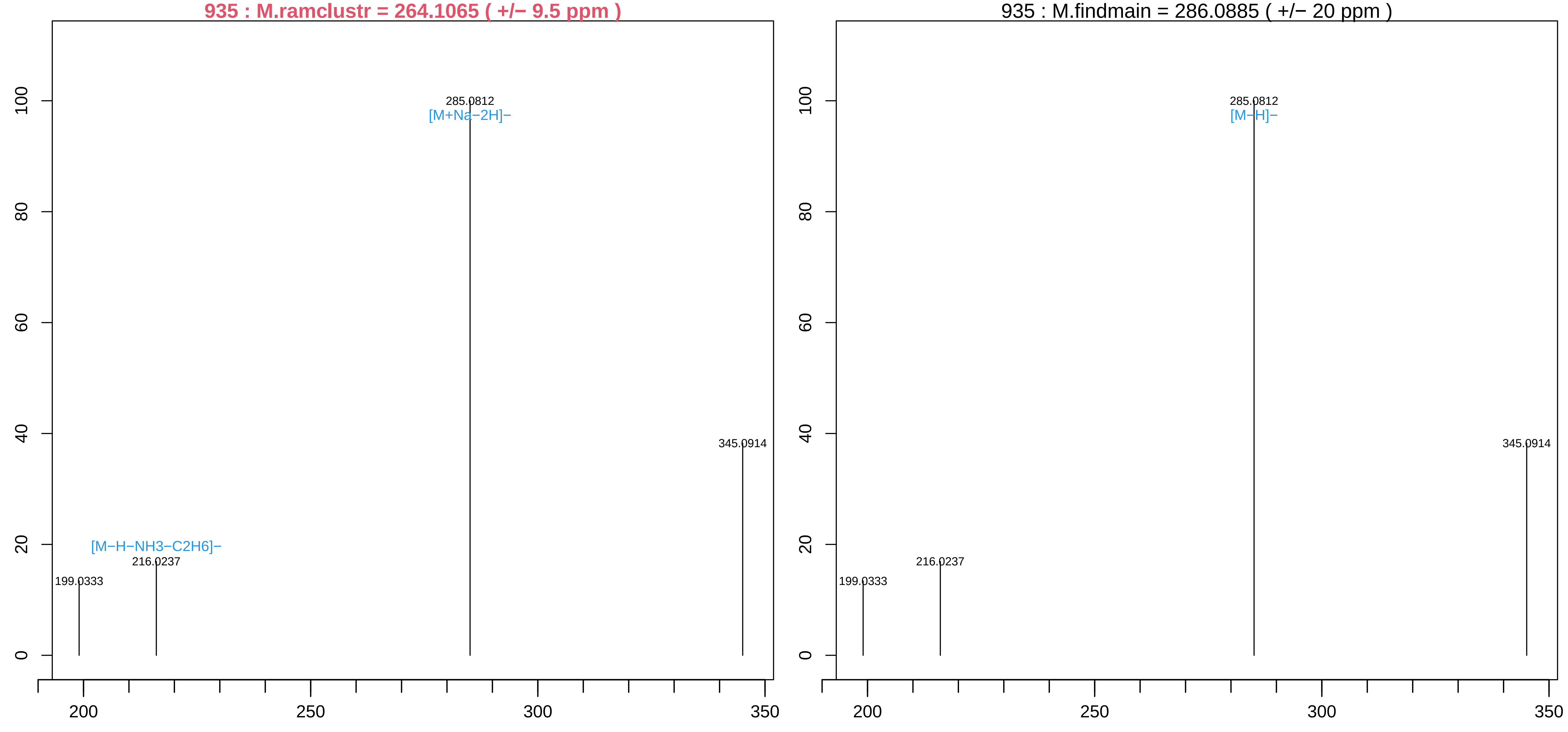

935 : M.ramclustr = 264.1065 (+/− 9.5 ppm)
285.0812
[M+Na−2H]−
345.0914
[M−H−NH3−C2H6]−
216.0237
199.0333
935 : M.findmain = 286.0885 (+/− 20 ppm)
285.0812
[M−H]−
345.0914
216.0237
199.0333

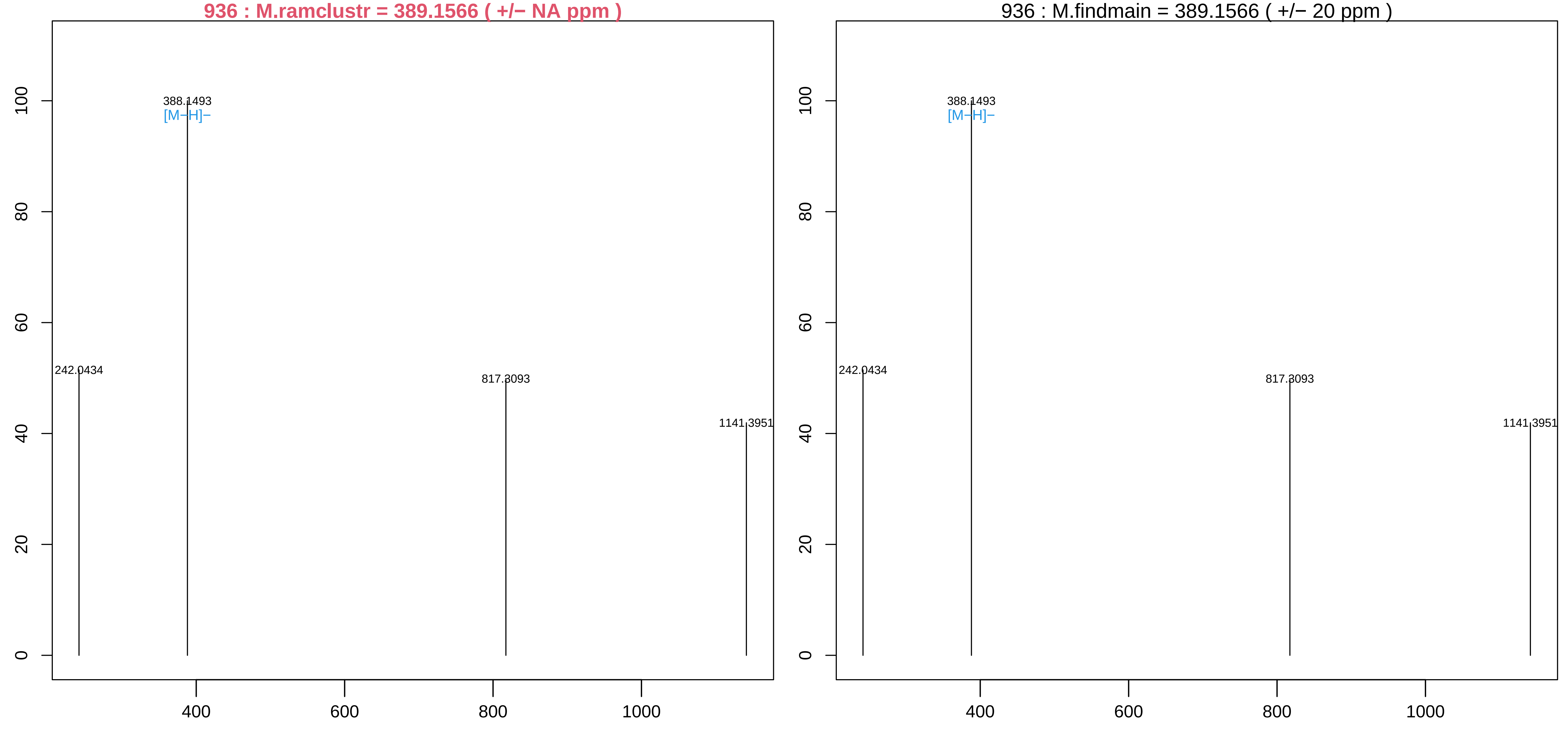
936 : M.ramclustr = 389.1566 (+/− NA ppm)
388.1493
[M−H]−
242.0434
817.3093
1141.3951
0
20
40
60
80
100
400
600
800
1000
936 : M.findmain = 389.1566 (+/− 20 ppm)
388.1493
[M−H]−
242.0434
817.3093
1141.3951
0
20
40
60
80
100
400
600
800
1000

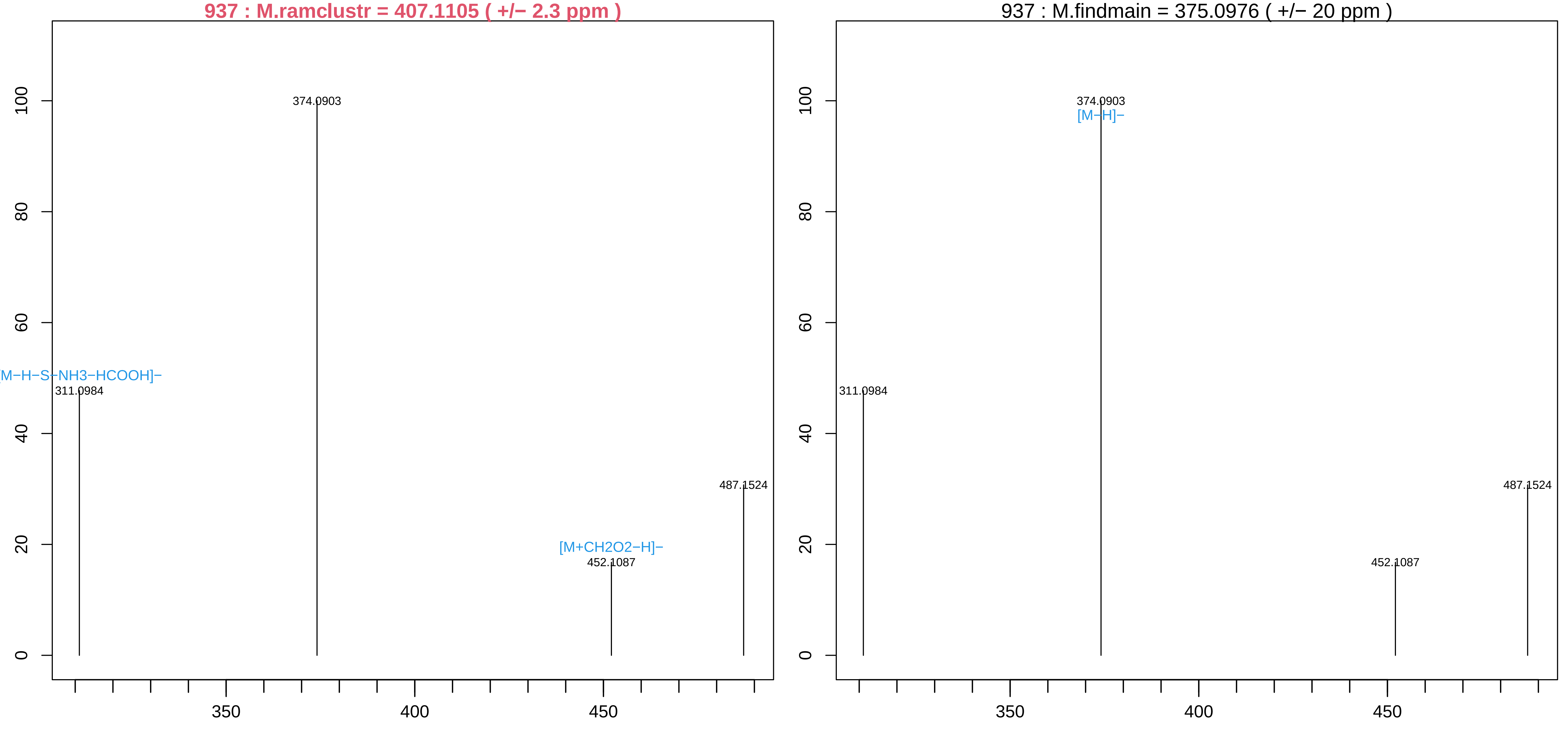
937 : M.ramclustr = 407.1105 (+/− 2.3 ppm)
374.0903
[M−H−S−NH3−HCOOH]−
311.0984
[M+CH2O2−H]−
452.1087
487.1524
0
20
40
60
80
100
350
400
450
937 : M.findmain = 375.0976 (+/− 20 ppm)
374.0903
[M−H]−
311.0984
452.1087
487.1524
0
20
40
60
80
100
350
400
450

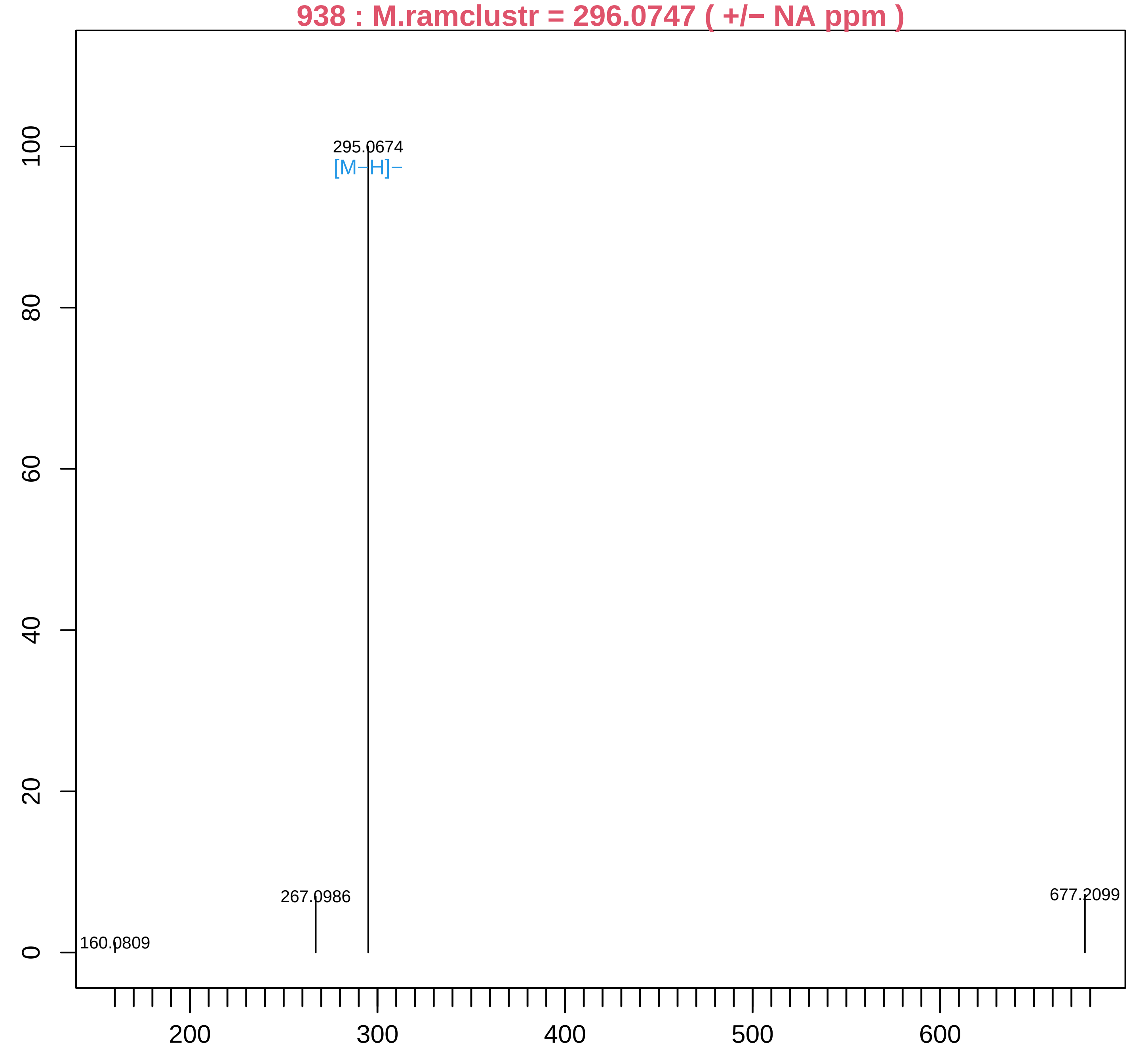
938 : M.ramclustr = 296.0747 (+/− NA ppm)
295.0674
[M−H]−
267.0986
677.2099
160.0809
0
20
40
60
80
100
200
300
400
500
600

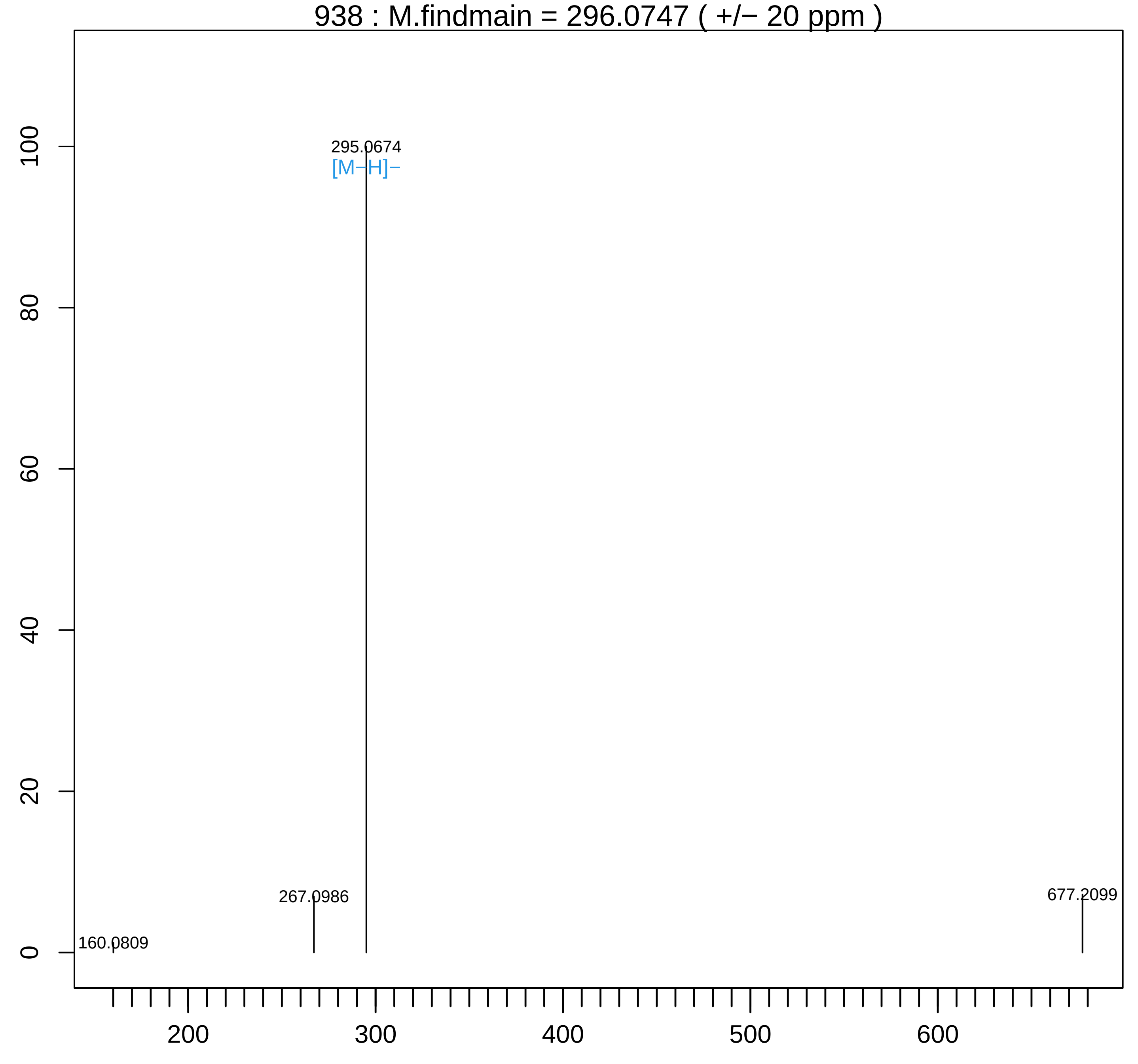
938 : M.findmain = 296.0747 (+/− 20 ppm)
295.0674
[M−H]−
267.0986
677.2099
160.0809
0
20
40
60
80
100
200
300
400
500
600

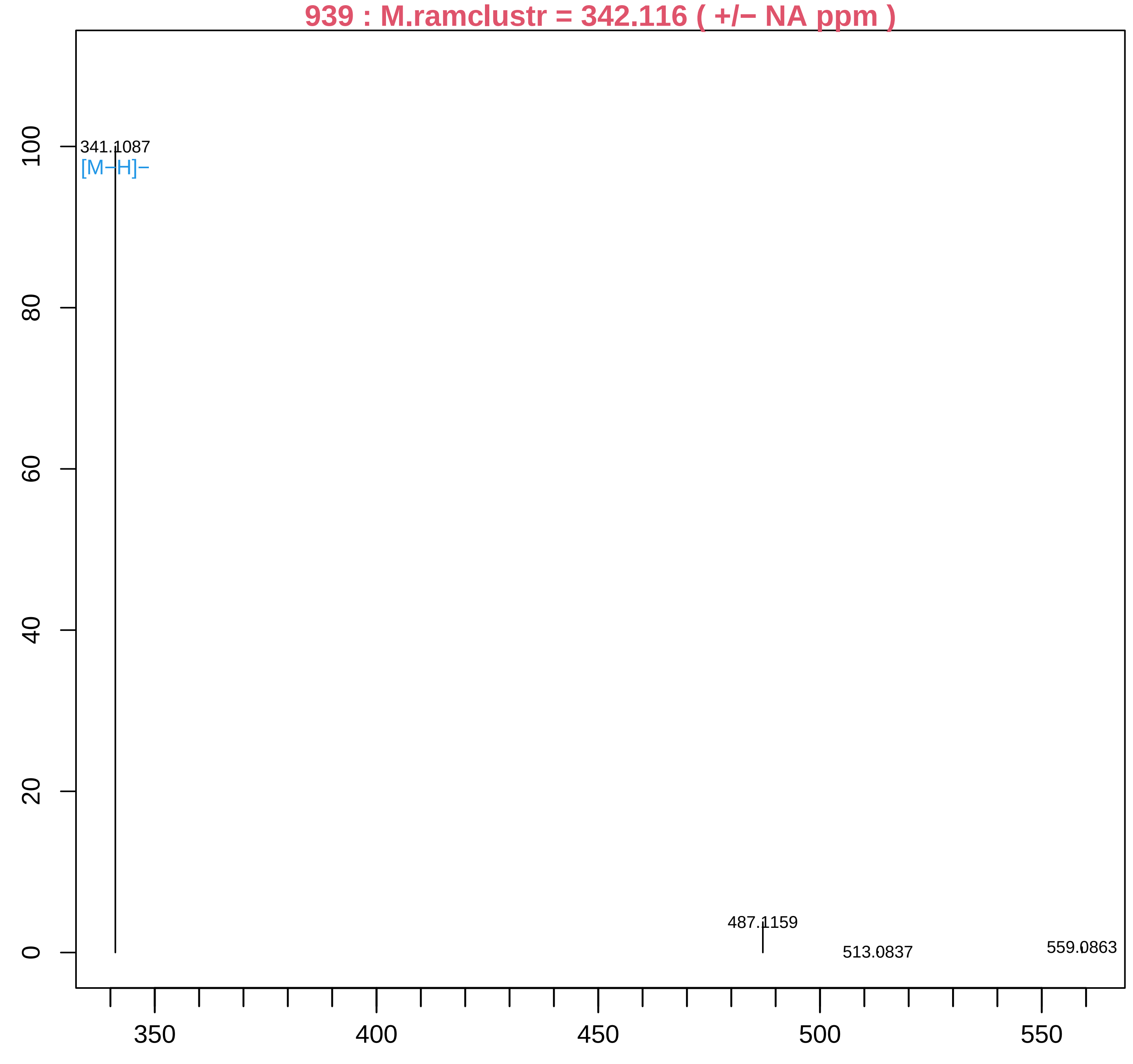
939 : M.ramclustr = 342.116 (+/− NA ppm)
341.1087
[M−H]−
487.1159
513.0837
559.0863
0
20
40
60
80
100
350
400
450
500
550

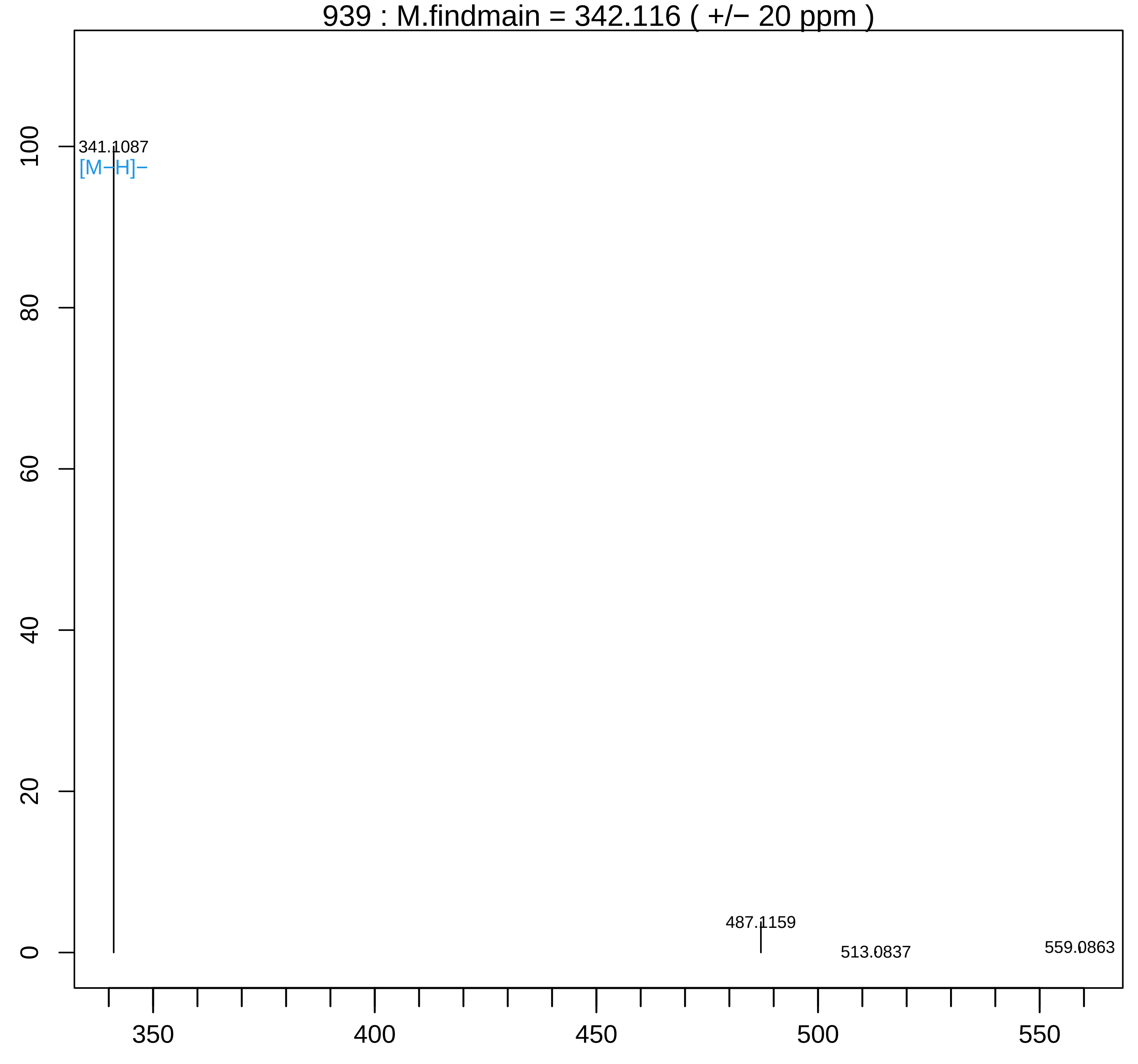
939 : M.findmain = 342.116 (+/− 20 ppm)
341.1087
[M−H]−
487.1159
513.0837
559.0863
0
20
40
60
80
100
350
400
450
500
550

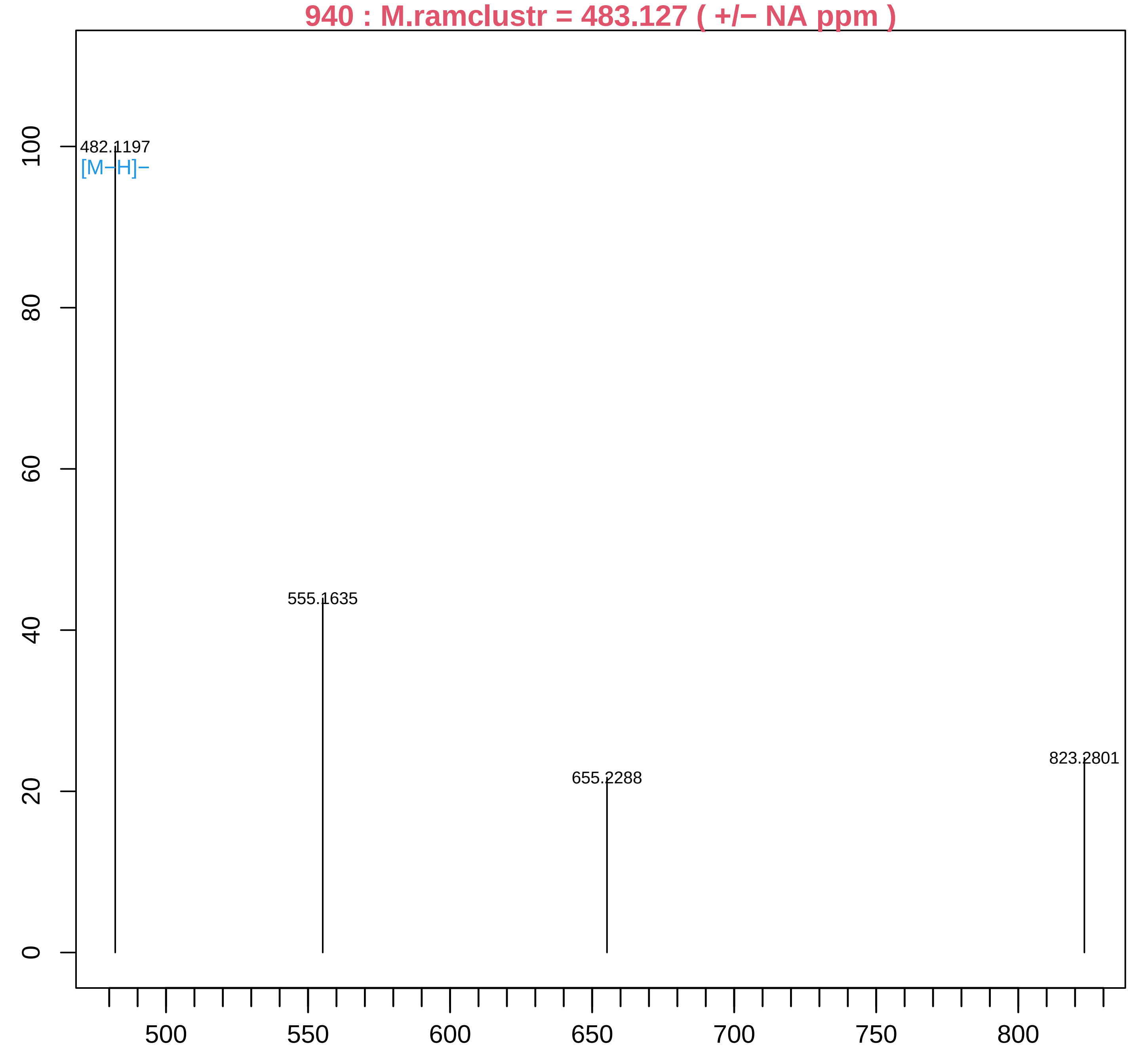
940 : M.ramclustr = 483.127 (+/− NA ppm)
482.1197
[M−H]−
555.1635
655.2288
823.2801
0
20
40
60
80
100
500
550
600
650
700
750
800

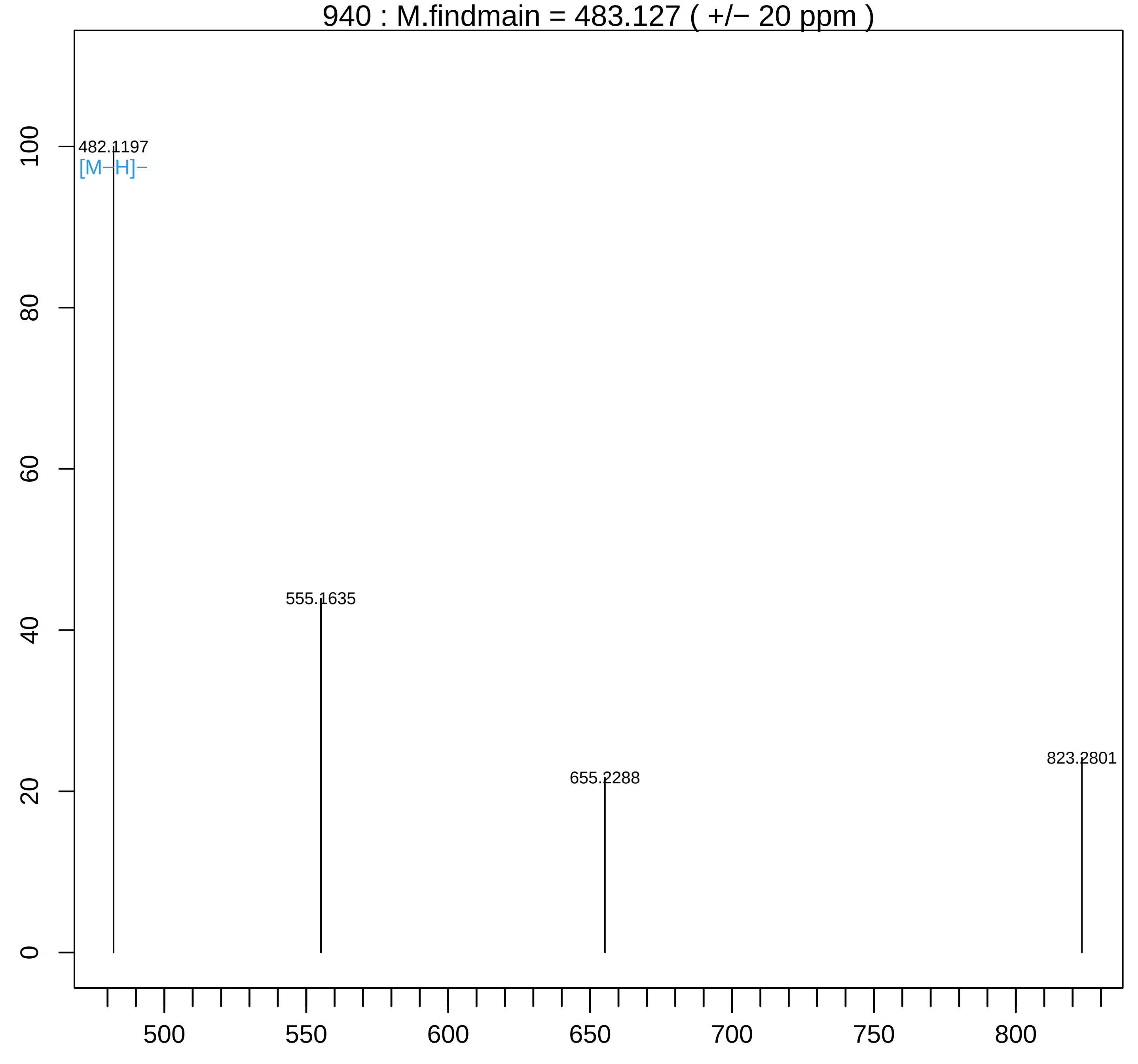
940 : M.findmain = 483.127 (+/− 20 ppm)
482.1197
[M−H]−
555.1635
655.2288
823.2801
0
20
40
60
80
100
500
550
600
650
700
750
800

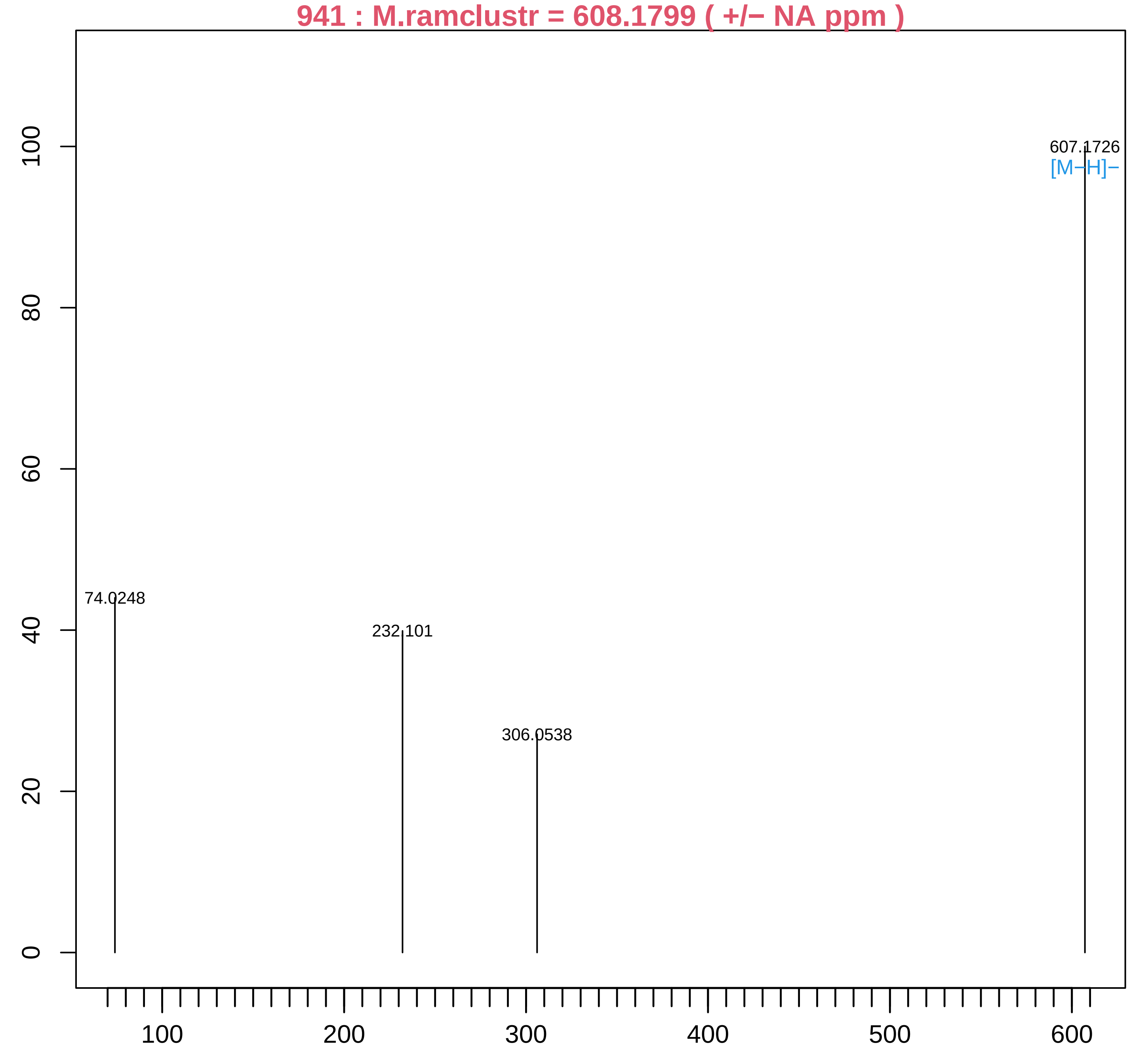
941 : M.ramclustr = 608.1799 (+/− NA ppm)
607.1726
[M−H]−
74.0248
232.101
306.0538
0
20
40
60
80
100
100
200
300
400
500
600

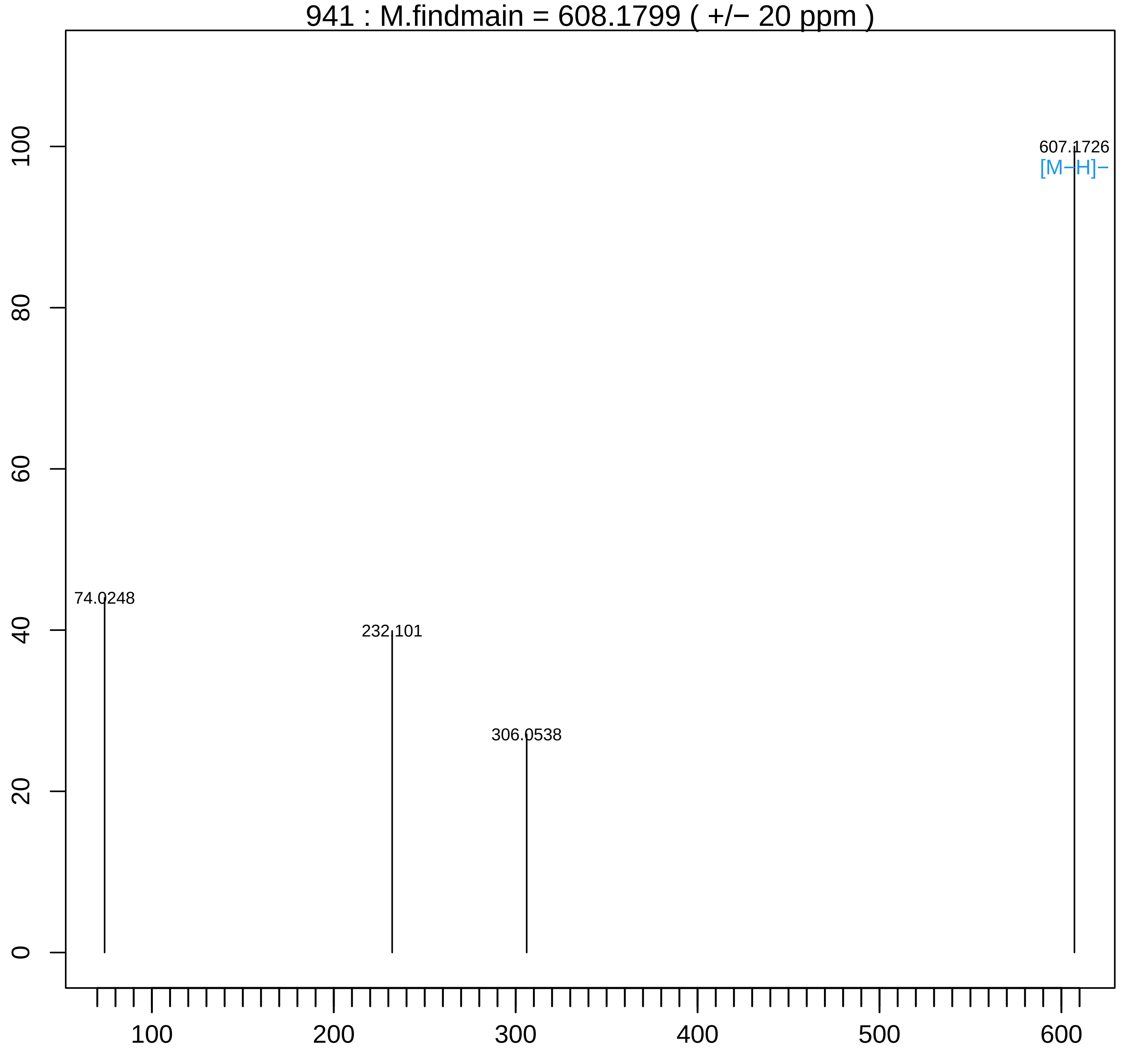
941 : M.findmain = 608.1799 (+/− 20 ppm)
607.1726
[M−H]−
74.0248
232.101
306.0538
0
20
40
60
80
100
100
200
300
400
500
600

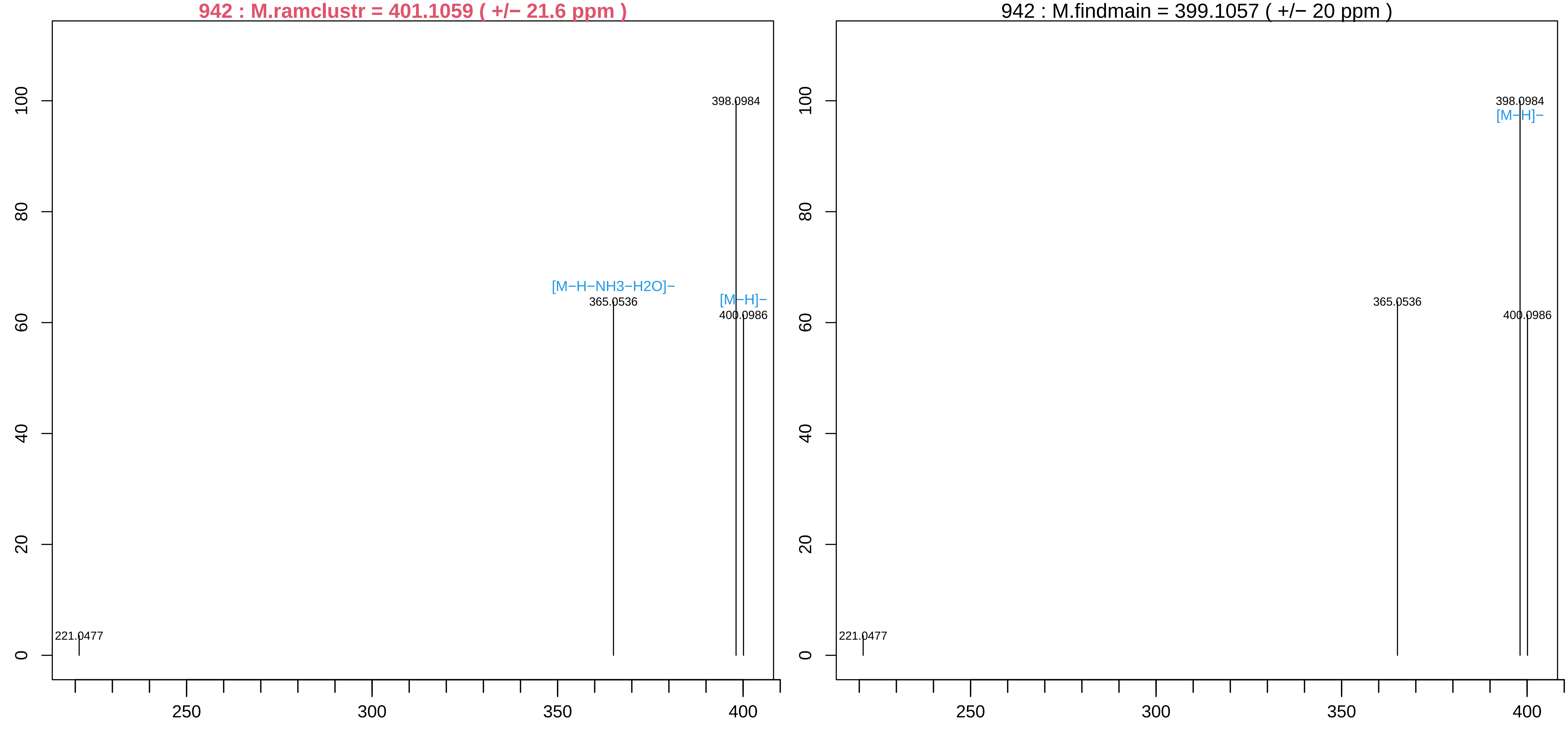

942 : M.ramclustr = 401.1059 (+/− 21.6 ppm)
398.0984
[M−H−NH3−H2O]−
365.0536
[M−H]−
400.0986
221.0477
942 : M.findmain = 399.1057 (+/− 20 ppm)
398.0984
[M−H]−
365.0536
400.0986
221.0477
0
20
40
60
80
100
250
300
350
400

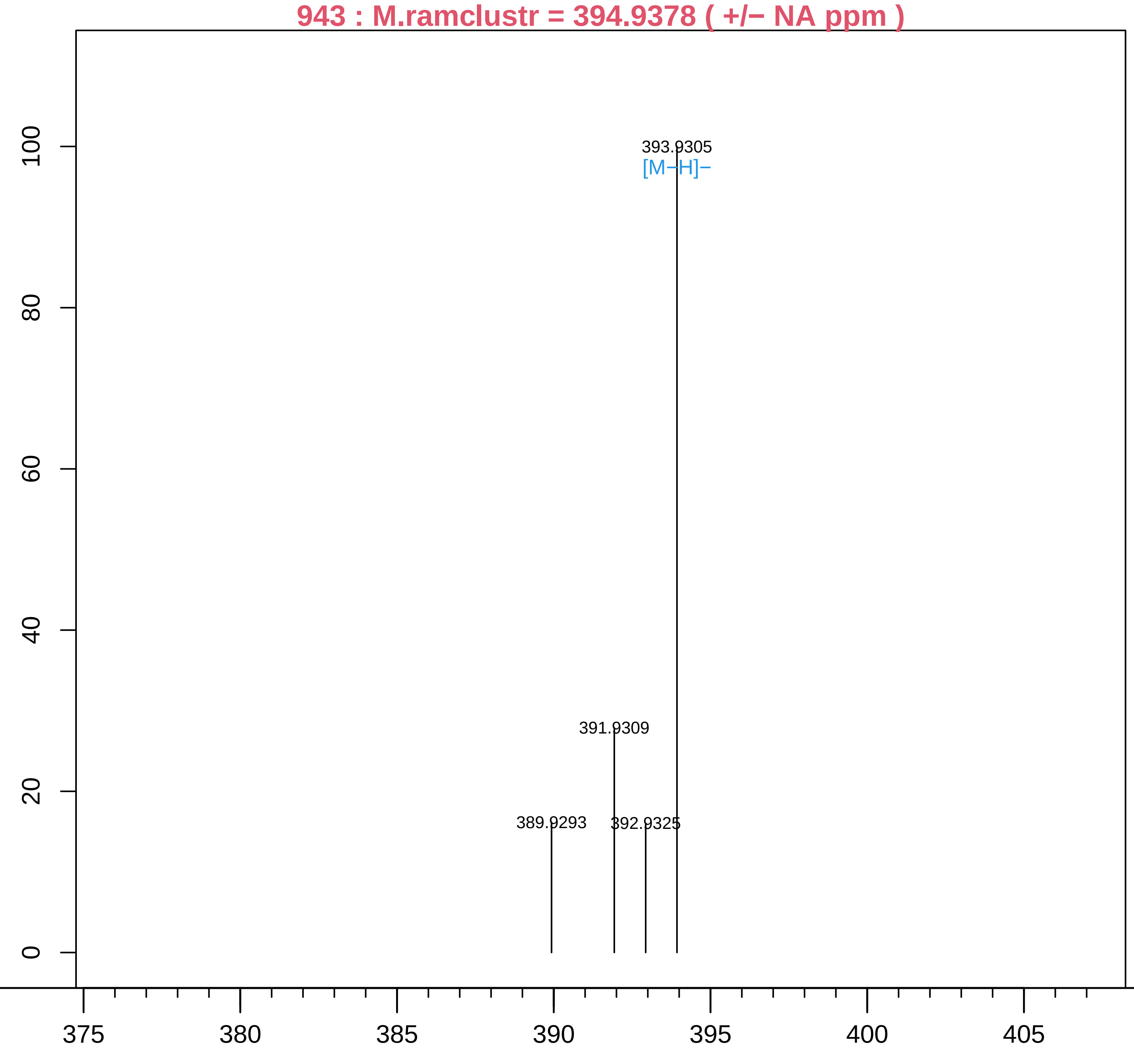
943 : M.ramclustr = 394.9378 (+/− NA ppm)
393.9305
[M−H]−
391.9309
389.9293
392.9325
0
20
40
60
80
100
375
380
385
390
395
400
405

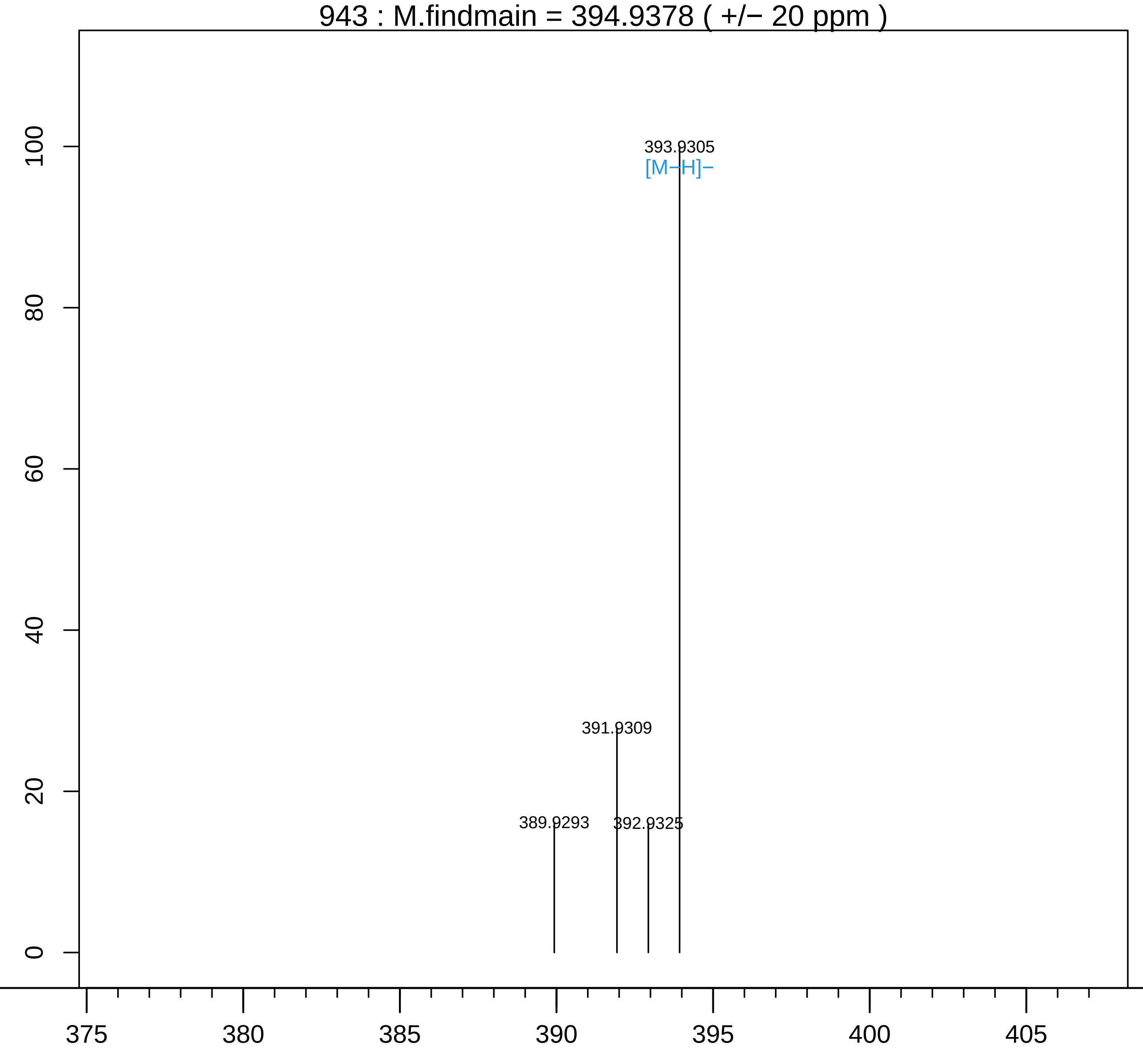
943 : M.findmain = 394.9378 (+/− 20 ppm)
393.9305
[M−H]−
391.9309
389.9293
392.9325
0
20
40
60
80
100
375
380
385
390
395
400
405

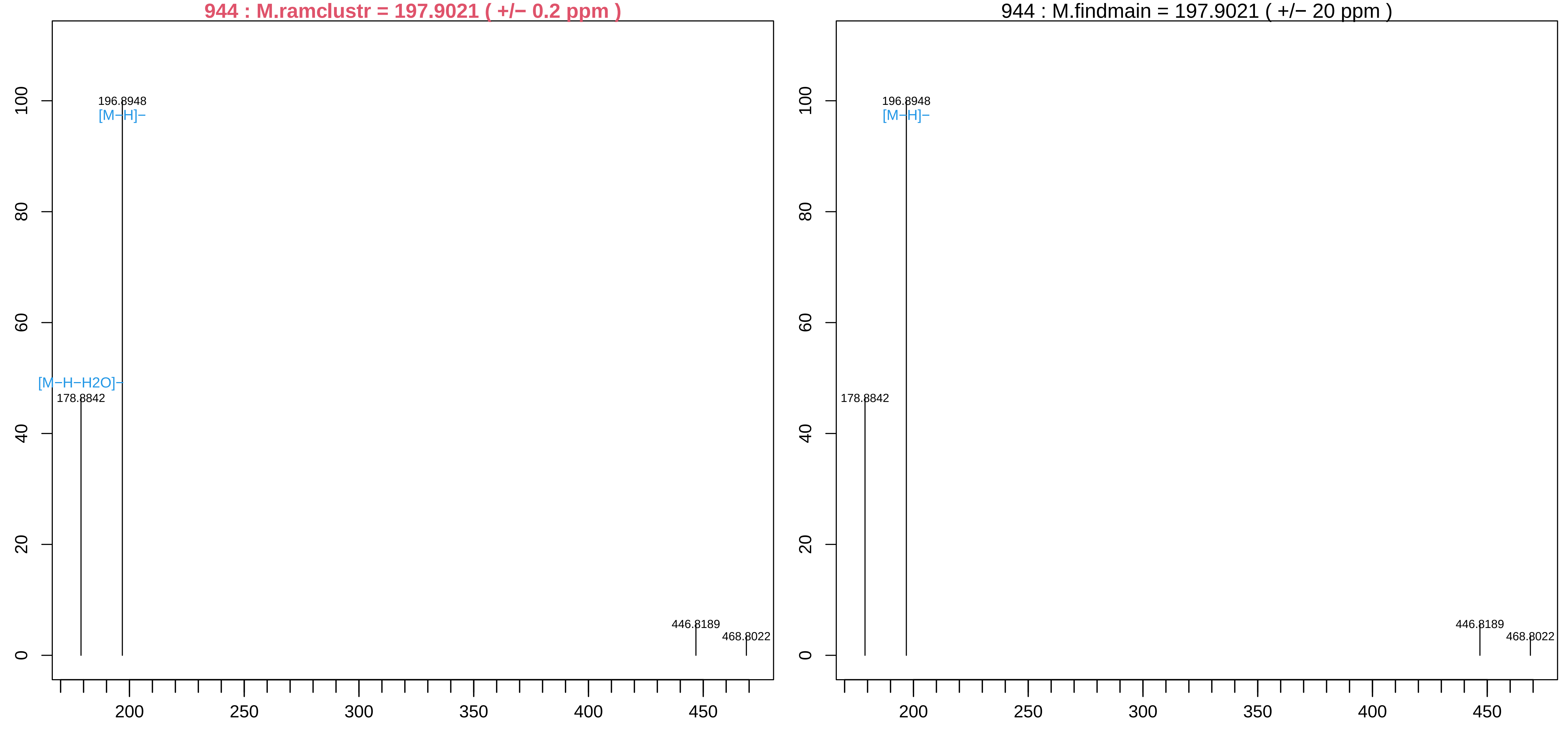
944 : M.ramclustr = 197.9021 (+/− 0.2 ppm)
196.8948
[M−H]−
[M−H−H2O]−
178.8842
446.8189
468.8022
944 : M.findmain = 197.9021 (+/− 20 ppm)
196.8948
[M−H]−
178.8842
446.8189
468.8022
0
20
40
60
80
100
200
250
300
350
400
450

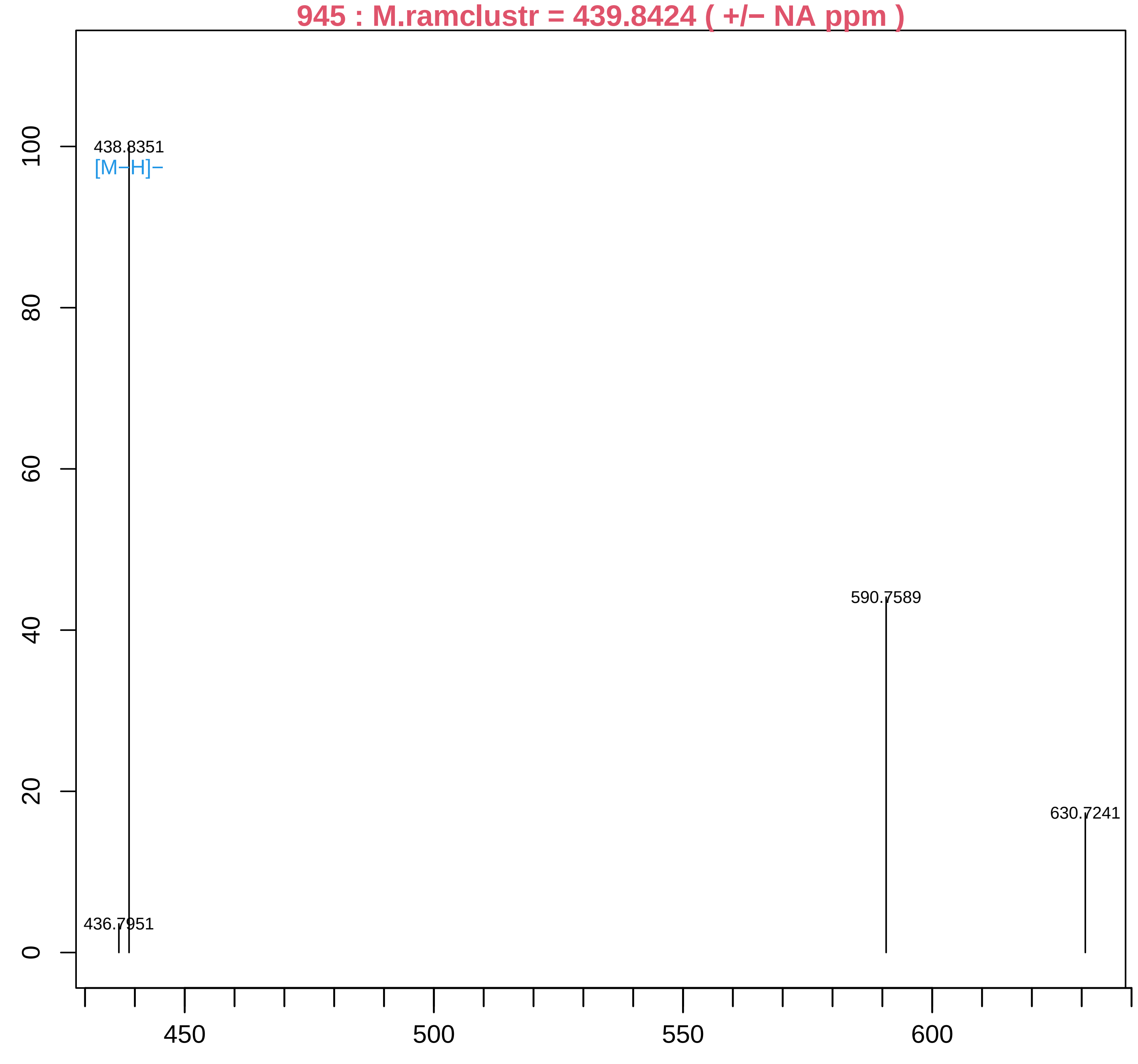

945 : M.ramclustr = 439.8424 (+/− NA ppm)
438.8351
[M−H]−
590.7589
630.7241
436.7951
0
20
40
60
80
100
450
500
550
600

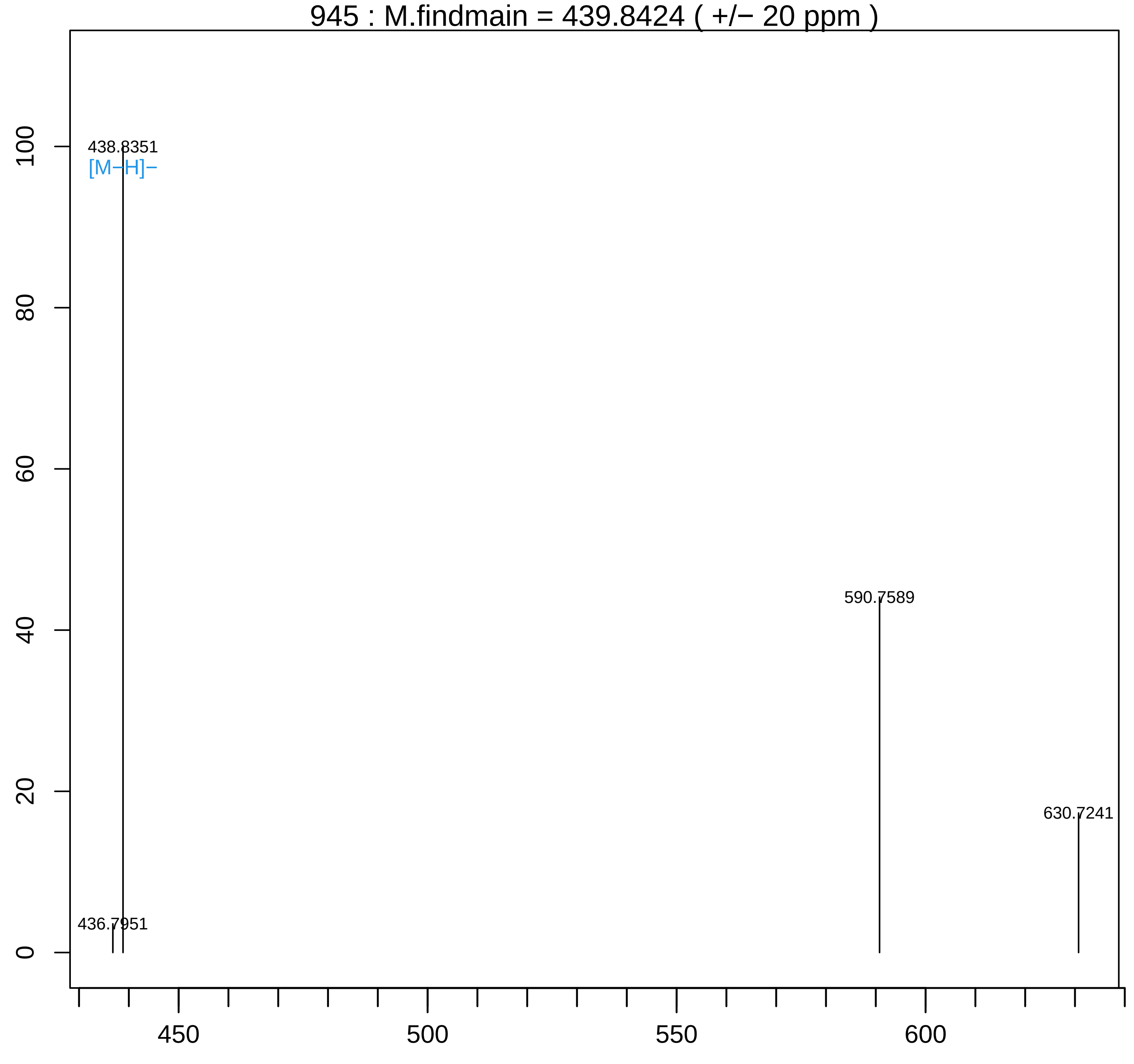

945 : M.findmain = 439.8424 (+/− 20 ppm)
438.8351
[M−H]−
590.7589
630.7241
436.7951
0
20
40
60
80
100
450
500
550
600

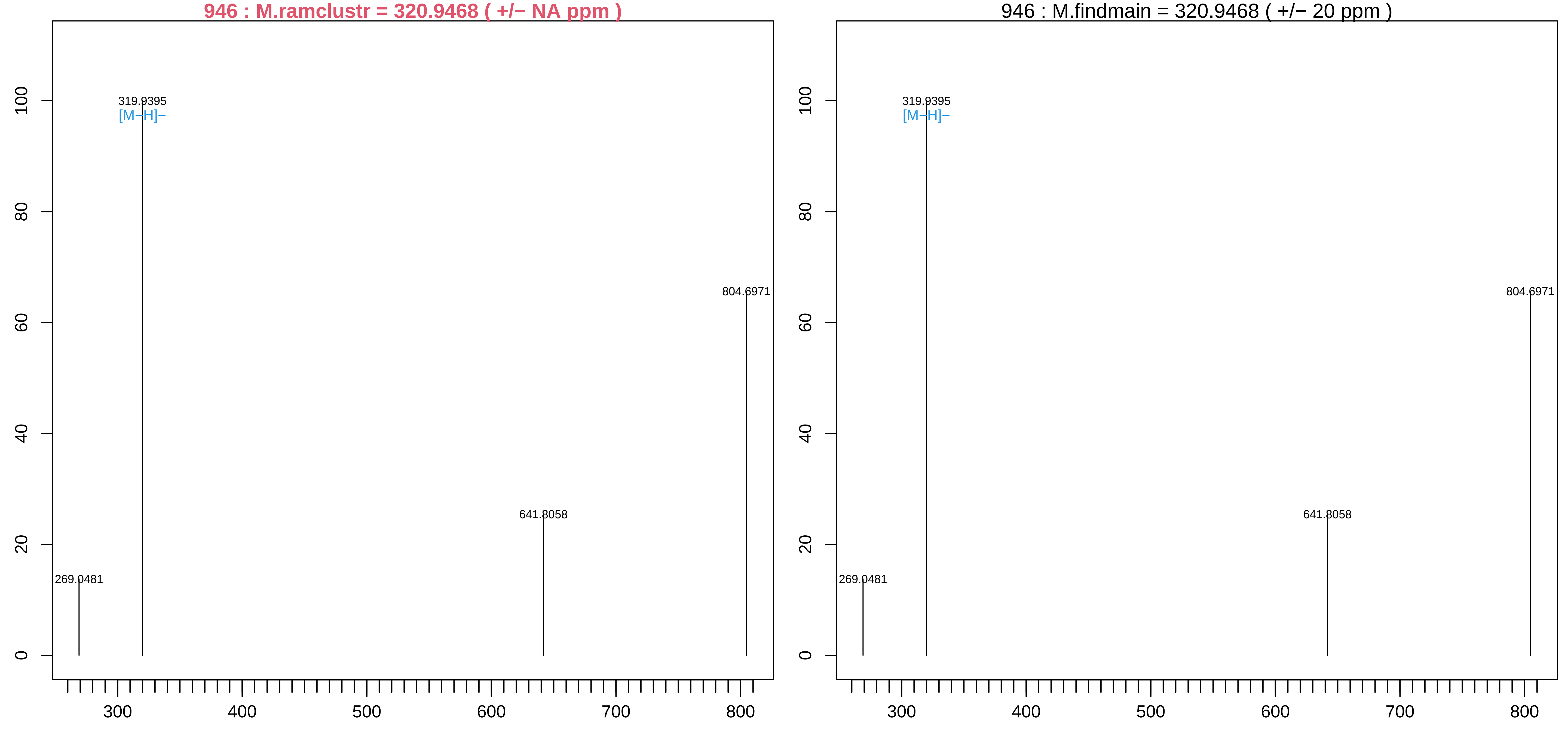

946 : M.ramclustr = 320.9468 (+/− NA ppm)
946 : M.findmain = 320.9468 (+/− 20 ppm)
319.9395
[M−H]−
804.6971
641.8058
269.0481
0
20
40
60
80
100
300
400
500
600
700
800

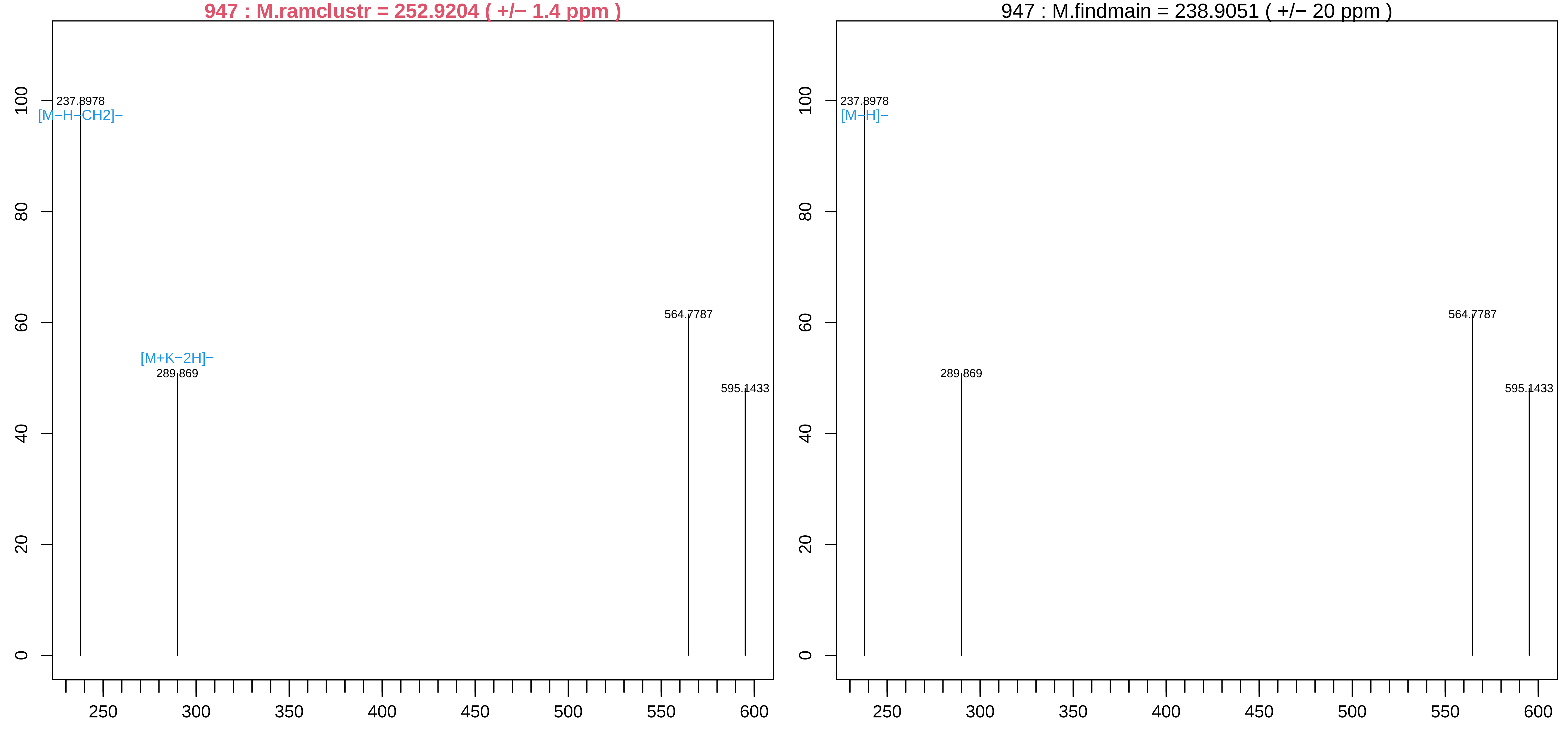

947 : M.ramclustr = 252.9204 (+/− 1.4 ppm)
237.8978
[M−H−CH2]−
[M+K−2H]−
289.869
564.7787
595.1433
0
20
40
60
80
100
250
300
350
400
450
500
550
600
947 : M.findmain = 238.9051 (+/− 20 ppm)
237.8978
[M−H]−
289.869
564.7787
595.1433

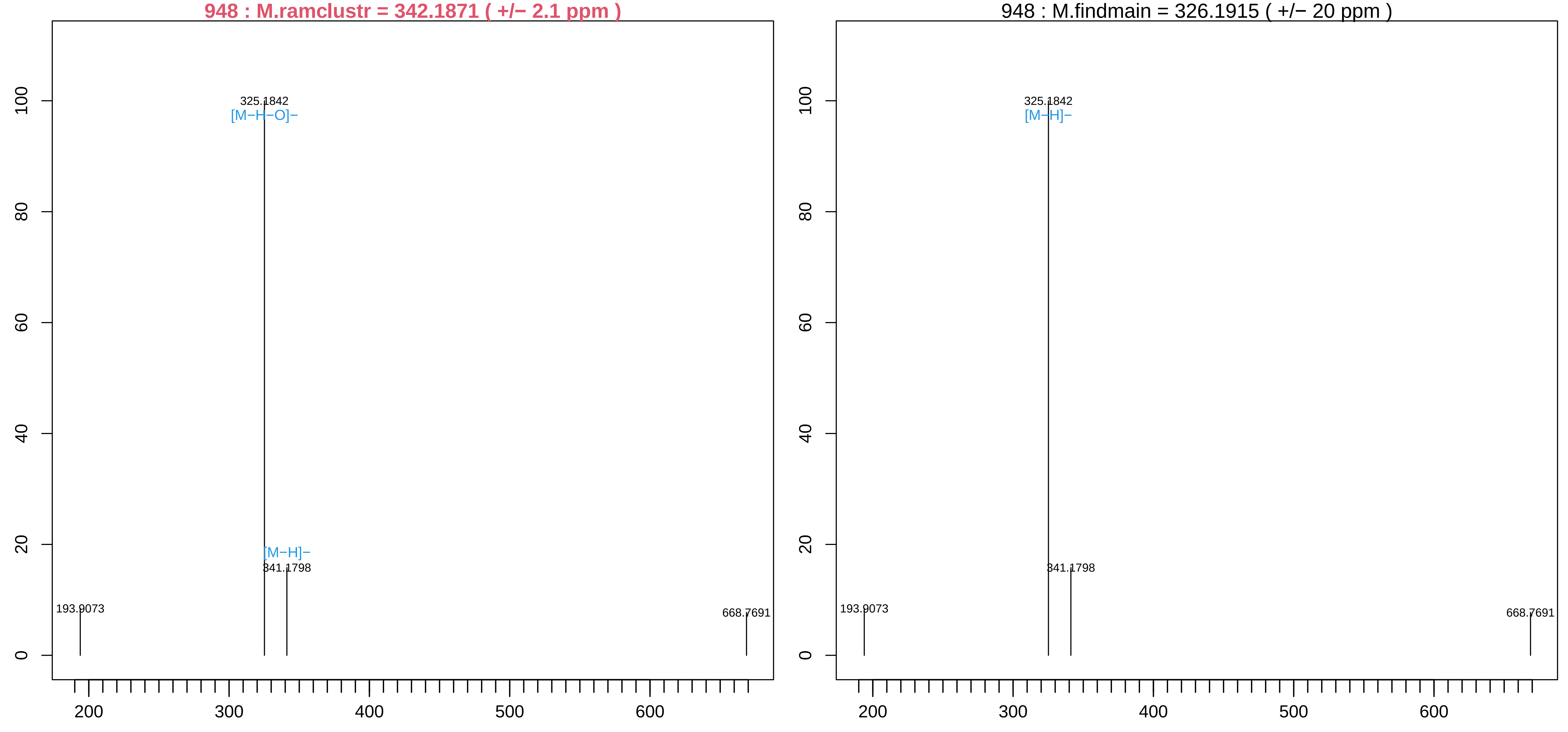

948 : M.ramclustr = 342.1871 (+/− 2.1 ppm)
325.1842
[M−H−O]−
[M−H]−
341.1798
193.9073
668.7691
0
20
40
60
80
100
200
300
400
500
600
948 : M.findmain = 326.1915 (+/− 20 ppm)
325.1842
[M−H]−
341.1798
193.9073
668.7691
0
20
40
60
80
100
200
300
400
500
600

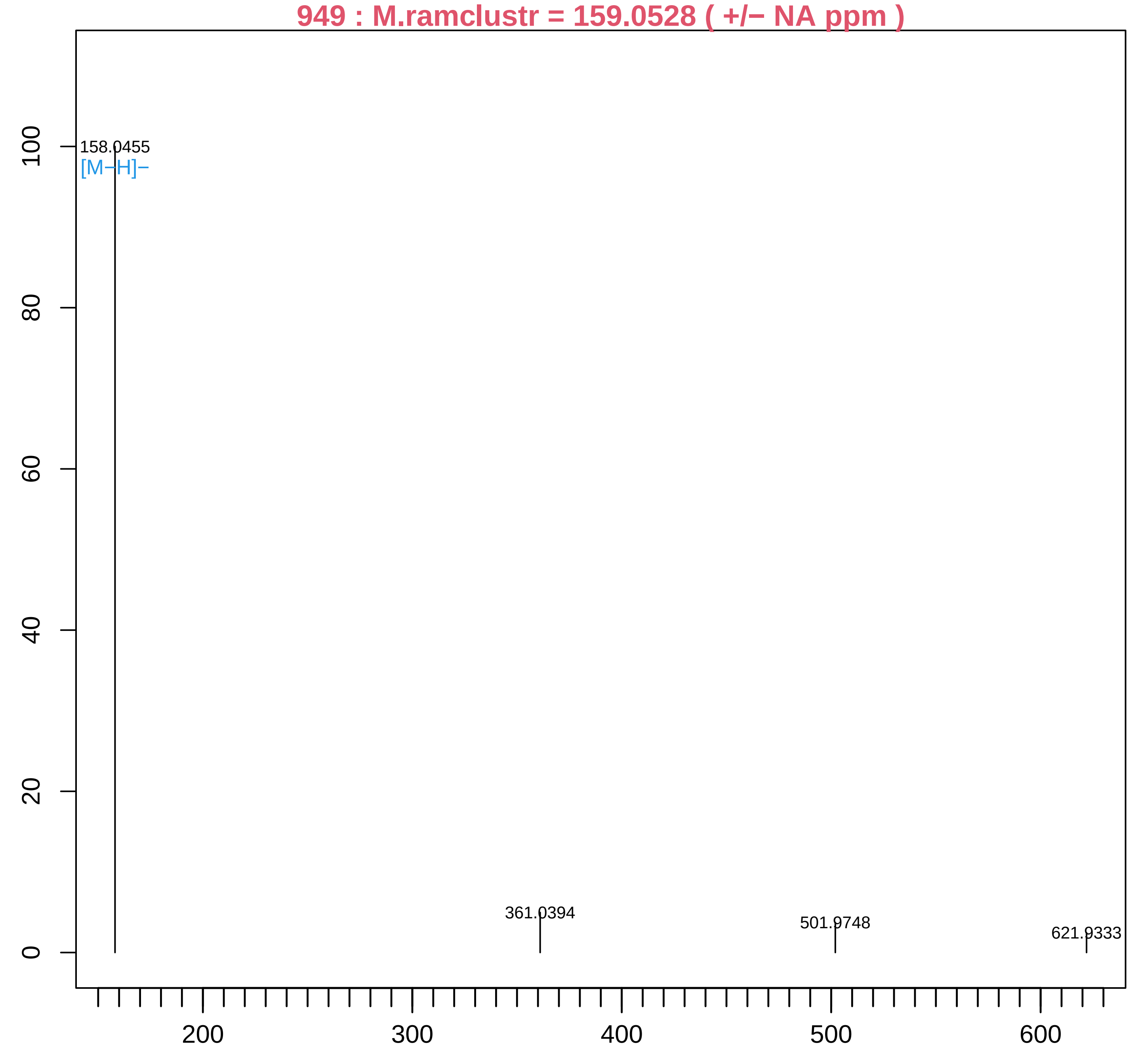

949 : M.ramclustr = 159.0528 (+/− NA ppm)
158.0455
[M−H]−
361.0394
501.9748
621.9333
0
20
40
60
80
100
200
300
400
500
600

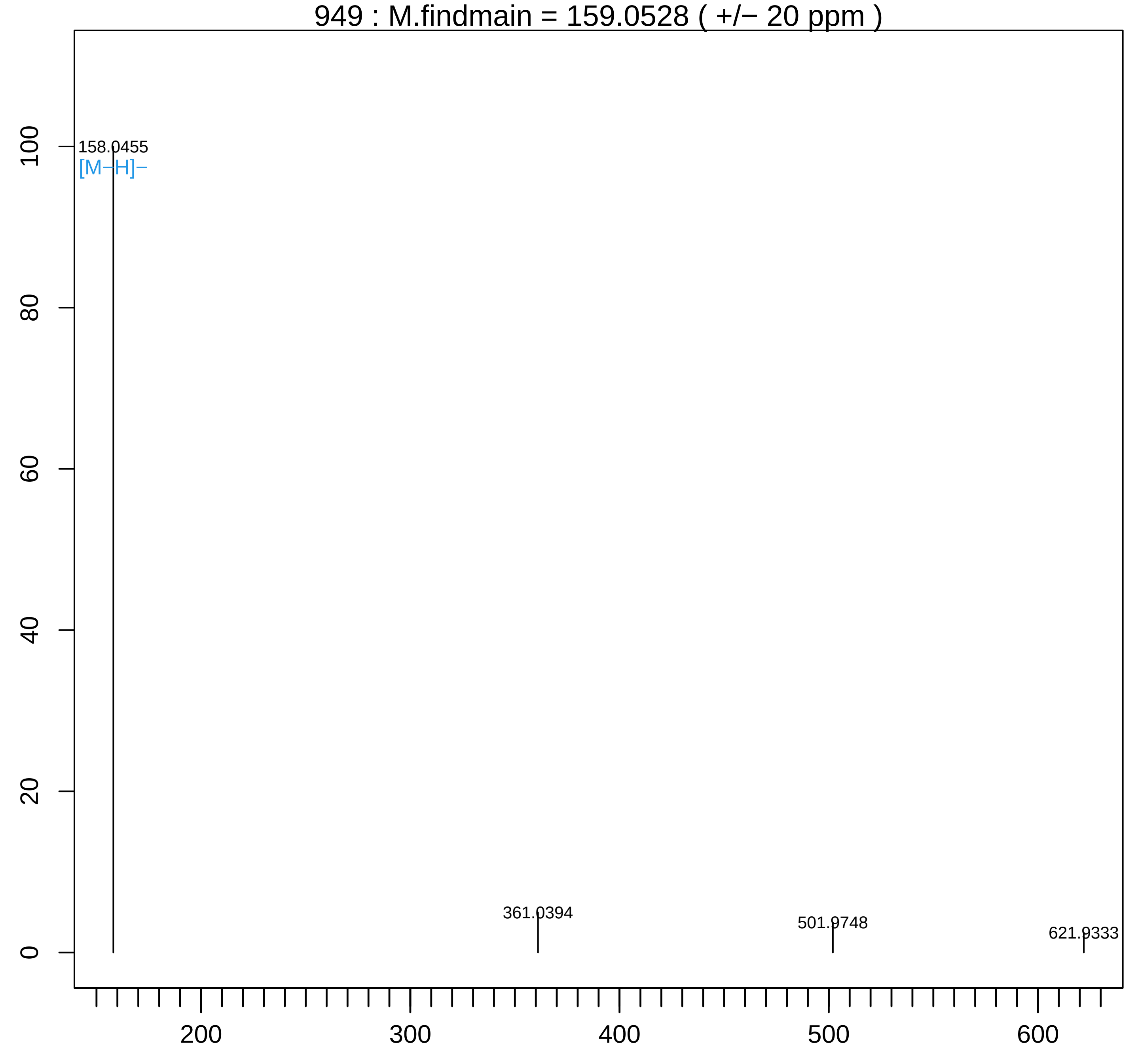

949 : M.findmain = 159.0528 (+/− 20 ppm)
158.0455
[M−H]−
361.0394
501.9748
621.9333
0
20
40
60
80
100
200
300
400
500
600

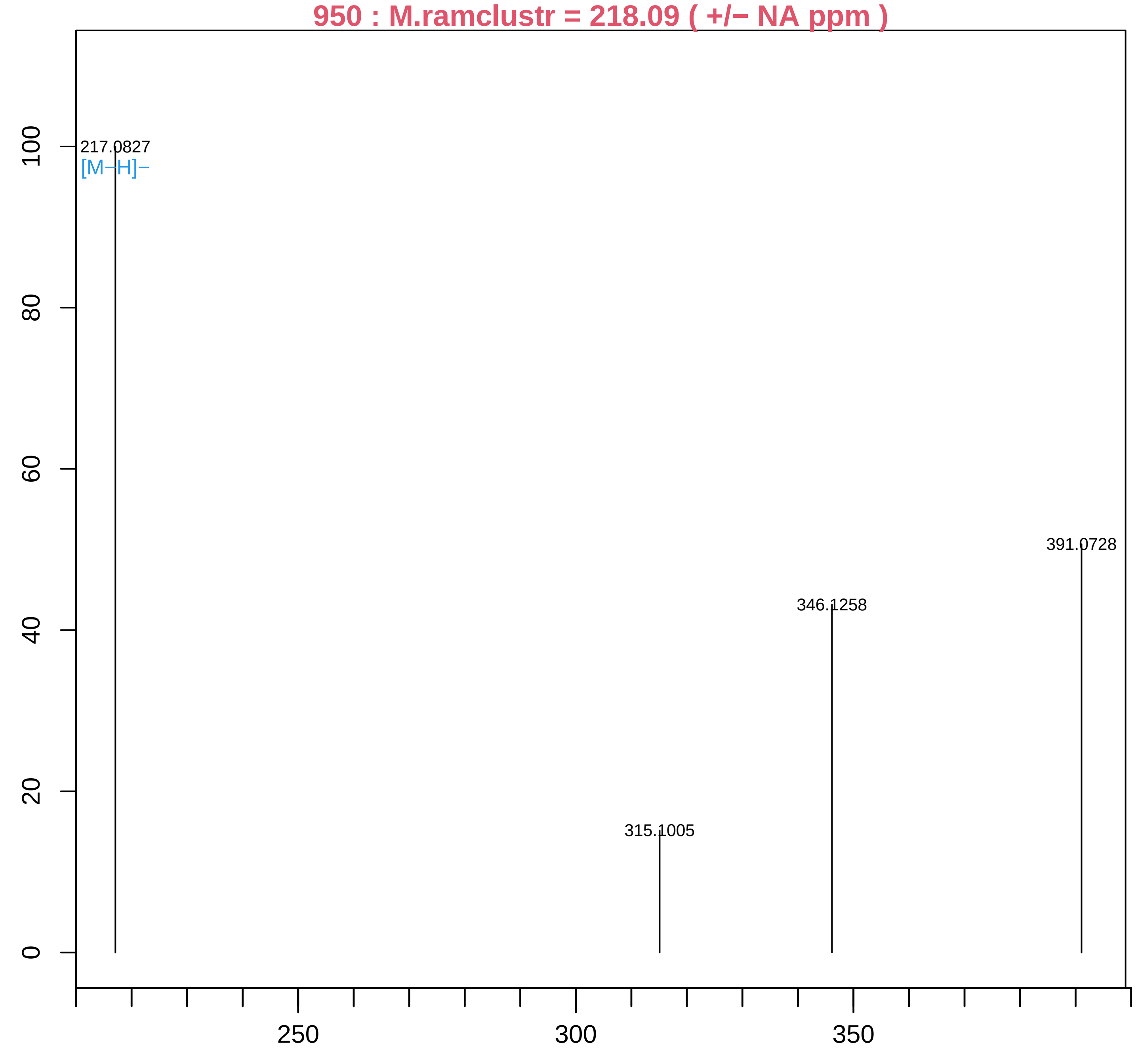
950 : M.ramclustr = 218.09 (+/− NA ppm)
217.0827
[M−H]−
315.1005
346.1258
391.0728
0
20
40
60
80
100
250
300
350

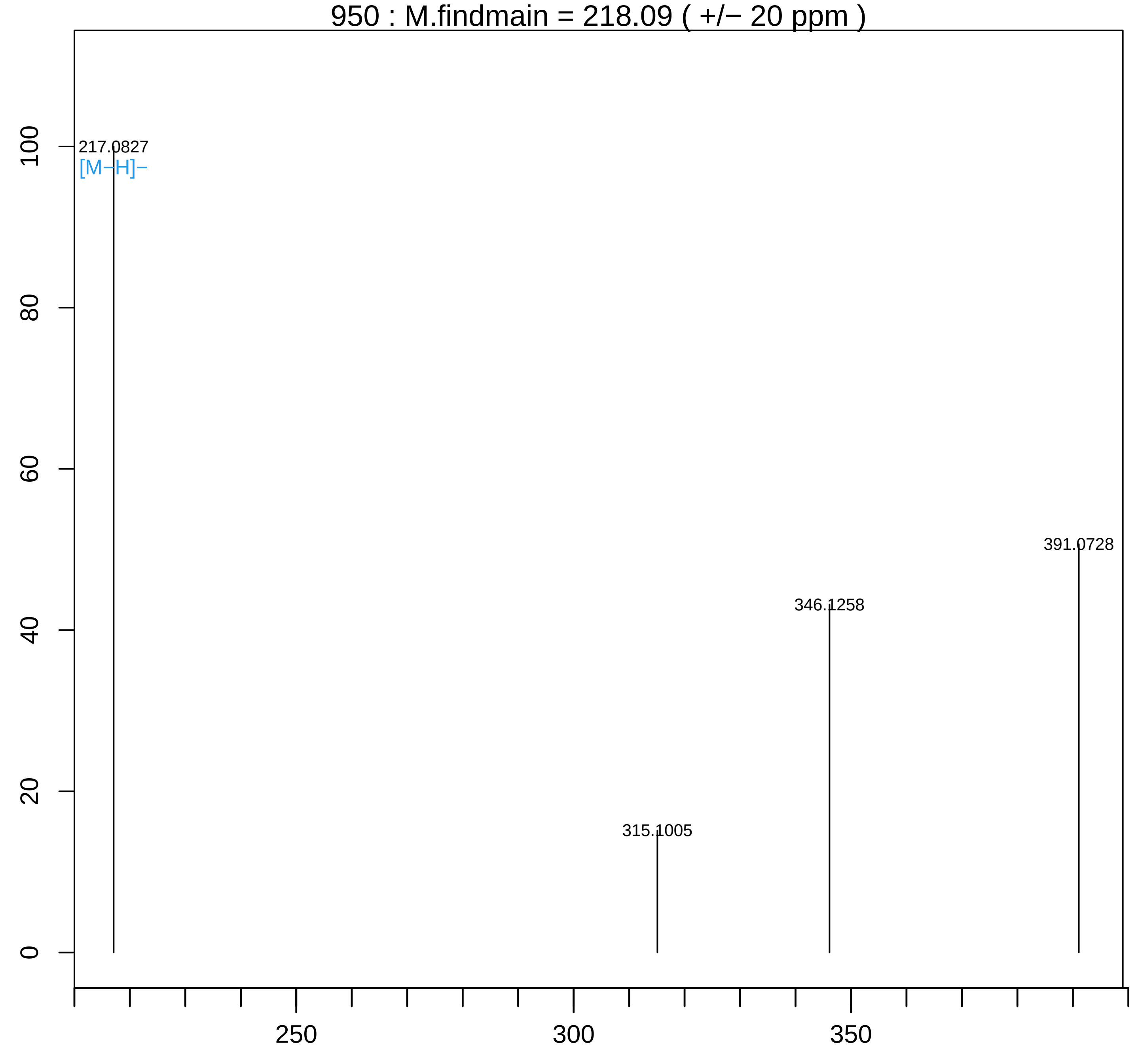
950 : M.findmain = 218.09 (+/− 20 ppm)
217.0827
[M−H]−
315.1005
346.1258
391.0728
0
20
40
60
80
100
250
300
350

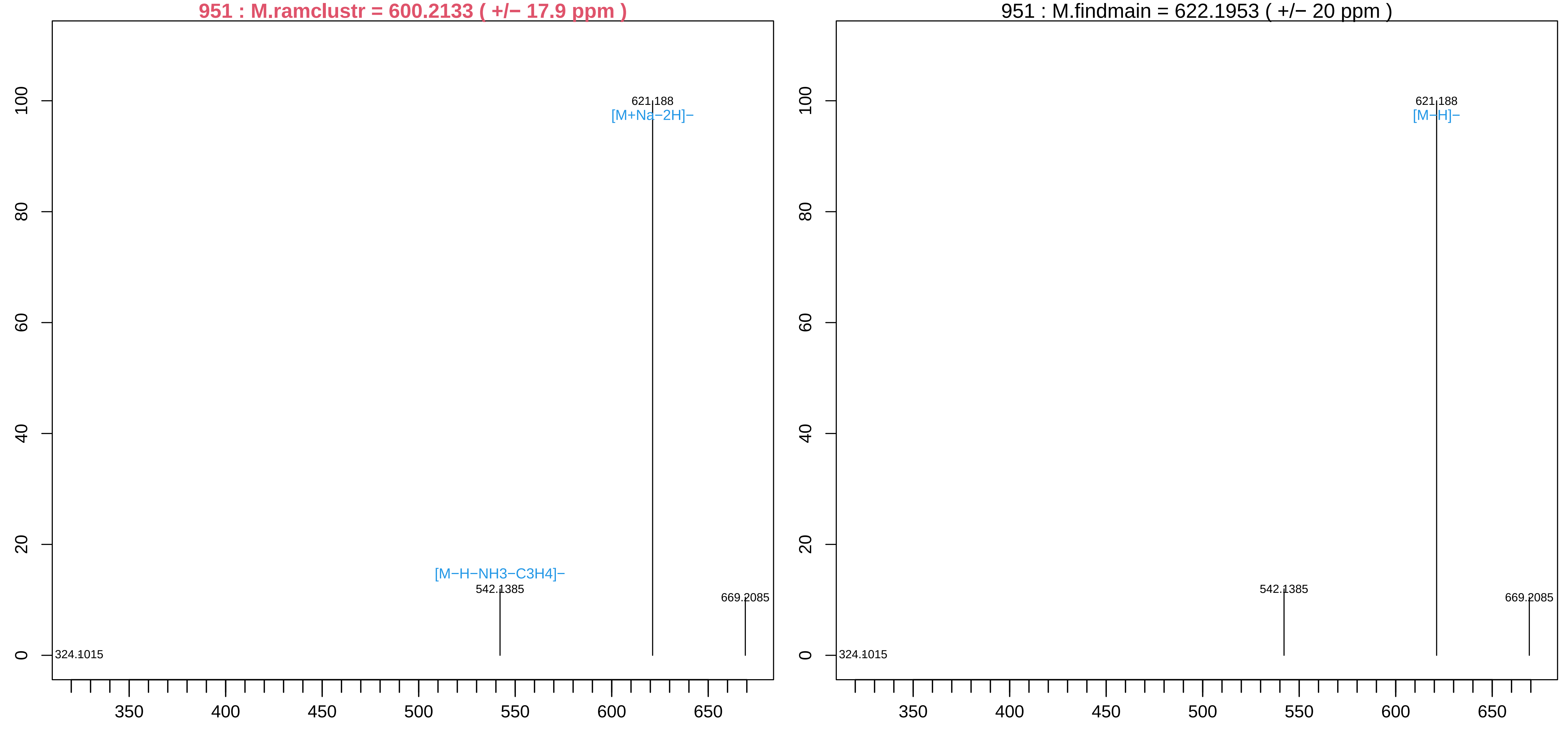
951 : M.ramclustr = 600.2133 (+/− 17.9 ppm)
621.188
[M+Na−2H]−
[M−H−NH3−C3H4]−
542.1385
669.2085
324.1015
951 : M.findmain = 622.1953 (+/− 20 ppm)
621.188
[M−H]−
542.1385
669.2085
324.1015

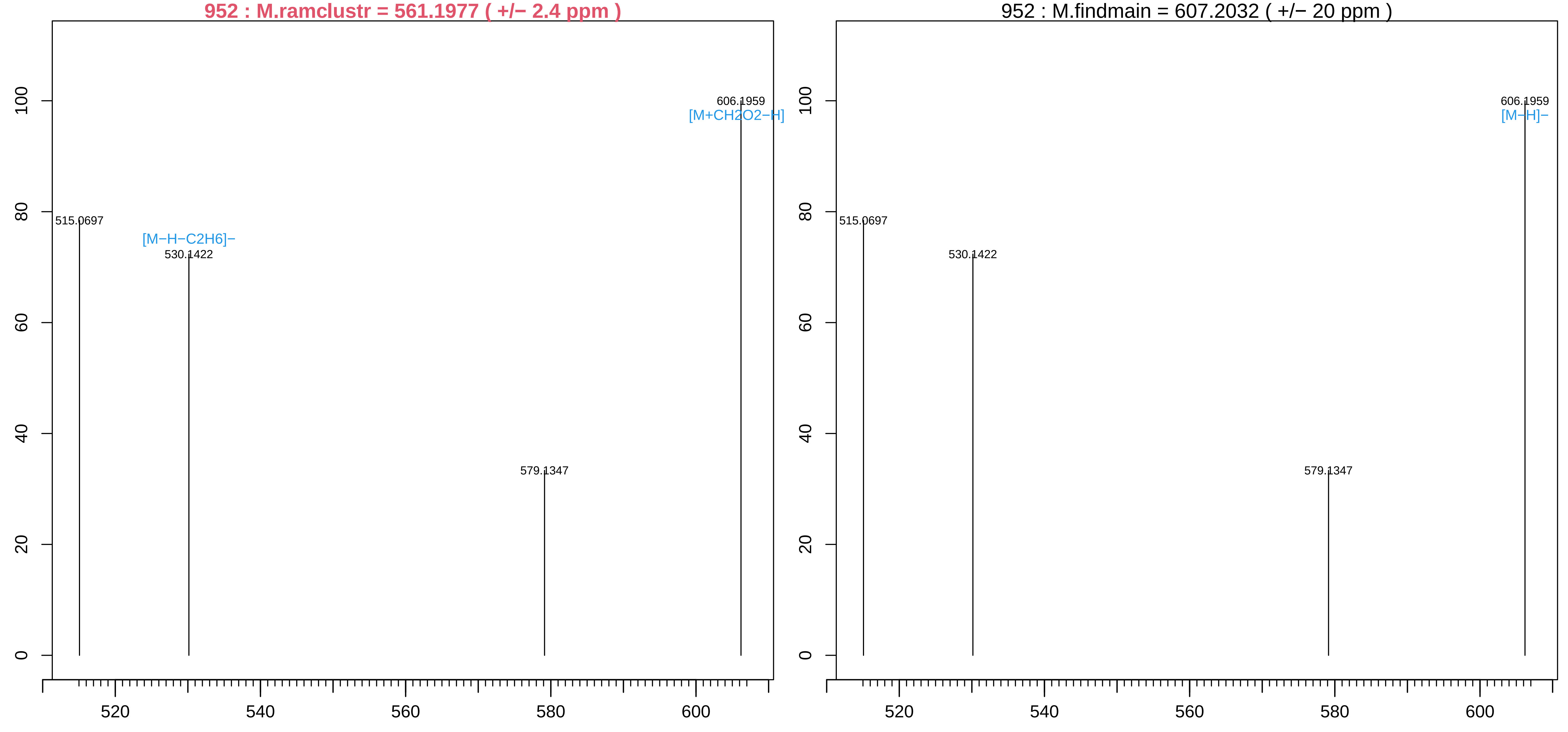
952 : M.ramclustr = 561.1977 (+/− 2.4 ppm)
515.0697
[M−H−C2H6]−
530.1422
579.1347
606.1959
[M+CH2O2−H]
952 : M.findmain = 607.2032 (+/− 20 ppm)
515.0697
530.1422
579.1347
606.1959
[M−H]−
0
20
40
60
80
100
520
540
560
580
600

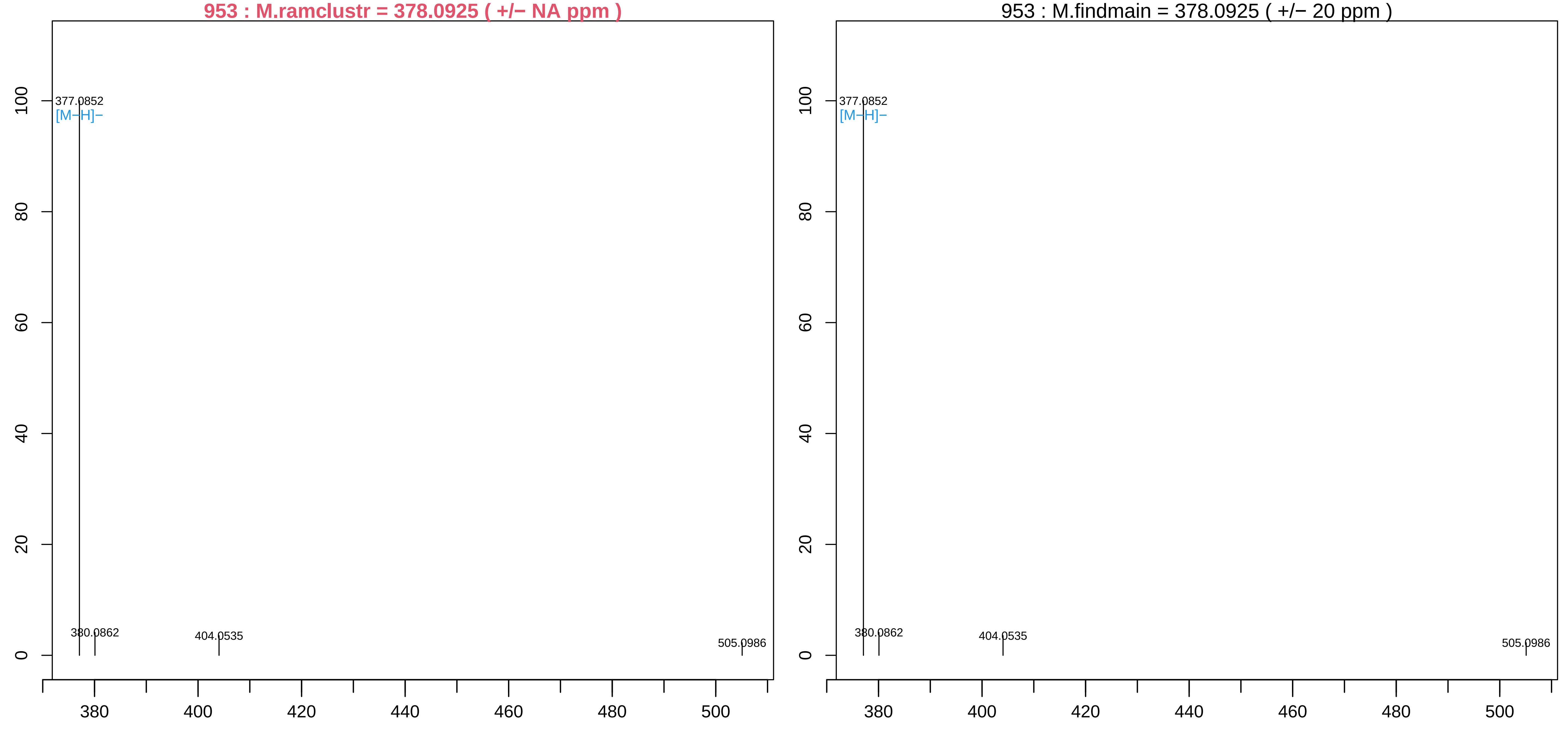
953 : M.ramclustr = 378.0925 (+/− NA ppm)
377.0852
[M−H]−
380.0862
404.0535
505.0986
953 : M.findmain = 378.0925 (+/− 20 ppm)
377.0852
[M−H]−
380.0862
404.0535
505.0986

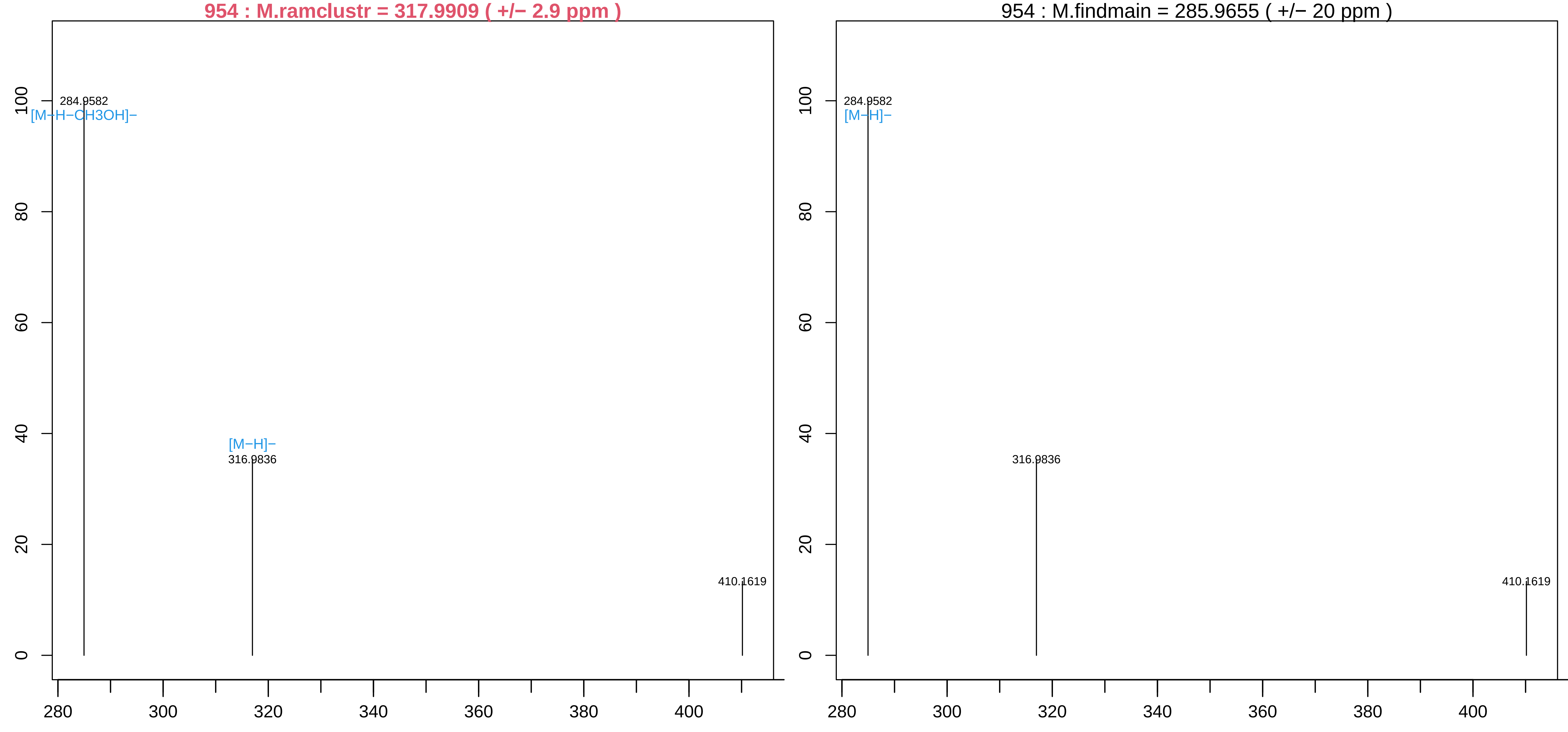
954 : M.ramclustr = 317.9909 (+/− 2.9 ppm)
284.9582
[M−H−CH3OH]−
[M−H]−
316.9836
410.1619
954 : M.findmain = 285.9655 (+/− 20 ppm)
284.9582
[M−H]−
316.9836
410.1619

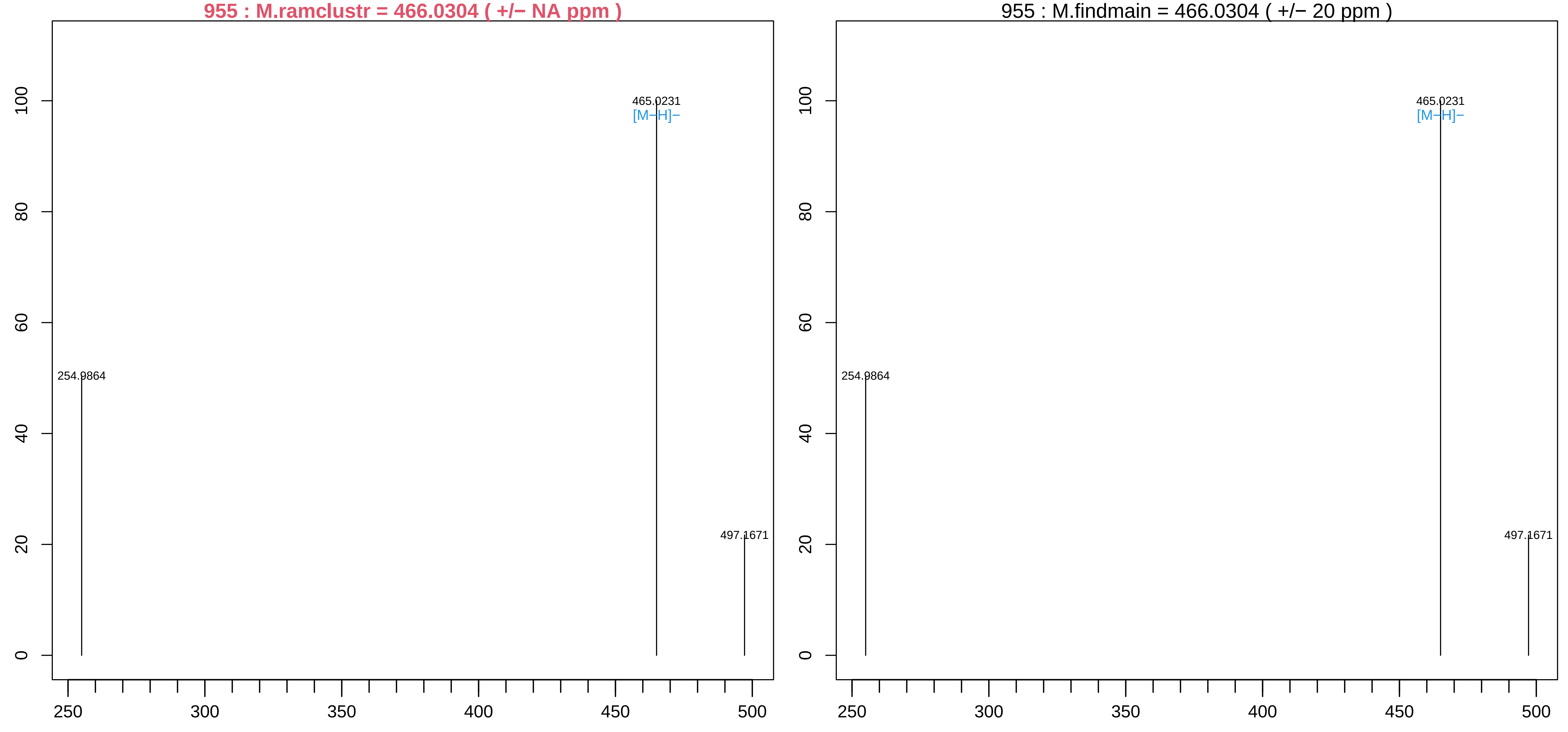

955 : M.ramclustr = 466.0304 (+/− NA ppm)
955 : M.findmain = 466.0304 (+/− 20 ppm)
465.0231
[M−H]−
254.9864
497.1671
0
20
40
60
80
100
250
300
350
400
450
500

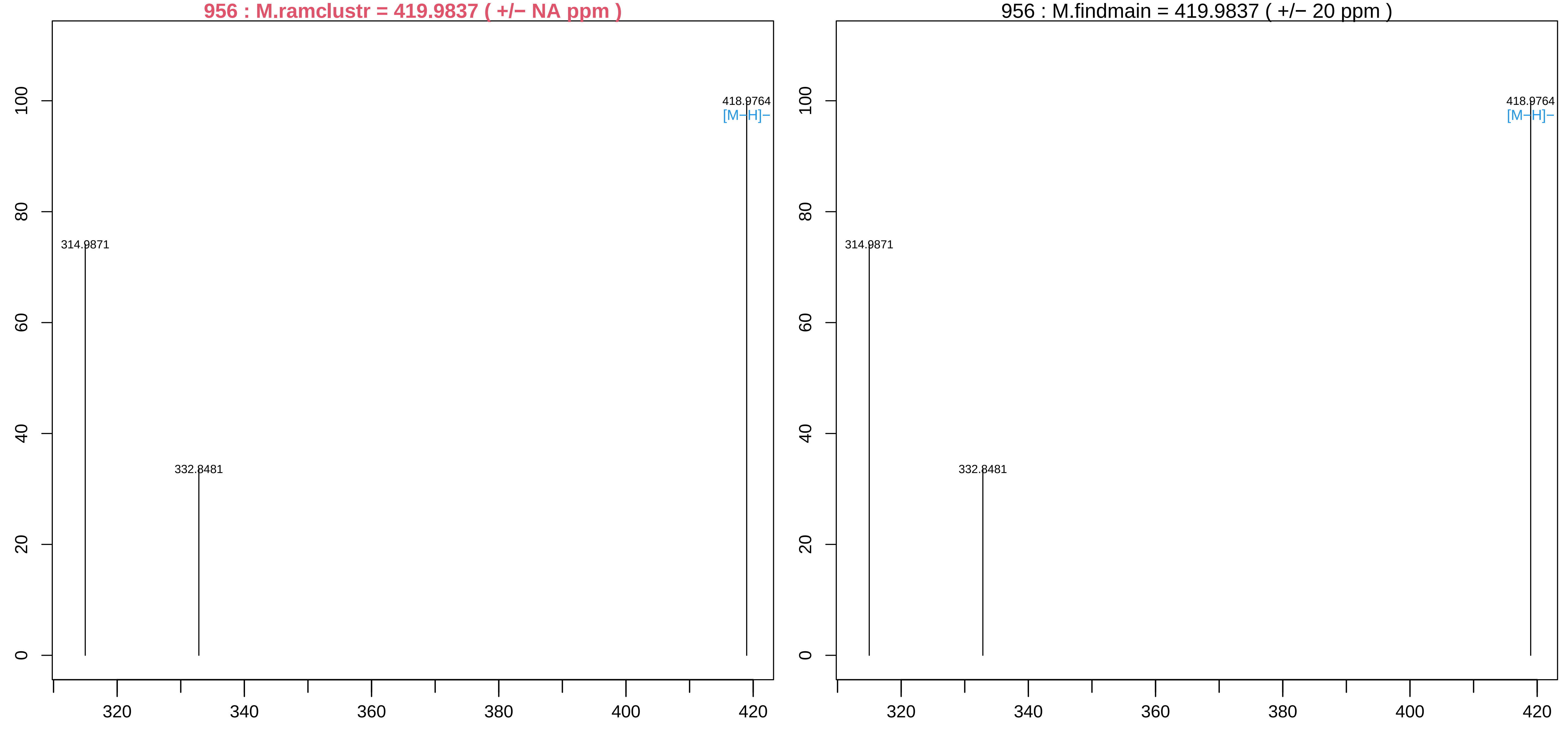
956 : M.ramclustr = 419.9837 (+/− NA ppm)
956 : M.findmain = 419.9837 (+/− 20 ppm)
314.9871
332.8481
418.9764
[M−H]−
0
20
40
60
80
100
320
340
360
380
400
420

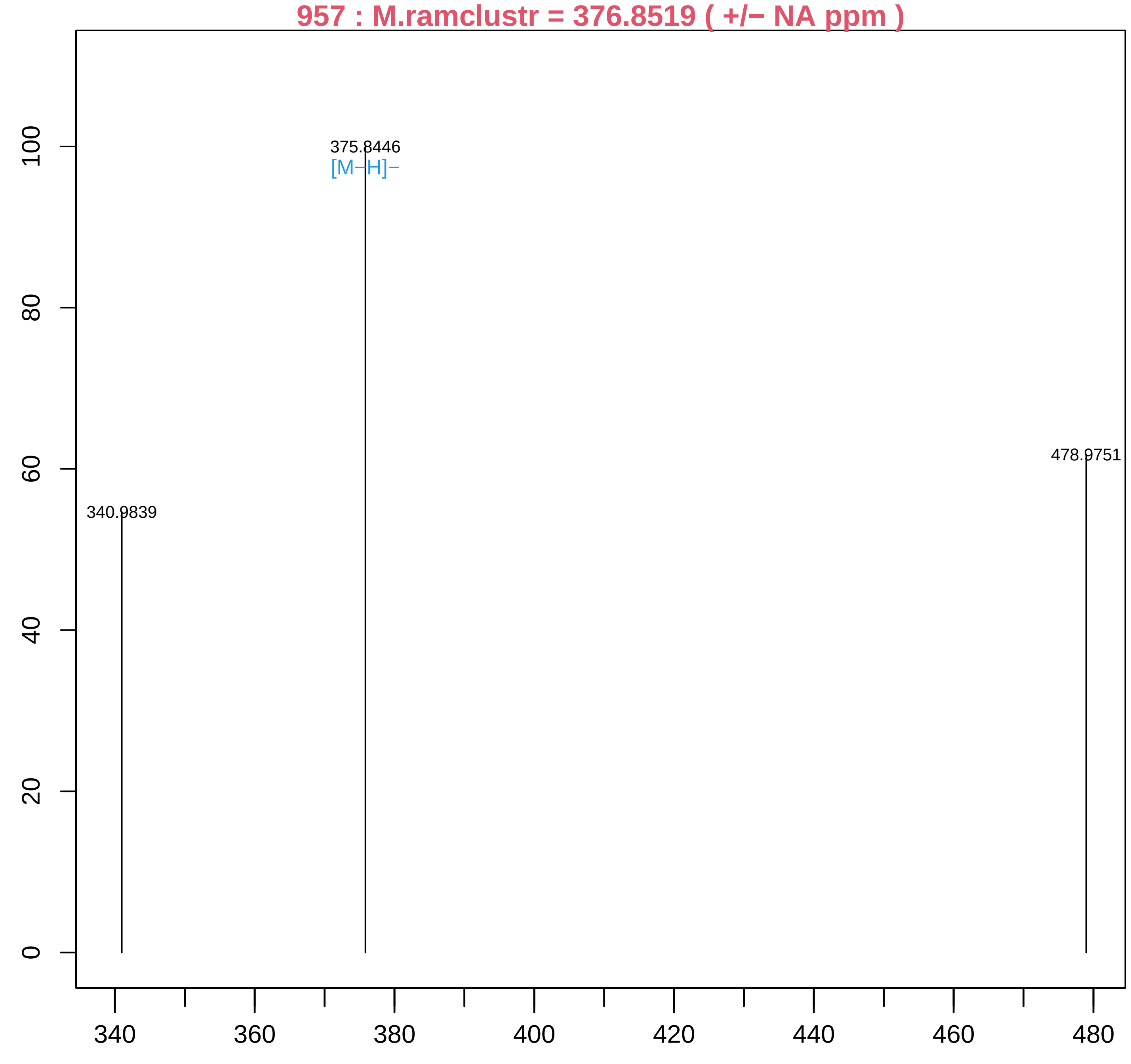

957 : M.ramclustr = 376.8519 (+/− NA ppm)
375.8446
[M−H]−
478.9751
340.9839

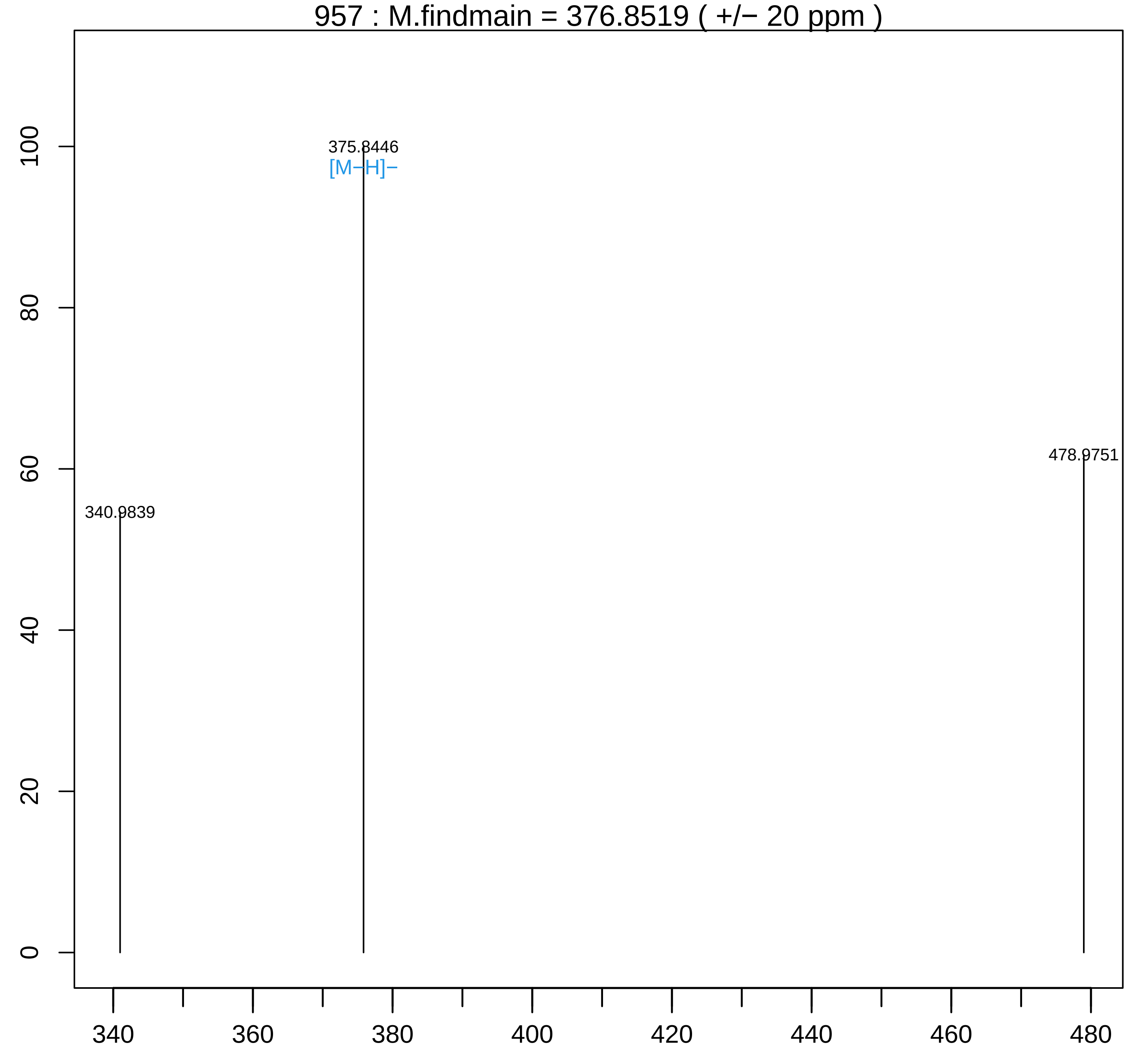

957 : M.findmain = 376.8519 (+/− 20 ppm)
375.8446
[M−H]−
478.9751
340.9839

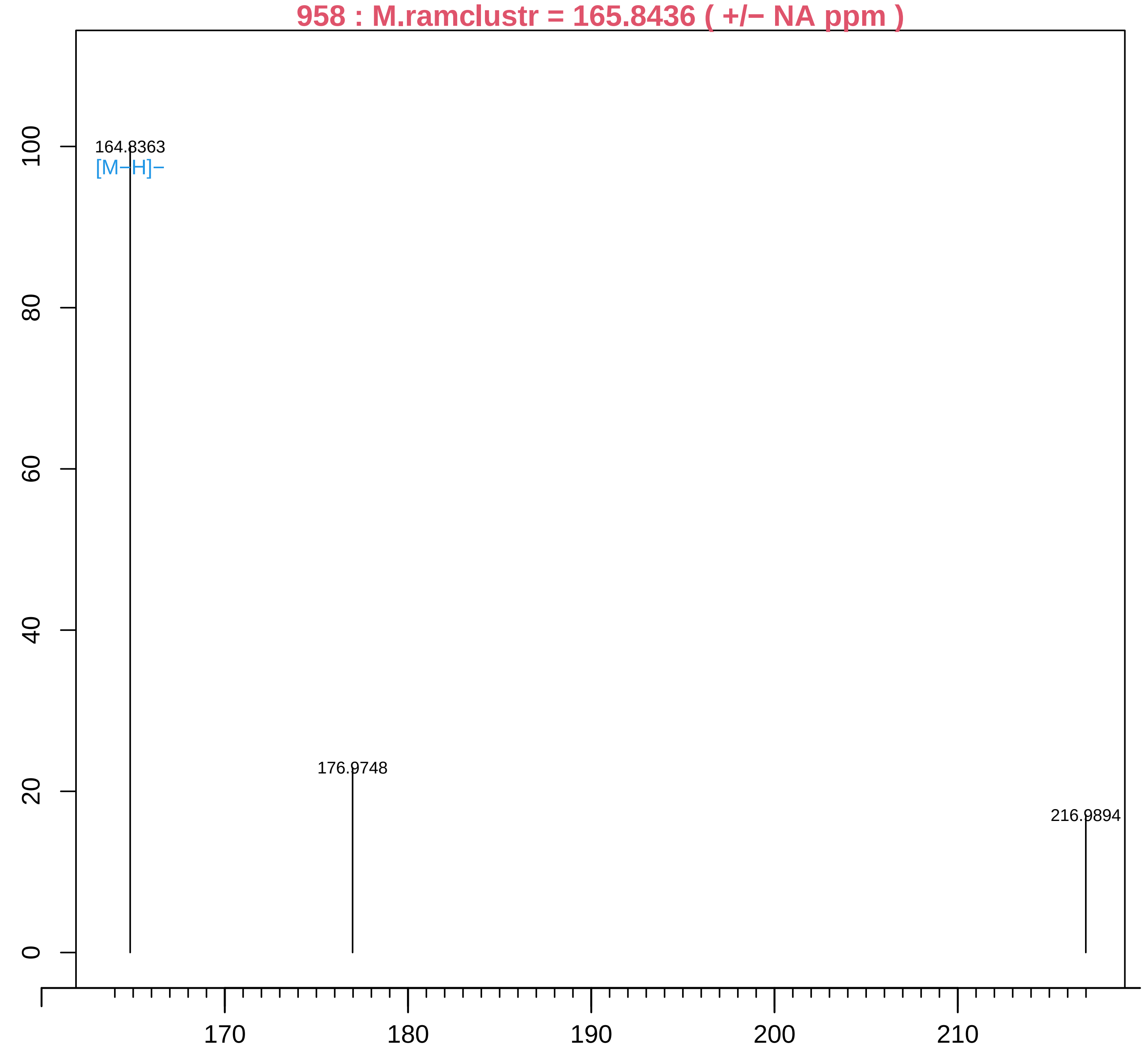
958 : M.ramclustr = 165.8436 (+/− NA ppm)
164.8363
[M−H]−
176.9748
216.9894
0
20
40
60
80
100
170
180
190
200
210

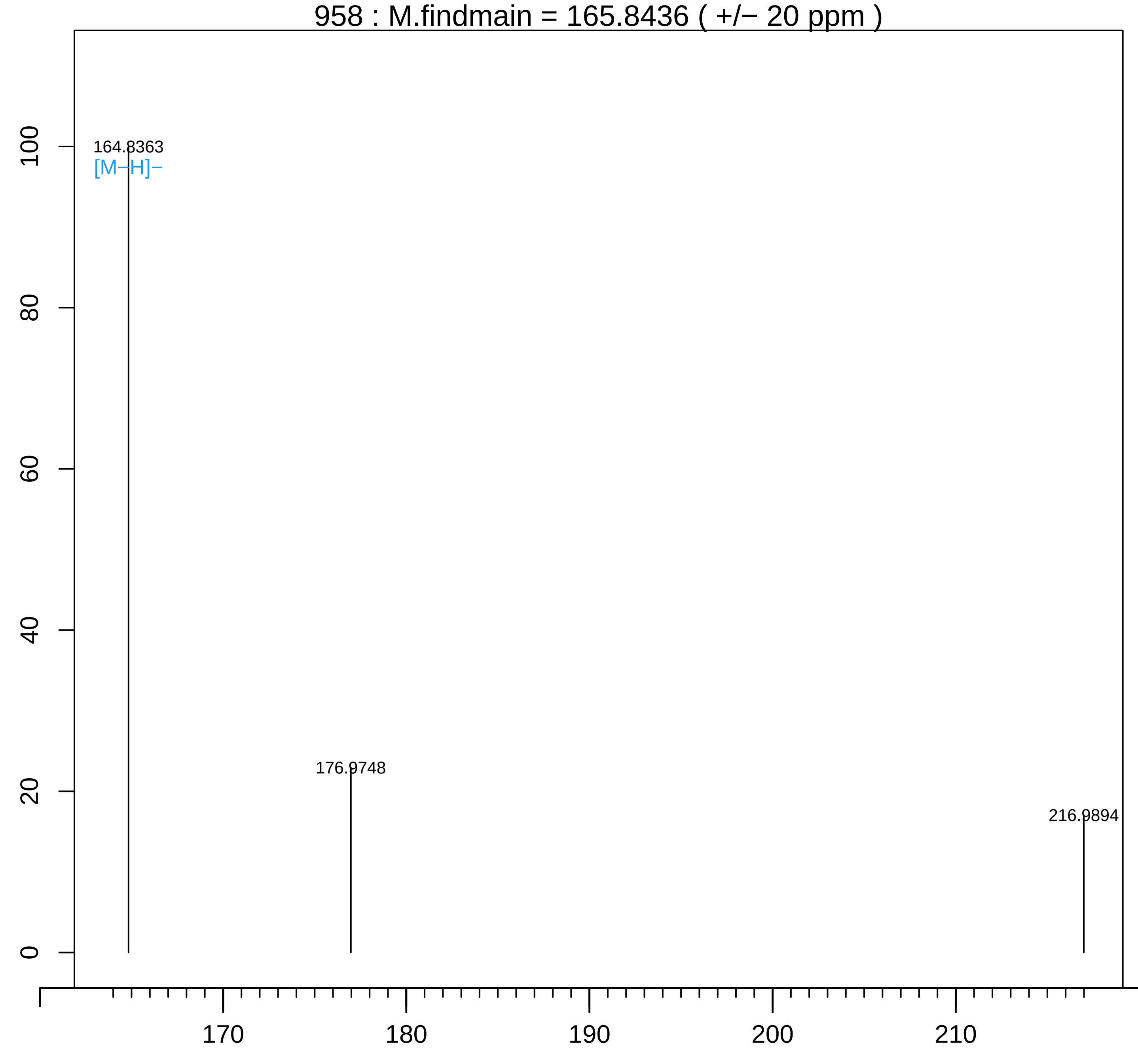
958 : M.findmain = 165.8436 (+/− 20 ppm)
164.8363
[M−H]−
176.9748
216.9894
0
20
40
60
80
100
170
180
190
200
210

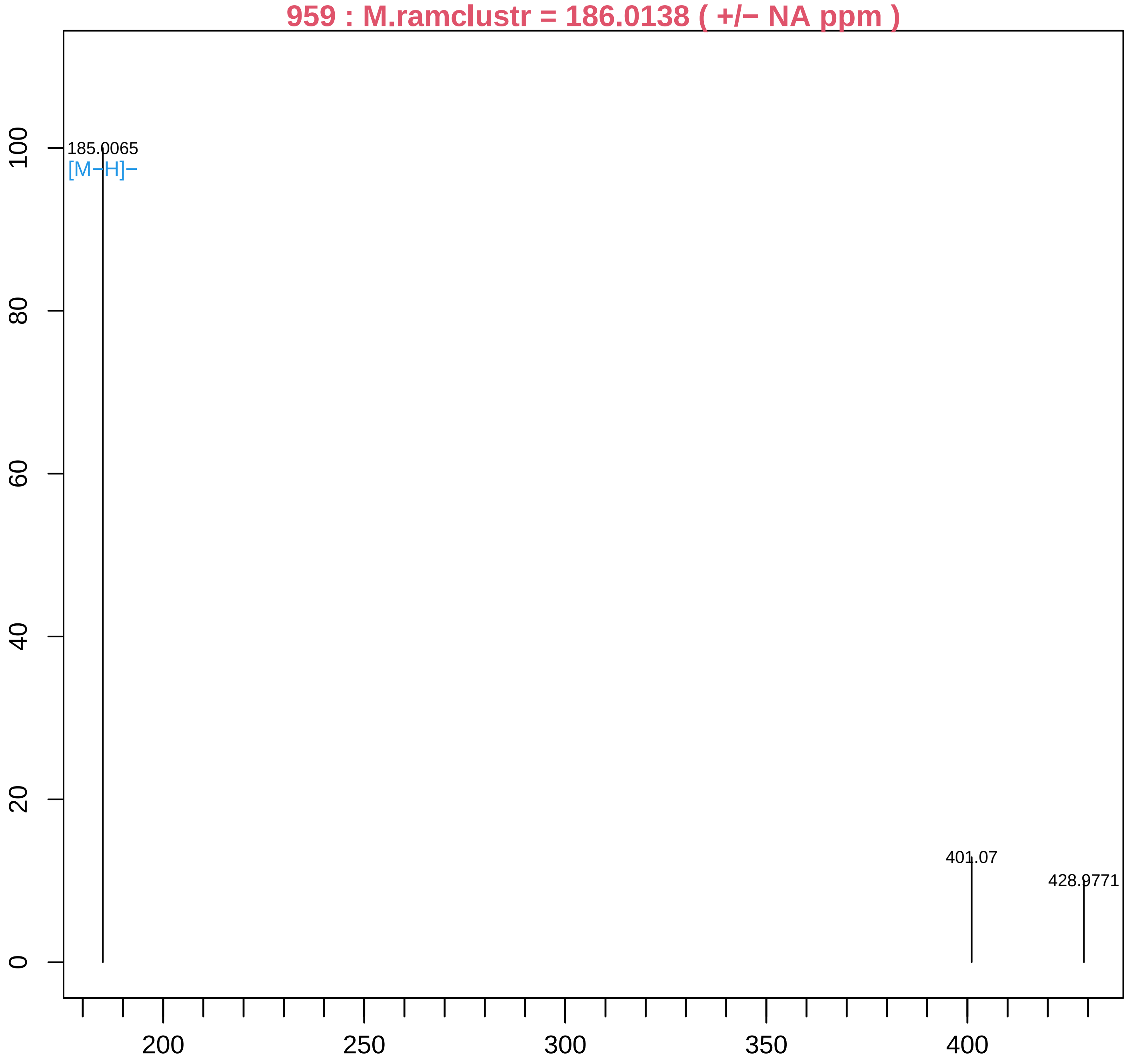
959 : M.ramclustr = 186.0138 (+/− NA ppm)
185.0065
[M−H]−
401.07
428.9771
0
20
40
60
80
100
200
250
300
350
400

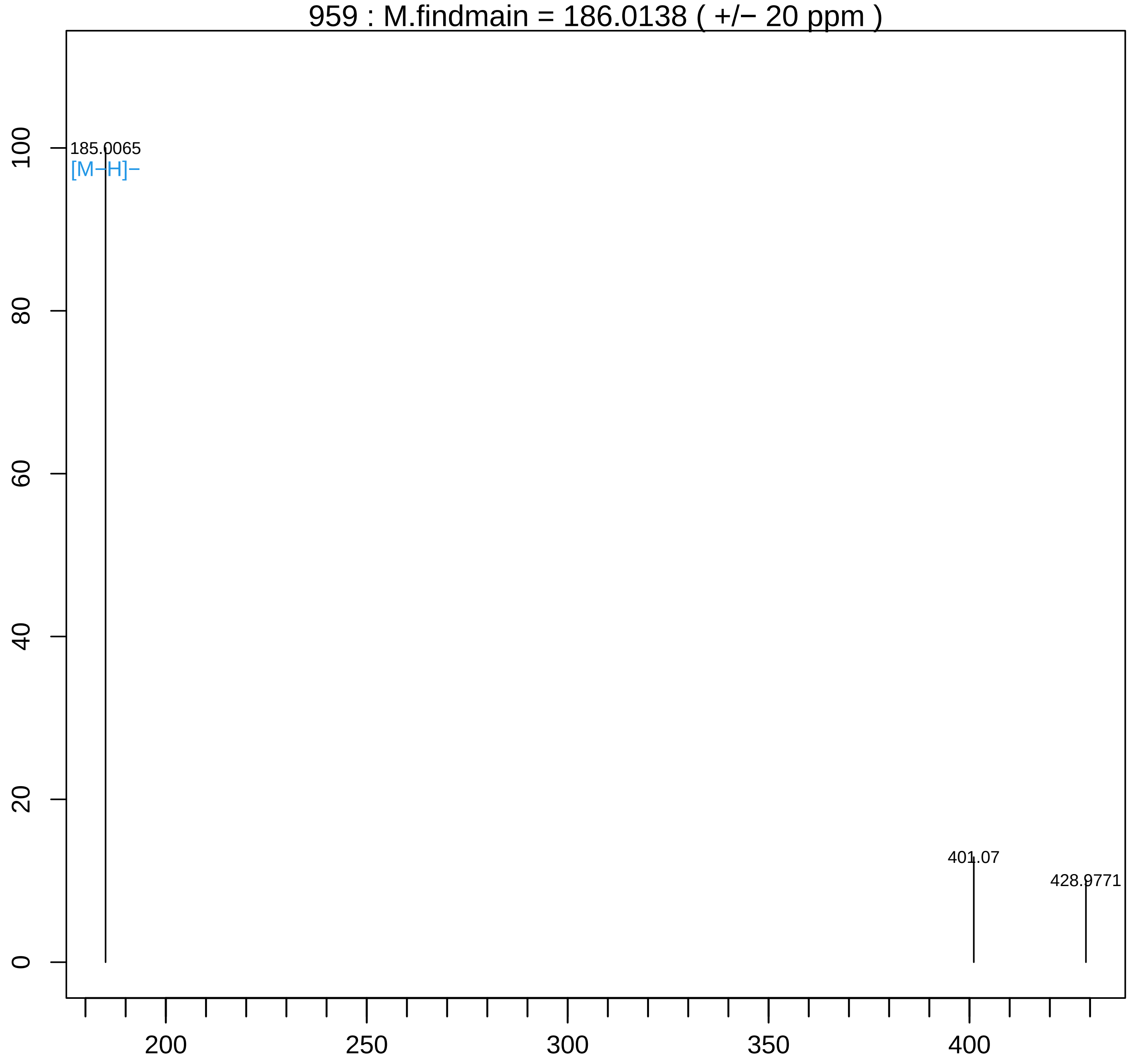
959 : M.findmain = 186.0138 (+/− 20 ppm)
185.0065
[M−H]−
401.07
428.9771
0
20
40
60
80
100
200
250
300
350
400

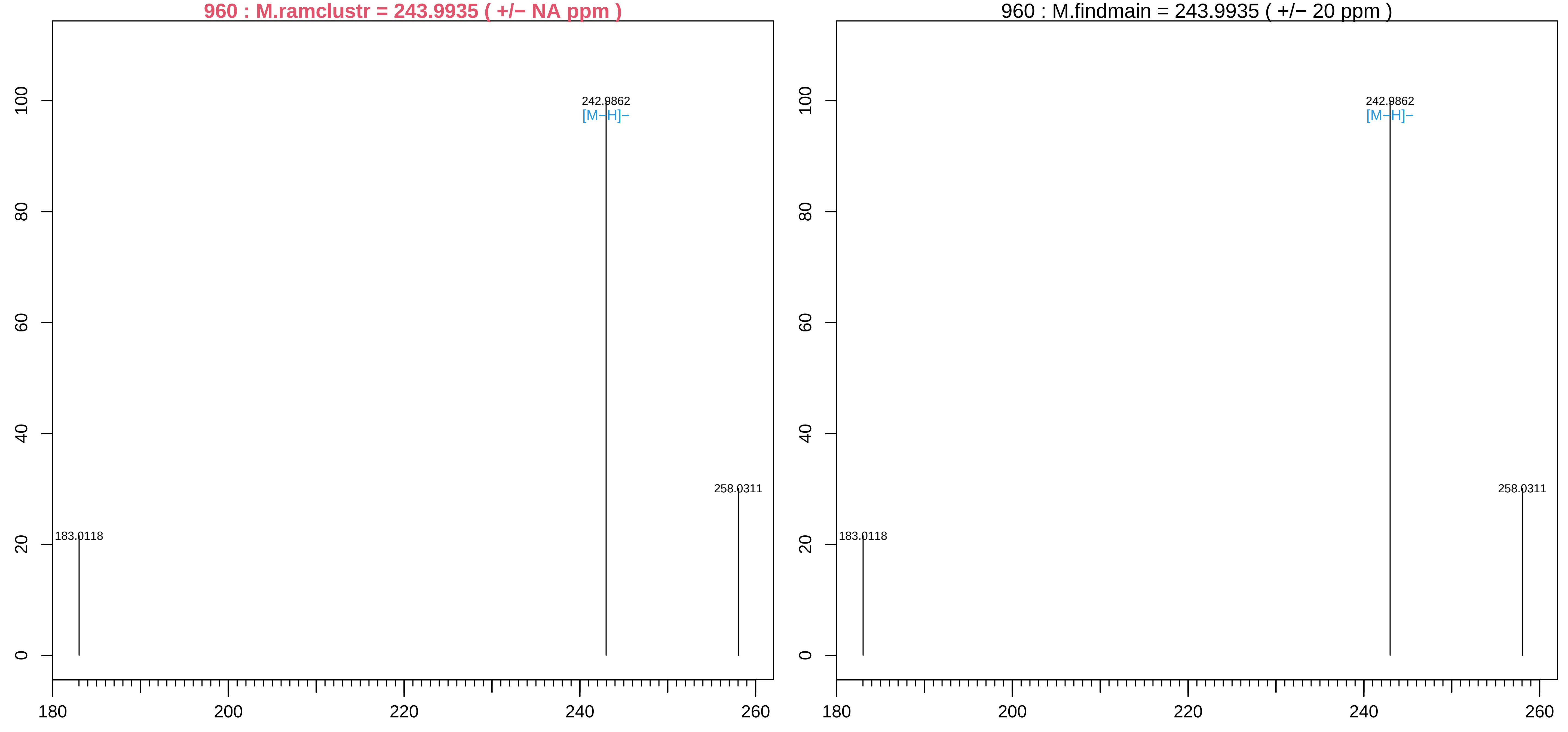
960 : M.ramclustr = 243.9935 (+/− NA ppm)
960 : M.findmain = 243.9935 (+/− 20 ppm)
242.9862
[M−H]−
258.0311
183.0118
242.9862
[M−H]−
258.0311
183.0118
0
20
40
60
80
100
180
200
220
240
260

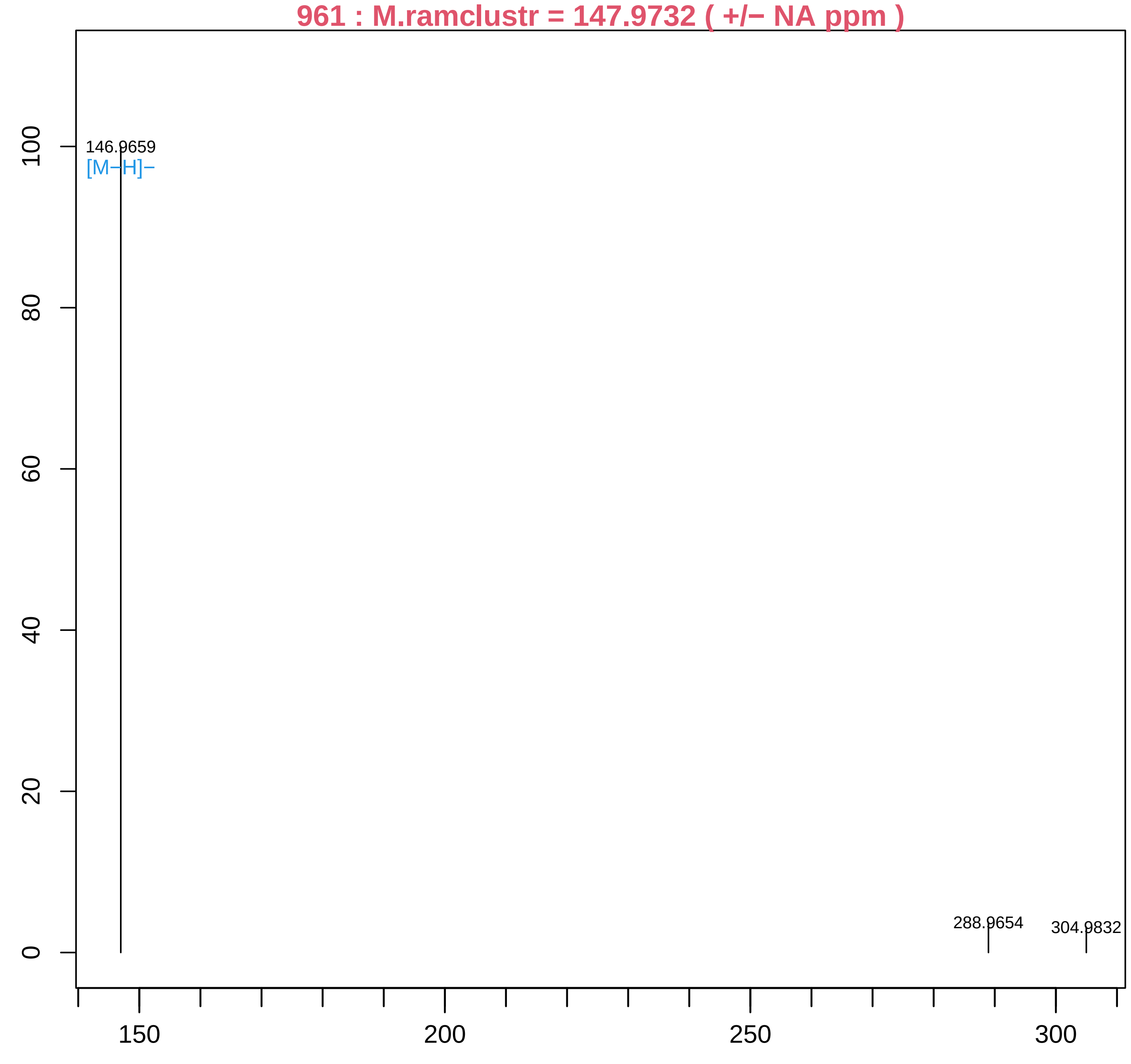
961 : M.ramclustr = 147.9732 (+/− NA ppm)
146.9659
[M−H]−
288.9654
304.9832
0
20
40
60
80
100
150
200
250
300

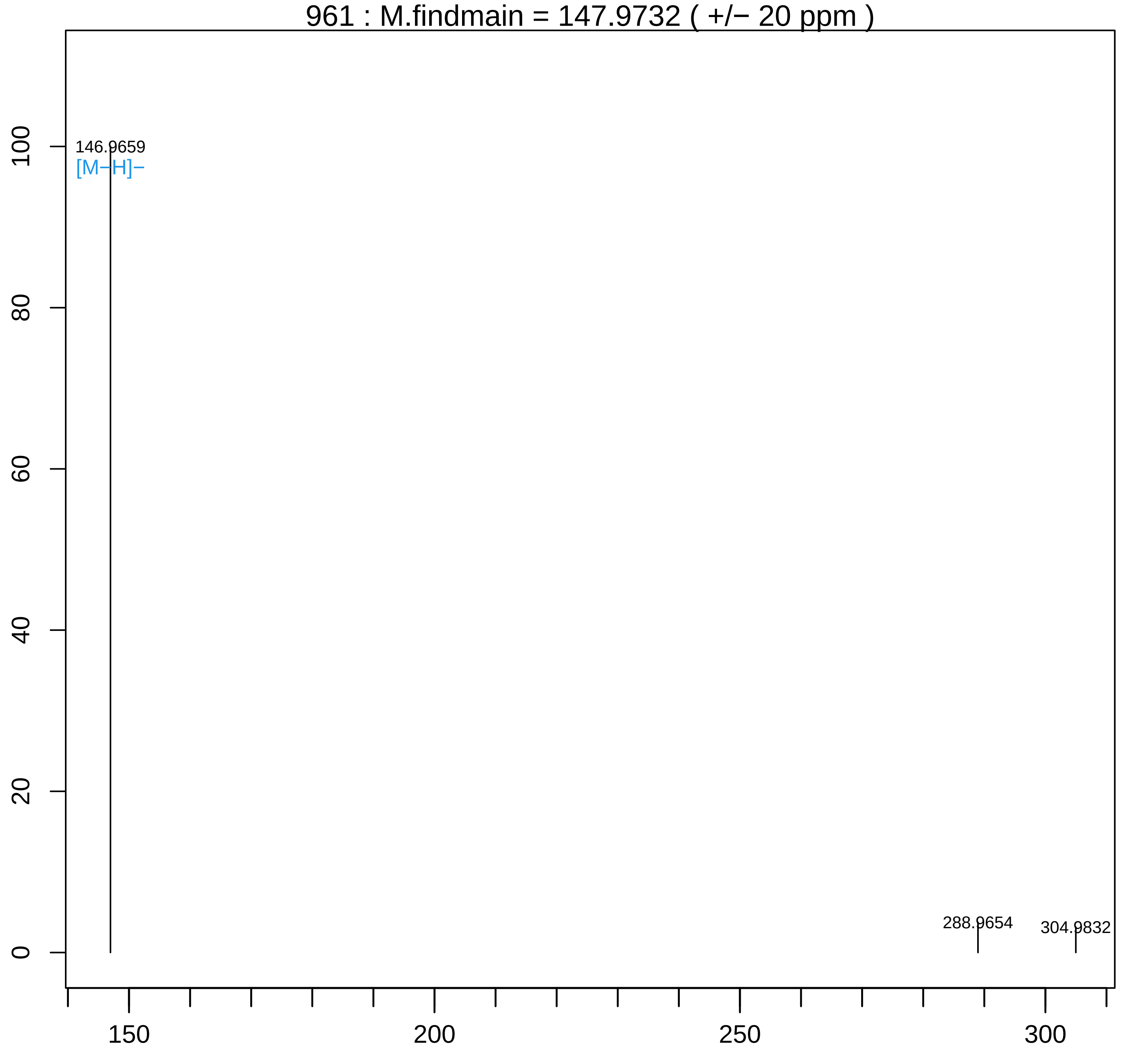
961 : M.findmain = 147.9732 (+/− 20 ppm)
146.9659
[M−H]−
288.9654
304.9832
0
20
40
60
80
100
150
200
250
300

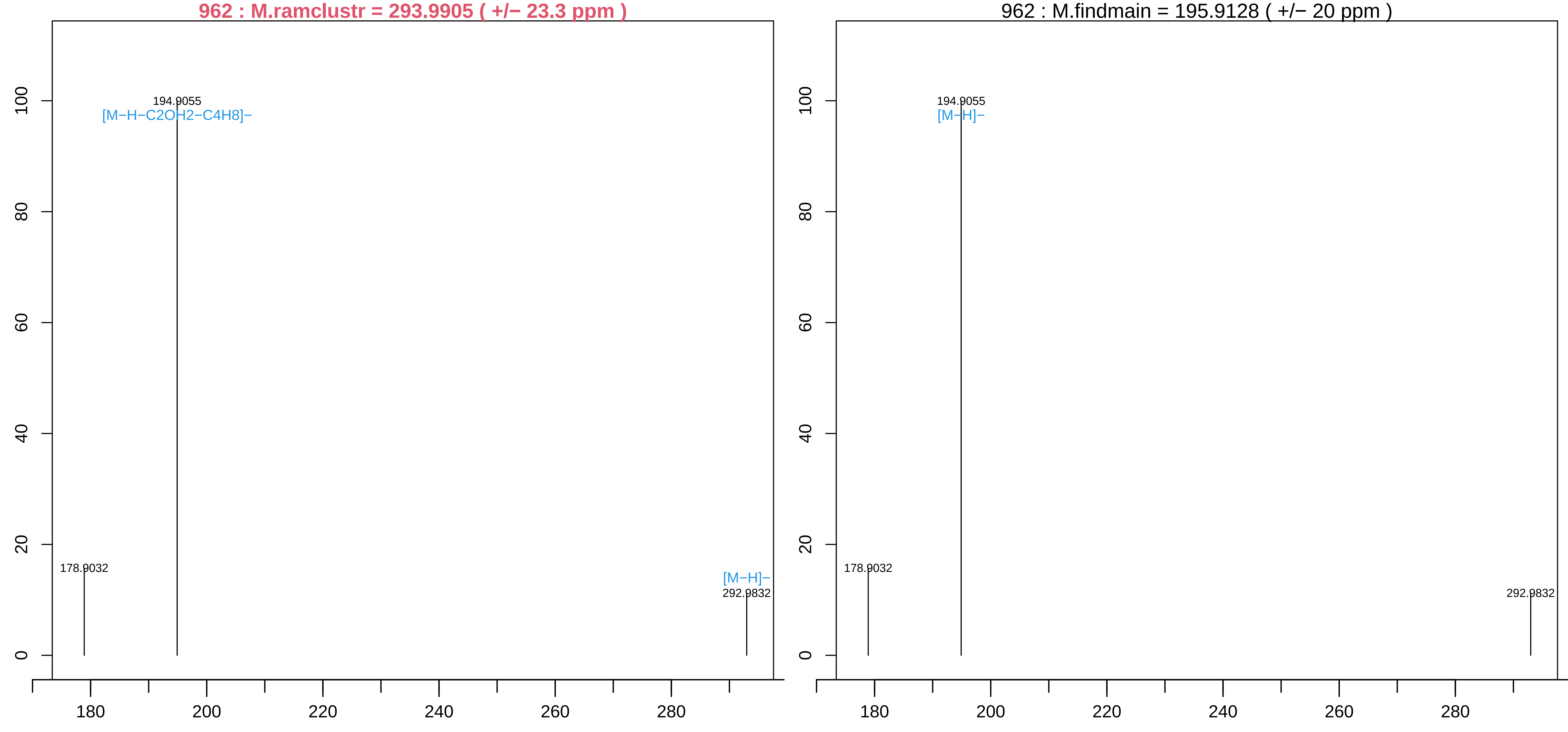

962 : M.ramclustr = 293.9905 (+/− 23.3 ppm)
194.9055
[M−H−C2OH2−C4H8]−
178.9032
[M−H]−
292.9832
962 : M.findmain = 195.9128 (+/− 20 ppm)
194.9055
[M−H]−
178.9032
292.9832

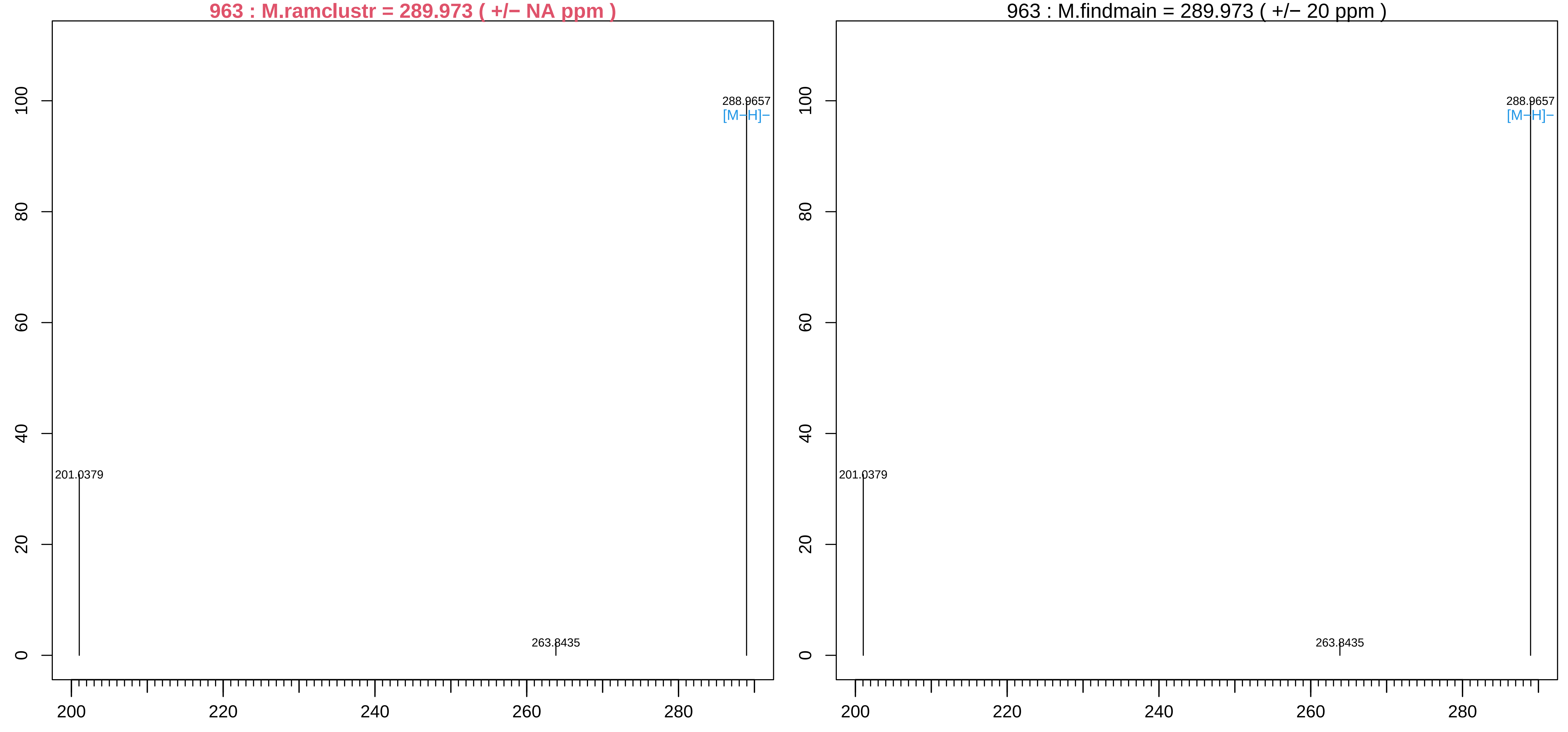
963 : M.ramclustr = 289.973 (+/− NA ppm)
963 : M.findmain = 289.973 (+/− 20 ppm)
288.9657
[M−H]−
201.0379
263.8435
288.9657
[M−H]−
201.0379
263.8435
0
20
40
60
80
100
200
220
240
260
280

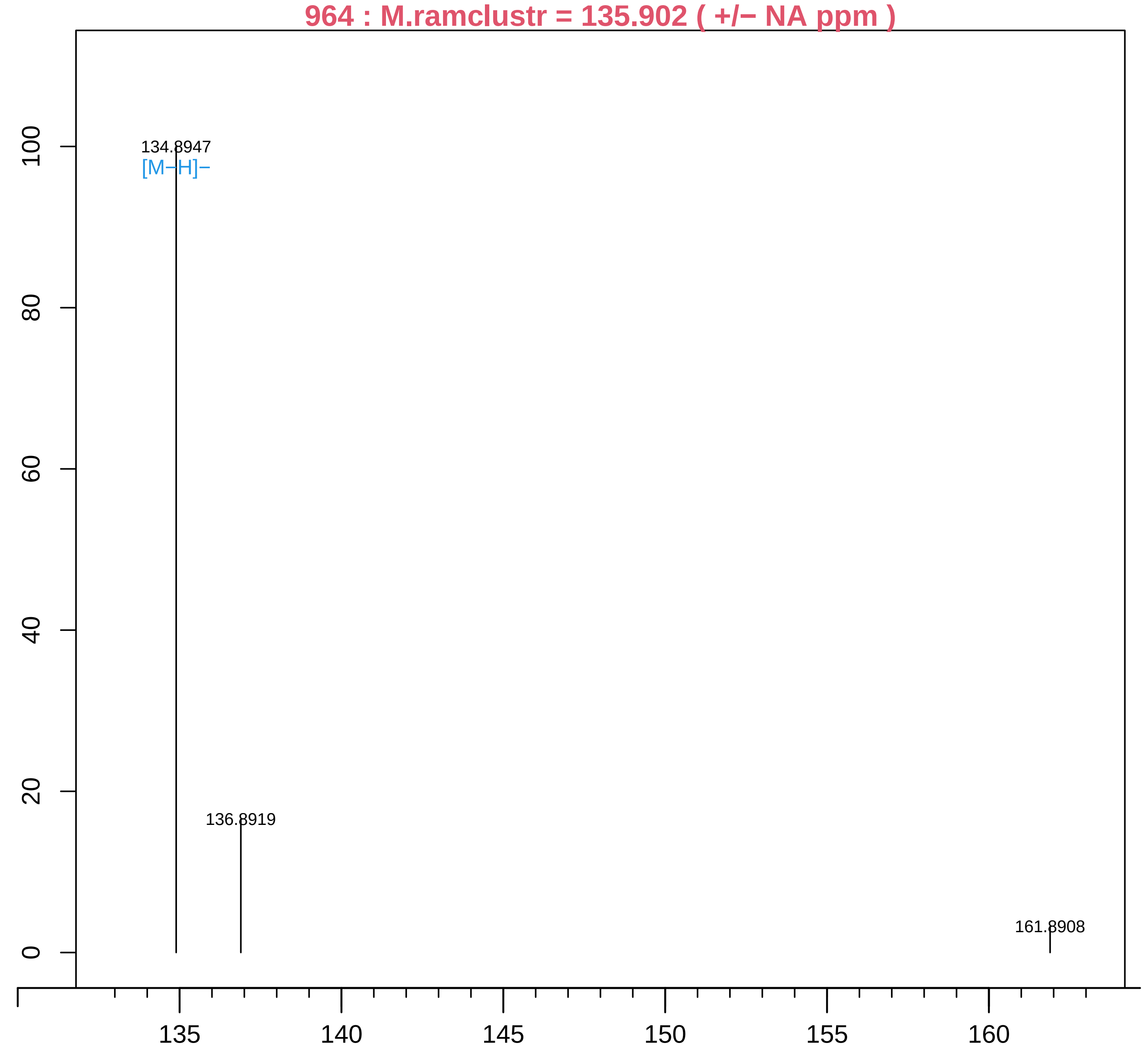
964 : M.ramclustr = 135.902 (+/− NA ppm)
134.8947
[M−H]−
136.8919
161.8908
0
20
40
60
80
100
135
140
145
150
155
160

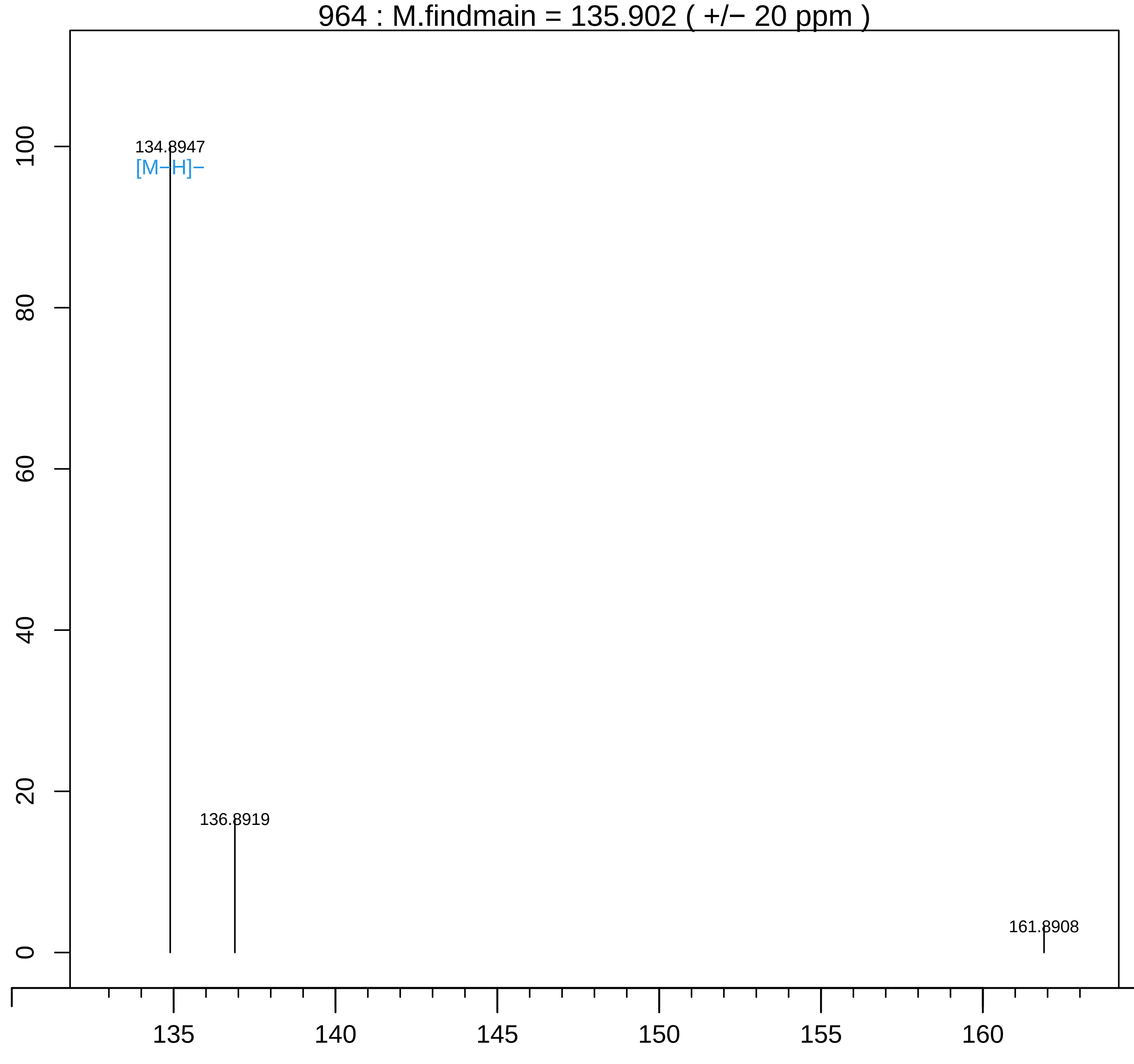
964 : M.findmain = 135.902 (+/− 20 ppm)
134.8947
[M−H]−
136.8919
161.8908
0
20
40
60
80
100
135
140
145
150
155
160

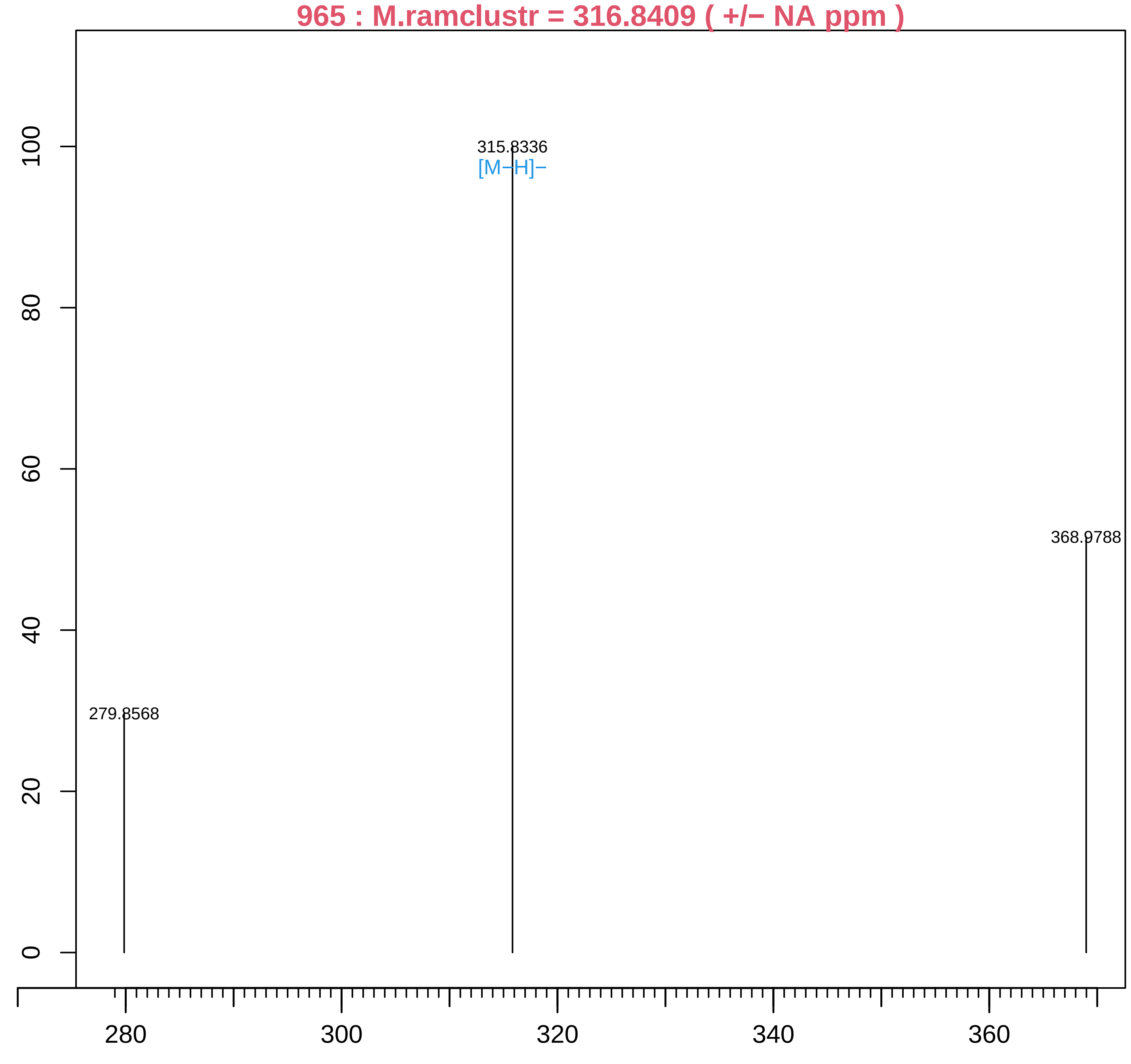
965 : M.ramclustr = 316.8409 (+/− NA ppm)
315.8336
[M−H]−
368.9788
279.8568
0
20
40
60
80
100
280
300
320
340
360

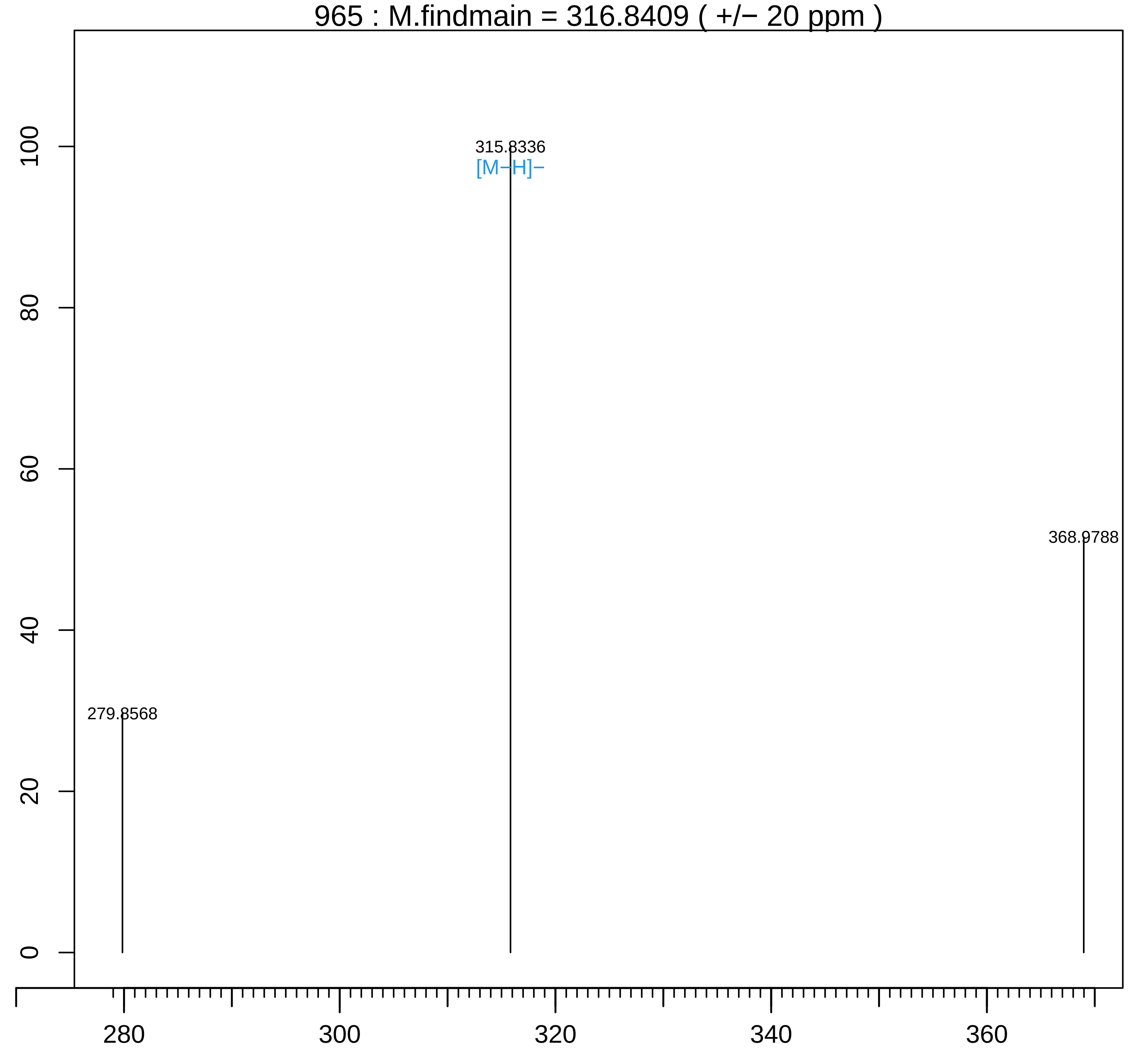
965 : M.findmain = 316.8409 (+/− 20 ppm)
315.8336
[M−H]−
368.9788
279.8568
0
20
40
60
80
100
280
300
320
340
360

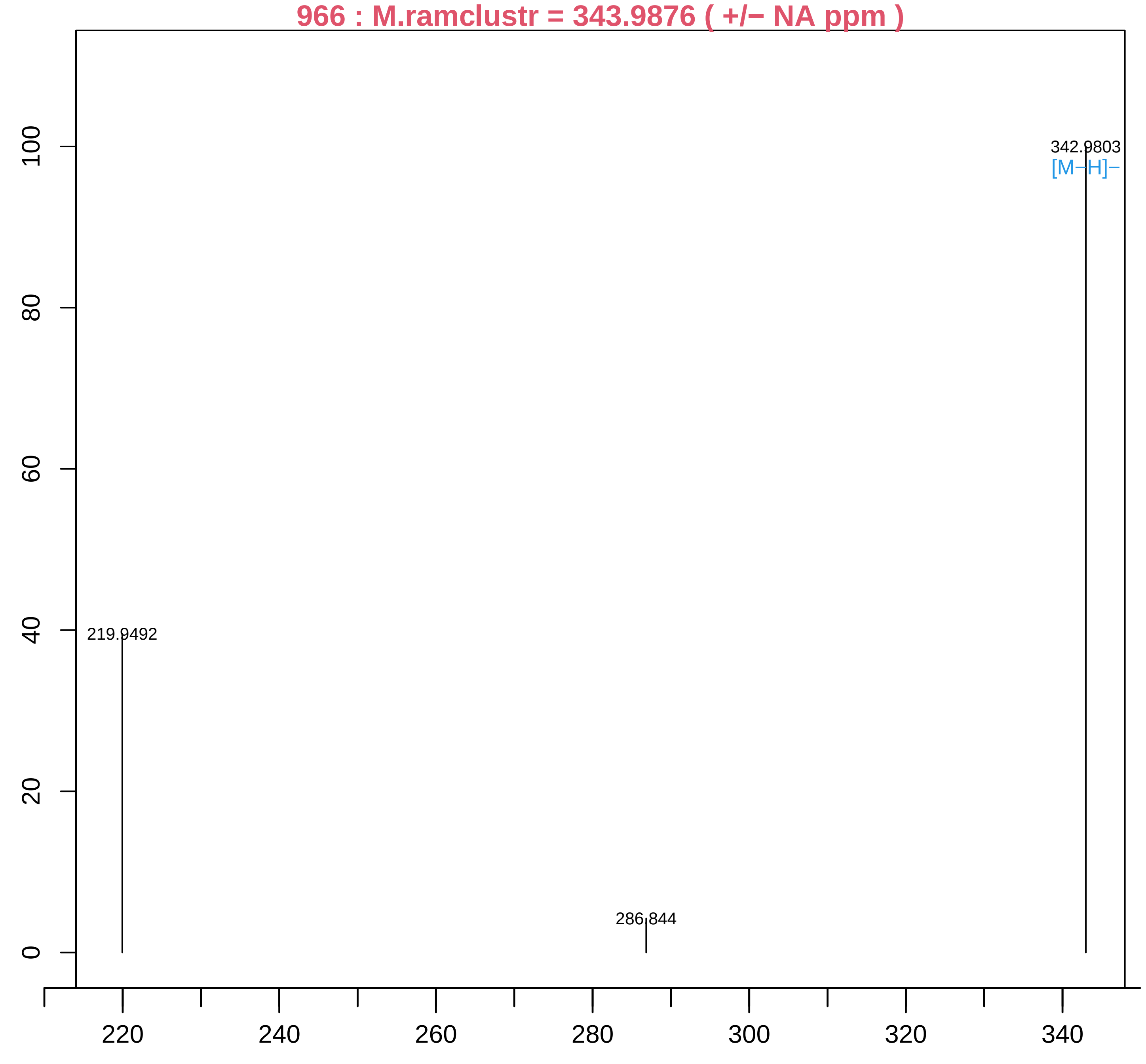
966 : M.ramclustr = 343.9876 (+/− NA ppm)
342.9803
[M−H]−
219.9492
286.844
0
20
40
60
80
100
220
240
260
280
300
320
340
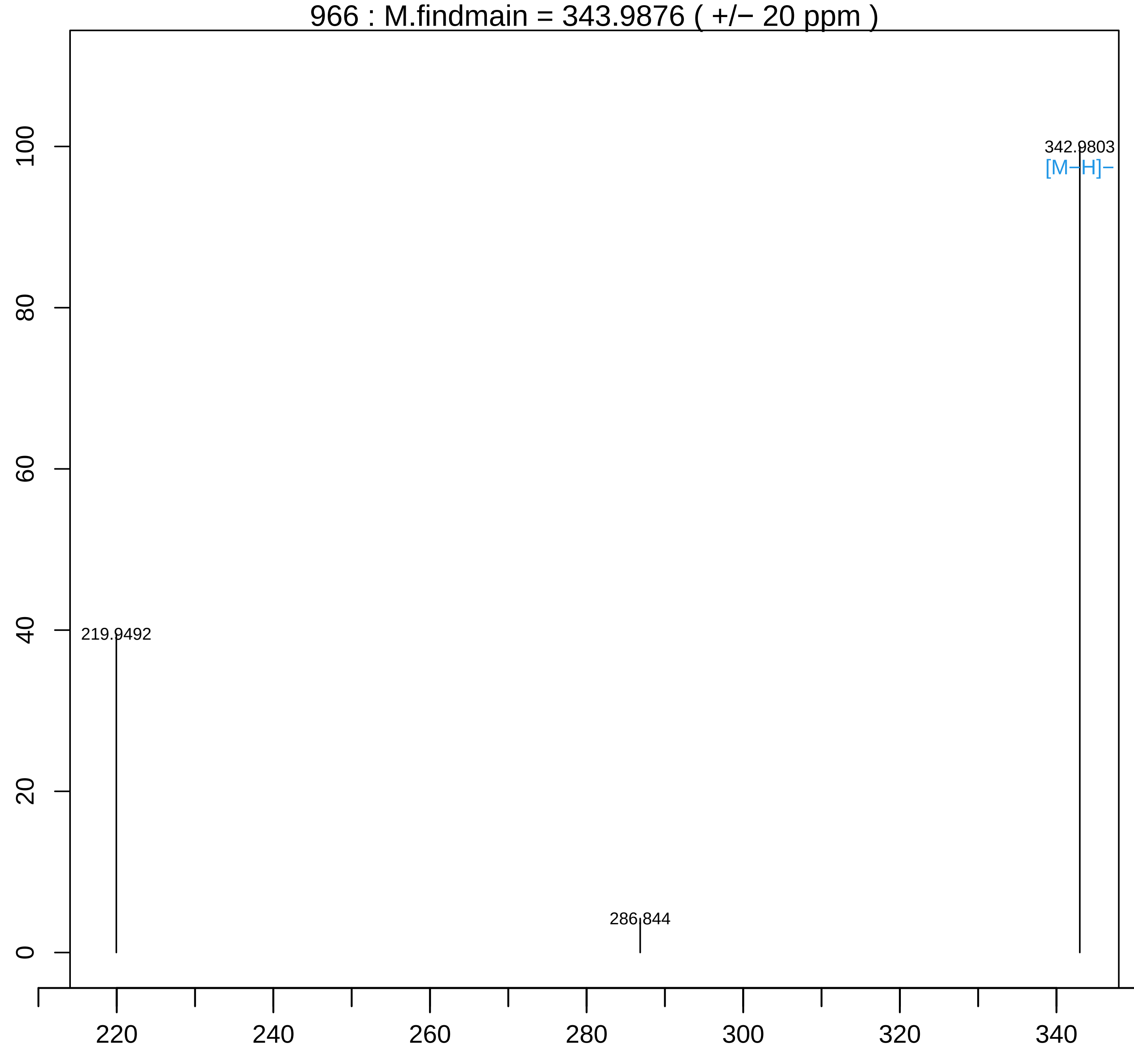
966 : M.findmain = 343.9876 (+/− 20 ppm)
342.9803
[M−H]−
219.9492
286.844
0
20
40
60
80
100
220
240
260
280
300
320
340

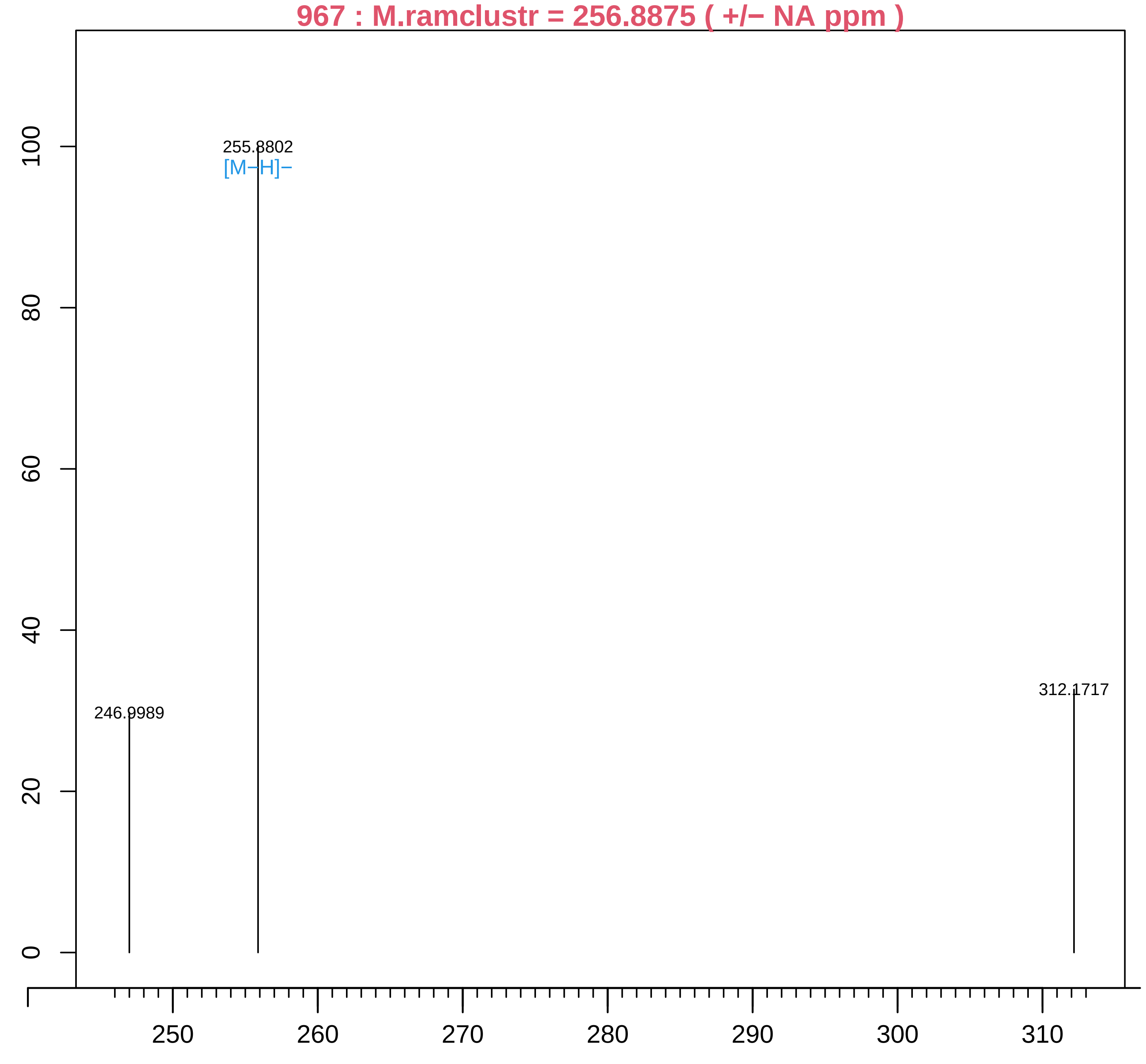
967 : M.ramclustr = 256.8875 (+/− NA ppm)
255.8802
[M−H]−
246.9989
312.1717
0
20
40
60
80
100
250
260
270
280
290
300
310

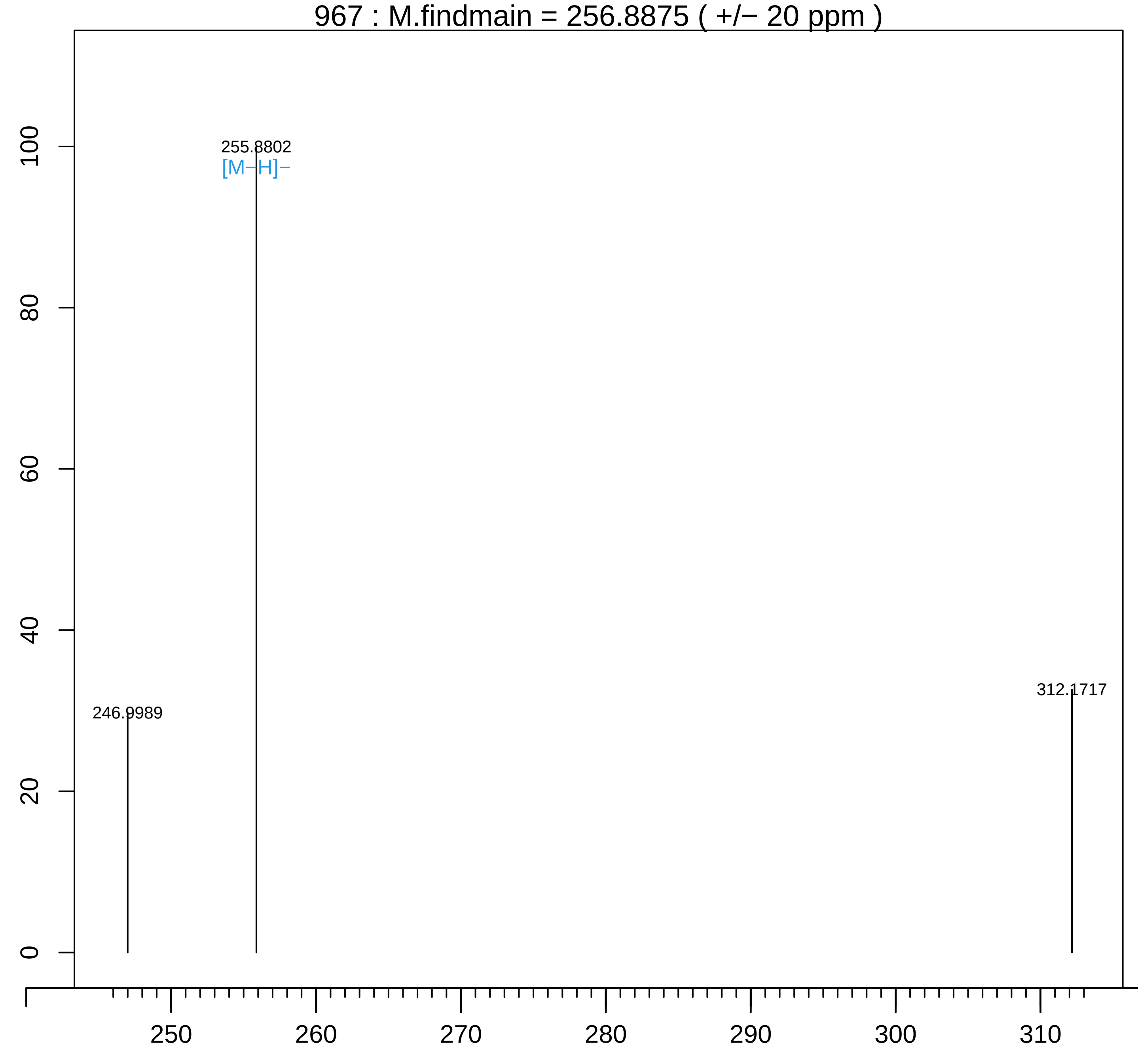
967 : M.findmain = 256.8875 (+/− 20 ppm)
255.8802
[M−H]−
246.9989
312.1717
0
20
40
60
80
100
250
260
270
280
290
300
310

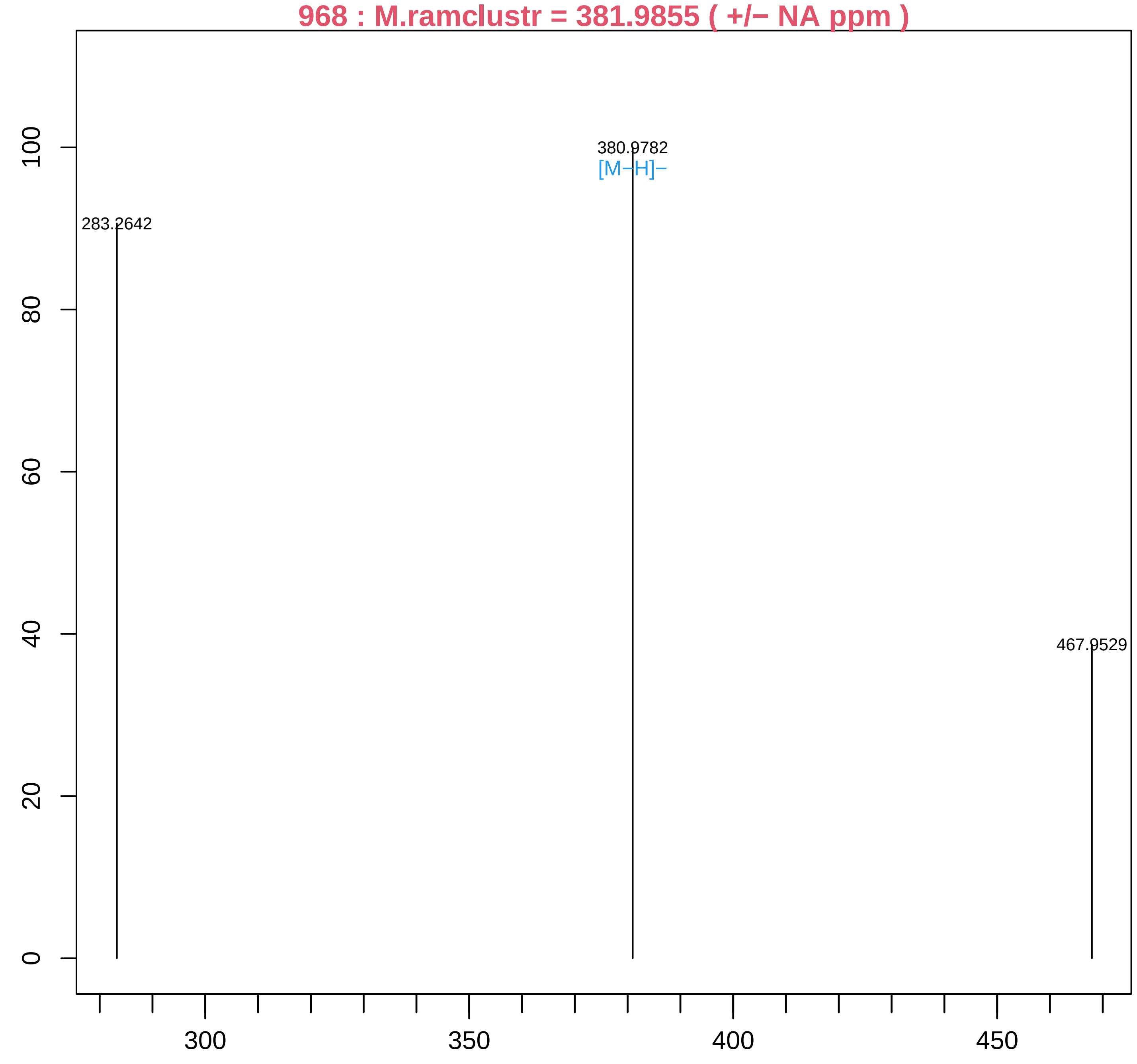
968 : M.ramclustr = 381.9855 (+/− NA ppm)
283.2642
380.9782
[M−H]−
467.9529
0
20
40
60
80
100
300
350
400
450

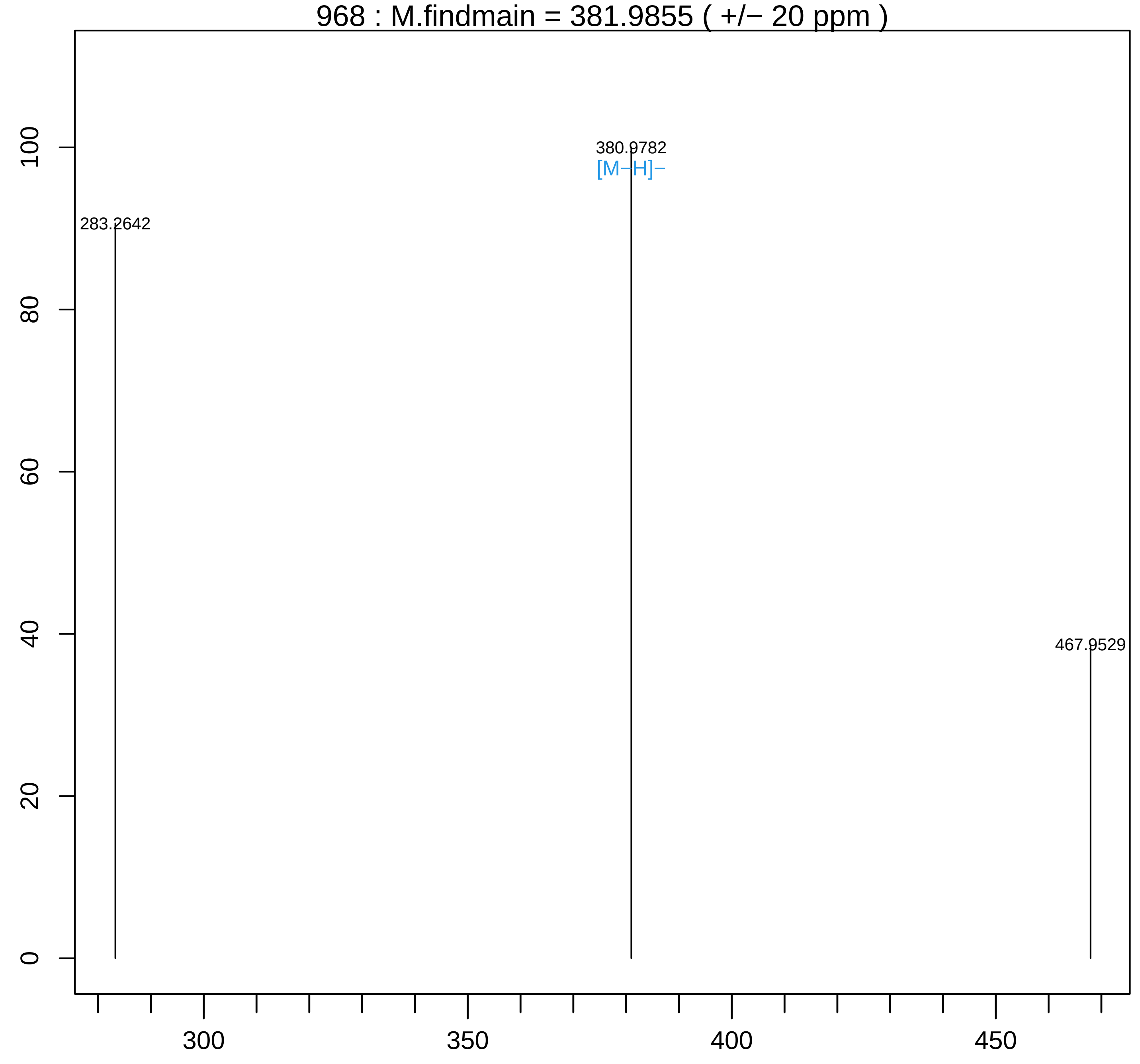
968 : M.findmain = 381.9855 (+/− 20 ppm)
283.2642
380.9782
[M−H]−
467.9529
0
20
40
60
80
100
300
350
400
450

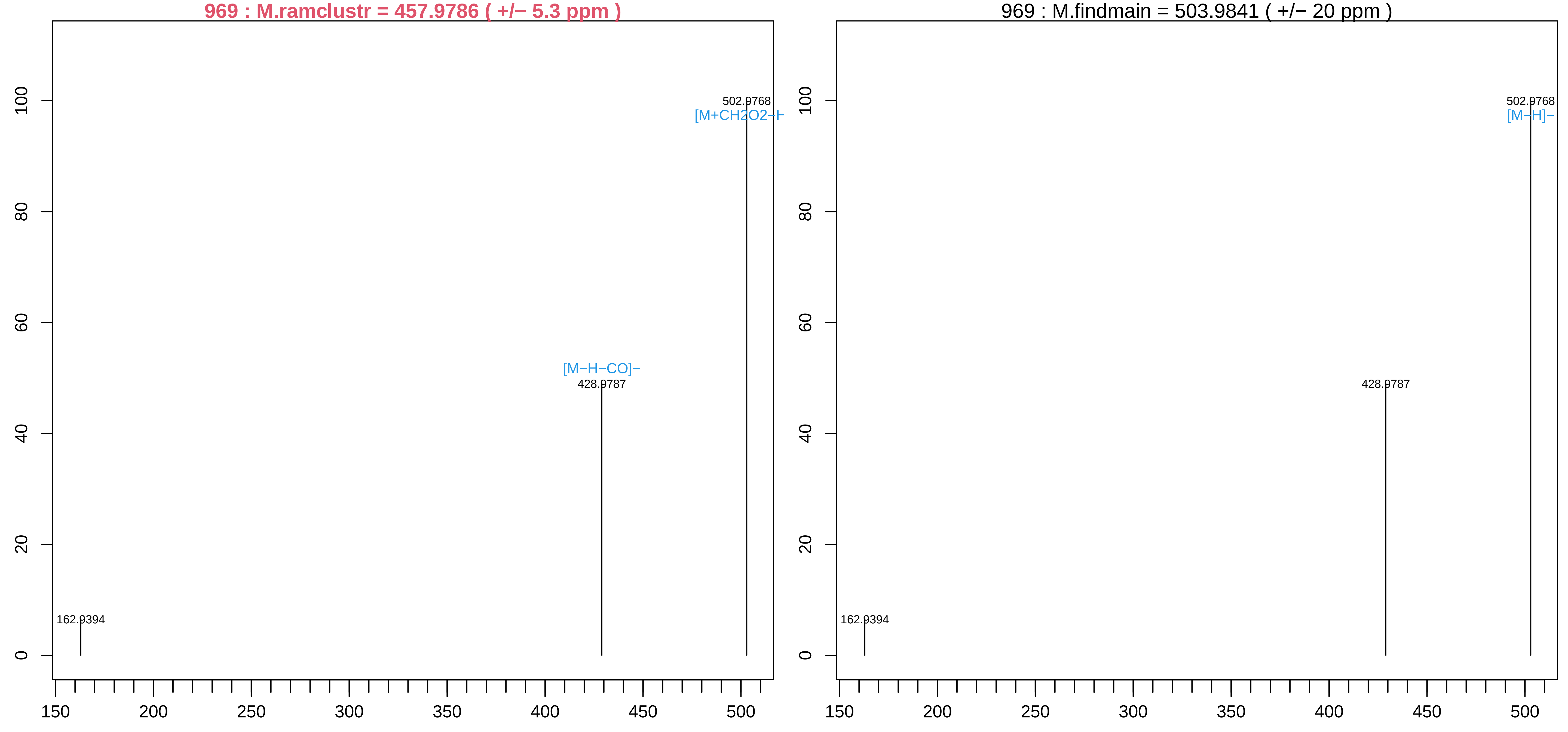
969 : M.ramclustr = 457.9786 (+/− 5.3 ppm)
502.9768
[M+CH2O2−H]−
[M−H−CO]−
428.9787
162.9394
969 : M.findmain = 503.9841 (+/− 20 ppm)
502.9768
[M−H]−
428.9787
162.9394
0
20
40
60
80
100
150
200
250
300
350
400
450
500

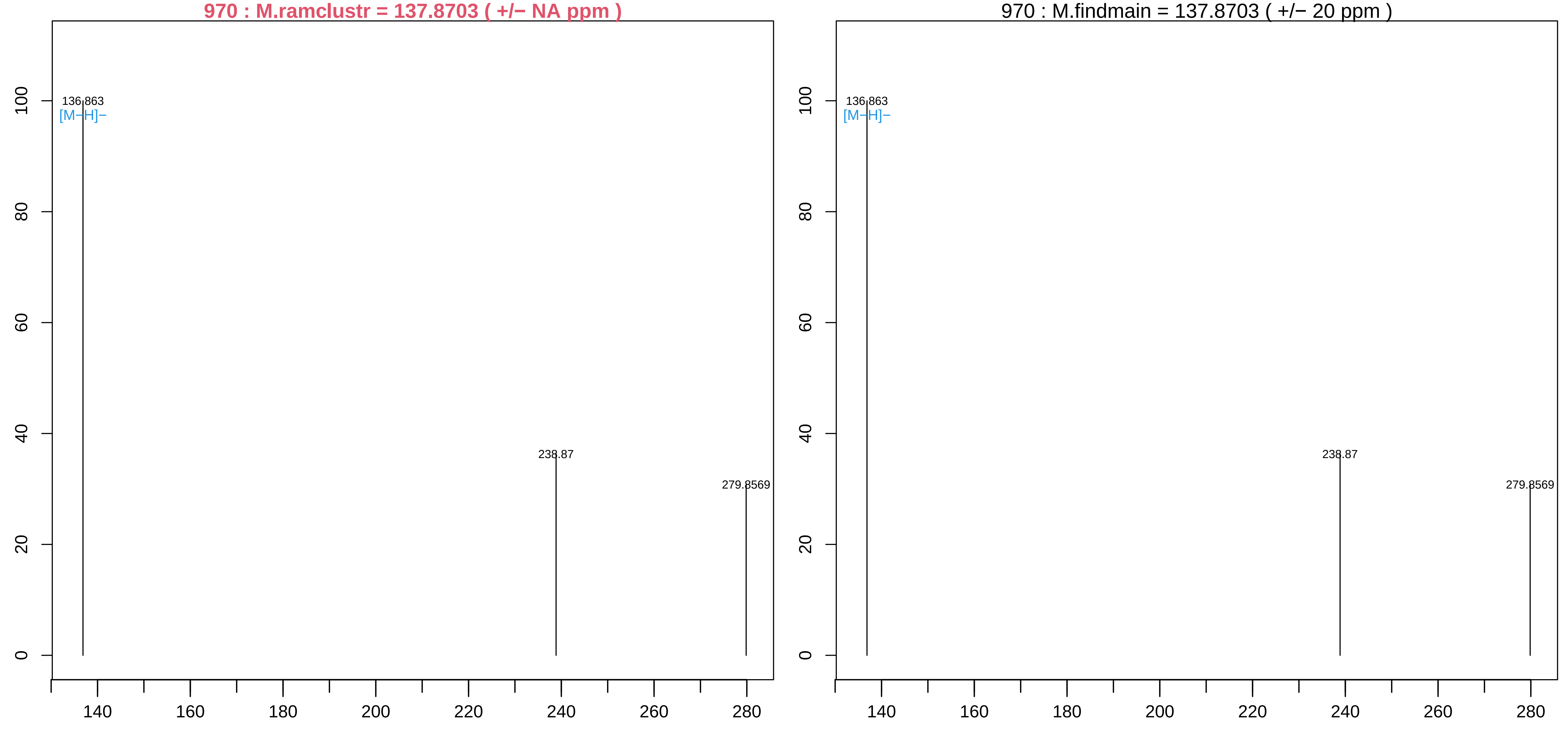

970 : M.ramclustr = 137.8703 (+/− NA ppm)
136.863
[M−H]−
238.87
279.8569
0
20
40
60
80
100
140
160
180
200
220
240
260
280
970 : M.findmain = 137.8703 (+/− 20 ppm)
136.863
[M−H]−
238.87
279.8569
0
20
40
60
80
100
140
160
180
200
220
240
260
280

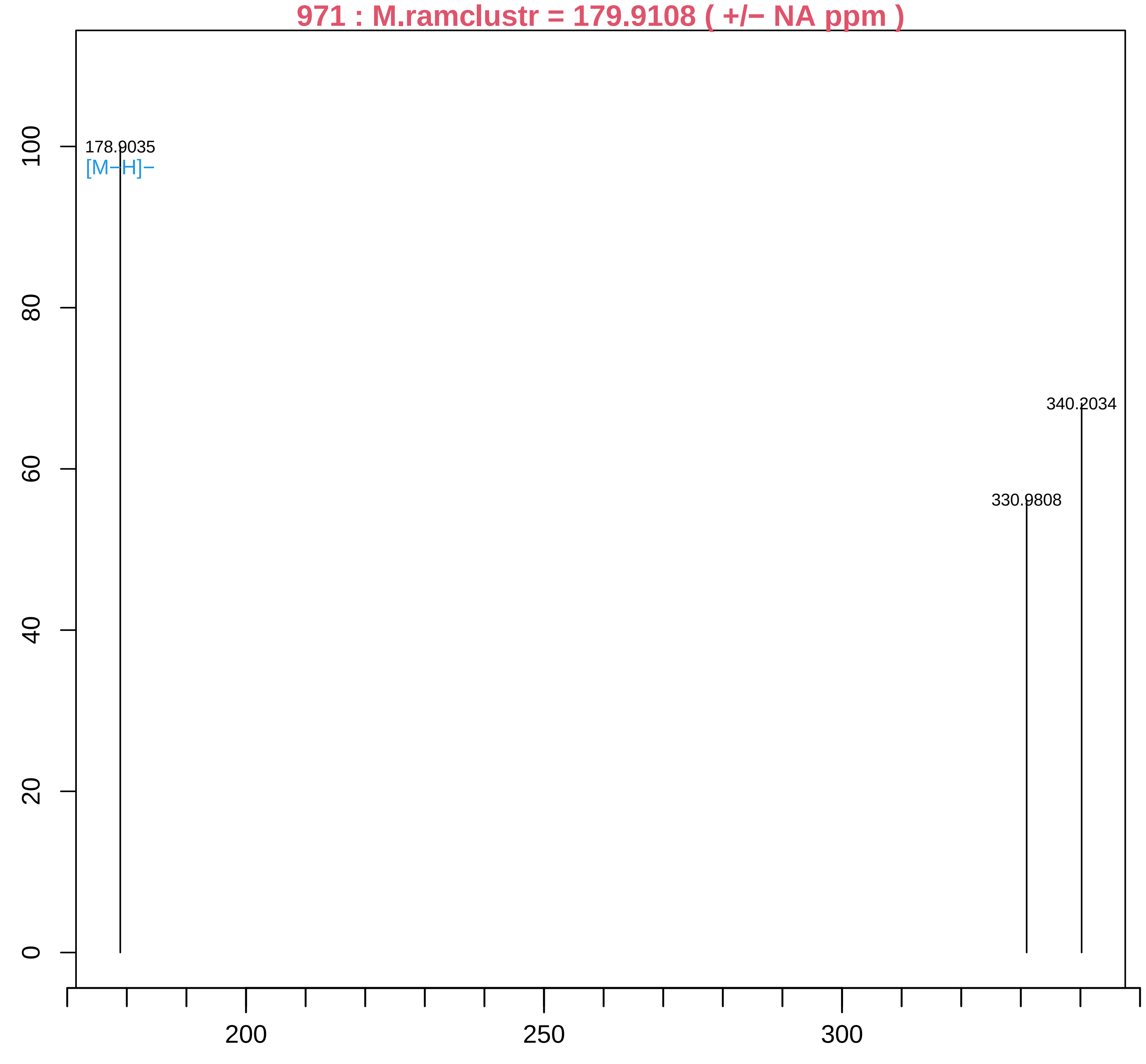

971 : M.ramclustr = 179.9108 (+/− NA ppm)
178.9035
[M−H]−
340.2034
330.9808
0
20
40
60
80
100
200
250
300

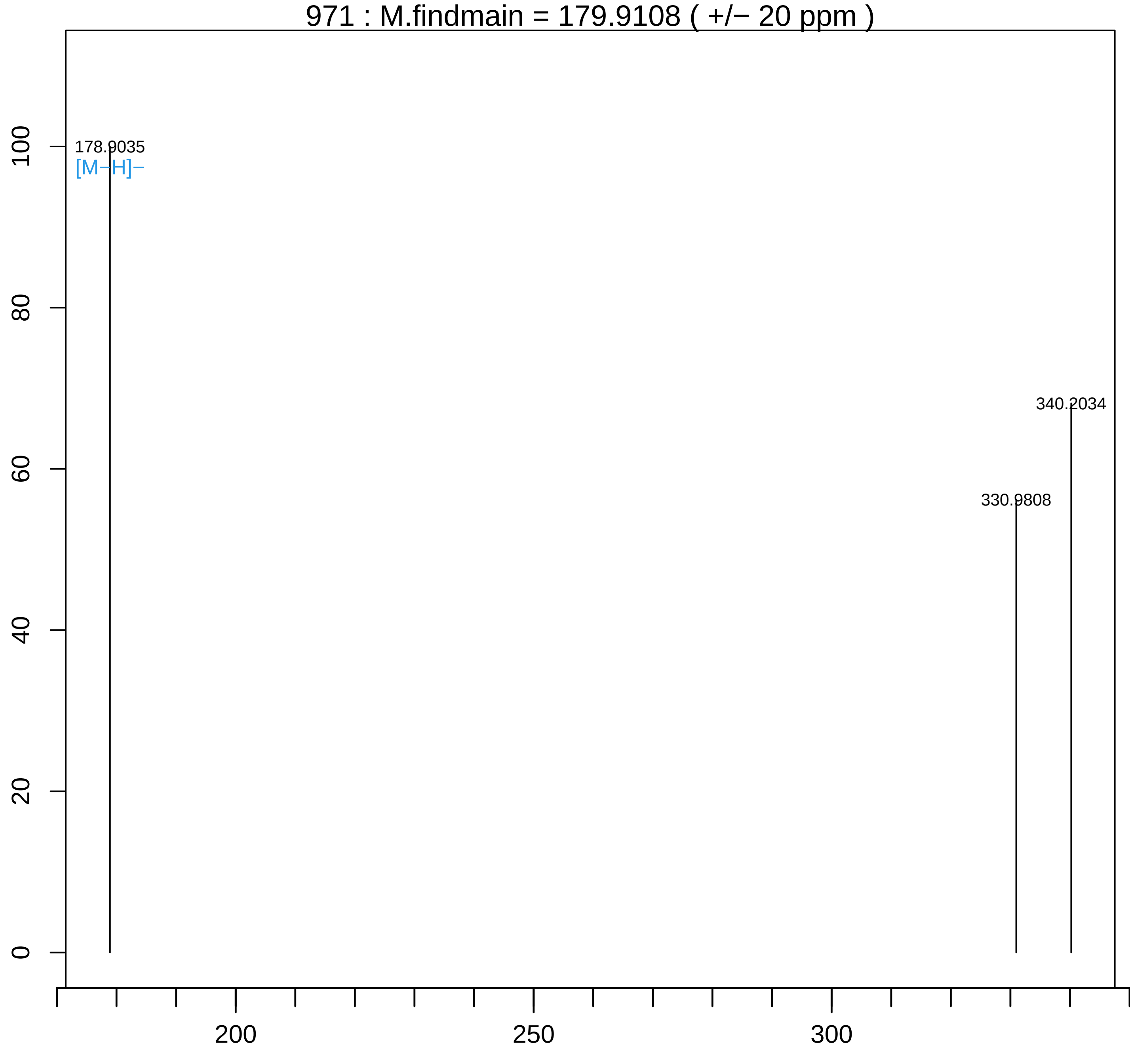

971 : M.findmain = 179.9108 (+/− 20 ppm)
178.9035
[M−H]−
340.2034
330.9808
0
20
40
60
80
100
200
250
300

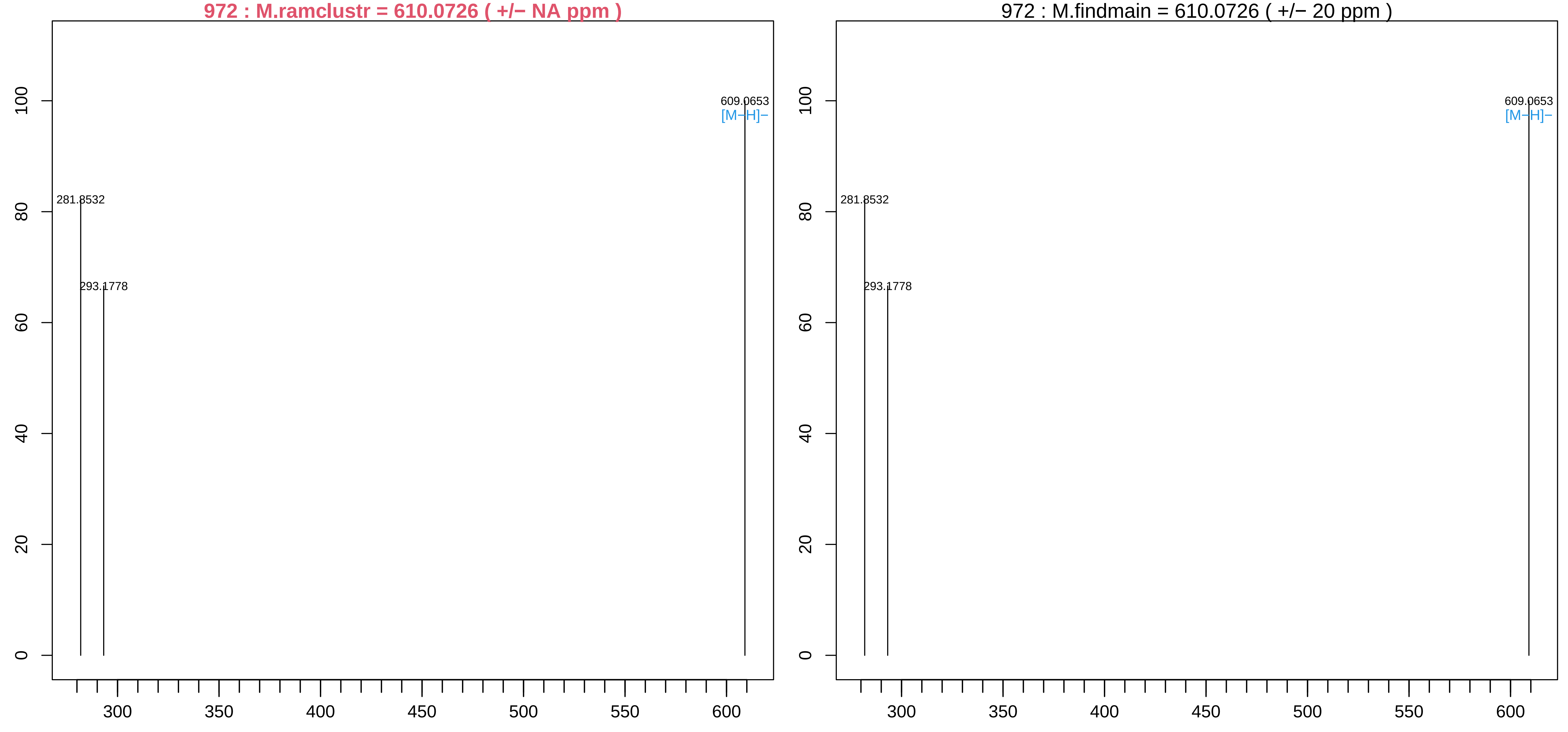
972 : M.ramclustr = 610.0726 (+/− NA ppm)
609.0653
[M−H]−
281.8532
293.1778
972 : M.findmain = 610.0726 (+/− 20 ppm)
609.0653
[M−H]−
281.8532
293.1778
0
20
40
60
80
100
300
350
400
450
500
550
600

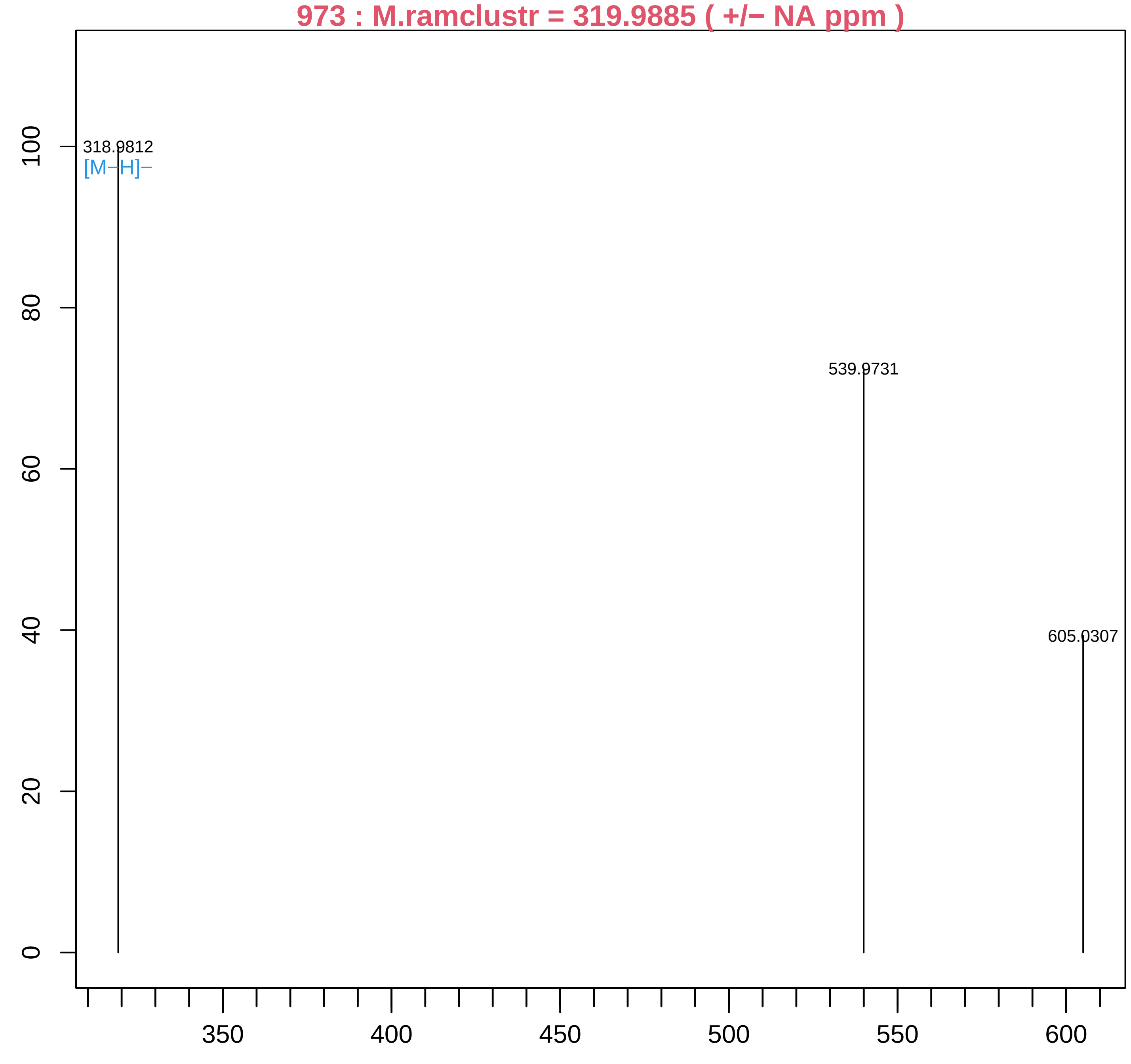
973 : M.ramclustr = 319.9885 (+/− NA ppm)
318.9812
[M−H]−
539.9731
605.0307
0
20
40
60
80
100
350
400
450
500
550
600

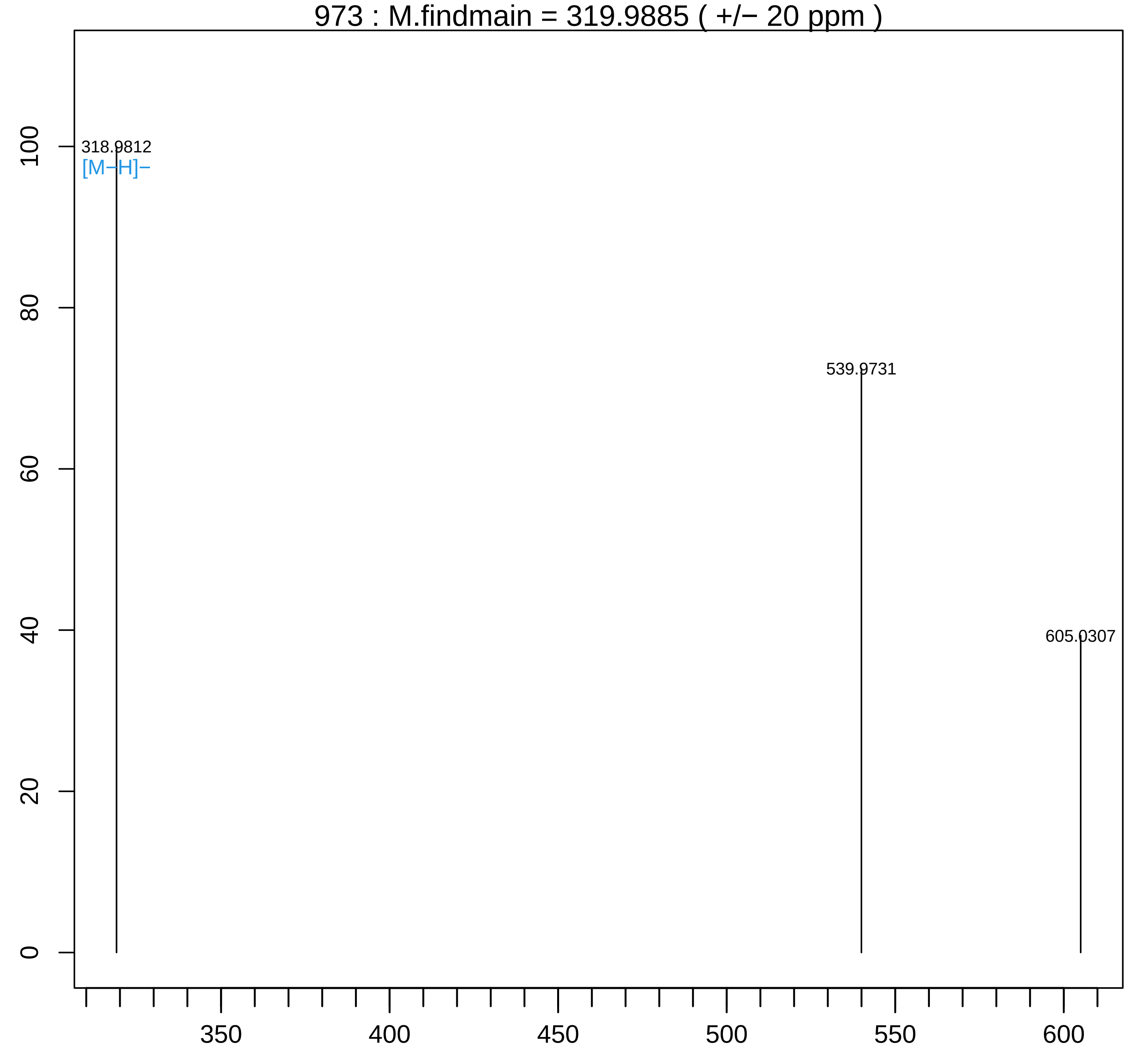
973 : M.findmain = 319.9885 (+/− 20 ppm)
318.9812
[M−H]−
539.9731
605.0307
0
20
40
60
80
100
350
400
450
500
550
600

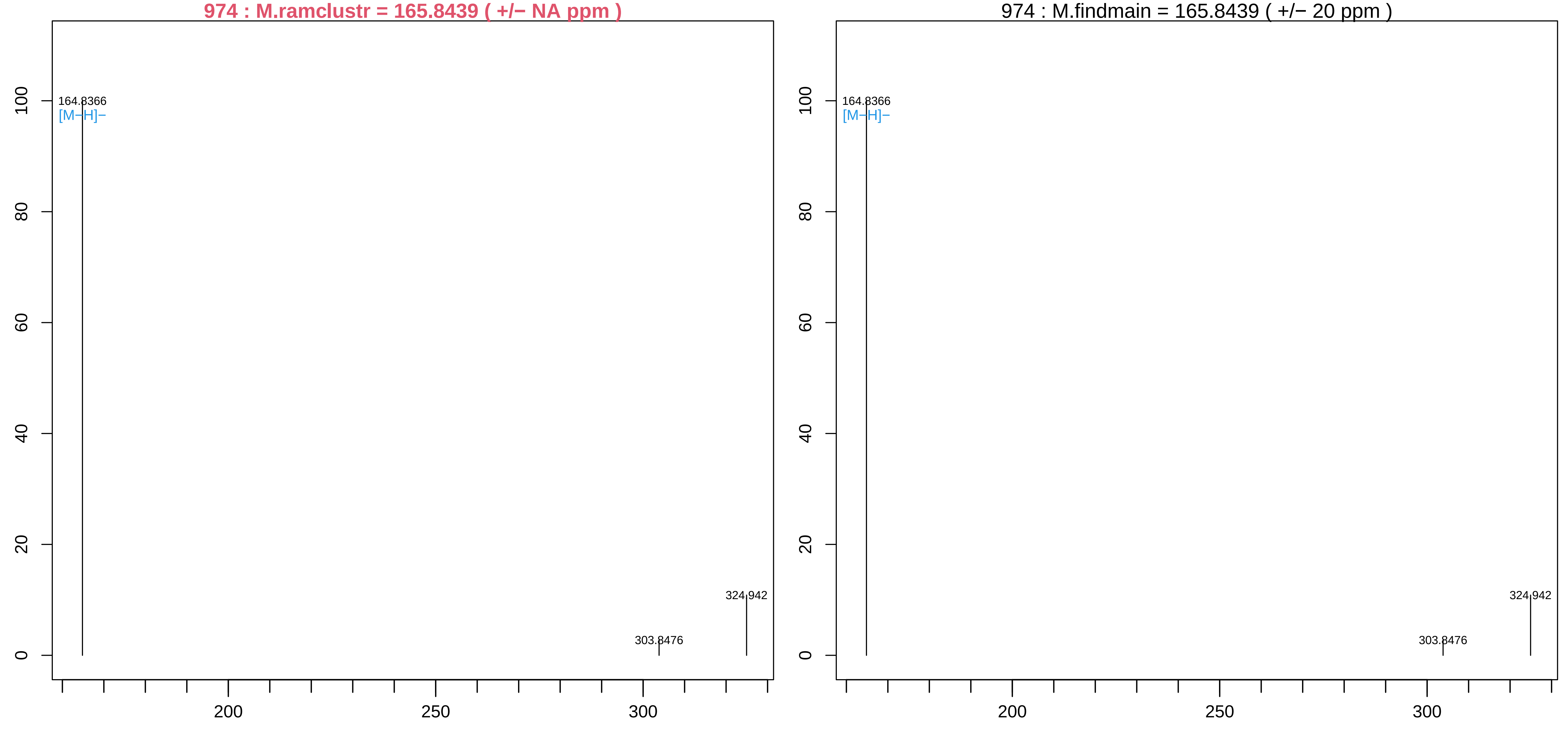
974 : M.ramclustr = 165.8439 (+/− NA ppm)
164.8366
[M−H]−
303.8476
324.942
0
20
40
60
80
100
200
250
300
974 : M.findmain = 165.8439 (+/− 20 ppm)
164.8366
[M−H]−
303.8476
324.942
0
20
40
60
80
100
200
250
300

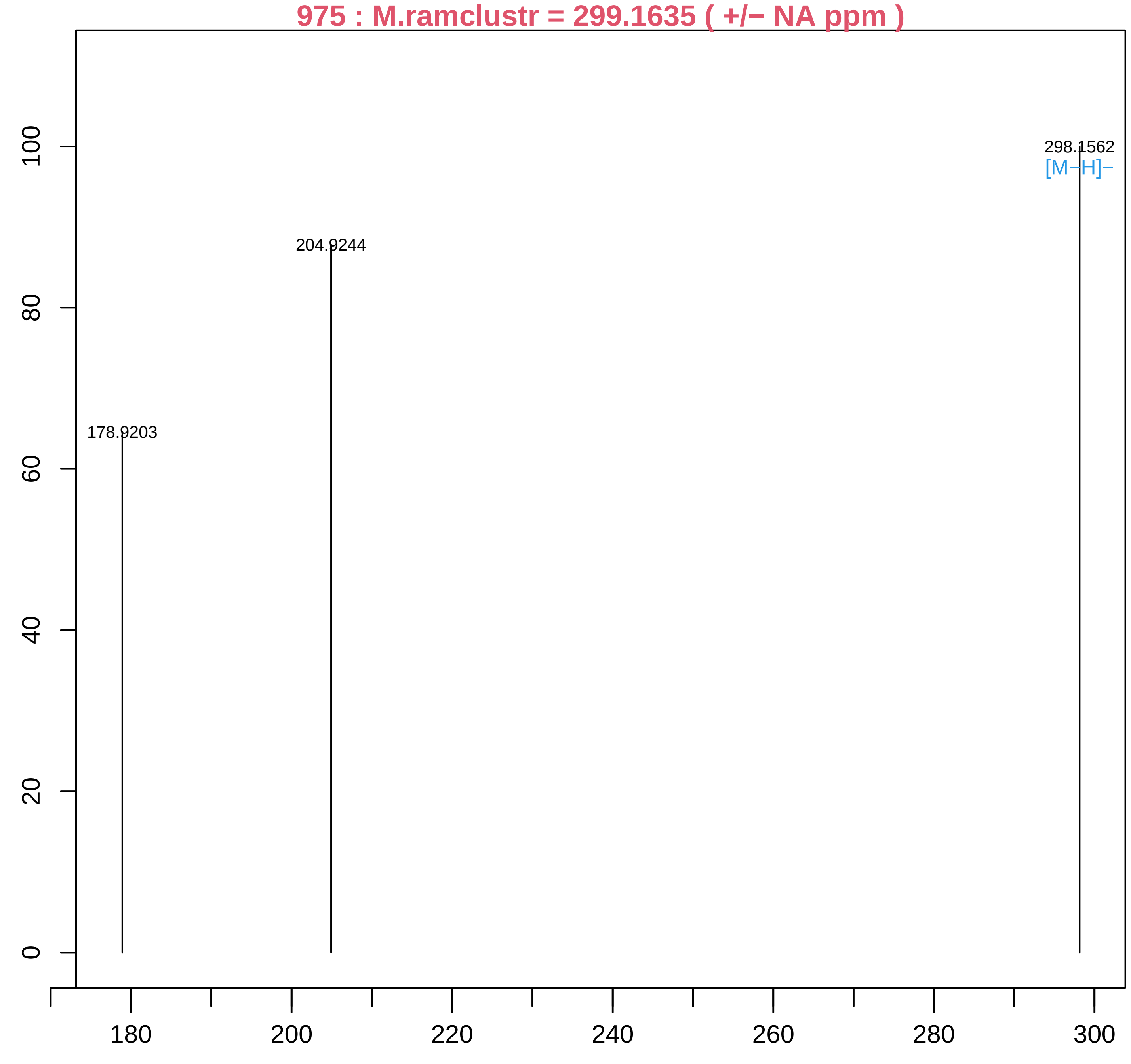
975 : M.ramclustr = 299.1635 (+/− NA ppm)
298.1562
[M−H]−
204.9244
178.9203
0
20
40
60
80
100
180
200
220
240
260
280
300

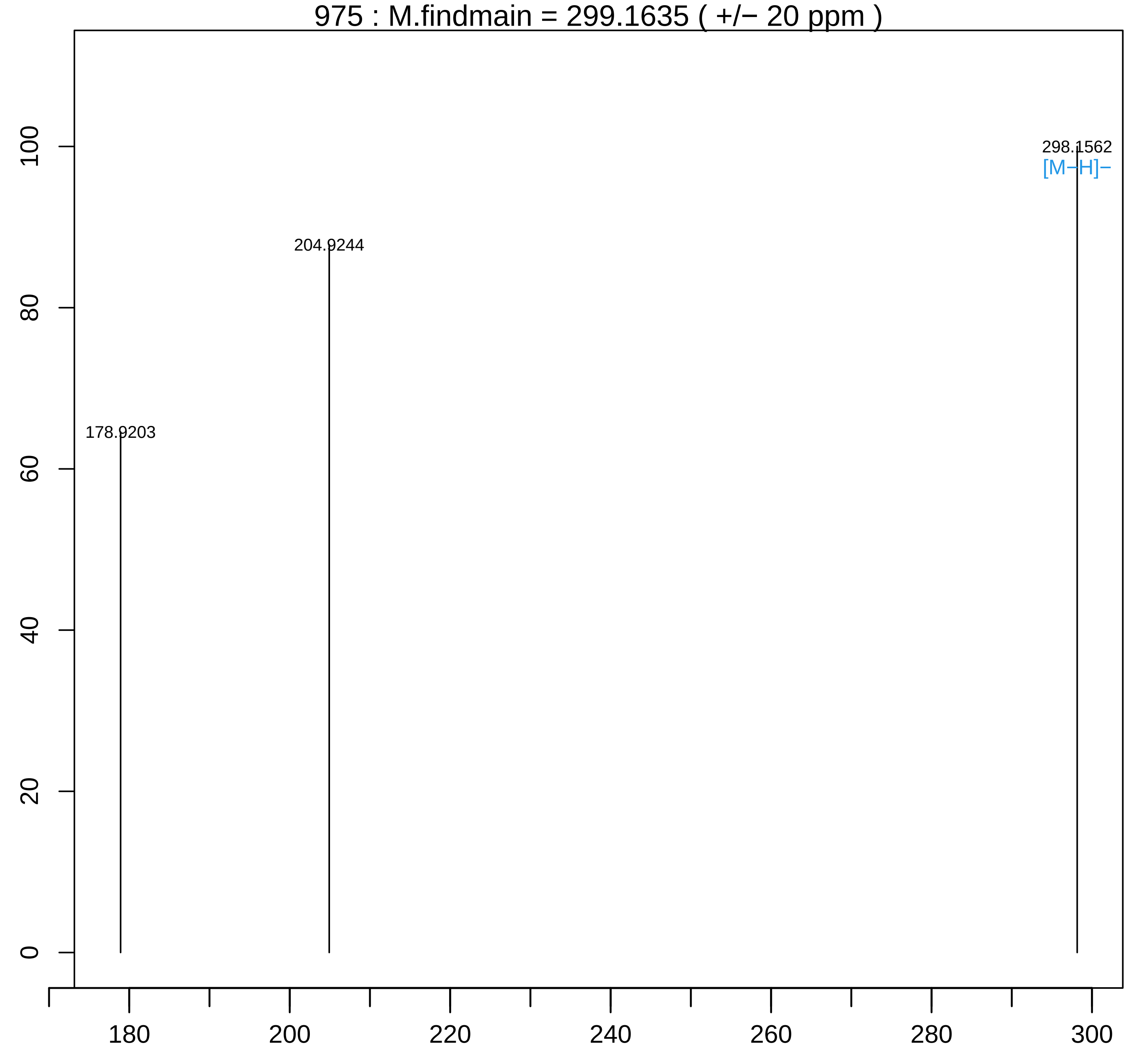
975 : M.findmain = 299.1635 (+/− 20 ppm)
298.1562
[M−H]−
204.9244
178.9203
0
20
40
60
80
100
180
200
220
240
260
280
300

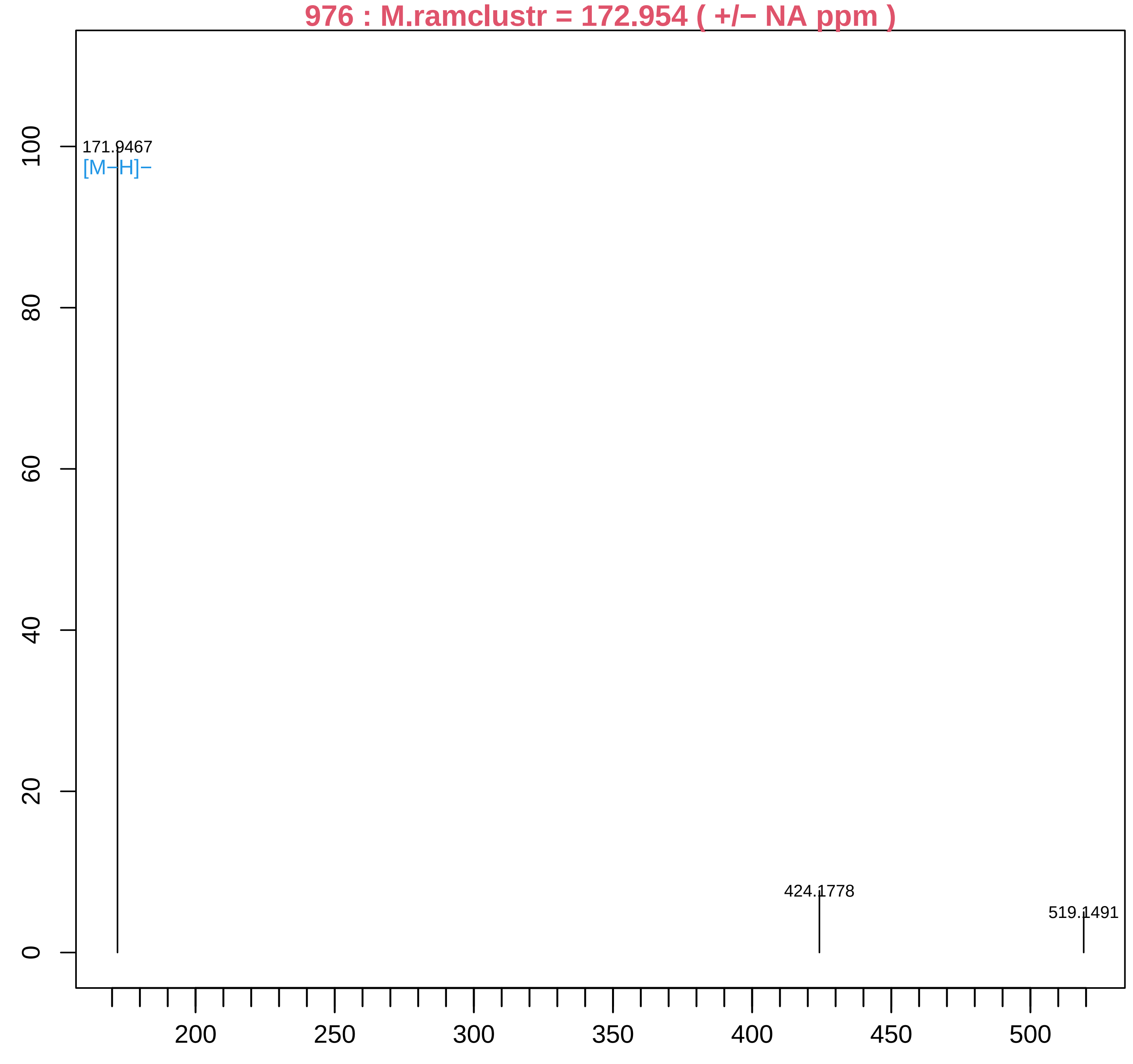

976 : M.ramclustr = 172.954 (+/− NA ppm)
171.9467
[M−H]−
424.1778
519.1491
0
20
40
60
80
100
200
250
300
350
400
450
500

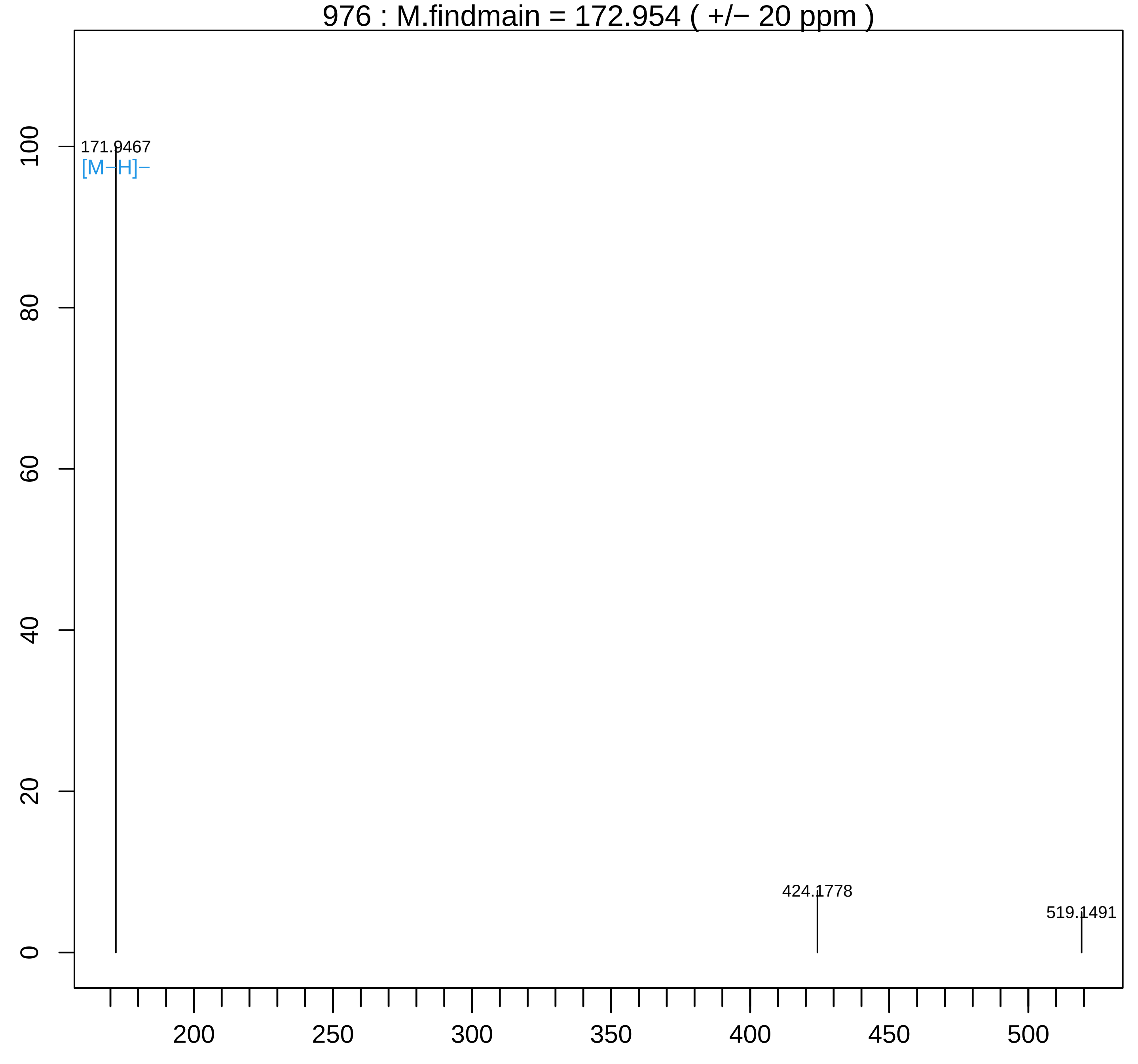

976 : M.findmain = 172.954 (+/− 20 ppm)
171.9467
[M−H]−
424.1778
519.1491
0
20
40
60
80
100
200
250
300
350
400
450
500

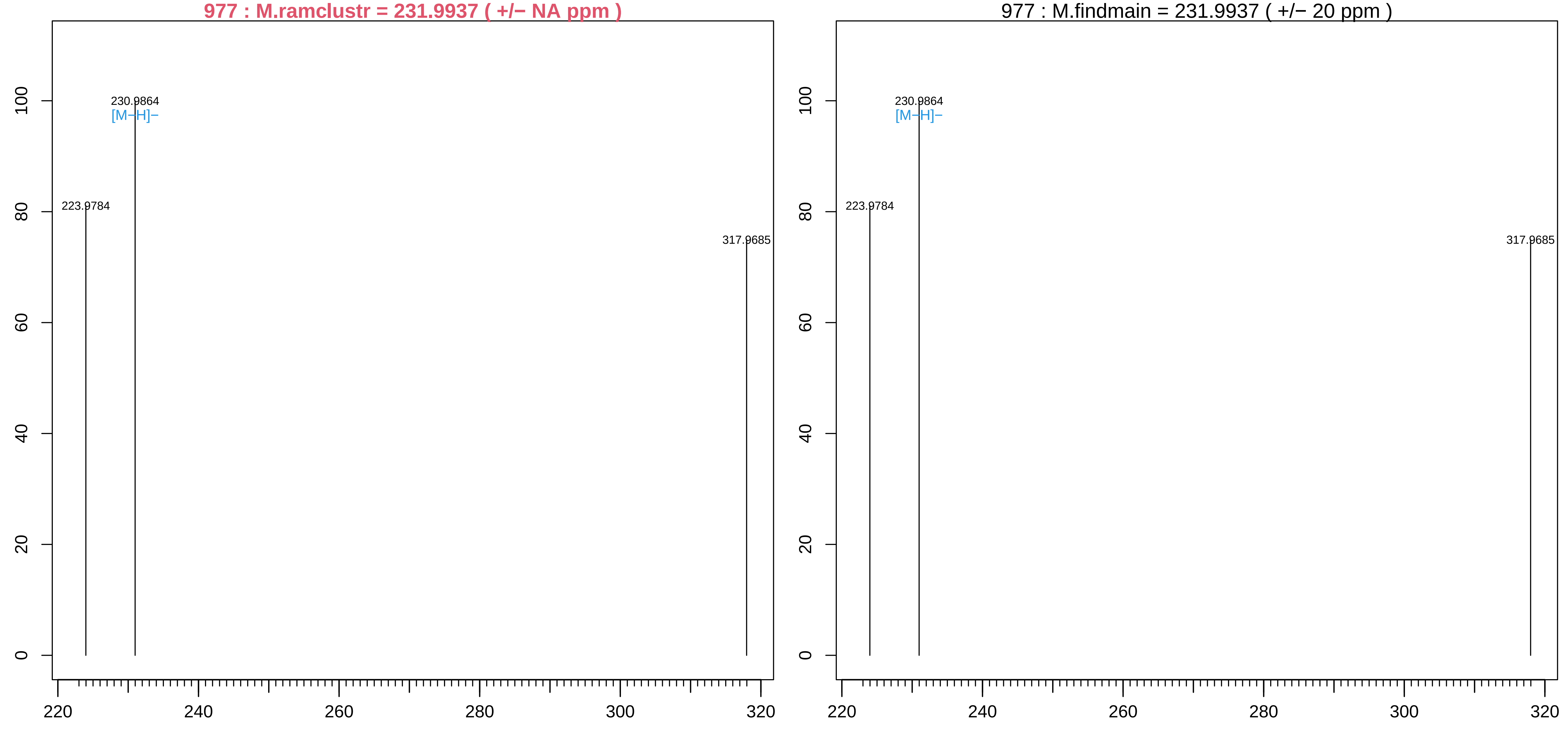
977 : M.ramclustr = 231.9937 (+/− NA ppm)
230.9864
[M−H]−
223.9784
317.9685
977 : M.findmain = 231.9937 (+/− 20 ppm)
230.9864
[M−H]−
223.9784
317.9685
0
20
40
60
80
100
220
240
260
280
300
320

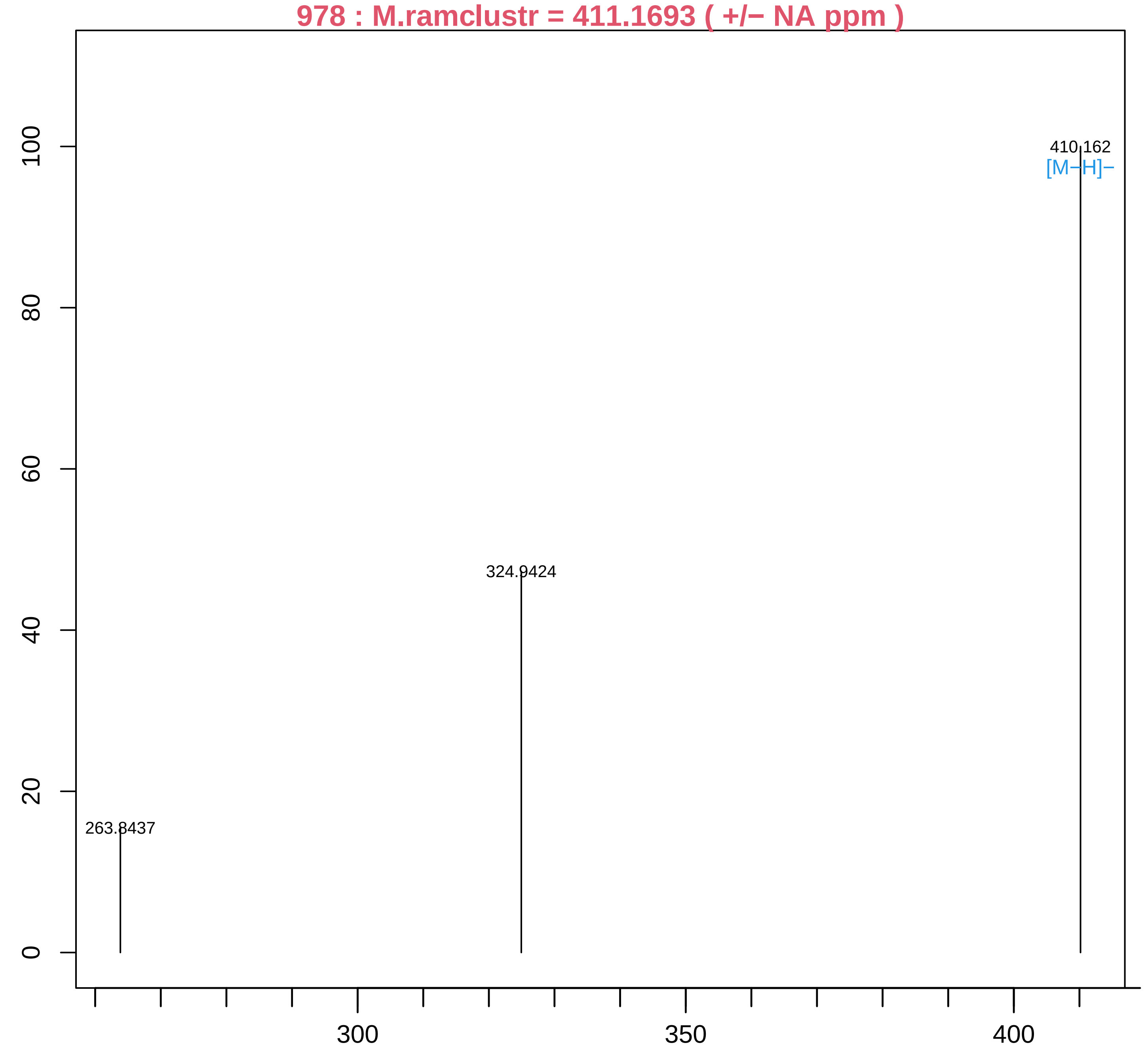
978 : M.ramclustr = 411.1693 (+/− NA ppm)
410.162
[M−H]−
324.9424
263.8437
0
20
40
60
80
100
300
350
400

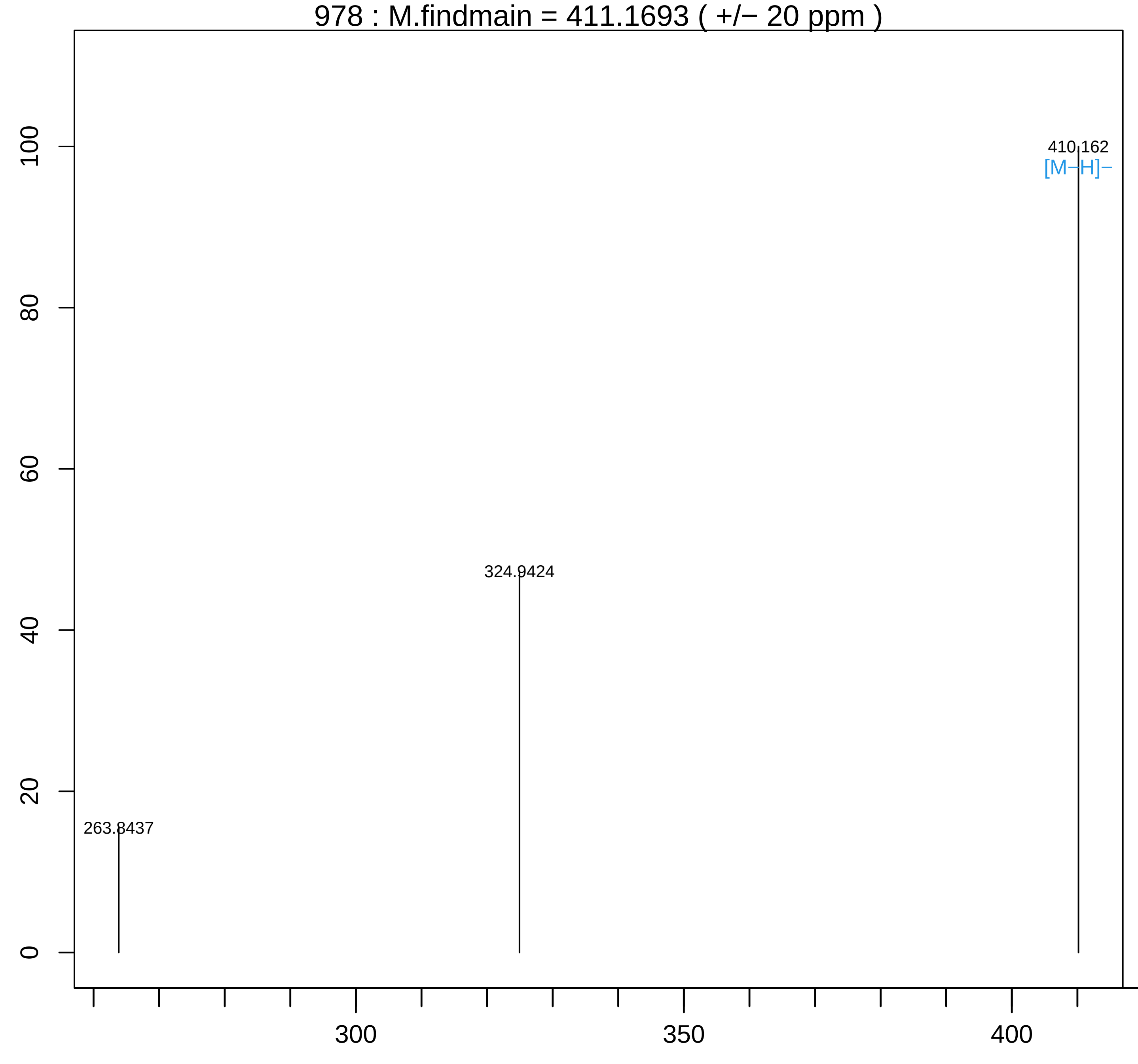
978 : M.findmain = 411.1693 (+/− 20 ppm)
410.162
[M−H]−
324.9424
263.8437
0
20
40
60
80
100
300
350
400

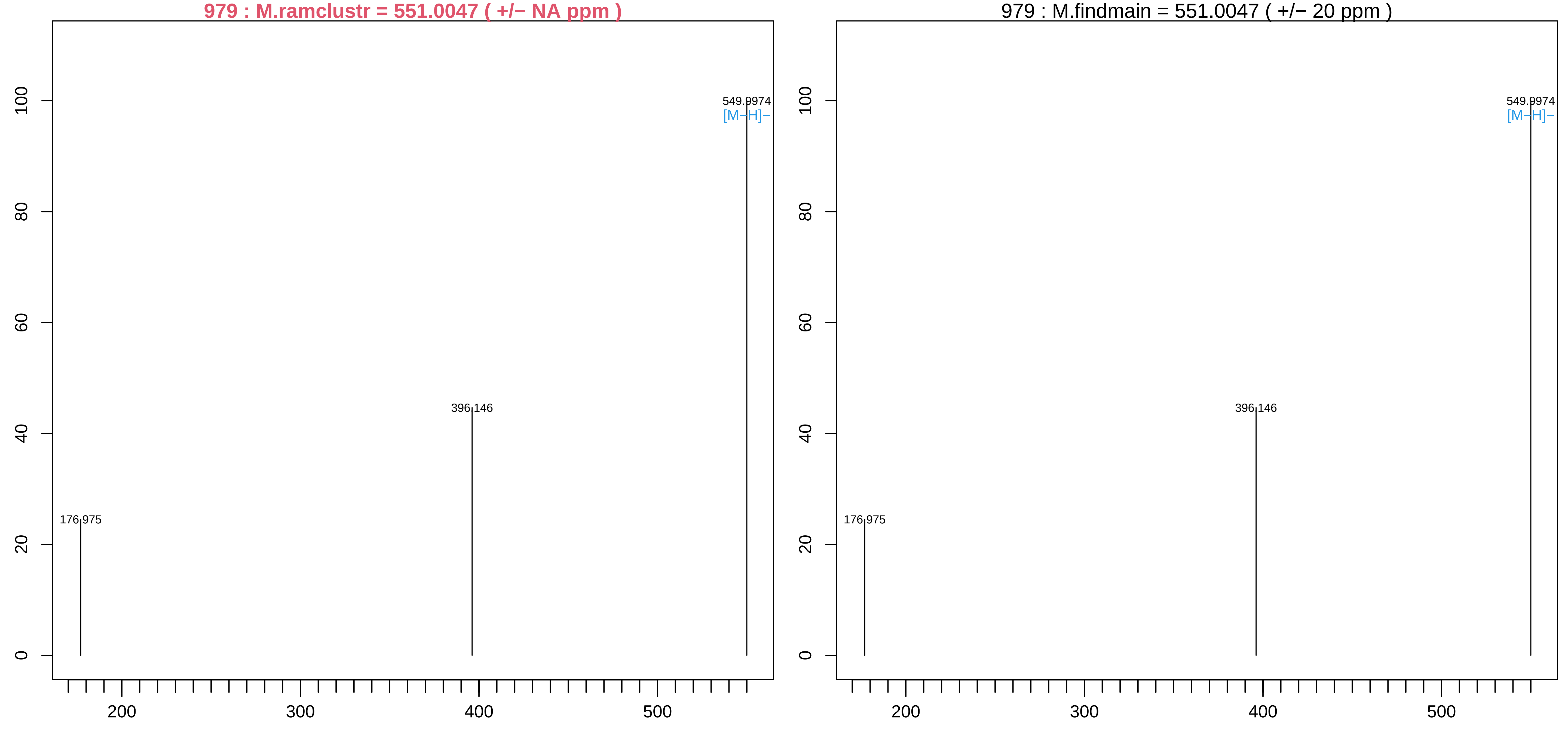

979 : M.ramclustr = 551.0047 (+/− NA ppm)
979 : M.findmain = 551.0047 (+/− 20 ppm)
549.9974
[M−H]−
396.146
176.975
549.9974
[M−H]−
396.146
176.975

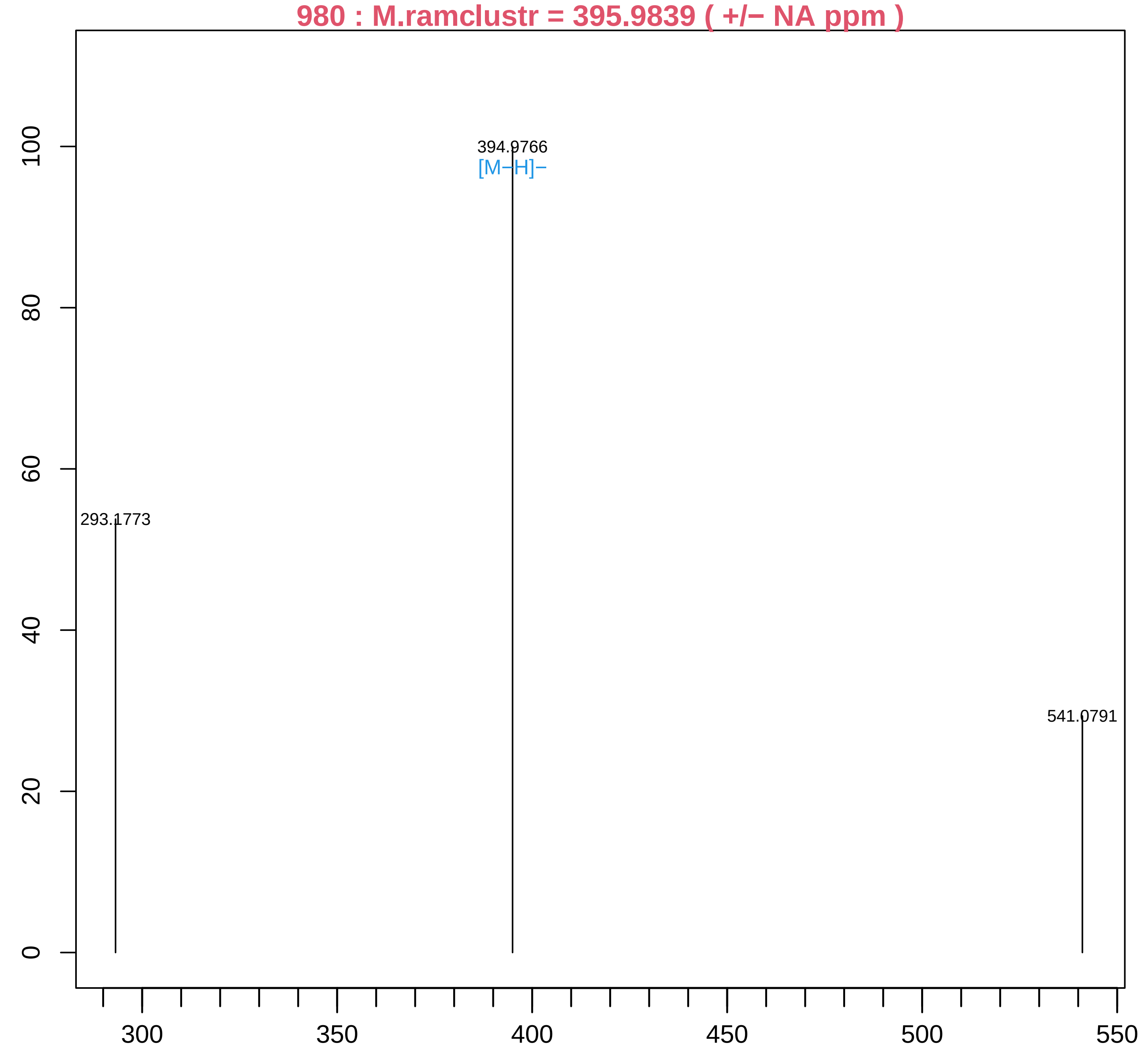
980 : M.ramclustr = 395.9839 (+/− NA ppm)
394.9766
[M−H]−
293.1773
541.0791
0
20
40
60
80
100
300
350
400
450
500
550

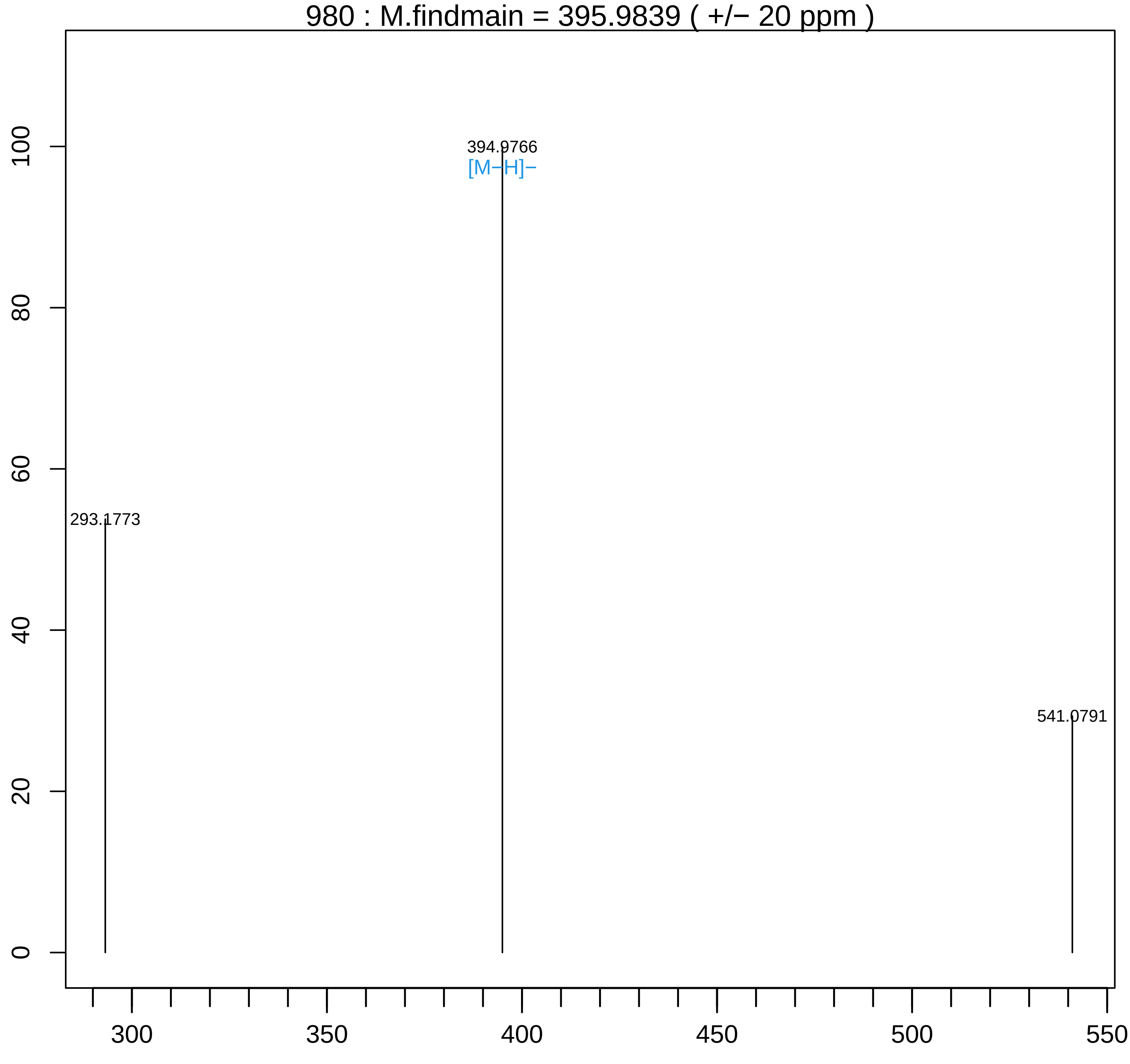
980 : M.findmain = 395.9839 (+/− 20 ppm)
394.9766
[M−H]−
293.1773
541.0791
0
20
40
60
80
100
300
350
400
450
500
550

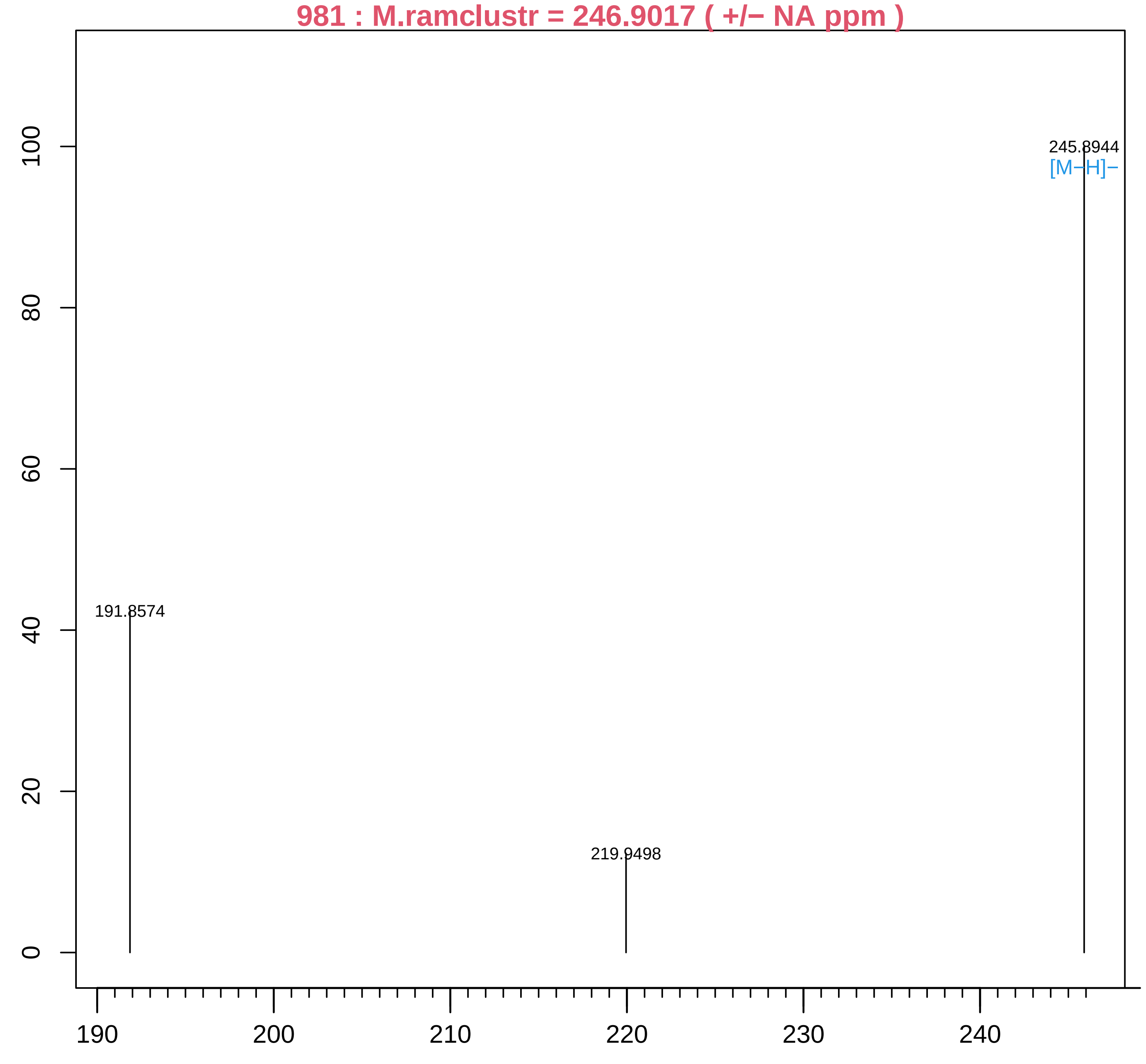

981 : M.ramclustr = 246.9017 (+/− NA ppm)
245.8944
[M−H]−
191.8574
219.9498
0
20
40
60
80
100
190
200
210
220
230
240

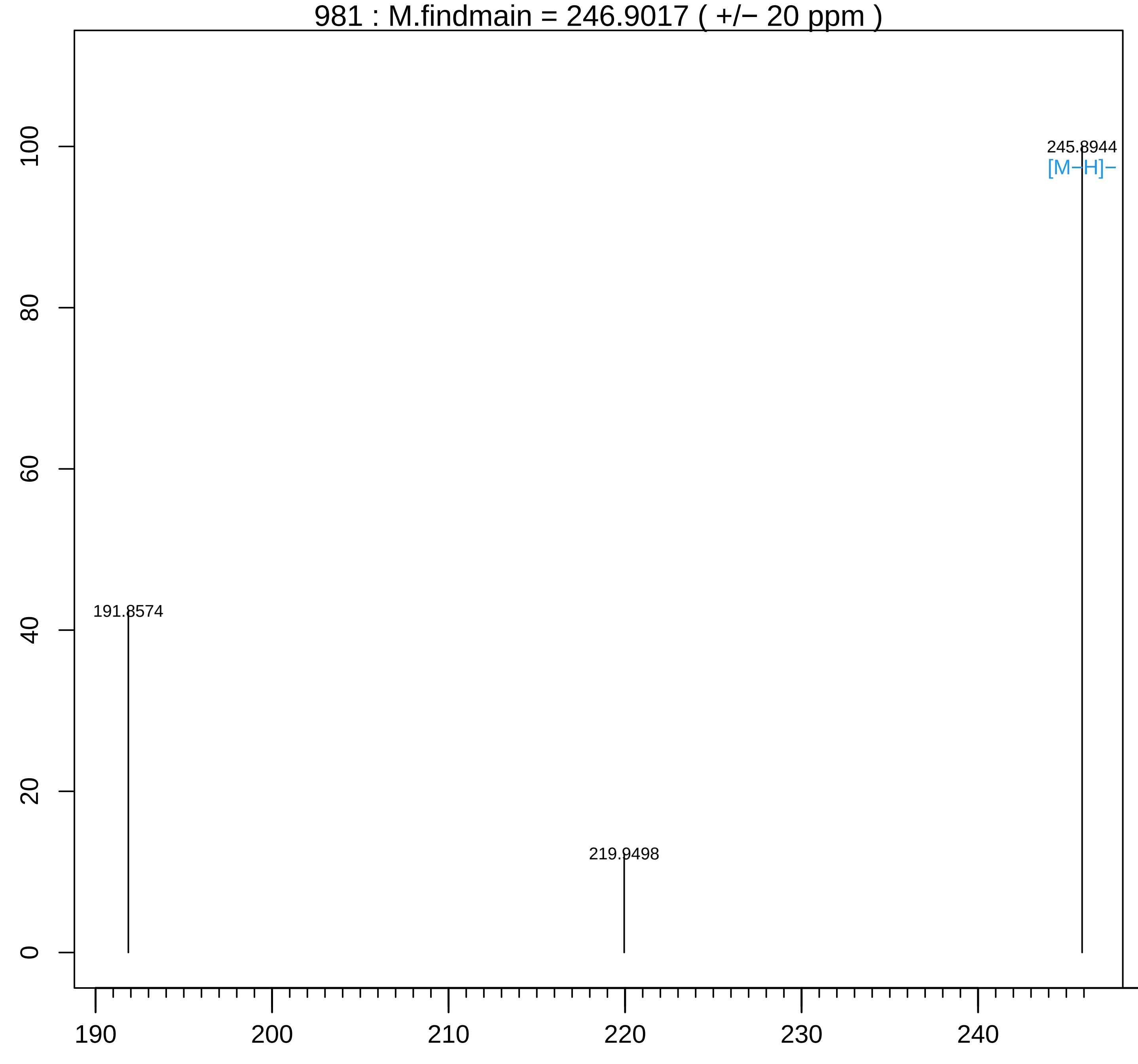

981 : M.findmain = 246.9017 (+/− 20 ppm)
245.8944
[M−H]−
191.8574
219.9498
0
20
40
60
80
100
190
200
210
220
230
240

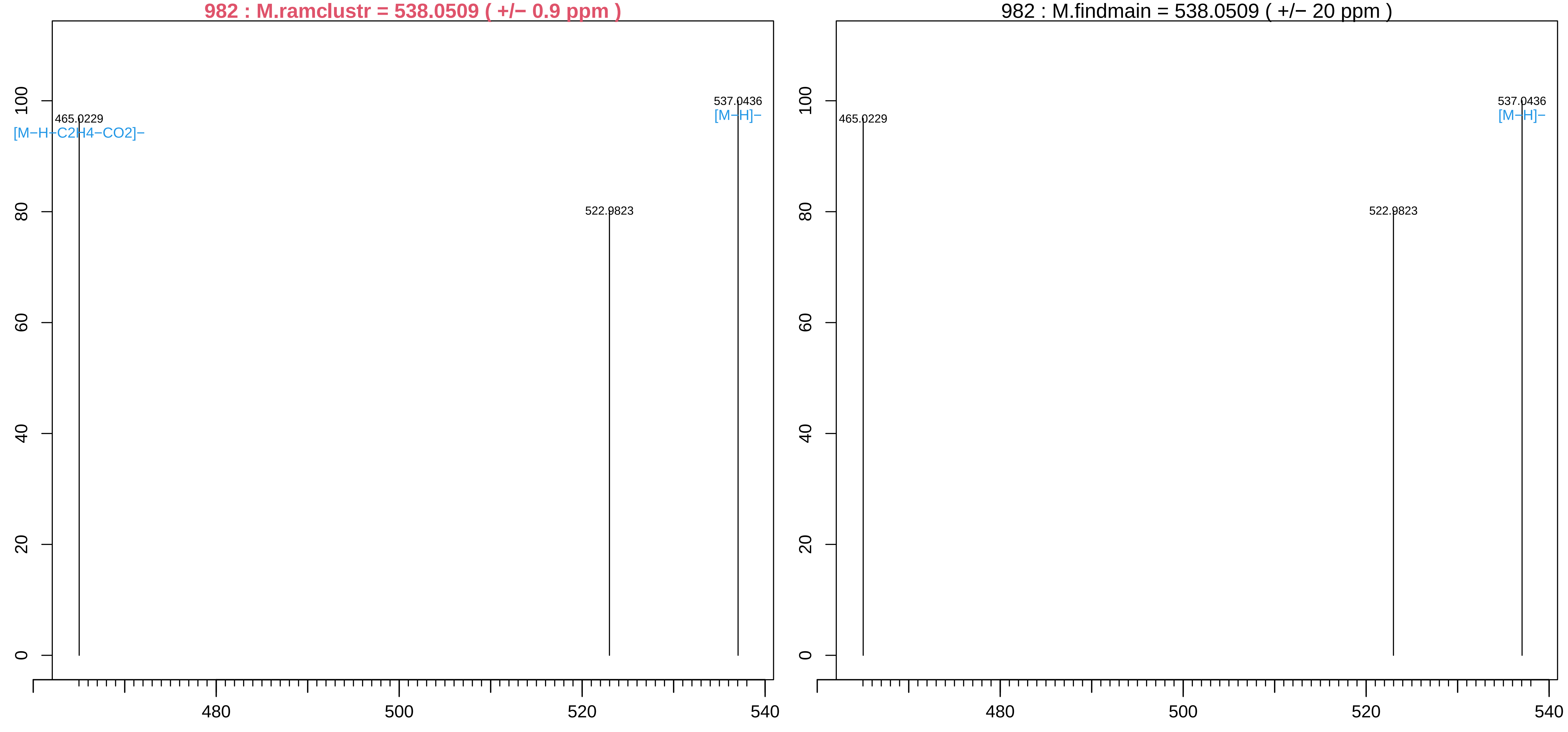
982 : M.ramclustr = 538.0509 (+/− 0.9 ppm)
465.0229
[M−H−C2H4−CO2]−
522.9823
537.0436
[M−H]−
982 : M.findmain = 538.0509 (+/− 20 ppm)
465.0229
522.9823
537.0436
[M−H]−
0
20
40
60
80
100
480
500
520
540

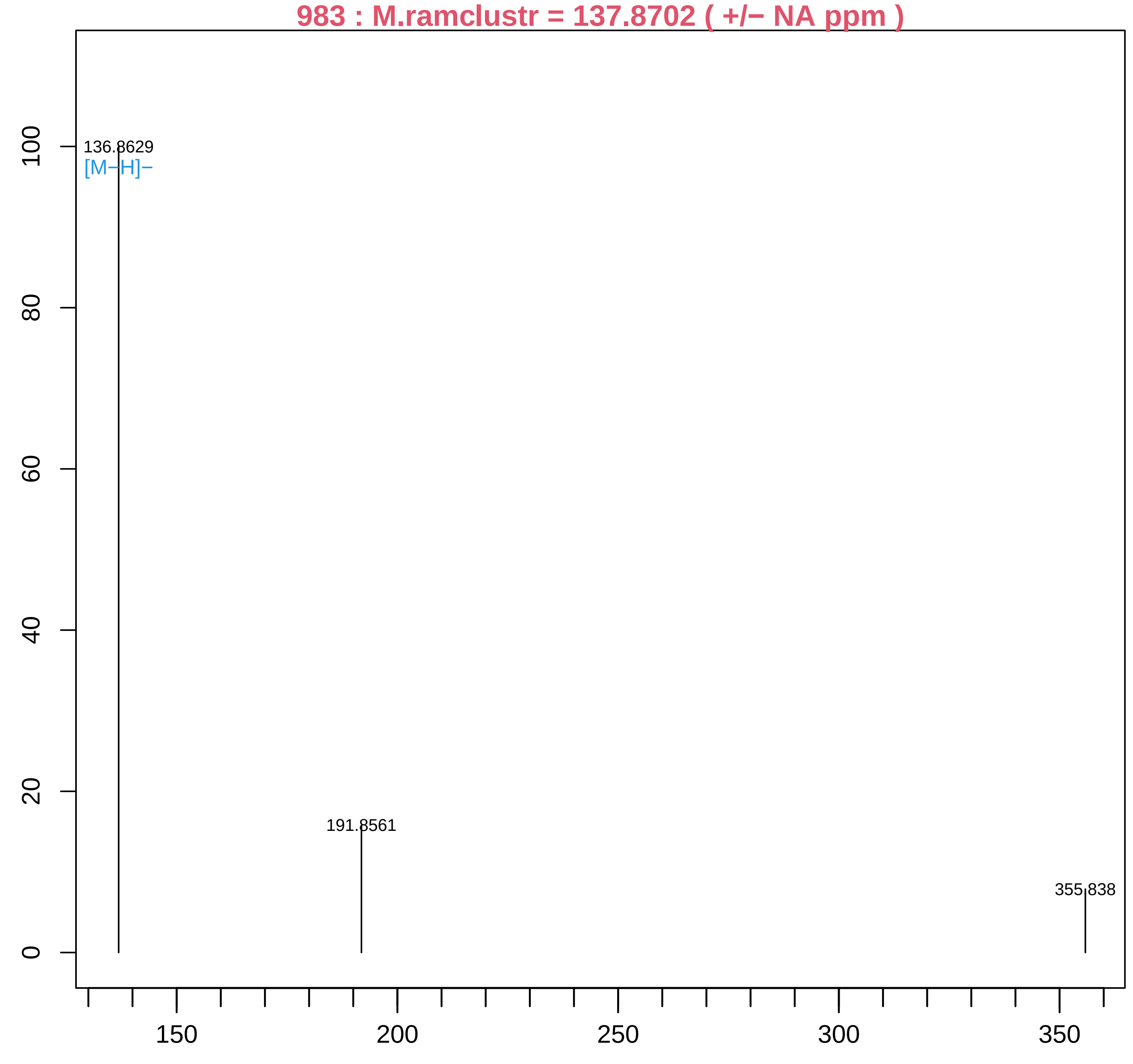
983 : M.ramclustr = 137.8702 (+/− NA ppm)
136.8629
[M−H]−
191.8561
355.838
0
20
40
60
80
100
150
200
250
300
350
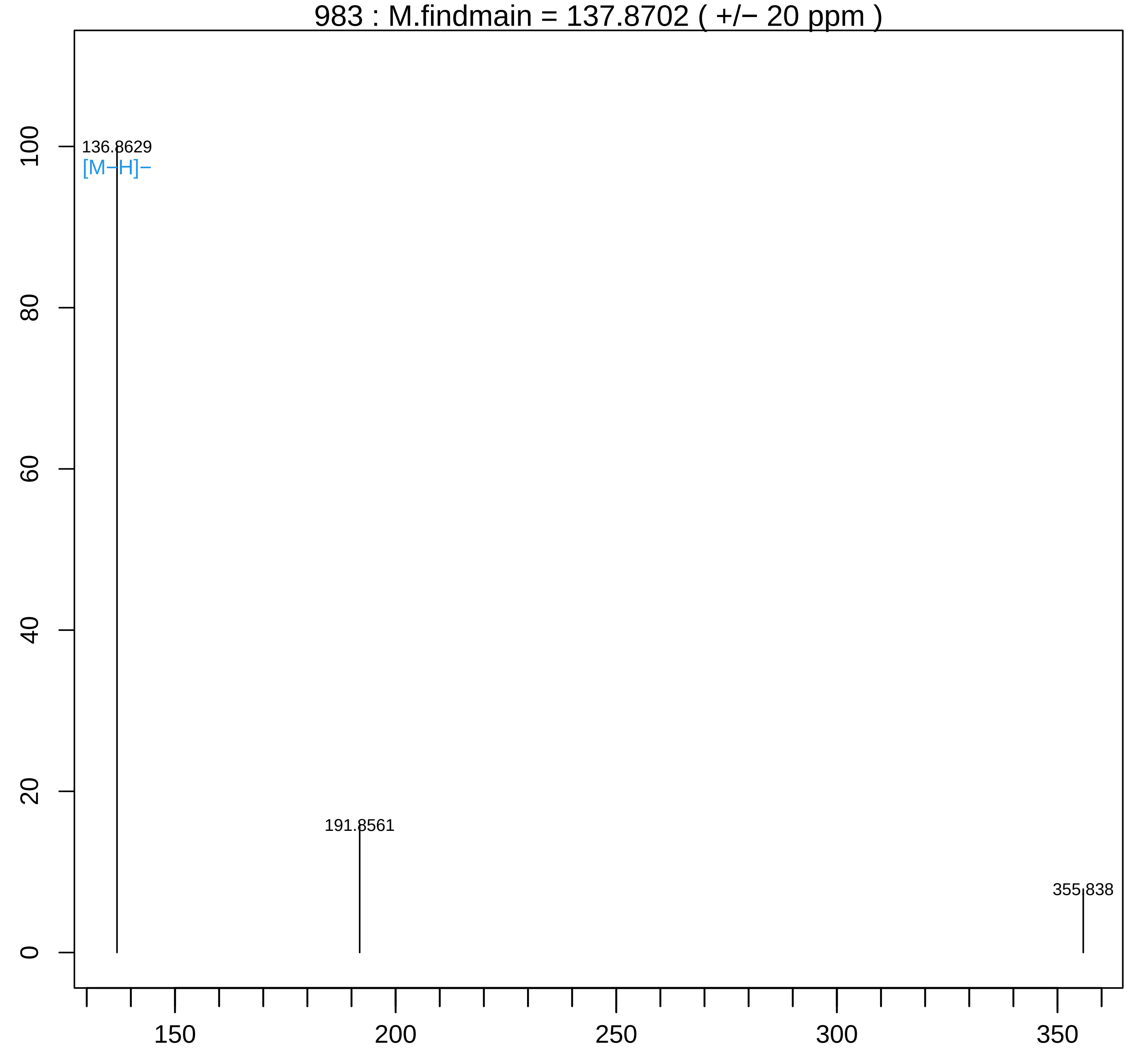
983 : M.findmain = 137.8702 (+/− 20 ppm)
136.8629
[M−H]−
191.8561
355.838
0
20
40
60
80
100
150
200
250
300
350

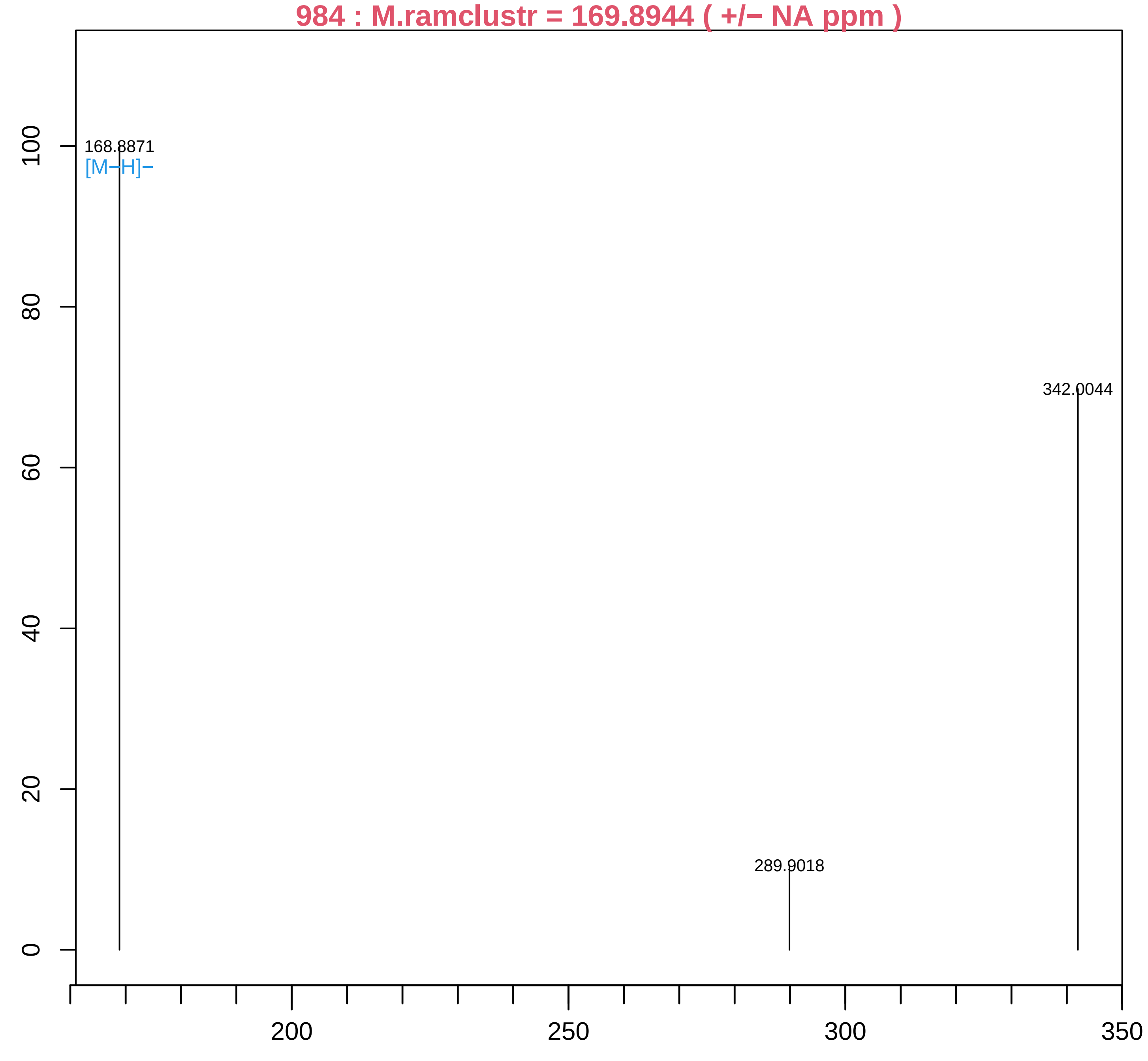

984 : M.ramclustr = 169.8944 (+/− NA ppm)
168.8871
[M−H]−
342.0044
289.9018
0
20
40
60
80
100
200
250
300
350

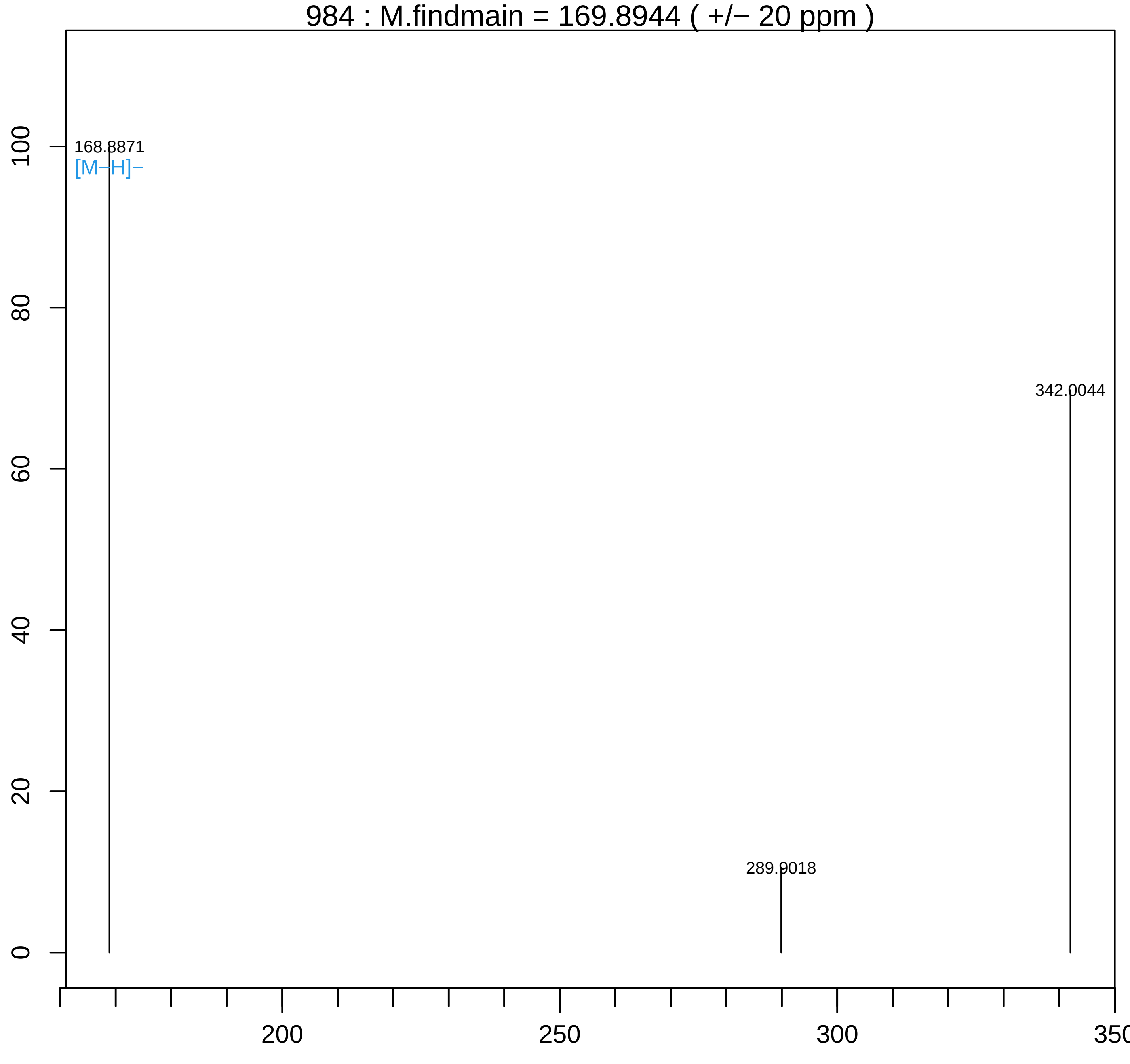

984 : M.findmain = 169.8944 (+/− 20 ppm)
168.8871
[M−H]−
342.0044
289.9018
0
20
40
60
80
100
200
250
300
350

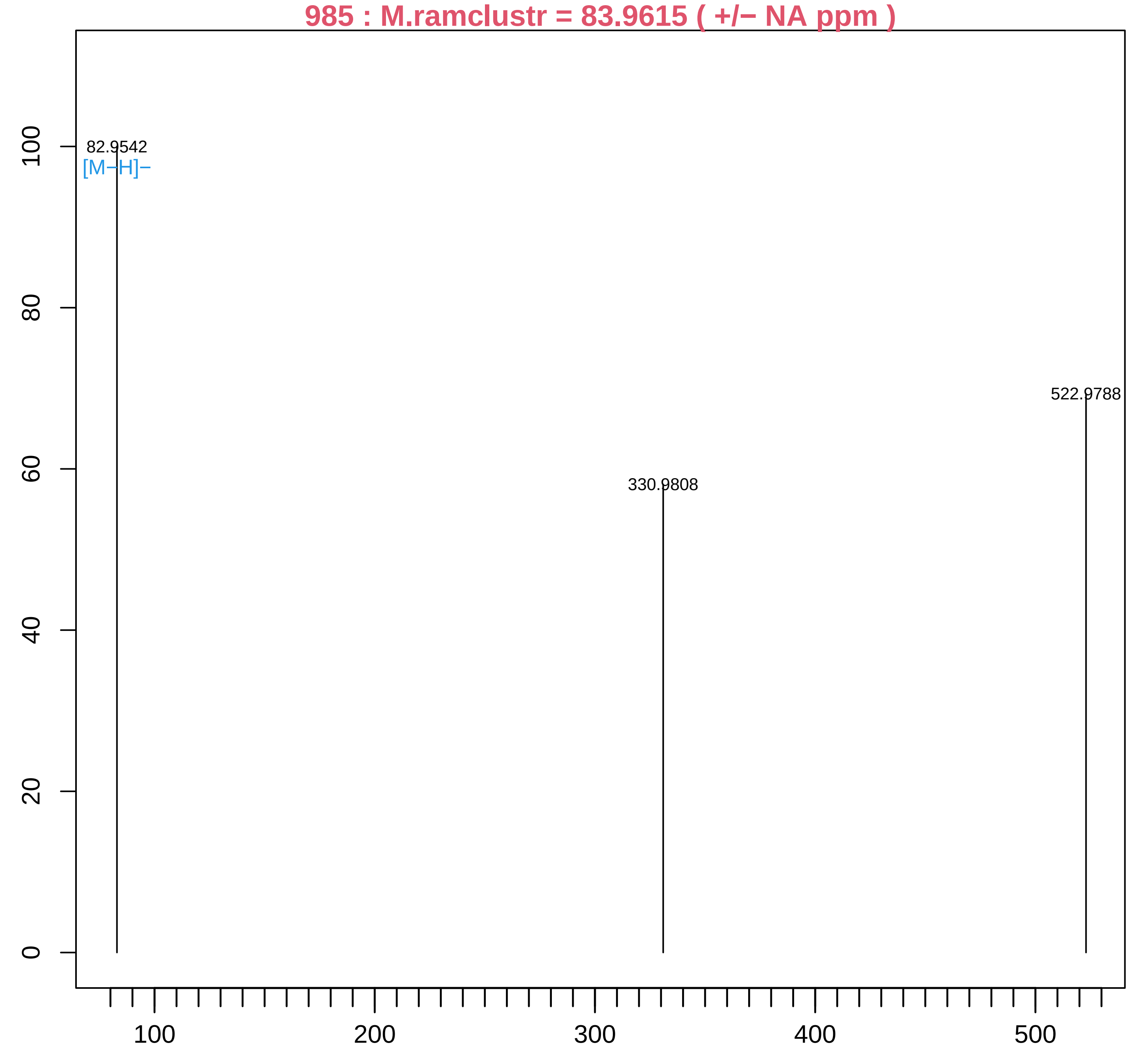

985 : M.ramclustr = 83.9615 (+/− NA ppm)
82.9542
[M−H]−
330.9808
522.9788
0
20
40
60
80
100
100
200
300
400
500

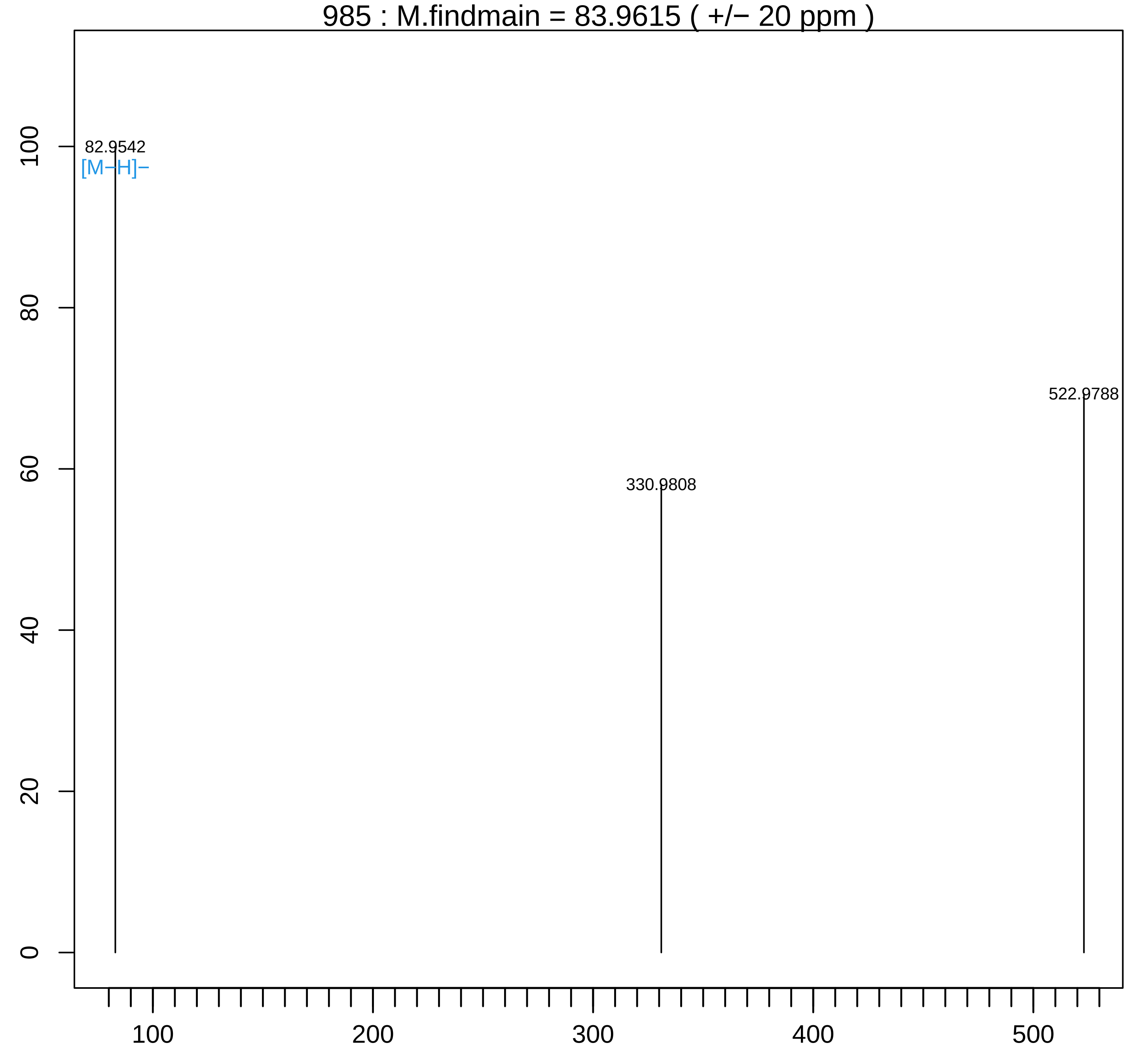

985 : M.findmain = 83.9615 (+/− 20 ppm)
82.9542
[M−H]−
330.9808
522.9788
0
20
40
60
80
100
100
200
300
400
500

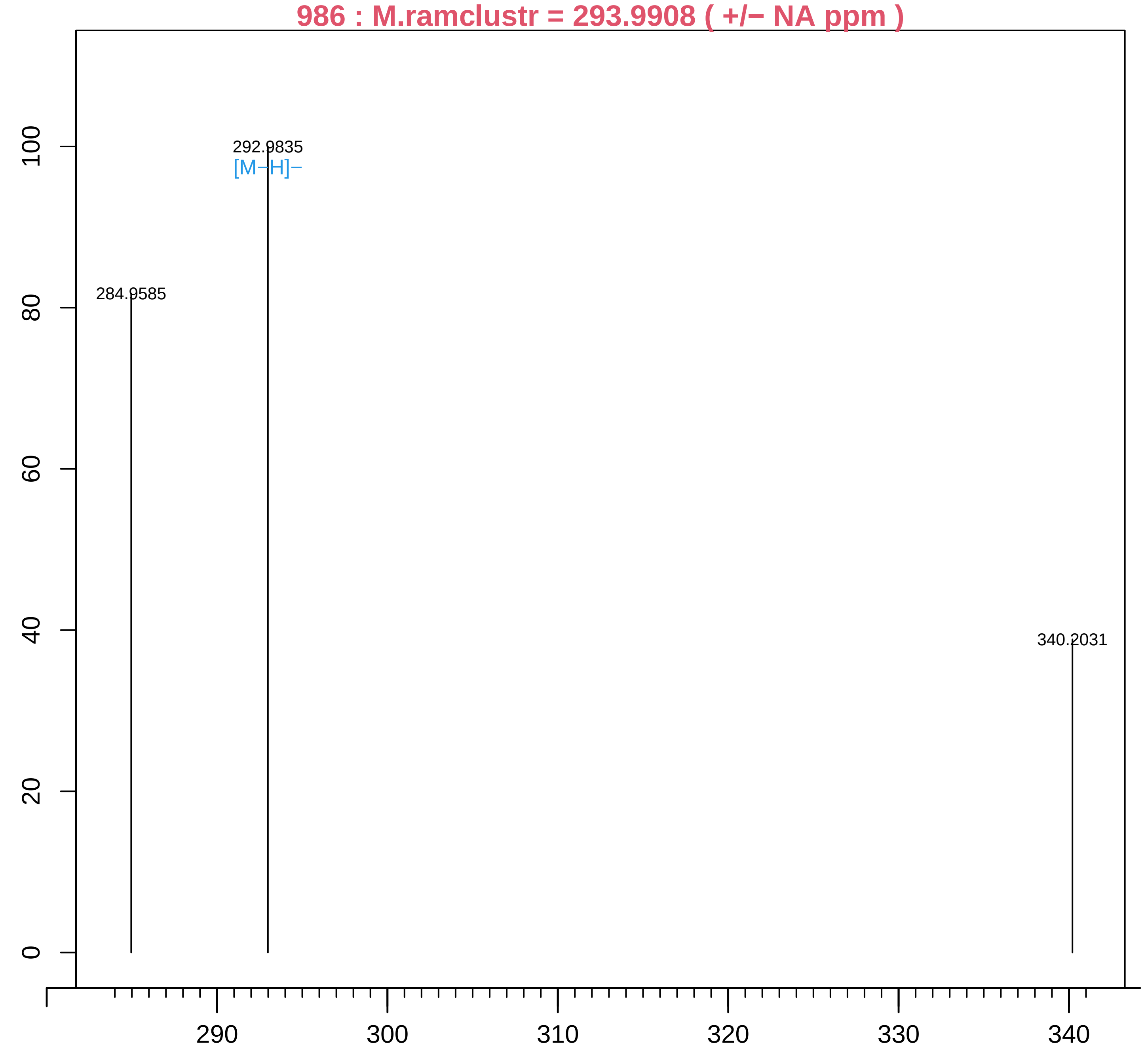

986 : M.ramclustr = 293.9908 (+/− NA ppm)
284.9585
292.9835
[M−H]−
340.2031
0
20
40
60
80
100
290
300
310
320
330
340

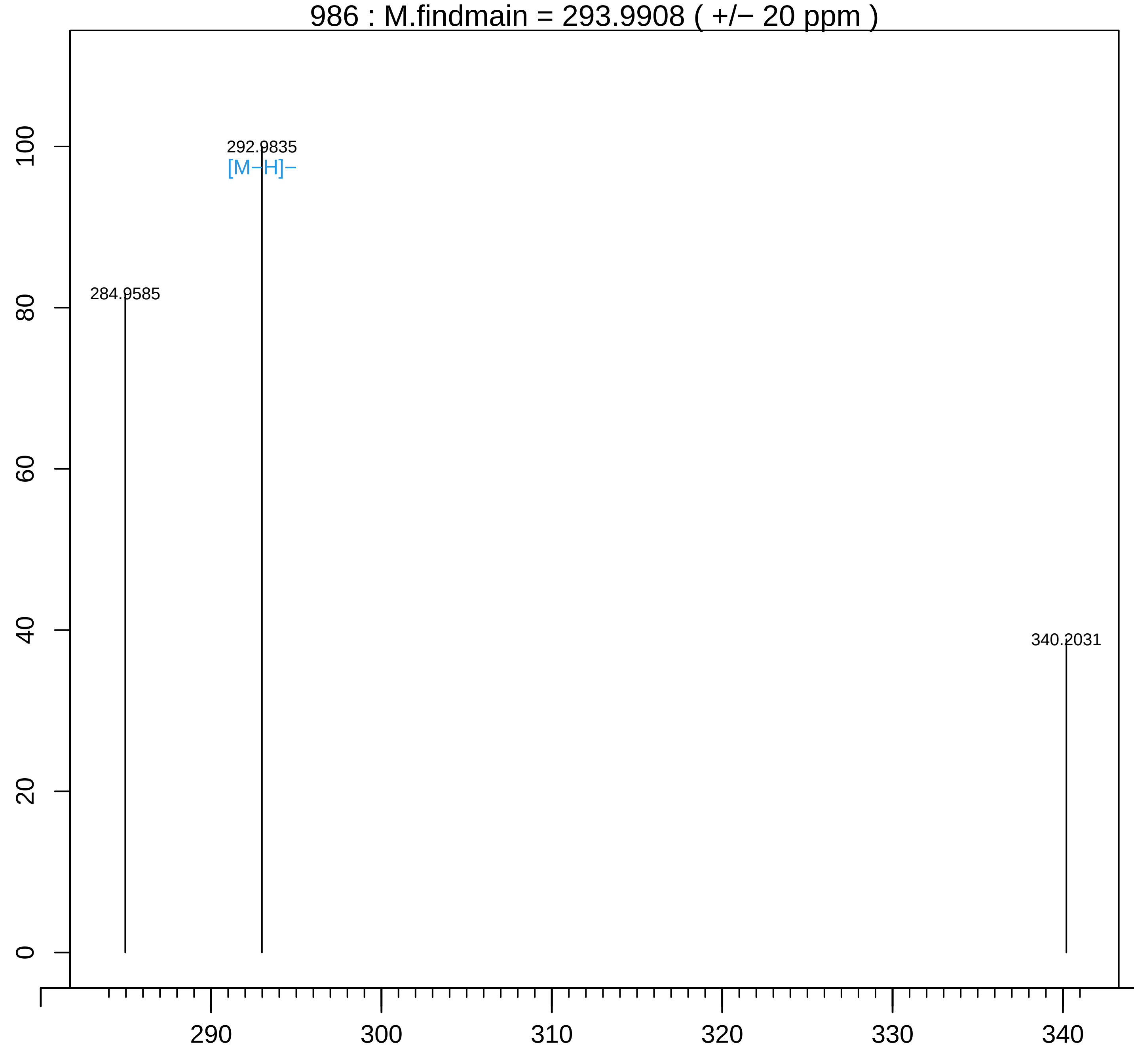

986 : M.findmain = 293.9908 (+/− 20 ppm)
284.9585
292.9835
[M−H]−
340.2031
0
20
40
60
80
100
290
300
310
320
330
340

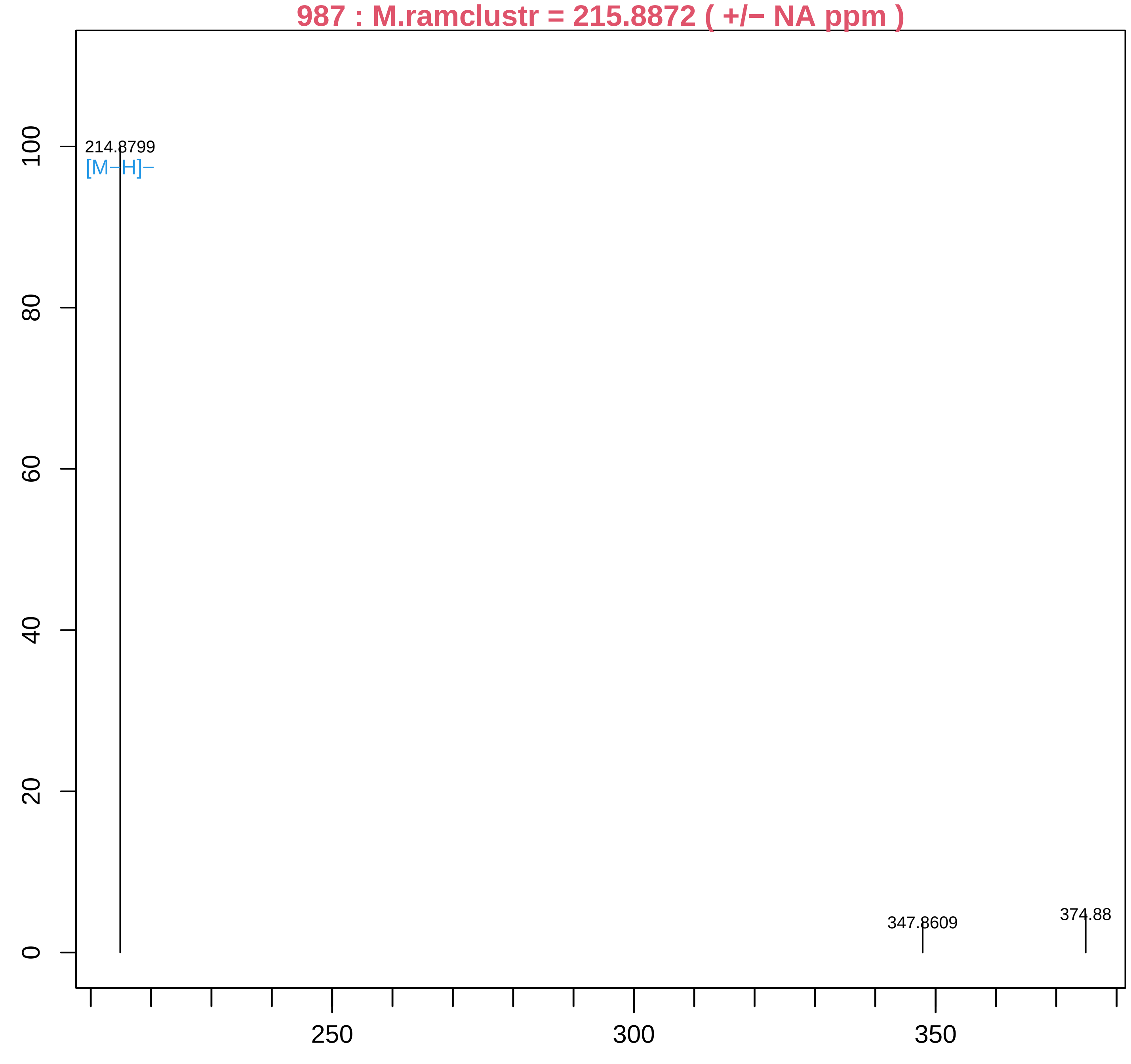
987 : M.ramclustr = 215.8872 (+/− NA ppm)
214.8799
[M−H]−
347.8609
374.88
0
20
40
60
80
100
250
300
350

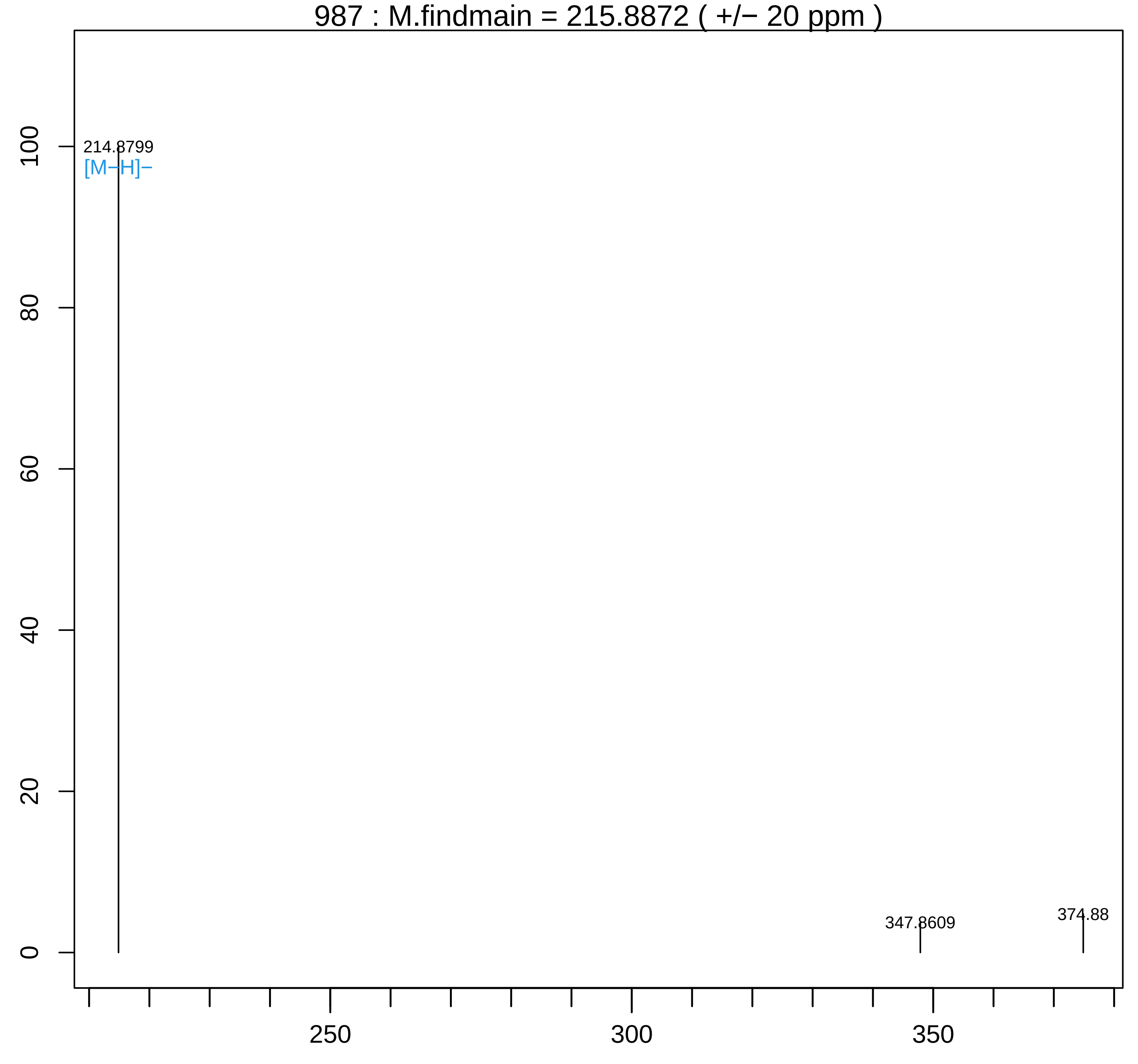
987 : M.findmain = 215.8872 (+/− 20 ppm)
214.8799
[M−H]−
347.8609
374.88
0
20
40
60
80
100
250
300
350

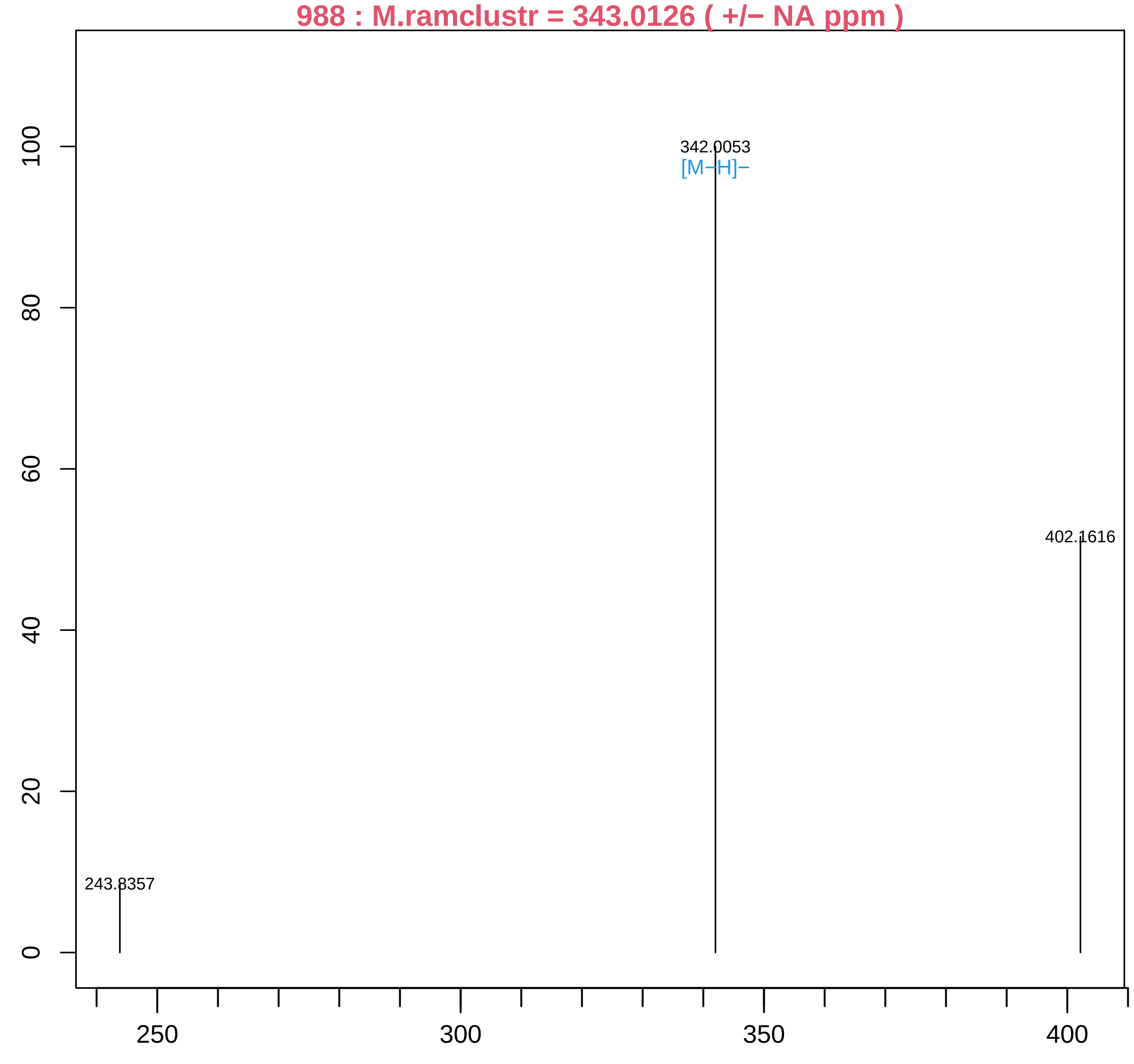
988 : M.ramclustr = 343.0126 (+/− NA ppm)
342.0053
[M−H]−
402.1616
243.8357
0
20
40
60
80
100
250
300
350
400

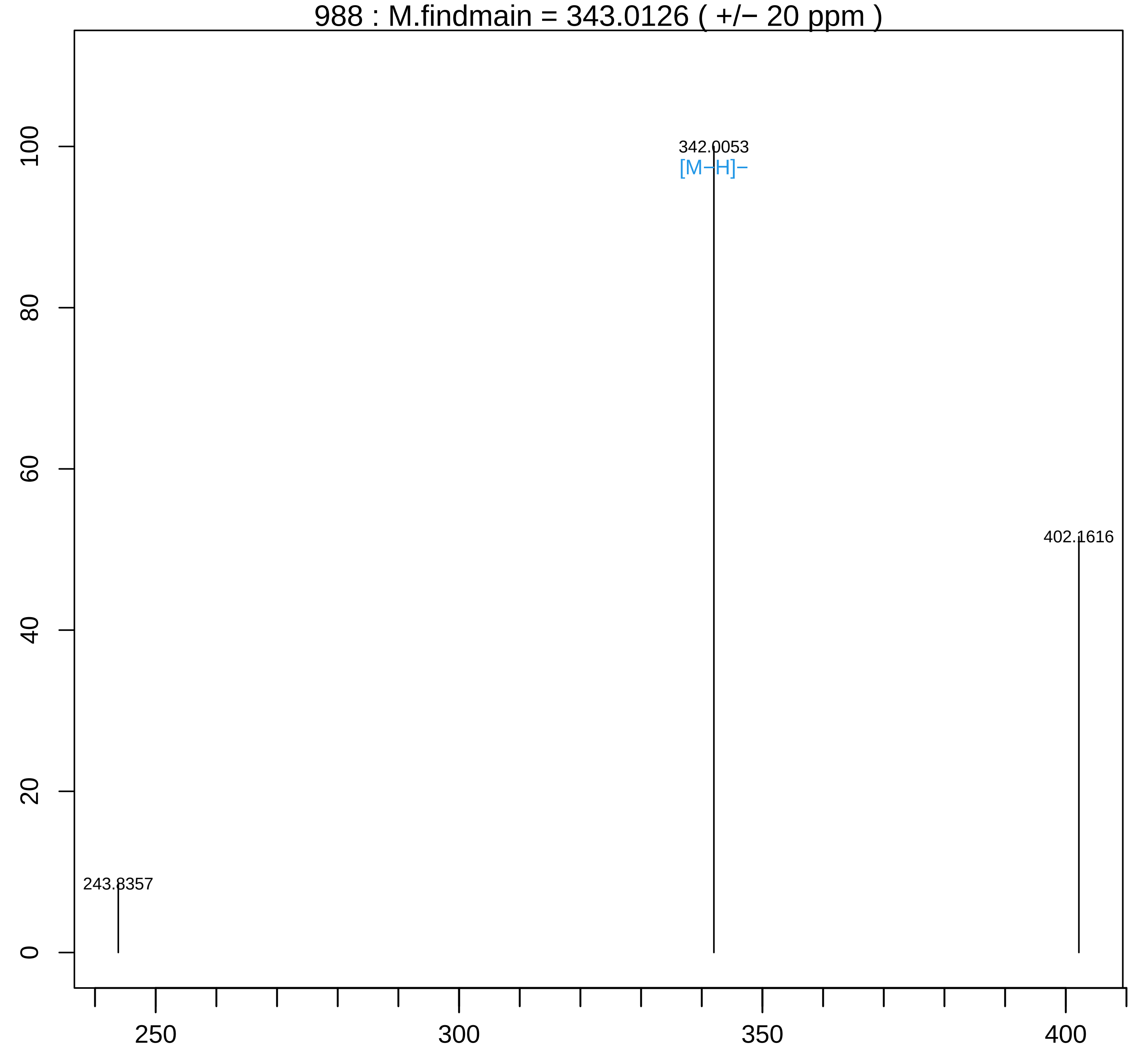
988 : M.findmain = 343.0126 (+/− 20 ppm)
342.0053
[M−H]−
402.1616
243.8357
0
20
40
60
80
100
250
300
350
400

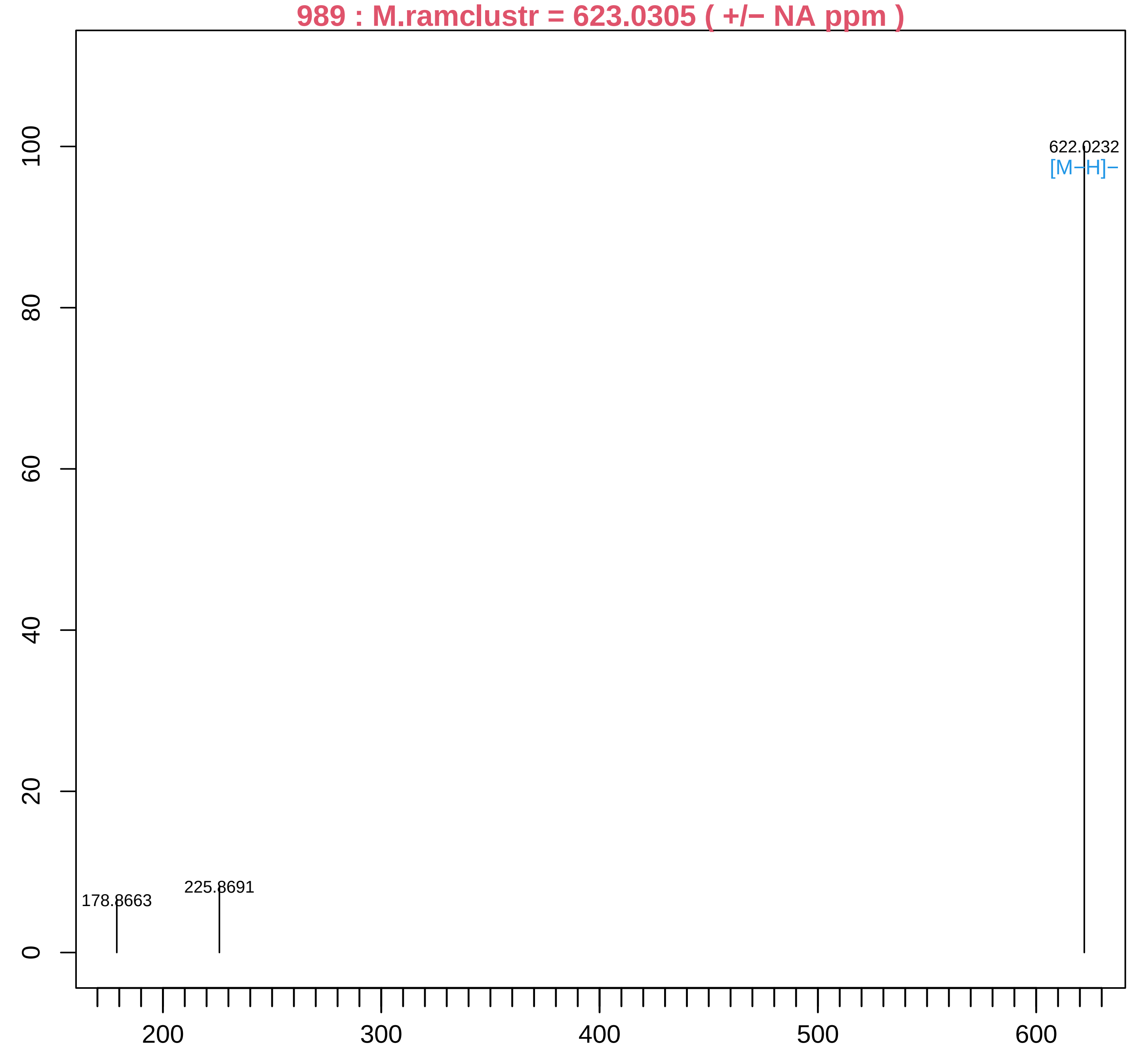
989 : M.ramclustr = 623.0305 (+/− NA ppm)
622.0232
[M−H]−
178.8663
225.8691
0
20
40
60
80
100
200
300
400
500
600
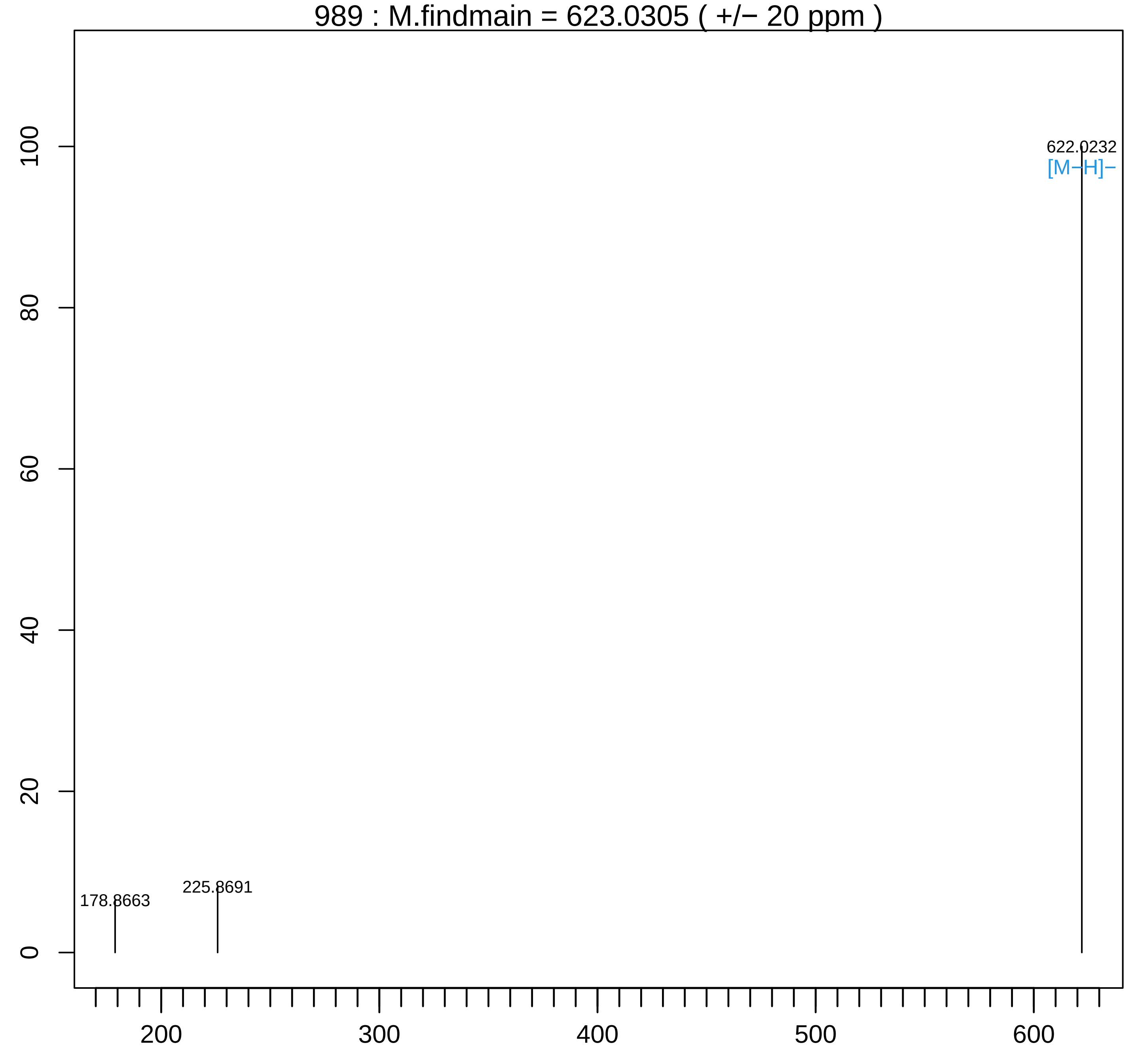
989 : M.findmain = 623.0305 (+/− 20 ppm)
622.0232
[M−H]−
178.8663
225.8691
0
20
40
60
80
100
200
300
400
500
600

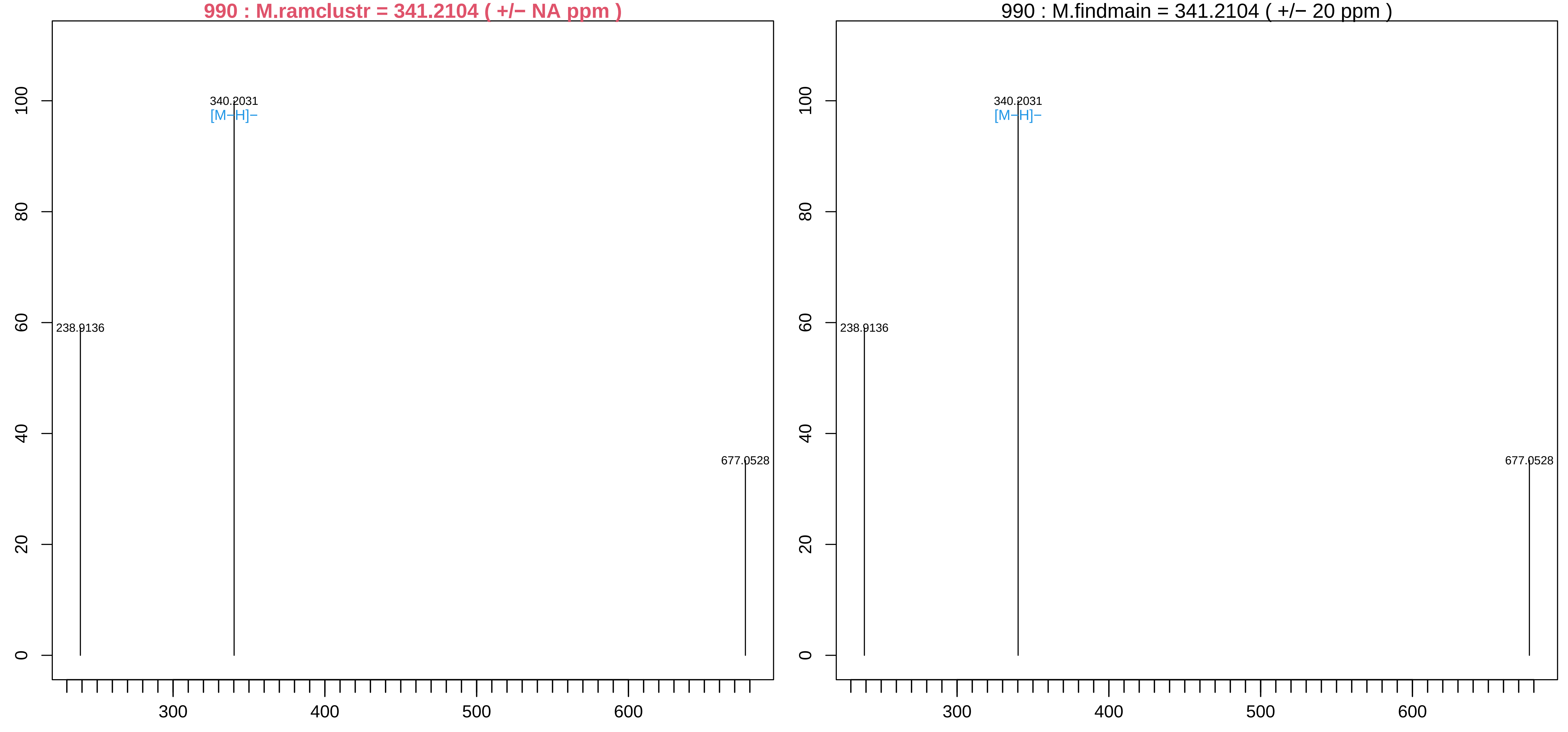
990 : M.ramclustr = 341.2104 (+/− NA ppm)
340.2031
[M−H]−
238.9136
677.0528
990 : M.findmain = 341.2104 (+/− 20 ppm)
340.2031
[M−H]−
238.9136
677.0528
0
20
40
60
80
100
300
400
500
600

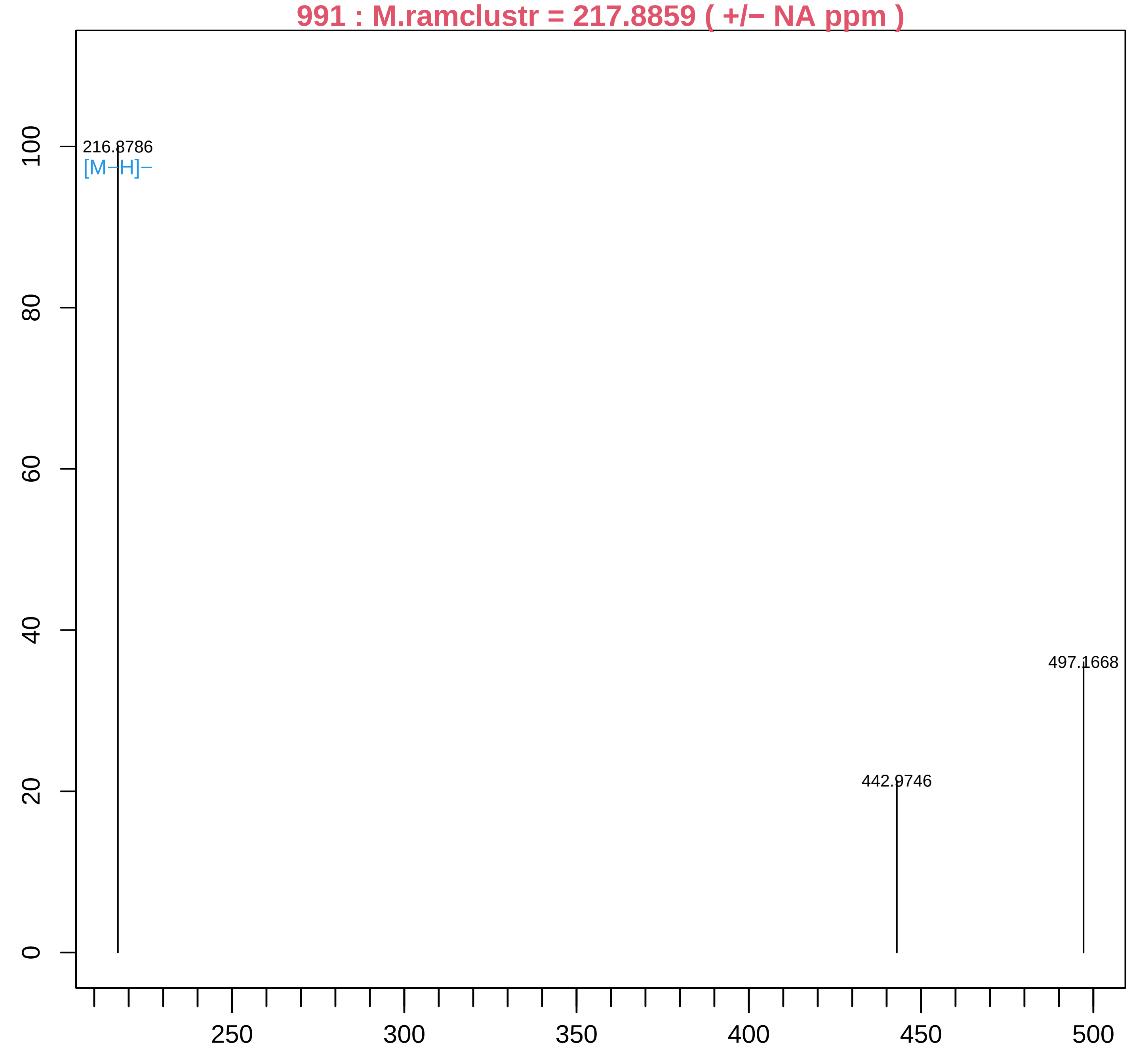
991 : M.ramclustr = 217.8859 (+/− NA ppm)
216.8786
[M−H]−
442.9746
497.1668
0
20
40
60
80
100
250
300
350
400
450
500

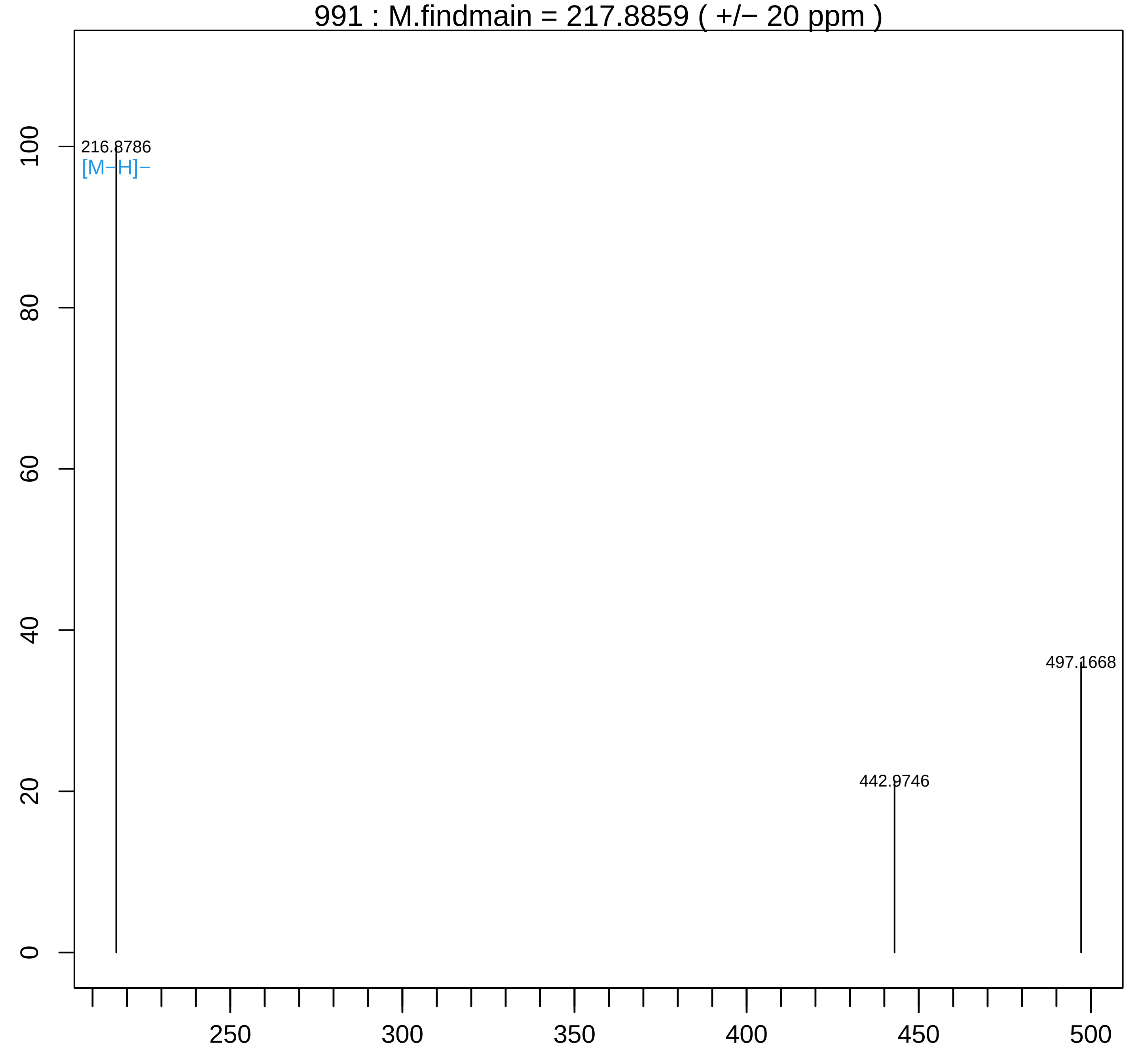
991 : M.findmain = 217.8859 (+/− 20 ppm)
216.8786
[M−H]−
442.9746
497.1668
0
20
40
60
80
100
250
300
350
400
450
500

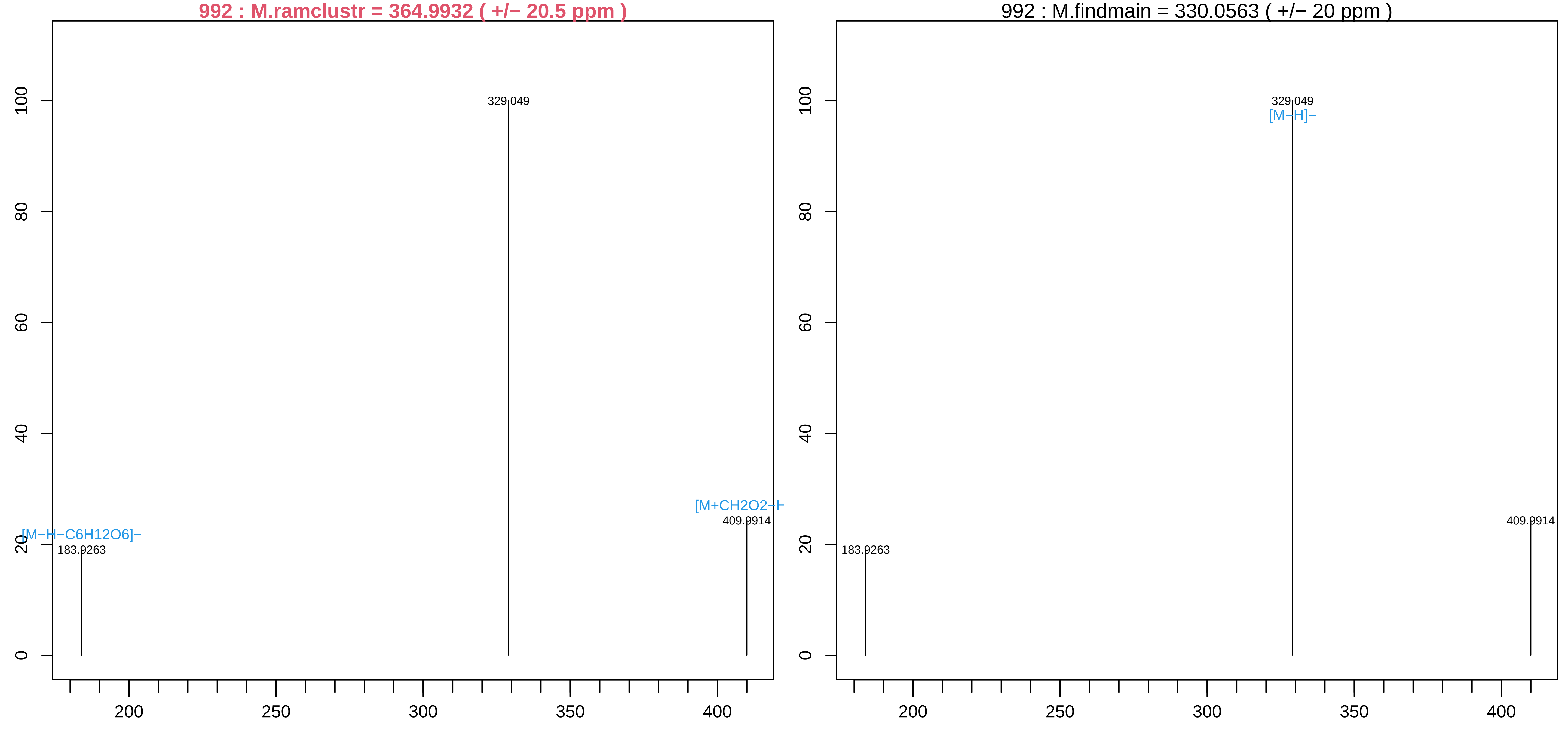
992 : M.ramclustr = 364.9932 (+/− 20.5 ppm)
329.049
[M+CH2O2−H
409.9914
[M−H−C6H12O6]−
183.9263
0
20
40
60
80
100
200
250
300
350
400
992 : M.findmain = 330.0563 (+/− 20 ppm)
329.049
[M−H]−
409.9914
183.9263
0
20
40
60
80
100
200
250
300
350
400

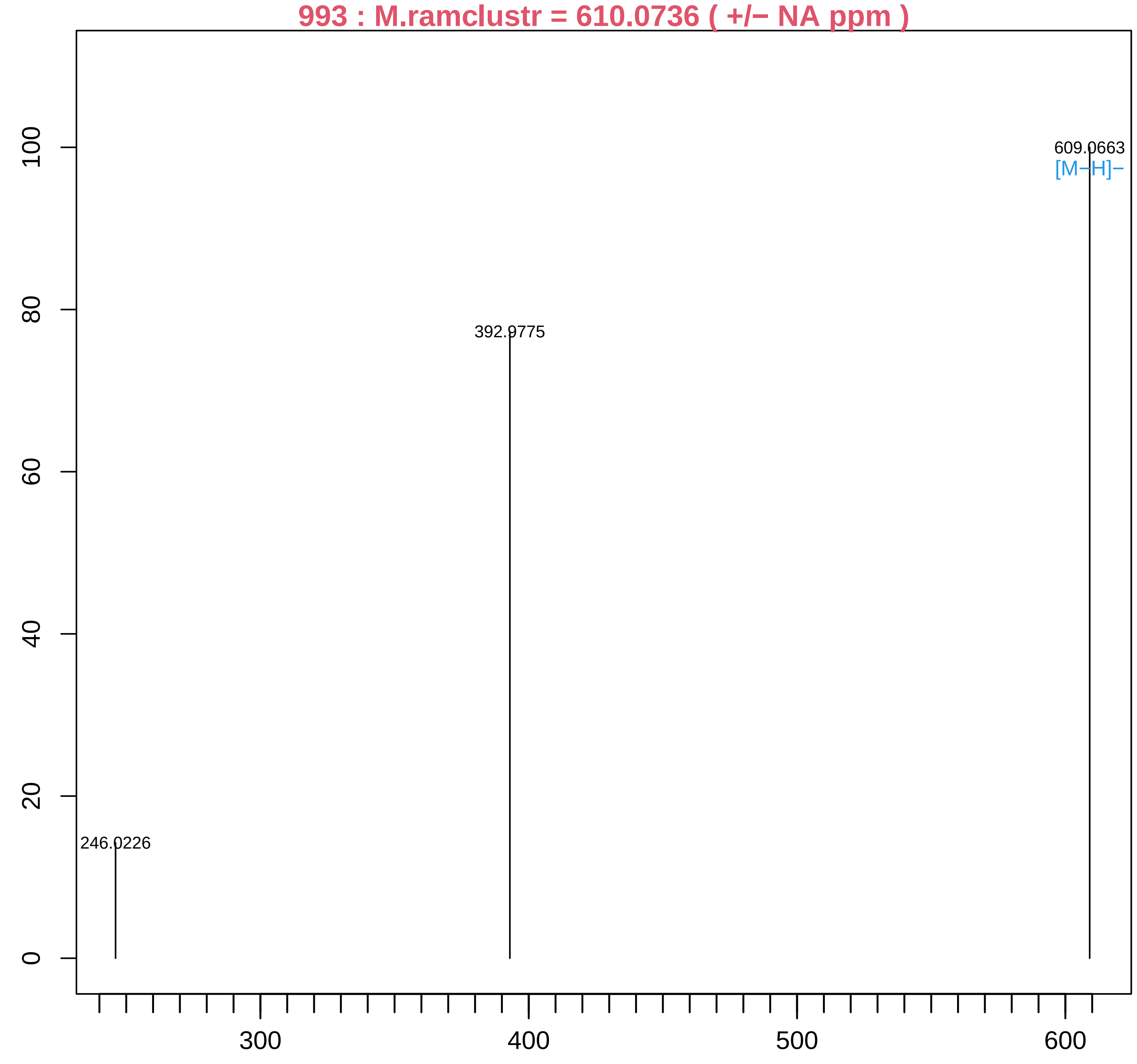
993 : M.ramclustr = 610.0736 (+/− NA ppm)
609.0663
[M−H]−
392.9775
246.0226
0
20
40
60
80
100
300
400
500
600

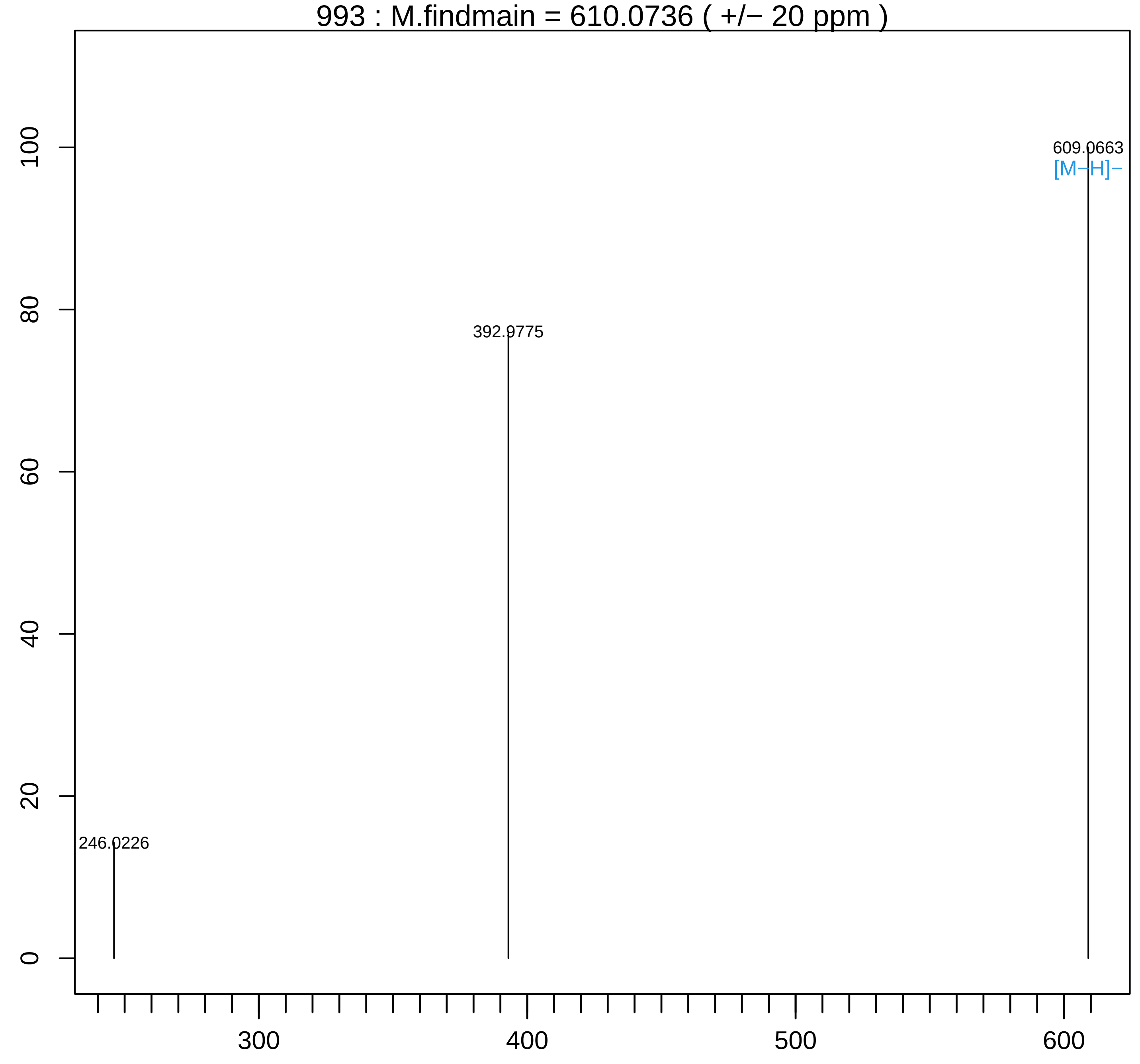
993 : M.findmain = 610.0736 (+/− 20 ppm)
609.0663
[M−H]−
392.9775
246.0226
0
20
40
60
80
100
300
400
500
600

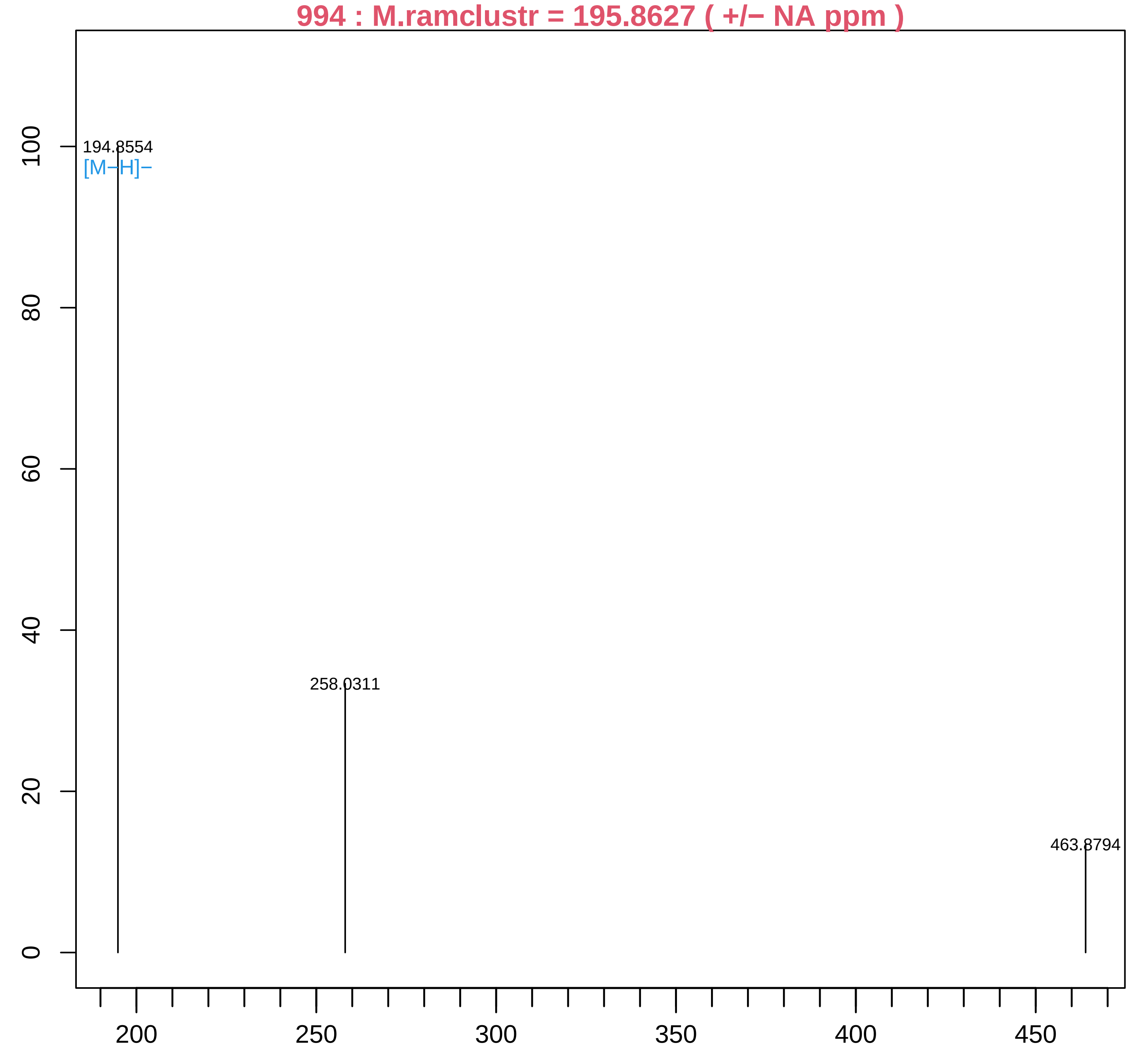
994 : M.ramclustr = 195.8627 (+/− NA ppm)
194.8554
[M−H]−
258.0311
463.8794
0
20
40
60
80
100
200
250
300
350
400
450

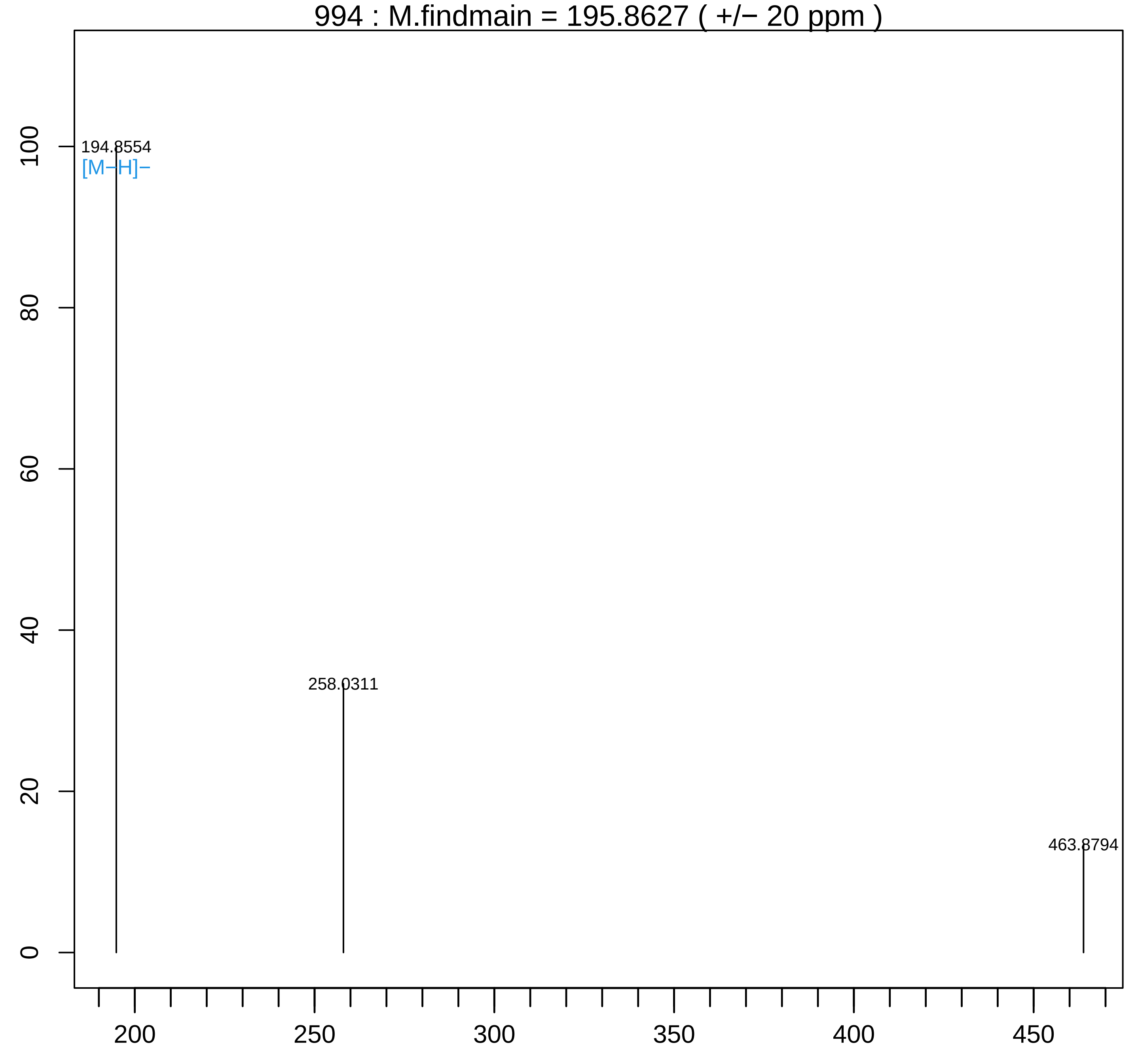
994 : M.findmain = 195.8627 (+/− 20 ppm)
194.8554
[M−H]−
258.0311
463.8794
0
20
40
60
80
100
200
250
300
350
400
450

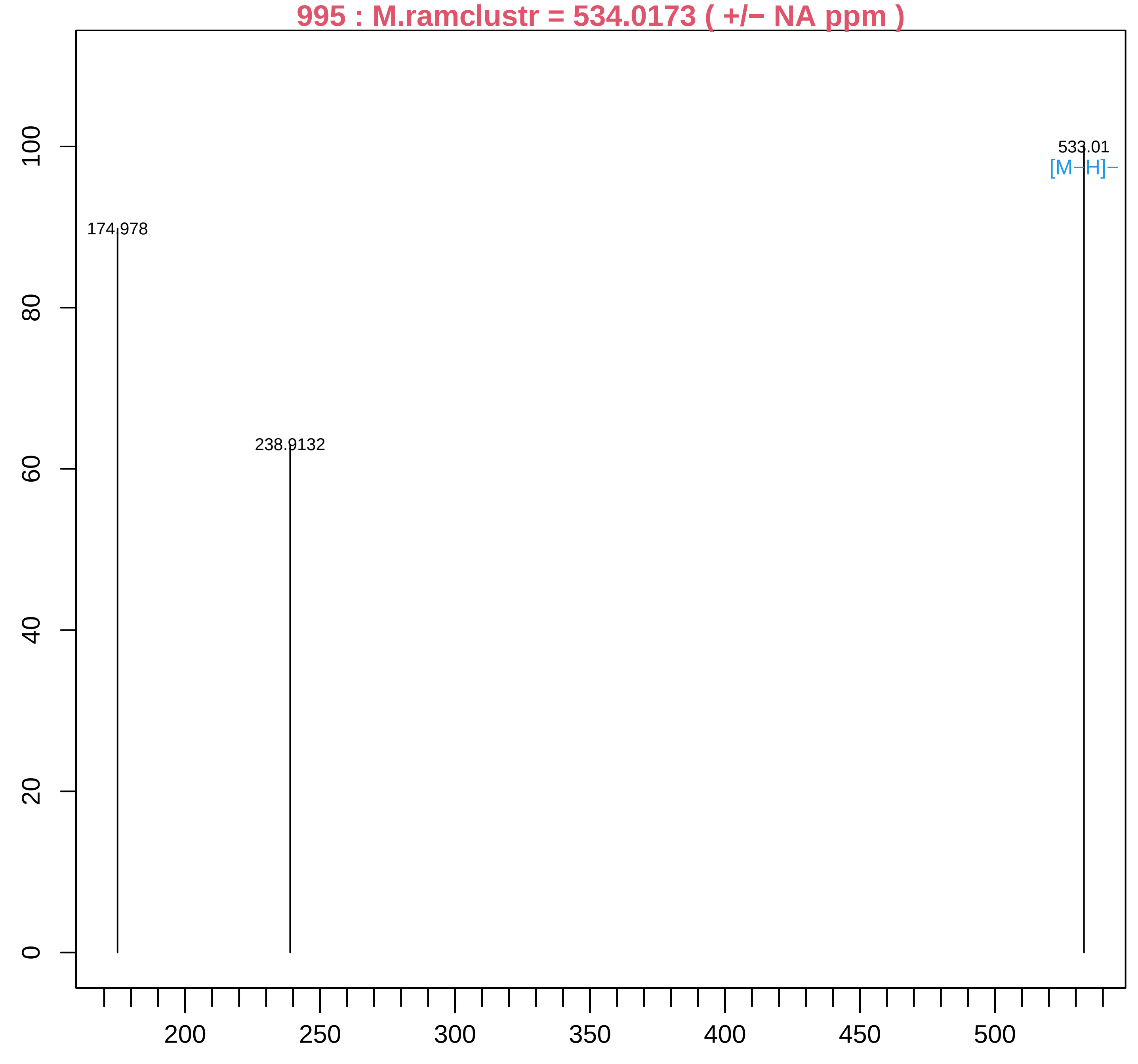
995 : M.ramclustr = 534.0173 (+/− NA ppm)
174.978
238.9132
533.01
[M−H]−
0
20
40
60
80
100
200
250
300
350
400
450
500
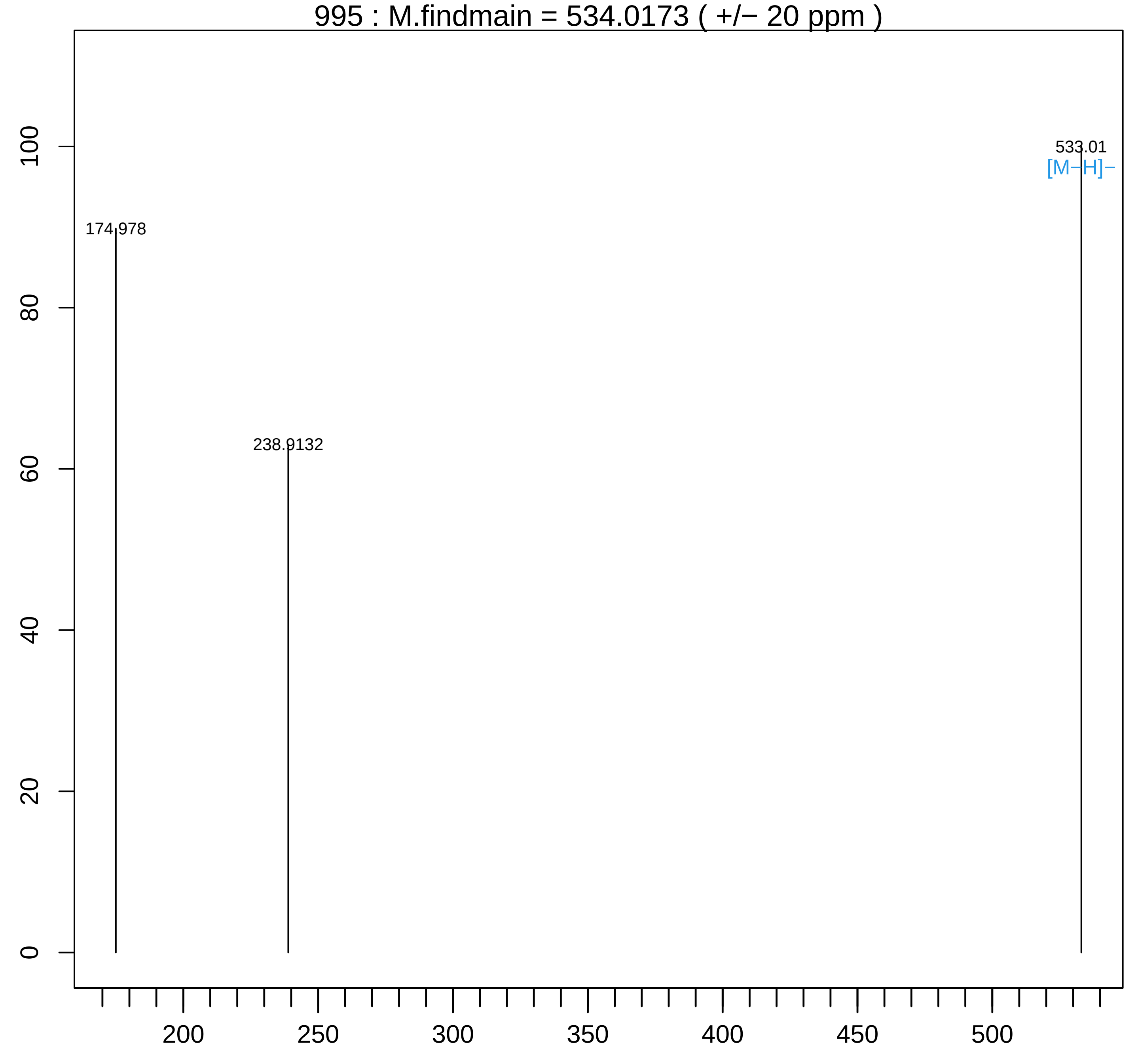
995 : M.findmain = 534.0173 (+/− 20 ppm)
174.978
238.9132
533.01
[M−H]−
0
20
40
60
80
100
200
250
300
350
400
450
500

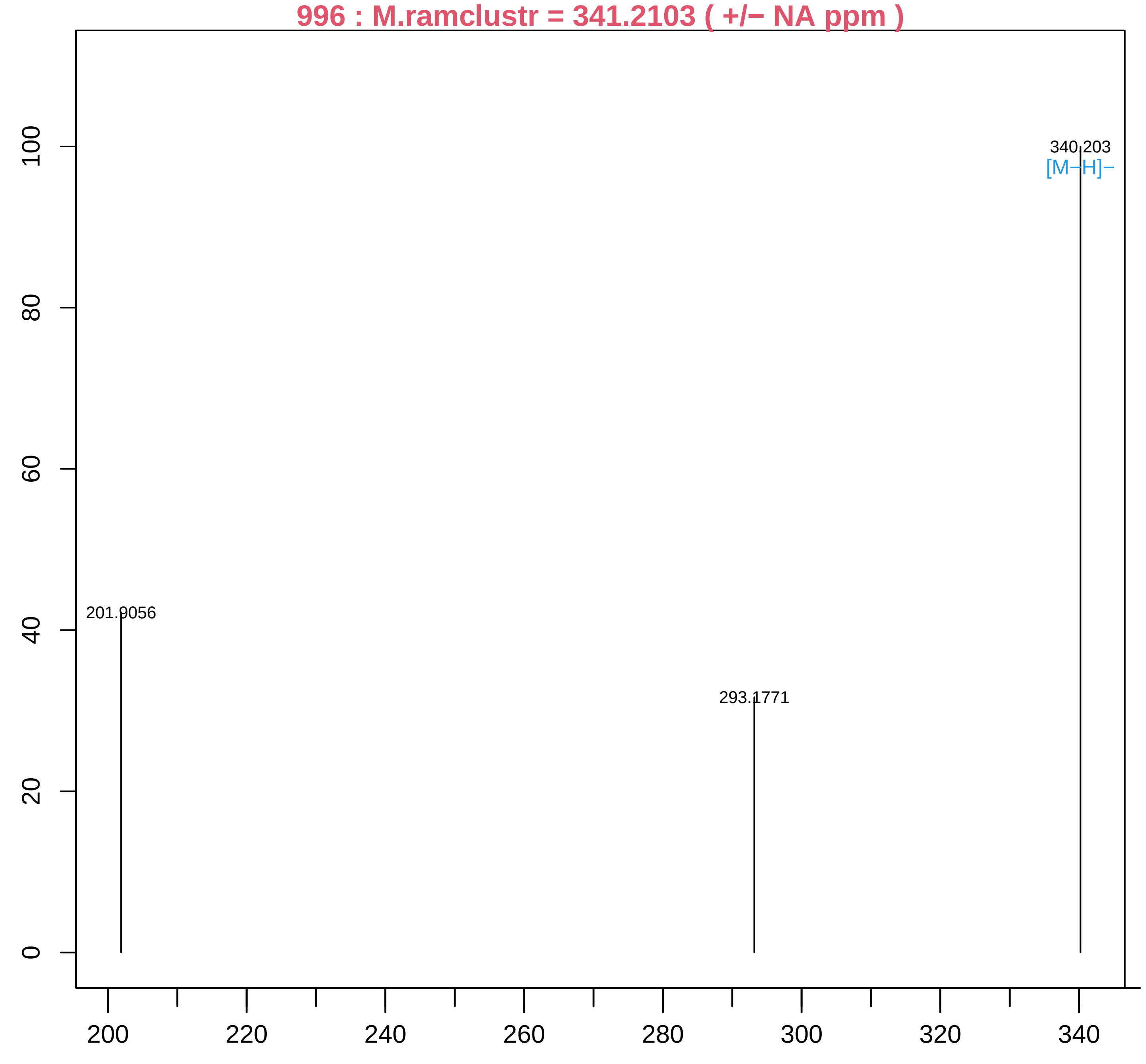

996 : M.ramclustr = 341.2103 (+/− NA ppm)
340.203
[M−H]−
201.9056
293.1771
0
20
40
60
80
100
200
220
240
260
280
300
320
340

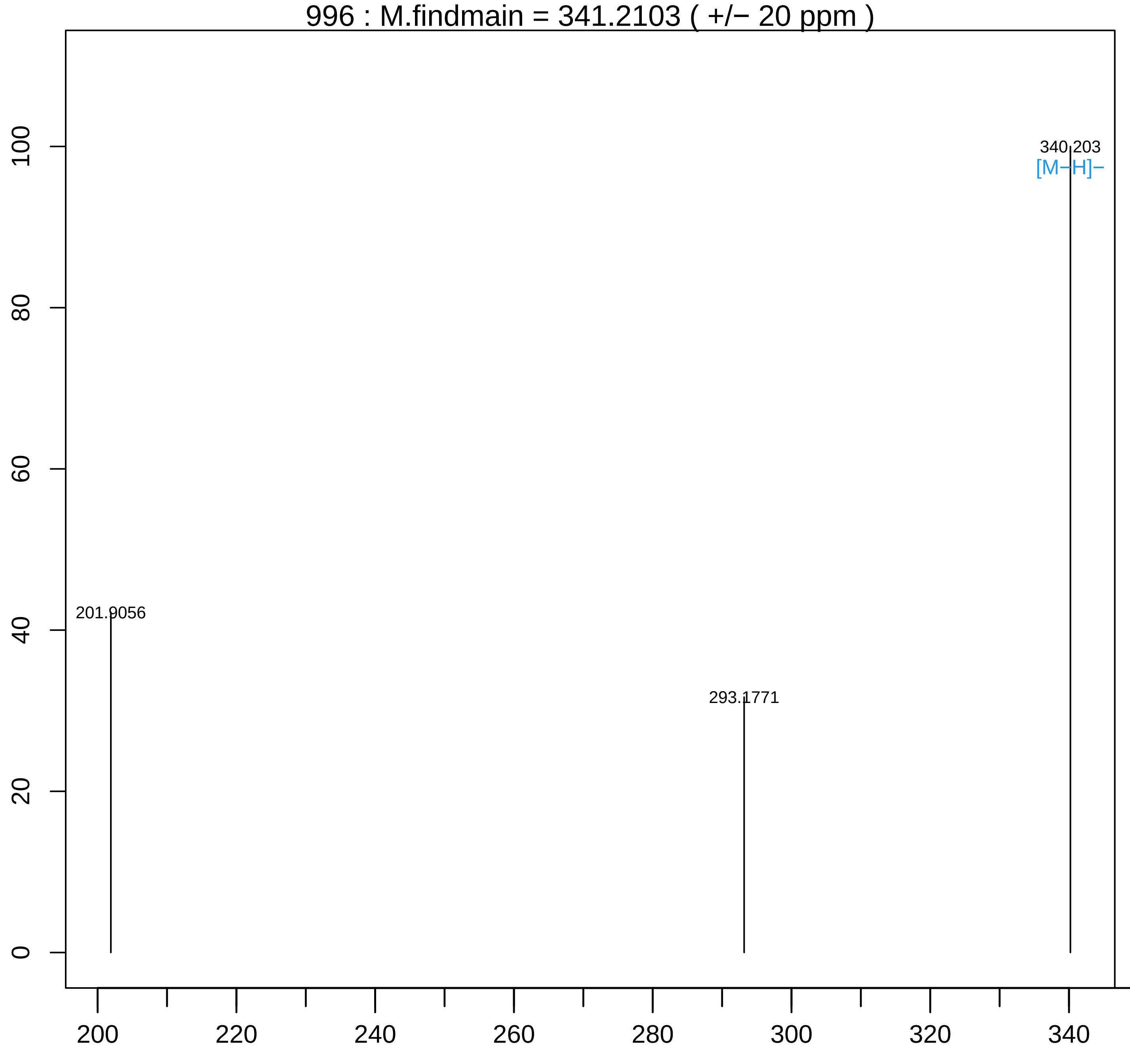

996 : M.findmain = 341.2103 (+/− 20 ppm)
340.203
[M−H]−
201.9056
293.1771
0
20
40
60
80
100
200
220
240
260
280
300
320
340

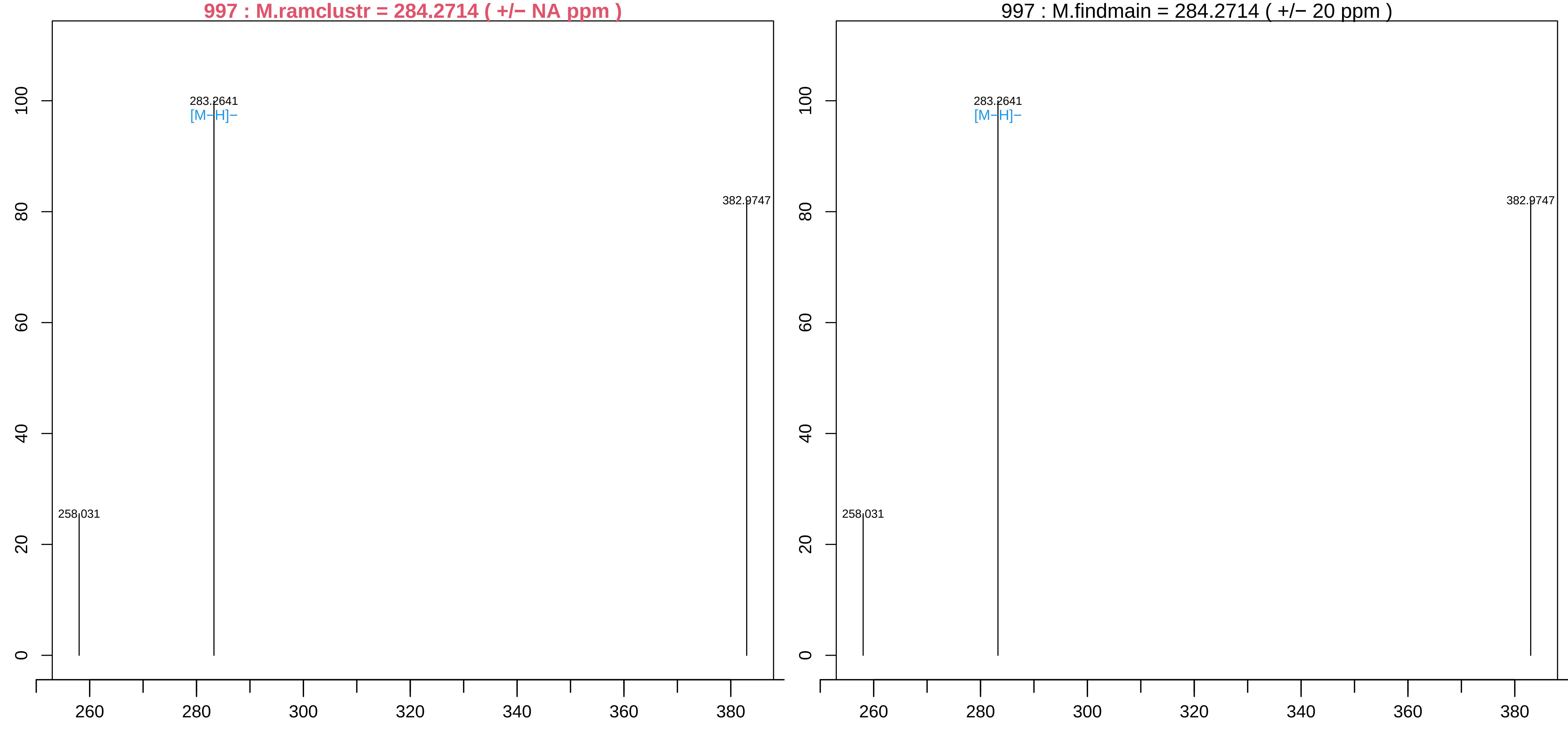
997 : M.ramclustr = 284.2714 (+/− NA ppm)
997 : M.findmain = 284.2714 (+/− 20 ppm)
283.2641
[M−H]−
382.9747
258.031
0
20
40
60
80
100
260
280
300
320
340
360
380

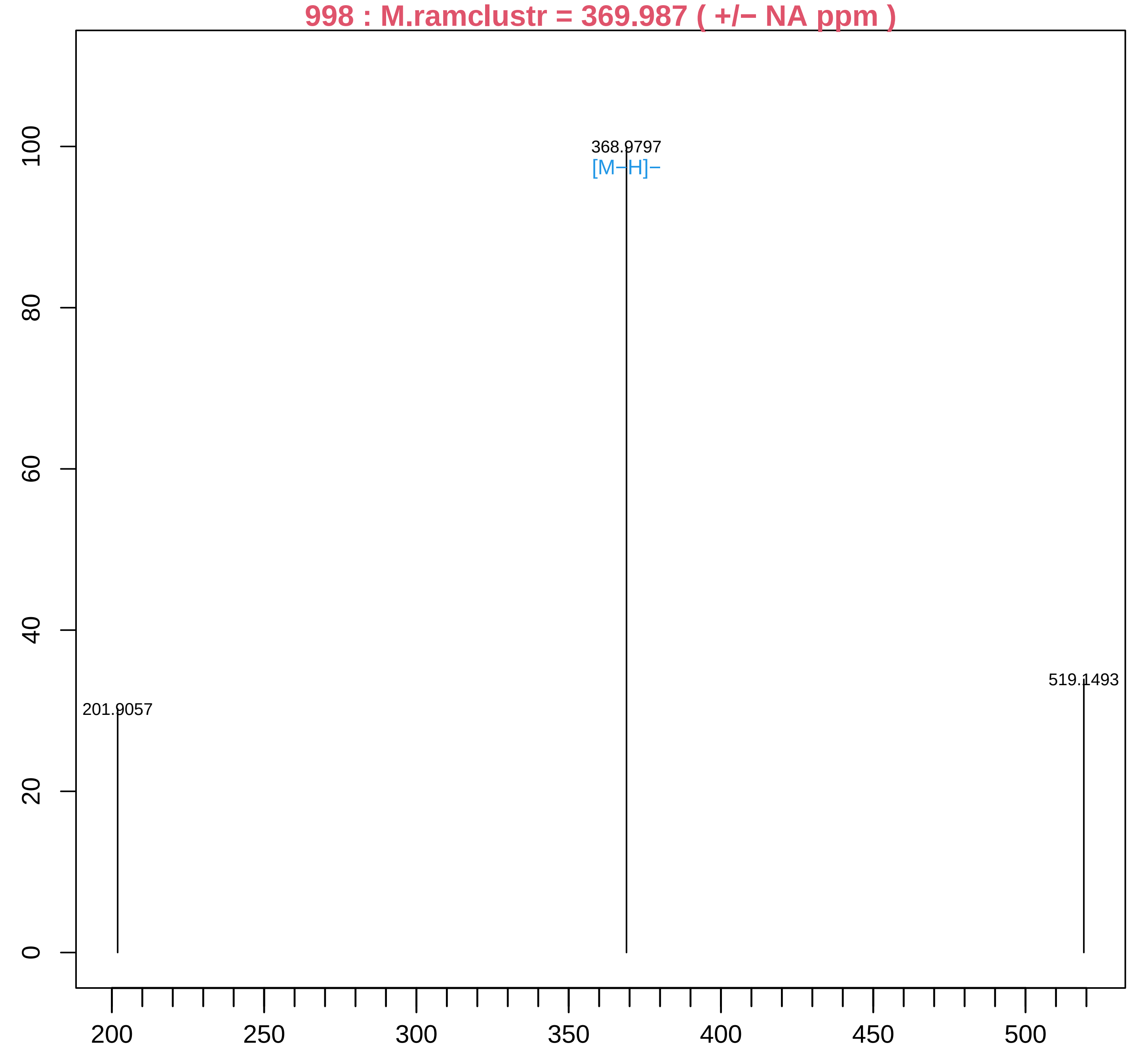
998 : M.ramclustr = 369.987 (+/− NA ppm)
368.9797
[M−H]−
201.9057
519.1493
0
20
40
60
80
100
200
250
300
350
400
450
500

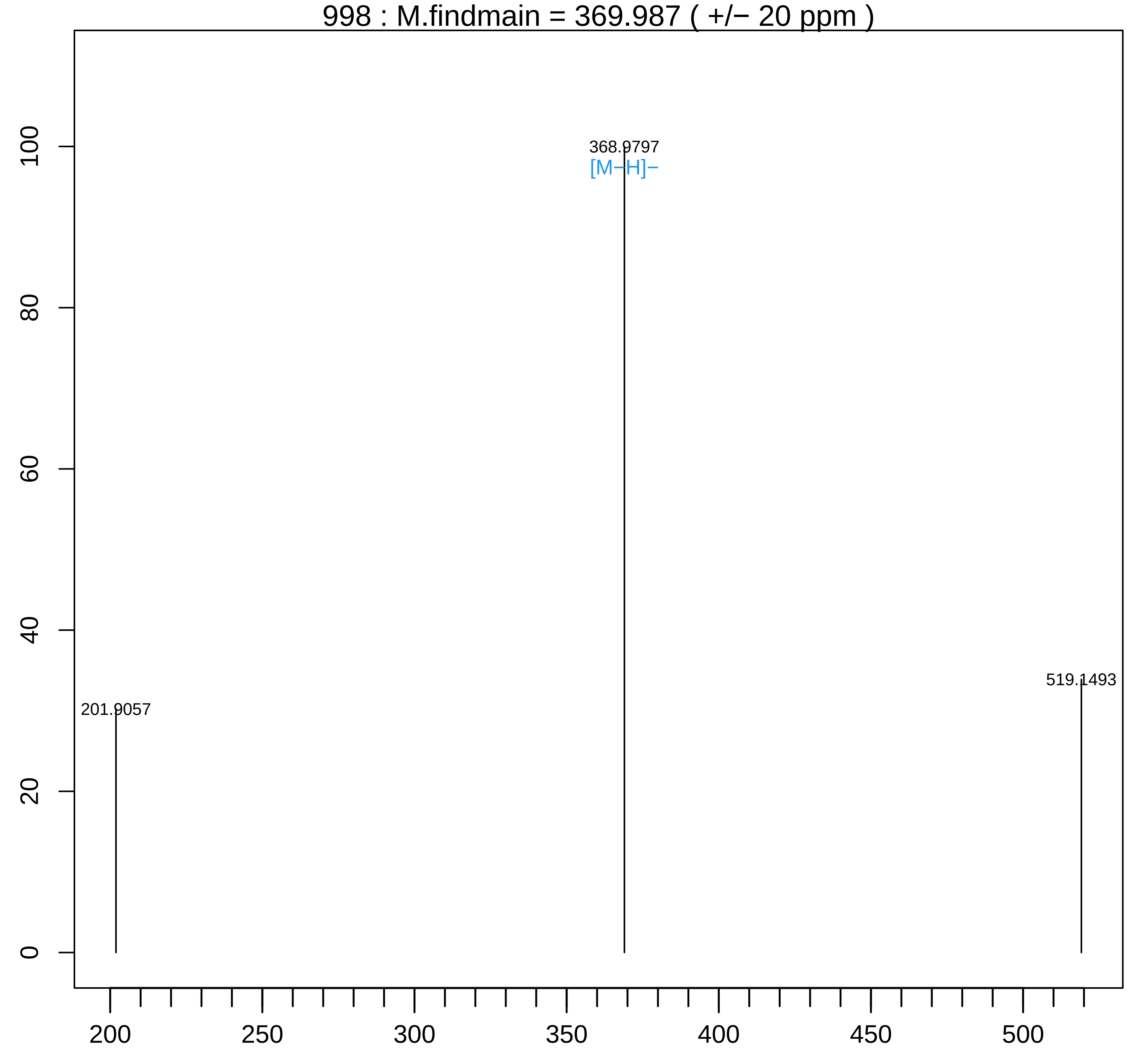
998 : M.findmain = 369.987 (+/− 20 ppm)
368.9797
[M−H]−
201.9057
519.1493
0
20
40
60
80
100
200
250
300
350
400
450
500

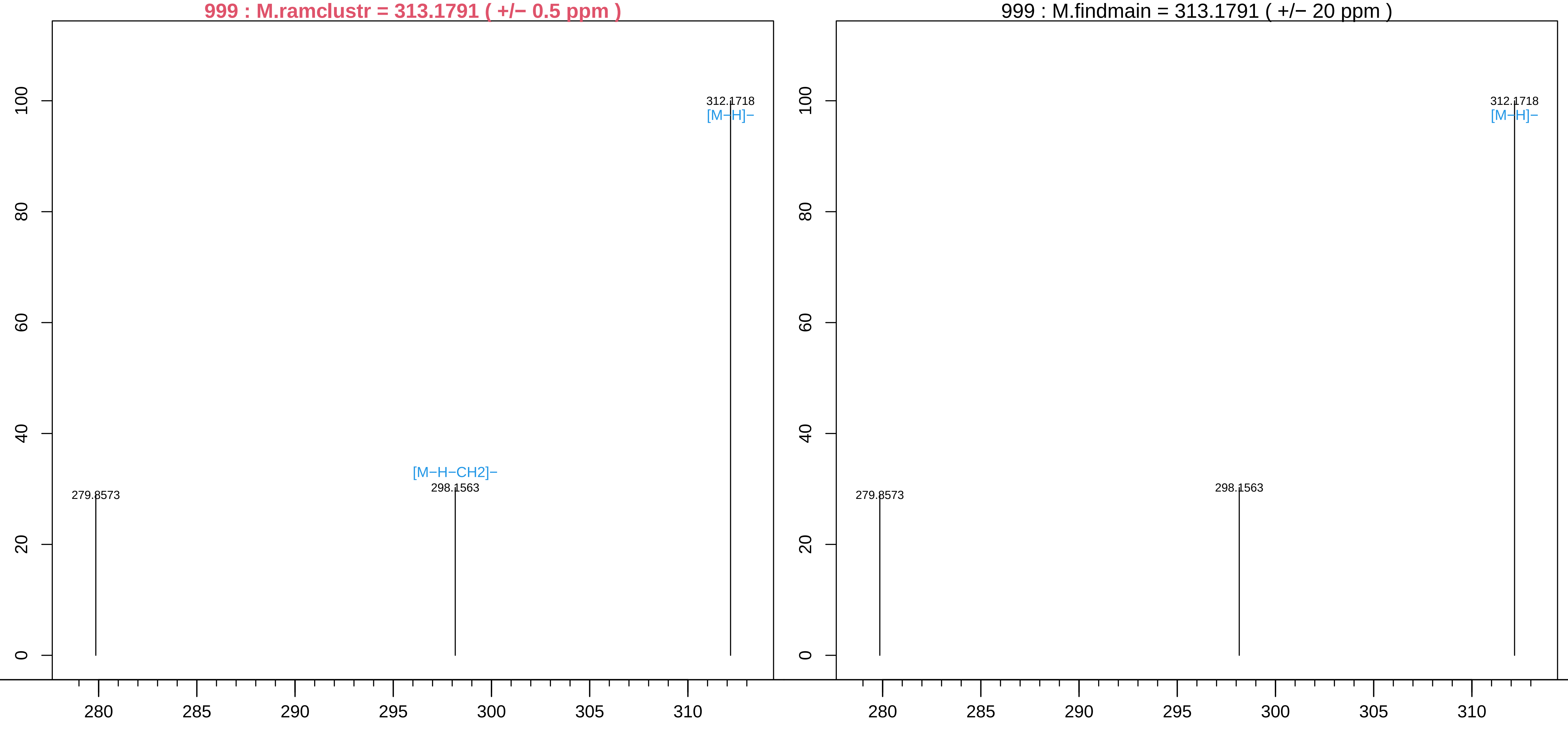
999 : M.ramclustr = 313.1791 (+/− 0.5 ppm)
312.1718
[M−H]−
[M−H−CH2]−
298.1563
279.8573
0
20
40
60
80
100
280
285
290
295
300
305
310
999 : M.findmain = 313.1791 (+/− 20 ppm)
312.1718
[M−H]−
298.1563
279.8573

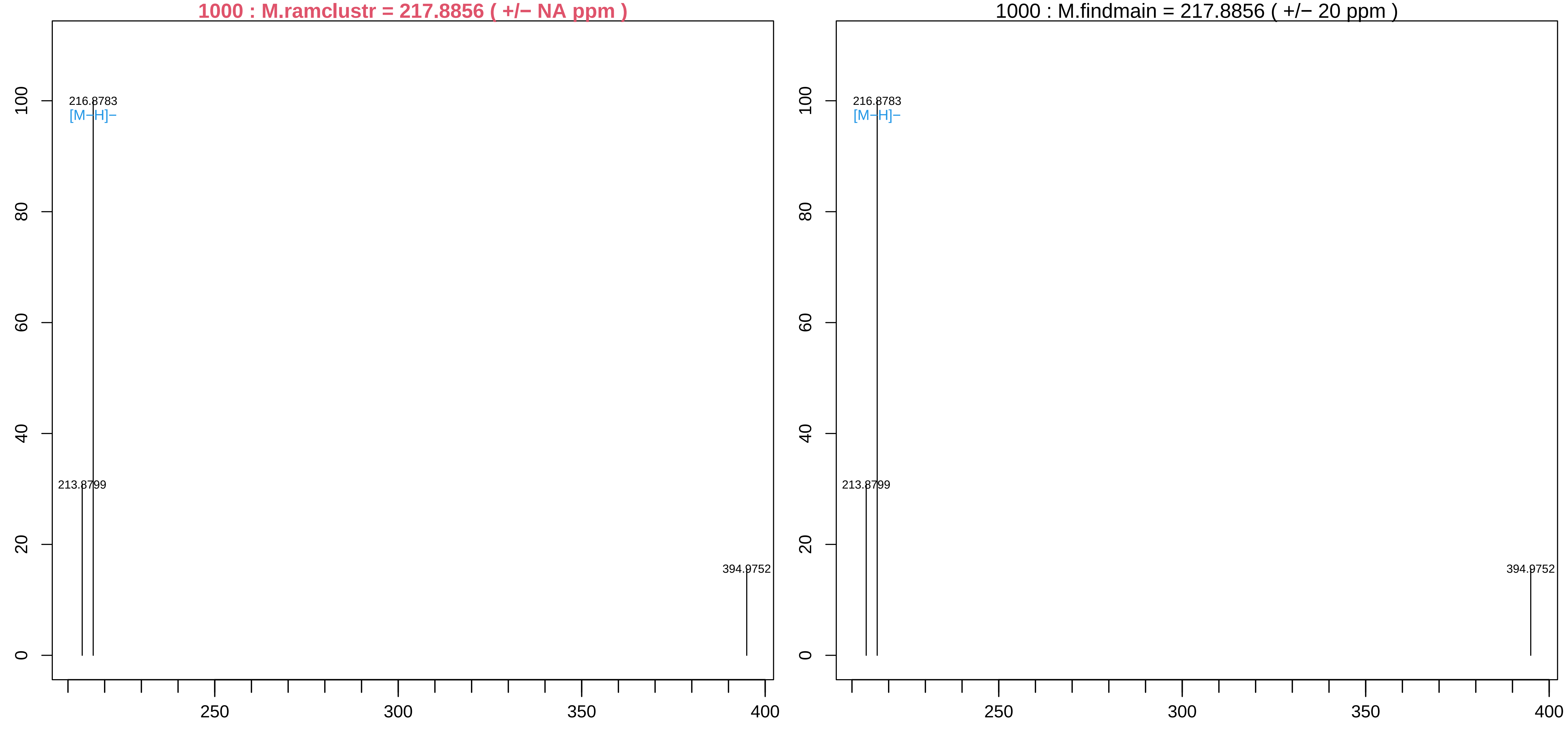
1000 : M.ramclustr = 217.8856 (+/− NA ppm)
216.8783
[M−H]−
213.8799
394.9752
1000 : M.findmain = 217.8856 (+/− 20 ppm)
216.8783
[M−H]−
213.8799
394.9752
0
20
40
60
80
100
250
300
350
400

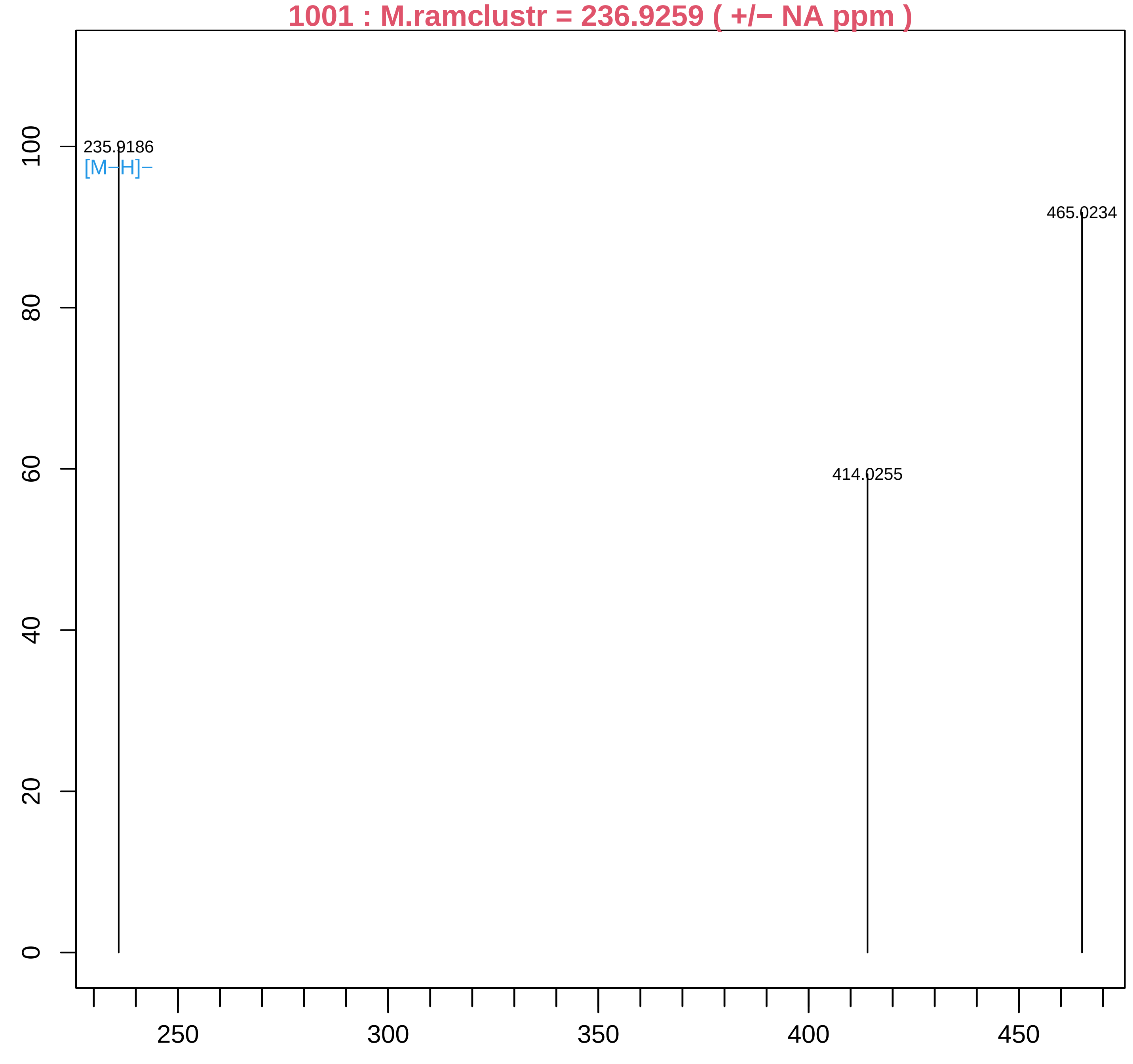

1001 : M.ramclustr = 236.9259 (+/− NA ppm)
235.9186
[M−H]−
465.0234
414.0255
0
20
40
60
80
100
250
300
350
400
450

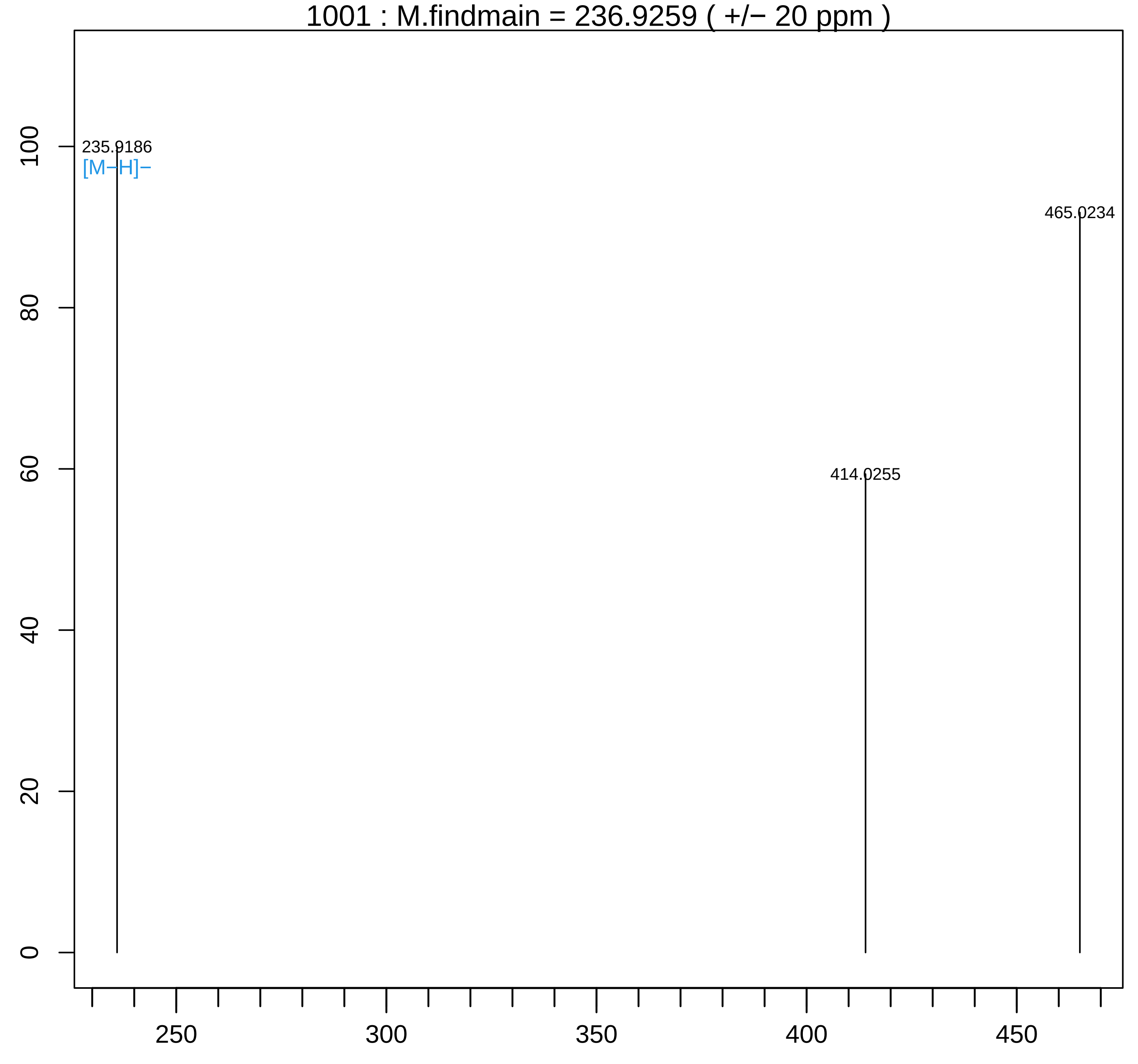

1001 : M.findmain = 236.9259 (+/− 20 ppm)
235.9186
[M−H]−
465.0234
414.0255
0
20
40
60
80
100
250
300
350
400
450

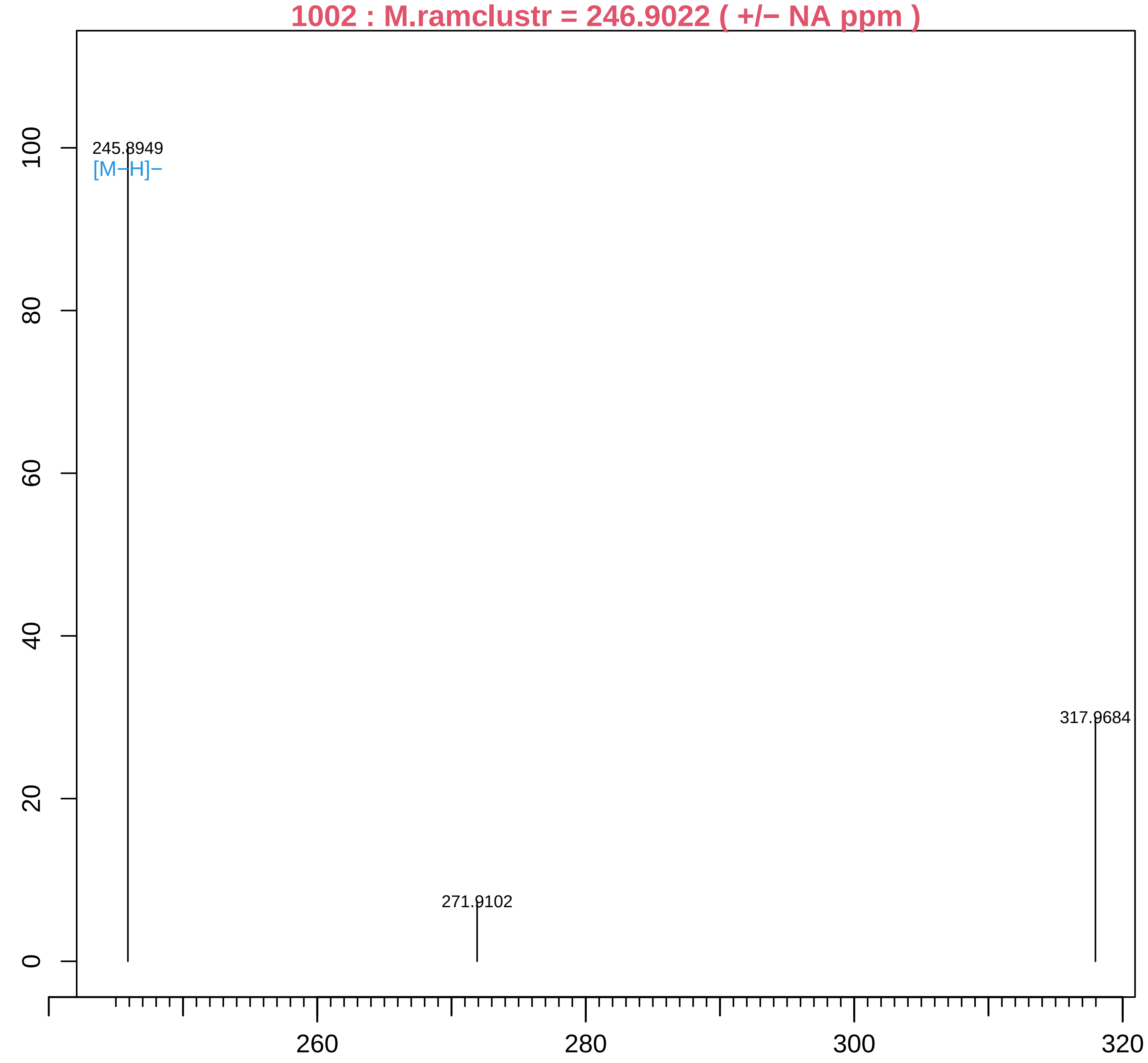
1002 : M.ramclustr = 246.9022 (+/− NA ppm)
245.8949
[M−H]−
271.9102
317.9684
0
20
40
60
80
100
260
280
300
320

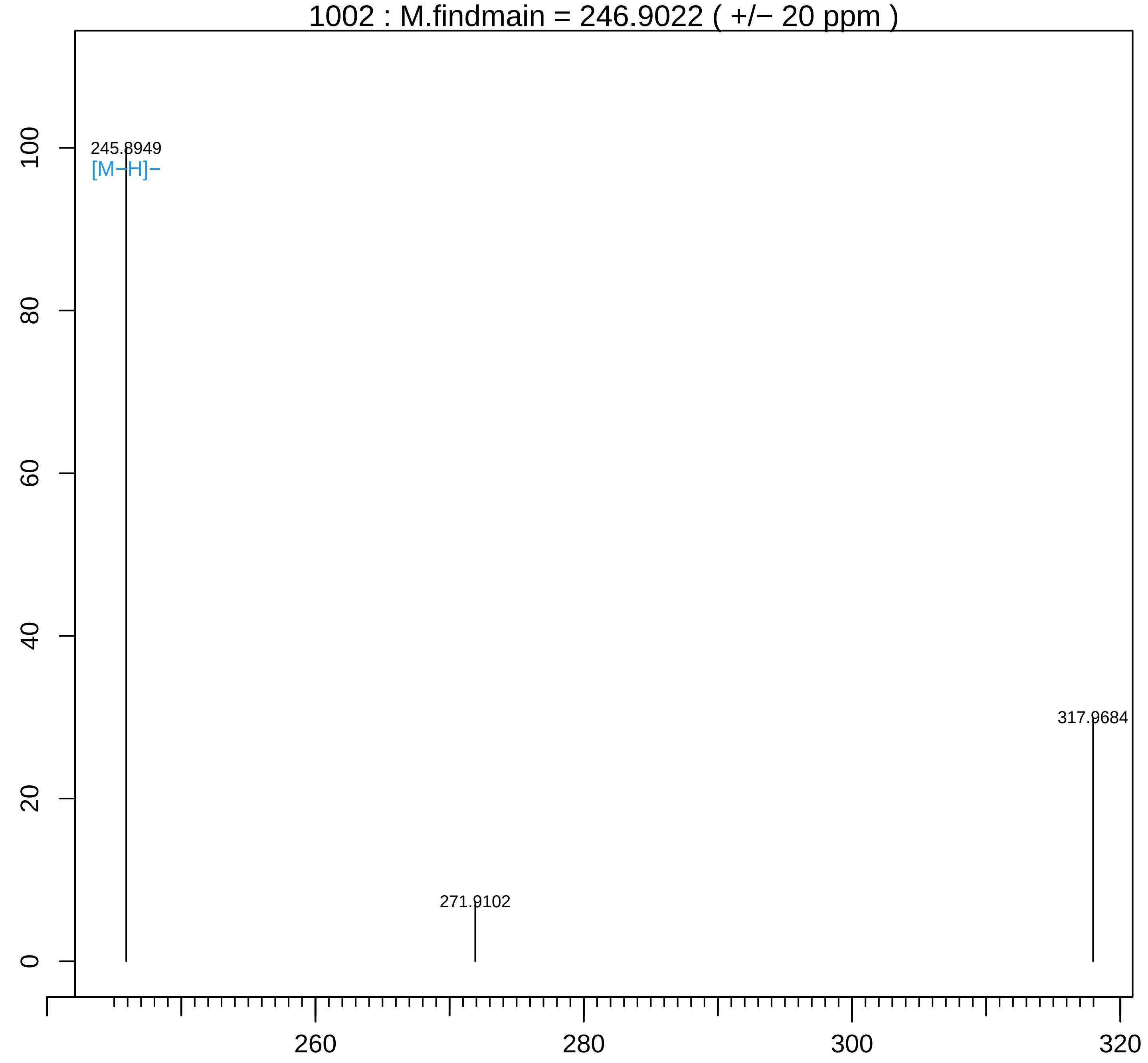
1002 : M.findmain = 246.9022 (+/− 20 ppm)
245.8949
[M−H]−
271.9102
317.9684
0
20
40
60
80
100
260
280
300
320

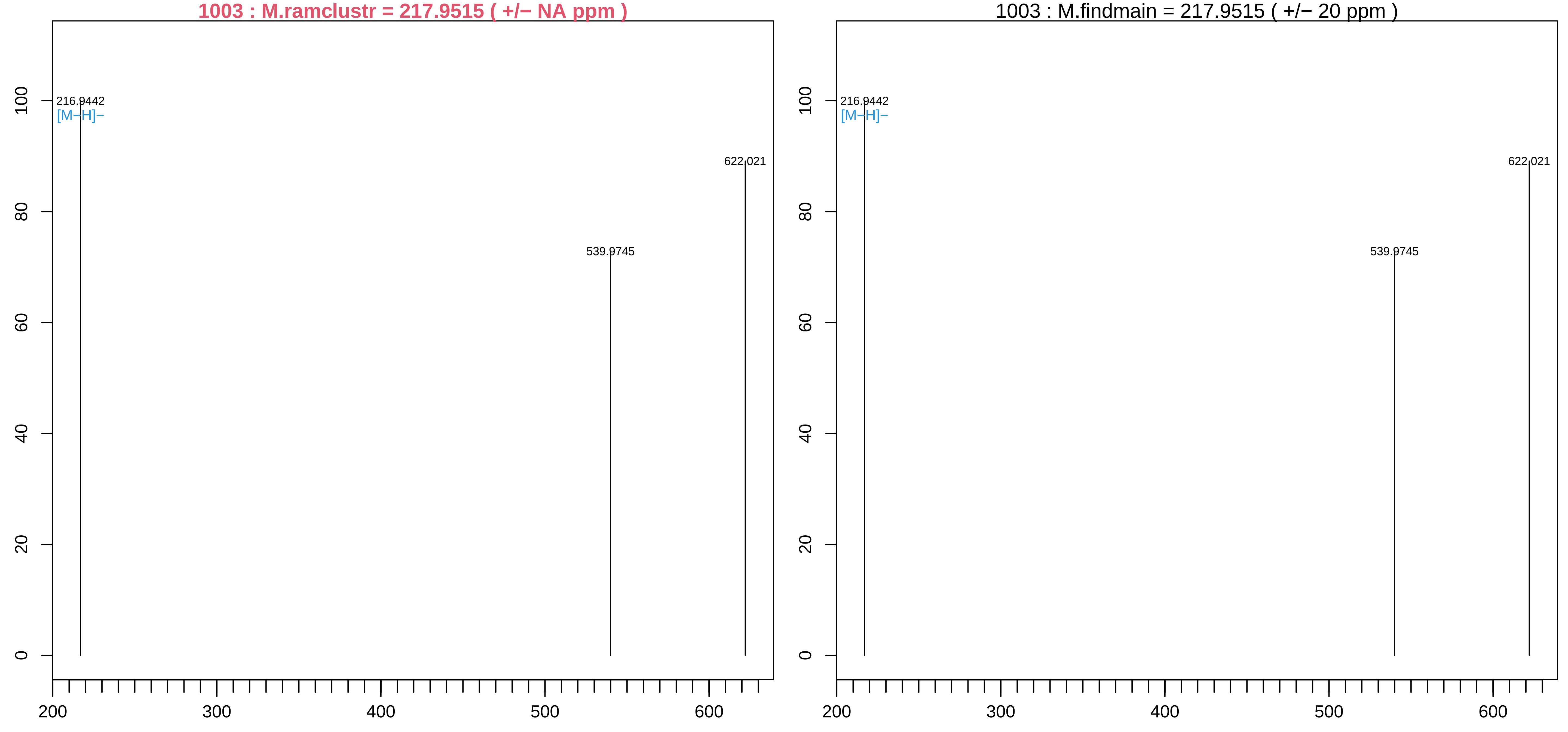

1003 : M.ramclustr = 217.9515 (+/− NA ppm)
216.9442
[M−H]−
539.9745
622.021
1003 : M.findmain = 217.9515 (+/− 20 ppm)
216.9442
[M−H]−
539.9745
622.021
0
20
40
60
80
100
200
300
400
500
600

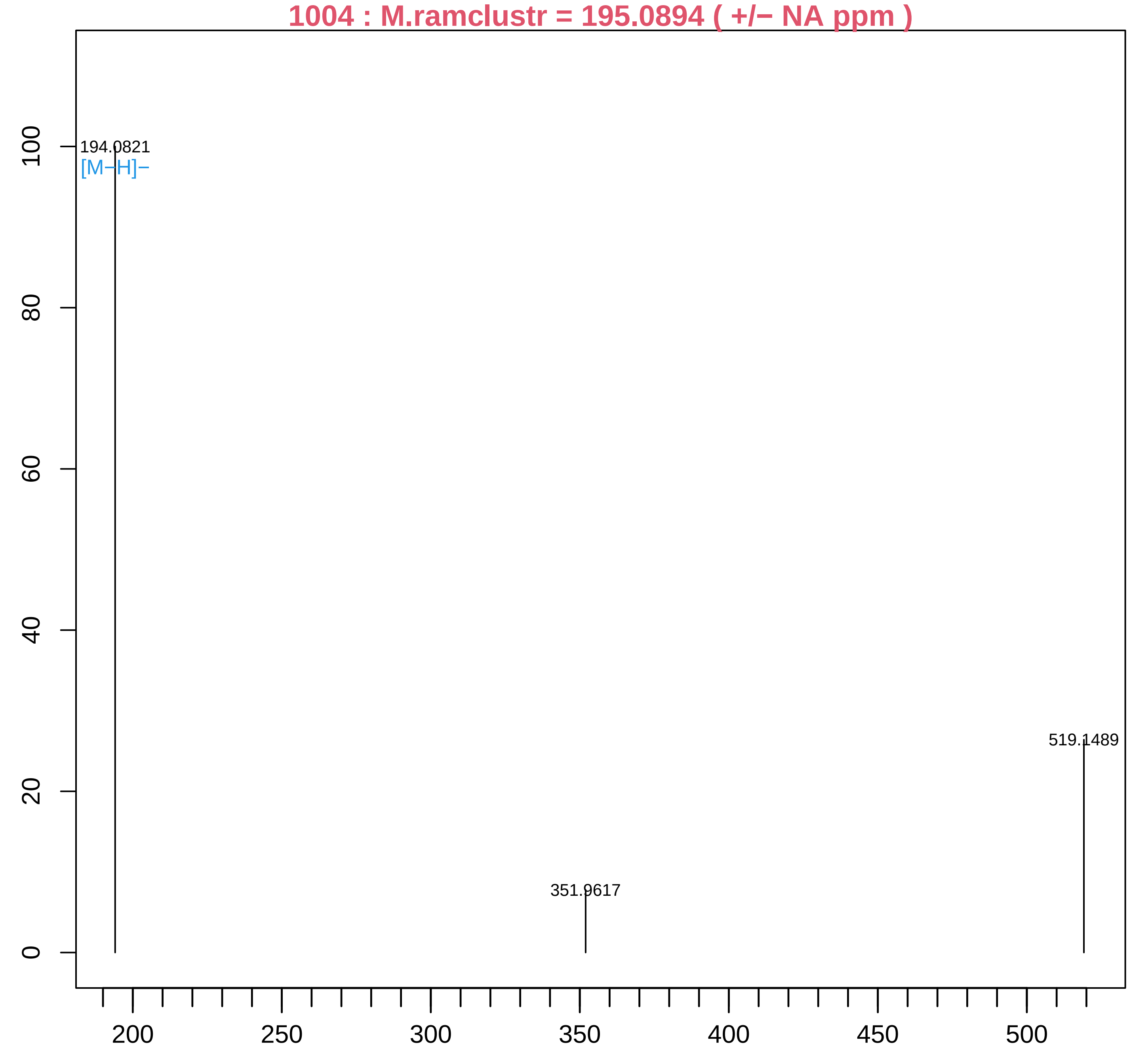
1004 : M.ramclustr = 195.0894 (+/− NA ppm)
194.0821
[M−H]−
351.9617
519.1489
0
20
40
60
80
100
200
250
300
350
400
450
500

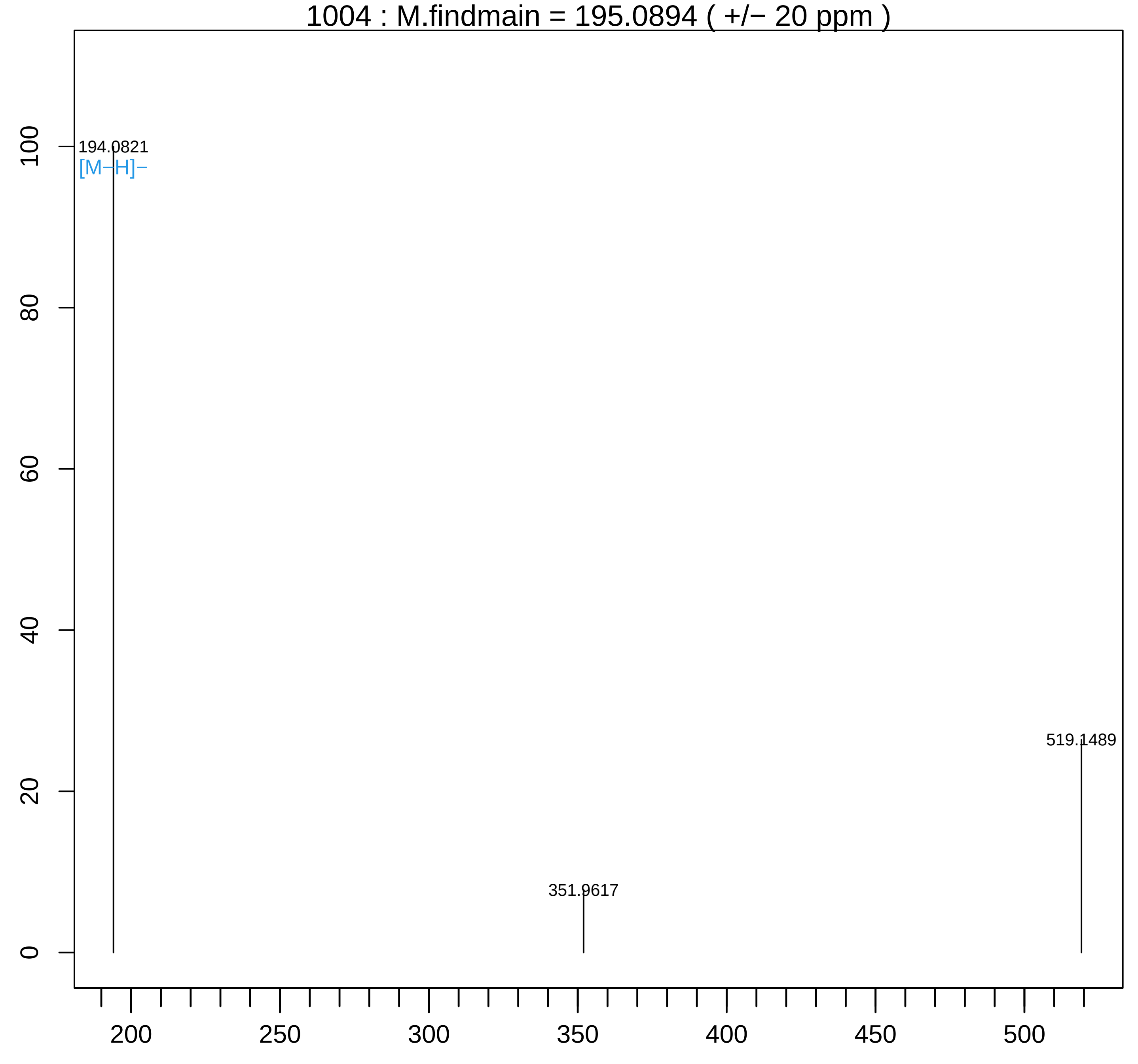
1004 : M.findmain = 195.0894 (+/− 20 ppm)
194.0821
[M−H]−
351.9617
519.1489
0
20
40
60
80
100
200
250
300
350
400
450
500

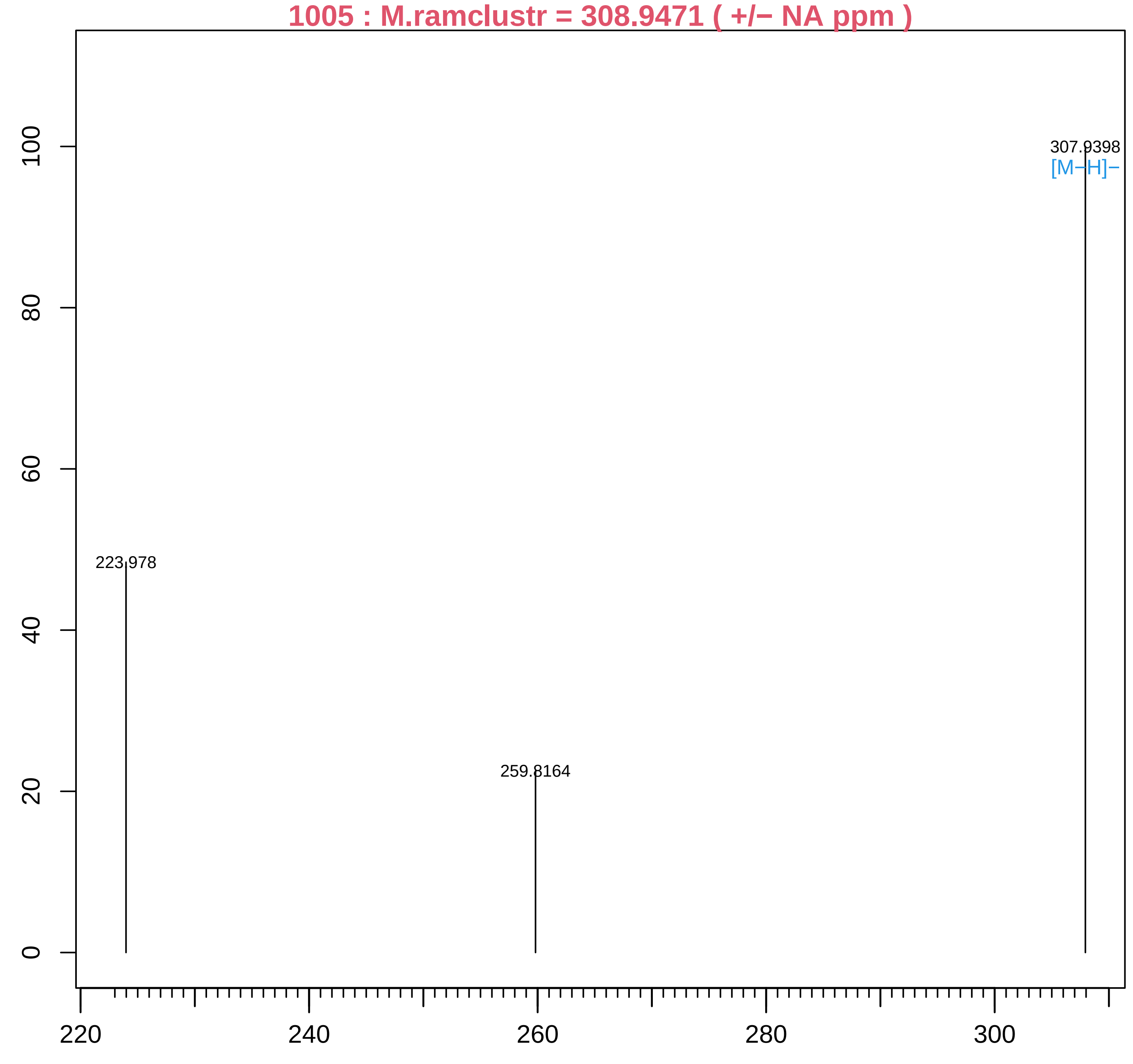
1005 : M.ramclustr = 308.9471 (+/− NA ppm)
307.9398
[M−H]−
223.978
259.8164
0
20
40
60
80
100
220
240
260
280
300

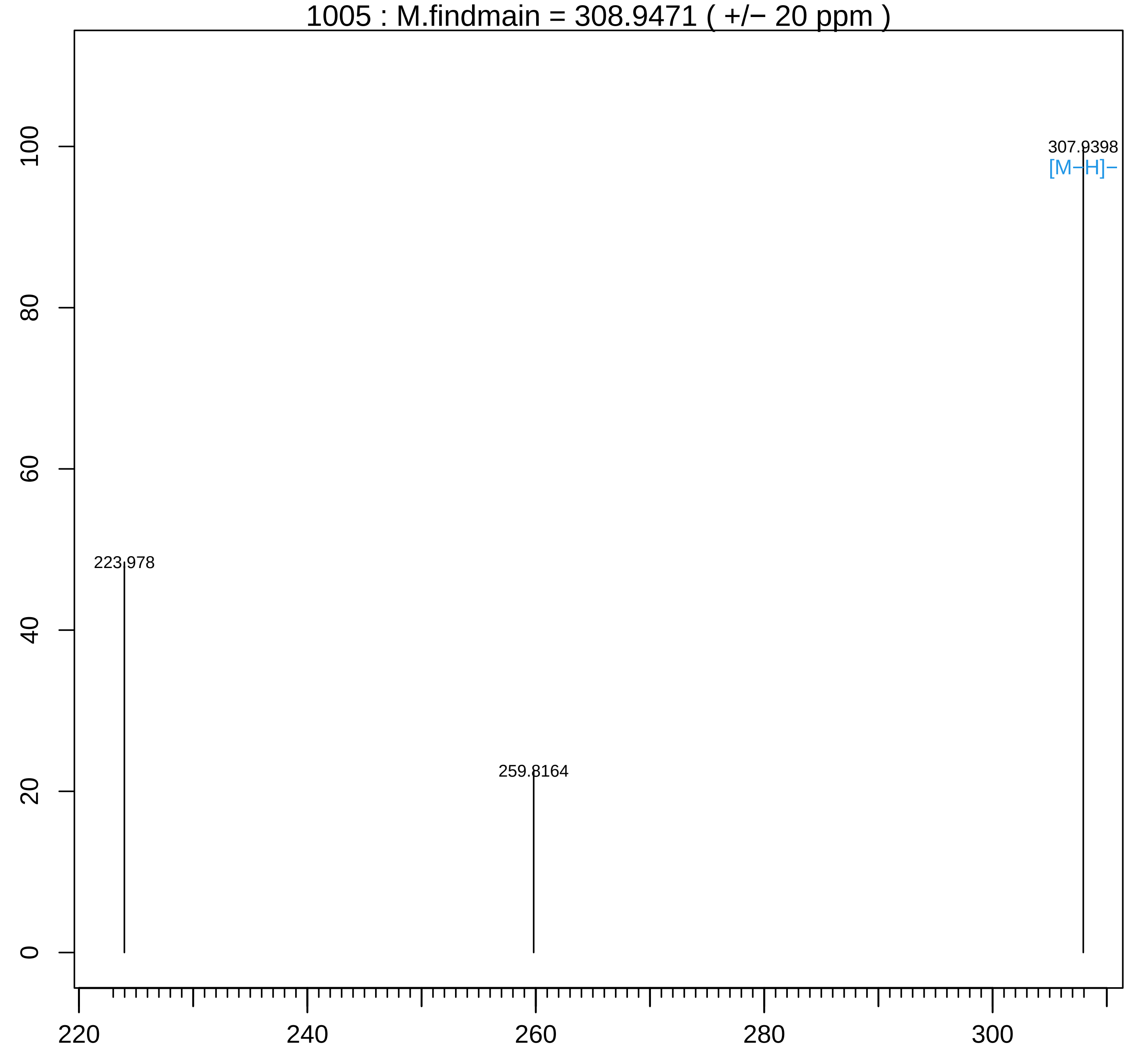
1005 : M.findmain = 308.9471 (+/− 20 ppm)
307.9398
[M−H]−
223.978
259.8164
0
20
40
60
80
100
220
240
260
280
300

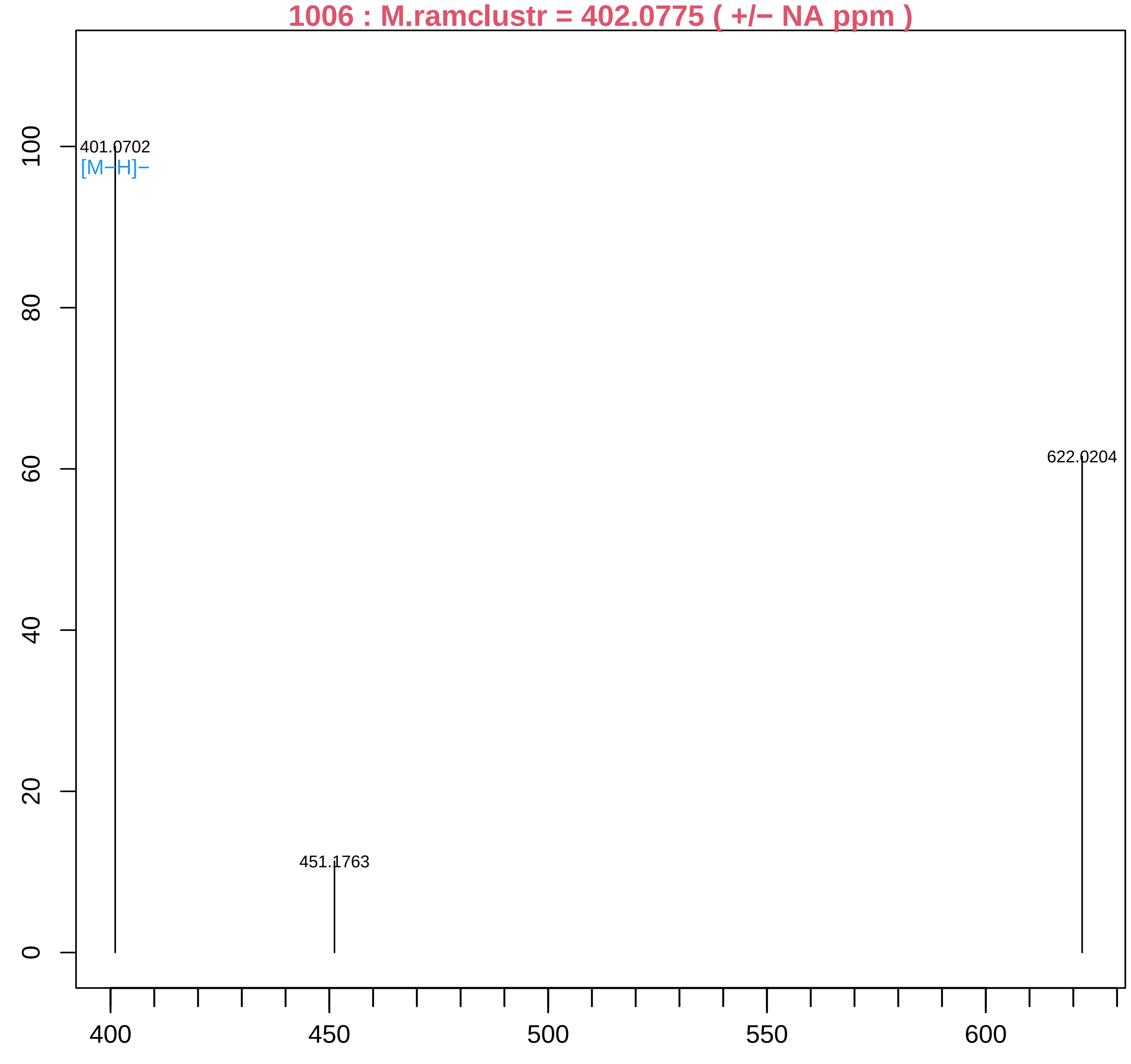

1006 : M.ramclustr = 402.0775 (+/− NA ppm)
401.0702
[M−H]−
451.1763
622.0204
0
20
40
60
80
100
400
450
500
550
600

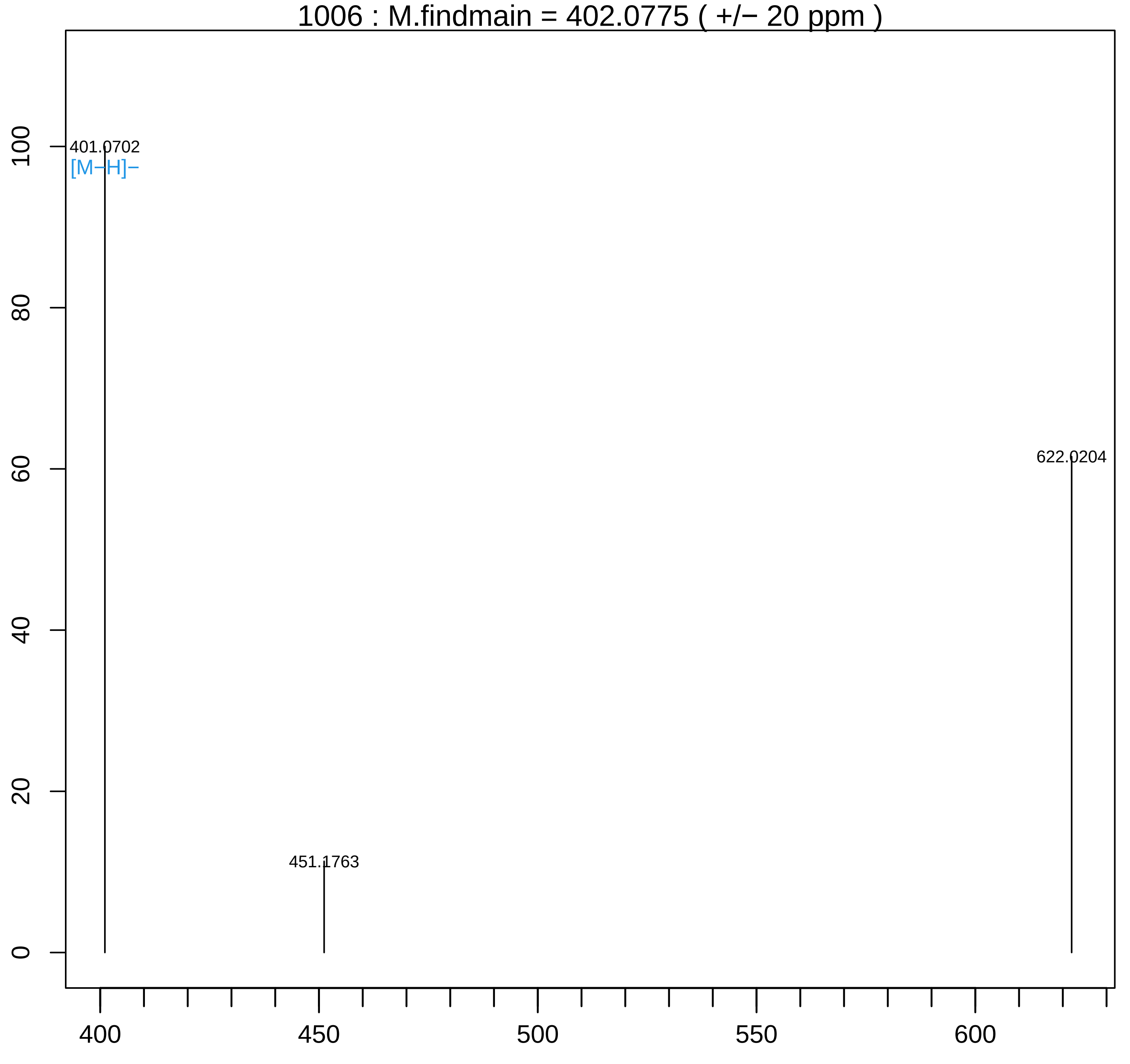

1006 : M.findmain = 402.0775 (+/− 20 ppm)
401.0702
[M−H]−
451.1763
622.0204
0
20
40
60
80
100
400
450
500
550
600

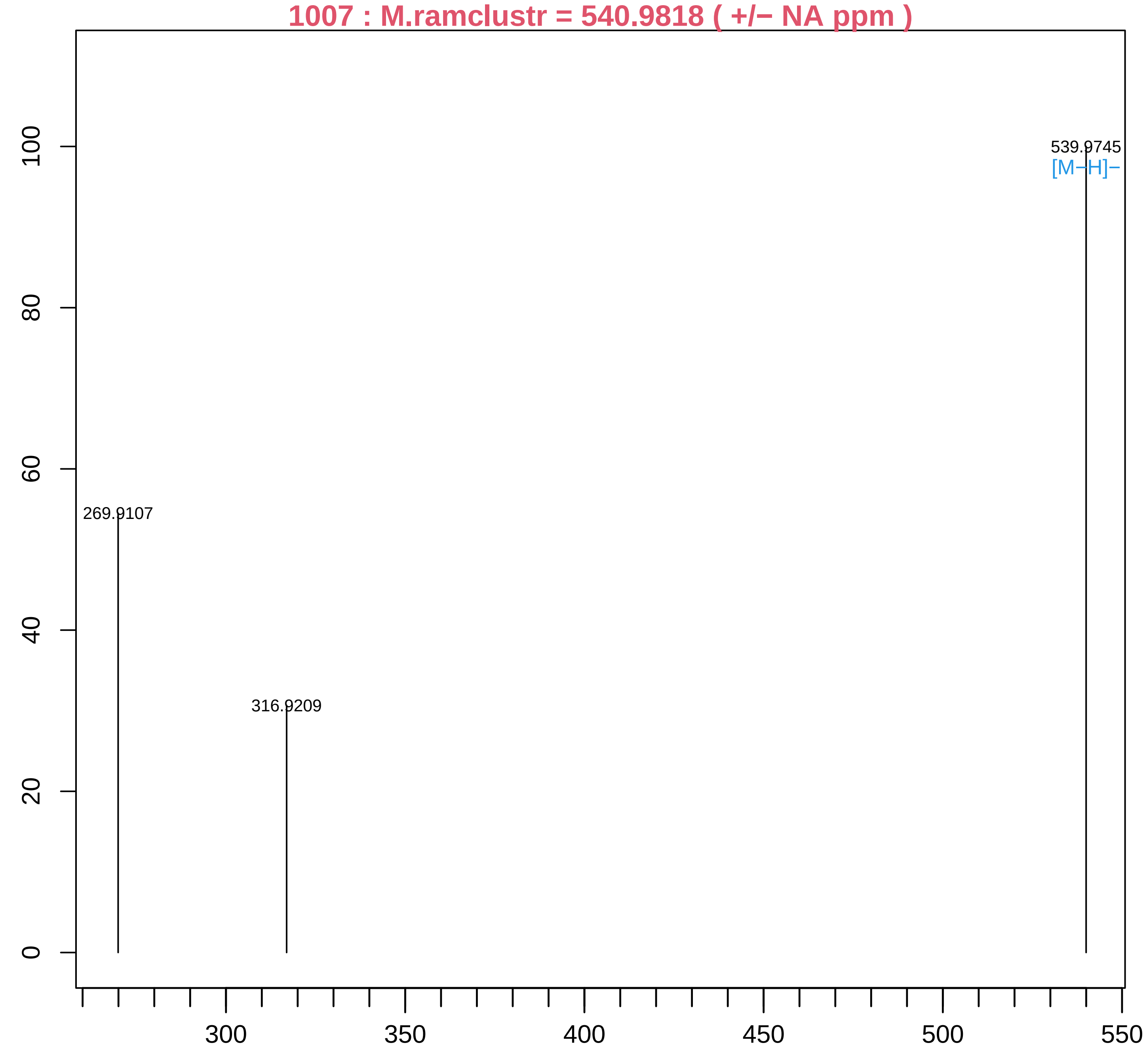
1007 : M.ramclustr = 540.9818 (+/− NA ppm)
539.9745
[M−H]−
269.9107
316.9209
0
20
40
60
80
100
300
350
400
450
500
550

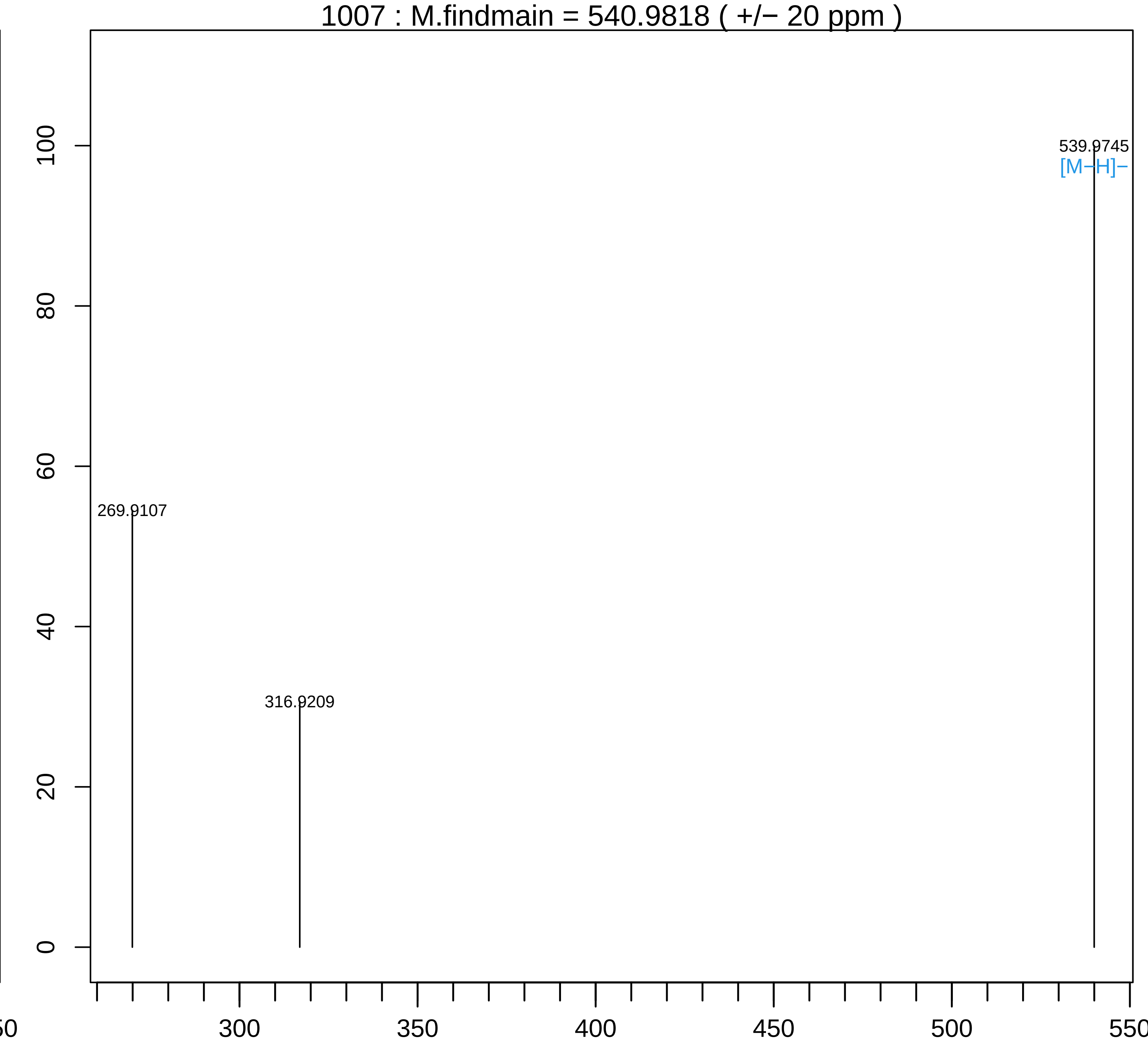
1007 : M.findmain = 540.9818 (+/− 20 ppm)
539.9745
[M−H]−
269.9107
316.9209
0
20
40
60
80
100
300
350
400
450
500
550

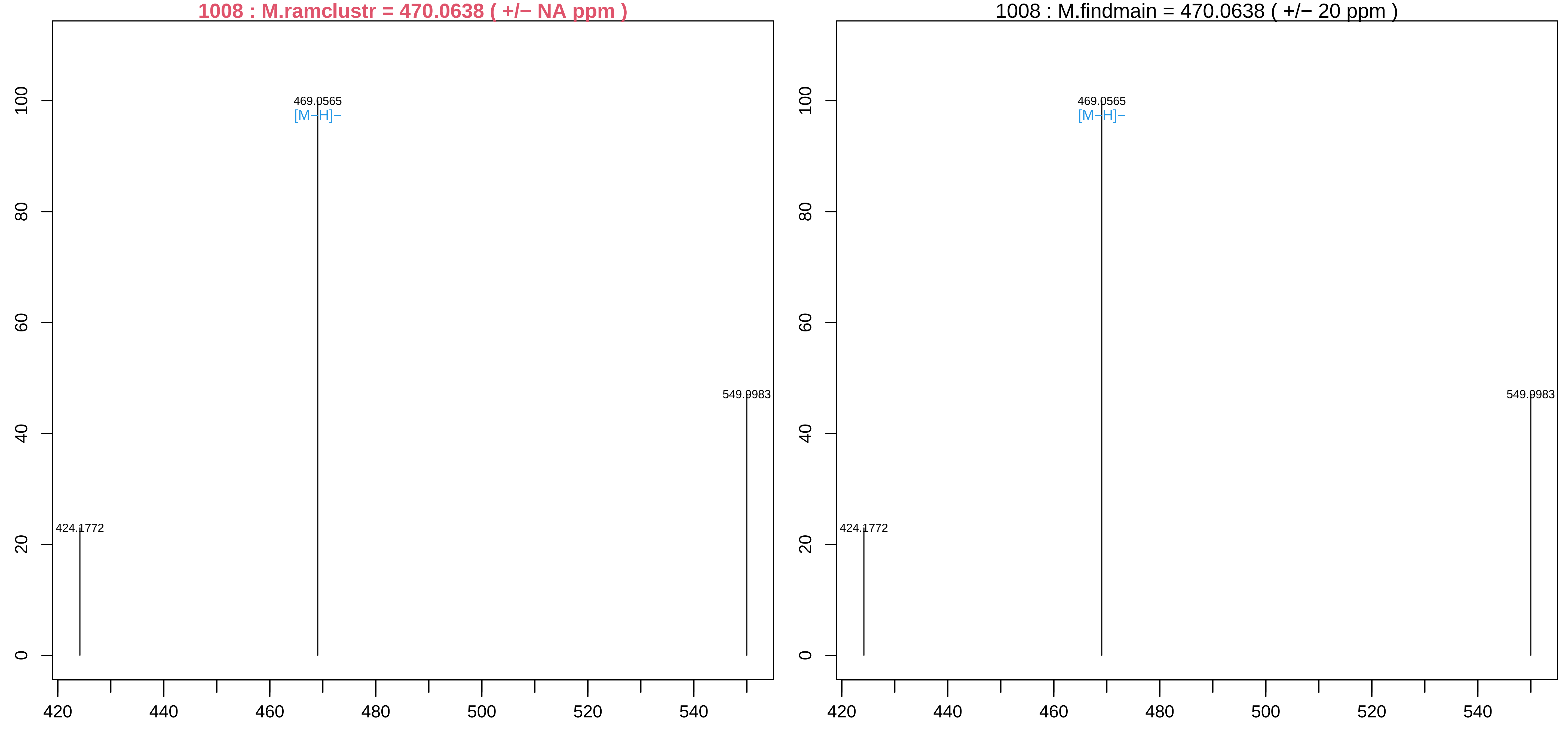
1008 : M.ramclustr = 470.0638 (+/− NA ppm)
469.0565
[M−H]−
549.9983
424.1772
1008 : M.findmain = 470.0638 (+/− 20 ppm)
469.0565
[M−H]−
549.9983
424.1772

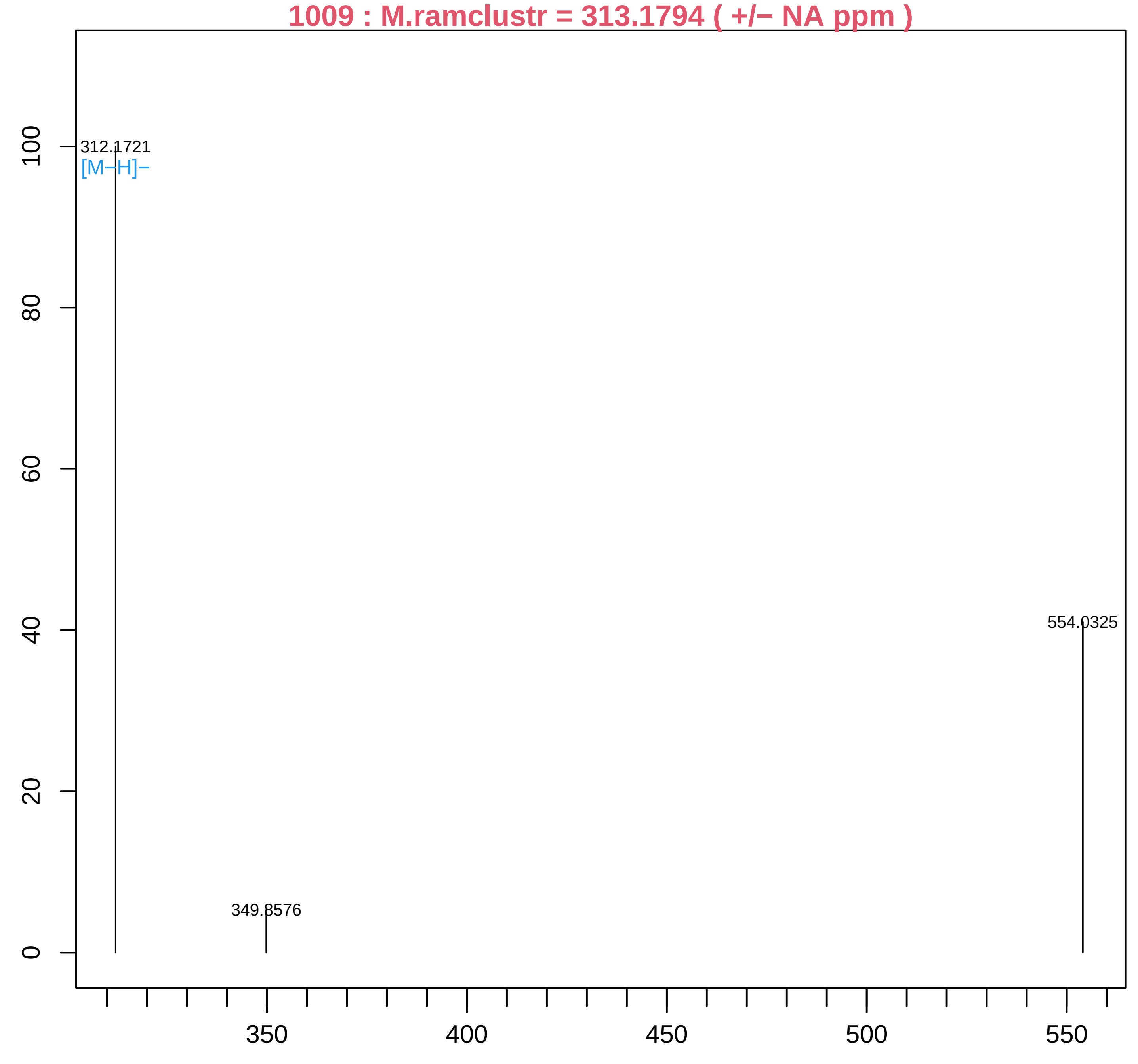
1009 : M.ramclustr = 313.1794 (+/− NA ppm)
312.1721
[M−H]−
349.8576
554.0325
0
20
40
60
80
100
350
400
450
500
550

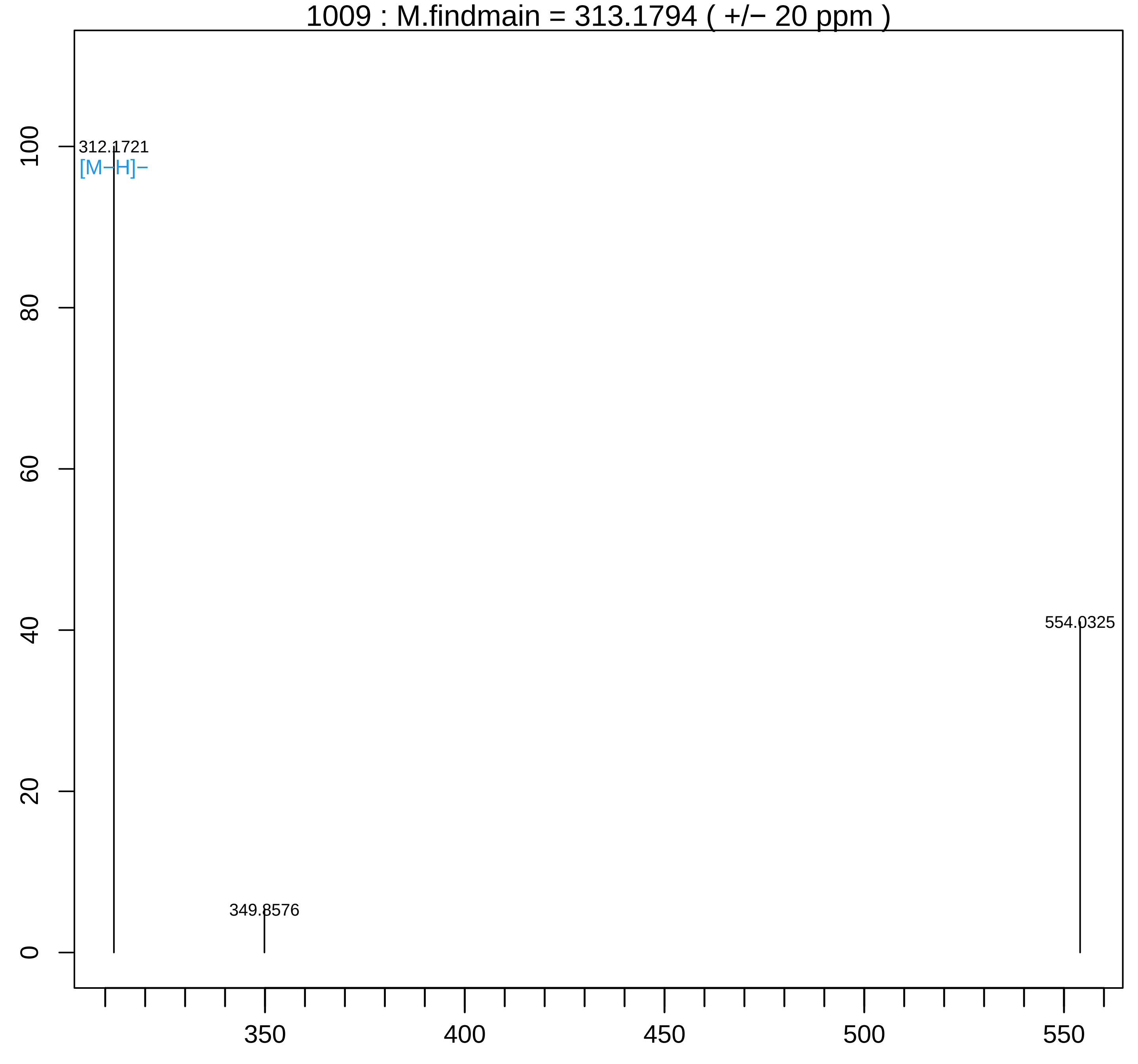
1009 : M.findmain = 313.1794 (+/− 20 ppm)
312.1721
[M−H]−
349.8576
554.0325
0
20
40
60
80
100
350
400
450
500
550

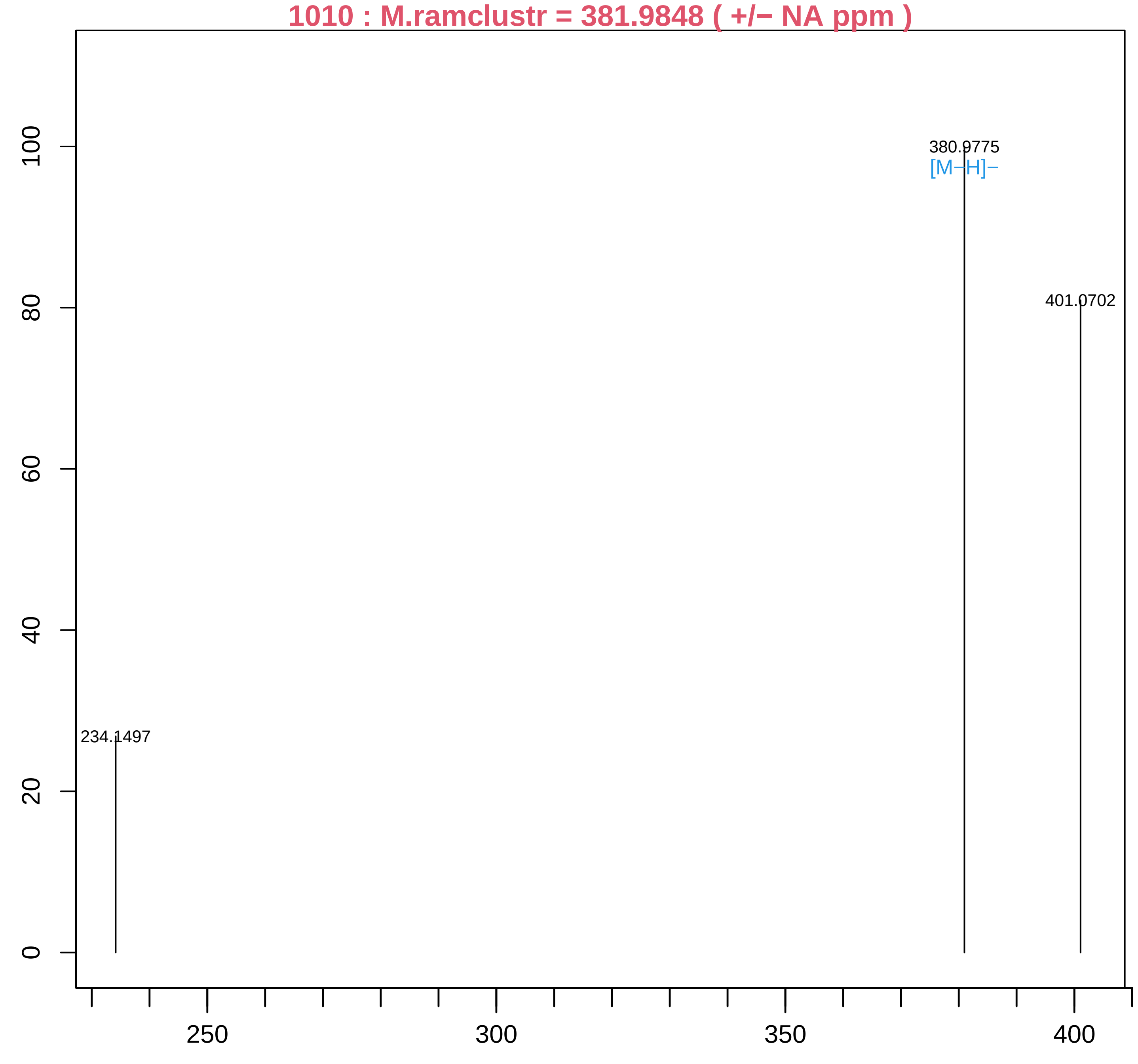
1010 : M.ramclustr = 381.9848 (+/− NA ppm)
380.9775
[M−H]−
401.0702
234.1497
0
20
40
60
80
100
250
300
350
400

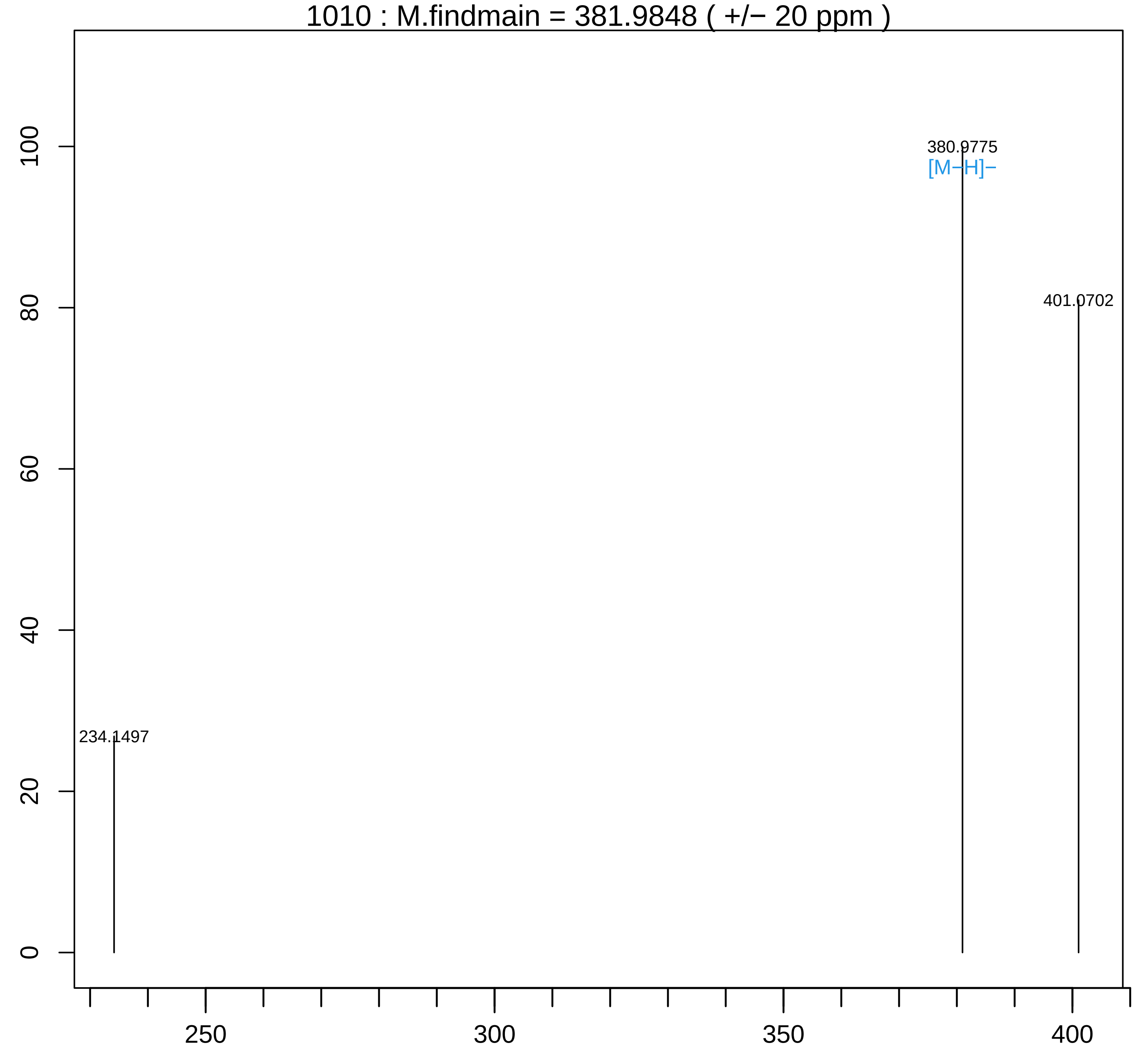
1010 : M.findmain = 381.9848 (+/− 20 ppm)
380.9775
[M−H]−
401.0702
234.1497
0
20
40
60
80
100
250
300
350
400

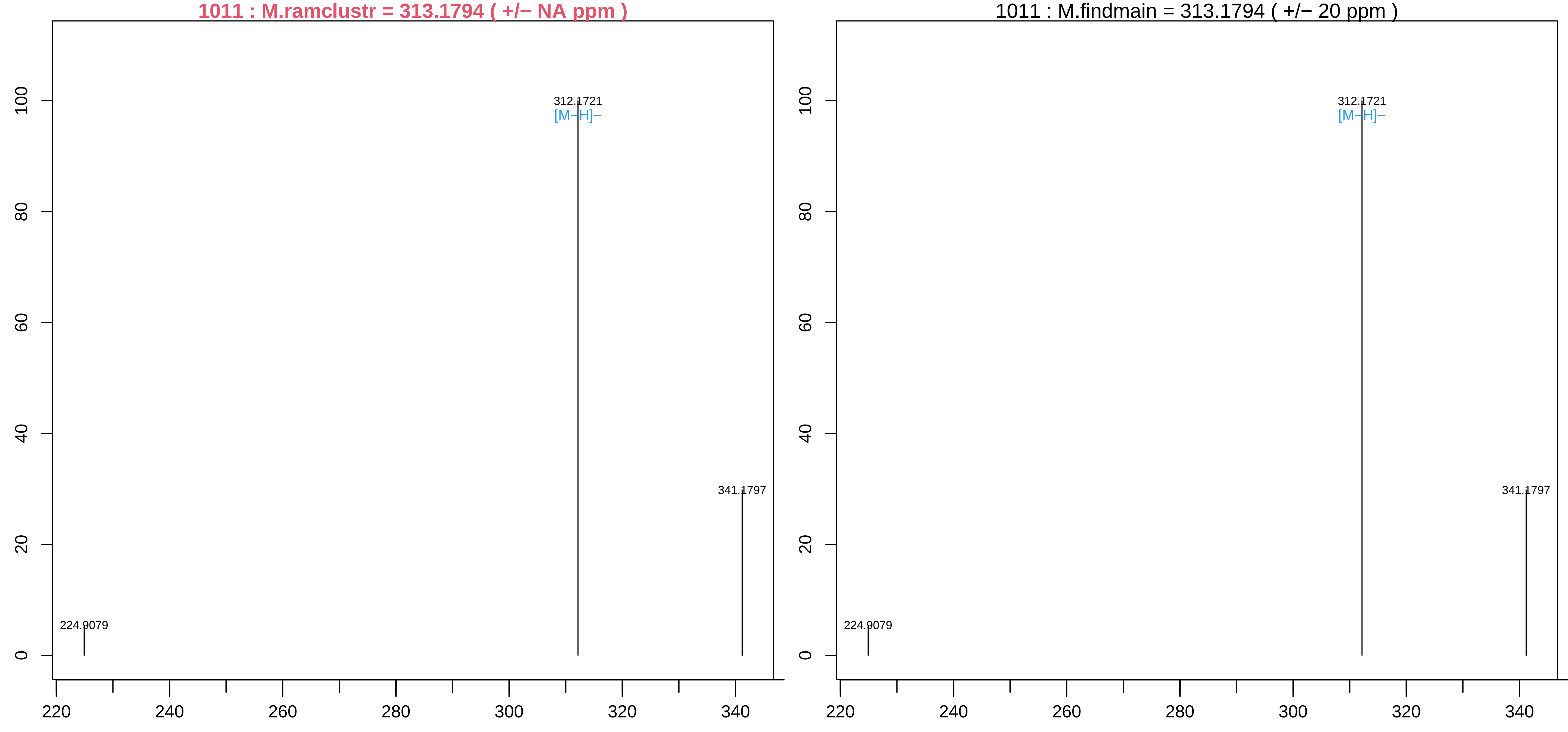

1011 : M.ramclustr = 313.1794 (+/− NA ppm)
312.1721
[M−H]−
341.1797
224.9079
0
20
40
60
80
100
220
240
260
280
300
320
340
1011 : M.findmain = 313.1794 (+/− 20 ppm)
312.1721
[M−H]−
341.1797
224.9079
0
20
40
60
80
100
220
240
260
280
300
320
340

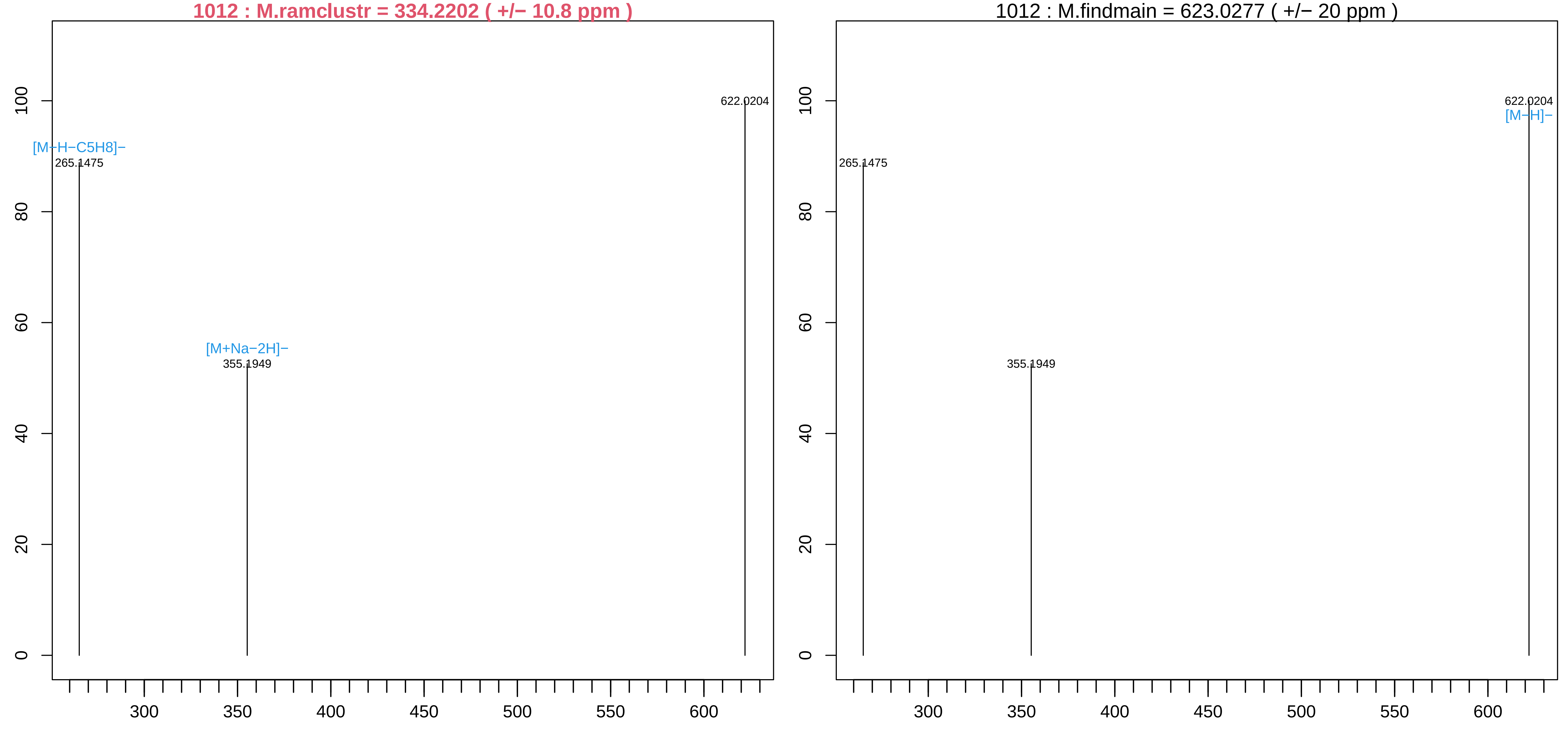
1012 : M.ramclustr = 334.2202 (+/− 10.8 ppm)
[M−H−C5H8]−
265.1475
[M+Na−2H]−
355.1949
622.0204
1012 : M.findmain = 623.0277 (+/− 20 ppm)
265.1475
355.1949
622.0204
[M−H]−
0
20
40
60
80
100
300
350
400
450
500
550
600

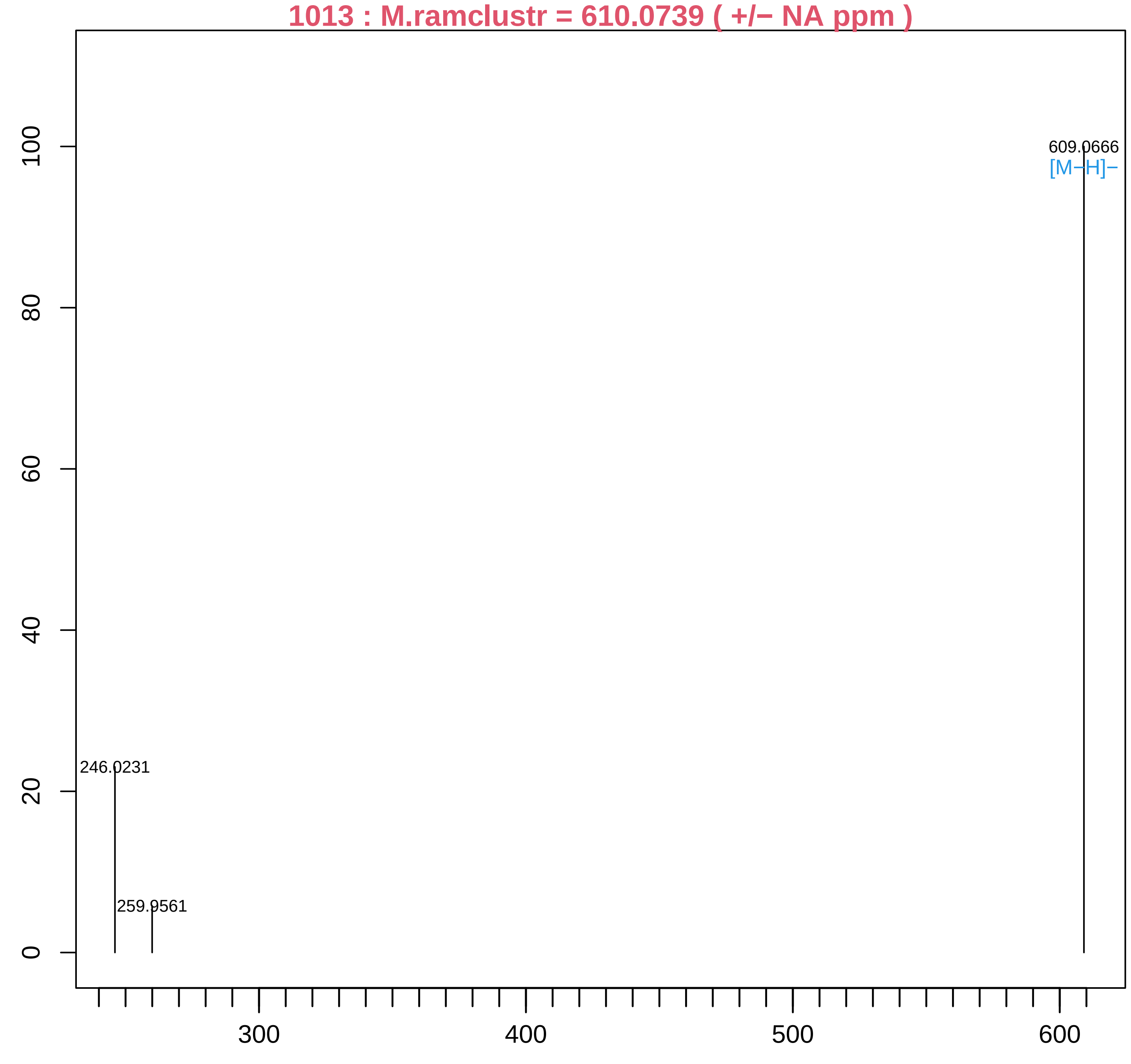
1013 : M.ramclustr = 610.0739 (+/− NA ppm)
609.0666
[M−H]−
246.0231
259.9561
0
20
40
60
80
100
300
400
500
600

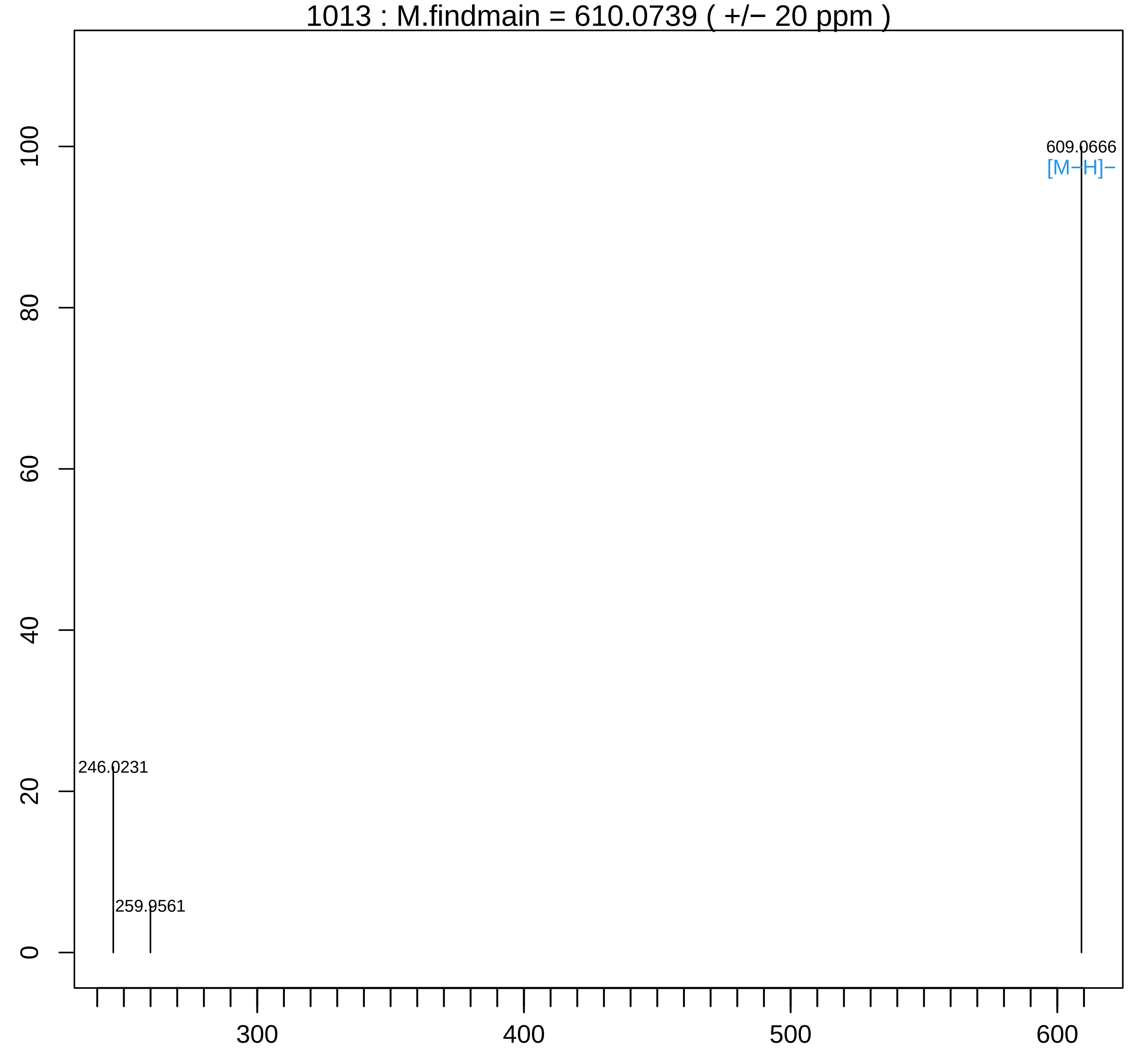
1013 : M.findmain = 610.0739 (+/− 20 ppm)
609.0666
[M−H]−
246.0231
259.9561
0
20
40
60
80
100
300
400
500
600

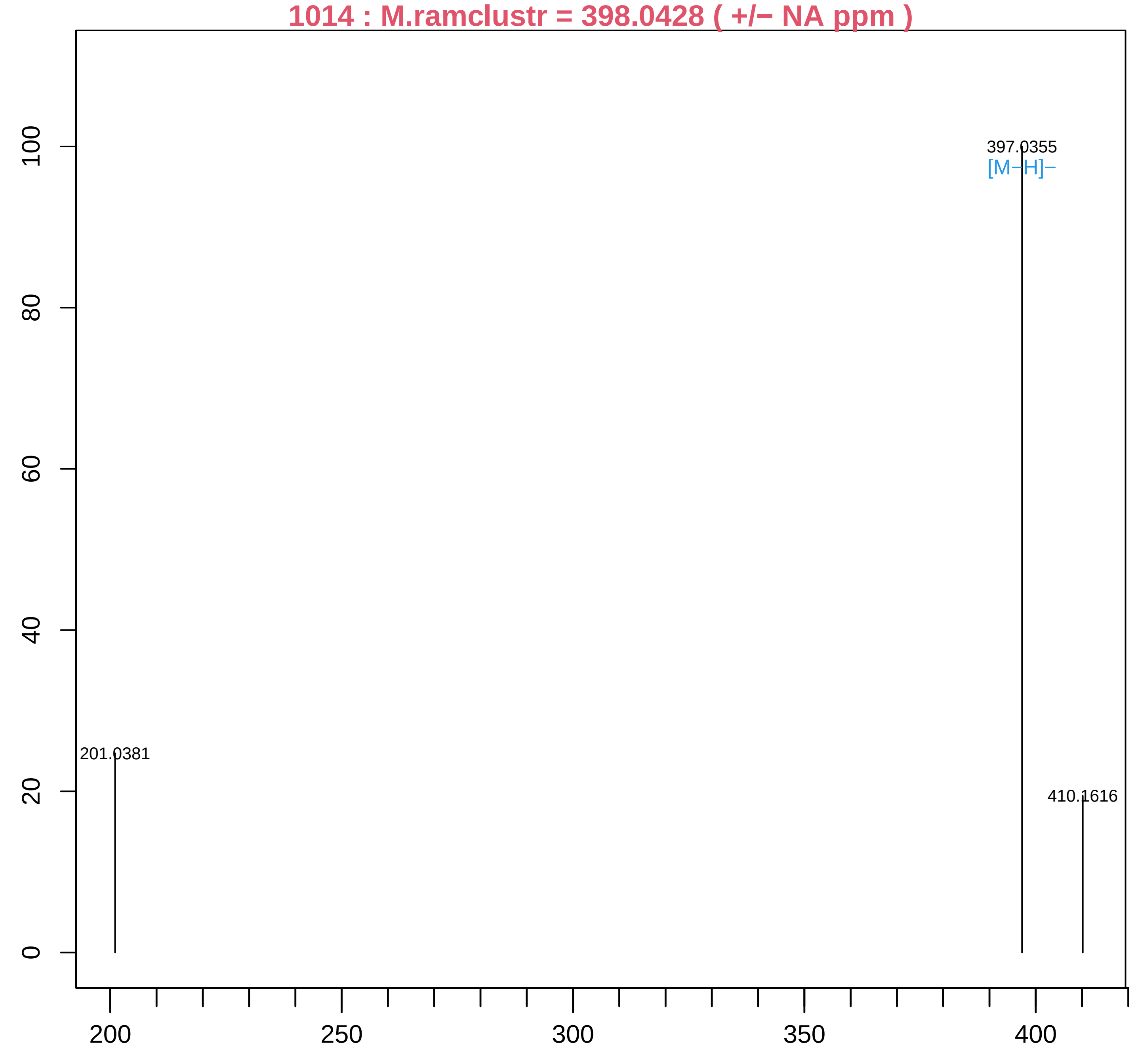
1014 : M.ramclustr = 398.0428 (+/− NA ppm)
397.0355
[M−H]−
201.0381
410.1616
0
20
40
60
80
100
200
250
300
350
400

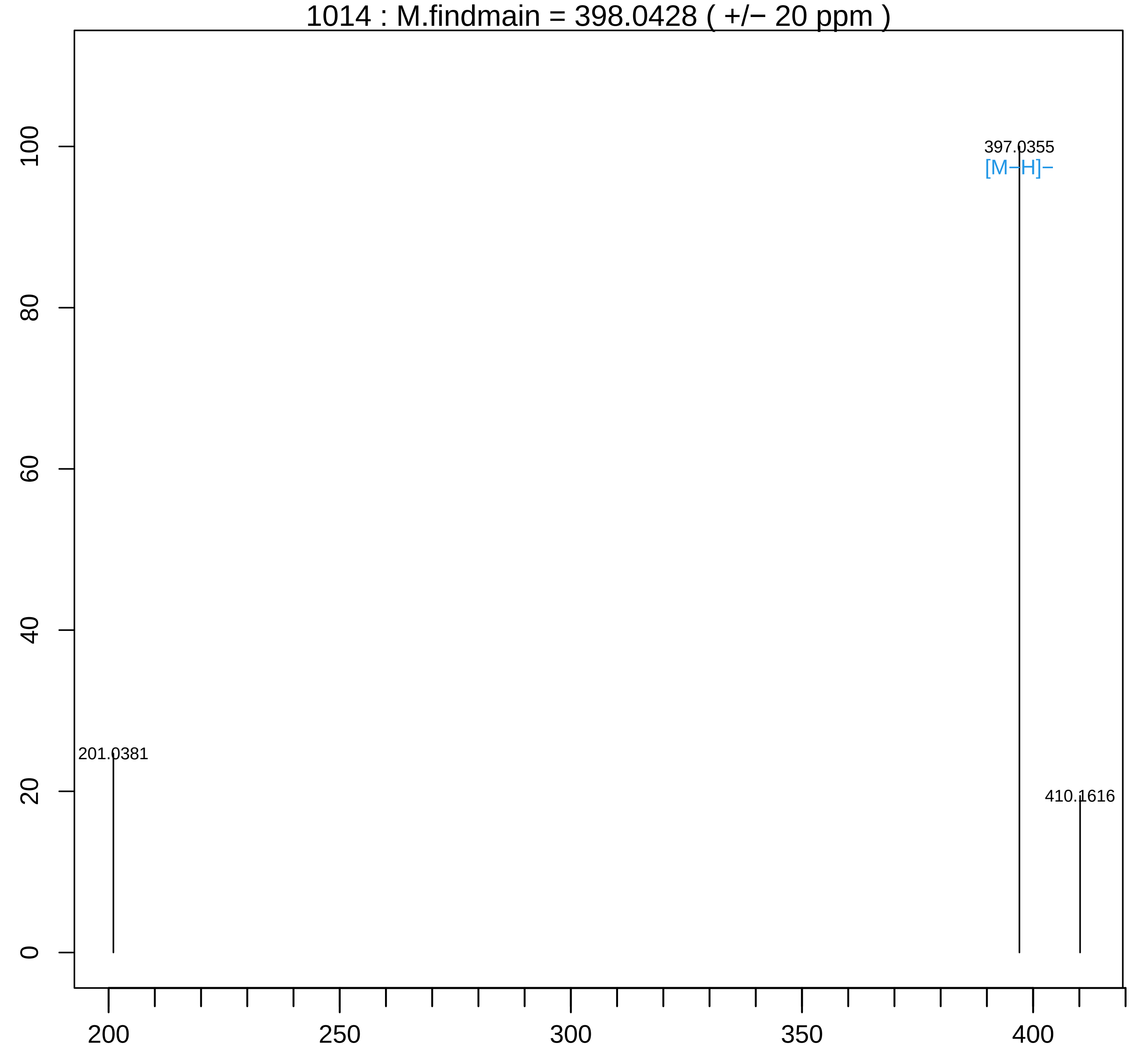
1014 : M.findmain = 398.0428 (+/− 20 ppm)
397.0355
[M−H]−
201.0381
410.1616
0
20
40
60
80
100
200
250
300
350
400

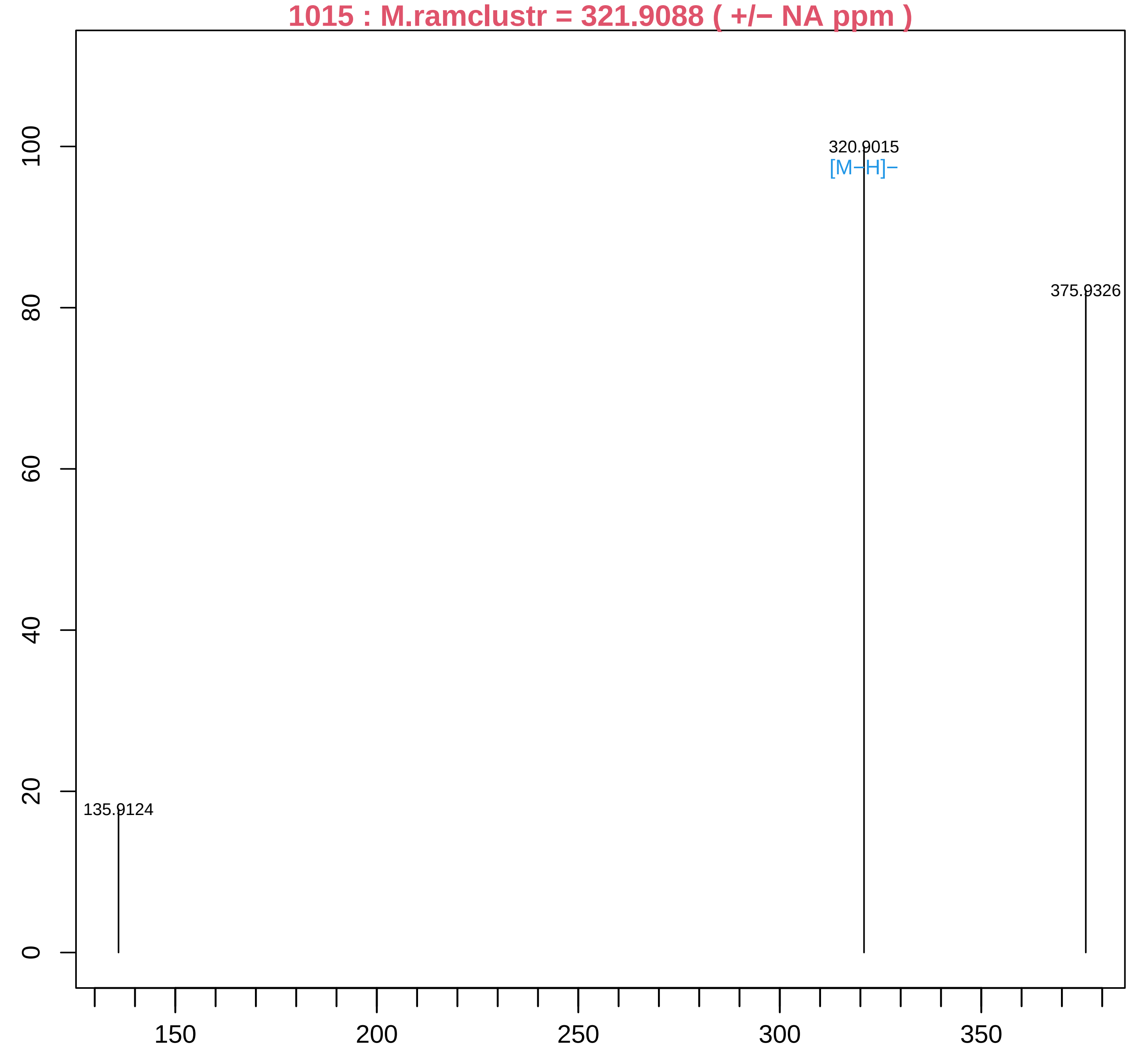
1015 : M.ramclustr = 321.9088 (+/− NA ppm)
320.9015
[M−H]−
375.9326
135.9124
0
20
40
60
80
100
150
200
250
300
350

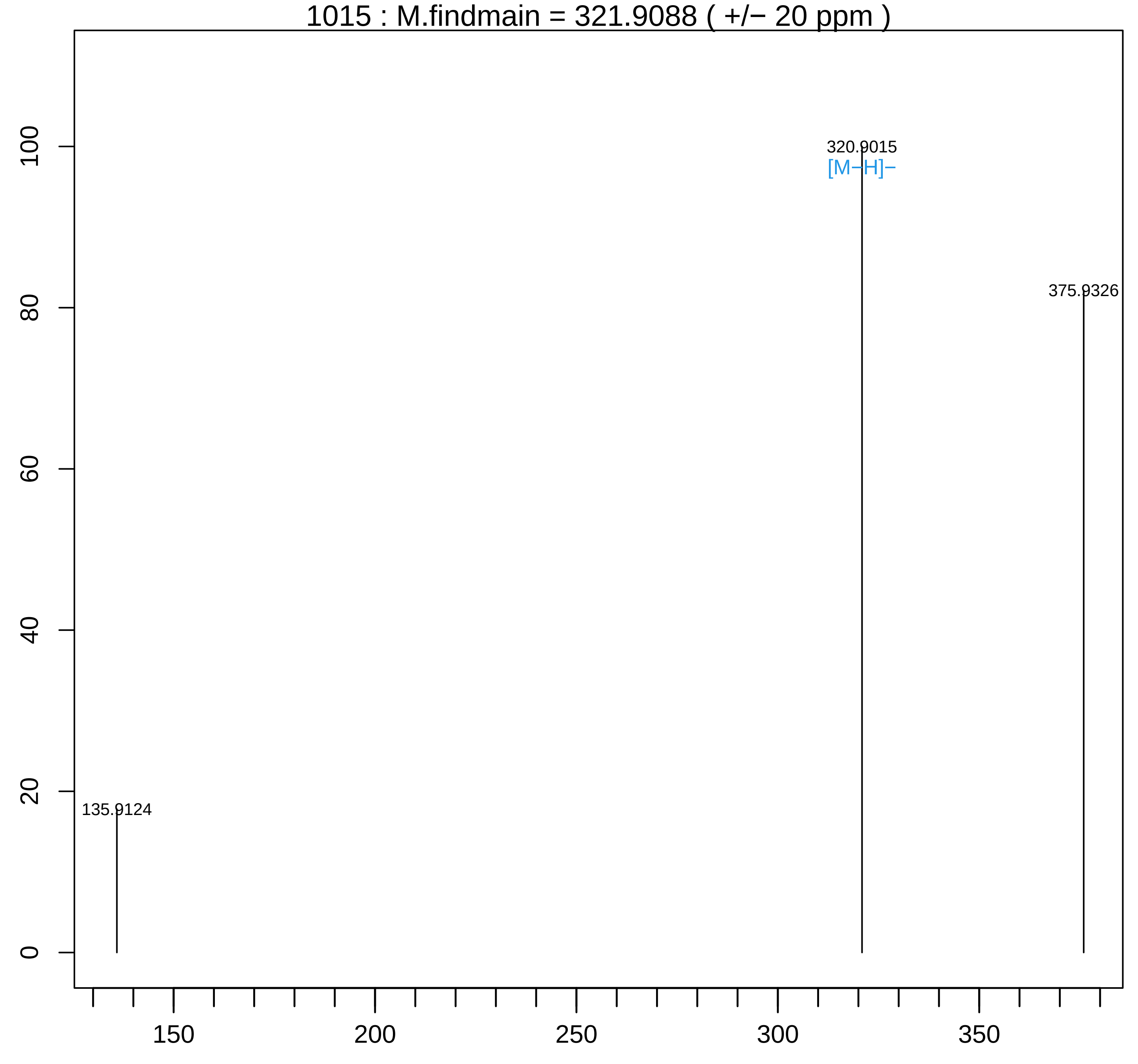
1015 : M.findmain = 321.9088 (+/− 20 ppm)
320.9015
[M−H]−
375.9326
135.9124
0
20
40
60
80
100
150
200
250
300
350

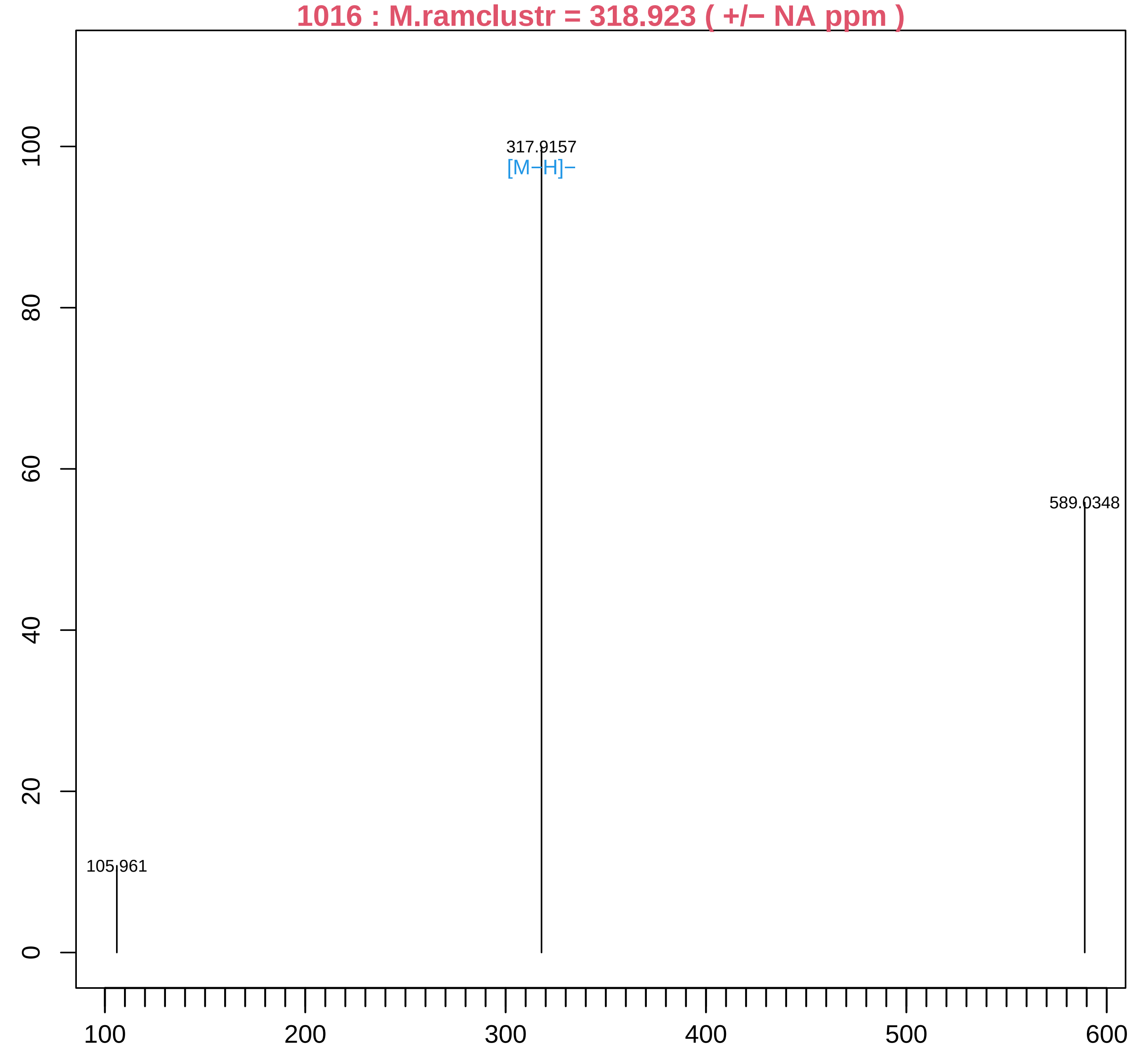
1016 : M.ramclustr = 318.923 (+/− NA ppm)
317.9157
[M−H]−
589.0348
105.961
0
20
40
60
80
100
100
200
300
400
500
600

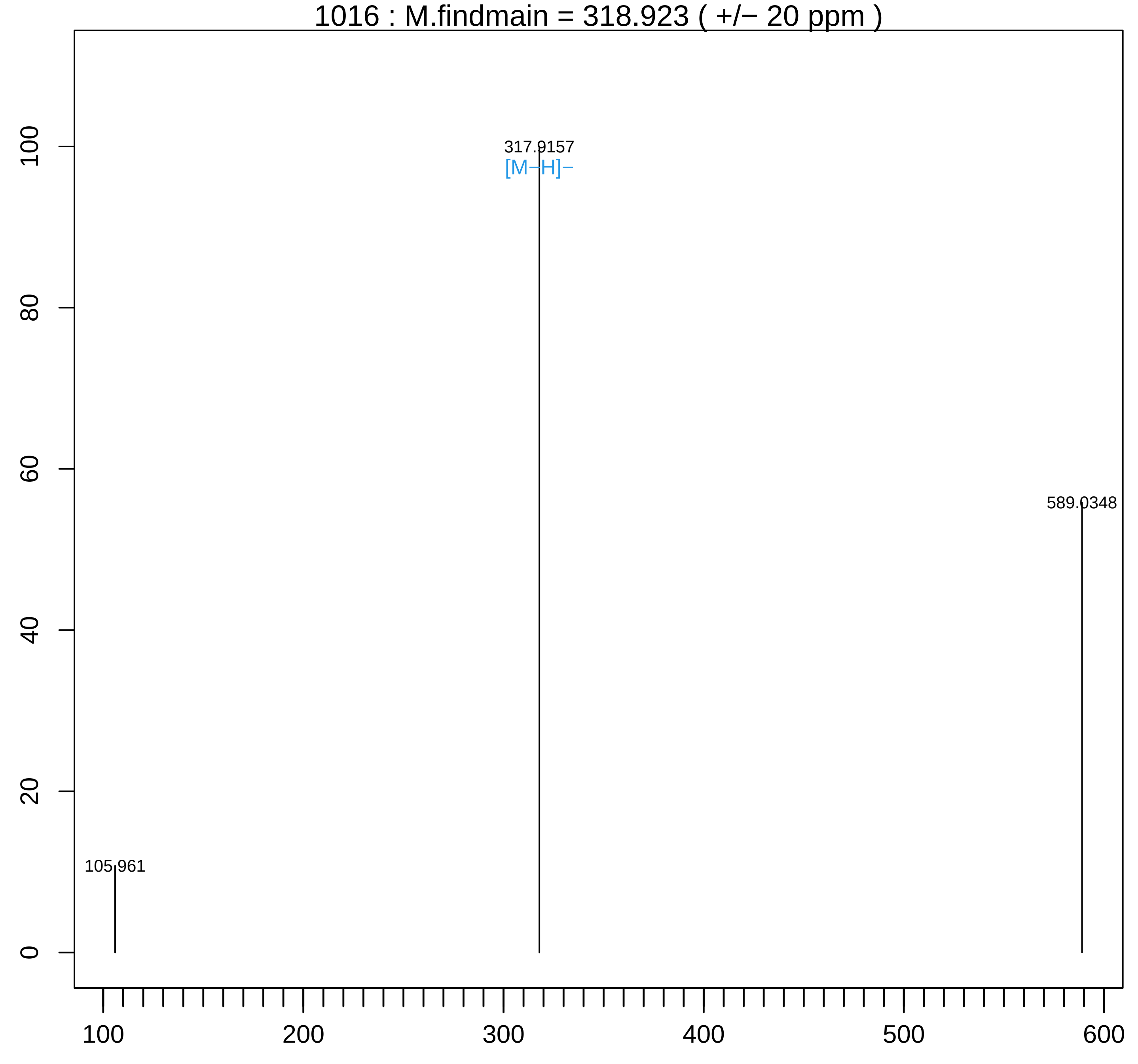
1016 : M.findmain = 318.923 (+/− 20 ppm)
317.9157
[M−H]−
589.0348
105.961
0
20
40
60
80
100
100
200
300
400
500
600

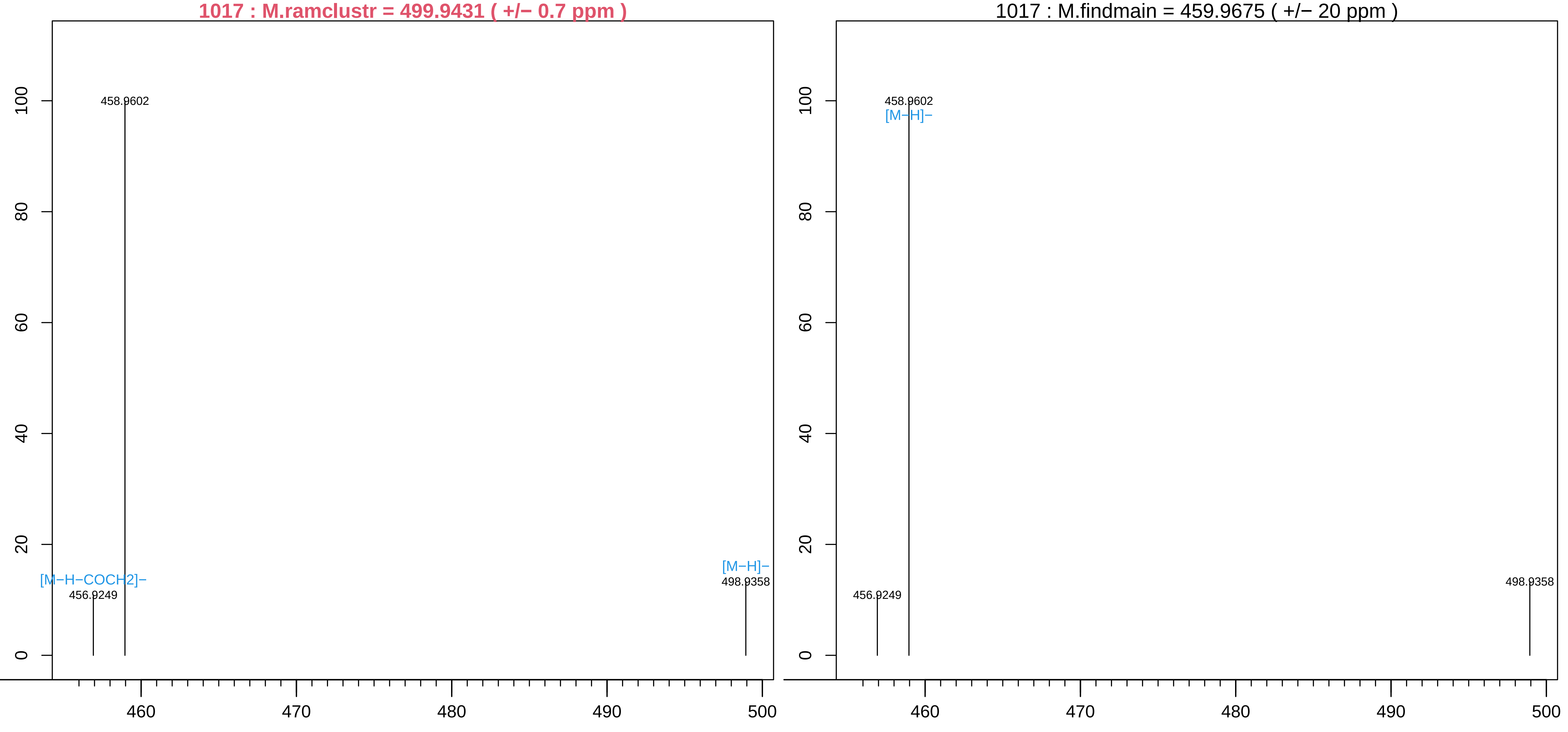

1017 : M.ramclustr = 499.9431 (+/− 0.7 ppm)
458.9602
[M−H−COCH2]−
456.9249
[M−H]−
498.9358
1017 : M.findmain = 459.9675 (+/− 20 ppm)
458.9602
[M−H]−
456.9249
498.9358
0
20
40
60
80
100
460
470
480
490
500

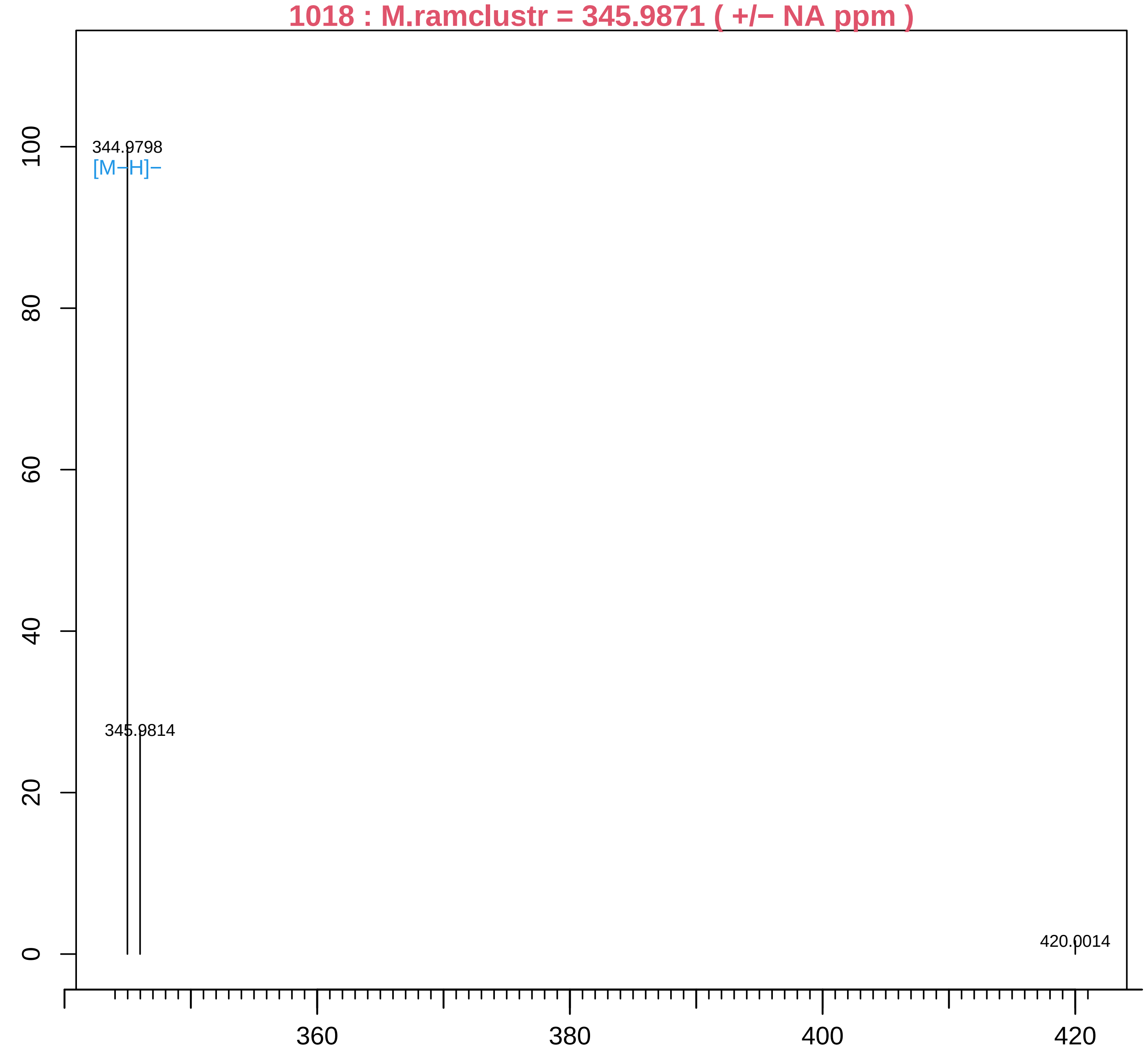

1018 : M.ramclustr = 345.9871 (+/− NA ppm)
344.9798
[M−H]−
345.9814
420.0014
0
20
40
60
80
100
360
380
400
420

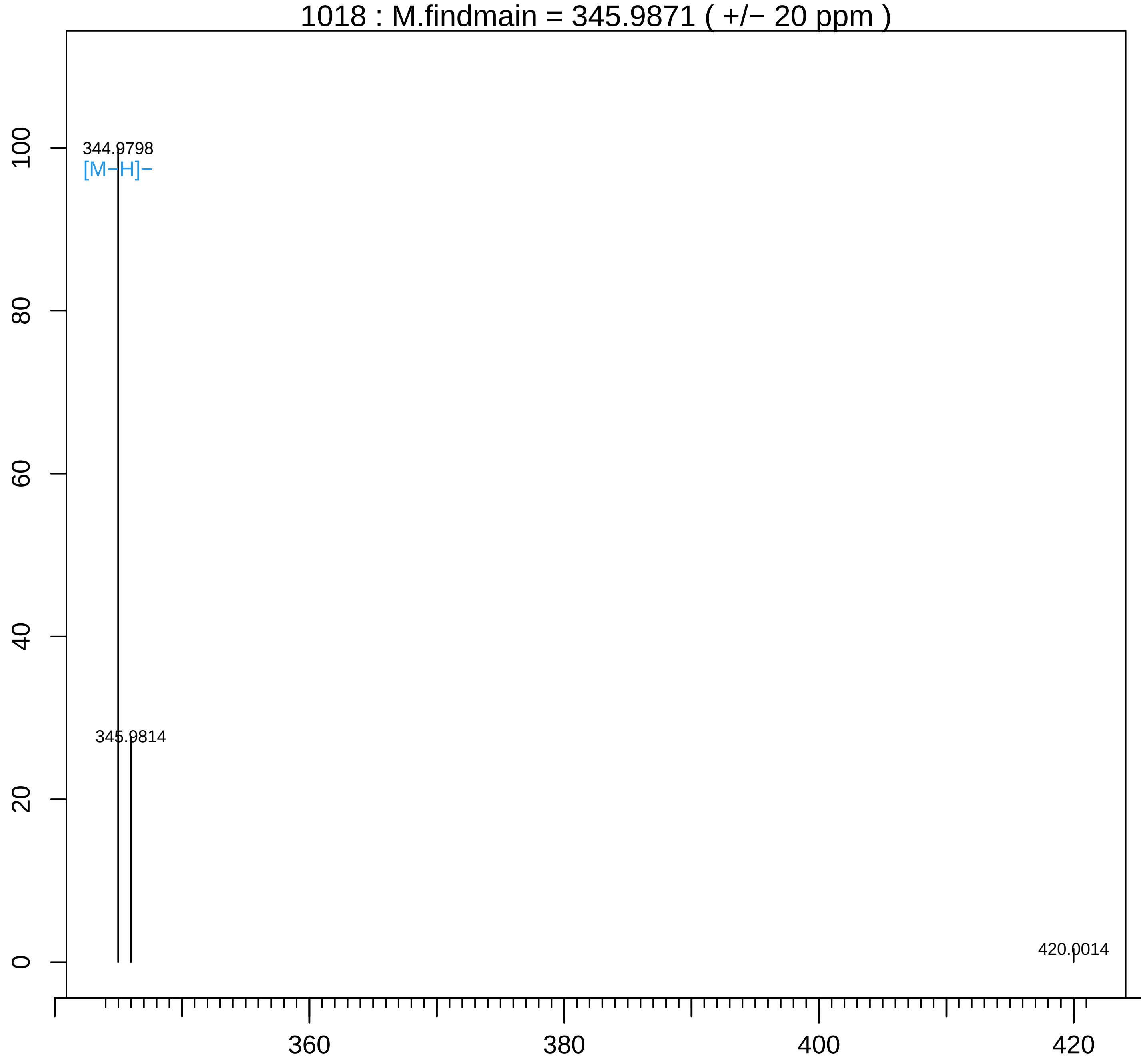

1018 : M.findmain = 345.9871 (+/− 20 ppm)
344.9798
[M−H]−
345.9814
420.0014
0
20
40
60
80
100
360
380
400
420

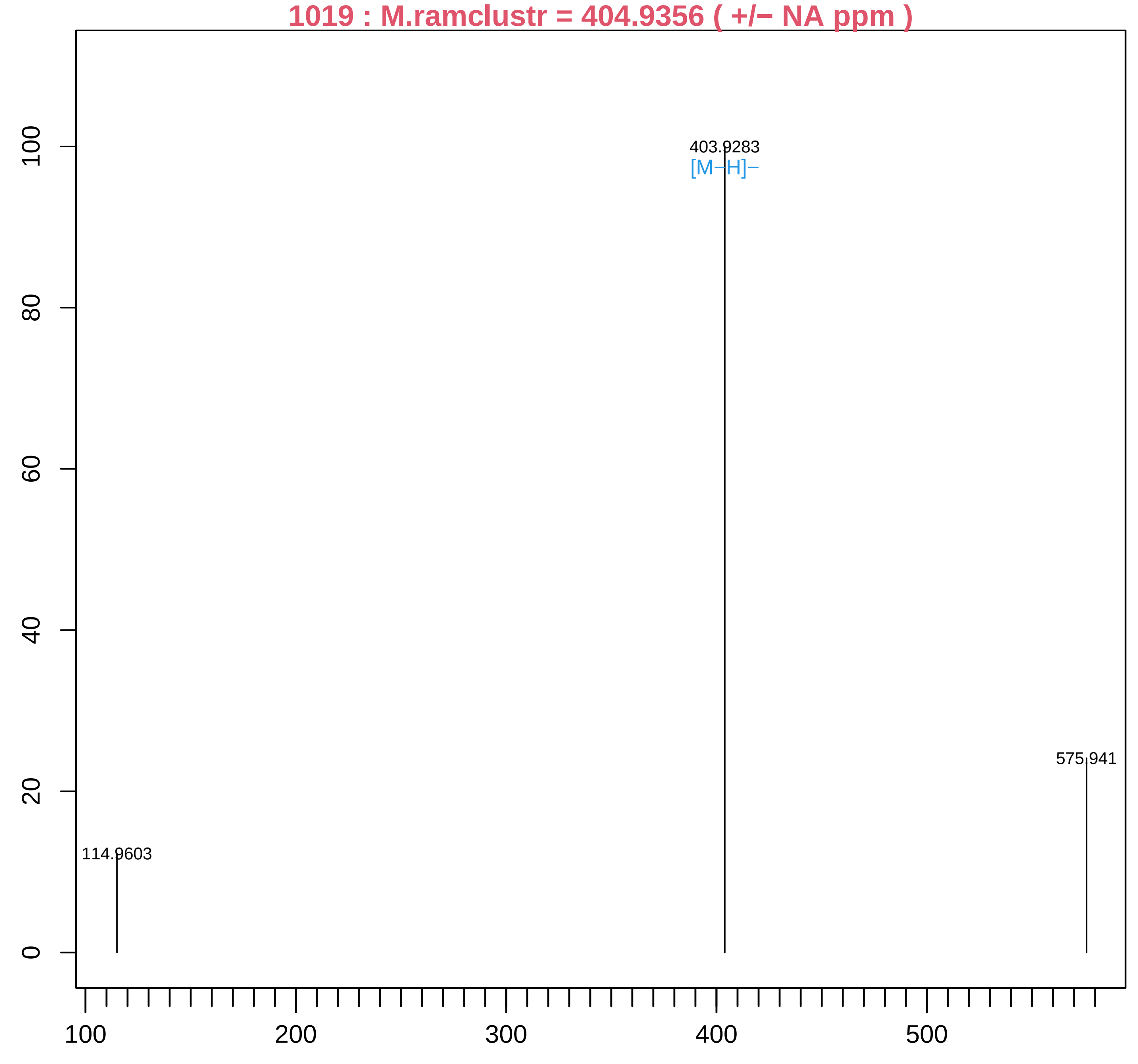
1019 : M.ramclustr = 404.9356 (+/− NA ppm)
403.9283
[M−H]−
575.941
114.9603
0
20
40
60
80
100
100
200
300
400
500

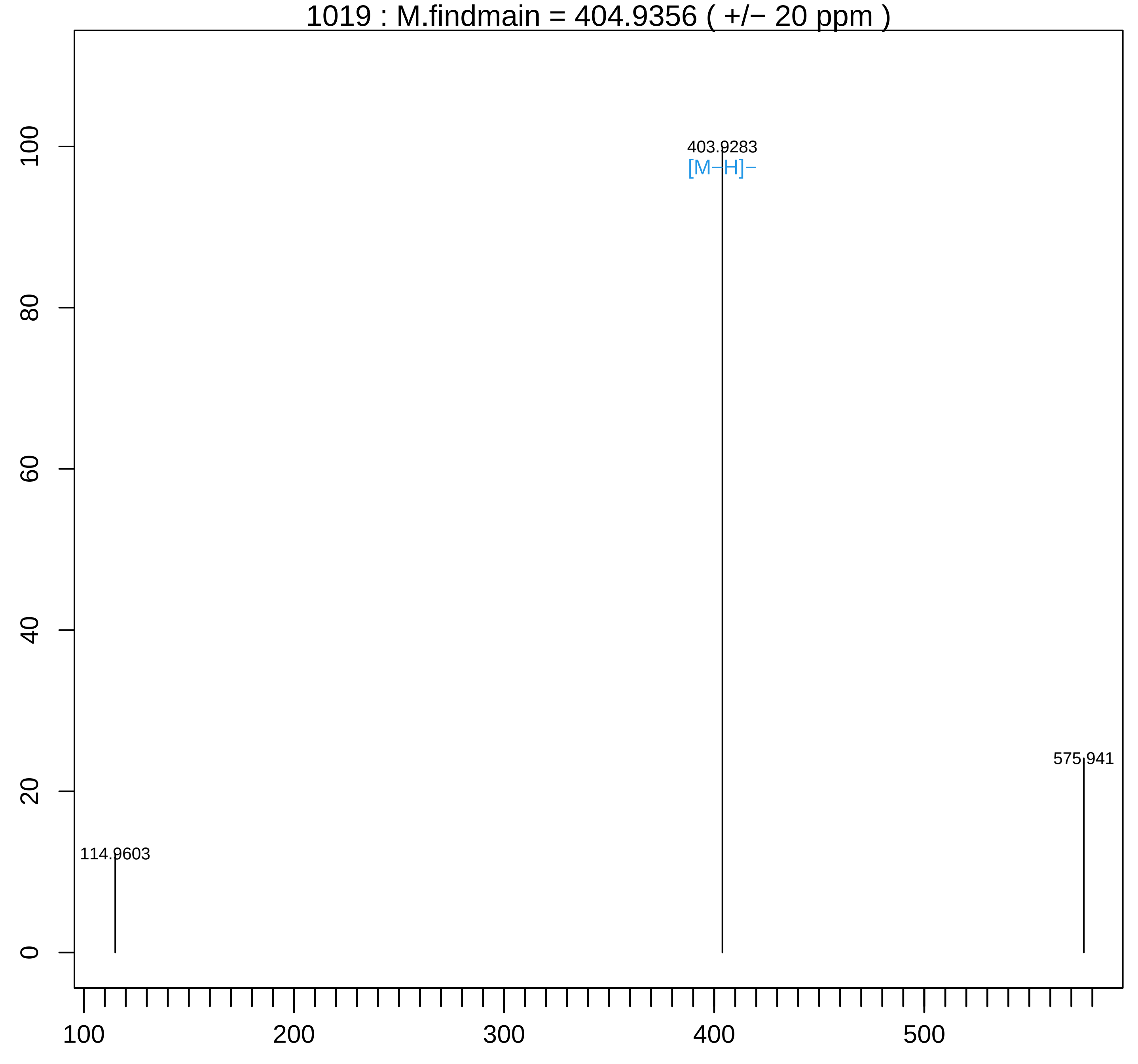
1019 : M.findmain = 404.9356 (+/− 20 ppm)
403.9283
[M−H]−
575.941
114.9603
0
20
40
60
80
100
100
200
300
400
500

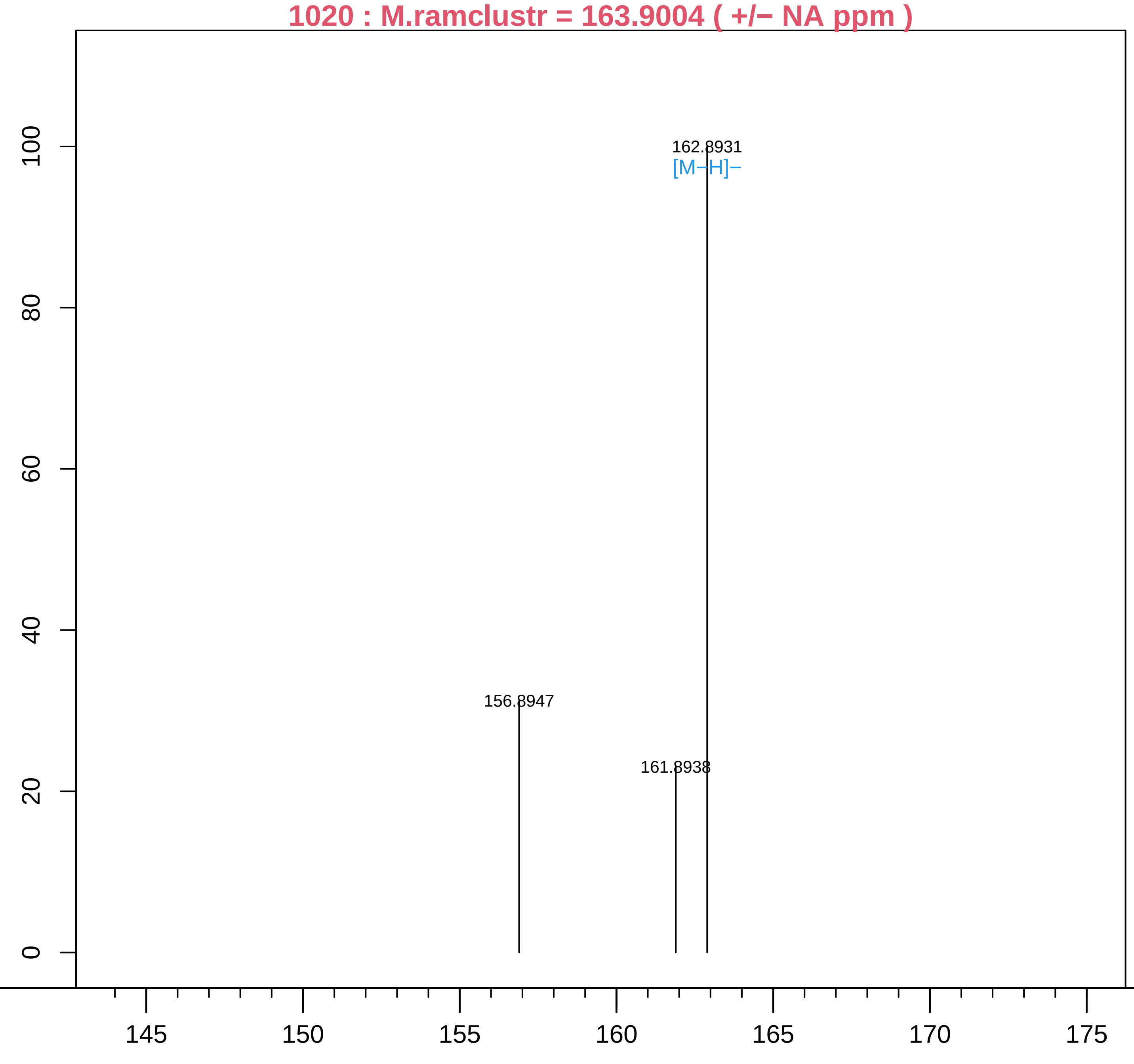
1020 : M.ramclustr = 163.9004 (+/− NA ppm)
162.8931
[M−H]−
156.8947
161.8938
0
20
40
60
80
100
145
150
155
160
165
170
175

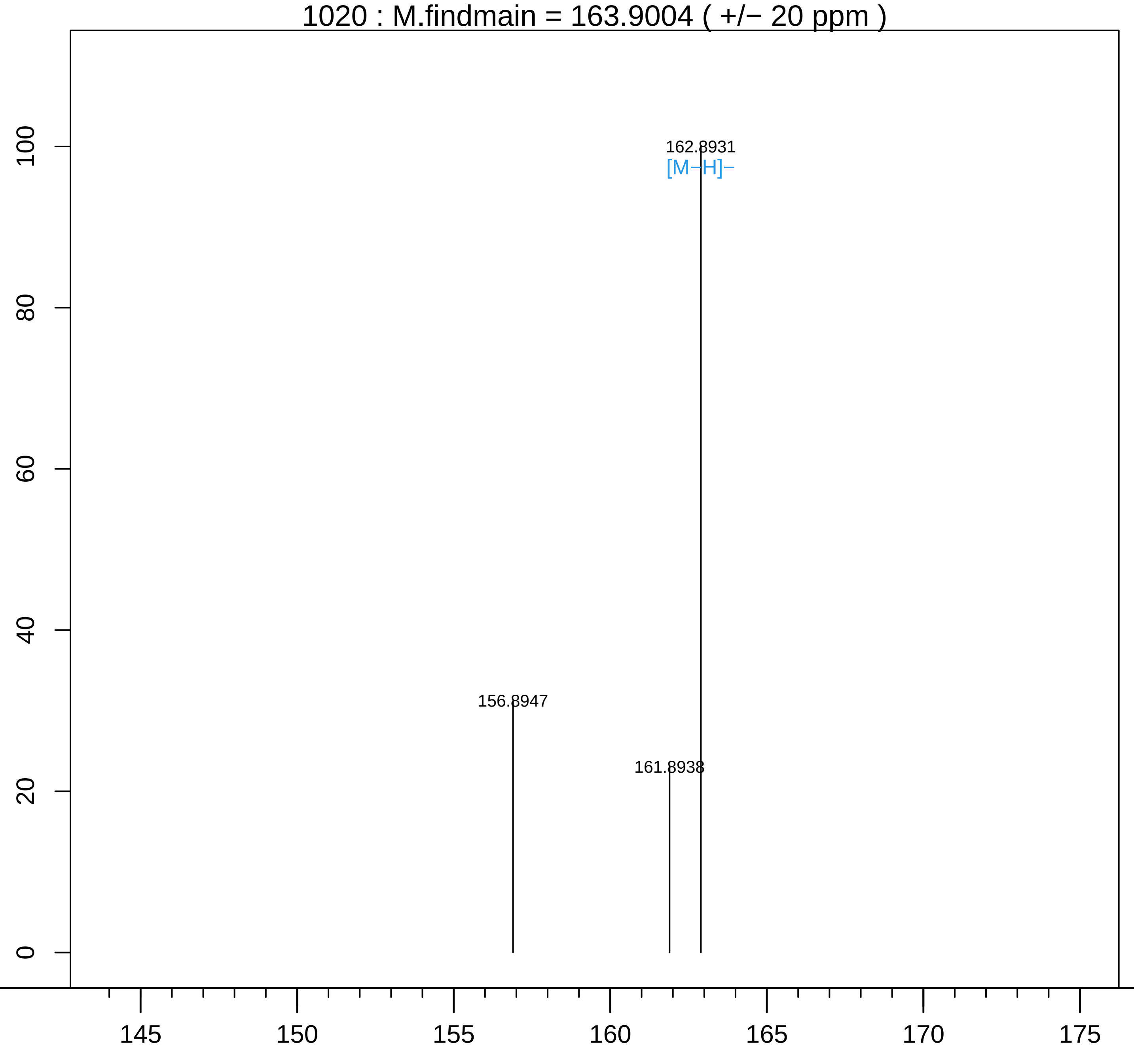
1020 : M.findmain = 163.9004 (+/− 20 ppm)
162.8931
[M−H]−
156.8947
161.8938
0
20
40
60
80
100
145
150
155
160
165
170
175

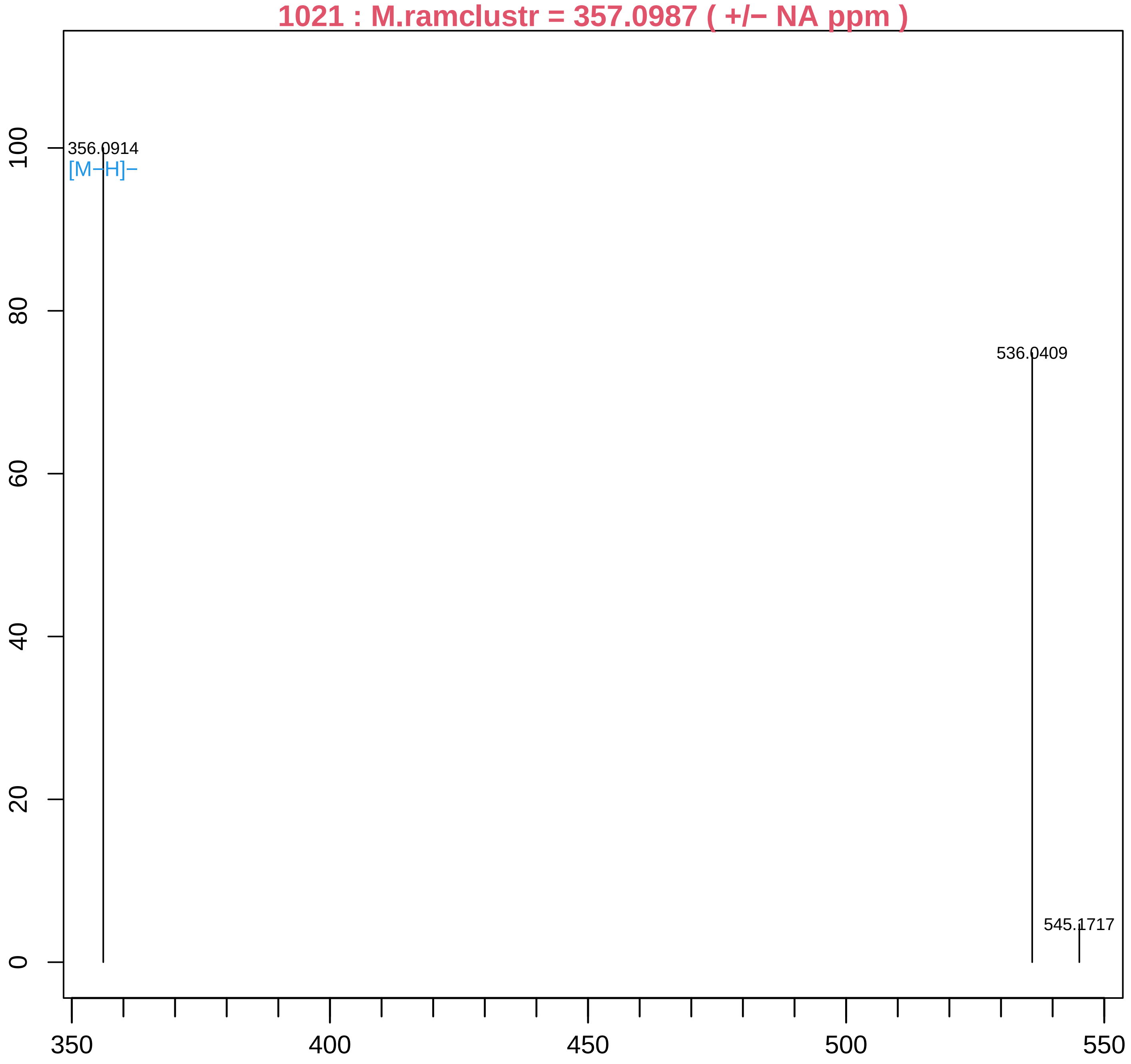
1021 : M.ramclustr = 357.0987 (+/− NA ppm)
356.0914
[M−H]−
536.0409
545.1717
0
20
40
60
80
100
350
400
450
500
550

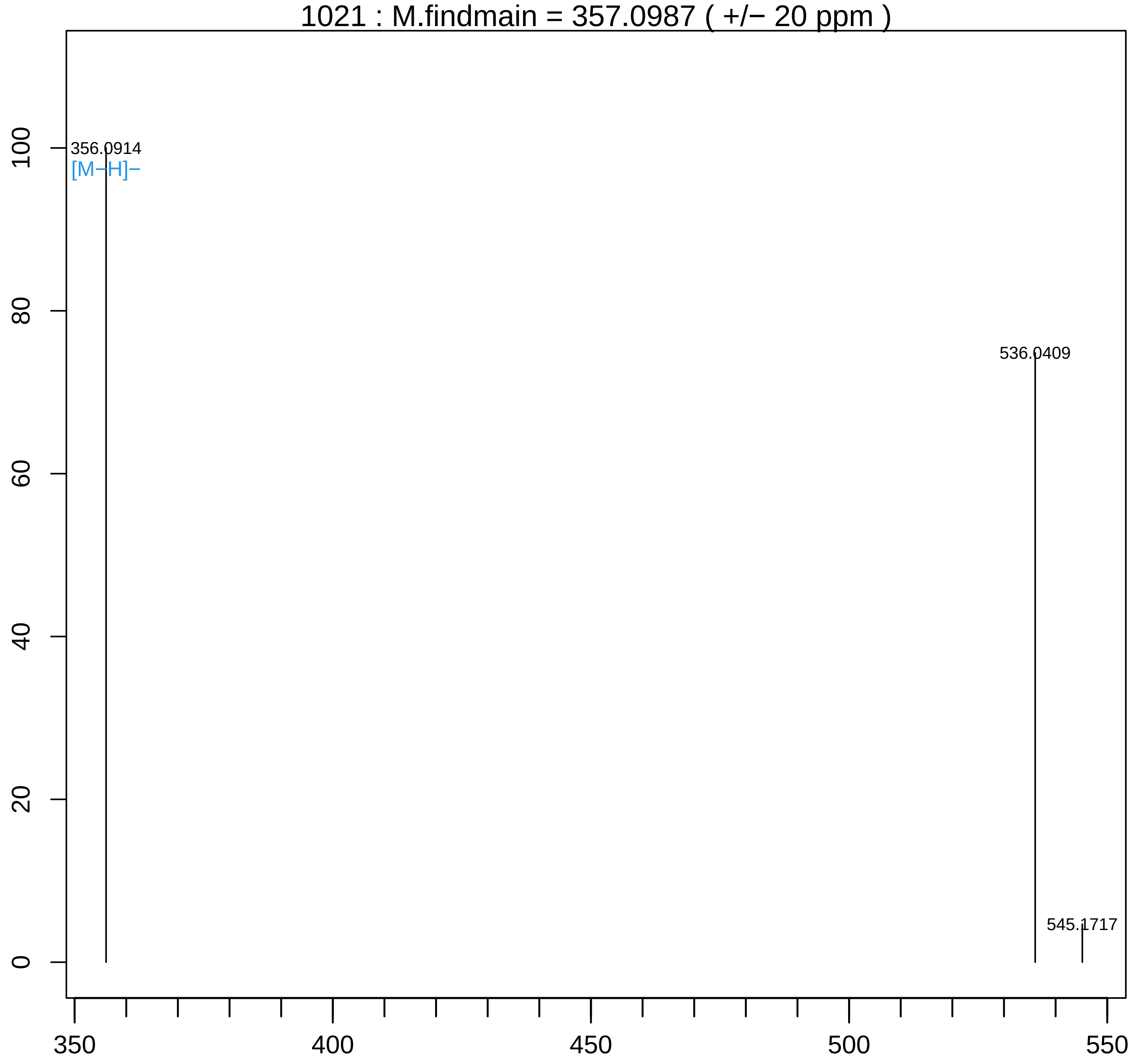
1021 : M.findmain = 357.0987 (+/− 20 ppm)
356.0914
[M−H]−
536.0409
545.1717
0
20
40
60
80
100
350
400
450
500
550

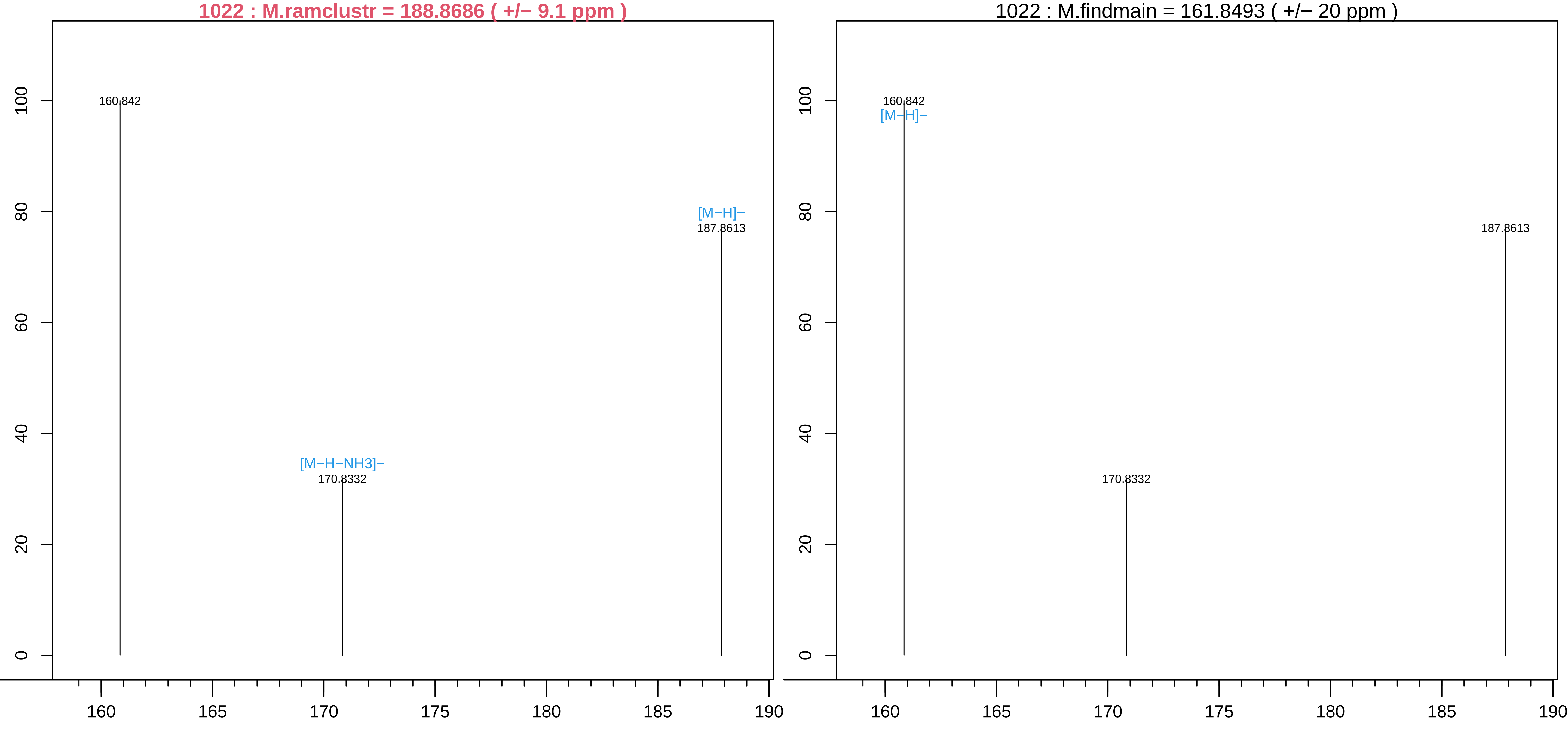

1022 : M.ramclustr = 188.8686 (+/− 9.1 ppm)
160.842
[M−H−NH3]−
170.8332
[M−H]−
187.8613
0
20
40
60
80
100
160
165
170
175
180
185
190
1022 : M.findmain = 161.8493 (+/− 20 ppm)
160.842
[M−H]−
170.8332
187.8613

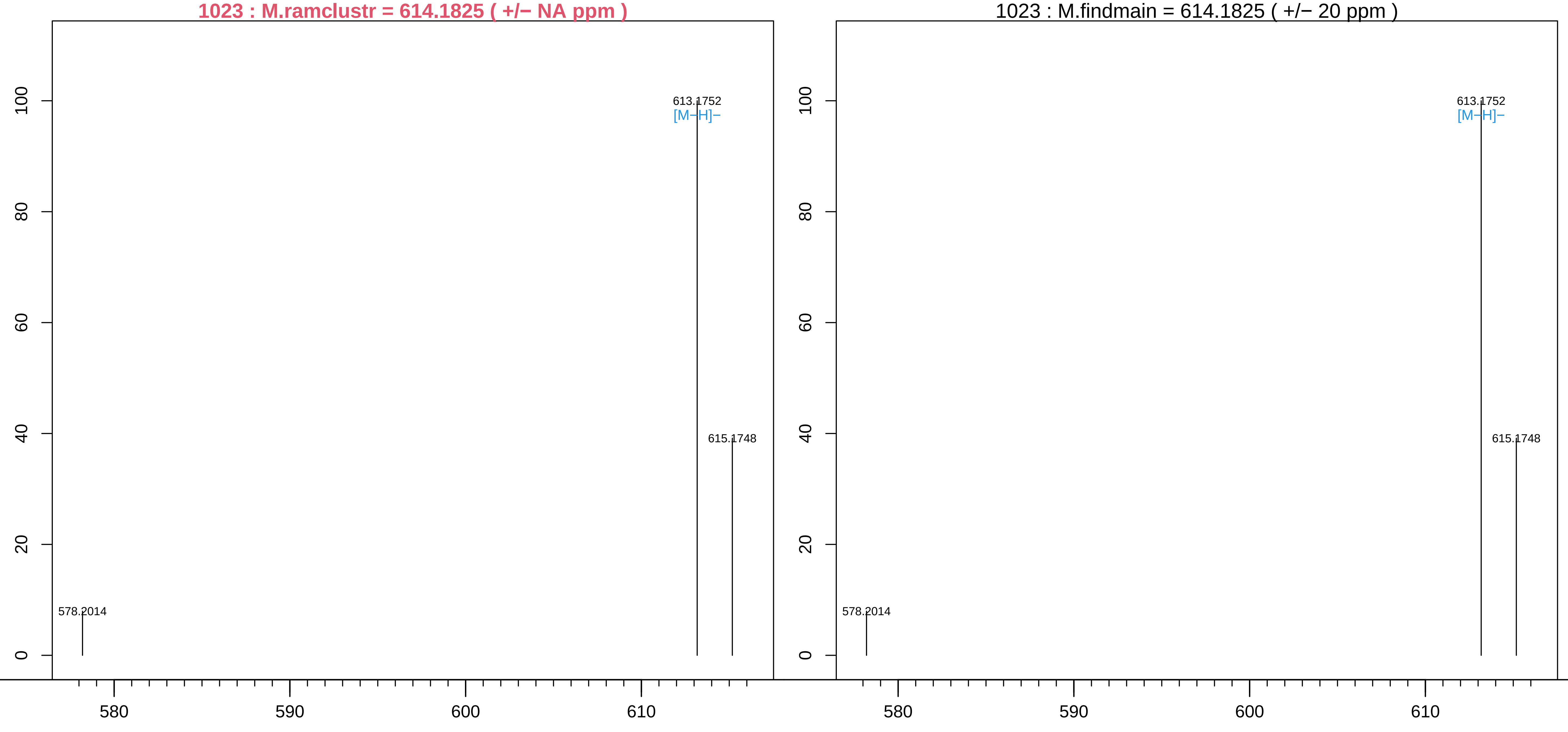
1023 : M.ramclustr = 614.1825 (+/− NA ppm)
1023 : M.findmain = 614.1825 (+/− 20 ppm)
613.1752
[M−H]−
615.1748
578.2014
0
20
40
60
80
100
580
590
600
610

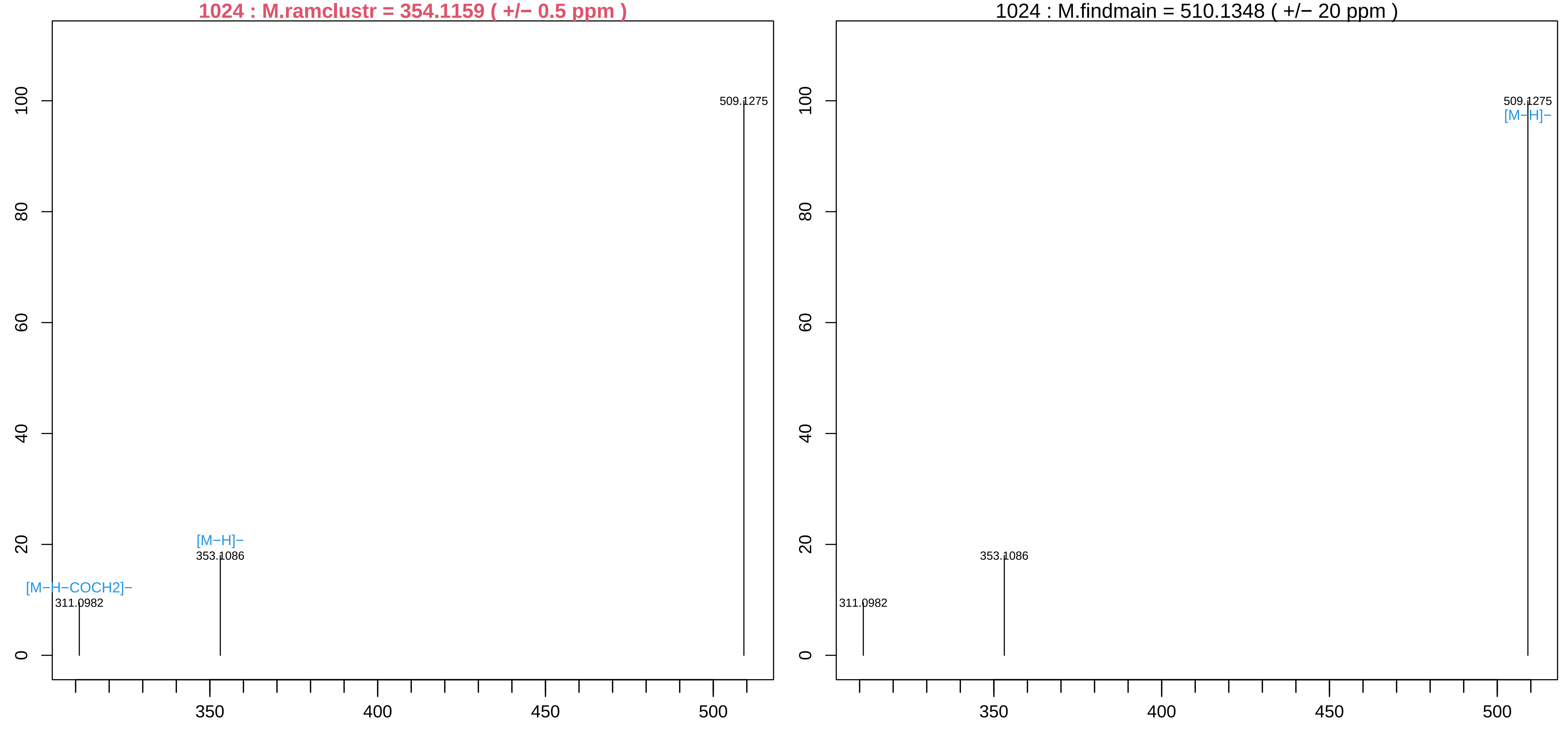
1024 : M.ramclustr = 354.1159 (+/− 0.5 ppm)
509.1275
[M−H]−
353.1086
[M−H−COCH2]−
311.0982
0
20
40
60
80
100
350
400
450
500
1024 : M.findmain = 510.1348 (+/− 20 ppm)
509.1275
[M−H]−
353.1086
311.0982
0
20
40
60
80
100
350
400
450
500

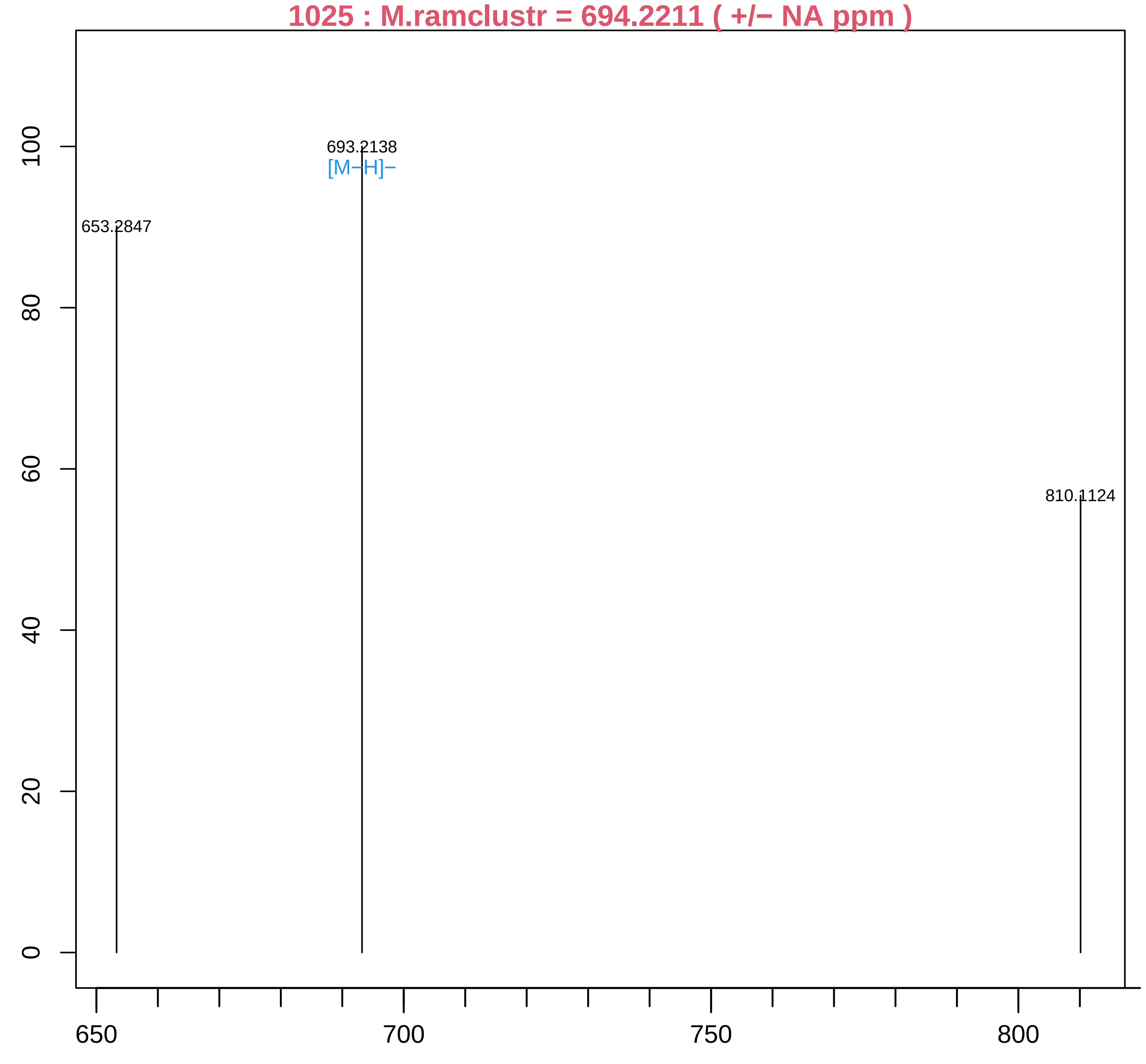

1025 : M.ramclustr = 694.2211 (+/− NA ppm)
693.2138
[M−H]−
653.2847
810.1124
0
20
40
60
80
100
650
700
750
800

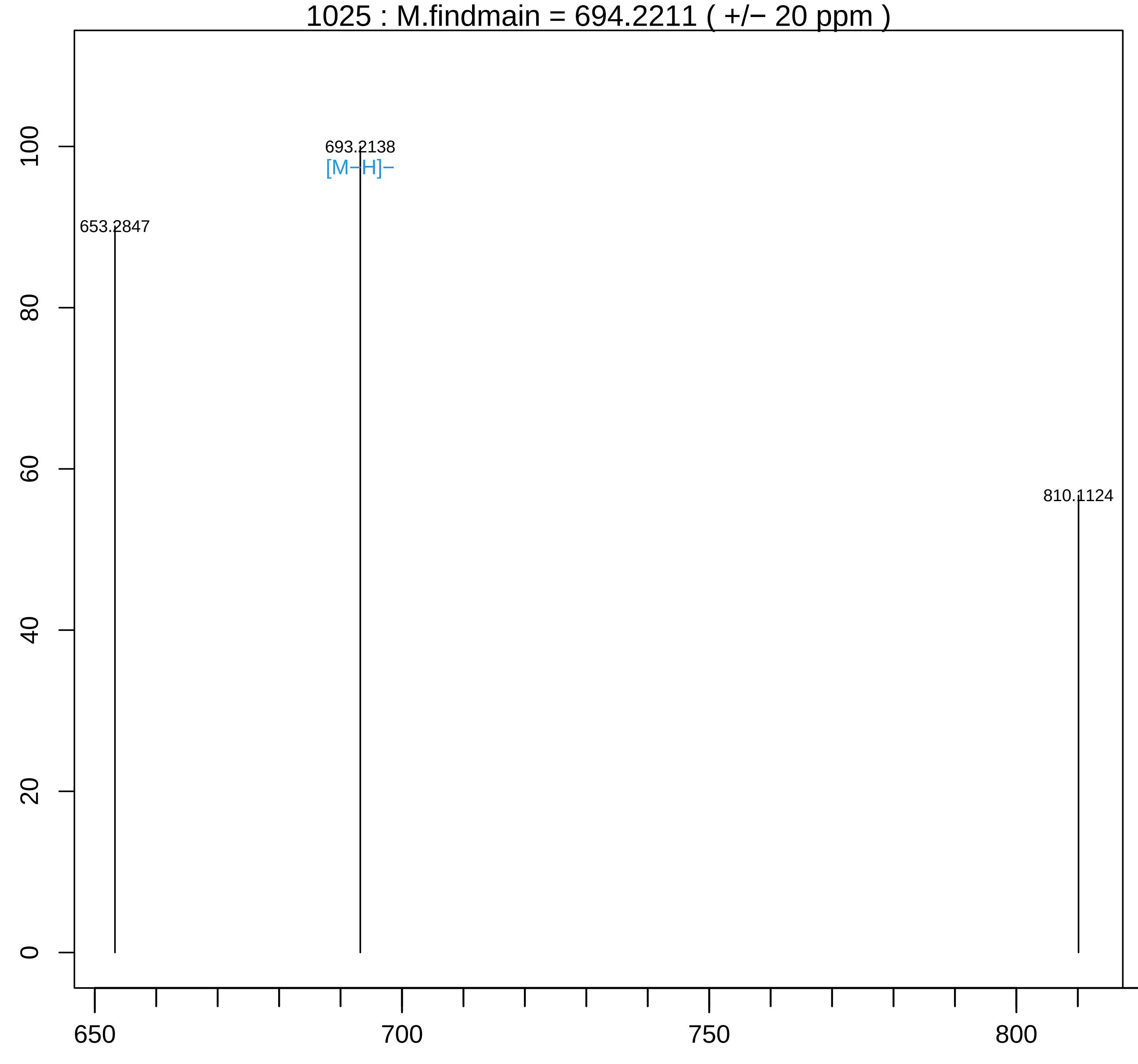

1025 : M.findmain = 694.2211 (+/− 20 ppm)
693.2138
[M−H]−
653.2847
810.1124
0
20
40
60
80
100
650
700
750
800

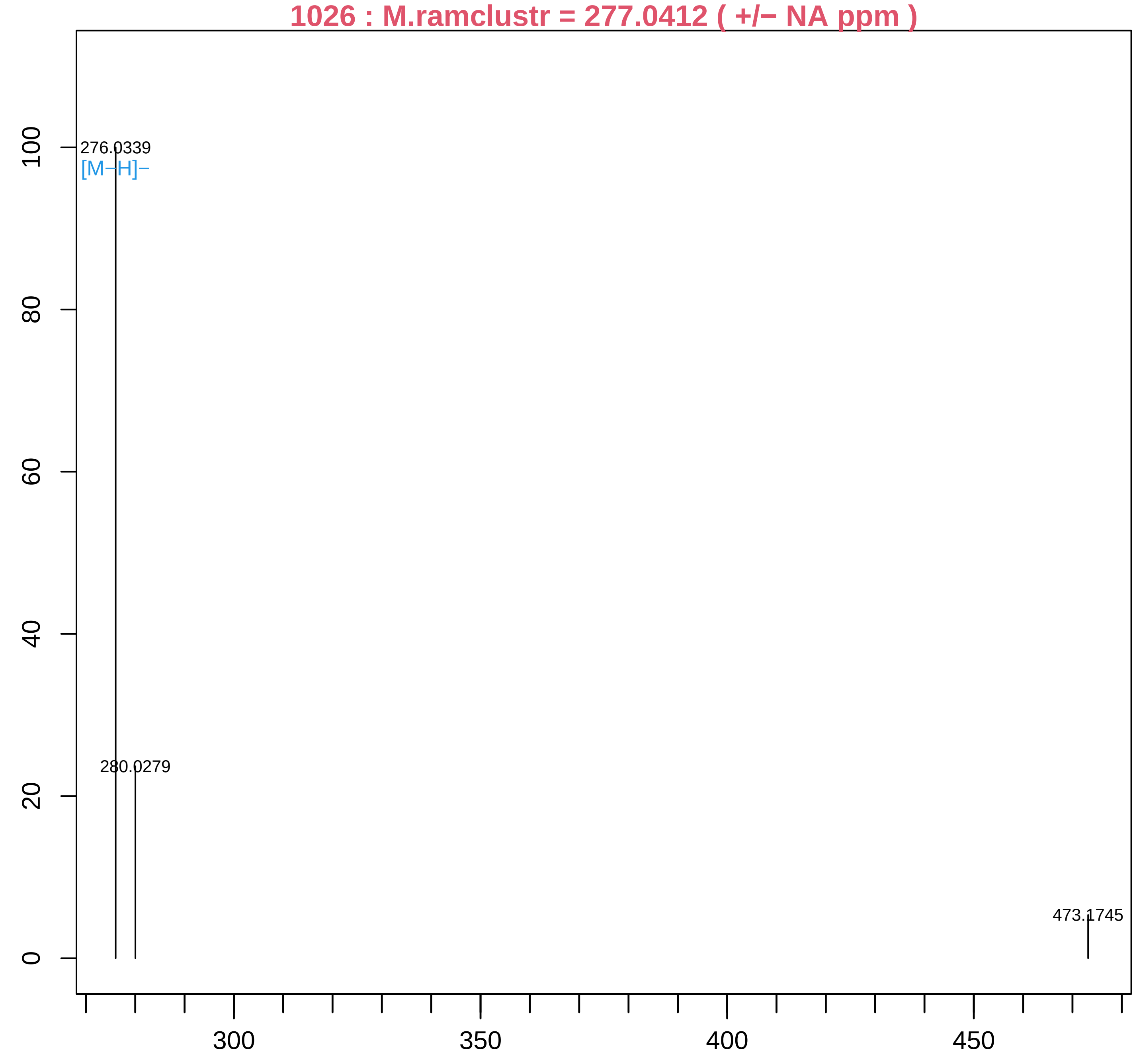
1026 : M.ramclustr = 277.0412 (+/− NA ppm)
276.0339
[M−H]−
280.0279
473.1745
0
20
40
60
80
100
300
350
400
450

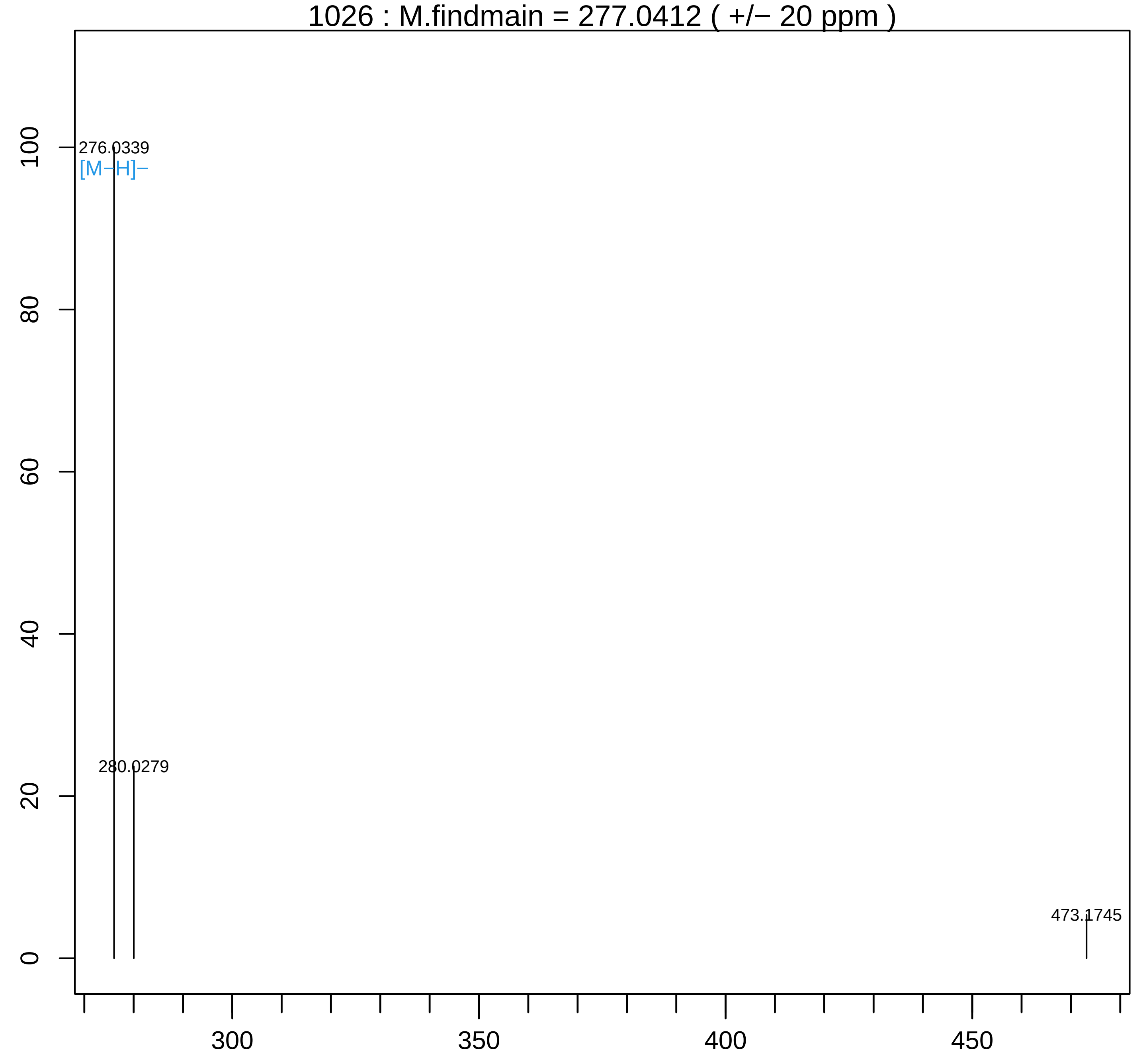
1026 : M.findmain = 277.0412 (+/− 20 ppm)
276.0339
[M−H]−
280.0279
473.1745
0
20
40
60
80
100
300
350
400
450

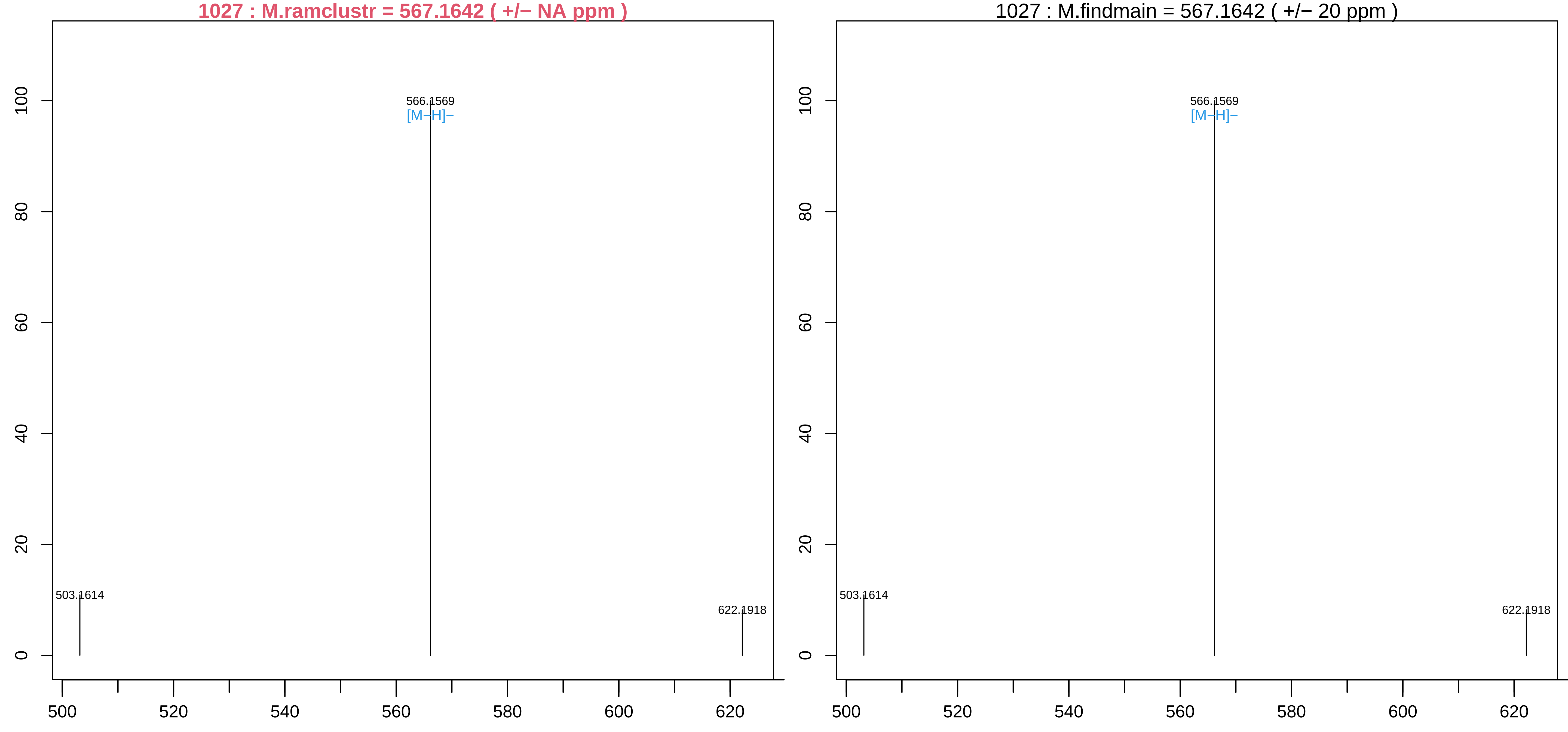

1027 : M.ramclustr = 567.1642 (+/− NA ppm)
566.1569
[M−H]−
503.1614
622.1918
1027 : M.findmain = 567.1642 (+/− 20 ppm)
566.1569
[M−H]−
503.1614
622.1918
0
20
40
60
80
100
500
520
540
560
580
600
620

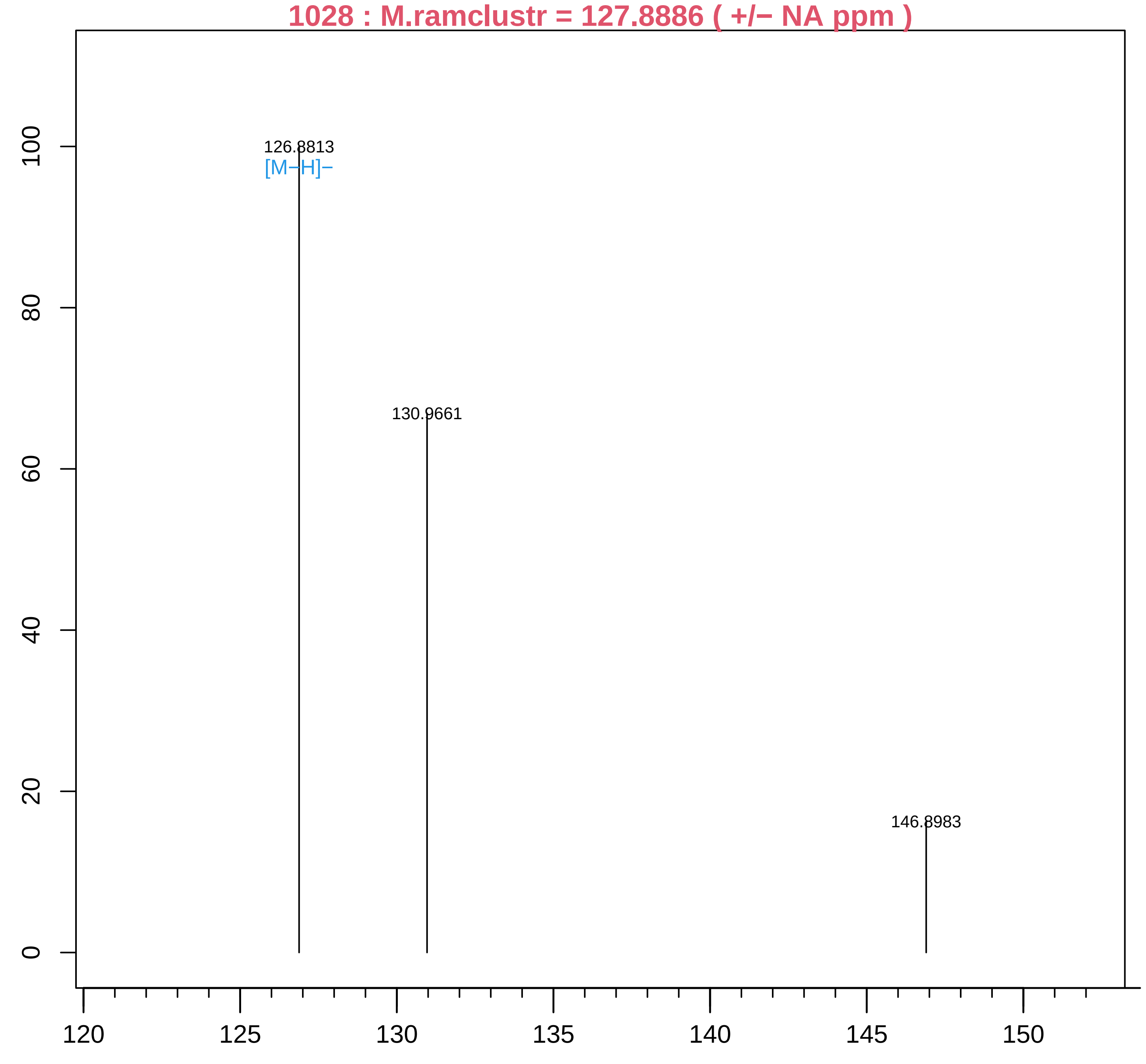

1028 : M.ramclustr = 127.8886 (+/− NA ppm)
126.8813
[M−H]−
130.9661
146.8983
0
20
40
60
80
100
120
125
130
135
140
145
150

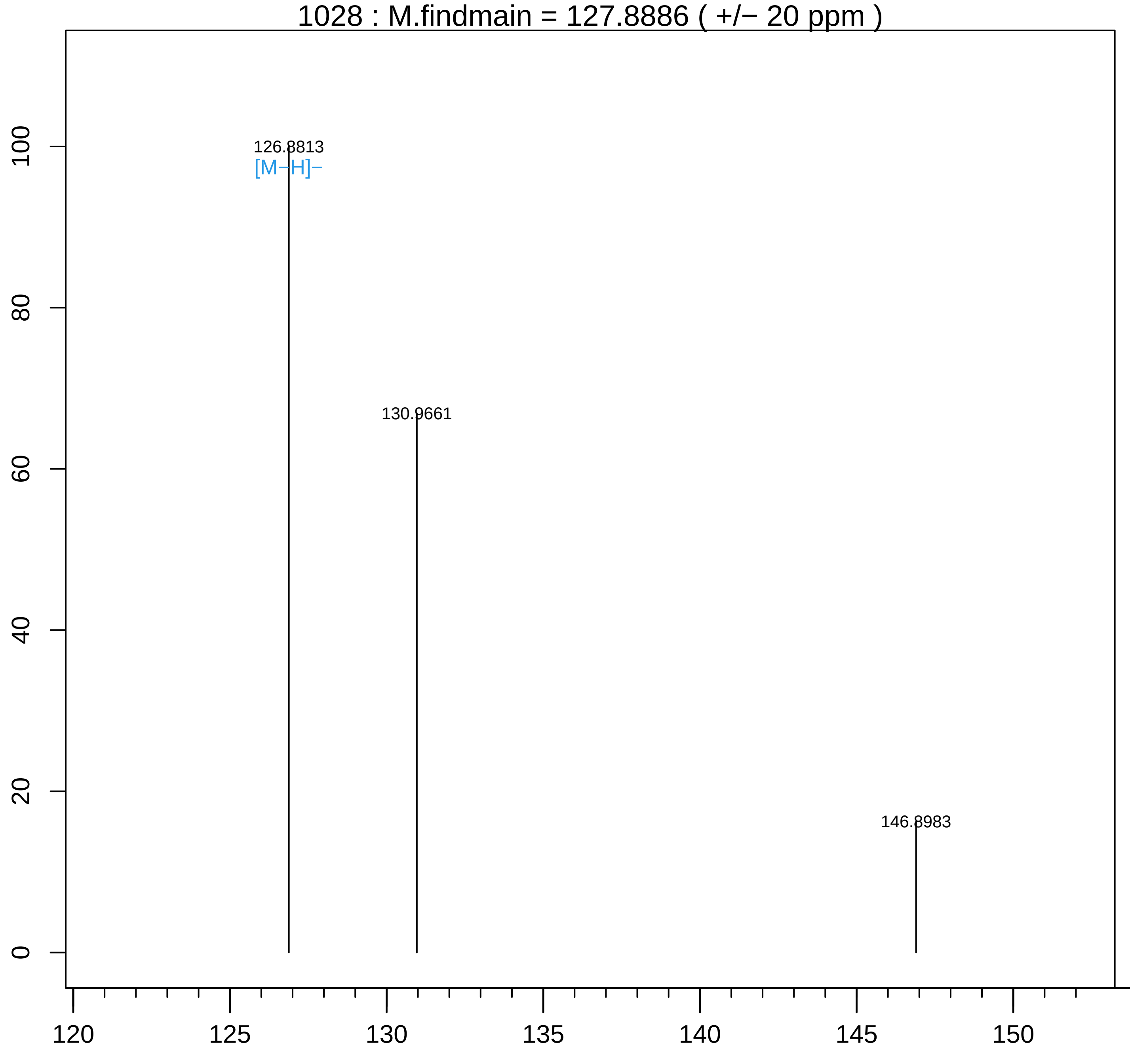

1028 : M.findmain = 127.8886 (+/− 20 ppm)
126.8813
[M−H]−
130.9661
146.8983
0
20
40
60
80
100
120
125
130
135
140
145
150

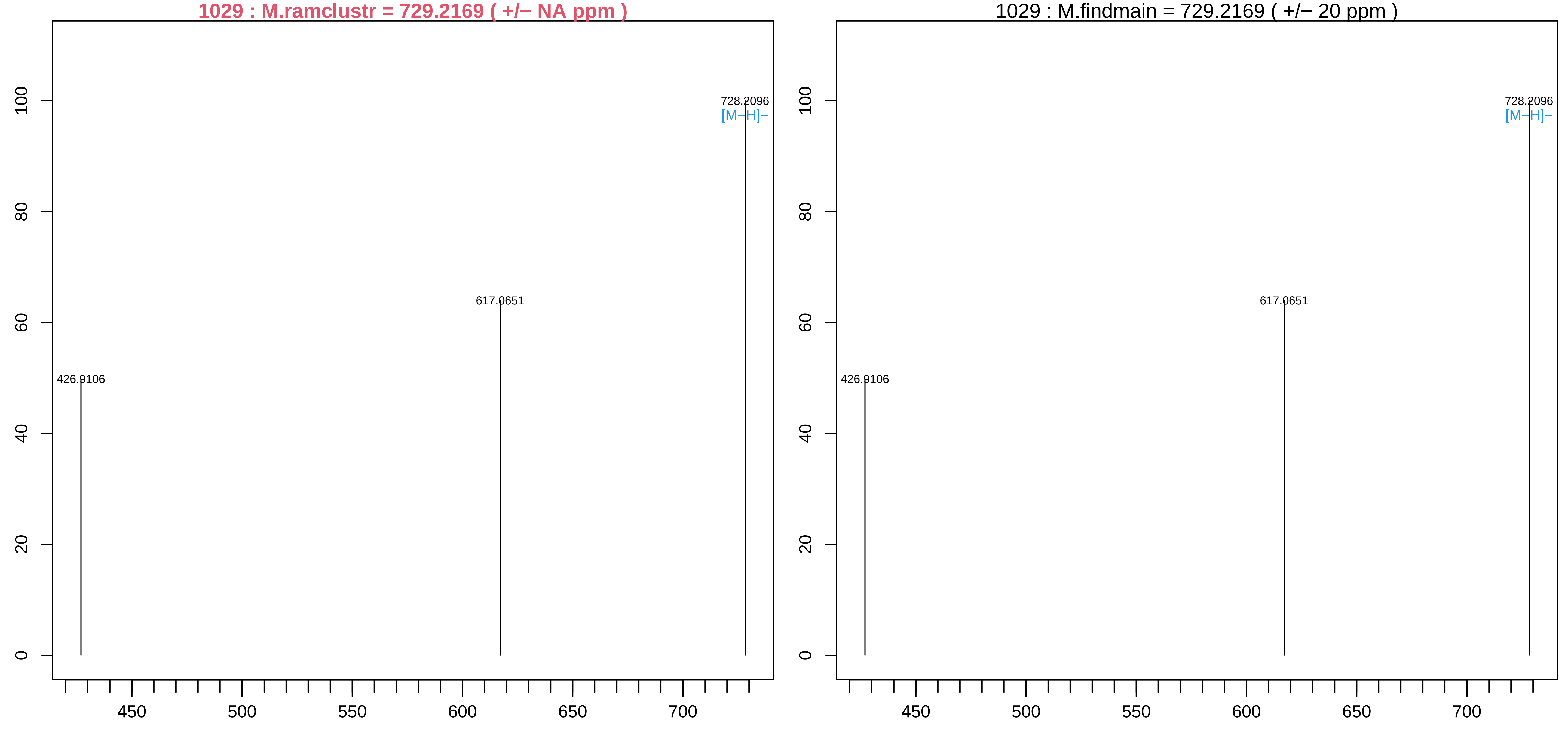
1029 : M.ramclustr = 729.2169 (+/− NA ppm)
728.2096
[M−H]−
617.0651
426.9106
1029 : M.findmain = 729.2169 (+/− 20 ppm)
728.2096
[M−H]−
617.0651
426.9106
0
20
40
60
80
100
450
500
550
600
650
700

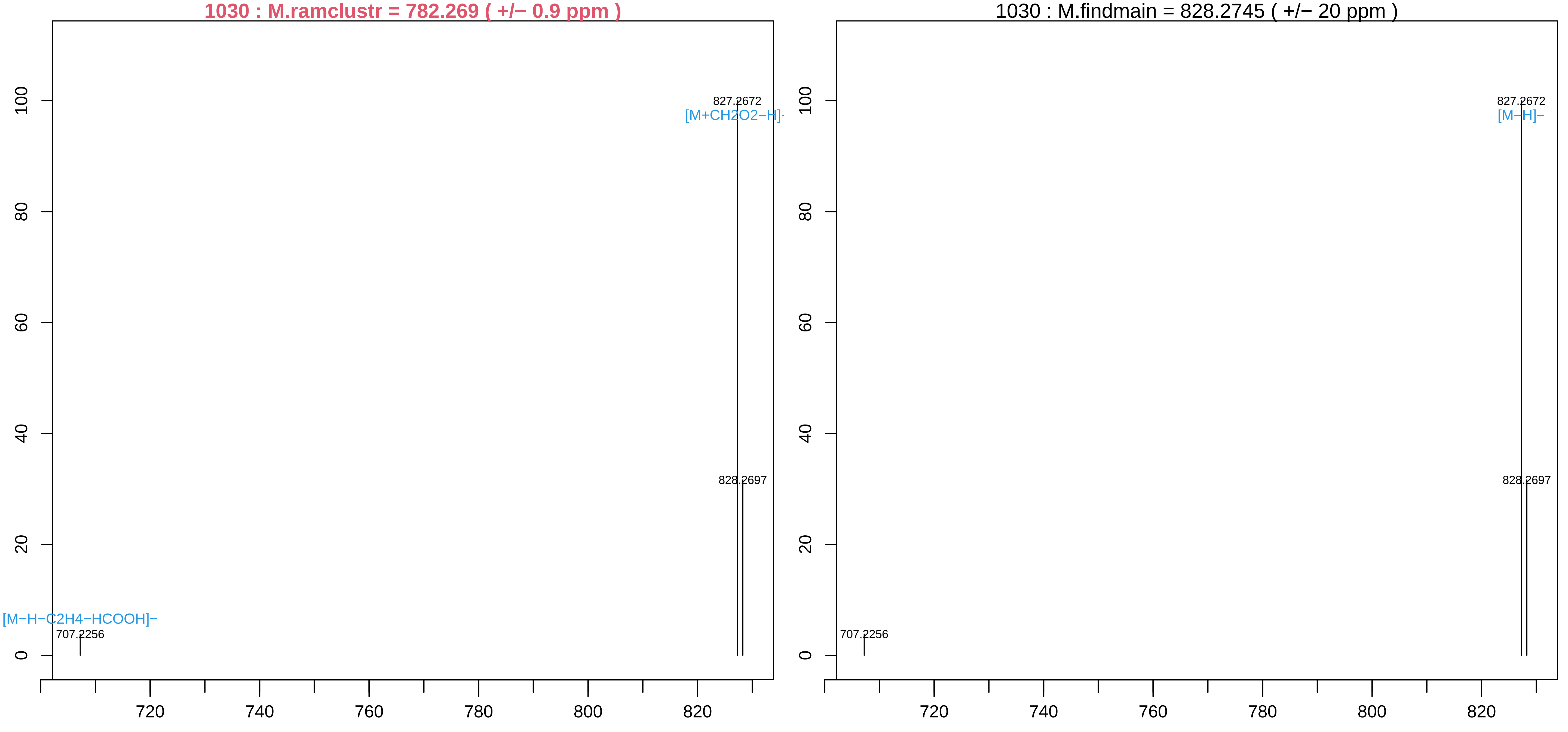
1030 : M.ramclustr = 782.269 (+/− 0.9 ppm)
827.2672
[M+CH2O2−H]−
828.2697
[M−H−C2H4−HCOOH]−
707.2256
0
20
40
60
80
100
720
740
760
780
800
820
1030 : M.findmain = 828.2745 (+/− 20 ppm)
827.2672
[M−H]−
828.2697
707.2256
0
20
40
60
80
100
720
740
760
780
800
820

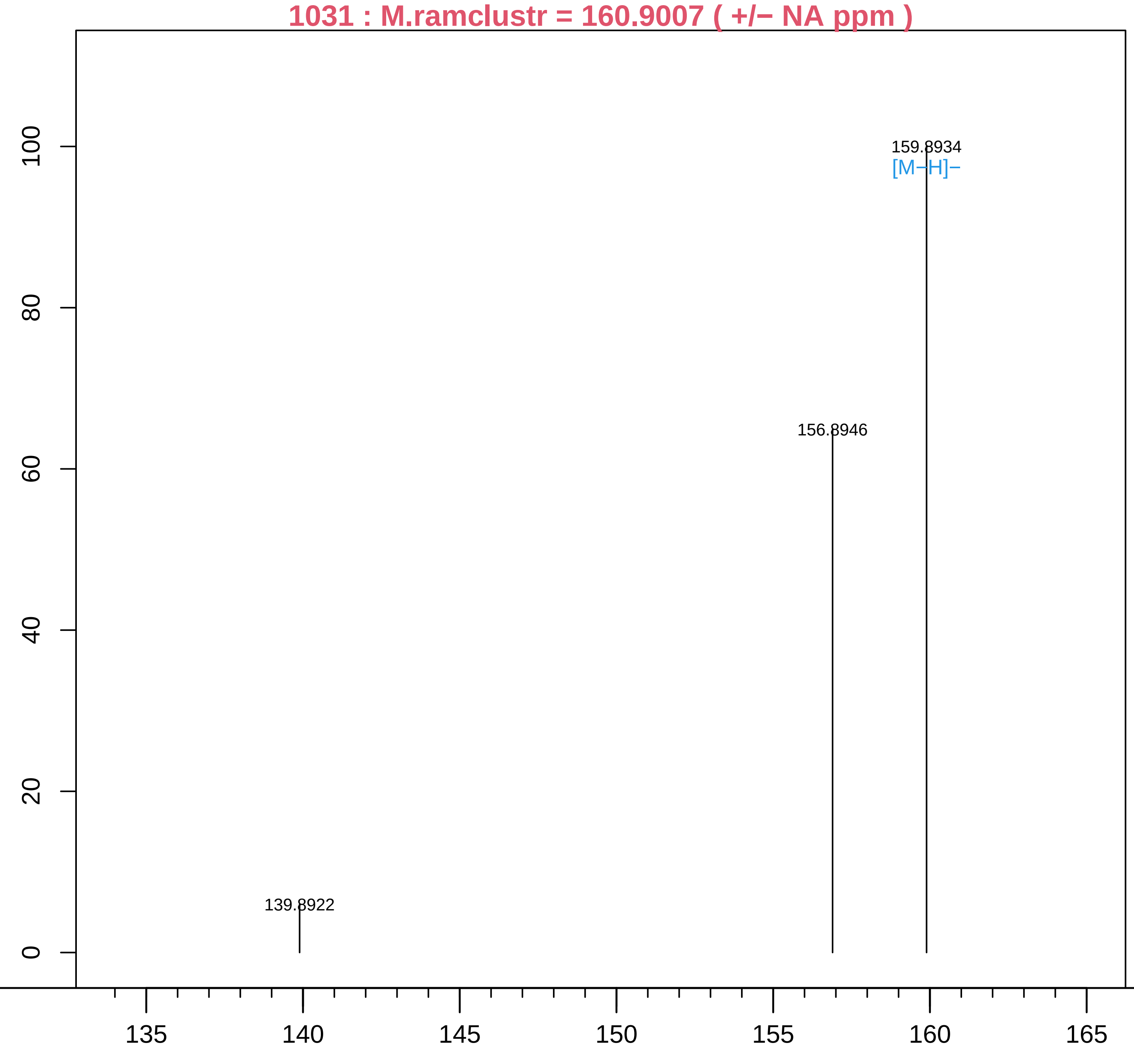
1031 : M.ramclustr = 160.9007 (+/− NA ppm)
159.8934
[M−H]−
156.8946
139.8922
0
20
40
60
80
100
135
140
145
150
155
160
165

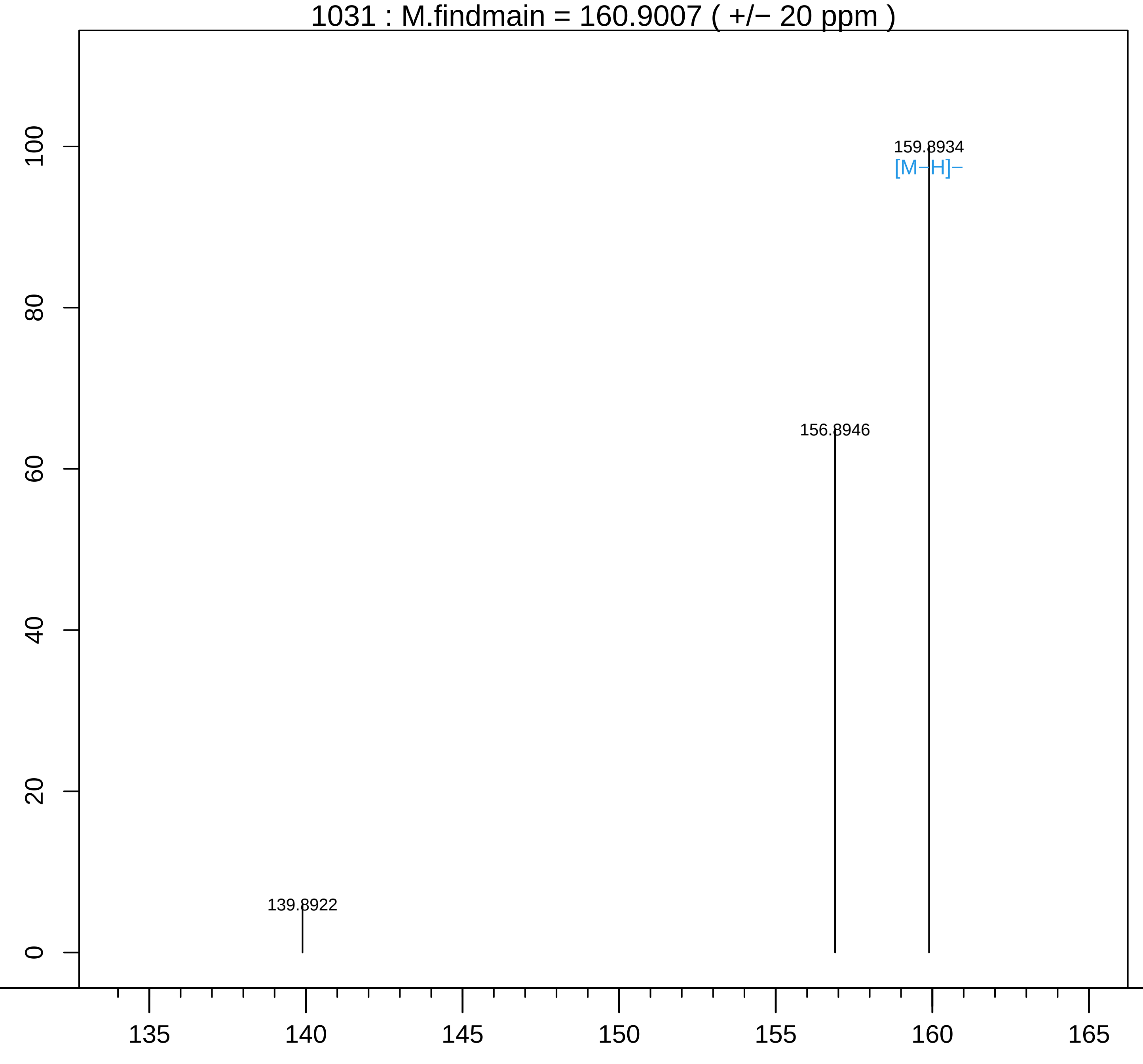
1031 : M.findmain = 160.9007 (+/− 20 ppm)
159.8934
[M−H]−
156.8946
139.8922
0
20
40
60
80
100
135
140
145
150
155
160
165

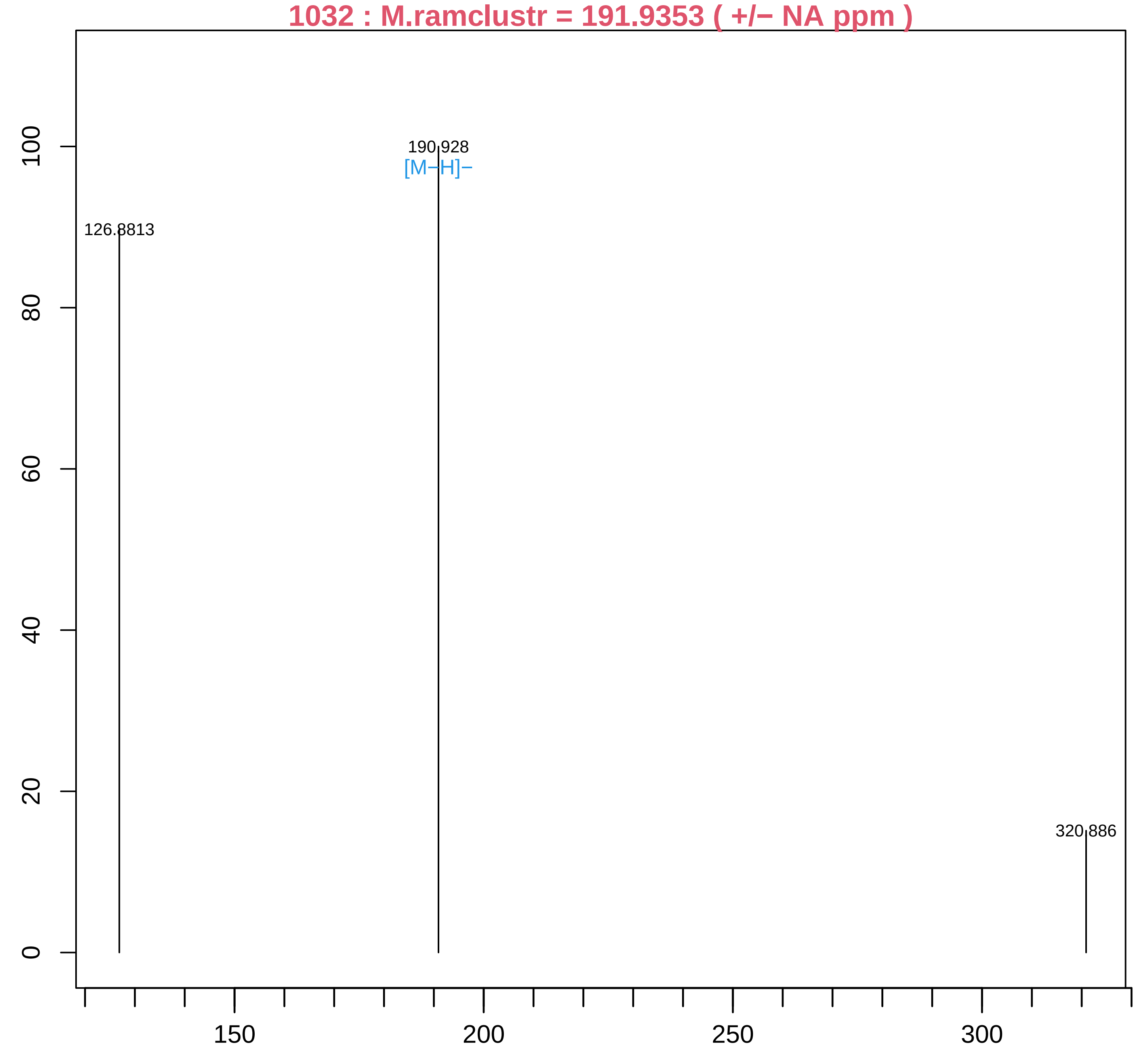
1032 : M.ramclustr = 191.9353 (+/− NA ppm)
126.8813
190.928
[M−H]−
320.886
0
20
40
60
80
100
150
200
250
300

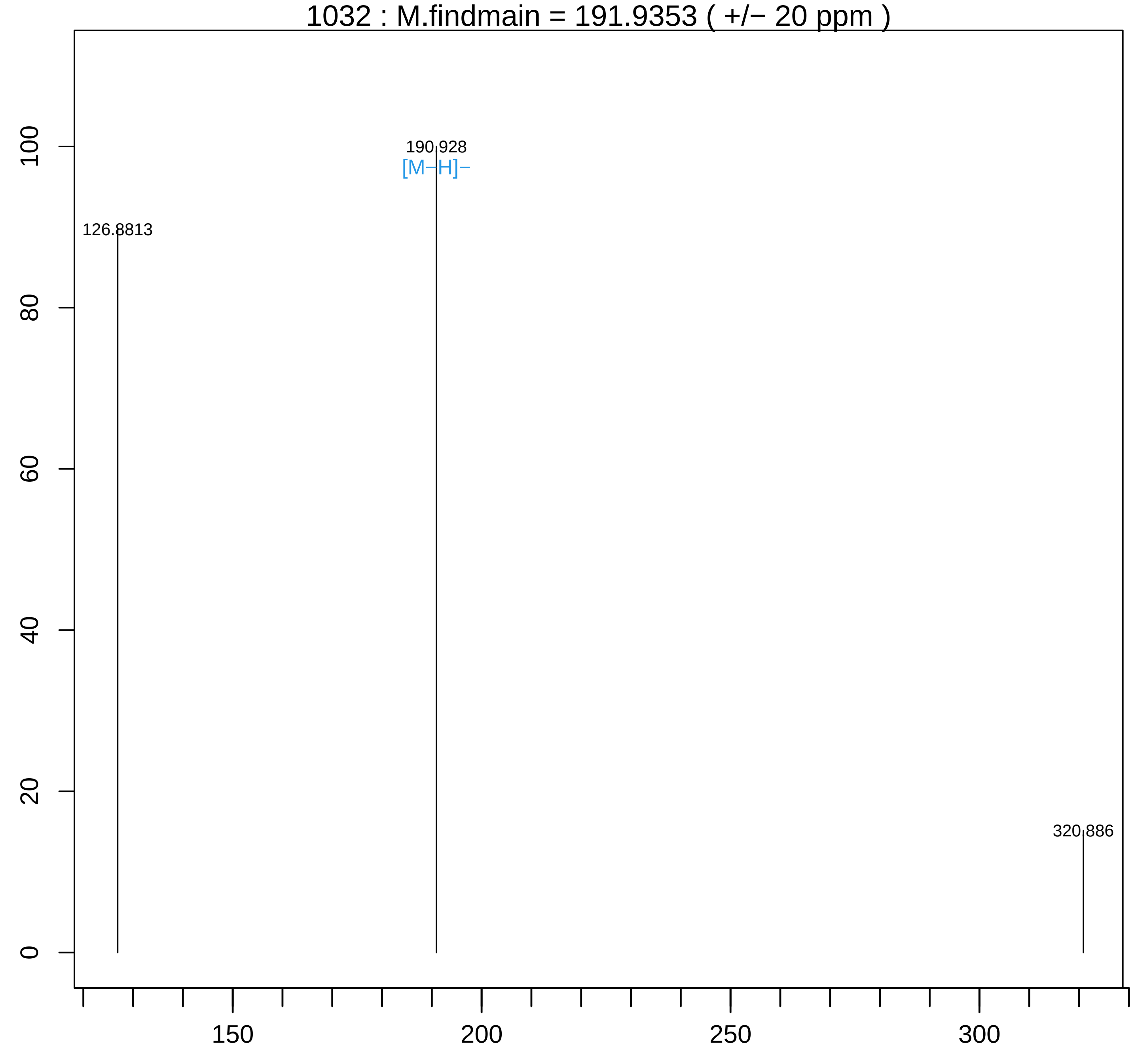
1032 : M.findmain = 191.9353 (+/− 20 ppm)
126.8813
190.928
[M−H]−
320.886
0
20
40
60
80
100
150
200
250
300

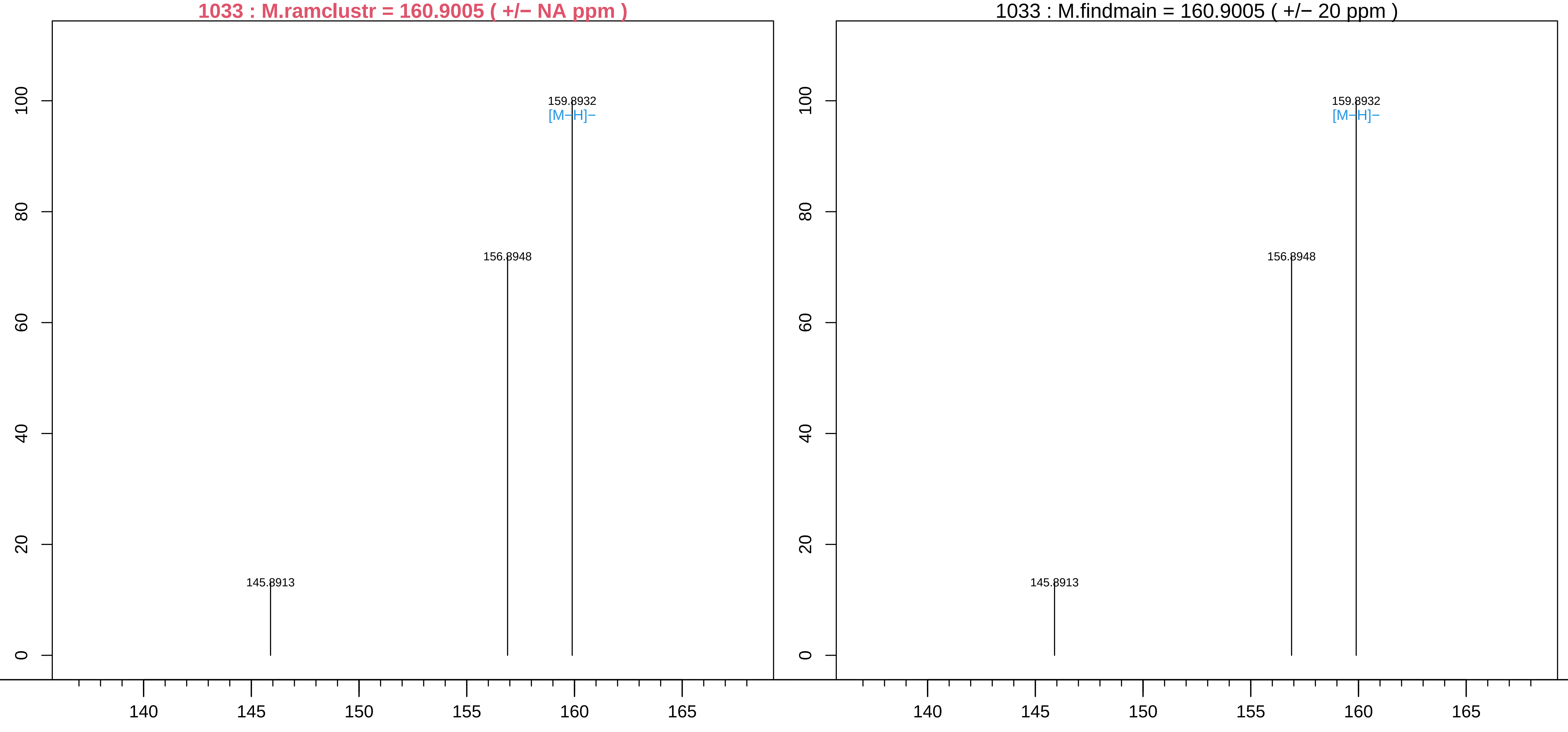

1033 : M.ramclustr = 160.9005 (+/− NA ppm)
159.8932
[M−H]−
156.8948
145.8913
1033 : M.findmain = 160.9005 (+/− 20 ppm)
159.8932
[M−H]−
156.8948
145.8913

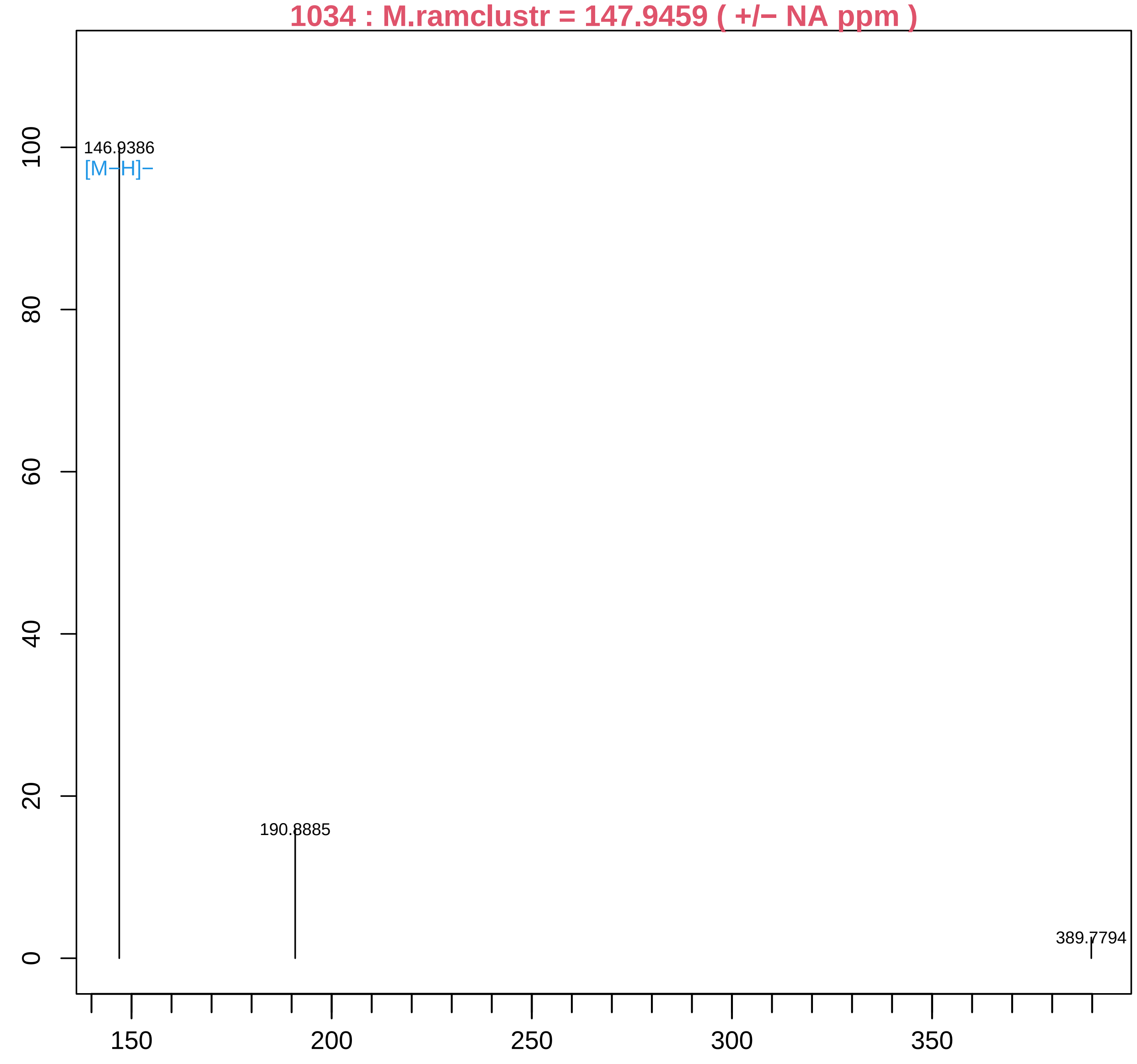

1034 : M.ramclustr = 147.9459 (+/− NA ppm)
146.9386
[M−H]−
190.8885
389.7794
0
20
40
60
80
100
150
200
250
300
350

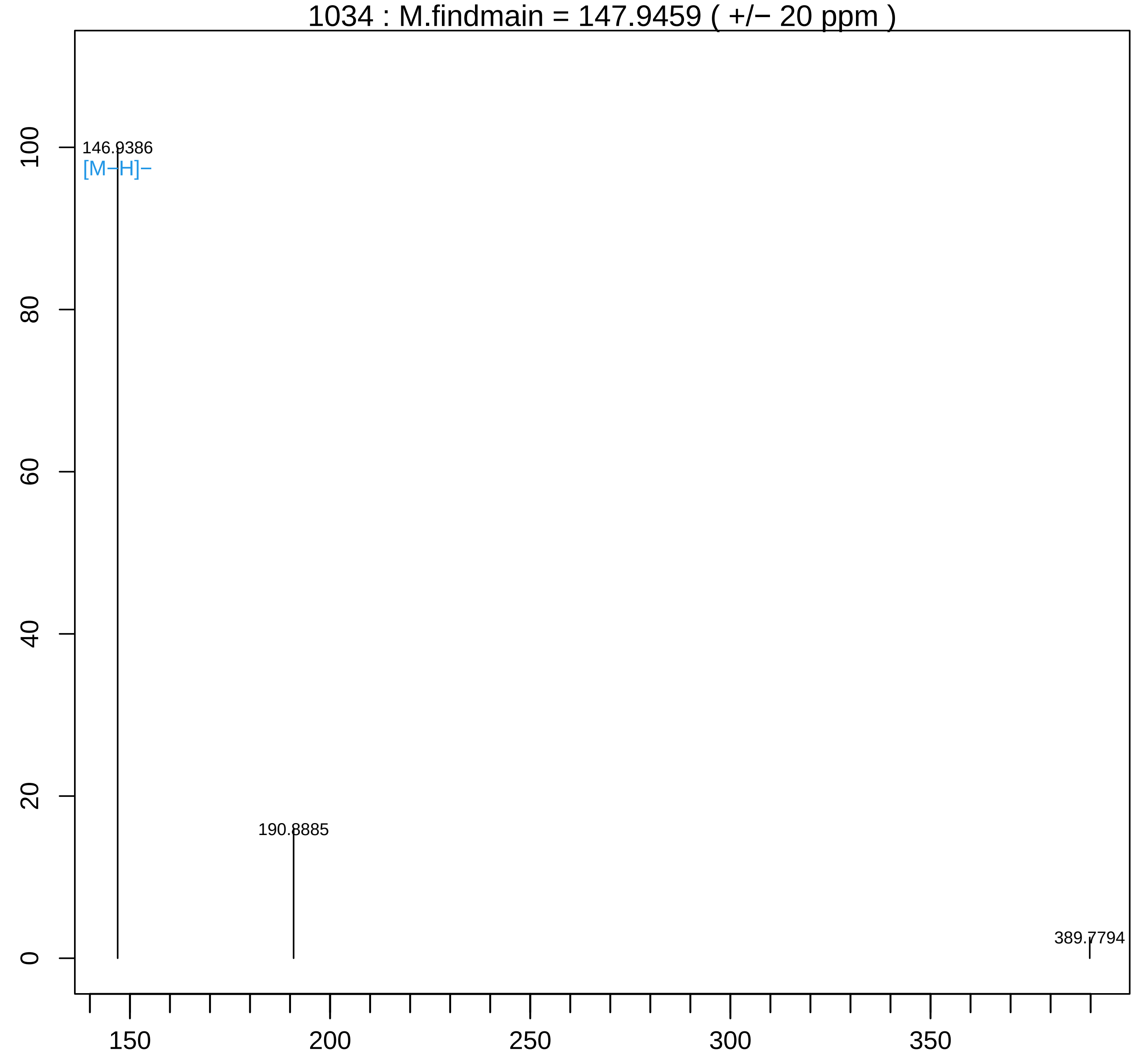

1034 : M.findmain = 147.9459 (+/− 20 ppm)
146.9386
[M−H]−
190.8885
389.7794
0
20
40
60
80
100
150
200
250
300
350

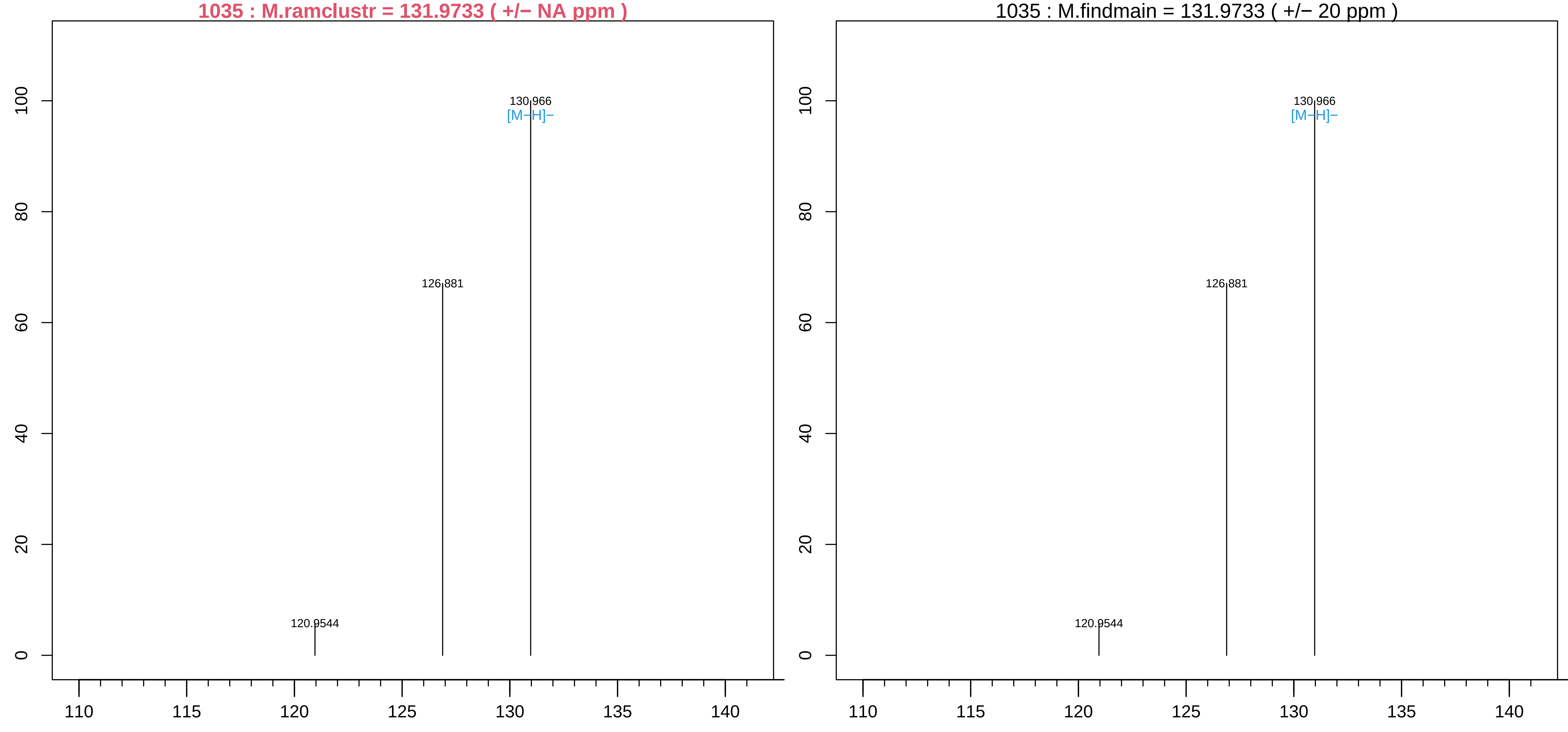
1035 : M.ramclustr = 131.9733 (+/− NA ppm)
130.966
[M−H]−
126.881
120.9544
1035 : M.findmain = 131.9733 (+/− 20 ppm)
130.966
[M−H]−
126.881
120.9544
0
20
40
60
80
100
110
115
120
125
130
135
140

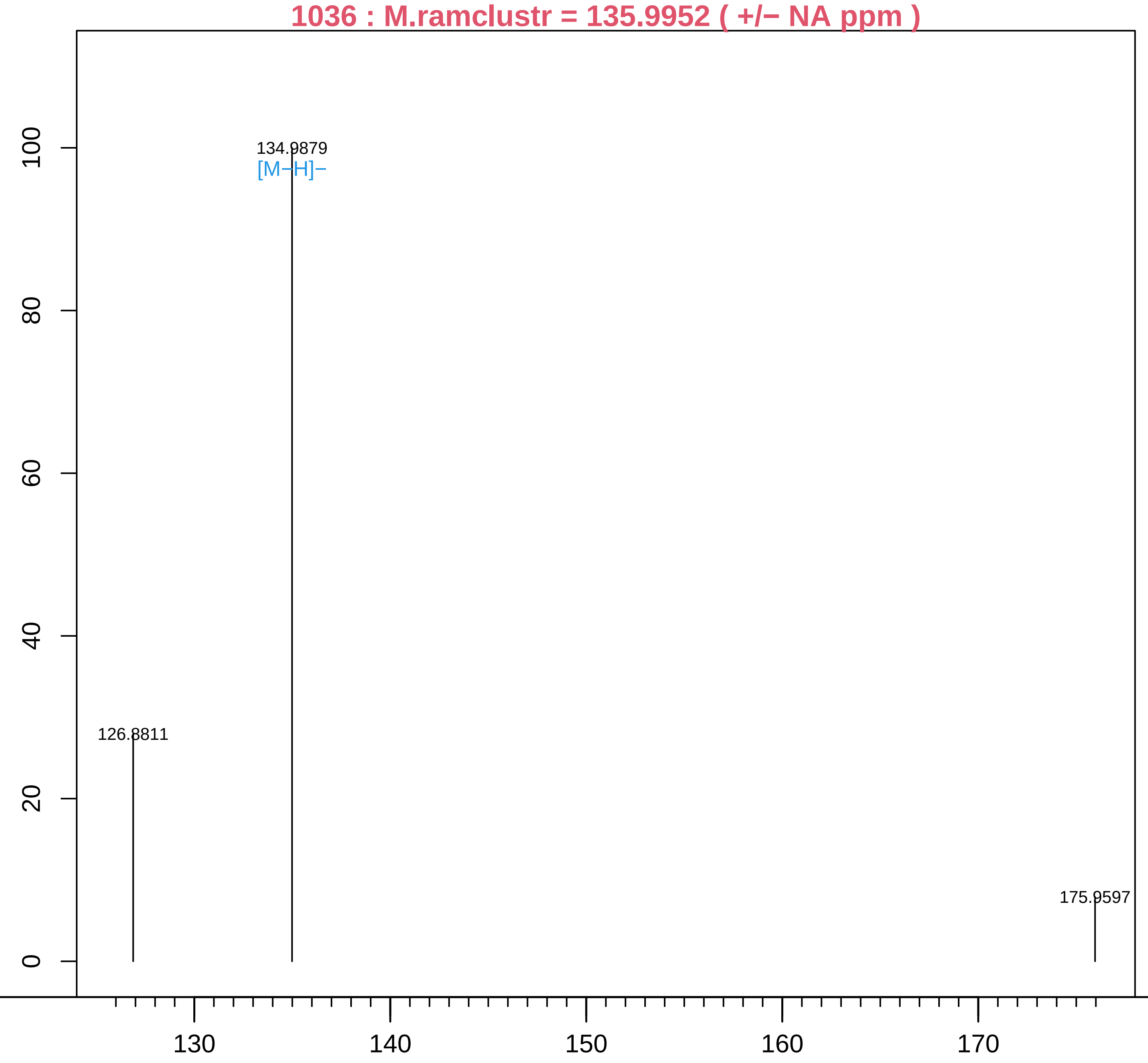
1036 : M.ramclustr = 135.9952 (+/− NA ppm)
134.9879
[M−H]−
126.8811
175.9597
0
20
40
60
80
100
130
140
150
160
170

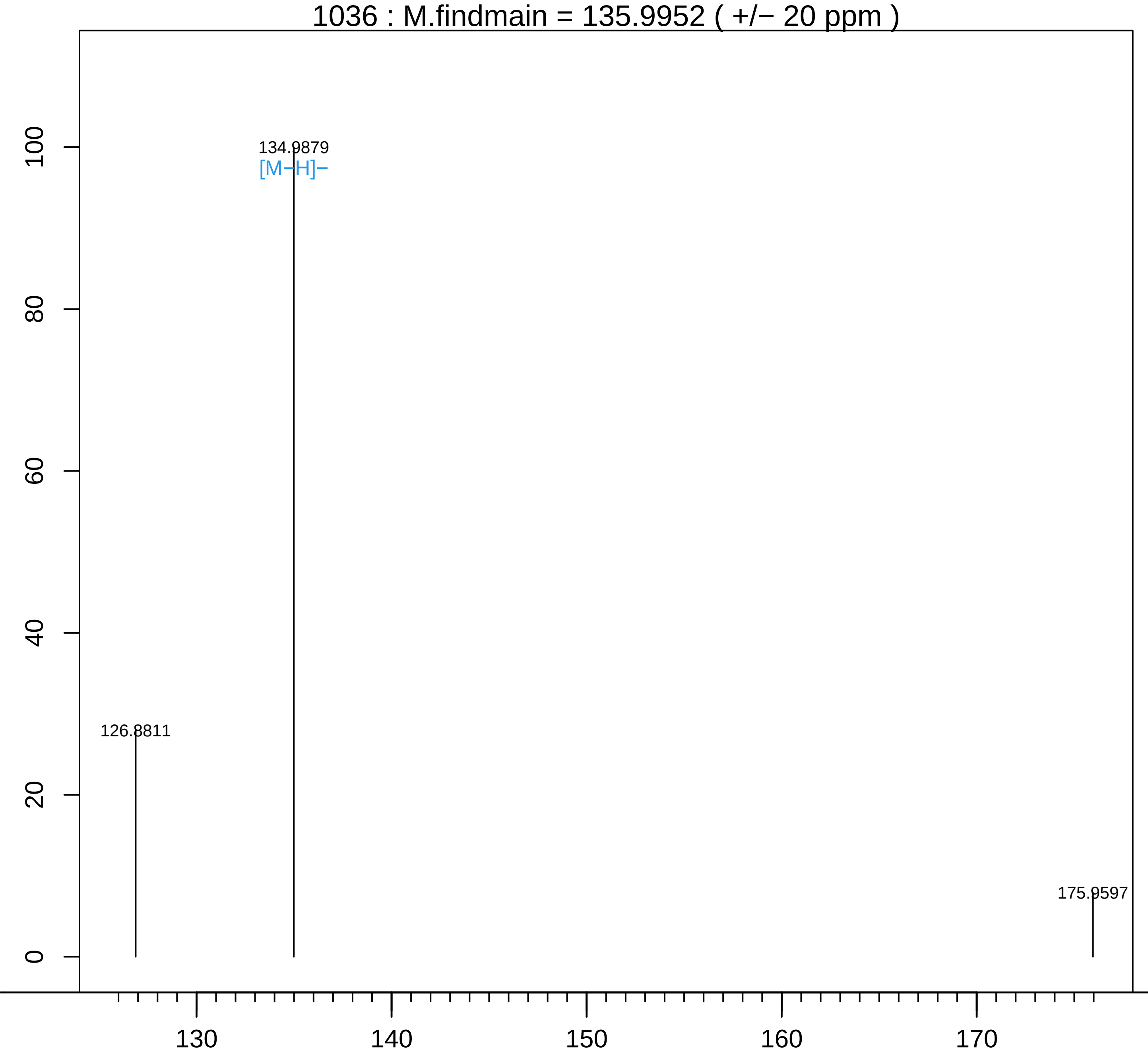
1036 : M.findmain = 135.9952 (+/− 20 ppm)
134.9879
[M−H]−
126.8811
175.9597
0
20
40
60
80
100
130
140
150
160
170

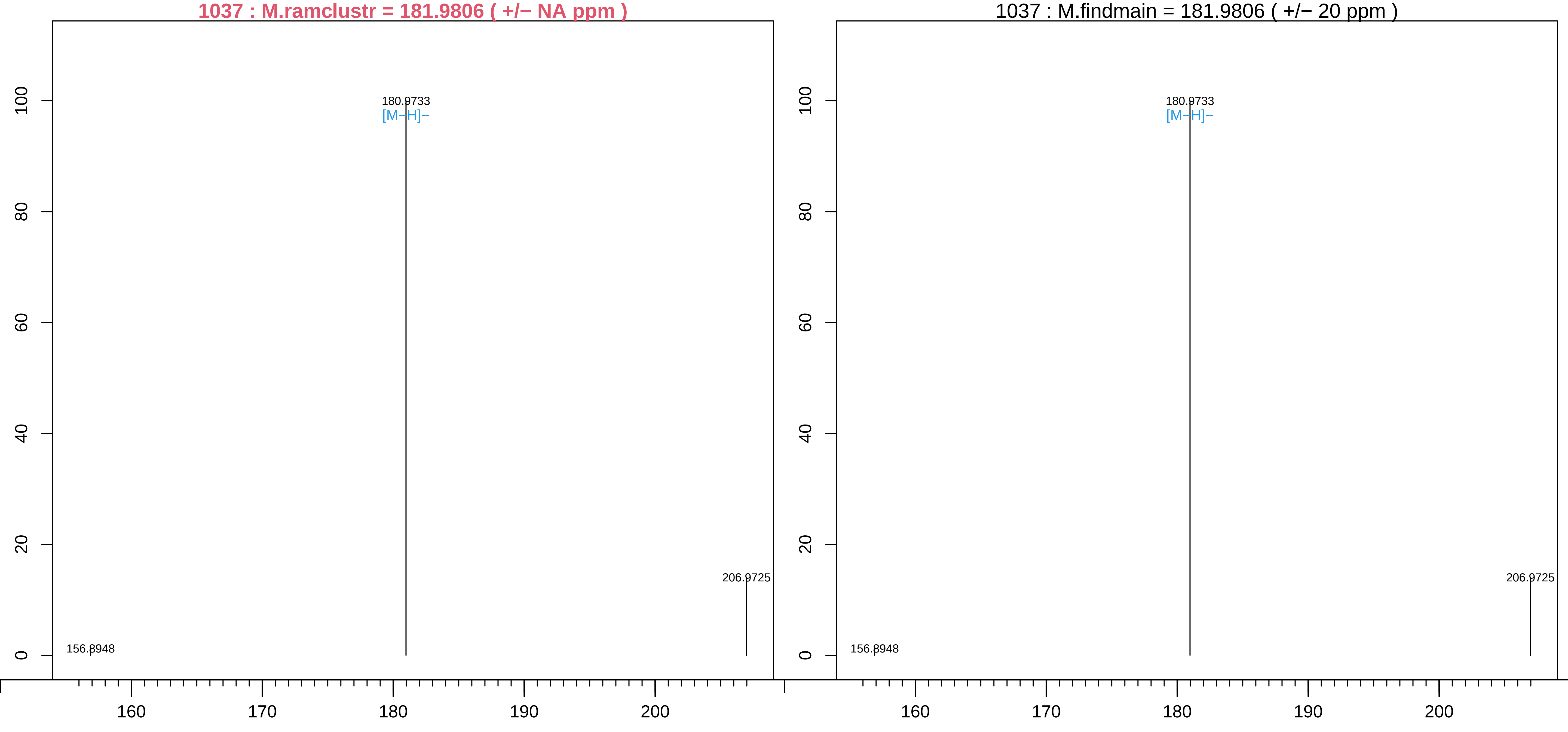
1037 : M.ramclustr = 181.9806 (+/− NA ppm)
180.9733
[M−H]−
206.9725
156.8948
0
20
40
60
80
100
160
170
180
190
200
1037 : M.findmain = 181.9806 (+/− 20 ppm)
180.9733
[M−H]−
206.9725
156.8948

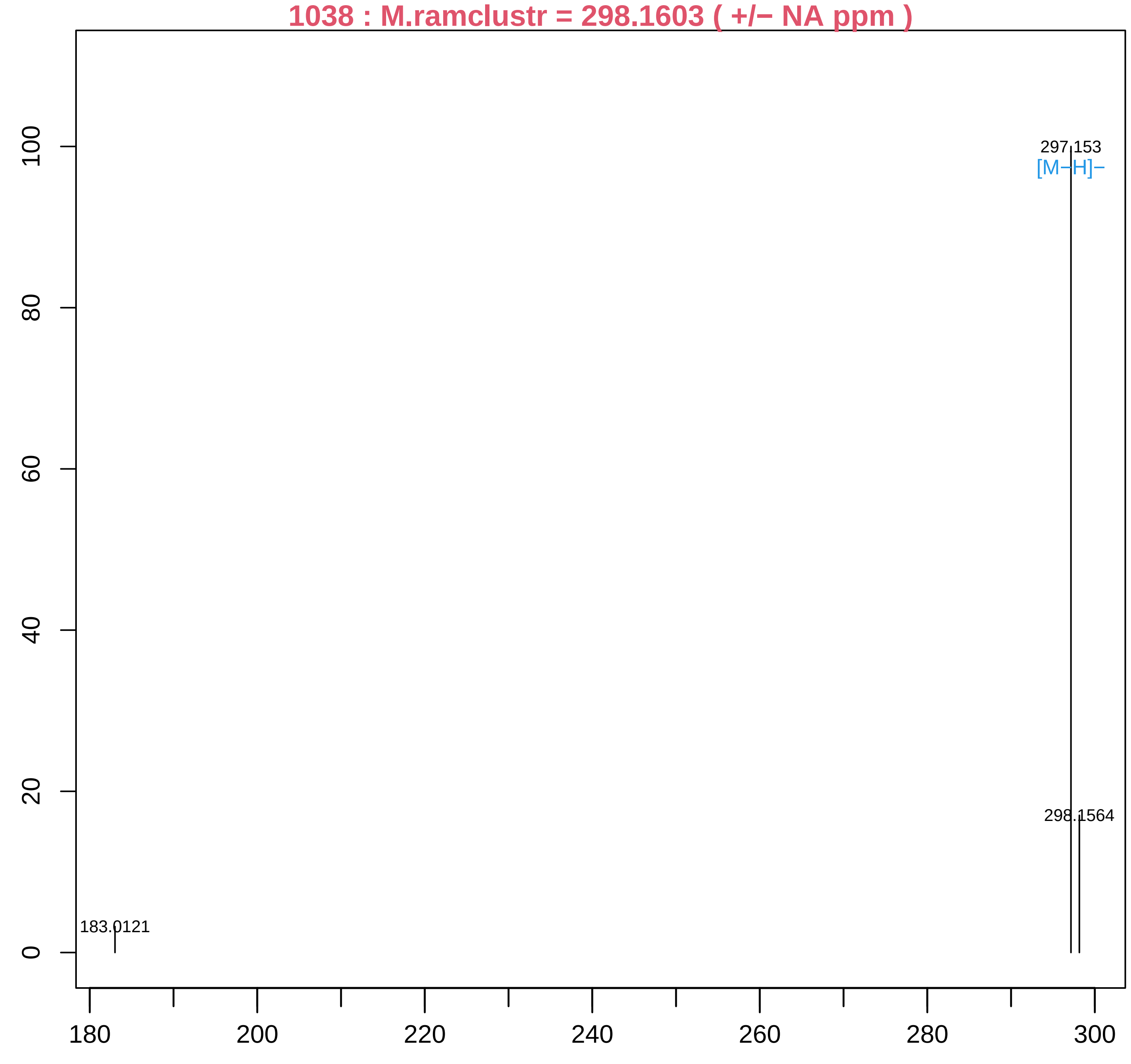
1038 : M.ramclustr = 298.1603 (+/− NA ppm)
297.153
[M−H]−
298.1564
183.0121
0
20
40
60
80
100
180
200
220
240
260
280
300

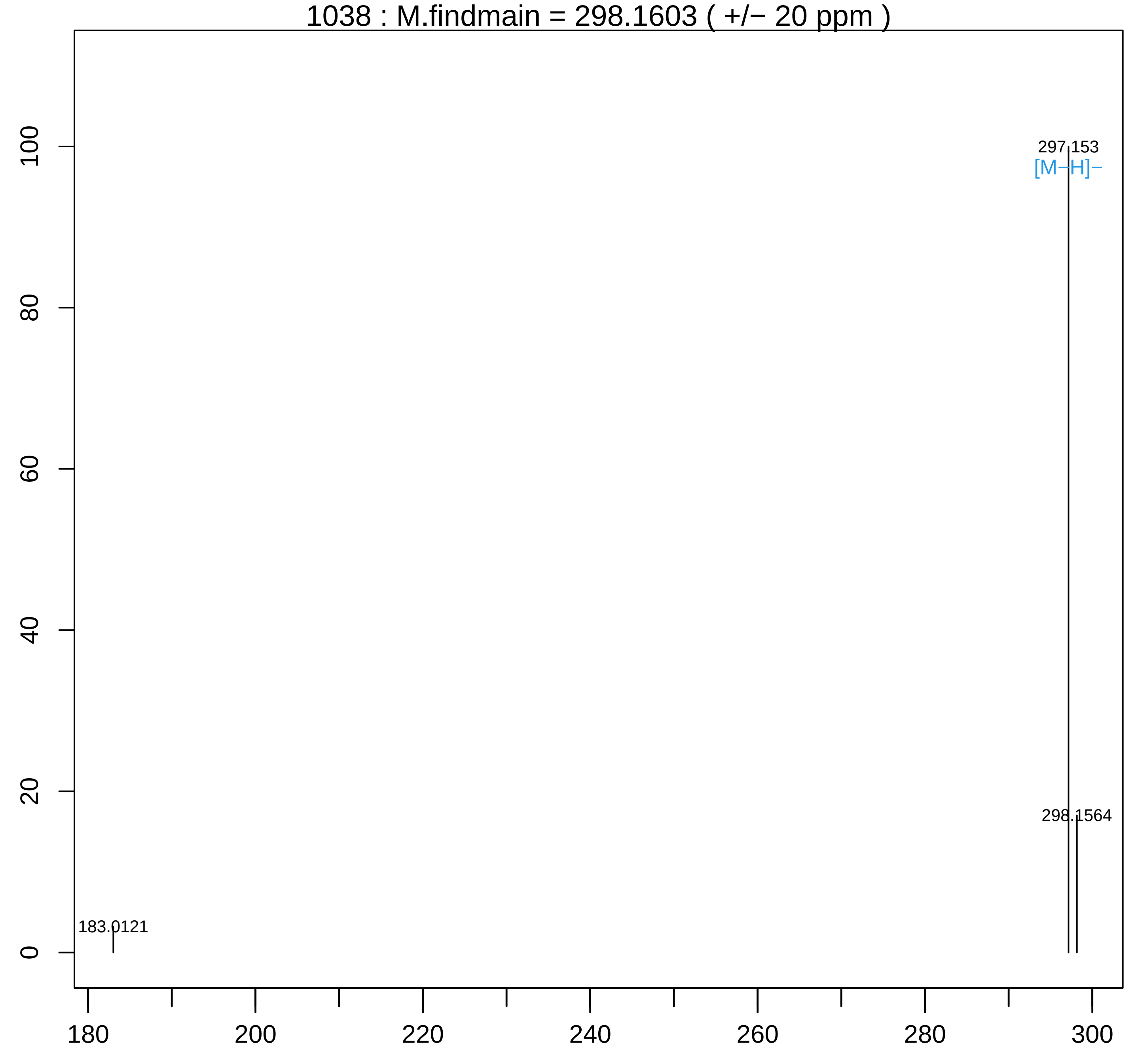
1038 : M.findmain = 298.1603 (+/− 20 ppm)
297.153
[M−H]−
298.1564
183.0121
0
20
40
60
80
100
180
200
220
240
260
280
300

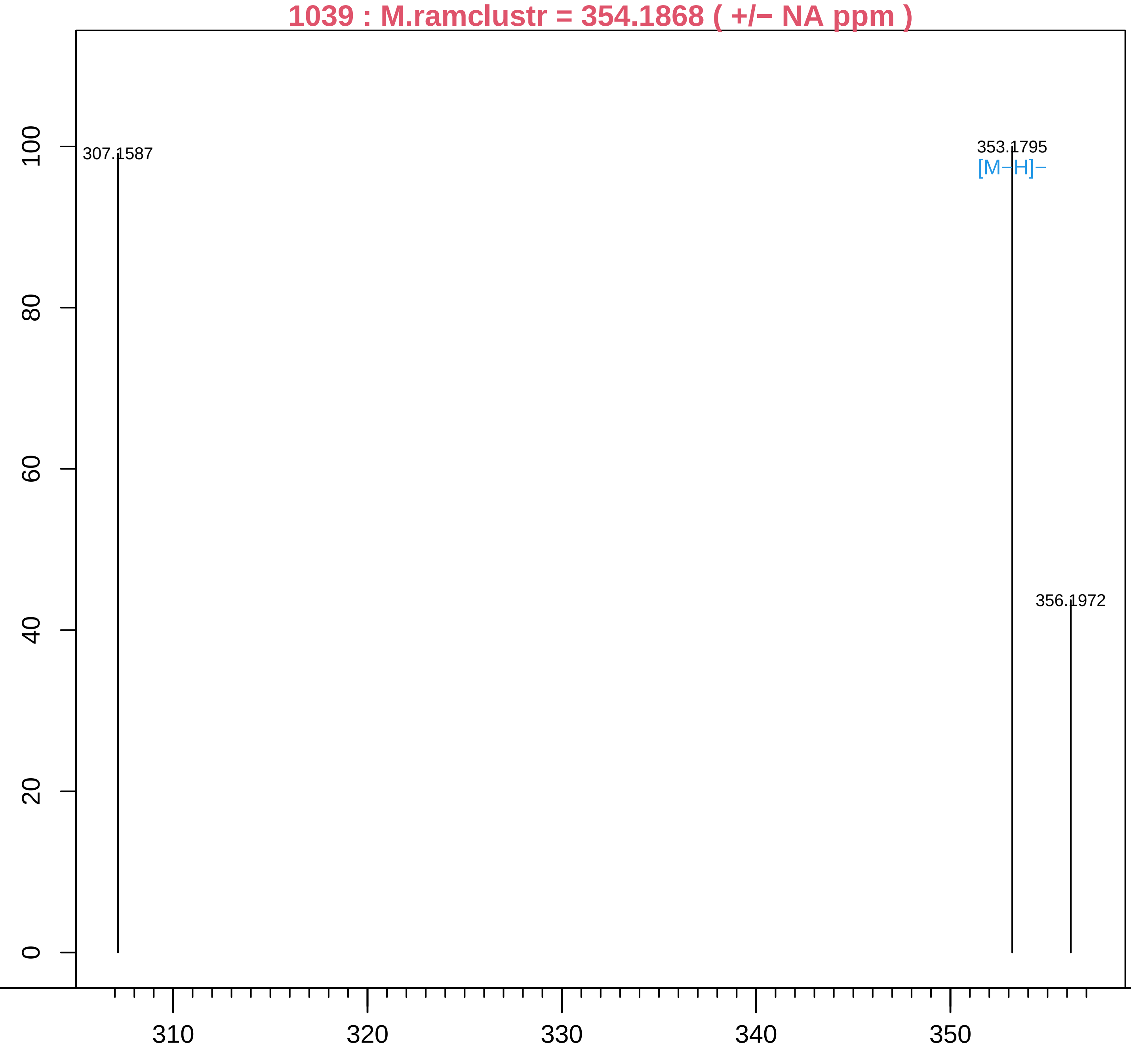
1039 : M.ramclustr = 354.1868 (+/− NA ppm)
307.1587
353.1795
[M−H]−
356.1972
0
20
40
60
80
100
310
320
330
340
350

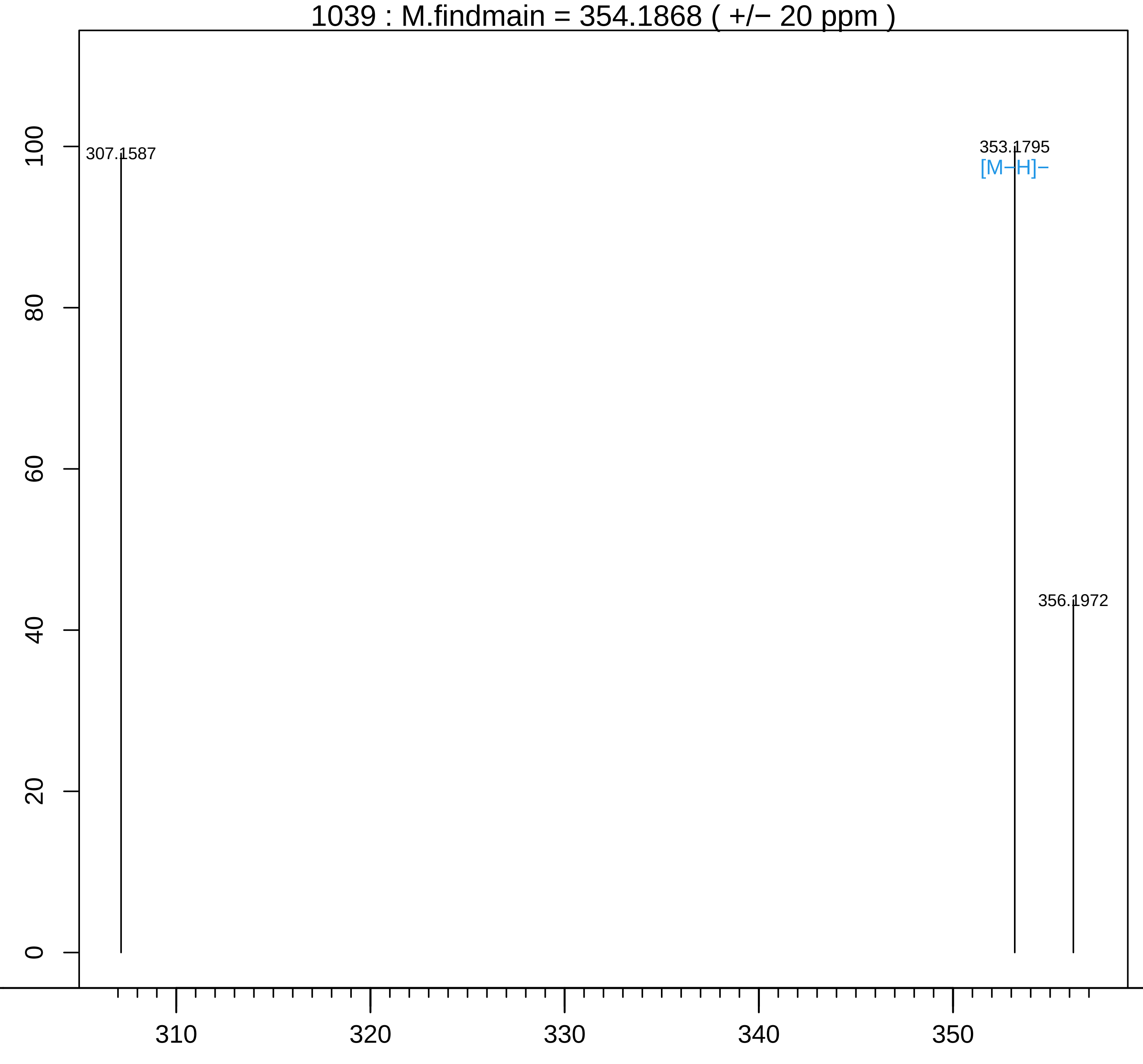
1039 : M.findmain = 354.1868 (+/− 20 ppm)
307.1587
353.1795
[M−H]−
356.1972
0
20
40
60
80
100
310
320
330
340
350

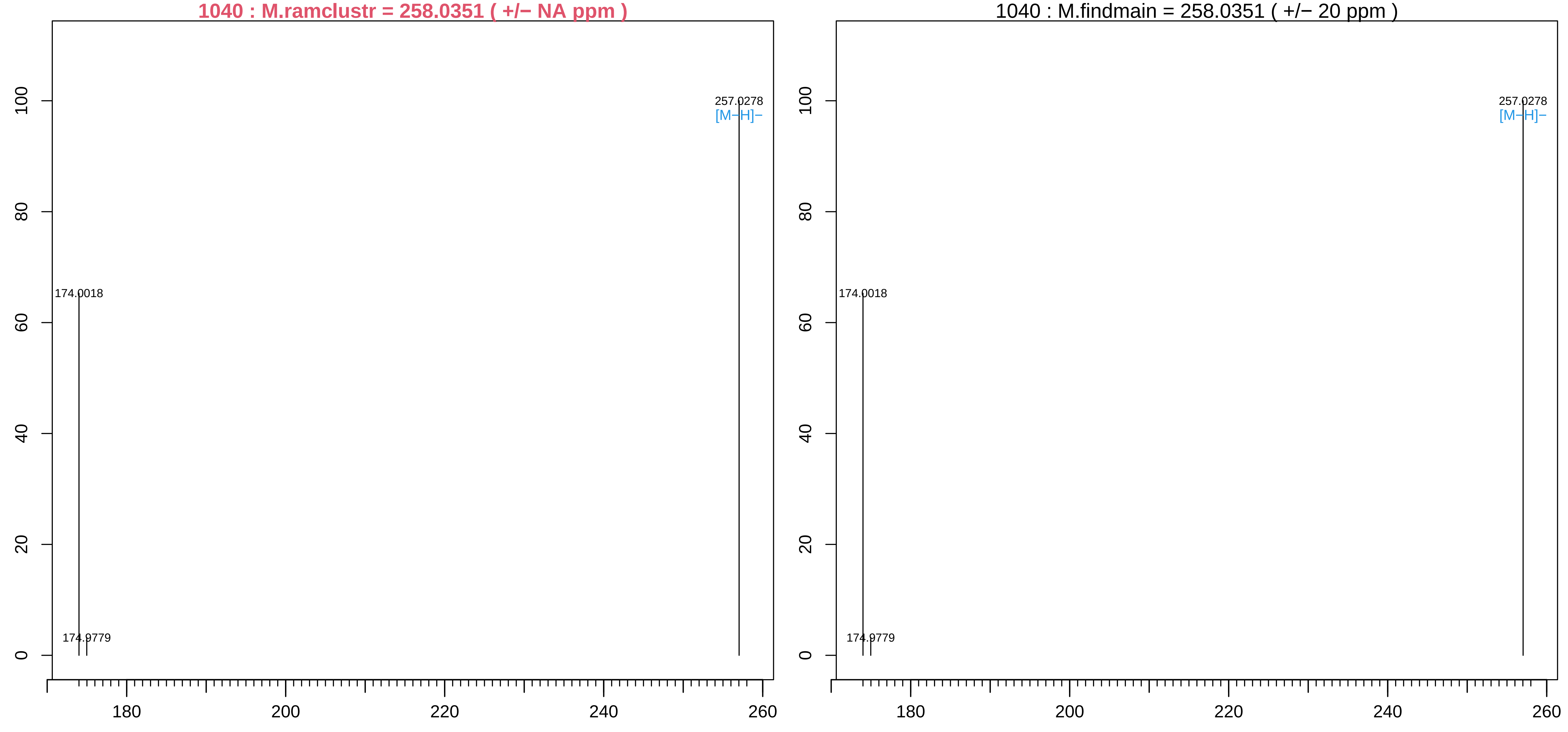
1040 : M.ramclustr = 258.0351 (+/− NA ppm)
1040 : M.findmain = 258.0351 (+/− 20 ppm)
257.0278
[M−H]−
174.0018
174.9779
257.0278
[M−H]−
174.0018
174.9779
0
20
40
60
80
100
180
200
220
240
260

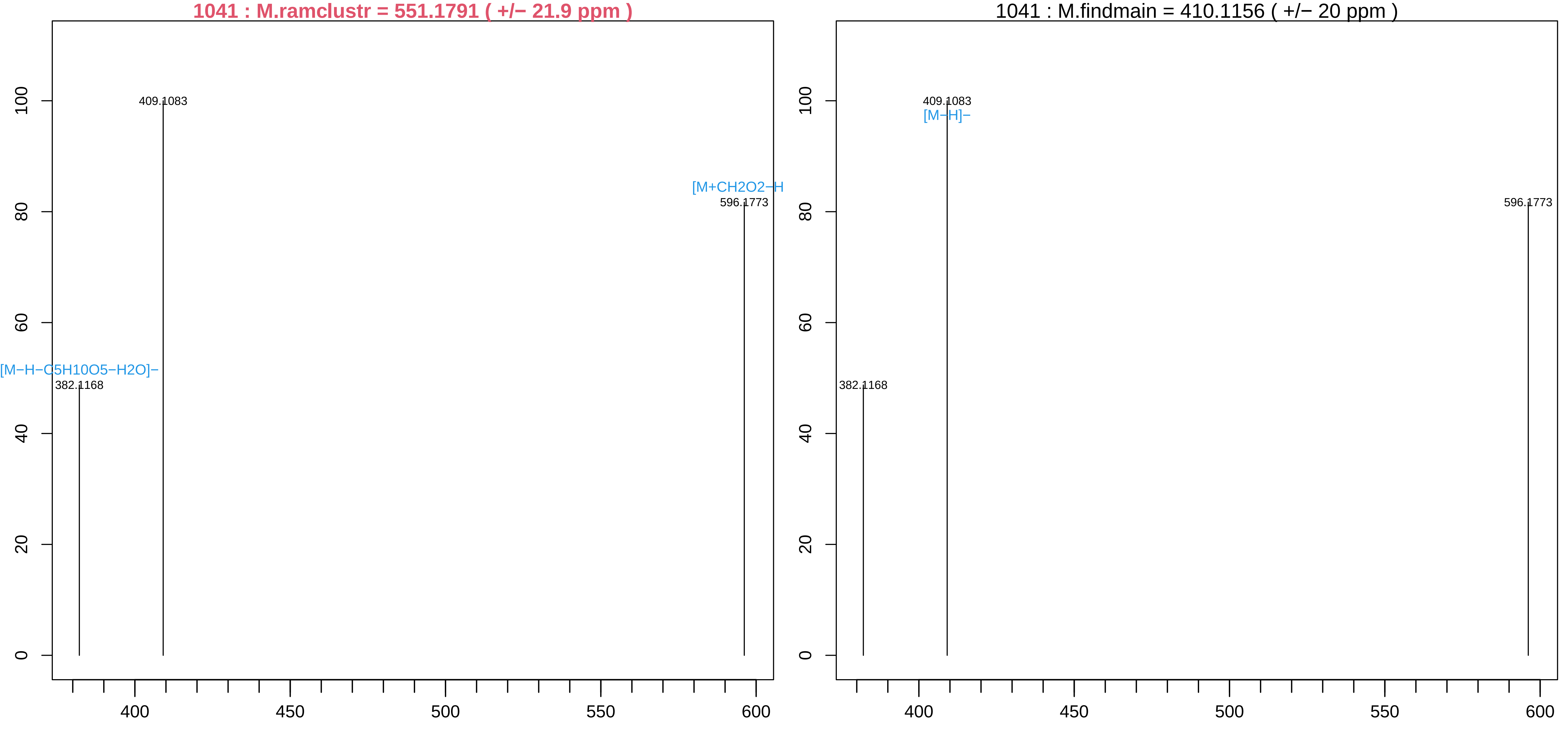

1041 : M.ramclustr = 551.1791 (+/− 21.9 ppm)
409.1083
[M+CH2O2−H
596.1773
[M−H−C5H10O5−H2O]−
382.1168
1041 : M.findmain = 410.1156 (+/− 20 ppm)
409.1083
[M−H]−
596.1773
382.1168

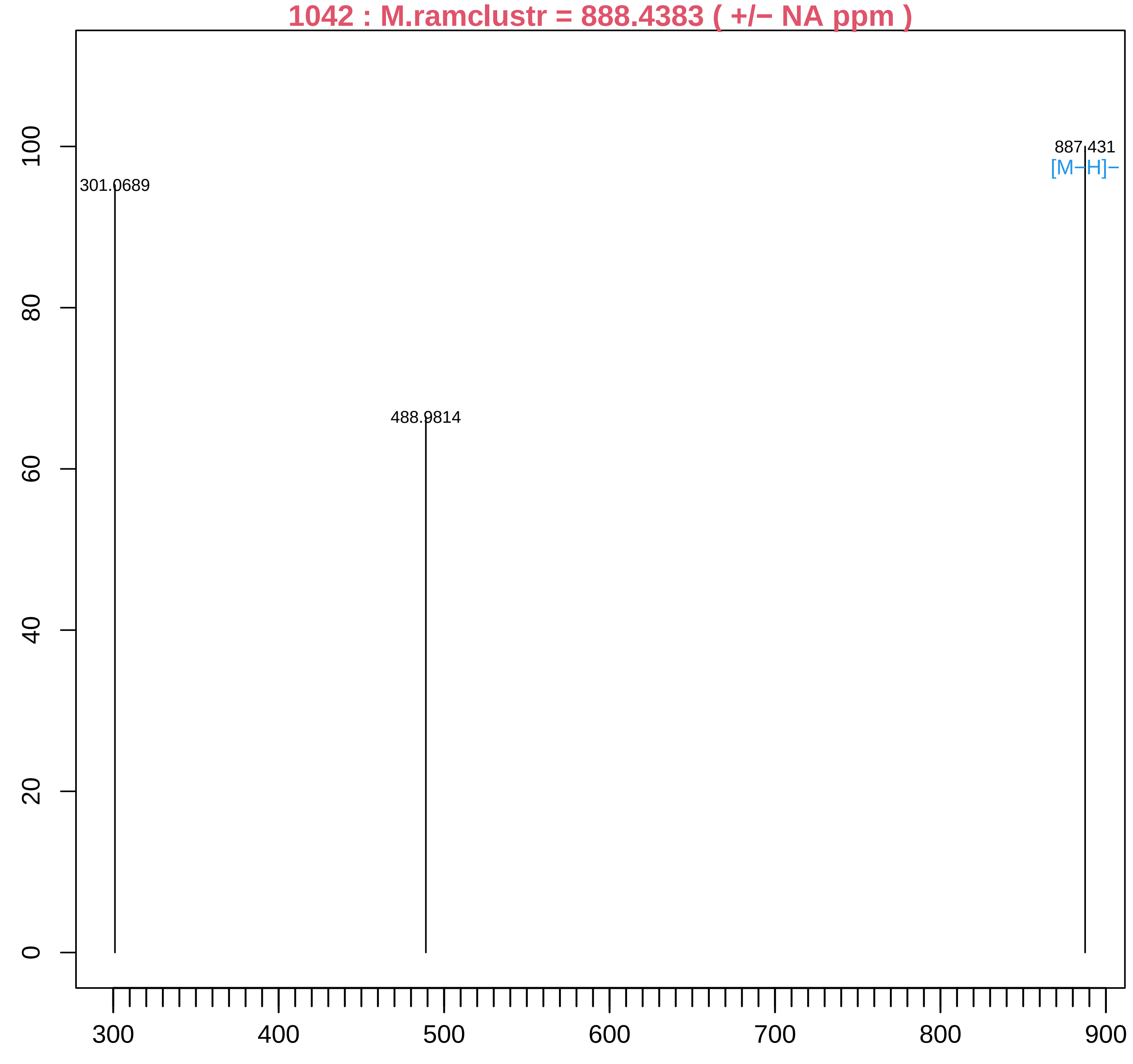
1042 : M.ramclustr = 888.4383 (+/− NA ppm)
301.0689
488.9814
887.431
[M−H]−
100
80
60
40
20
0
300
400
500
600
700
800
900

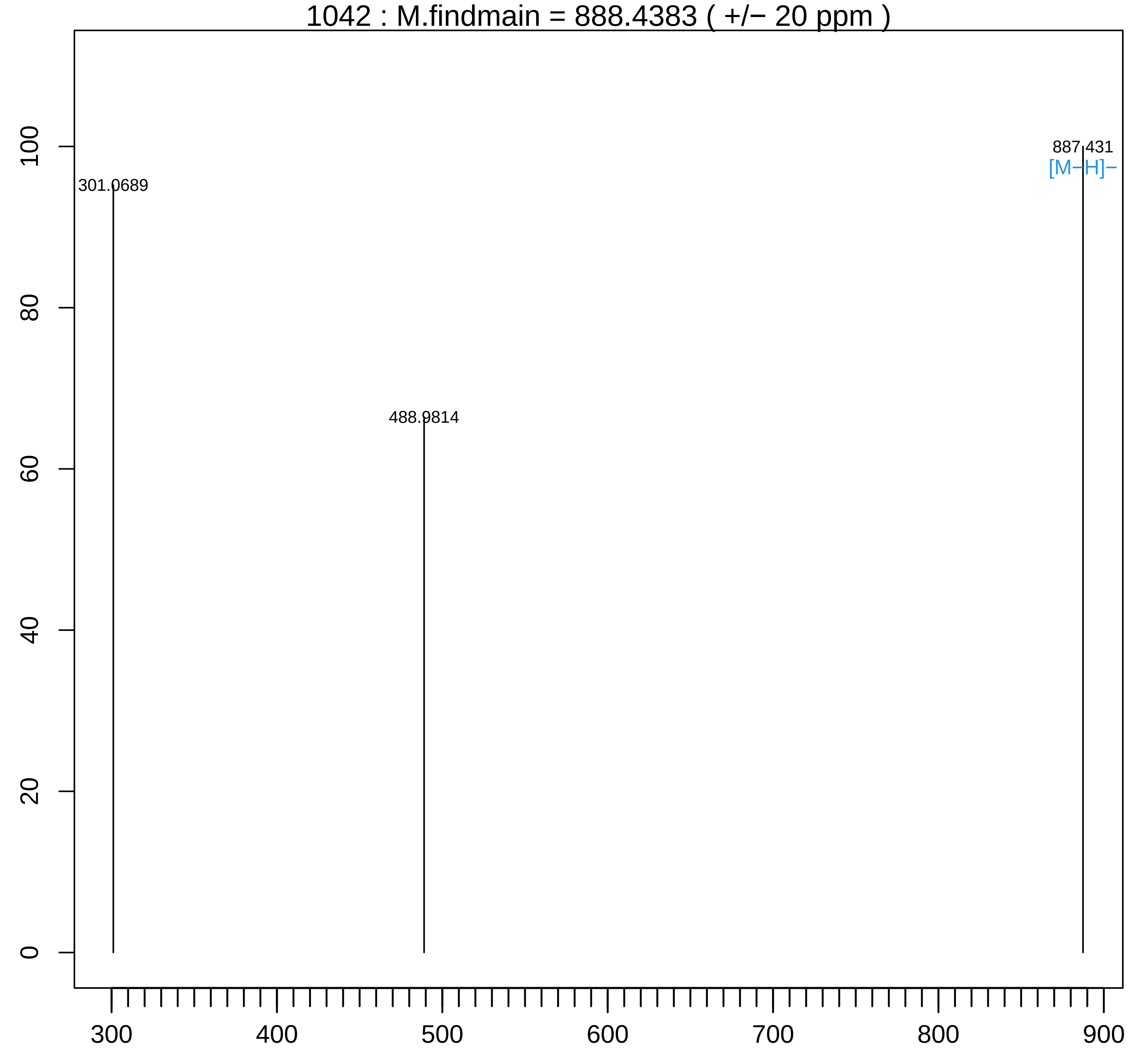
1042 : M.findmain = 888.4383 (+/− 20 ppm)
301.0689
488.9814
887.431
[M−H]−
100
80
60
40
20
0
300
400
500
600
700
800
900

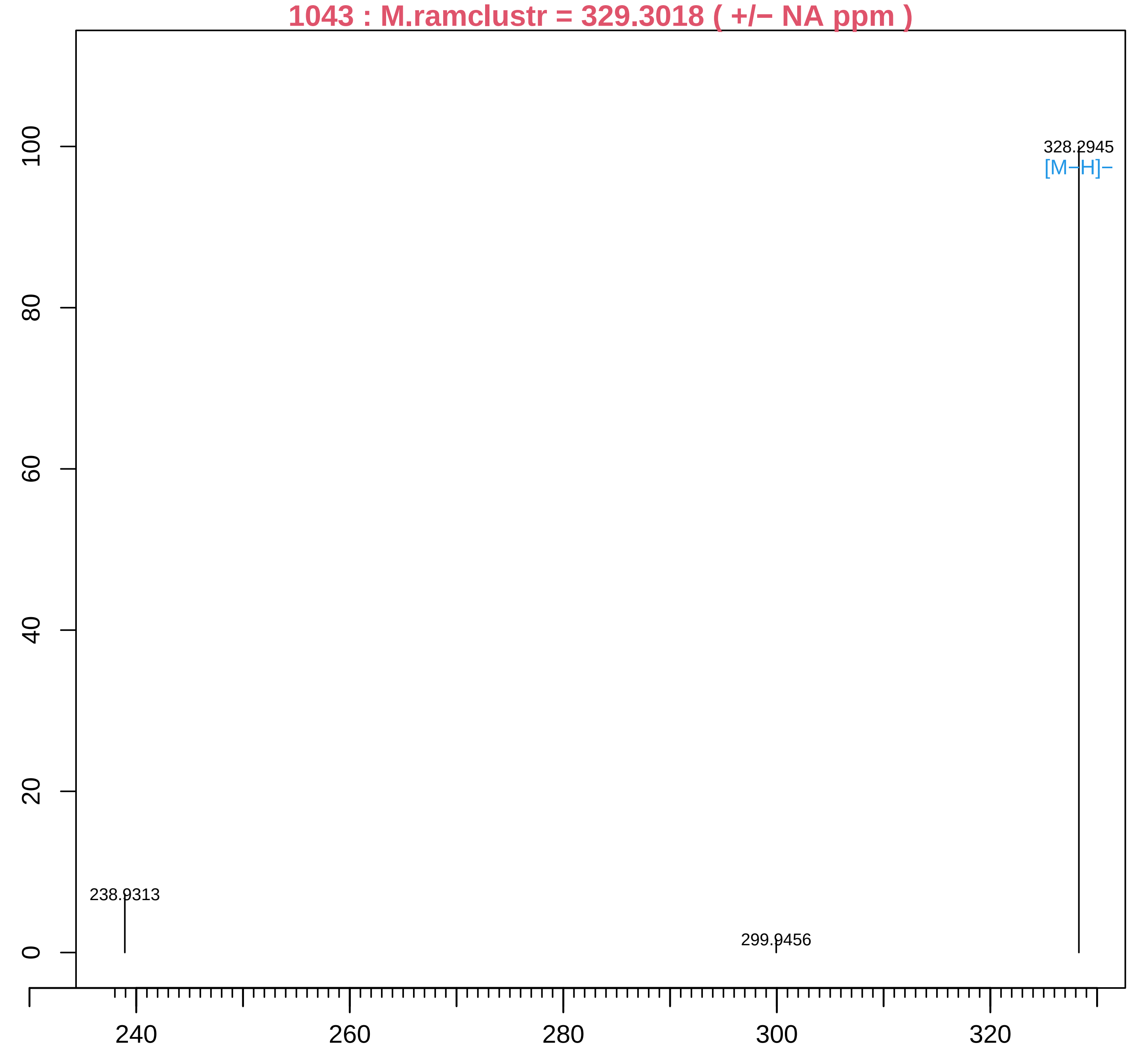
1043 : M.ramclustr = 329.3018 (+/− NA ppm)
328.2945
[M−H]−
238.9313
299.9456
0
20
40
60
80
100
240
260
280
300
320

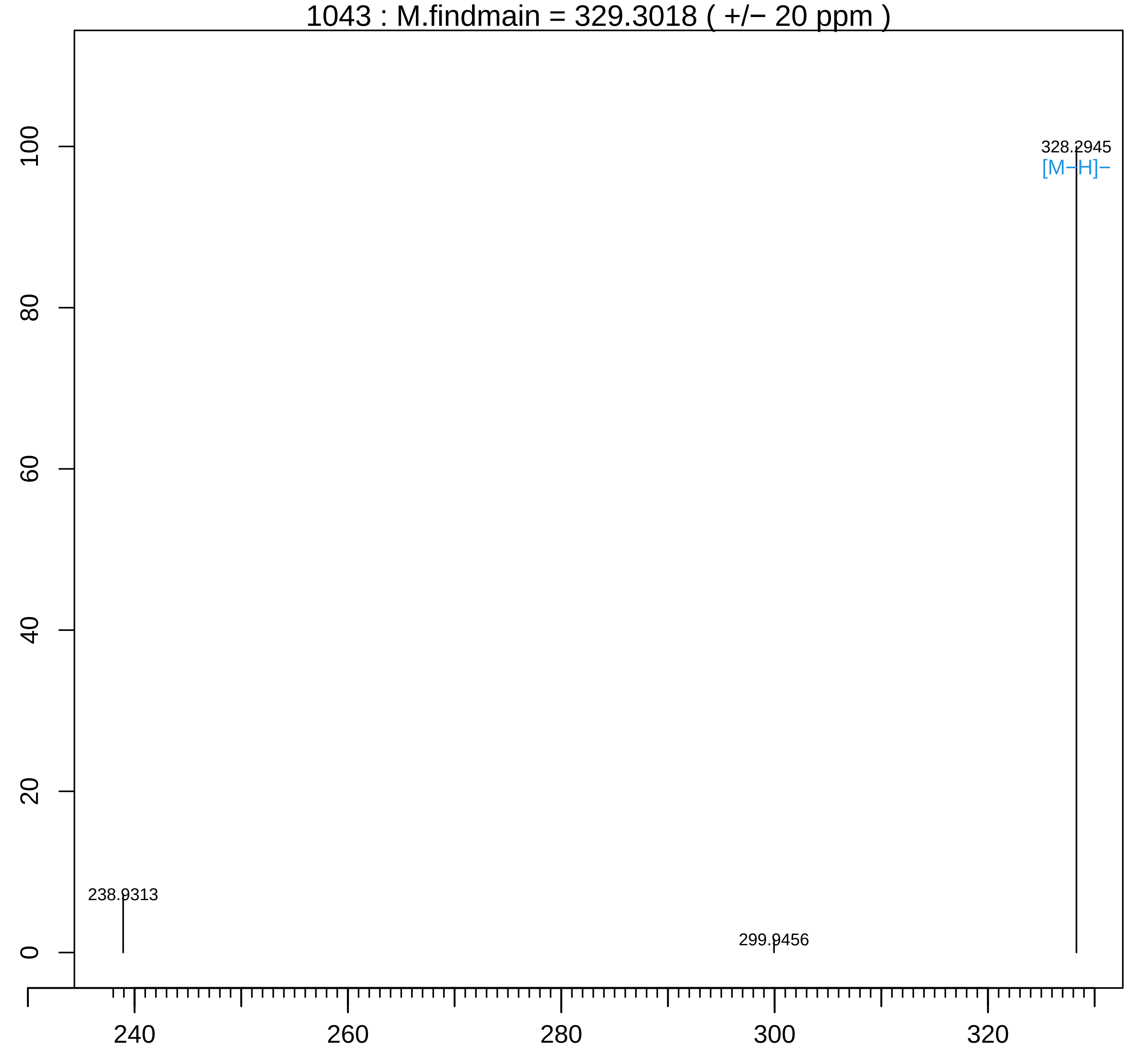
1043 : M.findmain = 329.3018 (+/− 20 ppm)
328.2945
[M−H]−
238.9313
299.9456
0
20
40
60
80
100
240
260
280
300
320

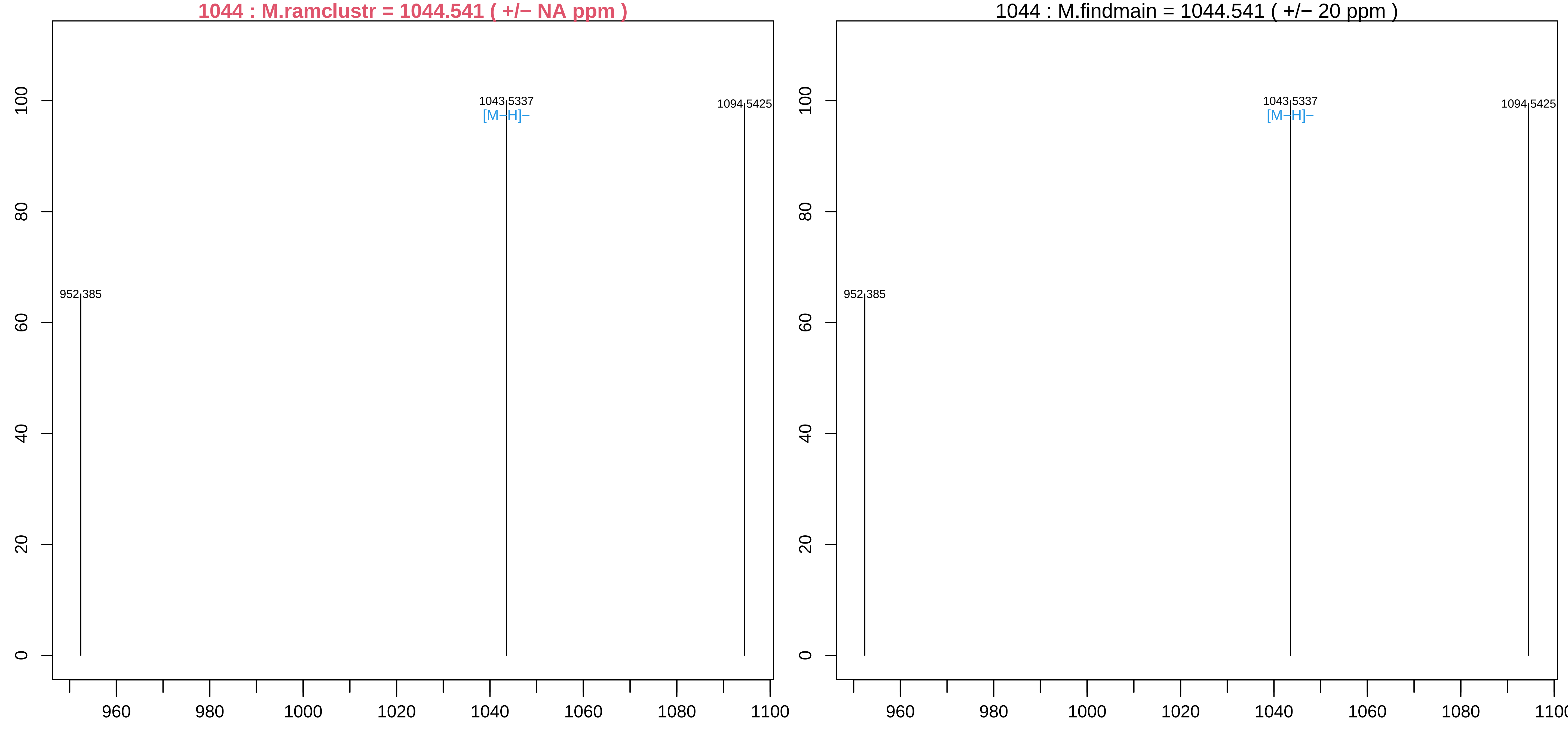
1044 : M.ramclustr = 1044.541 (+/− NA ppm)
1044 : M.findmain = 1044.541 (+/− 20 ppm)
952.385
1043.5337
[M−H]−
1094.5425
0
20
40
60
80
100
960
980
1000
1020
1040
1060
1080
1100

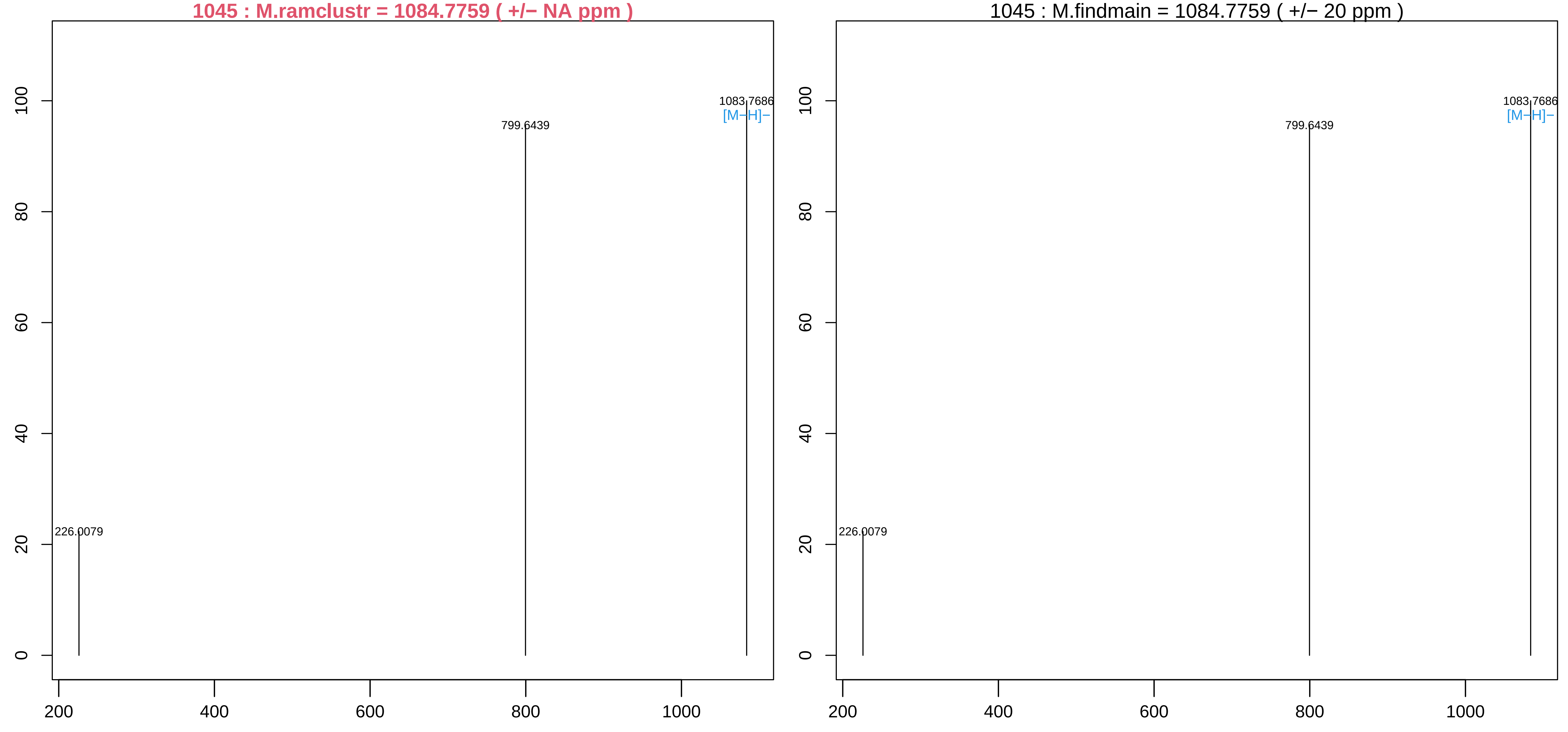
1045 : M.ramclustr = 1084.7759 (+/− NA ppm)
1083.7686
[M−H]−
799.6439
226.0079
1045 : M.findmain = 1084.7759 (+/− 20 ppm)
1083.7686
[M−H]−
799.6439
226.0079

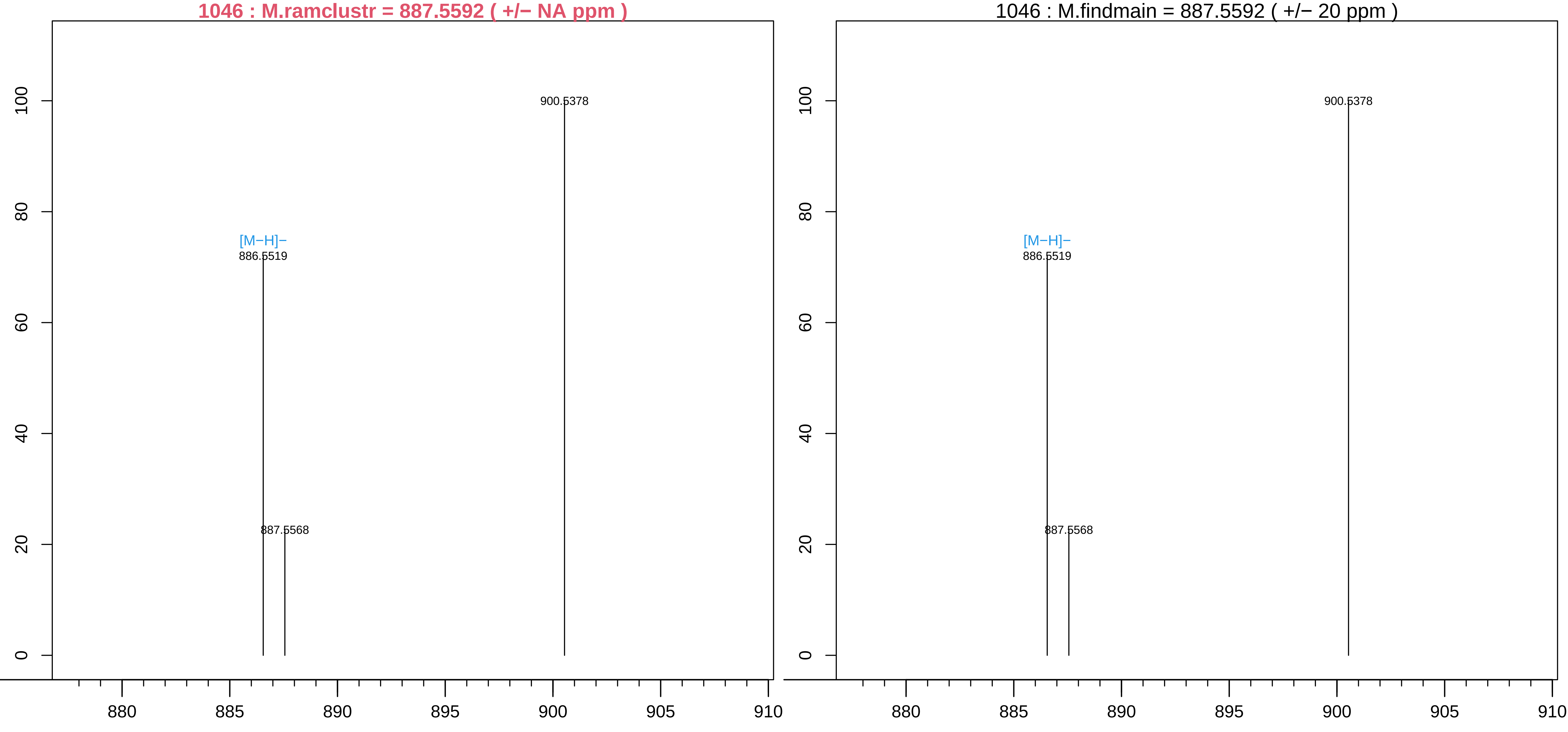
1046 : M.ramclustr = 887.5592 (+/− NA ppm)
[M−H]−
886.5519
887.5568
900.5378
1046 : M.findmain = 887.5592 (+/− 20 ppm)
[M−H]−
886.5519
887.5568
900.5378

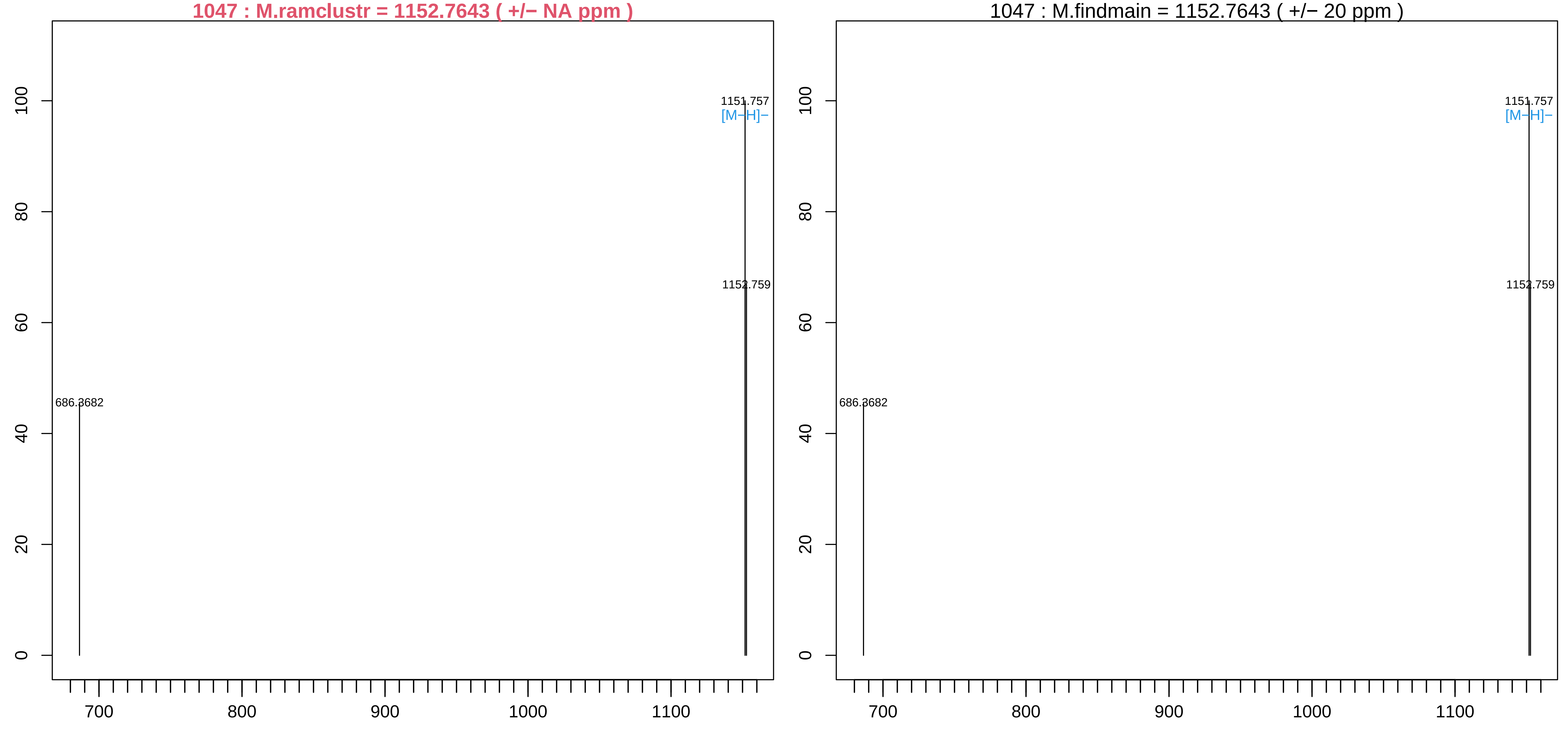
1047 : M.ramclustr = 1152.7643 (+/− NA ppm)
1151.757
[M−H]−
1152.759
686.3682
1047 : M.findmain = 1152.7643 (+/− 20 ppm)
1151.757
[M−H]−
1152.759
686.3682

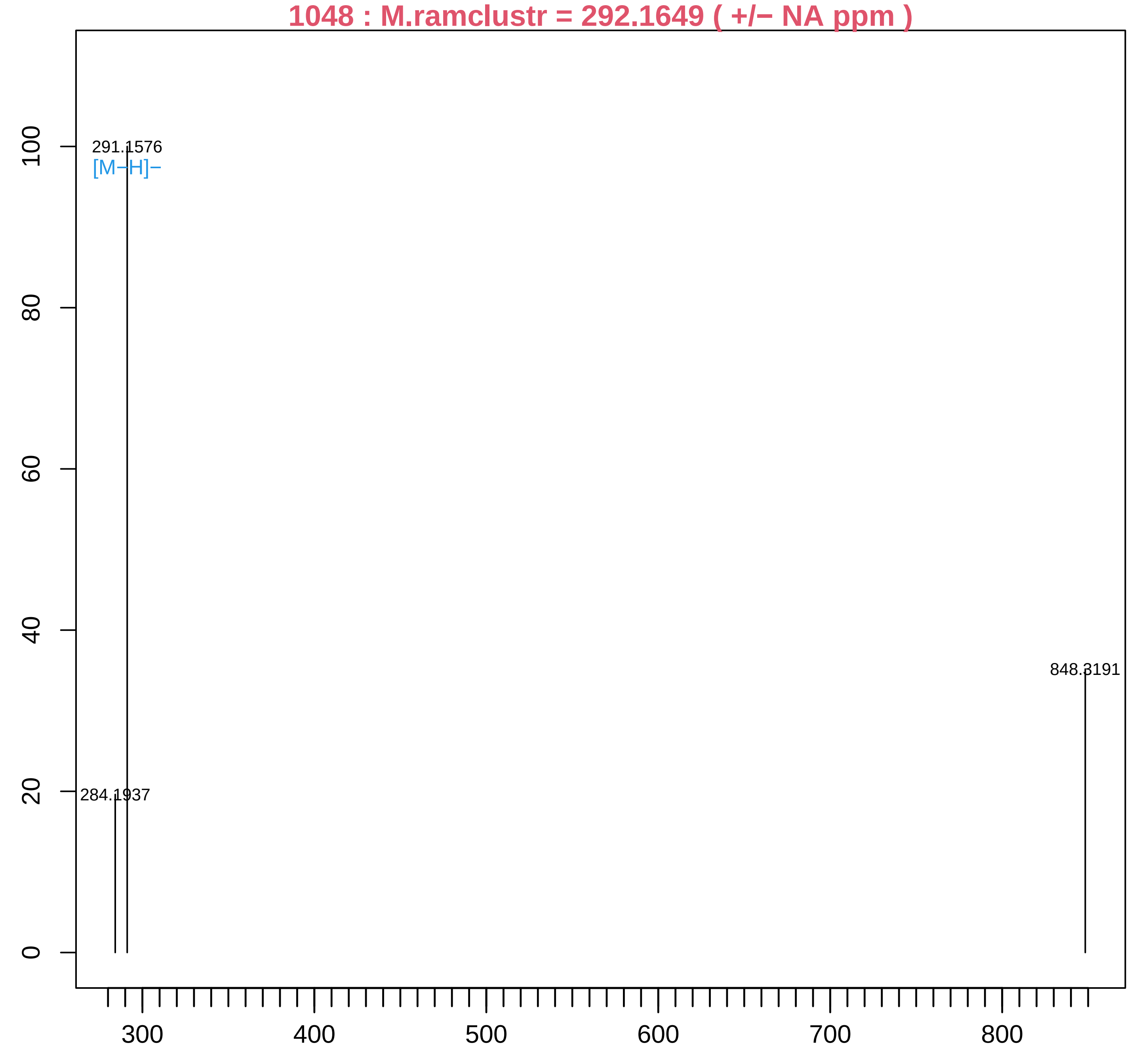

1048 : M.ramclustr = 292.1649 (+/− NA ppm)
291.1576
[M−H]−
284.1937
848.3191
0
20
40
60
80
100
300
400
500
600
700
800

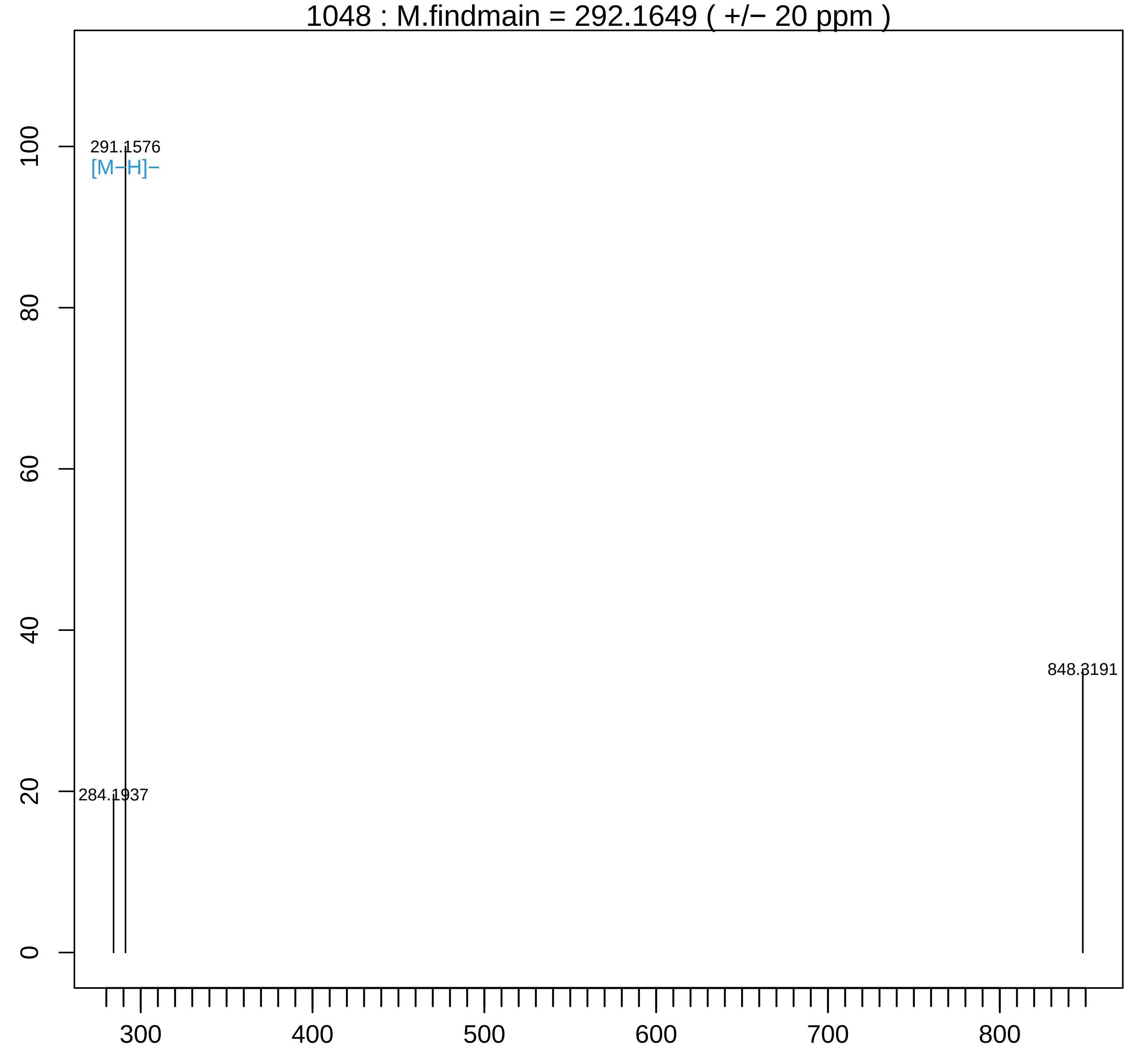

1048 : M.findmain = 292.1649 (+/− 20 ppm)
291.1576
[M−H]−
284.1937
848.3191
0
20
40
60
80
100
300
400
500
600
700
800

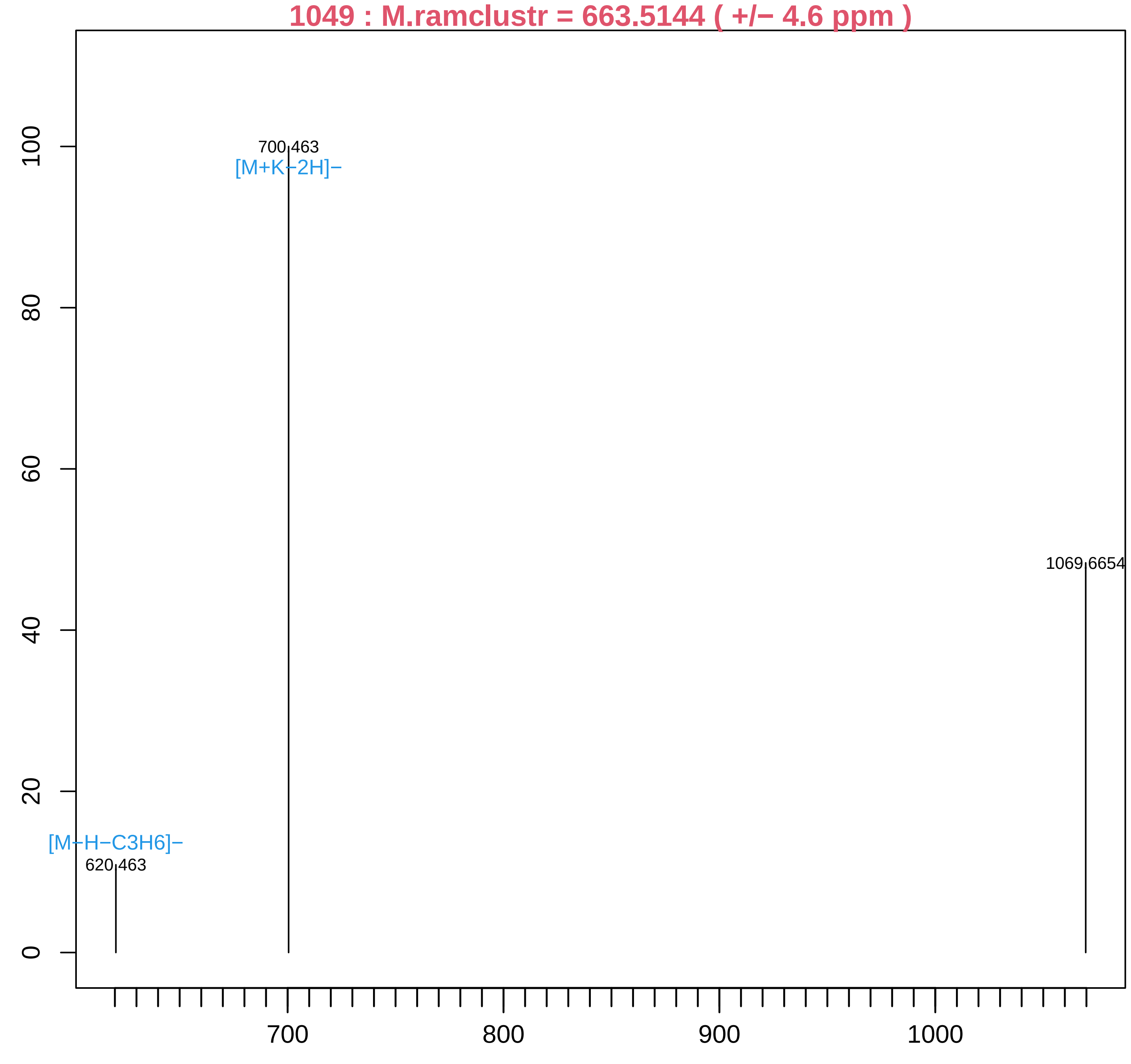

1049 : M.ramclustr = 663.5144 (+/− 4.6 ppm)
700.463
[M+K−2H]−
1069.6654
[M−H−C3H6]−
620.463
0
20
40
60
80
100
700
800
900
1000

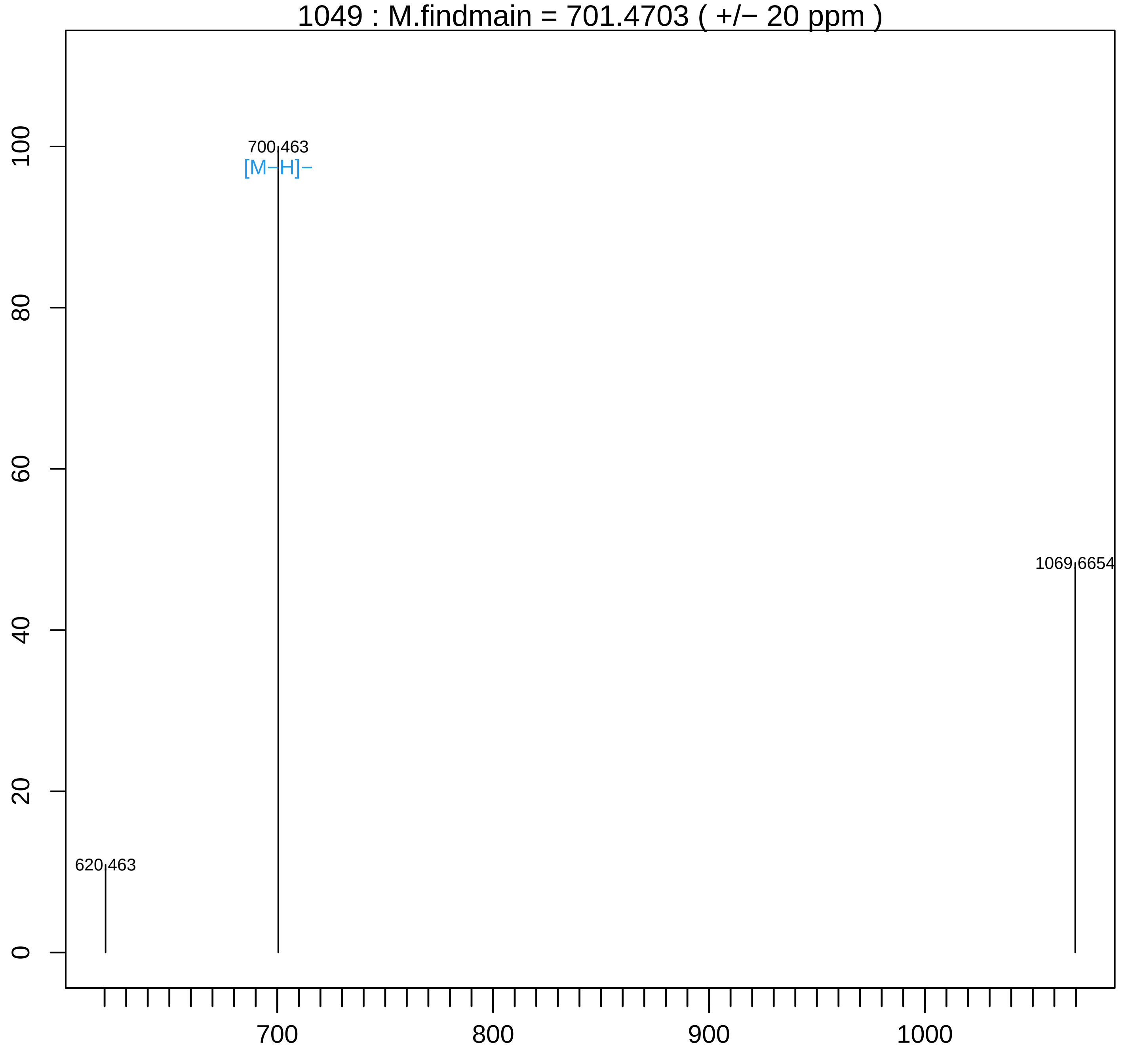

1049 : M.findmain = 701.4703 (+/− 20 ppm)
700.463
[M−H]−
1069.6654
620.463
0
20
40
60
80
100
700
800
900
1000

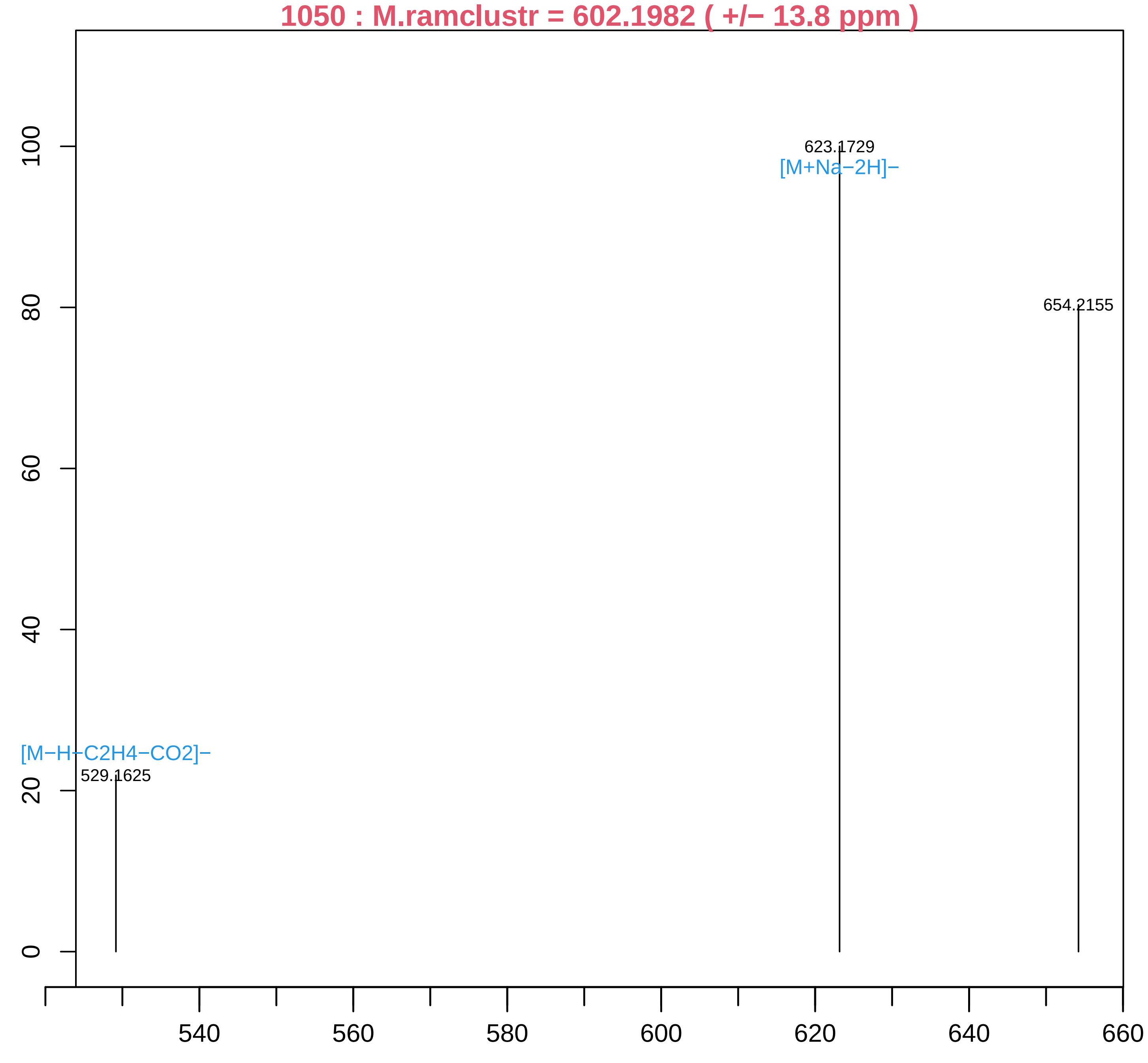
1050 : M.ramclustr = 602.1982 (+/− 13.8 ppm)
623.1729
[M+Na−2H]−
654.2155
[M−H−C2H4−CO2]−
529.1625
0
20
40
60
80
100
540
560
580
600
620
640
660

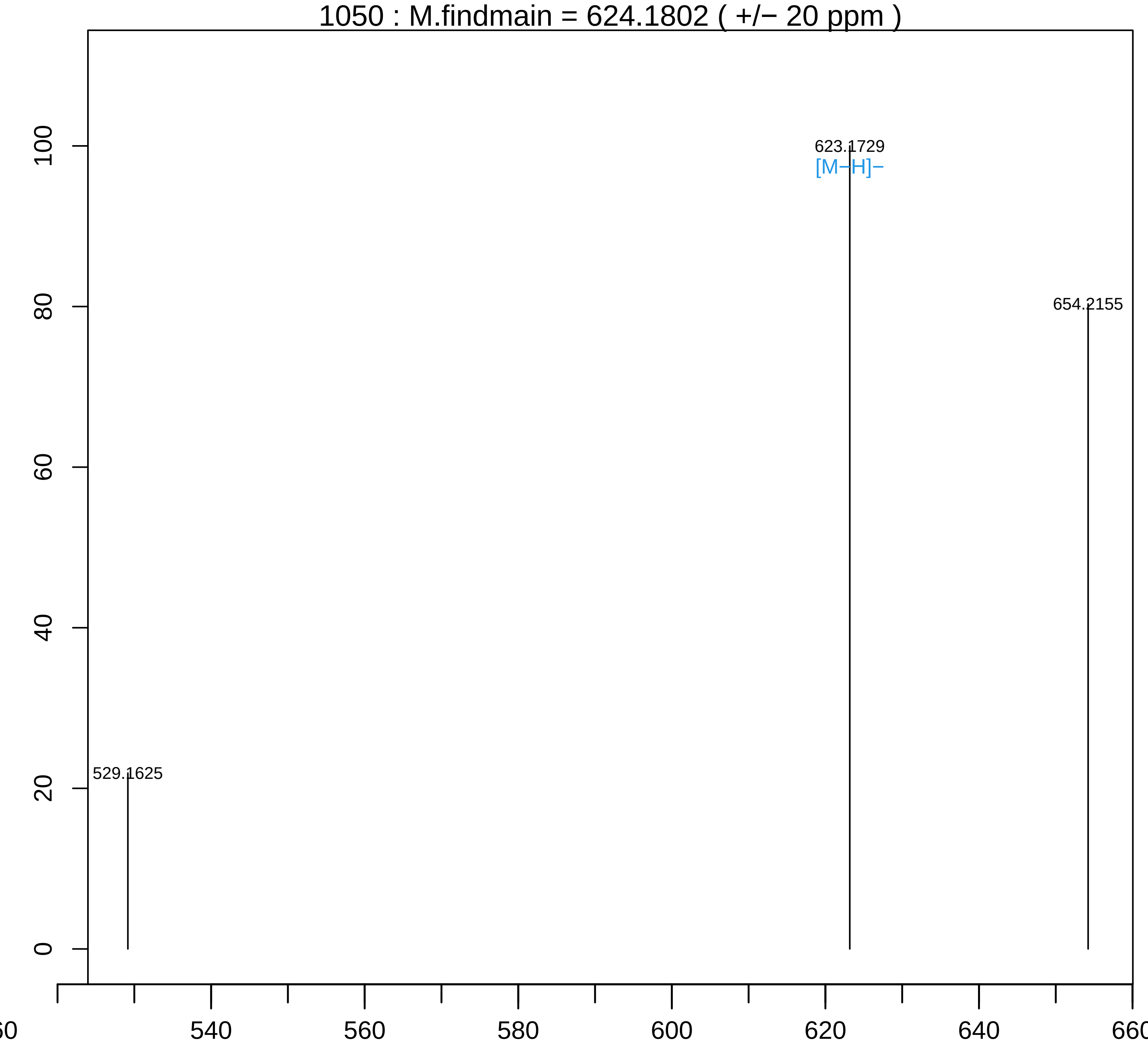
1050 : M.findmain = 624.1802 (+/− 20 ppm)
623.1729
[M−H]−
654.2155
529.1625
0
20
40
60
80
100
540
560
580
600
620
640
660

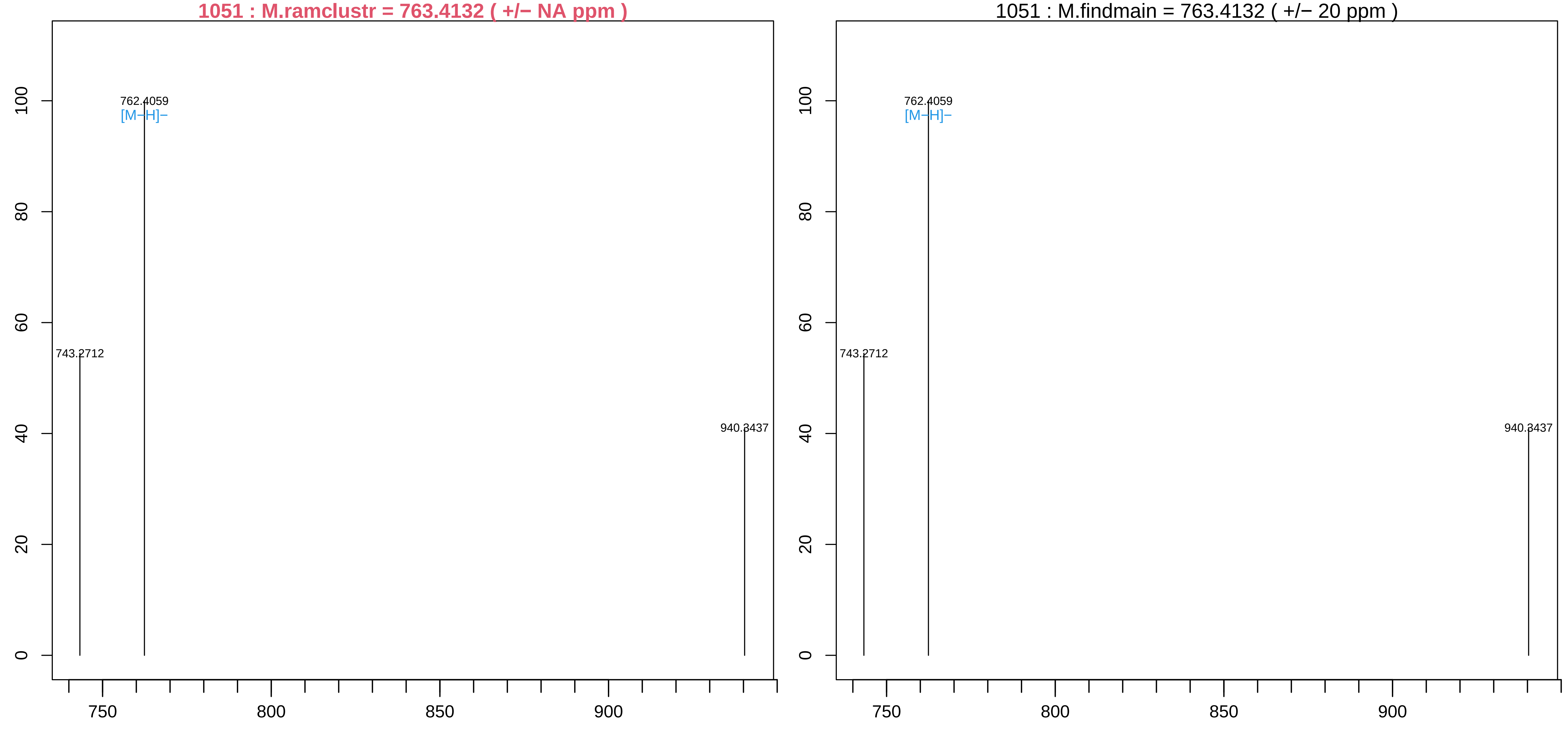
1051 : M.ramclustr = 763.4132 (+/− NA ppm)
762.4059
[M−H]−
743.2712
940.3437
1051 : M.findmain = 763.4132 (+/− 20 ppm)
762.4059
[M−H]−
743.2712
940.3437
0
20
40
60
80
100
750
800
850
900

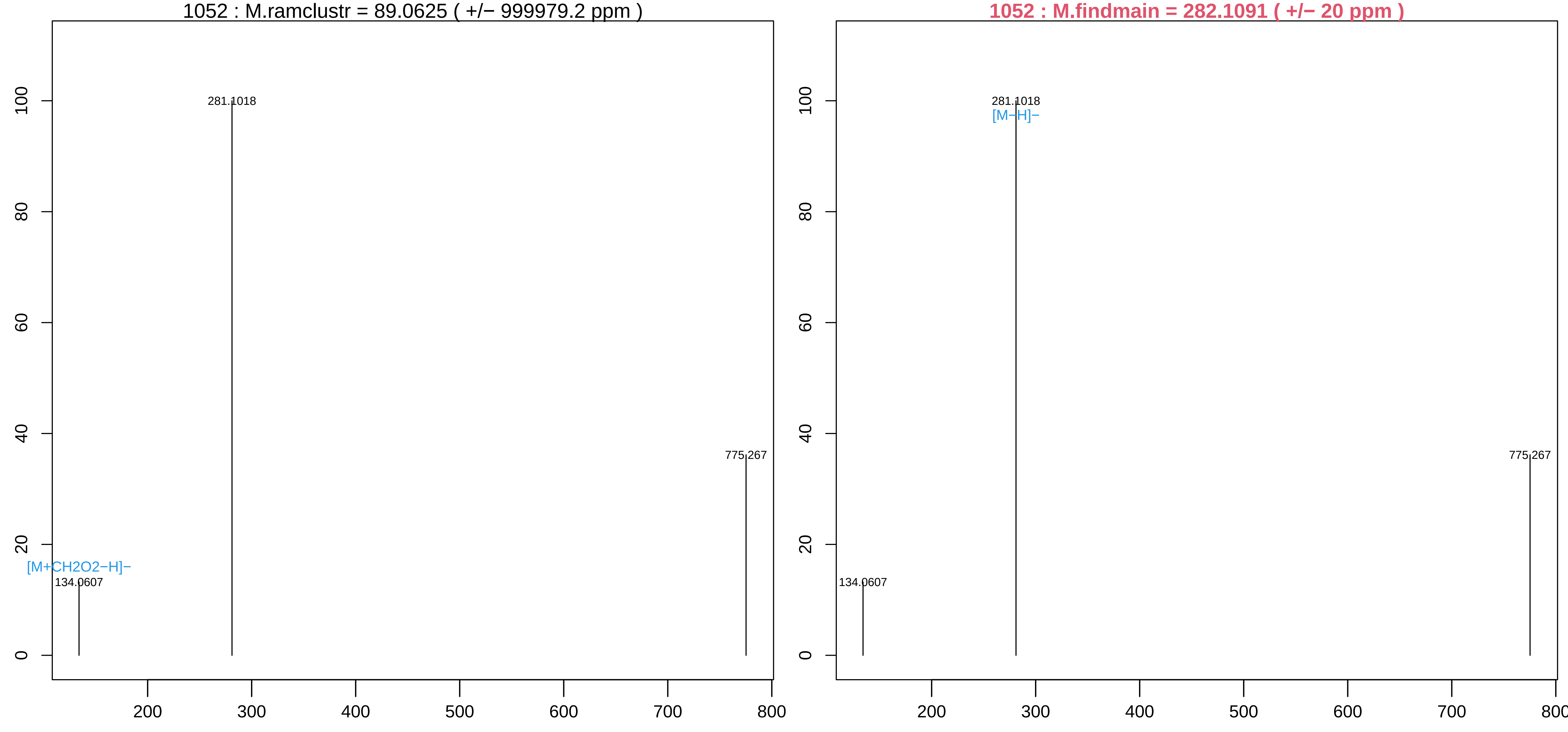
1052 : M.ramclustr = 89.0625 (+/− 999979.2 ppm)
281.1018
775.267
[M+CH2O2−H]−
134.0607
1052 : M.findmain = 282.1091 (+/− 20 ppm)
281.1018
[M−H]−
775.267
134.0607
0
20
40
60
80
100
200
300
400
500
600
700
800

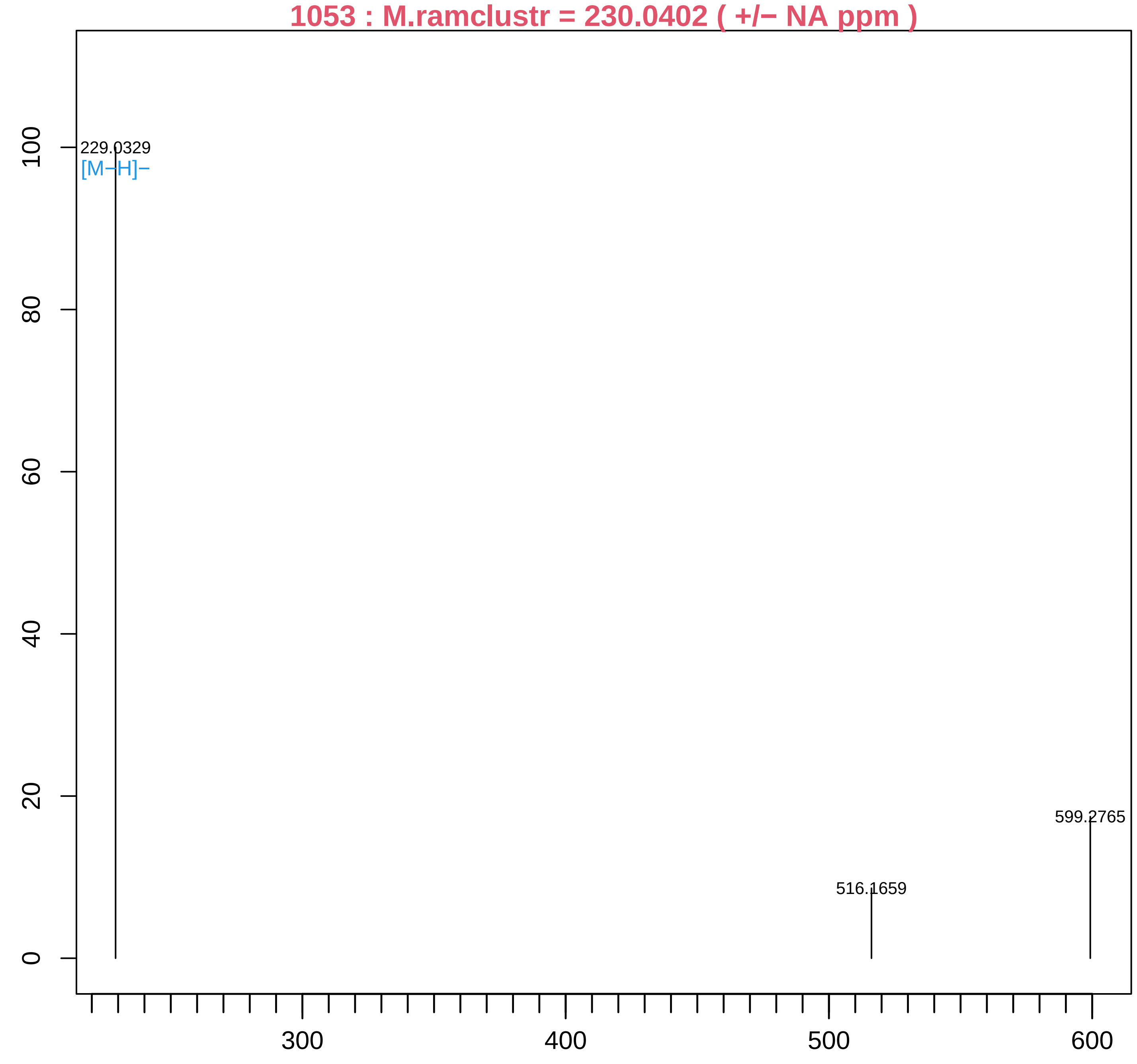
1053 : M.ramclustr = 230.0402 (+/− NA ppm)
229.0329
[M−H]−
516.1659
599.2765
0
20
40
60
80
100
300
400
500
600

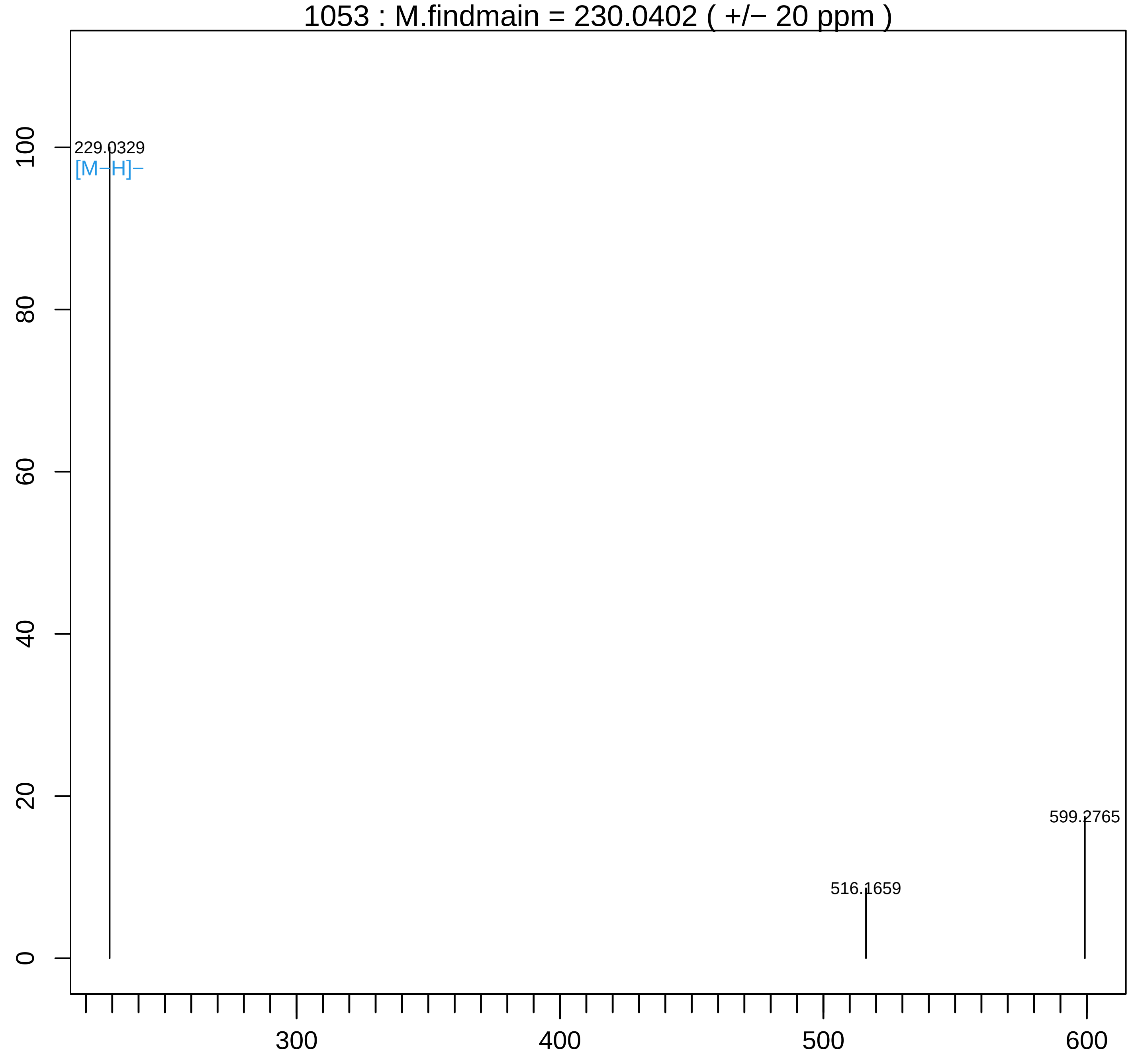
1053 : M.findmain = 230.0402 (+/− 20 ppm)
229.0329
[M−H]−
516.1659
599.2765
0
20
40
60
80
100
300
400
500
600

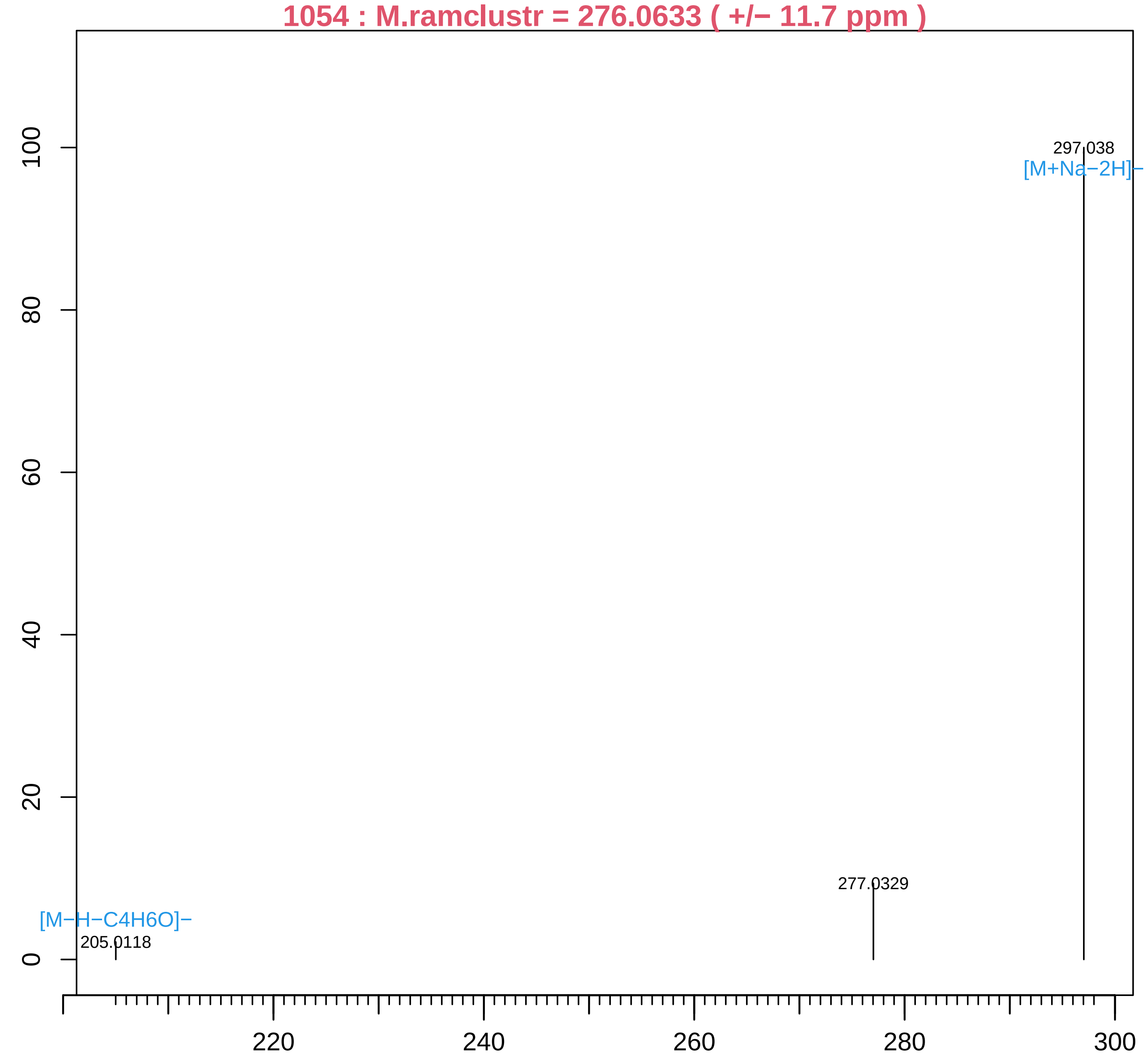
1054 : M.ramclustr = 276.0633 (+/− 11.7 ppm)
297.038
[M+Na−2H]−
277.0329
[M−H−C4H6O]−
205.0118
0
20
40
60
80
100
220
240
260
280
300

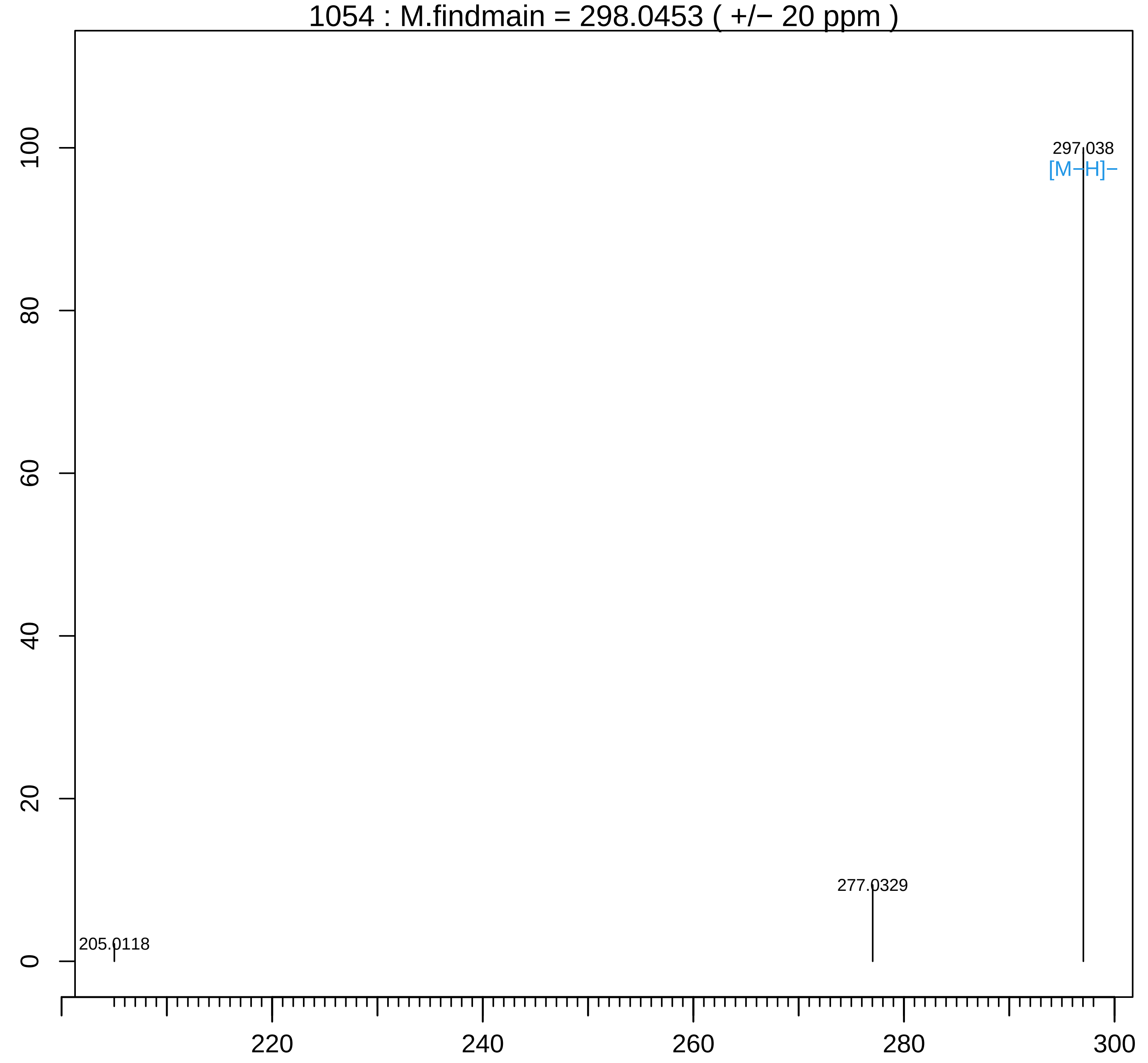
1054 : M.findmain = 298.0453 (+/− 20 ppm)
297.038
[M−H]−
277.0329
205.0118
0
20
40
60
80
100
220
240
260
280
300

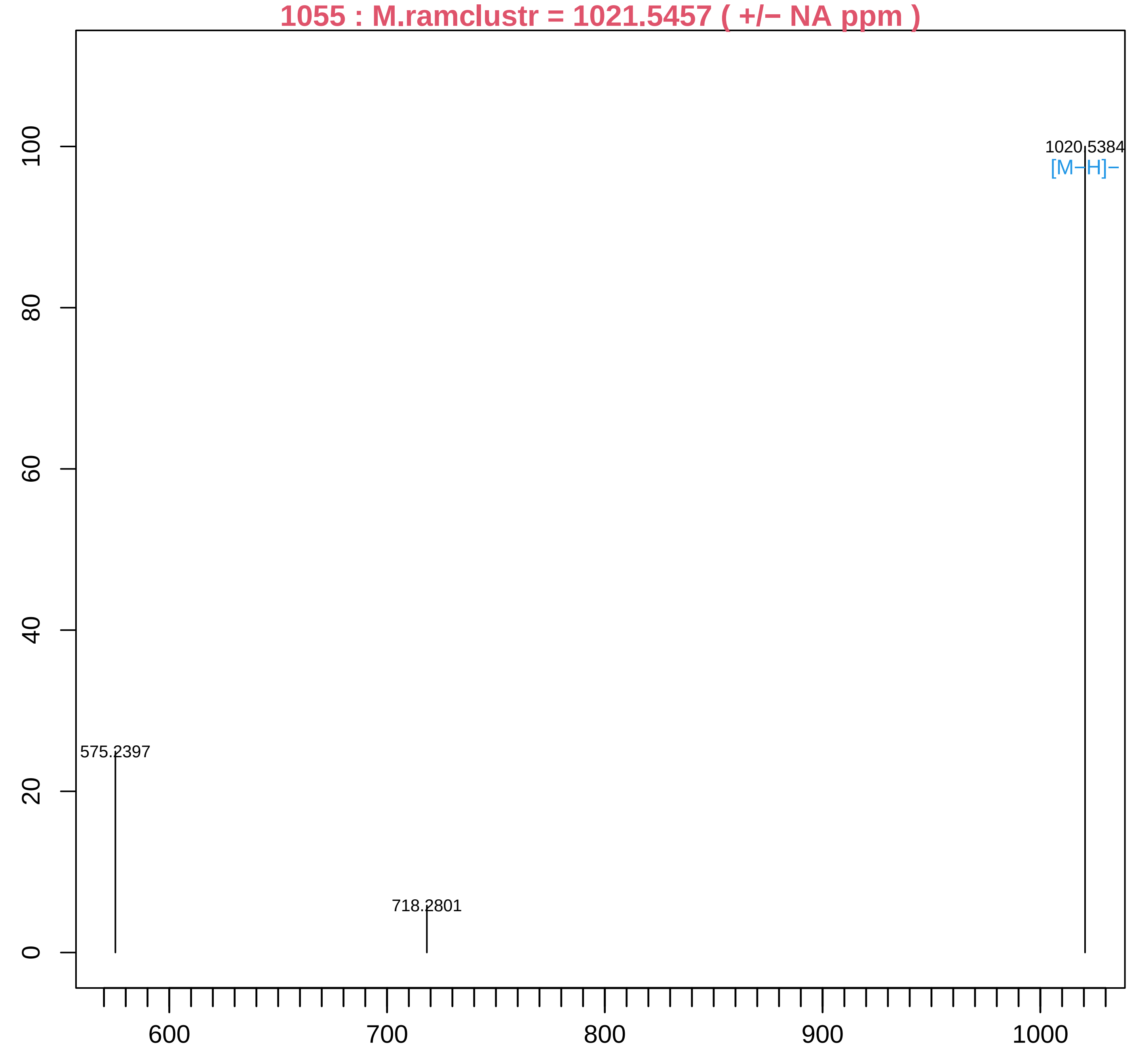
1055 : M.ramclustr = 1021.5457 (+/− NA ppm)
1020.5384
[M−H]−
575.2397
718.2801
0
20
40
60
80
100
600
700
800
900
1000

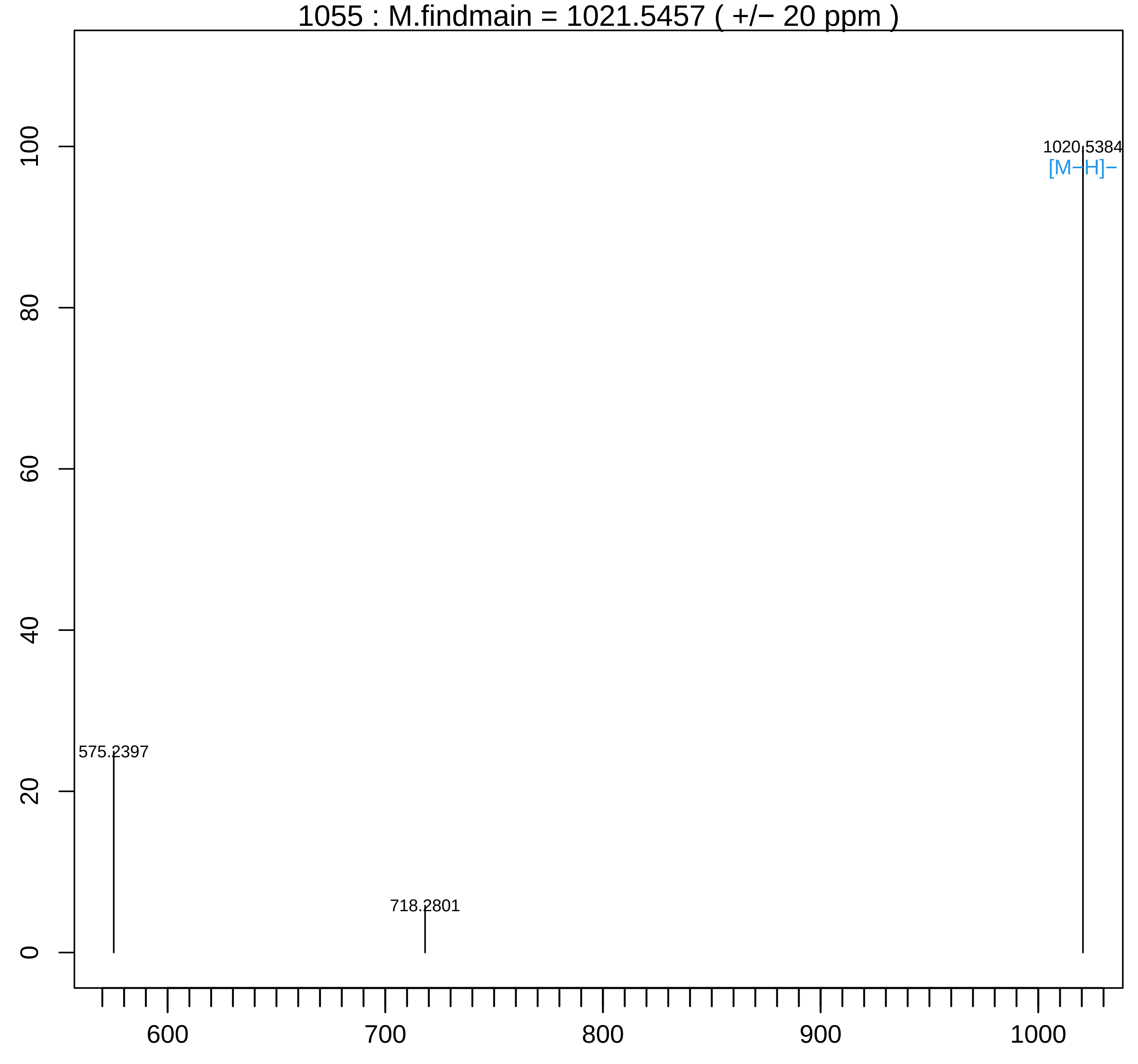
1055 : M.findmain = 1021.5457 (+/− 20 ppm)
1020.5384
[M−H]−
575.2397
718.2801
0
20
40
60
80
100
600
700
800
900
1000

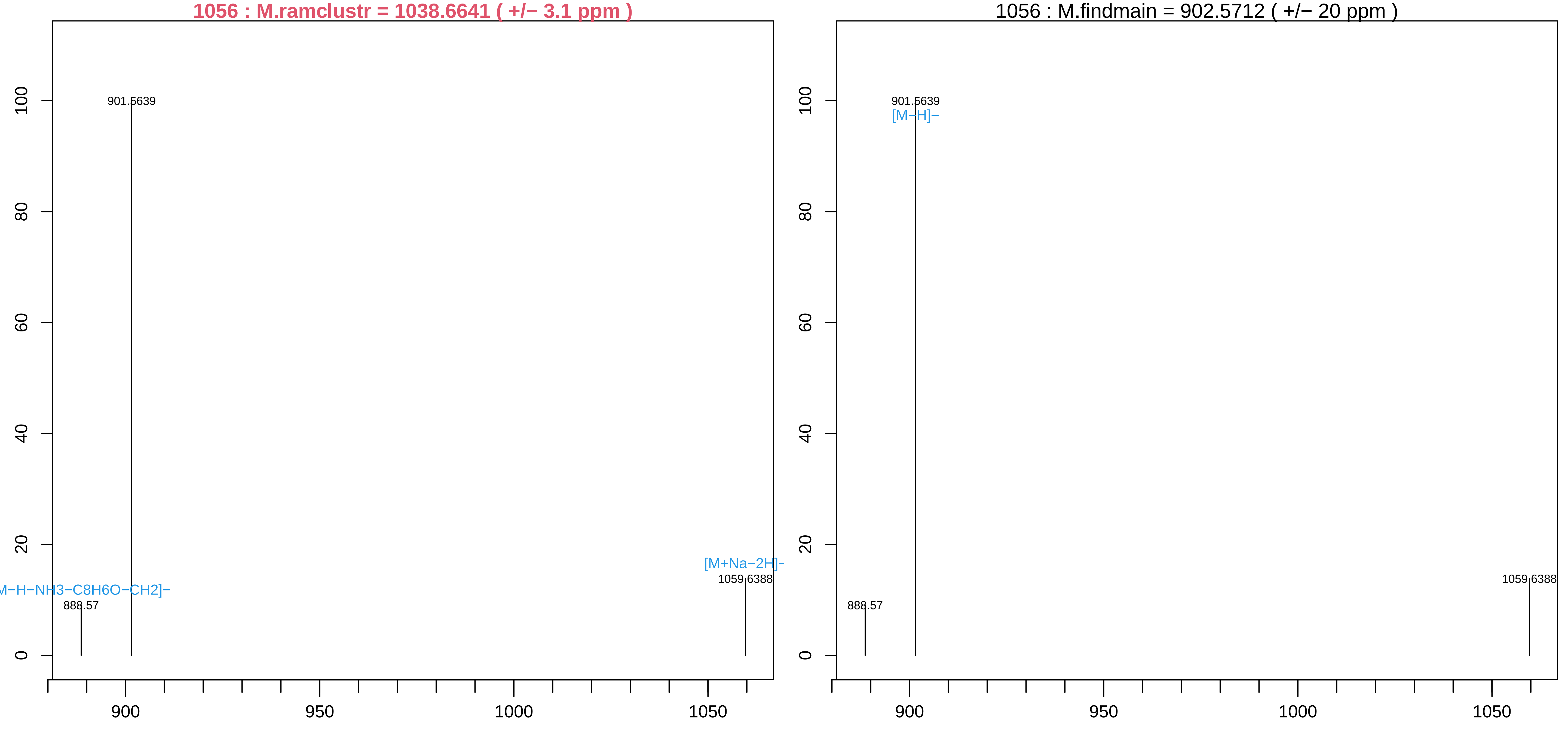

1056 : M.ramclustr = 1038.6641 (+/− 3.1 ppm)
901.5639
888.57
[M−H−NH3−C8H6O−CH2]−
1059.6388
[M+Na−2H]−
1056 : M.findmain = 902.5712 (+/− 20 ppm)
901.5639
[M−H]−
888.57
1059.6388
0
20
40
60
80
100
900
950
1000
1050

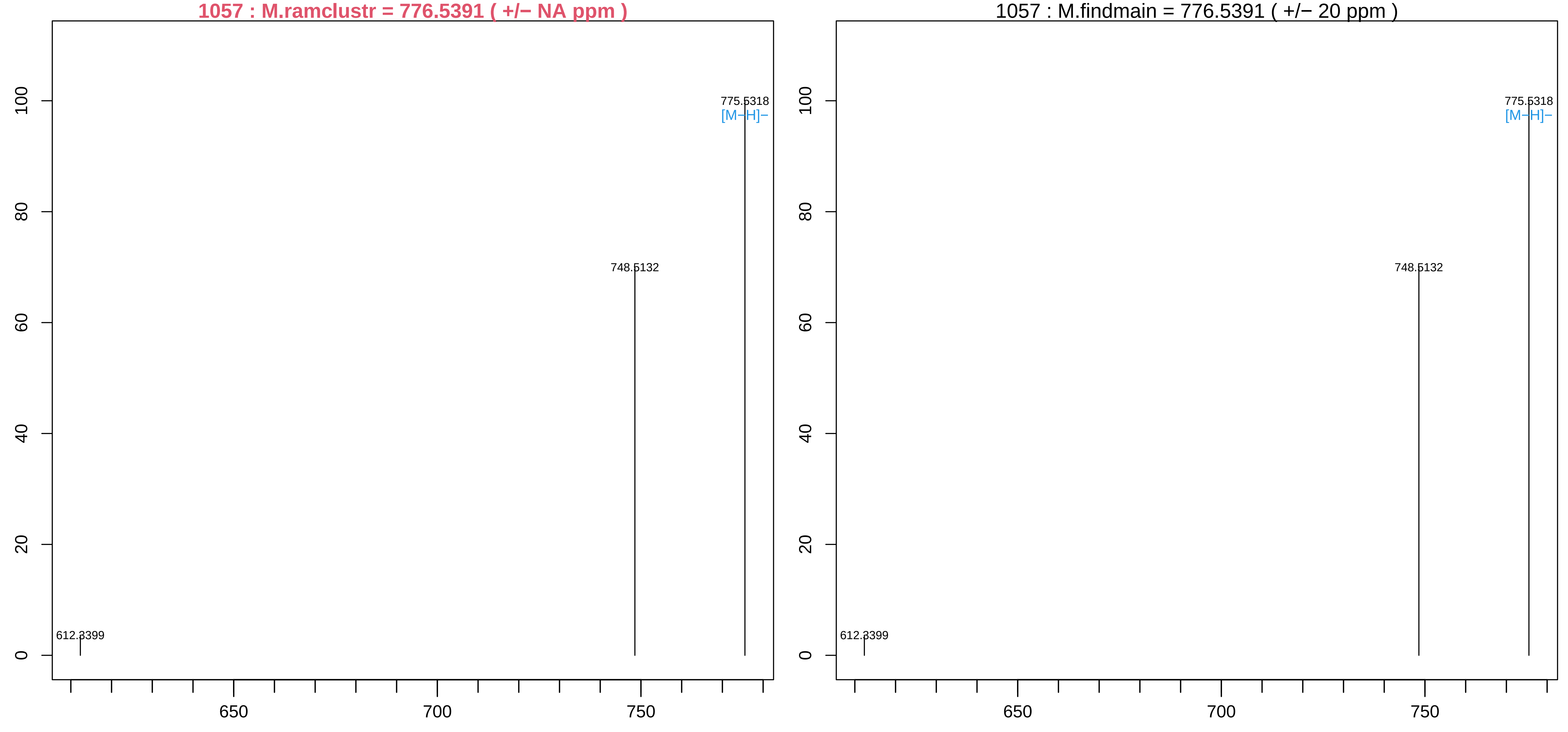

1057 : M.ramclustr = 776.5391 (+/− NA ppm)
1057 : M.findmain = 776.5391 (+/− 20 ppm)
775.5318
[M−H]−
748.5132
612.3399
0
20
40
60
80
100
650
700
750

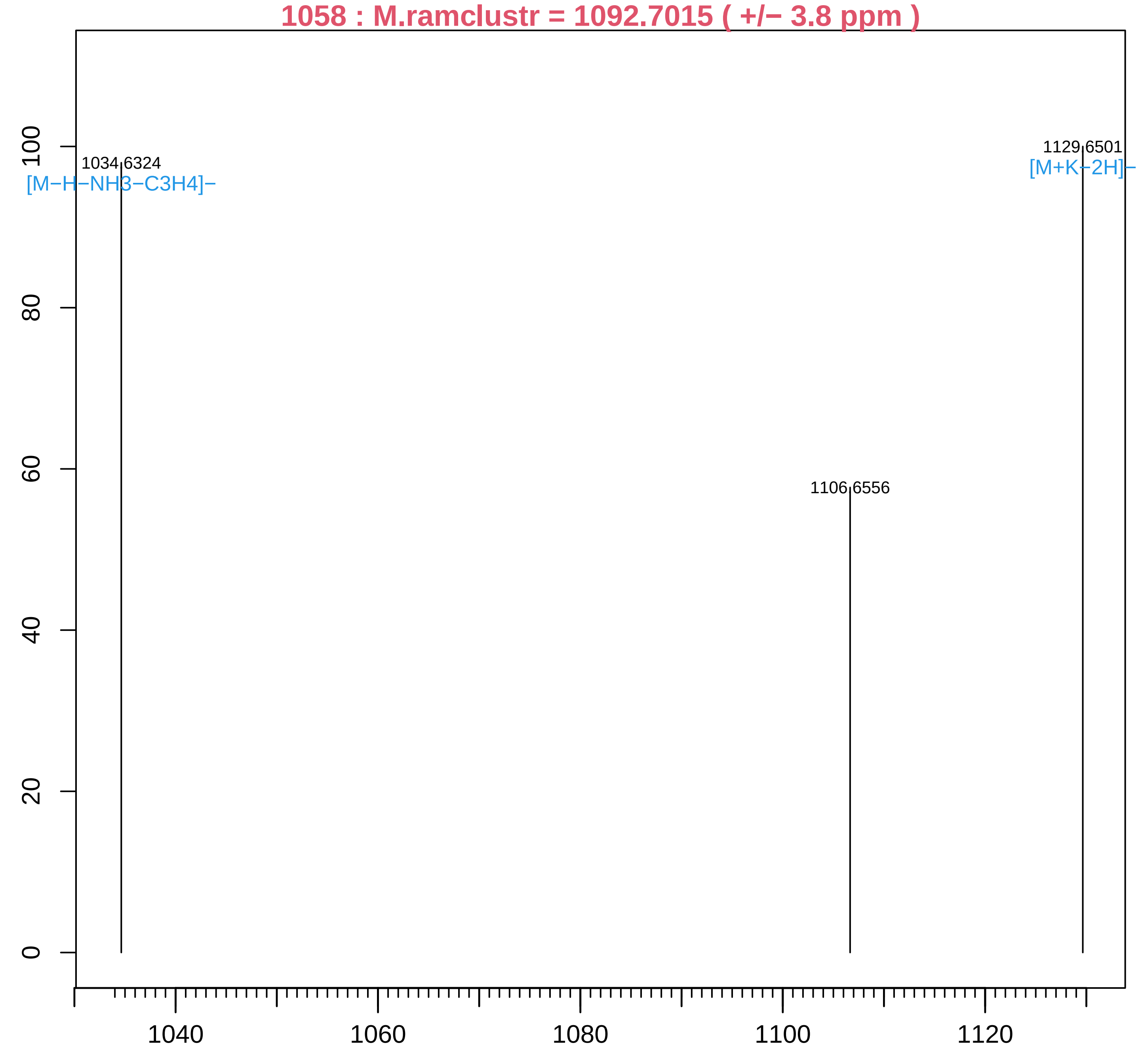

1058 : M.ramclustr = 1092.7015 (+/− 3.8 ppm)
1034.6324
[M−H−NH3−C3H4]−
1106.6556
1129.6501
[M+K−2H]−
0
20
40
60
80
100
1040
1060
1080
1100
1120

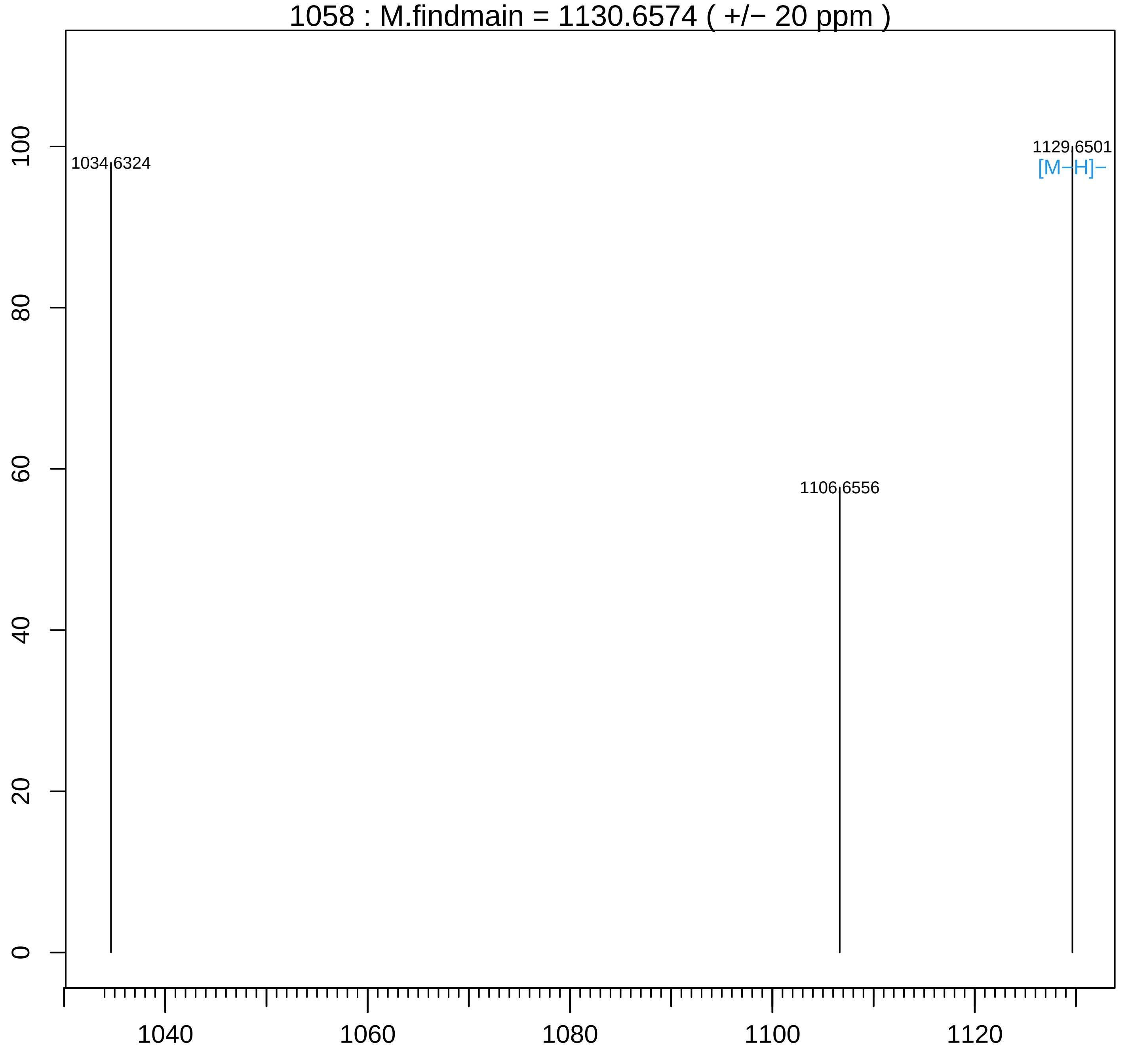

1058 : M.findmain = 1130.6574 (+/− 20 ppm)
1034.6324
1106.6556
1129.6501
[M−H]−
0
20
40
60
80
100
1040
1060
1080
1100
1120

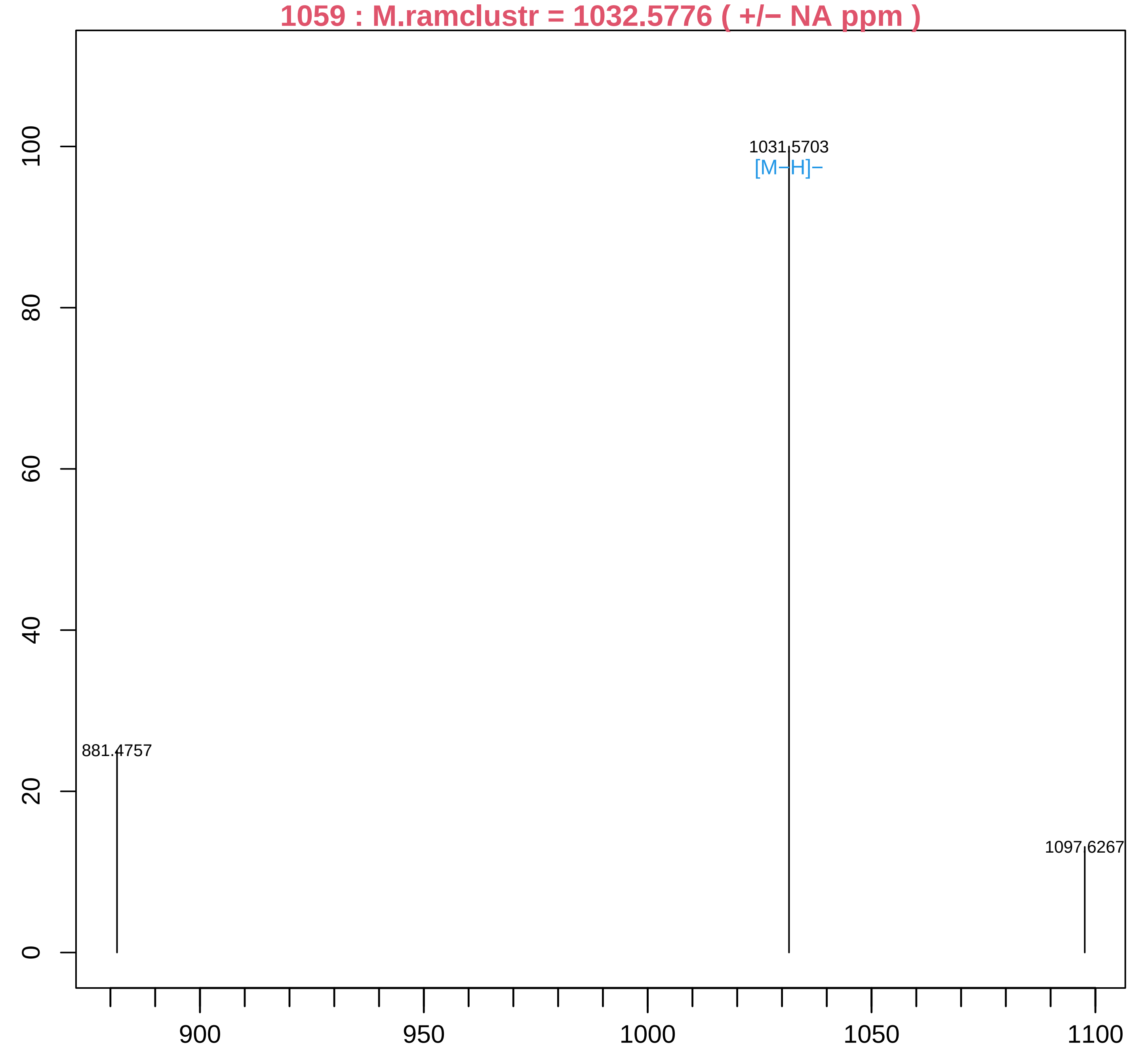
1059 : M.ramclustr = 1032.5776 (+/− NA ppm)
1031.5703
[M−H]−
881.4757
1097.6267
0
20
40
60
80
100
900
950
1000
1050
1100

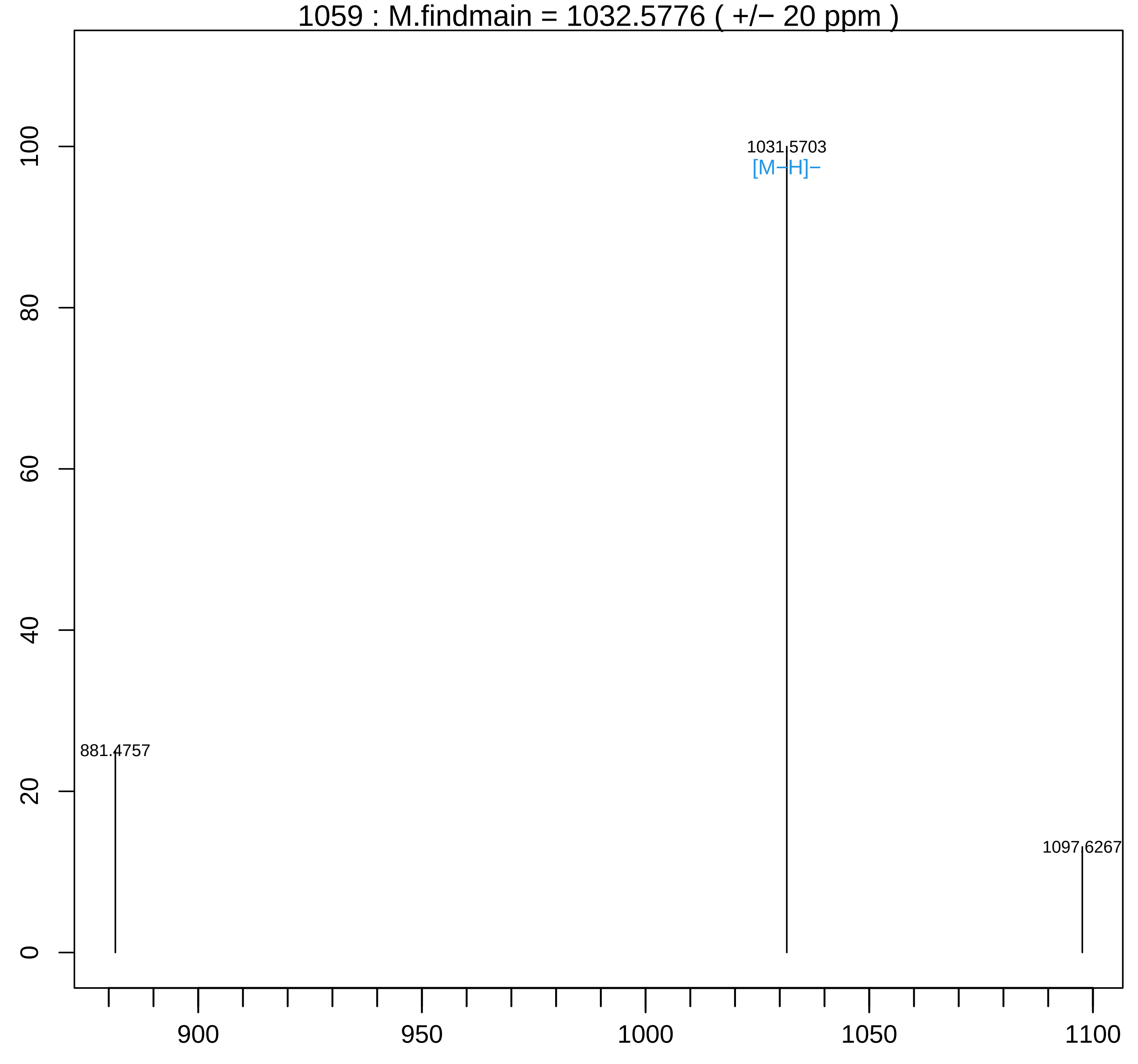
1059 : M.findmain = 1032.5776 (+/− 20 ppm)
1031.5703
[M−H]−
881.4757
1097.6267
0
20
40
60
80
100
900
950
1000
1050
1100

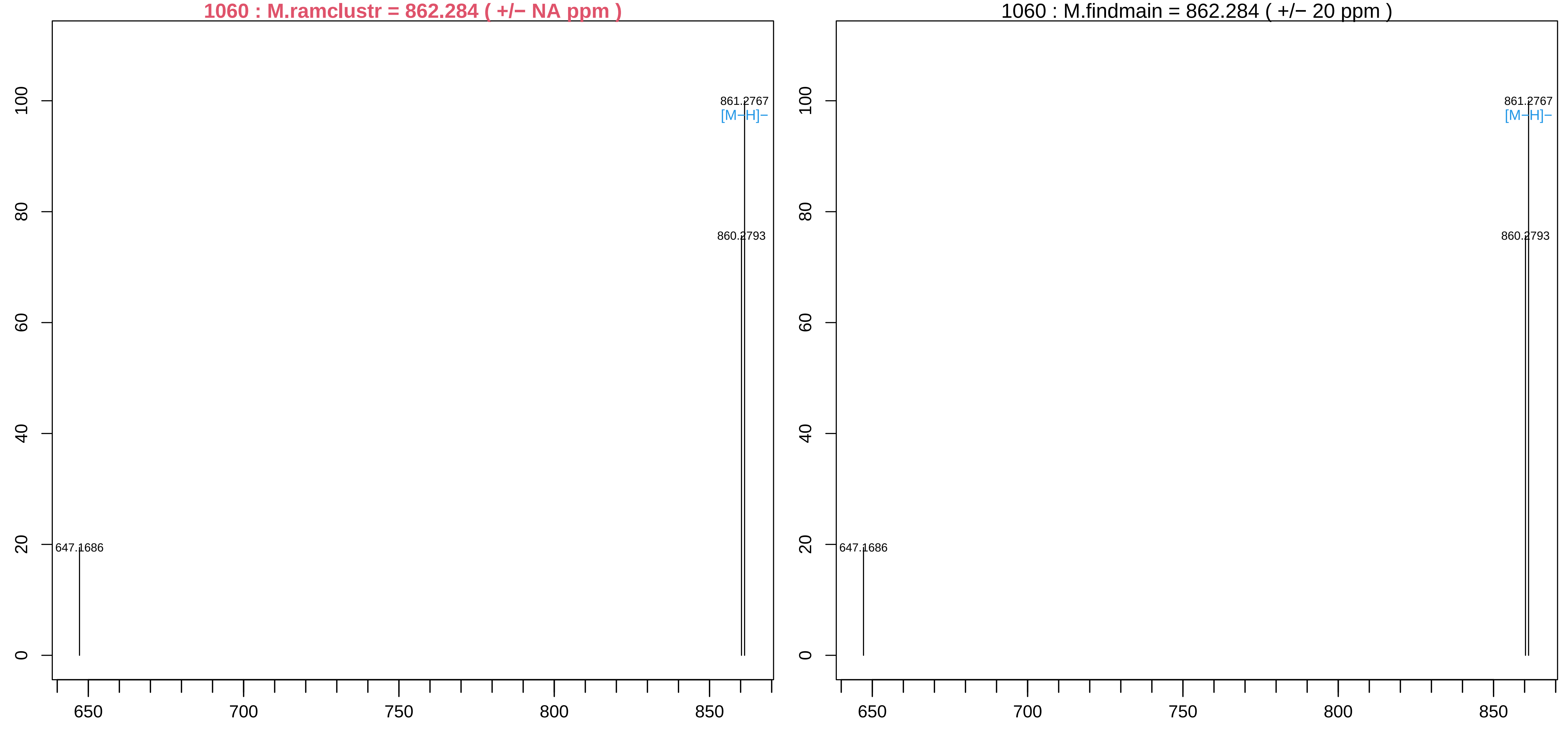

1060 : M.ramclustr = 862.284 (+/− NA ppm)
861.2767
[M−H]−
860.2793
647.1686
0
20
40
60
80
100
650
700
750
800
850
1060 : M.findmain = 862.284 (+/− 20 ppm)
861.2767
[M−H]−
860.2793
647.1686
0
20
40
60
80
100
650
700
750
800
850

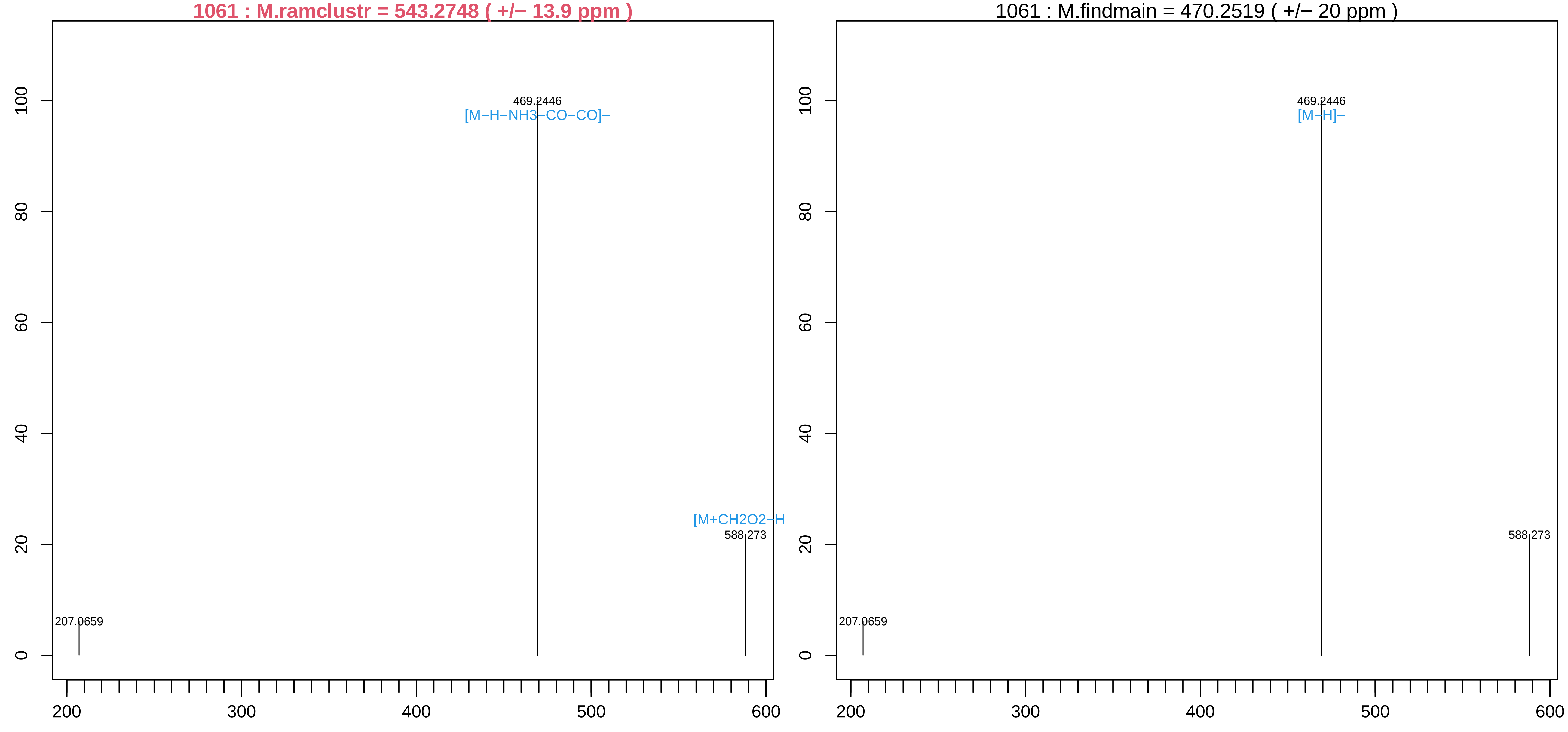
1061 : M.ramclustr = 543.2748 (+/− 13.9 ppm)
469.2446
[M−H−NH3−CO−CO]−
[M+CH2O2−H
588.273
207.0659
1061 : M.findmain = 470.2519 (+/− 20 ppm)
469.2446
[M−H]−
588.273
207.0659
0
20
40
60
80
100
200
300
400
500
600

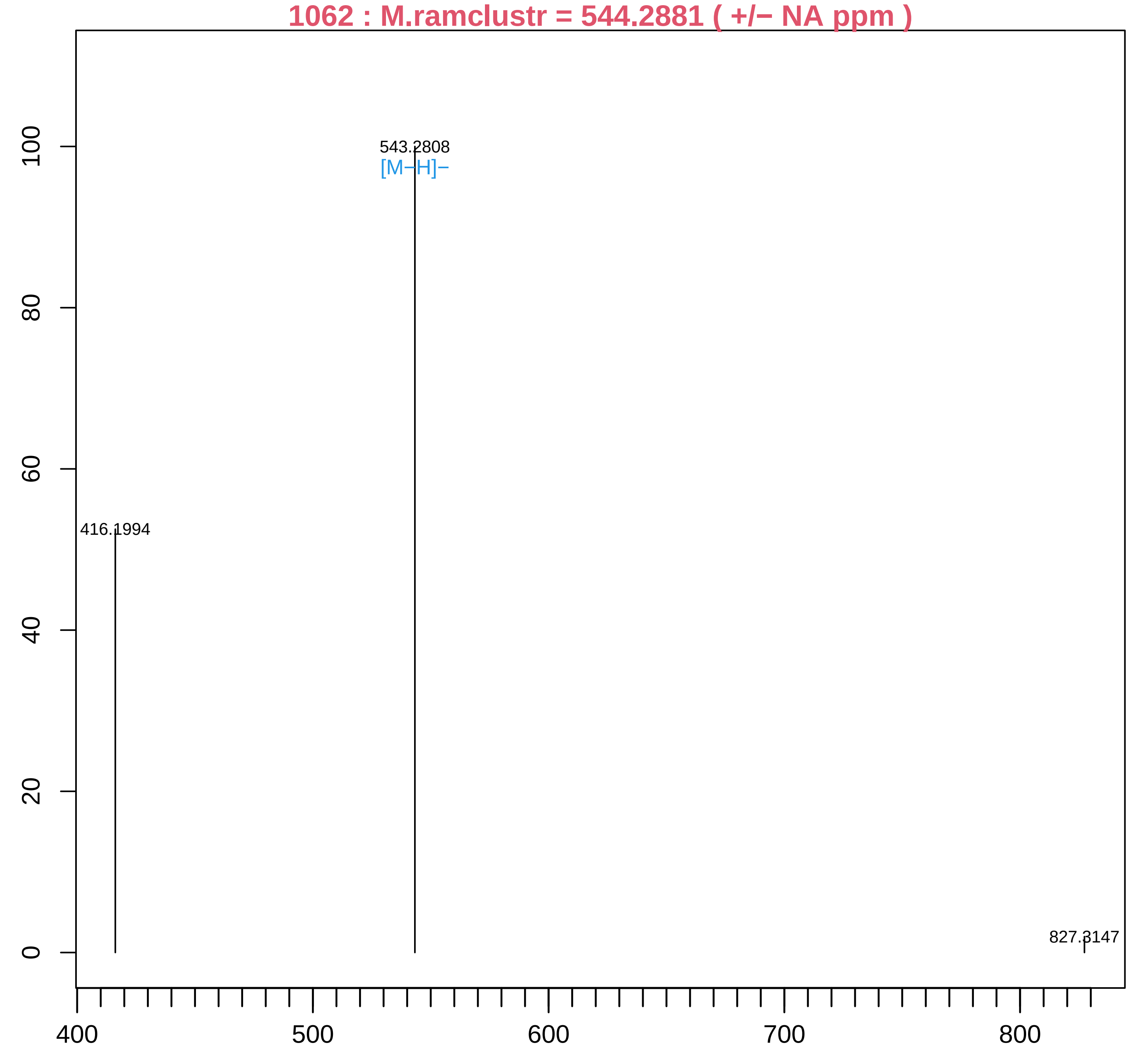
1062 : M.ramclustr = 544.2881 (+/− NA ppm)
543.2808
[M−H]−
416.1994
827.3147
0
20
40
60
80
100
400
500
600
700
800

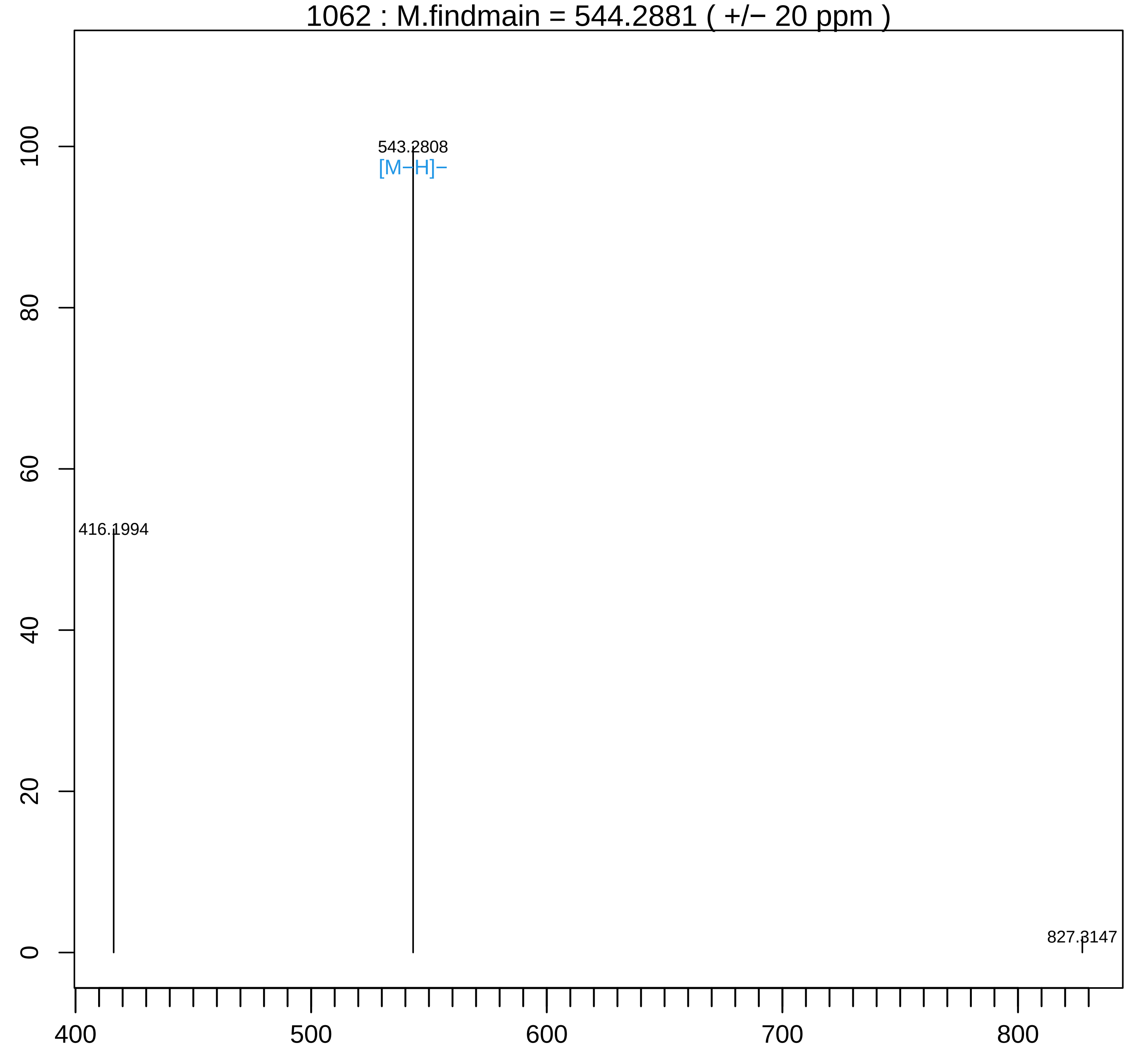
1062 : M.findmain = 544.2881 (+/− 20 ppm)
543.2808
[M−H]−
416.1994
827.3147
0
20
40
60
80
100
400
500
600
700
800

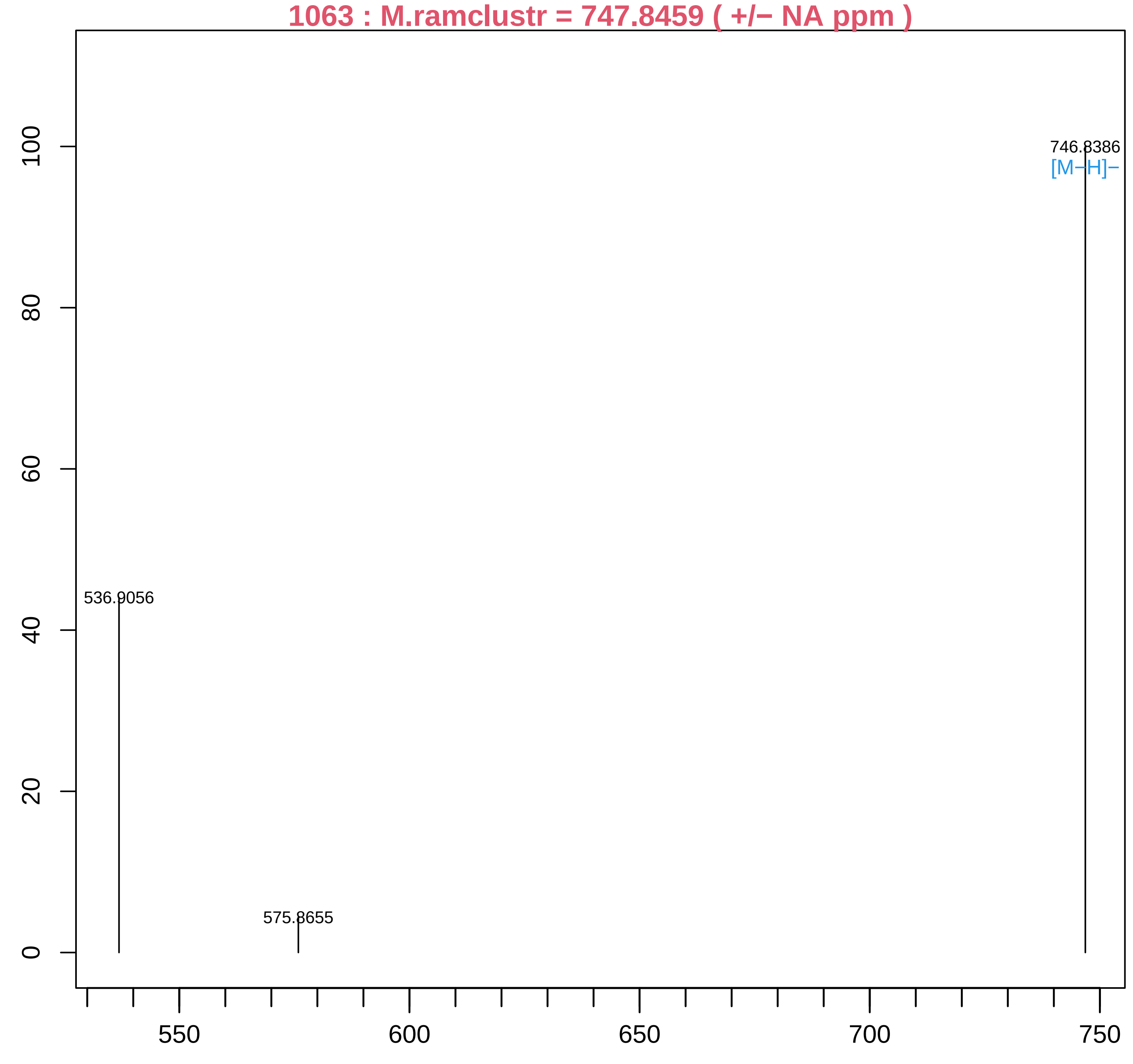
1063 : M.ramclustr = 747.8459 (+/− NA ppm)
746.8386
[M−H]−
536.9056
575.8655
0
20
40
60
80
100
550
600
650
700
750

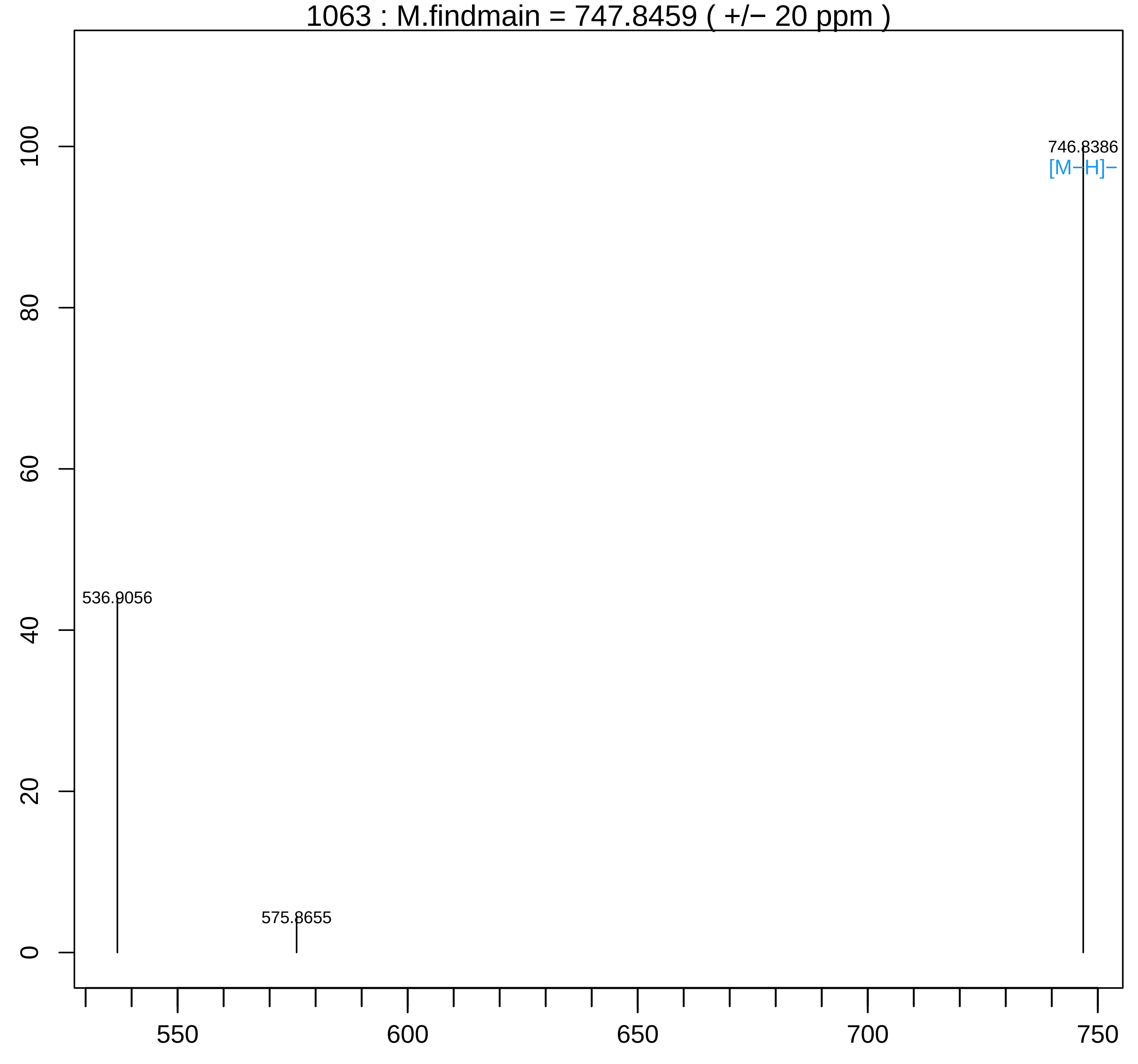
1063 : M.findmain = 747.8459 (+/− 20 ppm)
746.8386
[M−H]−
536.9056
575.8655
0
20
40
60
80
100
550
600
650
700
750

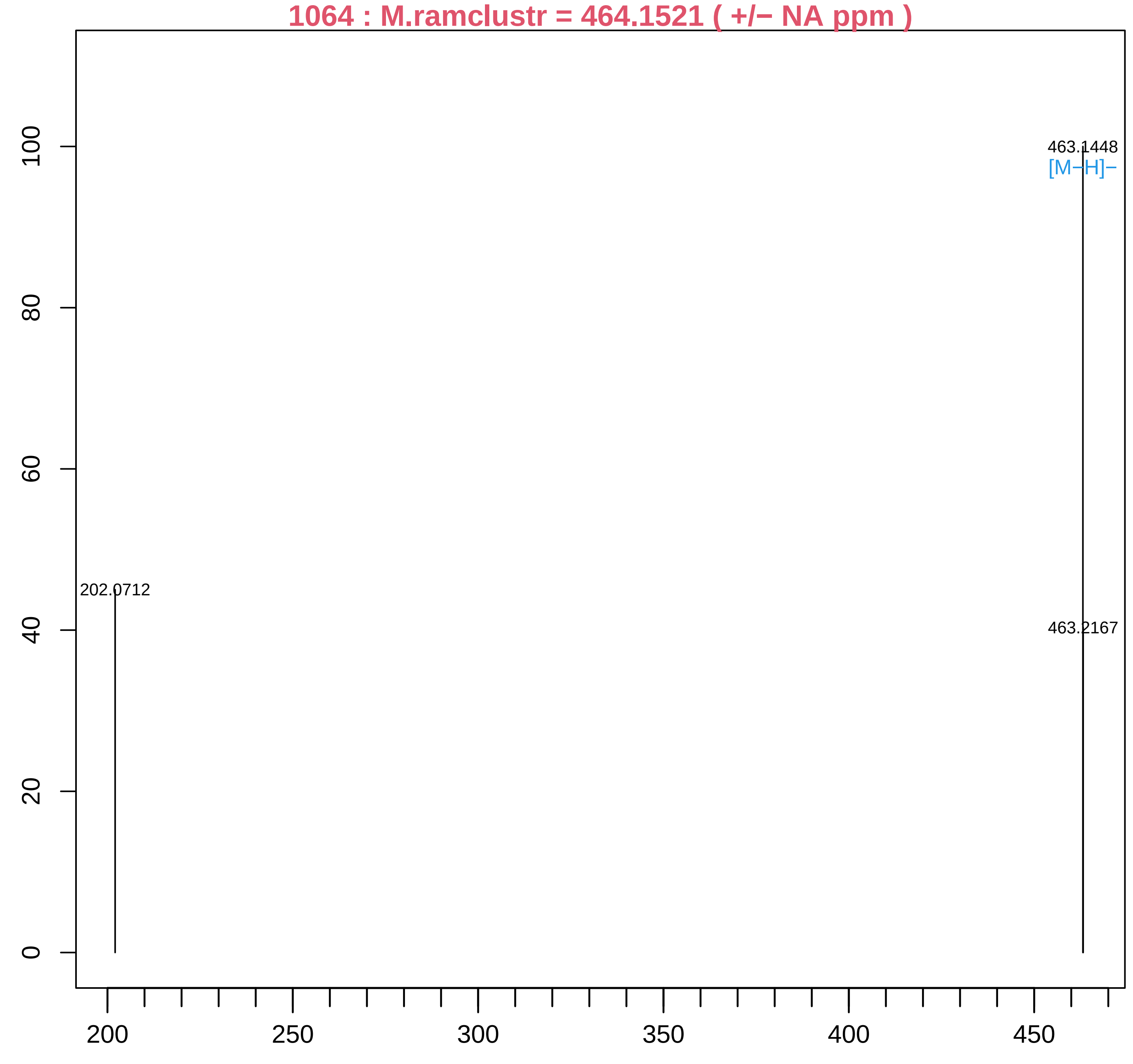
1064 : M.ramclustr = 464.1521 (+/− NA ppm)
463.1448
[M−H]−
202.0712
463.2167
0
20
40
60
80
100
200
250
300
350
400
450

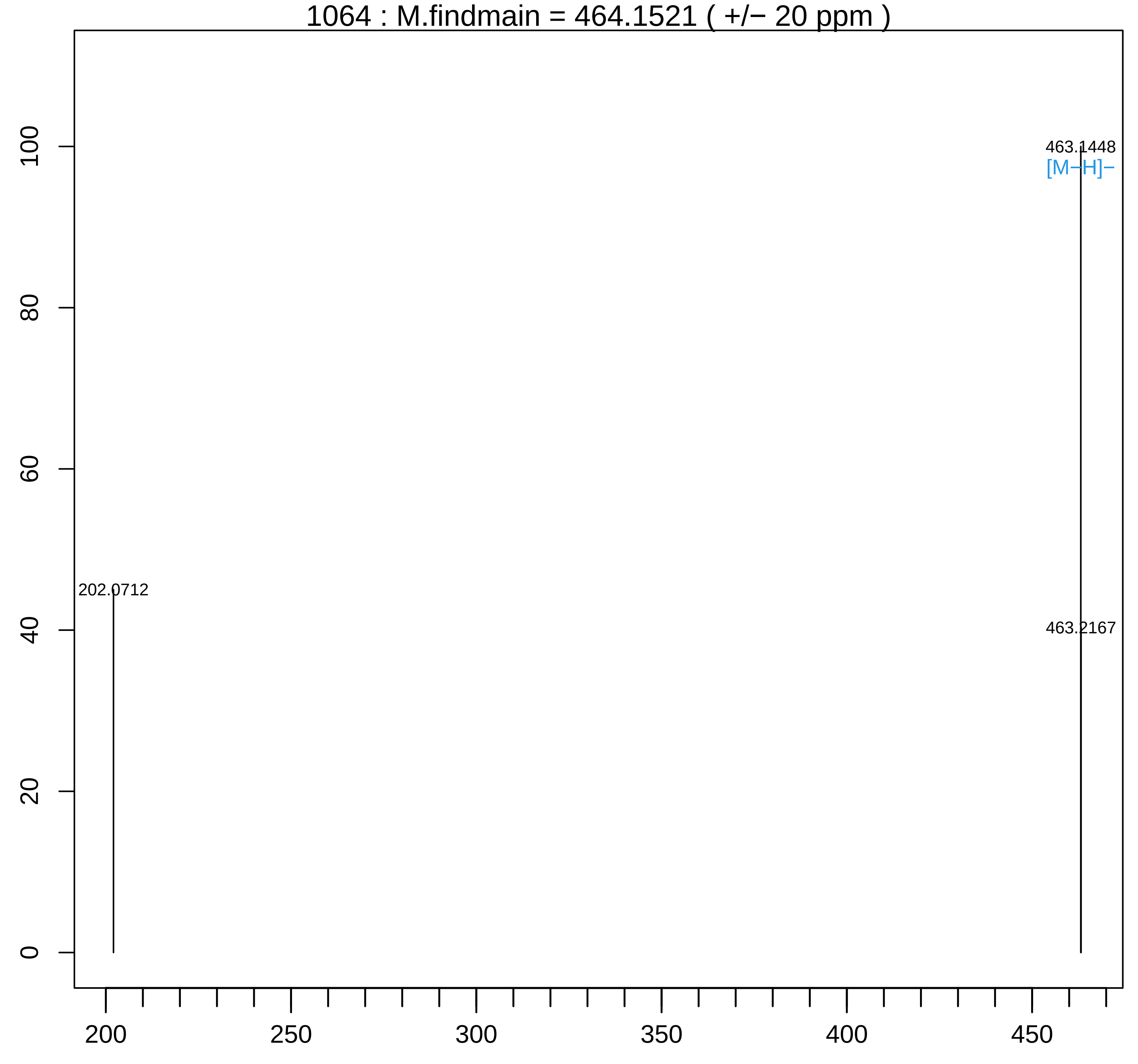
1064 : M.findmain = 464.1521 (+/− 20 ppm)
463.1448
[M−H]−
202.0712
463.2167
0
20
40
60
80
100
200
250
300
350
400
450

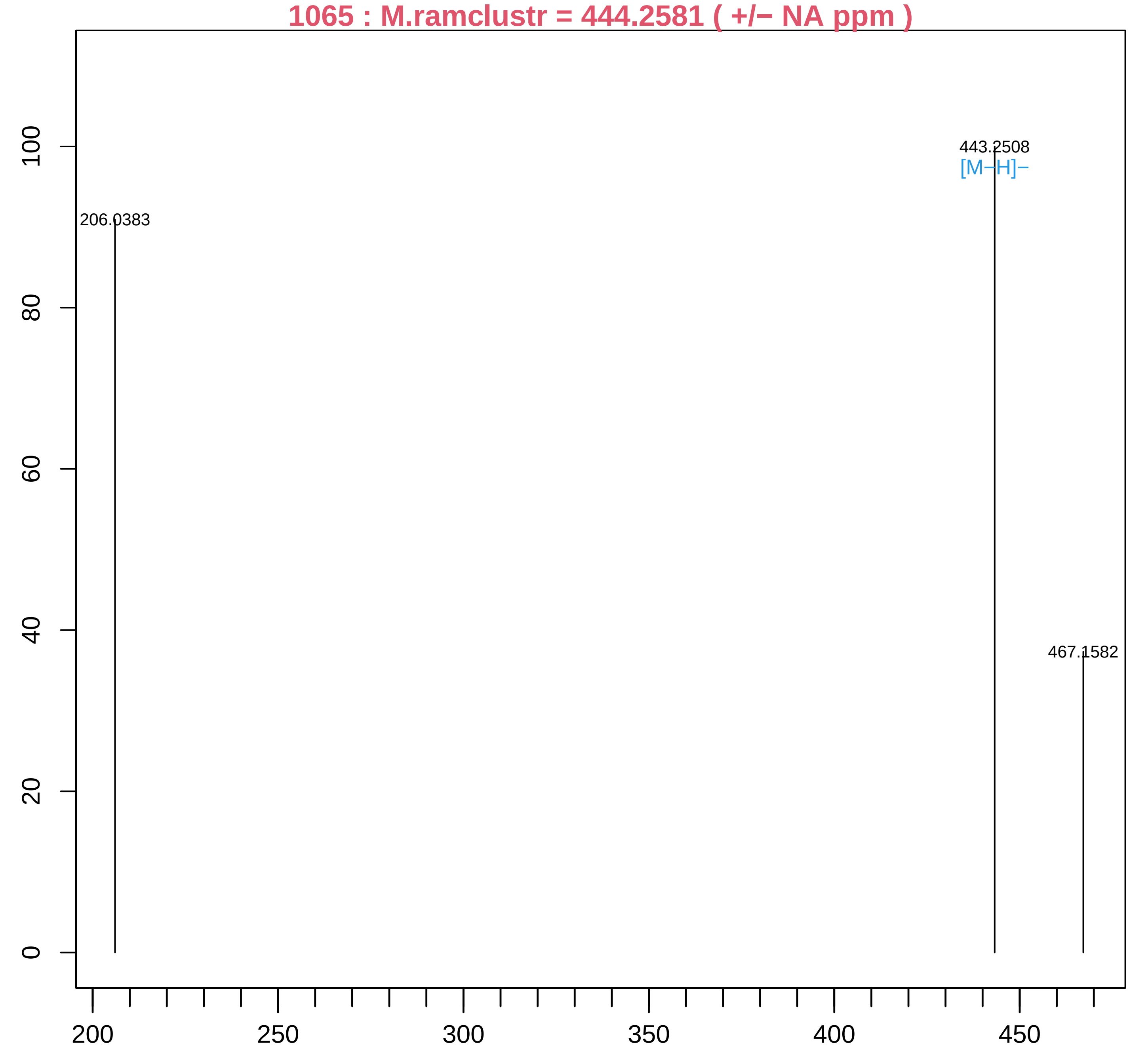
1065 : M.ramclustr = 444.2581 (+/− NA ppm)
206.0383
443.2508
[M−H]−
467.1582
0
20
40
60
80
100
200
250
300
350
400
450

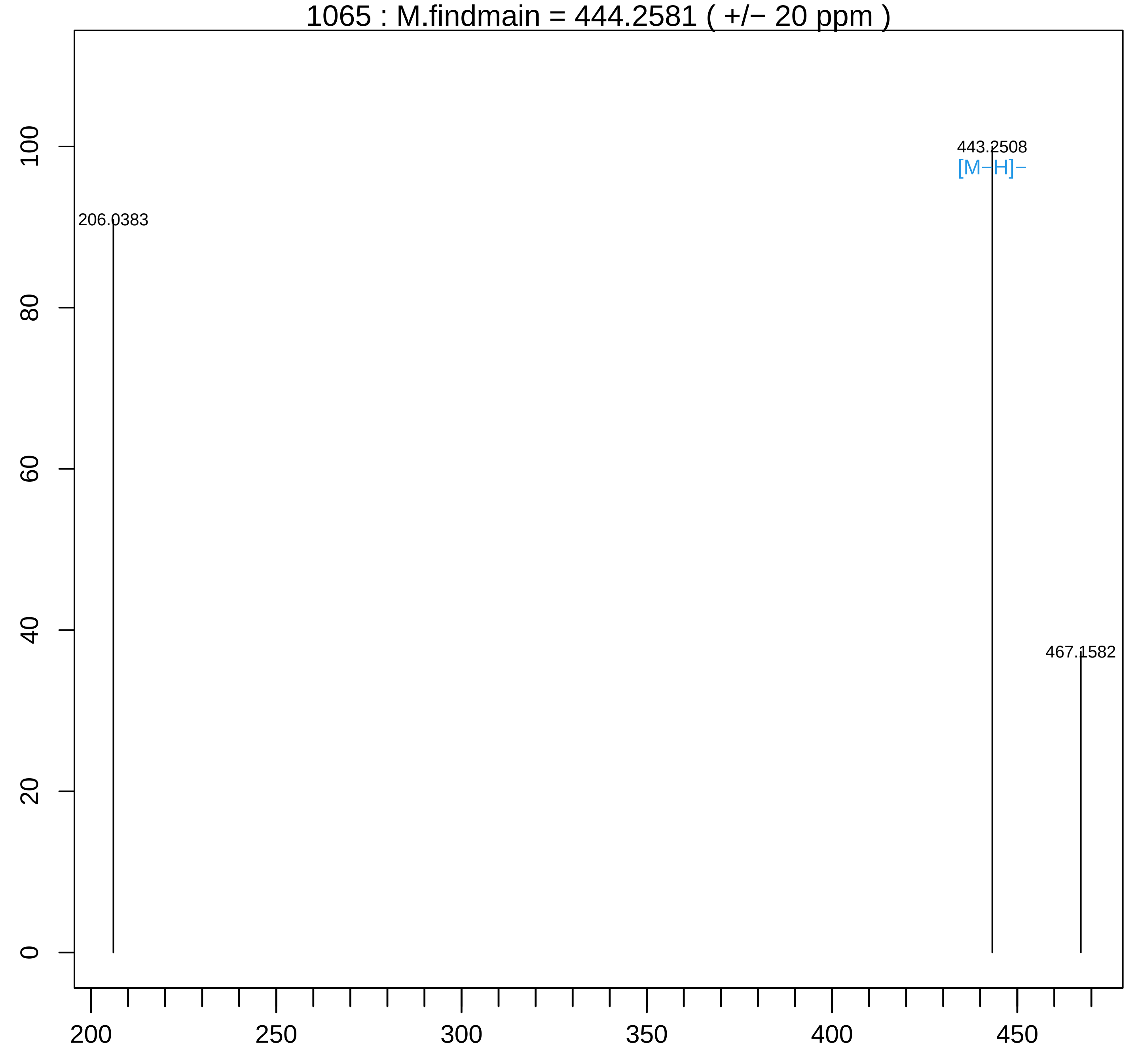
1065 : M.findmain = 444.2581 (+/− 20 ppm)
206.0383
443.2508
[M−H]−
467.1582
0
20
40
60
80
100
200
250
300
350
400
450

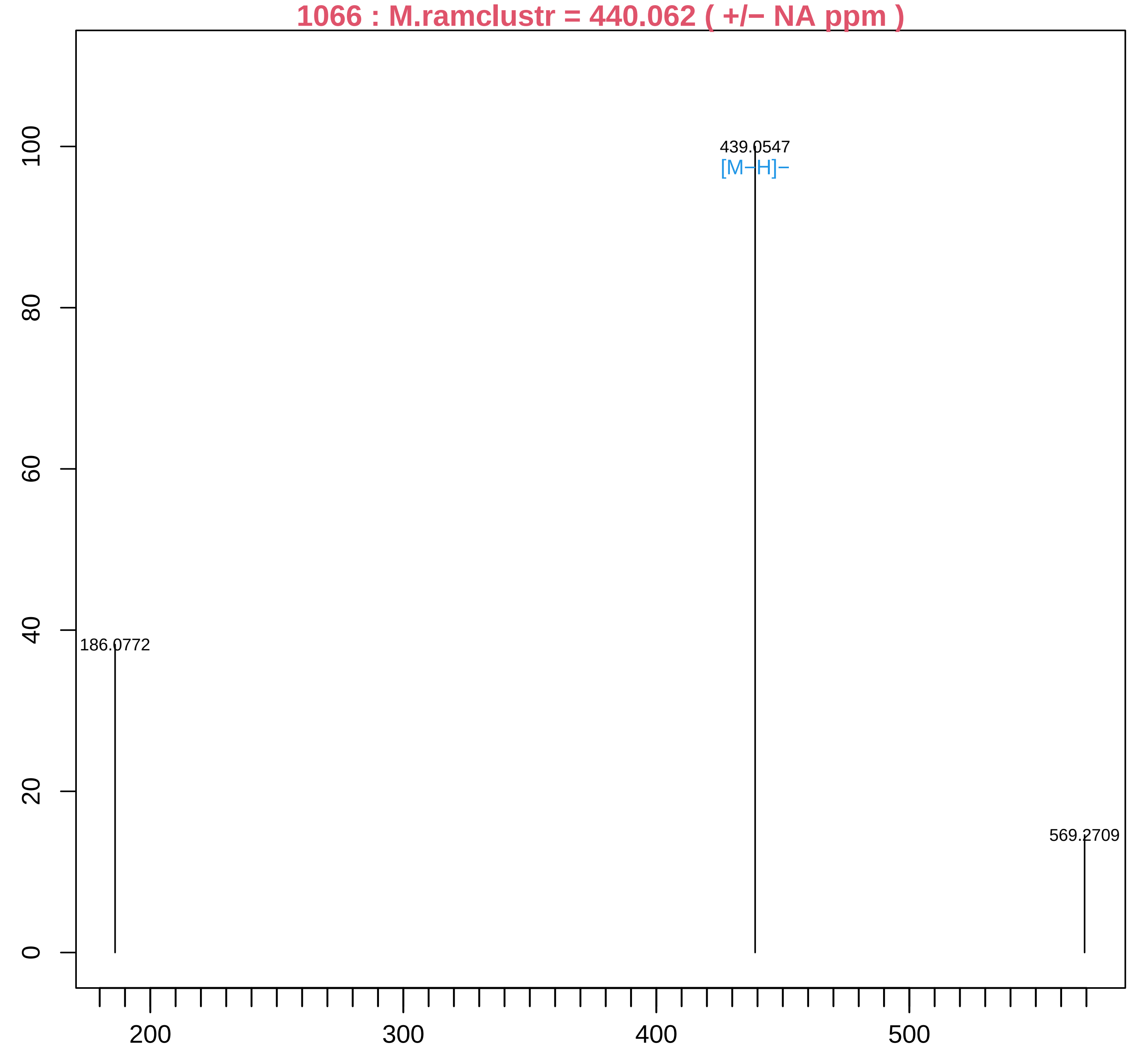
1066 : M.ramclustr = 440.062 (+/− NA ppm)
439.0547
[M−H]−
186.0772
569.2709
0
20
40
60
80
100
200
300
400
500

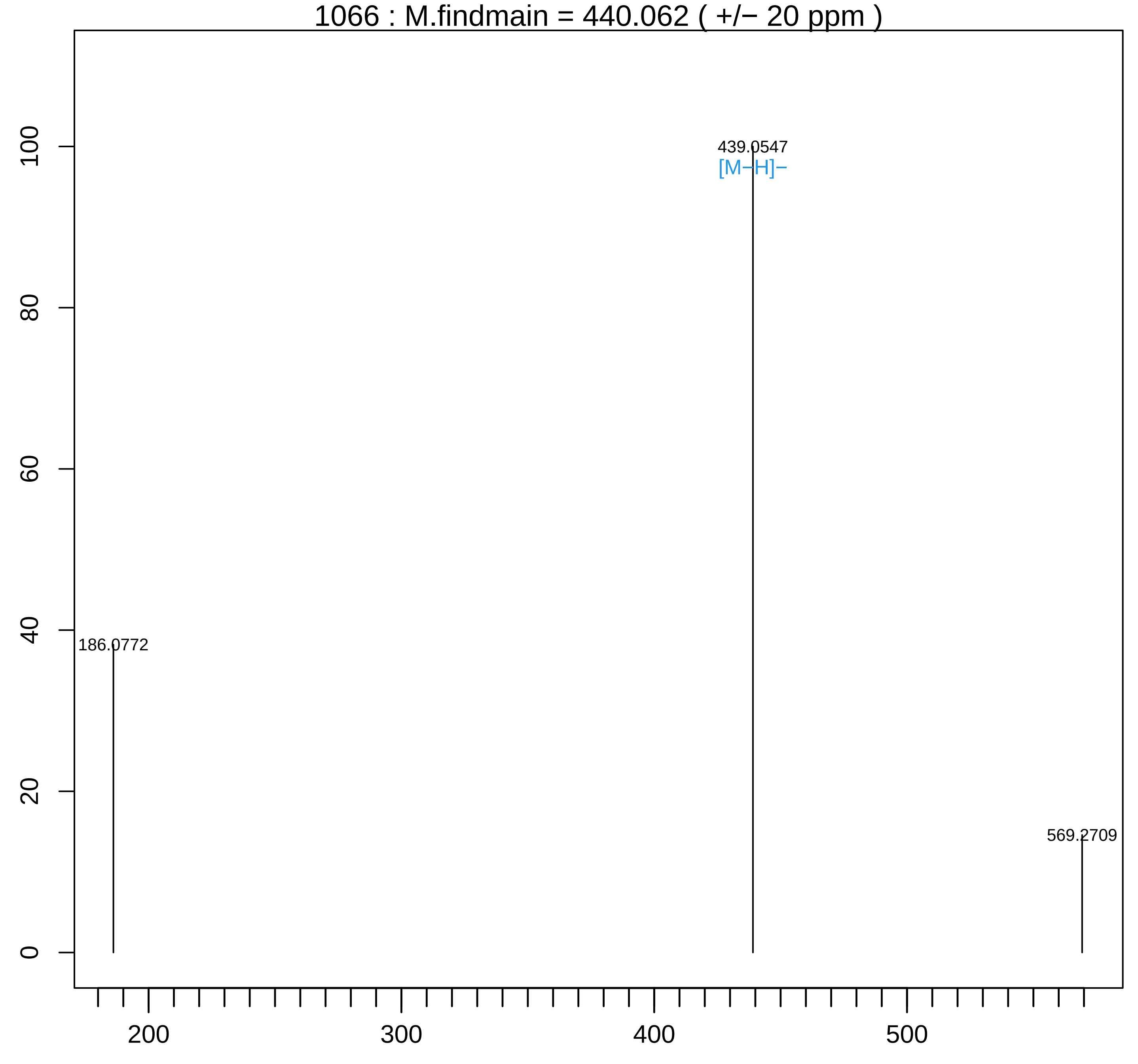
1066 : M.findmain = 440.062 (+/− 20 ppm)
439.0547
[M−H]−
186.0772
569.2709
0
20
40
60
80
100
200
300
400
500

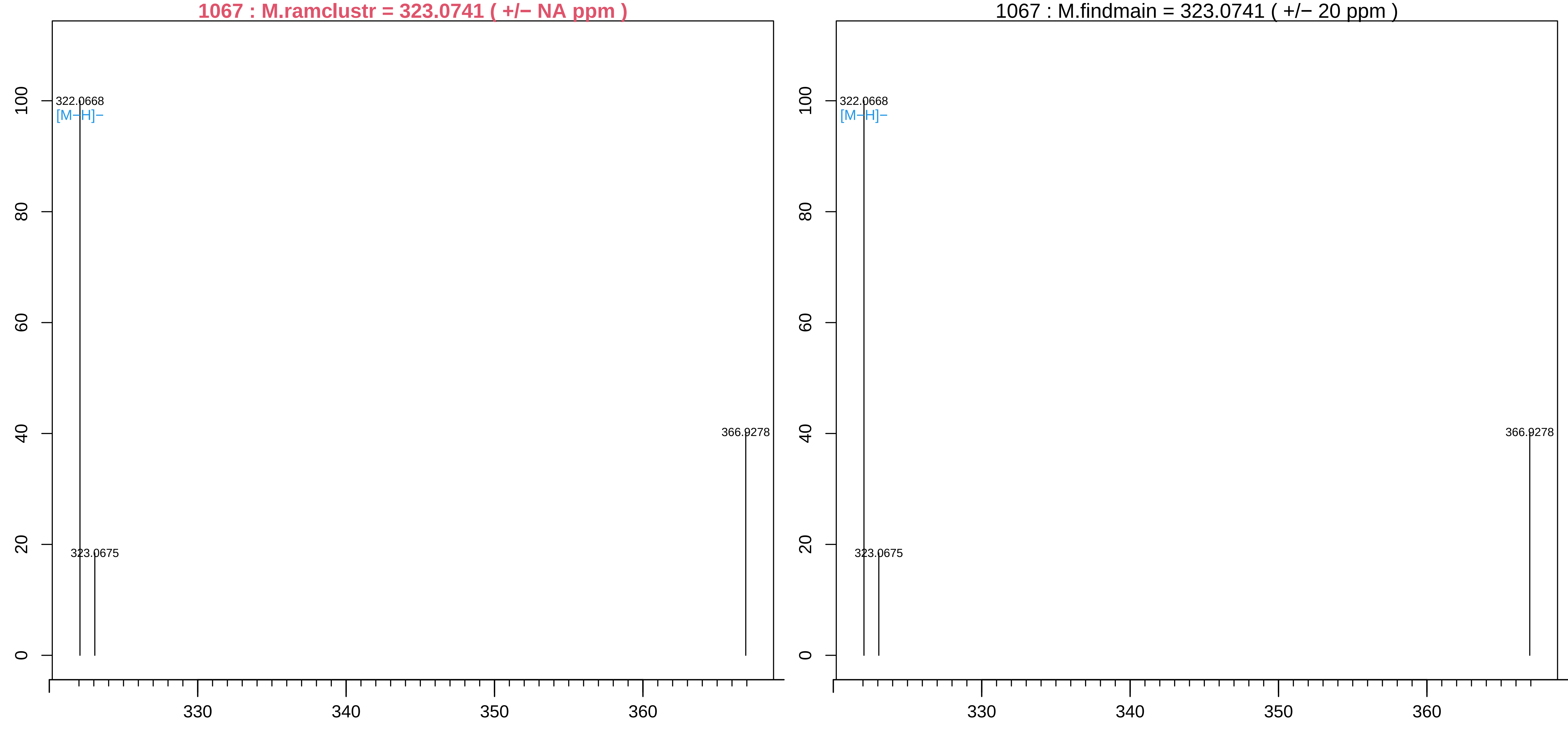
1067 : M.ramclustr = 323.0741 (+/− NA ppm)
322.0668
[M−H]−
323.0675
366.9278
0
20
40
60
80
100
330
340
350
360
1067 : M.findmain = 323.0741 (+/− 20 ppm)
322.0668
[M−H]−
323.0675
366.9278
0
20
40
60
80
100
330
340
350
360

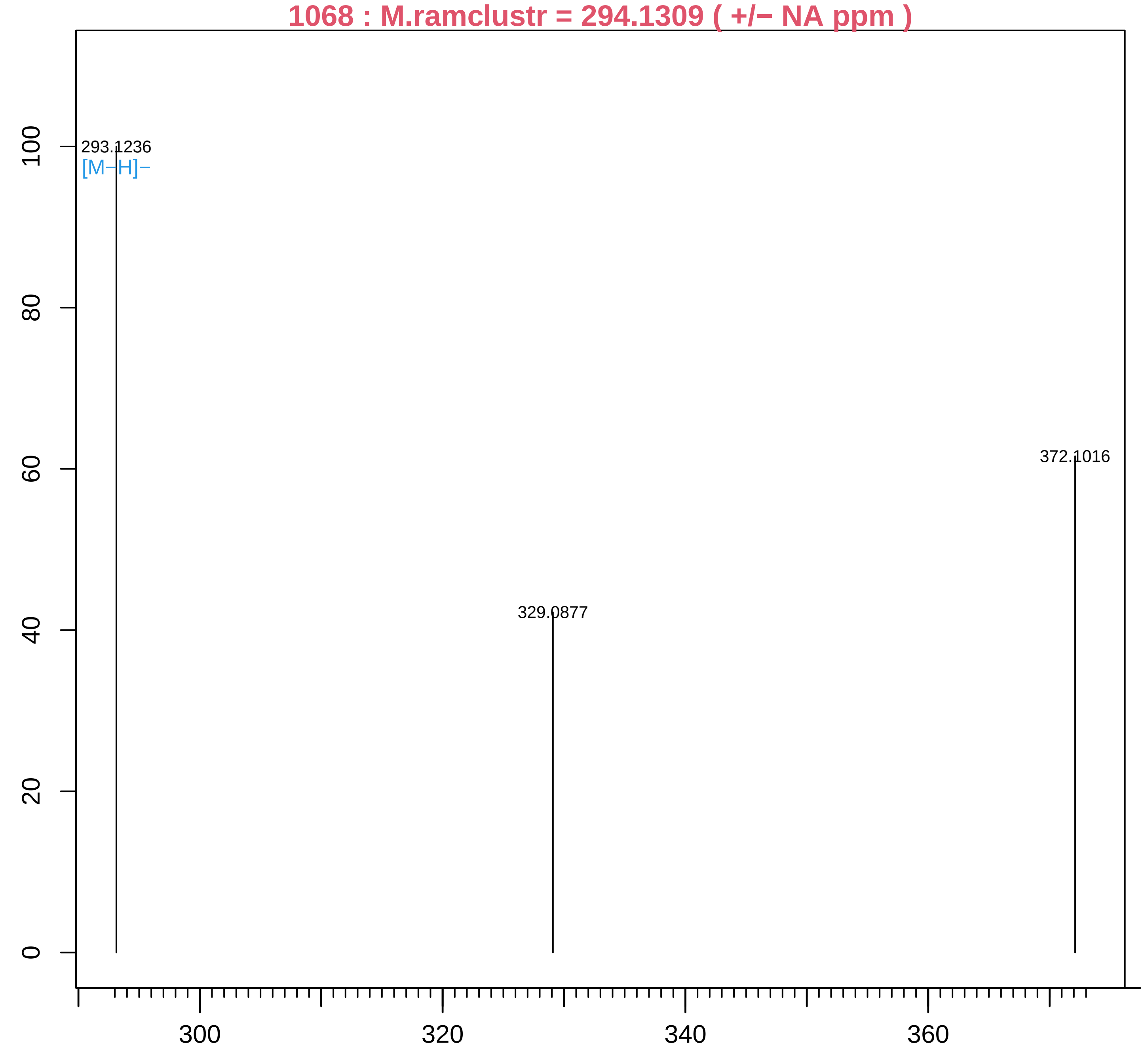
1068 : M.ramclustr = 294.1309 (+/− NA ppm)
293.1236
[M−H]−
329.0877
372.1016
0
20
40
60
80
100
300
320
340
360

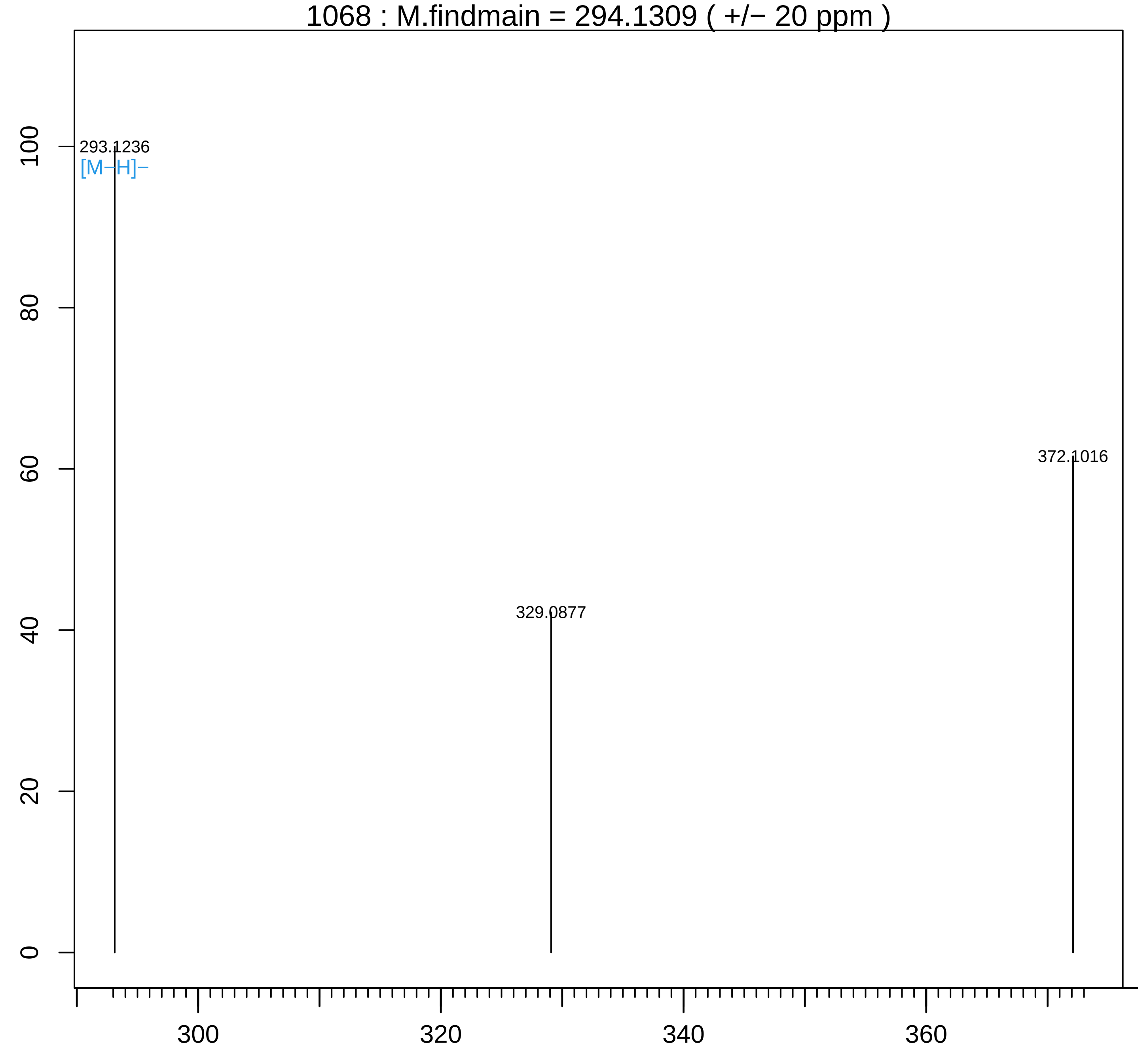
1068 : M.findmain = 294.1309 (+/− 20 ppm)
293.1236
[M−H]−
329.0877
372.1016
0
20
40
60
80
100
300
320
340
360

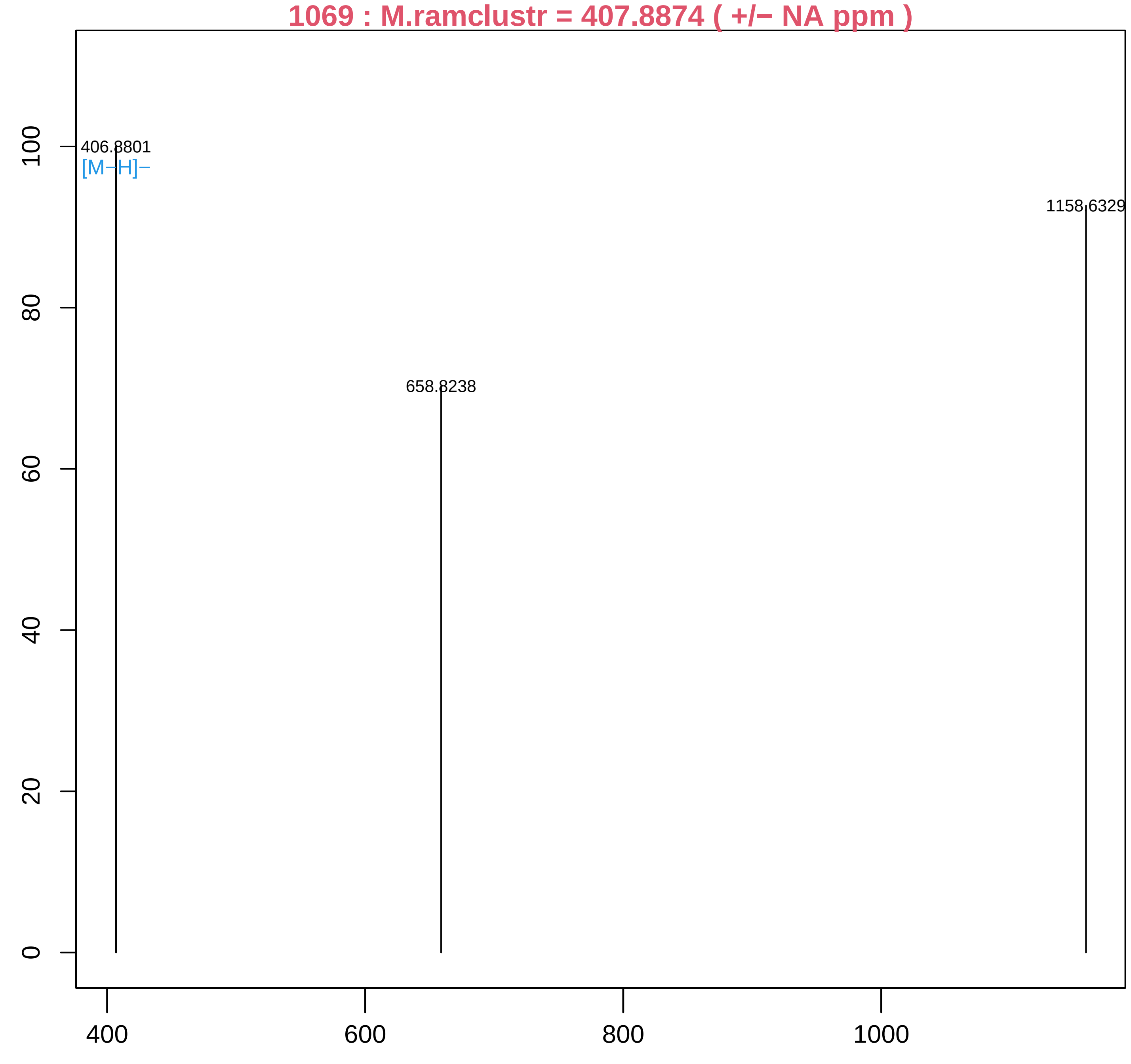
1069 : M.ramclustr = 407.8874 (+/− NA ppm)
406.8801
[M−H]−
658.8238
1158.6329
0
20
40
60
80
100
400
600
800
1000

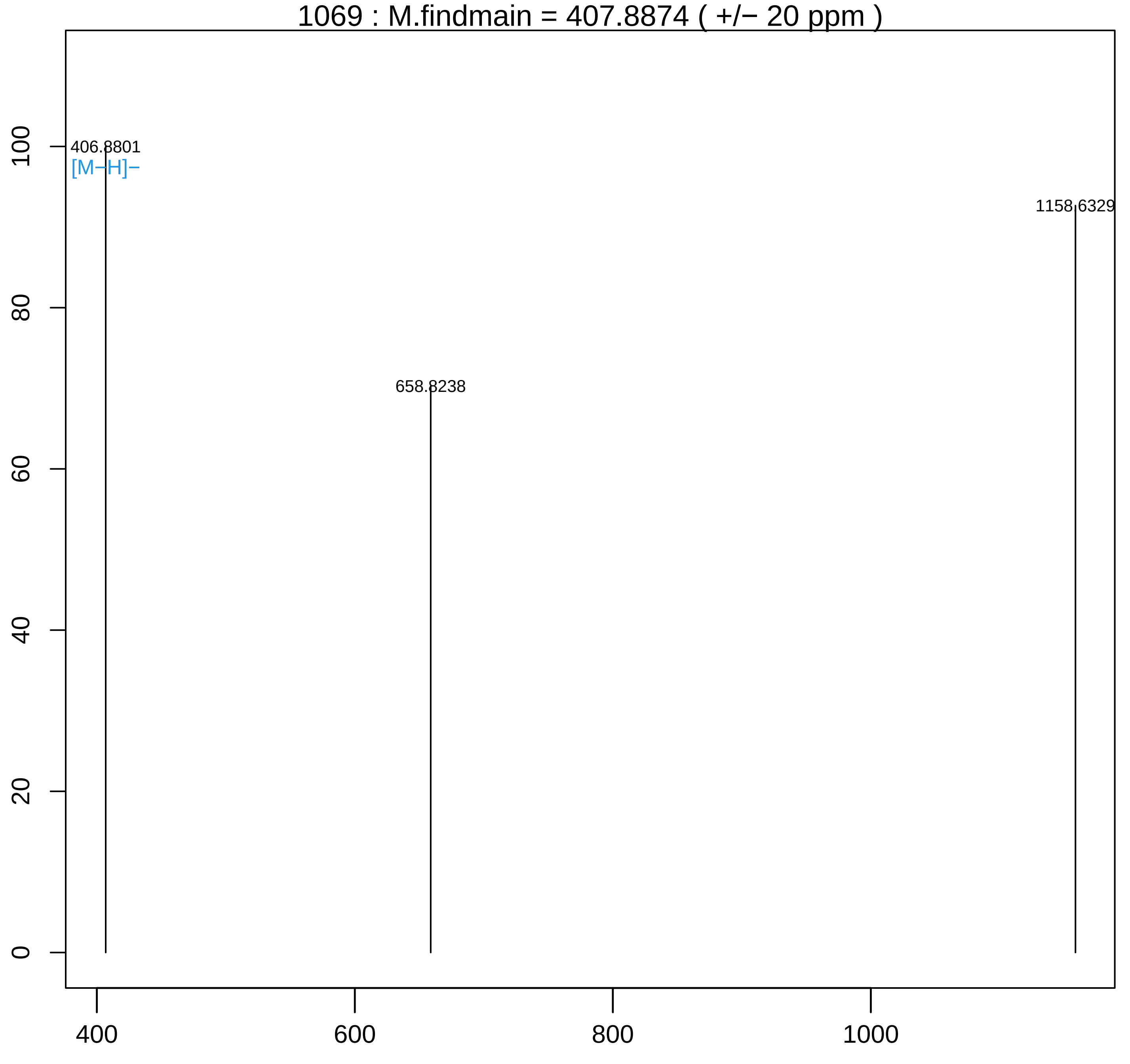
1069 : M.findmain = 407.8874 (+/− 20 ppm)
406.8801
[M−H]−
658.8238
1158.6329
0
20
40
60
80
100
400
600
800
1000

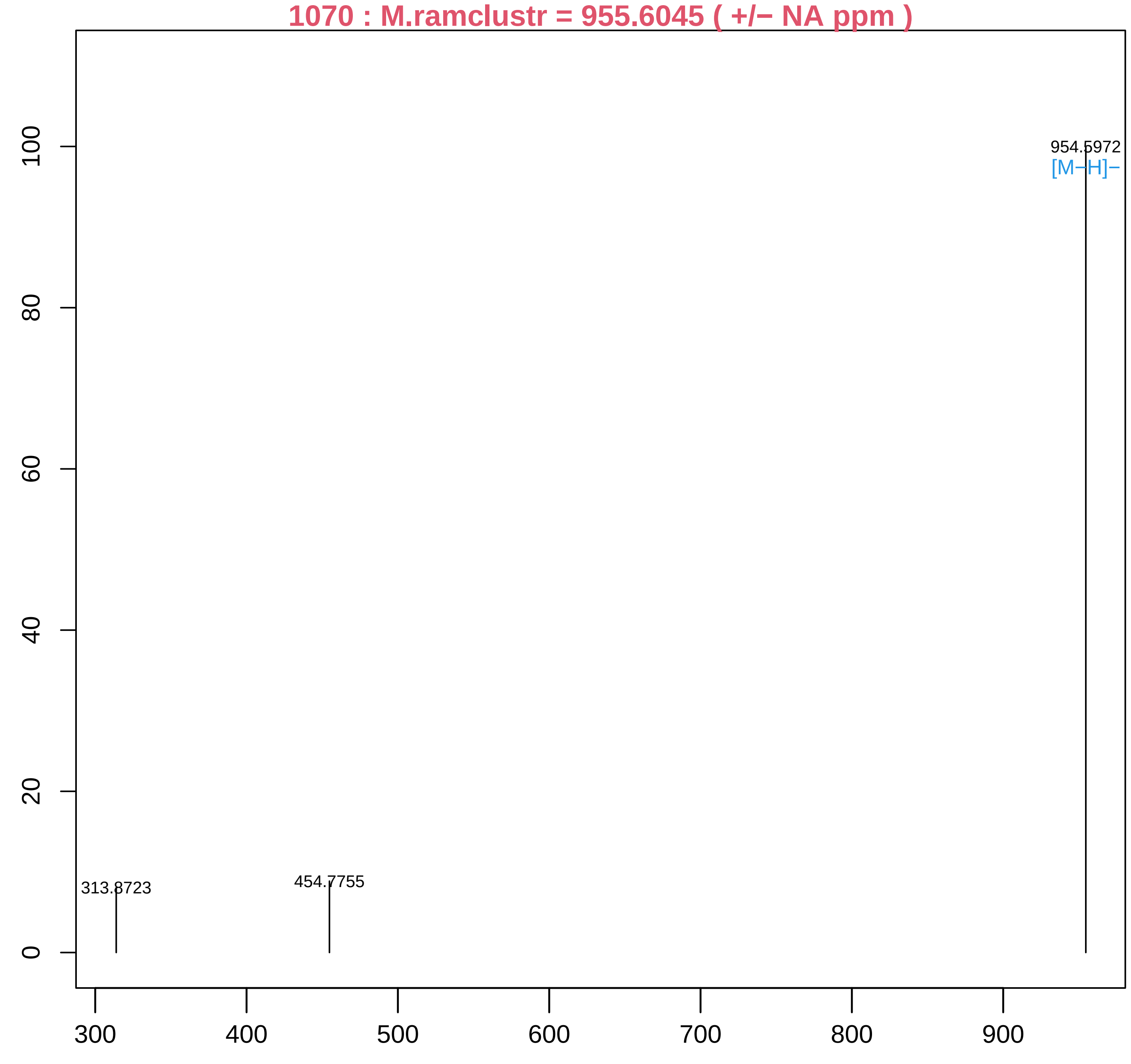
1070 : M.ramclustr = 955.6045 (+/− NA ppm)
954.5972
[M−H]−
313.8723
454.7755
0
20
40
60
80
100
300
400
500
600
700
800
900

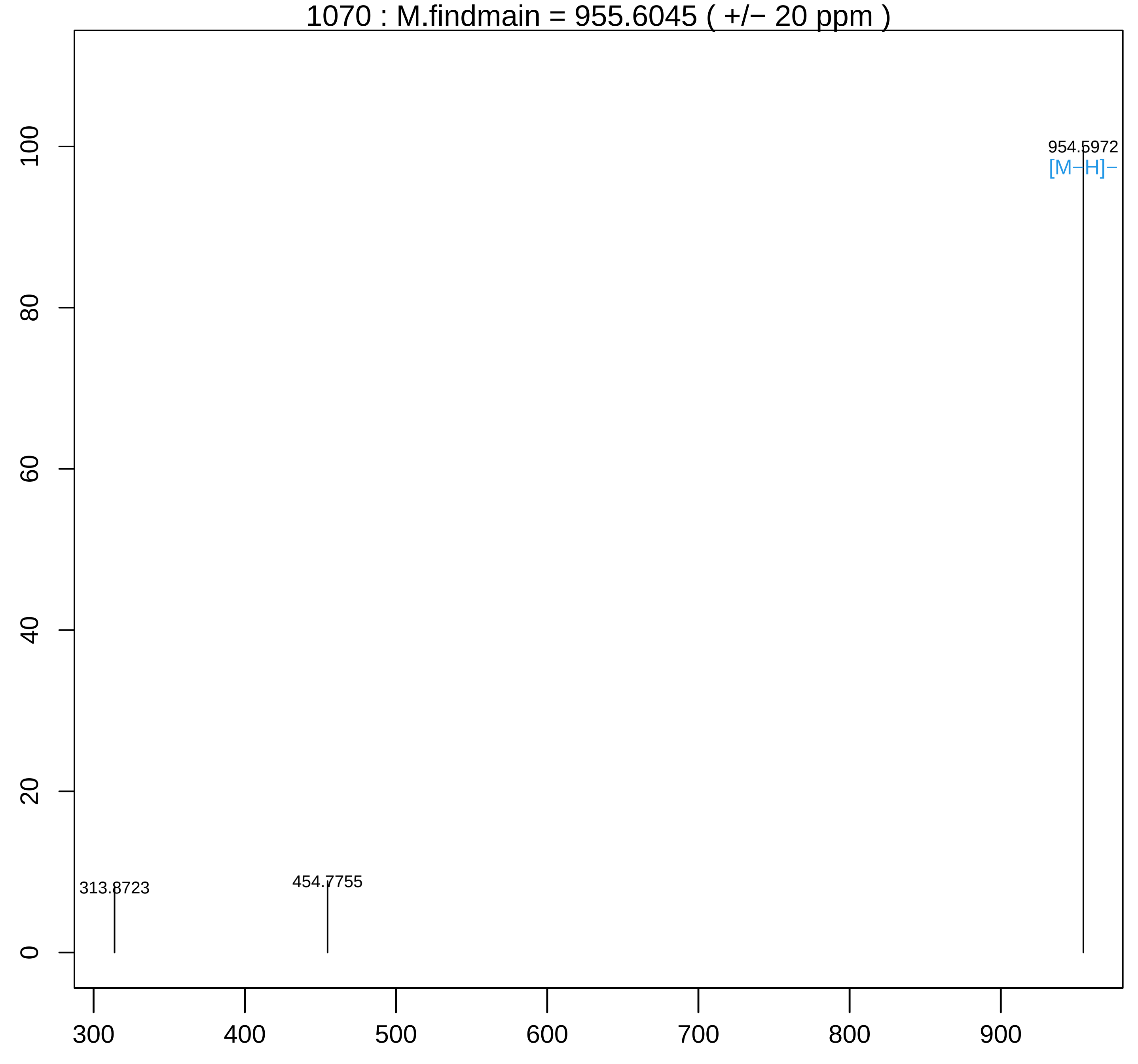
1070 : M.findmain = 955.6045 (+/− 20 ppm)
954.5972
[M−H]−
313.8723
454.7755
0
20
40
60
80
100
300
400
500
600
700
800
900

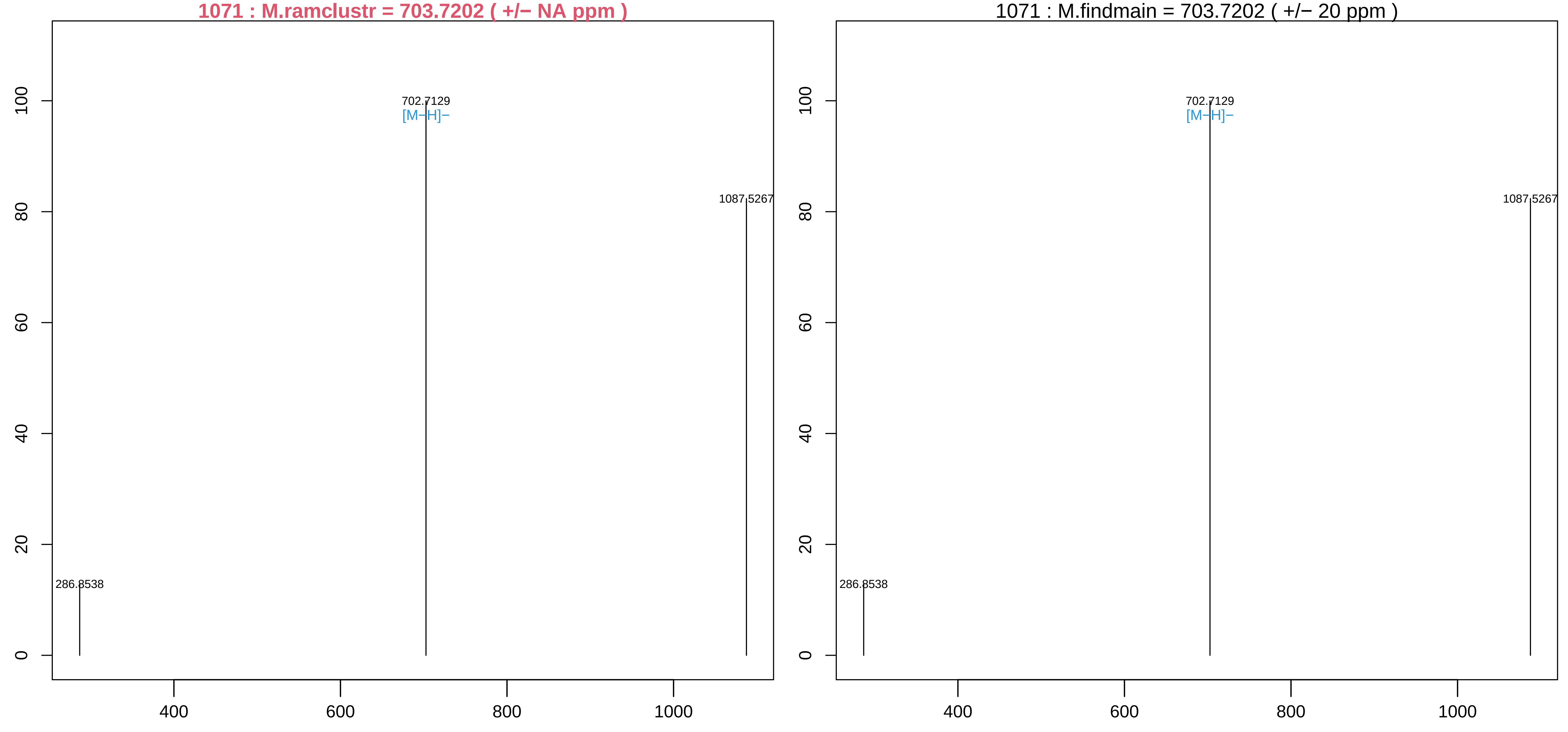
1071 : M.ramclustr = 703.7202 (+/− NA ppm)
702.7129
[M−H]−
1087.5267
286.8538
0
20
40
60
80
100
400
600
800
1000
1071 : M.findmain = 703.7202 (+/− 20 ppm)
702.7129
[M−H]−
1087.5267
286.8538
0
20
40
60
80
100
400
600
800
1000

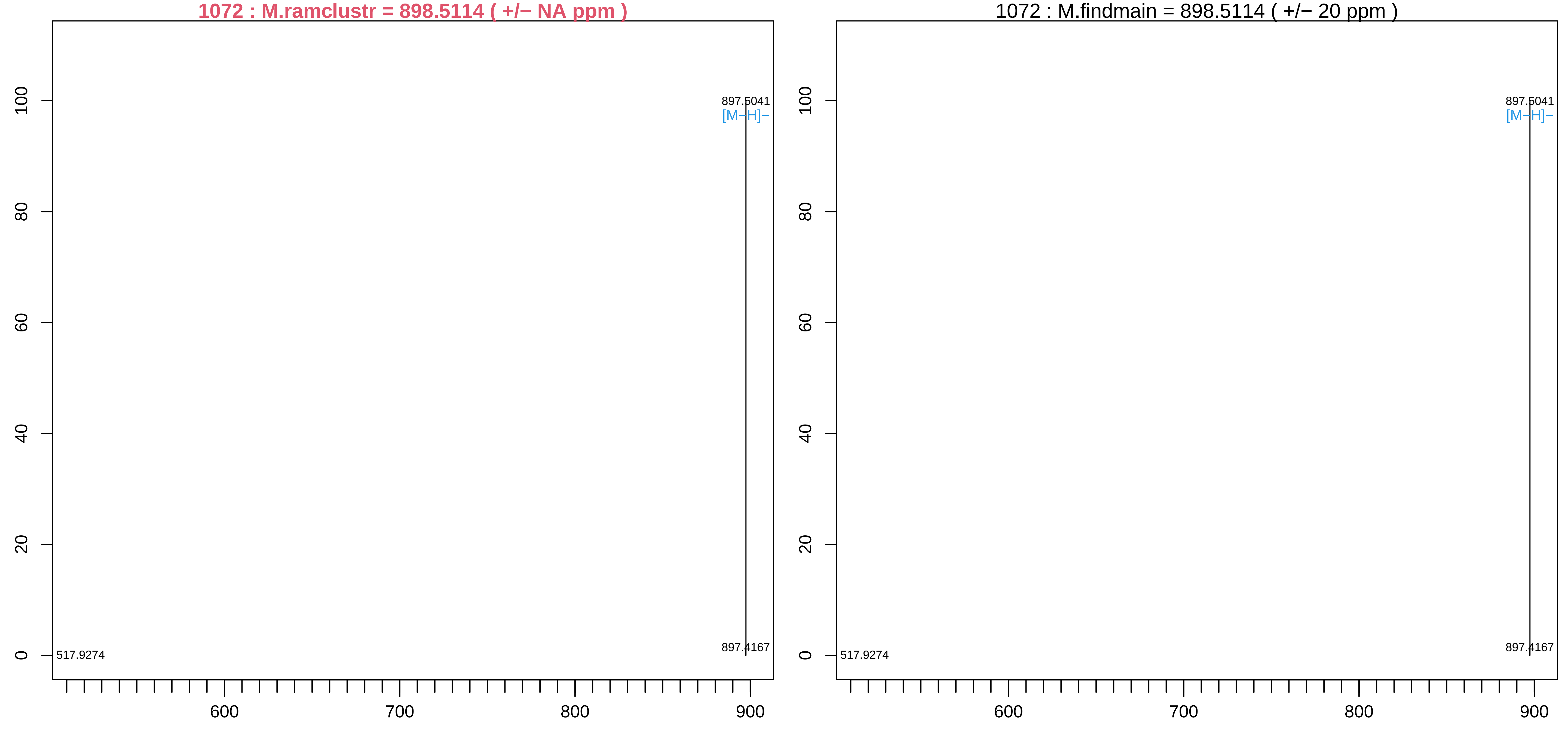
1072 : M.ramclustr = 898.5114 (+/− NA ppm)
897.5041
[M−H]−
517.9274
897.4167
1072 : M.findmain = 898.5114 (+/− 20 ppm)
897.5041
[M−H]−
517.9274
897.4167

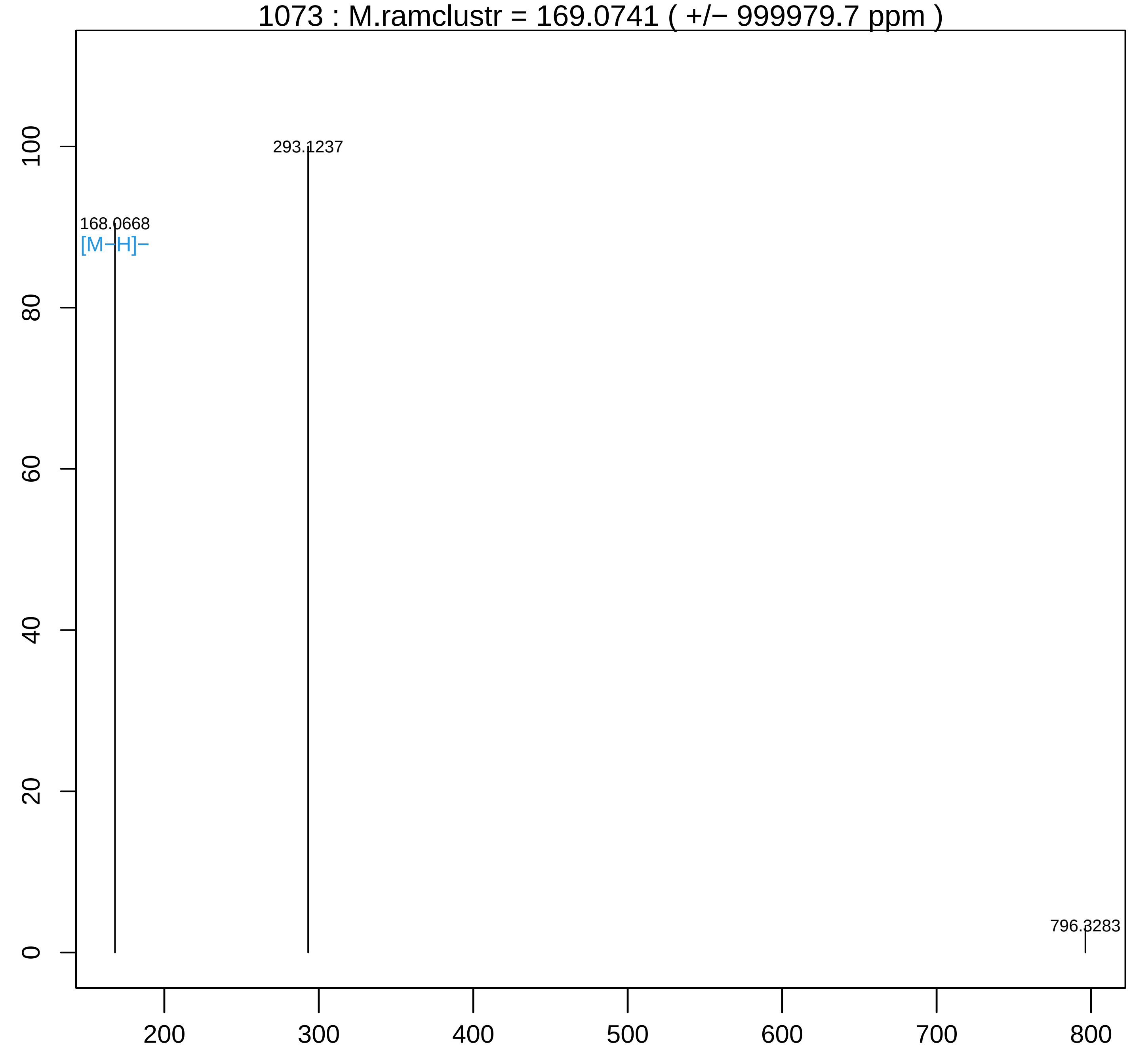
1073 : M.ramclustr = 169.0741 (+/− 999979.7 ppm)
293.1237
168.0668
[M−H]−
796.3283
0
20
40
60
80
100
200
300
400
500
600
700
800
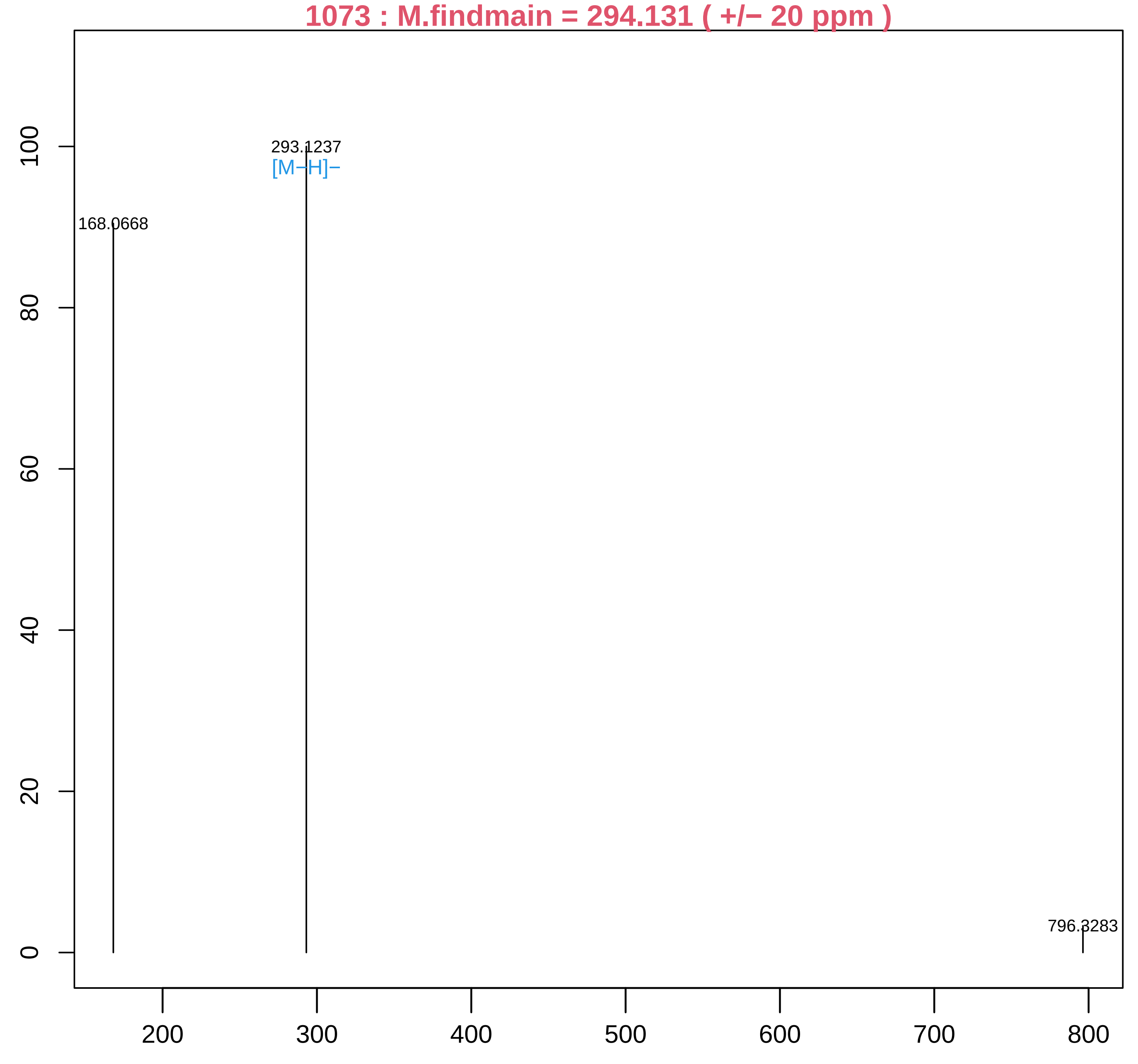
1073 : M.findmain = 294.131 (+/− 20 ppm)
293.1237
[M−H]−
168.0668
796.3283
0
20
40
60
80
100
200
300
400
500
600
700
800

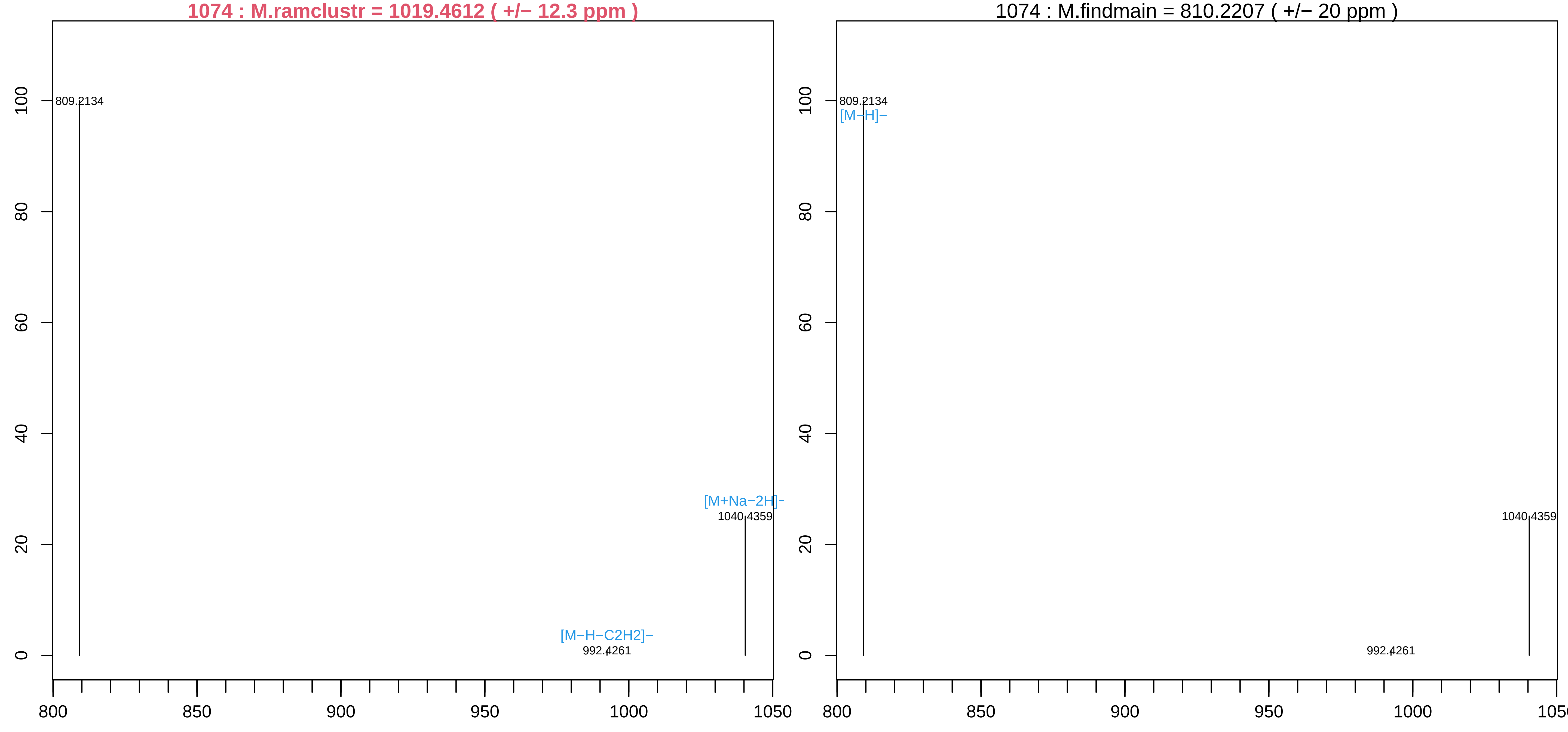

1074 : M.ramclustr = 1019.4612 (+/− 12.3 ppm)
809.2134
[M+Na−2H]−
1040.4359
[M−H−C2H2]−
992.4261
1074 : M.findmain = 810.2207 (+/− 20 ppm)
809.2134
[M−H]−
1040.4359
992.4261

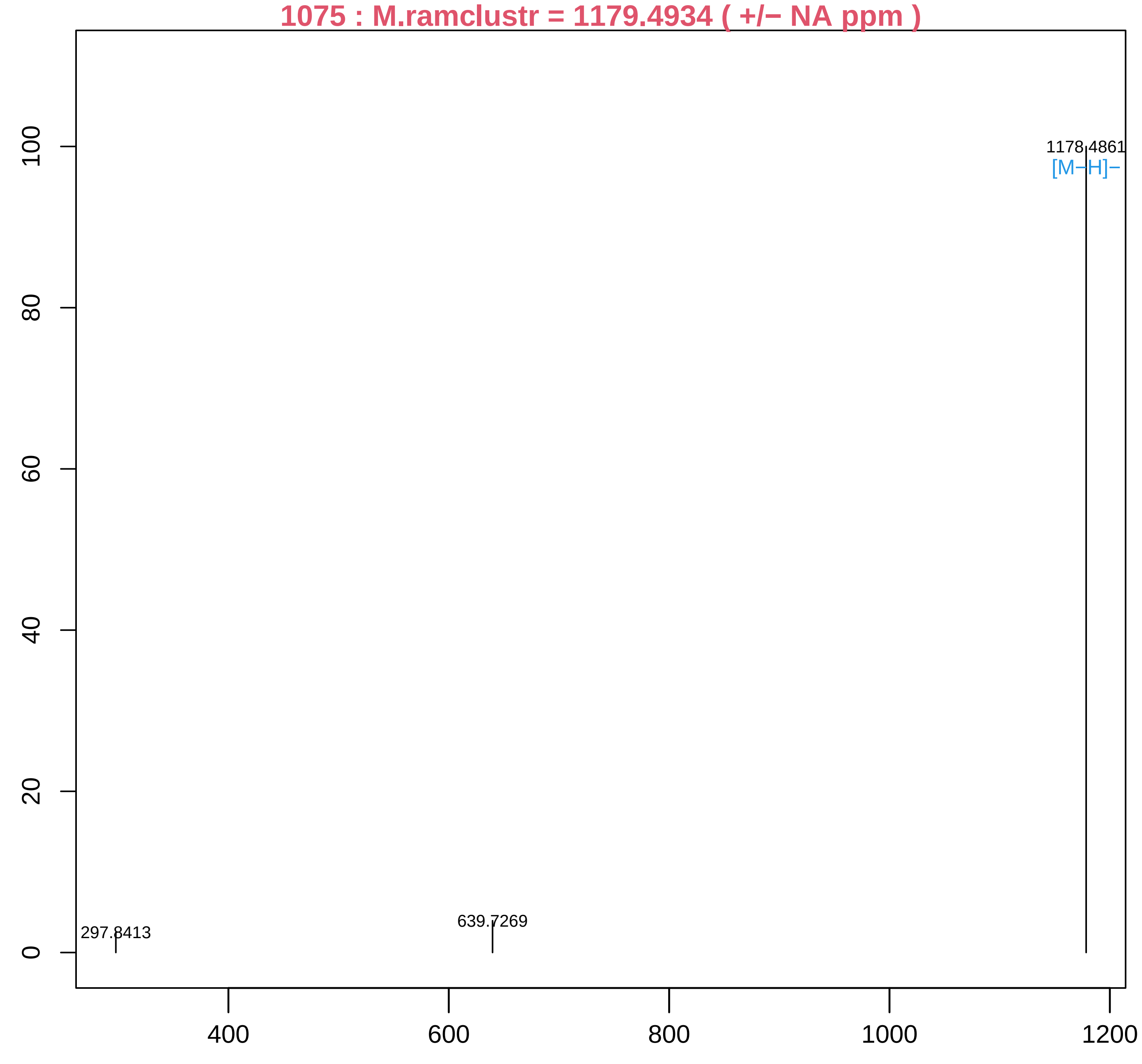
1075 : M.ramclustr = 1179.4934 (+/− NA ppm)
1178.4861
[M−H]−
639.7269
297.8413
0
20
40
60
80
100
400
600
800
1000
1200

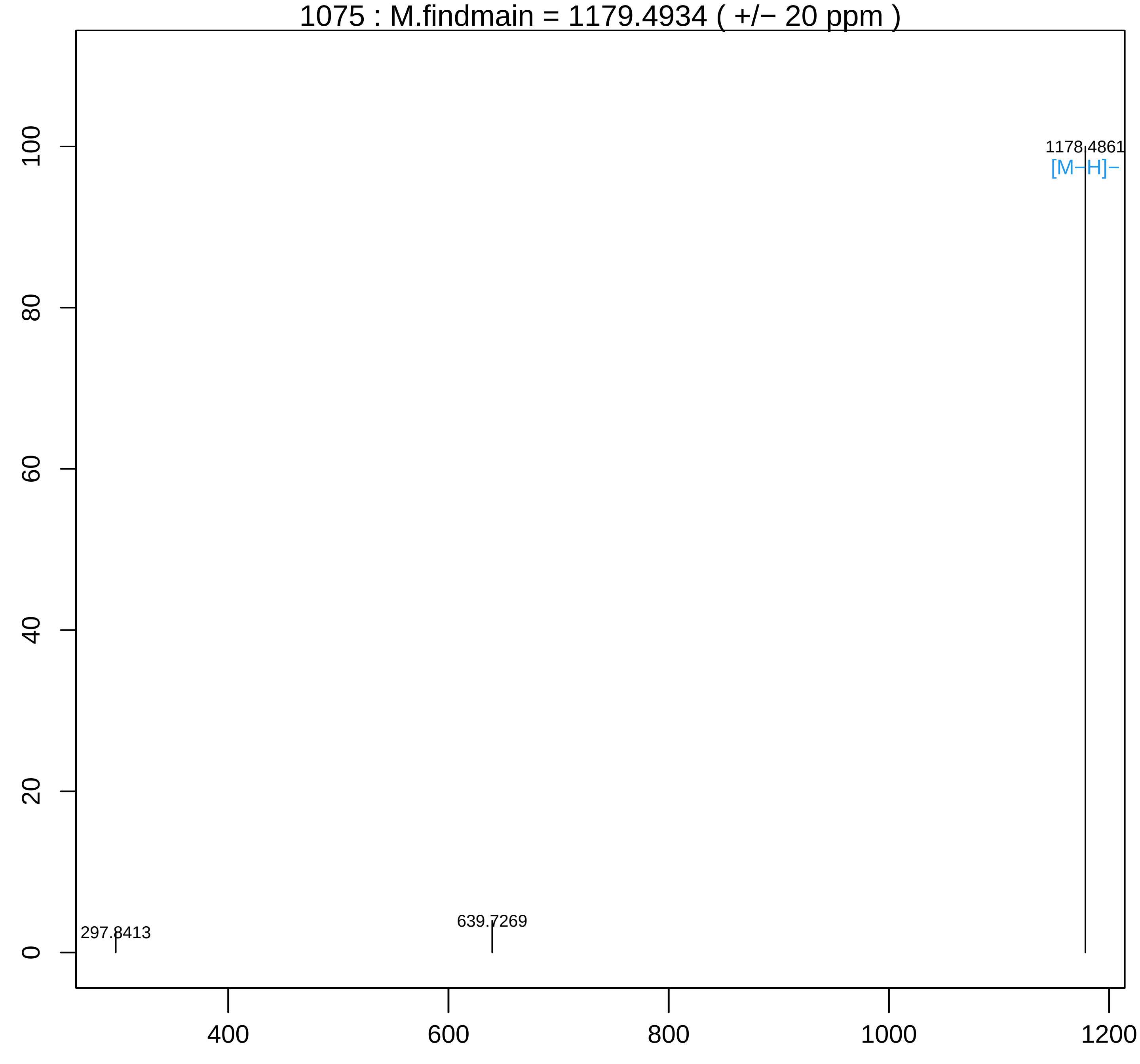
1075 : M.findmain = 1179.4934 (+/− 20 ppm)
1178.4861
[M−H]−
639.7269
297.8413
0
20
40
60
80
100
400
600
800
1000
1200

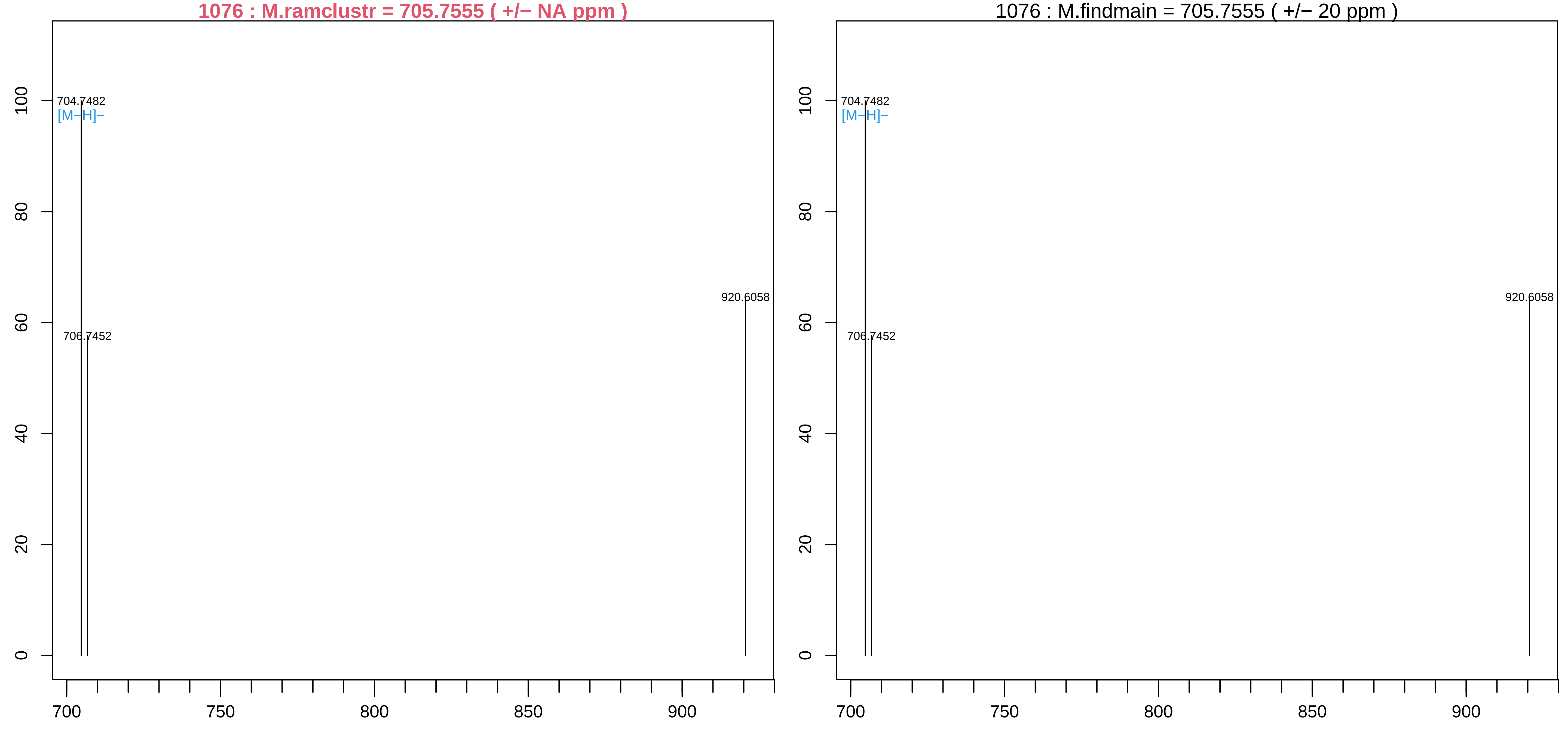
1076 : M.ramclustr = 705.7555 (+/− NA ppm)
704.7482
[M−H]−
706.7452
920.6058
0
20
40
60
80
100
700
750
800
850
900
1076 : M.findmain = 705.7555 (+/− 20 ppm)
704.7482
[M−H]−
706.7452
920.6058
0
20
40
60
80
100
700
750
800
850
900

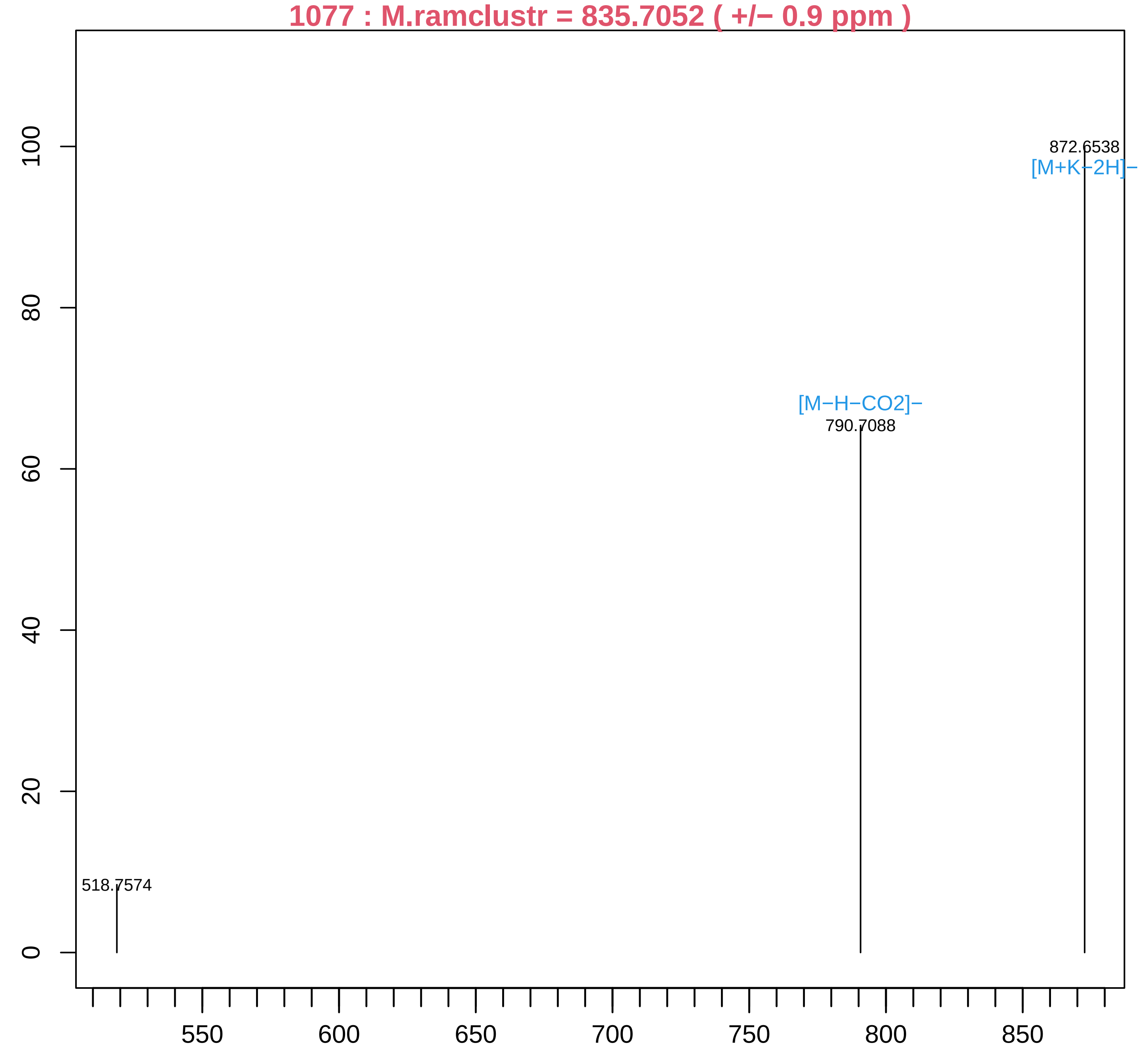
1077 : M.ramclustr = 835.7052 (+/− 0.9 ppm)
872.6538
[M+K−2H]−
[M−H−CO2]−
790.7088
518.7574
0
20
40
60
80
100
550
600
650
700
750
800
850

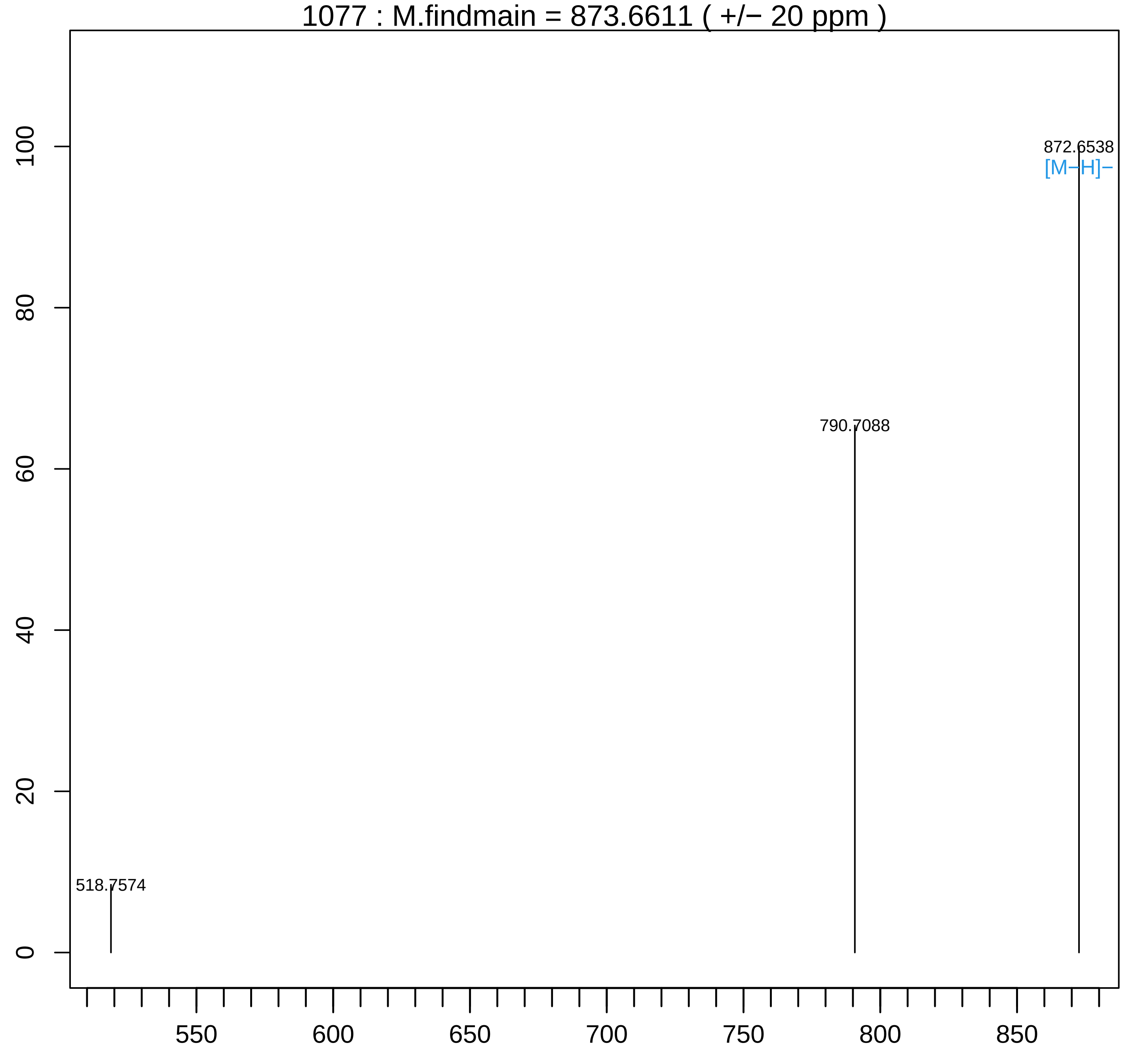
1077 : M.findmain = 873.6611 (+/− 20 ppm)
872.6538
[M−H]−
790.7088
518.7574
0
20
40
60
80
100
550
600
650
700
750
800
850

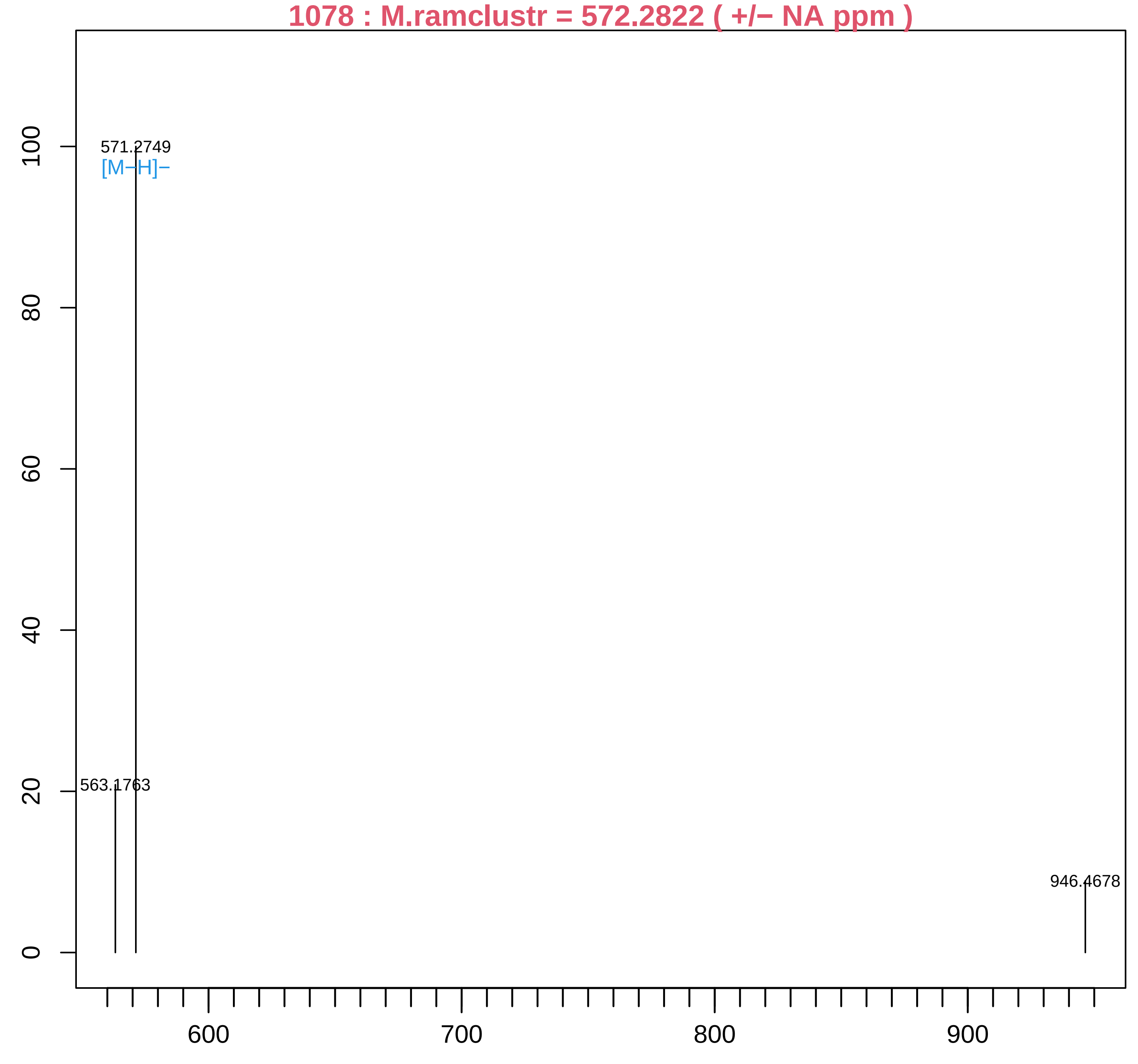
1078 : M.ramclustr = 572.2822 (+/− NA ppm)
571.2749
[M−H]−
563.1763
946.4678
0
20
40
60
80
100
600
700
800
900

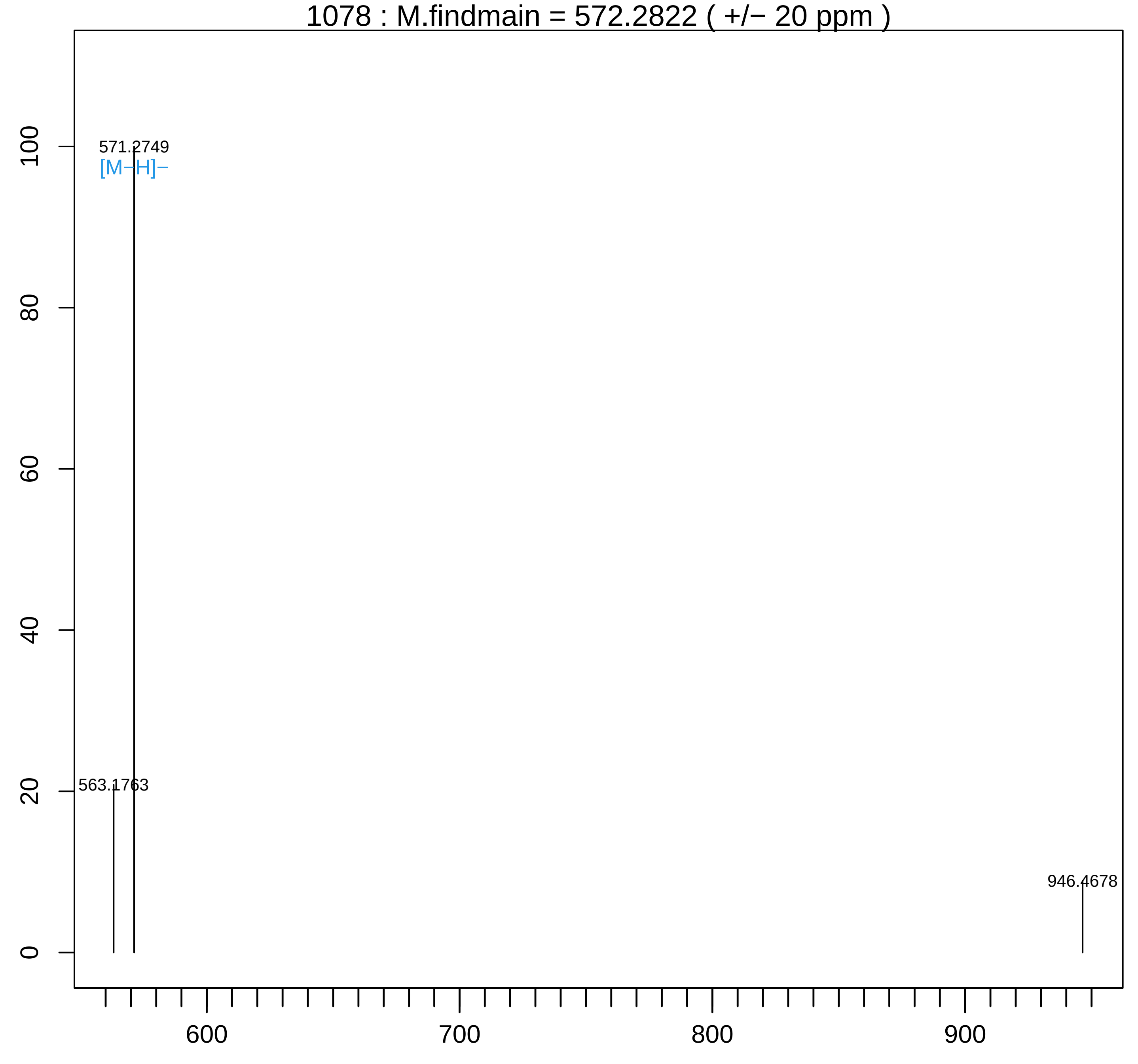
1078 : M.findmain = 572.2822 (+/− 20 ppm)
571.2749
[M−H]−
563.1763
946.4678
0
20
40
60
80
100
600
700
800
900

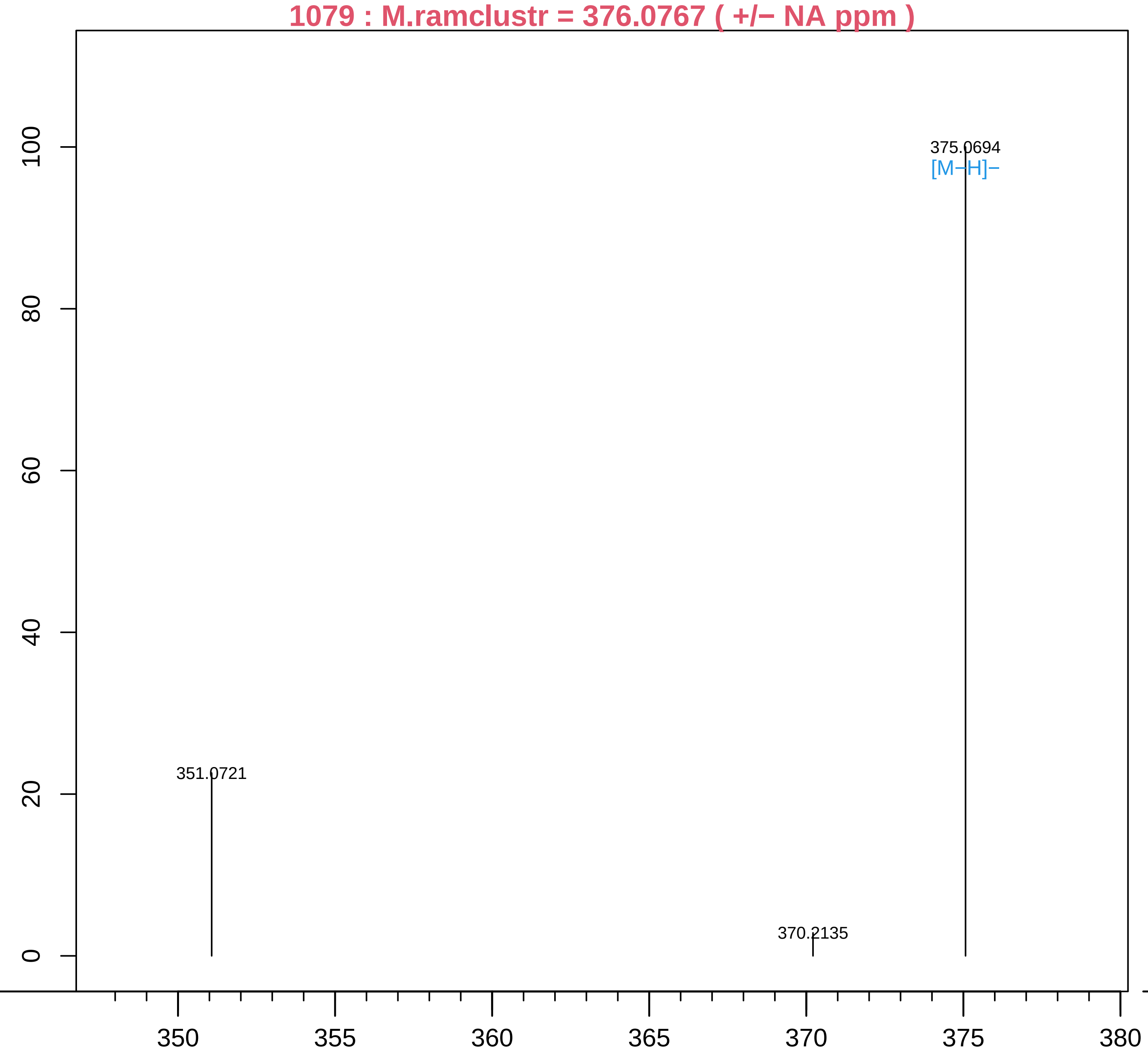
1079 : M.ramclustr = 376.0767 (+/− NA ppm)
375.0694
[M−H]−
351.0721
370.2135
0
20
40
60
80
100
350
355
360
365
370
375
380

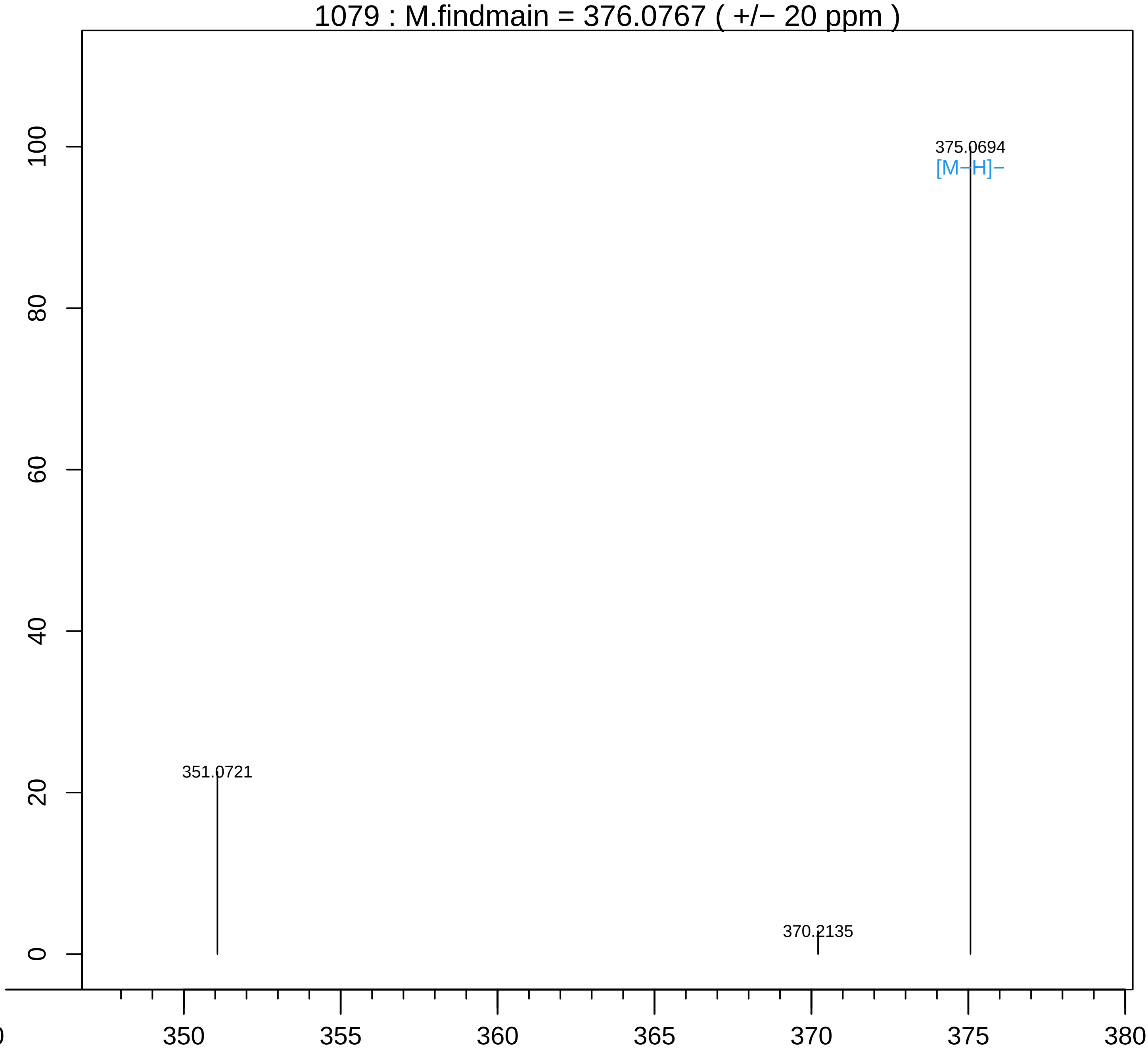
1079 : M.findmain = 376.0767 (+/− 20 ppm)
375.0694
[M−H]−
351.0721
370.2135
0
20
40
60
80
100
350
355
360
365
370
375
380

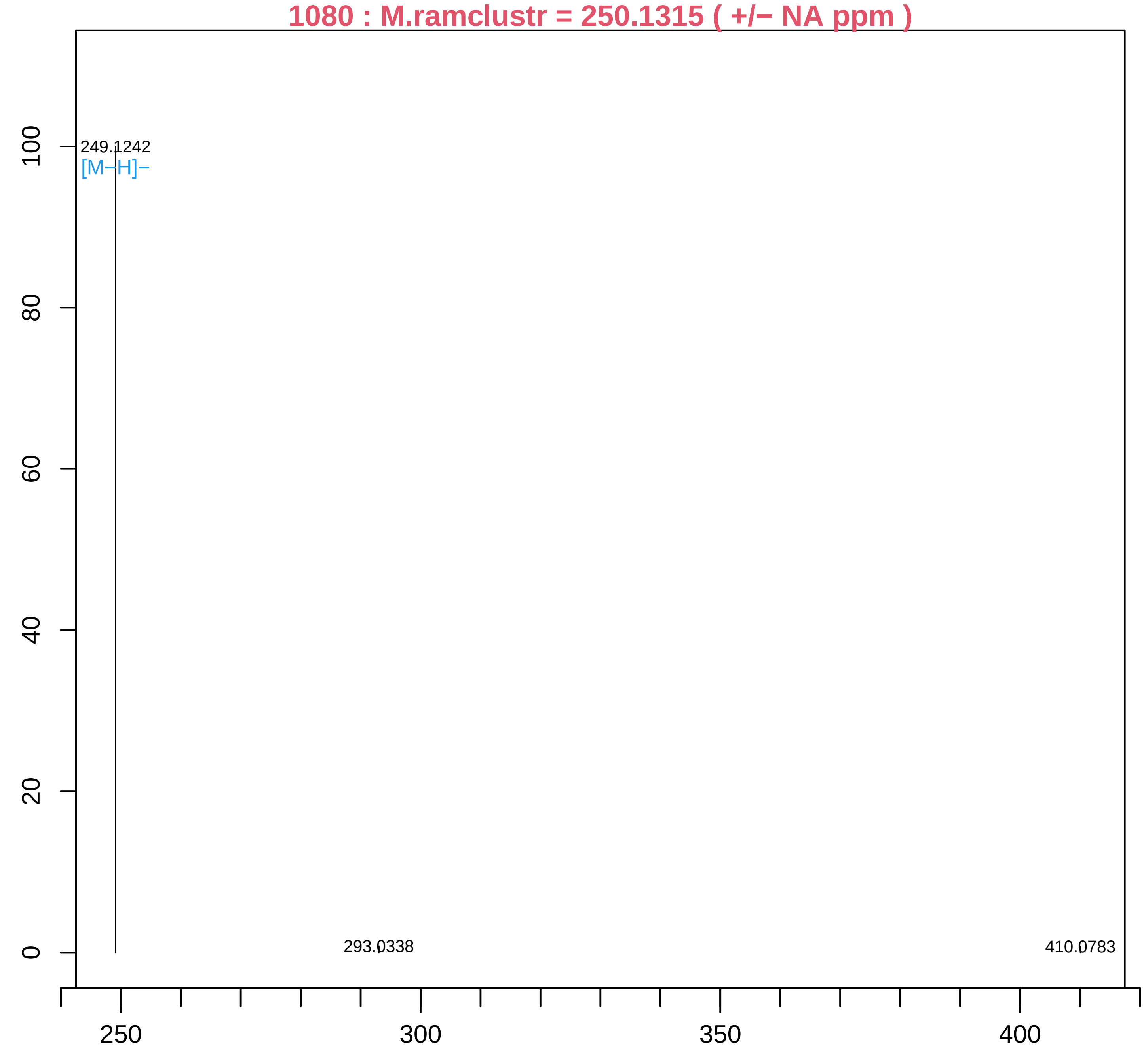
1080 : M.ramclustr = 250.1315 (+/− NA ppm)
249.1242
[M−H]−
293.0338
410.0783
0
20
40
60
80
100
250
300
350
400

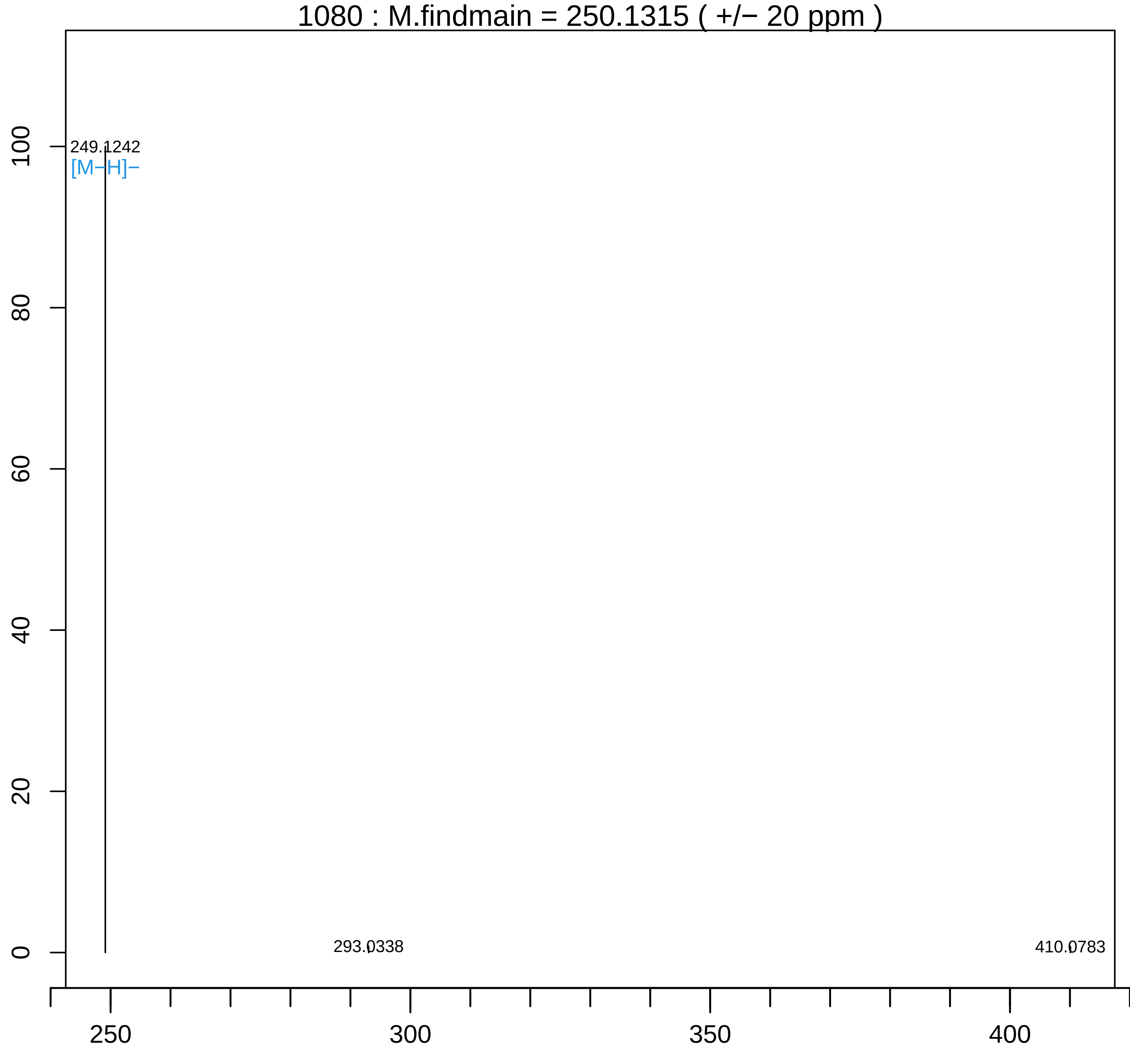
1080 : M.findmain = 250.1315 (+/− 20 ppm)
249.1242
[M−H]−
293.0338
410.0783
0
20
40
60
80
100
250
300
350
400

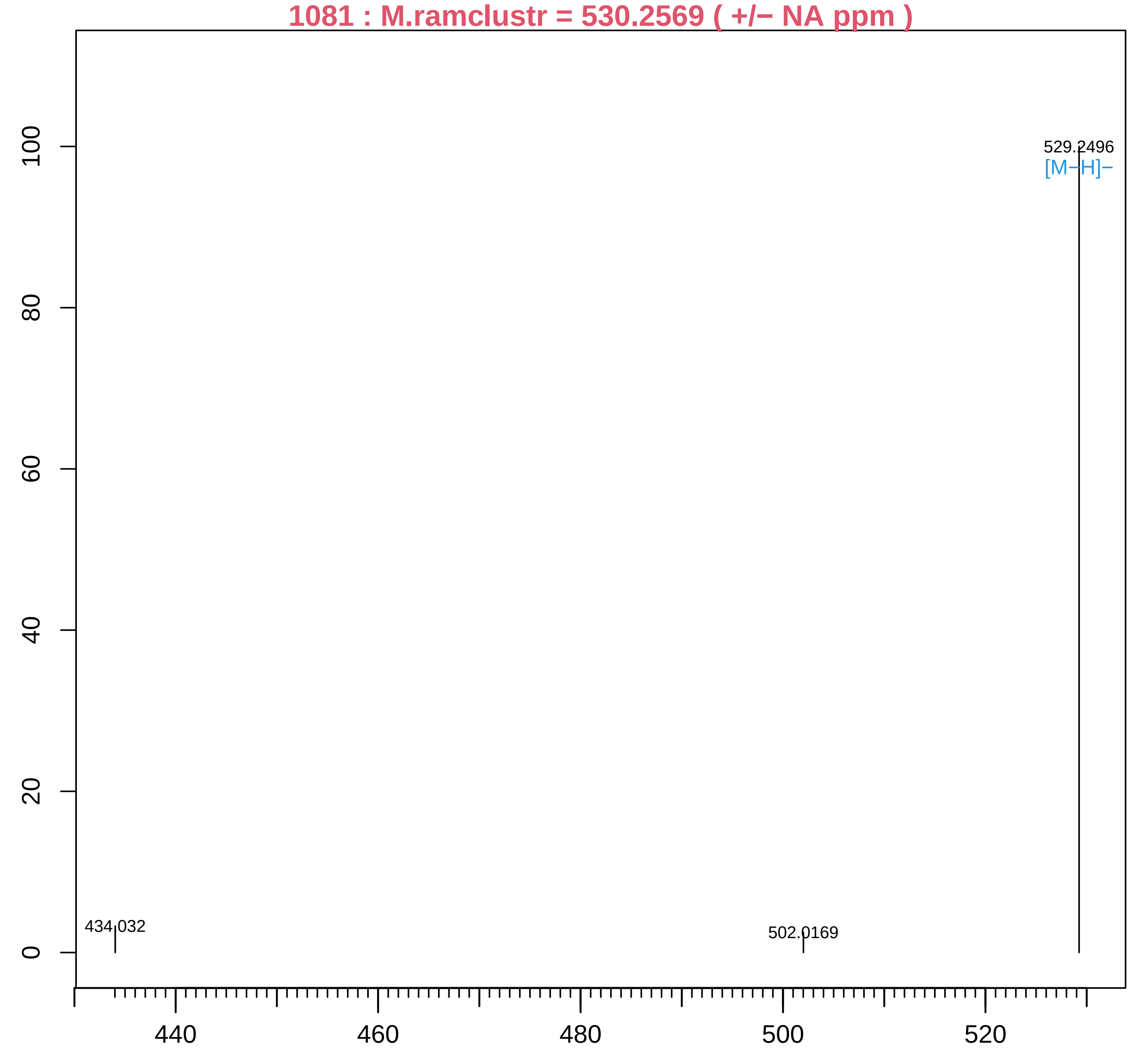

1081 : M.ramclustr = 530.2569 (+/− NA ppm)
529.2496
[M−H]−
434.032
502.0169
0
20
40
60
80
100
440
460
480
500
520

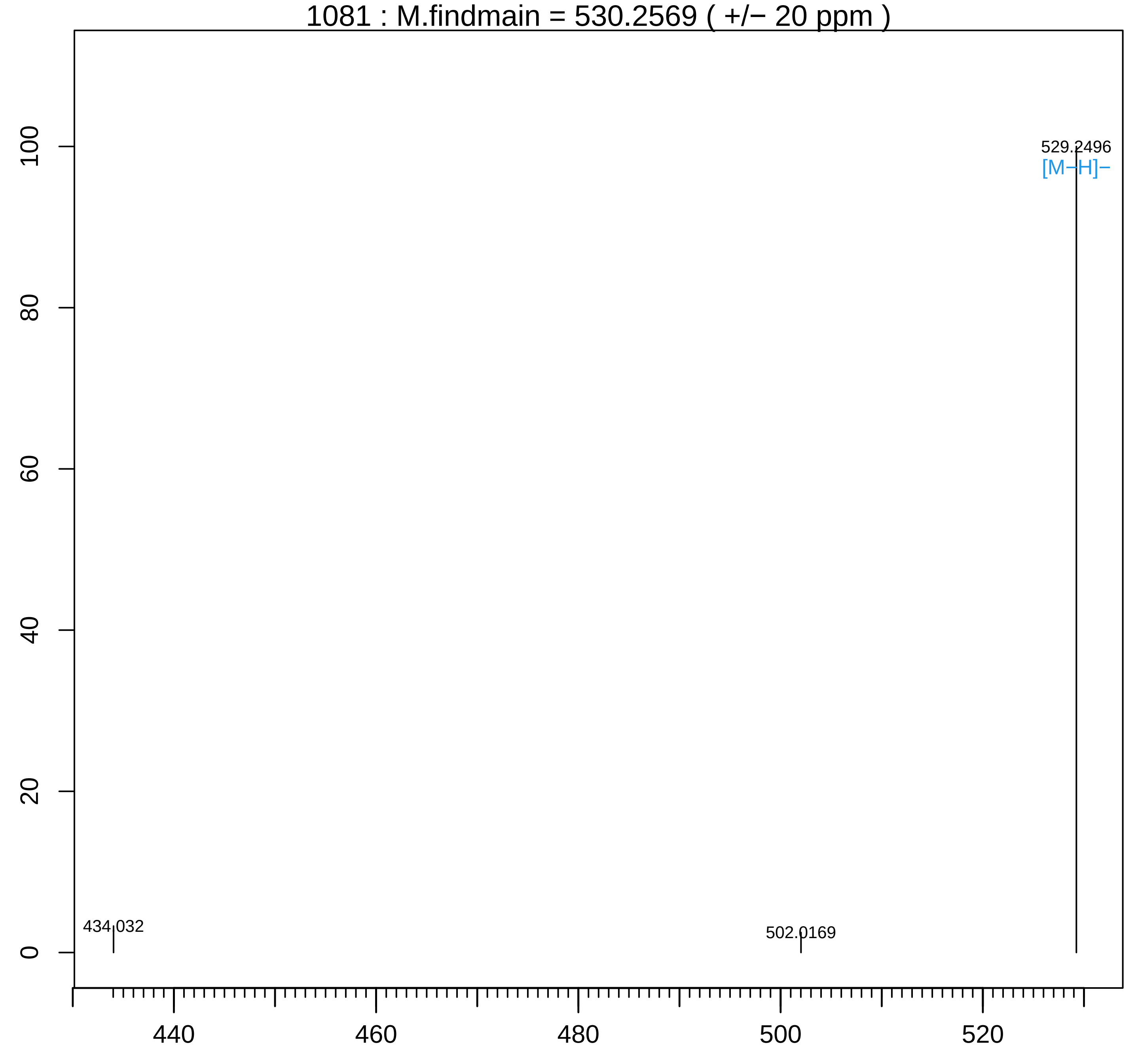

1081 : M.findmain = 530.2569 (+/− 20 ppm)
529.2496
[M−H]−
434.032
502.0169
0
20
40
60
80
100
440
460
480
500
520

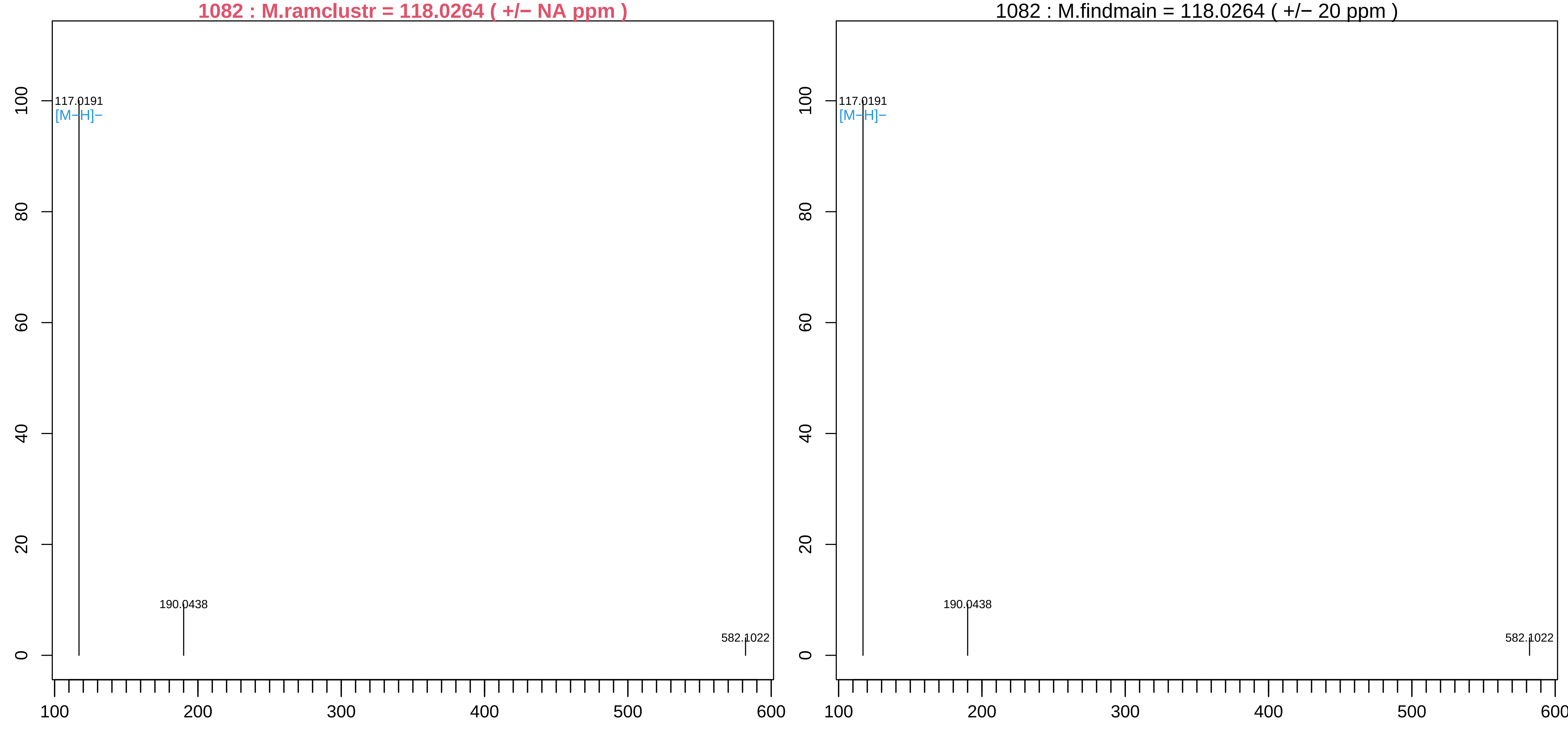
1082 : M.ramclustr = 118.0264 (+/− NA ppm)
117.0191
[M−H]−
190.0438
582.1022
1082 : M.findmain = 118.0264 (+/− 20 ppm)
117.0191
[M−H]−
190.0438
582.1022
0
20
40
60
80
100
100
200
300
400
500
600

1083 : M.ramclustr = 422.0829 (+/− NA ppm)

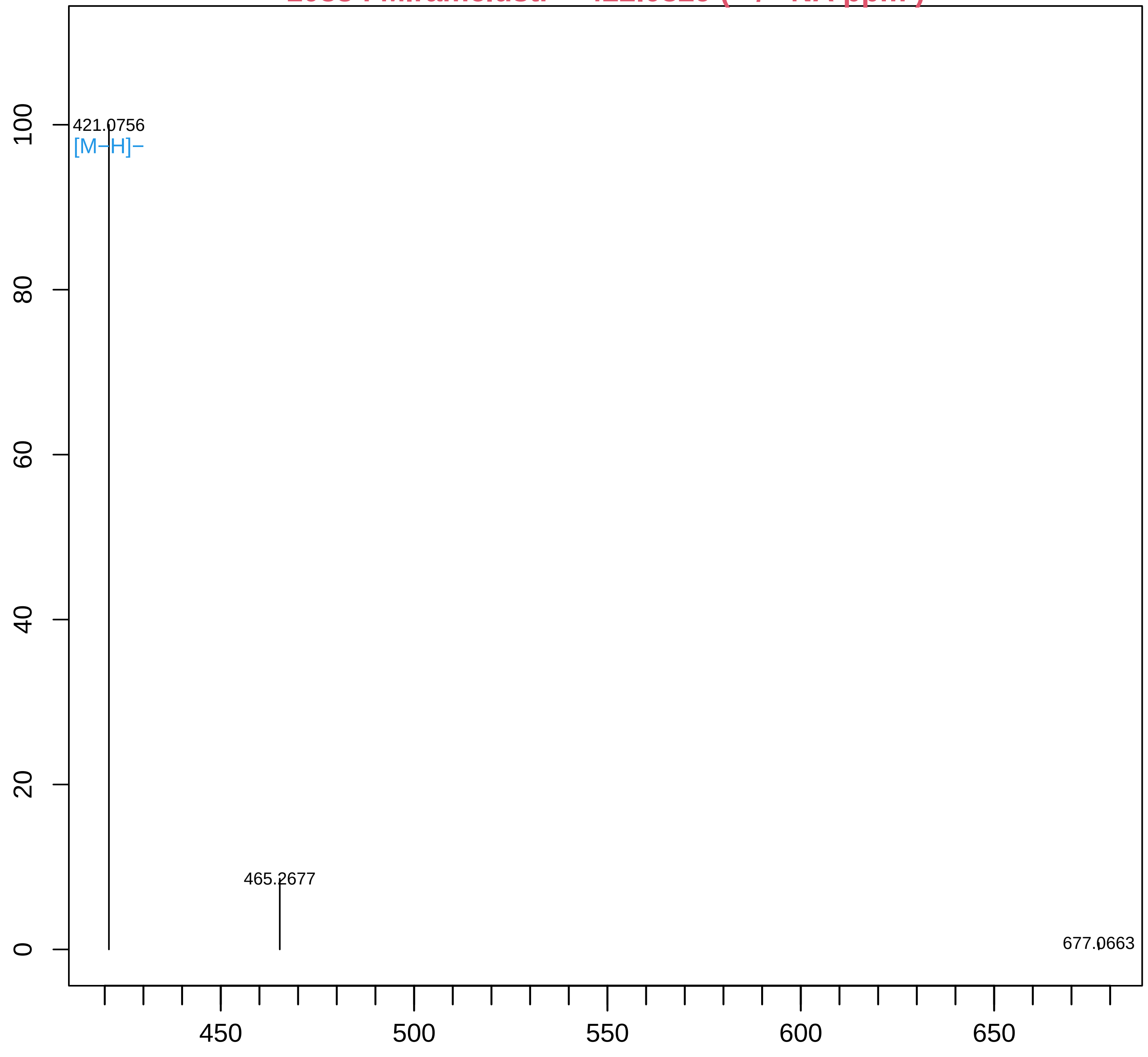

1083 : M.findmain = 422.0829 (+/− 20 ppm)

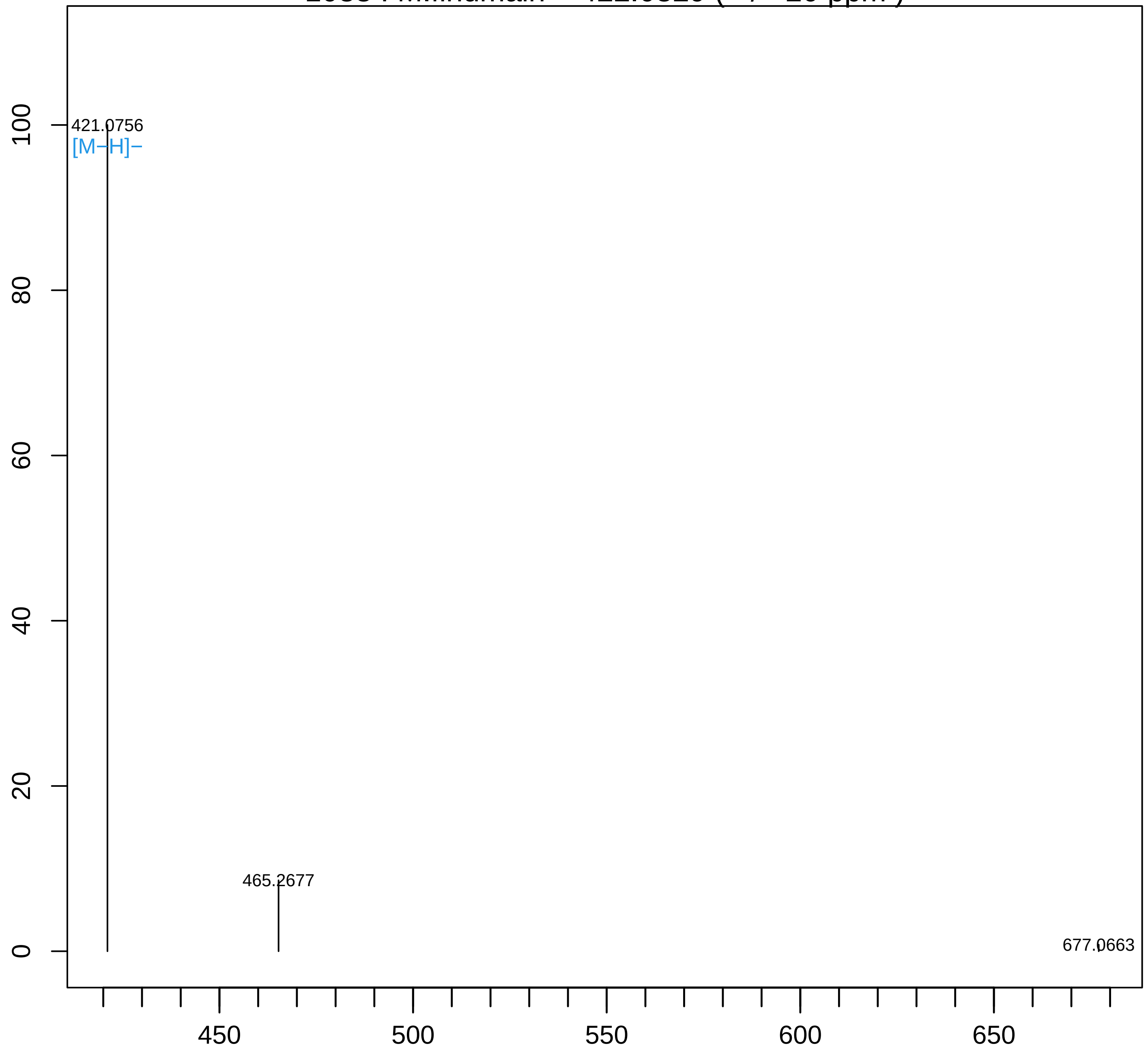

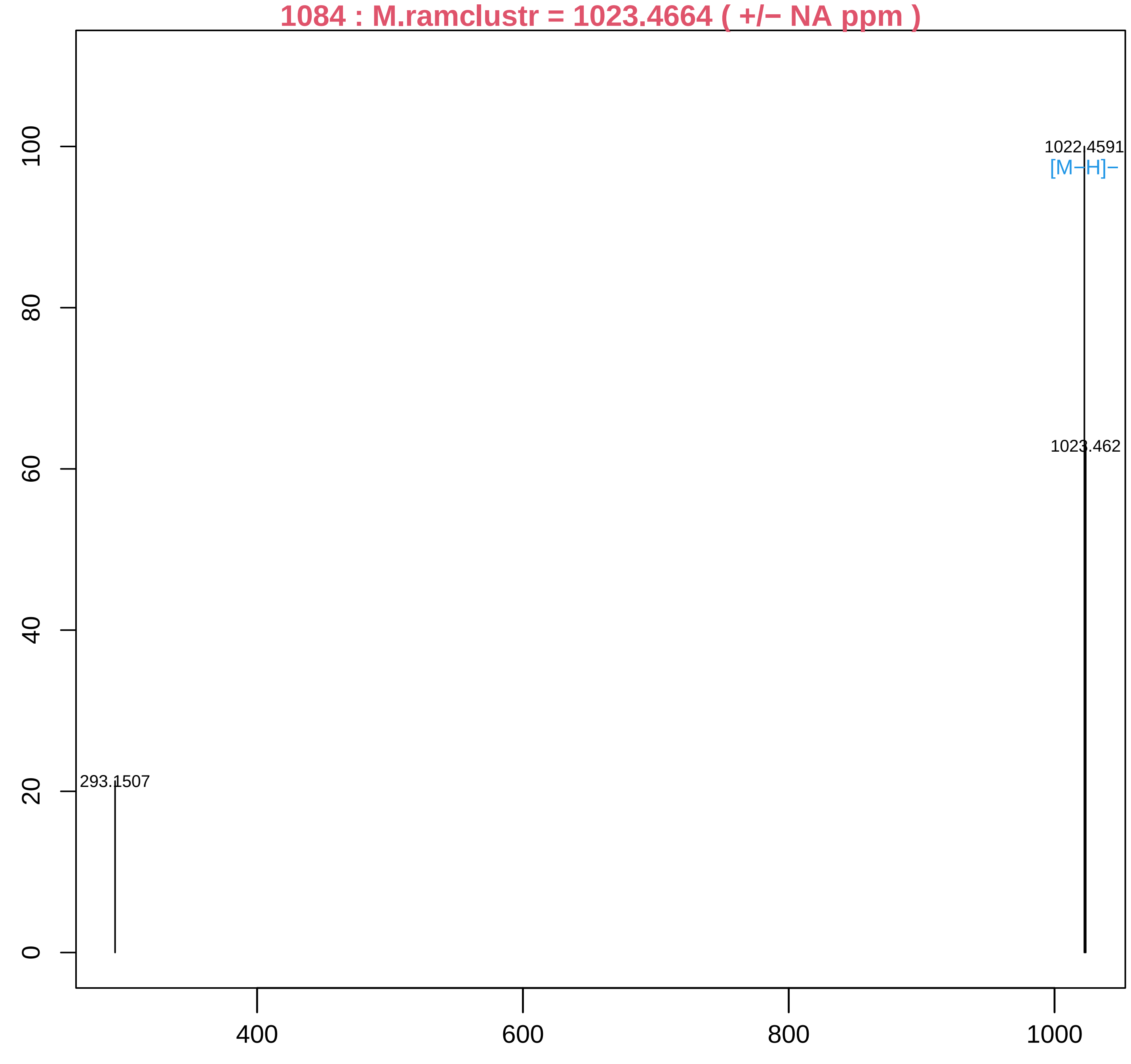
1084 : M.ramclustr = 1023.4664 (+/− NA ppm)
1022.4591
[M−H]−
1023.462
293.1507
0
20
40
60
80
100
400
600
800
1000

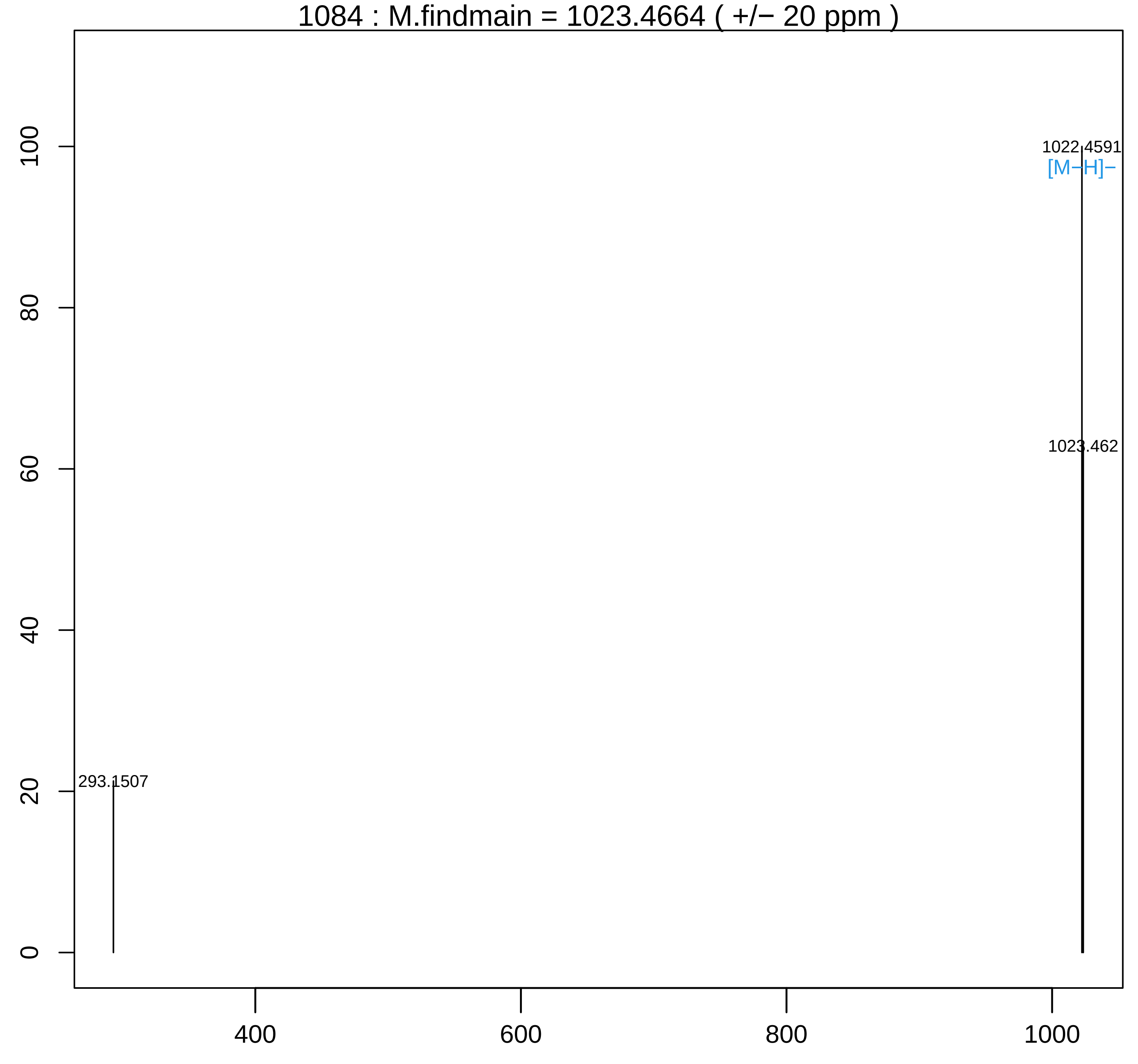
1084 : M.findmain = 1023.4664 (+/− 20 ppm)
1022.4591
[M−H]−
1023.462
293.1507
0
20
40
60
80
100
400
600
800
1000

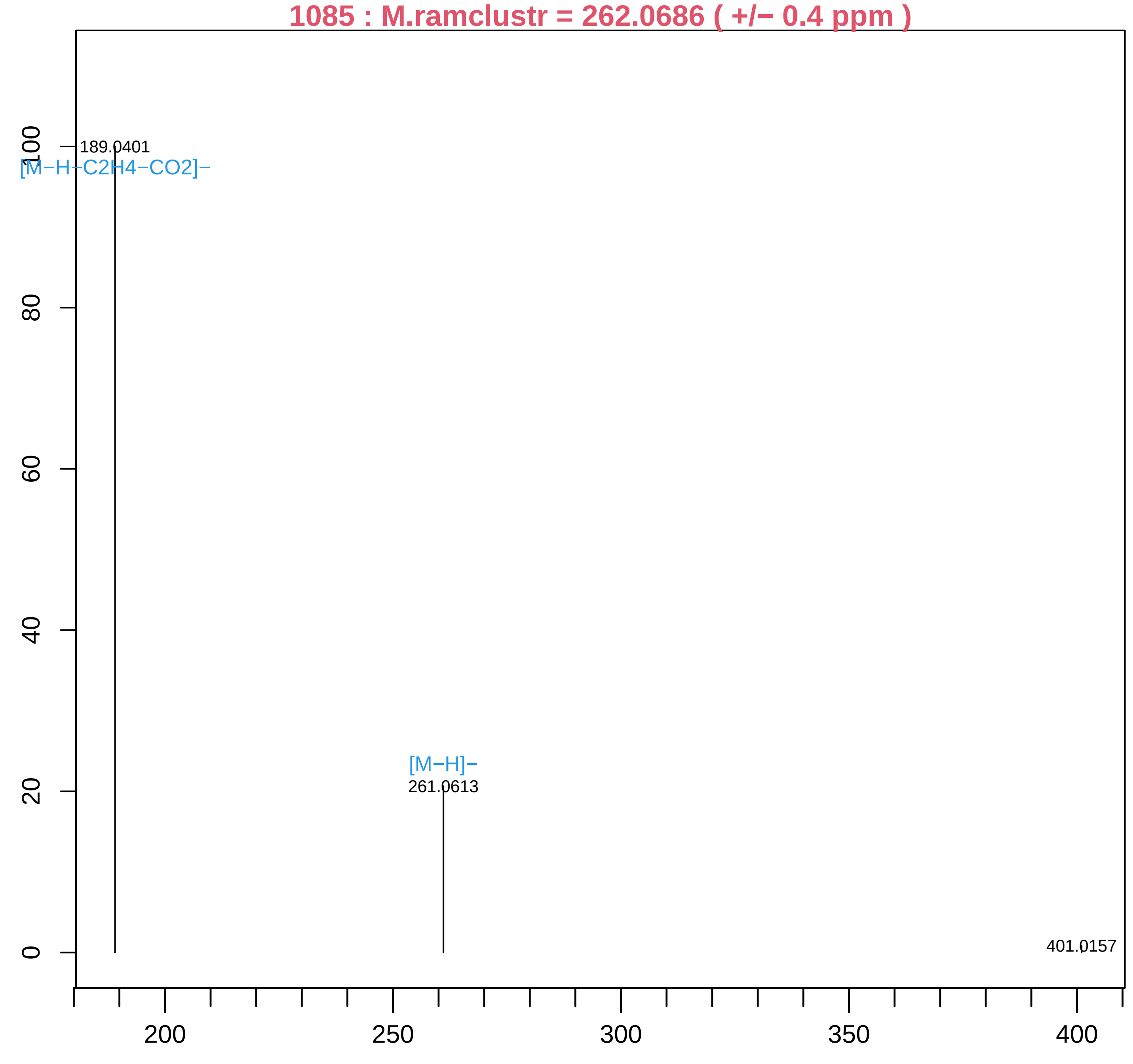
1085 : M.ramclustr = 262.0686 (+/− 0.4 ppm)
189.0401
[M−H−C2H4−CO2]−
[M−H]−
261.0613
401.0157
0
20
40
60
80
100
200
250
300
350
400

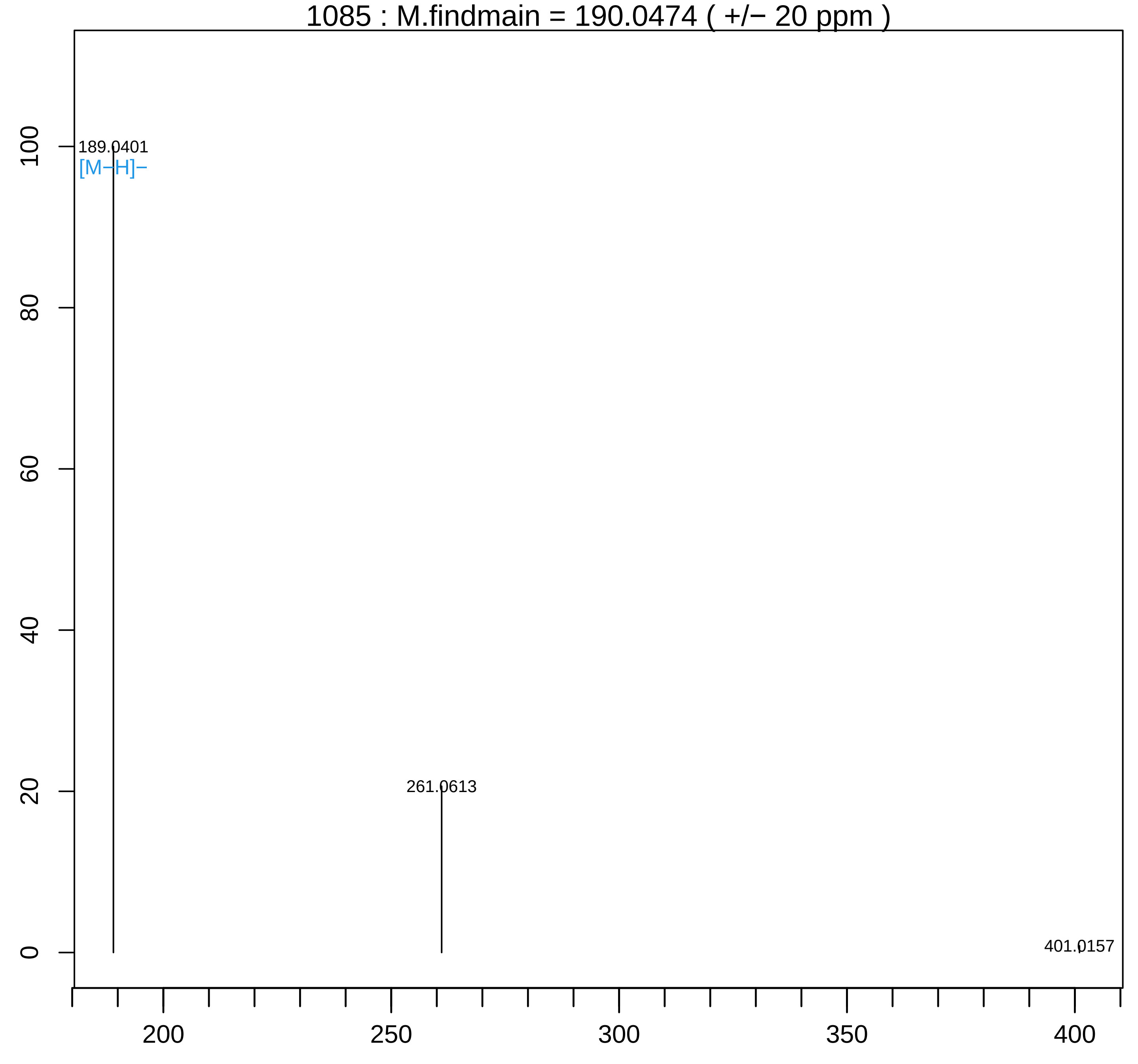
1085 : M.findmain = 190.0474 (+/− 20 ppm)
189.0401
[M−H]−
261.0613
401.0157
0
20
40
60
80
100
200
250
300
350
400

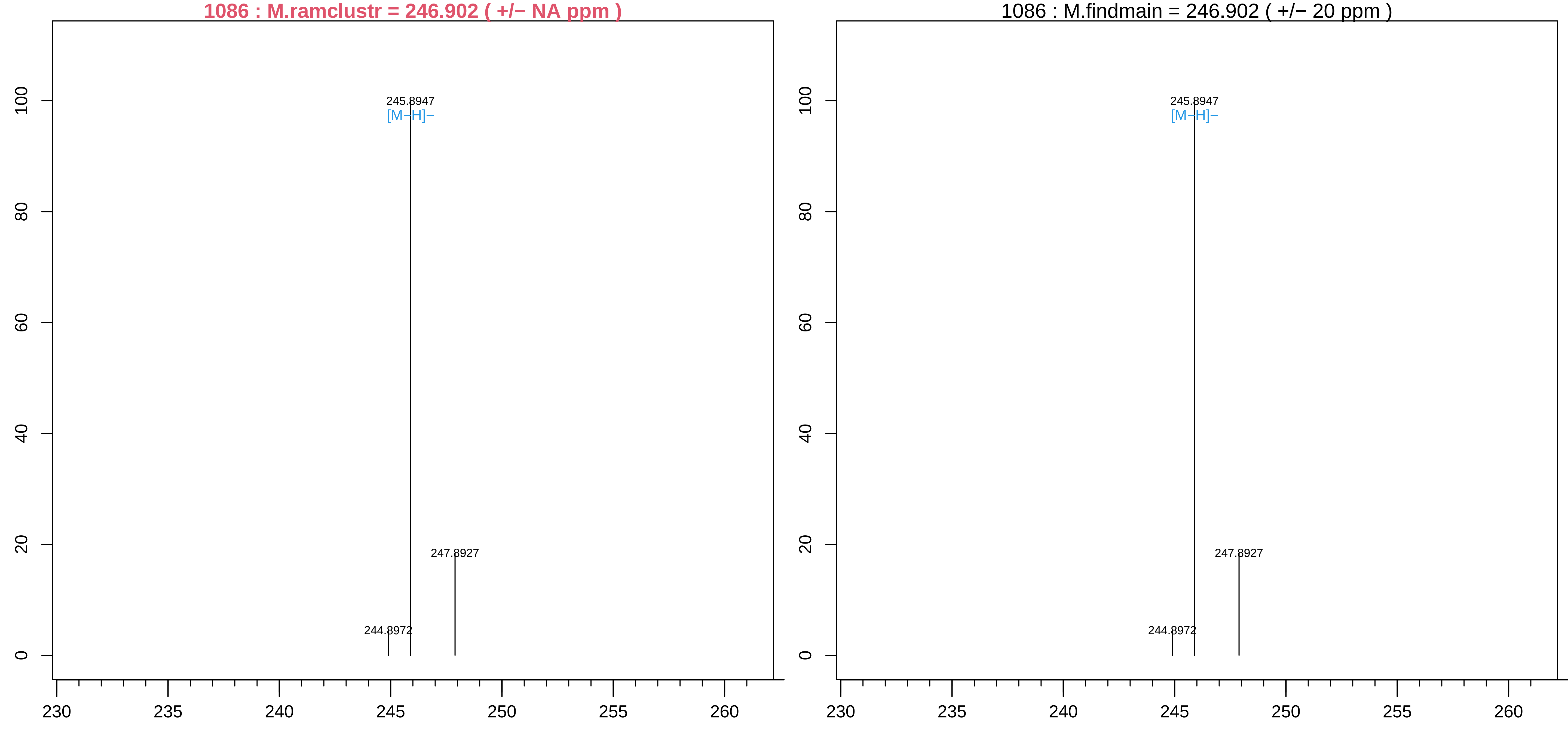
1086 : M.ramclustr = 246.902 (+/− NA ppm)
245.8947
[M−H]−
247.8927
244.8972
1086 : M.findmain = 246.902 (+/− 20 ppm)
245.8947
[M−H]−
247.8927
244.8972
0
20
40
60
80
100
230
235
240
245
250
255
260

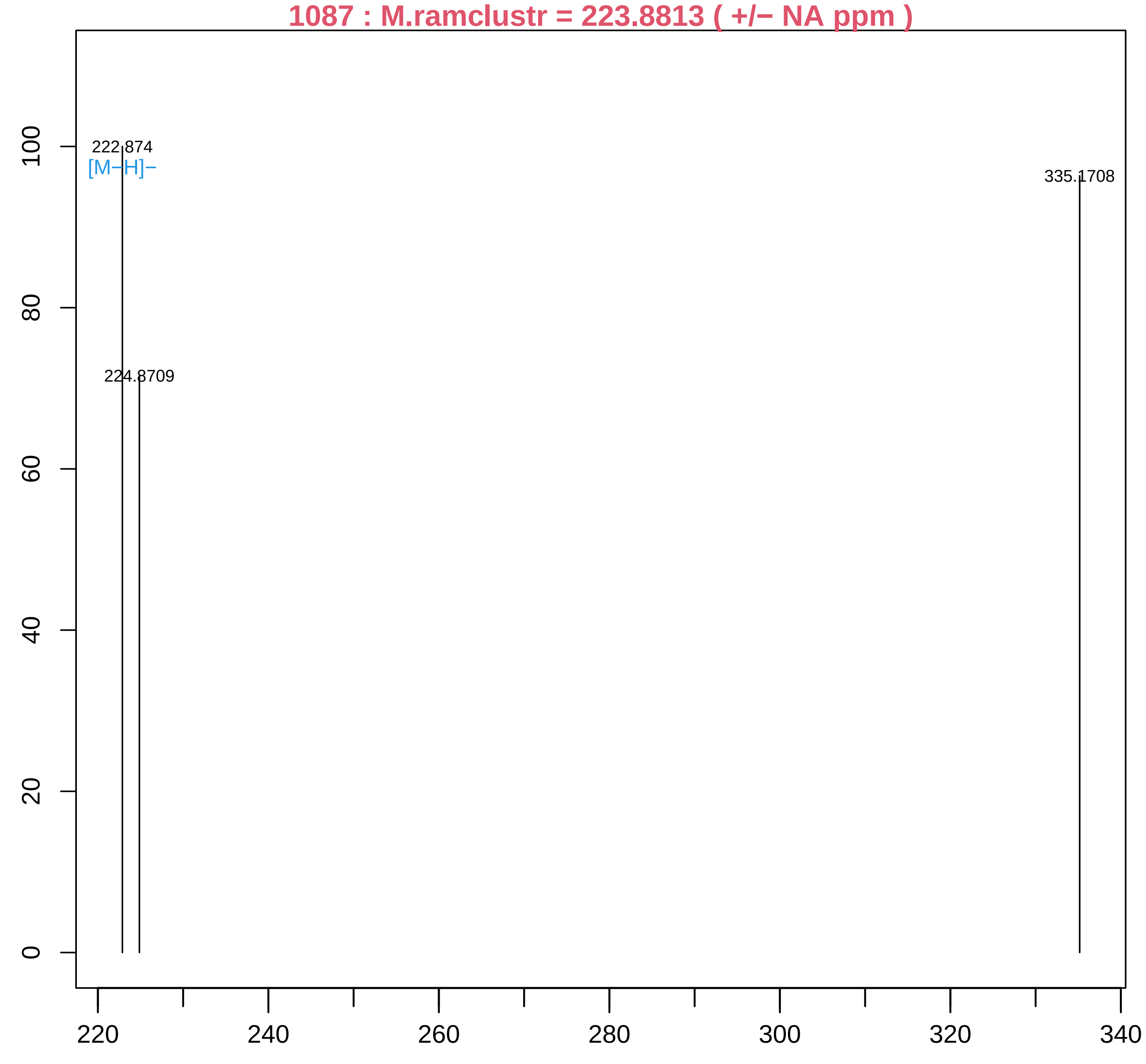
1087 : M.ramclustr = 223.8813 (+/− NA ppm)
222.874
[M−H]−
224.8709
335.1708
0
20
40
60
80
100
220
240
260
280
300
320
340
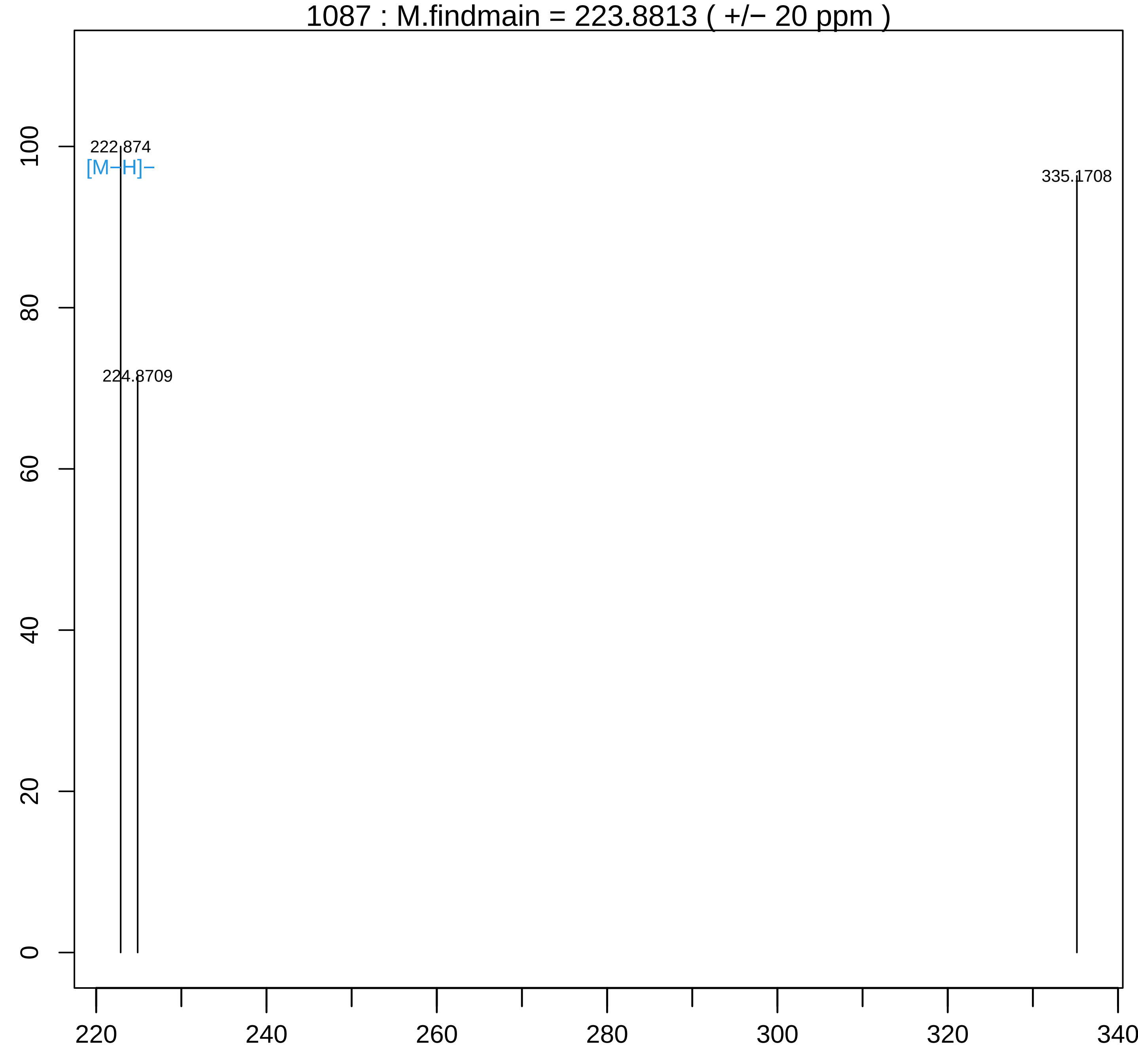
1087 : M.findmain = 223.8813 (+/− 20 ppm)
222.874
[M−H]−
224.8709
335.1708
0
20
40
60
80
100
220
240
260
280
300
320
340

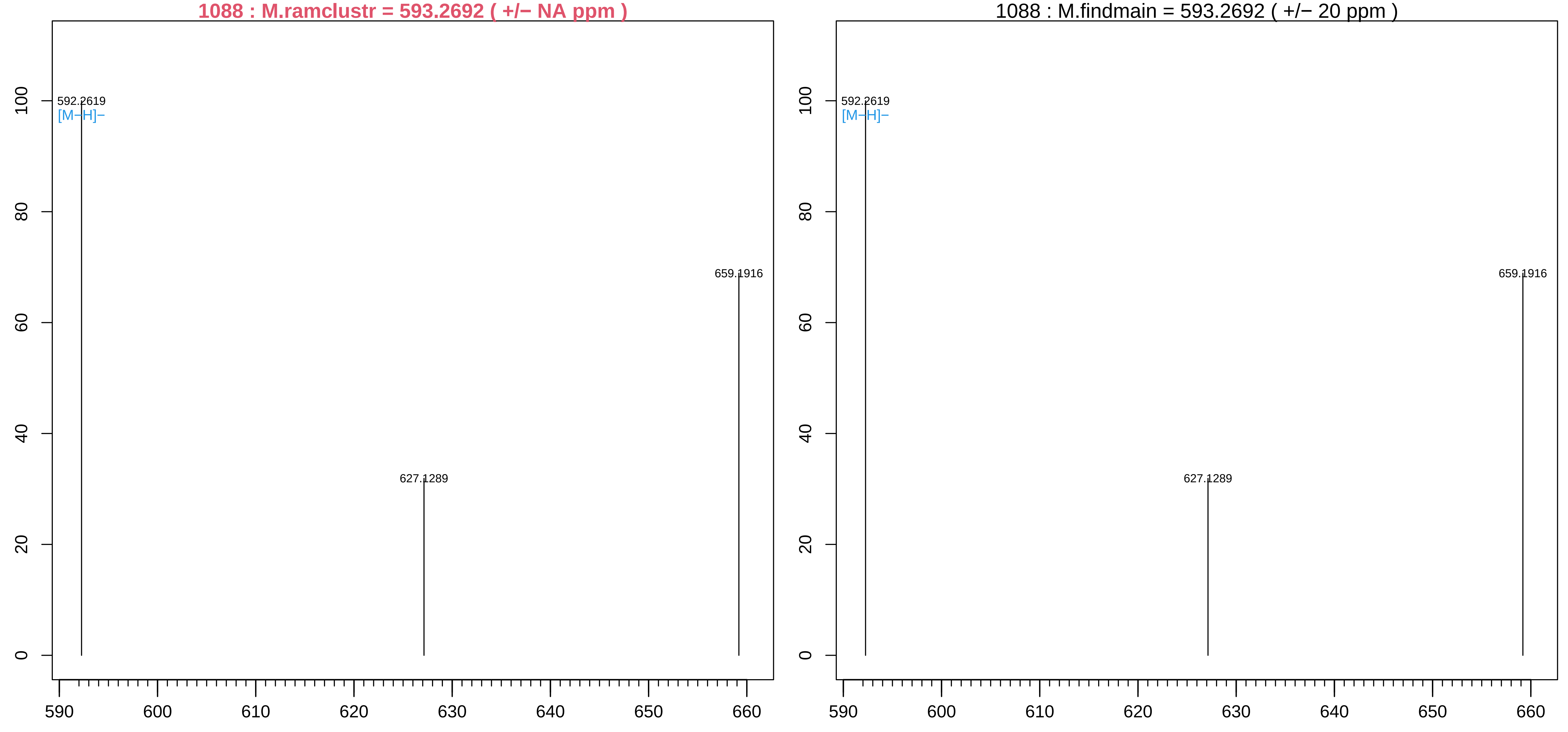
1088 : M.ramclustr = 593.2692 (+/− NA ppm)
592.2619
[M−H]−
627.1289
659.1916
0
20
40
60
80
100
590
600
610
620
630
640
650
660
1088 : M.findmain = 593.2692 (+/− 20 ppm)
592.2619
[M−H]−
627.1289
659.1916
0
20
40
60
80
100
590
600
610
620
630
640
650
660

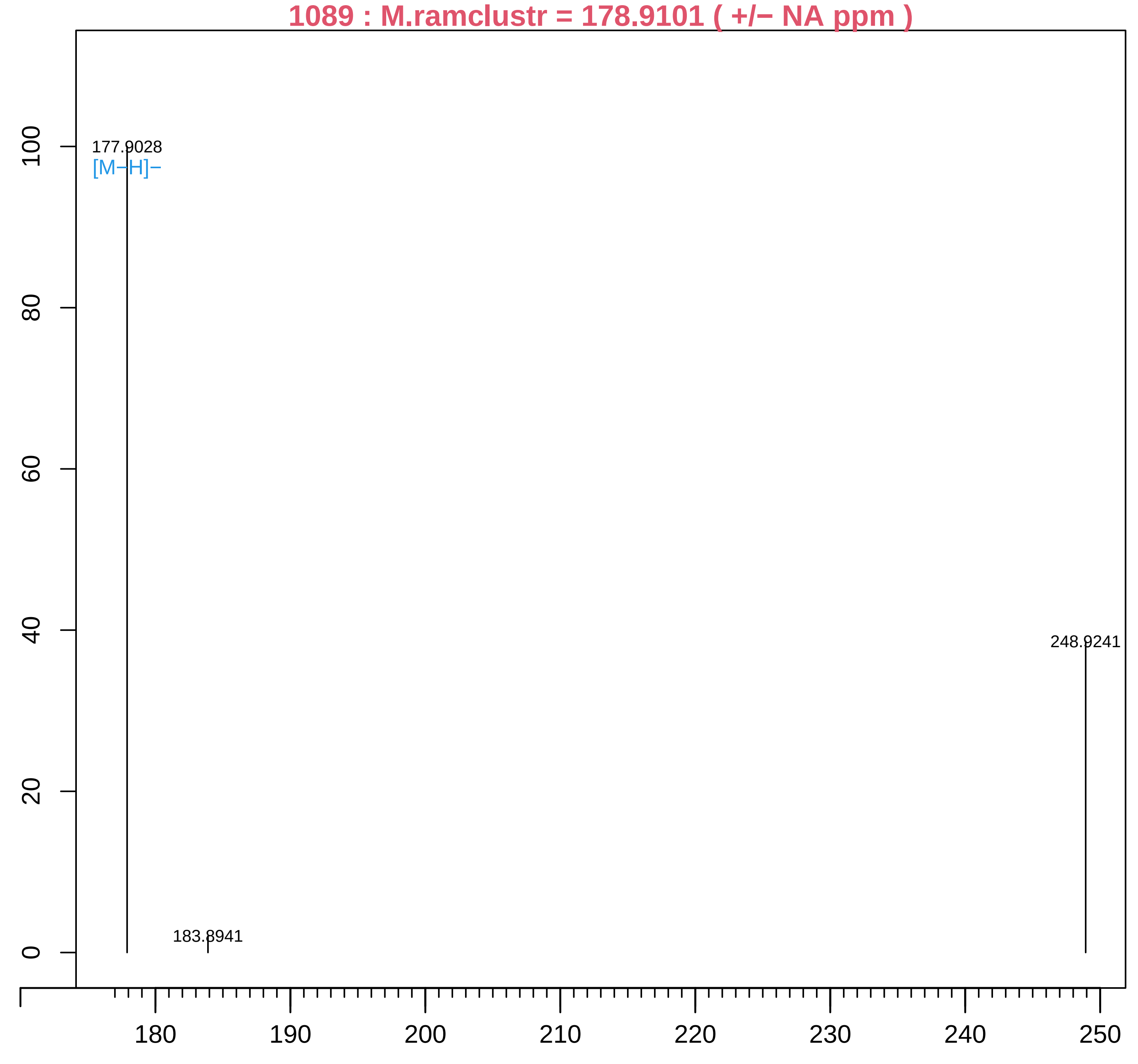
1089 : M.ramclustr = 178.9101 (+/− NA ppm)
177.9028
[M−H]−
183.8941
248.9241
0
20
40
60
80
100
180
190
200
210
220
230
240
250
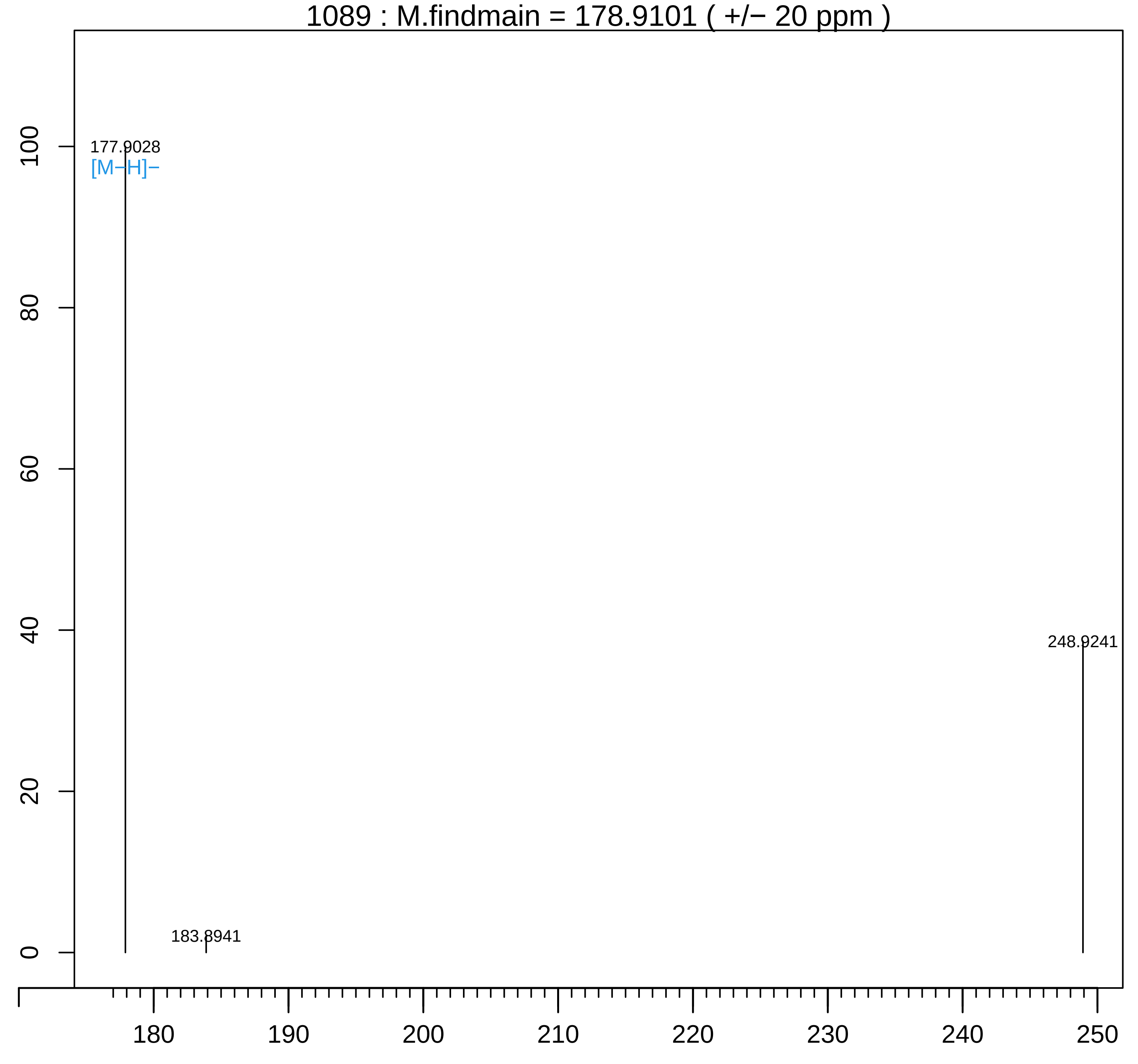
1089 : M.findmain = 178.9101 (+/− 20 ppm)
177.9028
[M−H]−
183.8941
248.9241
0
20
40
60
80
100
180
190
200
210
220
230
240
250

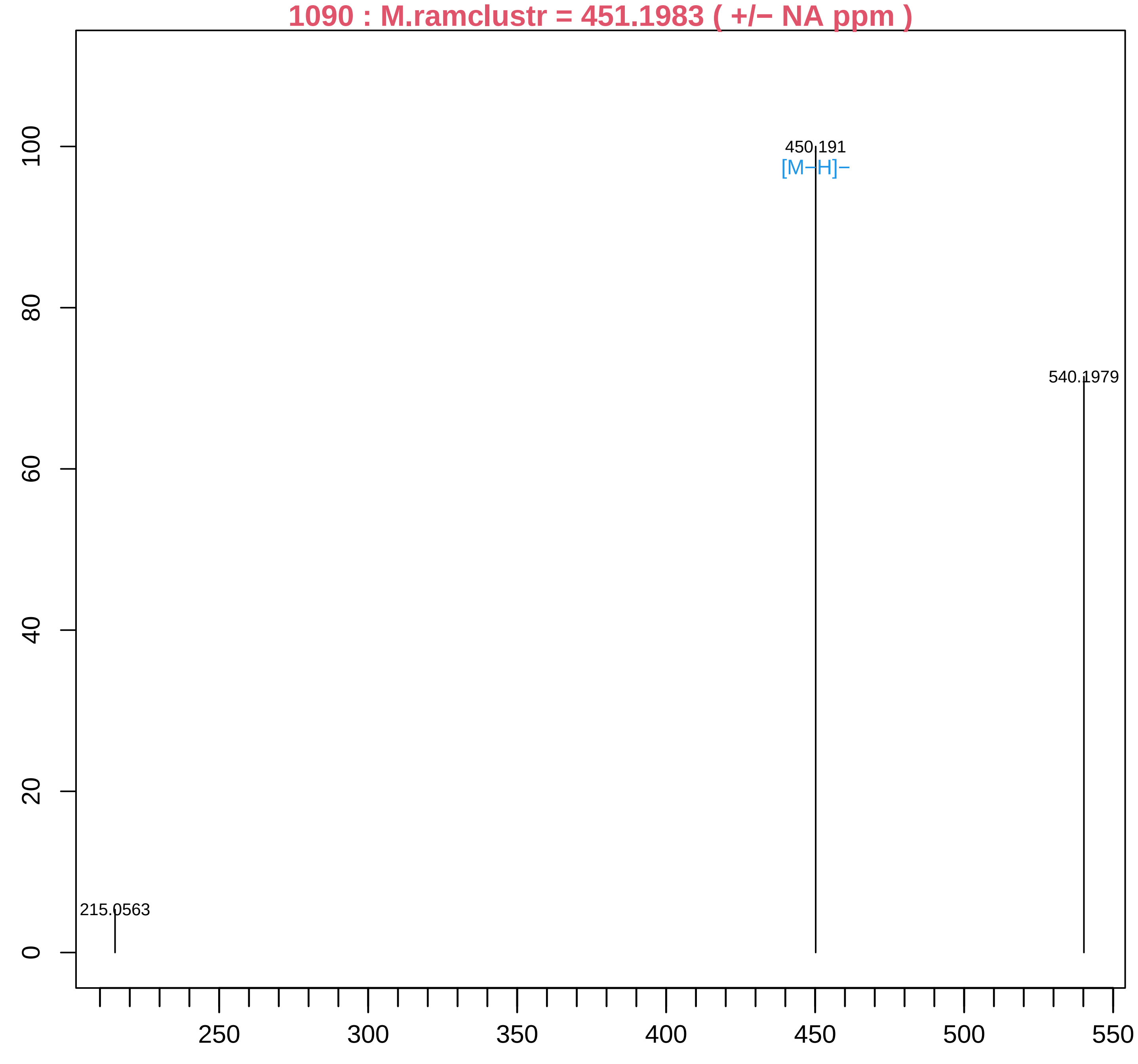

1090 : M.ramclustr = 451.1983 (+/− NA ppm)
450.191
[M−H]−
540.1979
215.0563
0
20
40
60
80
100
250
300
350
400
450
500
550

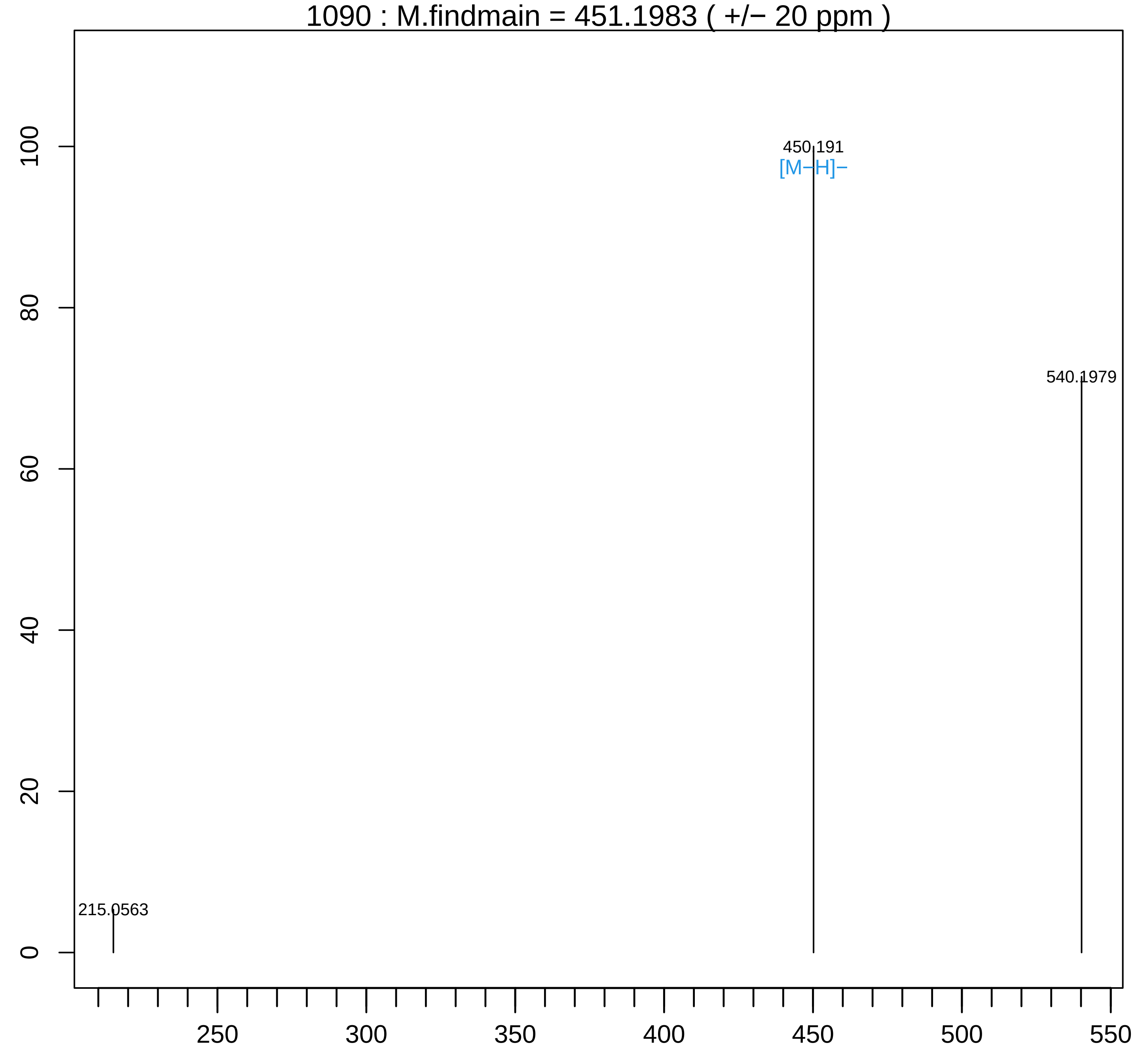

1090 : M.findmain = 451.1983 (+/− 20 ppm)
450.191
[M−H]−
540.1979
215.0563
0
20
40
60
80
100
250
300
350
400
450
500
550

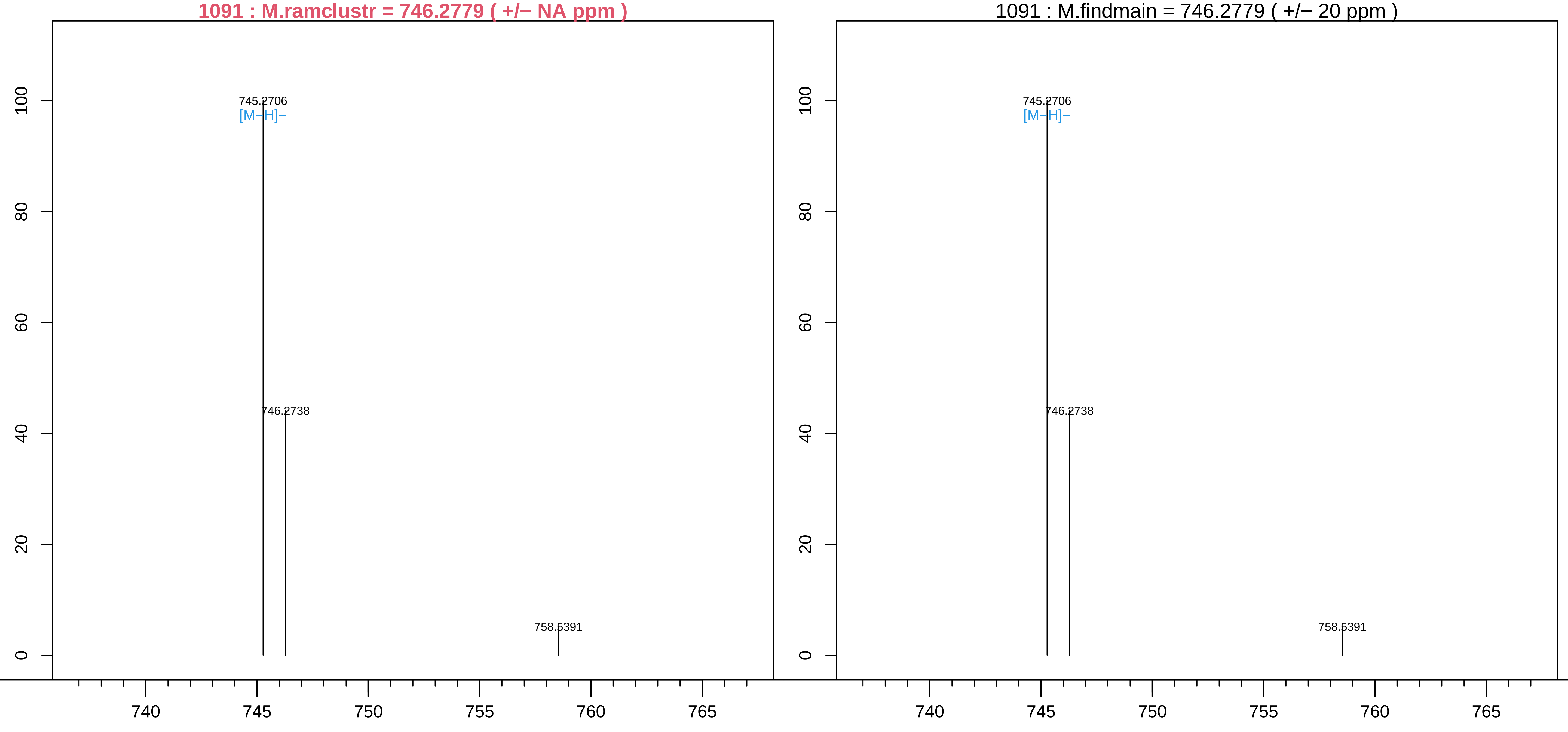

1091 : M.ramclustr = 746.2779 (+/− NA ppm)
745.2706
[M−H]−
746.2738
758.5391
1091 : M.findmain = 746.2779 (+/− 20 ppm)
745.2706
[M−H]−
746.2738
758.5391
0
20
40
60
80
100
740
745
750
755
760
765

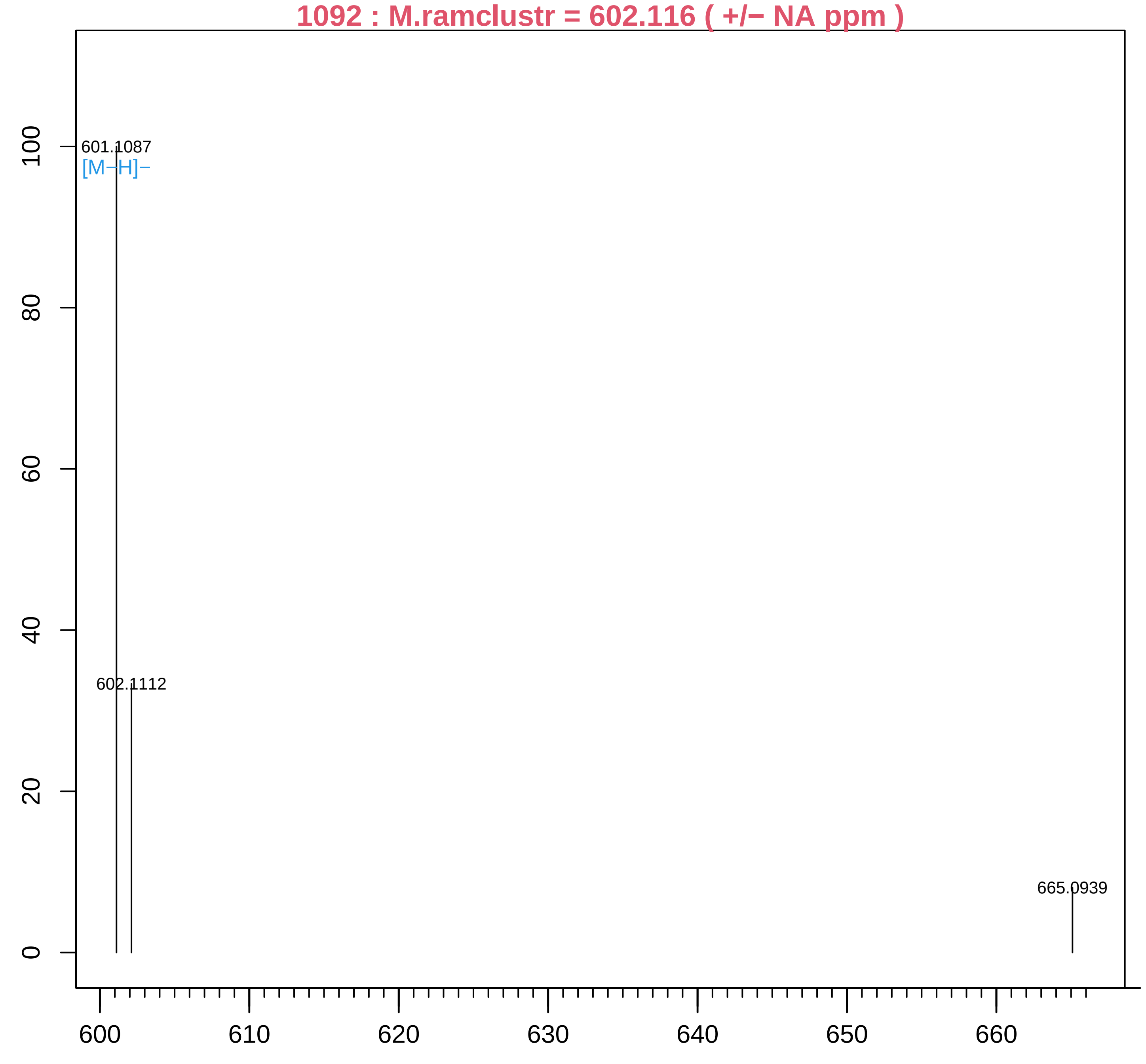
1092 : M.ramclustr = 602.116 (+/− NA ppm)
601.1087
[M−H]−
602.1112
665.0939
0
20
40
60
80
100
600
610
620
630
640
650
660

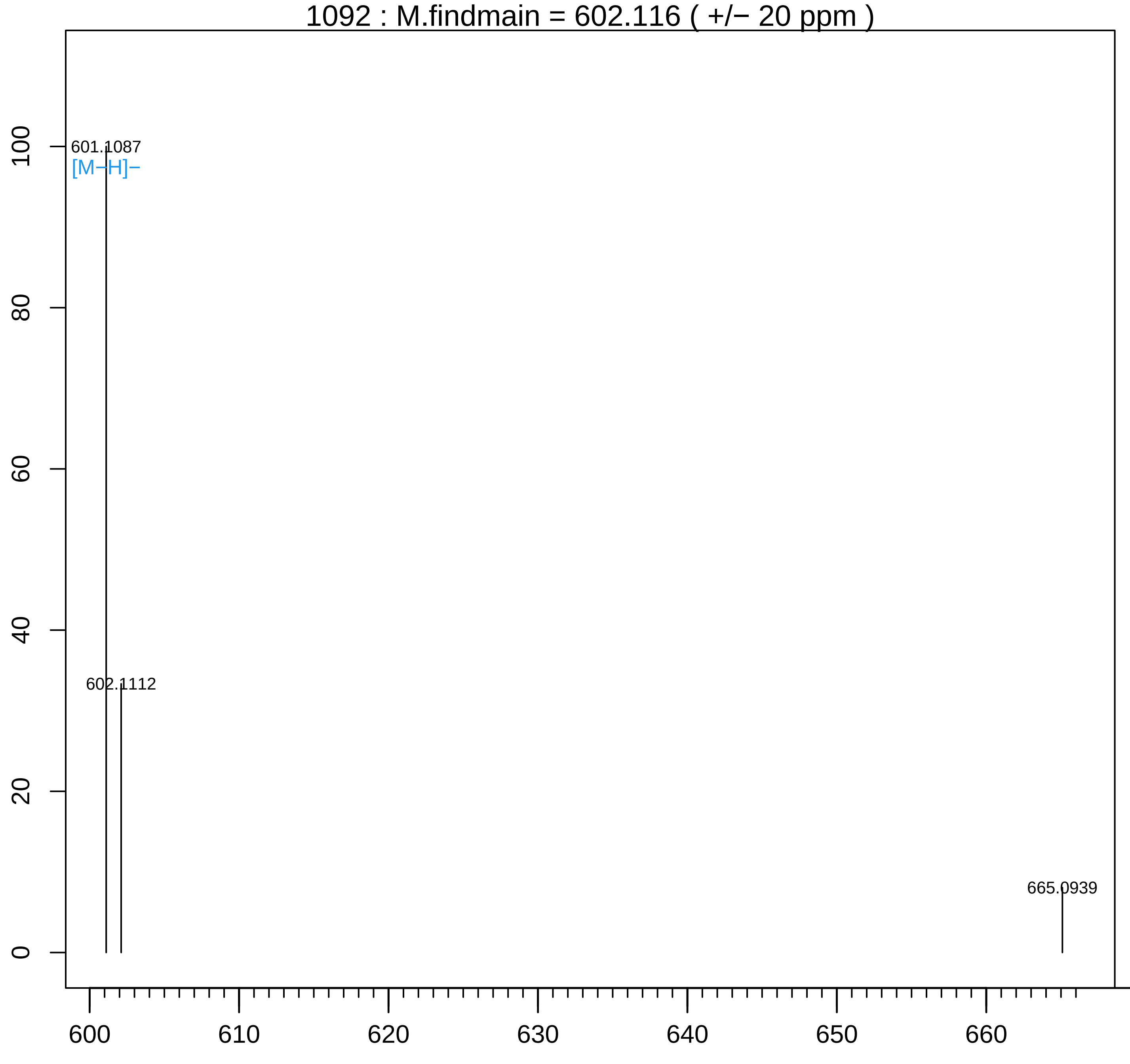
1092 : M.findmain = 602.116 (+/− 20 ppm)
601.1087
[M−H]−
602.1112
665.0939
0
20
40
60
80
100
600
610
620
630
640
650
660

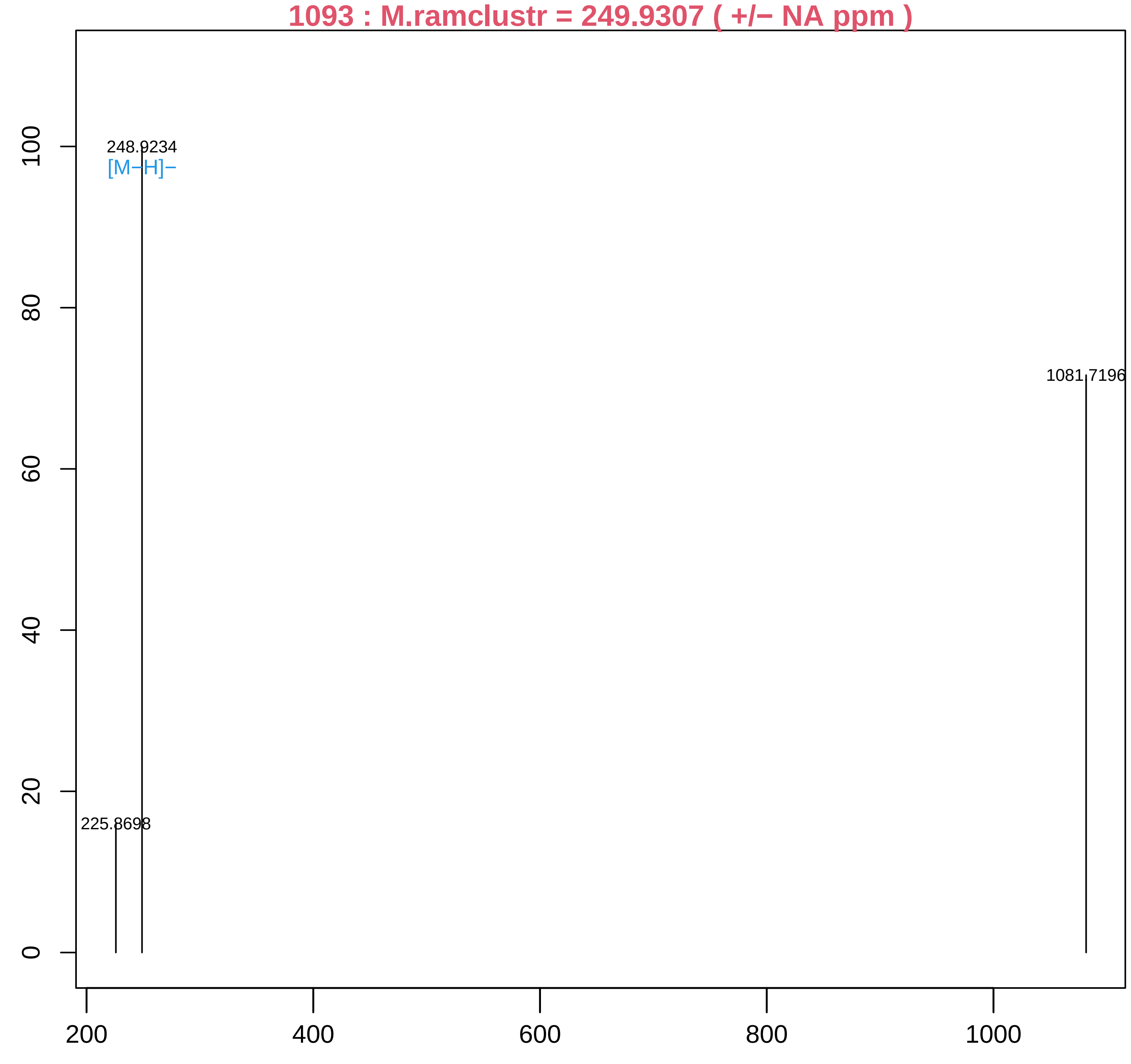
1093 : M.ramclustr = 249.9307 (+/− NA ppm)
248.9234
[M−H]−
1081.7196
225.8698
0
20
40
60
80
100
200
400
600
800
1000

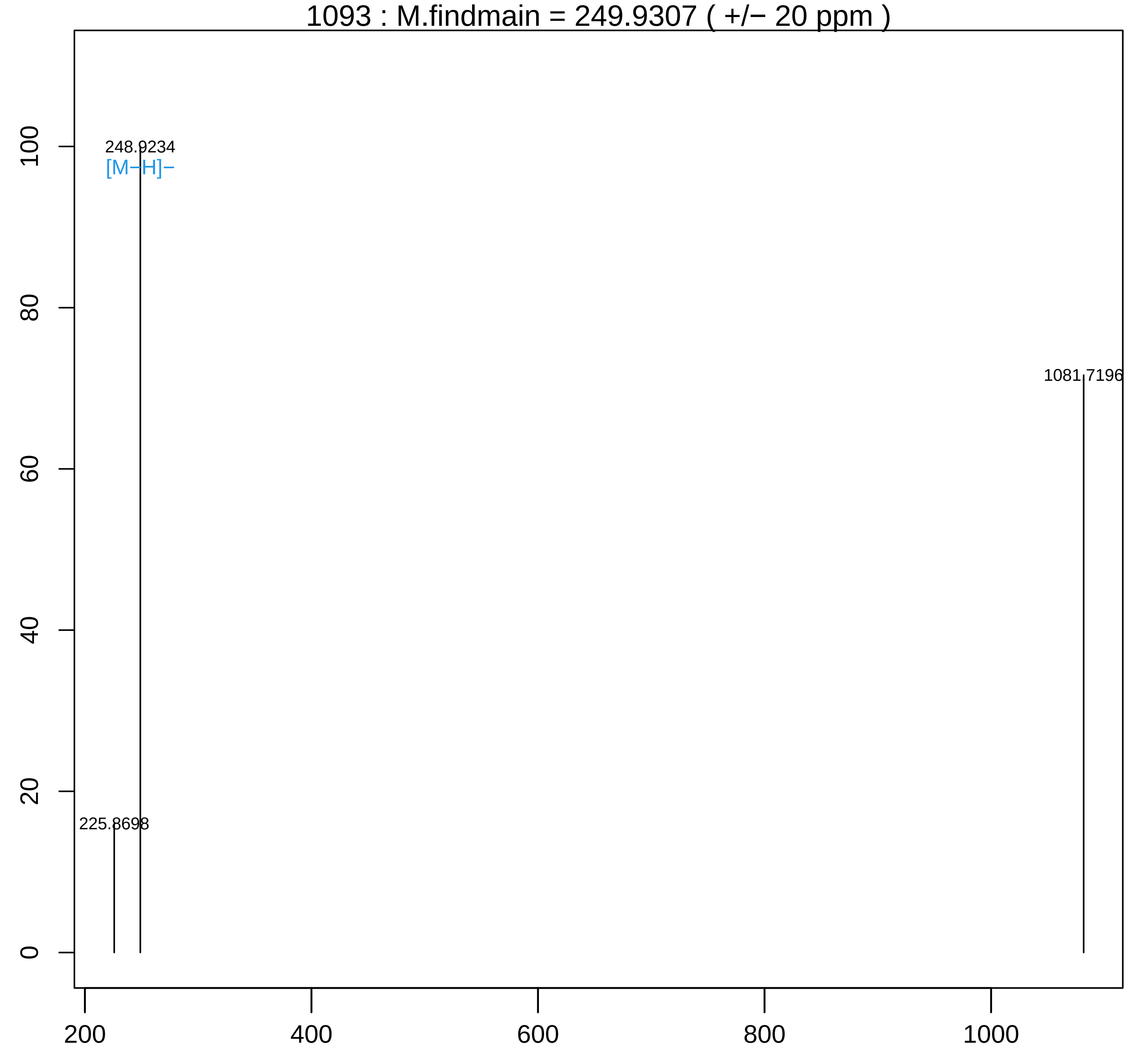
1093 : M.findmain = 249.9307 (+/− 20 ppm)
248.9234
[M−H]−
1081.7196
225.8698
0
20
40
60
80
100
200
400
600
800
1000

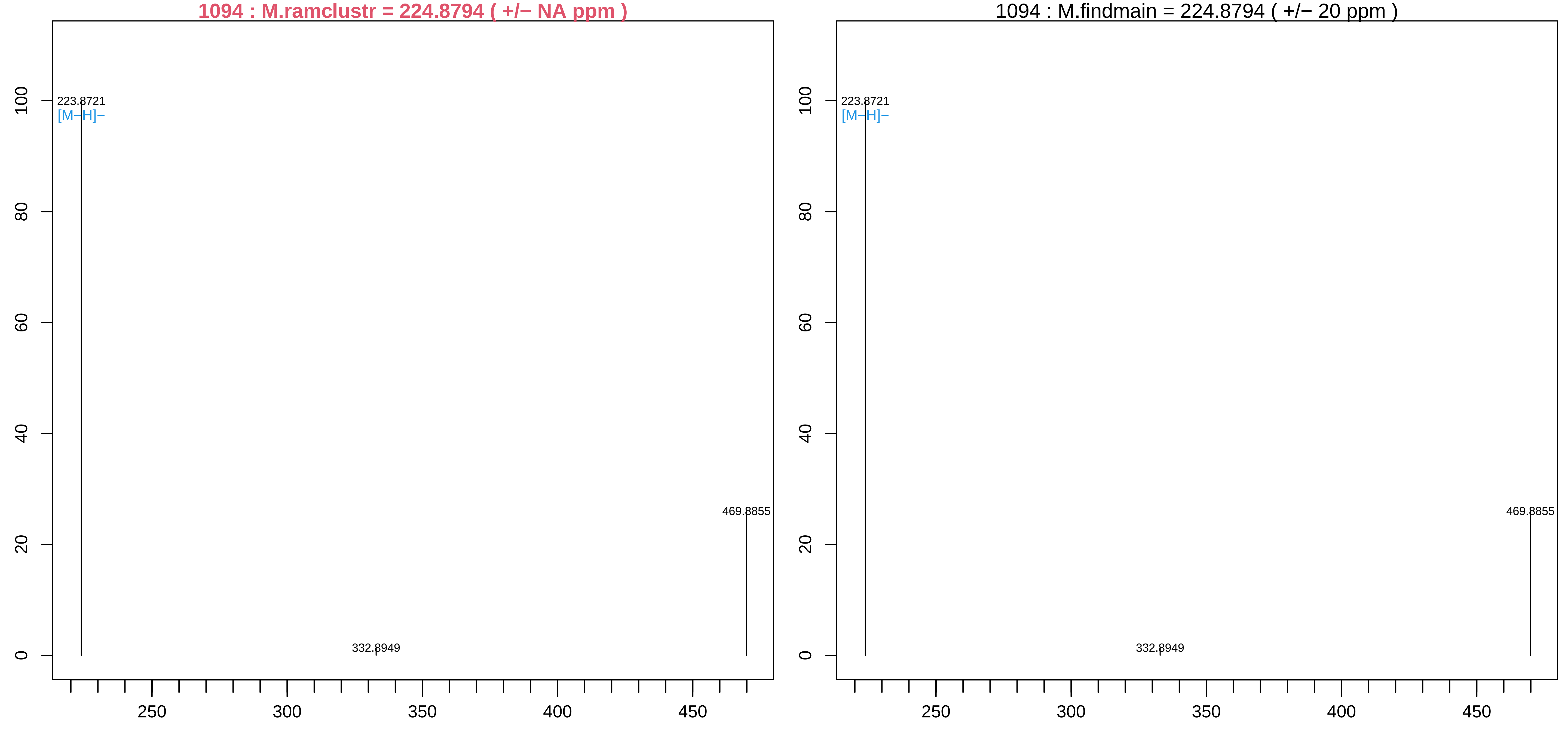

1094 : M.ramclustr = 224.8794 (+/− NA ppm)
223.8721
[M−H]−
332.8949
469.8855
0
20
40
60
80
100
250
300
350
400
450
1094 : M.findmain = 224.8794 (+/− 20 ppm)
223.8721
[M−H]−
332.8949
469.8855
0
20
40
60
80
100
250
300
350
400
450

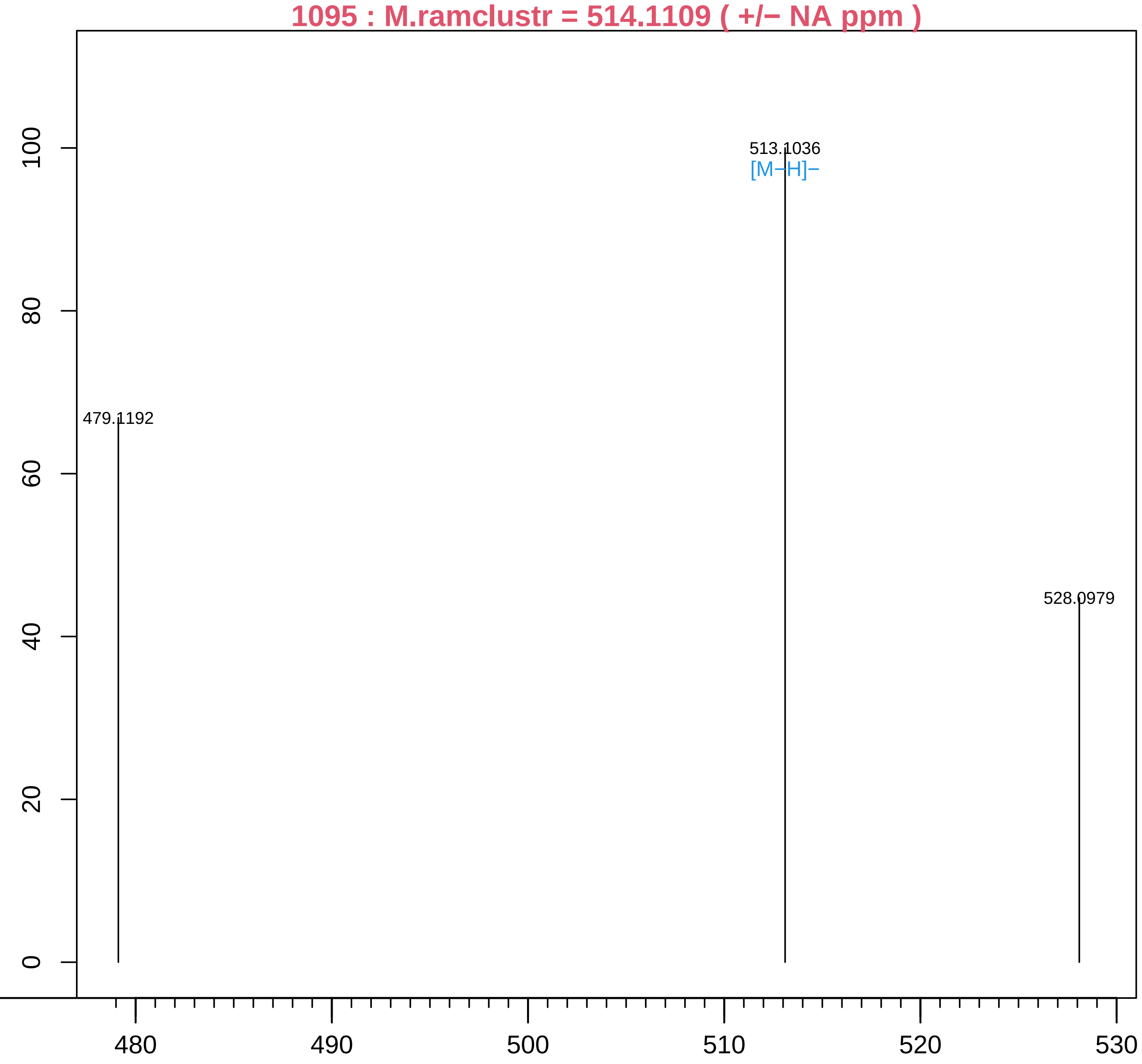
1095 : M.ramclustr = 514.1109 (+/− NA ppm)
513.1036
[M−H]−
479.1192
528.0979
0
20
40
60
80
100
480
490
500
510
520
530

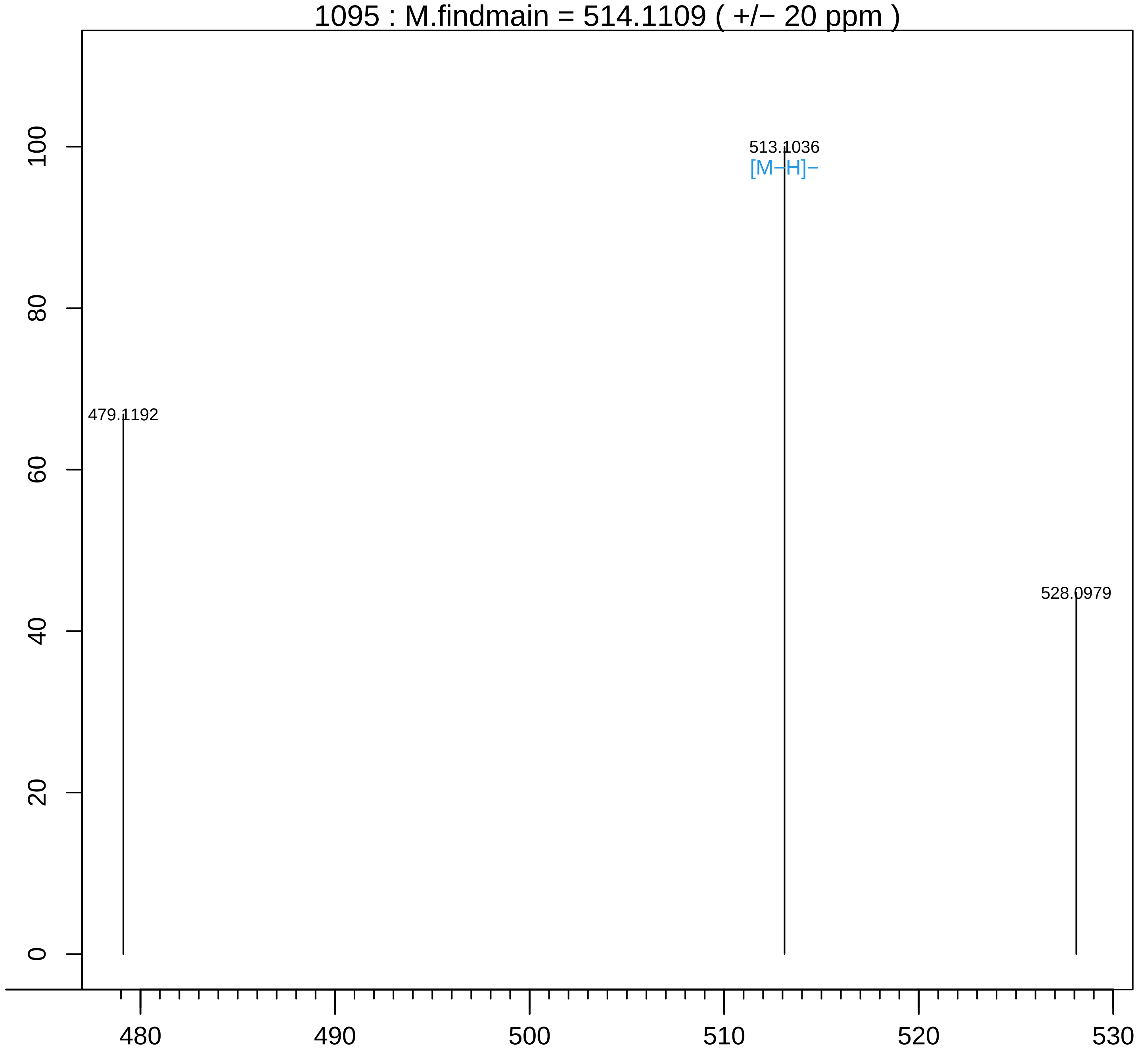
1095 : M.findmain = 514.1109 (+/− 20 ppm)
513.1036
[M−H]−
479.1192
528.0979
0
20
40
60
80
100
480
490
500
510
520
530

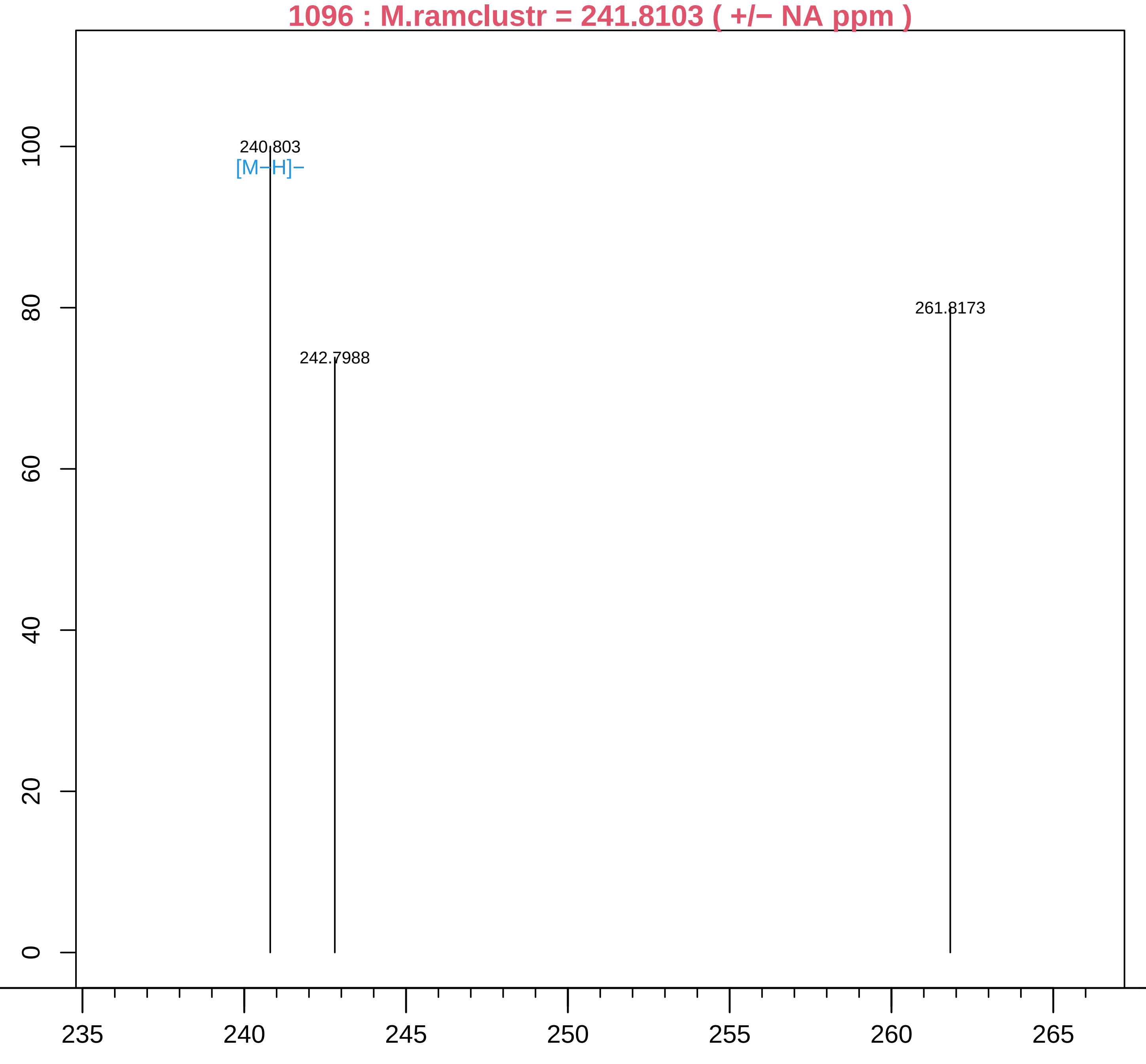
1096 : M.ramclustr = 241.8103 (+/− NA ppm)
240.803
[M−H]−
242.7988
261.8173
0
20
40
60
80
100
235
240
245
250
255
260
265
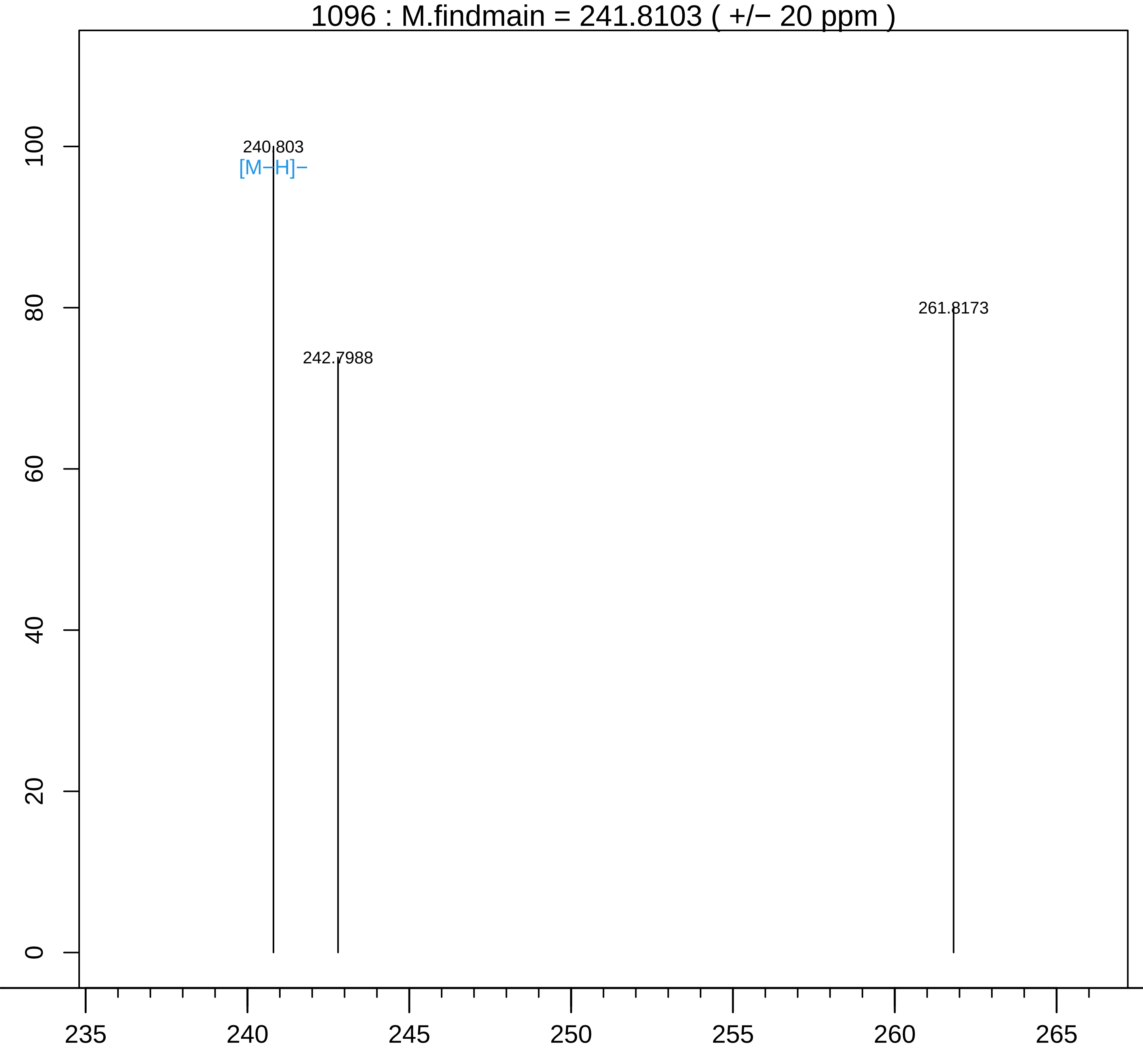
1096 : M.findmain = 241.8103 (+/− 20 ppm)
240.803
[M−H]−
242.7988
261.8173
0
20
40
60
80
100
235
240
245
250
255
260
265

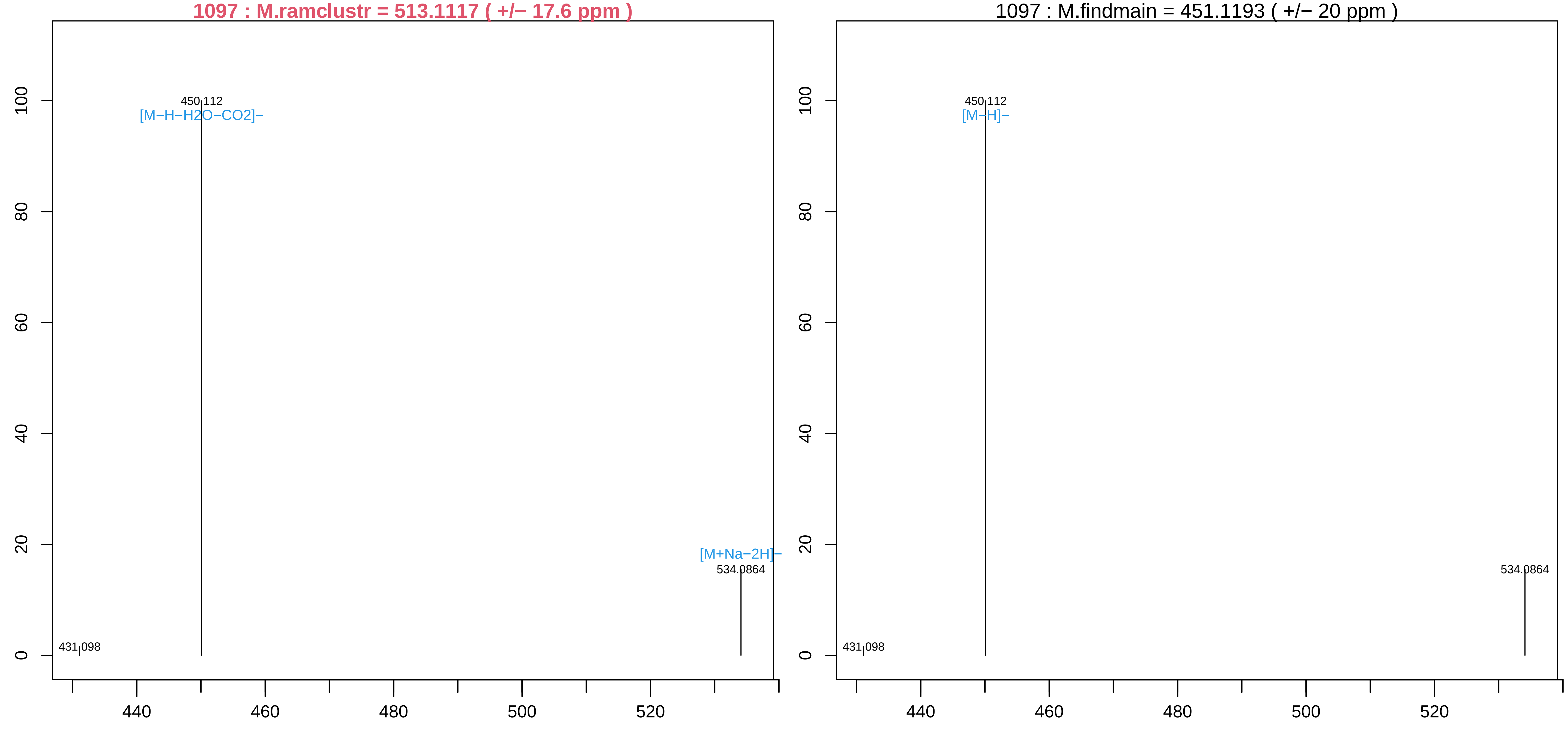
1097 : M.ramclustr = 513.1117 (+/− 17.6 ppm)
450.112
[M−H−H2O−CO2]−
[M+Na−2H]−
534.0864
431.098
0
20
40
60
80
100
440
460
480
500
520
1097 : M.findmain = 451.1193 (+/− 20 ppm)
450.112
[M−H]−
534.0864
431.098
0
20
40
60
80
100
440
460
480
500
520

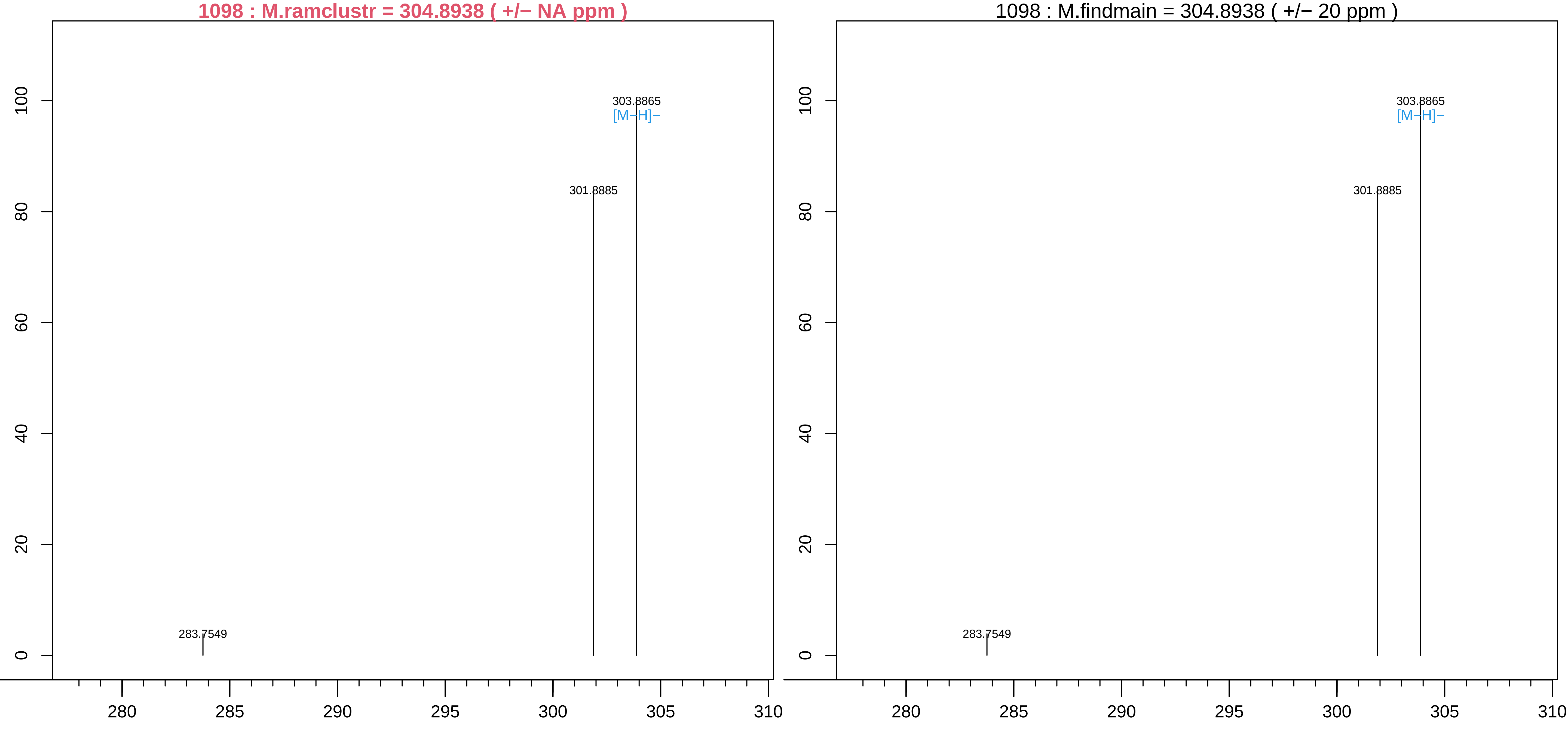

1098 : M.ramclustr = 304.8938 (+/− NA ppm)
303.8865
[M−H]−
301.8885
283.7549
0
20
40
60
80
100
280
285
290
295
300
305
310
1098 : M.findmain = 304.8938 (+/− 20 ppm)
303.8865
[M−H]−
301.8885
283.7549
0
20
40
60
80
100
280
285
290
295
300
305
310

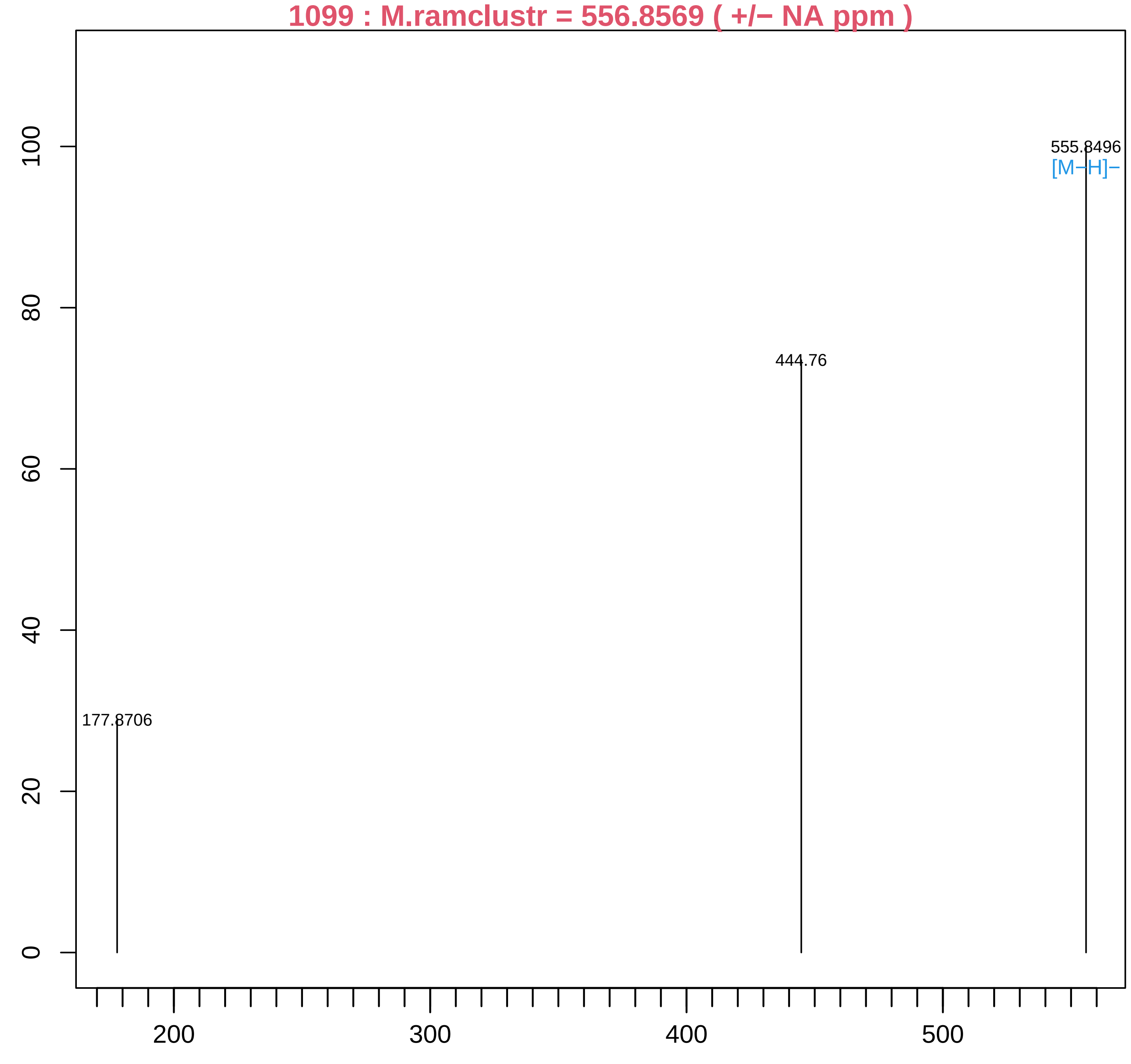
1099 : M.ramclustr = 556.8569 (+/− NA ppm)
555.8496
[M−H]−
444.76
177.8706
0
20
40
60
80
100
200
300
400
500

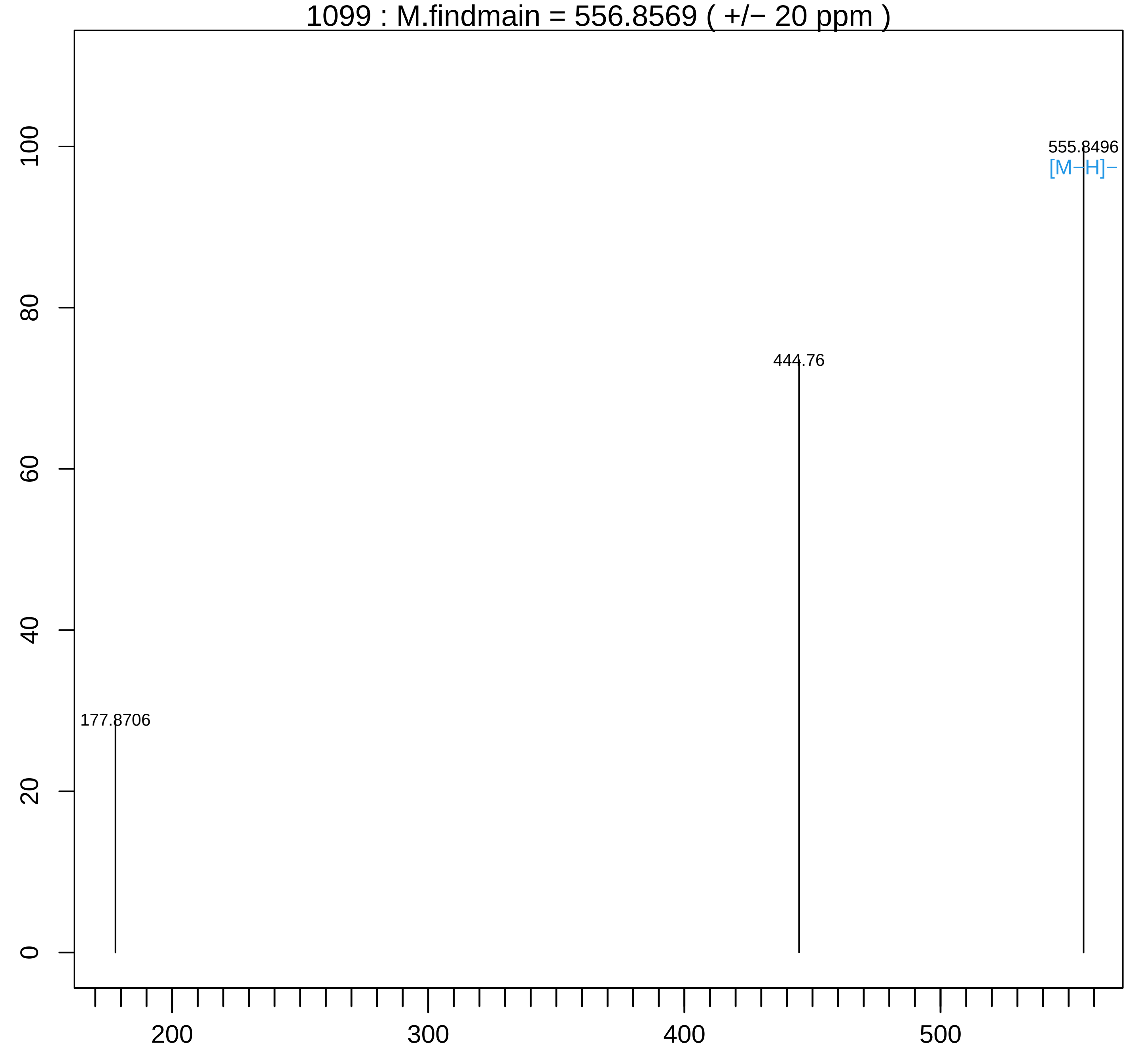
1099 : M.findmain = 556.8569 (+/− 20 ppm)
555.8496
[M−H]−
444.76
177.8706
0
20
40
60
80
100
200
300
400
500

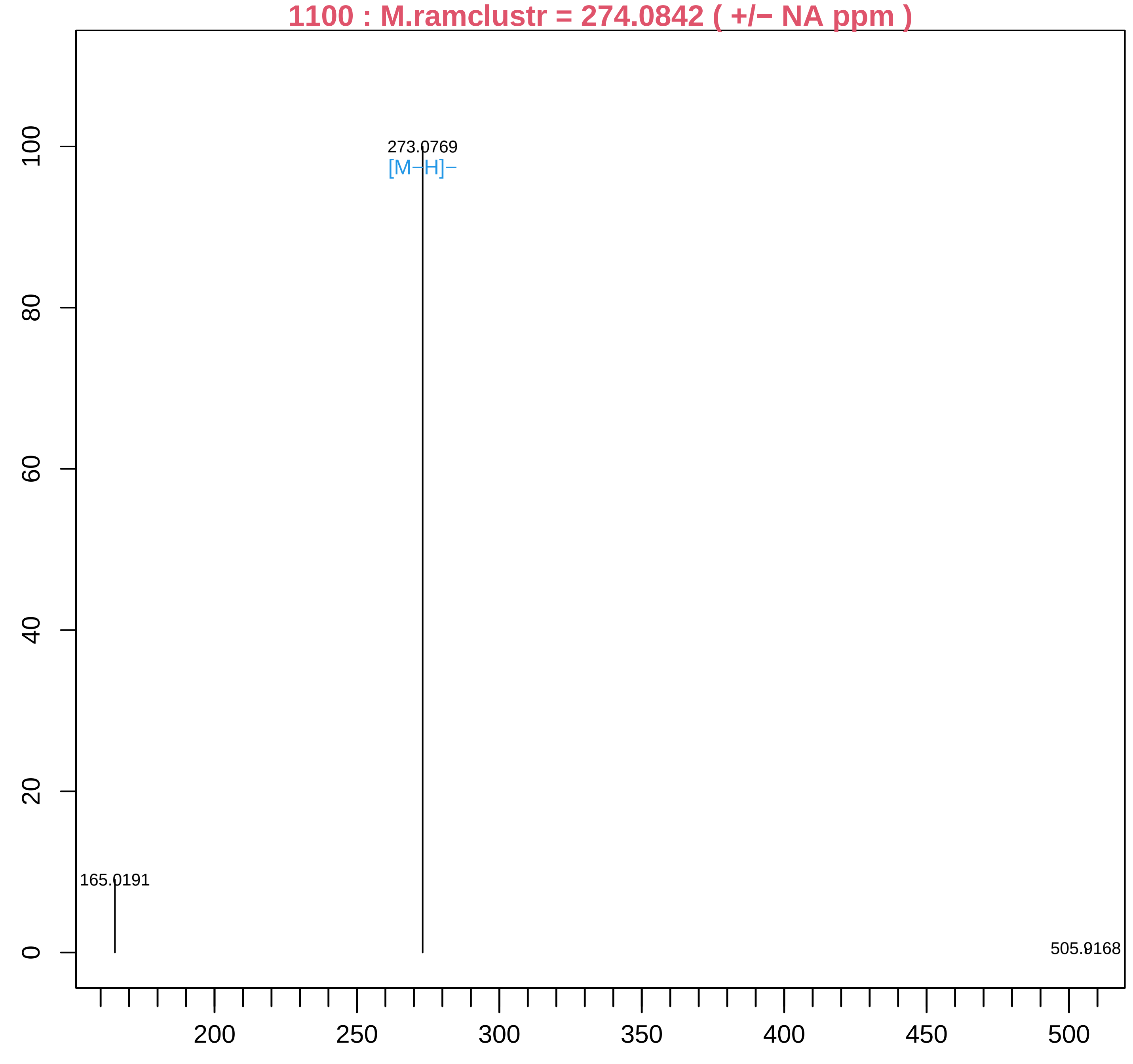
1100 : M.ramclustr = 274.0842 (+/− NA ppm)
273.0769
[M−H]−
165.0191
505.9168
0
20
40
60
80
100
200
250
300
350
400
450
500

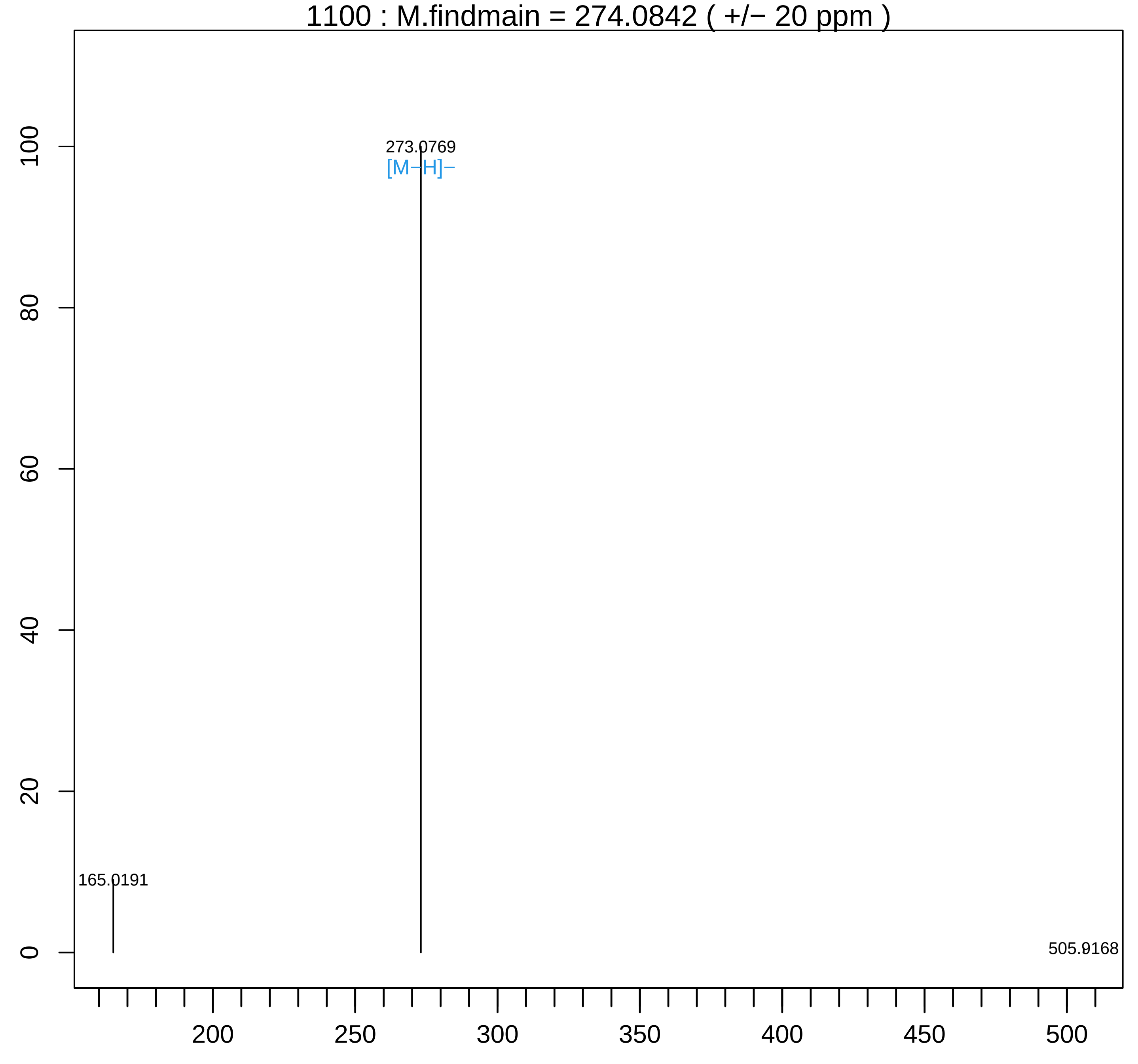
1100 : M.findmain = 274.0842 (+/− 20 ppm)
273.0769
[M−H]−
165.0191
505.9168
0
20
40
60
80
100
200
250
300
350
400
450
500

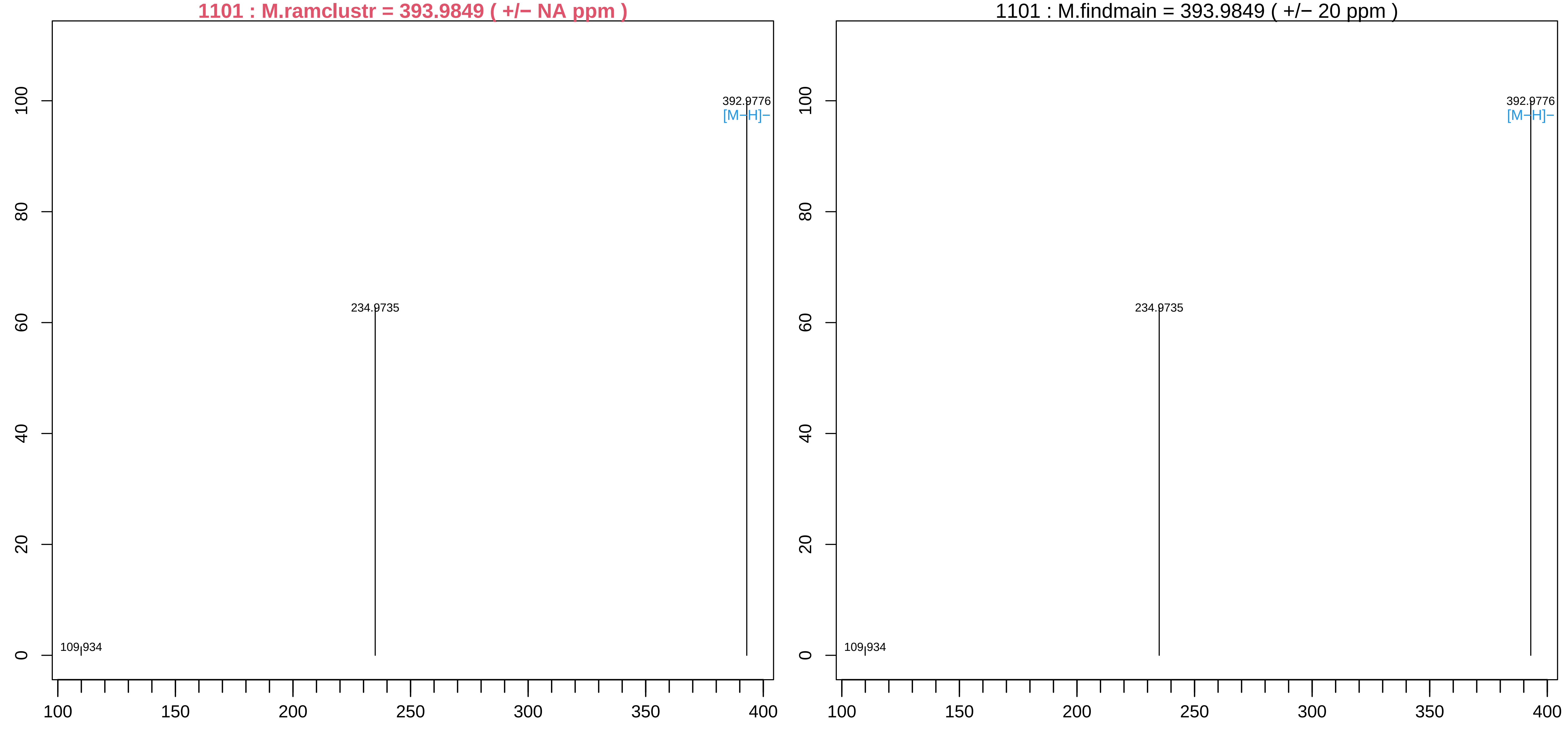
1101 : M.ramclustr = 393.9849 (+/− NA ppm)
392.9776
[M−H]−
234.9735
109.934
1101 : M.findmain = 393.9849 (+/− 20 ppm)
392.9776
[M−H]−
234.9735
109.934
0
20
40
60
80
100
100
150
200
250
300
350
400

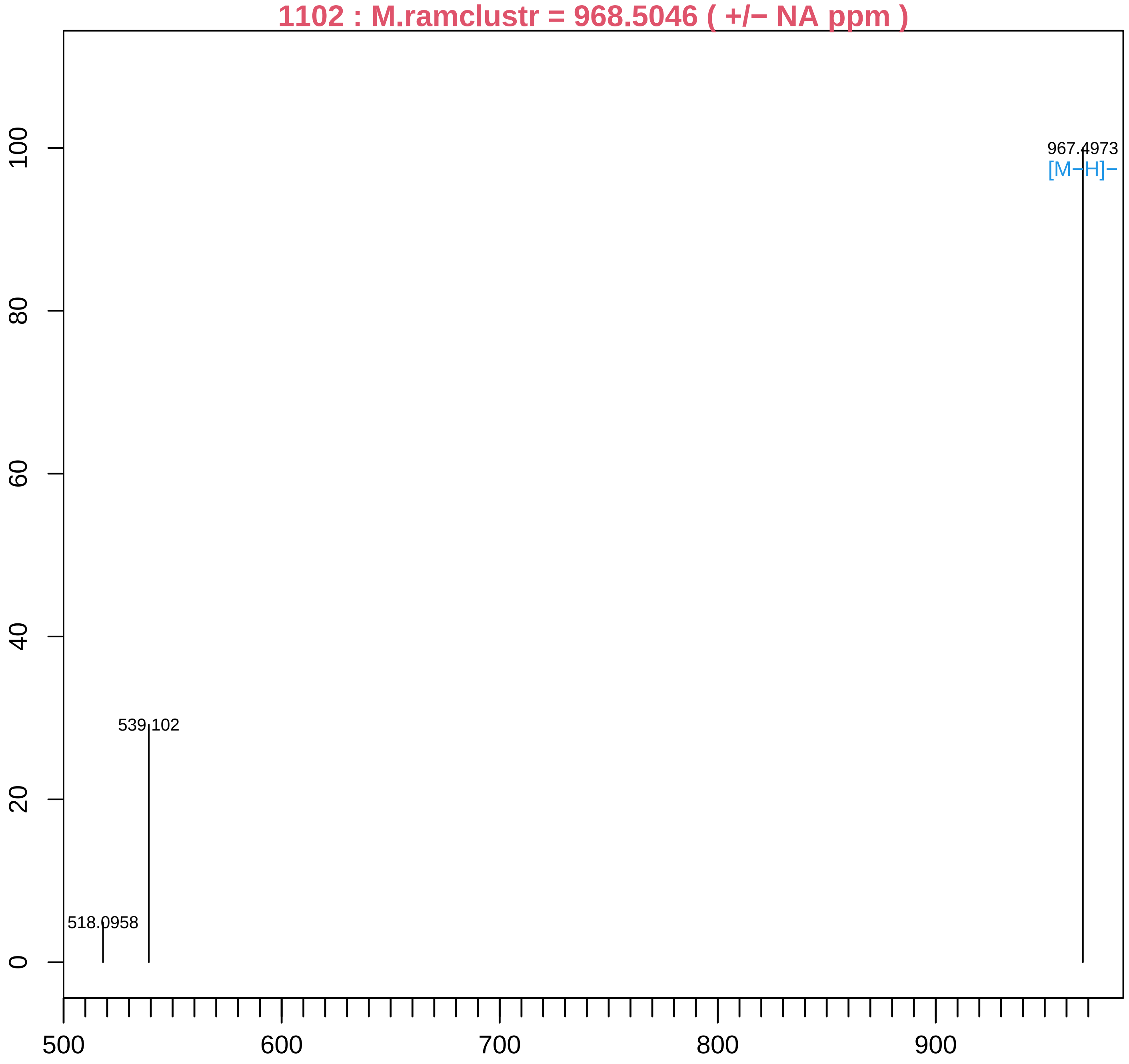

1102 : M.ramclustr = 968.5046 (+/− NA ppm)
967.4973
[M−H]−
539.102
518.0958
0
20
40
60
80
100
500
600
700
800
900

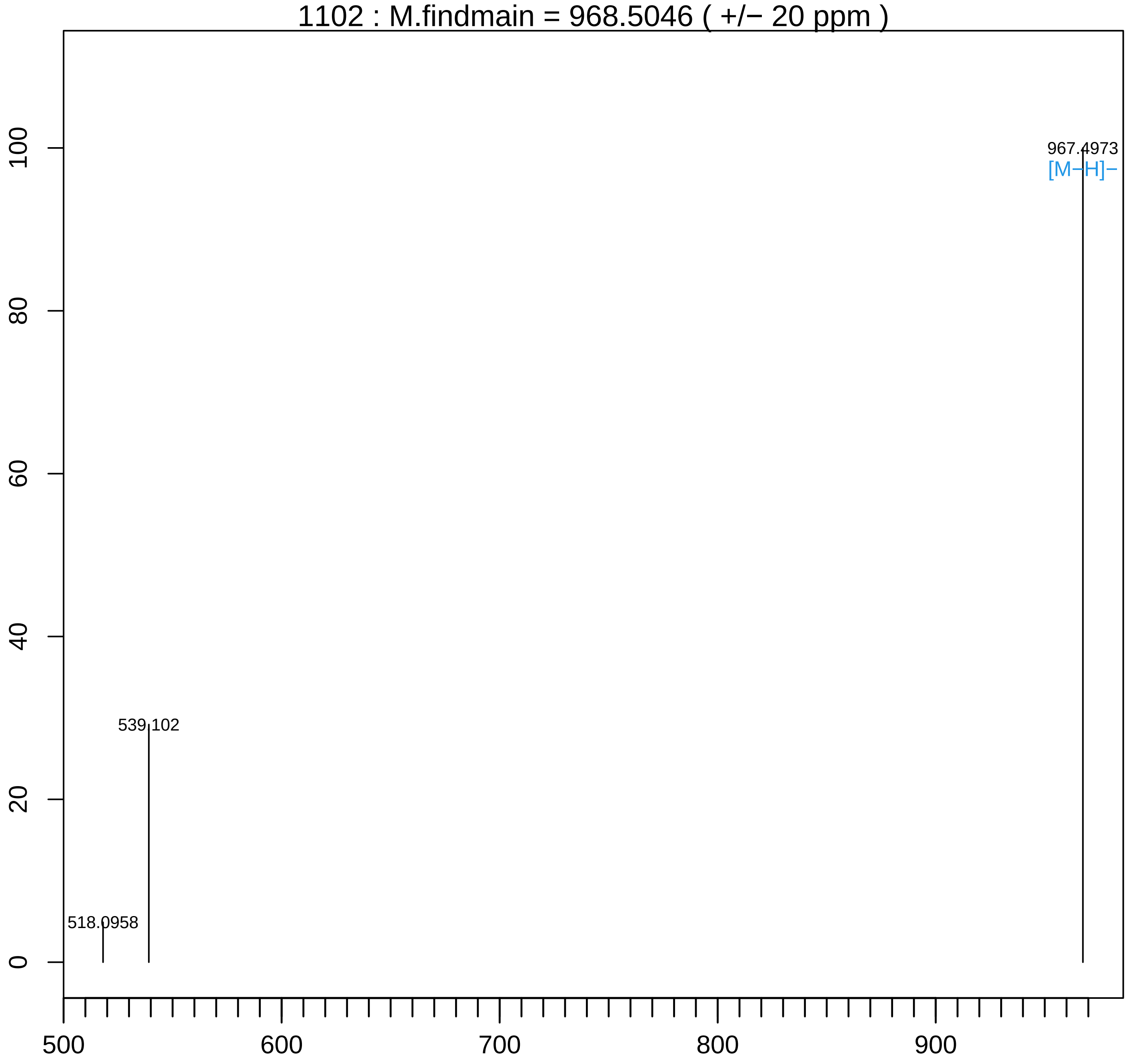

1102 : M.findmain = 968.5046 (+/− 20 ppm)
967.4973
[M−H]−
539.102
518.0958
0
20
40
60
80
100
500
600
700
800
900

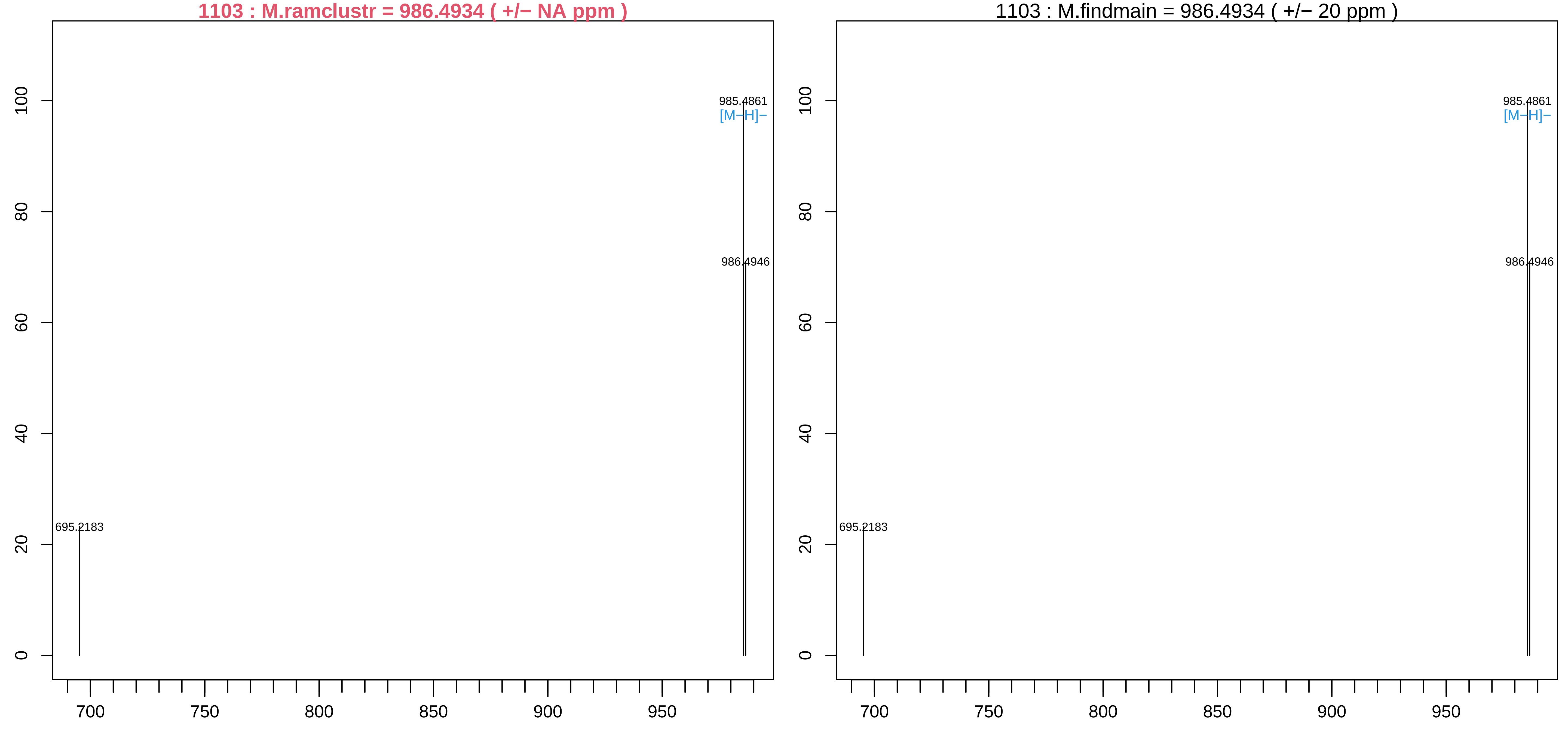
1103 : M.ramclustr = 986.4934 (+/− NA ppm)
985.4861
[M−H]−
986.4946
695.2183
1103 : M.findmain = 986.4934 (+/− 20 ppm)
985.4861
[M−H]−
986.4946
695.2183
0
20
40
60
80
100
700
750
800
850
900
950

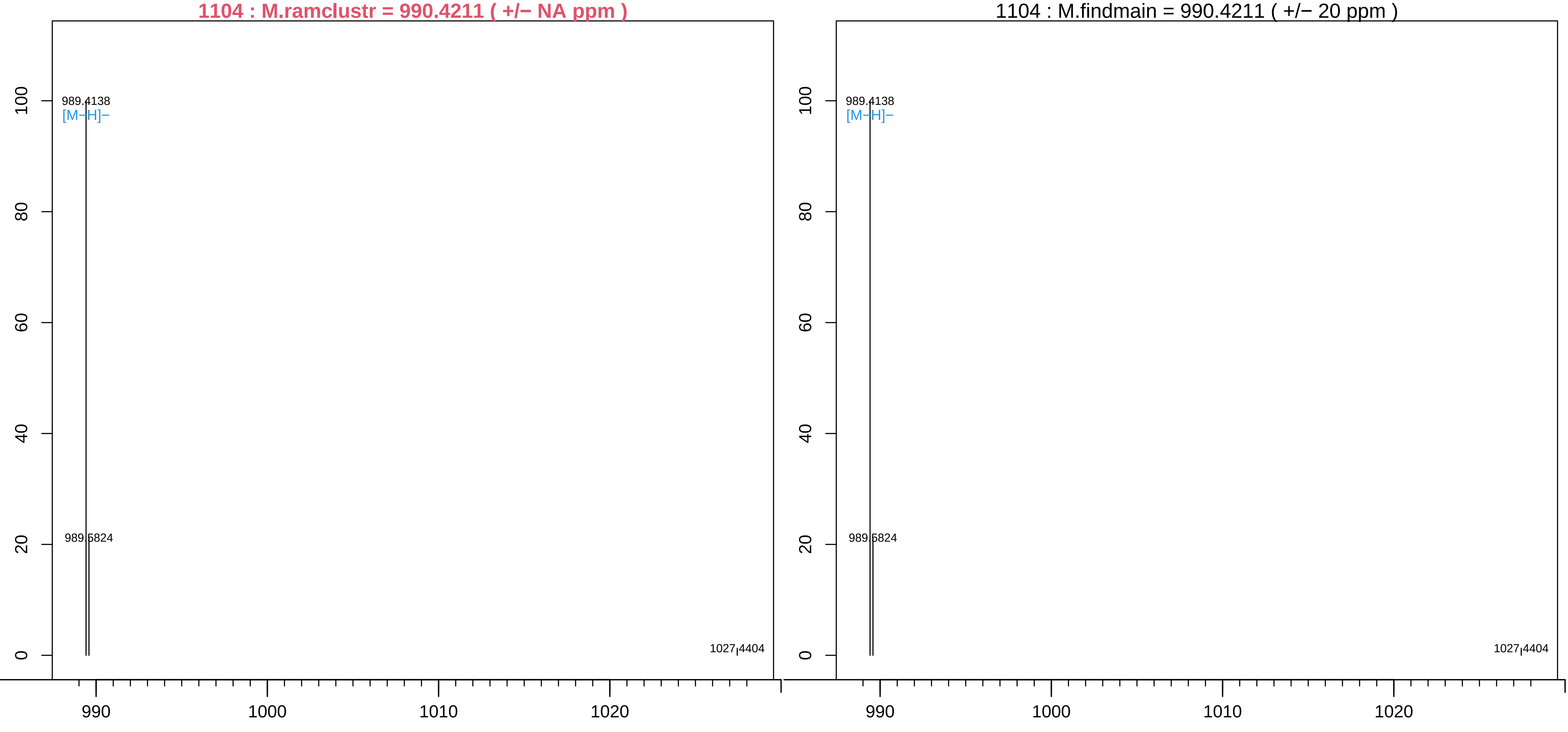

1104 : M.ramclustr = 990.4211 (+/− NA ppm)
989.4138
[M−H]−
989.5824
1027.4404
0
20
40
60
80
100
990
1000
1010
1020
1104 : M.findmain = 990.4211 (+/− 20 ppm)
989.4138
[M−H]−
989.5824
1027.4404
0
20
40
60
80
100
990
1000
1010
1020

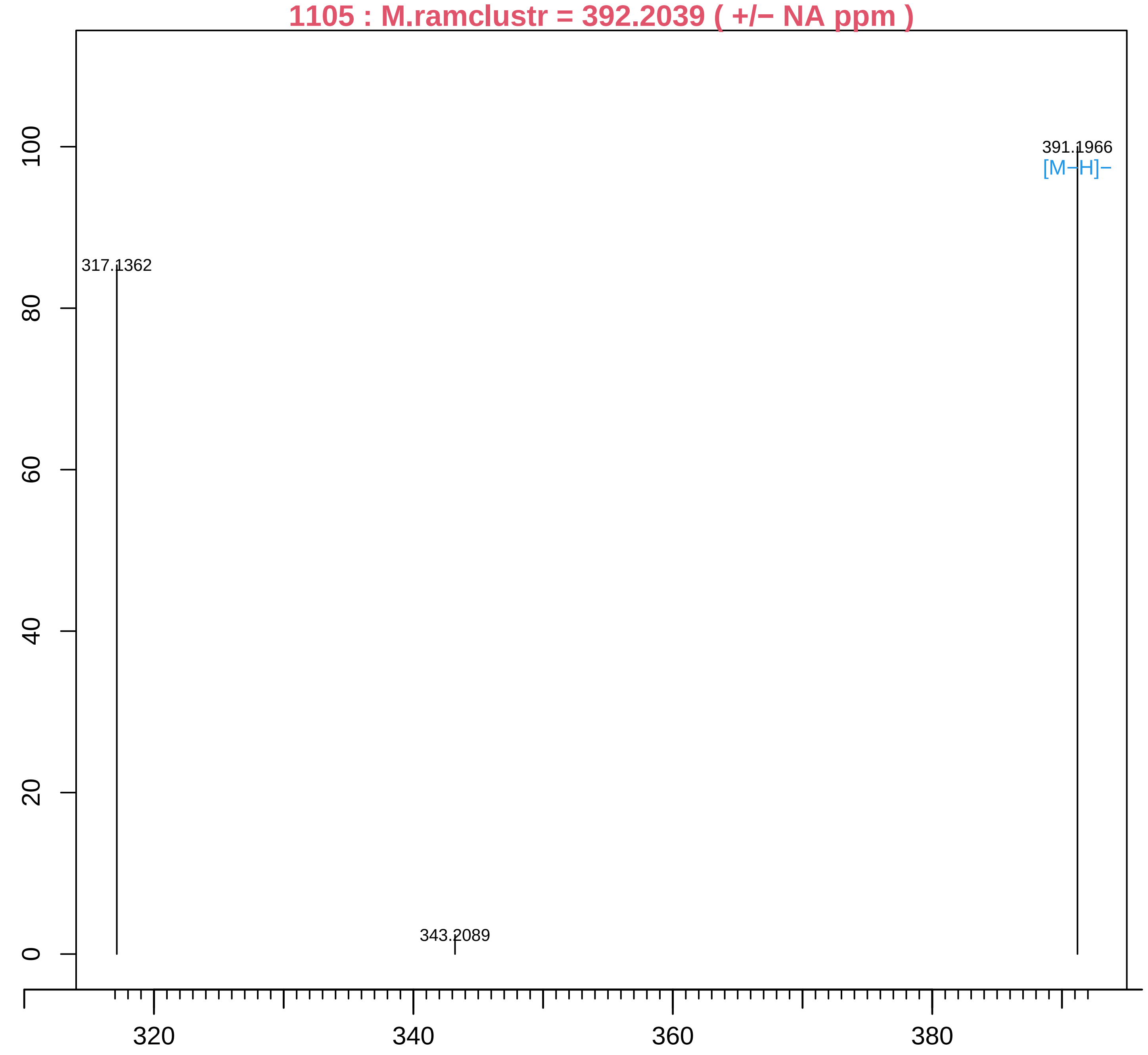

1105 : M.ramclustr = 392.2039 (+/− NA ppm)
391.1966
[M−H]−
317.1362
343.2089
0
20
40
60
80
100
320
340
360
380

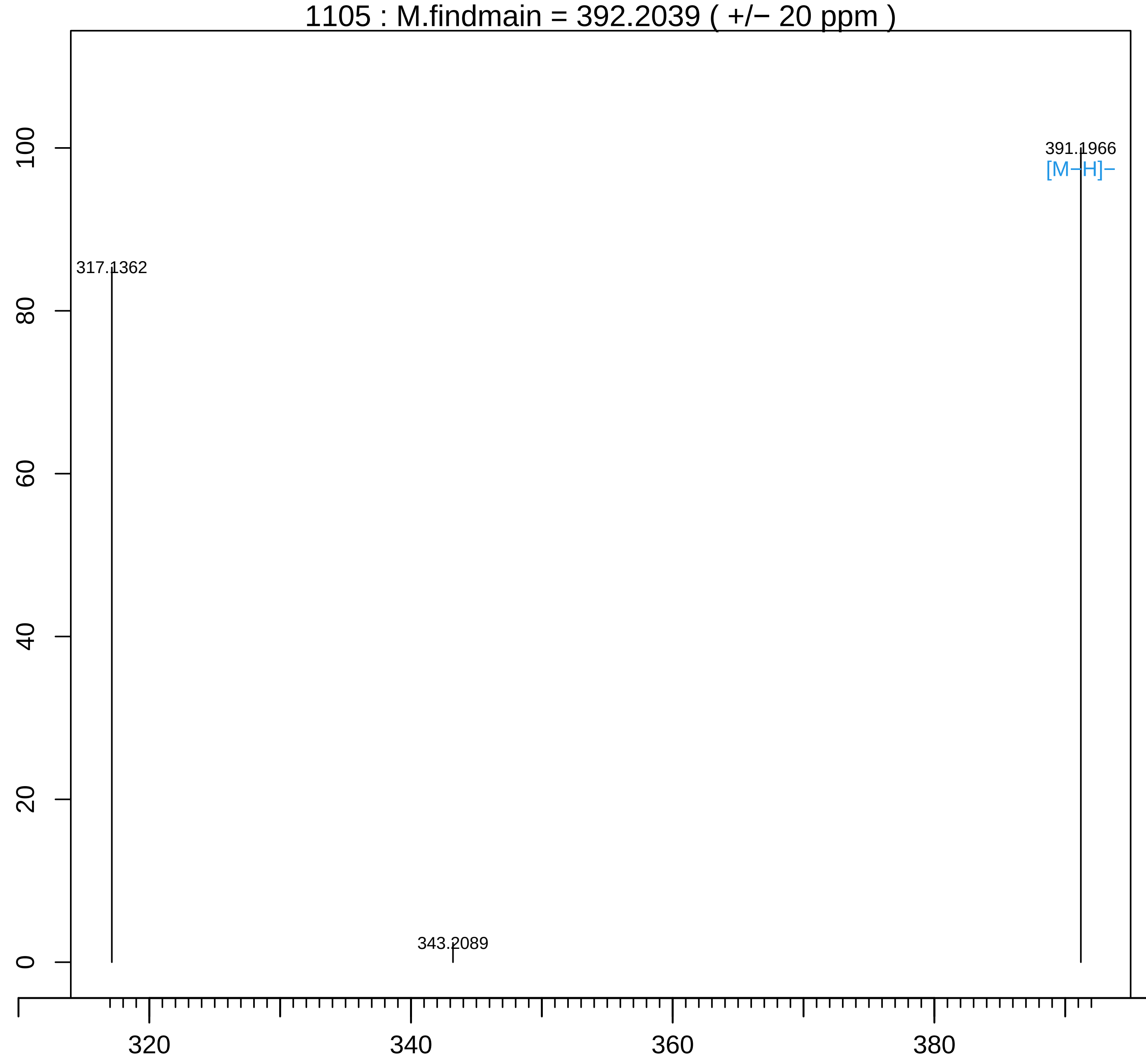

1105 : M.findmain = 392.2039 (+/− 20 ppm)
391.1966
[M−H]−
317.1362
343.2089
0
20
40
60
80
100
320
340
360
380

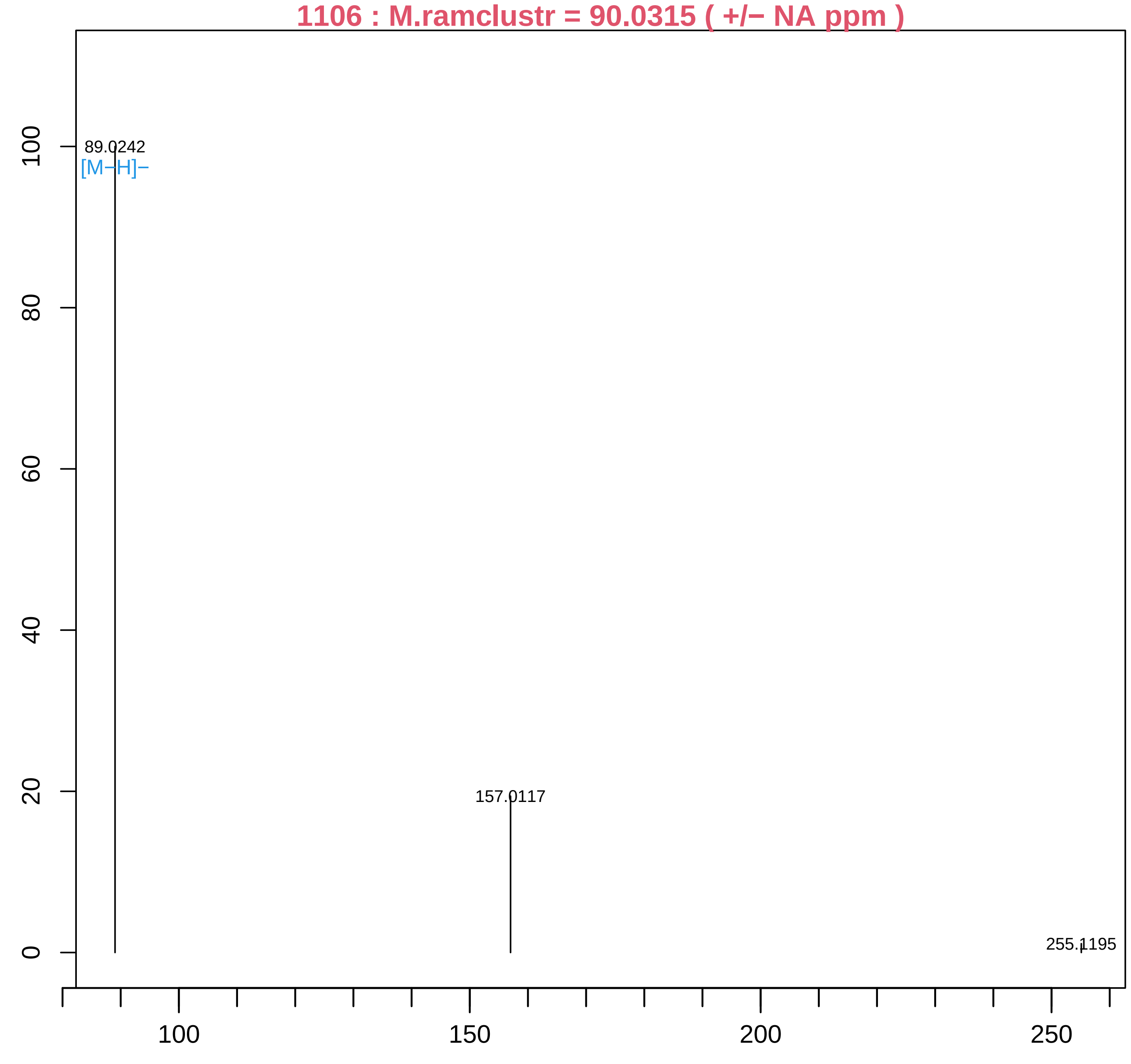

1106 : M.ramclustr = 90.0315 (+/− NA ppm)
89.0242
[M−H]−
157.0117
255.1195
0
20
40
60
80
100
100
150
200
250

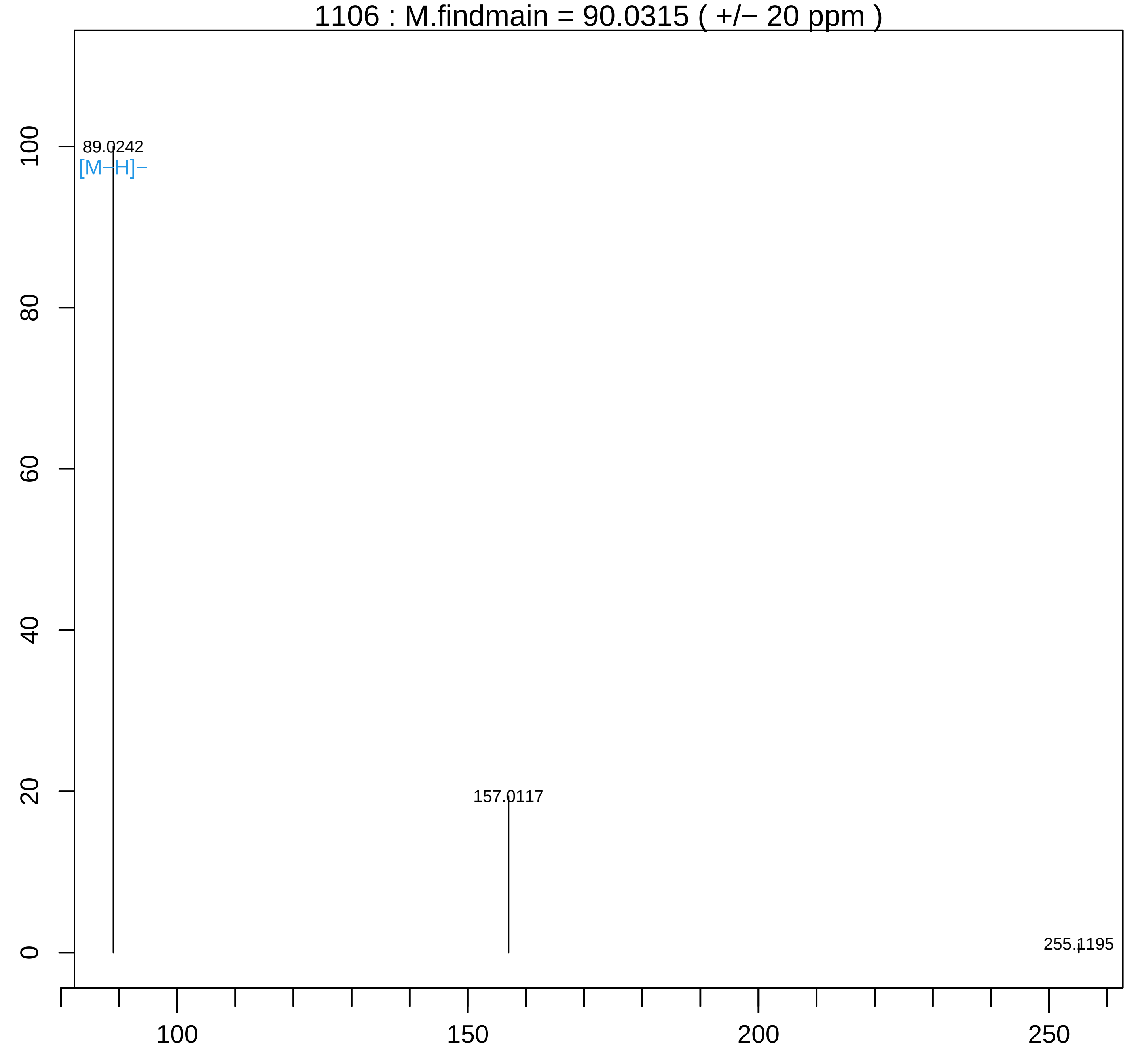

1106 : M.findmain = 90.0315 (+/− 20 ppm)
89.0242
[M−H]−
157.0117
255.1195
0
20
40
60
80
100
100
150
200
250

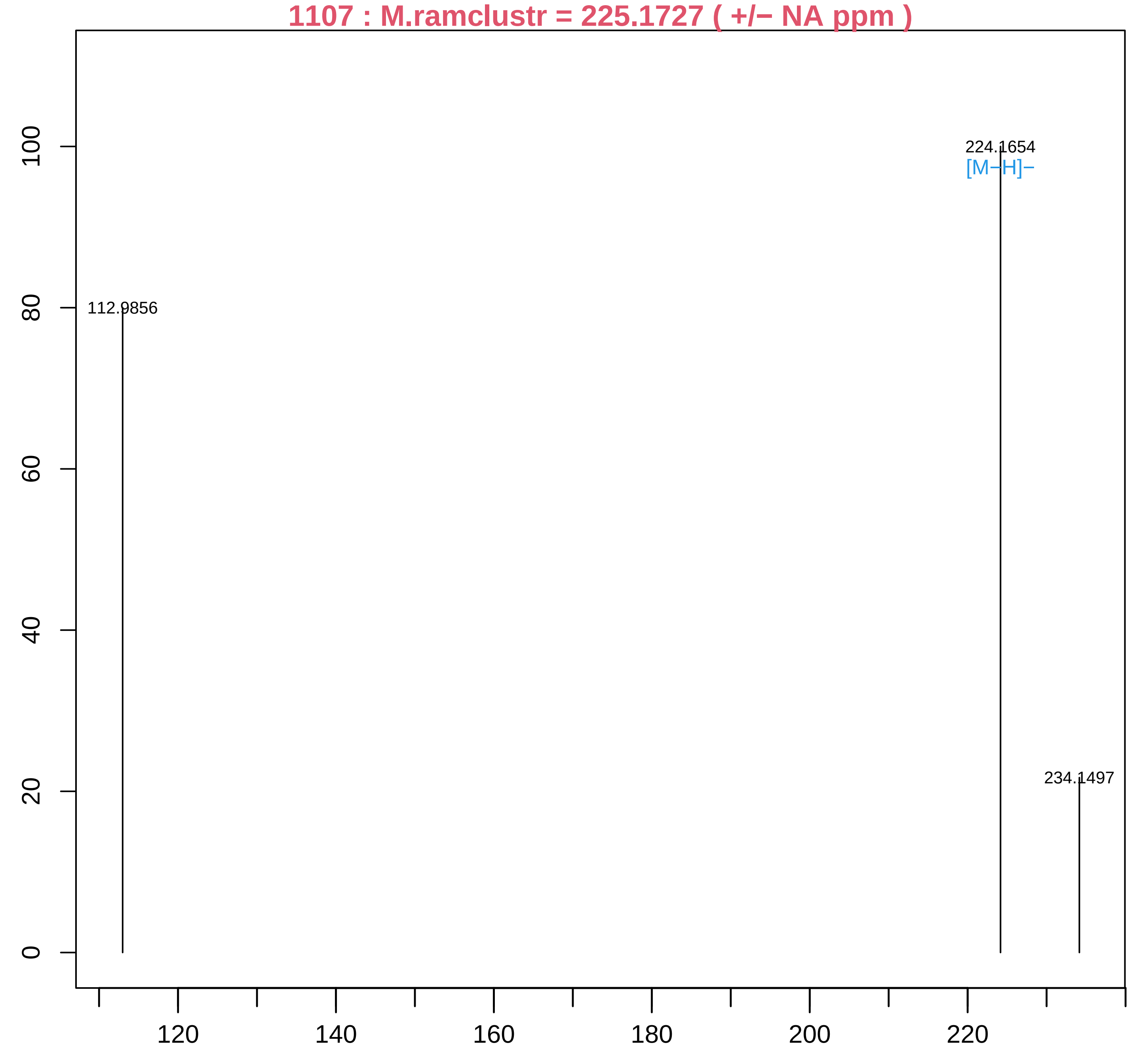
1107 : M.ramclustr = 225.1727 (+/− NA ppm)
224.1654
[M−H]−
112.9856
234.1497
0
20
40
60
80
100
120
140
160
180
200
220

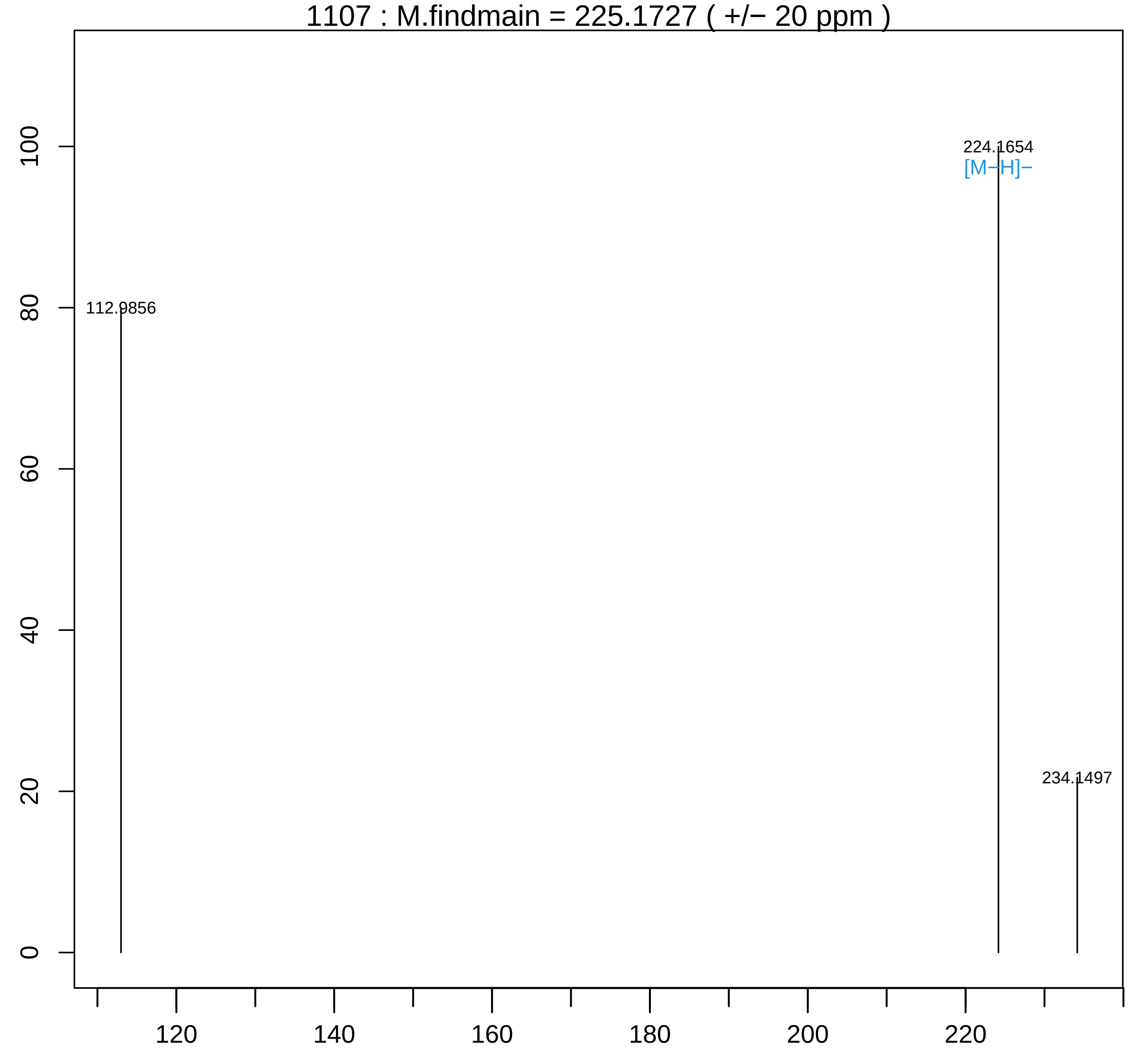
1107 : M.findmain = 225.1727 (+/− 20 ppm)
224.1654
[M−H]−
112.9856
234.1497
0
20
40
60
80
100
120
140
160
180
200
220

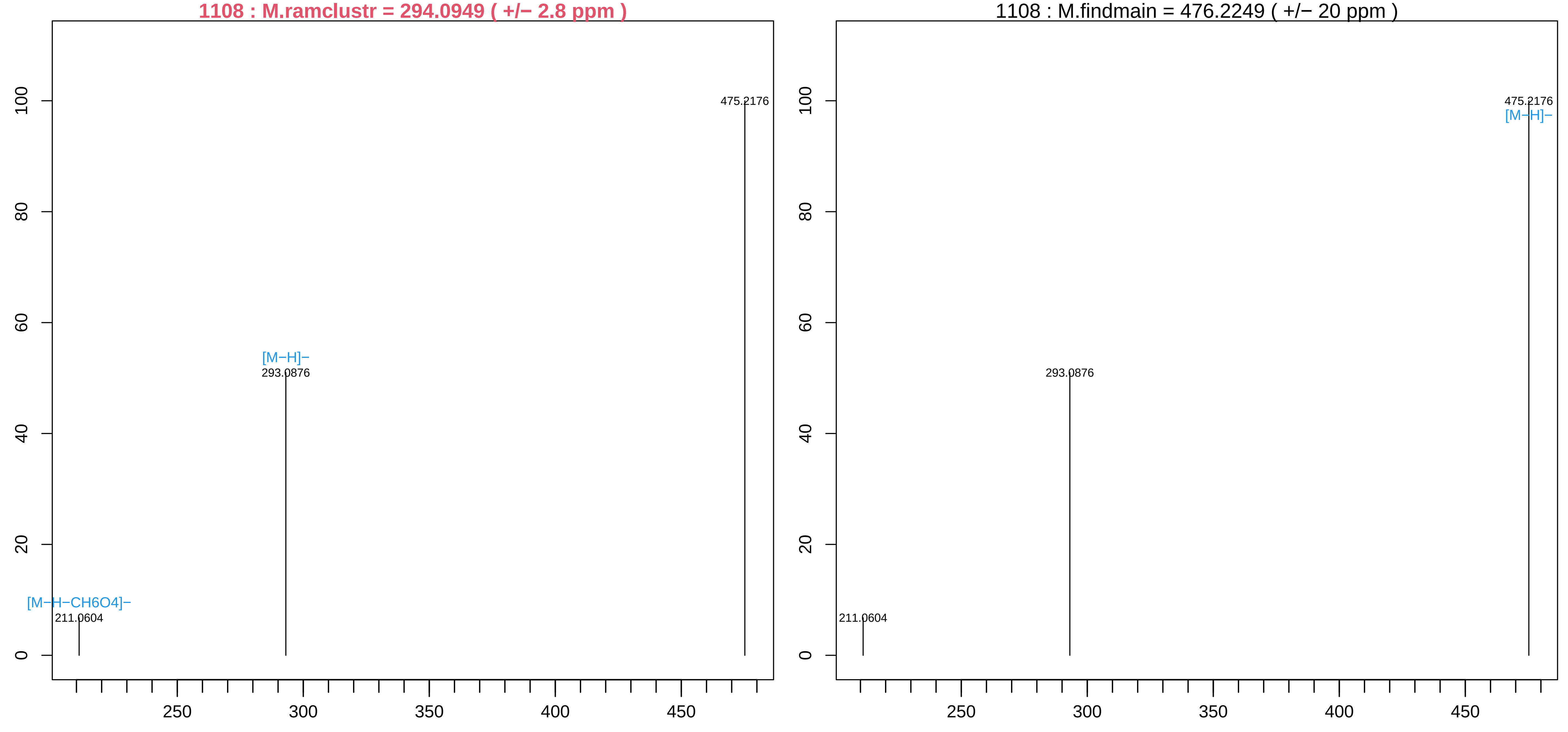

1108 : M.ramclustr = 294.0949 (+/− 2.8 ppm)
475.2176
[M−H]−
293.0876
[M−H−CH6O4]−
211.0604
0
20
40
60
80
100
250
300
350
400
450
1108 : M.findmain = 476.2249 (+/− 20 ppm)
475.2176
[M−H]−
293.0876
211.0604

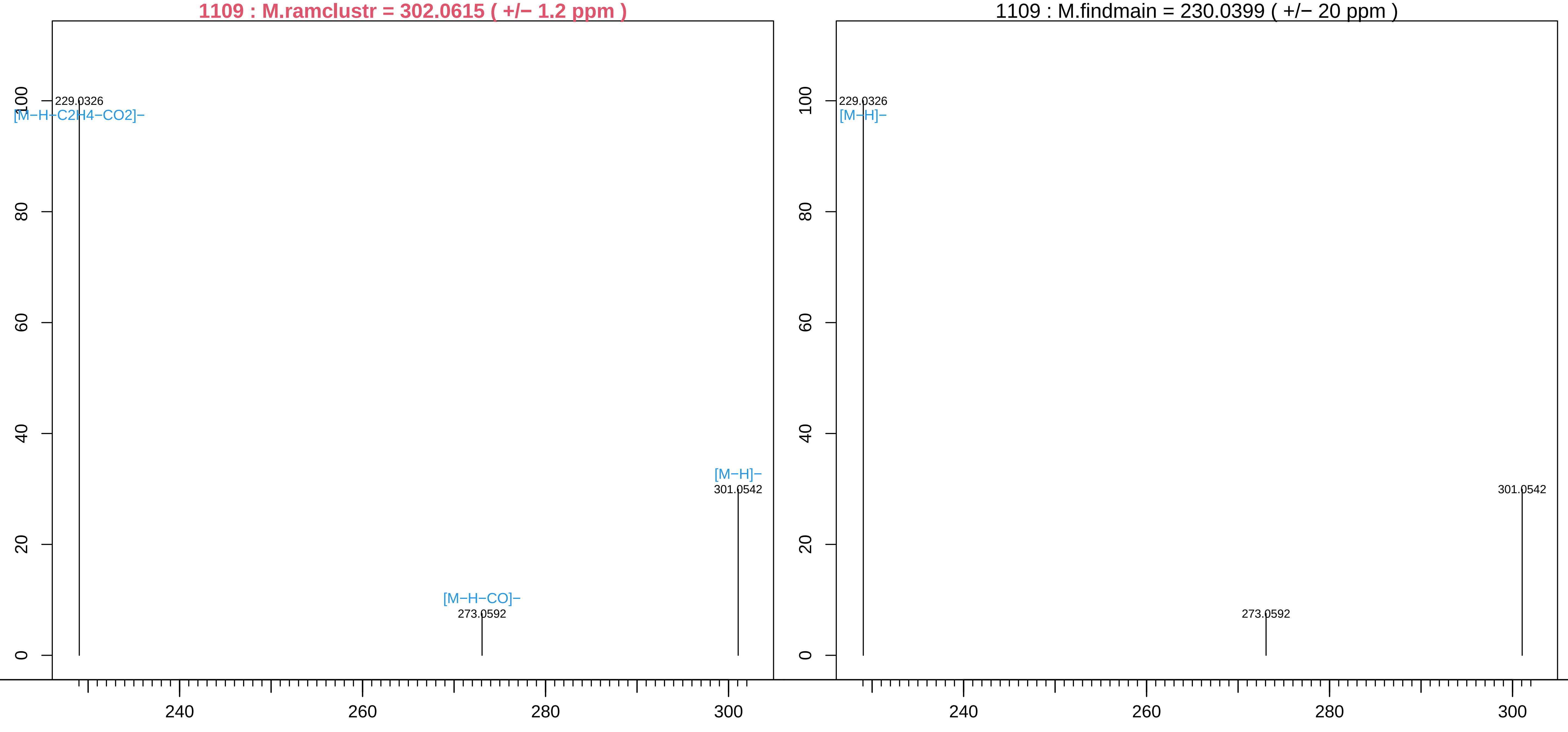
1109 : M.ramclustr = 302.0615 (+/− 1.2 ppm)
229.0326
[M−H−C2H4−CO2]−
[M−H−CO]−
273.0592
[M−H]−
301.0542
0
20
40
60
80
100
240
260
280
300
1109 : M.findmain = 230.0399 (+/− 20 ppm)
229.0326
[M−H]−
273.0592
301.0542
0
20
40
60
80
100
240
260
280
300

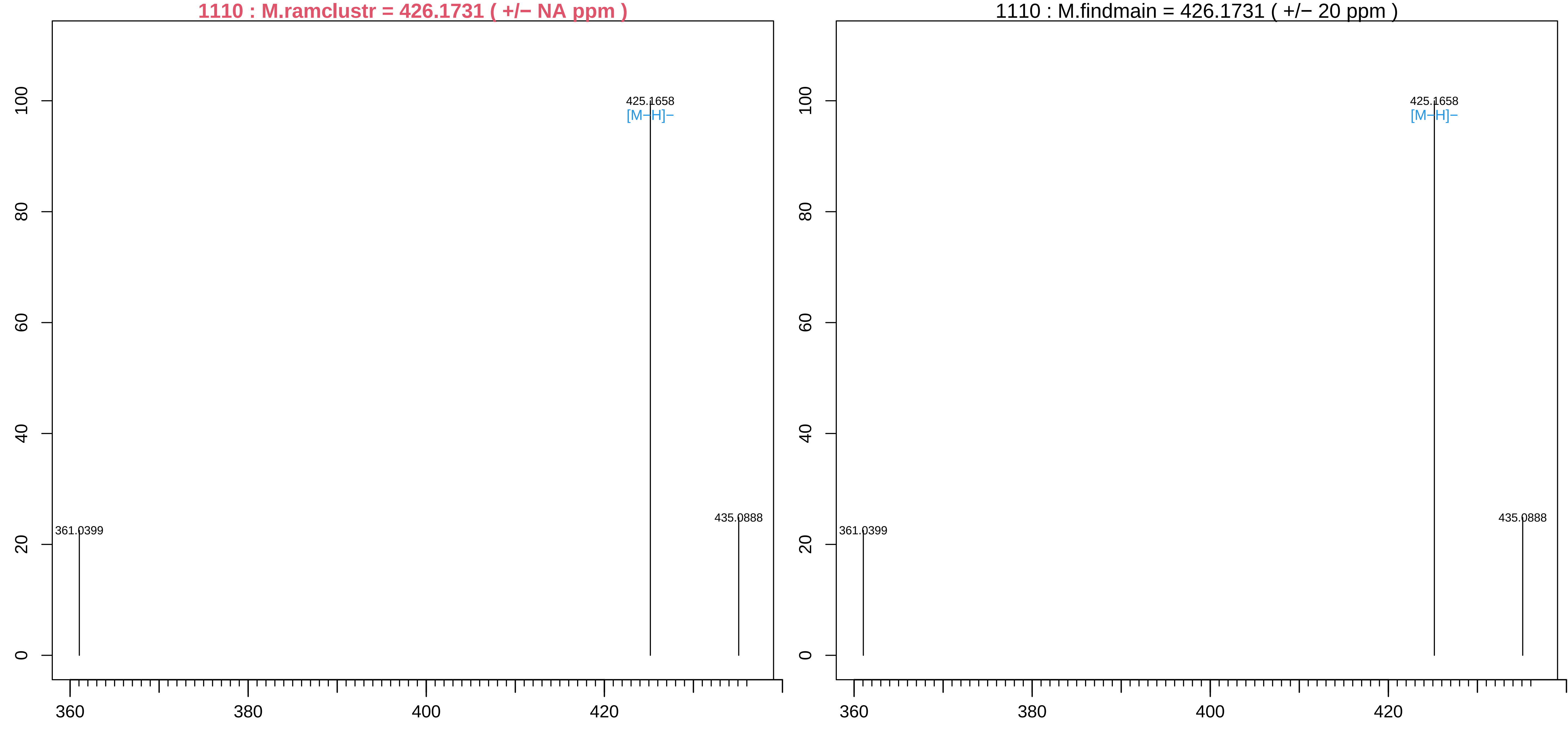

1110 : M.ramclustr = 426.1731 (+/− NA ppm)
425.1658
[M−H]−
361.0399
435.0888
1110 : M.findmain = 426.1731 (+/− 20 ppm)
425.1658
[M−H]−
361.0399
435.0888
0
20
40
60
80
100
360
380
400
420

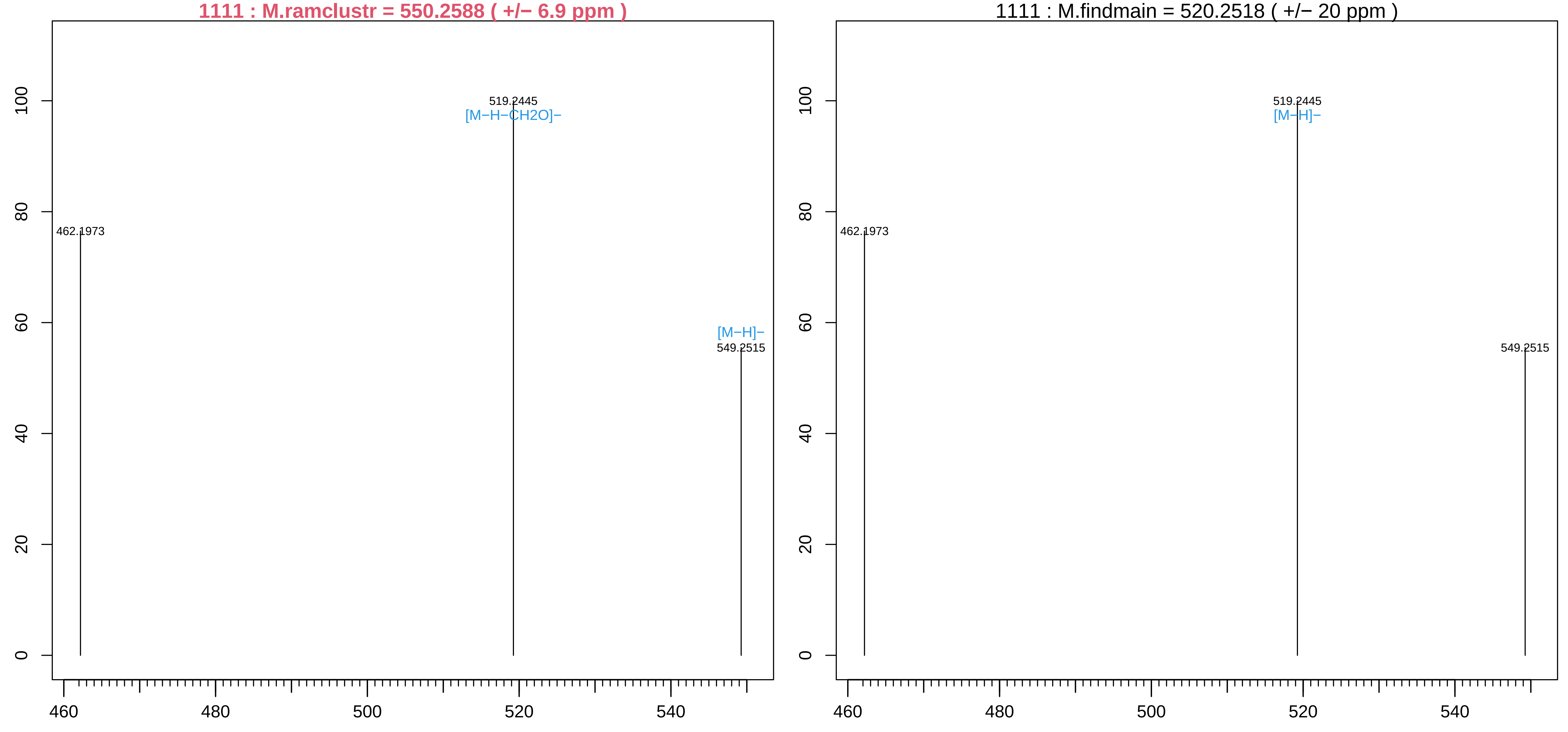

1111 : M.ramclustr = 550.2588 (+/− 6.9 ppm)
519.2445
[M−H−CH2O]−
462.1973
[M−H]−
549.2515
1111 : M.findmain = 520.2518 (+/− 20 ppm)
519.2445
[M−H]−
462.1973
549.2515

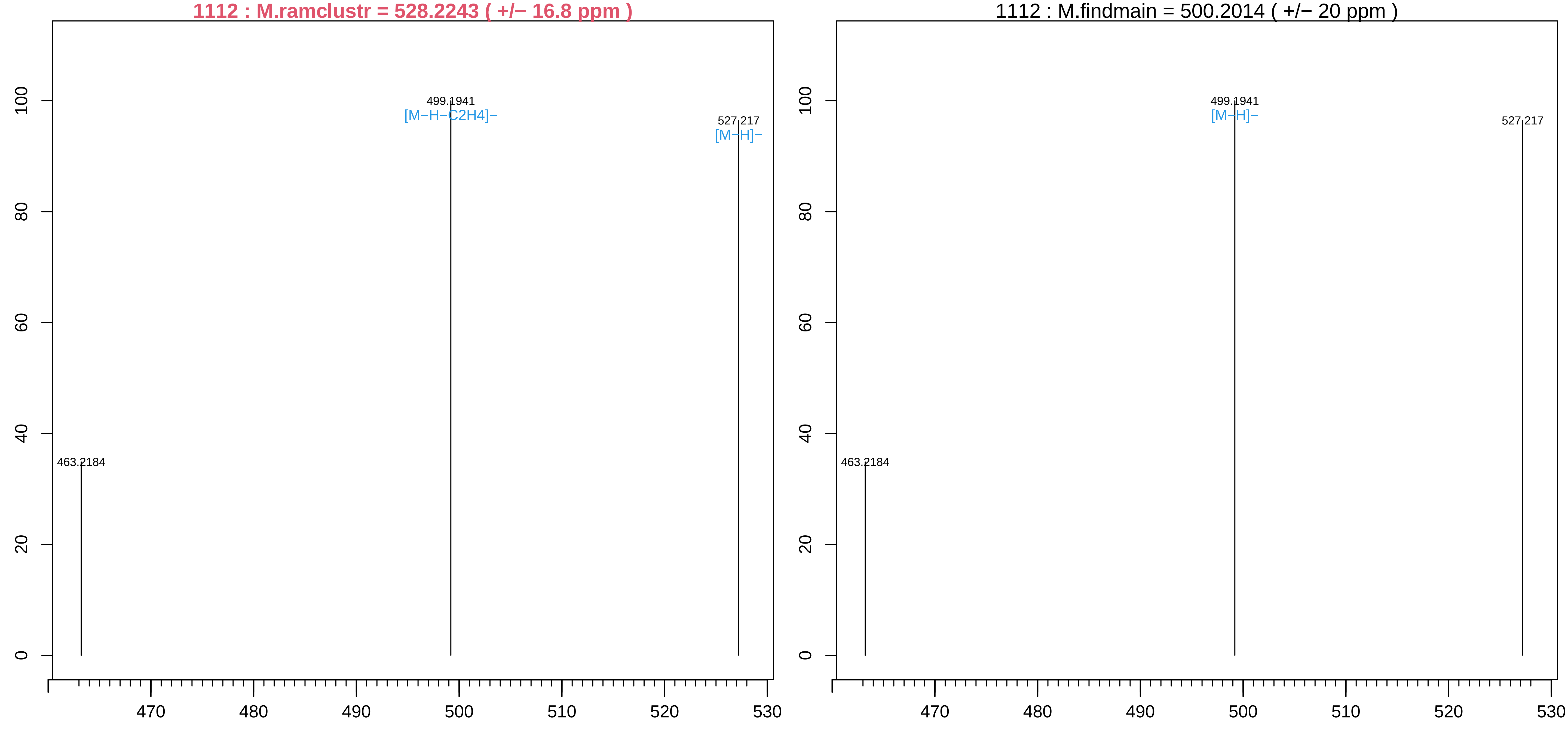

1112 : M.ramclustr = 528.2243 (+/− 16.8 ppm)
499.1941
[M−H−C2H4]−
527.217
[M−H]−
463.2184
1112 : M.findmain = 500.2014 (+/− 20 ppm)
499.1941
[M−H]−
527.217
463.2184
0
20
40
60
80
100
470
480
490
500
510
520
530

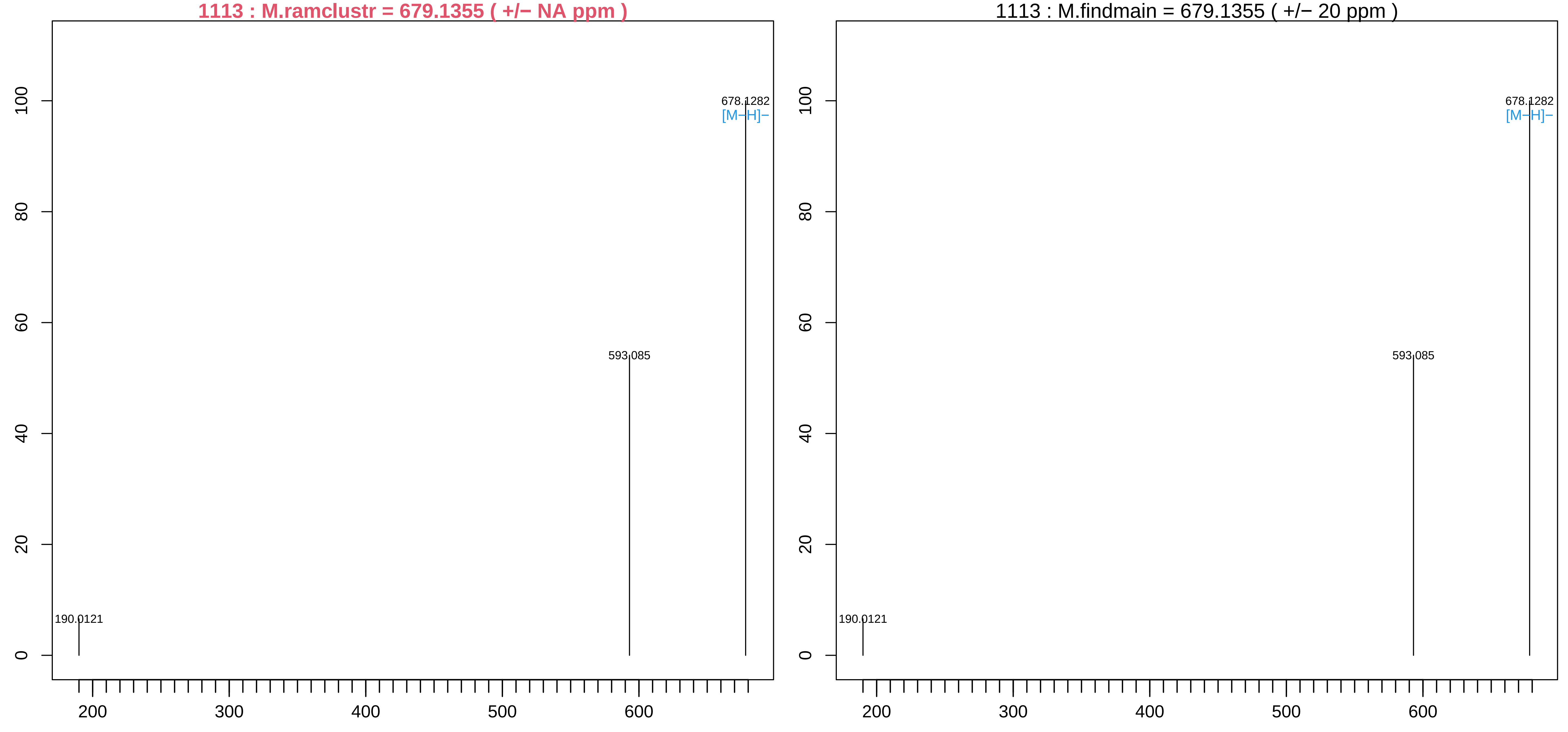
1113 : M.ramclustr = 679.1355 (+/− NA ppm)
1113 : M.findmain = 679.1355 (+/− 20 ppm)
678.1282
[M−H]−
593.085
190.0121
0
20
40
60
80
100
200
300
400
500
600

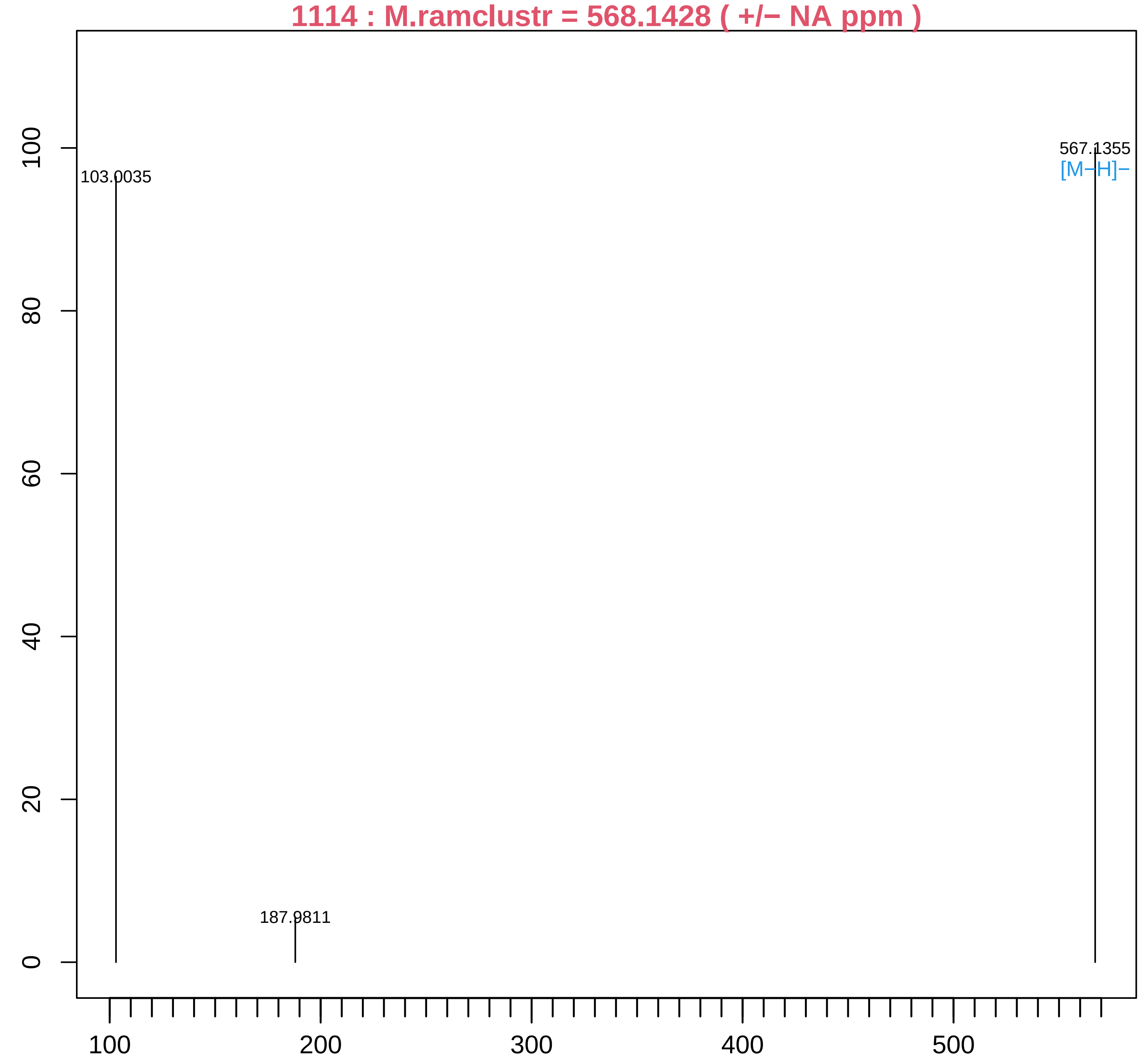
1114 : M.ramclustr = 568.1428 (+/− NA ppm)
103.0035
187.9811
567.1355
[M−H]−
0
20
40
60
80
100
100
200
300
400
500

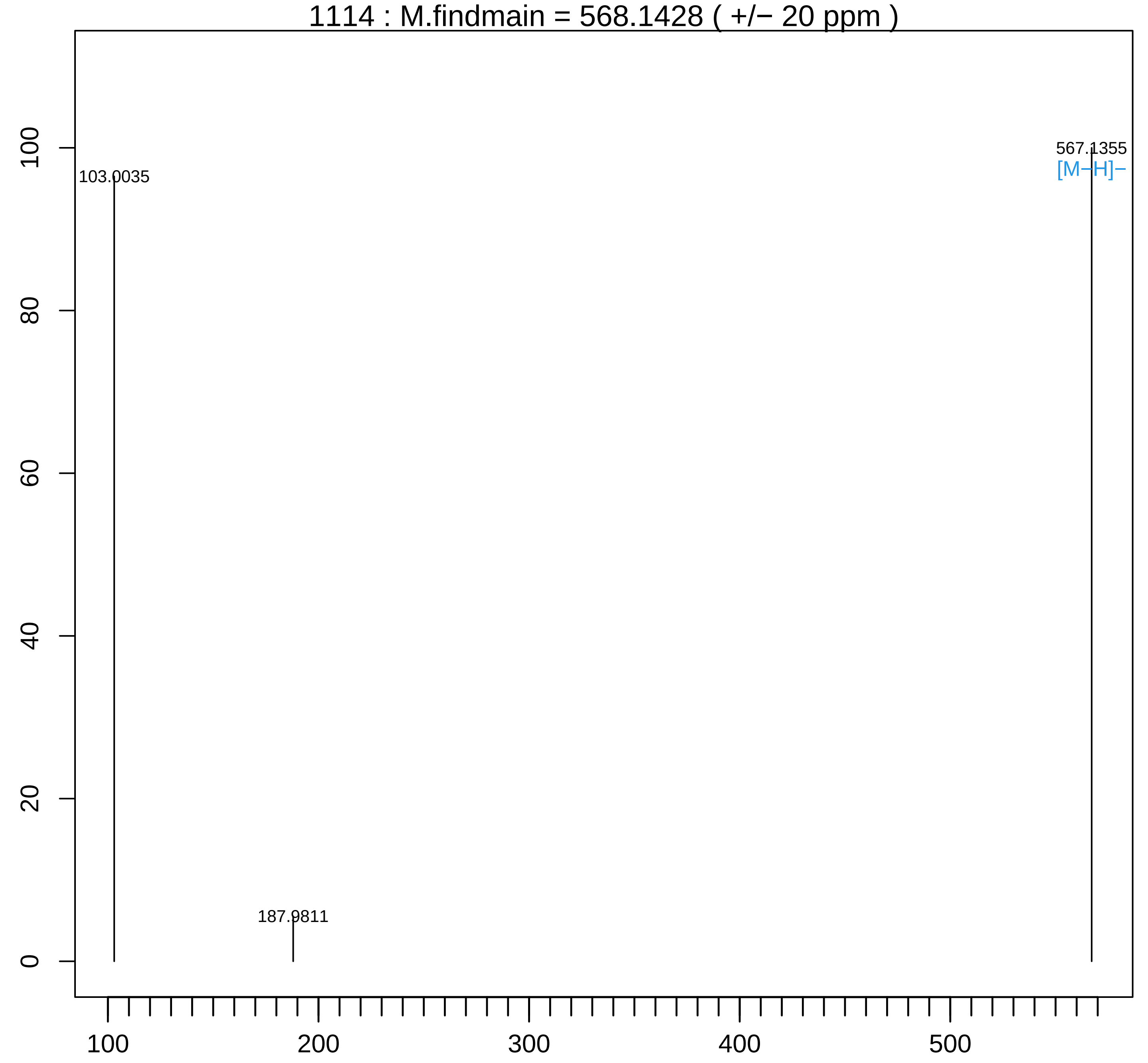
1114 : M.findmain = 568.1428 (+/− 20 ppm)
103.0035
187.9811
567.1355
[M−H]−
0
20
40
60
80
100
100
200
300
400
500

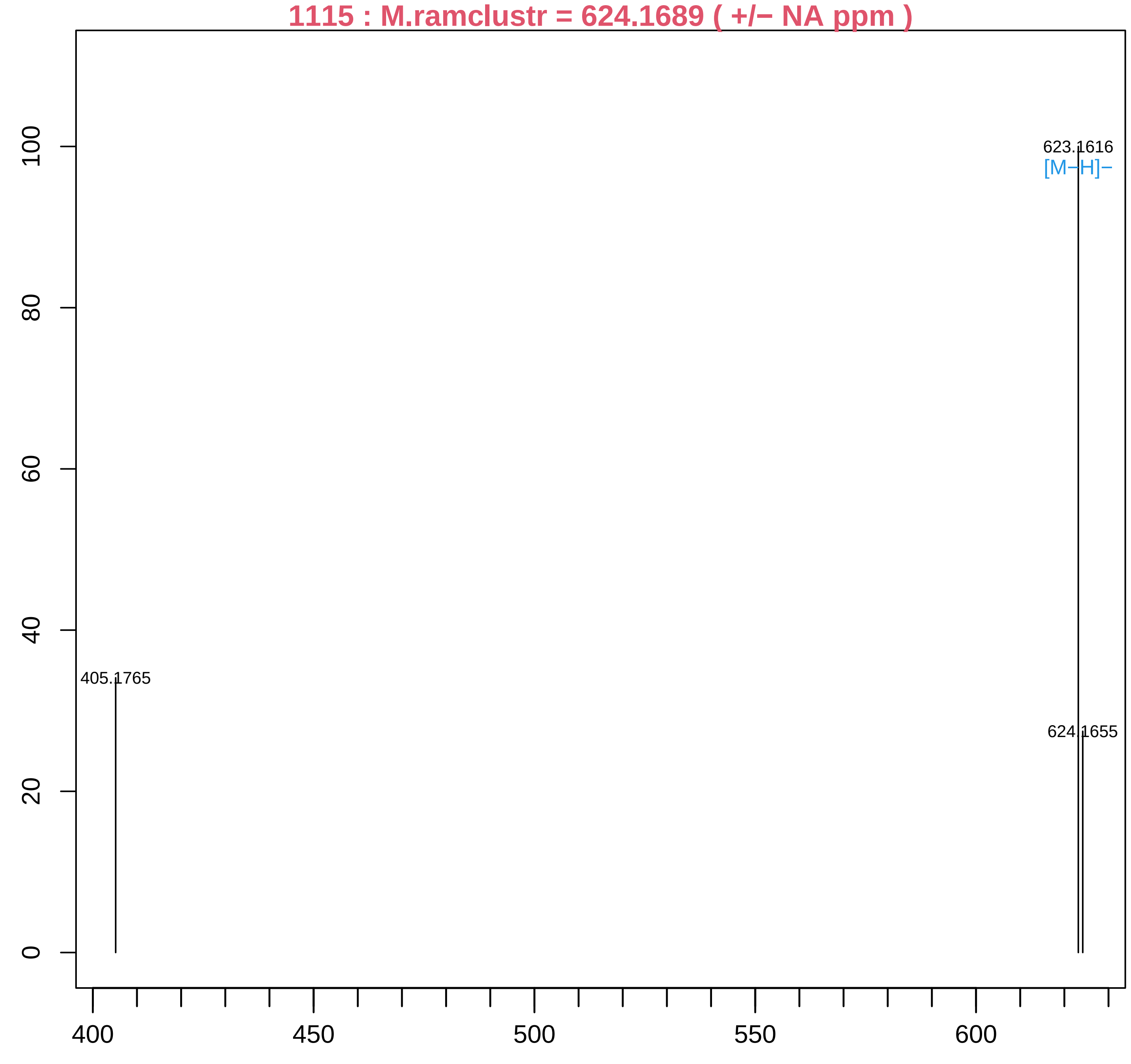

1115 : M.ramclustr = 624.1689 (+/− NA ppm)
623.1616
[M−H]−
405.1765
624.1655
0
20
40
60
80
100
400
450
500
550
600

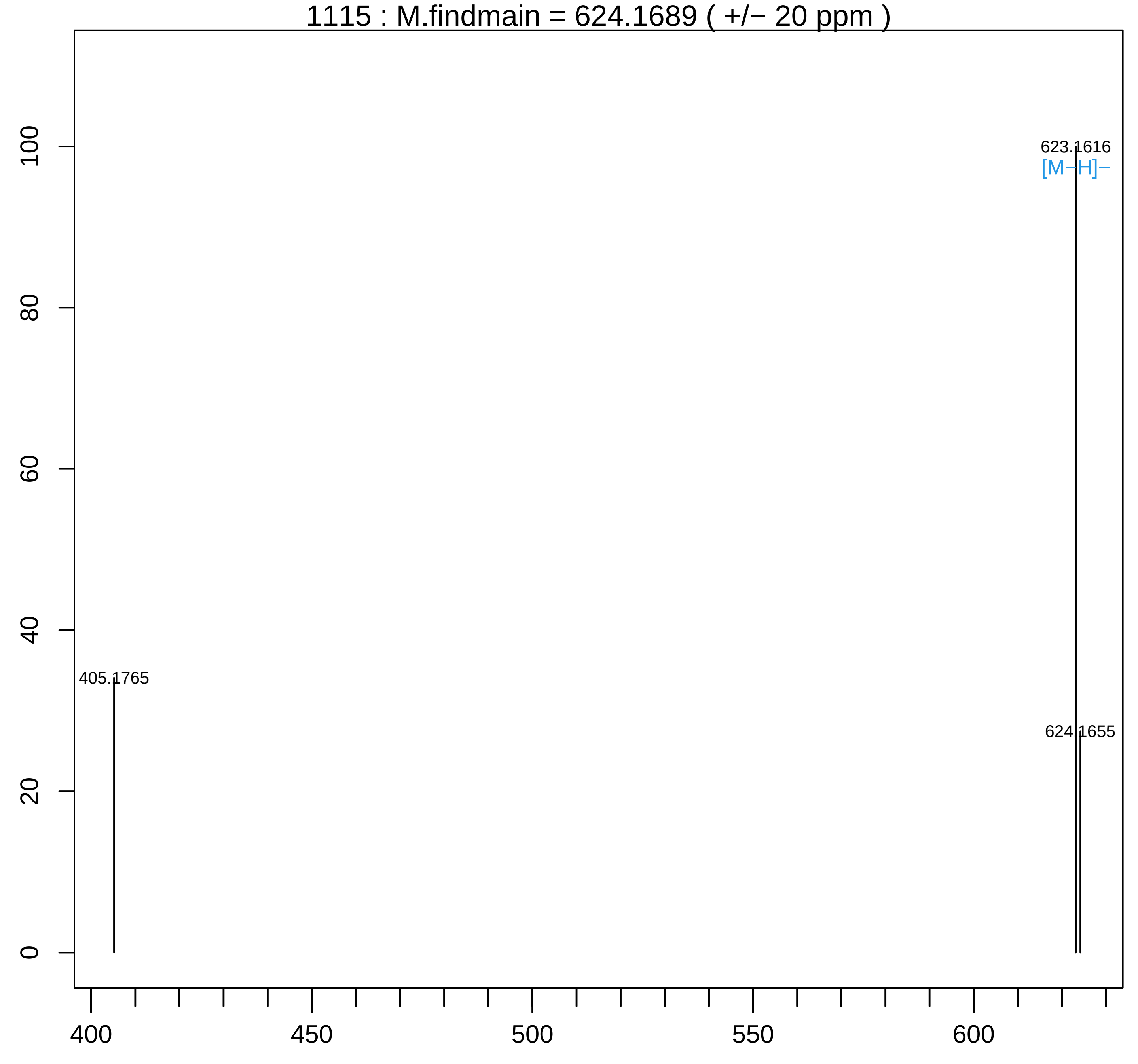

1115 : M.findmain = 624.1689 (+/− 20 ppm)
623.1616
[M−H]−
405.1765
624.1655
0
20
40
60
80
100
400
450
500
550
600

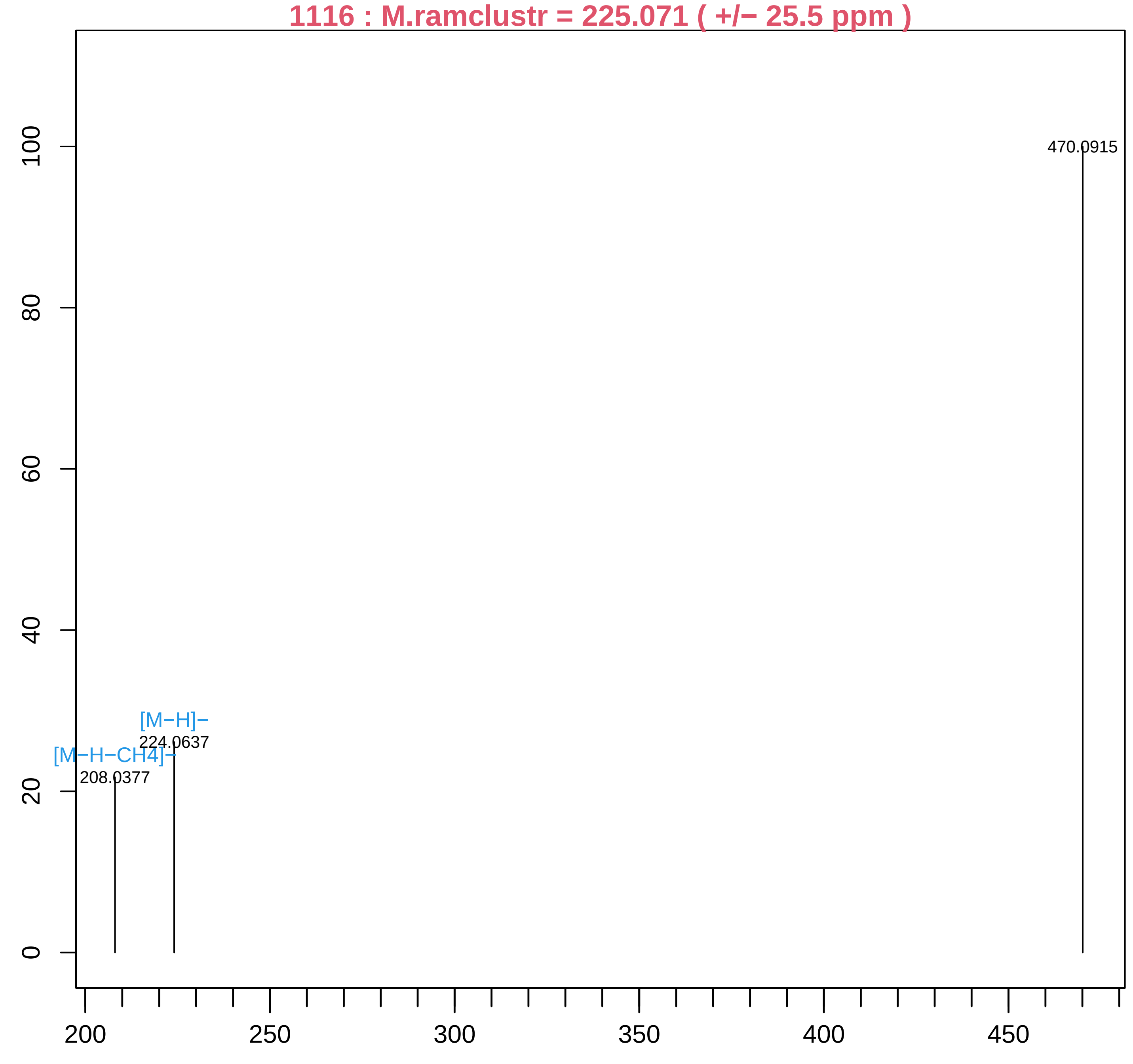
1116 : M.ramclustr = 225.071 (+/− 25.5 ppm)
470.0915
[M−H]−
224.0637
[M−H−CH4]−
208.0377
0
20
40
60
80
100
200
250
300
350
400
450

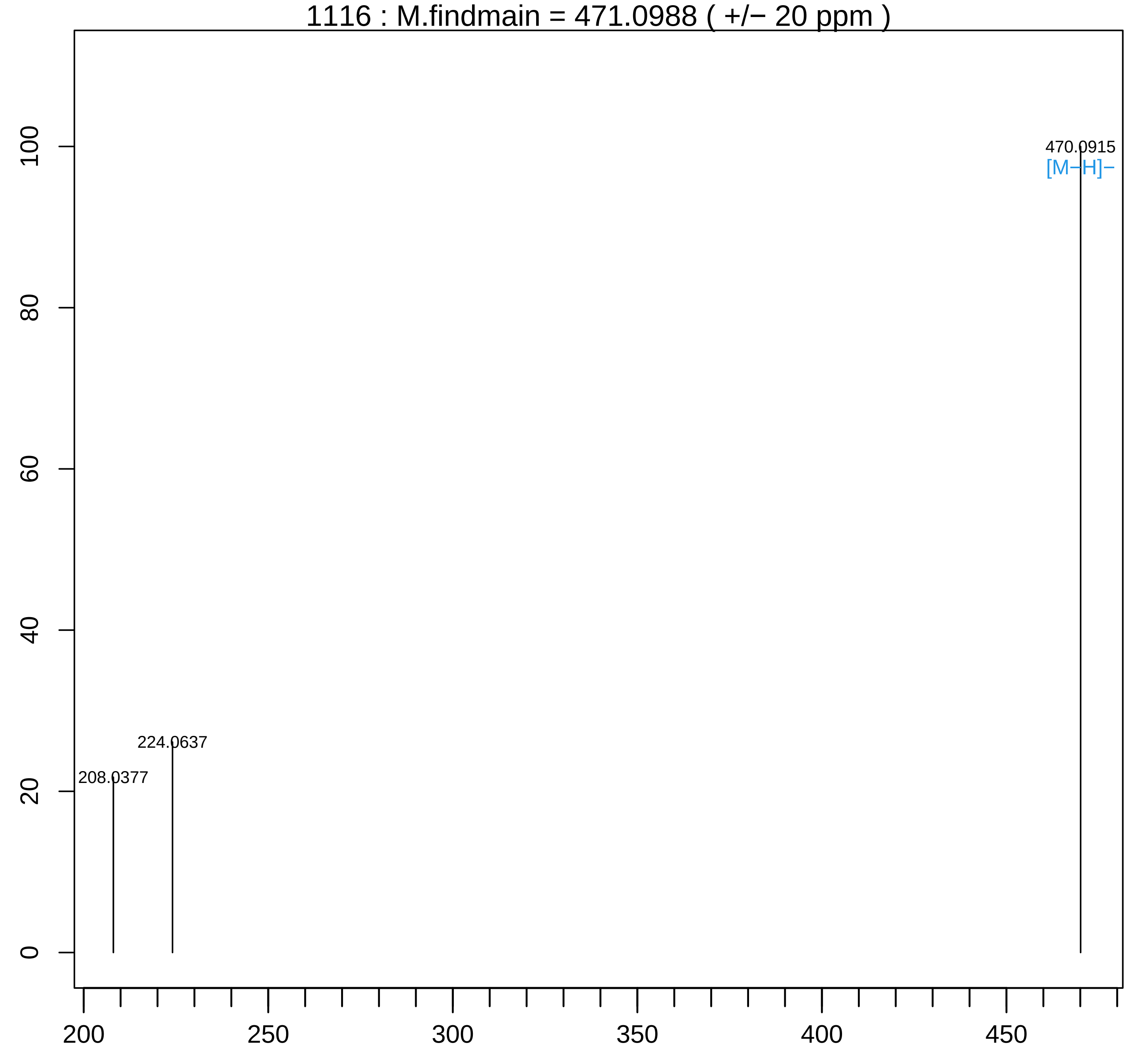
1116 : M.findmain = 471.0988 (+/− 20 ppm)
470.0915
[M−H]−
224.0637
208.0377
0
20
40
60
80
100
200
250
300
350
400
450

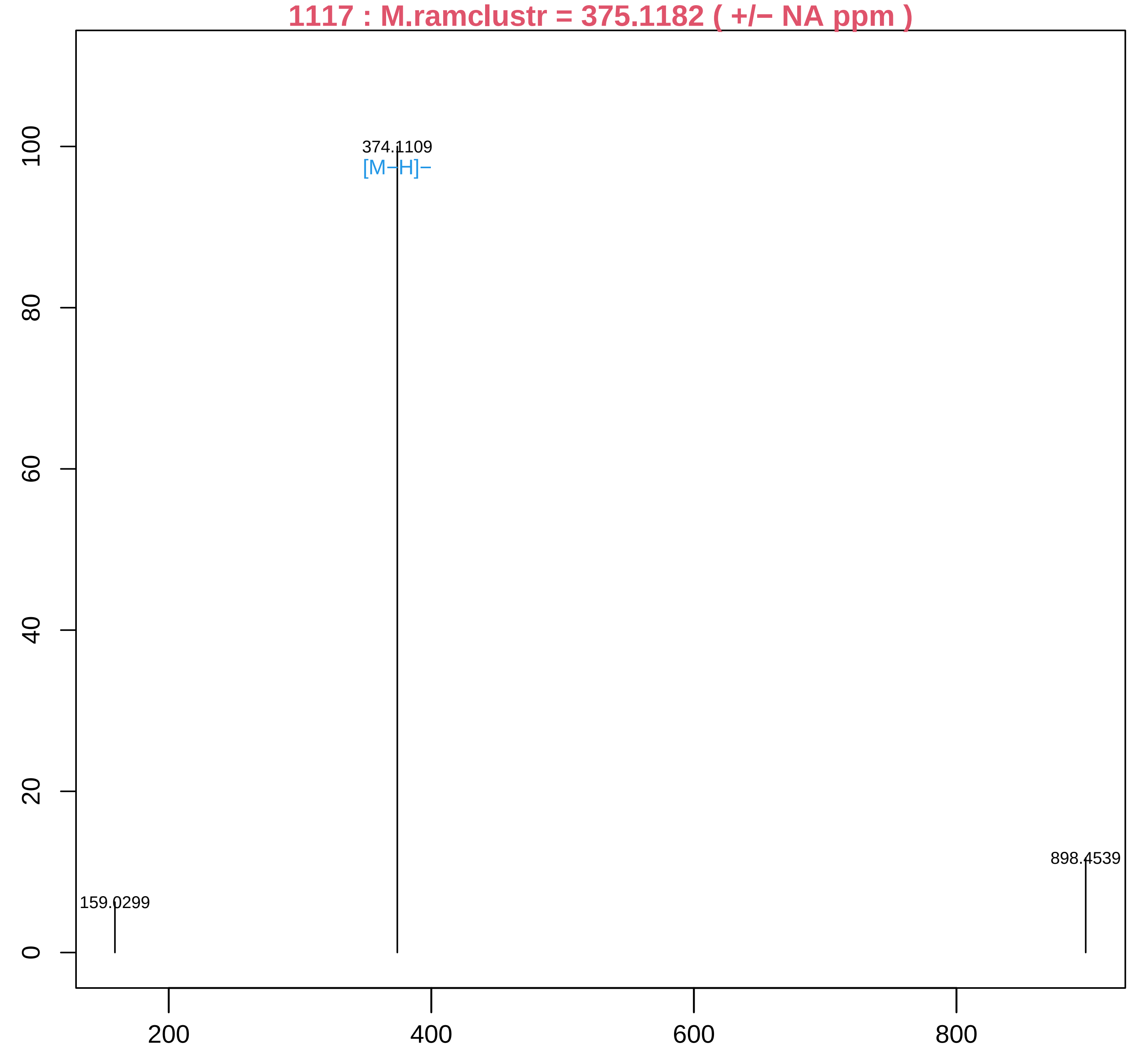
1117 : M.ramclustr = 375.1182 (+/− NA ppm)
374.1109
[M−H]−
898.4539
159.0299
0
20
40
60
80
100
200
400
600
800

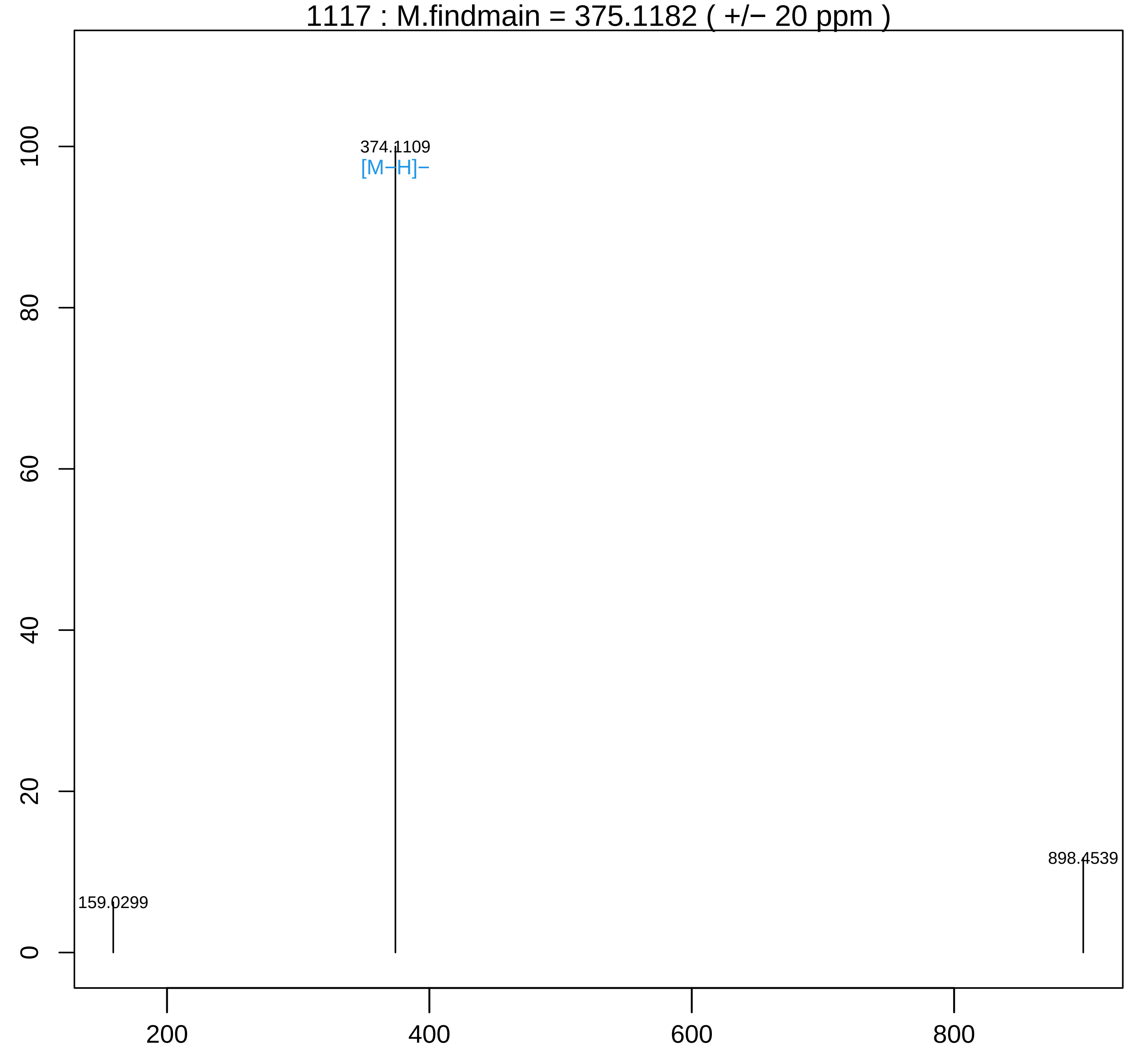
1117 : M.findmain = 375.1182 (+/− 20 ppm)
374.1109
[M−H]−
898.4539
159.0299
0
20
40
60
80
100
200
400
600
800

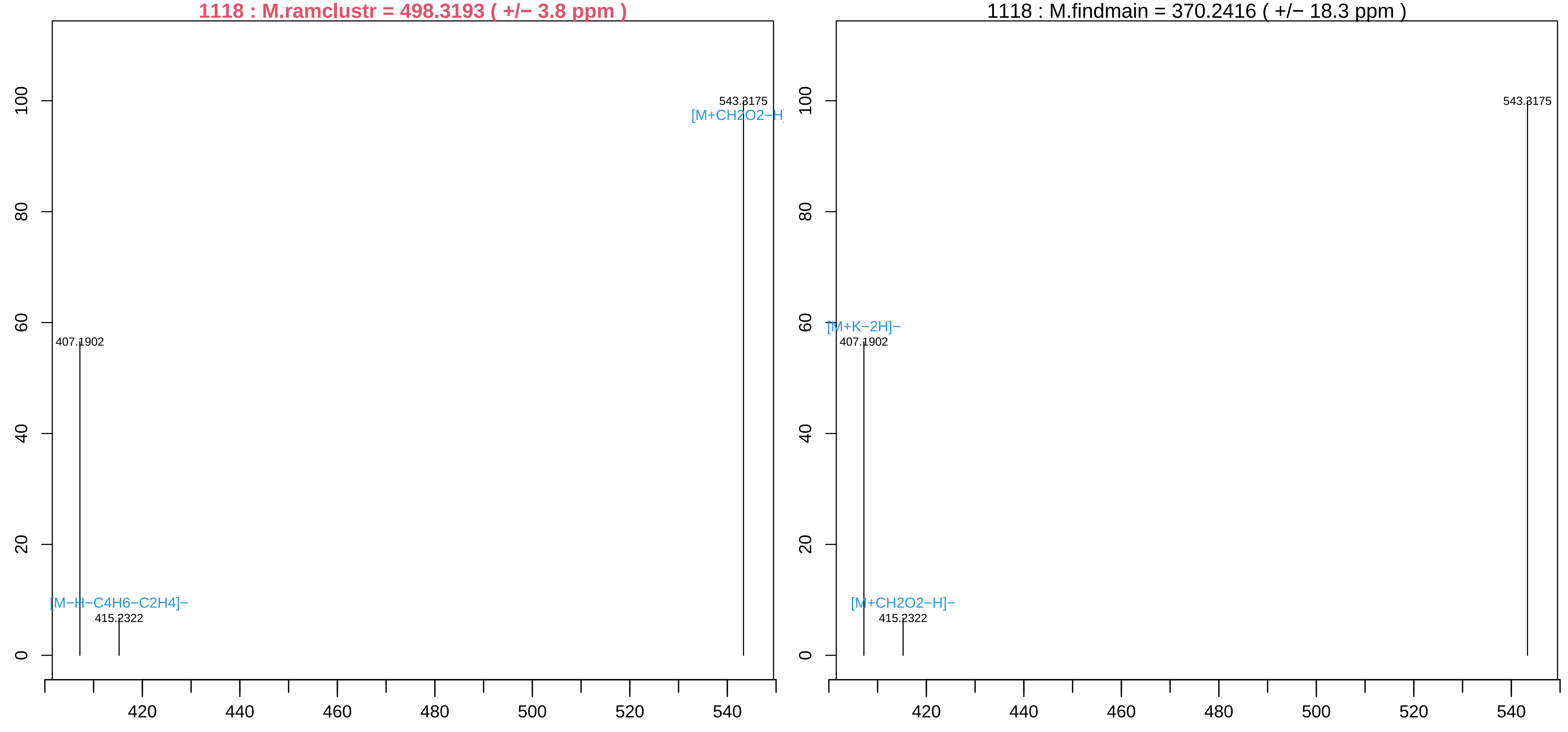

1118 : M.ramclustr = 498.3193 (+/− 3.8 ppm)
543.3175
[M+CH2O2−H]
407.1902
[M−H−C4H6−C2H4]−
415.2322
1118 : M.findmain = 370.2416 (+/− 18.3 ppm)
543.3175
[M+K−2H]−
407.1902
[M+CH2O2−H]−
415.2322
0
20
40
60
80
100
420
440
460
480
500
520
540

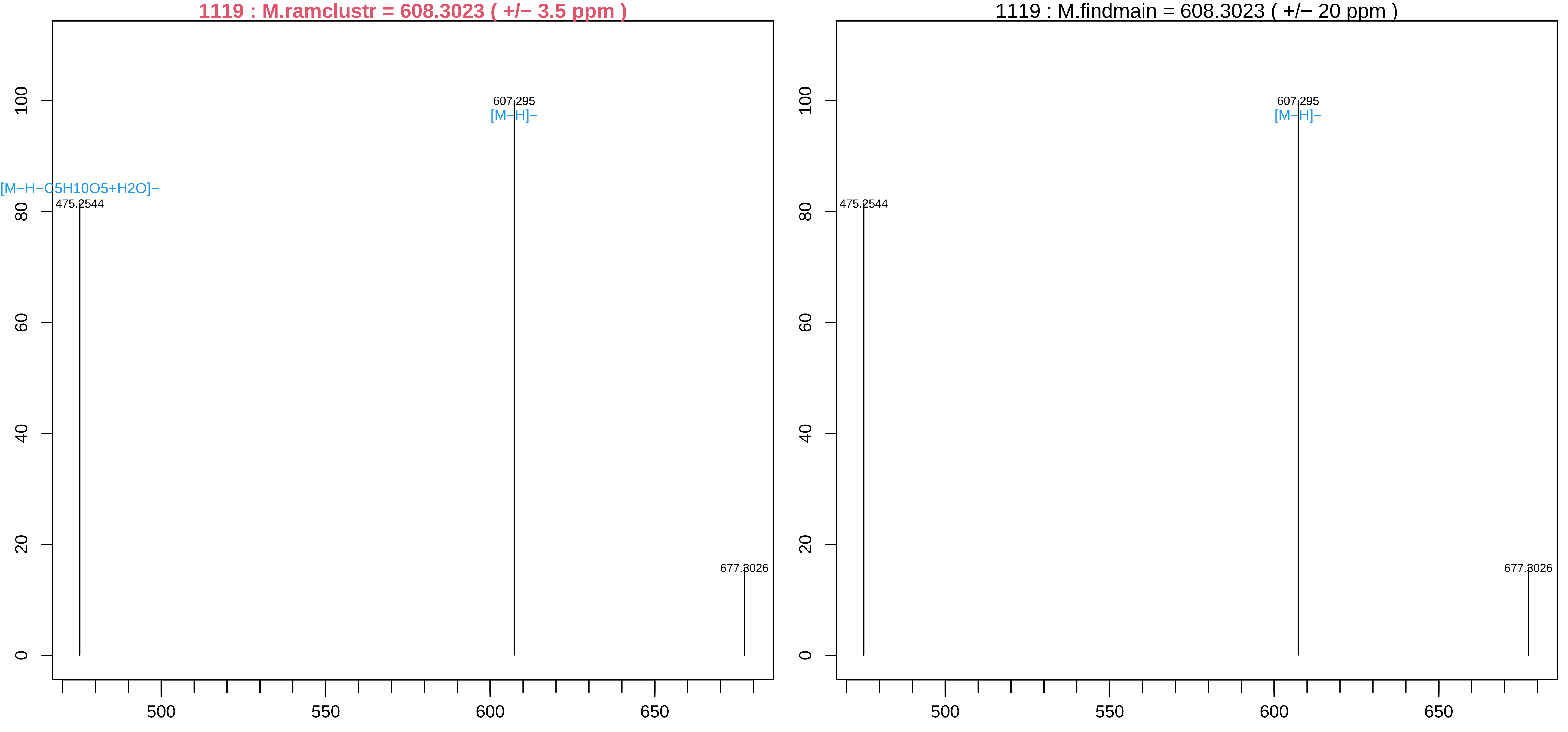
1119 : M.ramclustr = 608.3023 (+/− 3.5 ppm)
1119 : M.findmain = 608.3023 (+/− 20 ppm)
607.295
[M−H]−
[M−H−C5H10O5+H2O]−
475.2544
677.3026
0
20
40
60
80
100
500
550
600
650

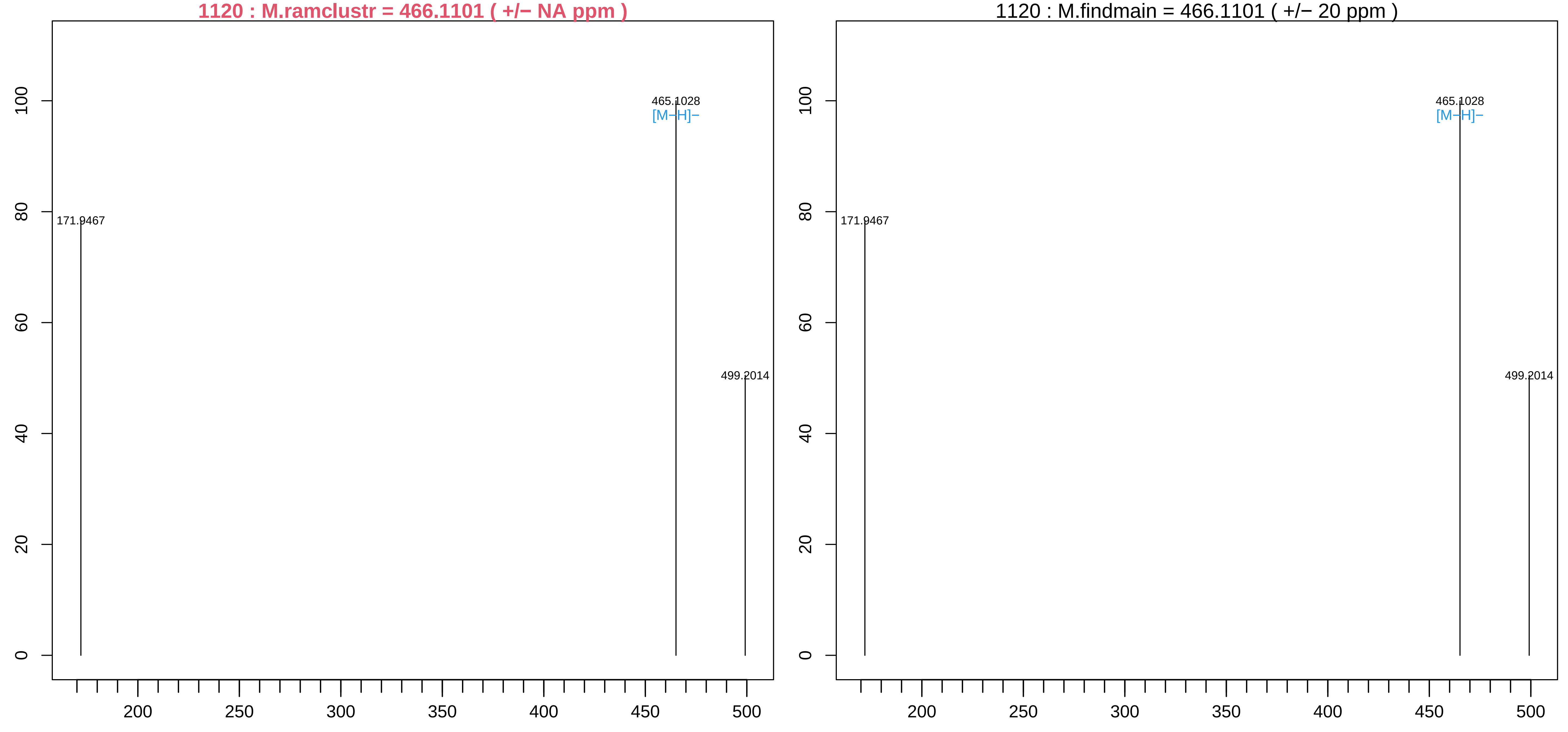
1120 : M.ramclustr = 466.1101 (+/− NA ppm)
171.9467
465.1028
[M−H]−
499.2014
1120 : M.findmain = 466.1101 (+/− 20 ppm)
171.9467
465.1028
[M−H]−
499.2014
0
20
40
60
80
100
200
250
300
350
400
450
500

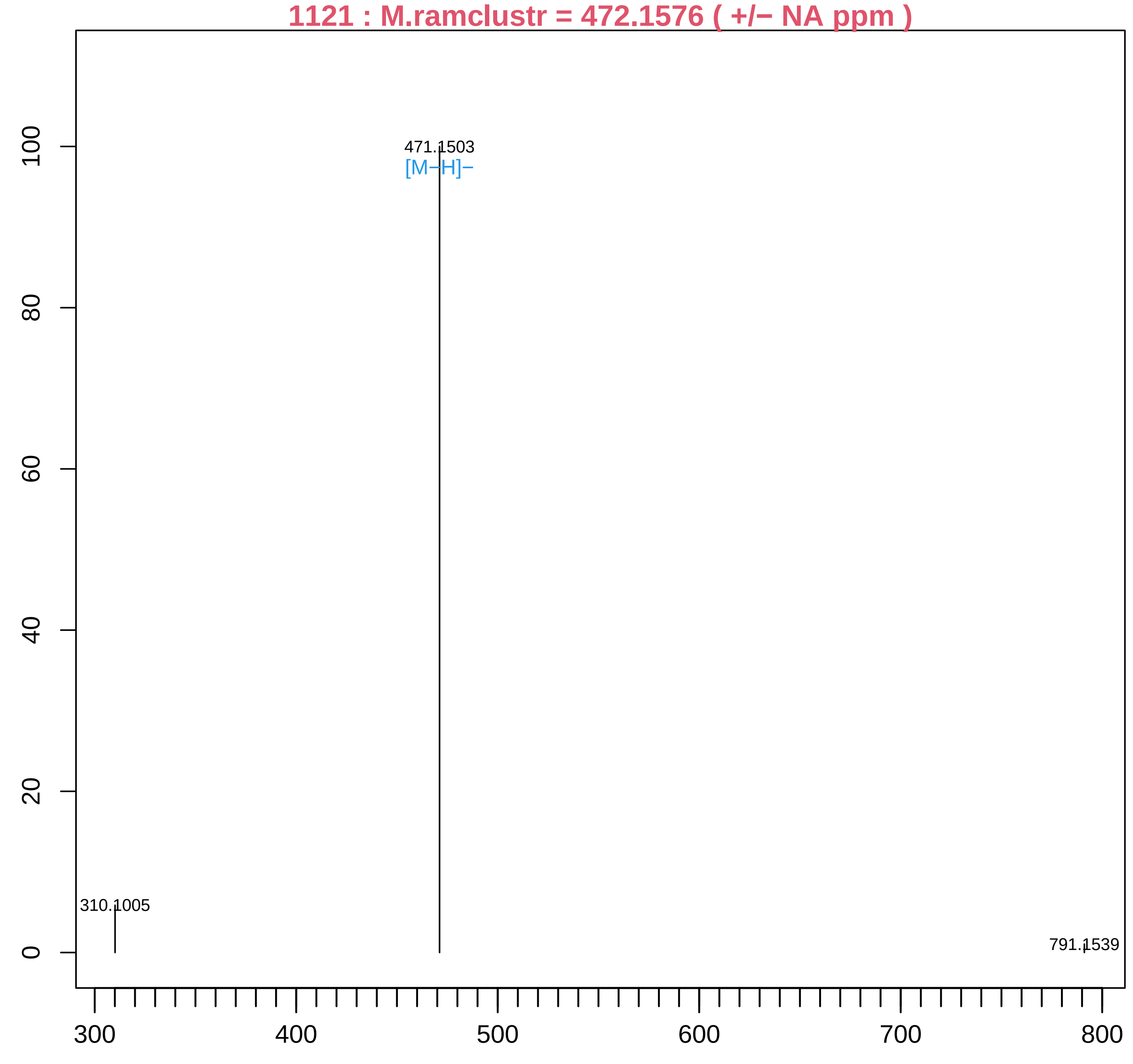
1121 : M.ramclustr = 472.1576 (+/− NA ppm)
471.1503
[M−H]−
310.1005
791.1539
0
20
40
60
80
100
300
400
500
600
700
800

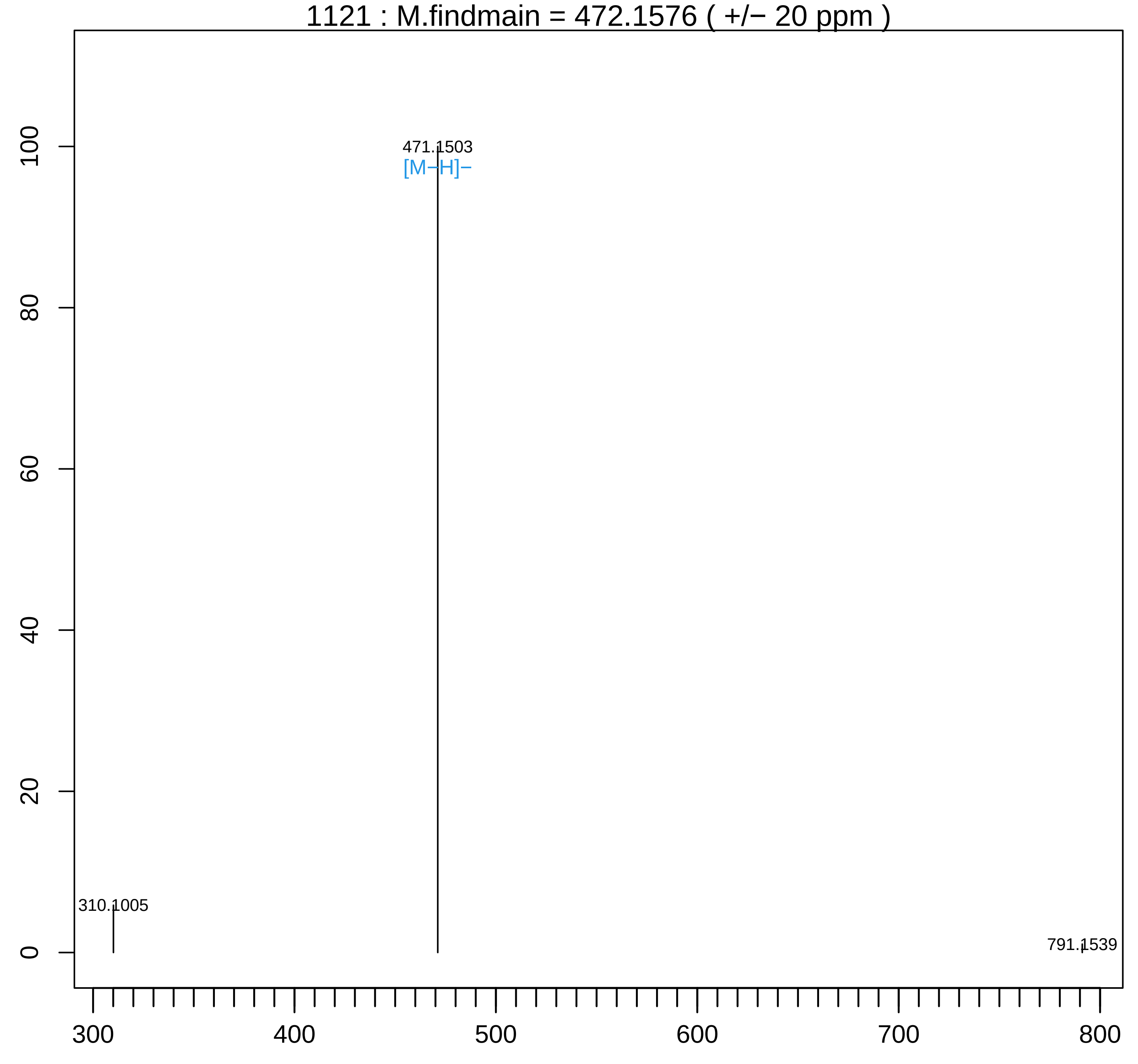
1121 : M.findmain = 472.1576 (+/− 20 ppm)
471.1503
[M−H]−
310.1005
791.1539
0
20
40
60
80
100
300
400
500
600
700
800

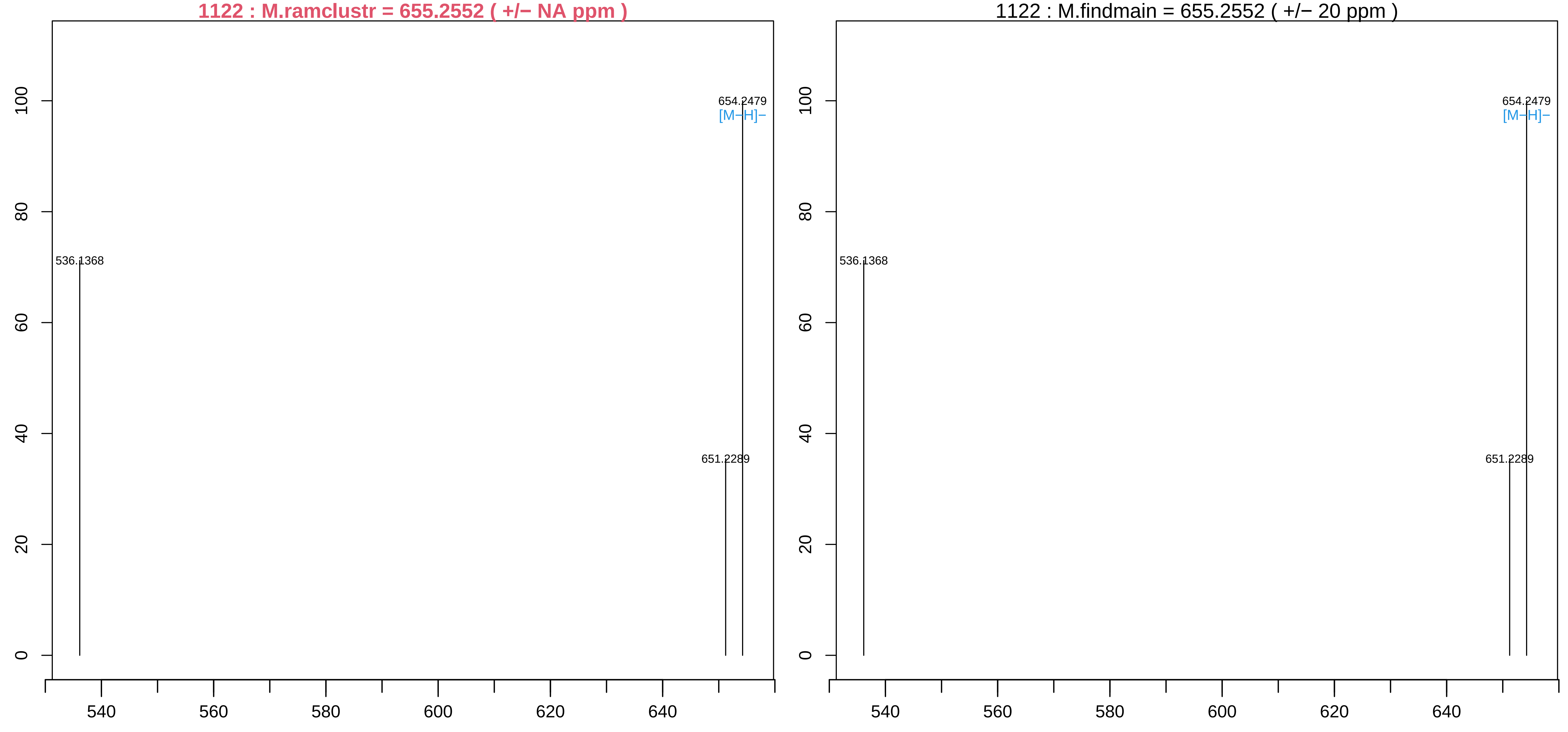
1122 : M.ramclustr = 655.2552 (+/− NA ppm)
654.2479
[M−H]−
536.1368
651.2289
1122 : M.findmain = 655.2552 (+/− 20 ppm)
654.2479
[M−H]−
536.1368
651.2289
0
20
40
60
80
100
540
560
580
600
620
640

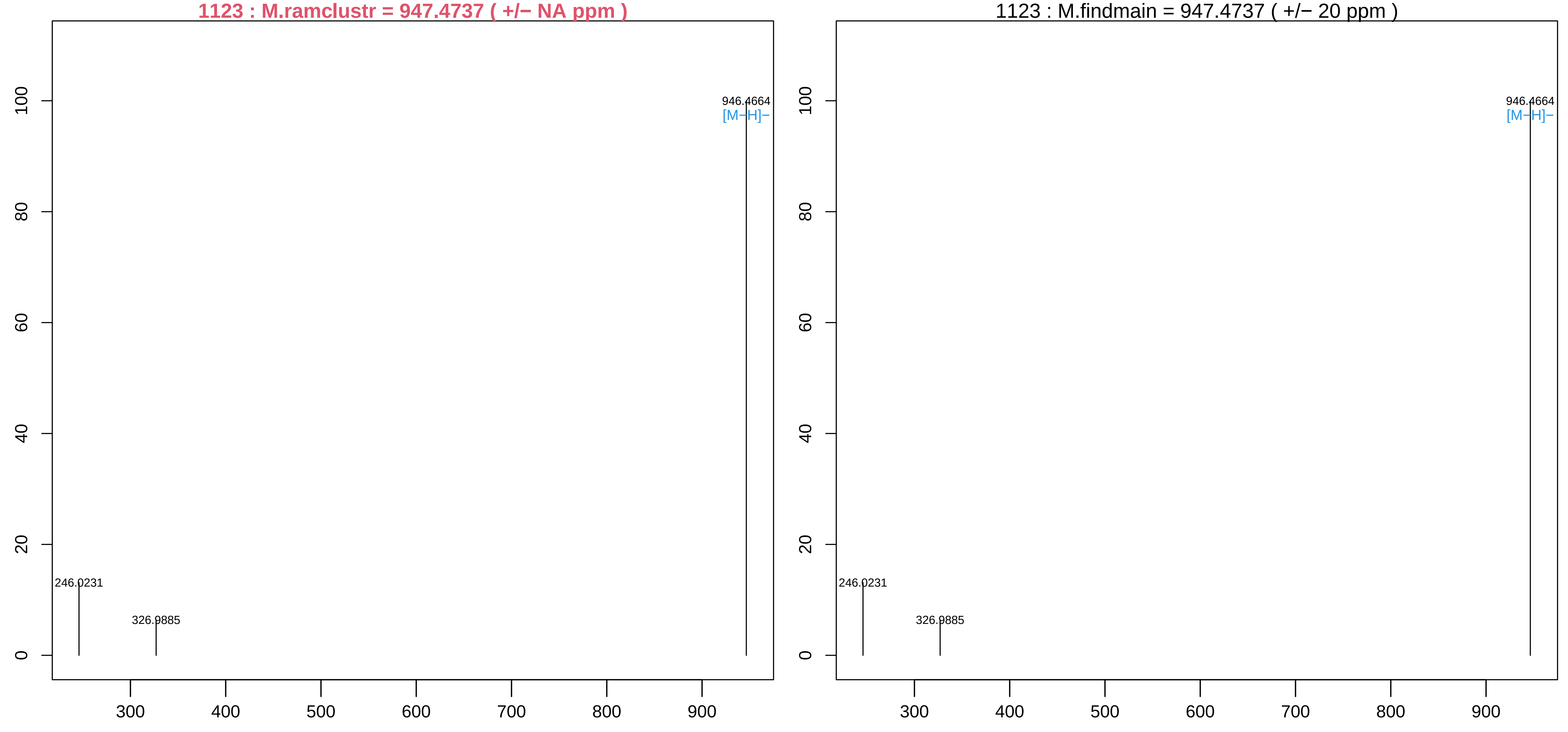
1123 : M.ramclustr = 947.4737 (+/− NA ppm)
946.4664
[M−H]−
246.0231
326.9885
0
20
40
60
80
100
300
400
500
600
700
800
900
1123 : M.findmain = 947.4737 (+/− 20 ppm)
946.4664
[M−H]−
246.0231
326.9885
0
20
40
60
80
100
300
400
500
600
700
800
900

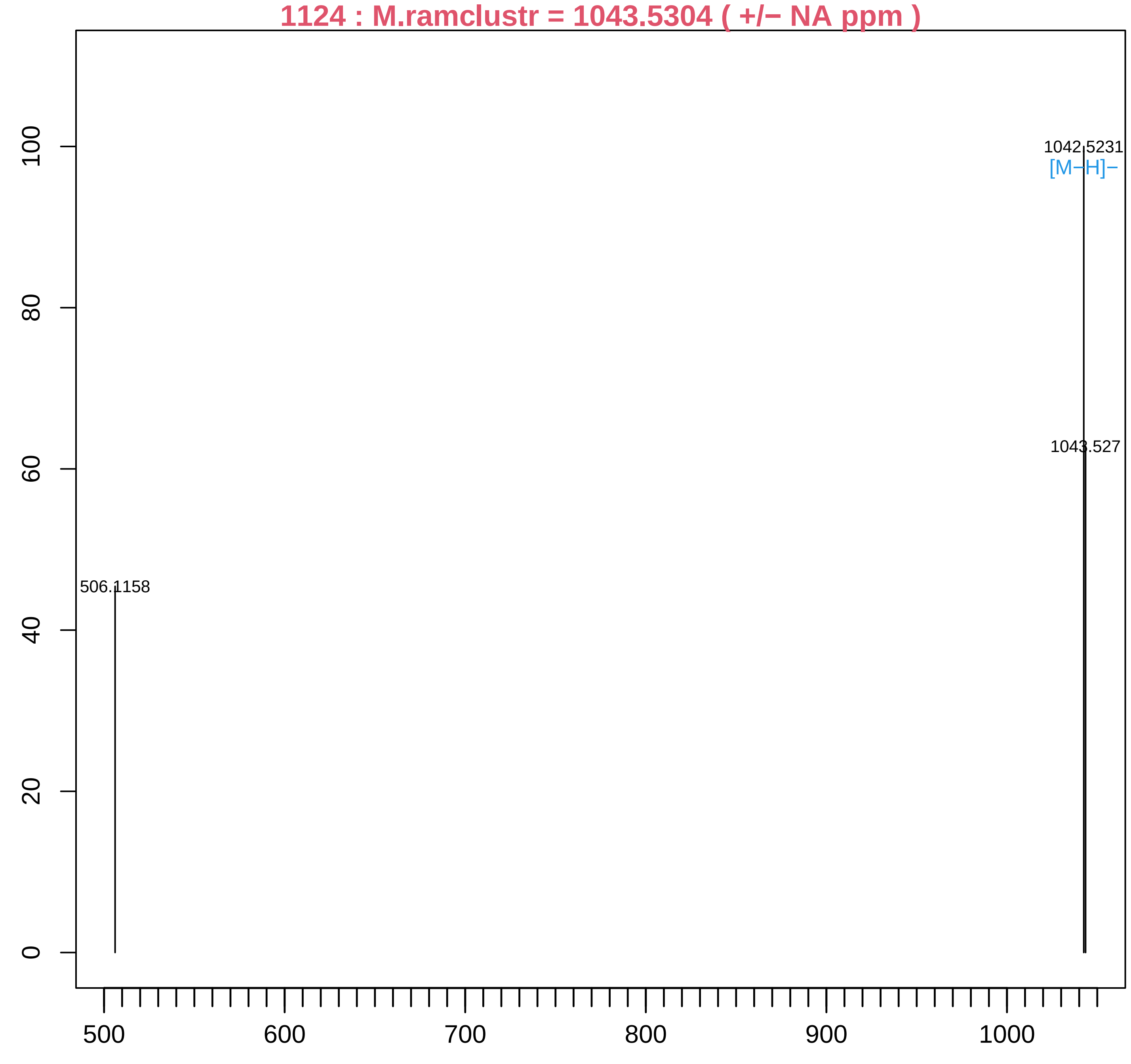
1124 : M.ramclustr = 1043.5304 (+/− NA ppm)
1042.5231
[M−H]−
1043.527
506.1158
0
20
40
60
80
100
500
600
700
800
900
1000

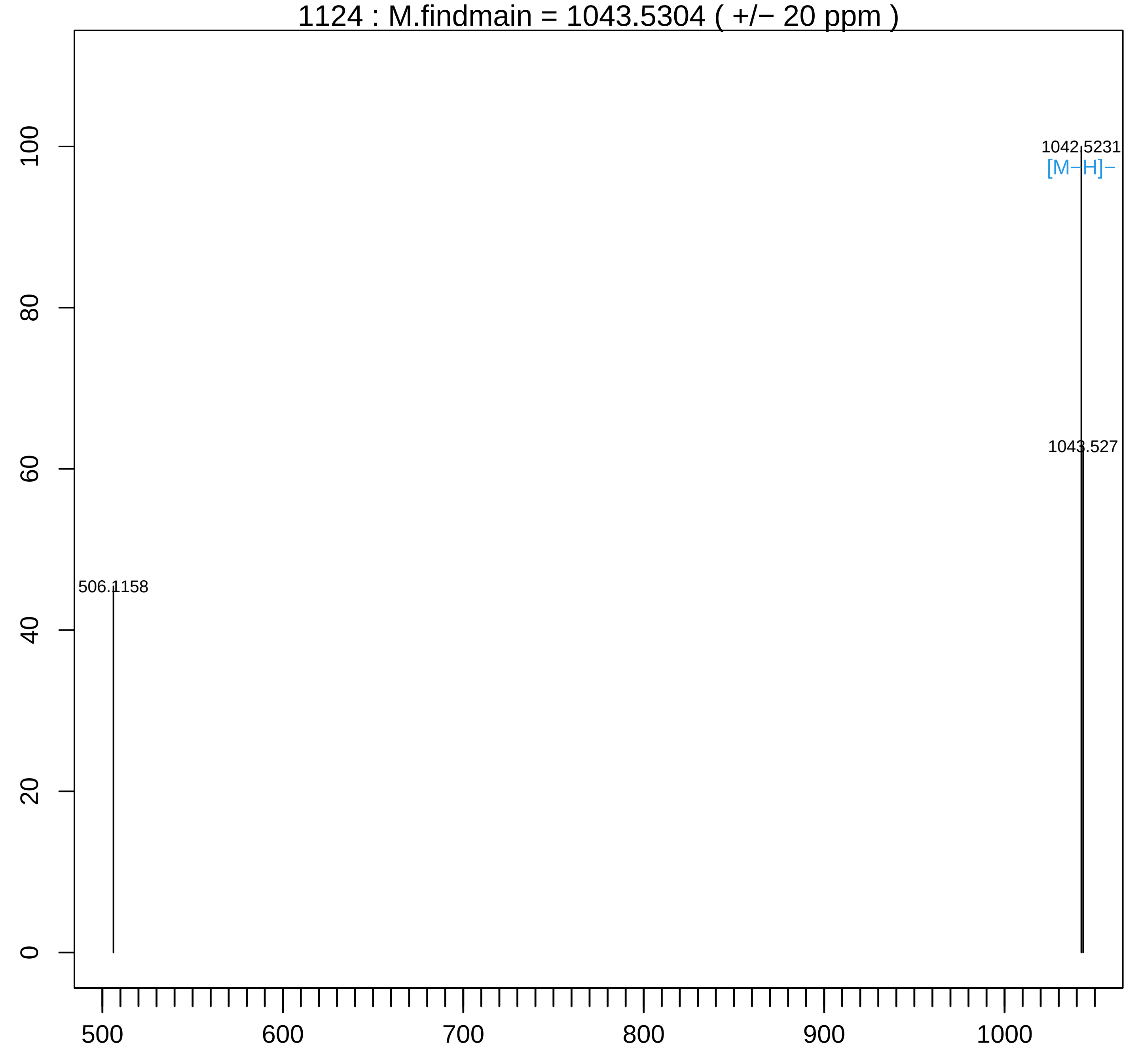
1124 : M.findmain = 1043.5304 (+/− 20 ppm)
1042.5231
[M−H]−
1043.527
506.1158
0
20
40
60
80
100
500
600
700
800
900
1000

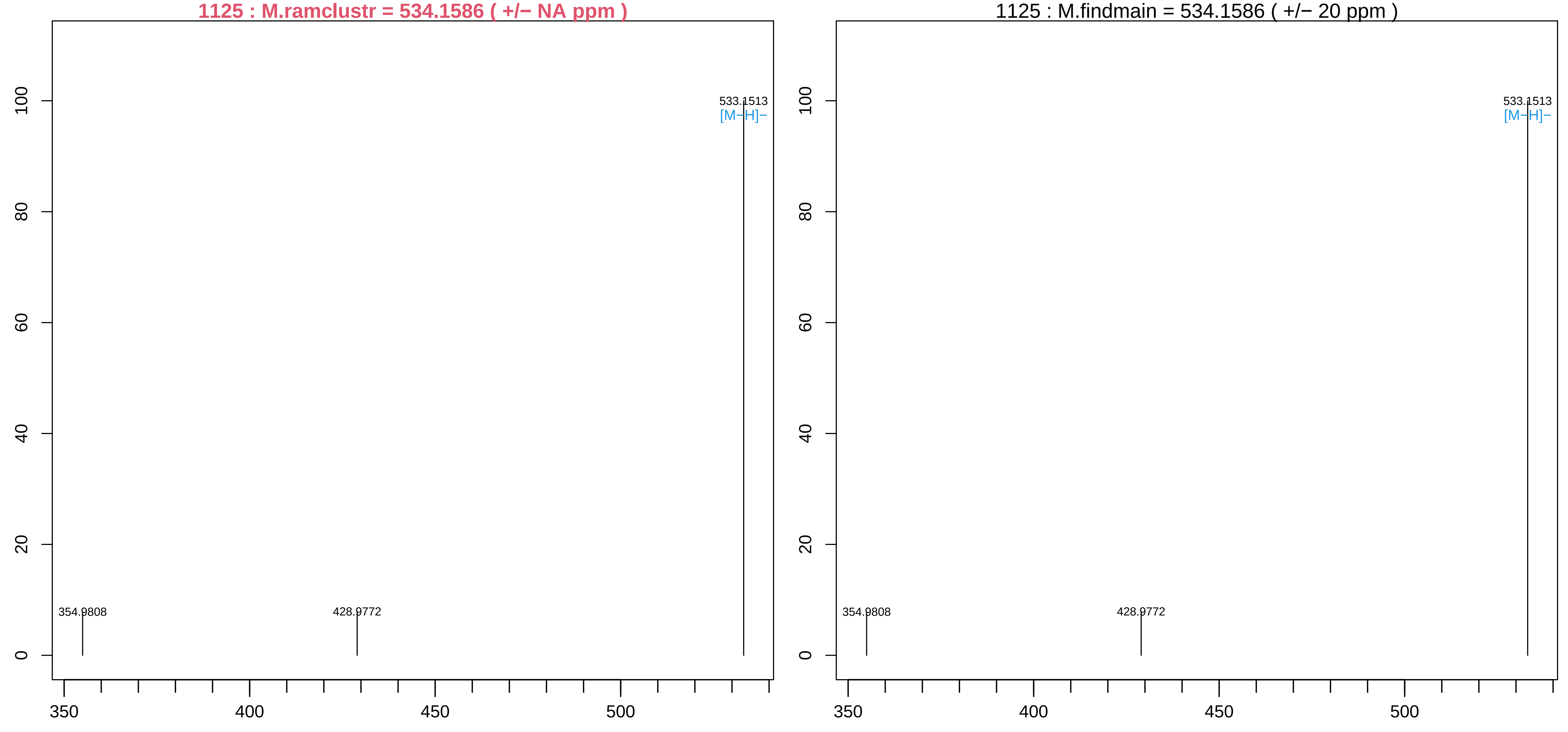
1125 : M.ramclustr = 534.1586 (+/− NA ppm)
1125 : M.findmain = 534.1586 (+/− 20 ppm)
533.1513
[M−H]−
354.9808
428.9772
0
20
40
60
80
100
350
400
450
500

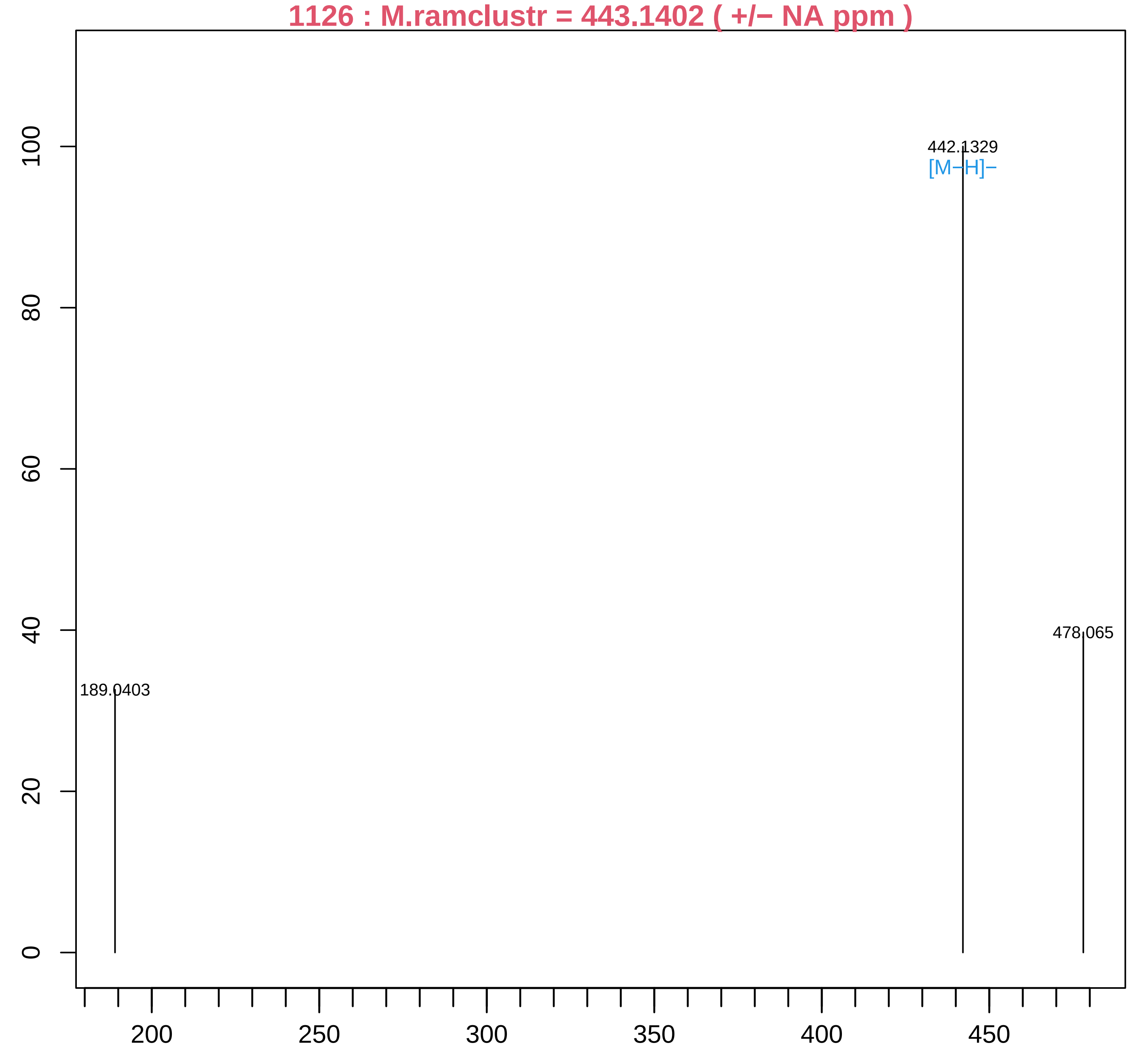
1126 : M.ramclustr = 443.1402 (+/− NA ppm)
442.1329
[M−H]−
478.065
189.0403
0
20
40
60
80
100
200
250
300
350
400
450
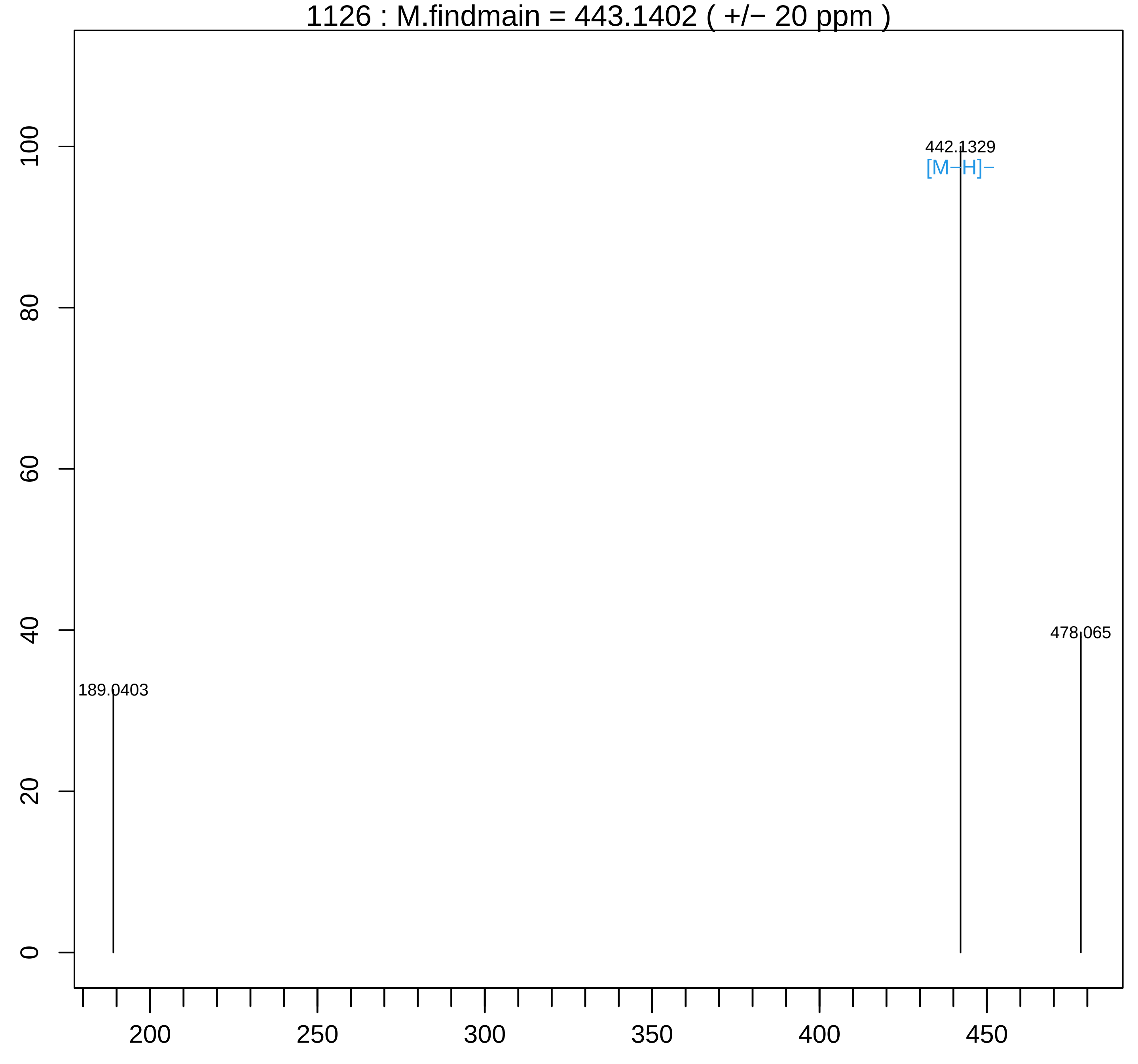
1126 : M.findmain = 443.1402 (+/− 20 ppm)
442.1329
[M−H]−
478.065
189.0403
0
20
40
60
80
100
200
250
300
350
400
450

1127 : M.ramclustr = 901.4752 (+/− NA ppm)

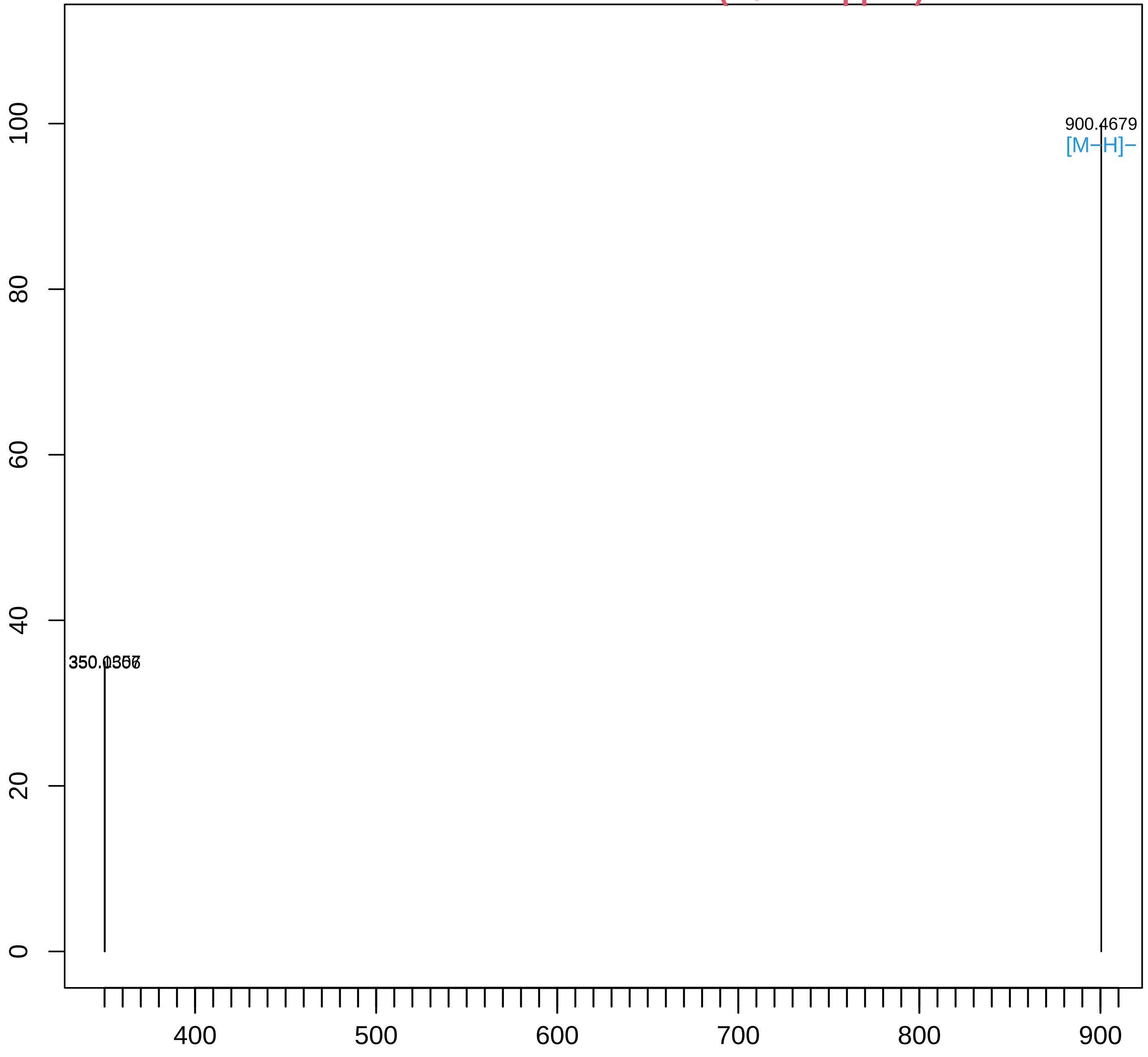

1127 : M.findmain = 901.4752 (+/− 20 ppm)

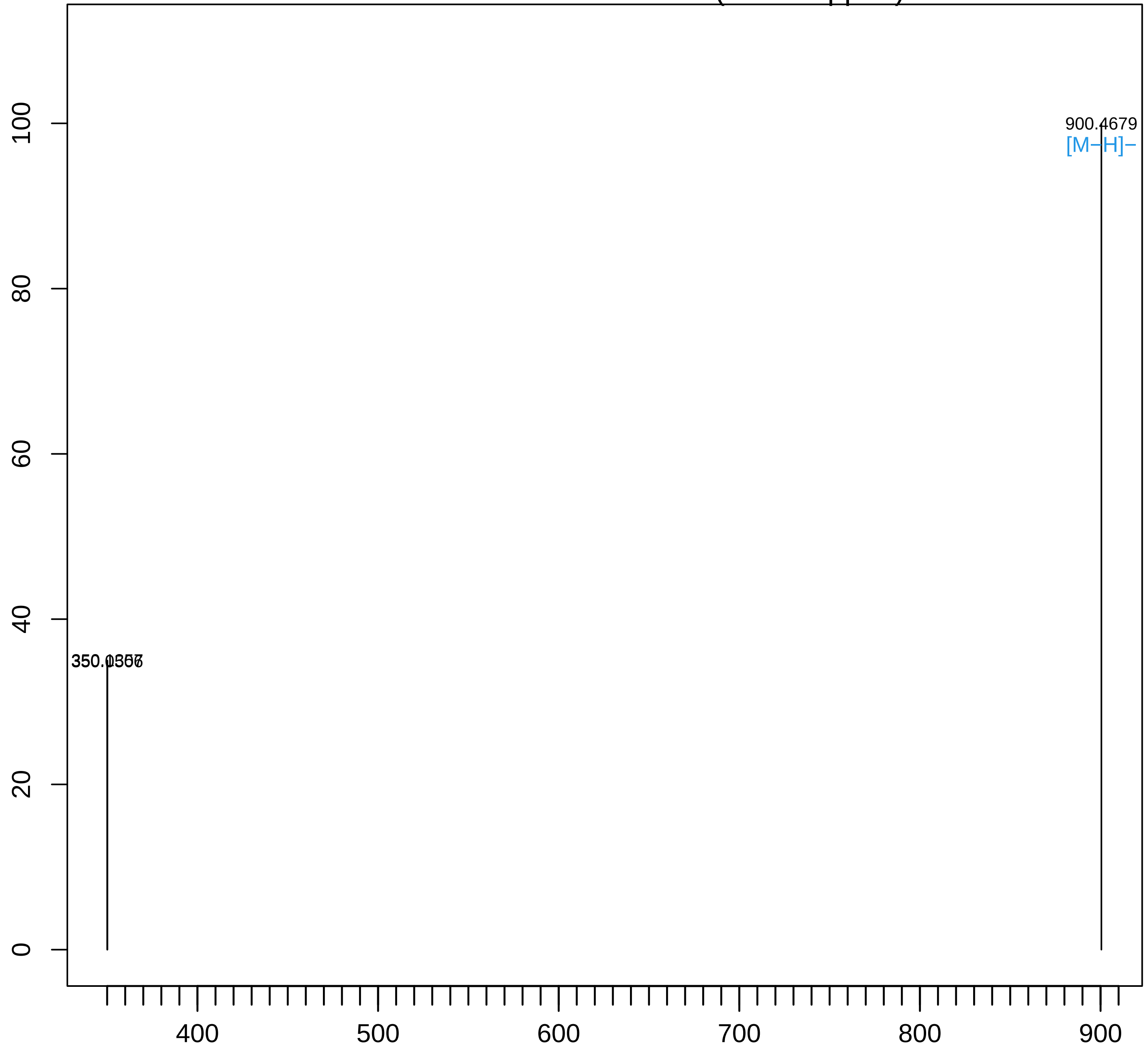

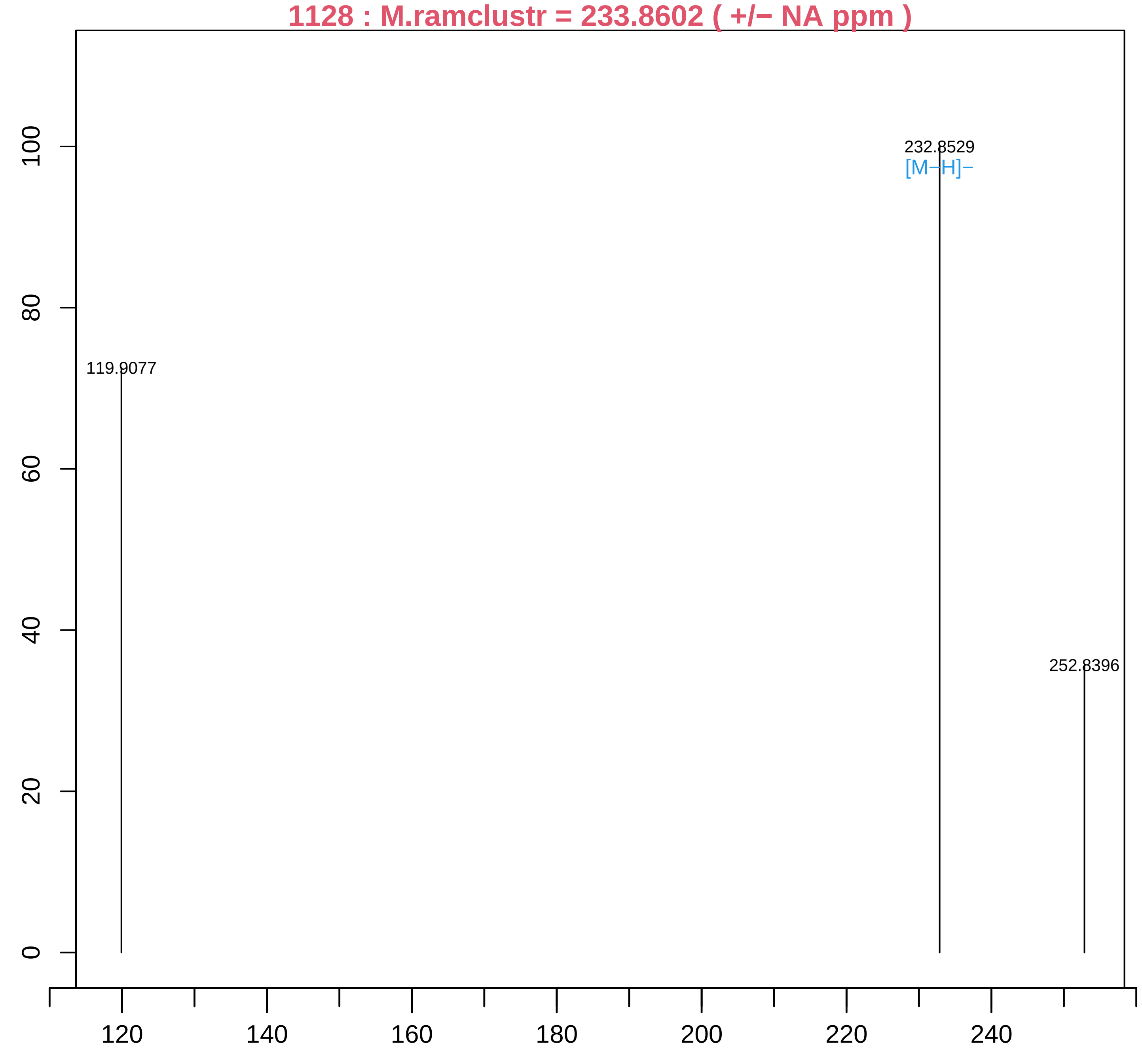

1128 : M.ramclustr = 233.8602 (+/− NA ppm)
232.8529
[M−H]−
119.9077
252.8396

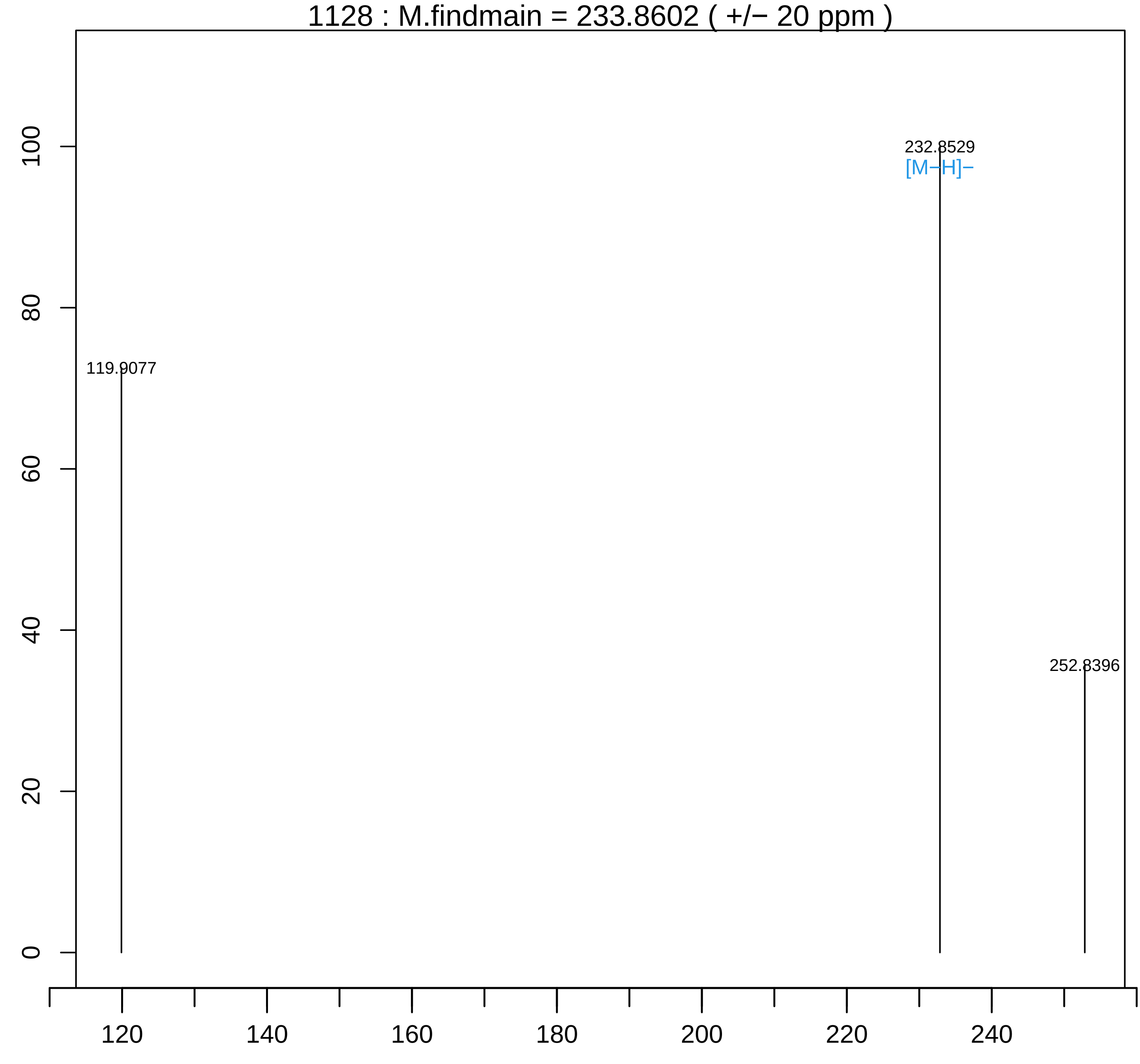

1128 : M.findmain = 233.8602 (+/− 20 ppm)
232.8529
[M−H]−
119.9077
252.8396

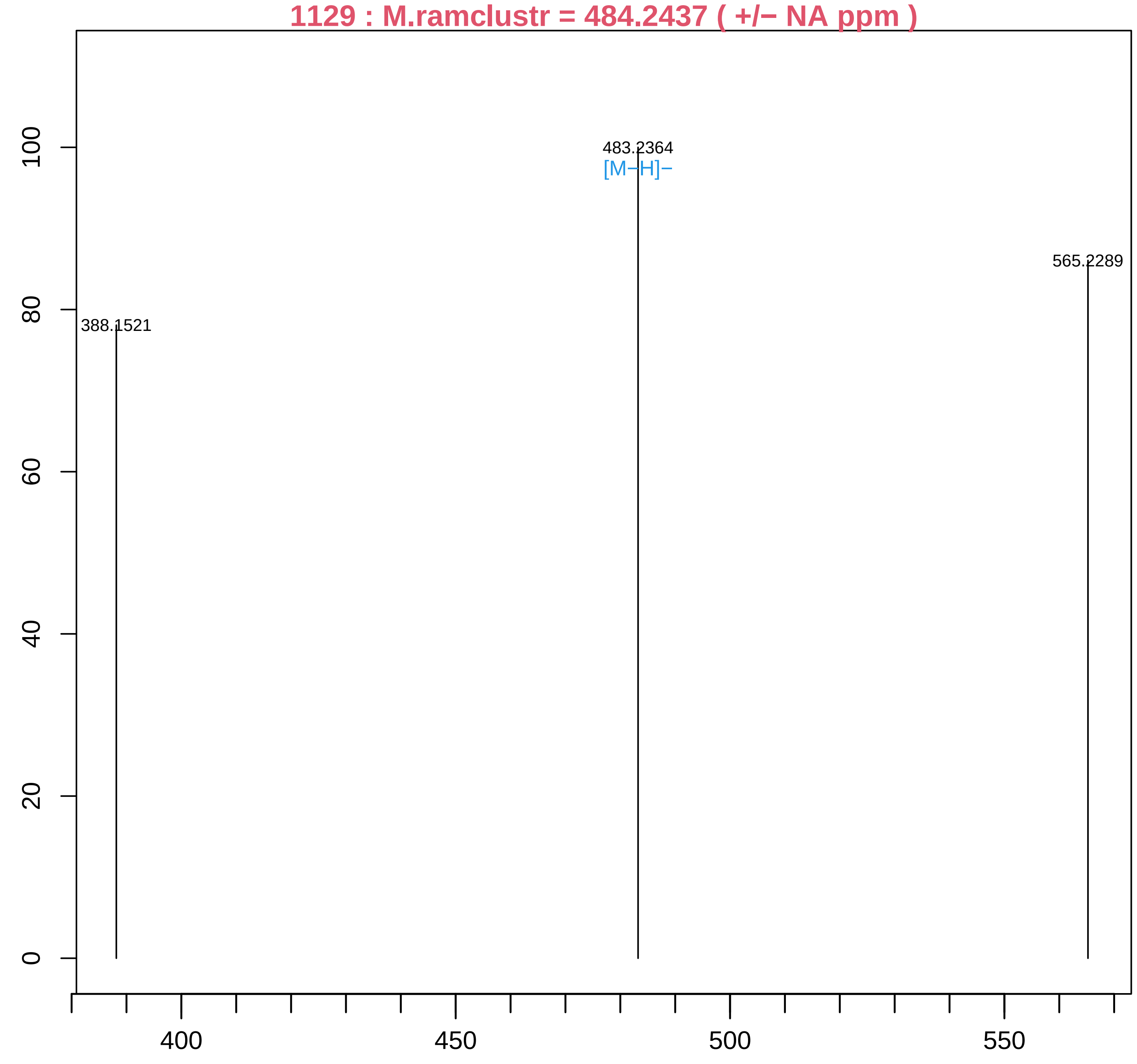
1129 : M.ramclustr = 484.2437 (+/− NA ppm)
483.2364
[M−H]−
565.2289
388.1521
0
20
40
60
80
100
400
450
500
550

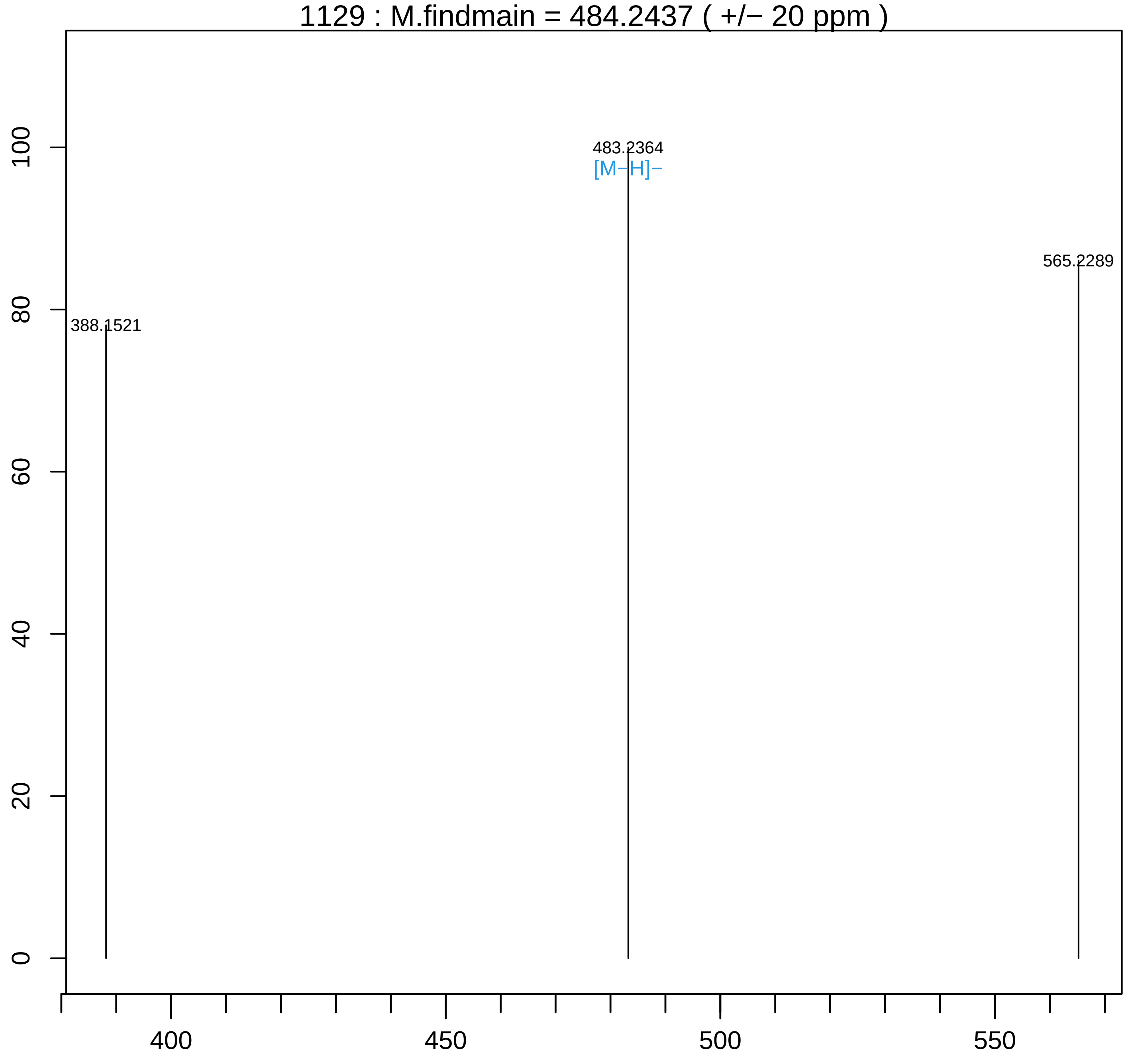
1129 : M.findmain = 484.2437 (+/− 20 ppm)
483.2364
[M−H]−
565.2289
388.1521
0
20
40
60
80
100
400
450
500
550

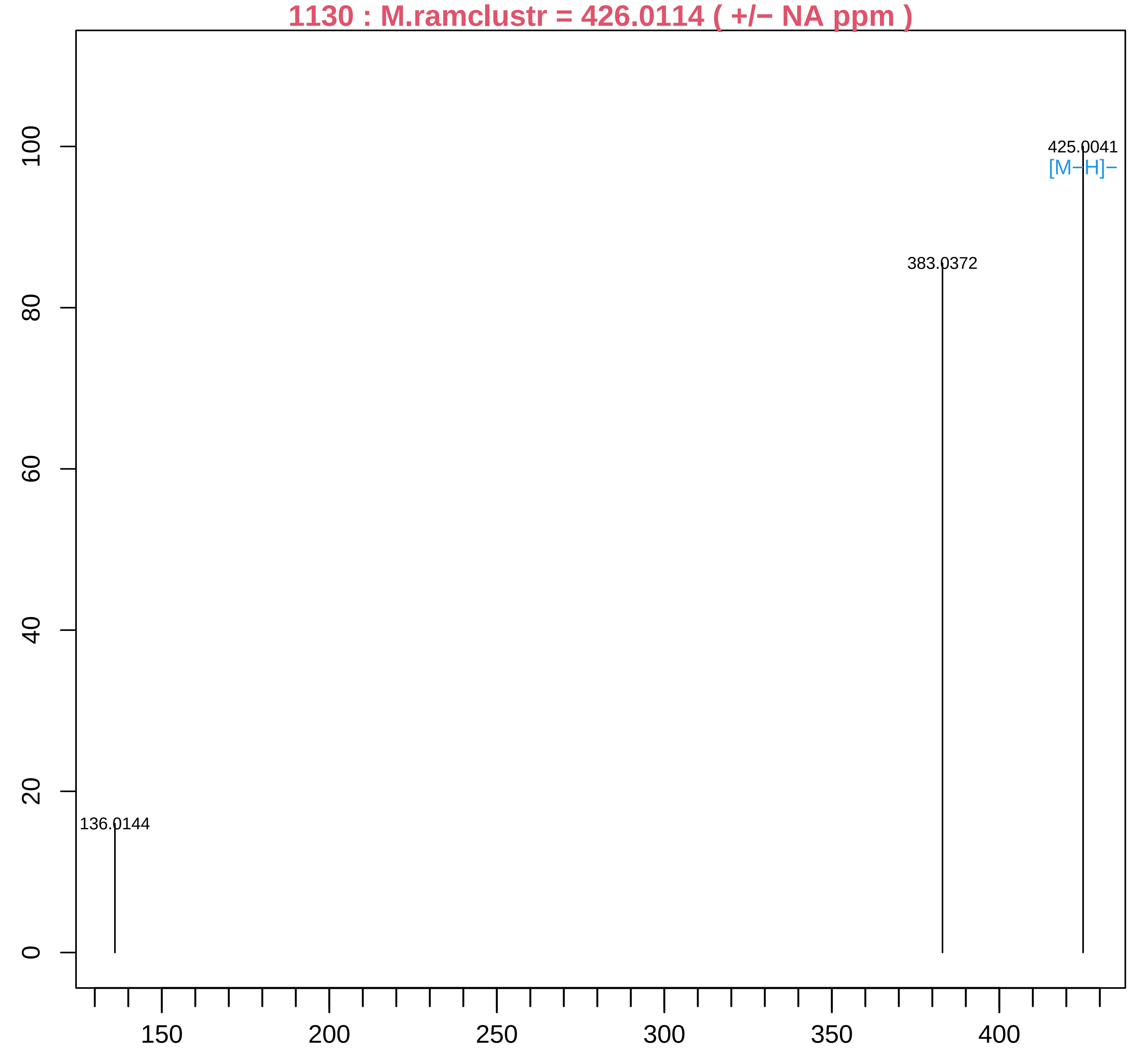
1130 : M.ramclustr = 426.0114 (+/− NA ppm)
425.0041
[M−H]−
383.0372
136.0144
0
20
40
60
80
100
150
200
250
300
350
400

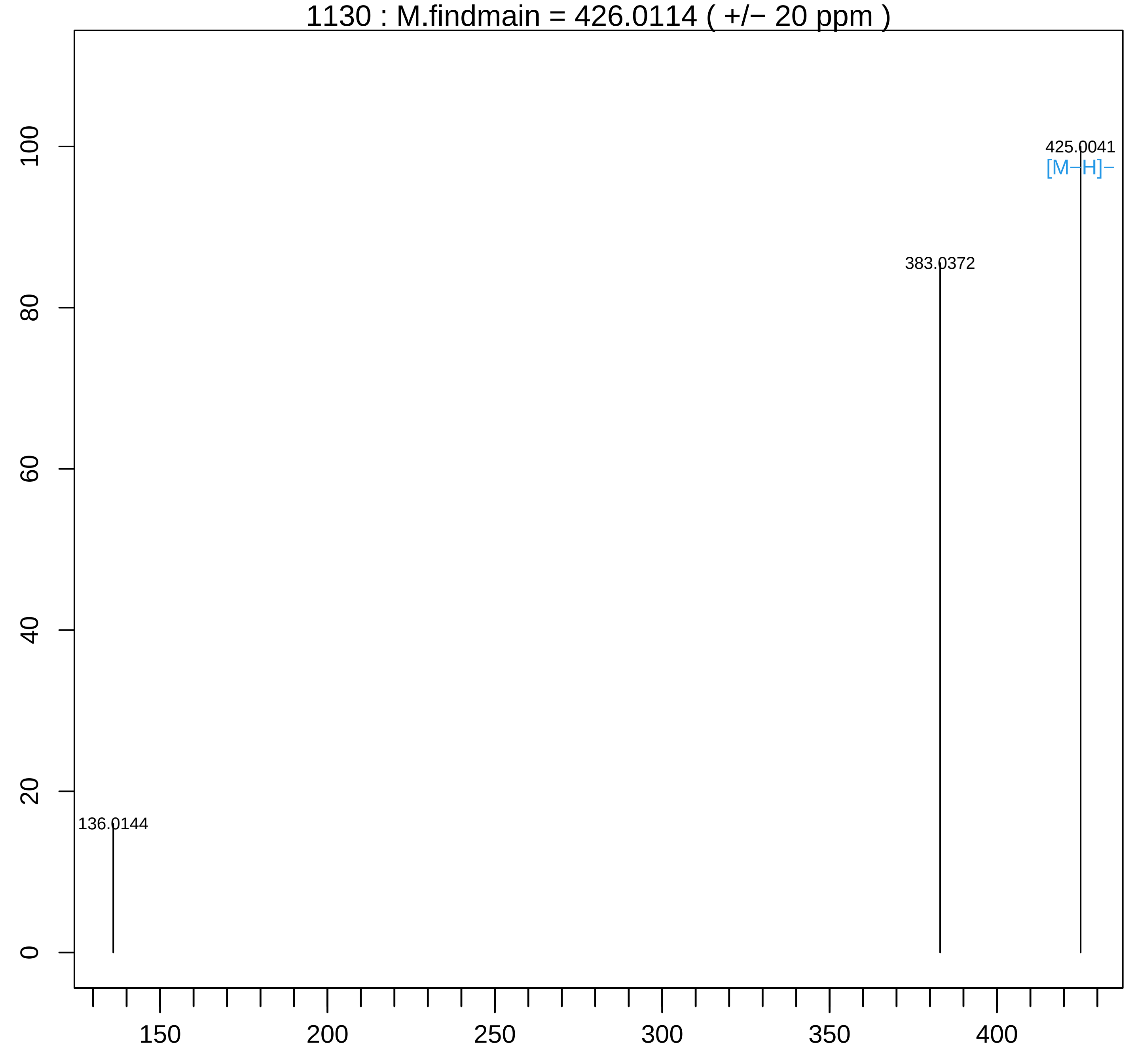
1130 : M.findmain = 426.0114 (+/− 20 ppm)
425.0041
[M−H]−
383.0372
136.0144
0
20
40
60
80
100
150
200
250
300
350
400

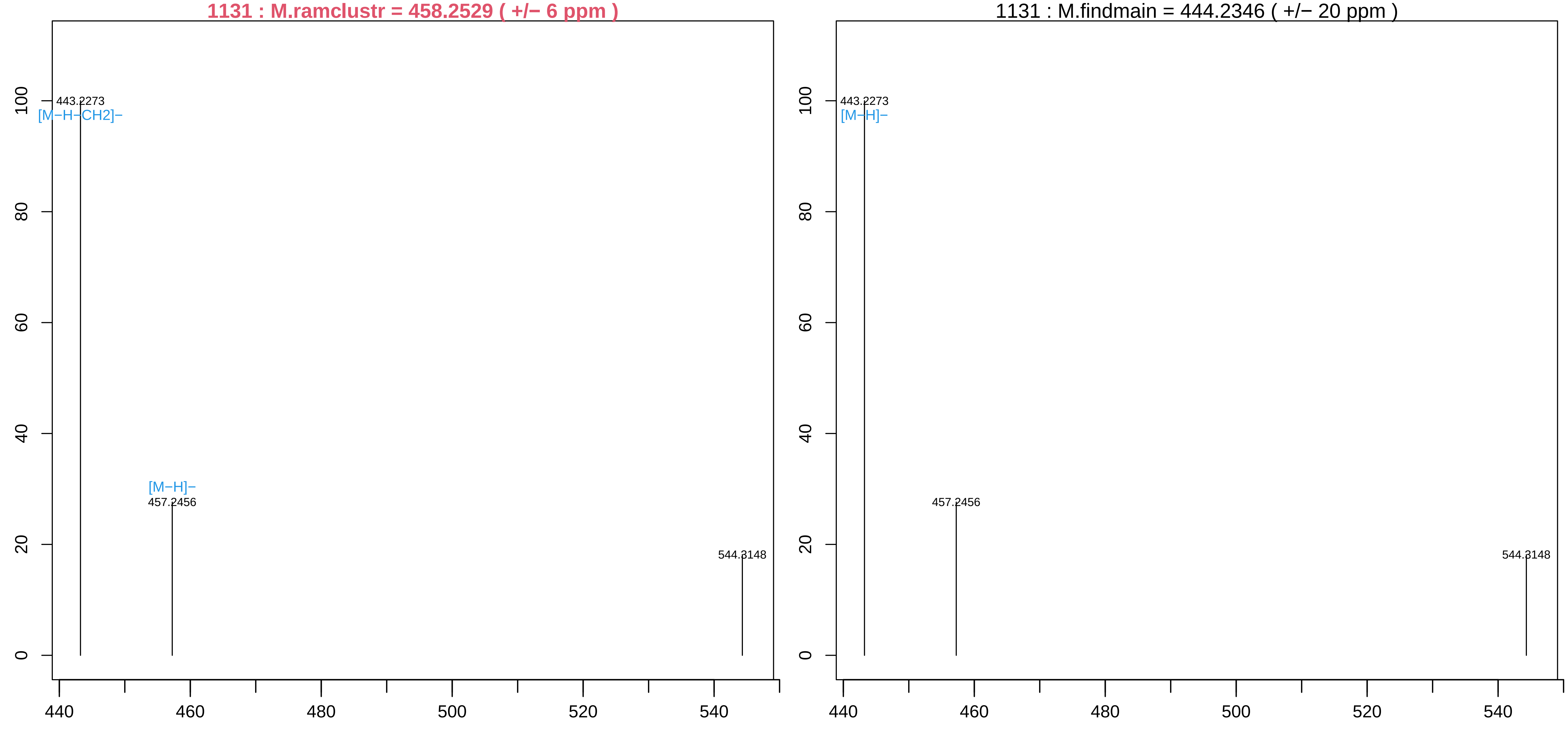

1131 : M.ramclustr = 458.2529 (+/− 6 ppm)
443.2273
[M−H−CH2]−
[M−H]−
457.2456
544.3148
1131 : M.findmain = 444.2346 (+/− 20 ppm)
443.2273
[M−H]−
457.2456
544.3148

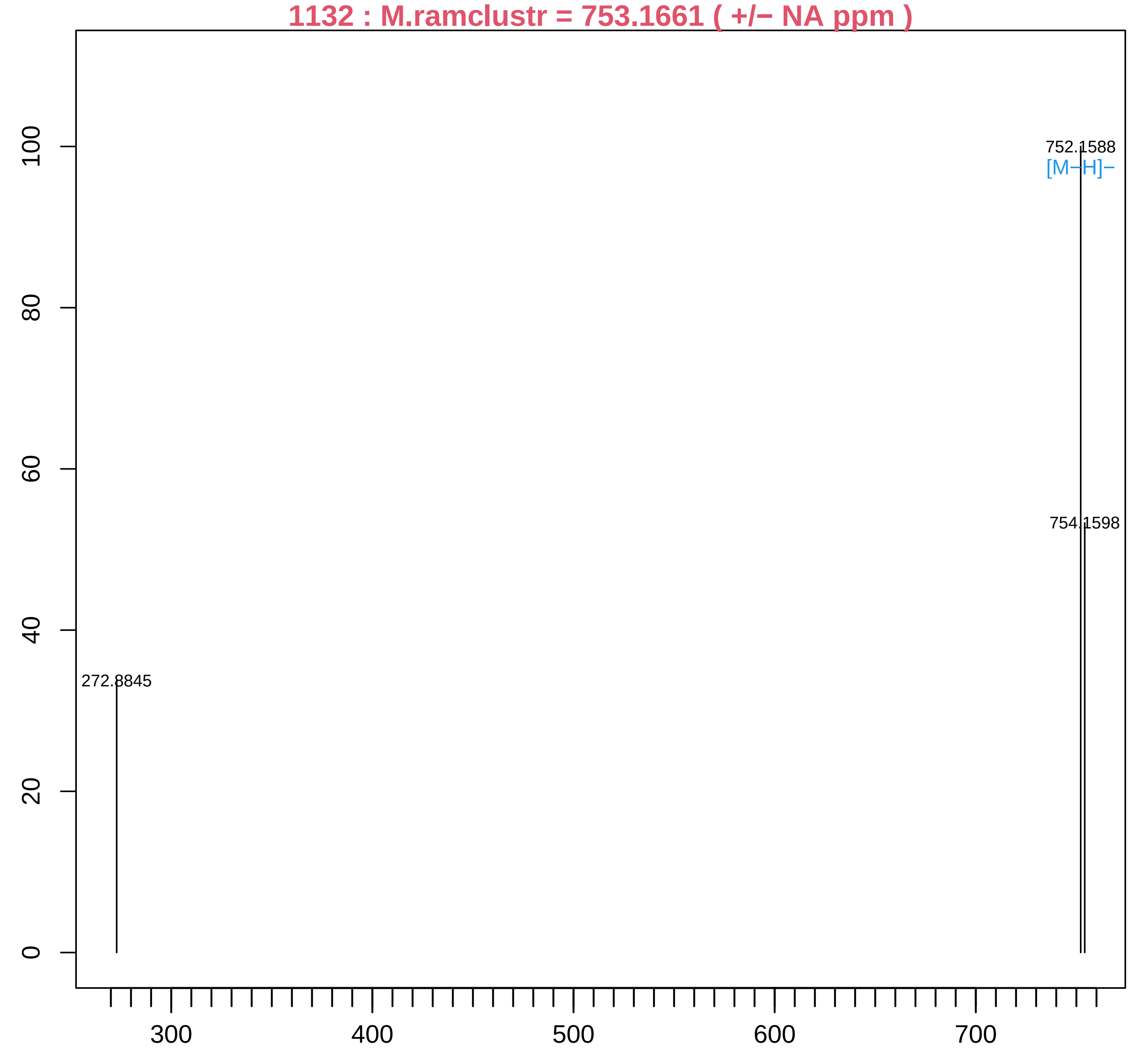

1132 : M.ramclustr = 753.1661 (+/− NA ppm)
752.1588
[M−H]−
754.1598
272.8845
0
20
40
60
80
100
300
400
500
600
700

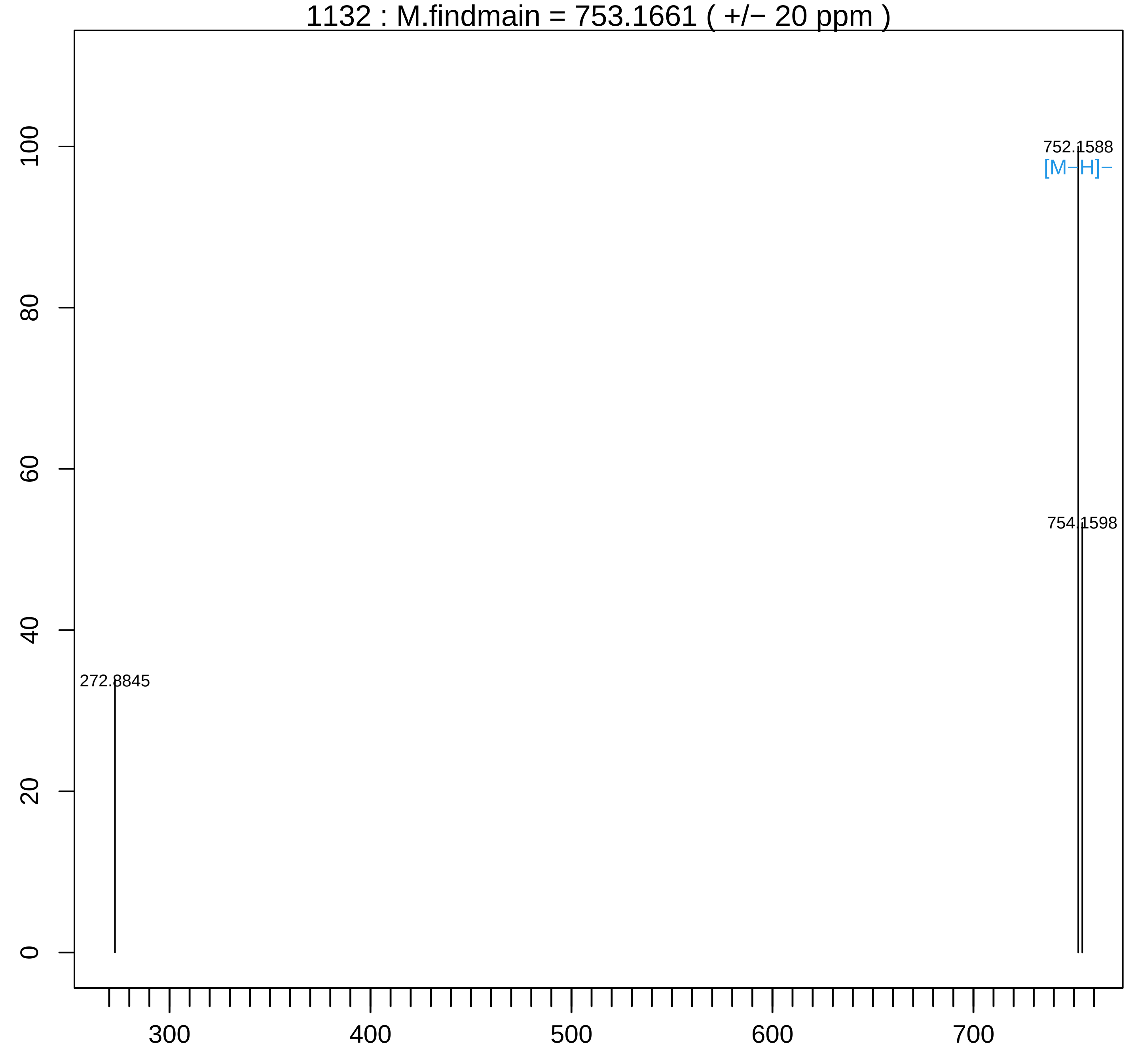

1132 : M.findmain = 753.1661 (+/− 20 ppm)
752.1588
[M−H]−
754.1598
272.8845
0
20
40
60
80
100
300
400
500
600
700

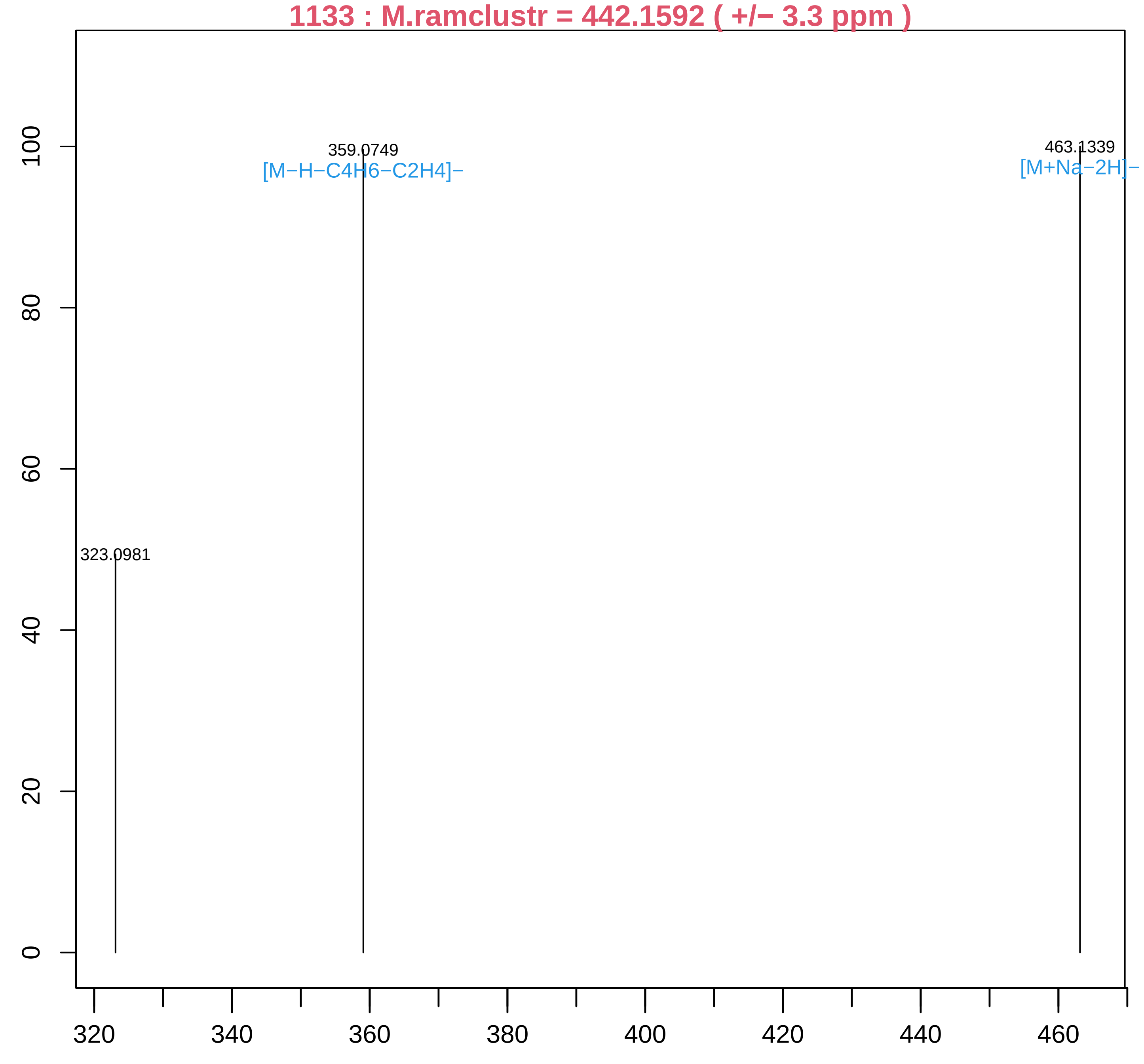
1133 : M.ramclustr = 442.1592 (+/− 3.3 ppm)
323.0981
359.0749
[M−H−C4H6−C2H4]−
463.1339
[M+Na−2H]−

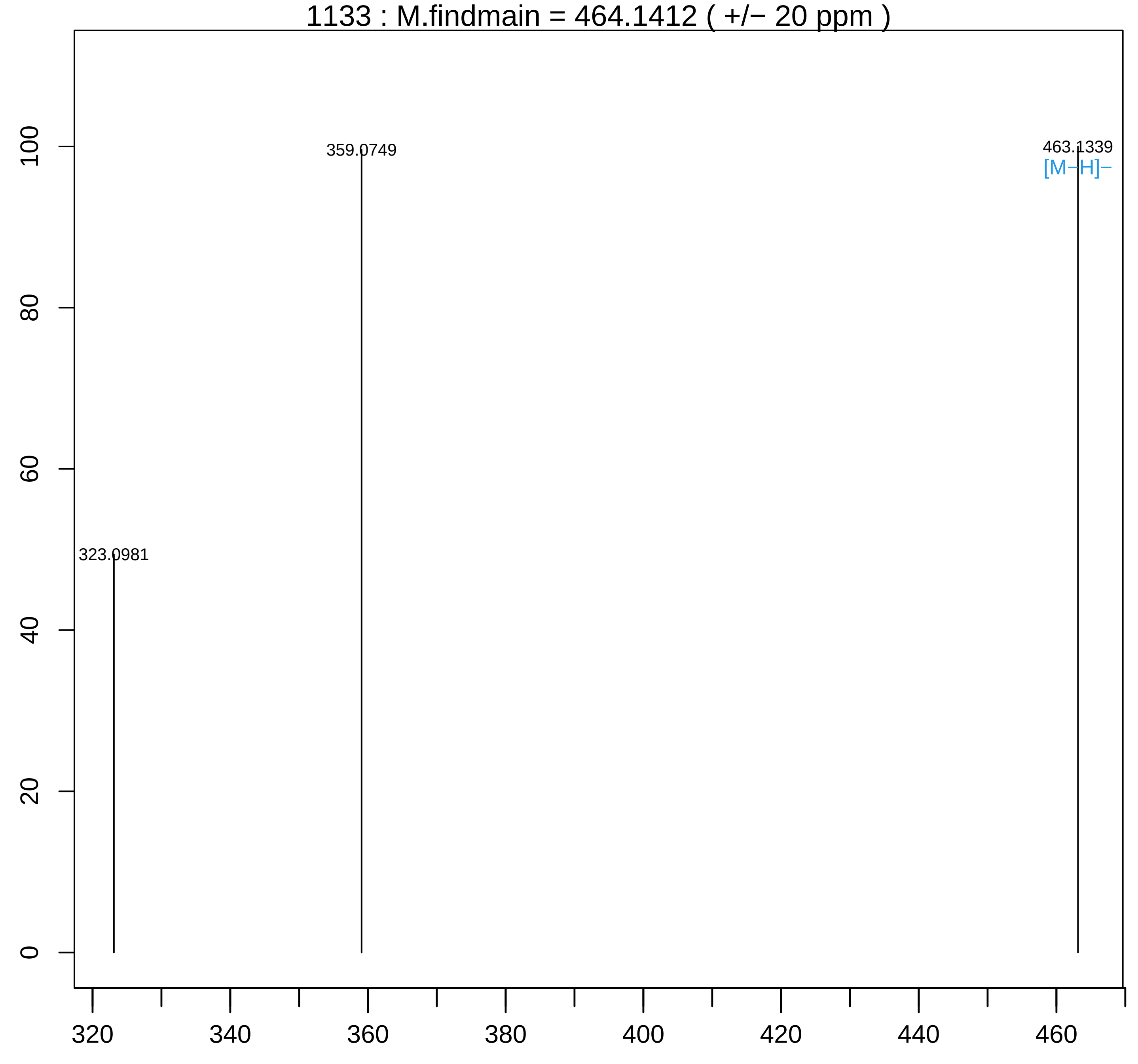
1133 : M.findmain = 464.1412 (+/− 20 ppm)
323.0981
359.0749
463.1339
[M−H]−

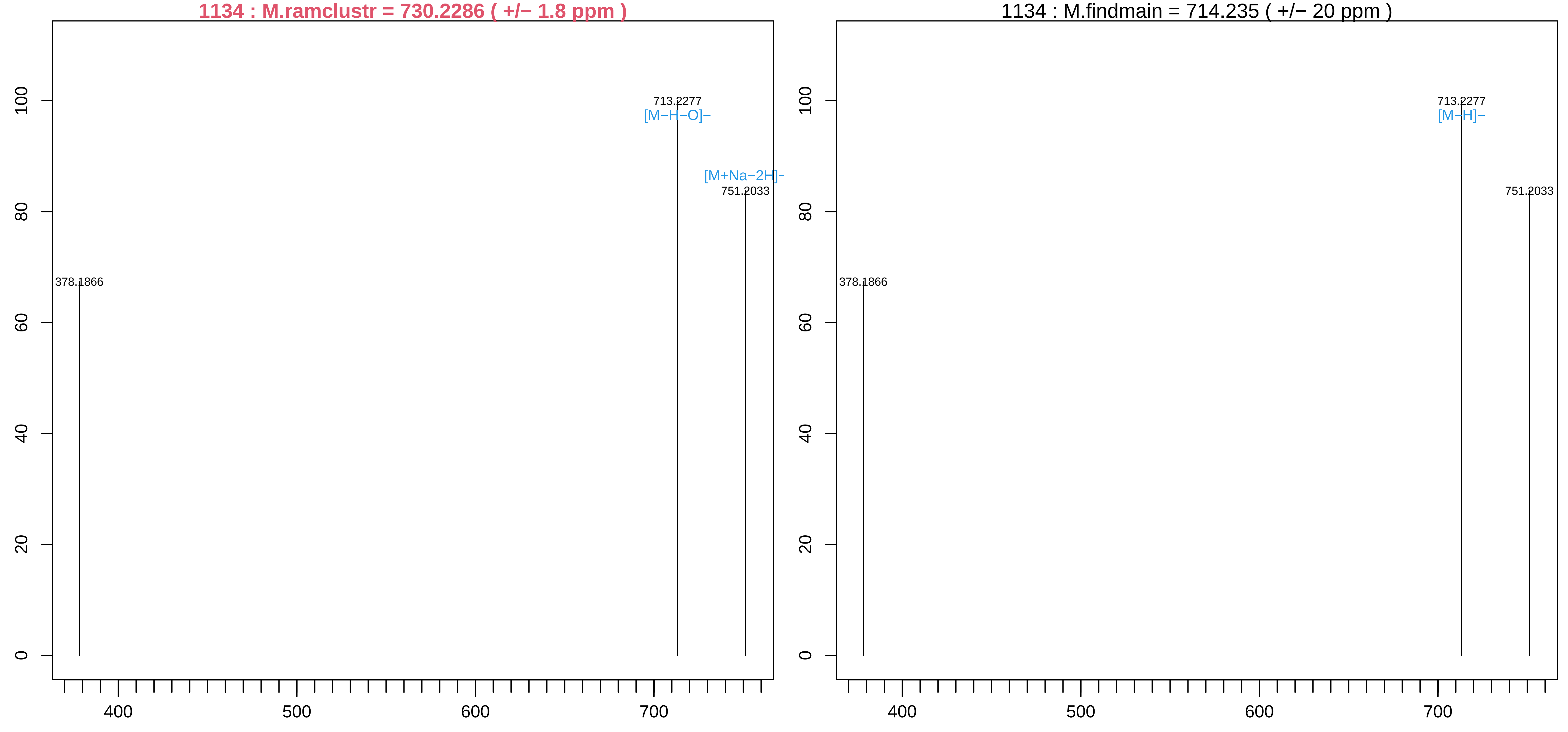
1134 : M.ramclustr = 730.2286 (+/− 1.8 ppm)
713.2277
[M−H−O]−
[M+Na−2H]−
751.2033
378.1866
0
20
40
60
80
100
400
500
600
700
1134 : M.findmain = 714.235 (+/− 20 ppm)
713.2277
[M−H]−
751.2033
378.1866
0
20
40
60
80
100
400
500
600
700

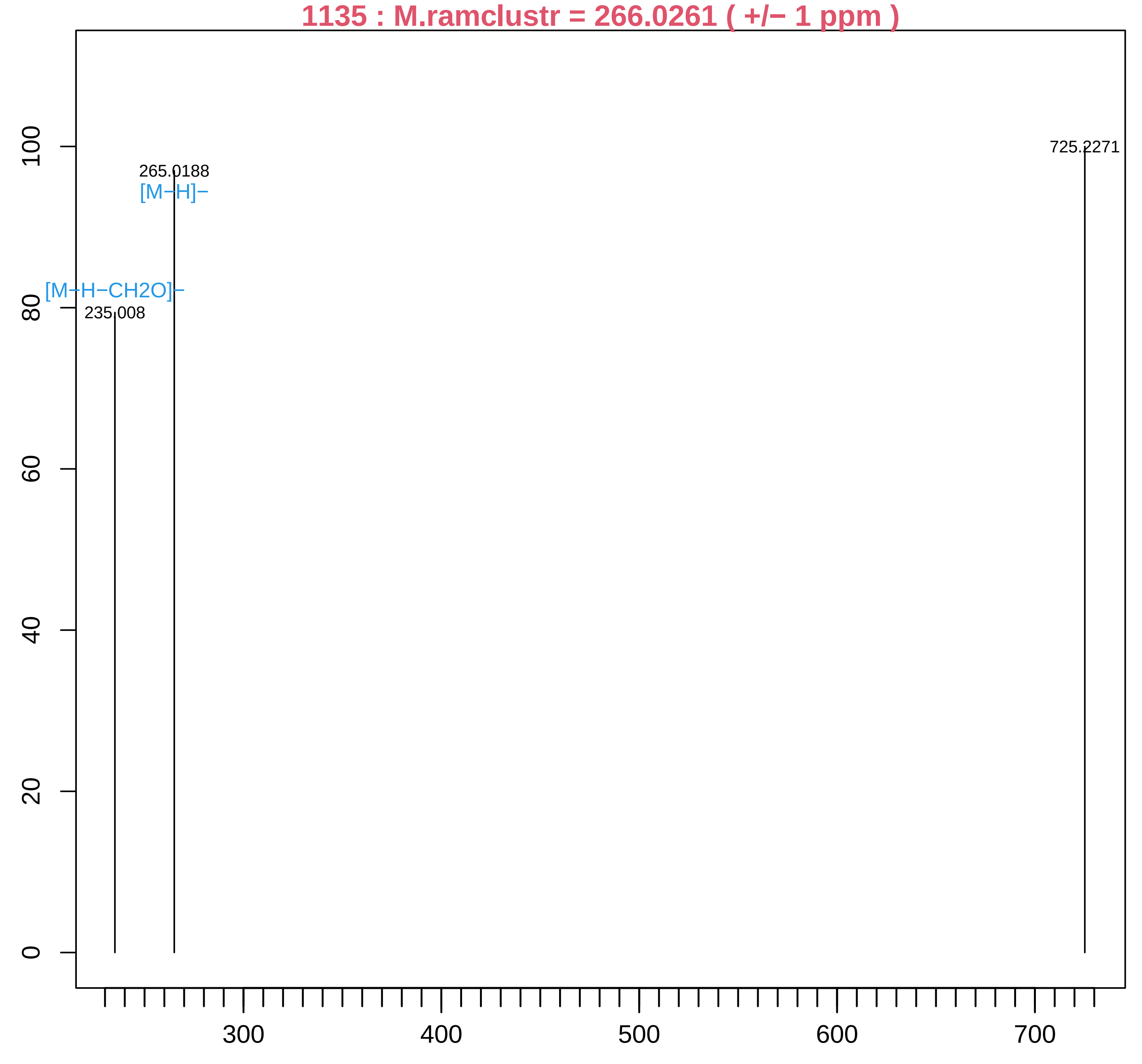
1135 : M.ramclustr = 266.0261 (+/− 1 ppm)
725.2271
265.0188
[M−H]−
[M−H−CH2O]−
235.008
0
20
40
60
80
100
300
400
500
600
700
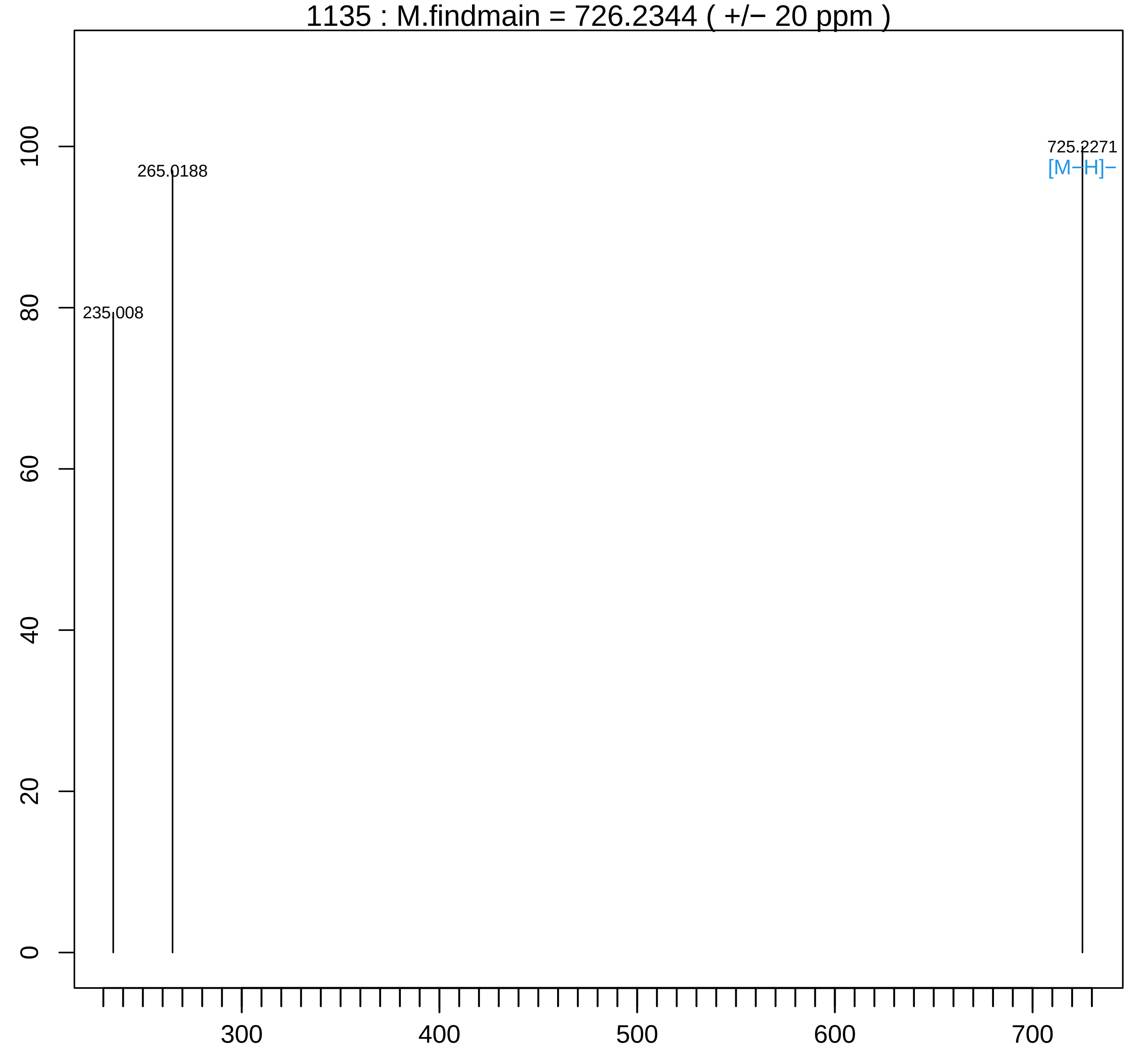
1135 : M.findmain = 726.2344 (+/− 20 ppm)
725.2271
[M−H]−
265.0188
235.008
0
20
40
60
80
100
300
400
500
600
700

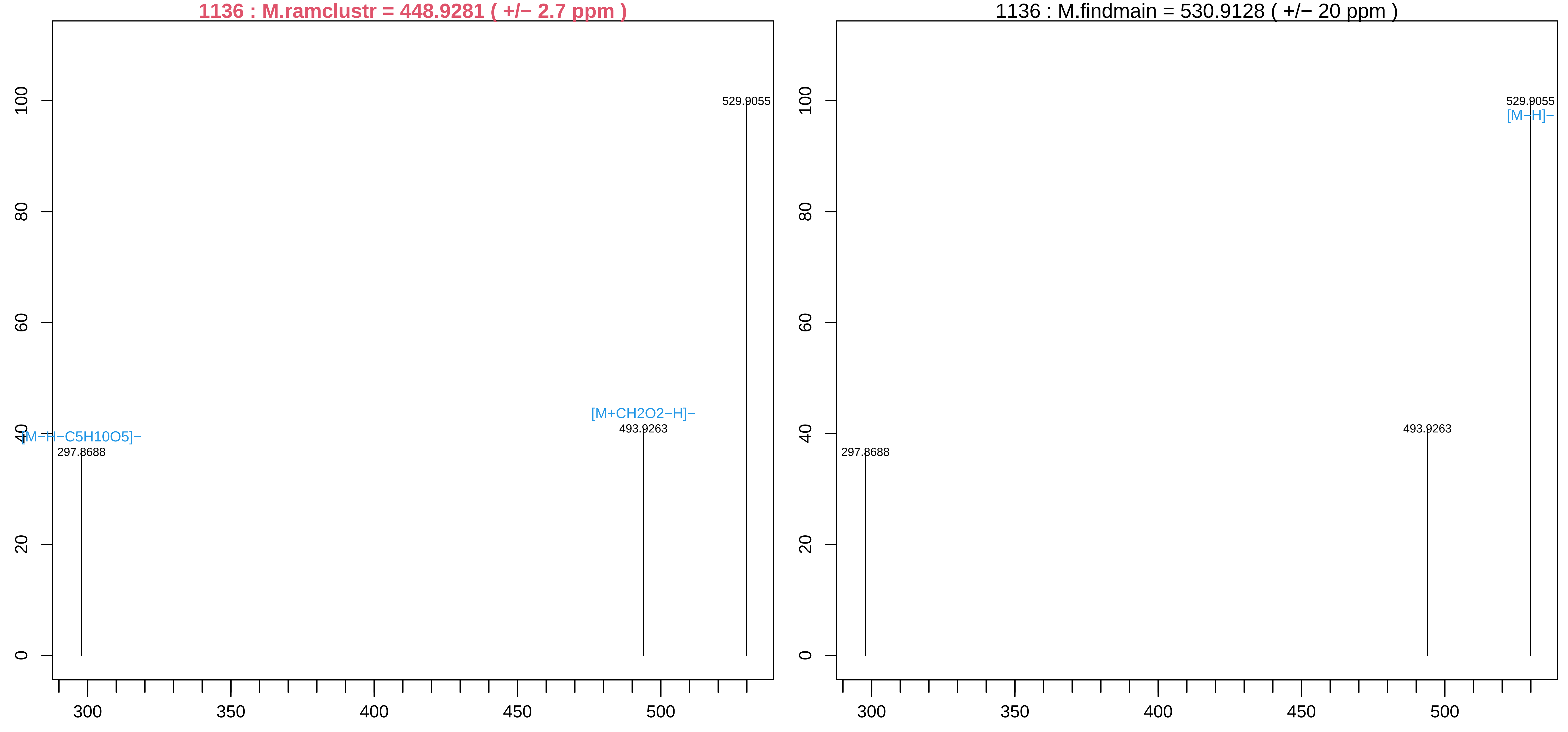

1136 : M.ramclustr = 448.9281 (+/− 2.7 ppm)
529.9055
[M+CH2O2−H]−
493.9263
[M−H−C5H10O5]−
297.8688
1136 : M.findmain = 530.9128 (+/− 20 ppm)
529.9055
[M−H]−
493.9263
297.8688
0
20
40
60
80
100
300
350
400
450
500

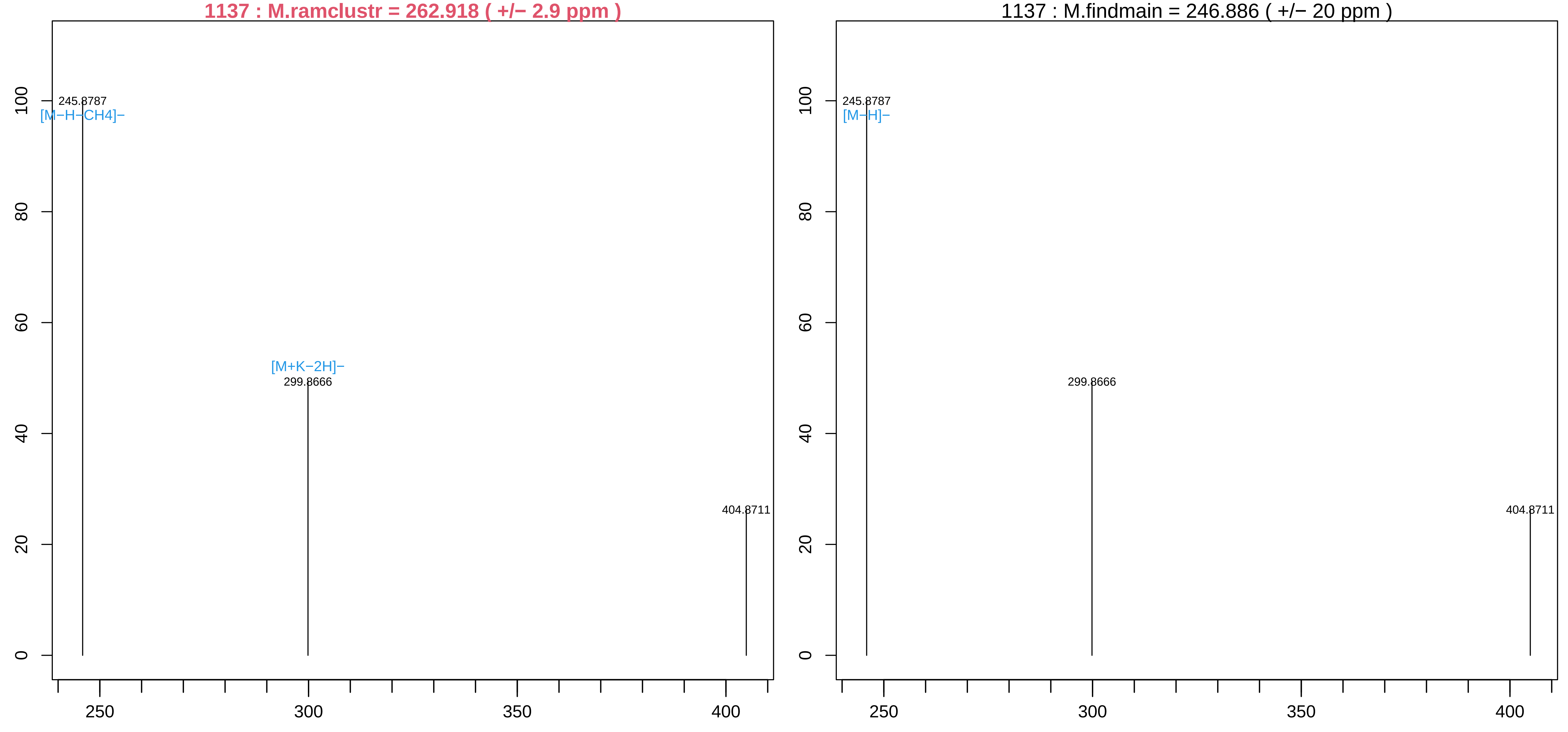
1137 : M.ramclustr = 262.918 (+/− 2.9 ppm)
245.8787
[M−H−CH4]−
[M+K−2H]−
299.8666
404.8711
1137 : M.findmain = 246.886 (+/− 20 ppm)
245.8787
[M−H]−
299.8666
404.8711
0
20
40
60
80
100
250
300
350
400

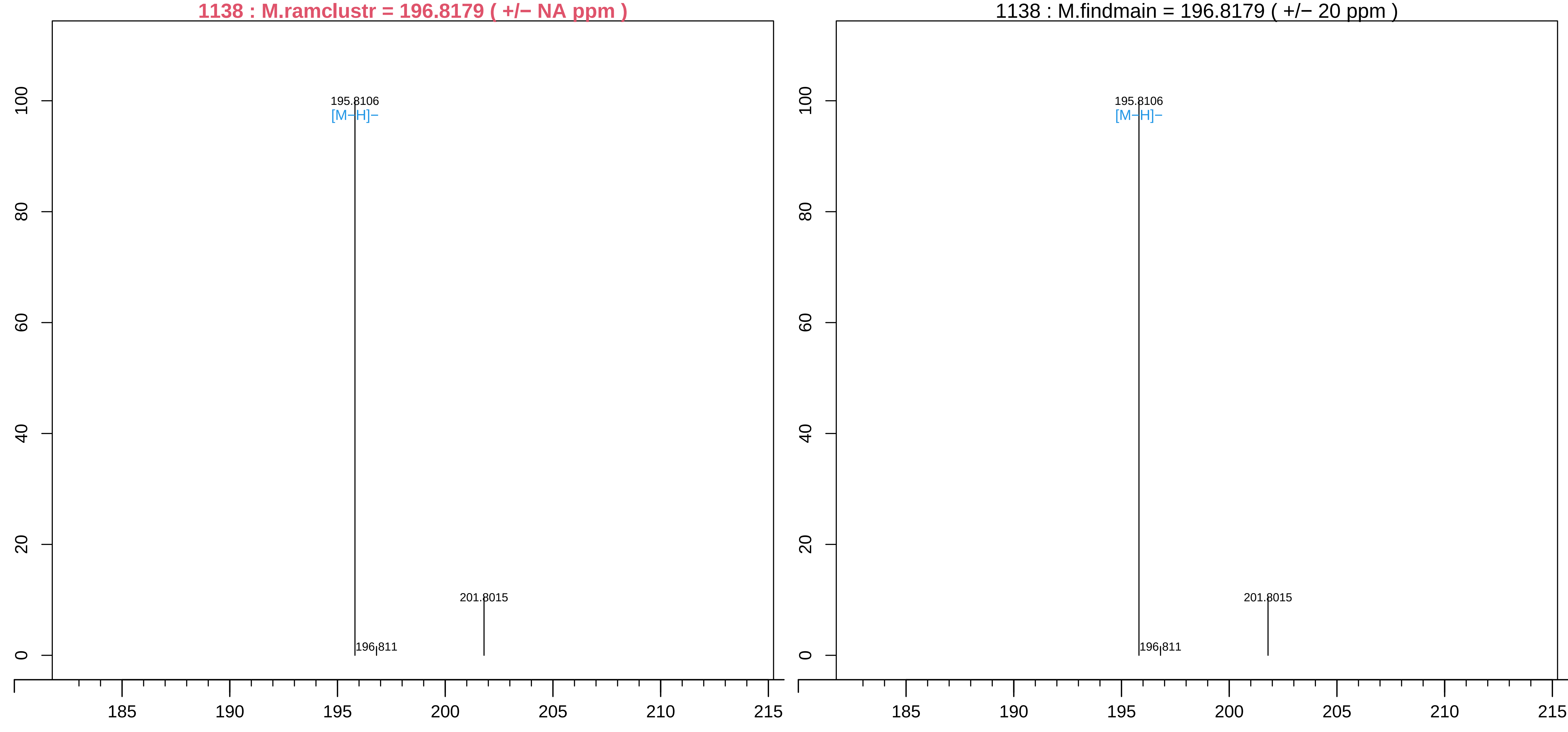
1138 : M.ramclustr = 196.8179 (+/− NA ppm)
1138 : M.findmain = 196.8179 (+/− 20 ppm)
195.8106
[M−H]−
196.811
201.8015
0
20
40
60
80
100
185
190
195
200
205
210
215

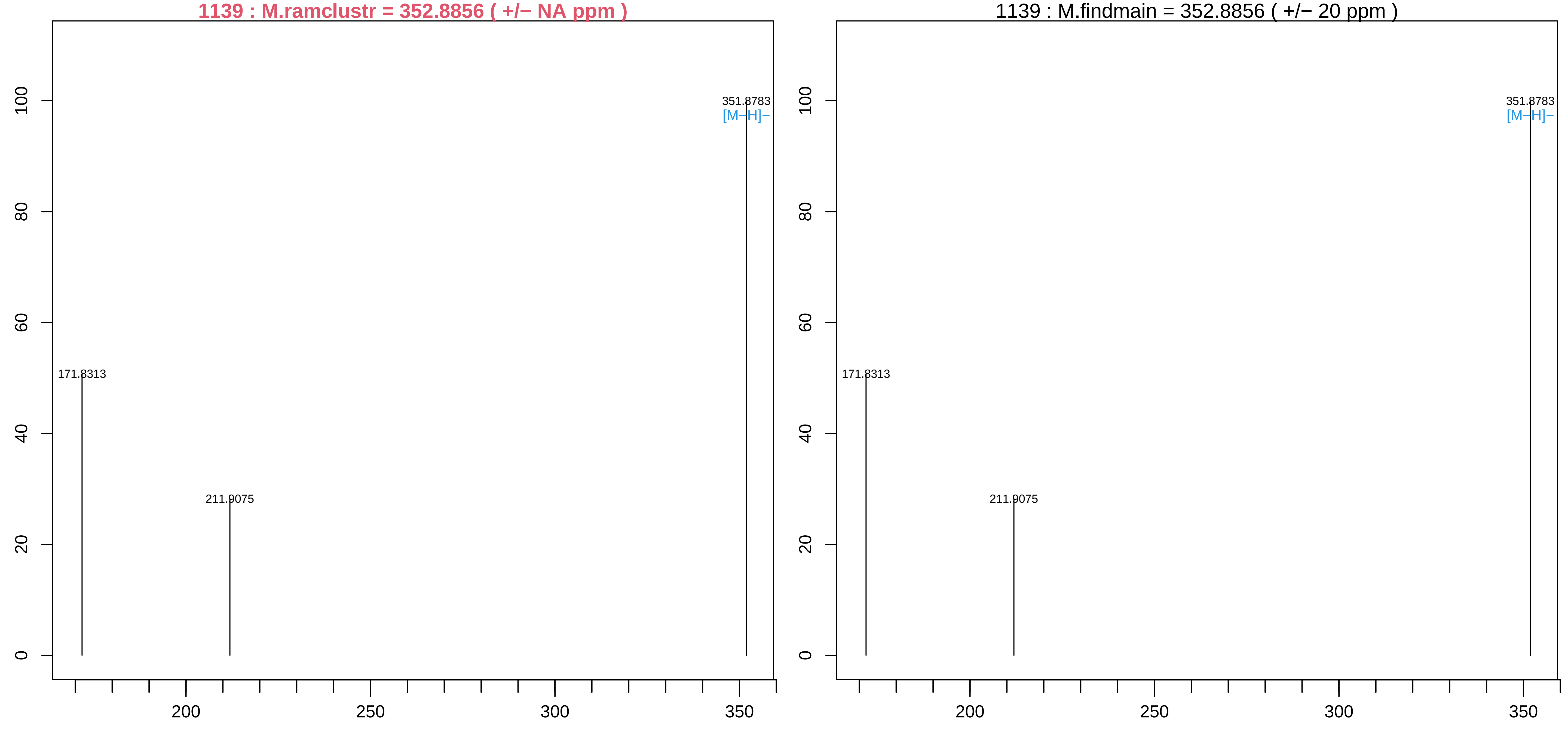
1139 : M.ramclustr = 352.8856 (+/− NA ppm)
351.8783
[M−H]−
171.8313
211.9075
1139 : M.findmain = 352.8856 (+/− 20 ppm)
351.8783
[M−H]−
171.8313
211.9075
0
20
40
60
80
100
200
250
300
350

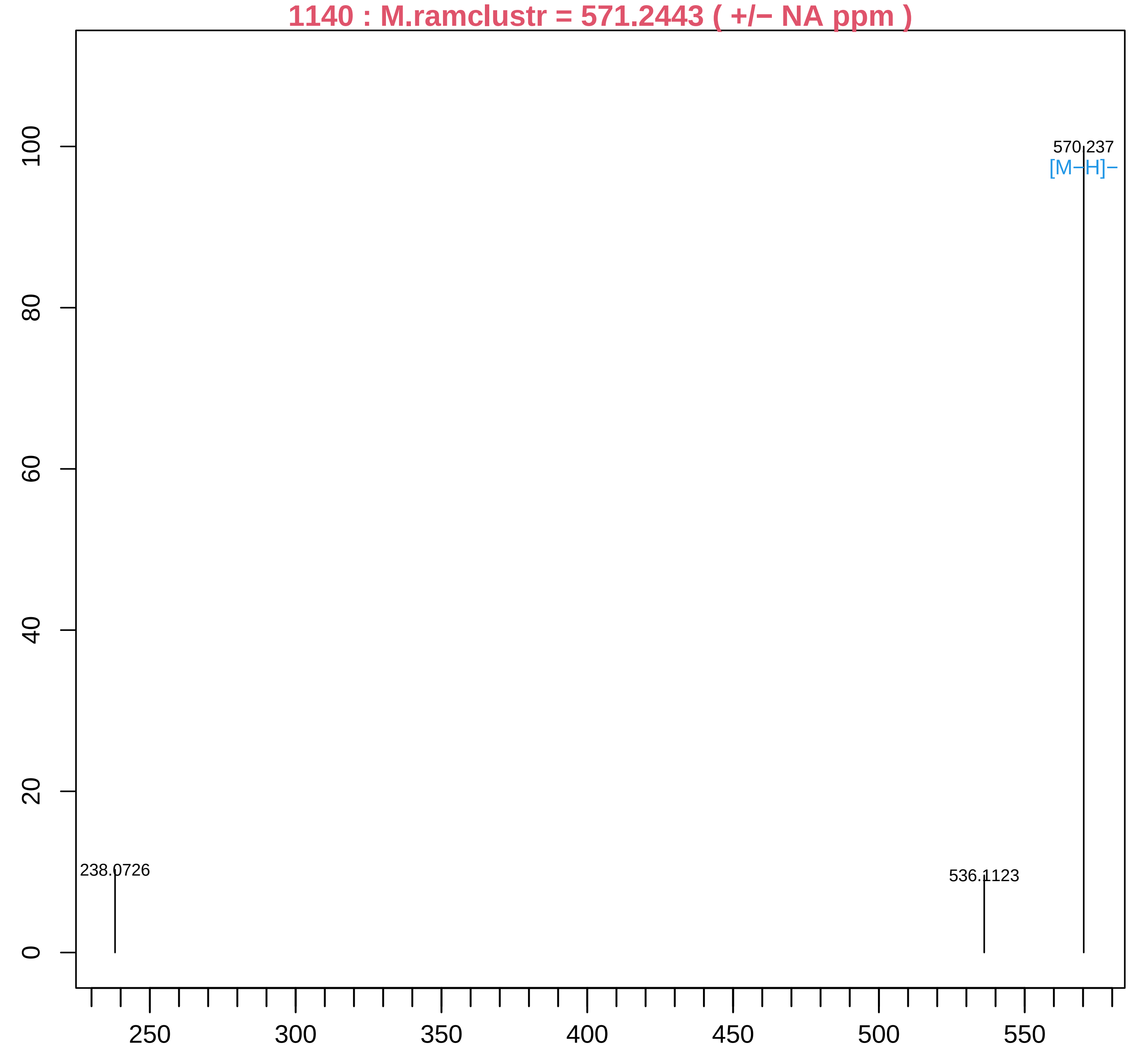

1140 : M.ramclustr = 571.2443 (+/− NA ppm)
570.237
[M−H]−
238.0726
536.1123

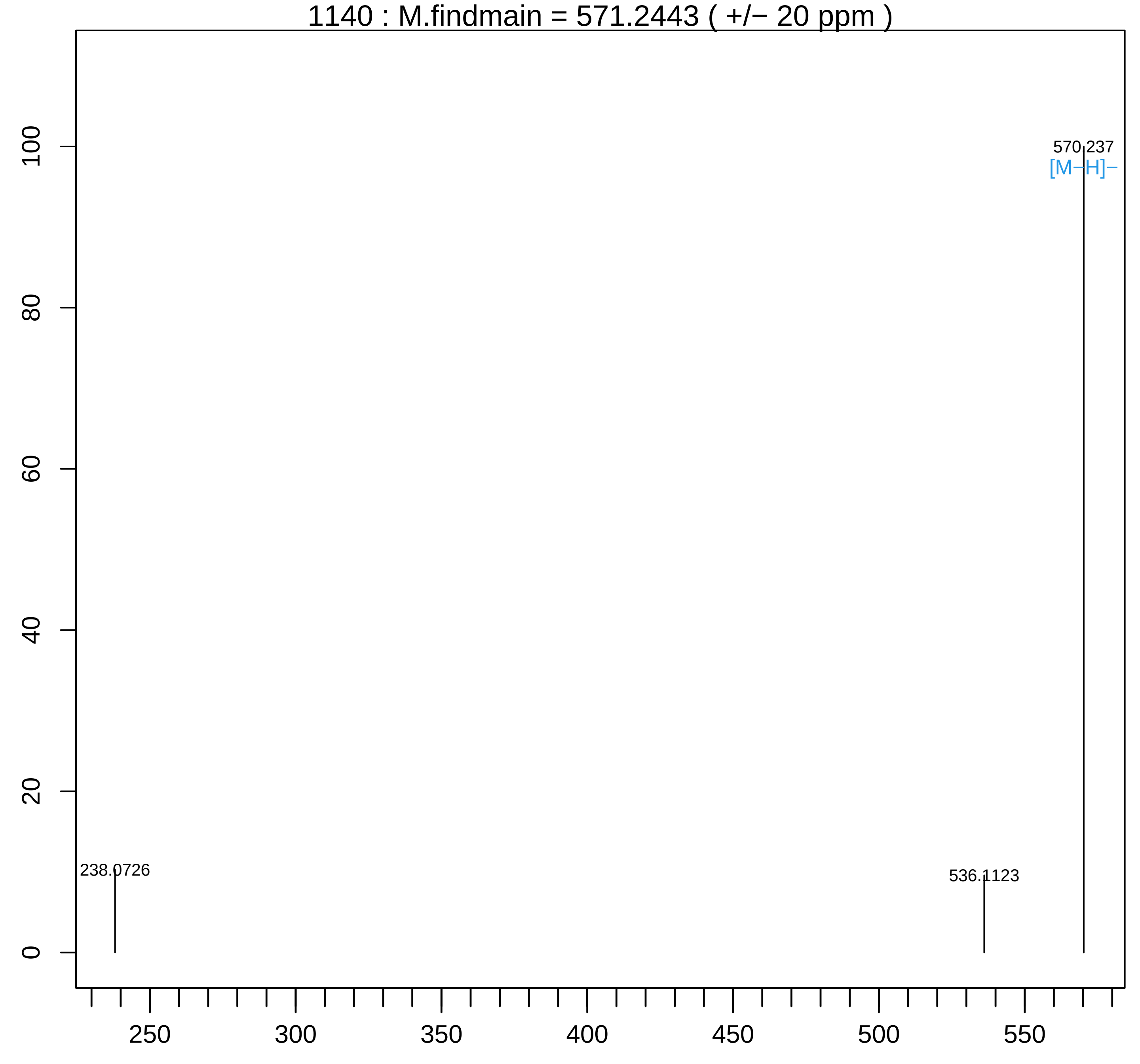

1140 : M.findmain = 571.2443 (+/− 20 ppm)
570.237
[M−H]−
238.0726
536.1123

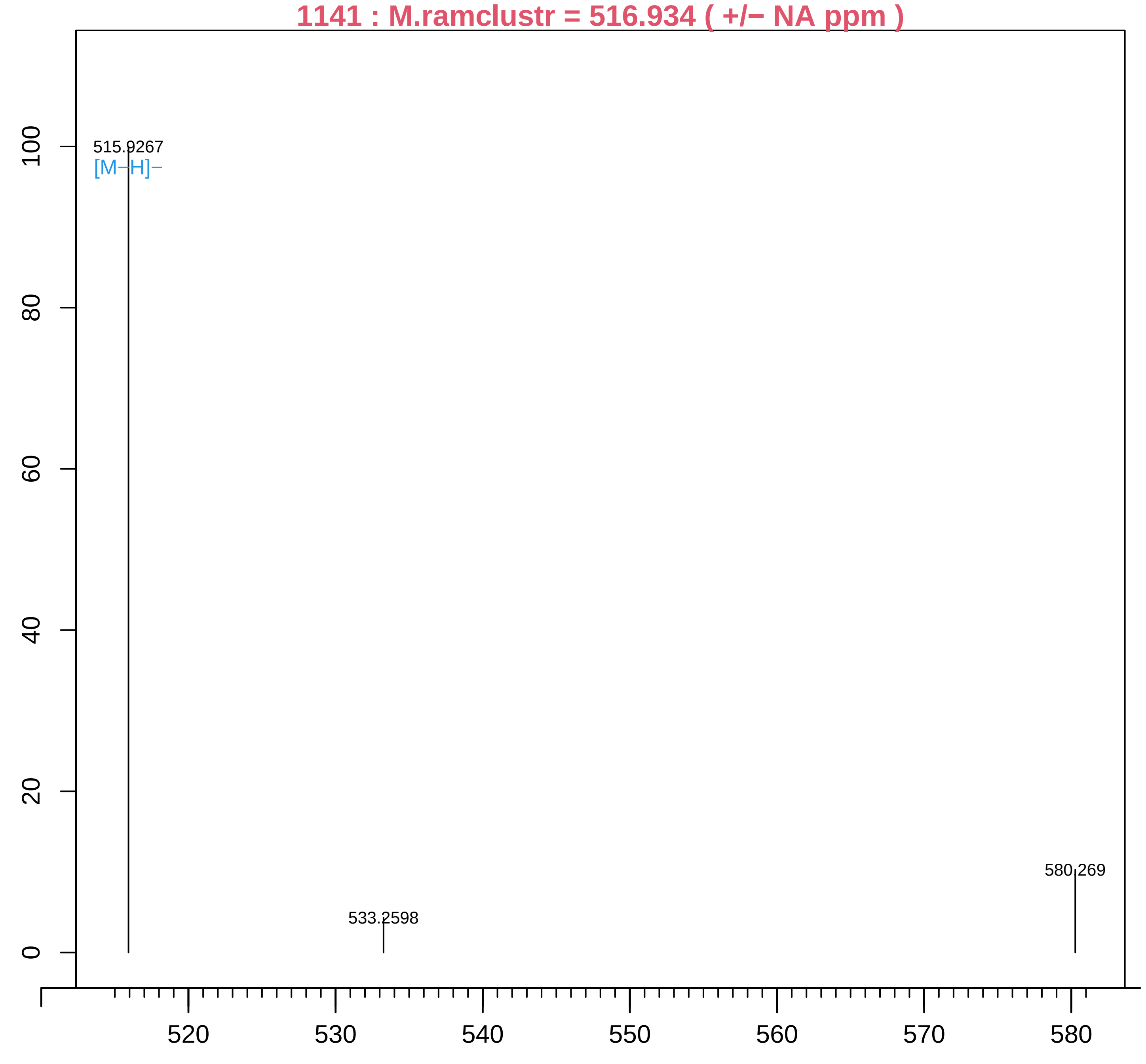
1141 : M.ramclustr = 516.934 (+/− NA ppm)
515.9267
[M−H]−
533.2598
580.269
0
20
40
60
80
100
520
530
540
550
560
570
580

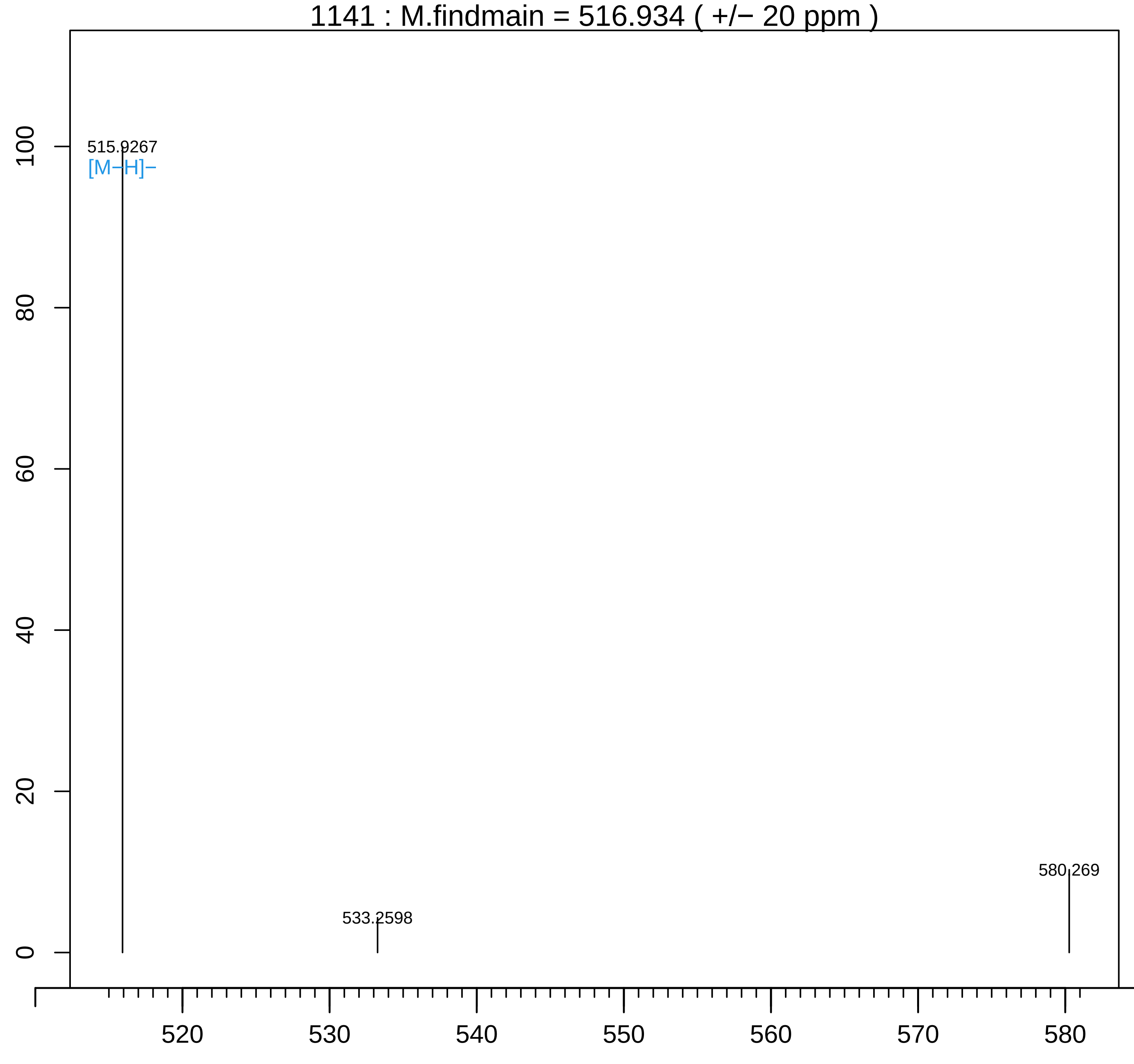
1141 : M.findmain = 516.934 (+/− 20 ppm)
515.9267
[M−H]−
533.2598
580.269
0
20
40
60
80
100
520
530
540
550
560
570
580

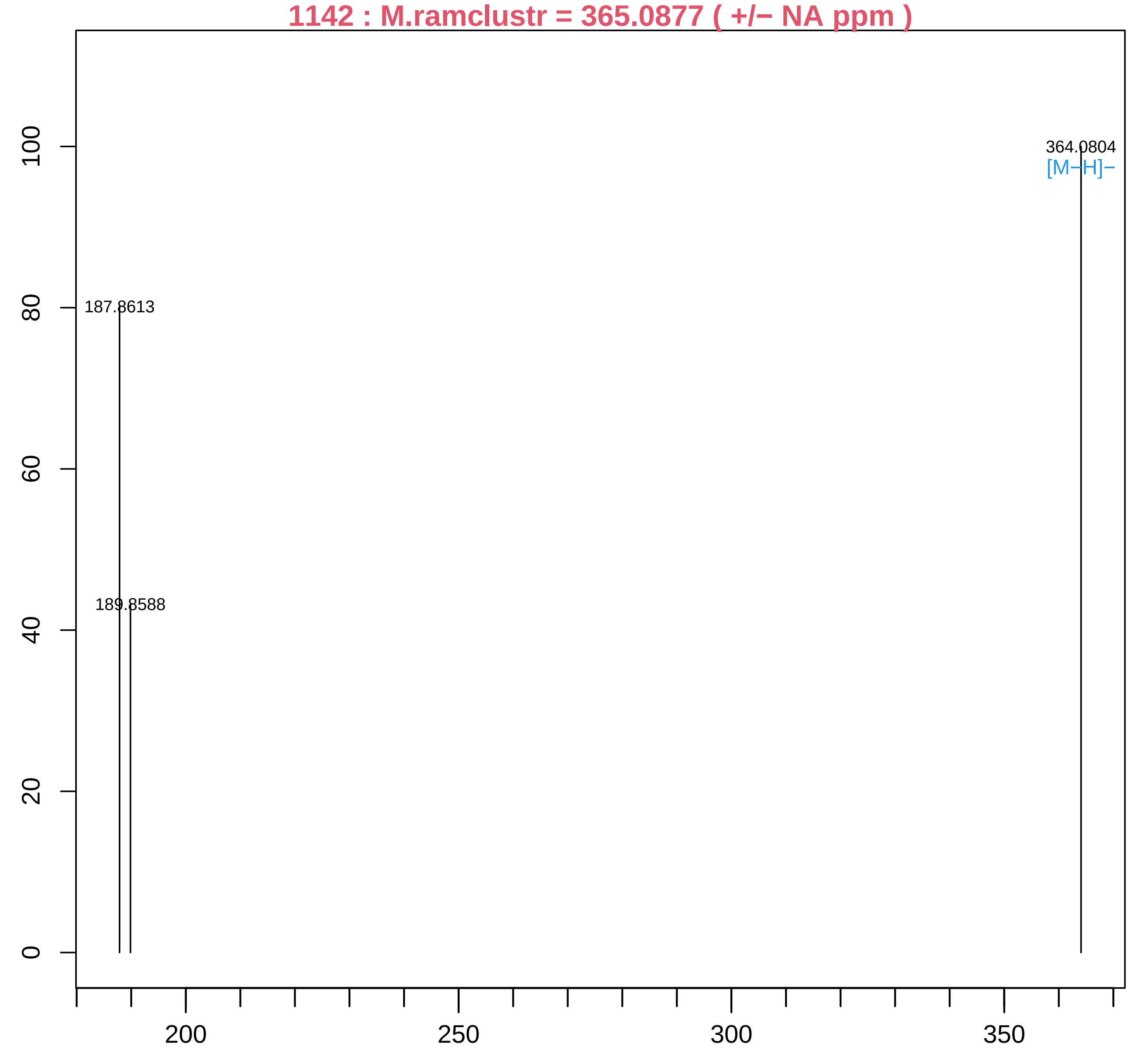
1142 : M.ramclustr = 365.0877 (+/− NA ppm)
364.0804
[M−H]−
187.8613
189.8588
0
20
40
60
80
100
200
250
300
350

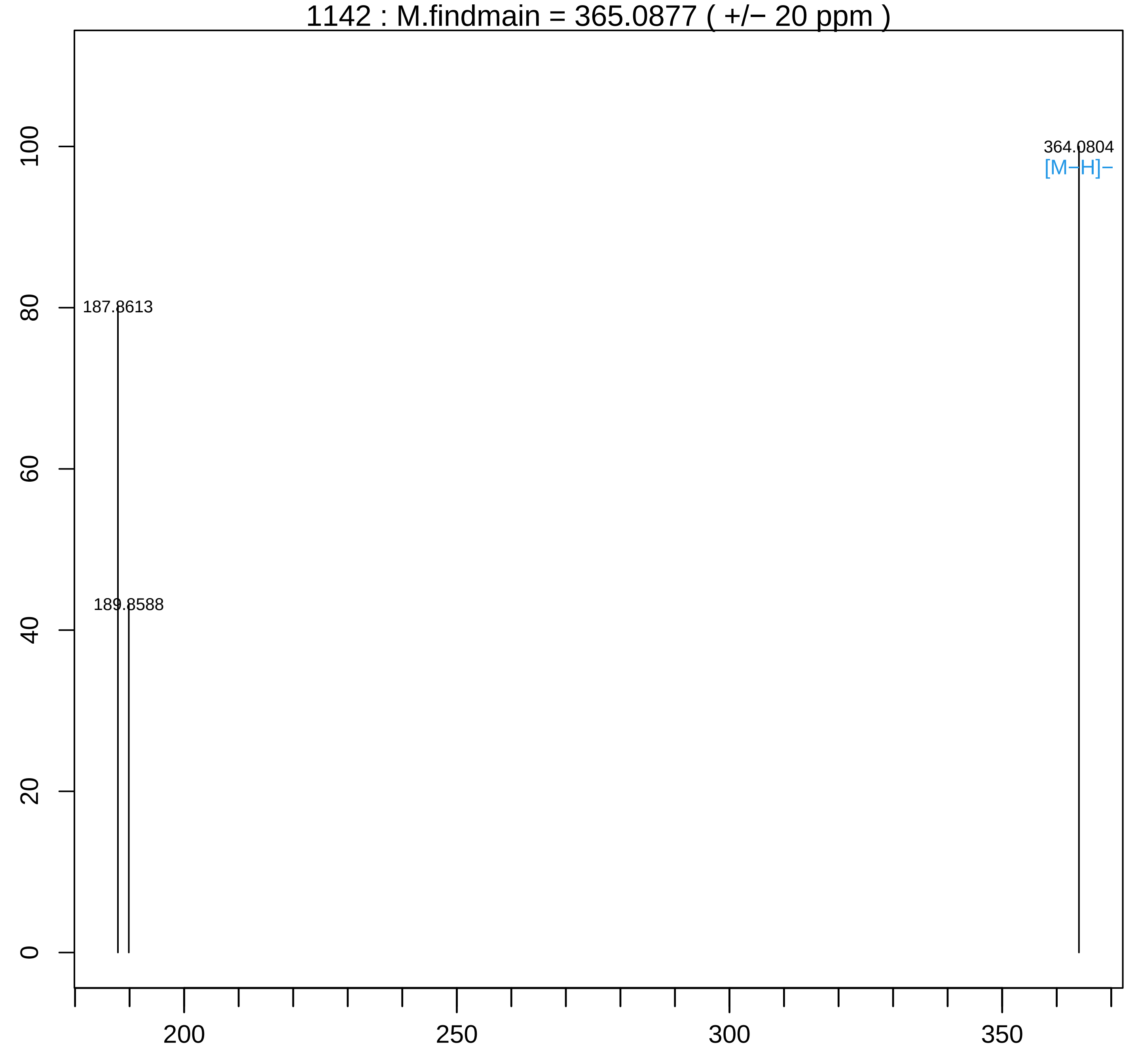
1142 : M.findmain = 365.0877 (+/− 20 ppm)
364.0804
[M−H]−
187.8613
189.8588
0
20
40
60
80
100
200
250
300
350

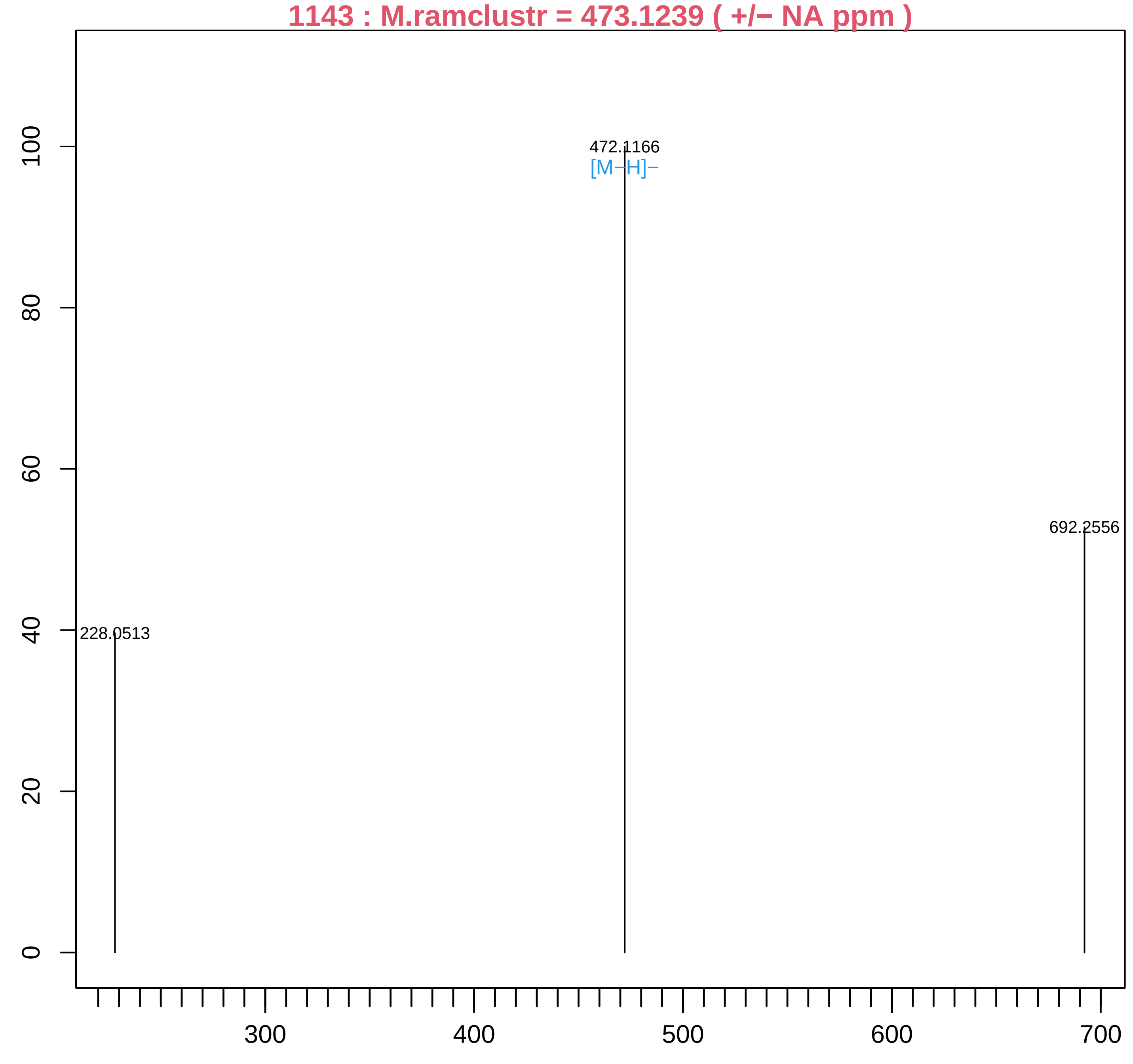
1143 : M.ramclustr = 473.1239 (+/− NA ppm)
472.1166
[M−H]−
692.2556
228.0513
0
20
40
60
80
100
300
400
500
600
700

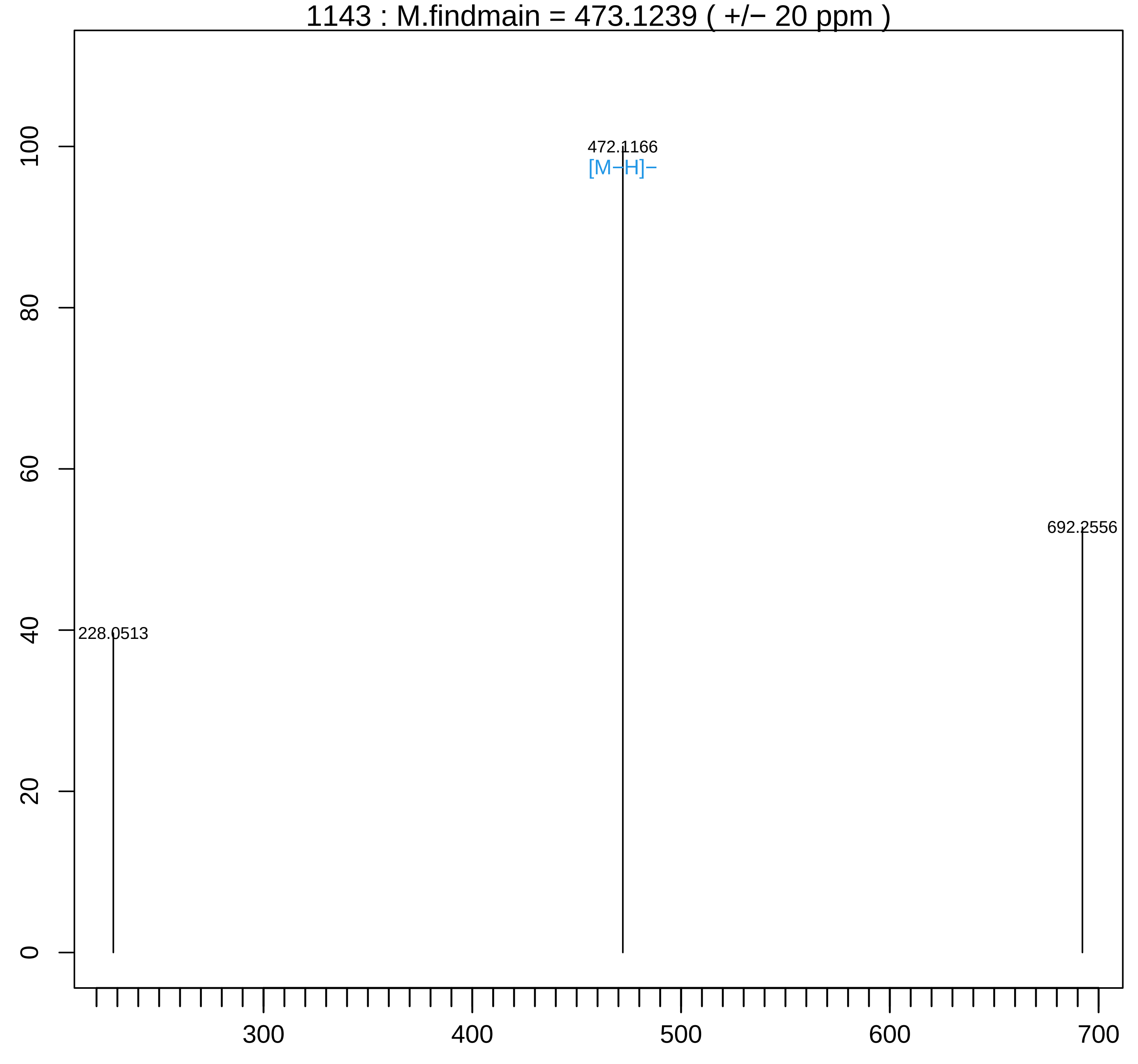
1143 : M.findmain = 473.1239 (+/− 20 ppm)
472.1166
[M−H]−
692.2556
228.0513
0
20
40
60
80
100
300
400
500
600
700

1144 : M.ramclustr = 903.4726 (+/− NA ppm)

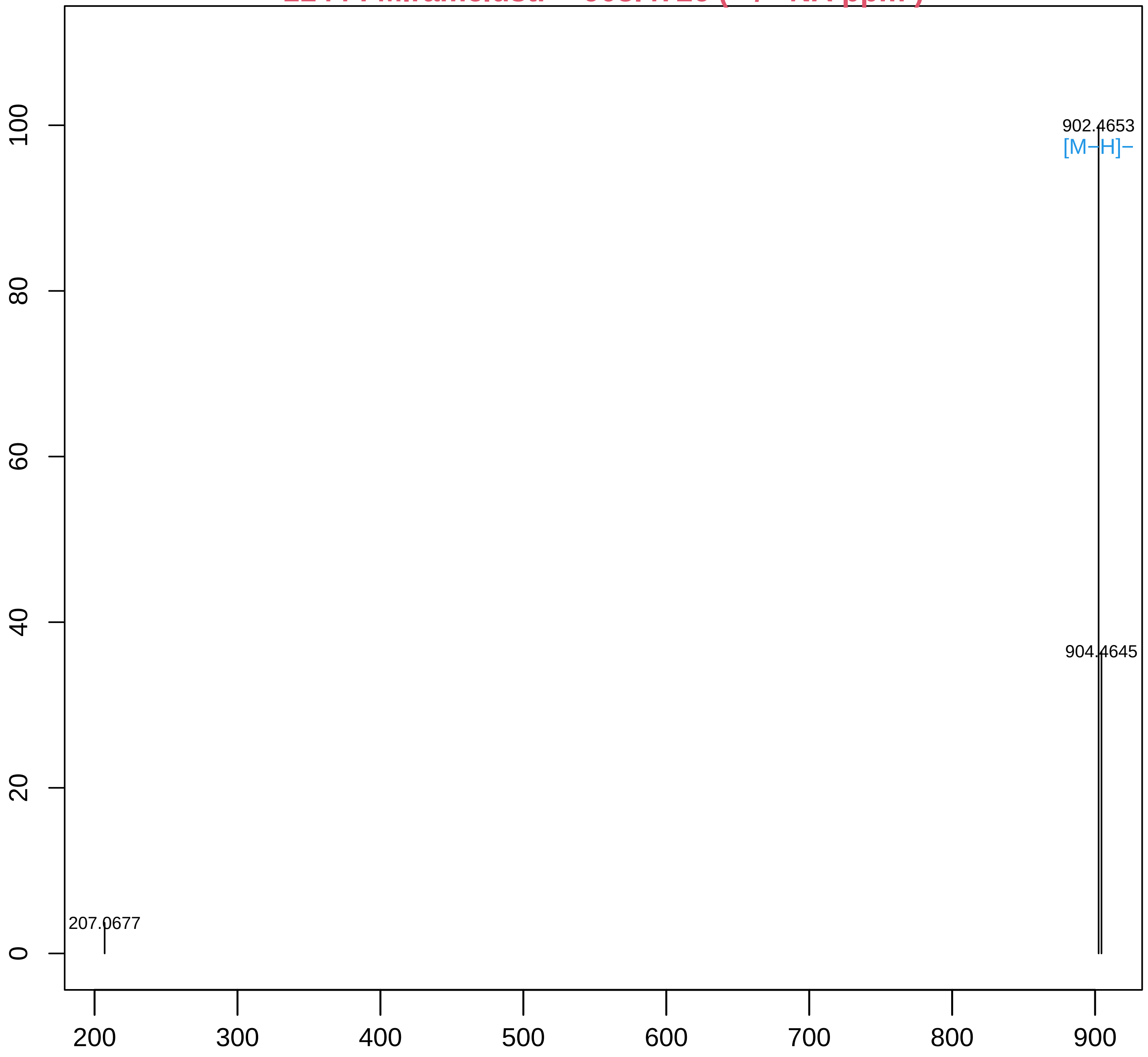

1144 : M.findmain = 903.4726 (+/− 20 ppm)

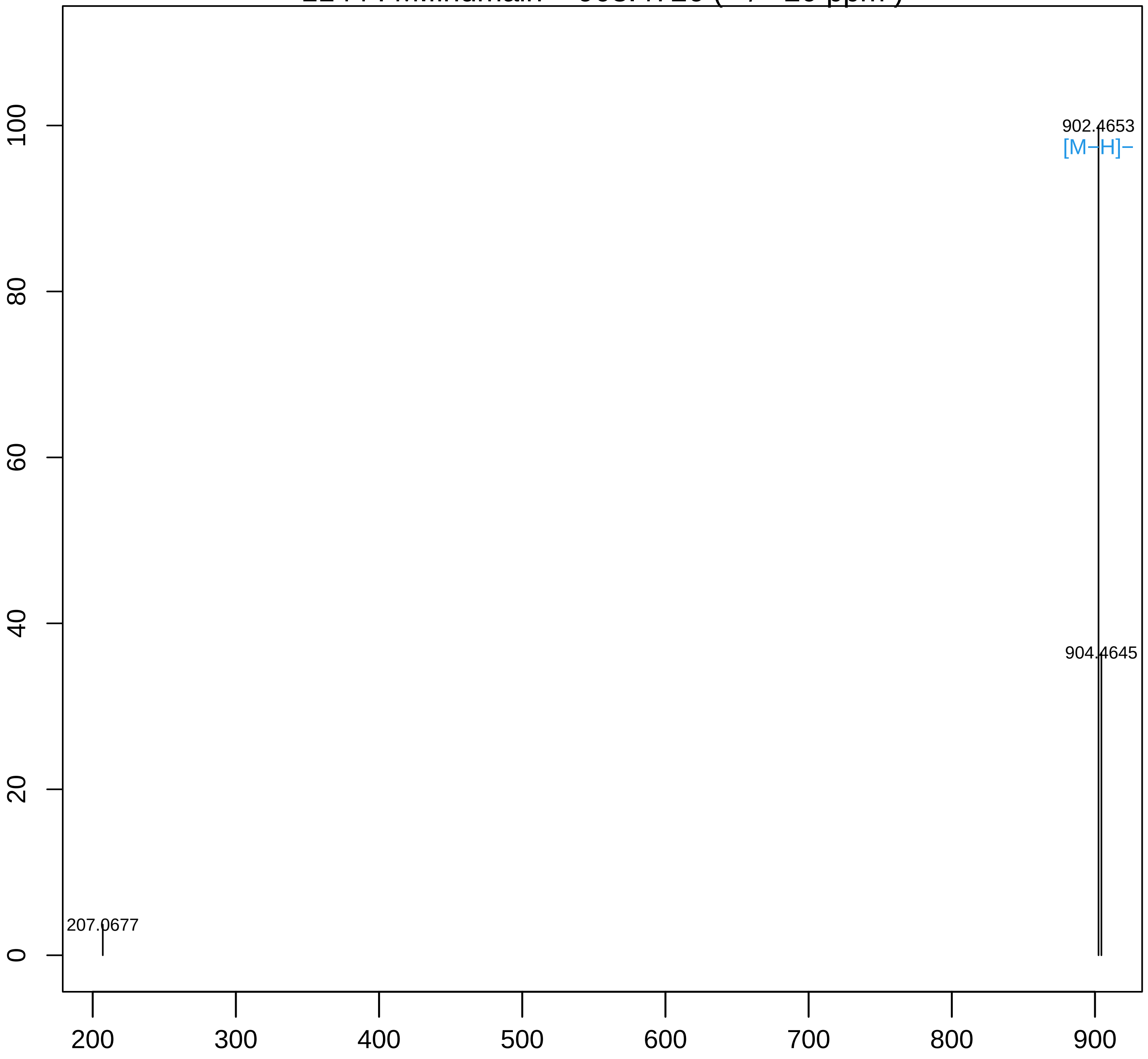

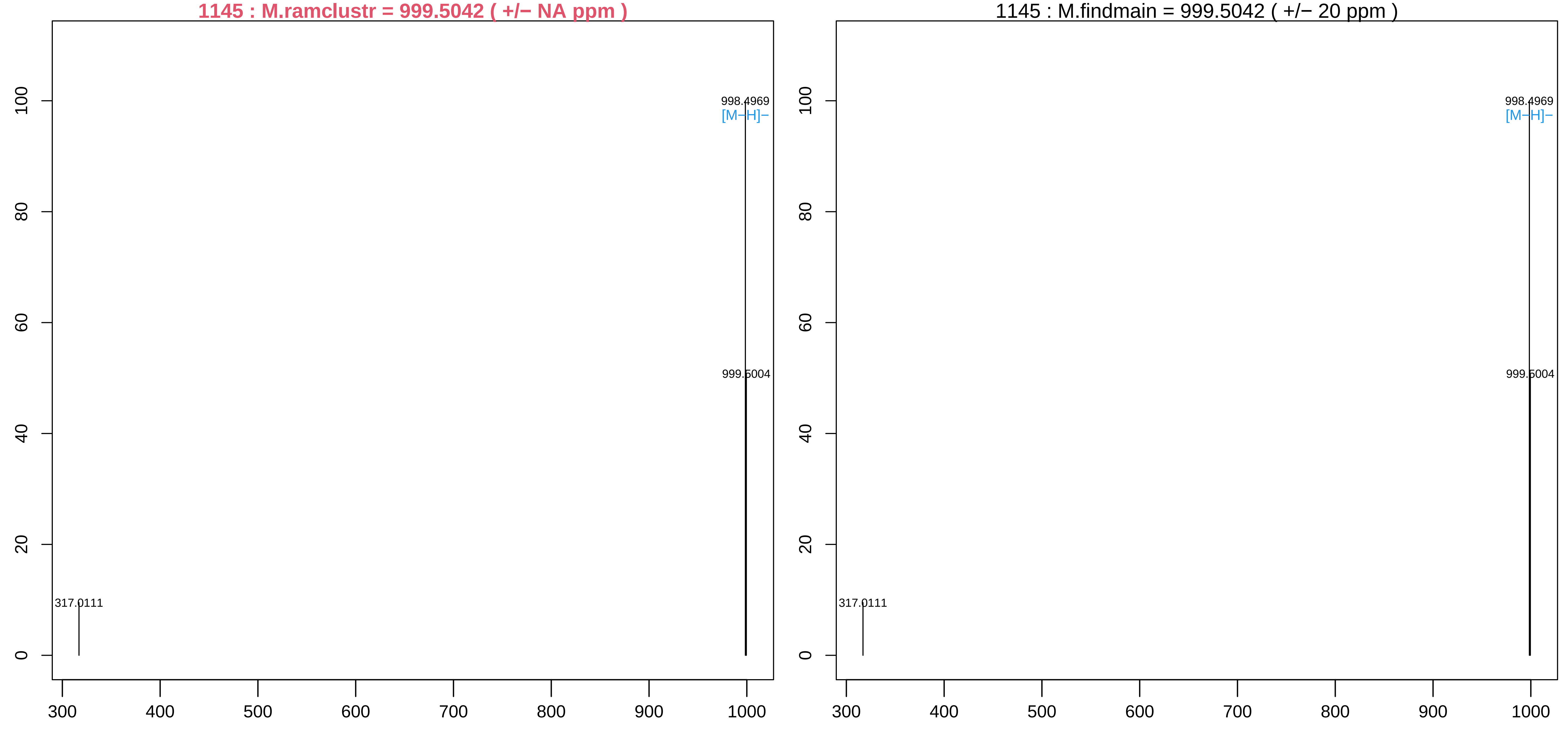
1145 : M.ramclustr = 999.5042 (+/− NA ppm)
998.4969
[M−H]−
999.5004
317.0111
1145 : M.findmain = 999.5042 (+/− 20 ppm)
998.4969
[M−H]−
999.5004
317.0111

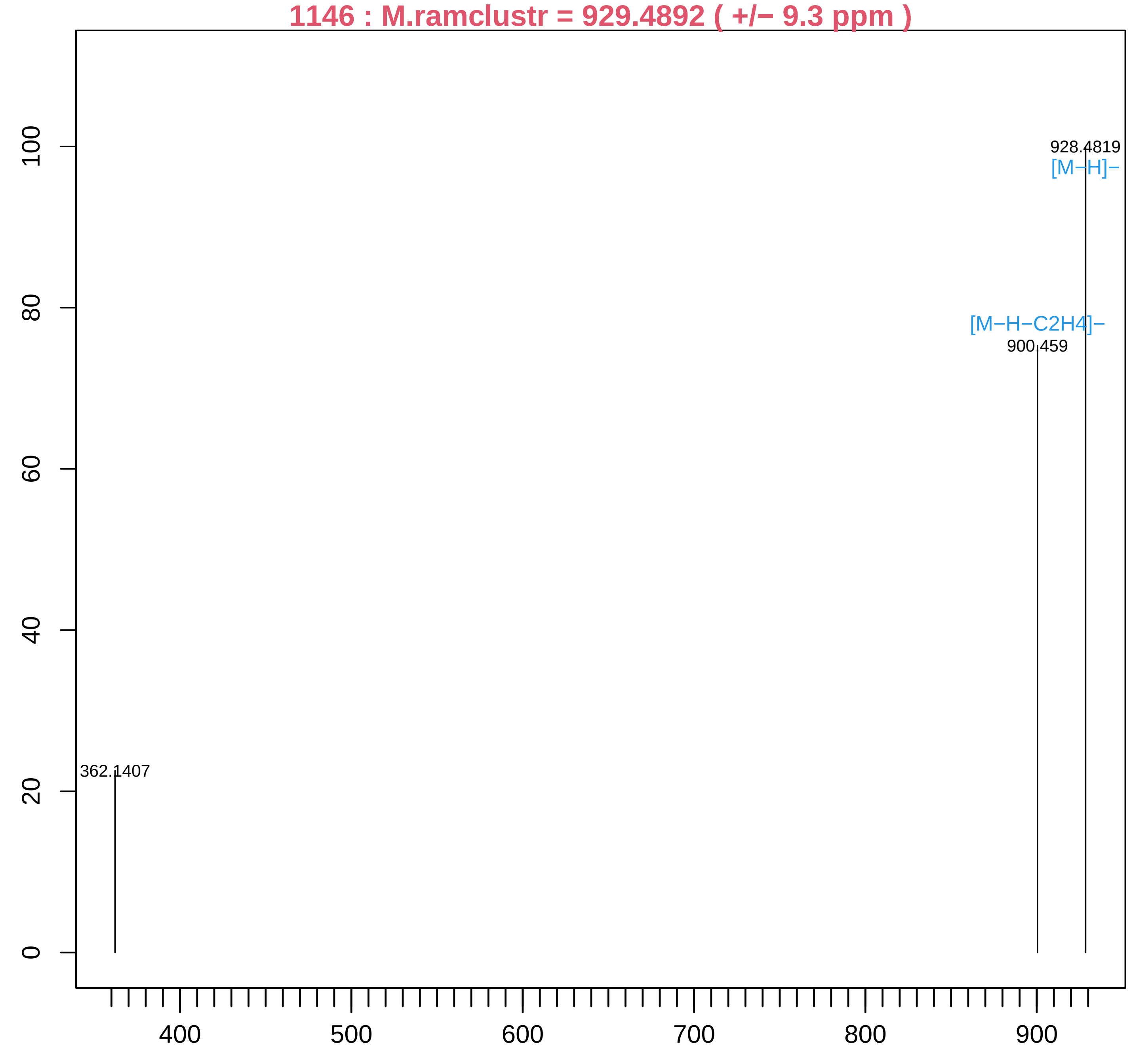

1146 : M.ramclustr = 929.4892 (+/− 9.3 ppm)
928.4819
[M−H]−
[M−H−C2H4]−
900.459
362.1407
0
20
40
60
80
100
400
500
600
700
800
900

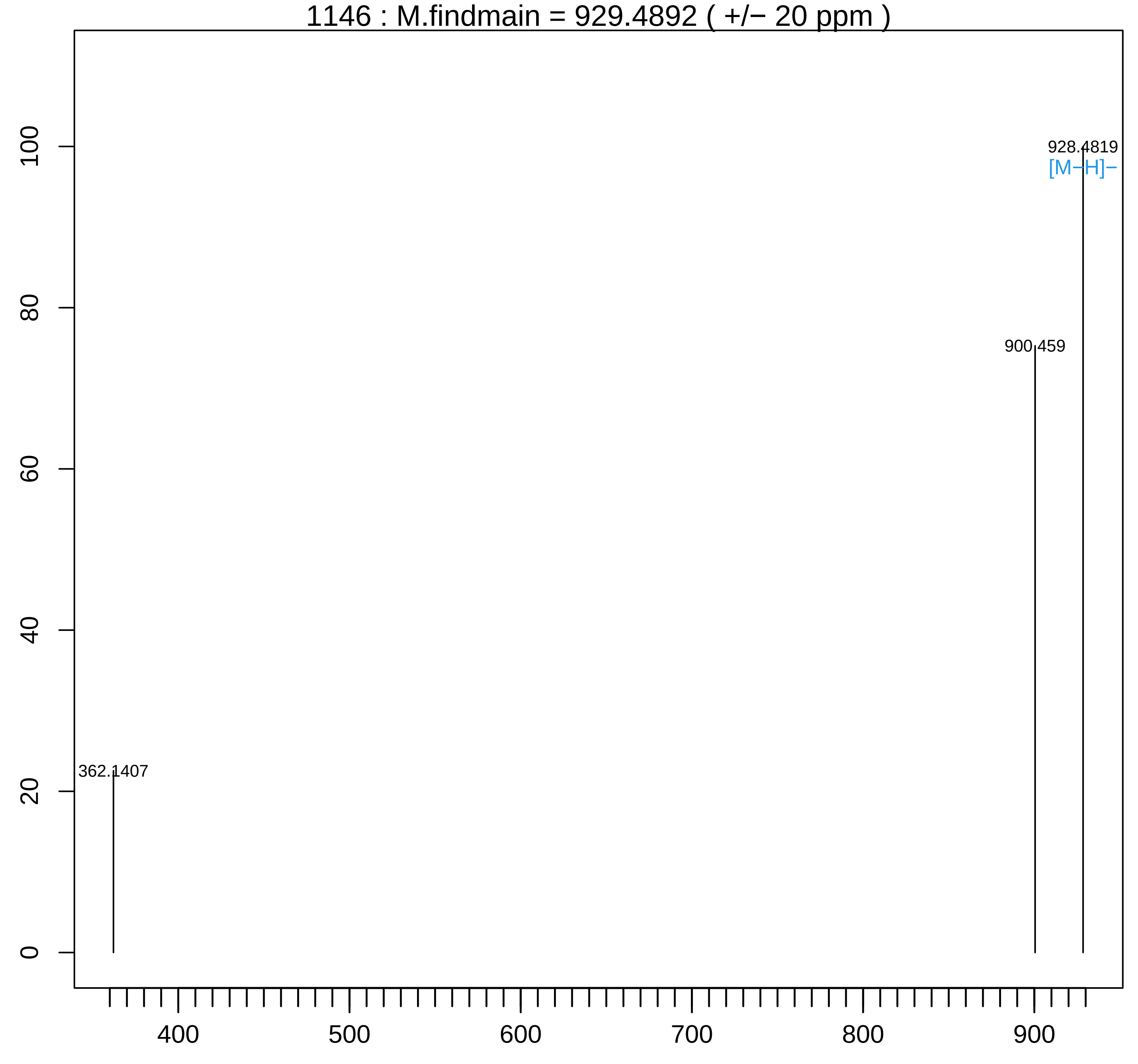

1146 : M.findmain = 929.4892 (+/− 20 ppm)
928.4819
[M−H]−
900.459
362.1407
0
20
40
60
80
100
400
500
600
700
800
900

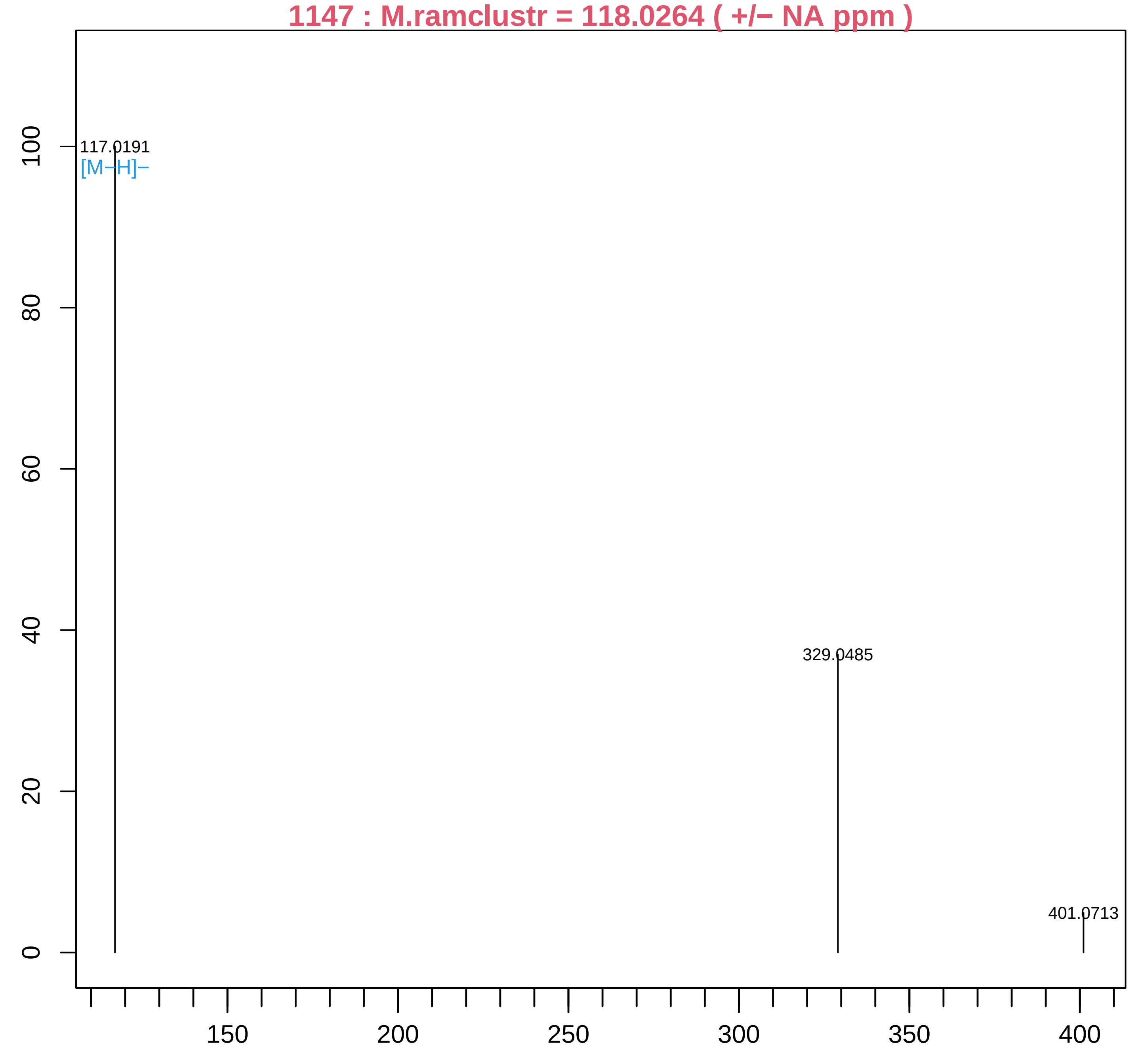

1147 : M.ramclustr = 118.0264 (+/− NA ppm)
117.0191
[M−H]−
329.0485
401.0713
0
20
40
60
80
100
150
200
250
300
350
400

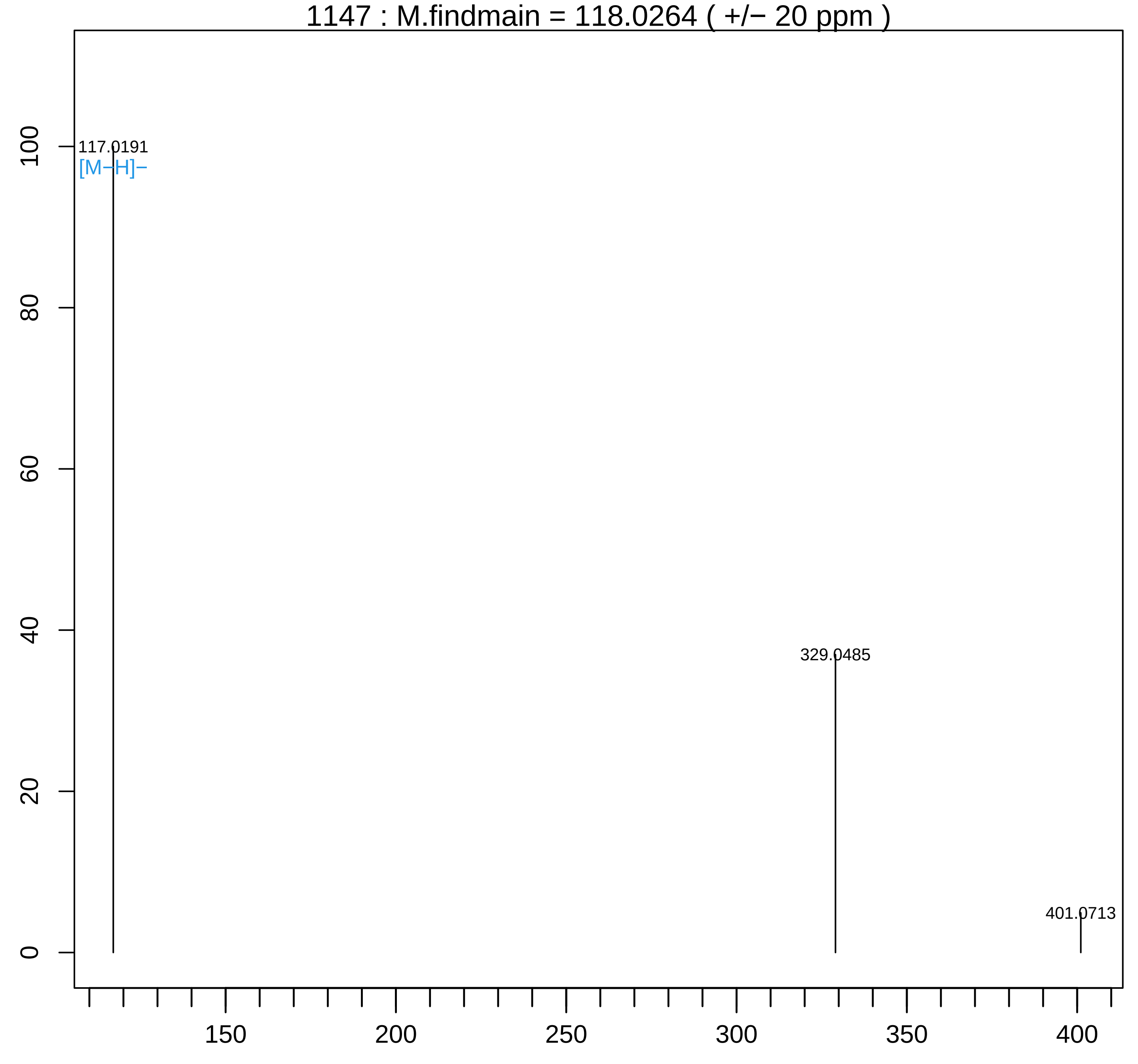

1147 : M.findmain = 118.0264 (+/− 20 ppm)
117.0191
[M−H]−
329.0485
401.0713
0
20
40
60
80
100
150
200
250
300
350
400

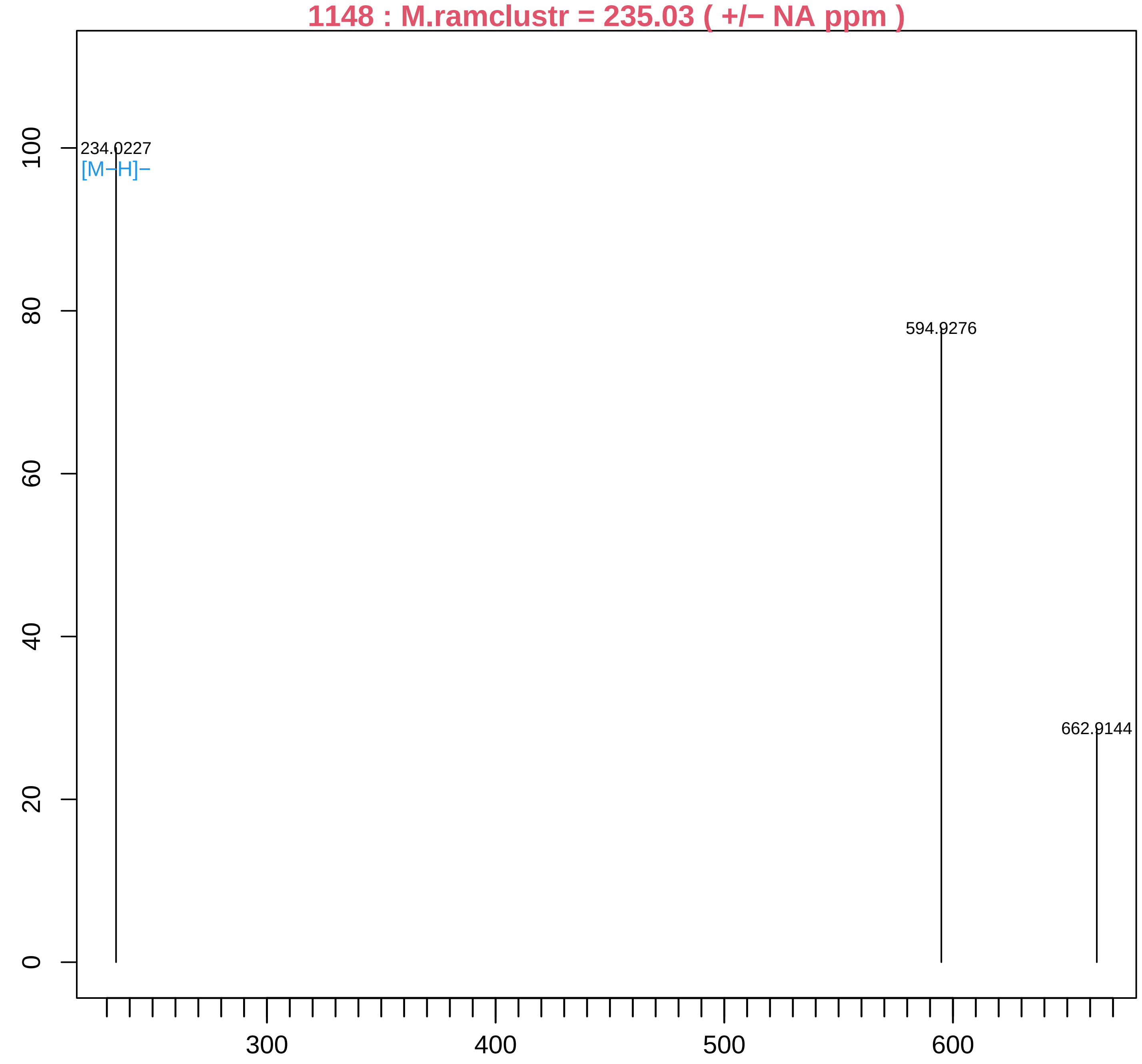

1148 : M.ramclustr = 235.03 (+/− NA ppm)
234.0227
[M−H]−
594.9276
662.9144
0
20
40
60
80
100
300
400
500
600

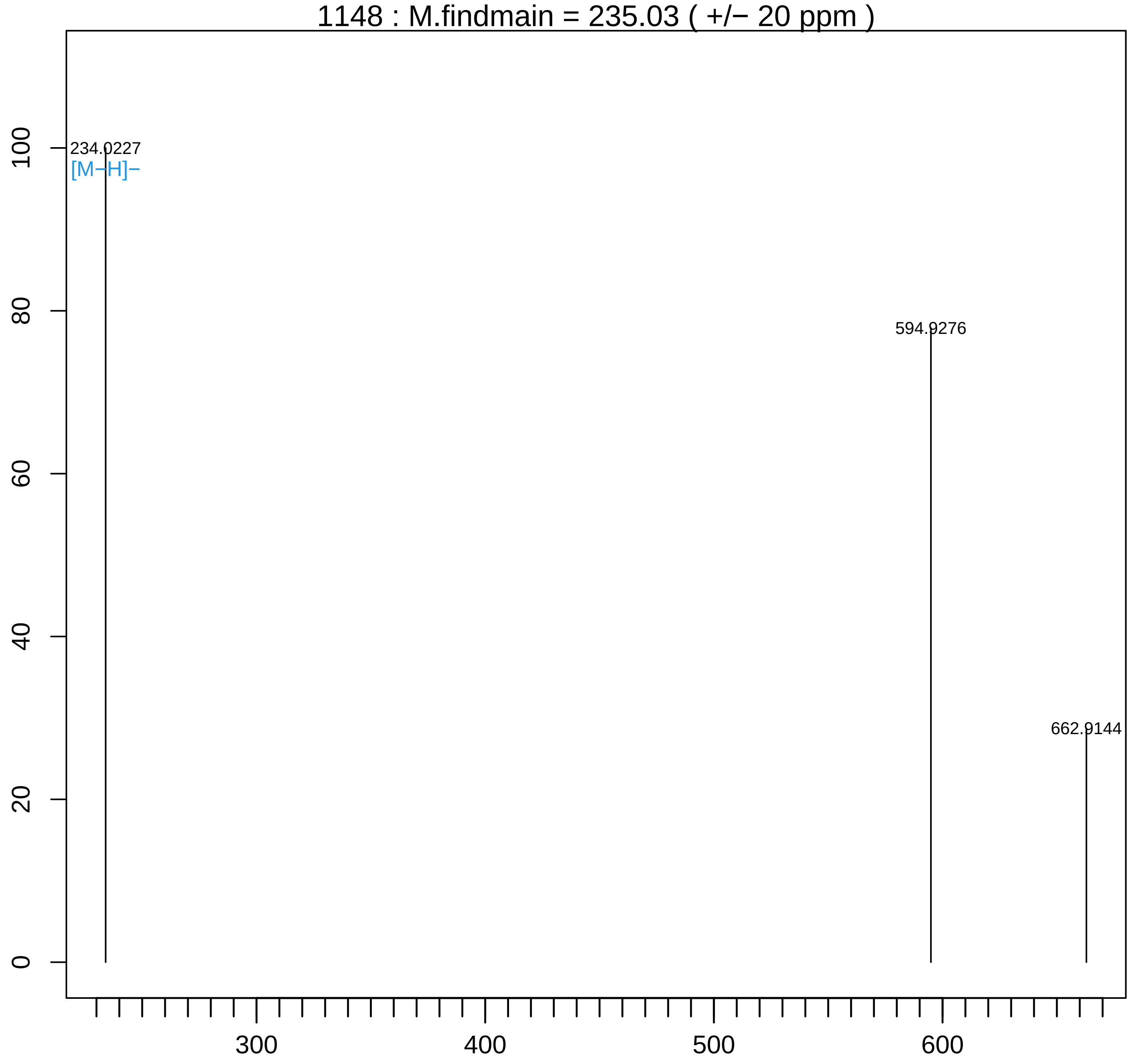

1148 : M.findmain = 235.03 (+/− 20 ppm)
234.0227
[M−H]−
594.9276
662.9144
0
20
40
60
80
100
300
400
500
600

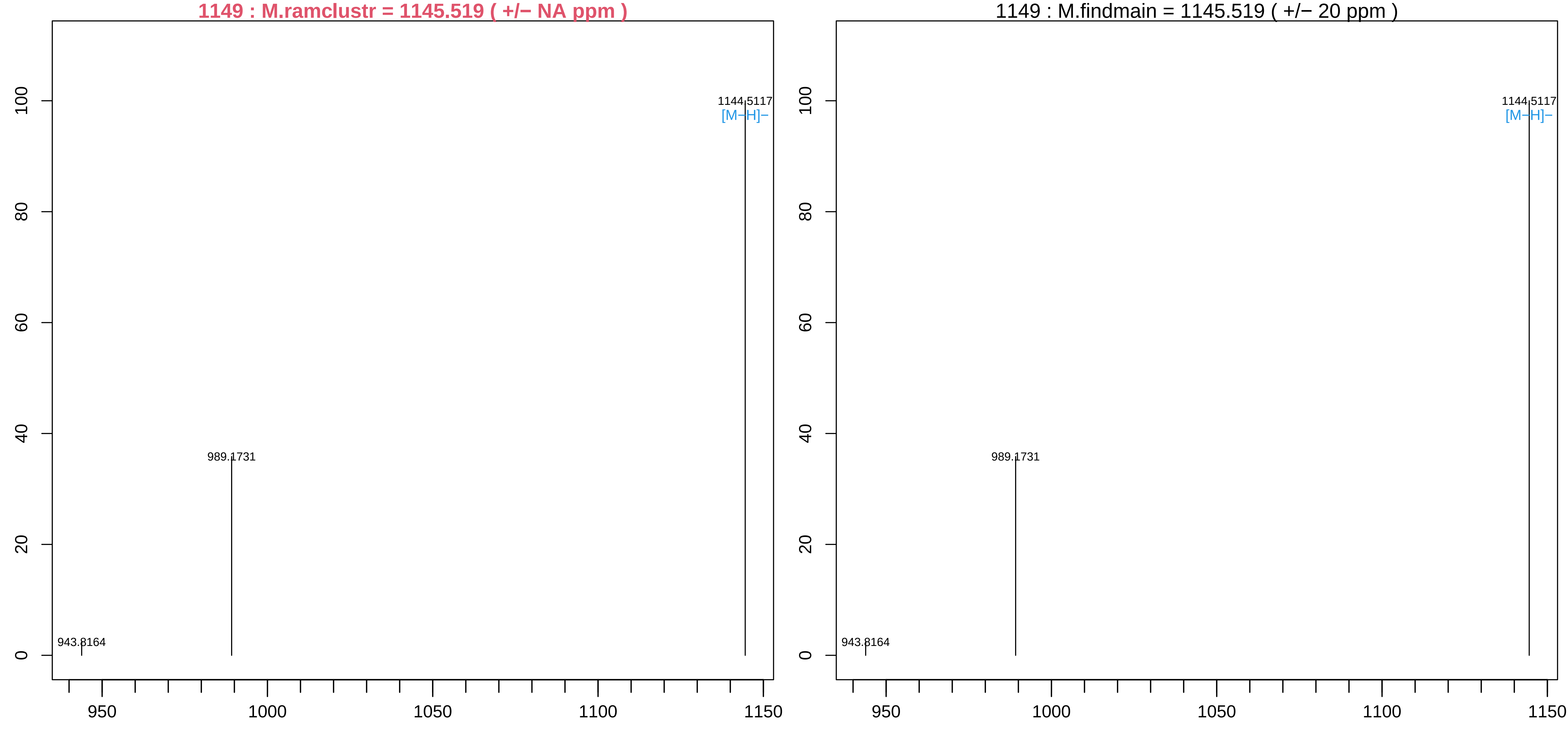
1149 : M.ramclustr = 1145.519 (+/− NA ppm)
1144.5117
[M−H]−
989.1731
943.8164
1149 : M.findmain = 1145.519 (+/− 20 ppm)
1144.5117
[M−H]−
989.1731
943.8164
0
20
40
60
80
100
950
1000
1050
1100
1150

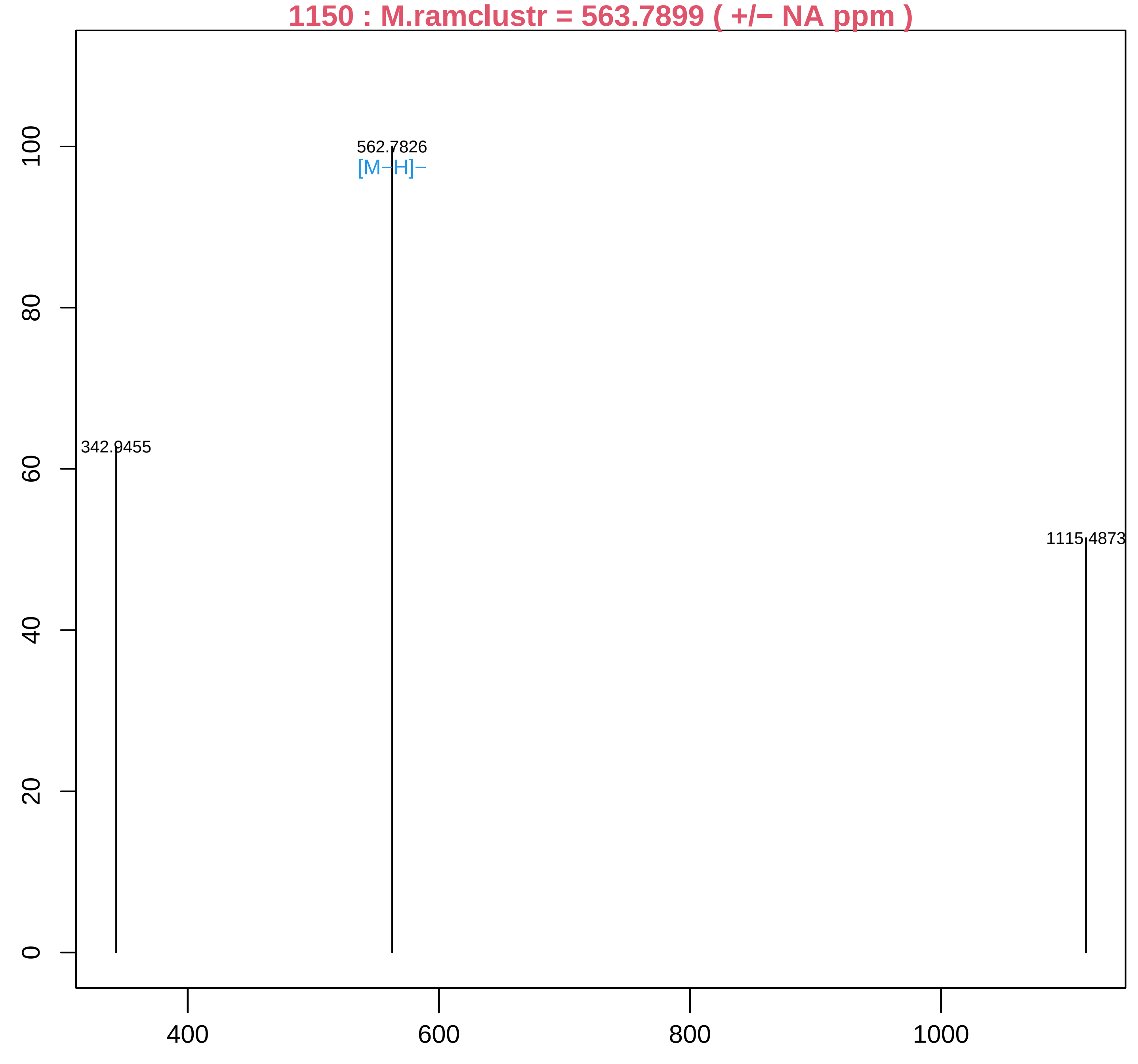
1150 : M.ramclustr = 563.7899 (+/− NA ppm)
562.7826
[M−H]−
342.9455
1115.4873
0
20
40
60
80
100
400
600
800
1000

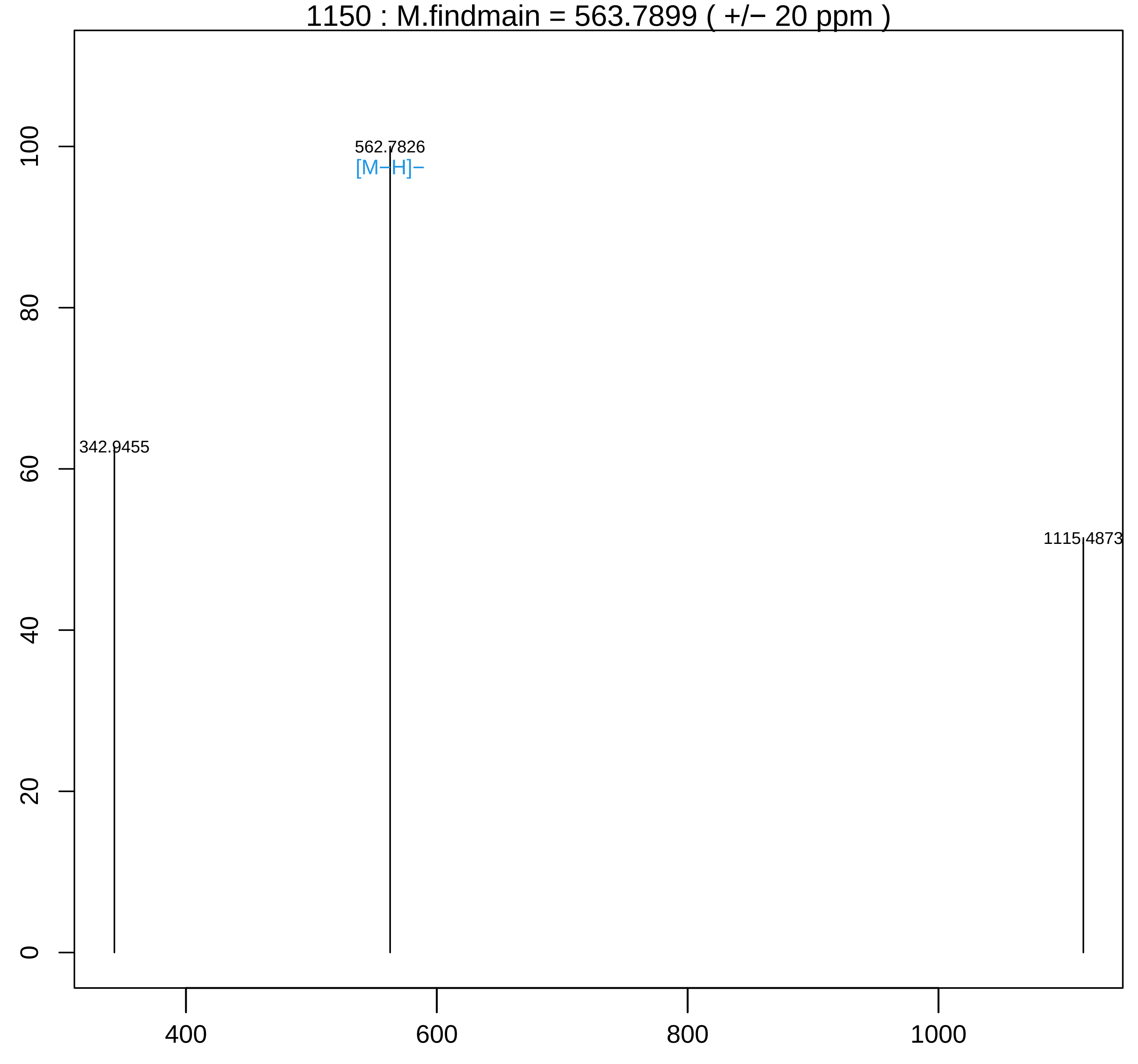
1150 : M.findmain = 563.7899 (+/− 20 ppm)
562.7826
[M−H]−
342.9455
1115.4873
0
20
40
60
80
100
400
600
800
1000

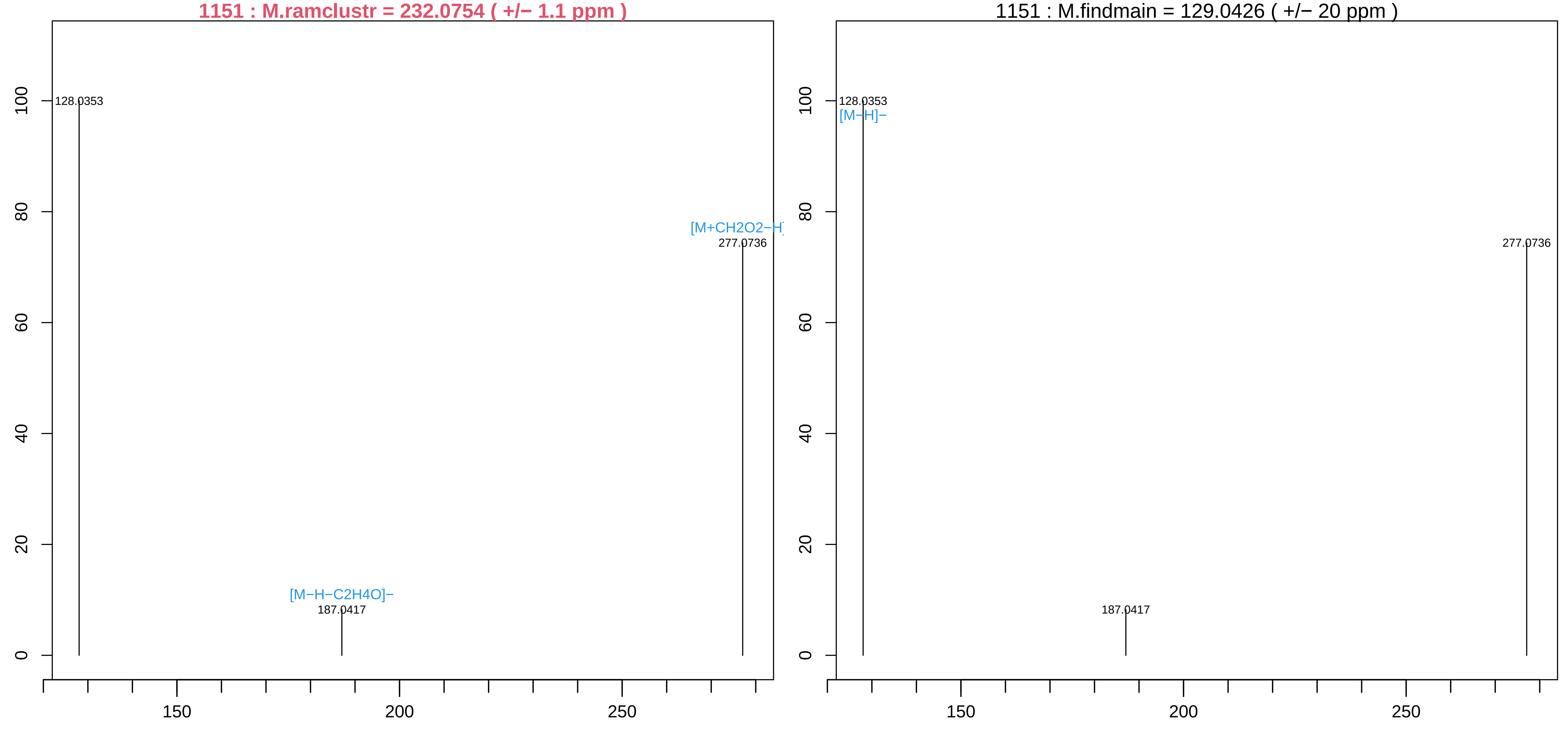
1151 : M.ramclustr = 232.0754 (+/− 1.1 ppm)
128.0353
[M−H−C2H4O]−
187.0417
[M+CH2O2−H]
277.0736
1151 : M.findmain = 129.0426 (+/− 20 ppm)
128.0353
[M−H]−
187.0417
277.0736
0
20
40
60
80
100
150
200
250

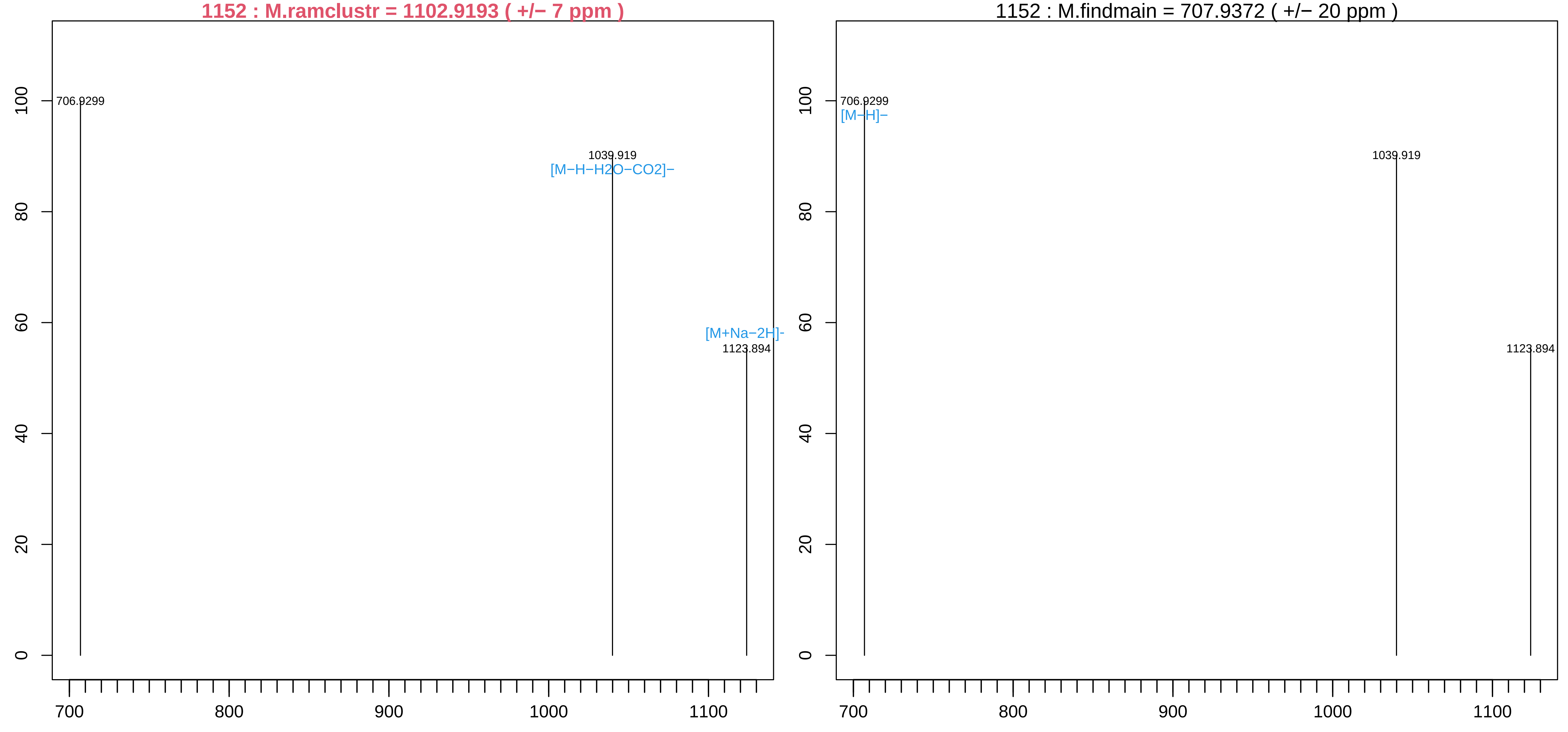
1152 : M.ramclustr = 1102.9193 (+/− 7 ppm)
706.9299
1039.919
[M−H−H2O−CO2]−
[M+Na−2H]-
1123.894
0
20
40
60
80
100
700
800
900
1000
1100
1152 : M.findmain = 707.9372 (+/− 20 ppm)
706.9299
[M−H]−
1039.919
1123.894
0
20
40
60
80
100
700
800
900
1000
1100

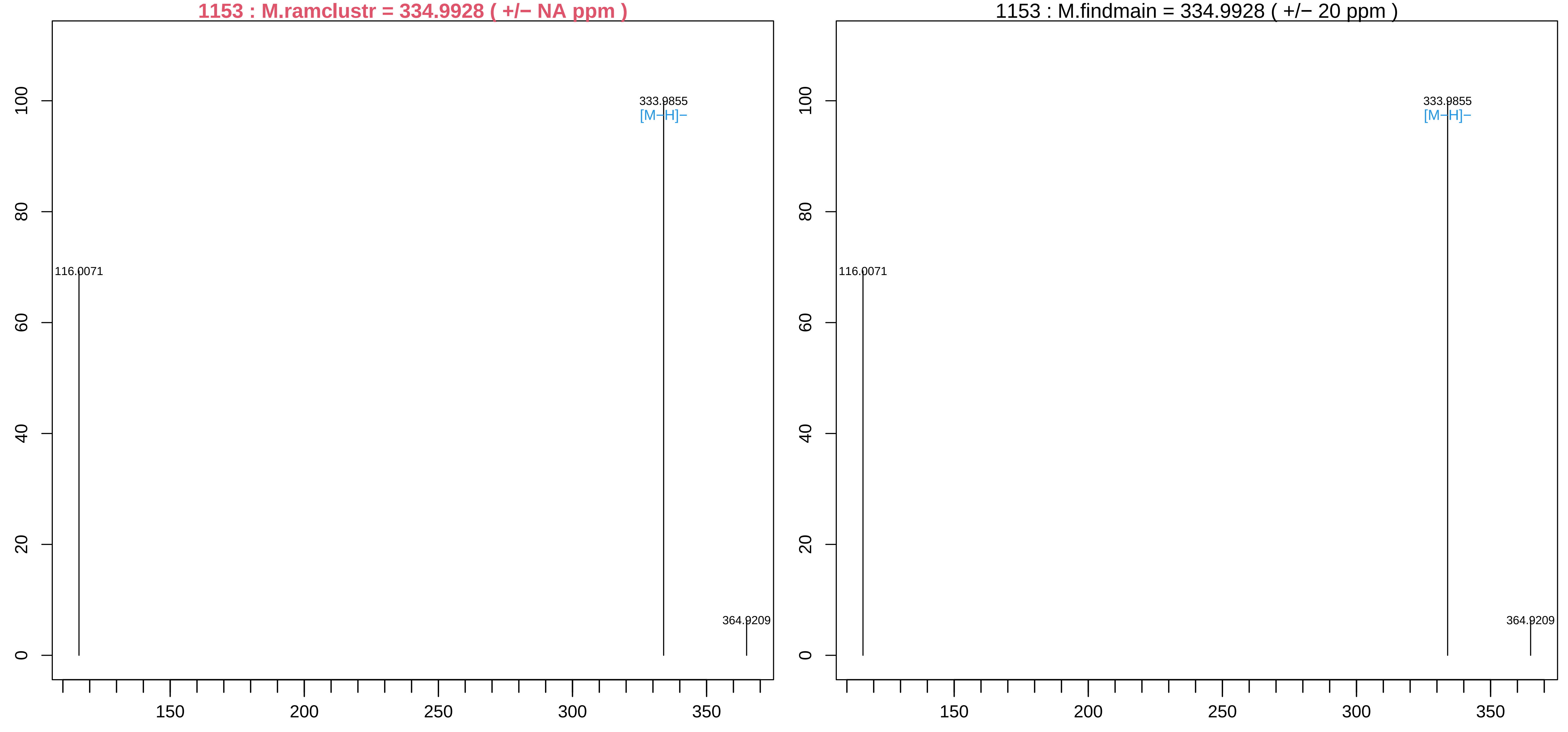

1153 : M.ramclustr = 334.9928 (+/− NA ppm)
1153 : M.findmain = 334.9928 (+/− 20 ppm)
116.0071
333.9855
[M−H]−
364.9209
116.0071
333.9855
[M−H]−
364.9209
0
20
40
60
80
100
150
200
250
300
350

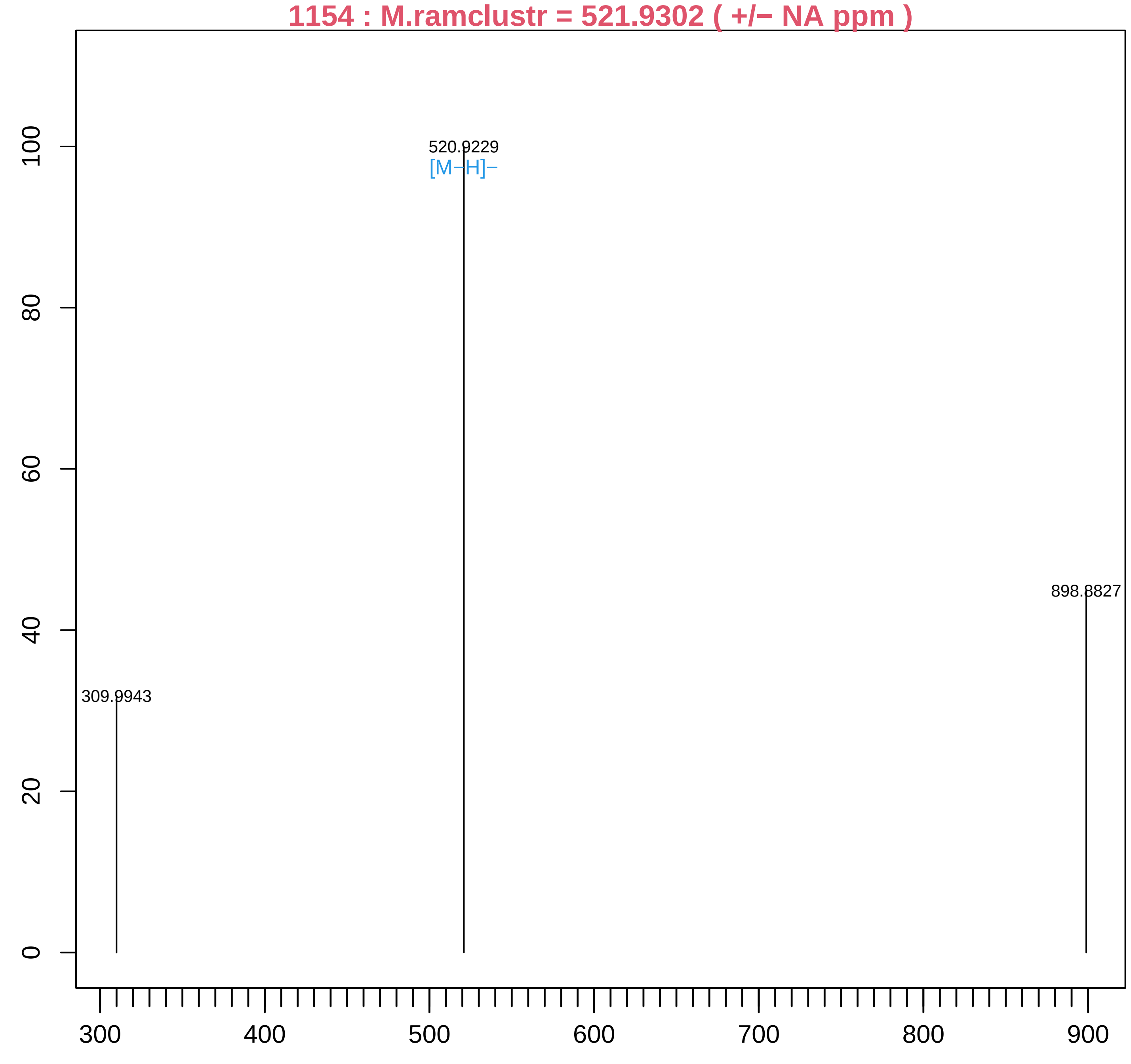
1154 : M.ramclustr = 521.9302 (+/− NA ppm)
520.9229
[M−H]−
898.8827
309.9943
0
20
40
60
80
100
300
400
500
600
700
800
900

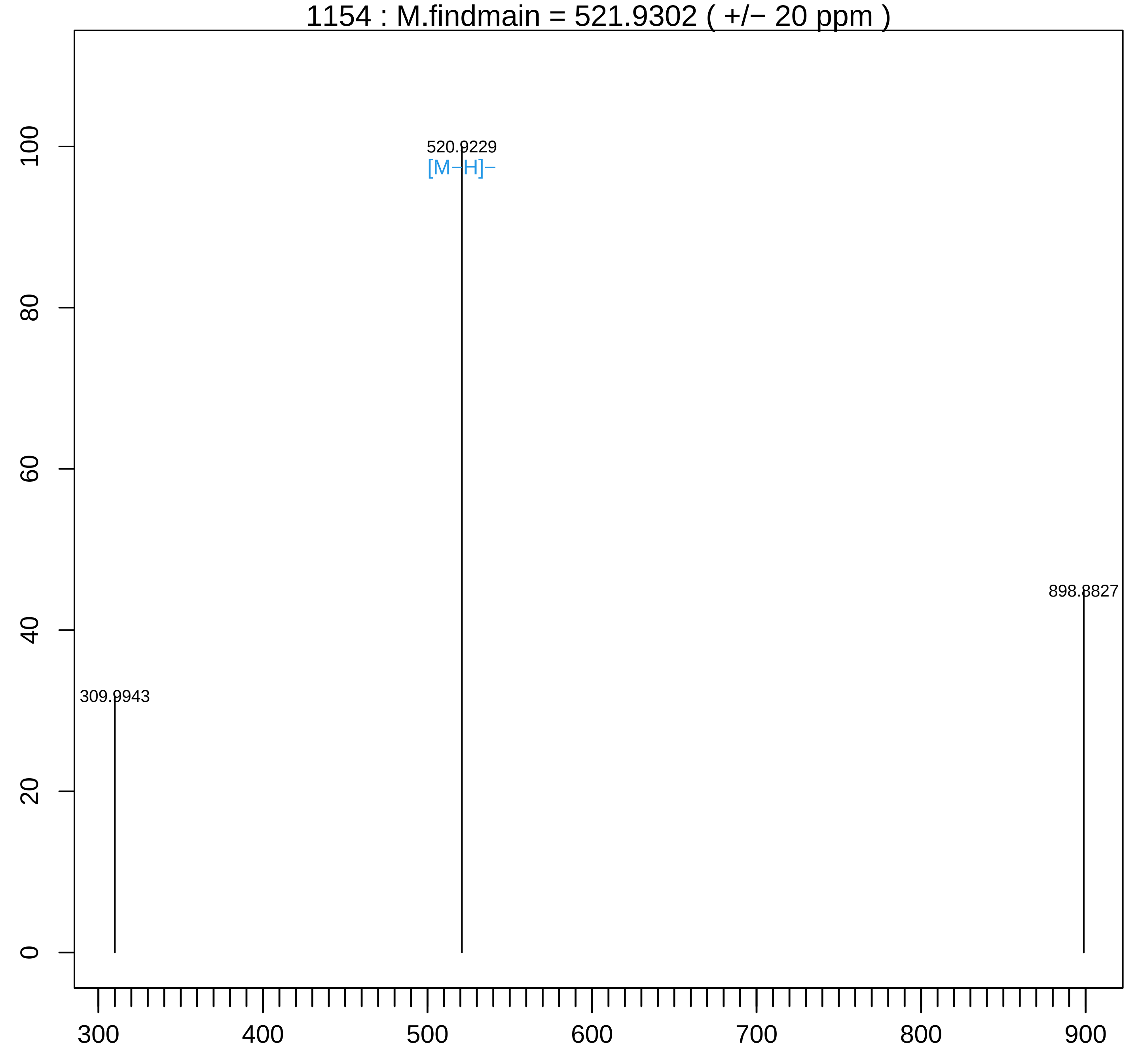
1154 : M.findmain = 521.9302 (+/− 20 ppm)
520.9229
[M−H]−
898.8827
309.9943
0
20
40
60
80
100
300
400
500
600
700
800
900

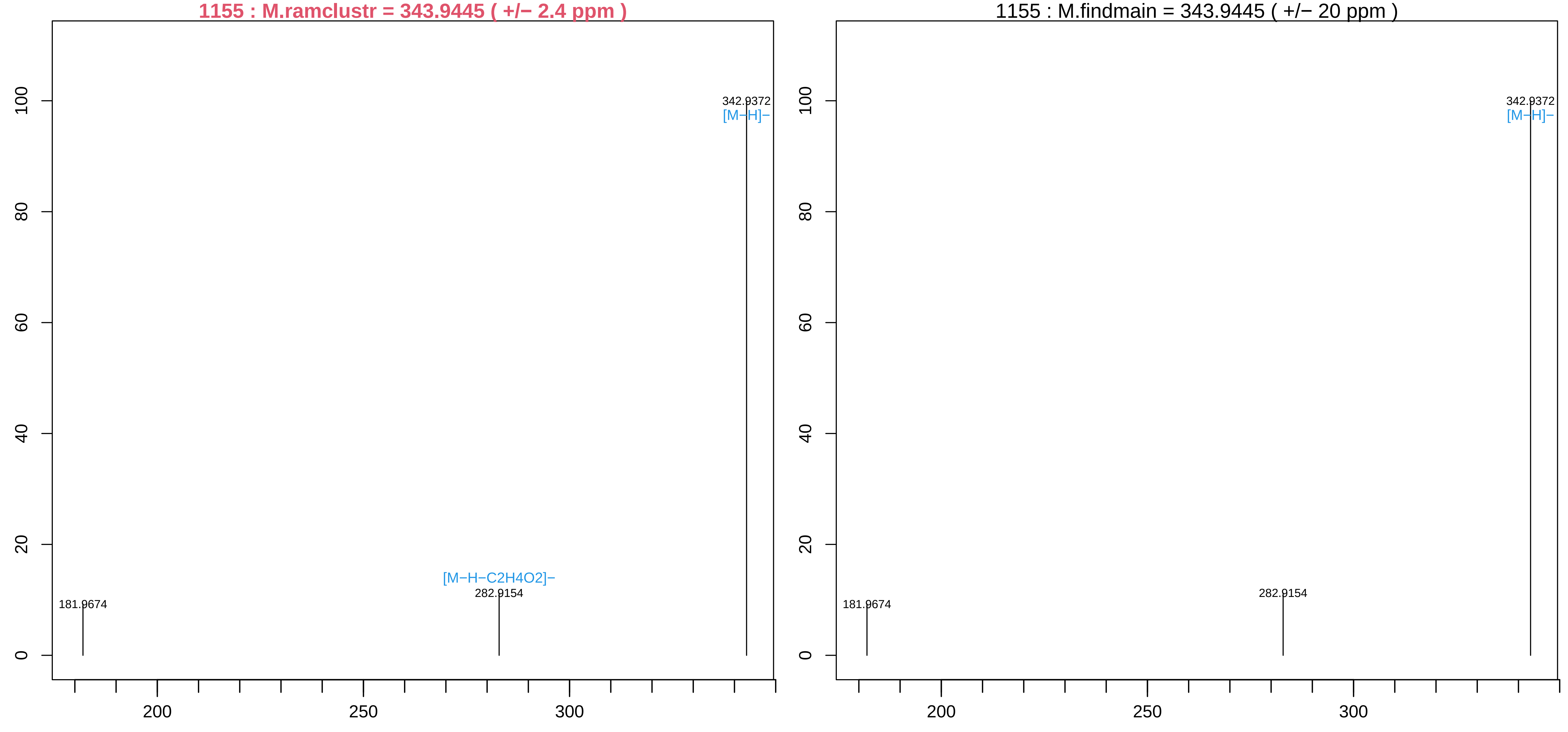

1155 : M.ramclustr = 343.9445 (+/− 2.4 ppm)
342.9372
[M−H]−
[M−H−C2H4O2]−
282.9154
181.9674
1155 : M.findmain = 343.9445 (+/− 20 ppm)
342.9372
[M−H]−
282.9154
181.9674
0
20
40
60
80
100
200
250
300

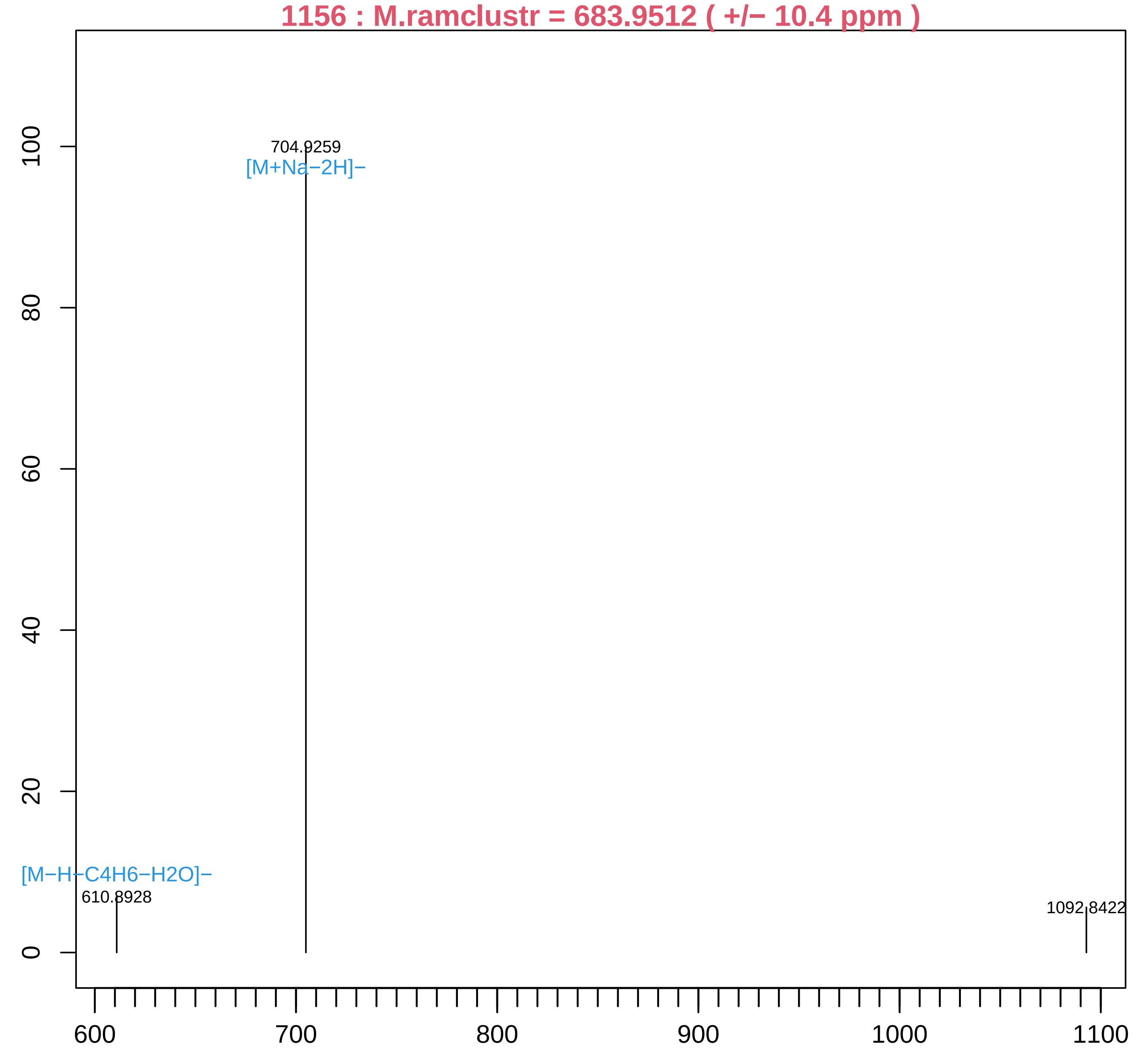
1156 : M.ramclustr = 683.9512 (+/− 10.4 ppm)
704.9259
[M+Na−2H]−
[M−H−C4H6−H2O]−
610.8928
1092.8422
0
20
40
60
80
100
600
700
800
900
1000
1100

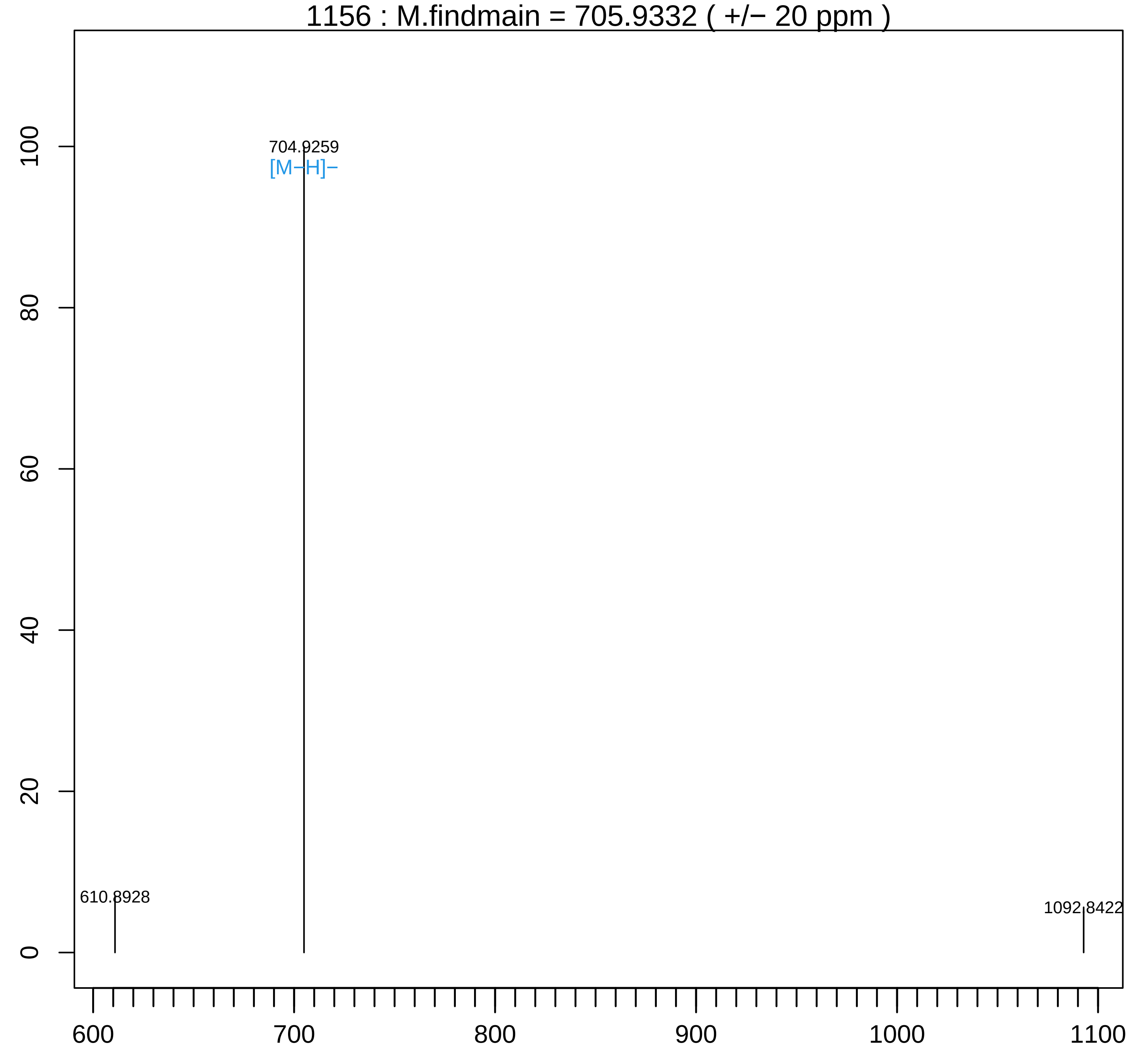
1156 : M.findmain = 705.9332 (+/− 20 ppm)
704.9259
[M−H]−
610.8928
1092.8422
0
20
40
60
80
100
600
700
800
900
1000
1100

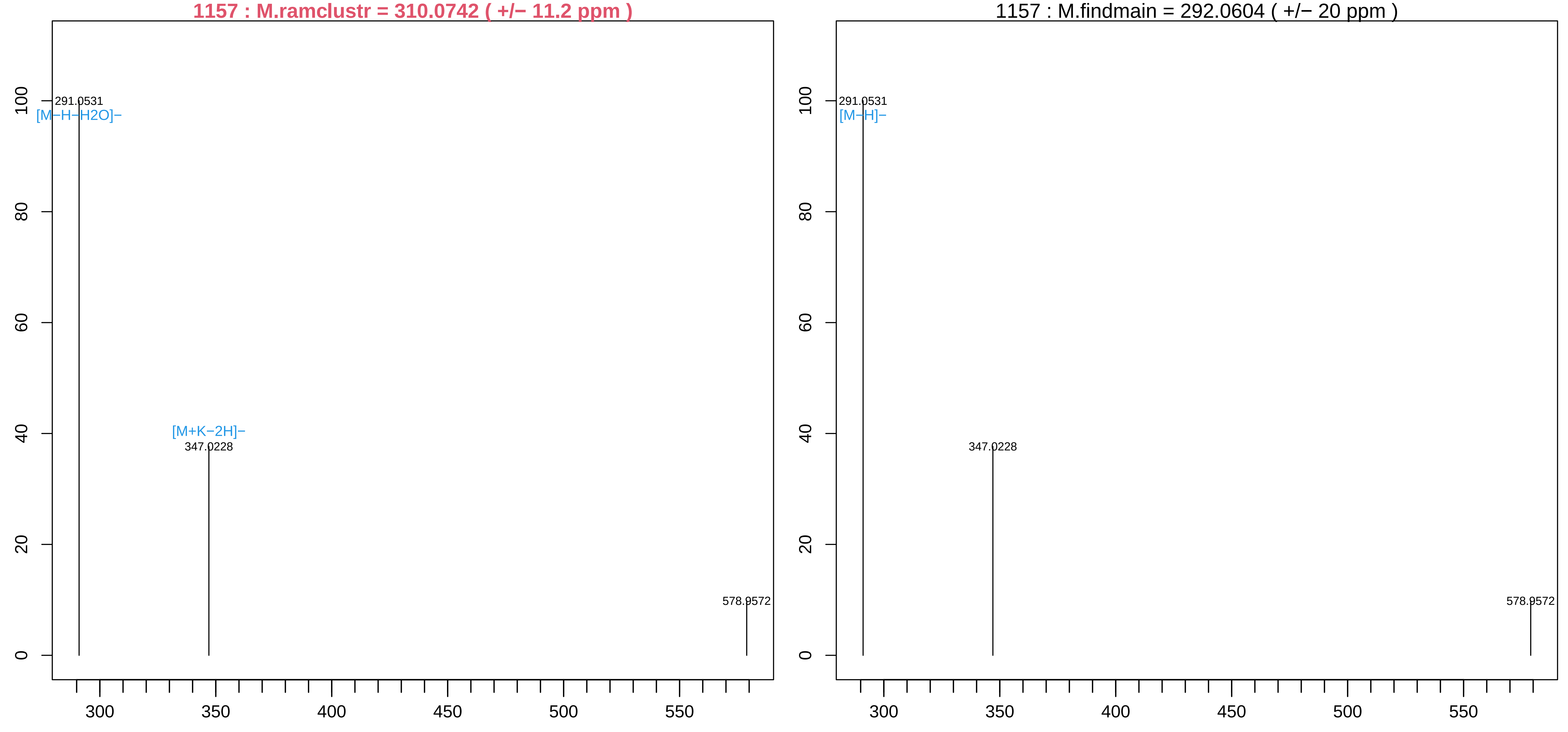
1157 : M.ramclustr = 310.0742 (+/− 11.2 ppm)
291.0531
[M−H−H2O]−
[M+K−2H]−
347.0228
578.9572
1157 : M.findmain = 292.0604 (+/− 20 ppm)
291.0531
[M−H]−
347.0228
578.9572
0
20
40
60
80
100
300
350
400
450
500
550

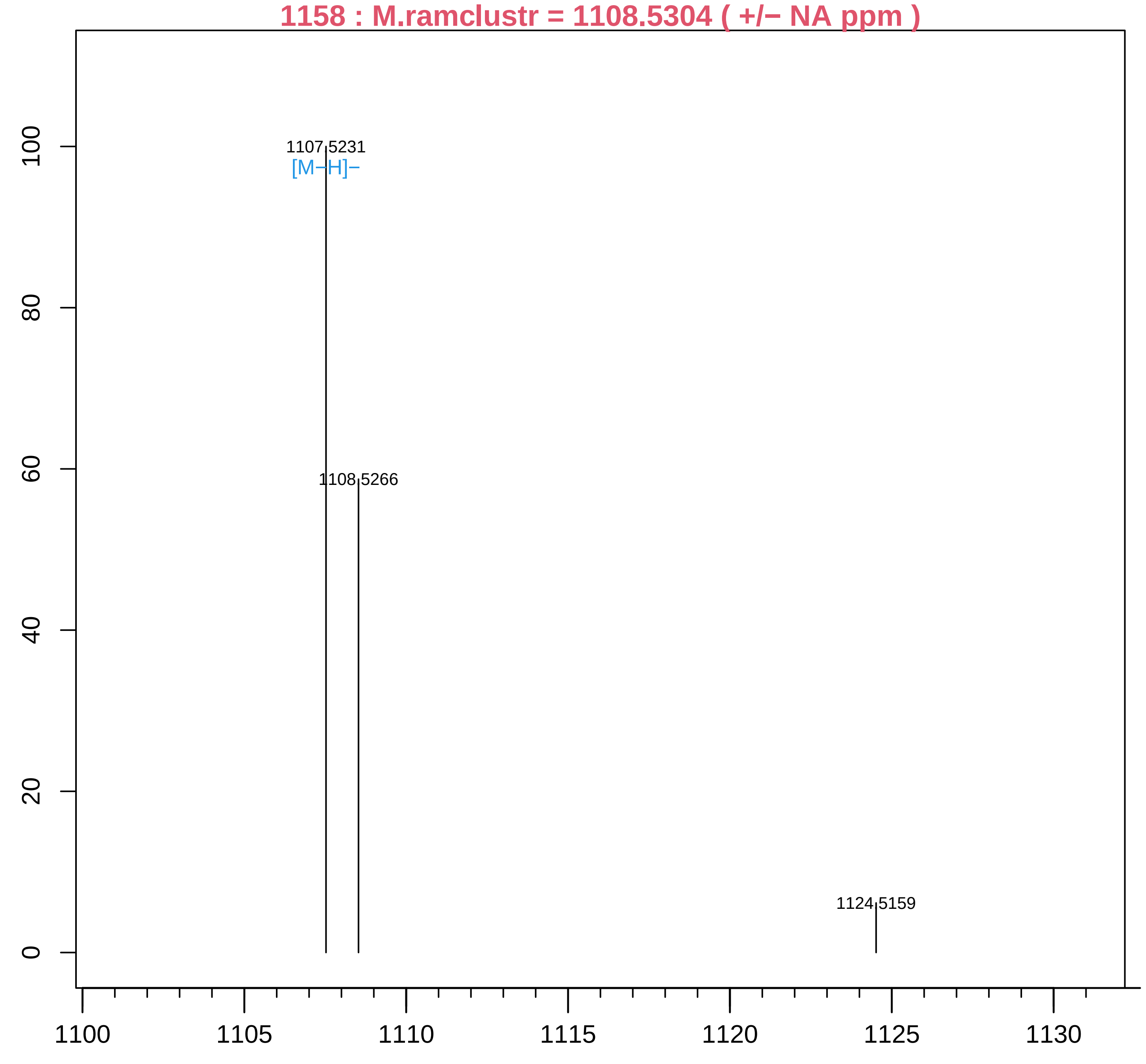

1158 : M.ramclustr = 1108.5304 (+/− NA ppm)
1107.5231
[M−H]−
1108.5266
1124.5159
0
20
40
60
80
100
1100
1105
1110
1115
1120
1125
1130

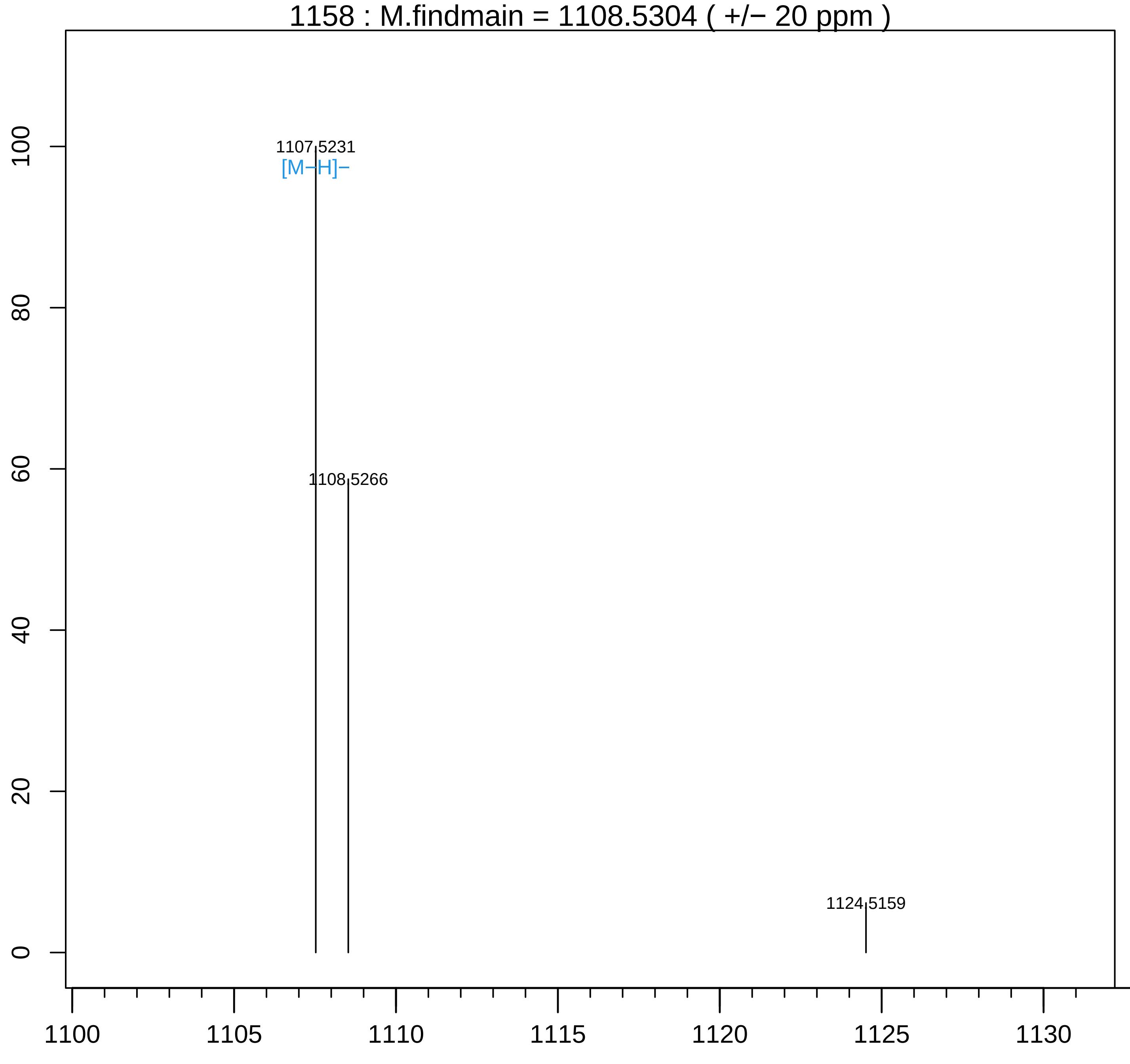

1158 : M.findmain = 1108.5304 (+/− 20 ppm)
1107.5231
[M−H]−
1108.5266
1124.5159
0
20
40
60
80
100
1100
1105
1110
1115
1120
1125
1130

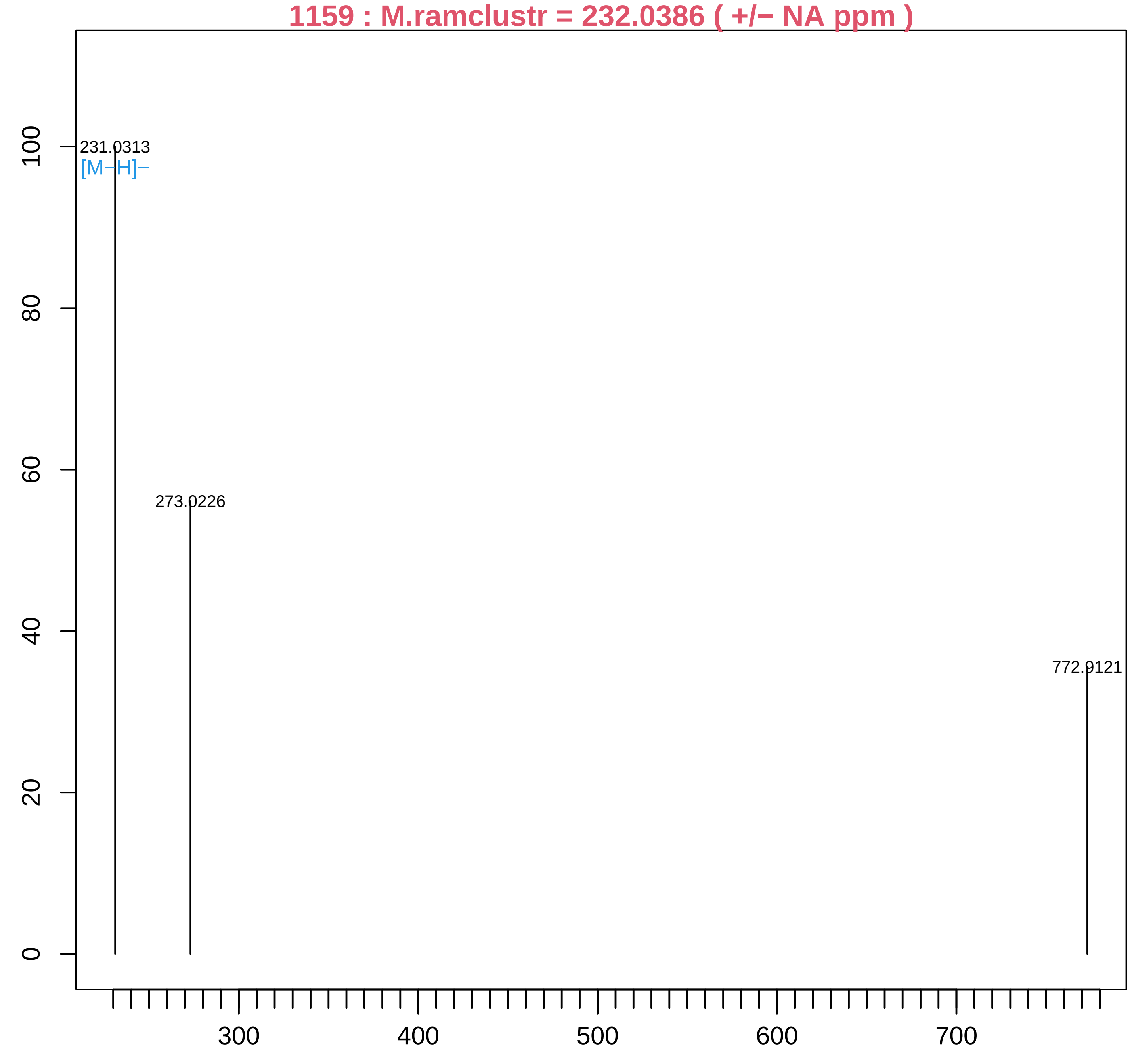

1159 : M.ramclustr = 232.0386 (+/− NA ppm)
231.0313
[M−H]−
273.0226
772.9121
0
20
40
60
80
100
300
400
500
600
700

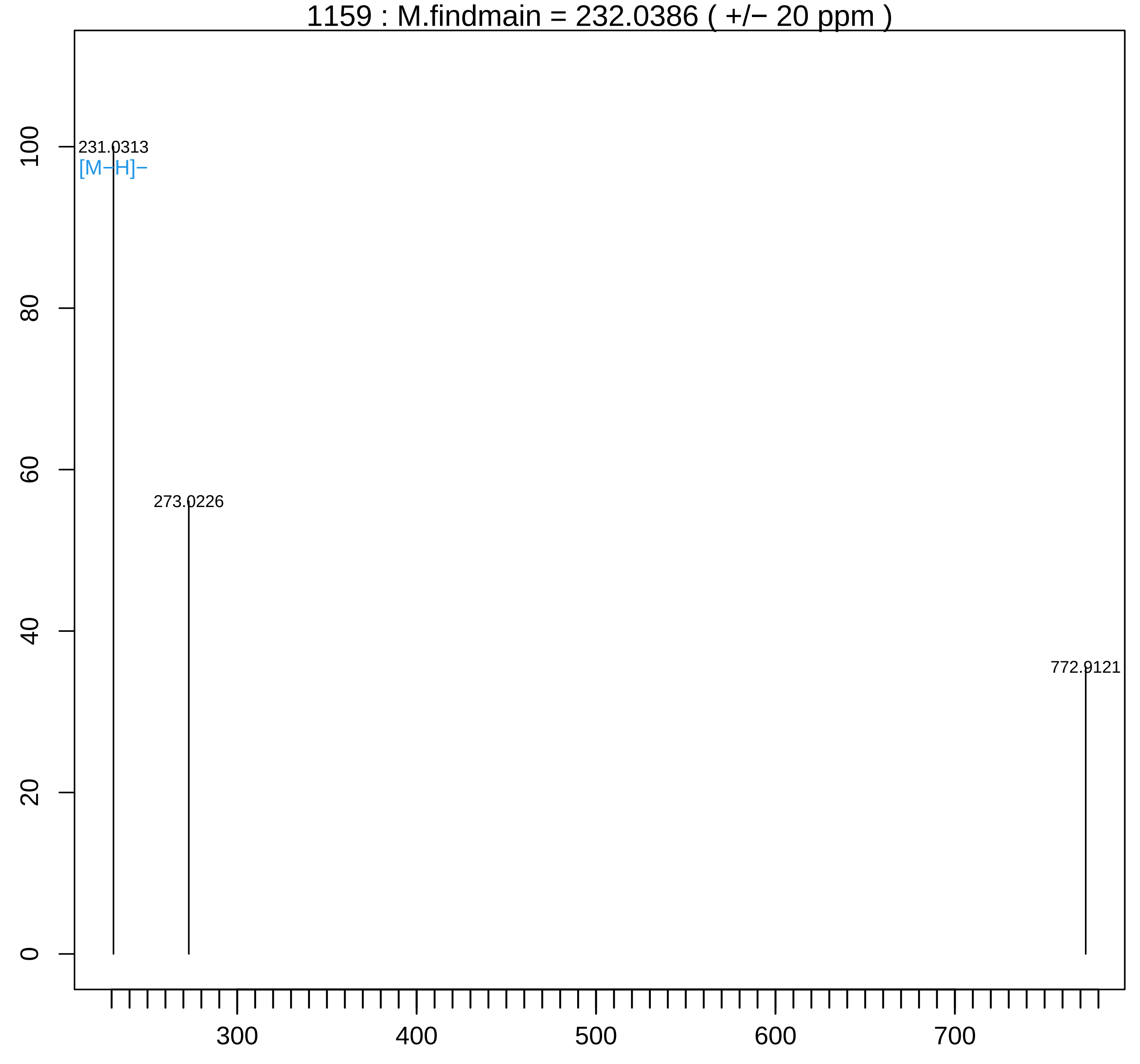

1159 : M.findmain = 232.0386 (+/− 20 ppm)
231.0313
[M−H]−
273.0226
772.9121
0
20
40
60
80
100
300
400
500
600
700

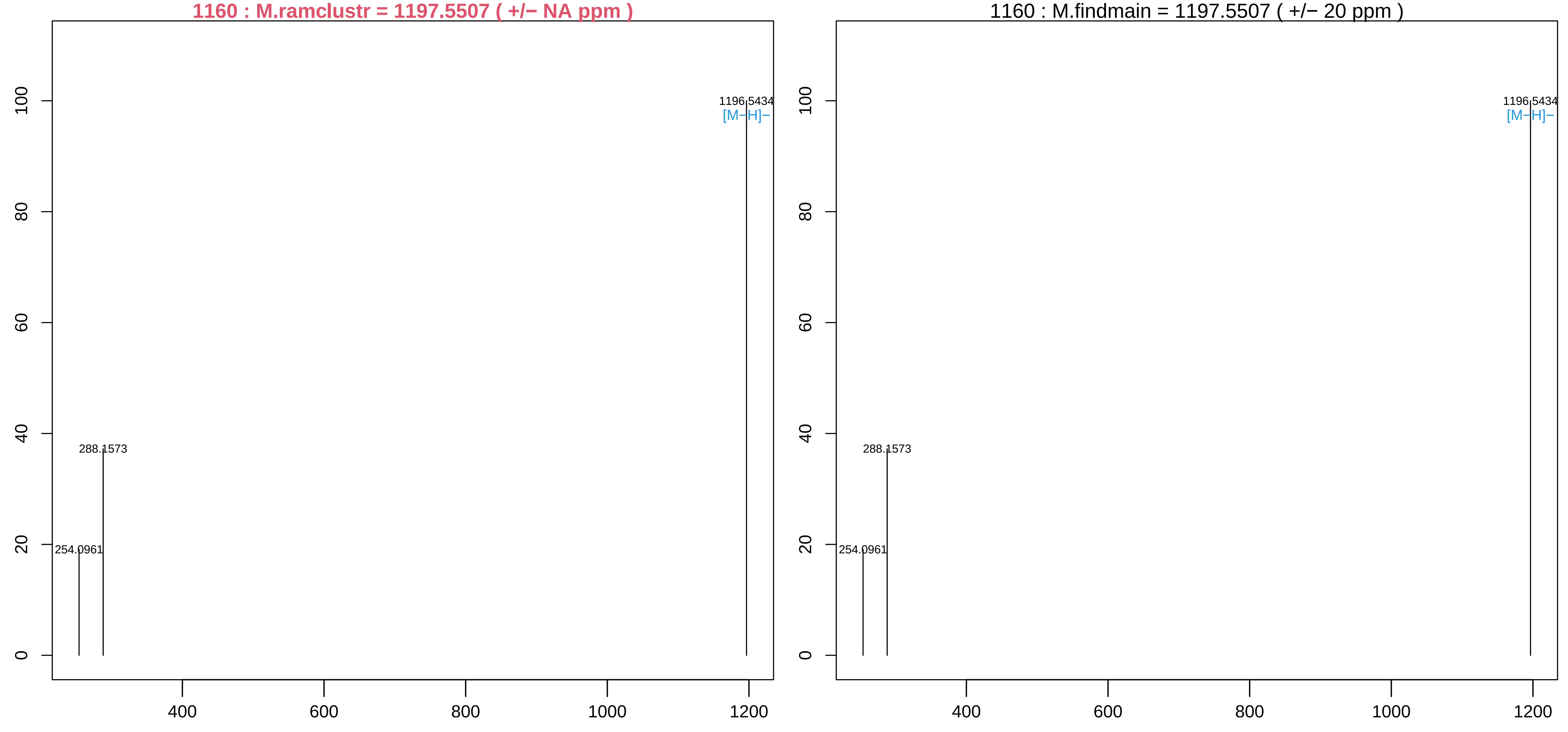
1160 : M.ramclustr = 1197.5507 (+/− NA ppm)
1160 : M.findmain = 1197.5507 (+/− 20 ppm)
1196.5434
[M−H]−
288.1573
254.0961
0
20
40
60
80
100
400
600
800
1000
1200

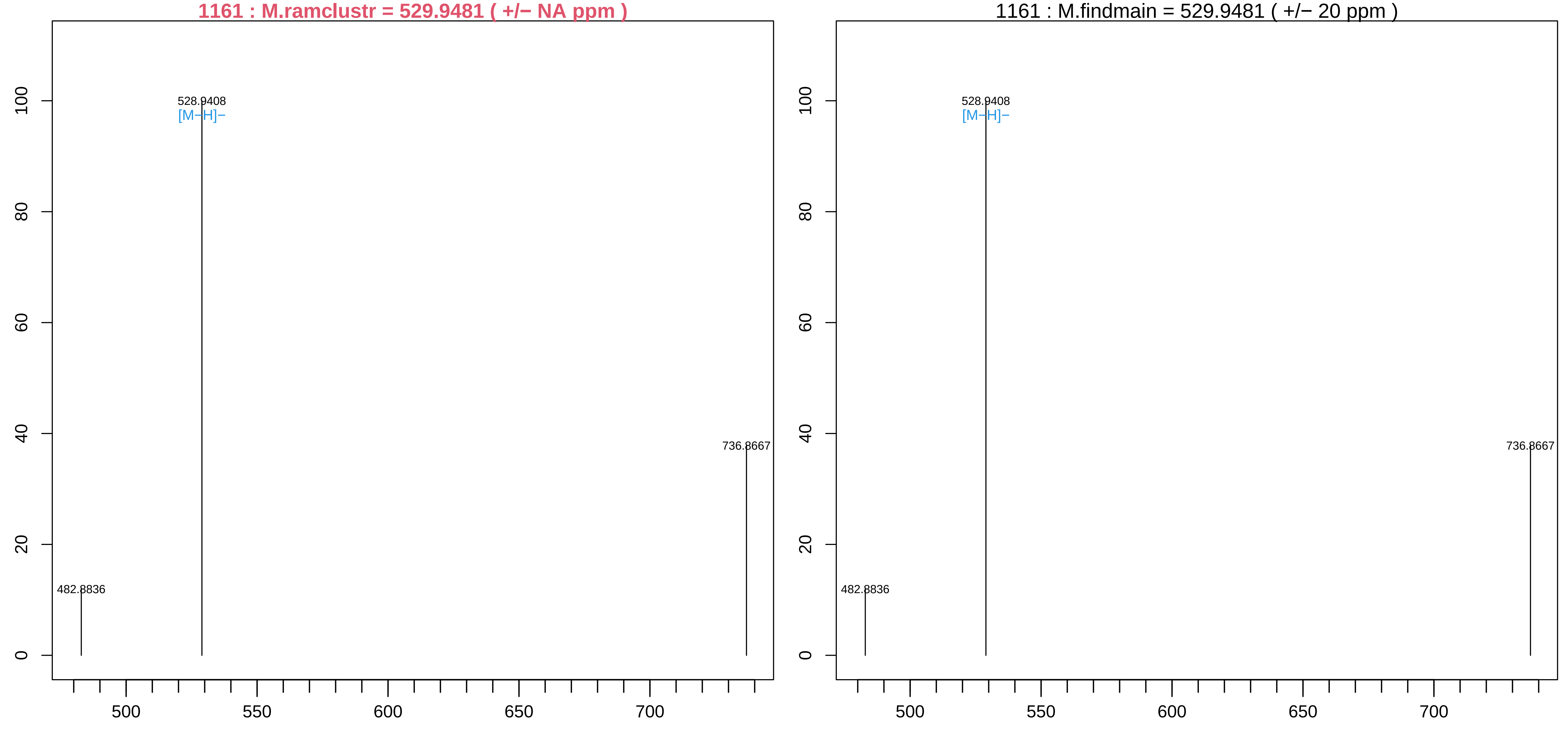
1161 : M.ramclustr = 529.9481 (+/− NA ppm)
528.9408
[M−H]−
736.8667
482.8836
1161 : M.findmain = 529.9481 (+/− 20 ppm)
528.9408
[M−H]−
736.8667
482.8836
0
20
40
60
80
100
500
550
600
650
700

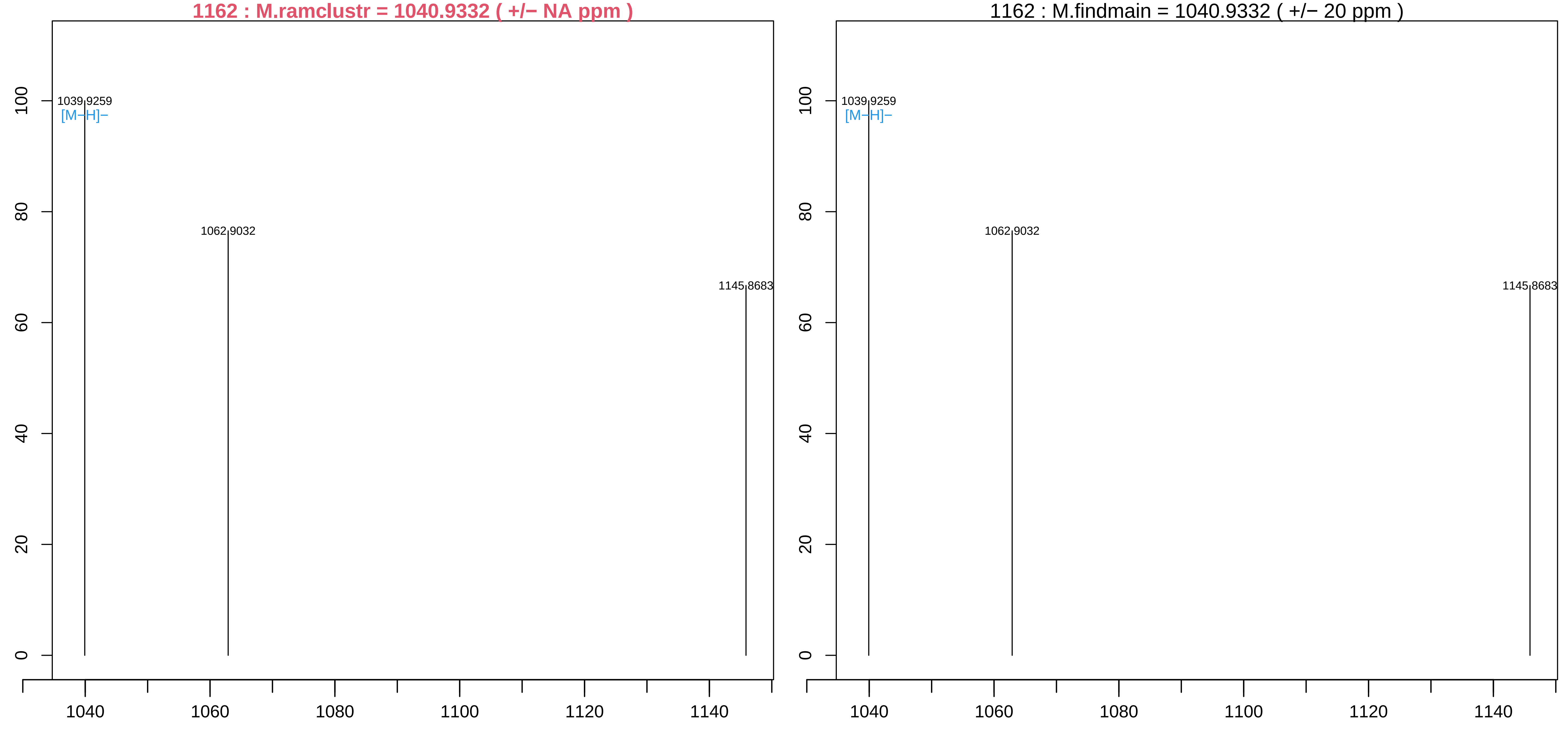

1162 : M.ramclustr = 1040.9332 (+/− NA ppm)
1039.9259
[M−H]−
1062.9032
1145.8683
0
20
40
60
80
100
1040
1060
1080
1100
1120
1140
1162 : M.findmain = 1040.9332 (+/− 20 ppm)
1039.9259
[M−H]−
1062.9032
1145.8683
0
20
40
60
80
100
1040
1060
1080
1100
1120
1140

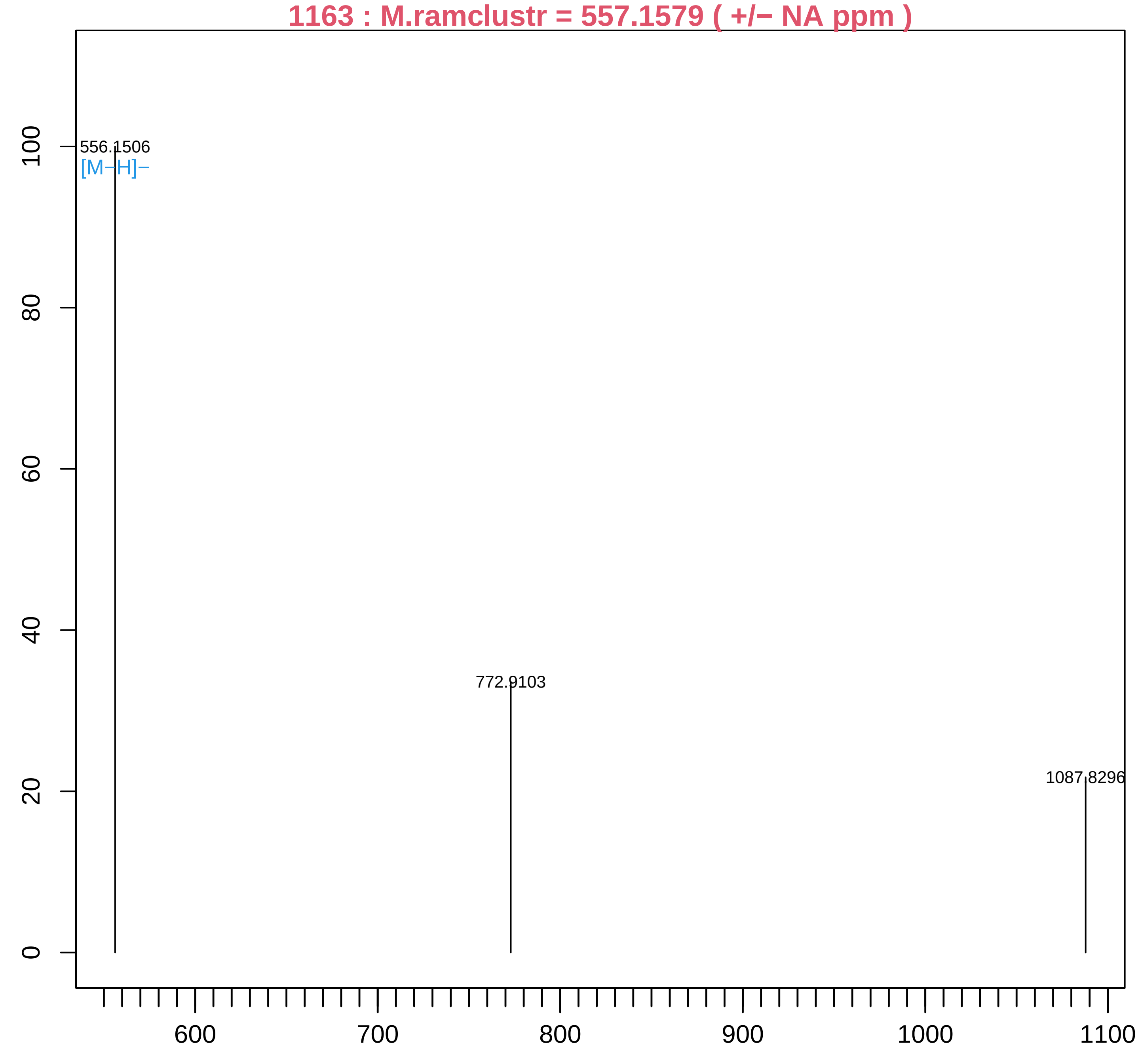
1163 : M.ramclustr = 557.1579 (+/− NA ppm)
556.1506
[M−H]−
772.9103
1087.8296
0
20
40
60
80
100
600
700
800
900
1000
1100
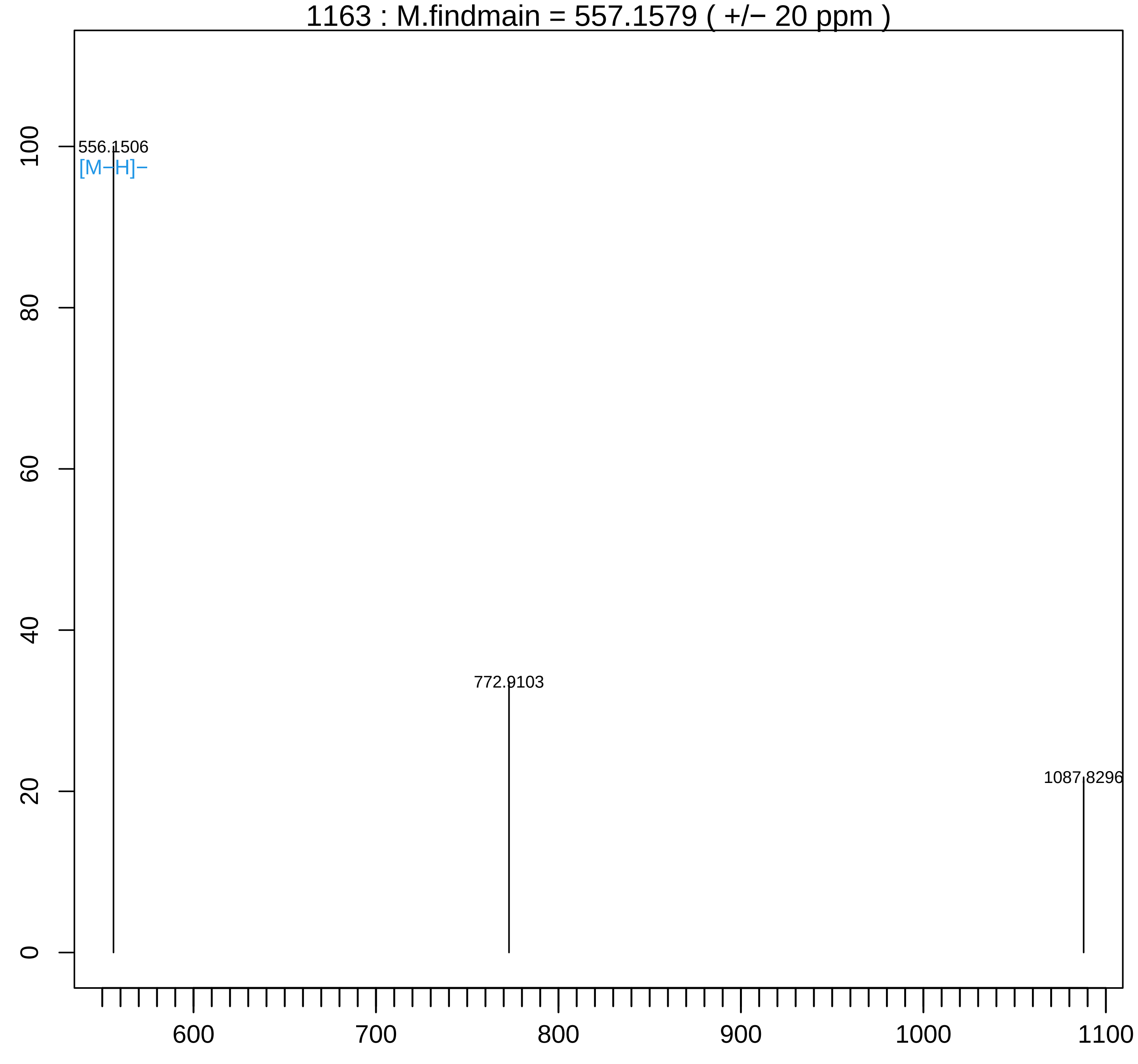
1163 : M.findmain = 557.1579 (+/− 20 ppm)
556.1506
[M−H]−
772.9103
1087.8296
0
20
40
60
80
100
600
700
800
900
1000
1100

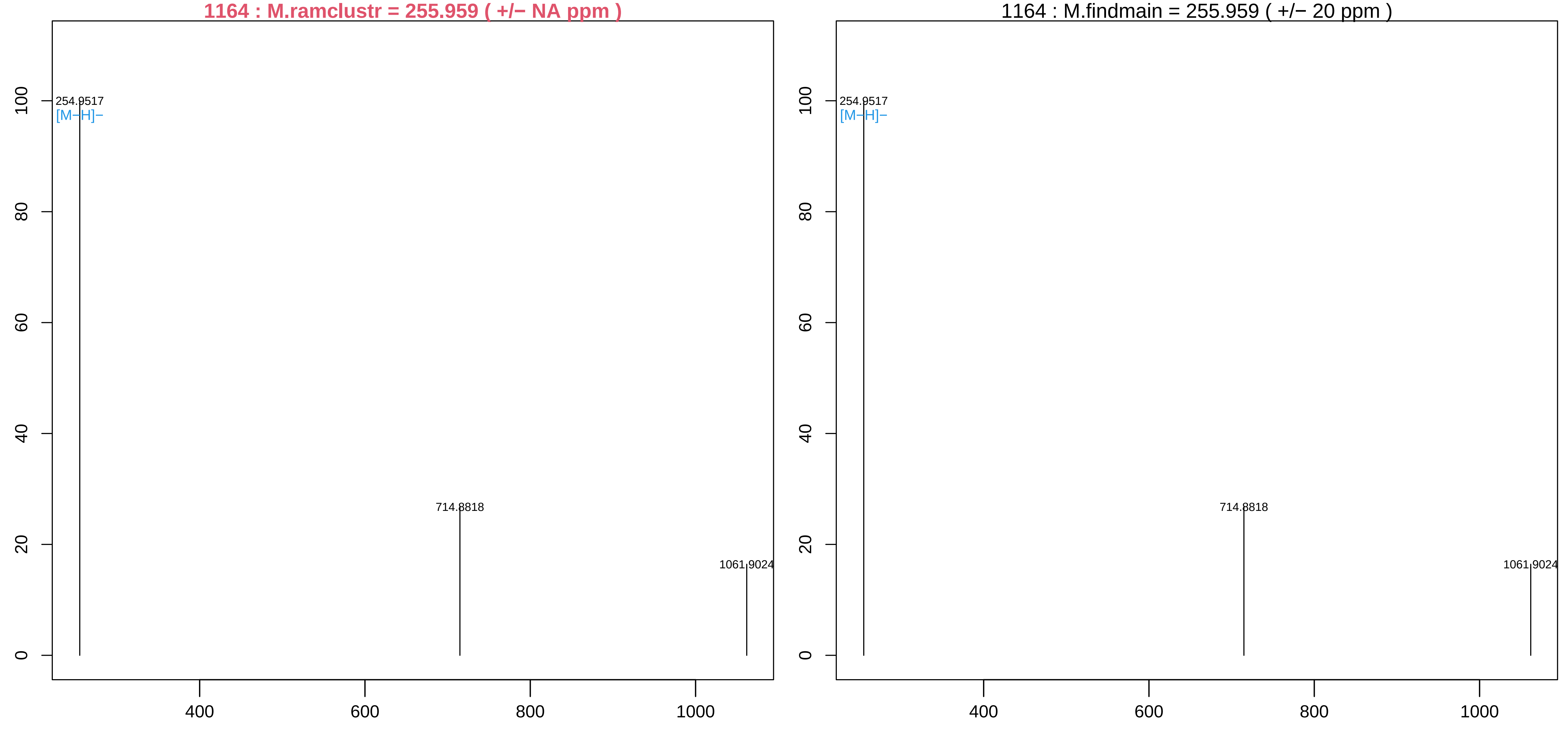
1164 : M.ramclustr = 255.959 (+/− NA ppm)
254.9517
[M−H]−
714.8818
1061.9024
1164 : M.findmain = 255.959 (+/− 20 ppm)
254.9517
[M−H]−
714.8818
1061.9024

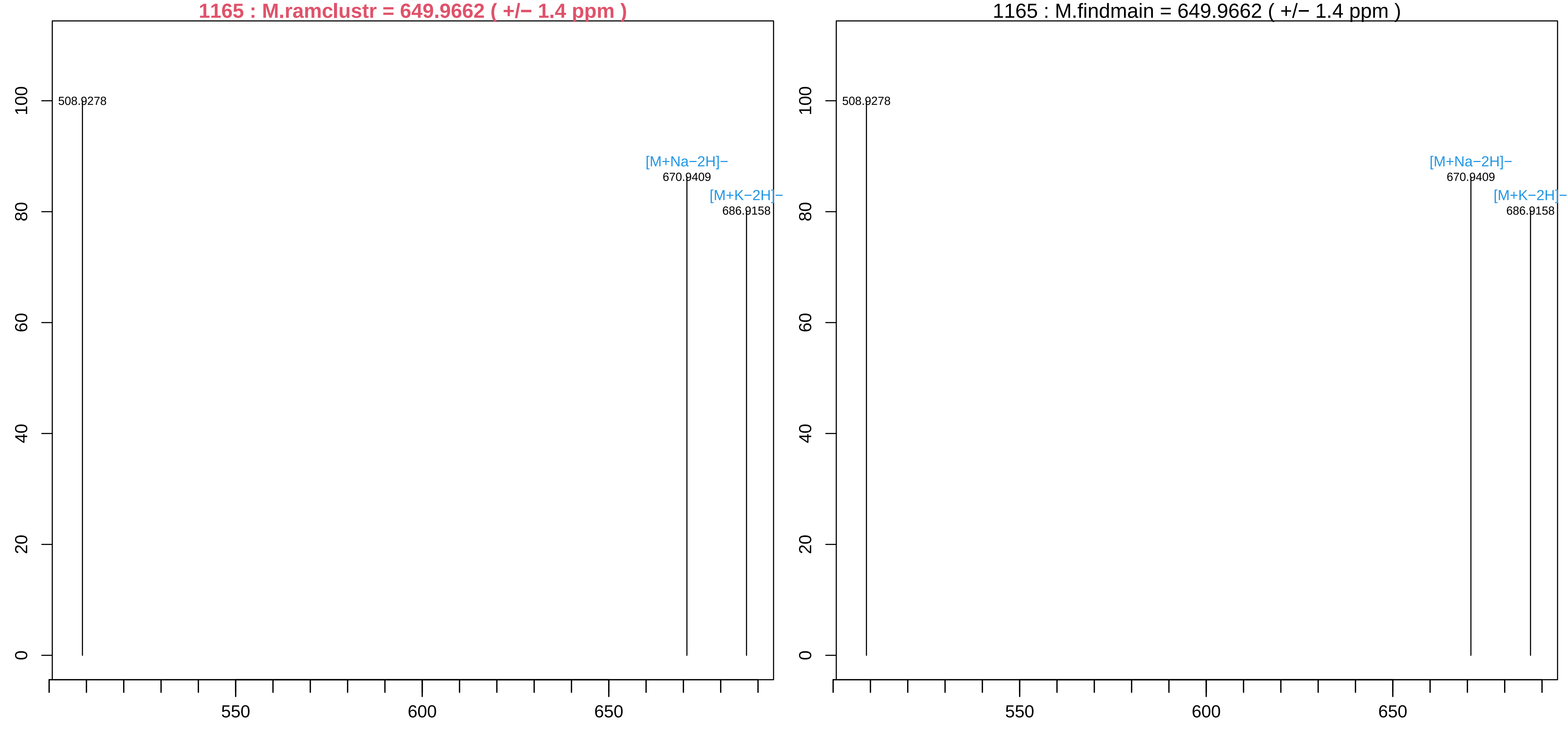
1165 : M.ramclustr = 649.9662 (+/− 1.4 ppm)
508.9278
[M+Na−2H]−
670.9409
[M+K−2H]−
686.9158
0
20
40
60
80
100
550
600
650
1165 : M.findmain = 649.9662 (+/− 1.4 ppm)
508.9278
[M+Na−2H]−
670.9409
[M+K−2H]−
686.9158
0
20
40
60
80
100
550
600
650

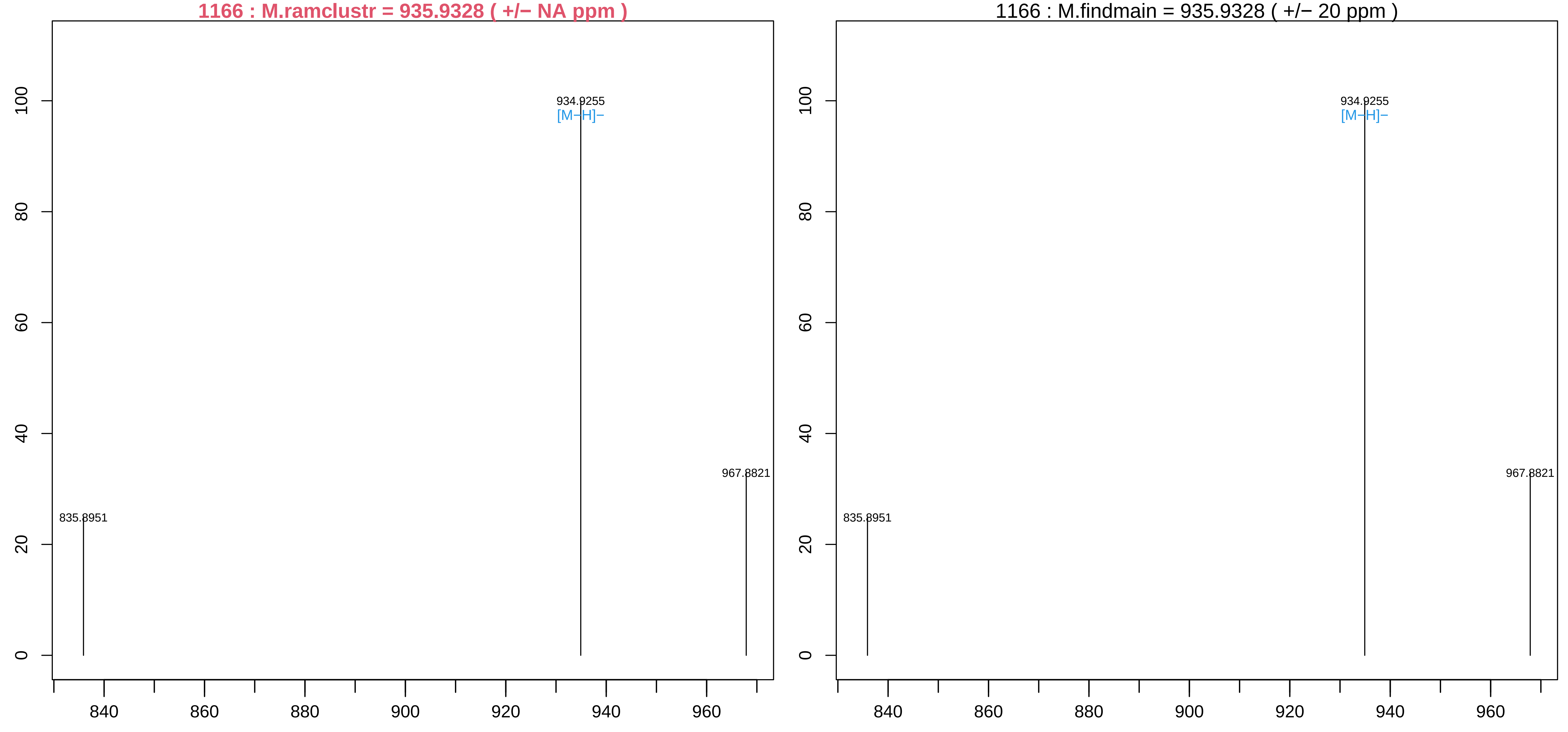
1166 : M.ramclustr = 935.9328 (+/− NA ppm)
934.9255
[M−H]−
967.8821
835.8951
1166 : M.findmain = 935.9328 (+/− 20 ppm)
934.9255
[M−H]−
967.8821
835.8951
0
20
40
60
80
100
840
860
880
900
920
940
960

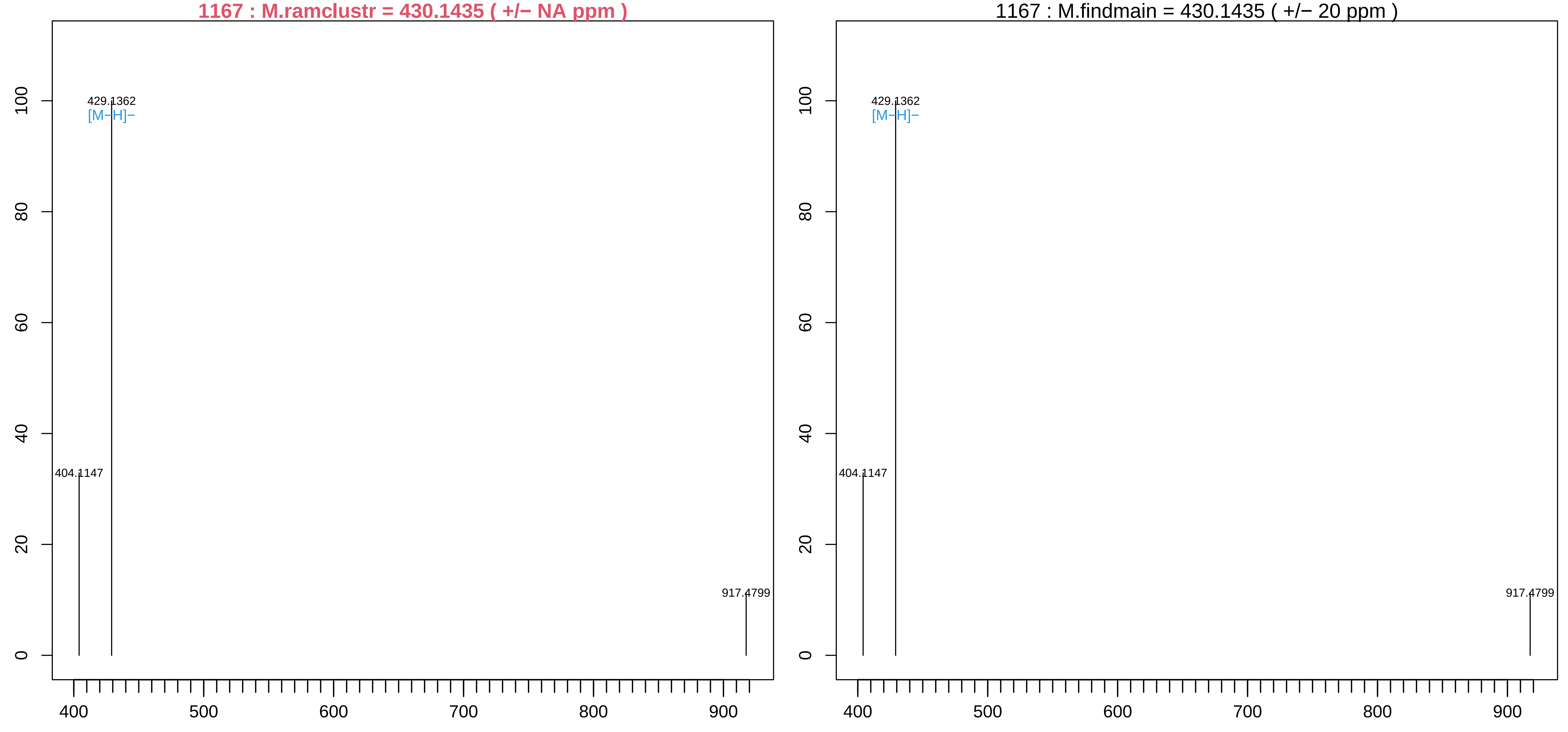

1167 : M.ramclustr = 430.1435 (+/− NA ppm)
429.1362
[M−H]−
404.1147
917.4799
1167 : M.findmain = 430.1435 (+/− 20 ppm)
429.1362
[M−H]−
404.1147
917.4799

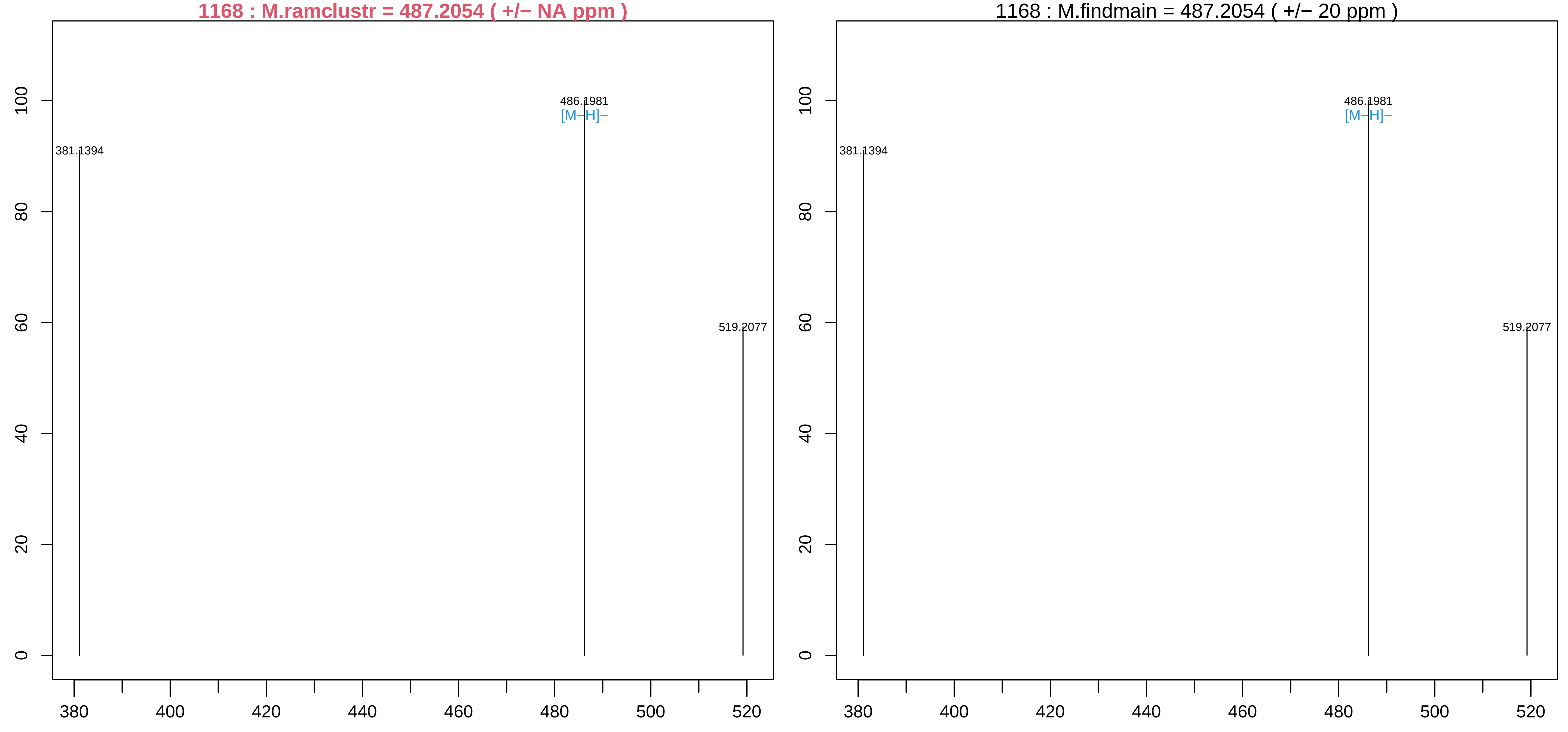
1168 : M.ramclustr = 487.2054 (+/− NA ppm)
1168 : M.findmain = 487.2054 (+/− 20 ppm)
381.1394
486.1981
[M−H]−
519.2077
0
20
40
60
80
100
380
400
420
440
460
480
500
520

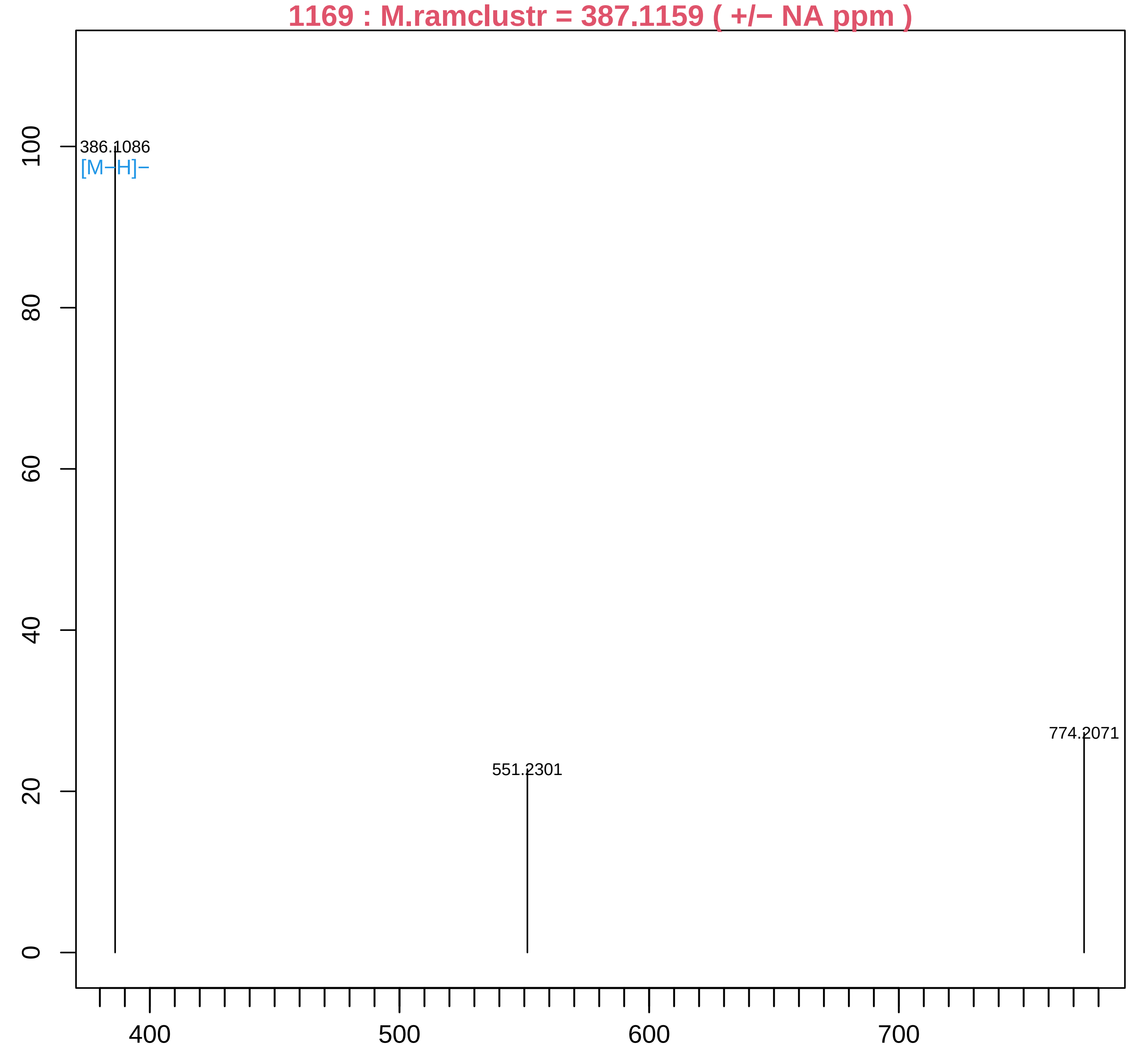
1169 : M.ramclustr = 387.1159 (+/− NA ppm)
386.1086
[M−H]−
551.2301
774.2071
0
20
40
60
80
100
400
500
600
700

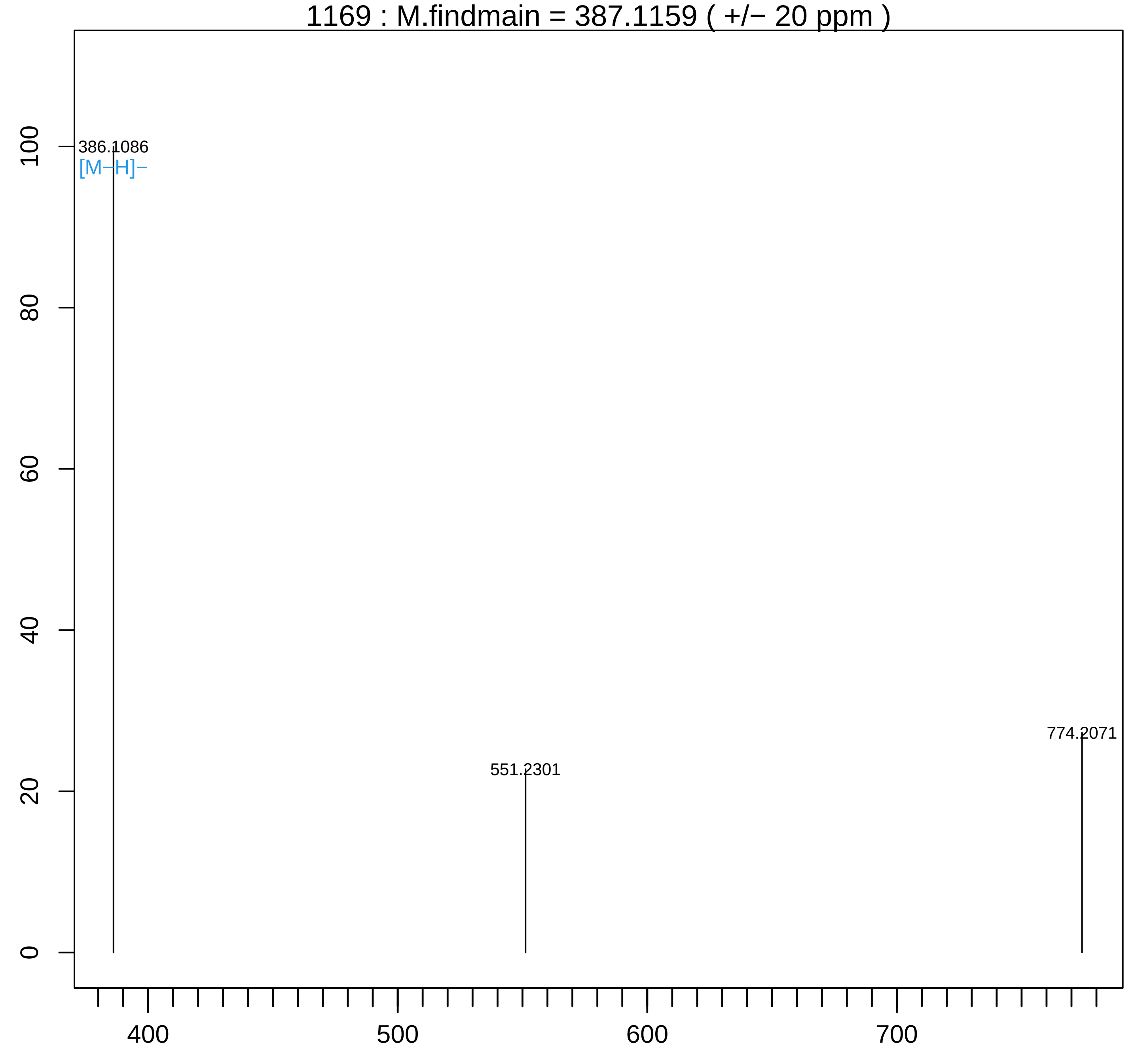
1169 : M.findmain = 387.1159 (+/− 20 ppm)
386.1086
[M−H]−
551.2301
774.2071
0
20
40
60
80
100
400
500
600
700

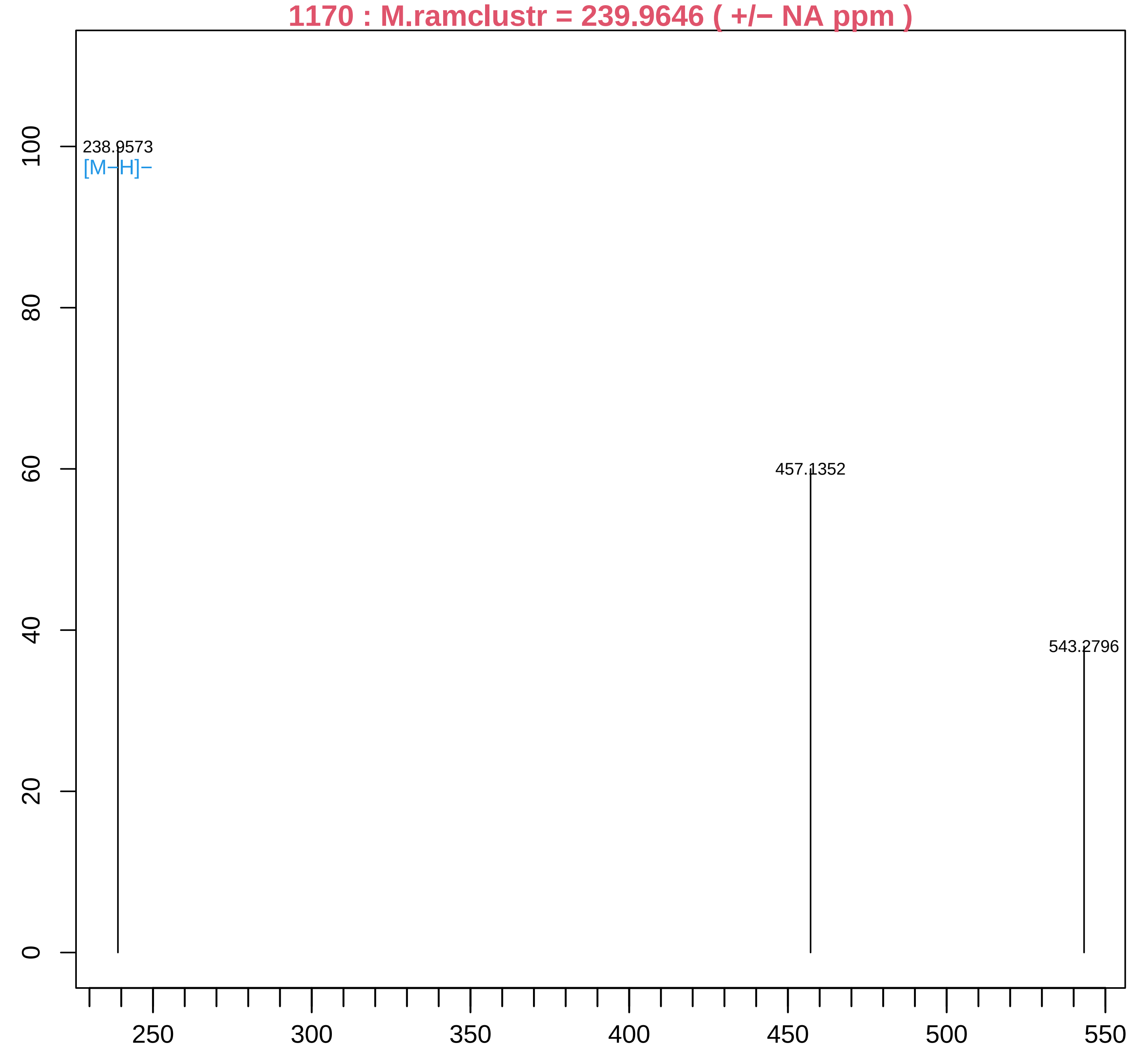
1170 : M.ramclustr = 239.9646 (+/− NA ppm)
238.9573
[M−H]−
457.1352
543.2796
0
20
40
60
80
100
250
300
350
400
450
500
550

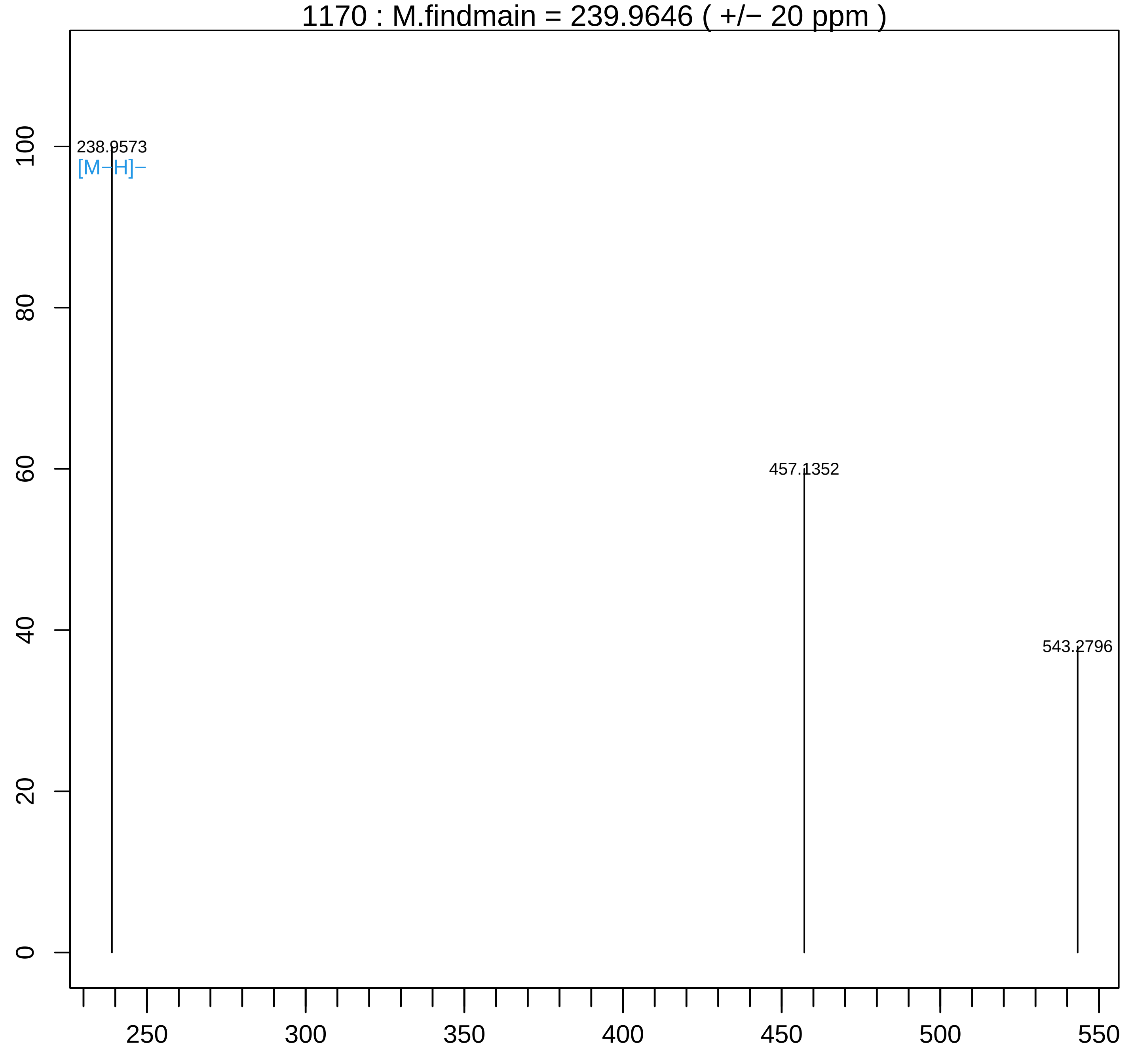
1170 : M.findmain = 239.9646 (+/− 20 ppm)
238.9573
[M−H]−
457.1352
543.2796
0
20
40
60
80
100
250
300
350
400
450
500
550

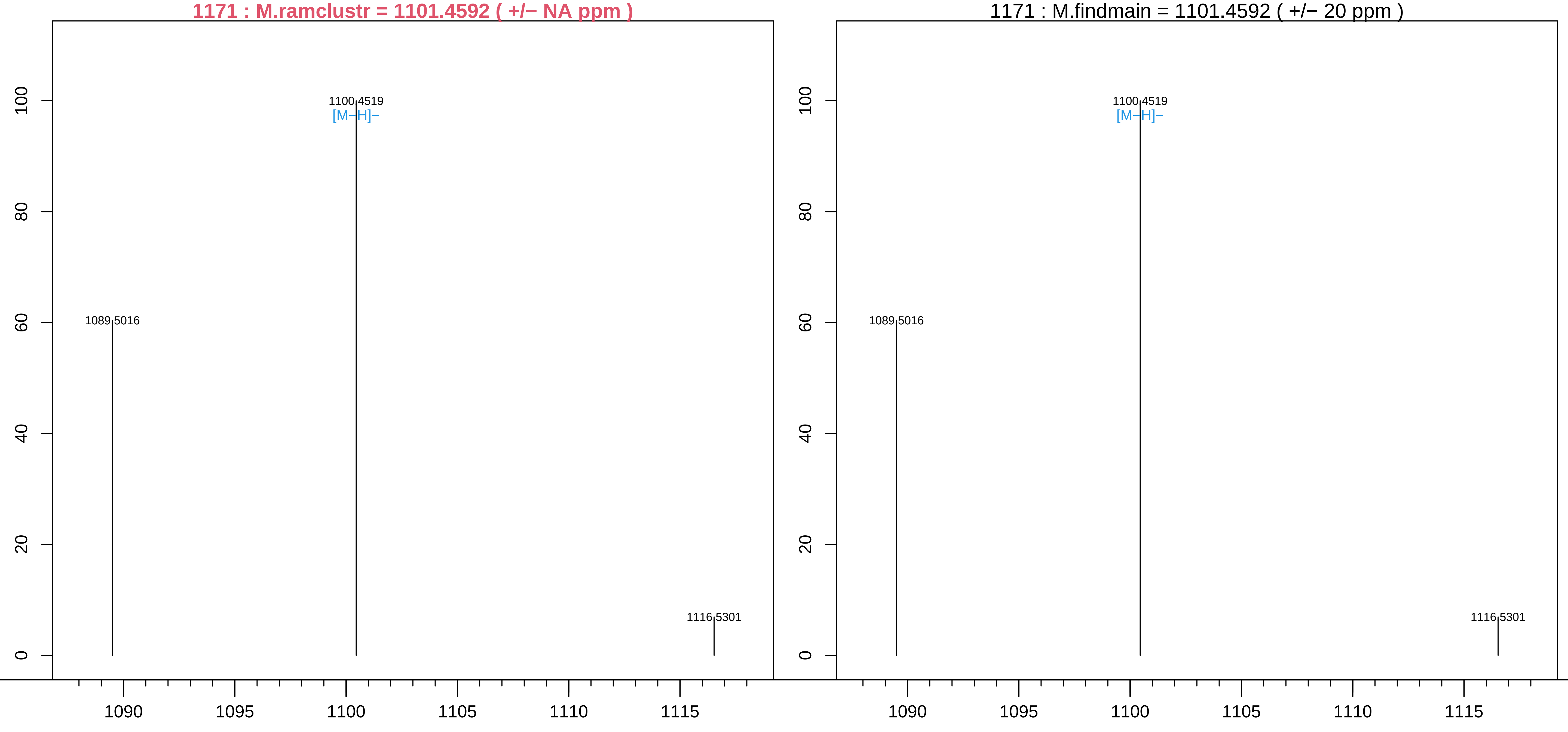
1171 : M.ramclustr = 1101.4592 (+/− NA ppm)
1171 : M.findmain = 1101.4592 (+/− 20 ppm)
1100.4519
[M−H]−
1089.5016
1116.5301
0
20
40
60
80
100
1090
1095
1100
1105
1110
1115

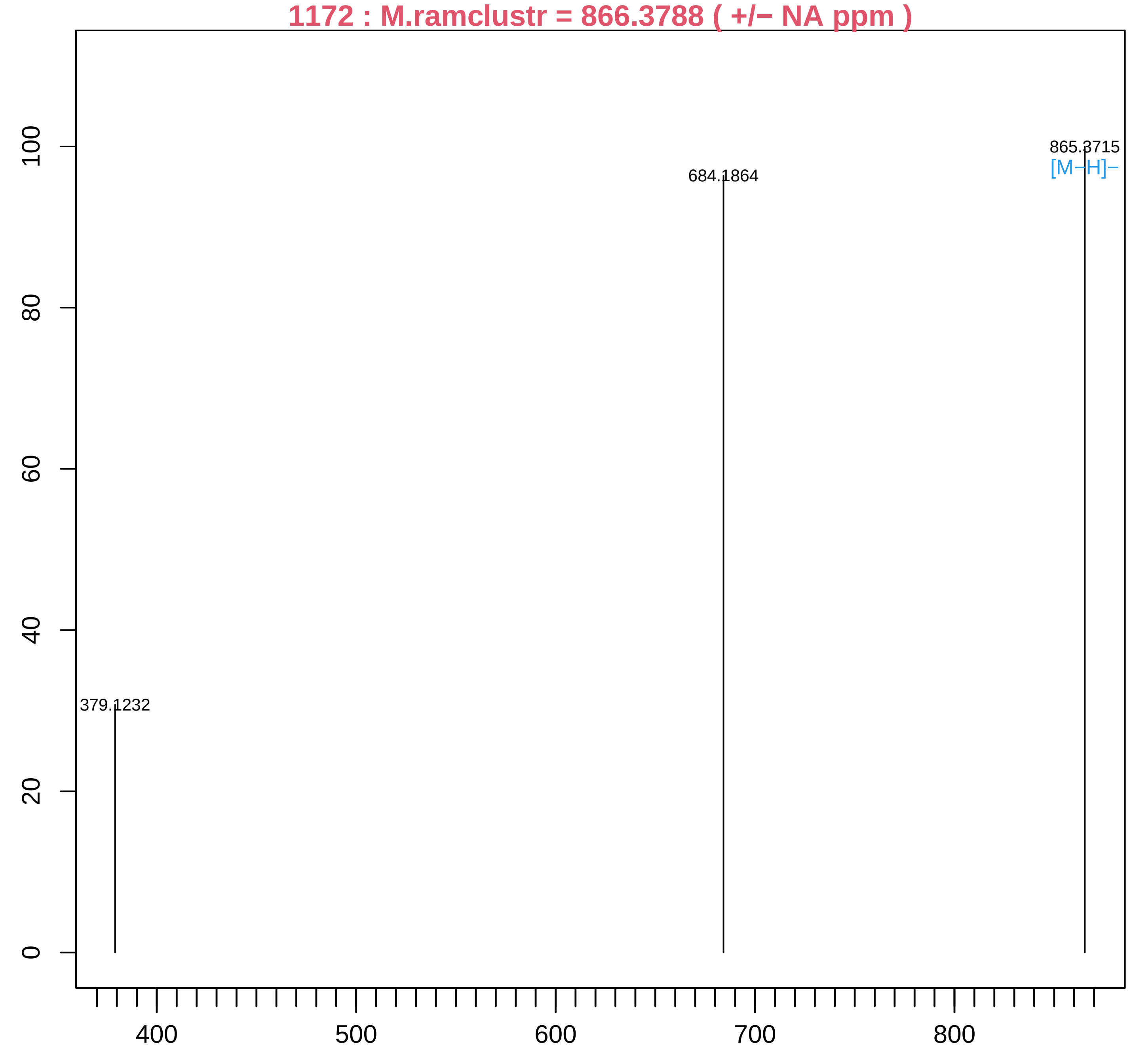

1172 : M.ramclustr = 866.3788 (+/− NA ppm)
865.3715
[M−H]−
684.1864
379.1232
0
20
40
60
80
100
400
500
600
700
800

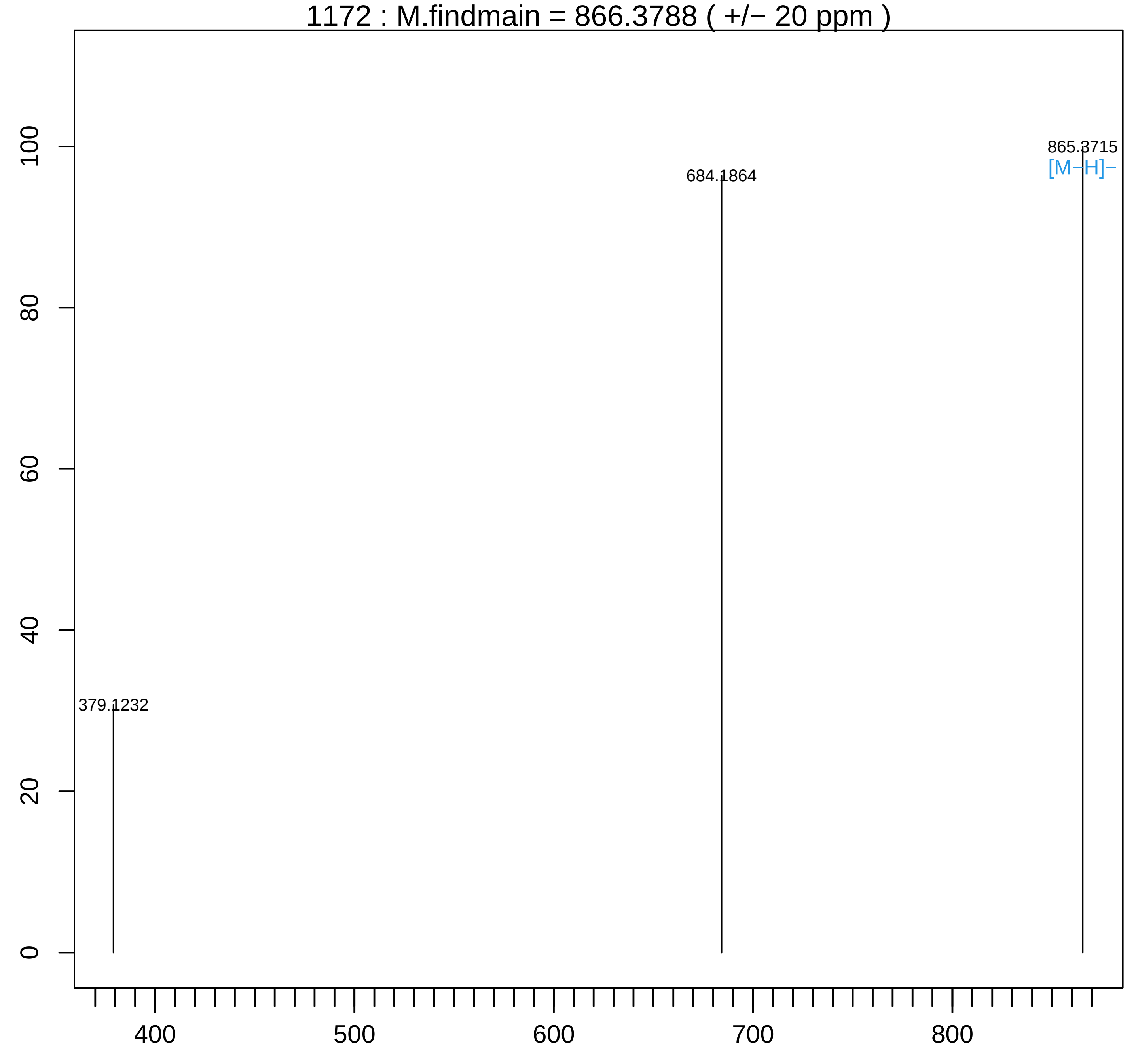

1172 : M.findmain = 866.3788 (+/− 20 ppm)
865.3715
[M−H]−
684.1864
379.1232
0
20
40
60
80
100
400
500
600
700
800

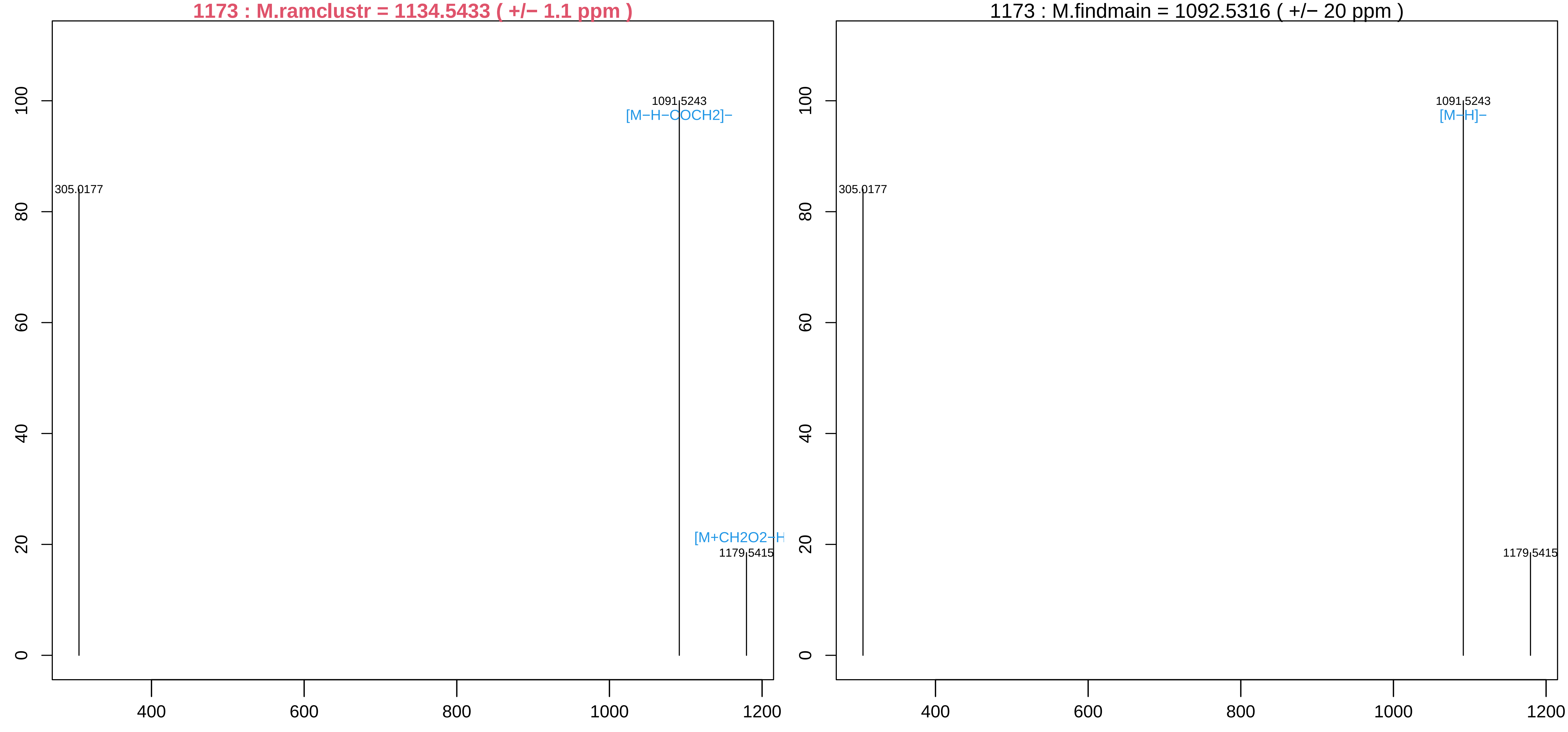

1173 : M.ramclustr = 1134.5433 (+/− 1.1 ppm)
1091.5243
[M−H−COCH2]−
305.0177
[M+CH2O2−H
1179.5415
1173 : M.findmain = 1092.5316 (+/− 20 ppm)
1091.5243
[M−H]−
305.0177
1179.5415

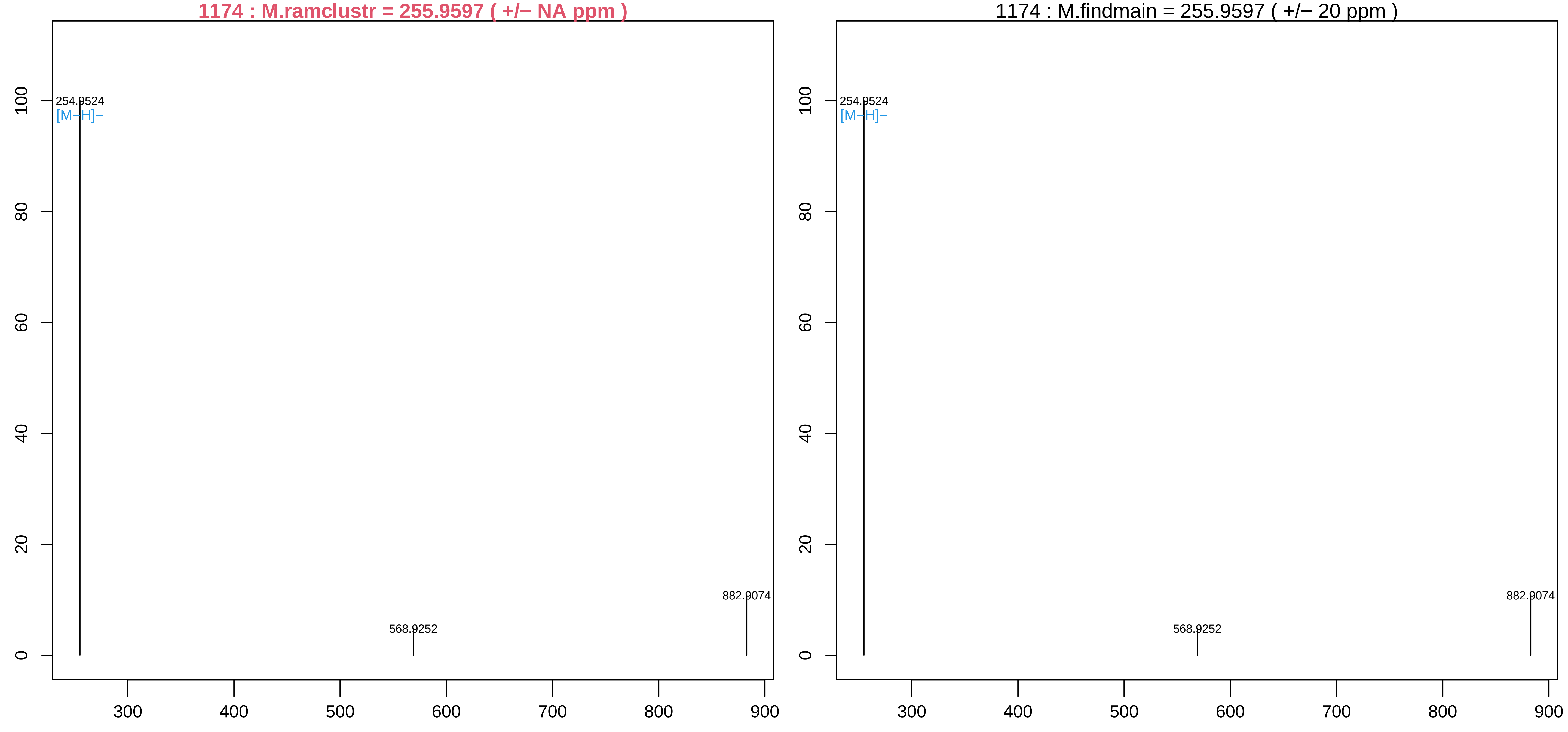

1174 : M.ramclustr = 255.9597 (+/− NA ppm)
254.9524
[M−H]−
568.9252
882.9074
1174 : M.findmain = 255.9597 (+/− 20 ppm)
254.9524
[M−H]−
568.9252
882.9074

1175 : M.ramclustr = 407.1129 (+/− NA ppm)

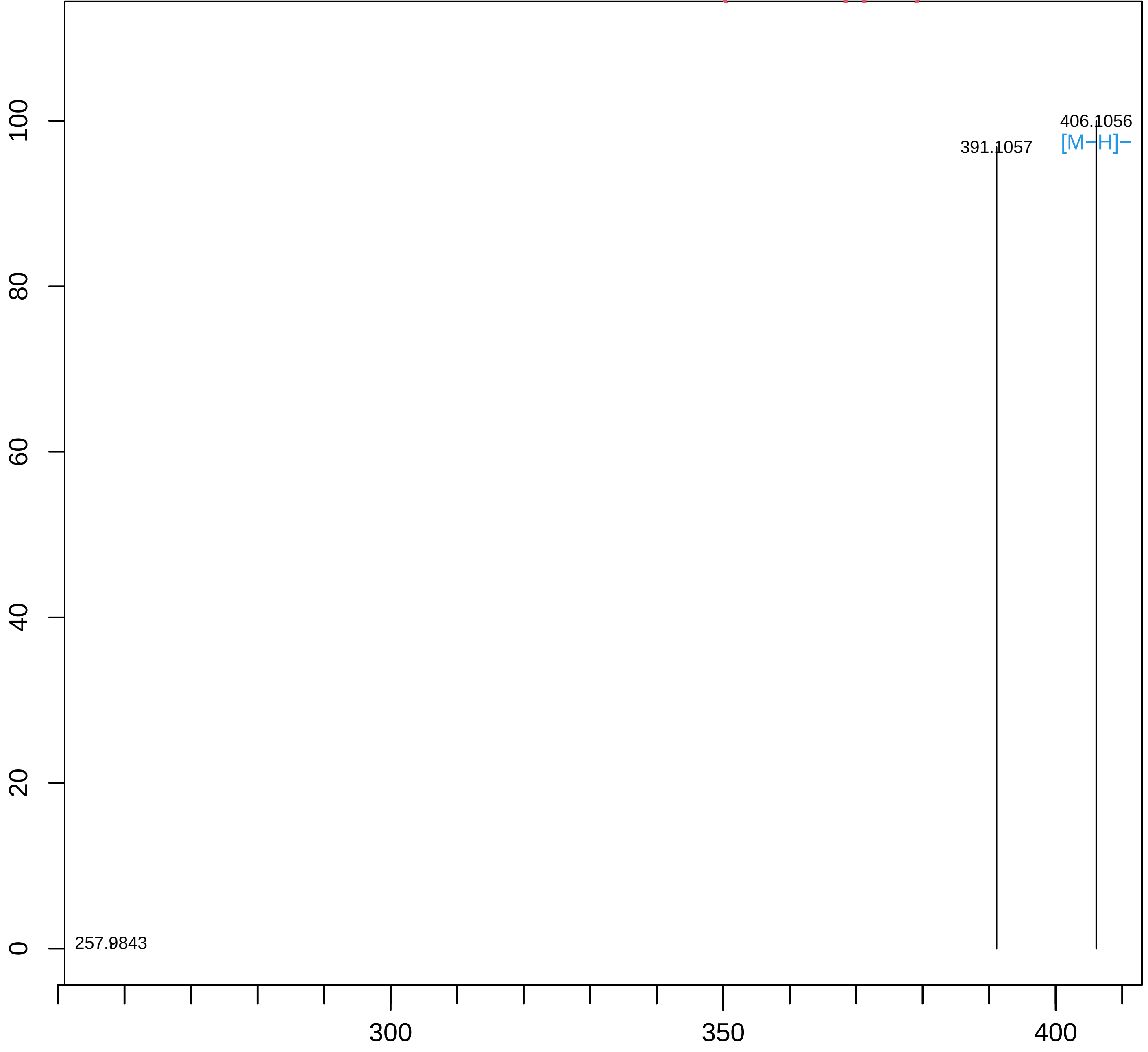

1175 : M.findmain = 407.1129 (+/− 20 ppm)

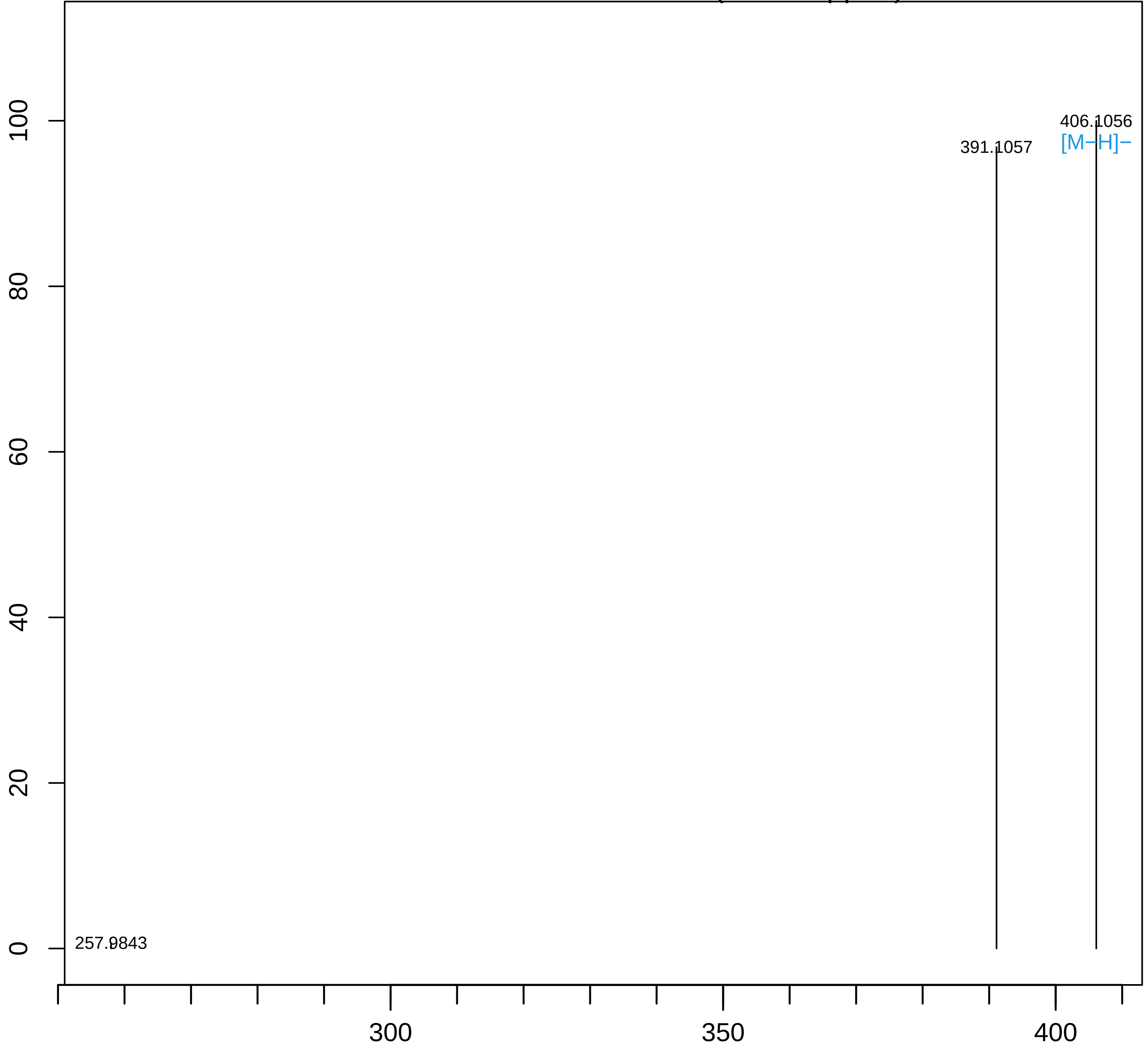

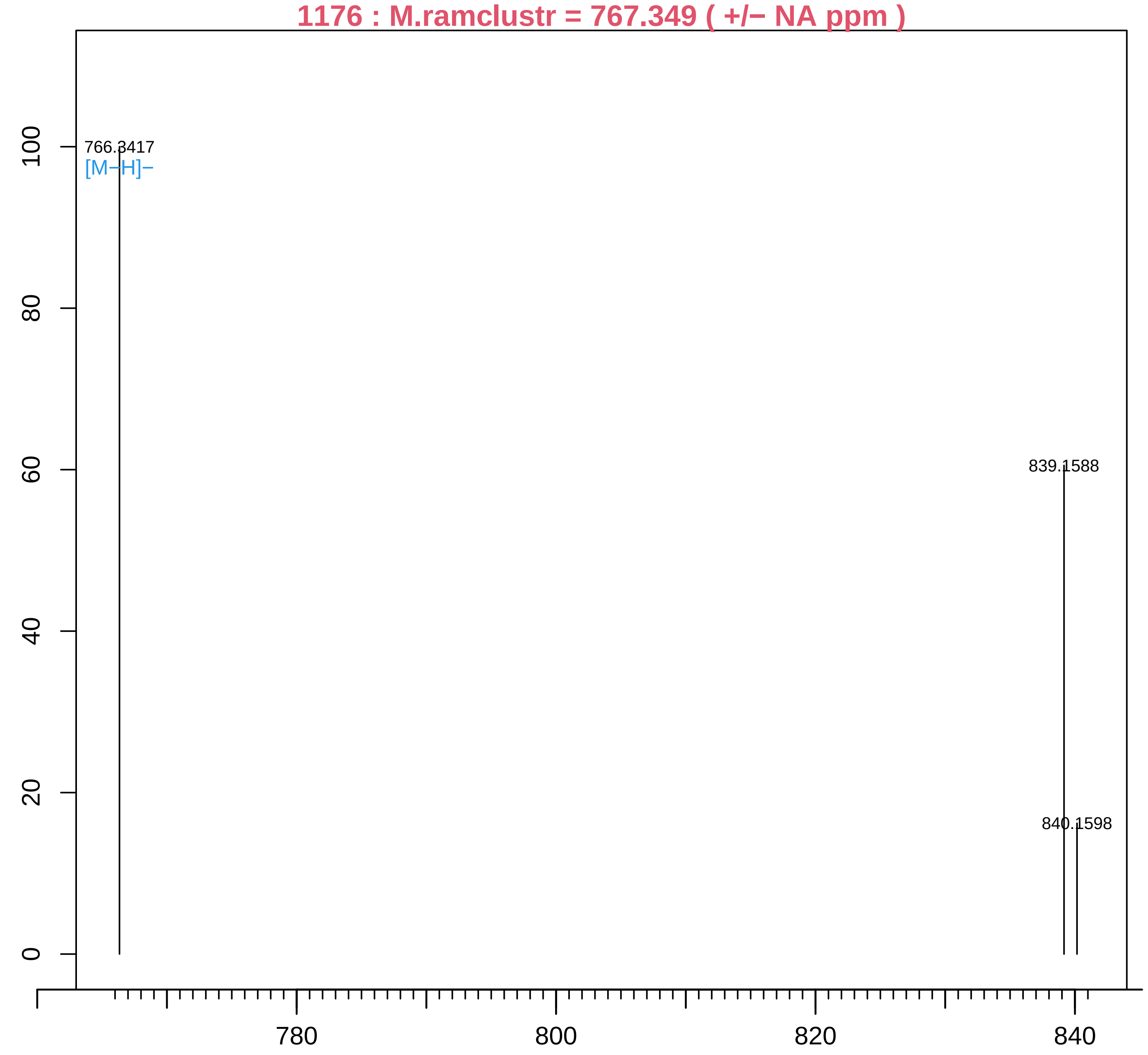

1176 : M.ramclustr = 767.349 (+/− NA ppm)
766.3417
[M−H]−
839.1588
840.1598
0
20
40
60
80
100
780
800
820
840

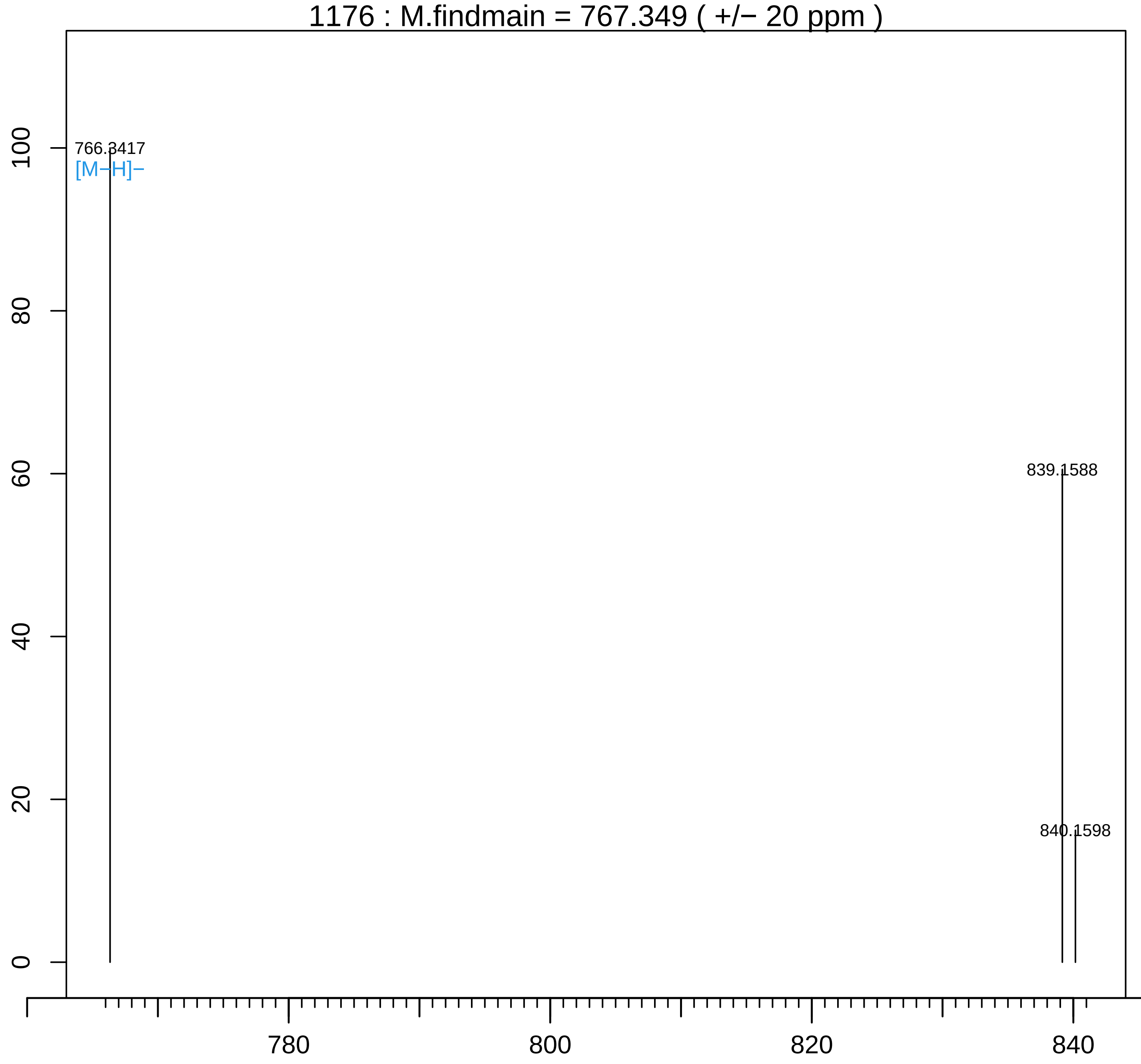

1176 : M.findmain = 767.349 (+/− 20 ppm)
766.3417
[M−H]−
839.1588
840.1598
0
20
40
60
80
100
780
800
820
840

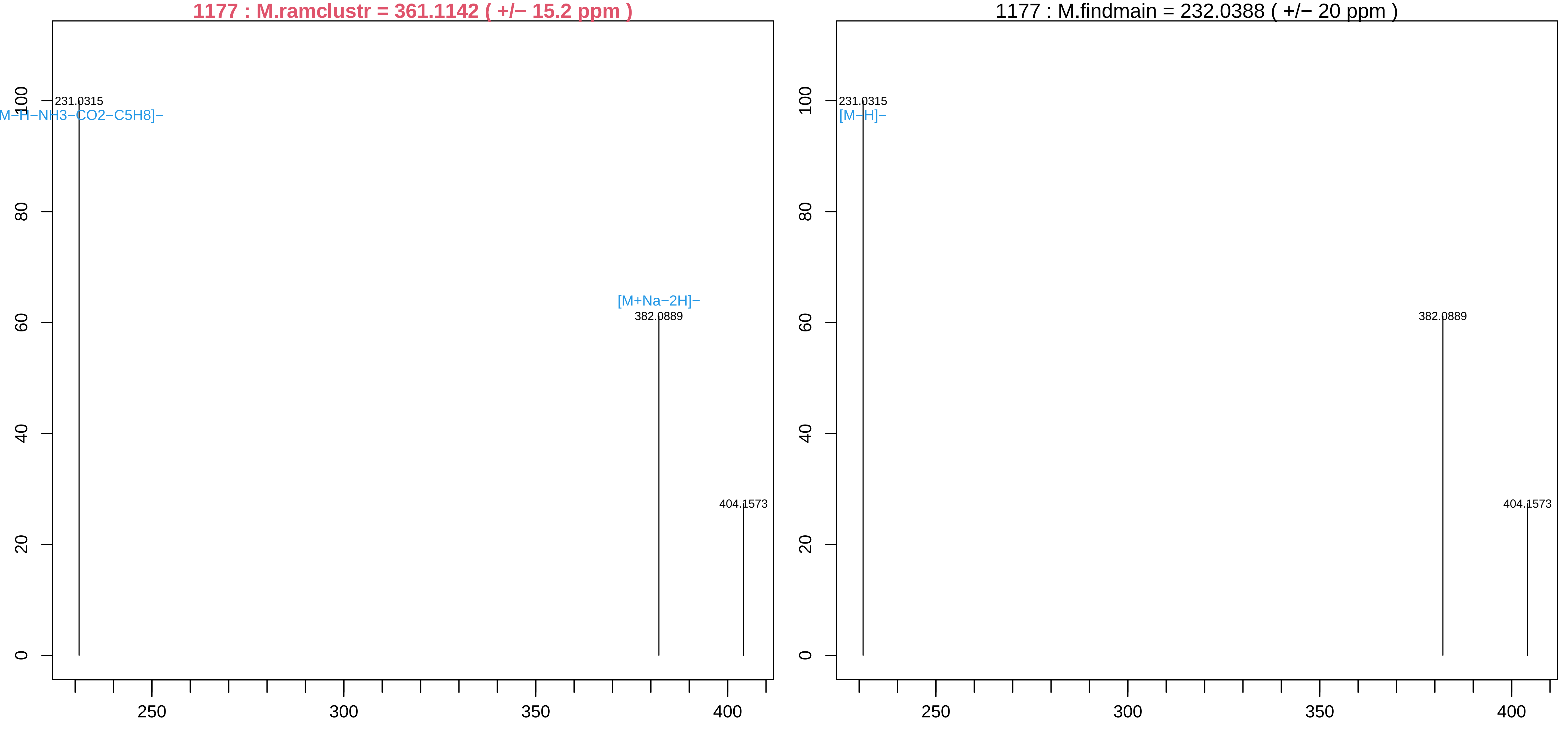
1177 : M.ramclustr = 361.1142 (+/− 15.2 ppm)
231.0315
M−H−NH3−CO2−C5H8]−
[M+Na−2H]−
382.0889
404.1573
1177 : M.findmain = 232.0388 (+/− 20 ppm)
231.0315
[M−H]−
382.0889
404.1573
0
20
40
60
80
100
250
300
350
400

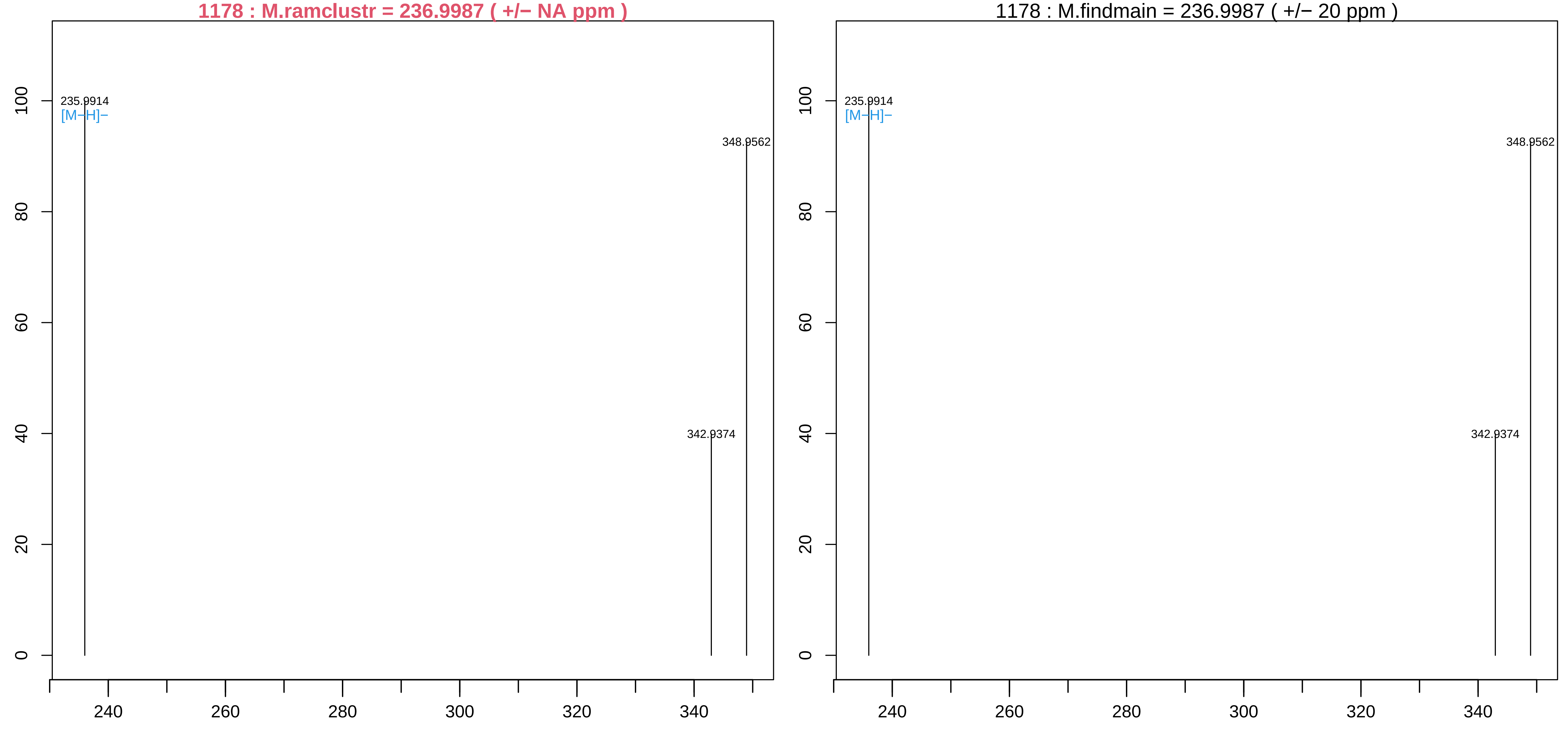
1178 : M.ramclustr = 236.9987 (+/− NA ppm)
235.9914
[M−H]−
348.9562
342.9374
1178 : M.findmain = 236.9987 (+/− 20 ppm)
235.9914
[M−H]−
348.9562
342.9374

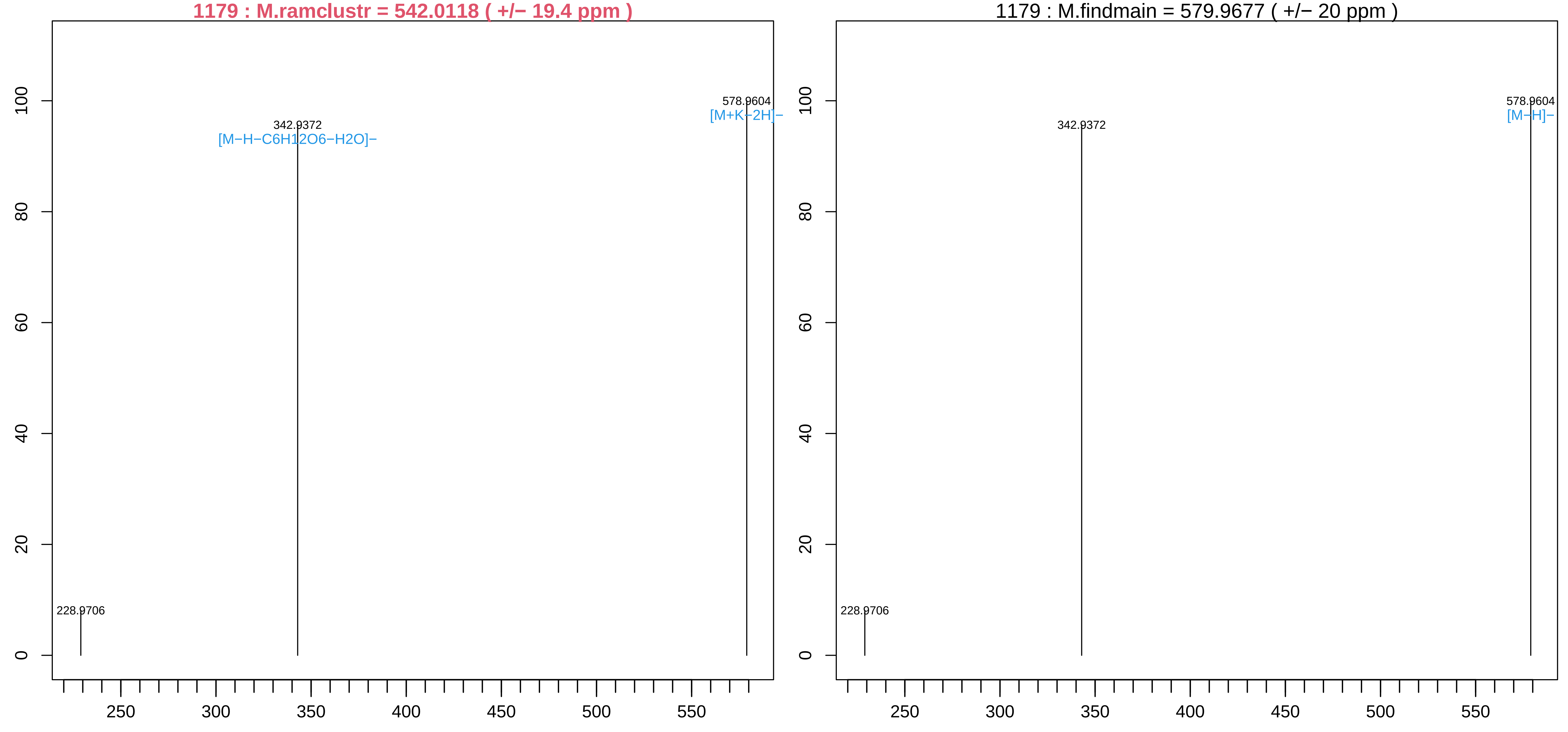

1179 : M.ramclustr = 542.0118 (+/− 19.4 ppm)
578.9604
[M+K−2H]−
342.9372
[M−H−C6H12O6−H2O]−
228.9706
1179 : M.findmain = 579.9677 (+/− 20 ppm)
578.9604
[M−H]−
342.9372
228.9706

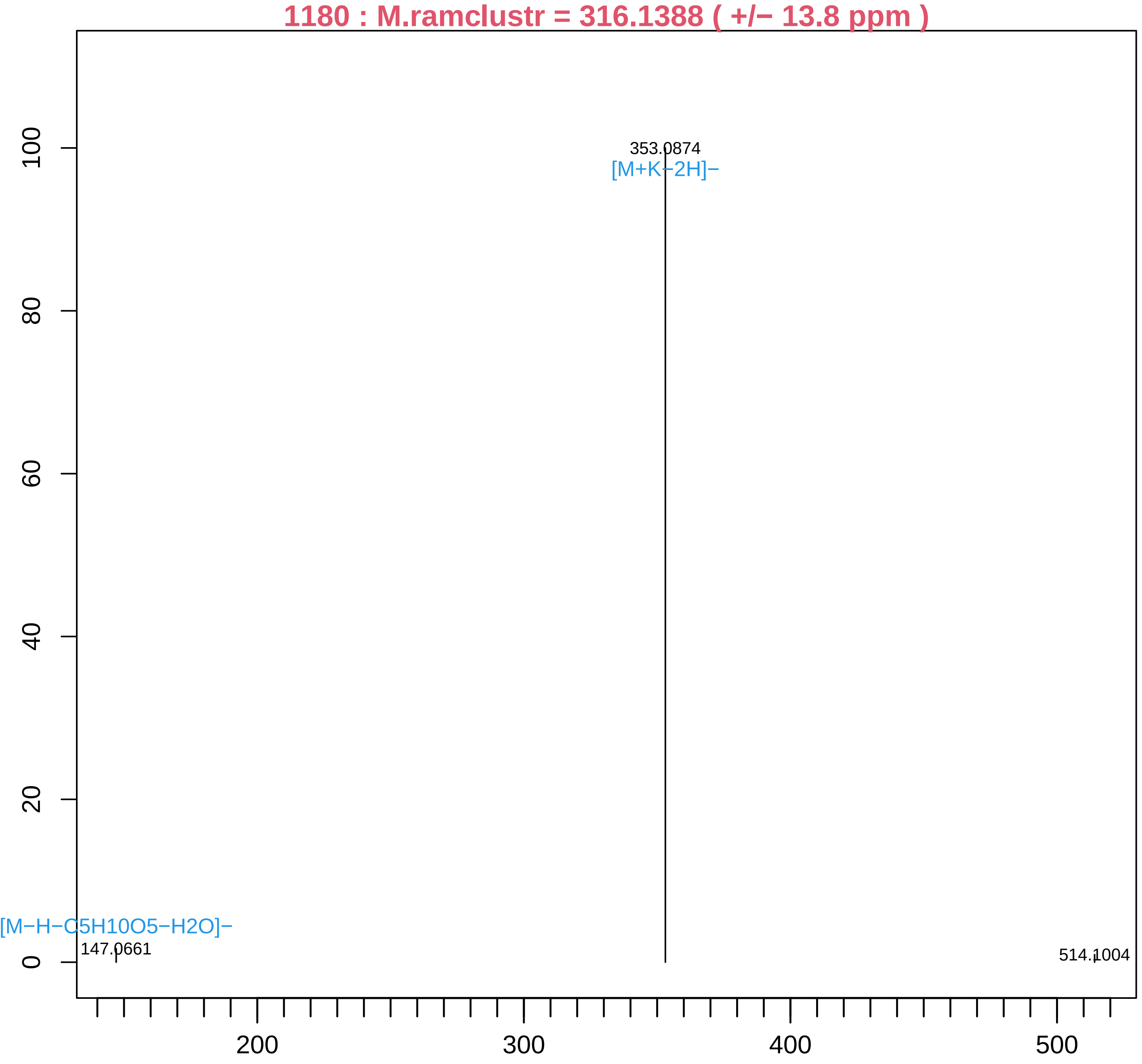
1180 : M.ramclustr = 316.1388 (+/− 13.8 ppm)
353.0874
[M+K−2H]−
[M−H−C5H10O5−H2O]−
147.0661
514.1004
0
20
40
60
80
100
200
300
400
500

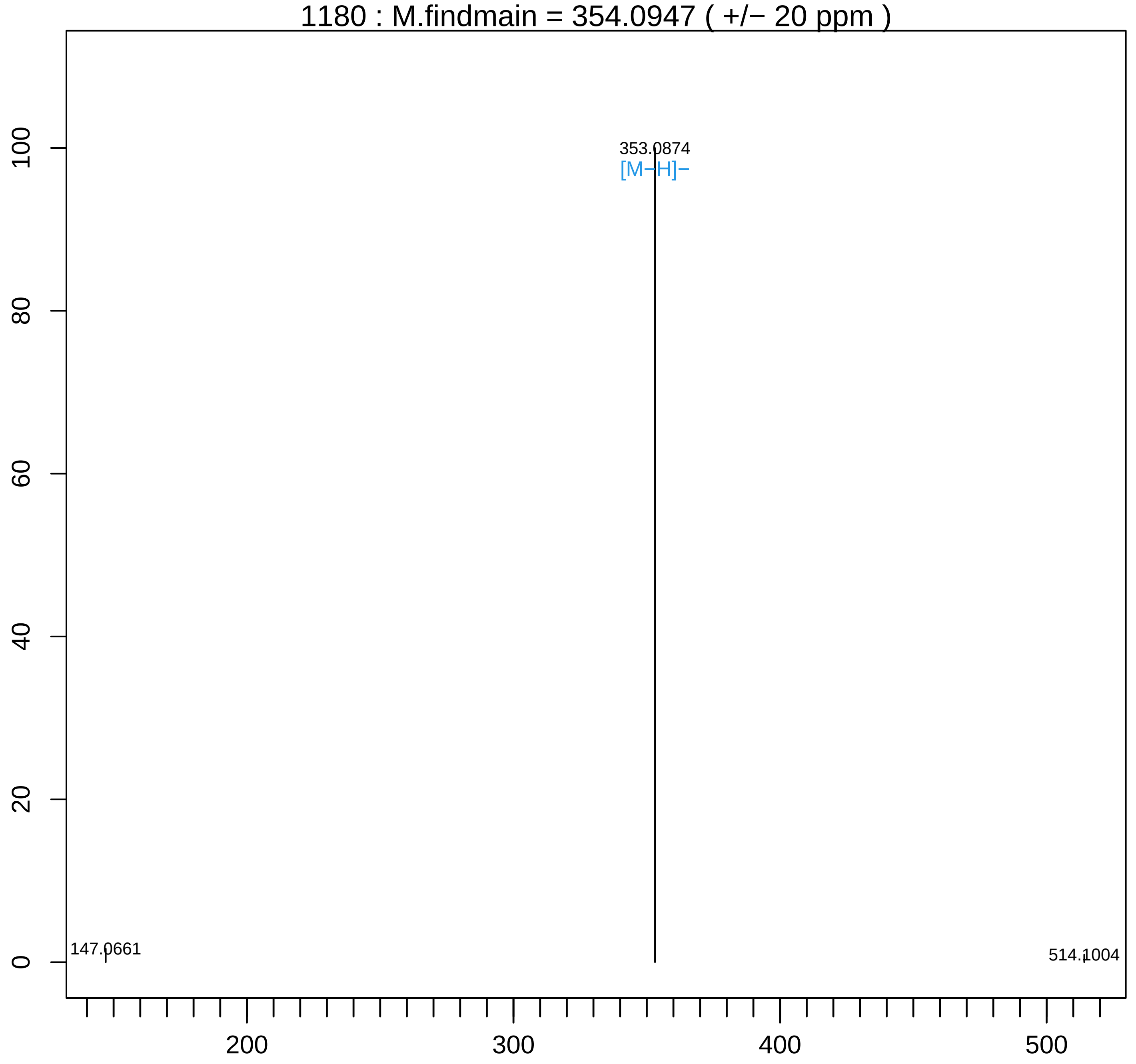
1180 : M.findmain = 354.0947 (+/− 20 ppm)
353.0874
[M−H]−
147.0661
514.1004
0
20
40
60
80
100
200
300
400
500

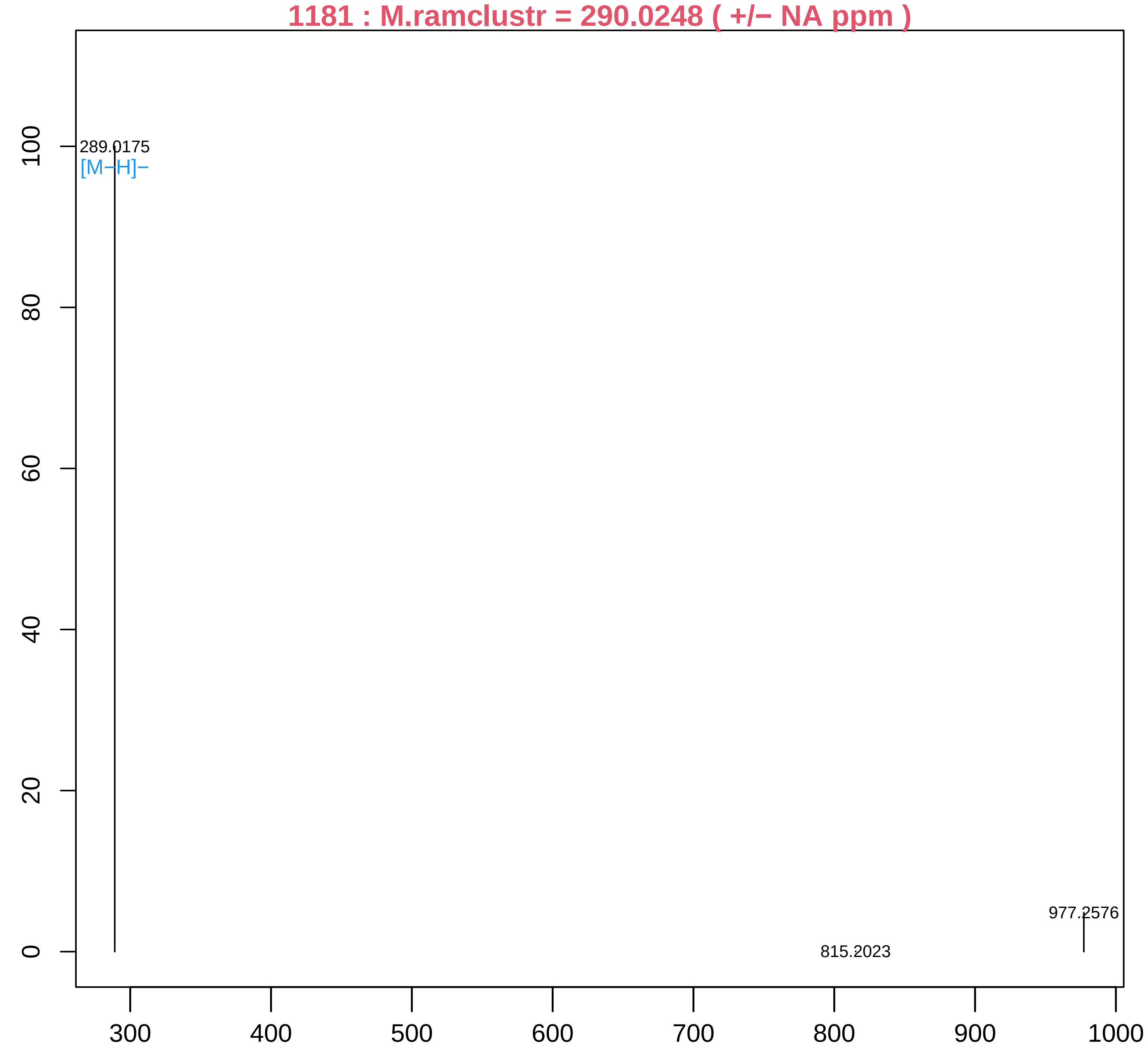
1181 : M.ramclustr = 290.0248 (+/− NA ppm)
289.0175
[M−H]−
977.2576
815.2023
0
20
40
60
80
100
300
400
500
600
700
800
900
1000

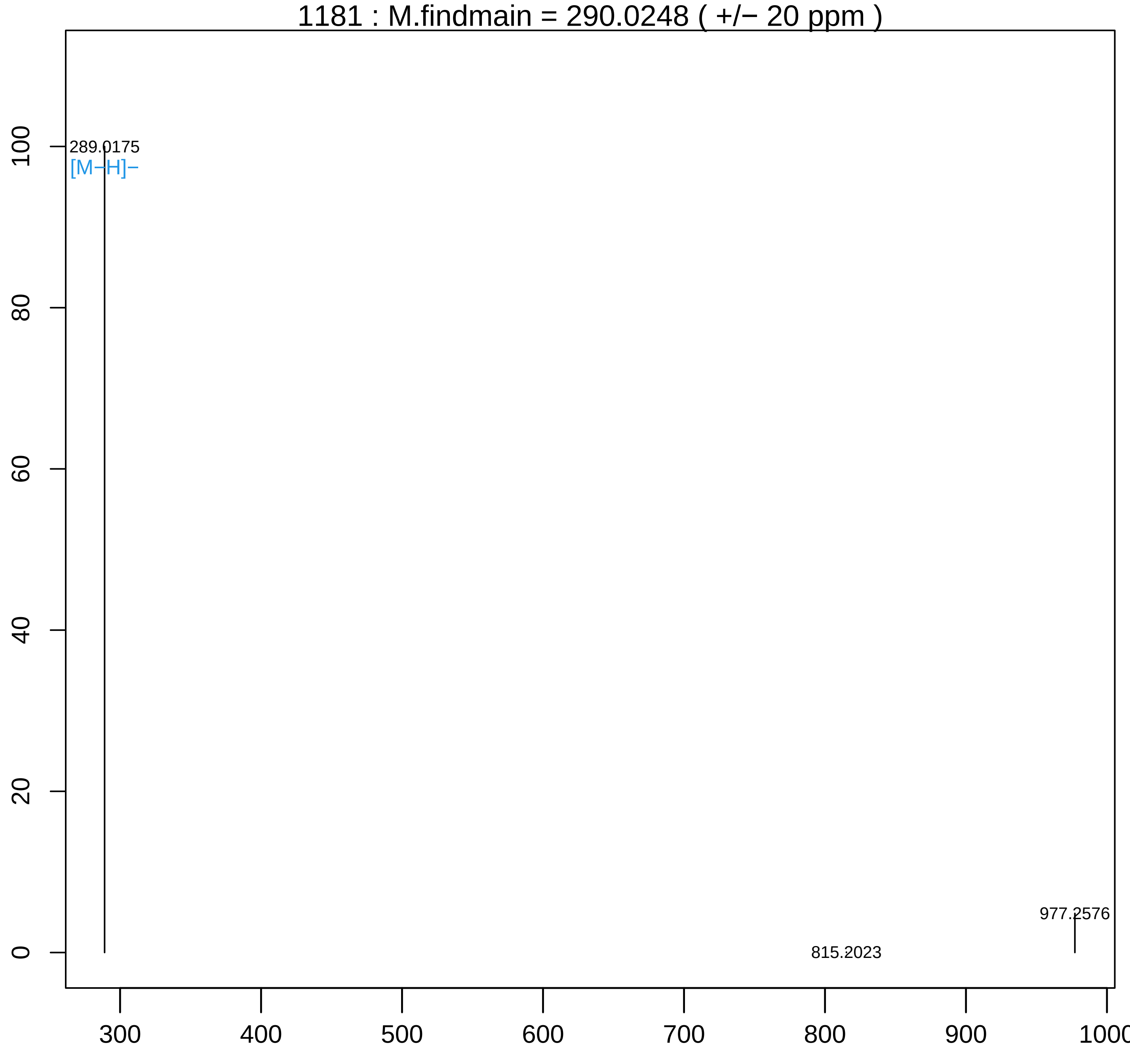
1181 : M.findmain = 290.0248 (+/− 20 ppm)
289.0175
[M−H]−
977.2576
815.2023
0
20
40
60
80
100
300
400
500
600
700
800
900
1000

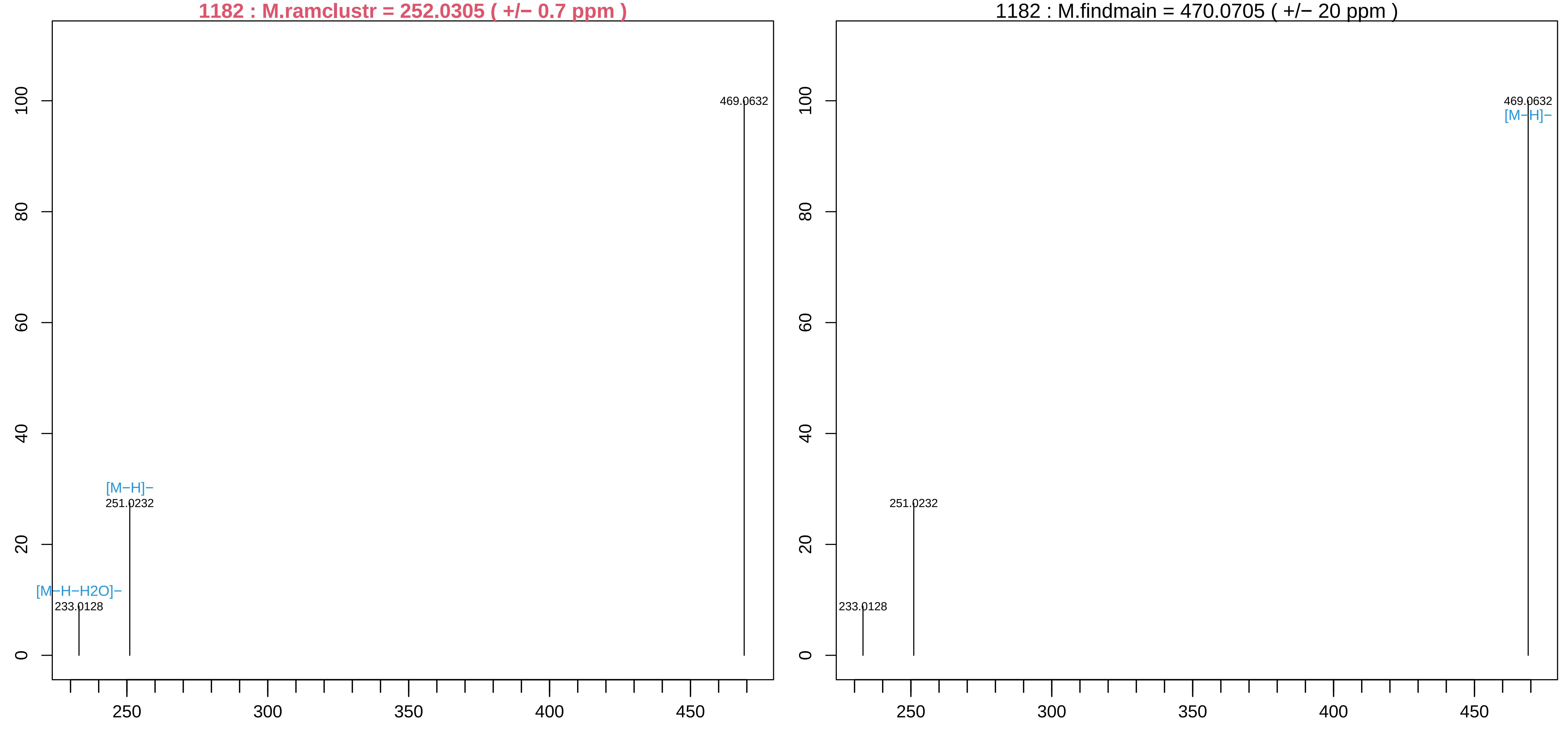

1182 : M.ramclustr = 252.0305 (+/− 0.7 ppm)
469.0632
[M−H]−
251.0232
[M−H−H2O]−
233.0128
1182 : M.findmain = 470.0705 (+/− 20 ppm)
469.0632
[M−H]−
251.0232
233.0128
0
20
40
60
80
100
250
300
350
400
450

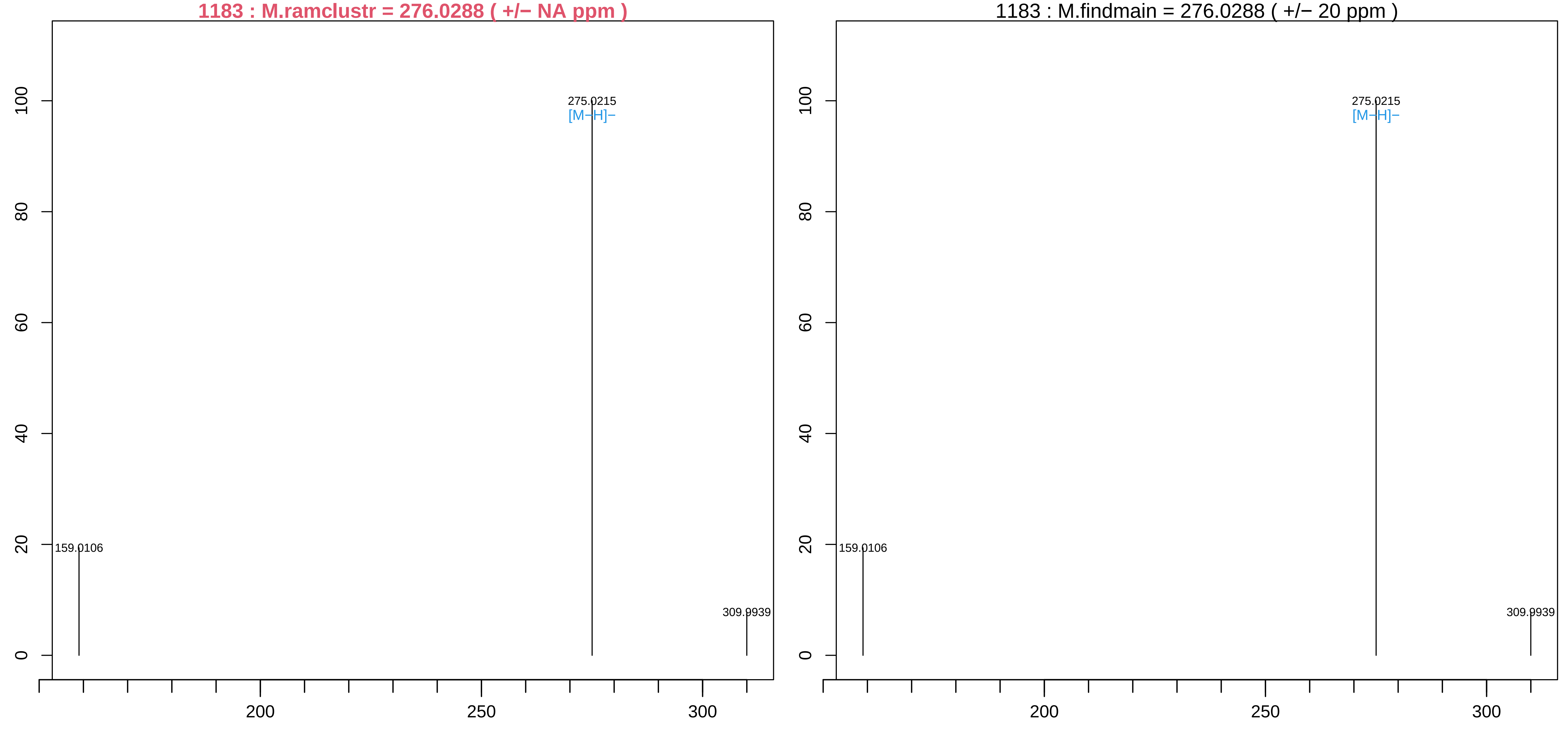

1183 : M.ramclustr = 276.0288 (+/− NA ppm)
1183 : M.findmain = 276.0288 (+/− 20 ppm)
275.0215
[M−H]−
159.0106
309.9939
275.0215
[M−H]−
159.0106
309.9939
0
20
40
60
80
100
200
250
300

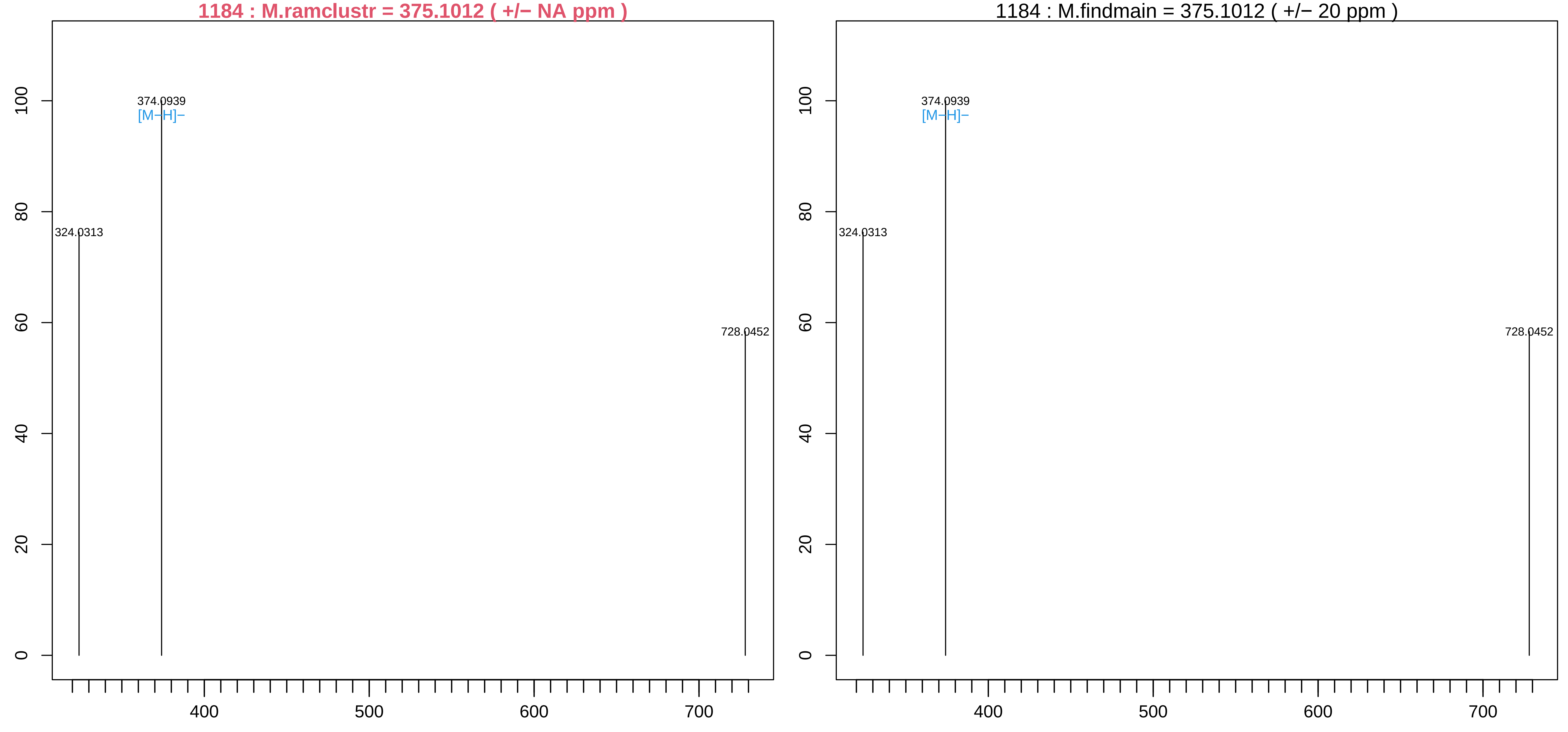
1184 : M.ramclustr = 375.1012 (+/− NA ppm)
1184 : M.findmain = 375.1012 (+/− 20 ppm)
374.0939
[M−H]−
324.0313
728.0452
0
20
40
60
80
100
400
500
600
700

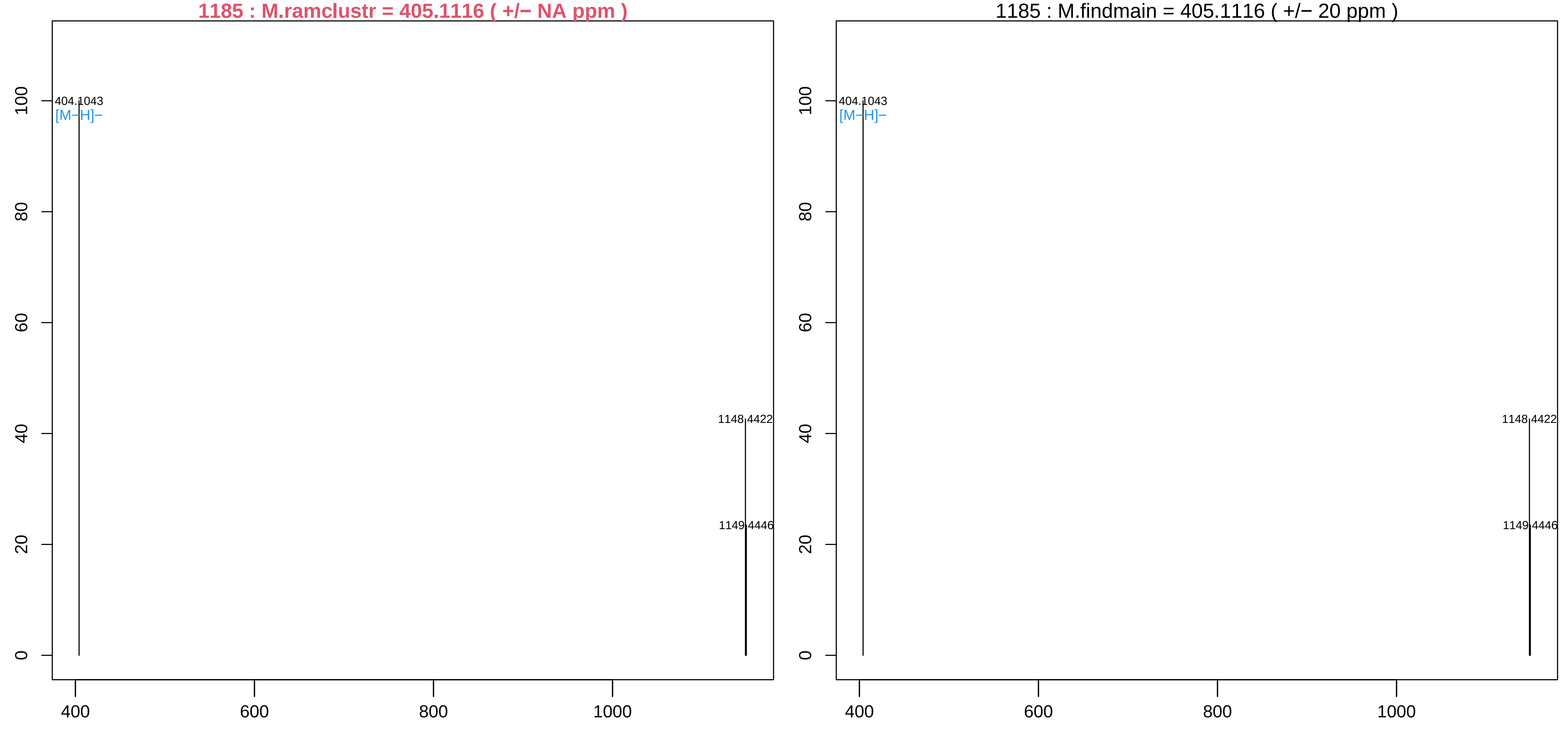

1185 : M.ramclustr = 405.1116 (+/− NA ppm)
404.1043
[M−H]−
1148.4422
1149.4446
0
20
40
60
80
100
400
600
800
1000
1185 : M.findmain = 405.1116 (+/− 20 ppm)
404.1043
[M−H]−
1148.4422
1149.4446
0
20
40
60
80
100
400
600
800
1000

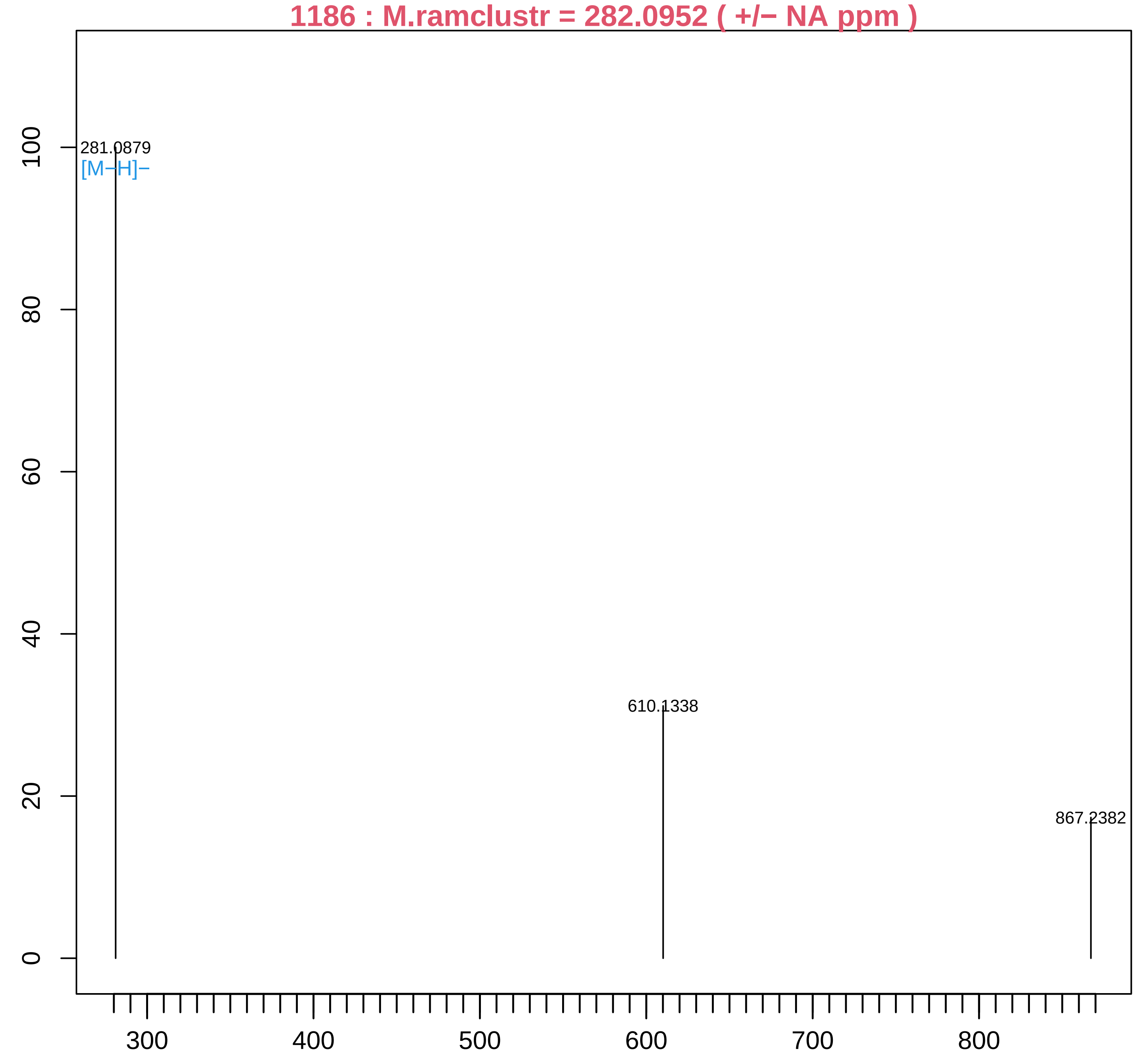

1186 : M.ramclustr = 282.0952 (+/− NA ppm)
281.0879
[M−H]−
610.1338
867.2382
0
20
40
60
80
100
300
400
500
600
700
800

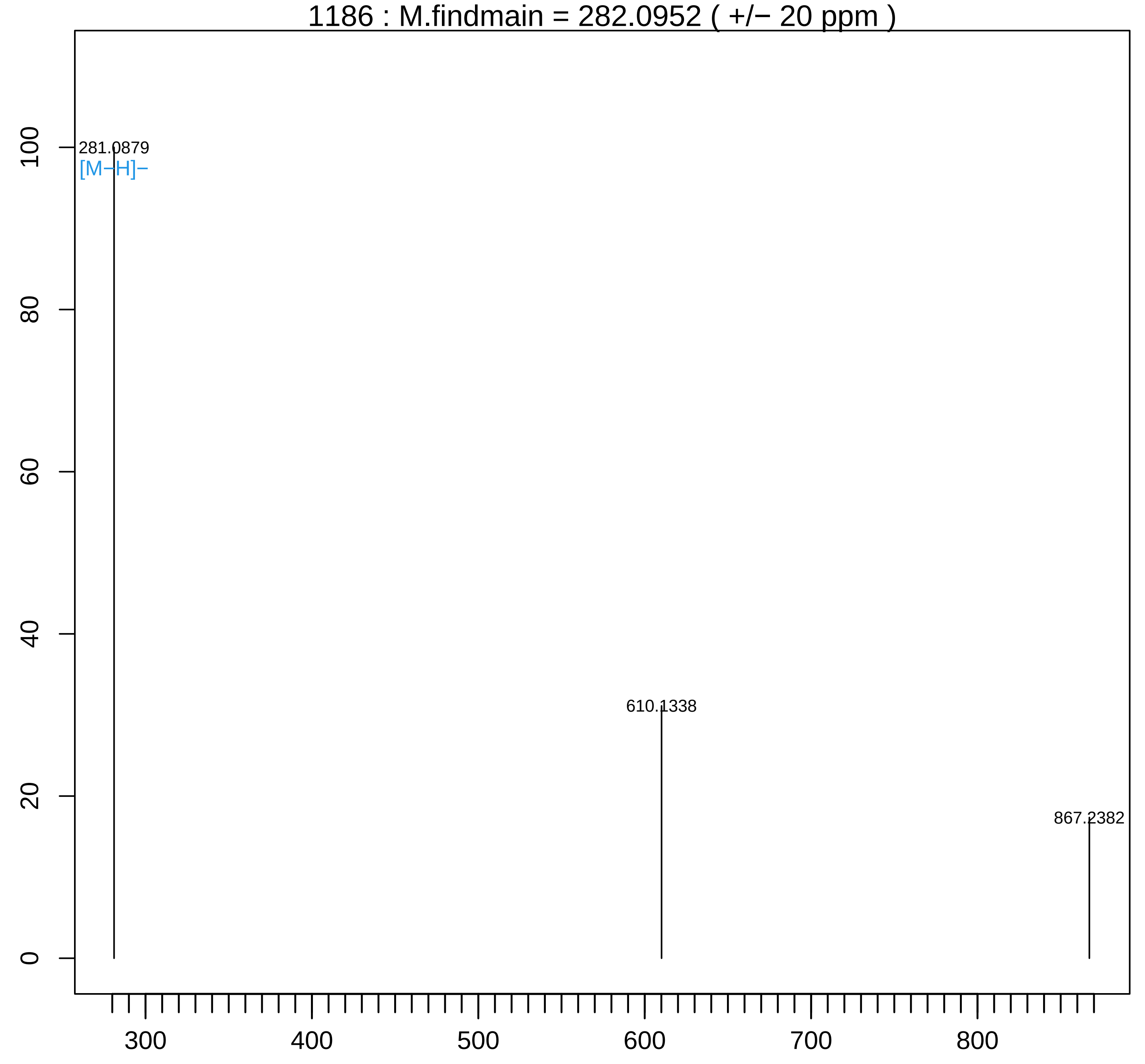

1186 : M.findmain = 282.0952 (+/− 20 ppm)
281.0879
[M−H]−
610.1338
867.2382
0
20
40
60
80
100
300
400
500
600
700
800

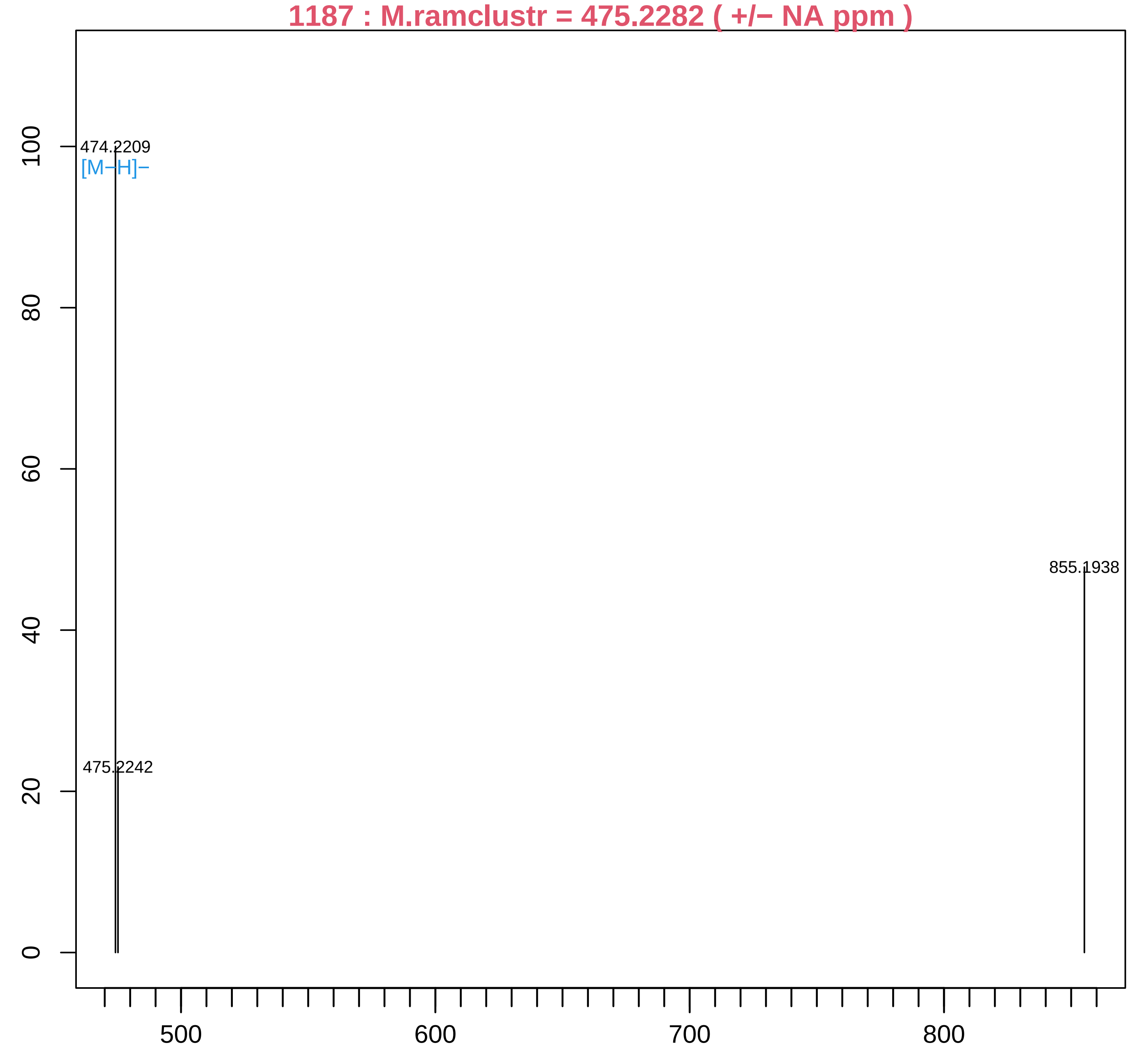
1187 : M.ramclustr = 475.2282 (+/− NA ppm)
474.2209
[M−H]−
475.2242
855.1938
0
20
40
60
80
100
500
600
700
800

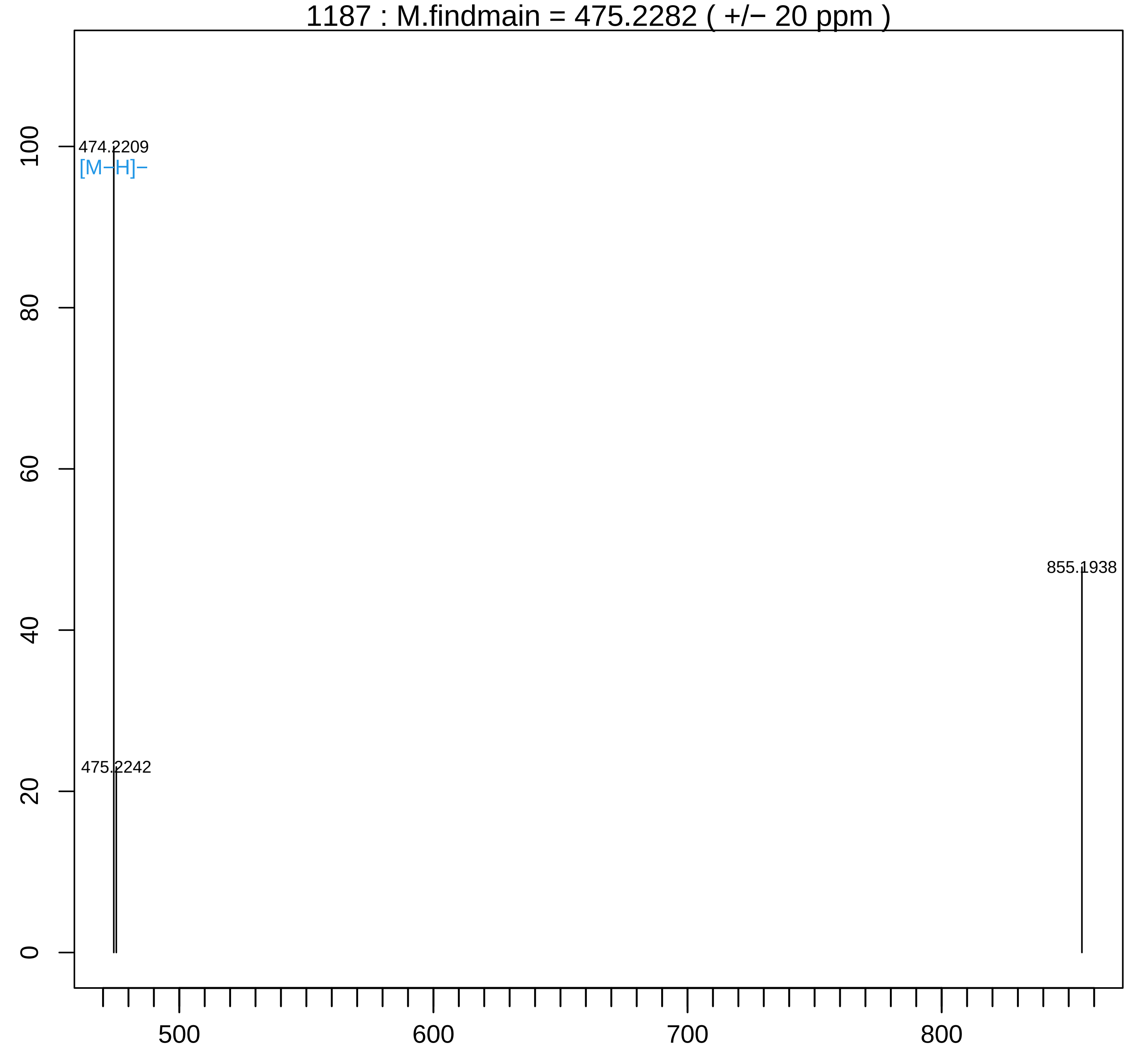
1187 : M.findmain = 475.2282 (+/− 20 ppm)
474.2209
[M−H]−
475.2242
855.1938
0
20
40
60
80
100
500
600
700
800

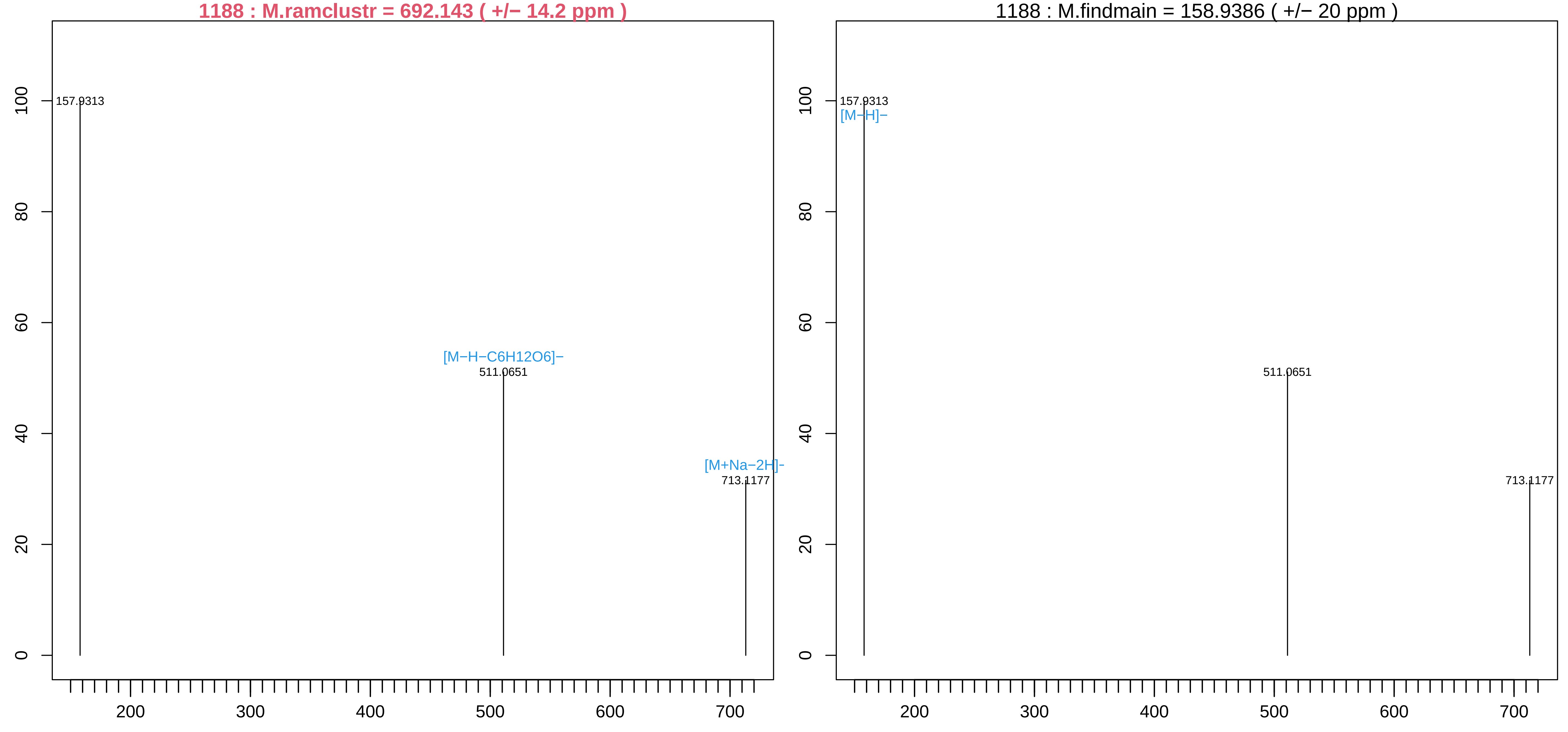
1188 : M.ramclustr = 692.143 (+/− 14.2 ppm)
157.9313
[M−H−C6H12O6]−
511.0651
[M+Na−2H]-
713.1177
0
20
40
60
80
100
200
300
400
500
600
700
1188 : M.findmain = 158.9386 (+/− 20 ppm)
157.9313
[M−H]−
511.0651
713.1177
0
20
40
60
80
100
200
300
400
500
600
700

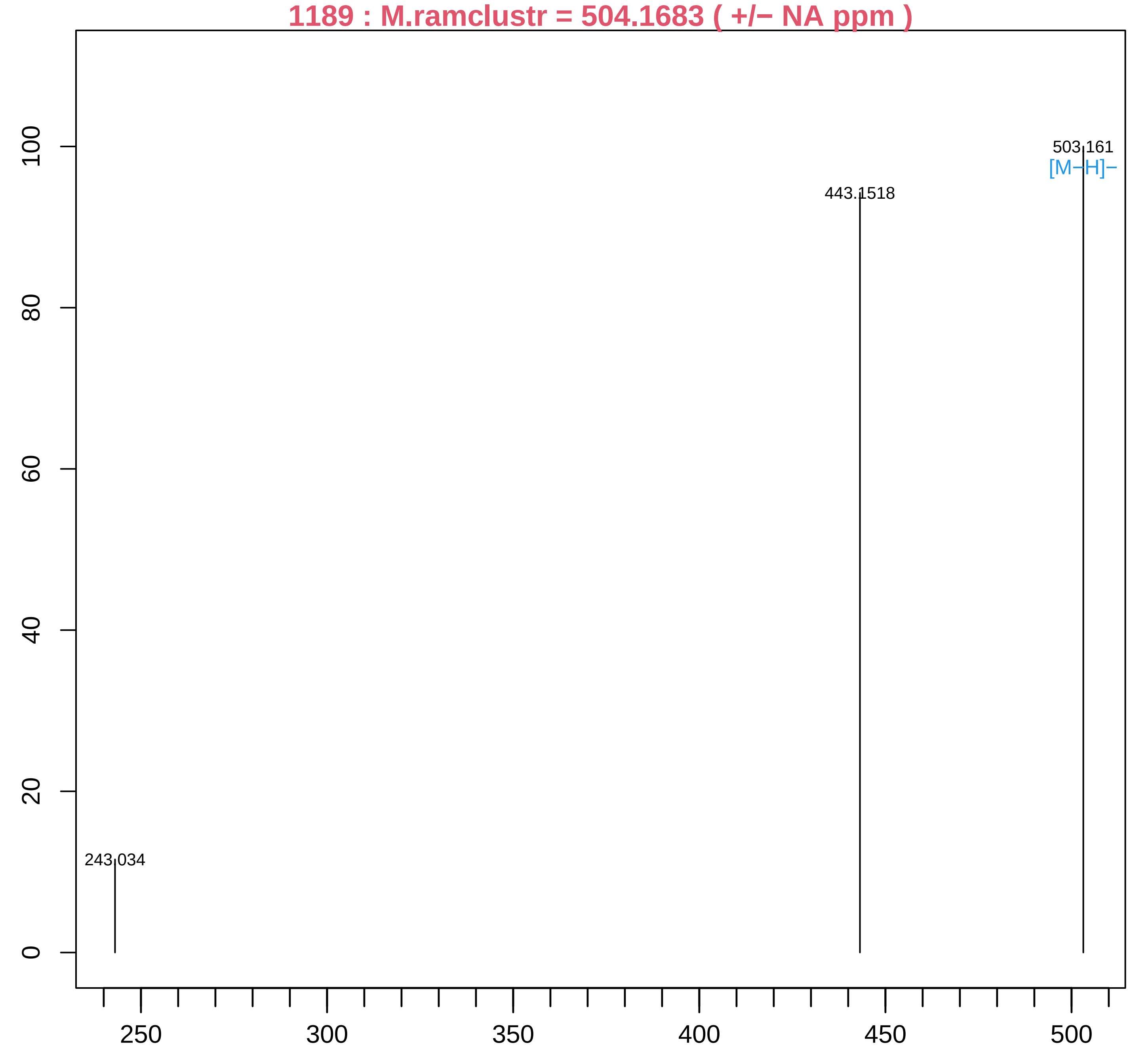
1189 : M.ramclustr = 504.1683 (+/− NA ppm)
503.161
[M−H]−
443.1518
243.034
0
20
40
60
80
100
250
300
350
400
450
500

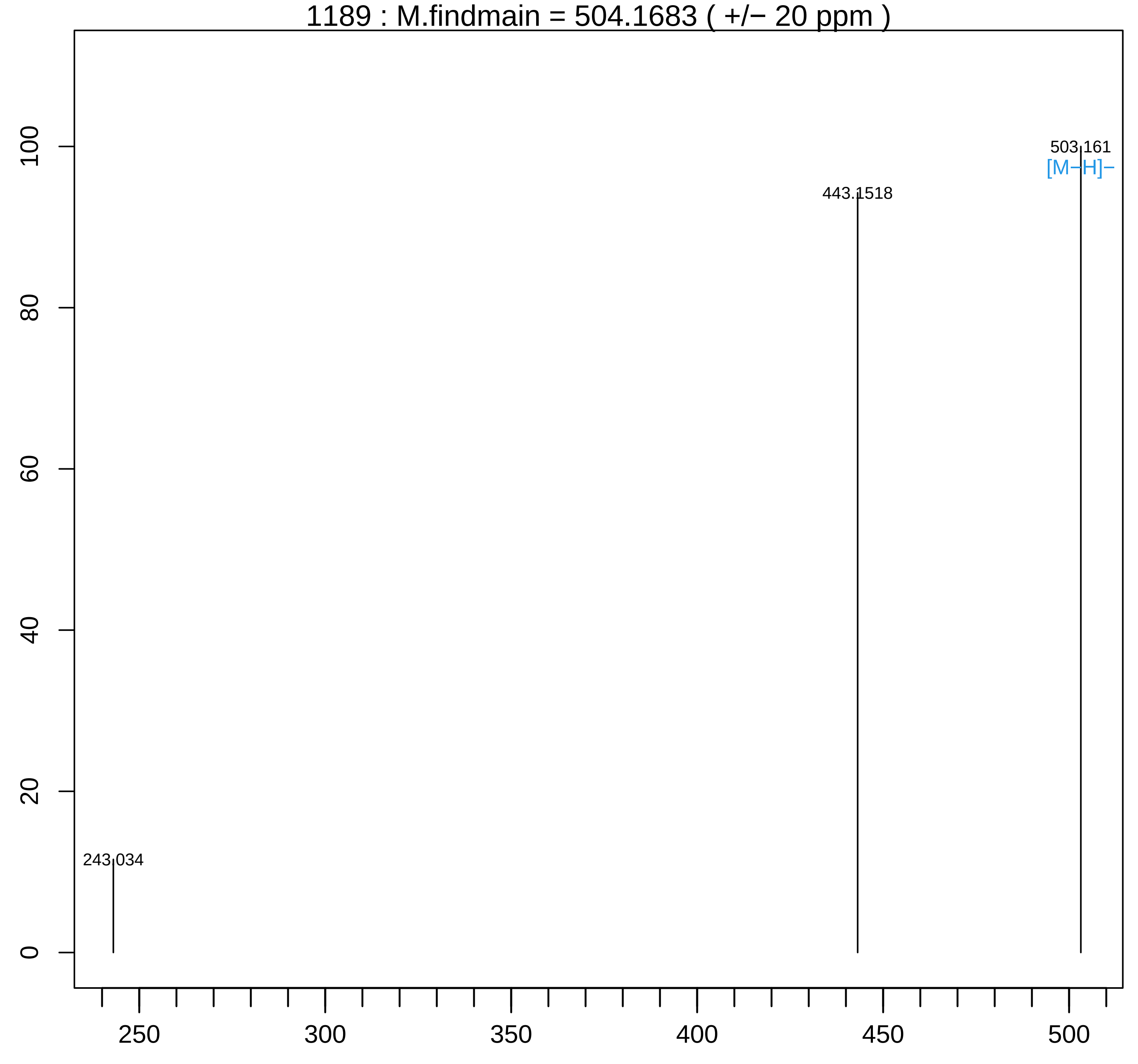
1189 : M.findmain = 504.1683 (+/− 20 ppm)
503.161
[M−H]−
443.1518
243.034
0
20
40
60
80
100
250
300
350
400
450
500

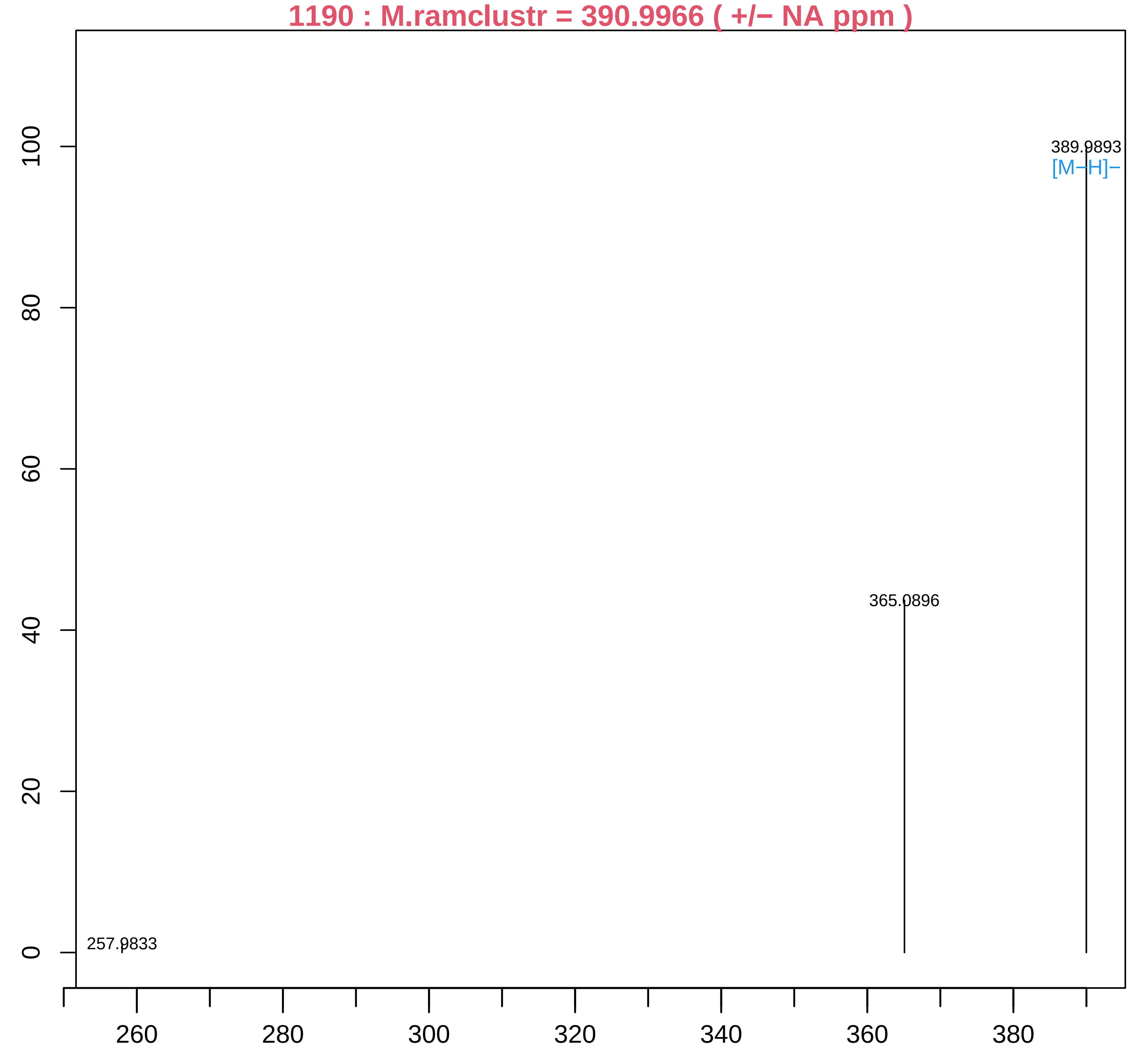
1190 : M.ramclustr = 390.9966 (+/− NA ppm)
389.9893
[M−H]−
365.0896
257.9833
0
20
40
60
80
100
260
280
300
320
340
360
380

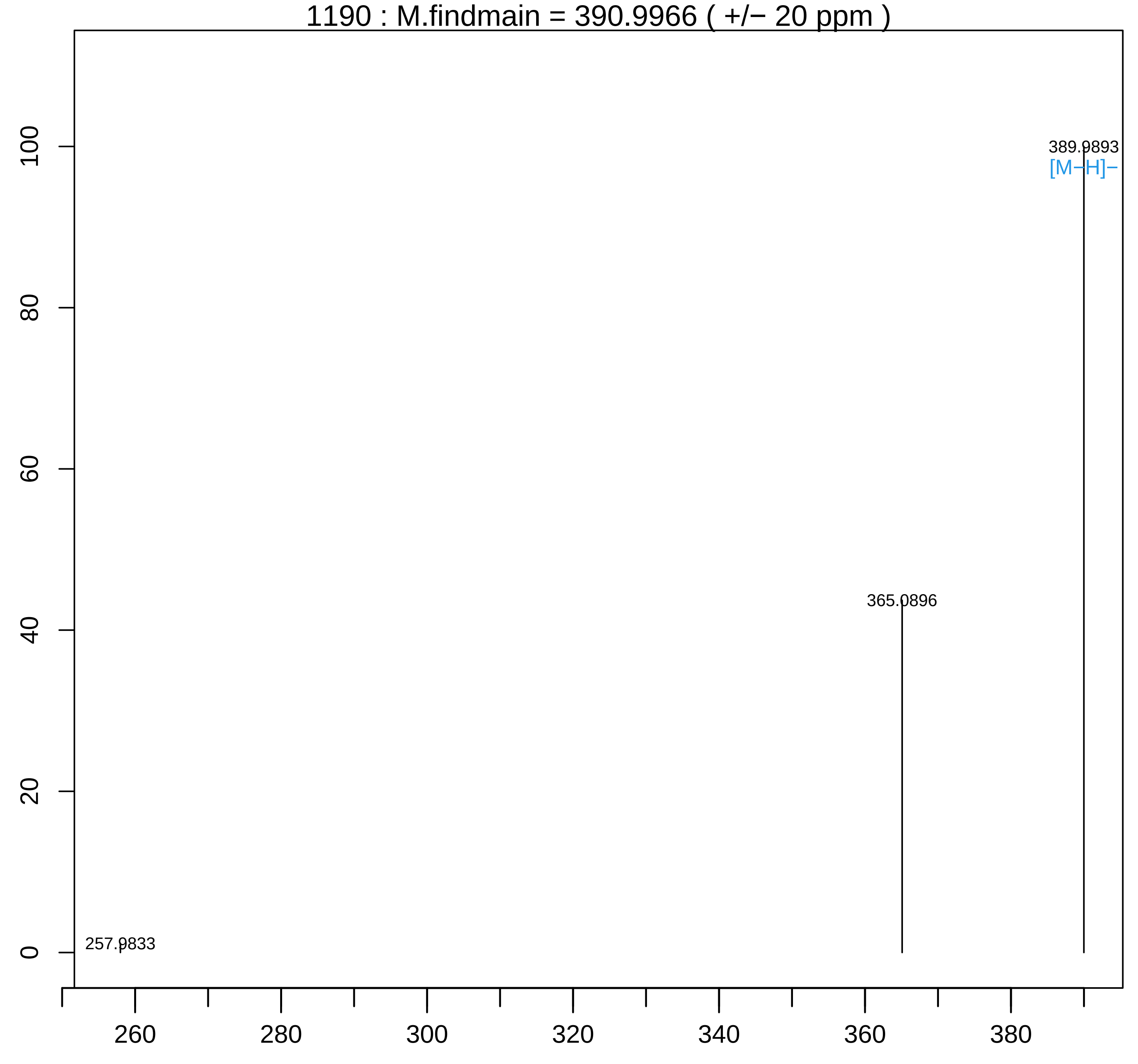
1190 : M.findmain = 390.9966 (+/− 20 ppm)
389.9893
[M−H]−
365.0896
257.9833
0
20
40
60
80
100
260
280
300
320
340
360
380

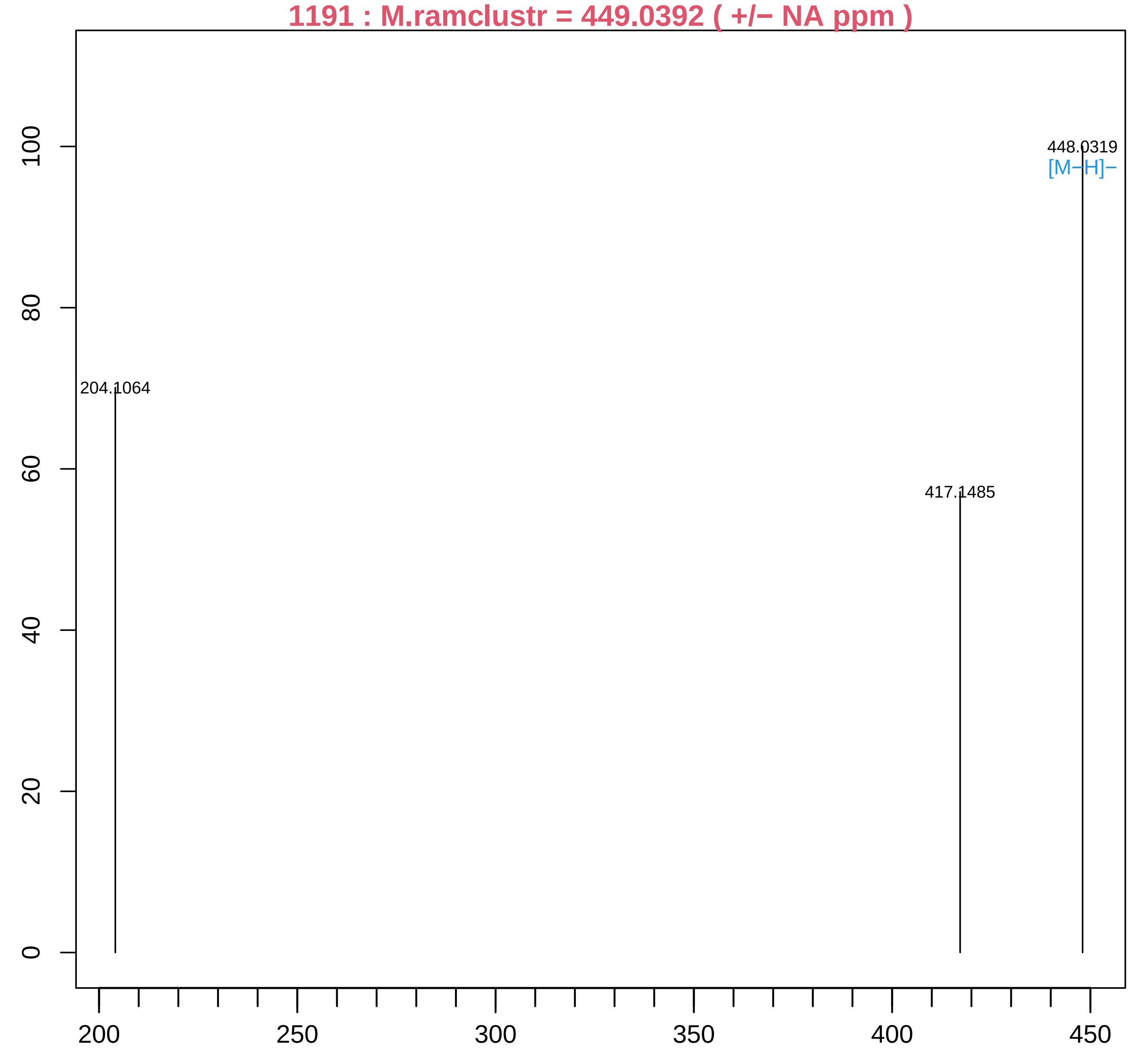

1191 : M.ramclustr = 449.0392 (+/− NA ppm)
448.0319
[M−H]−
204.1064
417.1485
0
20
40
60
80
100
200
250
300
350
400
450

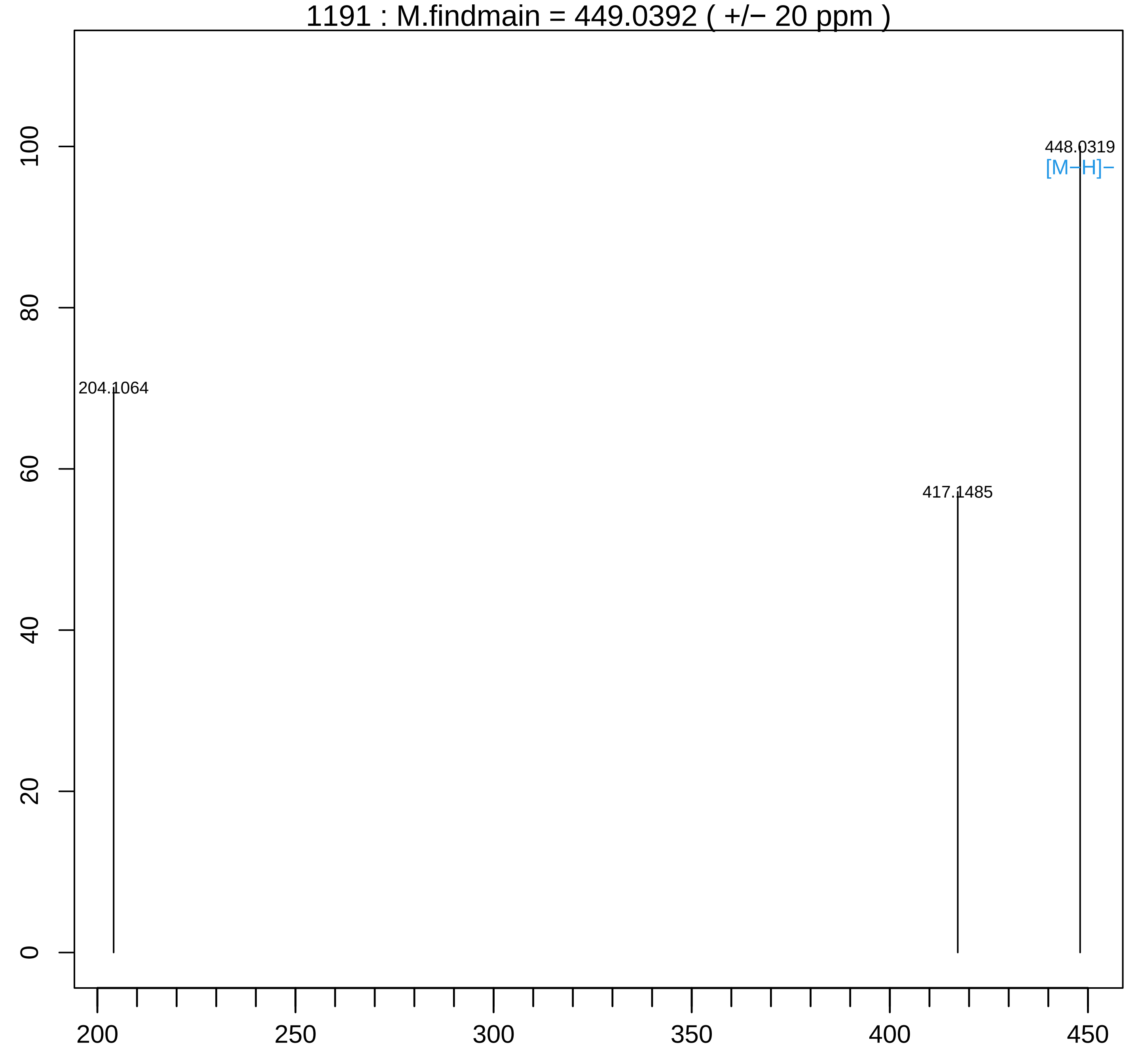

1191 : M.findmain = 449.0392 (+/− 20 ppm)
448.0319
[M−H]−
204.1064
417.1485
0
20
40
60
80
100
200
250
300
350
400
450

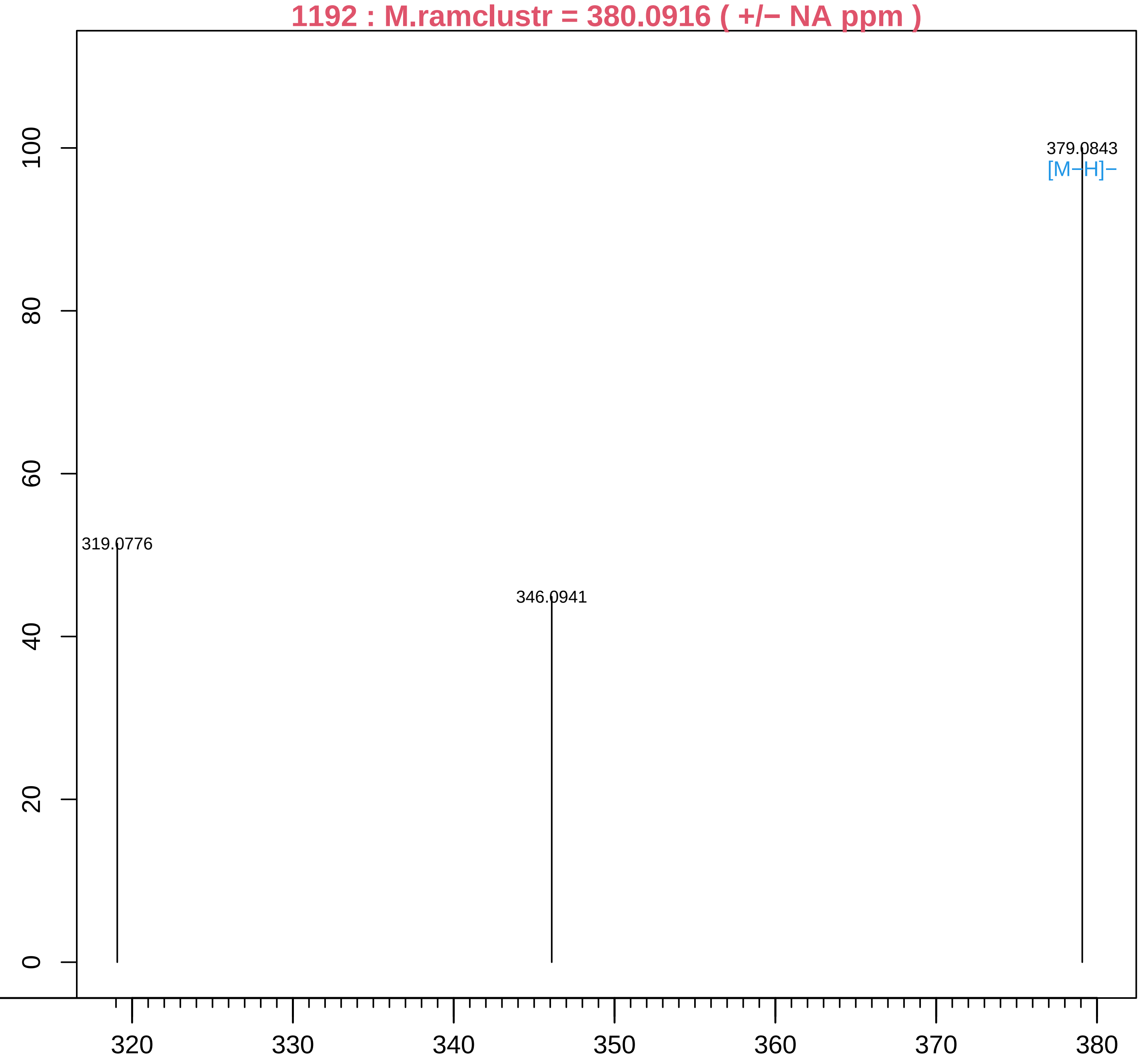
1192 : M.ramclustr = 380.0916 (+/− NA ppm)
379.0843
[M−H]−
319.0776
346.0941
0
20
40
60
80
100
320
330
340
350
360
370
380

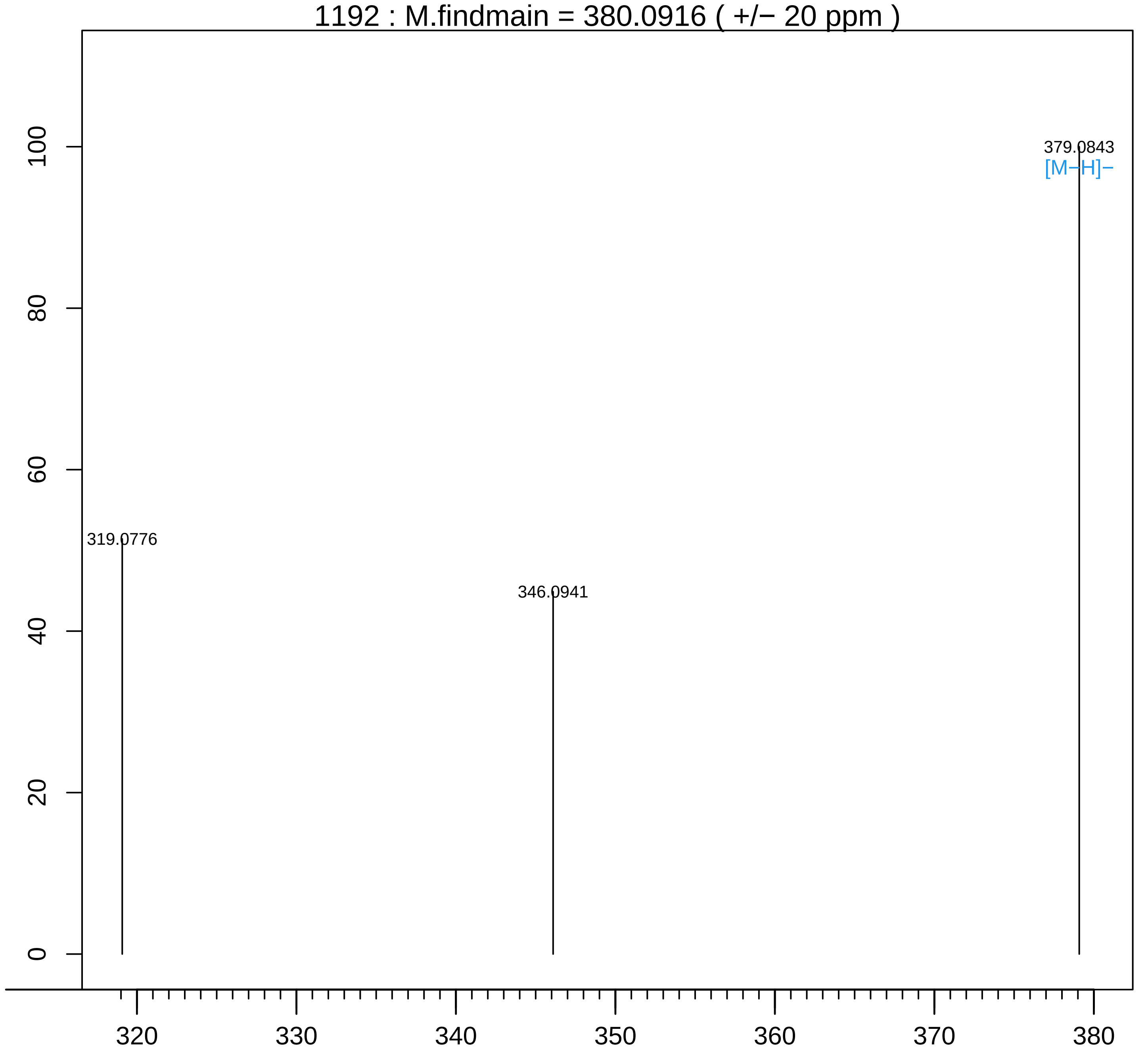
1192 : M.findmain = 380.0916 (+/− 20 ppm)
379.0843
[M−H]−
319.0776
346.0941
0
20
40
60
80
100
320
330
340
350
360
370
380

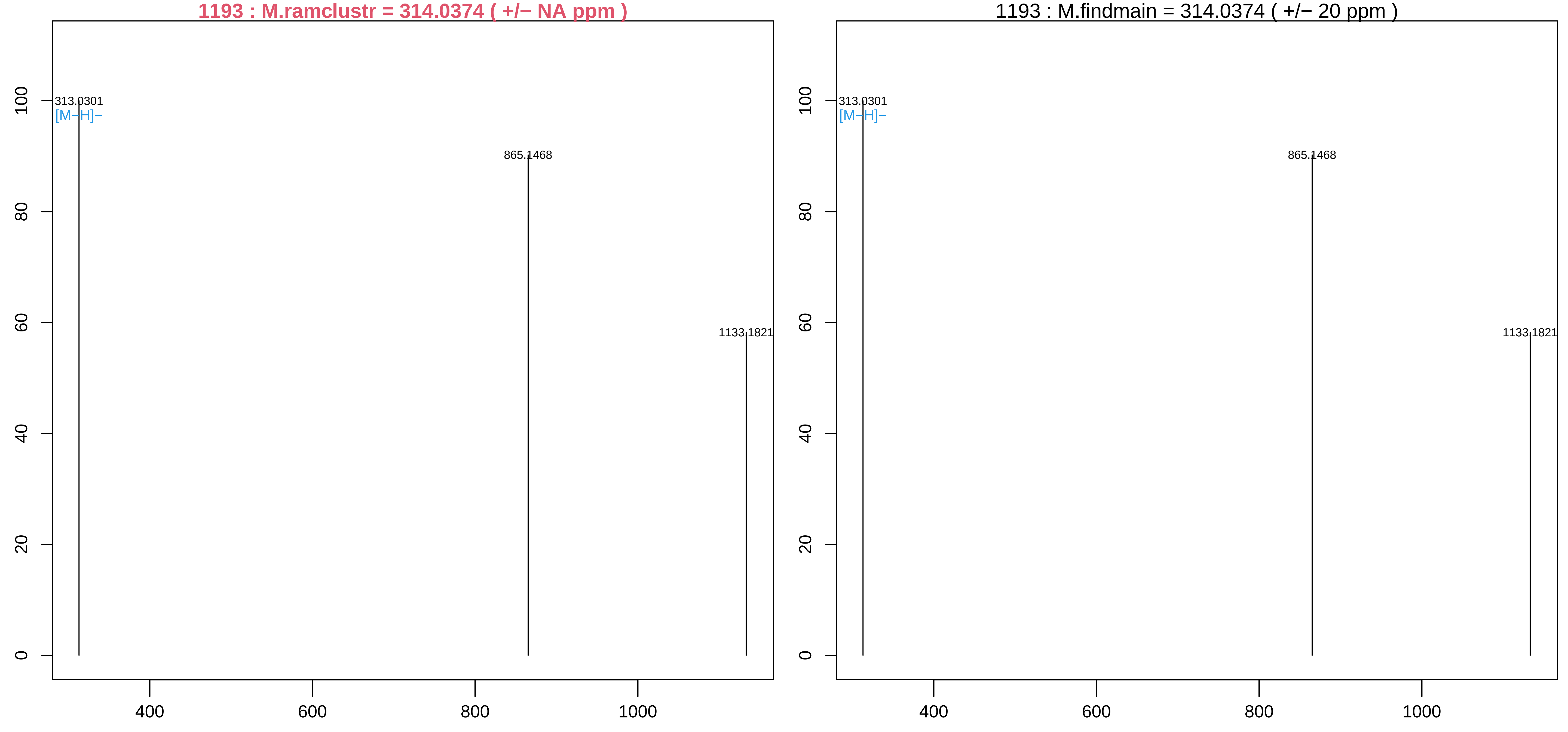
1193 : M.ramclustr = 314.0374 (+/− NA ppm)
1193 : M.findmain = 314.0374 (+/− 20 ppm)
313.0301
[M−H]−
865.1468
1133.1821
0
20
40
60
80
100
400
600
800
1000

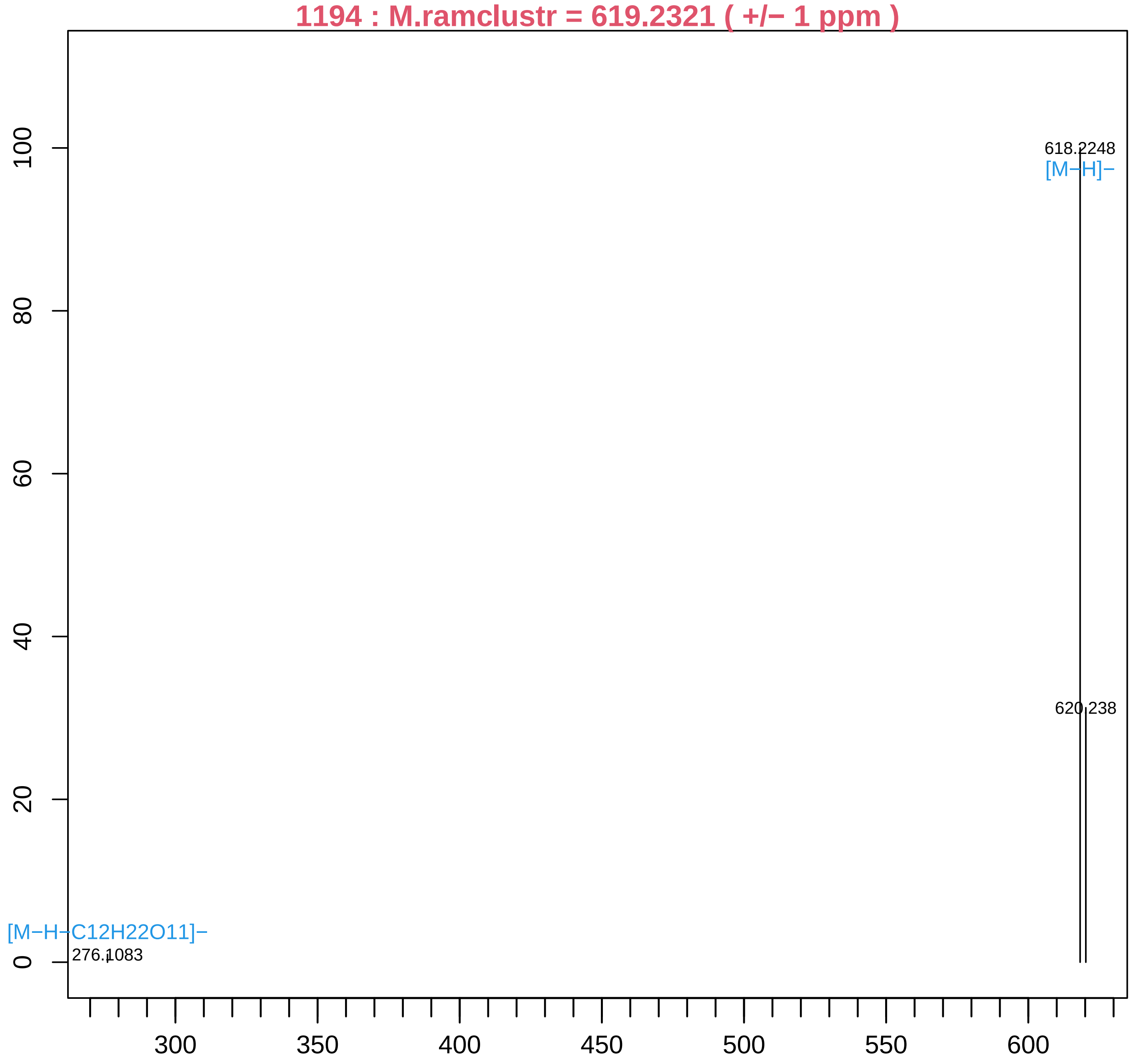
1194 : M.ramclustr = 619.2321 (+/− 1 ppm)
618.2248
[M−H]−
620.238
[M−H−C12H22O11]−
276.1083
0
20
40
60
80
100
300
350
400
450
500
550
600

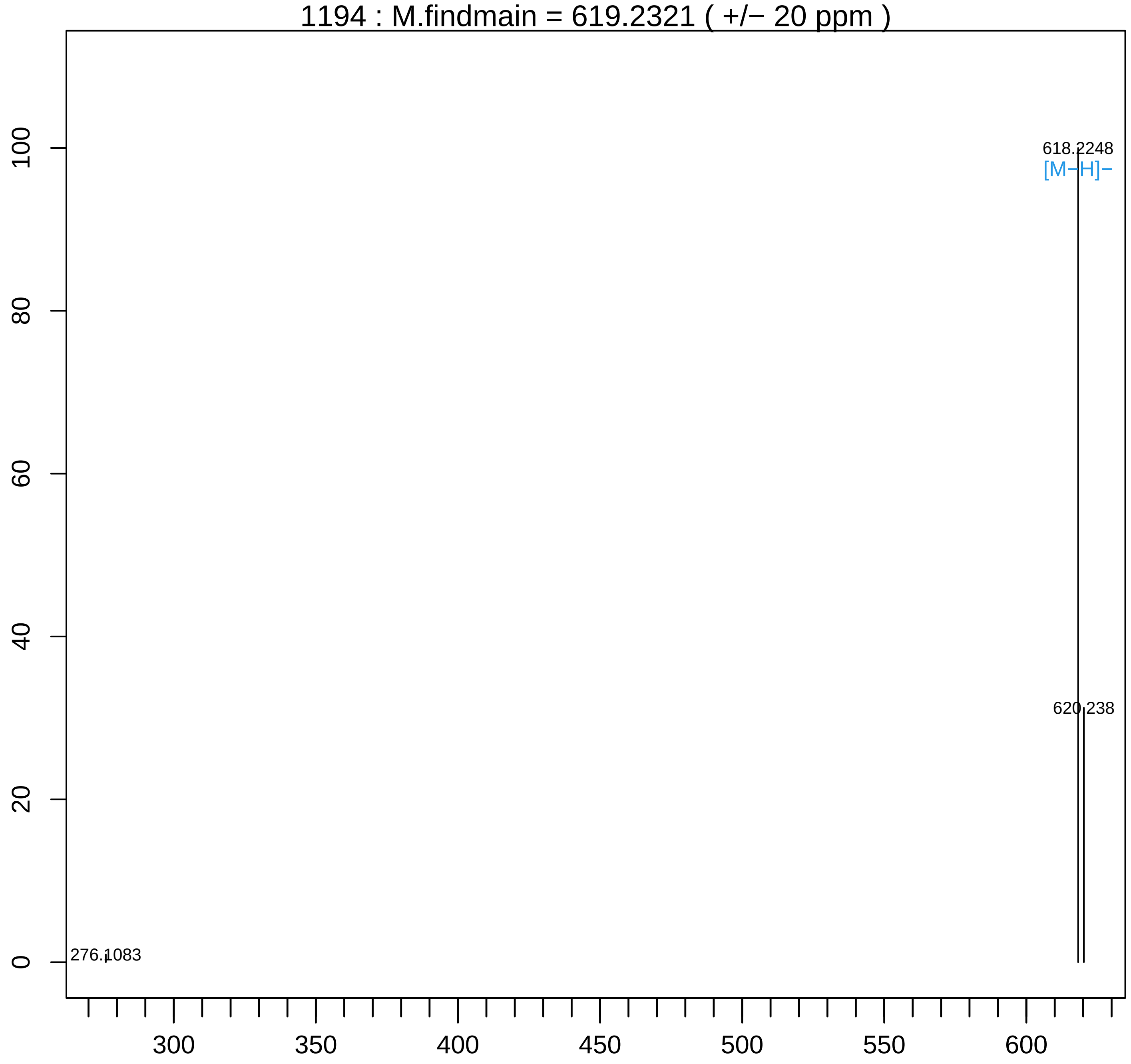
1194 : M.findmain = 619.2321 (+/− 20 ppm)
618.2248
[M−H]−
620.238
276.1083
0
20
40
60
80
100
300
350
400
450
500
550
600

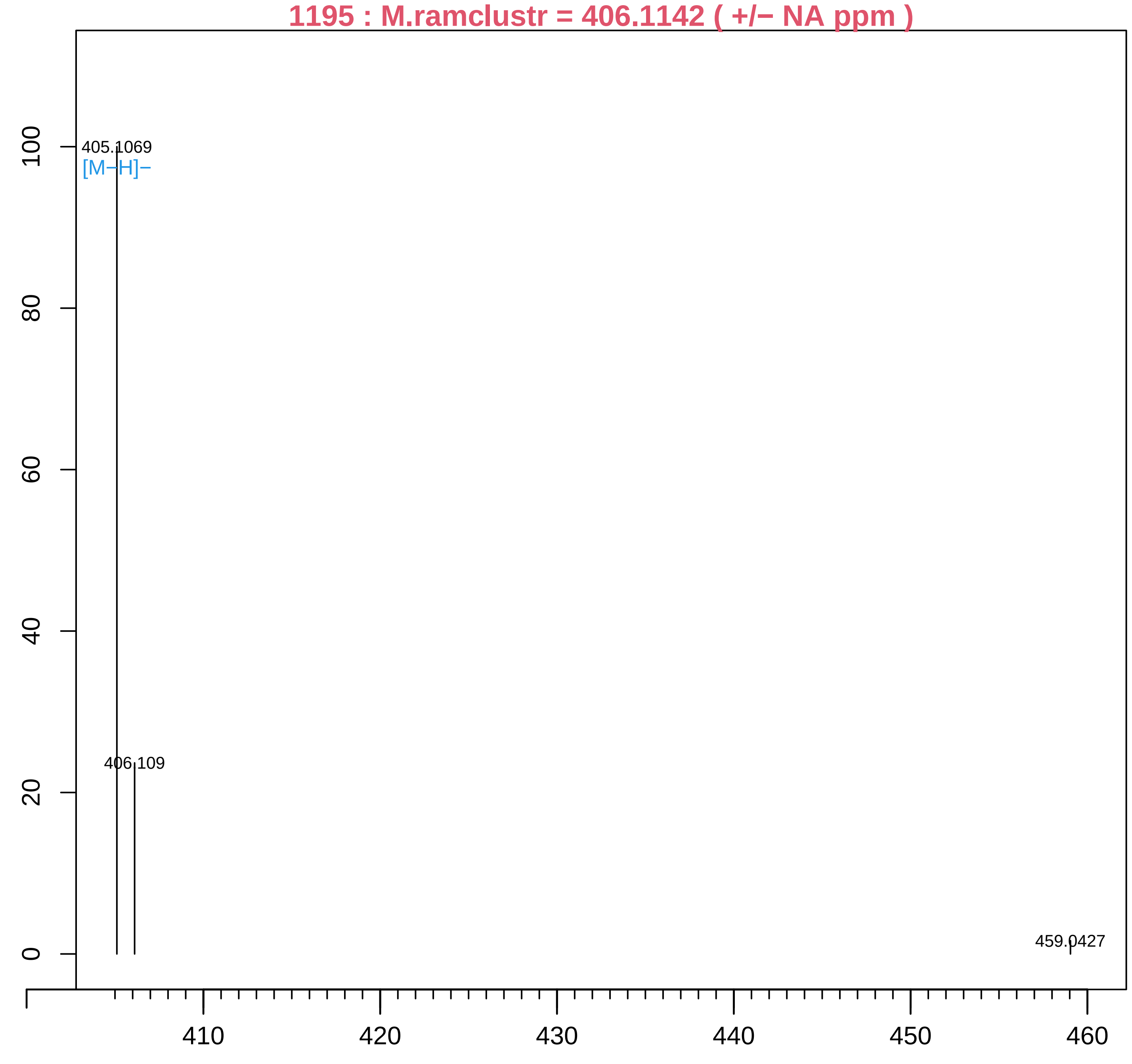
1195 : M.ramclustr = 406.1142 (+/− NA ppm)
405.1069
[M−H]−
406.109
459.0427
0
20
40
60
80
100
410
420
430
440
450
460

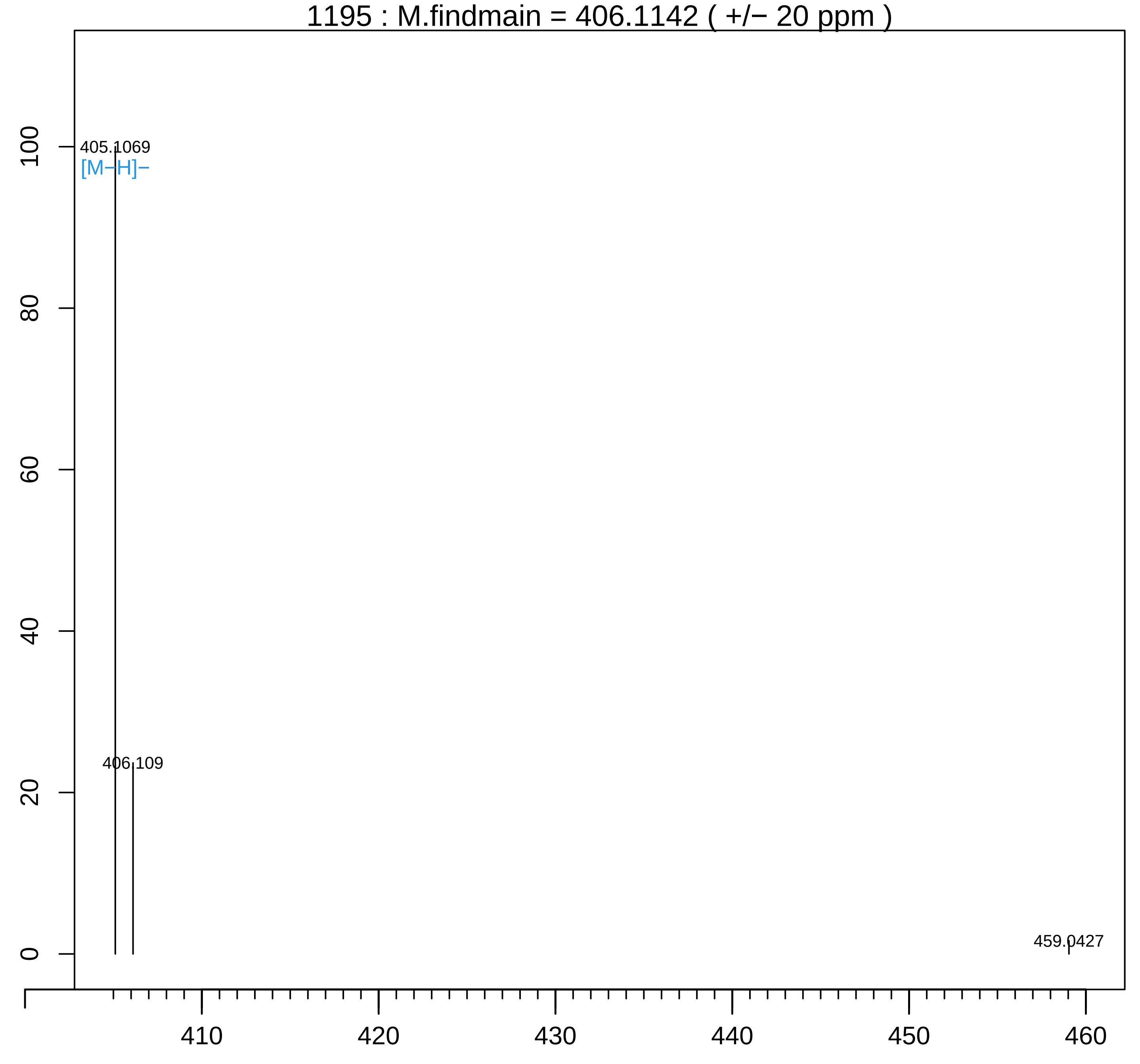
1195 : M.findmain = 406.1142 (+/− 20 ppm)
405.1069
[M−H]−
406.109
459.0427
0
20
40
60
80
100
410
420
430
440
450
460

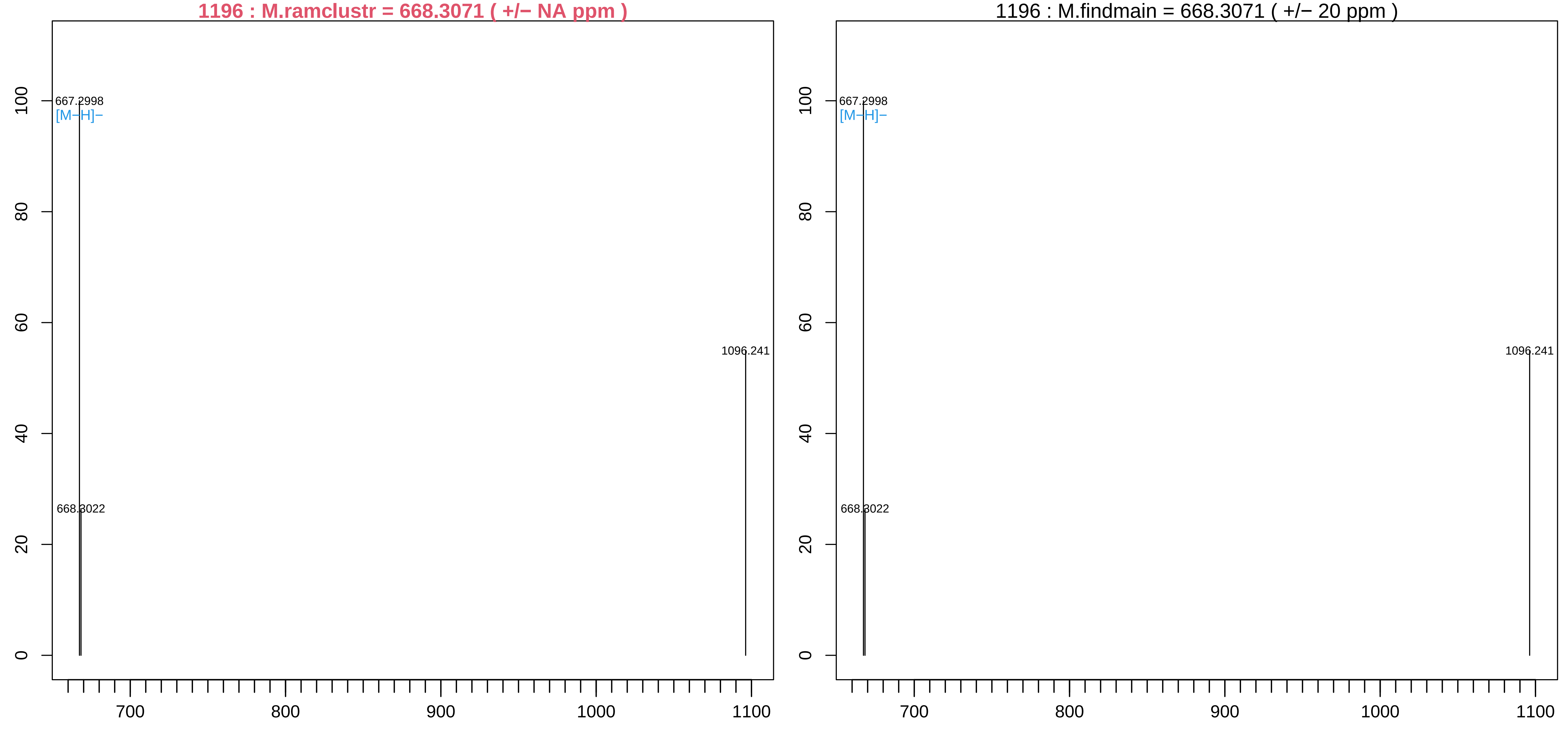
1196 : M.ramclustr = 668.3071 (+/− NA ppm)
667.2998
[M−H]−
668.3022
1096.241
1196 : M.findmain = 668.3071 (+/− 20 ppm)
667.2998
[M−H]−
668.3022
1096.241
0
20
40
60
80
100
700
800
900
1000
1100

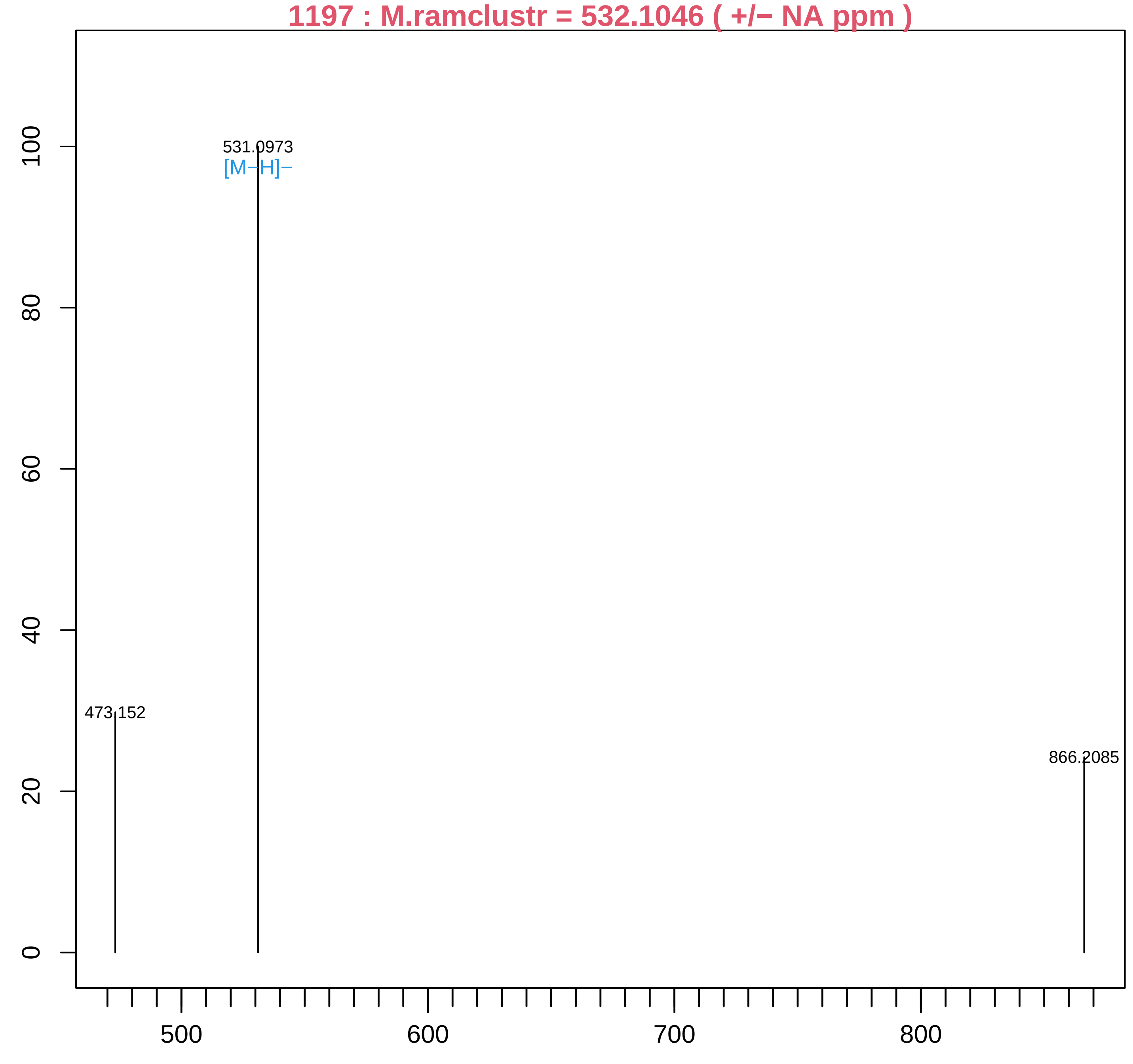
1197 : M.ramclustr = 532.1046 (+/− NA ppm)
531.0973
[M−H]−
473.152
866.2085
0
20
40
60
80
100
500
600
700
800

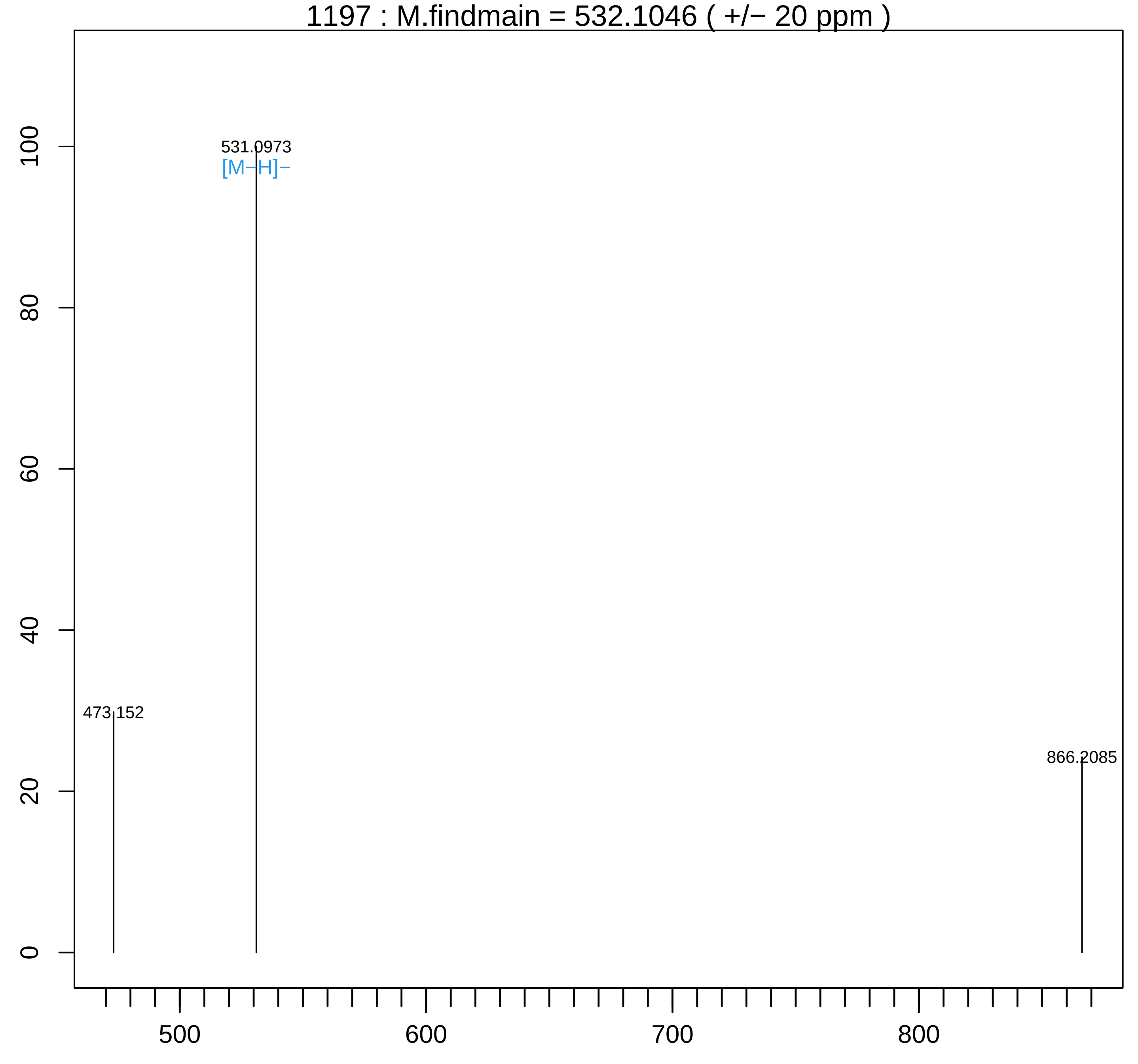
1197 : M.findmain = 532.1046 (+/− 20 ppm)
531.0973
[M−H]−
473.152
866.2085
0
20
40
60
80
100
500
600
700
800

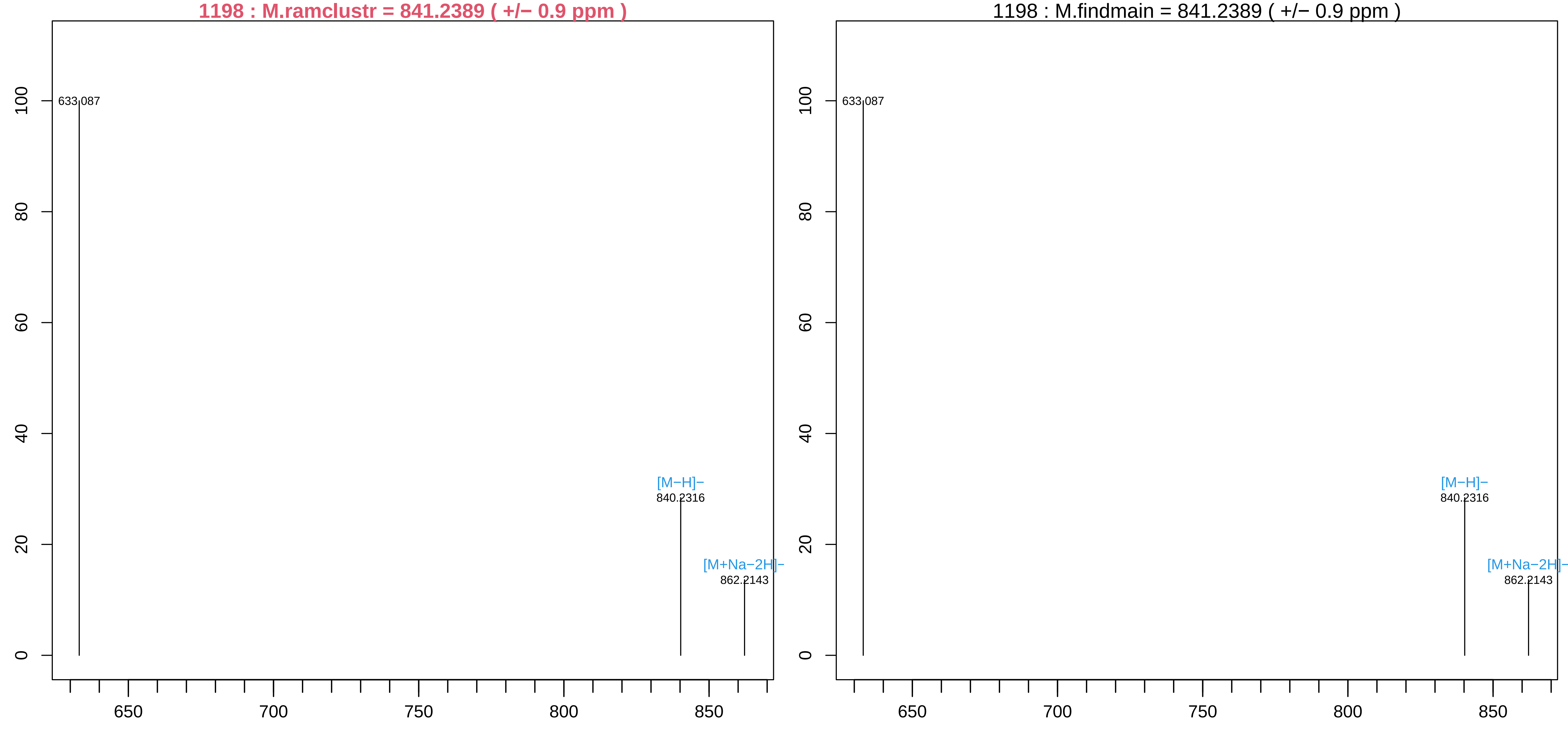

1198 : M.ramclustr = 841.2389 (+/− 0.9 ppm)
1198 : M.findmain = 841.2389 (+/− 0.9 ppm)
633.087
[M−H]−
840.2316
[M+Na−2H]−
862.2143
0
20
40
60
80
100
650
700
750
800
850

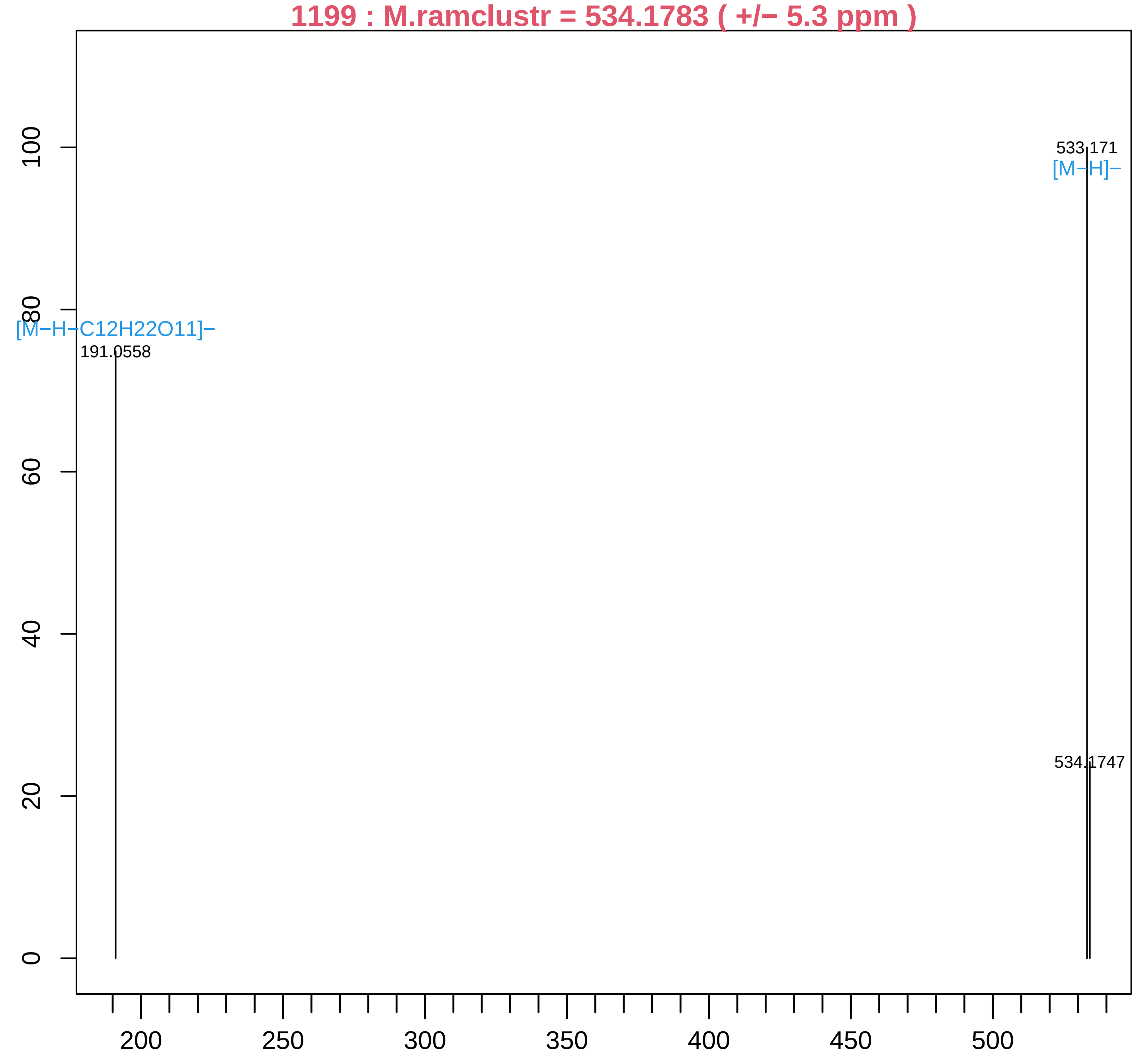
1199 : M.ramclustr = 534.1783 (+/− 5.3 ppm)
533.171
[M−H]−
[M−H−C12H22O11]−
191.0558
534.1747
0
20
40
60
80
100
200
250
300
350
400
450
500

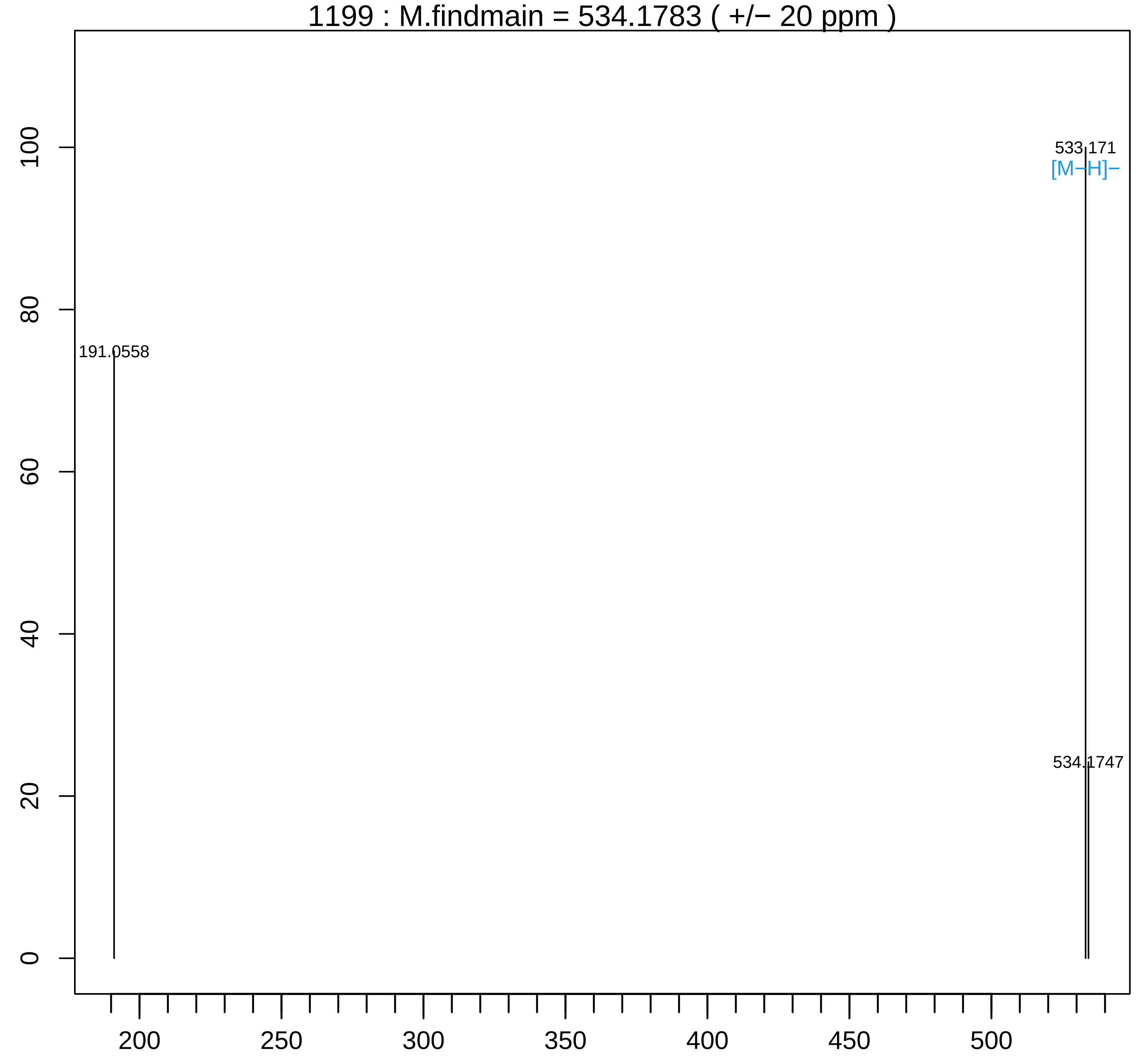
1199 : M.findmain = 534.1783 (+/− 20 ppm)
533.171
[M−H]−
191.0558
534.1747
0
20
40
60
80
100
200
250
300
350
400
450
500

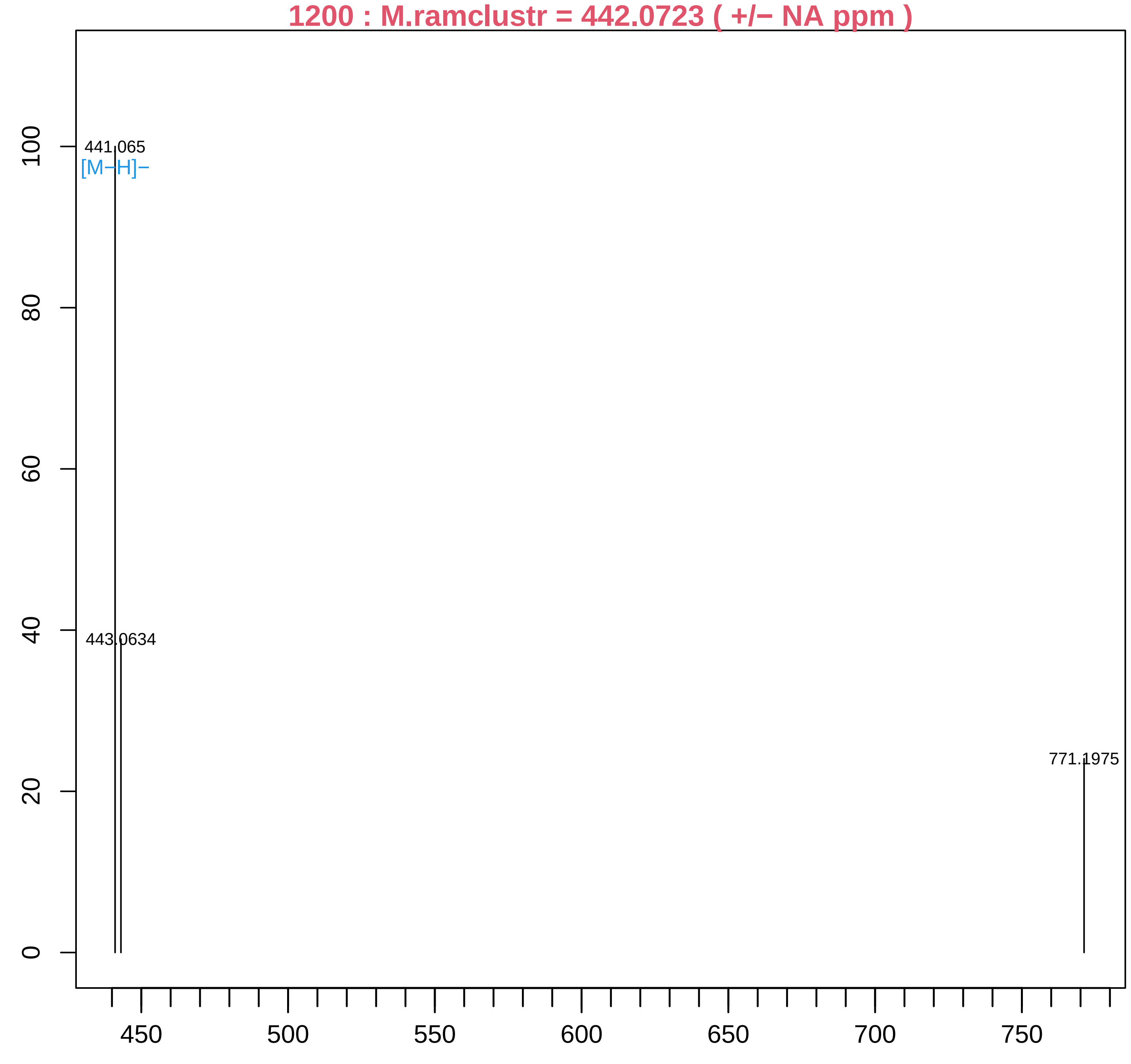
1200 : M.ramclustr = 442.0723 (+/− NA ppm)
441.065
[M−H]−
443.0634
771.1975
0
20
40
60
80
100
450
500
550
600
650
700
750

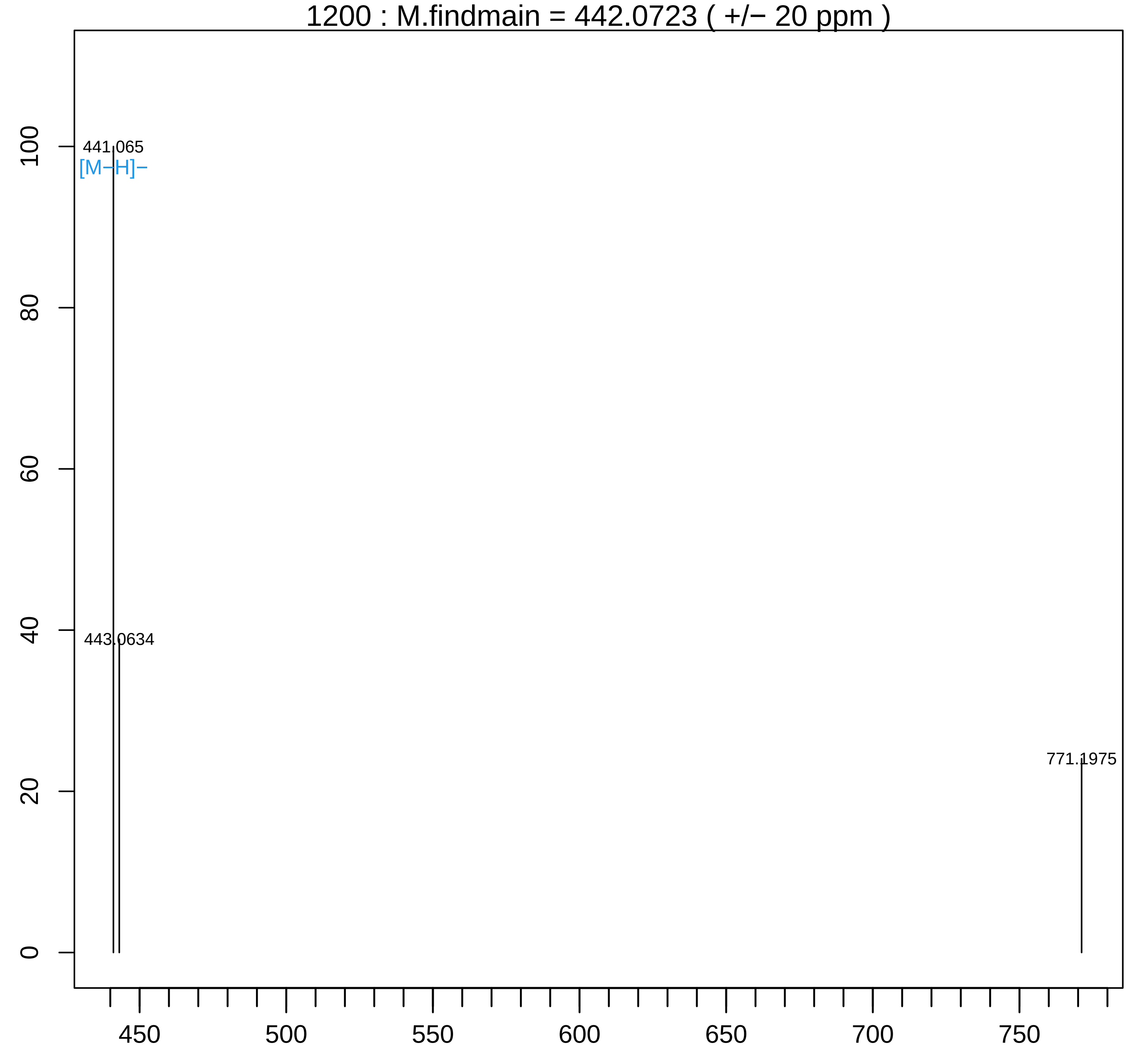
1200 : M.findmain = 442.0723 (+/− 20 ppm)
441.065
[M−H]−
443.0634
771.1975
0
20
40
60
80
100
450
500
550
600
650
700
750

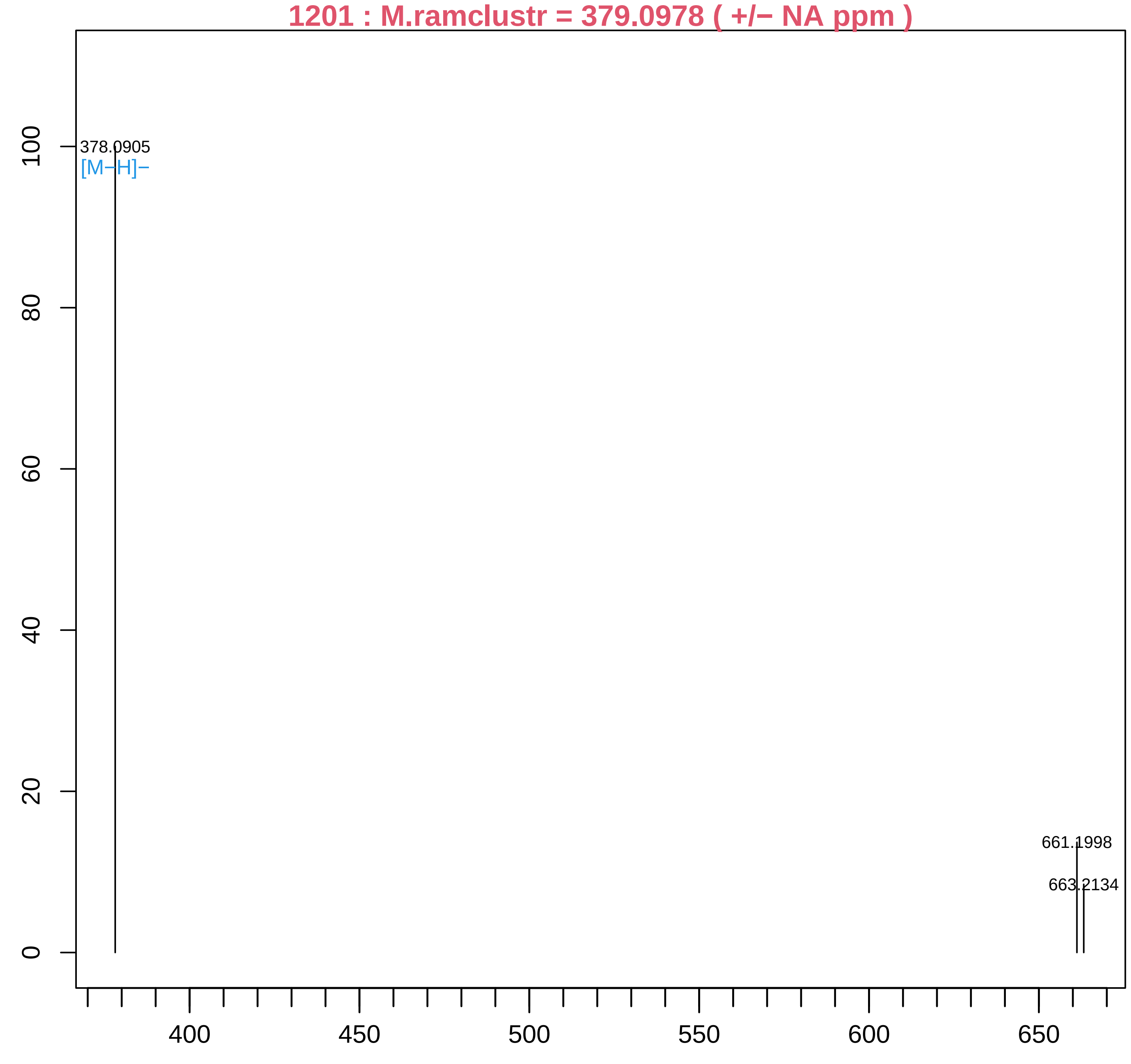

1201 : M.ramclustr = 379.0978 (+/− NA ppm)
378.0905
[M−H]−
661.1998
663.2134
0
20
40
60
80
100
400
450
500
550
600
650

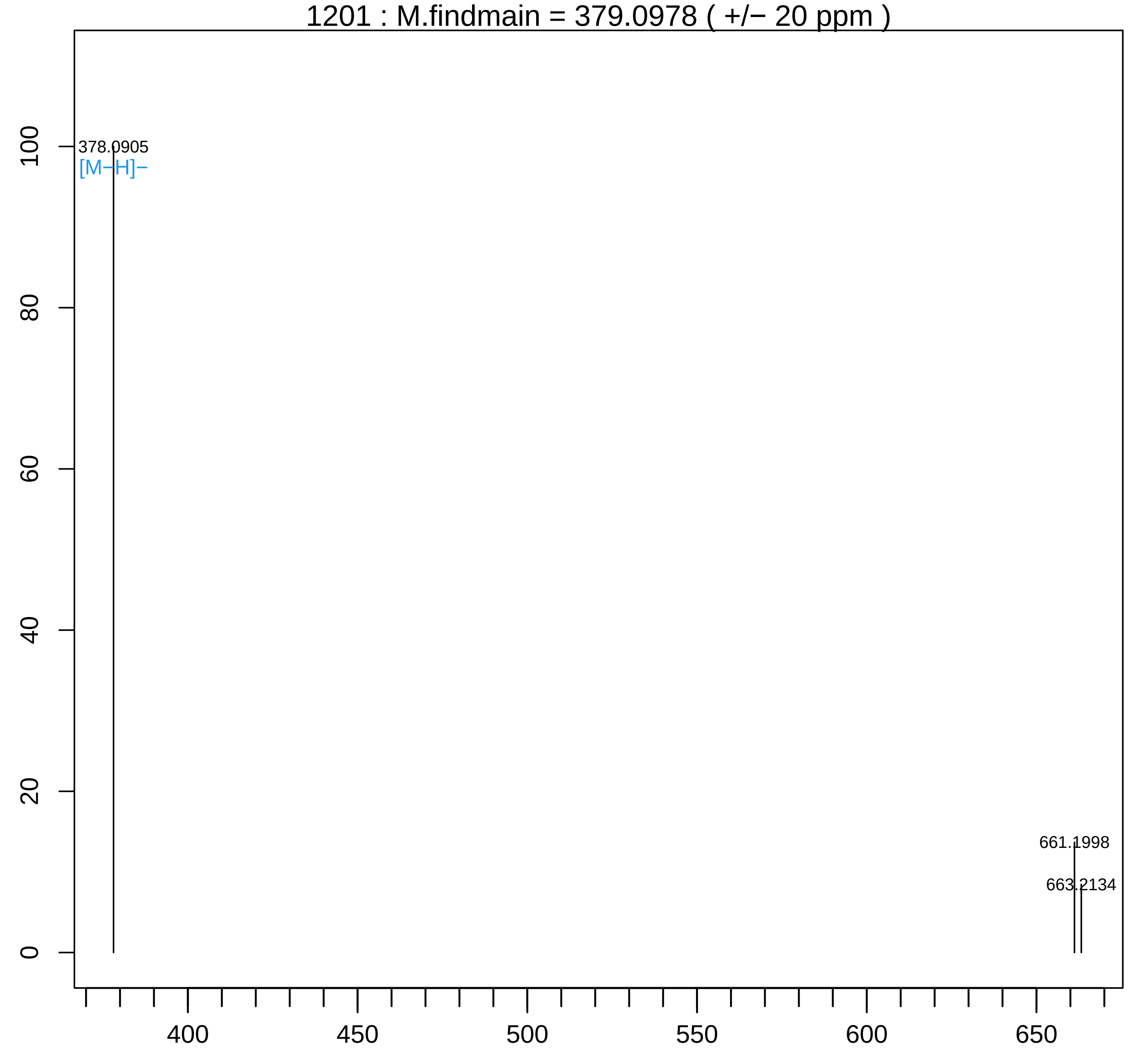

1201 : M.findmain = 379.0978 (+/− 20 ppm)
378.0905
[M−H]−
661.1998
663.2134
0
20
40
60
80
100
400
450
500
550
600
650

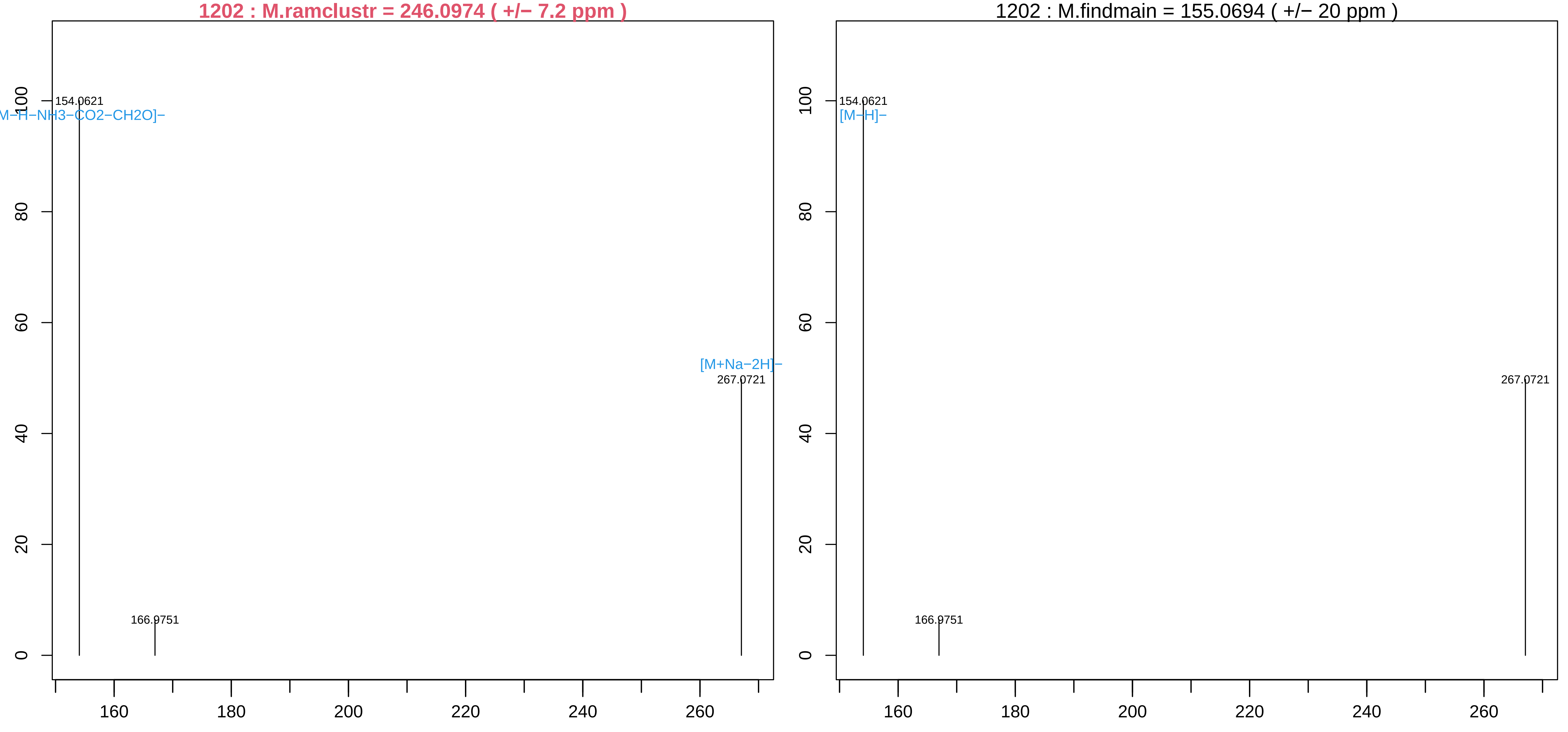

1202 : M.ramclustr = 246.0974 (+/− 7.2 ppm)
154.0621
M−H−NH3−CO2−CH2O]−
166.9751
[M+Na−2H]−
267.0721
1202 : M.findmain = 155.0694 (+/− 20 ppm)
154.0621
[M−H]−
166.9751
267.0721
0
20
40
60
80
100
160
180
200
220
240
260

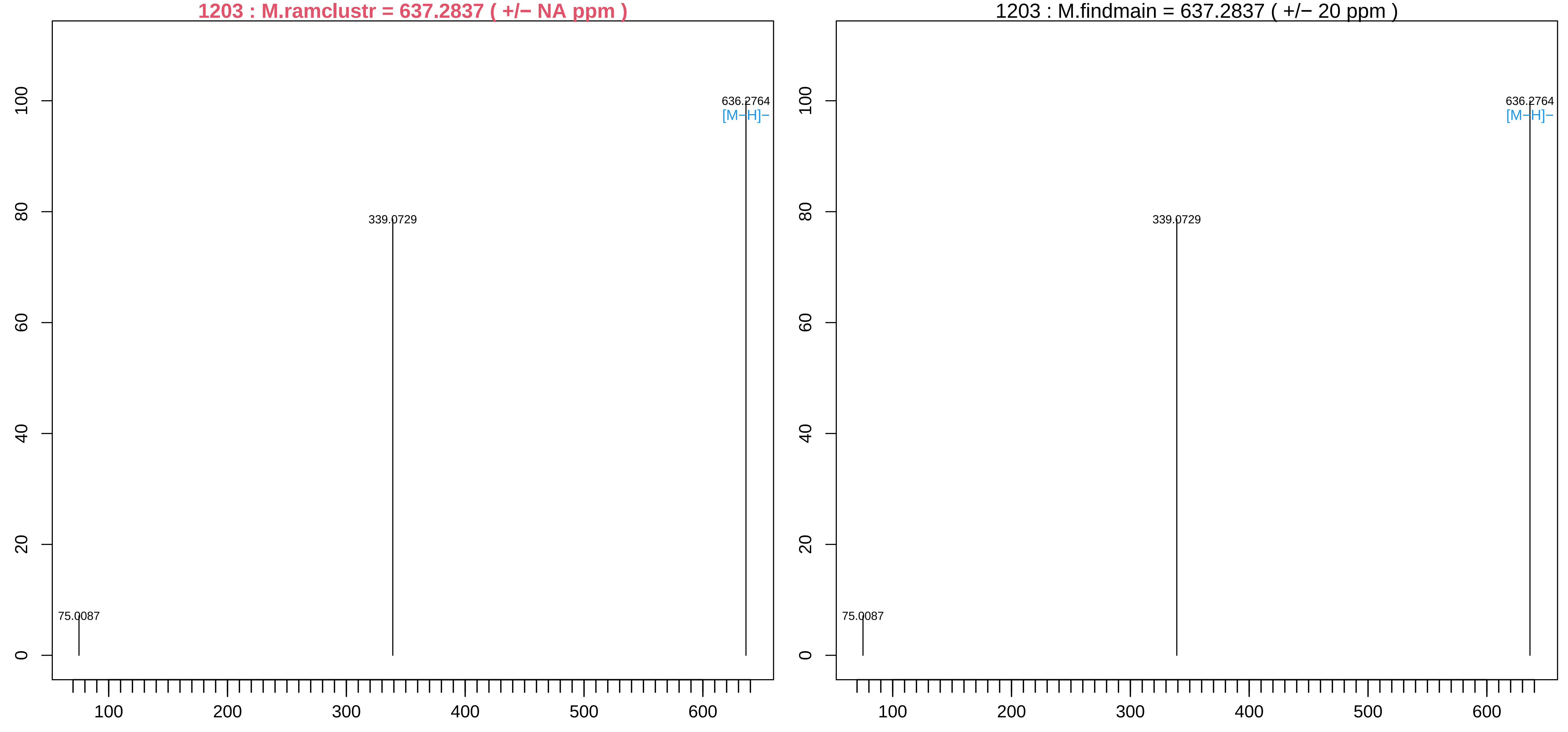
1203 : M.ramclustr = 637.2837 (+/− NA ppm)
1203 : M.findmain = 637.2837 (+/− 20 ppm)
636.2764
[M−H]−
339.0729
75.0087
0
20
40
60
80
100
100
200
300
400
500
600

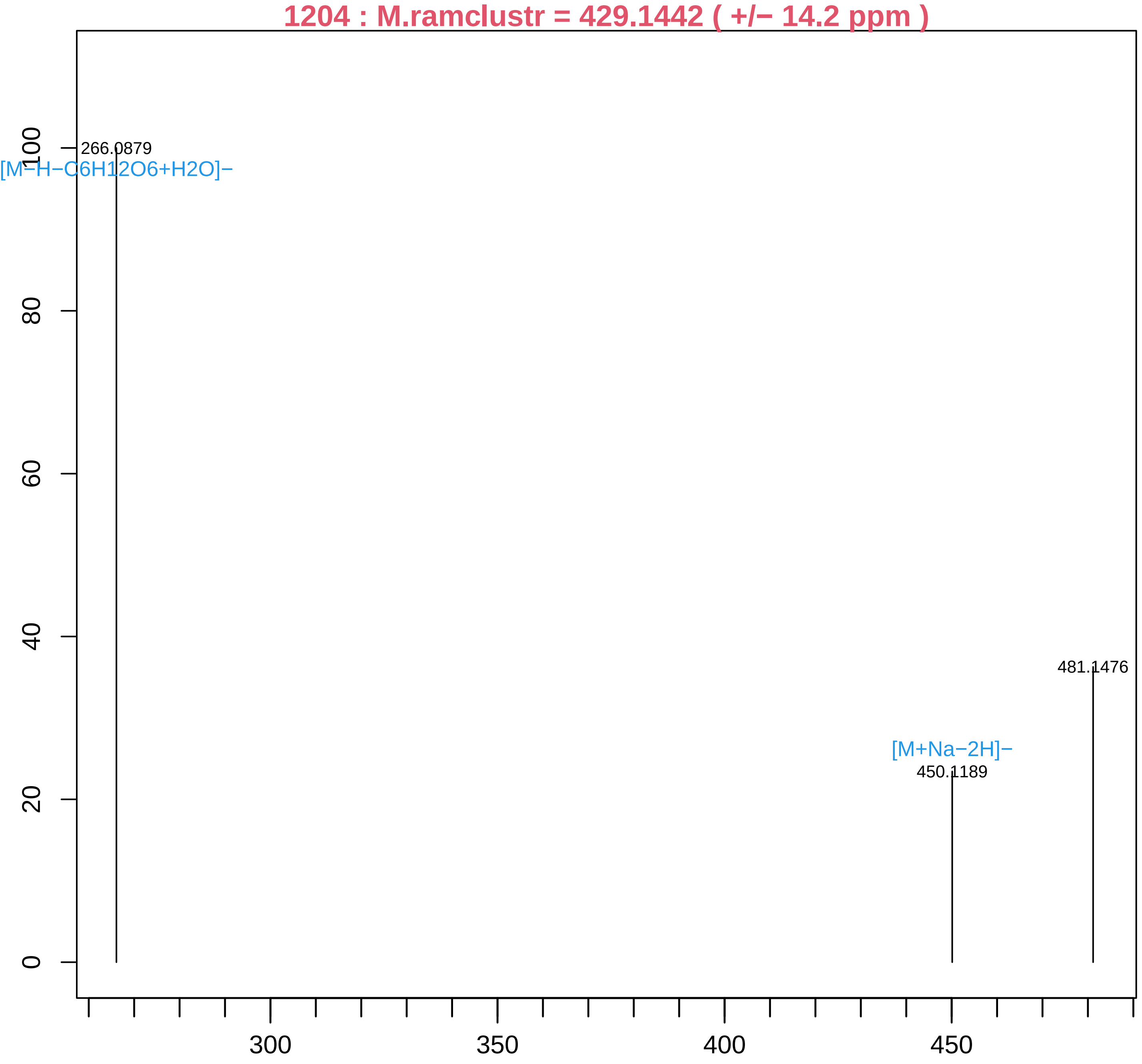

1204 : M.ramclustr = 429.1442 (+/− 14.2 ppm)
266.0879
[M−H−C6H12O6+H2O]−
[M+Na−2H]−
450.1189
481.1476
0
20
40
60
80
100
300
350
400
450

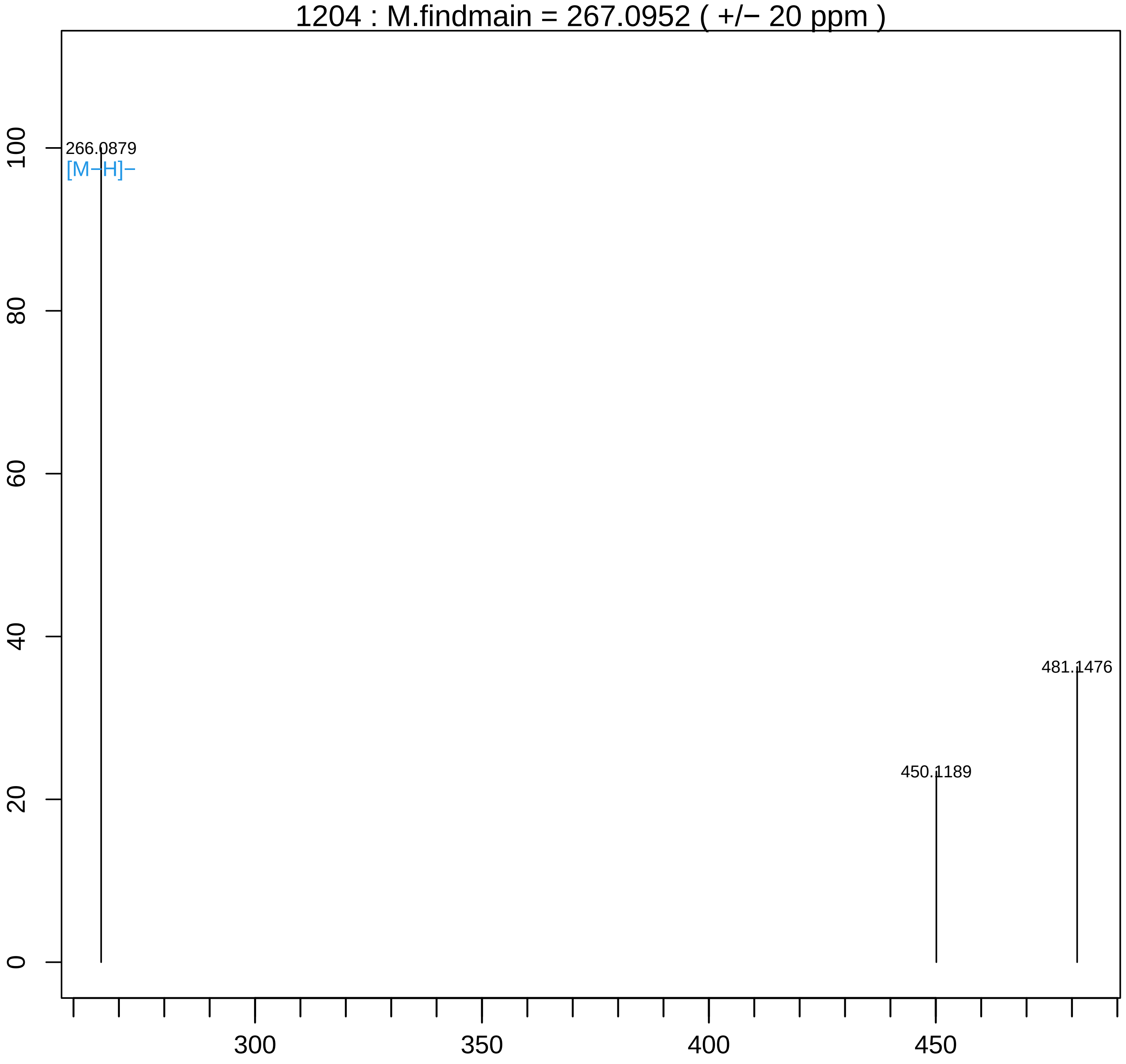

1204 : M.findmain = 267.0952 (+/− 20 ppm)
266.0879
[M−H]−
450.1189
481.1476
0
20
40
60
80
100
300
350
400
450

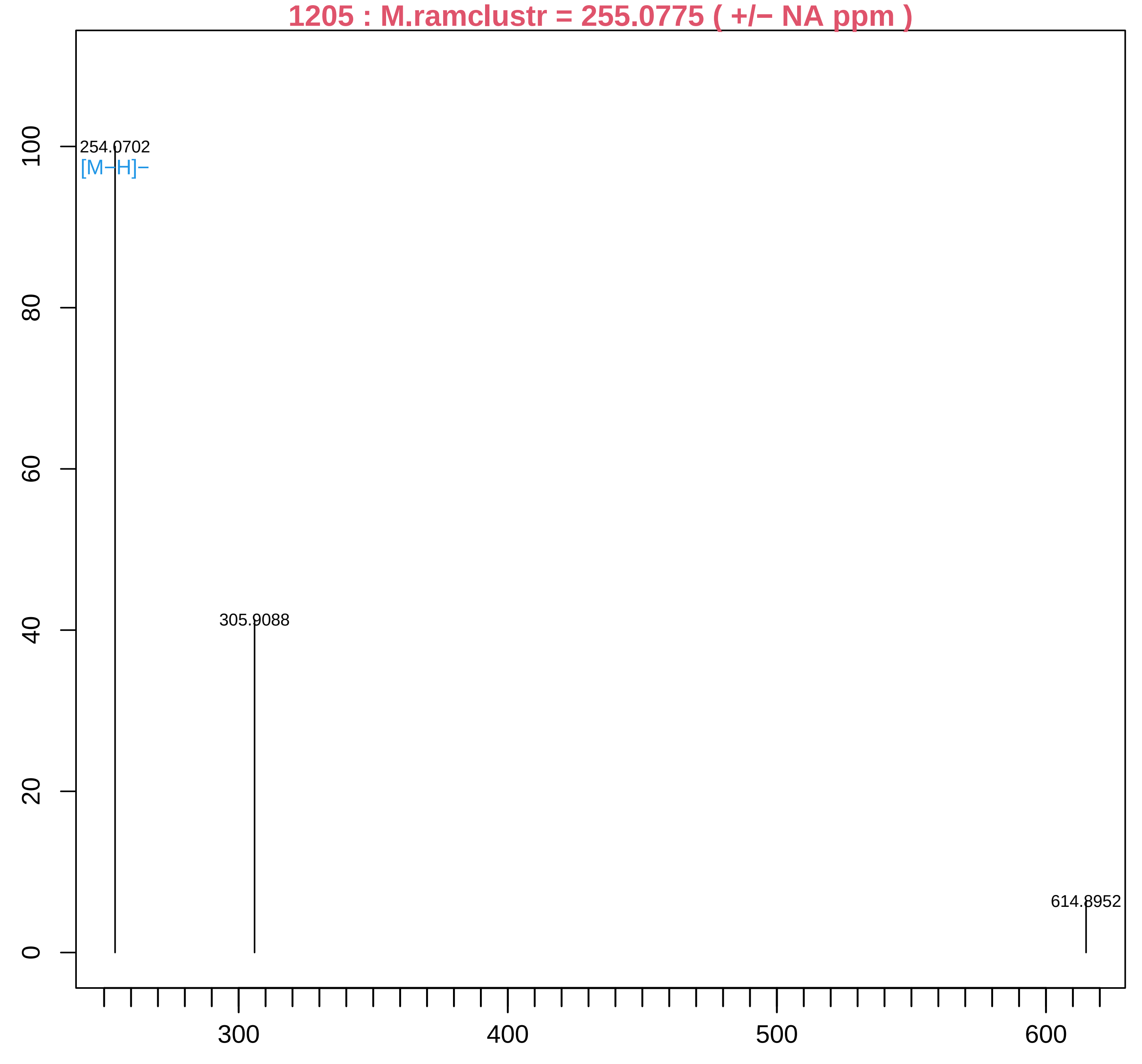
1205 : M.ramclustr = 255.0775 (+/− NA ppm)
254.0702
[M−H]−
305.9088
614.8952
0
20
40
60
80
100
300
400
500
600

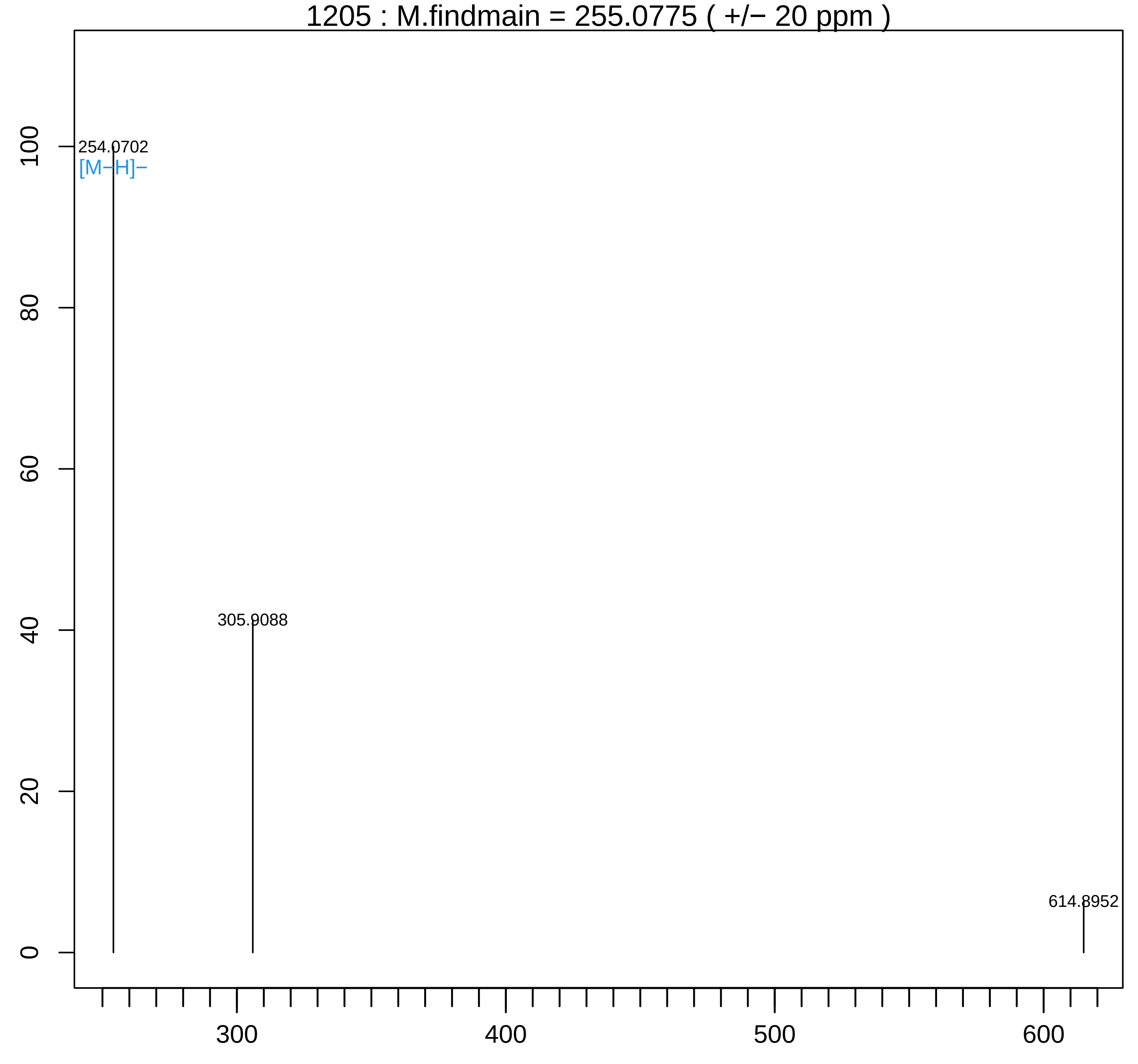
1205 : M.findmain = 255.0775 (+/− 20 ppm)
254.0702
[M−H]−
305.9088
614.8952
0
20
40
60
80
100
300
400
500
600

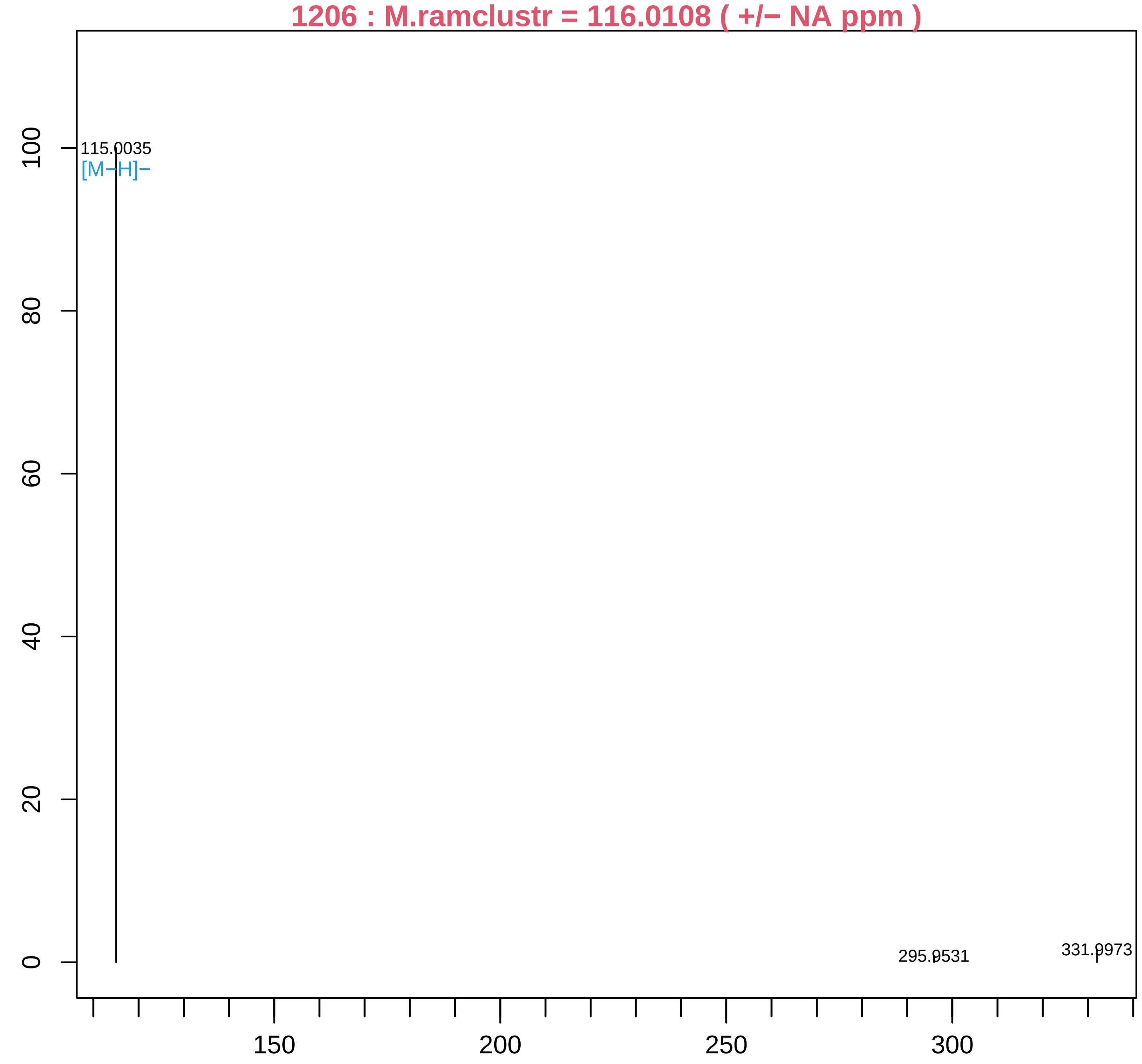
1206 : M.ramclustr = 116.0108 (+/− NA ppm)
115.0035
[M−H]−
295.9531
331.9973
0
20
40
60
80
100
150
200
250
300

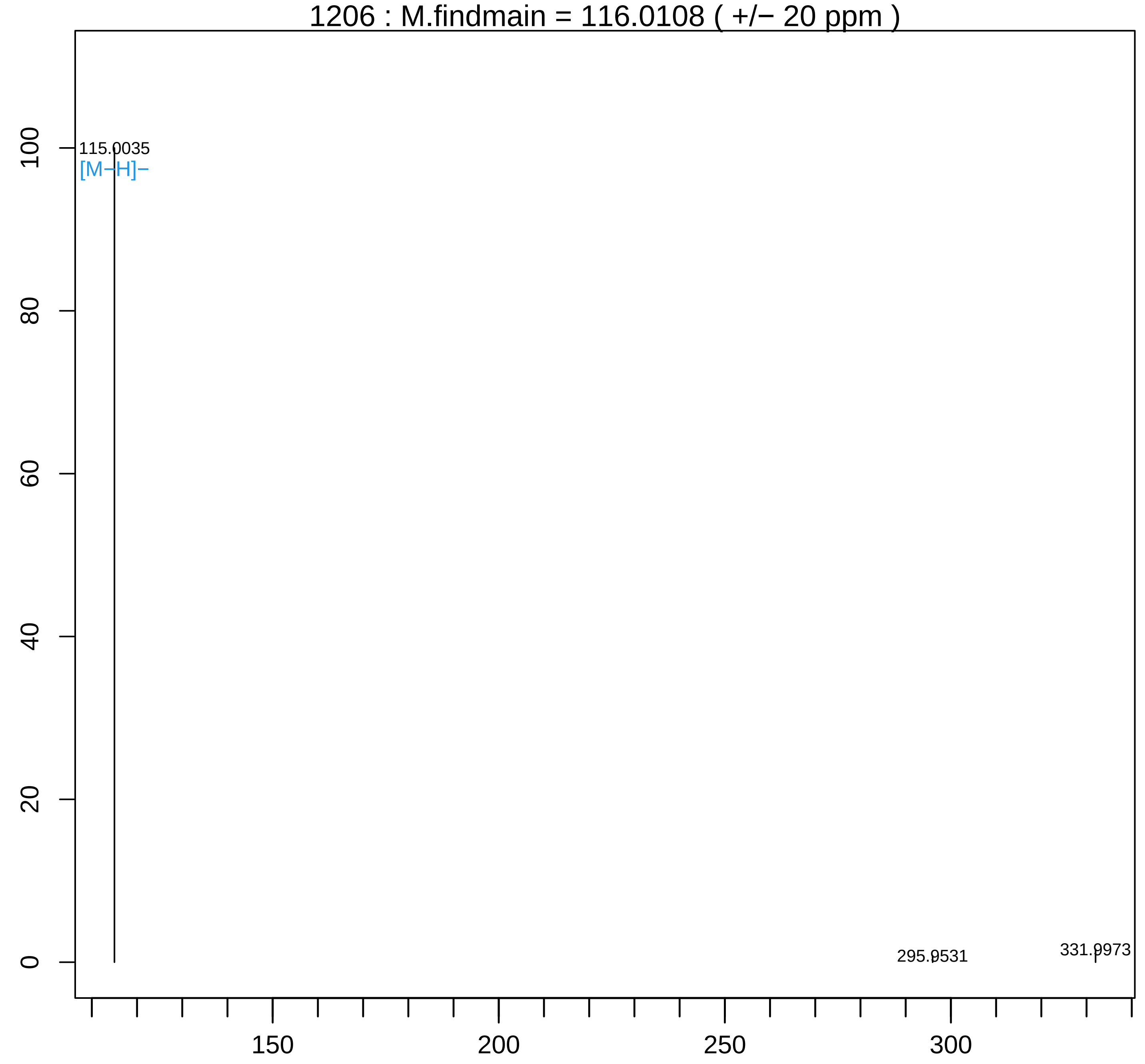
1206 : M.findmain = 116.0108 (+/− 20 ppm)
115.0035
[M−H]−
295.9531
331.9973
0
20
40
60
80
100
150
200
250
300

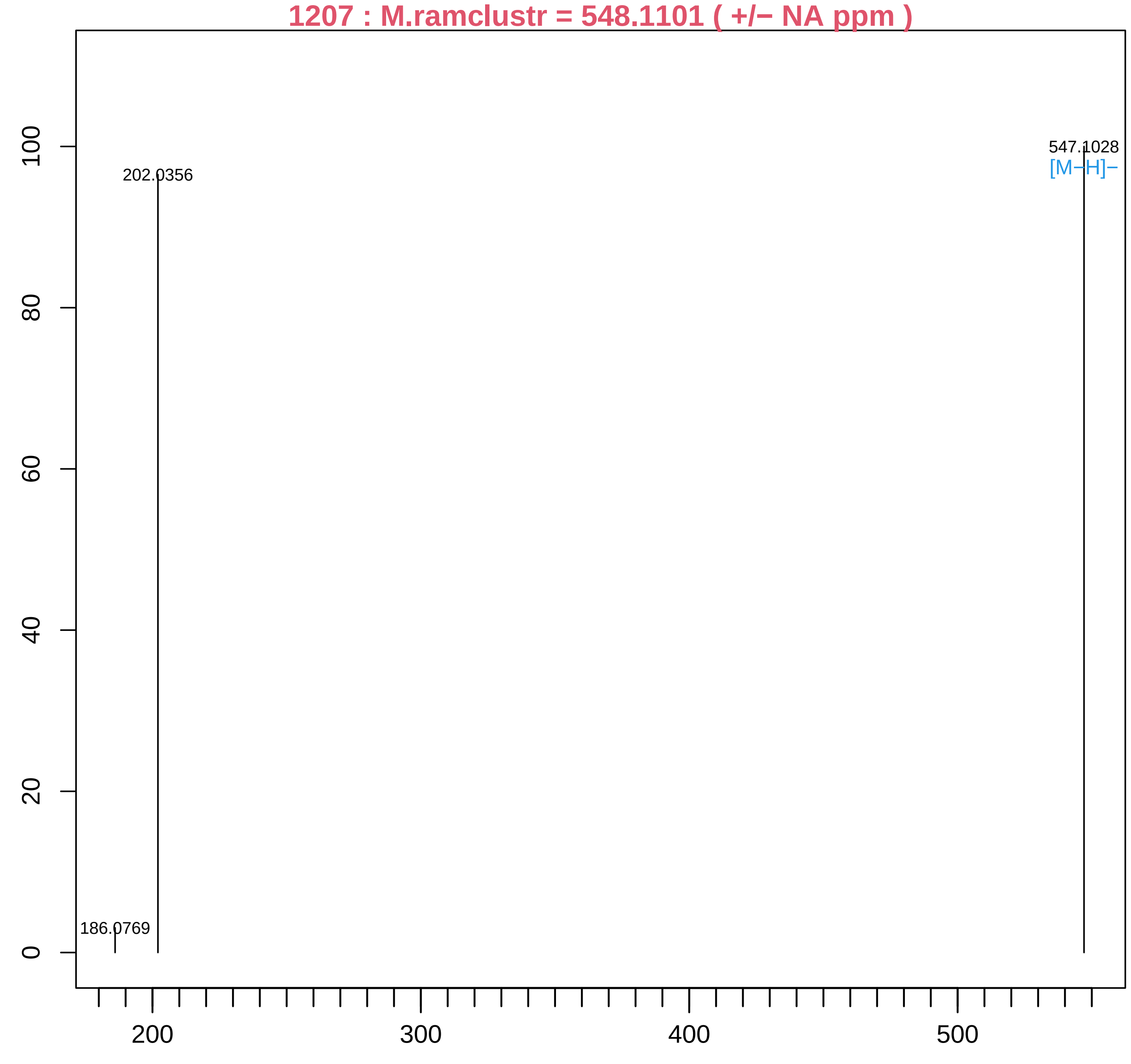

1207 : M.ramclustr = 548.1101 (+/− NA ppm)
547.1028
[M−H]−
202.0356
186.0769
0
20
40
60
80
100
200
300
400
500

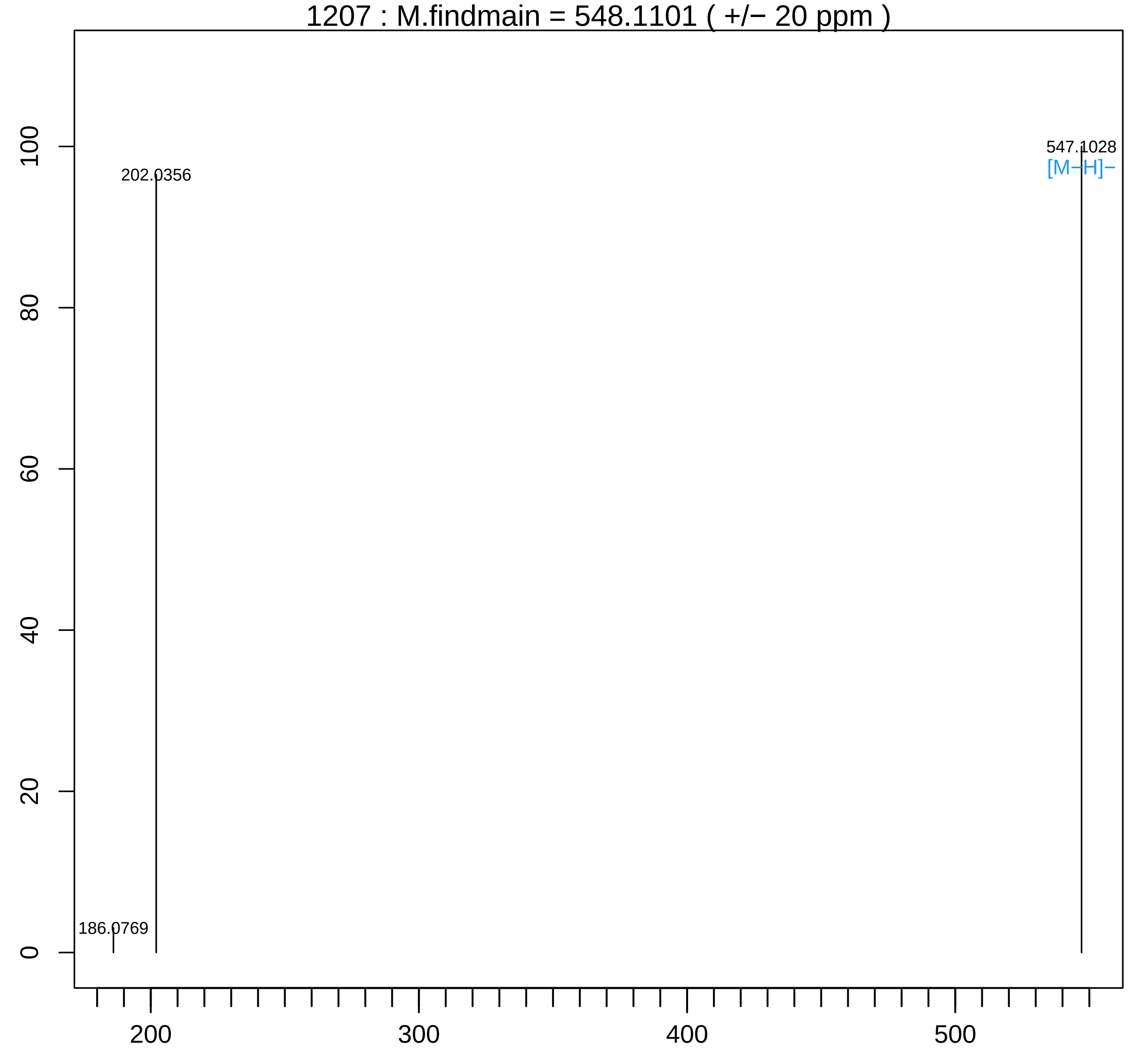

1207 : M.findmain = 548.1101 (+/− 20 ppm)
547.1028
[M−H]−
202.0356
186.0769
0
20
40
60
80
100
200
300
400
500

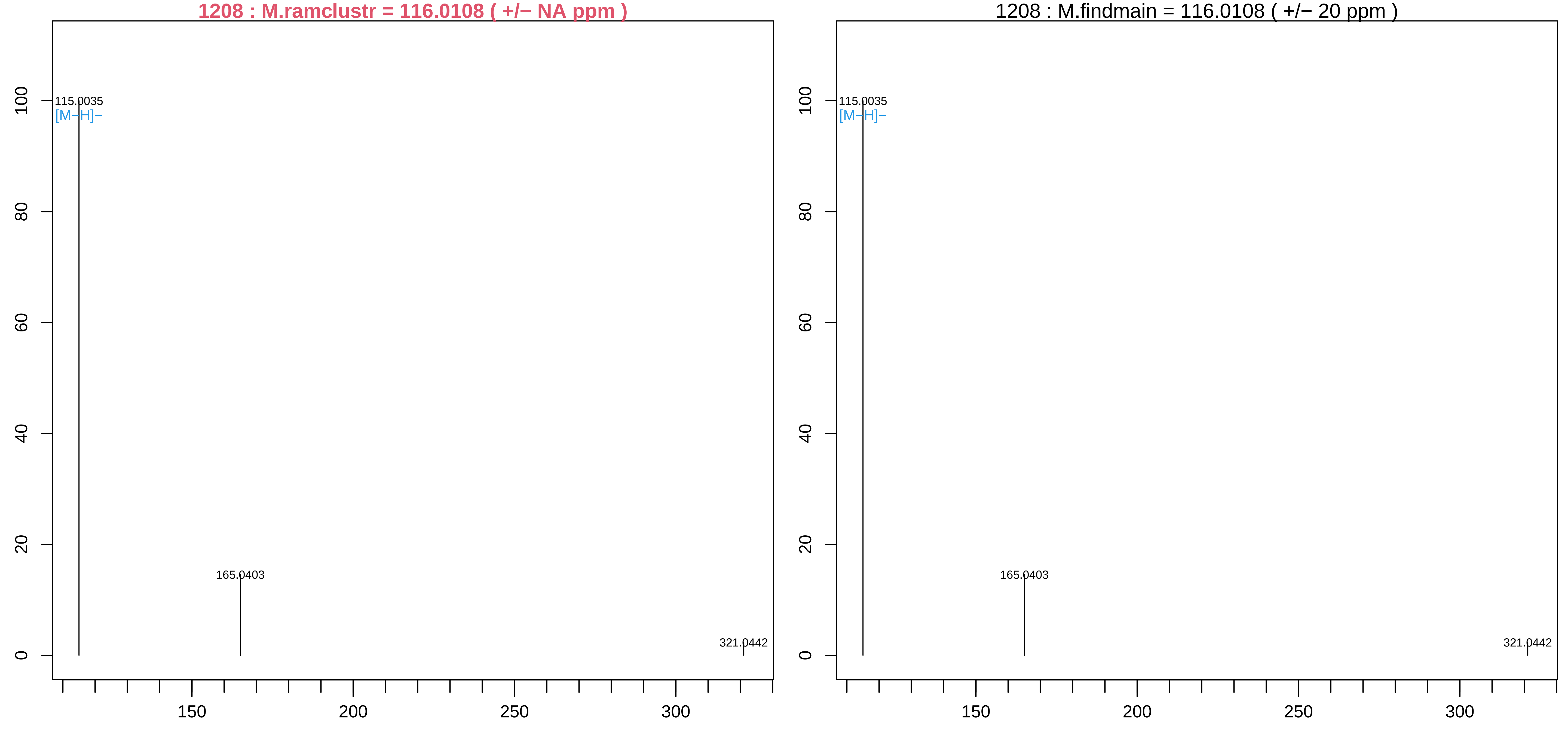
1208 : M.ramclustr = 116.0108 (+/− NA ppm)
115.0035
[M−H]−
165.0403
321.0442
0
20
40
60
80
100
150
200
250
300
1208 : M.findmain = 116.0108 (+/− 20 ppm)
115.0035
[M−H]−
165.0403
321.0442
0
20
40
60
80
100
150
200
250
300

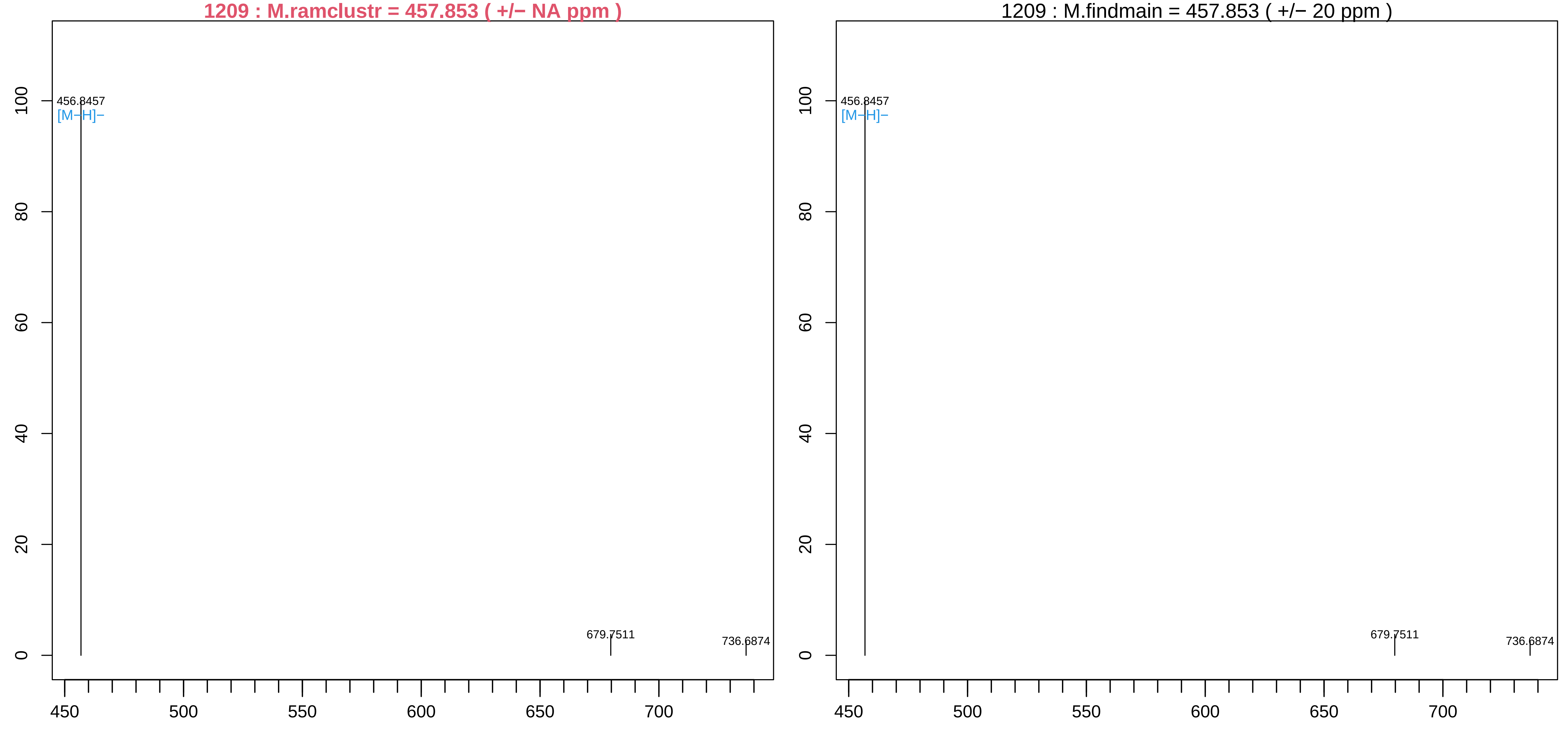

1209 : M.ramclustr = 457.853 (+/− NA ppm)
456.8457
[M−H]−
679.7511
736.6874
1209 : M.findmain = 457.853 (+/− 20 ppm)
456.8457
[M−H]−
679.7511
736.6874

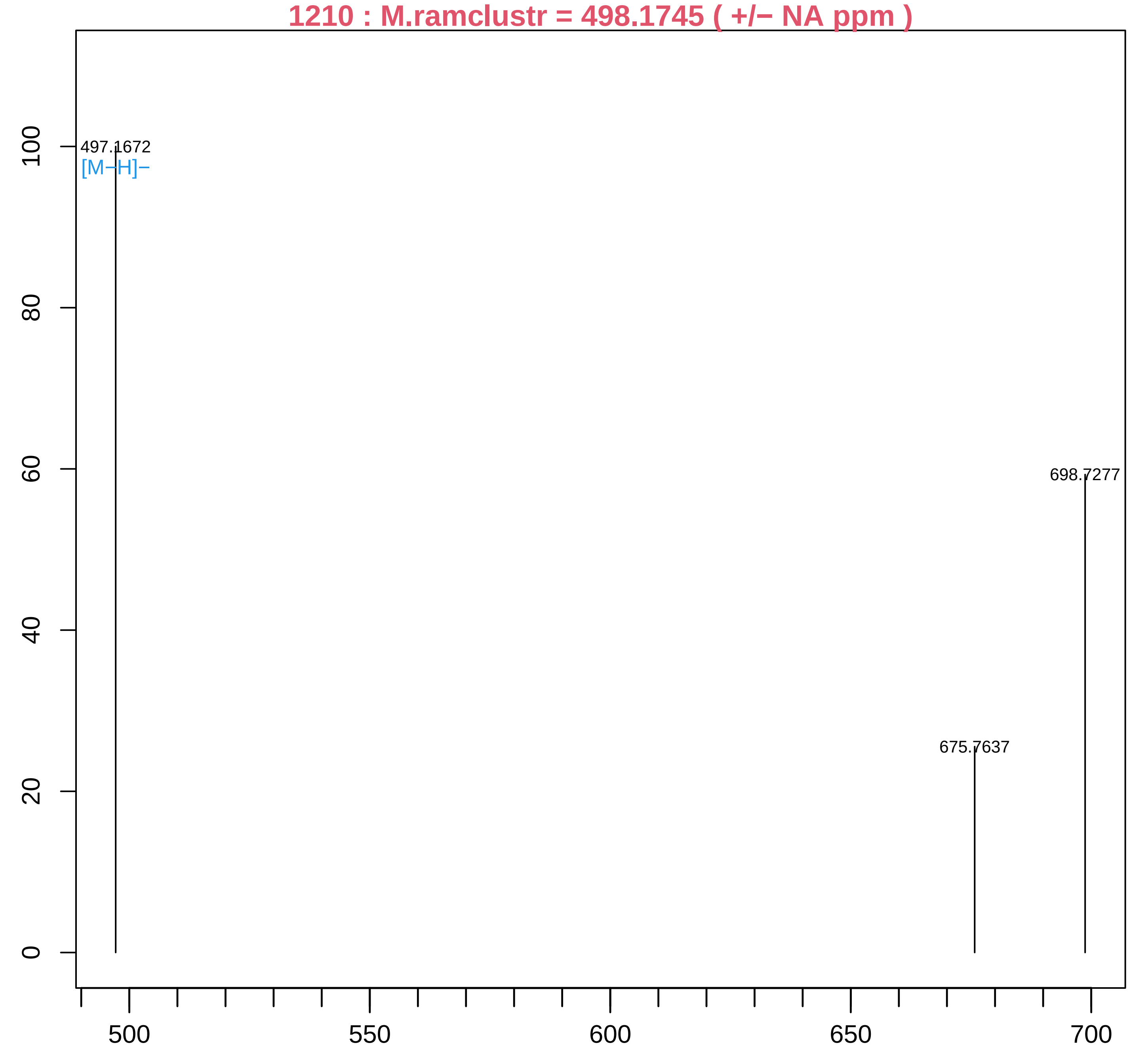
1210 : M.ramclustr = 498.1745 (+/− NA ppm)
497.1672
[M−H]−
698.7277
675.7637
0
20
40
60
80
100
500
550
600
650
700

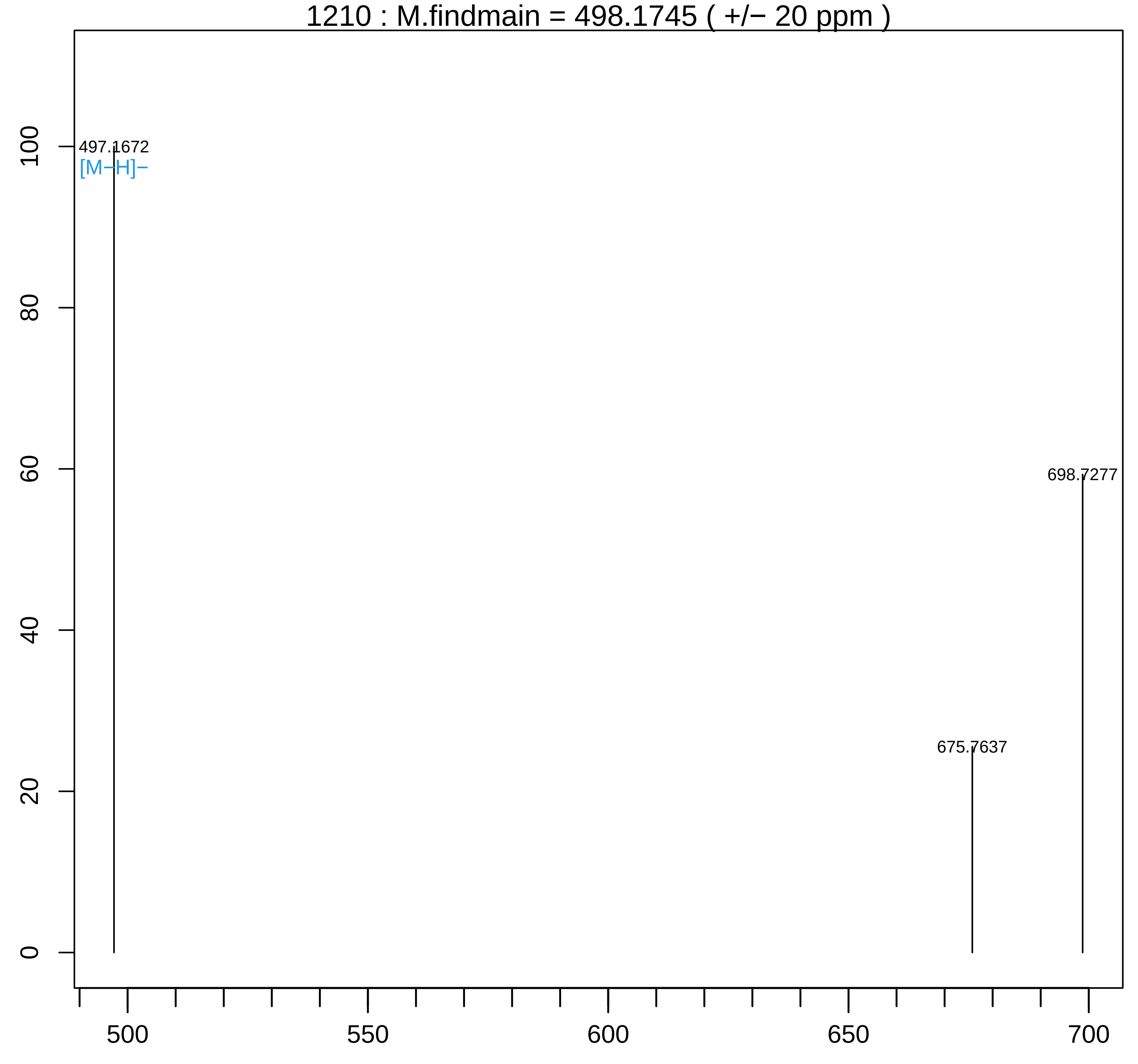
1210 : M.findmain = 498.1745 (+/− 20 ppm)
497.1672
[M−H]−
698.7277
675.7637
0
20
40
60
80
100
500
550
600
650
700

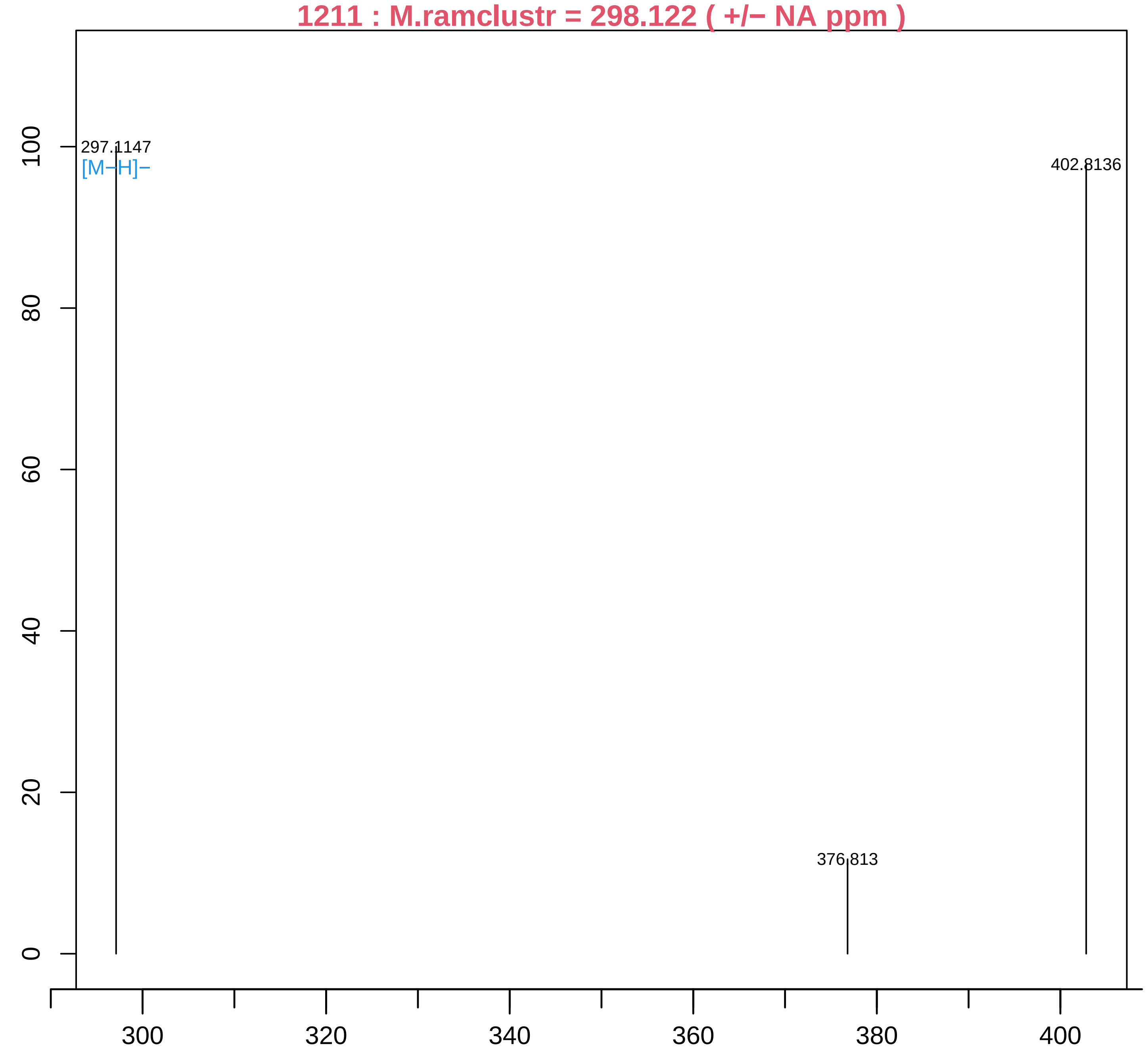

1211 : M.ramclustr = 298.122 (+/− NA ppm)
297.1147
[M−H]−
402.8136
376.813
0
20
40
60
80
100
300
320
340
360
380
400

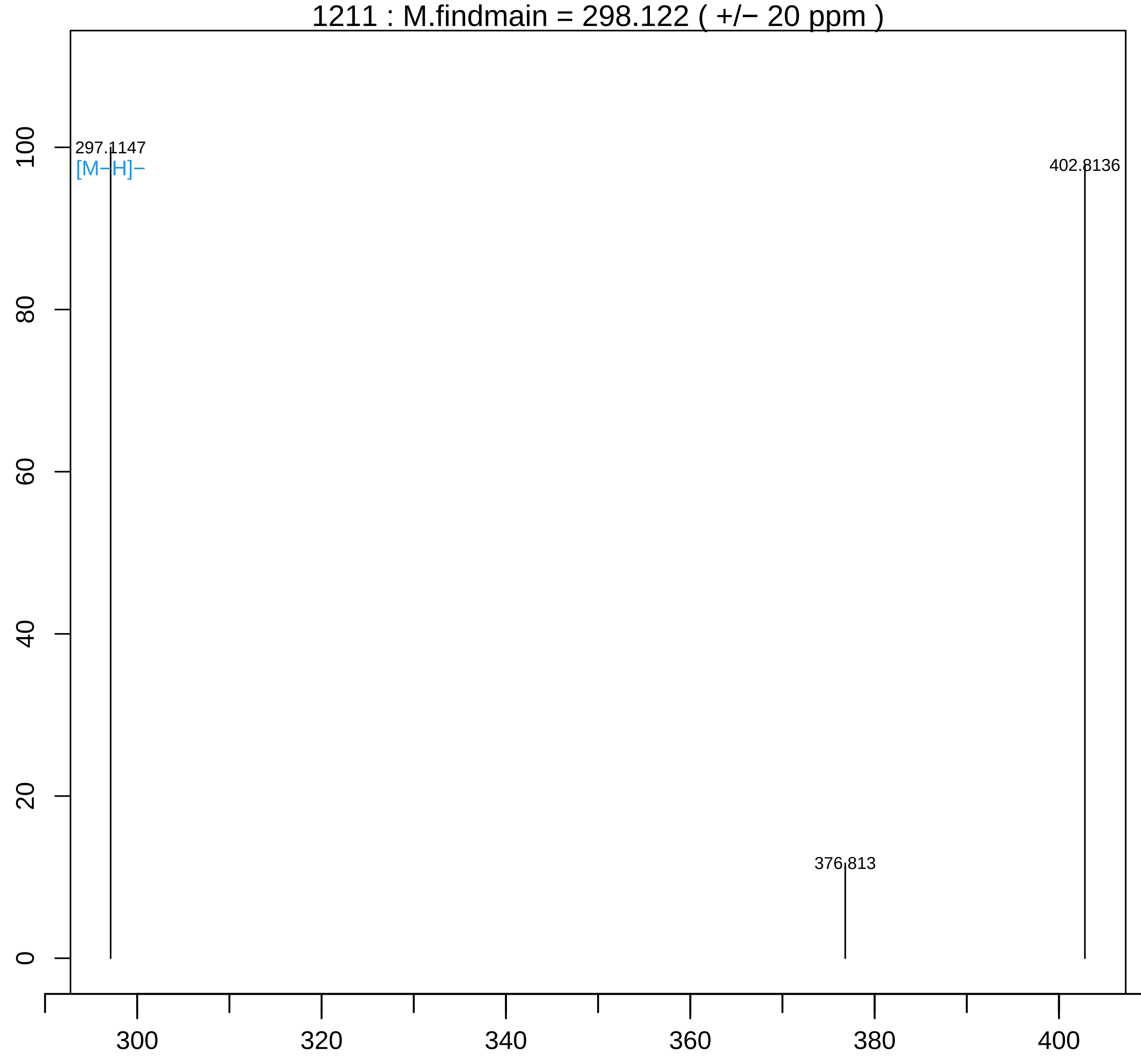

1211 : M.findmain = 298.122 (+/− 20 ppm)
297.1147
[M−H]−
402.8136
376.813
0
20
40
60
80
100
300
320
340
360
380
400

1212 : M.ramclustr = 244.8707 (+/− NA ppm)

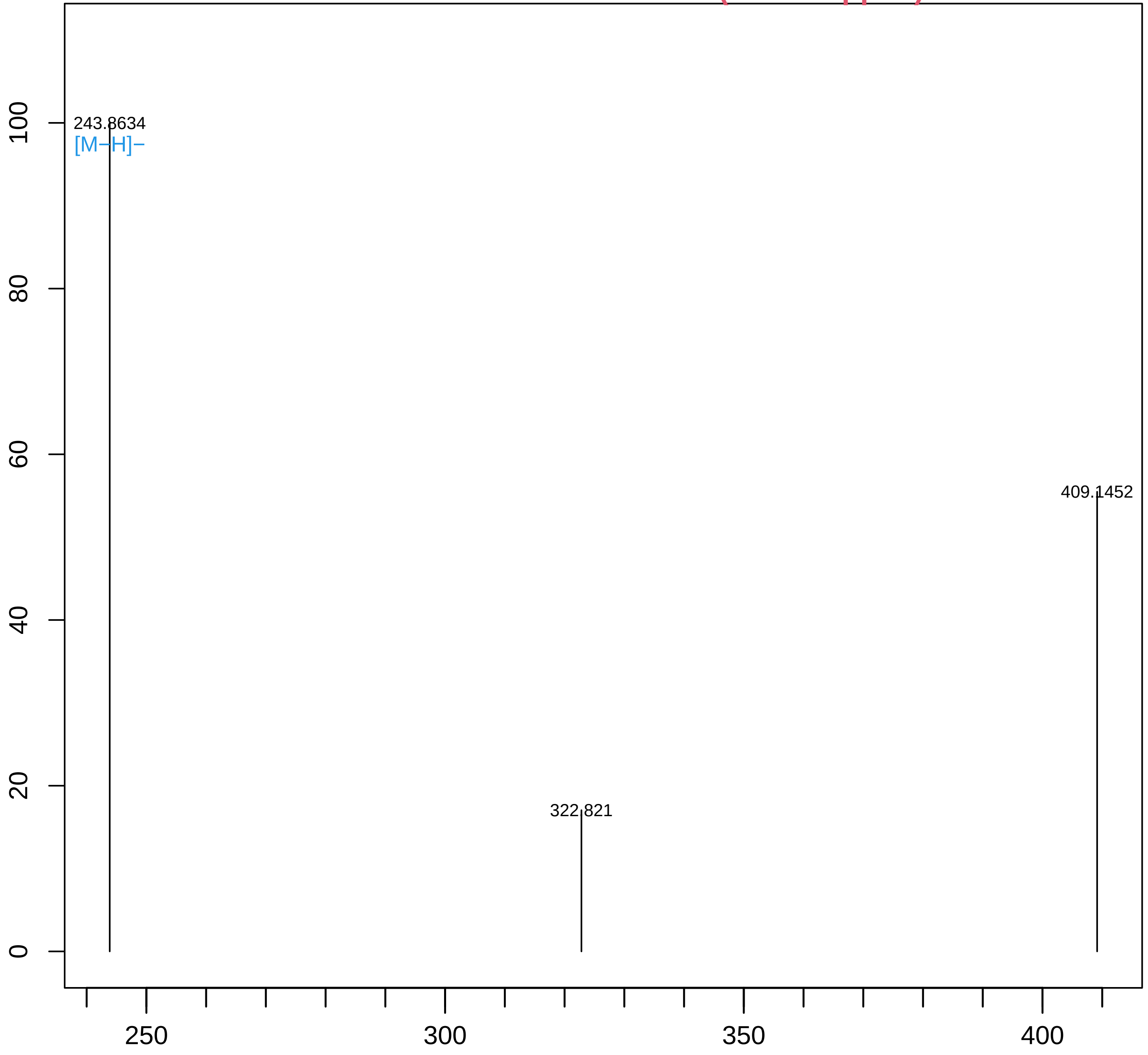

1212 : M.findmain = 244.8707 (+/− 20 ppm)

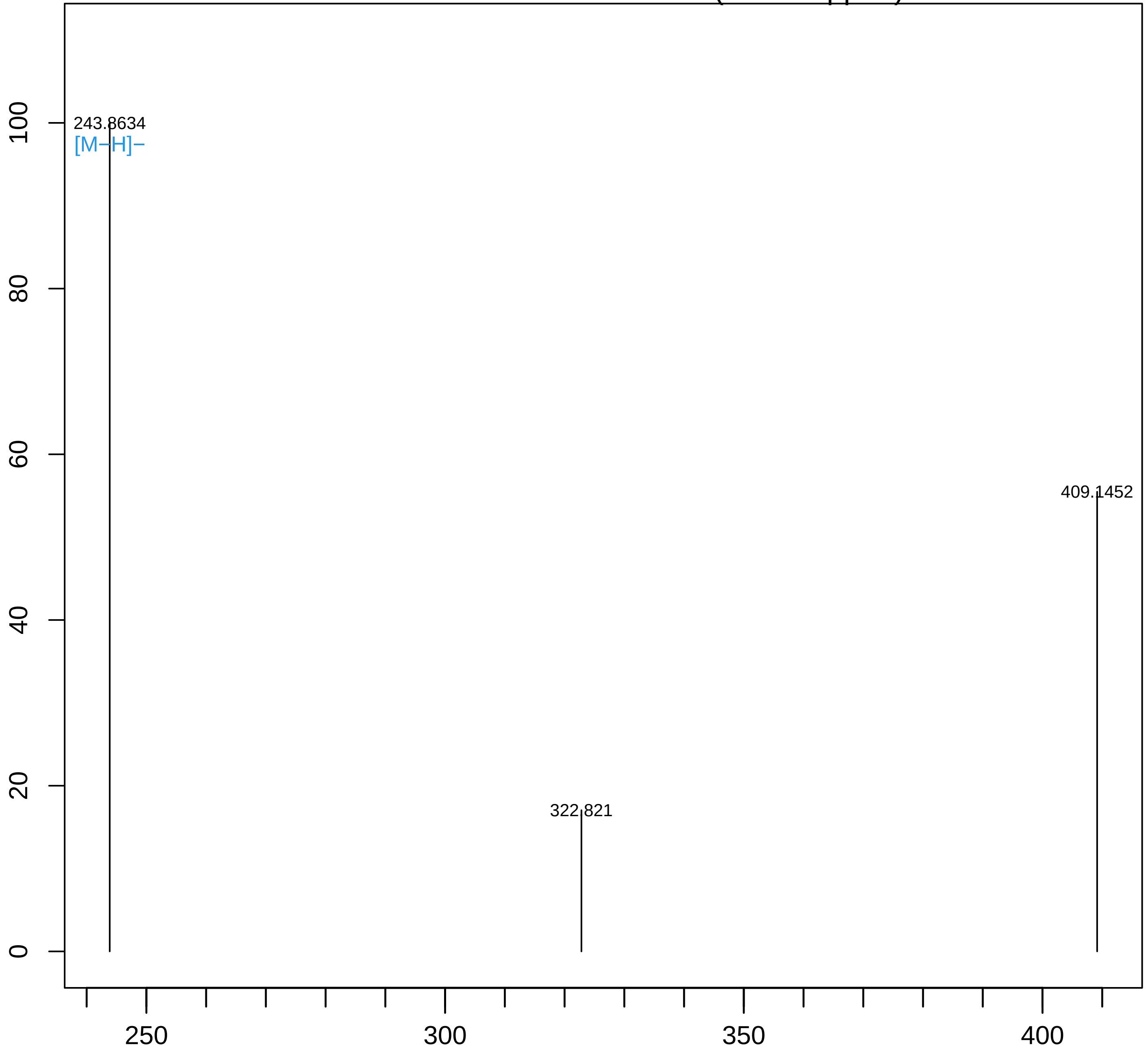

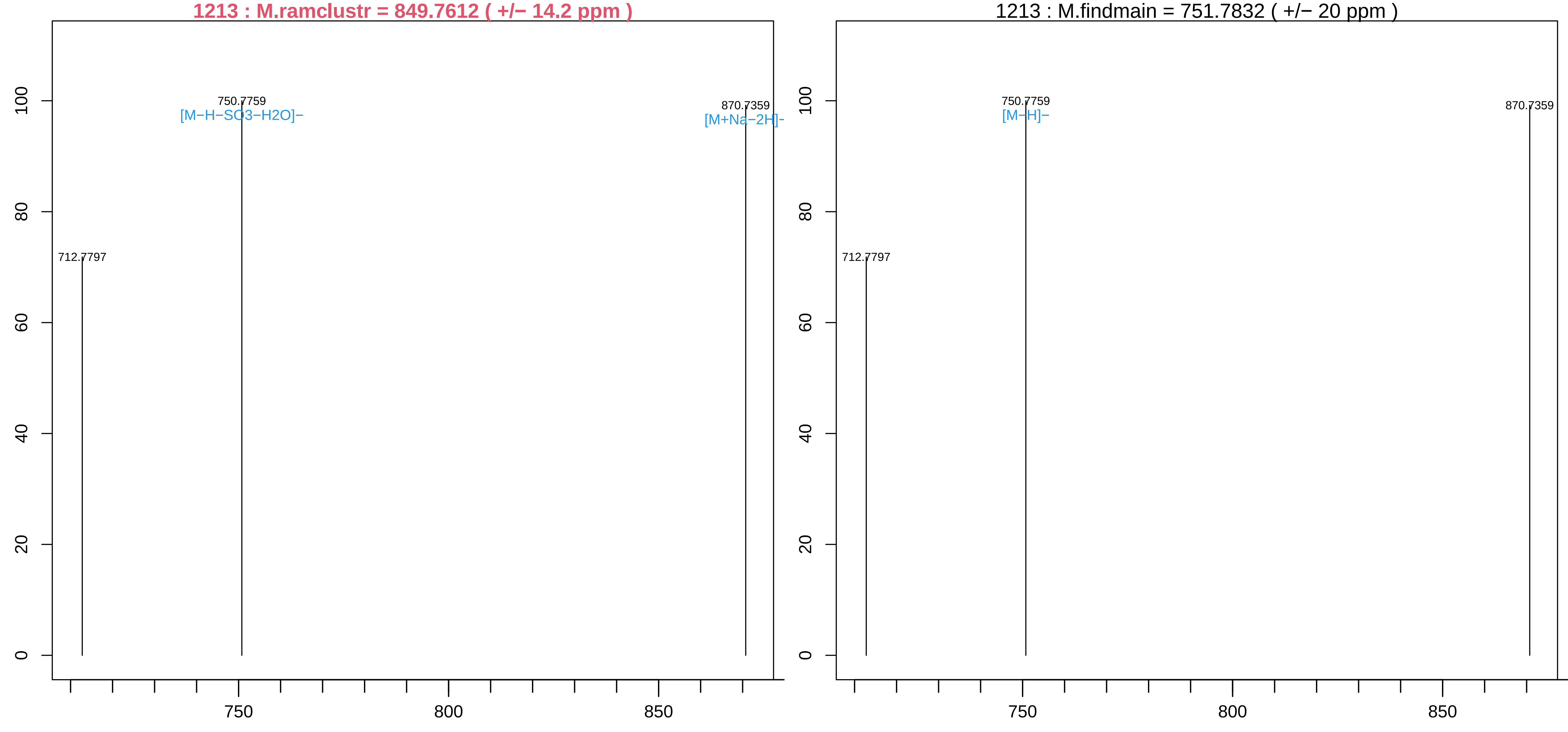

1213 : M.ramclustr = 849.7612 (+/− 14.2 ppm)
750.7759
[M−H−SO3−H2O]−
870.7359
[M+Na−2H]−
712.7797
1213 : M.findmain = 751.7832 (+/− 20 ppm)
750.7759
[M−H]−
870.7359
712.7797
0
20
40
60
80
100
750
800
850

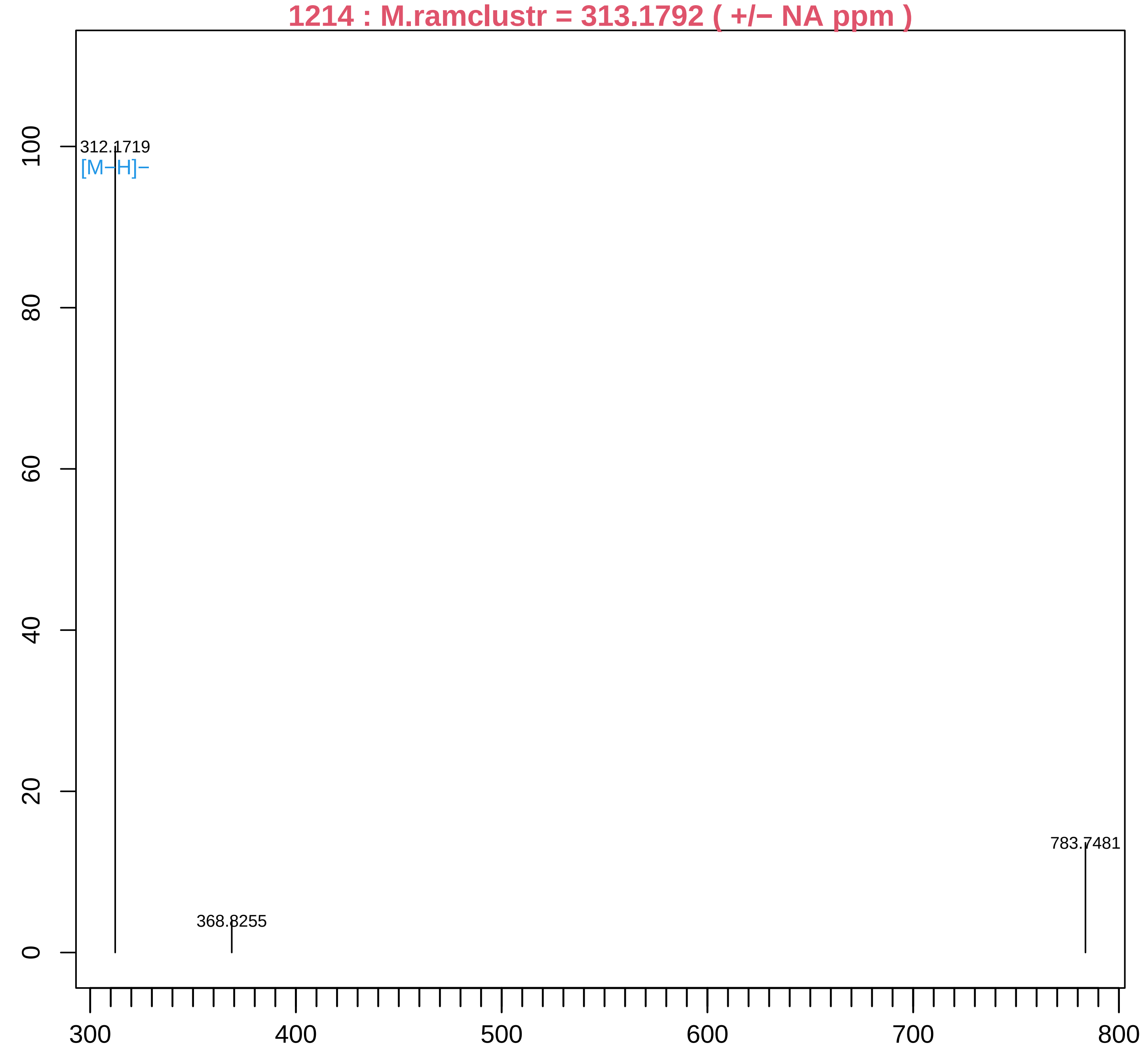
1214 : M.ramclustr = 313.1792 (+/− NA ppm)
312.1719
[M−H]−
368.8255
783.7481
0
20
40
60
80
100
300
400
500
600
700
800

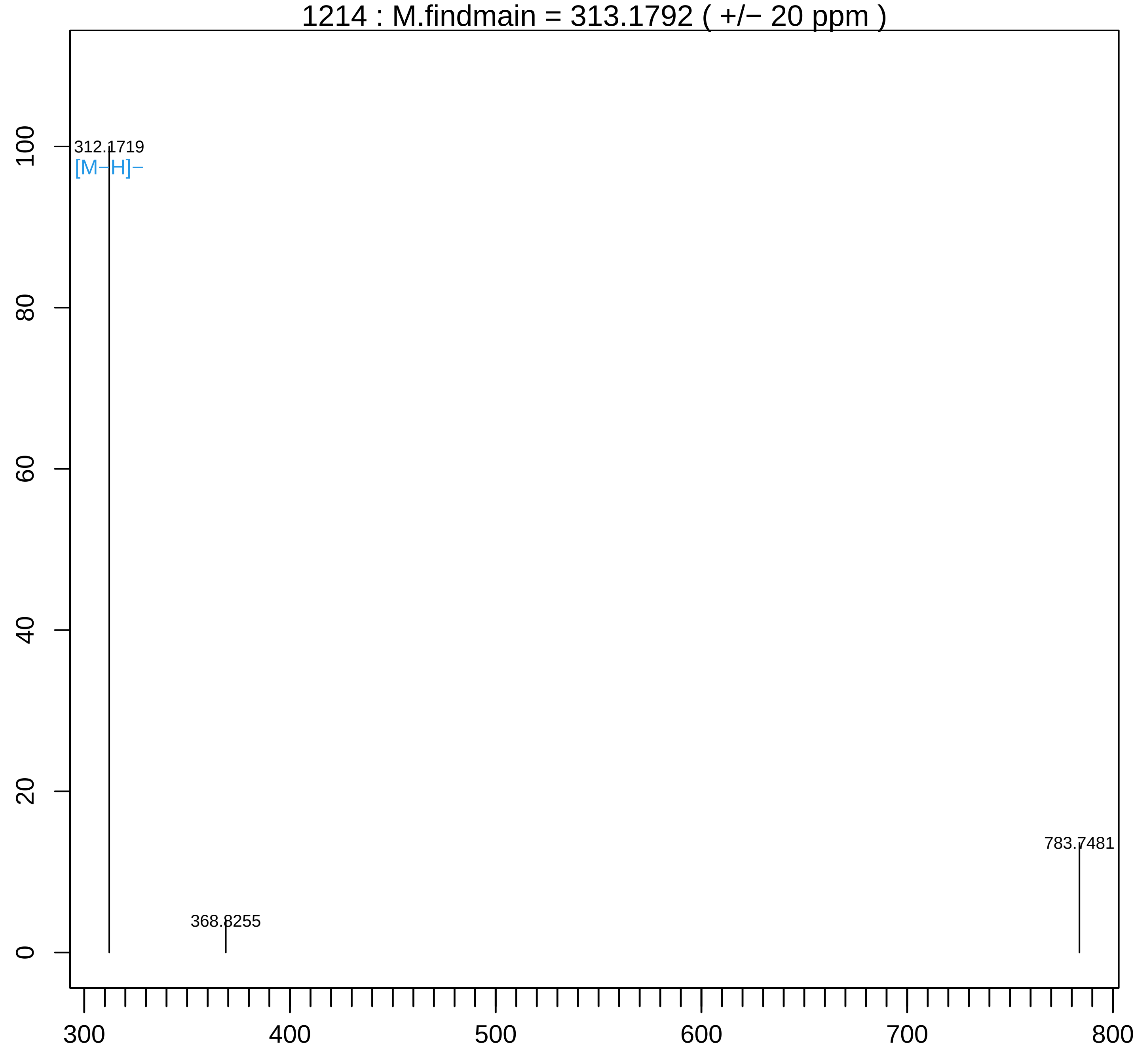
1214 : M.findmain = 313.1792 (+/− 20 ppm)
312.1719
[M−H]−
368.8255
783.7481
0
20
40
60
80
100
300
400
500
600
700
800

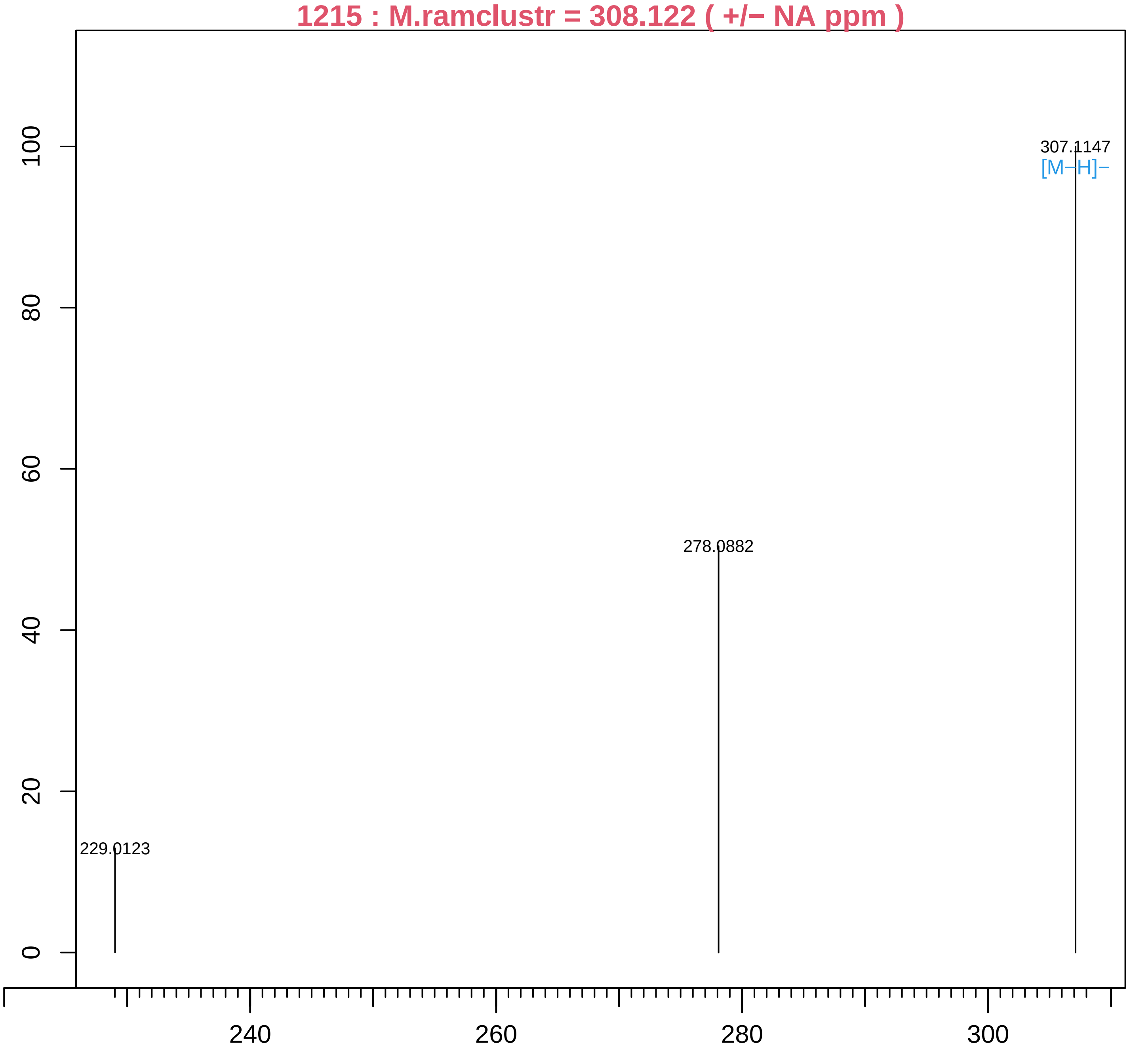
1215 : M.ramclustr = 308.122 (+/− NA ppm)
307.1147
[M−H]−
278.0882
229.0123
0
20
40
60
80
100
240
260
280
300

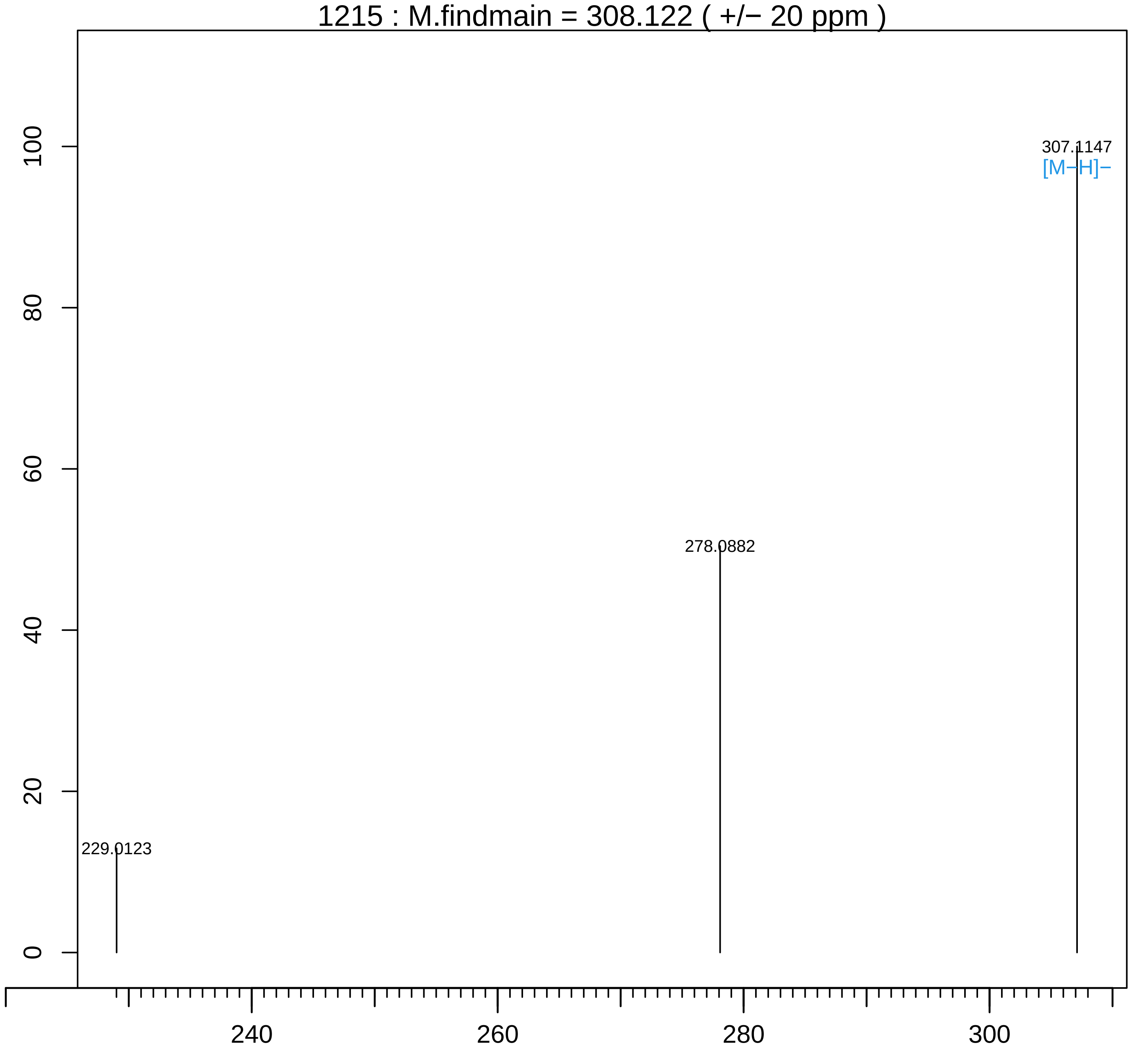
1215 : M.findmain = 308.122 (+/− 20 ppm)
307.1147
[M−H]−
278.0882
229.0123
0
20
40
60
80
100
240
260
280
300

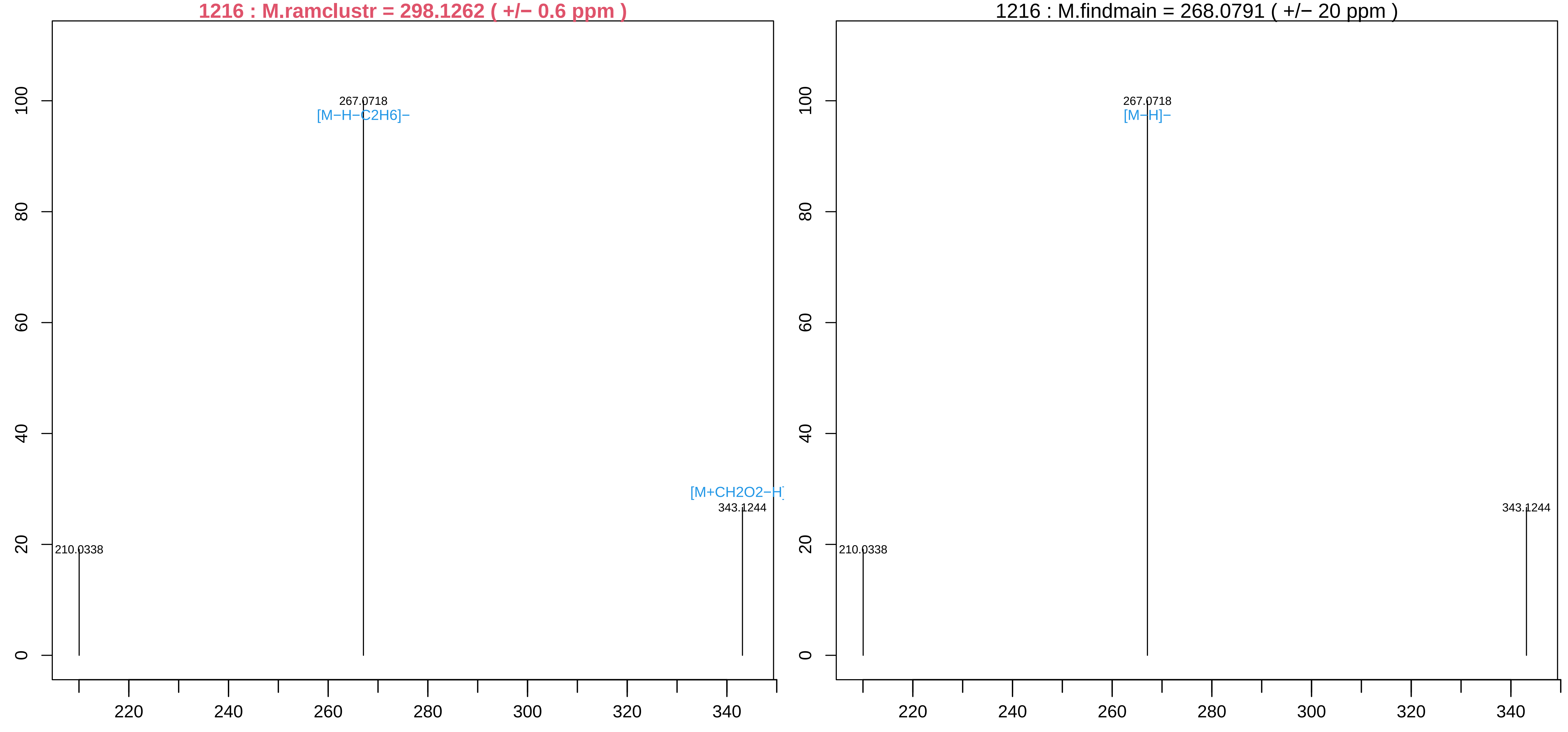

1216 : M.ramclustr = 298.1262 (+/− 0.6 ppm)
267.0718
[M−H−C2H6]−
[M+CH2O2−H]
343.1244
210.0338
1216 : M.findmain = 268.0791 (+/− 20 ppm)
267.0718
[M−H]−
343.1244
210.0338

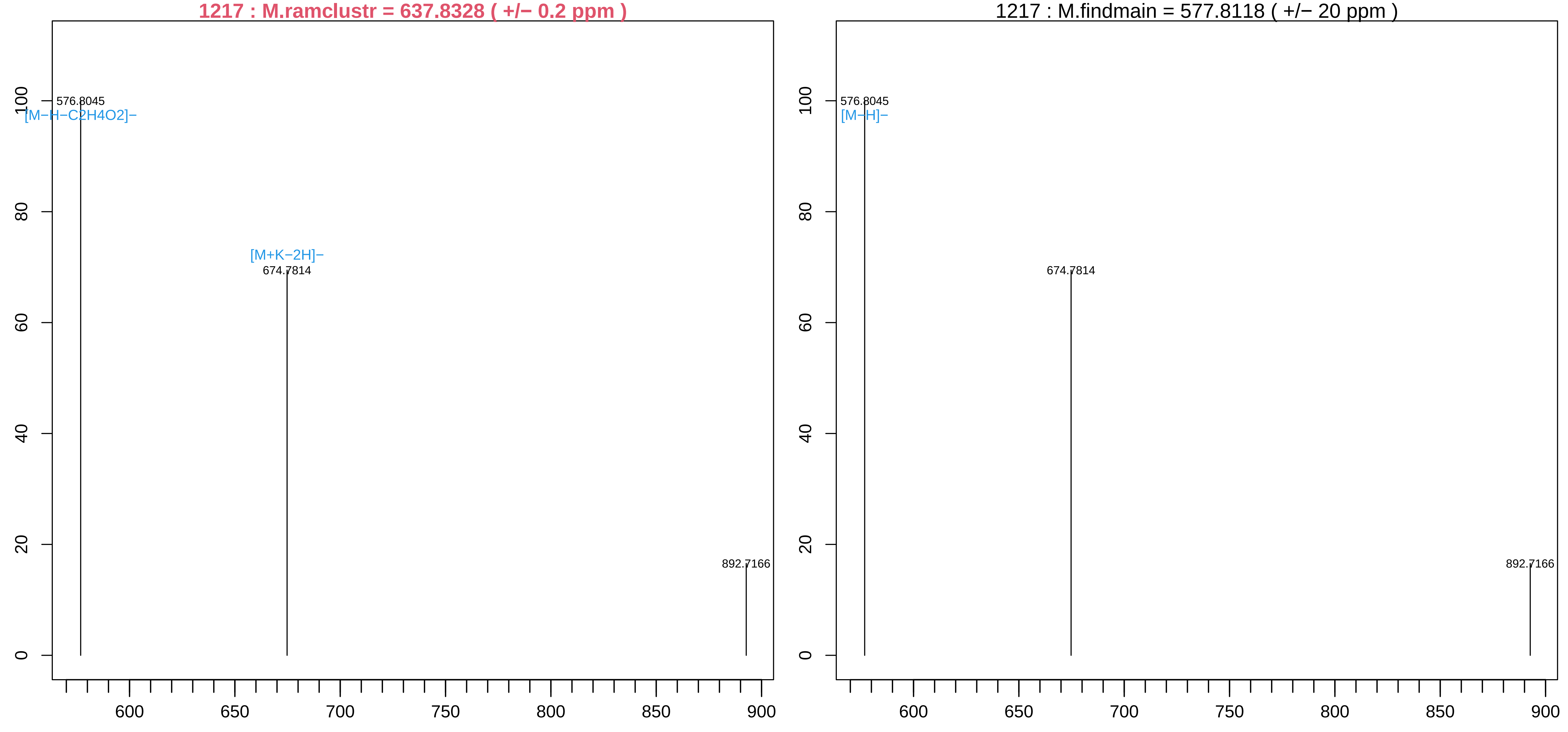

1217 : M.ramclustr = 637.8328 (+/− 0.2 ppm)
576.8045
[M−H−C2H4O2]−
[M+K−2H]−
674.7814
892.7166
1217 : M.findmain = 577.8118 (+/− 20 ppm)
576.8045
[M−H]−
674.7814
892.7166

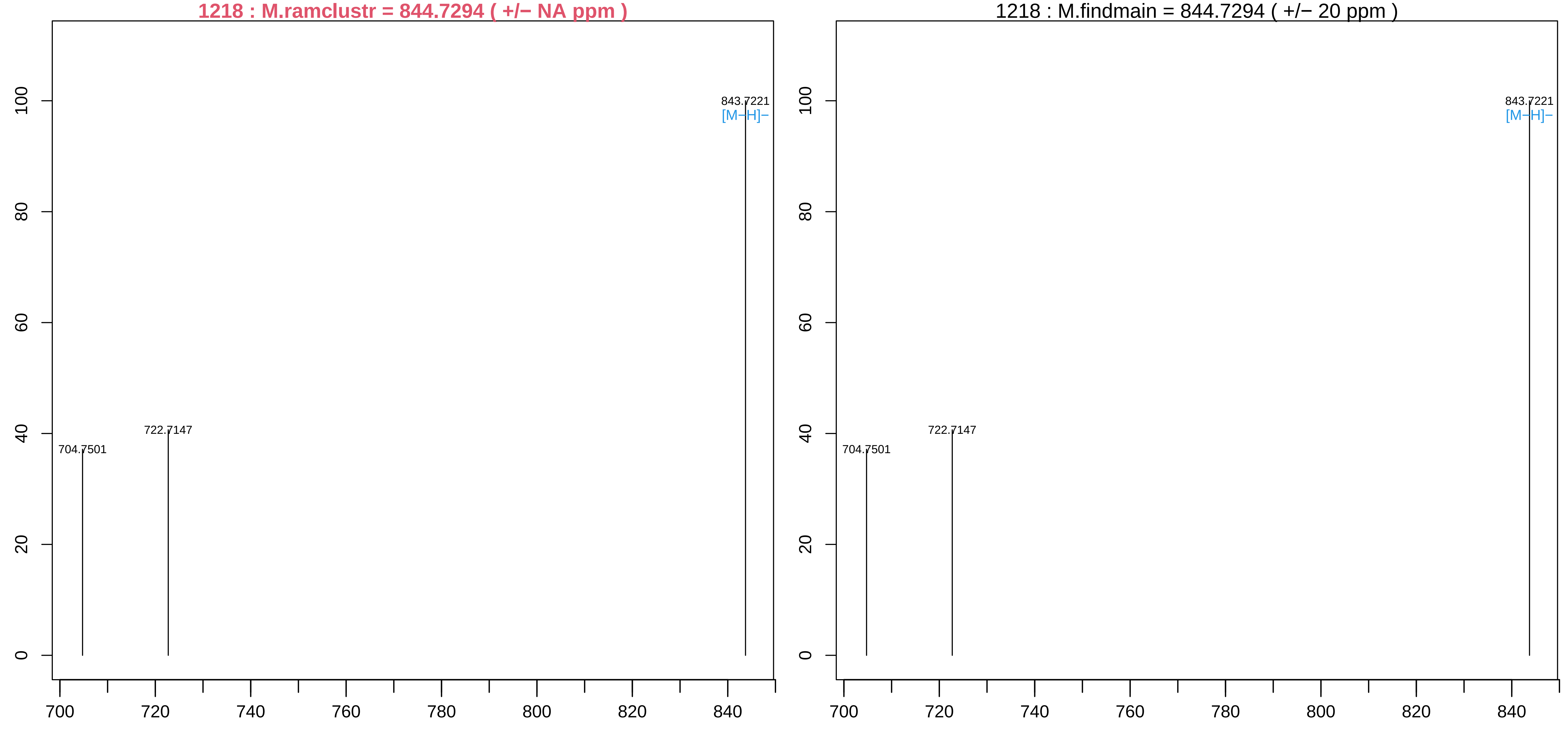

1218 : M.ramclustr = 844.7294 (+/− NA ppm)
1218 : M.findmain = 844.7294 (+/− 20 ppm)
843.7221
[M−H]−
722.7147
704.7501
0
20
40
60
80
100
700
720
740
760
780
800
820
840

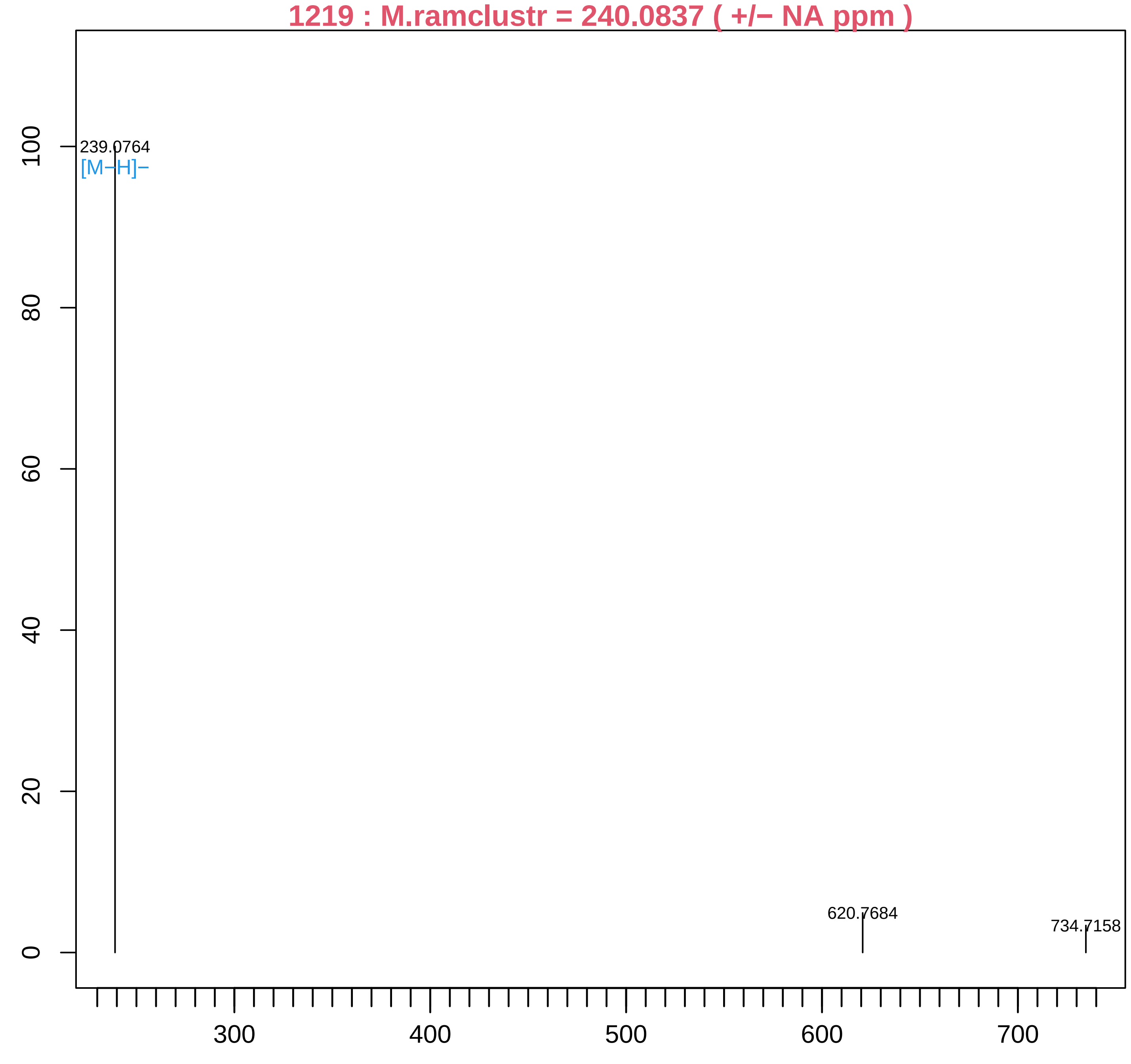

1219 : M.ramclustr = 240.0837 (+/− NA ppm)
239.0764
[M−H]−
620.7684
734.7158
0
20
40
60
80
100
300
400
500
600
700

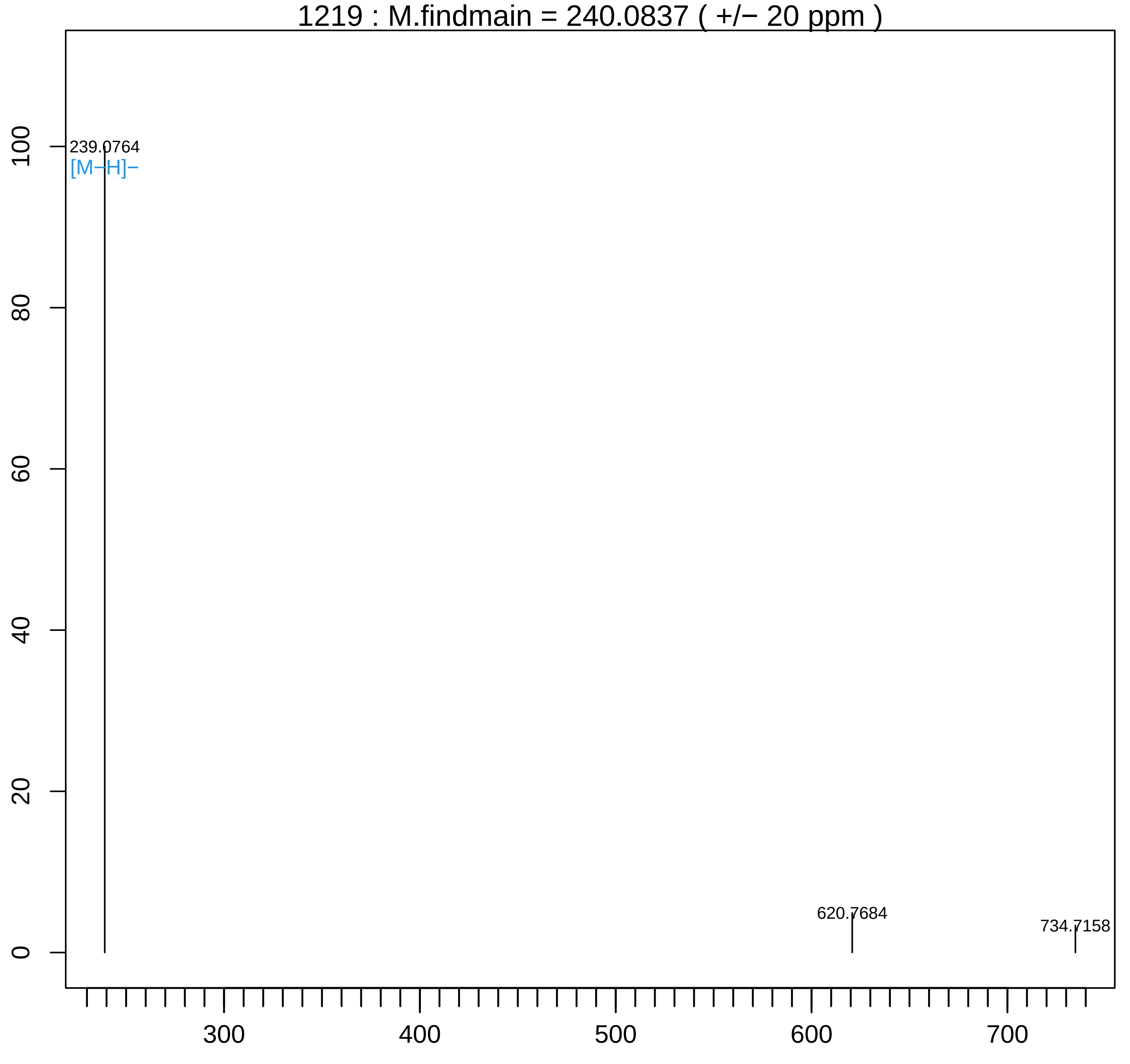

1219 : M.findmain = 240.0837 (+/− 20 ppm)
239.0764
[M−H]−
620.7684
734.7158
0
20
40
60
80
100
300
400
500
600
700

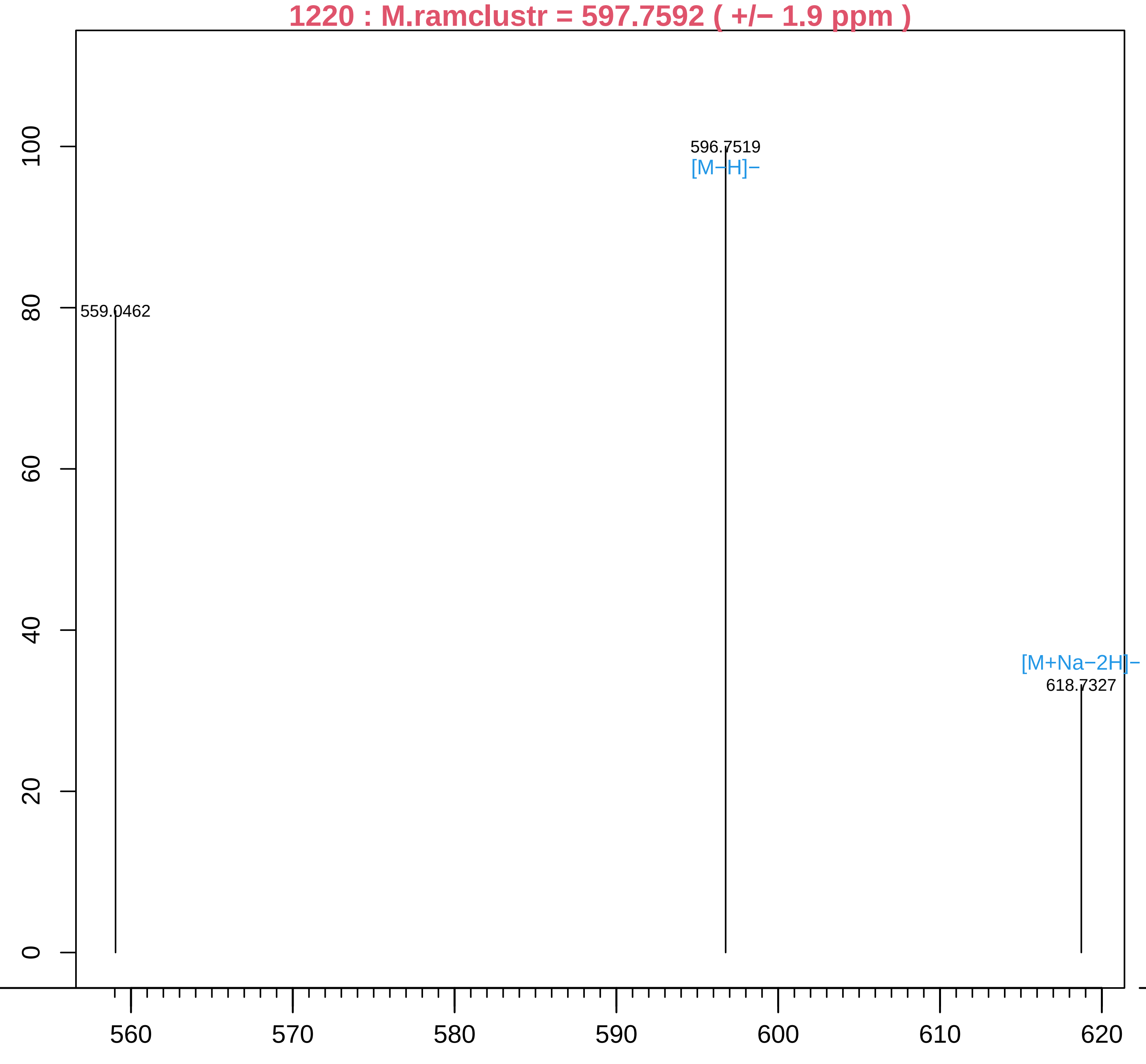
1220 : M.ramclustr = 597.7592 (+/− 1.9 ppm)
559.0462
596.7519
[M−H]−
[M+Na−2H]−
618.7327
0
20
40
60
80
100
560
570
580
590
600
610
620

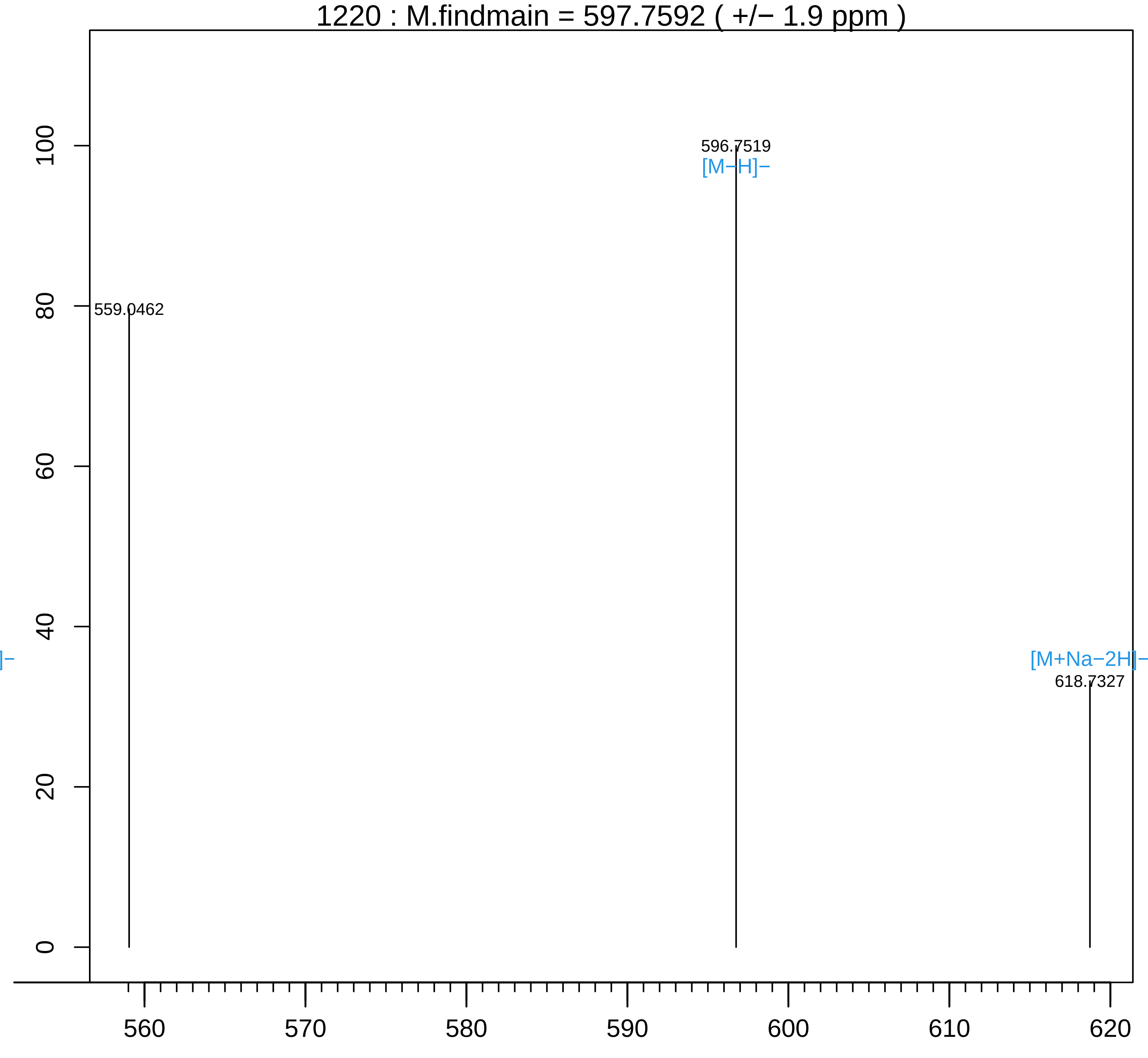
1220 : M.findmain = 597.7592 (+/− 1.9 ppm)
559.0462
596.7519
[M−H]−
[M+Na−2H]−
618.7327
0
20
40
60
80
100
560
570
580
590
600
610
620

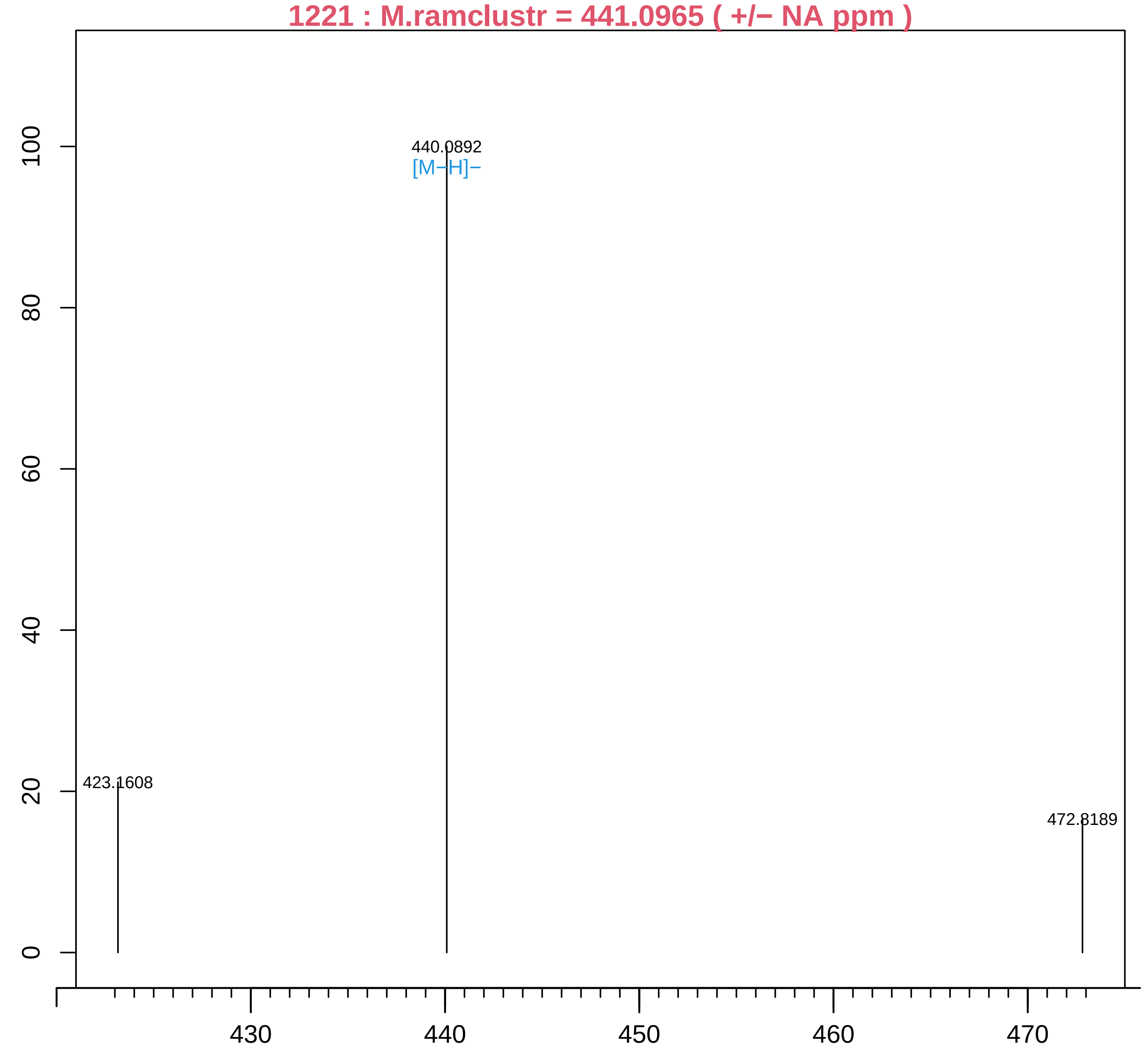
1221 : M.ramclustr = 441.0965 (+/− NA ppm)
440.0892
[M−H]−
423.1608
472.8189
0
20
40
60
80
100
430
440
450
460
470

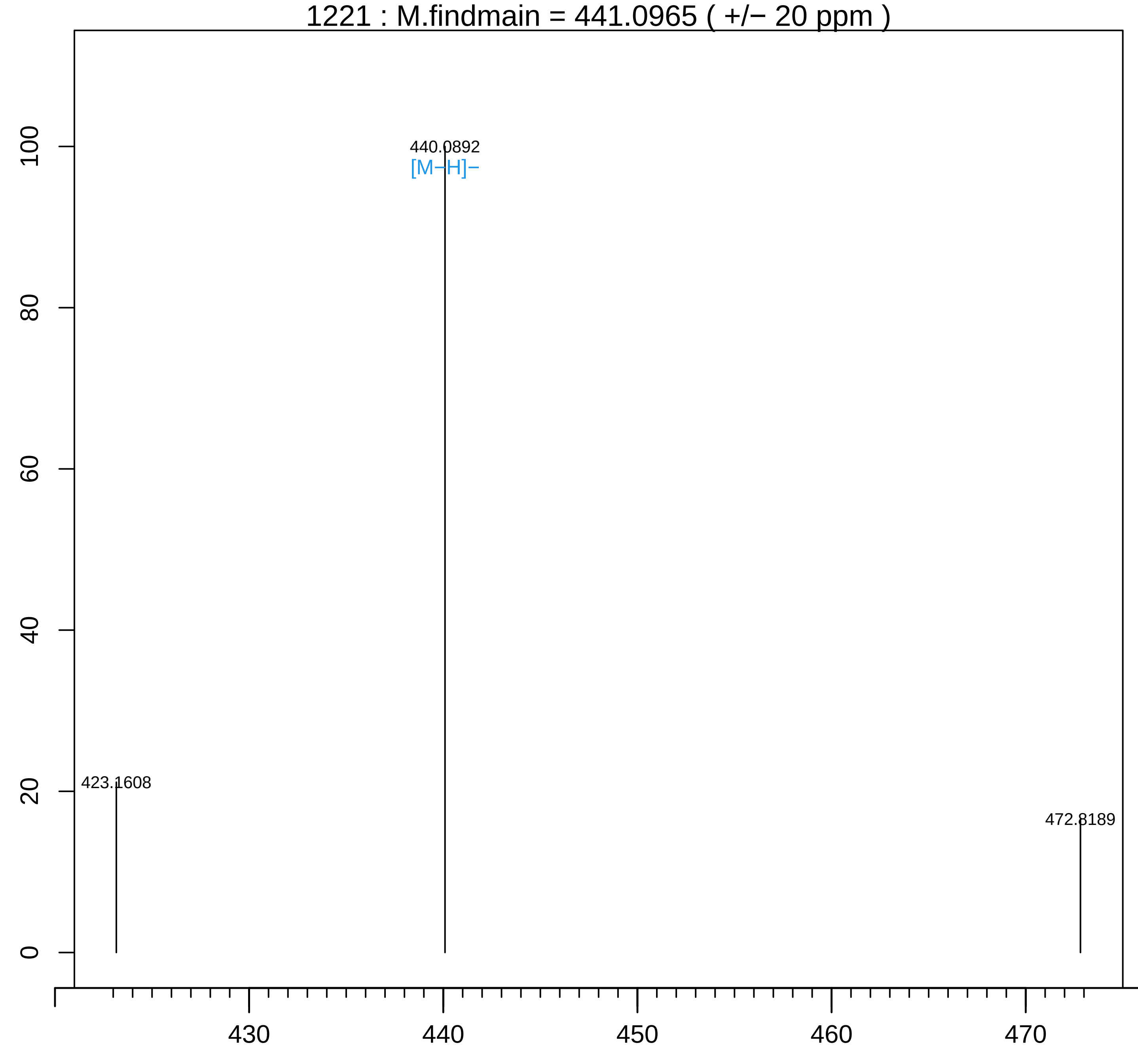
1221 : M.findmain = 441.0965 (+/− 20 ppm)
440.0892
[M−H]−
423.1608
472.8189
0
20
40
60
80
100
430
440
450
460
470

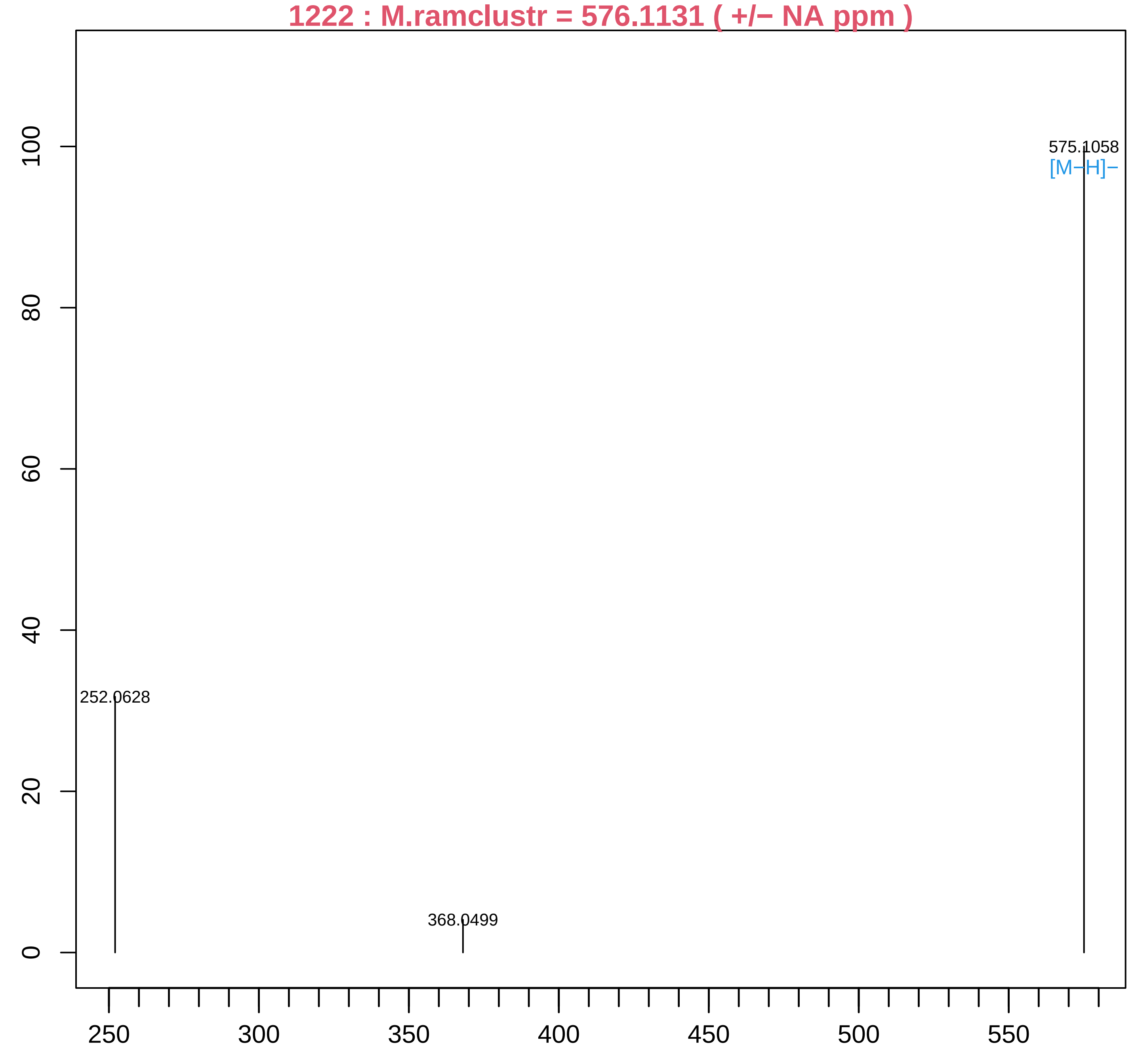
1222 : M.ramclustr = 576.1131 (+/− NA ppm)
575.1058
[M−H]−
252.0628
368.0499
0
20
40
60
80
100
250
300
350
400
450
500
550
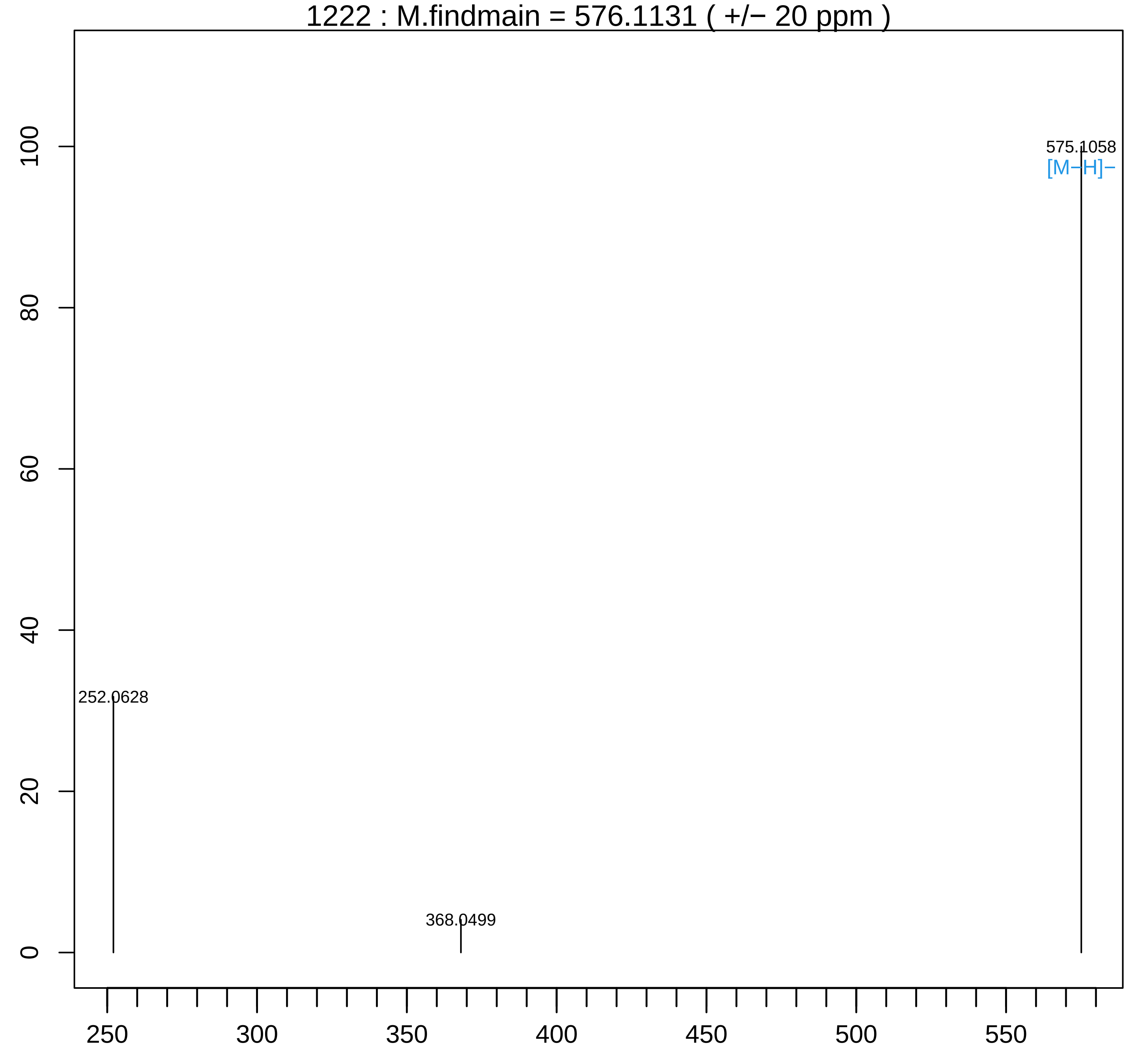
1222 : M.findmain = 576.1131 (+/− 20 ppm)
575.1058
[M−H]−
252.0628
368.0499
0
20
40
60
80
100
250
300
350
400
450
500
550

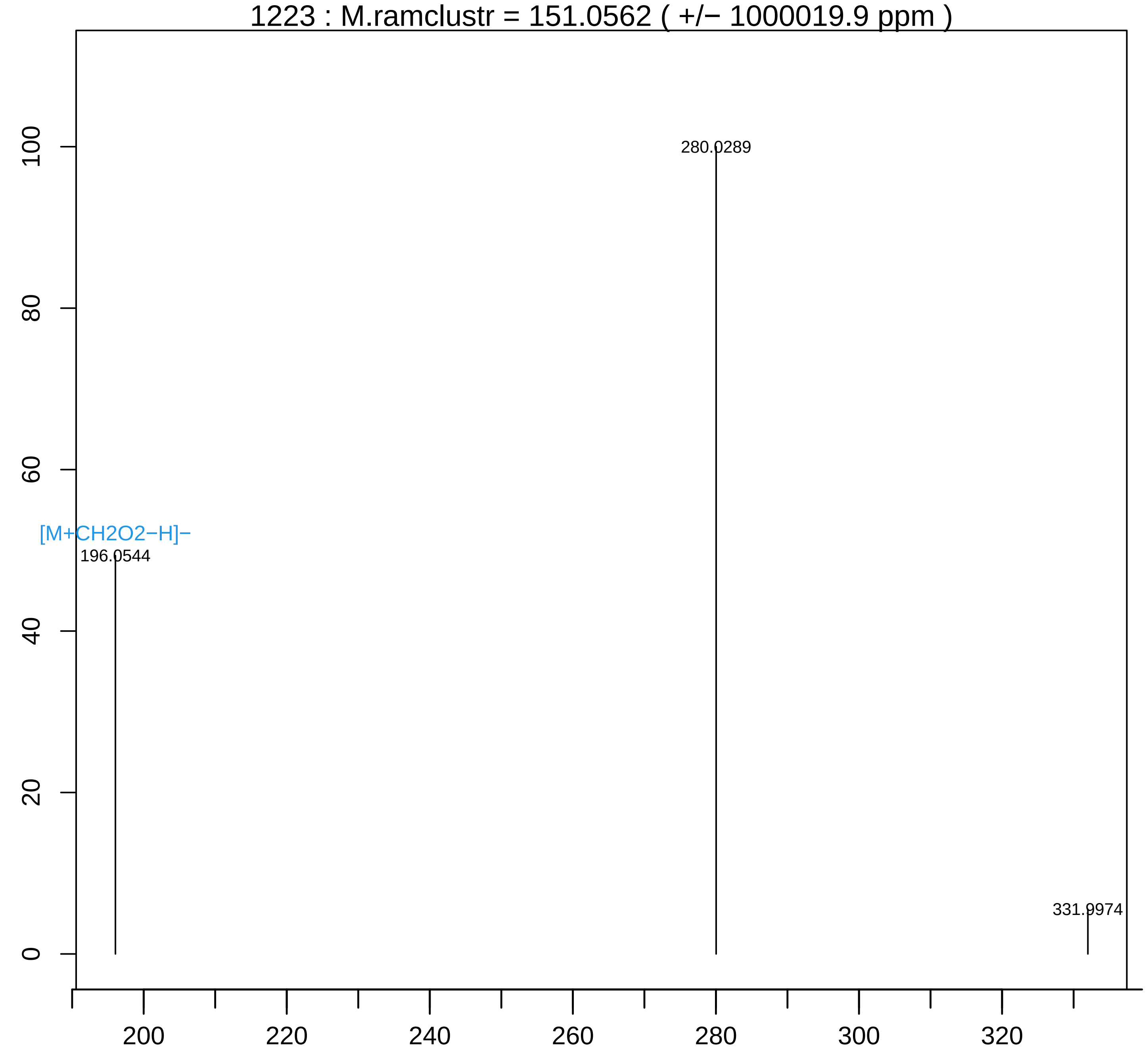
1223 : M.ramclustr = 151.0562 (+/− 1000019.9 ppm)
280.0289
[M+CH2O2−H]−
196.0544
331.9974
0
20
40
60
80
100
200
220
240
260
280
300
320

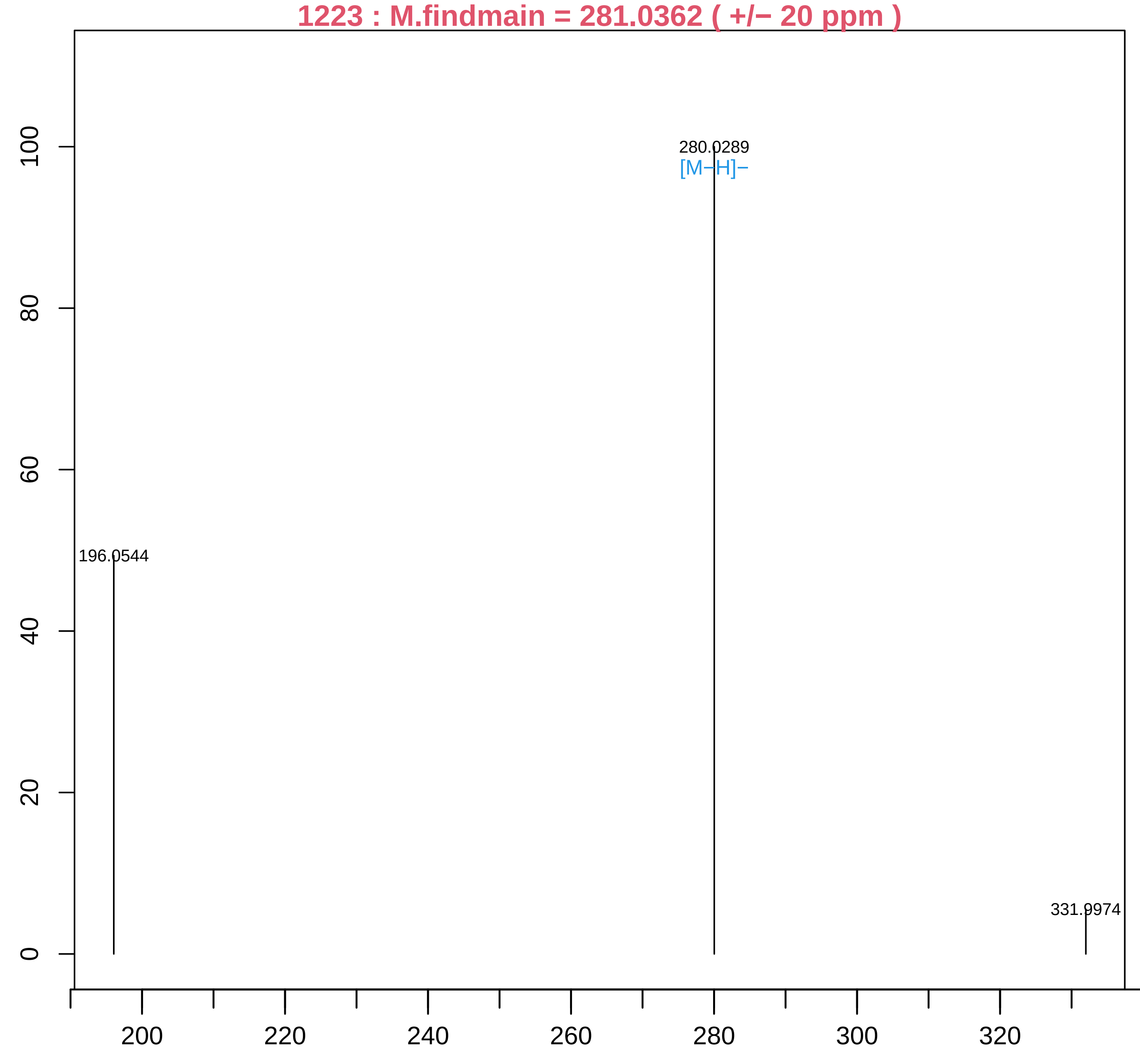
1223 : M.findmain = 281.0362 (+/− 20 ppm)
280.0289
[M−H]−
196.0544
331.9974
0
20
40
60
80
100
200
220
240
260
280
300
320

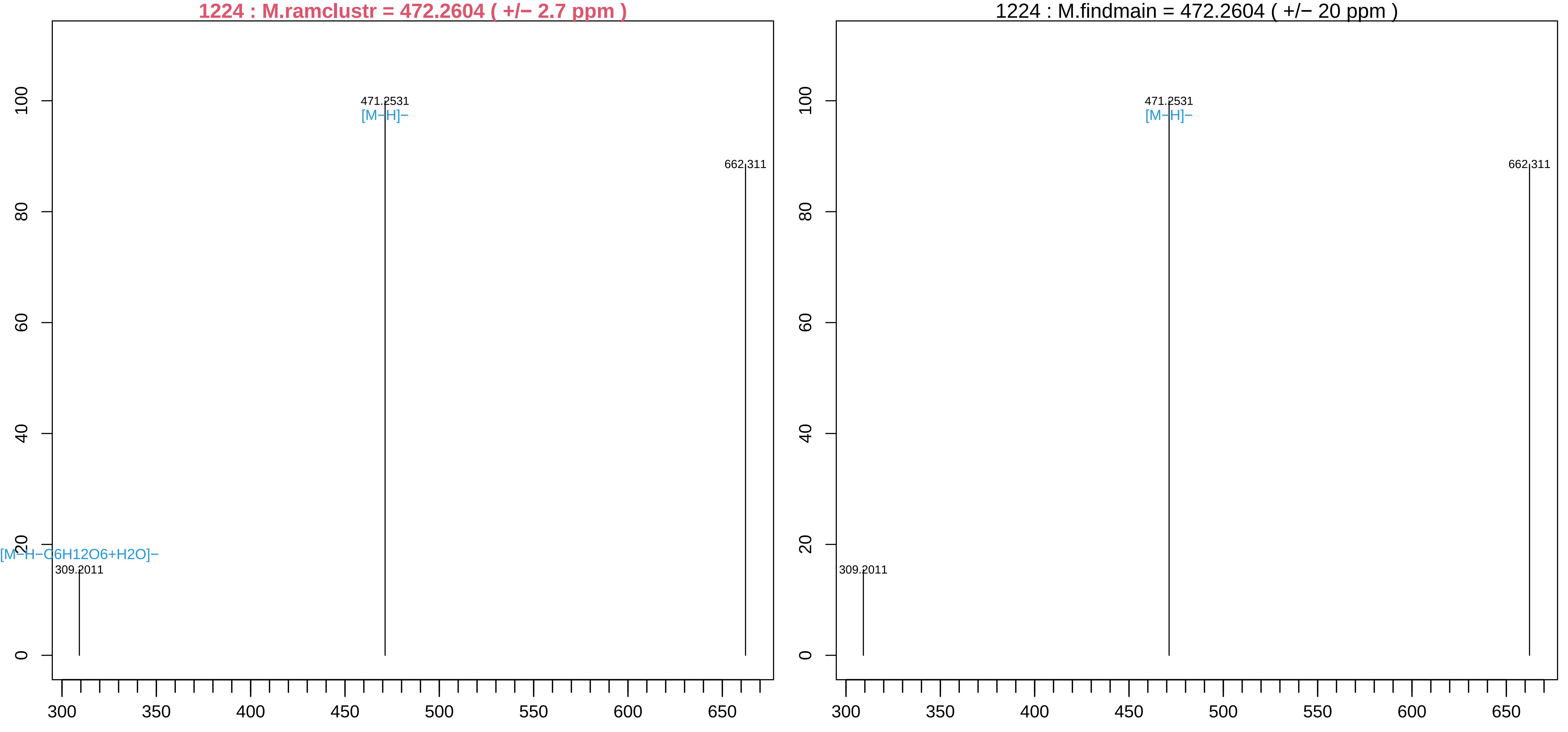
1224 : M.ramclustr = 472.2604 (+/− 2.7 ppm)
471.2531
[M−H]−
662.311
[M−H−C6H12O6+H2O]−
309.2011
1224 : M.findmain = 472.2604 (+/− 20 ppm)
471.2531
[M−H]−
662.311
309.2011
0
20
40
60
80
100
300
350
400
450
500
550
600
650

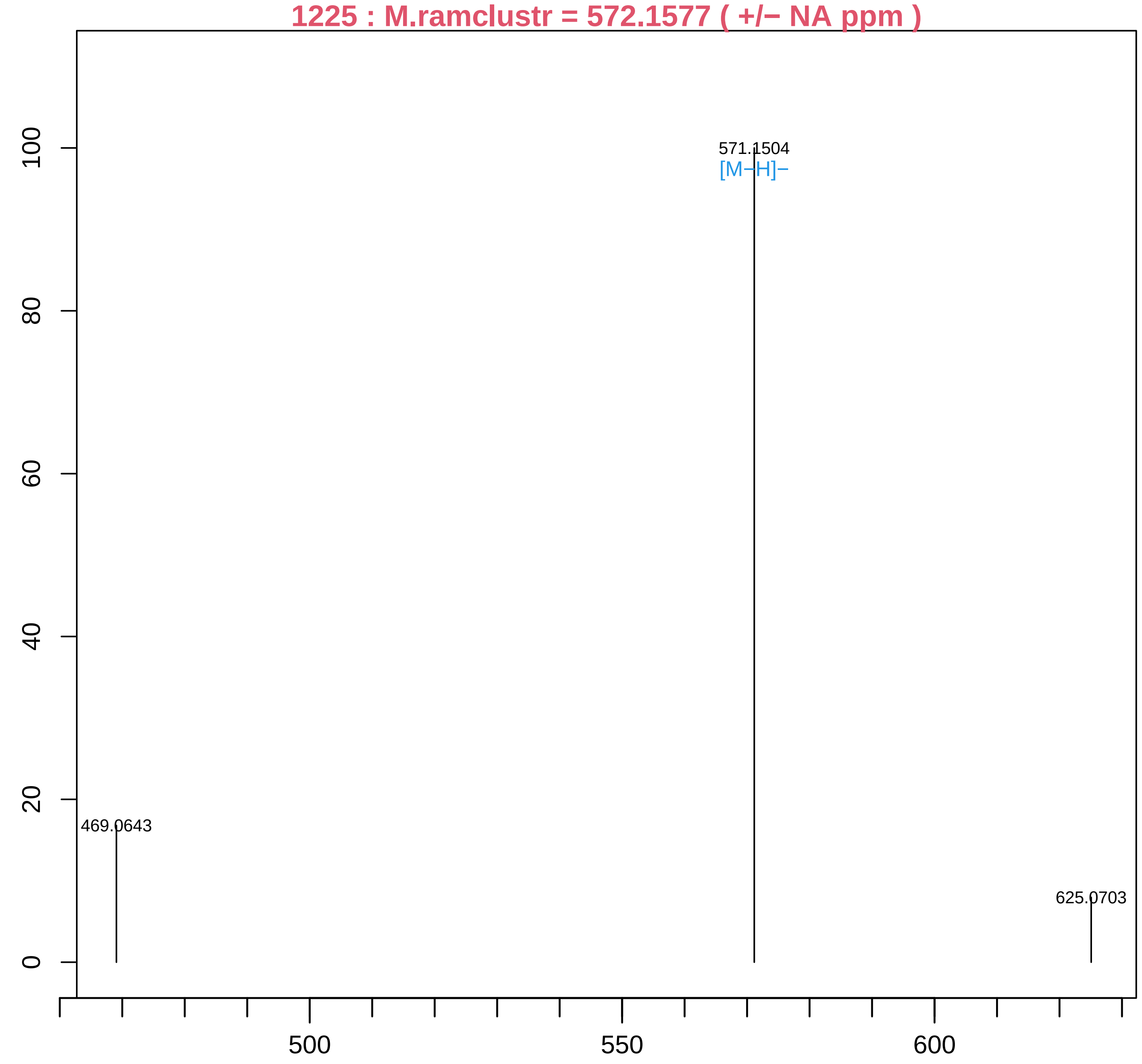

1225 : M.ramclustr = 572.1577 (+/− NA ppm)
571.1504
[M−H]−
469.0643
625.0703
0
20
40
60
80
100
500
550
600

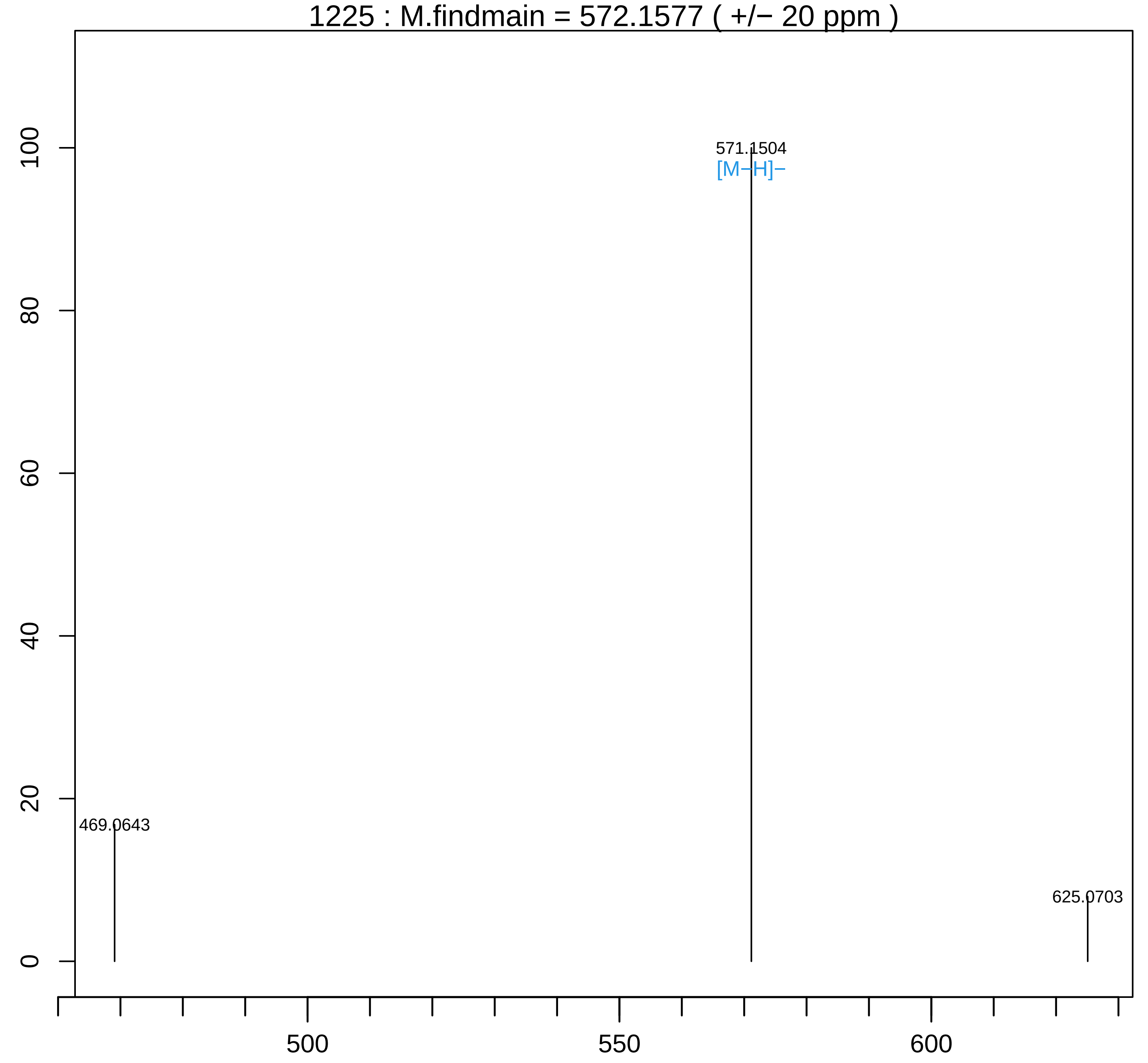

1225 : M.findmain = 572.1577 (+/− 20 ppm)
571.1504
[M−H]−
469.0643
625.0703
0
20
40
60
80
100
500
550
600

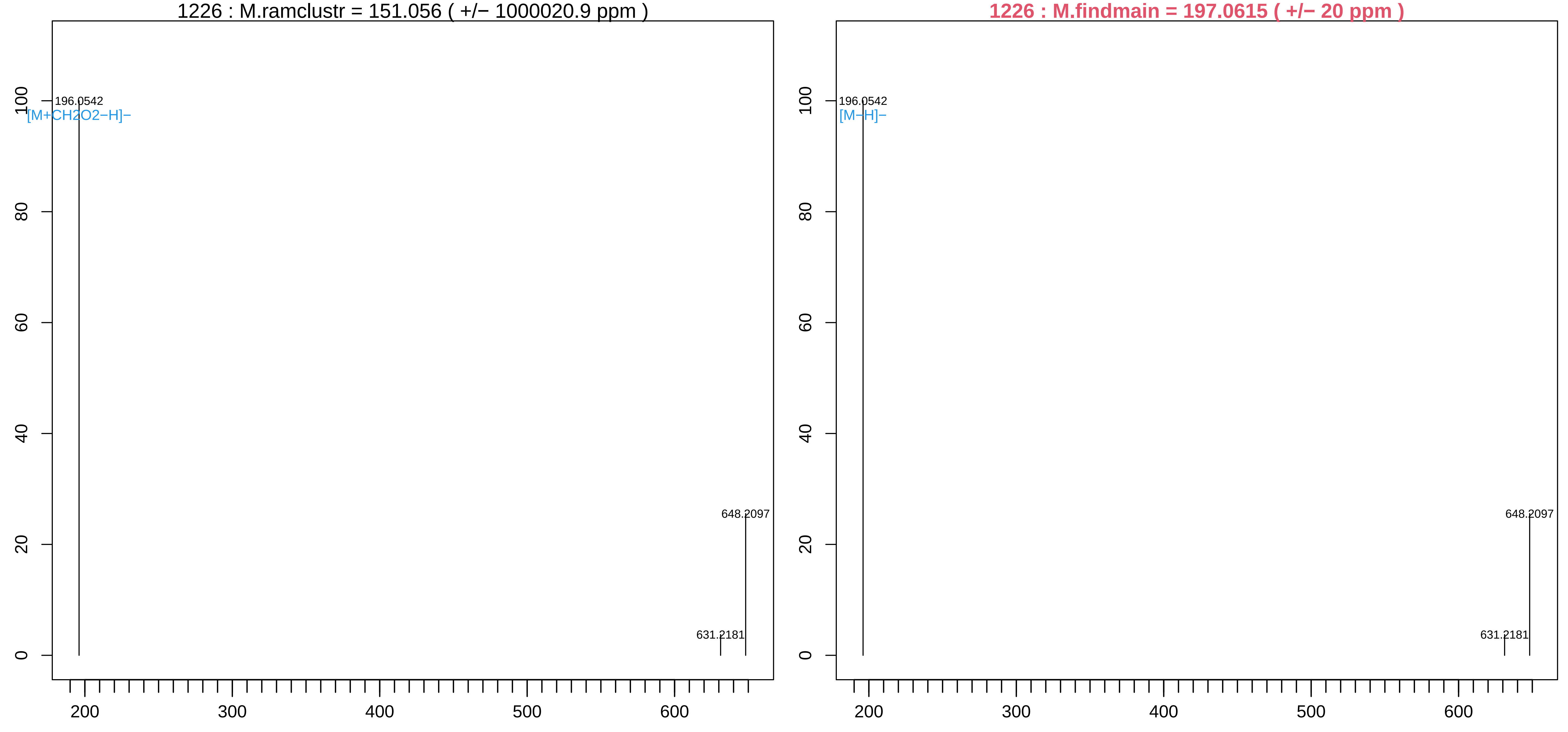
1226 : M.ramclustr = 151.056 (+/− 1000020.9 ppm)
196.0542
[M+CH2O2−H]−
648.2097
631.2181
1226 : M.findmain = 197.0615 (+/− 20 ppm)
196.0542
[M−H]−
648.2097
631.2181

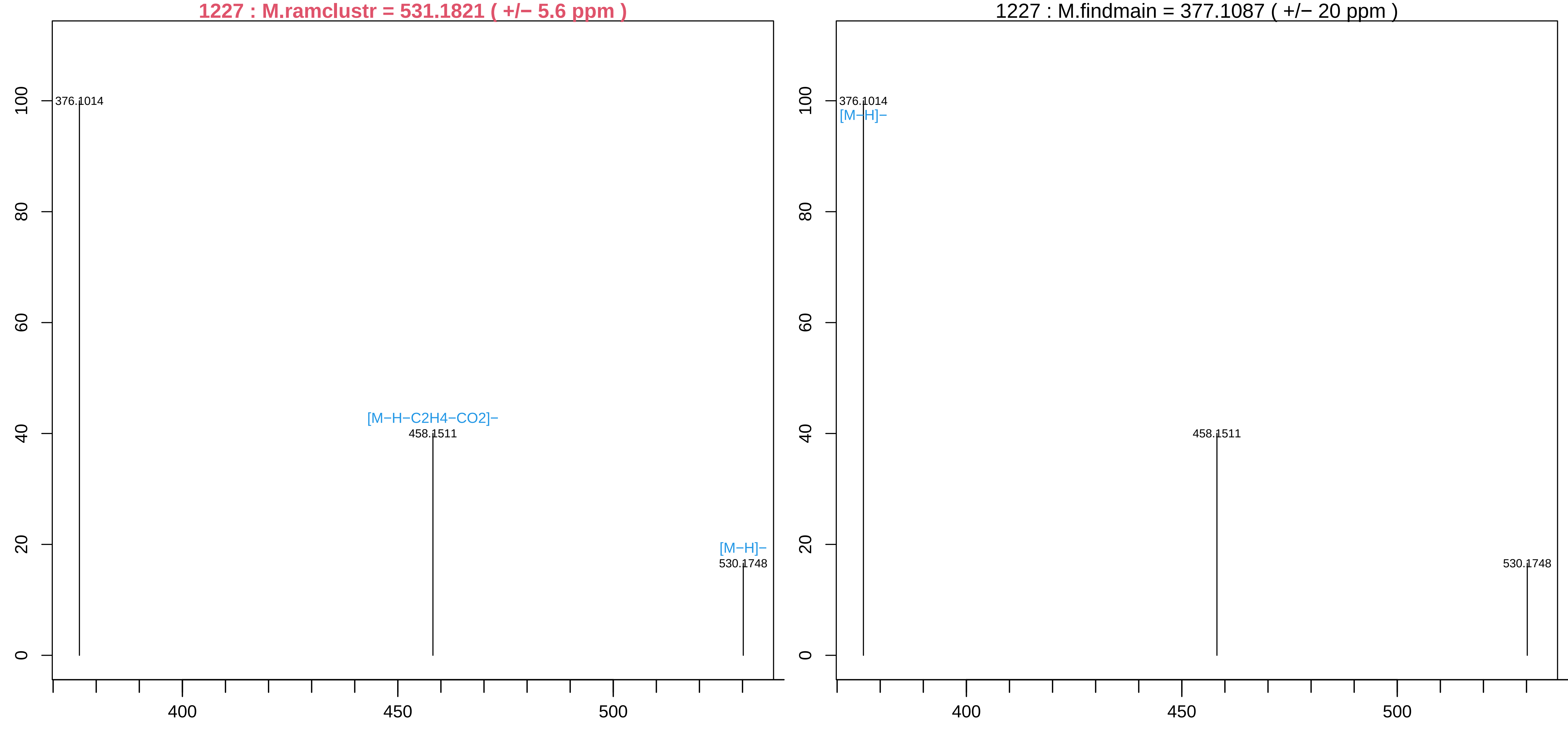

1227 : M.ramclustr = 531.1821 (+/− 5.6 ppm)
376.1014
[M−H−C2H4−CO2]−
458.1511
[M−H]−
530.1748
1227 : M.findmain = 377.1087 (+/− 20 ppm)
376.1014
[M−H]−
458.1511
530.1748
0
20
40
60
80
100
400
450
500

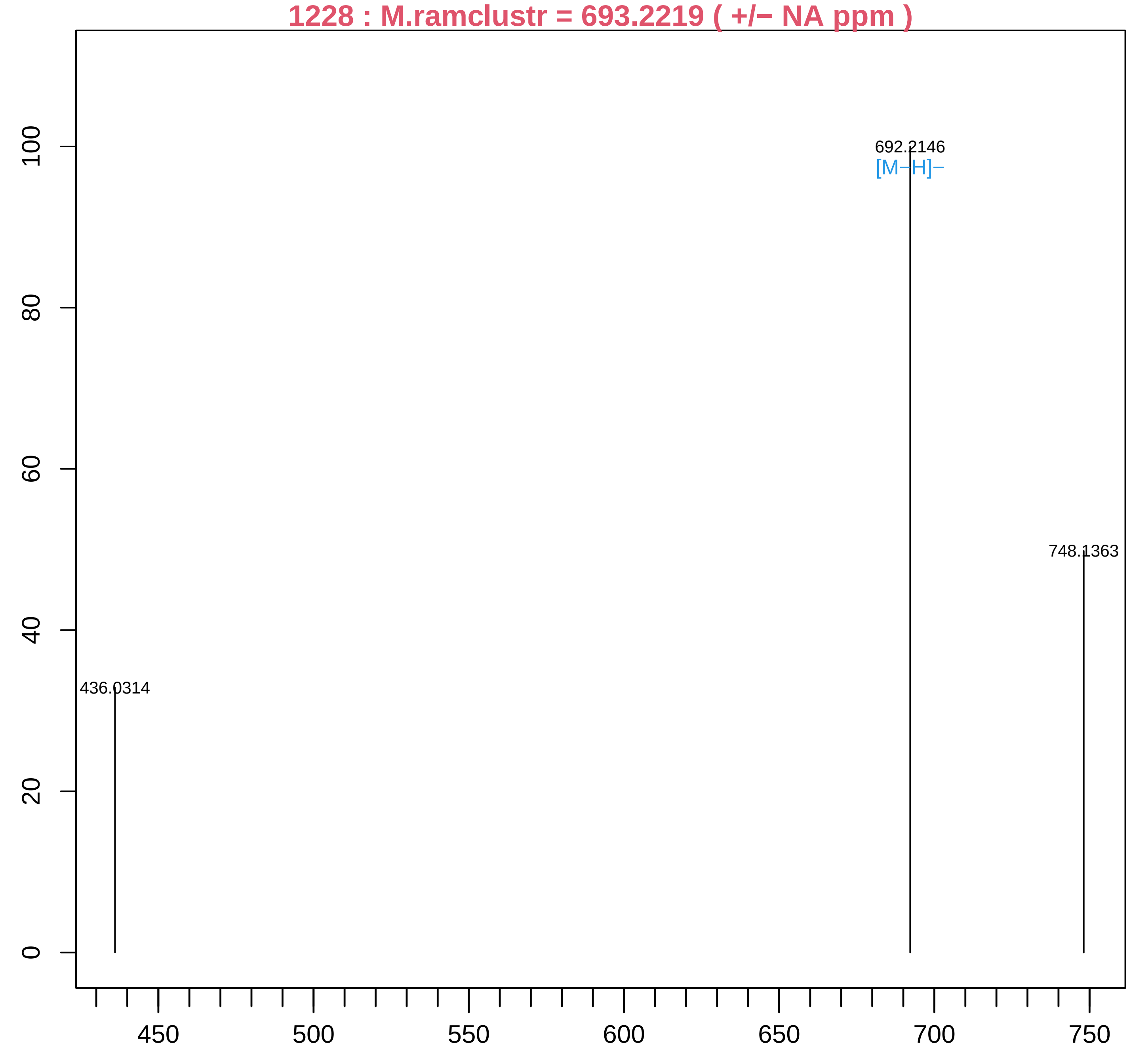
1228 : M.ramclustr = 693.2219 (+/− NA ppm)
692.2146
[M−H]−
748.1363
436.0314
0
20
40
60
80
100
450
500
550
600
650
700
750

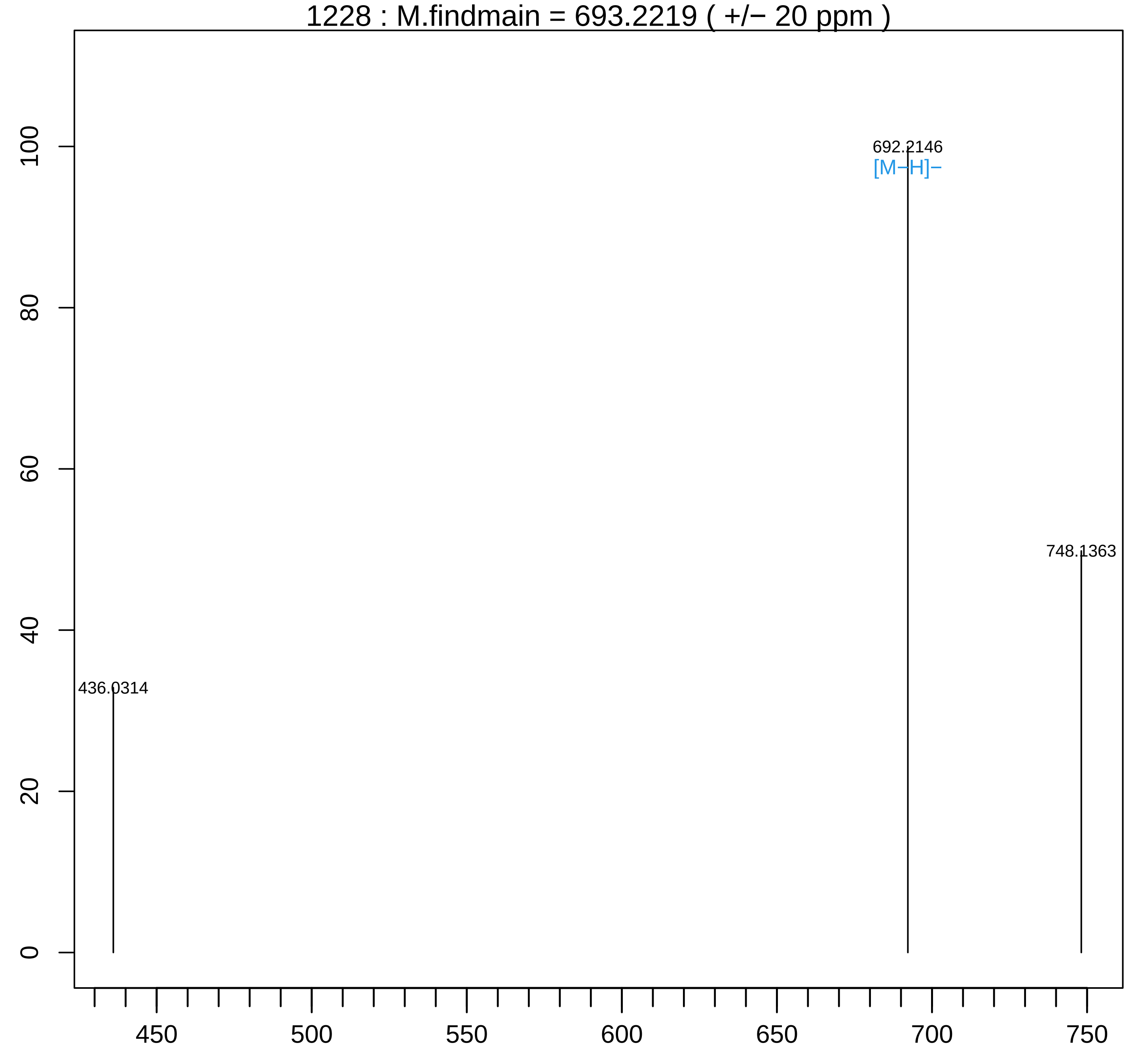
1228 : M.findmain = 693.2219 (+/− 20 ppm)
692.2146
[M−H]−
748.1363
436.0314
0
20
40
60
80
100
450
500
550
600
650
700
750

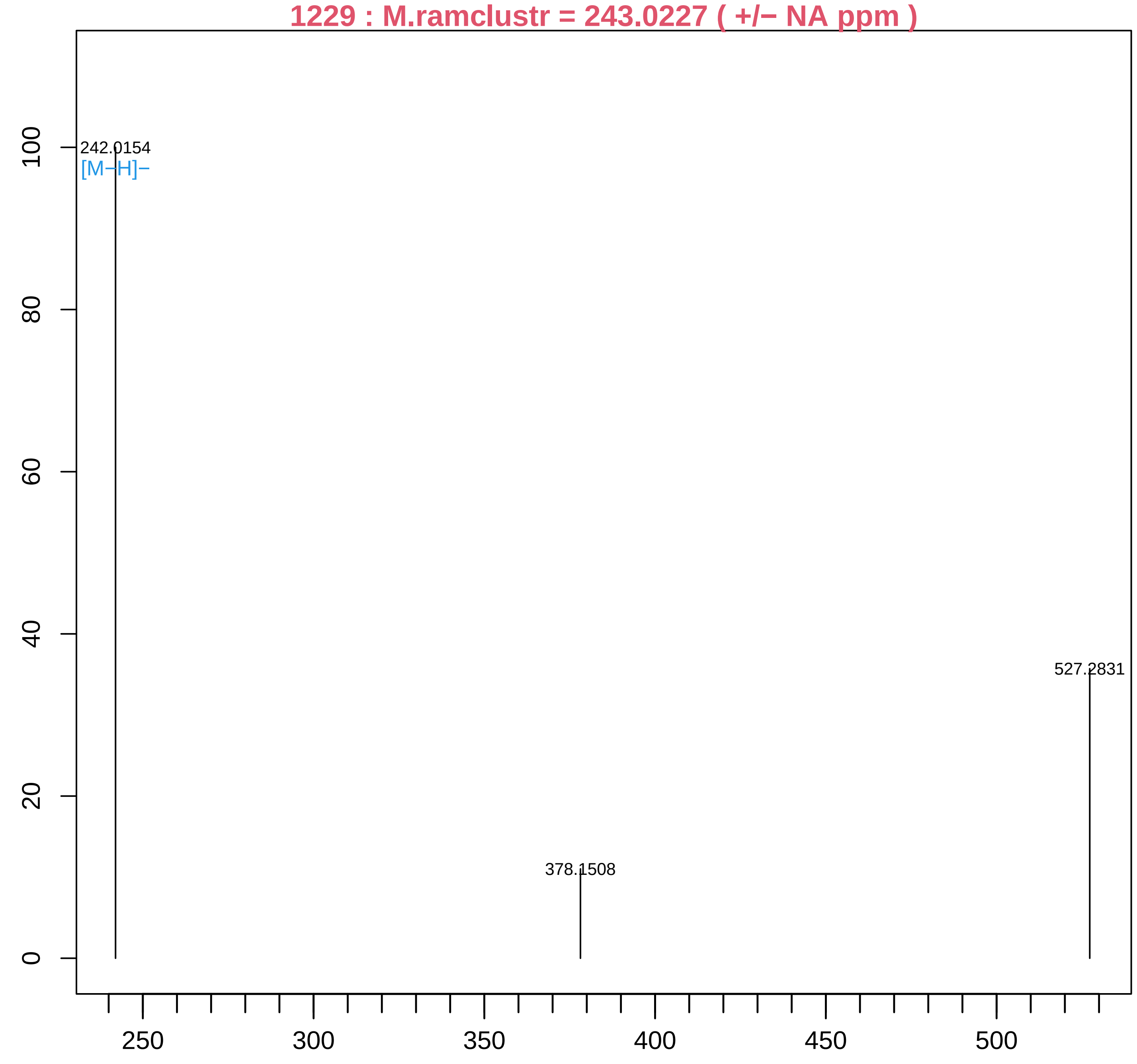
1229 : M.ramclustr = 243.0227 (+/− NA ppm)
242.0154
[M−H]−
378.1508
527.2831
0
20
40
60
80
100
250
300
350
400
450
500

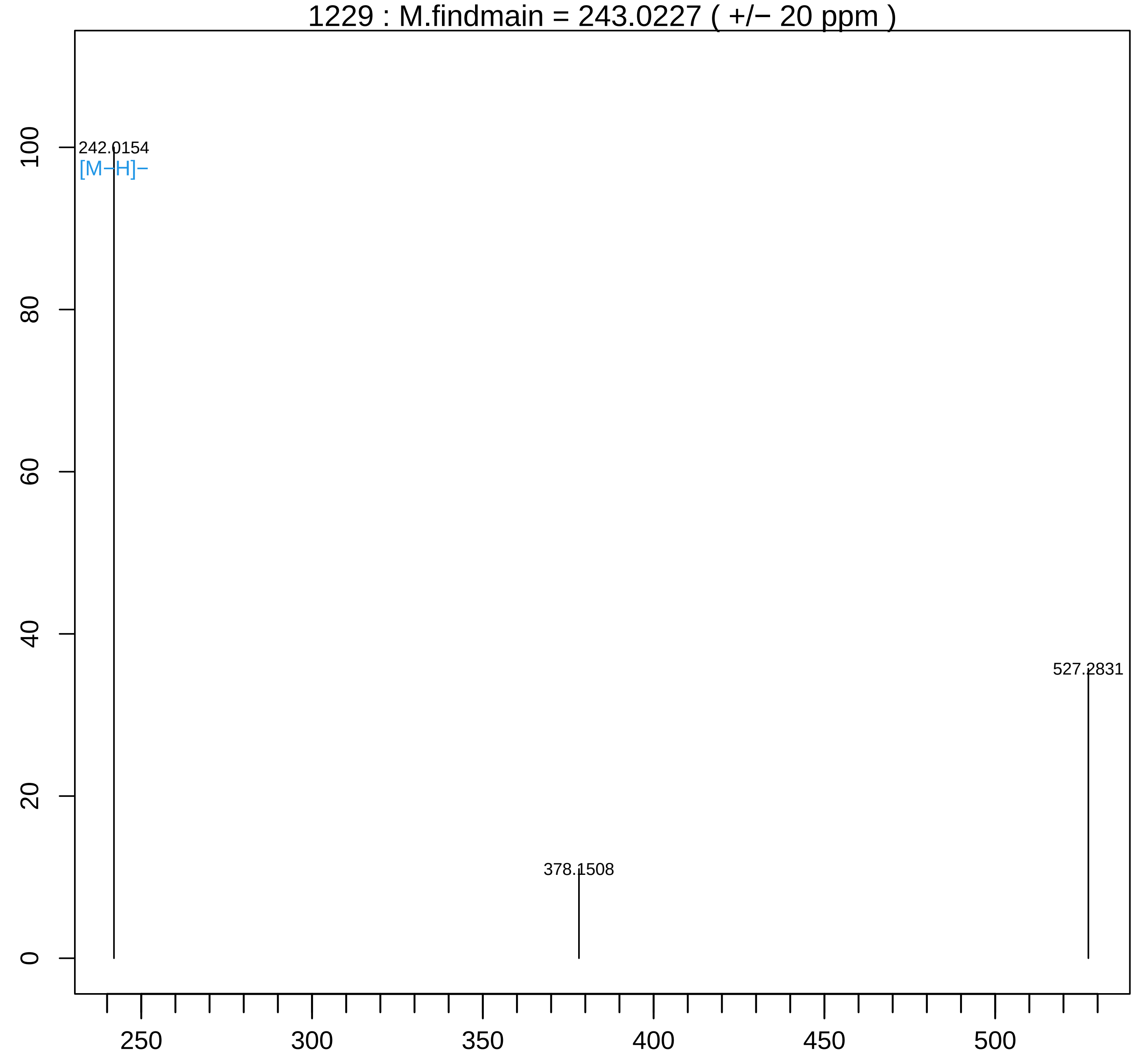
1229 : M.findmain = 243.0227 (+/− 20 ppm)
242.0154
[M−H]−
378.1508
527.2831
0
20
40
60
80
100
250
300
350
400
450
500

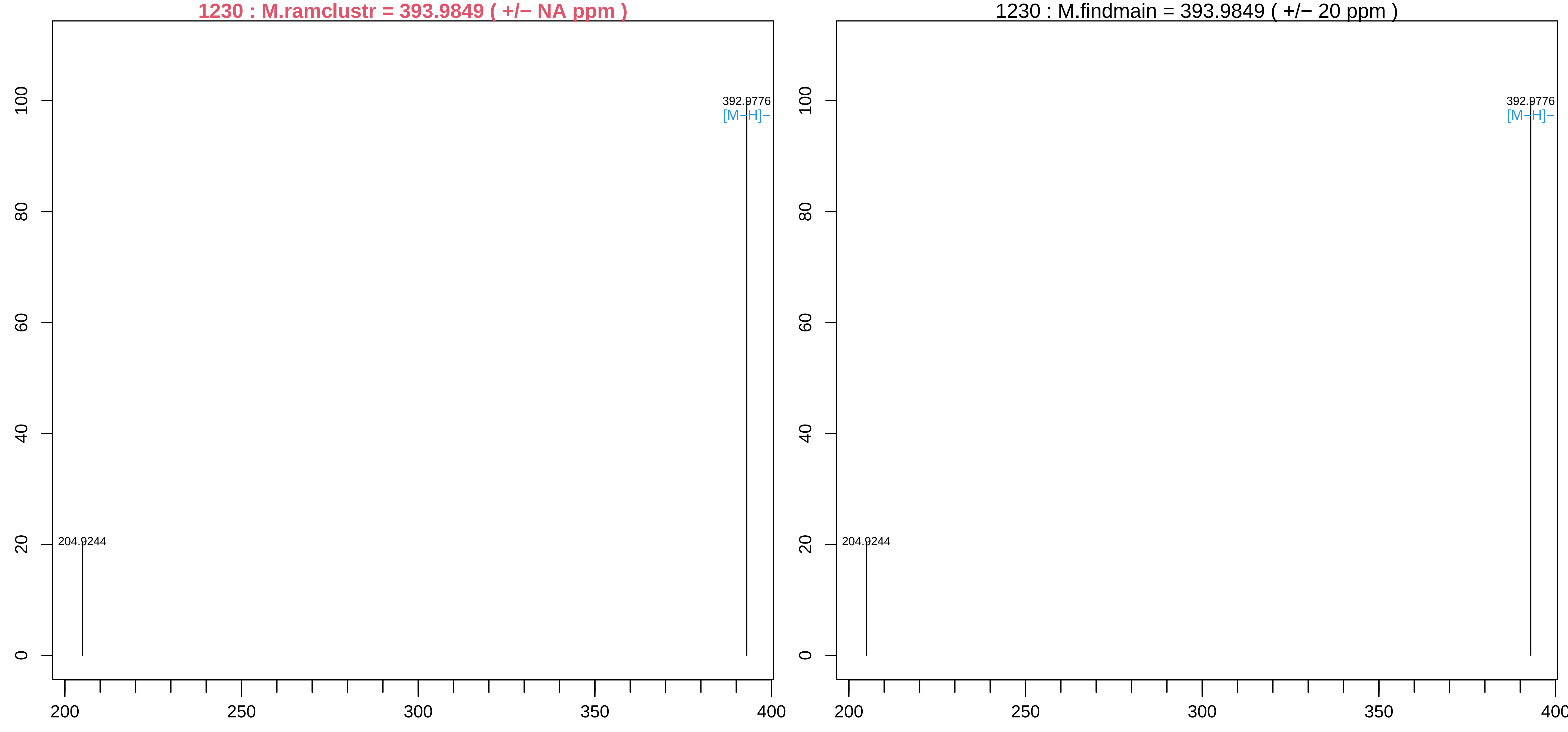

1230 : M.ramclustr = 393.9849 (+/− NA ppm)
1230 : M.findmain = 393.9849 (+/− 20 ppm)
392.9776
[M−H]−
204.9244
0
20
40
60
80
100
200
250
300
350
400

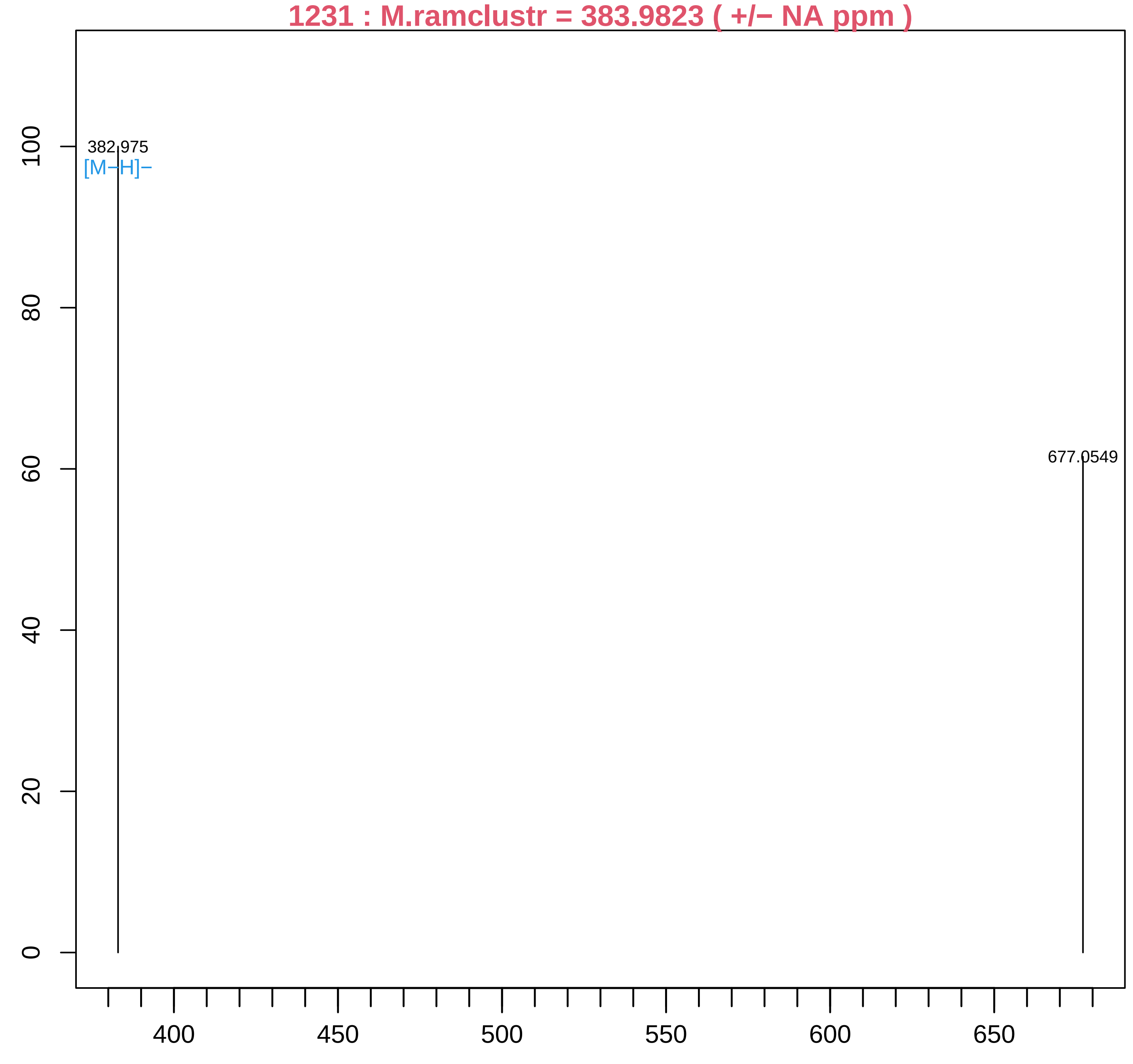
1231 : M.ramclustr = 383.9823 (+/− NA ppm)
382.975
[M−H]−
677.0549
0
20
40
60
80
100
400
450
500
550
600
650

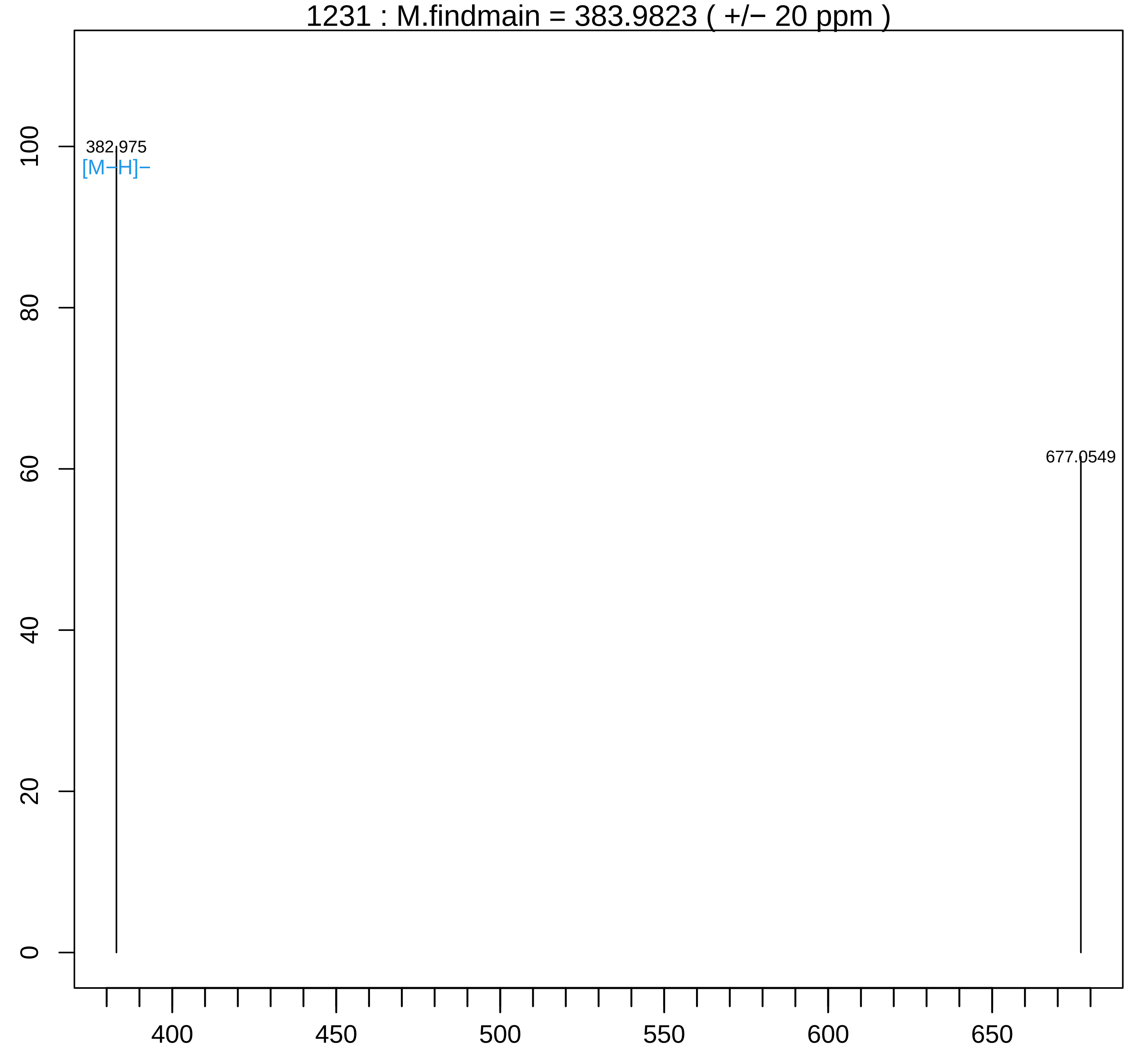
1231 : M.findmain = 383.9823 (+/− 20 ppm)
382.975
[M−H]−
677.0549
0
20
40
60
80
100
400
450
500
550
600
650

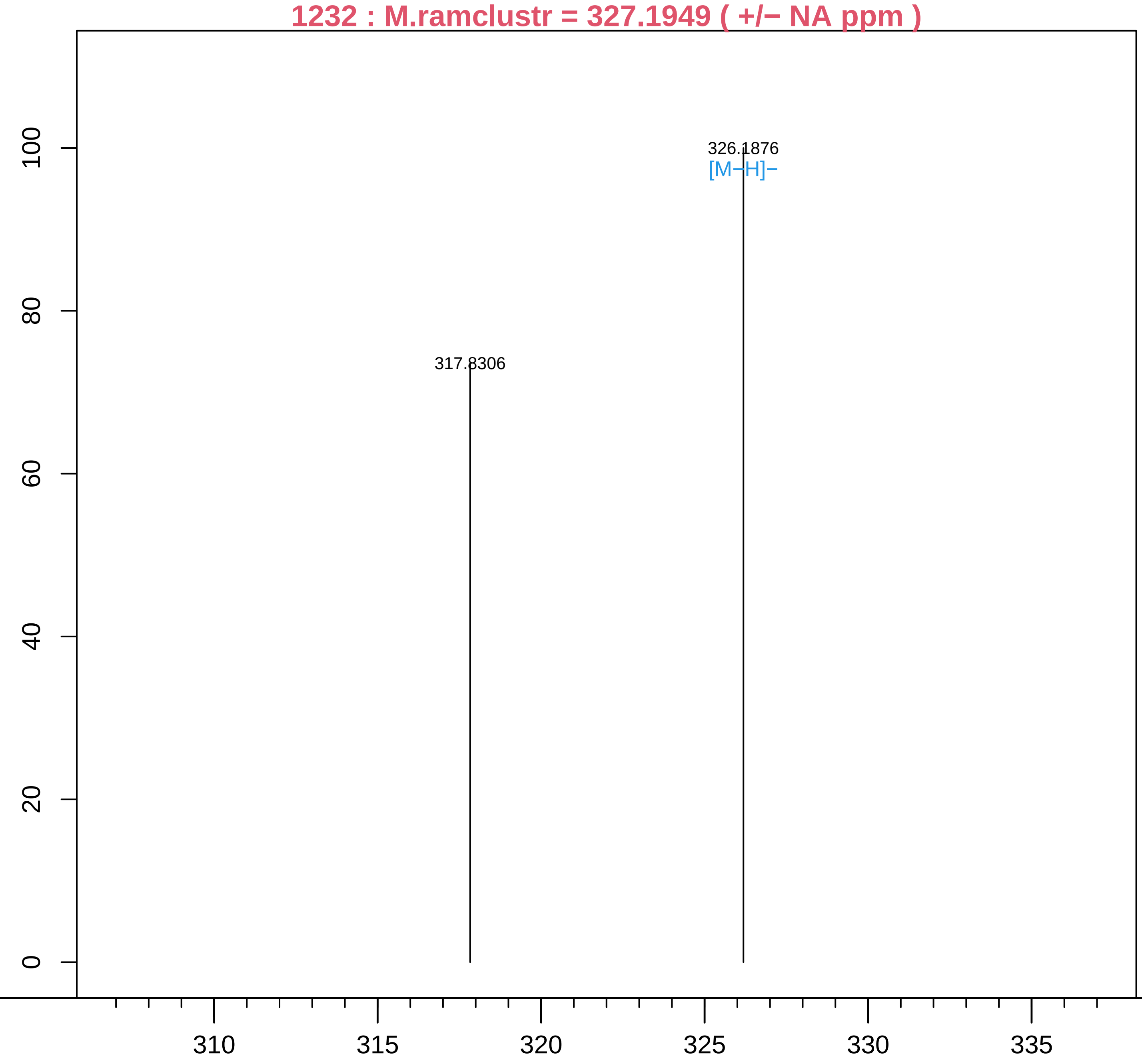
1232 : M.ramclustr = 327.1949 (+/− NA ppm)
326.1876
[M−H]−
317.8306
0
20
40
60
80
100
310
315
320
325
330
335

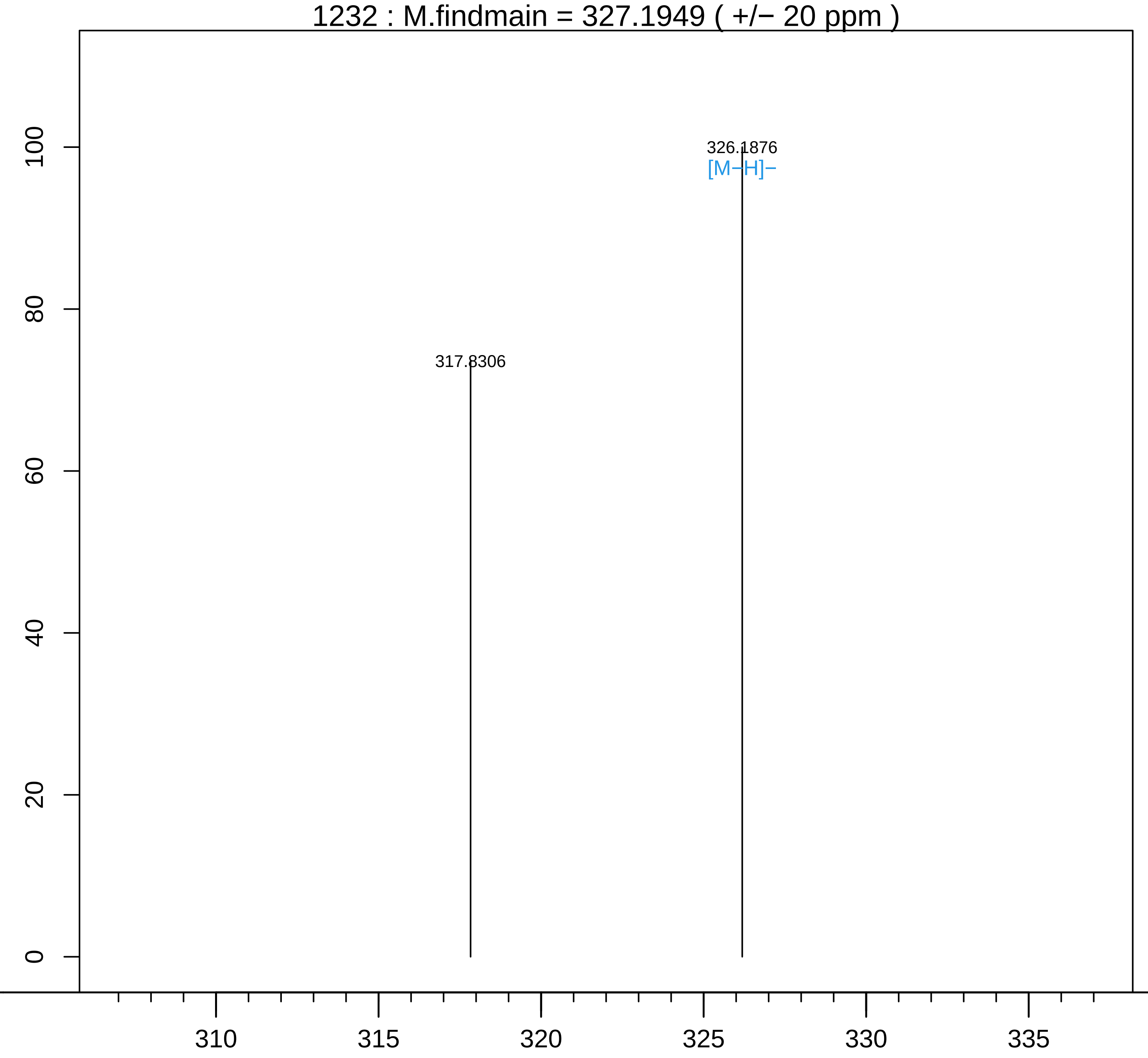
1232 : M.findmain = 327.1949 (+/− 20 ppm)
326.1876
[M−H]−
317.8306
0
20
40
60
80
100
310
315
320
325
330
335

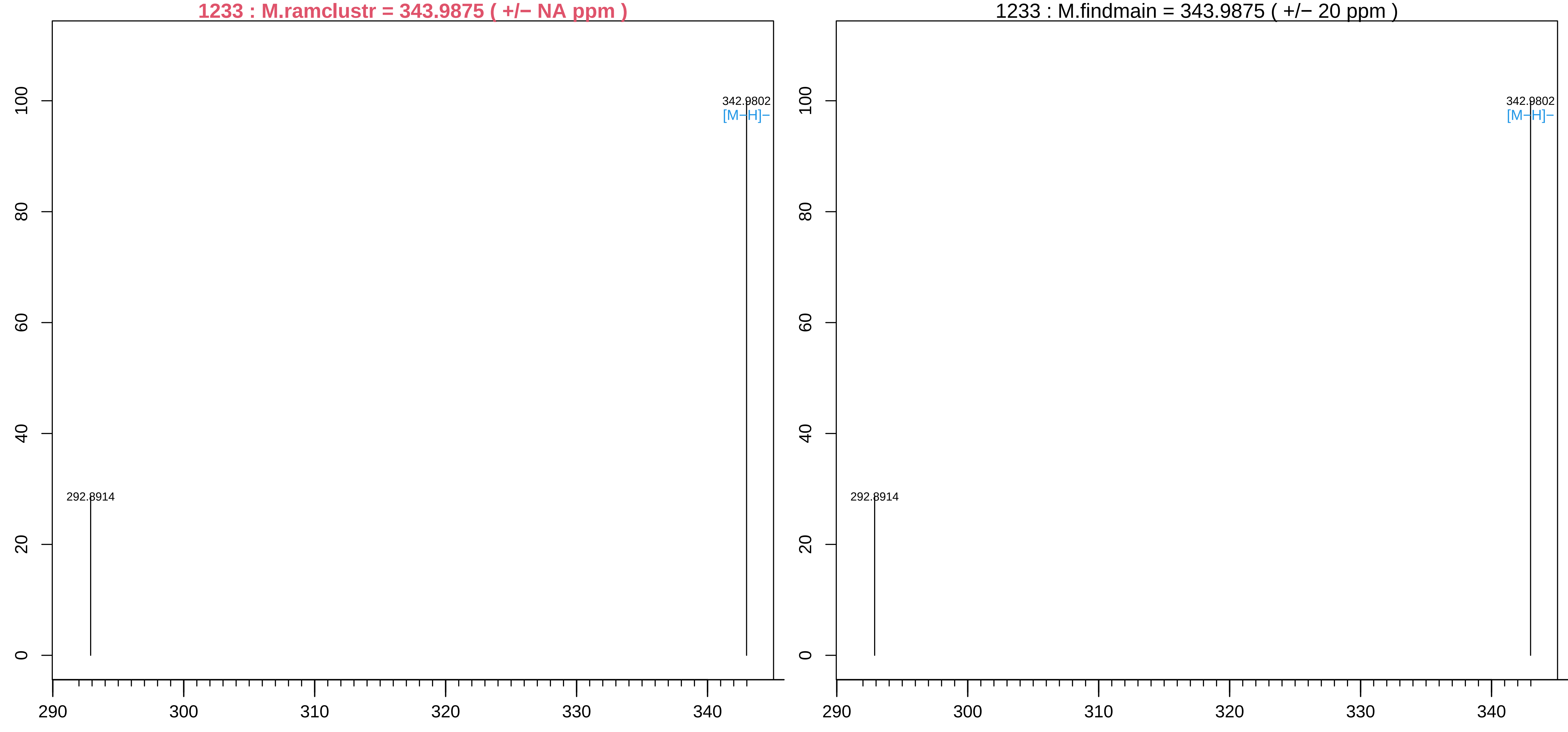
1233 : M.ramclustr = 343.9875 (+/− NA ppm)
1233 : M.findmain = 343.9875 (+/− 20 ppm)
342.9802
[M−H]−
292.8914
0
20
40
60
80
100
290
300
310
320
330
340

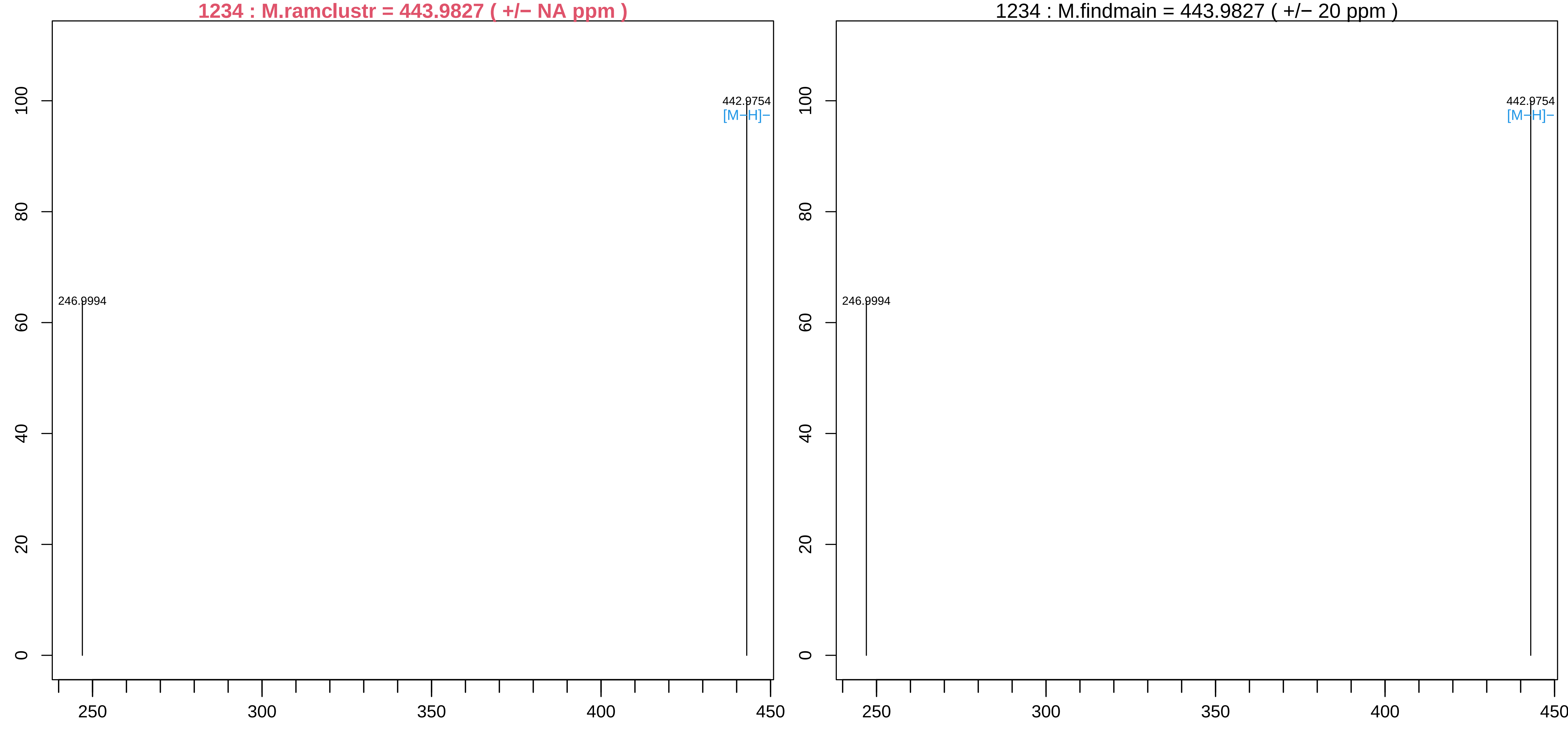
1234 : M.ramclustr = 443.9827 (+/− NA ppm)
442.9754
[M−H]−
246.9994
1234 : M.findmain = 443.9827 (+/− 20 ppm)
442.9754
[M−H]−
246.9994

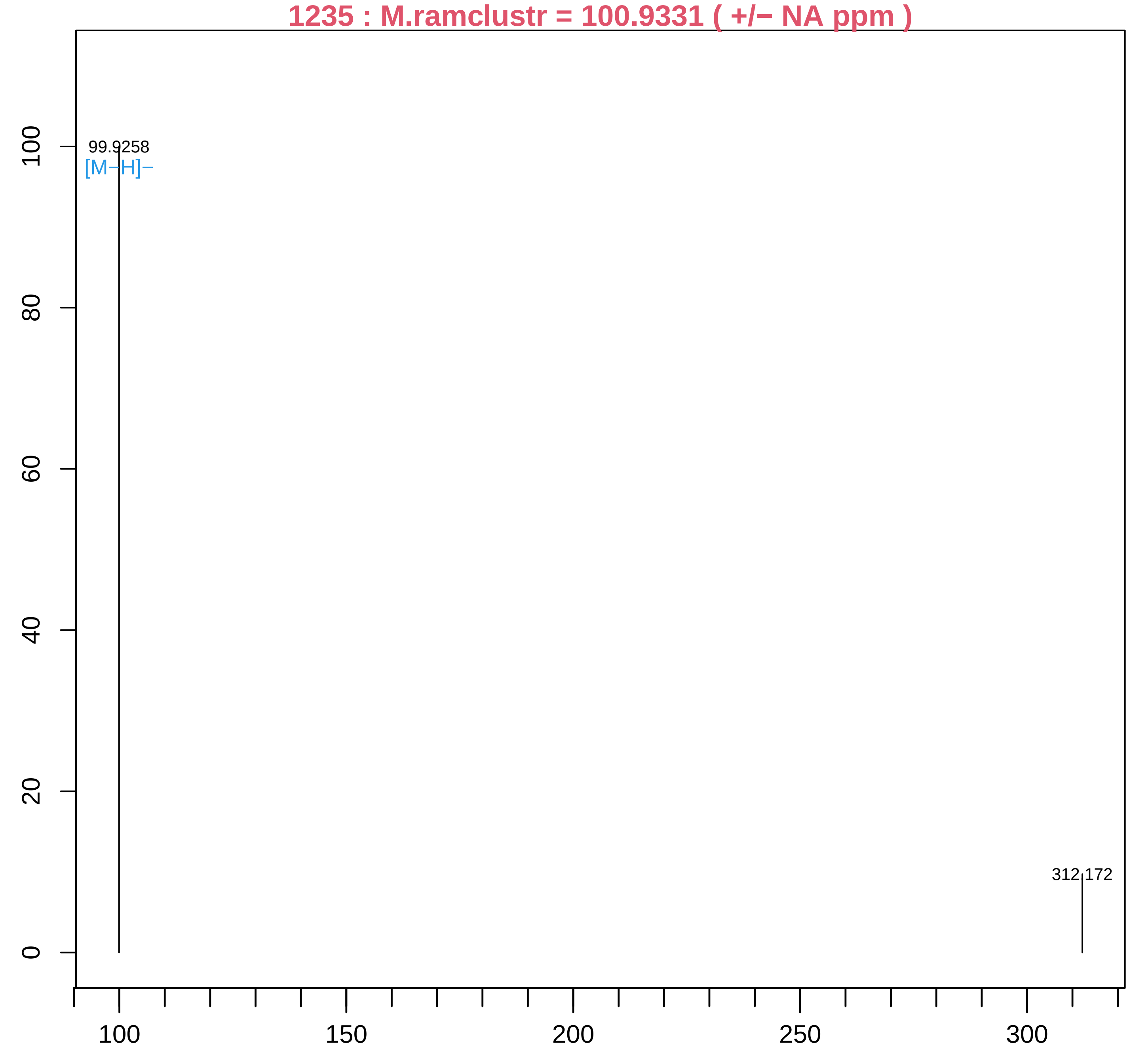
1235 : M.ramclustr = 100.9331 (+/− NA ppm)
99.9258
[M−H]−
312.172
0
20
40
60
80
100
150
200
250
300

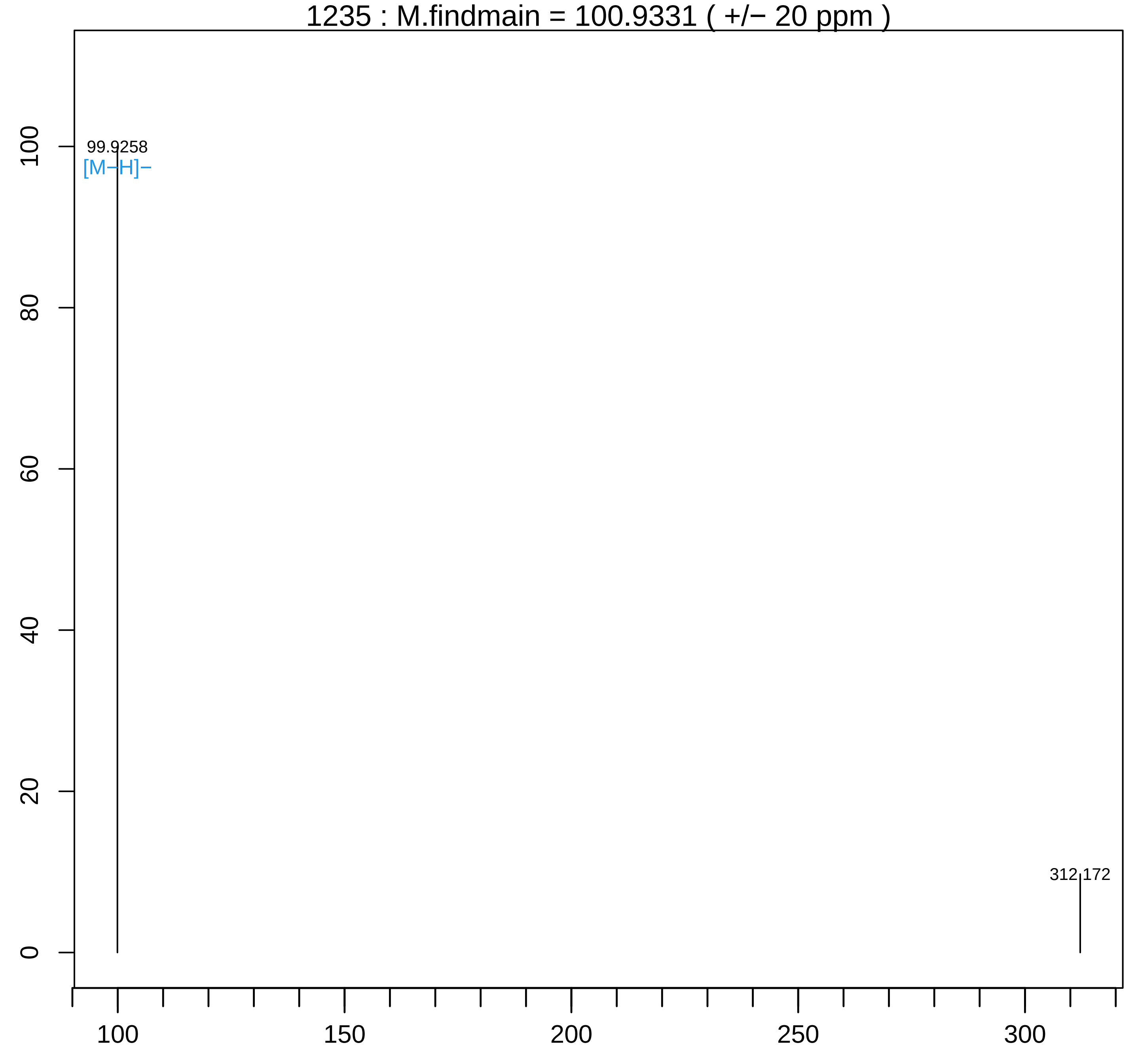
1235 : M.findmain = 100.9331 (+/− 20 ppm)
99.9258
[M−H]−
312.172
0
20
40
60
80
100
150
200
250
300

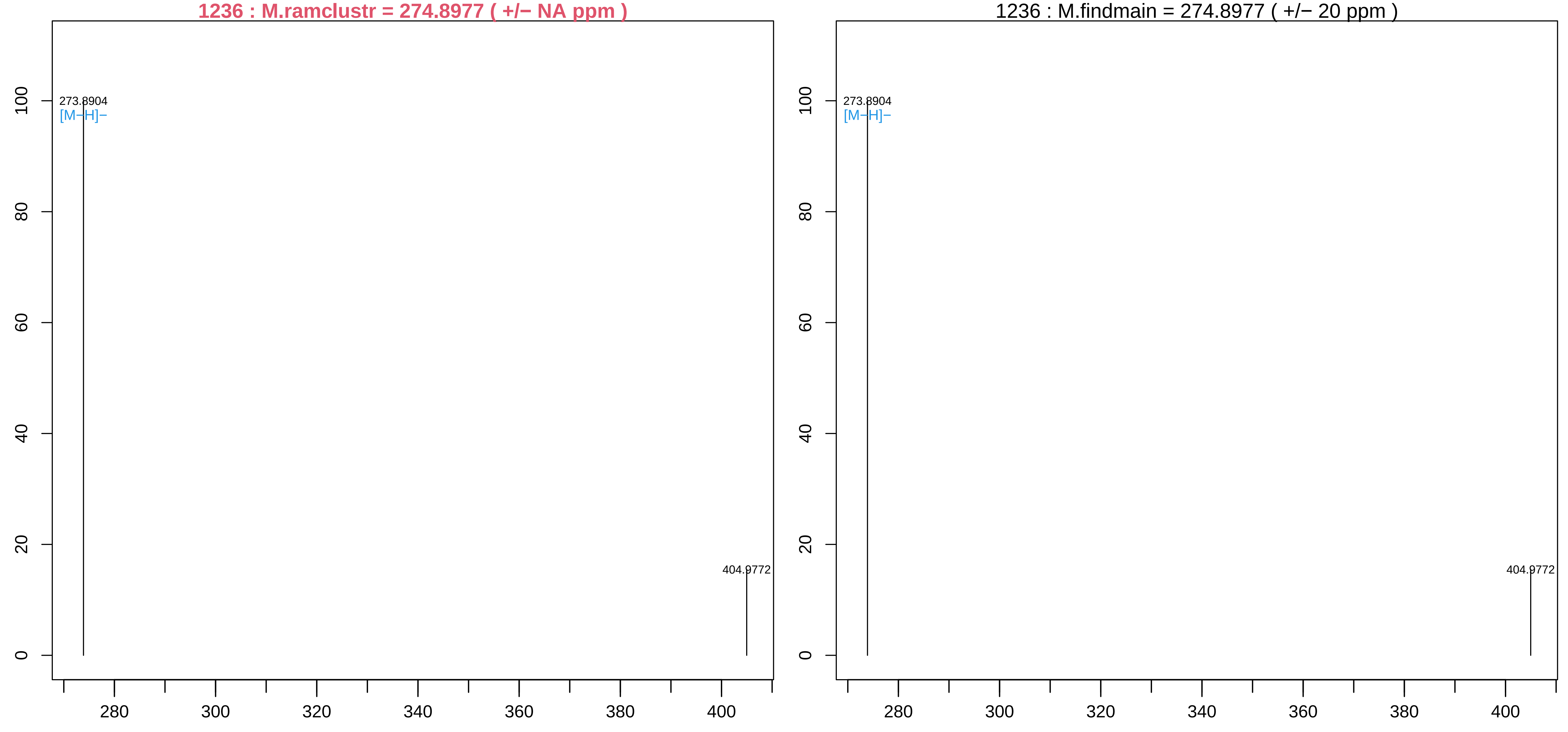
1236 : M.ramclustr = 274.8977 (+/− NA ppm)
273.8904
[M−H]−
404.9772
0
20
40
60
80
100
280
300
320
340
360
380
400
1236 : M.findmain = 274.8977 (+/− 20 ppm)
273.8904
[M−H]−
404.9772
0
20
40
60
80
100
280
300
320
340
360
380
400

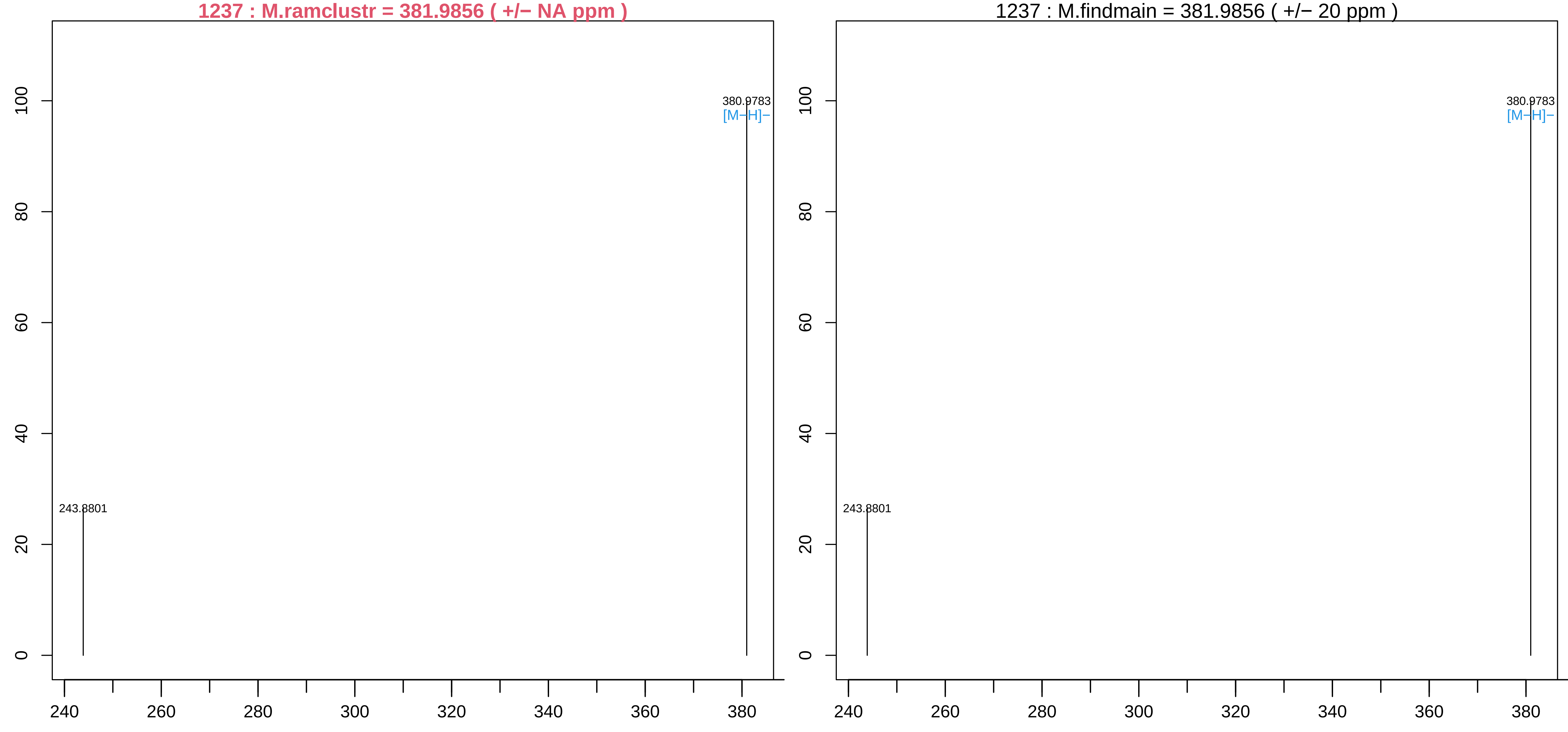
1237 : M.ramclustr = 381.9856 (+/− NA ppm)
380.9783
[M−H]−
243.8801
1237 : M.findmain = 381.9856 (+/− 20 ppm)
380.9783
[M−H]−
243.8801
0
20
40
60
80
100
240
260
280
300
320
340
360
380

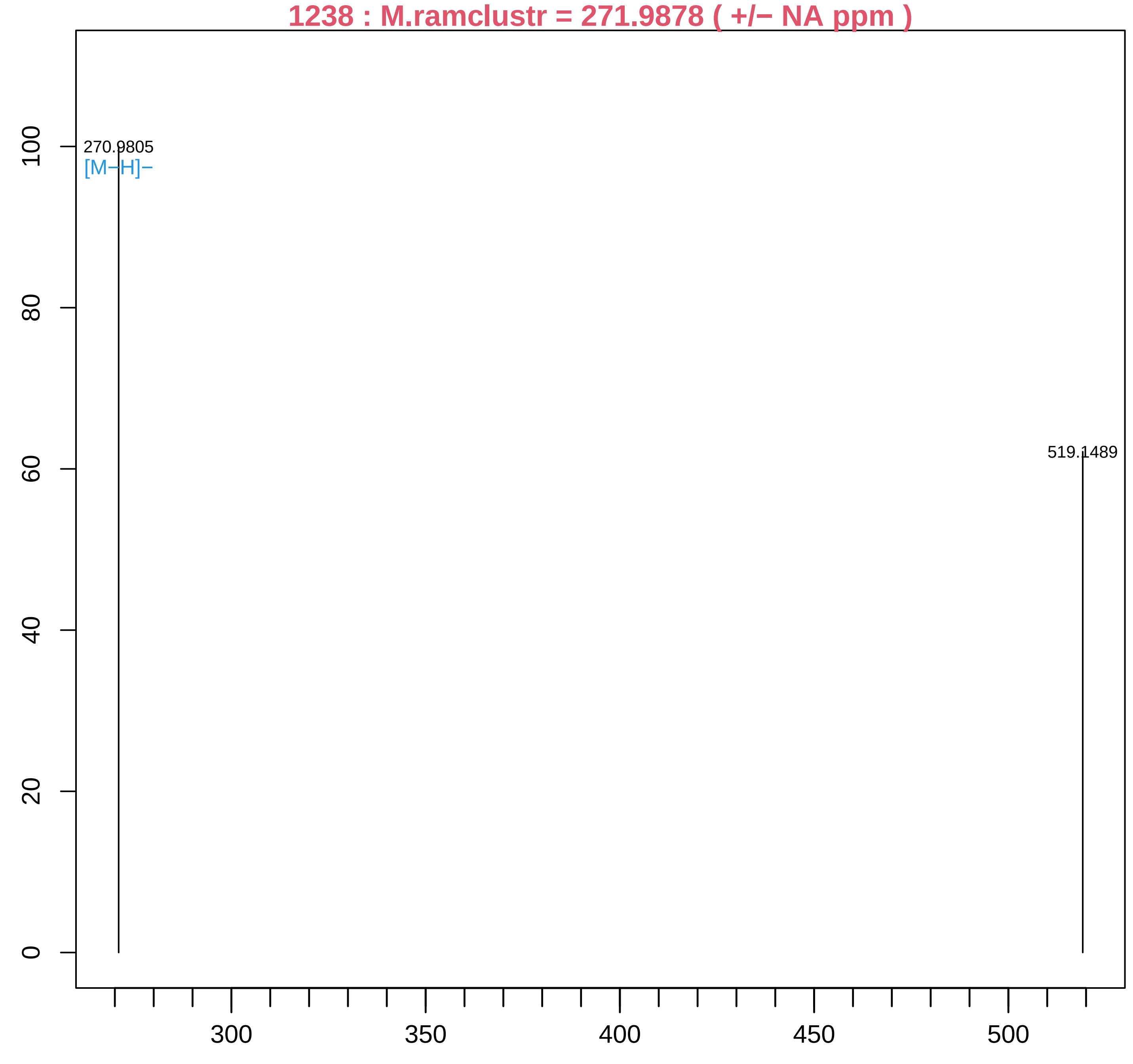
1238 : M.ramclustr = 271.9878 (+/− NA ppm)
270.9805
[M−H]−
519.1489
0
20
40
60
80
100
300
350
400
450
500

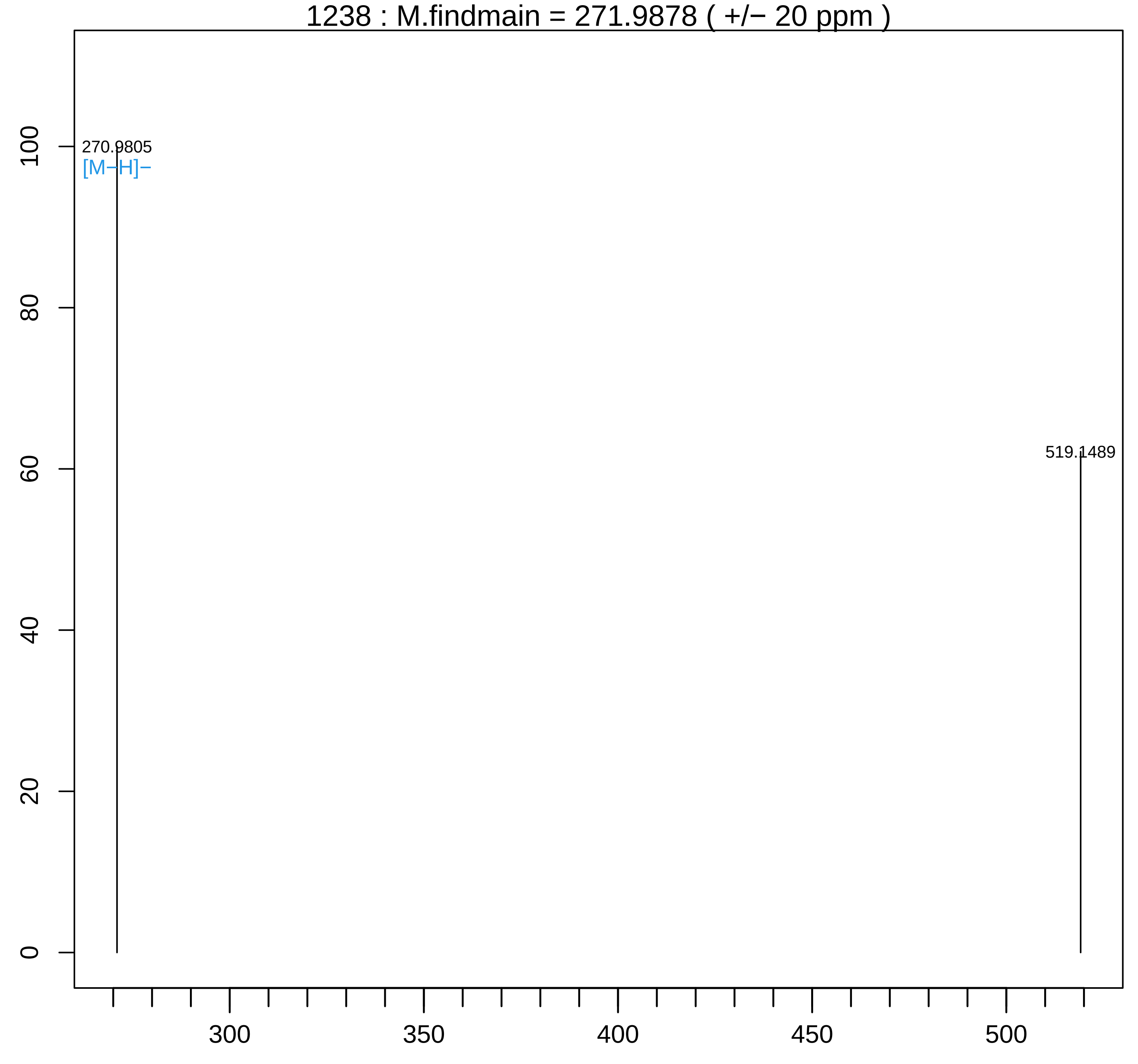
1238 : M.findmain = 271.9878 (+/− 20 ppm)
270.9805
[M−H]−
519.1489
0
20
40
60
80
100
300
350
400
450
500

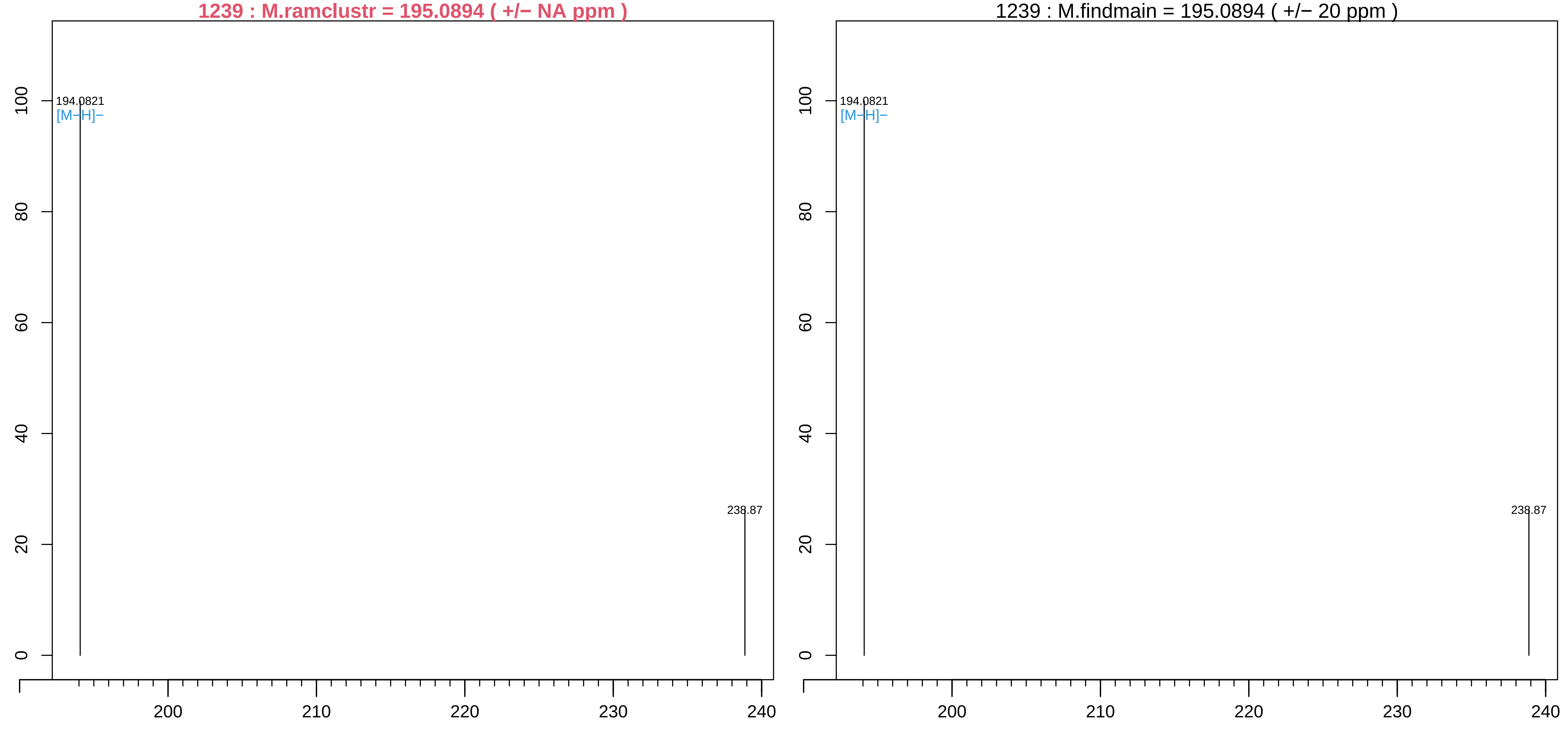
1239 : M.ramclustr = 195.0894 (+/− NA ppm)
194.0821
[M−H]−
238.87
0
20
40
60
80
100
200
210
220
230
240
1239 : M.findmain = 195.0894 (+/− 20 ppm)
194.0821
[M−H]−
238.87
0
20
40
60
80
100
200
210
220
230
240

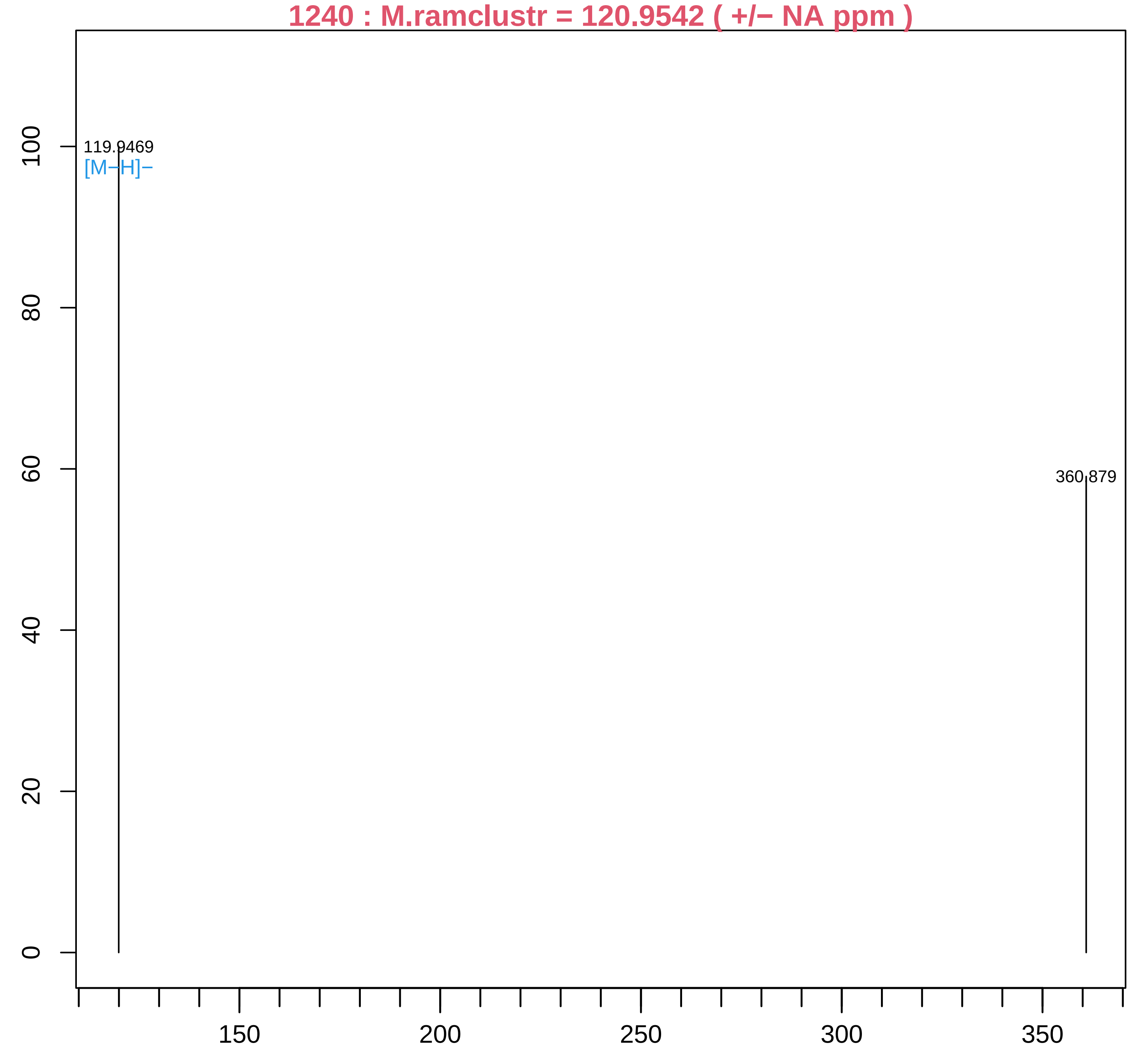

1240 : M.ramclustr = 120.9542 (+/− NA ppm)
119.9469
[M−H]−
360.879
0
20
40
60
80
100
150
200
250
300
350

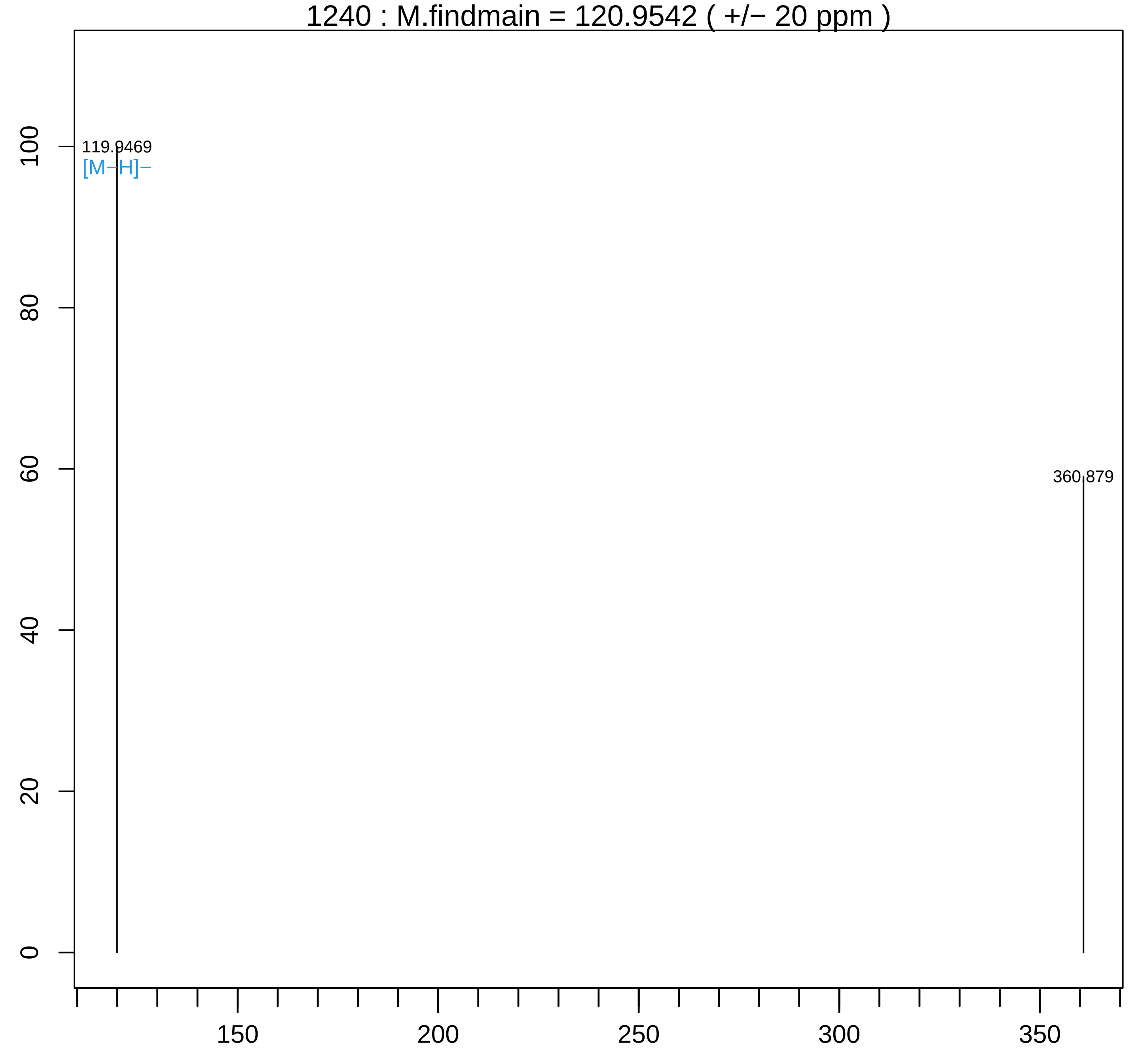

1240 : M.findmain = 120.9542 (+/− 20 ppm)
119.9469
[M−H]−
360.879
0
20
40
60
80
100
150
200
250
300
350

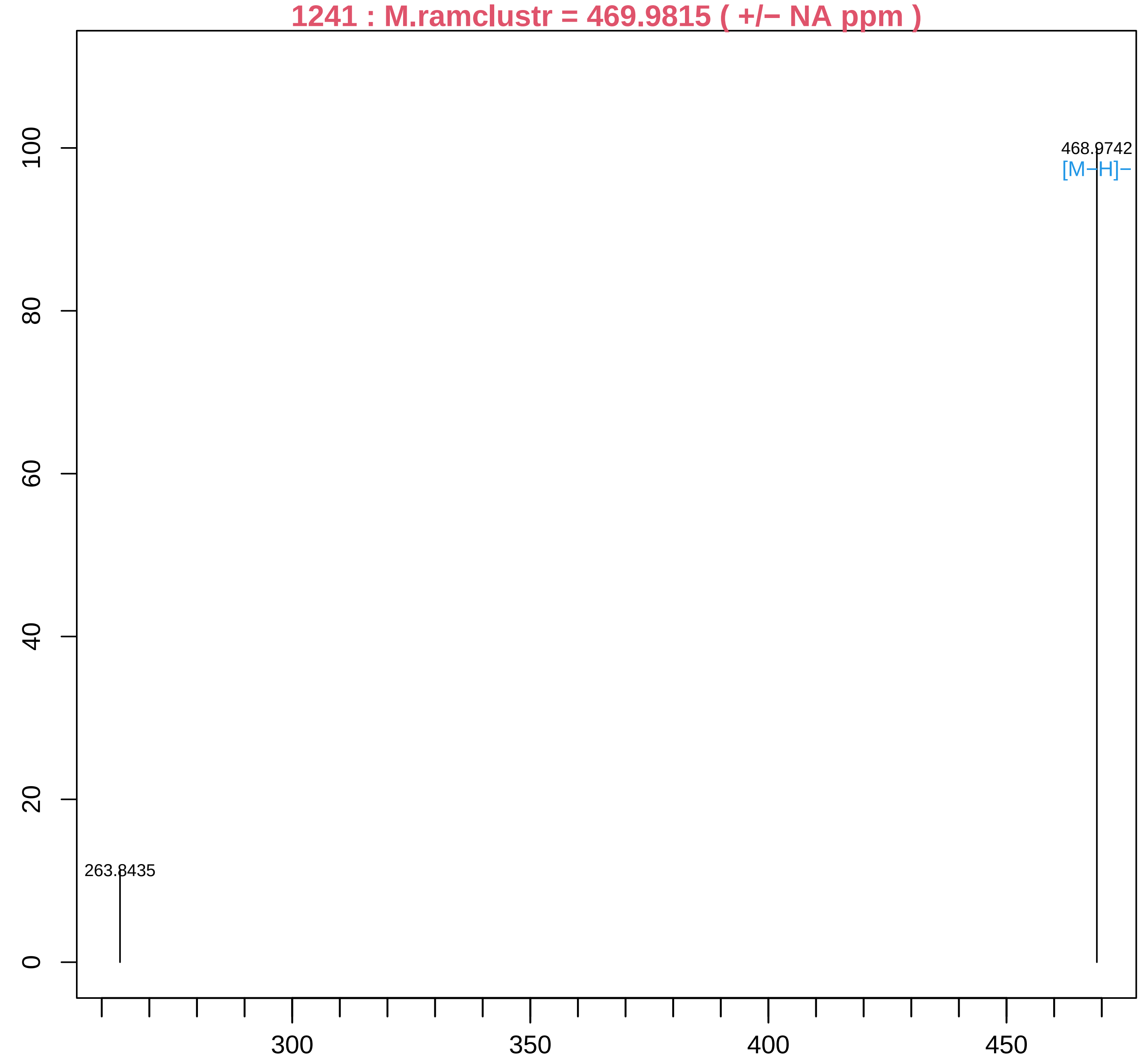
1241 : M.ramclustr = 469.9815 (+/− NA ppm)
468.9742
[M−H]−
263.8435
0
20
40
60
80
100
300
350
400
450

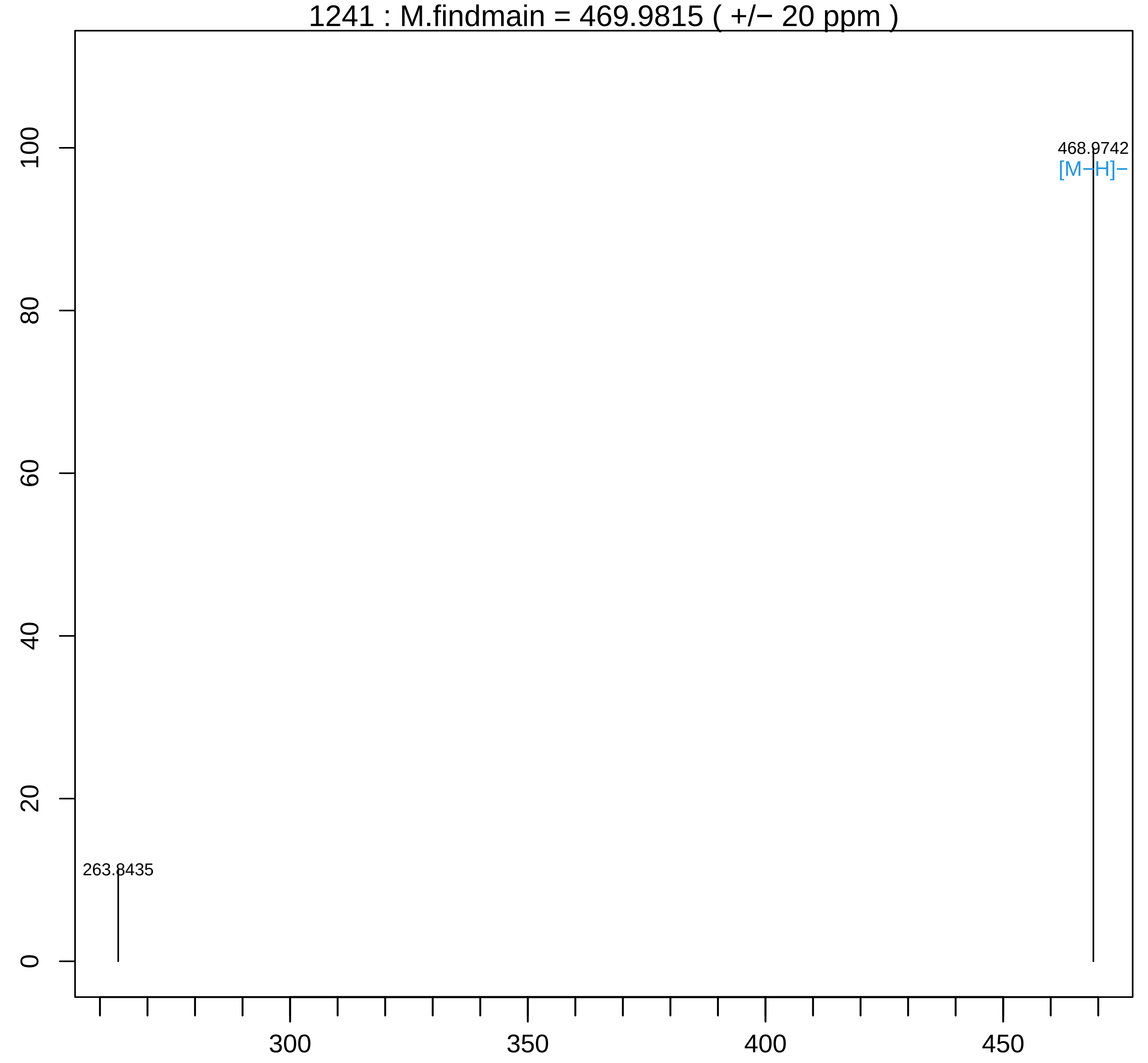
1241 : M.findmain = 469.9815 (+/− 20 ppm)
468.9742
[M−H]−
263.8435
0
20
40
60
80
100
300
350
400
450

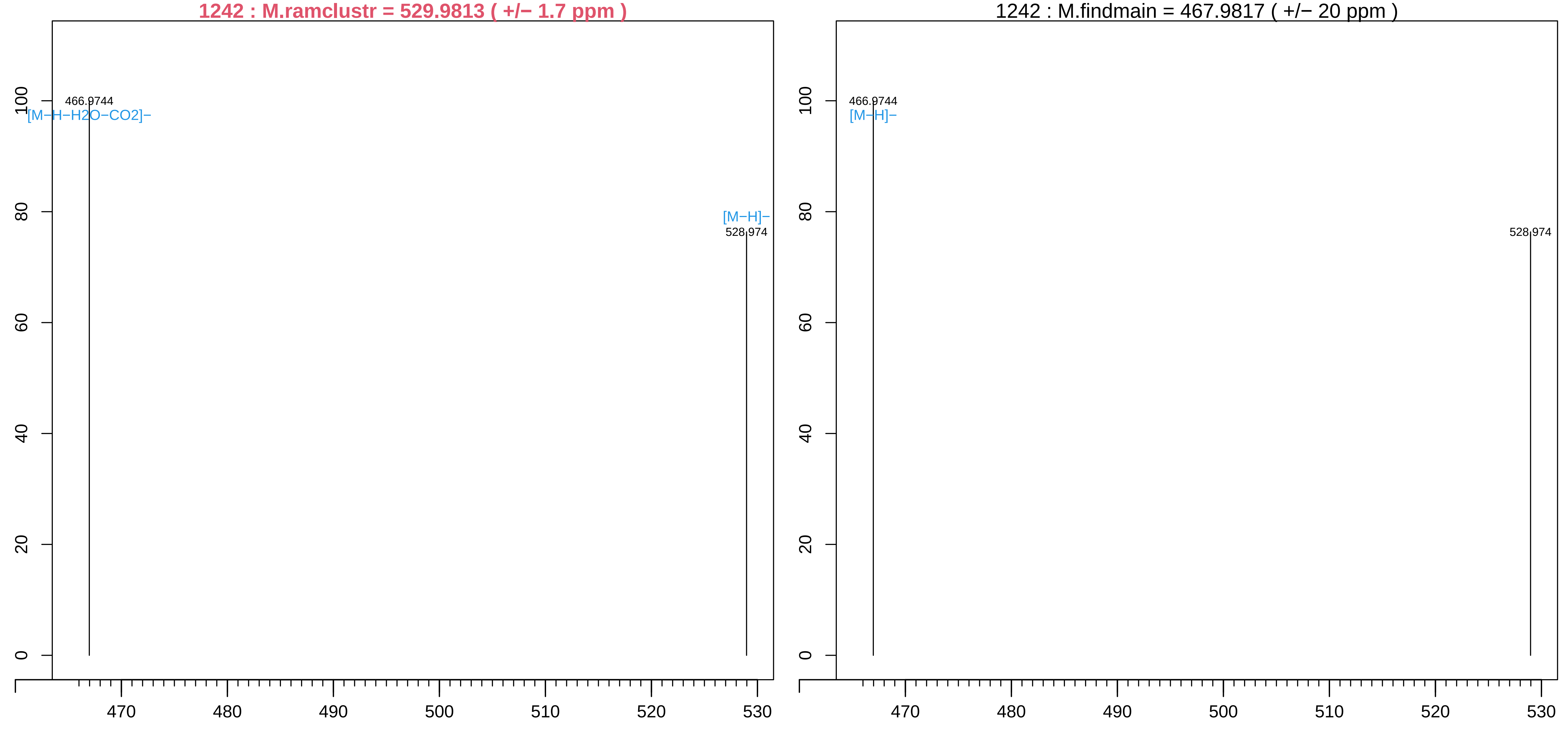
1242 : M.ramclustr = 529.9813 (+/− 1.7 ppm)
466.9744
[M−H−H2O−CO2]−
[M−H]−
528.974
1242 : M.findmain = 467.9817 (+/− 20 ppm)
466.9744
[M−H]−
528.974

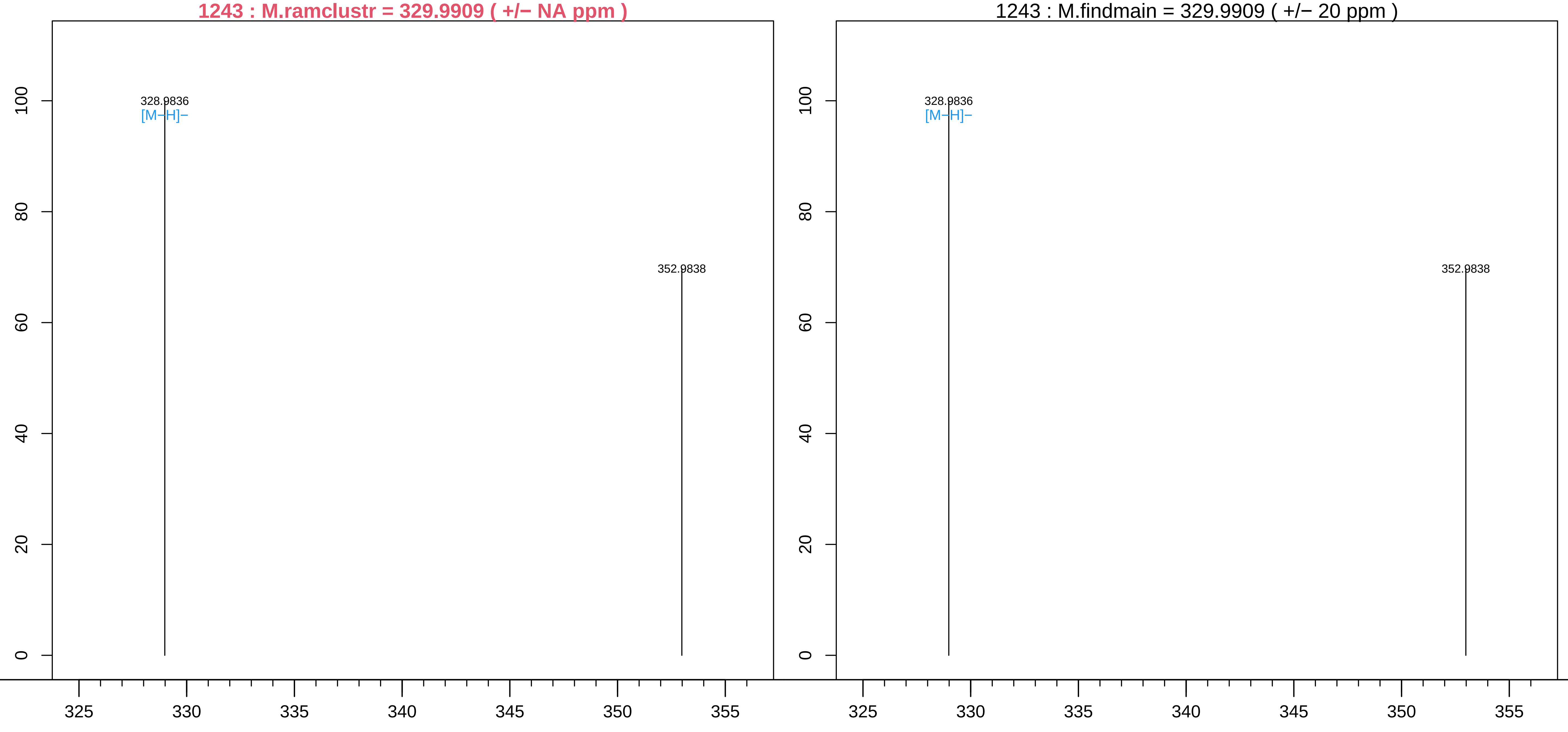
1243 : M.ramclustr = 329.9909 (+/− NA ppm)
328.9836
[M−H]−
352.9838
1243 : M.findmain = 329.9909 (+/− 20 ppm)
328.9836
[M−H]−
352.9838

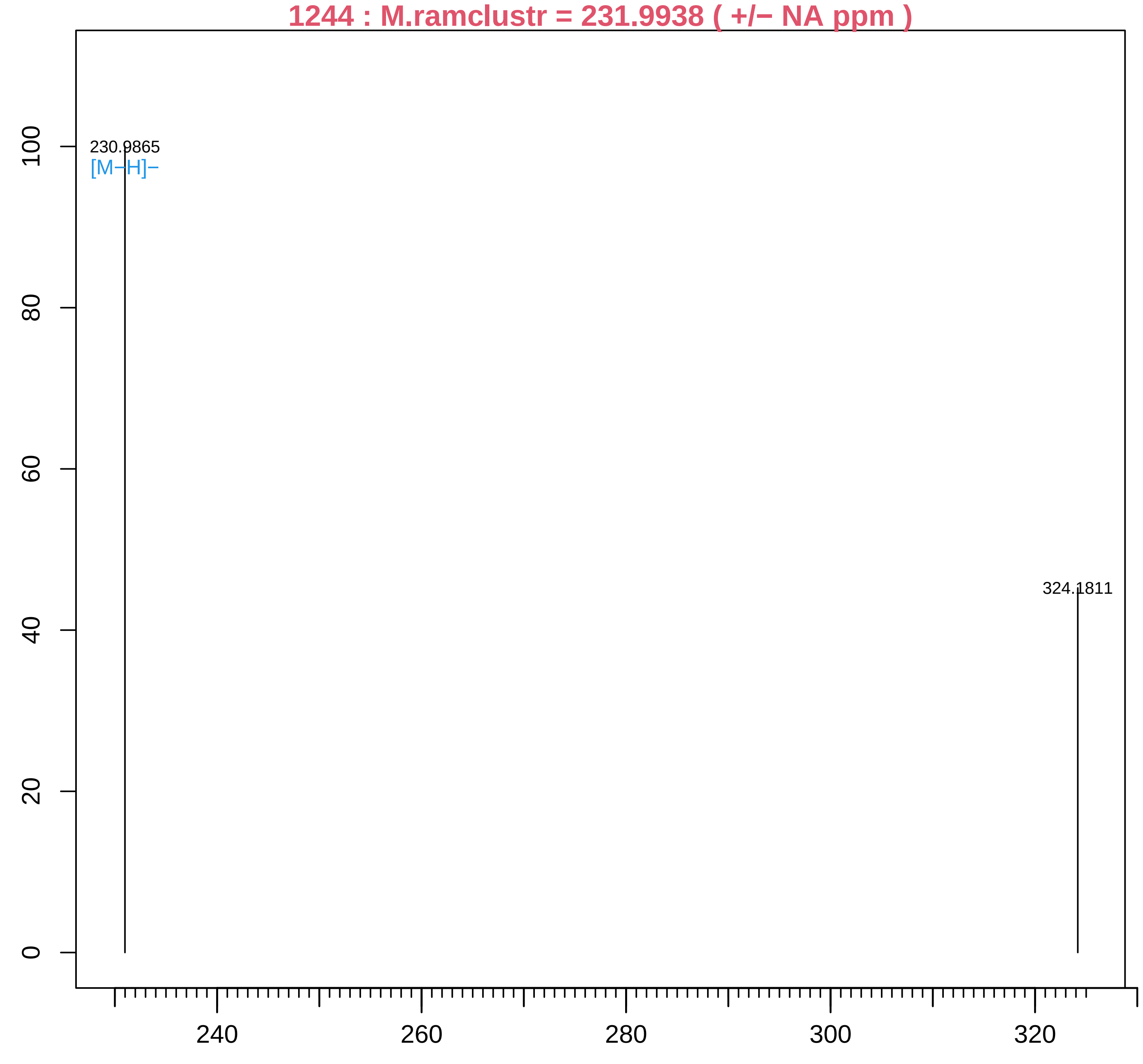

1244 : M.ramclustr = 231.9938 (+/− NA ppm)
230.9865
[M−H]−
324.1811
0
20
40
60
80
100
240
260
280
300
320

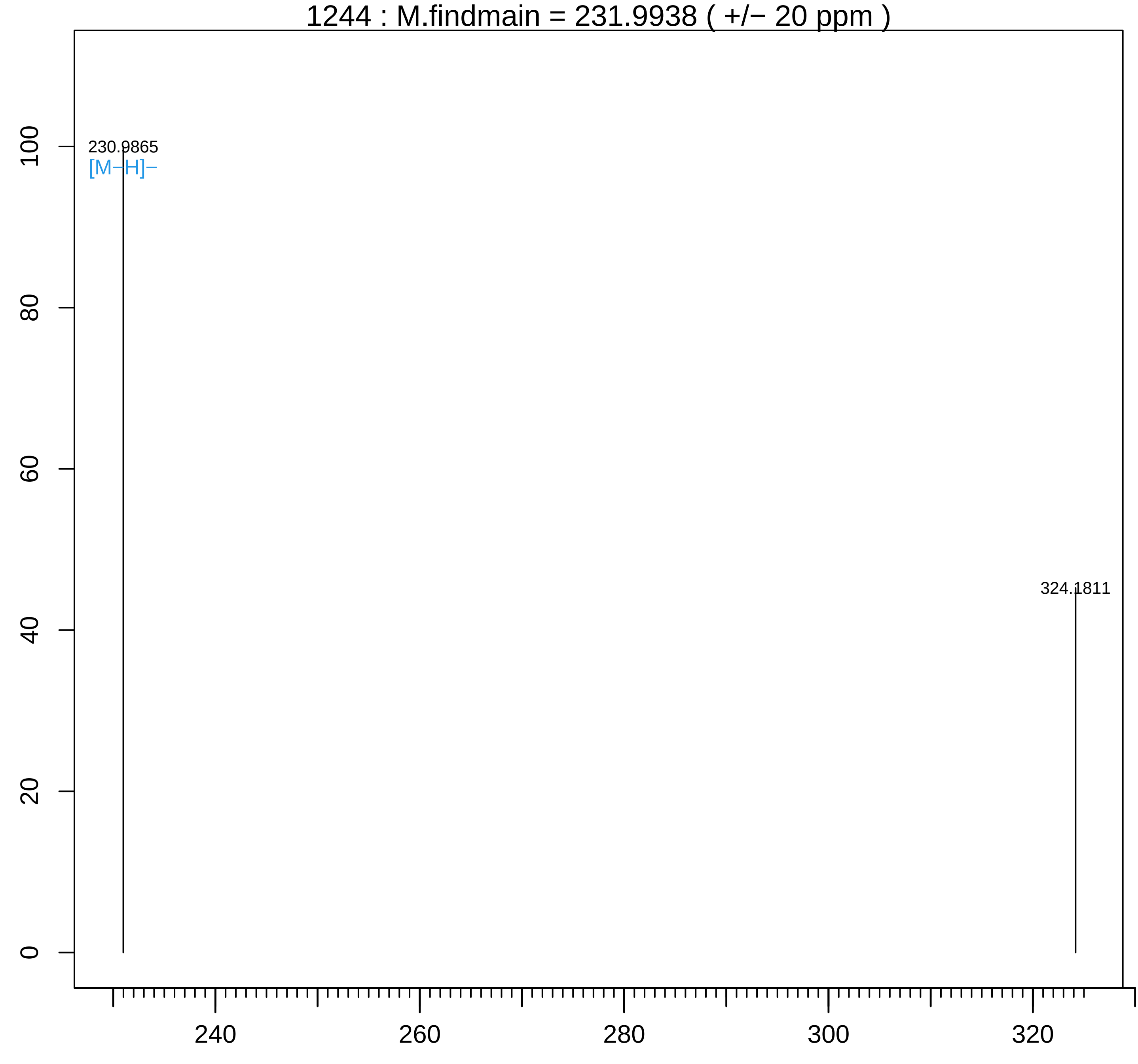

1244 : M.findmain = 231.9938 (+/− 20 ppm)
230.9865
[M−H]−
324.1811
0
20
40
60
80
100
240
260
280
300
320

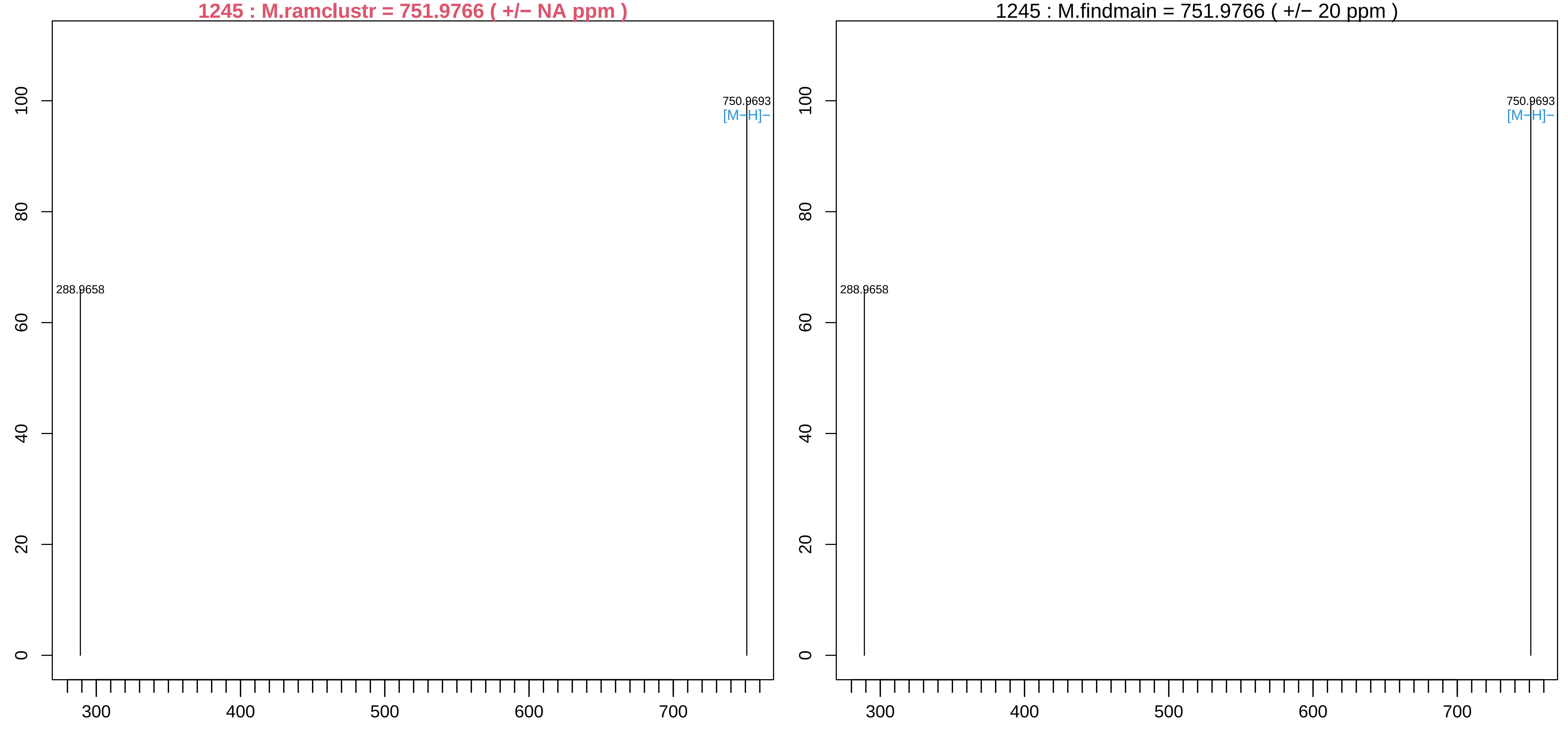

1245 : M.ramclustr = 751.9766 (+/− NA ppm)
1245 : M.findmain = 751.9766 (+/− 20 ppm)
750.9693
[M−H]−
288.9658
0
20
40
60
80
100
300
400
500
600
700

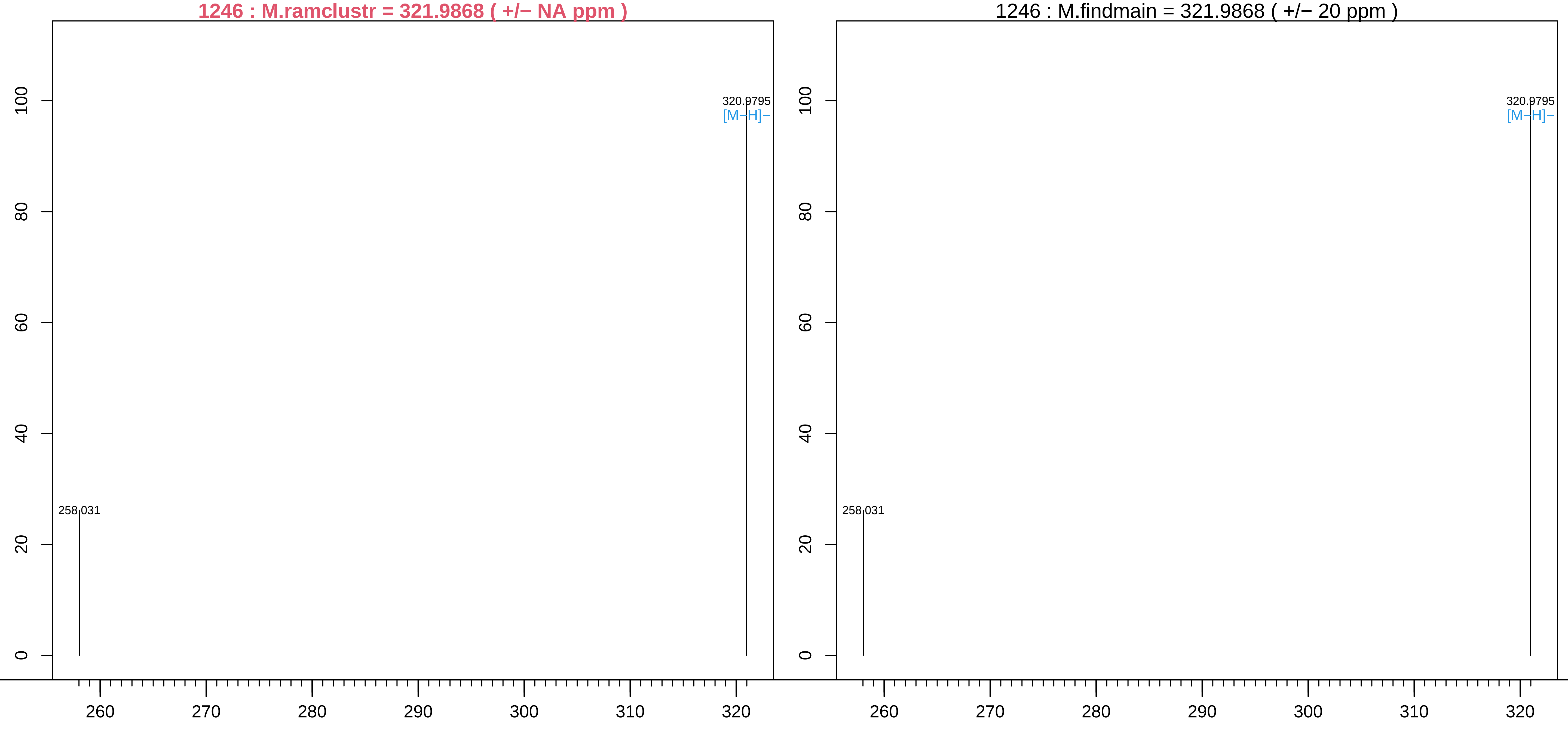
1246 : M.ramclustr = 321.9868 (+/− NA ppm)
1246 : M.findmain = 321.9868 (+/− 20 ppm)
320.9795
[M−H]−
258.031
0
20
40
60
80
100
260
270
280
290
300
310
320

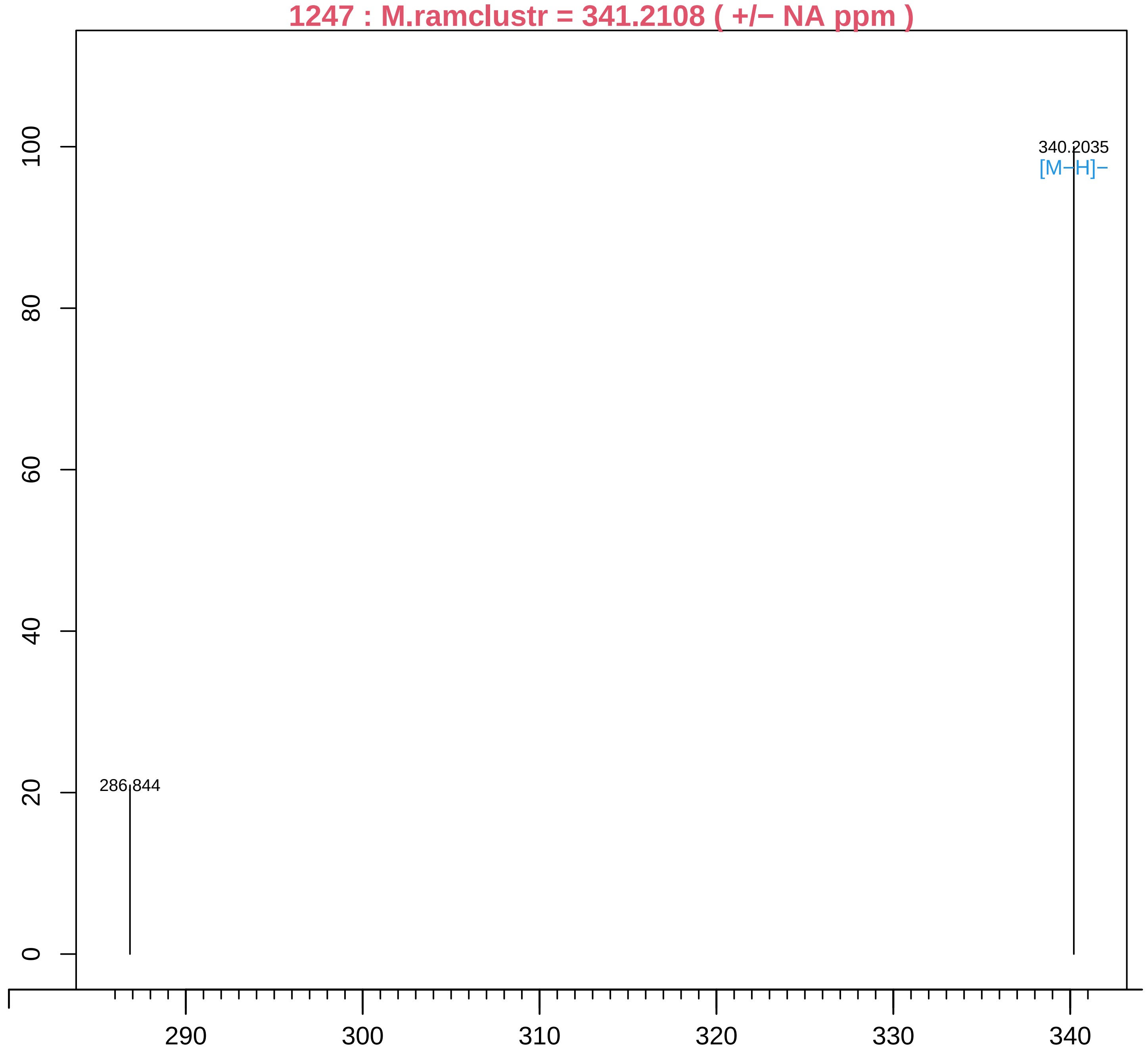
1247 : M.ramclustr = 341.2108 (+/− NA ppm)
340.2035
[M−H]−
286.844
0
20
40
60
80
100
290
300
310
320
330
340

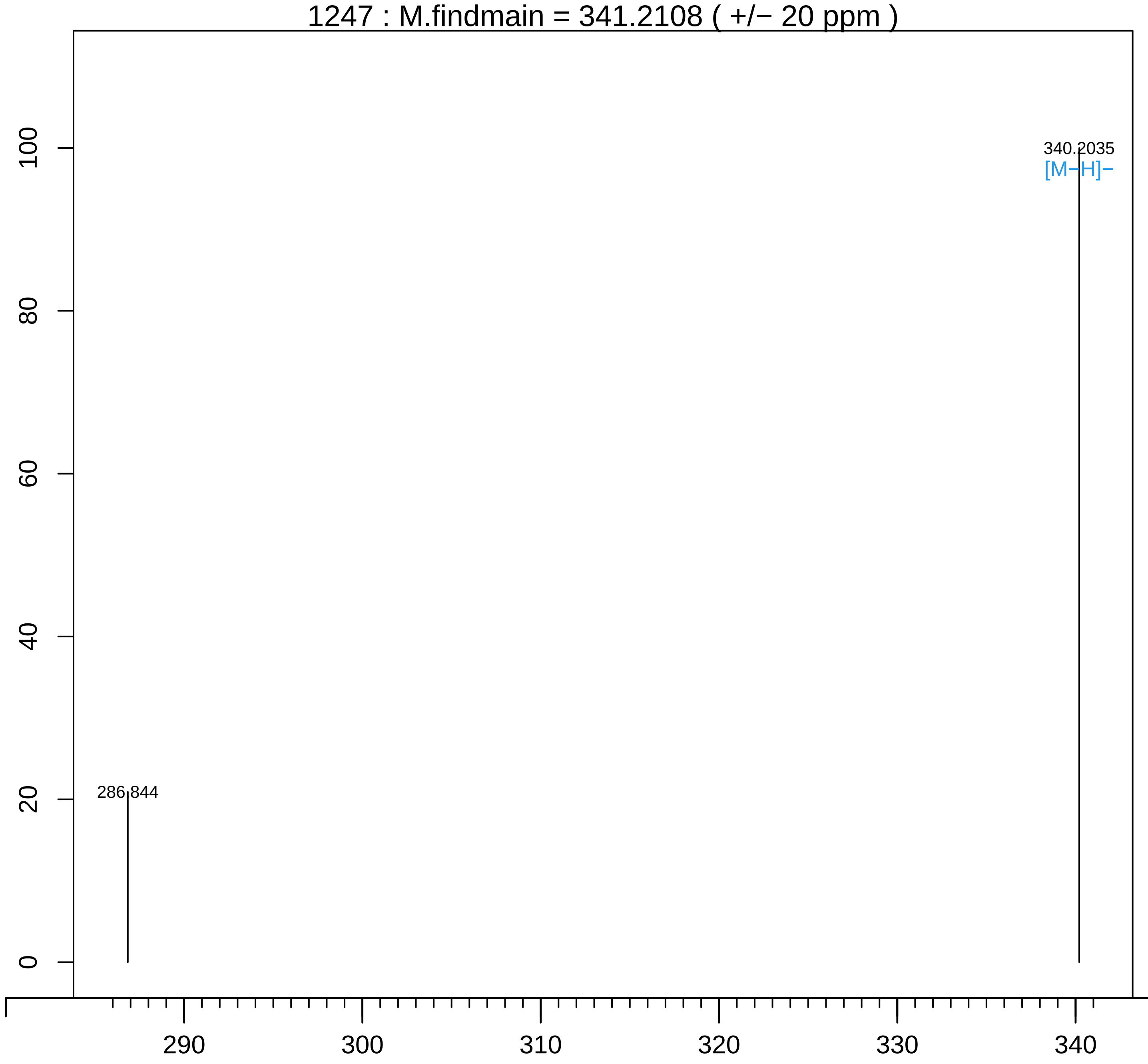
1247 : M.findmain = 341.2108 (+/− 20 ppm)
340.2035
[M−H]−
286.844
0
20
40
60
80
100
290
300
310
320
330
340

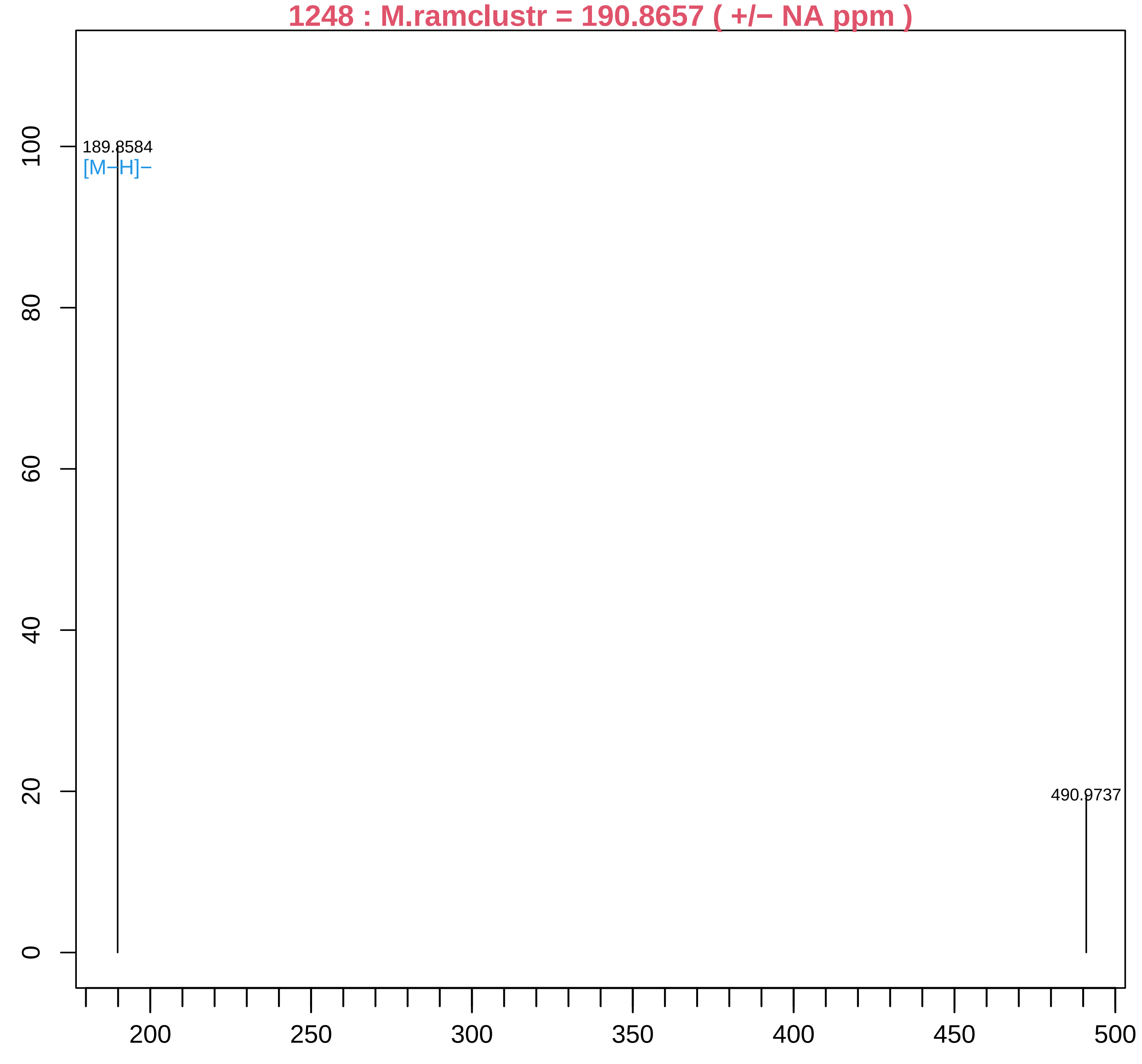
1248 : M.ramclustr = 190.8657 (+/− NA ppm)
189.8584
[M−H]−
490.9737
0
20
40
60
80
100
200
250
300
350
400
450
500

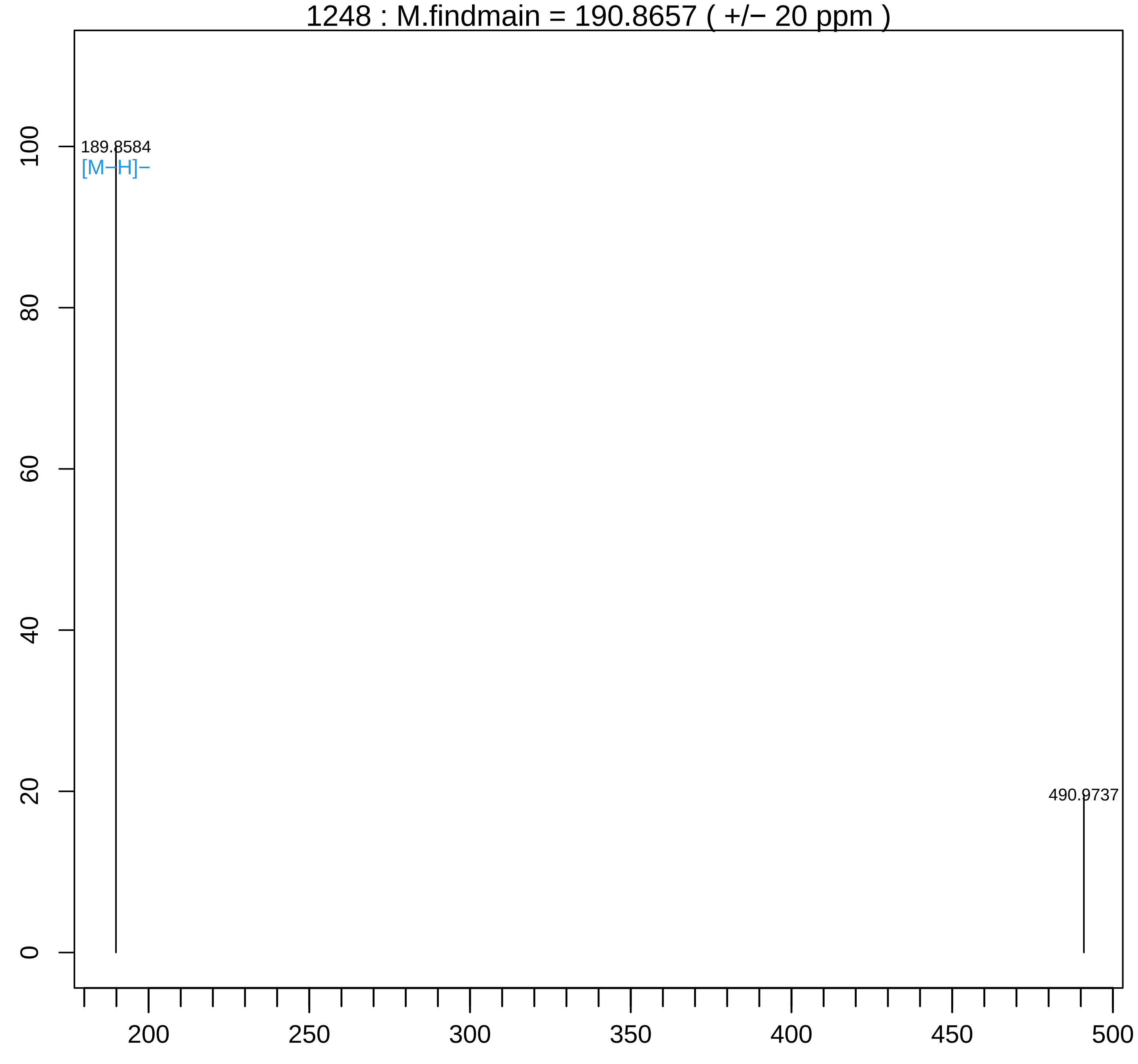
1248 : M.findmain = 190.8657 (+/− 20 ppm)
189.8584
[M−H]−
490.9737
0
20
40
60
80
100
200
250
300
350
400
450
500

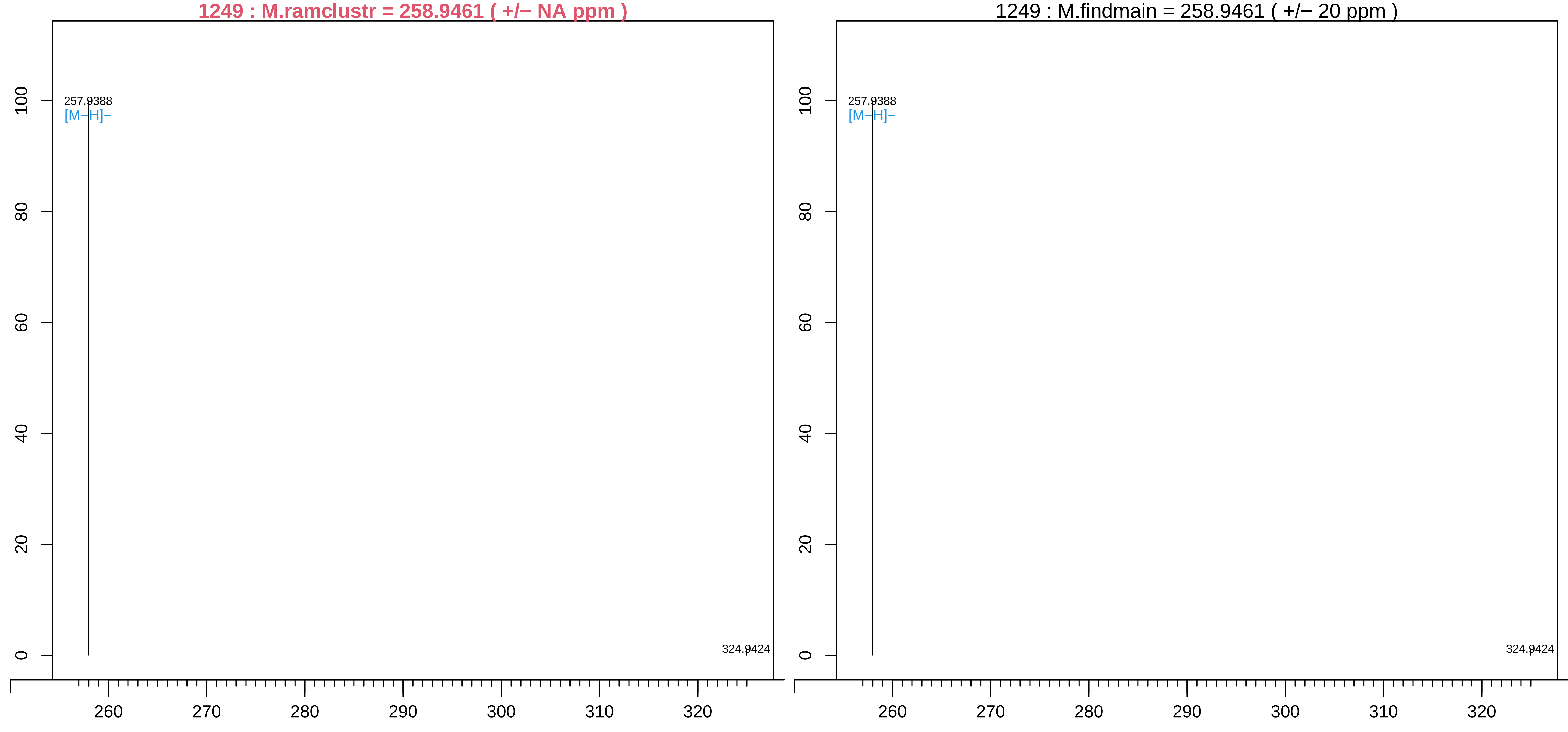

1249 : M.ramclustr = 258.9461 (+/− NA ppm)
257.9388
[M−H]−
324.9424
1249 : M.findmain = 258.9461 (+/− 20 ppm)
257.9388
[M−H]−
324.9424

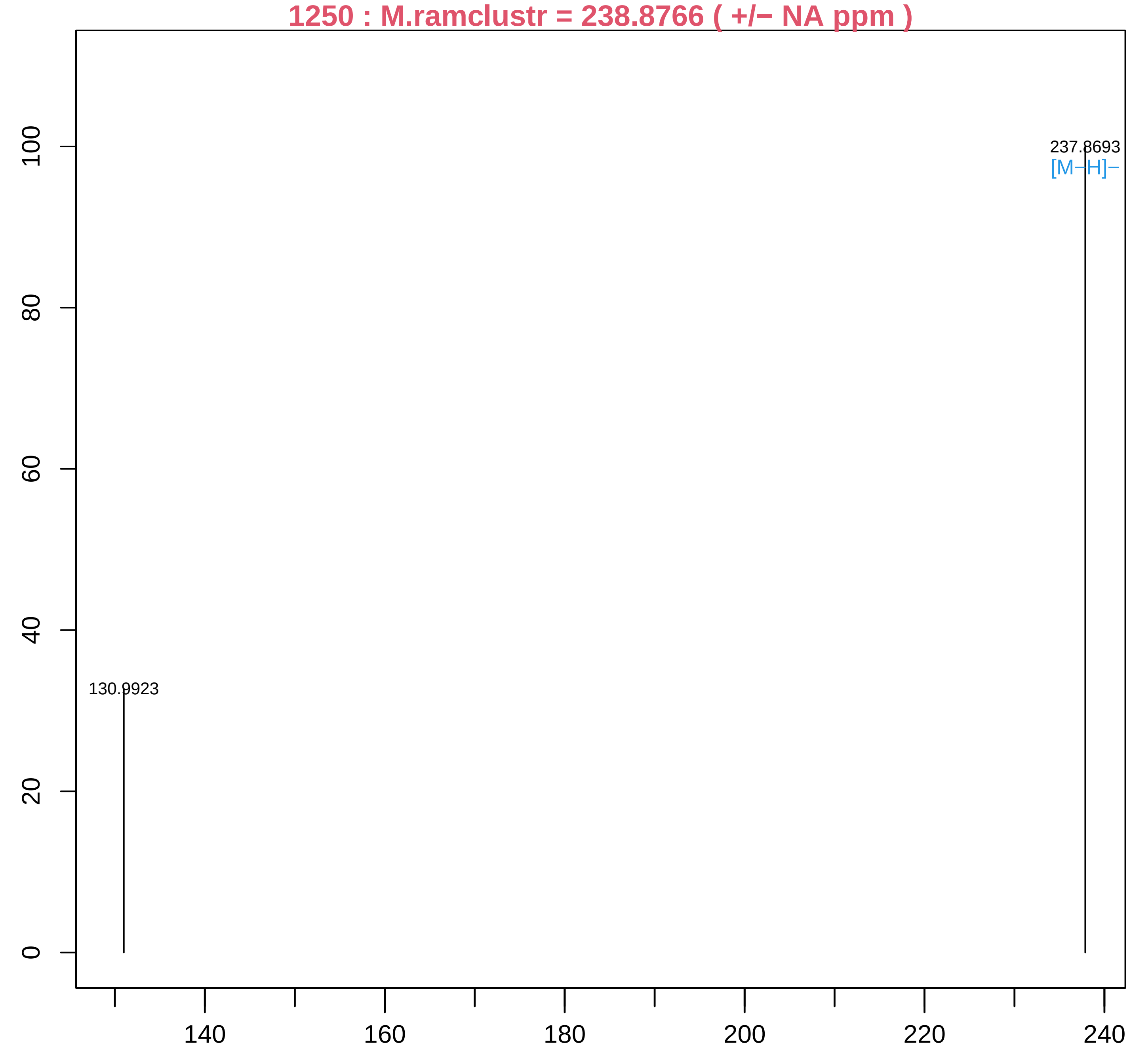

1250 : M.ramclustr = 238.8766 (+/− NA ppm)
237.8693
[M−H]−
130.9923
0
20
40
60
80
100
140
160
180
200
220
240

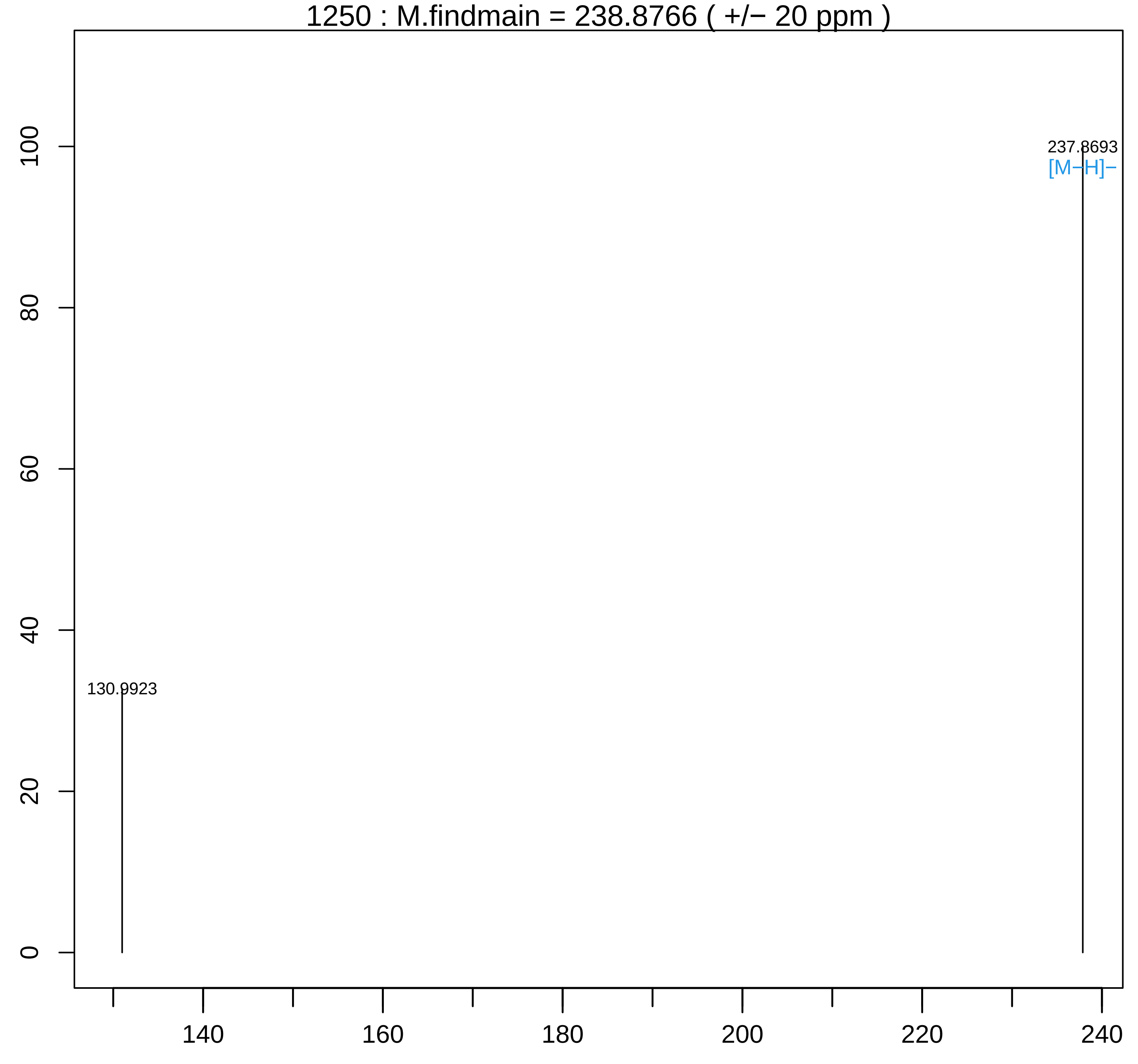

1250 : M.findmain = 238.8766 (+/− 20 ppm)
237.8693
[M−H]−
130.9923
0
20
40
60
80
100
140
160
180
200
220
240

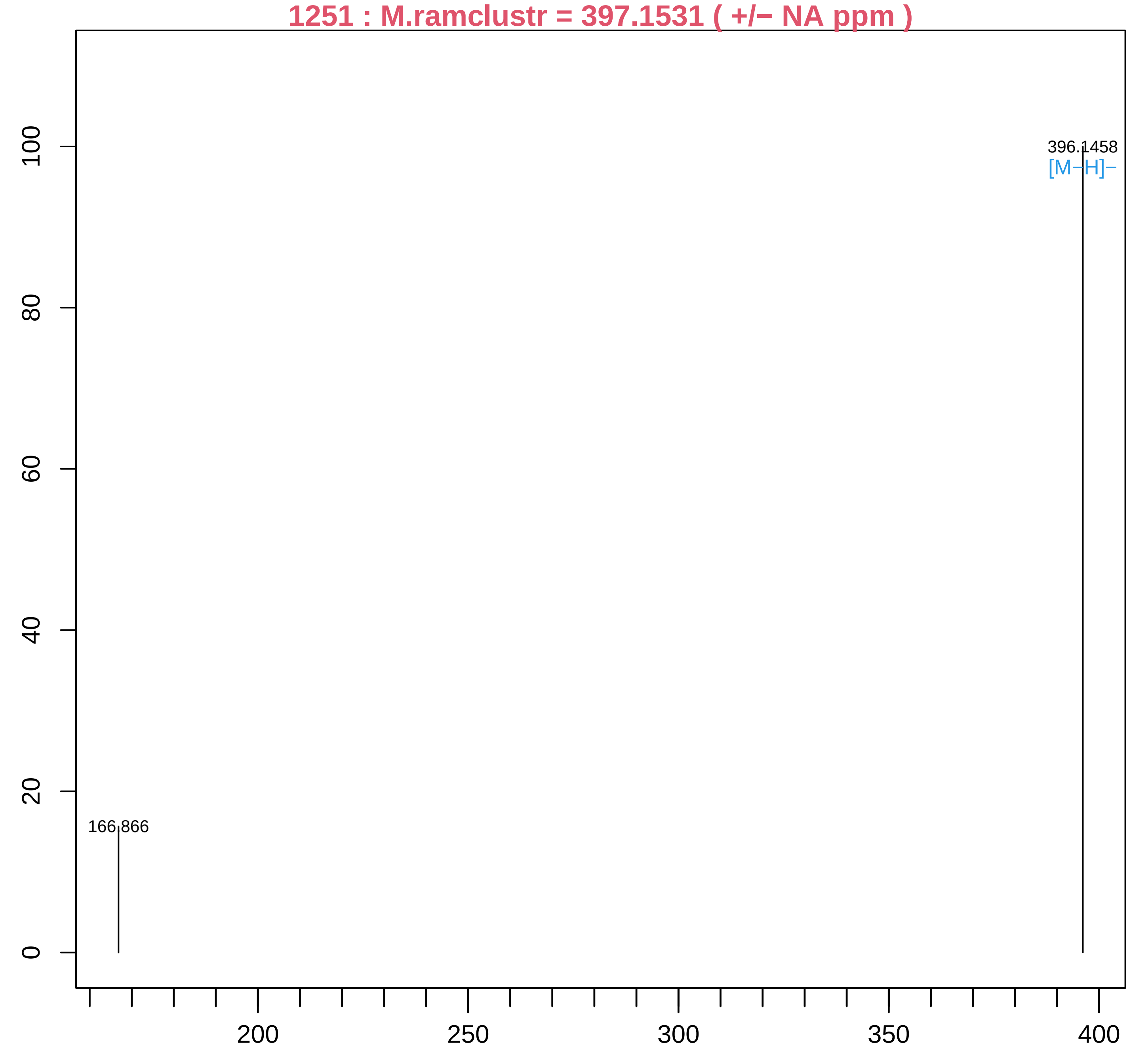
1251 : M.ramclustr = 397.1531 (+/− NA ppm)
396.1458
[M−H]−
166.866
0
20
40
60
80
100
200
250
300
350
400

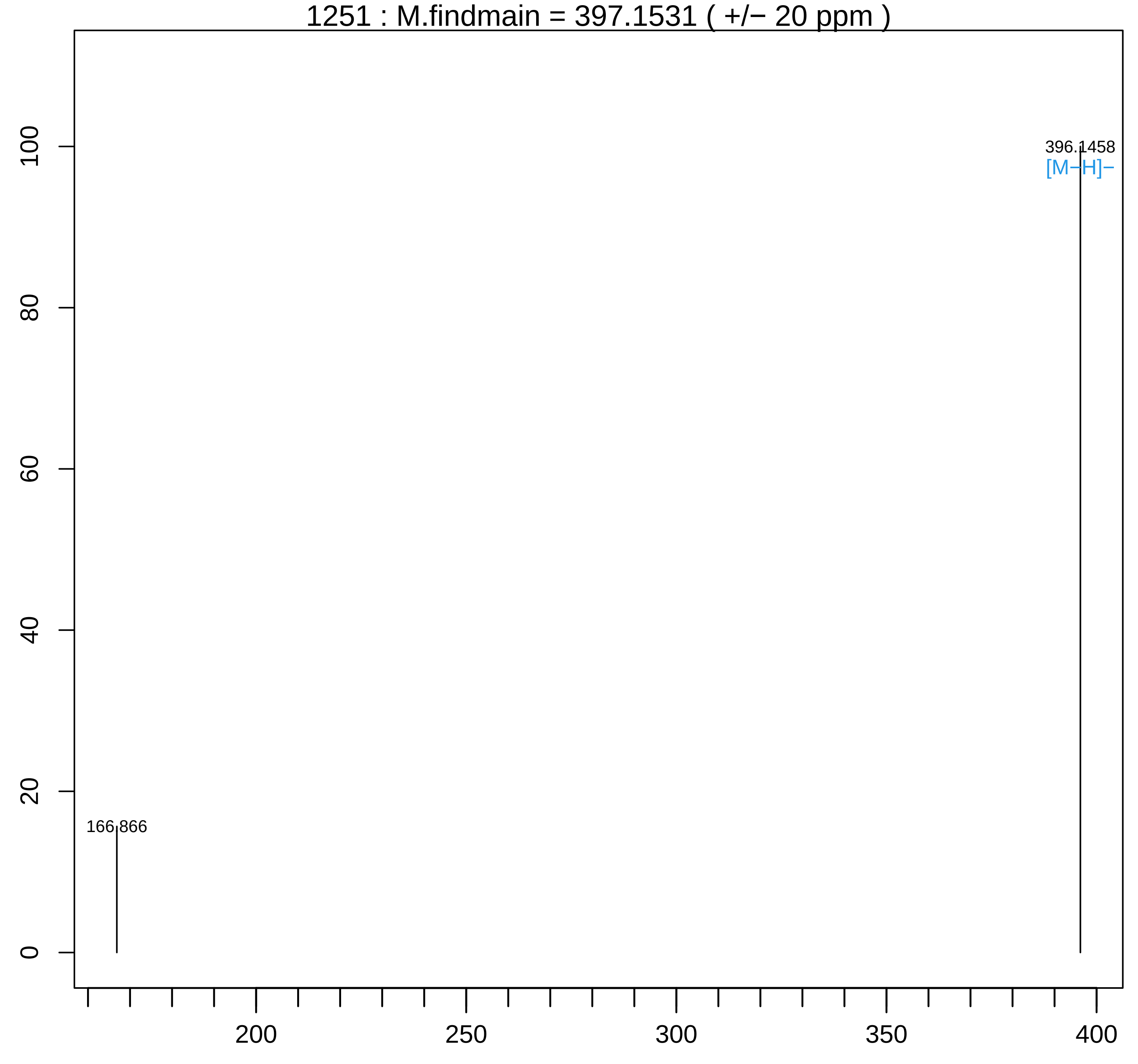
1251 : M.findmain = 397.1531 (+/− 20 ppm)
396.1458
[M−H]−
166.866
0
20
40
60
80
100
200
250
300
350
400

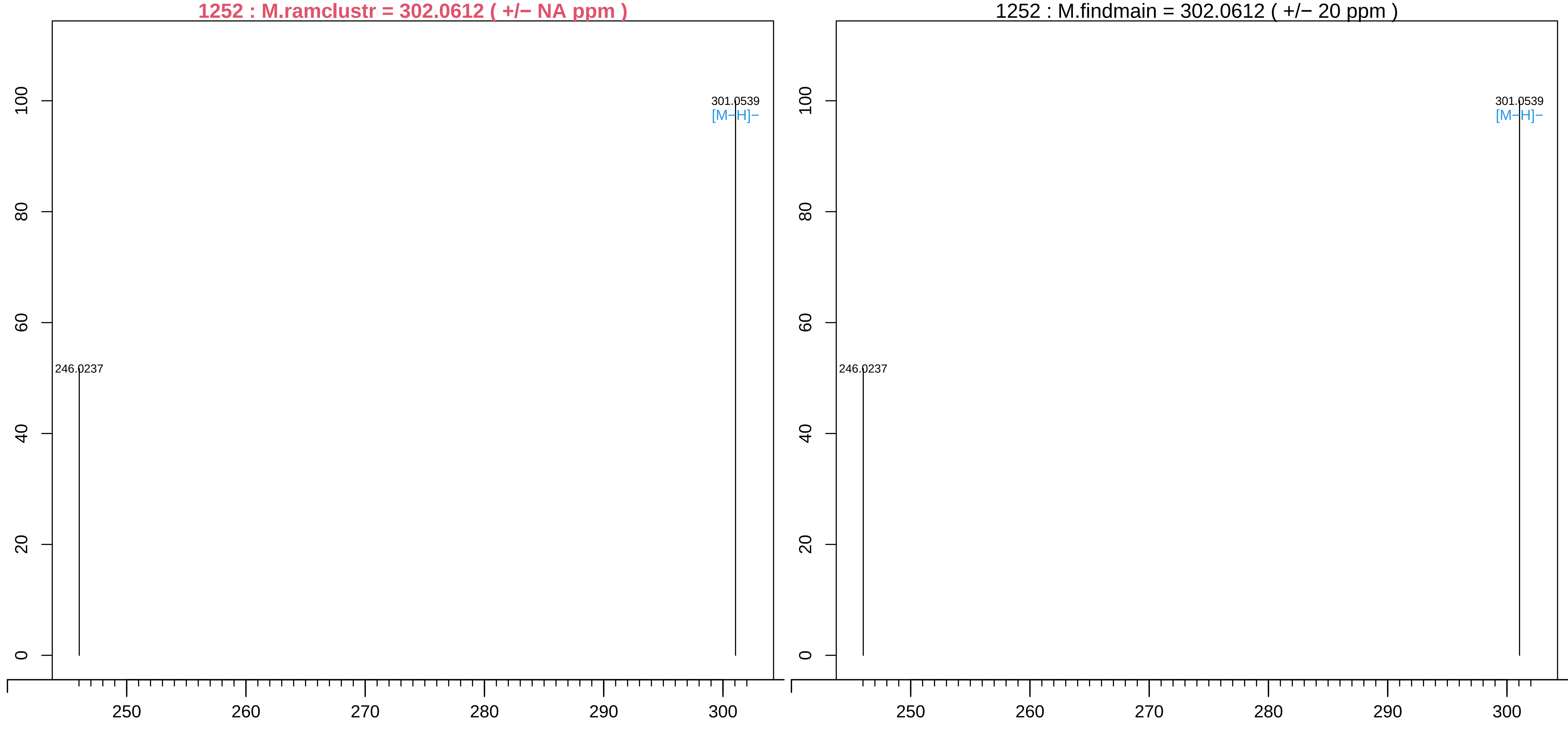
1252 : M.ramclustr = 302.0612 (+/− NA ppm)
1252 : M.findmain = 302.0612 (+/− 20 ppm)
301.0539
[M−H]−
246.0237
0
20
40
60
80
100
250
260
270
280
290
300

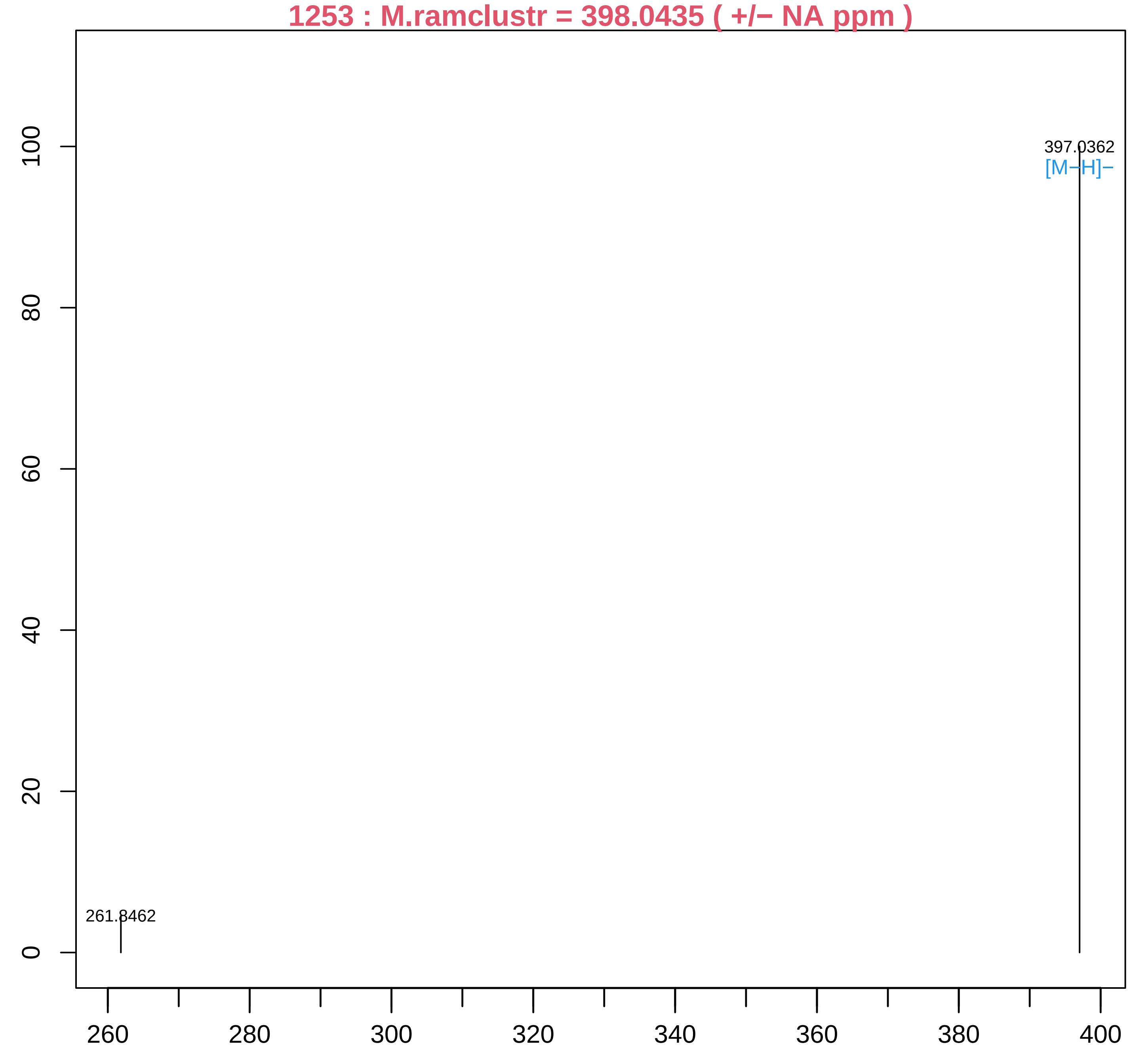
1253 : M.ramclustr = 398.0435 (+/− NA ppm)
397.0362
[M−H]−
261.8462
0
20
40
60
80
100
260
280
300
320
340
360
380
400

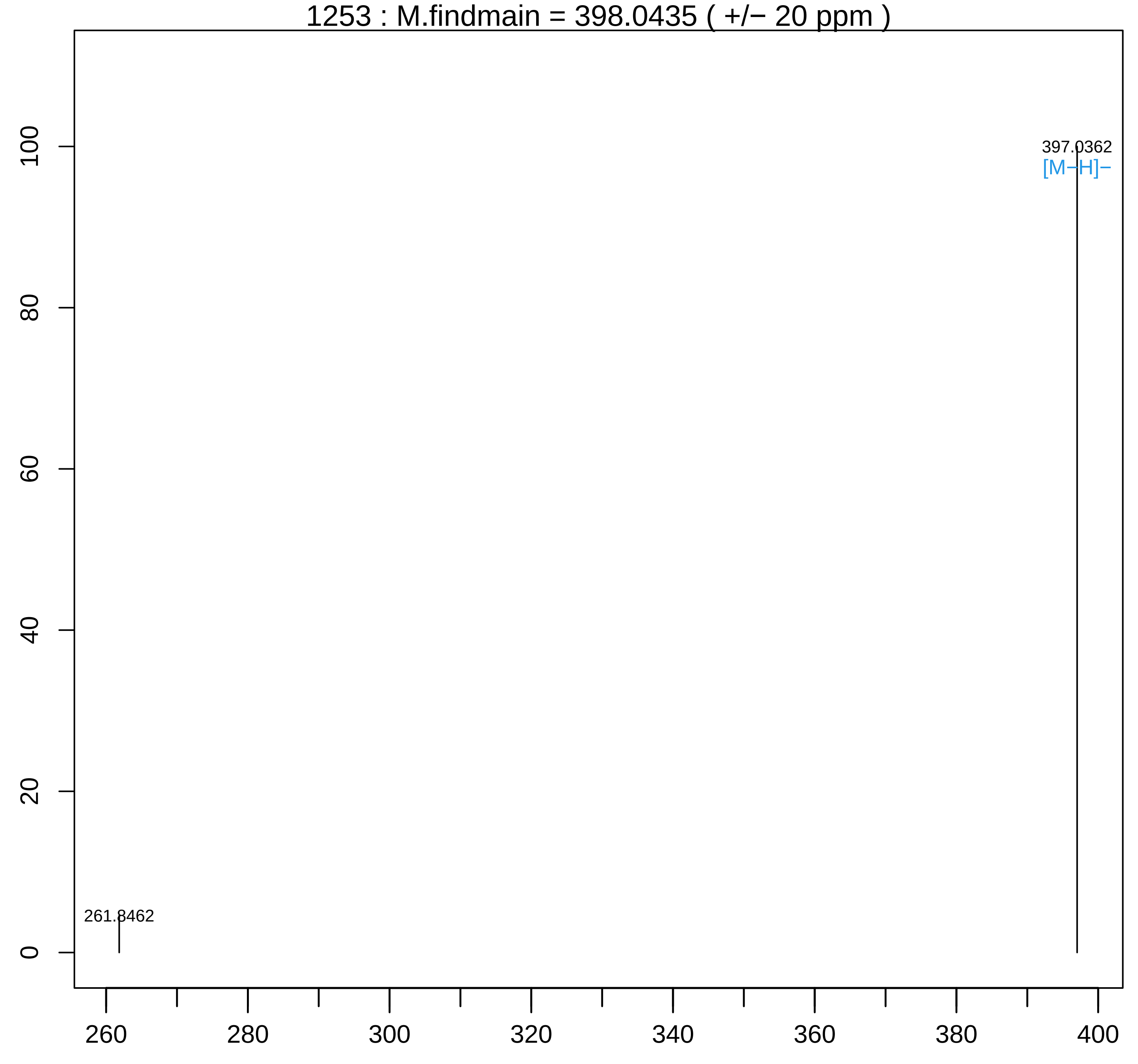
1253 : M.findmain = 398.0435 (+/− 20 ppm)
397.0362
[M−H]−
261.8462
0
20
40
60
80
100
260
280
300
320
340
360
380
400

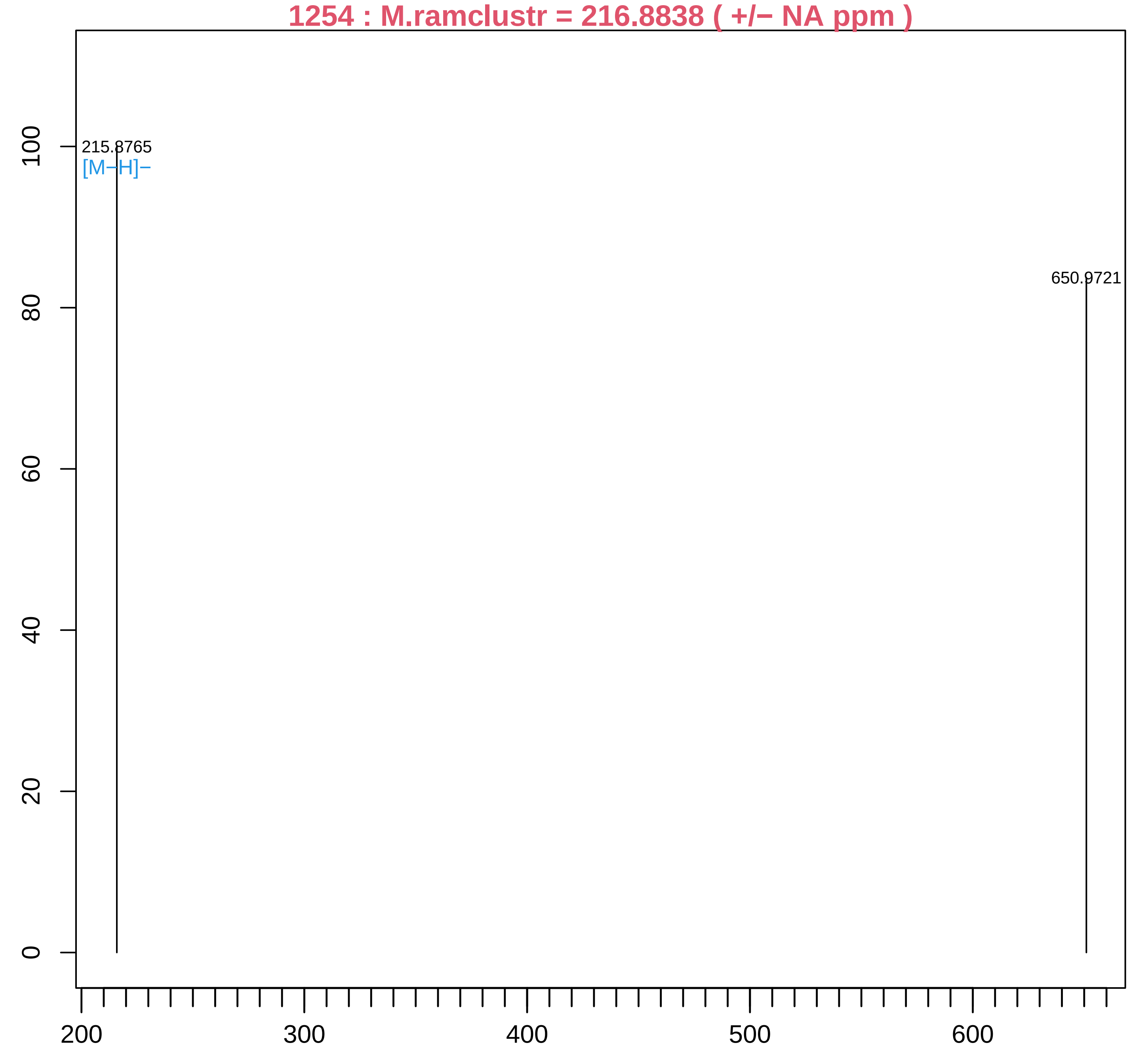
1254 : M.ramclustr = 216.8838 (+/− NA ppm)
215.8765
[M−H]−
650.9721
0
20
40
60
80
100
200
300
400
500
600

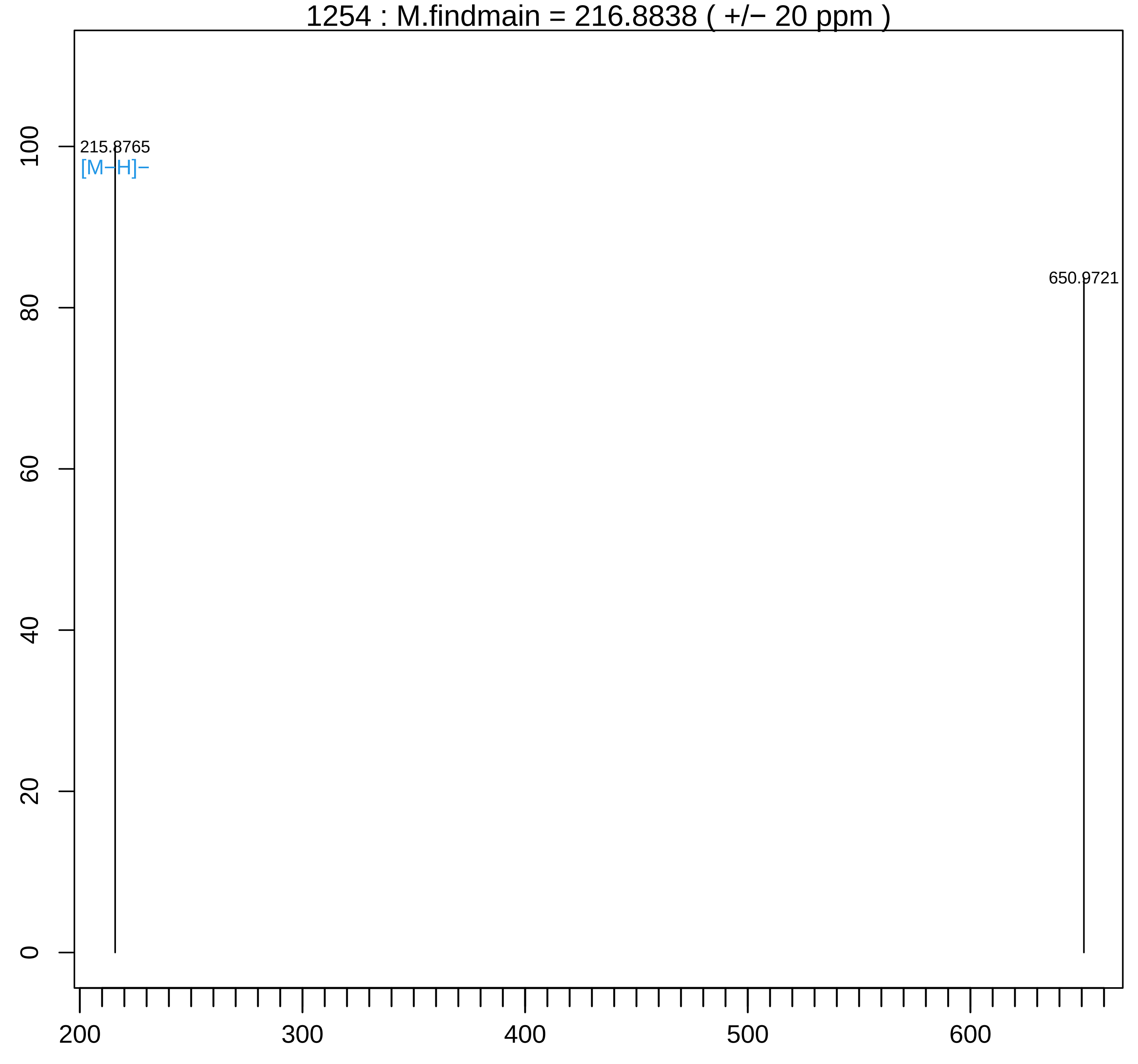
1254 : M.findmain = 216.8838 (+/− 20 ppm)
215.8765
[M−H]−
650.9721
0
20
40
60
80
100
200
300
400
500
600

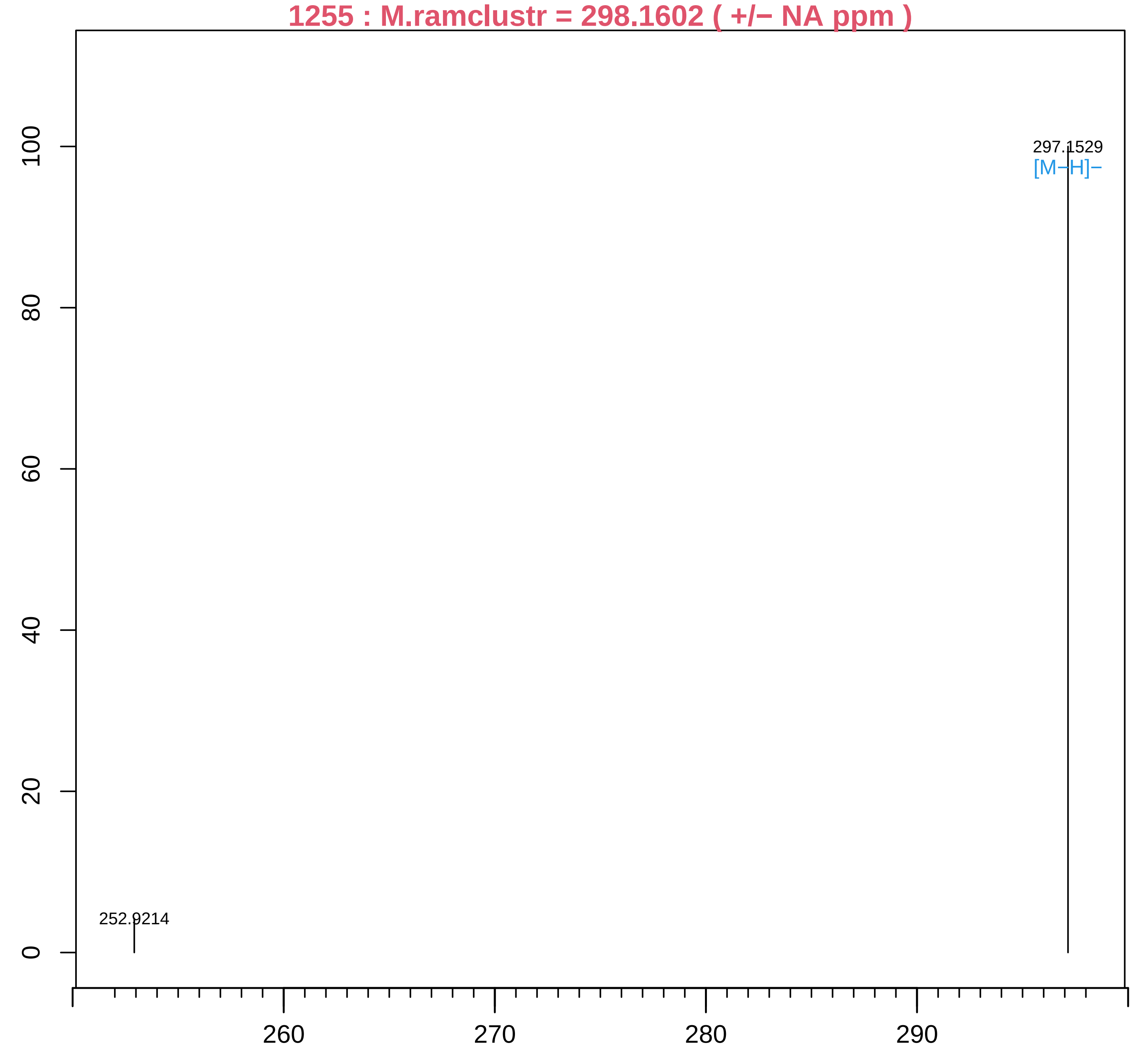
1255 : M.ramclustr = 298.1602 (+/− NA ppm)
297.1529
[M−H]−
252.9214
0
20
40
60
80
100
260
270
280
290

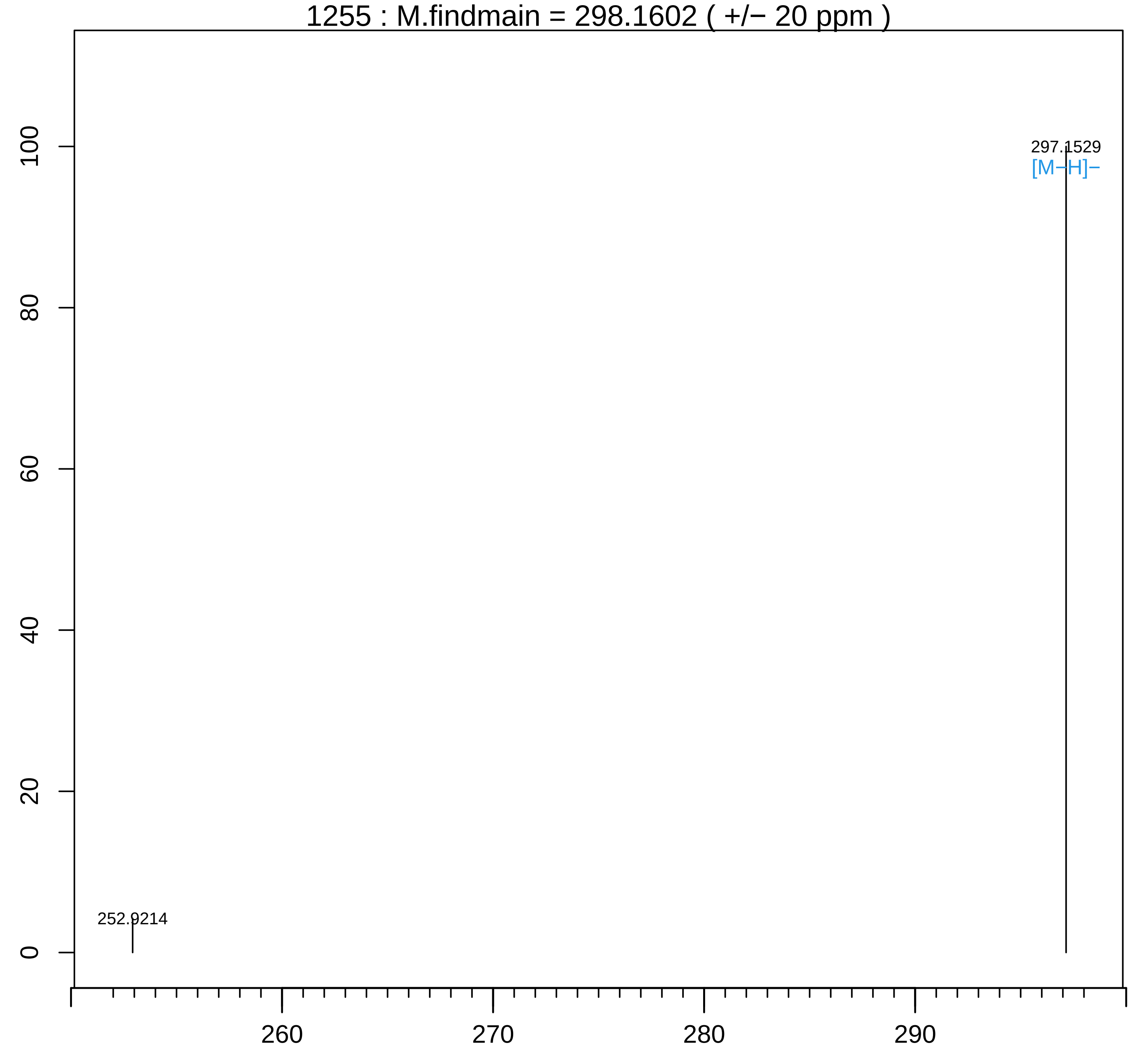
1255 : M.findmain = 298.1602 (+/− 20 ppm)
297.1529
[M−H]−
252.9214
0
20
40
60
80
100
260
270
280
290

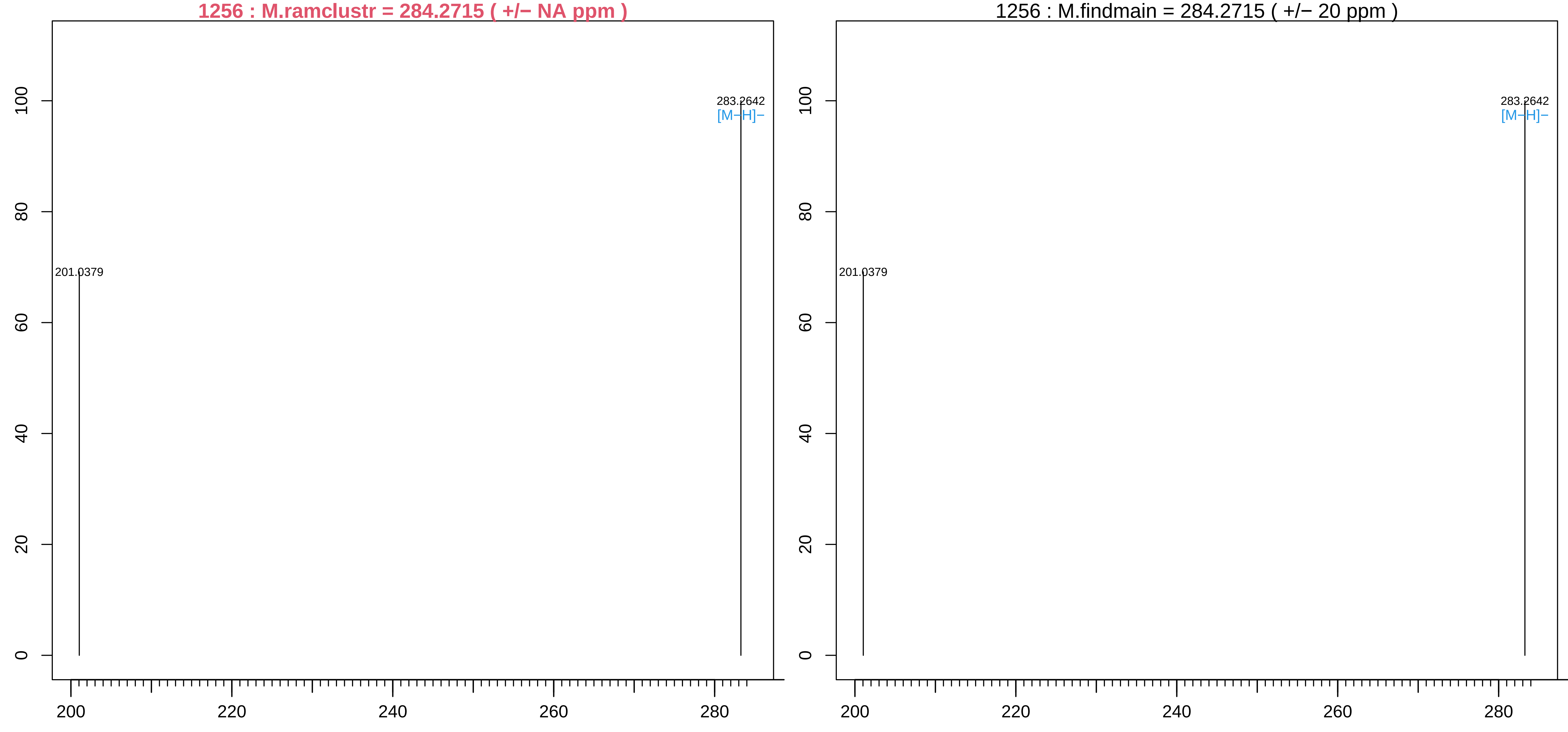
1256 : M.ramclustr = 284.2715 (+/− NA ppm)
283.2642
[M−H]−
201.0379
1256 : M.findmain = 284.2715 (+/− 20 ppm)
283.2642
[M−H]−
201.0379
0
20
40
60
80
100
200
220
240
260
280

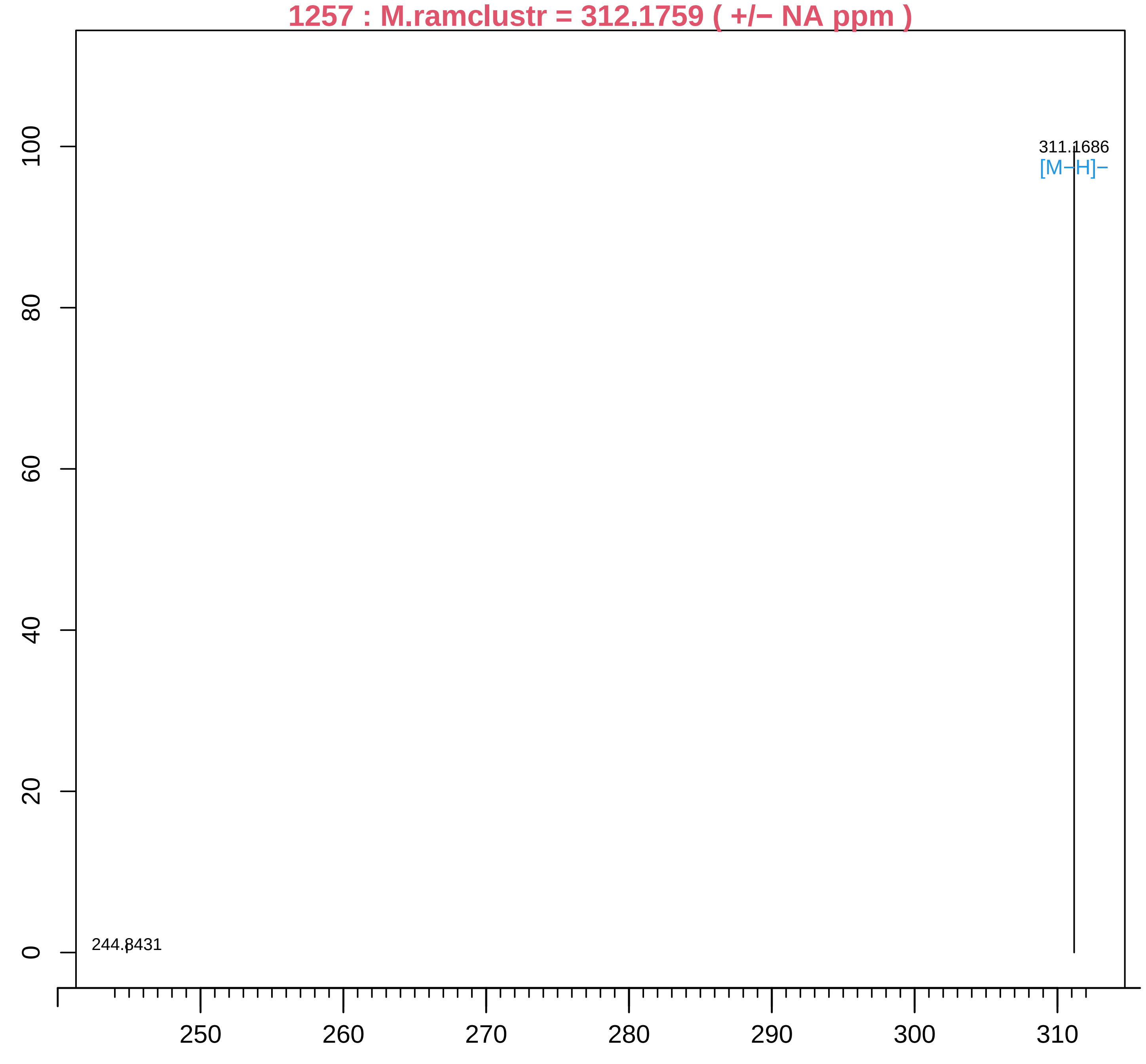

1257 : M.ramclustr = 312.1759 (+/− NA ppm)
311.1686
[M−H]−
244.8431
0
20
40
60
80
100
250
260
270
280
290
300
310

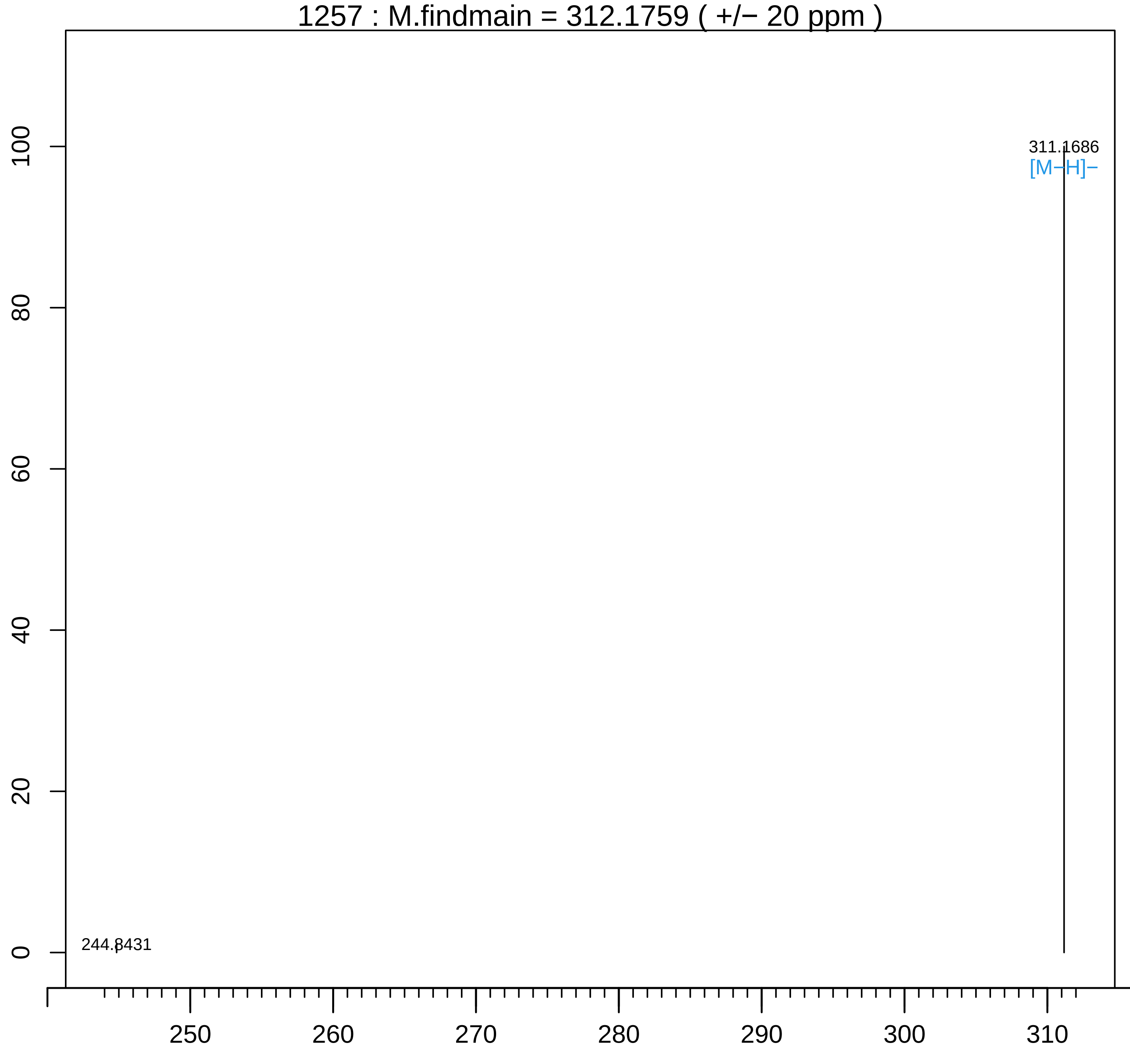

1257 : M.findmain = 312.1759 (+/− 20 ppm)
311.1686
[M−H]−
244.8431
0
20
40
60
80
100
250
260
270
280
290
300
310

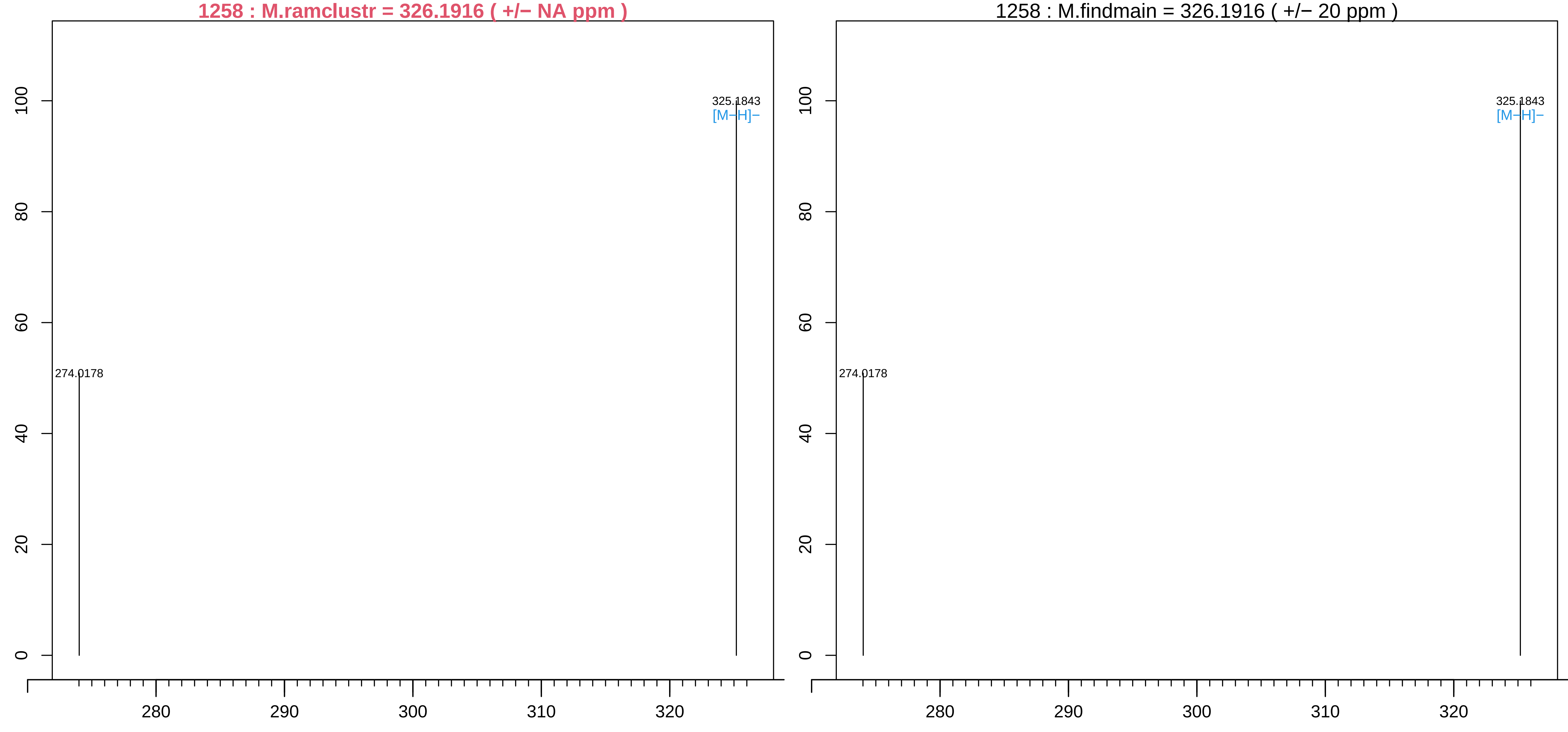

1258 : M.ramclustr = 326.1916 (+/− NA ppm)
325.1843
[M−H]−
274.0178
1258 : M.findmain = 326.1916 (+/− 20 ppm)
325.1843
[M−H]−
274.0178
0
20
40
60
80
100
280
290
300
310
320

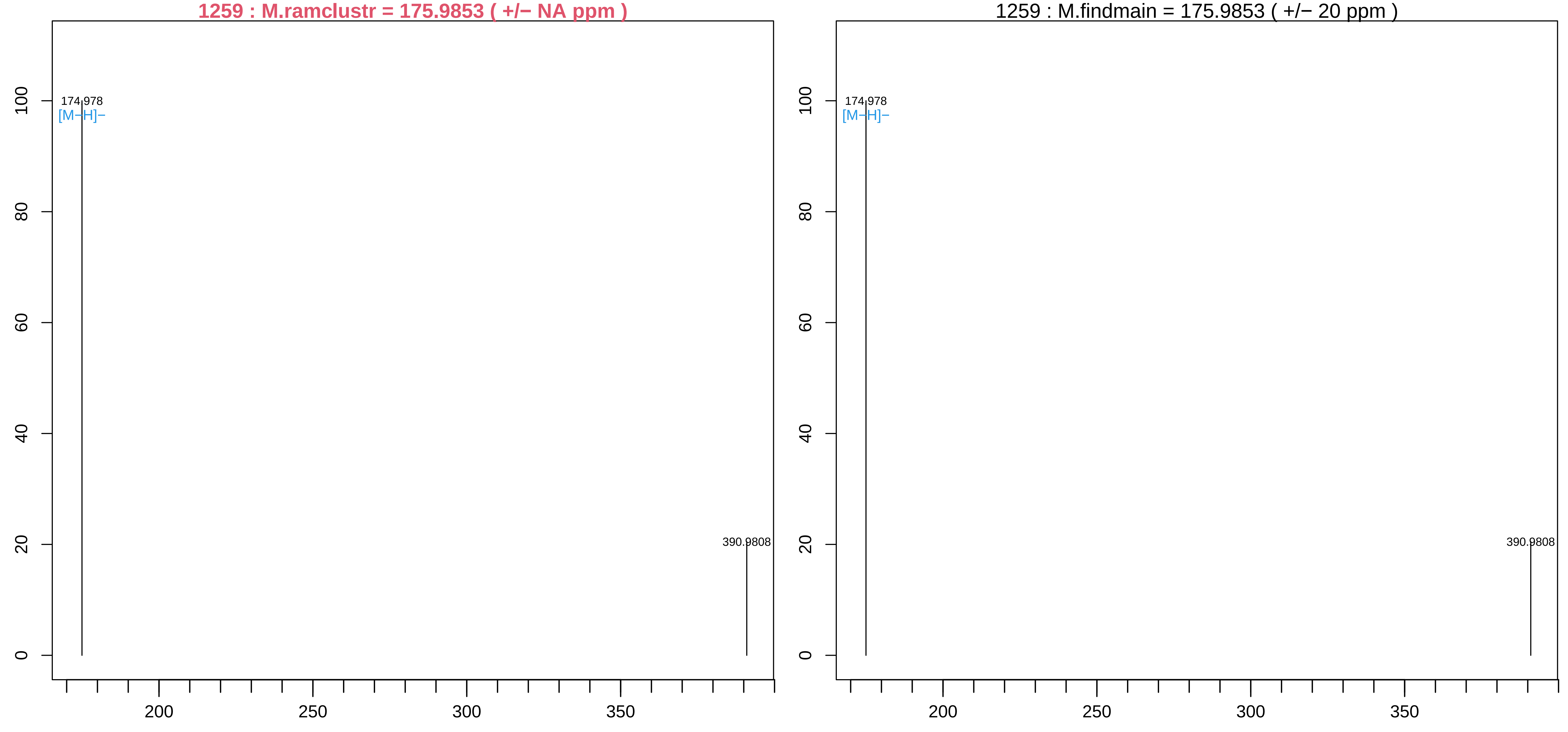
1259 : M.ramclustr = 175.9853 (+/− NA ppm)
174.978
[M−H]−
390.9808
0
20
40
60
80
100
200
250
300
350
1259 : M.findmain = 175.9853 (+/− 20 ppm)
174.978
[M−H]−
390.9808
0
20
40
60
80
100
200
250
300
350

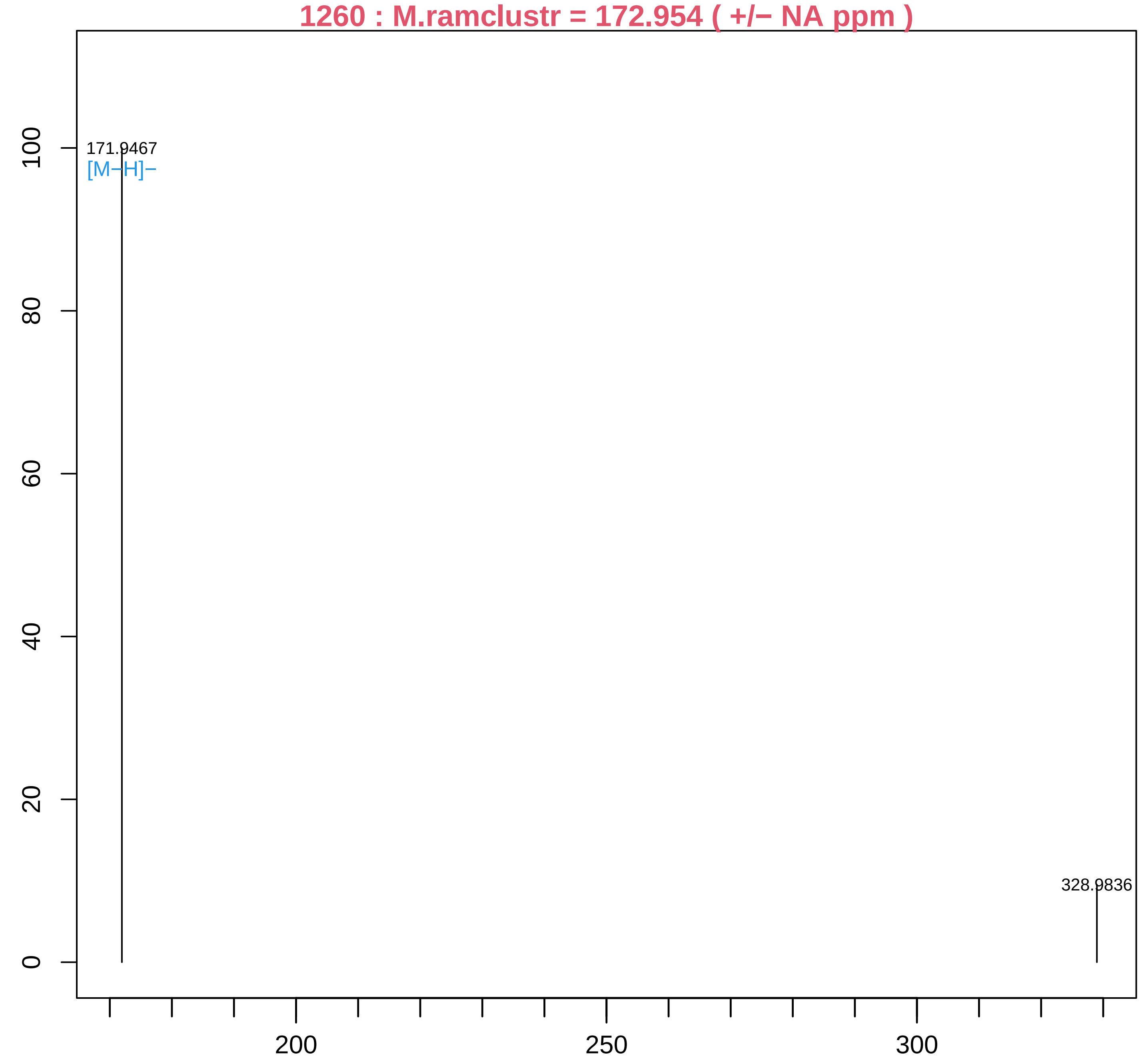

1260 : M.ramclustr = 172.954 (+/− NA ppm)
171.9467
[M−H]−
328.9836
0
20
40
60
80
100
200
250
300

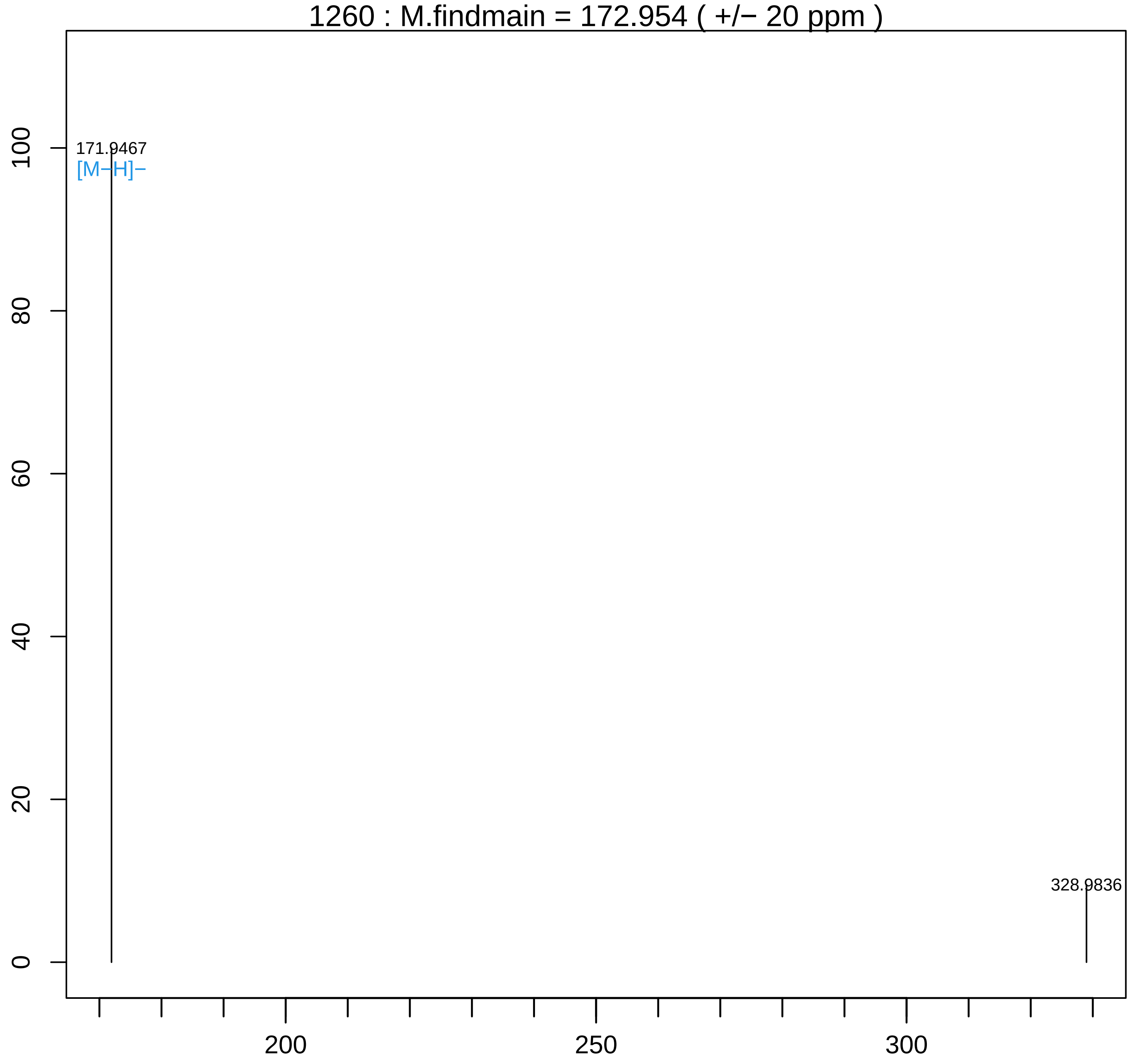

1260 : M.findmain = 172.954 (+/− 20 ppm)
171.9467
[M−H]−
328.9836
0
20
40
60
80
100
200
250
300

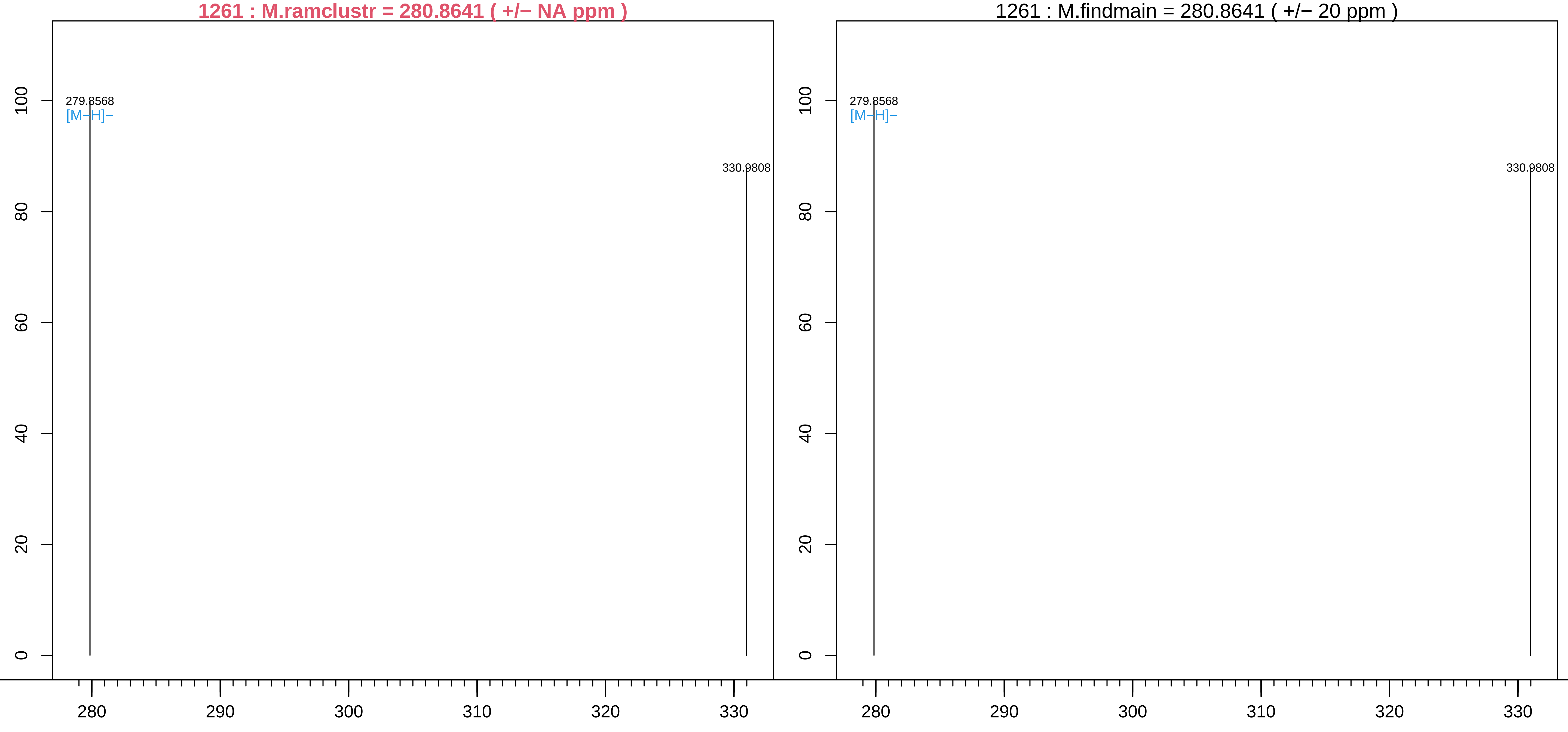
1261 : M.ramclustr = 280.8641 (+/− NA ppm)
279.8568
[M−H]−
330.9808
1261 : M.findmain = 280.8641 (+/− 20 ppm)
279.8568
[M−H]−
330.9808
0
20
40
60
80
100
280
290
300
310
320
330

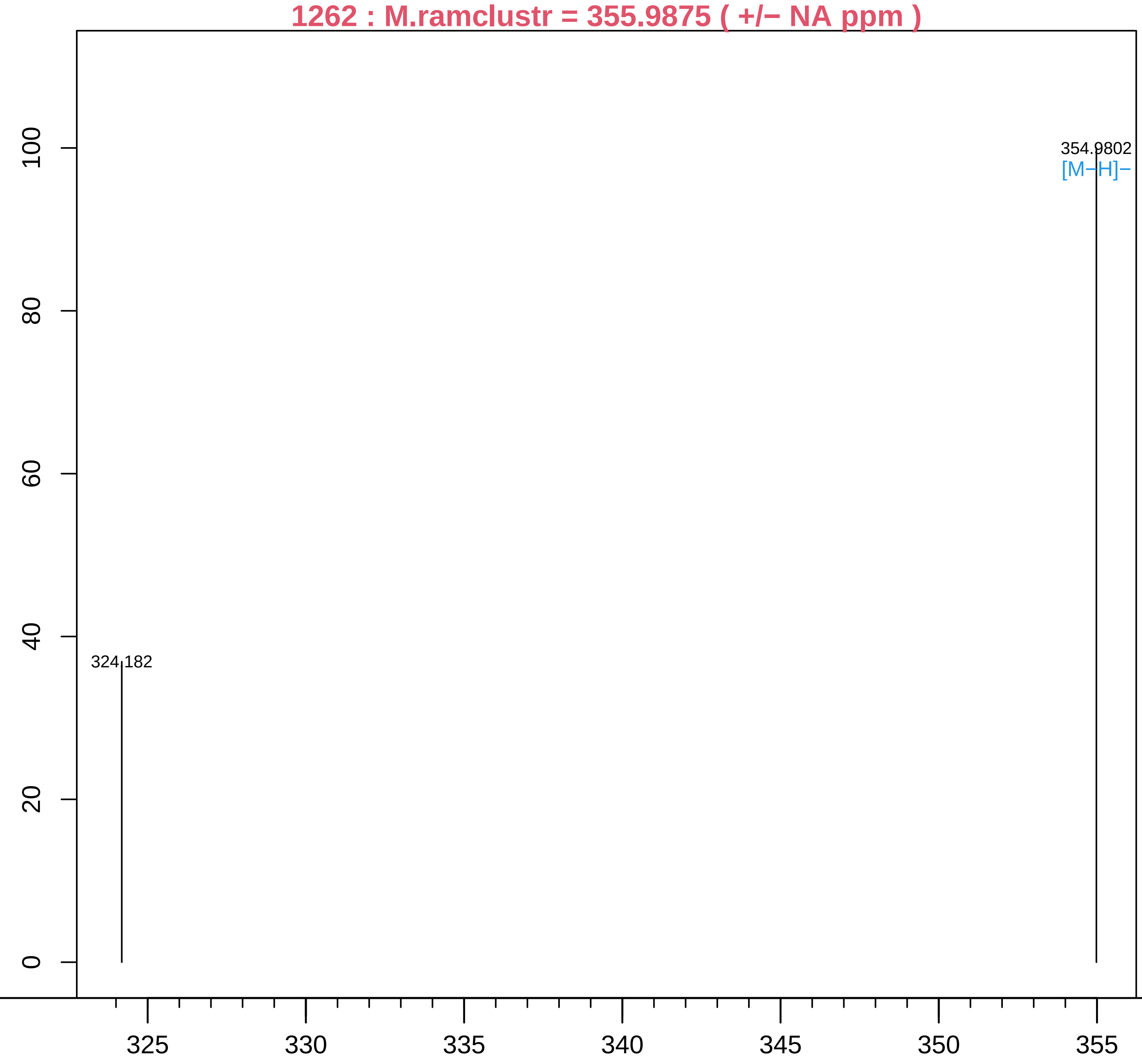

1262 : M.ramclustr = 355.9875 (+/− NA ppm)
354.9802
[M−H]−
324.182
0
20
40
60
80
100
325
330
335
340
345
350
355

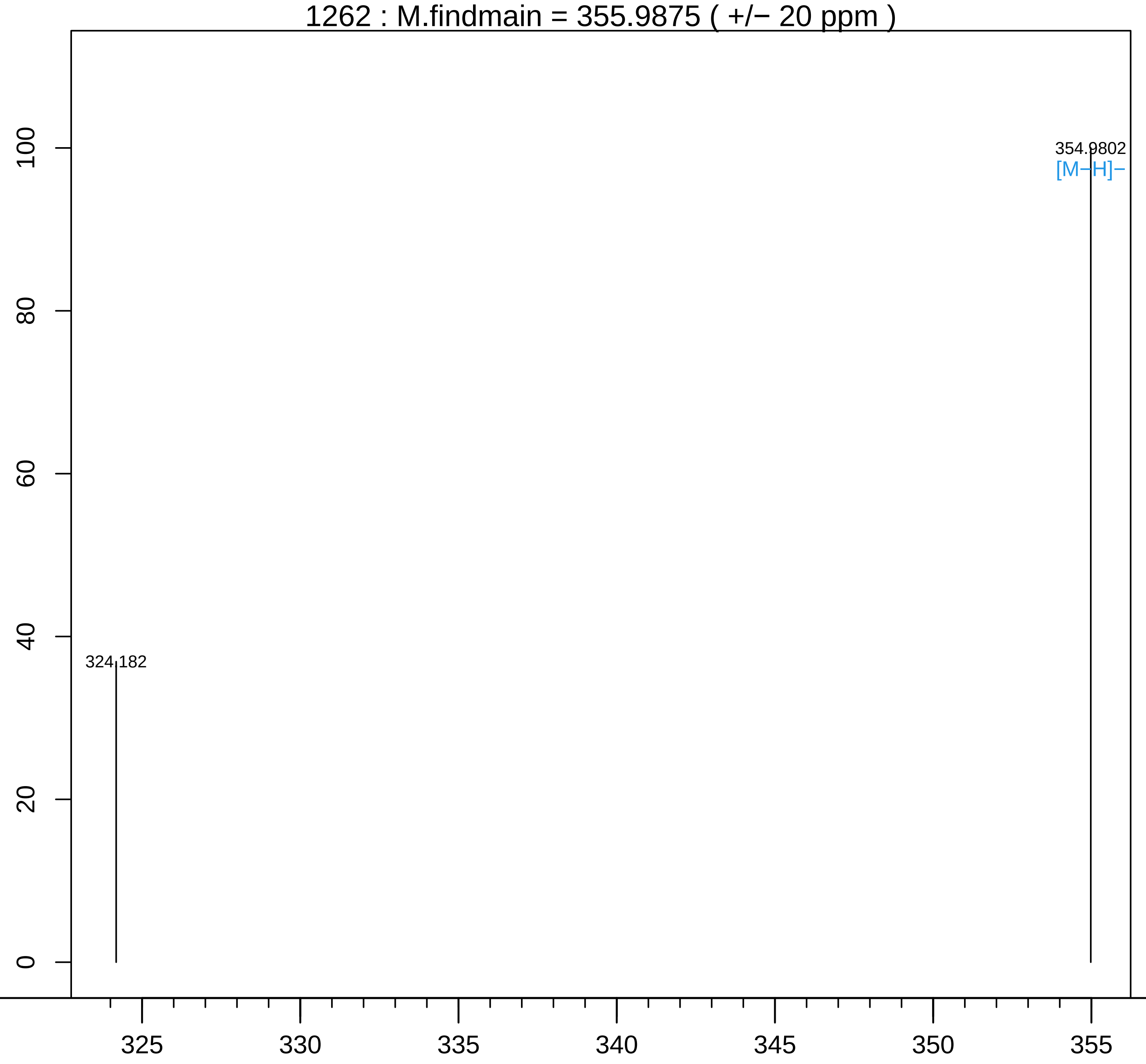

1262 : M.findmain = 355.9875 (+/− 20 ppm)
354.9802
[M−H]−
324.182
0
20
40
60
80
100
325
330
335
340
345
350
355

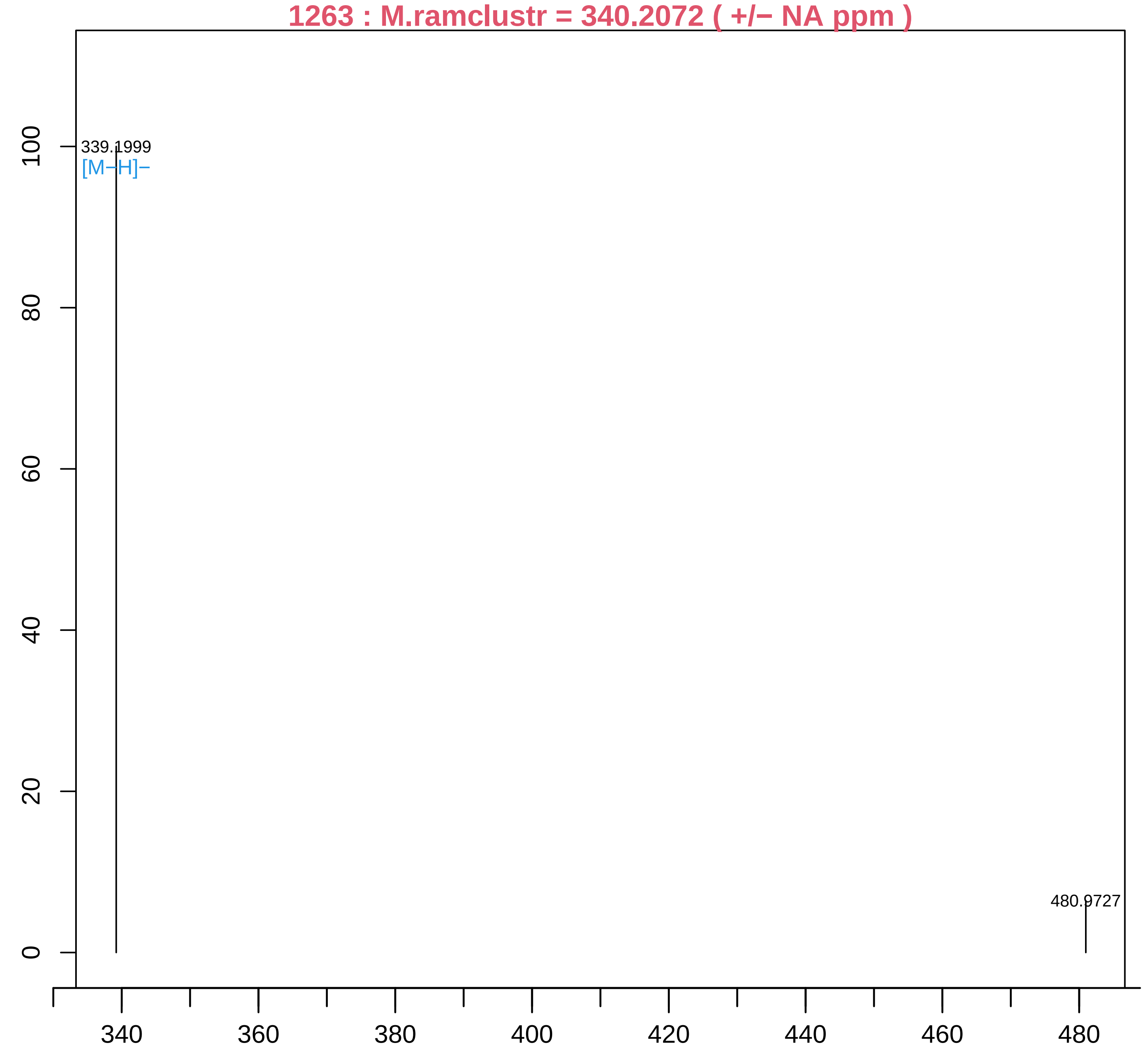
1263 : M.ramclustr = 340.2072 (+/− NA ppm)
339.1999
[M−H]−
480.9727
0
20
40
60
80
100
340
360
380
400
420
440
460
480

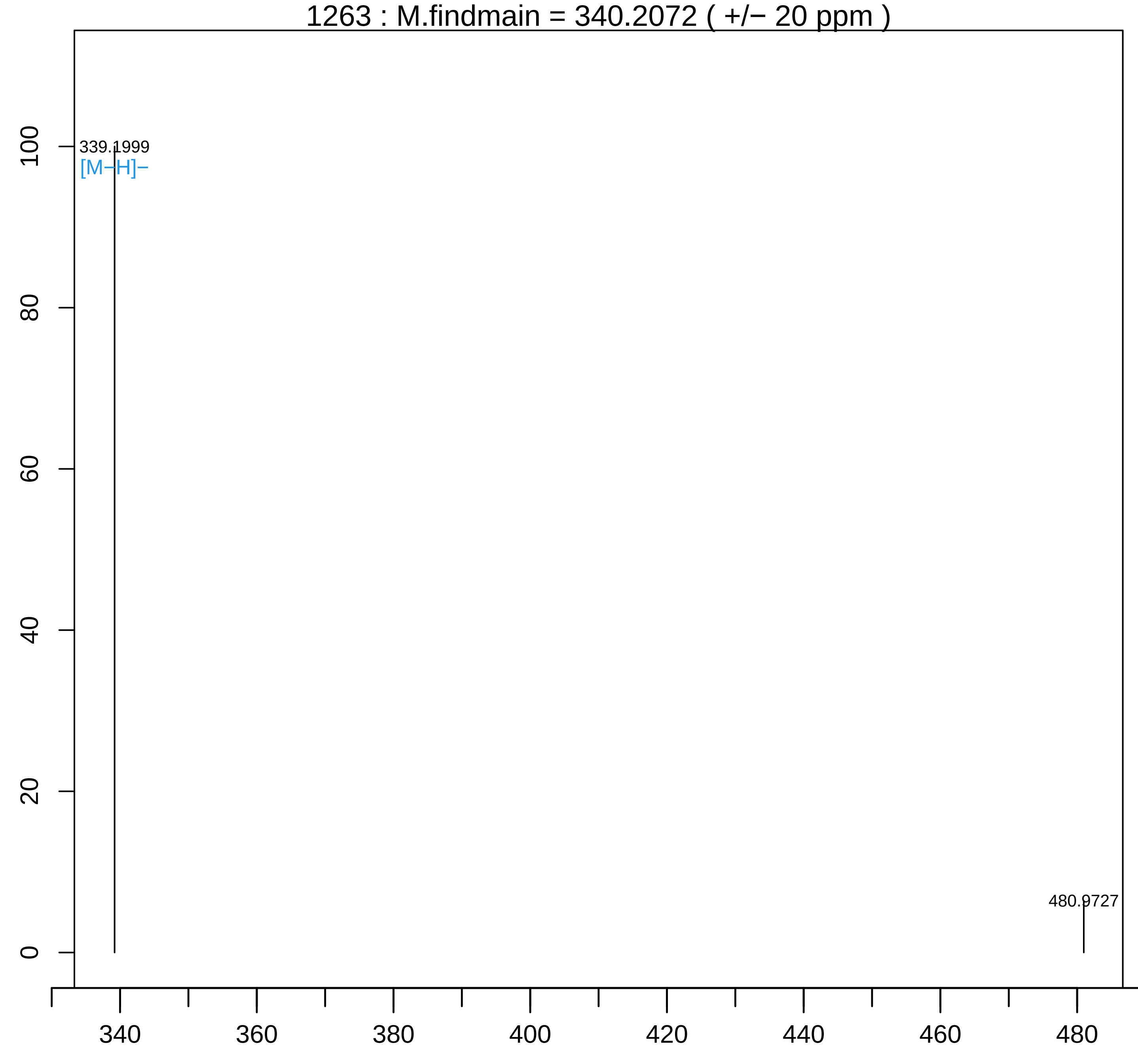
1263 : M.findmain = 340.2072 (+/− 20 ppm)
339.1999
[M−H]−
480.9727
0
20
40
60
80
100
340
360
380
400
420
440
460
480

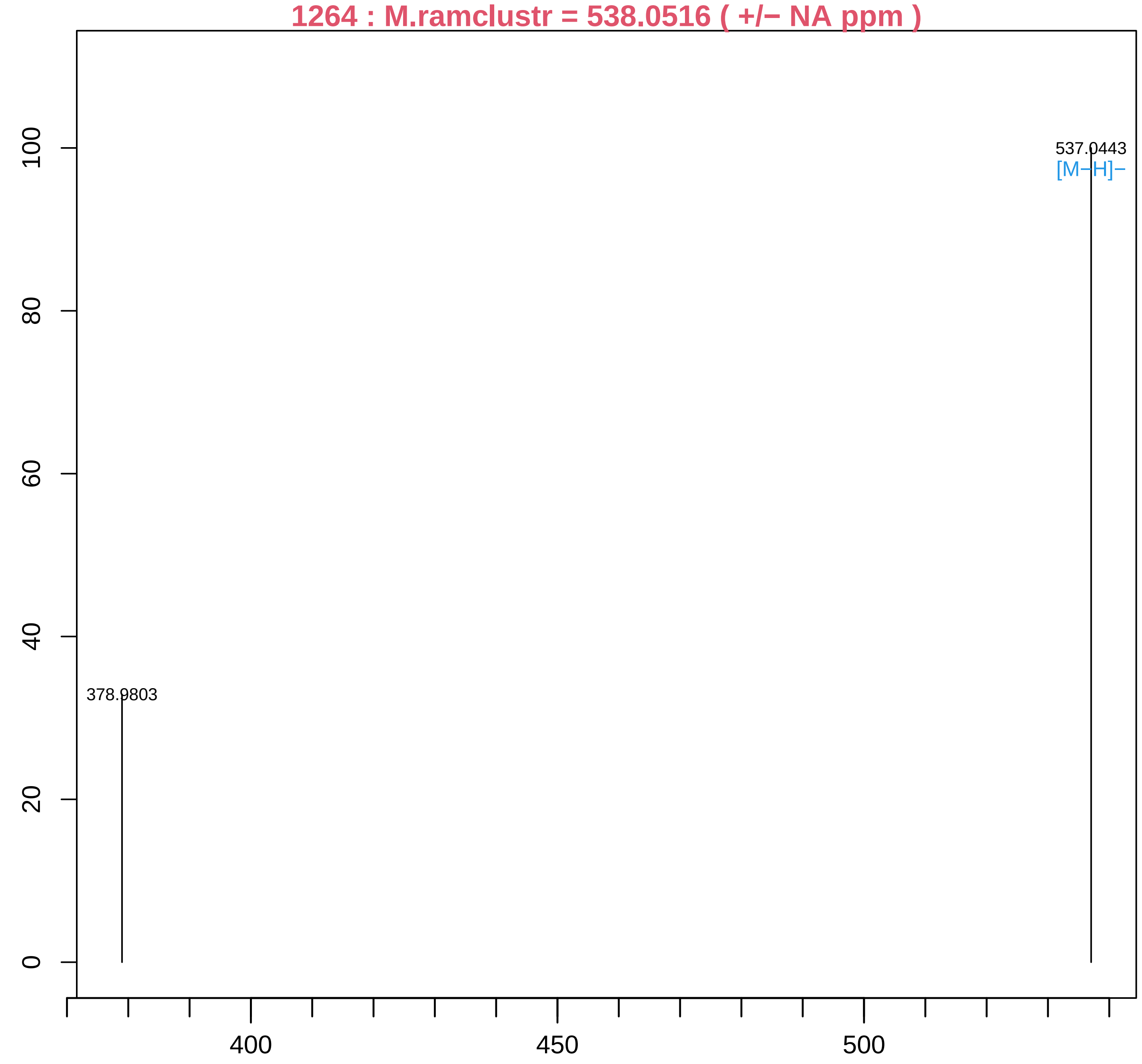
1264 : M.ramclustr = 538.0516 (+/− NA ppm)
537.0443
[M−H]−
378.9803
0
20
40
60
80
100
400
450
500

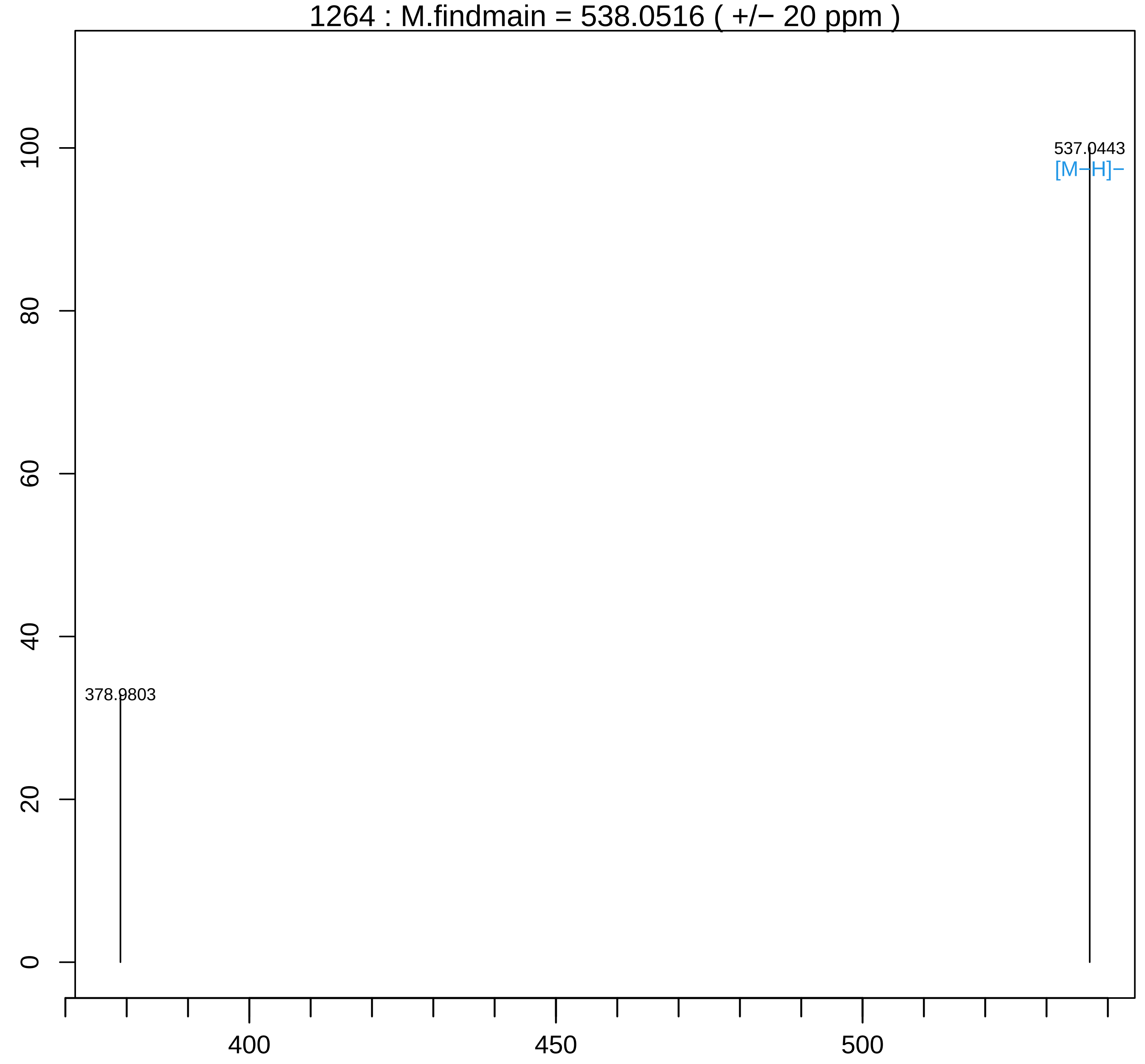
1264 : M.findmain = 538.0516 (+/− 20 ppm)
537.0443
[M−H]−
378.9803
0
20
40
60
80
100
400
450
500

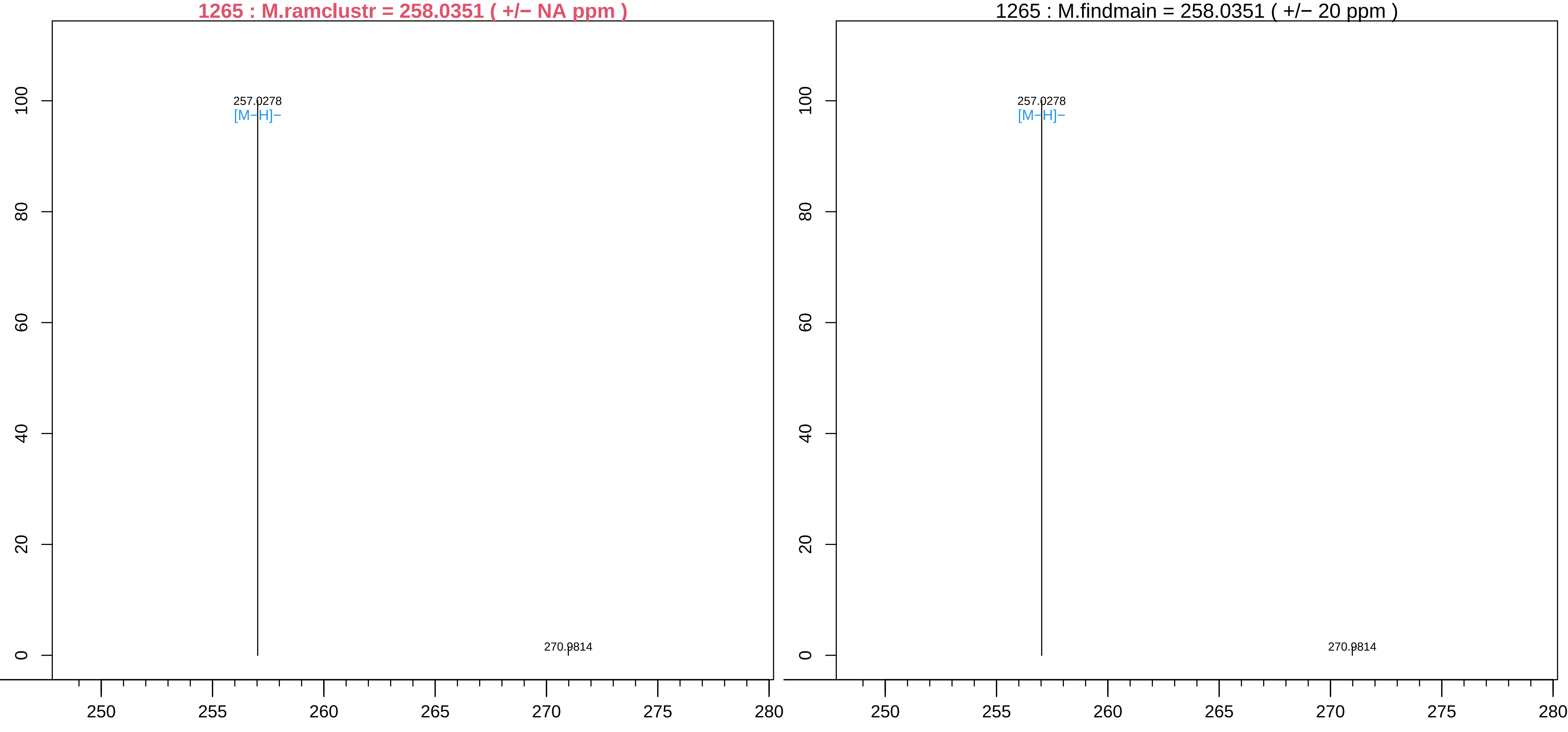
1265 : M.ramclustr = 258.0351 (+/− NA ppm)
257.0278
[M−H]−
270.9814
1265 : M.findmain = 258.0351 (+/− 20 ppm)
257.0278
[M−H]−
270.9814
0
20
40
60
80
100
250
255
260
265
270
275
280

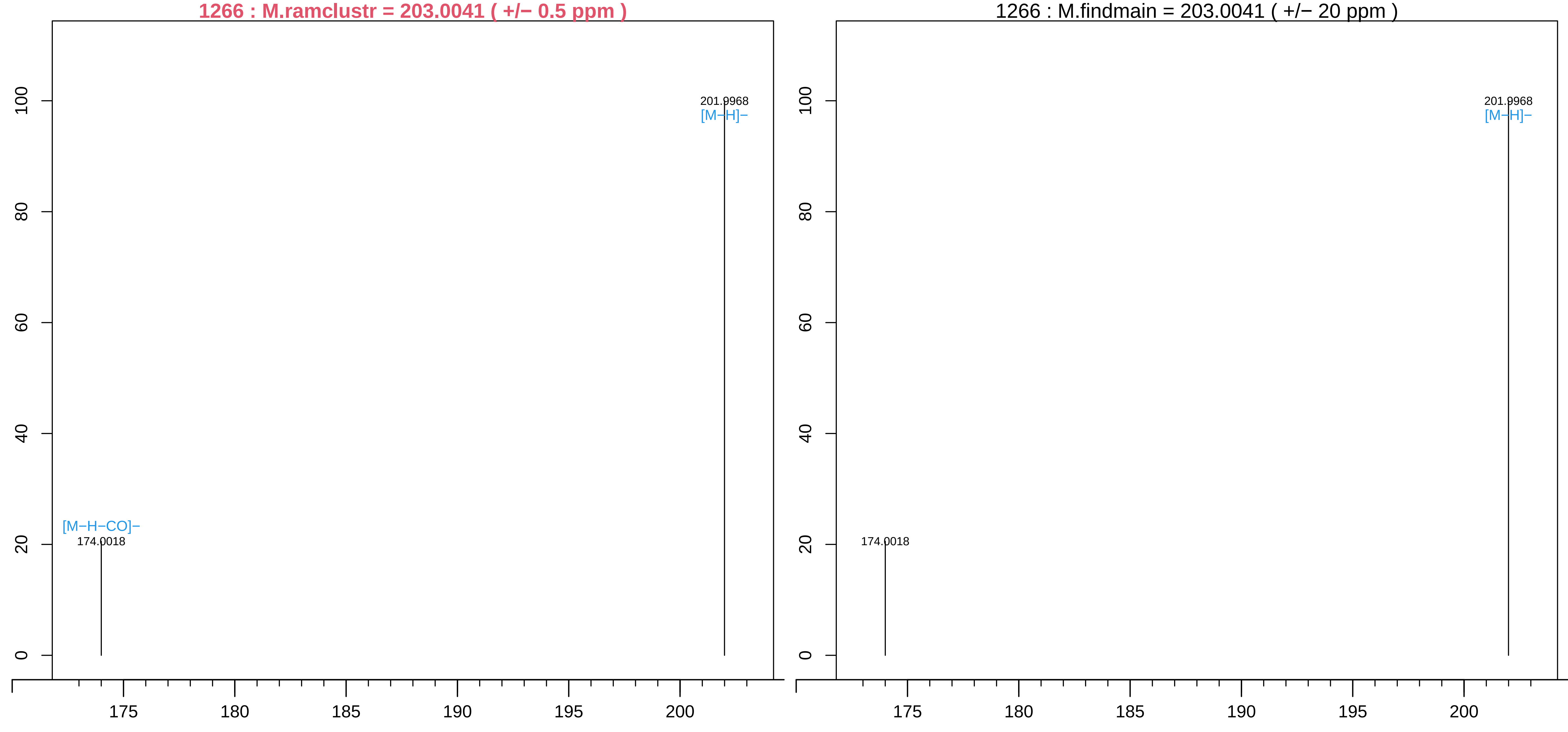
1266 : M.ramclustr = 203.0041 (+/− 0.5 ppm)
201.9968
[M−H]−
[M−H−CO]−
174.0018
1266 : M.findmain = 203.0041 (+/− 20 ppm)
201.9968
[M−H]−
174.0018
0
20
40
60
80
100
175
180
185
190
195
200

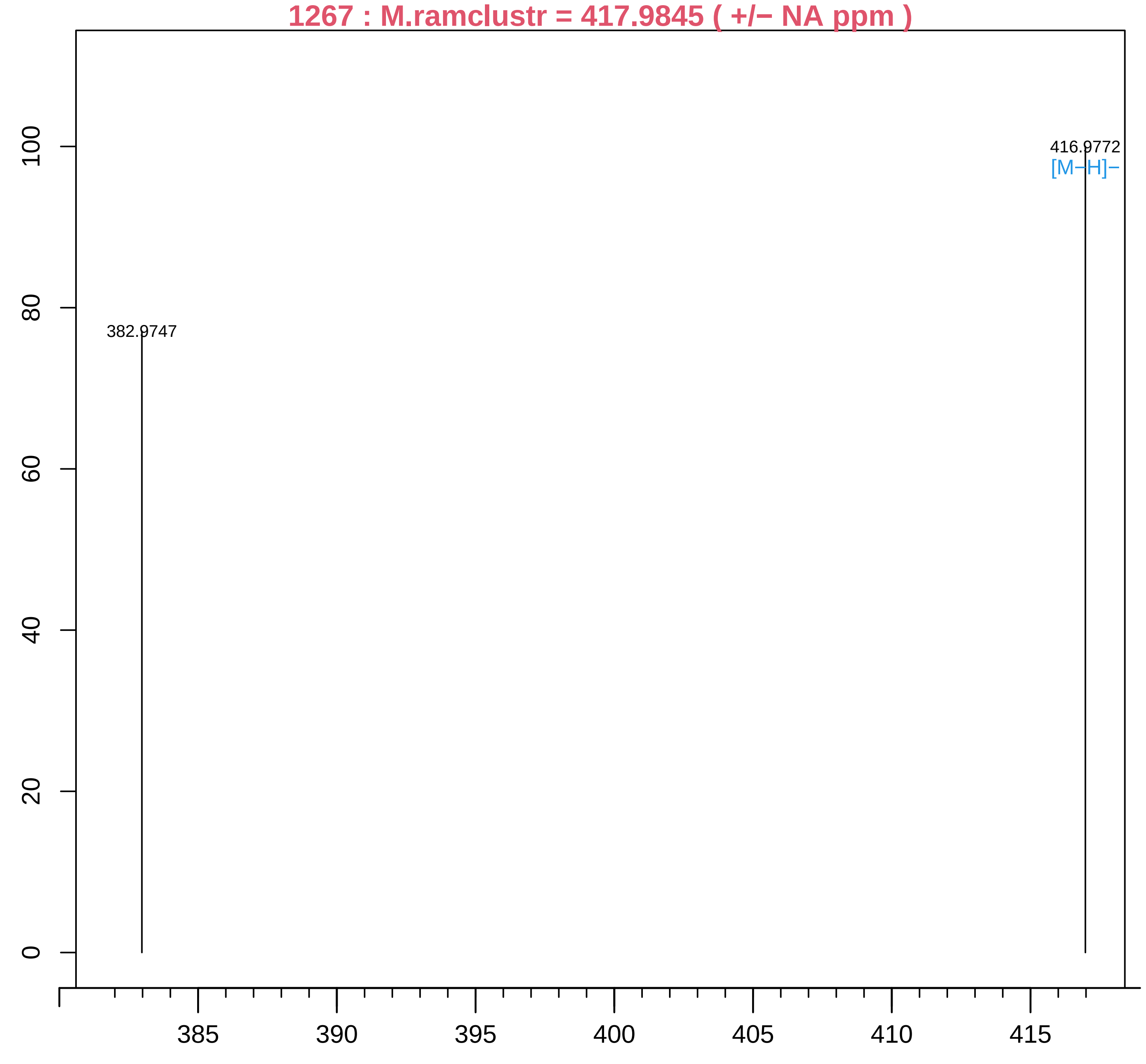

1267 : M.ramclustr = 417.9845 (+/− NA ppm)
382.9747
416.9772
[M−H]−
0
20
40
60
80
100
385
390
395
400
405
410
415

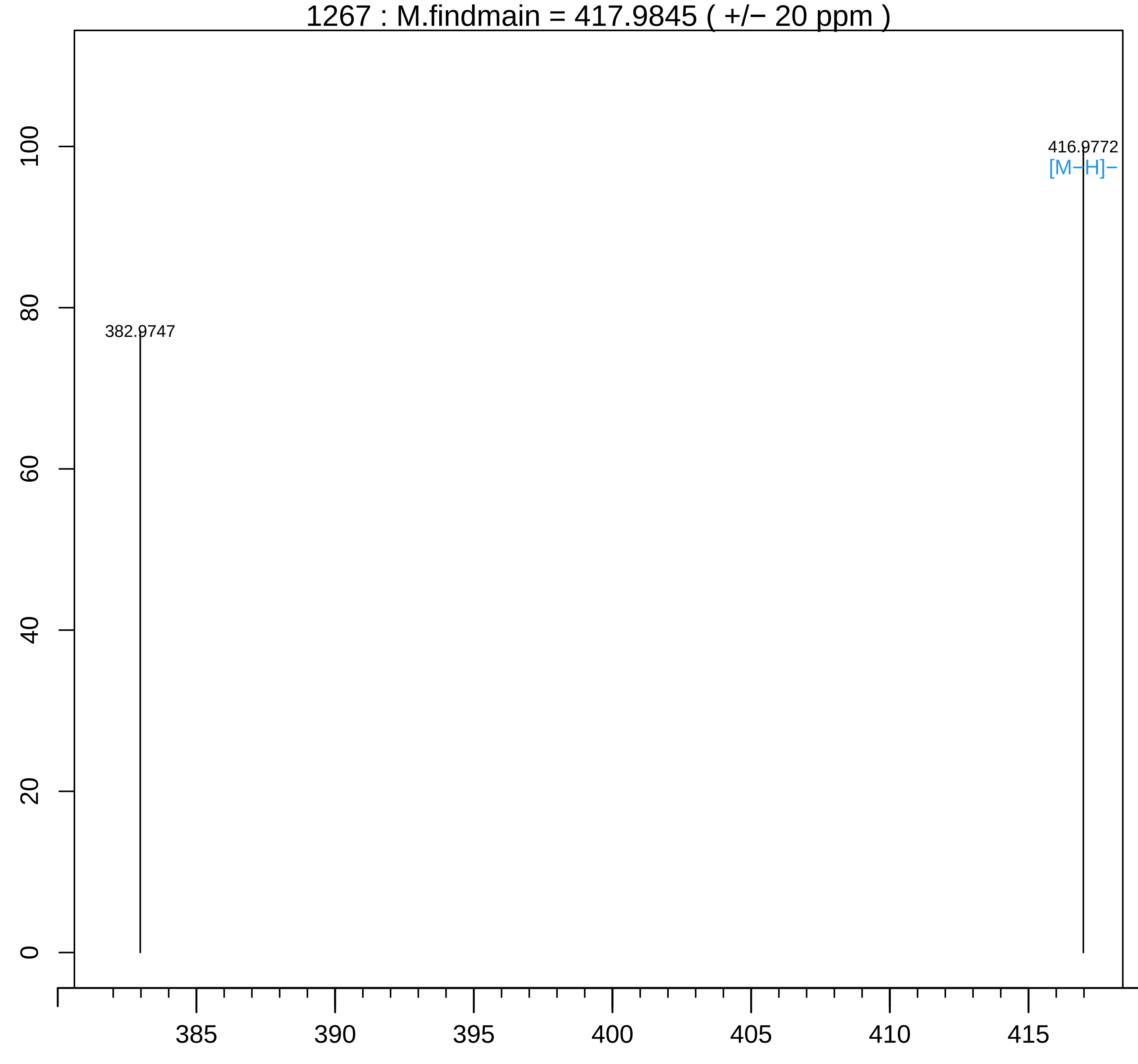

1267 : M.findmain = 417.9845 (+/− 20 ppm)
382.9747
416.9772
[M−H]−
0
20
40
60
80
100
385
390
395
400
405
410
415

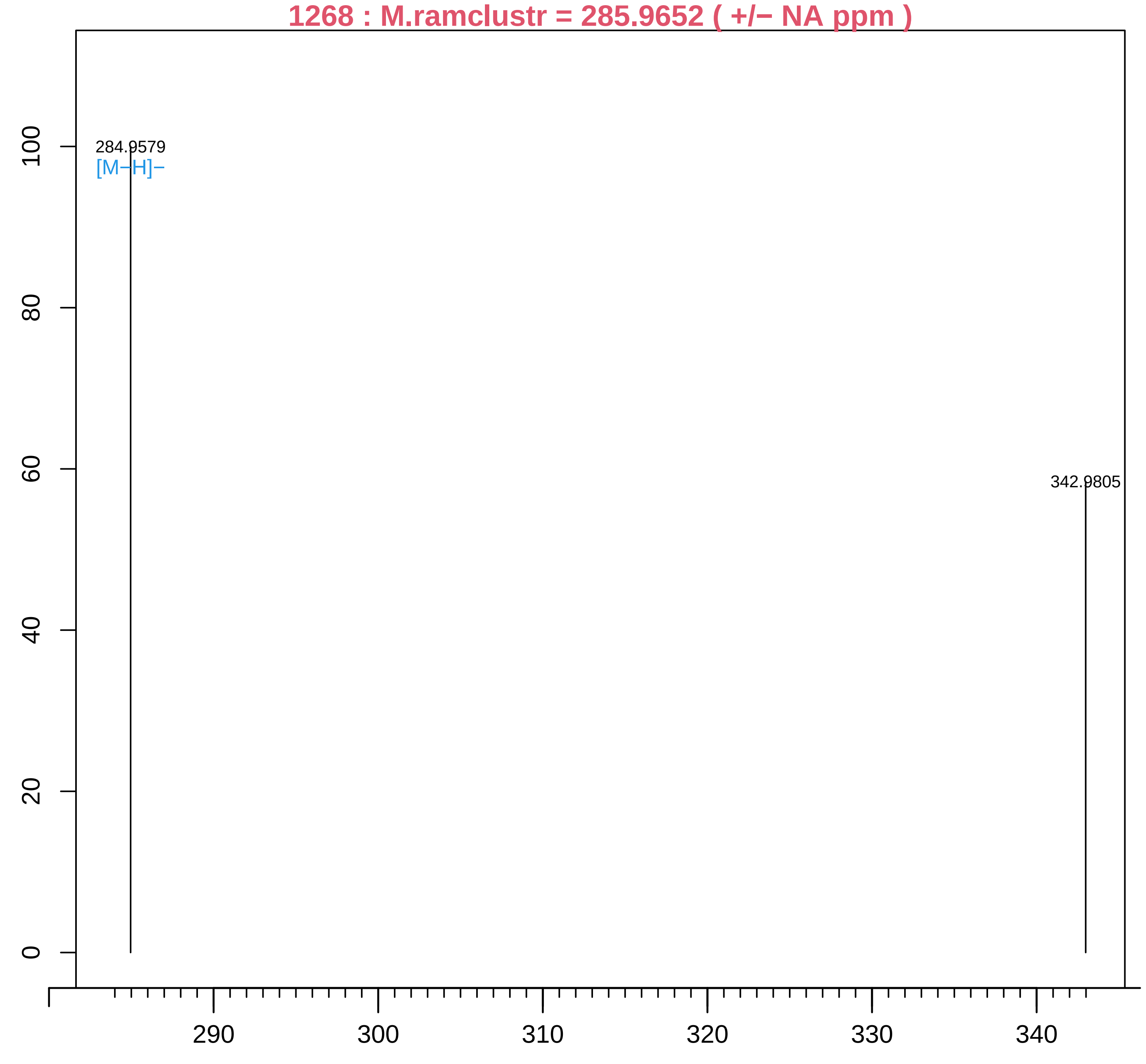
1268 : M.ramclustr = 285.9652 (+/− NA ppm)
284.9579
[M−H]−
342.9805

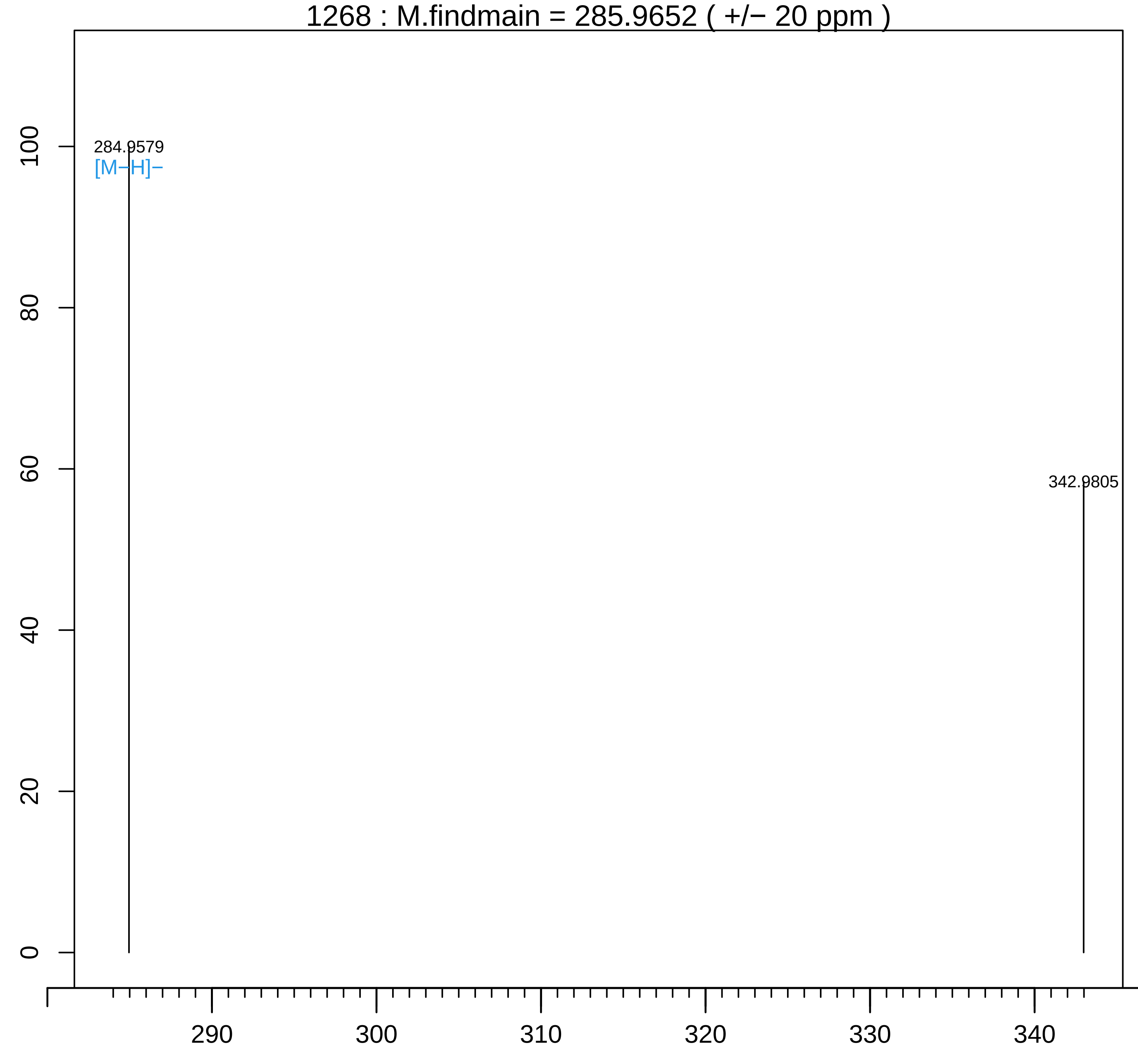
1268 : M.findmain = 285.9652 (+/− 20 ppm)
284.9579
[M−H]−
342.9805

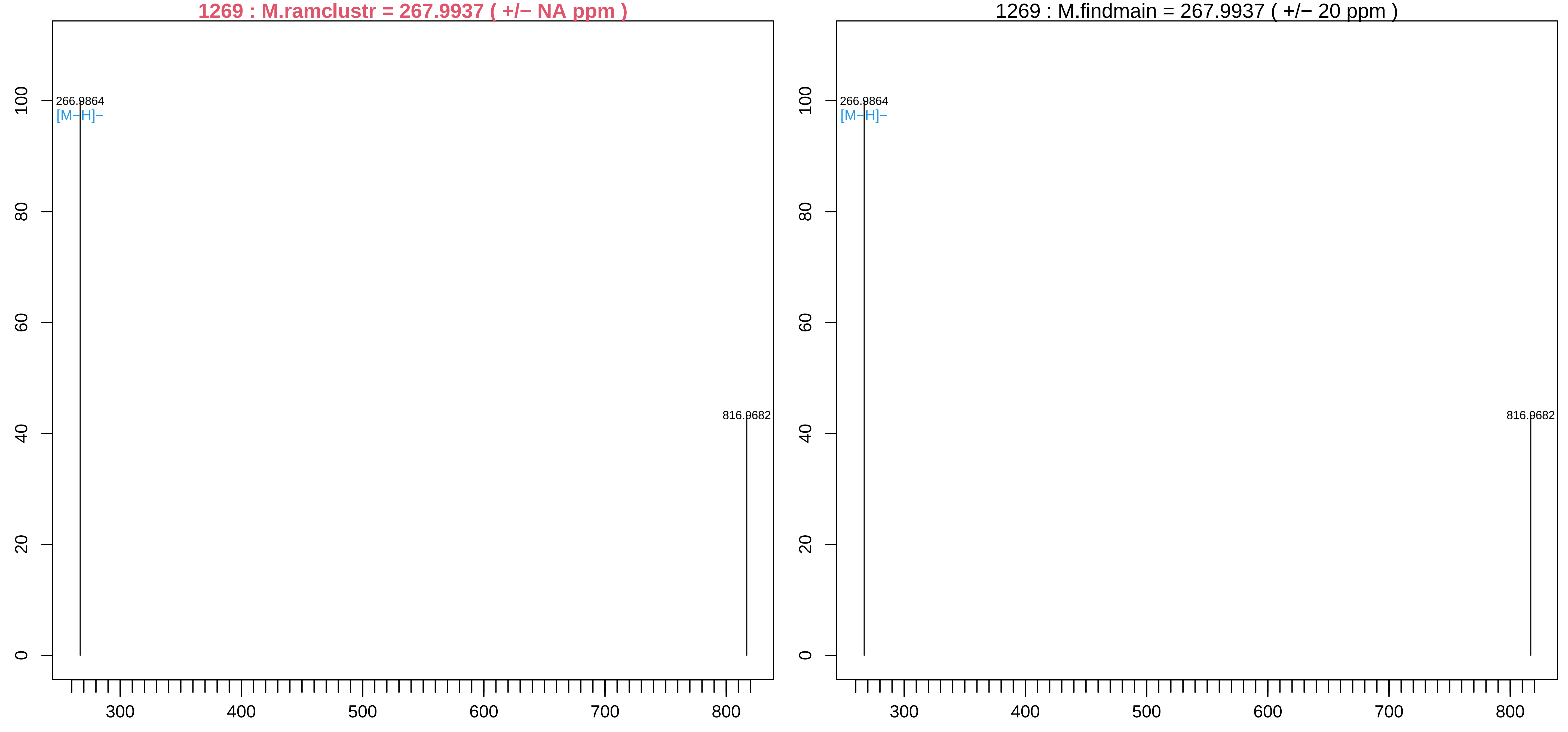
1269 : M.ramclustr = 267.9937 (+/− NA ppm)
266.9864
[M−H]−
816.9682
1269 : M.findmain = 267.9937 (+/− 20 ppm)
266.9864
[M−H]−
816.9682
0
20
40
60
80
100
300
400
500
600
700
800

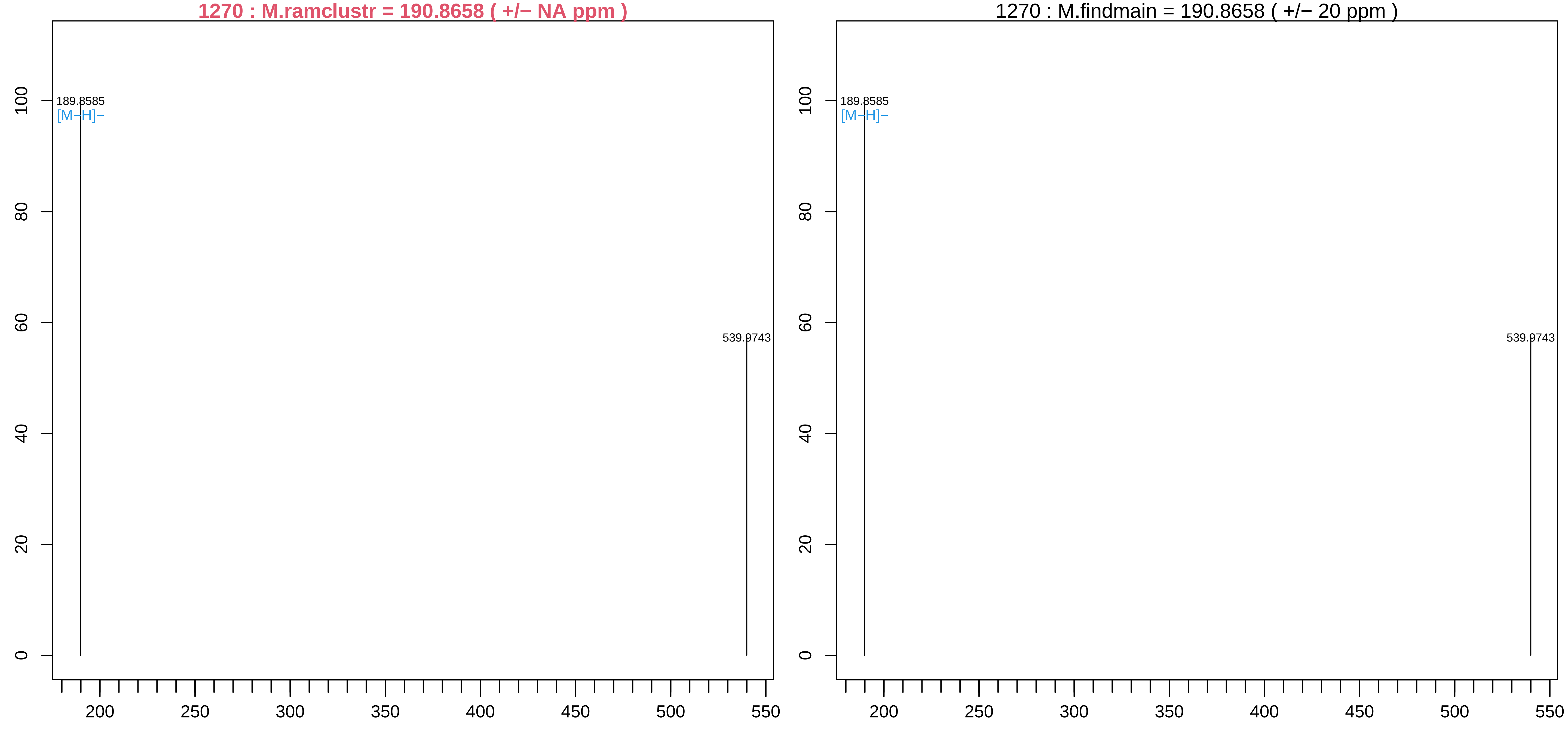

1270 : M.ramclustr = 190.8658 (+/− NA ppm)
189.8585
[M−H]−
539.9743
0
20
40
60
80
100
200
250
300
350
400
450
500
550
1270 : M.findmain = 190.8658 (+/− 20 ppm)
189.8585
[M−H]−
539.9743
0
20
40
60
80
100
200
250
300
350
400
450
500
550

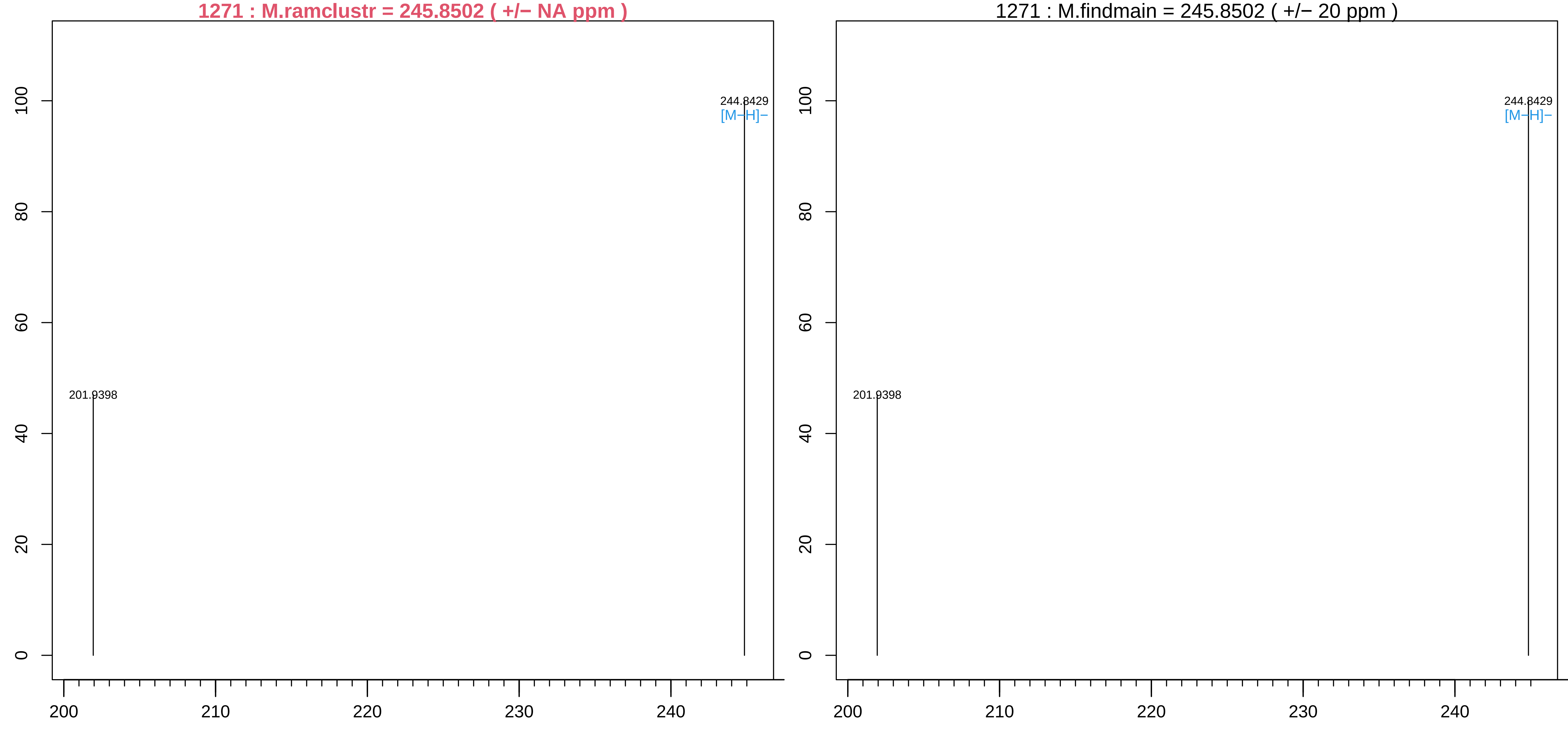

1271 : M.ramclustr = 245.8502 (+/− NA ppm)
244.8429
[M−H]−
201.9398
0
20
40
60
80
100
200
210
220
230
240
1271 : M.findmain = 245.8502 (+/− 20 ppm)
244.8429
[M−H]−
201.9398
0
20
40
60
80
100
200
210
220
230
240

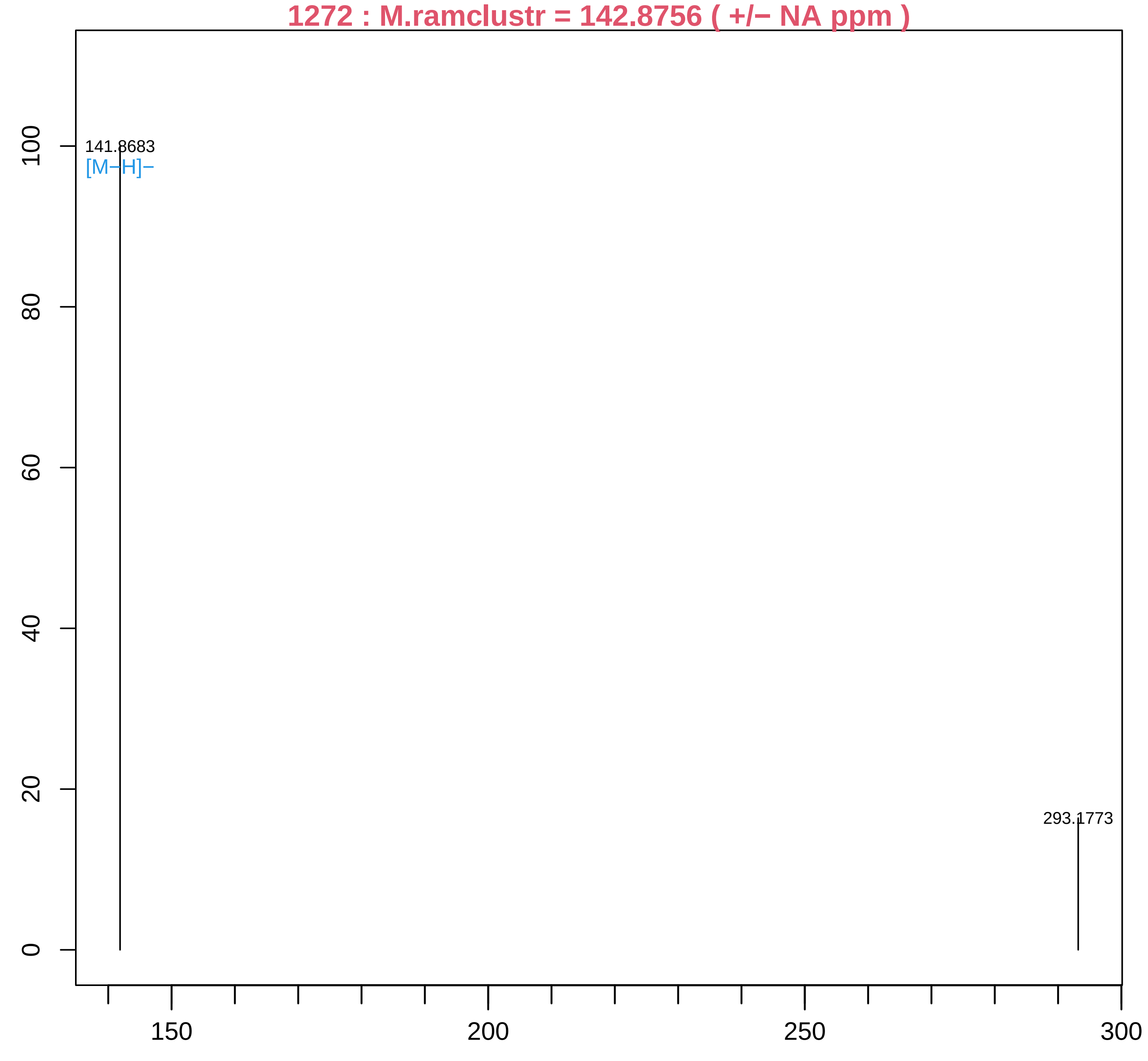
1272 : M.ramclustr = 142.8756 (+/− NA ppm)
141.8683
[M−H]−
293.1773
0
20
40
60
80
100
150
200
250
300

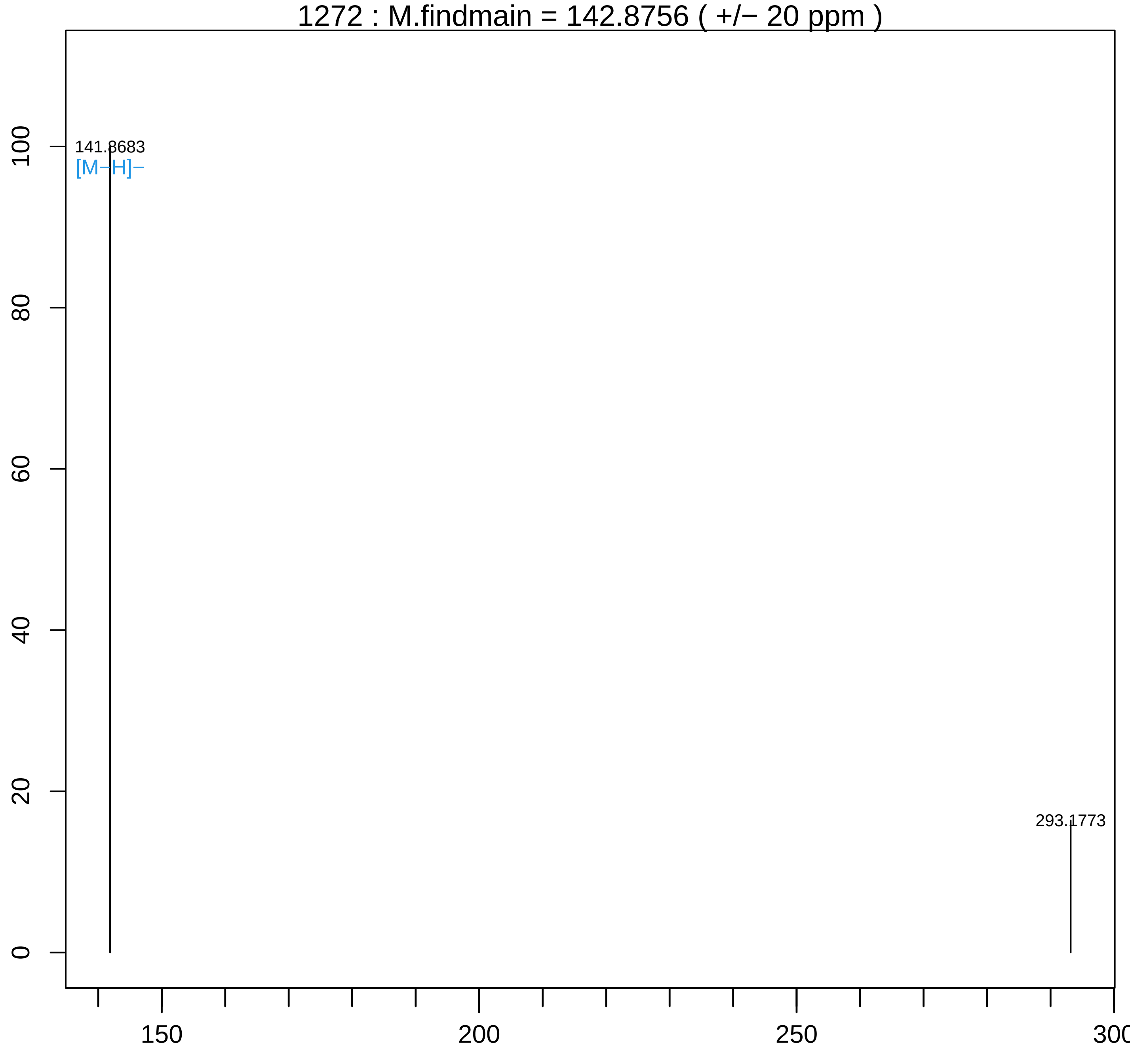
1272 : M.findmain = 142.8756 (+/− 20 ppm)
141.8683
[M−H]−
293.1773
0
20
40
60
80
100
150
200
250
300

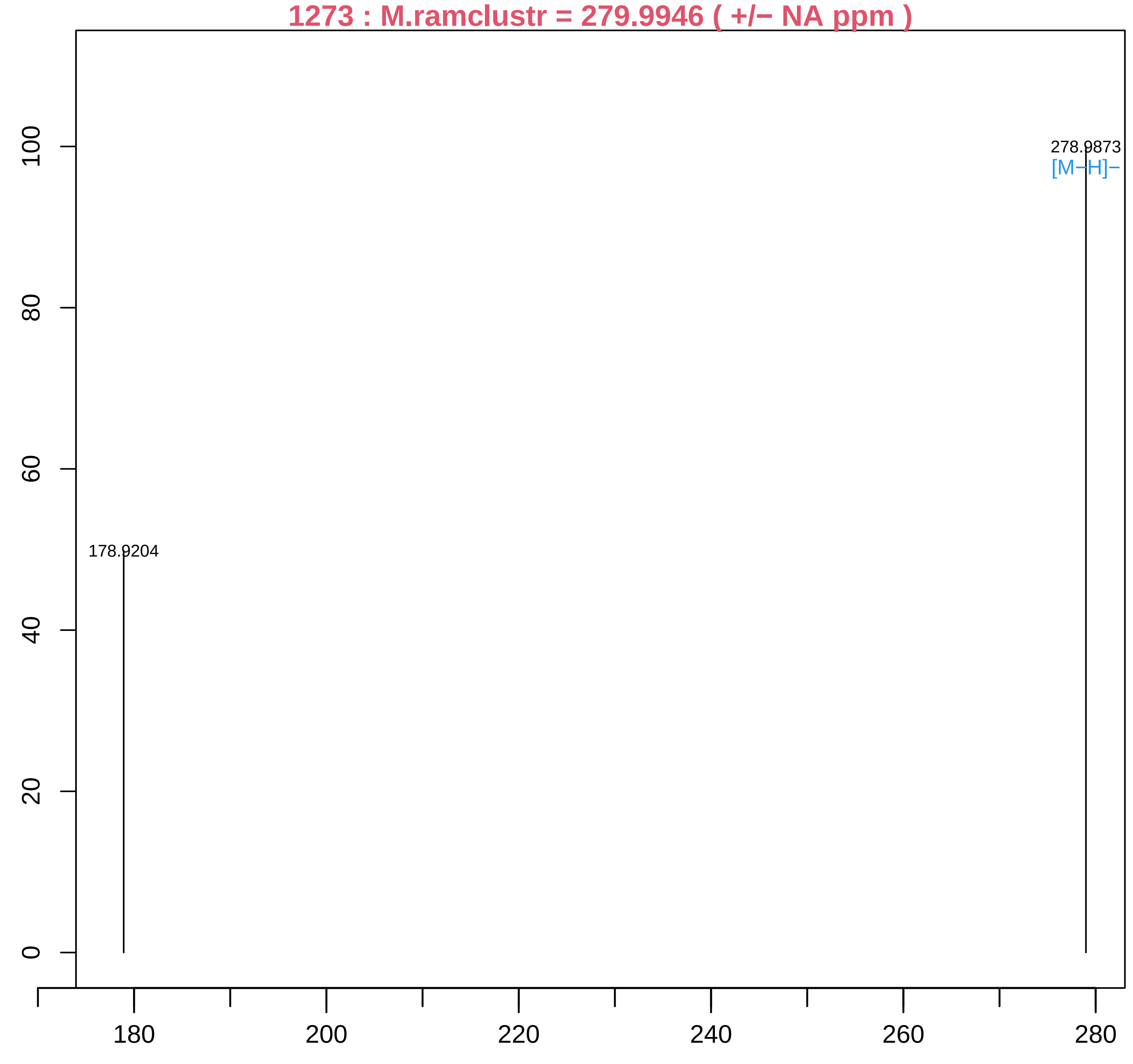

1273 : M.ramclustr = 279.9946 (+/− NA ppm)
278.9873
[M−H]−
178.9204
0
20
40
60
80
100
180
200
220
240
260
280

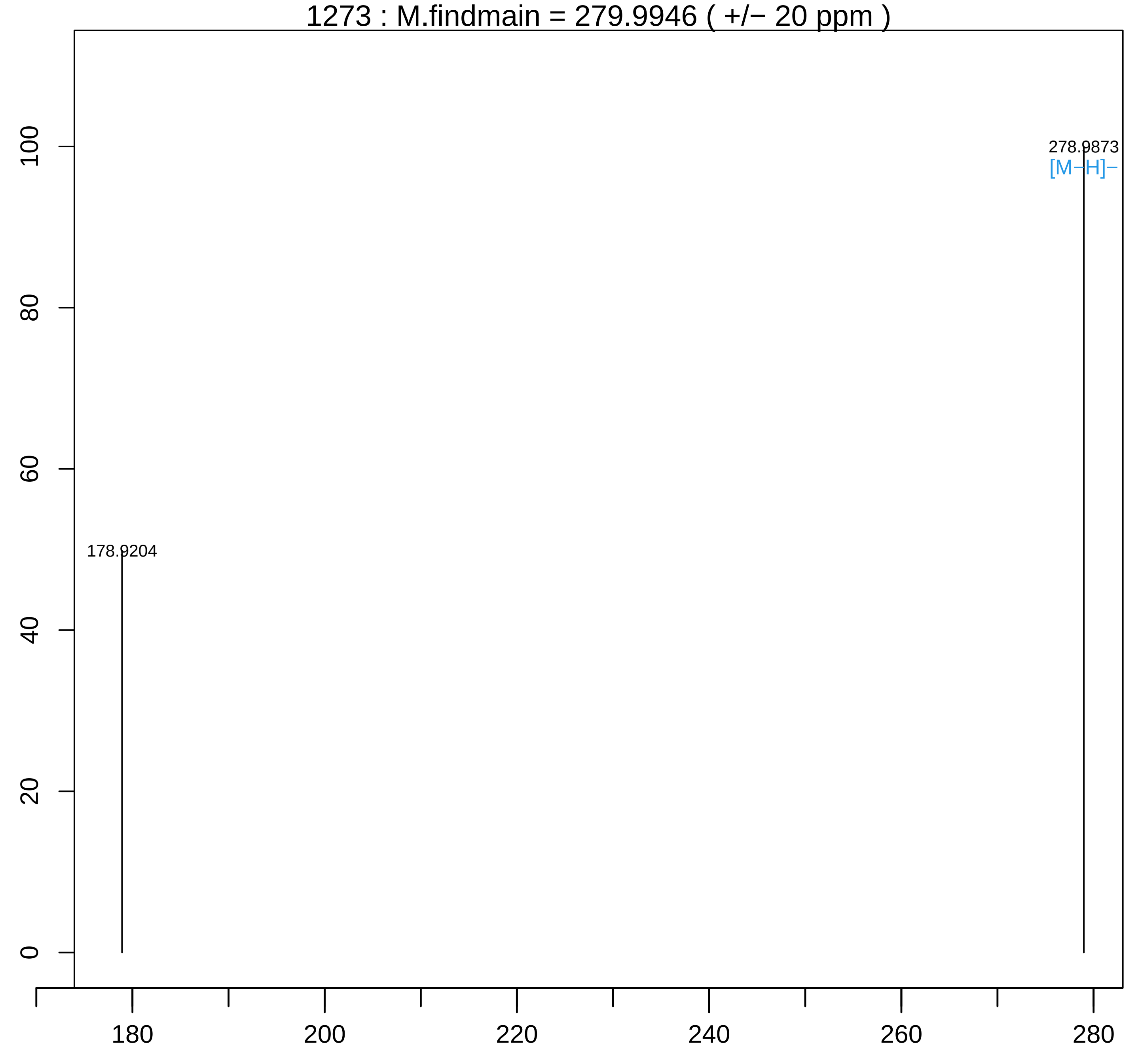

1273 : M.findmain = 279.9946 (+/− 20 ppm)
278.9873
[M−H]−
178.9204
0
20
40
60
80
100
180
200
220
240
260
280

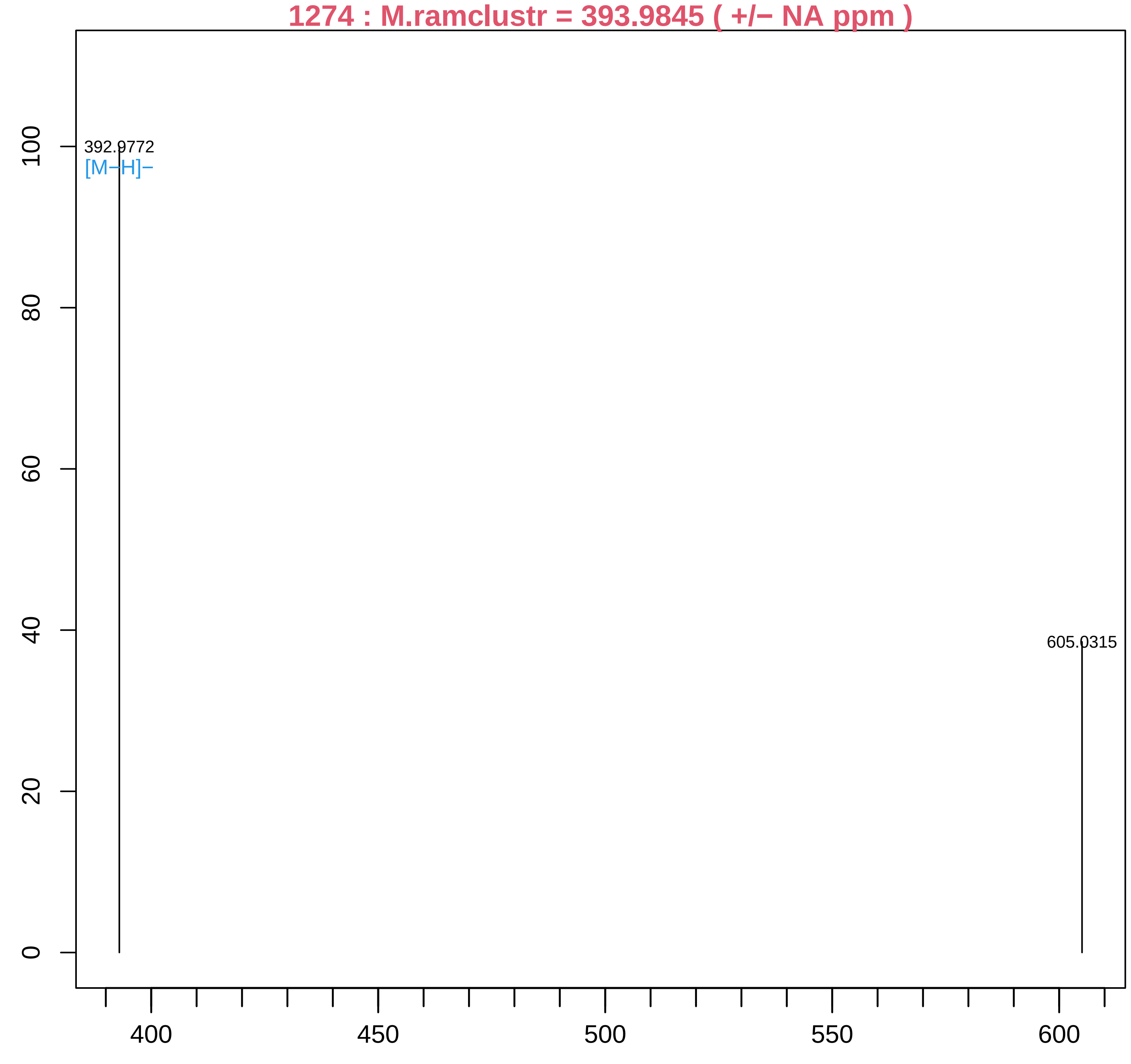
1274 : M.ramclustr = 393.9845 (+/− NA ppm)
392.9772
[M−H]−
605.0315
0
20
40
60
80
100
400
450
500
550
600

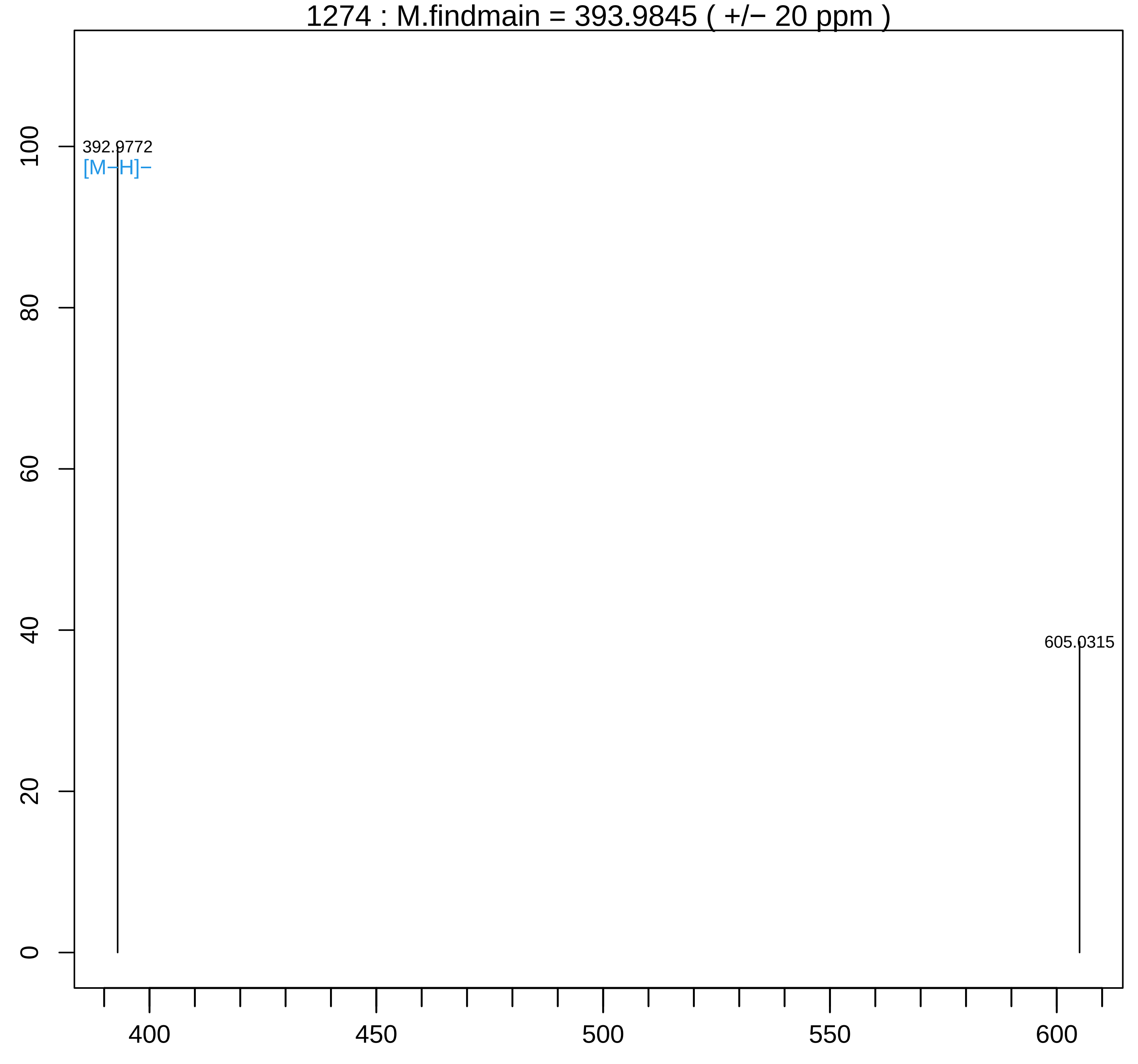
1274 : M.findmain = 393.9845 (+/− 20 ppm)
392.9772
[M−H]−
605.0315
0
20
40
60
80
100
400
450
500
550
600

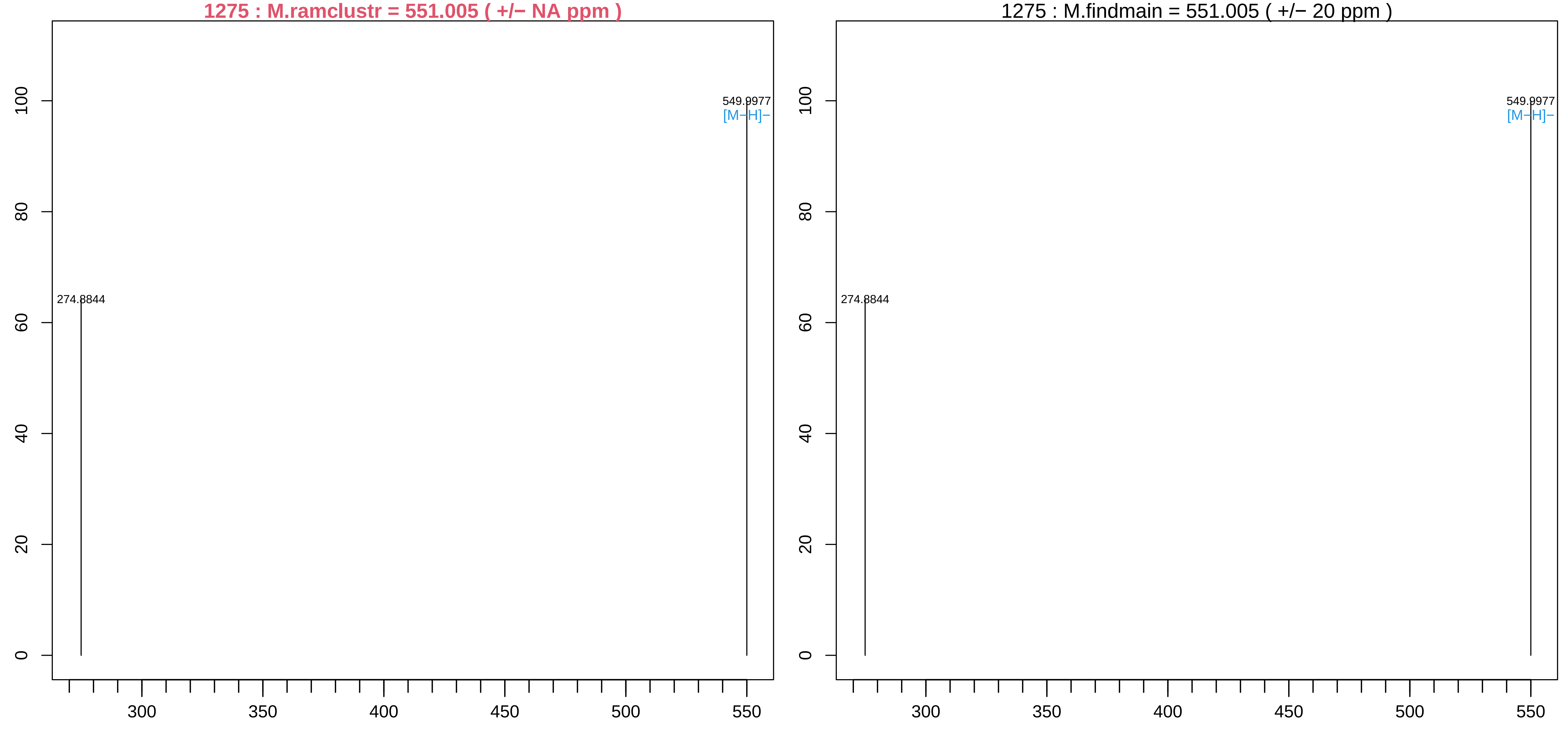
1275 : M.ramclustr = 551.005 (+/− NA ppm)
1275 : M.findmain = 551.005 (+/− 20 ppm)
274.8844
549.9977
[M−H]−
274.8844
549.9977
[M−H]−
0
20
40
60
80
100
300
350
400
450
500
550

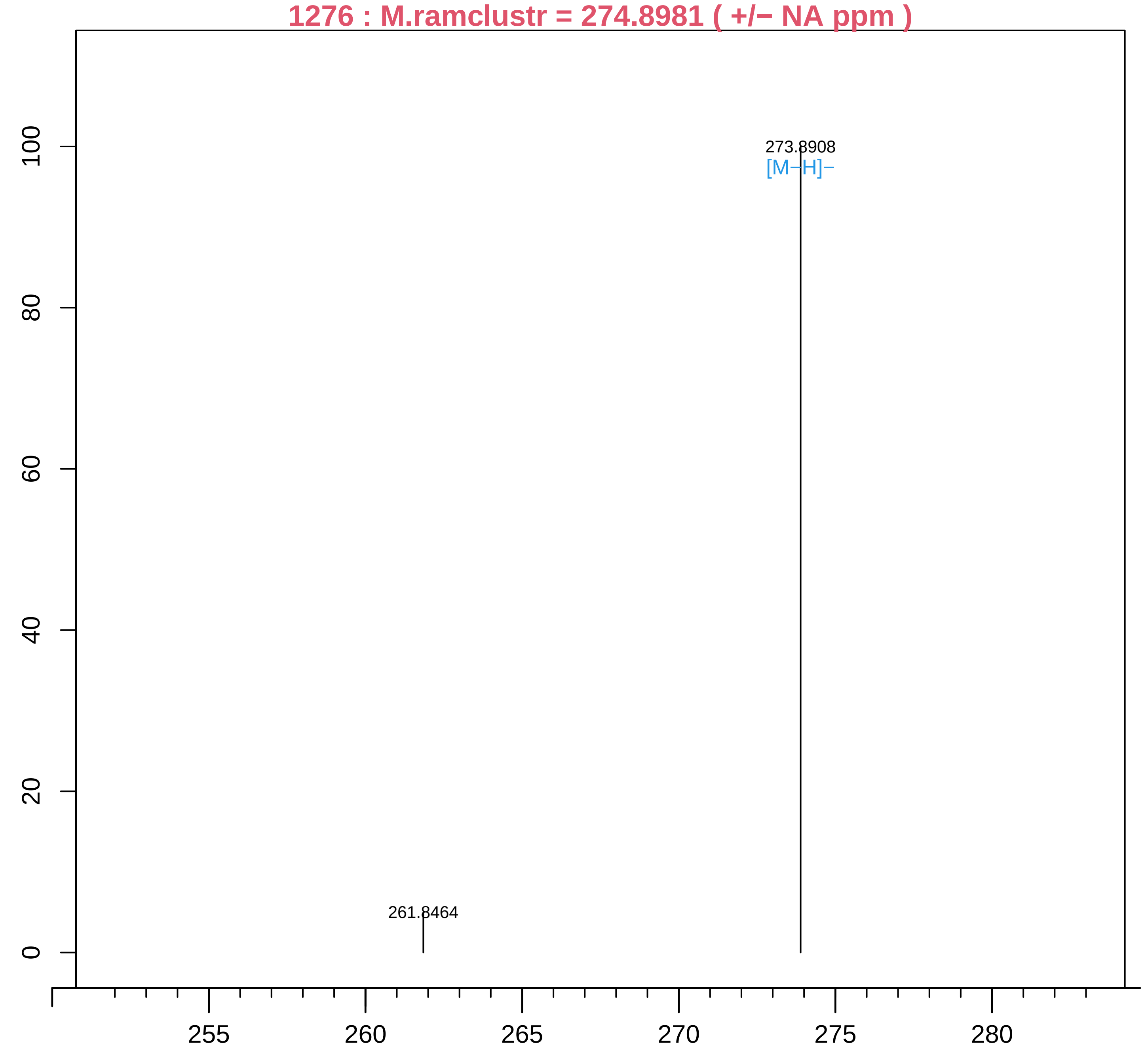
1276 : M.ramclustr = 274.8981 (+/− NA ppm)
273.8908
[M−H]−
261.8464
0
20
40
60
80
100
255
260
265
270
275
280

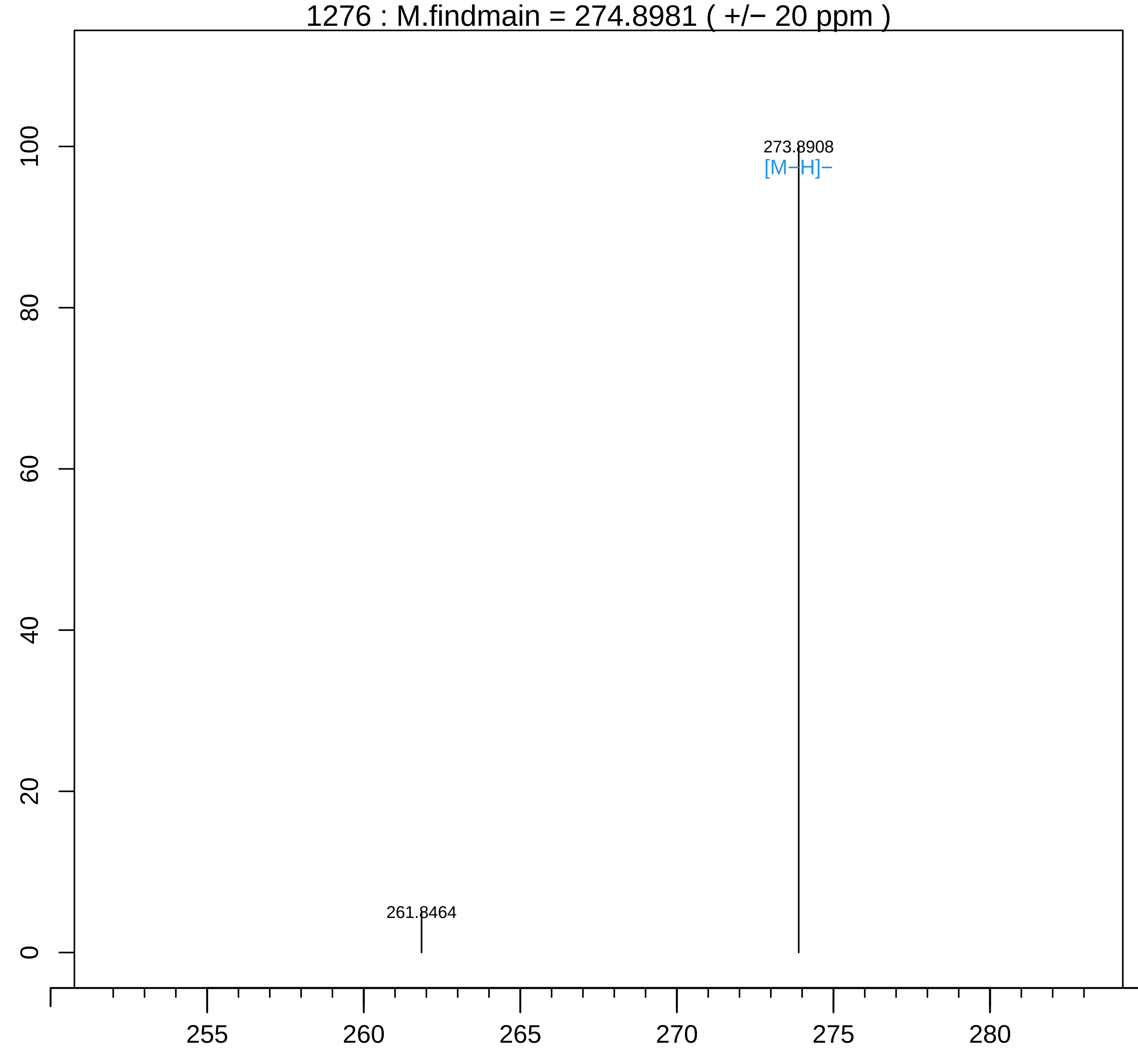
1276 : M.findmain = 274.8981 (+/− 20 ppm)
273.8908
[M−H]−
261.8464
0
20
40
60
80
100
255
260
265
270
275
280

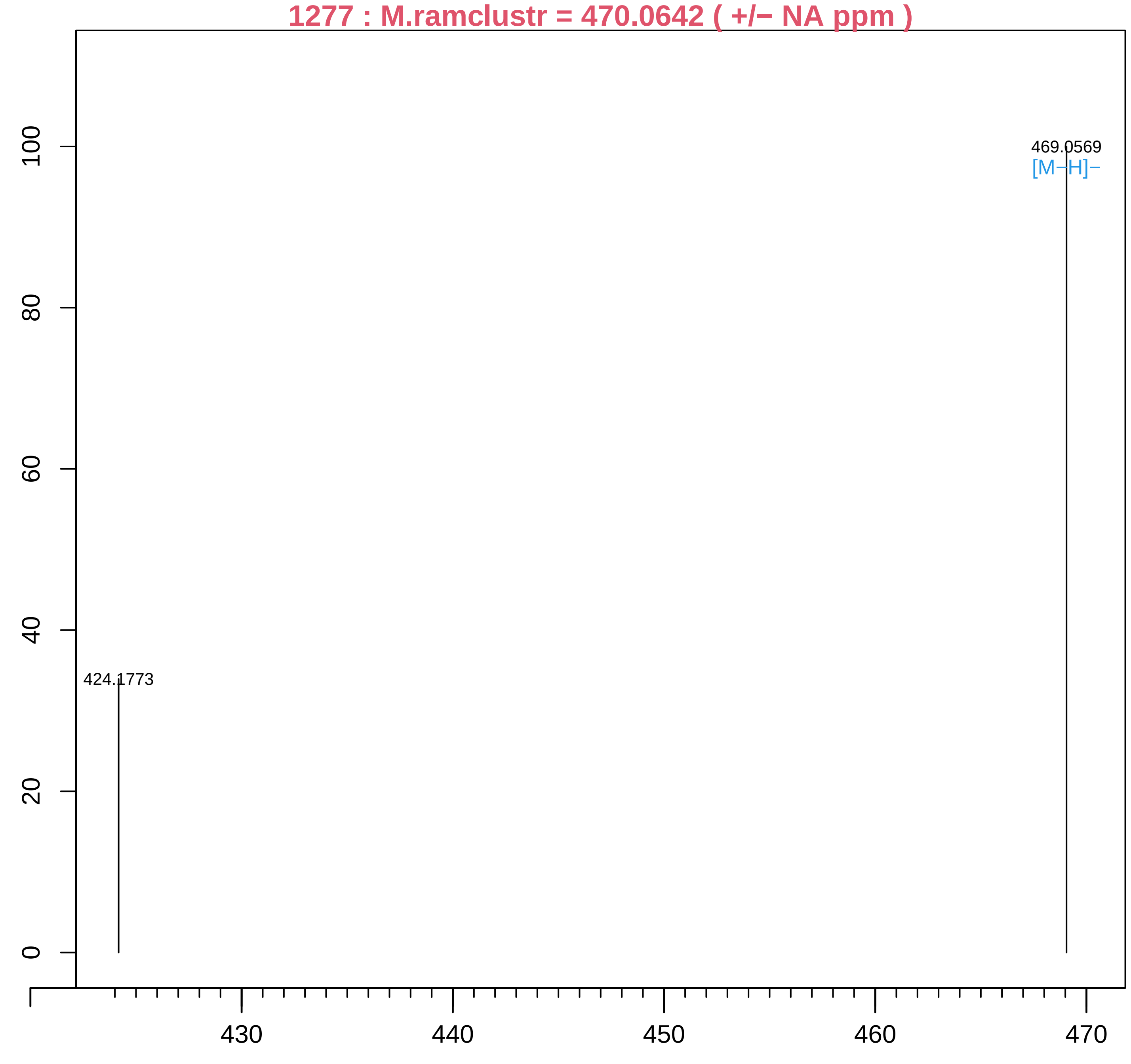
1277 : M.ramclustr = 470.0642 (+/− NA ppm)
469.0569
[M−H]−
424.1773
0
20
40
60
80
100
430
440
450
460
470

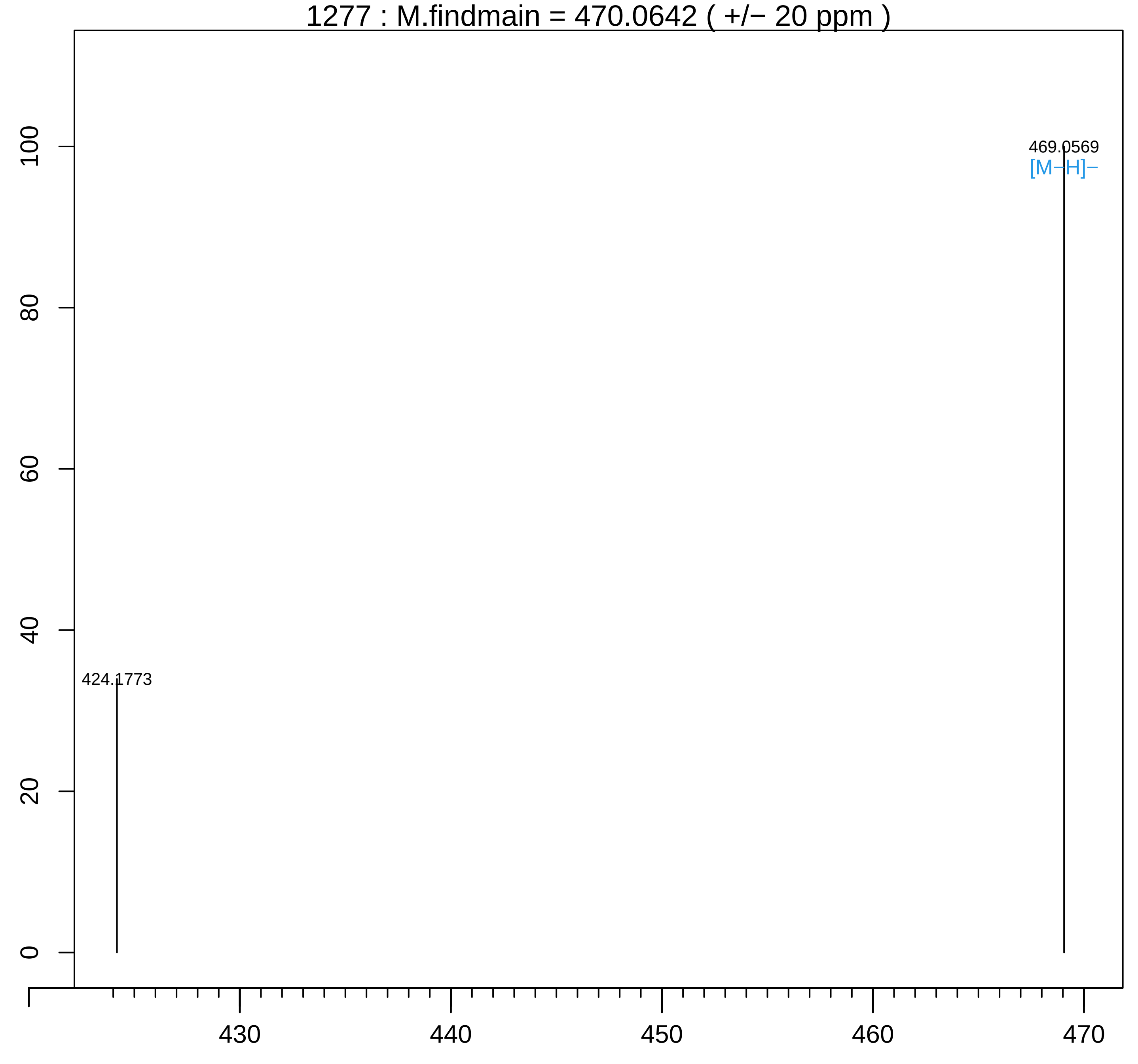
1277 : M.findmain = 470.0642 (+/− 20 ppm)
469.0569
[M−H]−
424.1773
0
20
40
60
80
100
430
440
450
460
470

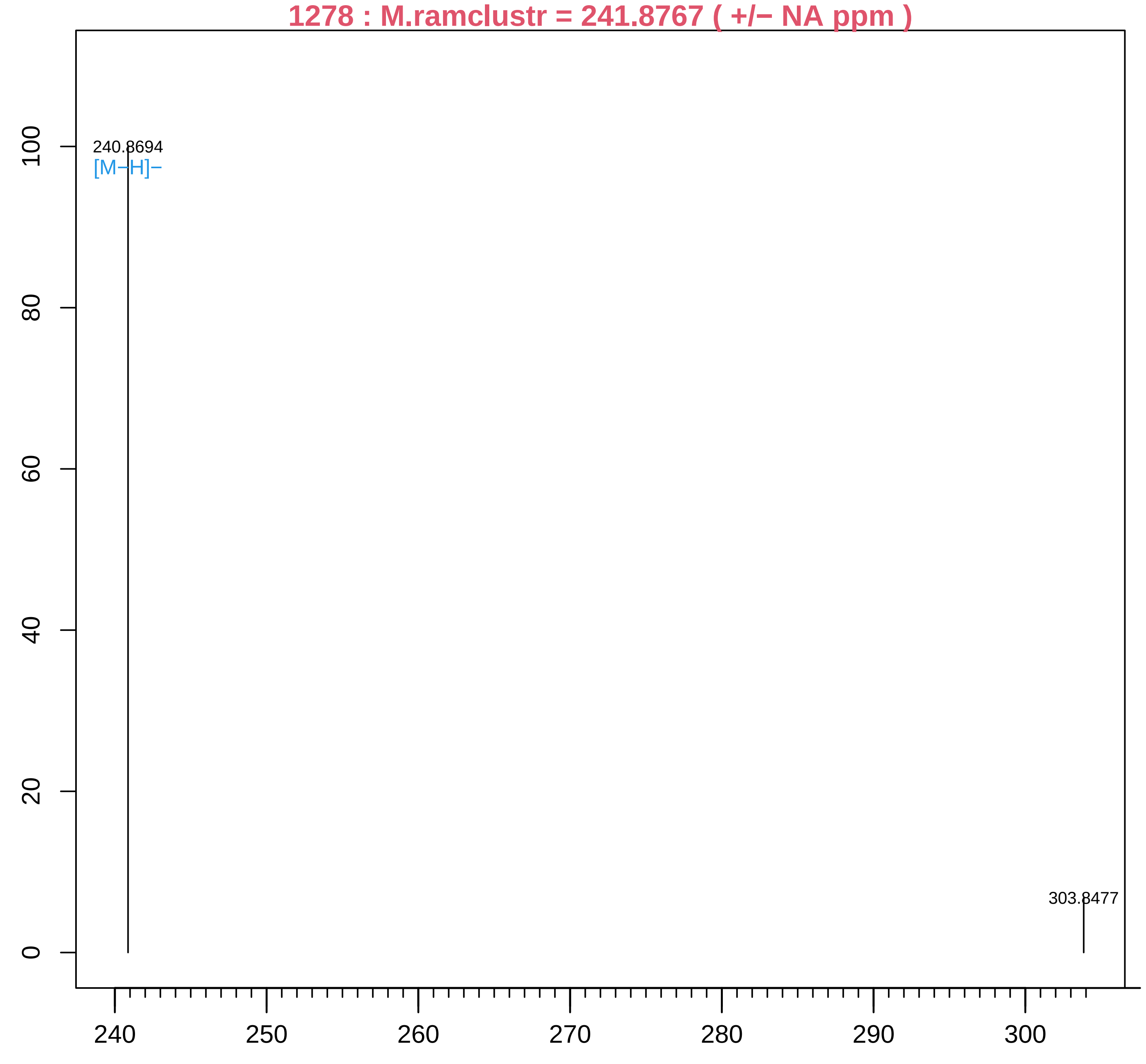
1278 : M.ramclustr = 241.8767 (+/− NA ppm)
240.8694
[M−H]−
303.8477
0
20
40
60
80
100
240
250
260
270
280
290
300
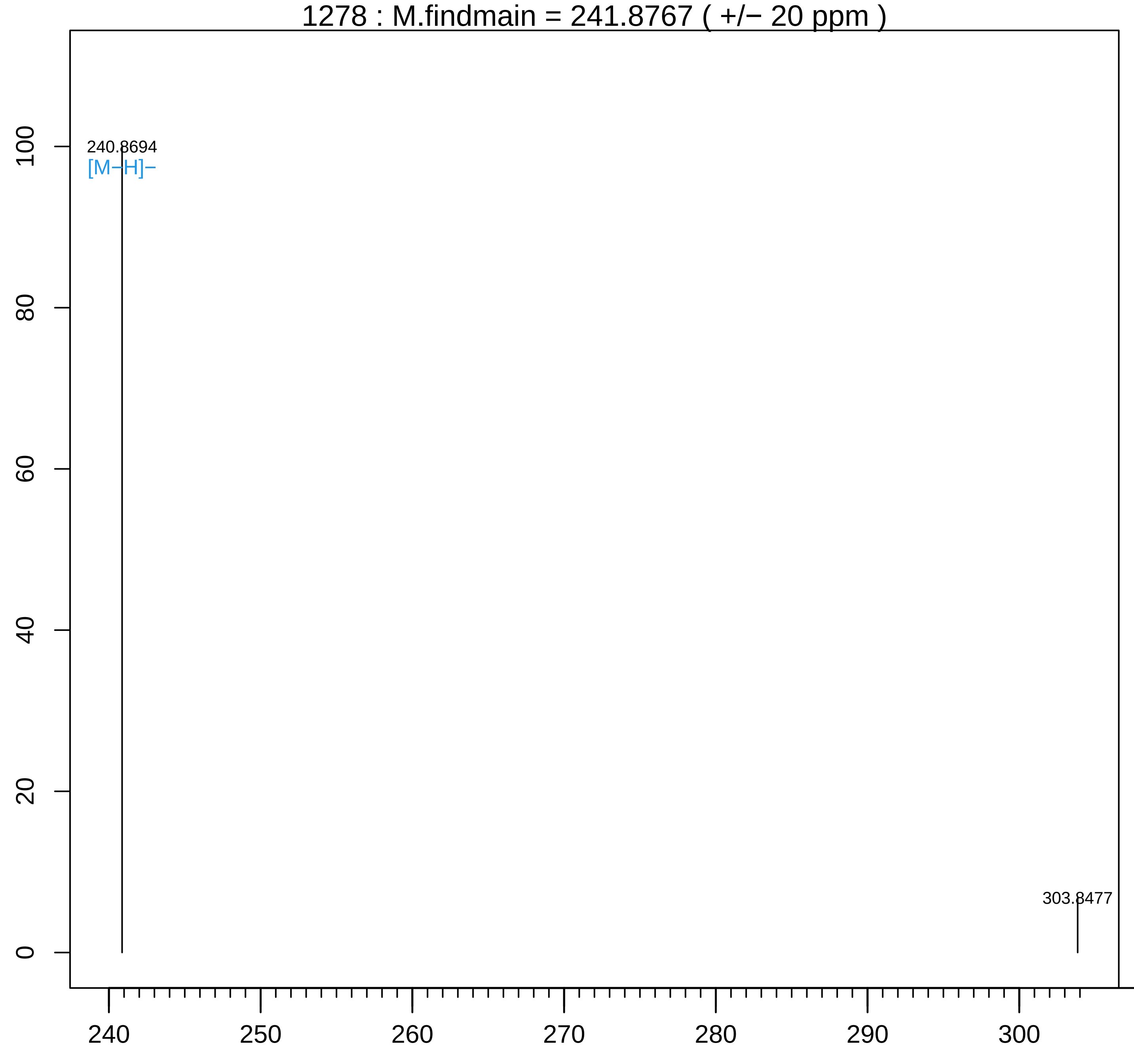
1278 : M.findmain = 241.8767 (+/− 20 ppm)
240.8694
[M−H]−
303.8477
0
20
40
60
80
100
240
250
260
270
280
290
300

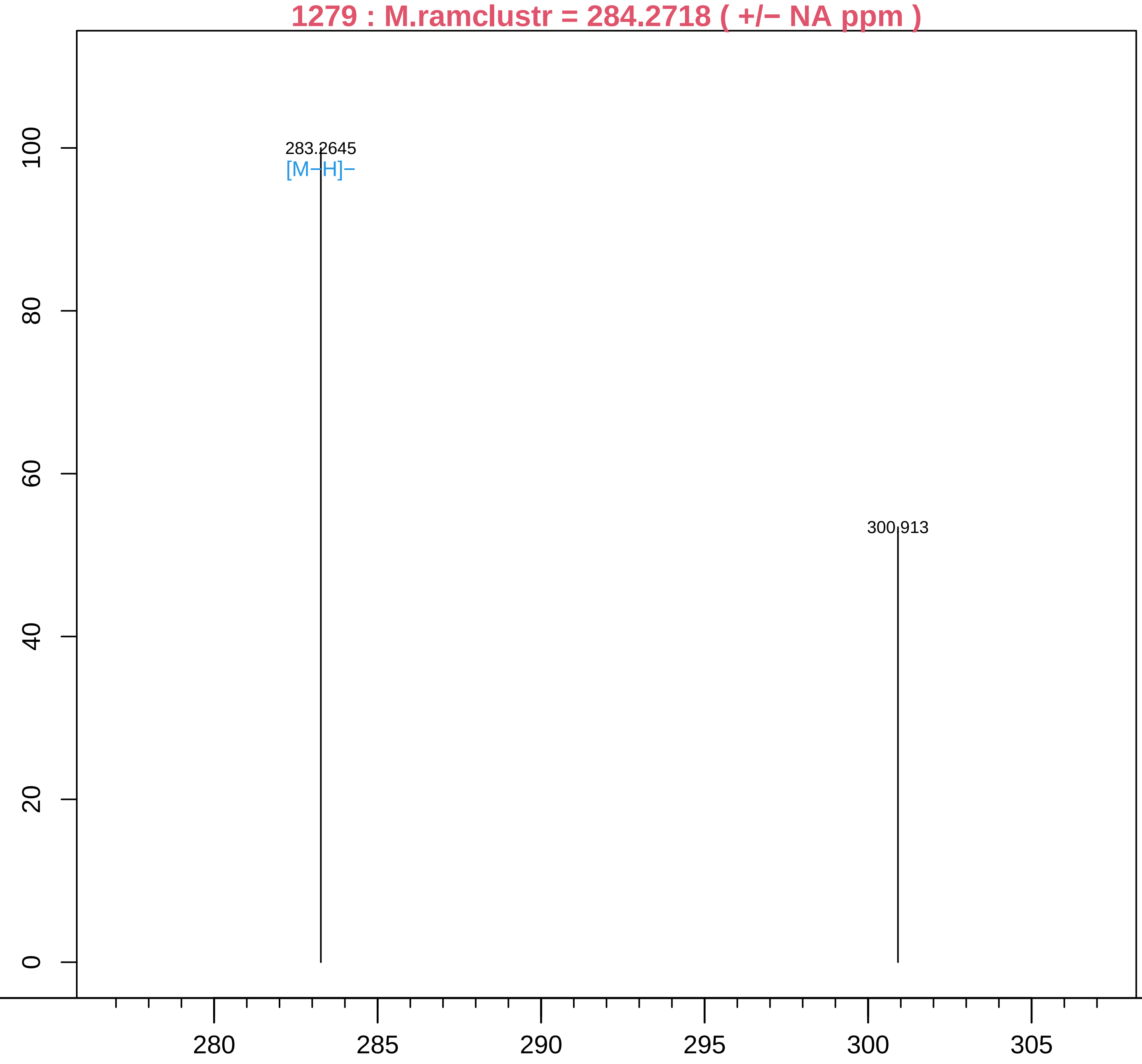
1279 : M.ramclustr = 284.2718 (+/− NA ppm)
283.2645
[M−H]−
300.913
0
20
40
60
80
100
280
285
290
295
300
305

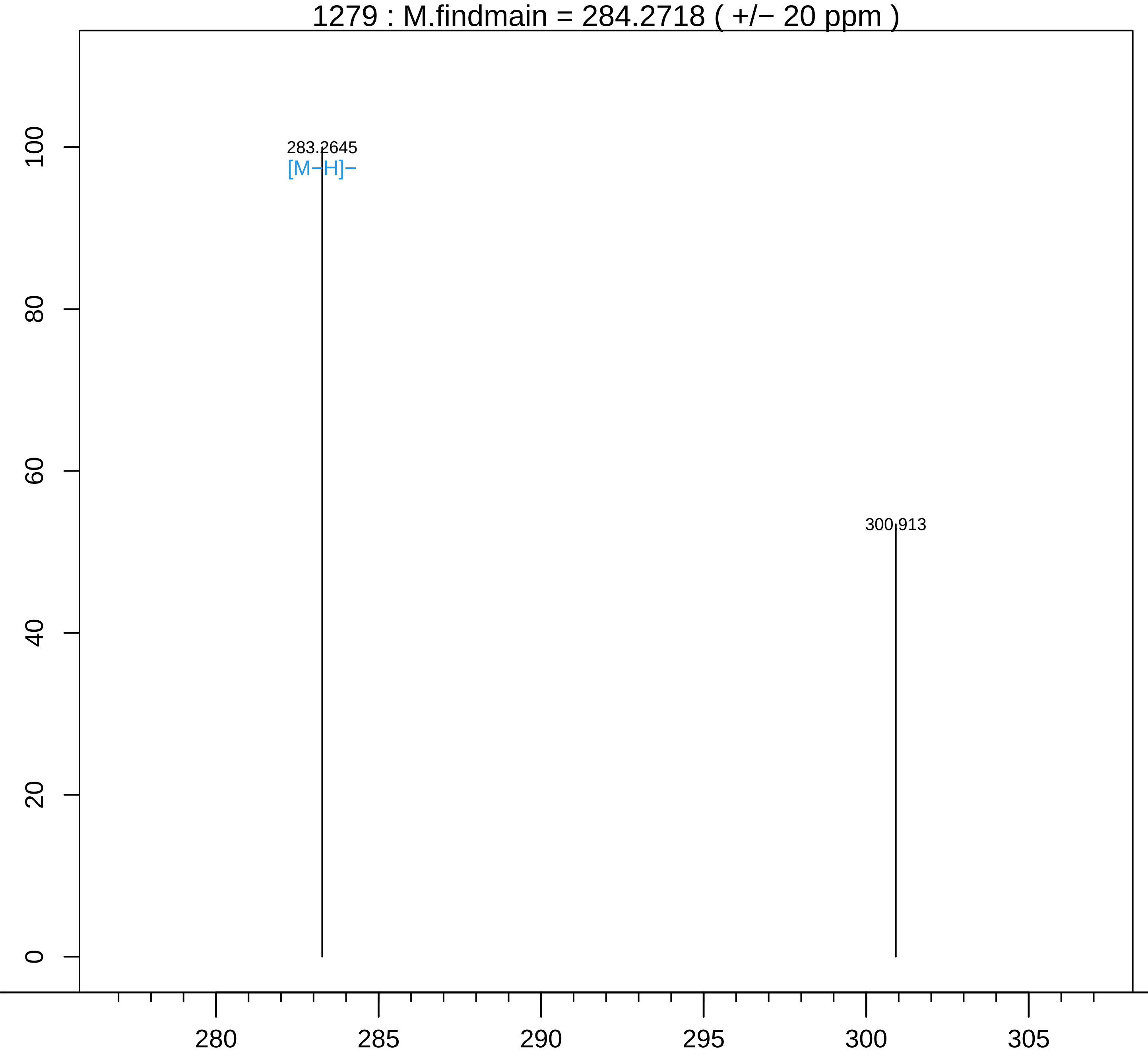
1279 : M.findmain = 284.2718 (+/− 20 ppm)
283.2645
[M−H]−
300.913
0
20
40
60
80
100
280
285
290
295
300
305

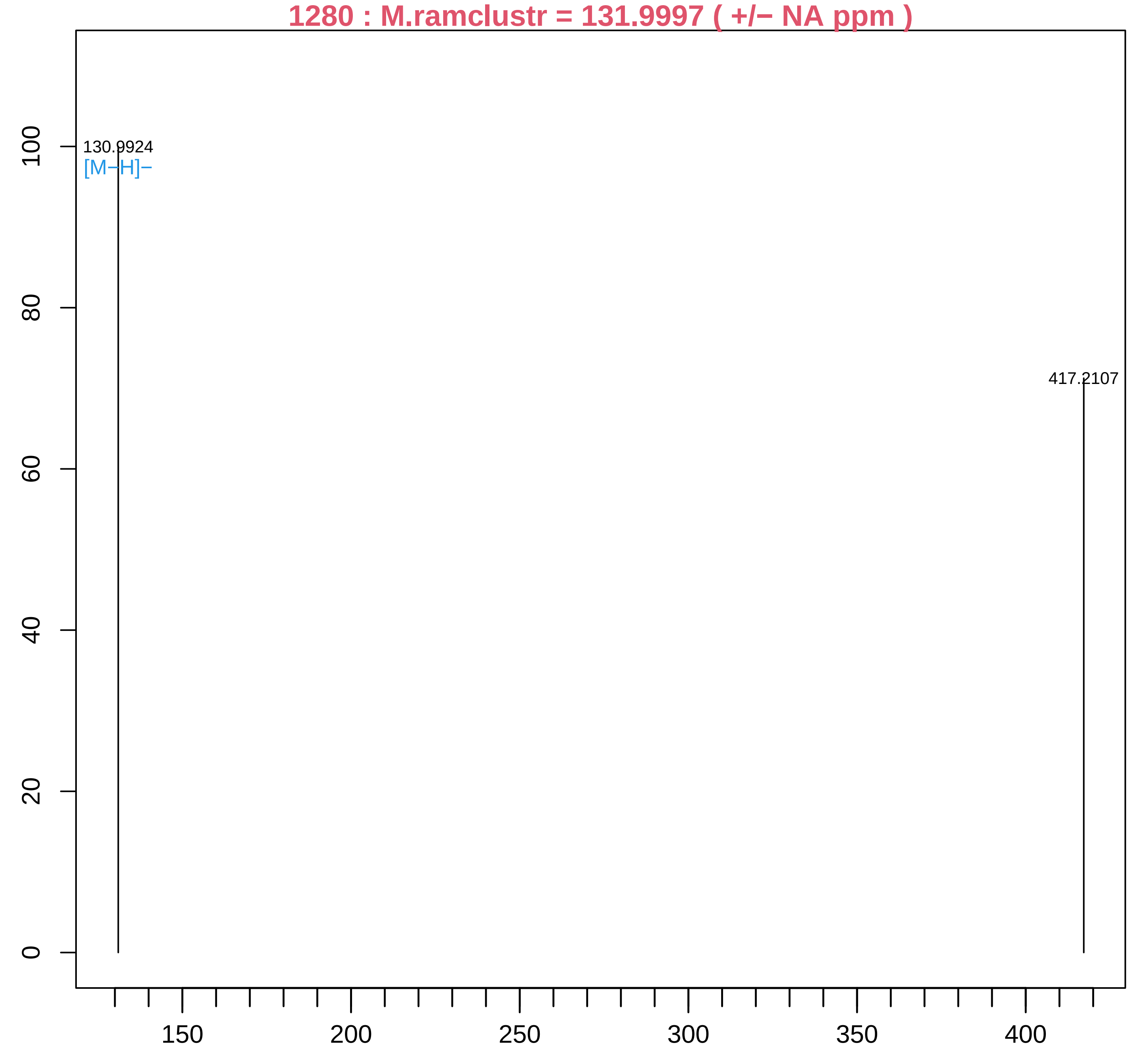
1280 : M.ramclustr = 131.9997 (+/− NA ppm)
130.9924
[M−H]−
417.2107
0
20
40
60
80
100
150
200
250
300
350
400

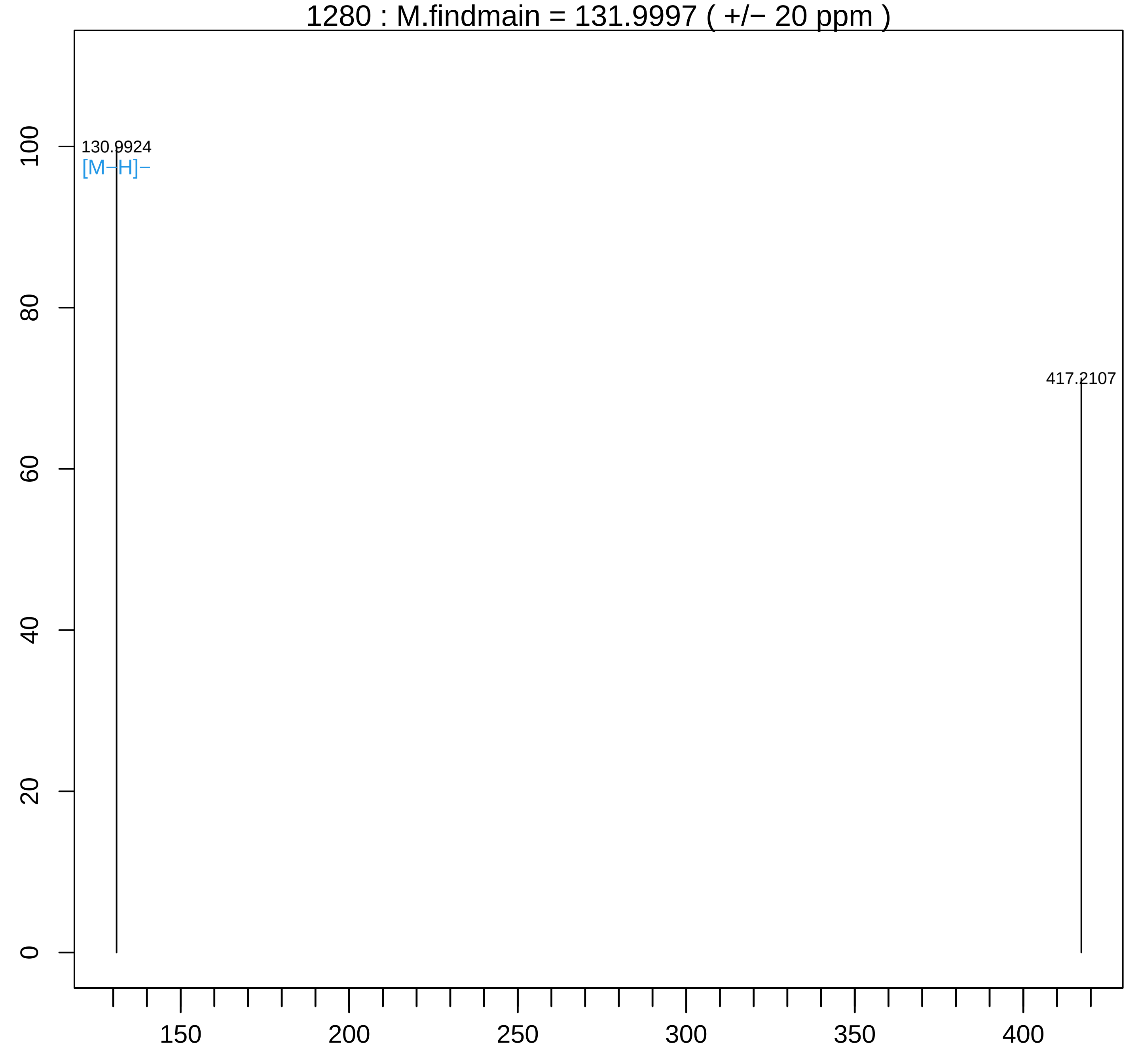
1280 : M.findmain = 131.9997 (+/− 20 ppm)
130.9924
[M−H]−
417.2107
0
20
40
60
80
100
150
200
250
300
350
400

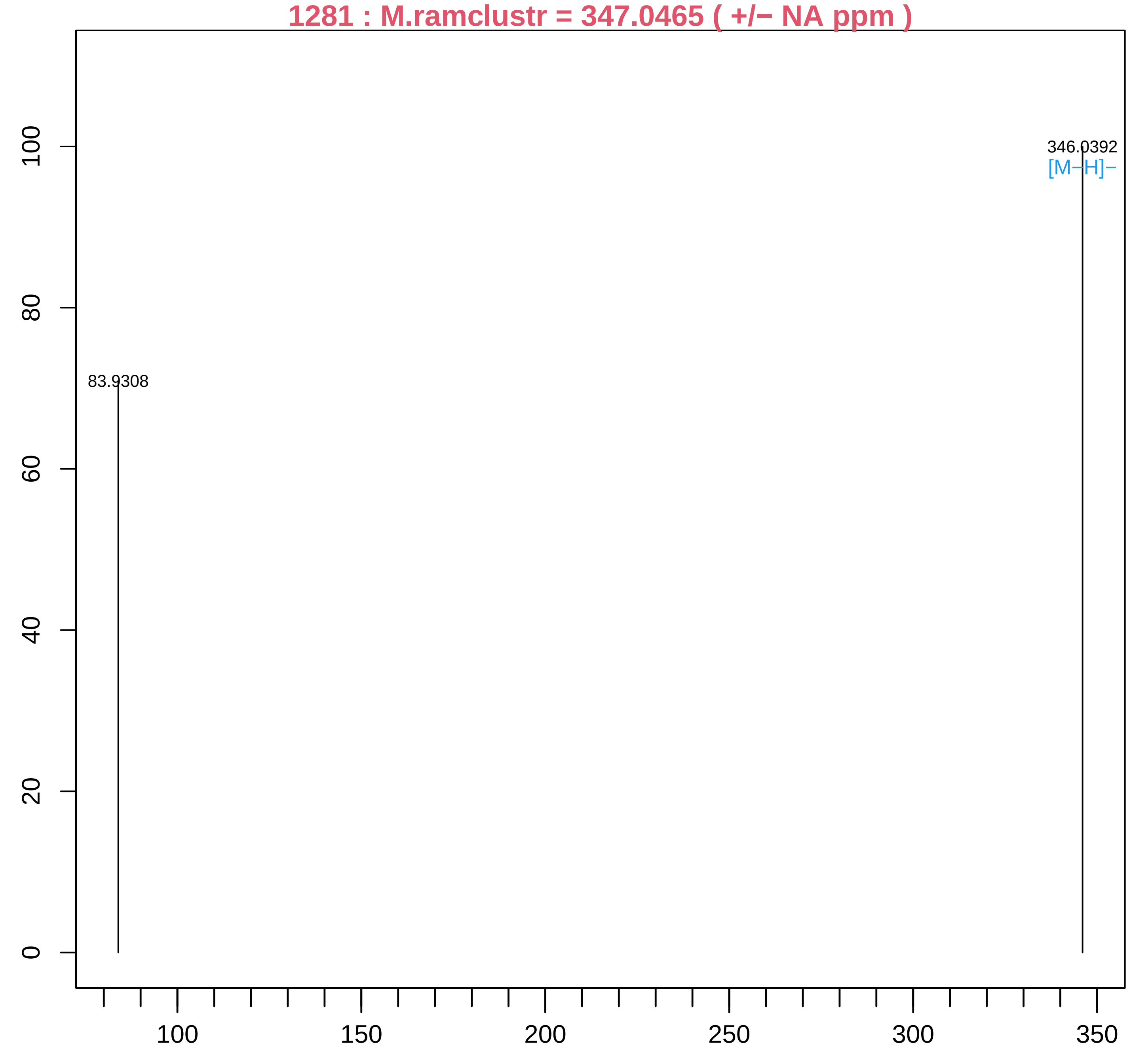
1281 : M.ramclustr = 347.0465 (+/− NA ppm)
346.0392
[M−H]−
83.9308
0
20
40
60
80
100
100
150
200
250
300
350

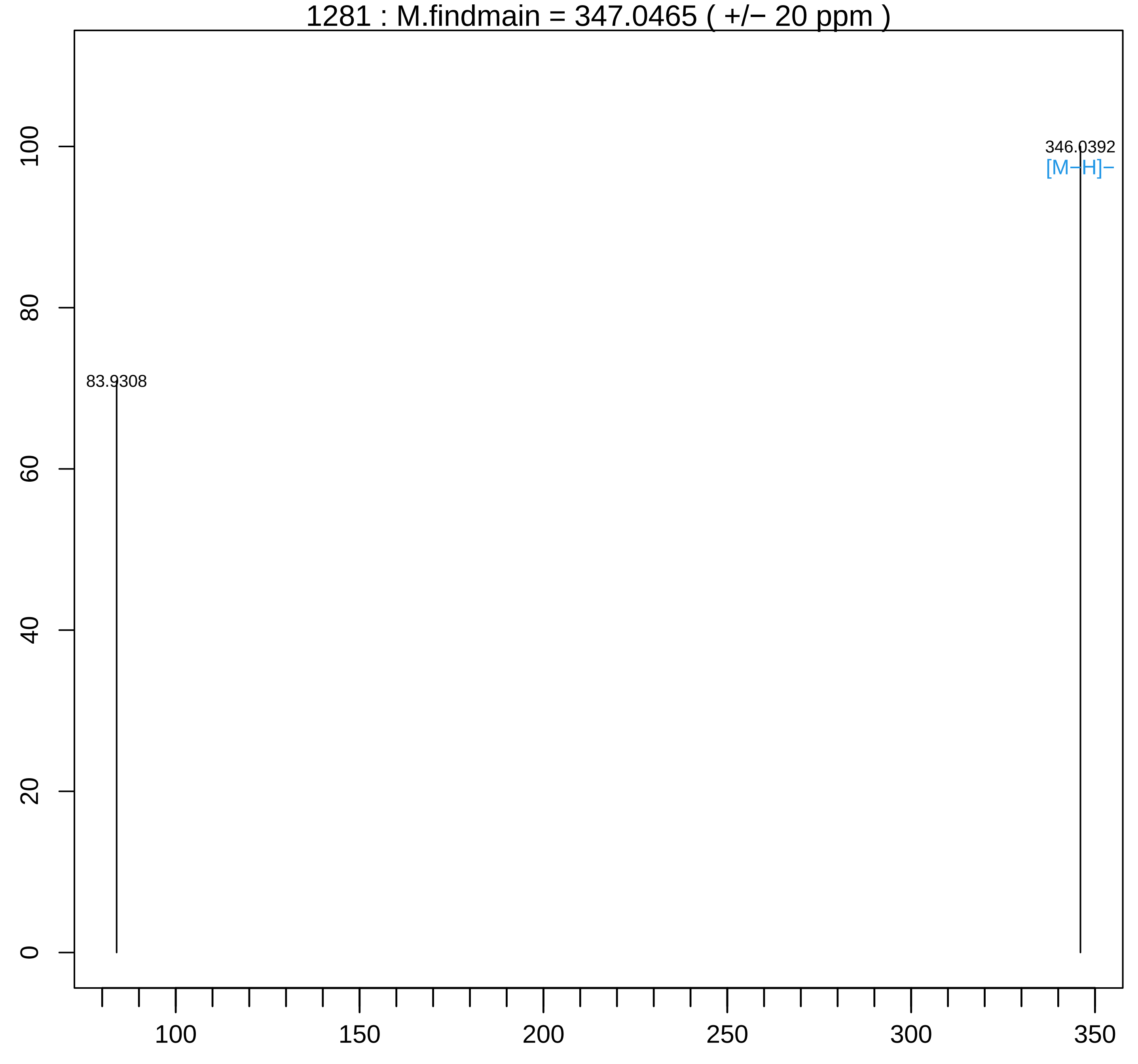
1281 : M.findmain = 347.0465 (+/− 20 ppm)
346.0392
[M−H]−
83.9308
0
20
40
60
80
100
100
150
200
250
300
350

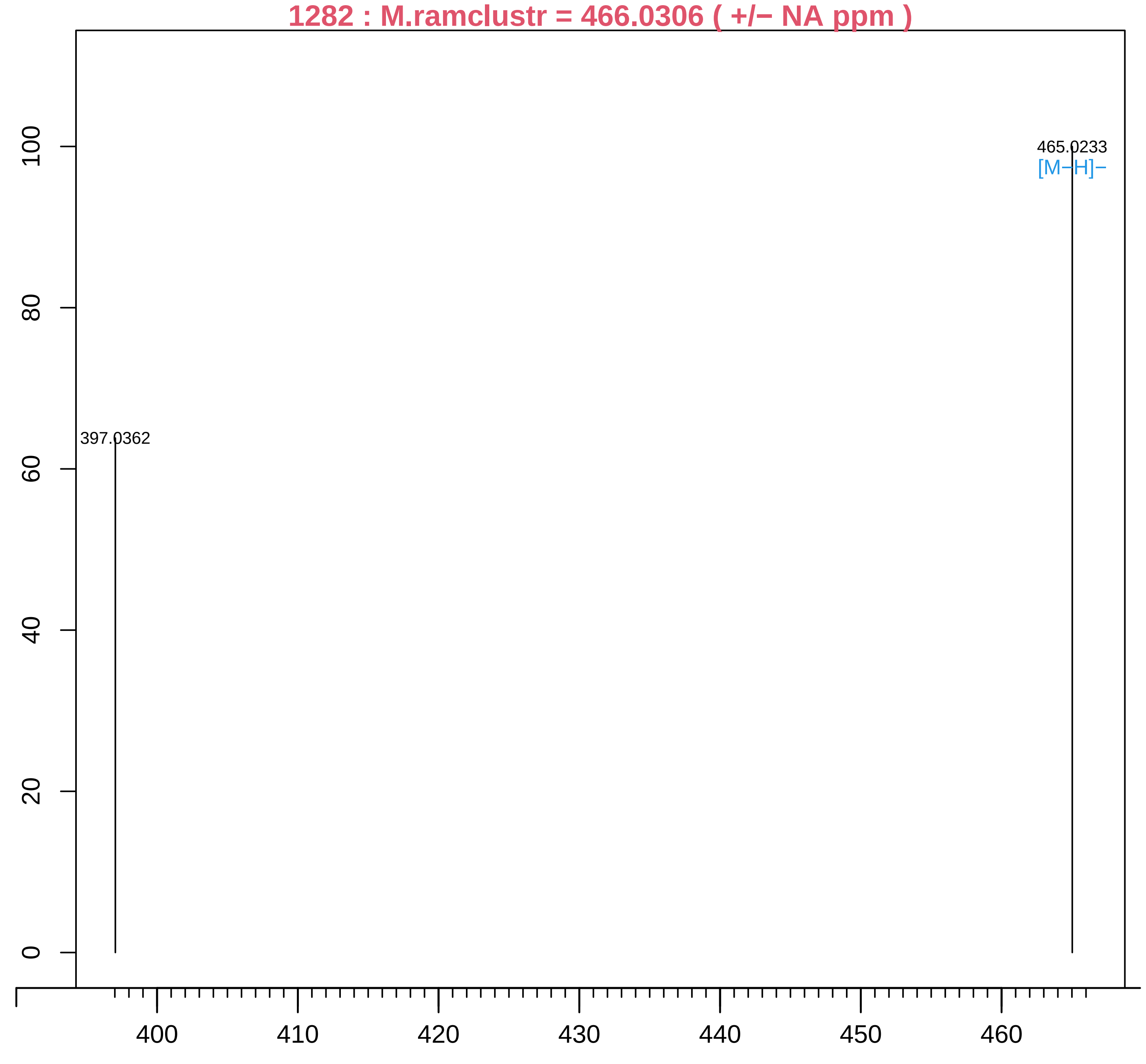
1282 : M.ramclustr = 466.0306 (+/− NA ppm)
465.0233
[M−H]−
397.0362
0
20
40
60
80
100
400
410
420
430
440
450
460

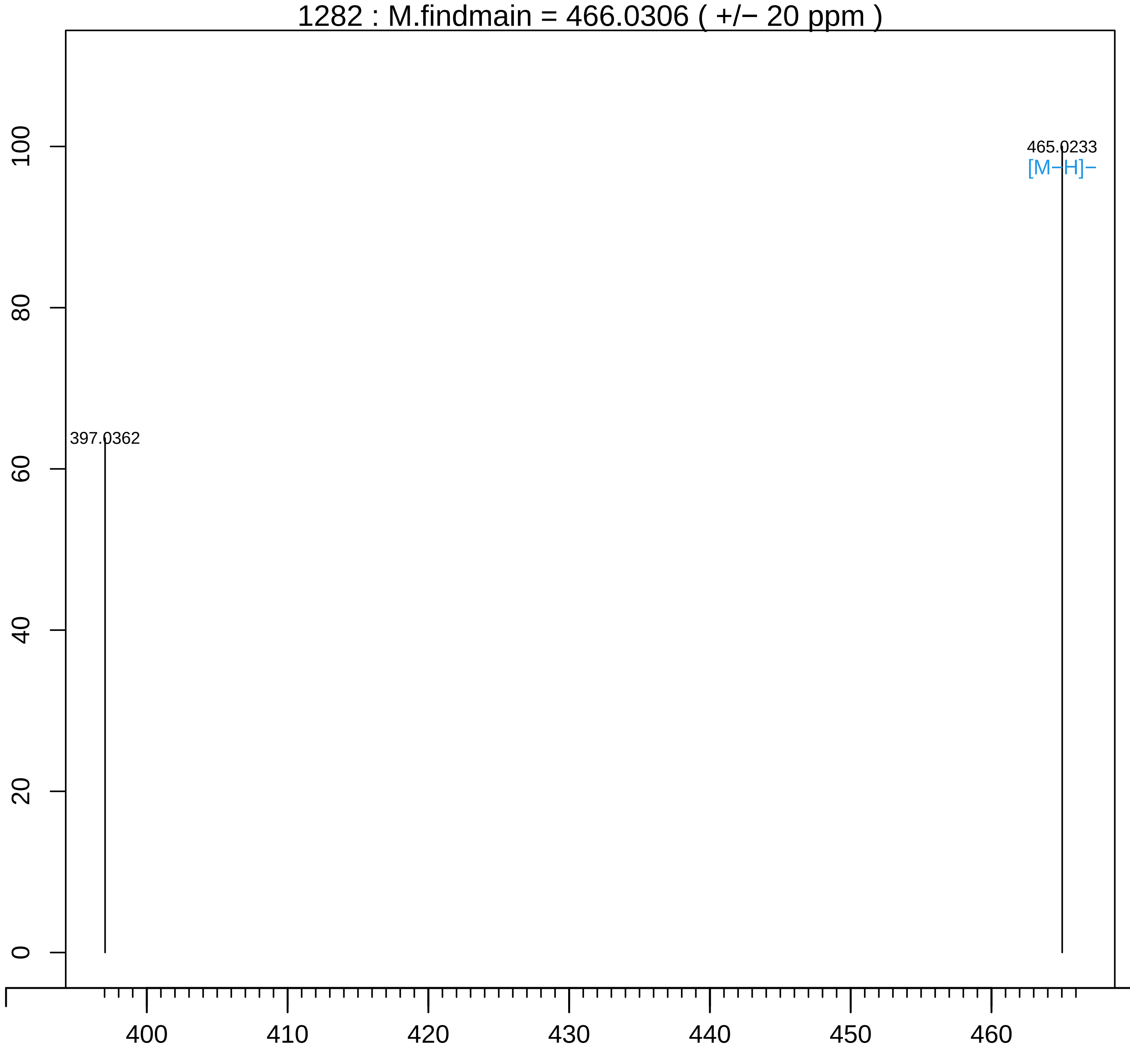
1282 : M.findmain = 466.0306 (+/− 20 ppm)
465.0233
[M−H]−
397.0362
0
20
40
60
80
100
400
410
420
430
440
450
460

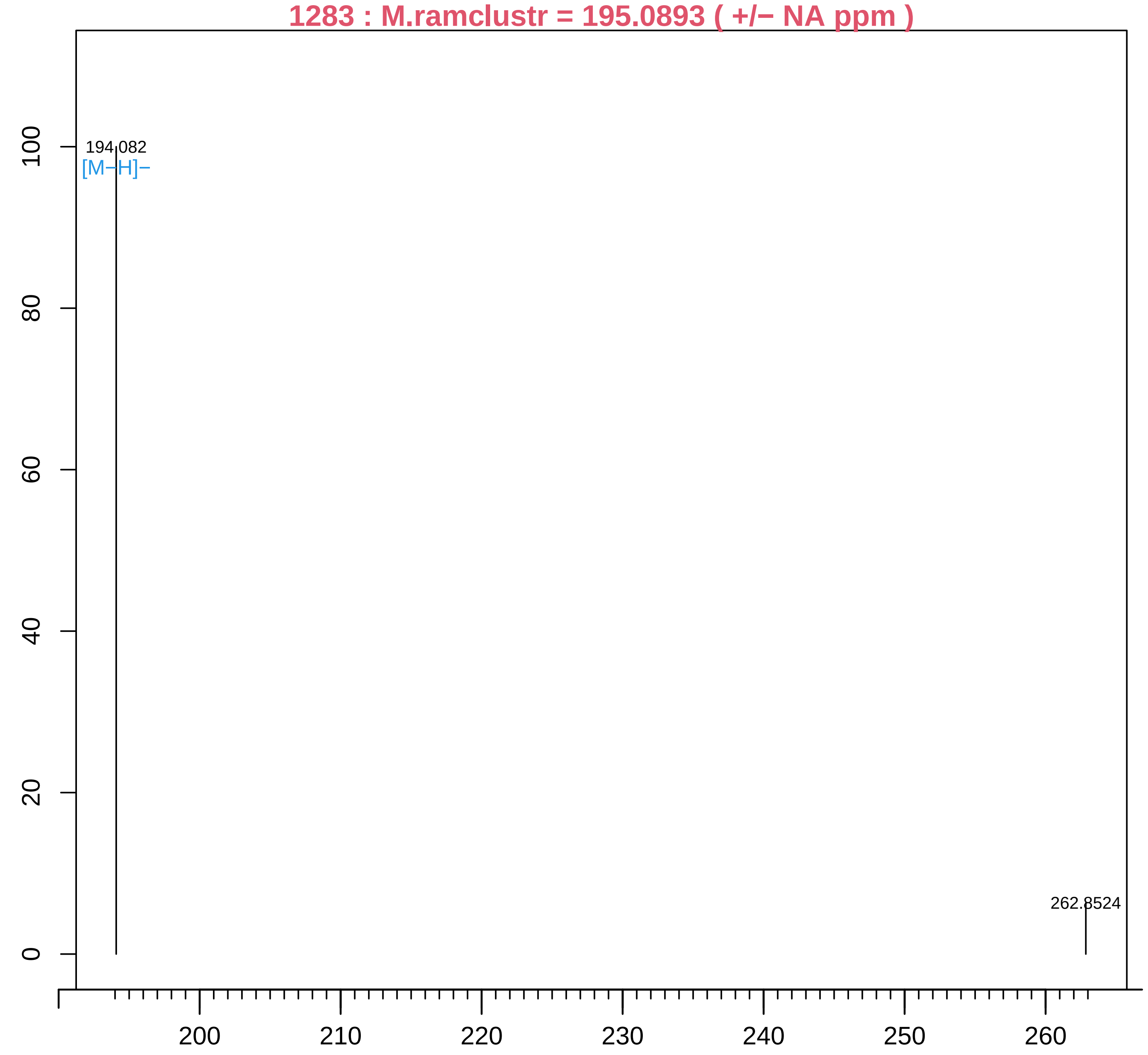
1283 : M.ramclustr = 195.0893 (+/− NA ppm)
194.082
[M−H]−
262.8524
0
20
40
60
80
100
200
210
220
230
240
250
260

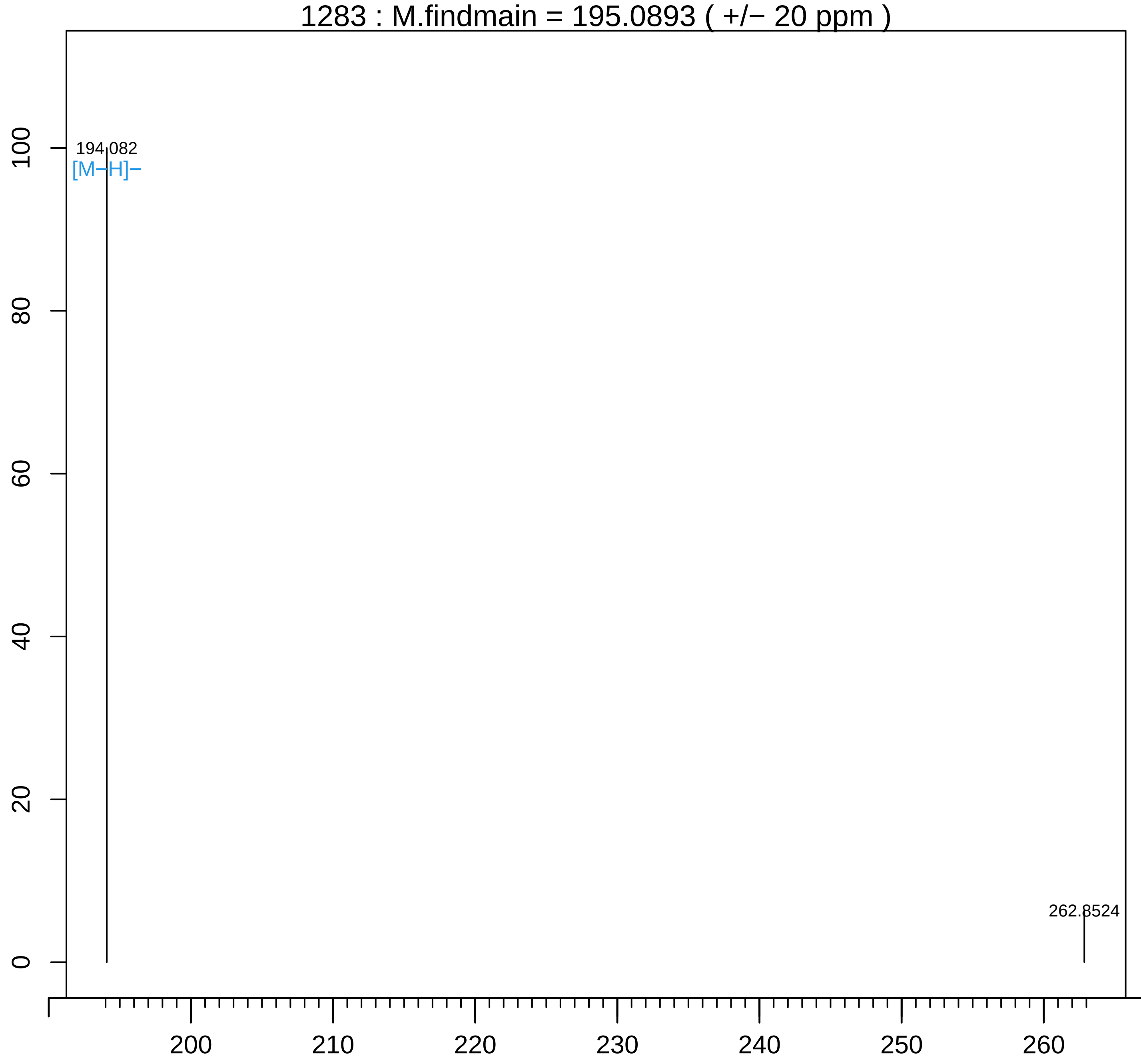
1283 : M.findmain = 195.0893 (+/− 20 ppm)
194.082
[M−H]−
262.8524
0
20
40
60
80
100
200
210
220
230
240
250
260

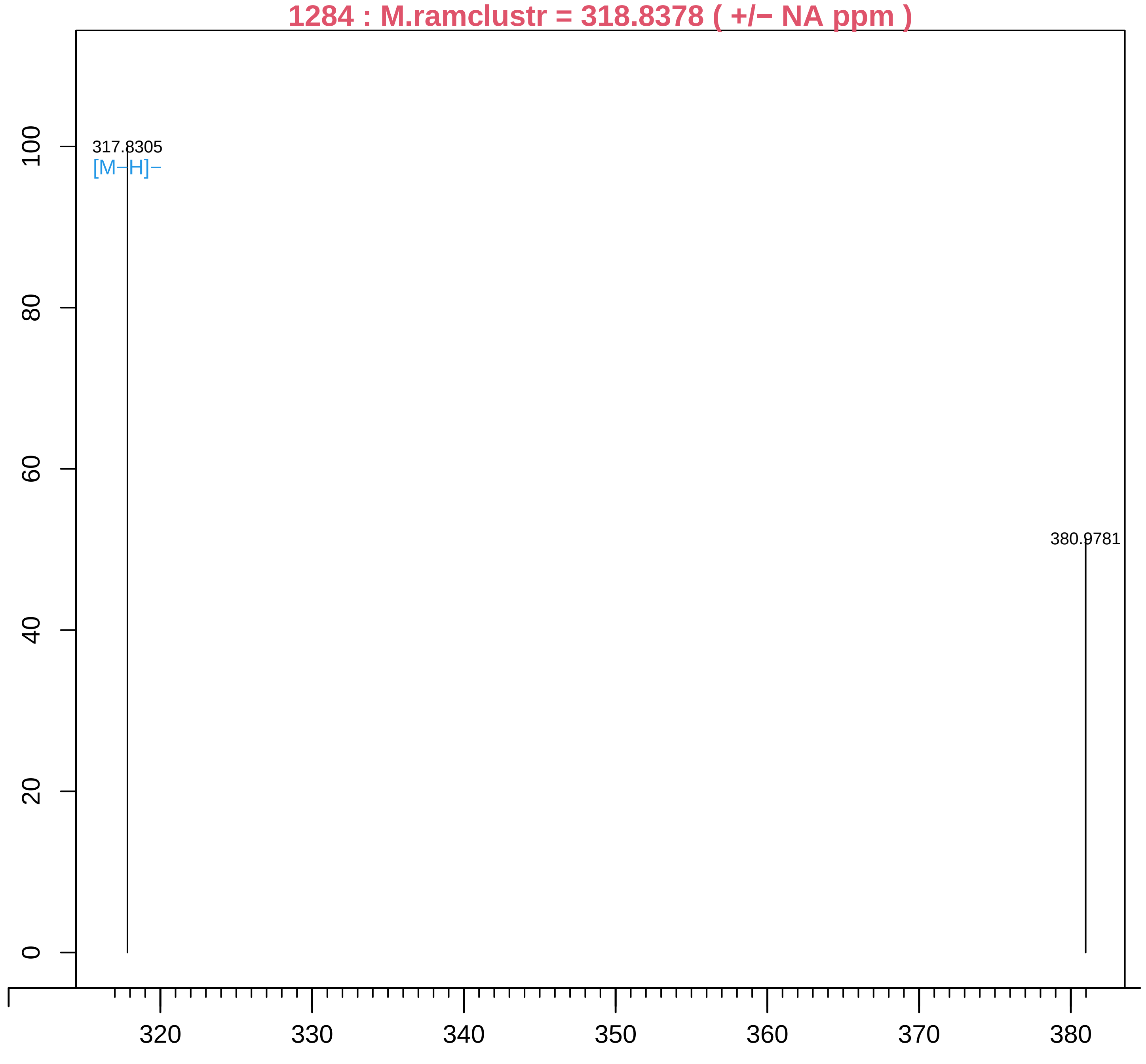
1284 : M.ramclustr = 318.8378 (+/− NA ppm)
317.8305
[M−H]−
380.9781
0
20
40
60
80
100
320
330
340
350
360
370
380
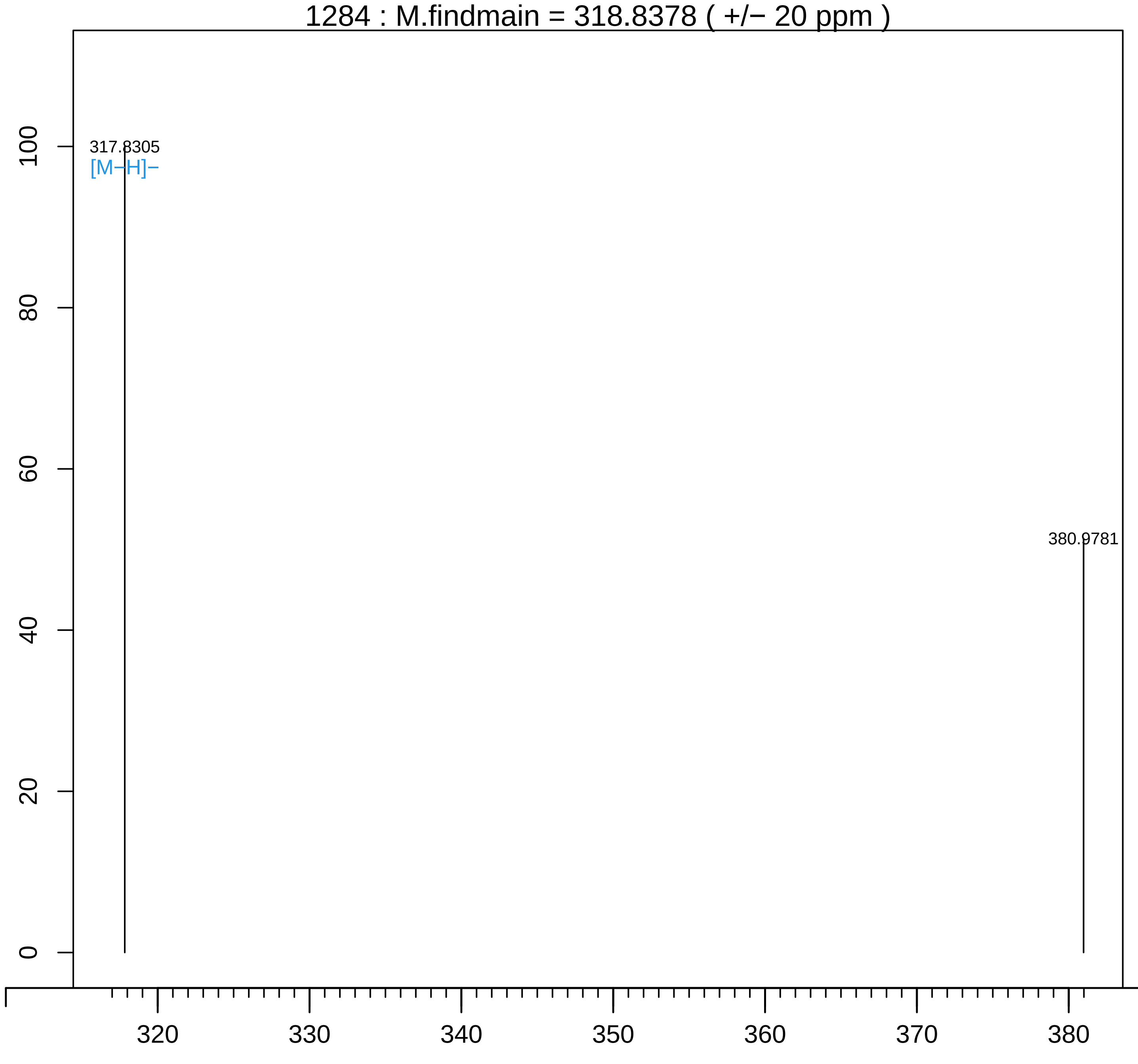
1284 : M.findmain = 318.8378 (+/− 20 ppm)
317.8305
[M−H]−
380.9781
0
20
40
60
80
100
320
330
340
350
360
370
380

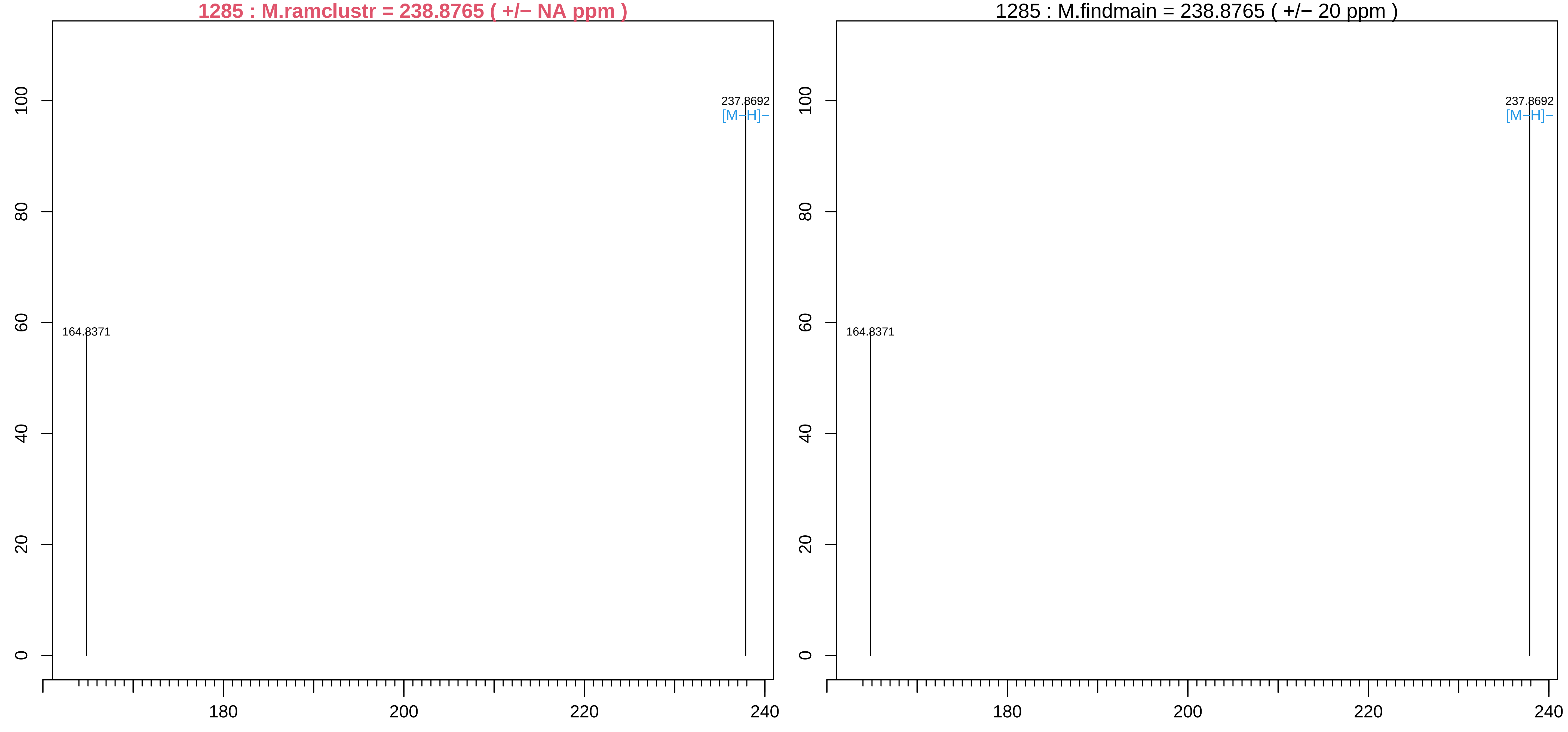

1285 : M.ramclustr = 238.8765 (+/− NA ppm)
237.8692
[M−H]−
164.8371
1285 : M.findmain = 238.8765 (+/− 20 ppm)
237.8692
[M−H]−
164.8371
0
20
40
60
80
100
180
200
220
240

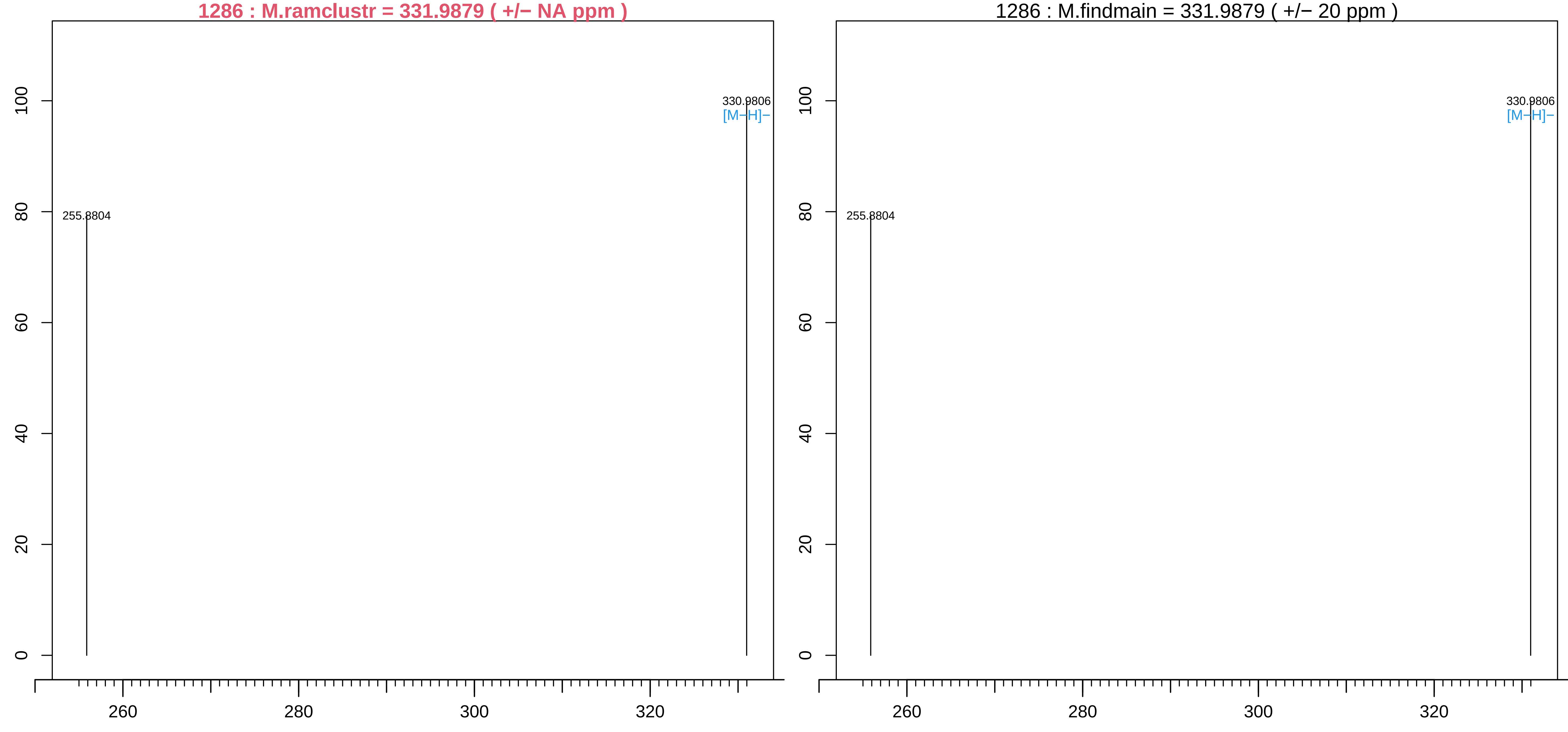
1286 : M.ramclustr = 331.9879 (+/− NA ppm)
1286 : M.findmain = 331.9879 (+/− 20 ppm)
330.9806
[M−H]−
255.8804
0
20
40
60
80
100
260
280
300
320

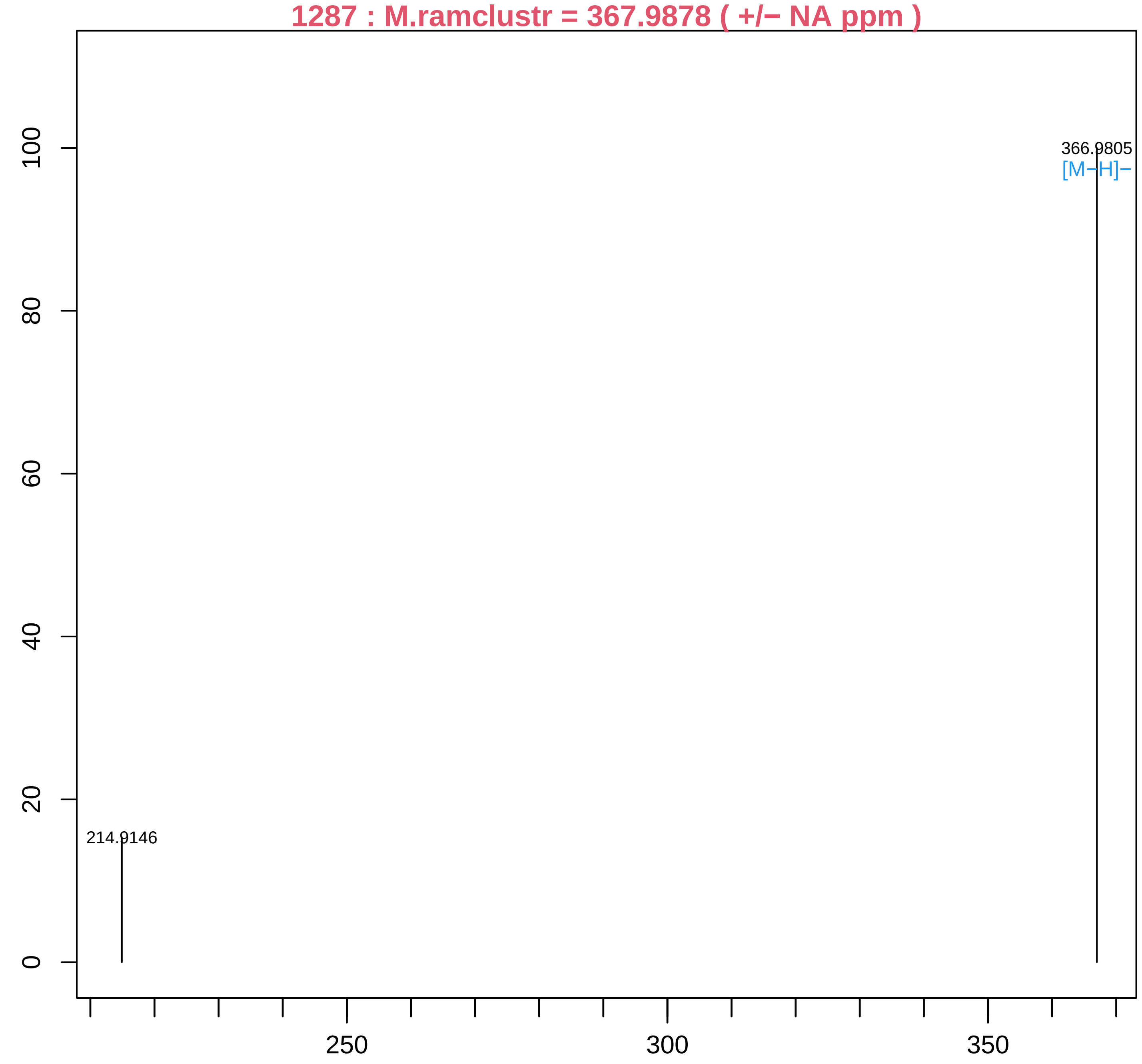
1287 : M.ramclustr = 367.9878 (+/− NA ppm)
366.9805
[M−H]−
214.9146
0
20
40
60
80
100
250
300
350

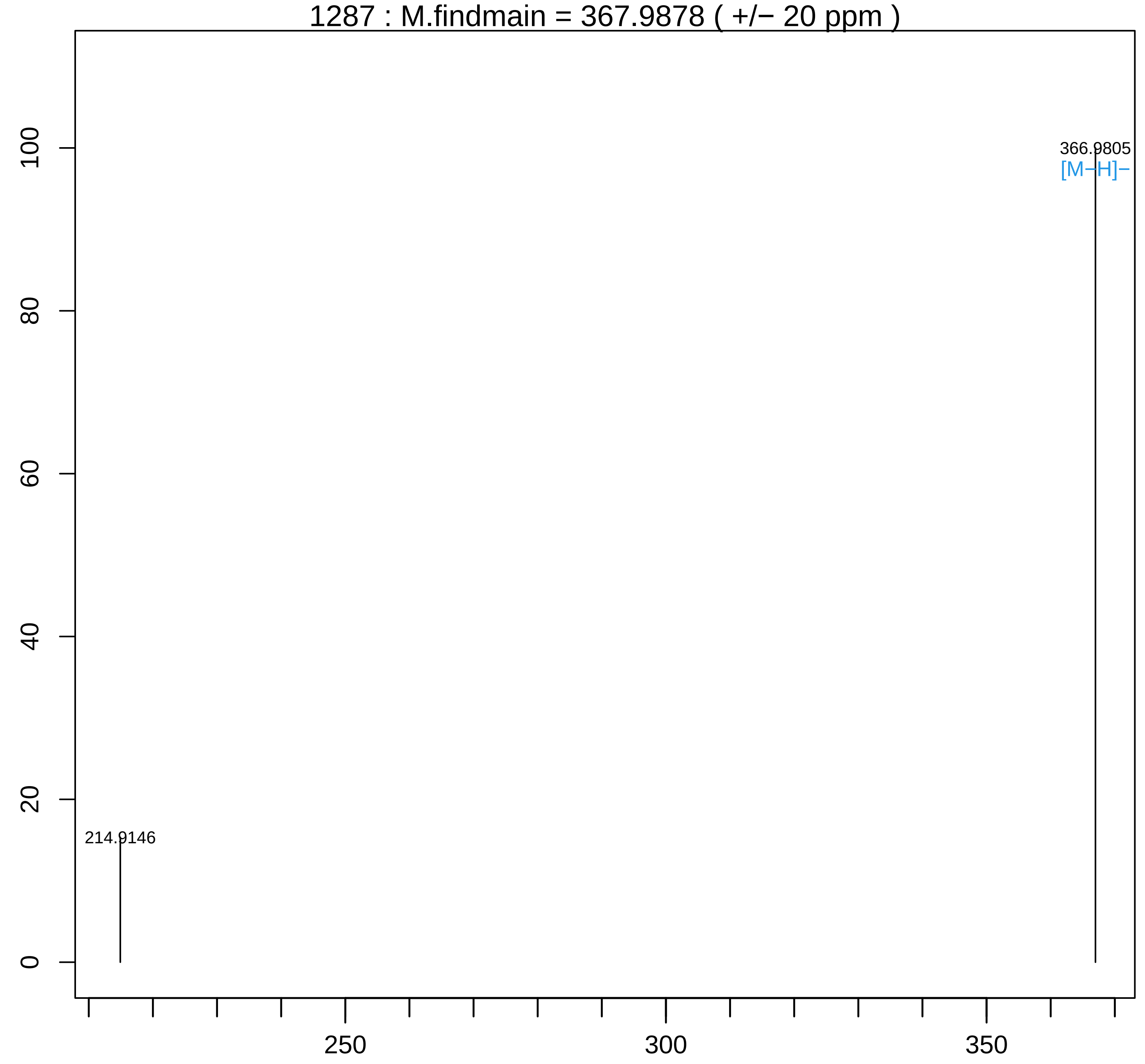
1287 : M.findmain = 367.9878 (+/− 20 ppm)
366.9805
[M−H]−
214.9146
0
20
40
60
80
100
250
300
350

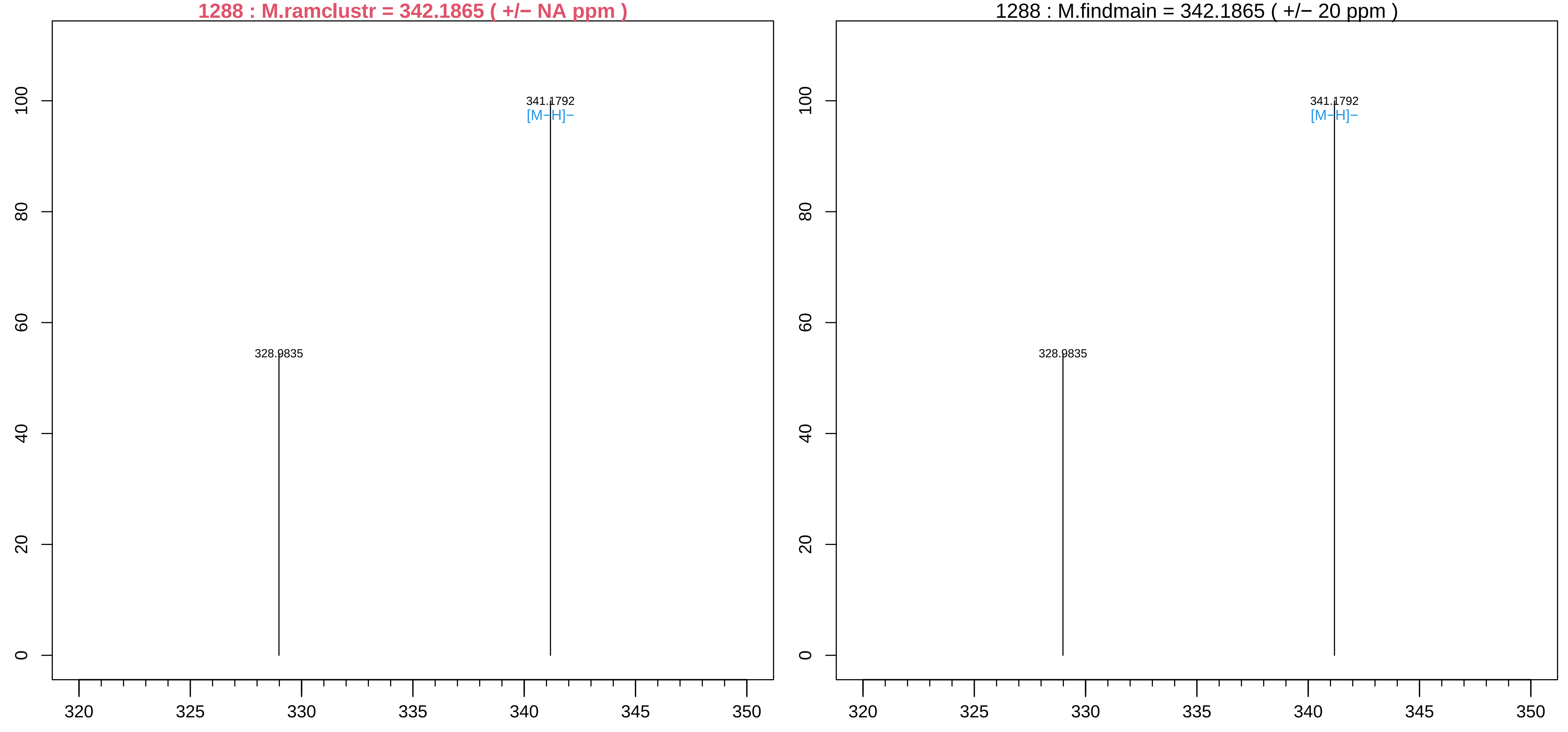
1288 : M.ramclustr = 342.1865 (+/− NA ppm)
1288 : M.findmain = 342.1865 (+/− 20 ppm)
341.1792
[M−H]−
328.9835
0
20
40
60
80
100
320
325
330
335
340
345
350

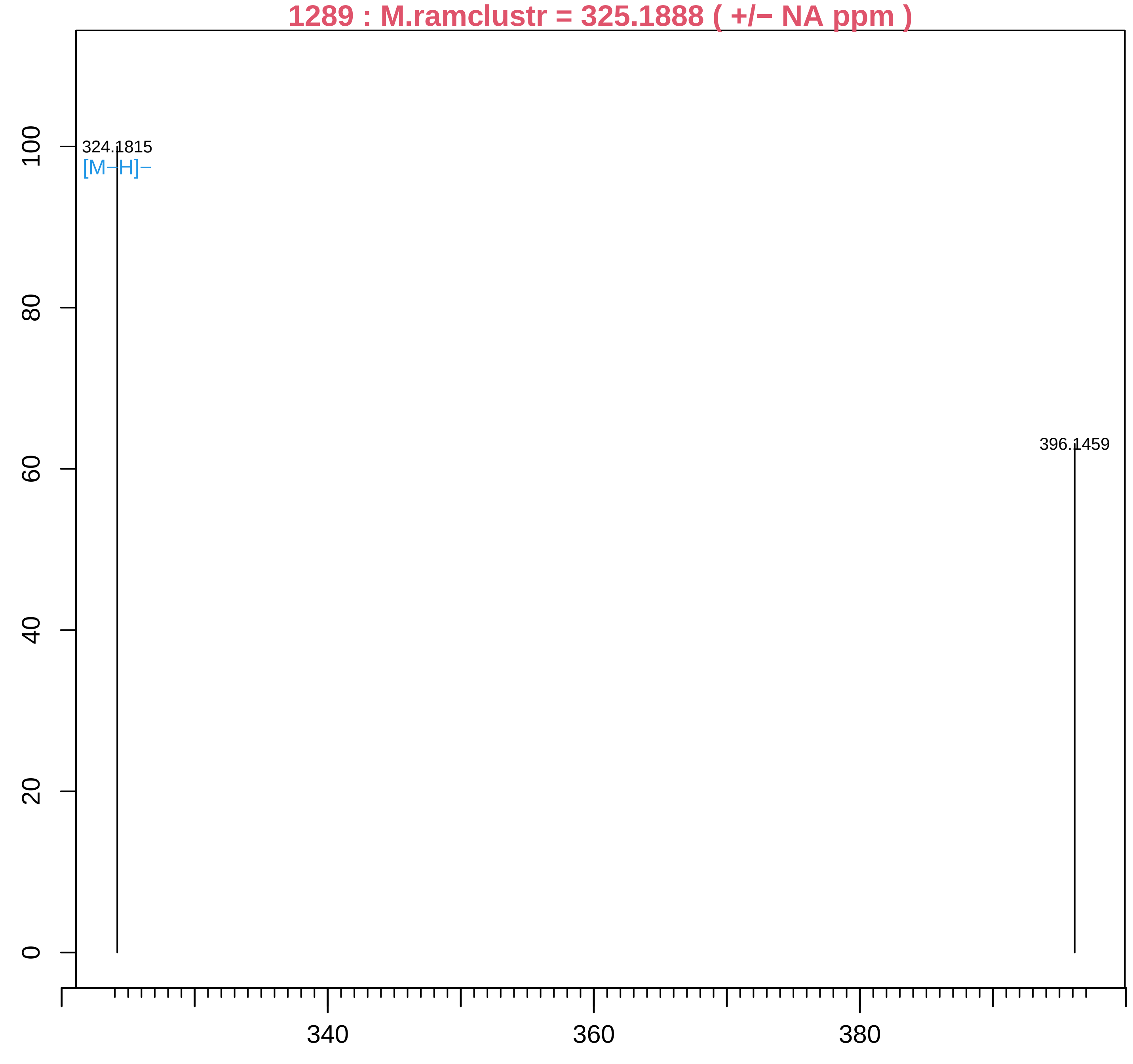
1289 : M.ramclustr = 325.1888 (+/− NA ppm)
324.1815
[M−H]−
396.1459
0
20
40
60
80
100
340
360
380
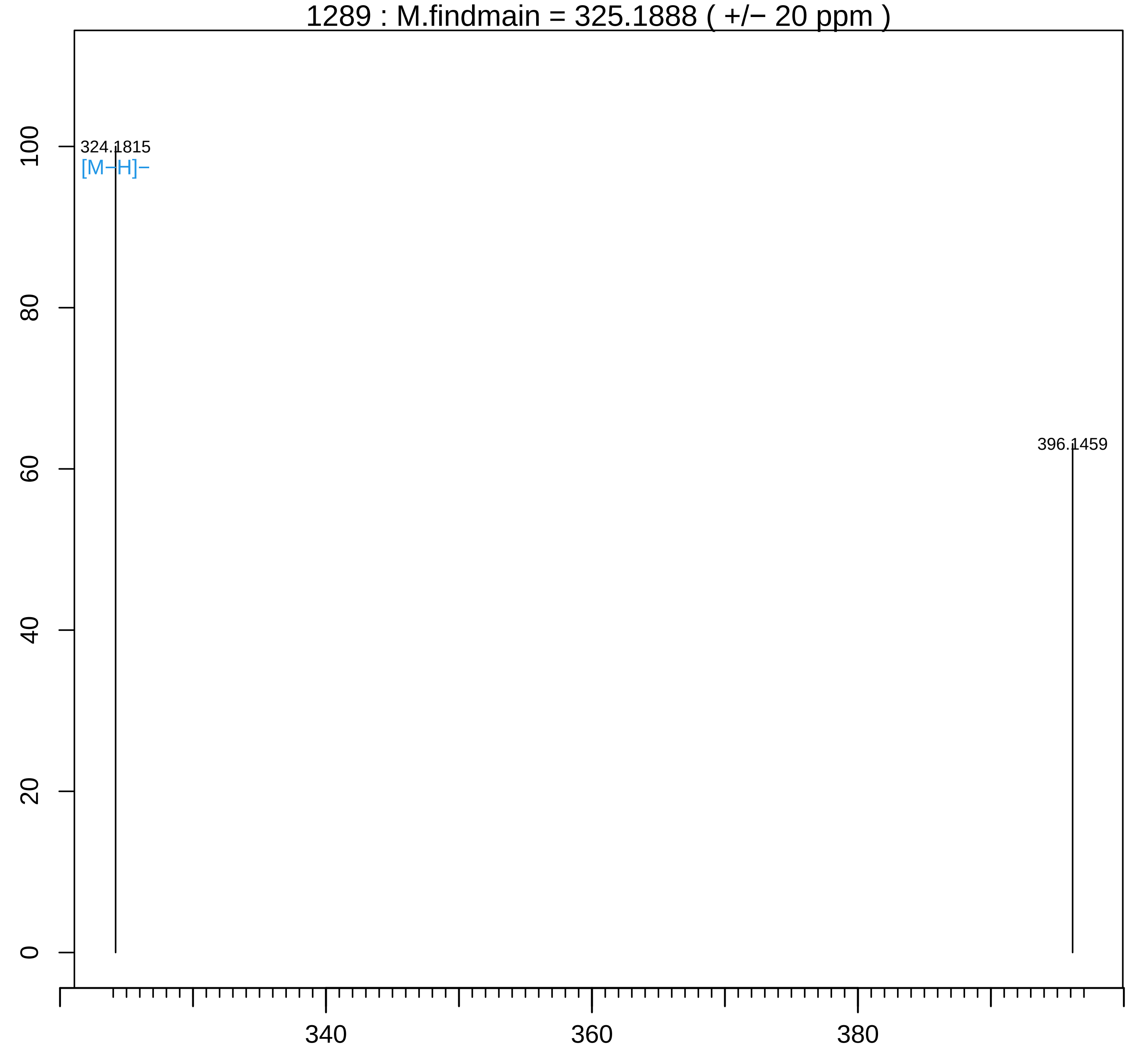
1289 : M.findmain = 325.1888 (+/− 20 ppm)
324.1815
[M−H]−
396.1459
0
20
40
60
80
100
340
360
380

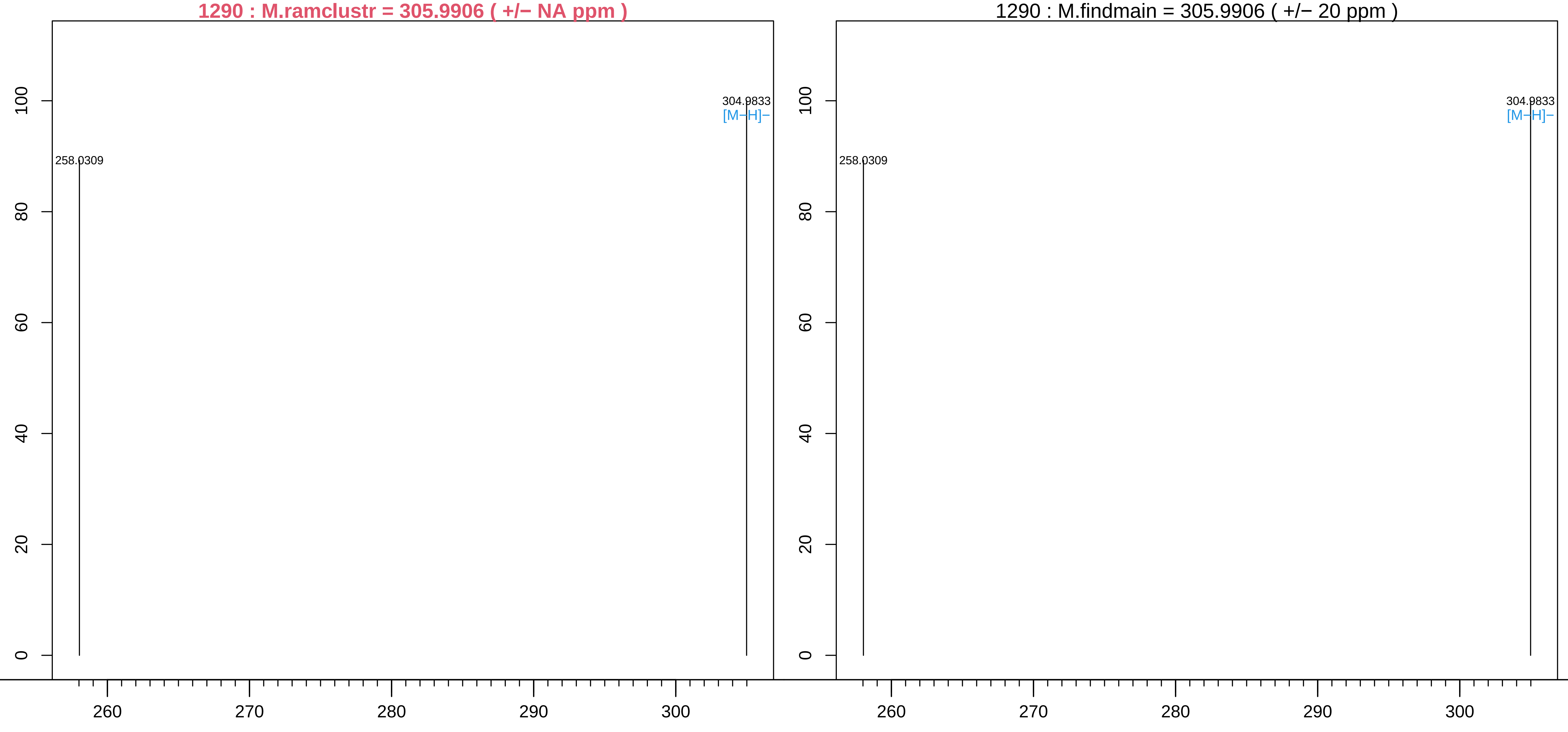
1290 : M.ramclustr = 305.9906 (+/− NA ppm)
304.9833
[M−H]−
258.0309
1290 : M.findmain = 305.9906 (+/− 20 ppm)
304.9833
[M−H]−
258.0309
0
20
40
60
80
100
260
270
280
290
300

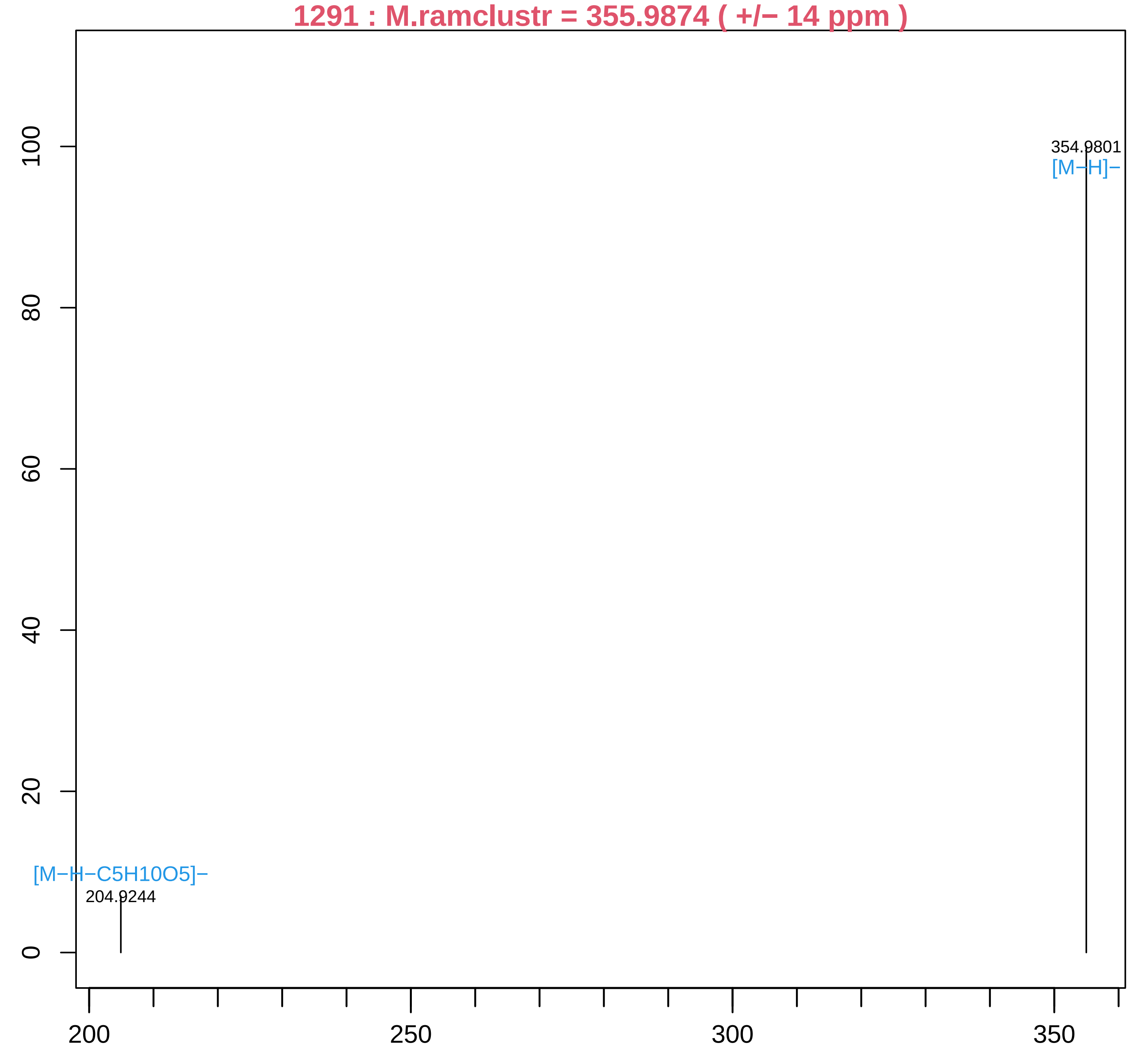

1291 : M.ramclustr = 355.9874 (+/− 14 ppm)
354.9801
[M−H]−
[M−H−C5H10O5]−
204.9244
0
20
40
60
80
100
200
250
300
350

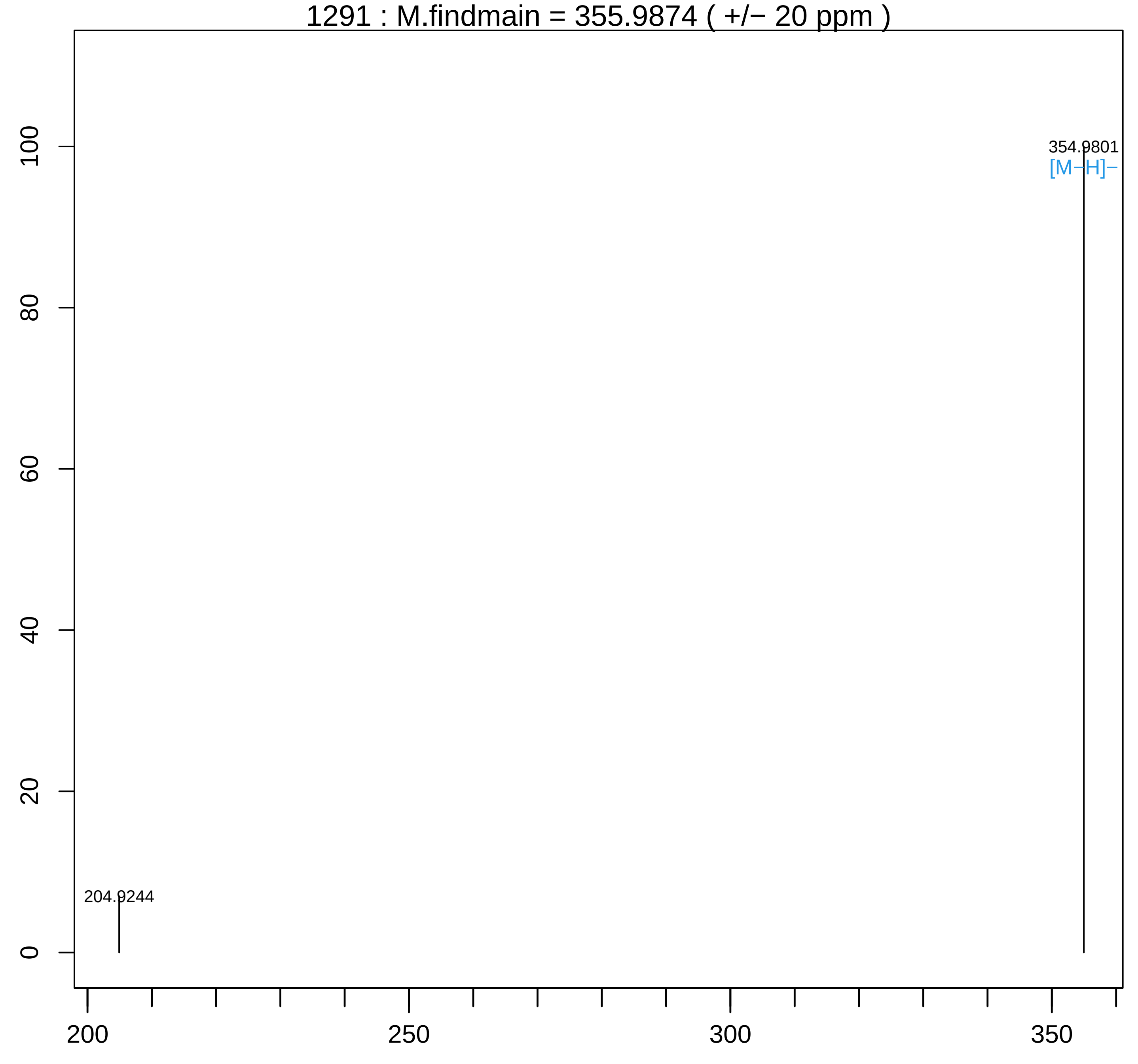

1291 : M.findmain = 355.9874 (+/− 20 ppm)
354.9801
[M−H]−
204.9244
0
20
40
60
80
100
200
250
300
350

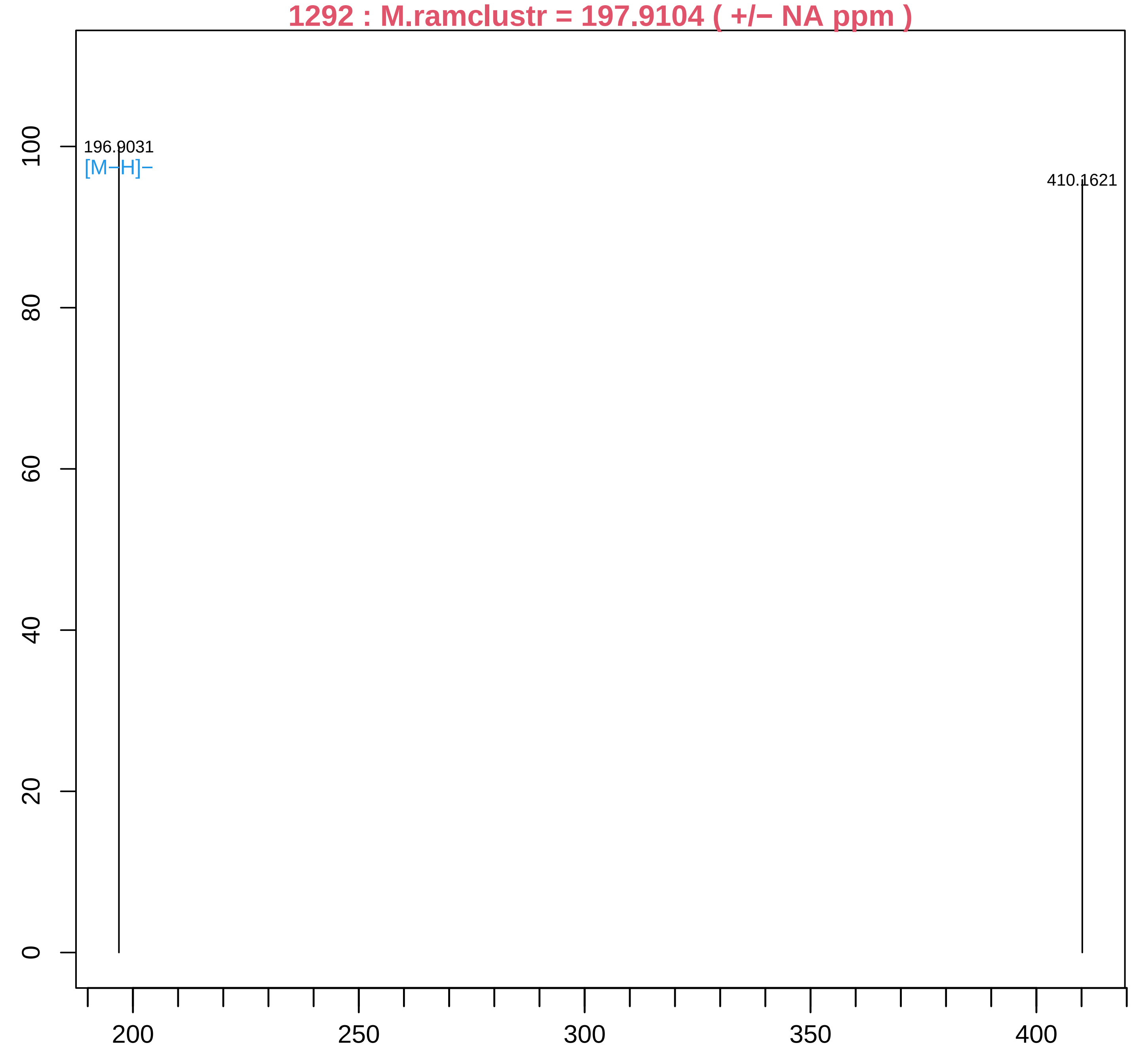

1292 : M.ramclustr = 197.9104 (+/− NA ppm)
196.9031
[M−H]−
410.1621
0
20
40
60
80
100
200
250
300
350
400

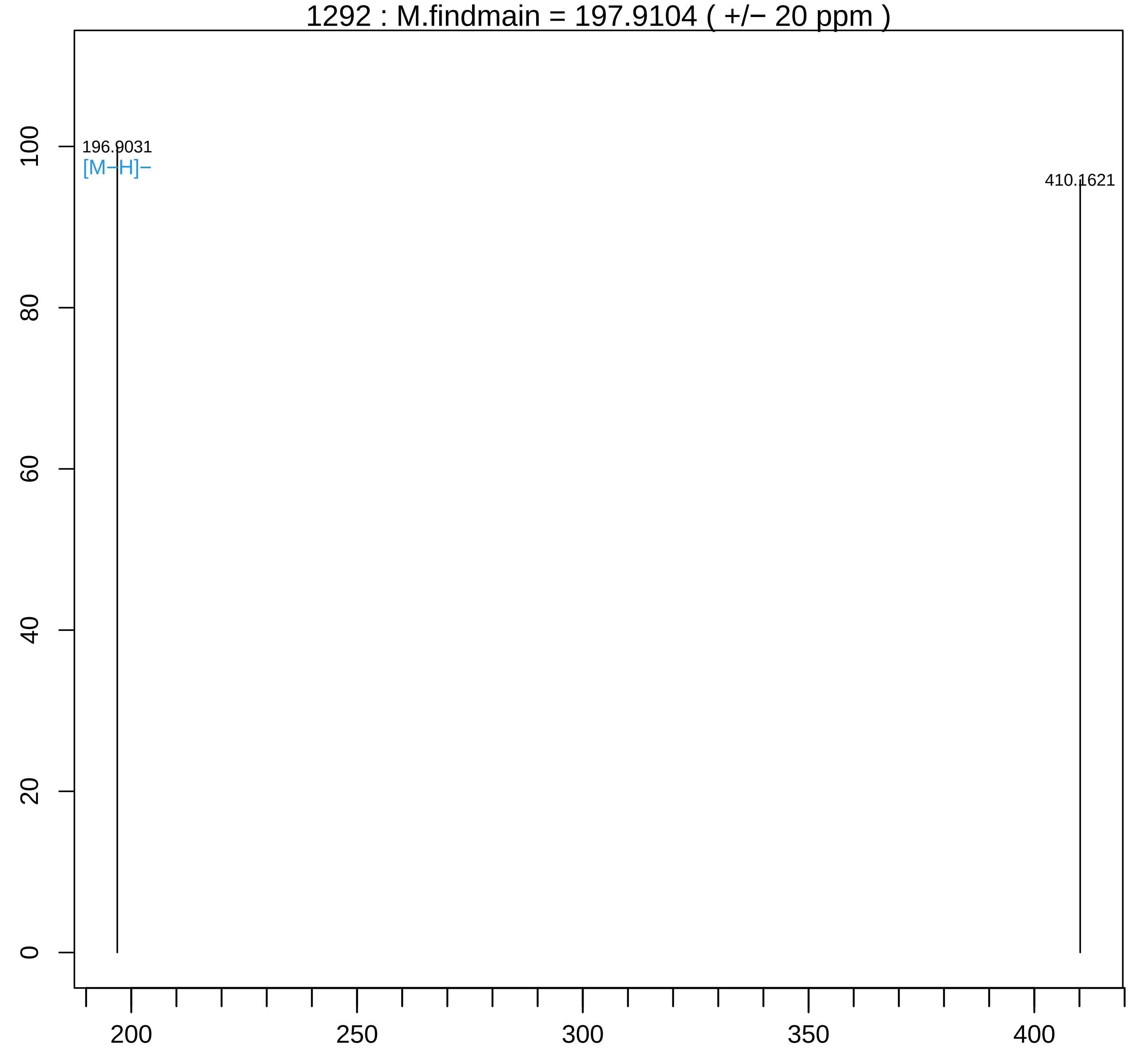

1292 : M.findmain = 197.9104 (+/− 20 ppm)
196.9031
[M−H]−
410.1621
0
20
40
60
80
100
200
250
300
350
400

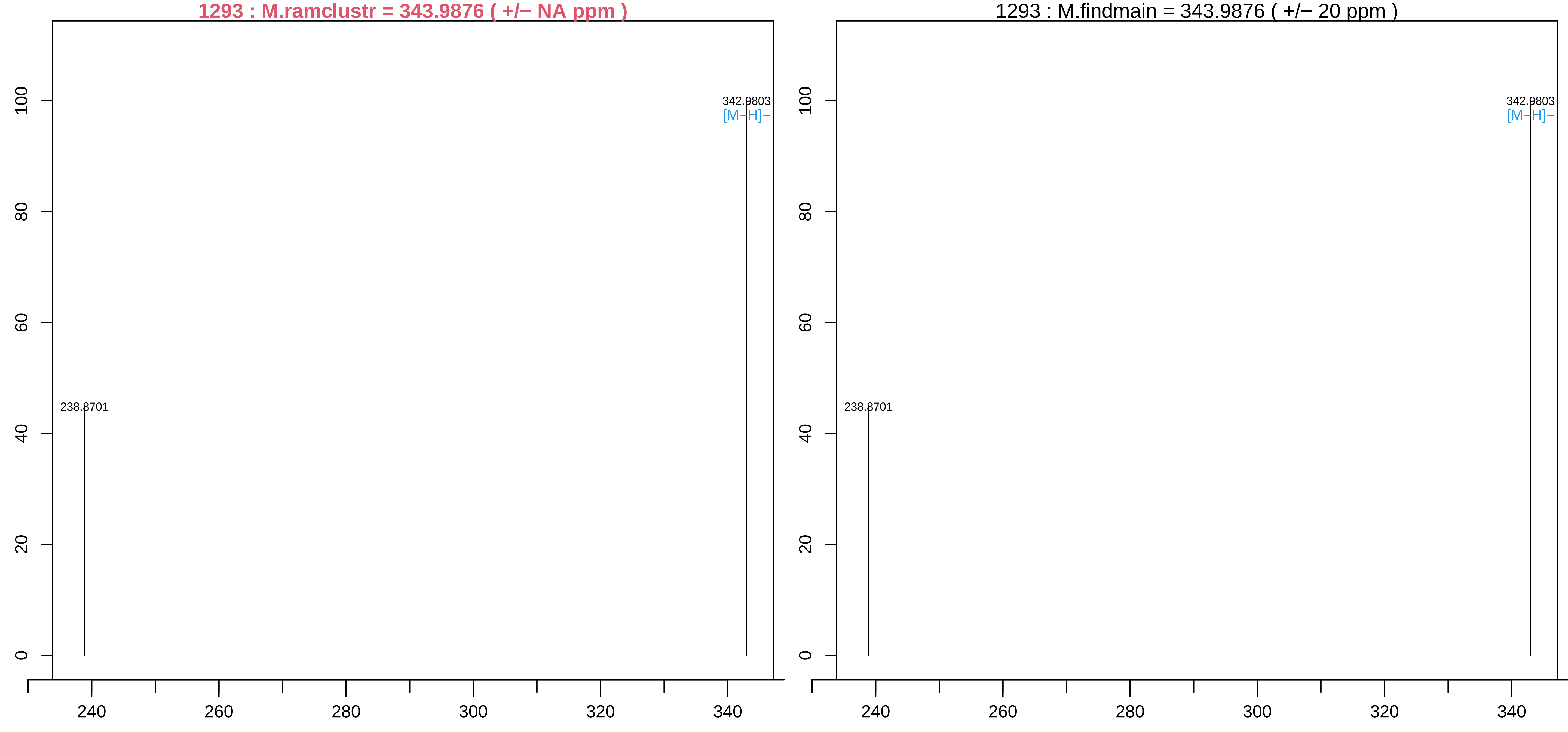
1293 : M.ramclustr = 343.9876 (+/− NA ppm)
342.9803
[M−H]−
238.8701
1293 : M.findmain = 343.9876 (+/− 20 ppm)
342.9803
[M−H]−
238.8701
0
20
40
60
80
100
240
260
280
300
320
340

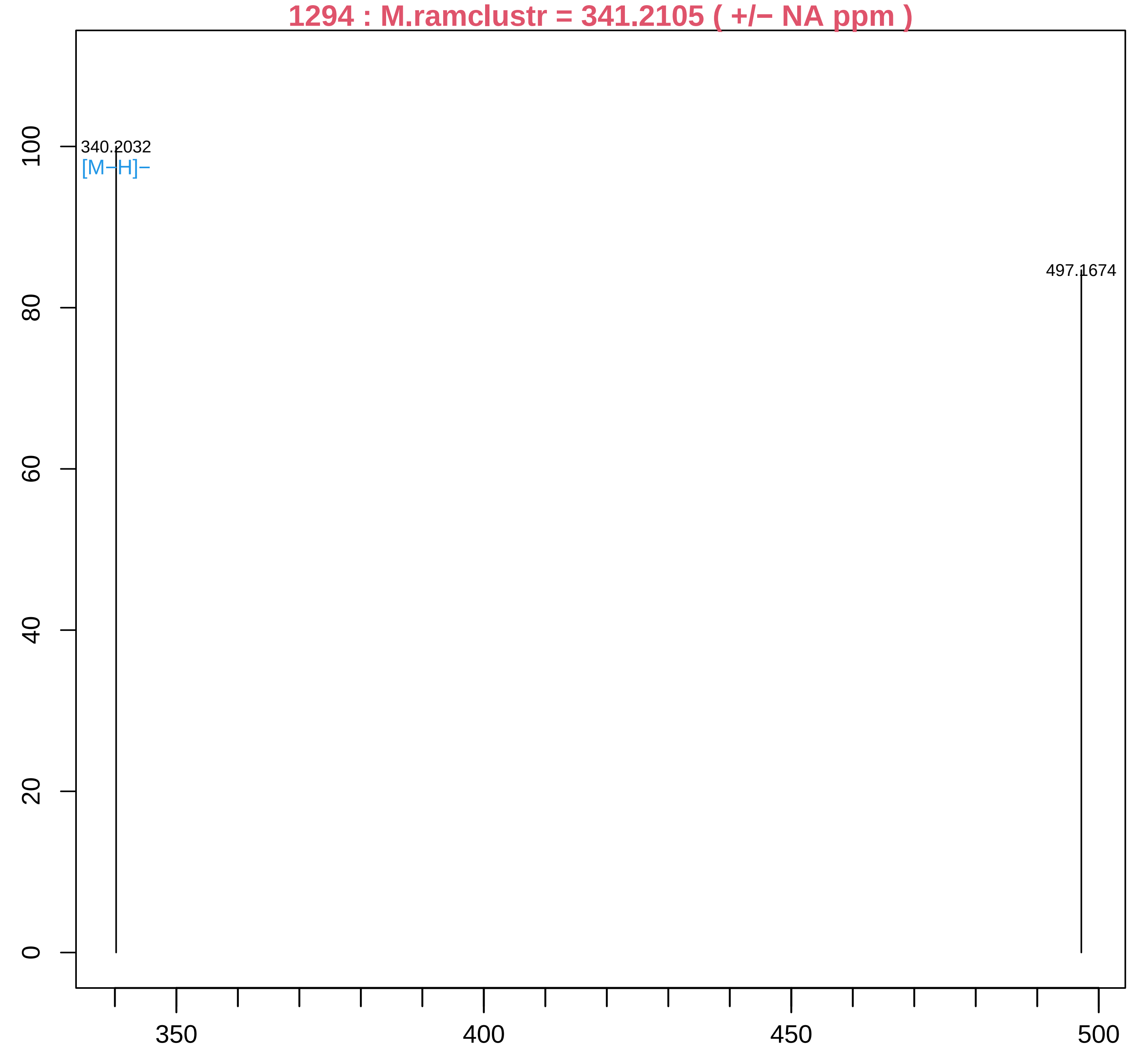

1294 : M.ramclustr = 341.2105 (+/− NA ppm)
340.2032
[M−H]−
497.1674
0
20
40
60
80
100
350
400
450
500

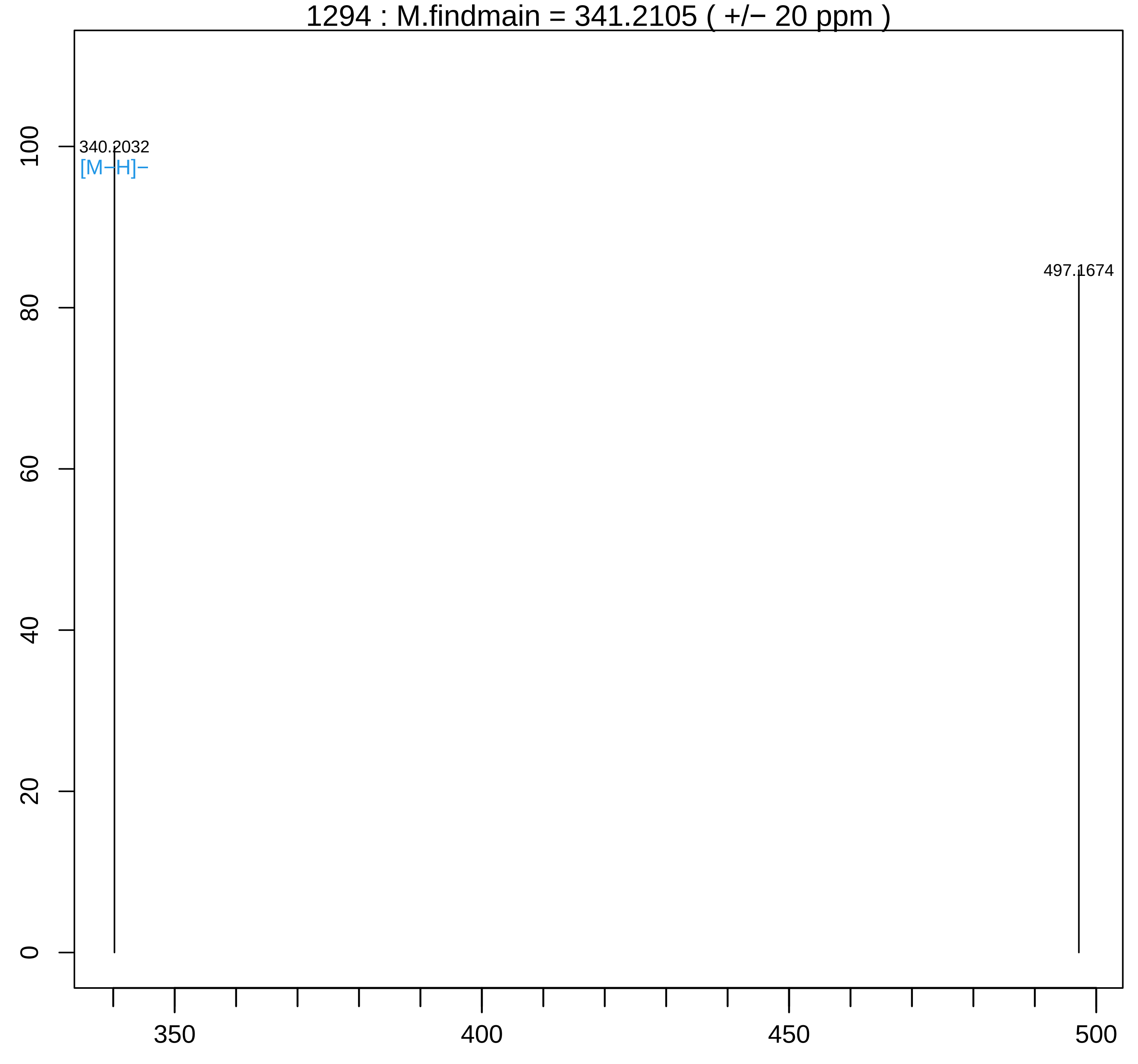

1294 : M.findmain = 341.2105 (+/− 20 ppm)
340.2032
[M−H]−
497.1674
0
20
40
60
80
100
350
400
450
500

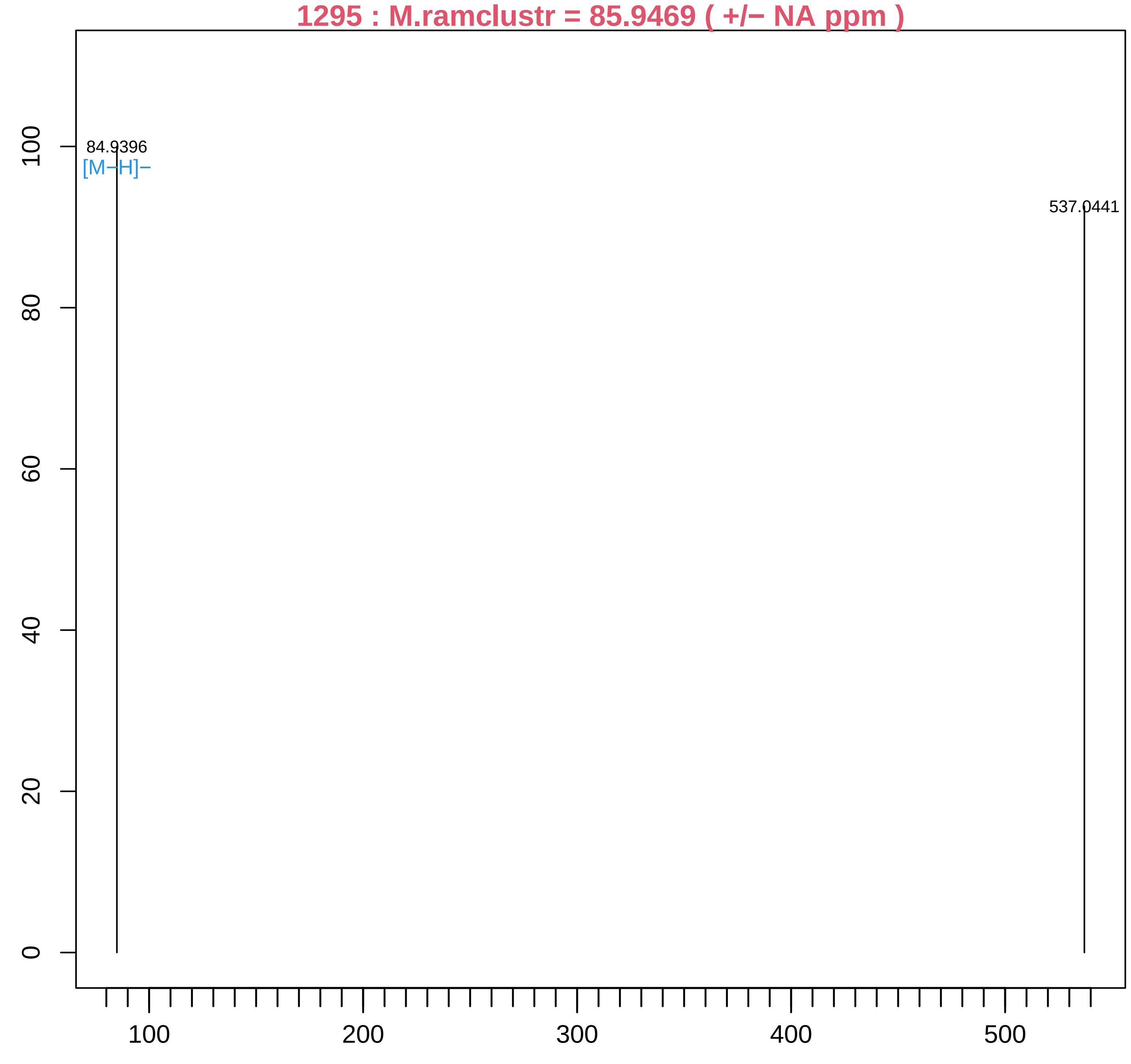
1295 : M.ramclustr = 85.9469 (+/− NA ppm)
84.9396
[M−H]−
537.0441
0
20
40
60
80
100
100
200
300
400
500

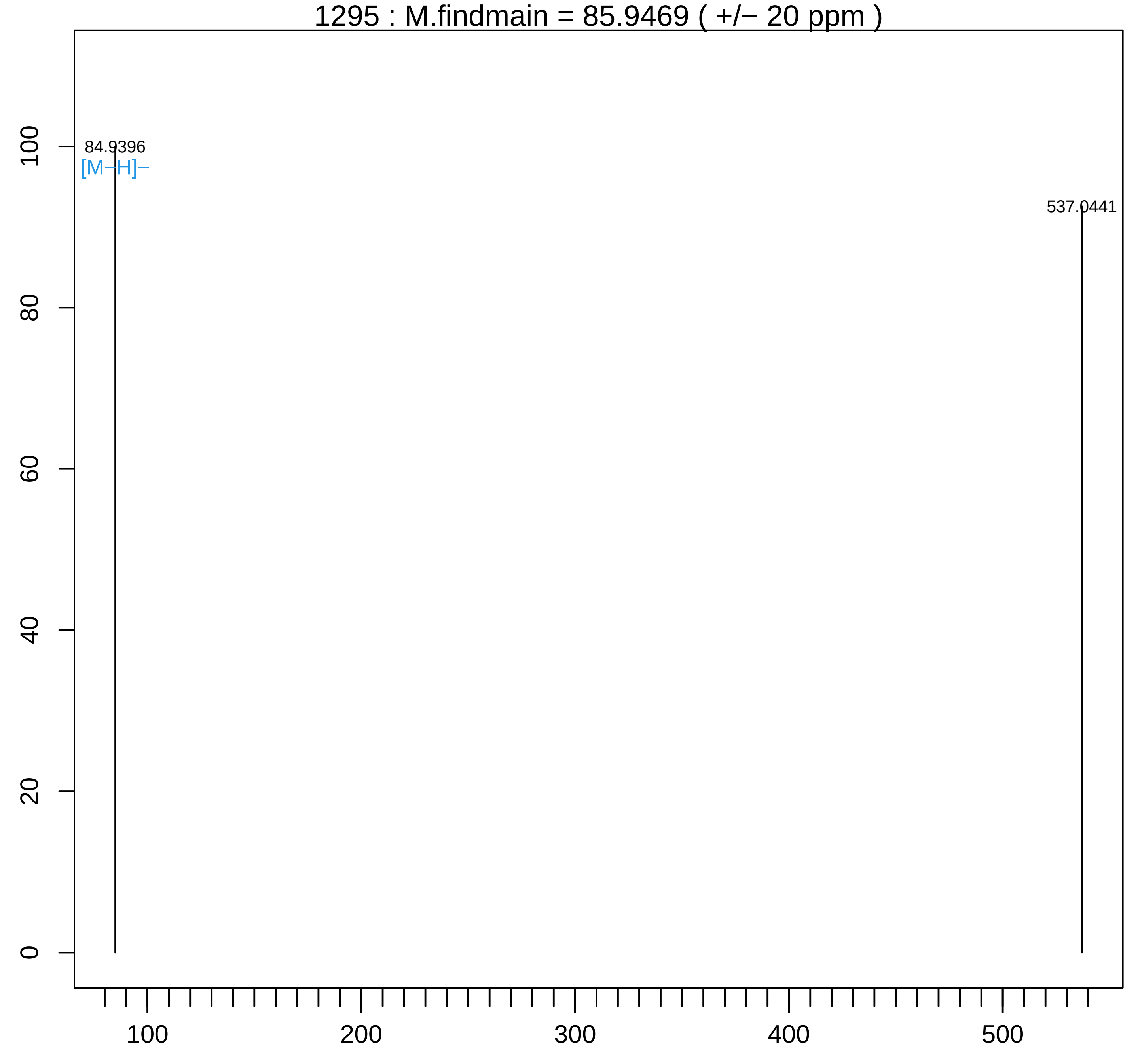
1295 : M.findmain = 85.9469 (+/− 20 ppm)
84.9396
[M−H]−
537.0441
0
20
40
60
80
100
100
200
300
400
500

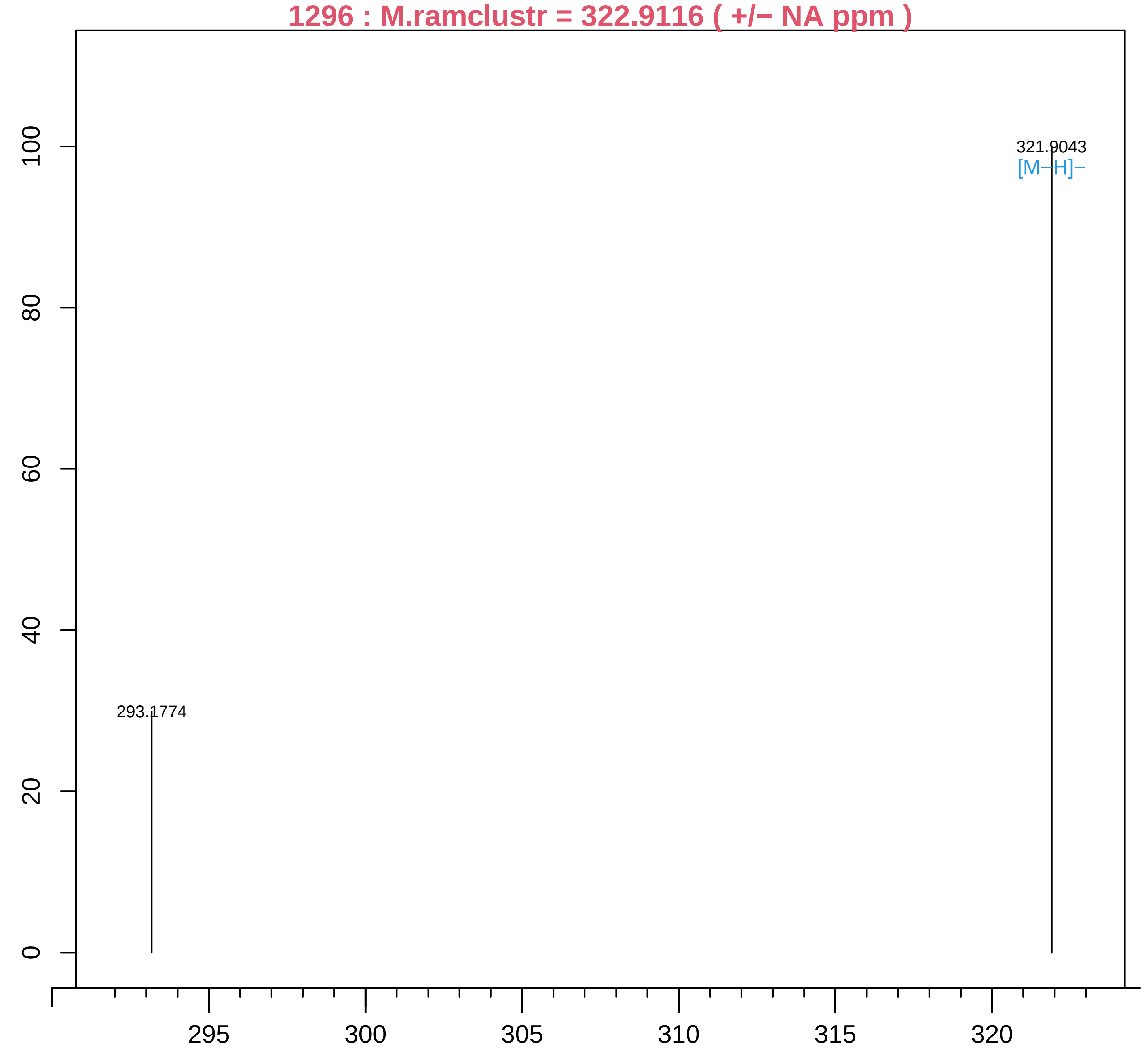
1296 : M.ramclustr = 322.9116 (+/− NA ppm)
321.9043
[M−H]−
293.1774
0
20
40
60
80
100
295
300
305
310
315
320

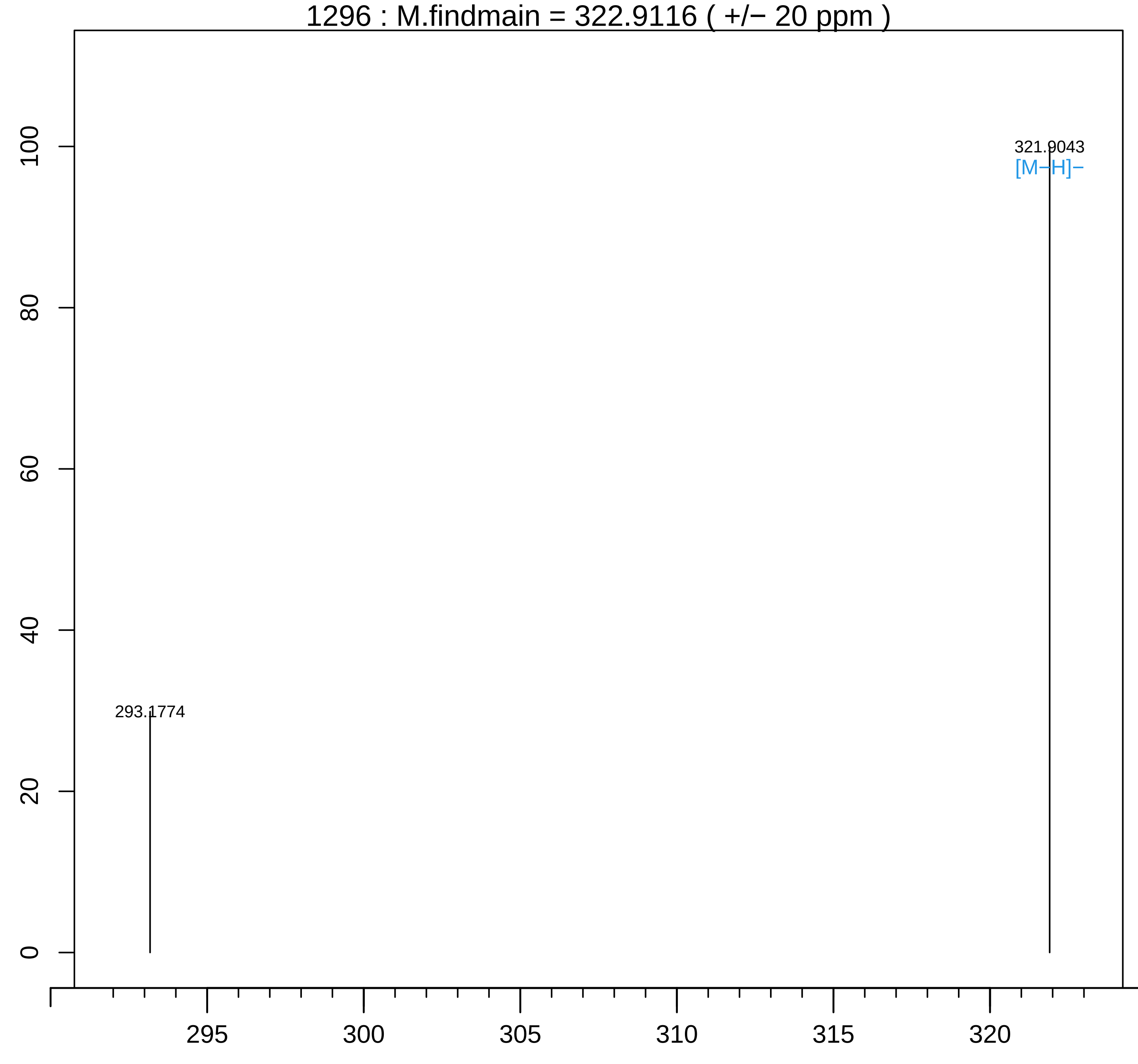
1296 : M.findmain = 322.9116 (+/− 20 ppm)
321.9043
[M−H]−
293.1774
0
20
40
60
80
100
295
300
305
310
315
320

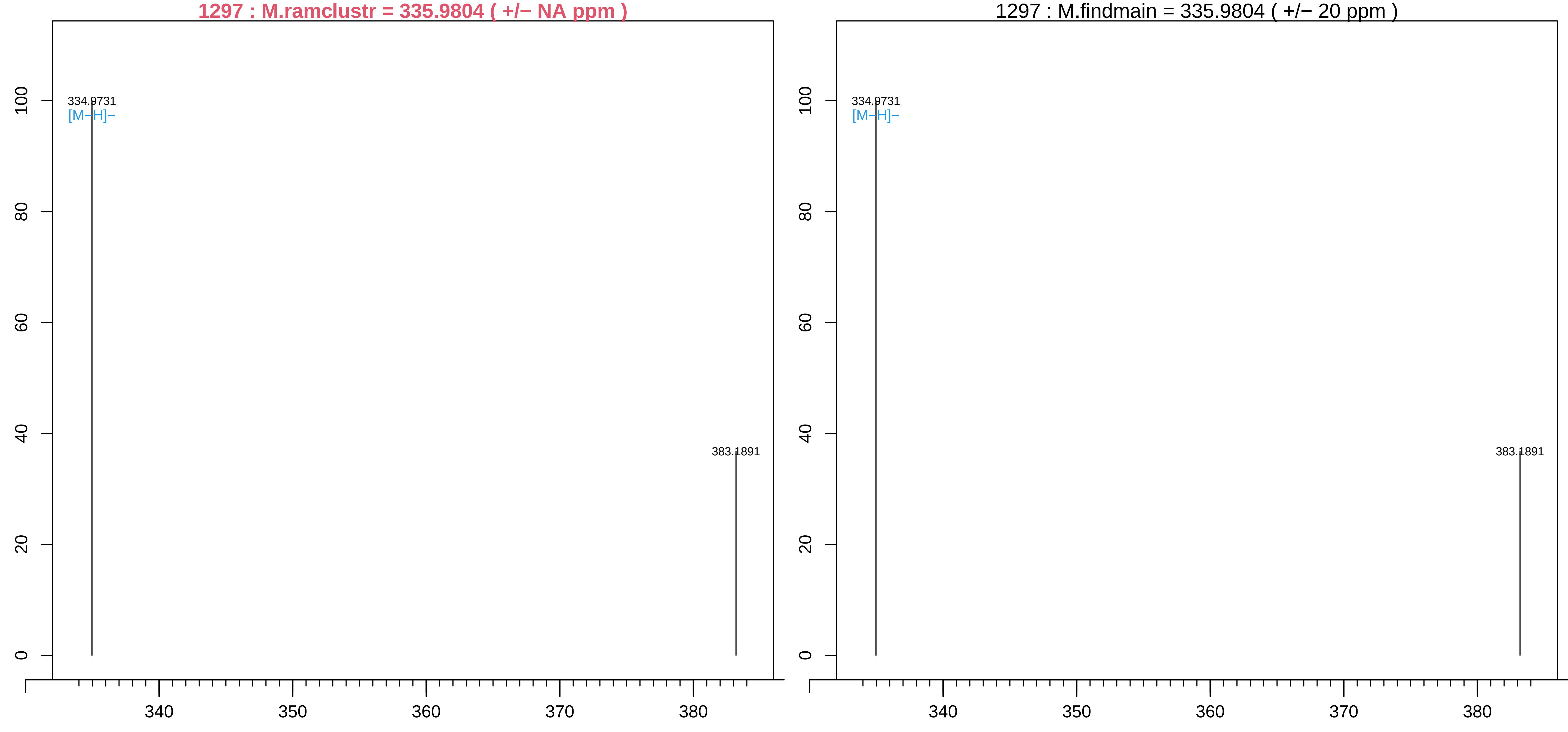
1297 : M.ramclustr = 335.9804 (+/− NA ppm)
334.9731
[M−H]−
383.1891
0
20
40
60
80
100
340
350
360
370
380
1297 : M.findmain = 335.9804 (+/− 20 ppm)
334.9731
[M−H]−
383.1891
0
20
40
60
80
100
340
350
360
370
380

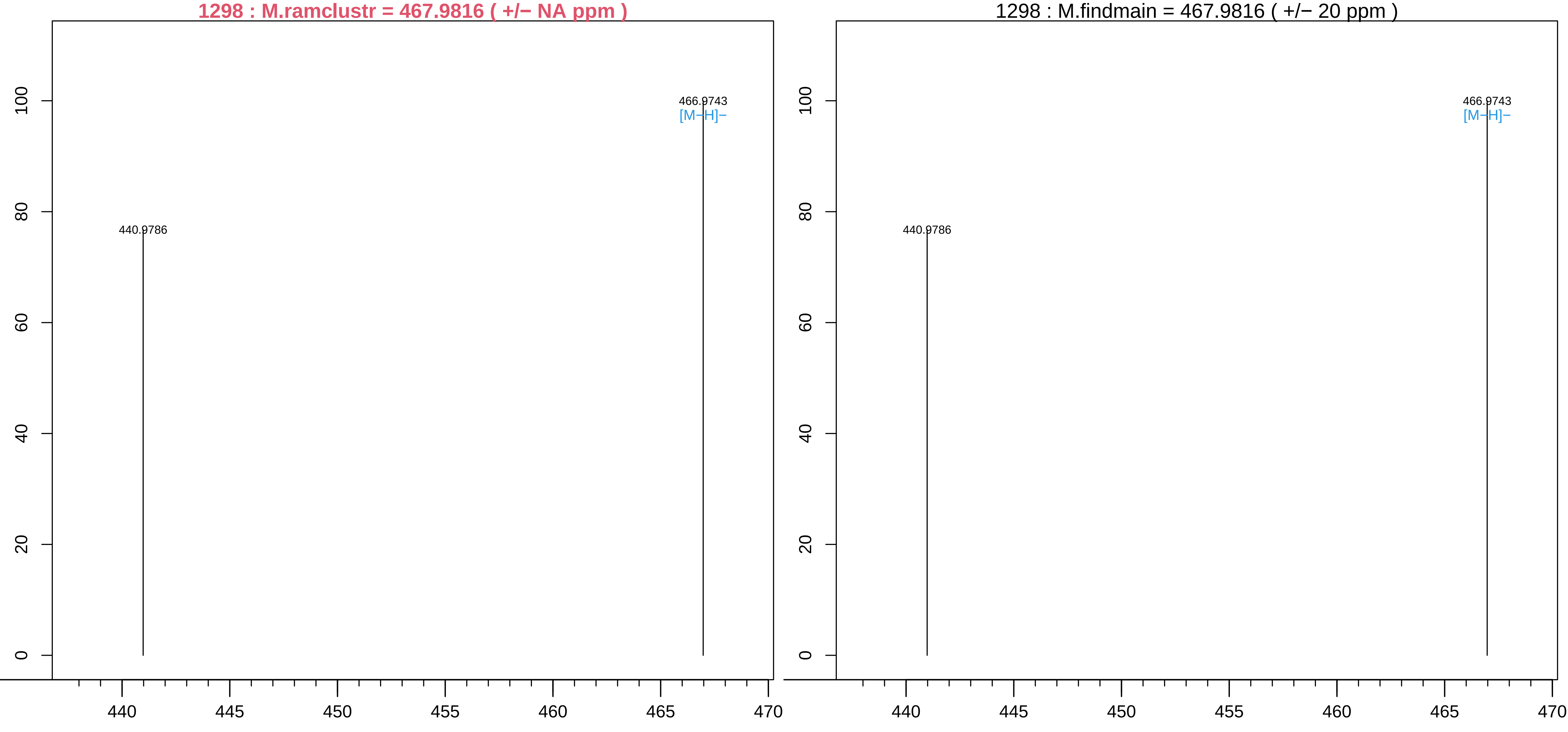
1298 : M.ramclustr = 467.9816 (+/− NA ppm)
466.9743
[M−H]−
440.9786
1298 : M.findmain = 467.9816 (+/− 20 ppm)
466.9743
[M−H]−
440.9786
0
20
40
60
80
100
440
445
450
455
460
465
470

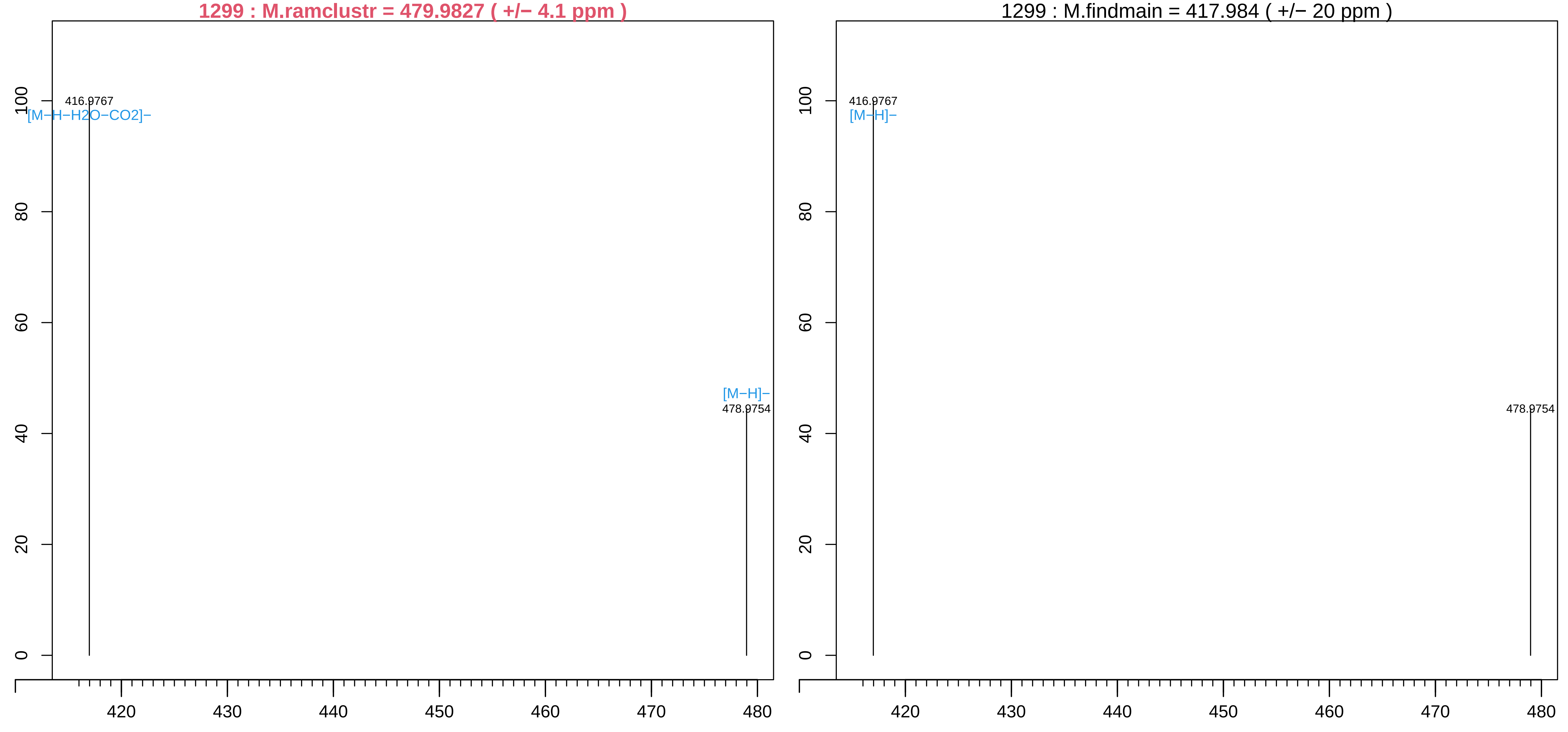

1299 : M.ramclustr = 479.9827 (+/− 4.1 ppm)
416.9767
[M−H−H2O−CO2]−
[M−H]−
478.9754
1299 : M.findmain = 417.984 (+/− 20 ppm)
416.9767
[M−H]−
478.9754

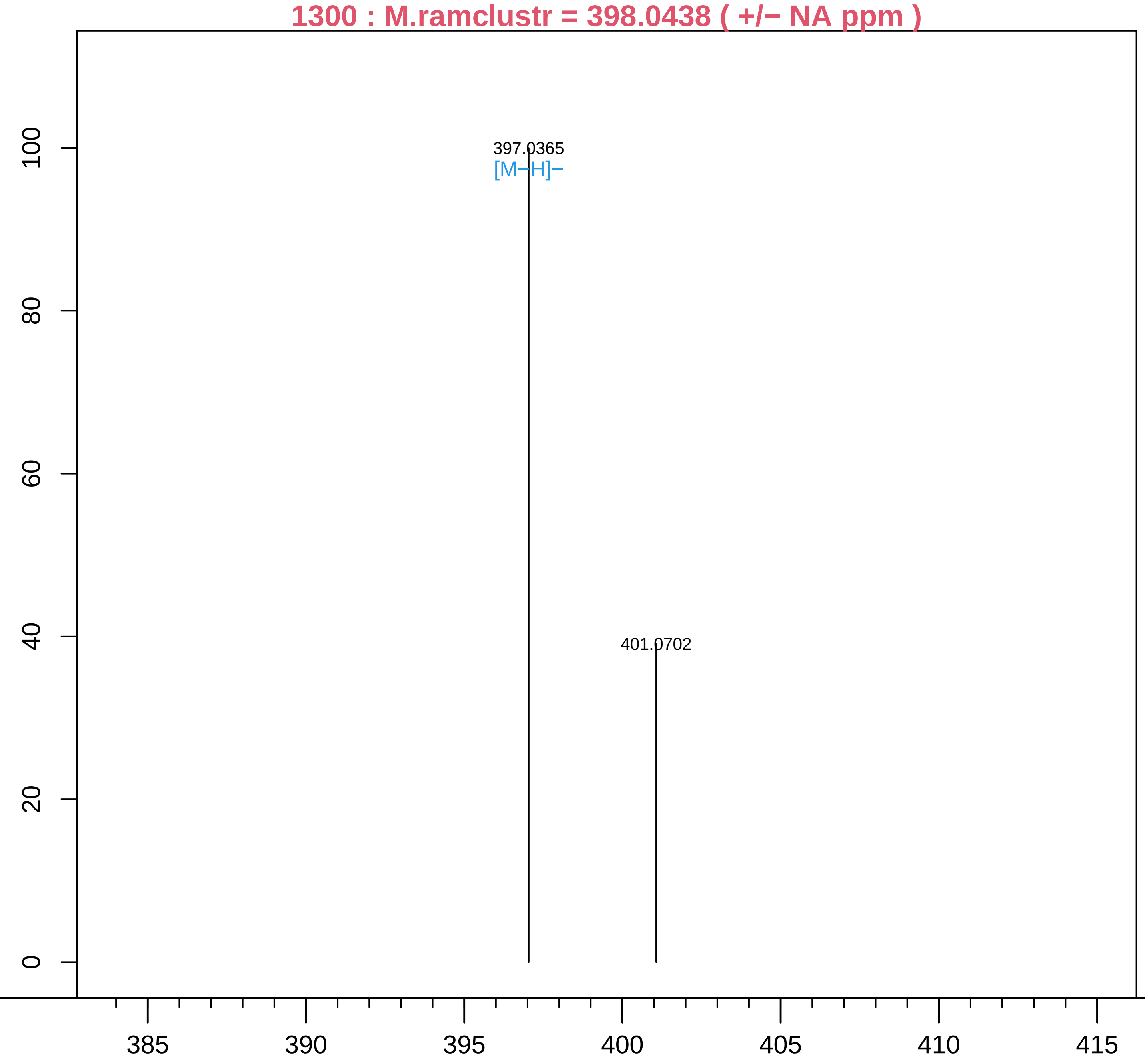
1300 : M.ramclustr = 398.0438 (+/− NA ppm)
397.0365
[M−H]−
401.0702
0
20
40
60
80
100
385
390
395
400
405
410
415

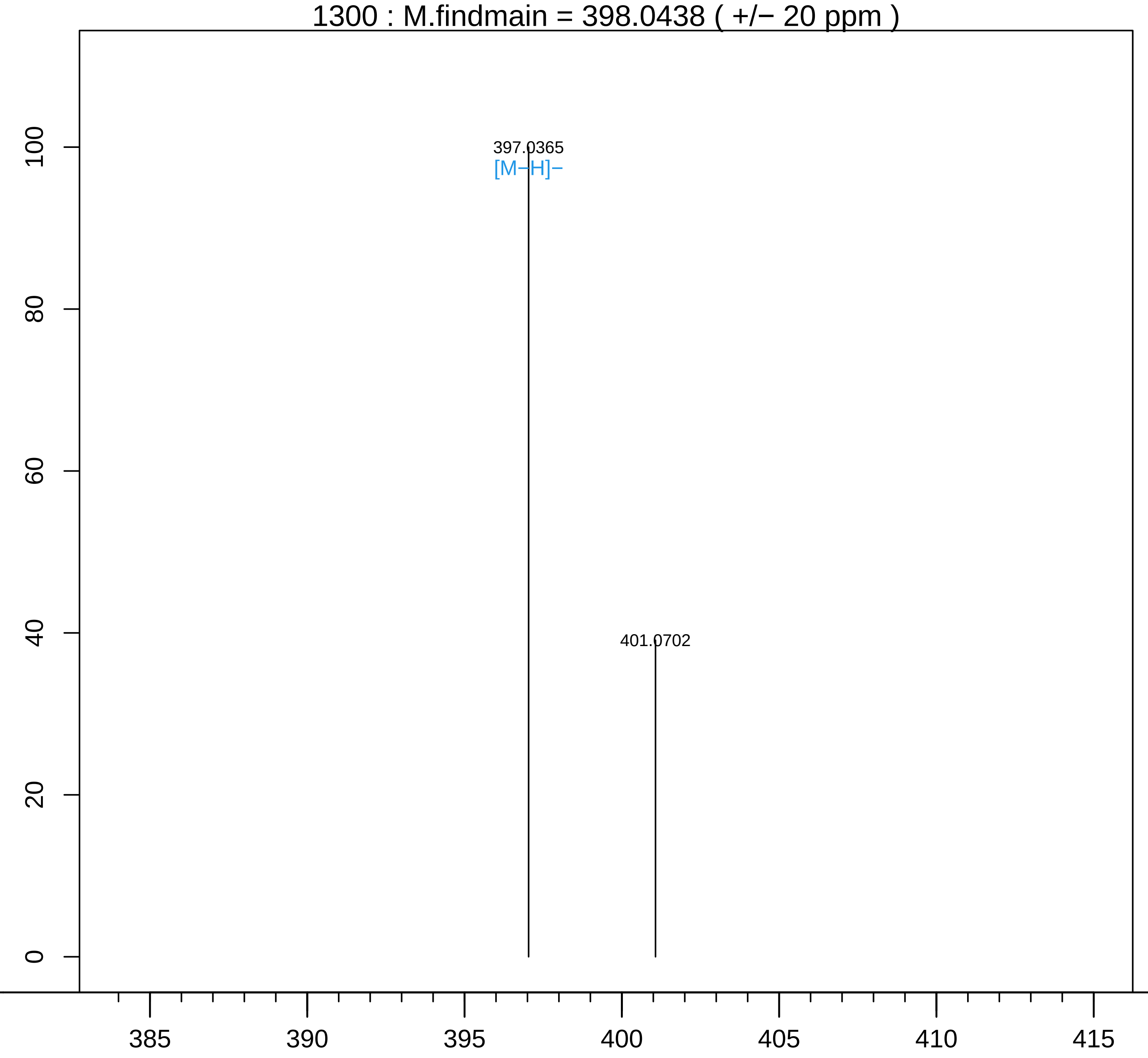
1300 : M.findmain = 398.0438 (+/− 20 ppm)
397.0365
[M−H]−
401.0702
0
20
40
60
80
100
385
390
395
400
405
410
415

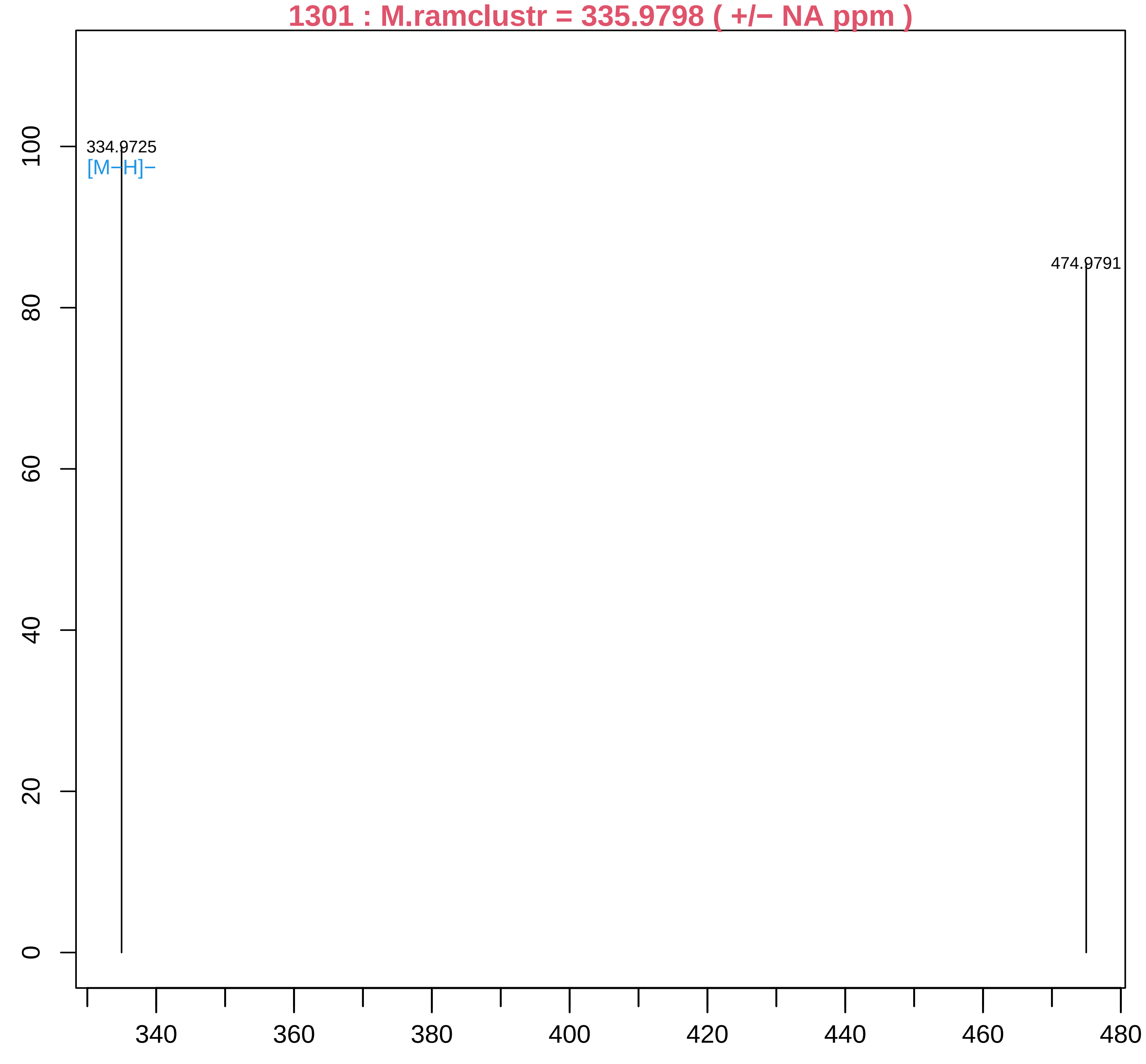
1301 : M.ramclustr = 335.9798 (+/− NA ppm)
334.9725
[M−H]−
474.9791
0
20
40
60
80
100
340
360
380
400
420
440
460
480

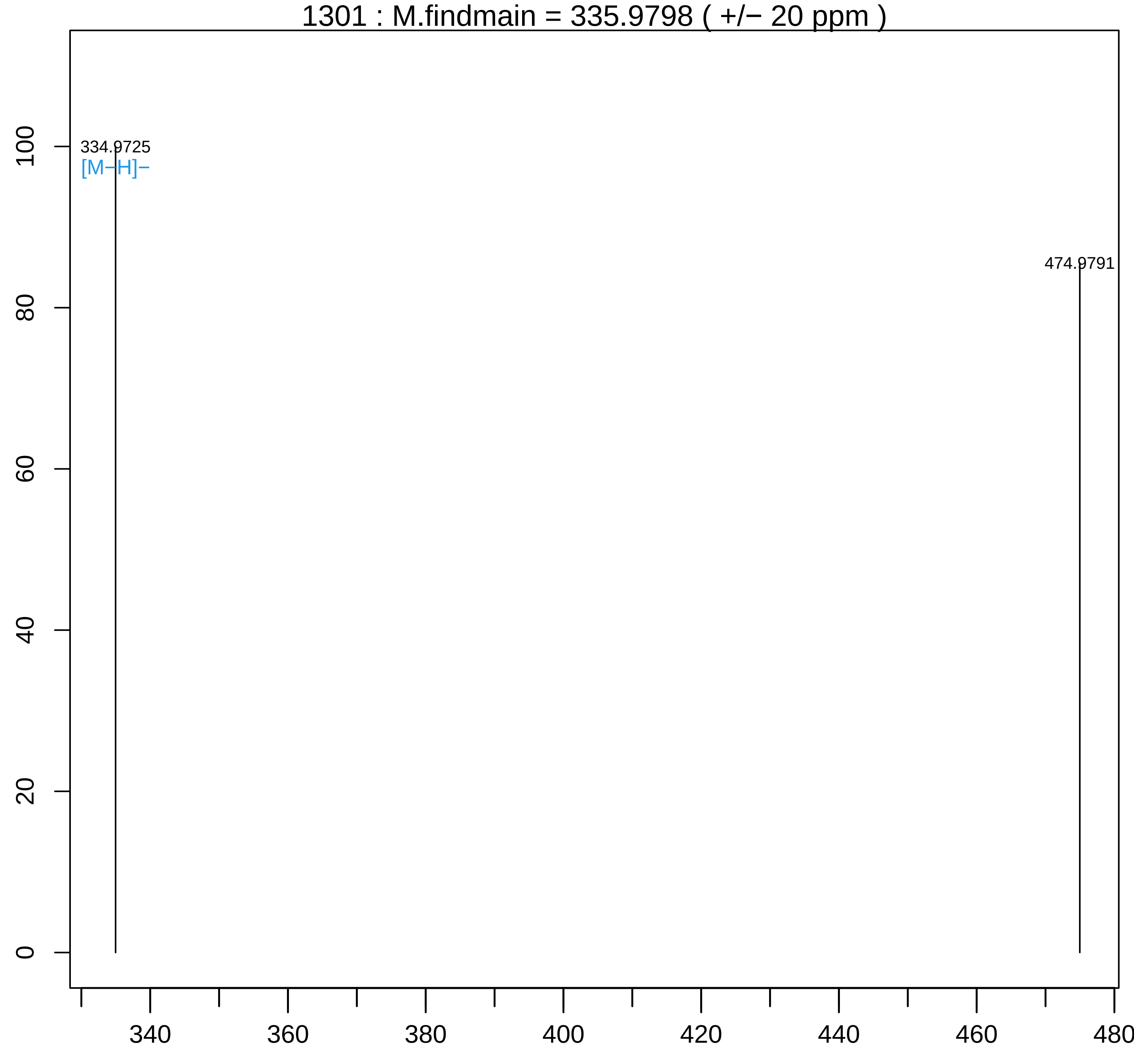
1301 : M.findmain = 335.9798 (+/− 20 ppm)
334.9725
[M−H]−
474.9791
0
20
40
60
80
100
340
360
380
400
420
440
460
480

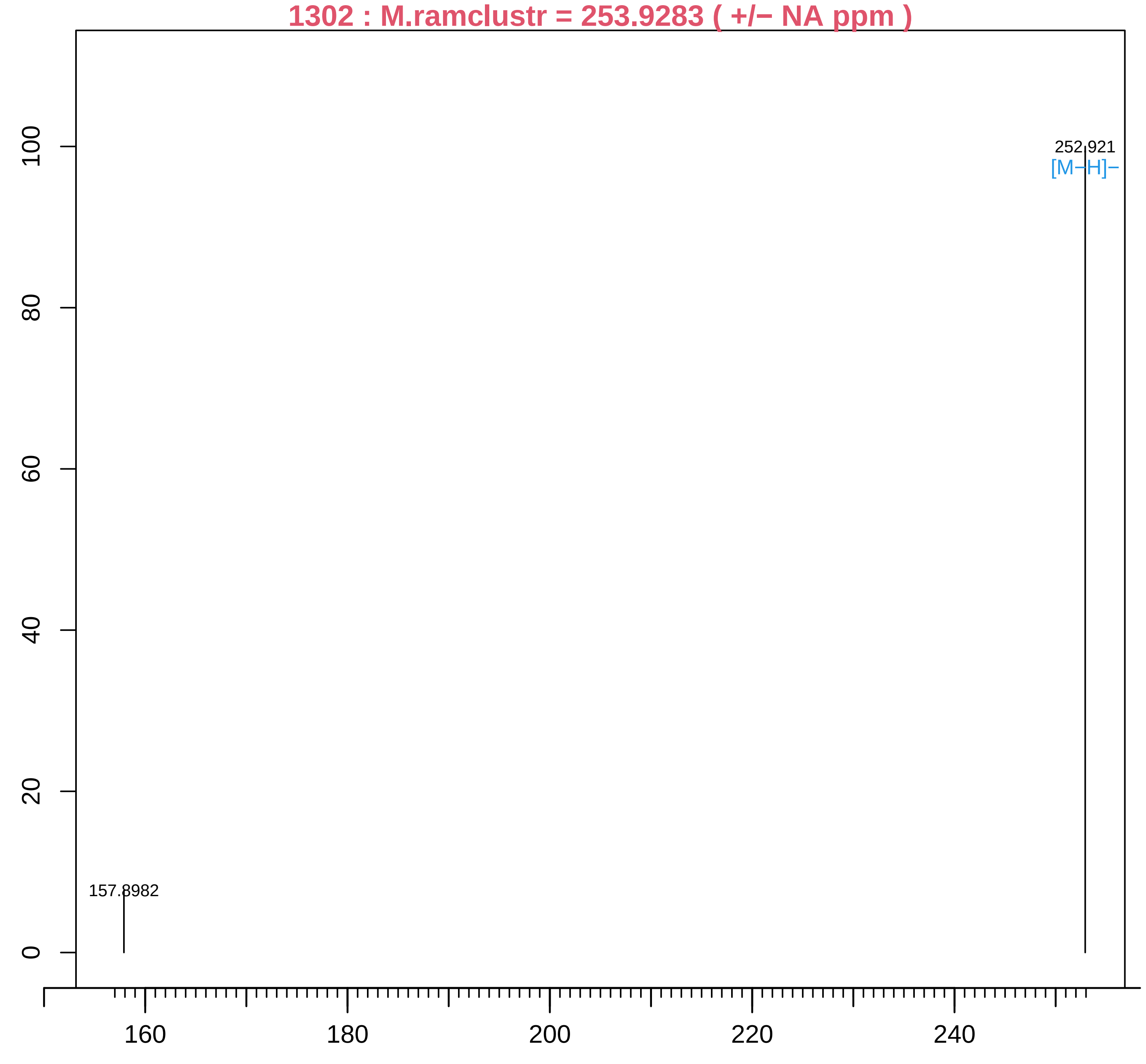
1302 : M.ramclustr = 253.9283 (+/− NA ppm)
252.921
[M−H]−
157.8982
0
20
40
60
80
100
160
180
200
220
240

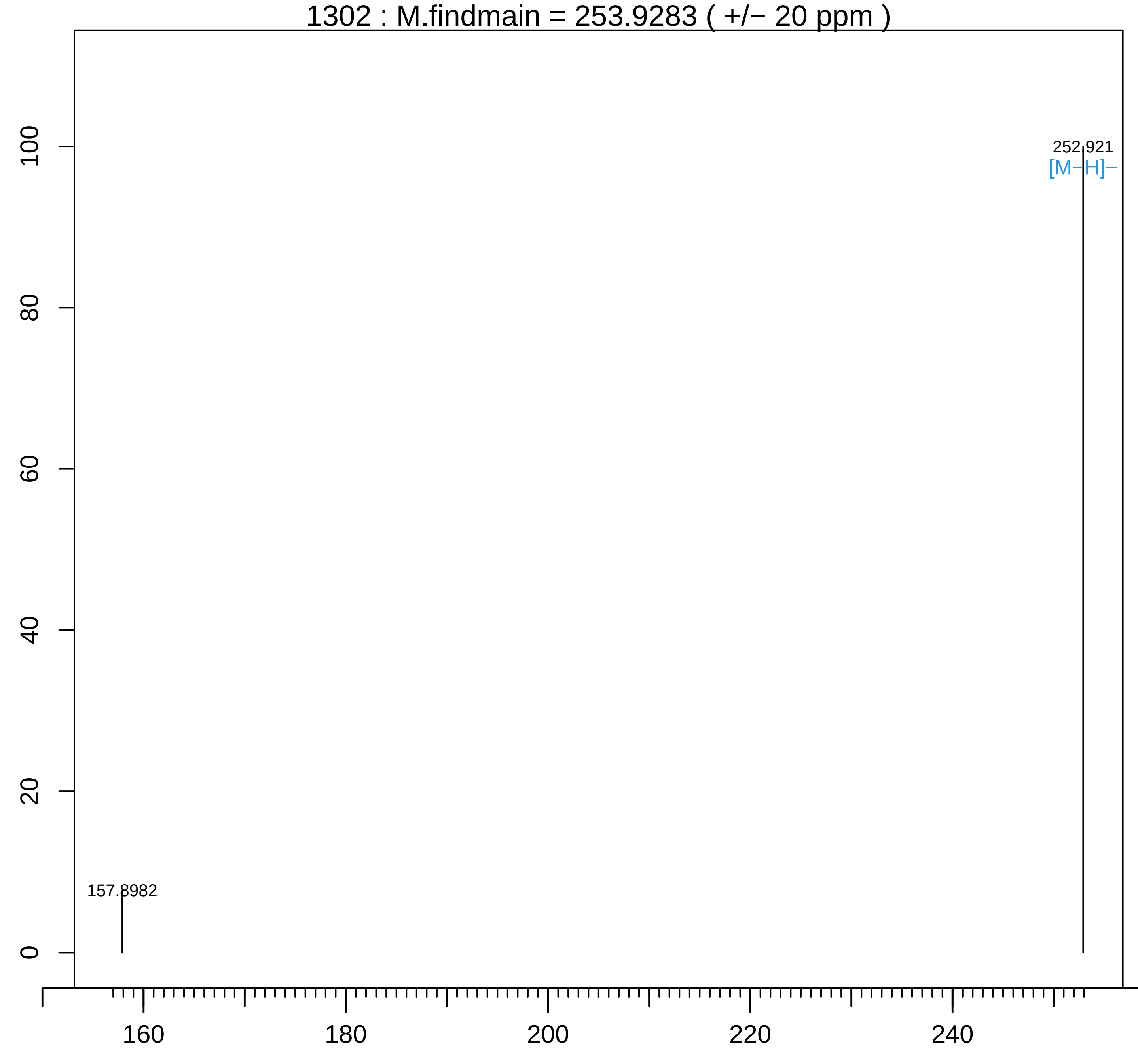
1302 : M.findmain = 253.9283 (+/− 20 ppm)
252.921
[M−H]−
157.8982
0
20
40
60
80
100
160
180
200
220
240

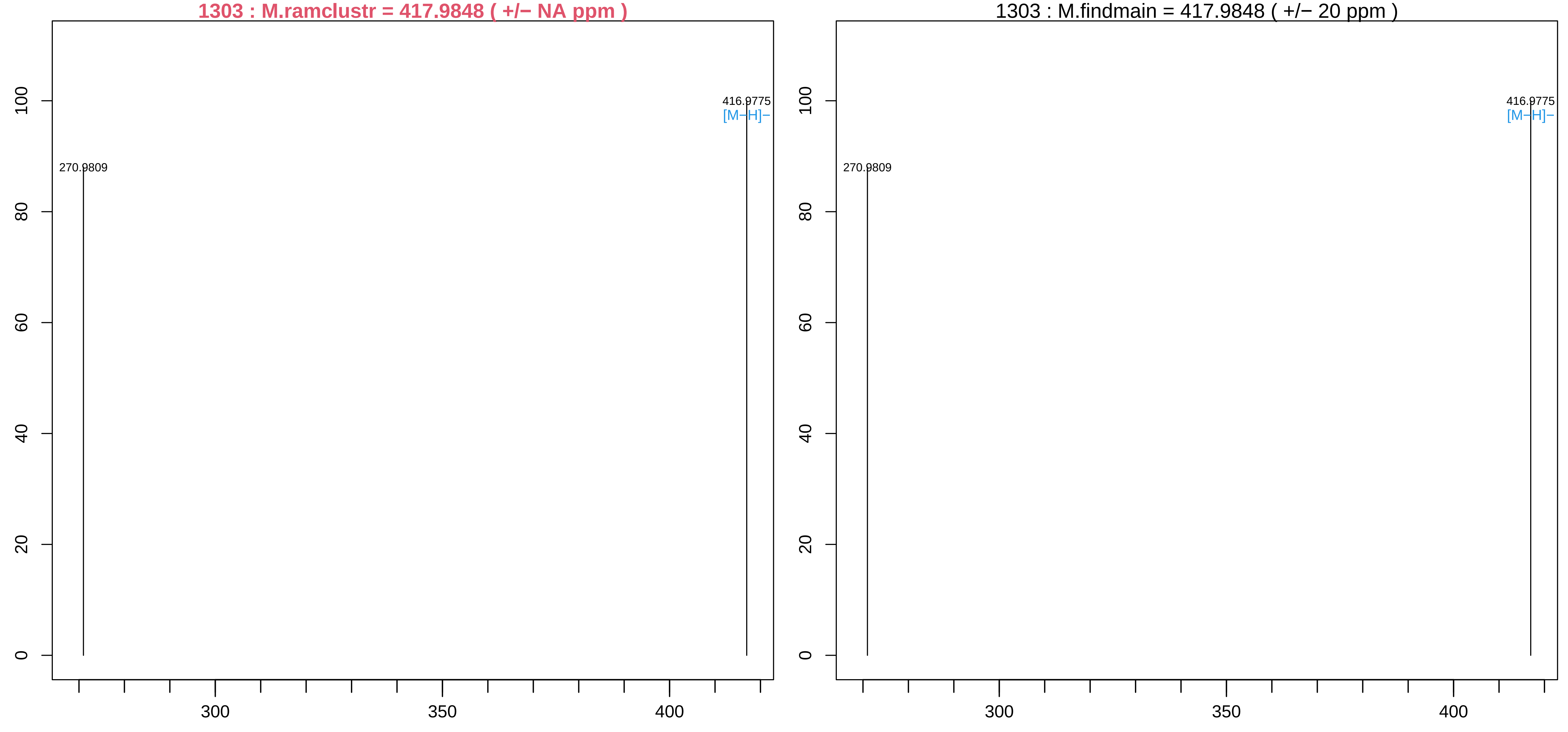
1303 : M.ramclustr = 417.9848 (+/− NA ppm)
270.9809
416.9775
[M−H]−
0
20
40
60
80
100
300
350
400
1303 : M.findmain = 417.9848 (+/− 20 ppm)
270.9809
416.9775
[M−H]−
0
20
40
60
80
100
300
350
400

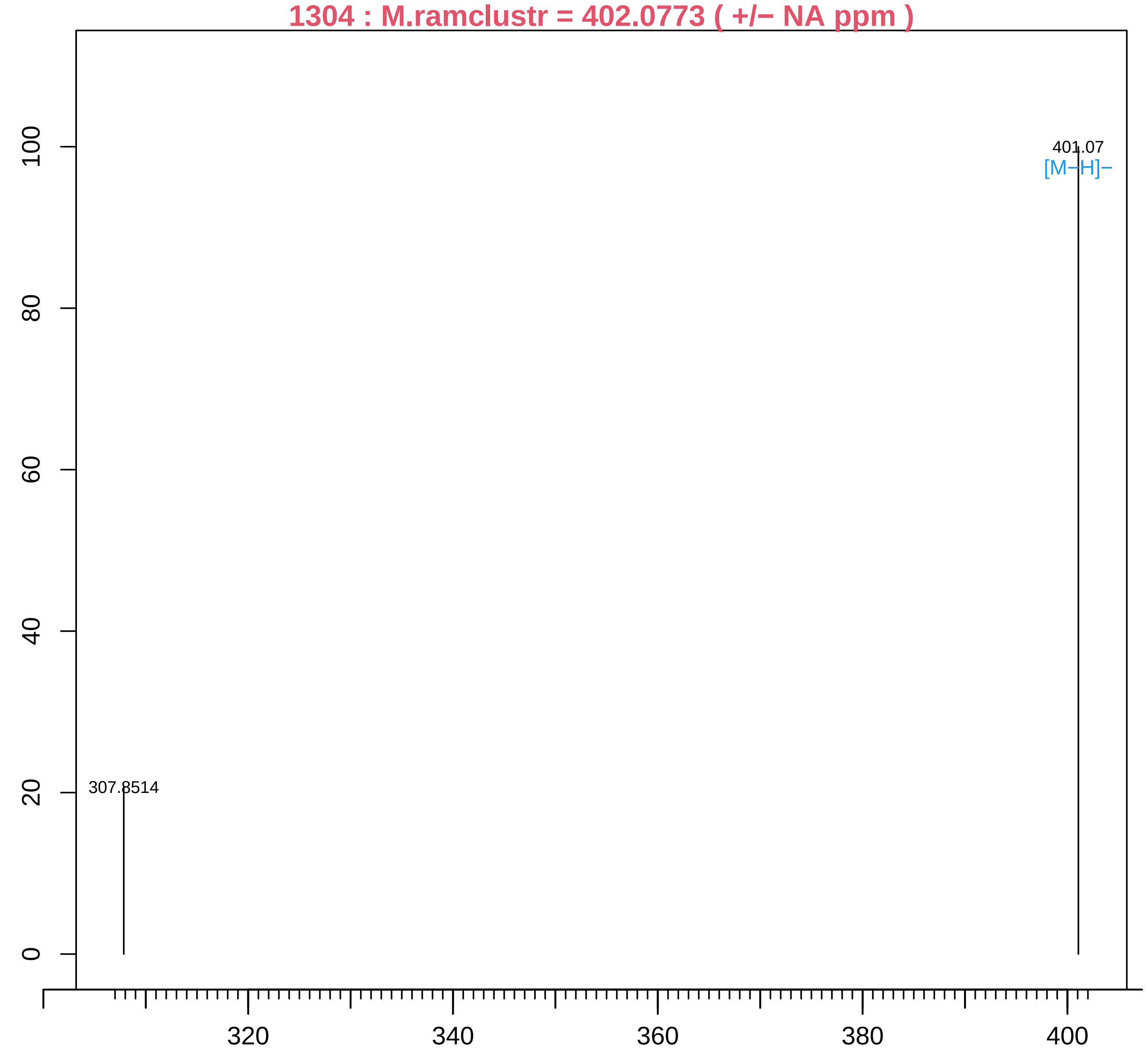

1304 : M.ramclustr = 402.0773 (+/− NA ppm)
401.07
[M−H]−
307.8514
0
20
40
60
80
100
320
340
360
380
400

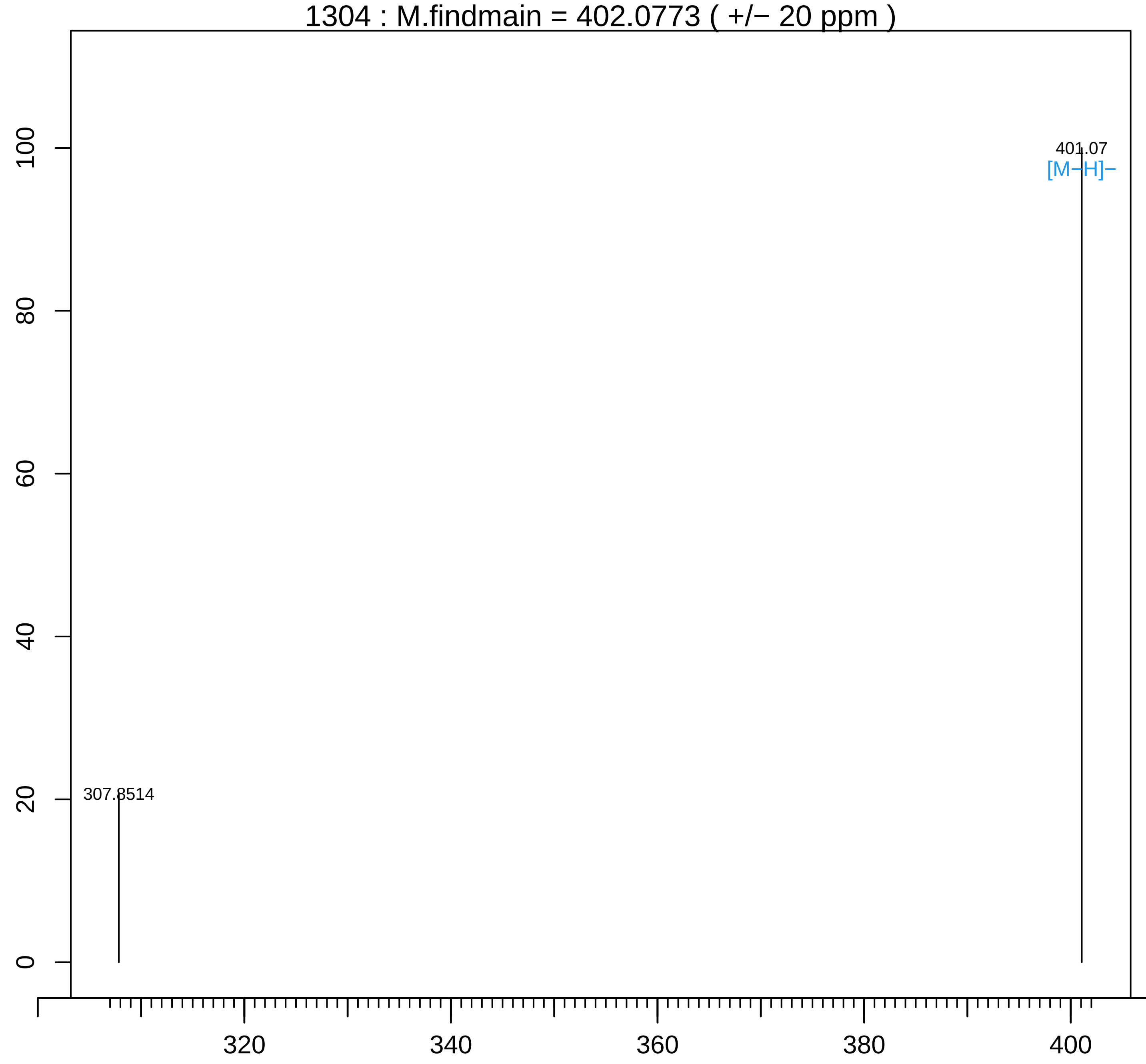

1304 : M.findmain = 402.0773 (+/− 20 ppm)
401.07
[M−H]−
307.8514
0
20
40
60
80
100
320
340
360
380
400

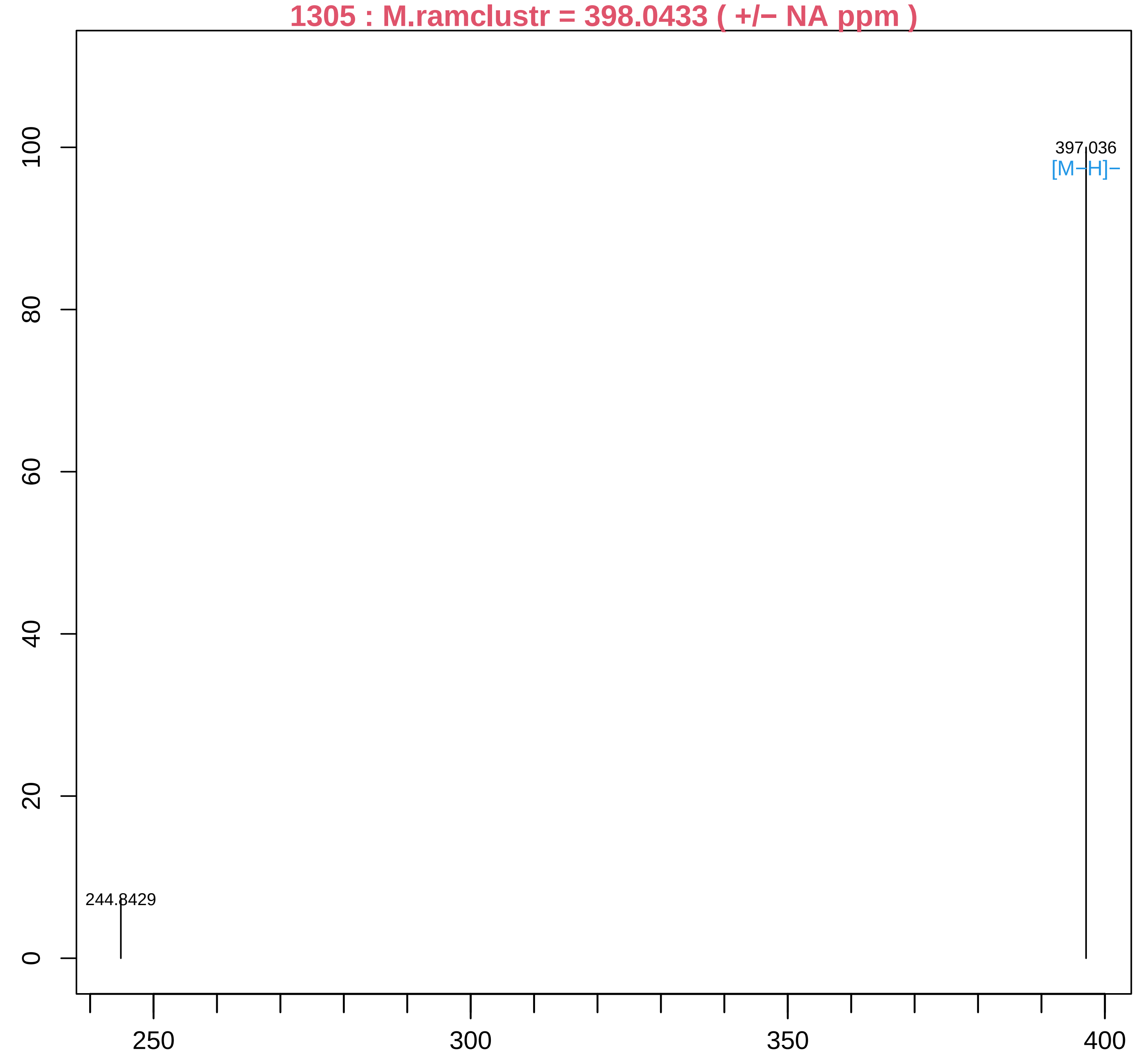

1305 : M.ramclustr = 398.0433 (+/− NA ppm)
397.036
[M−H]−
244.8429
0
20
40
60
80
100
250
300
350
400

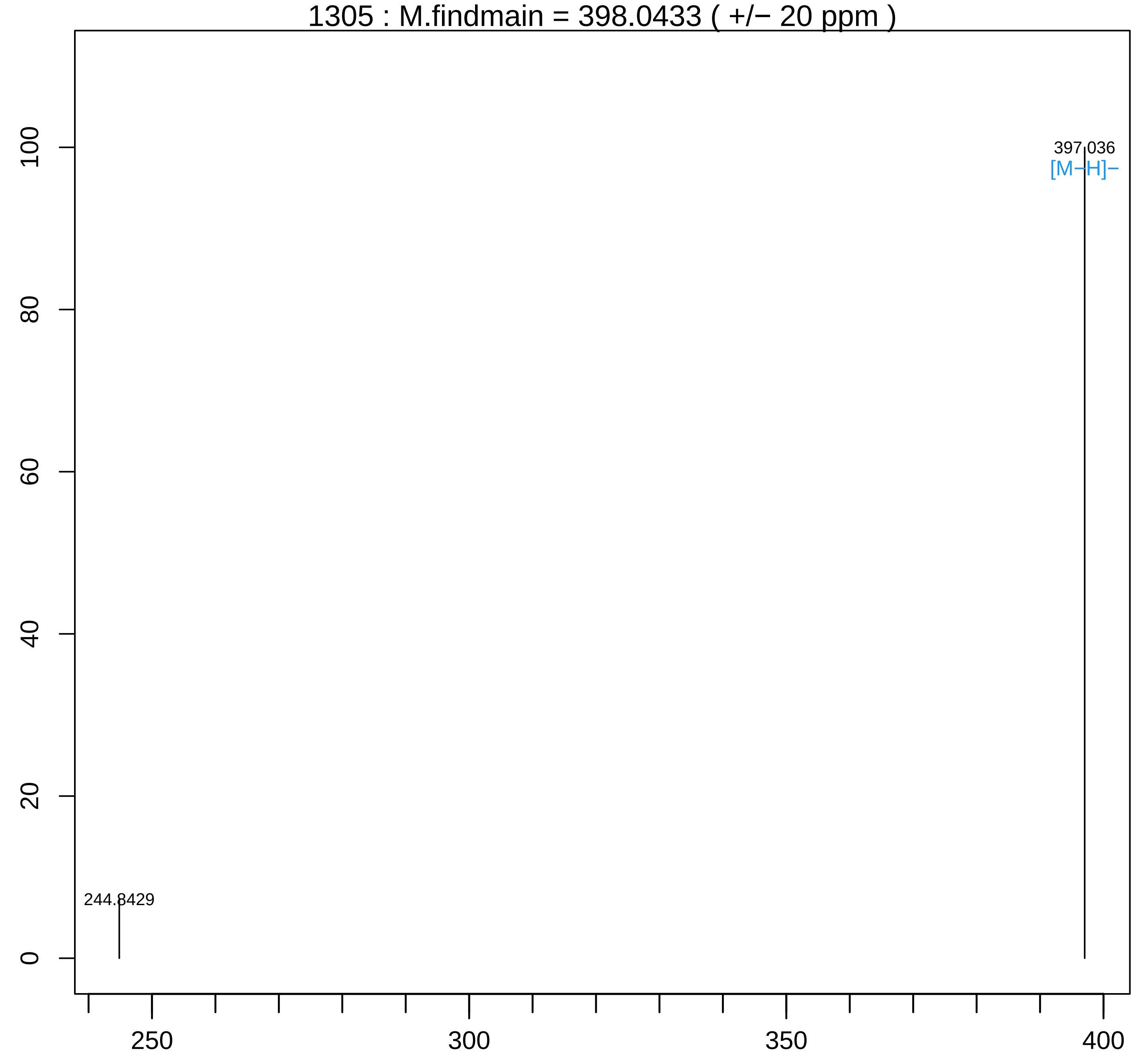

1305 : M.findmain = 398.0433 (+/− 20 ppm)
397.036
[M−H]−
244.8429
0
20
40
60
80
100
250
300
350
400

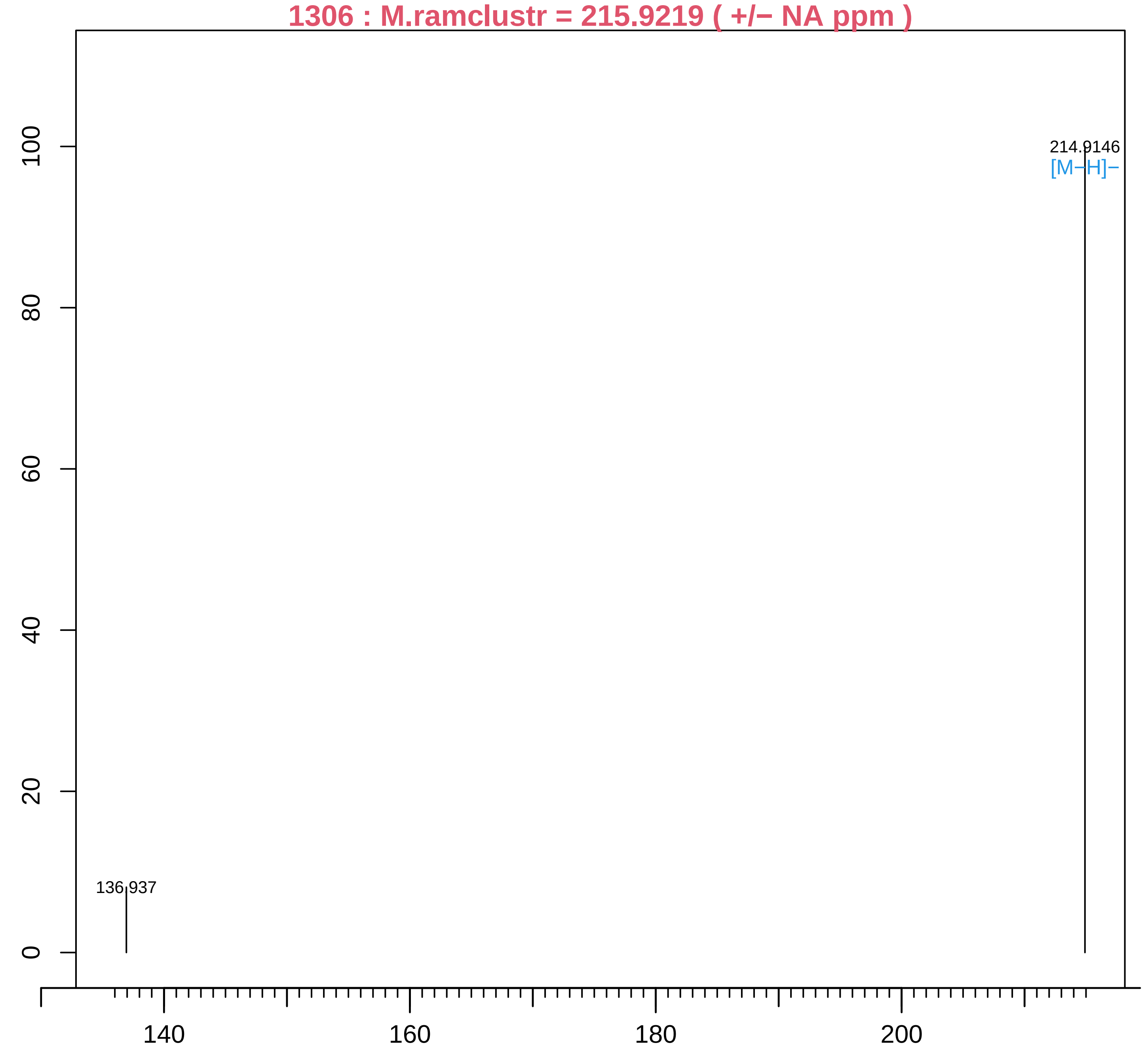
1306 : M.ramclustr = 215.9219 (+/− NA ppm)
214.9146
[M−H]−
136.937
0
20
40
60
80
100
140
160
180
200

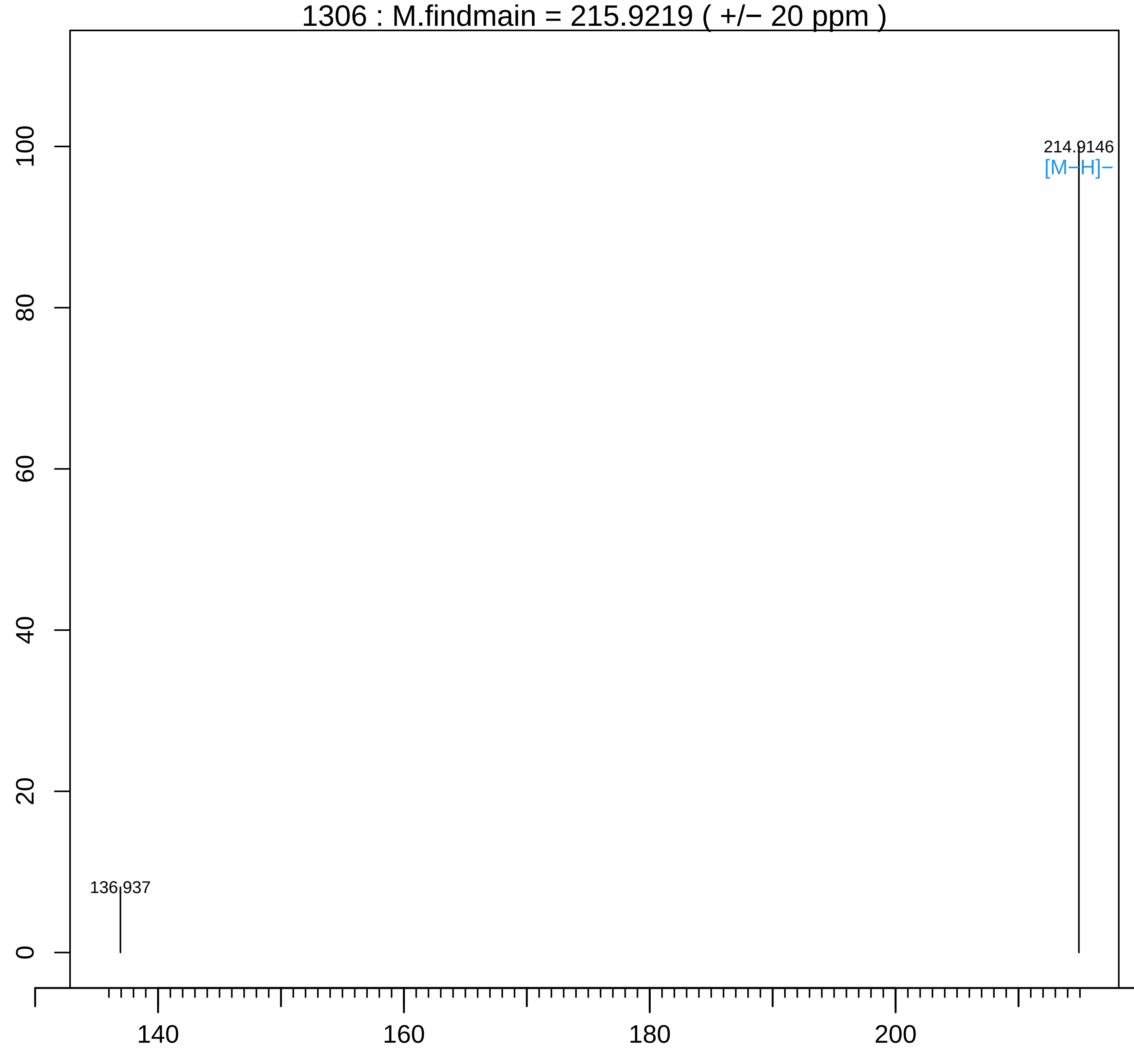
1306 : M.findmain = 215.9219 (+/− 20 ppm)
214.9146
[M−H]−
136.937
0
20
40
60
80
100
140
160
180
200

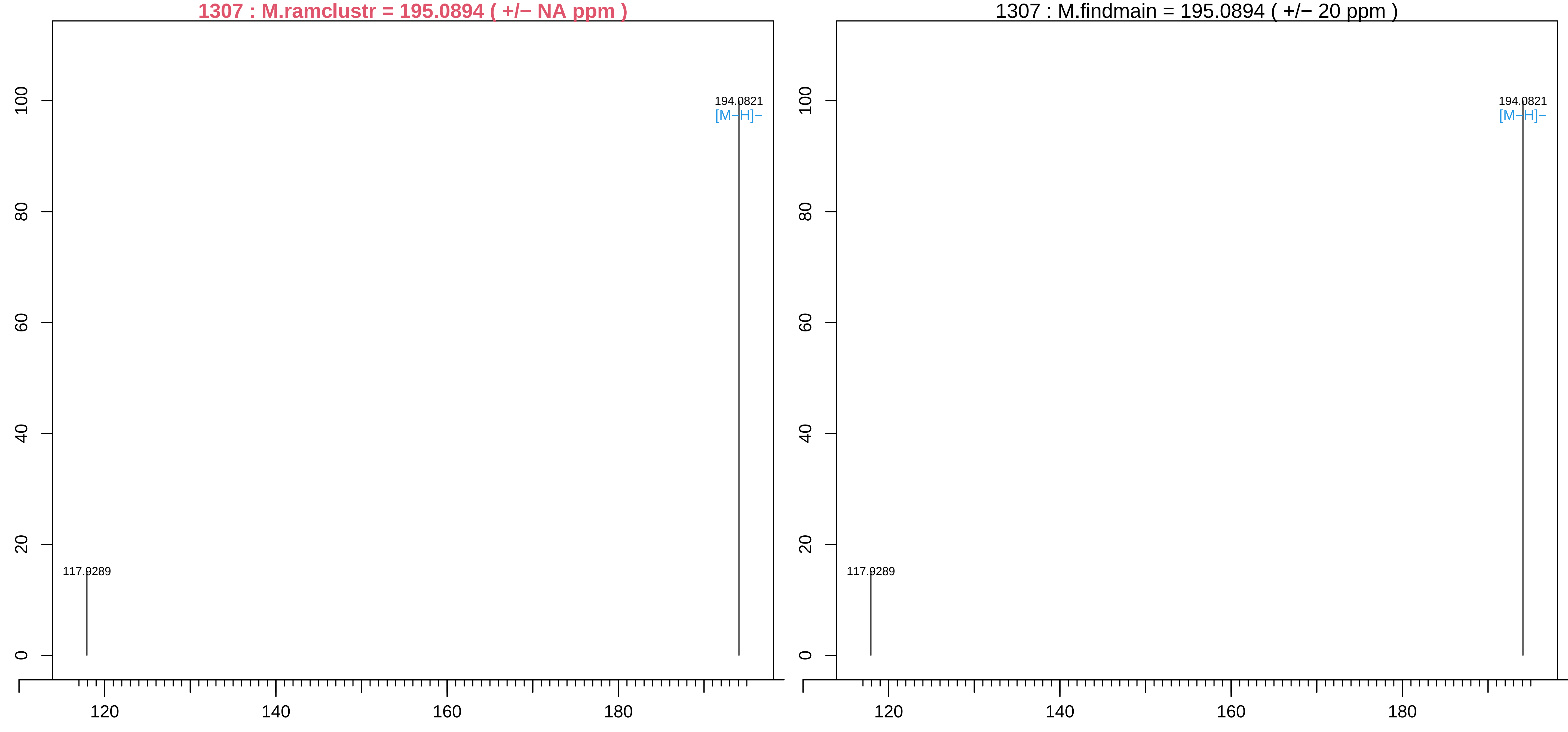
1307 : M.ramclustr = 195.0894 (+/− NA ppm)
194.0821
[M−H]−
117.9289
1307 : M.findmain = 195.0894 (+/− 20 ppm)
194.0821
[M−H]−
117.9289
0
20
40
60
80
100
120
140
160
180

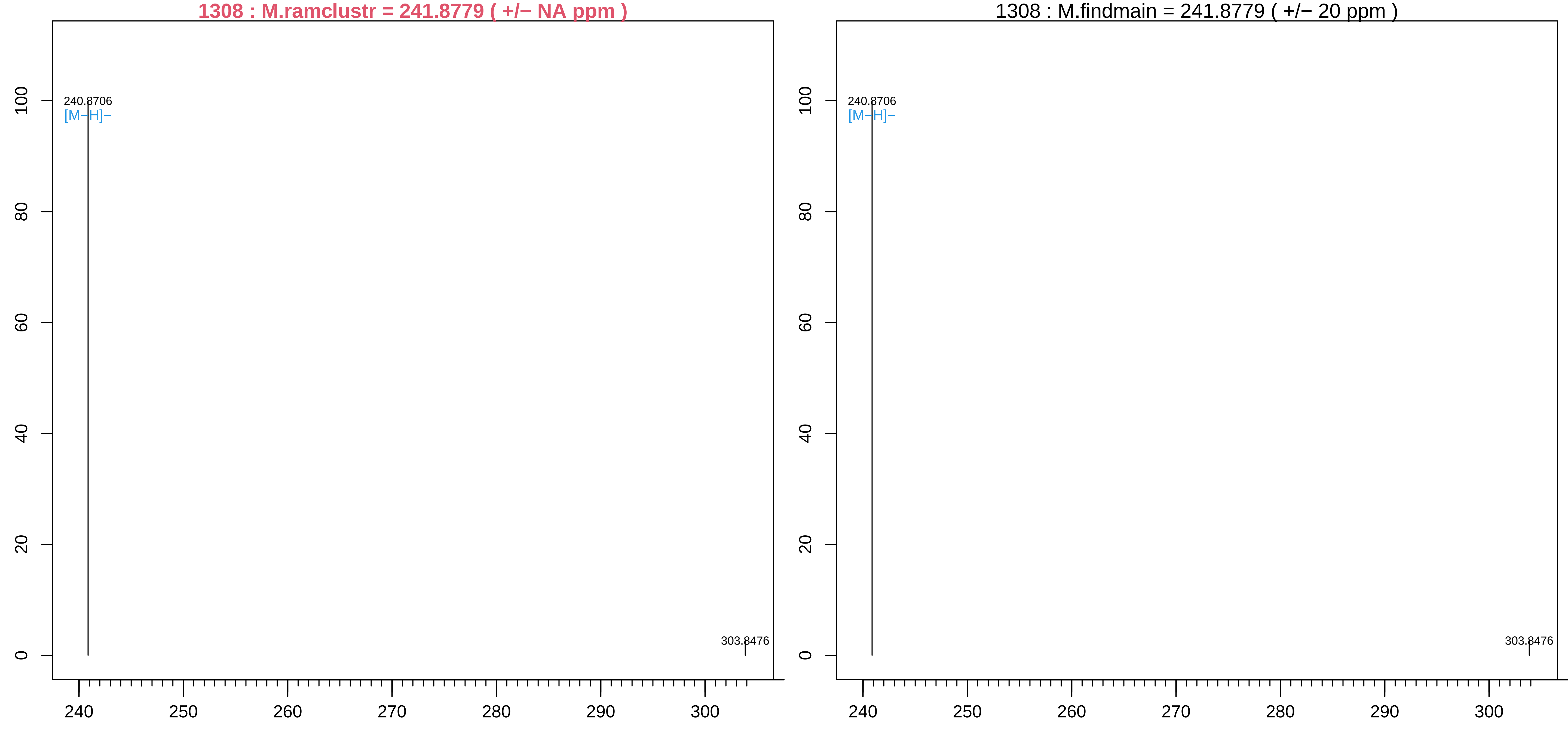
1308 : M.ramclustr = 241.8779 (+/− NA ppm)
240.8706
[M−H]−
303.8476
1308 : M.findmain = 241.8779 (+/− 20 ppm)
240.8706
[M−H]−
303.8476
0
20
40
60
80
100
240
250
260
270
280
290
300

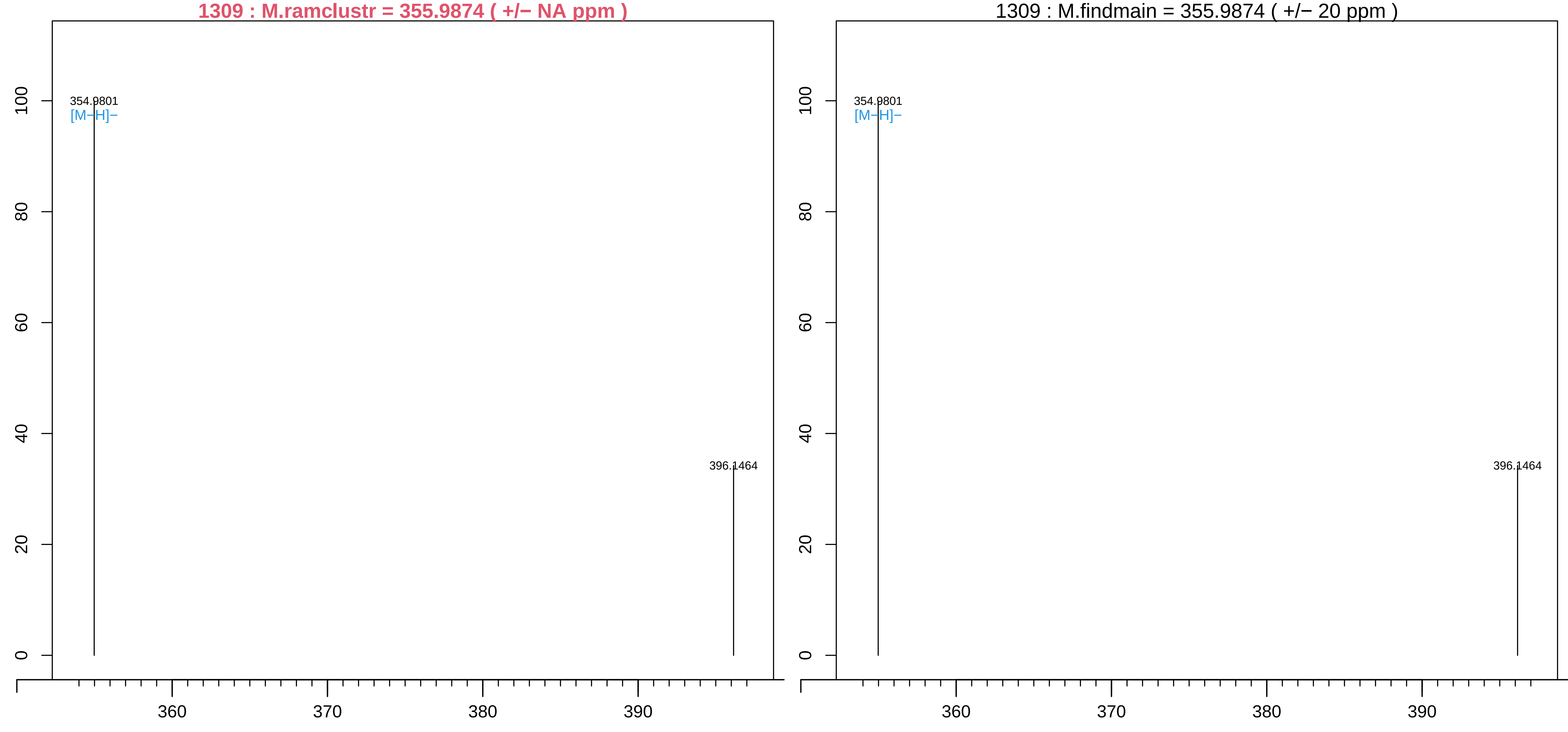
1309 : M.ramclustr = 355.9874 (+/− NA ppm)
354.9801
[M−H]−
396.1464
1309 : M.findmain = 355.9874 (+/− 20 ppm)
354.9801
[M−H]−
396.1464
0
20
40
60
80
100
360
370
380
390

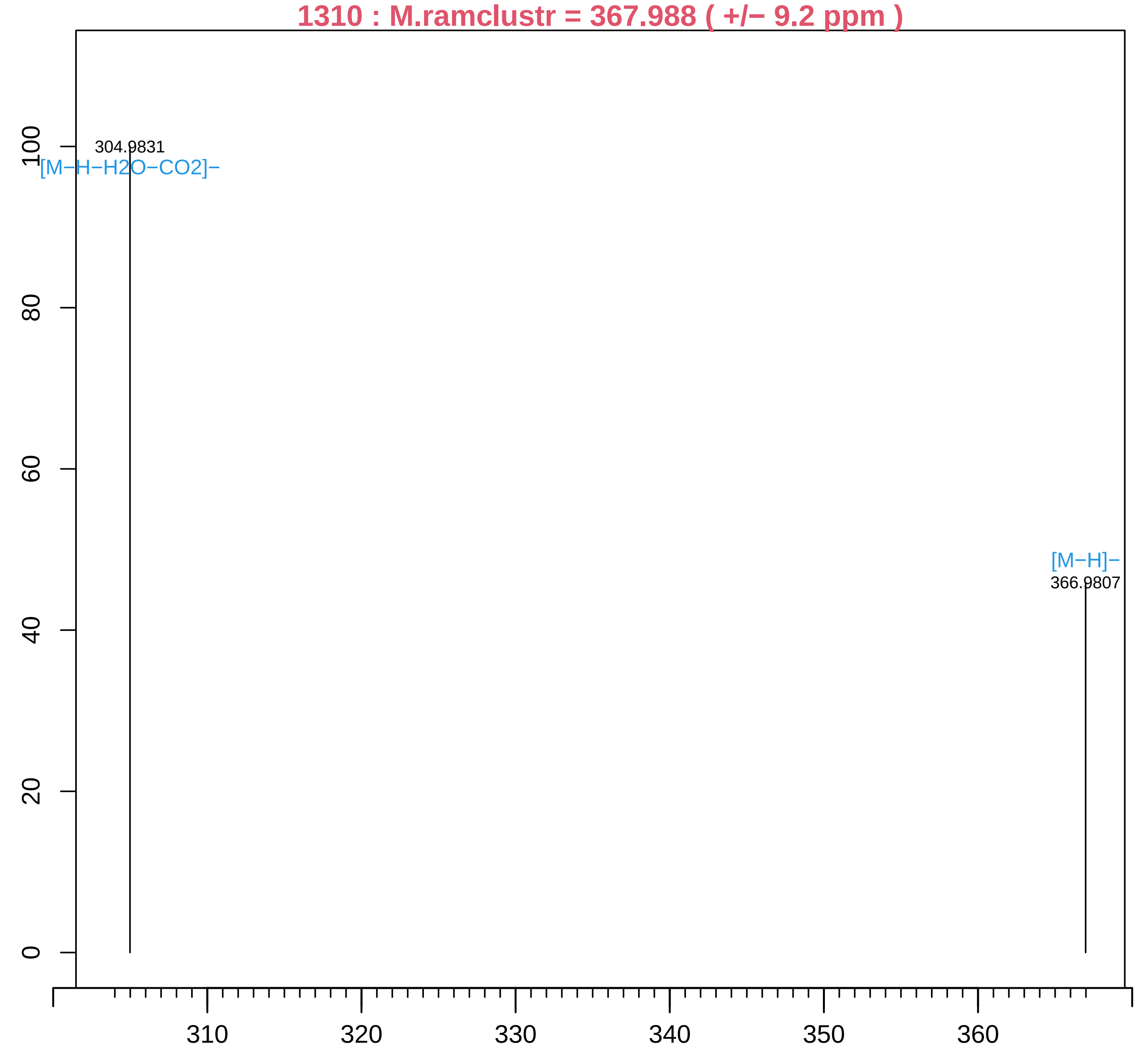
1310 : M.ramclustr = 367.988 (+/− 9.2 ppm)
304.9831
[M−H−H2O−CO2]−
[M−H]−
366.9807
0
20
40
60
80
100
310
320
330
340
350
360

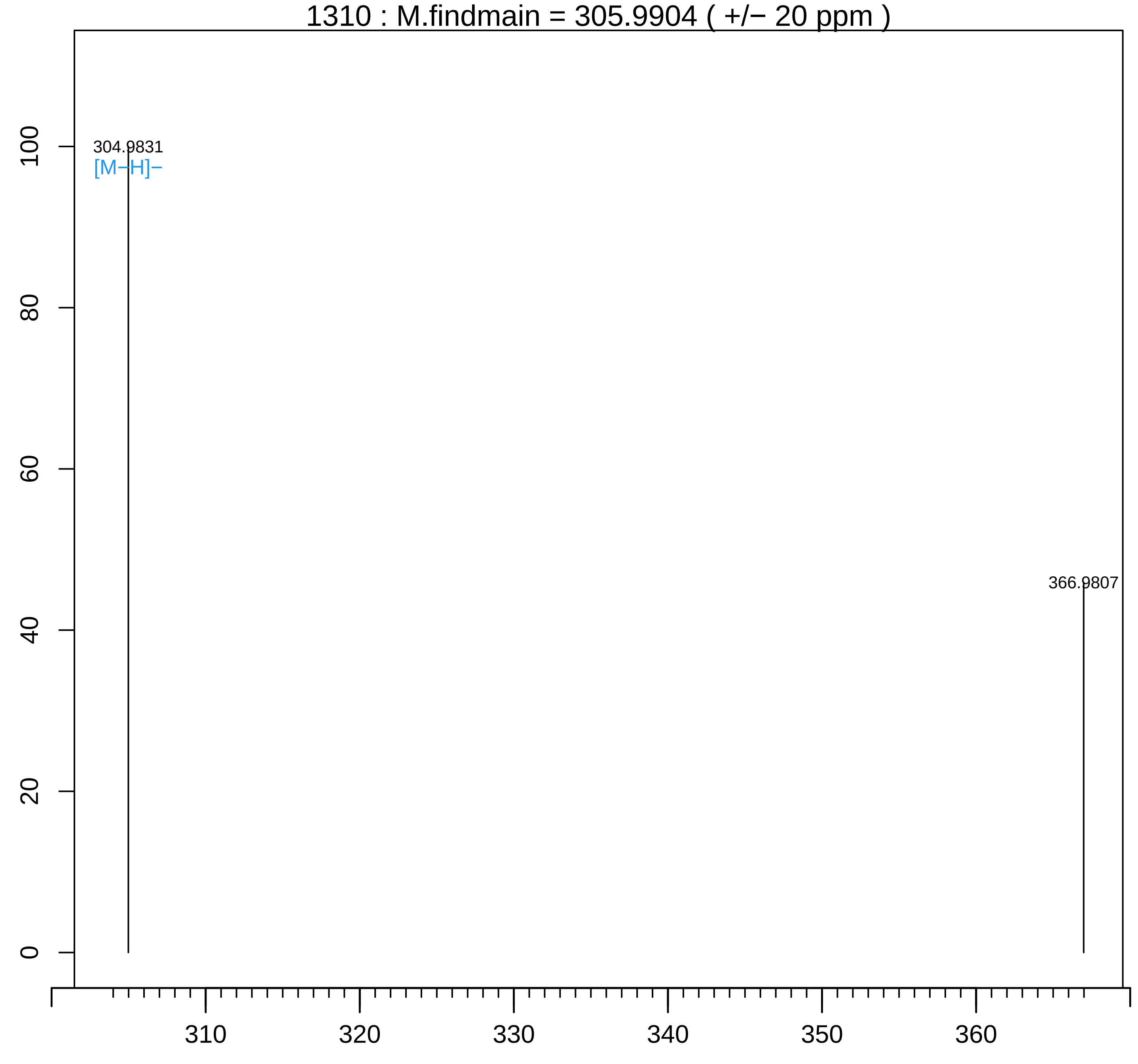
1310 : M.findmain = 305.9904 (+/− 20 ppm)
304.9831
[M−H]−
366.9807
0
20
40
60
80
100
310
320
330
340
350
360

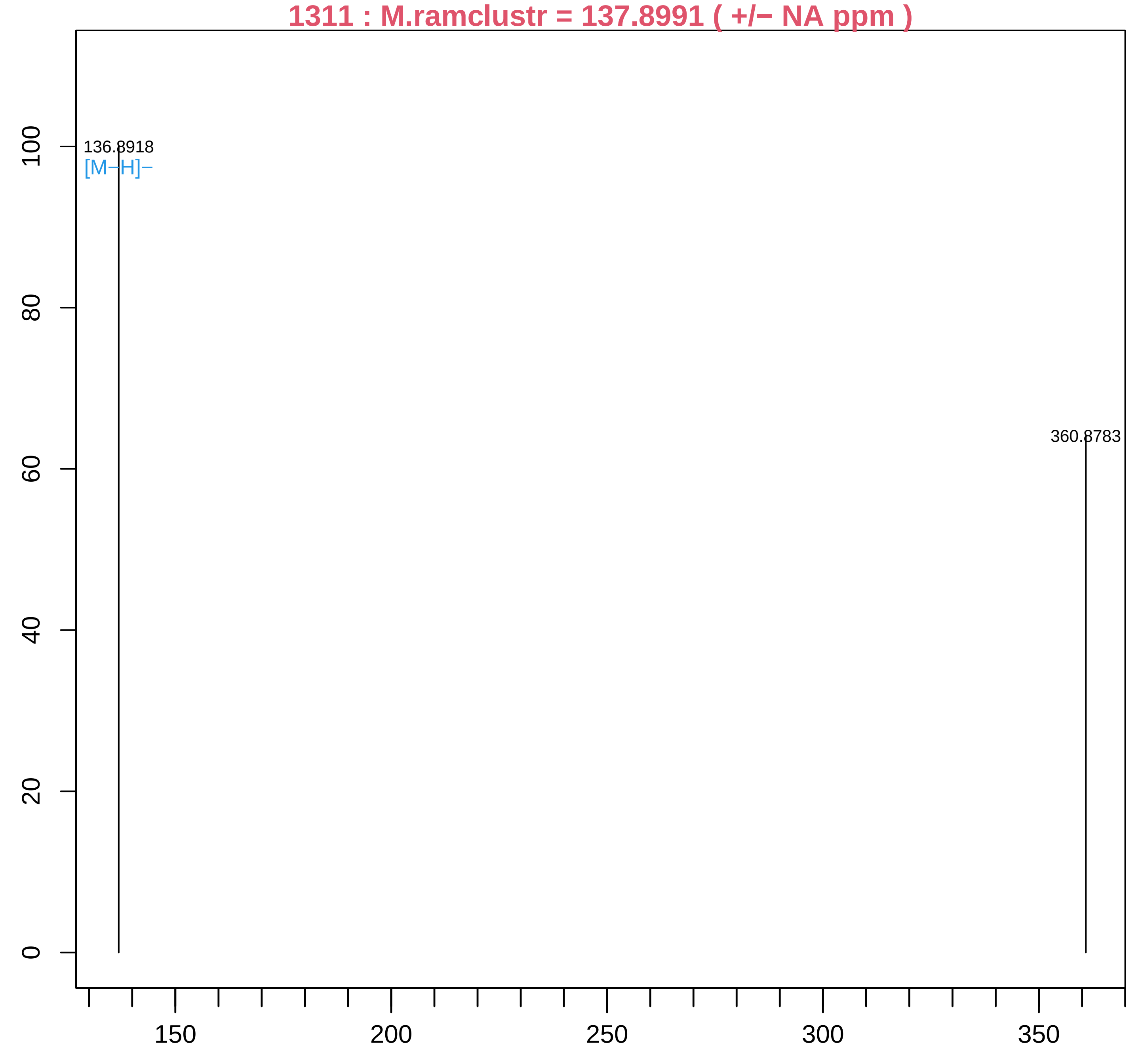
1311 : M.ramclustr = 137.8991 (+/− NA ppm)
136.8918
[M−H]−
360.8783
0
20
40
60
80
100
150
200
250
300
350

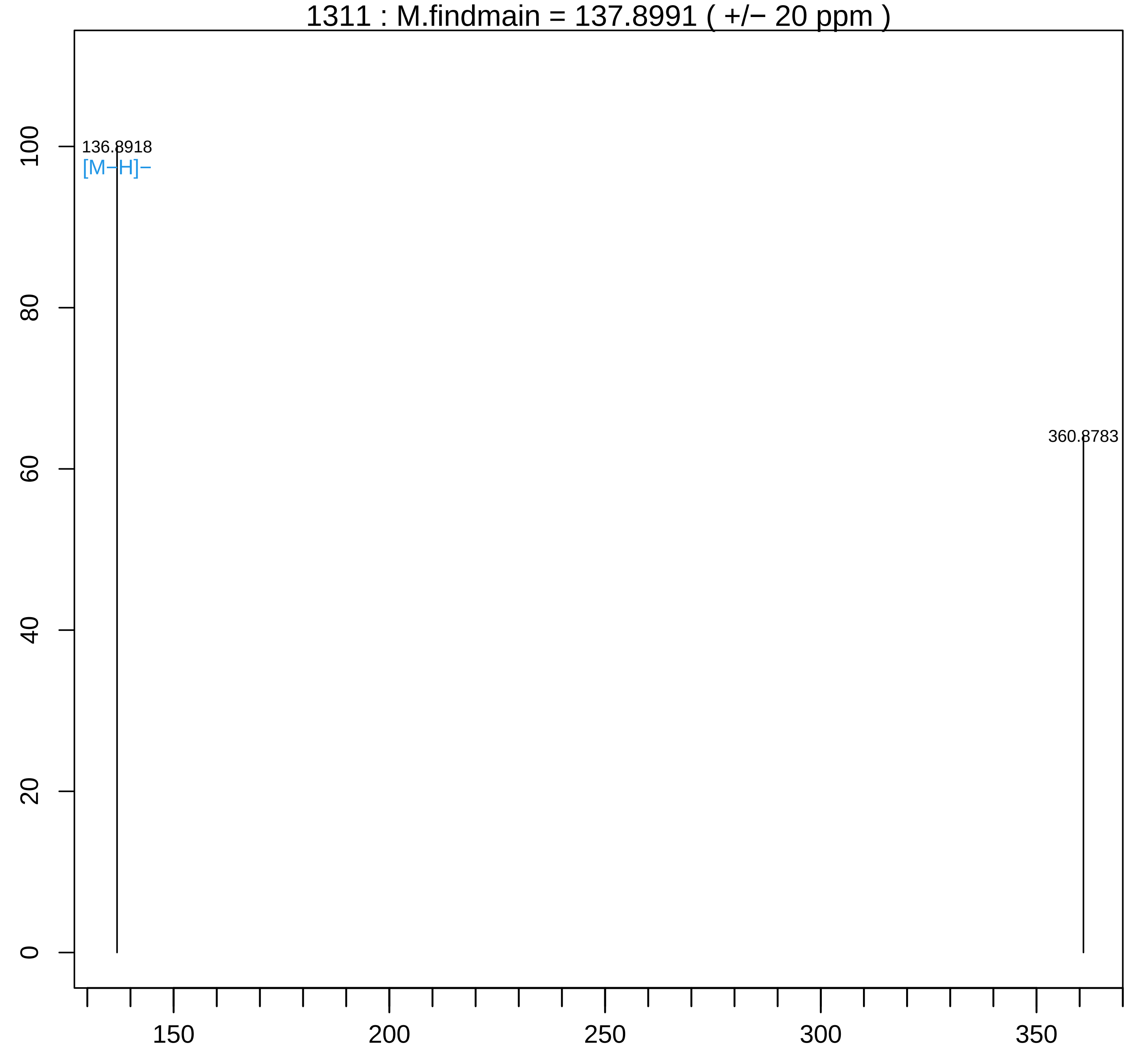
1311 : M.findmain = 137.8991 (+/− 20 ppm)
136.8918
[M−H]−
360.8783
0
20
40
60
80
100
150
200
250
300
350

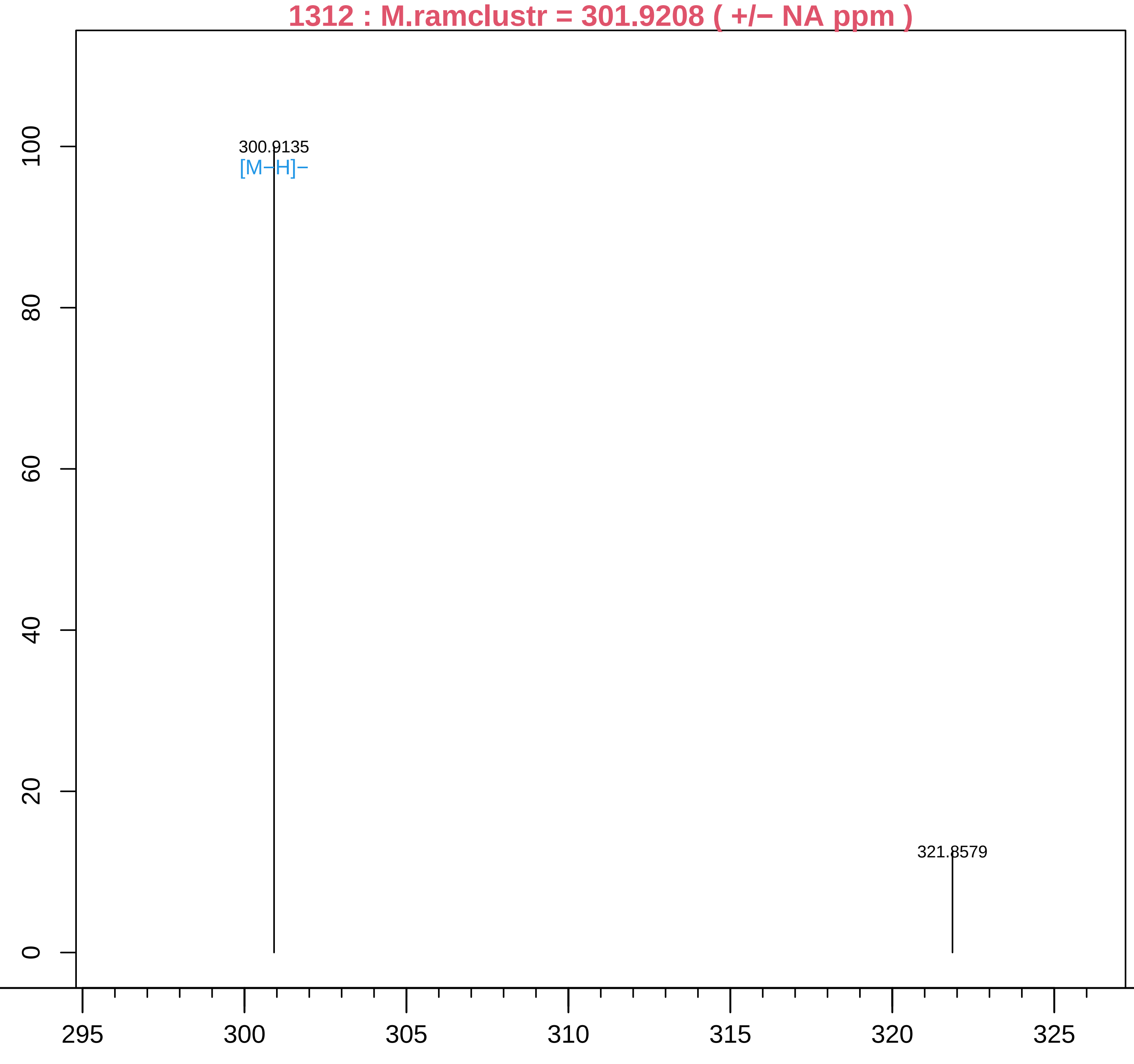
1312 : M.ramclustr = 301.9208 (+/− NA ppm)
300.9135
[M−H]−
321.8579
0
20
40
60
80
100
295
300
305
310
315
320
325

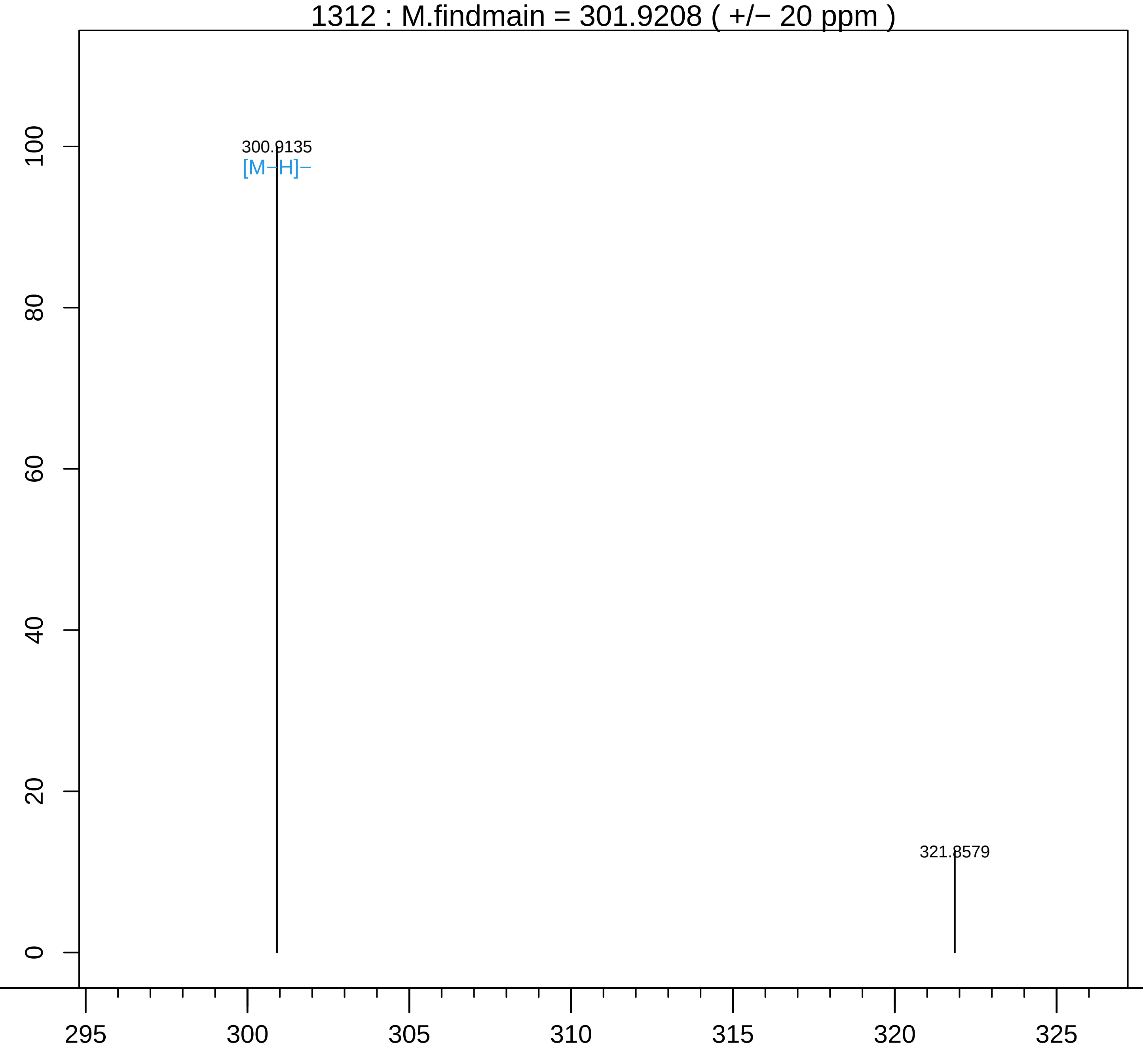
1312 : M.findmain = 301.9208 (+/− 20 ppm)
300.9135
[M−H]−
321.8579
0
20
40
60
80
100
295
300
305
310
315
320
325

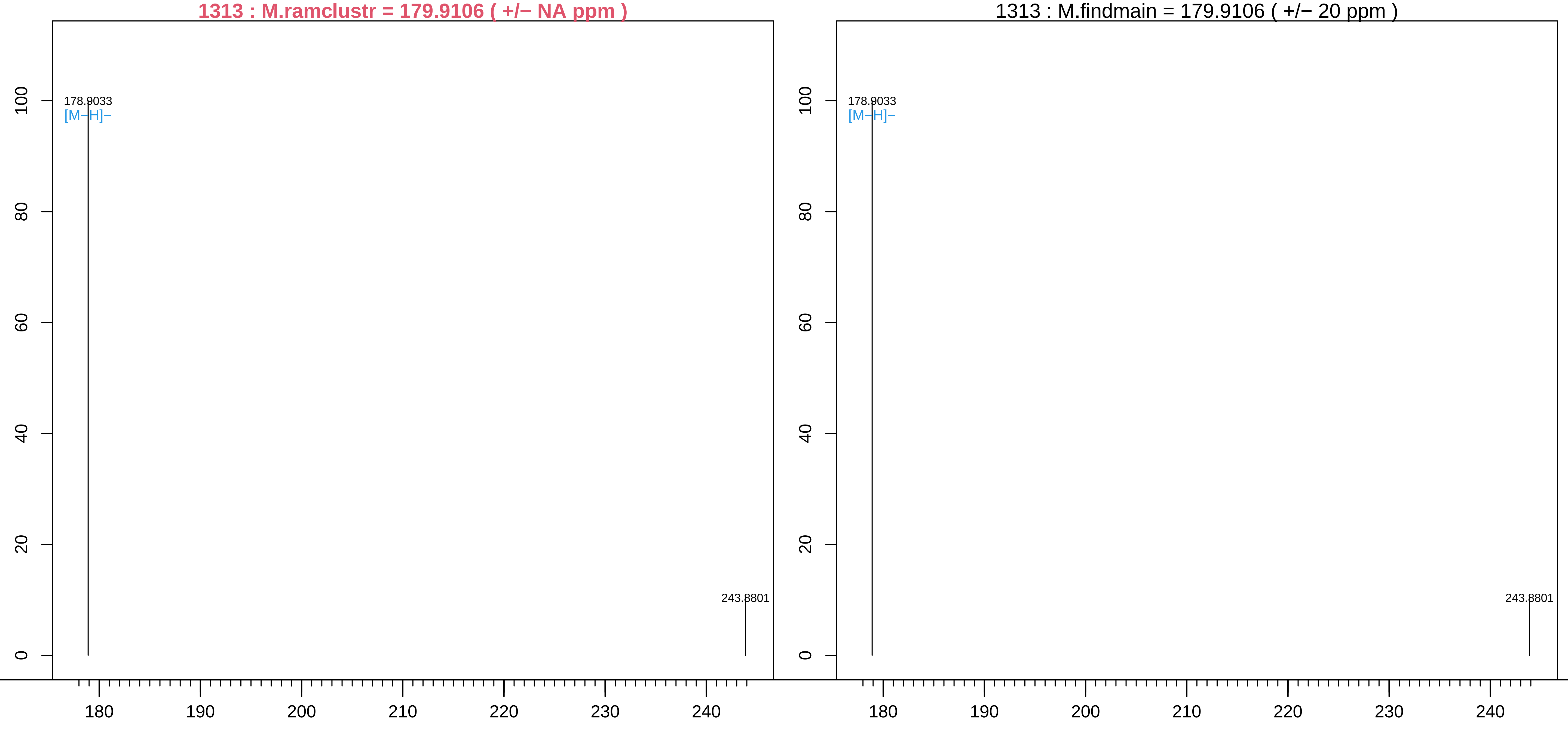
1313 : M.ramclustr = 179.9106 (+/− NA ppm)
178.9033
[M−H]−
243.8801
1313 : M.findmain = 179.9106 (+/− 20 ppm)
178.9033
[M−H]−
243.8801
0
20
40
60
80
100
180
190
200
210
220
230
240

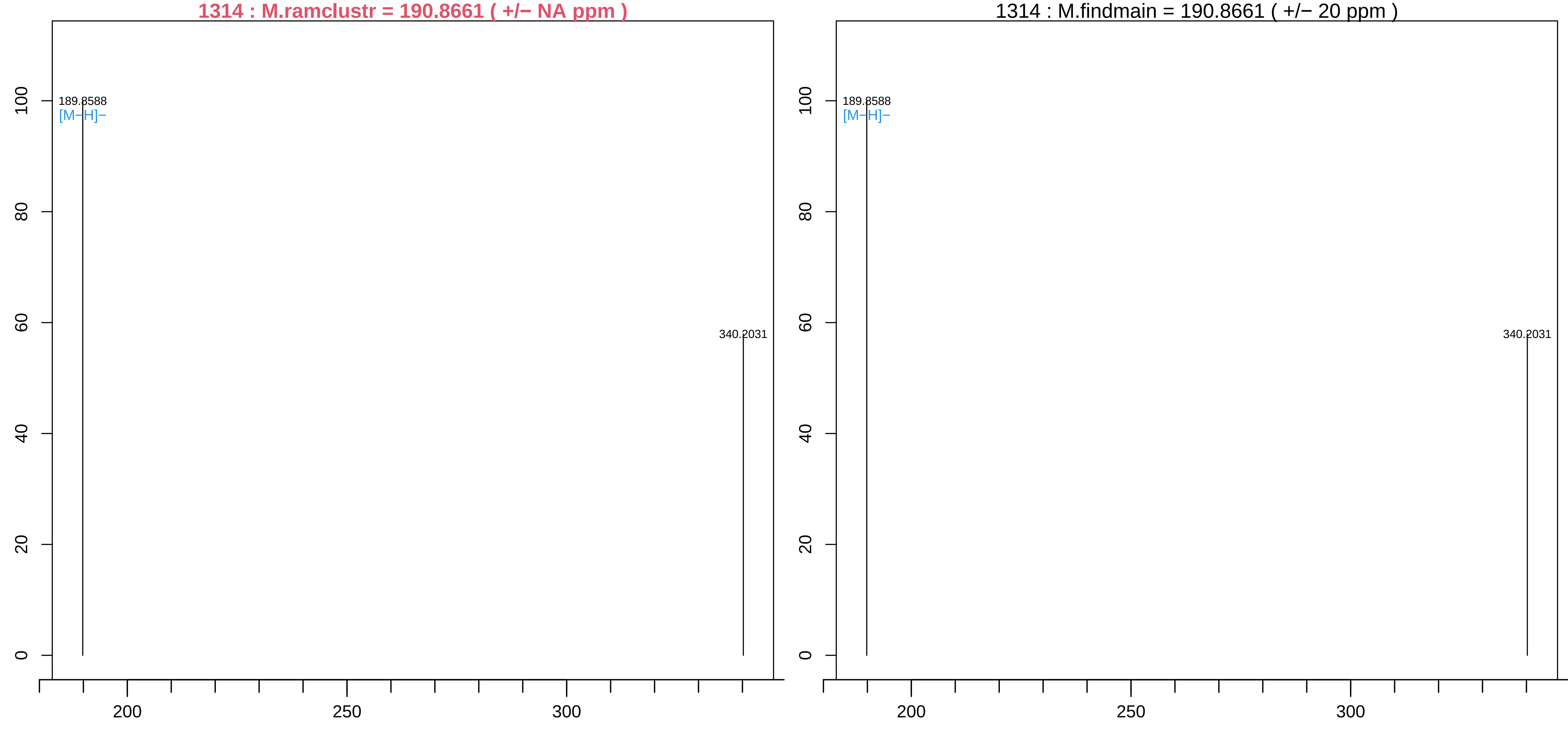
1314 : M.ramclustr = 190.8661 (+/− NA ppm)
189.8588
[M−H]−
340.2031
0
20
40
60
80
100
200
250
300
1314 : M.findmain = 190.8661 (+/− 20 ppm)
189.8588
[M−H]−
340.2031
0
20
40
60
80
100
200
250
300

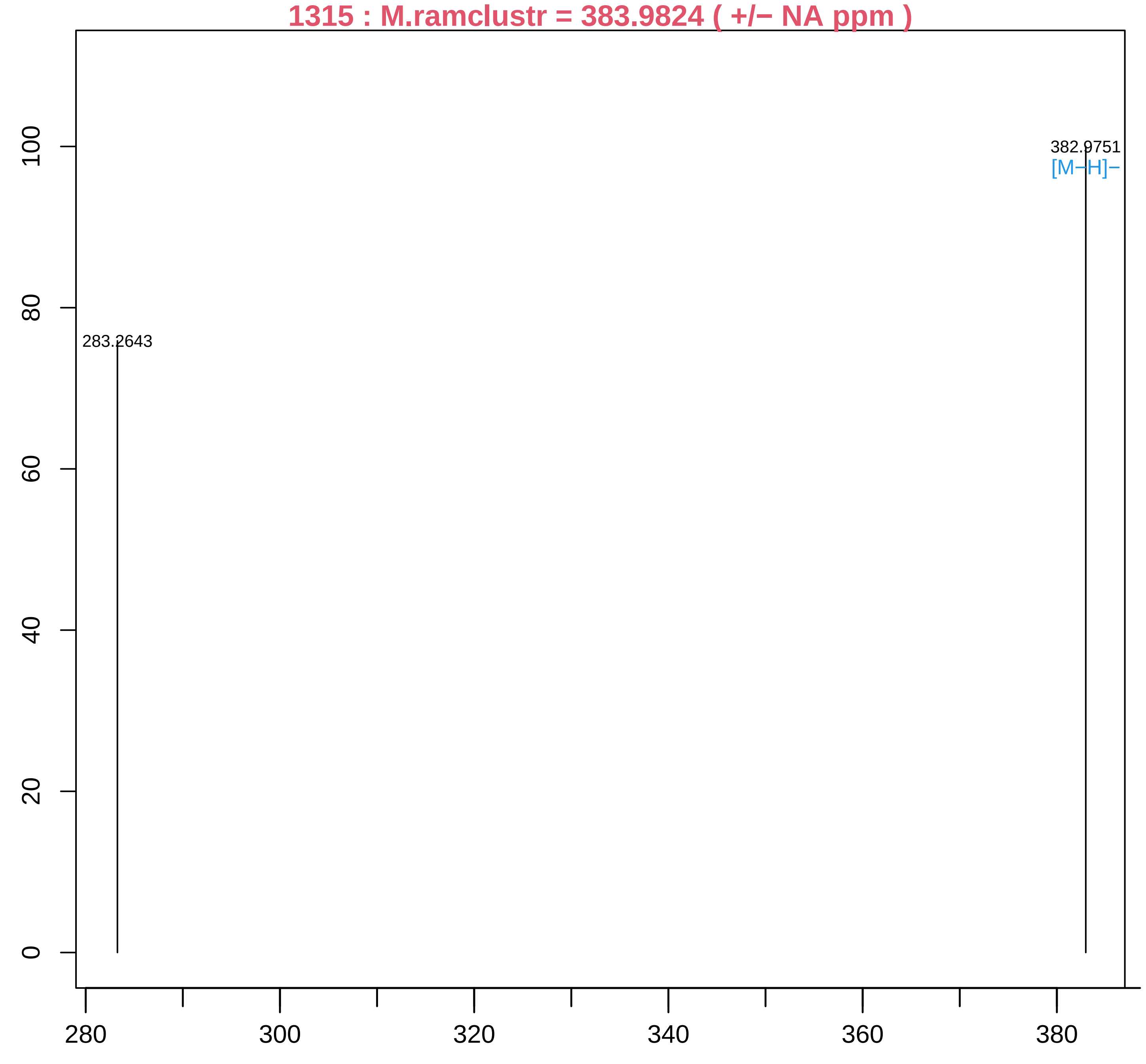
1315 : M.ramclustr = 383.9824 (+/− NA ppm)
382.9751
[M−H]−
283.2643
0
20
40
60
80
100
280
300
320
340
360
380

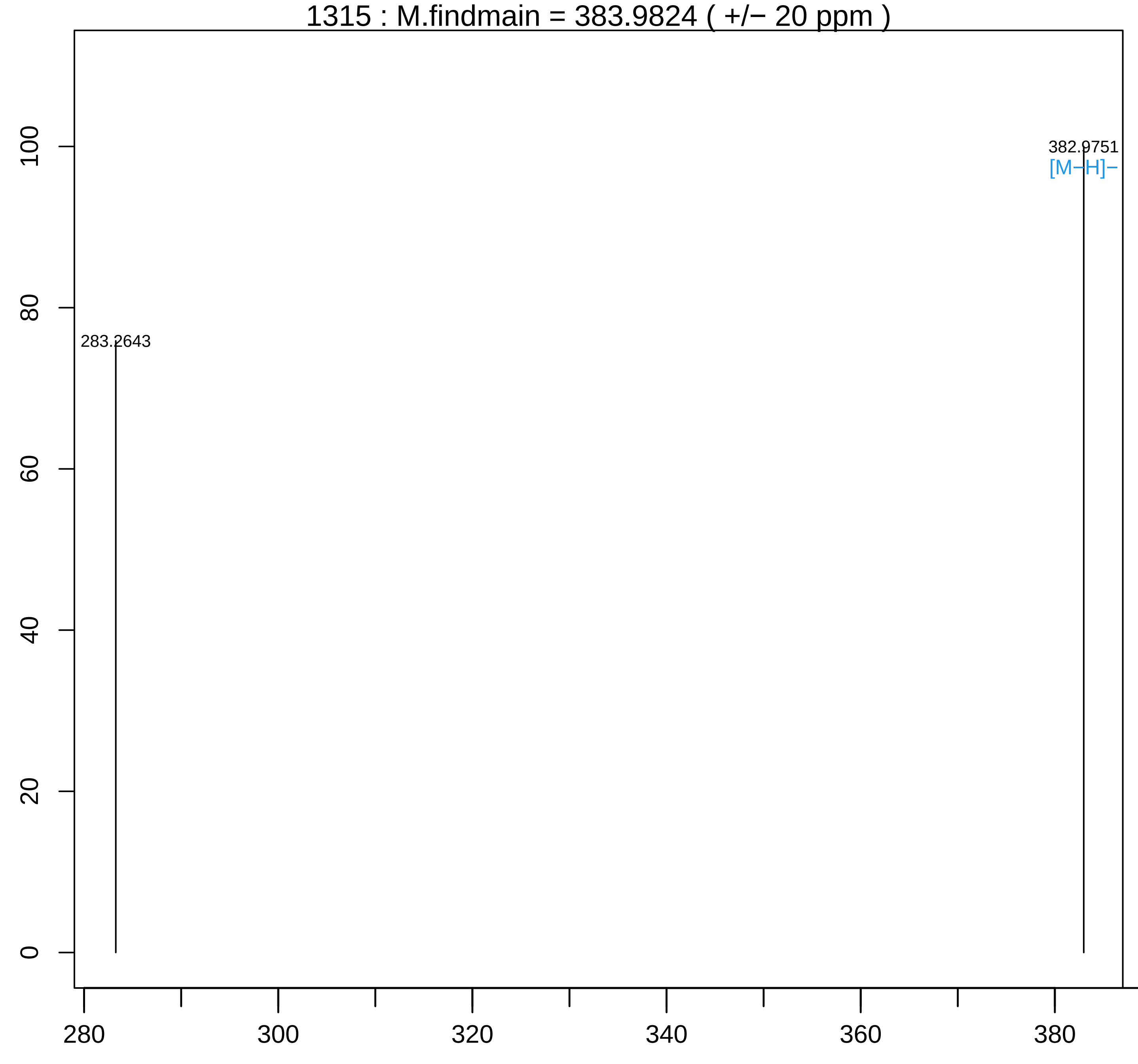
1315 : M.findmain = 383.9824 (+/− 20 ppm)
382.9751
[M−H]−
283.2643
0
20
40
60
80
100
280
300
320
340
360
380

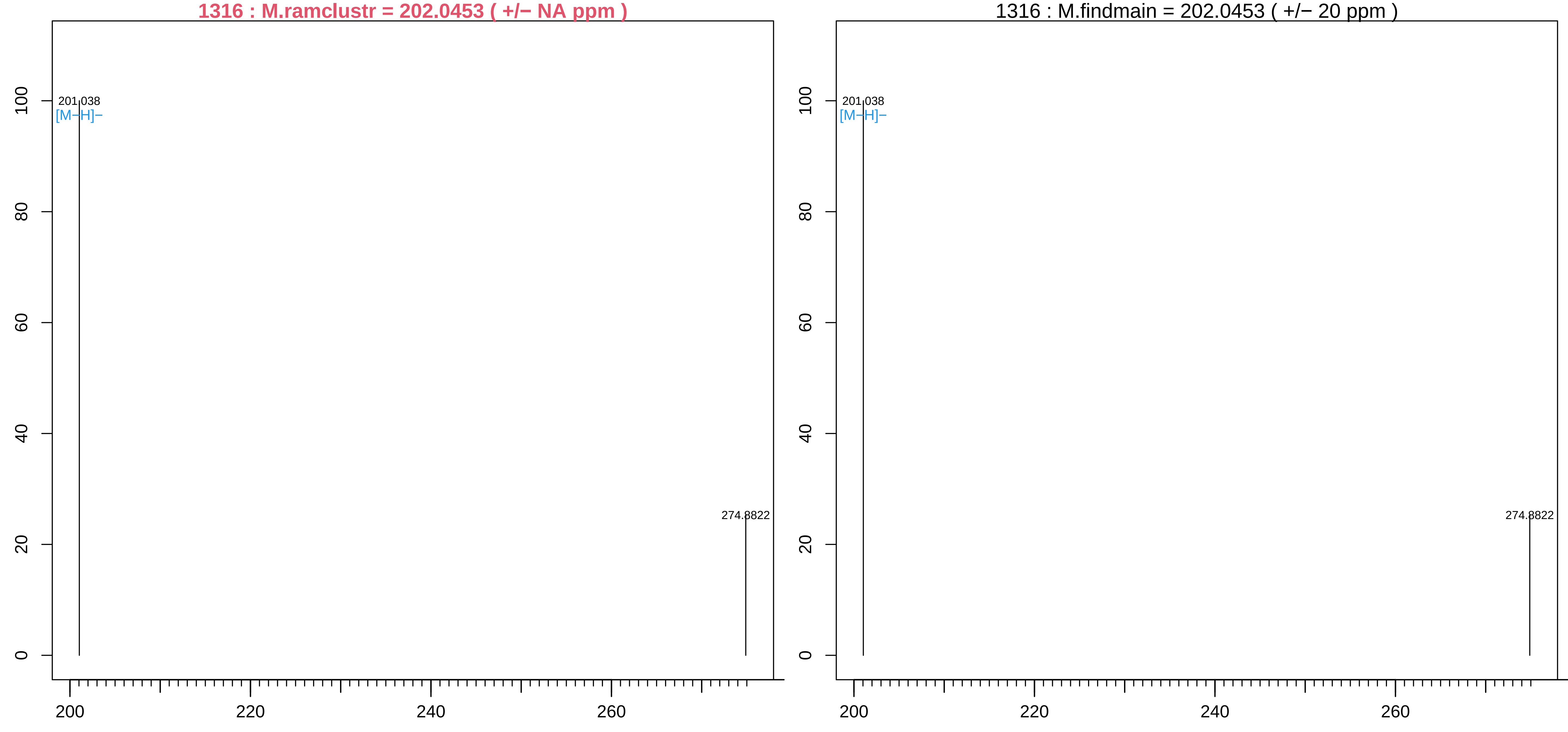
1316 : M.ramclustr = 202.0453 (+/− NA ppm)
201.038
[M−H]−
274.8822
1316 : M.findmain = 202.0453 (+/− 20 ppm)
201.038
[M−H]−
274.8822
0
20
40
60
80
100
200
220
240
260

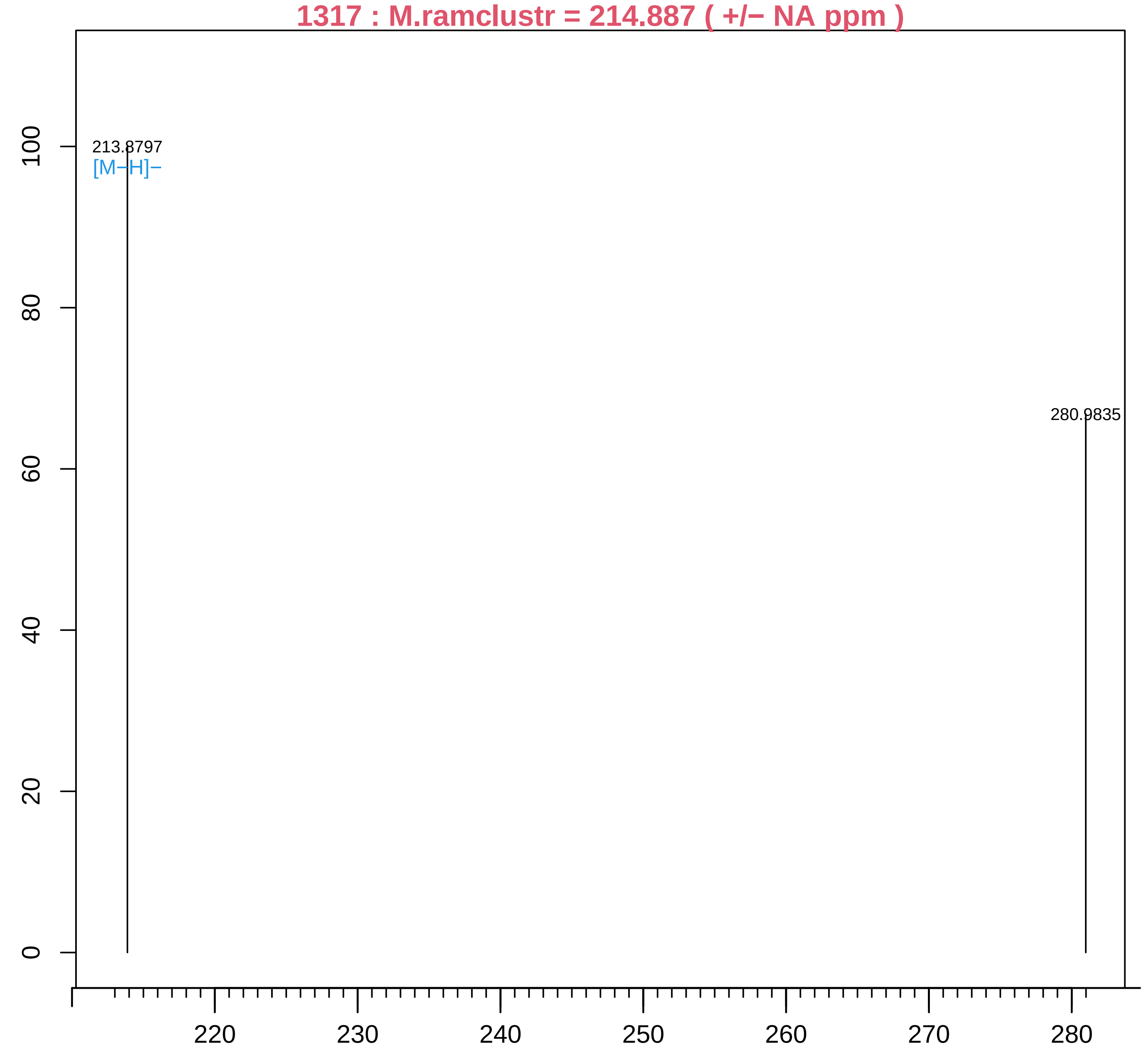
1317 : M.ramclustr = 214.887 (+/− NA ppm)
213.8797
[M−H]−
280.9835
0
20
40
60
80
100
220
230
240
250
260
270
280

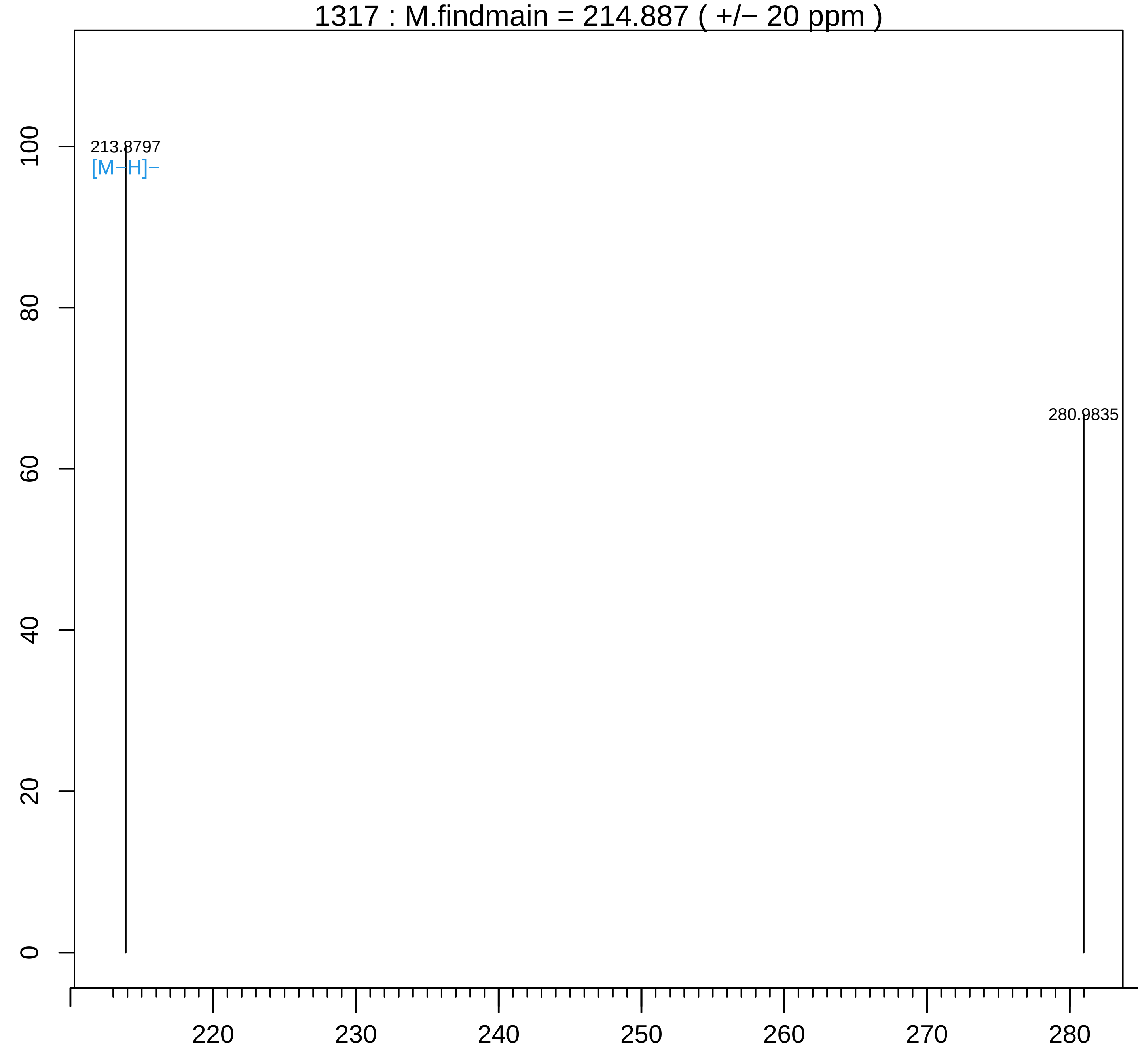
1317 : M.findmain = 214.887 (+/− 20 ppm)
213.8797
[M−H]−
280.9835
0
20
40
60
80
100
220
230
240
250
260
270
280

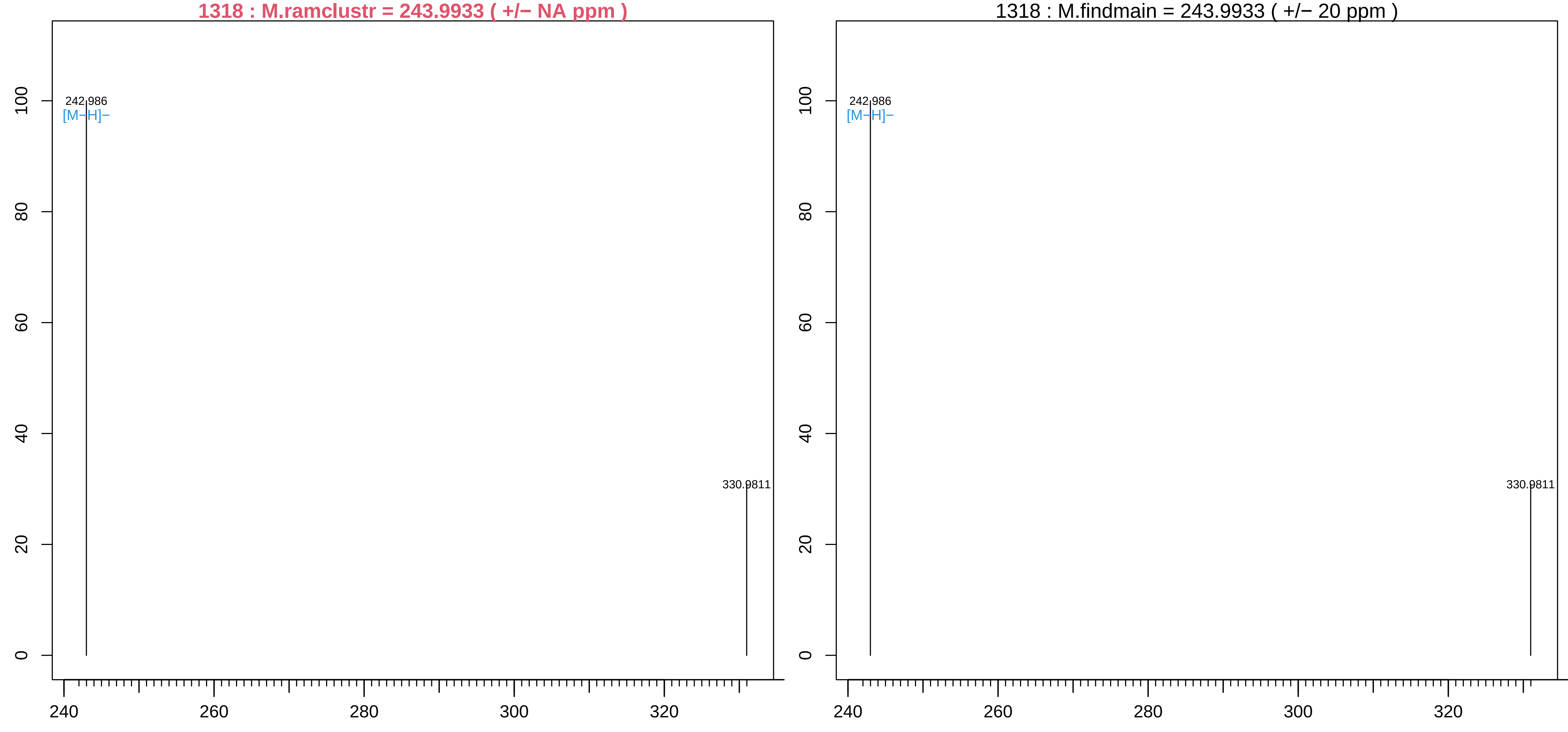
1318 : M.ramclustr = 243.9933 (+/− NA ppm)
242.986
[M−H]−
330.9811
1318 : M.findmain = 243.9933 (+/− 20 ppm)
242.986
[M−H]−
330.9811
0
20
40
60
80
100
240
260
280
300
320

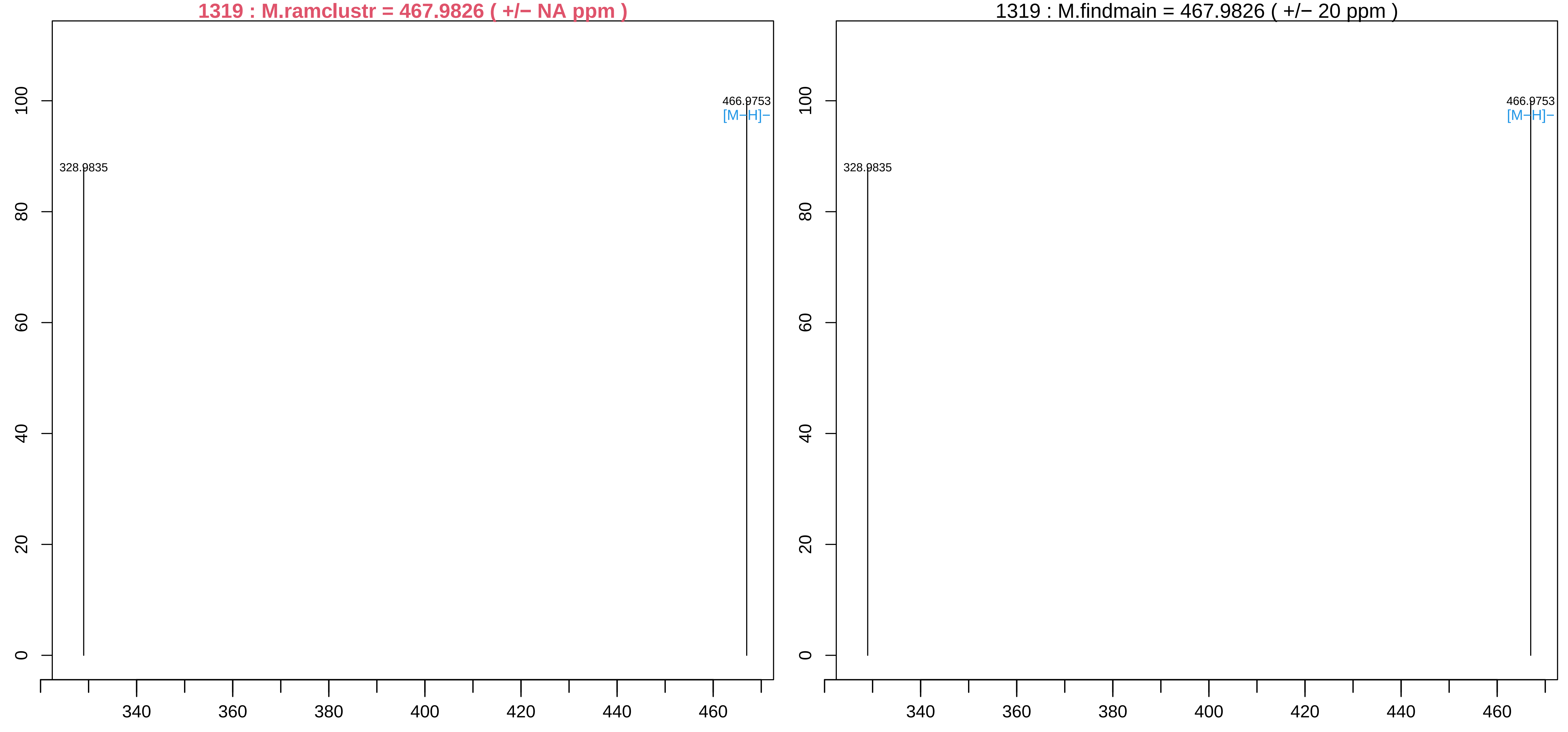

1319 : M.ramclustr = 467.9826 (+/− NA ppm)
466.9753
[M−H]−
328.9835
0
20
40
60
80
100
340
360
380
400
420
440
460
1319 : M.findmain = 467.9826 (+/− 20 ppm)
466.9753
[M−H]−
328.9835
0
20
40
60
80
100
340
360
380
400
420
440
460

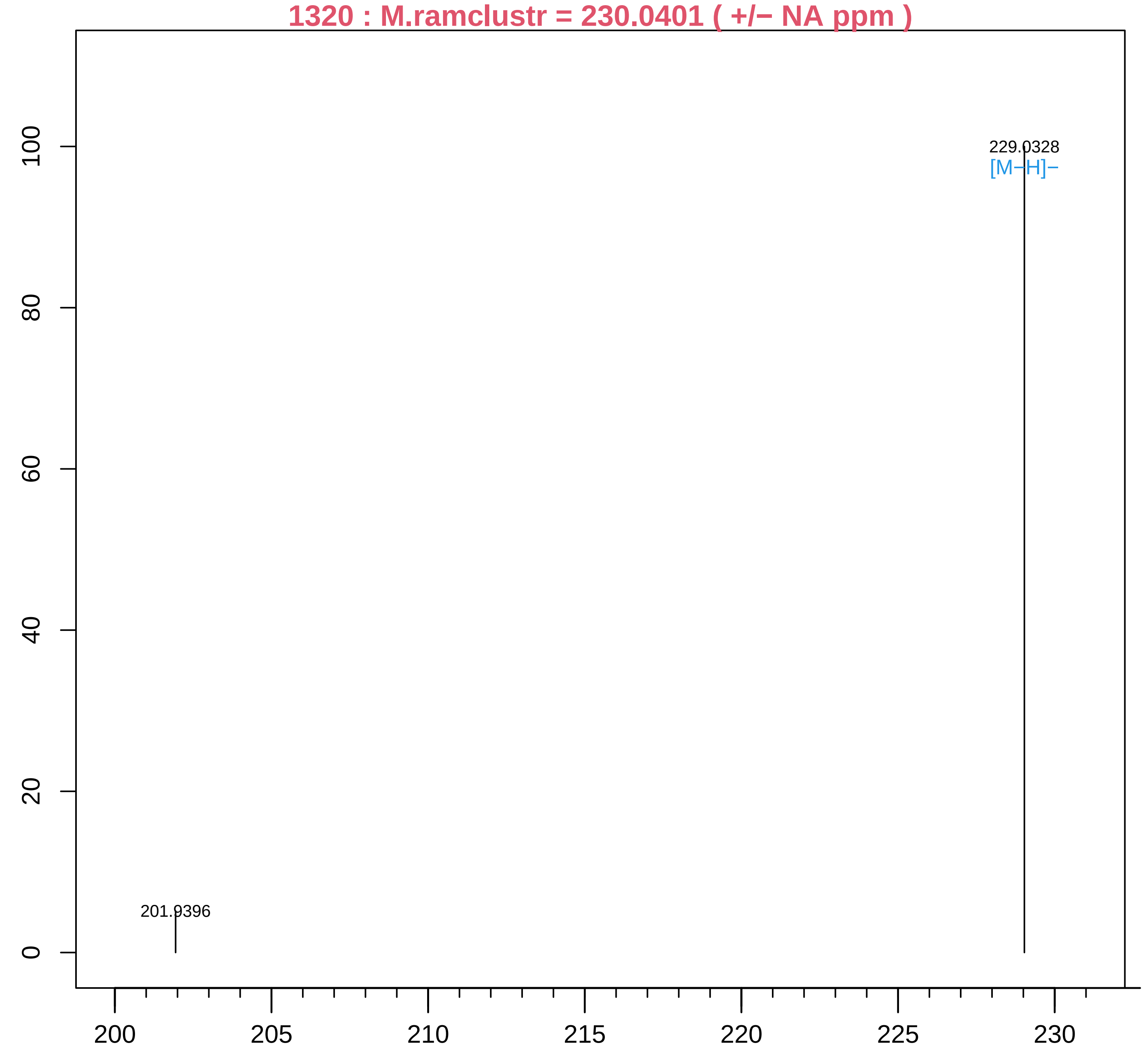
1320 : M.ramclustr = 230.0401 (+/− NA ppm)
229.0328
[M−H]−
201.9396
0
20
40
60
80
100
200
205
210
215
220
225
230

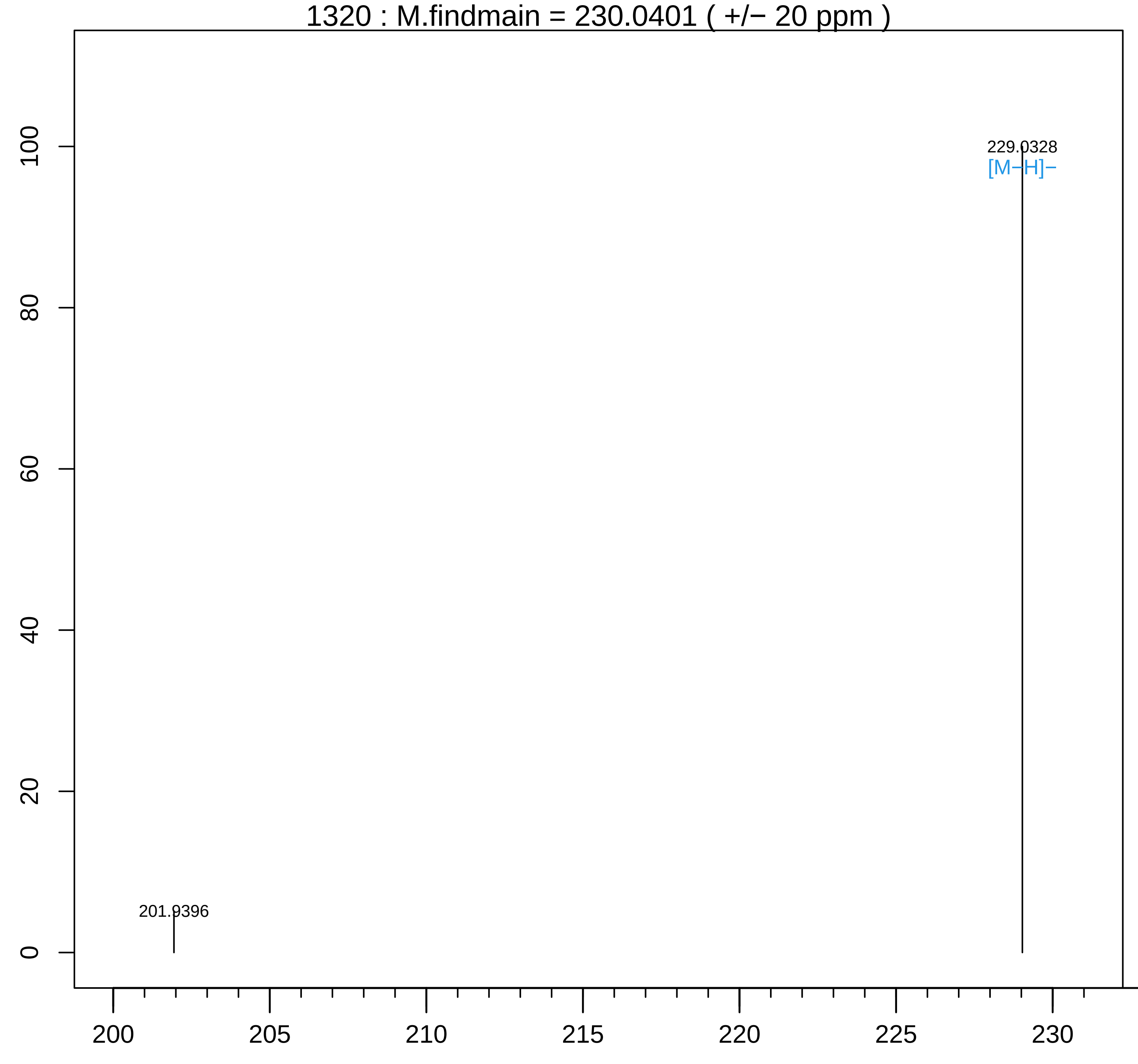
1320 : M.findmain = 230.0401 (+/− 20 ppm)
229.0328
[M−H]−
201.9396
0
20
40
60
80
100
200
205
210
215
220
225
230

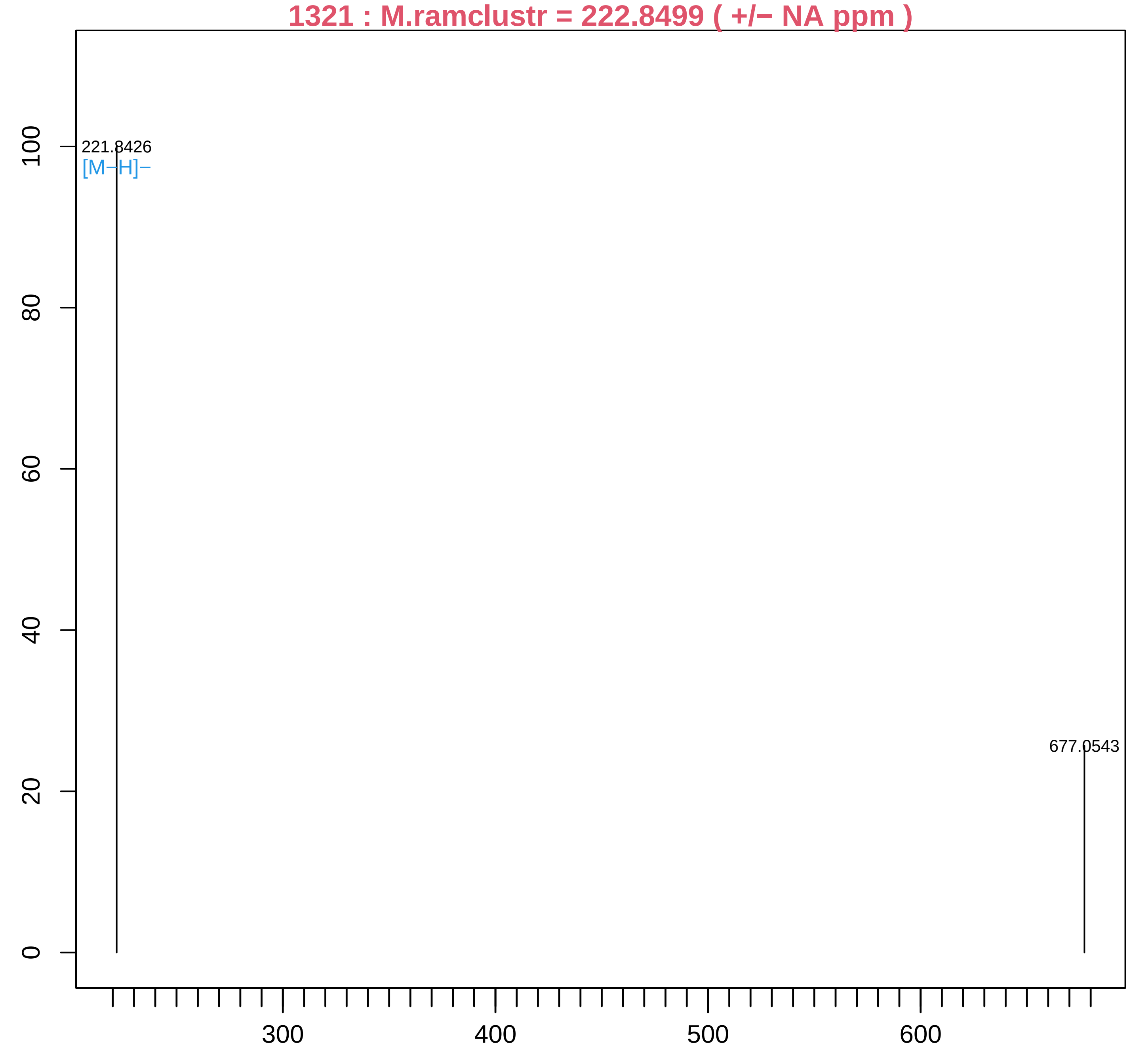
1321 : M.ramclustr = 222.8499 (+/− NA ppm)
221.8426
[M−H]−
677.0543
0
20
40
60
80
100
300
400
500
600

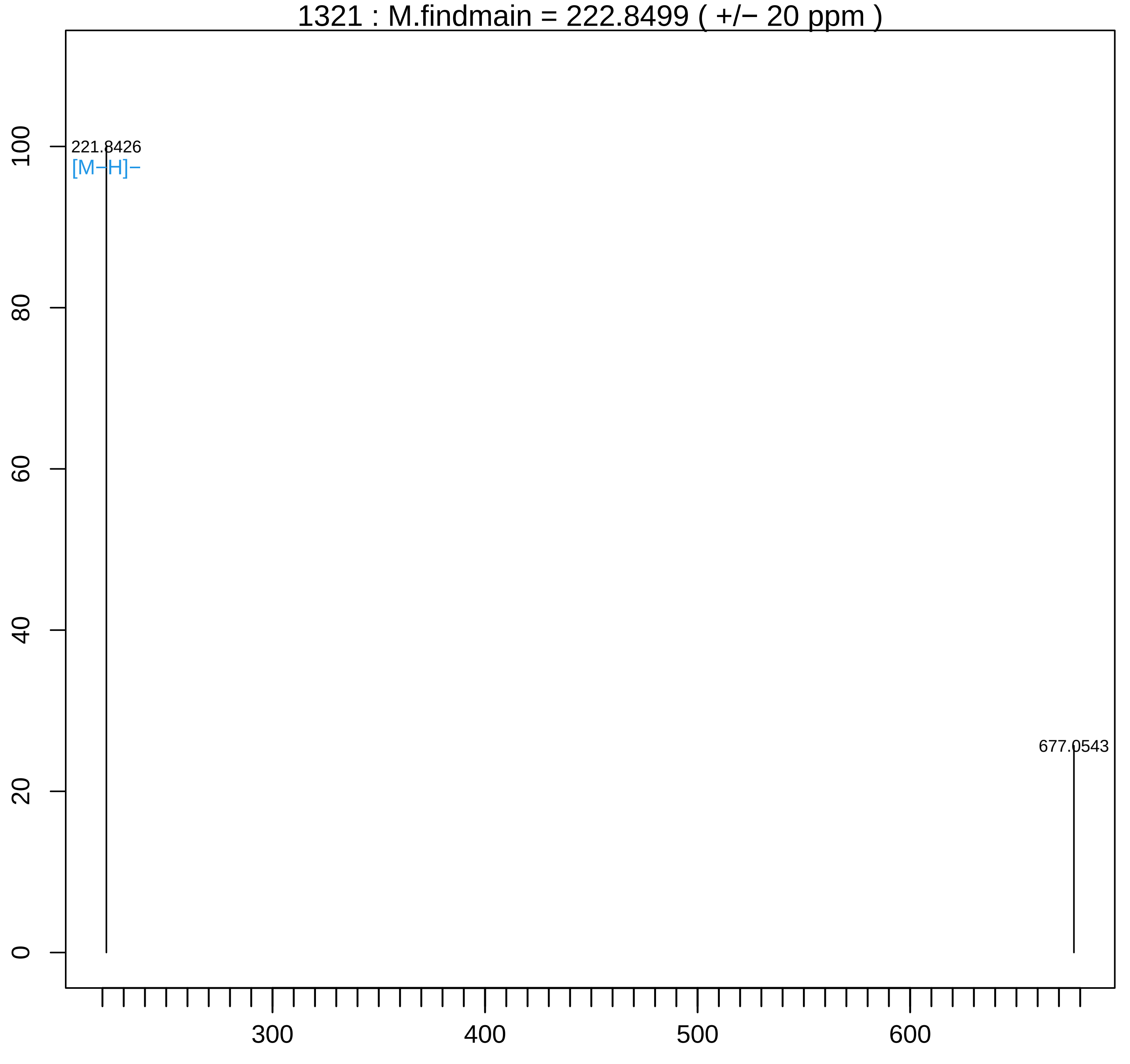
1321 : M.findmain = 222.8499 (+/− 20 ppm)
221.8426
[M−H]−
677.0543
0
20
40
60
80
100
300
400
500
600

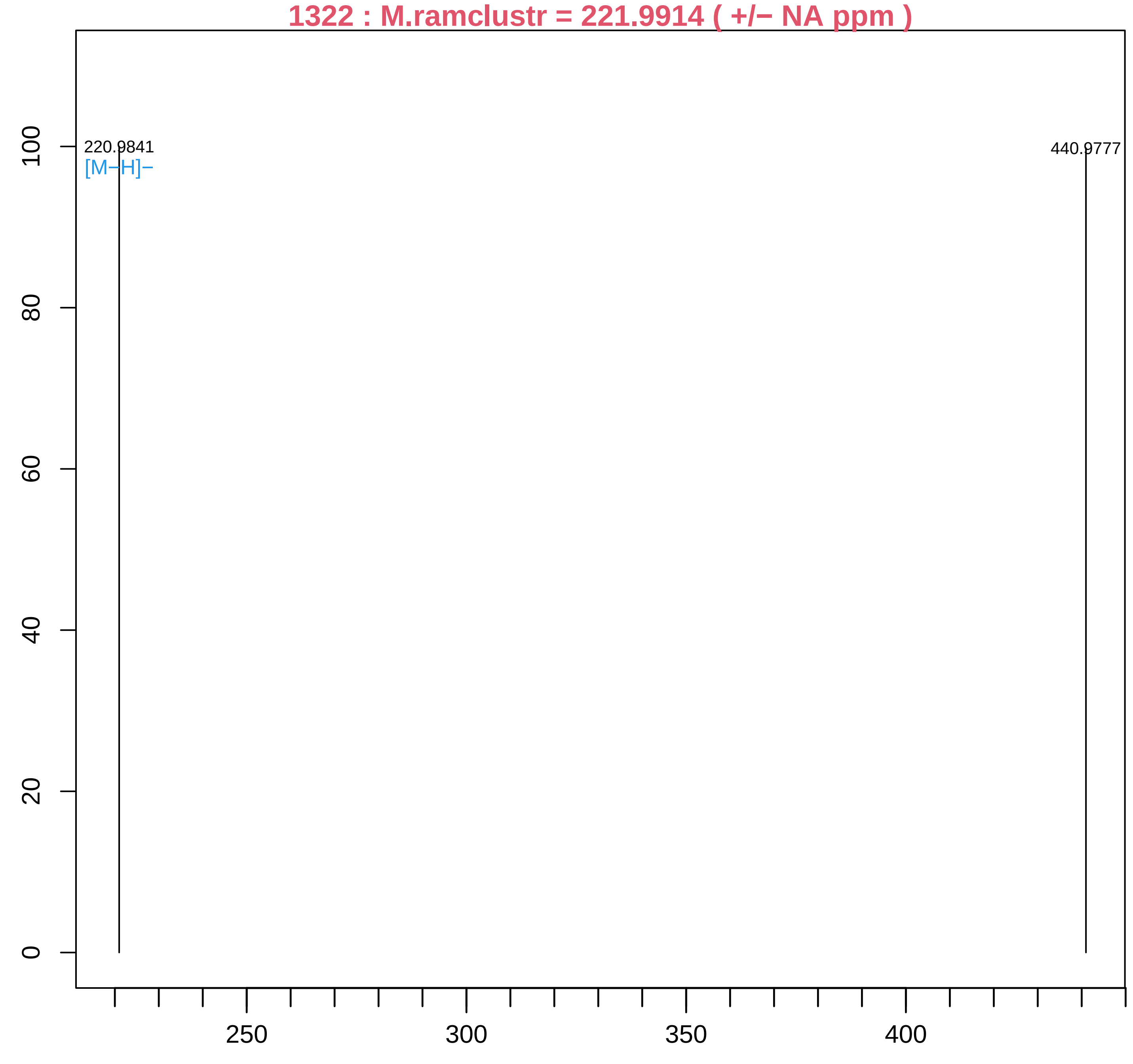

1322 : M.ramclustr = 221.9914 (+/− NA ppm)
220.9841
[M−H]−
440.9777
0
20
40
60
80
100
250
300
350
400

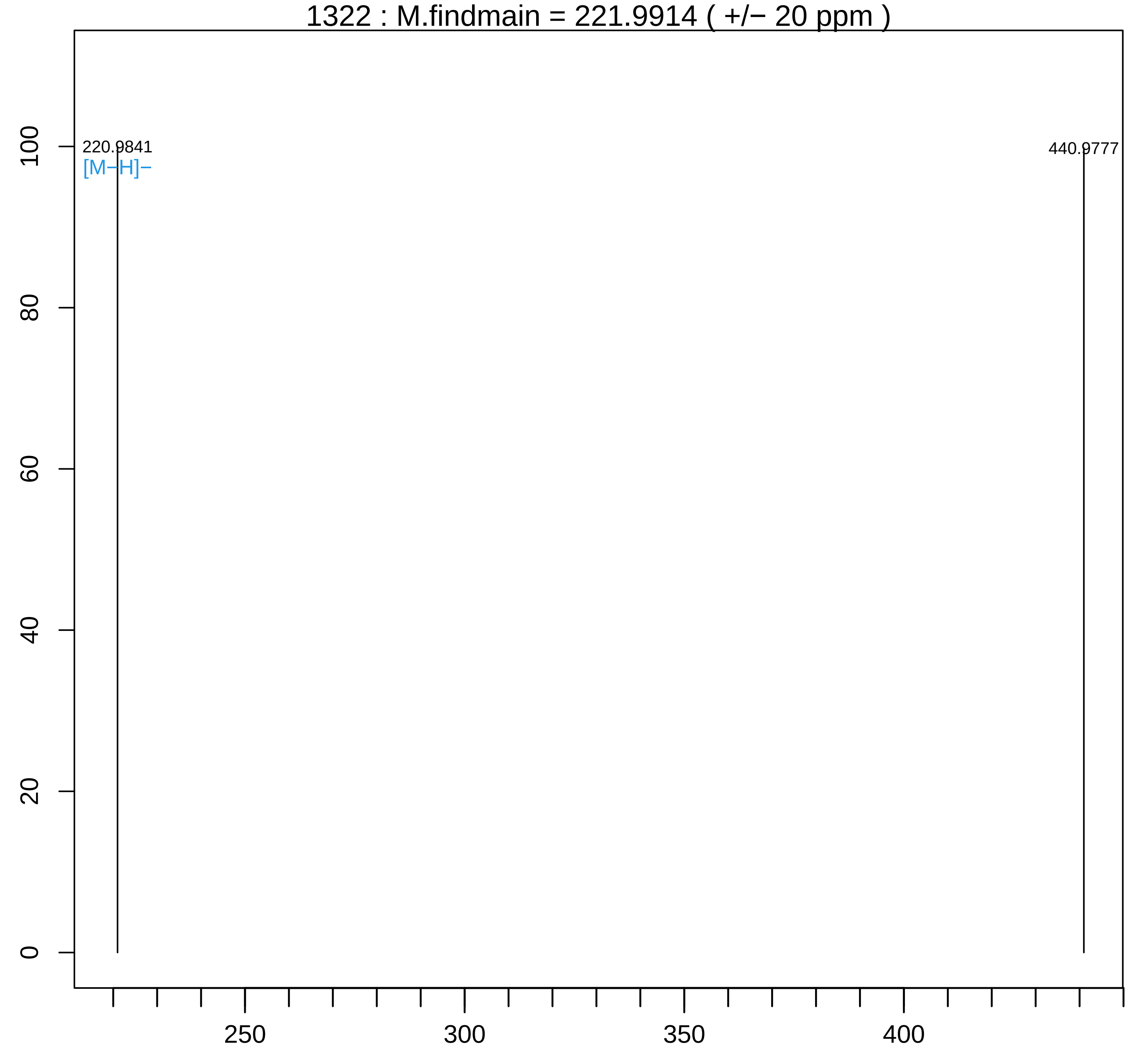

1322 : M.findmain = 221.9914 (+/− 20 ppm)
220.9841
[M−H]−
440.9777
0
20
40
60
80
100
250
300
350
400

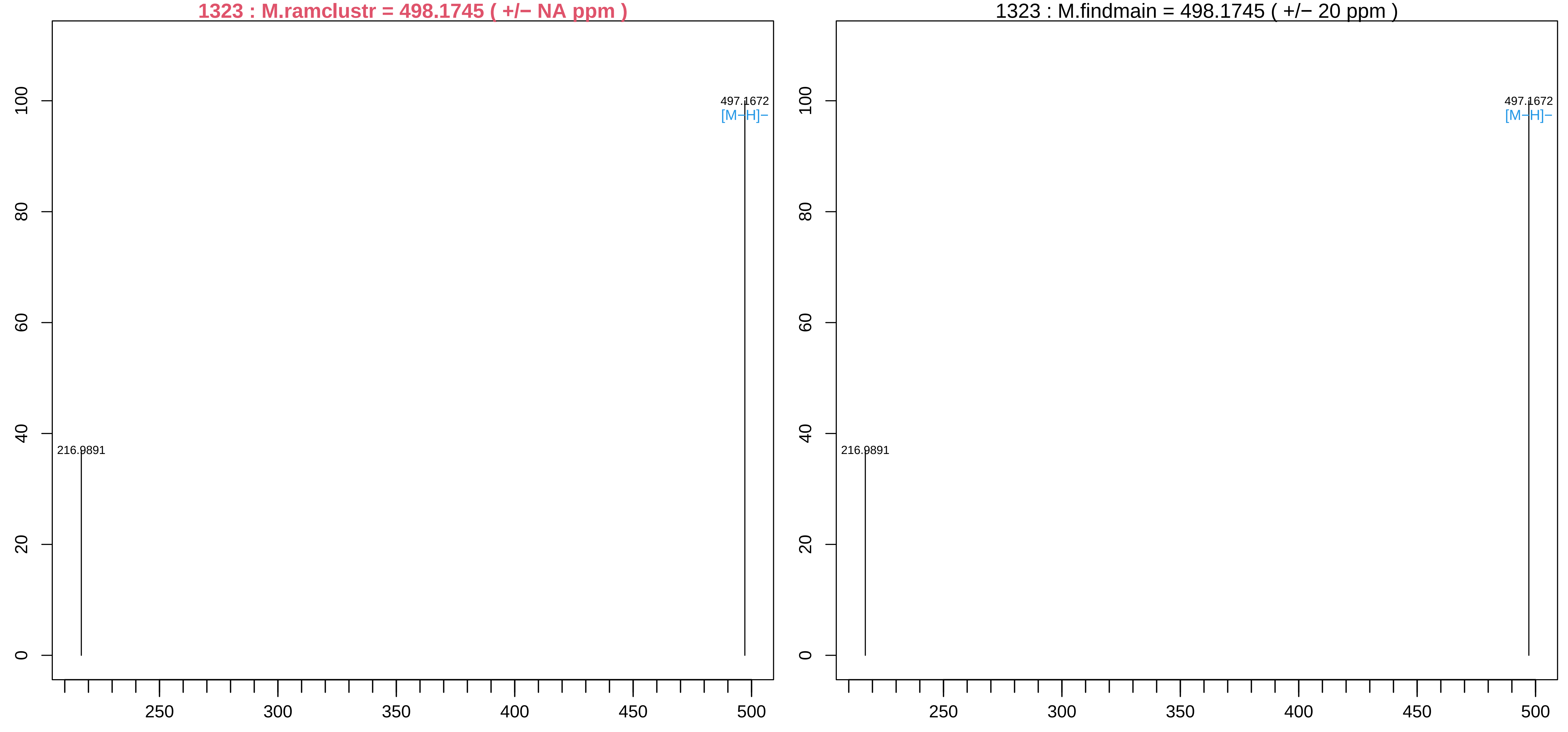
1323 : M.ramclustr = 498.1745 (+/− NA ppm)
497.1672
[M−H]−
216.9891
1323 : M.findmain = 498.1745 (+/− 20 ppm)
497.1672
[M−H]−
216.9891
0
20
40
60
80
100
250
300
350
400
450
500

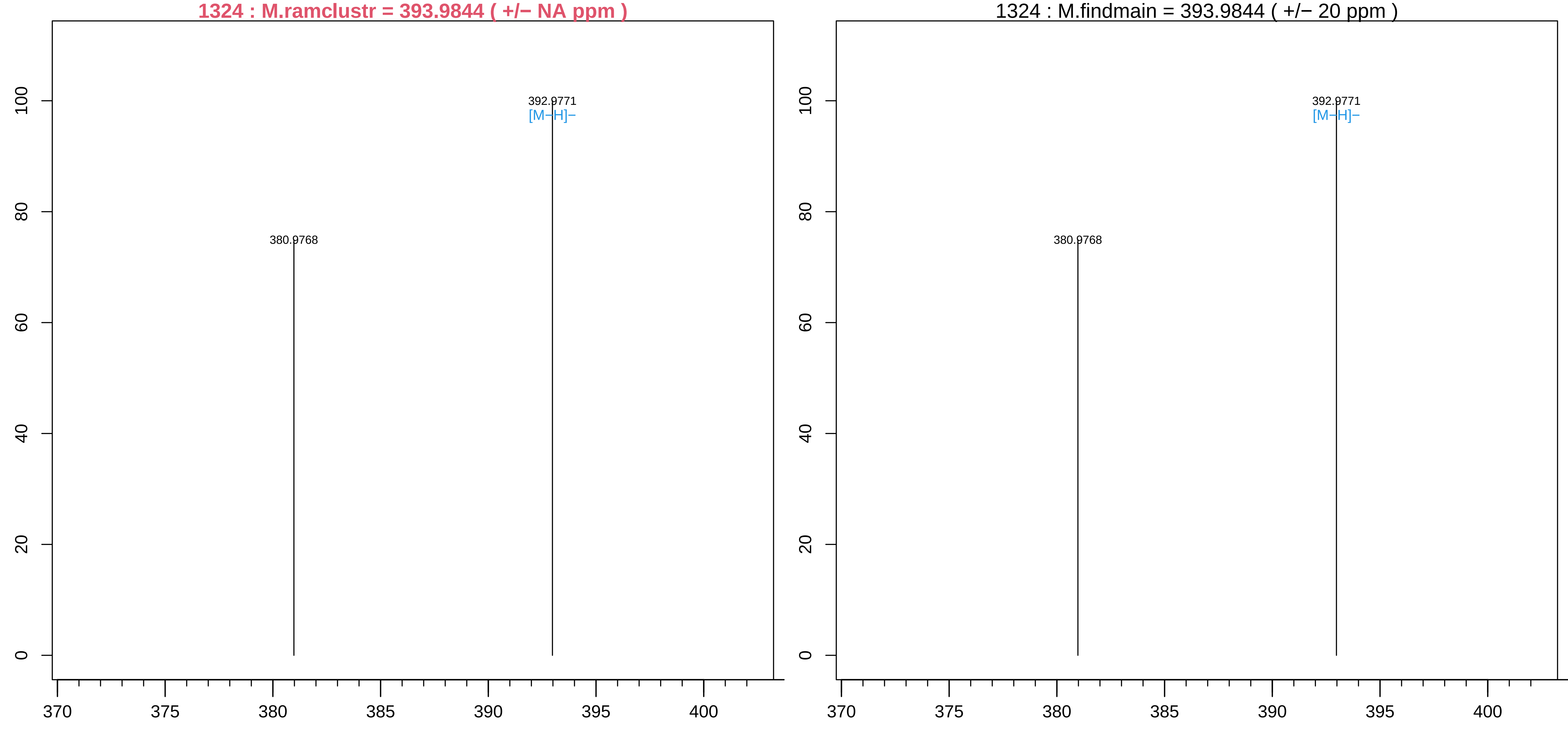

1324 : M.ramclustr = 393.9844 (+/− NA ppm)
392.9771
[M−H]−
380.9768
1324 : M.findmain = 393.9844 (+/− 20 ppm)
392.9771
[M−H]−
380.9768
0
20
40
60
80
100
370
375
380
385
390
395
400

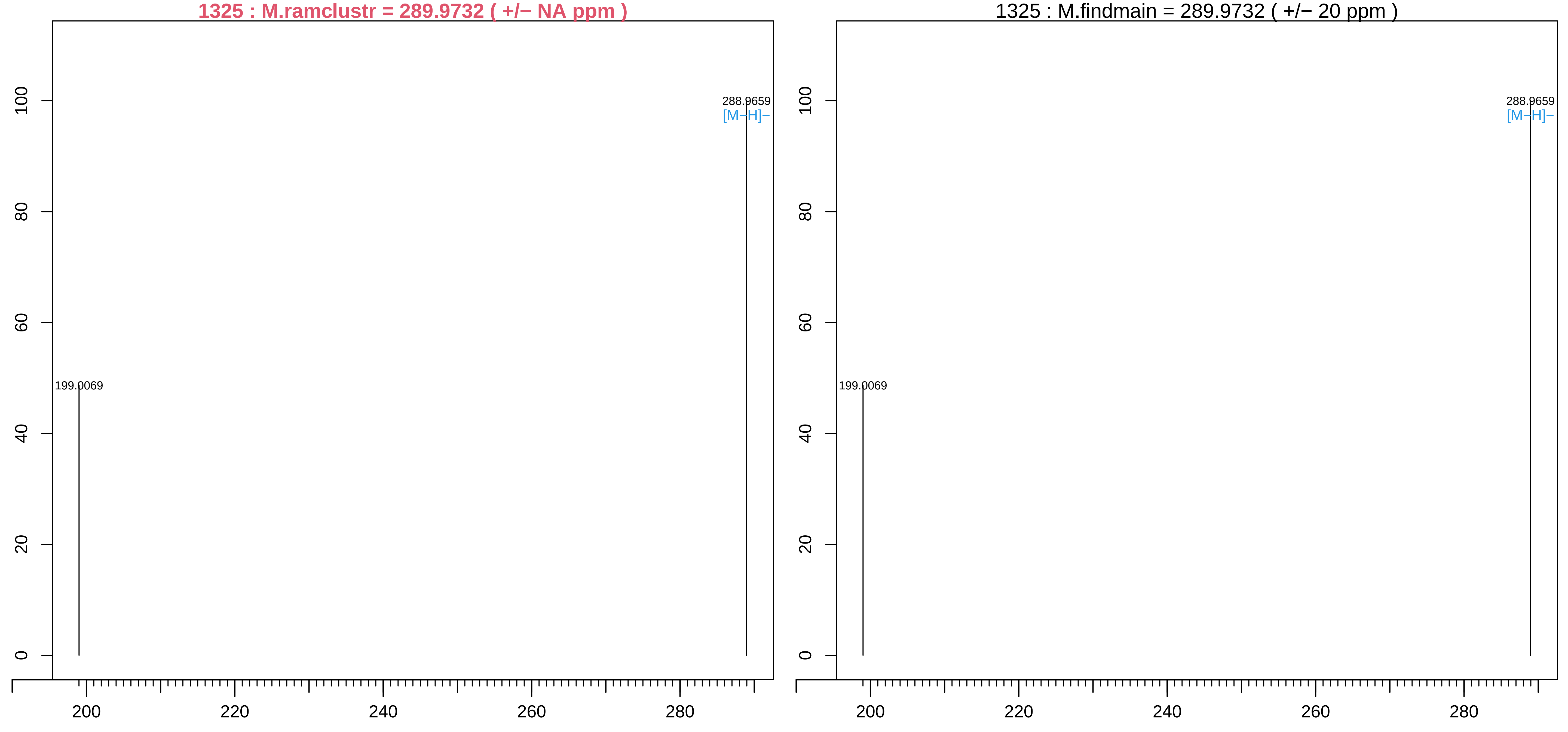
1325 : M.ramclustr = 289.9732 (+/− NA ppm)
288.9659
[M−H]−
199.0069
1325 : M.findmain = 289.9732 (+/− 20 ppm)
288.9659
[M−H]−
199.0069
0
20
40
60
80
100
200
220
240
260
280

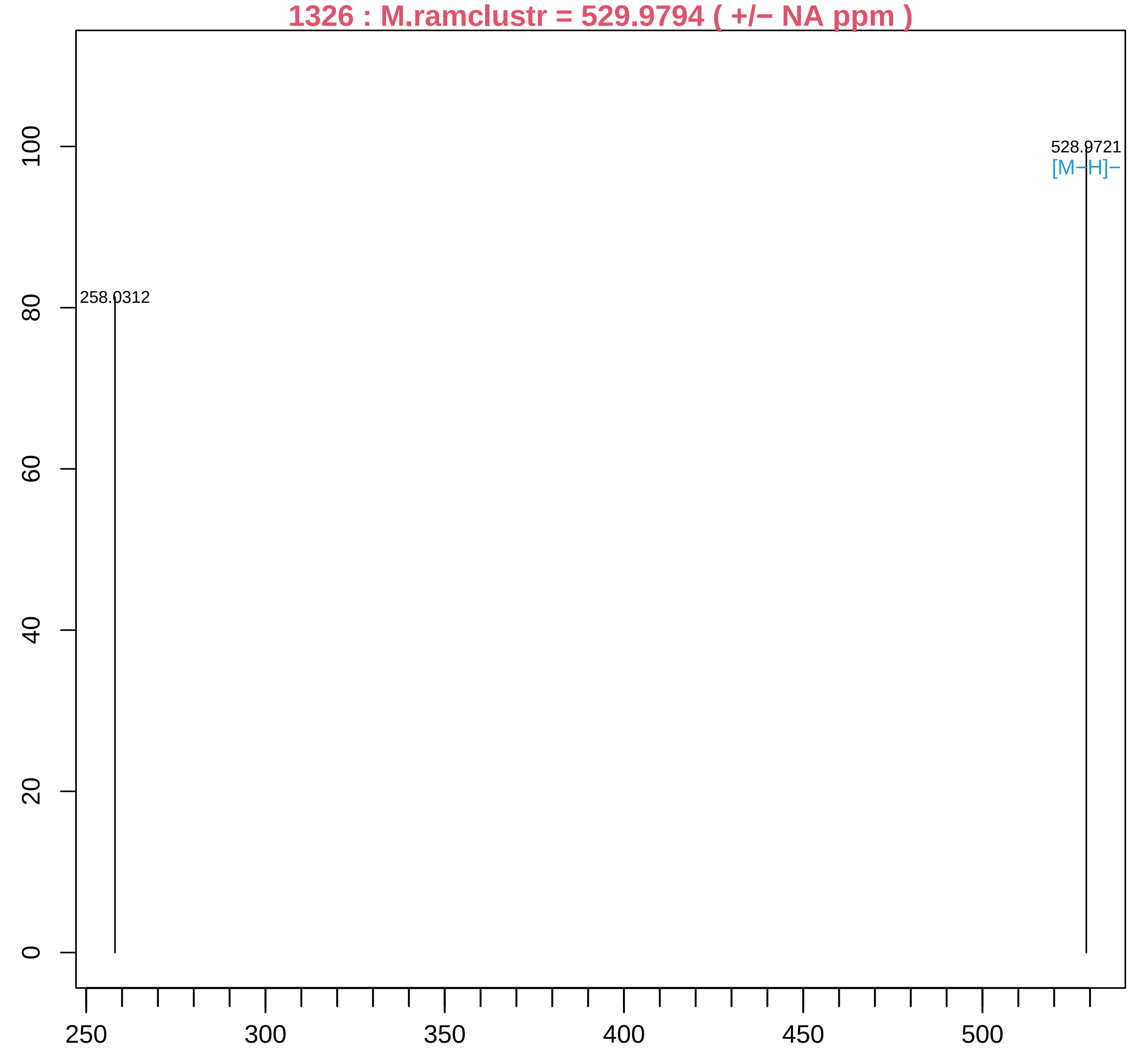
1326 : M.ramclustr = 529.9794 (+/− NA ppm)
528.9721
[M−H]−
258.0312
0
20
40
60
80
100
250
300
350
400
450
500

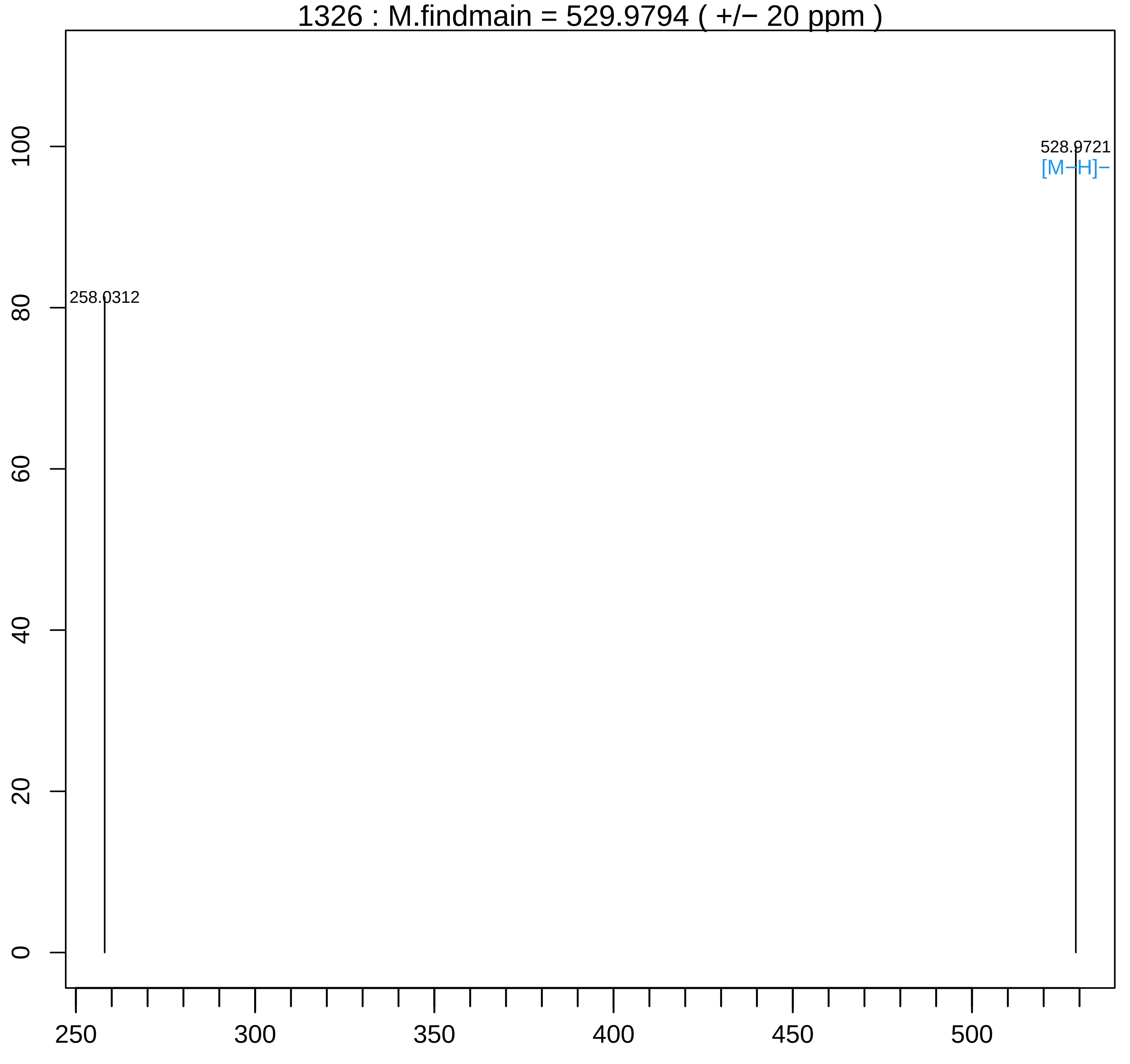
1326 : M.findmain = 529.9794 (+/− 20 ppm)
528.9721
[M−H]−
258.0312
0
20
40
60
80
100
250
300
350
400
450
500

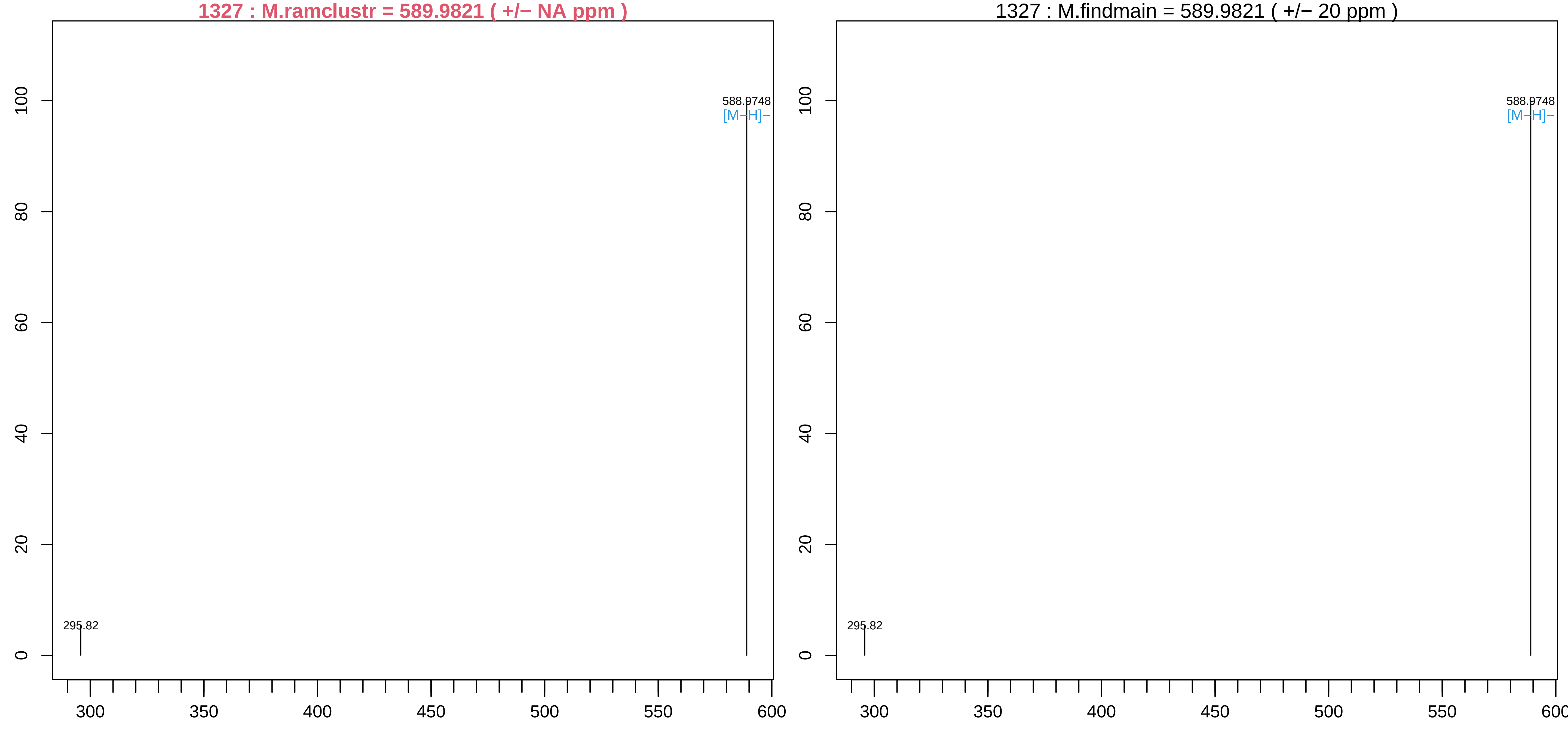
1327 : M.ramclustr = 589.9821 (+/− NA ppm)
588.9748
[M−H]−
295.82
1327 : M.findmain = 589.9821 (+/− 20 ppm)
588.9748
[M−H]−
295.82

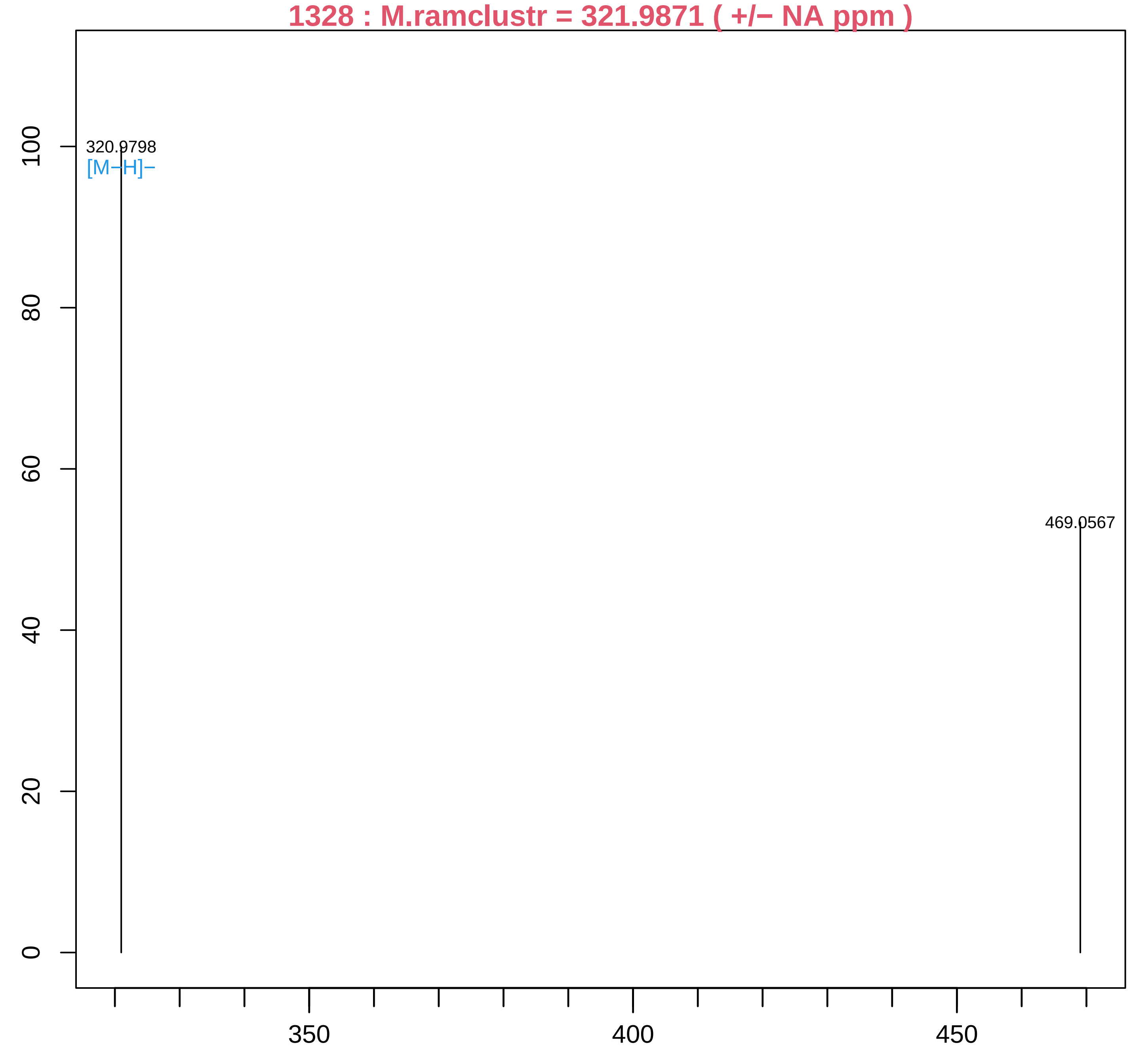
1328 : M.ramclustr = 321.9871 (+/− NA ppm)
320.9798
[M−H]−
469.0567
0
20
40
60
80
100
350
400
450

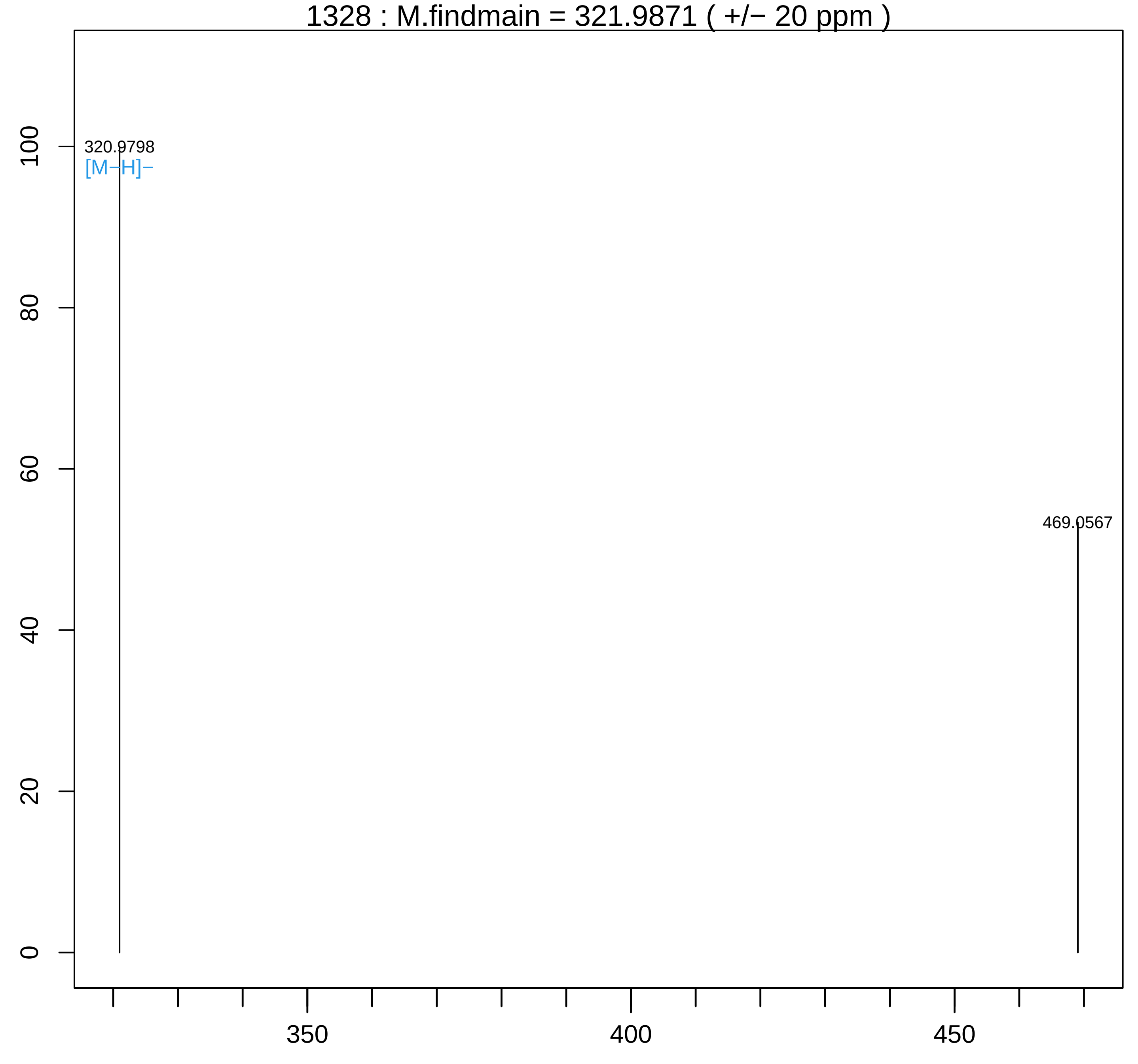
1328 : M.findmain = 321.9871 (+/− 20 ppm)
320.9798
[M−H]−
469.0567
0
20
40
60
80
100
350
400
450

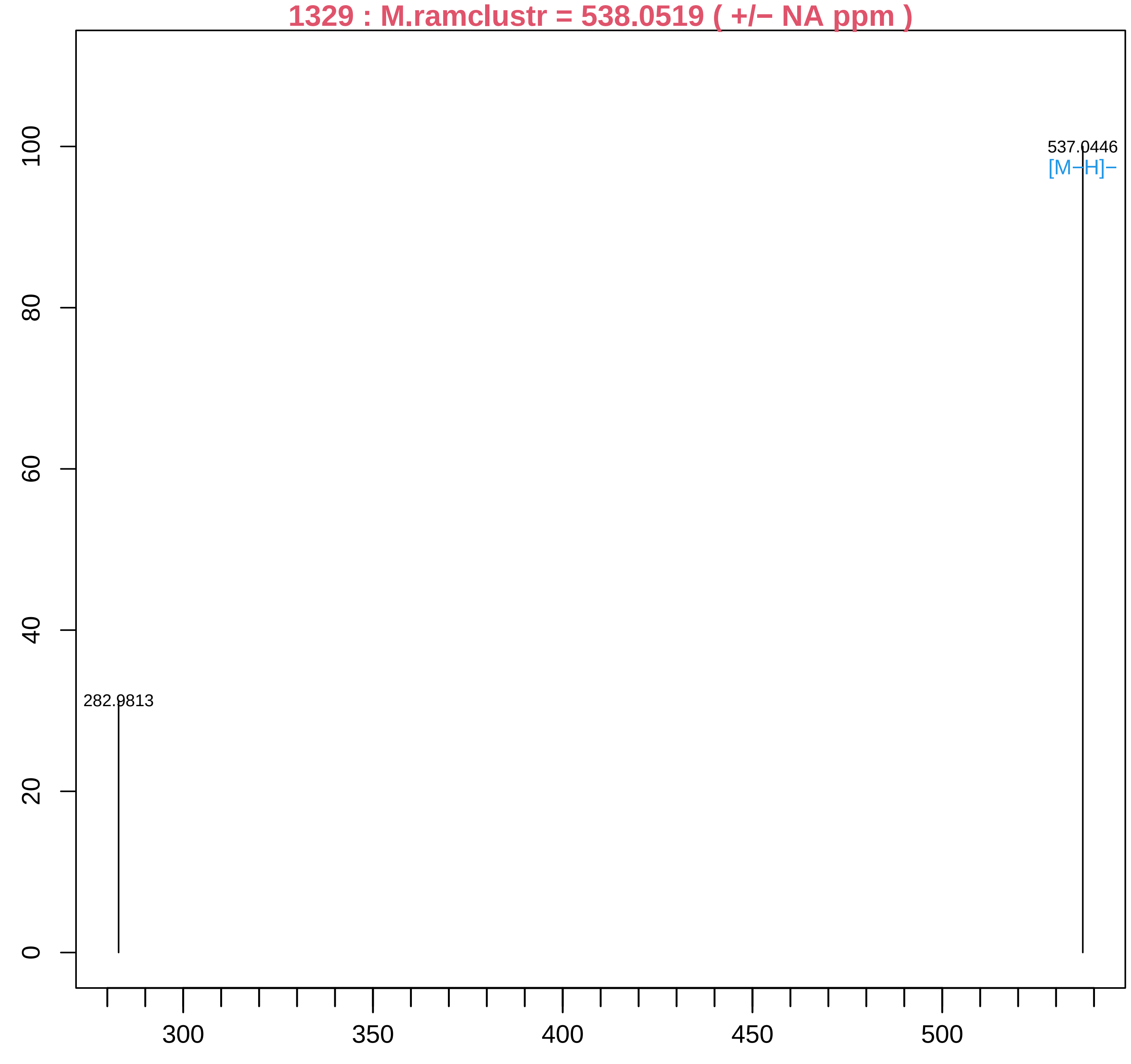
1329 : M.ramclustr = 538.0519 (+/− NA ppm)
537.0446
[M−H]−
282.9813
0
20
40
60
80
100
300
350
400
450
500

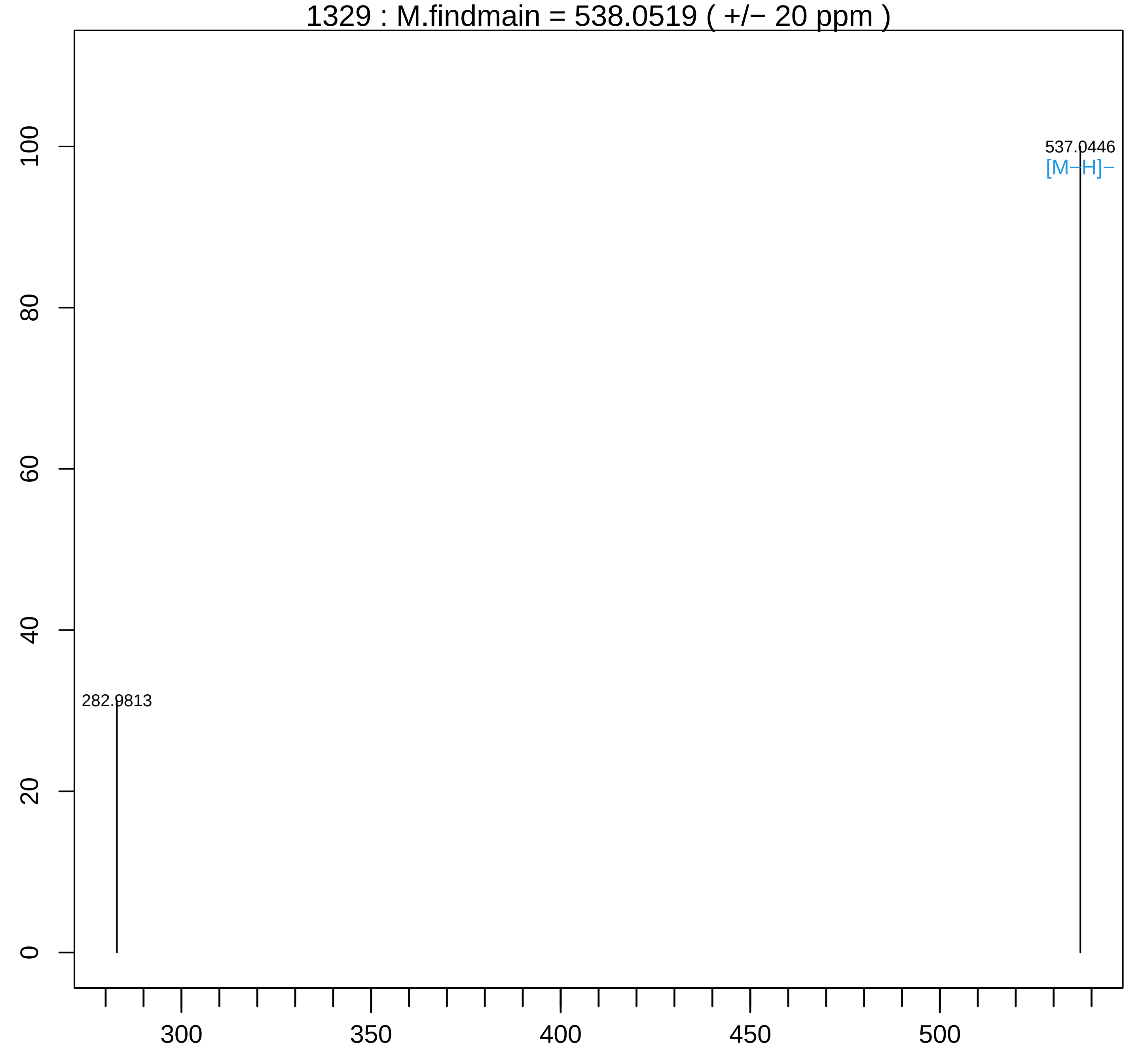
1329 : M.findmain = 538.0519 (+/− 20 ppm)
537.0446
[M−H]−
282.9813
0
20
40
60
80
100
300
350
400
450
500

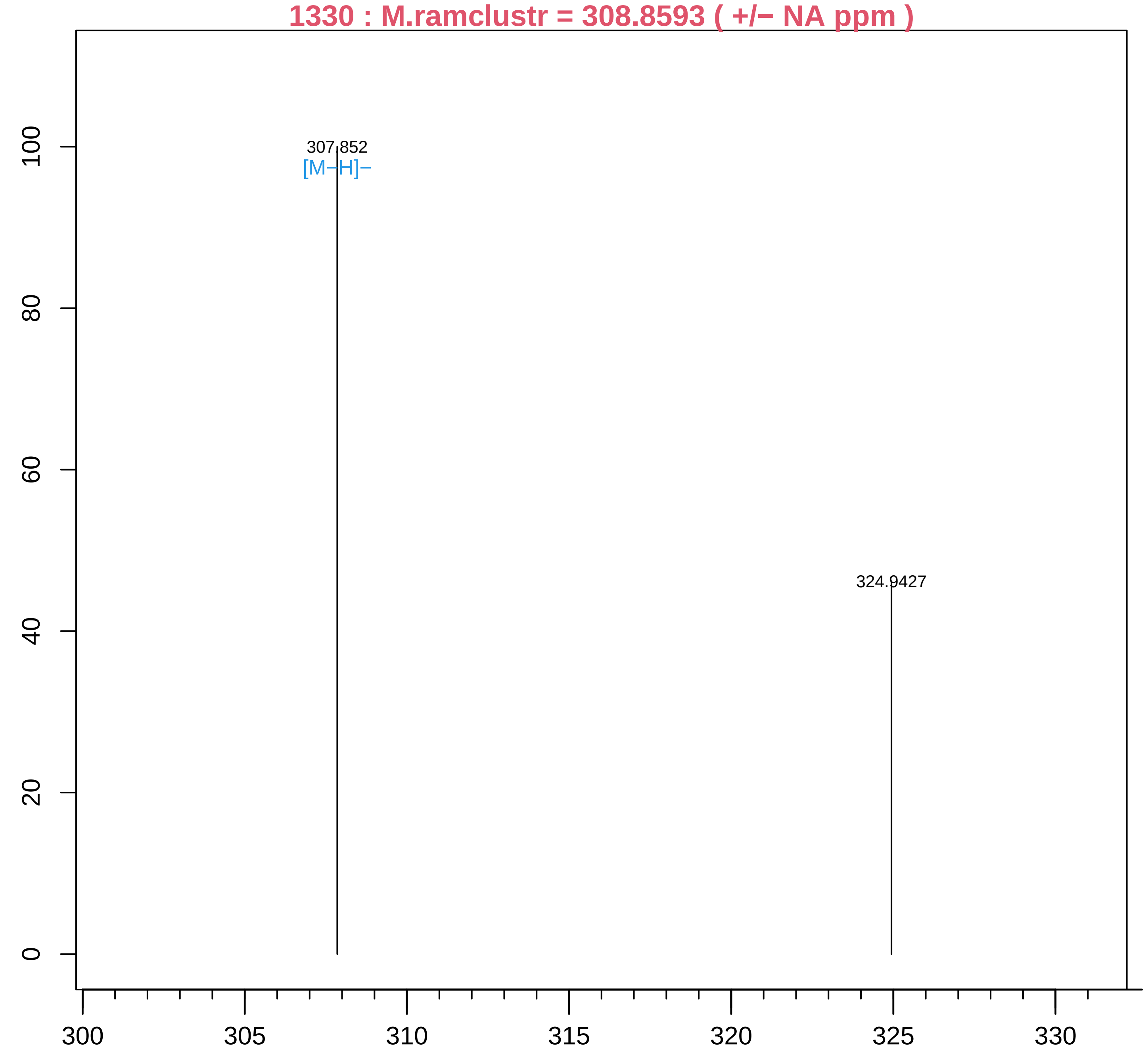
1330 : M.ramclustr = 308.8593 (+/− NA ppm)
307.852
[M−H]−
324.9427
0
20
40
60
80
100
300
305
310
315
320
325
330

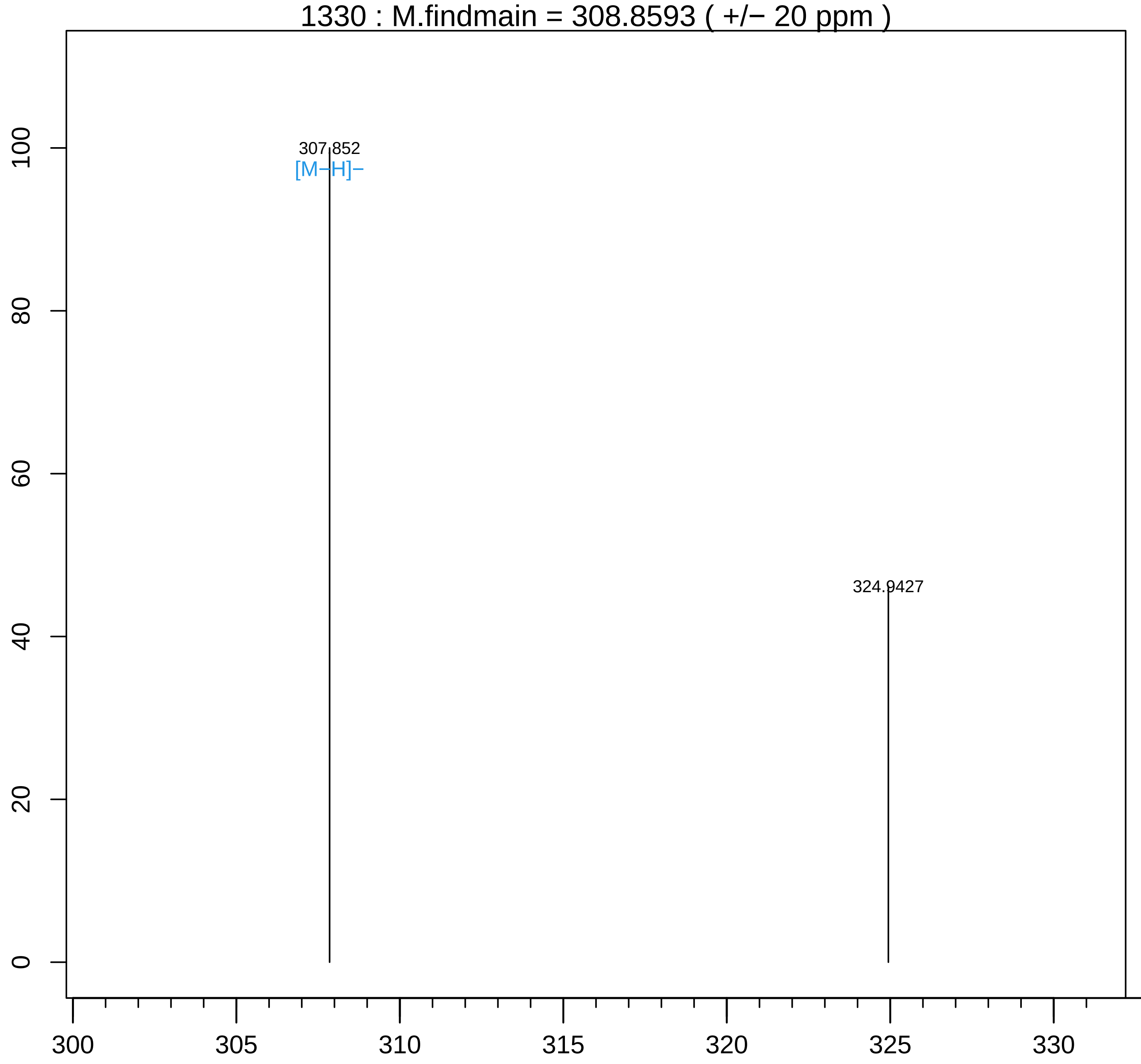
1330 : M.findmain = 308.8593 (+/− 20 ppm)
307.852
[M−H]−
324.9427
0
20
40
60
80
100
300
305
310
315
320
325
330

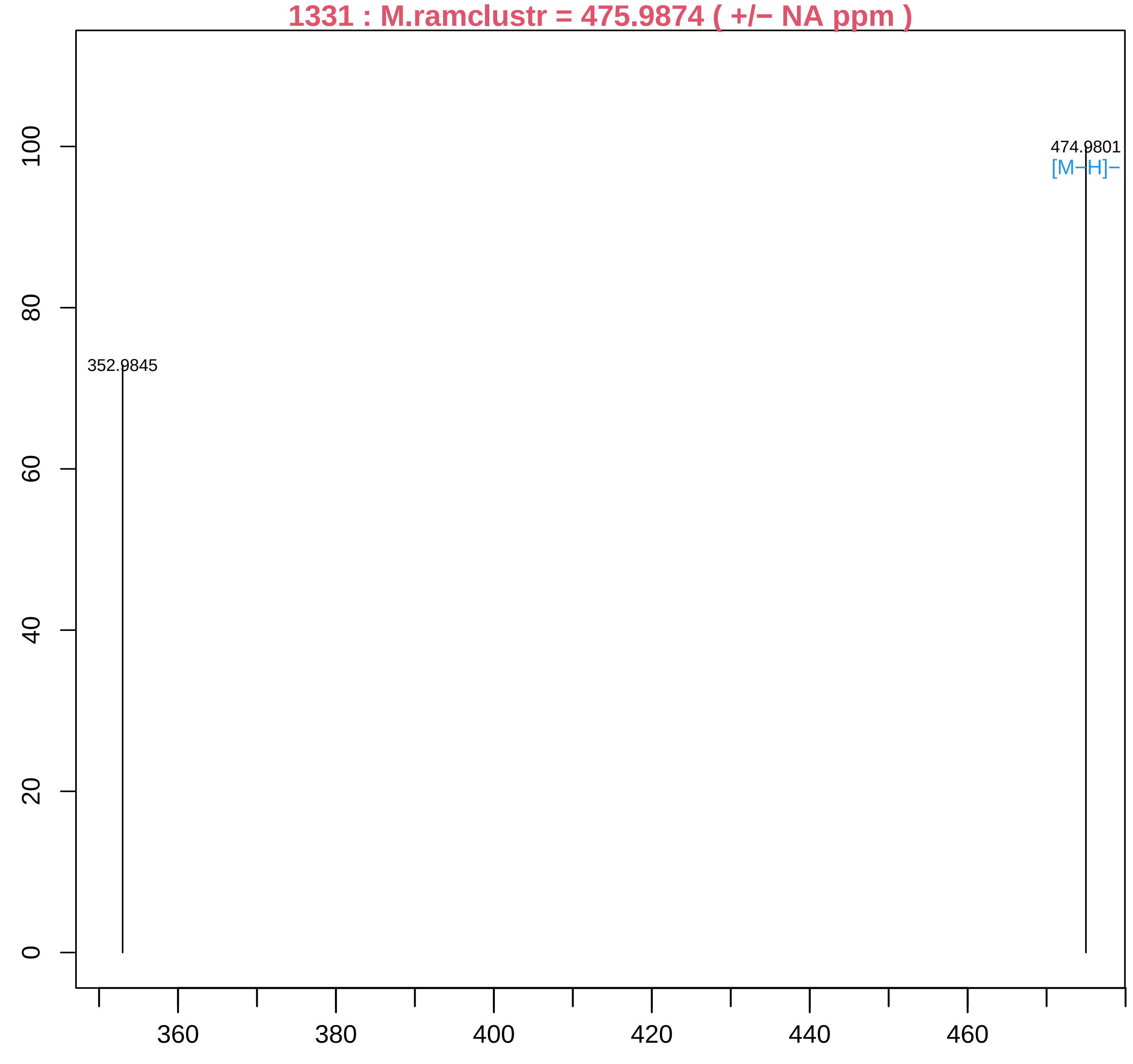
1331 : M.ramclustr = 475.9874 (+/− NA ppm)
474.9801
[M−H]−
352.9845
0
20
40
60
80
100
360
380
400
420
440
460

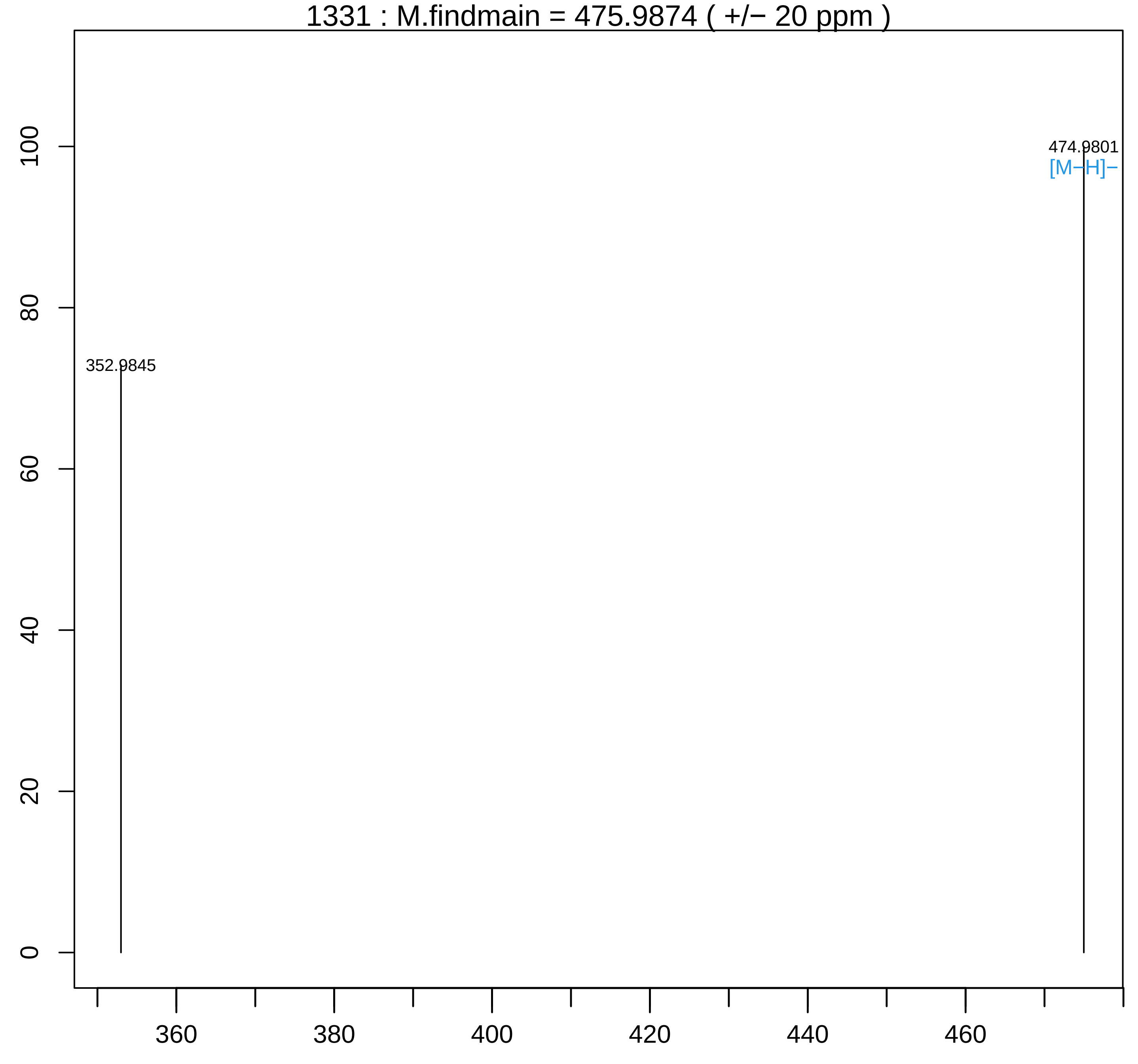
1331 : M.findmain = 475.9874 (+/− 20 ppm)
474.9801
[M−H]−
352.9845
0
20
40
60
80
100
360
380
400
420
440
460

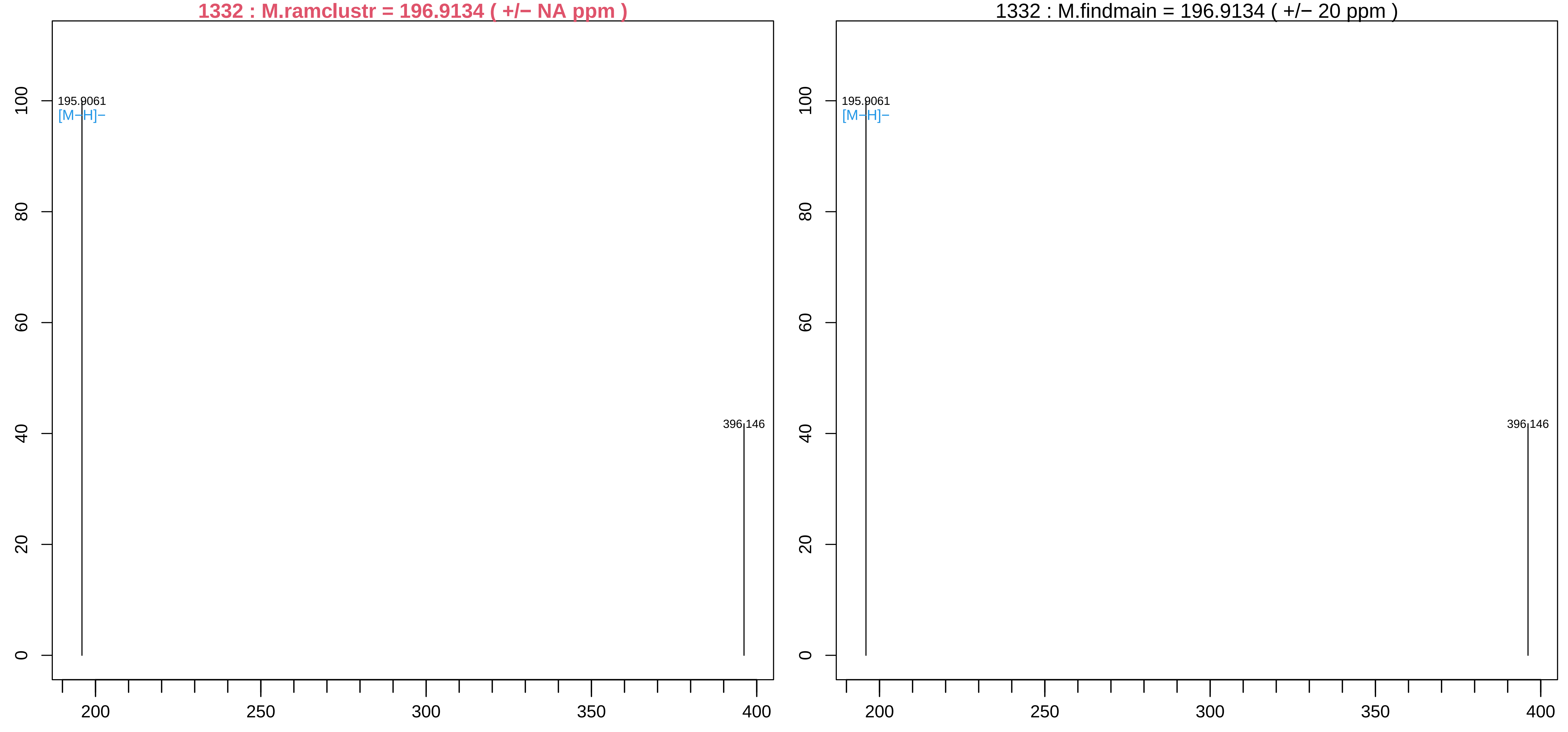

1332 : M.ramclustr = 196.9134 (+/− NA ppm)
195.9061
[M−H]−
396.146
0
20
40
60
80
100
200
250
300
350
400
1332 : M.findmain = 196.9134 (+/− 20 ppm)
195.9061
[M−H]−
396.146
0
20
40
60
80
100
200
250
300
350
400

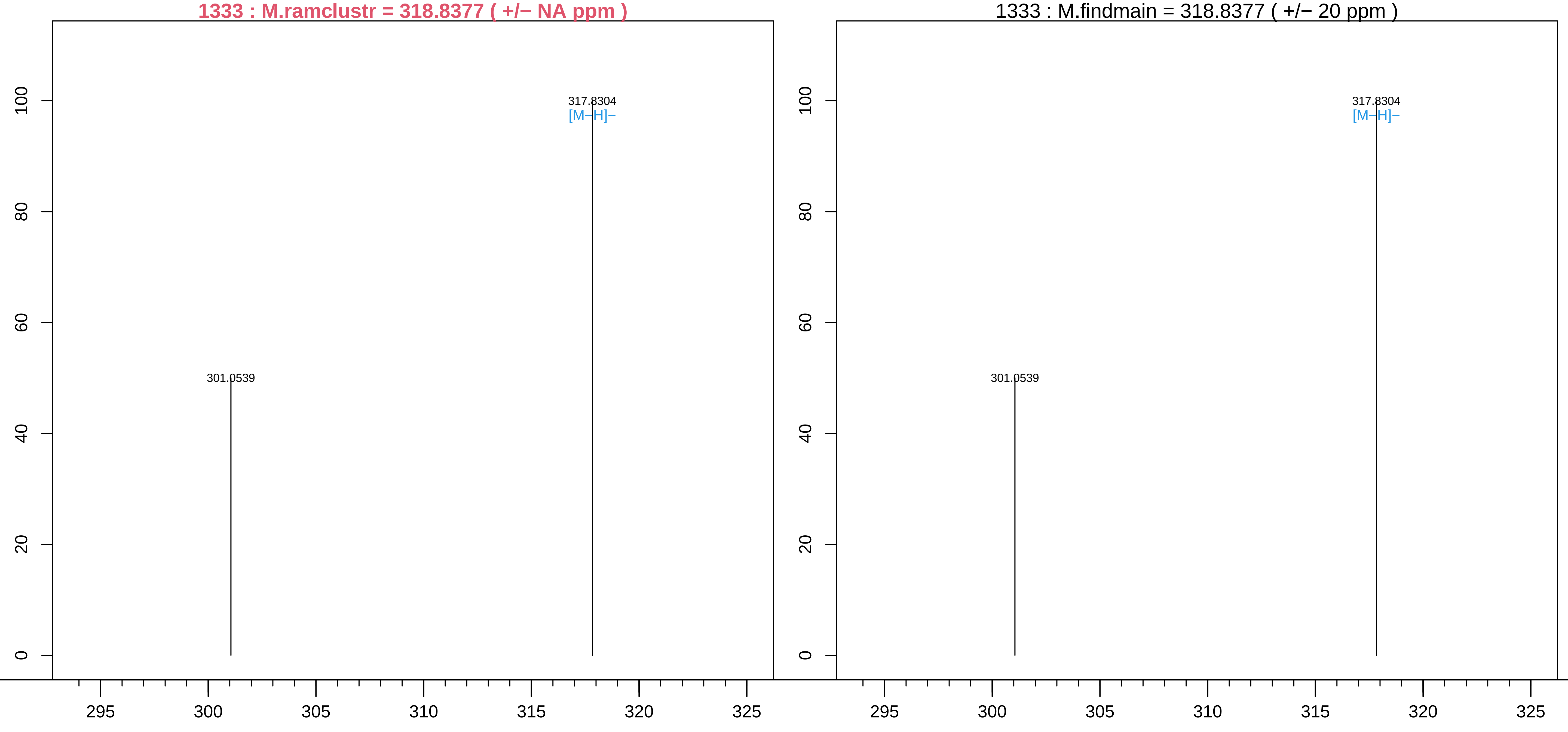
1333 : M.ramclustr = 318.8377 (+/− NA ppm)
1333 : M.findmain = 318.8377 (+/− 20 ppm)
317.8304
[M−H]−
301.0539
0
20
40
60
80
100
295
300
305
310
315
320
325

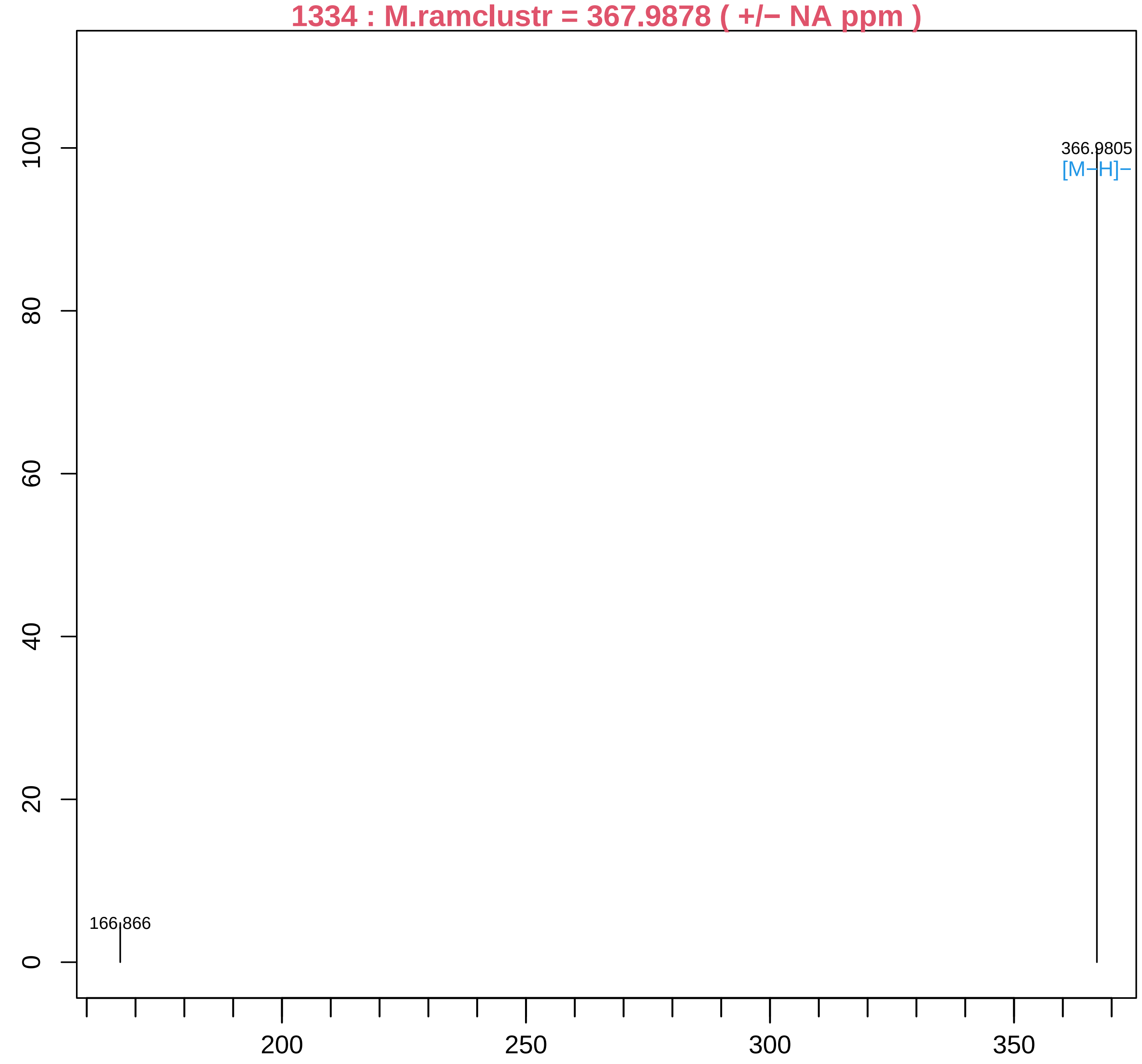
1334 : M.ramclustr = 367.9878 (+/− NA ppm)
366.9805
[M−H]−
166.866
0
20
40
60
80
100
200
250
300
350

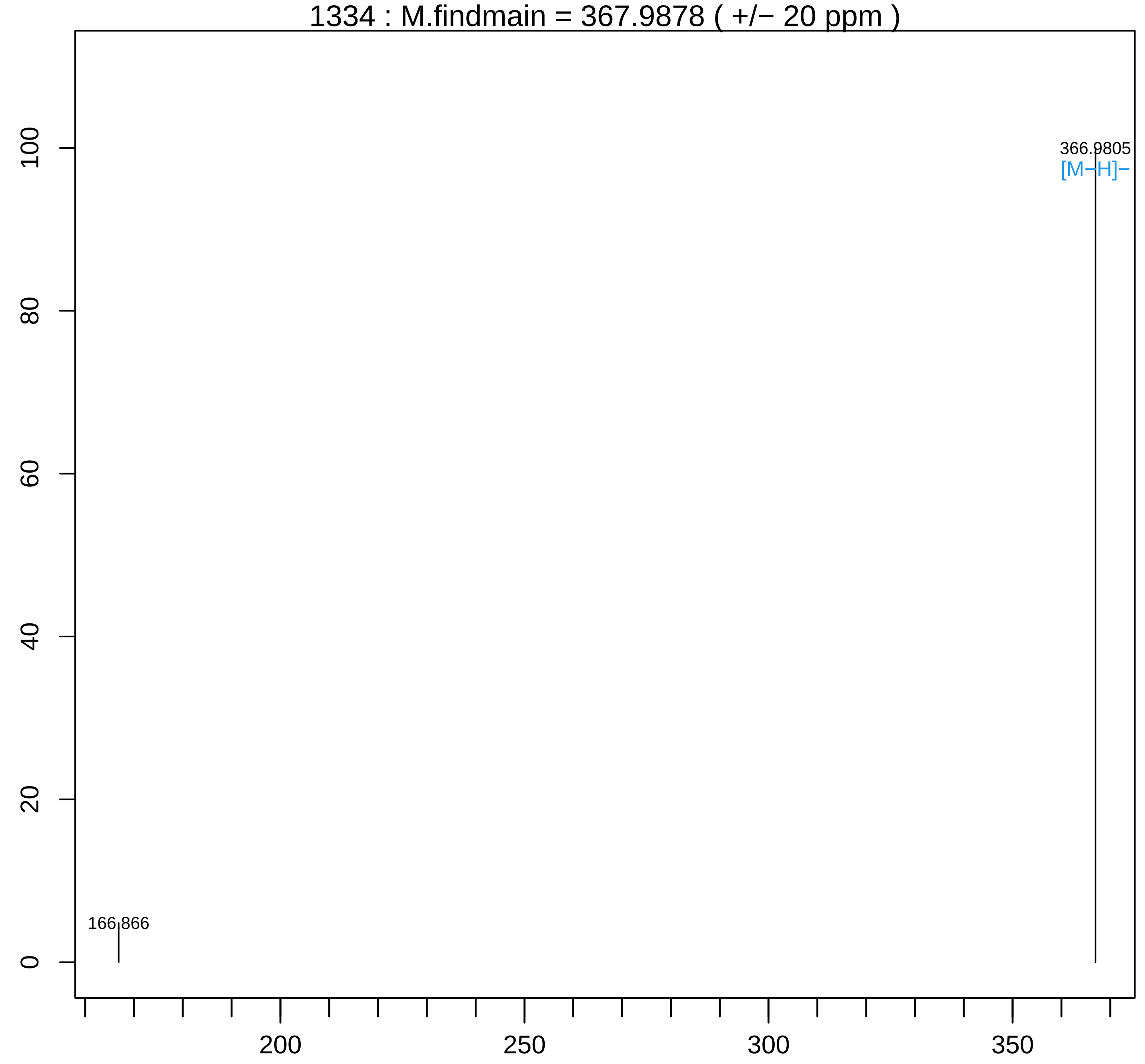
1334 : M.findmain = 367.9878 (+/− 20 ppm)
366.9805
[M−H]−
166.866
0
20
40
60
80
100
200
250
300
350

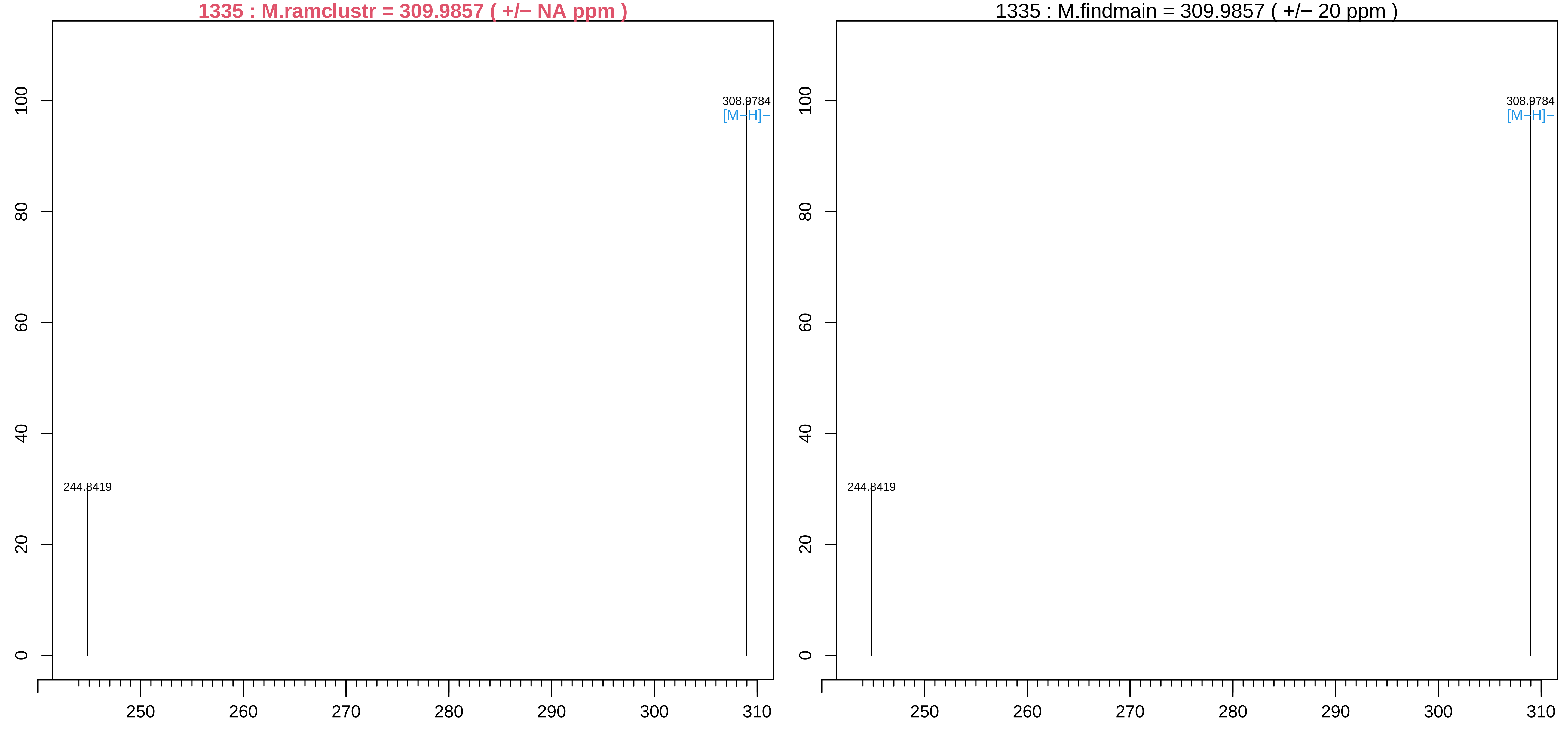
1335 : M.ramclustr = 309.9857 (+/− NA ppm)
308.9784
[M−H]−
244.8419
1335 : M.findmain = 309.9857 (+/− 20 ppm)
308.9784
[M−H]−
244.8419
0
20
40
60
80
100
250
260
270
280
290
300
310

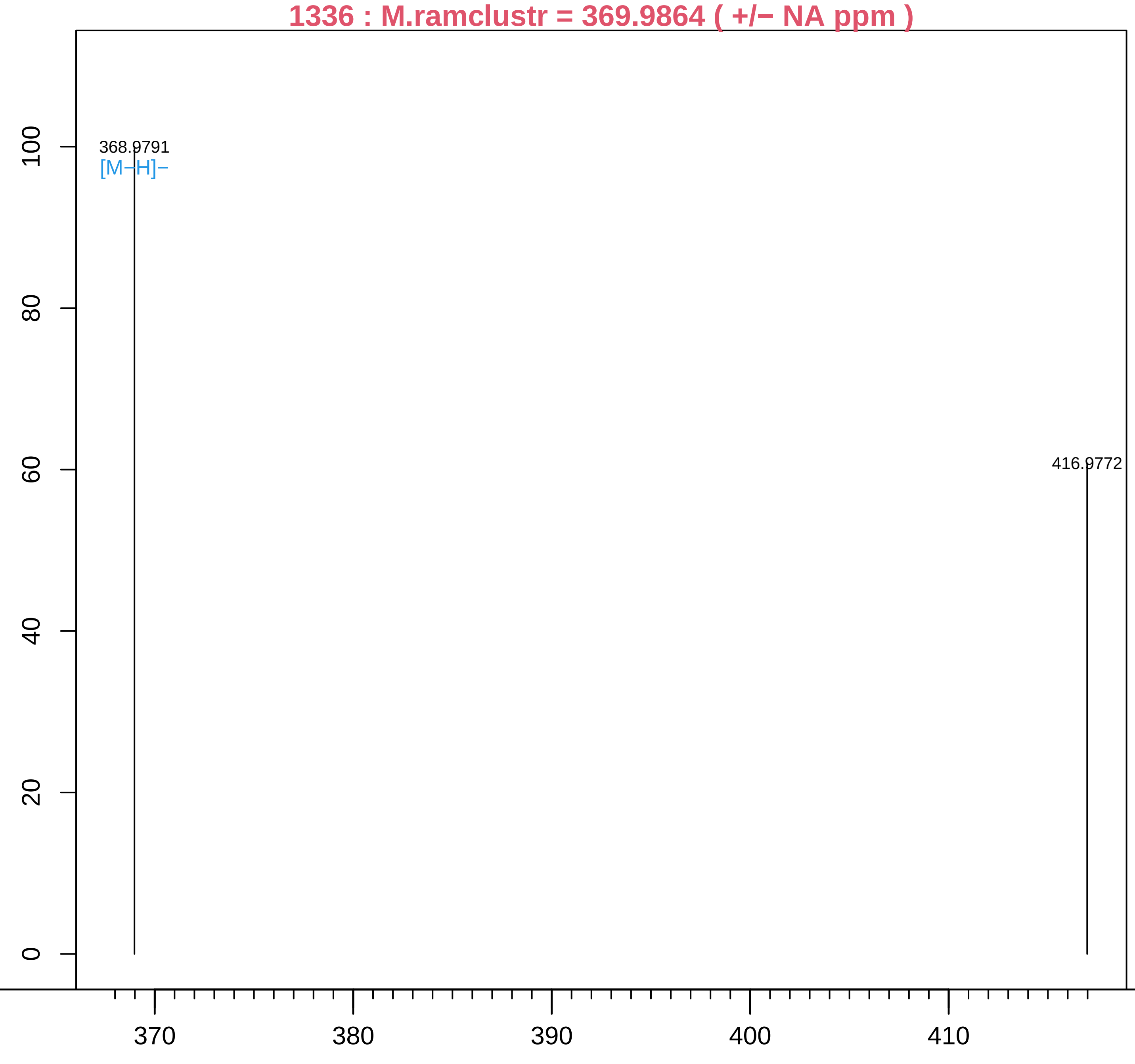
1336 : M.ramclustr = 369.9864 (+/− NA ppm)
368.9791
[M−H]−
416.9772
0
20
40
60
80
100
370
380
390
400
410
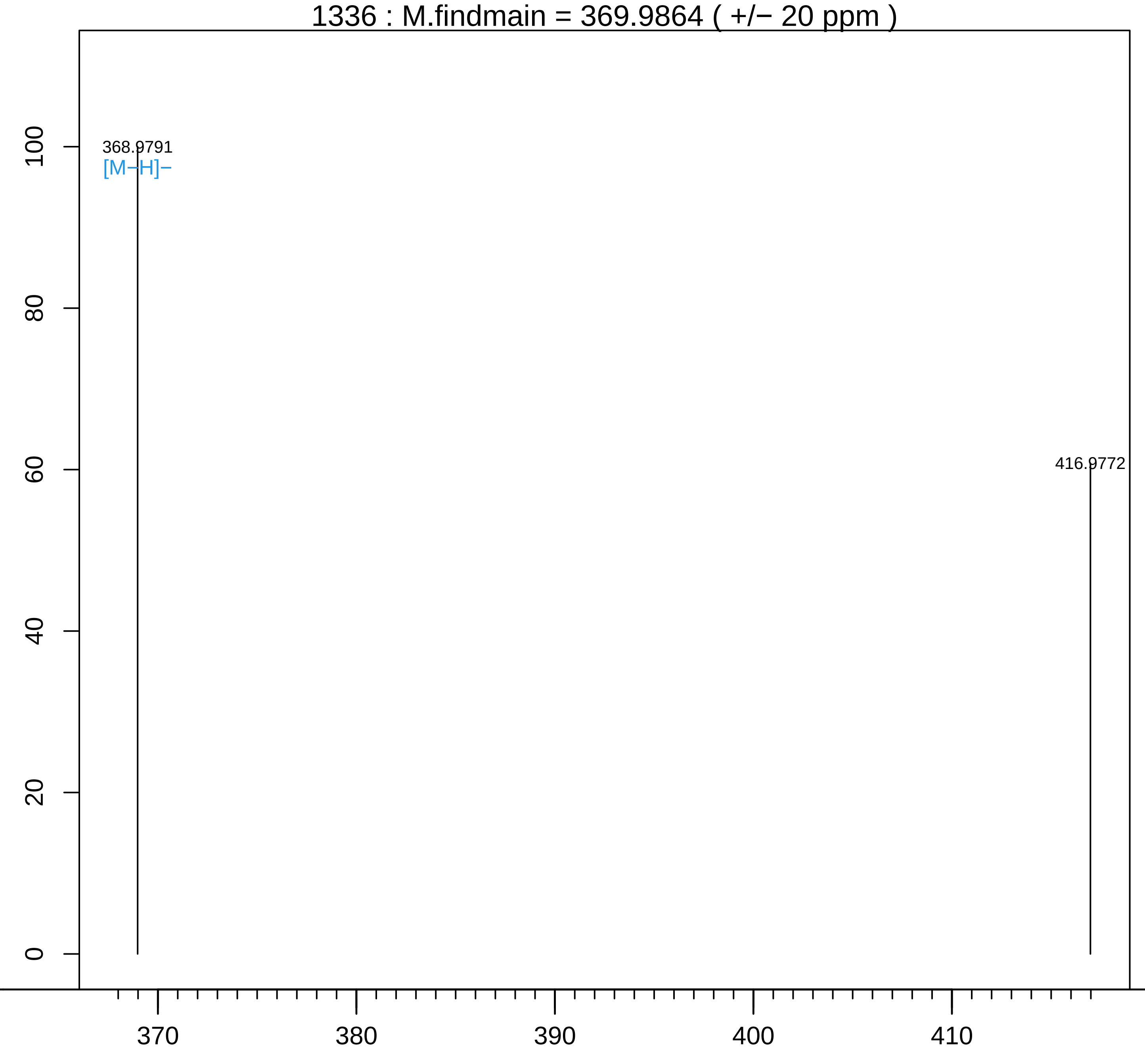
1336 : M.findmain = 369.9864 (+/− 20 ppm)
368.9791
[M−H]−
416.9772
0
20
40
60
80
100
370
380
390
400
410

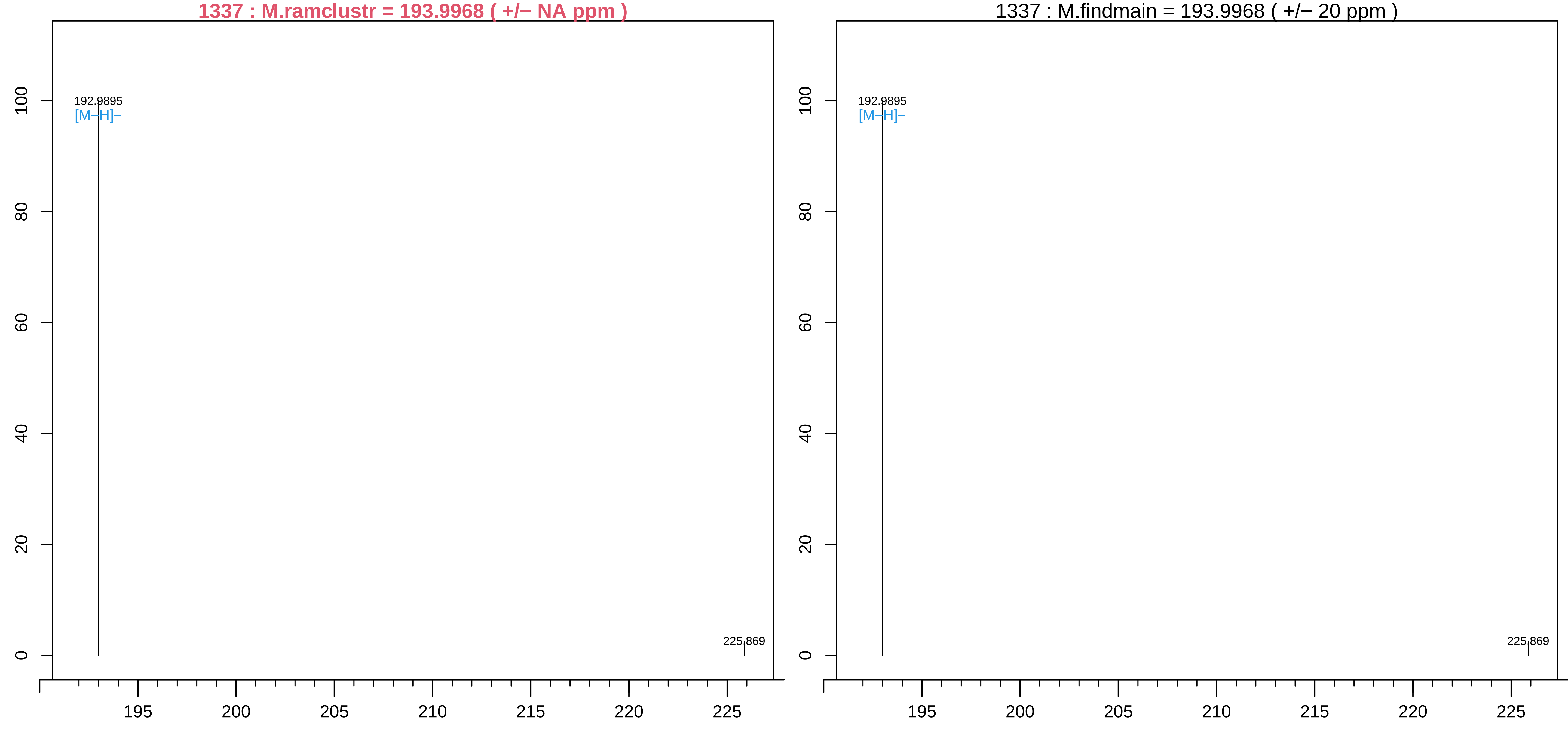
1337 : M.ramclustr = 193.9968 (+/− NA ppm)
192.9895
[M−H]−
225.869
1337 : M.findmain = 193.9968 (+/− 20 ppm)
192.9895
[M−H]−
225.869
0
20
40
60
80
100
195
200
205
210
215
220
225

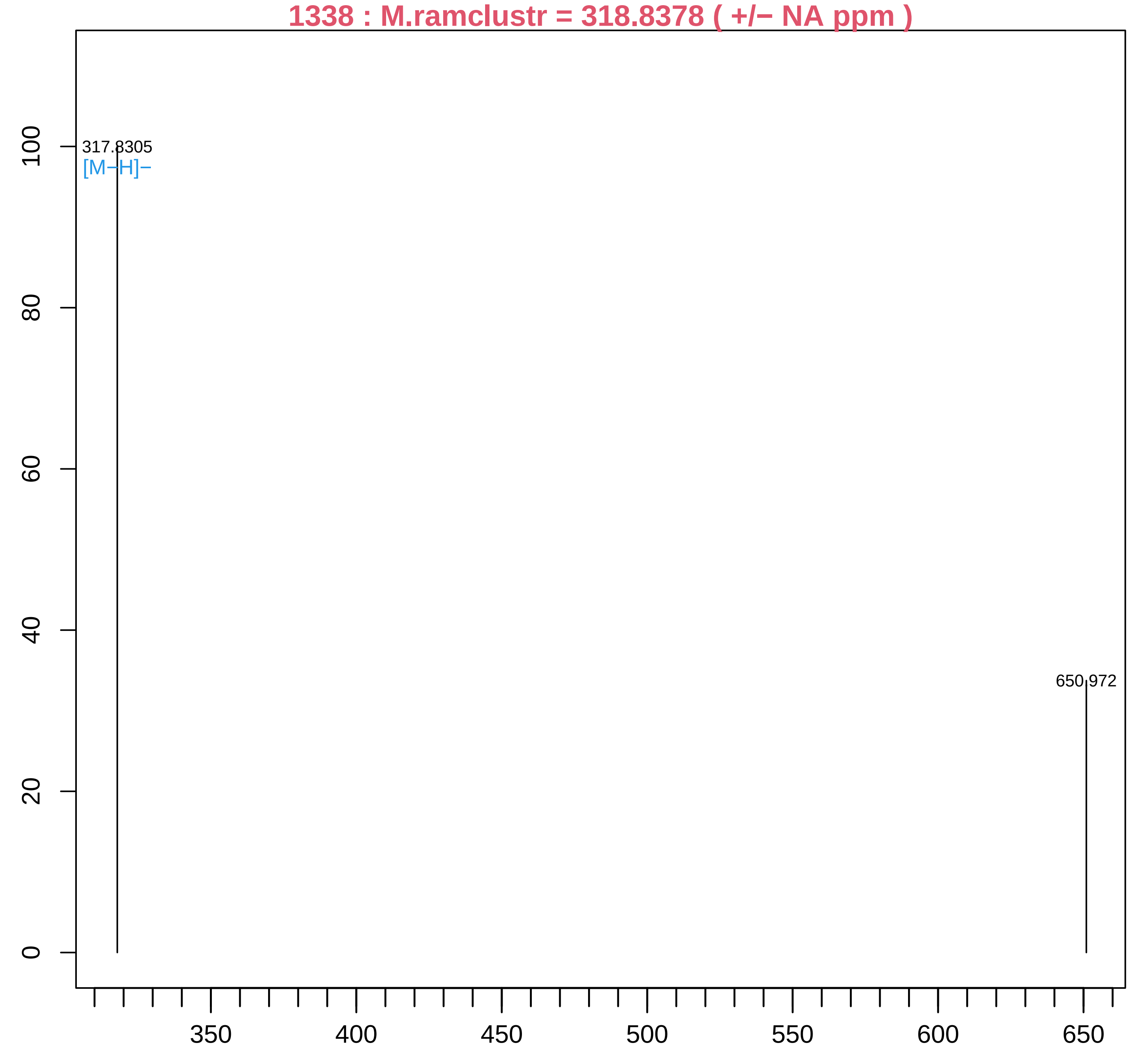
1338 : M.ramclustr = 318.8378 (+/− NA ppm)
317.8305
[M−H]−
650.972
0
20
40
60
80
100
350
400
450
500
550
600
650

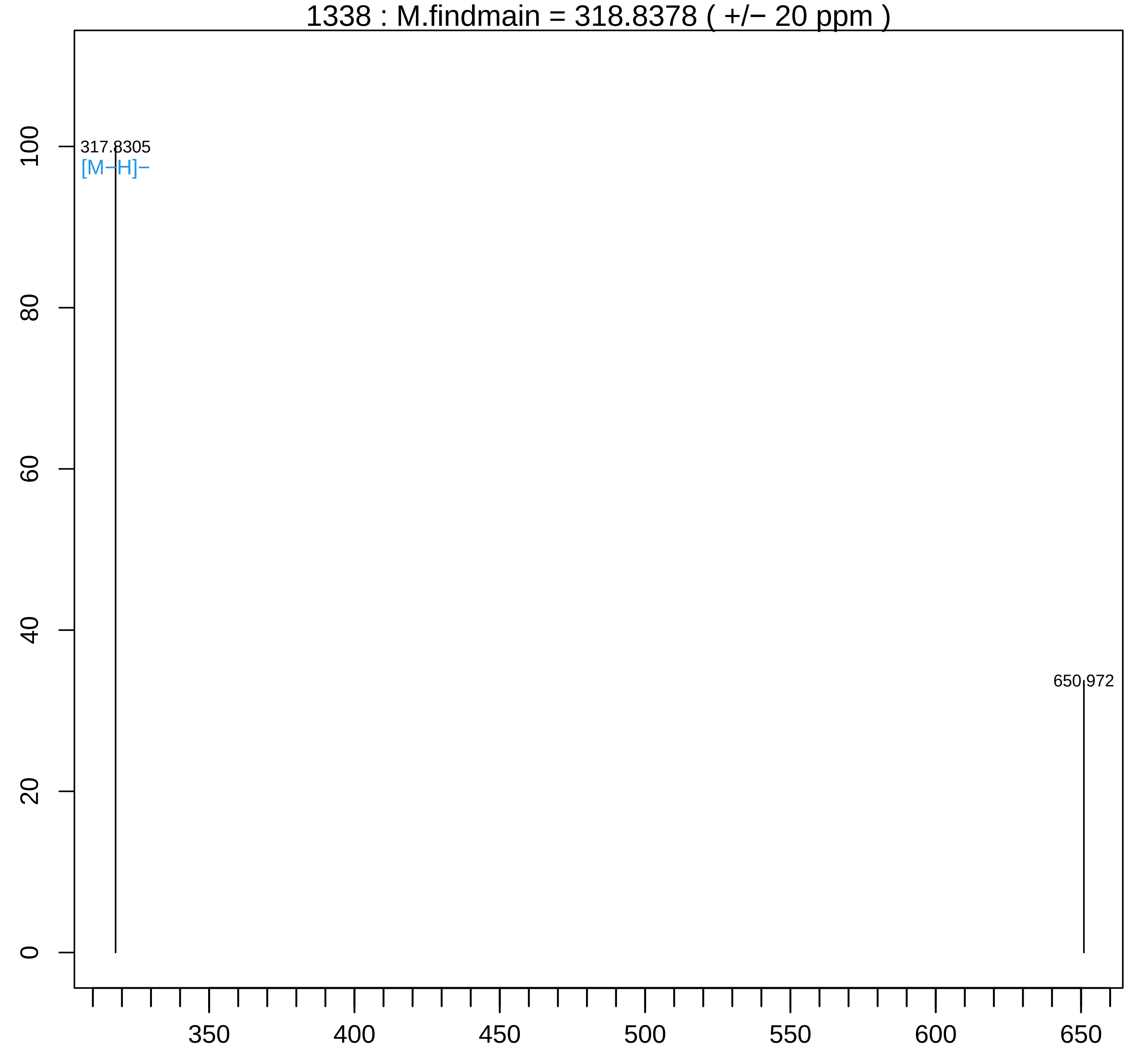
1338 : M.findmain = 318.8378 (+/− 20 ppm)
317.8305
[M−H]−
650.972
0
20
40
60
80
100
350
400
450
500
550
600
650

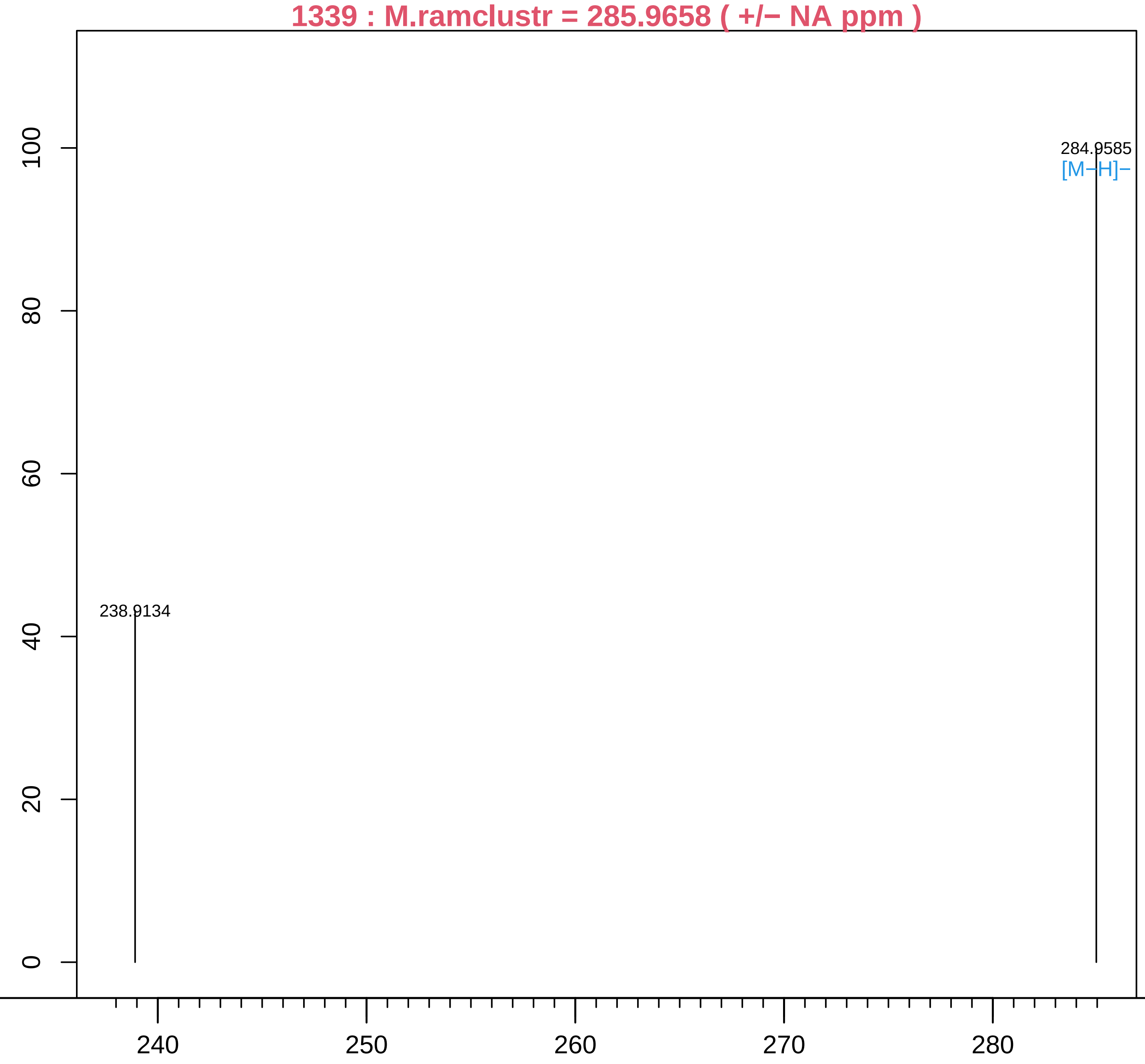

1339 : M.ramclustr = 285.9658 (+/− NA ppm)
284.9585
[M−H]−
238.9134
0
20
40
60
80
100
240
250
260
270
280

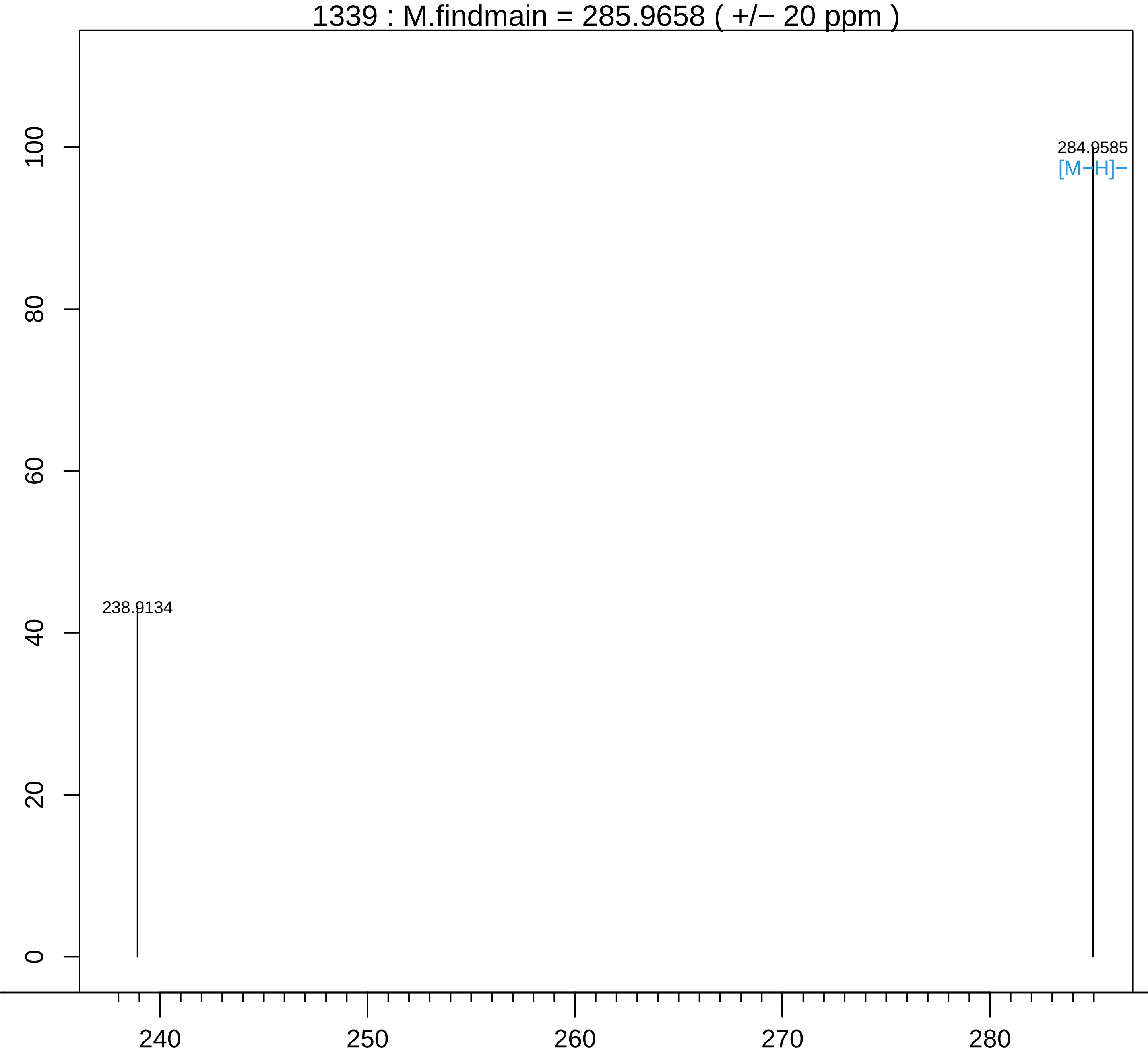

1339 : M.findmain = 285.9658 (+/− 20 ppm)
284.9585
[M−H]−
238.9134
0
20
40
60
80
100
240
250
260
270
280

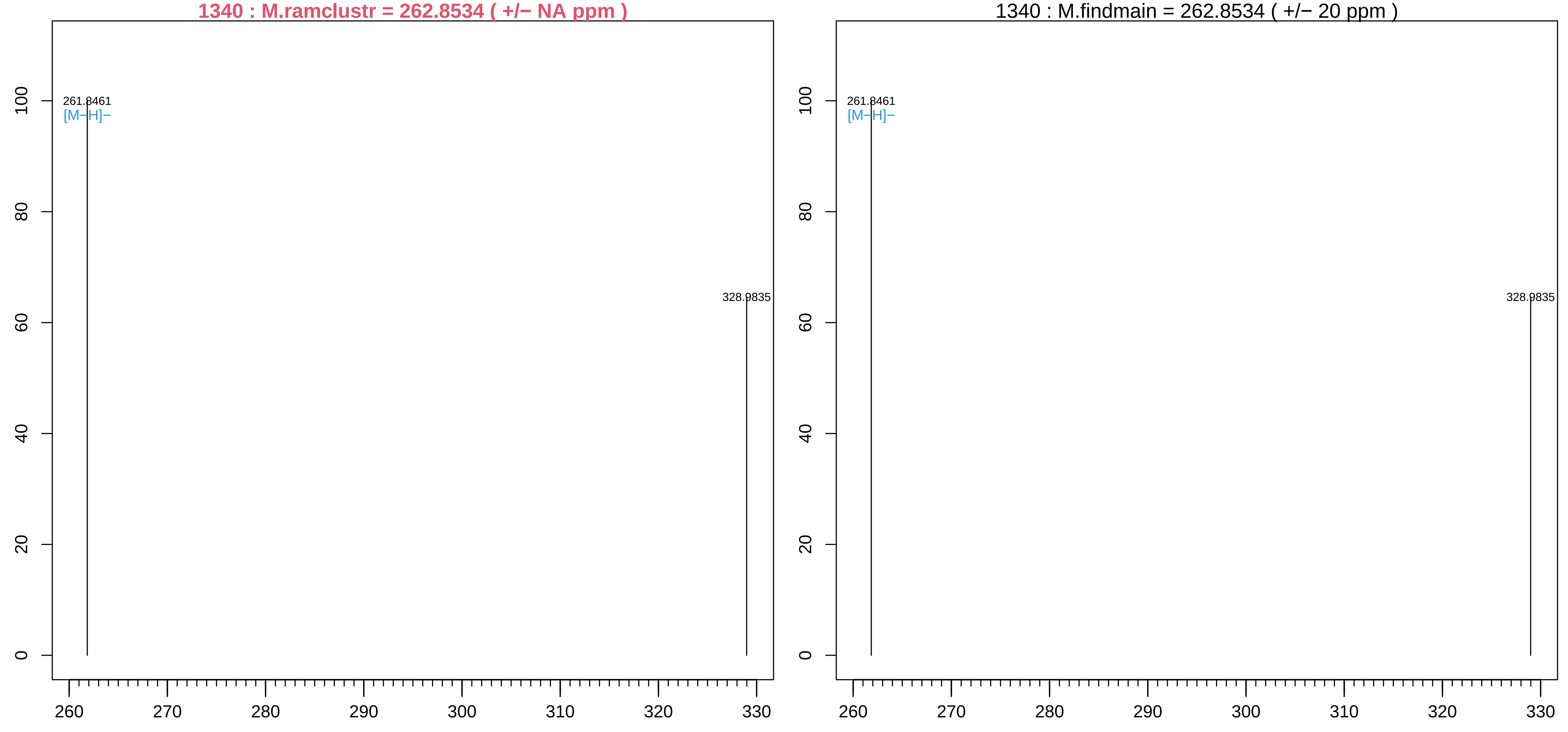

1340 : M.ramclustr = 262.8534 (+/− NA ppm)
261.8461
[M−H]−
328.9835
1340 : M.findmain = 262.8534 (+/− 20 ppm)
261.8461
[M−H]−
328.9835

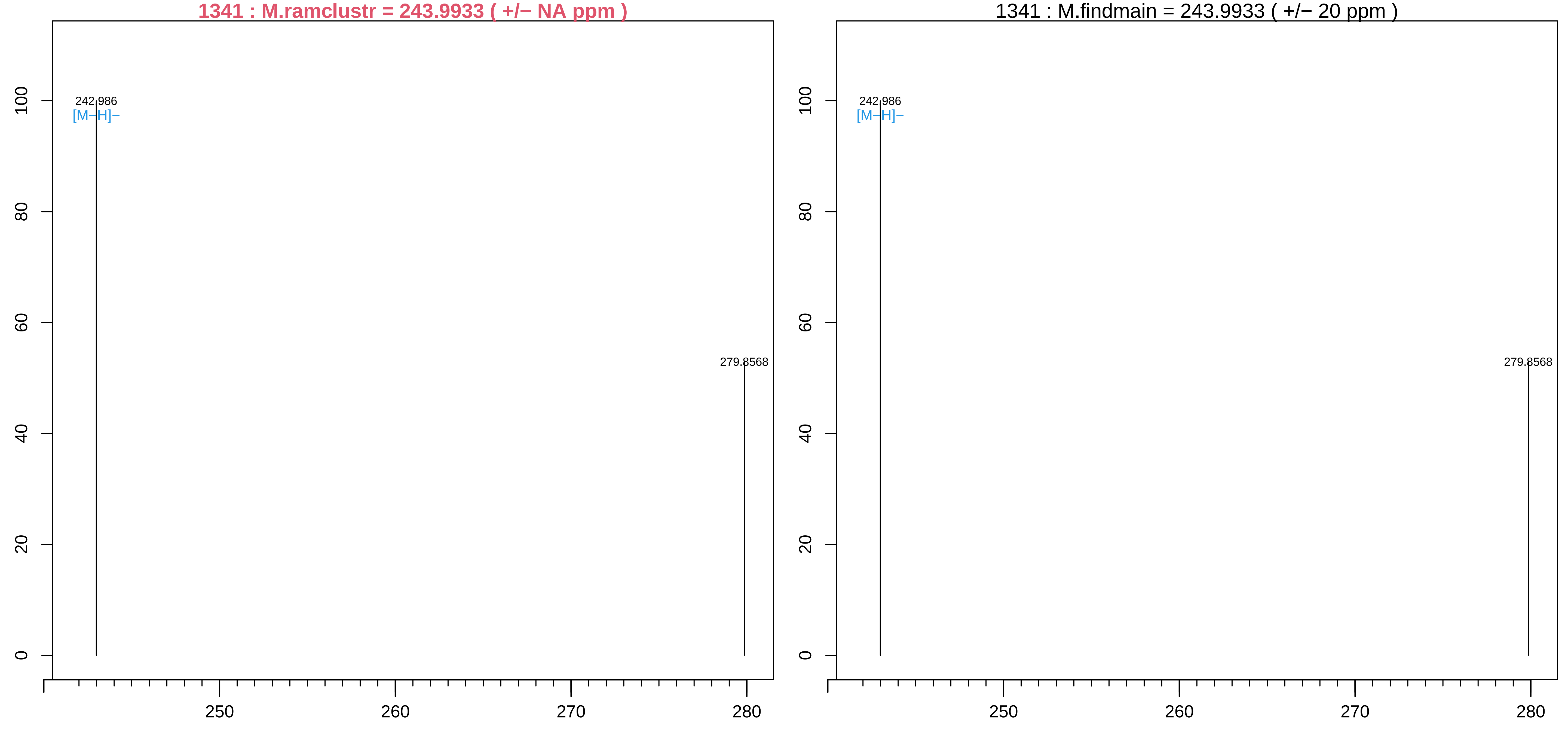

1341 : M.ramclustr = 243.9933 (+/− NA ppm)
242.986
[M−H]−
279.8568
1341 : M.findmain = 243.9933 (+/− 20 ppm)
242.986
[M−H]−
279.8568
0
20
40
60
80
100
250
260
270
280

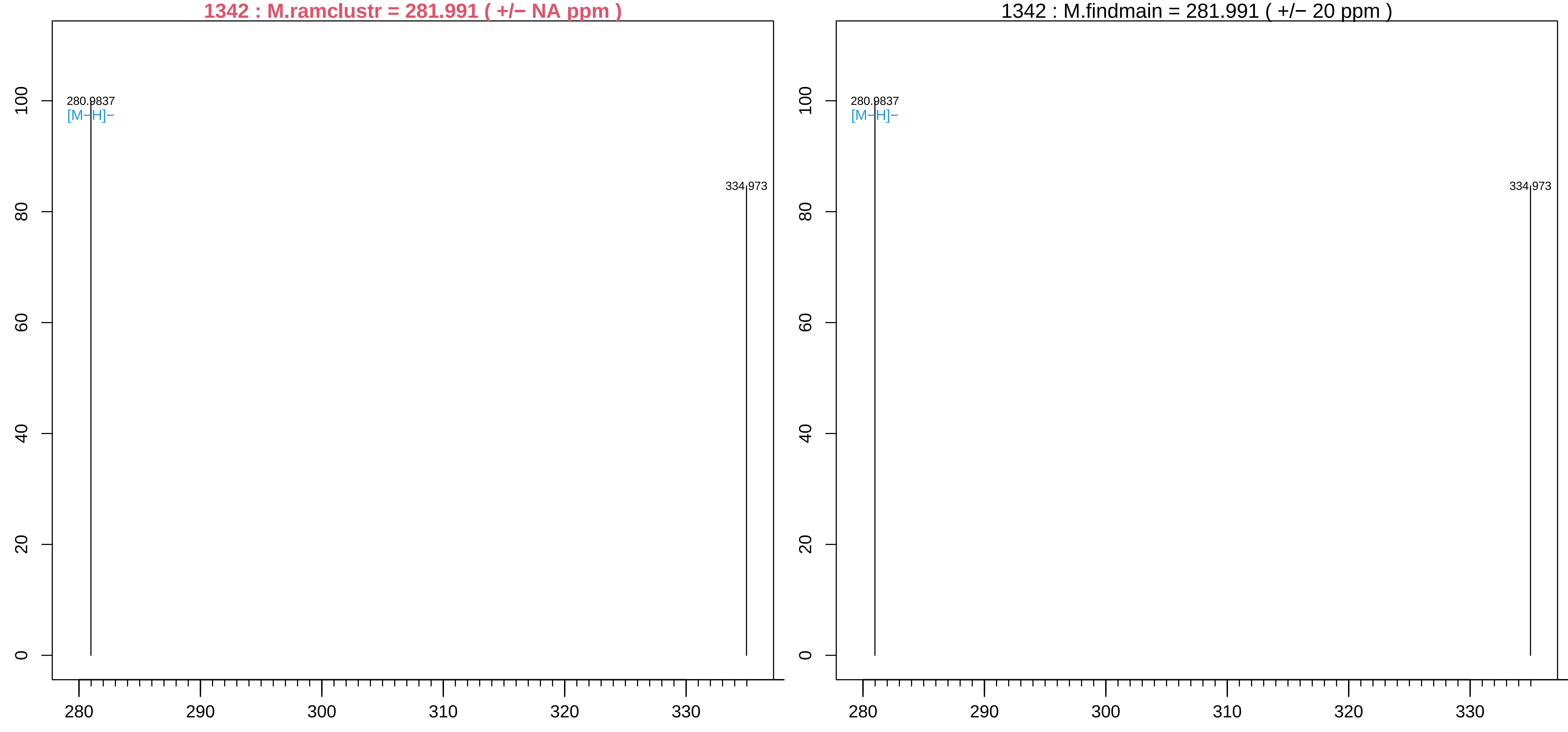
1342 : M.ramclustr = 281.991 (+/− NA ppm)
280.9837
[M−H]−
334.973
1342 : M.findmain = 281.991 (+/− 20 ppm)
280.9837
[M−H]−
334.973

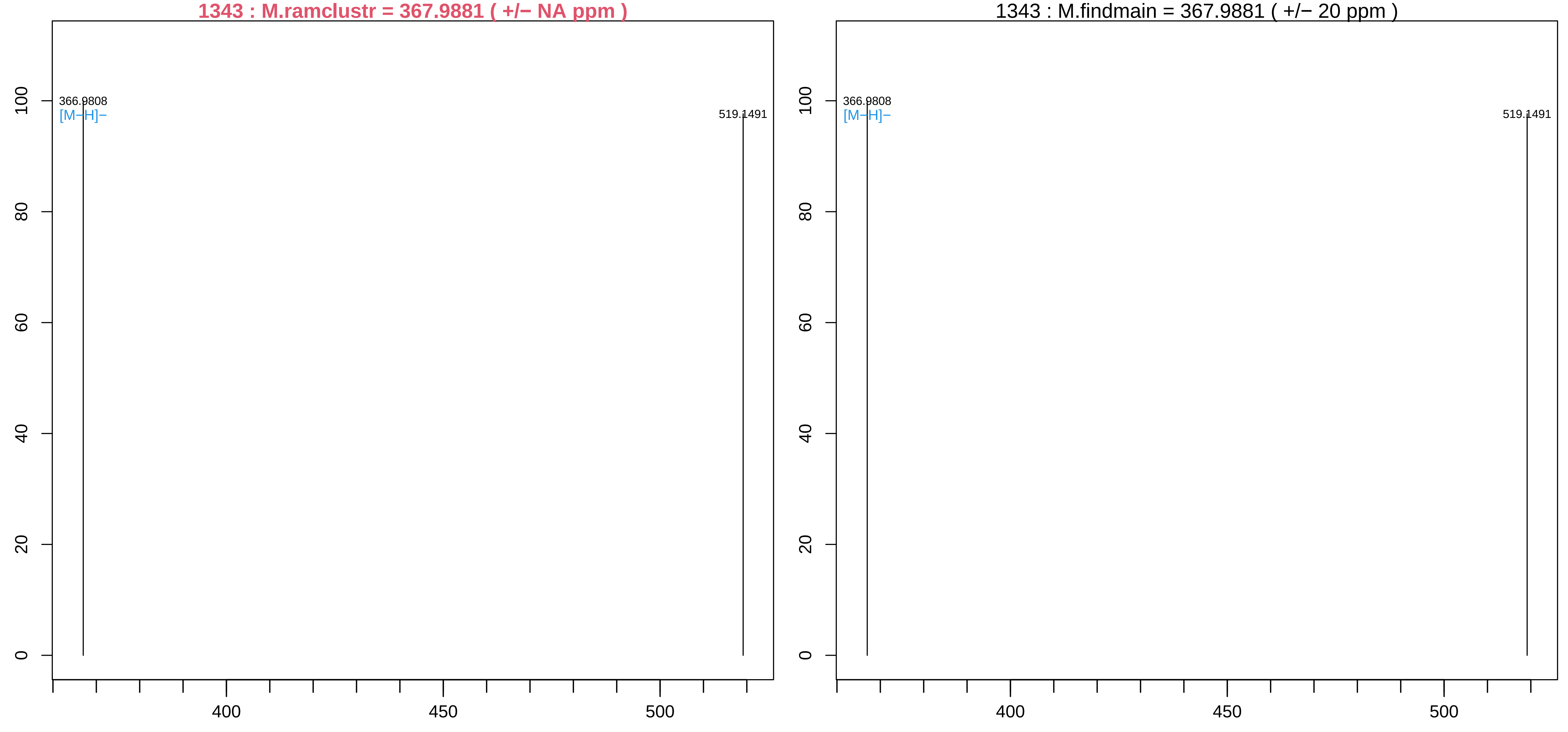
1343 : M.ramclustr = 367.9881 (+/− NA ppm)
366.9808
[M−H]−
519.1491
1343 : M.findmain = 367.9881 (+/− 20 ppm)
366.9808
[M−H]−
519.1491
0
20
40
60
80
100
400
450
500

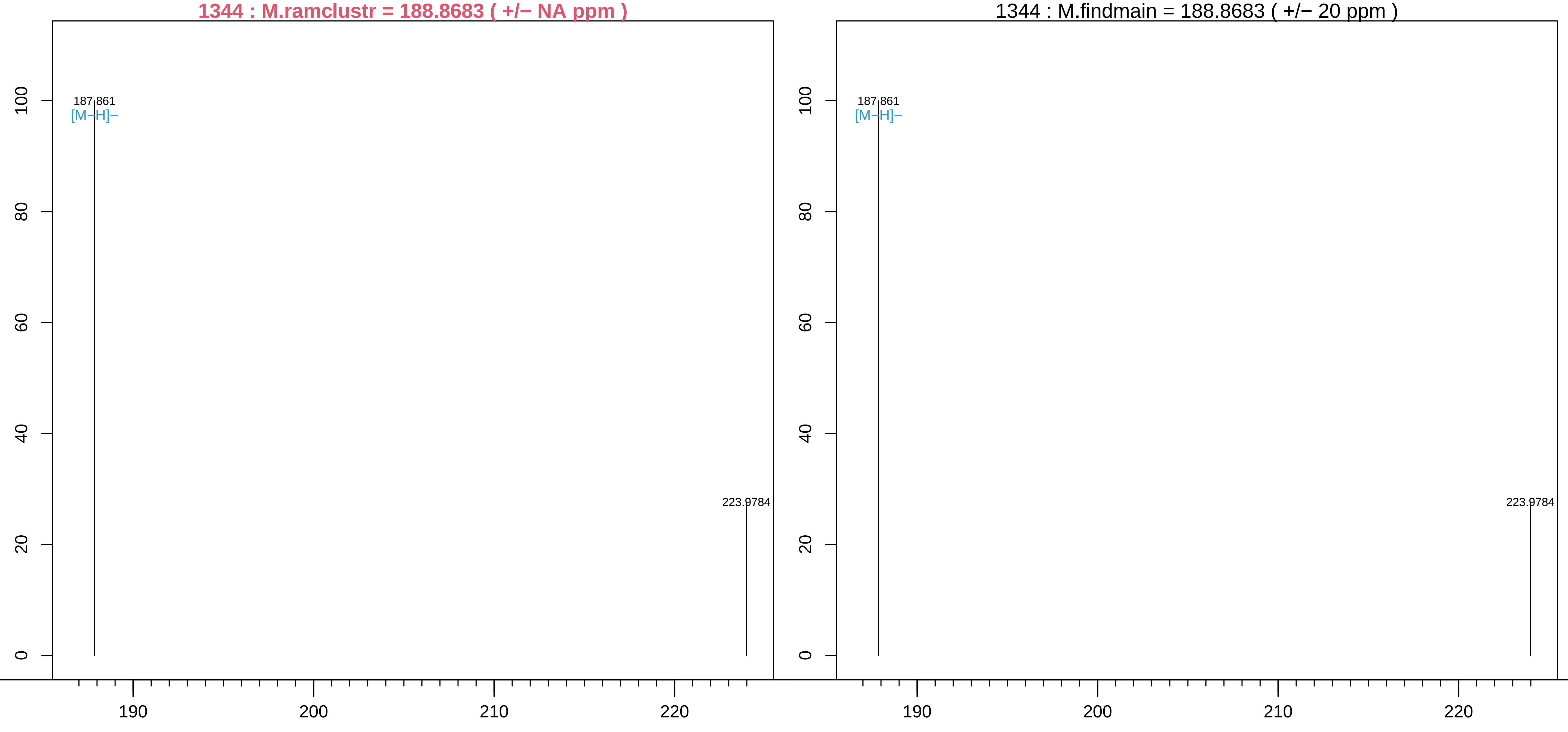

1344 : M.ramclustr = 188.8683 (+/− NA ppm)
187.861
[M−H]−
223.9784
1344 : M.findmain = 188.8683 (+/− 20 ppm)
187.861
[M−H]−
223.9784
0
20
40
60
80
100
190
200
210
220

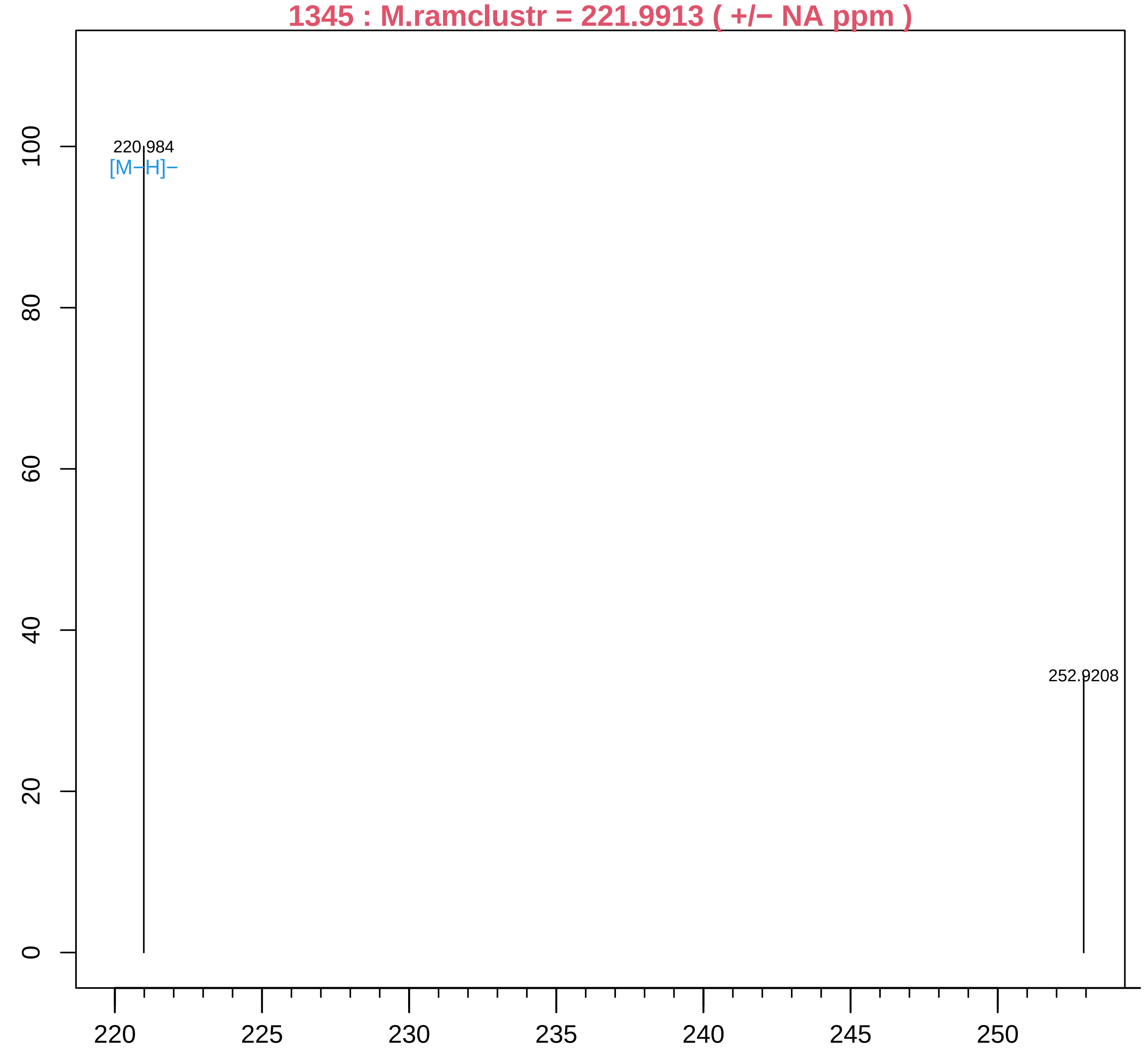
1345 : M.ramclustr = 221.9913 (+/− NA ppm)
220.984
[M−H]−
252.9208
0
20
40
60
80
100
220
225
230
235
240
245
250

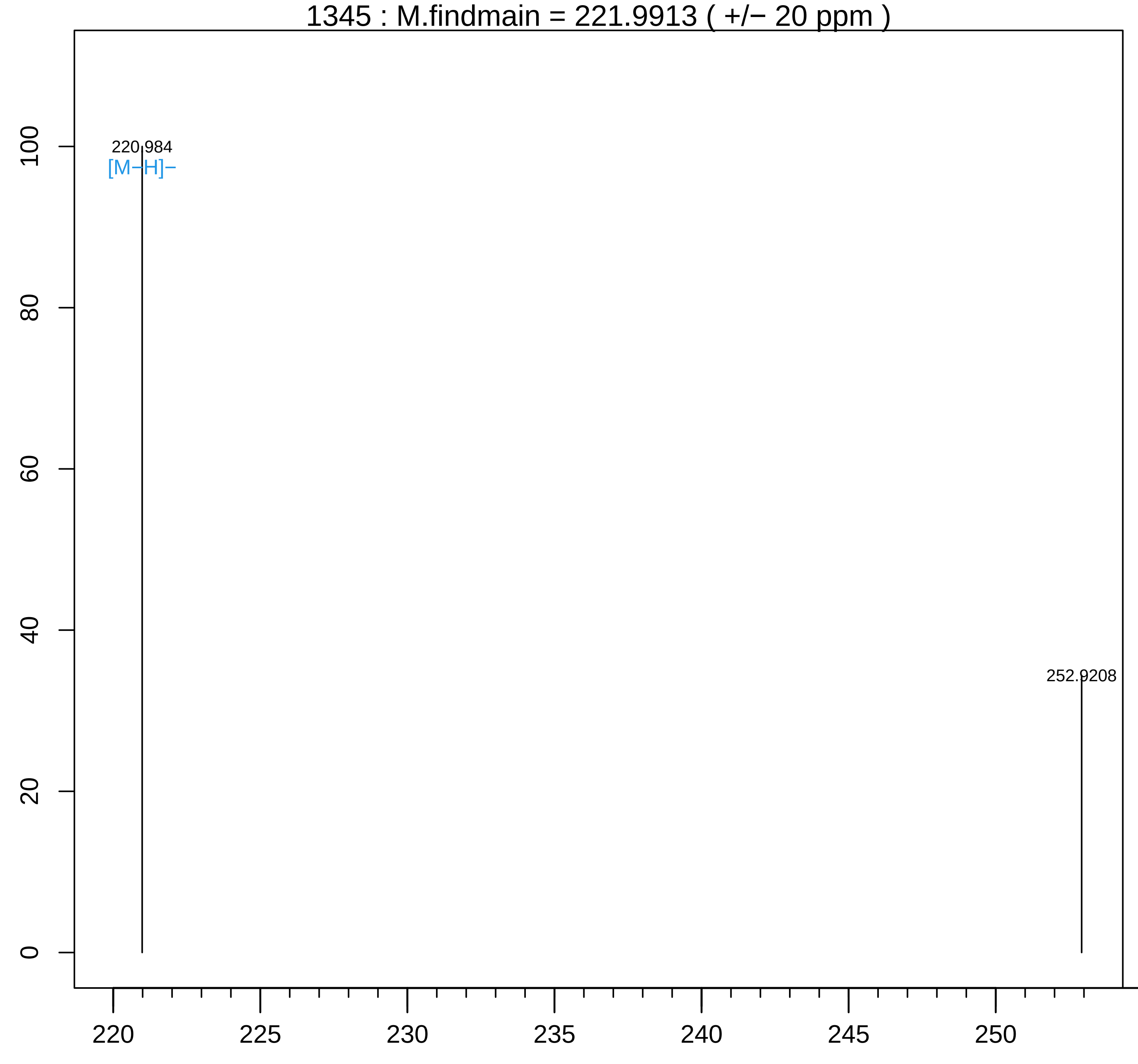
1345 : M.findmain = 221.9913 (+/− 20 ppm)
220.984
[M−H]−
252.9208
0
20
40
60
80
100
220
225
230
235
240
245
250

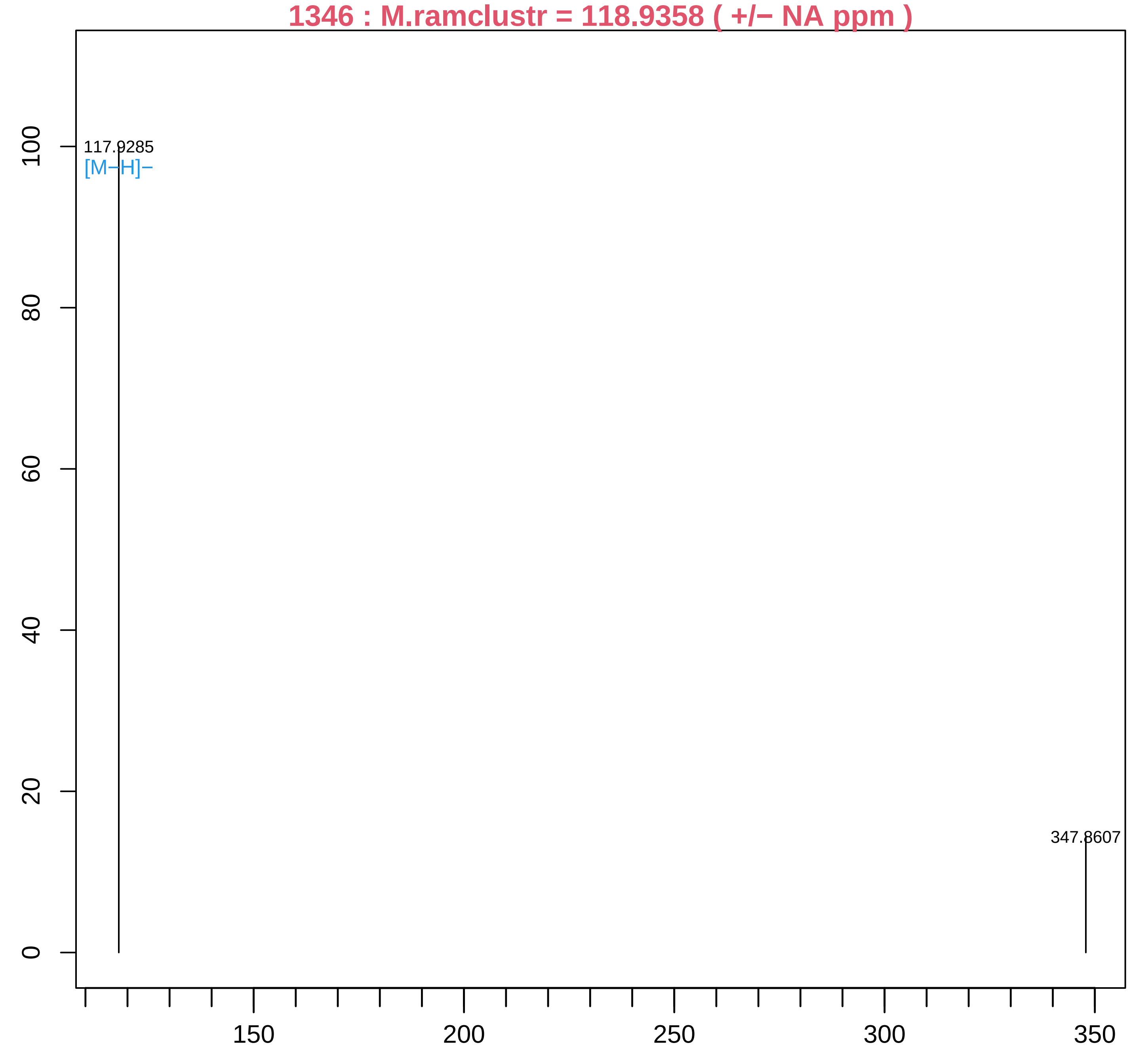
1346 : M.ramclustr = 118.9358 (+/− NA ppm)
117.9285
[M−H]−
347.8607
0
20
40
60
80
100
150
200
250
300
350

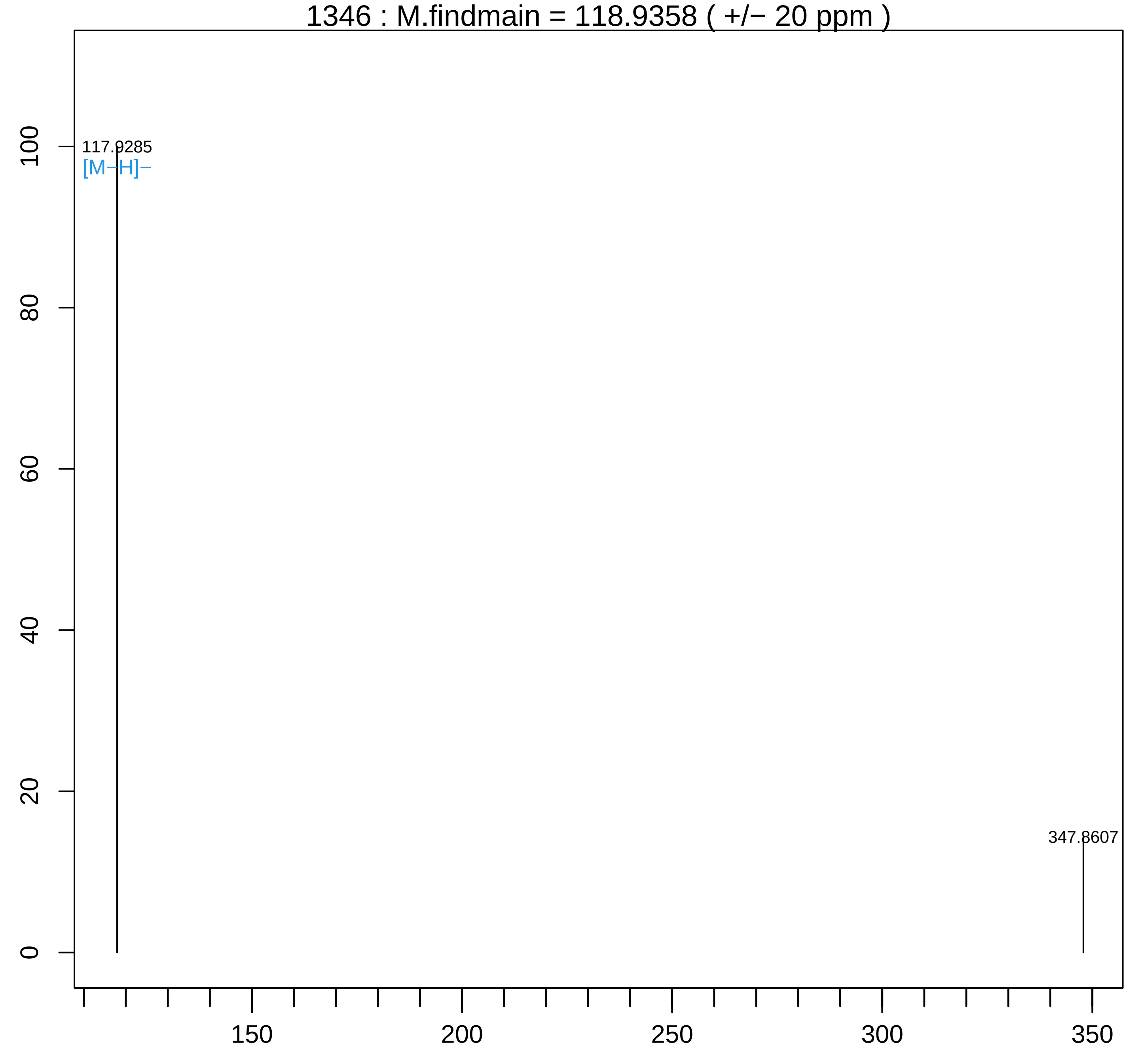
1346 : M.findmain = 118.9358 (+/− 20 ppm)
117.9285
[M−H]−
347.8607
0
20
40
60
80
100
150
200
250
300
350

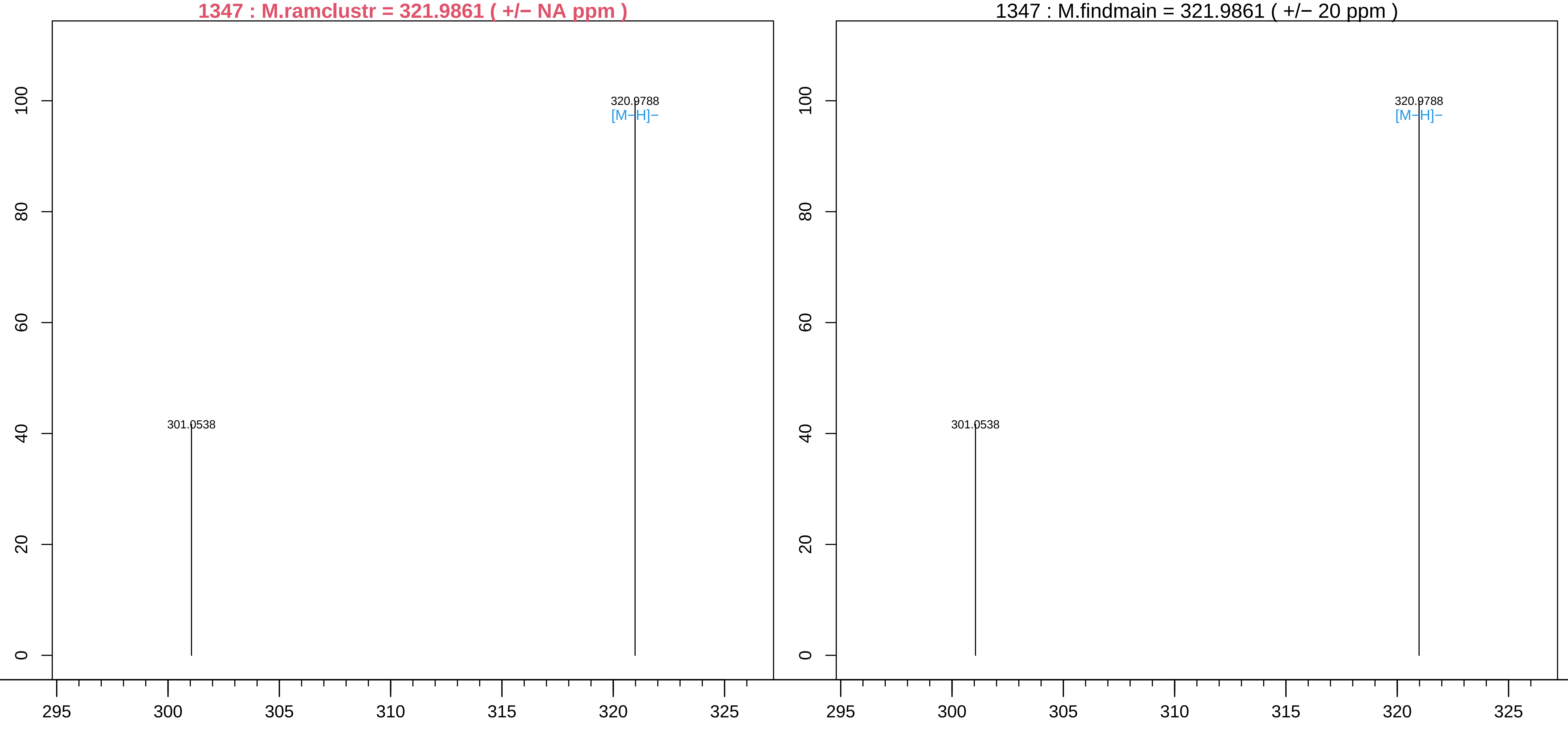
1347 : M.ramclustr = 321.9861 (+/− NA ppm)
1347 : M.findmain = 321.9861 (+/− 20 ppm)
320.9788
[M−H]−
301.0538
320.9788
[M−H]−
301.0538
0
20
40
60
80
100
295
300
305
310
315
320
325

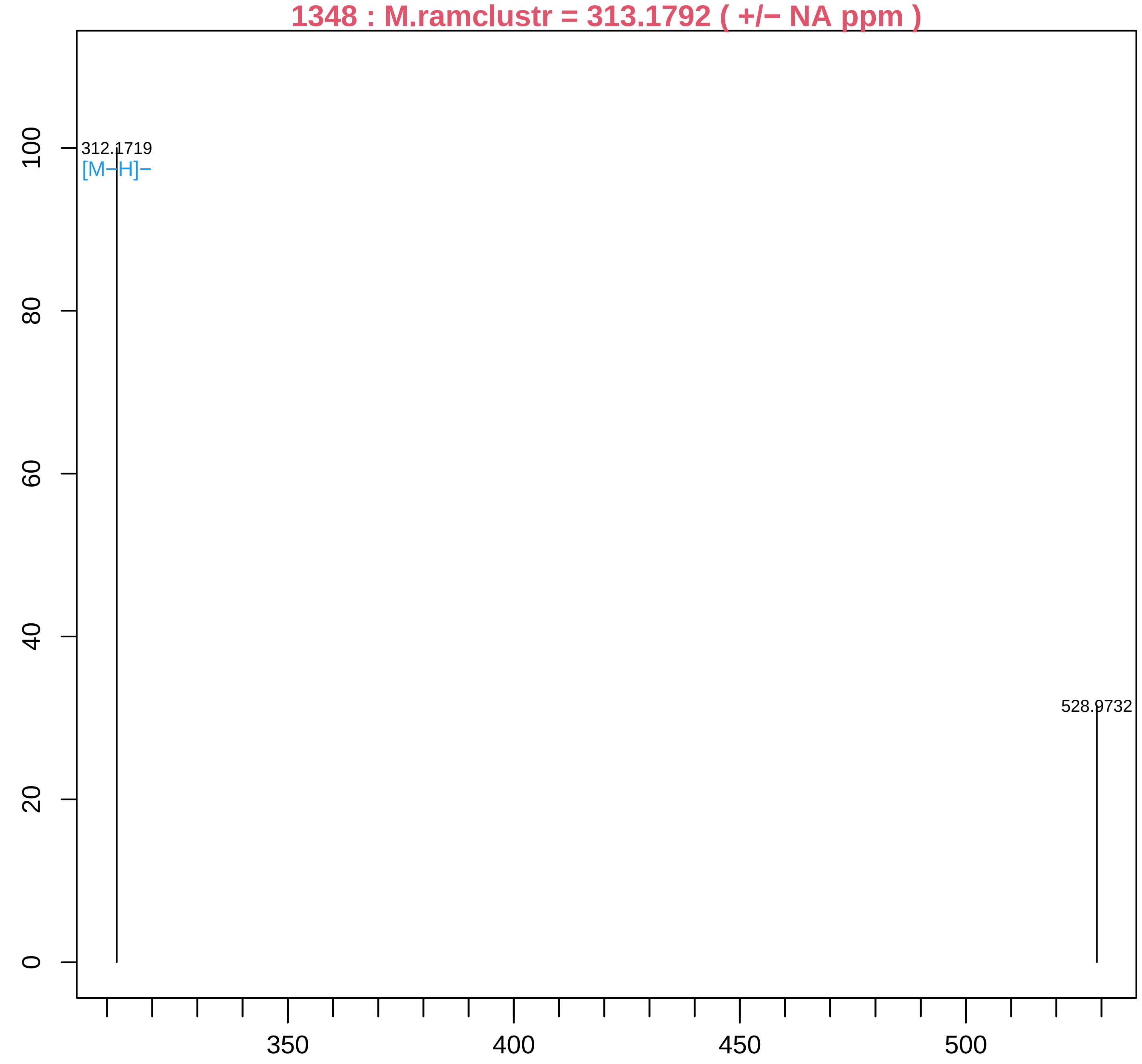
1348 : M.ramclustr = 313.1792 (+/− NA ppm)
312.1719
[M−H]−
528.9732
0
20
40
60
80
100
350
400
450
500

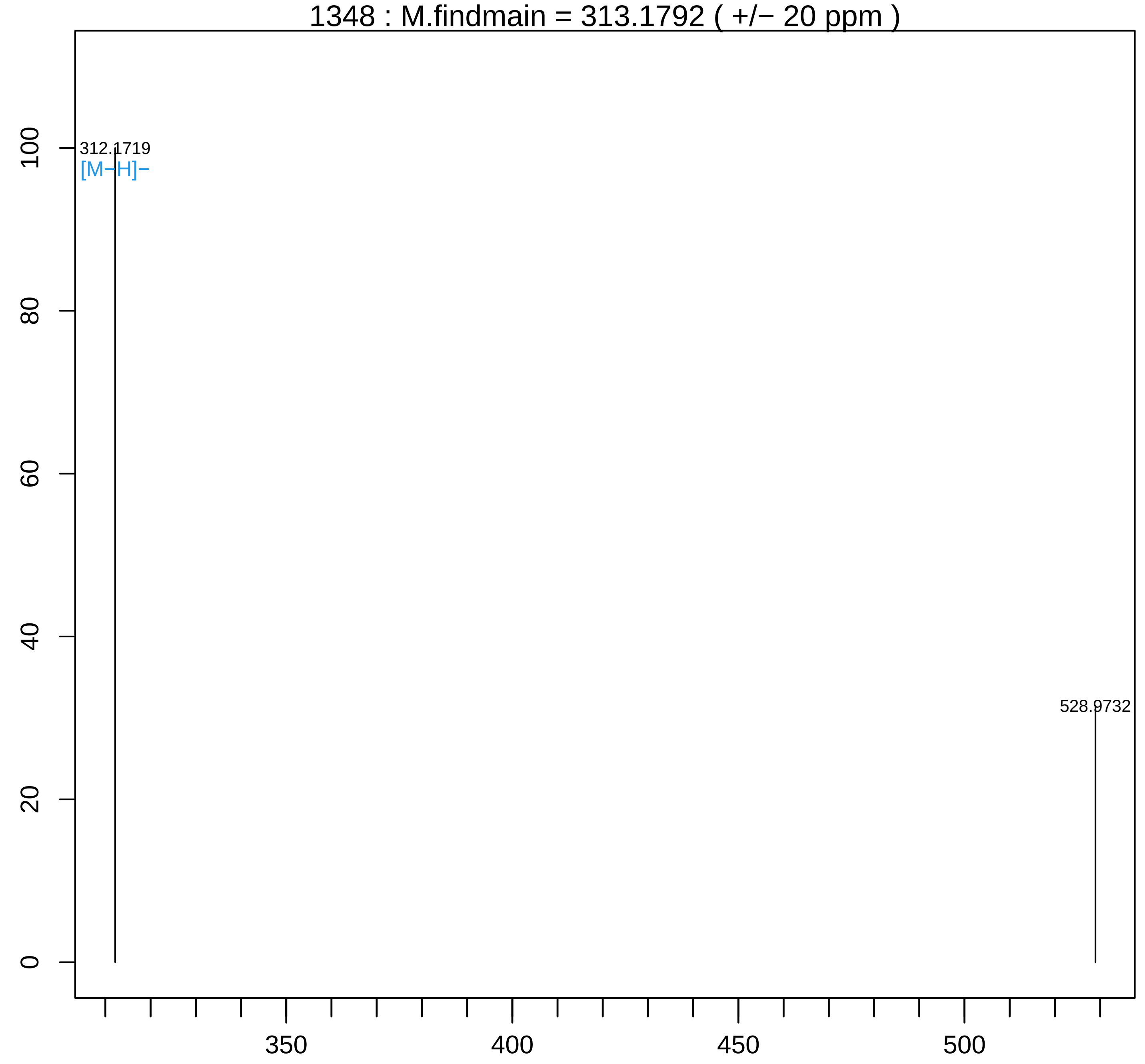
1348 : M.findmain = 313.1792 (+/− 20 ppm)
312.1719
[M−H]−
528.9732
0
20
40
60
80
100
350
400
450
500

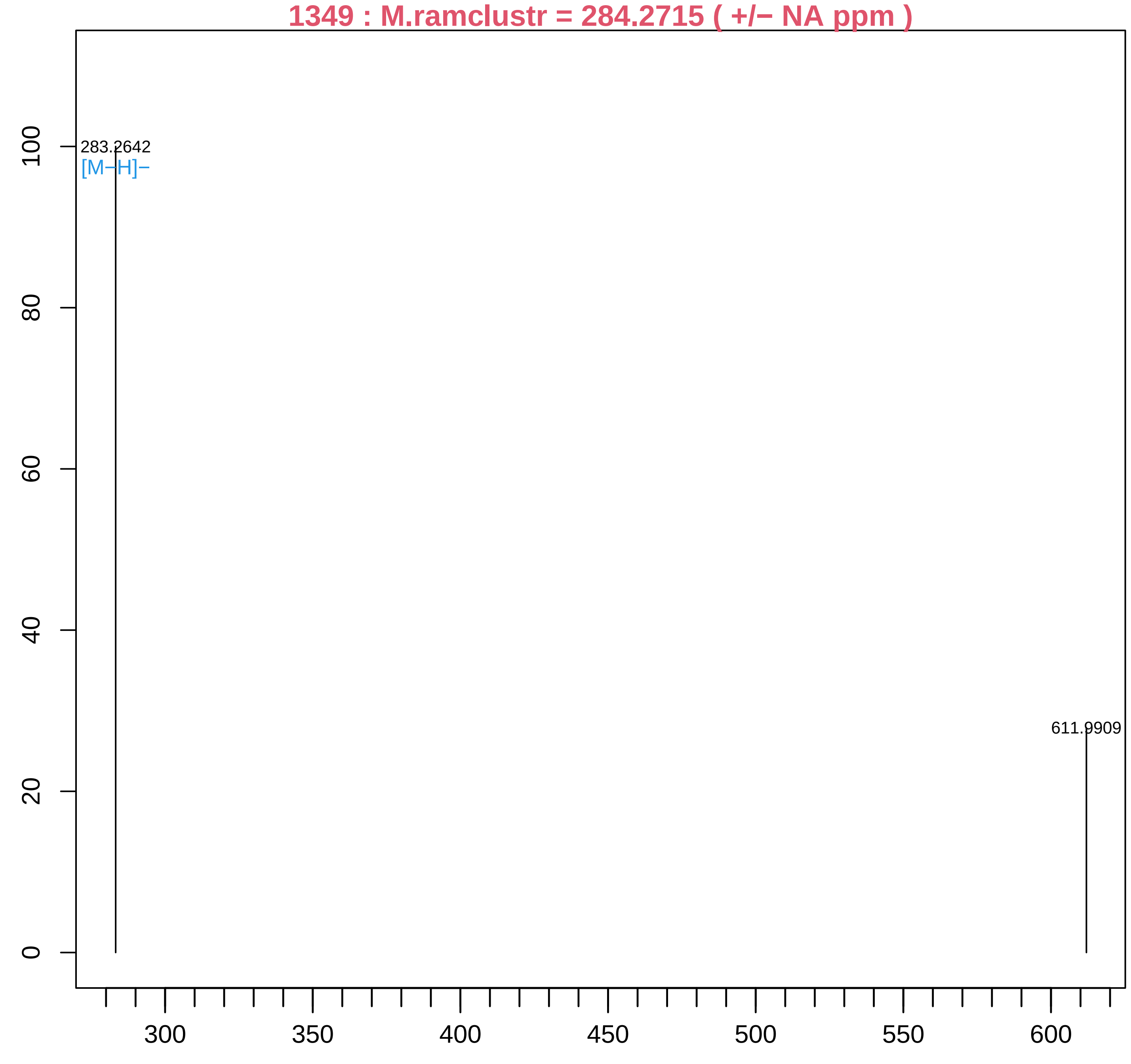
1349 : M.ramclustr = 284.2715 (+/− NA ppm)
283.2642
[M−H]−
611.9909
0
20
40
60
80
100
300
350
400
450
500
550
600

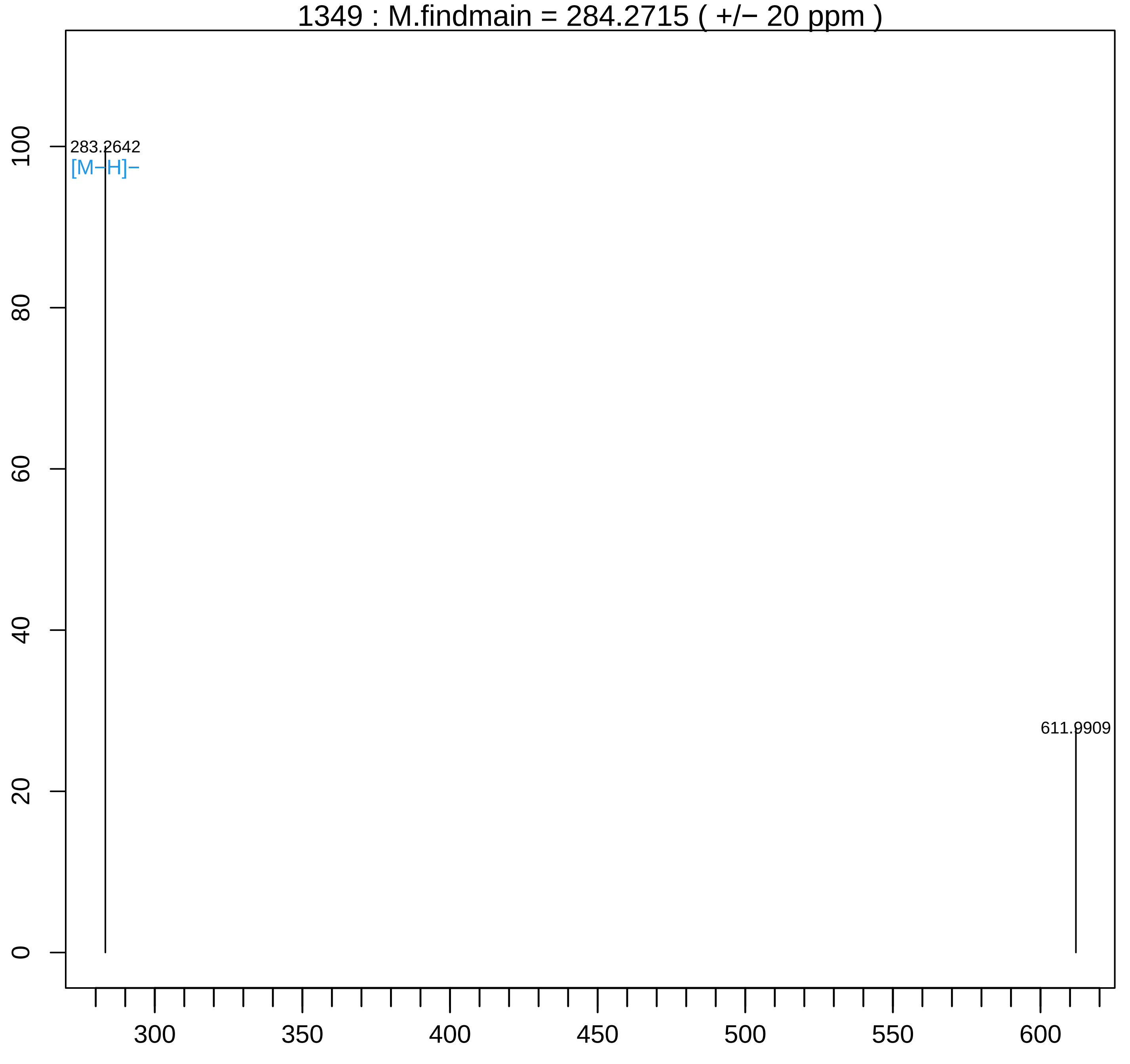
1349 : M.findmain = 284.2715 (+/− 20 ppm)
283.2642
[M−H]−
611.9909
0
20
40
60
80
100
300
350
400
450
500
550
600

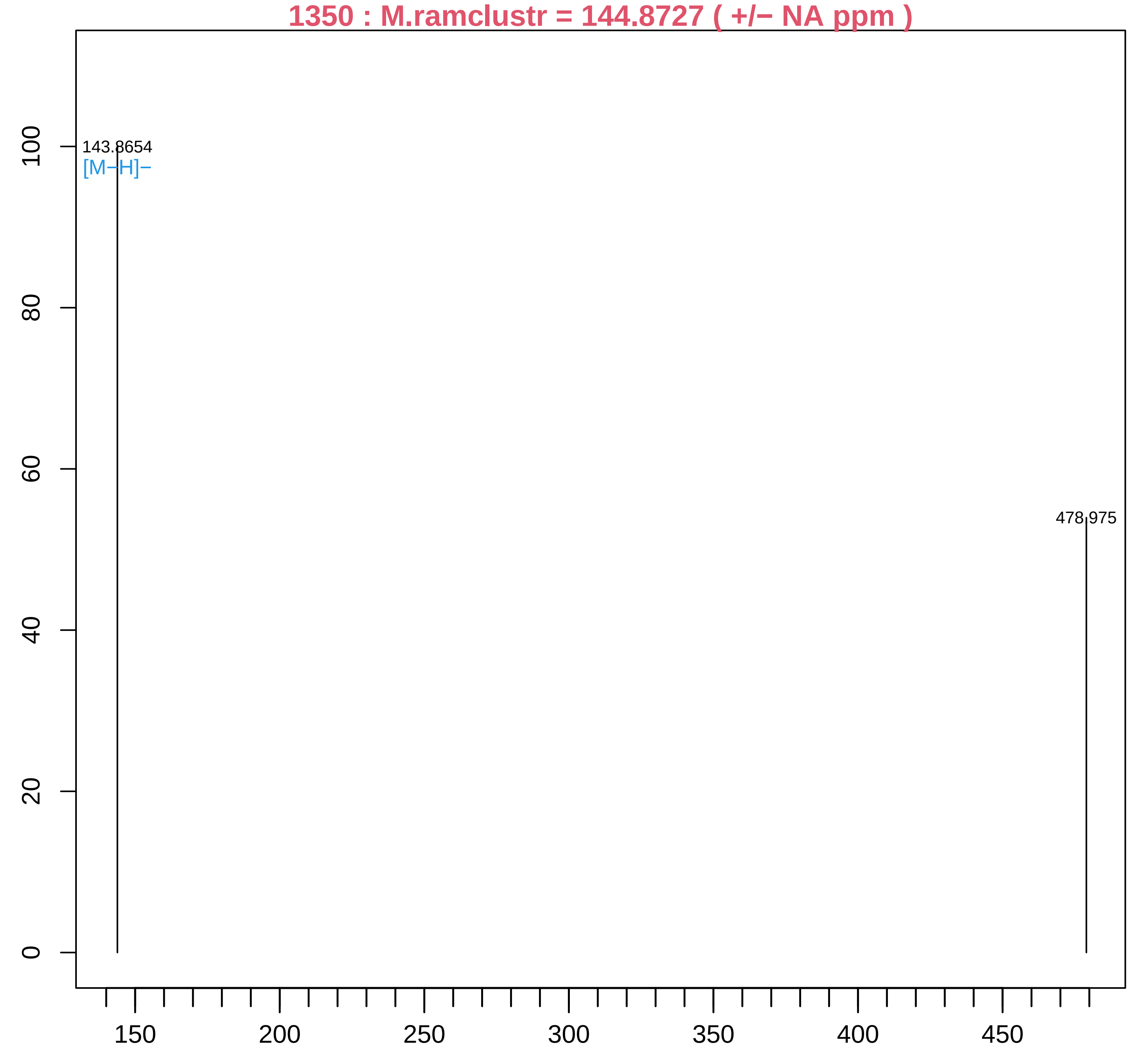

1350 : M.ramclustr = 144.8727 (+/− NA ppm)
143.8654
[M−H]−
478.975
0
20
40
60
80
100
150
200
250
300
350
400
450

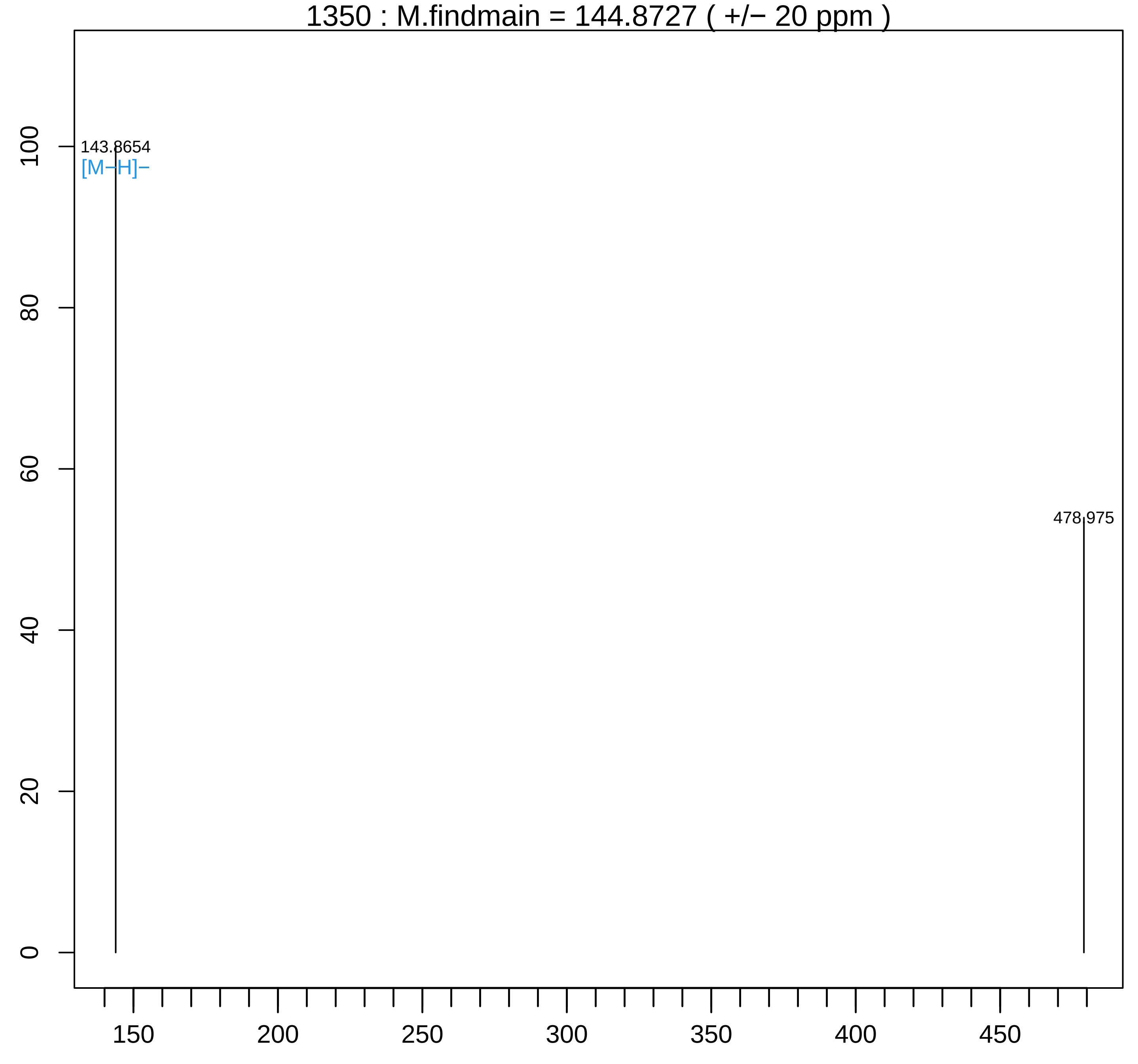

1350 : M.findmain = 144.8727 (+/− 20 ppm)
143.8654
[M−H]−
478.975
0
20
40
60
80
100
150
200
250
300
350
400
450

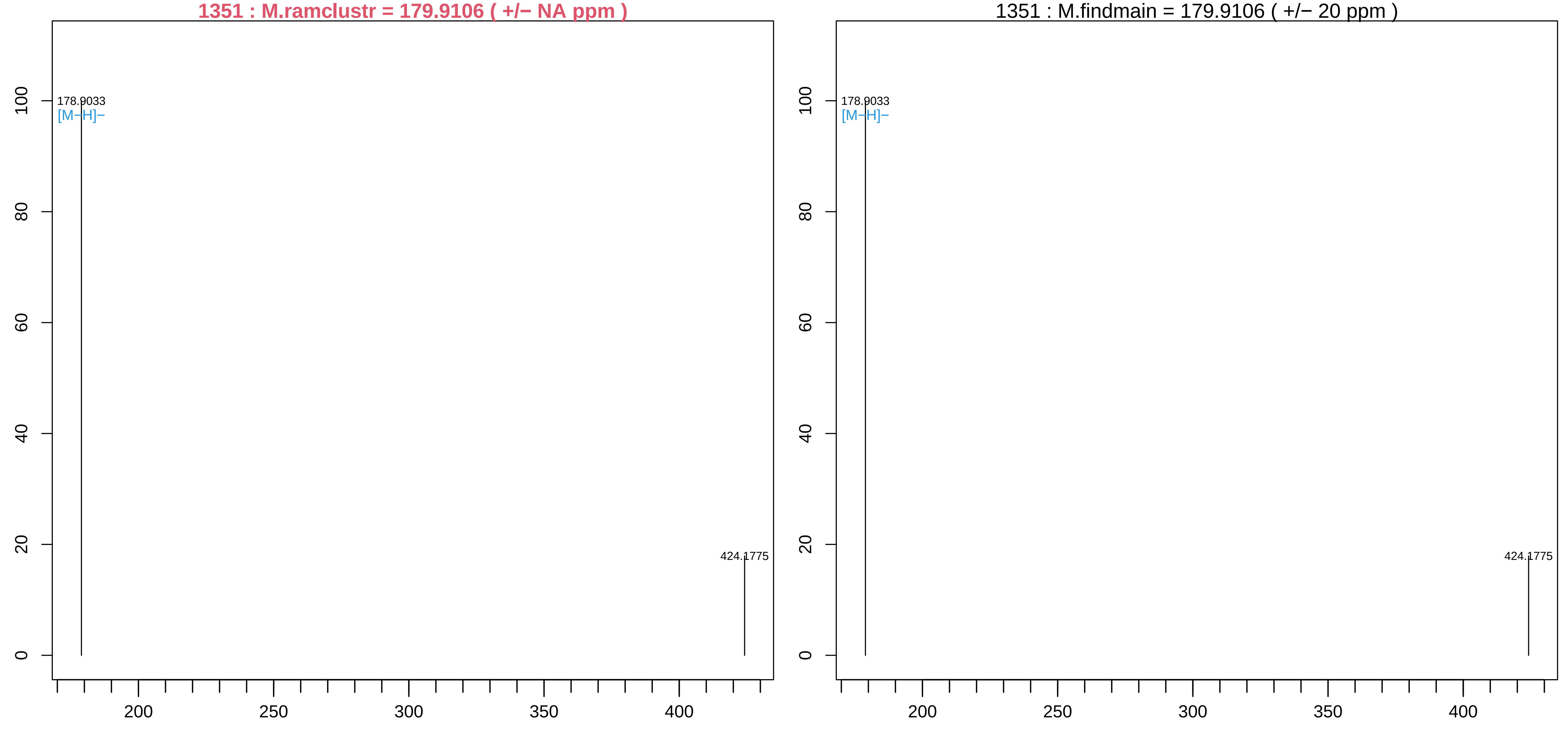
1351 : M.ramclustr = 179.9106 (+/− NA ppm)
178.9033
[M−H]−
424.1775
0
20
40
60
80
100
200
250
300
350
400
1351 : M.findmain = 179.9106 (+/− 20 ppm)
178.9033
[M−H]−
424.1775
0
20
40
60
80
100
200
250
300
350
400

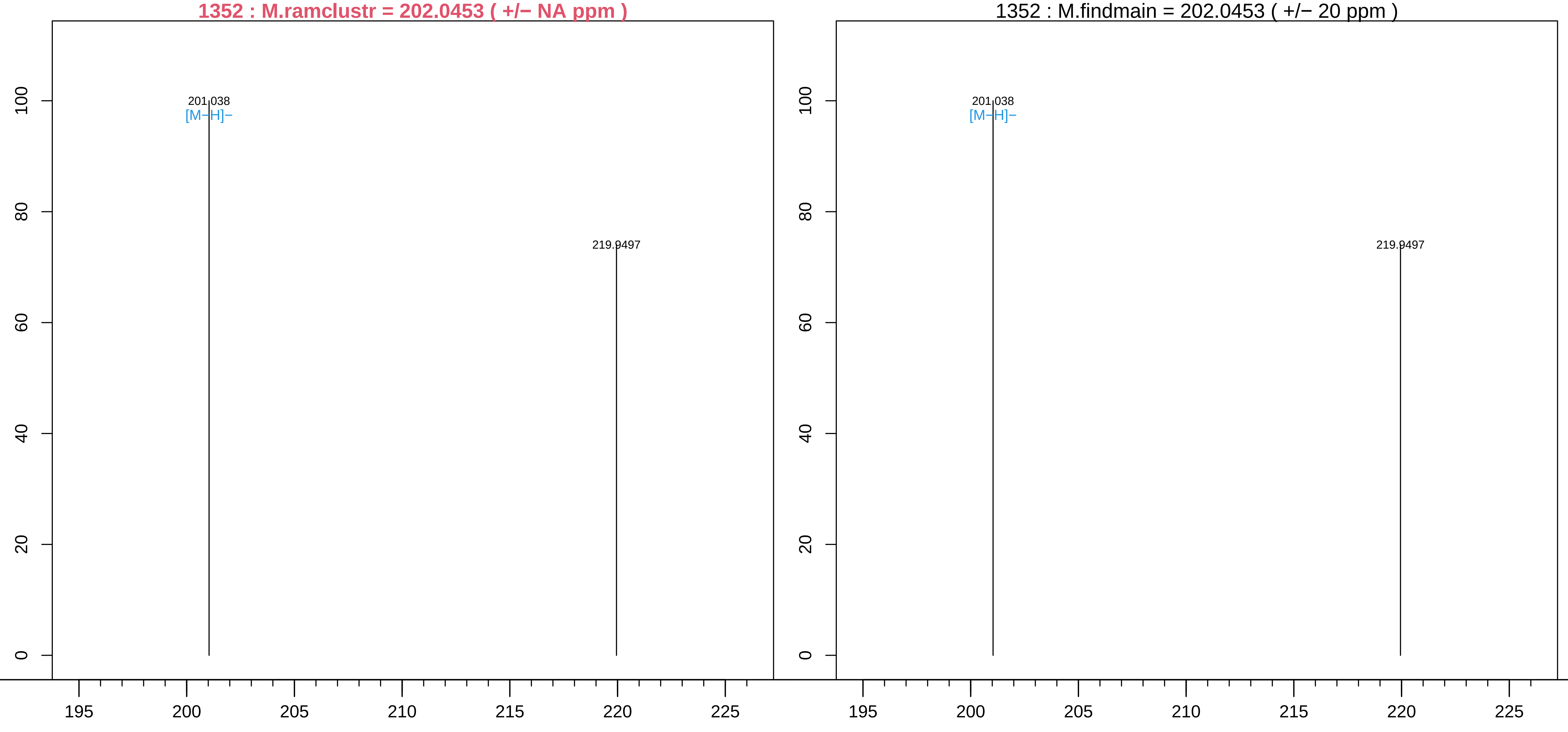
1352 : M.ramclustr = 202.0453 (+/− NA ppm)
201.038
[M−H]−
219.9497
1352 : M.findmain = 202.0453 (+/− 20 ppm)
201.038
[M−H]−
219.9497

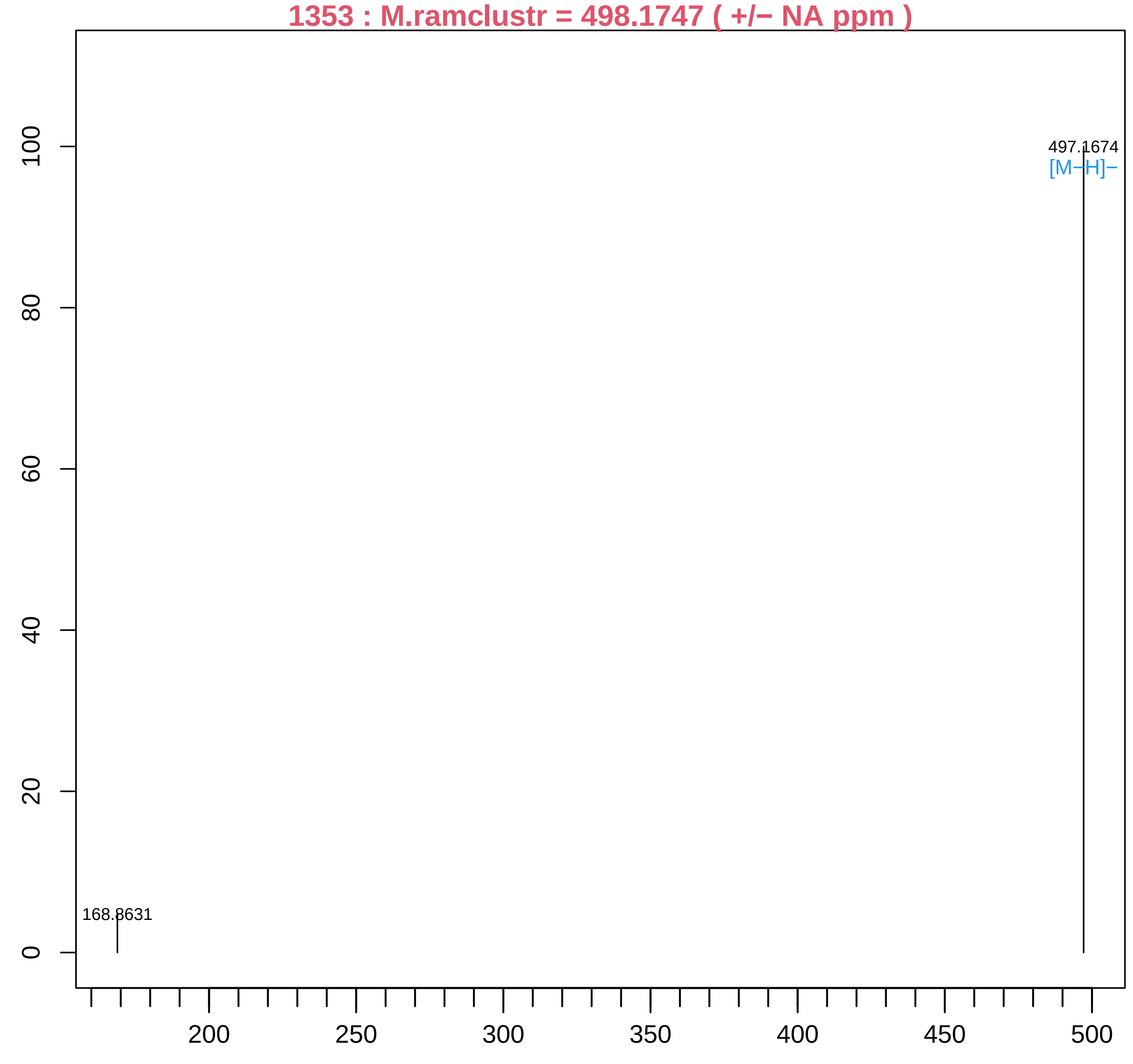
1353 : M.ramclustr = 498.1747 (+/− NA ppm)
497.1674
[M−H]−
168.8631
0
20
40
60
80
100
200
250
300
350
400
450
500

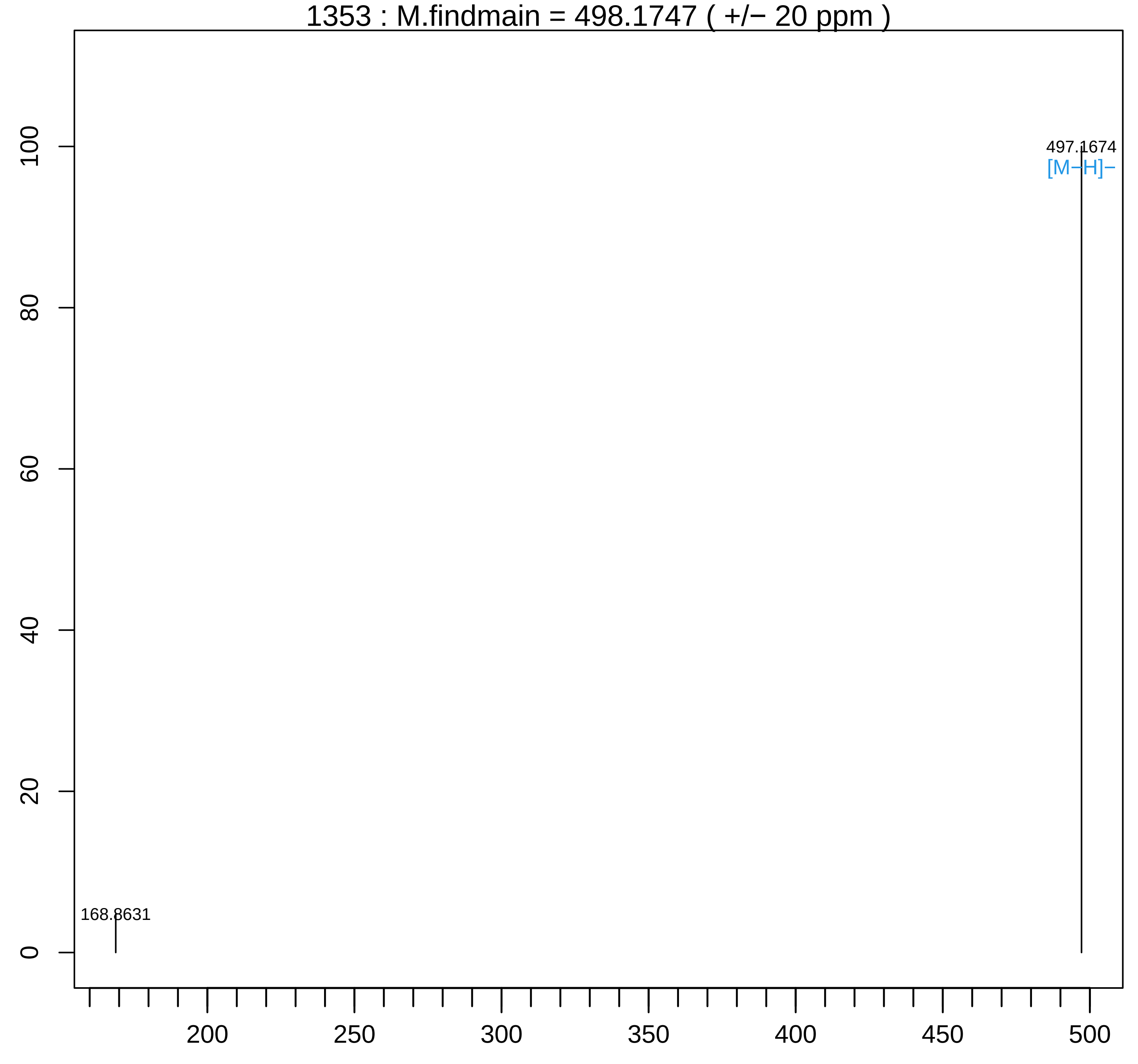
1353 : M.findmain = 498.1747 (+/− 20 ppm)
497.1674
[M−H]−
168.8631
0
20
40
60
80
100
200
250
300
350
400
450
500

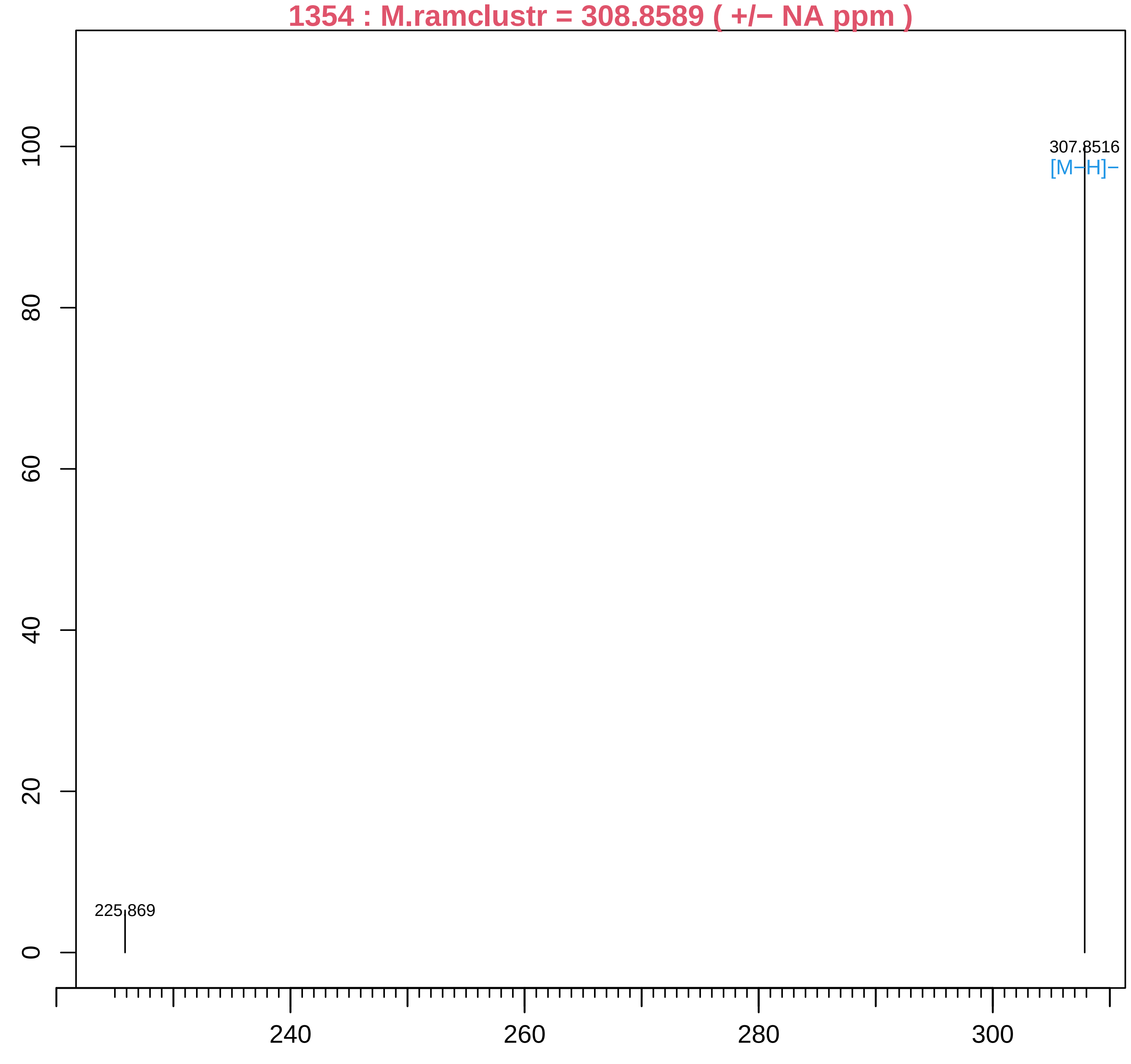

1354 : M.ramclustr = 308.8589 (+/− NA ppm)
307.8516
[M−H]−
225.869
0
20
40
60
80
100
240
260
280
300

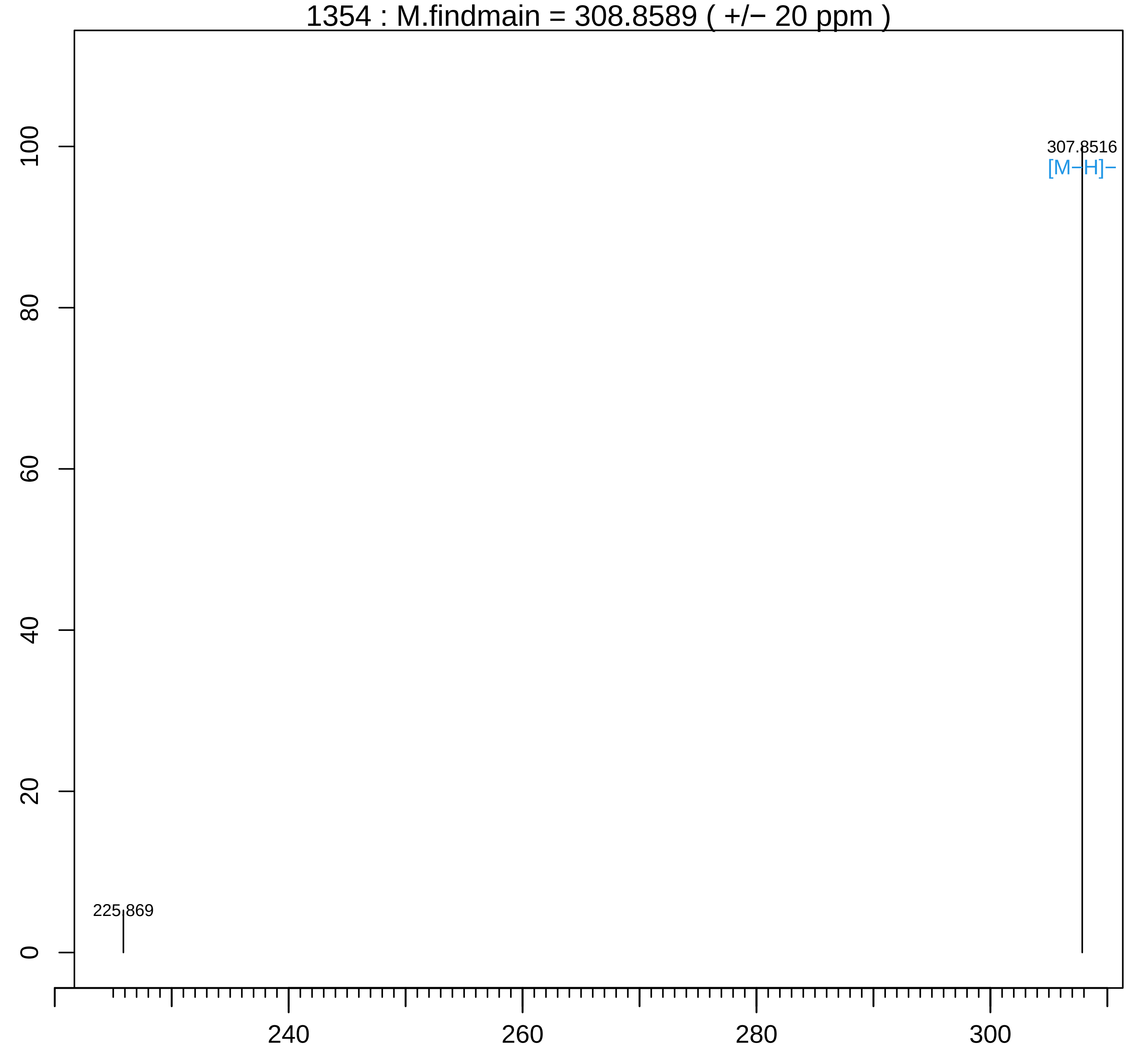

1354 : M.findmain = 308.8589 (+/− 20 ppm)
307.8516
[M−H]−
225.869
0
20
40
60
80
100
240
260
280
300

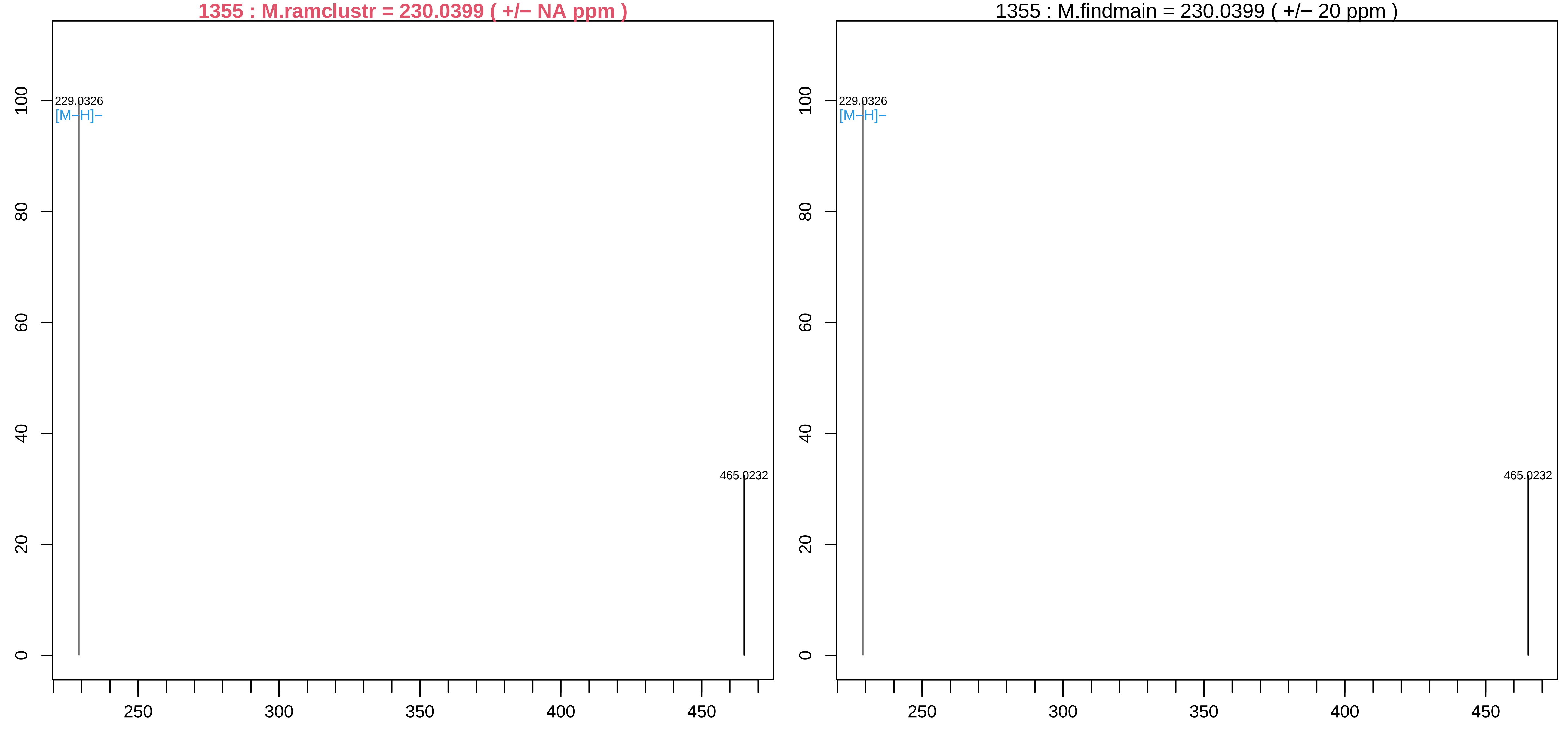
1355 : M.ramclustr = 230.0399 (+/− NA ppm)
229.0326
[M−H]−
465.0232
0
20
40
60
80
100
250
300
350
400
450
1355 : M.findmain = 230.0399 (+/− 20 ppm)
229.0326
[M−H]−
465.0232
0
20
40
60
80
100
250
300
350
400
450

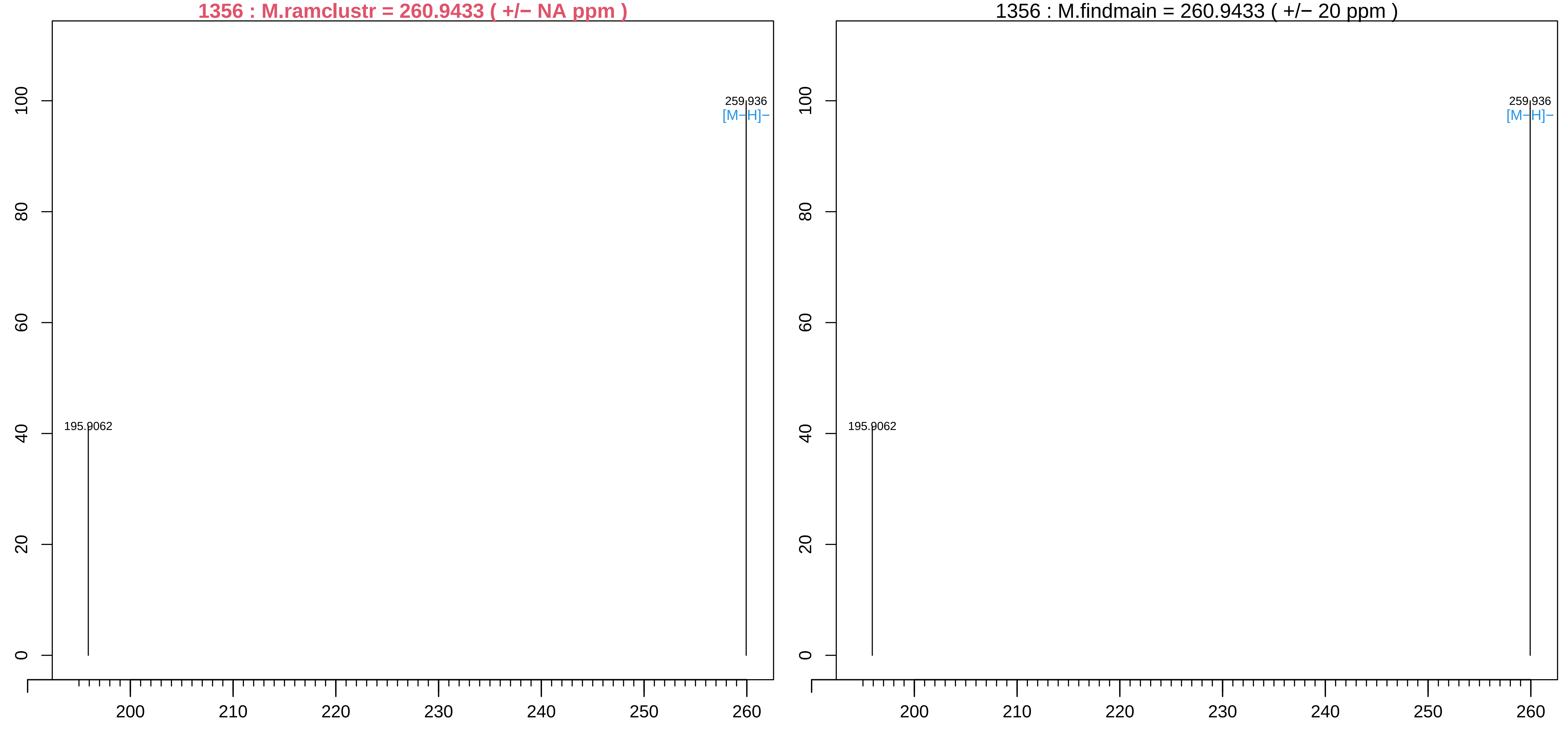
1356 : M.ramclustr = 260.9433 (+/− NA ppm)
259.936
[M−H]−
195.9062
1356 : M.findmain = 260.9433 (+/− 20 ppm)
259.936
[M−H]−
195.9062
0
20
40
60
80
100
200
210
220
230
240
250
260

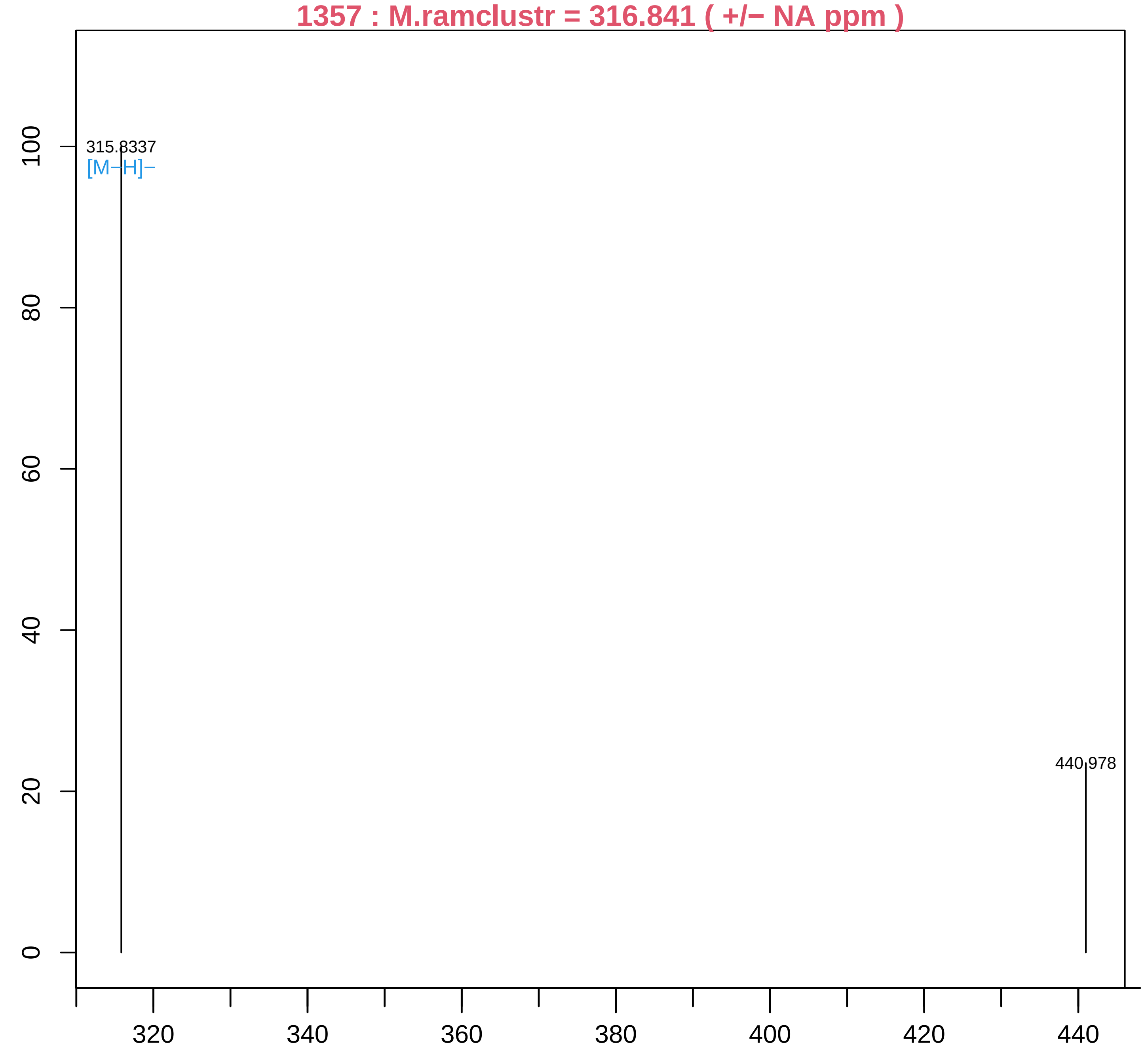
1357 : M.ramclustr = 316.841 (+/− NA ppm)
315.8337
[M−H]−
440.978
0
20
40
60
80
100
320
340
360
380
400
420
440

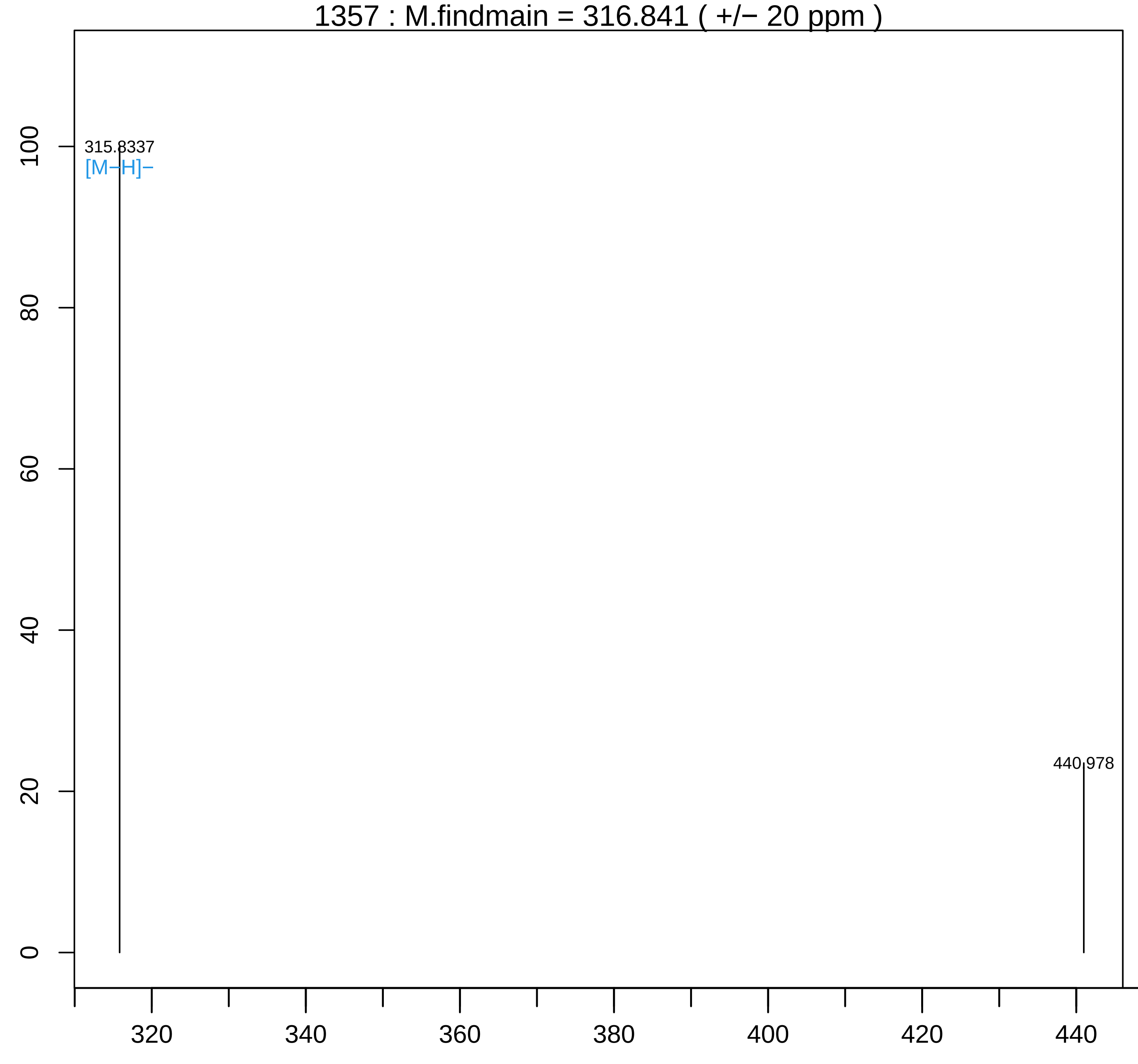
1357 : M.findmain = 316.841 (+/− 20 ppm)
315.8337
[M−H]−
440.978
0
20
40
60
80
100
320
340
360
380
400
420
440

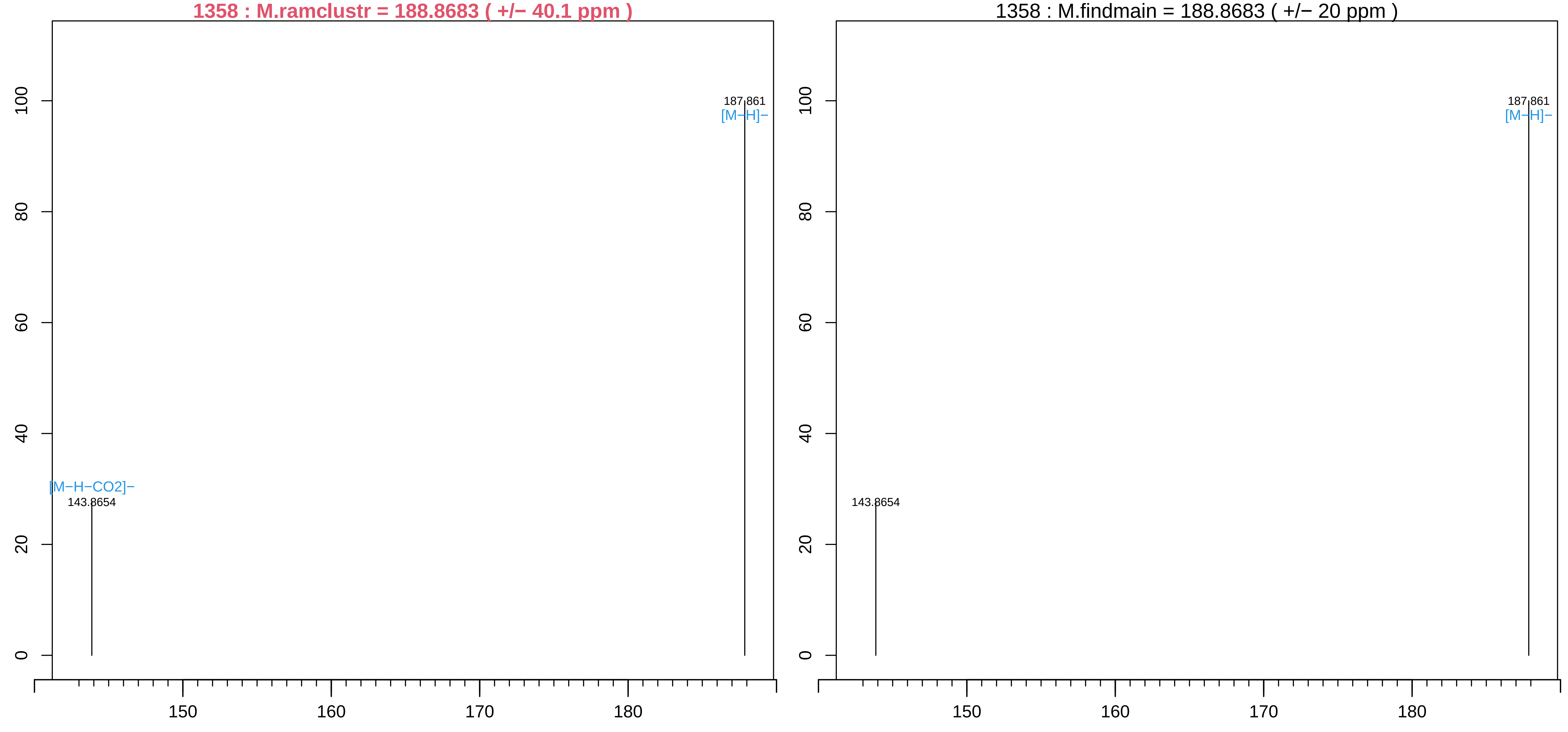

1358 : M.ramclustr = 188.8683 (+/− 40.1 ppm)
1358 : M.findmain = 188.8683 (+/− 20 ppm)
187.861
[M−H]−
[M−H−CO2]−
143.8654
187.861
[M−H]−
143.8654
0
20
40
60
80
100
150
160
170
180

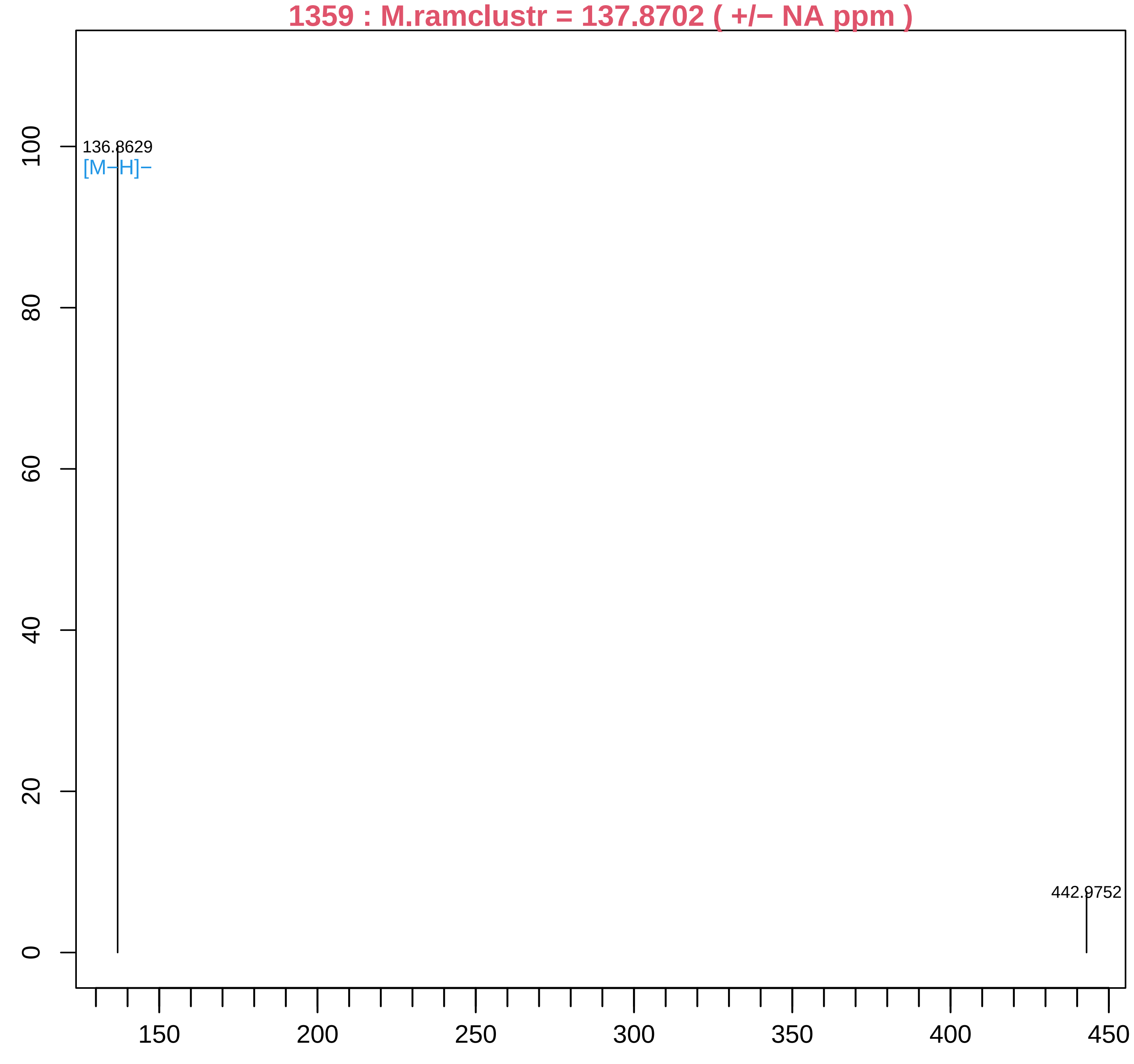
1359 : M.ramclustr = 137.8702 (+/− NA ppm)
136.8629
[M−H]−
442.9752
0
20
40
60
80
100
150
200
250
300
350
400
450

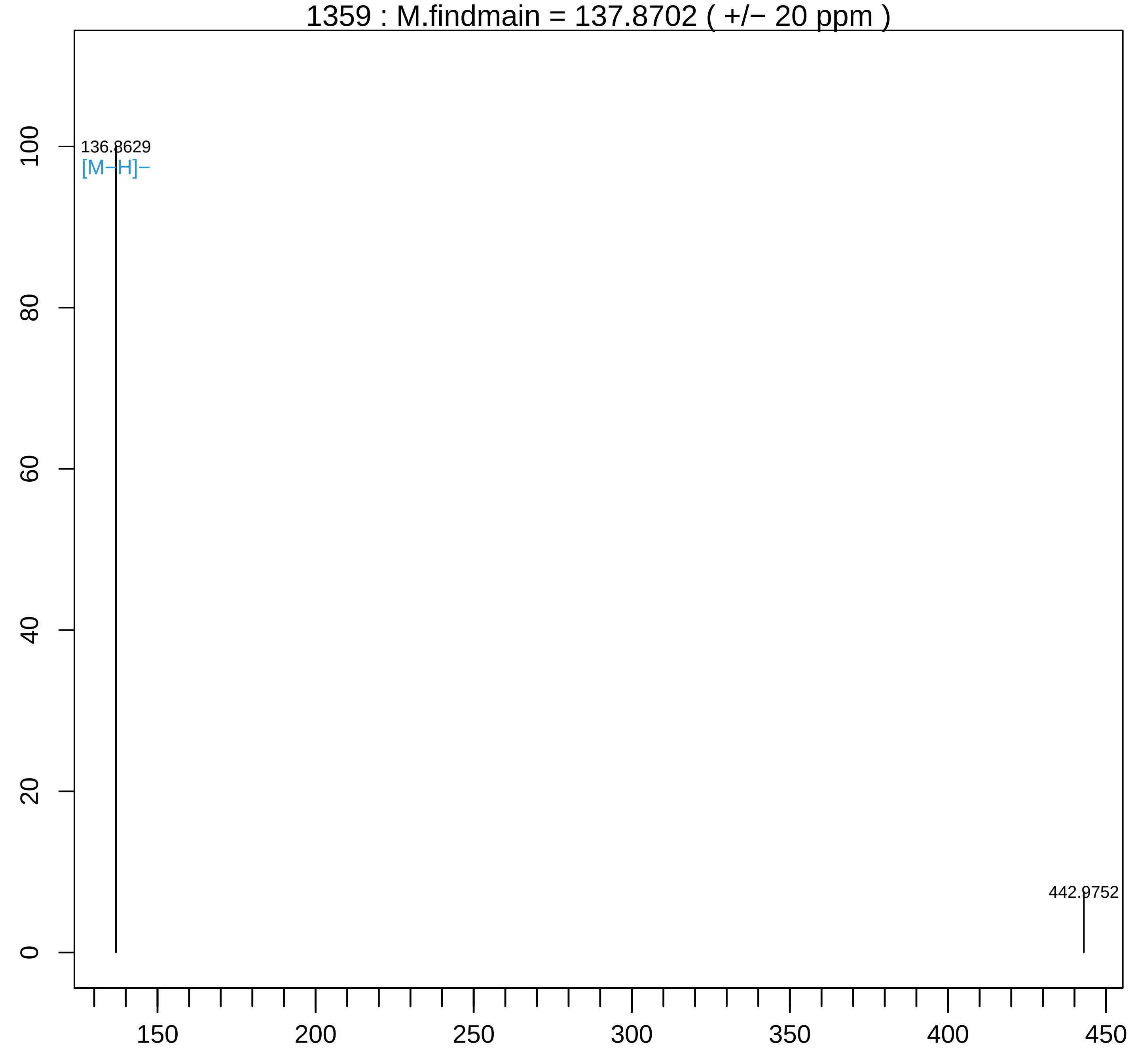
1359 : M.findmain = 137.8702 (+/− 20 ppm)
136.8629
[M−H]−
442.9752
0
20
40
60
80
100
150
200
250
300
350
400
450

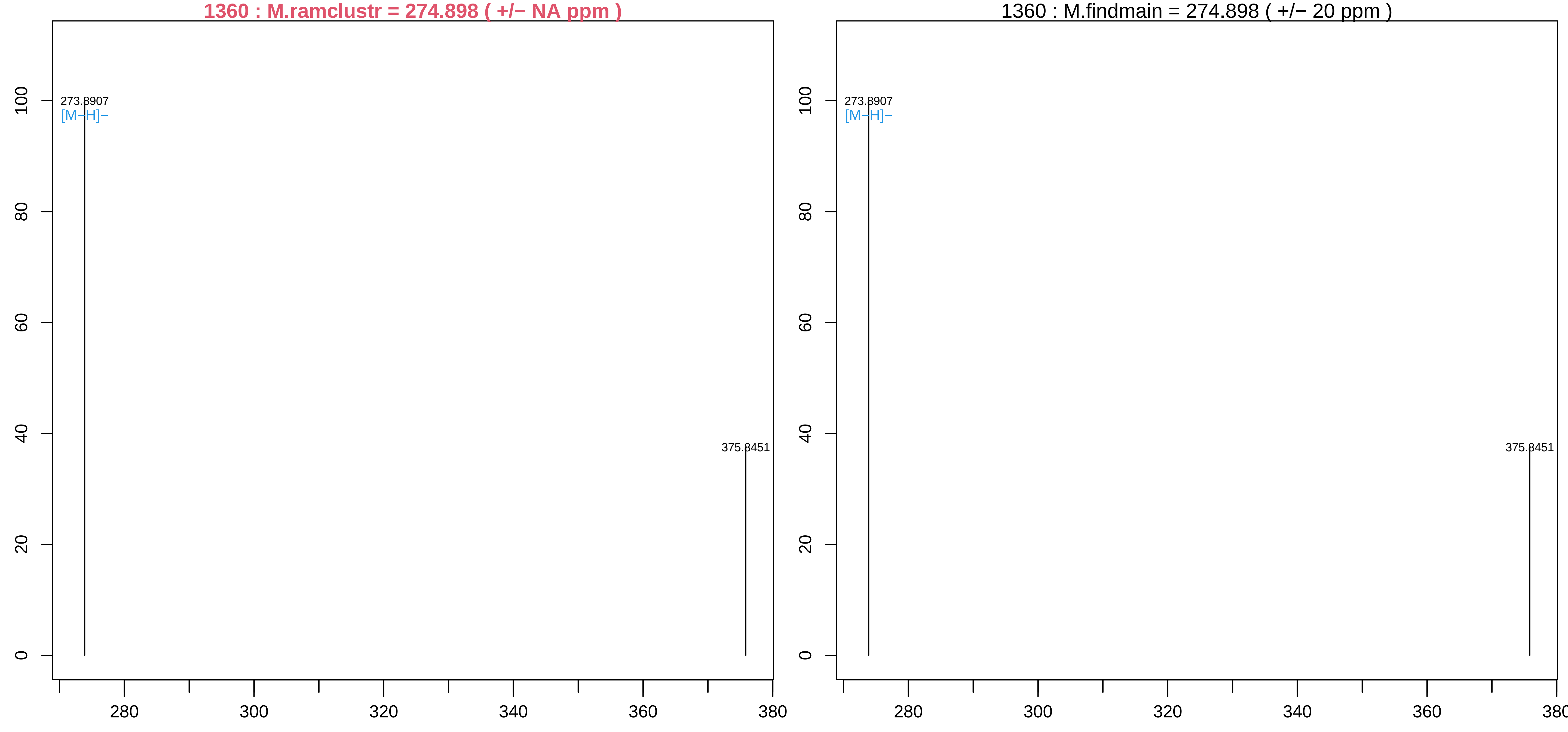
1360 : M.ramclustr = 274.898 (+/− NA ppm)
273.8907
[M−H]−
375.8451
0
20
40
60
80
100
280
300
320
340
360
380
1360 : M.findmain = 274.898 (+/− 20 ppm)
273.8907
[M−H]−
375.8451
0
20
40
60
80
100
280
300
320
340
360
380

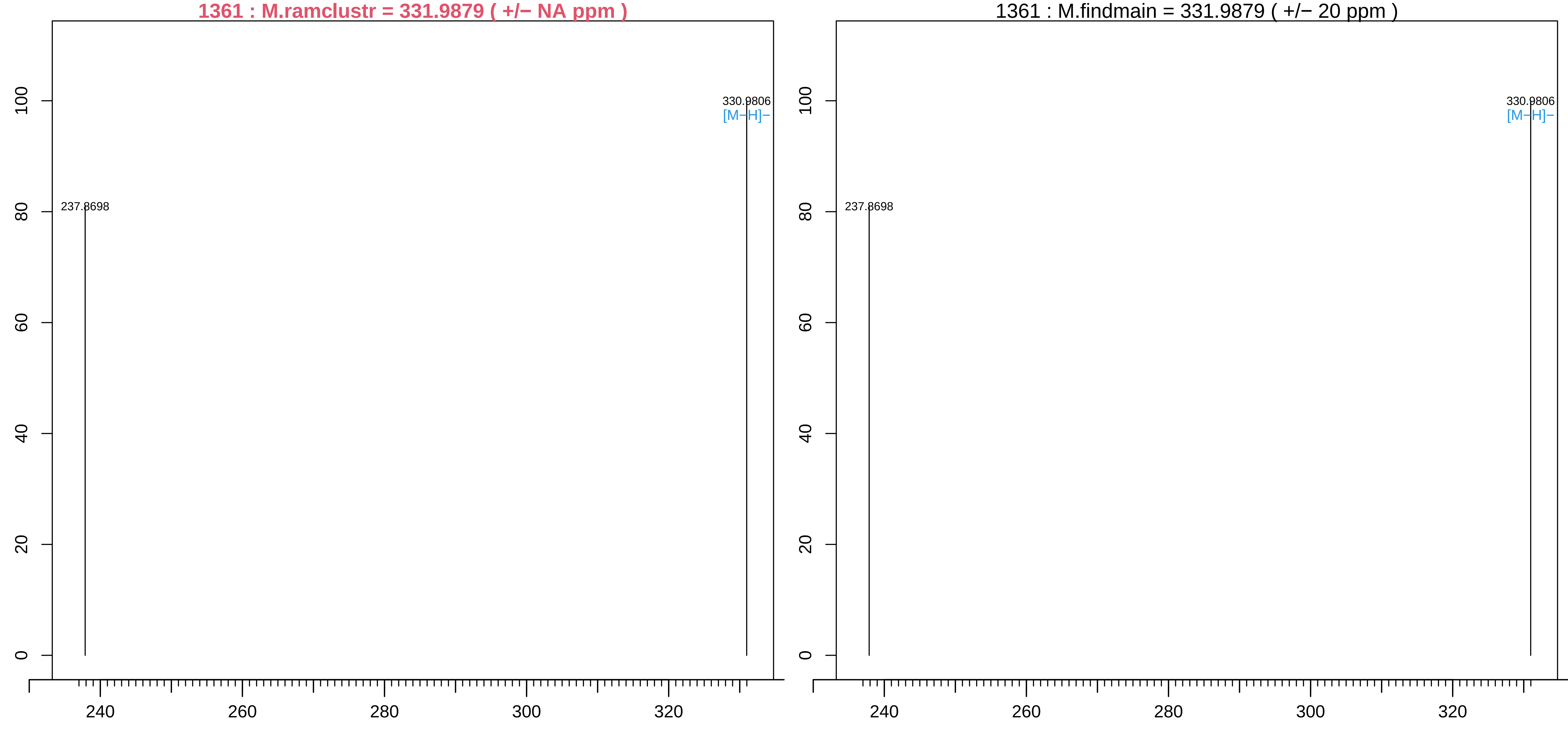
1361 : M.ramclustr = 331.9879 (+/− NA ppm)
330.9806
[M−H]−
237.8698
1361 : M.findmain = 331.9879 (+/− 20 ppm)
330.9806
[M−H]−
237.8698
0
20
40
60
80
100
240
260
280
300
320

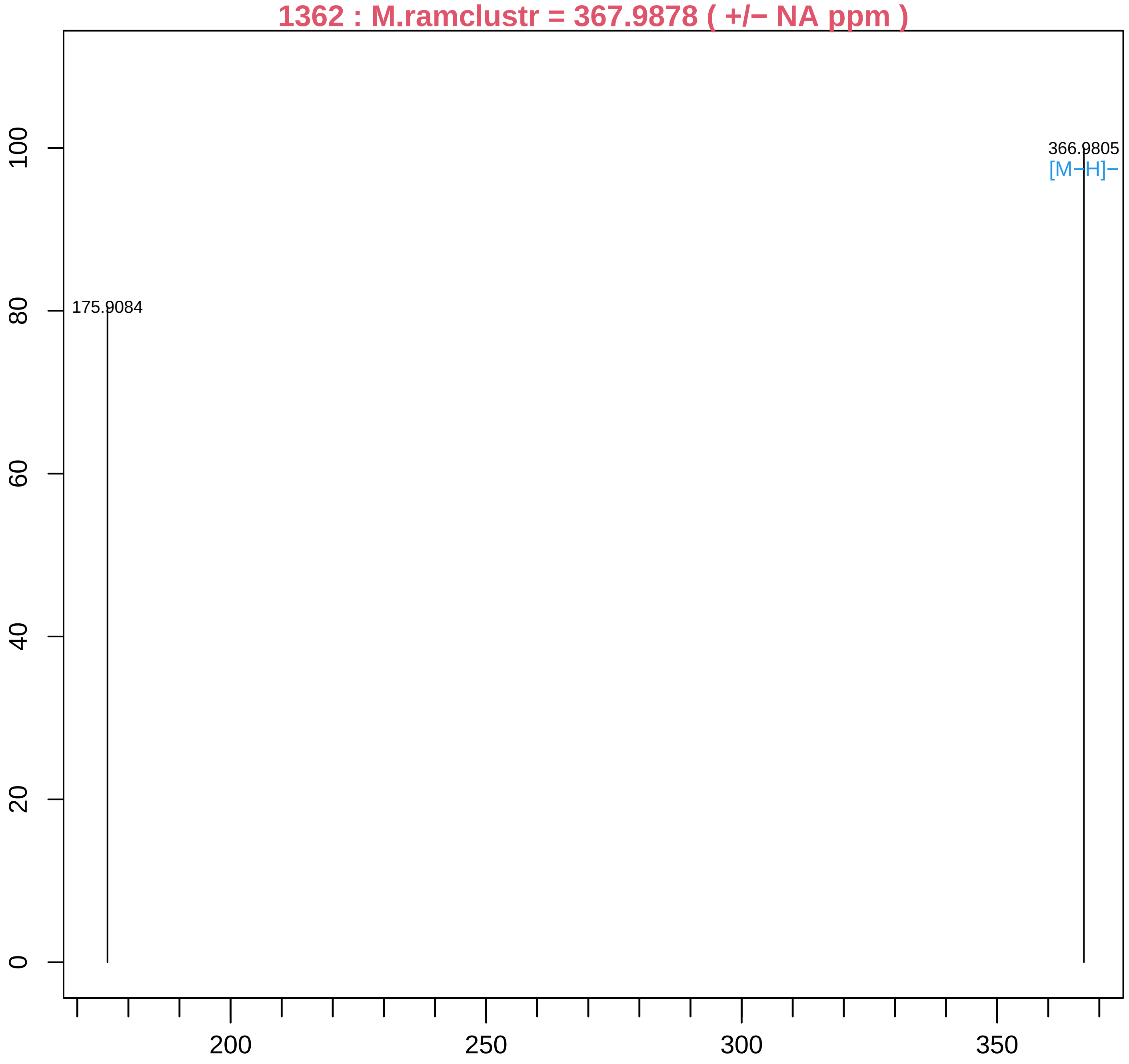
1362 : M.ramclustr = 367.9878 (+/− NA ppm)
366.9805
[M−H]−
175.9084
0
20
40
60
80
100
200
250
300
350

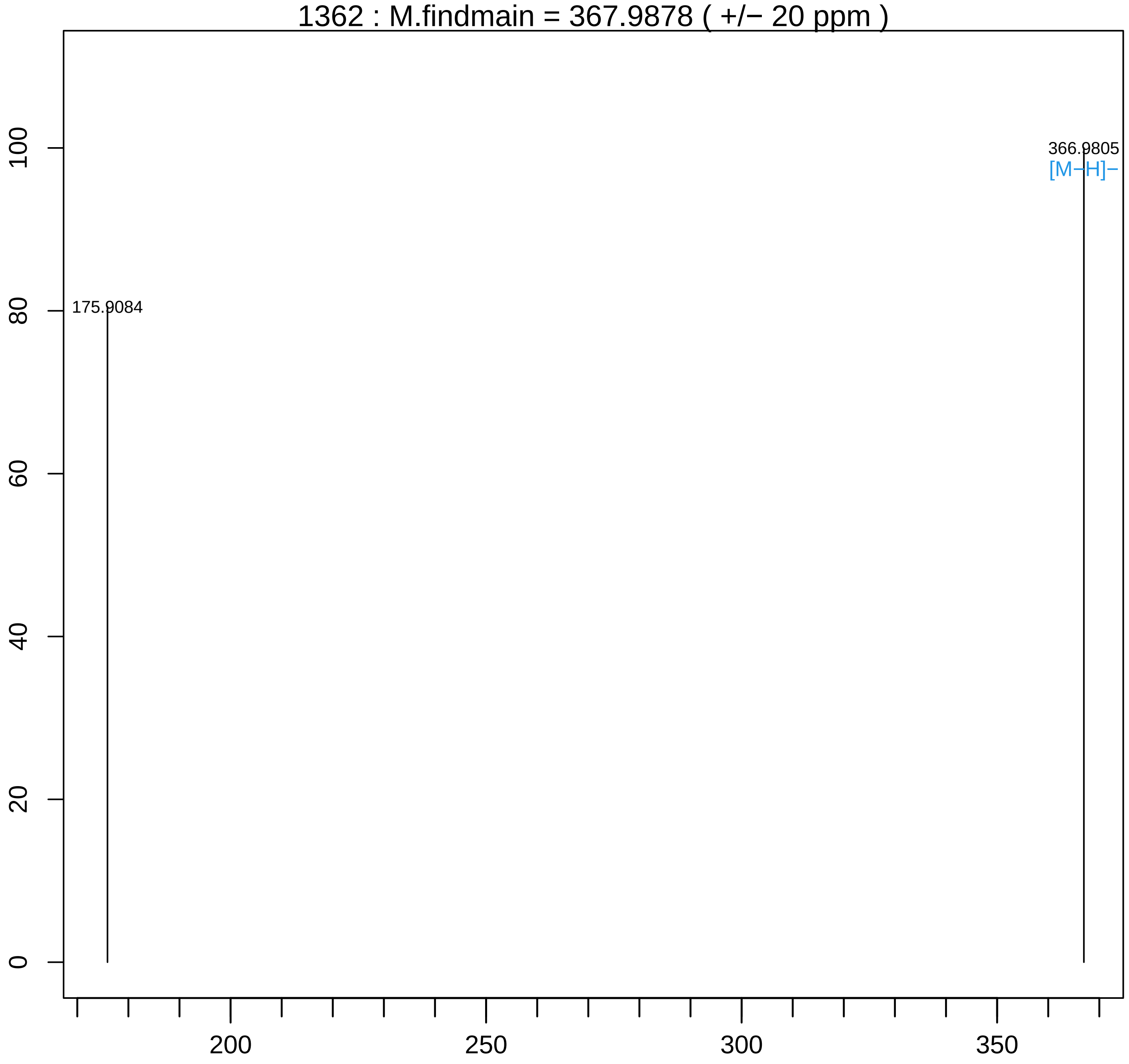
1362 : M.findmain = 367.9878 (+/− 20 ppm)
366.9805
[M−H]−
175.9084
0
20
40
60
80
100
200
250
300
350

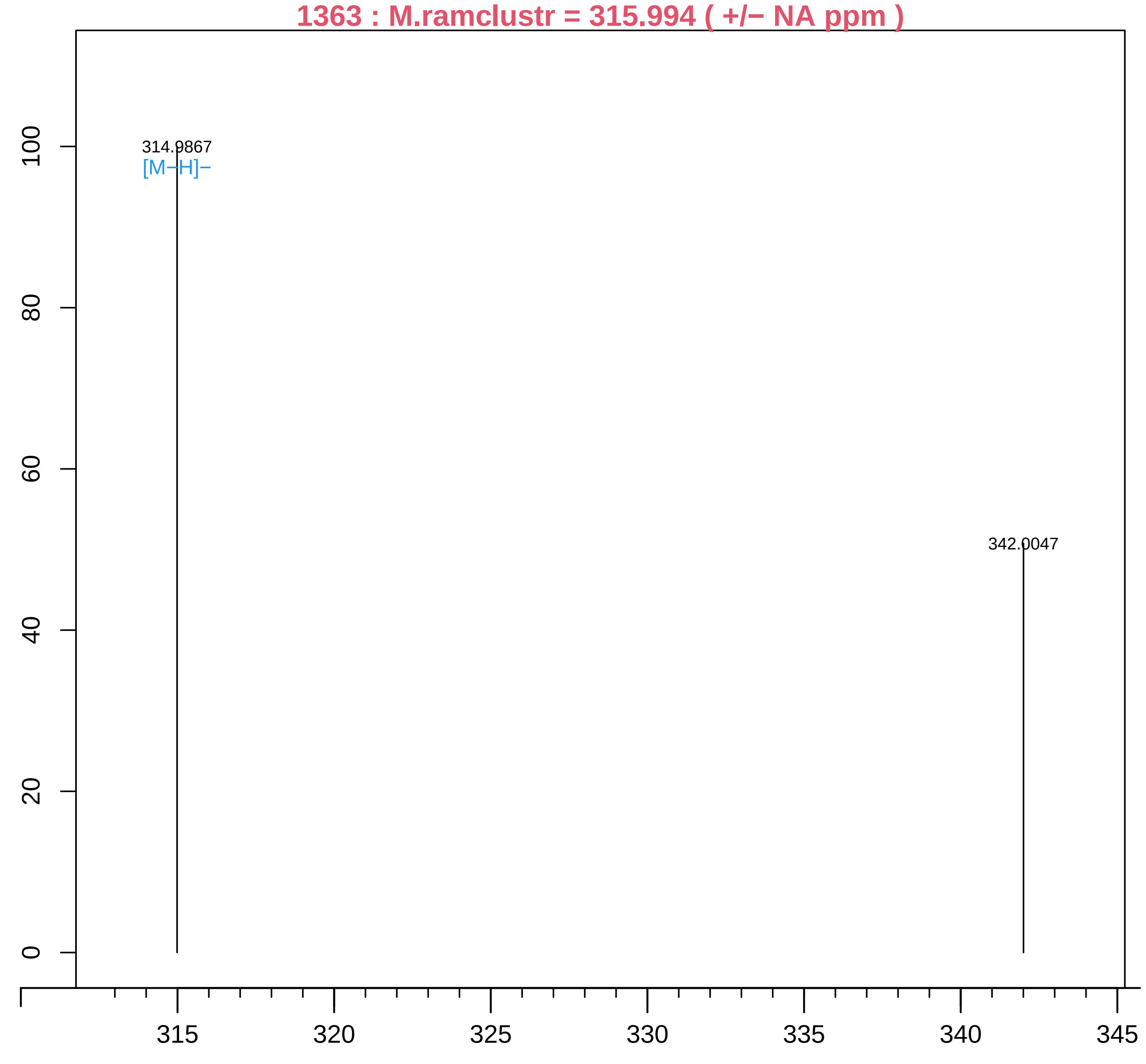

1363 : M.ramclustr = 315.994 (+/− NA ppm)
314.9867
[M−H]−
342.0047
0
20
40
60
80
100
315
320
325
330
335
340
345

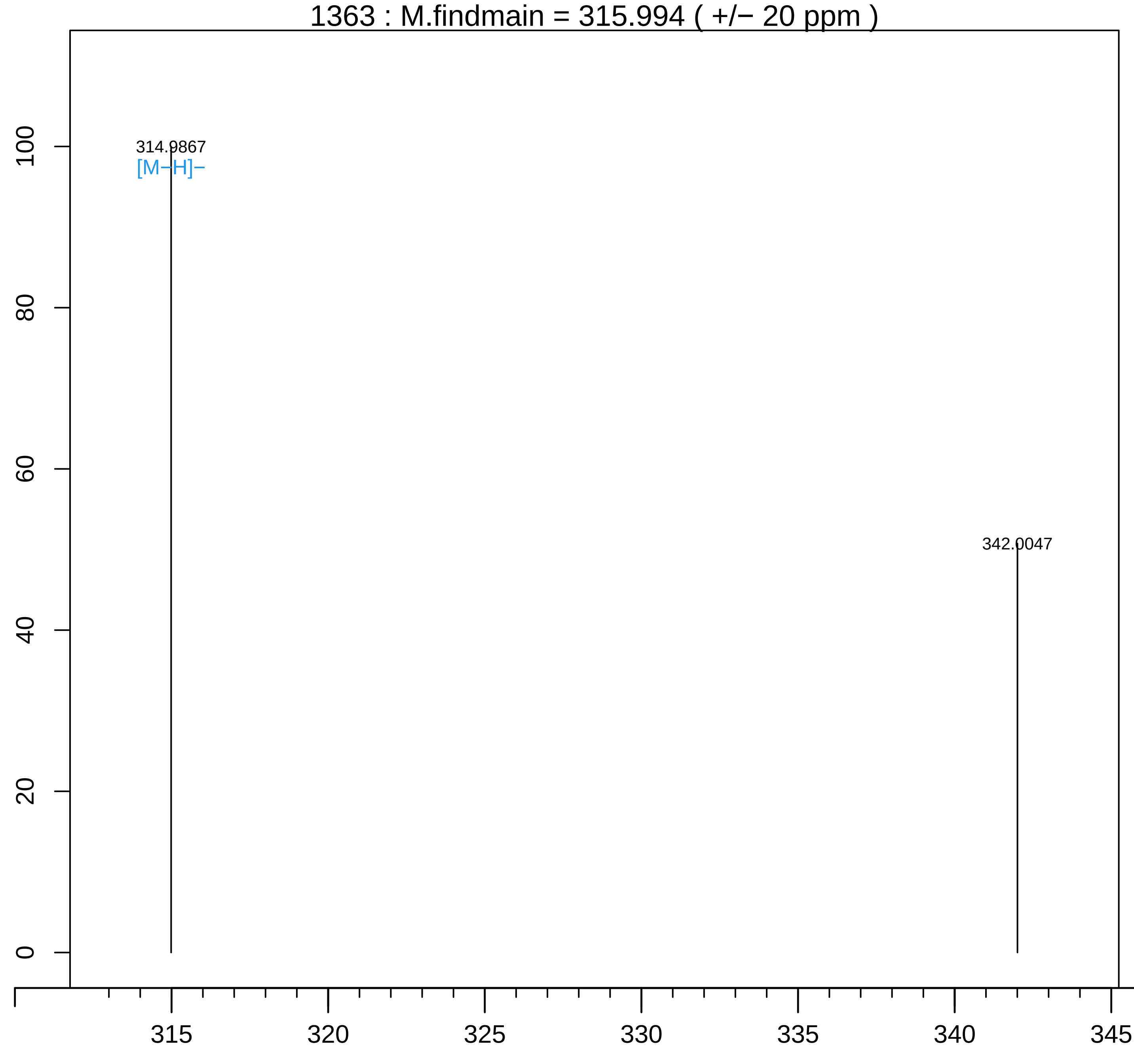

1363 : M.findmain = 315.994 (+/− 20 ppm)
314.9867
[M−H]−
342.0047
0
20
40
60
80
100
315
320
325
330
335
340
345

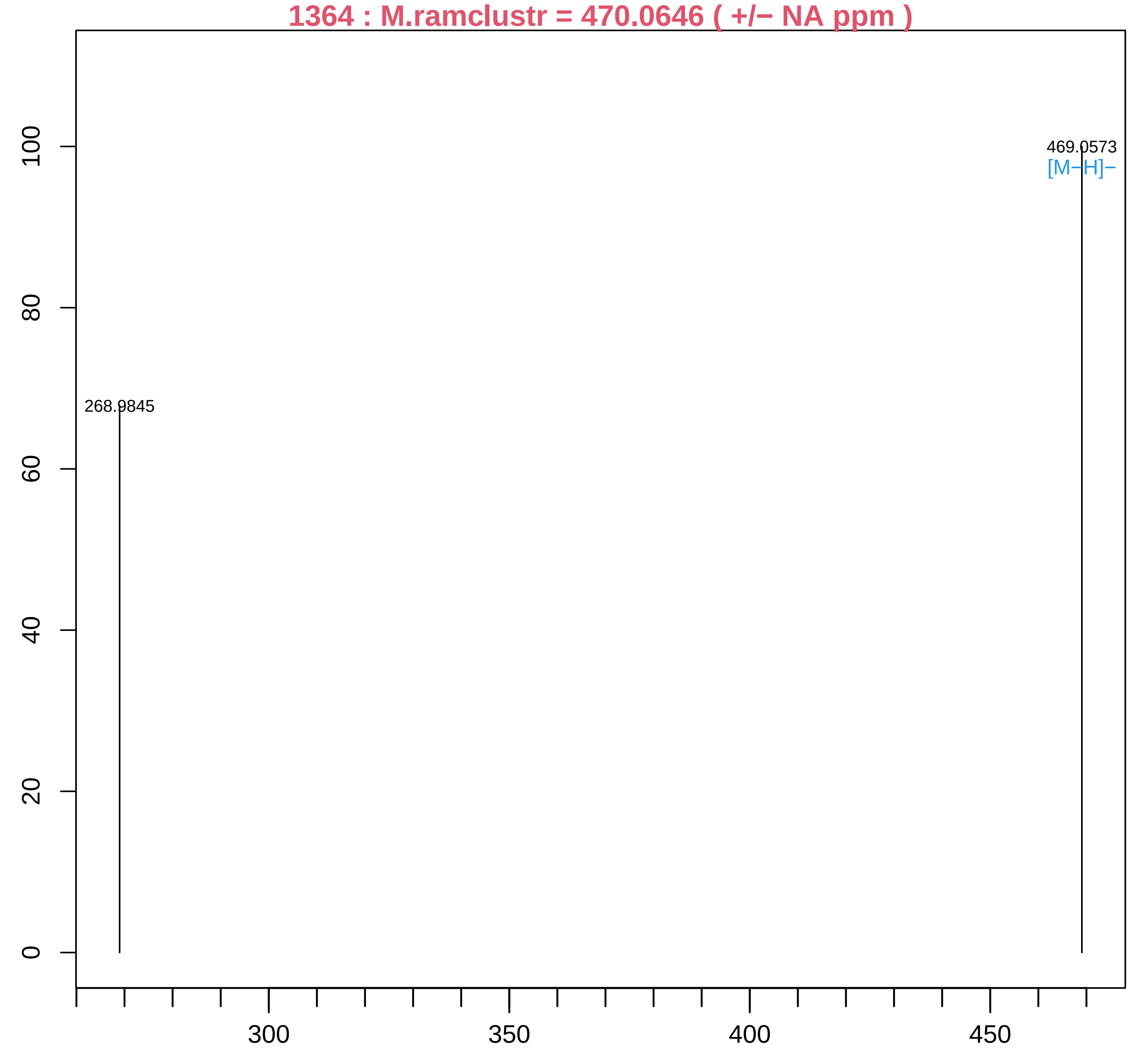

1364 : M.ramclustr = 470.0646 (+/− NA ppm)
469.0573
[M−H]−
268.9845
0
20
40
60
80
100
300
350
400
450

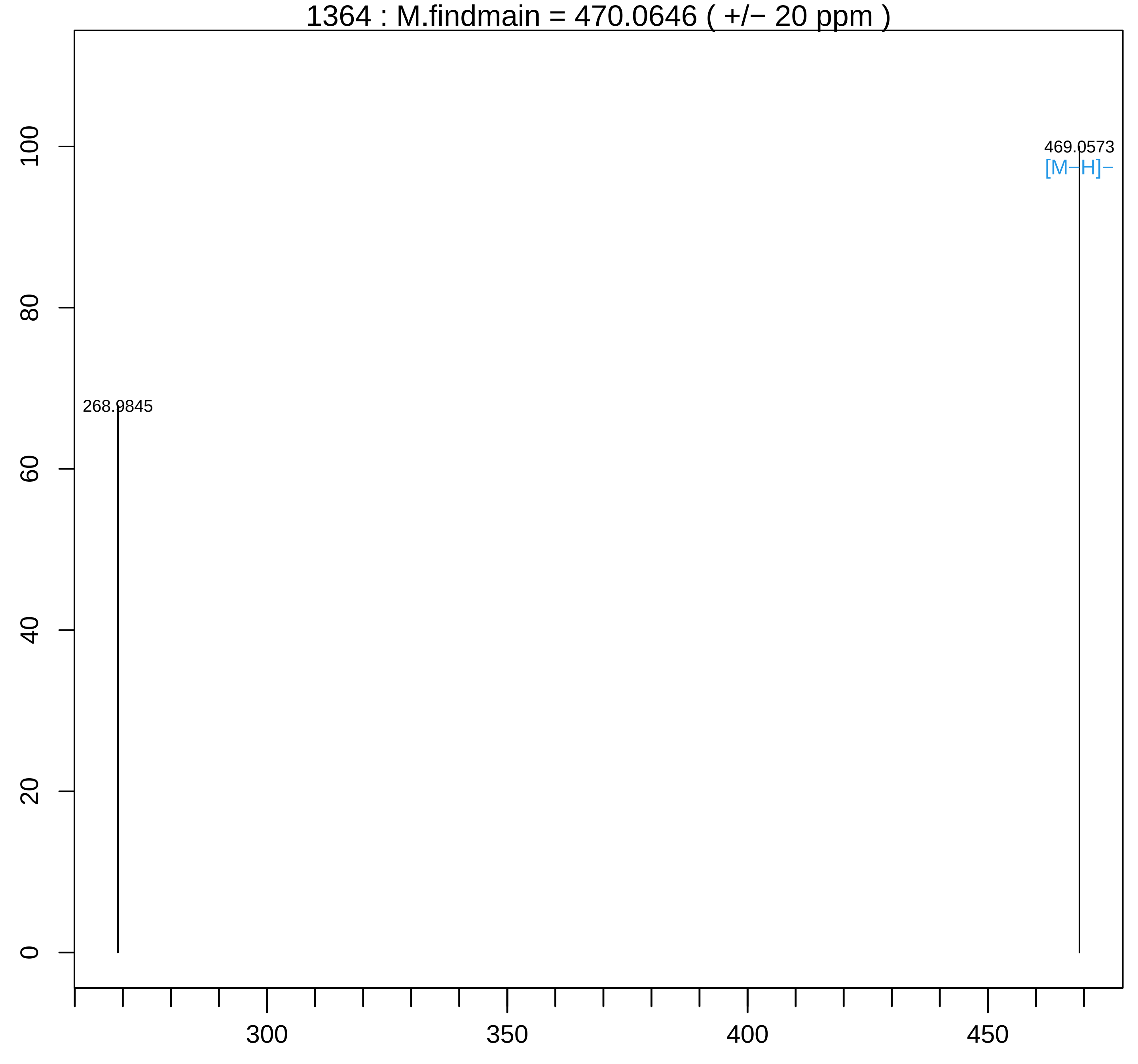

1364 : M.findmain = 470.0646 (+/− 20 ppm)
469.0573
[M−H]−
268.9845
0
20
40
60
80
100
300
350
400
450

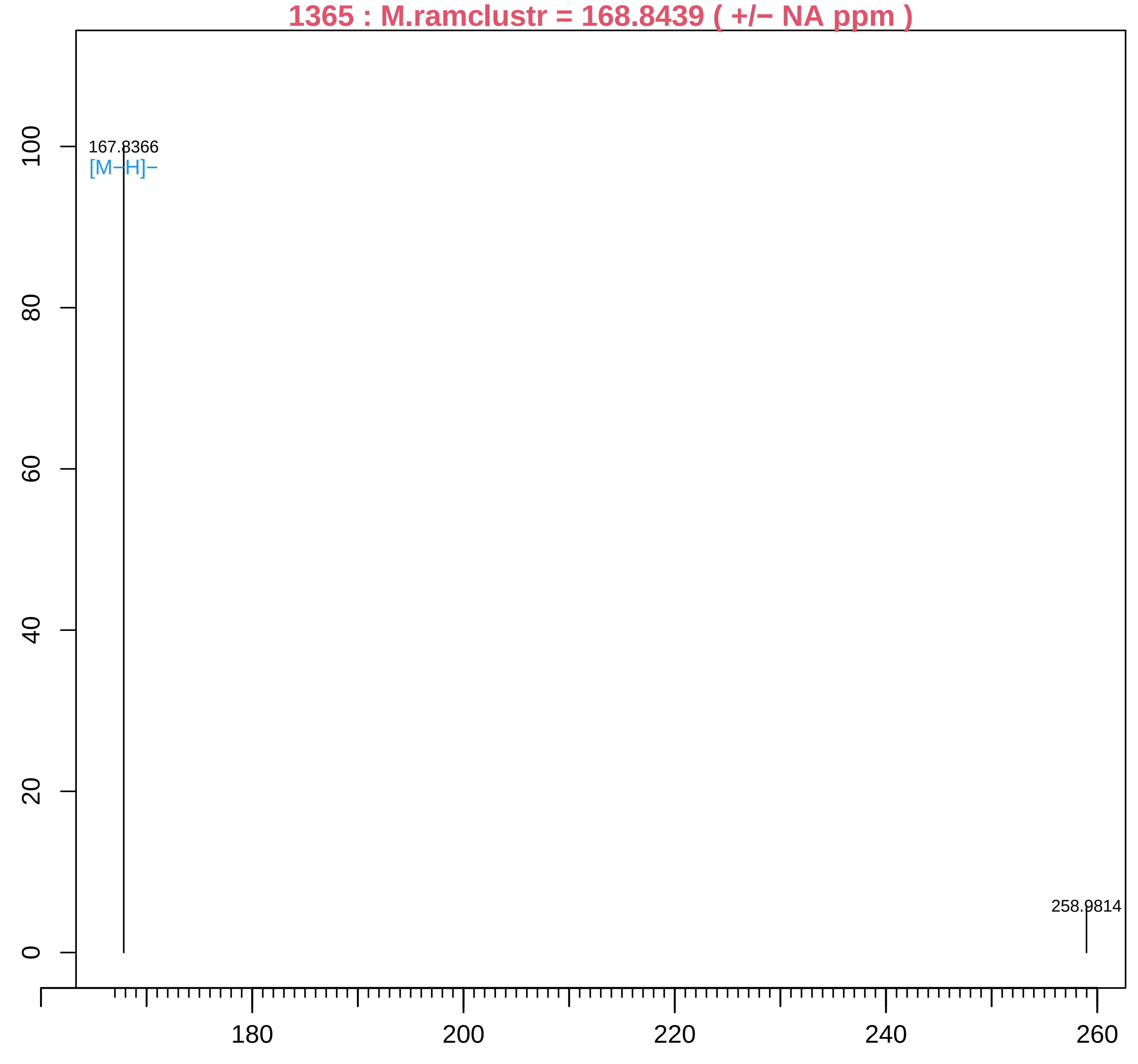
1365 : M.ramclustr = 168.8439 (+/− NA ppm)
167.8366
[M−H]−
258.9814
0
20
40
60
80
100
180
200
220
240
260

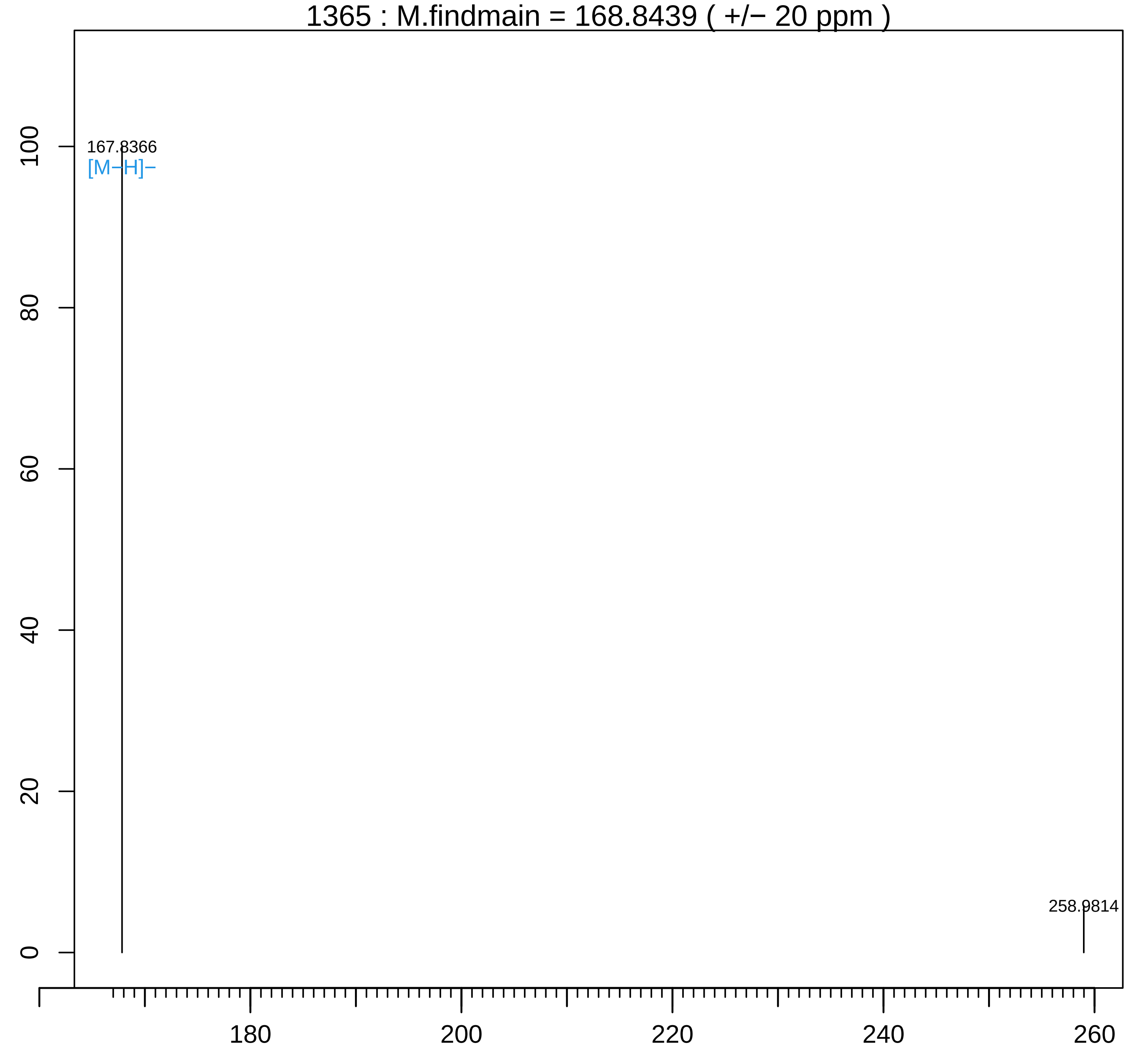
1365 : M.findmain = 168.8439 (+/− 20 ppm)
167.8366
[M−H]−
258.9814
0
20
40
60
80
100
180
200
220
240
260

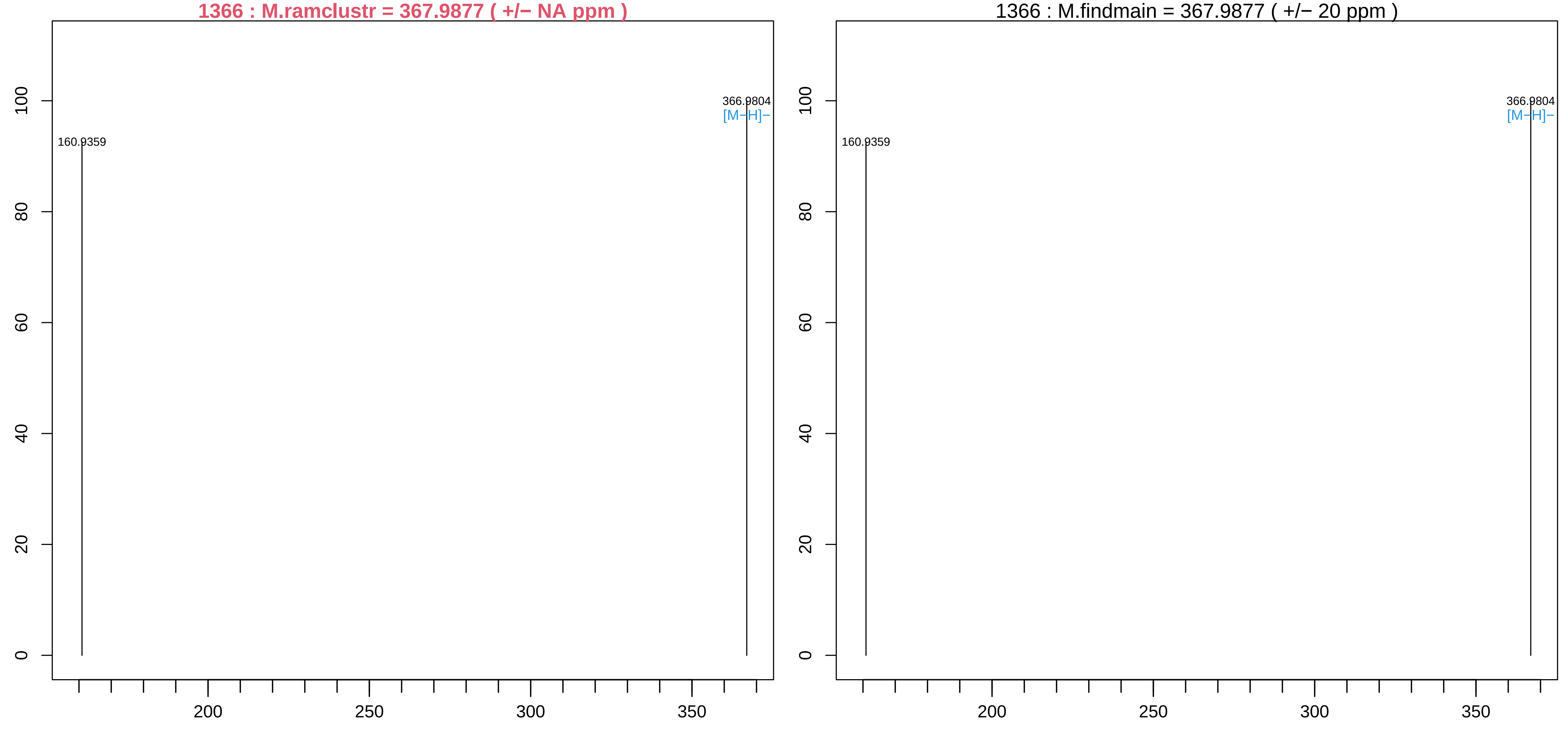

1366 : M.ramclustr = 367.9877 (+/− NA ppm)
160.9359
366.9804
[M−H]−
1366 : M.findmain = 367.9877 (+/− 20 ppm)
160.9359
366.9804
[M−H]−
0
20
40
60
80
100
200
250
300
350

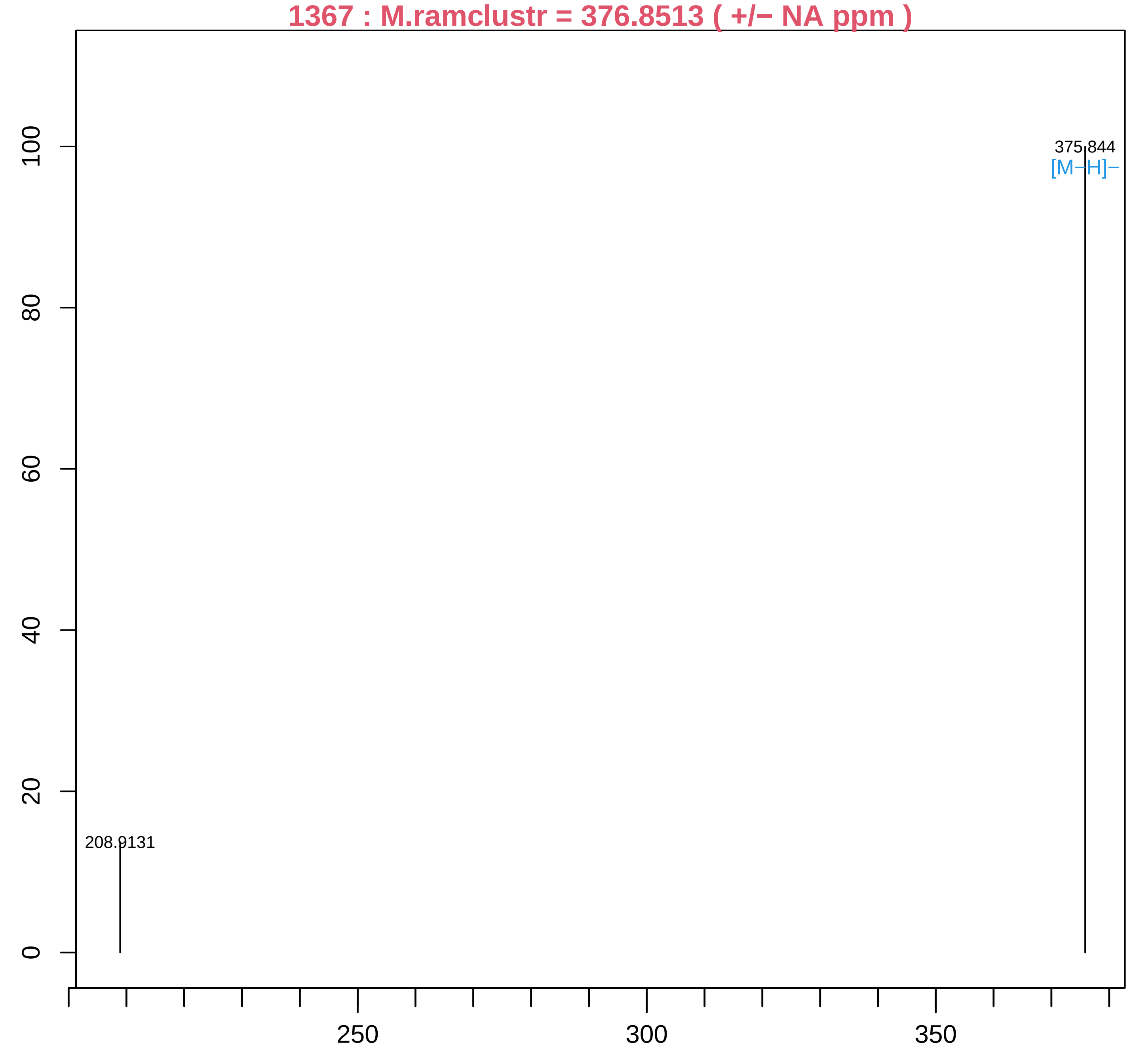
1367 : M.ramclustr = 376.8513 (+/− NA ppm)
375.844
[M−H]−
208.9131
0
20
40
60
80
100
250
300
350

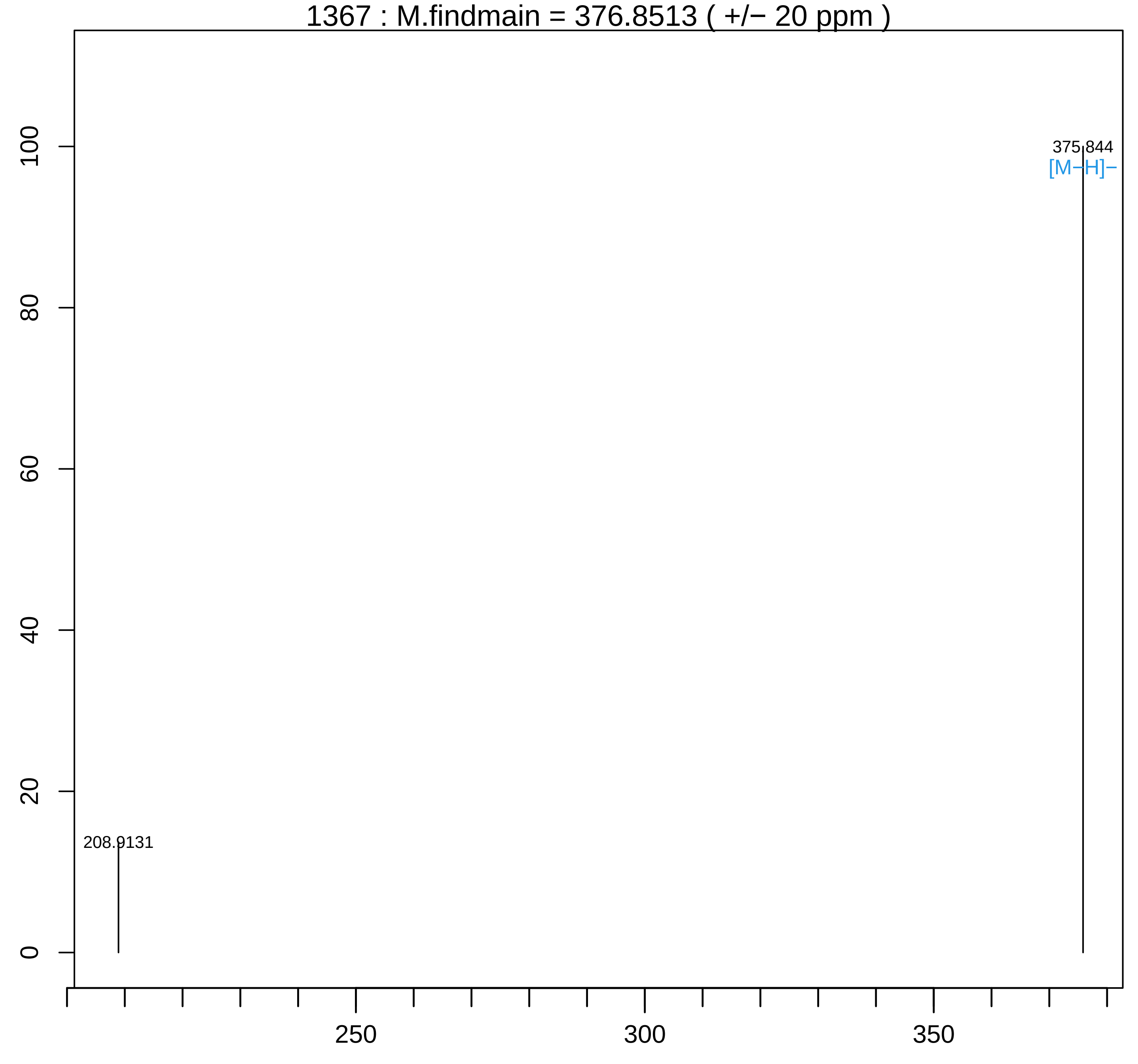
1367 : M.findmain = 376.8513 (+/− 20 ppm)
375.844
[M−H]−
208.9131
0
20
40
60
80
100
250
300
350

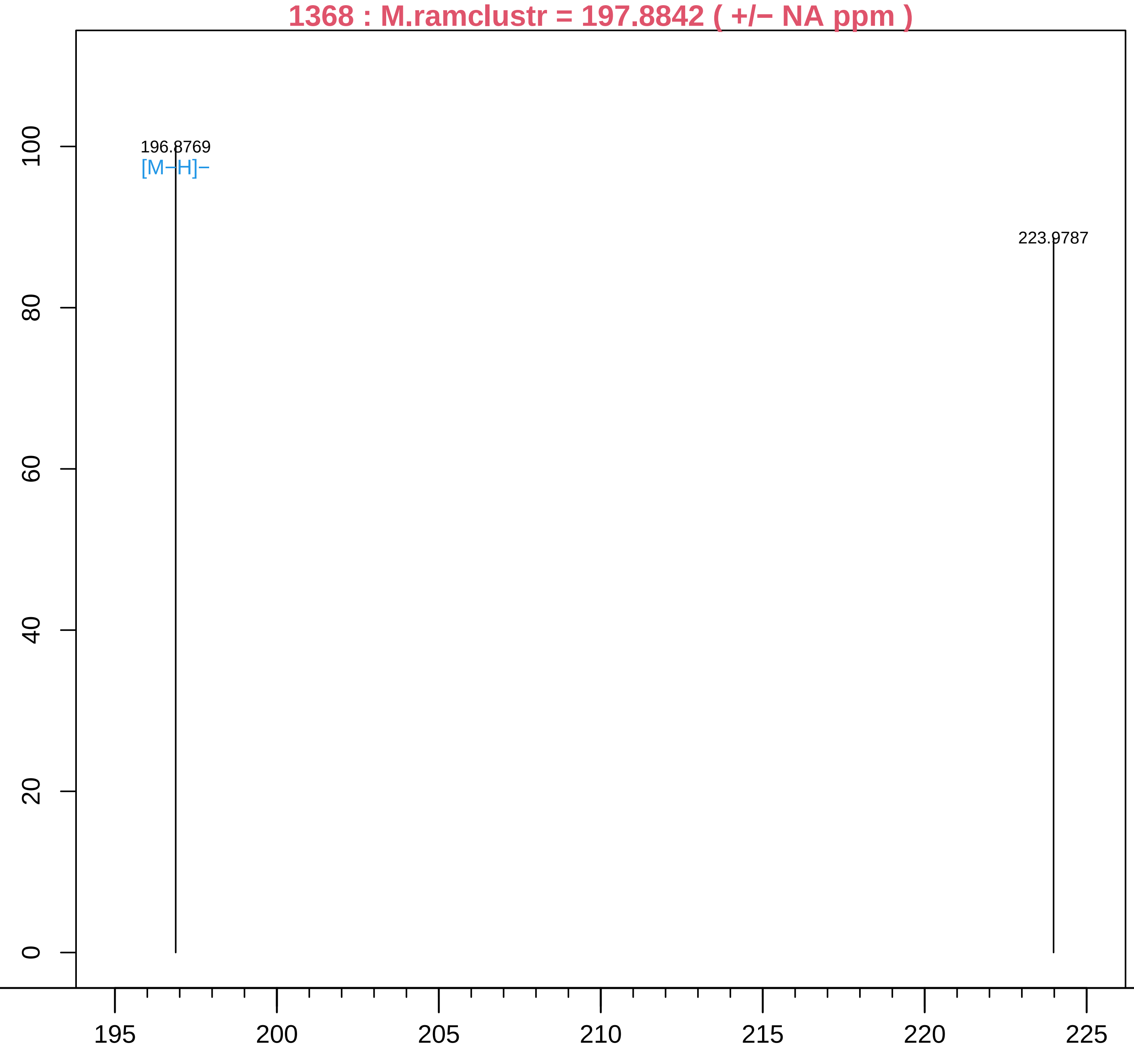
1368 : M.ramclustr = 197.8842 (+/− NA ppm)
196.8769
[M−H]−
223.9787
0
20
40
60
80
100
195
200
205
210
215
220
225

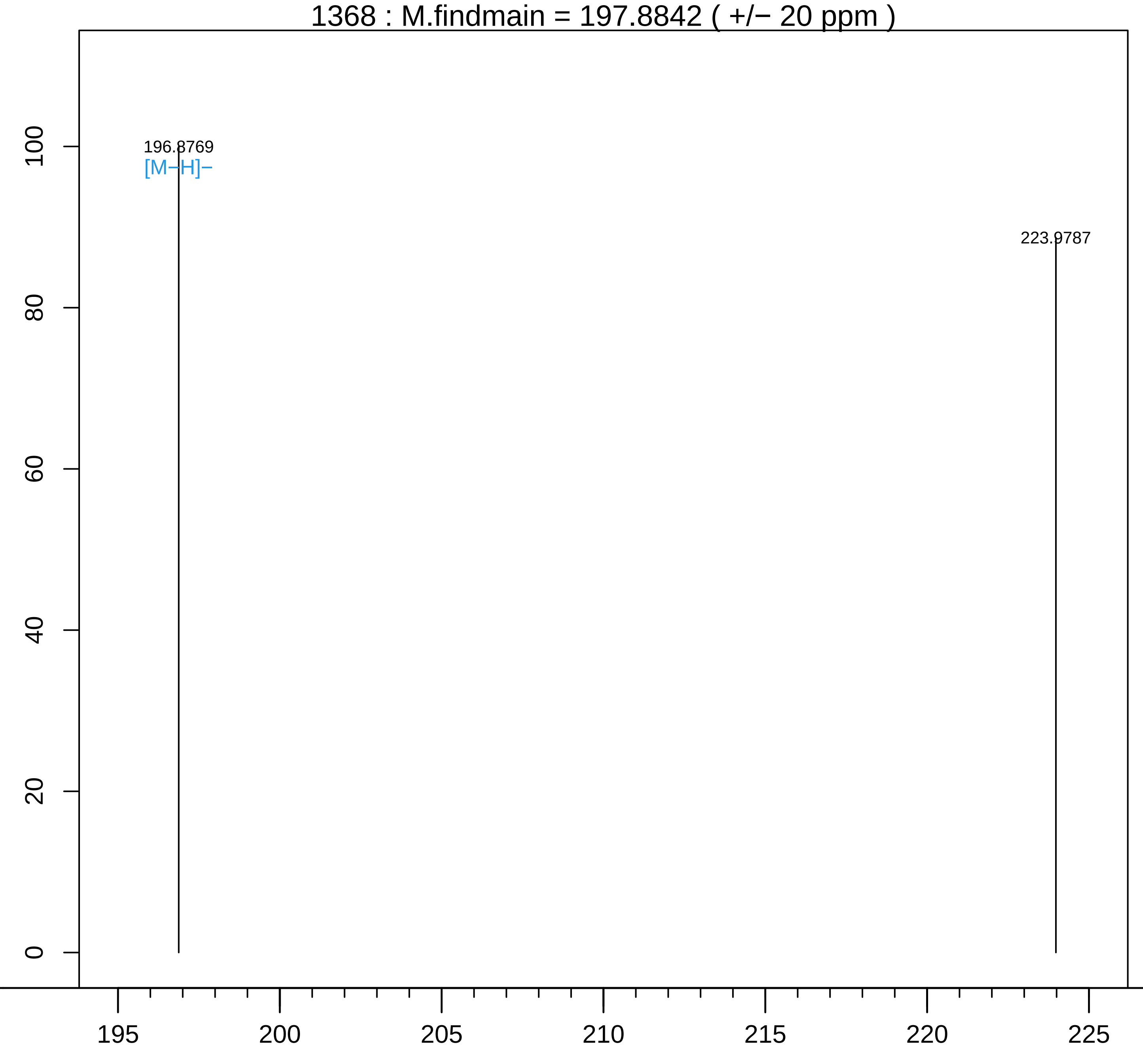
1368 : M.findmain = 197.8842 (+/− 20 ppm)
196.8769
[M−H]−
223.9787
0
20
40
60
80
100
195
200
205
210
215
220
225

1369 : M.ramclustr = 135.902 (+/− NA ppm)

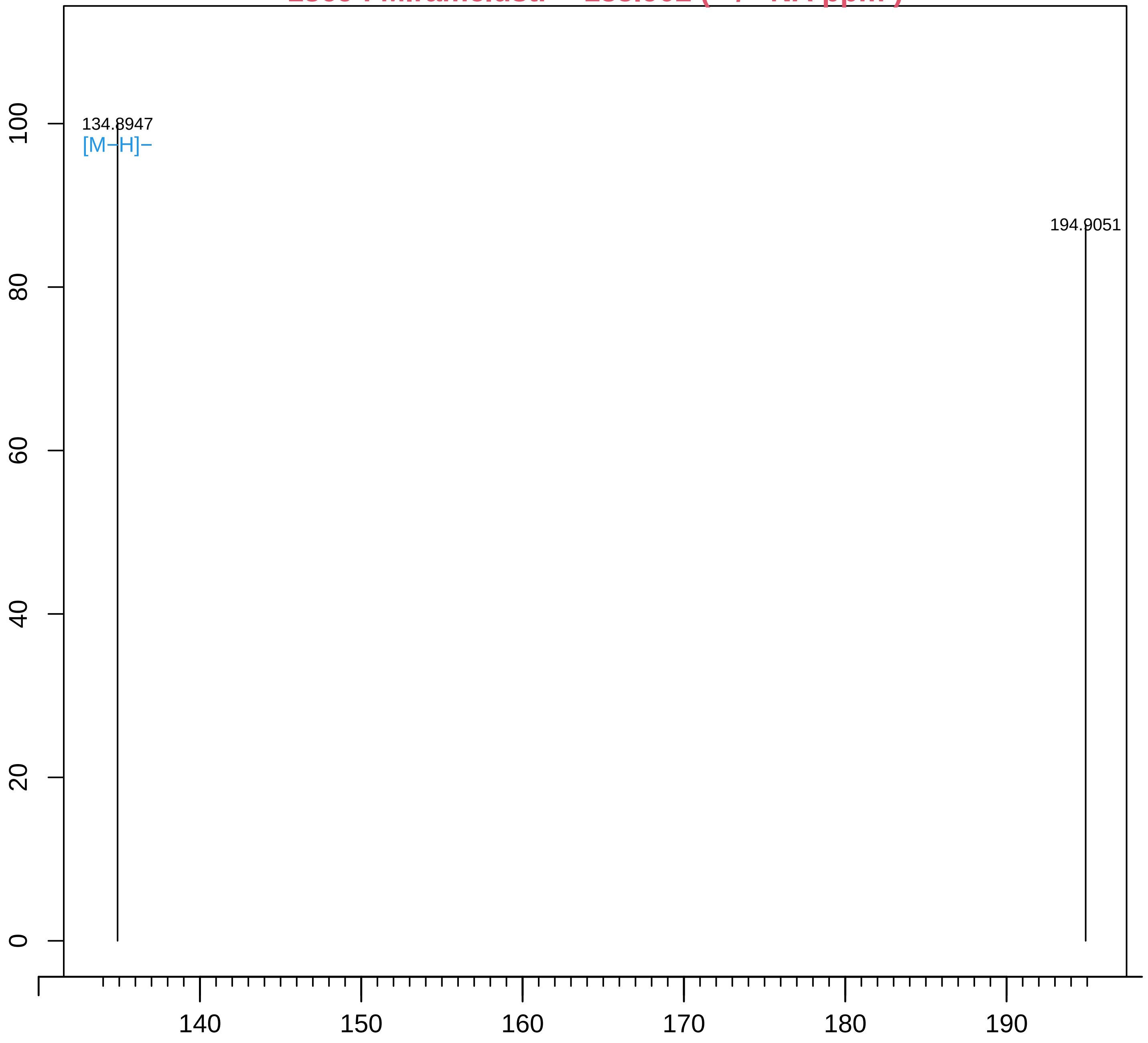

1369 : M.findmain = 135.902 (+/− 20 ppm)

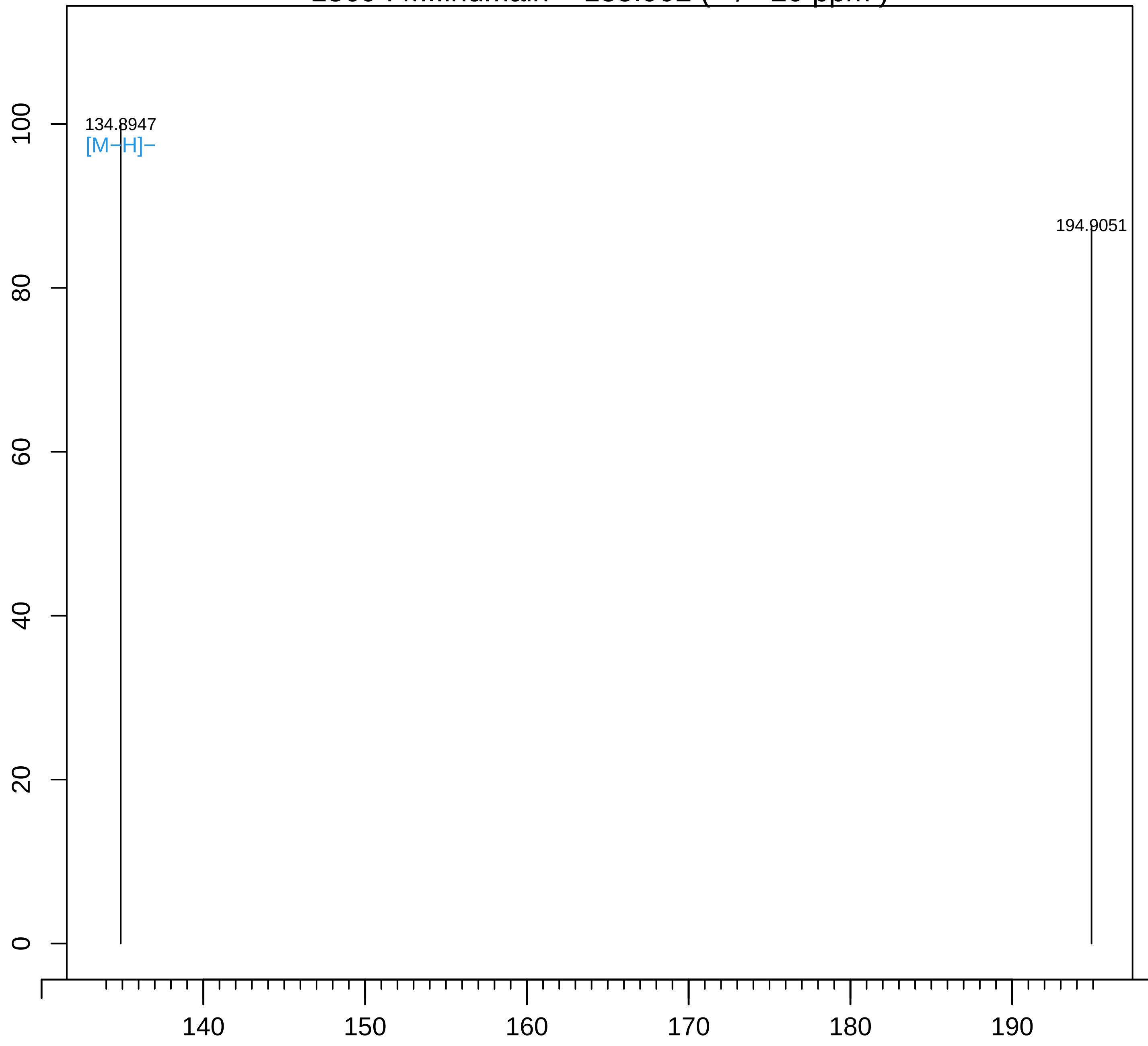

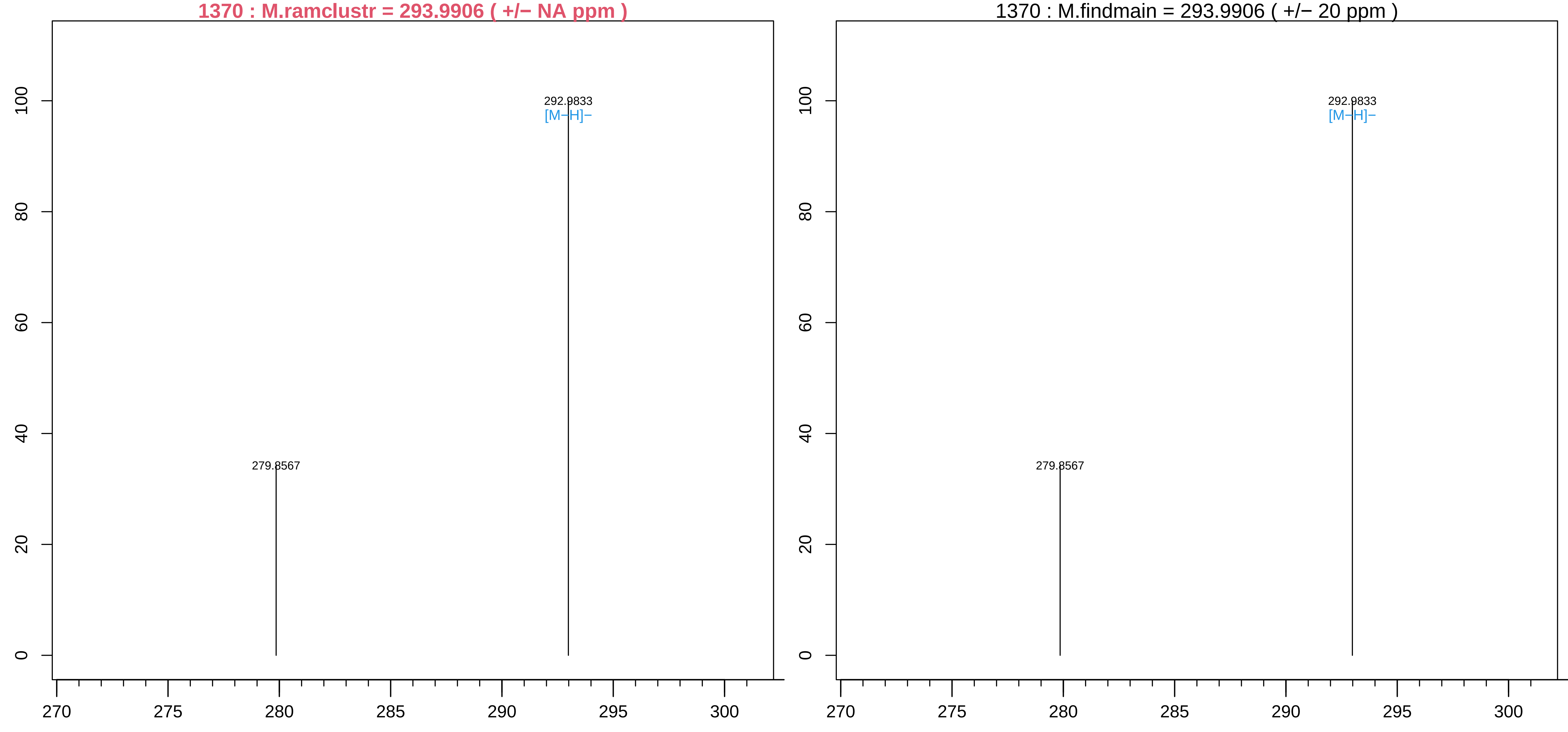
1370 : M.ramclustr = 293.9906 (+/− NA ppm)
292.9833
[M−H]−
279.8567
1370 : M.findmain = 293.9906 (+/− 20 ppm)
292.9833
[M−H]−
279.8567
0
20
40
60
80
100
270
275
280
285
290
295
300

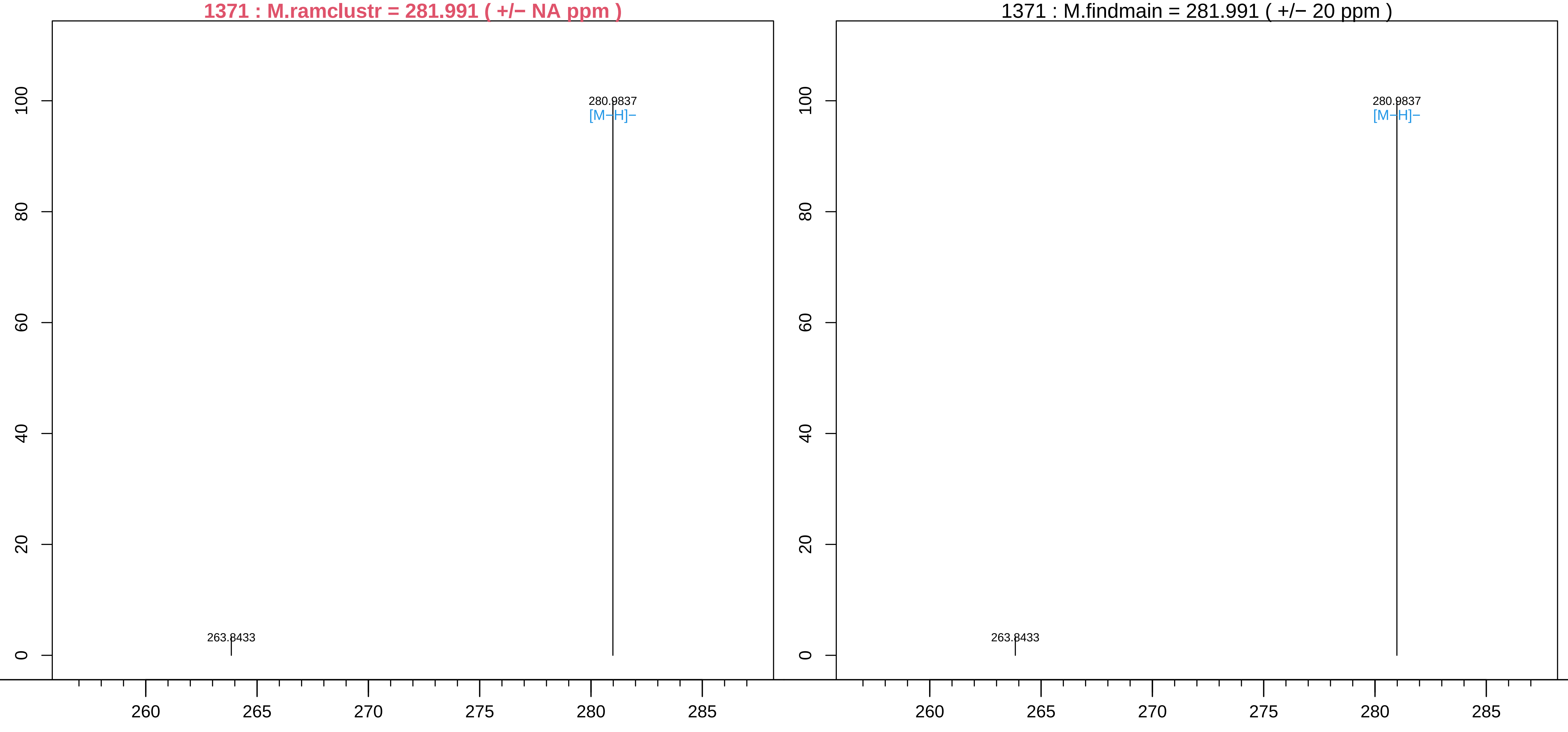
1371 : M.ramclustr = 281.991 (+/− NA ppm)
1371 : M.findmain = 281.991 (+/− 20 ppm)
280.9837
[M−H]−
263.8433
280.9837
[M−H]−
263.8433
0
20
40
60
80
100
260
265
270
275
280
285

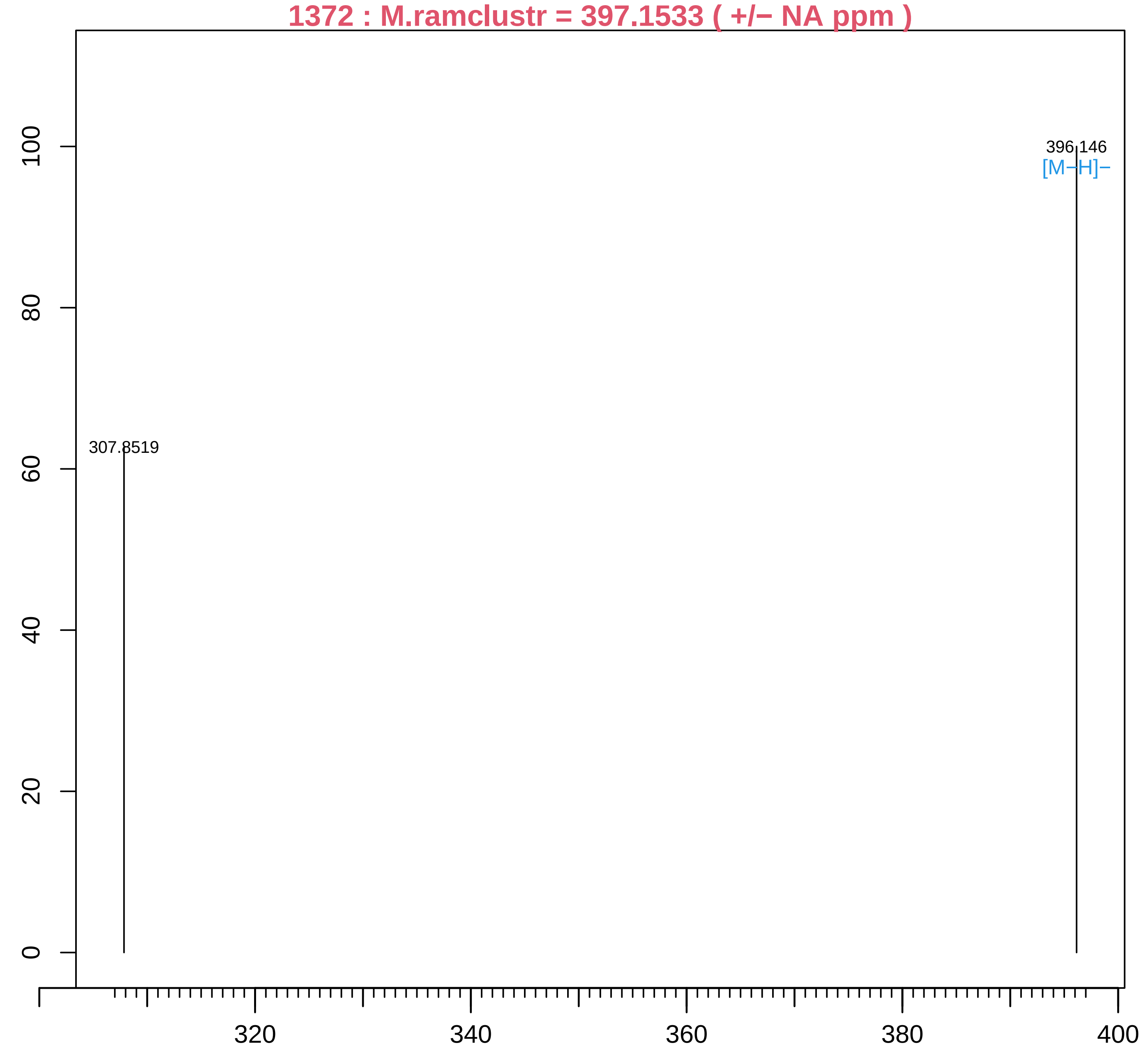

1372 : M.ramclustr = 397.1533 (+/− NA ppm)
307.8519
396.146
[M−H]−
0
20
40
60
80
100
320
340
360
380
400

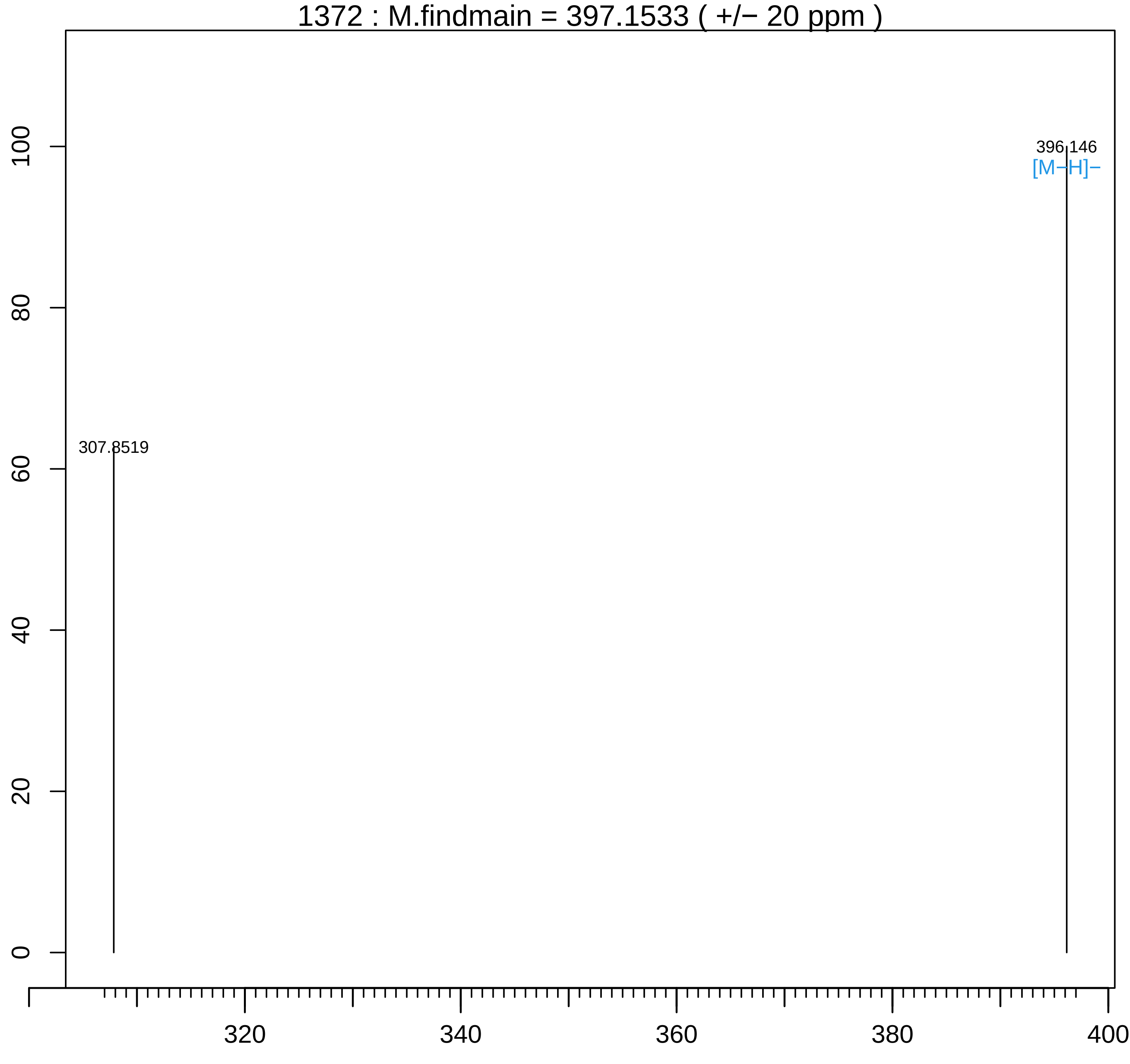

1372 : M.findmain = 397.1533 (+/− 20 ppm)
307.8519
396.146
[M−H]−
0
20
40
60
80
100
320
340
360
380
400

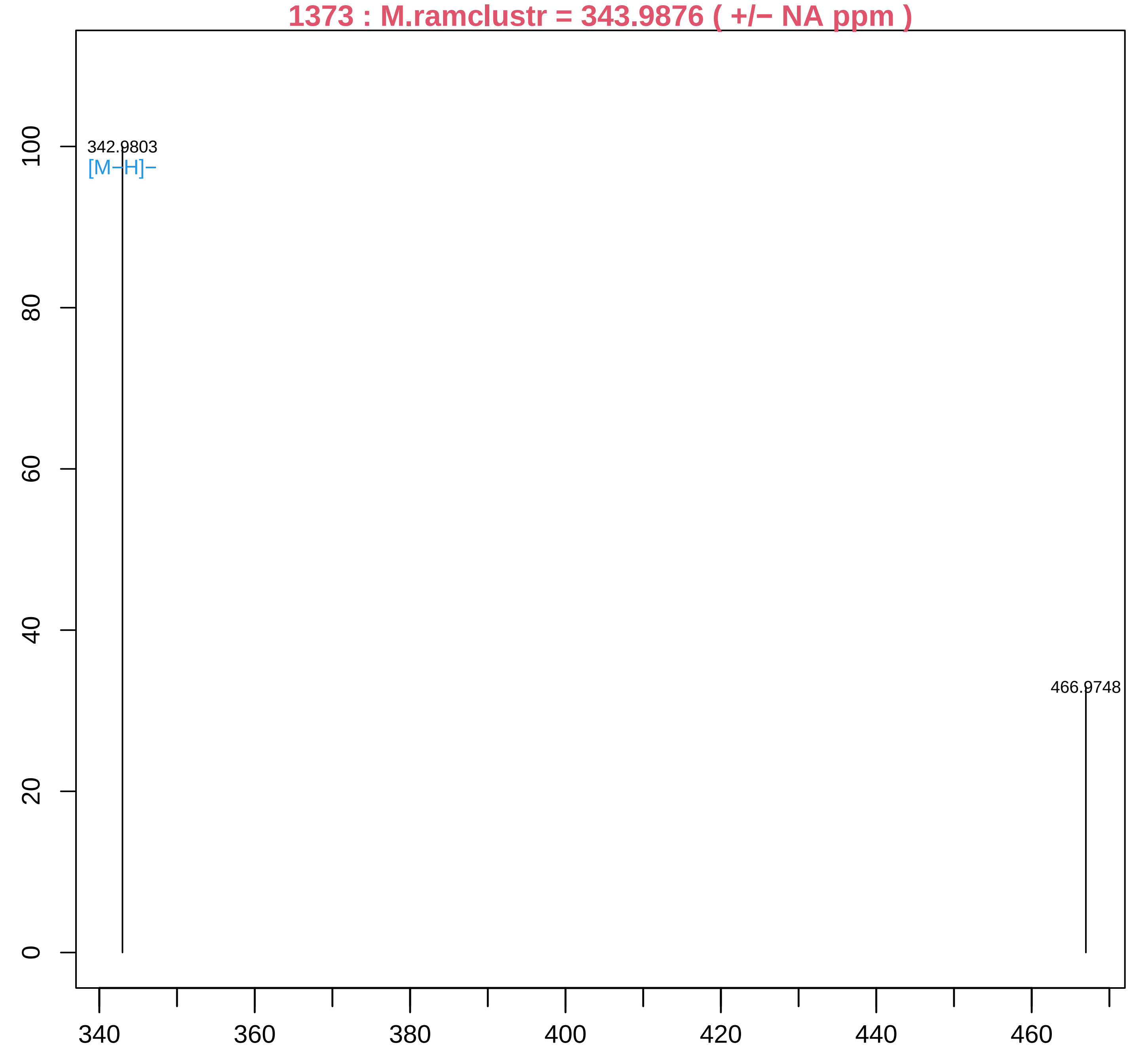
1373 : M.ramclustr = 343.9876 (+/− NA ppm)
342.9803
[M−H]−
466.9748
0
20
40
60
80
100
340
360
380
400
420
440
460

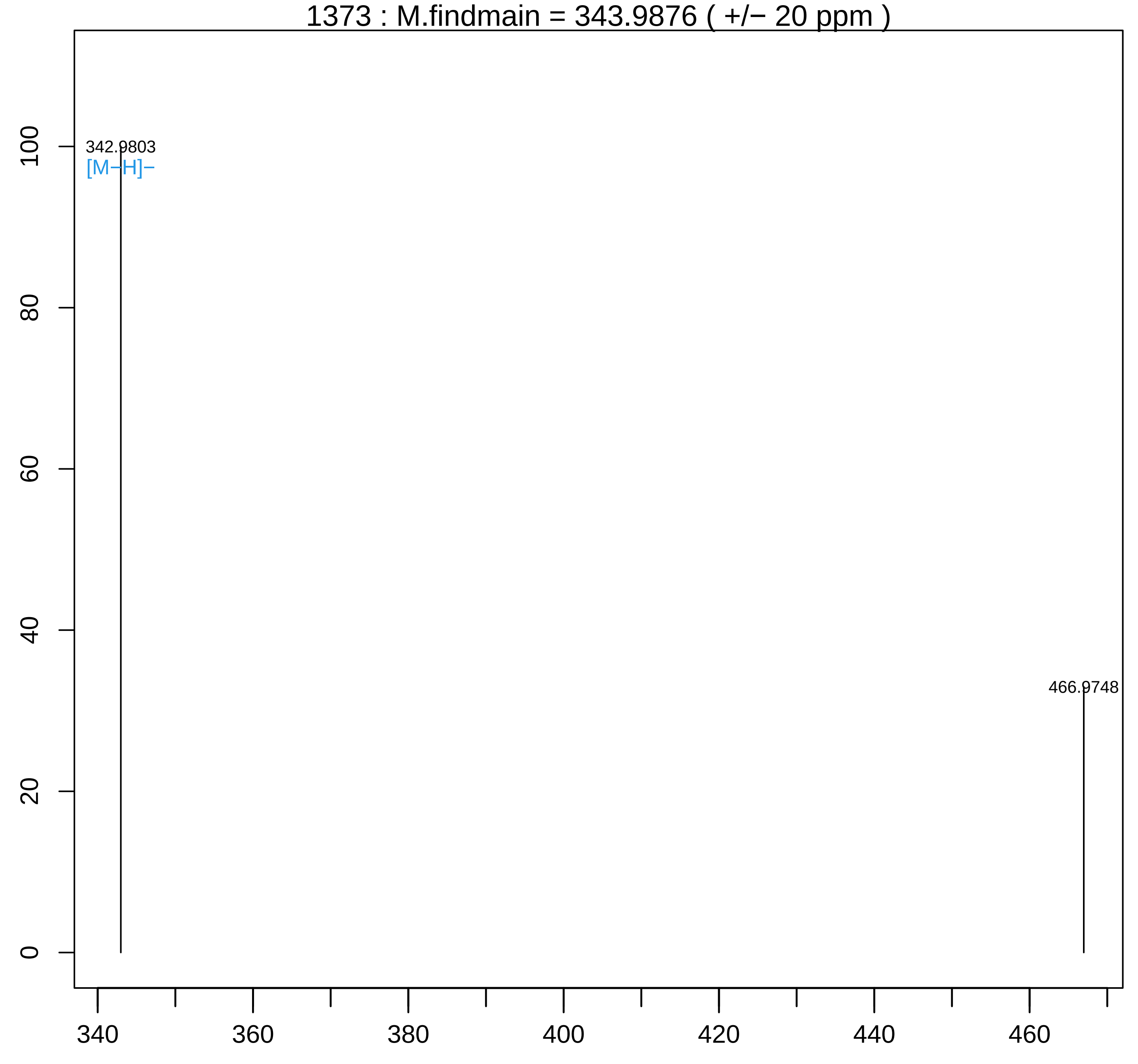
1373 : M.findmain = 343.9876 (+/− 20 ppm)
342.9803
[M−H]−
466.9748
0
20
40
60
80
100
340
360
380
400
420
440
460

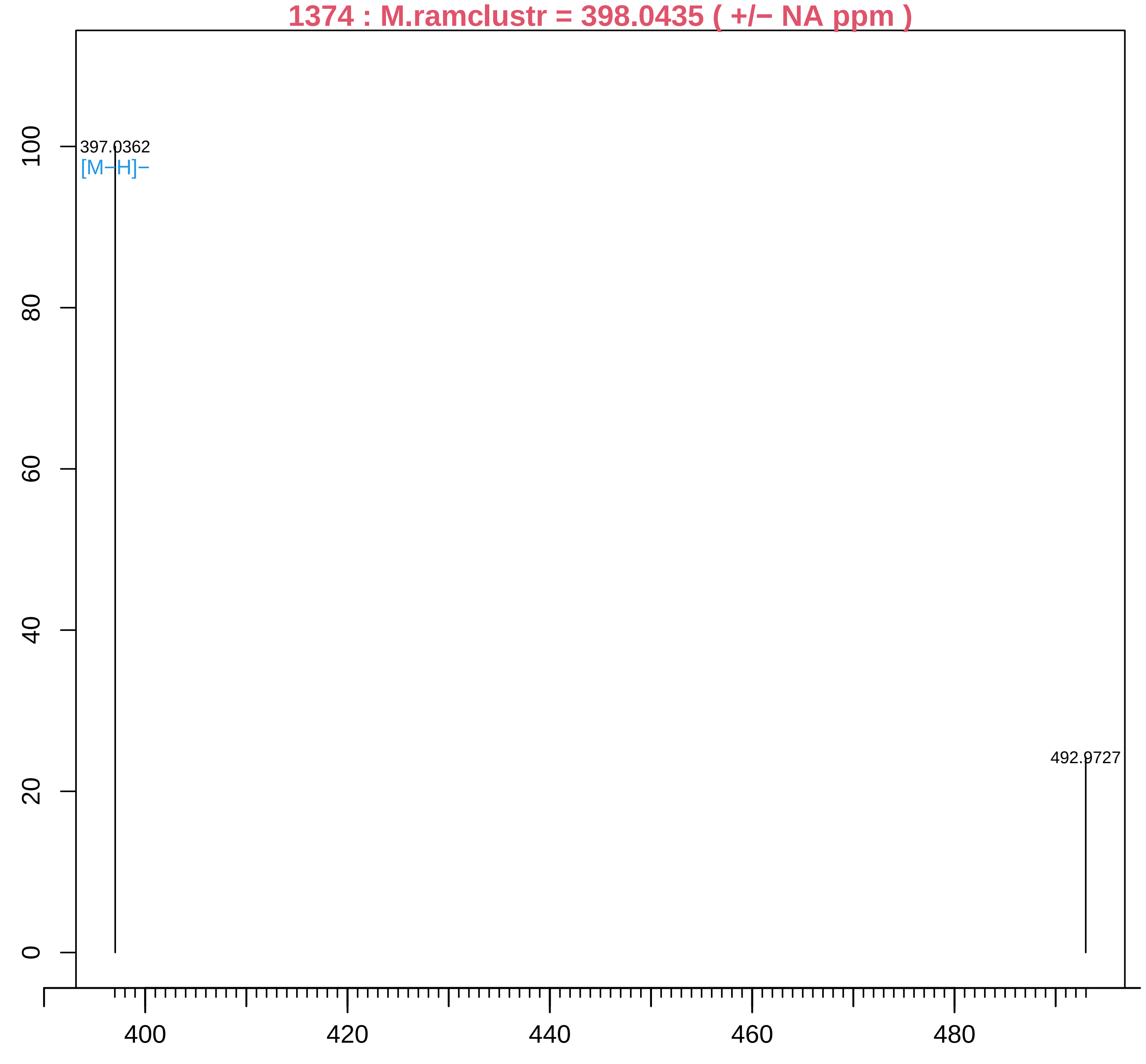

1374 : M.ramclustr = 398.0435 (+/− NA ppm)
397.0362
[M−H]−
492.9727
0
20
40
60
80
100
400
420
440
460
480

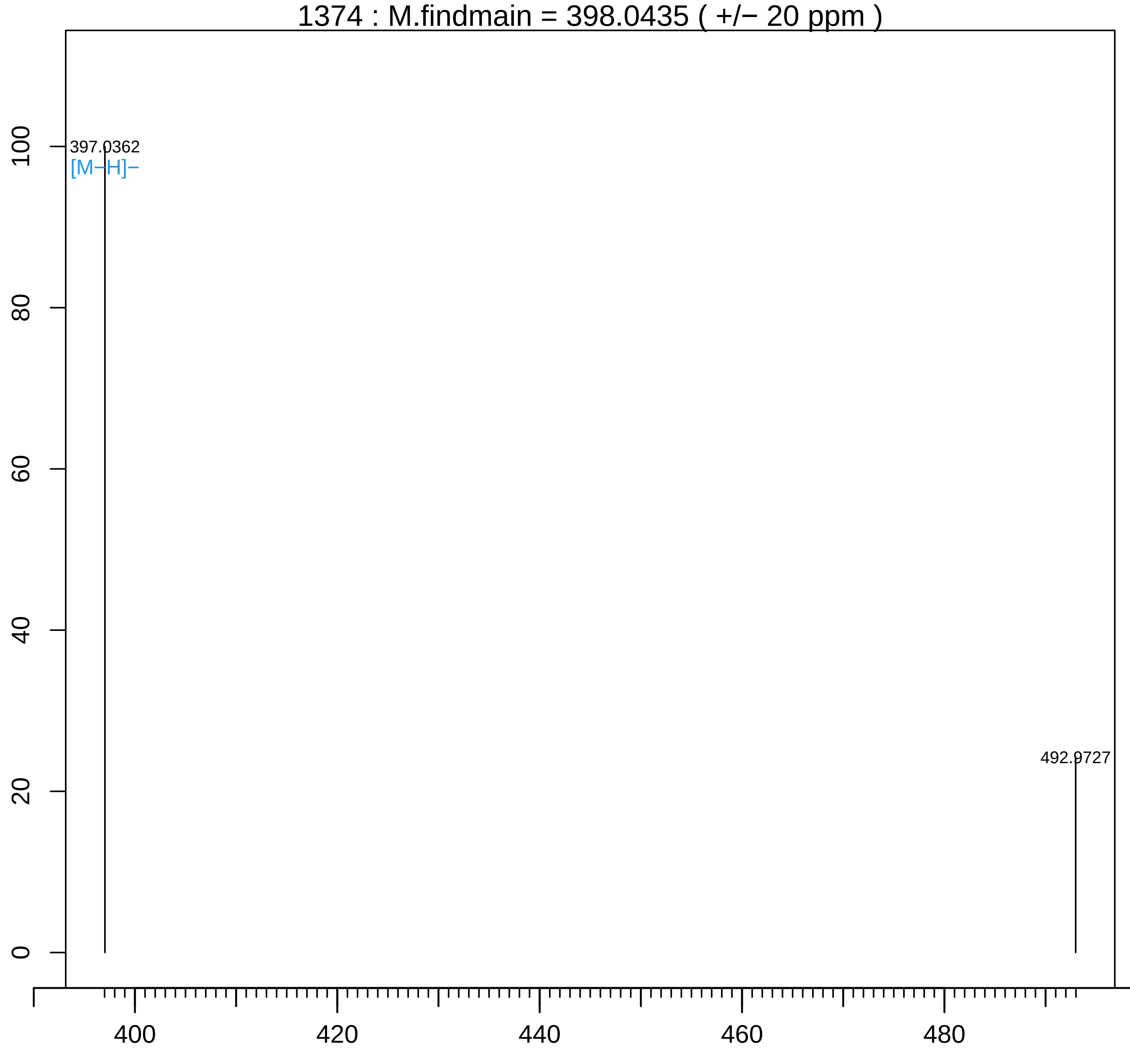

1374 : M.findmain = 398.0435 (+/− 20 ppm)
397.0362
[M−H]−
492.9727
0
20
40
60
80
100
400
420
440
460
480

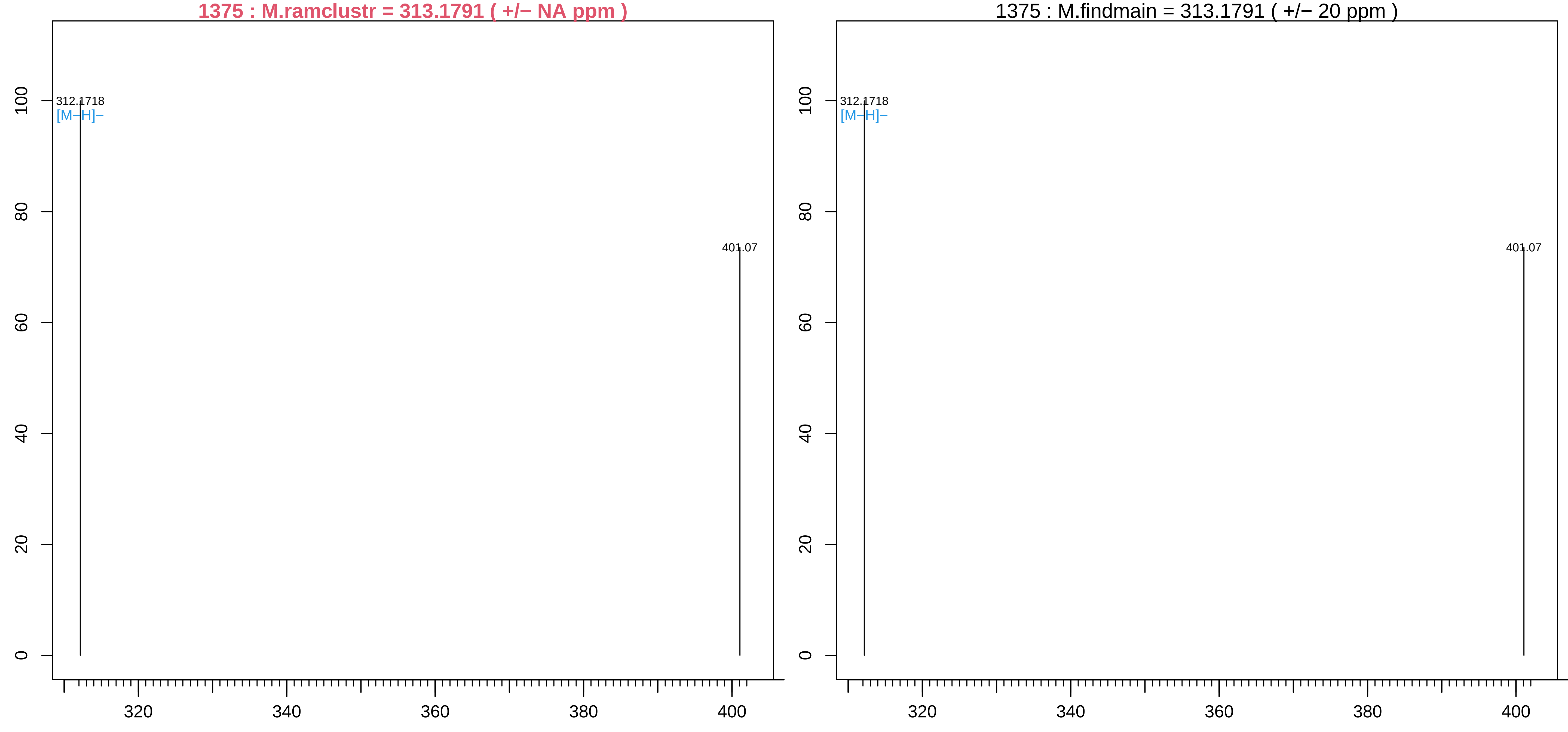
1375 : M.ramclustr = 313.1791 (+/− NA ppm)
312.1718
[M−H]−
401.07
1375 : M.findmain = 313.1791 (+/− 20 ppm)
312.1718
[M−H]−
401.07
0
20
40
60
80
100
320
340
360
380
400

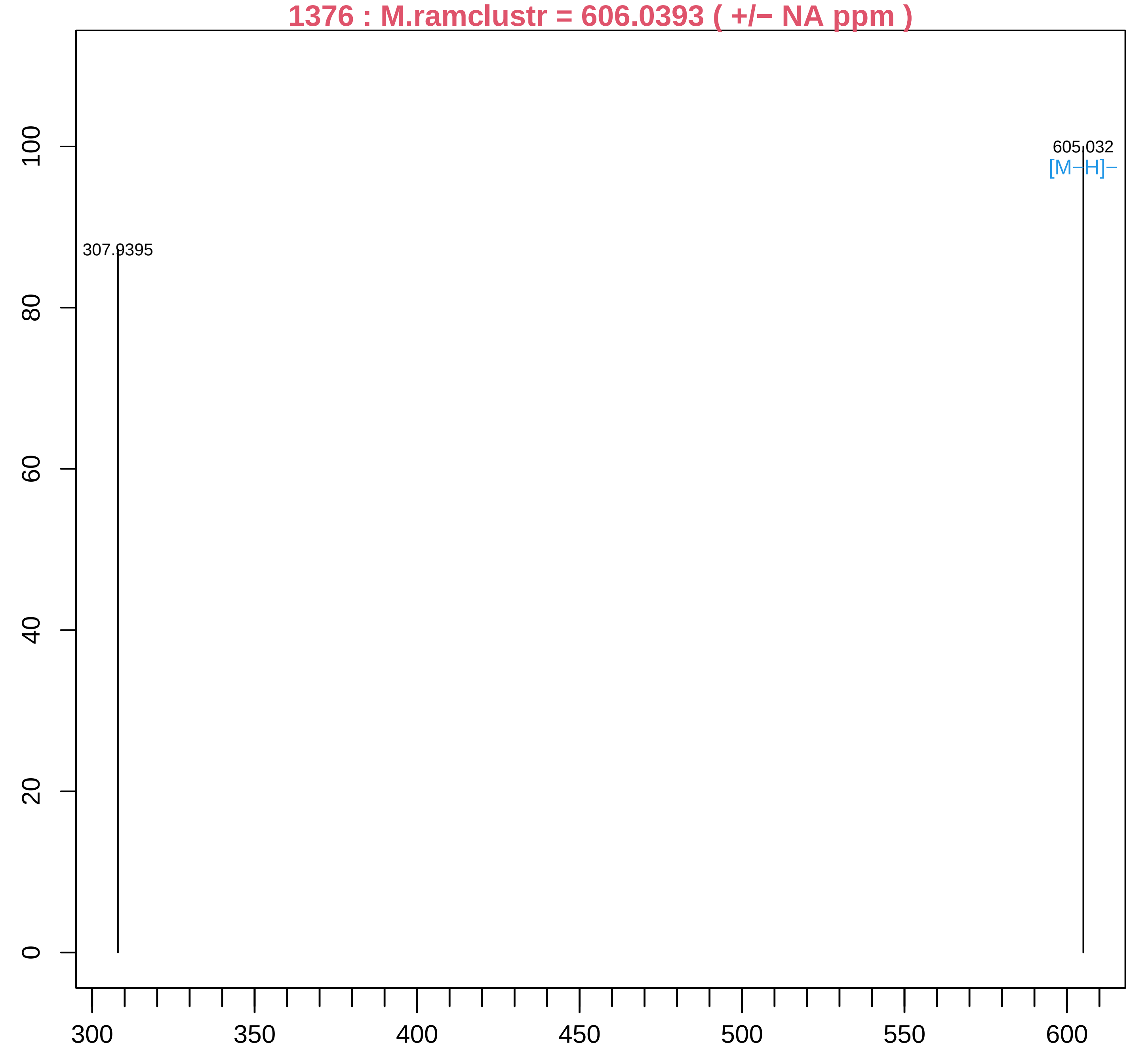
1376 : M.ramclustr = 606.0393 (+/− NA ppm)
605.032
[M−H]−
307.9395
0
20
40
60
80
100
300
350
400
450
500
550
600

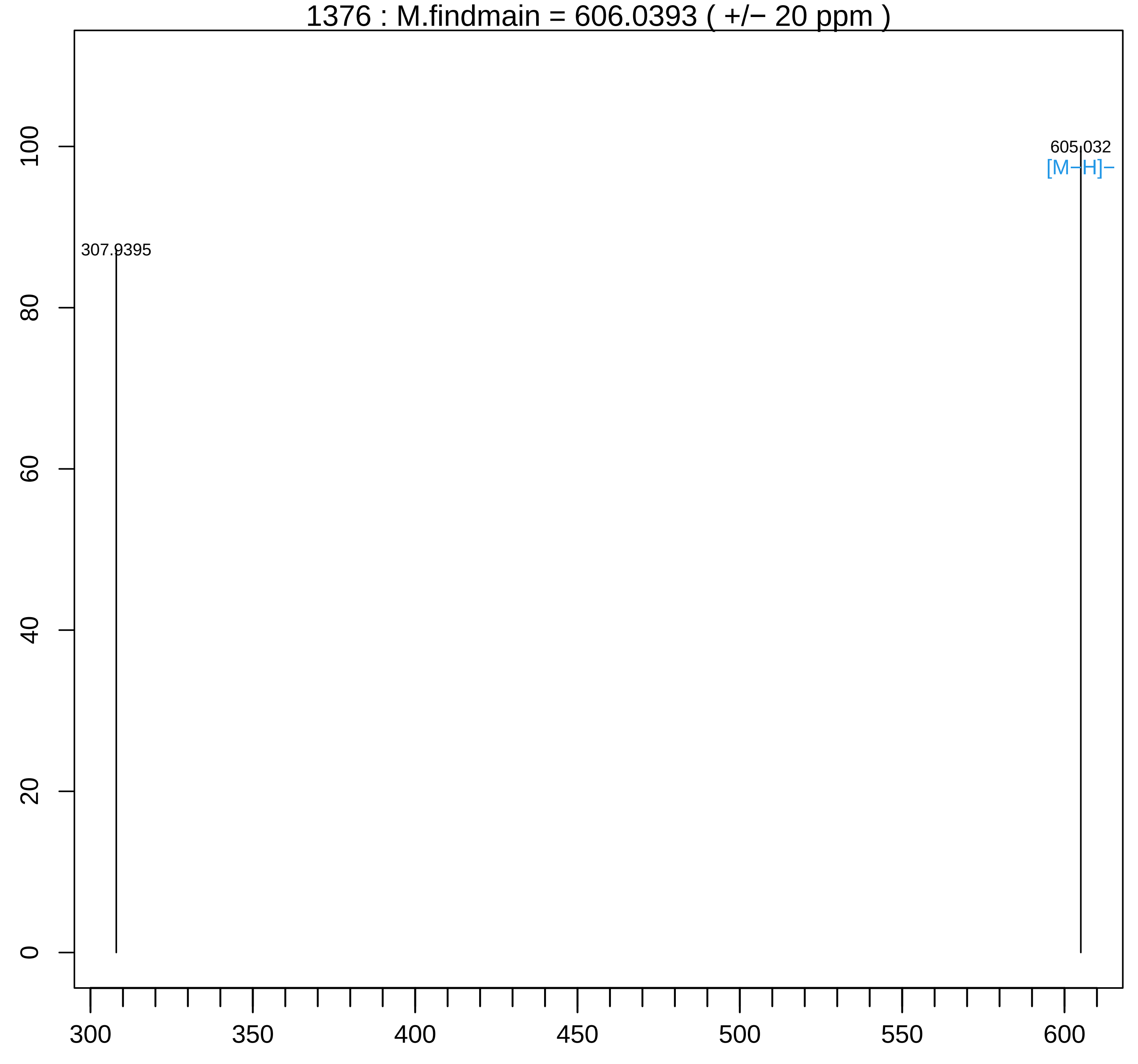
1376 : M.findmain = 606.0393 (+/− 20 ppm)
605.032
[M−H]−
307.9395
0
20
40
60
80
100
300
350
400
450
500
550
600

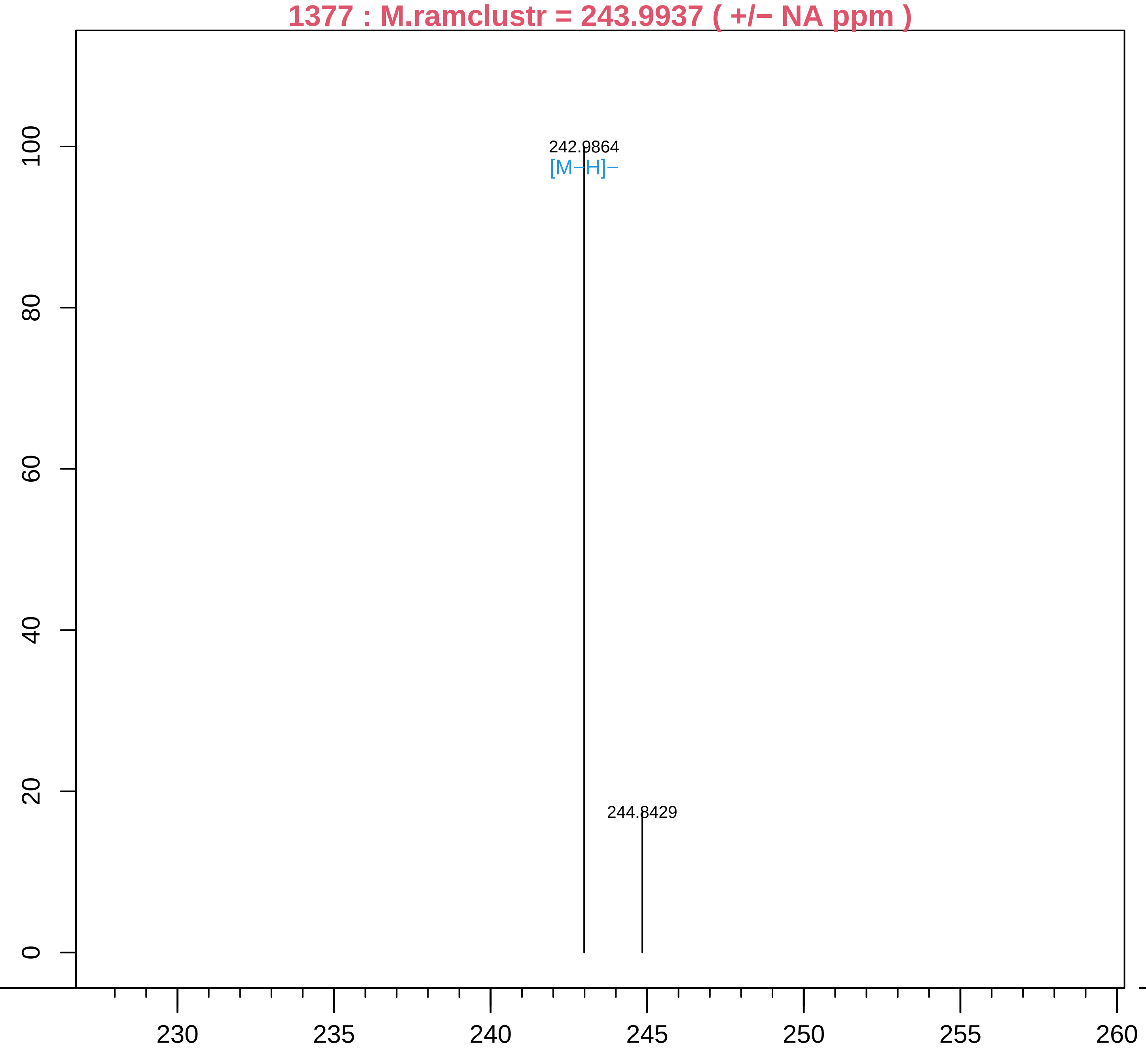

1377 : M.ramclustr = 243.9937 (+/− NA ppm)
242.9864
[M−H]−
244.8429
0
20
40
60
80
100
230
235
240
245
250
255
260

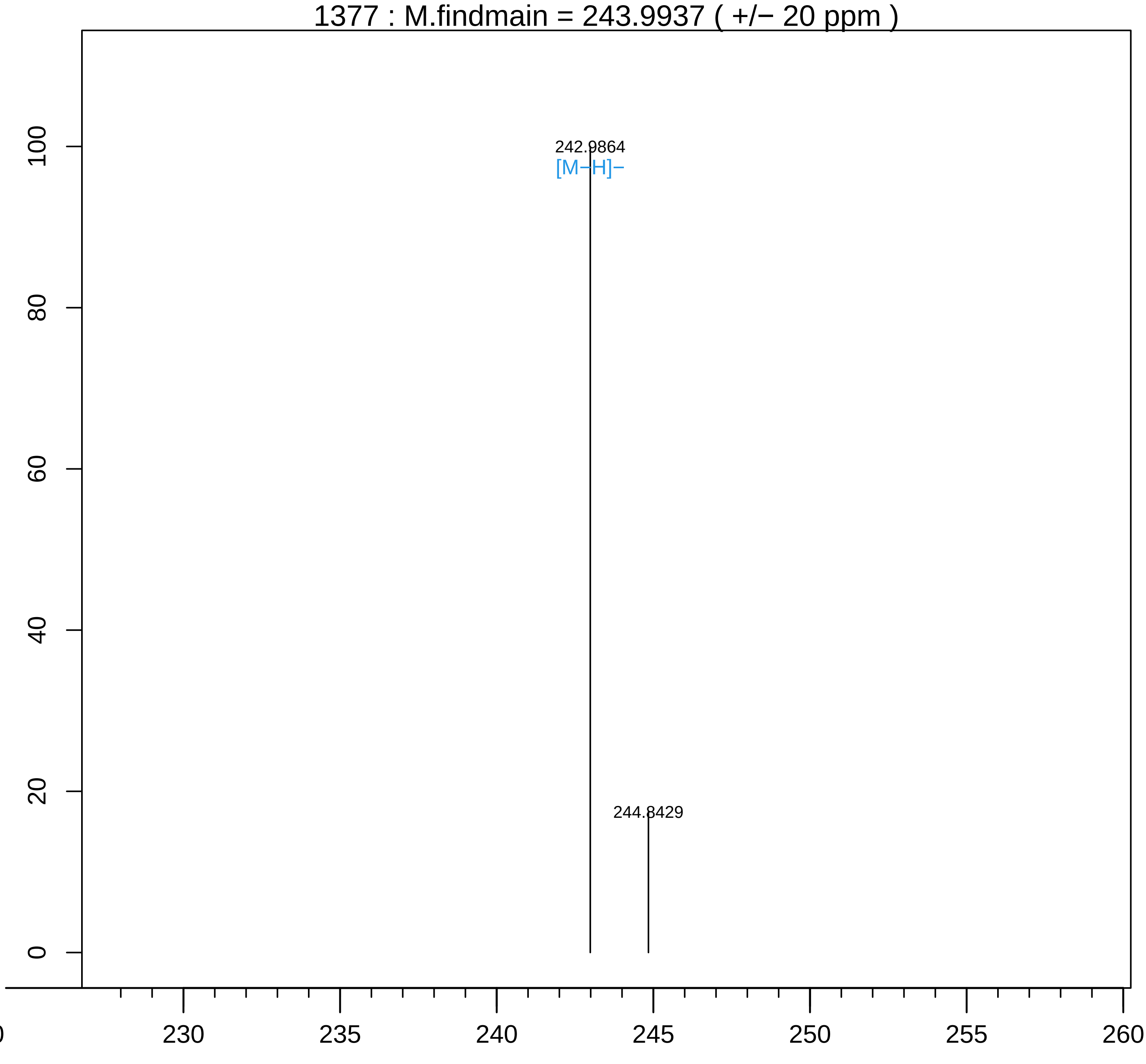

1377 : M.findmain = 243.9937 (+/− 20 ppm)
242.9864
[M−H]−
244.8429
0
20
40
60
80
100
230
235
240
245
250
255
260

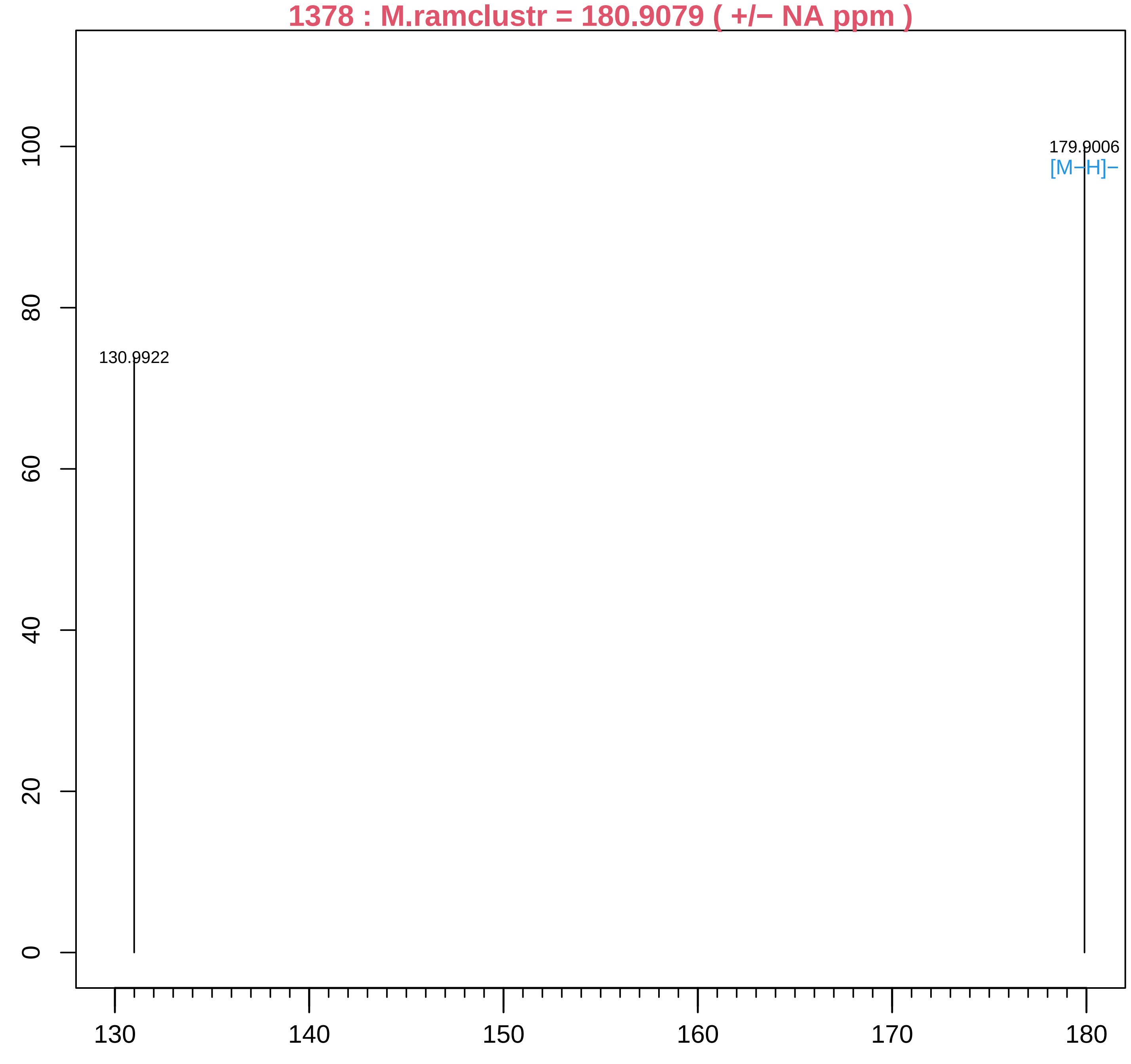

1378 : M.ramclustr = 180.9079 (+/− NA ppm)
179.9006
[M−H]−
130.9922
0
20
40
60
80
100
130
140
150
160
170
180

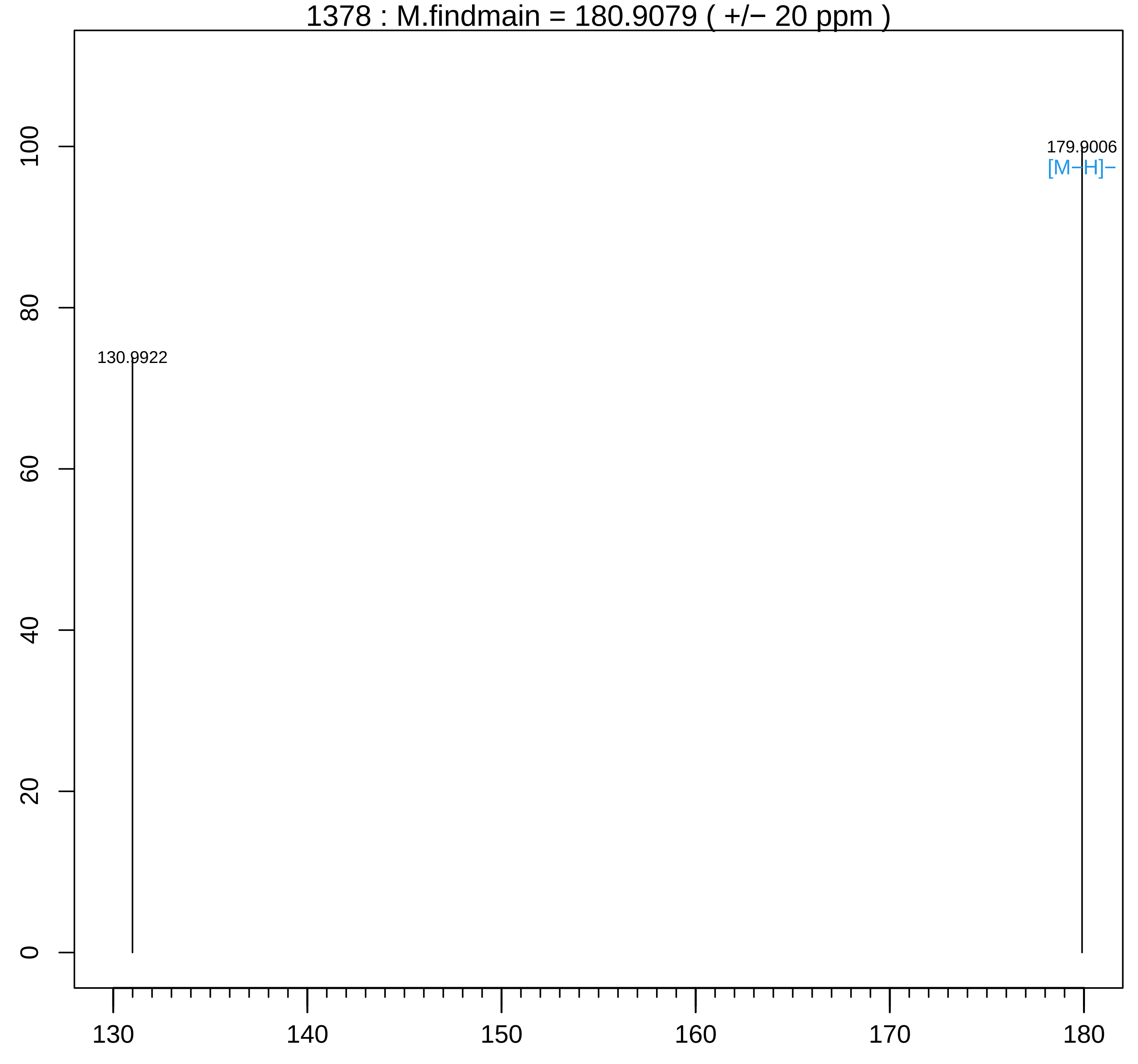

1378 : M.findmain = 180.9079 (+/− 20 ppm)
179.9006
[M−H]−
130.9922
0
20
40
60
80
100
130
140
150
160
170
180

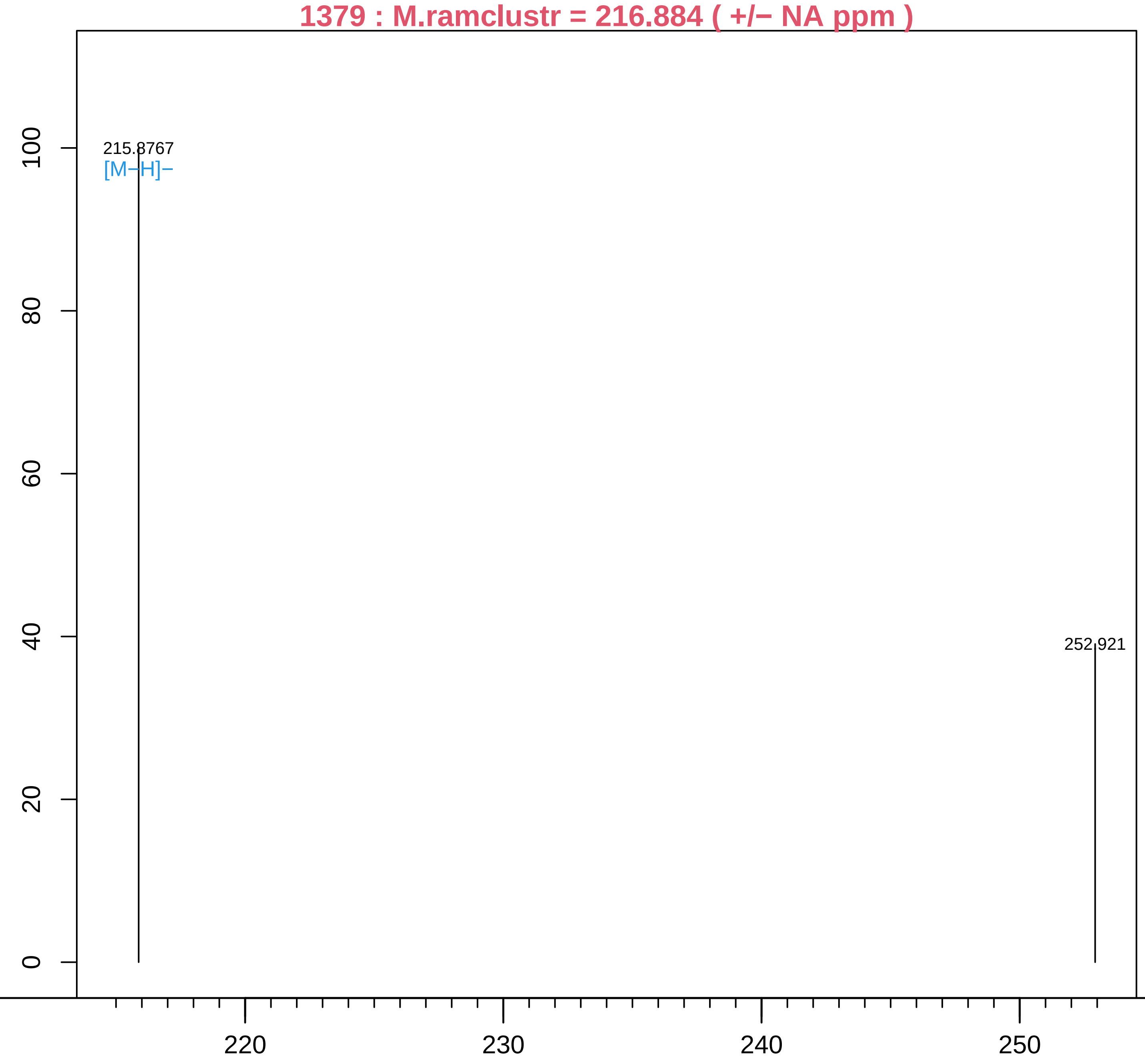

1379 : M.ramclustr = 216.884 (+/− NA ppm)
215.8767
[M−H]−
252.921
0
20
40
60
80
100
220
230
240
250

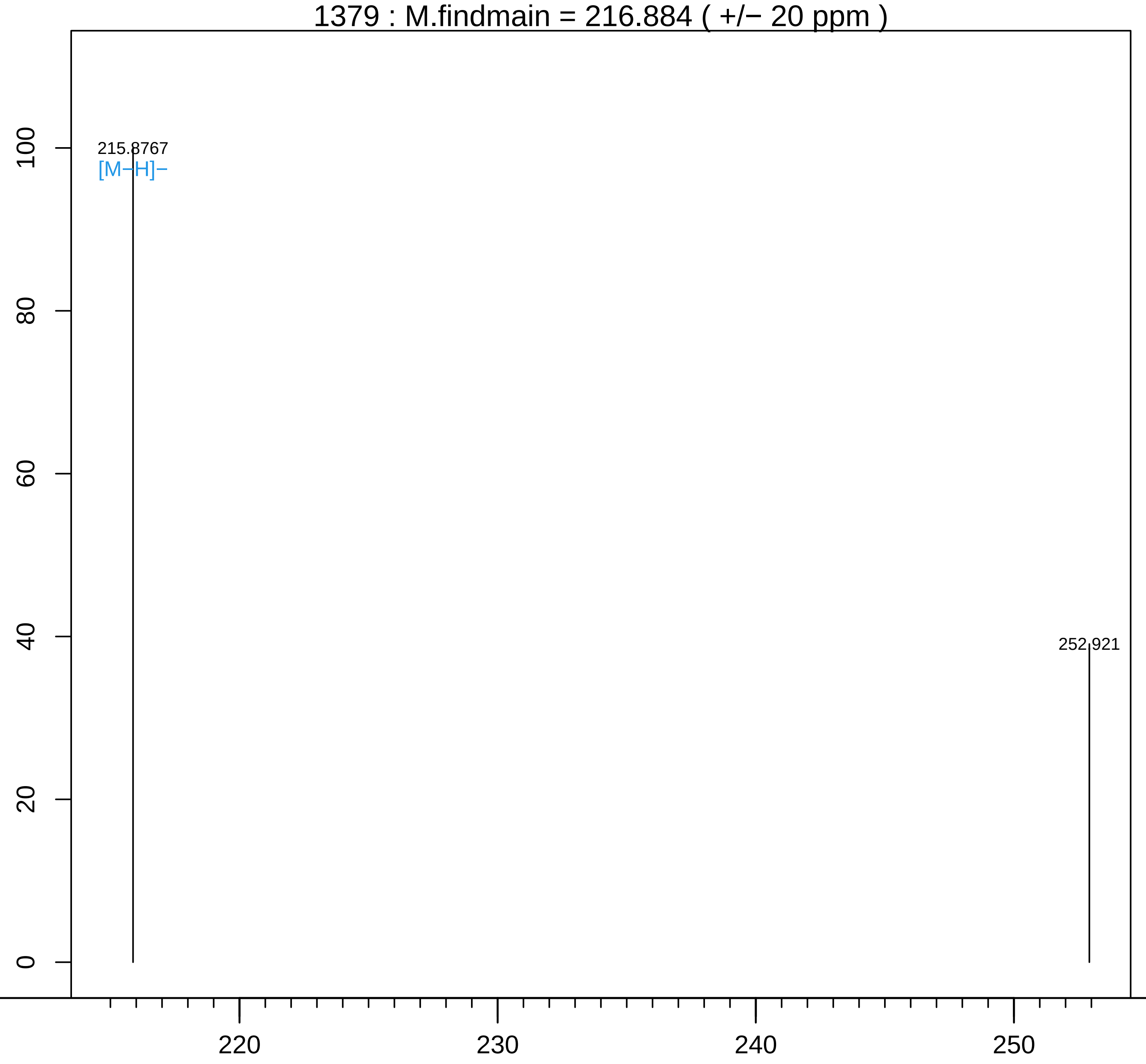

1379 : M.findmain = 216.884 (+/− 20 ppm)
215.8767
[M−H]−
252.921
0
20
40
60
80
100
220
230
240
250

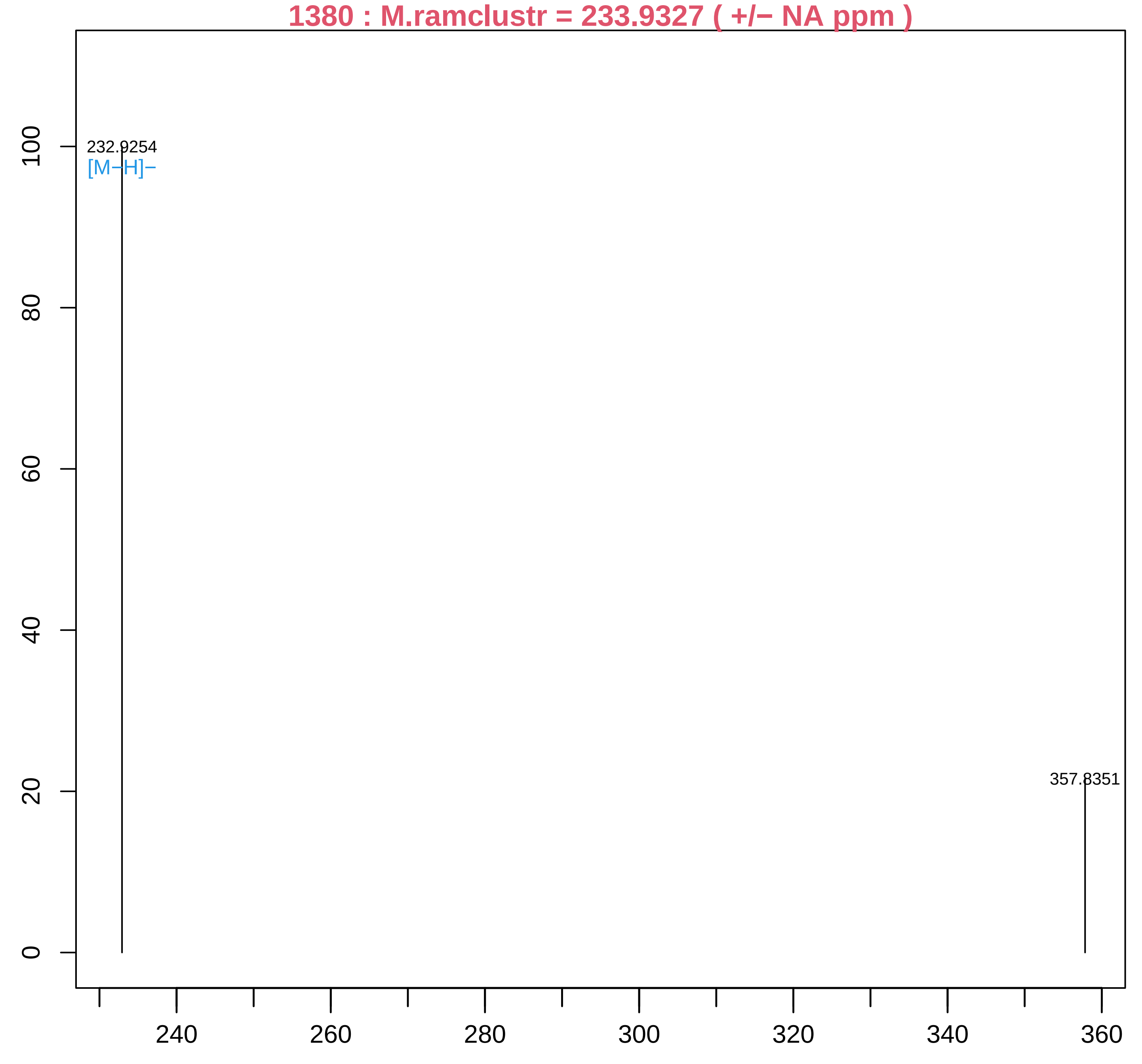

1380 : M.ramclustr = 233.9327 (+/− NA ppm)
232.9254
[M−H]−
357.8351
0
20
40
60
80
100
240
260
280
300
320
340
360

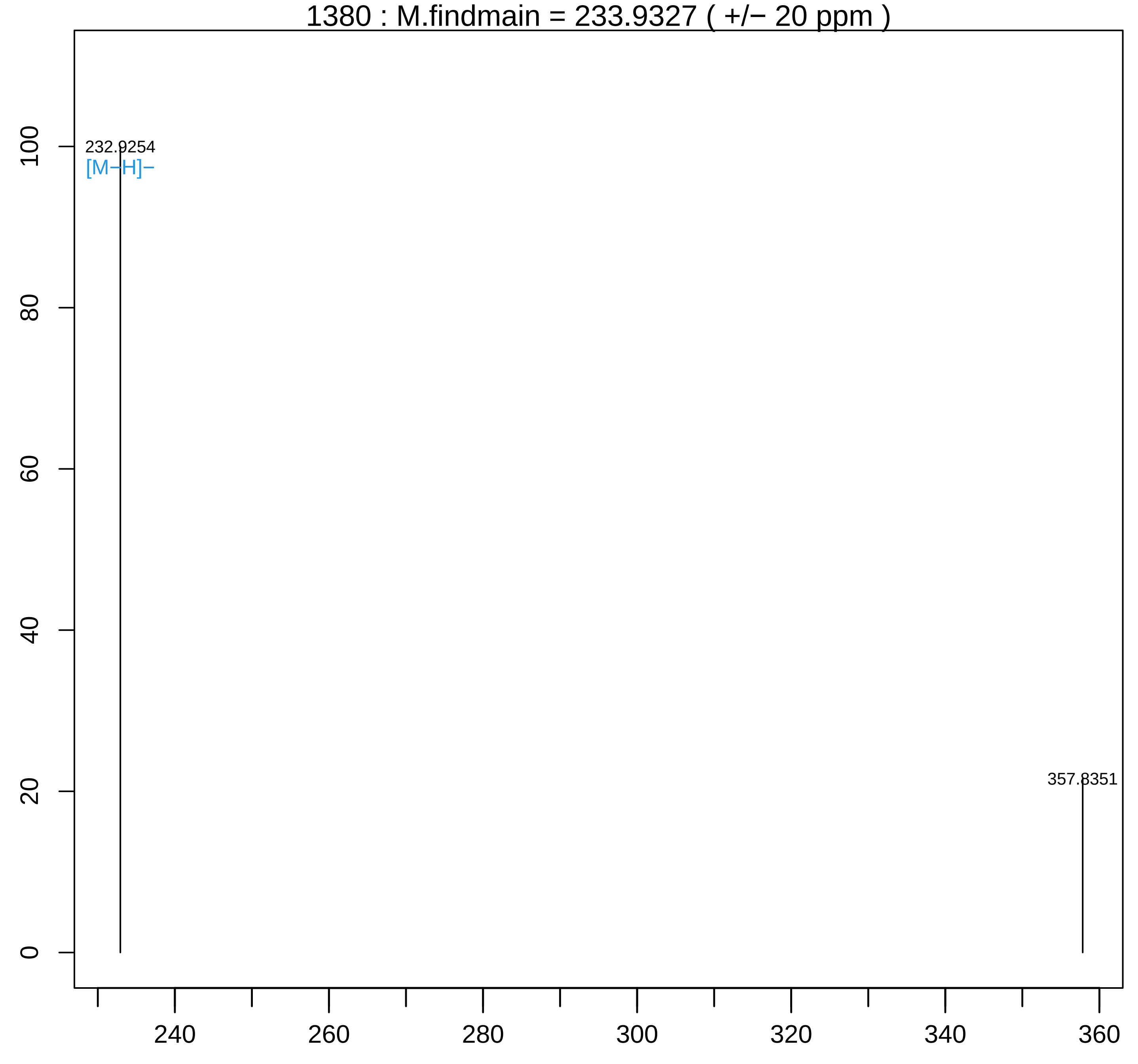

1380 : M.findmain = 233.9327 (+/− 20 ppm)
232.9254
[M−H]−
357.8351
0
20
40
60
80
100
240
260
280
300
320
340
360

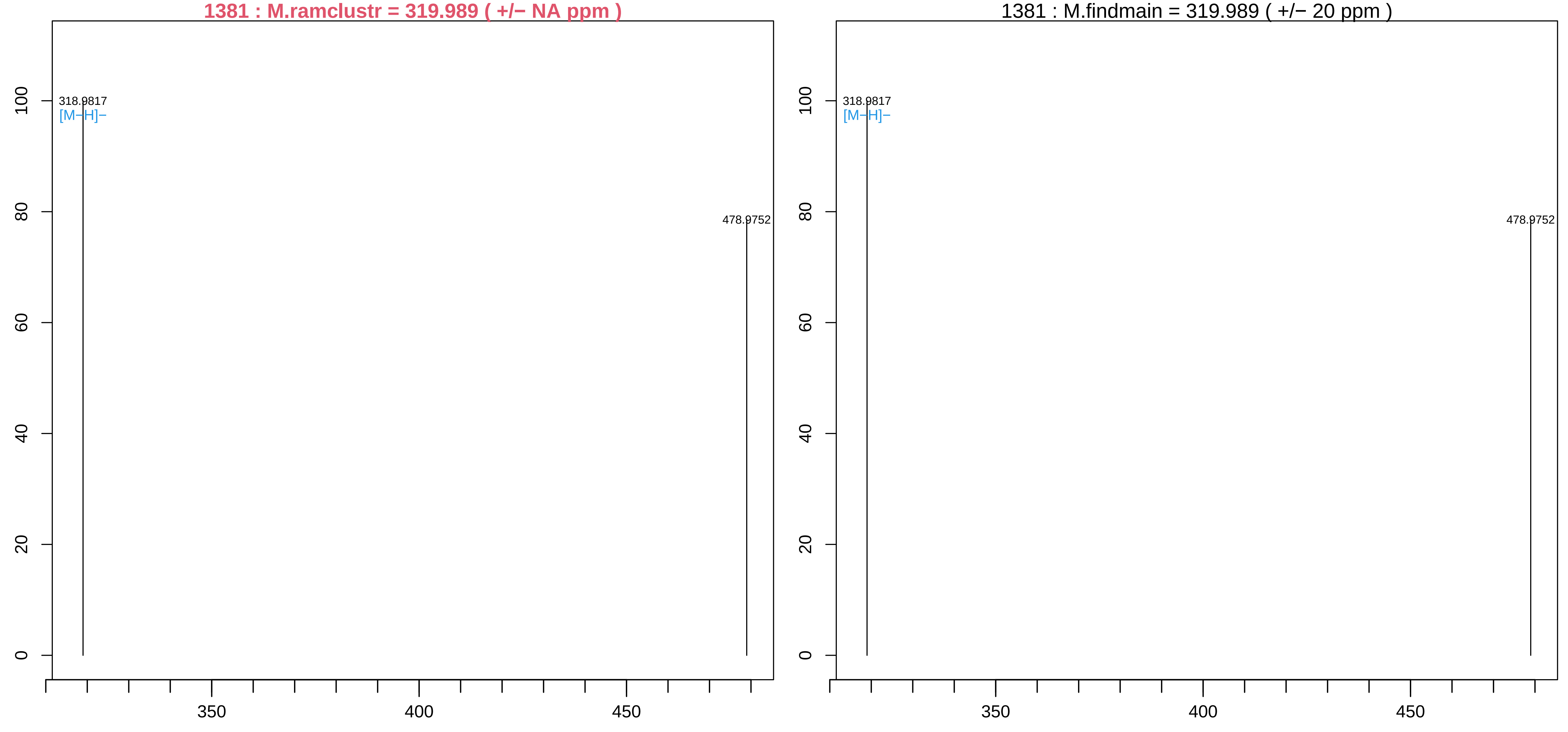
1381 : M.ramclustr = 319.989 (+/− NA ppm)
318.9817
[M−H]−
478.9752
1381 : M.findmain = 319.989 (+/− 20 ppm)
318.9817
[M−H]−
478.9752
0
20
40
60
80
100
350
400
450

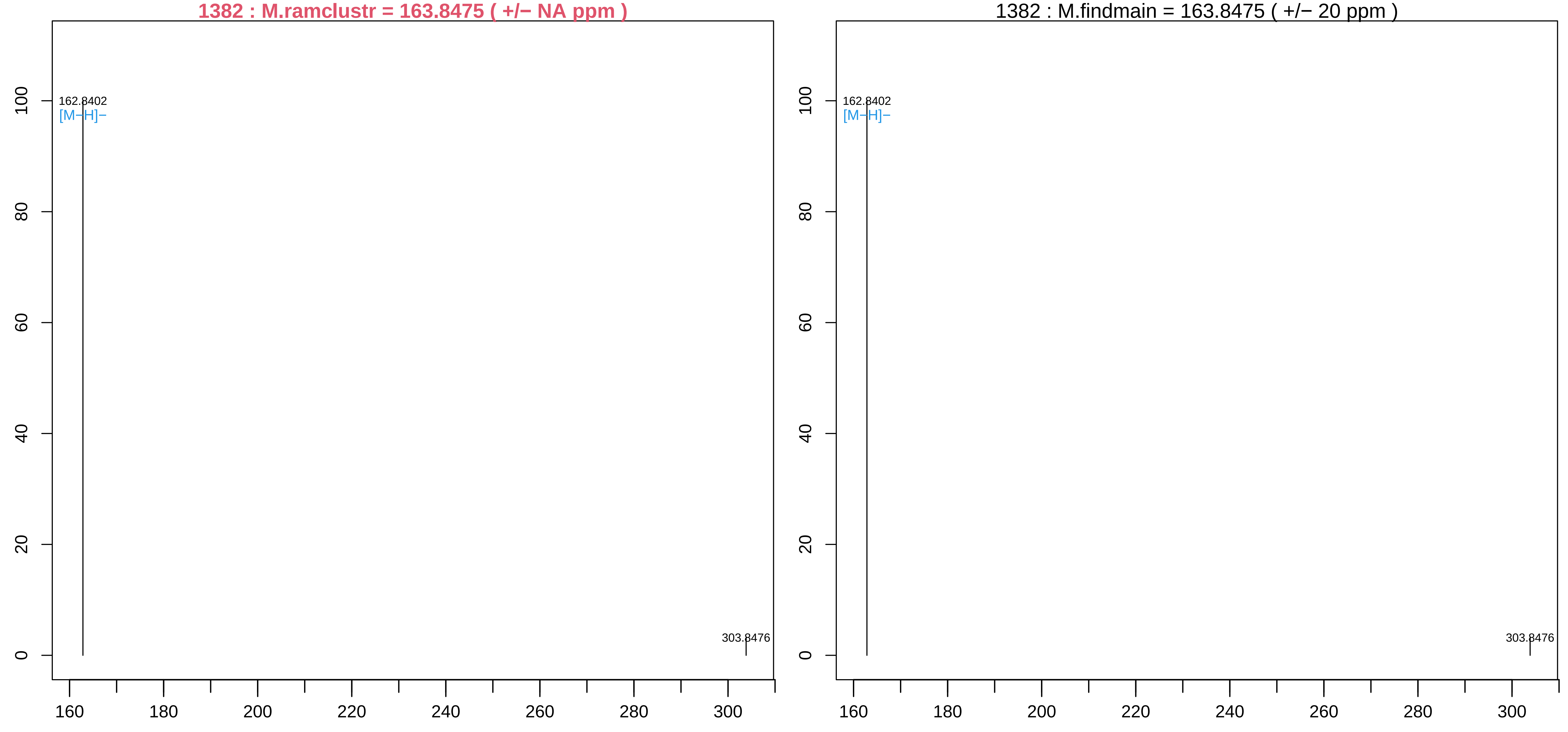

1382 : M.ramclustr = 163.8475 (+/− NA ppm)
162.8402
[M−H]−
303.8476
1382 : M.findmain = 163.8475 (+/− 20 ppm)
162.8402
[M−H]−
303.8476

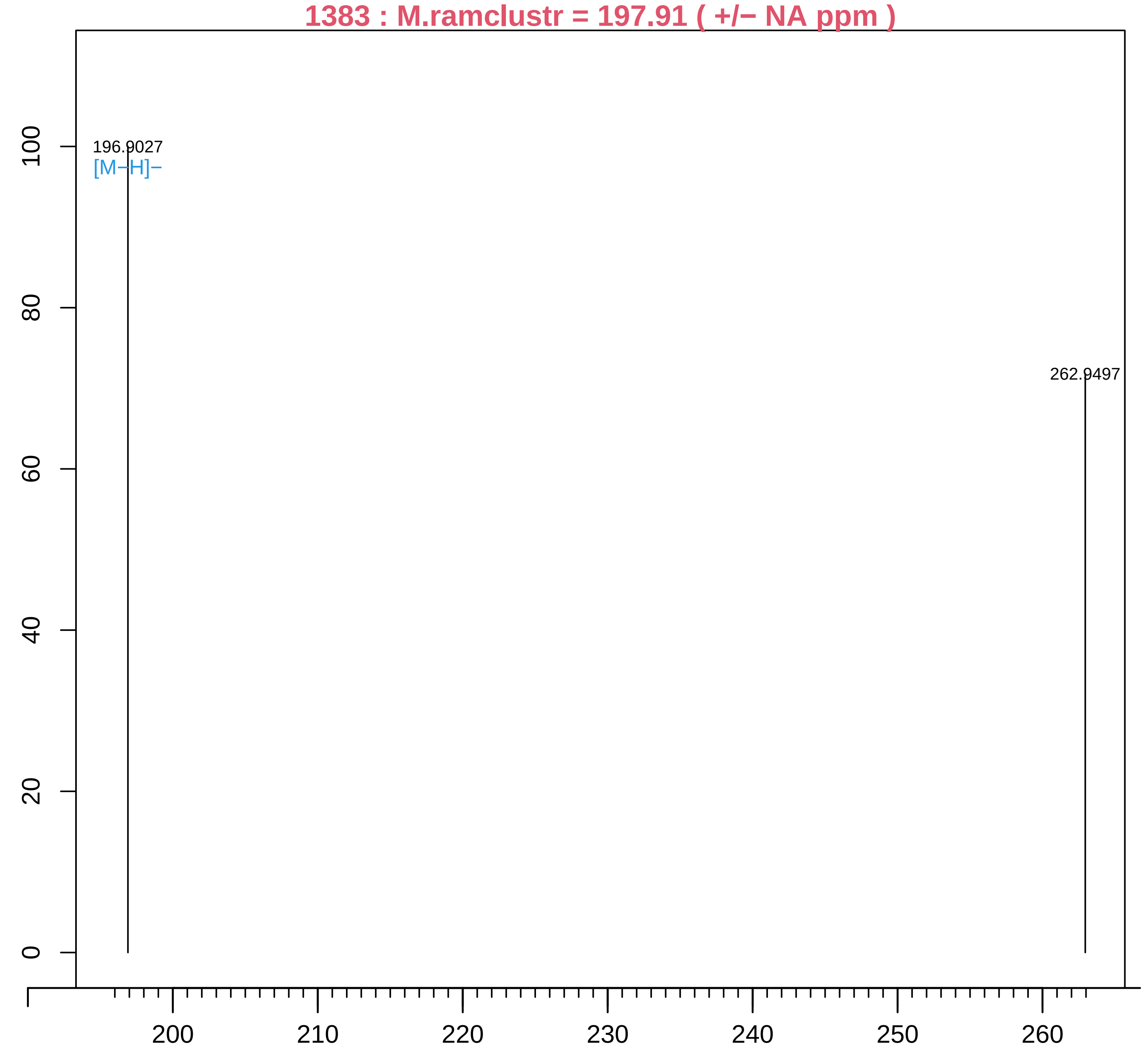
1383 : M.ramclustr = 197.91 (+/− NA ppm)
196.9027
[M−H]−
262.9497
0
20
40
60
80
100
200
210
220
230
240
250
260

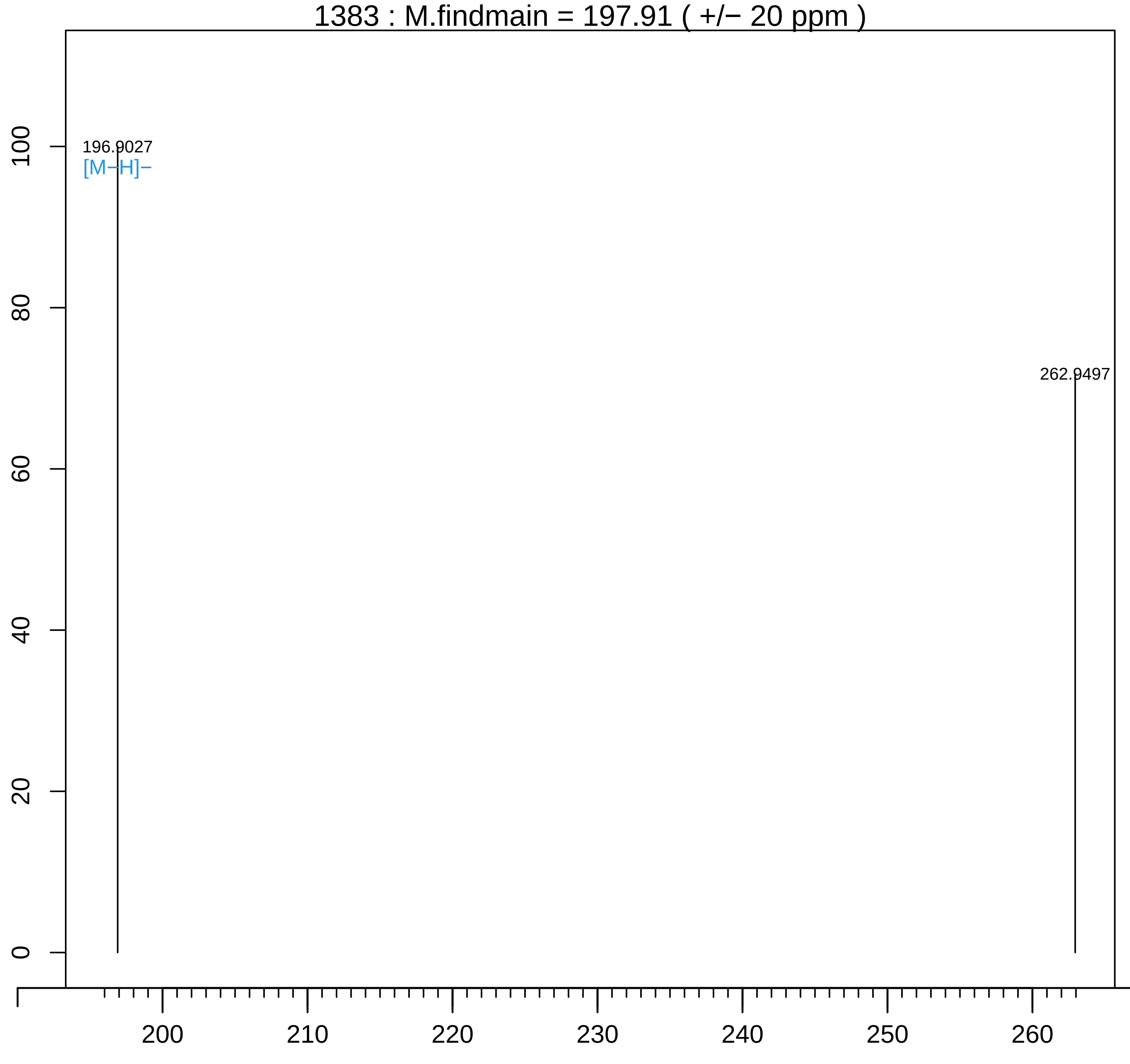
1383 : M.findmain = 197.91 (+/− 20 ppm)
196.9027
[M−H]−
262.9497
0
20
40
60
80
100
200
210
220
230
240
250
260

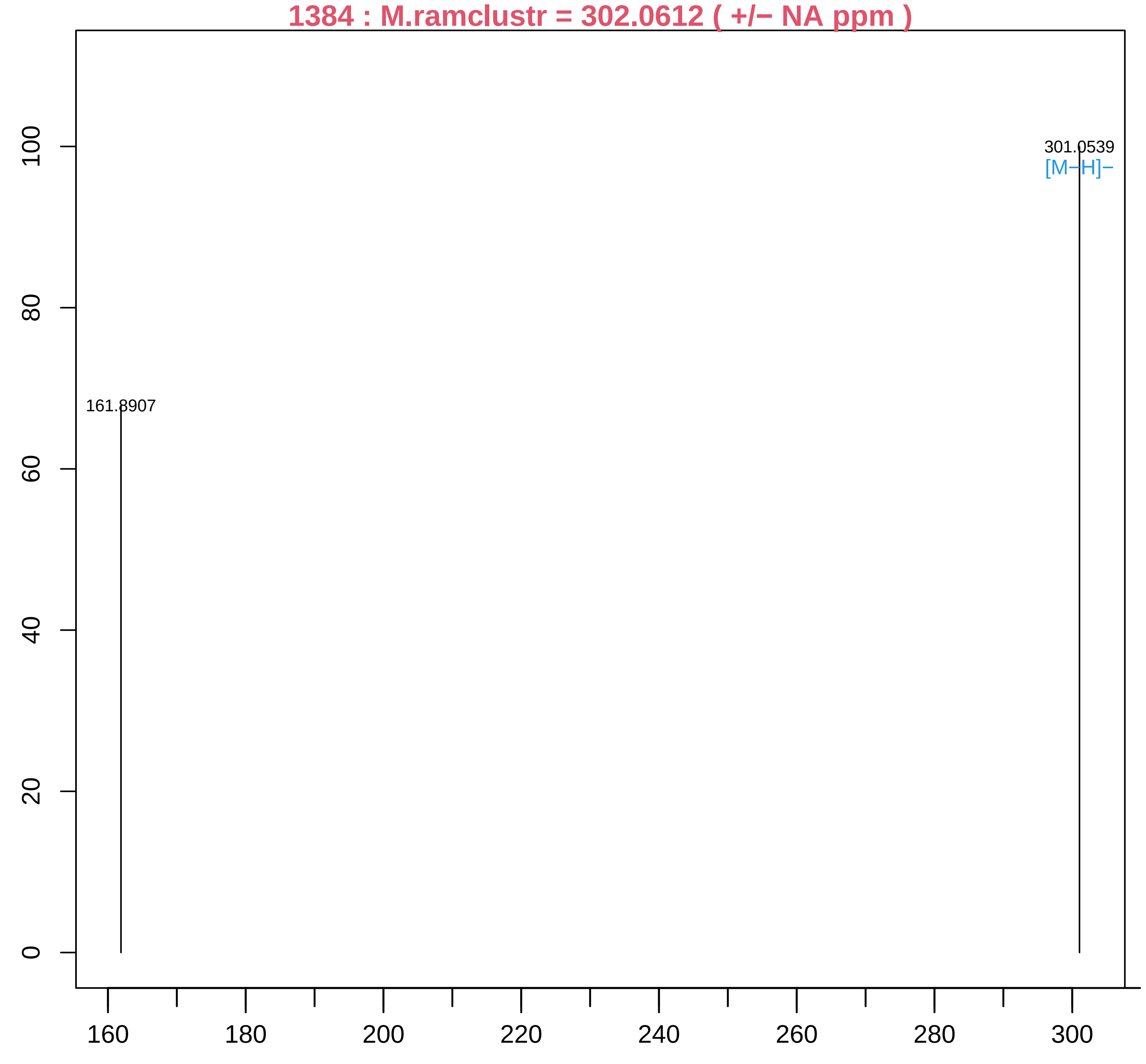
1384 : M.ramclustr = 302.0612 (+/− NA ppm)
301.0539
[M−H]−
161.8907
0
20
40
60
80
100
160
180
200
220
240
260
280
300

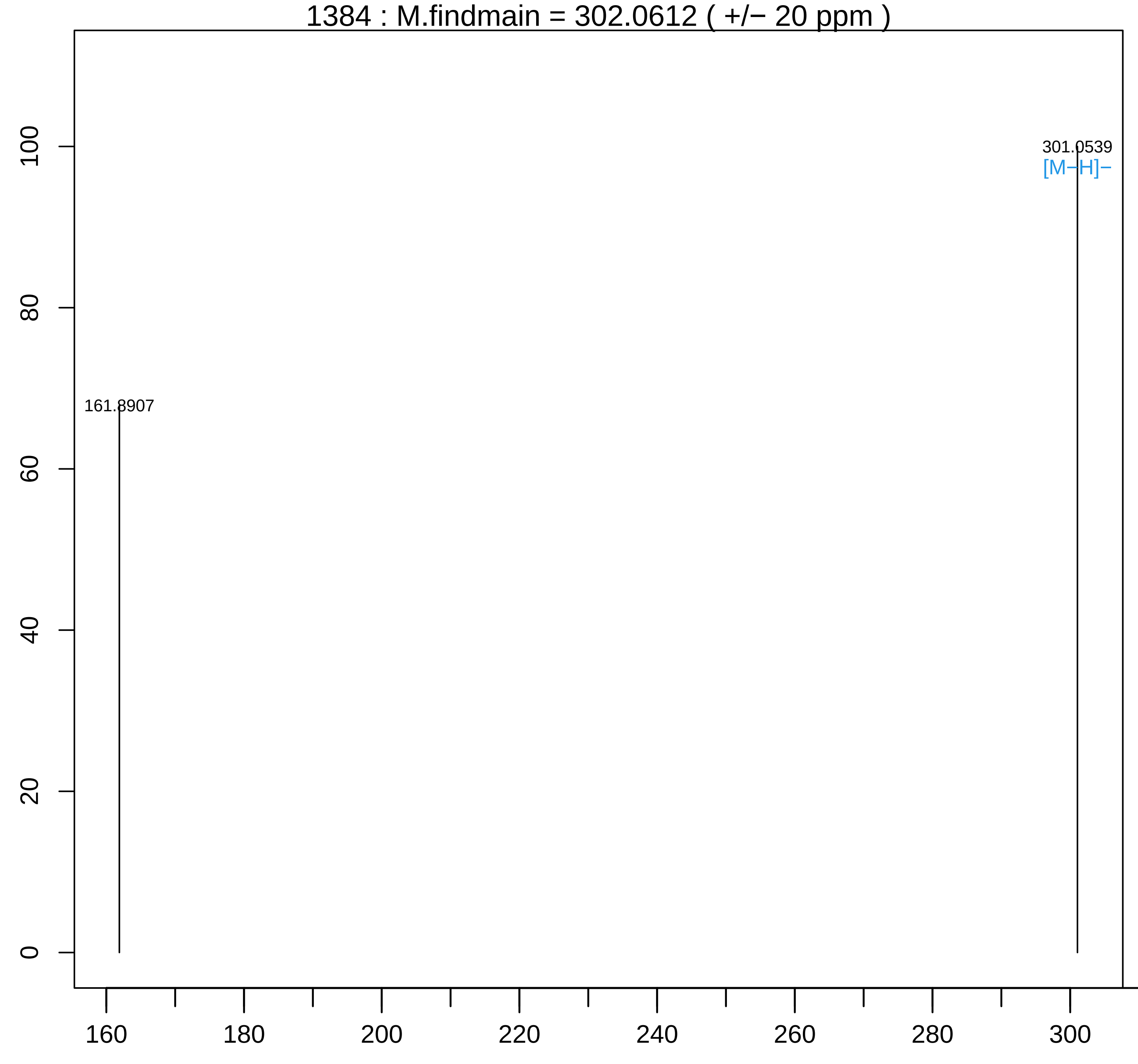
1384 : M.findmain = 302.0612 (+/− 20 ppm)
301.0539
[M−H]−
161.8907
0
20
40
60
80
100
160
180
200
220
240
260
280
300

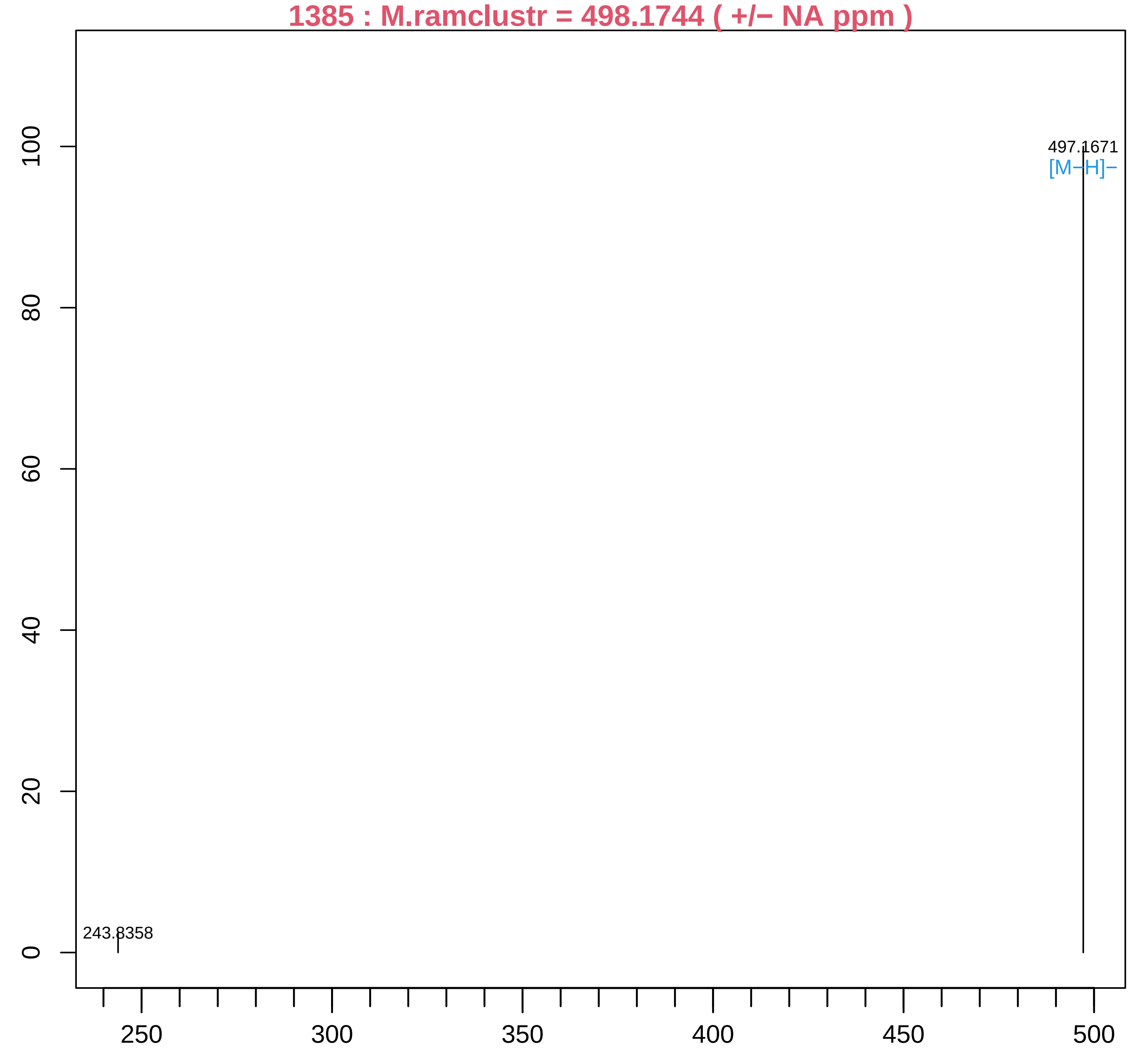
1385 : M.ramclustr = 498.1744 (+/− NA ppm)
497.1671
[M−H]−
243.8358
0
20
40
60
80
100
250
300
350
400
450
500

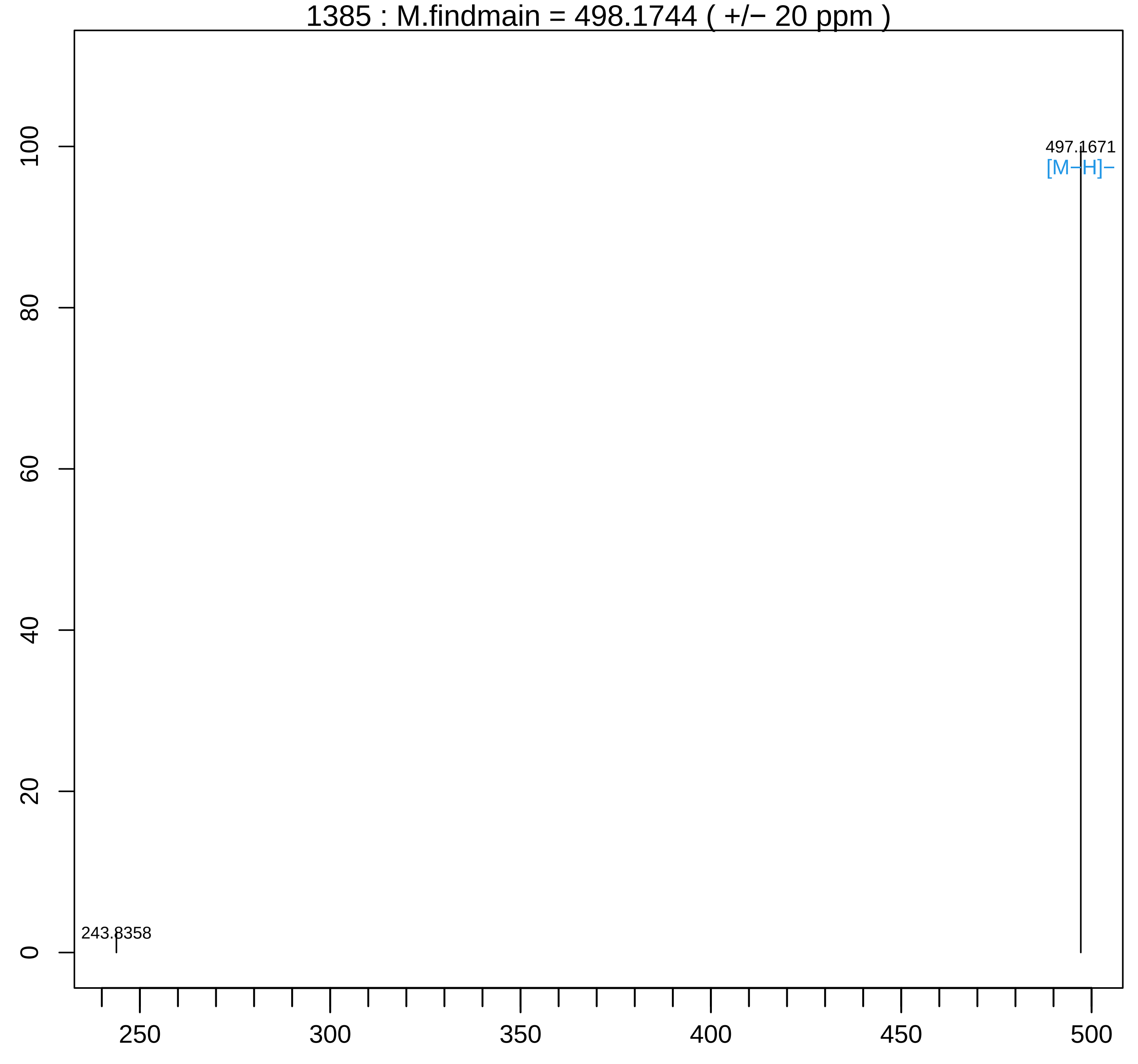
1385 : M.findmain = 498.1744 (+/− 20 ppm)
497.1671
[M−H]−
243.8358
0
20
40
60
80
100
250
300
350
400
450
500

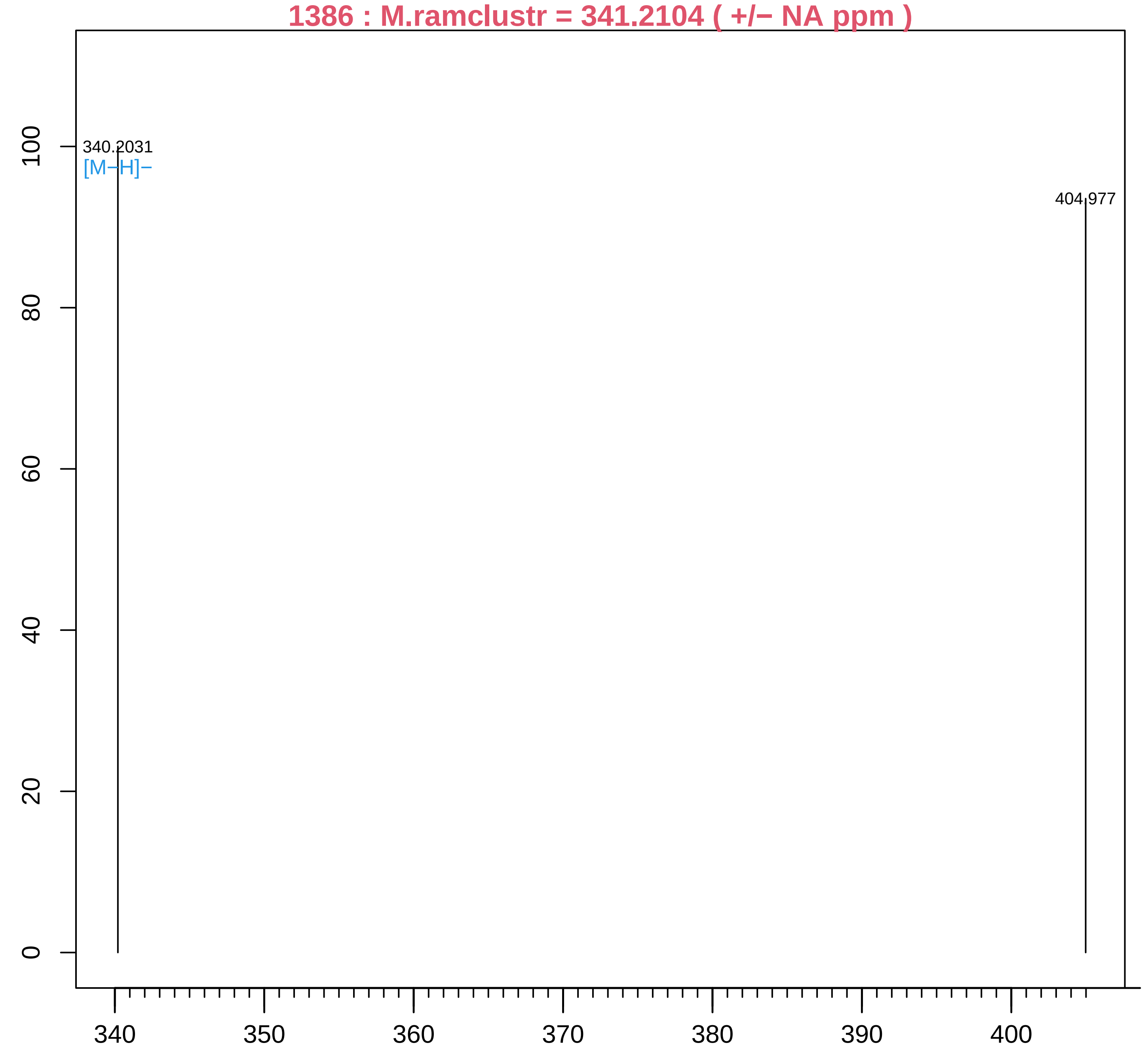
1386 : M.ramclustr = 341.2104 (+/− NA ppm)
340.2031
[M−H]−
404.977
0
20
40
60
80
100
340
350
360
370
380
390
400

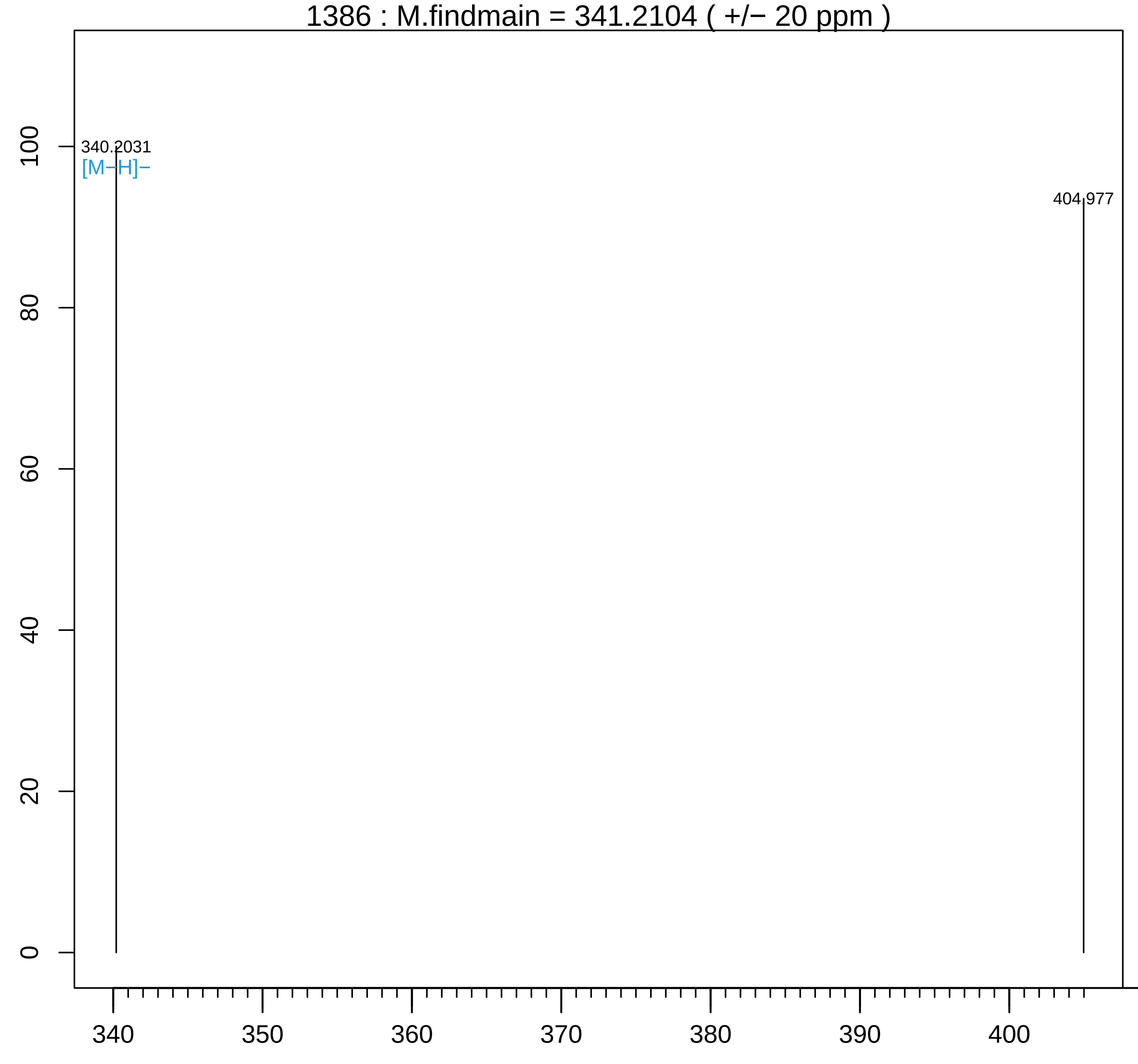
1386 : M.findmain = 341.2104 (+/− 20 ppm)
340.2031
[M−H]−
404.977
0
20
40
60
80
100
340
350
360
370
380
390
400

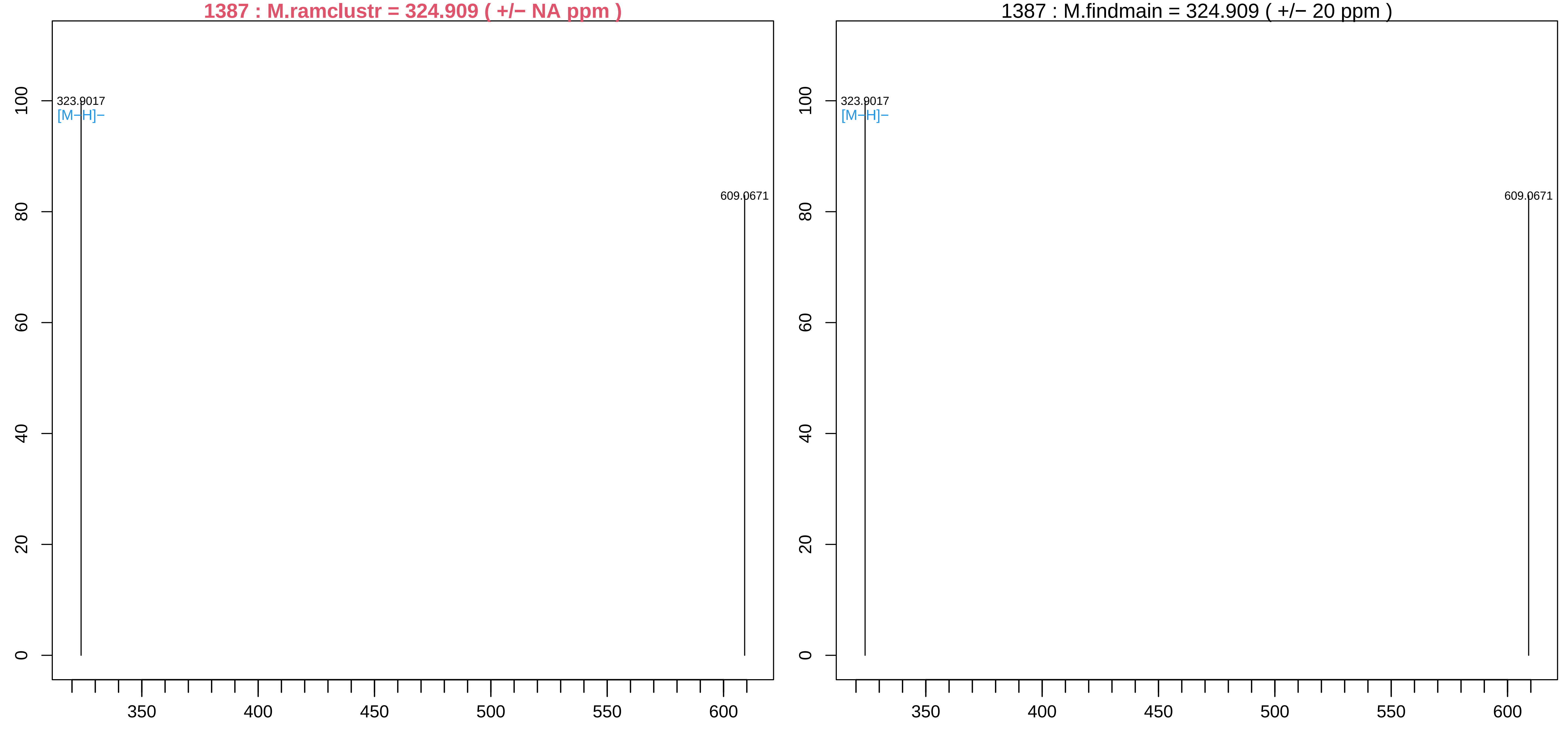
1387 : M.ramclustr = 324.909 (+/− NA ppm)
323.9017
[M−H]−
609.0671
1387 : M.findmain = 324.909 (+/− 20 ppm)
323.9017
[M−H]−
609.0671
0
20
40
60
80
100
350
400
450
500
550
600

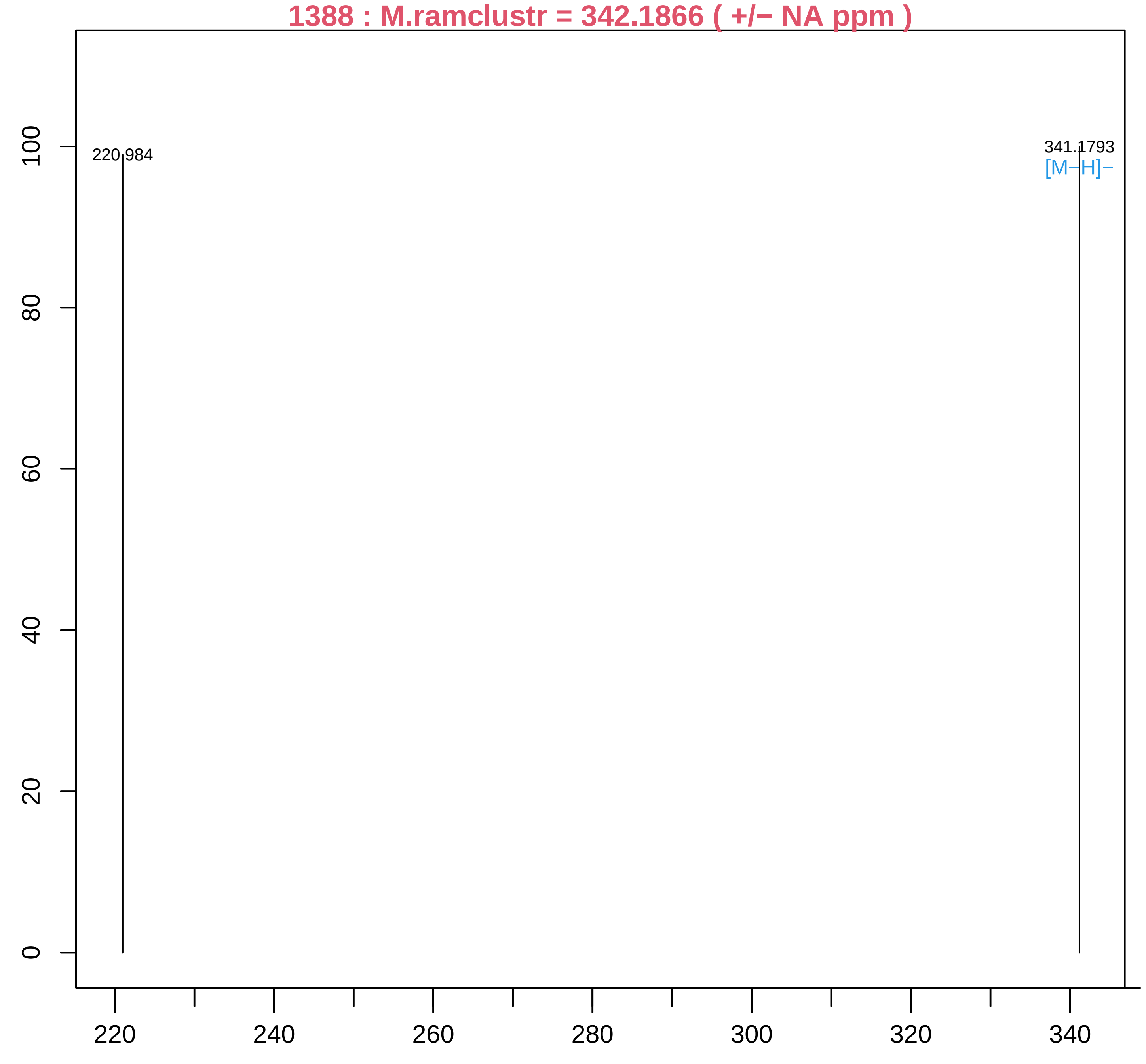
1388 : M.ramclustr = 342.1866 (+/− NA ppm)
220.984
341.1793
[M−H]−
0
20
40
60
80
100
220
240
260
280
300
320
340

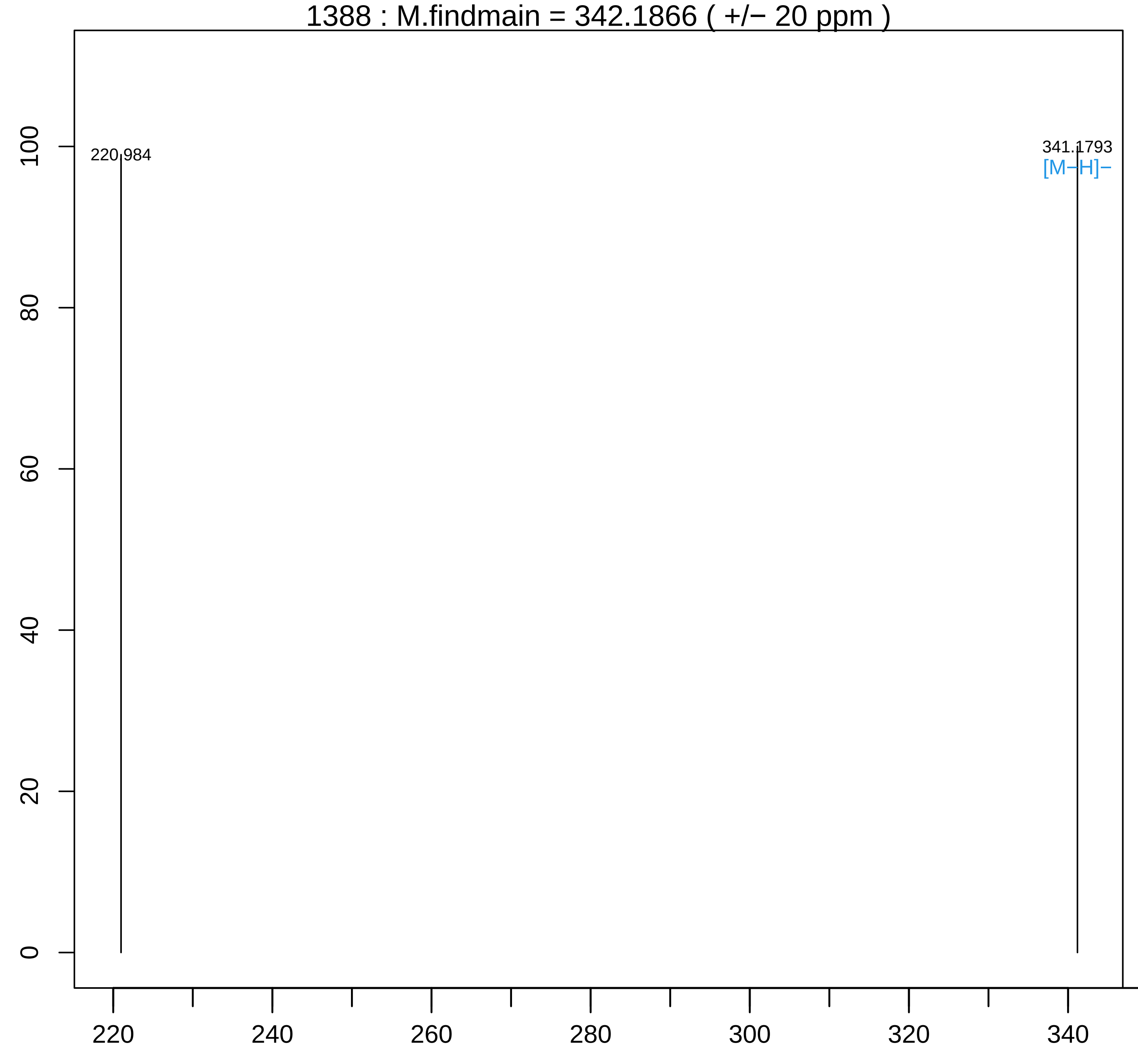
1388 : M.findmain = 342.1866 (+/− 20 ppm)
220.984
341.1793
[M−H]−
0
20
40
60
80
100
220
240
260
280
300
320
340

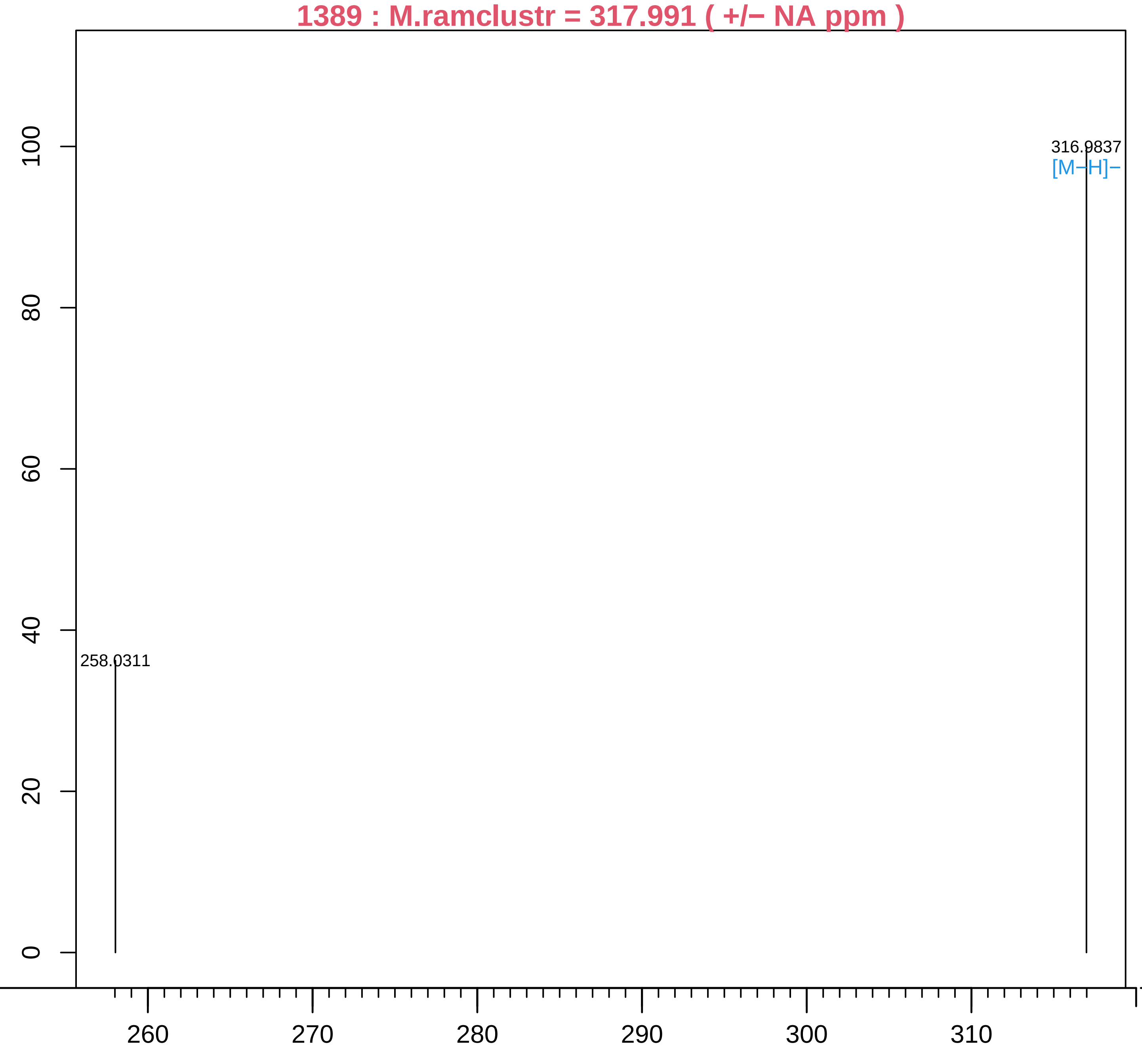
1389 : M.ramclustr = 317.991 (+/− NA ppm)
316.9837
[M−H]−
258.0311
0
20
40
60
80
100
260
270
280
290
300
310

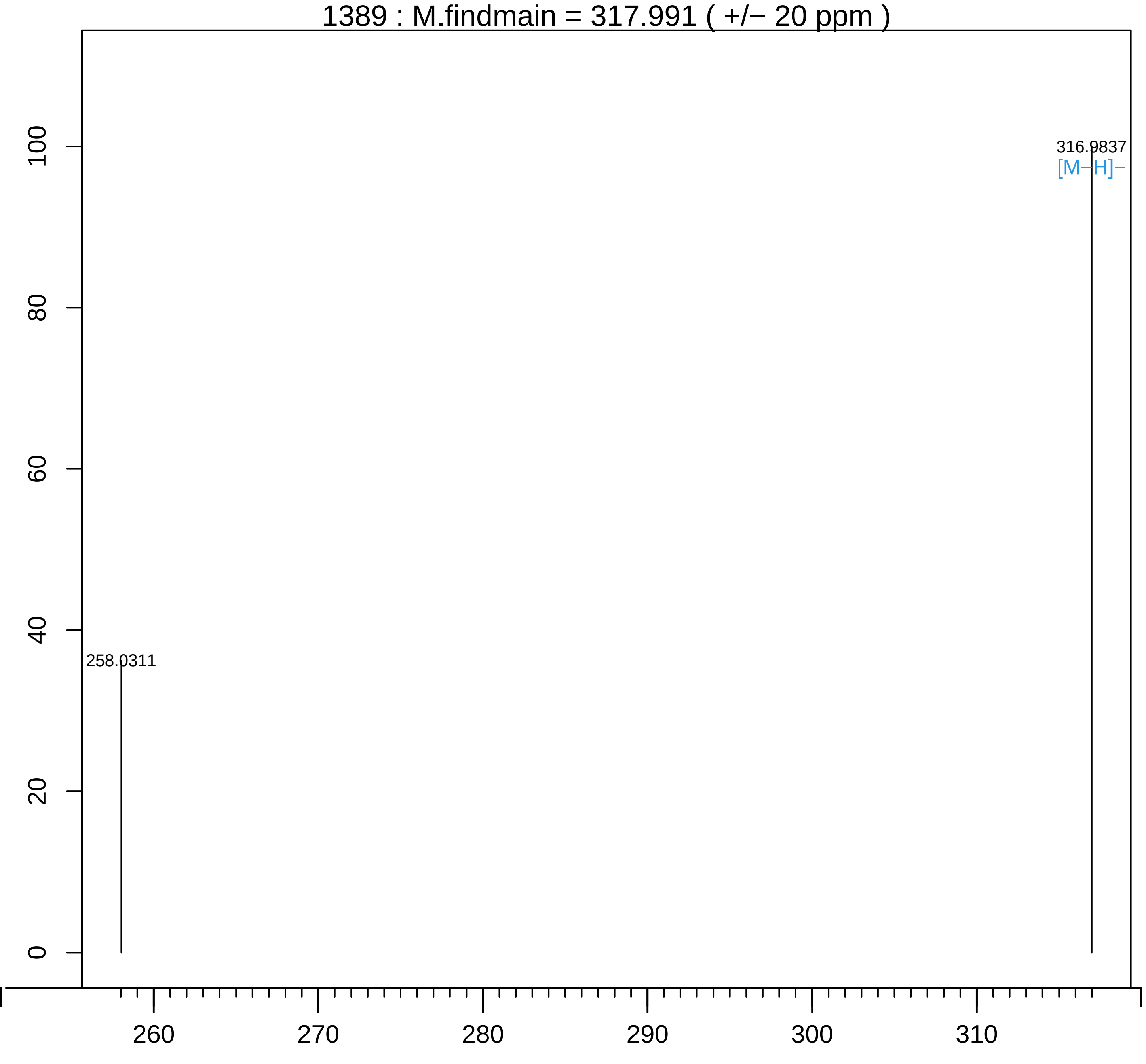
1389 : M.findmain = 317.991 (+/− 20 ppm)
316.9837
[M−H]−
258.0311
0
20
40
60
80
100
260
270
280
290
300
310

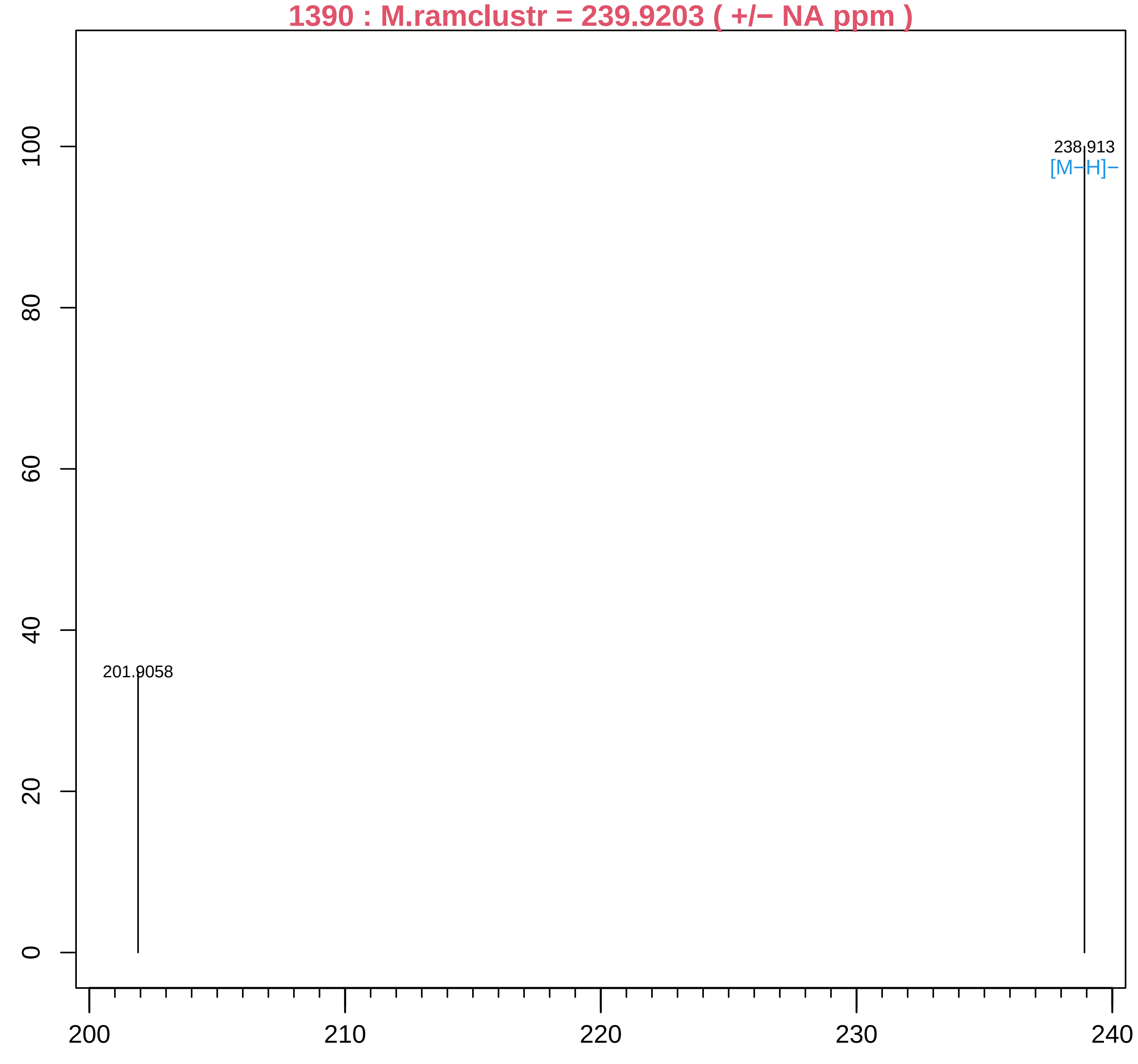

1390 : M.ramclustr = 239.9203 (+/− NA ppm)
238.913
[M−H]−
201.9058
0
20
40
60
80
100
200
210
220
230
240

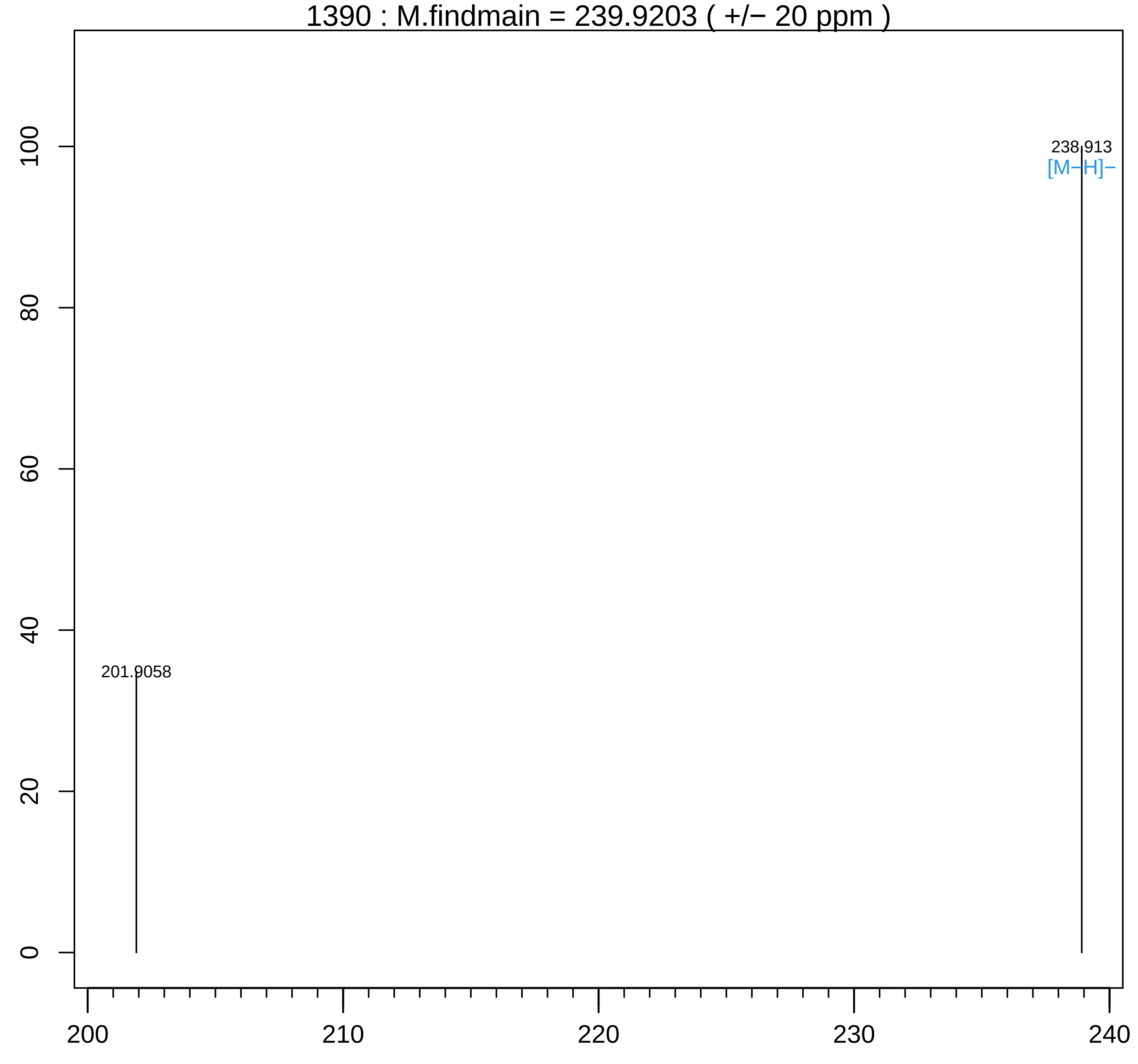

1390 : M.findmain = 239.9203 (+/− 20 ppm)
238.913
[M−H]−
201.9058
0
20
40
60
80
100
200
210
220
230
240

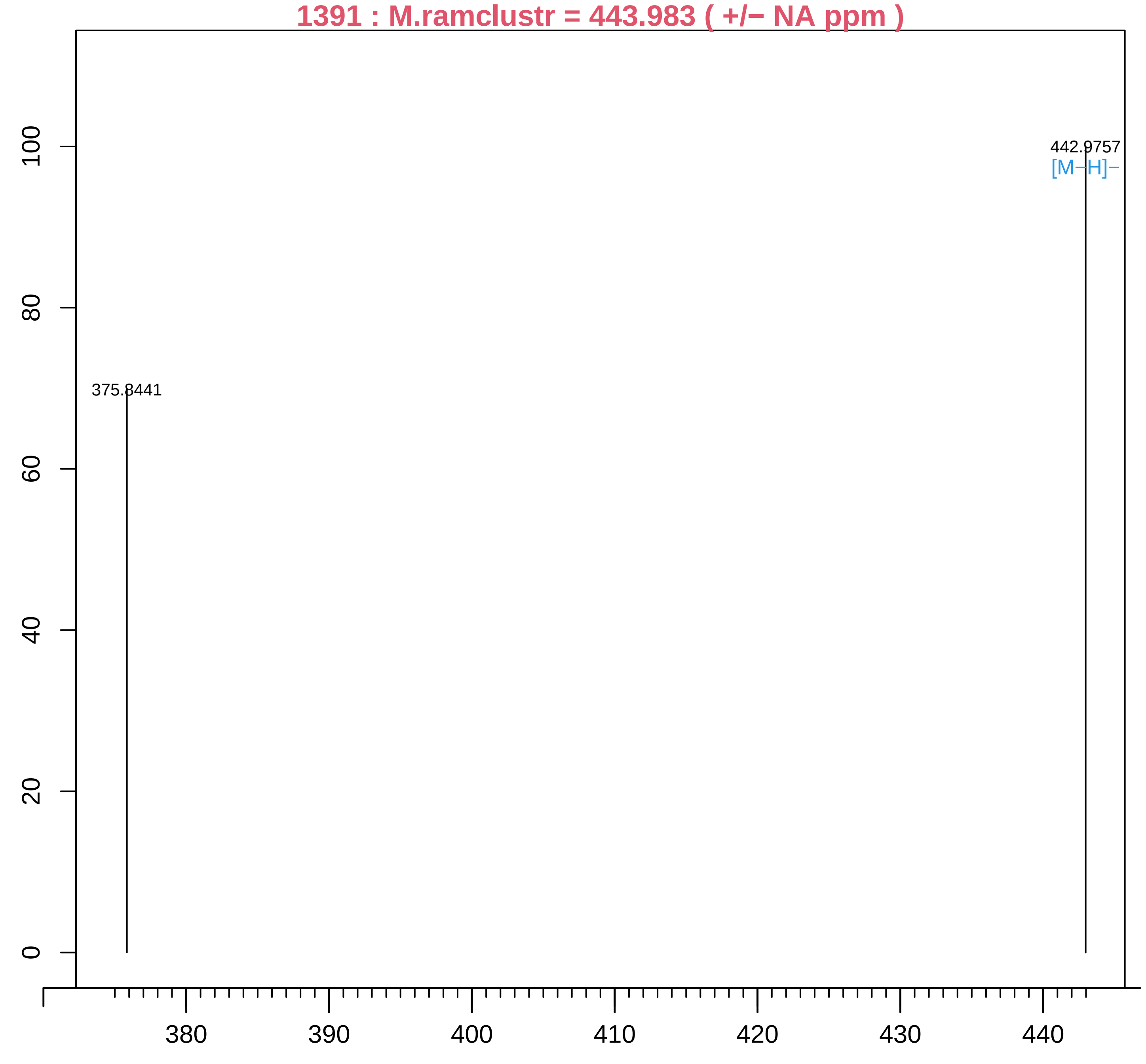
1391 : M.ramclustr = 443.983 (+/− NA ppm)
442.9757
[M−H]−
375.8441
0
20
40
60
80
100
380
390
400
410
420
430
440

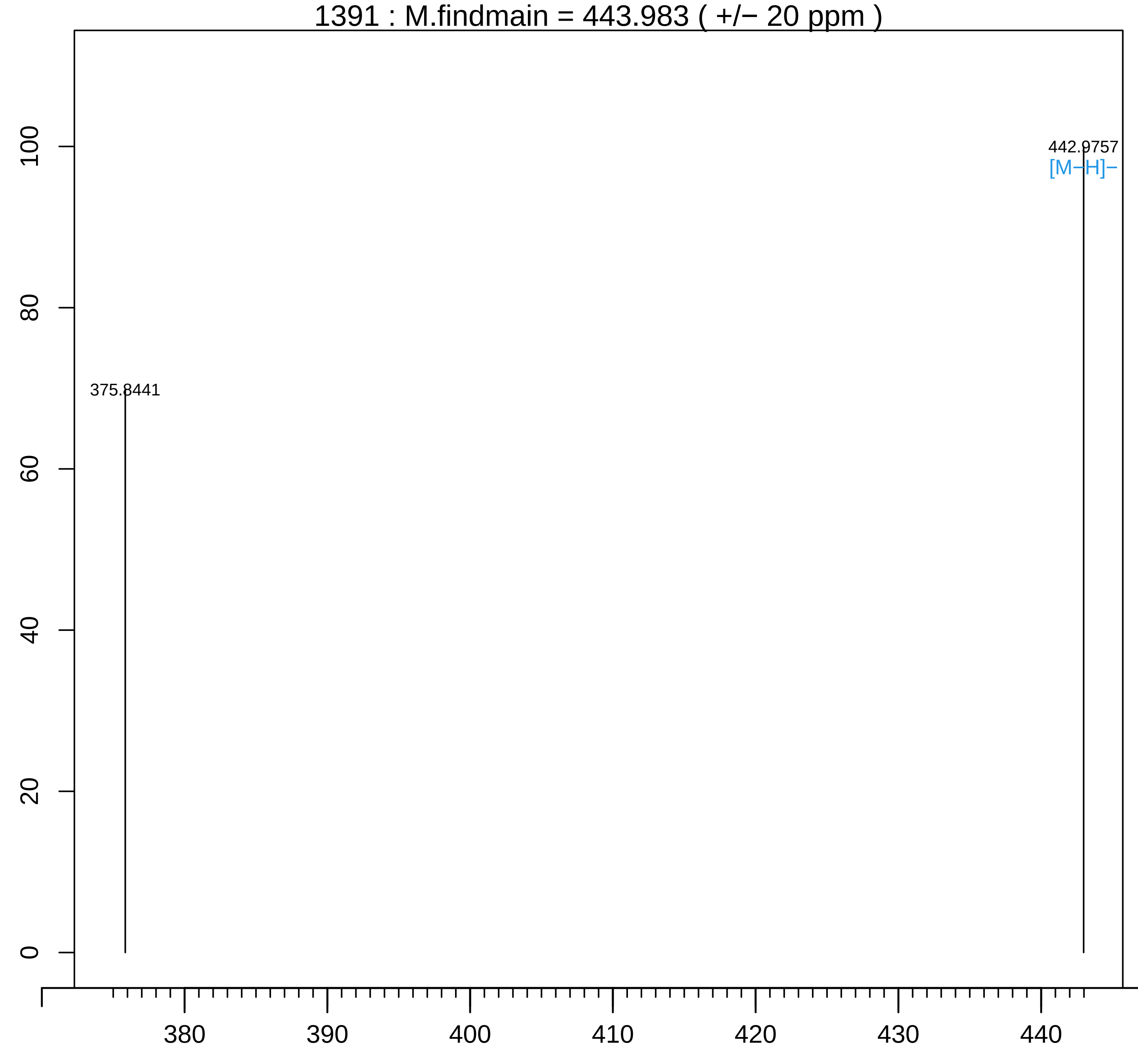
1391 : M.findmain = 443.983 (+/− 20 ppm)
442.9757
[M−H]−
375.8441
0
20
40
60
80
100
380
390
400
410
420
430
440

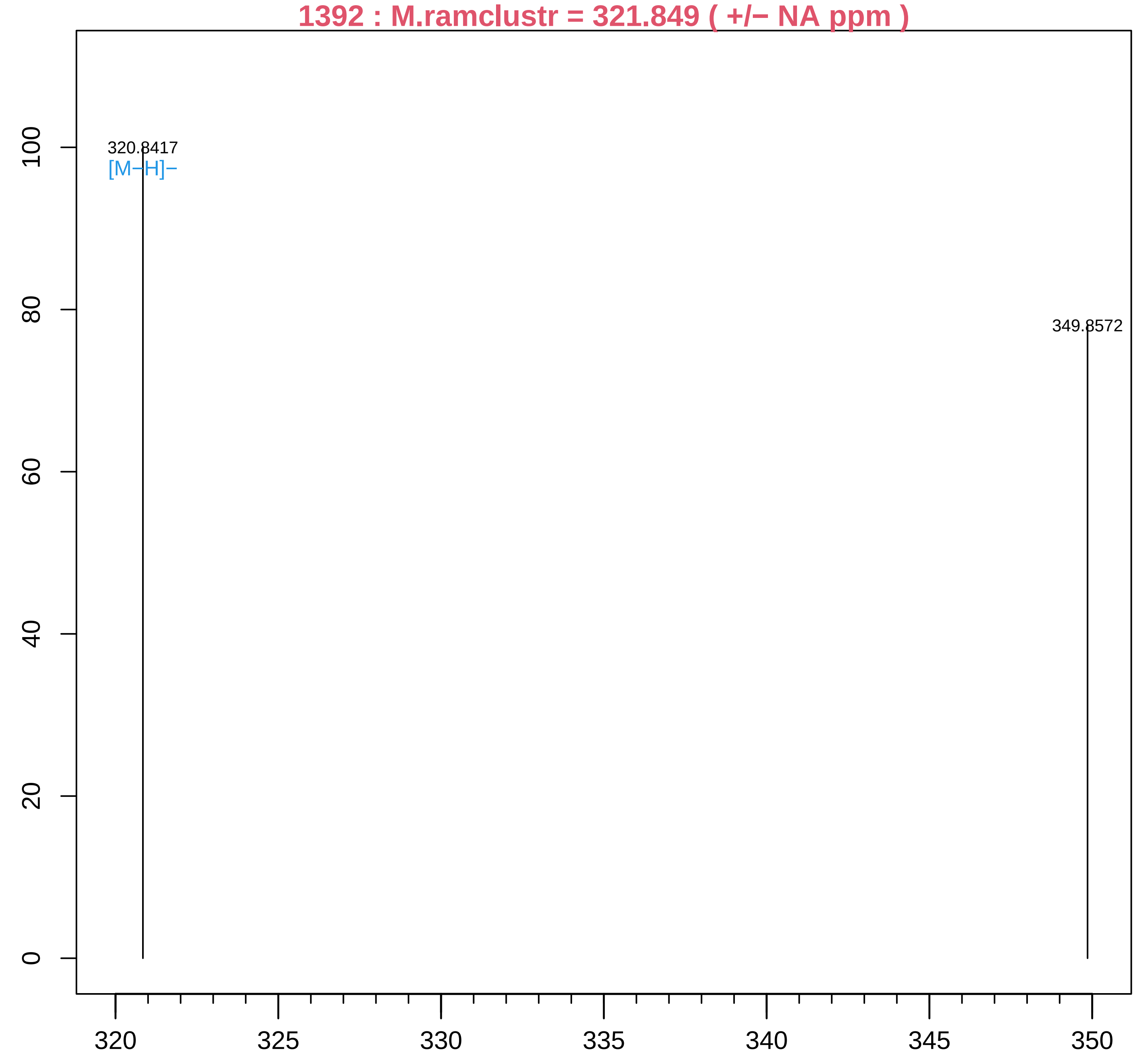
1392 : M.ramclustr = 321.849 (+/− NA ppm)
320.8417
[M−H]−
349.8572
0
20
40
60
80
100
320
325
330
335
340
345
350

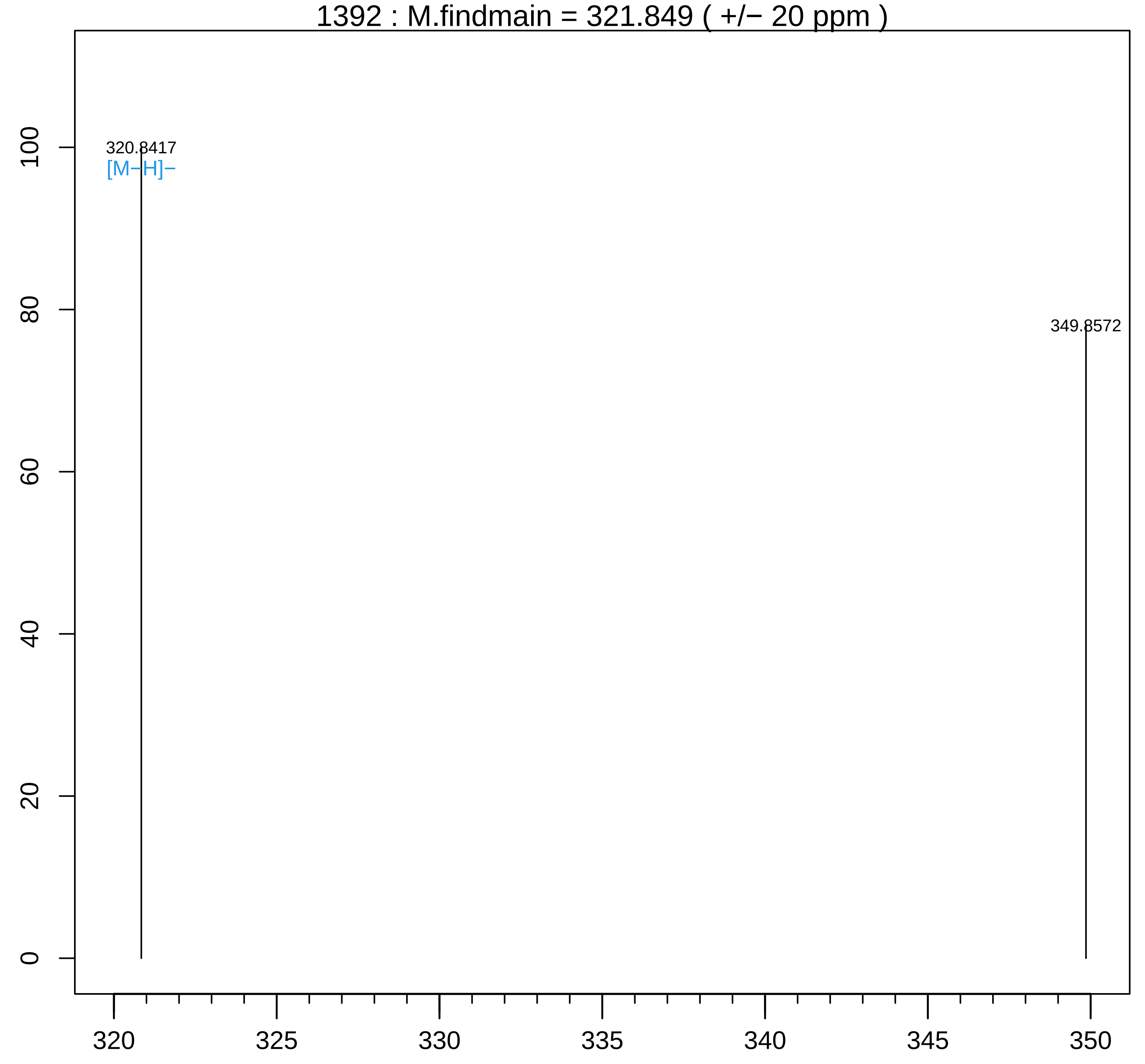
1392 : M.findmain = 321.849 (+/− 20 ppm)
320.8417
[M−H]−
349.8572
0
20
40
60
80
100
320
325
330
335
340
345
350

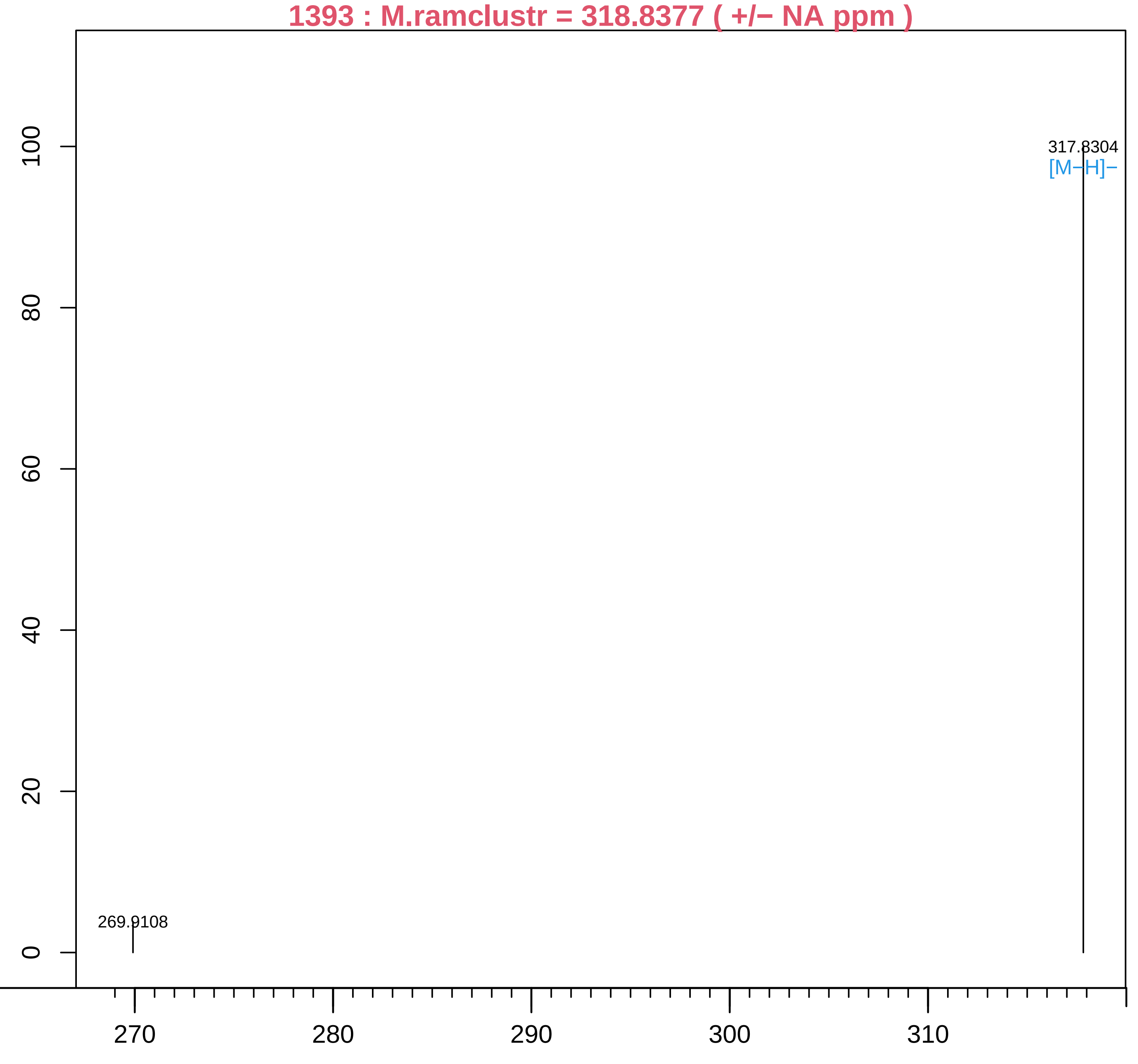
1393 : M.ramclustr = 318.8377 (+/− NA ppm)
317.8304
[M−H]−
269.9108
0
20
40
60
80
100
270
280
290
300
310

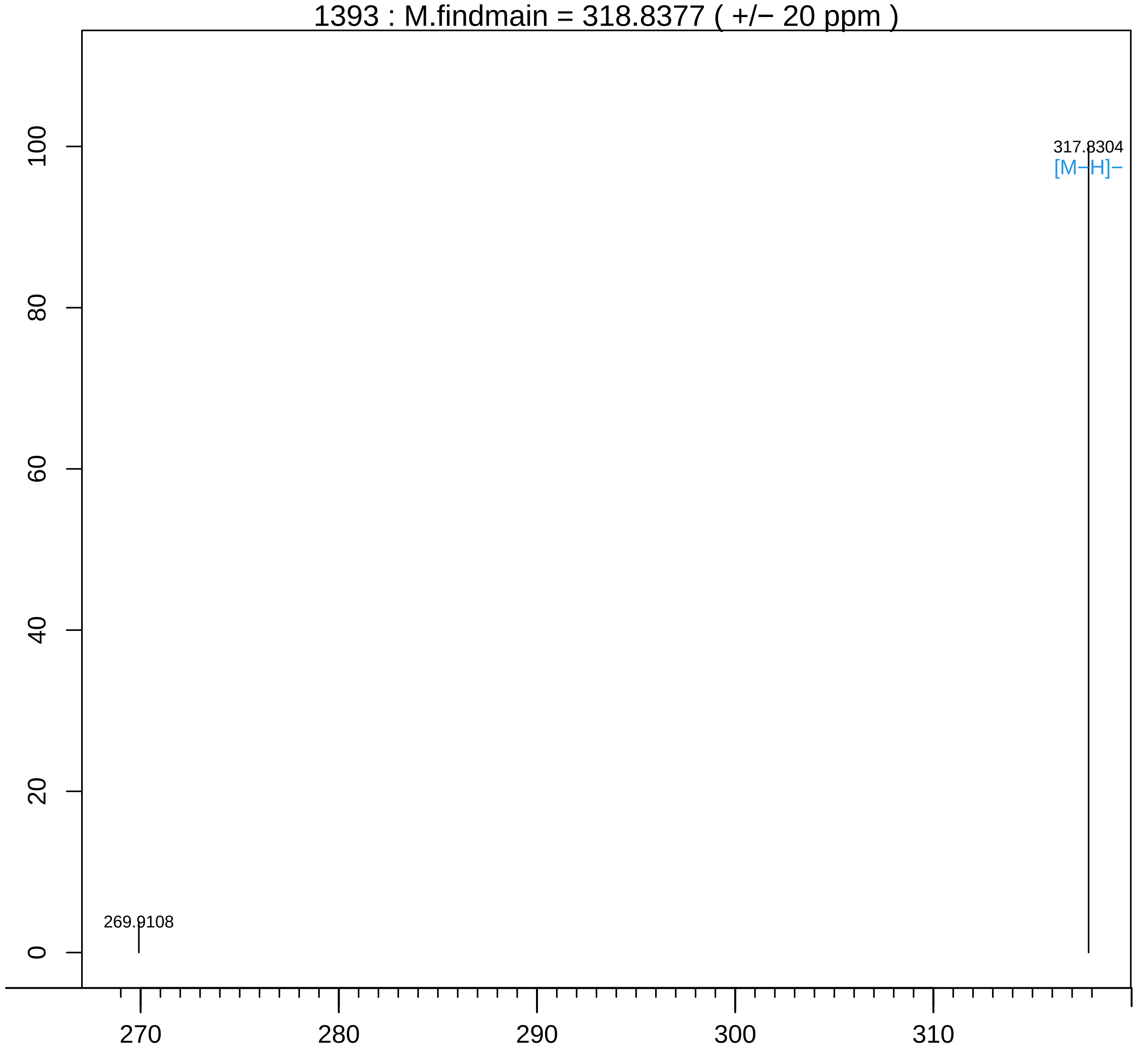
1393 : M.findmain = 318.8377 (+/− 20 ppm)
317.8304
[M−H]−
269.9108
0
20
40
60
80
100
270
280
290
300
310

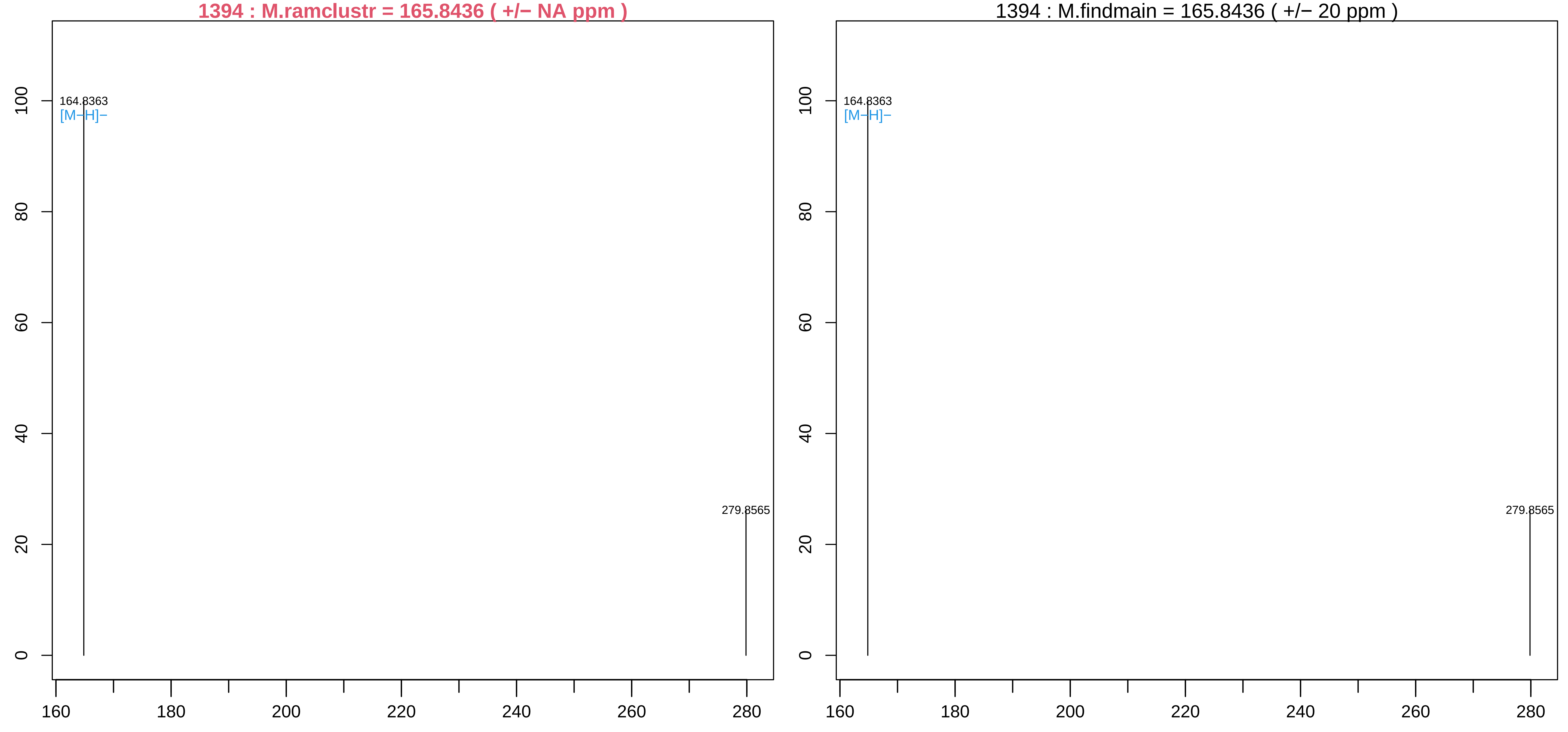

1394 : M.ramclustr = 165.8436 (+/− NA ppm)
1394 : M.findmain = 165.8436 (+/− 20 ppm)
164.8363
[M−H]−
279.8565
164.8363
[M−H]−
279.8565
0
20
40
60
80
100
160
180
200
220
240
260
280

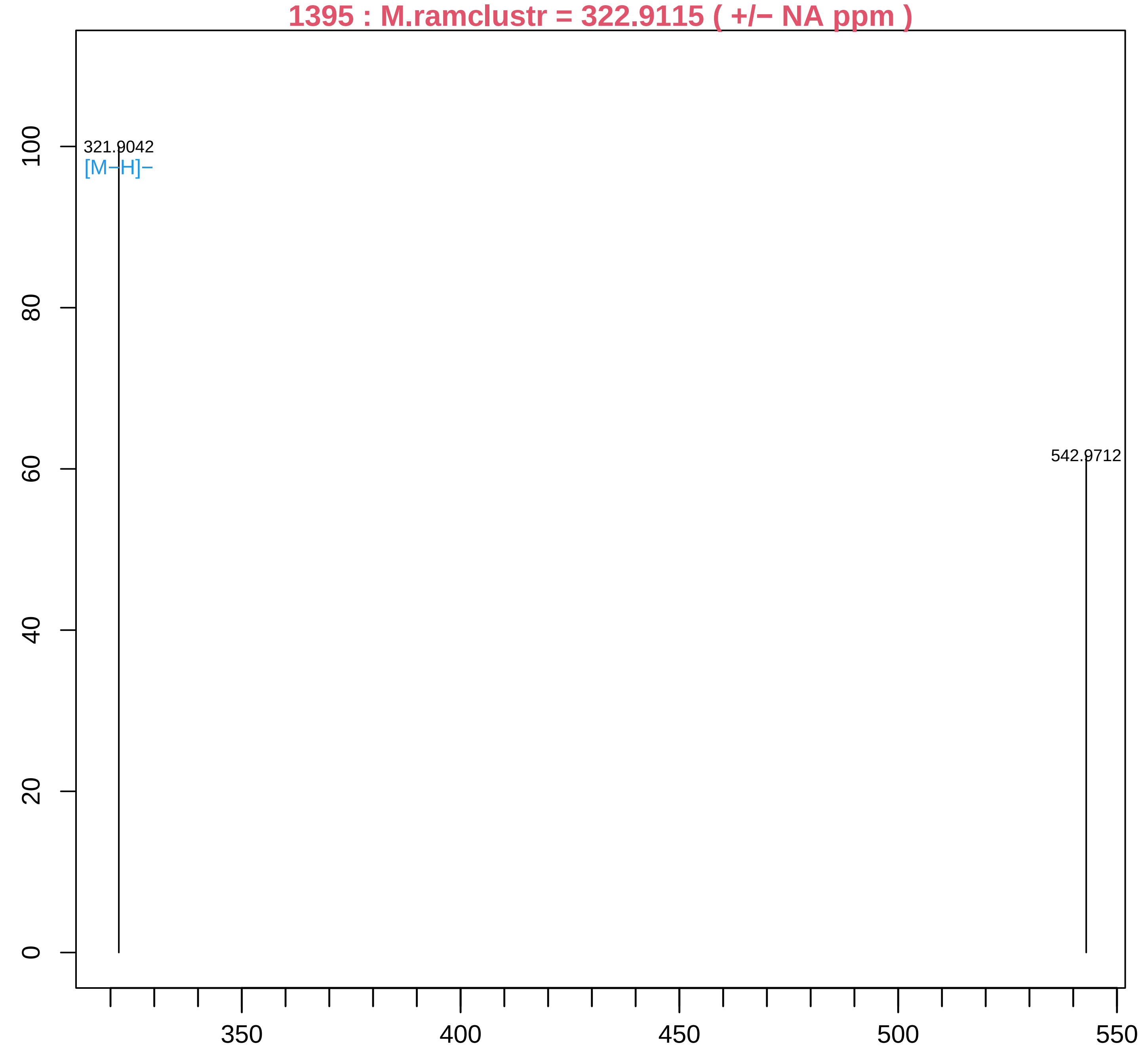
1395 : M.ramclustr = 322.9115 (+/− NA ppm)
321.9042
[M−H]−
542.9712
0
20
40
60
80
100
350
400
450
500
550

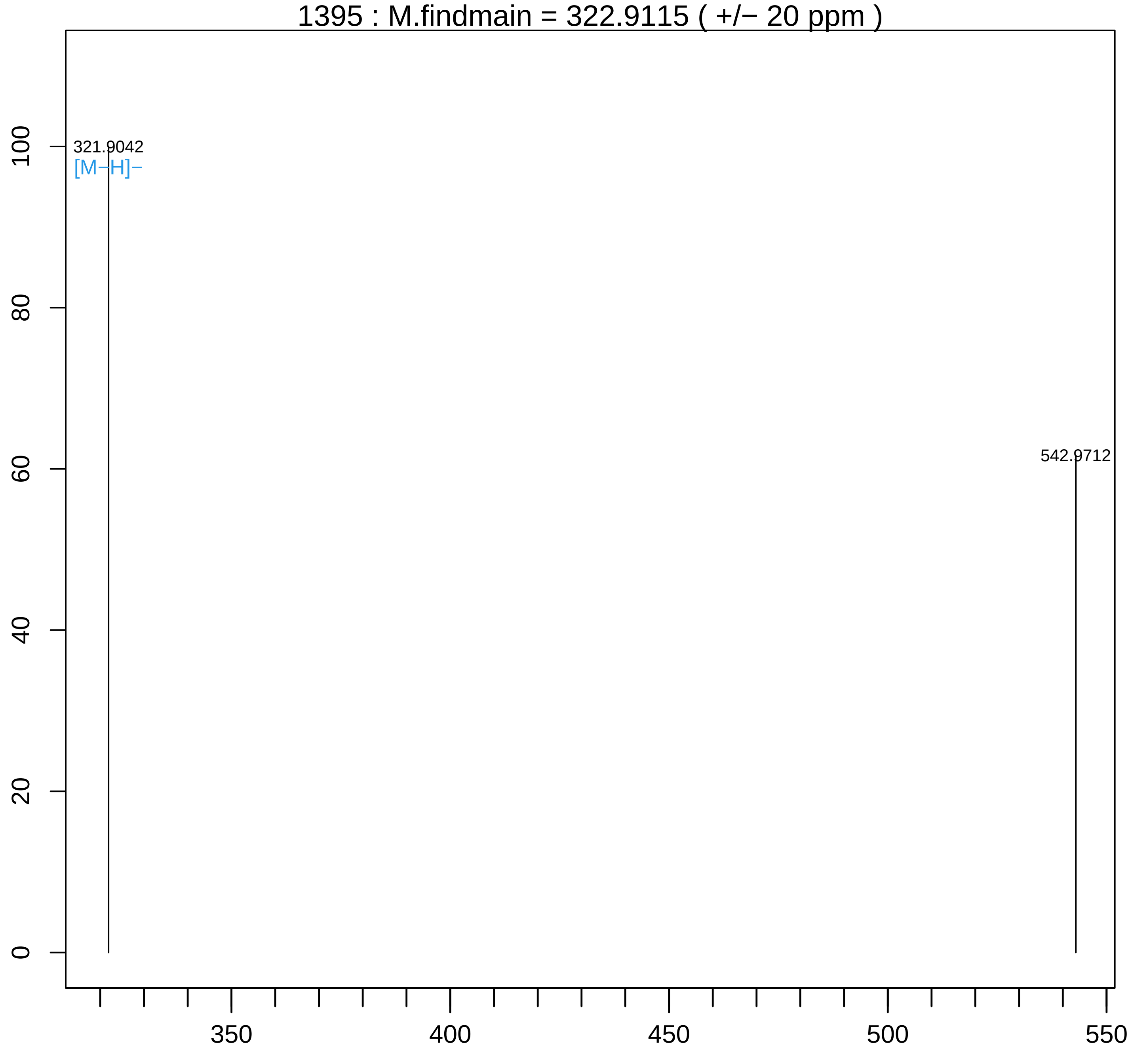
1395 : M.findmain = 322.9115 (+/− 20 ppm)
321.9042
[M−H]−
542.9712
0
20
40
60
80
100
350
400
450
500
550

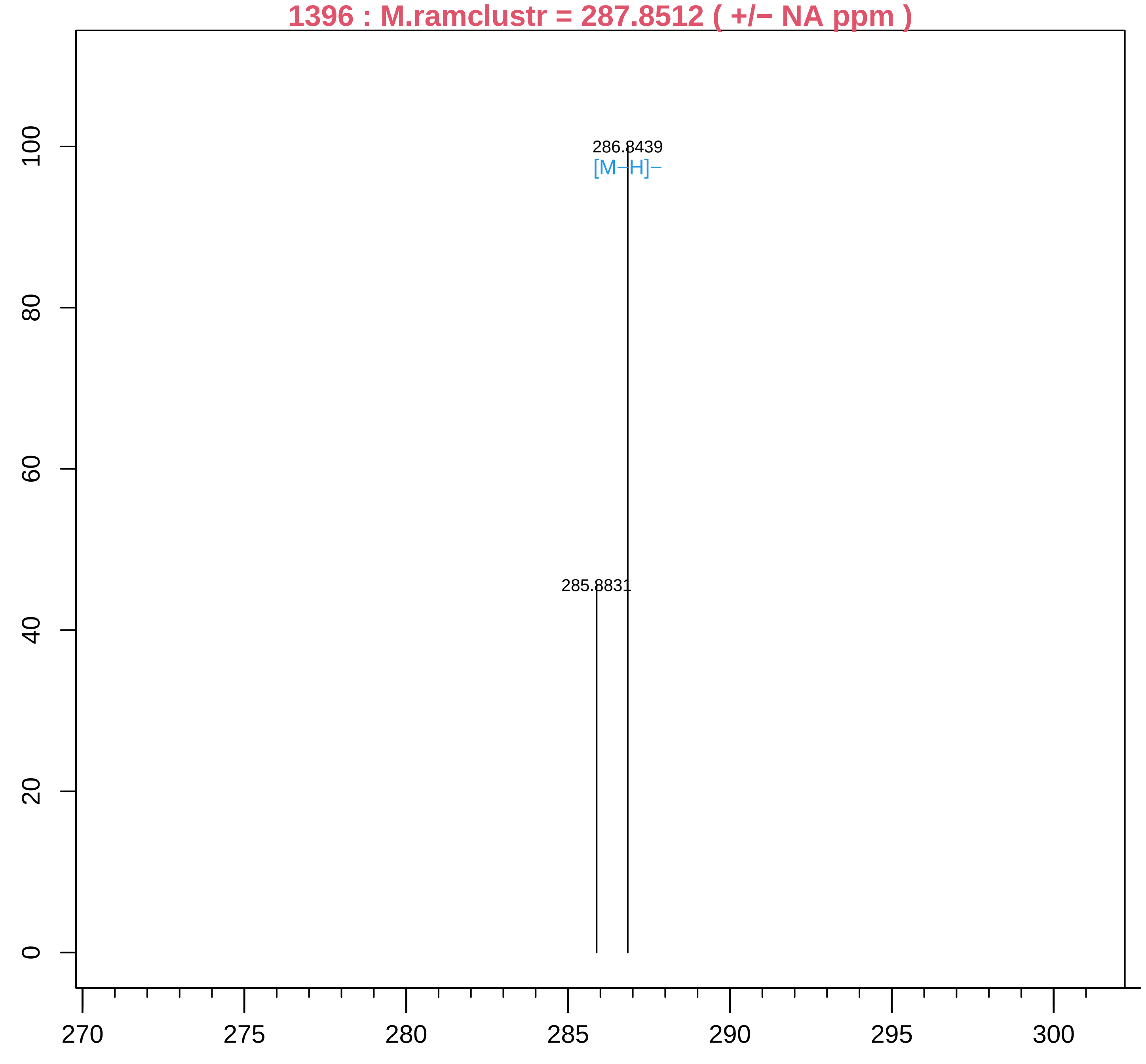
1396 : M.ramclustr = 287.8512 (+/− NA ppm)
286.8439
[M−H]−
285.8831
0
20
40
60
80
100
270
275
280
285
290
295
300

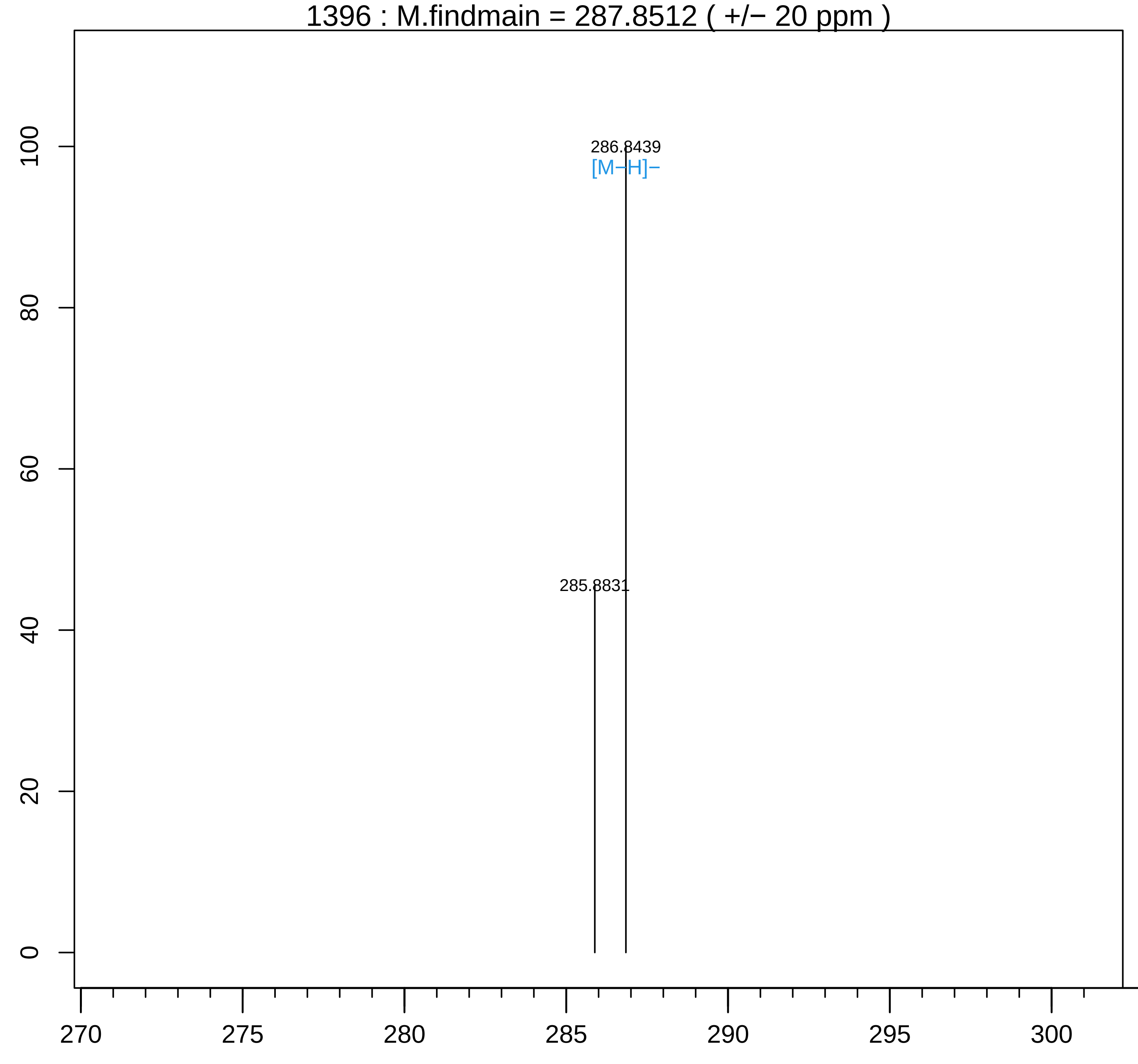
1396 : M.findmain = 287.8512 (+/− 20 ppm)
286.8439
[M−H]−
285.8831
0
20
40
60
80
100
270
275
280
285
290
295
300

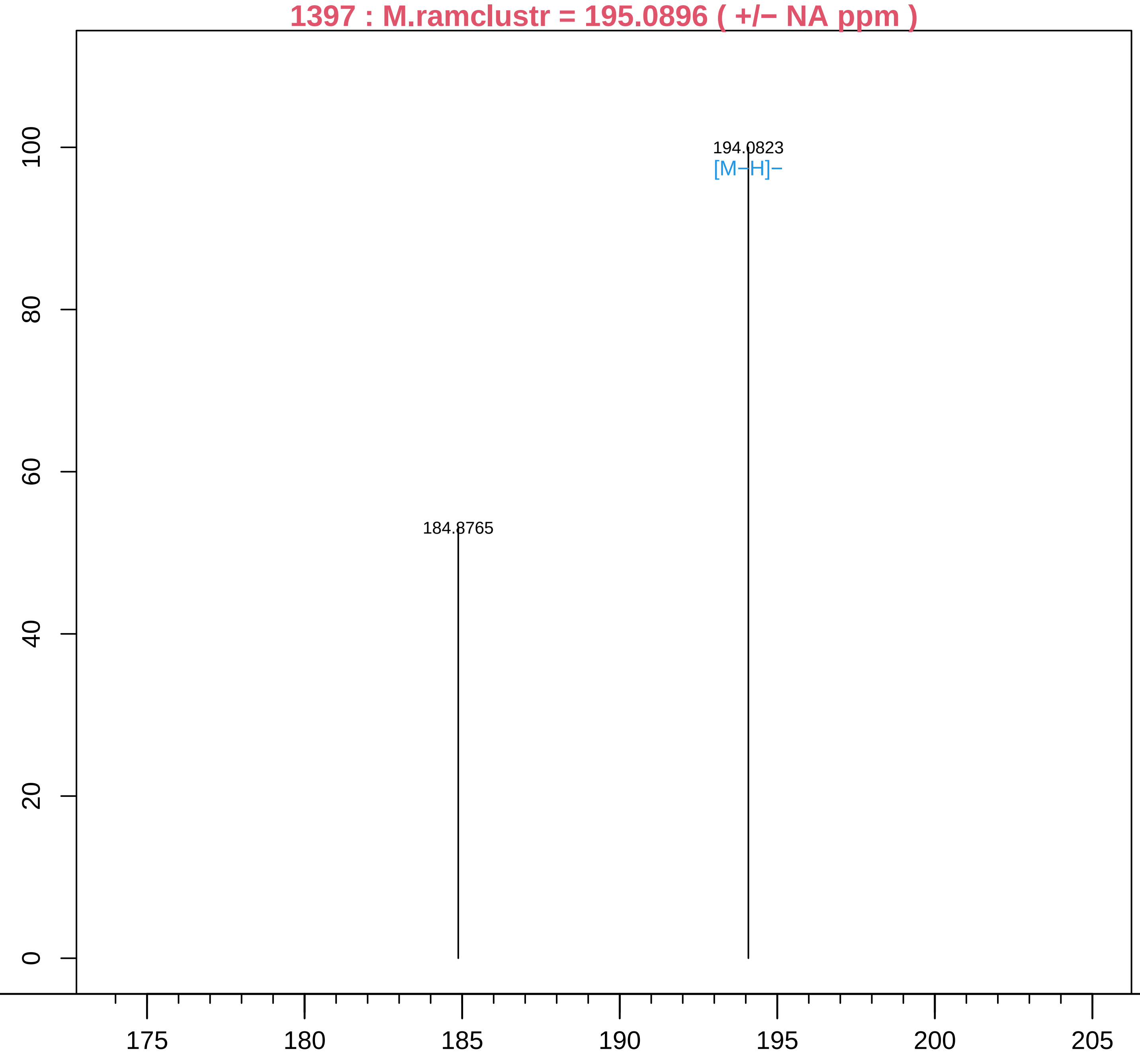
1397 : M.ramclustr = 195.0896 (+/− NA ppm)
194.0823
[M−H]−
184.8765
0
20
40
60
80
100
175
180
185
190
195
200
205

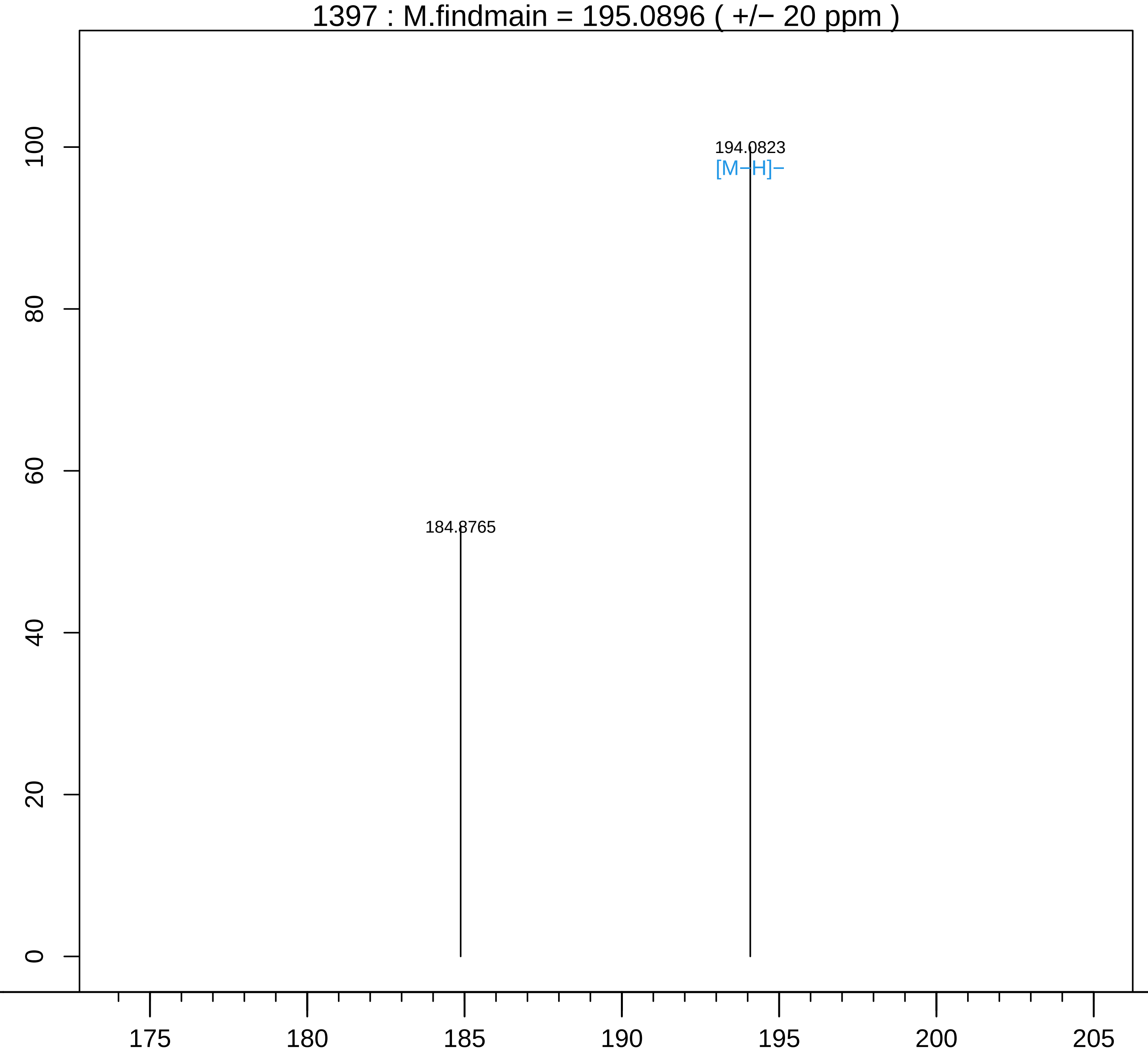
1397 : M.findmain = 195.0896 (+/− 20 ppm)
194.0823
[M−H]−
184.8765
0
20
40
60
80
100
175
180
185
190
195
200
205

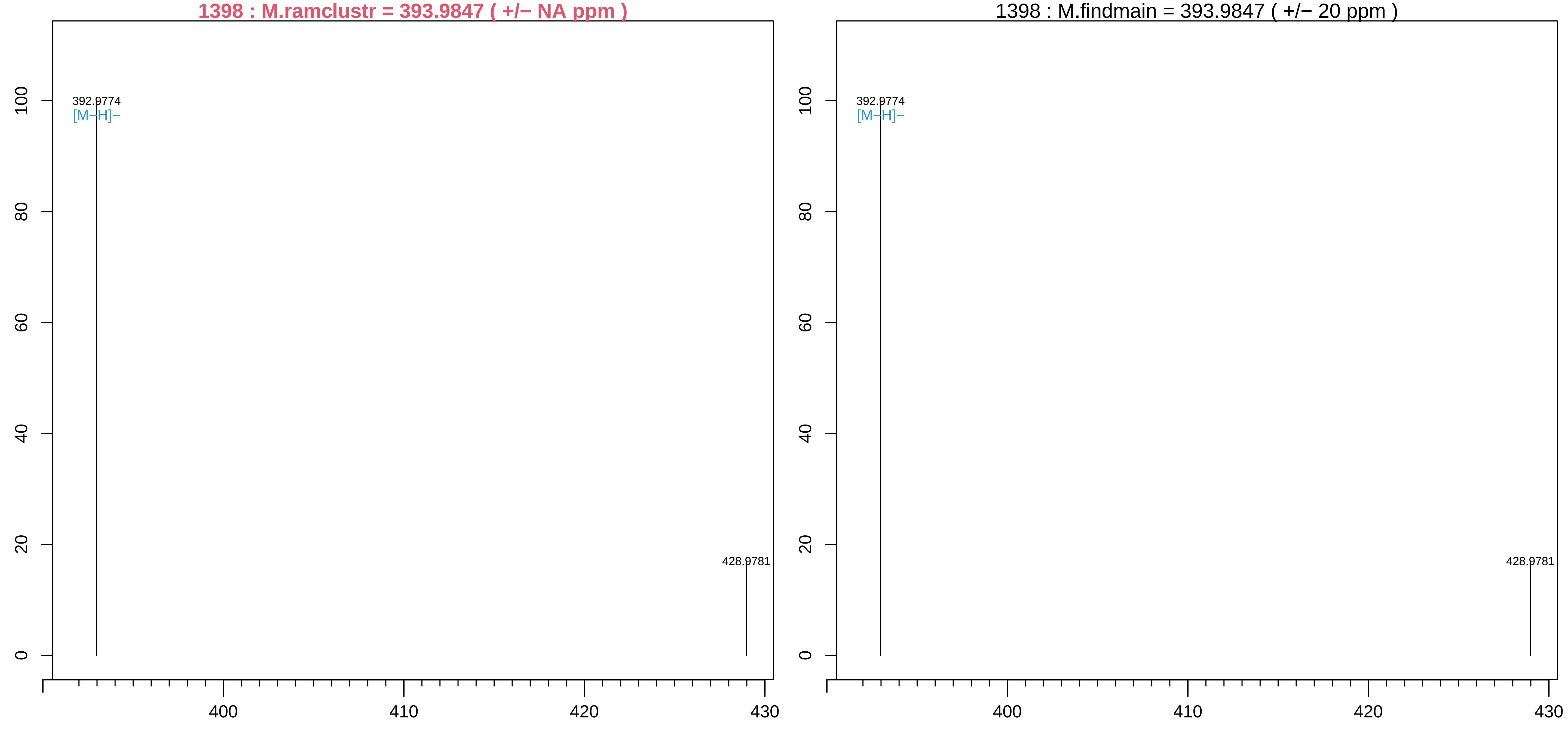
1398 : M.ramclustr = 393.9847 (+/− NA ppm)
392.9774
[M−H]−
428.9781
0
20
40
60
80
100
400
410
420
430
1398 : M.findmain = 393.9847 (+/− 20 ppm)
392.9774
[M−H]−
428.9781
0
20
40
60
80
100
400
410
420
430

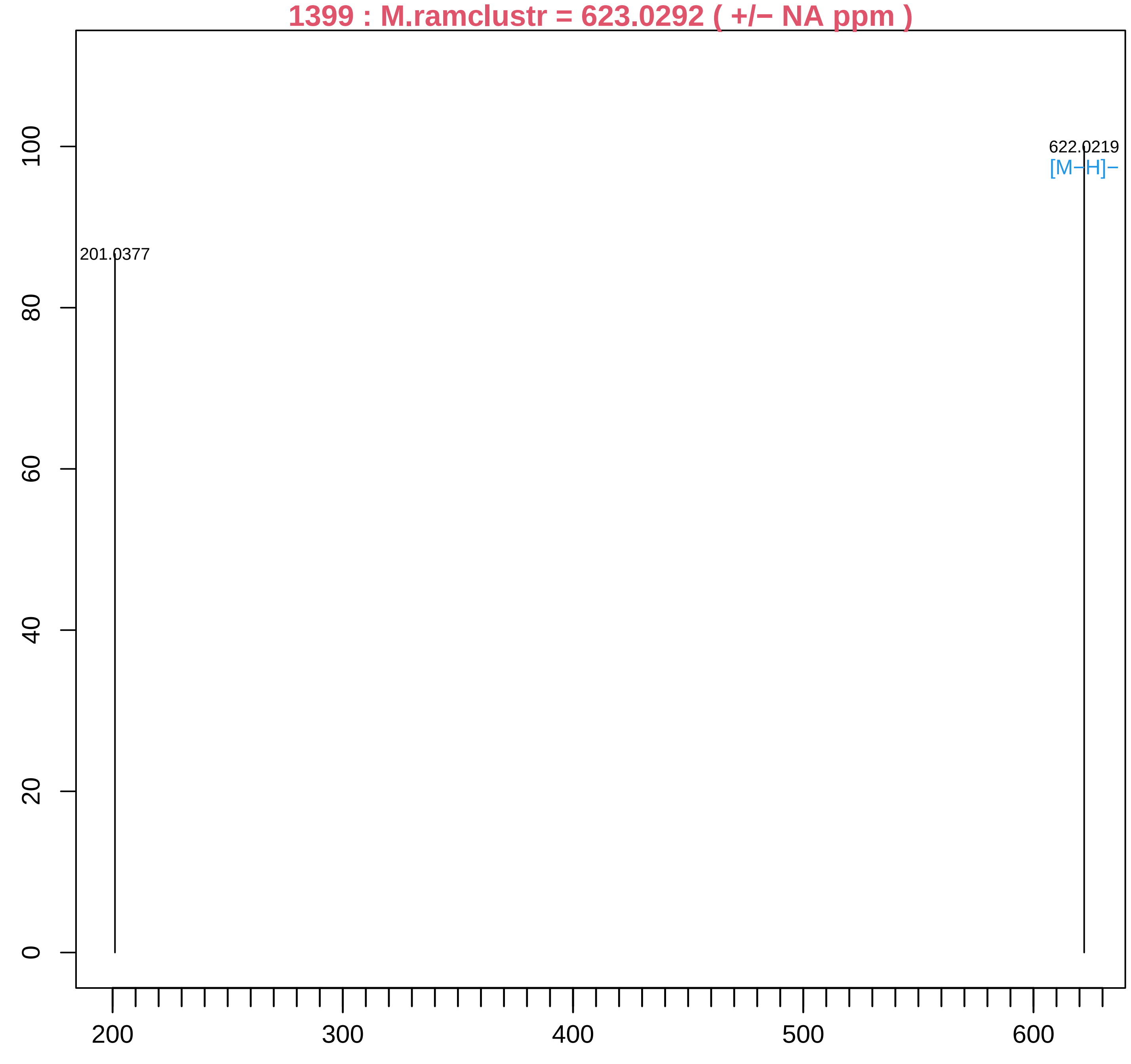
1399 : M.ramclustr = 623.0292 (+/− NA ppm)
622.0219
[M−H]−
201.0377
0
20
40
60
80
100
200
300
400
500
600

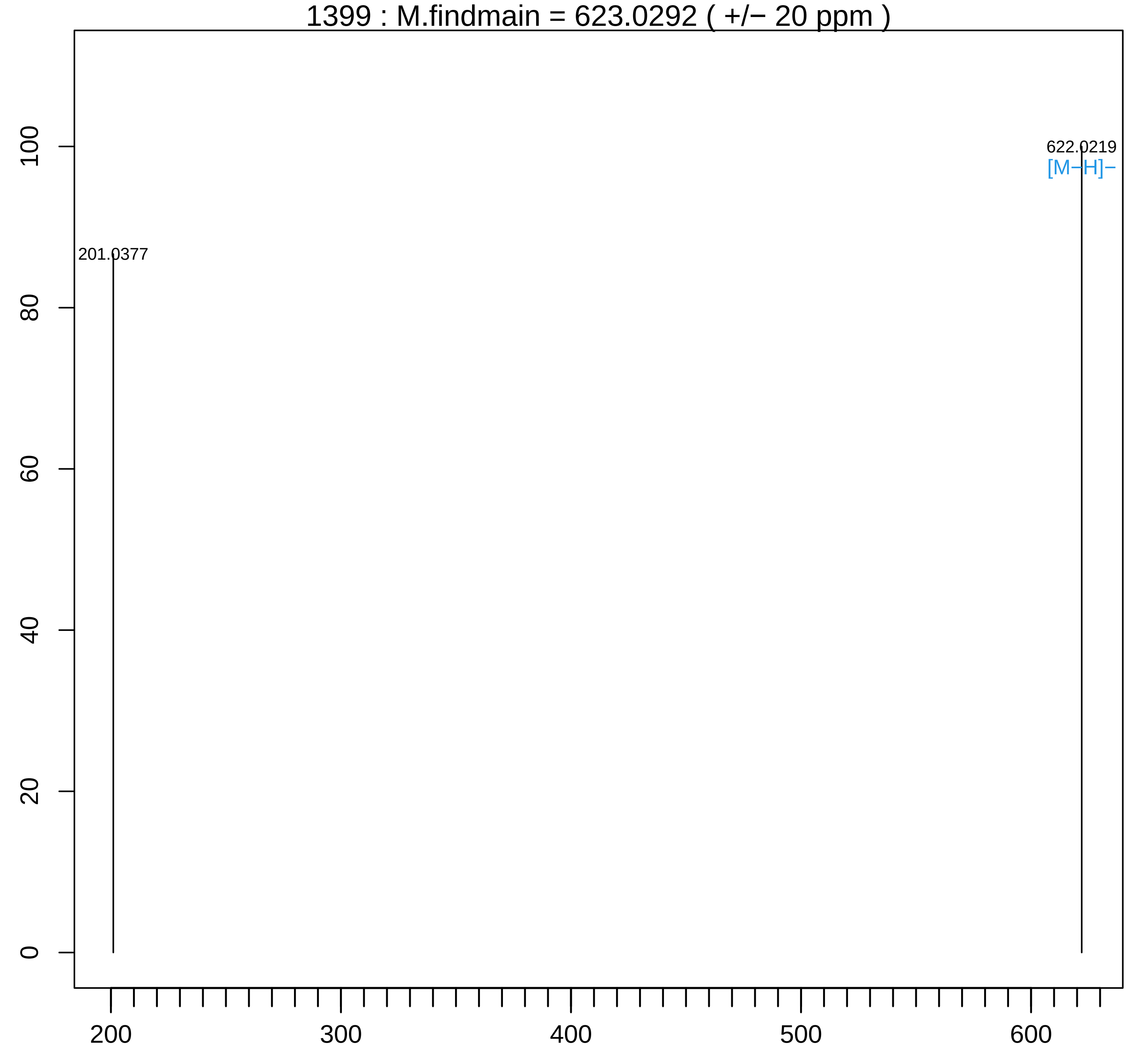
1399 : M.findmain = 623.0292 (+/− 20 ppm)
622.0219
[M−H]−
201.0377
0
20
40
60
80
100
200
300
400
500
600

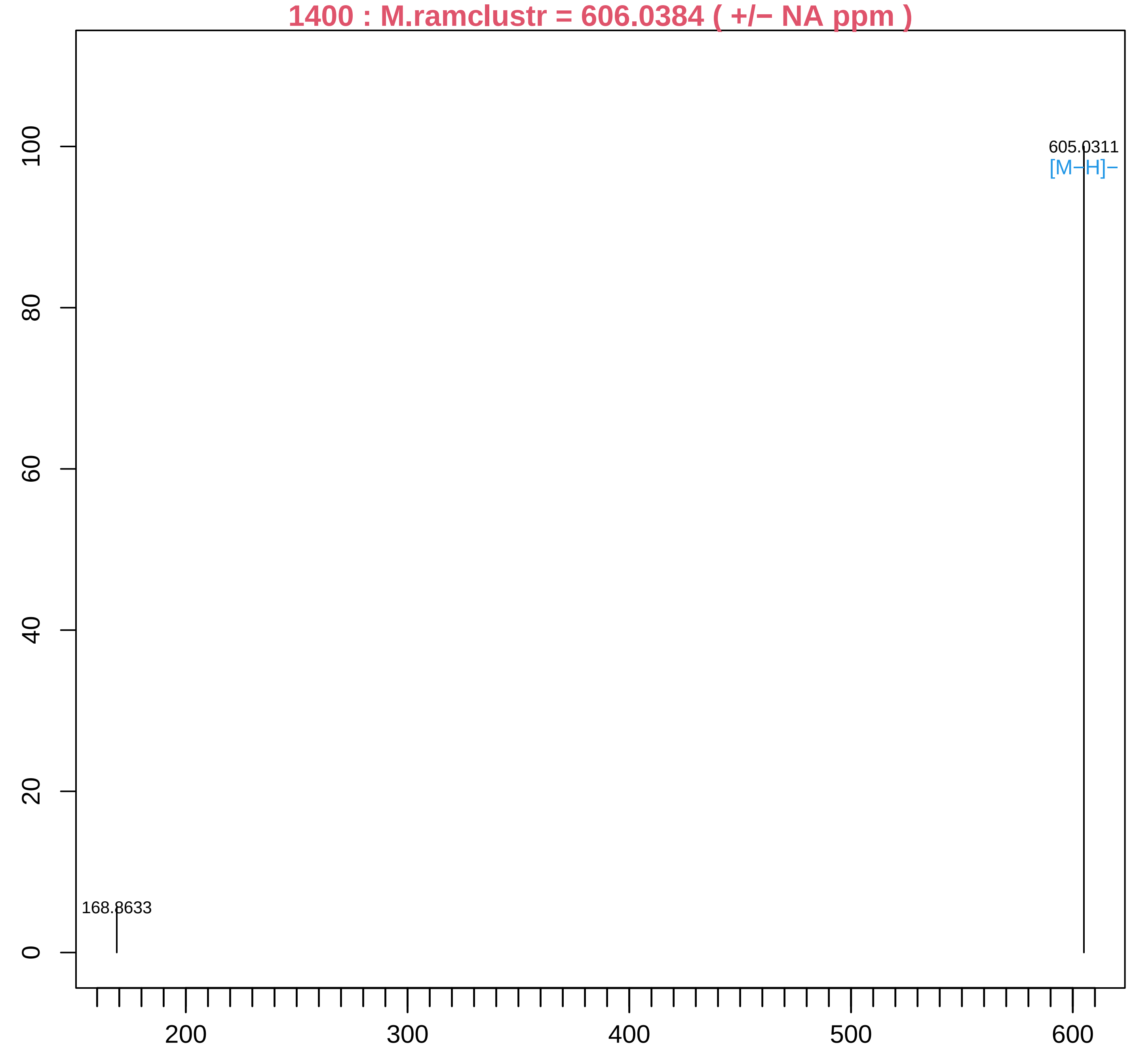

1400 : M.ramclustr = 606.0384 (+/− NA ppm)
605.0311
[M−H]−
168.8633
0
20
40
60
80
100
200
300
400
500
600

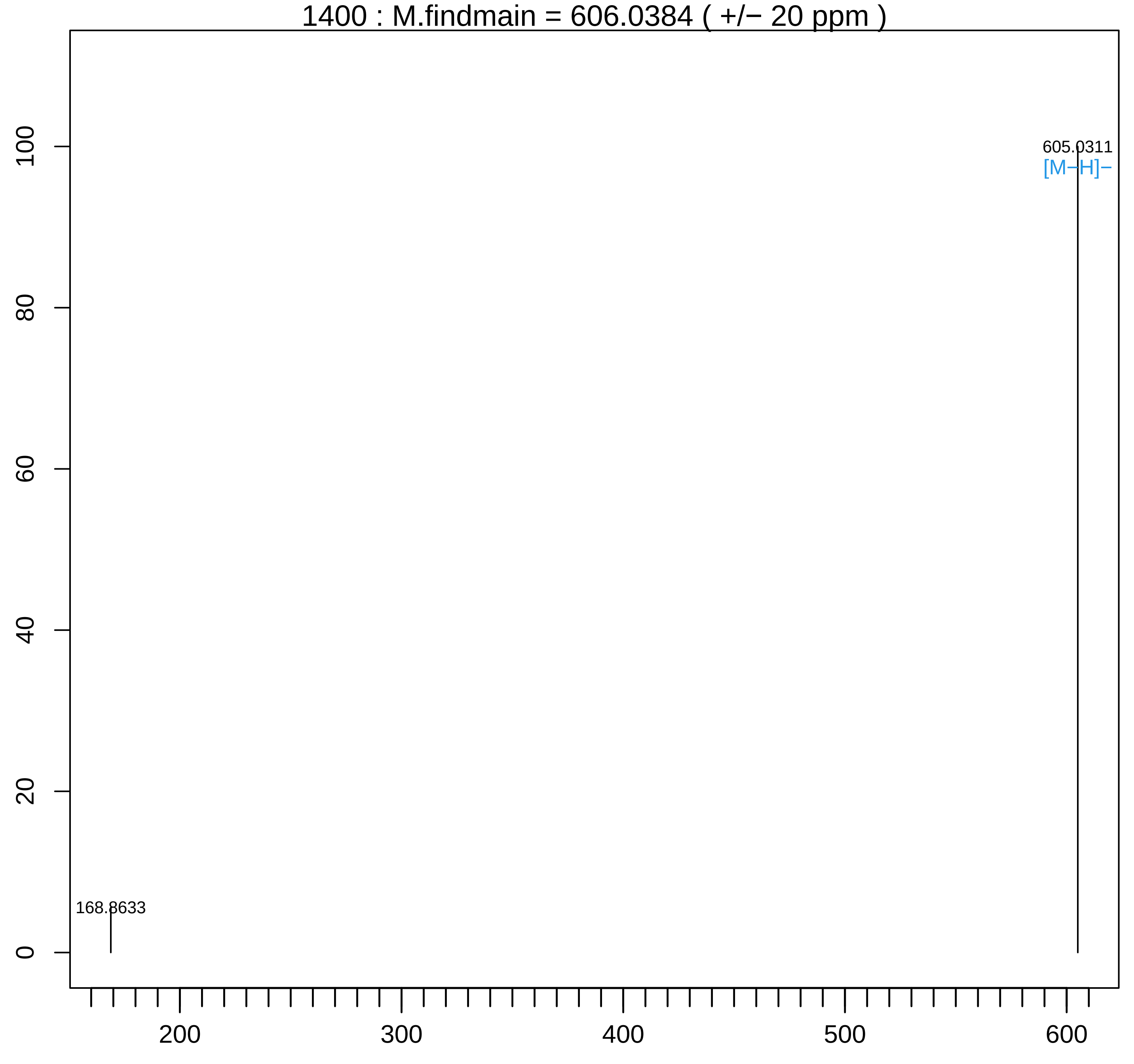

1400 : M.findmain = 606.0384 (+/− 20 ppm)
605.0311
[M−H]−
168.8633
0
20
40
60
80
100
200
300
400
500
600

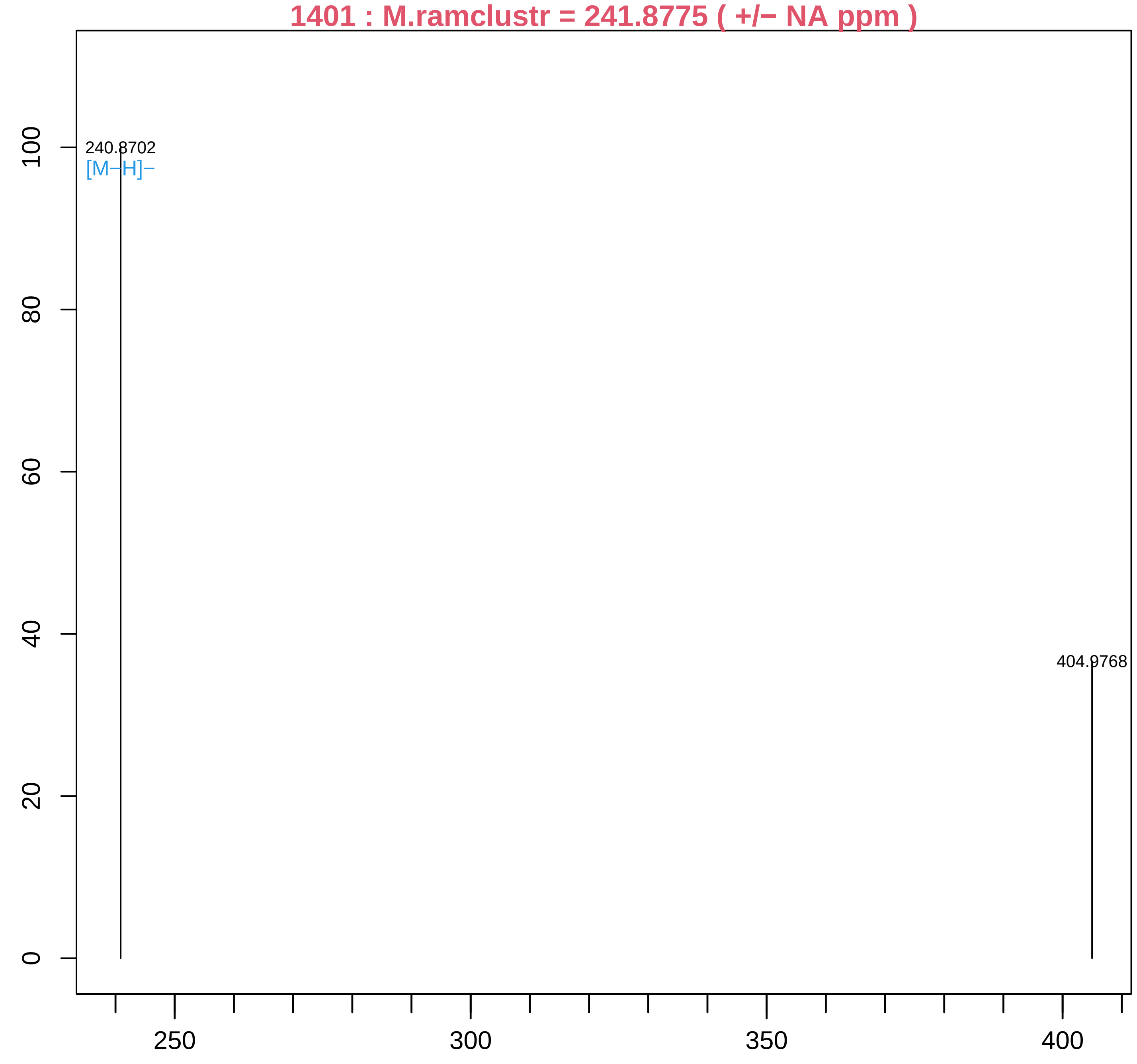
1401 : M.ramclustr = 241.8775 (+/− NA ppm)
240.8702
[M−H]−
404.9768
0
20
40
60
80
100
250
300
350
400

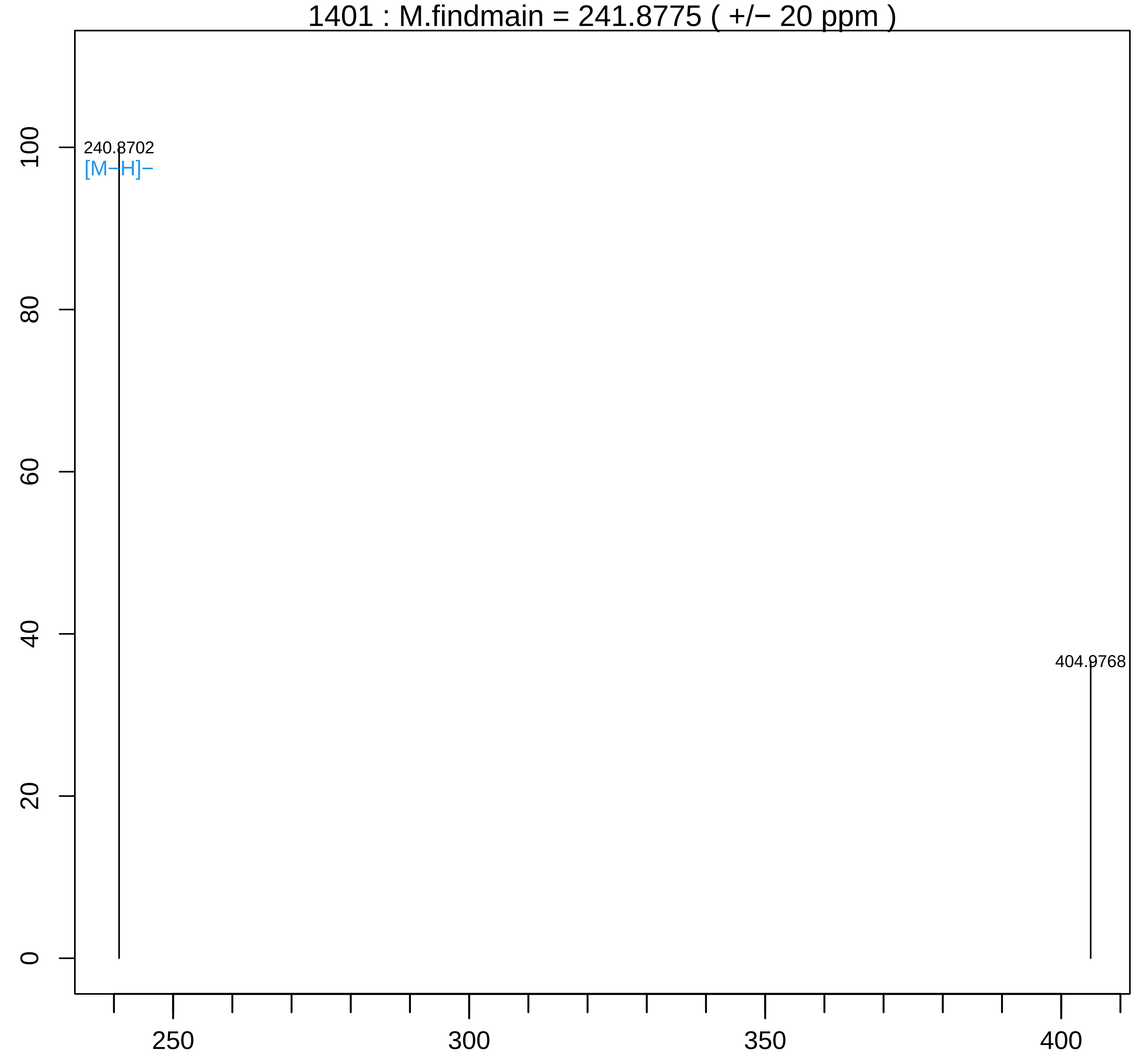
1401 : M.findmain = 241.8775 (+/− 20 ppm)
240.8702
[M−H]−
404.9768
0
20
40
60
80
100
250
300
350
400

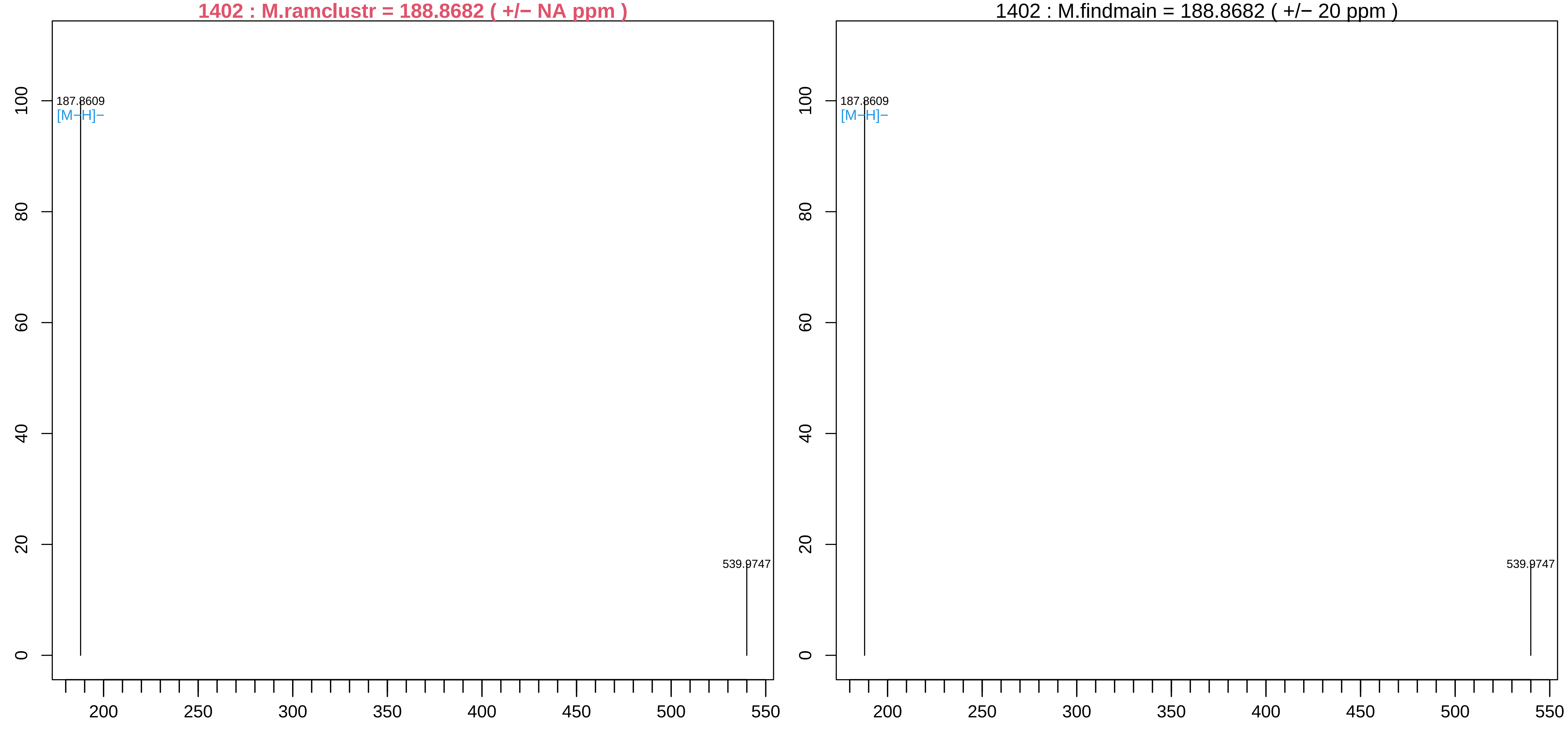
1402 : M.ramclustr = 188.8682 (+/− NA ppm)
187.8609
[M−H]−
539.9747
1402 : M.findmain = 188.8682 (+/− 20 ppm)
187.8609
[M−H]−
539.9747

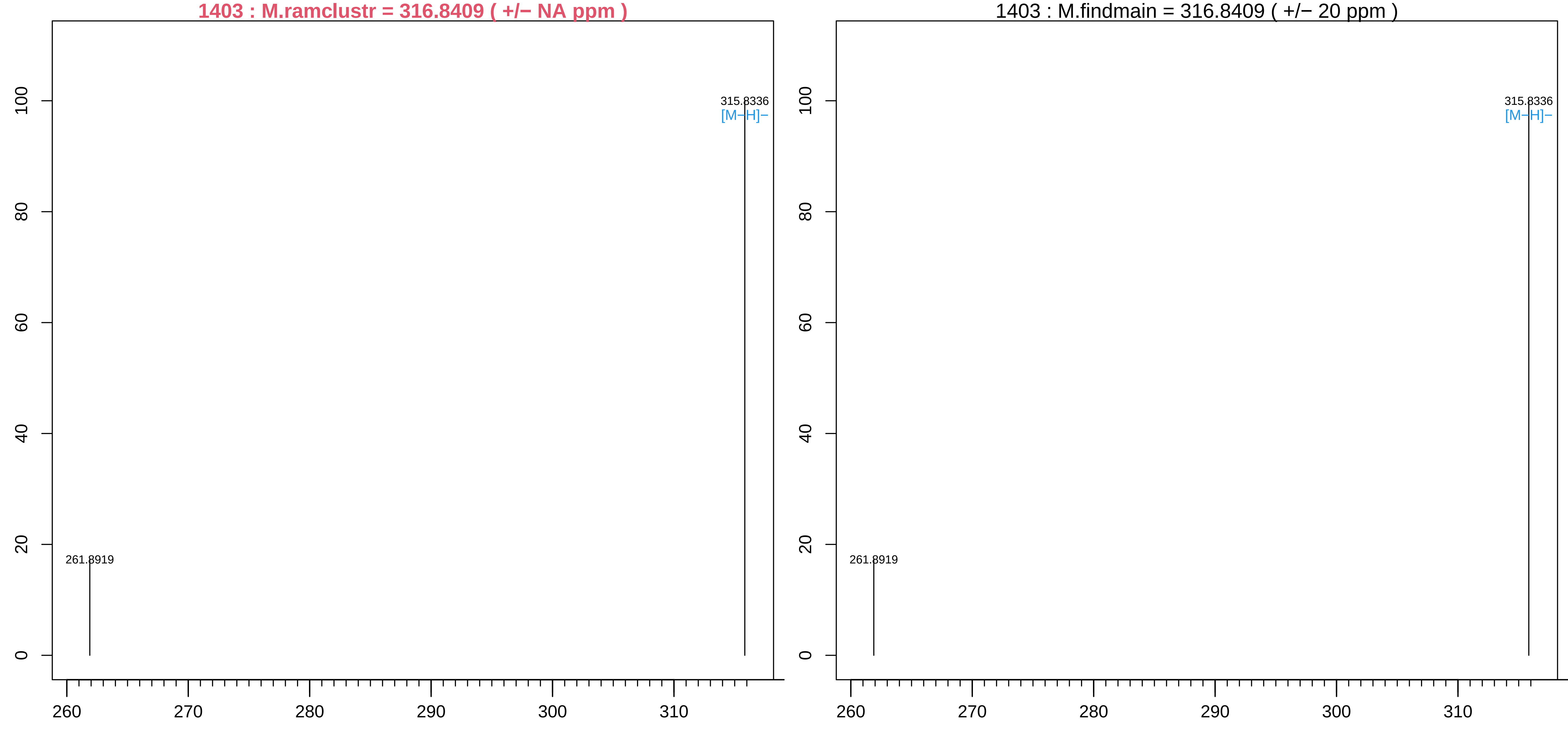
1403 : M.ramclustr = 316.8409 (+/− NA ppm)
315.8336
[M−H]−
261.8919
1403 : M.findmain = 316.8409 (+/− 20 ppm)
315.8336
[M−H]−
261.8919
0
20
40
60
80
100
260
270
280
290
300
310

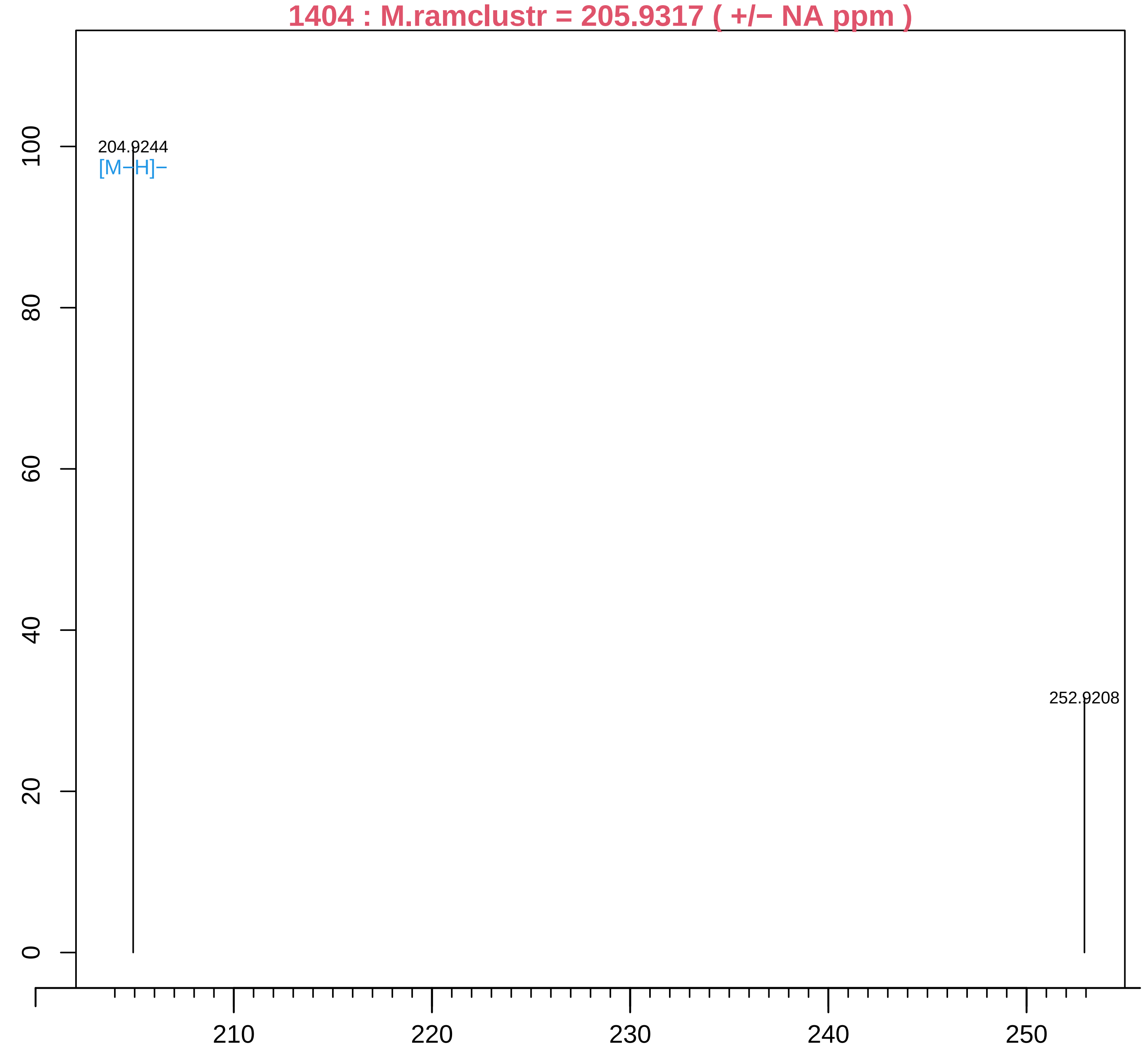
1404 : M.ramclustr = 205.9317 (+/− NA ppm)
204.9244
[M−H]−
252.9208
0
20
40
60
80
100
210
220
230
240
250

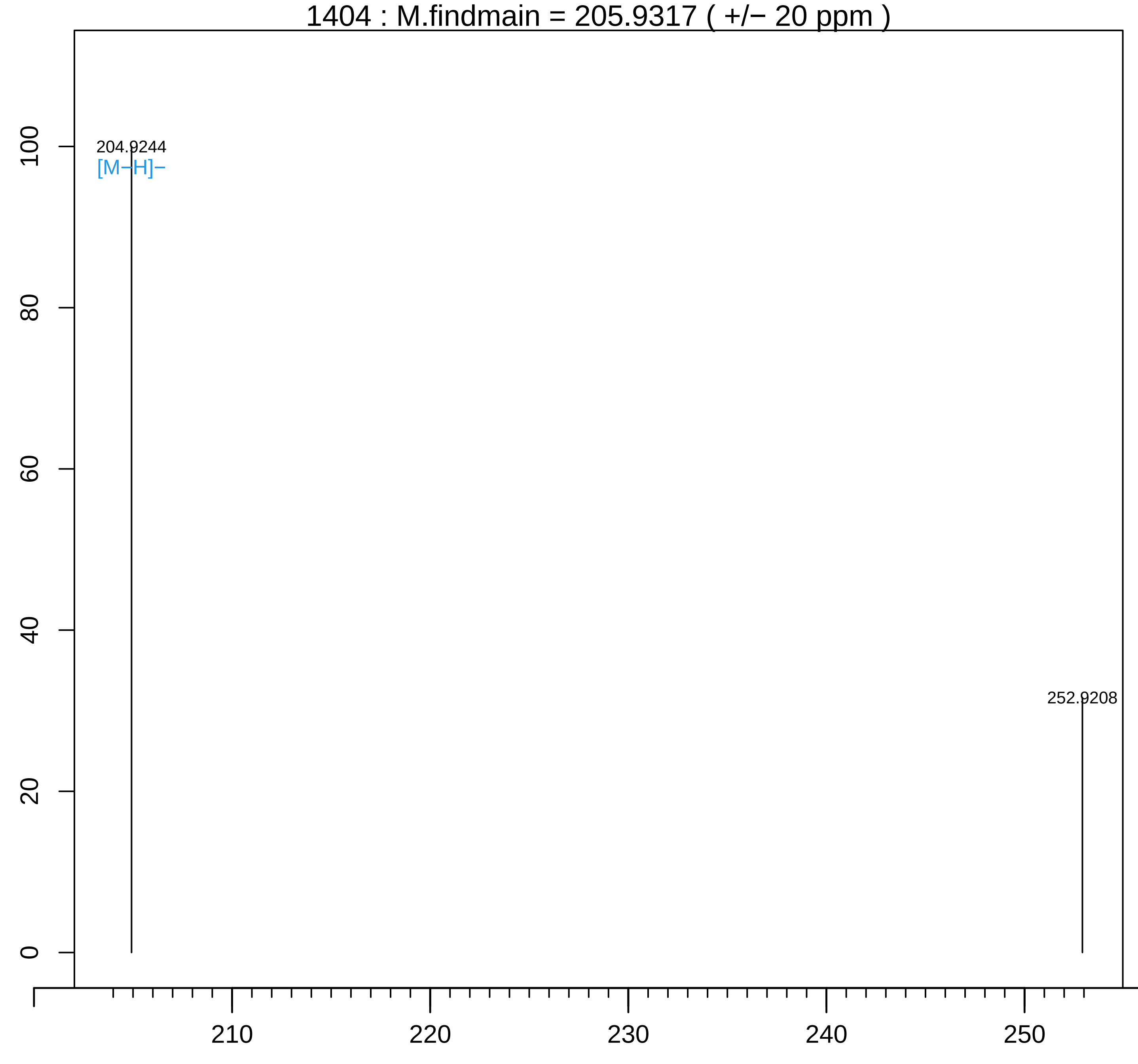
1404 : M.findmain = 205.9317 (+/− 20 ppm)
204.9244
[M−H]−
252.9208
0
20
40
60
80
100
210
220
230
240
250

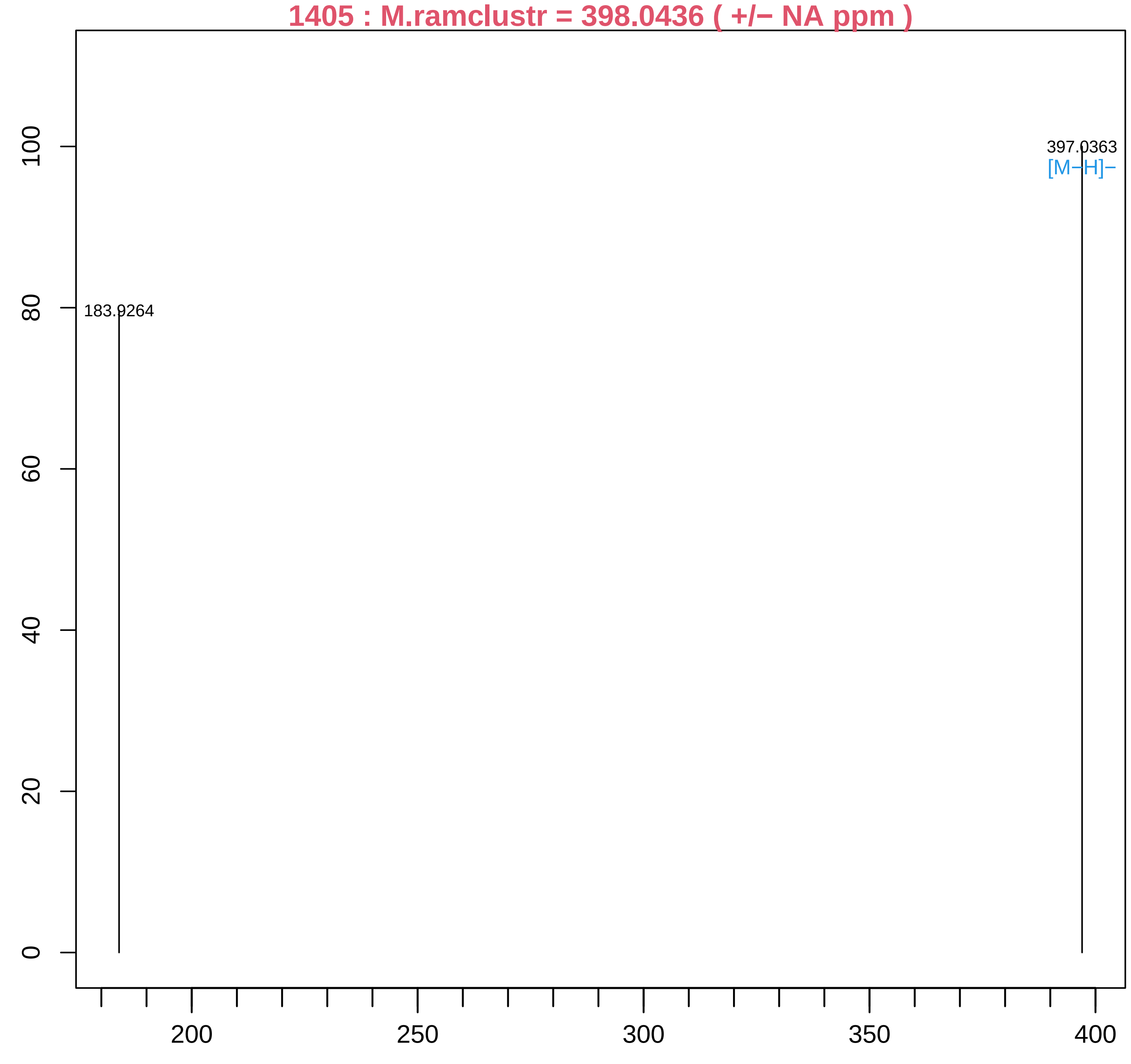

1405 : M.ramclustr = 398.0436 (+/− NA ppm)
397.0363
[M−H]−
183.9264
0
20
40
60
80
100
200
250
300
350
400

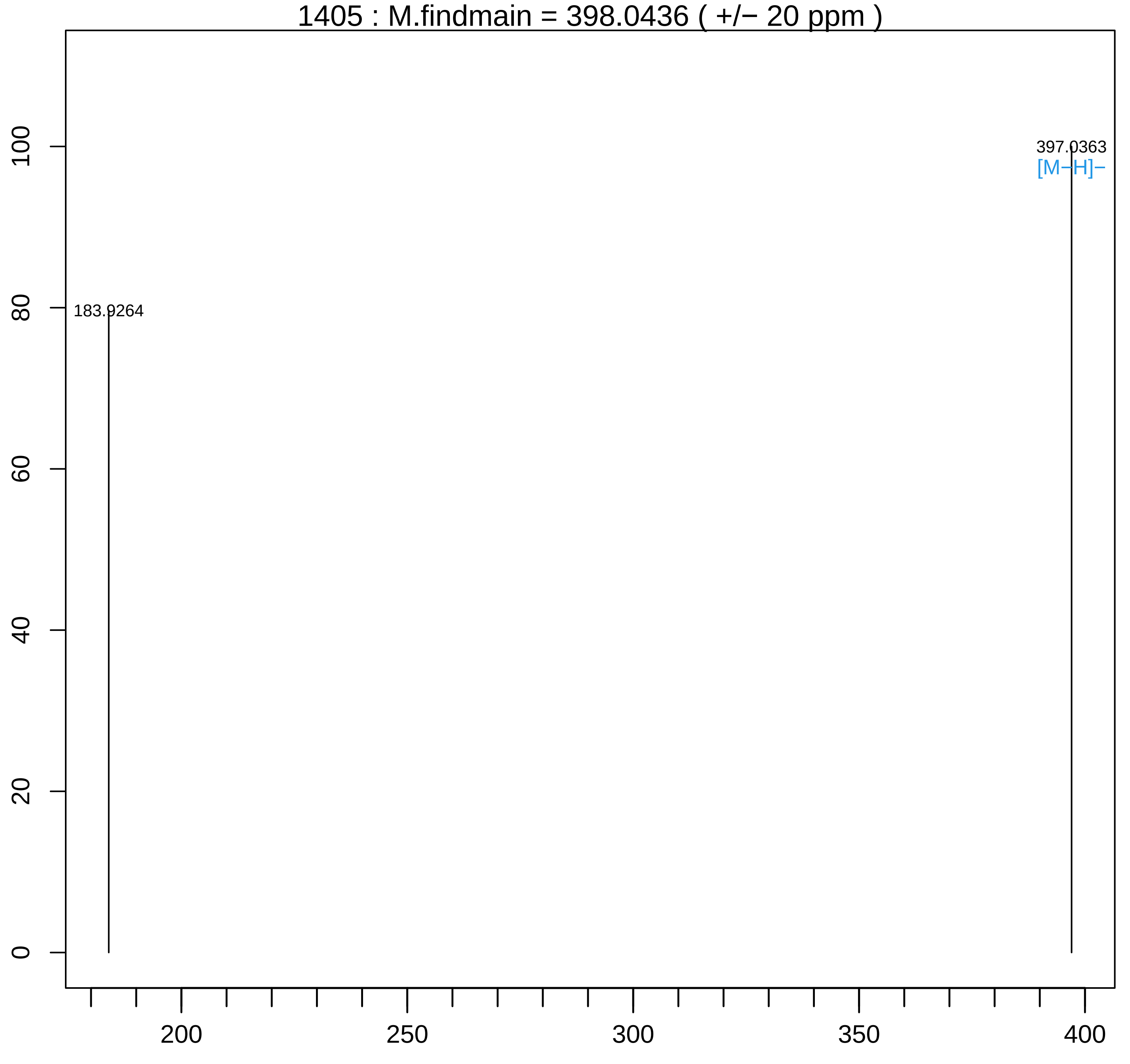

1405 : M.findmain = 398.0436 (+/− 20 ppm)
397.0363
[M−H]−
183.9264
0
20
40
60
80
100
200
250
300
350
400

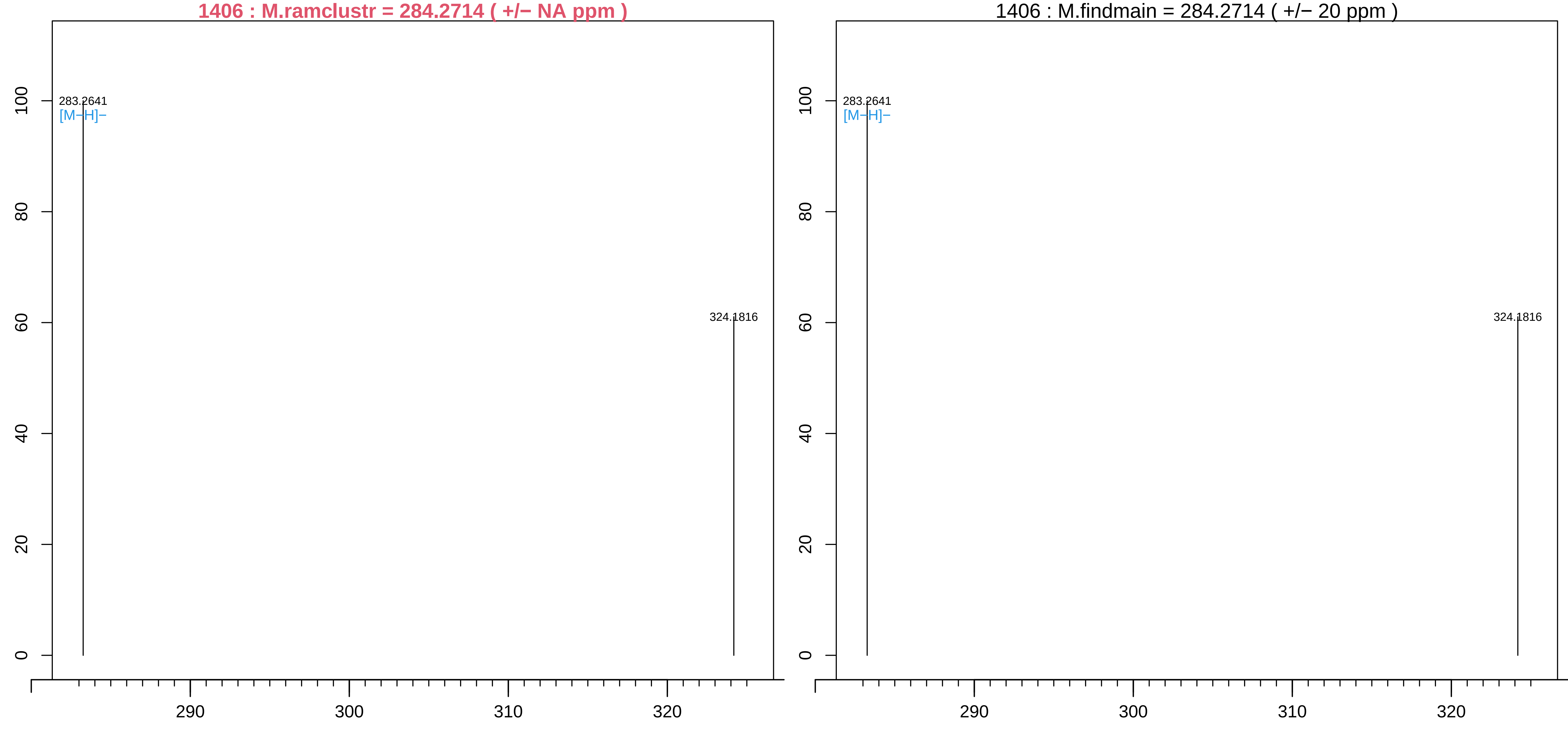

1406 : M.ramclustr = 284.2714 (+/− NA ppm)
283.2641
[M−H]−
324.1816
1406 : M.findmain = 284.2714 (+/− 20 ppm)
283.2641
[M−H]−
324.1816
0
20
40
60
80
100
290
300
310
320

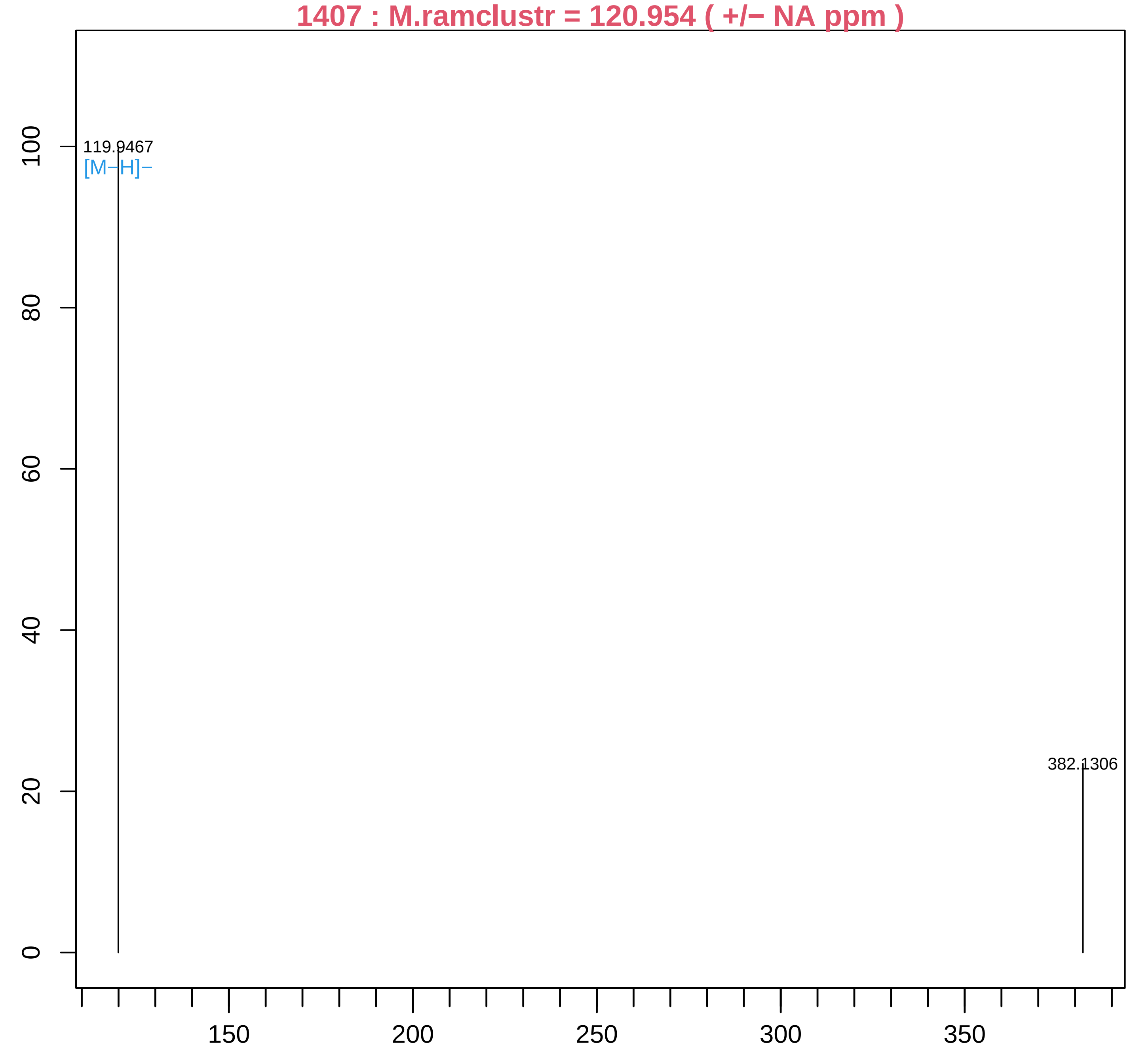
1407 : M.ramclustr = 120.954 (+/− NA ppm)
119.9467
[M−H]−
382.1306
0
20
40
60
80
100
150
200
250
300
350

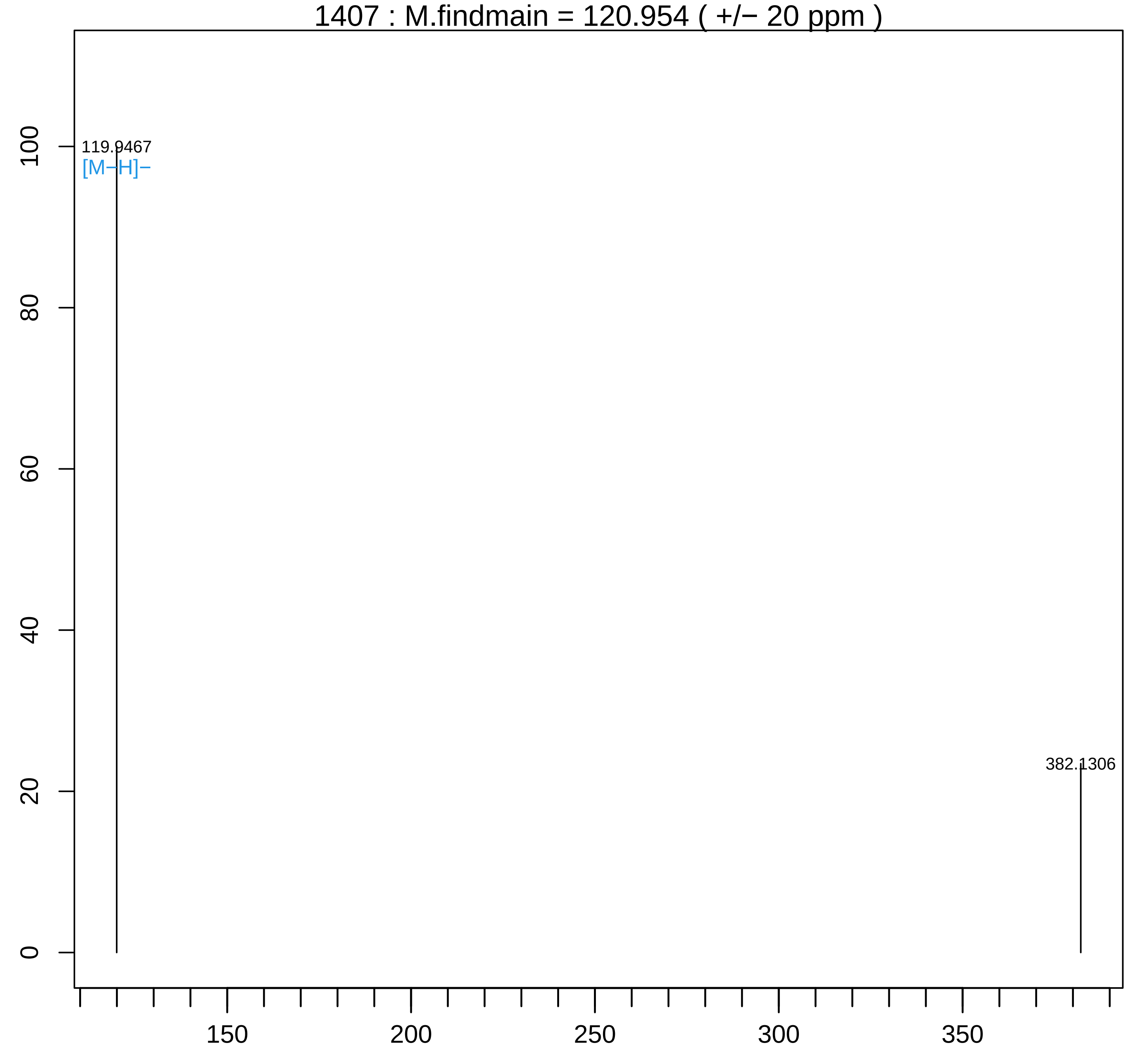
1407 : M.findmain = 120.954 (+/− 20 ppm)
119.9467
[M−H]−
382.1306
0
20
40
60
80
100
150
200
250
300
350

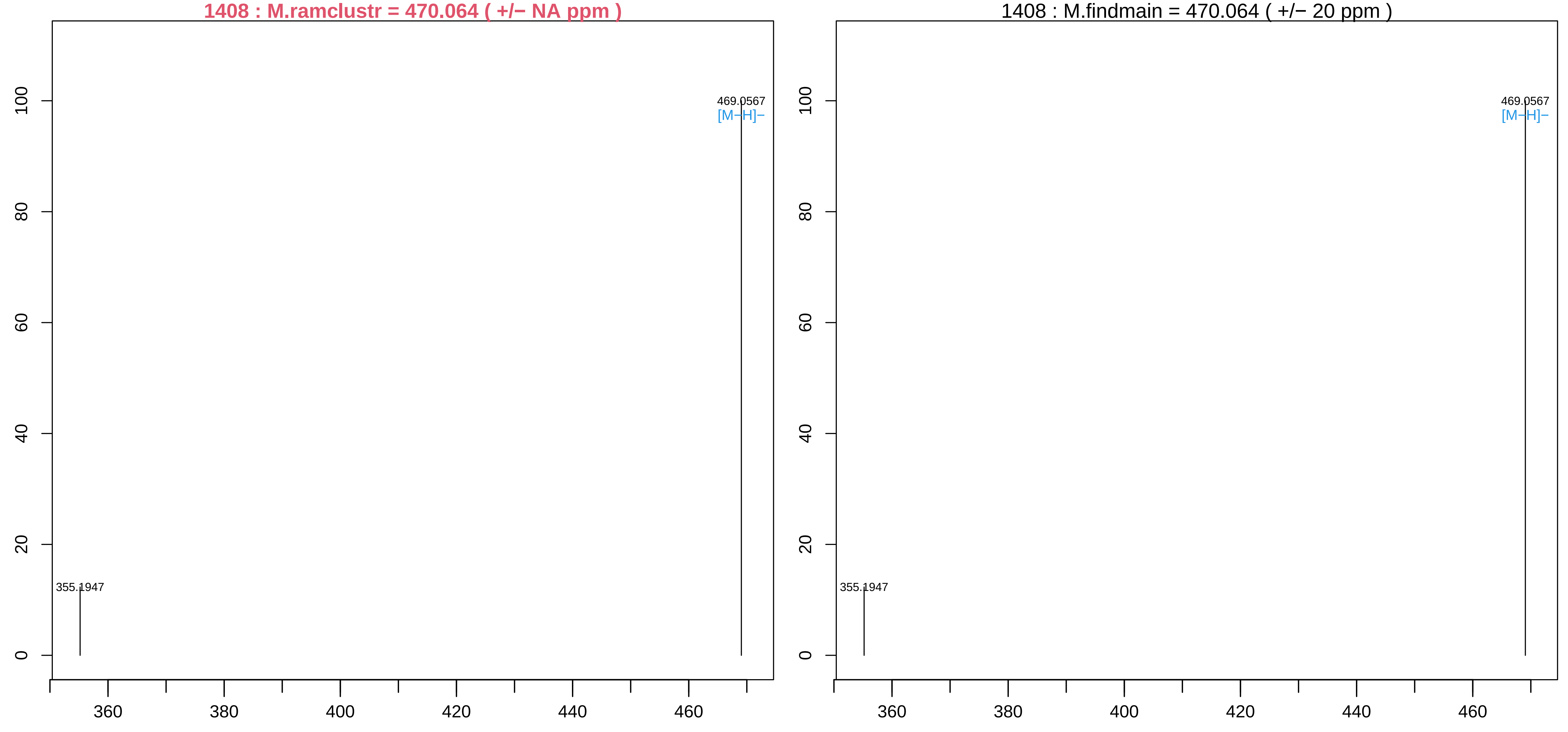

1408 : M.ramclustr = 470.064 (+/− NA ppm)
469.0567
[M−H]−
355.1947
0
20
40
60
80
100
360
380
400
420
440
460
1408 : M.findmain = 470.064 (+/− 20 ppm)
469.0567
[M−H]−
355.1947
0
20
40
60
80
100
360
380
400
420
440
460

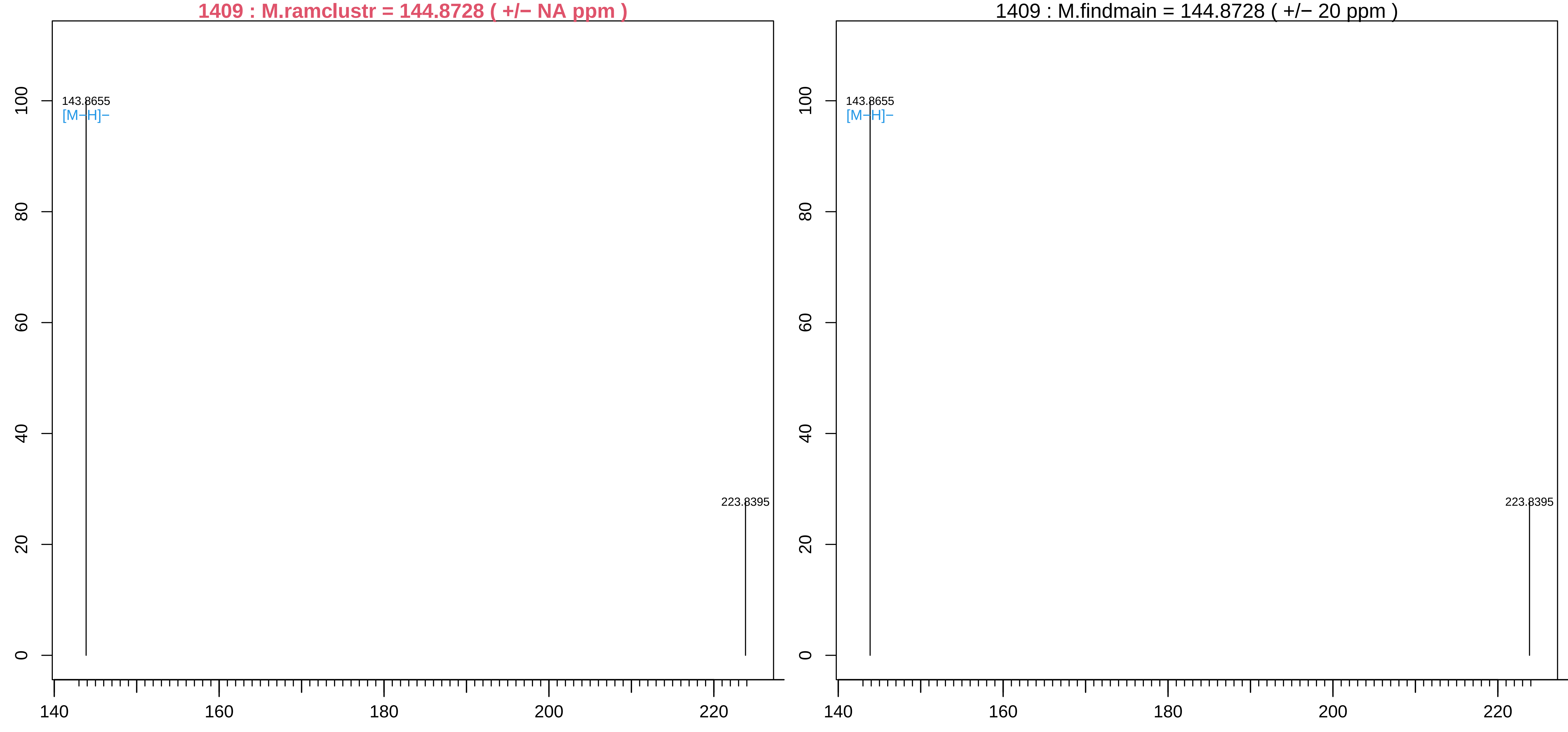
1409 : M.ramclustr = 144.8728 (+/− NA ppm)
143.8655
[M−H]−
223.8395
1409 : M.findmain = 144.8728 (+/− 20 ppm)
143.8655
[M−H]−
223.8395
0
20
40
60
80
100
140
160
180
200
220

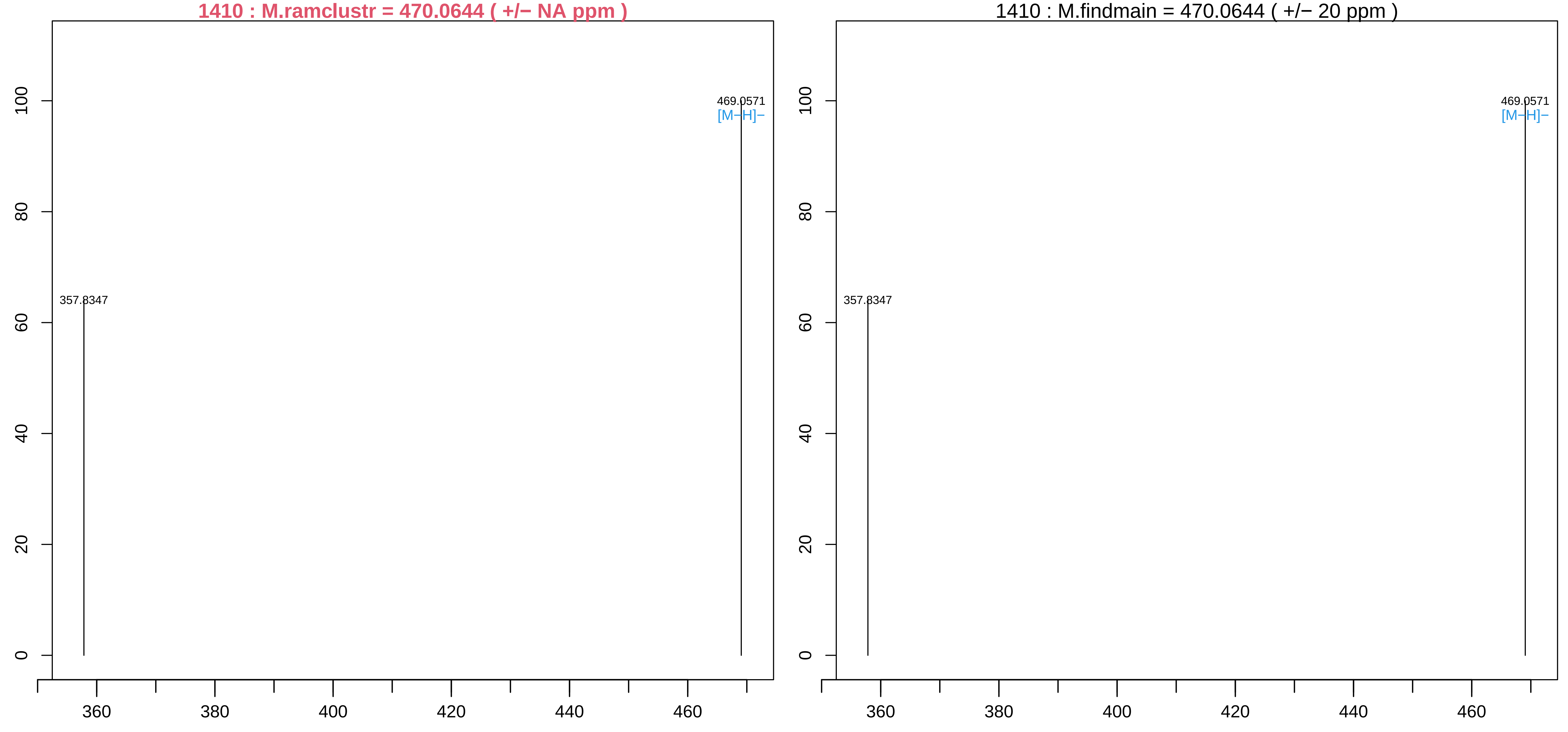

1410 : M.ramclustr = 470.0644 (+/− NA ppm)
469.0571
[M−H]−
357.8347
1410 : M.findmain = 470.0644 (+/− 20 ppm)
469.0571
[M−H]−
357.8347

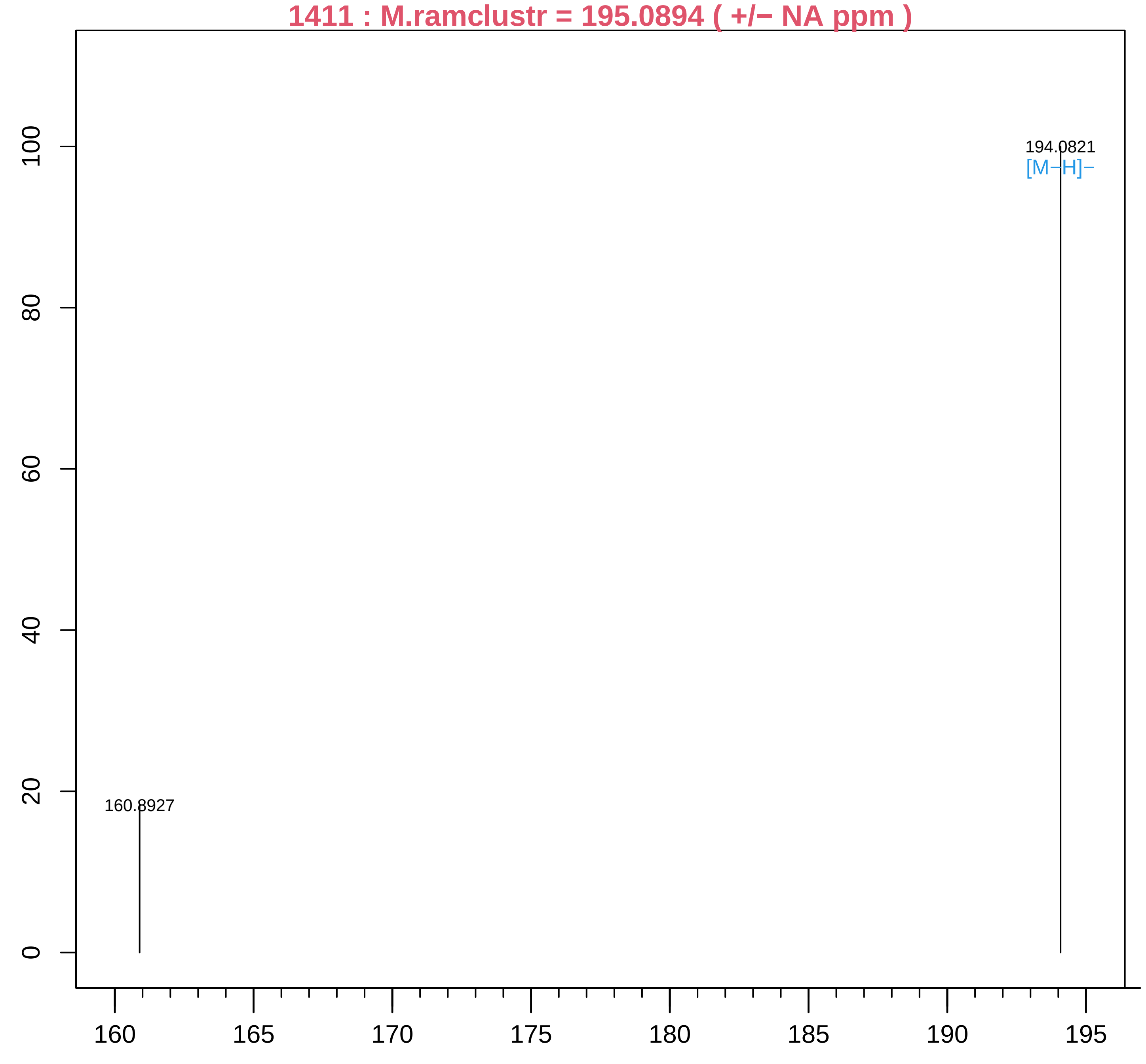

1411 : M.ramclustr = 195.0894 (+/− NA ppm)
194.0821
[M−H]−
160.8927
0
20
40
60
80
100
160
165
170
175
180
185
190
195

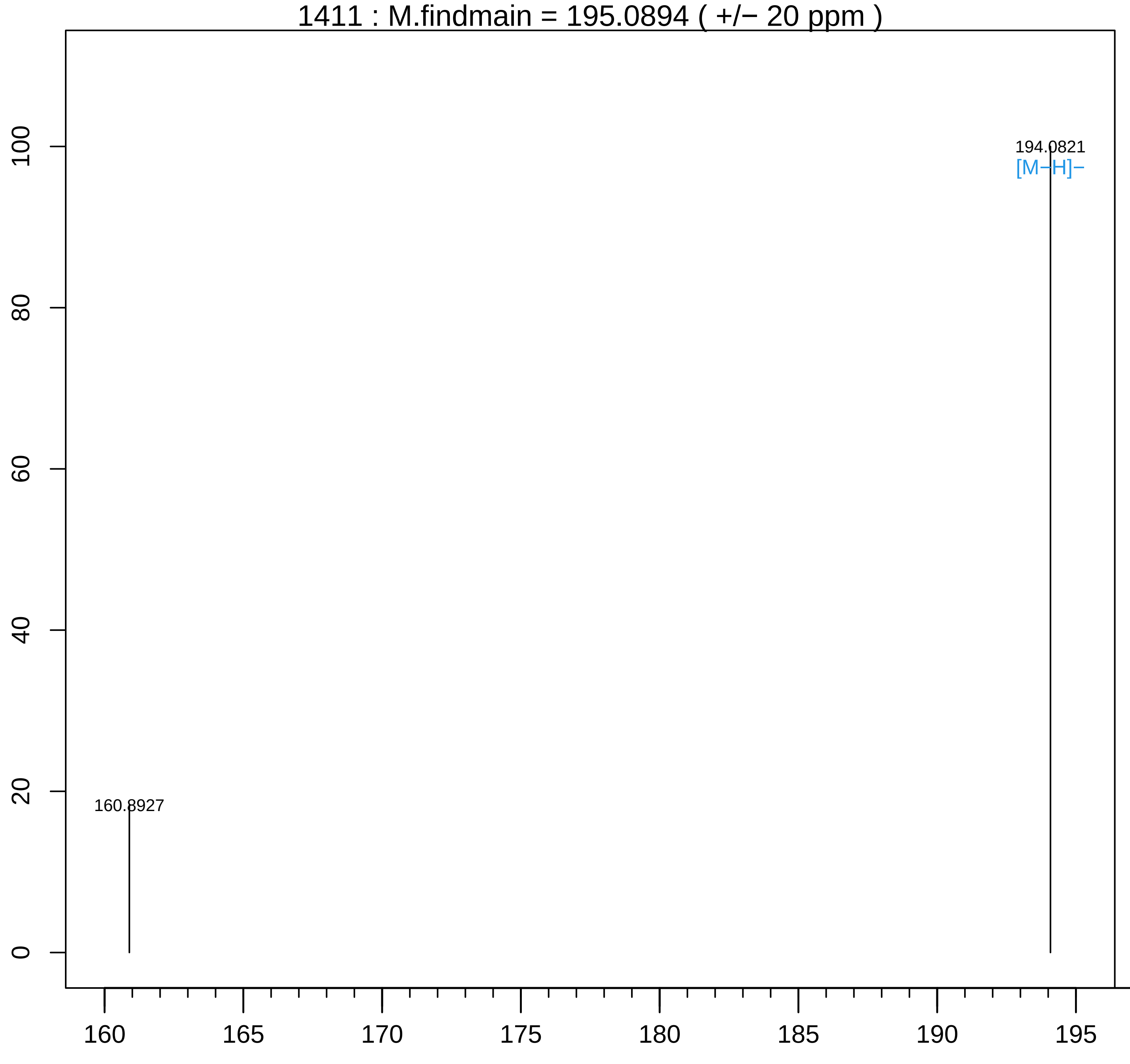

1411 : M.findmain = 195.0894 (+/− 20 ppm)
194.0821
[M−H]−
160.8927
0
20
40
60
80
100
160
165
170
175
180
185
190
195

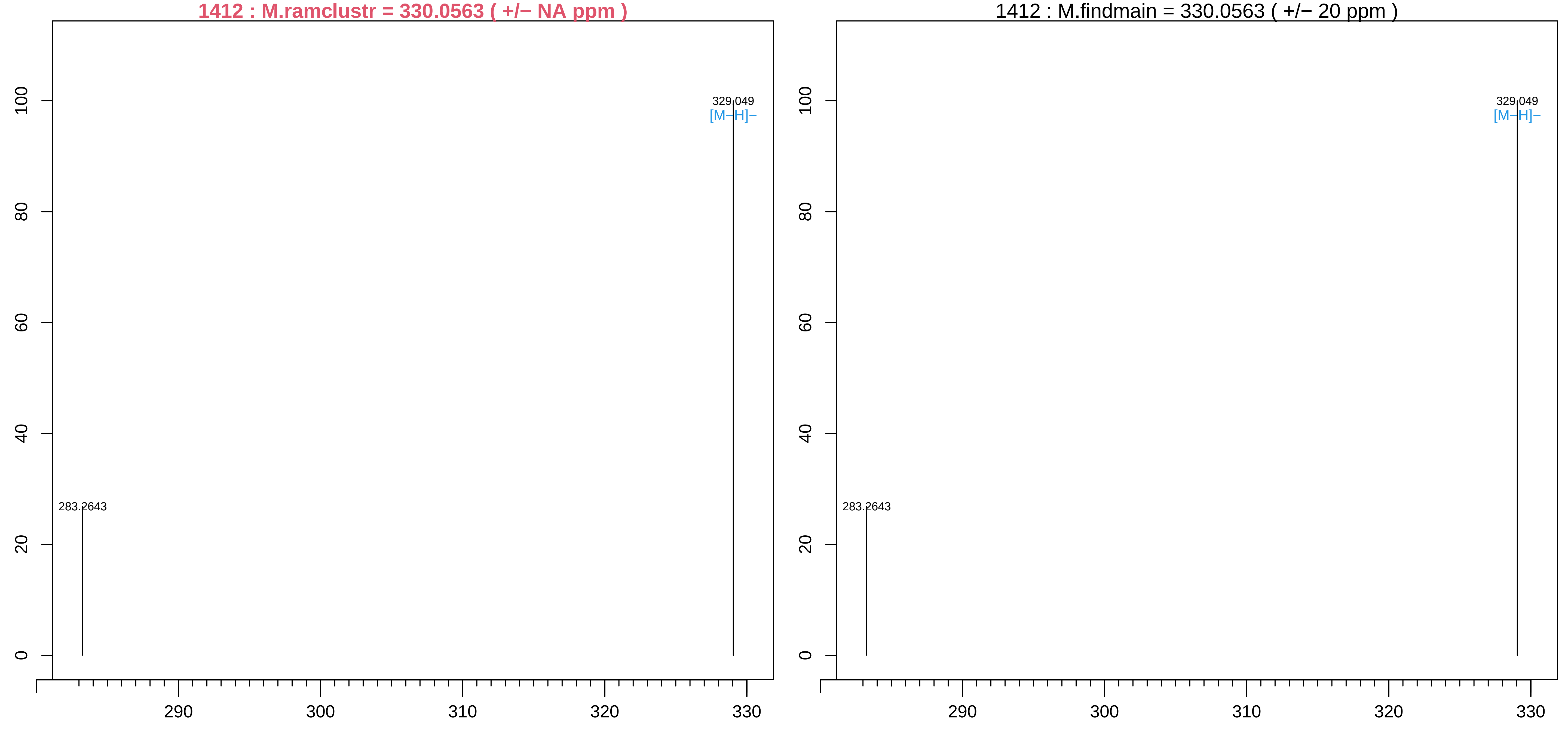
1412 : M.ramclustr = 330.0563 (+/− NA ppm)
1412 : M.findmain = 330.0563 (+/− 20 ppm)
329.049
[M−H]−
283.2643
329.049
[M−H]−
283.2643

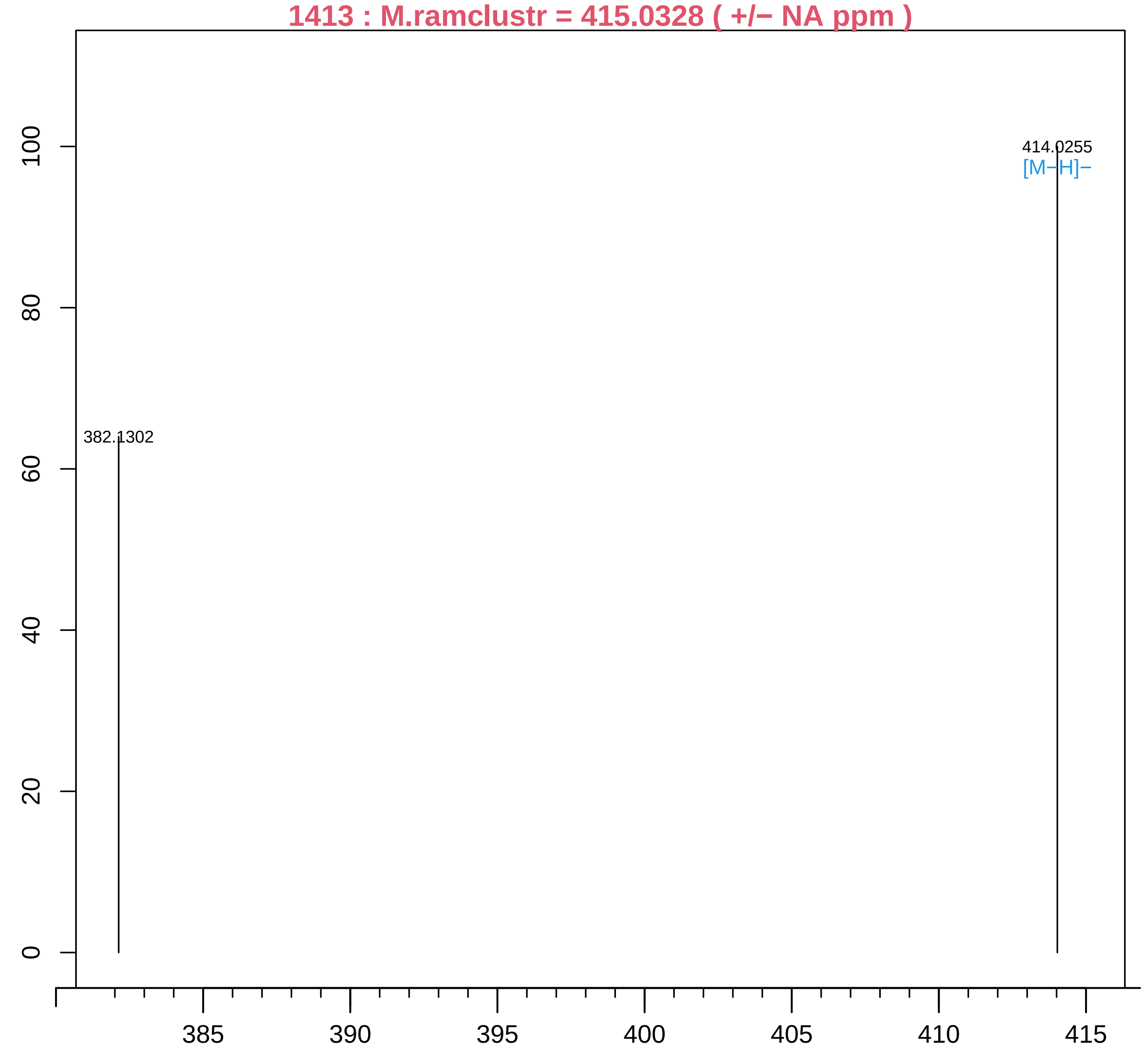
1413 : M.ramclustr = 415.0328 (+/− NA ppm)
414.0255
[M−H]−
382.1302
0
20
40
60
80
100
385
390
395
400
405
410
415

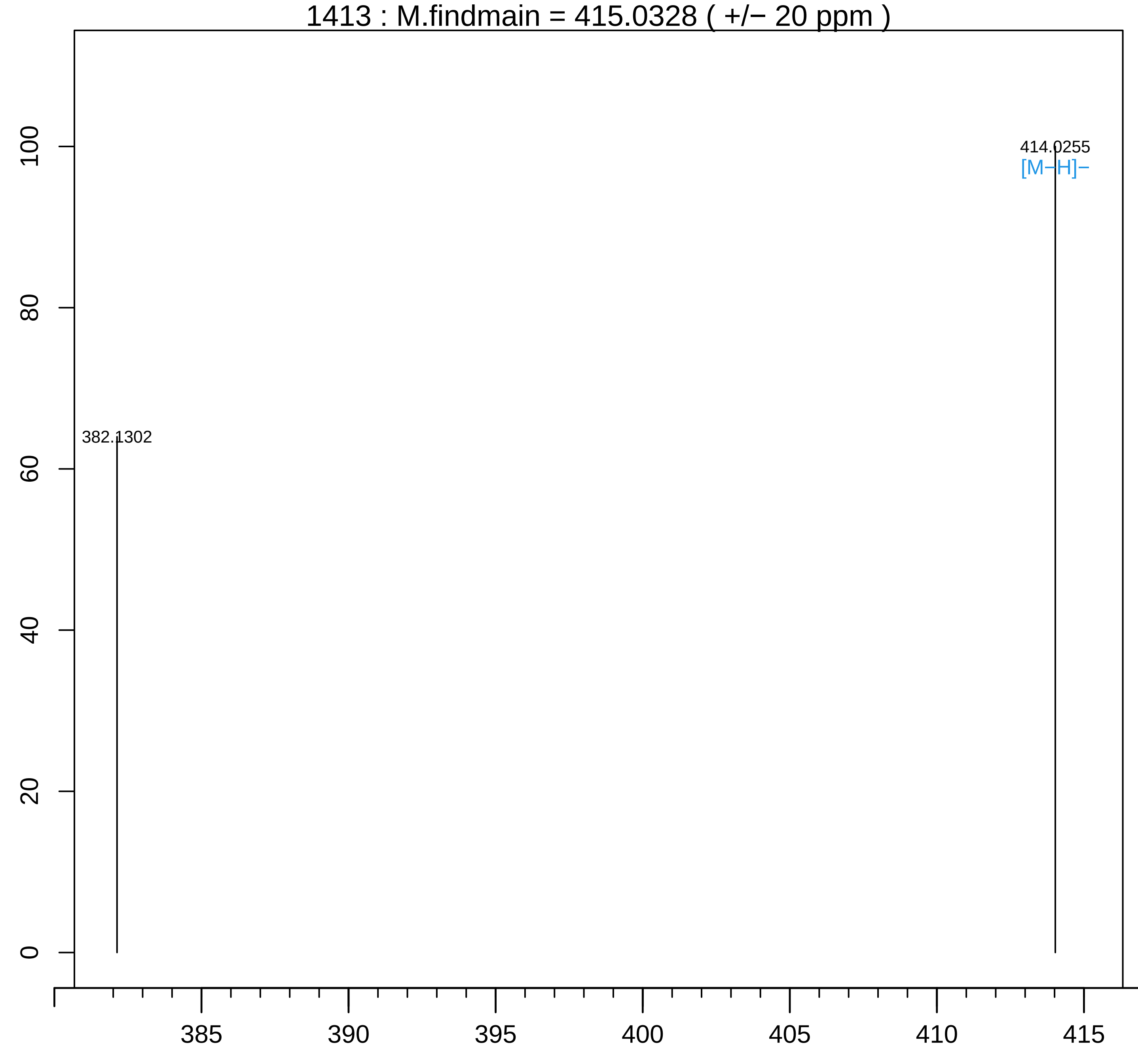
1413 : M.findmain = 415.0328 (+/− 20 ppm)
414.0255
[M−H]−
382.1302
0
20
40
60
80
100
385
390
395
400
405
410
415

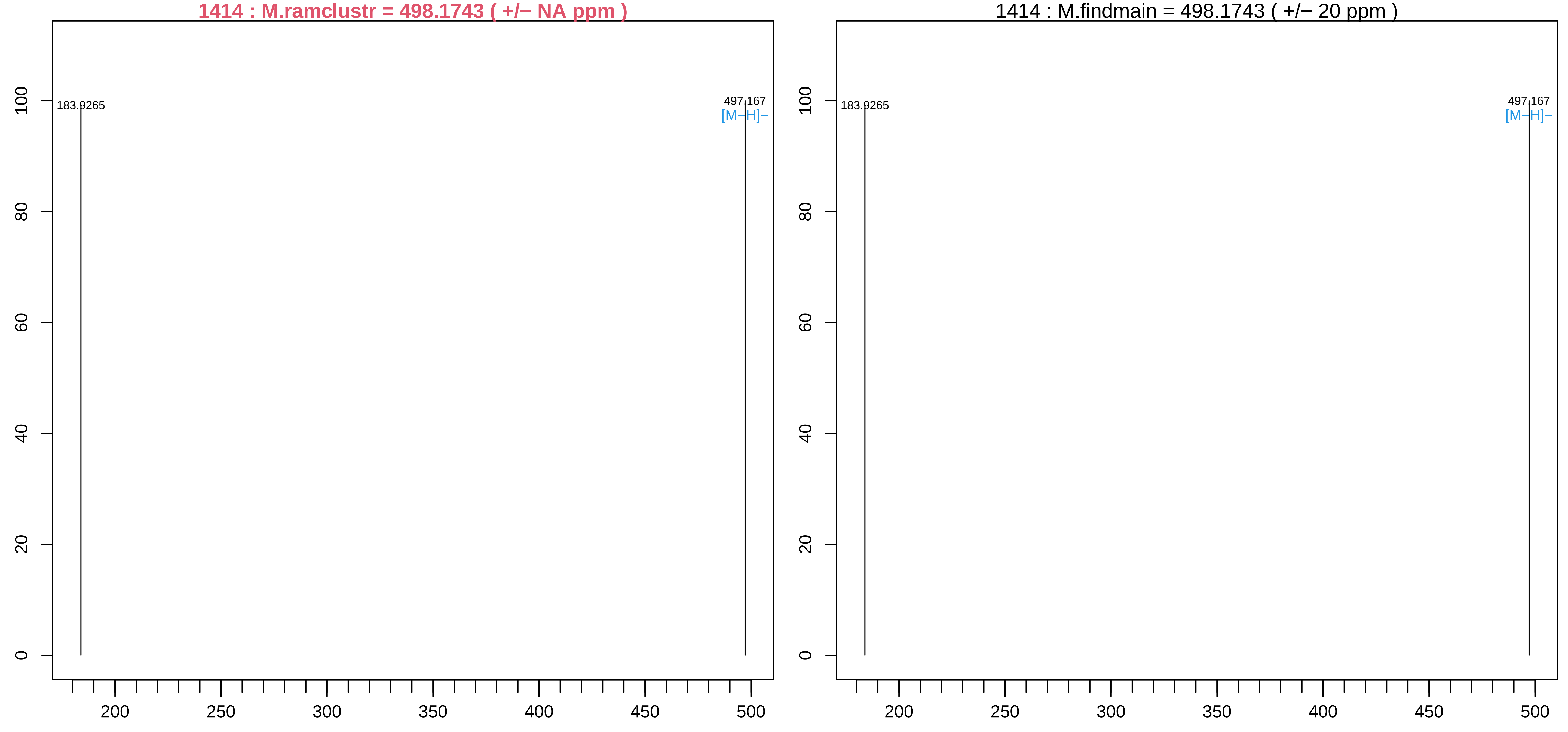
1414 : M.ramclustr = 498.1743 (+/− NA ppm)
183.9265
497.167
[M−H]−
1414 : M.findmain = 498.1743 (+/− 20 ppm)
183.9265
497.167
[M−H]−
0
20
40
60
80
100
200
250
300
350
400
450
500

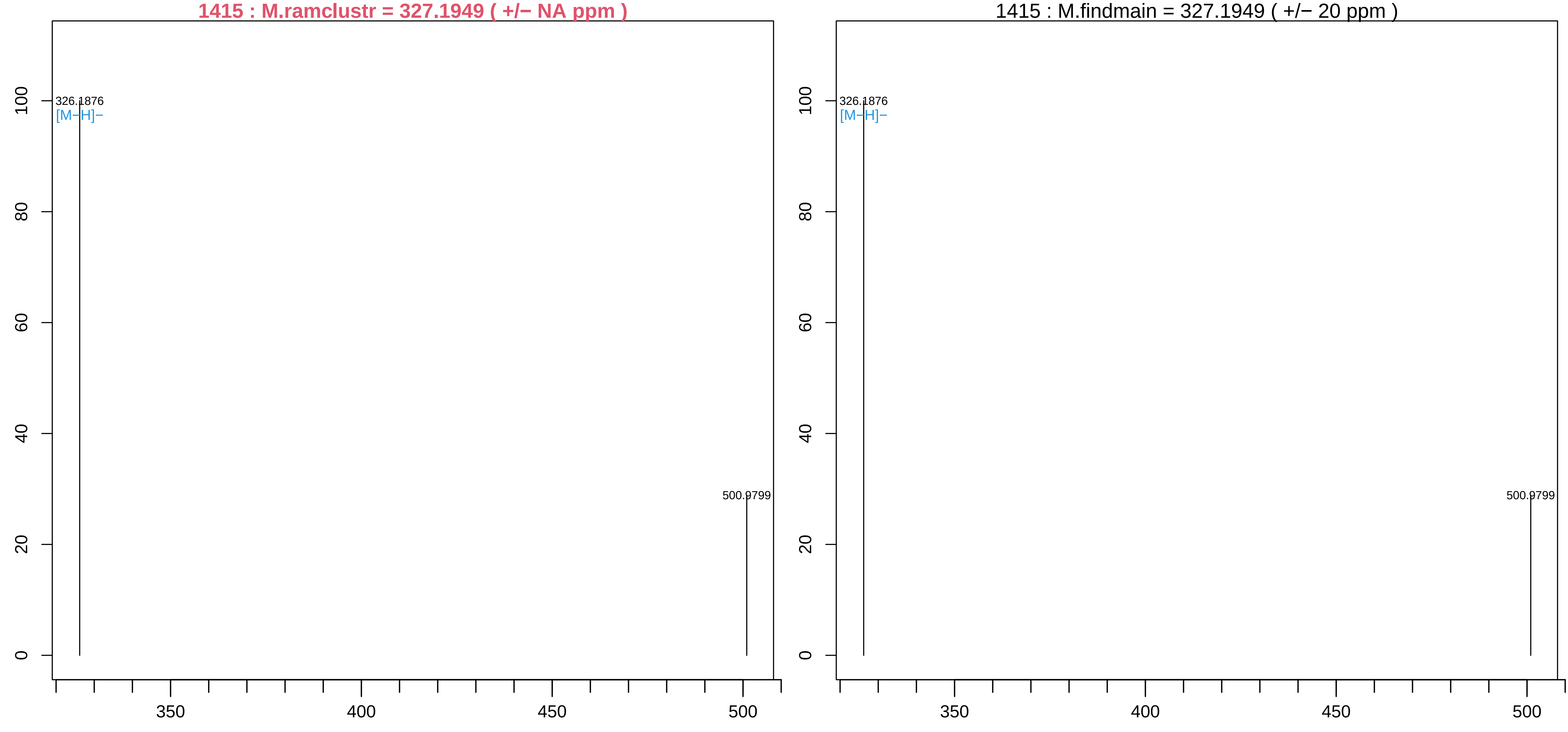
1415 : M.ramclustr = 327.1949 (+/− NA ppm)
326.1876
[M−H]−
500.9799
1415 : M.findmain = 327.1949 (+/− 20 ppm)
326.1876
[M−H]−
500.9799
0
20
40
60
80
100
350
400
450
500

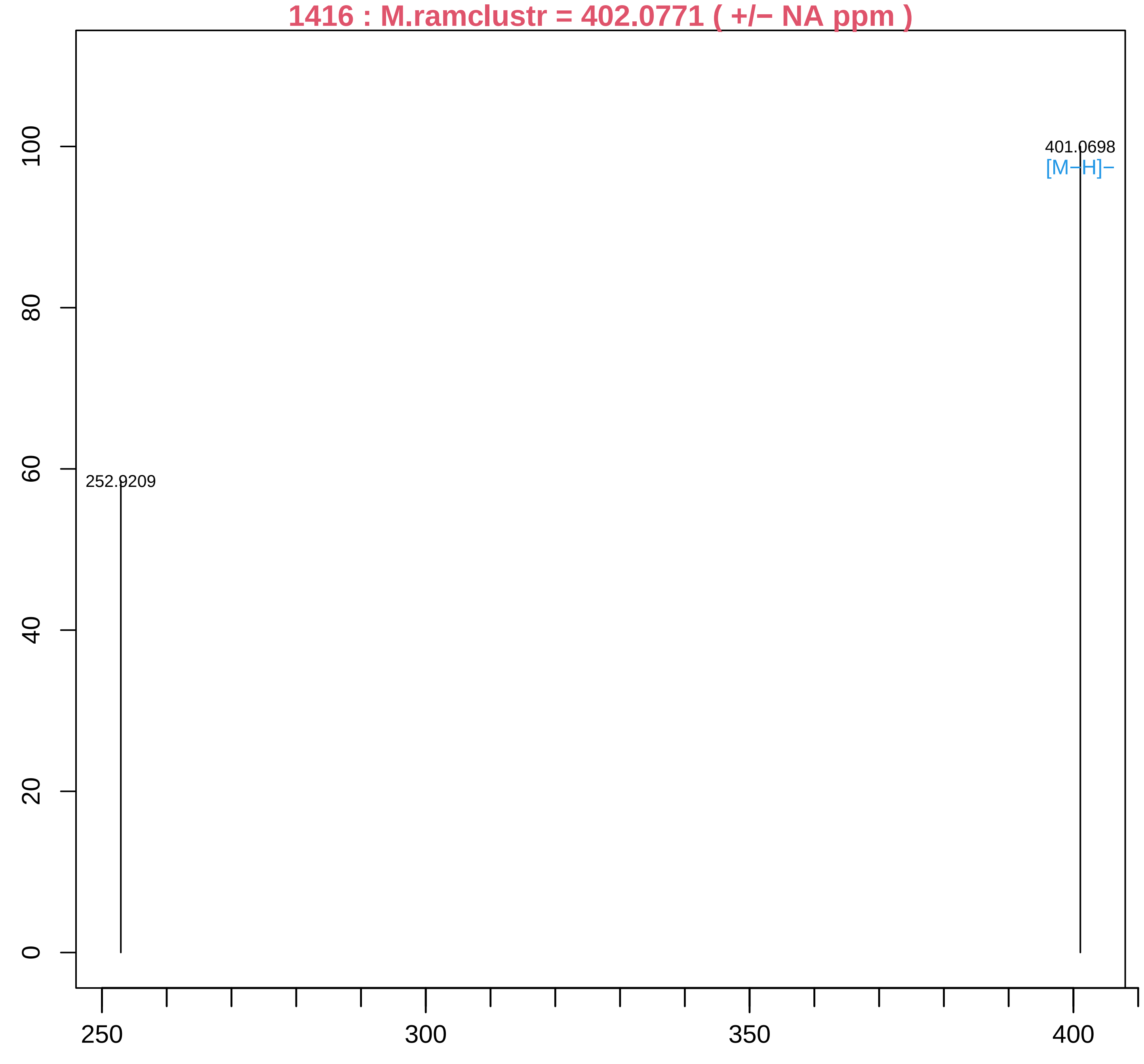
1416 : M.ramclustr = 402.0771 (+/− NA ppm)
401.0698
[M−H]−
252.9209
0
20
40
60
80
100
250
300
350
400

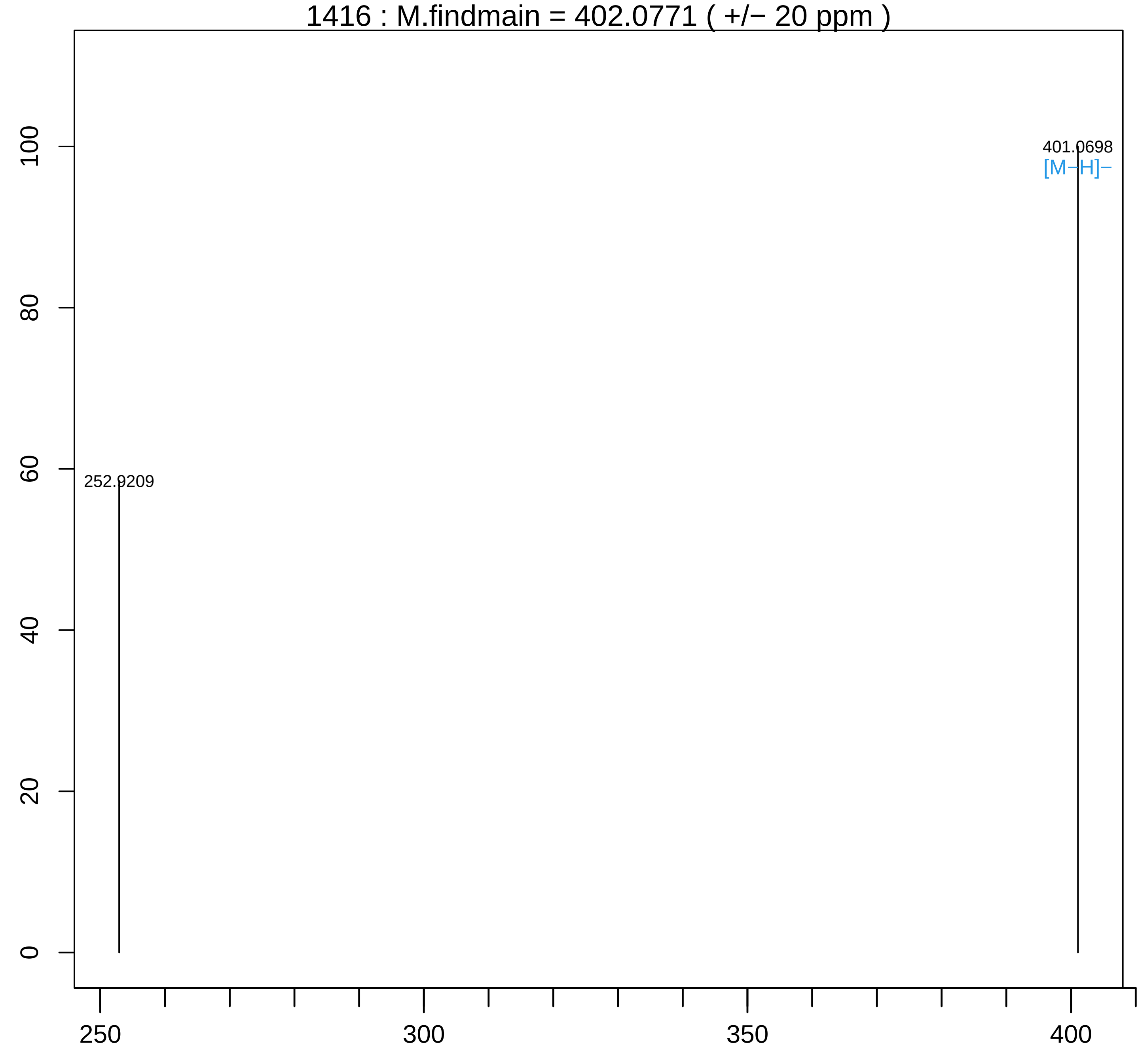
1416 : M.findmain = 402.0771 (+/− 20 ppm)
401.0698
[M−H]−
252.9209
0
20
40
60
80
100
250
300
350
400

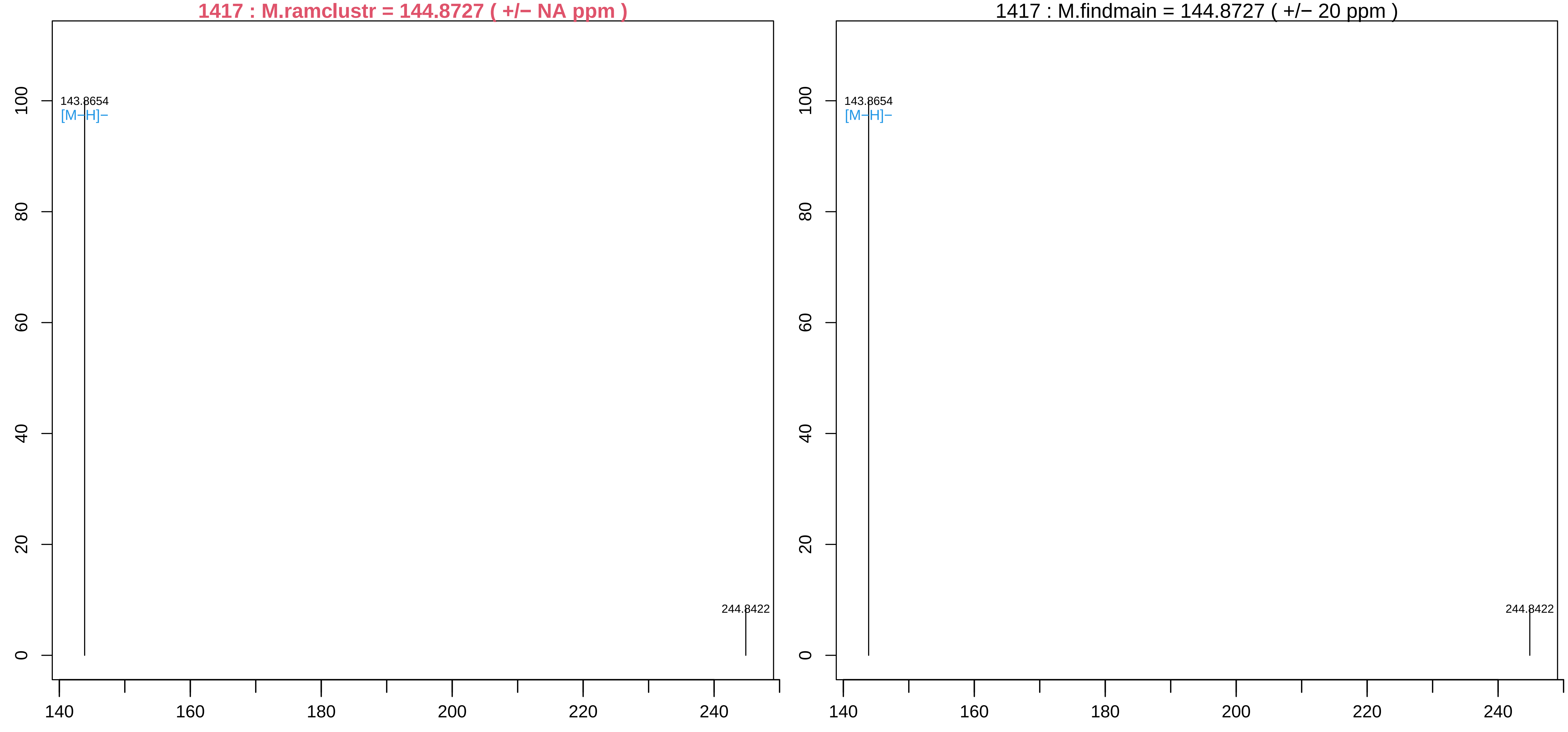
1417 : M.ramclustr = 144.8727 (+/− NA ppm)
143.8654
[M−H]−
244.8422
0
20
40
60
80
100
140
160
180
200
220
240
1417 : M.findmain = 144.8727 (+/− 20 ppm)
143.8654
[M−H]−
244.8422
0
20
40
60
80
100
140
160
180
200
220
240

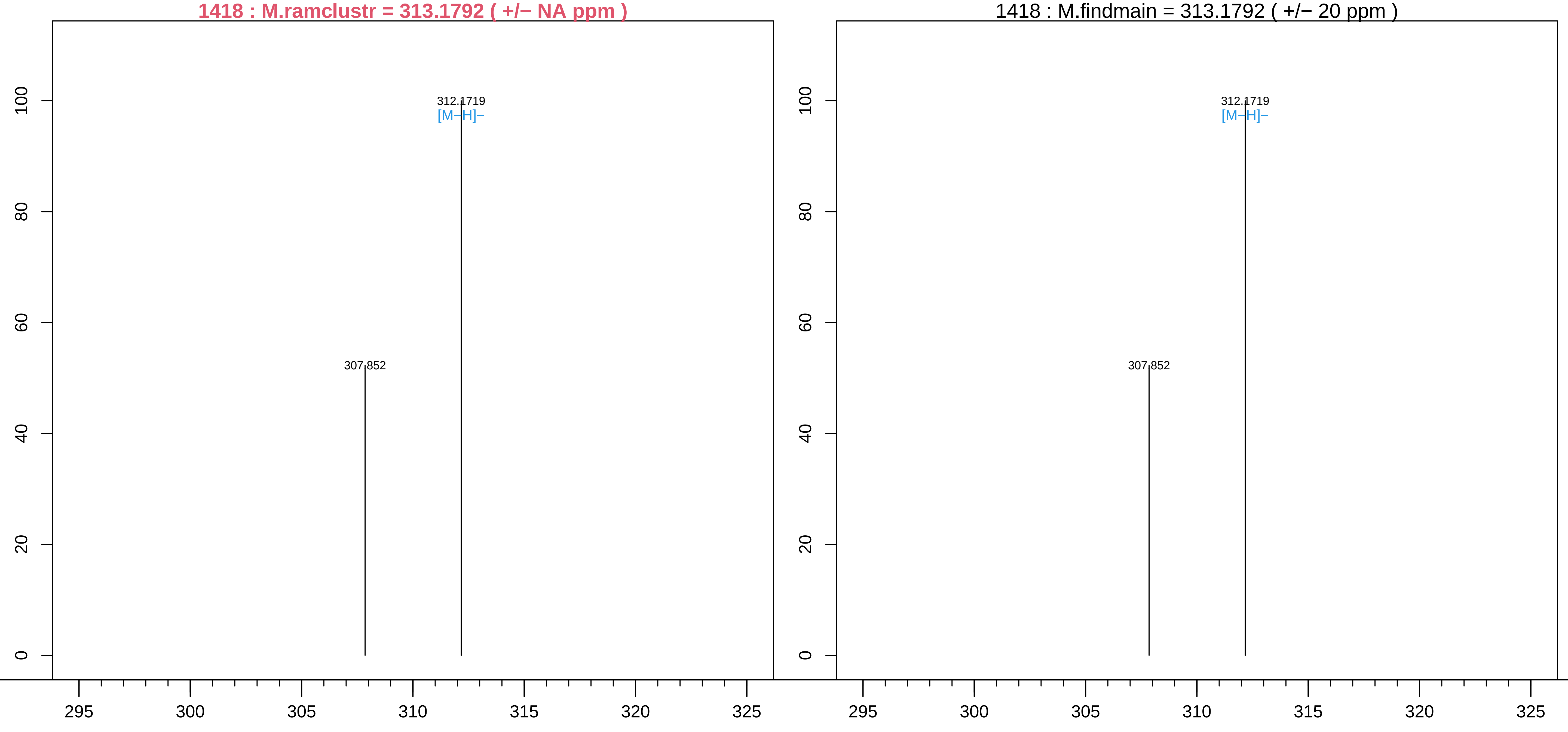
1418 : M.ramclustr = 313.1792 (+/− NA ppm)
312.1719
[M−H]−
307.852
1418 : M.findmain = 313.1792 (+/− 20 ppm)
312.1719
[M−H]−
307.852
0
20
40
60
80
100
295
300
305
310
315
320
325

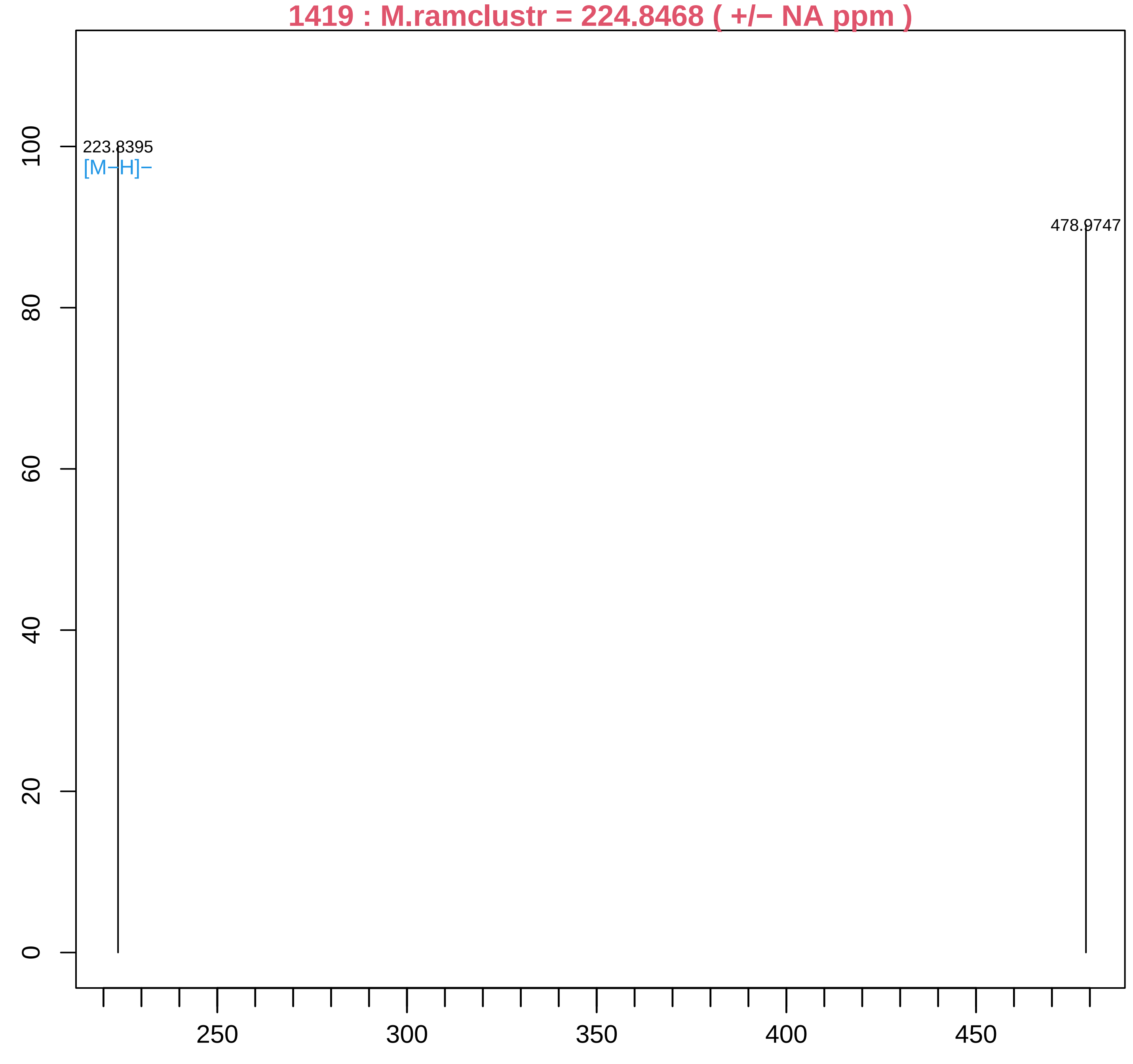
1419 : M.ramclustr = 224.8468 (+/− NA ppm)
223.8395
[M−H]−
478.9747
0
20
40
60
80
100
250
300
350
400
450

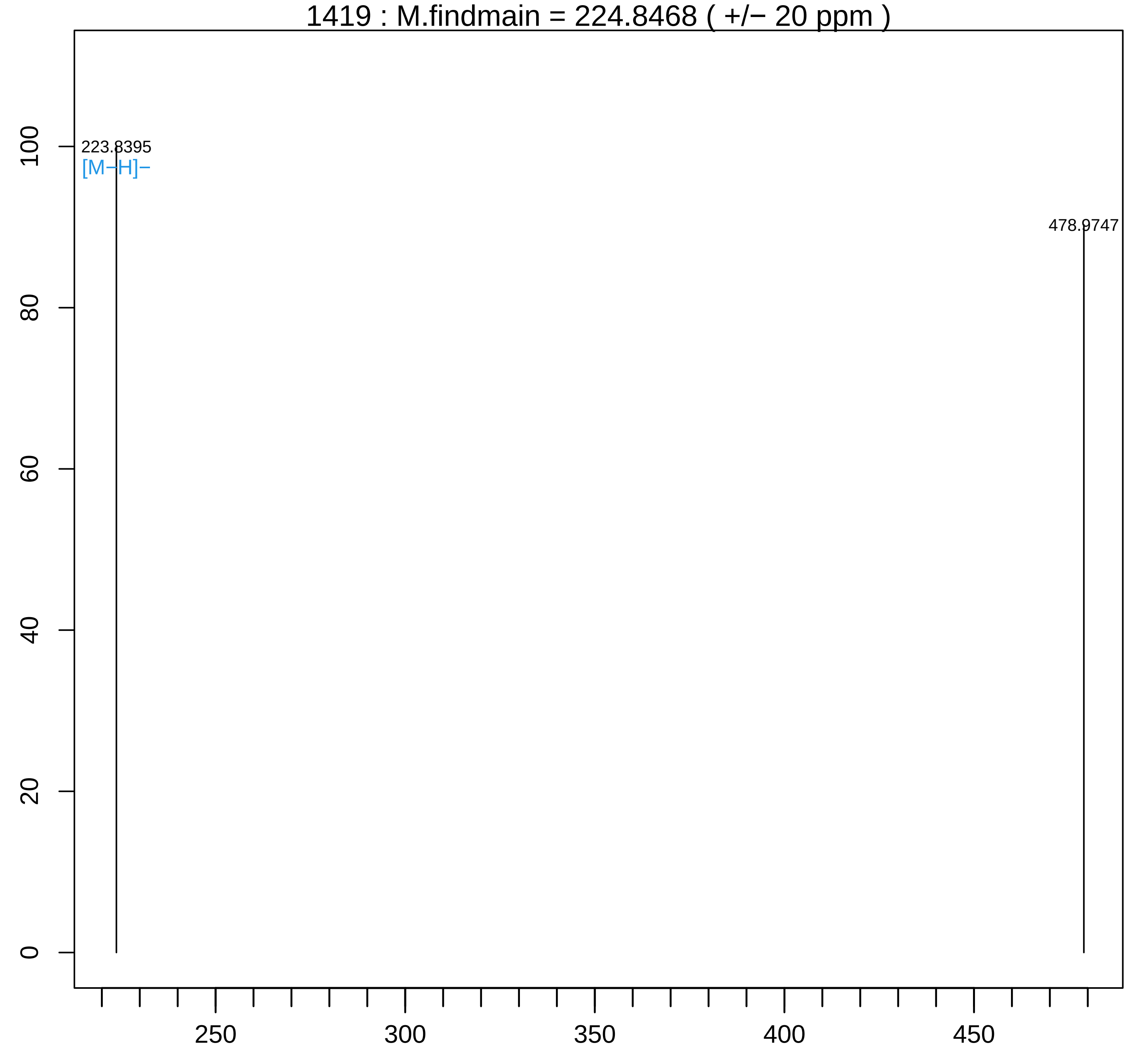
1419 : M.findmain = 224.8468 (+/− 20 ppm)
223.8395
[M−H]−
478.9747
0
20
40
60
80
100
250
300
350
400
450

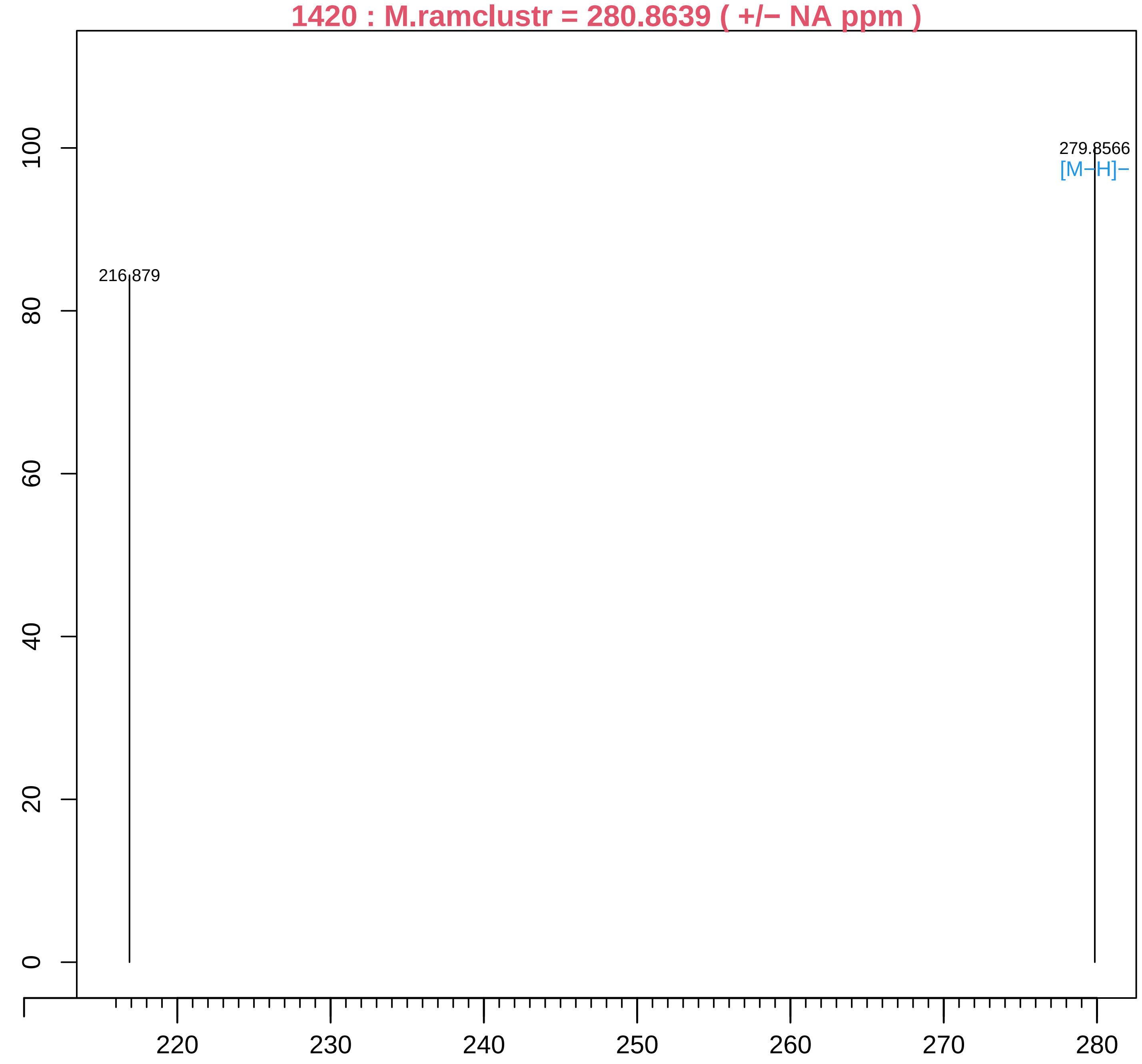
1420 : M.ramclustr = 280.8639 (+/− NA ppm)
279.8566
[M−H]−
216.879
0
20
40
60
80
100
220
230
240
250
260
270
280

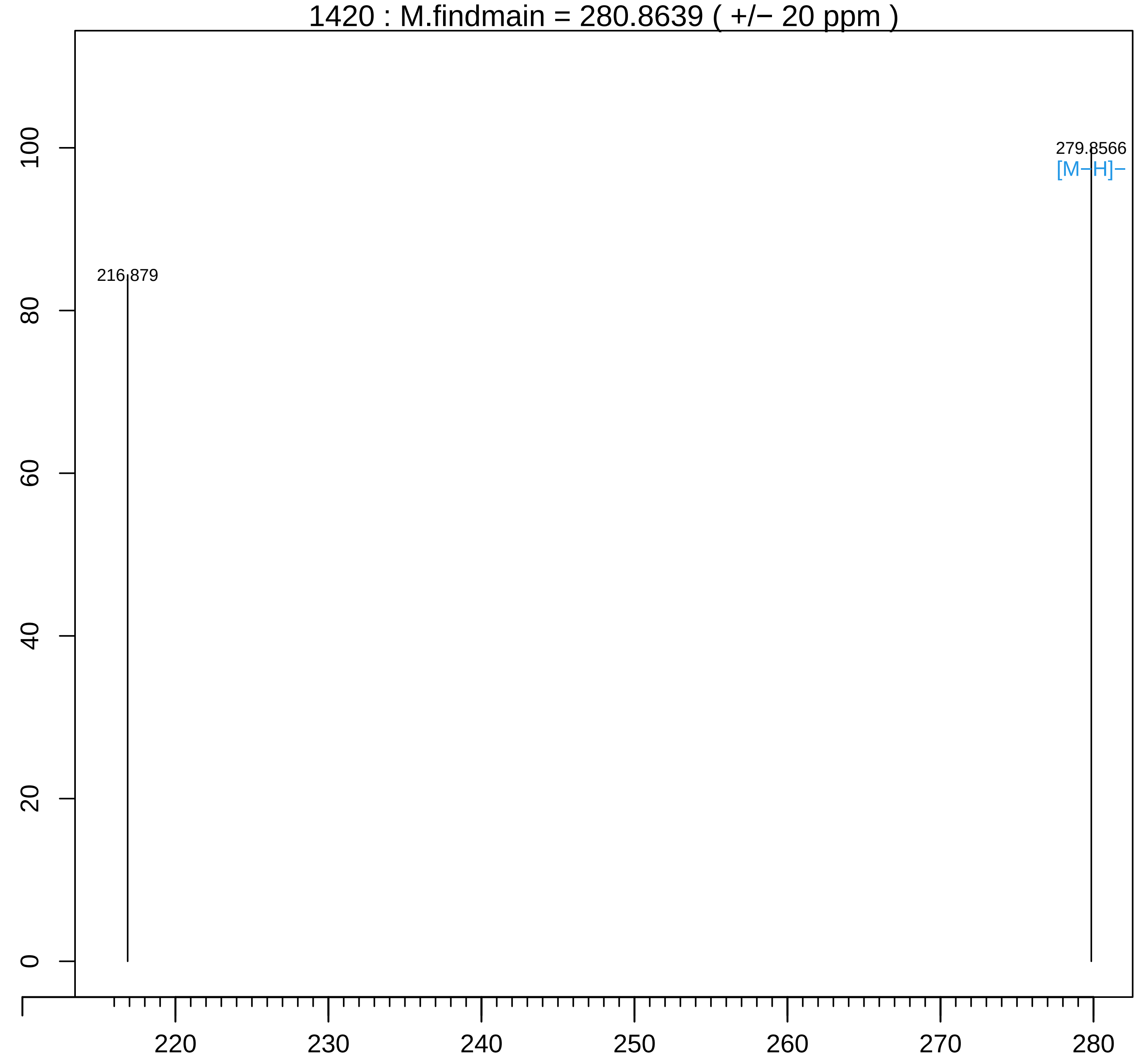
1420 : M.findmain = 280.8639 (+/− 20 ppm)
279.8566
[M−H]−
216.879
0
20
40
60
80
100
220
230
240
250
260
270
280

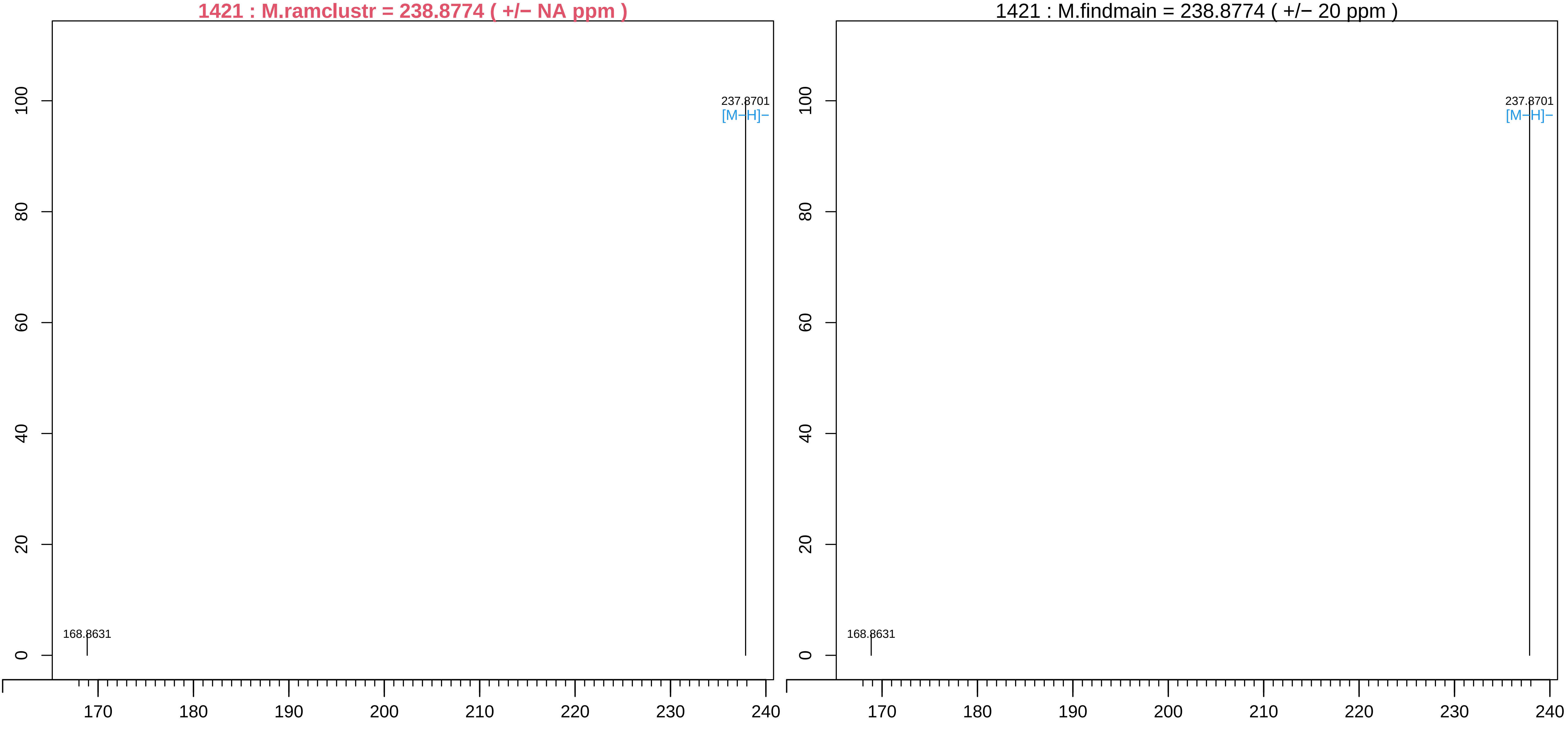

1421 : M.ramclustr = 238.8774 (+/− NA ppm)
237.8701
[M−H]−
168.8631
1421 : M.findmain = 238.8774 (+/− 20 ppm)
237.8701
[M−H]−
168.8631

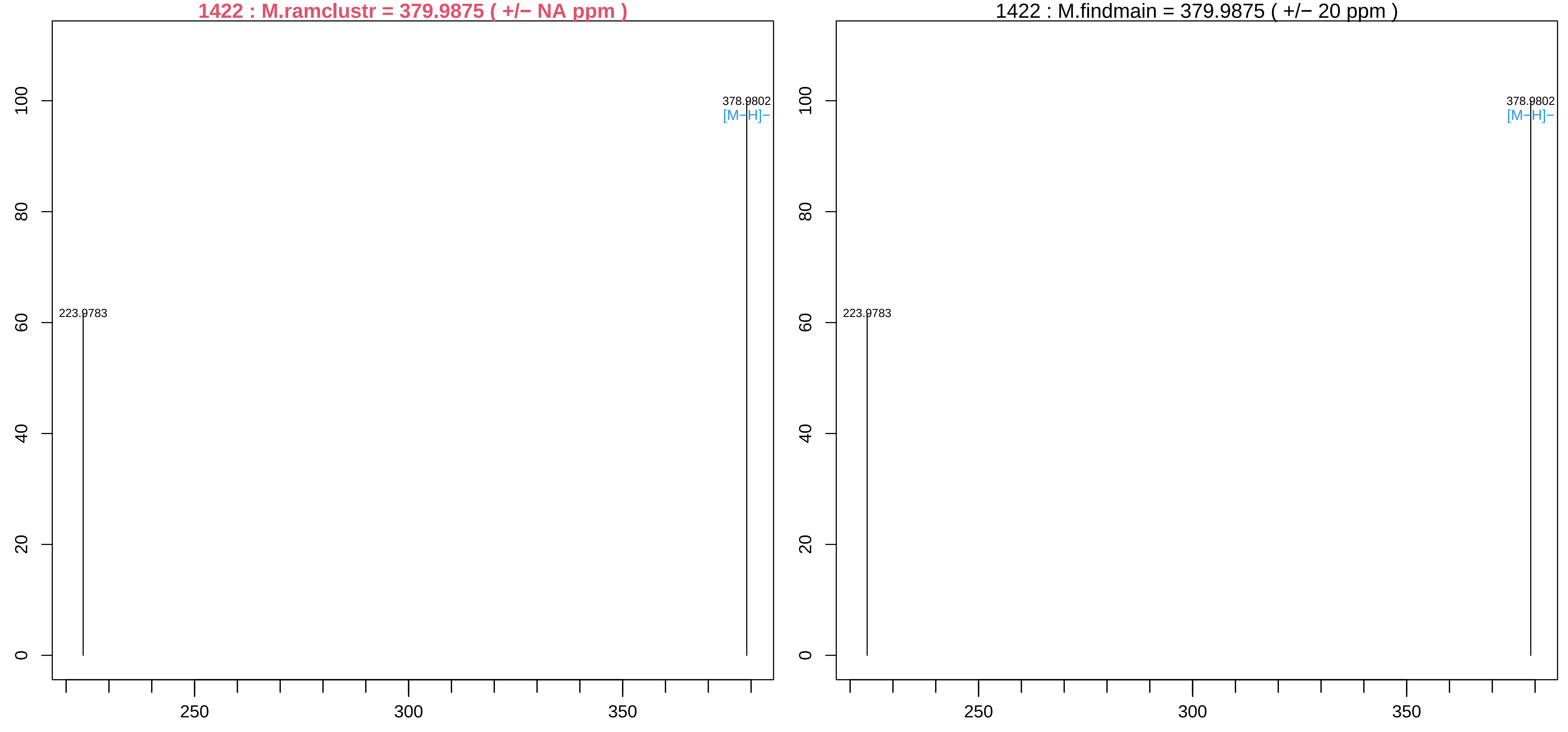
1422 : M.ramclustr = 379.9875 (+/− NA ppm)
1422 : M.findmain = 379.9875 (+/− 20 ppm)
378.9802
[M−H]−
223.9783
0
20
40
60
80
100
250
300
350

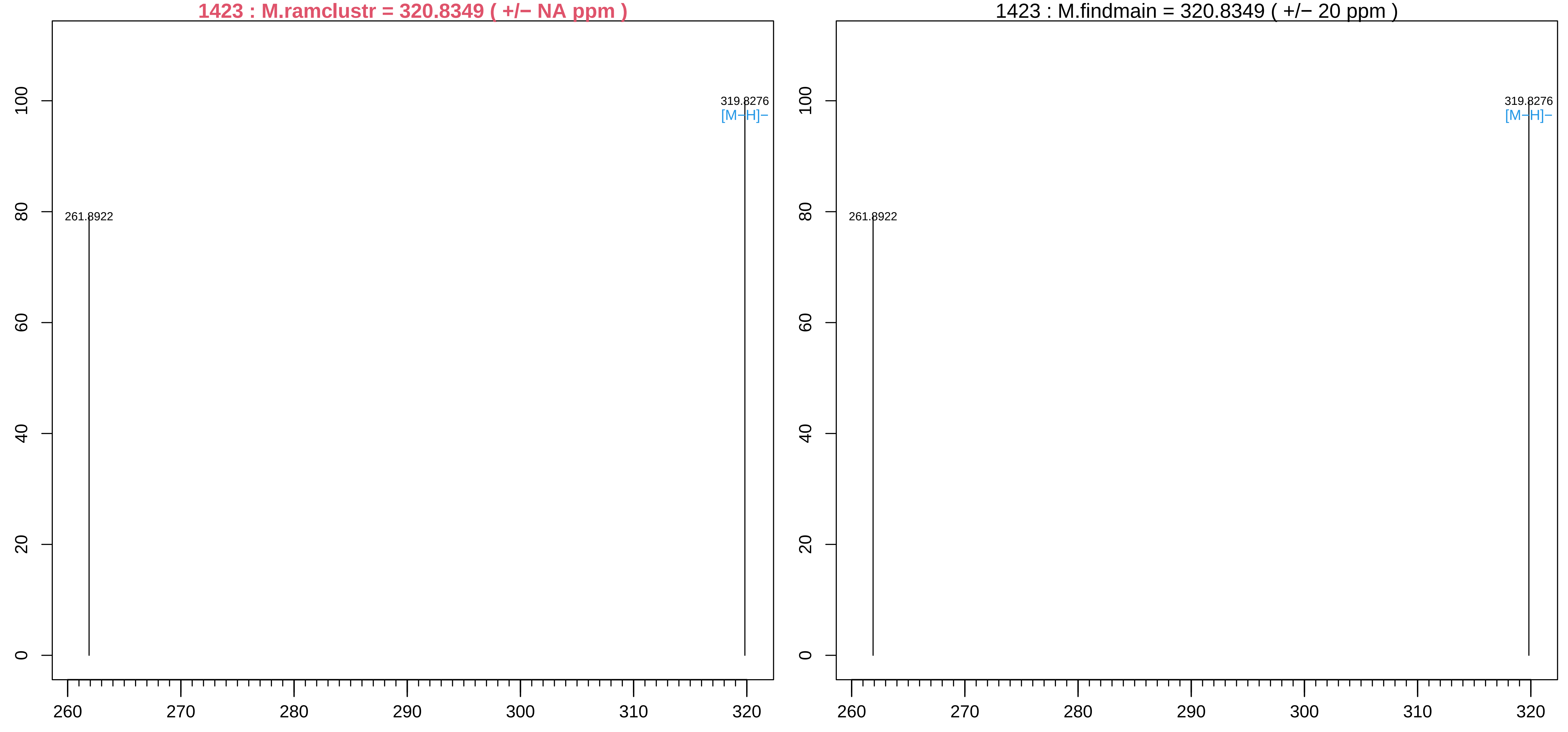
1423 : M.ramclustr = 320.8349 (+/− NA ppm)
261.8922
319.8276
[M−H]−
1423 : M.findmain = 320.8349 (+/− 20 ppm)
261.8922
319.8276
[M−H]−
0
20
40
60
80
100
260
270
280
290
300
310
320

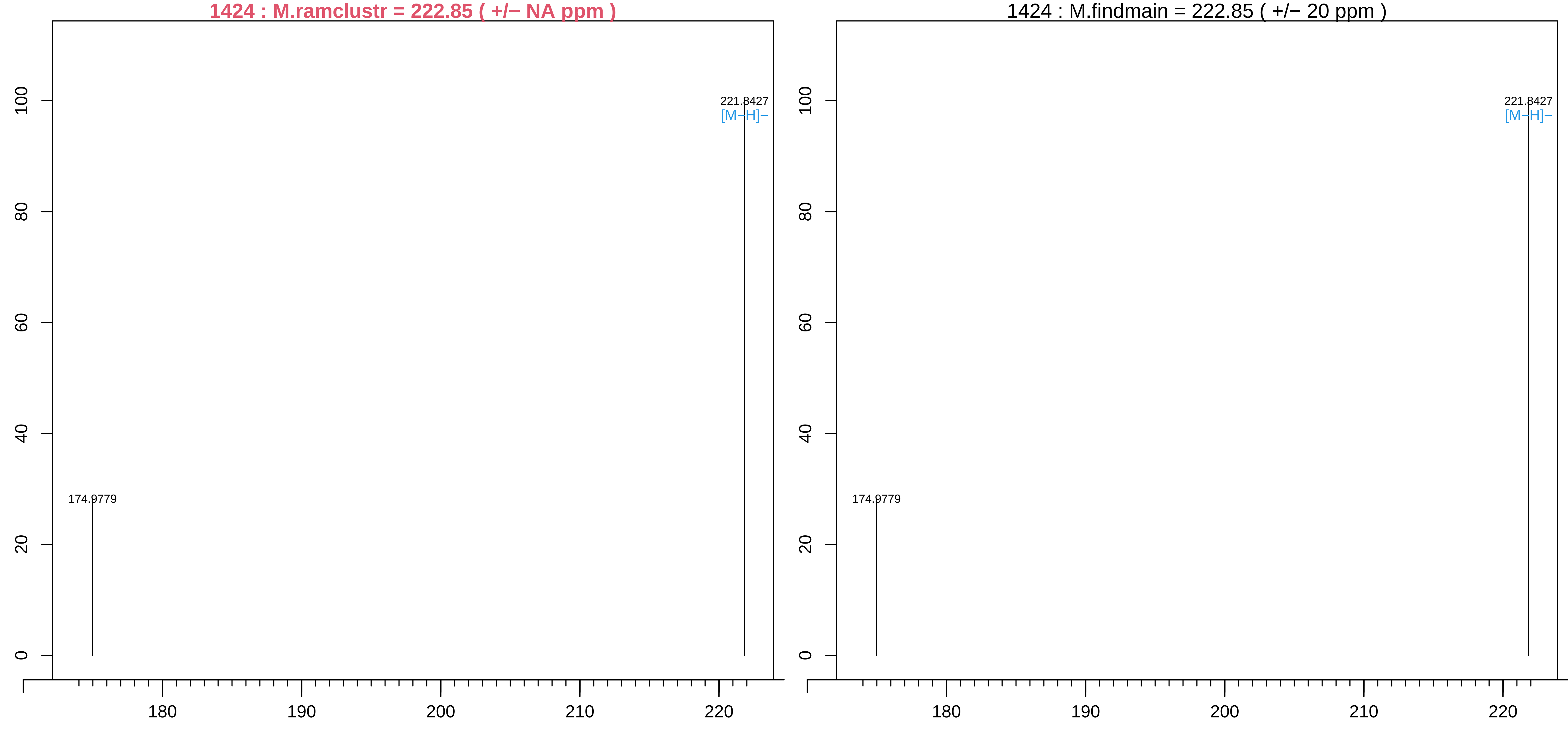
1424 : M.ramclustr = 222.85 (+/− NA ppm)
1424 : M.findmain = 222.85 (+/− 20 ppm)
221.8427
[M−H]−
174.9779
221.8427
[M−H]−
174.9779
0
20
40
60
80
100
180
190
200
210
220

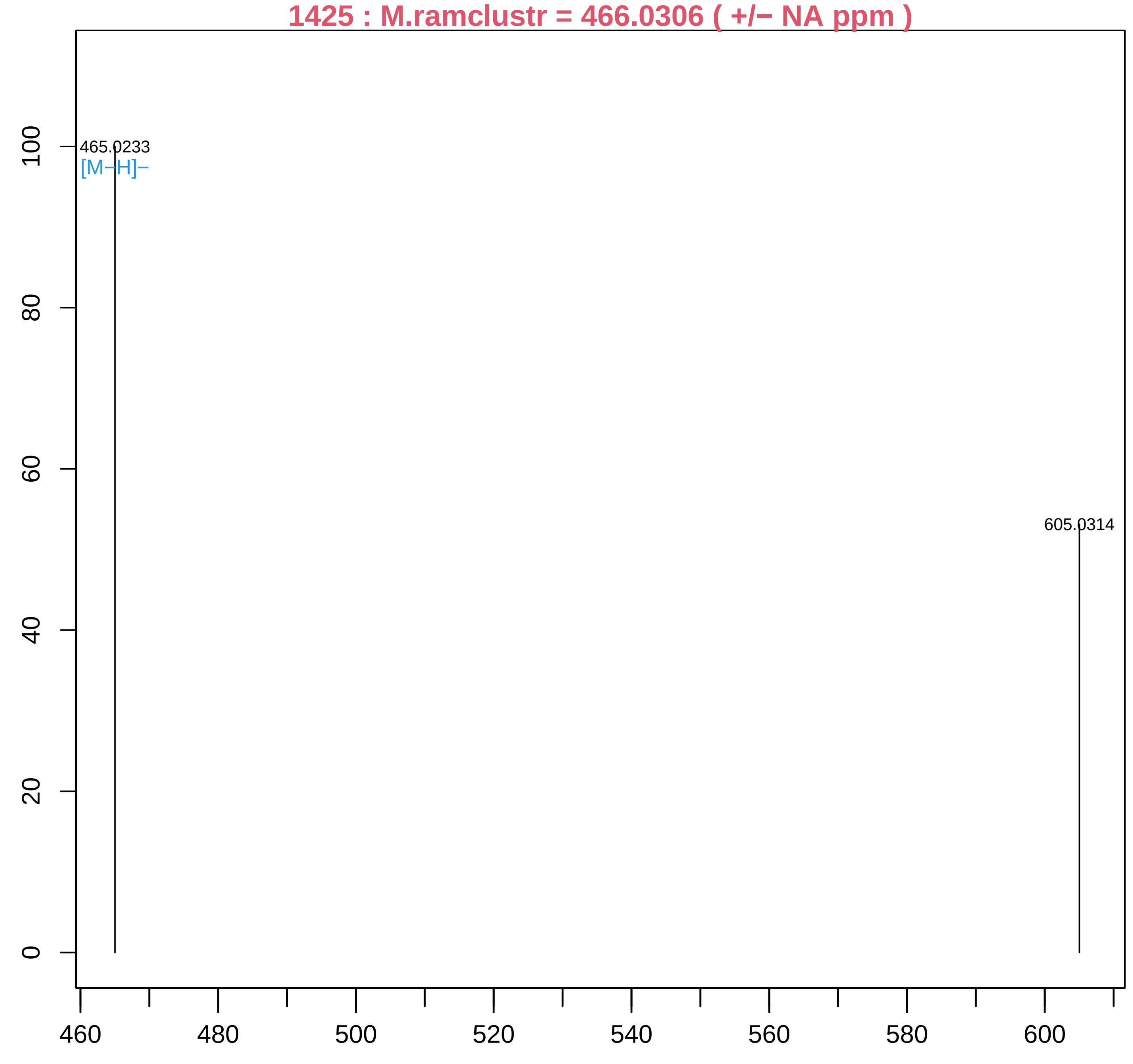

1425 : M.ramclustr = 466.0306 (+/− NA ppm)
465.0233
[M−H]−
605.0314

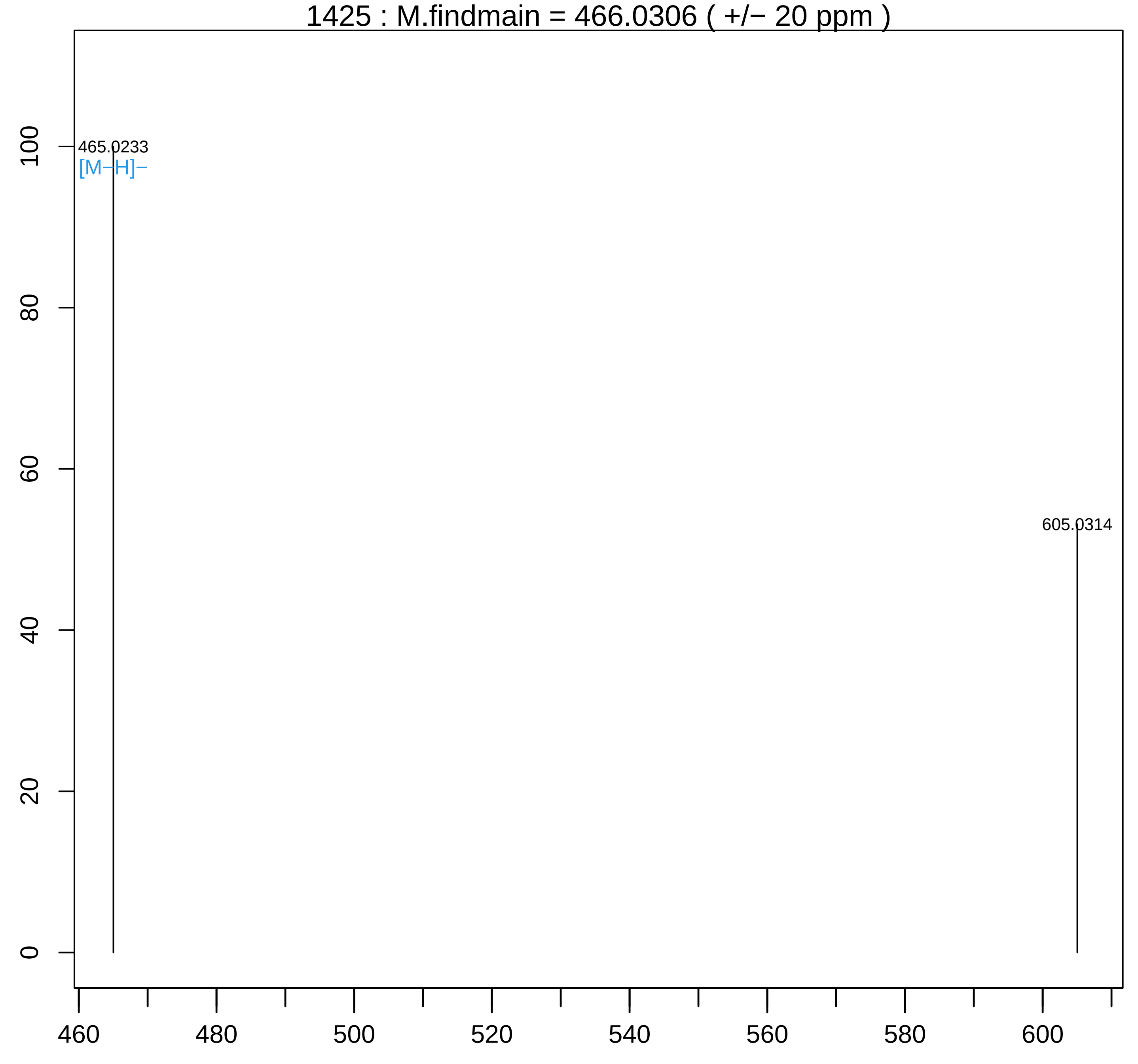

1425 : M.findmain = 466.0306 (+/− 20 ppm)
465.0233
[M−H]−
605.0314

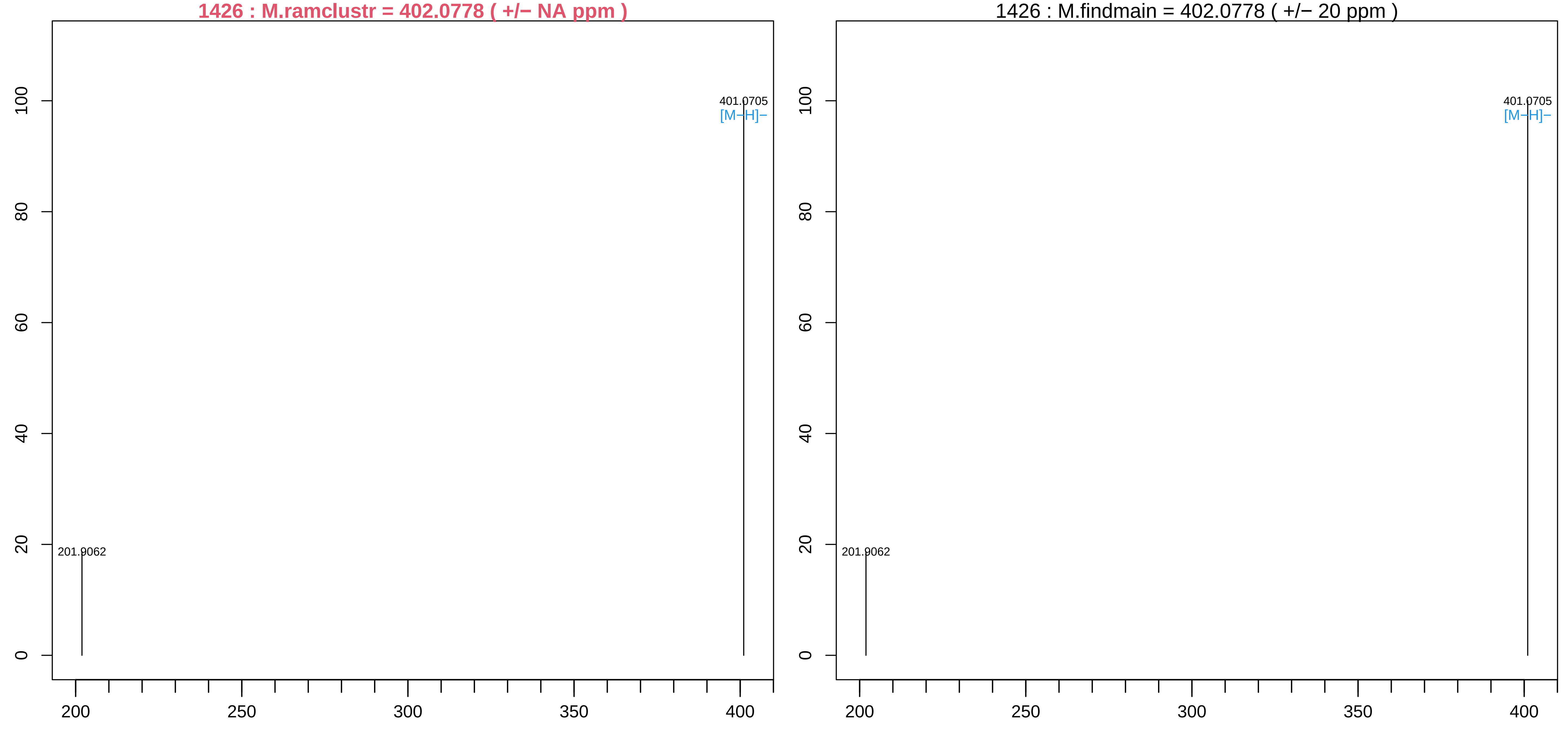
1426 : M.ramclustr = 402.0778 (+/− NA ppm)
401.0705
[M−H]−
201.9062
1426 : M.findmain = 402.0778 (+/− 20 ppm)
401.0705
[M−H]−
201.9062

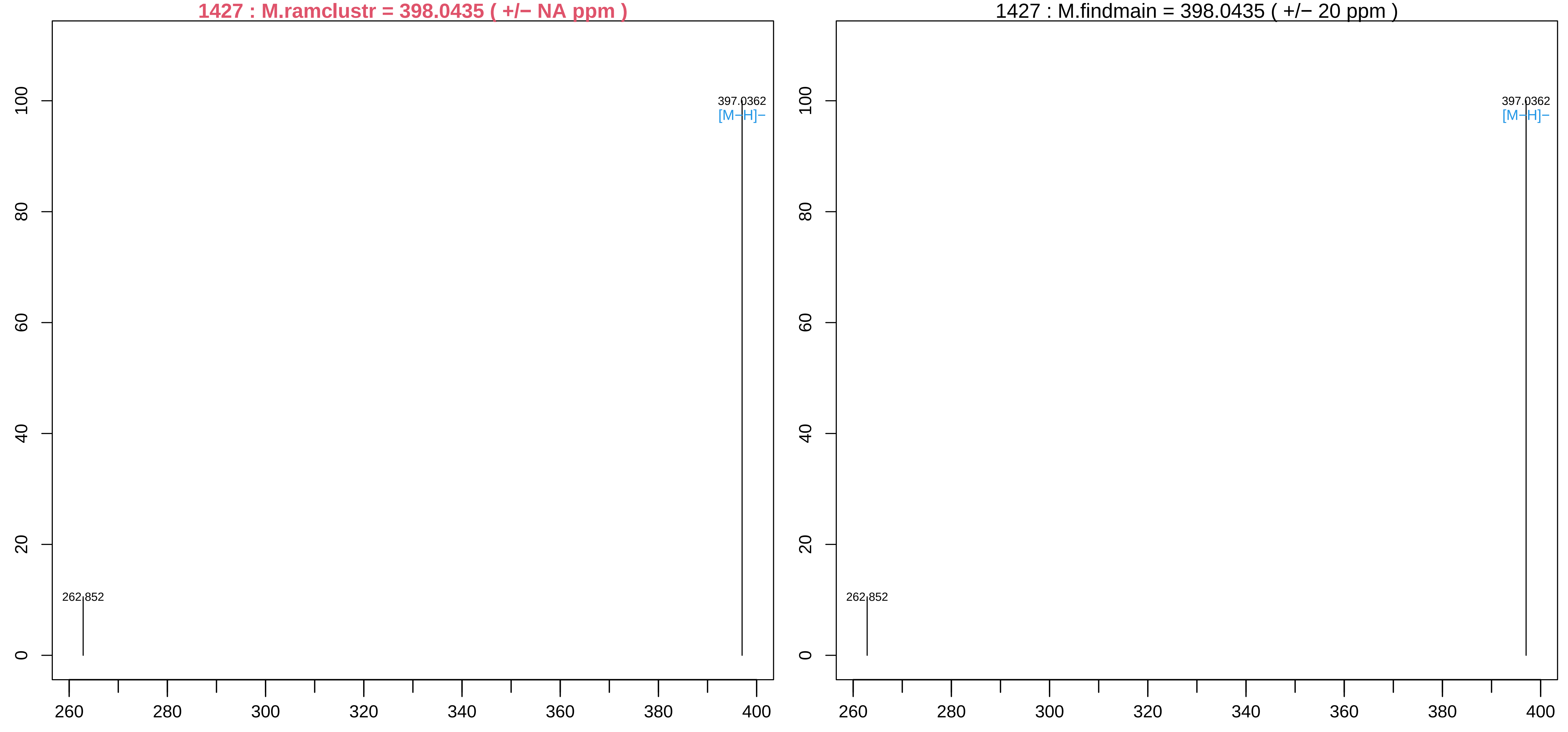
1427 : M.ramclustr = 398.0435 (+/− NA ppm)
397.0362
[M−H]−
262.852
1427 : M.findmain = 398.0435 (+/− 20 ppm)
397.0362
[M−H]−
262.852

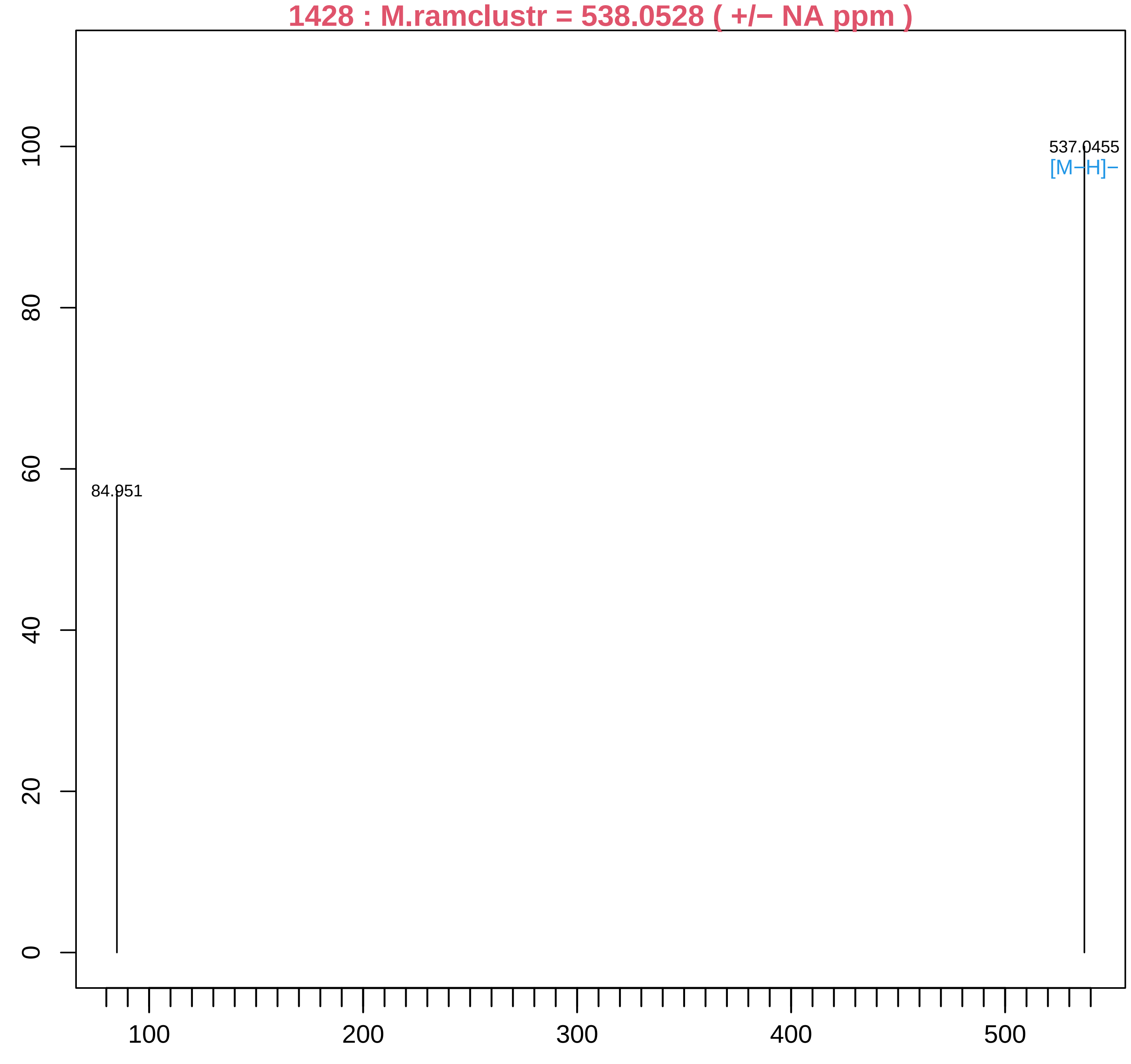
1428 : M.ramclustr = 538.0528 (+/− NA ppm)
537.0455
[M−H]−
84.951
0
20
40
60
80
100
100
200
300
400
500

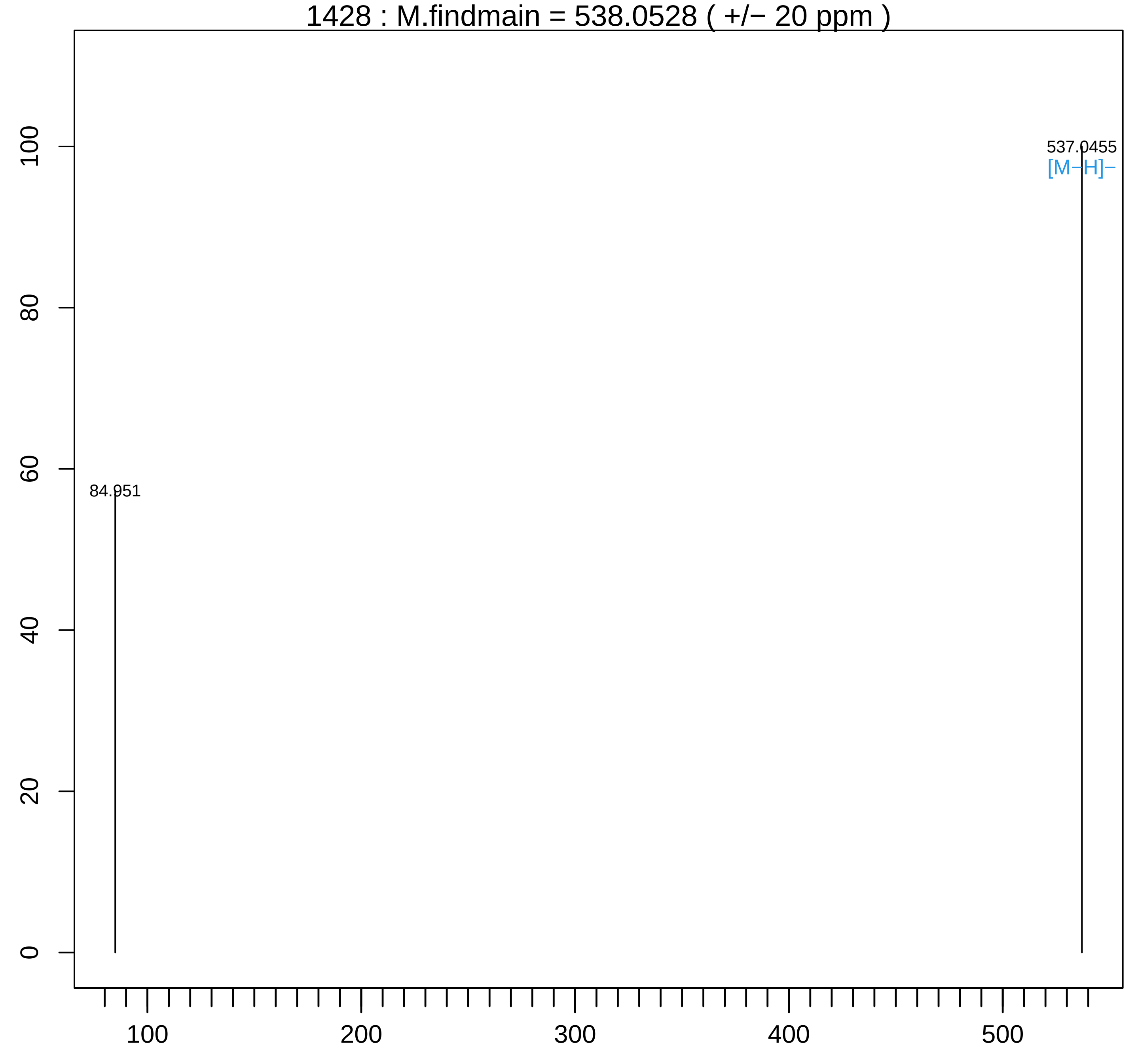
1428 : M.findmain = 538.0528 (+/− 20 ppm)
537.0455
[M−H]−
84.951
0
20
40
60
80
100
100
200
300
400
500

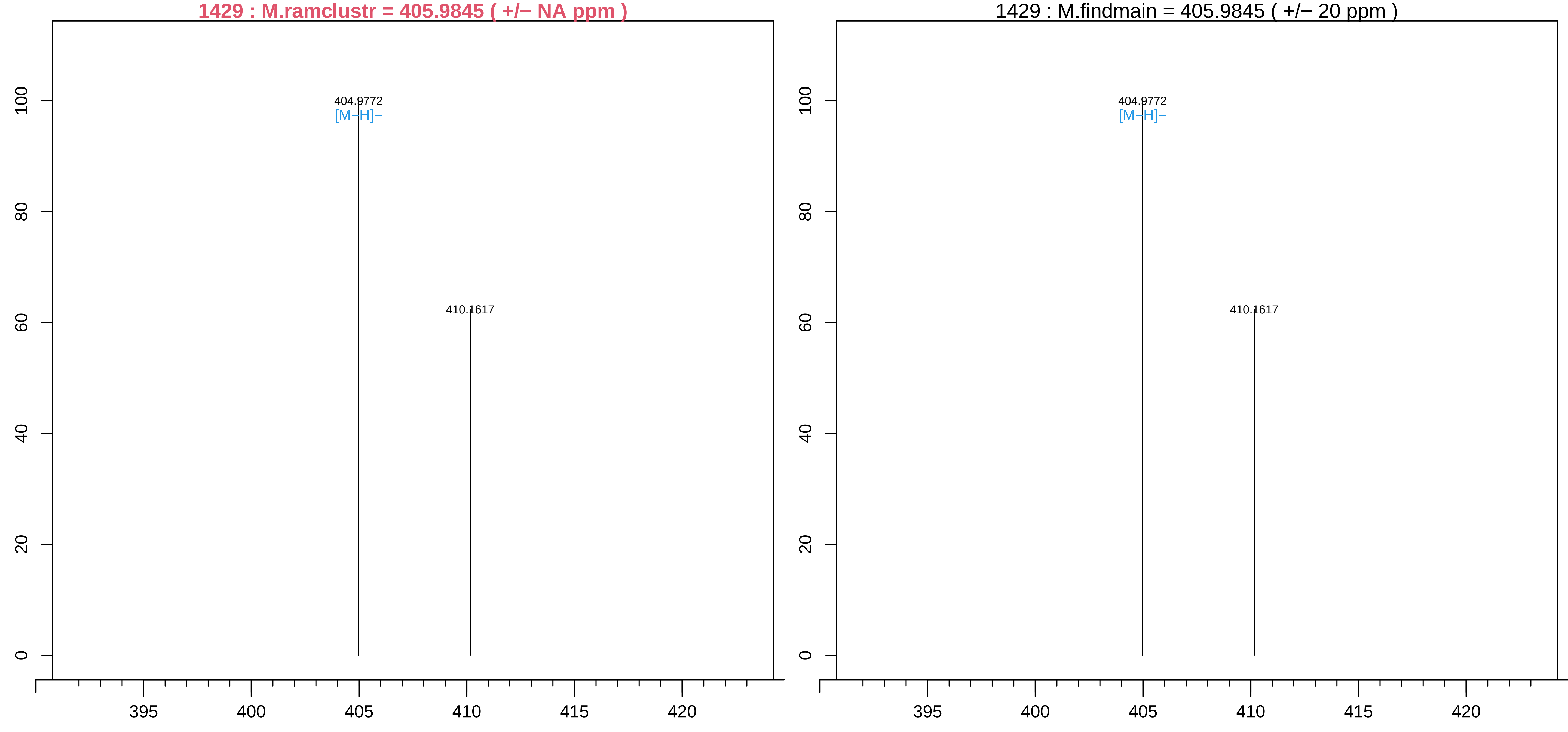
1429 : M.ramclustr = 405.9845 (+/− NA ppm)
404.9772
[M−H]−
410.1617
1429 : M.findmain = 405.9845 (+/− 20 ppm)
404.9772
[M−H]−
410.1617
0
20
40
60
80
100
395
400
405
410
415
420

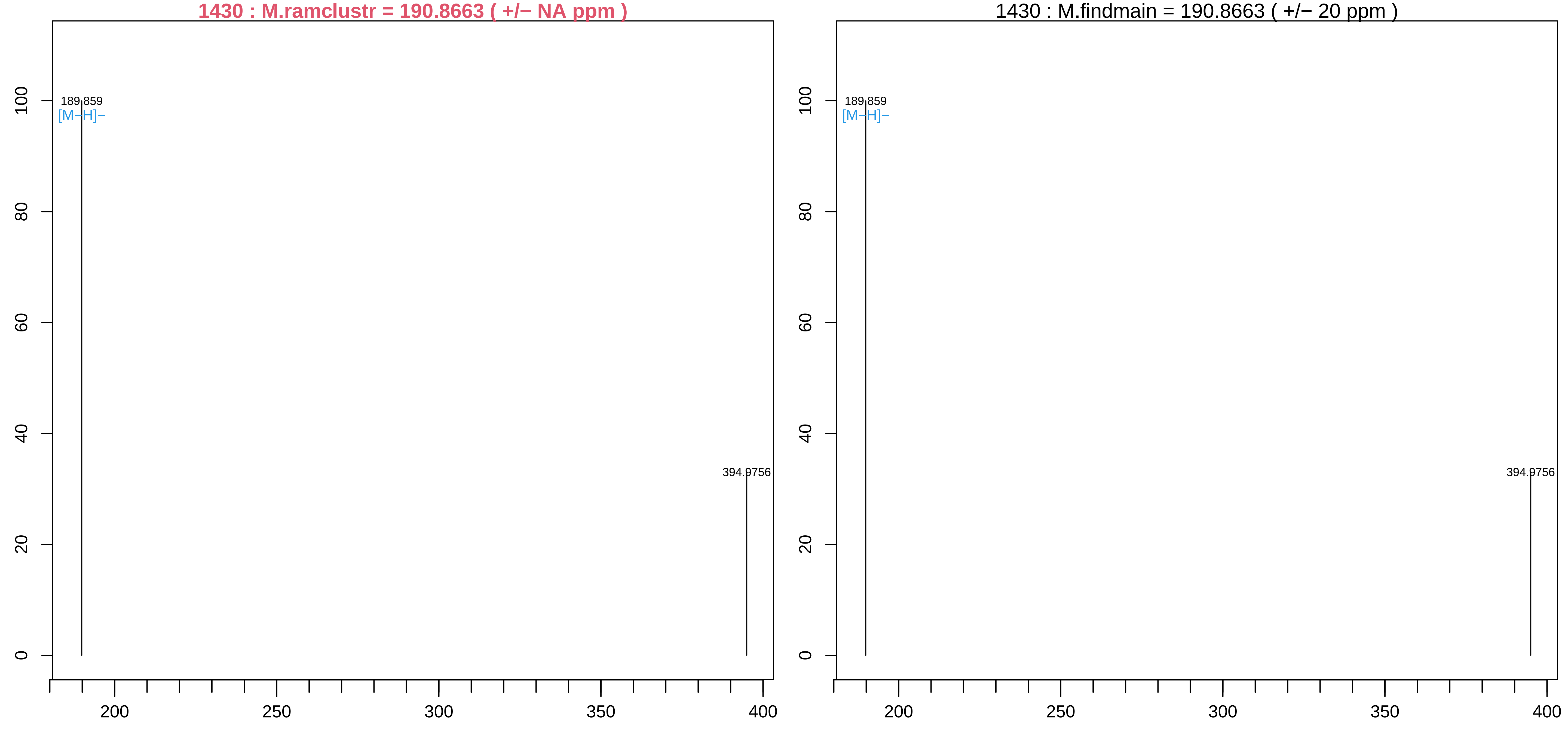

1430 : M.ramclustr = 190.8663 (+/− NA ppm)
189.859
[M−H]−
394.9756
0
20
40
60
80
100
200
250
300
350
400
1430 : M.findmain = 190.8663 (+/− 20 ppm)
189.859
[M−H]−
394.9756
0
20
40
60
80
100
200
250
300
350
400

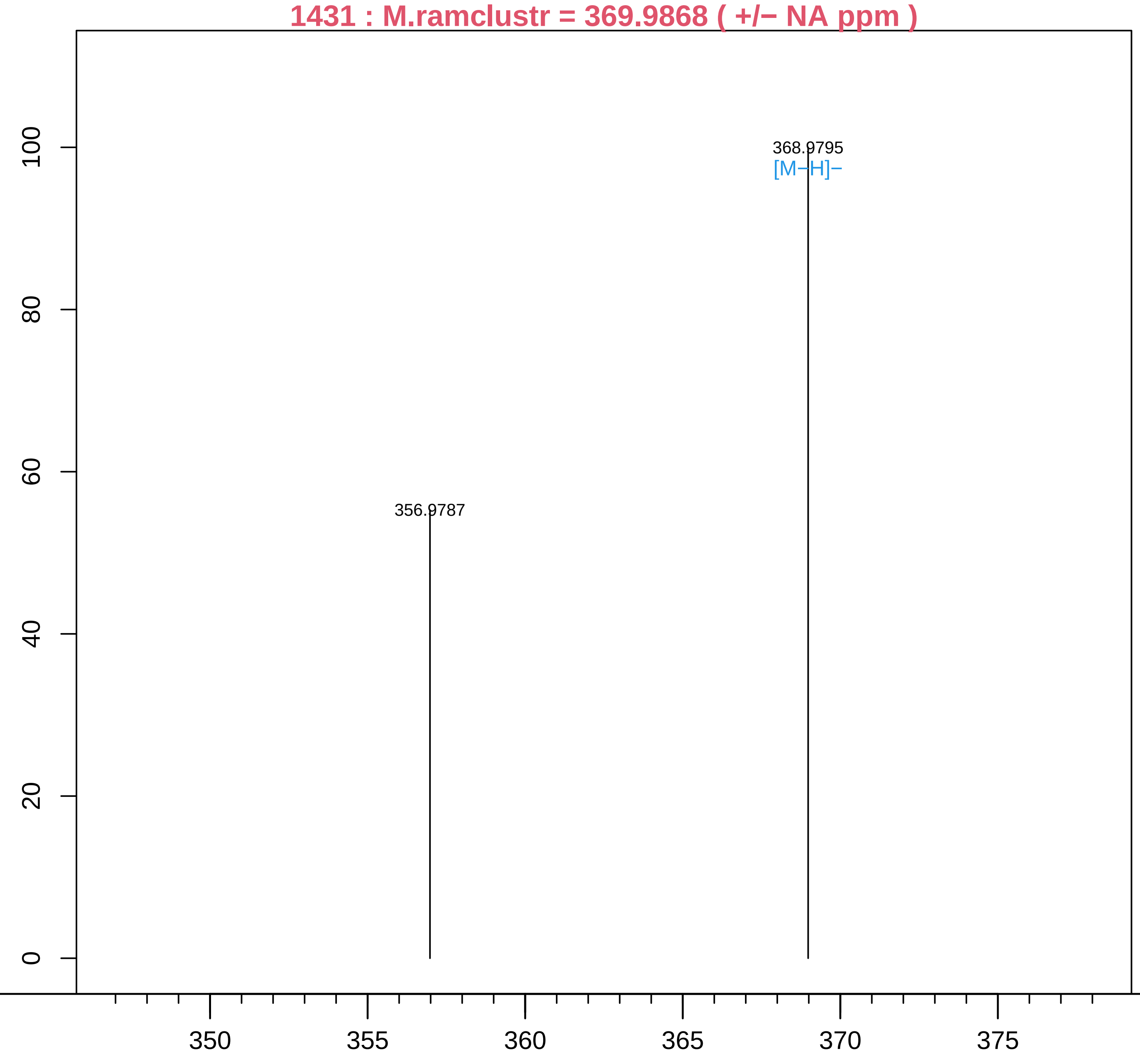
1431 : M.ramclustr = 369.9868 (+/− NA ppm)
368.9795
[M−H]−
356.9787
0
20
40
60
80
100
350
355
360
365
370
375

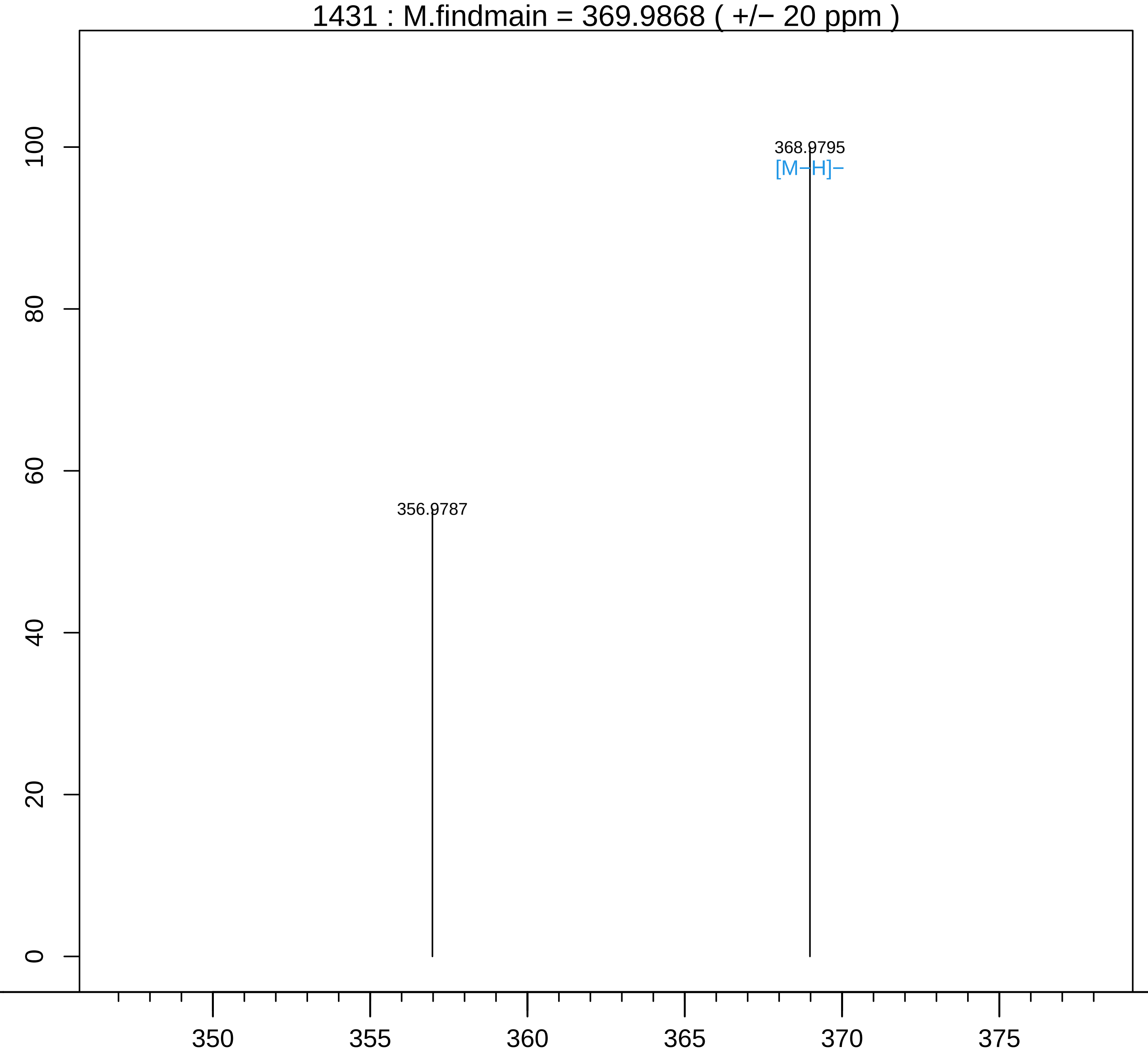
1431 : M.findmain = 369.9868 (+/− 20 ppm)
368.9795
[M−H]−
356.9787
0
20
40
60
80
100
350
355
360
365
370
375

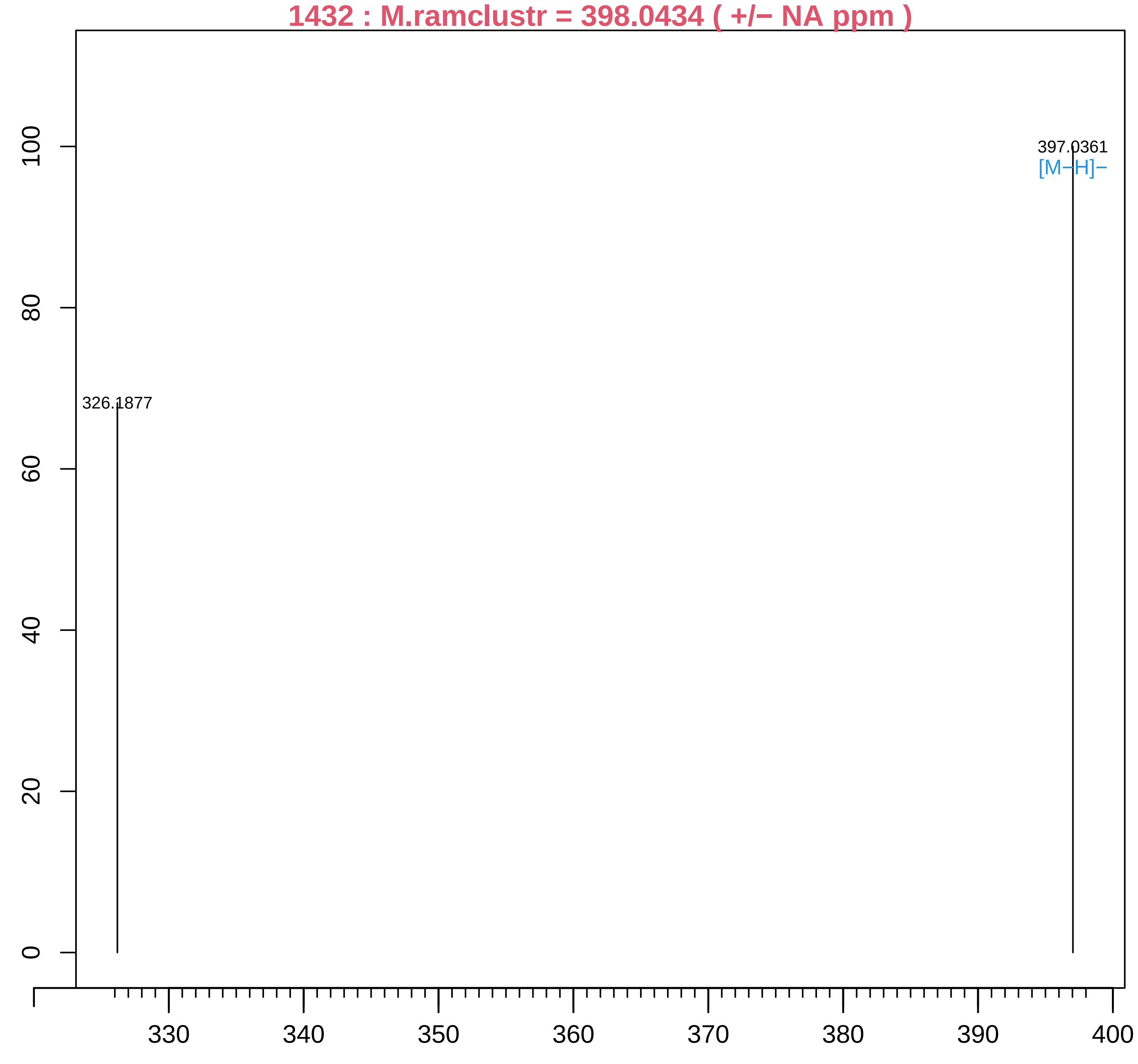
1432 : M.ramclustr = 398.0434 (+/− NA ppm)
397.0361
[M−H]−
326.1877
0
20
40
60
80
100
330
340
350
360
370
380
390
400

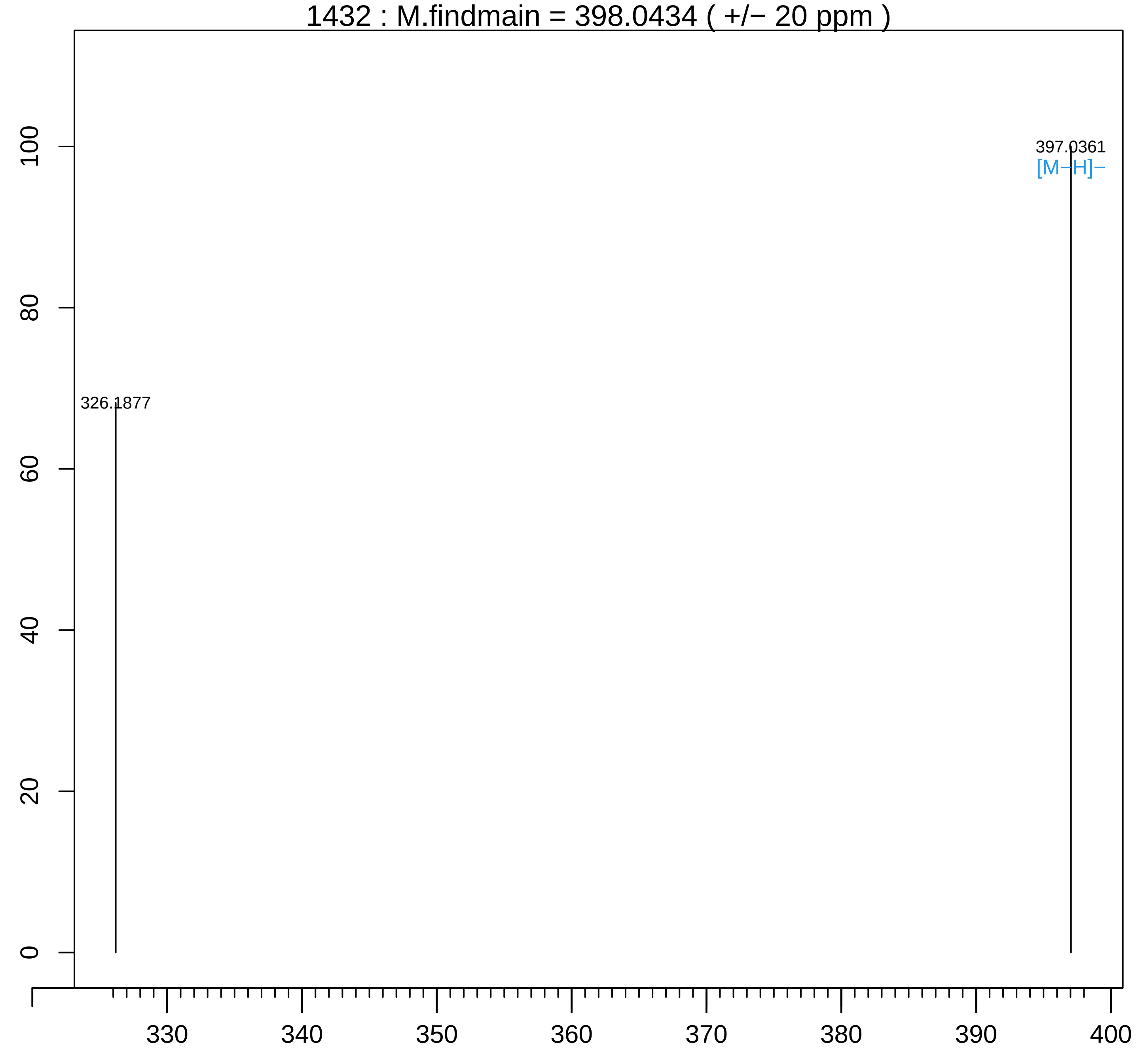
1432 : M.findmain = 398.0434 (+/− 20 ppm)
397.0361
[M−H]−
326.1877
0
20
40
60
80
100
330
340
350
360
370
380
390
400

1433 : M.ramclustr = 355.9872 (+/− NA ppm)

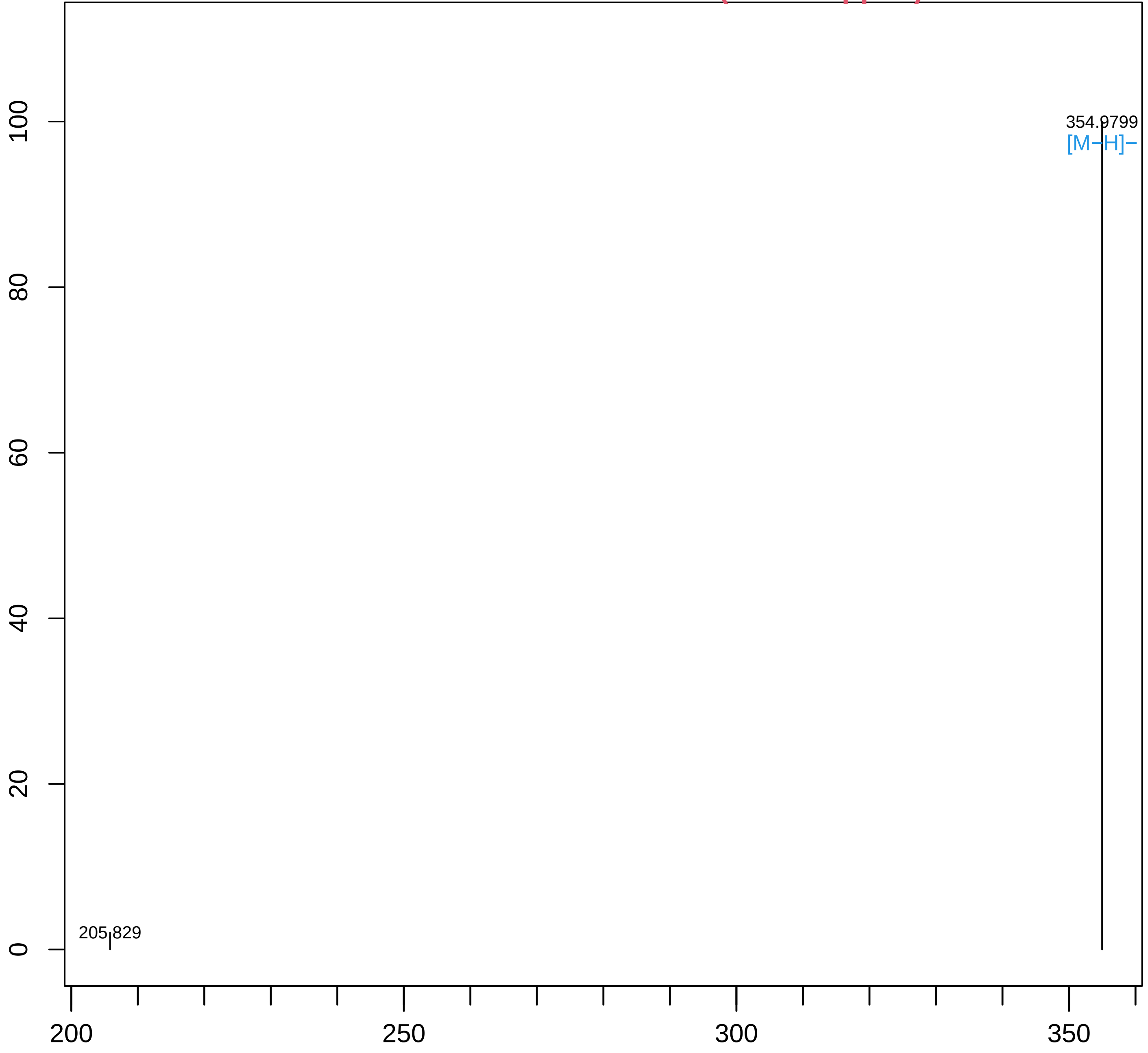

1433 : M.findmain = 355.9872 (+/− 20 ppm)

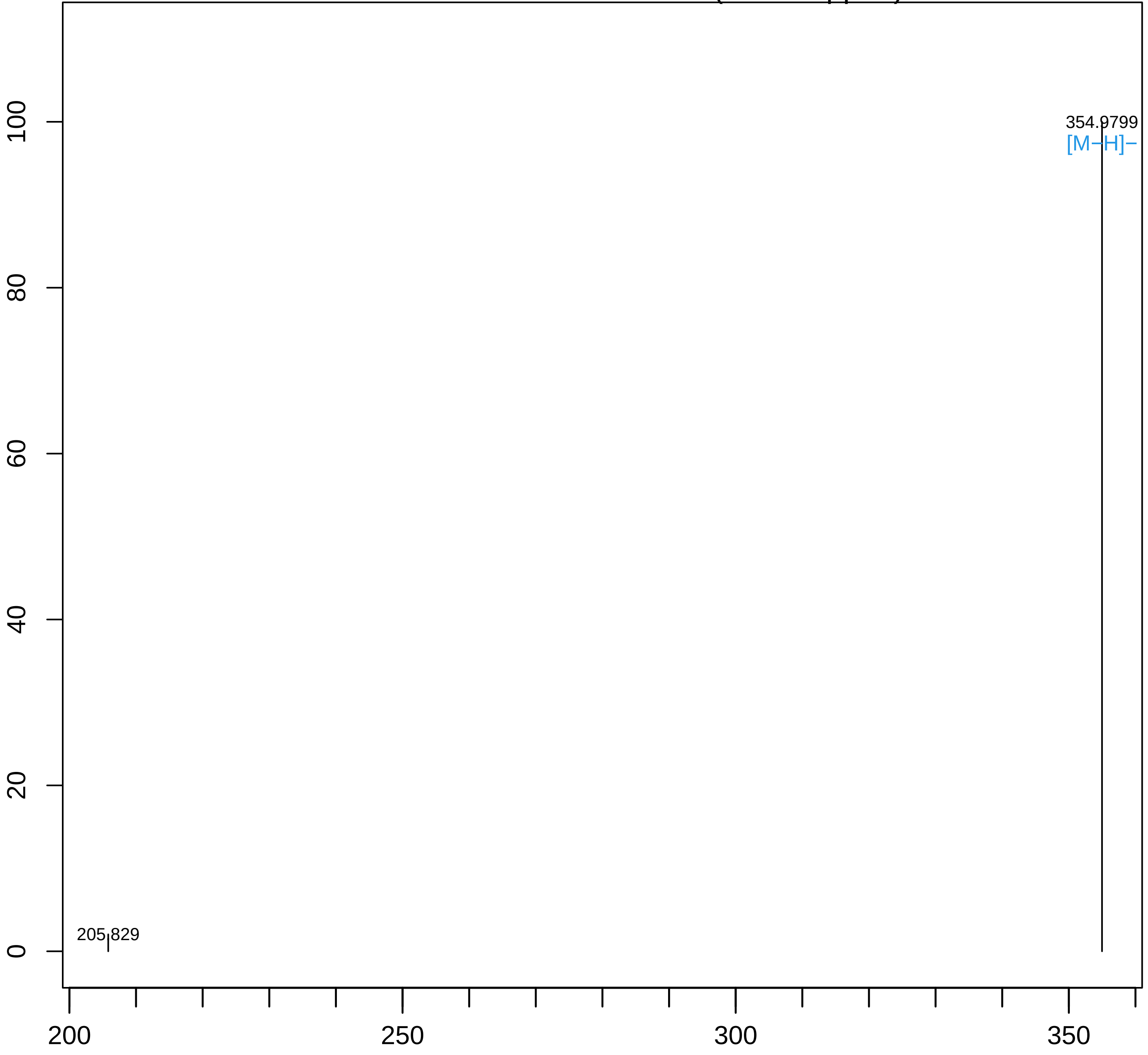

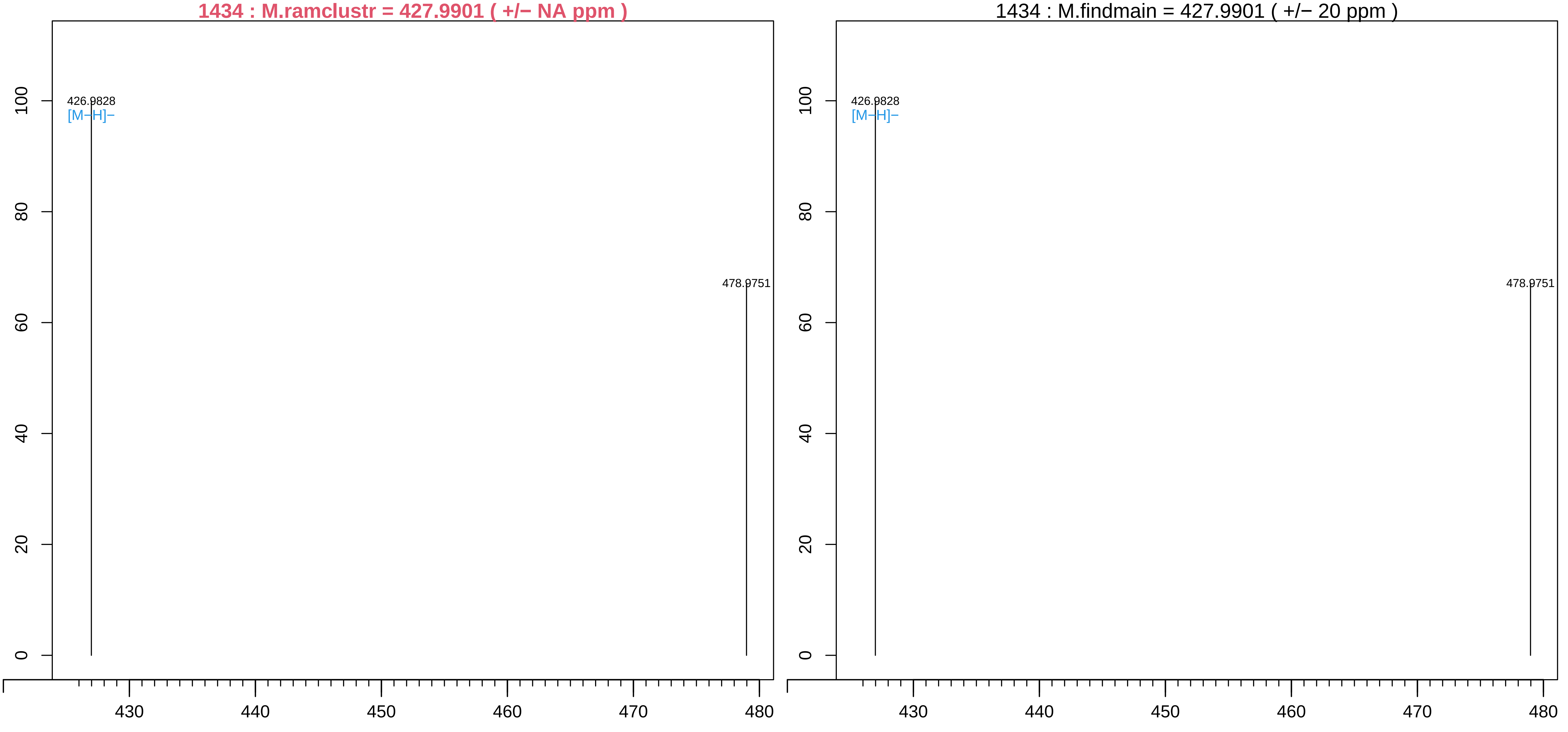
1434 : M.ramclustr = 427.9901 (+/− NA ppm)
426.9828
[M−H]−
478.9751
1434 : M.findmain = 427.9901 (+/− 20 ppm)
426.9828
[M−H]−
478.9751
0
20
40
60
80
100
430
440
450
460
470
480

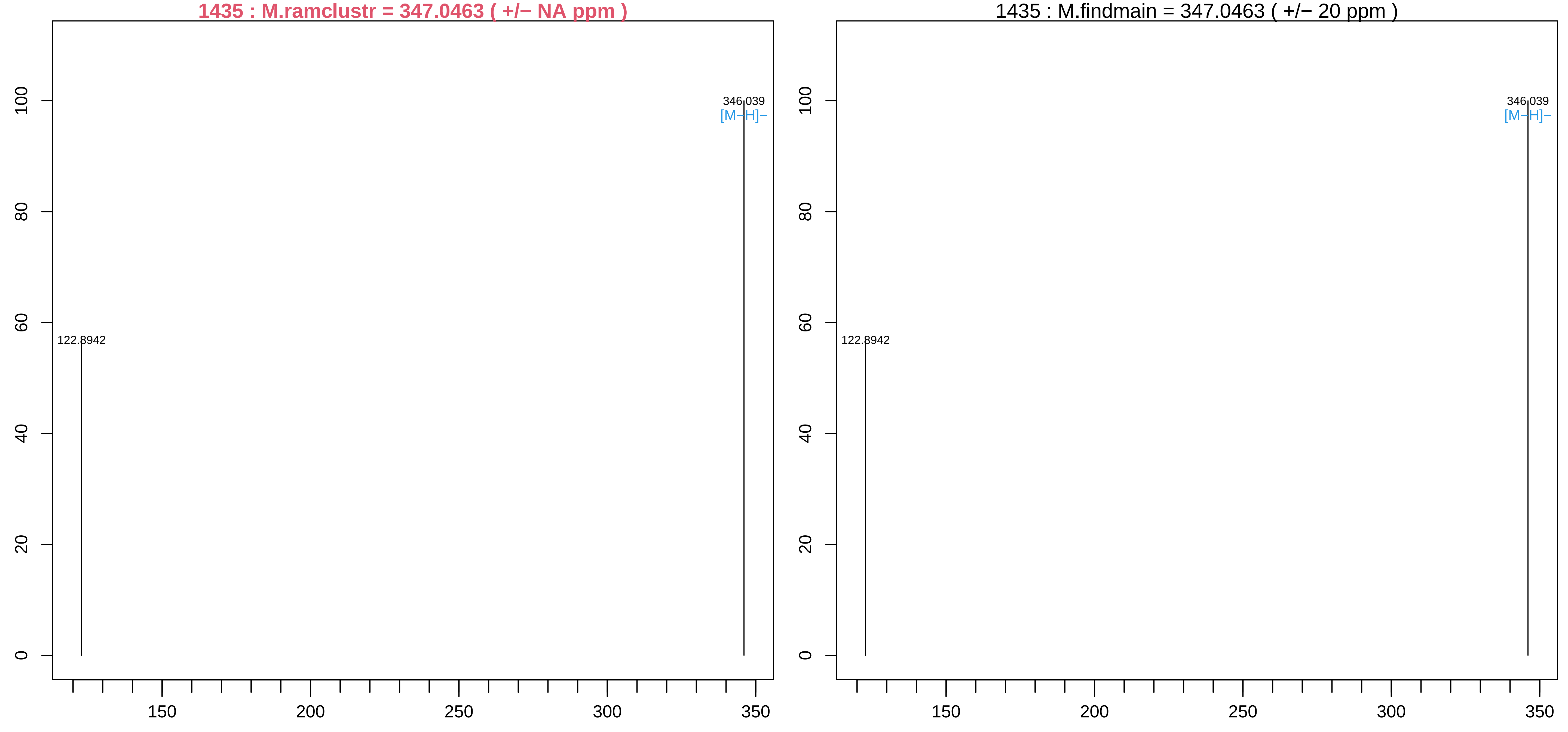

1435 : M.ramclustr = 347.0463 (+/− NA ppm)
346.039
[M−H]−
122.8942
1435 : M.findmain = 347.0463 (+/− 20 ppm)
346.039
[M−H]−
122.8942
0
20
40
60
80
100
150
200
250
300
350

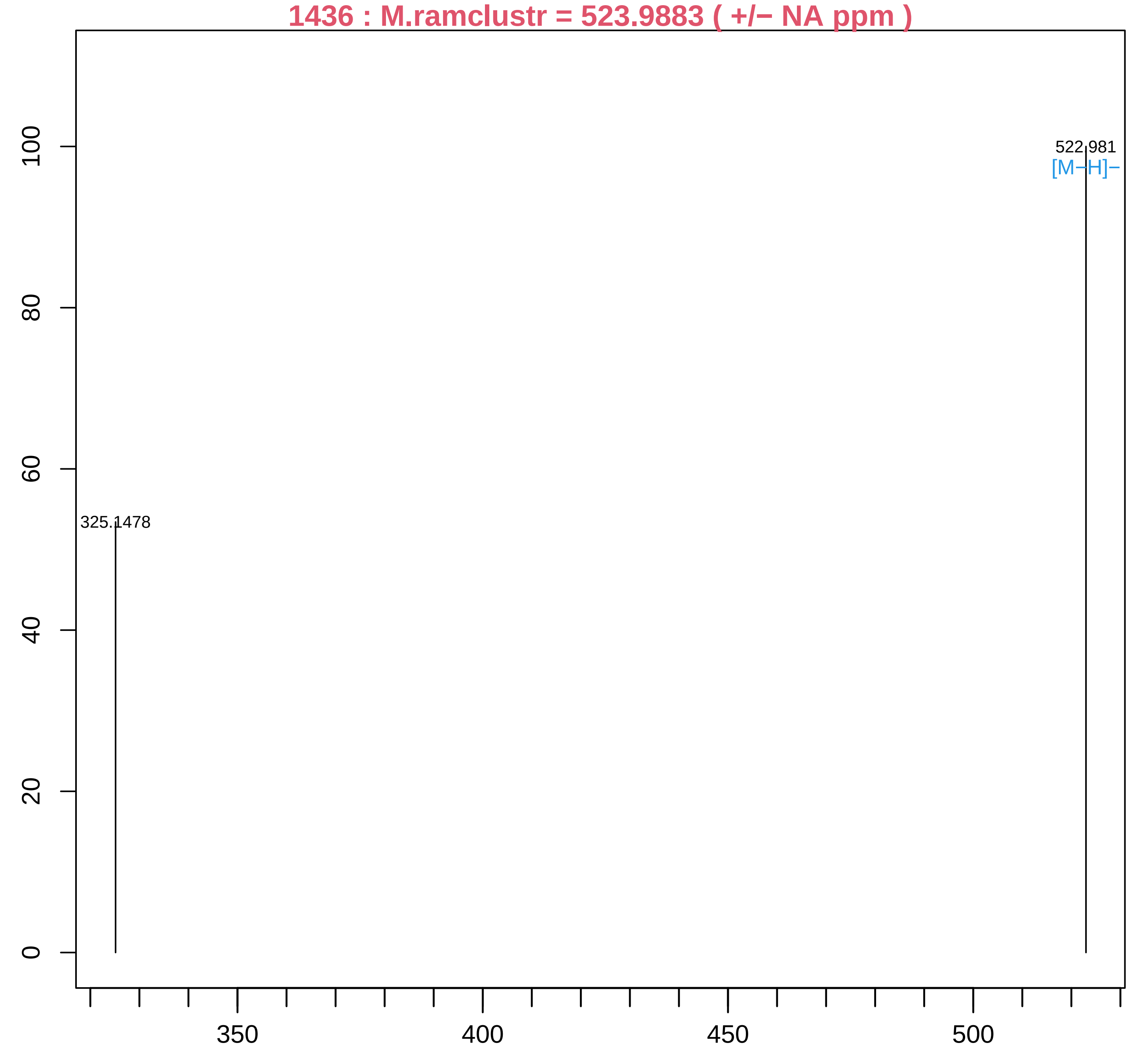
1436 : M.ramclustr = 523.9883 (+/− NA ppm)
100
80
60
40
20
0
350
400
450
500
522.981
[M−H]−
325.1478

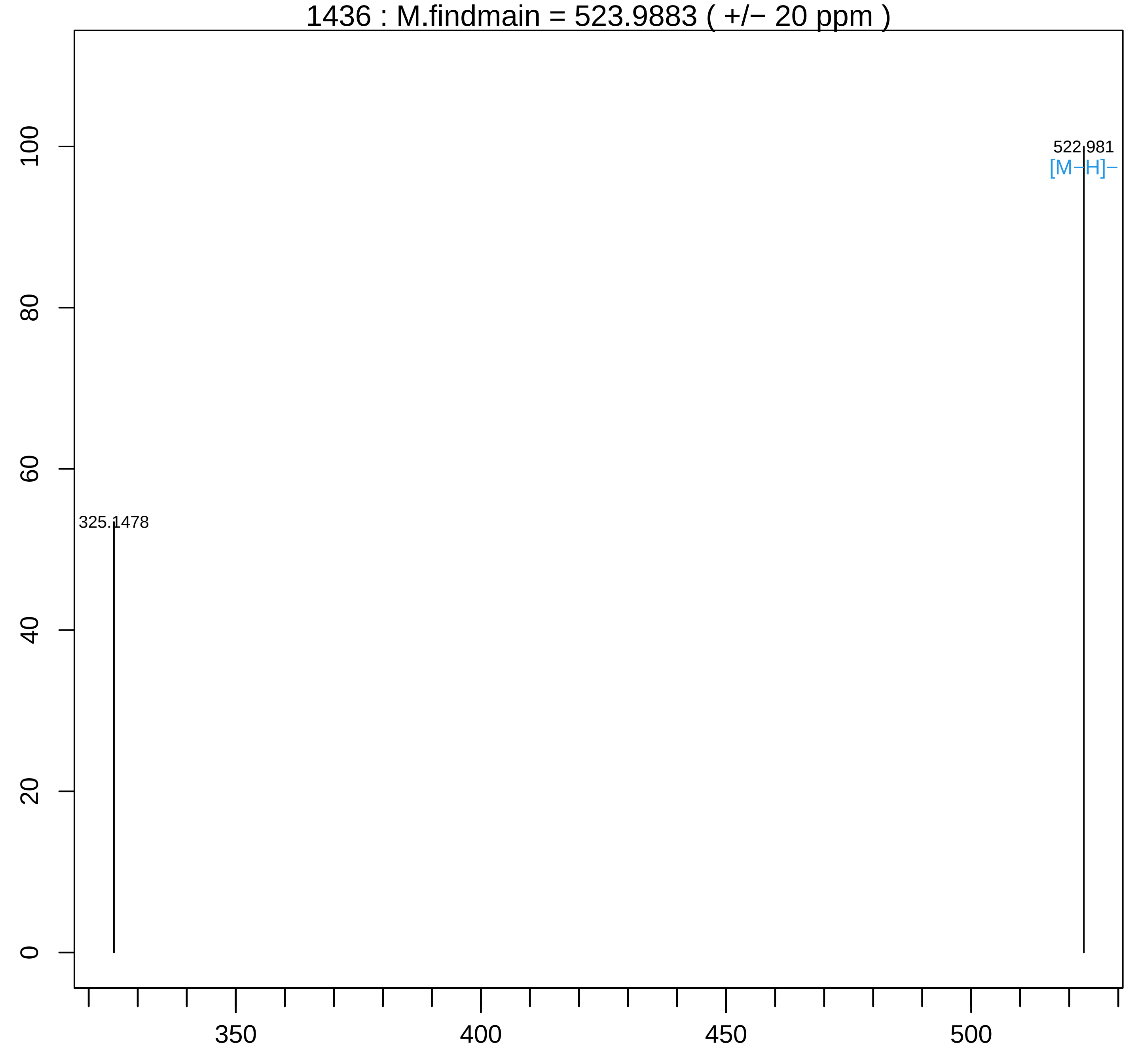
1436 : M.findmain = 523.9883 (+/− 20 ppm)
100
80
60
40
20
0
350
400
450
500
522.981
[M−H]−
325.1478

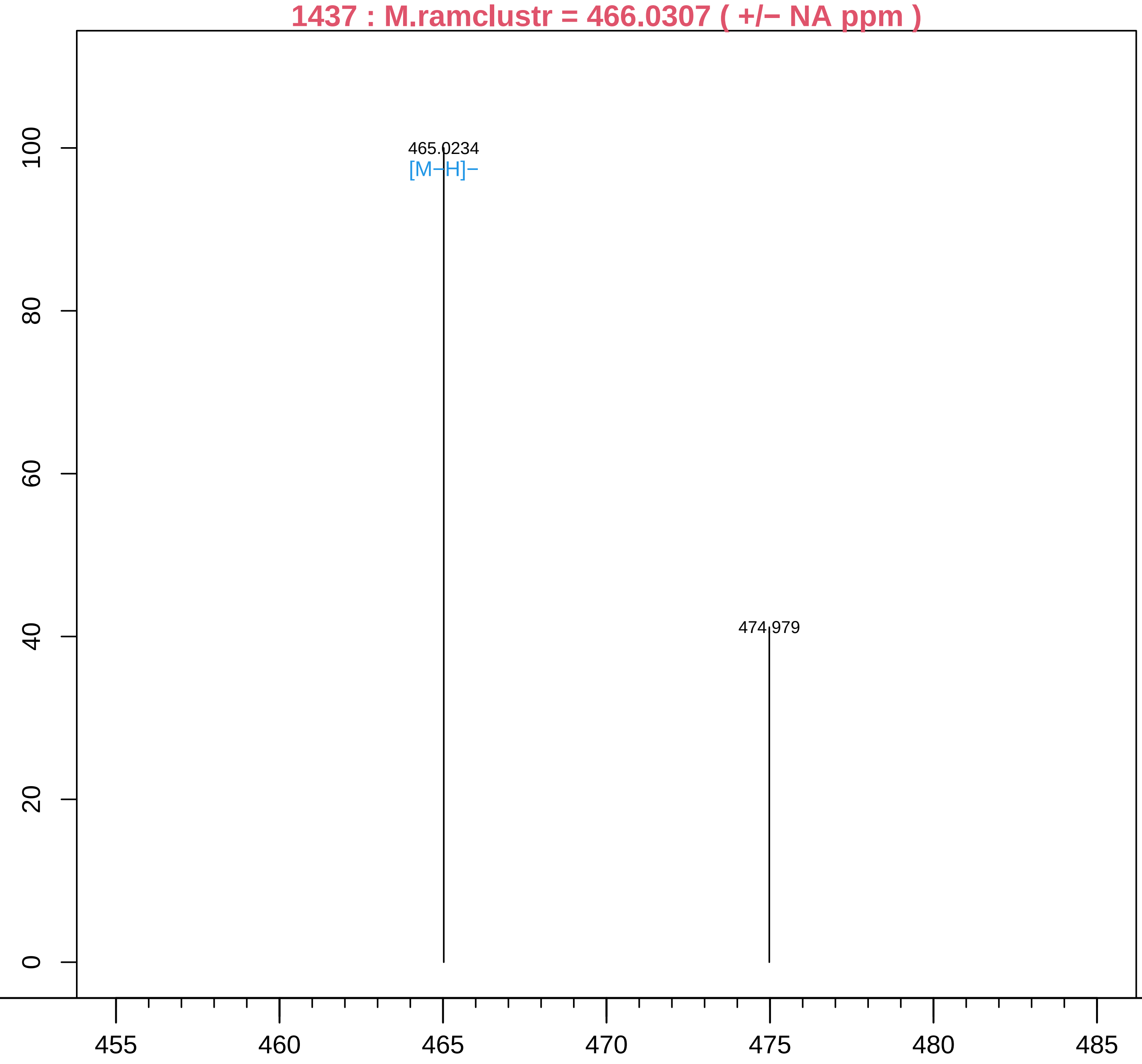
1437 : M.ramclustr = 466.0307 (+/− NA ppm)
465.0234
[M−H]−
474.979
0
20
40
60
80
100
455
460
465
470
475
480
485

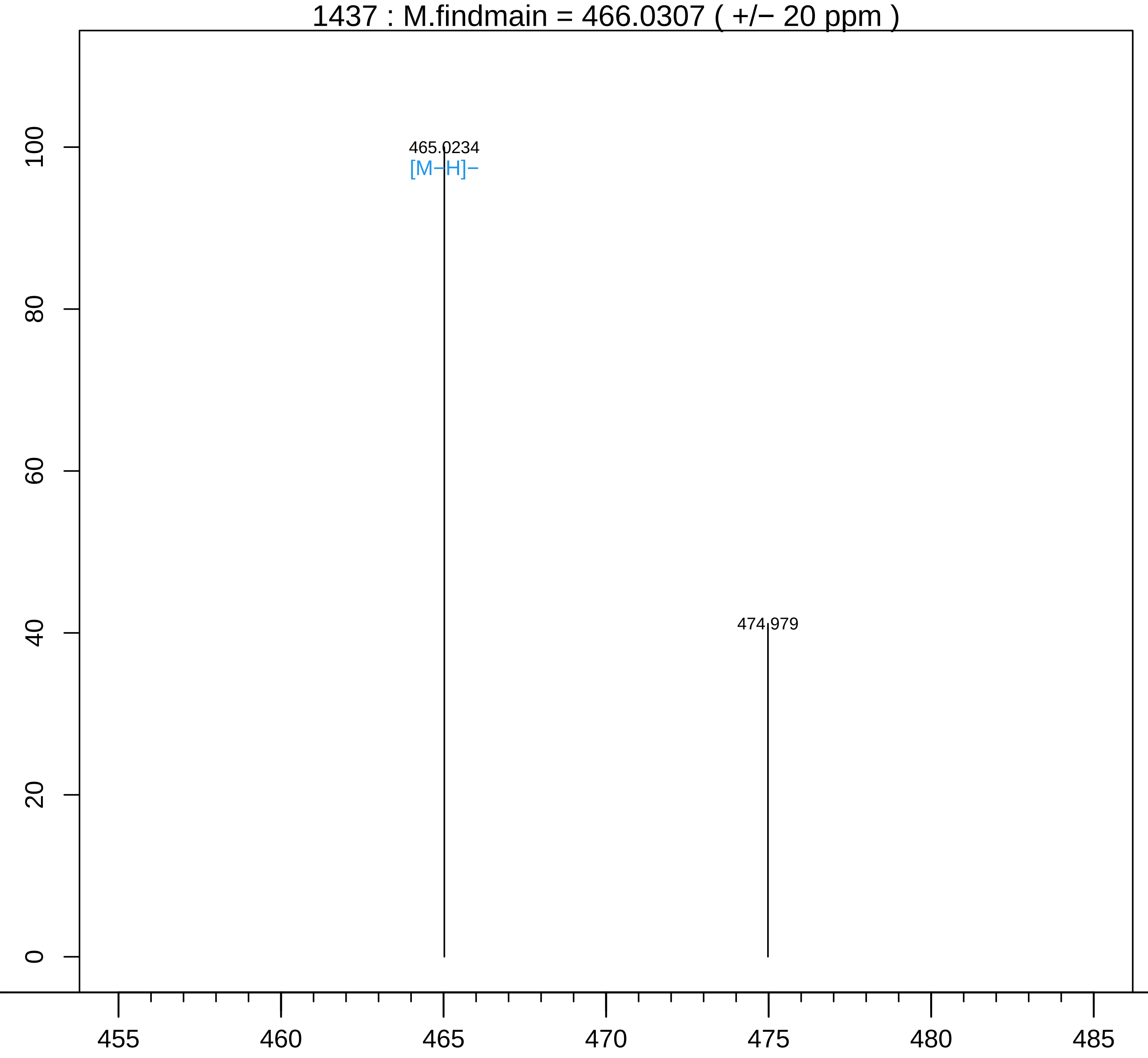
1437 : M.findmain = 466.0307 (+/− 20 ppm)
465.0234
[M−H]−
474.979
0
20
40
60
80
100
455
460
465
470
475
480
485

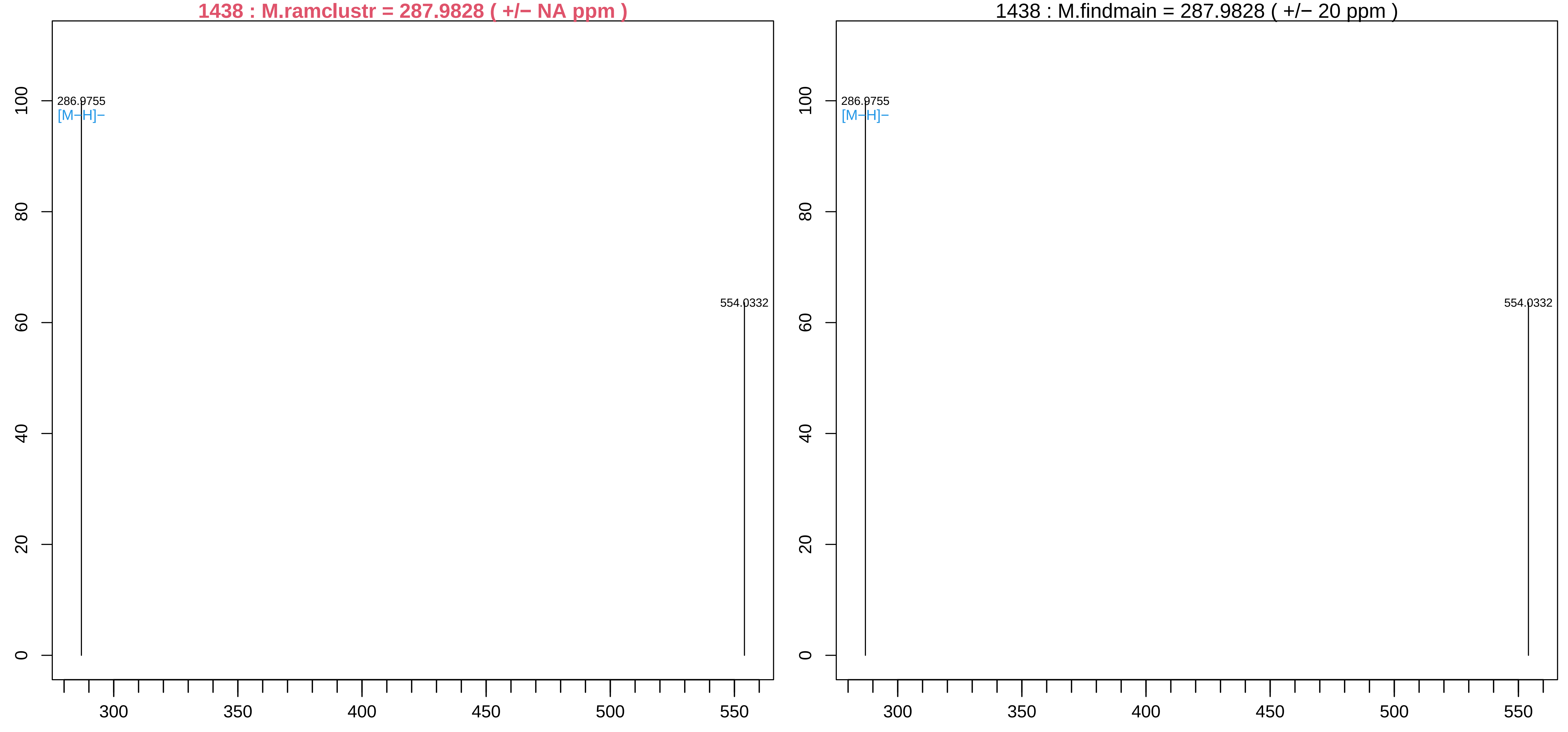

1438 : M.ramclustr = 287.9828 (+/− NA ppm)
286.9755
[M−H]−
554.0332
1438 : M.findmain = 287.9828 (+/− 20 ppm)
286.9755
[M−H]−
554.0332
0
20
40
60
80
100
300
350
400
450
500
550

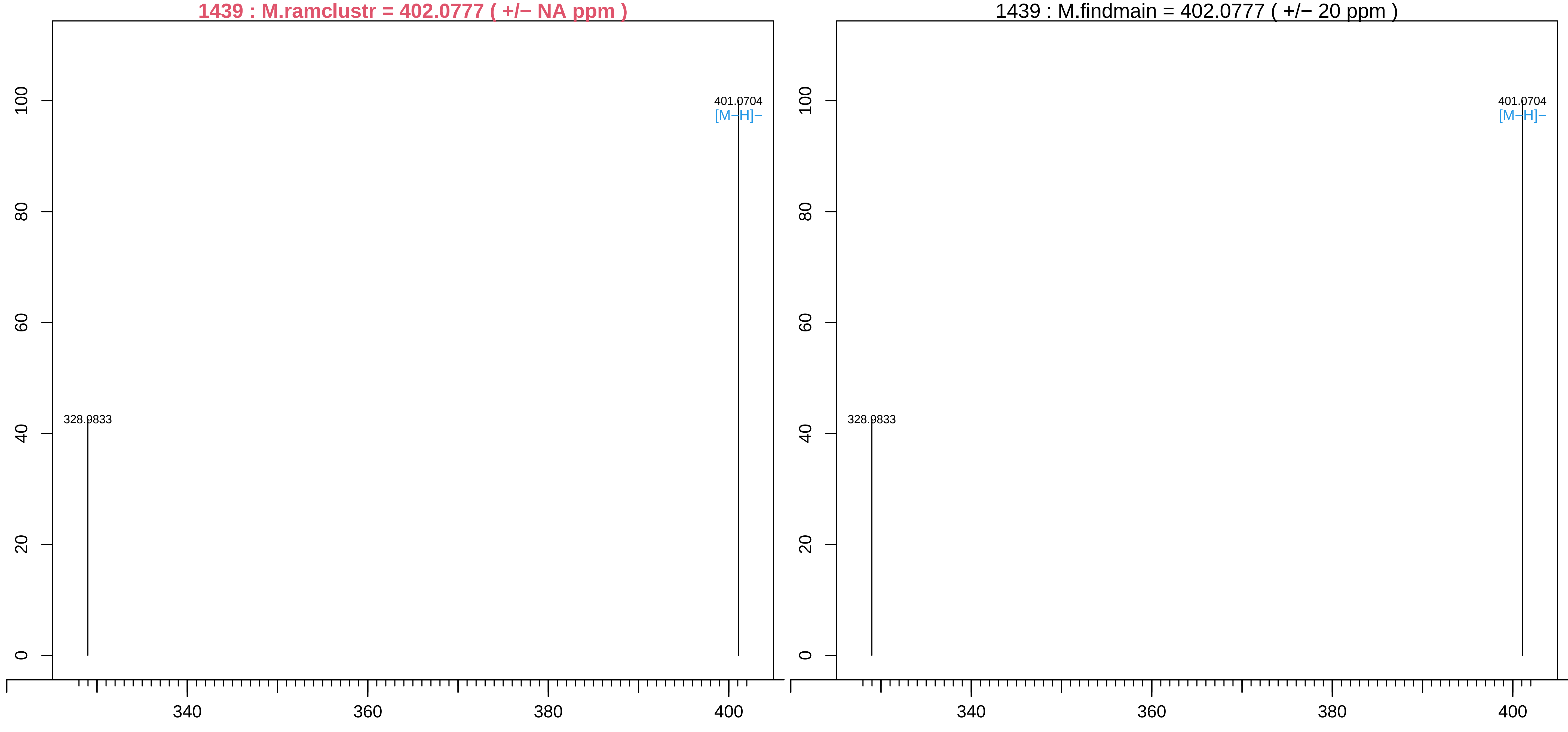

1439 : M.ramclustr = 402.0777 (+/− NA ppm)
401.0704
[M−H]−
328.9833
0
20
40
60
80
100
340
360
380
400
1439 : M.findmain = 402.0777 (+/− 20 ppm)
401.0704
[M−H]−
328.9833
0
20
40
60
80
100
340
360
380
400

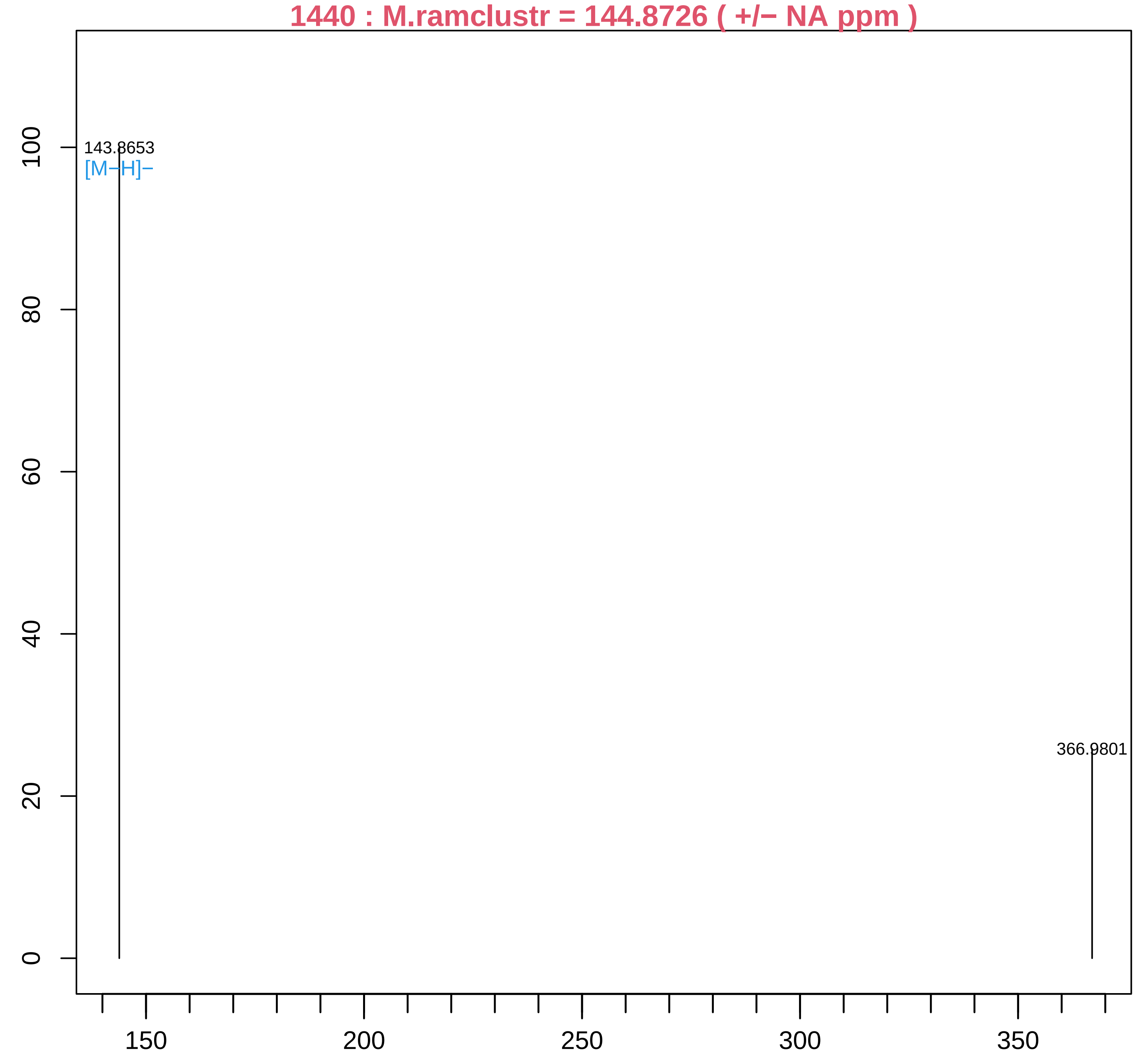
1440 : M.ramclustr = 144.8726 (+/− NA ppm)
143.8653
[M−H]−
366.9801
0
20
40
60
80
100
150
200
250
300
350

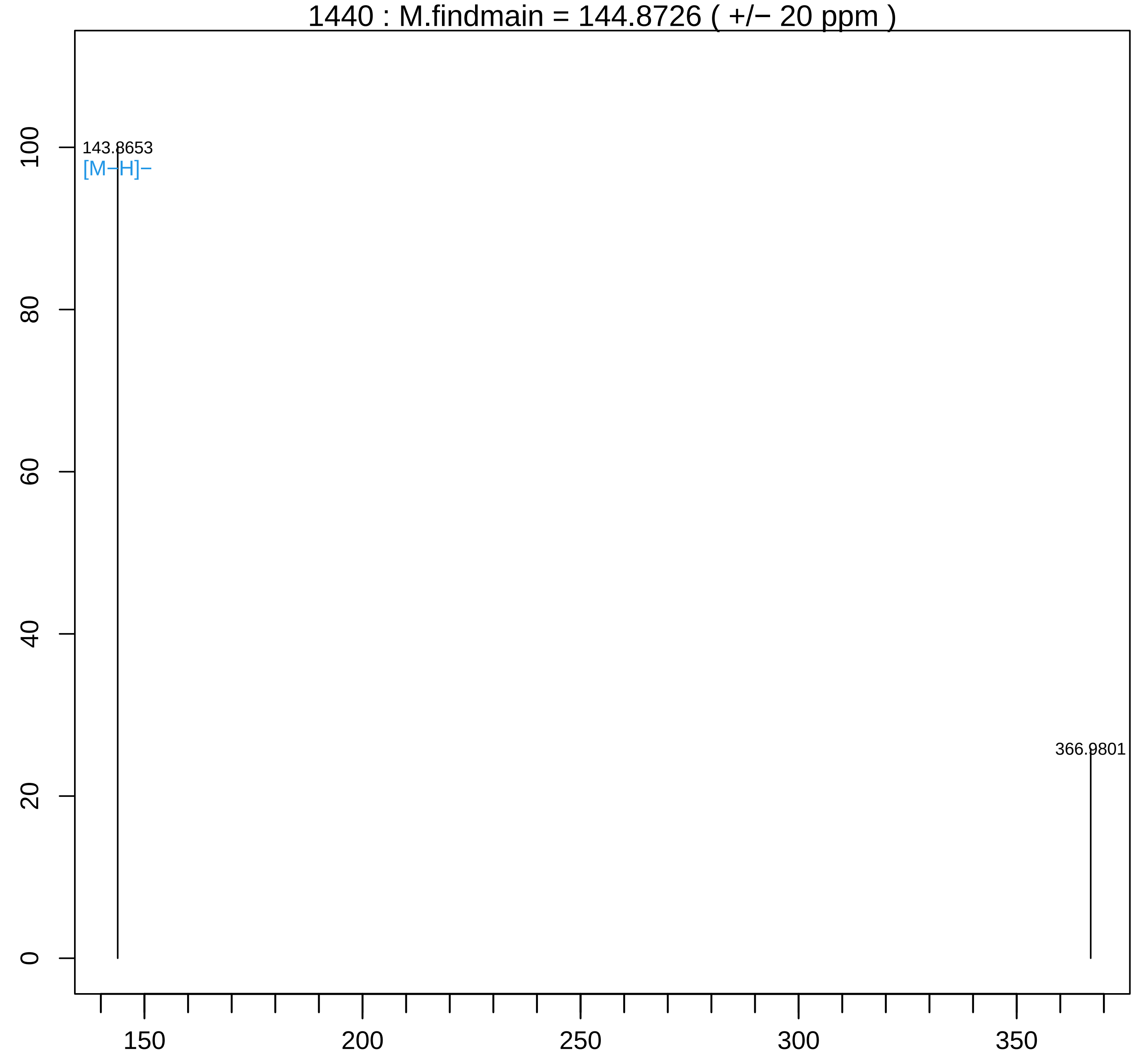
1440 : M.findmain = 144.8726 (+/− 20 ppm)
143.8653
[M−H]−
366.9801
0
20
40
60
80
100
150
200
250
300
350

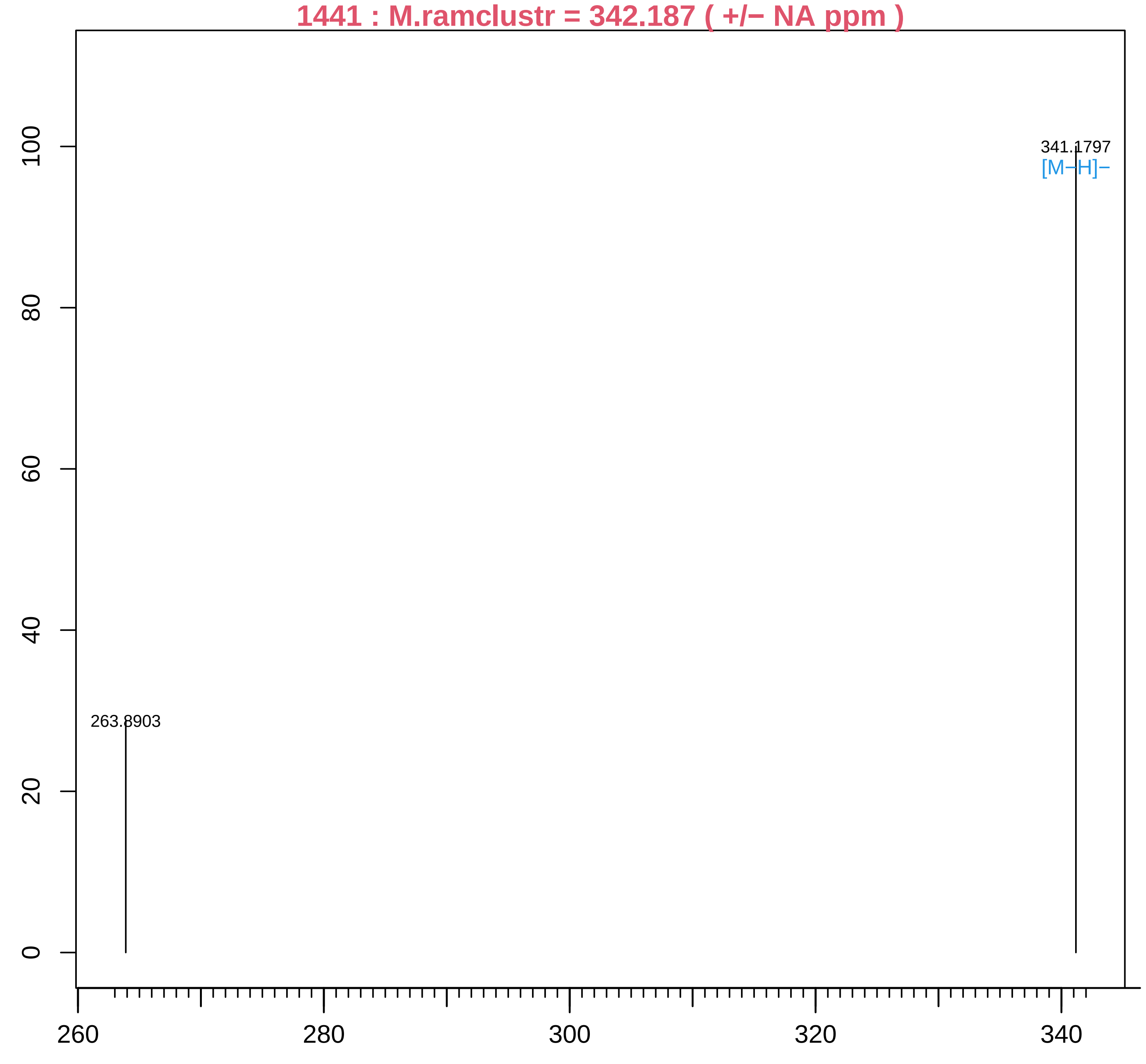
1441 : M.ramclustr = 342.187 (+/− NA ppm)
341.1797
[M−H]−
263.8903
0
20
40
60
80
100
260
280
300
320
340

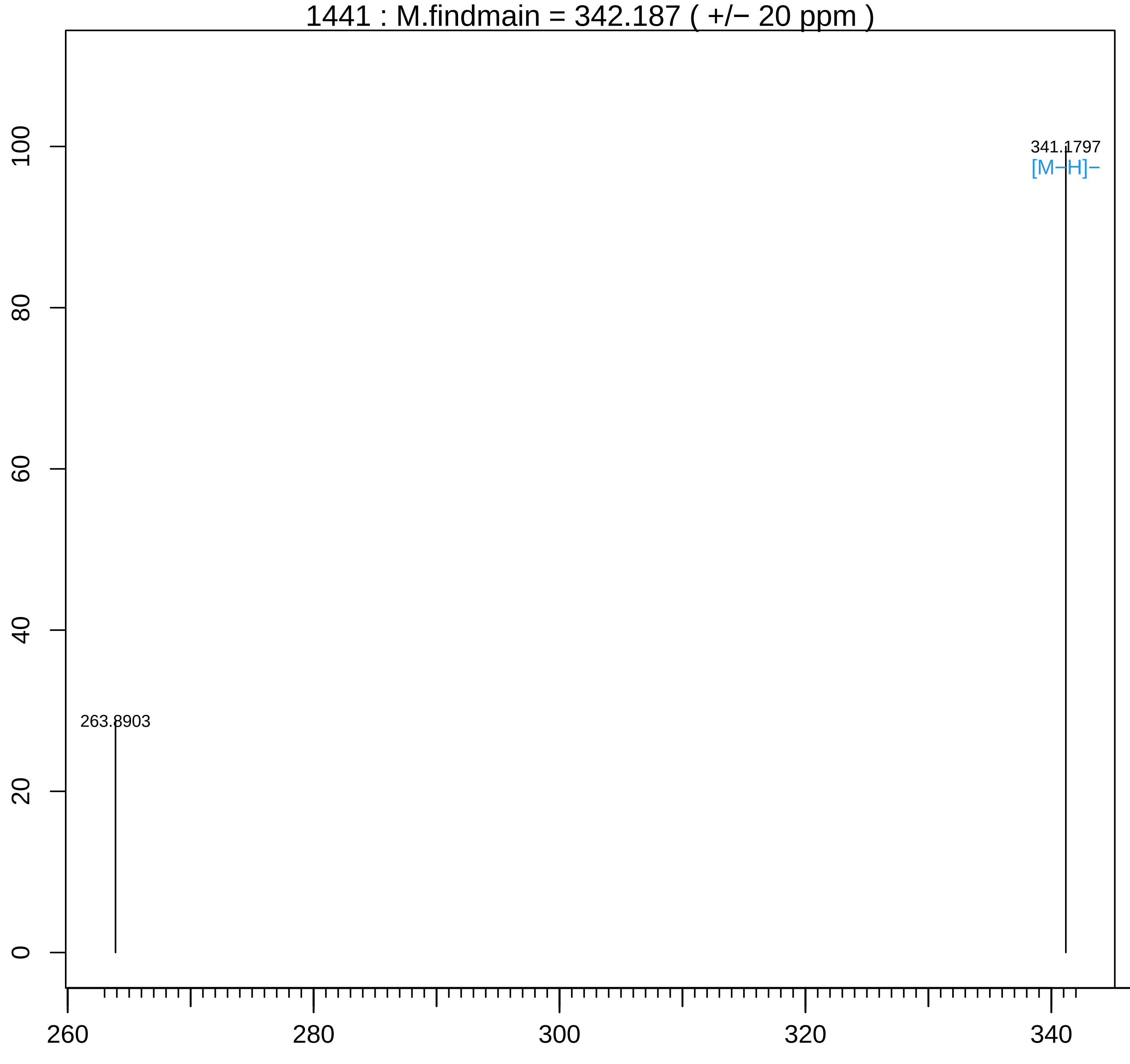
1441 : M.findmain = 342.187 (+/− 20 ppm)
341.1797
[M−H]−
263.8903
0
20
40
60
80
100
260
280
300
320
340

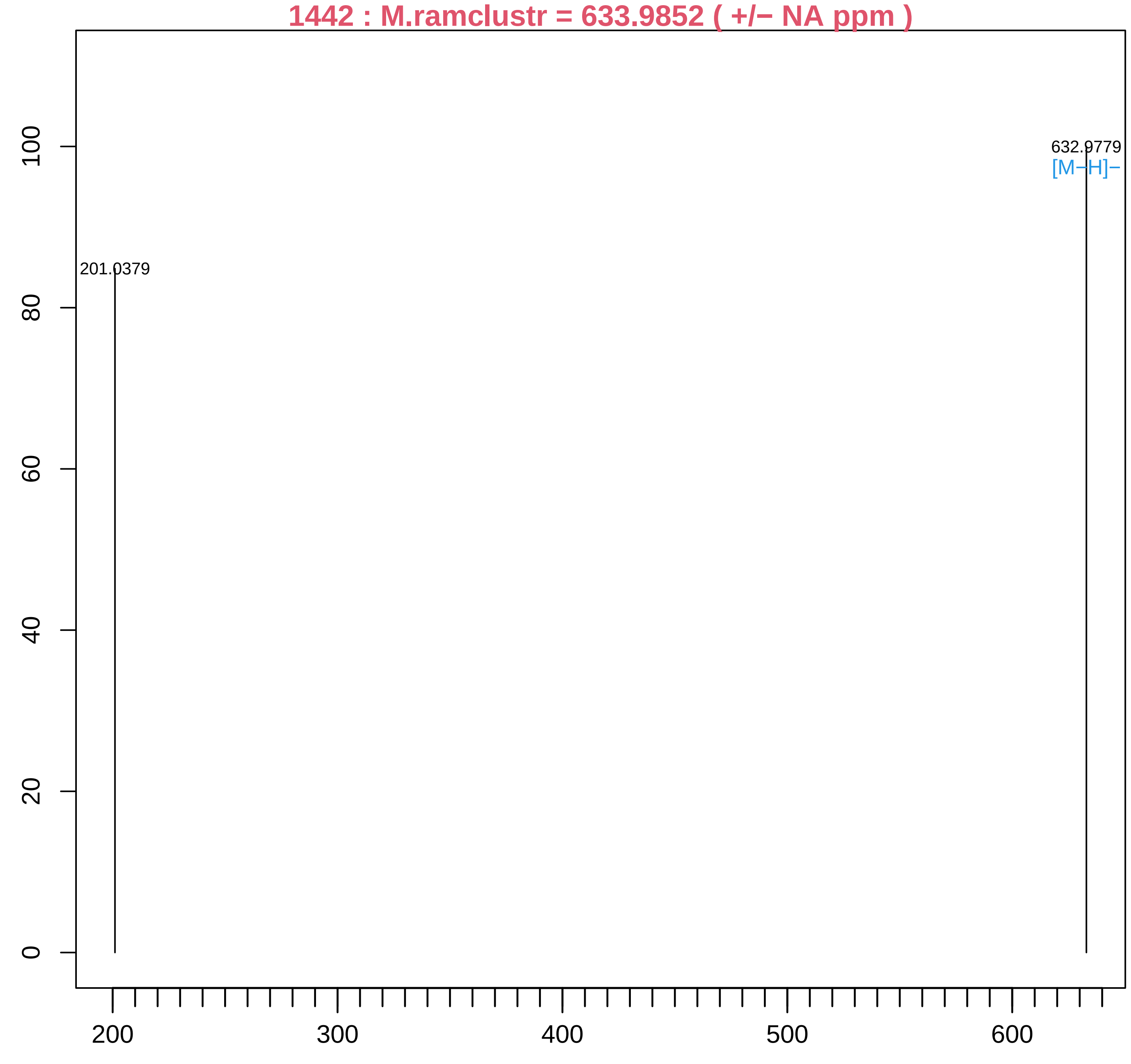
1442 : M.ramclustr = 633.9852 (+/− NA ppm)
632.9779
[M−H]−
201.0379
0
20
40
60
80
100
200
300
400
500
600

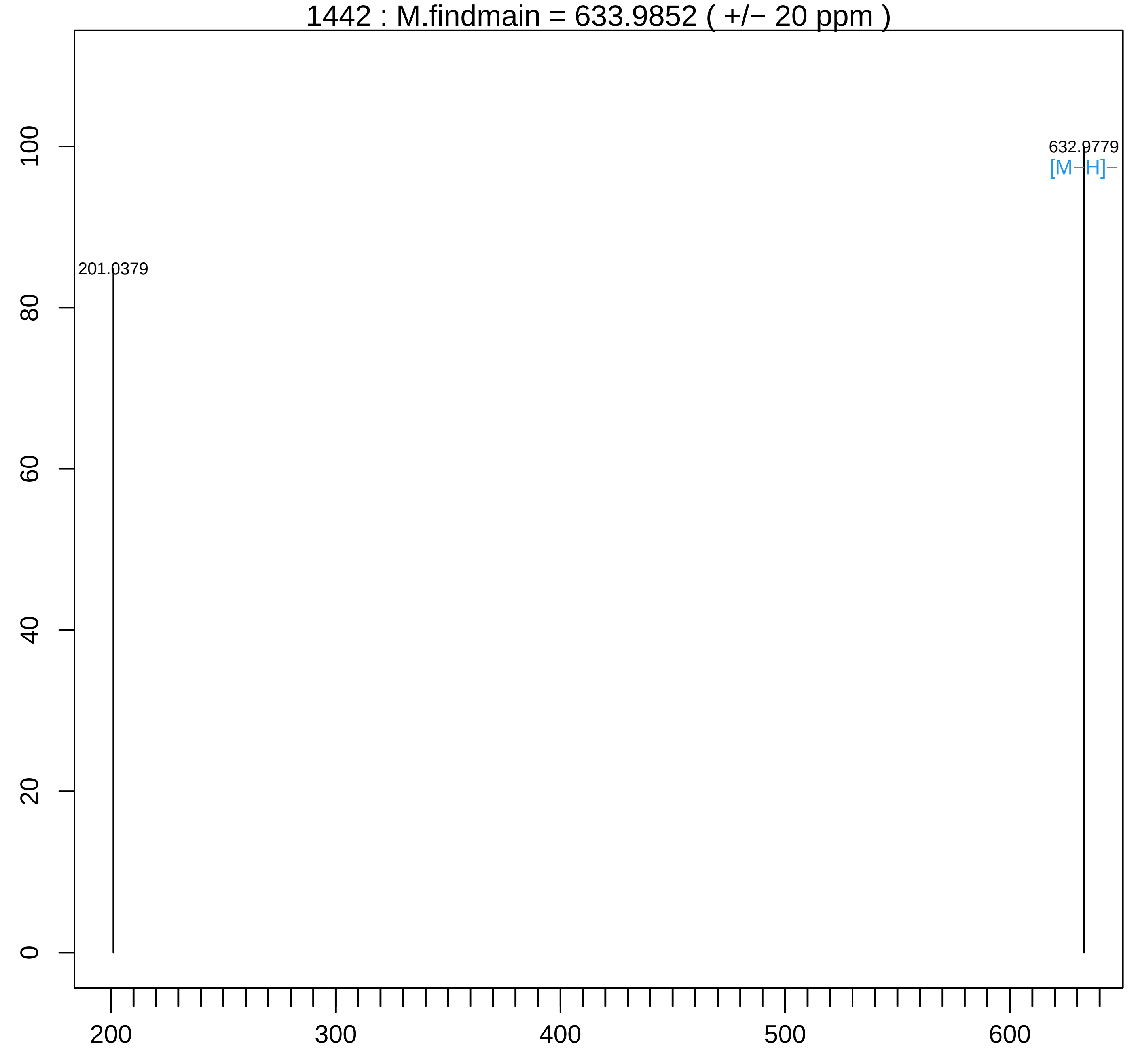
1442 : M.findmain = 633.9852 (+/− 20 ppm)
632.9779
[M−H]−
201.0379
0
20
40
60
80
100
200
300
400
500
600

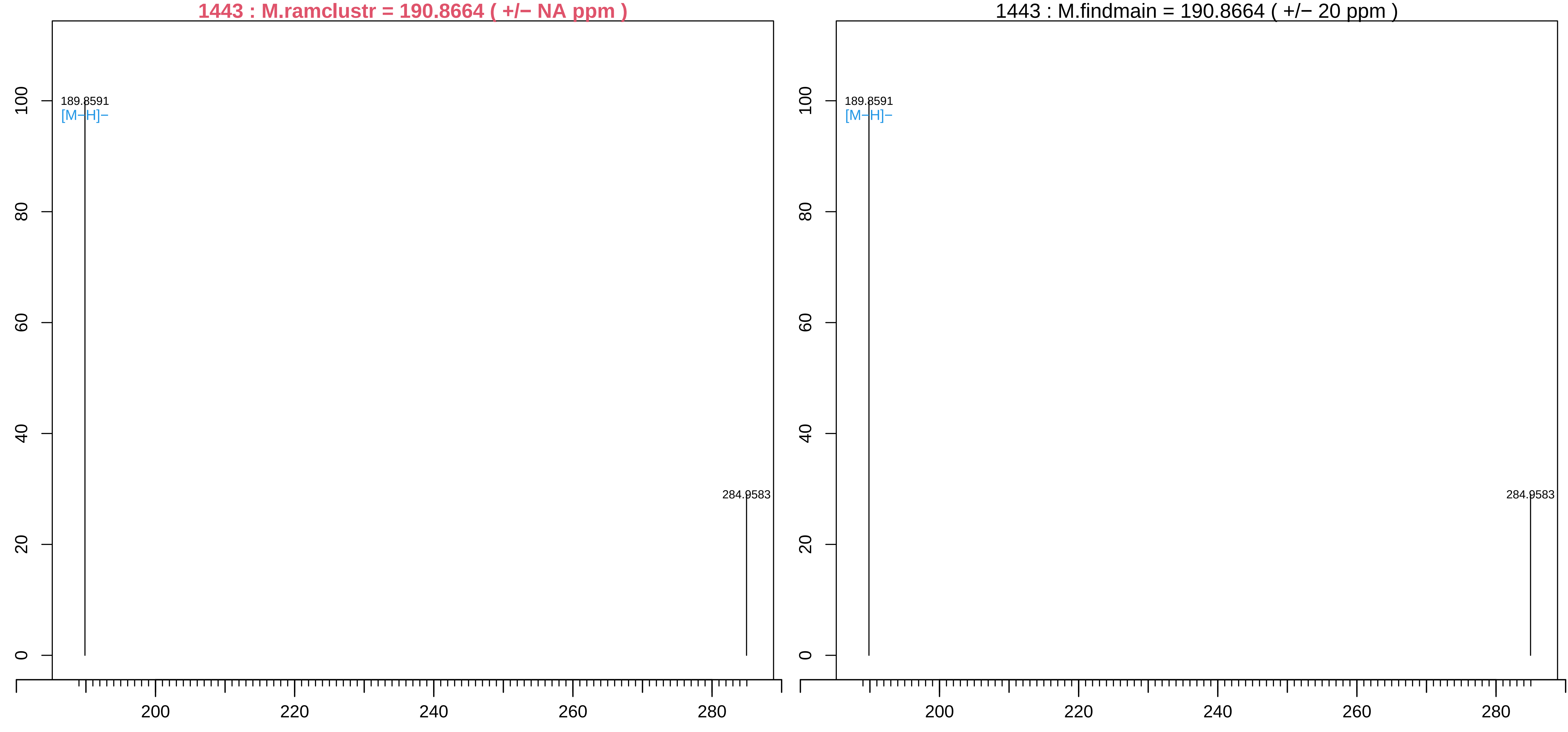
1443 : M.ramclustr = 190.8664 (+/− NA ppm)
1443 : M.findmain = 190.8664 (+/− 20 ppm)
189.8591
[M−H]−
284.9583
0
20
40
60
80
100
200
220
240
260
280

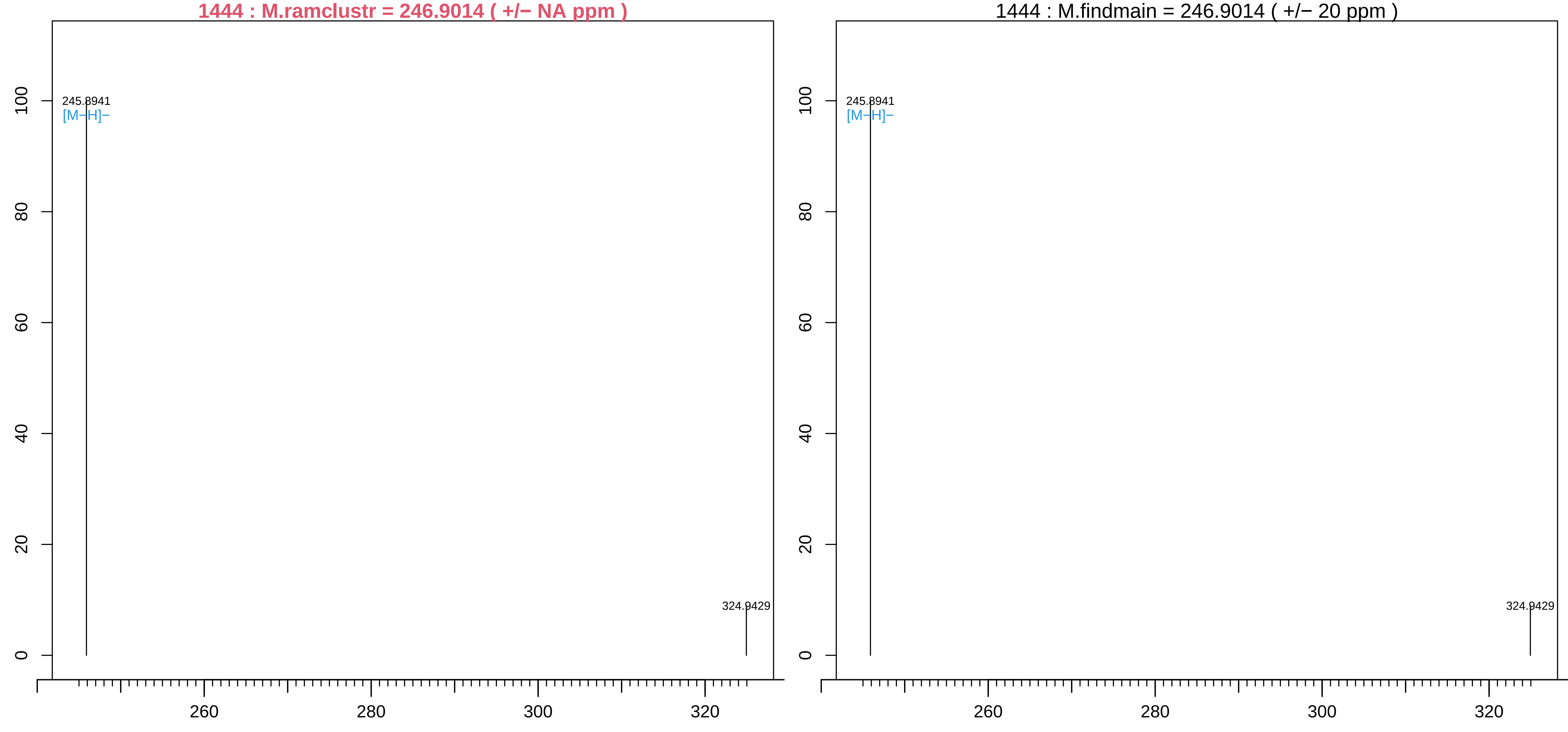
1444 : M.ramclustr = 246.9014 (+/− NA ppm)
245.8941
[M−H]−
324.9429
1444 : M.findmain = 246.9014 (+/− 20 ppm)
245.8941
[M−H]−
324.9429
0
20
40
60
80
100
260
280
300
320

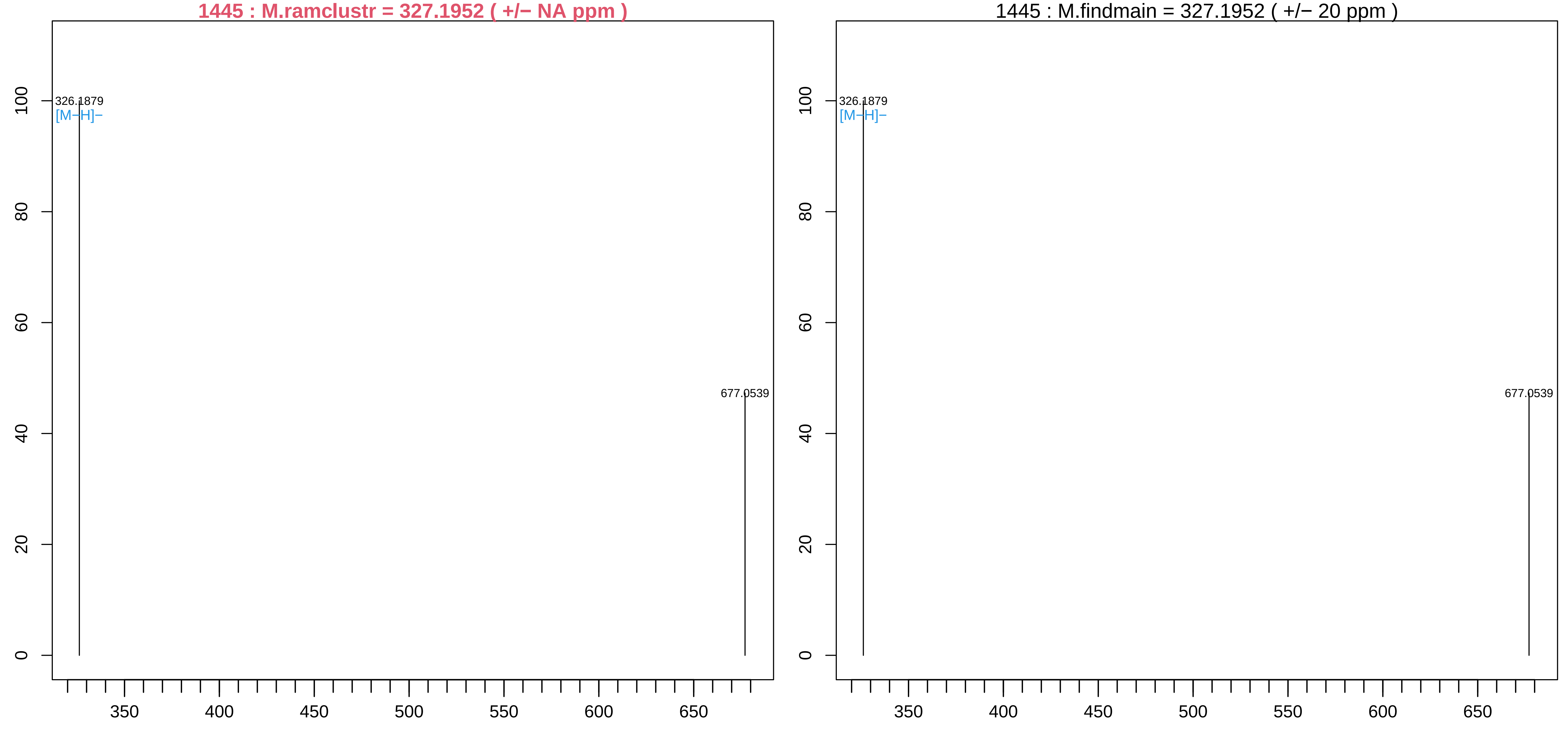
1445 : M.ramclustr = 327.1952 (+/− NA ppm)
326.1879
[M−H]−
677.0539
0
20
40
60
80
100
350
400
450
500
550
600
650
1445 : M.findmain = 327.1952 (+/− 20 ppm)
326.1879
[M−H]−
677.0539
0
20
40
60
80
100
350
400
450
500
550
600
650

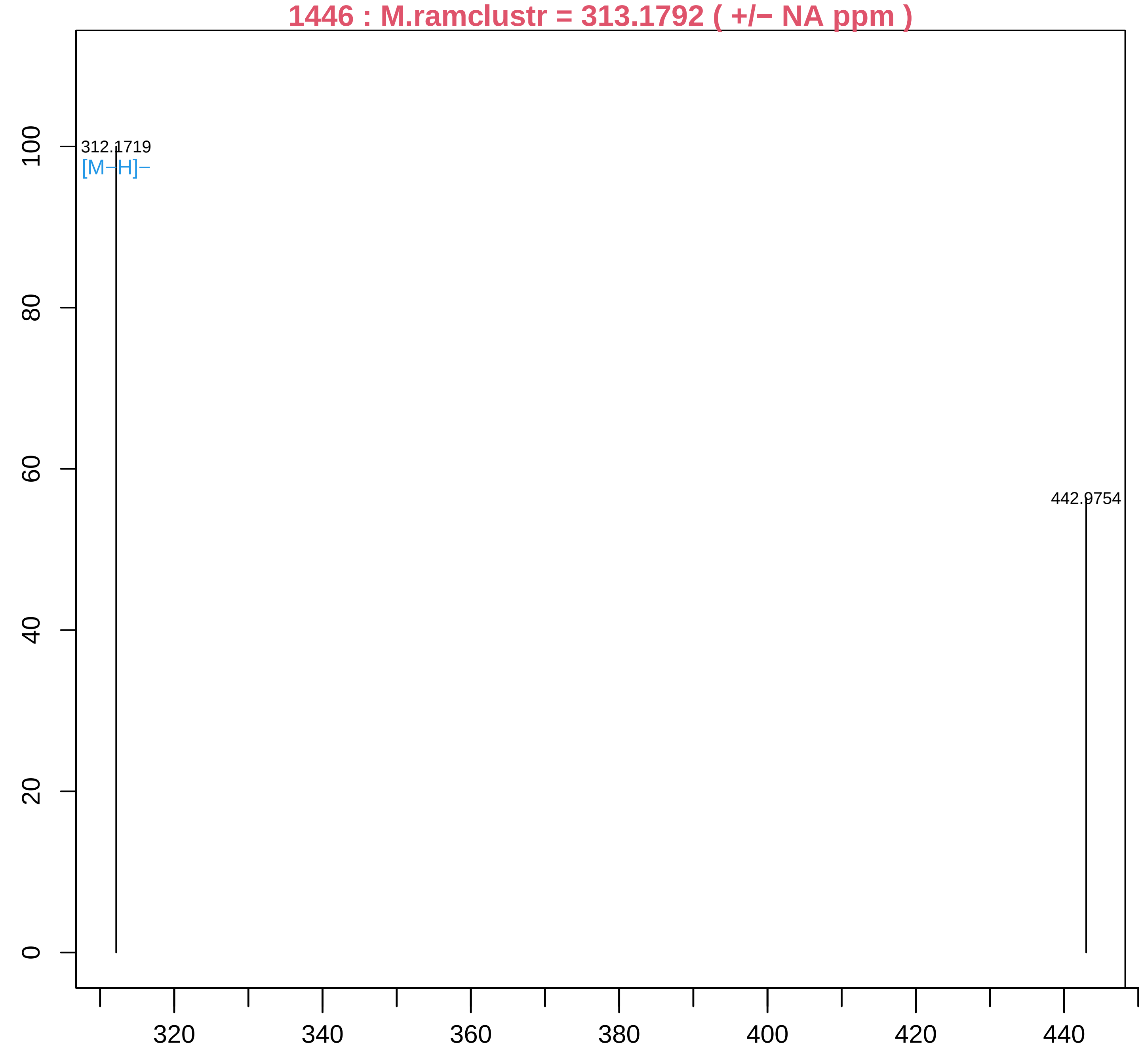
1446 : M.ramclustr = 313.1792 (+/− NA ppm)
312.1719
[M−H]−
442.9754
0
20
40
60
80
100
320
340
360
380
400
420
440
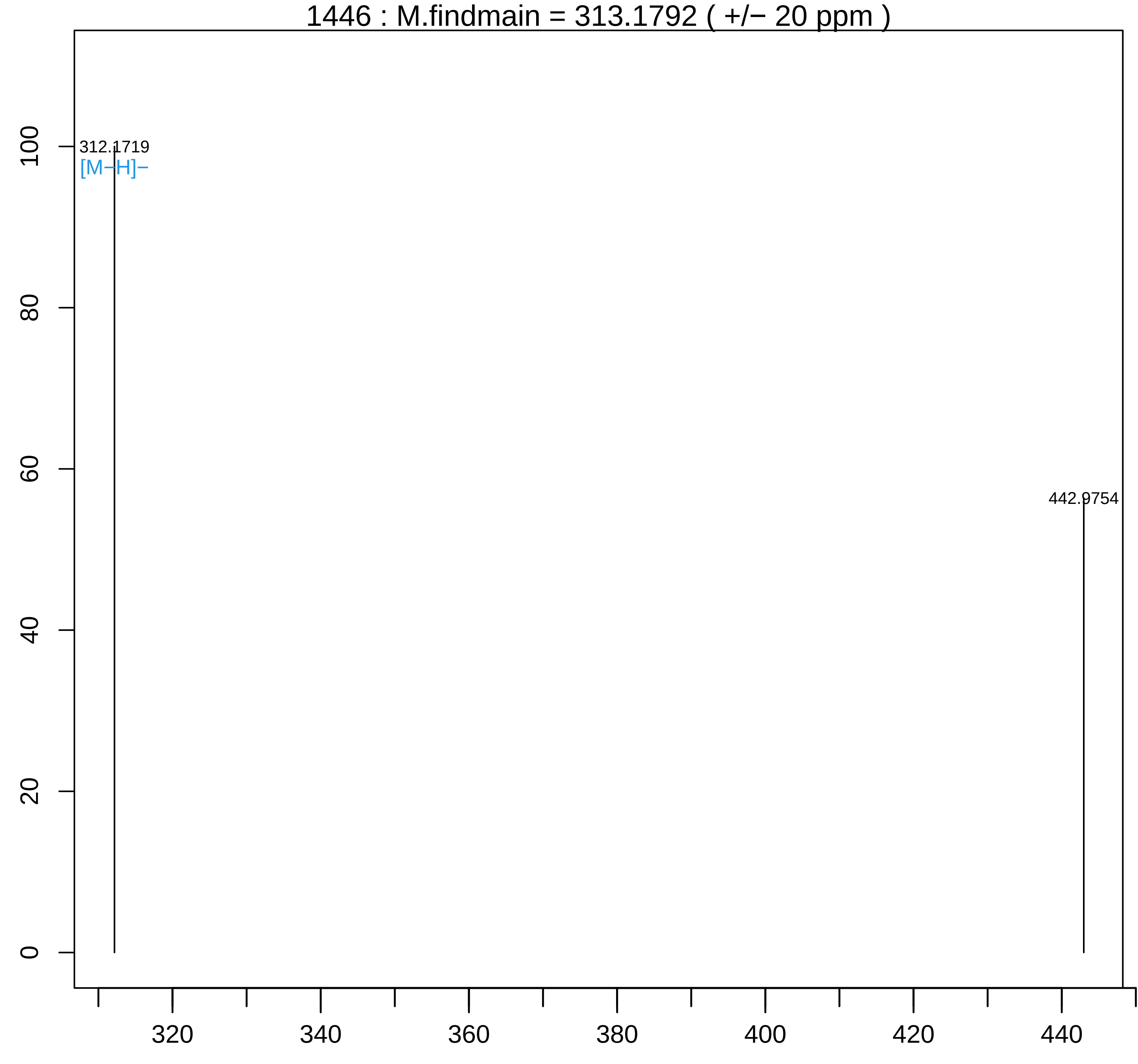
1446 : M.findmain = 313.1792 (+/− 20 ppm)
312.1719
[M−H]−
442.9754
0
20
40
60
80
100
320
340
360
380
400
420
440

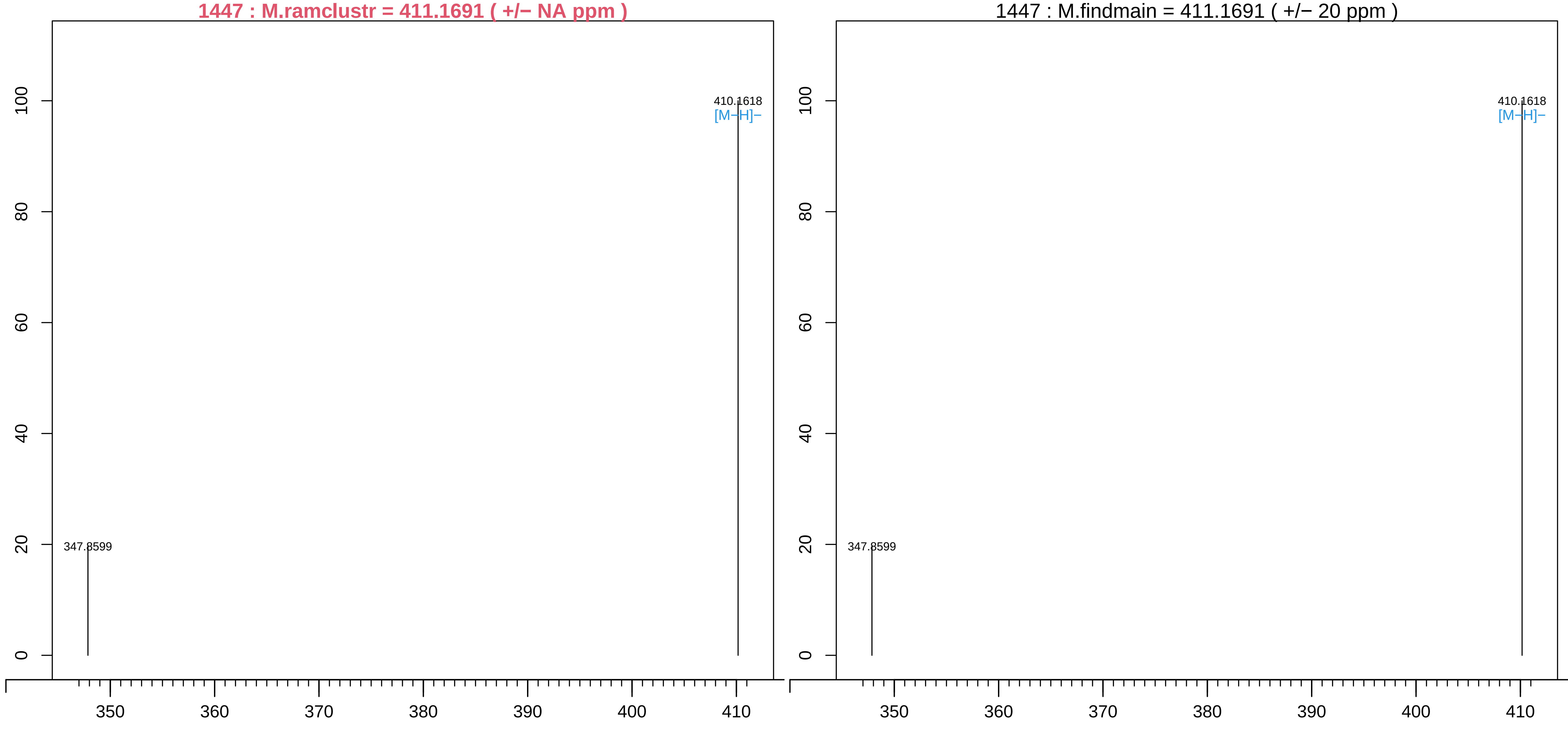

1447 : M.ramclustr = 411.1691 (+/− NA ppm)
410.1618
[M−H]−
347.8599
1447 : M.findmain = 411.1691 (+/− 20 ppm)
410.1618
[M−H]−
347.8599

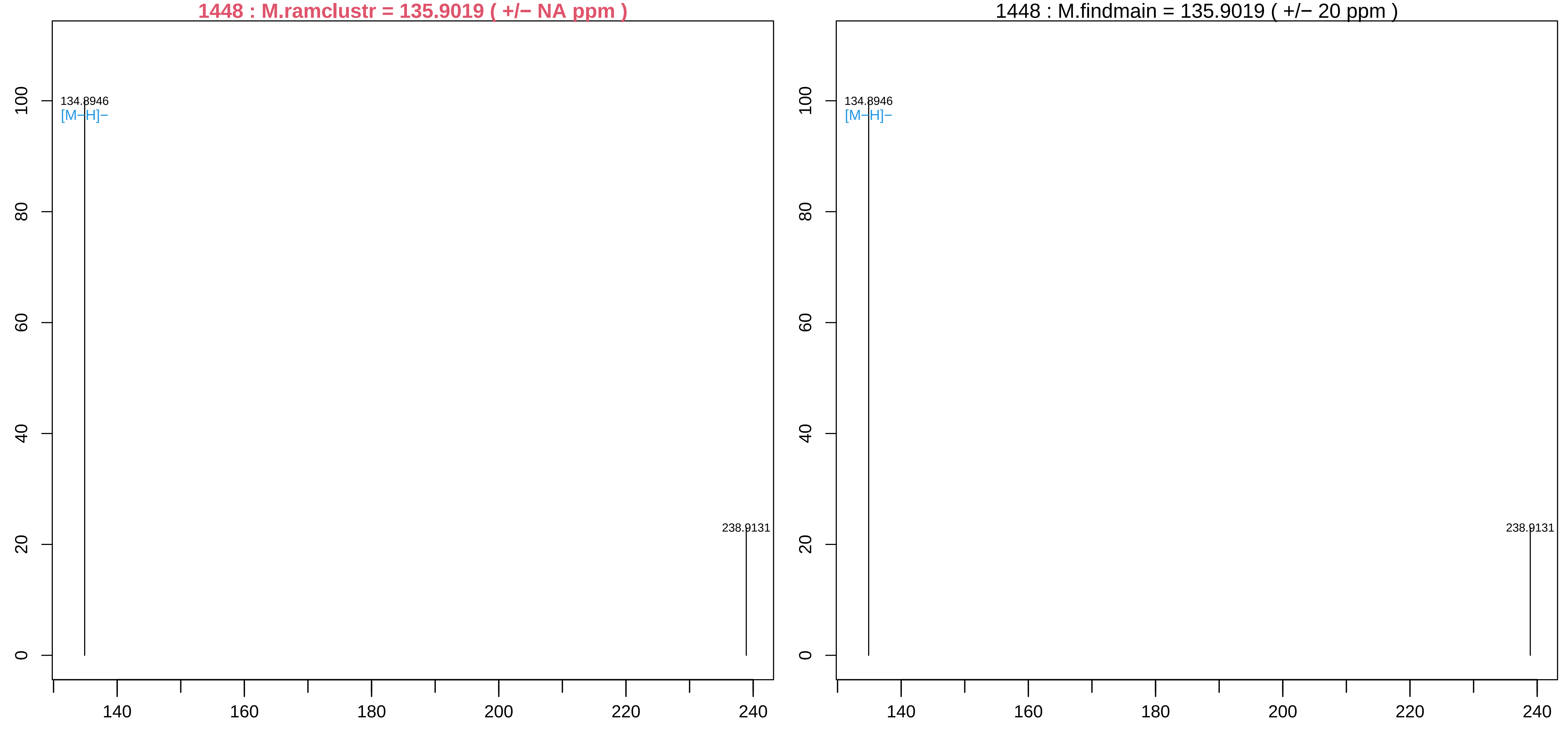
1448 : M.ramclustr = 135.9019 (+/− NA ppm)
1448 : M.findmain = 135.9019 (+/− 20 ppm)
134.8946
[M−H]−
238.9131
0
20
40
60
80
100
140
160
180
200
220
240

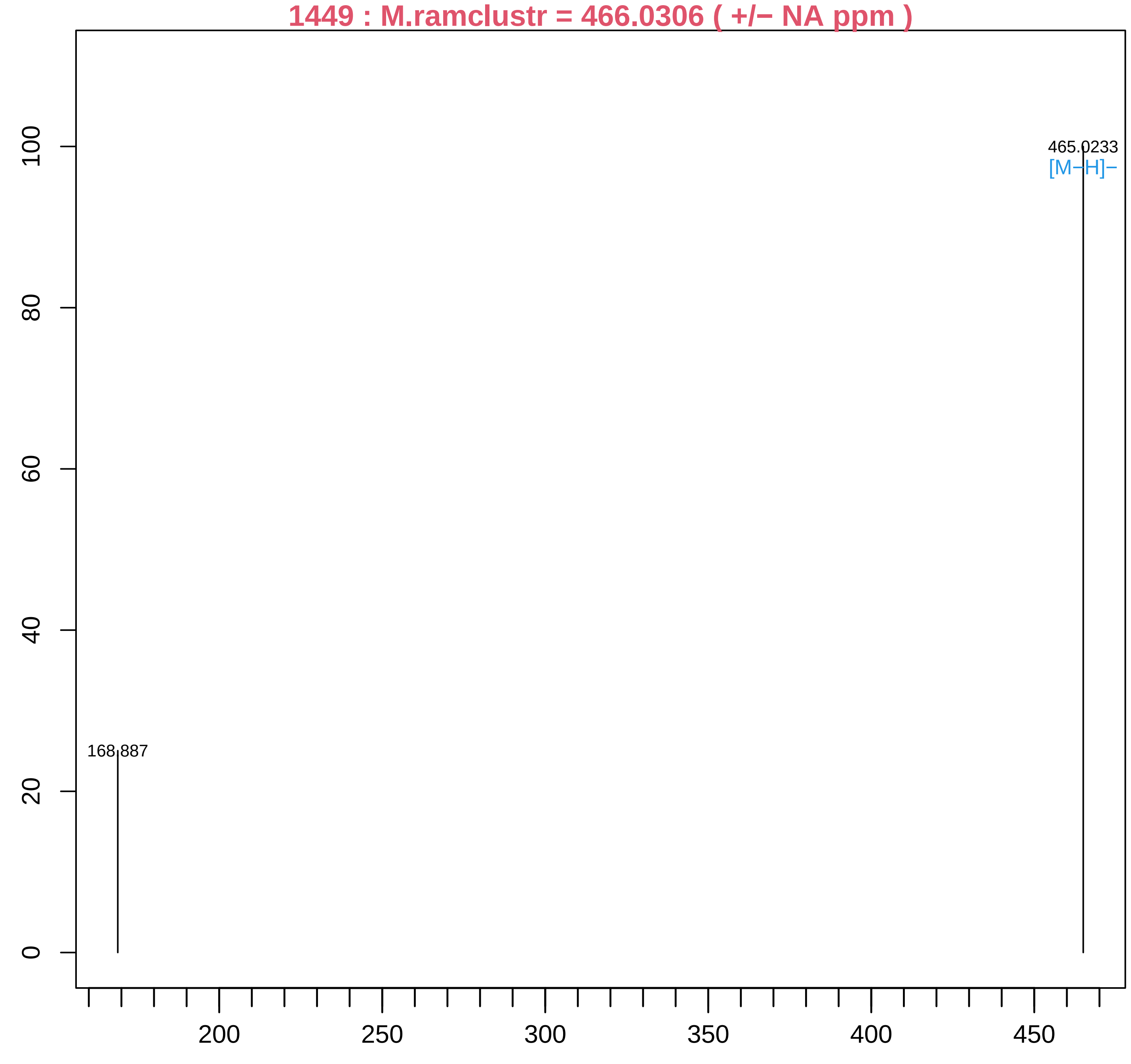

1449 : M.ramclustr = 466.0306 (+/− NA ppm)
465.0233
[M−H]−
168.887
0
20
40
60
80
100
200
250
300
350
400
450

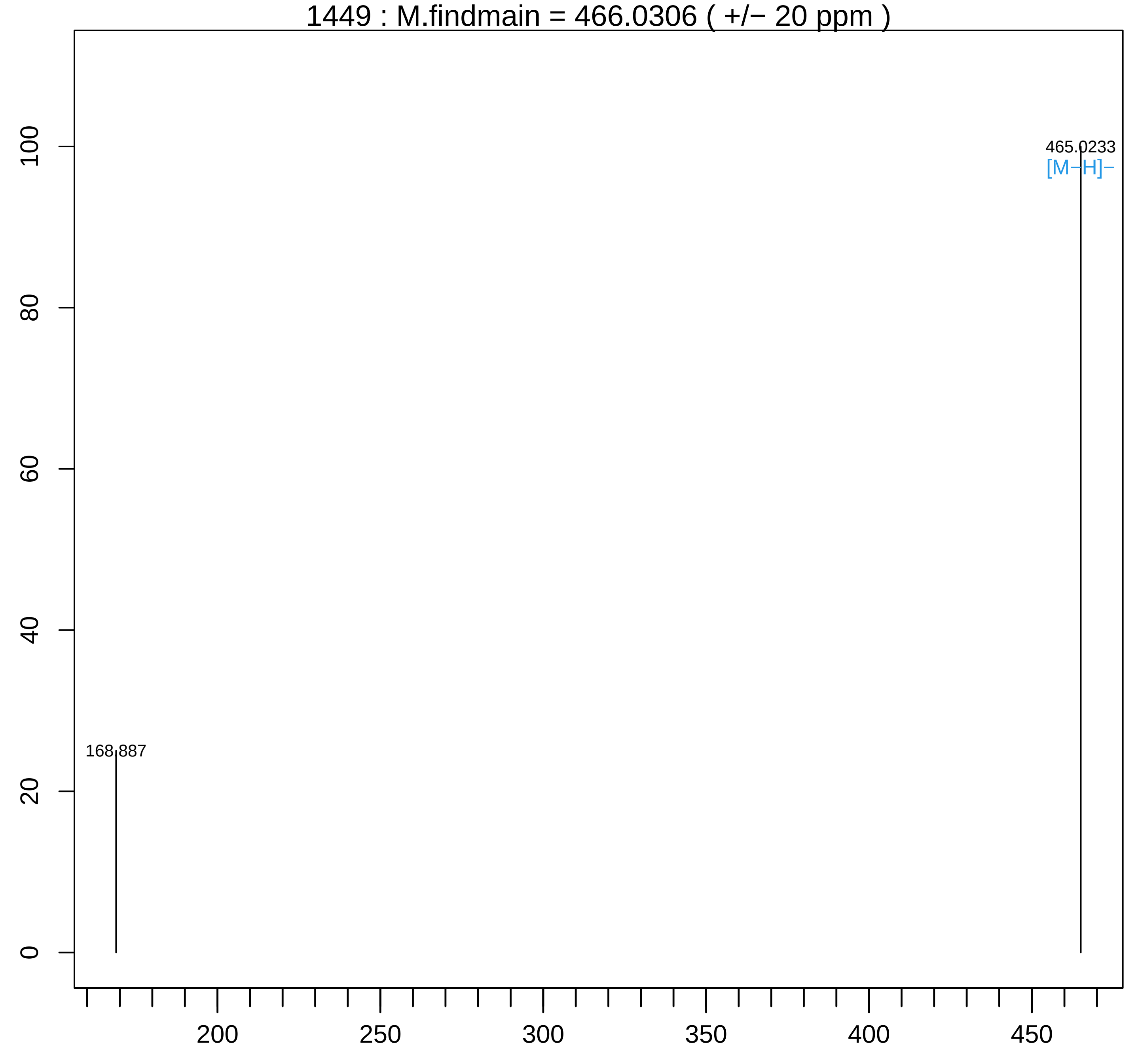

1449 : M.findmain = 466.0306 (+/− 20 ppm)
465.0233
[M−H]−
168.887
0
20
40
60
80
100
200
250
300
350
400
450

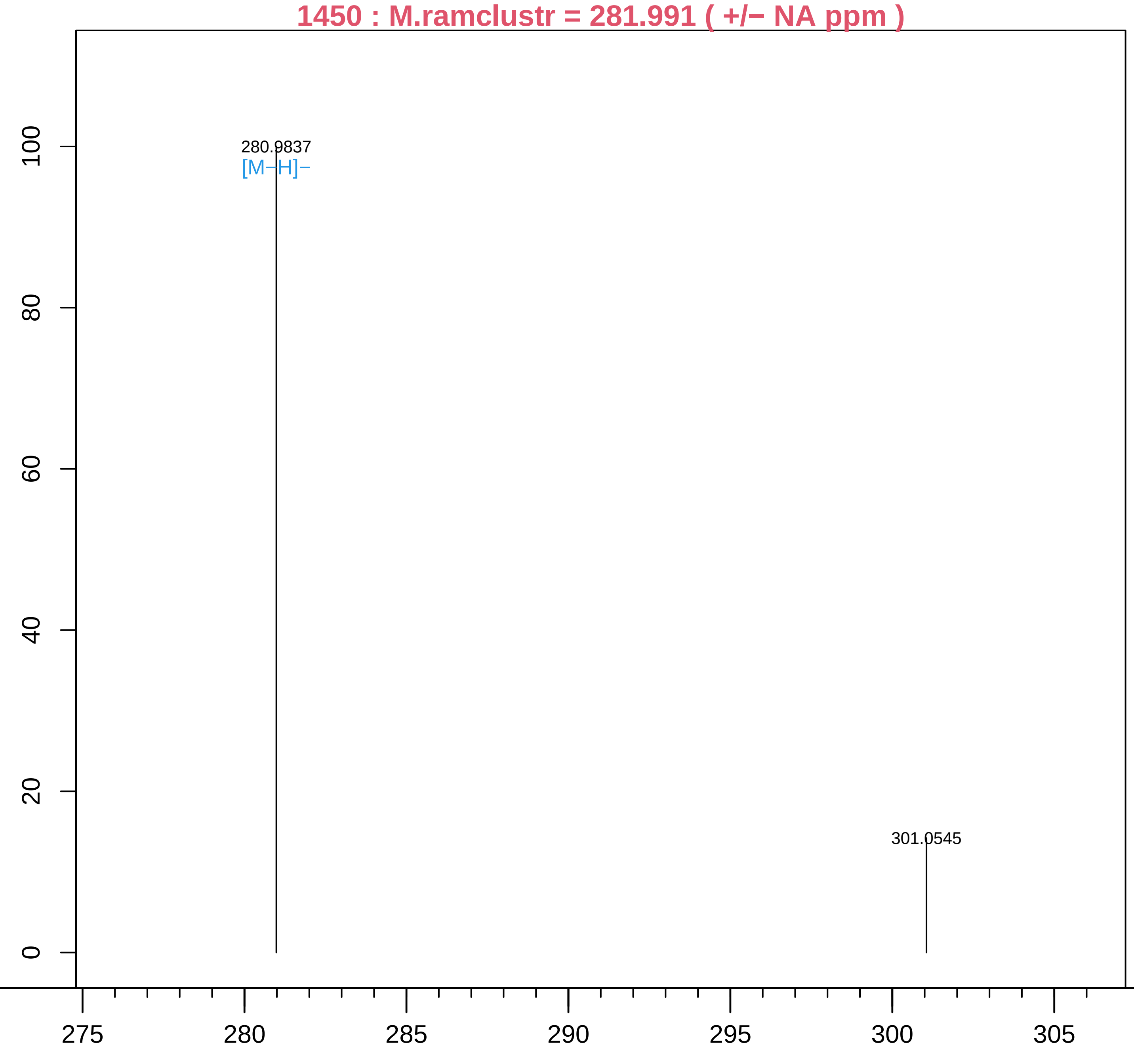
1450 : M.ramclustr = 281.991 (+/− NA ppm)
280.9837
[M−H]−
301.0545
0
20
40
60
80
100
275
280
285
290
295
300
305

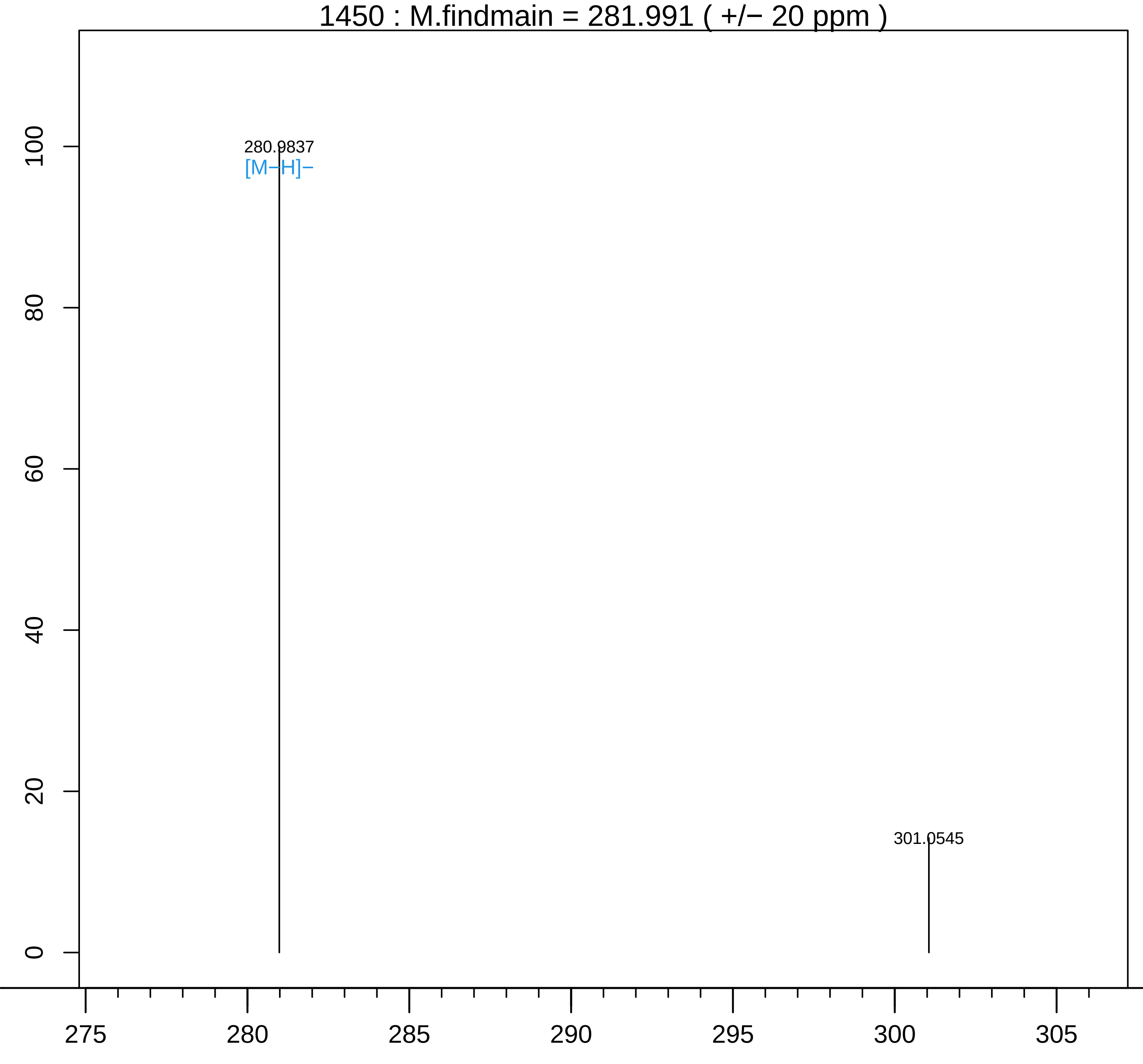
1450 : M.findmain = 281.991 (+/− 20 ppm)
280.9837
[M−H]−
301.0545
0
20
40
60
80
100
275
280
285
290
295
300
305

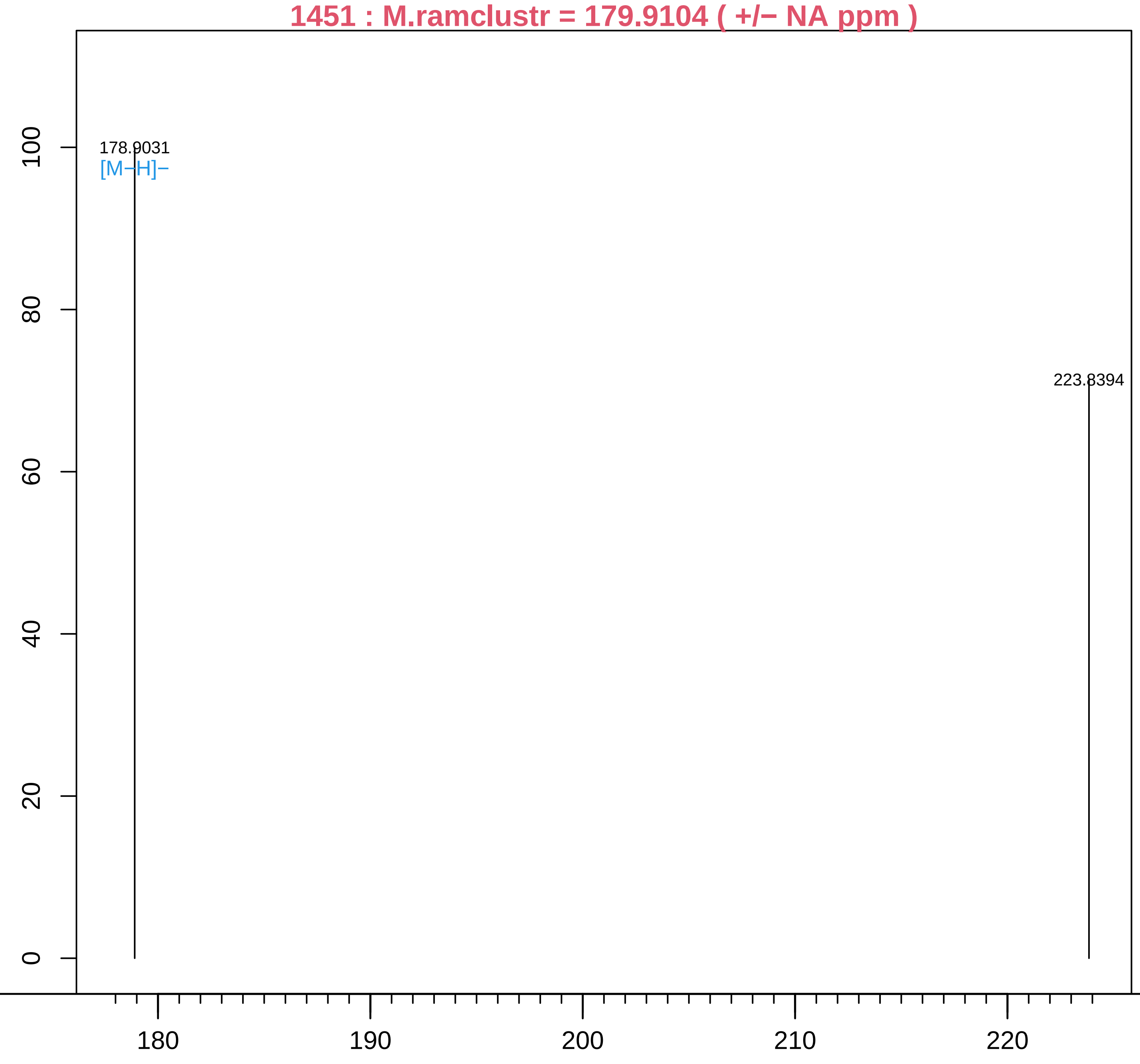
1451 : M.ramclustr = 179.9104 (+/− NA ppm)
178.9031
[M−H]−
223.8394
0
20
40
60
80
100
180
190
200
210
220

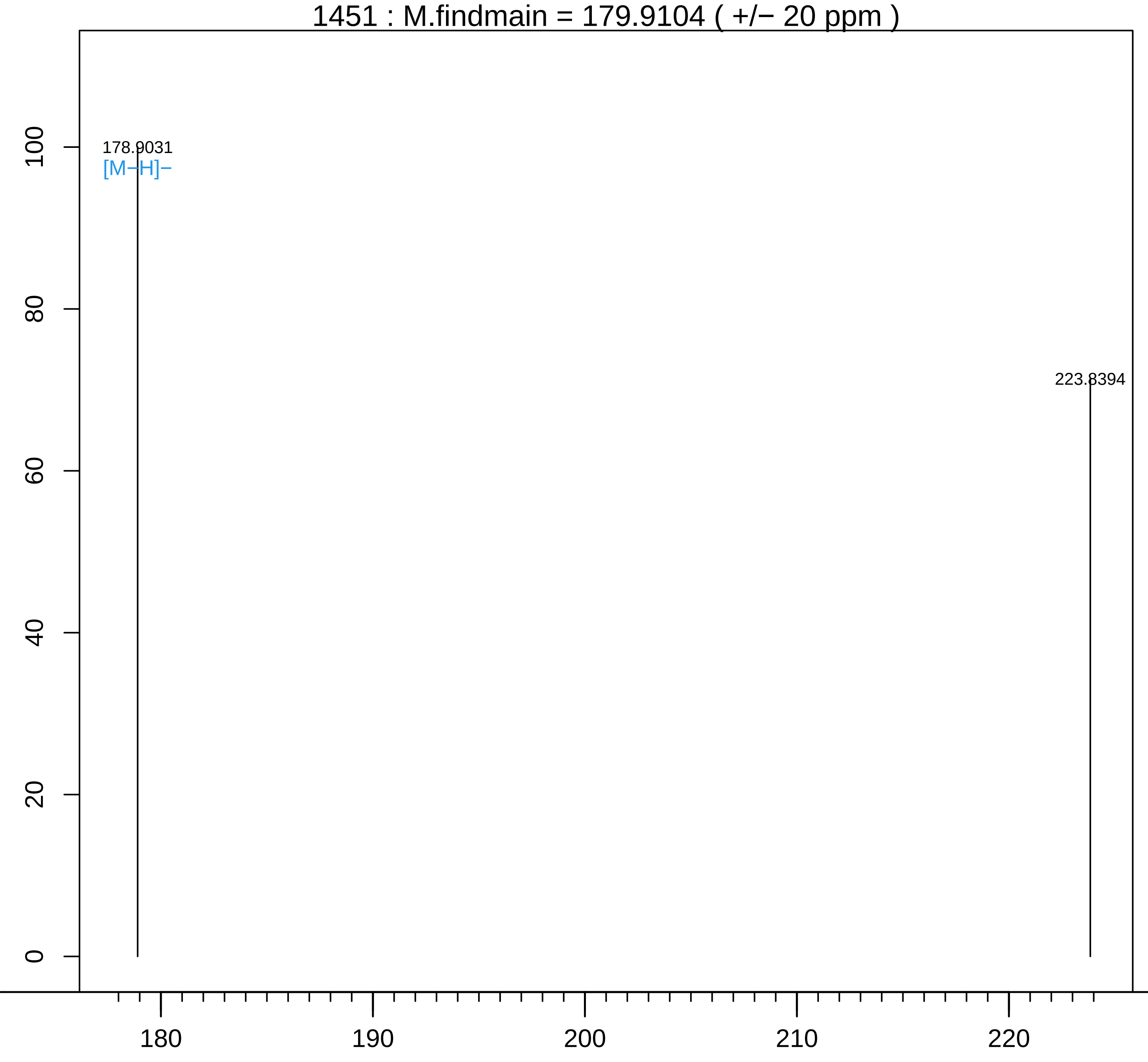
1451 : M.findmain = 179.9104 (+/− 20 ppm)
178.9031
[M−H]−
223.8394
0
20
40
60
80
100
180
190
200
210
220

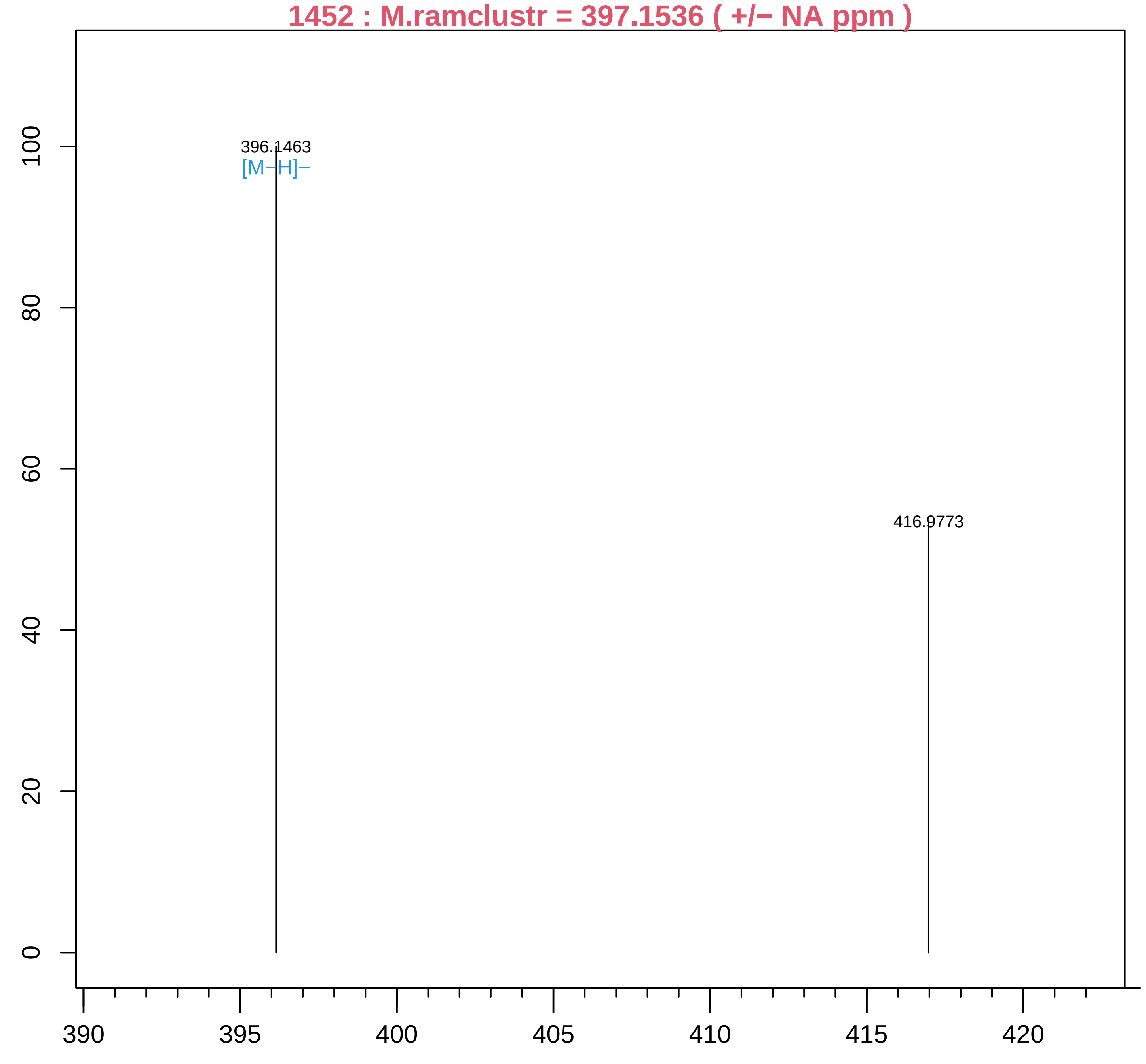
1452 : M.ramclustr = 397.1536 (+/− NA ppm)
396.1463
[M−H]−
416.9773
0
20
40
60
80
100
390
395
400
405
410
415
420
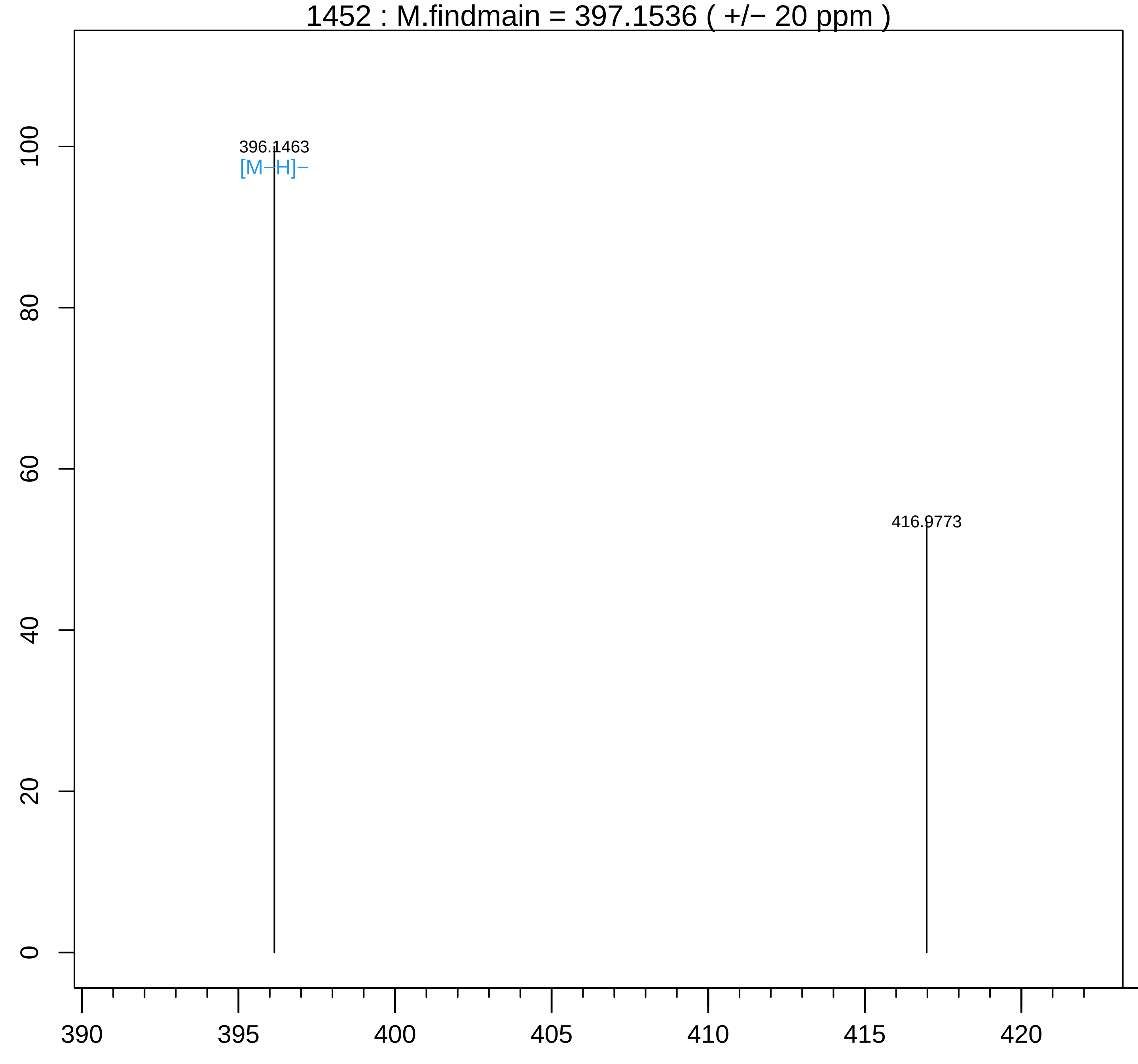
1452 : M.findmain = 397.1536 (+/− 20 ppm)
396.1463
[M−H]−
416.9773
0
20
40
60
80
100
390
395
400
405
410
415
420

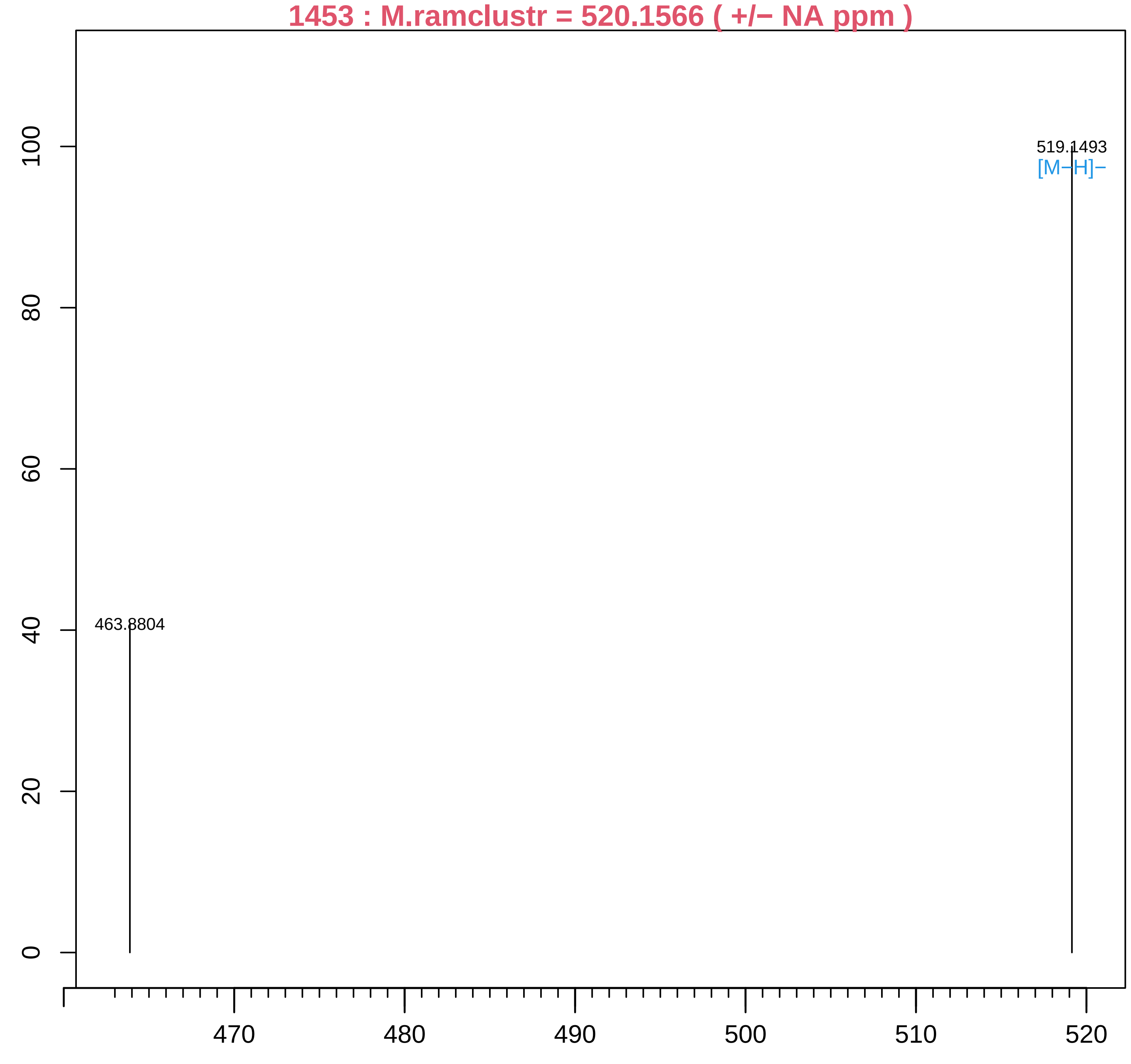
1453 : M.ramclustr = 520.1566 (+/− NA ppm)
519.1493
[M−H]−
463.8804
0
20
40
60
80
100
470
480
490
500
510
520

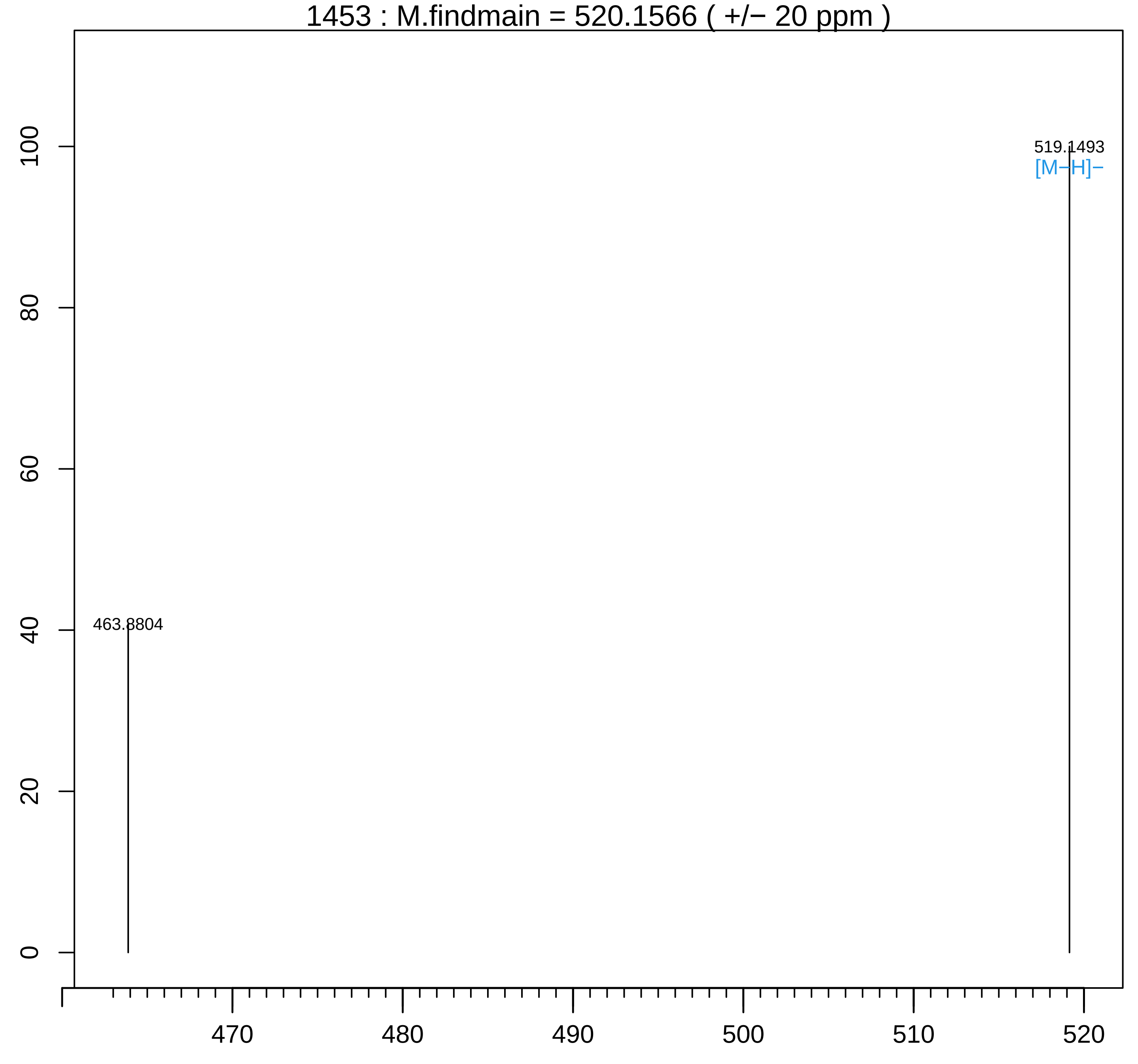
1453 : M.findmain = 520.1566 (+/− 20 ppm)
519.1493
[M−H]−
463.8804
0
20
40
60
80
100
470
480
490
500
510
520

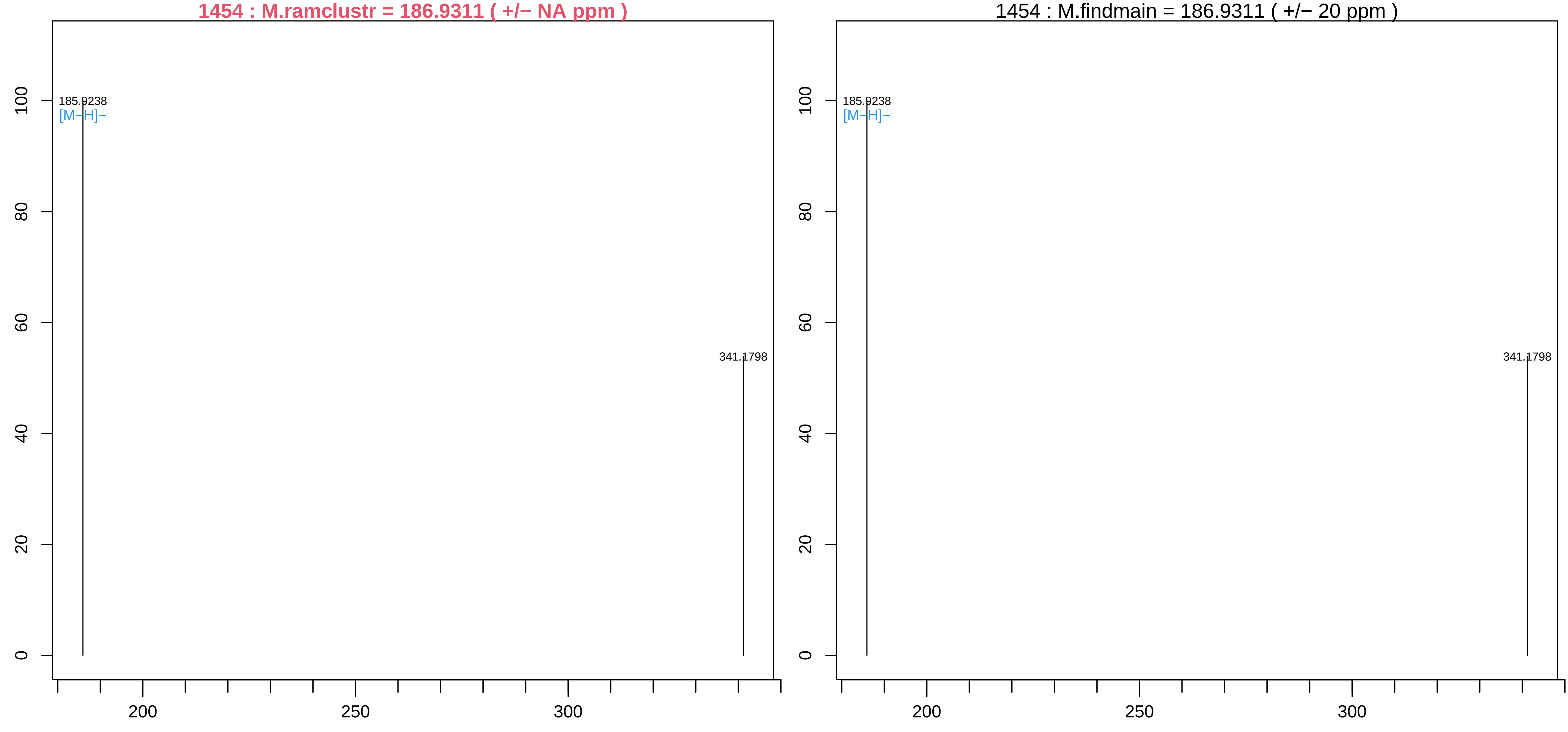
1454 : M.ramclustr = 186.9311 (+/− NA ppm)
185.9238
[M−H]−
341.1798
0
20
40
60
80
100
200
250
300
1454 : M.findmain = 186.9311 (+/− 20 ppm)
185.9238
[M−H]−
341.1798
0
20
40
60
80
100
200
250
300

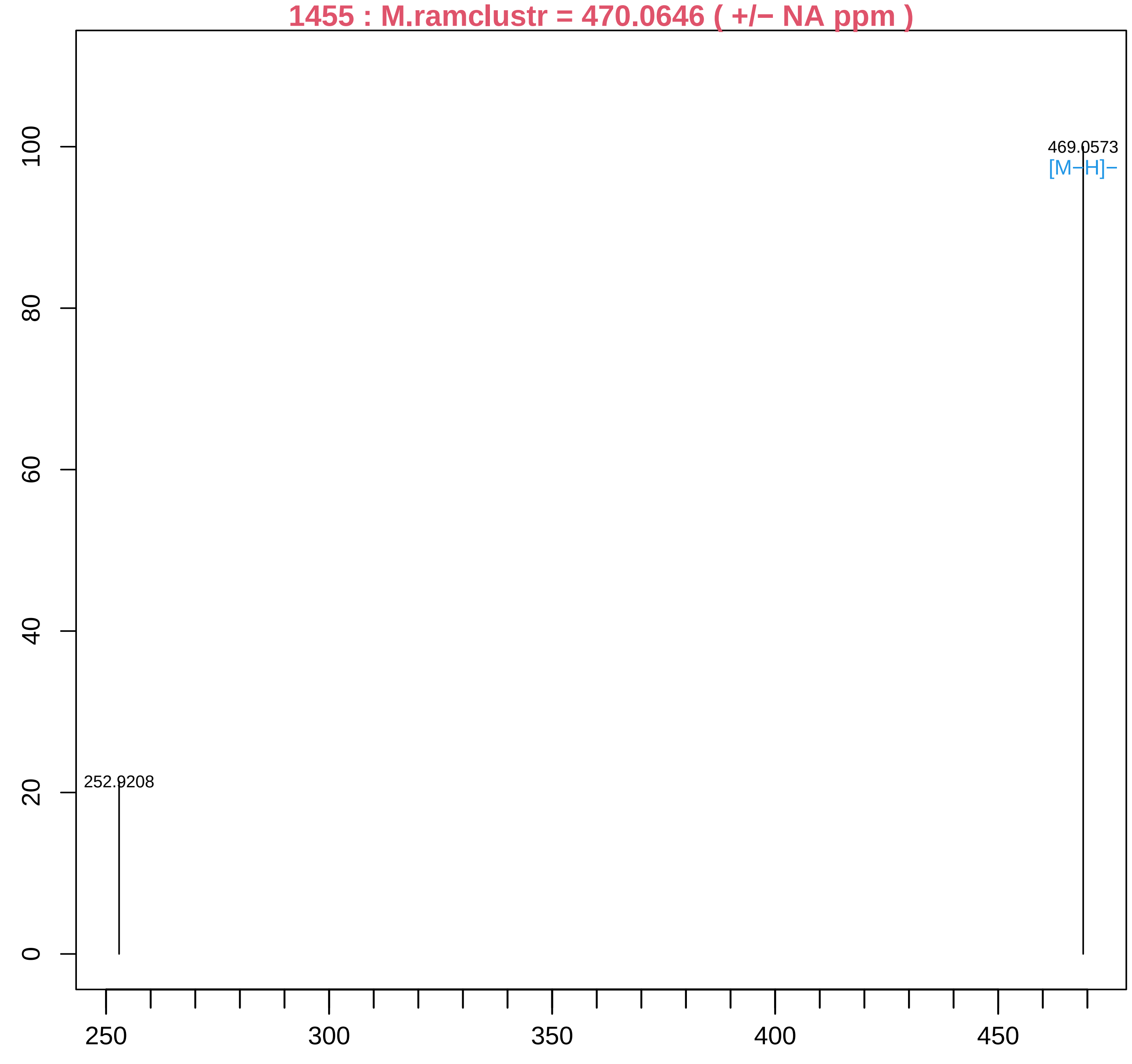
1455 : M.ramclustr = 470.0646 (+/− NA ppm)
469.0573
[M−H]−
252.9208
0
20
40
60
80
100
250
300
350
400
450

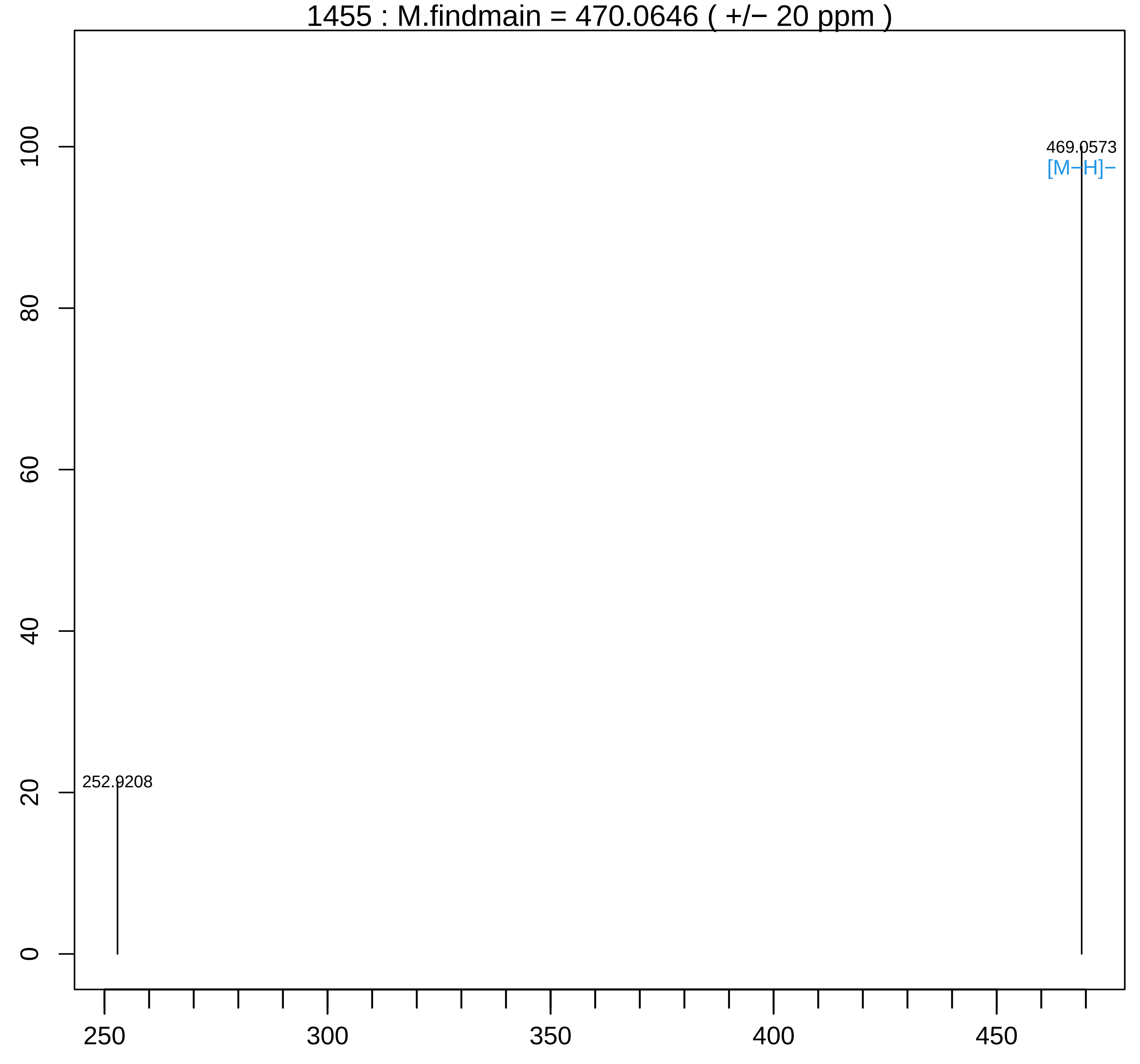
1455 : M.findmain = 470.0646 (+/− 20 ppm)
469.0573
[M−H]−
252.9208
0
20
40
60
80
100
250
300
350
400
450

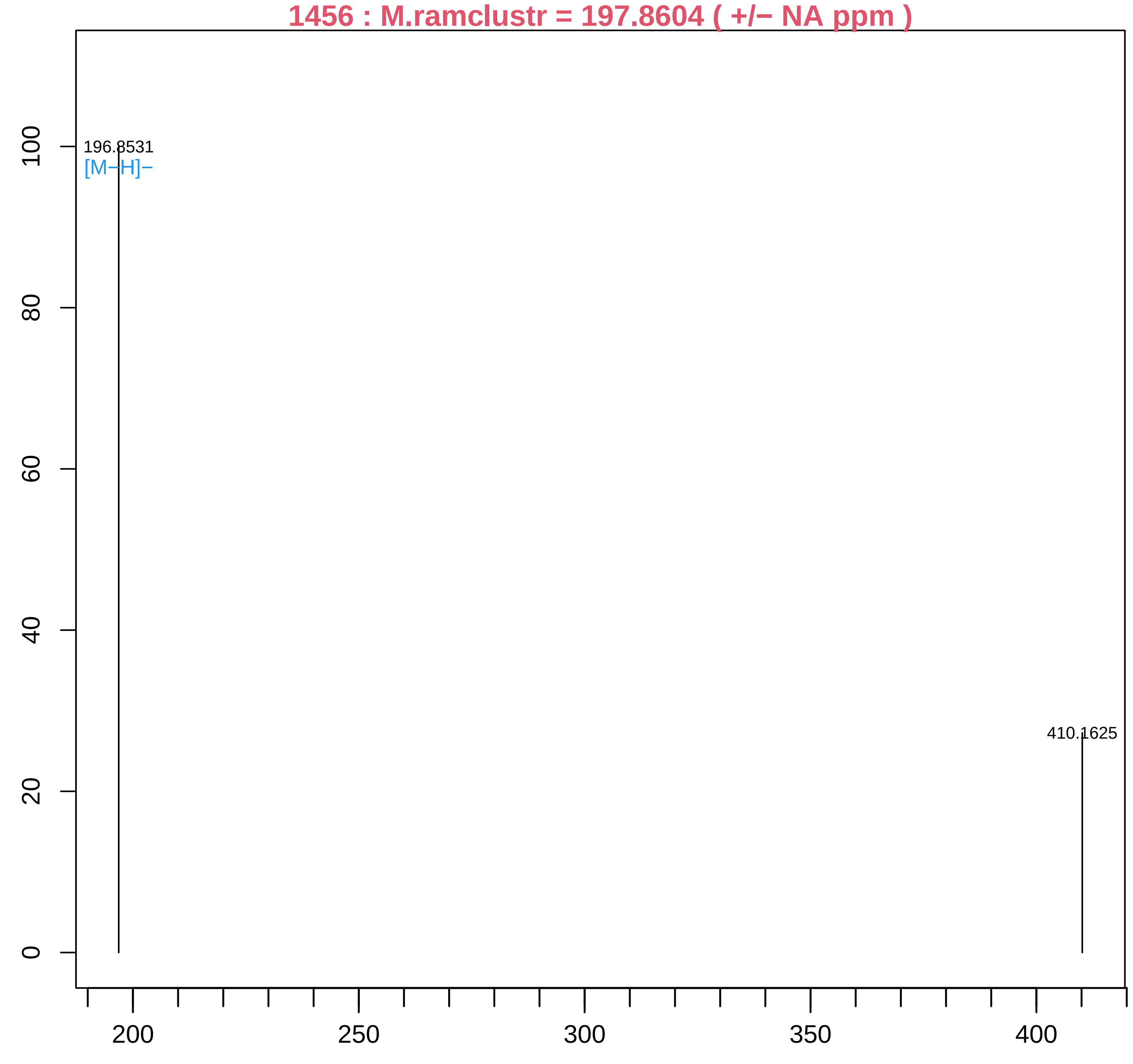
1456 : M.ramclustr = 197.8604 (+/− NA ppm)
196.8531
[M−H]−
410.1625
0
20
40
60
80
100
200
250
300
350
400

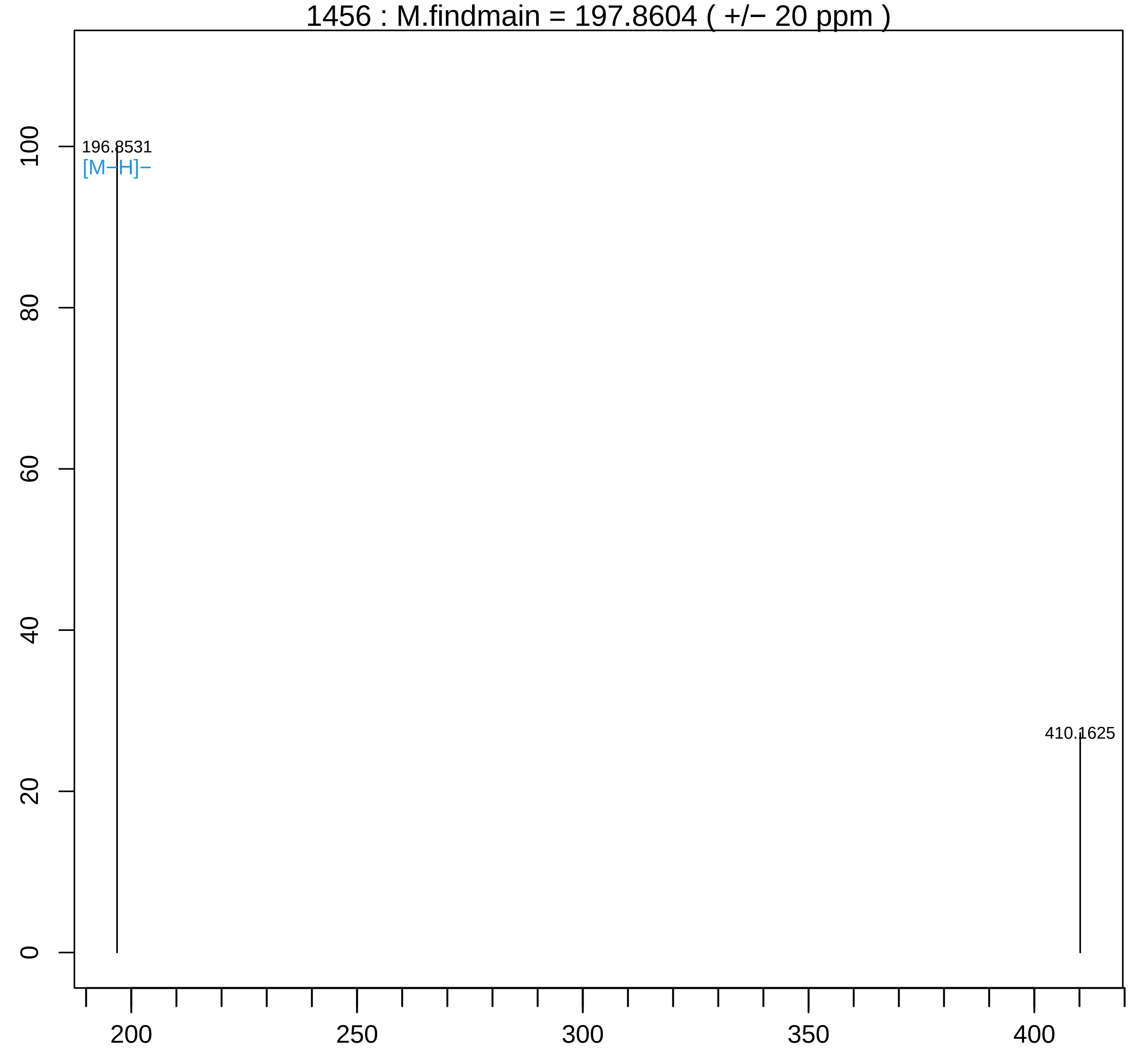
1456 : M.findmain = 197.8604 (+/− 20 ppm)
196.8531
[M−H]−
410.1625
0
20
40
60
80
100
200
250
300
350
400

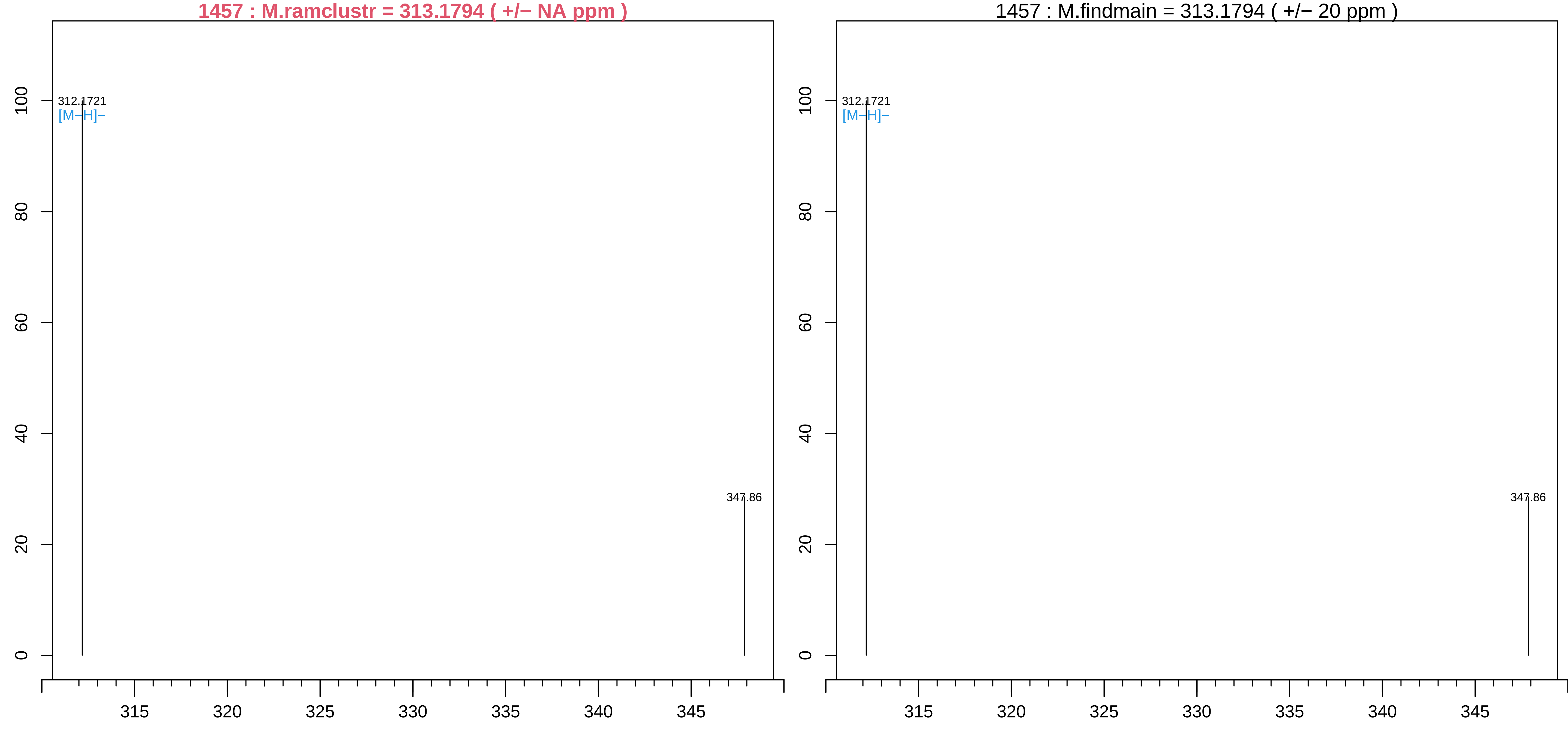

1457 : M.ramclustr = 313.1794 (+/− NA ppm)
312.1721
[M−H]−
347.86
1457 : M.findmain = 313.1794 (+/− 20 ppm)
312.1721
[M−H]−
347.86
0
20
40
60
80
100
315
320
325
330
335
340
345

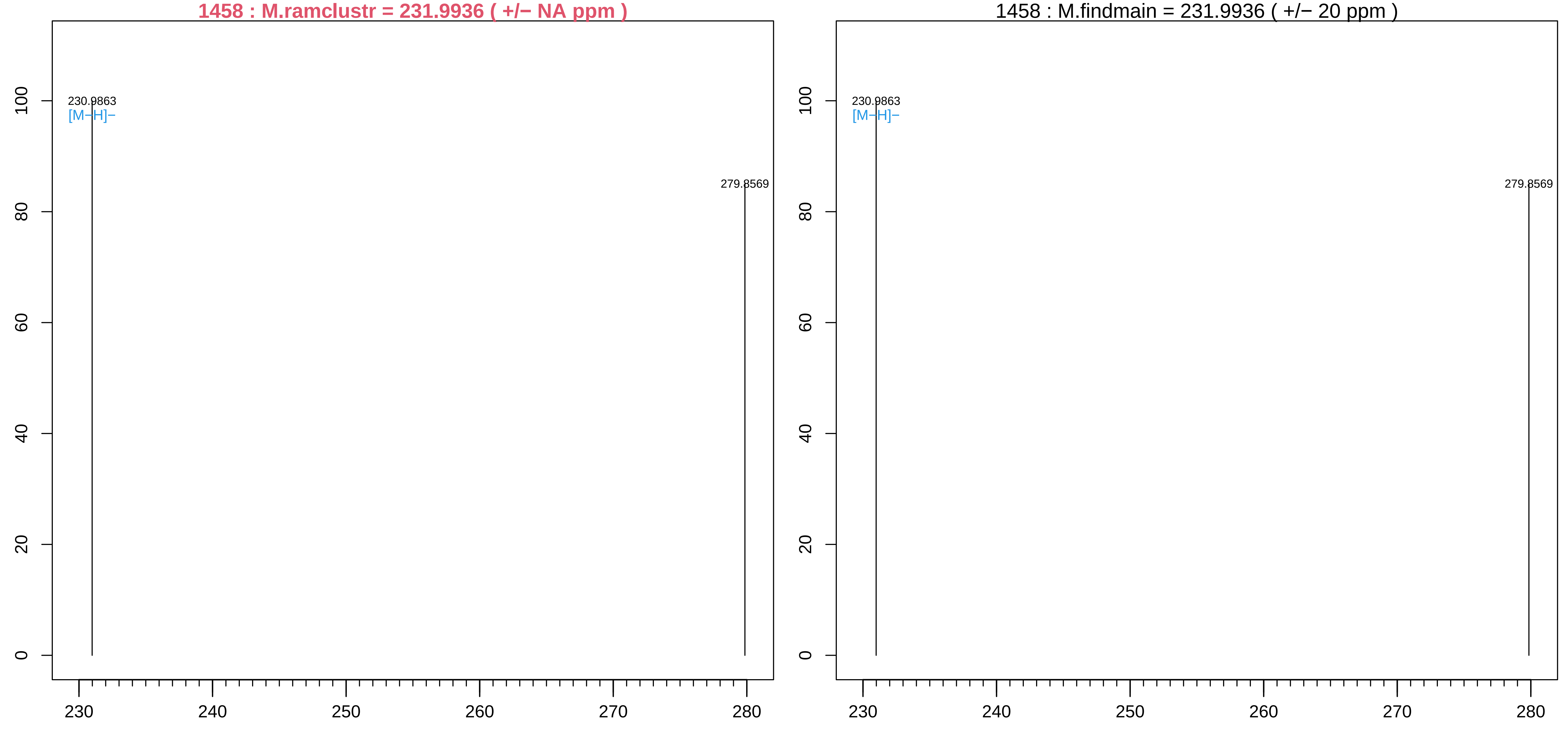
1458 : M.ramclustr = 231.9936 (+/− NA ppm)
230.9863
[M−H]−
279.8569
1458 : M.findmain = 231.9936 (+/− 20 ppm)
230.9863
[M−H]−
279.8569
0
20
40
60
80
100
230
240
250
260
270
280

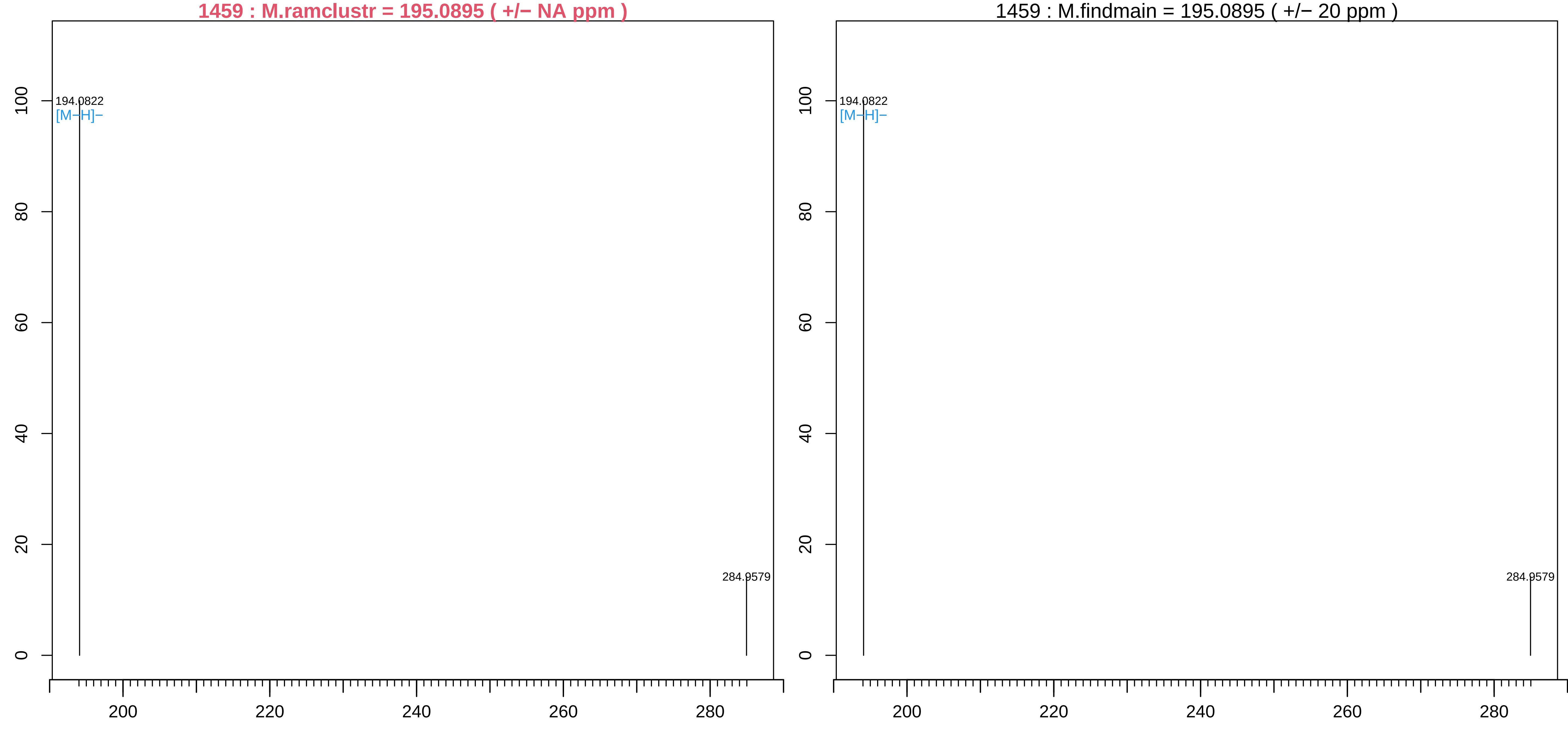

1459 : M.ramclustr = 195.0895 (+/− NA ppm)
194.0822
[M−H]−
284.9579
0
20
40
60
80
100
200
220
240
260
280
1459 : M.findmain = 195.0895 (+/− 20 ppm)
194.0822
[M−H]−
284.9579
0
20
40
60
80
100
200
220
240
260
280

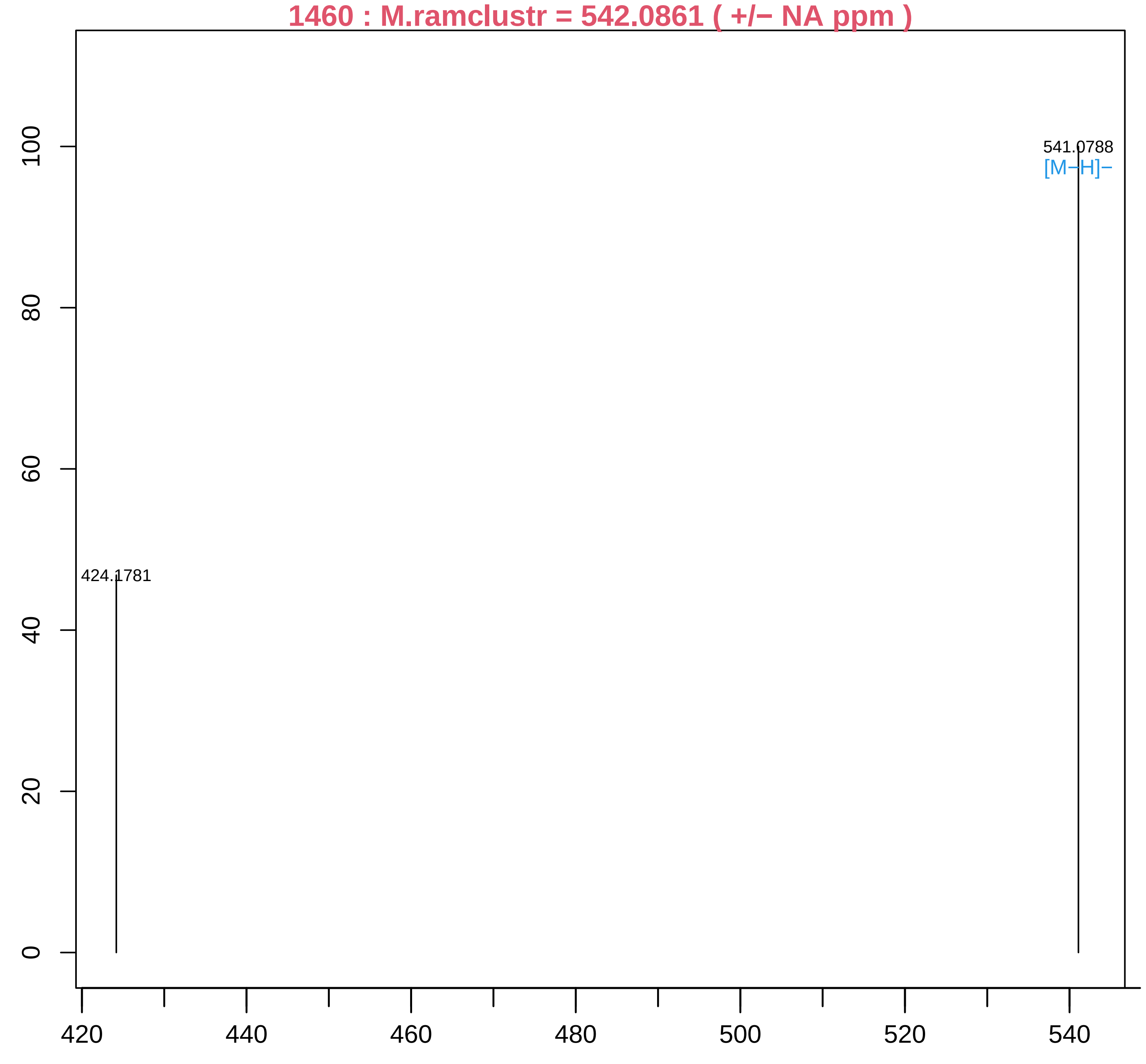
1460 : M.ramclustr = 542.0861 (+/− NA ppm)
541.0788
[M−H]−
424.1781
0
20
40
60
80
100
420
440
460
480
500
520
540

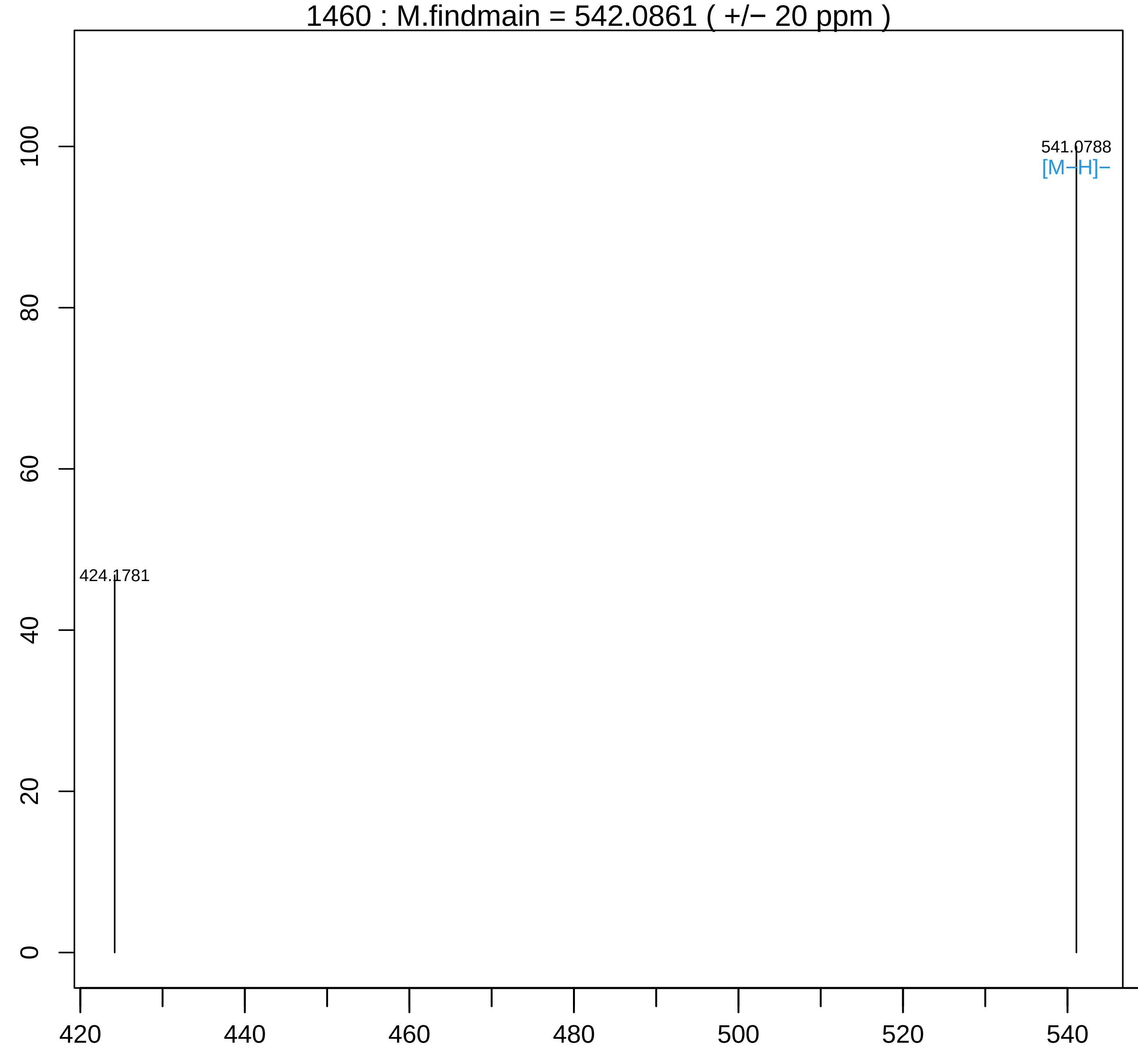
1460 : M.findmain = 542.0861 (+/− 20 ppm)
541.0788
[M−H]−
424.1781
0
20
40
60
80
100
420
440
460
480
500
520
540

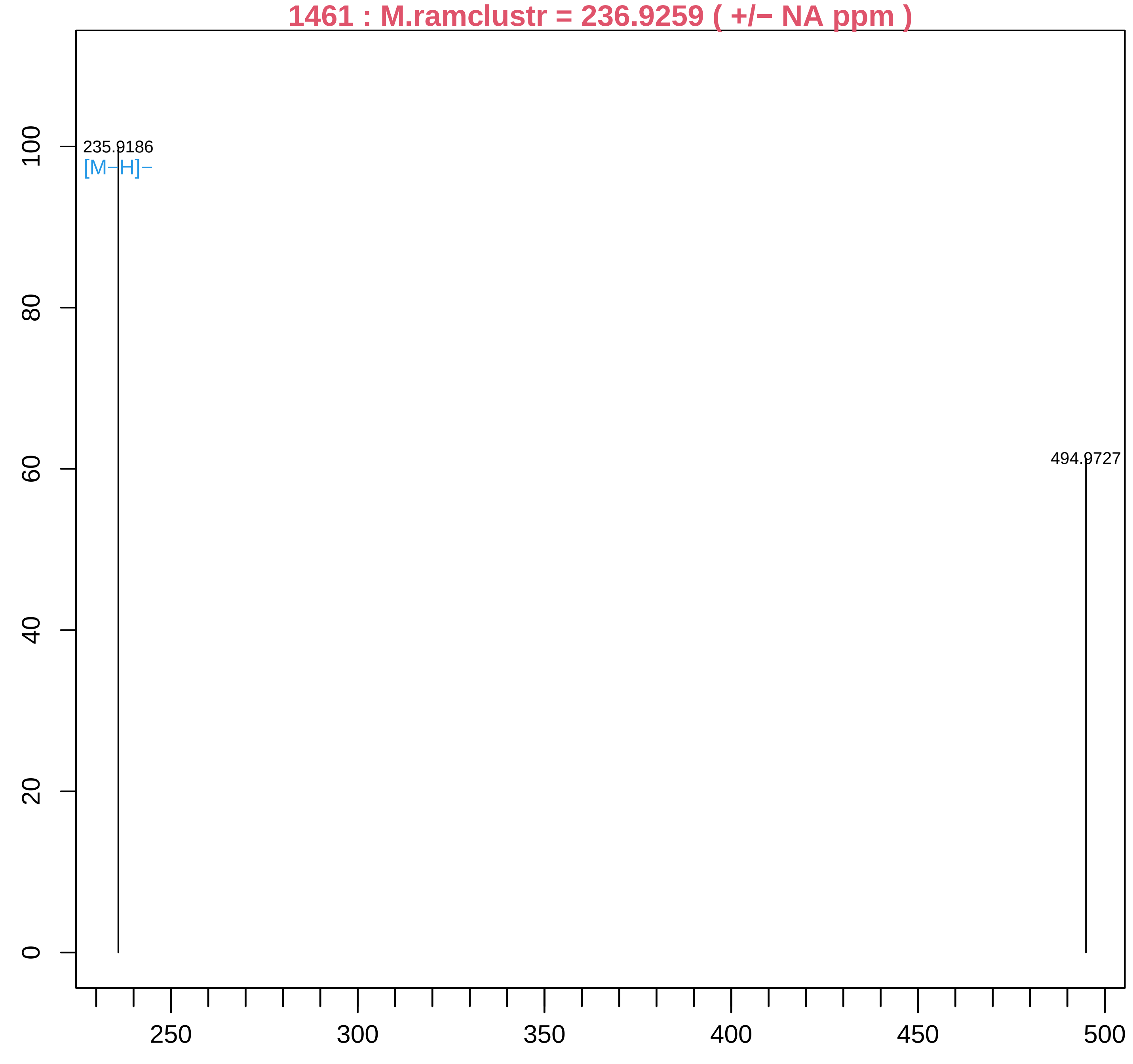
1461 : M.ramclustr = 236.9259 (+/− NA ppm)
235.9186
[M−H]−
494.9727
0
20
40
60
80
100
250
300
350
400
450
500

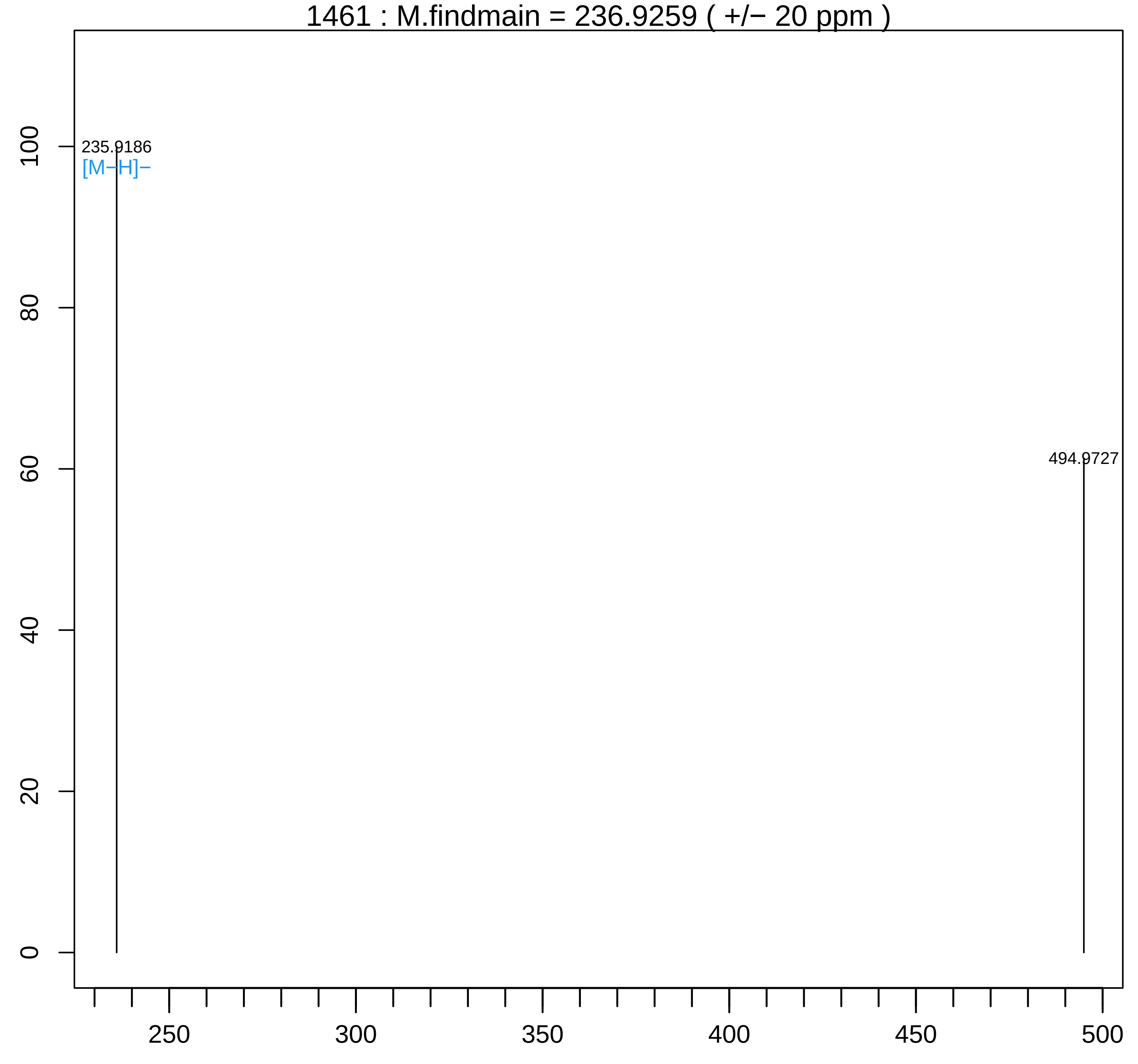
1461 : M.findmain = 236.9259 (+/− 20 ppm)
235.9186
[M−H]−
494.9727
0
20
40
60
80
100
250
300
350
400
450
500

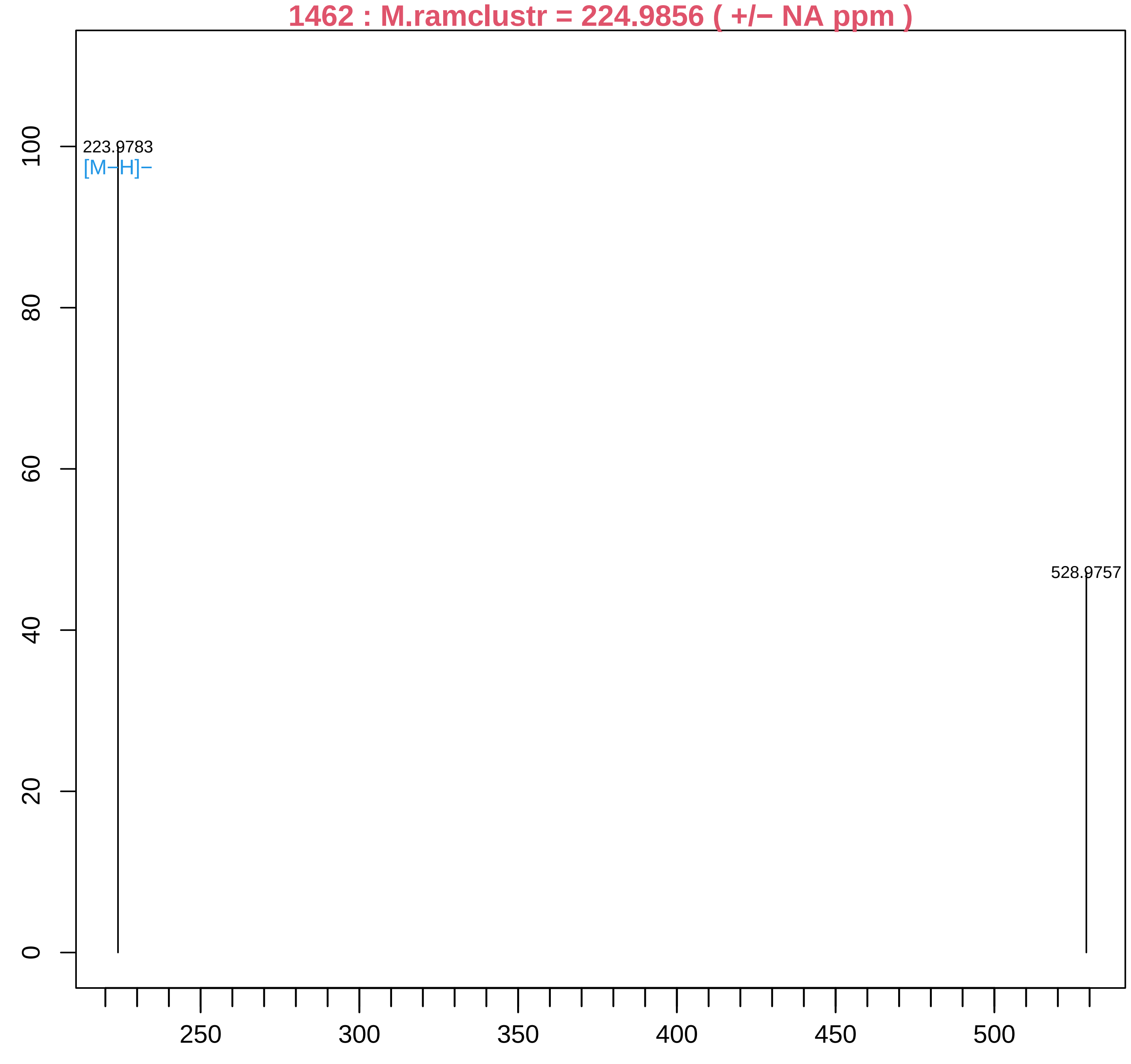

1462 : M.ramclustr = 224.9856 (+/− NA ppm)
223.9783
[M−H]−
528.9757
0
20
40
60
80
100
250
300
350
400
450
500

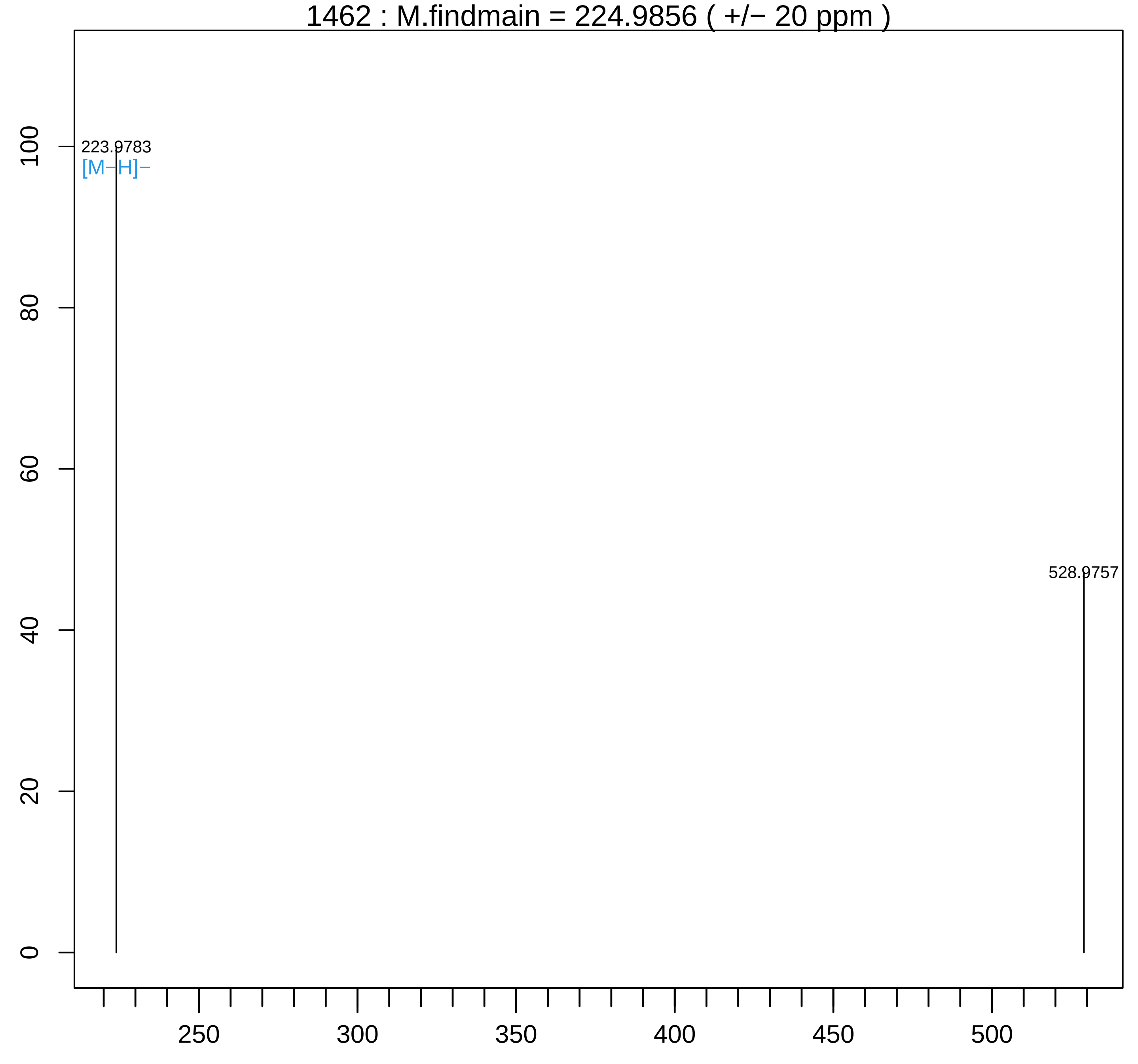

1462 : M.findmain = 224.9856 (+/− 20 ppm)
223.9783
[M−H]−
528.9757
0
20
40
60
80
100
250
300
350
400
450
500

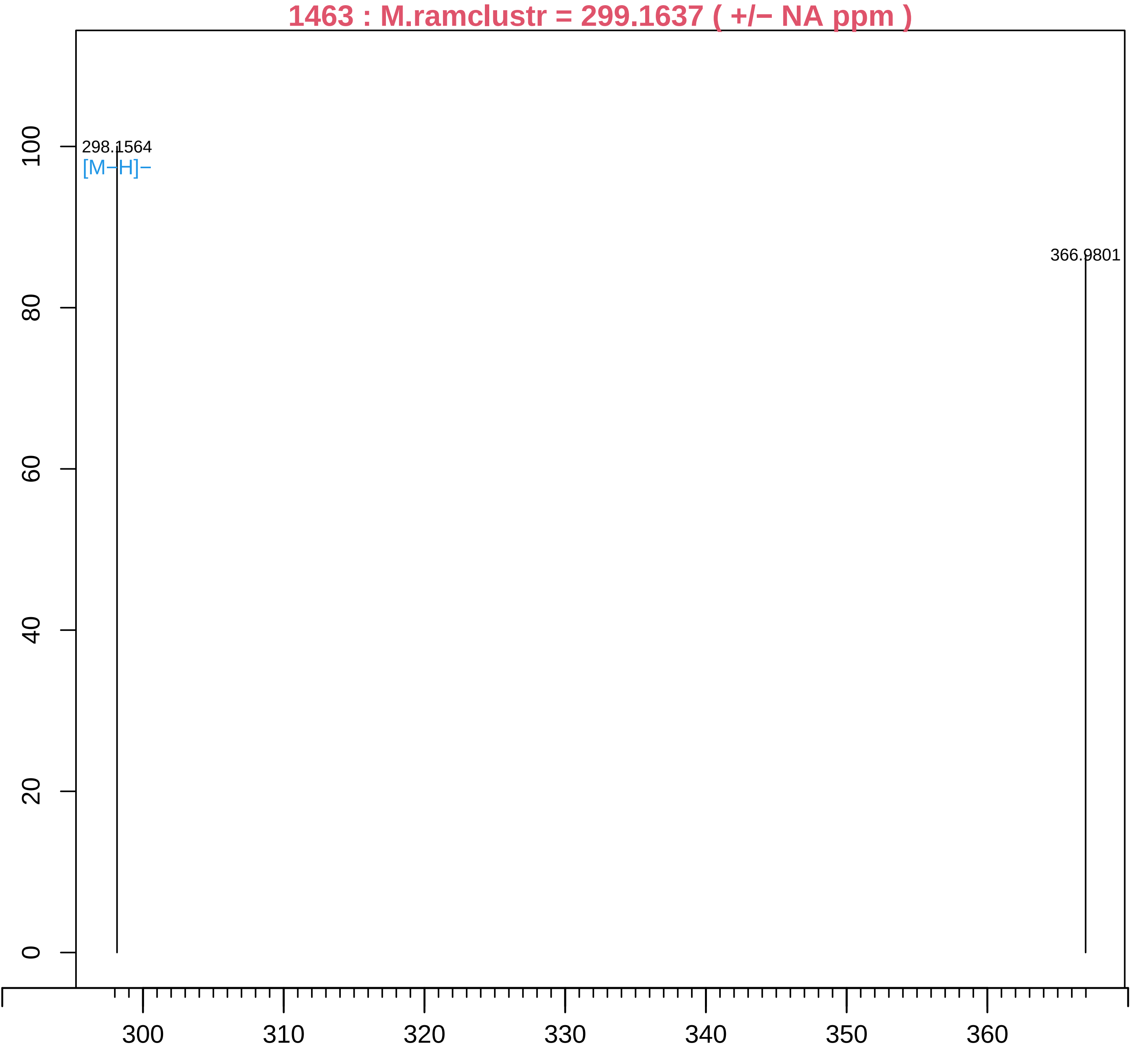
1463 : M.ramclustr = 299.1637 (+/− NA ppm)
298.1564
[M−H]−
366.9801
0
20
40
60
80
100
300
310
320
330
340
350
360

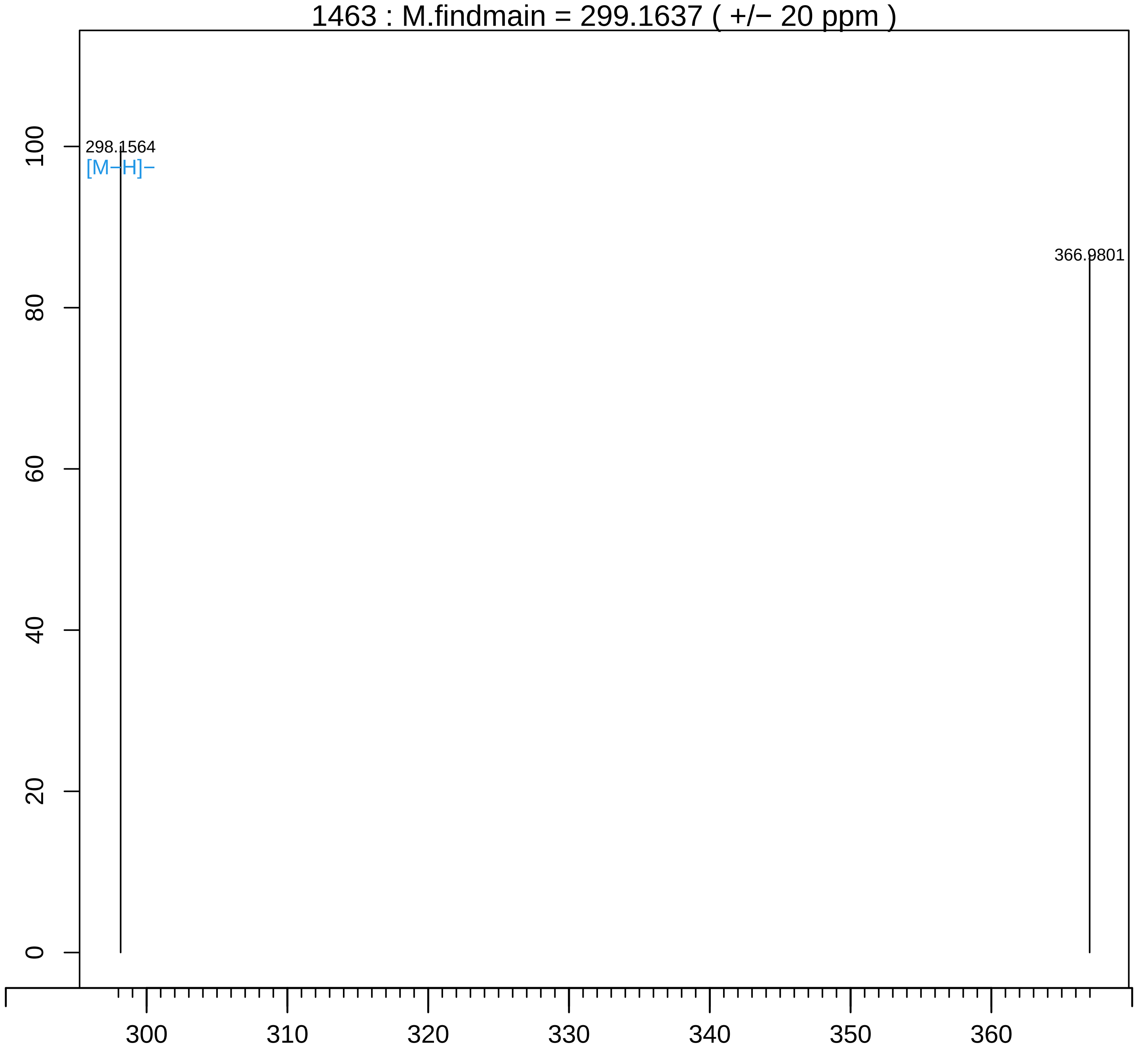
1463 : M.findmain = 299.1637 (+/− 20 ppm)
298.1564
[M−H]−
366.9801
0
20
40
60
80
100
300
310
320
330
340
350
360

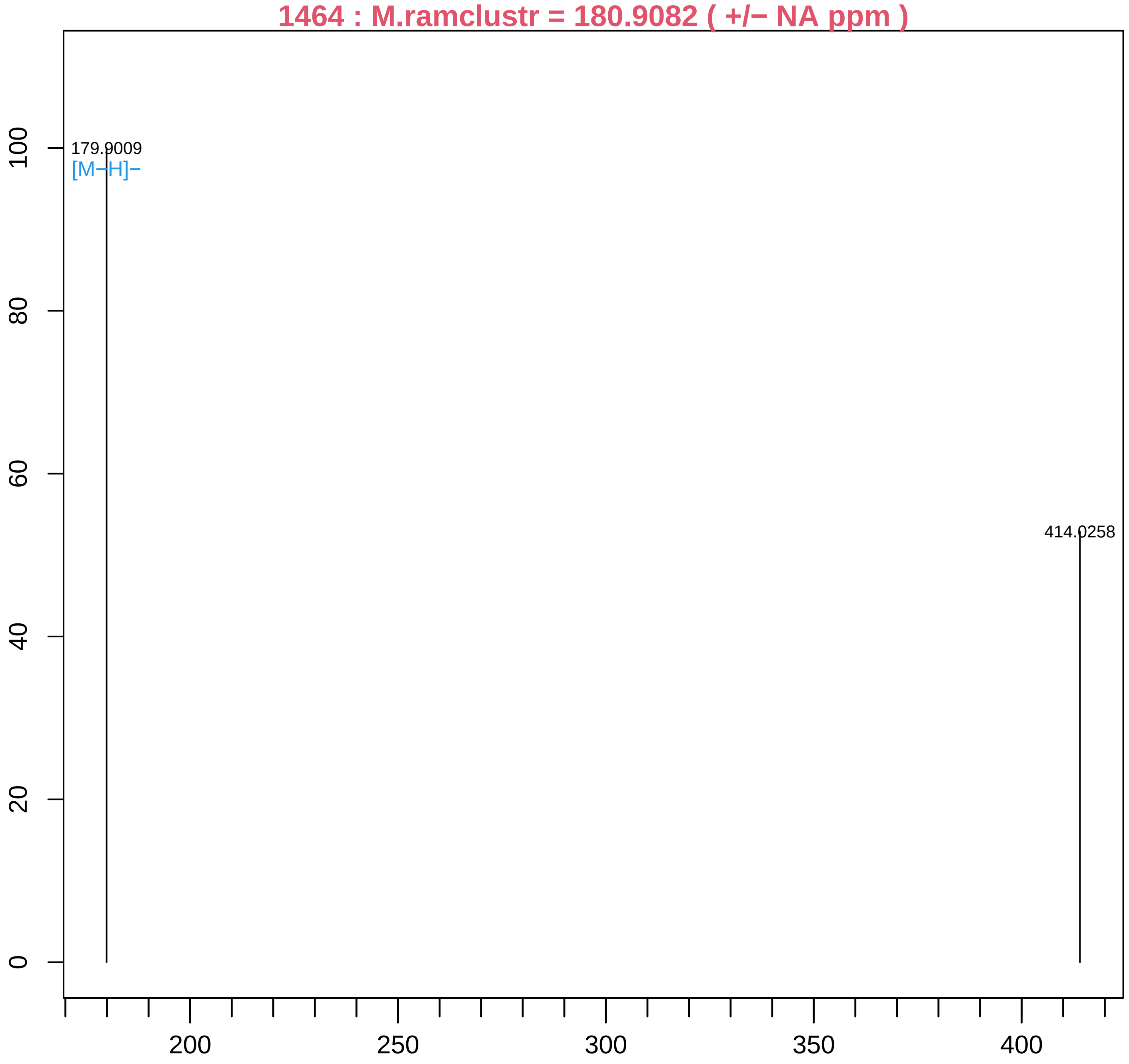
1464 : M.ramclustr = 180.9082 (+/− NA ppm)
179.9009
[M−H]−
414.0258
0
20
40
60
80
100
200
250
300
350
400

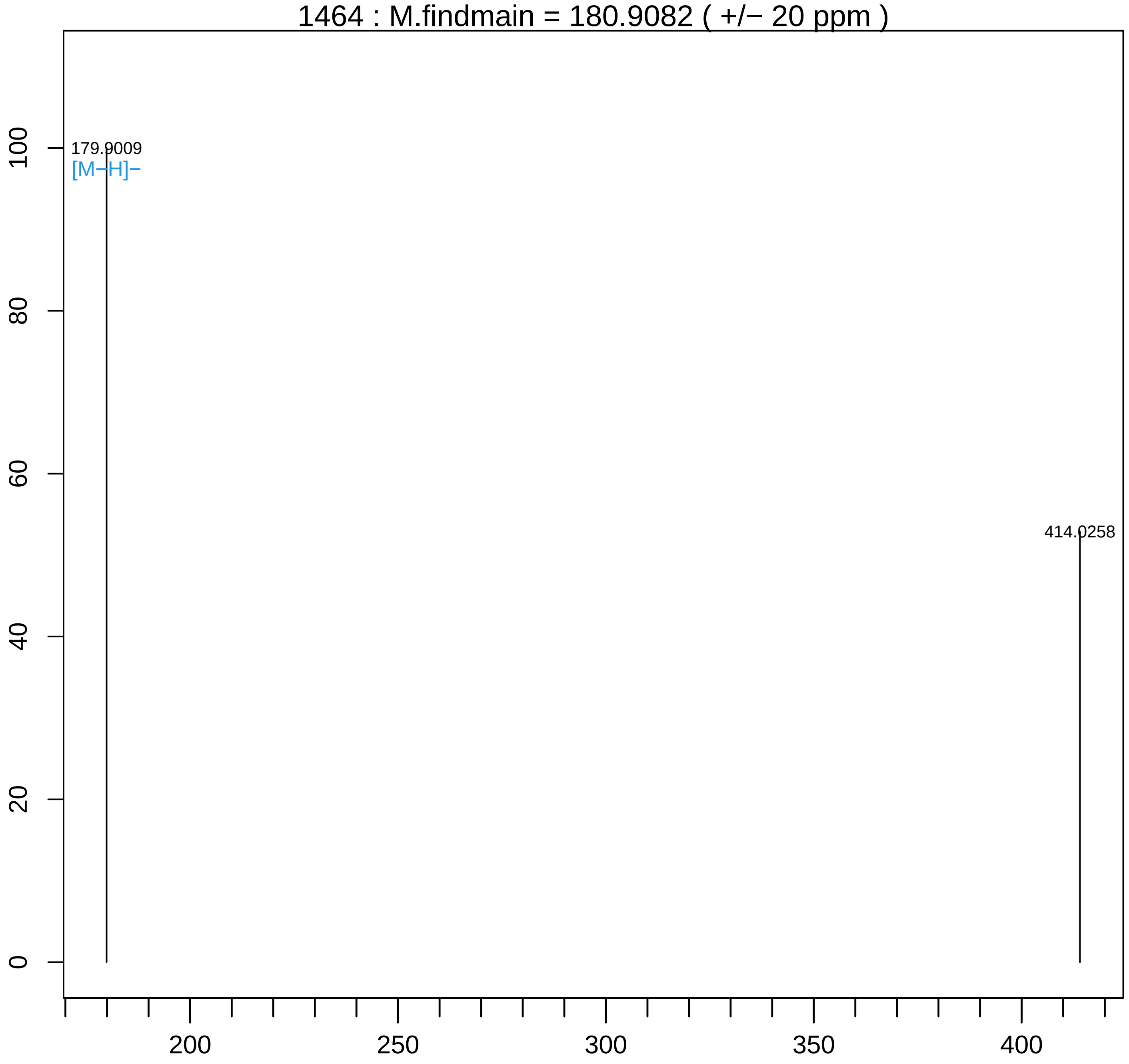
1464 : M.findmain = 180.9082 (+/− 20 ppm)
179.9009
[M−H]−
414.0258
0
20
40
60
80
100
200
250
300
350
400

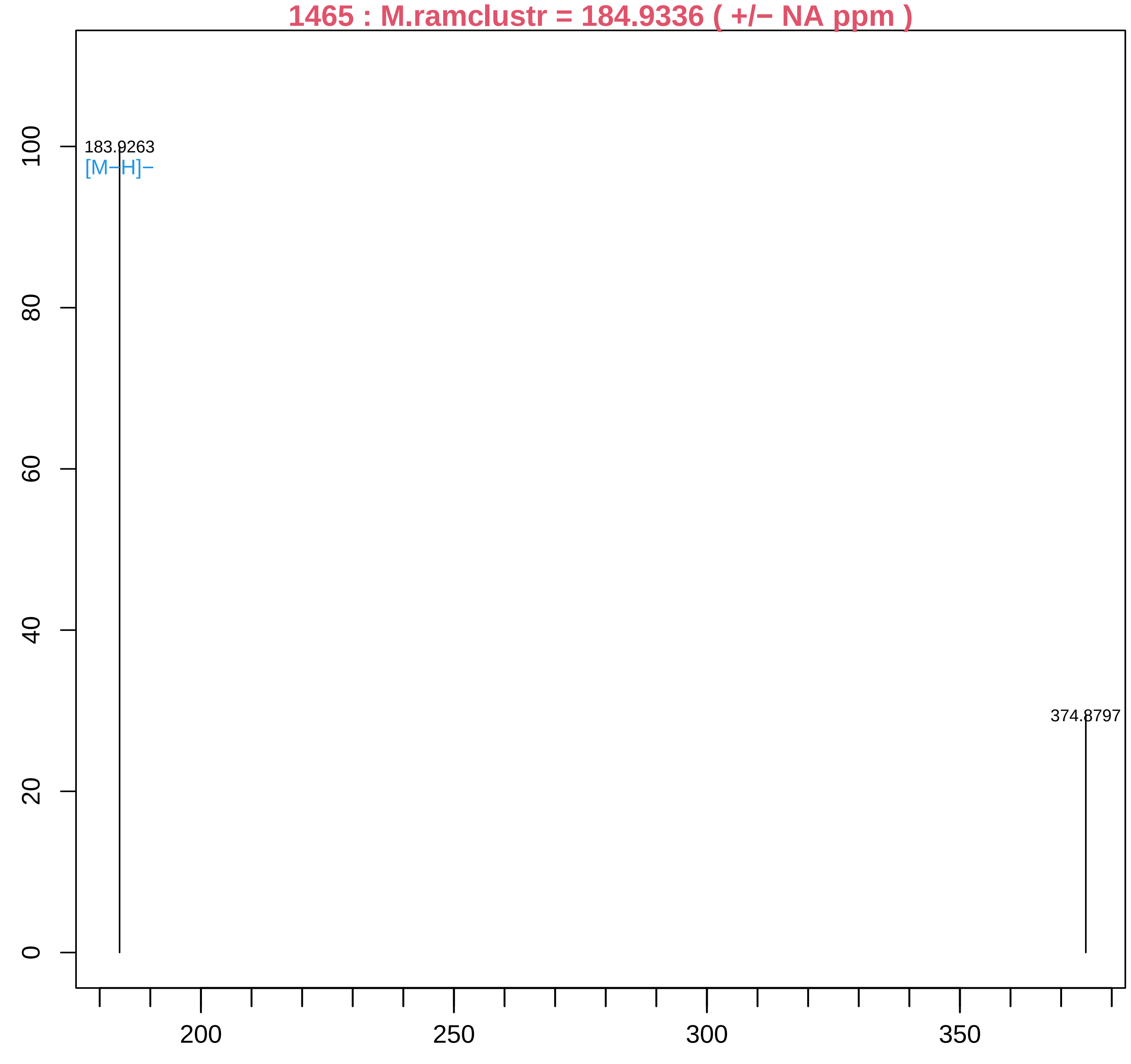
1465 : M.ramclustr = 184.9336 (+/− NA ppm)
183.9263
[M−H]−
374.8797
0
20
40
60
80
100
200
250
300
350

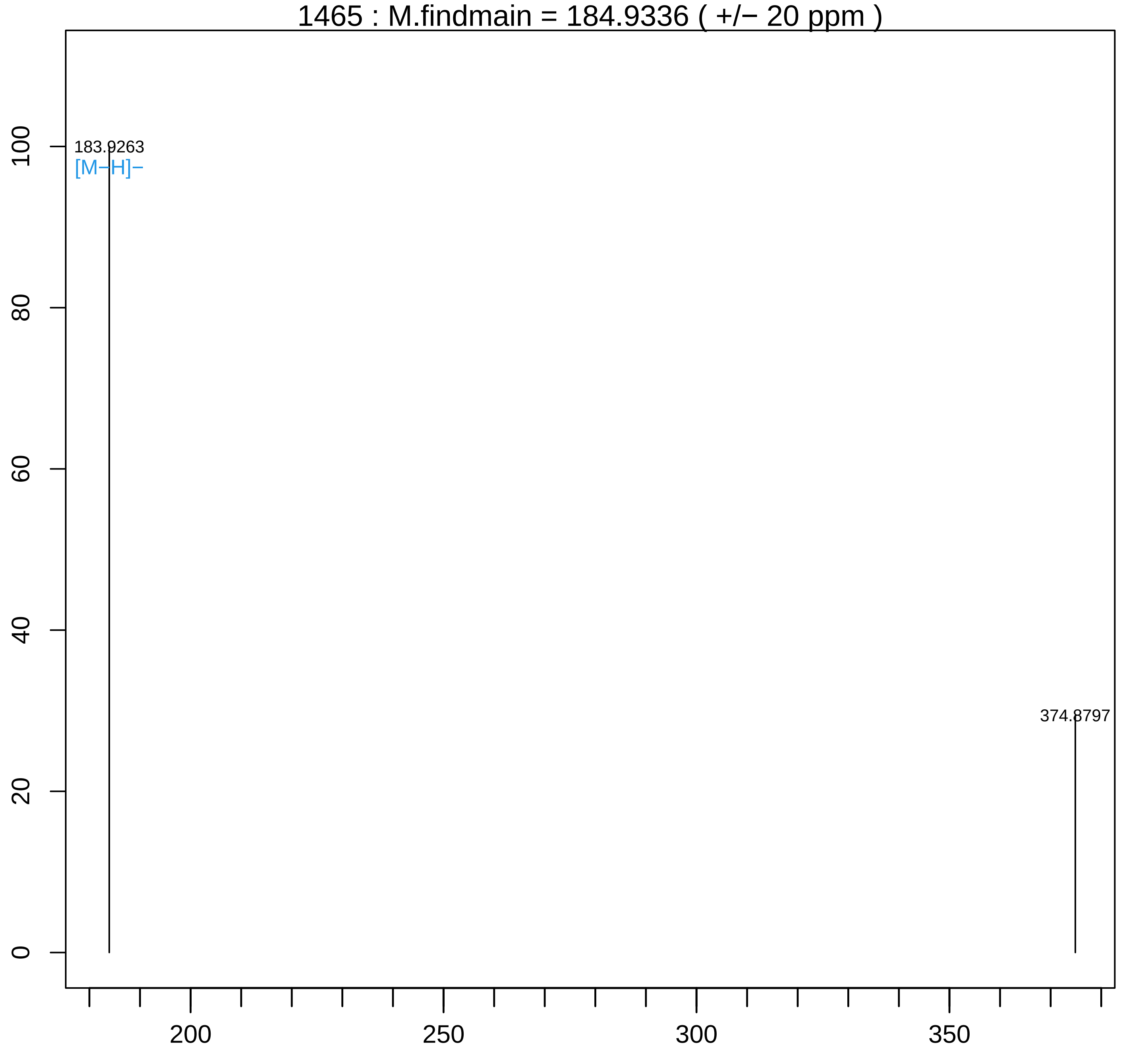
1465 : M.findmain = 184.9336 (+/− 20 ppm)
183.9263
[M−H]−
374.8797
0
20
40
60
80
100
200
250
300
350

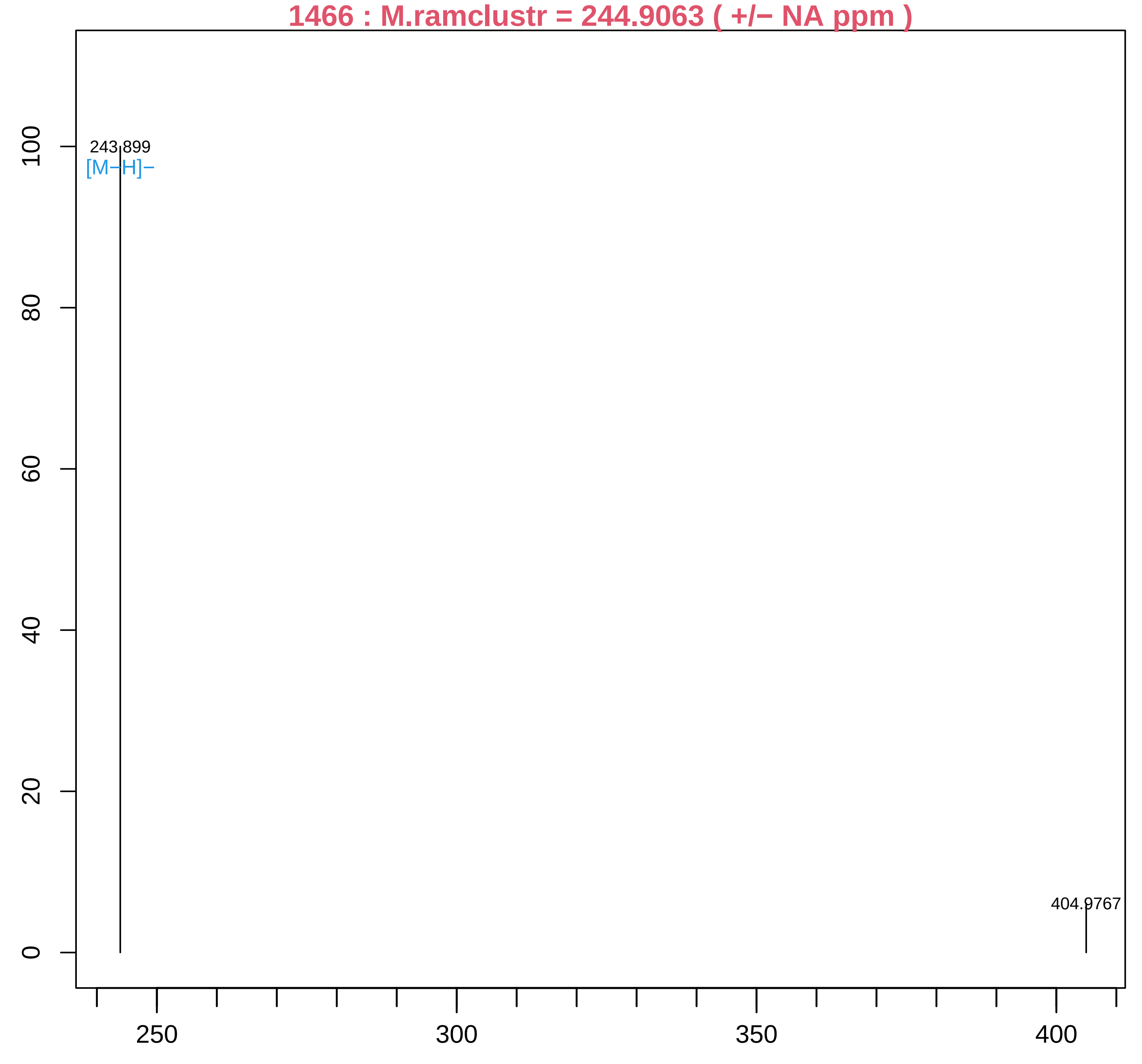
1466 : M.ramclustr = 244.9063 (+/− NA ppm)
243.899
[M−H]−
404.9767
0
20
40
60
80
100
250
300
350
400

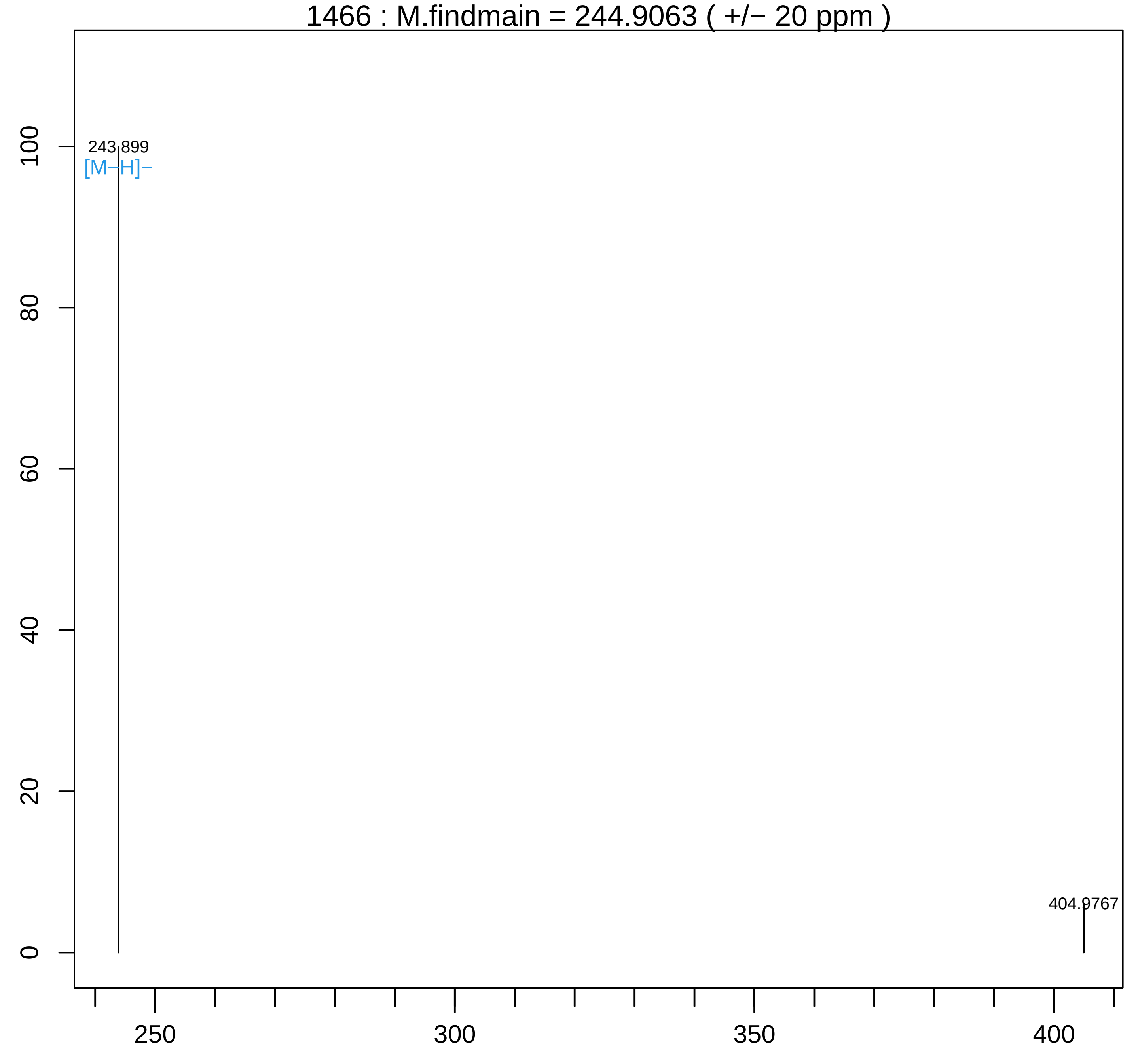
1466 : M.findmain = 244.9063 (+/− 20 ppm)
243.899
[M−H]−
404.9767
0
20
40
60
80
100
250
300
350
400

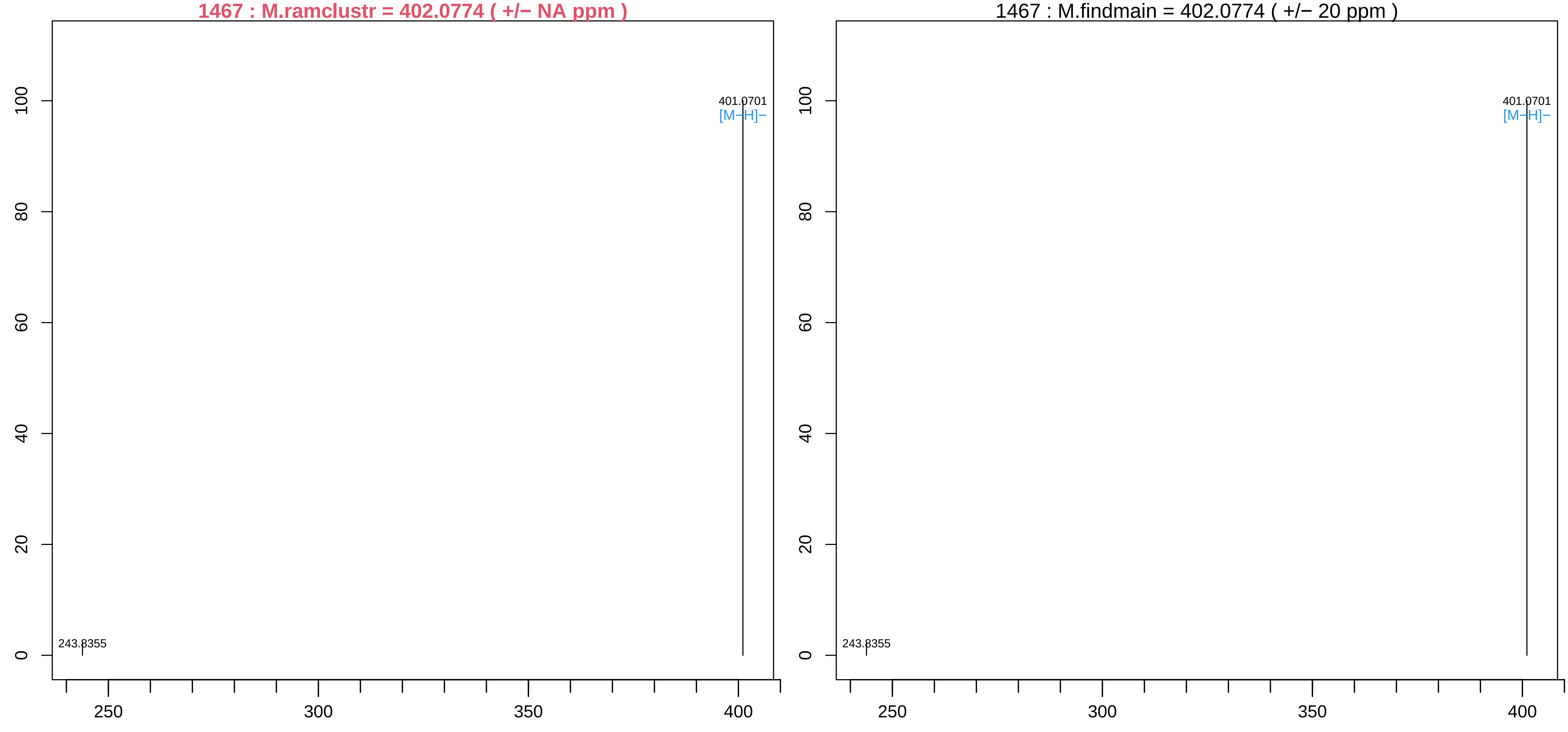
1467 : M.ramclustr = 402.0774 (+/− NA ppm)
401.0701
[M−H]−
243.8355
1467 : M.findmain = 402.0774 (+/− 20 ppm)
401.0701
[M−H]−
243.8355
0
20
40
60
80
100
250
300
350
400

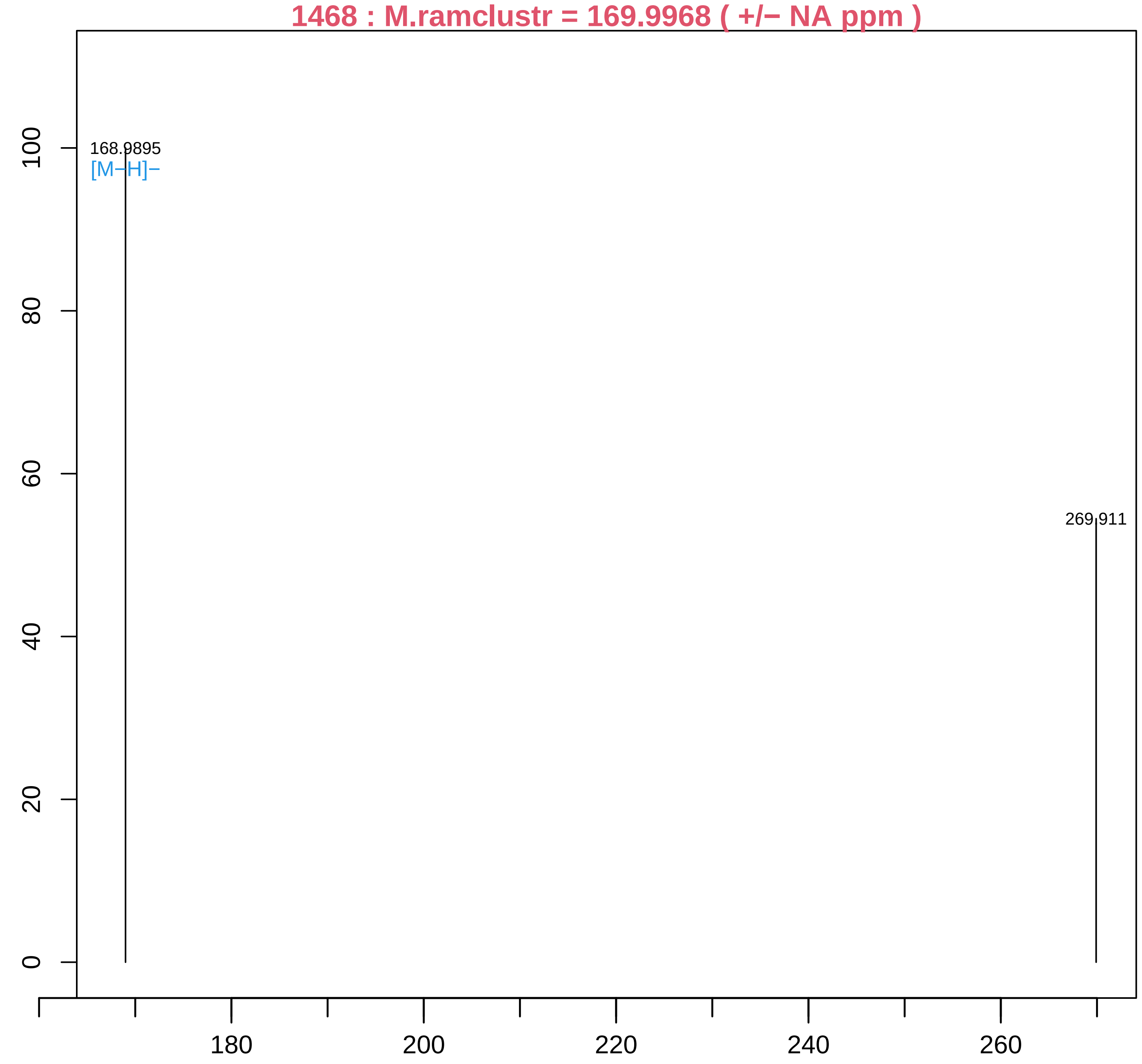
1468 : M.ramclustr = 169.9968 (+/− NA ppm)
168.9895
[M−H]−
269.911
0
20
40
60
80
100
180
200
220
240
260

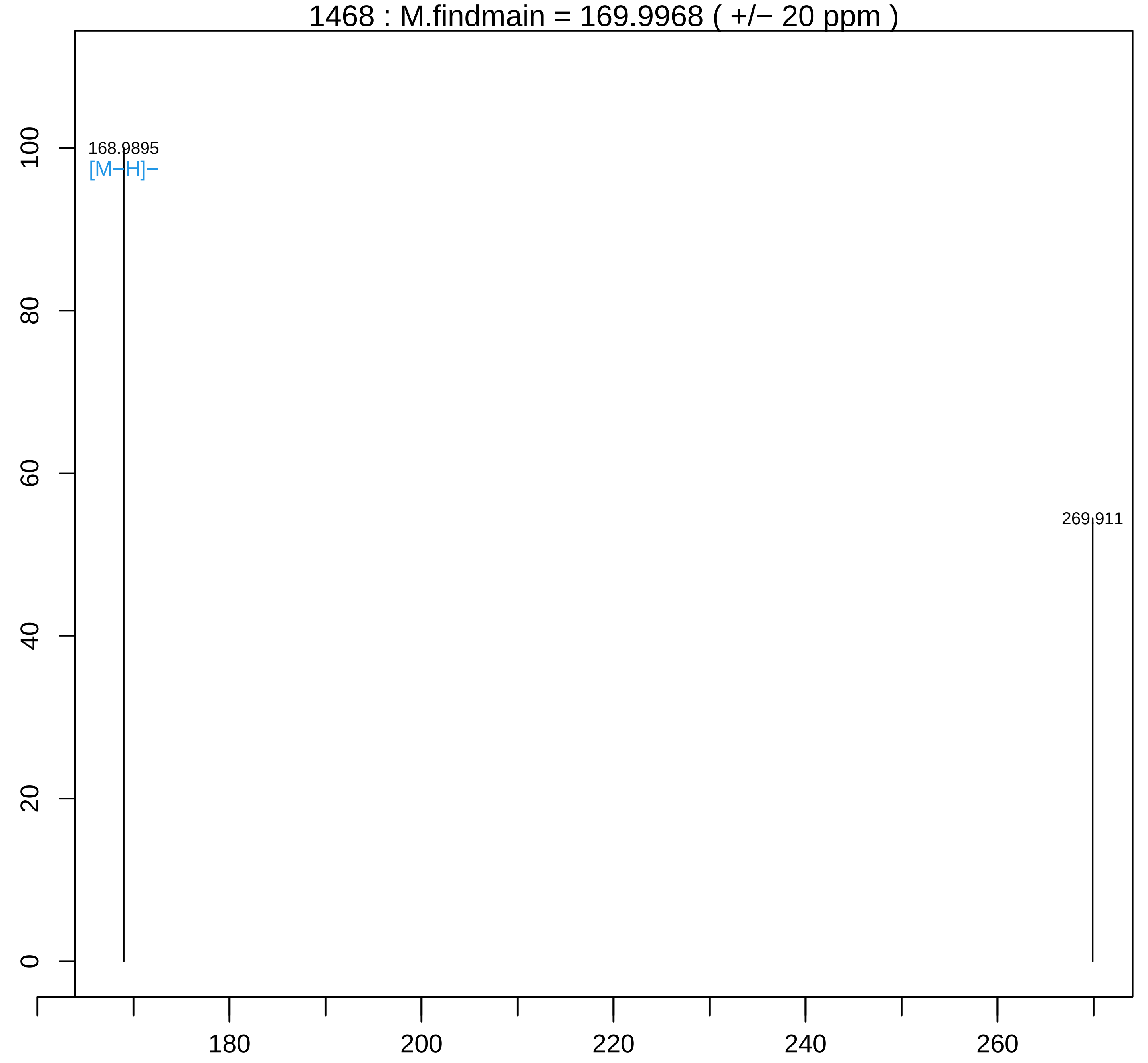
1468 : M.findmain = 169.9968 (+/− 20 ppm)
168.9895
[M−H]−
269.911
0
20
40
60
80
100
180
200
220
240
260

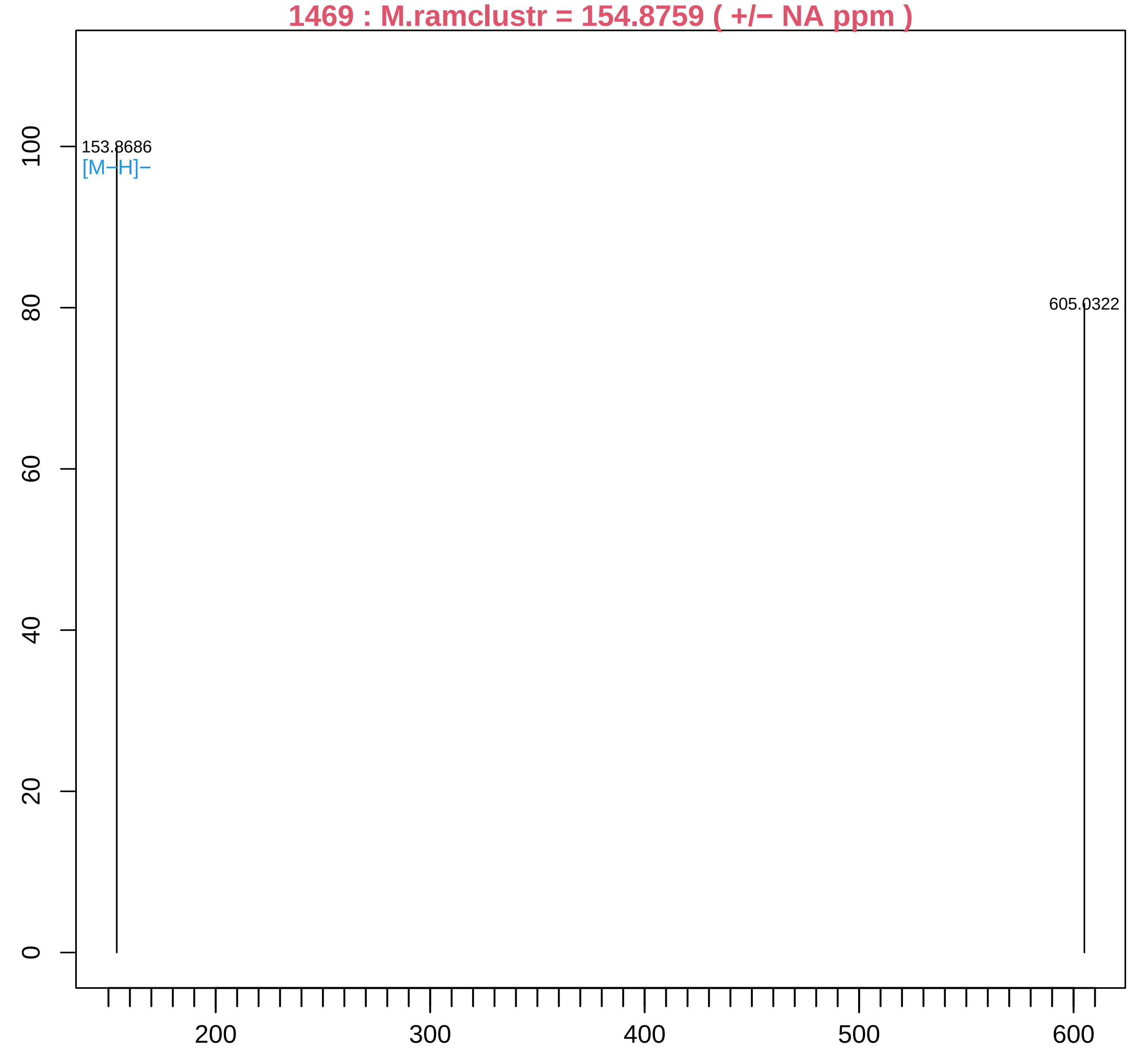
1469 : M.ramclustr = 154.8759 (+/− NA ppm)
153.8686
[M−H]−
605.0322
0
20
40
60
80
100
200
300
400
500
600

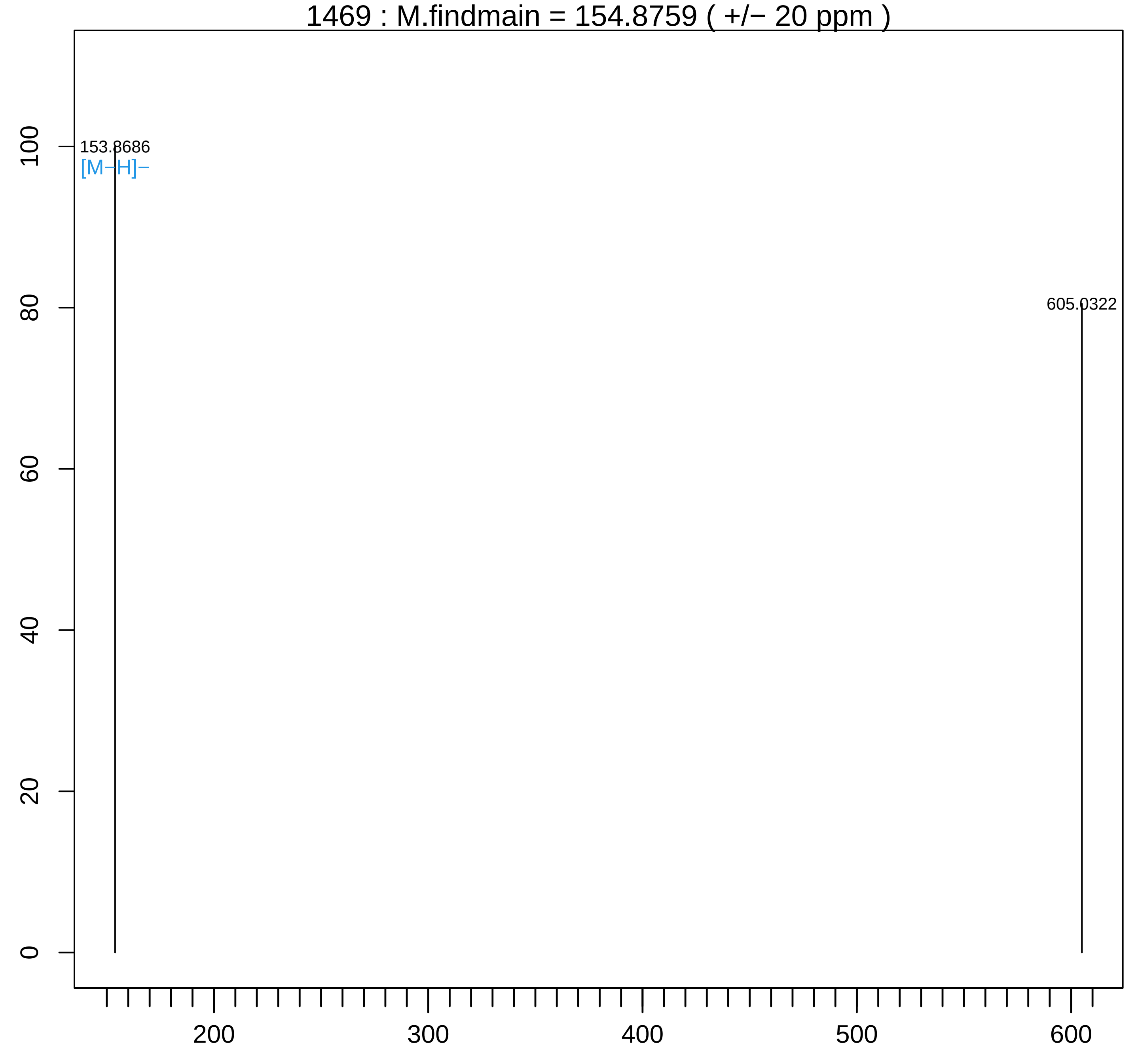
1469 : M.findmain = 154.8759 (+/− 20 ppm)
153.8686
[M−H]−
605.0322
0
20
40
60
80
100
200
300
400
500
600

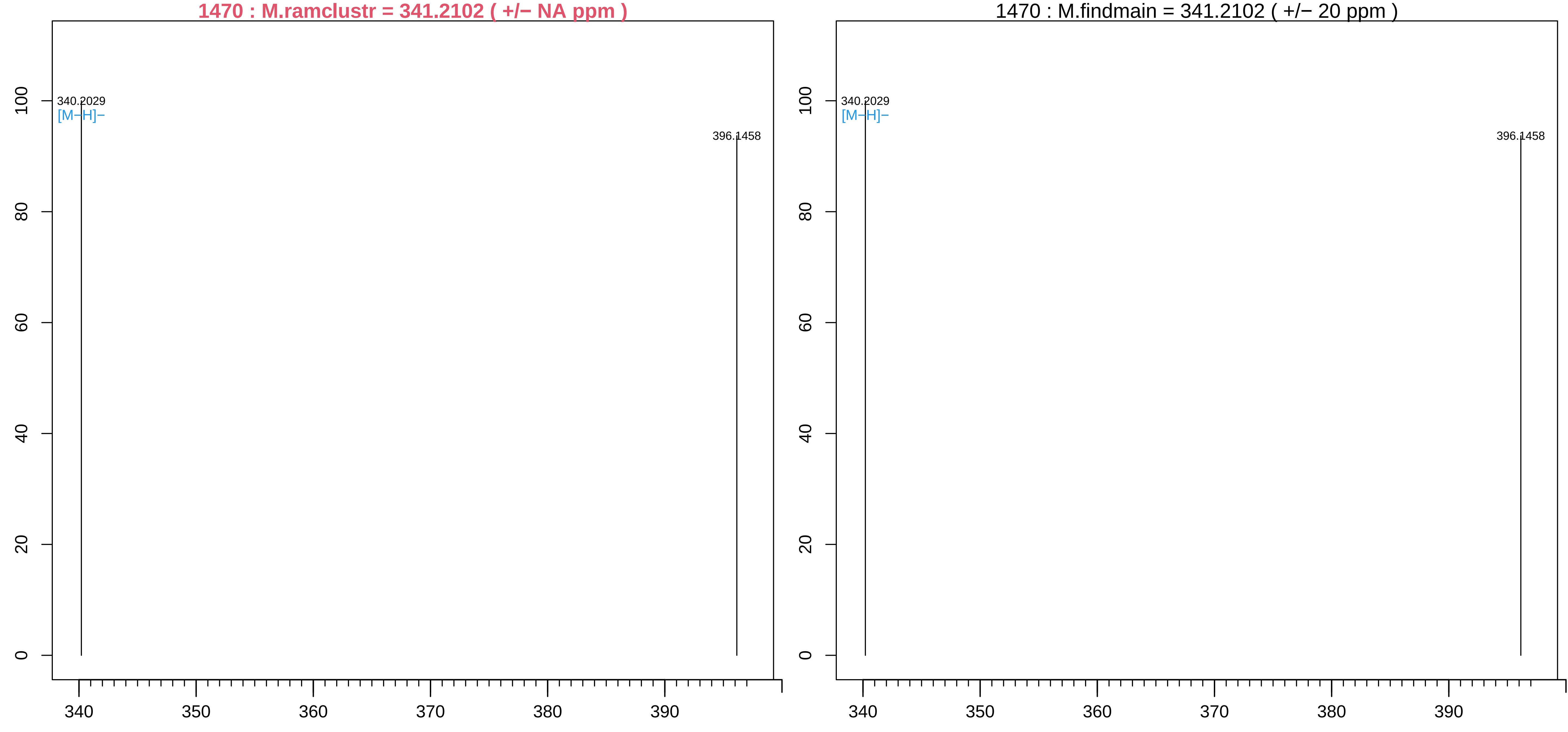

1470 : M.ramclustr = 341.2102 (+/− NA ppm)
340.2029
[M−H]−
396.1458
1470 : M.findmain = 341.2102 (+/− 20 ppm)
340.2029
[M−H]−
396.1458
0
20
40
60
80
100
340
350
360
370
380
390

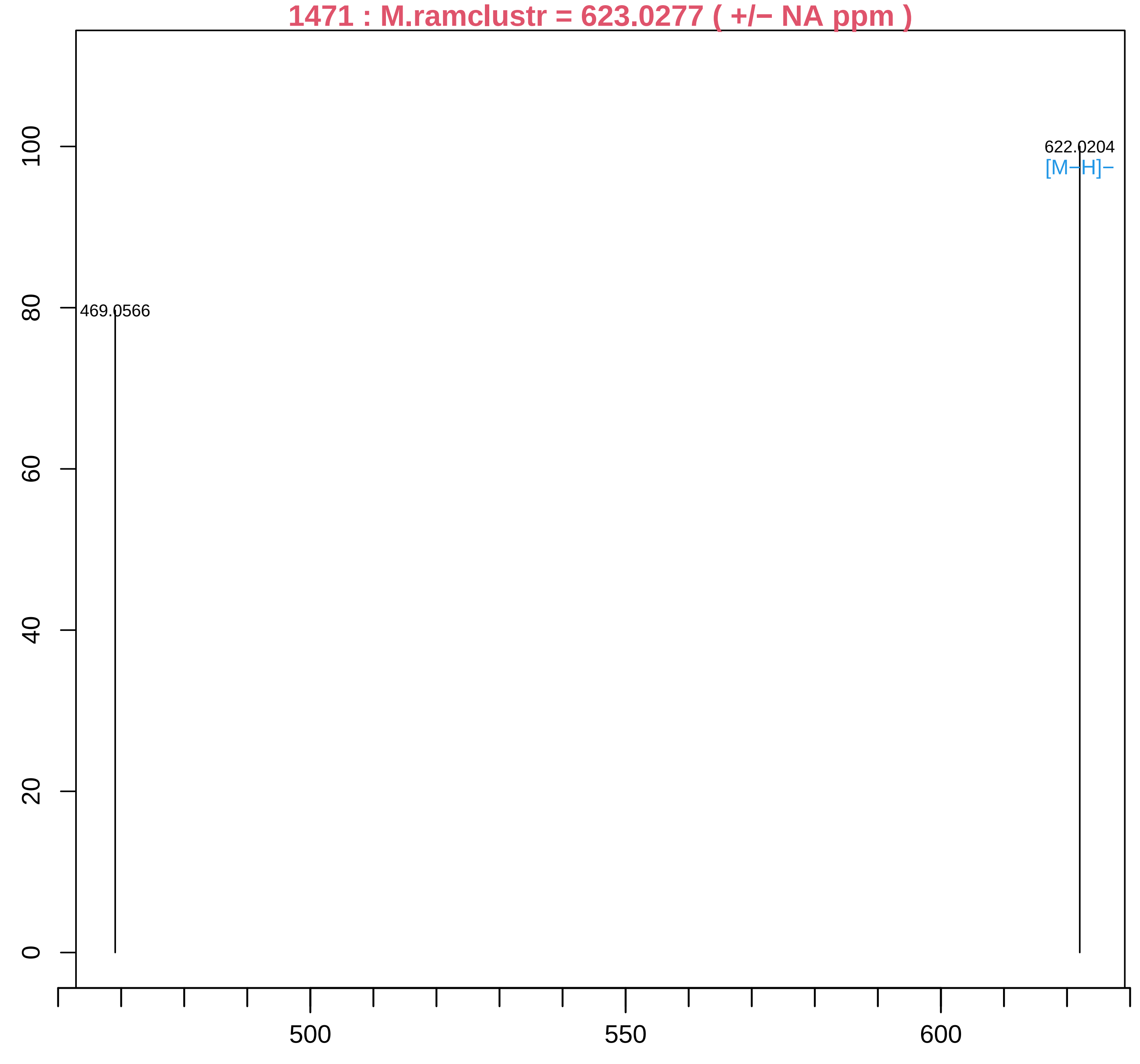
1471 : M.ramclustr = 623.0277 (+/− NA ppm)
622.0204
[M−H]−
469.0566
0
20
40
60
80
100
500
550
600

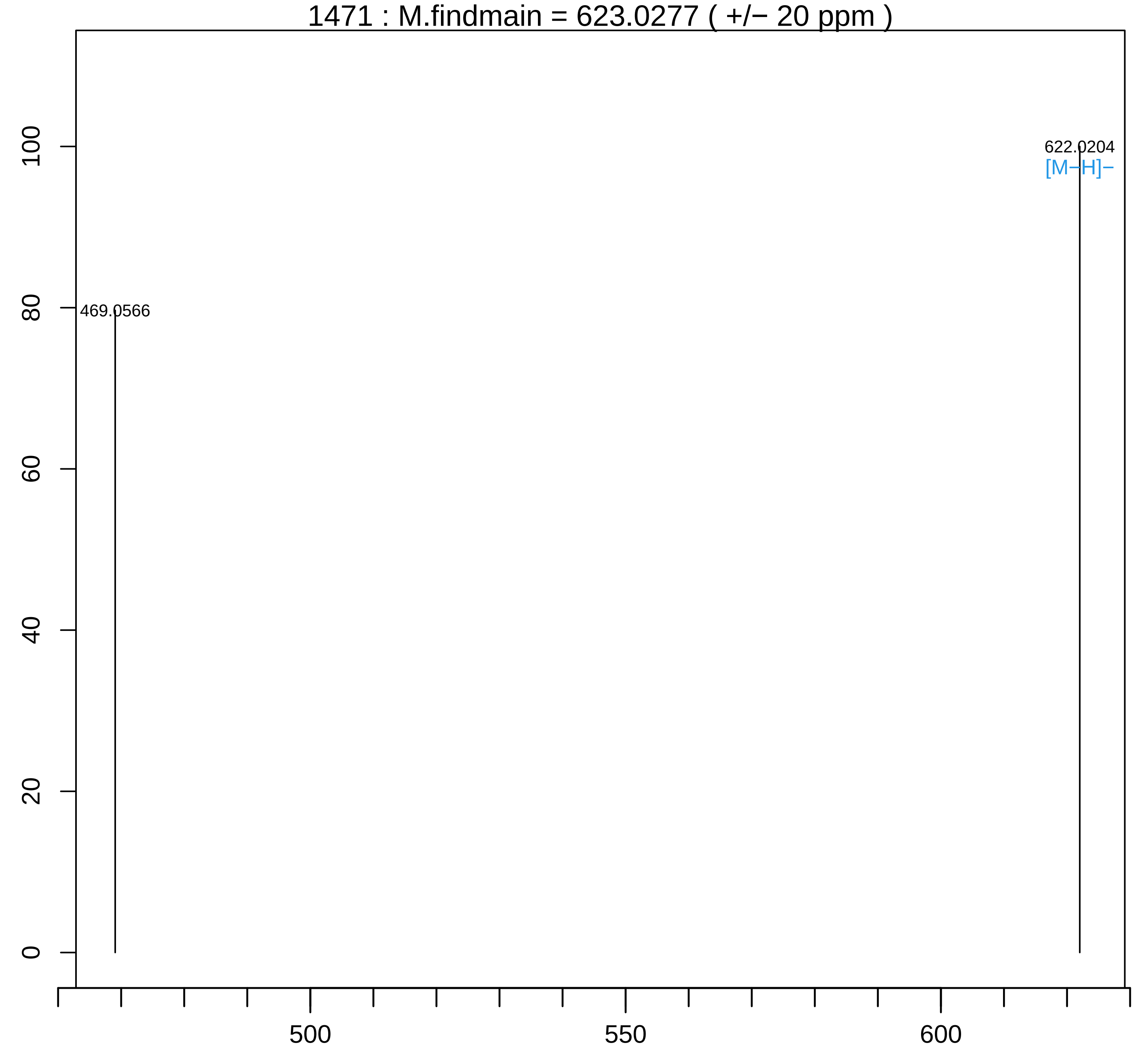
1471 : M.findmain = 623.0277 (+/− 20 ppm)
622.0204
[M−H]−
469.0566
0
20
40
60
80
100
500
550
600

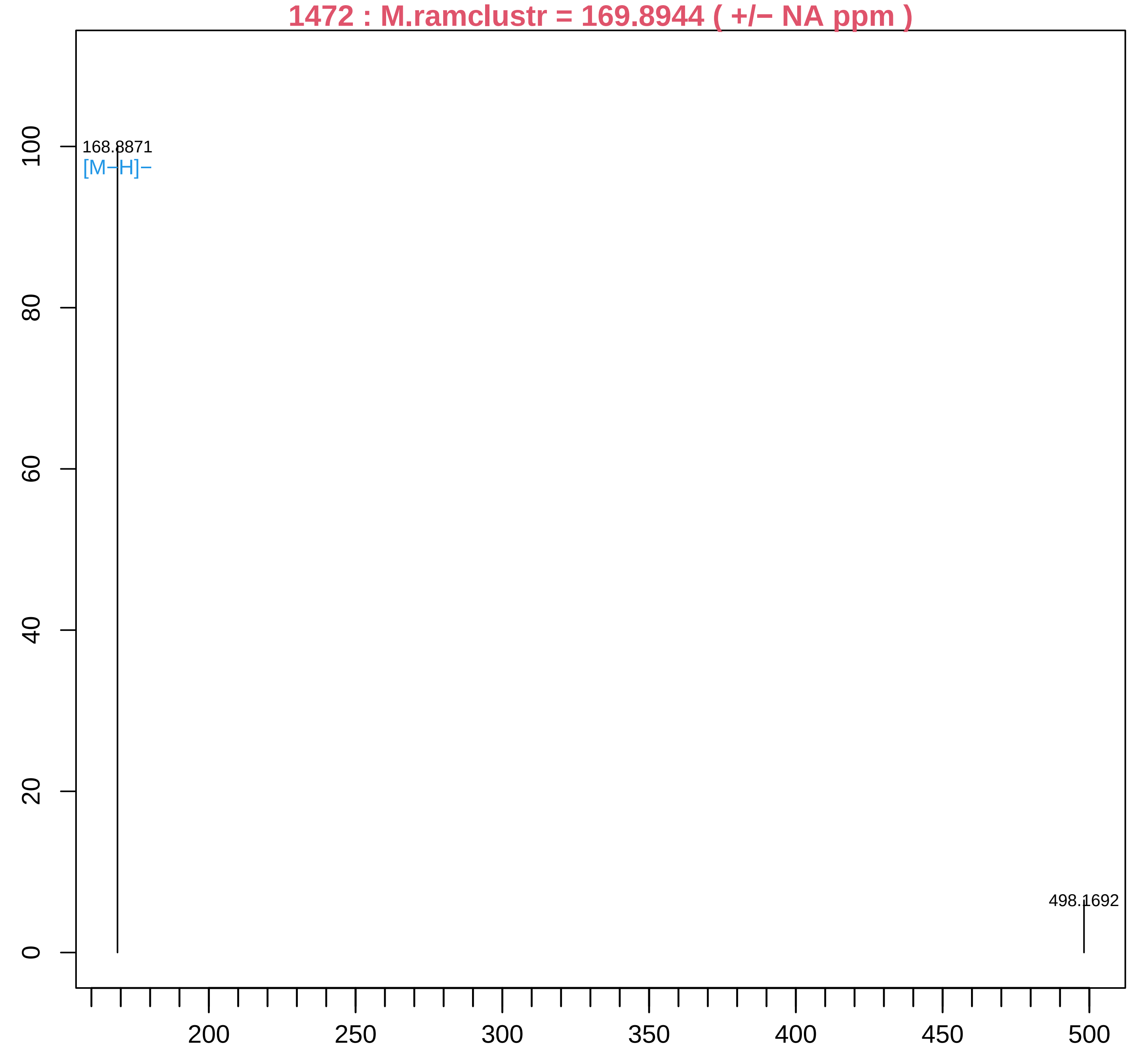

1472 : M.ramclustr = 169.8944 (+/− NA ppm)
168.8871
[M−H]−
498.1692
0
20
40
60
80
100
200
250
300
350
400
450
500

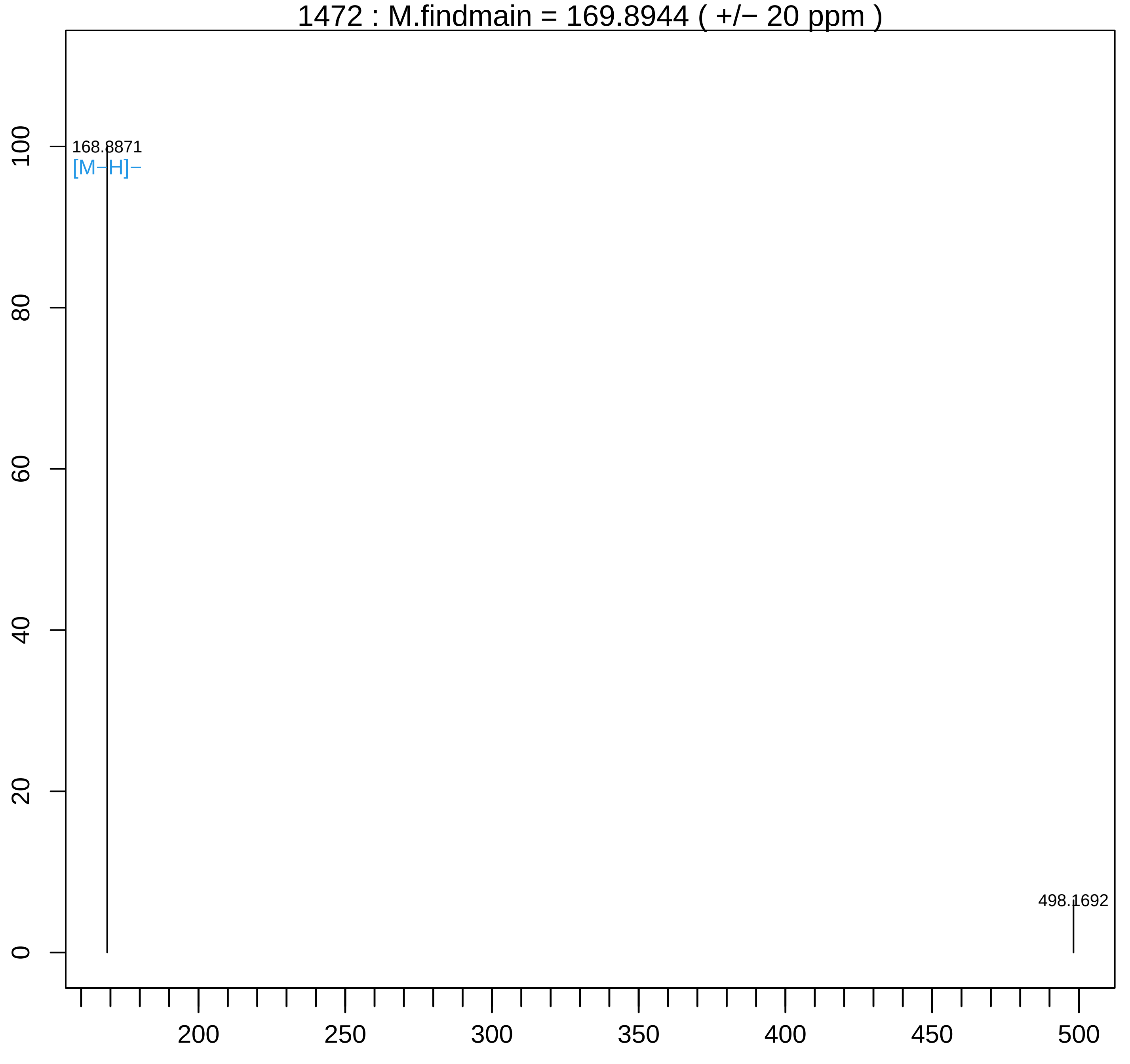

1472 : M.findmain = 169.8944 (+/− 20 ppm)
168.8871
[M−H]−
498.1692
0
20
40
60
80
100
200
250
300
350
400
450
500

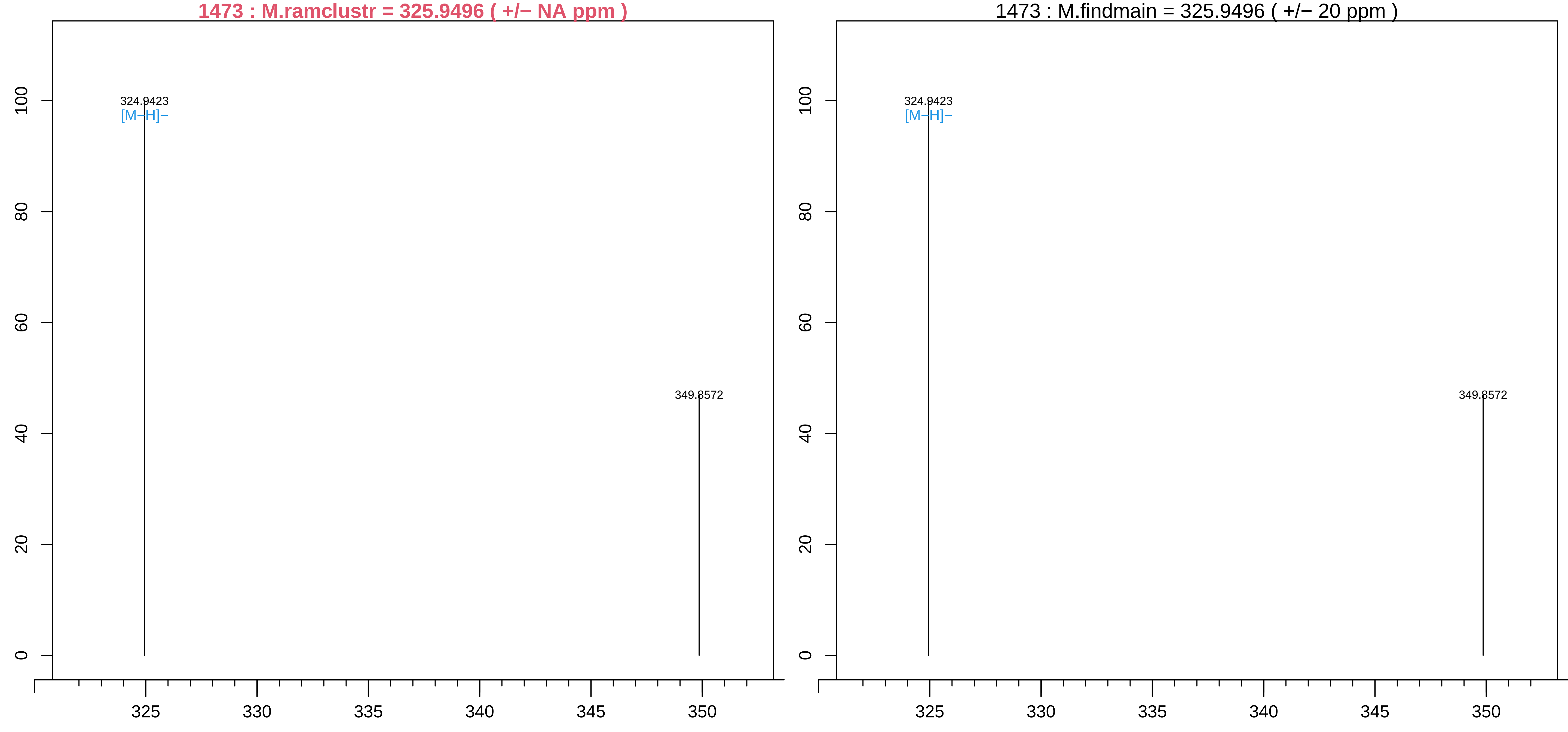

1473 : M.ramclustr = 325.9496 (+/− NA ppm)
324.9423
[M−H]−
349.8572
1473 : M.findmain = 325.9496 (+/− 20 ppm)
324.9423
[M−H]−
349.8572
0
20
40
60
80
100
325
330
335
340
345
350

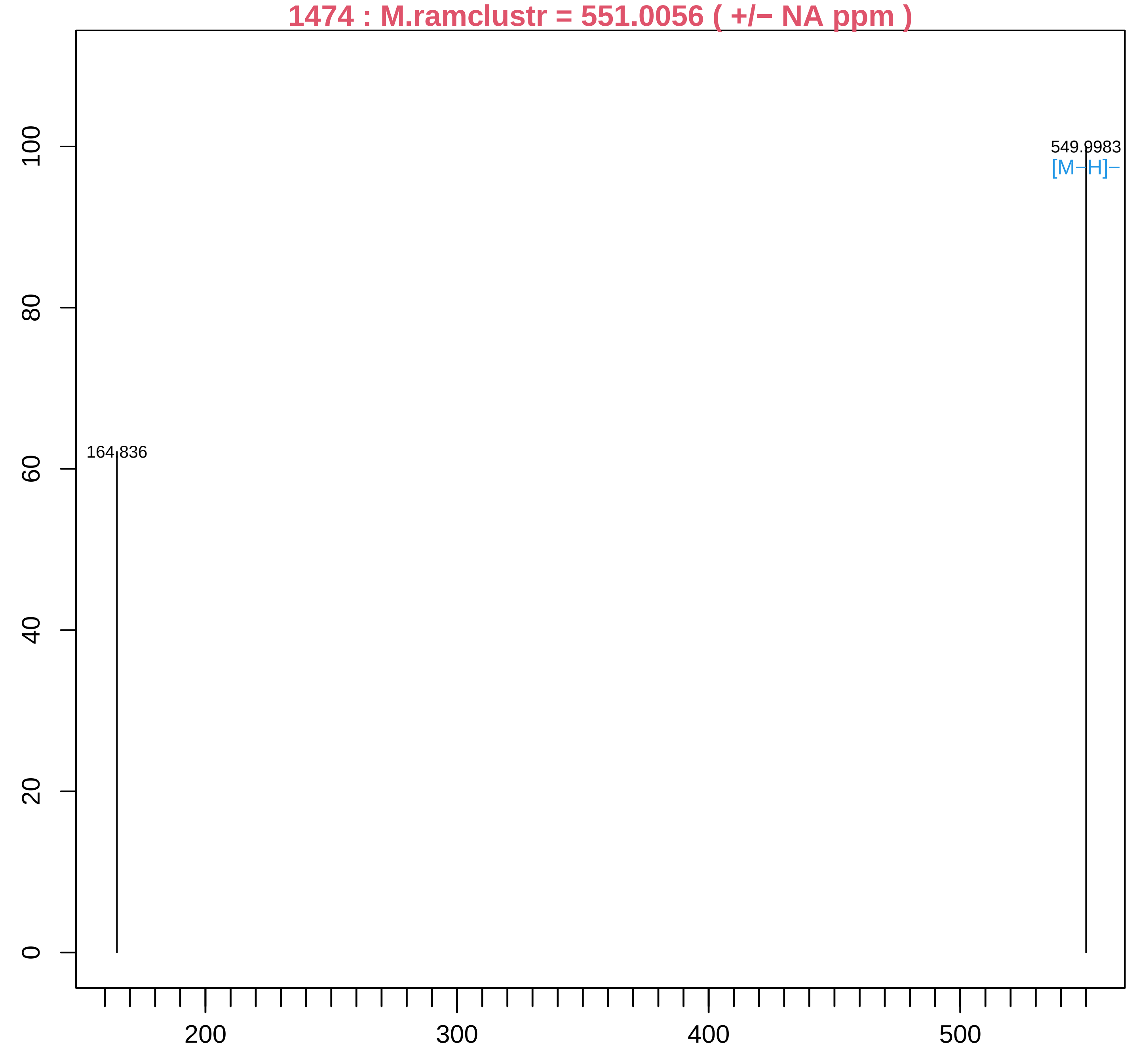
1474 : M.ramclustr = 551.0056 (+/− NA ppm)
549.9983
[M−H]−
164.836
0
20
40
60
80
100
200
300
400
500

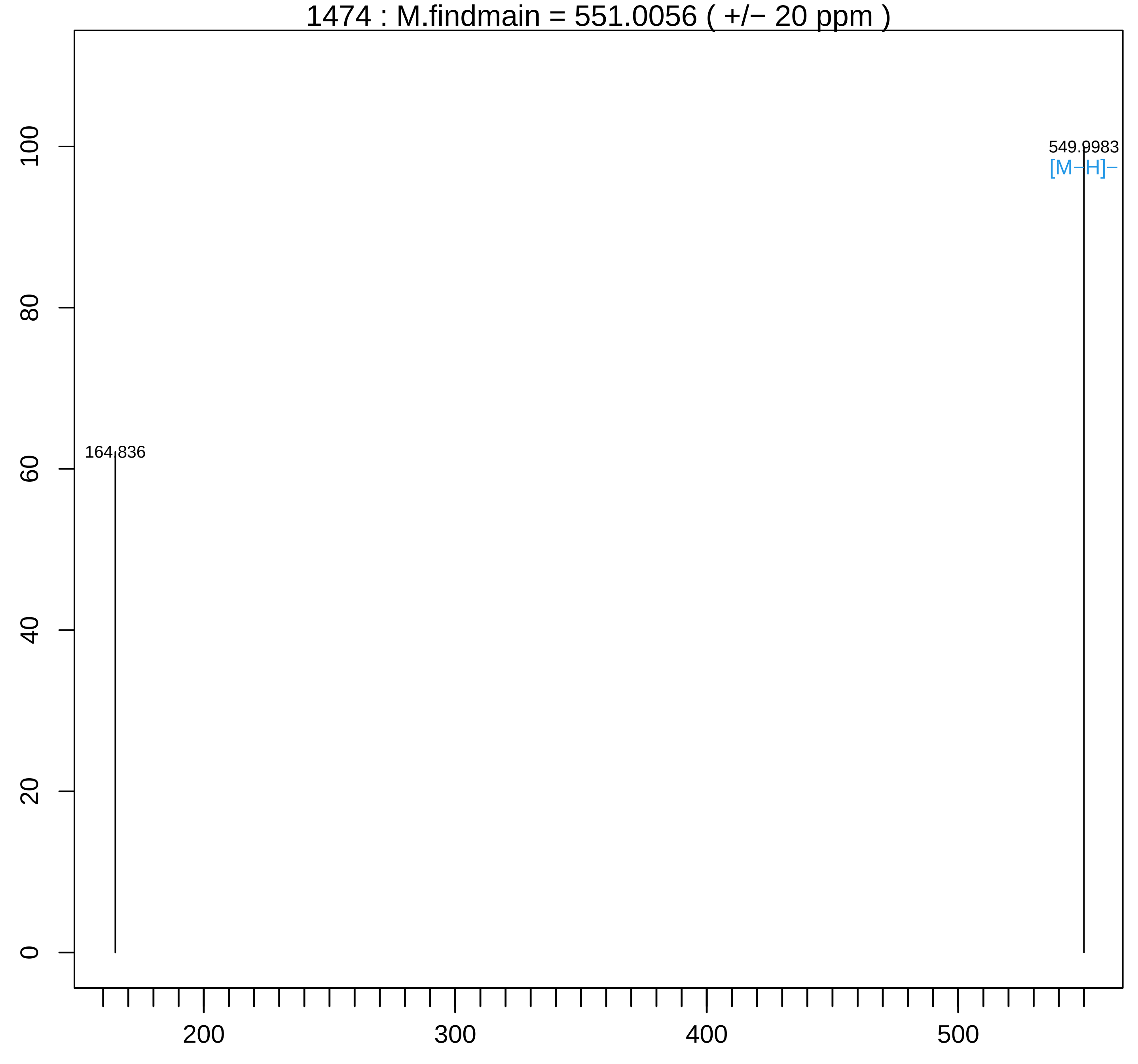
1474 : M.findmain = 551.0056 (+/− 20 ppm)
549.9983
[M−H]−
164.836
0
20
40
60
80
100
200
300
400
500

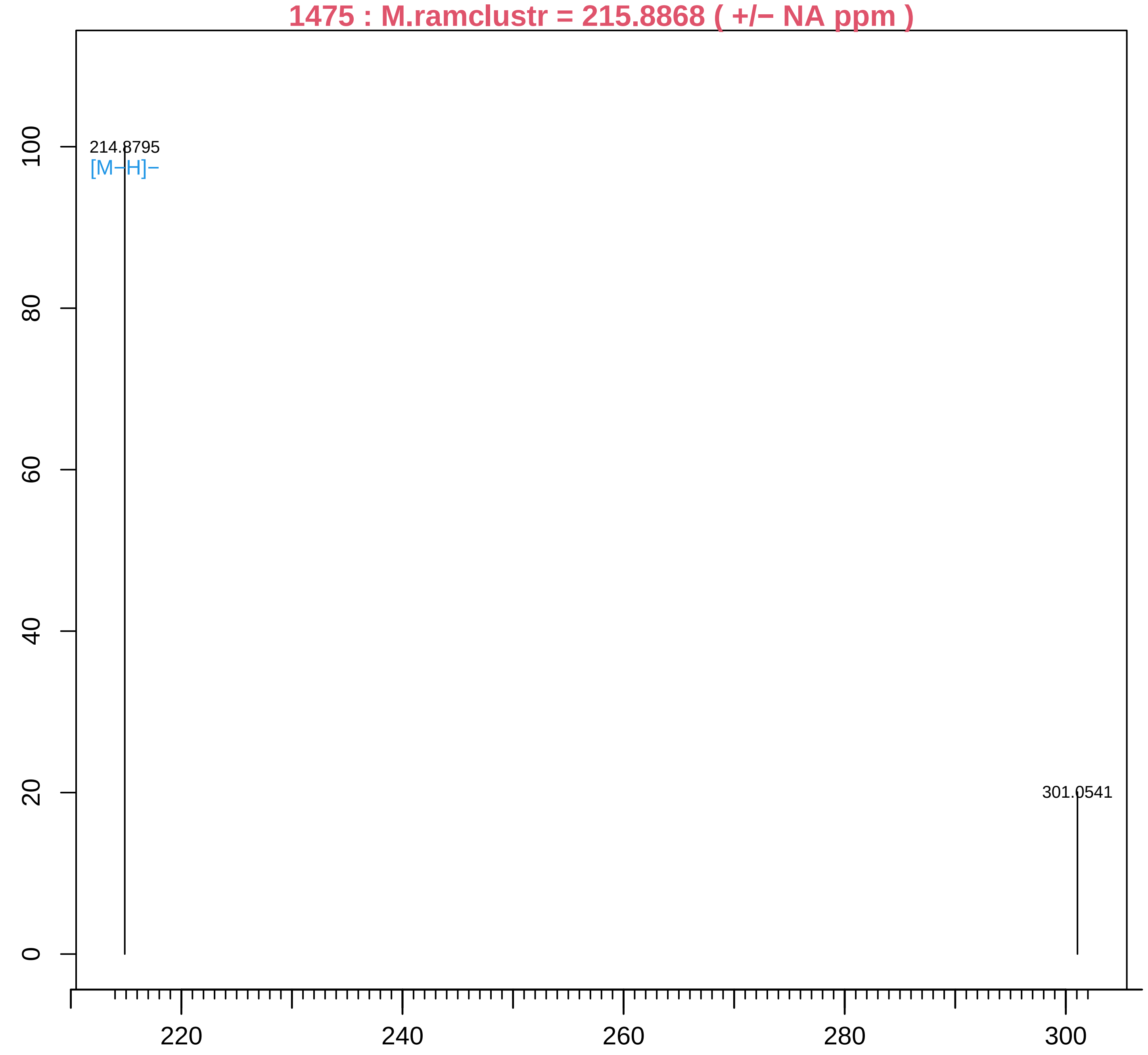

1475 : M.ramclustr = 215.8868 (+/− NA ppm)
214.8795
[M−H]−
301.0541
0
20
40
60
80
100
220
240
260
280
300

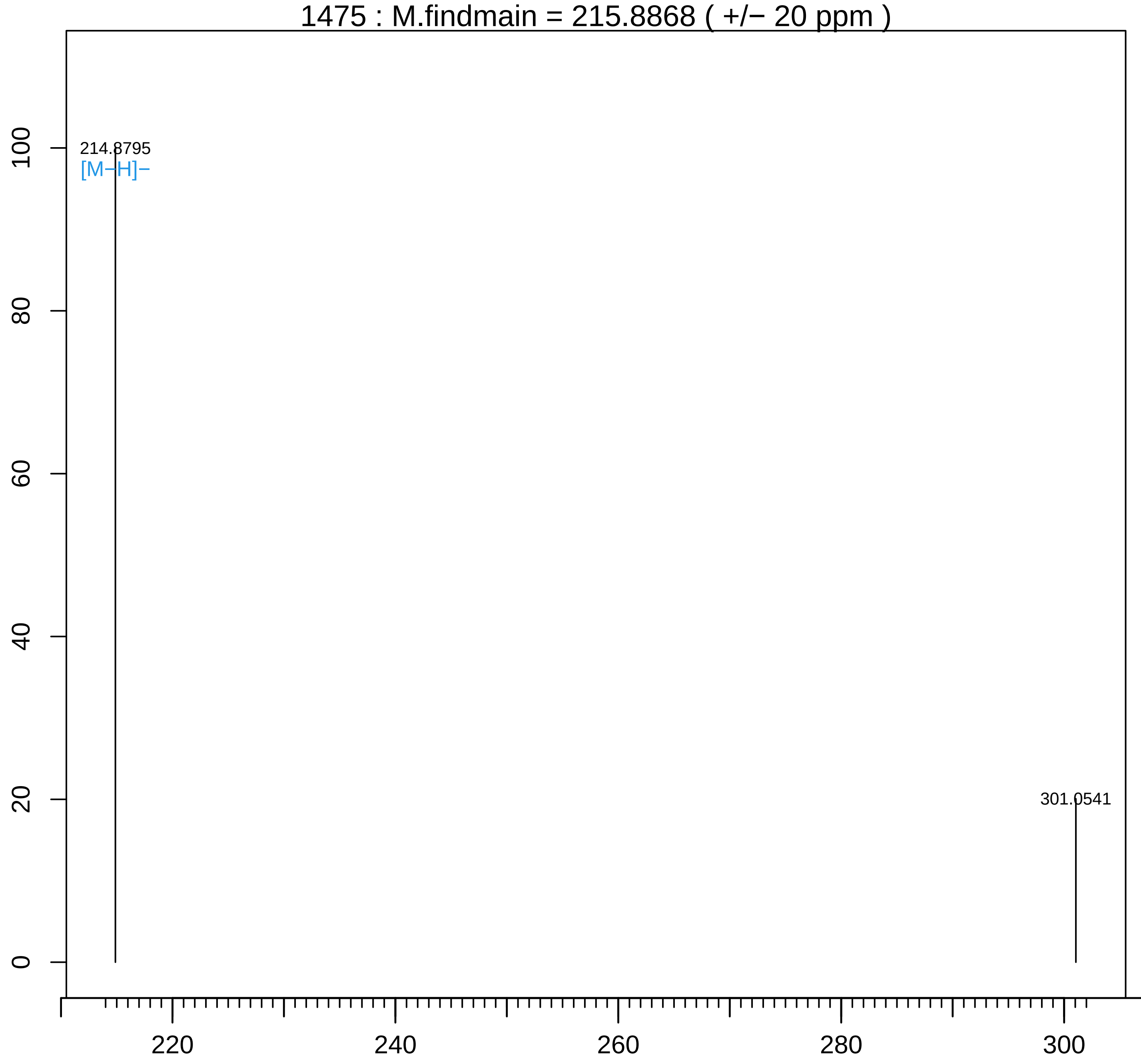

1475 : M.findmain = 215.8868 (+/− 20 ppm)
214.8795
[M−H]−
301.0541
0
20
40
60
80
100
220
240
260
280
300

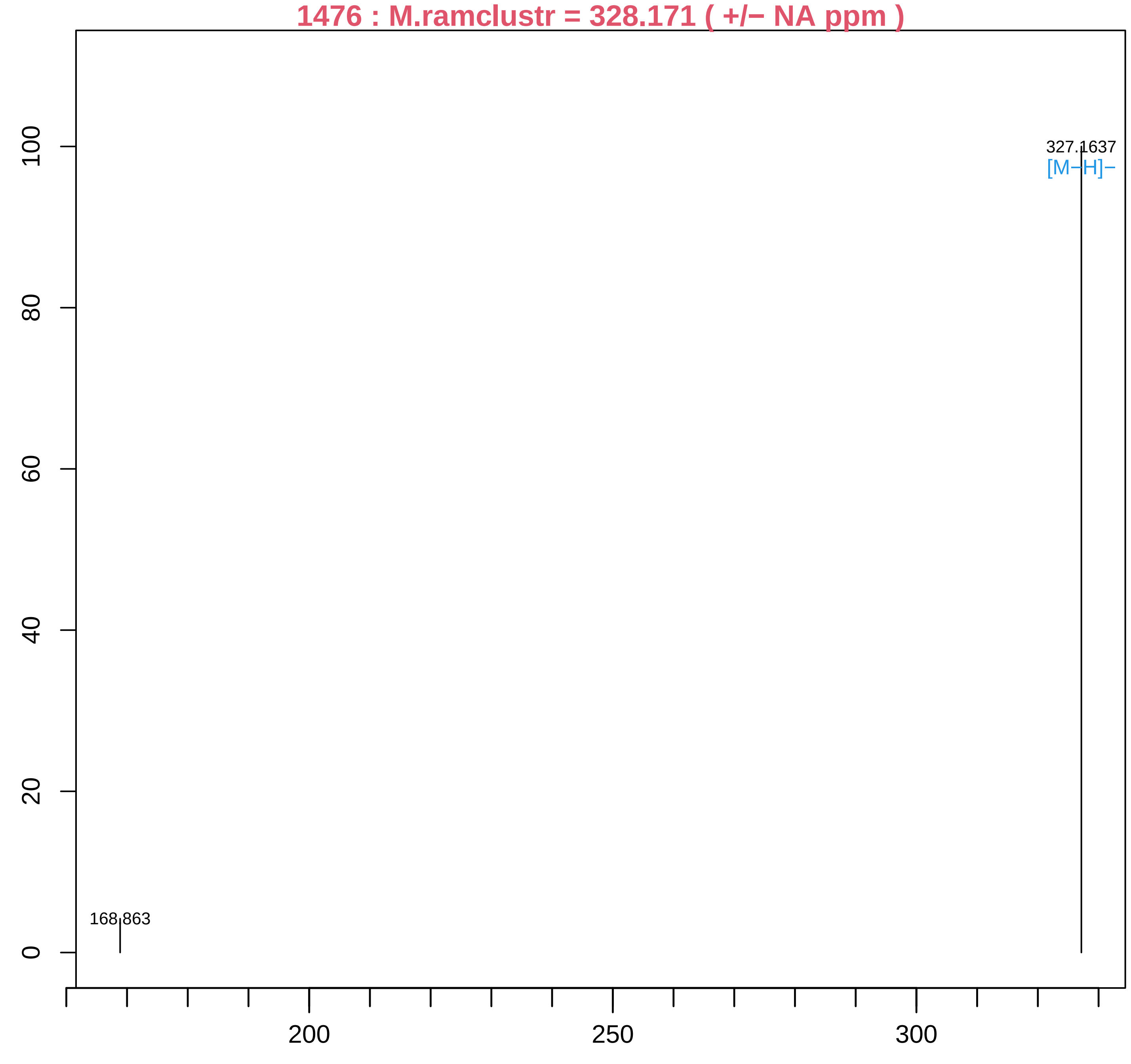

1476 : M.ramclustr = 328.171 (+/− NA ppm)
327.1637
[M−H]−
168.863
0
20
40
60
80
100
200
250
300

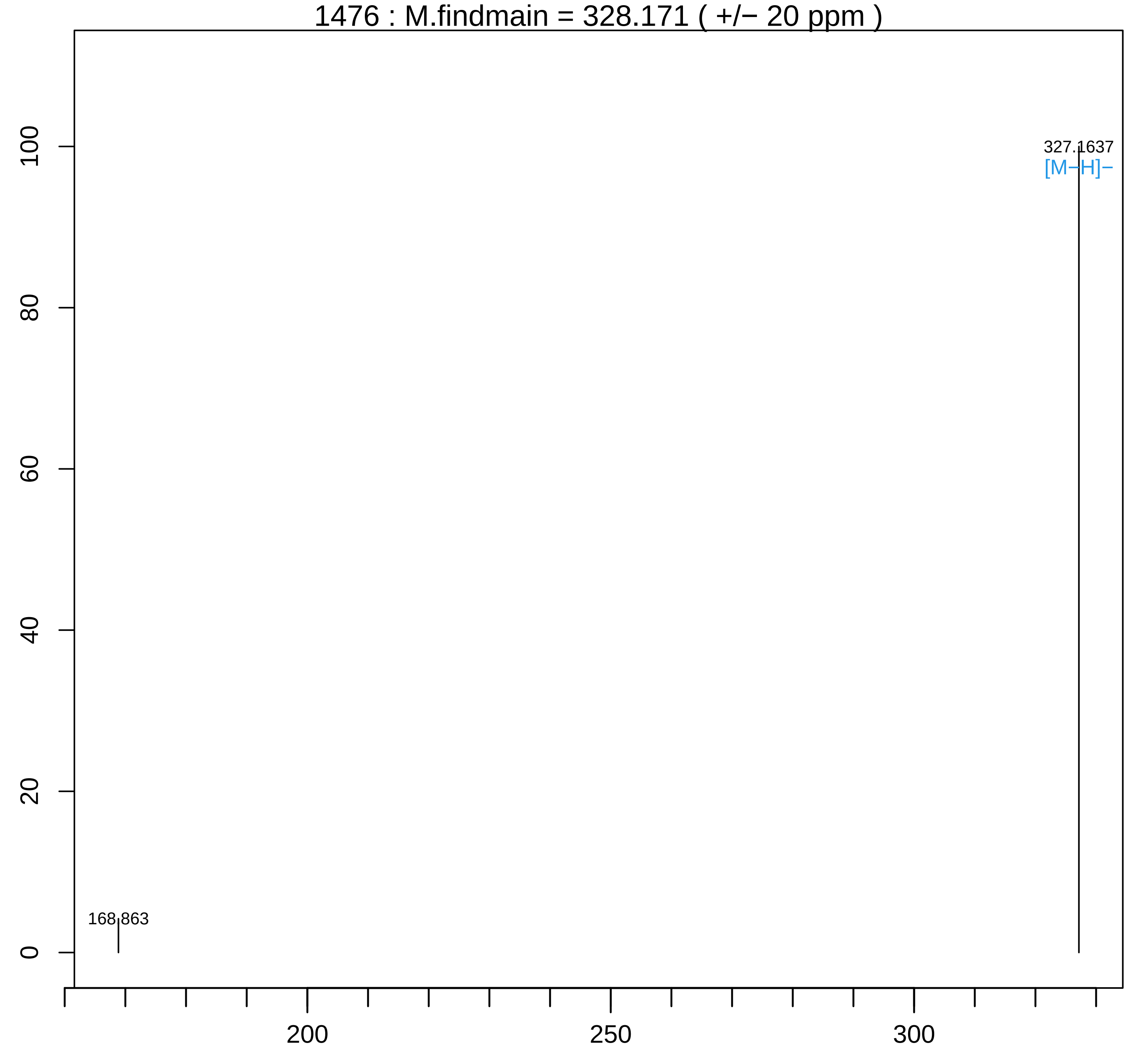

1476 : M.findmain = 328.171 (+/− 20 ppm)
327.1637
[M−H]−
168.863
0
20
40
60
80
100
200
250
300

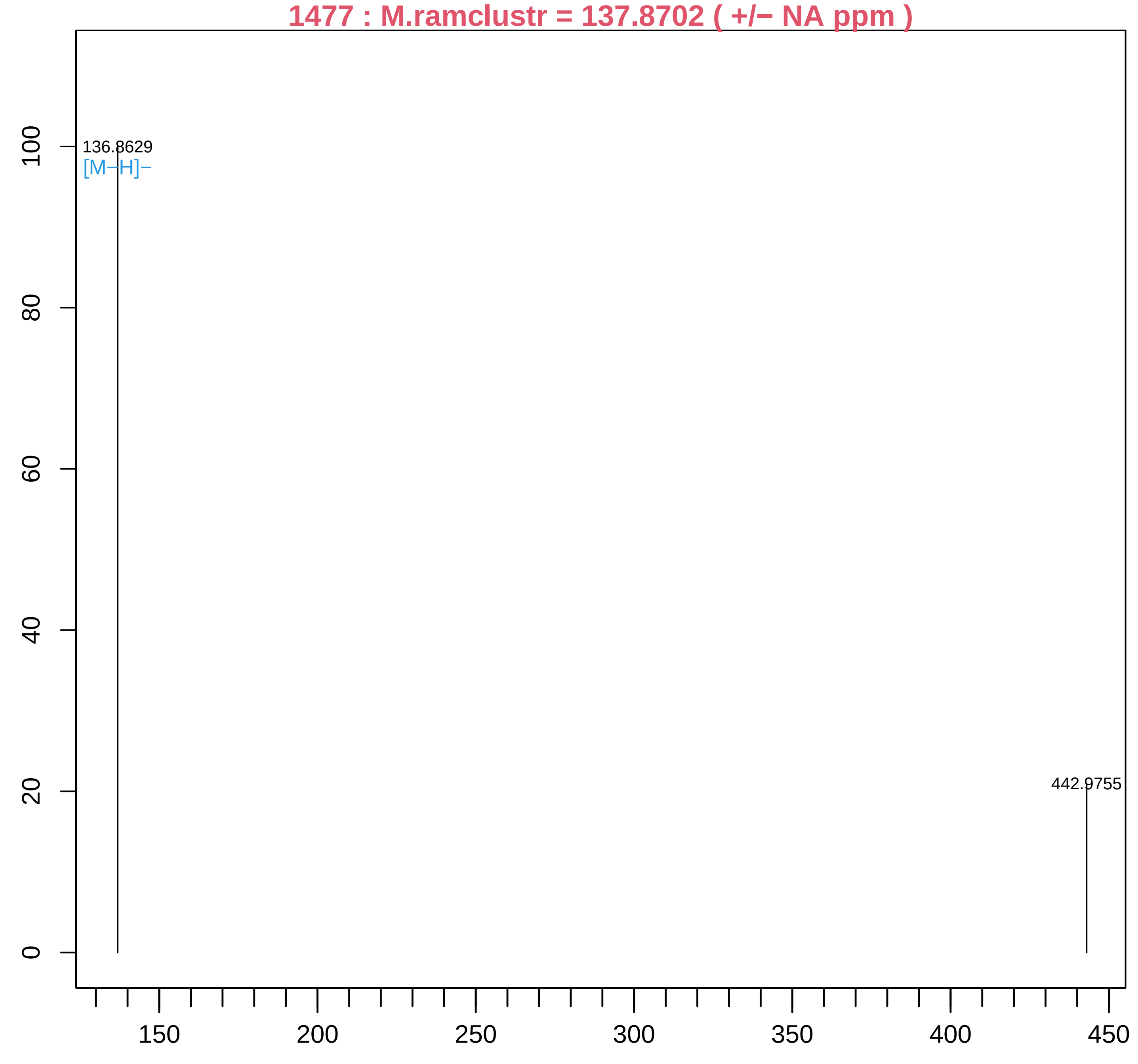
1477 : M.ramclustr = 137.8702 (+/− NA ppm)
136.8629
[M−H]−
442.9755
0
20
40
60
80
100
150
200
250
300
350
400
450

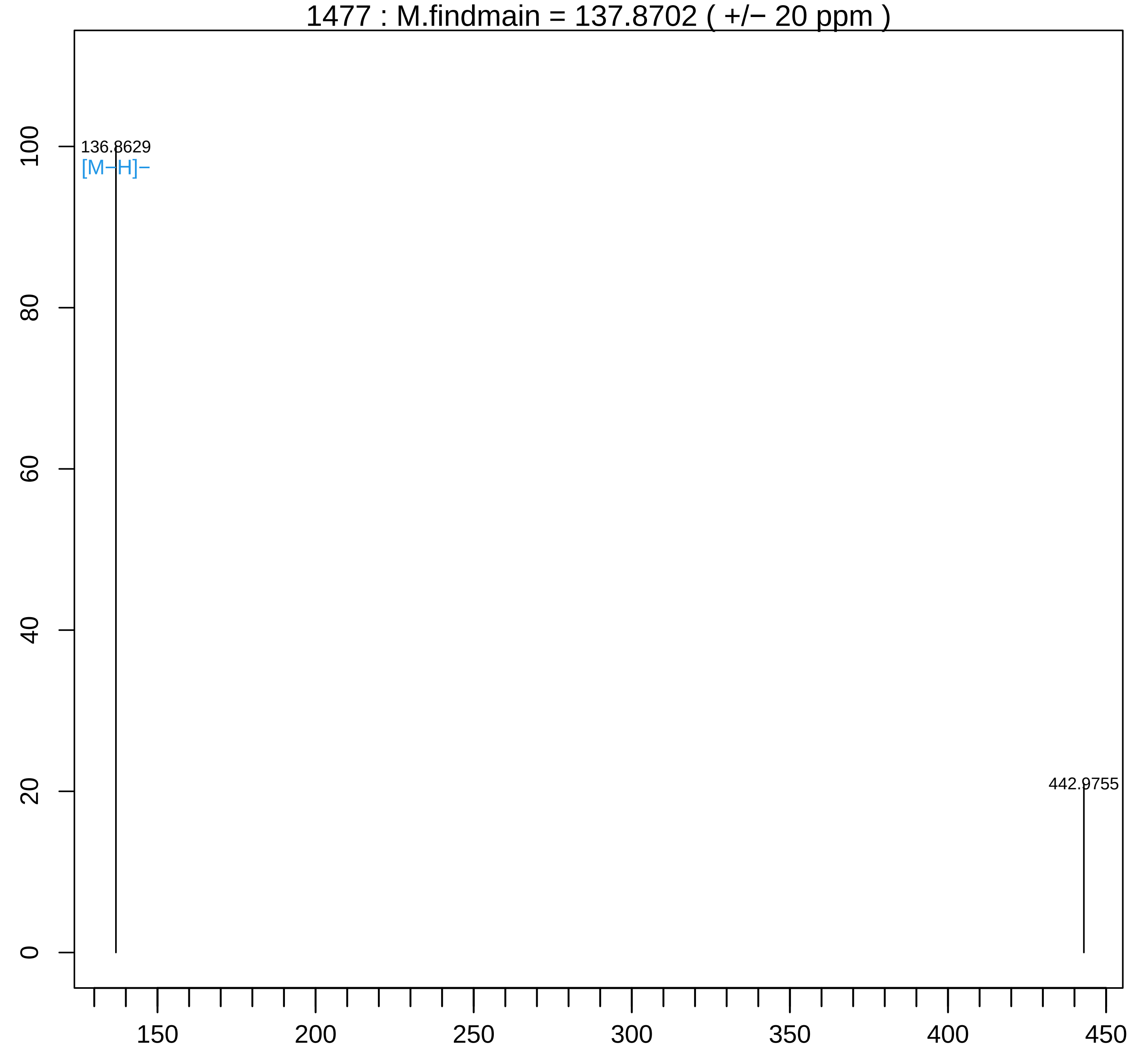
1477 : M.findmain = 137.8702 (+/− 20 ppm)
136.8629
[M−H]−
442.9755
0
20
40
60
80
100
150
200
250
300
350
400
450

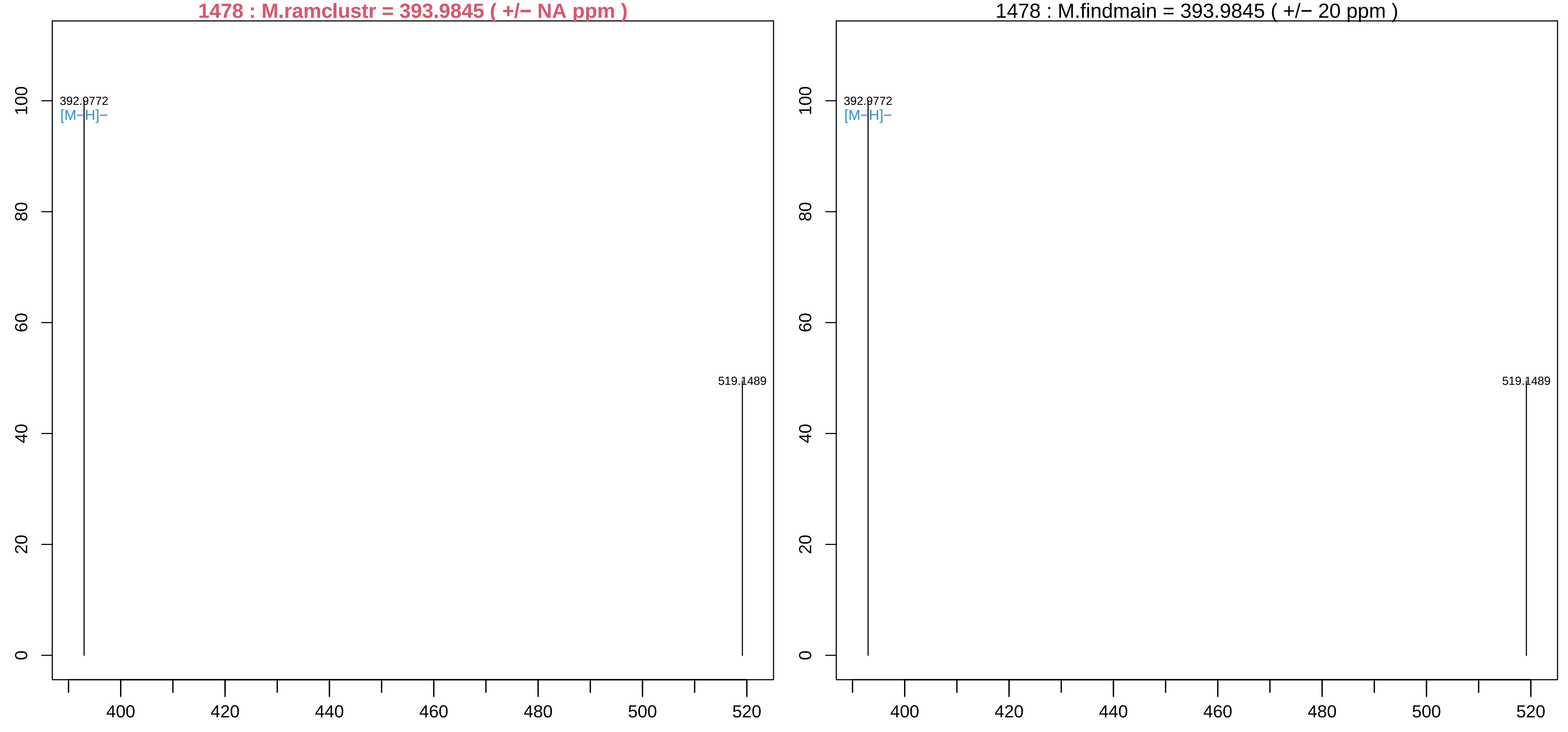
1478 : M.ramclustr = 393.9845 (+/− NA ppm)
392.9772
[M−H]−
519.1489
0
20
40
60
80
100
400
420
440
460
480
500
520
1478 : M.findmain = 393.9845 (+/− 20 ppm)
392.9772
[M−H]−
519.1489
0
20
40
60
80
100
400
420
440
460
480
500
520

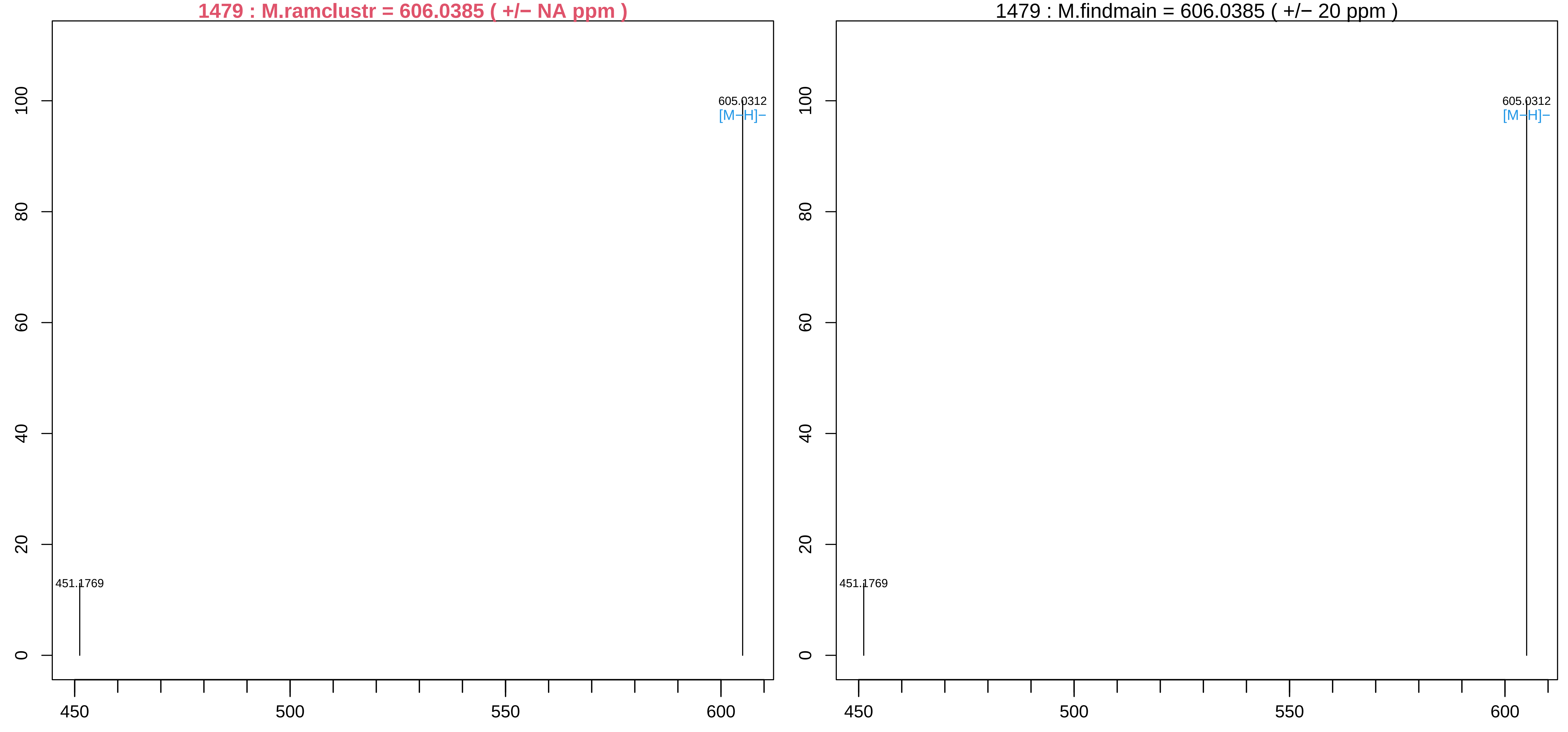
1479 : M.ramclustr = 606.0385 (+/− NA ppm)
605.0312
[M−H]−
451.1769
0
20
40
60
80
100
450
500
550
600
1479 : M.findmain = 606.0385 (+/− 20 ppm)
605.0312
[M−H]−
451.1769
0
20
40
60
80
100
450
500
550
600

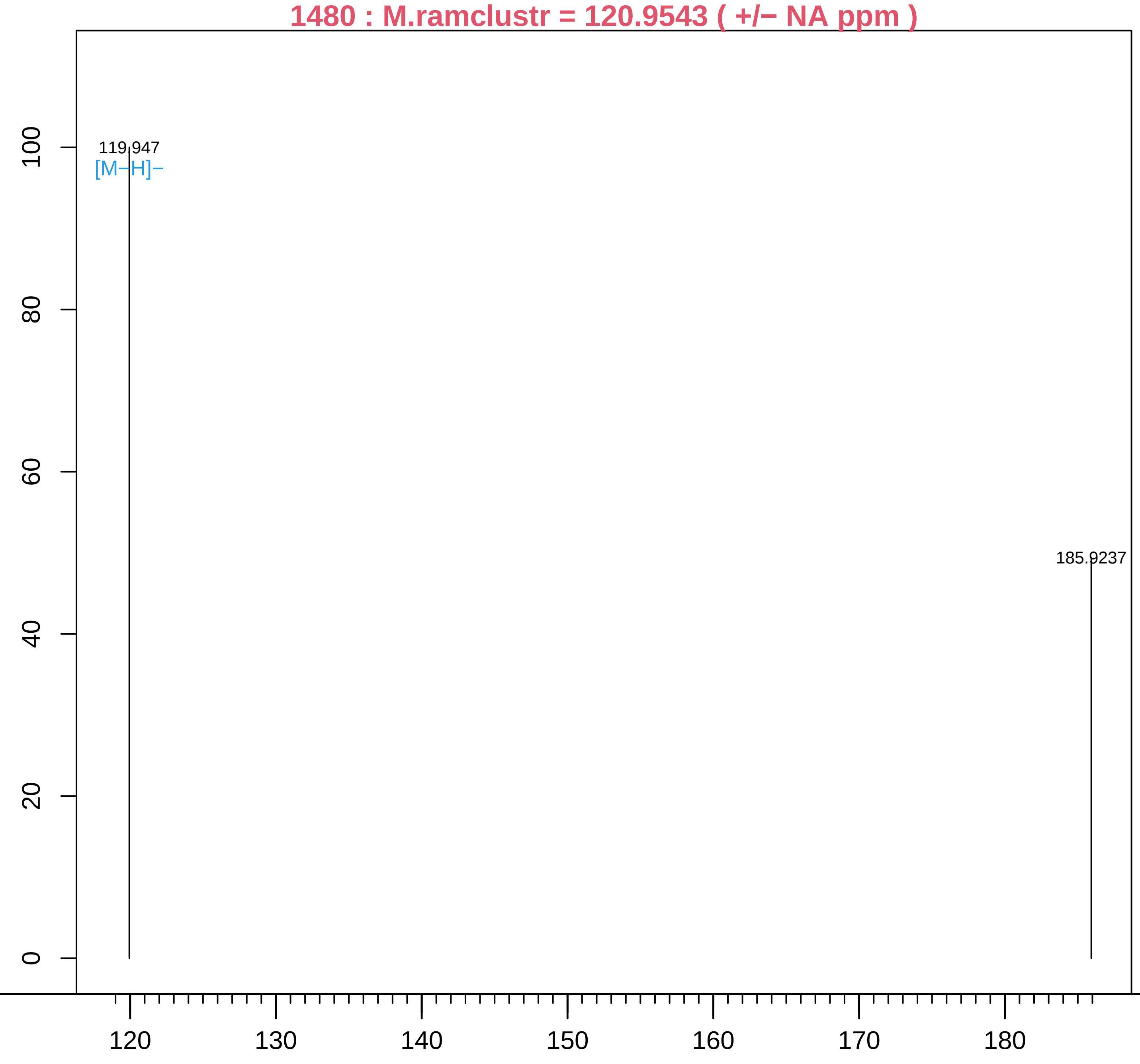
1480 : M.ramclustr = 120.9543 (+/− NA ppm)
119.947
[M−H]−
185.9237
0
20
40
60
80
100
120
130
140
150
160
170
180

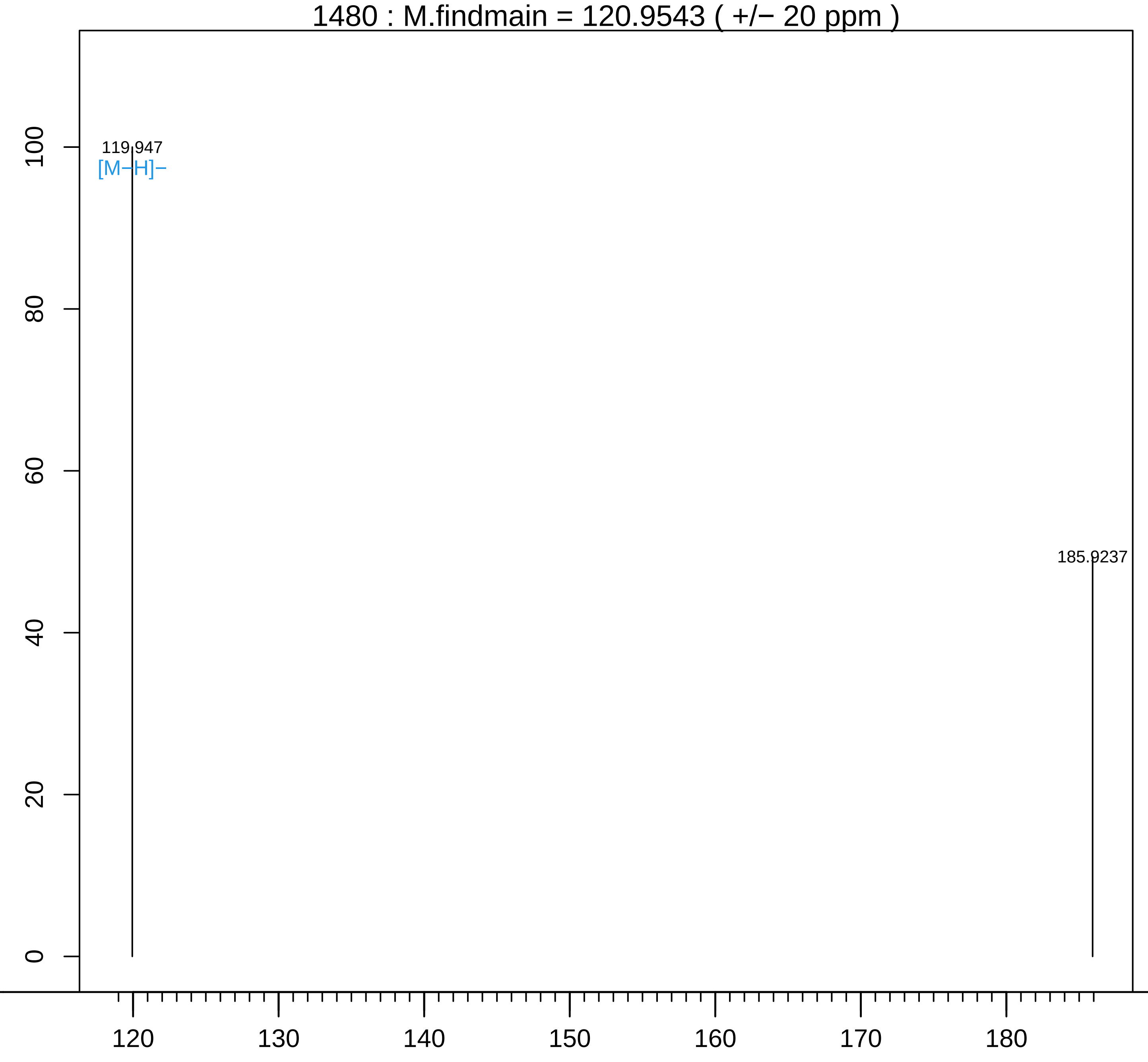
1480 : M.findmain = 120.9543 (+/− 20 ppm)
119.947
[M−H]−
185.9237
0
20
40
60
80
100
120
130
140
150
160
170
180

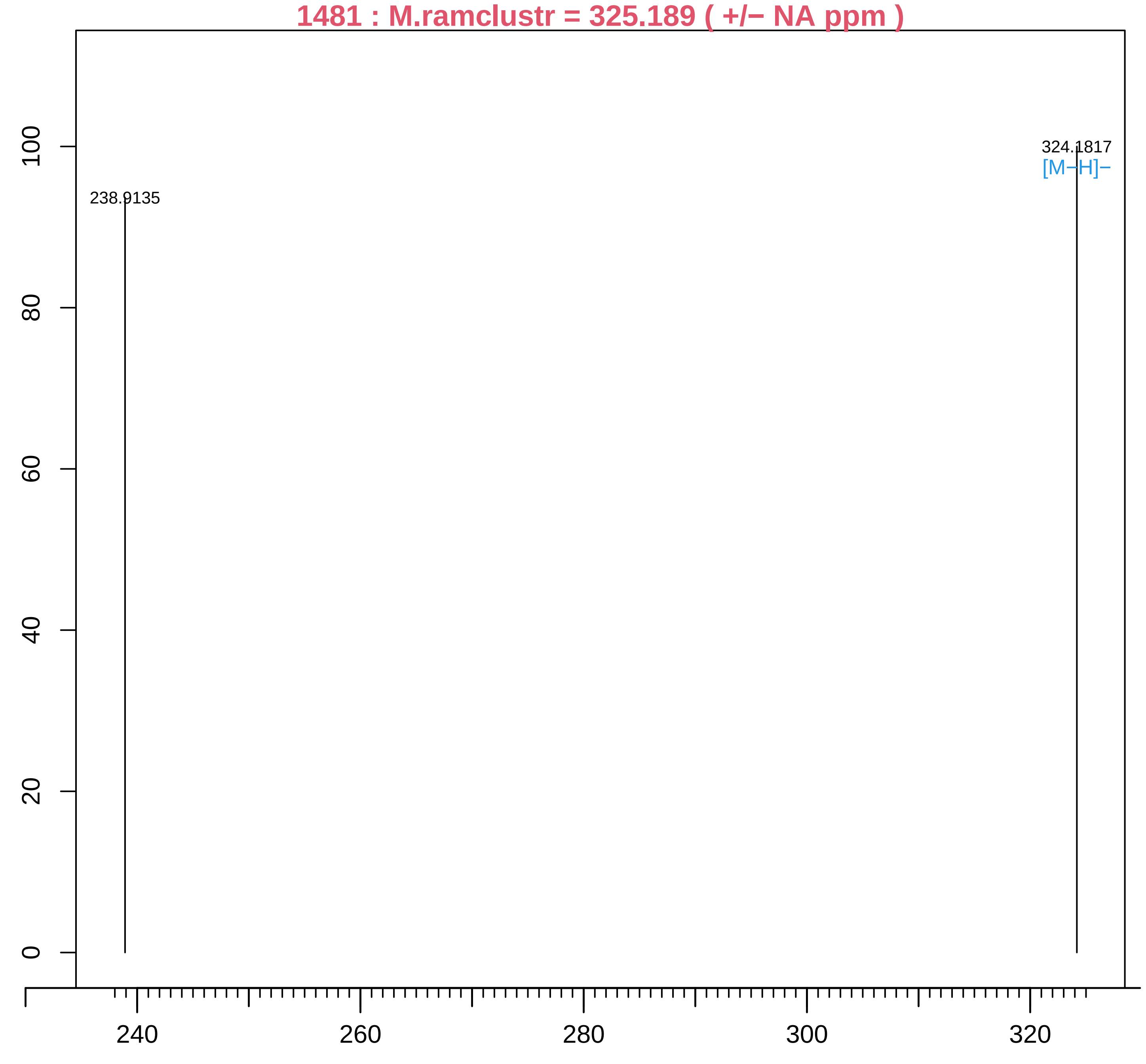
1481 : M.ramclustr = 325.189 (+/− NA ppm)
324.1817
[M−H]−
238.9135
0
20
40
60
80
100
240
260
280
300
320

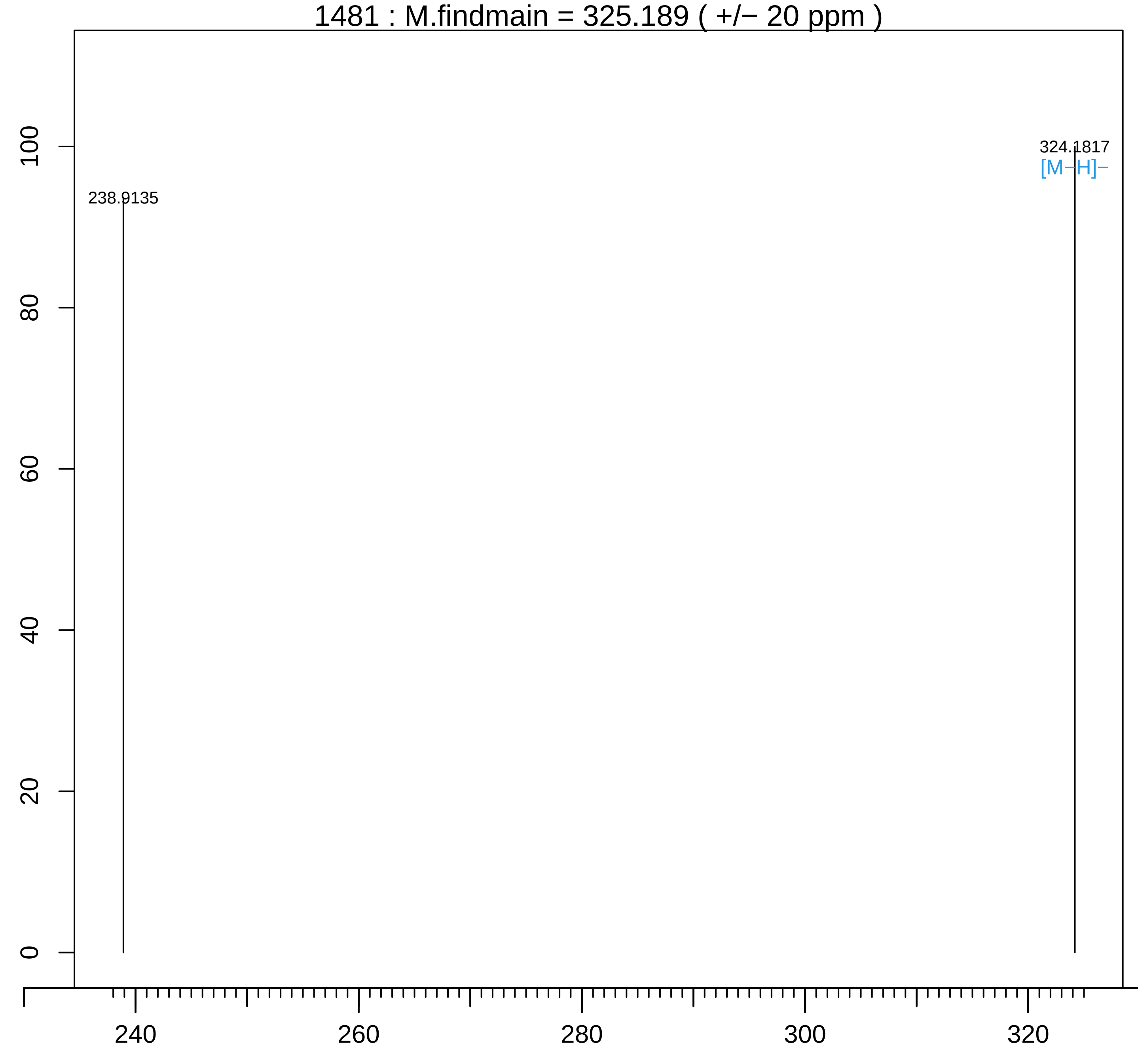
1481 : M.findmain = 325.189 (+/− 20 ppm)
324.1817
[M−H]−
238.9135
0
20
40
60
80
100
240
260
280
300
320

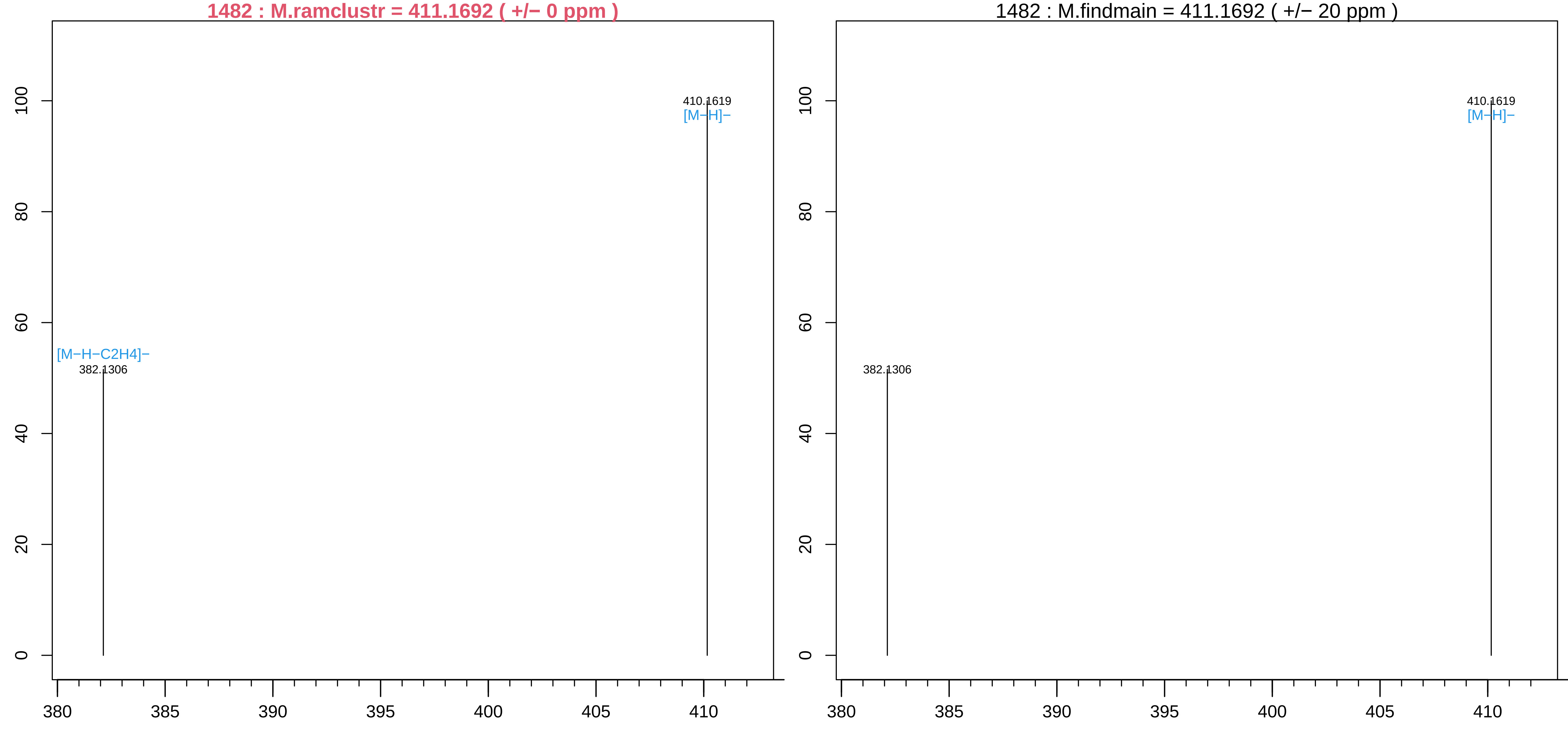
1482 : M.ramclustr = 411.1692 (+/− 0 ppm)
410.1619
[M−H]−
[M−H−C2H4]−
382.1306
1482 : M.findmain = 411.1692 (+/− 20 ppm)
410.1619
[M−H]−
382.1306
0
20
40
60
80
100
380
385
390
395
400
405
410

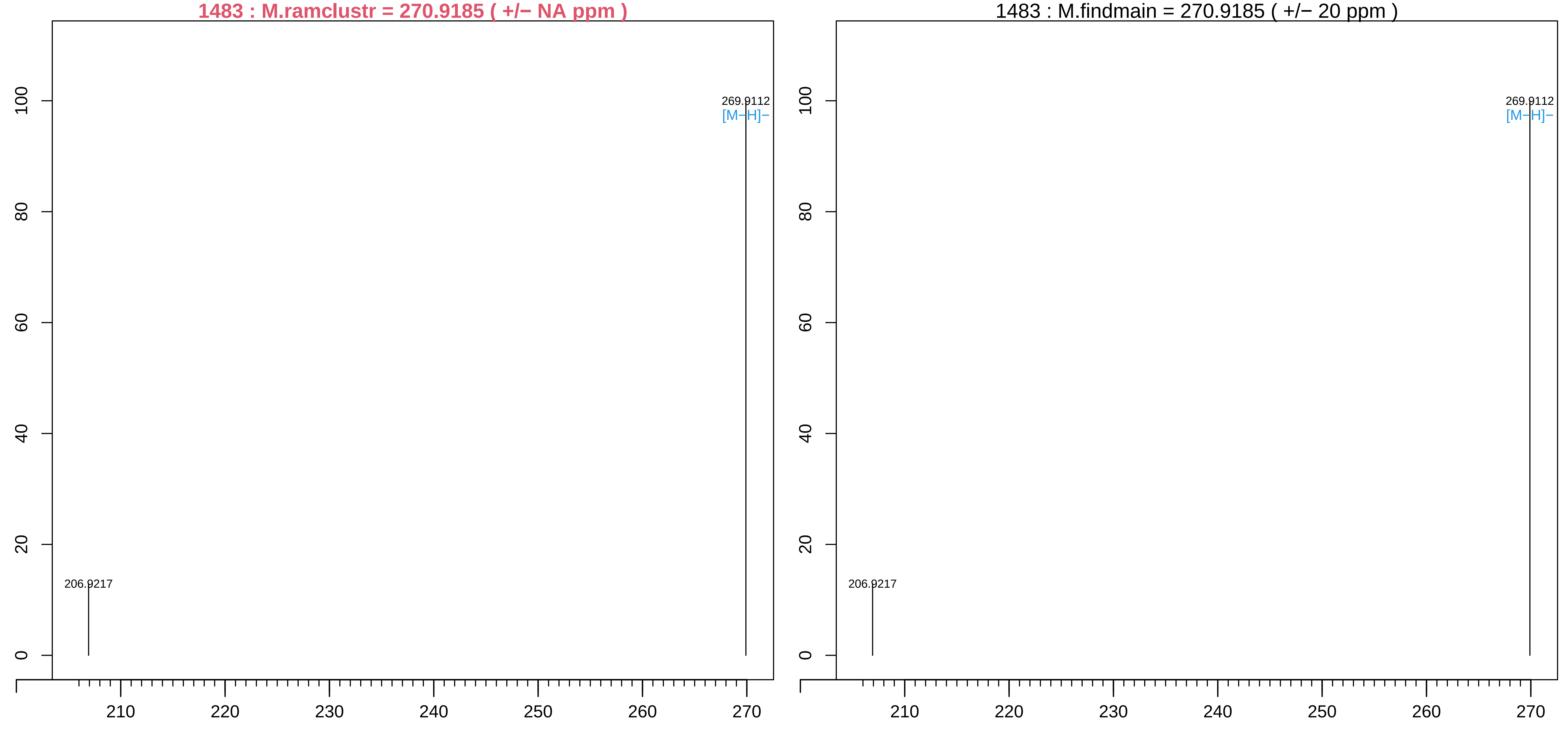
1483 : M.ramclustr = 270.9185 (+/− NA ppm)
269.9112
[M−H]−
206.9217
1483 : M.findmain = 270.9185 (+/− 20 ppm)
269.9112
[M−H]−
206.9217
0
20
40
60
80
100
210
220
230
240
250
260
270

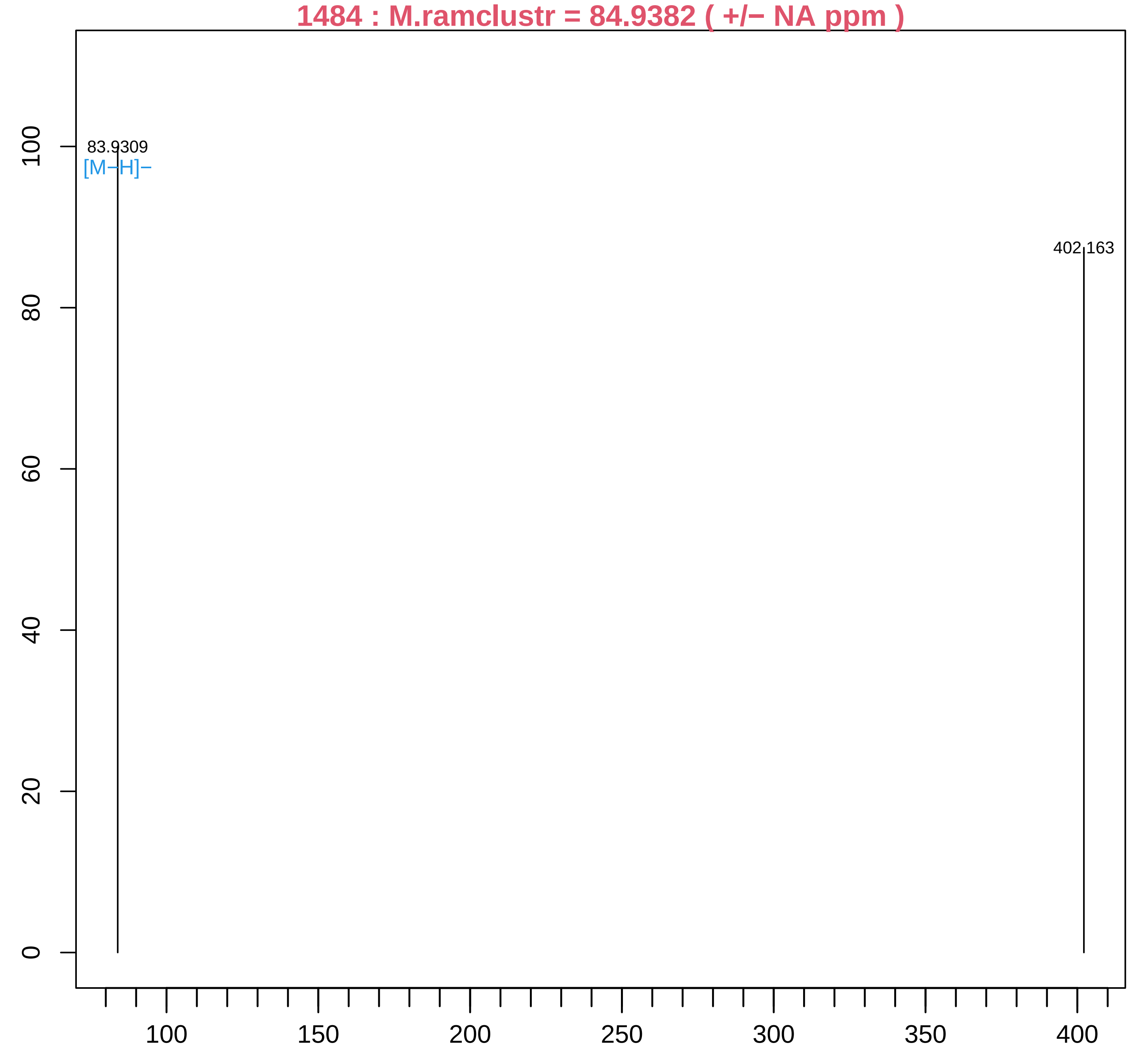
1484 : M.ramclustr = 84.9382 (+/− NA ppm)
83.9309
[M−H]−
402.163
0
20
40
60
80
100
100
150
200
250
300
350
400

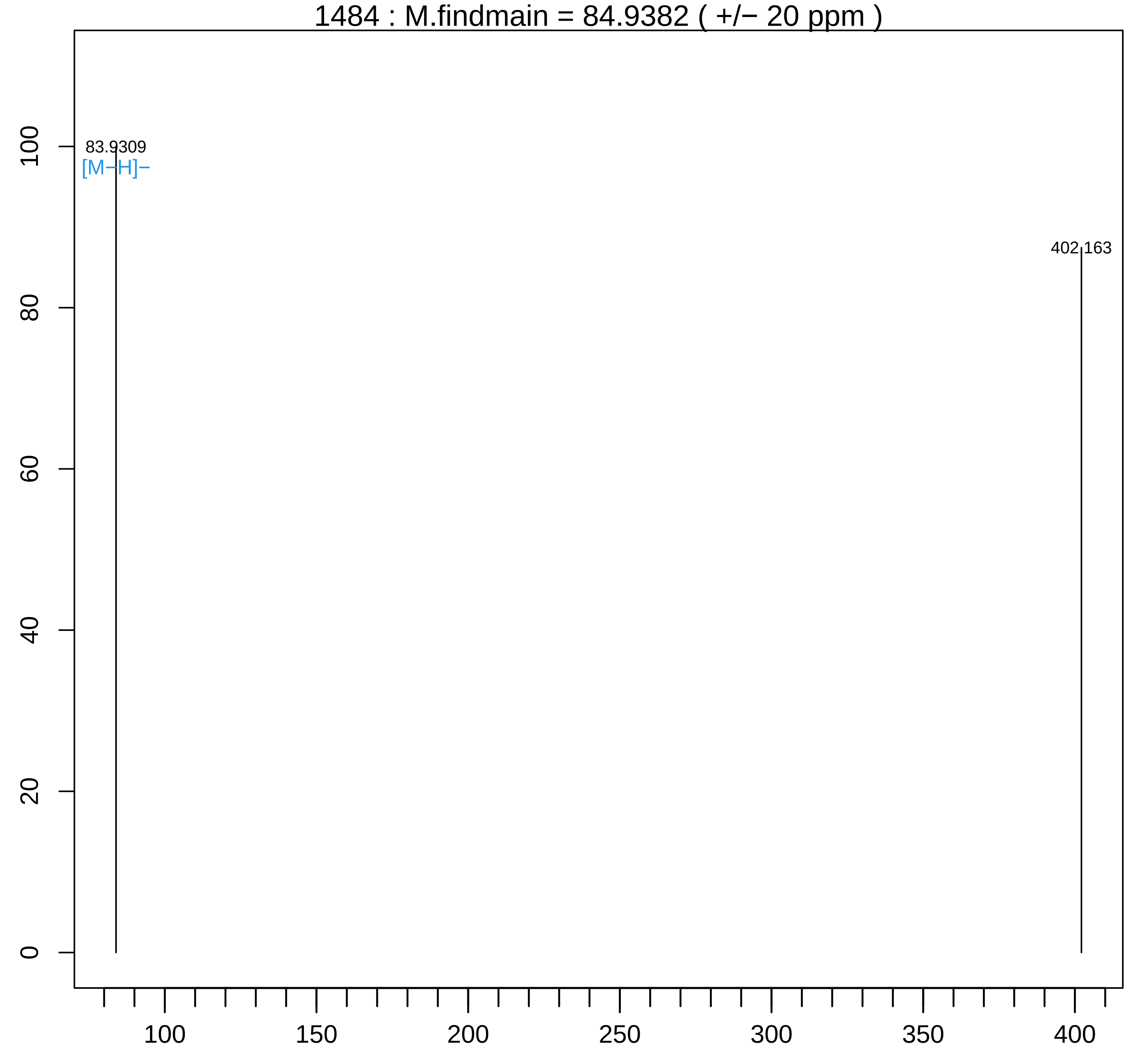
1484 : M.findmain = 84.9382 (+/− 20 ppm)
83.9309
[M−H]−
402.163
0
20
40
60
80
100
100
150
200
250
300
350
400

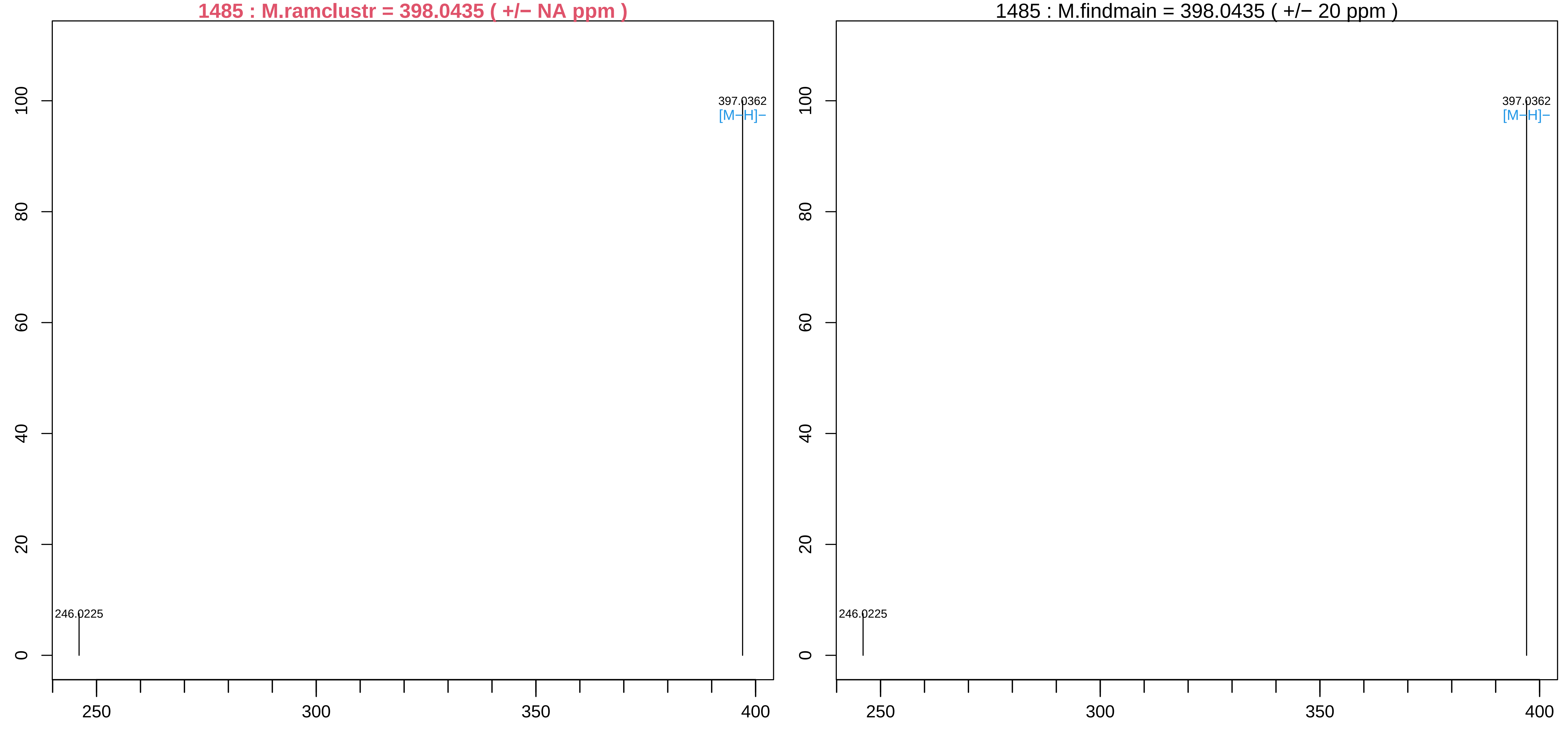

1485 : M.ramclustr = 398.0435 (+/− NA ppm)
397.0362
[M−H]−
246.0225
0
20
40
60
80
100
250
300
350
400
1485 : M.findmain = 398.0435 (+/− 20 ppm)
397.0362
[M−H]−
246.0225
0
20
40
60
80
100
250
300
350
400

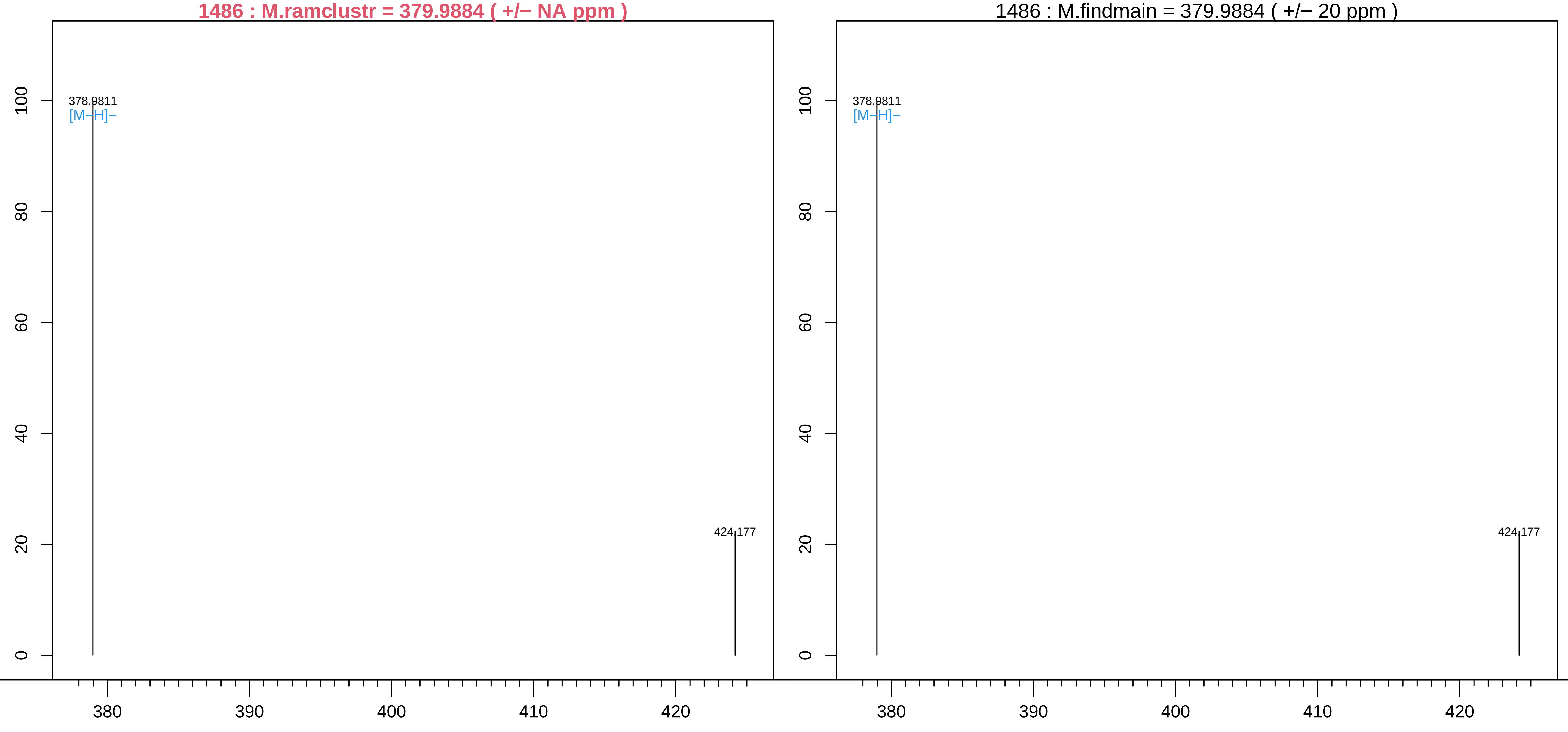
1486 : M.ramclustr = 379.9884 (+/− NA ppm)
378.9811
[M−H]−
424.177
1486 : M.findmain = 379.9884 (+/− 20 ppm)
378.9811
[M−H]−
424.177
0
20
40
60
80
100
380
390
400
410
420

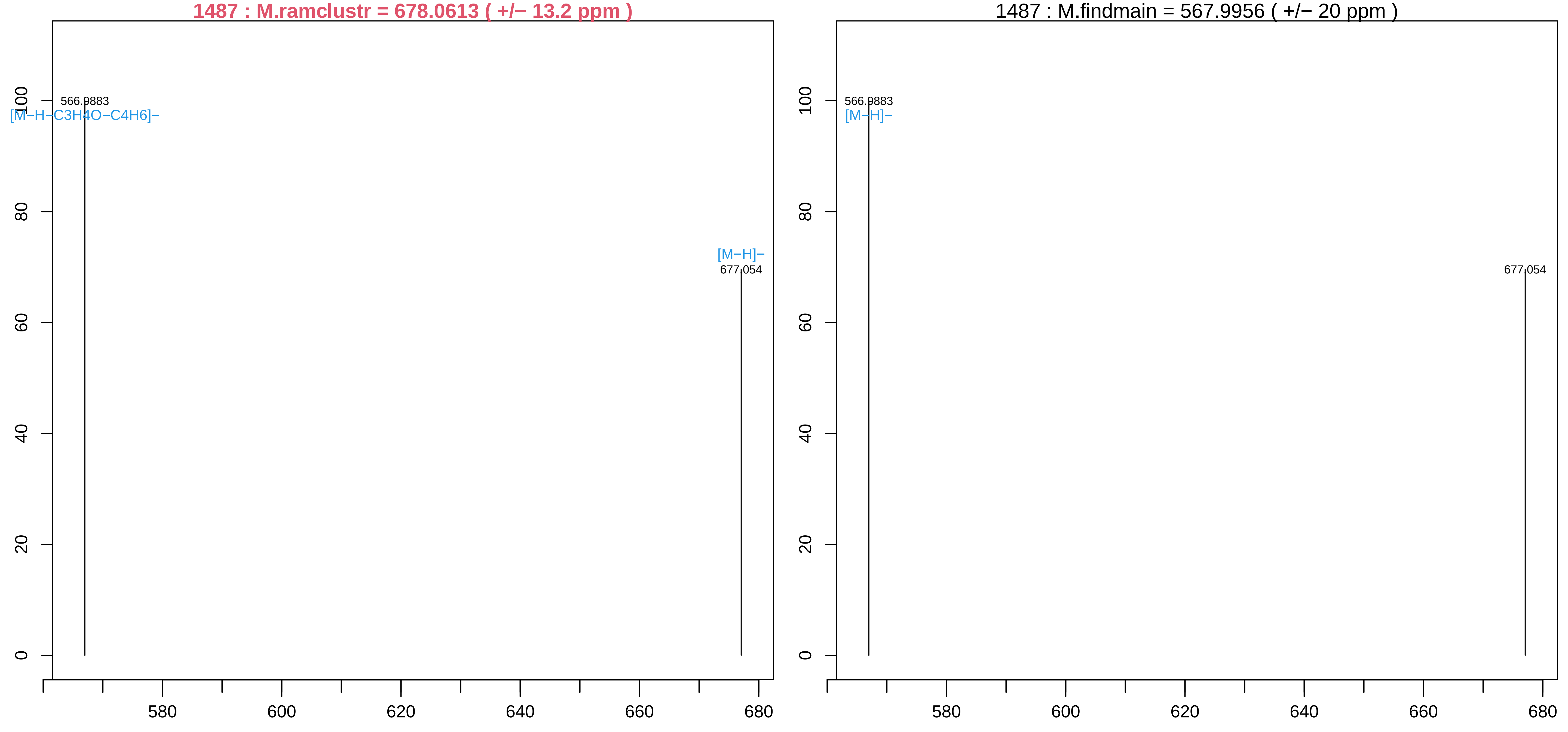

1487 : M.ramclustr = 678.0613 (+/− 13.2 ppm)
566.9883
[M−H−C3H4O−C4H6]−
[M−H]−
677.054
1487 : M.findmain = 567.9956 (+/− 20 ppm)
566.9883
[M−H]−
677.054

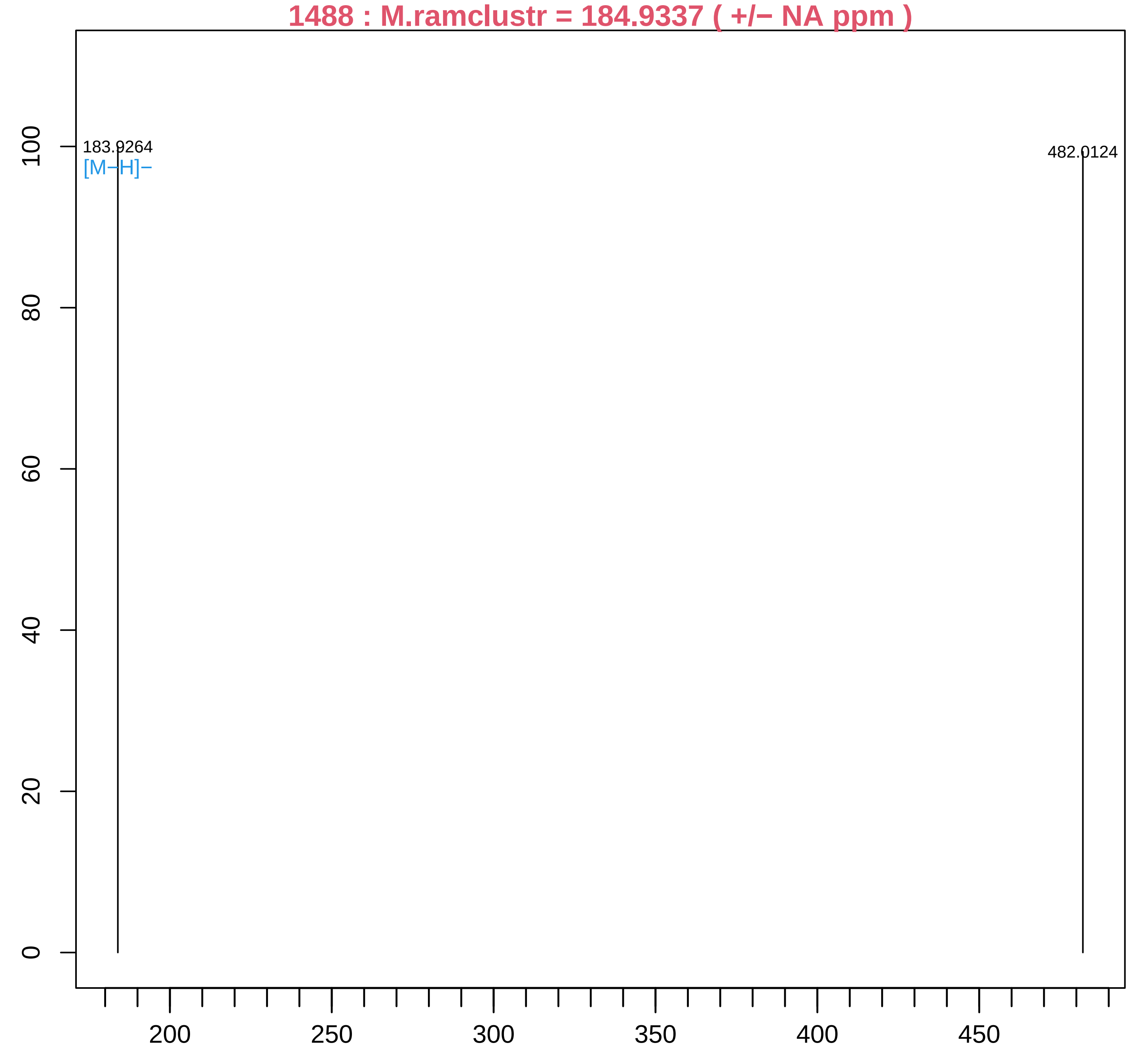
1488 : M.ramclustr = 184.9337 (+/− NA ppm)
183.9264
[M−H]−
482.0124
0
20
40
60
80
100
200
250
300
350
400
450

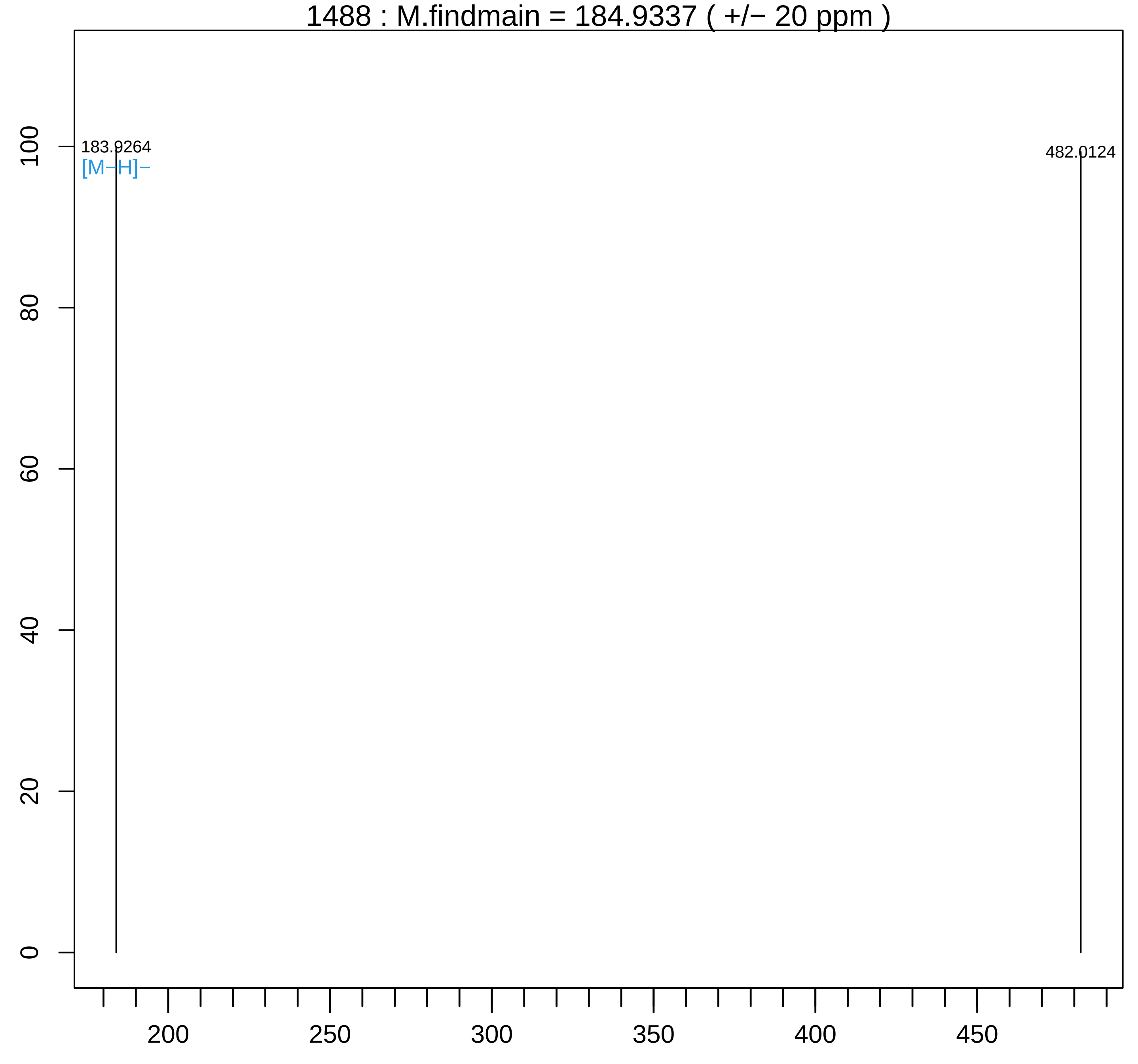
1488 : M.findmain = 184.9337 (+/− 20 ppm)
183.9264
[M−H]−
482.0124
0
20
40
60
80
100
200
250
300
350
400
450

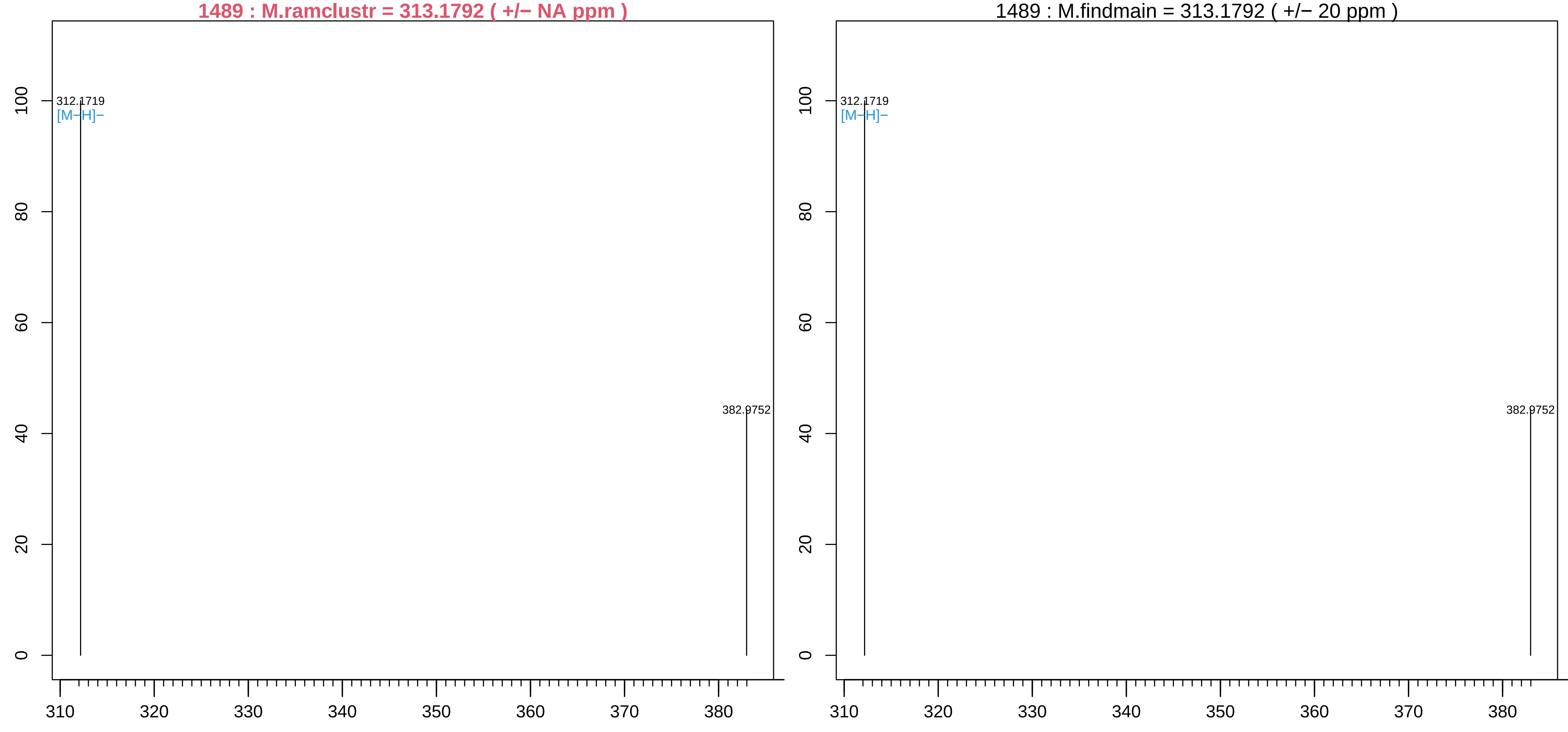

1489 : M.ramclustr = 313.1792 (+/− NA ppm)
312.1719
[M−H]−
382.9752
1489 : M.findmain = 313.1792 (+/− 20 ppm)
312.1719
[M−H]−
382.9752

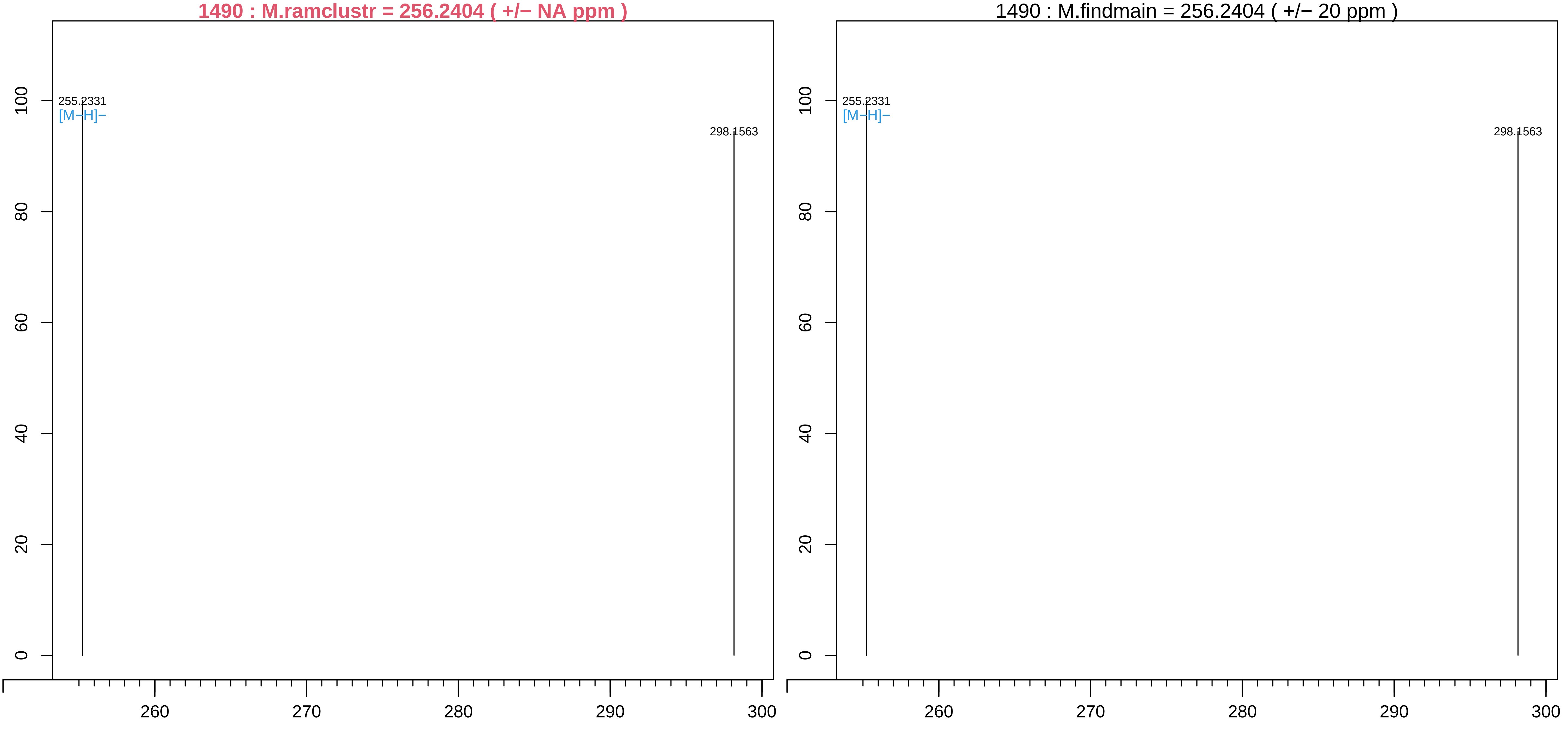

1490 : M.ramclustr = 256.2404 (+/− NA ppm)
255.2331
[M−H]−
298.1563
1490 : M.findmain = 256.2404 (+/− 20 ppm)
255.2331
[M−H]−
298.1563

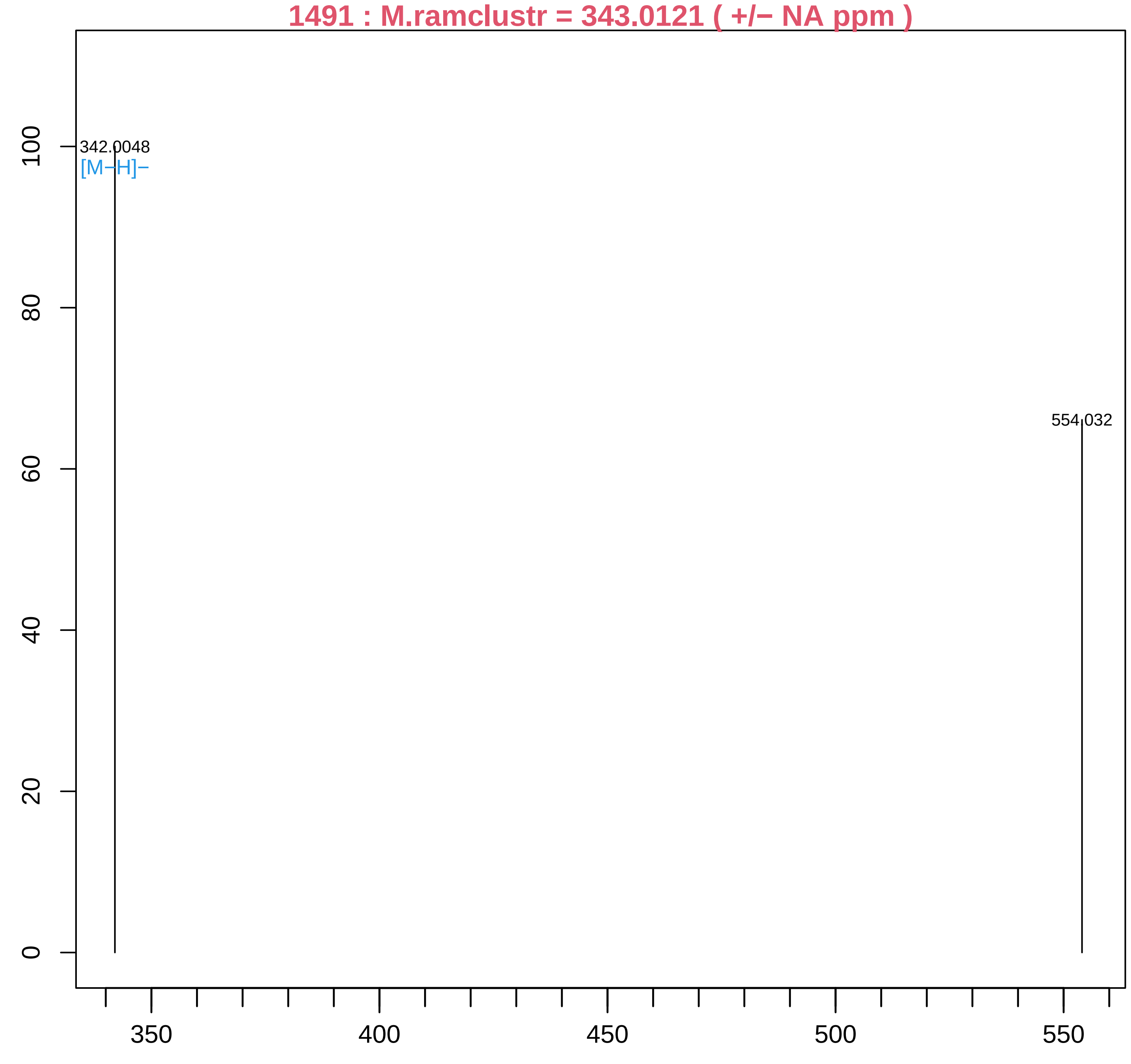

1491 : M.ramclustr = 343.0121 (+/− NA ppm)
342.0048
[M−H]−
554.032

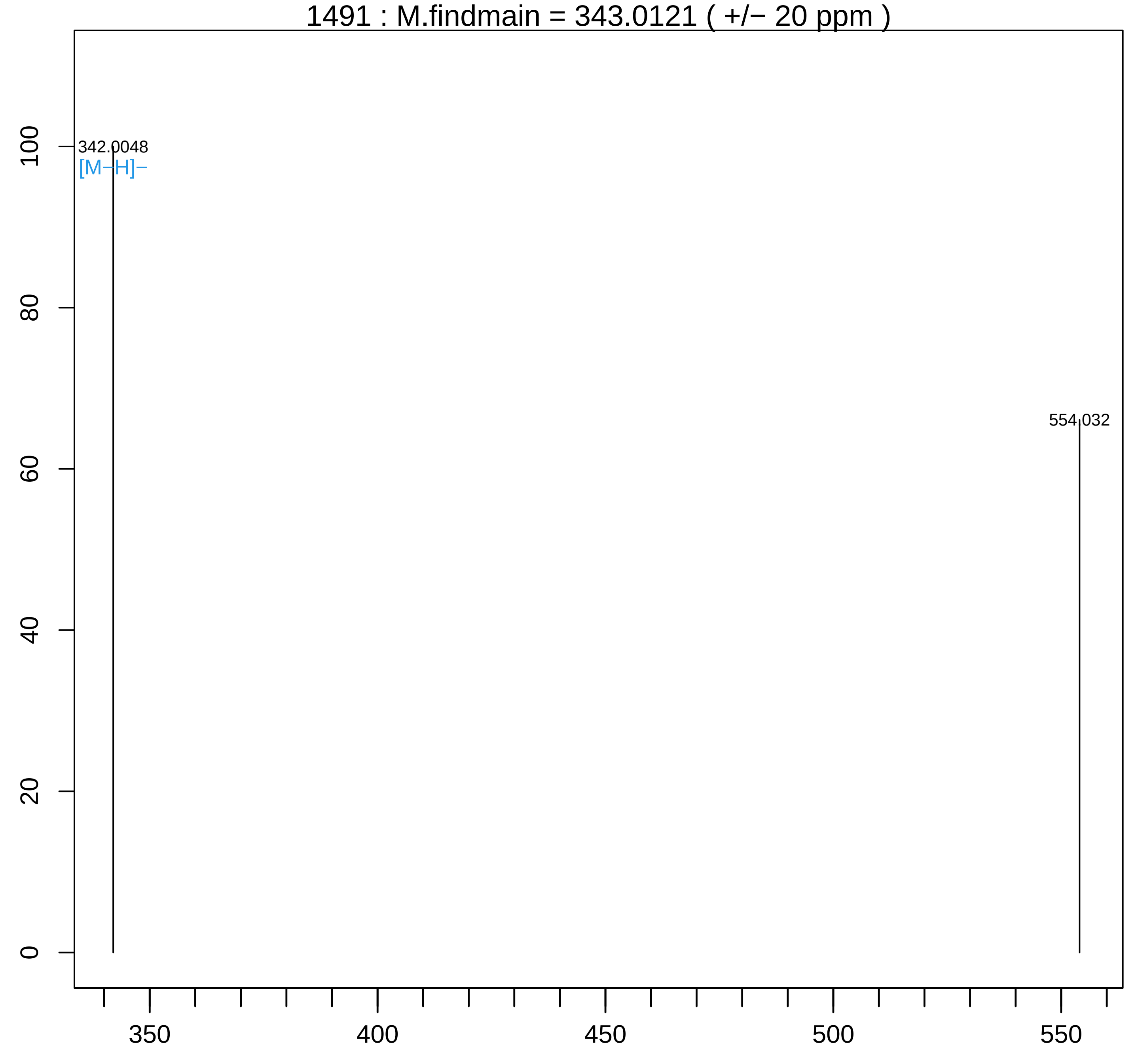

1491 : M.findmain = 343.0121 (+/− 20 ppm)
342.0048
[M−H]−
554.032

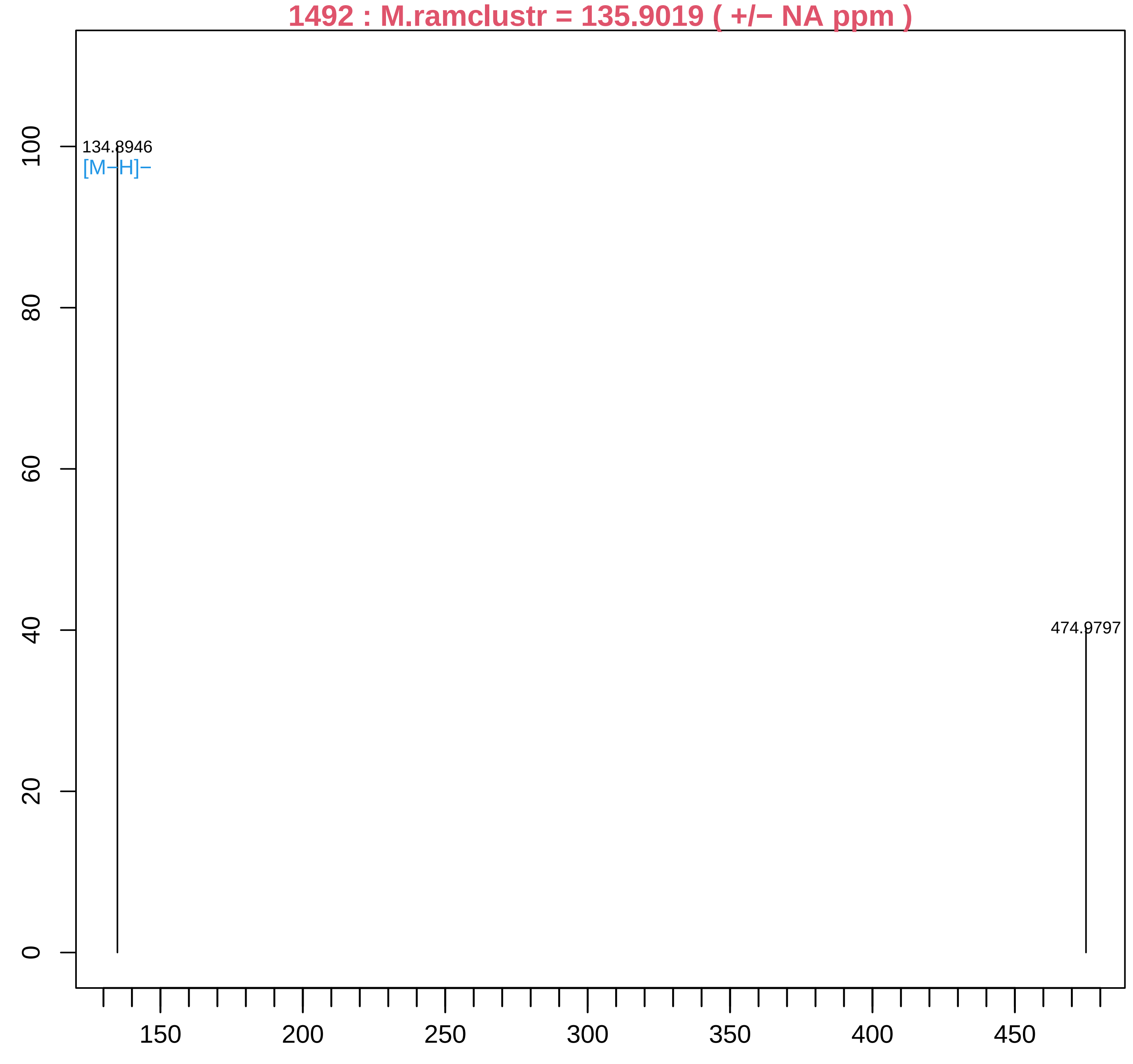

1492 : M.ramclustr = 135.9019 (+/− NA ppm)
134.8946
[M−H]−
474.9797
0
20
40
60
80
100
150
200
250
300
350
400
450

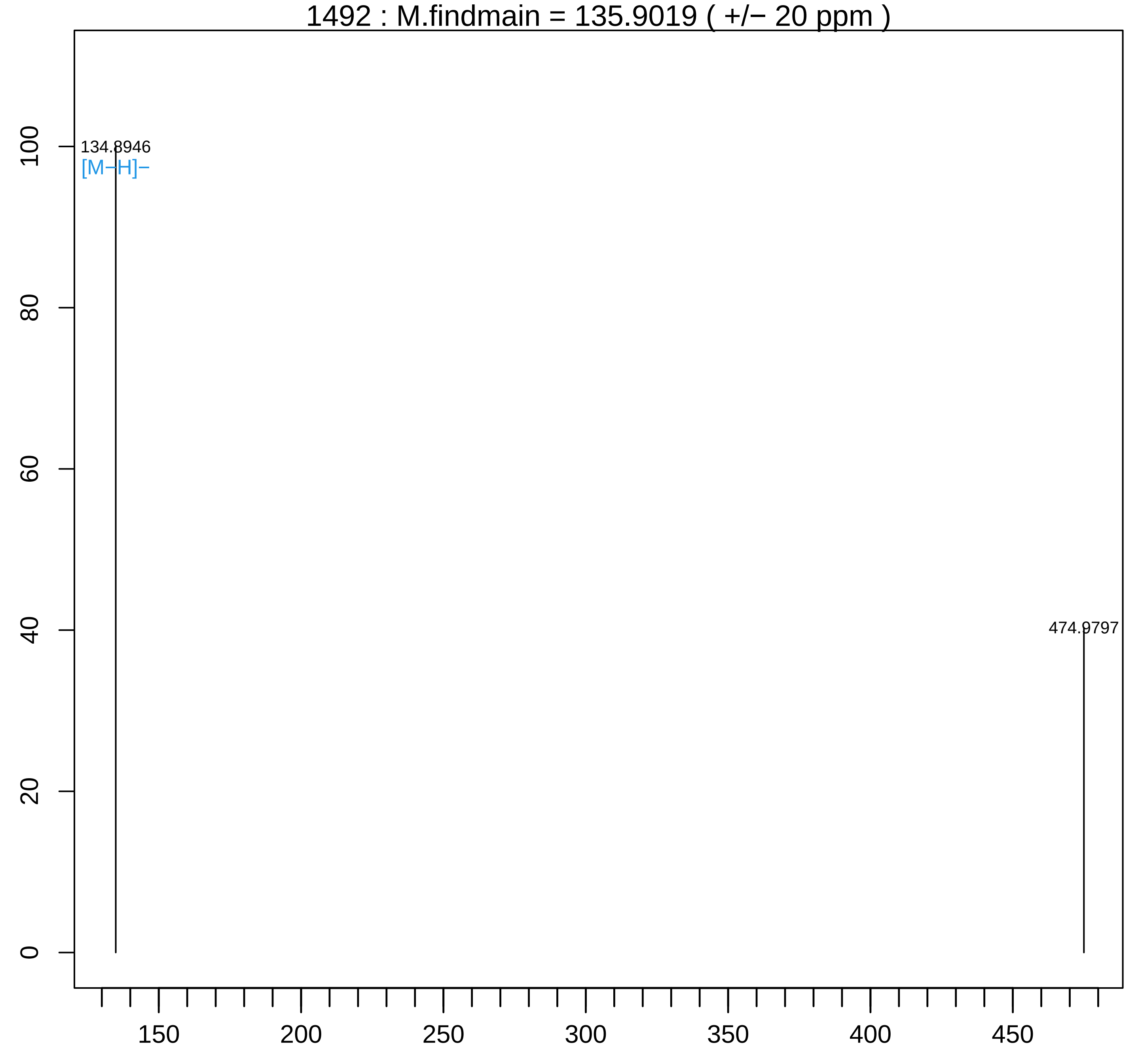

1492 : M.findmain = 135.9019 (+/− 20 ppm)
134.8946
[M−H]−
474.9797
0
20
40
60
80
100
150
200
250
300
350
400
450

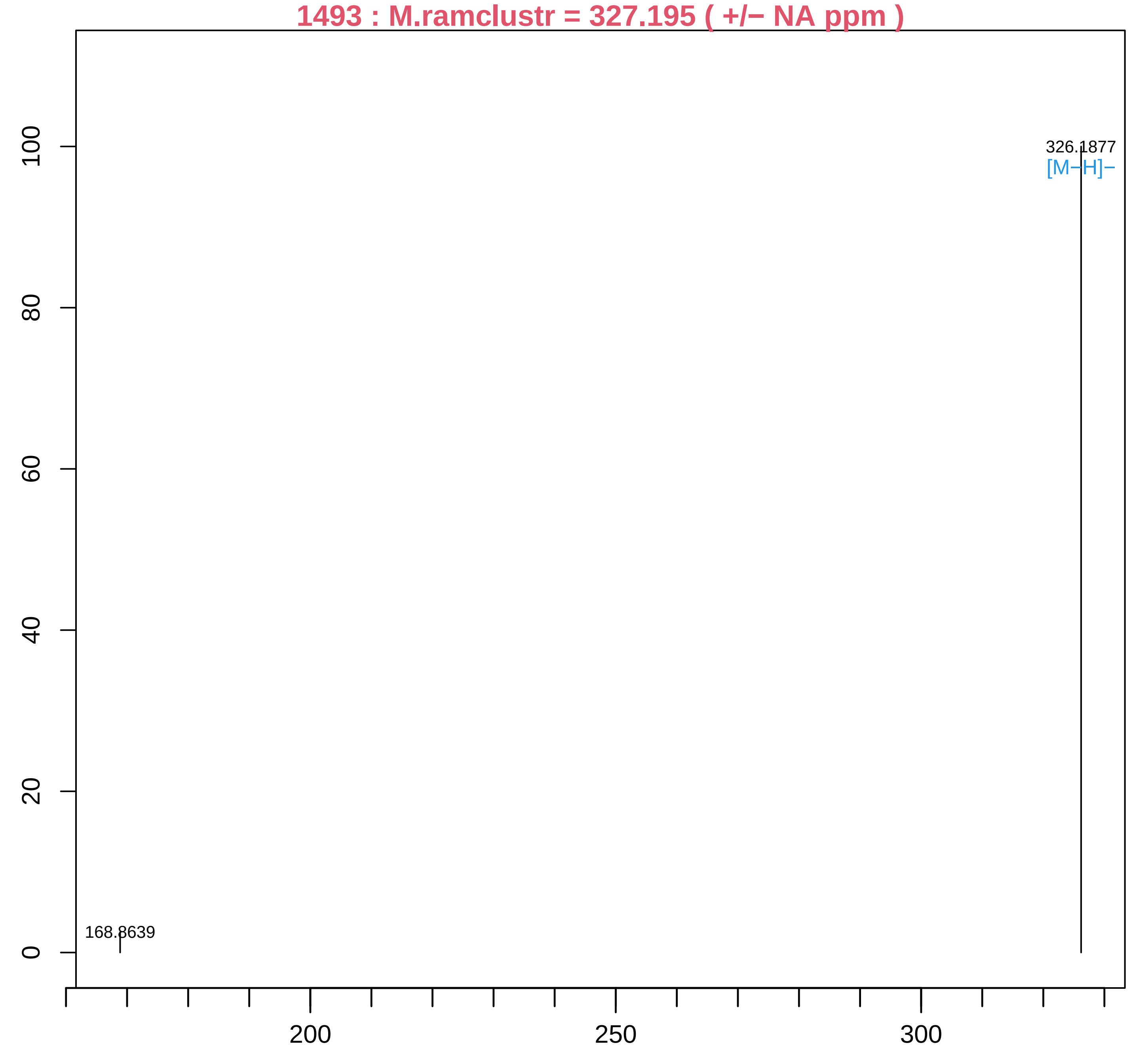
1493 : M.ramclustr = 327.195 (+/− NA ppm)
326.1877
[M−H]−
168.8639
0
20
40
60
80
100
200
250
300

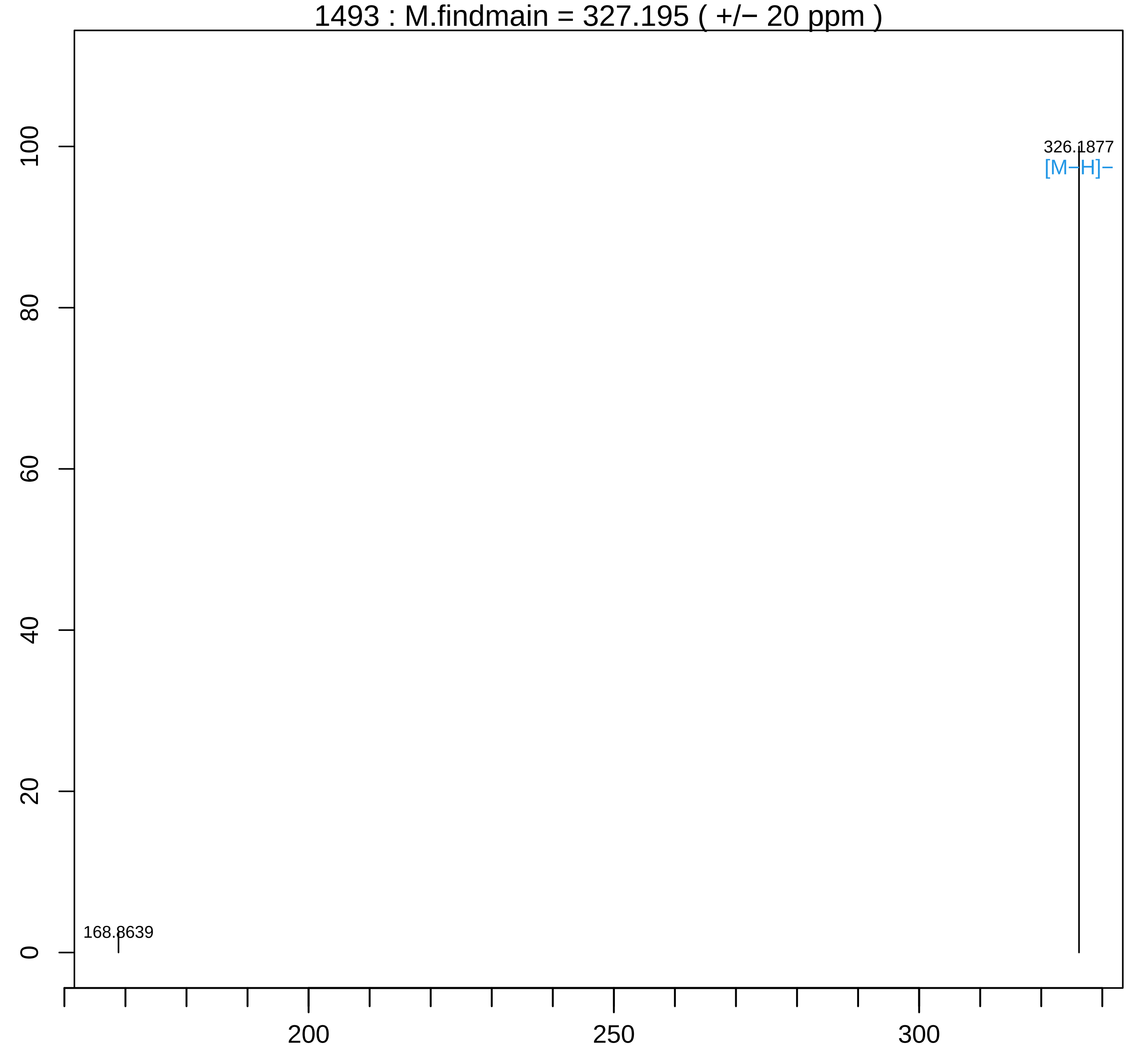
1493 : M.findmain = 327.195 (+/− 20 ppm)
326.1877
[M−H]−
168.8639
0
20
40
60
80
100
200
250
300

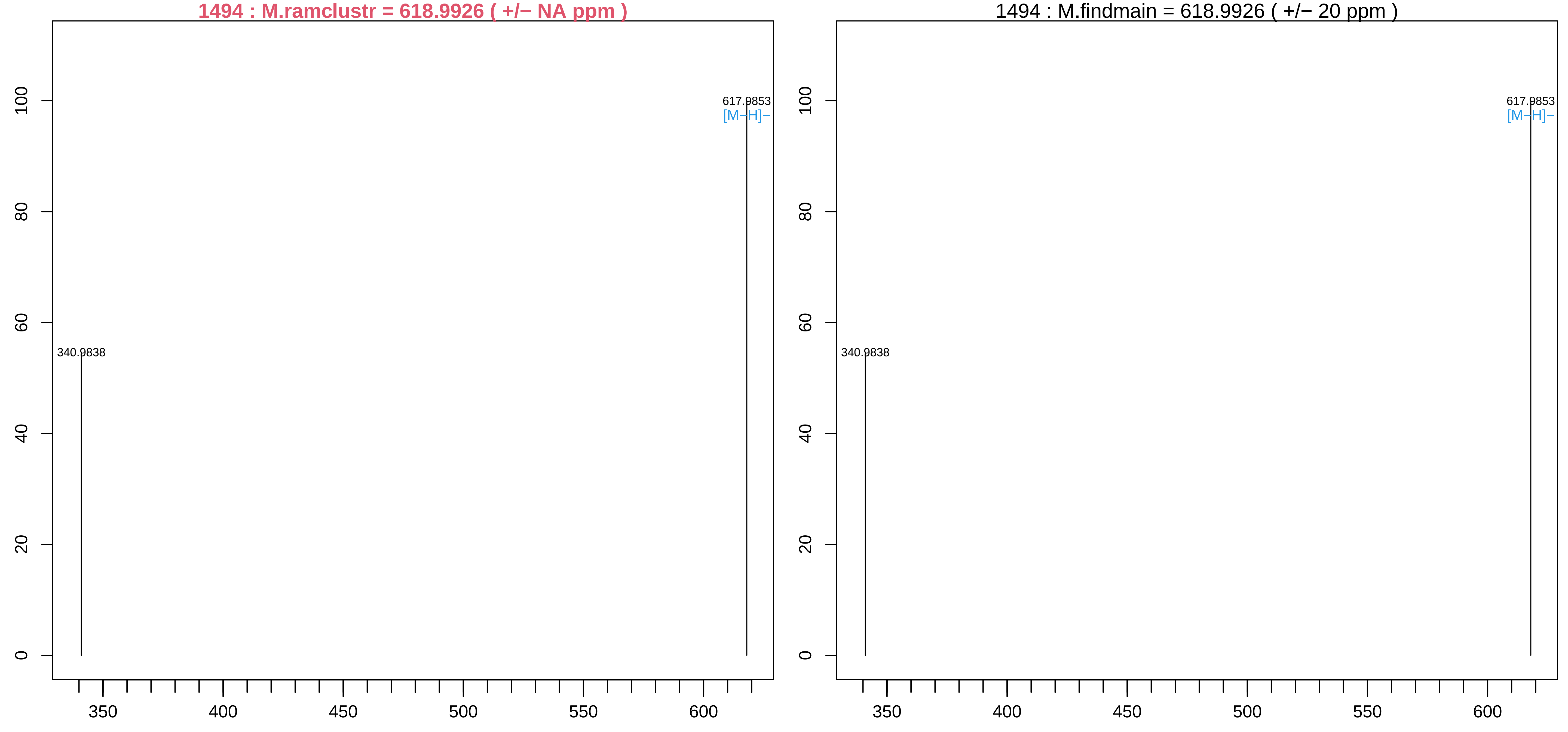
1494 : M.ramclustr = 618.9926 (+/− NA ppm)
1494 : M.findmain = 618.9926 (+/− 20 ppm)
617.9853
[M−H]−
340.9838
0
20
40
60
80
100
350
400
450
500
550
600

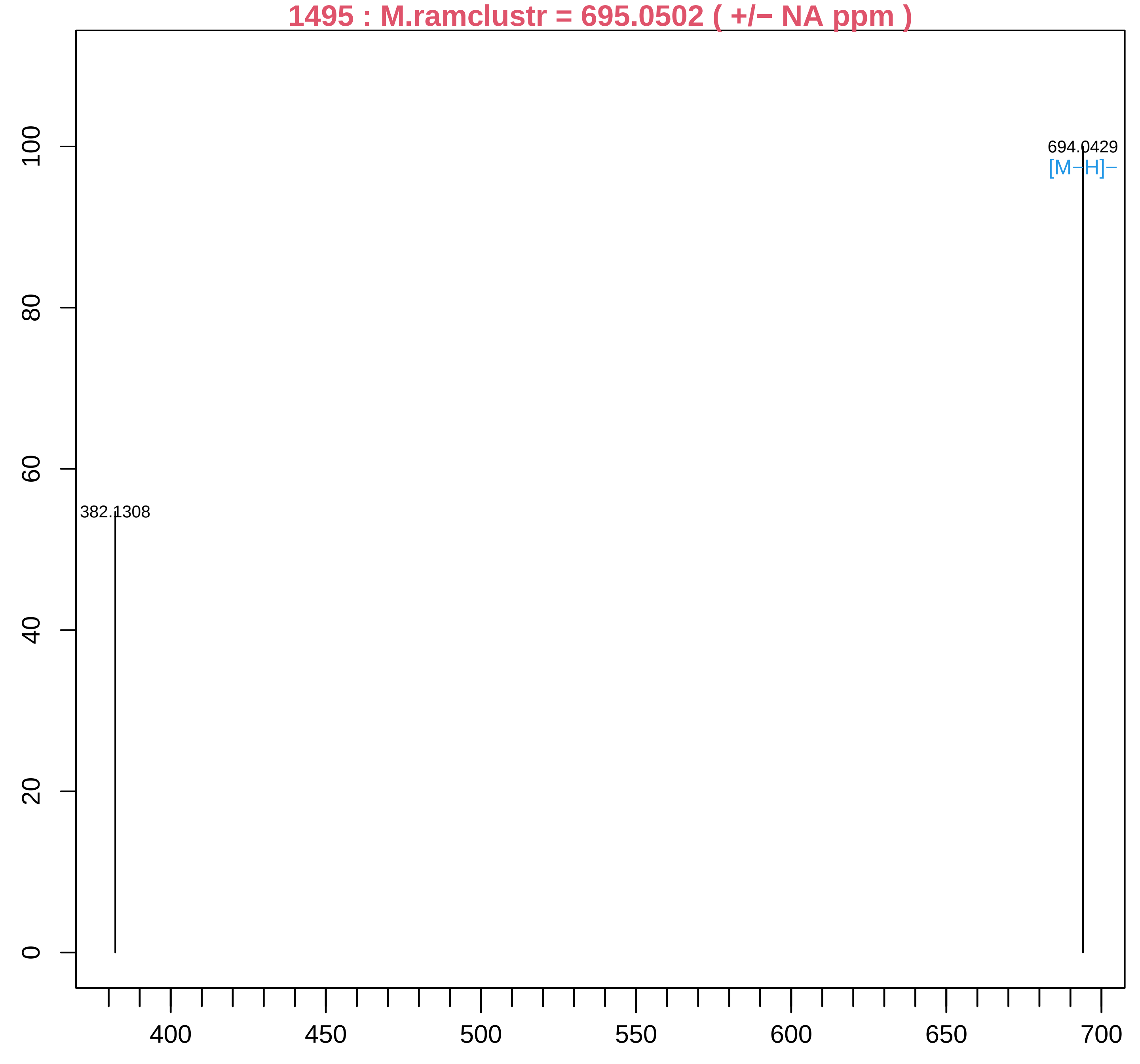

1495 : M.ramclustr = 695.0502 (+/− NA ppm)
694.0429
[M−H]−
382.1308
0
20
40
60
80
100
400
450
500
550
600
650
700

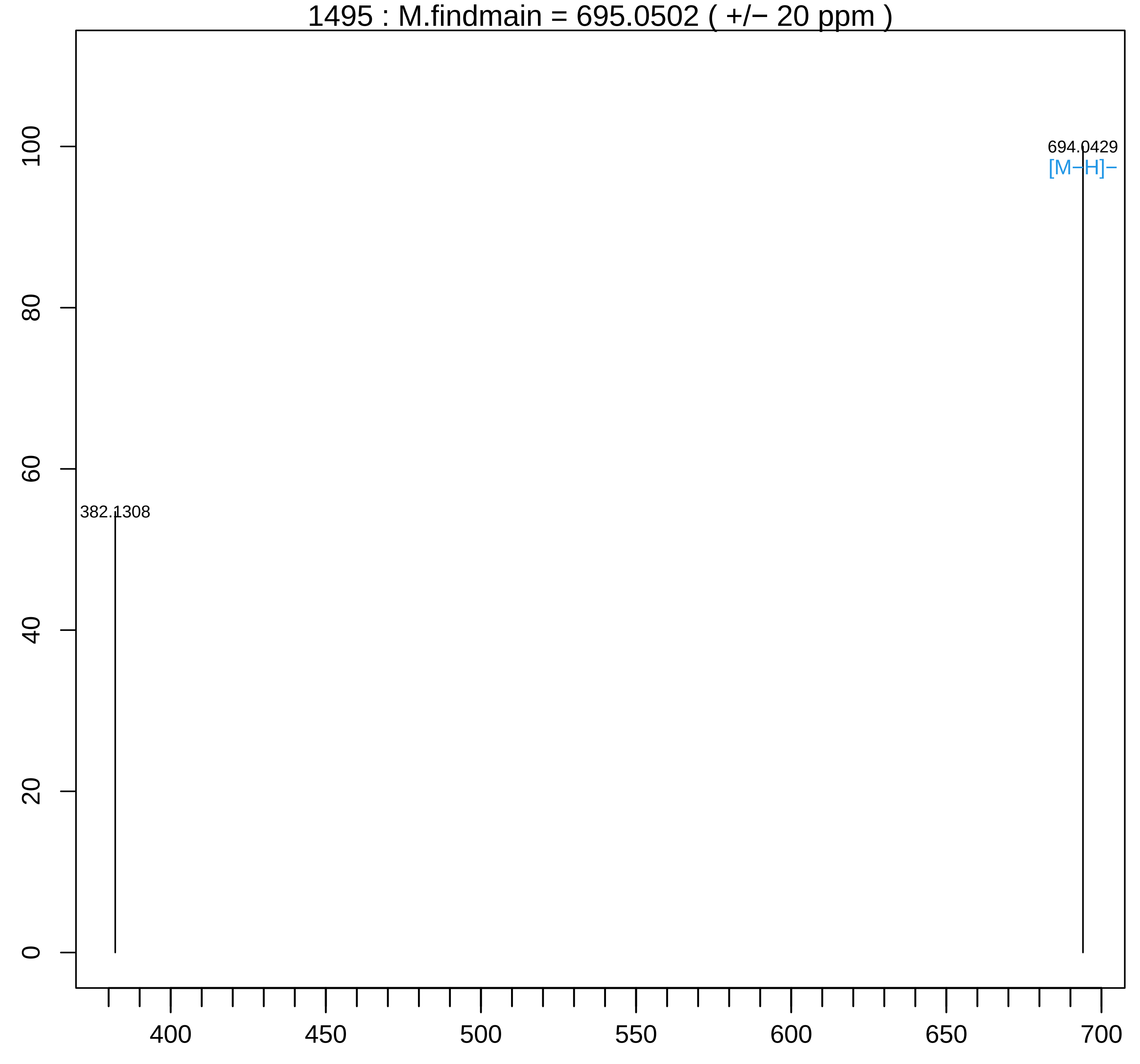

1495 : M.findmain = 695.0502 (+/− 20 ppm)
694.0429
[M−H]−
382.1308
0
20
40
60
80
100
400
450
500
550
600
650
700

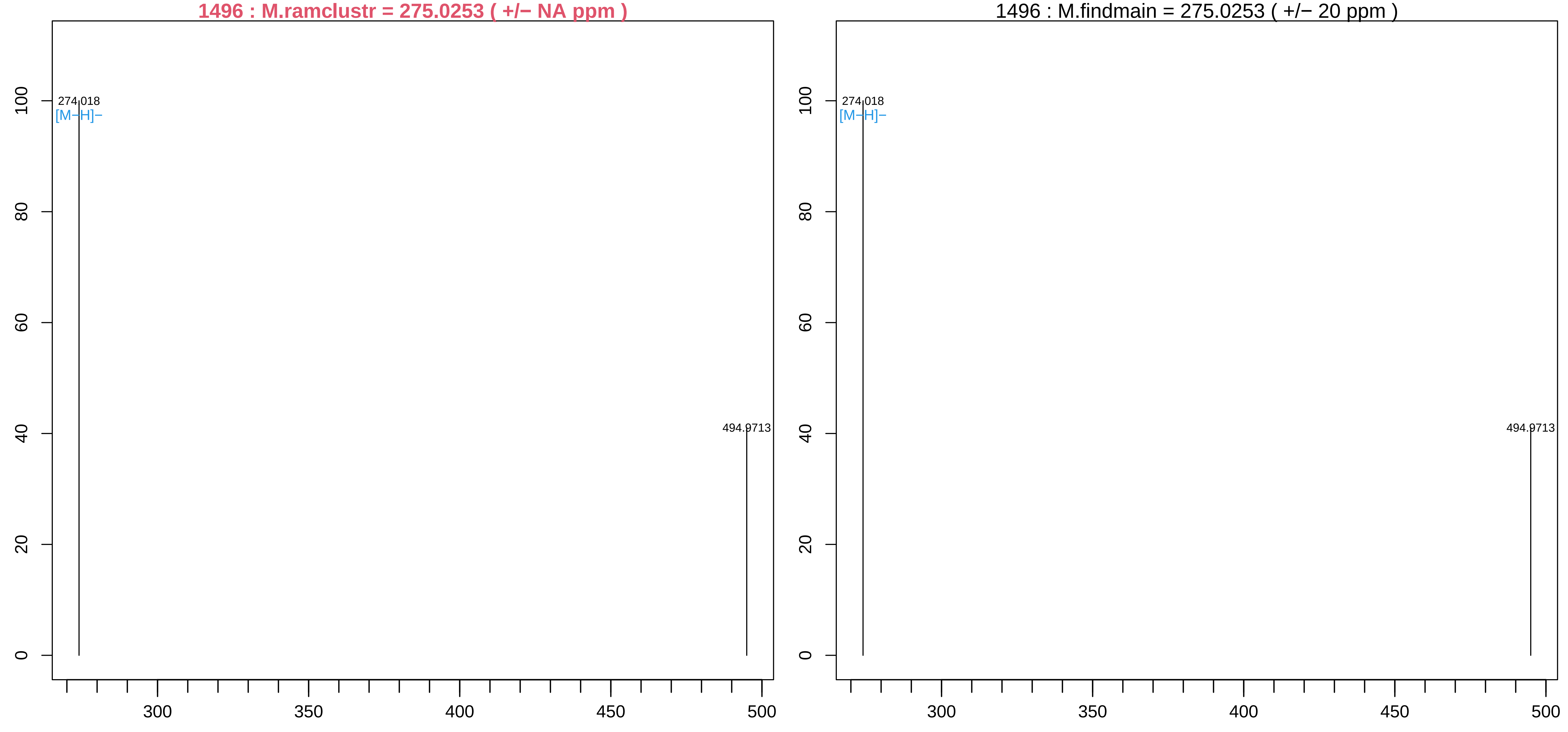
1496 : M.ramclustr = 275.0253 (+/− NA ppm)
274.018
[M−H]−
494.9713
1496 : M.findmain = 275.0253 (+/− 20 ppm)
274.018
[M−H]−
494.9713
0
20
40
60
80
100
300
350
400
450
500

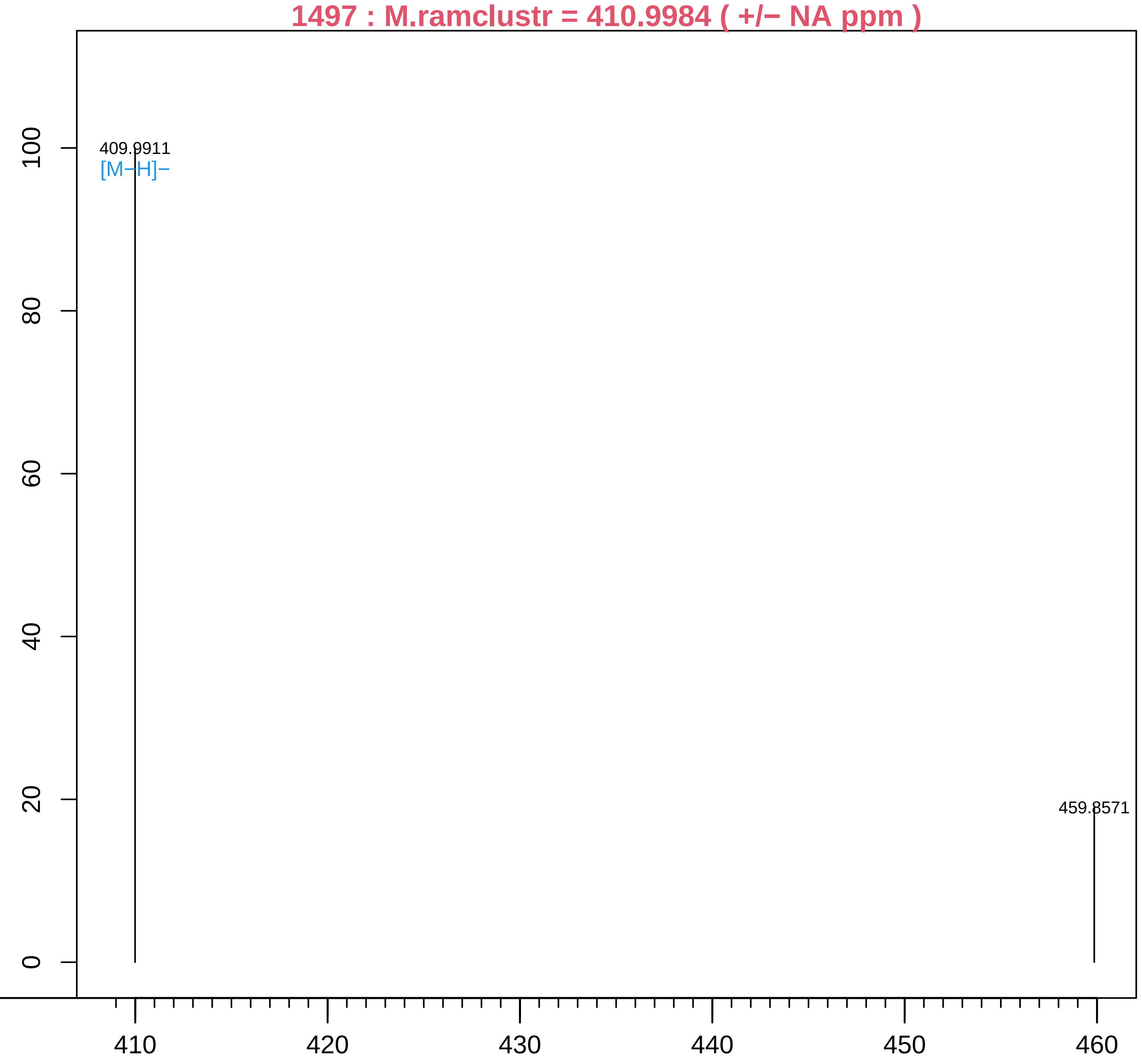
1497 : M.ramclustr = 410.9984 (+/− NA ppm)
409.9911
[M−H]−
459.8571
0
20
40
60
80
100
410
420
430
440
450
460

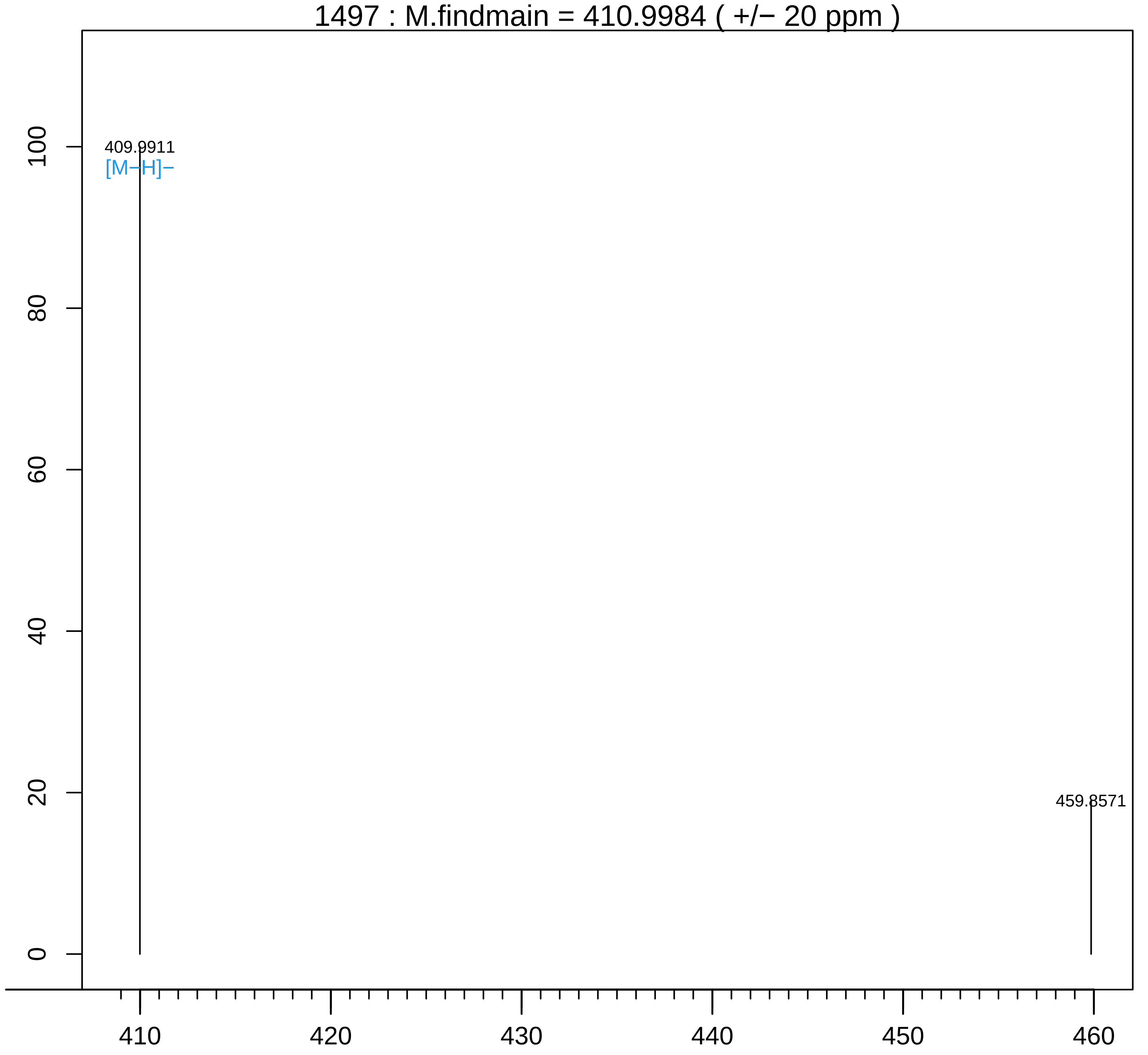
1497 : M.findmain = 410.9984 (+/− 20 ppm)
409.9911
[M−H]−
459.8571
0
20
40
60
80
100
410
420
430
440
450
460

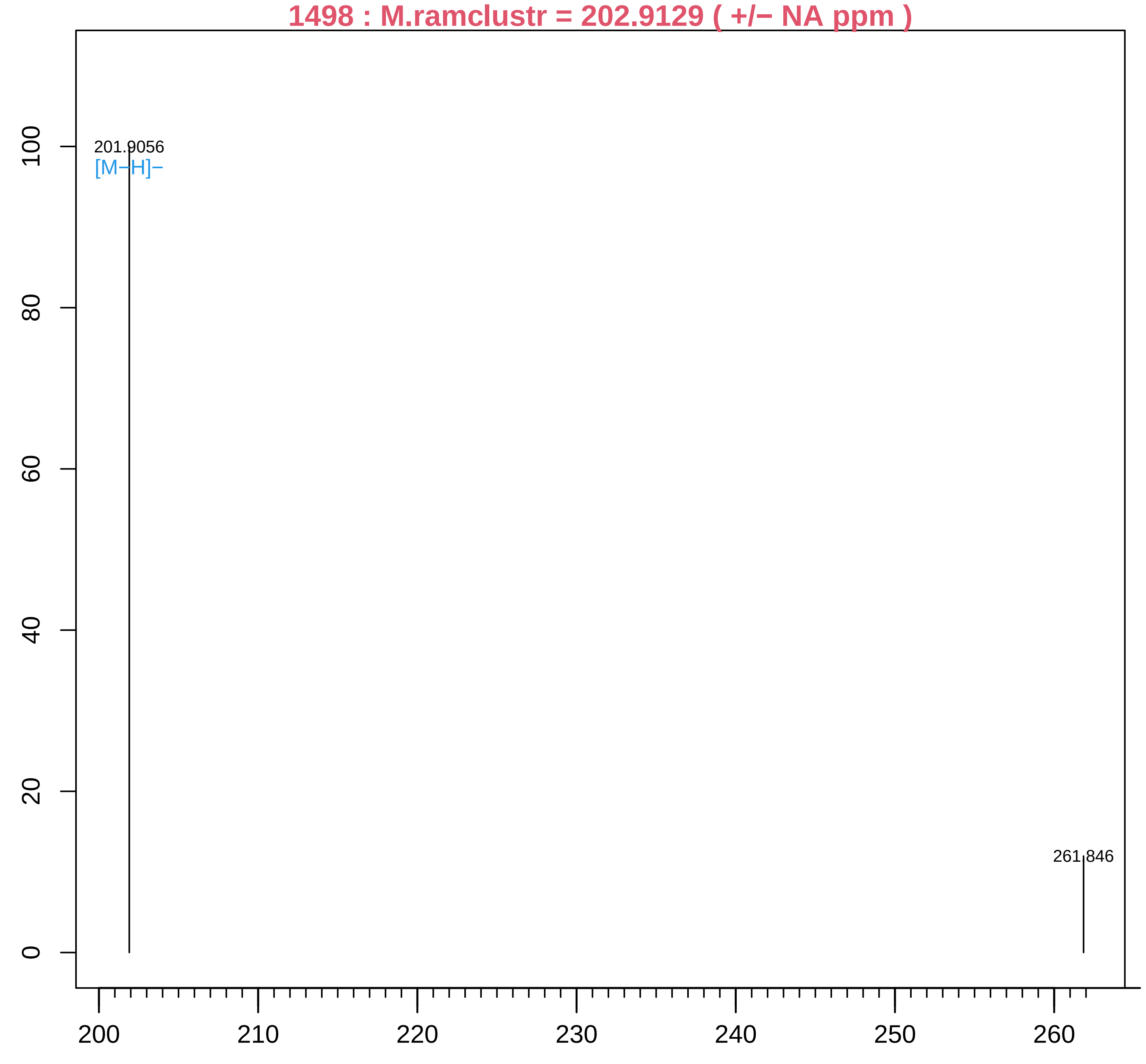
1498 : M.ramclustr = 202.9129 (+/− NA ppm)
201.9056
[M−H]−
261.846
0
20
40
60
80
100
200
210
220
230
240
250
260

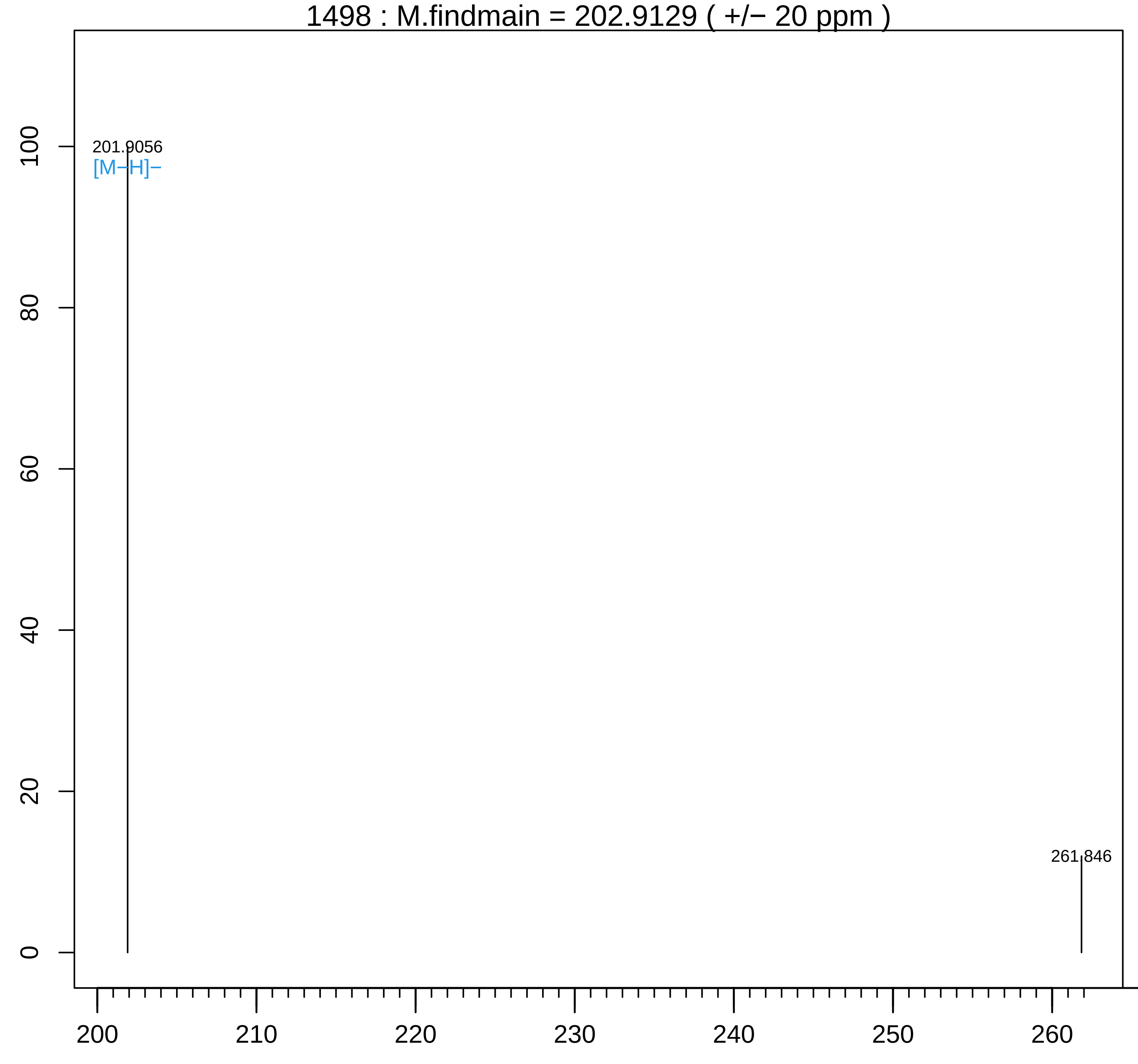
1498 : M.findmain = 202.9129 (+/− 20 ppm)
201.9056
[M−H]−
261.846
0
20
40
60
80
100
200
210
220
230
240
250
260

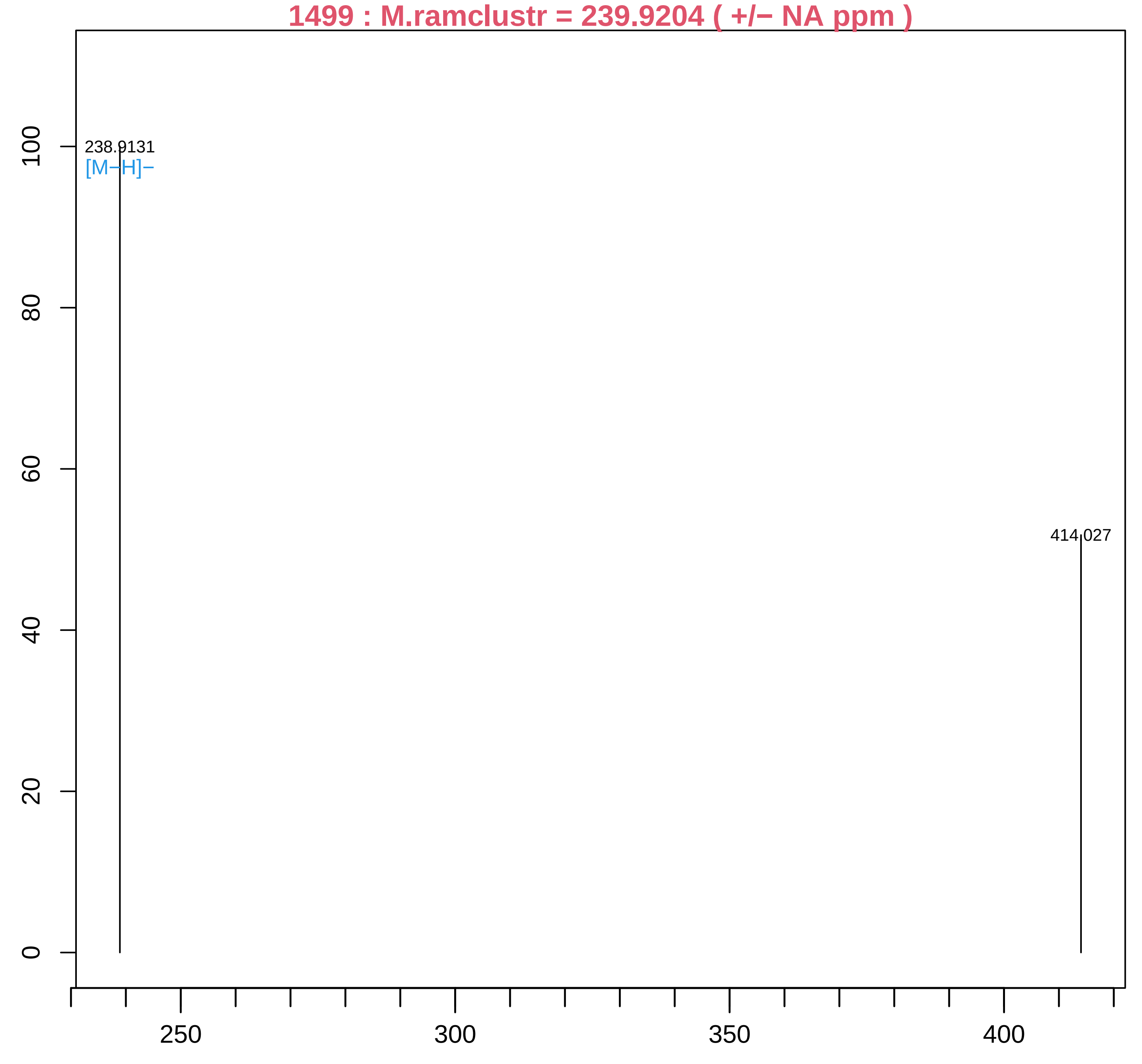
1499 : M.ramclustr = 239.9204 (+/− NA ppm)
238.9131
[M−H]−
414.027
0
20
40
60
80
100
250
300
350
400

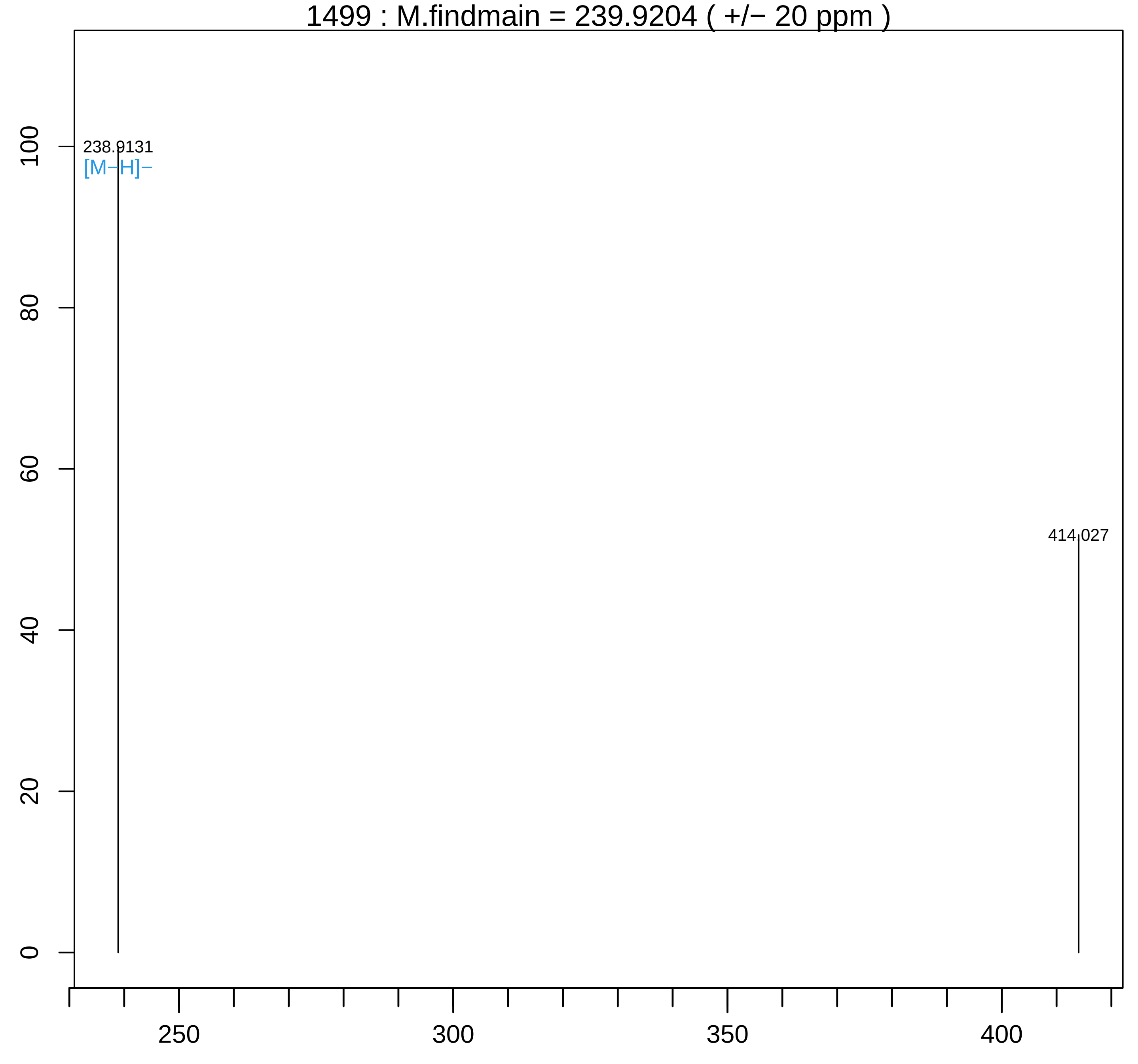
1499 : M.findmain = 239.9204 (+/− 20 ppm)
238.9131
[M−H]−
414.027
0
20
40
60
80
100
250
300
350
400

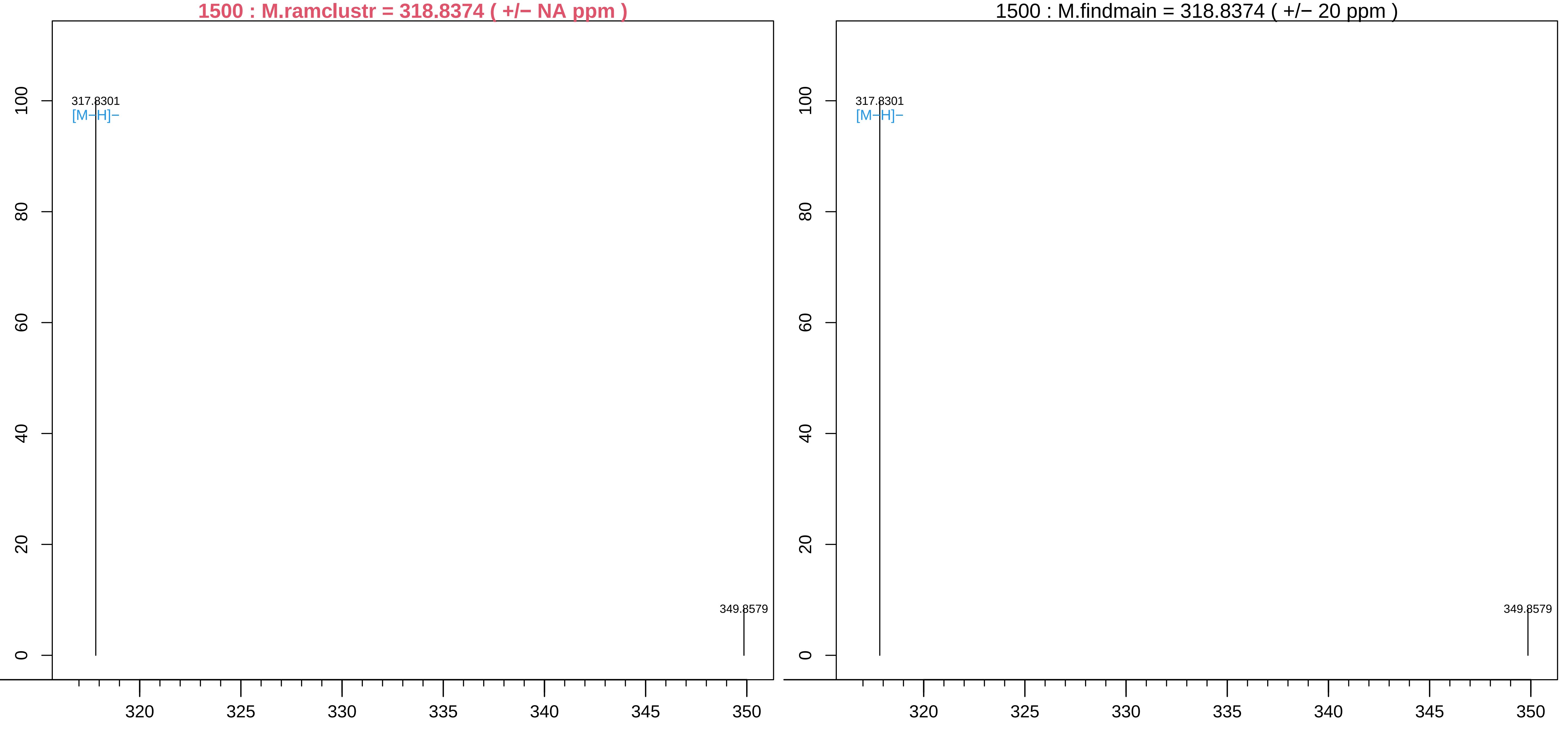
1500 : M.ramclustr = 318.8374 (+/− NA ppm)
317.8301
[M−H]−
349.8579
1500 : M.findmain = 318.8374 (+/− 20 ppm)
317.8301
[M−H]−
349.8579
0
20
40
60
80
100
320
325
330
335
340
345
350

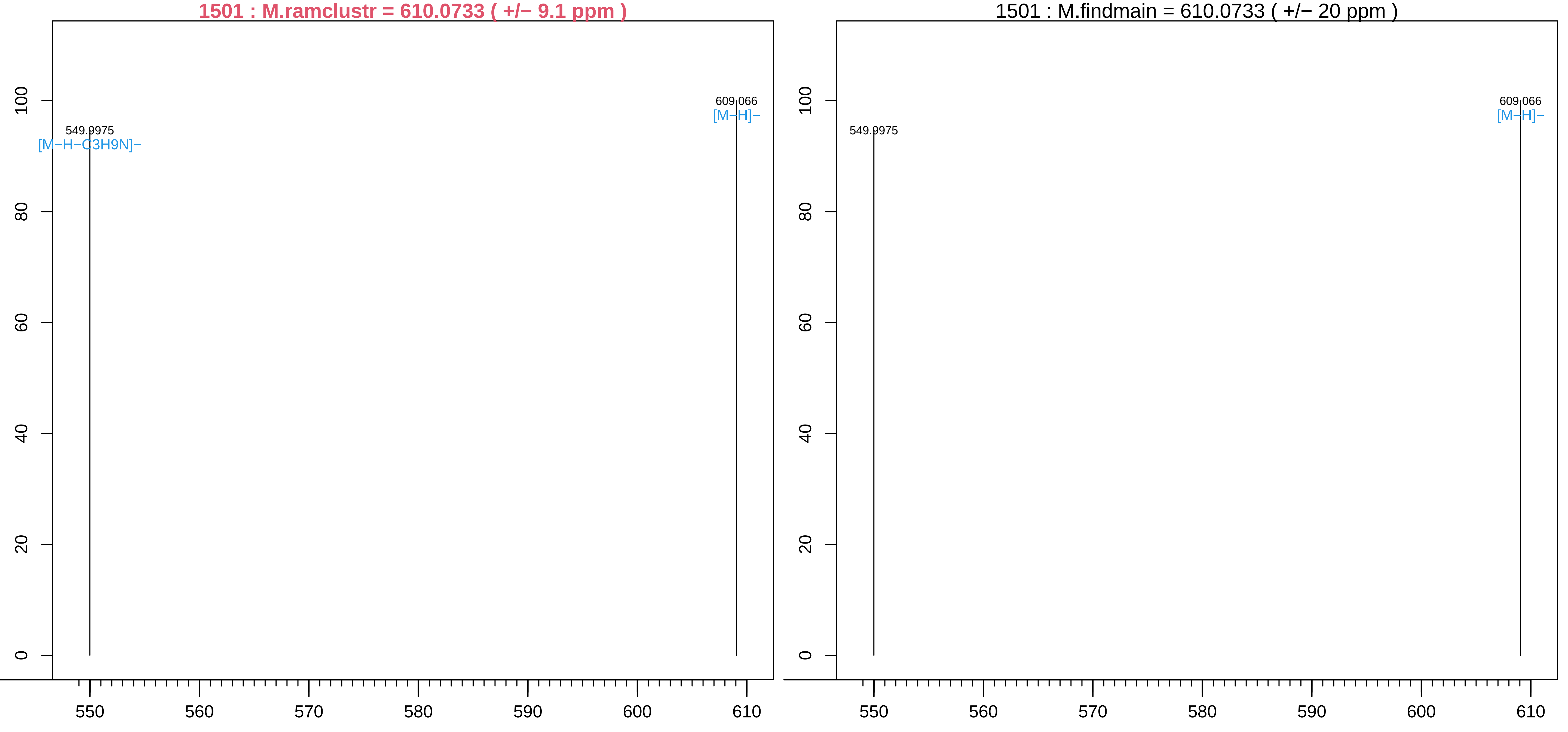
1501 : M.ramclustr = 610.0733 (+/− 9.1 ppm)
549.9975
[M−H−C3H9N]−
609.066
[M−H]−
1501 : M.findmain = 610.0733 (+/− 20 ppm)
549.9975
609.066
[M−H]−

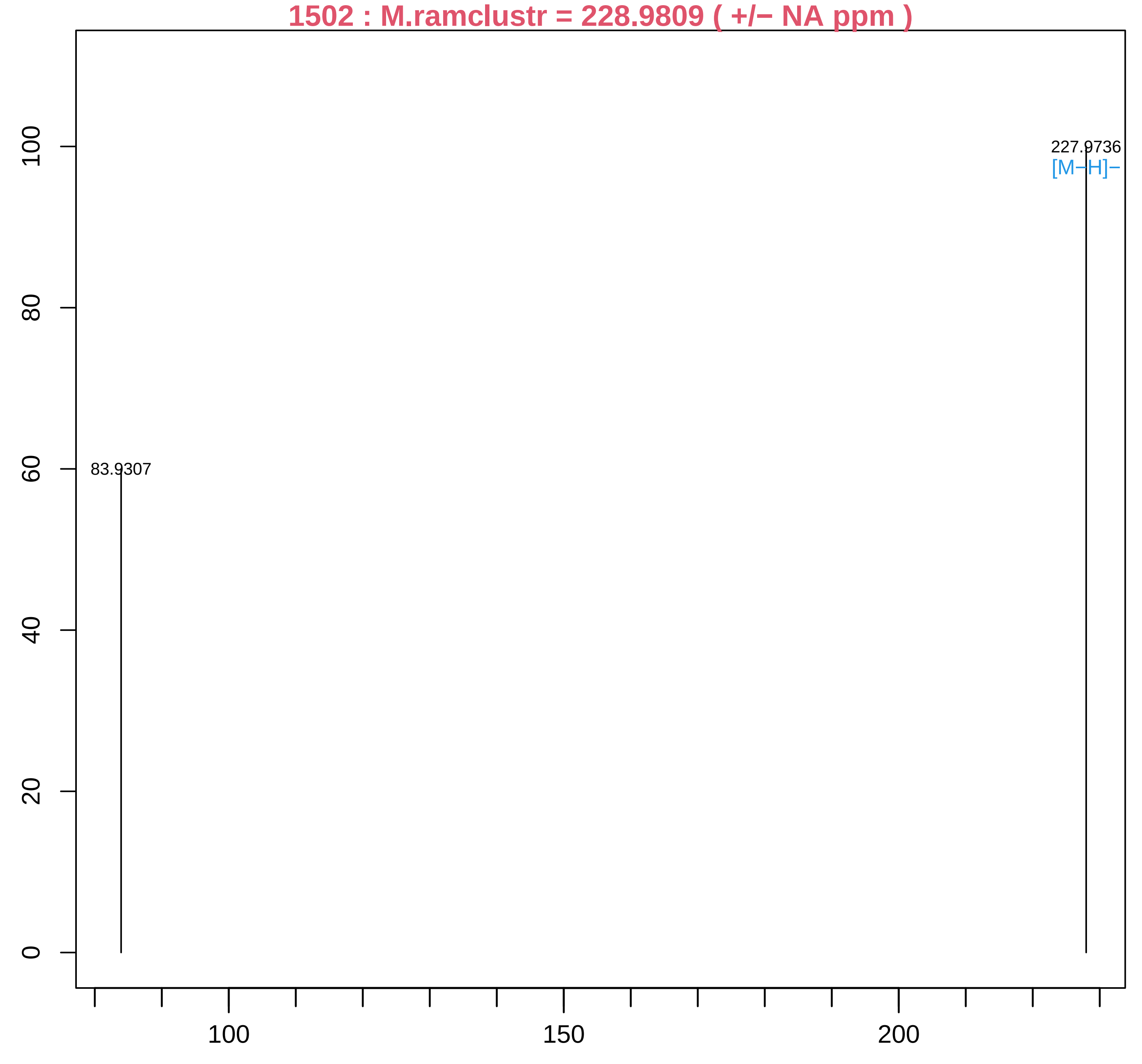
1502 : M.ramclustr = 228.9809 (+/− NA ppm)
227.9736
[M−H]−
83.9307
0
20
40
60
80
100
100
150
200

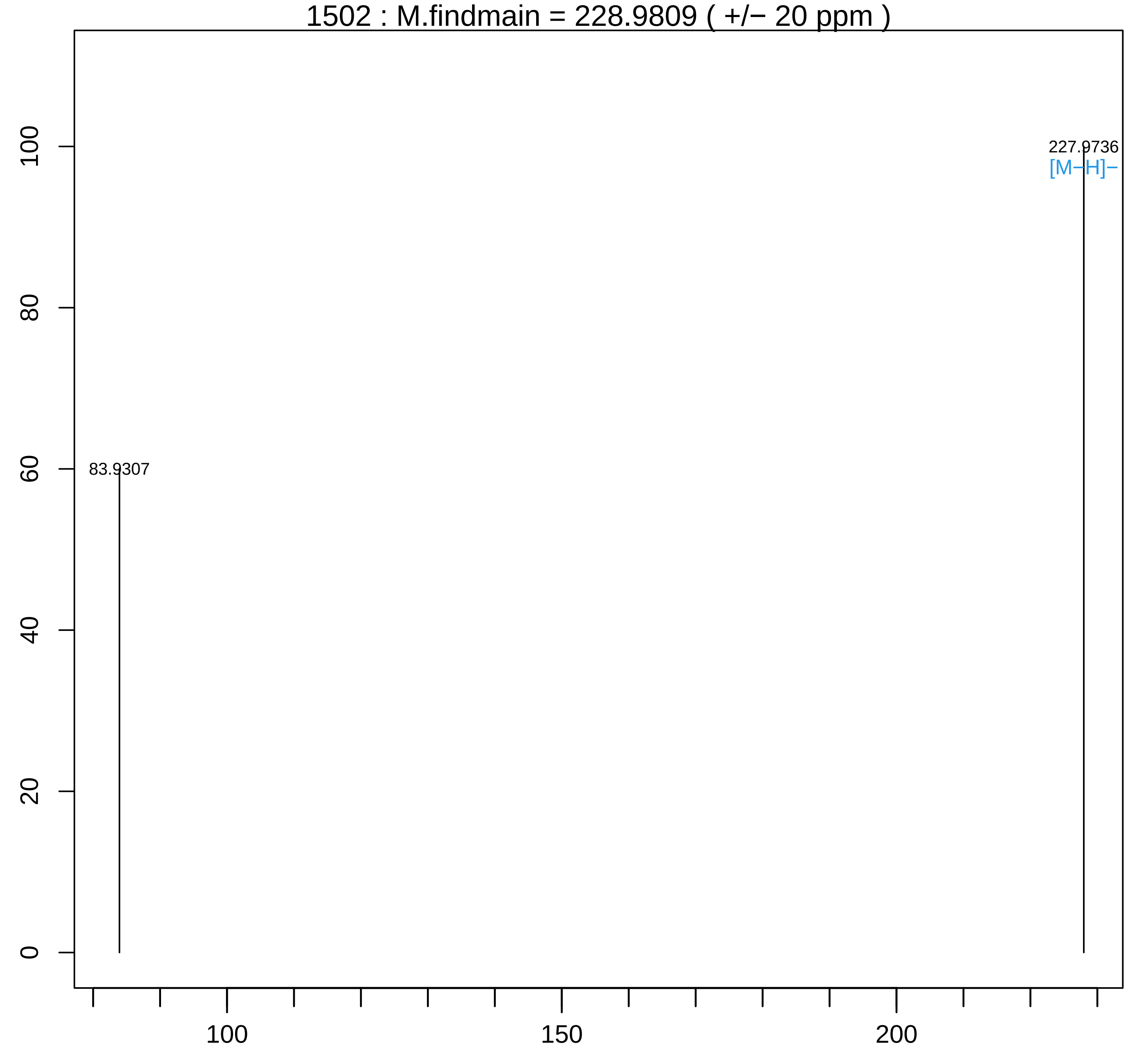
1502 : M.findmain = 228.9809 (+/− 20 ppm)
227.9736
[M−H]−
83.9307
0
20
40
60
80
100
100
150
200

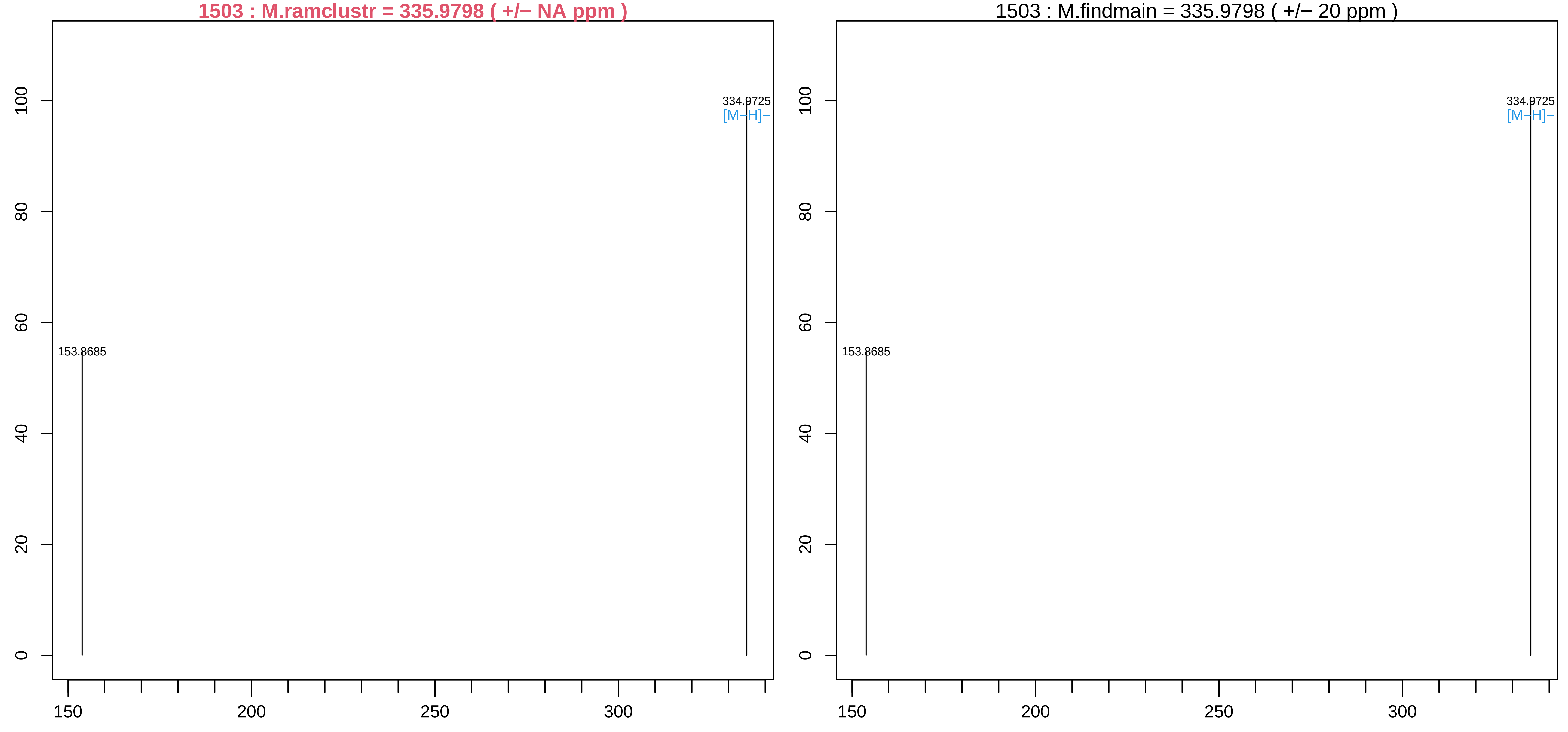
1503 : M.ramclustr = 335.9798 (+/− NA ppm)
334.9725
[M−H]−
153.8685
1503 : M.findmain = 335.9798 (+/− 20 ppm)
334.9725
[M−H]−
153.8685
0
20
40
60
80
100
150
200
250
300

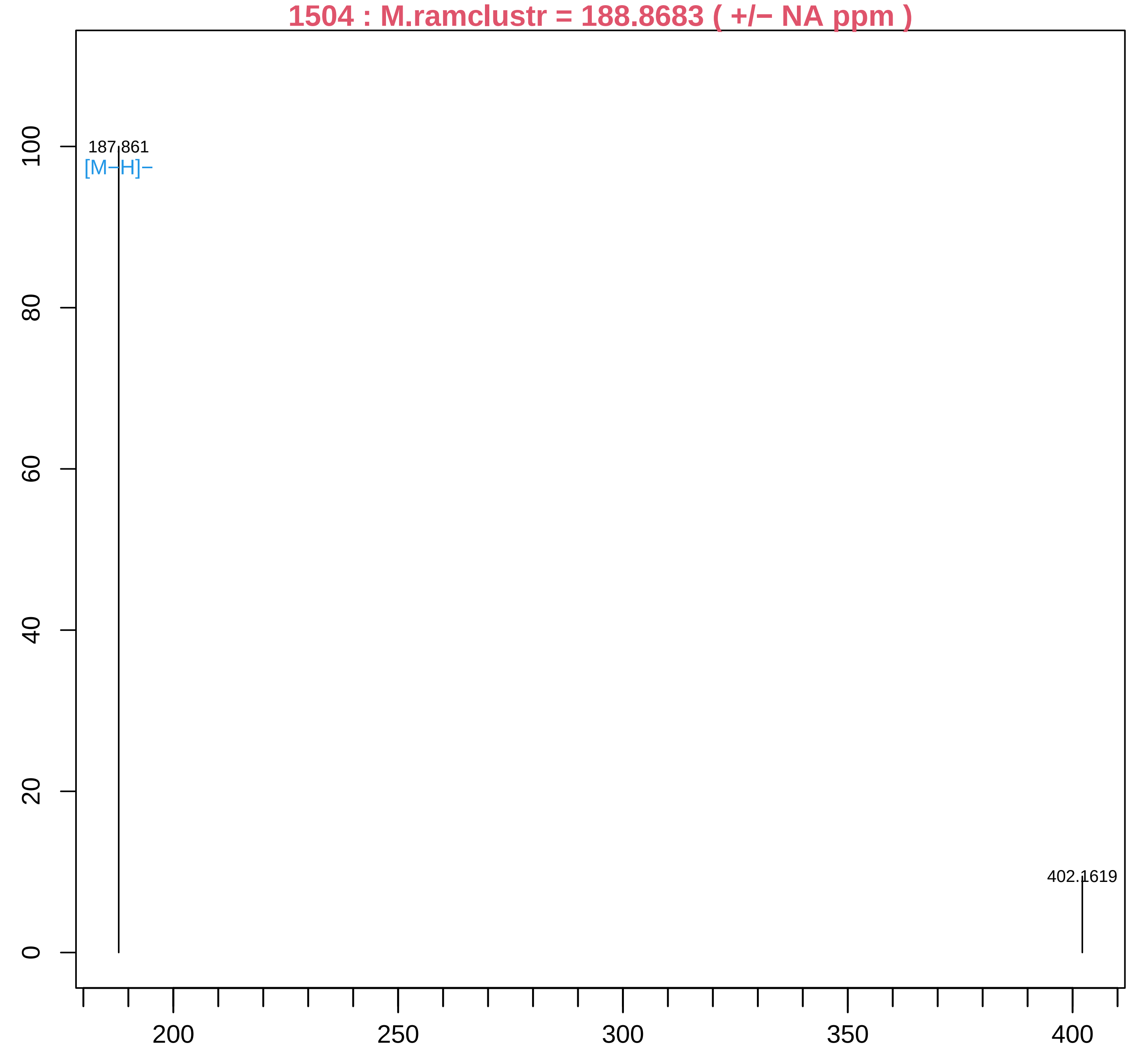
1504 : M.ramclustr = 188.8683 (+/− NA ppm)
187.861
[M−H]−
402.1619
0
20
40
60
80
100
200
250
300
350
400

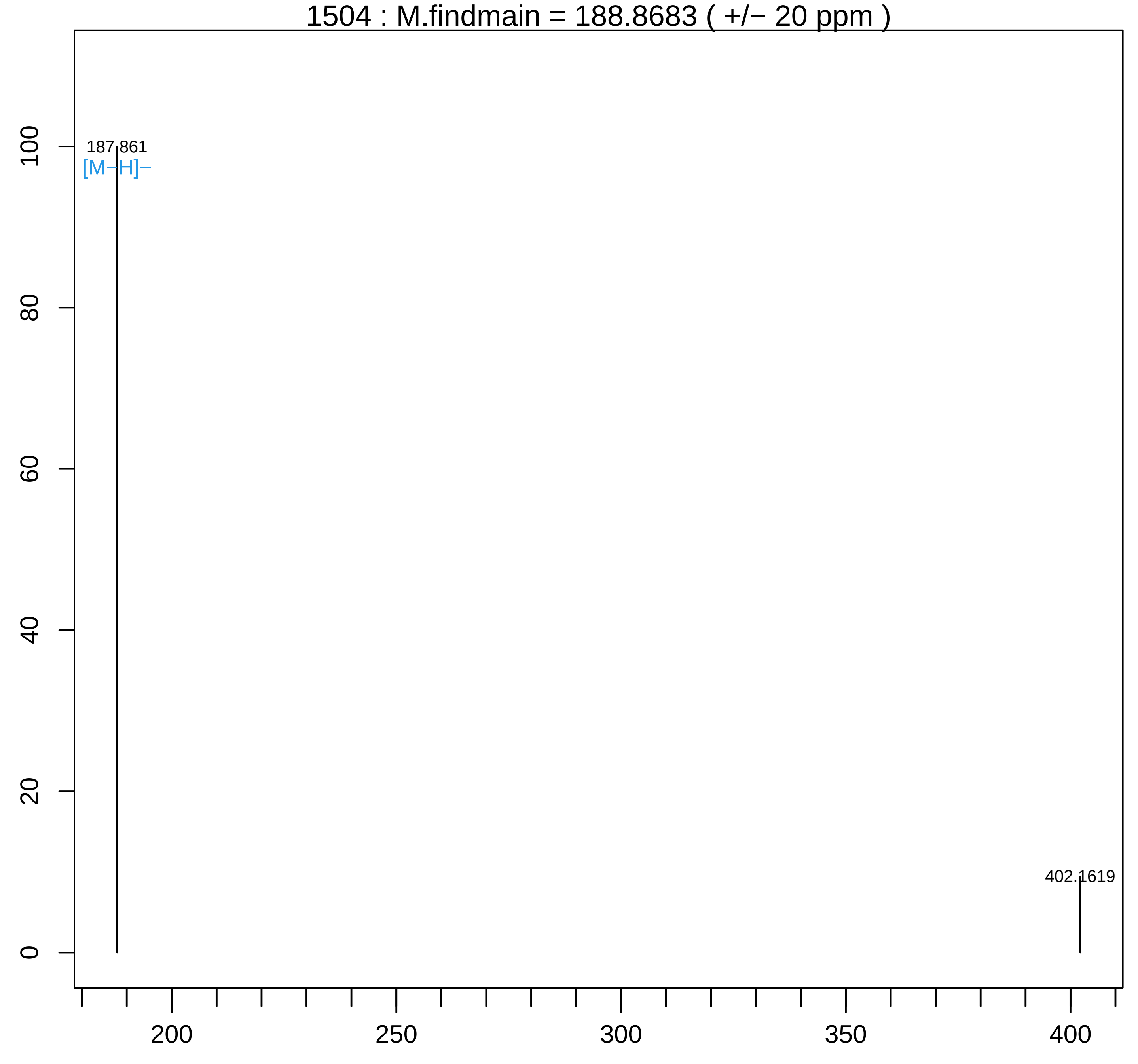
1504 : M.findmain = 188.8683 (+/− 20 ppm)
187.861
[M−H]−
402.1619
0
20
40
60
80
100
200
250
300
350
400

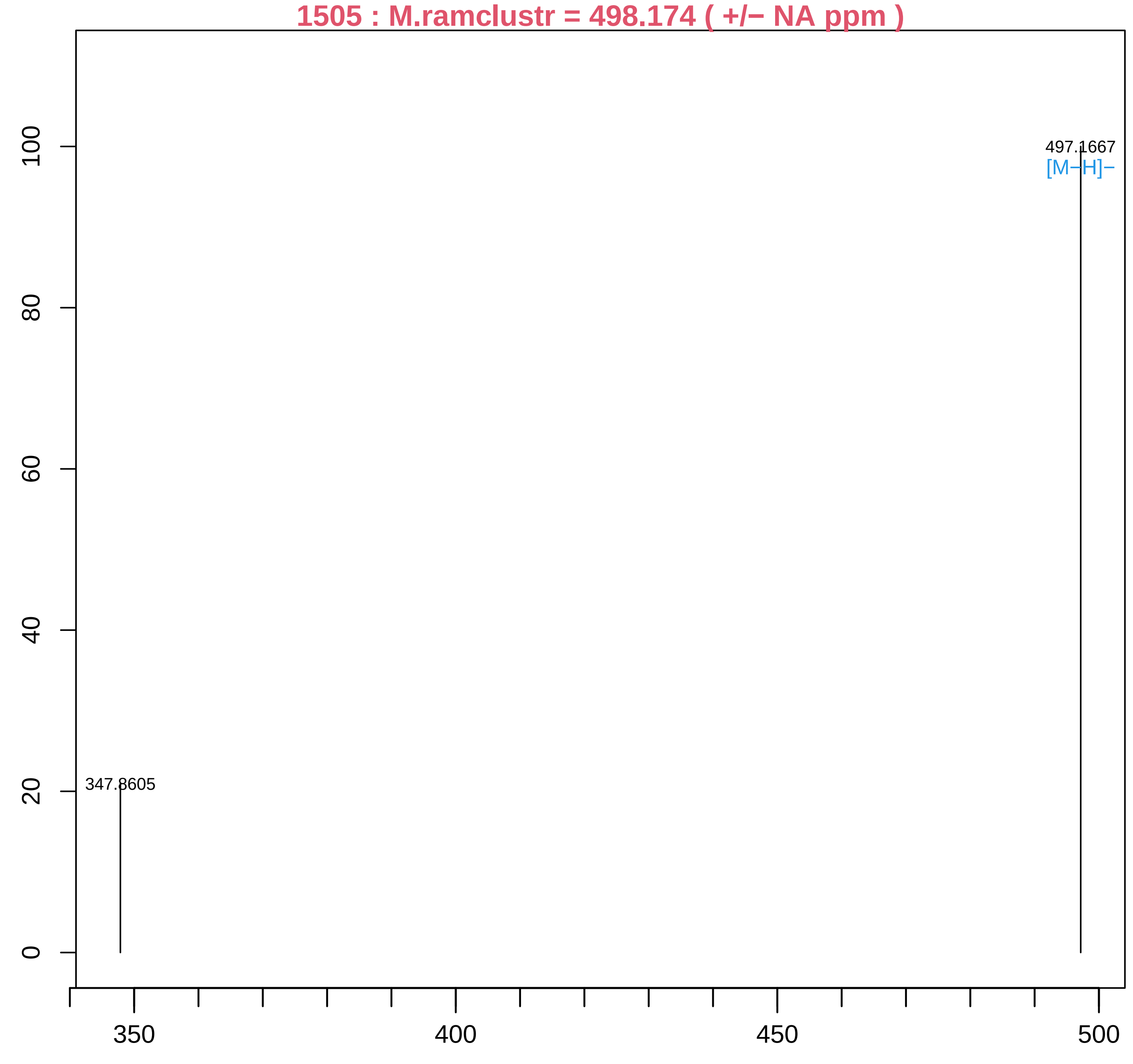
1505 : M.ramclustr = 498.174 (+/− NA ppm)
497.1667
[M−H]−
347.8605
0
20
40
60
80
100
350
400
450
500

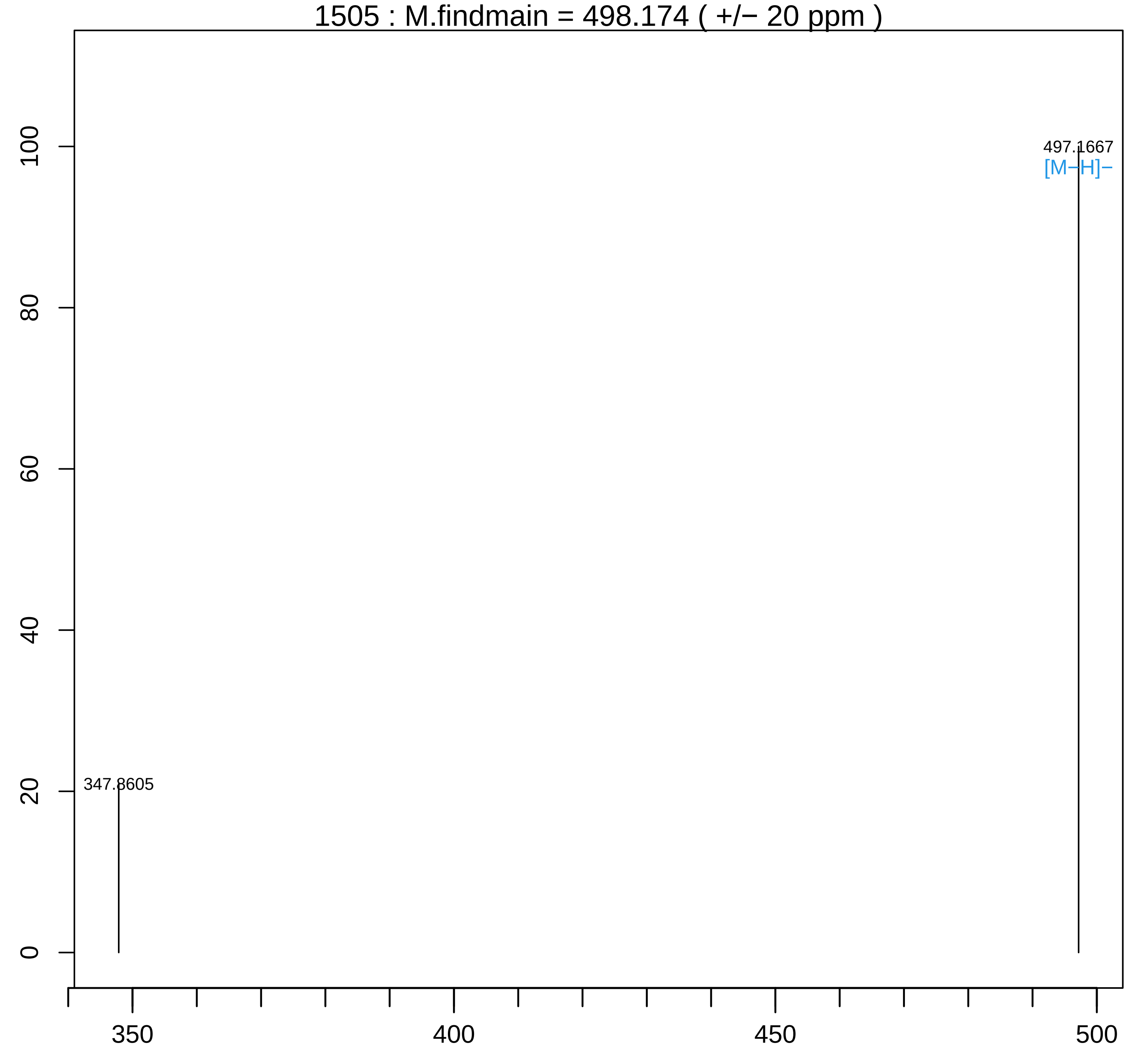
1505 : M.findmain = 498.174 (+/− 20 ppm)
497.1667
[M−H]−
347.8605
0
20
40
60
80
100
350
400
450
500

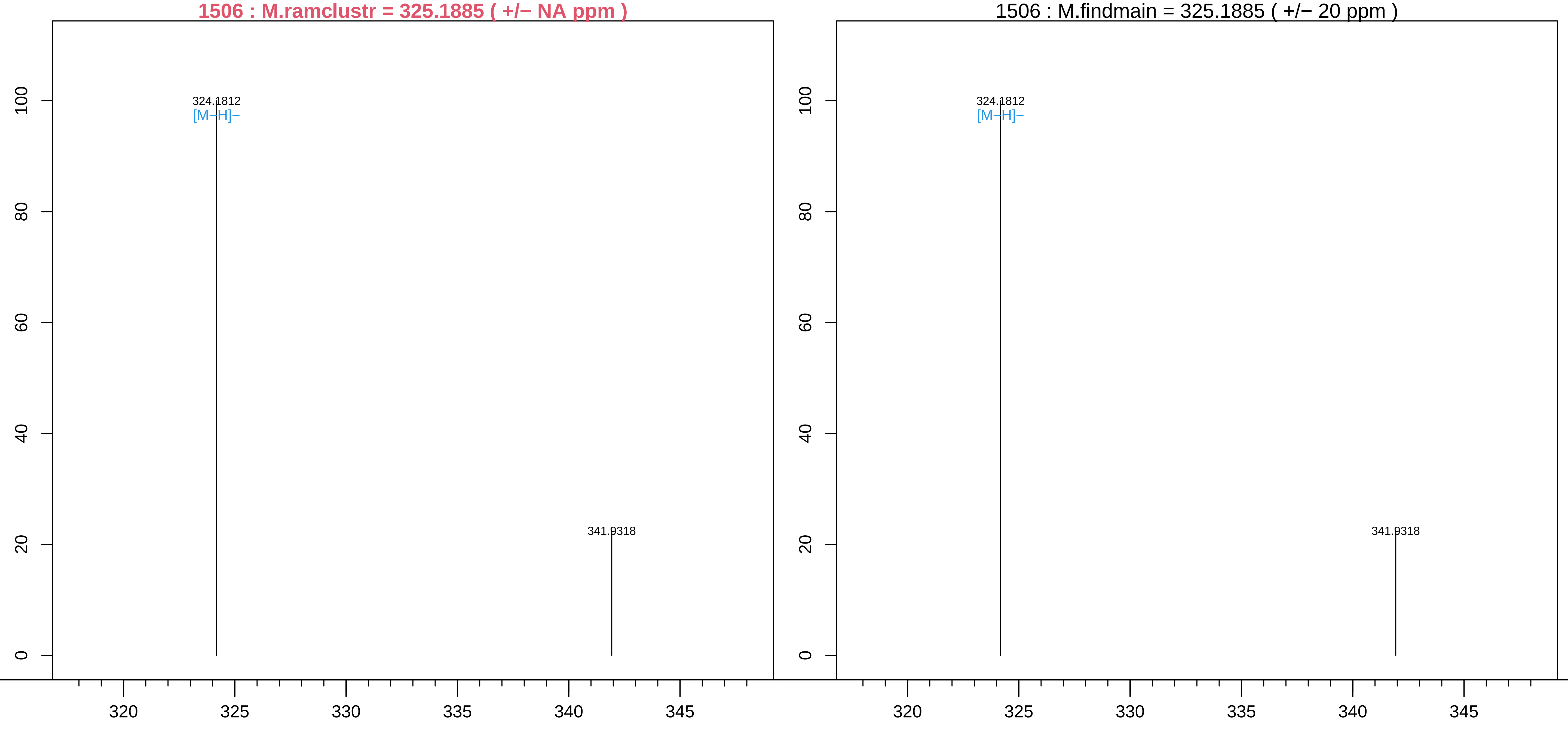

1506 : M.ramclustr = 325.1885 (+/− NA ppm)
324.1812
[M−H]−
341.9318
1506 : M.findmain = 325.1885 (+/− 20 ppm)
324.1812
[M−H]−
341.9318
0
20
40
60
80
100
320
325
330
335
340
345

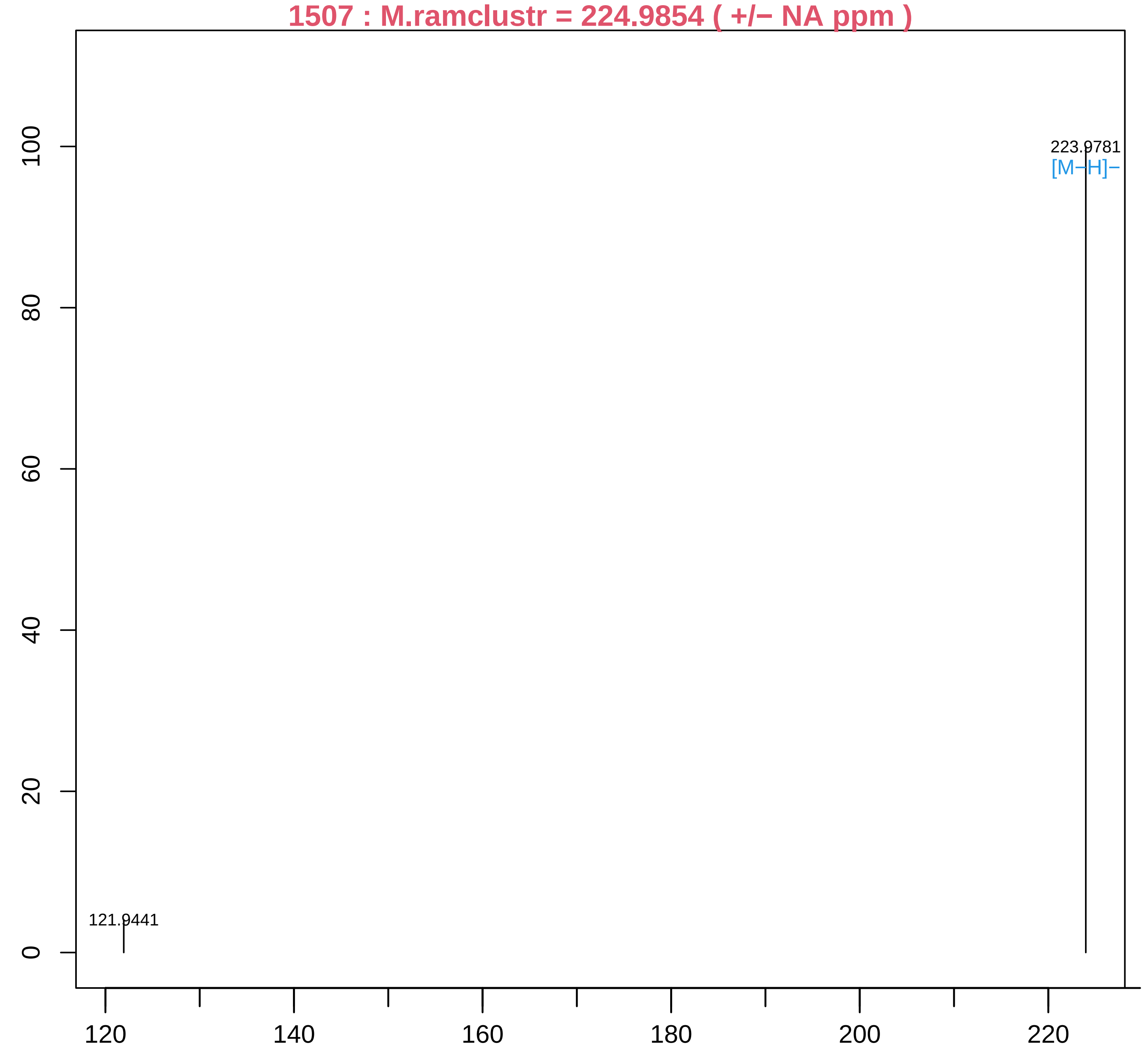
1507 : M.ramclustr = 224.9854 (+/− NA ppm)
223.9781
[M−H]−
121.9441
0
20
40
60
80
100
120
140
160
180
200
220

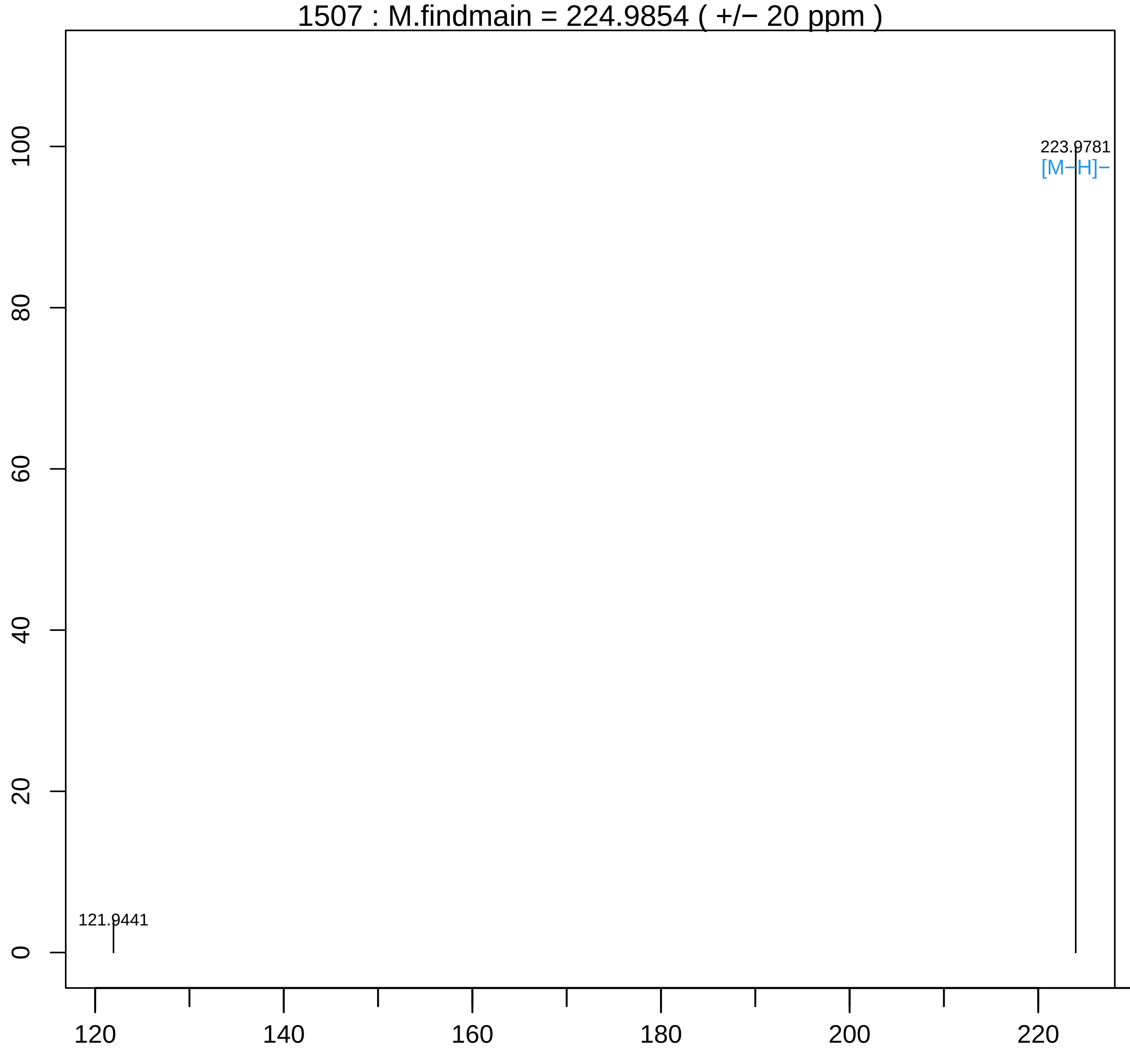
1507 : M.findmain = 224.9854 (+/− 20 ppm)
223.9781
[M−H]−
121.9441
0
20
40
60
80
100
120
140
160
180
200
220

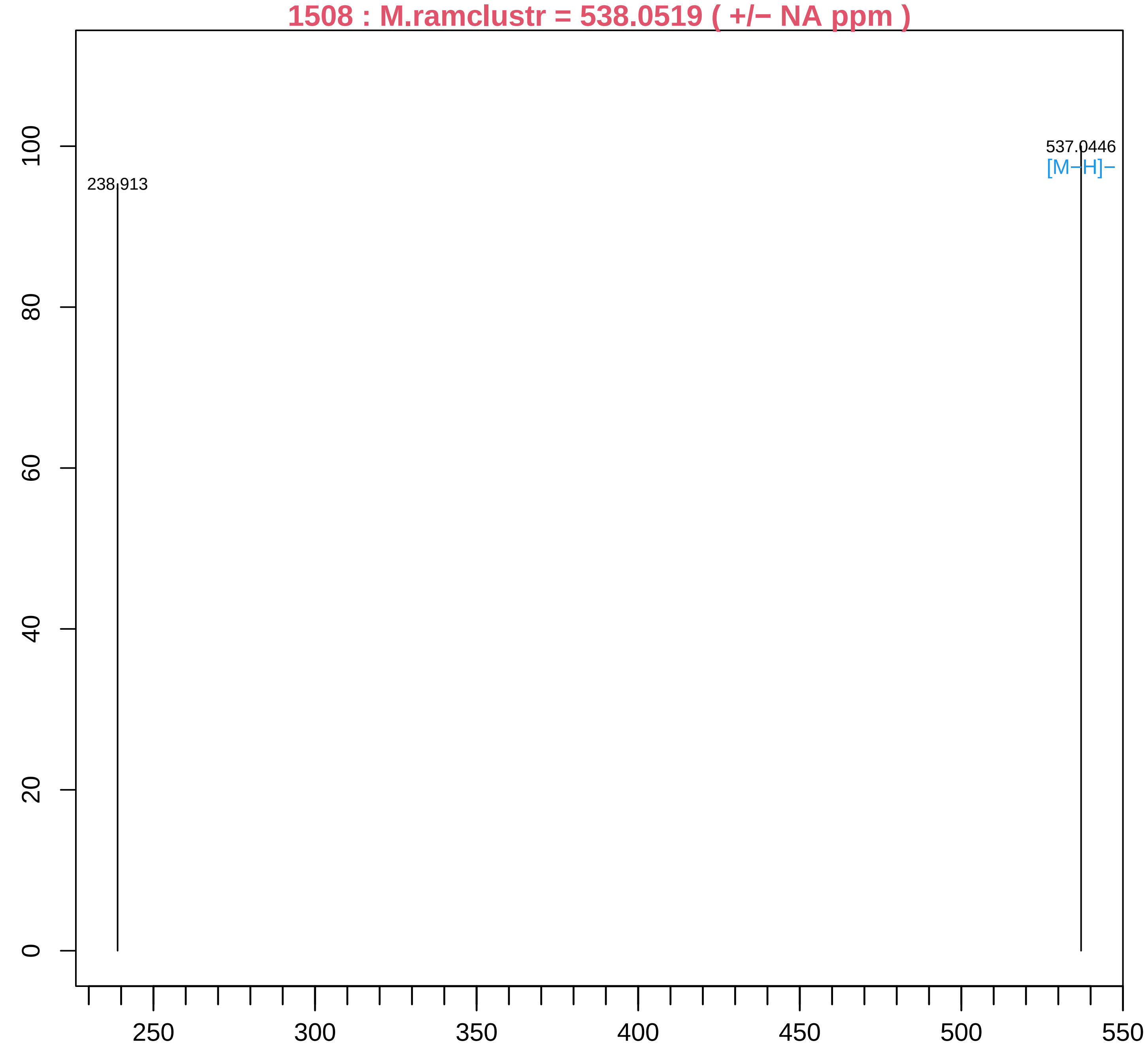

1508 : M.ramclustr = 538.0519 (+/− NA ppm)
238.913
537.0446
[M−H]−
0
20
40
60
80
100
250
300
350
400
450
500
550

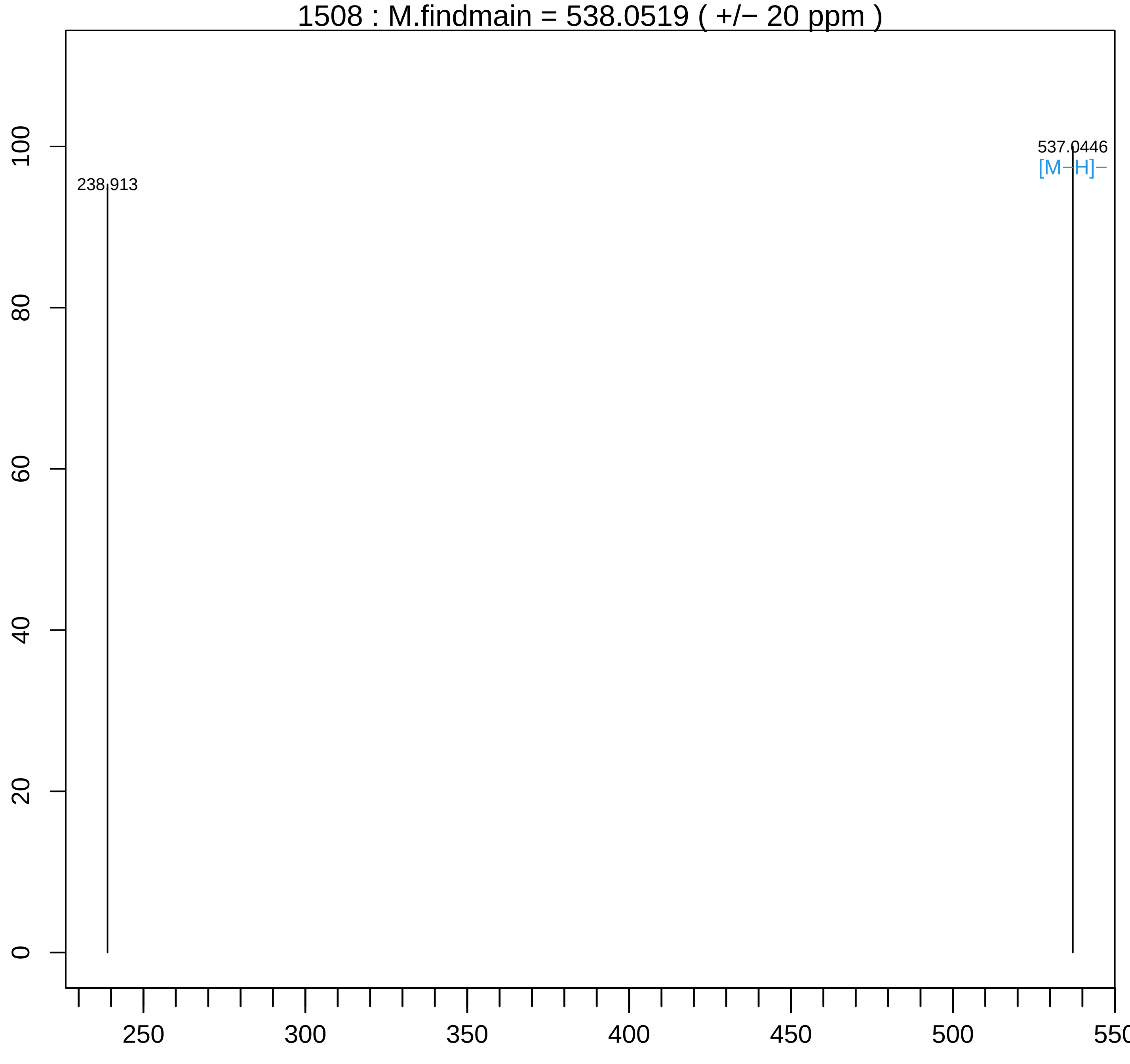

1508 : M.findmain = 538.0519 (+/− 20 ppm)
238.913
537.0446
[M−H]−
0
20
40
60
80
100
250
300
350
400
450
500

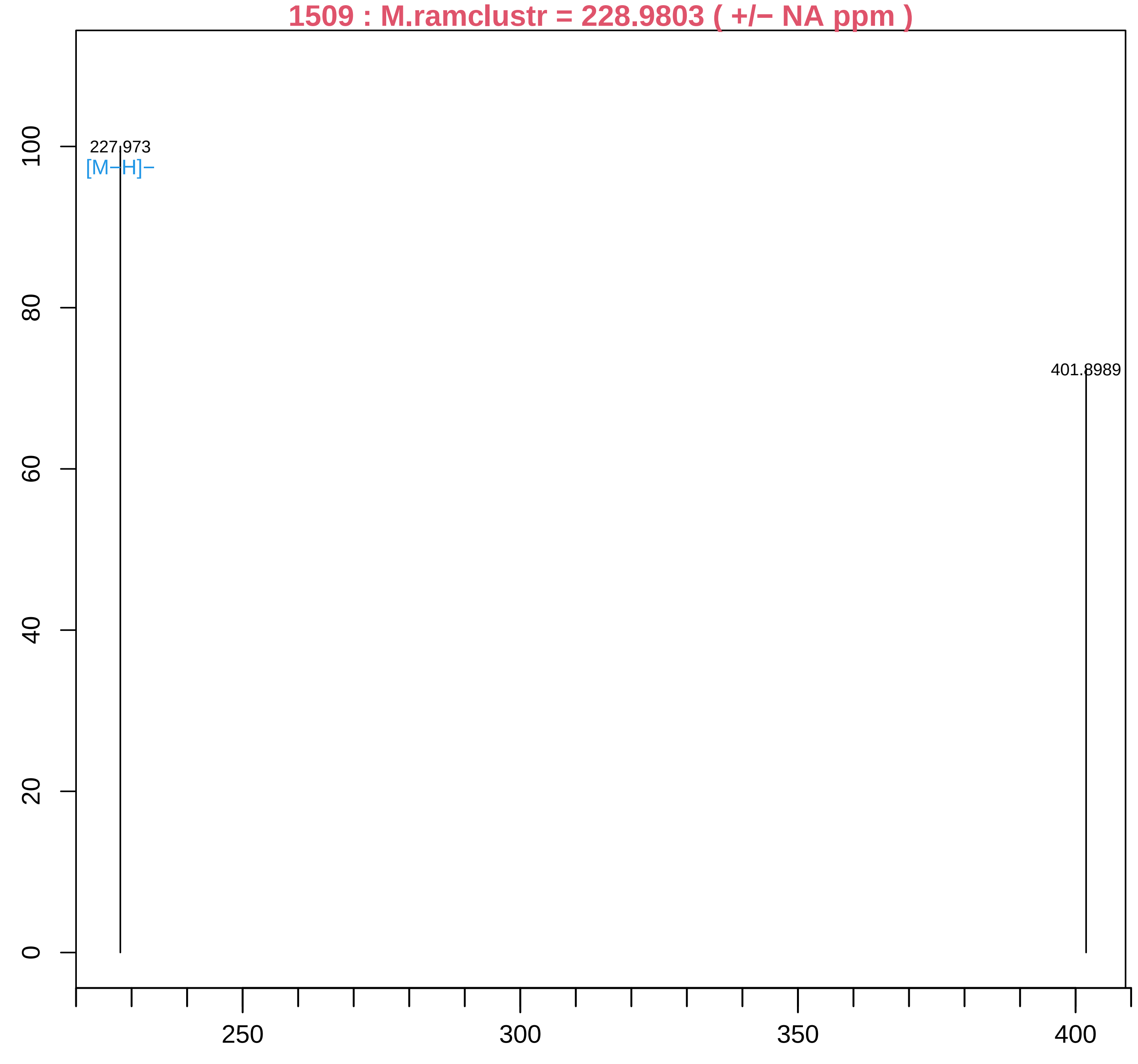
1509 : M.ramclustr = 228.9803 (+/− NA ppm)
227.973
[M−H]−
401.8989
0
20
40
60
80
100
250
300
350
400

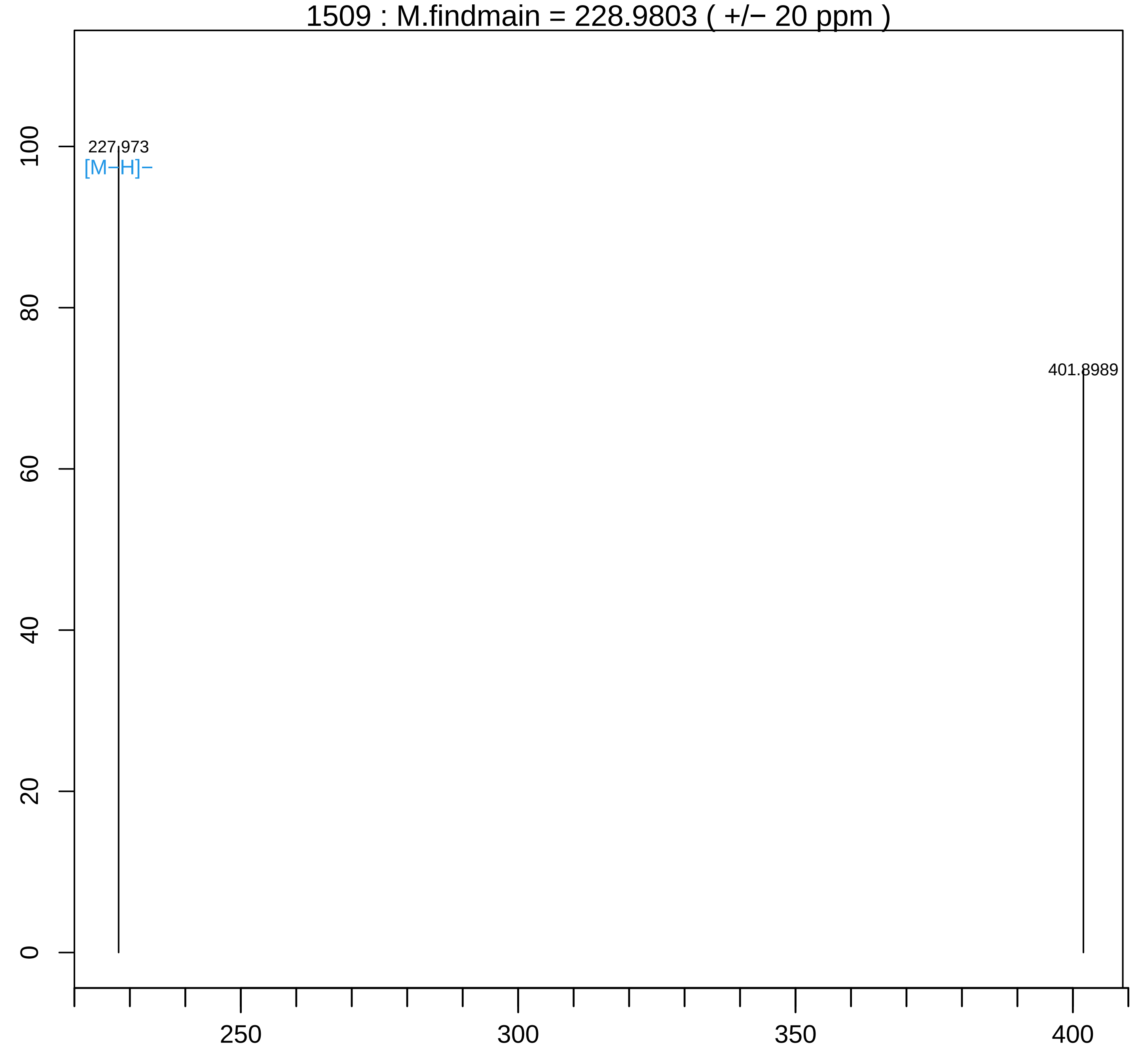
1509 : M.findmain = 228.9803 (+/− 20 ppm)
227.973
[M−H]−
401.8989
0
20
40
60
80
100
250
300
350
400

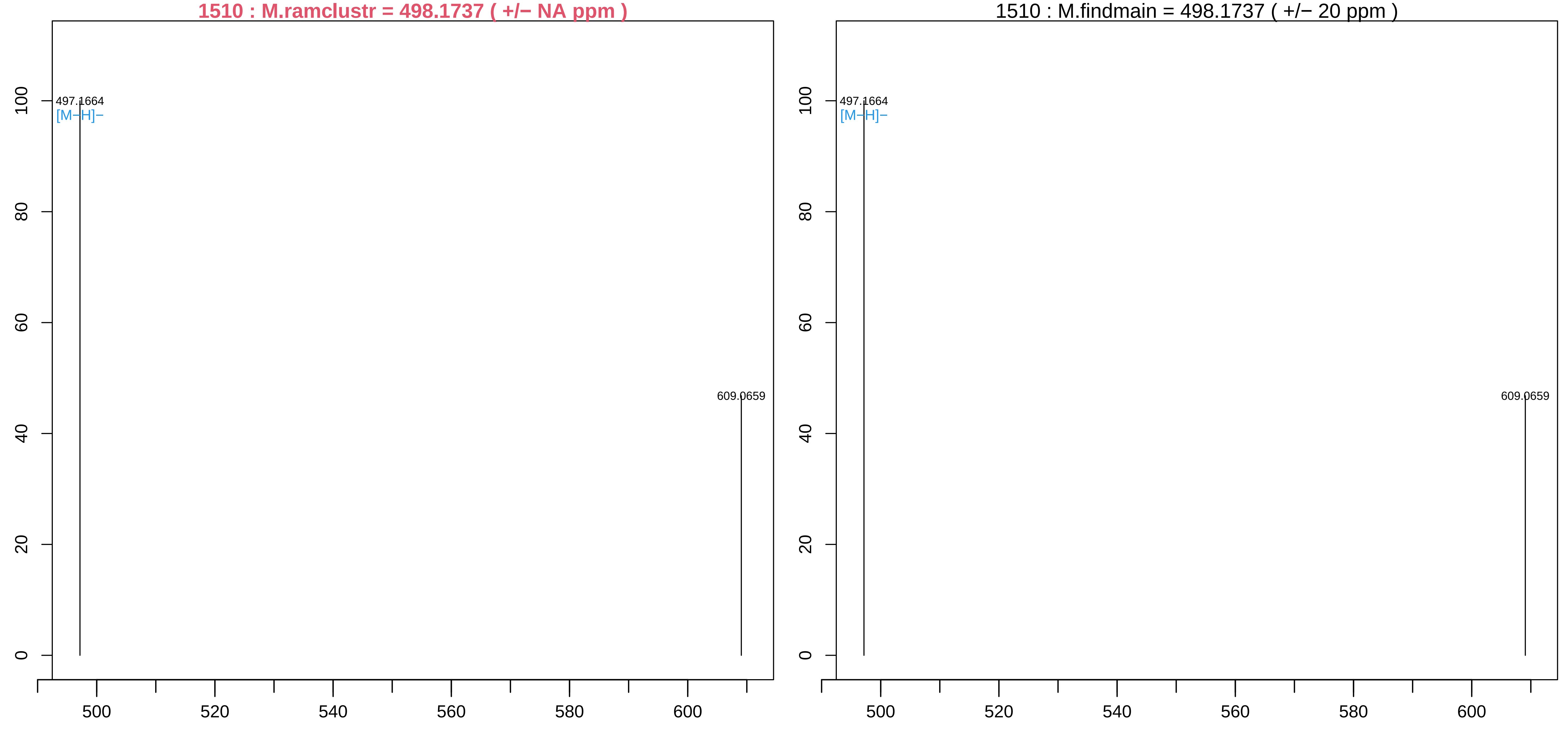
1510 : M.ramclustr = 498.1737 (+/− NA ppm)
497.1664
[M−H]−
609.0659
0
20
40
60
80
100
500
520
540
560
580
600
1510 : M.findmain = 498.1737 (+/− 20 ppm)
497.1664
[M−H]−
609.0659
0
20
40
60
80
100
500
520
540
560
580
600

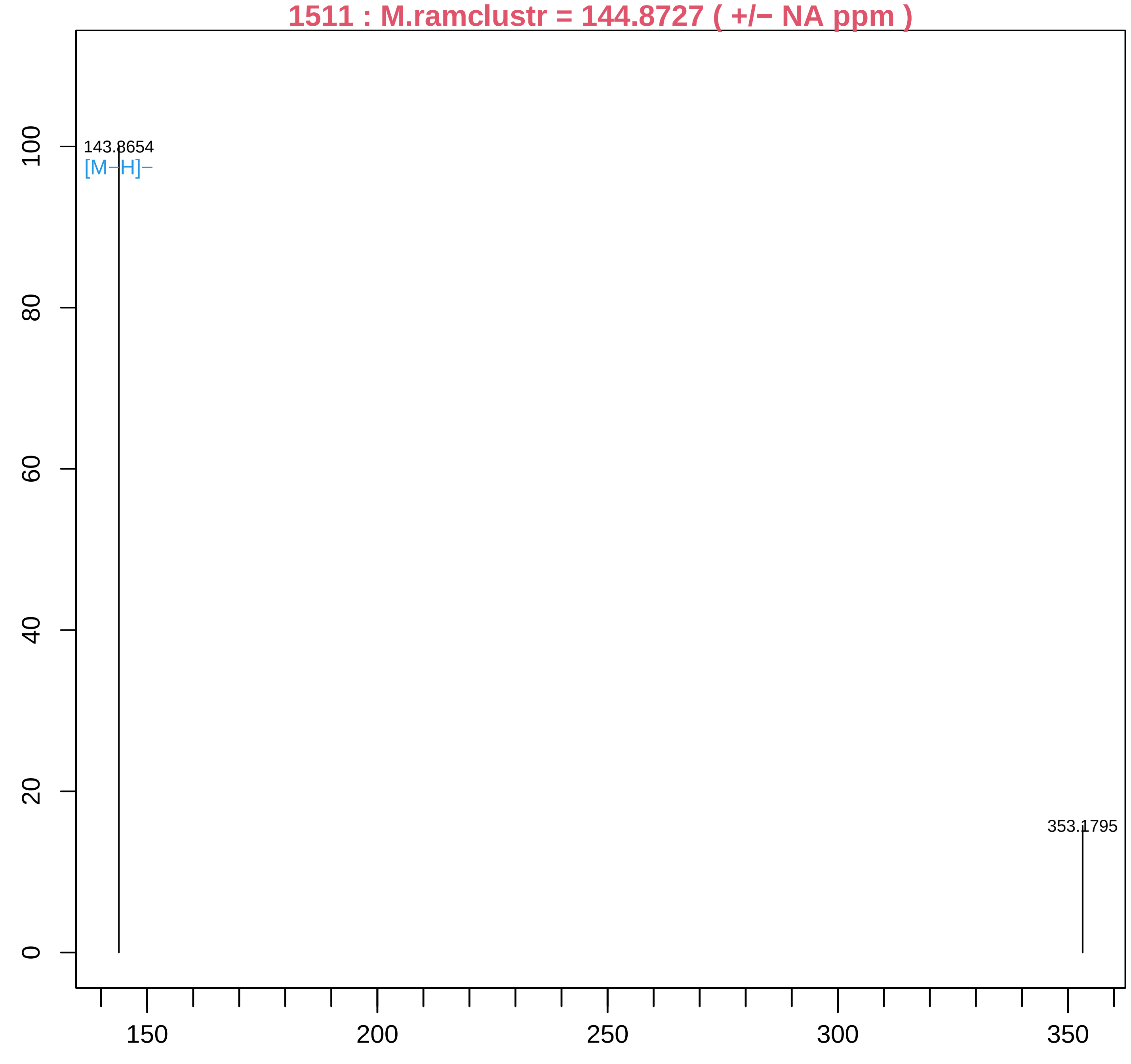
1511 : M.ramclustr = 144.8727 (+/− NA ppm)
143.8654
[M−H]−
353.1795
0
20
40
60
80
100
150
200
250
300
350

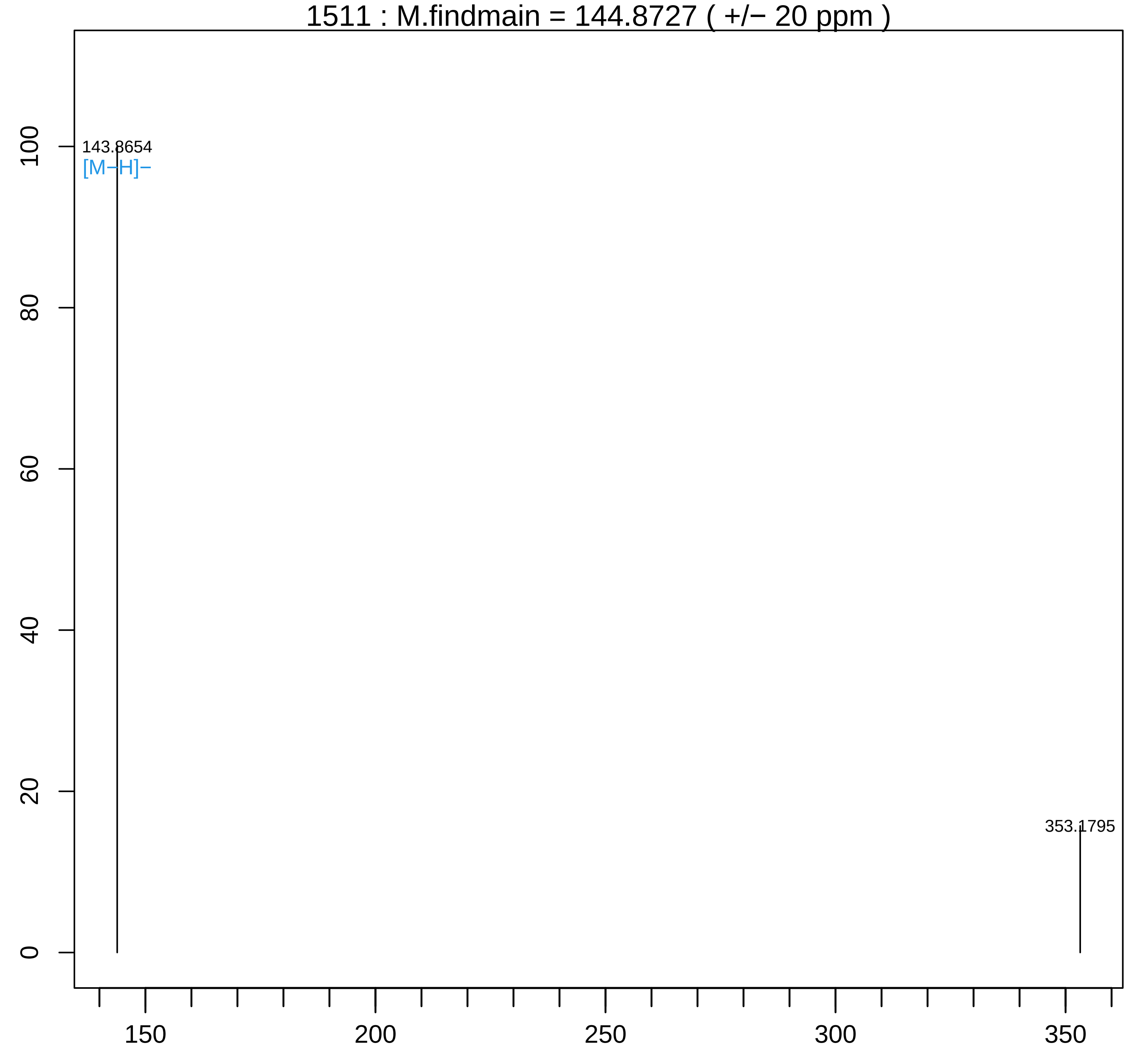
1511 : M.findmain = 144.8727 (+/− 20 ppm)
143.8654
[M−H]−
353.1795
0
20
40
60
80
100
150
200
250
300
350

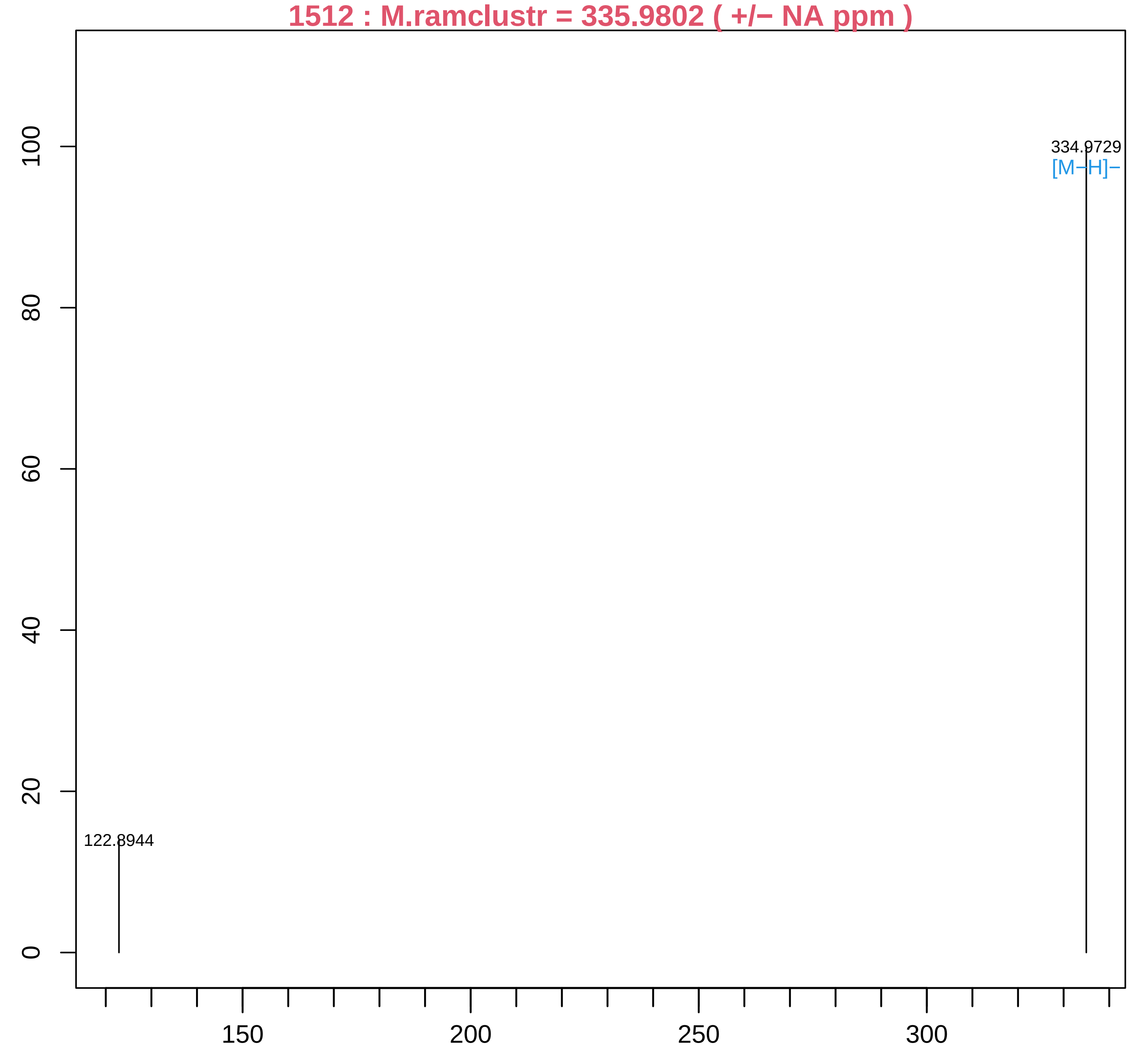

1512 : M.ramclustr = 335.9802 (+/− NA ppm)
334.9729
[M−H]−
122.8944
0
20
40
60
80
100
150
200
250
300

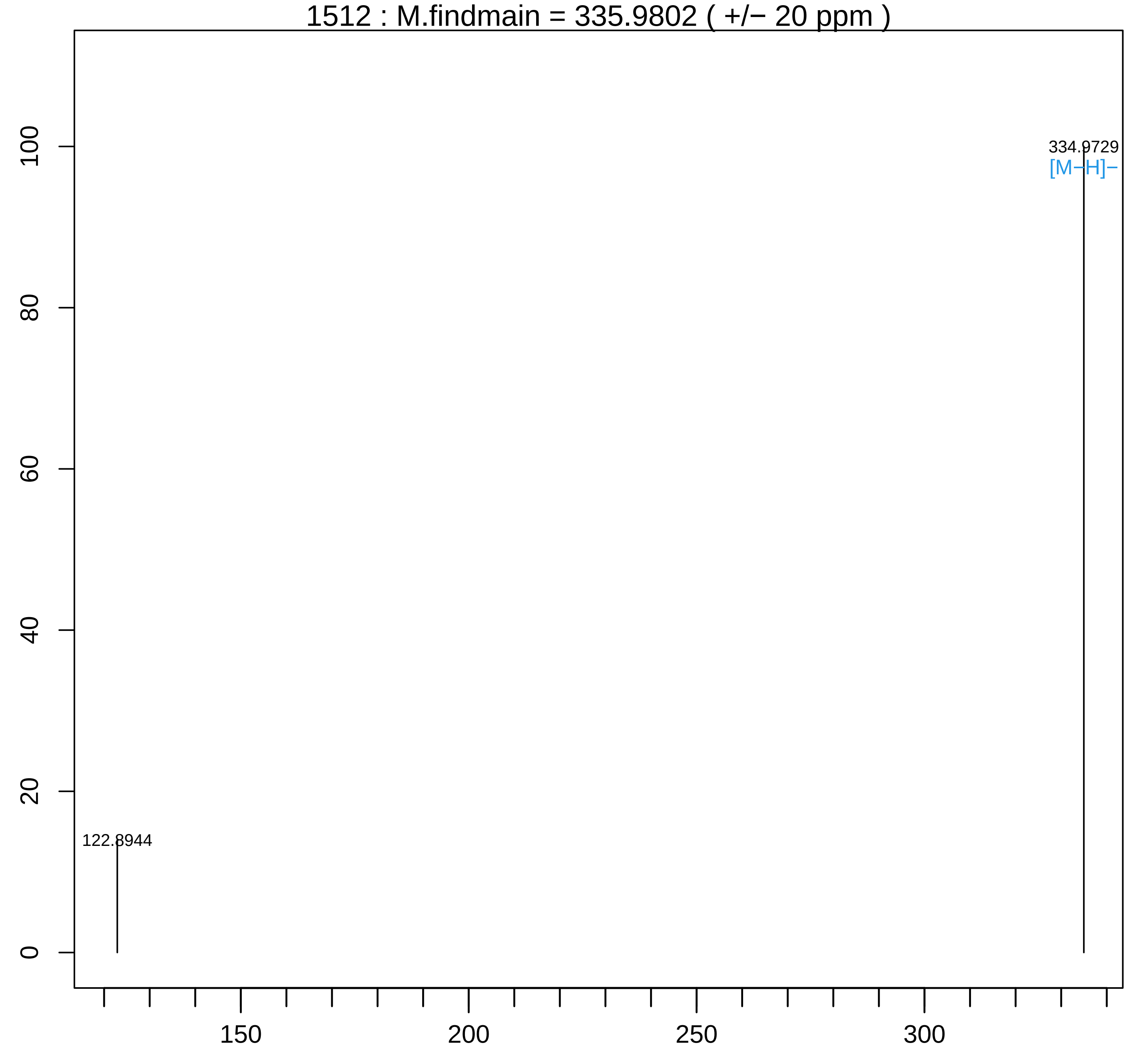

1512 : M.findmain = 335.9802 (+/− 20 ppm)
334.9729
[M−H]−
122.8944
0
20
40
60
80
100
150
200
250
300

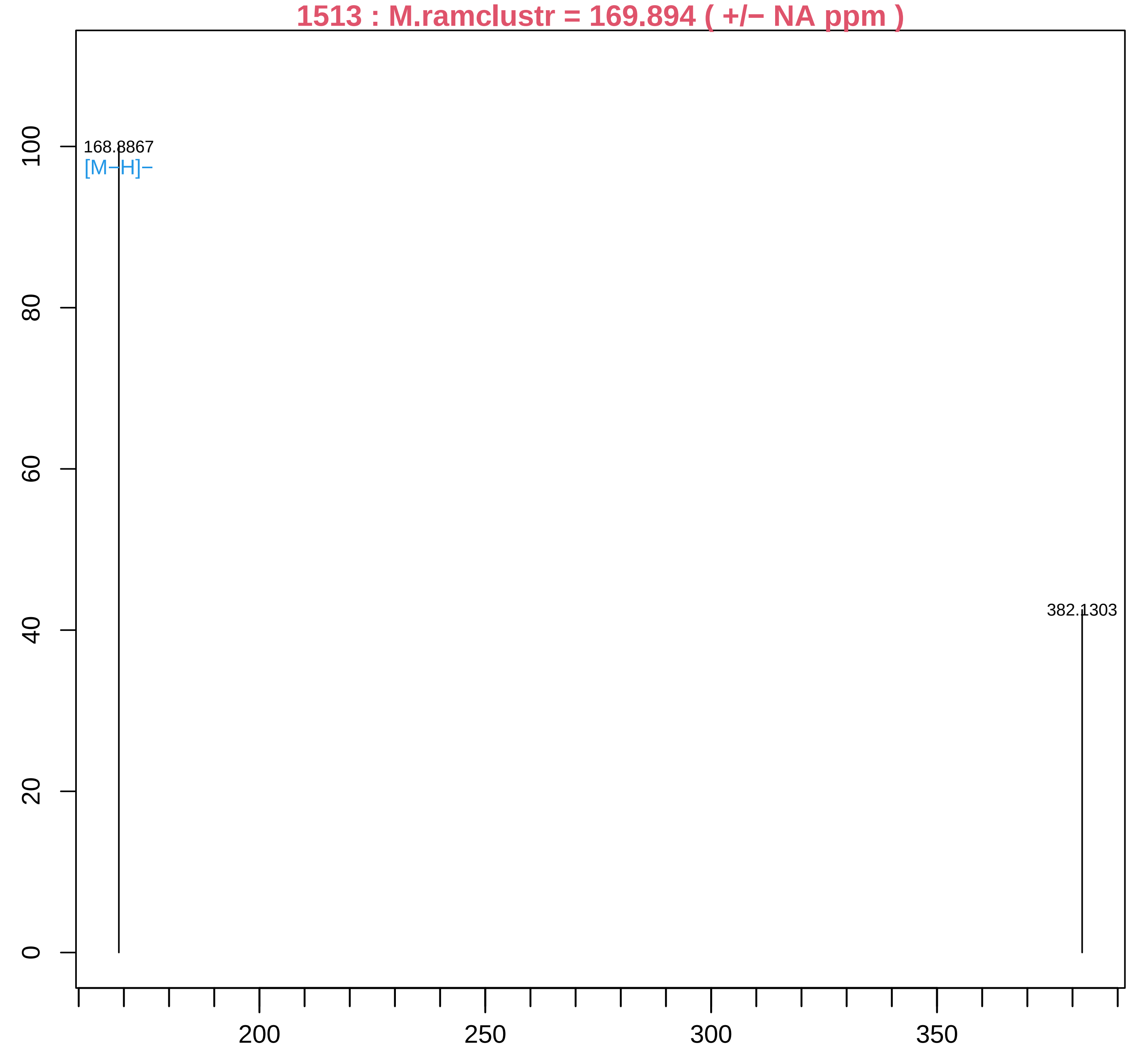
1513 : M.ramclustr = 169.894 (+/− NA ppm)
168.8867
[M−H]−
382.1303
0
20
40
60
80
100
200
250
300
350

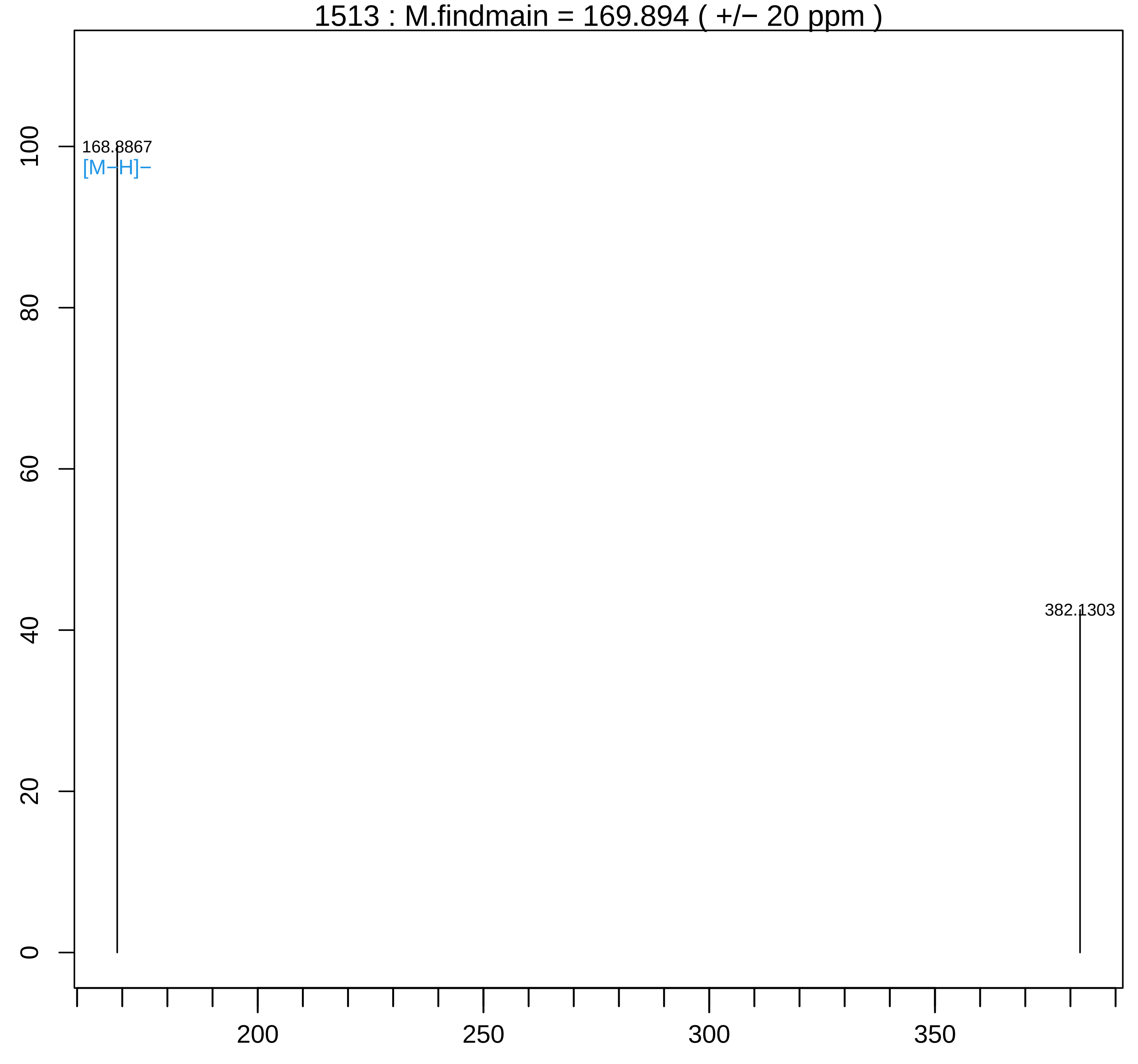
1513 : M.findmain = 169.894 (+/− 20 ppm)
168.8867
[M−H]−
382.1303
0
20
40
60
80
100
200
250
300
350

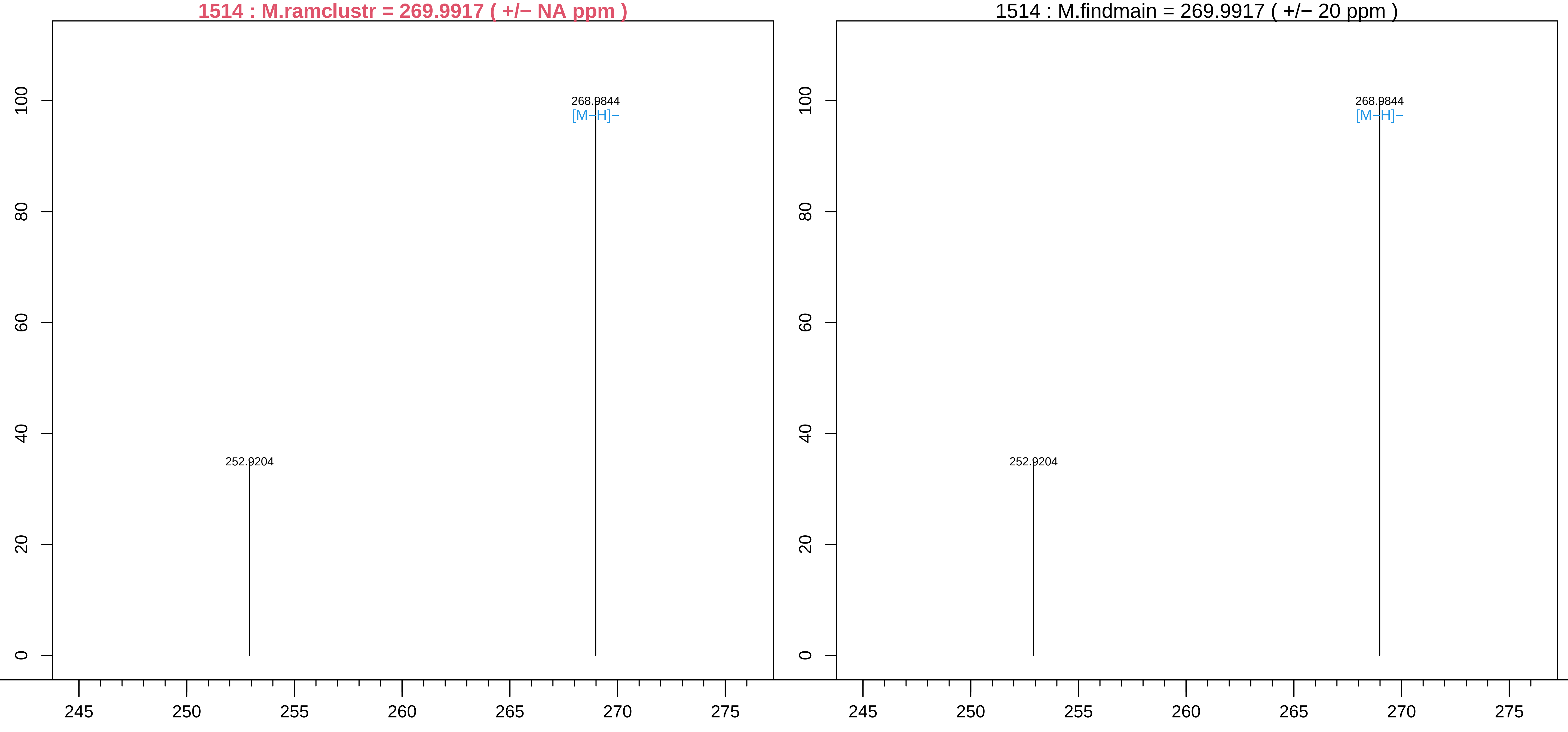

1514 : M.ramclustr = 269.9917 (+/− NA ppm)
1514 : M.findmain = 269.9917 (+/− 20 ppm)
268.9844
[M−H]−
252.9204
0
20
40
60
80
100
245
250
255
260
265
270
275

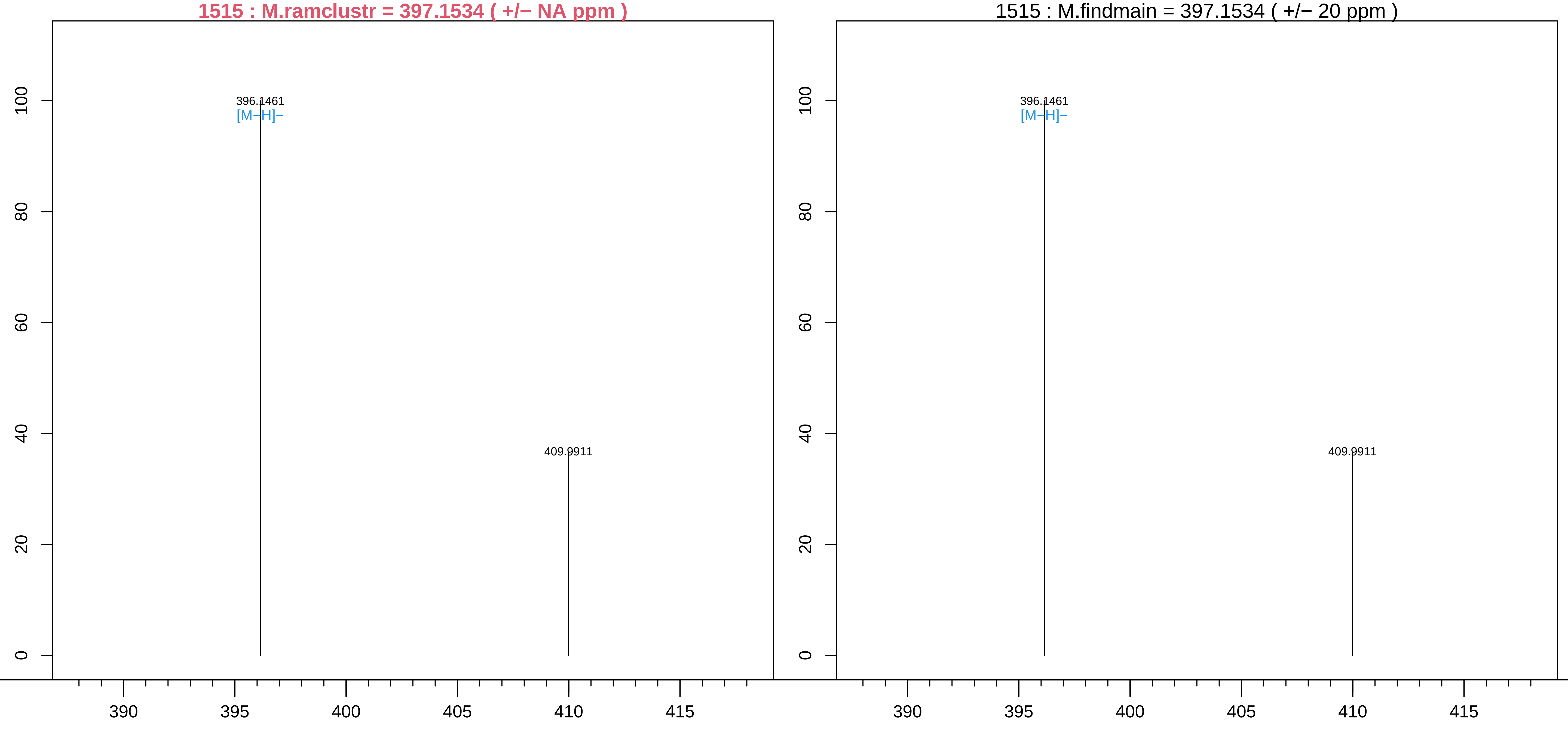
1515 : M.ramclustr = 397.1534 (+/− NA ppm)
396.1461
[M−H]−
409.9911
0
20
40
60
80
100
390
395
400
405
410
415
1515 : M.findmain = 397.1534 (+/− 20 ppm)
396.1461
[M−H]−
409.9911
0
20
40
60
80
100
390
395
400
405
410
415

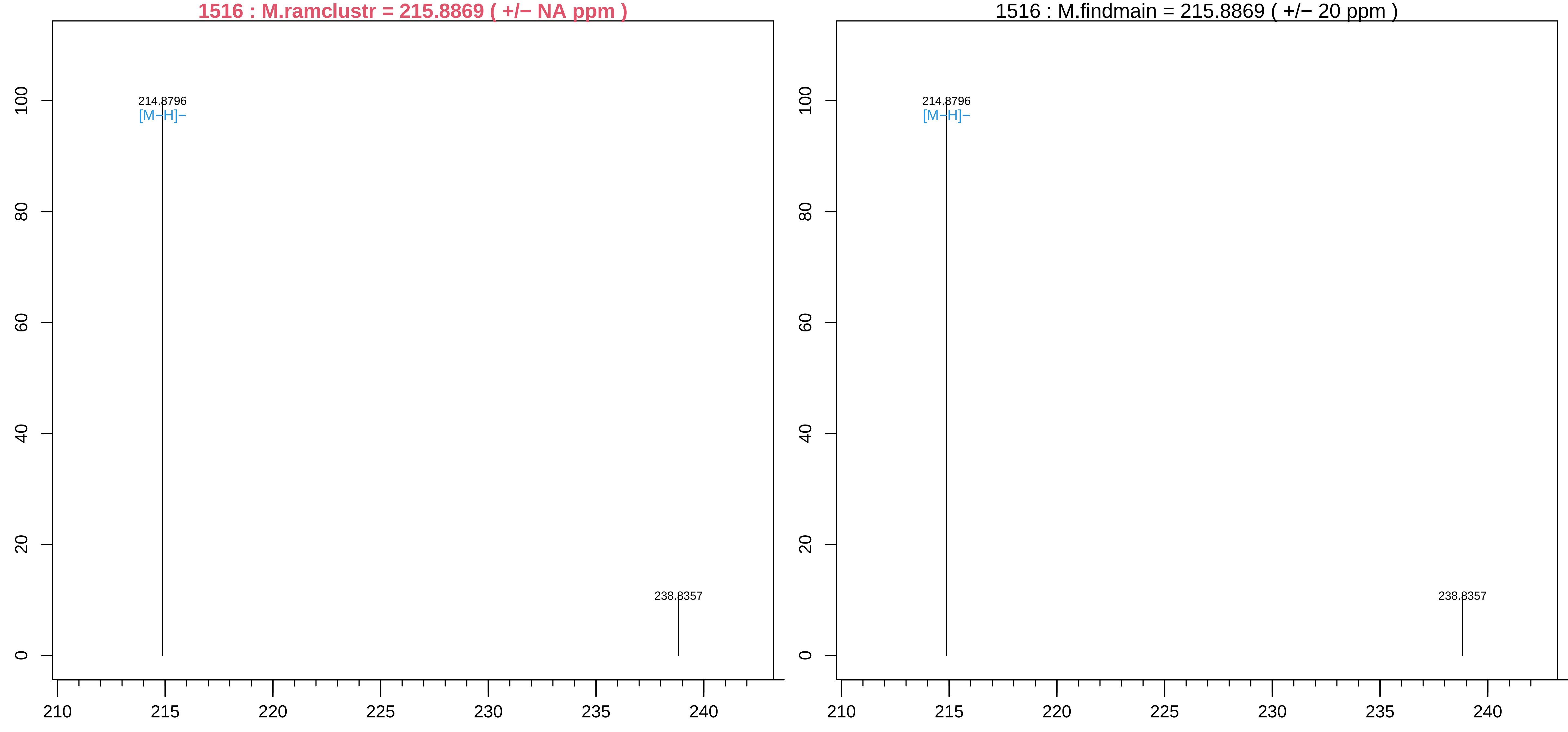
1516 : M.ramclustr = 215.8869 (+/− NA ppm)
214.8796
[M−H]−
238.8357
0
20
40
60
80
100
210
215
220
225
230
235
240
1516 : M.findmain = 215.8869 (+/− 20 ppm)
214.8796
[M−H]−
238.8357
0
20
40
60
80
100
210
215
220
225
230
235
240

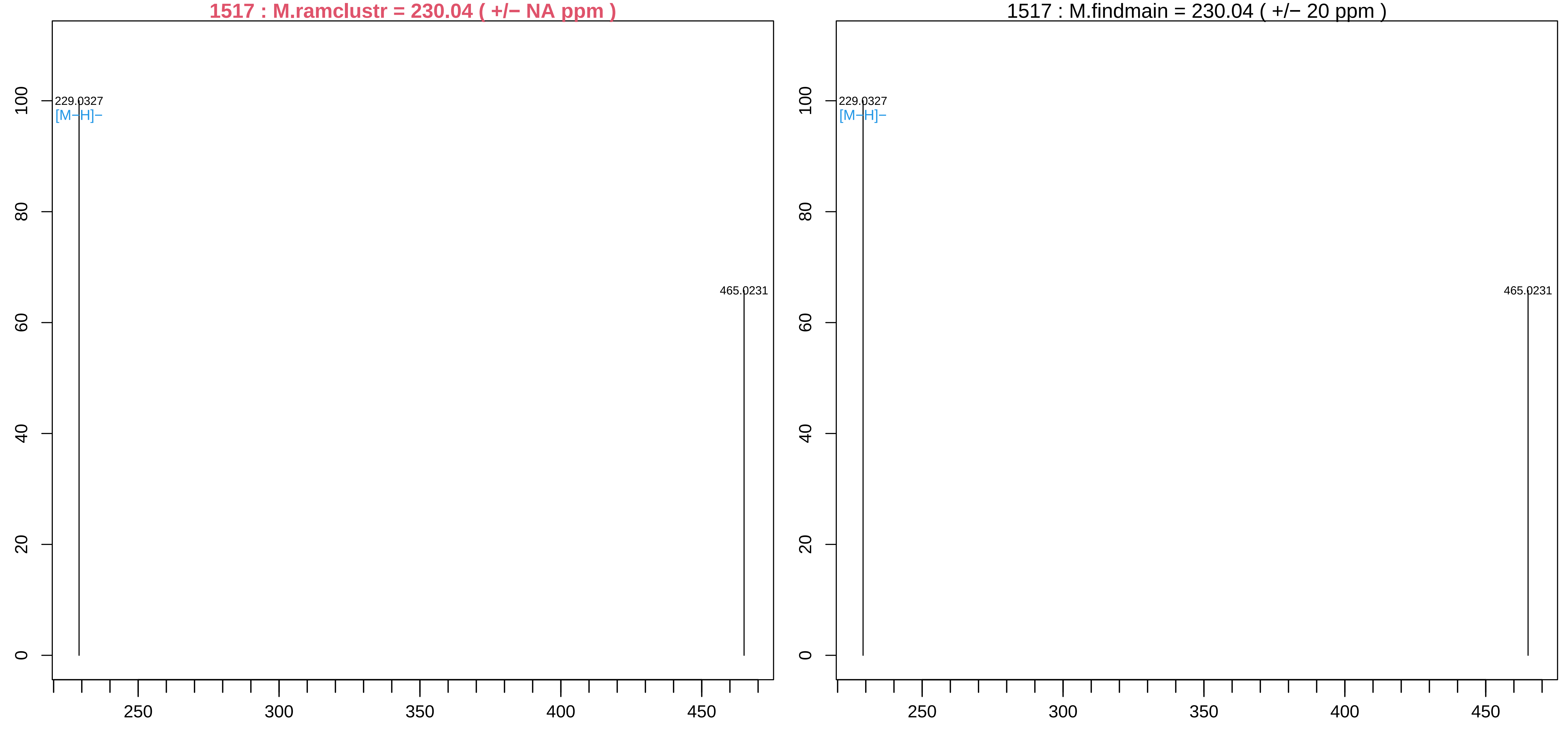

1517 : M.ramclustr = 230.04 (+/− NA ppm)
229.0327
[M−H]−
465.0231
0
20
40
60
80
100
250
300
350
400
450
1517 : M.findmain = 230.04 (+/− 20 ppm)
229.0327
[M−H]−
465.0231
0
20
40
60
80
100
250
300
350
400
450

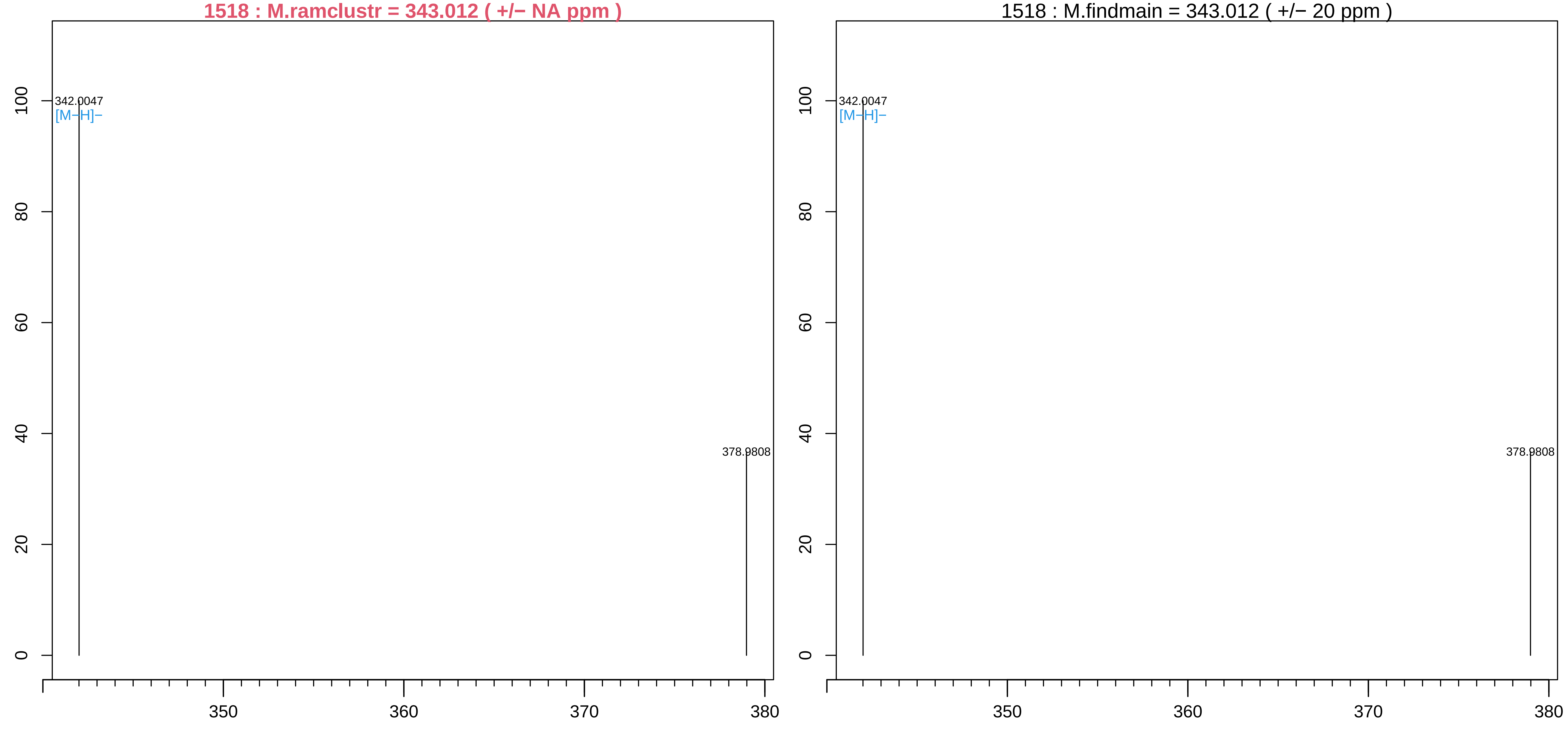
1518 : M.ramclustr = 343.012 (+/− NA ppm)
342.0047
[M−H]−
378.9808
0
20
40
60
80
100
350
360
370
380
1518 : M.findmain = 343.012 (+/− 20 ppm)
342.0047
[M−H]−
378.9808
0
20
40
60
80
100
350
360
370
380

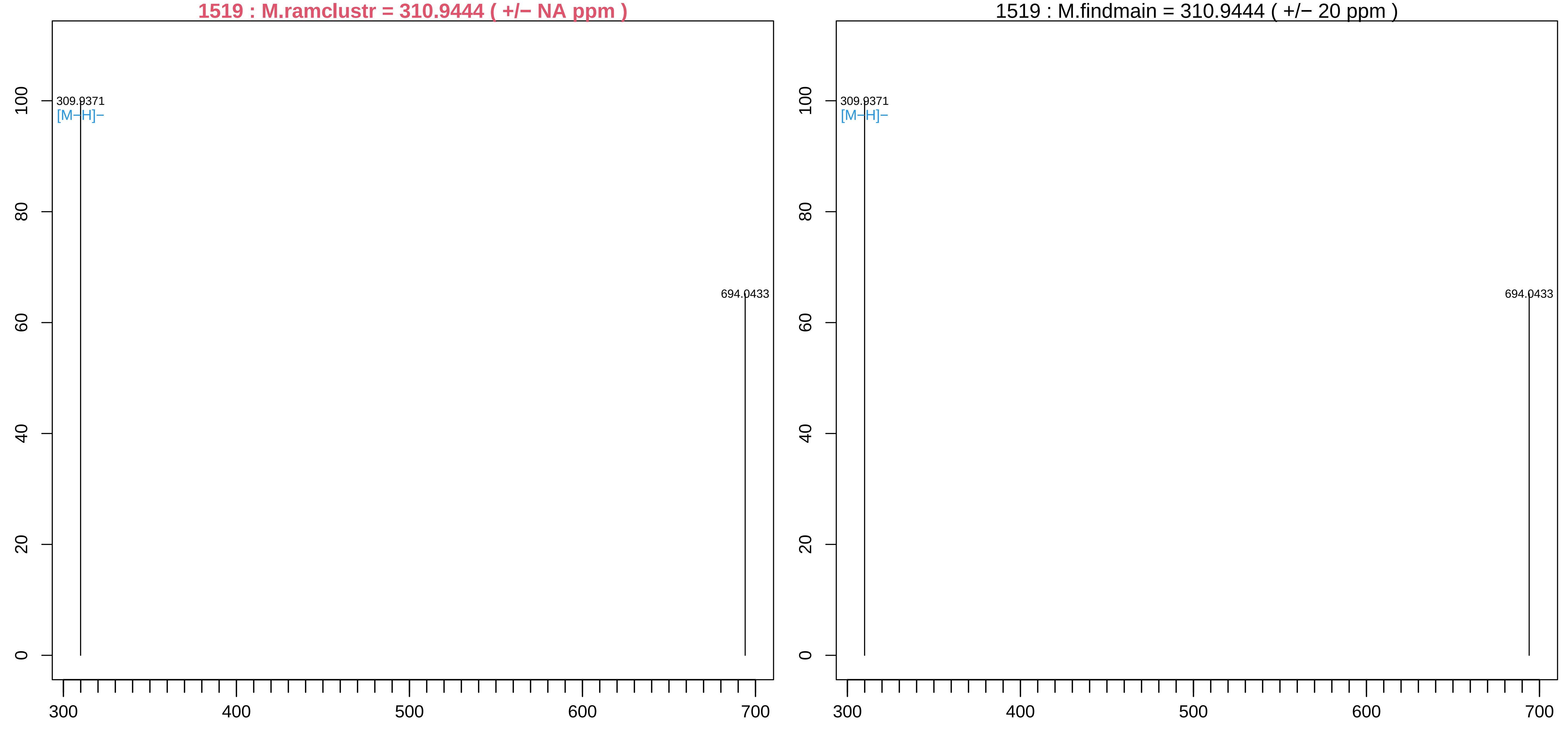
1519 : M.ramclustr = 310.9444 (+/− NA ppm)
309.9371
[M−H]−
694.0433
0
20
40
60
80
100
300
400
500
600
700
1519 : M.findmain = 310.9444 (+/− 20 ppm)
309.9371
[M−H]−
694.0433
0
20
40
60
80
100
300
400
500
600
700

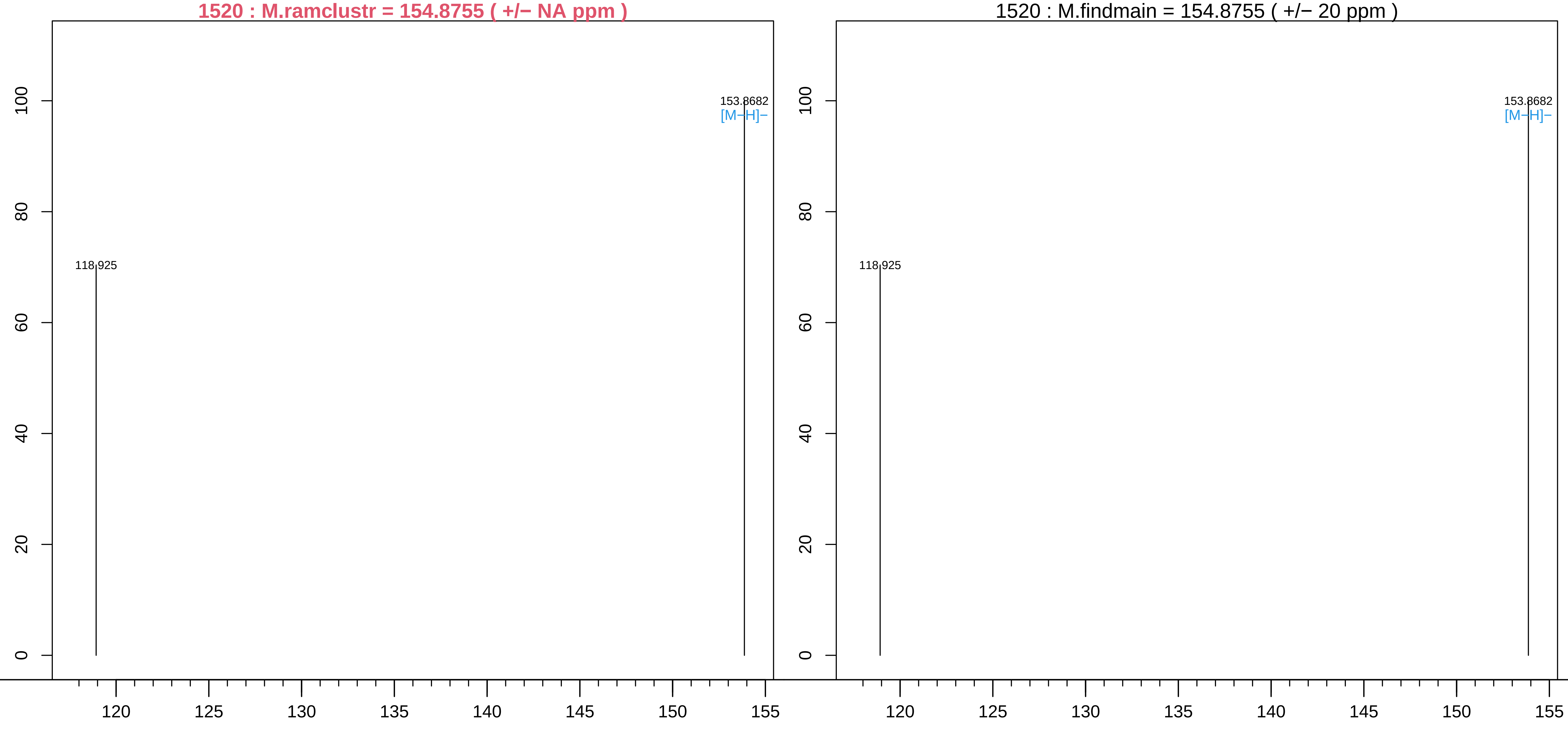
1520 : M.ramclustr = 154.8755 (+/− NA ppm)
153.8682
[M−H]−
118.925
1520 : M.findmain = 154.8755 (+/− 20 ppm)
153.8682
[M−H]−
118.925
0
20
40
60
80
100
120
125
130
135
140
145
150
155

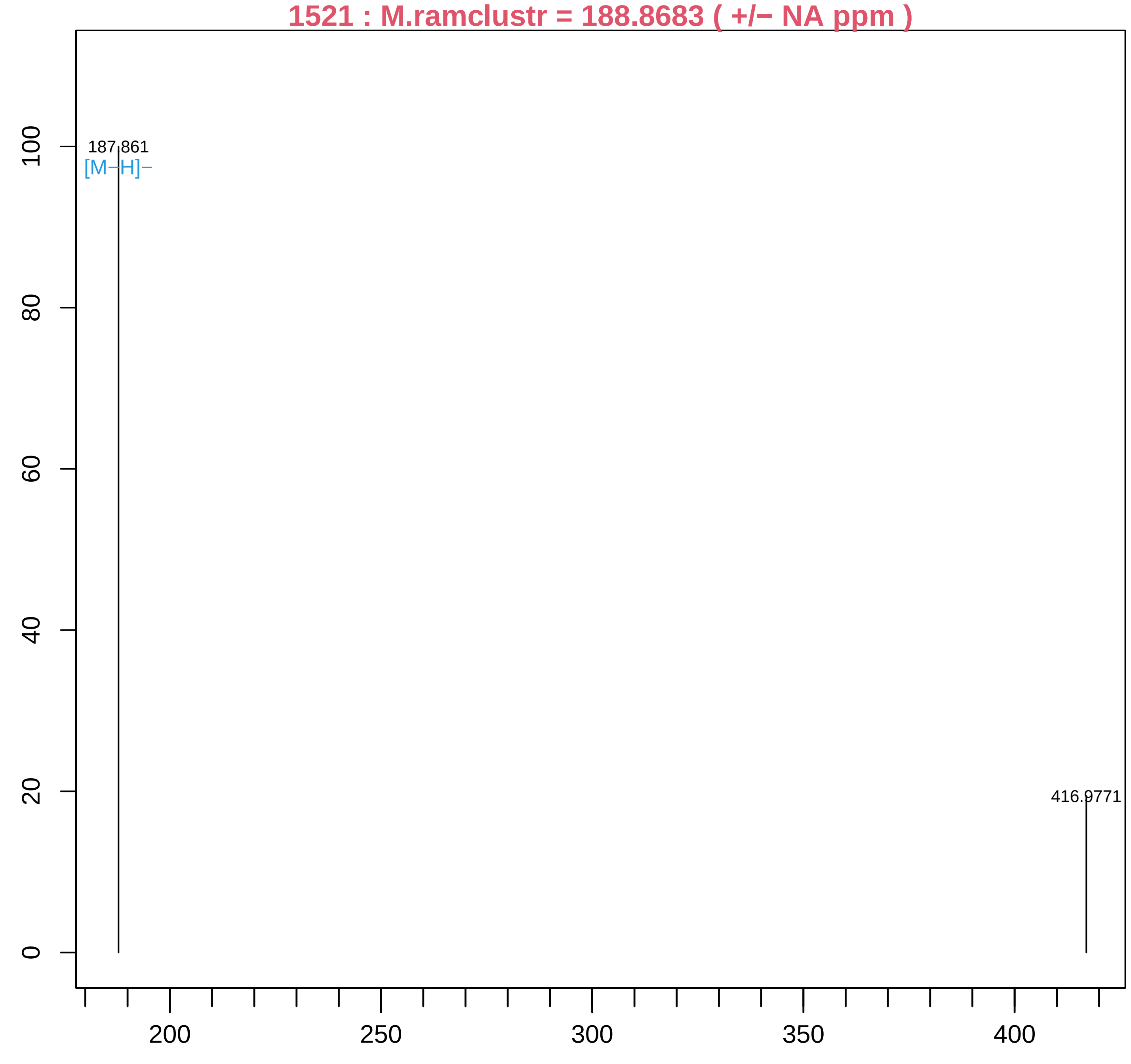
1521 : M.ramclustr = 188.8683 (+/− NA ppm)
187.861
[M−H]−
416.9771
0
20
40
60
80
100
200
250
300
350
400

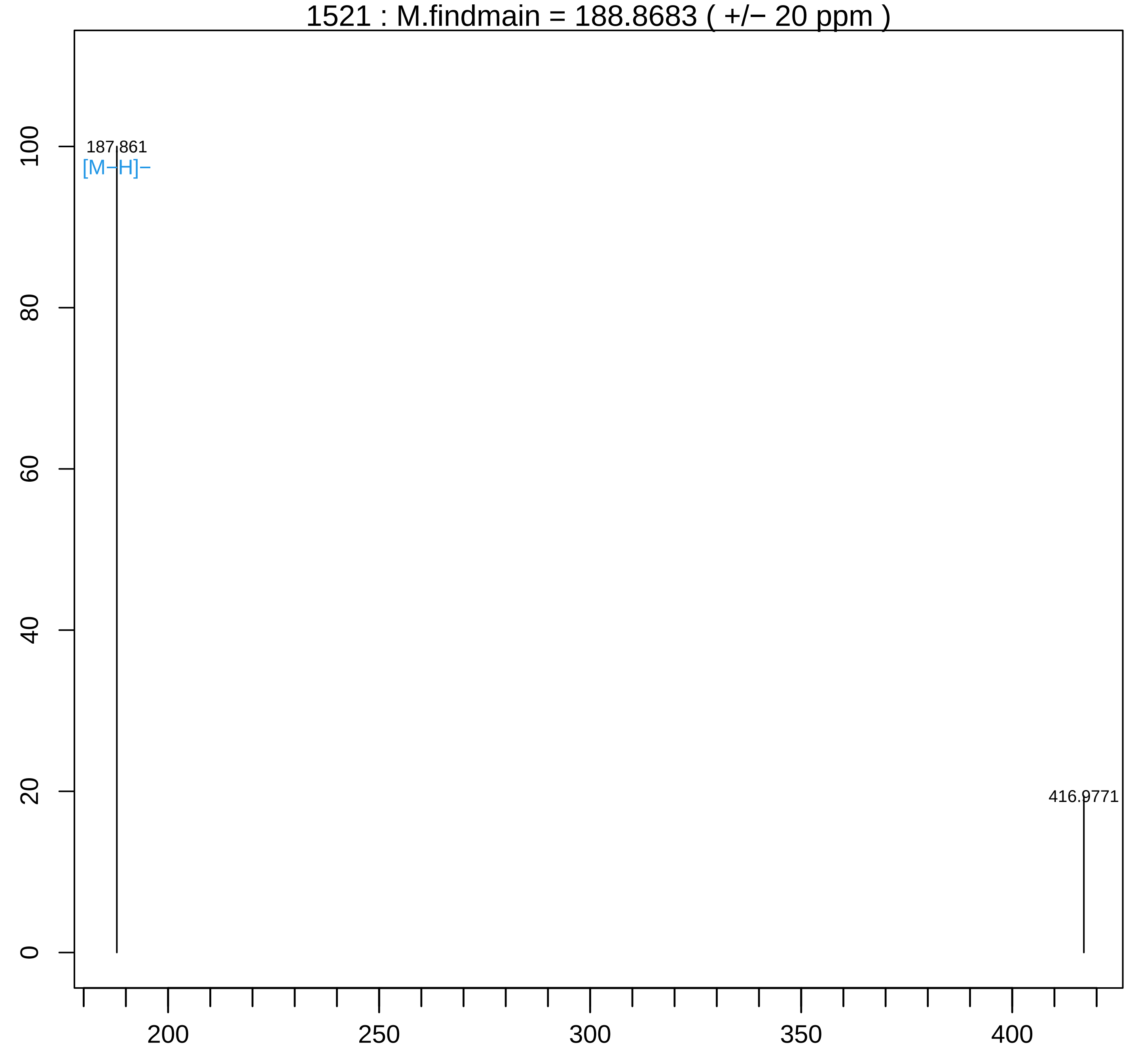
1521 : M.findmain = 188.8683 (+/− 20 ppm)
187.861
[M−H]−
416.9771
0
20
40
60
80
100
200
250
300
350
400

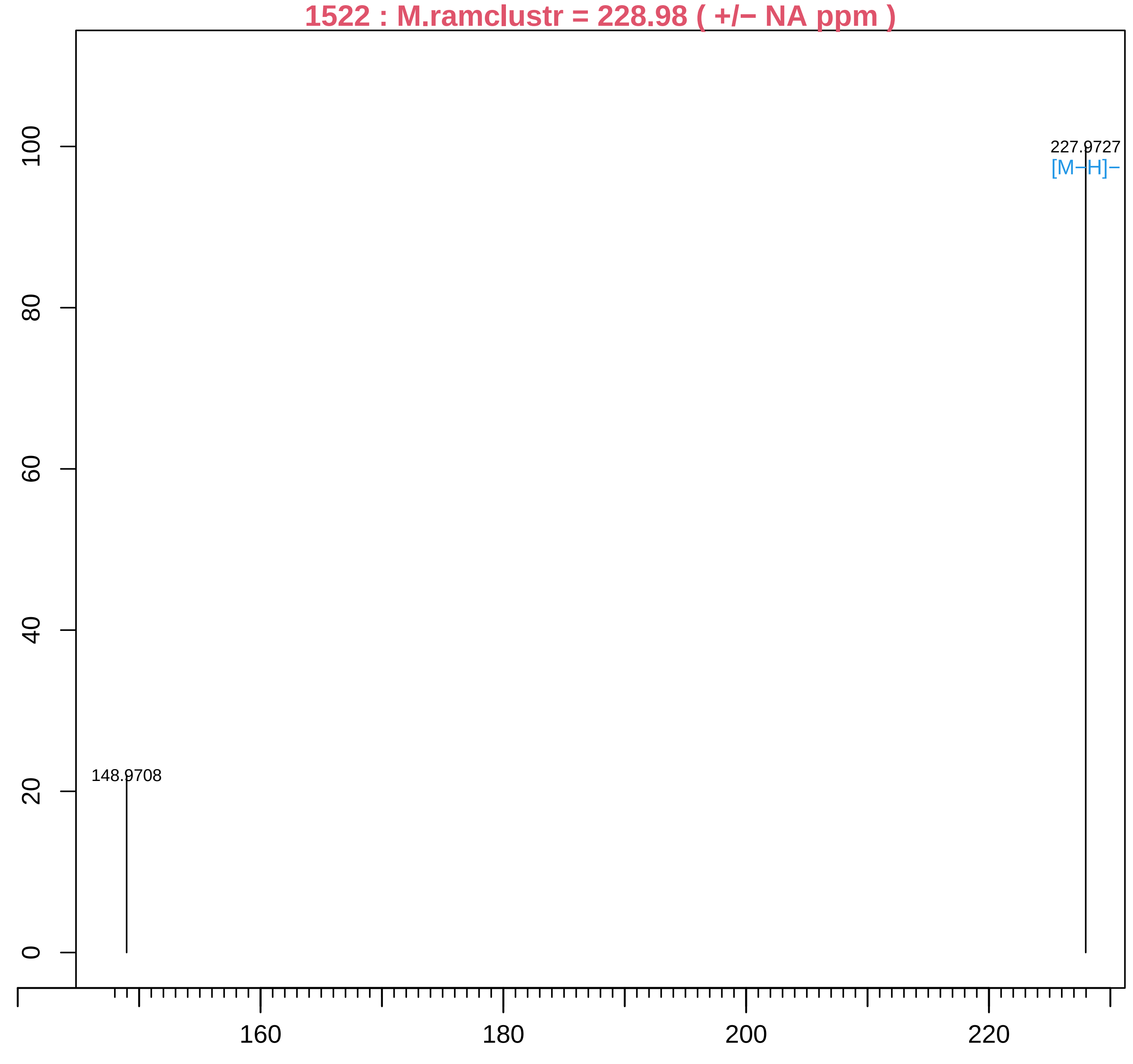

1522 : M.ramclustr = 228.98 (+/− NA ppm)
227.9727
[M−H]−
148.9708
0
20
40
60
80
100
160
180
200
220

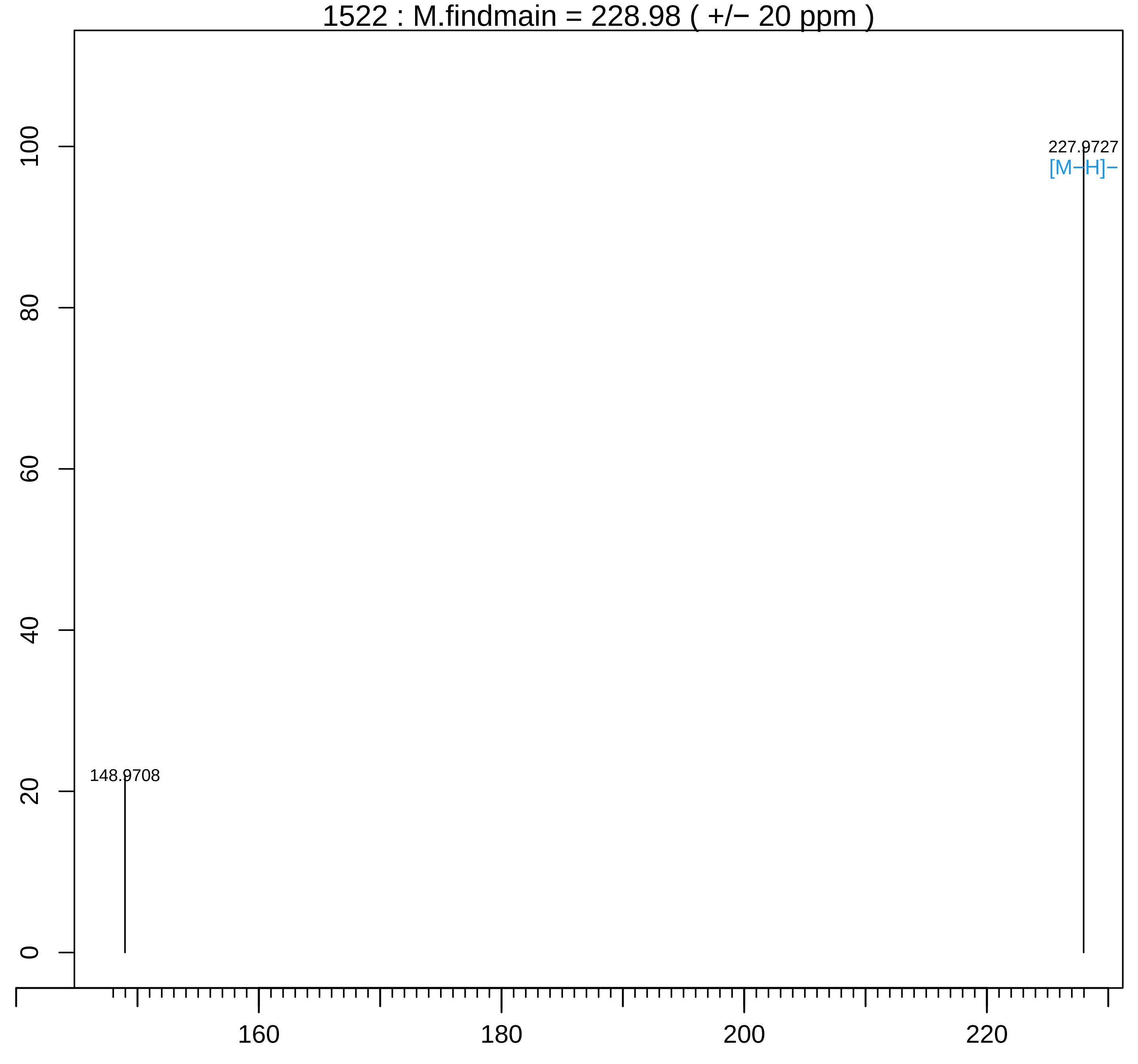

1522 : M.findmain = 228.98 (+/− 20 ppm)
227.9727
[M−H]−
148.9708
0
20
40
60
80
100
160
180
200
220

1523 : M.ramclustr = 538.0516 (+/− NA ppm)

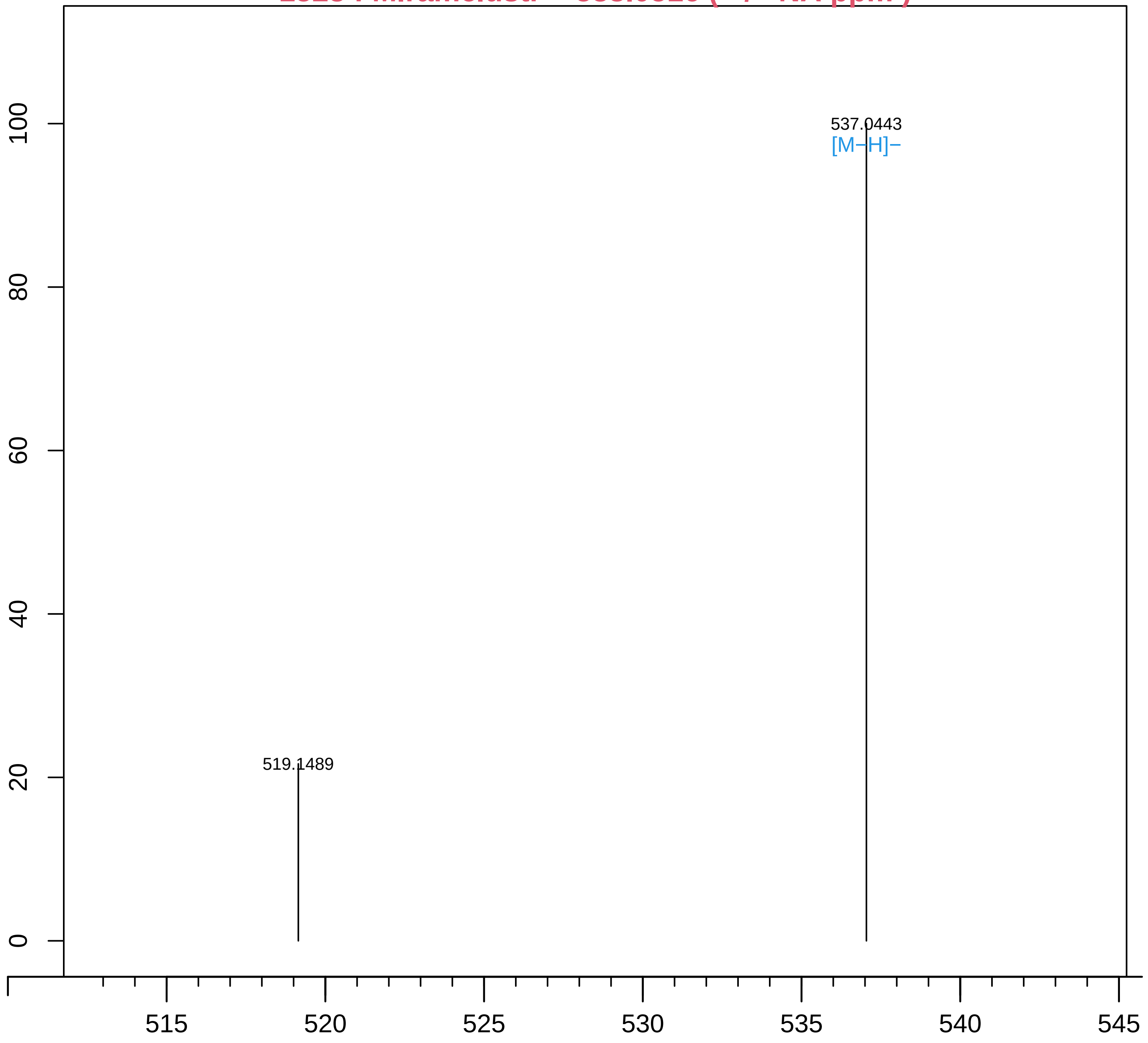

1523 : M.findmain = 538.0516 (+/− 20 ppm)

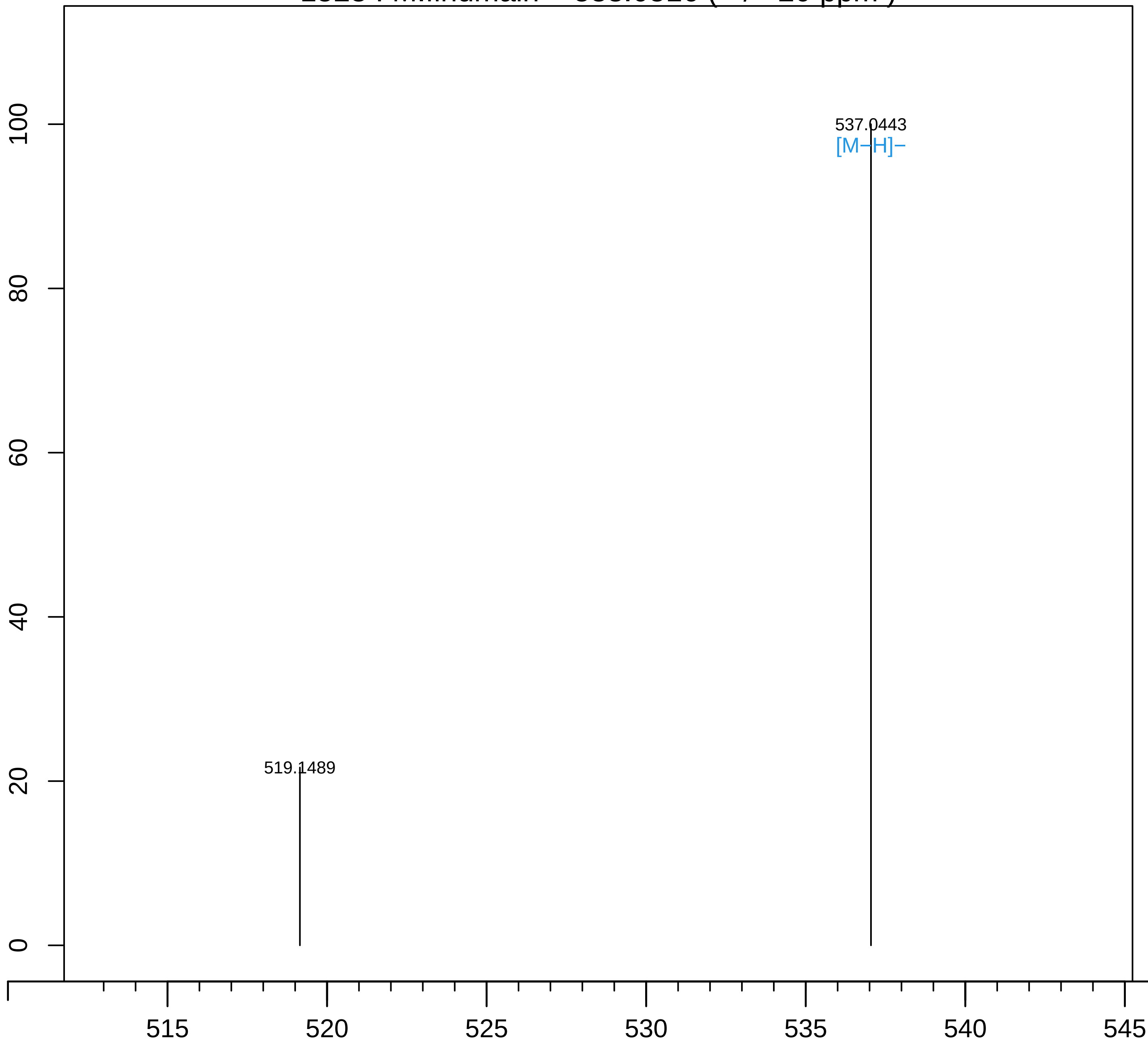

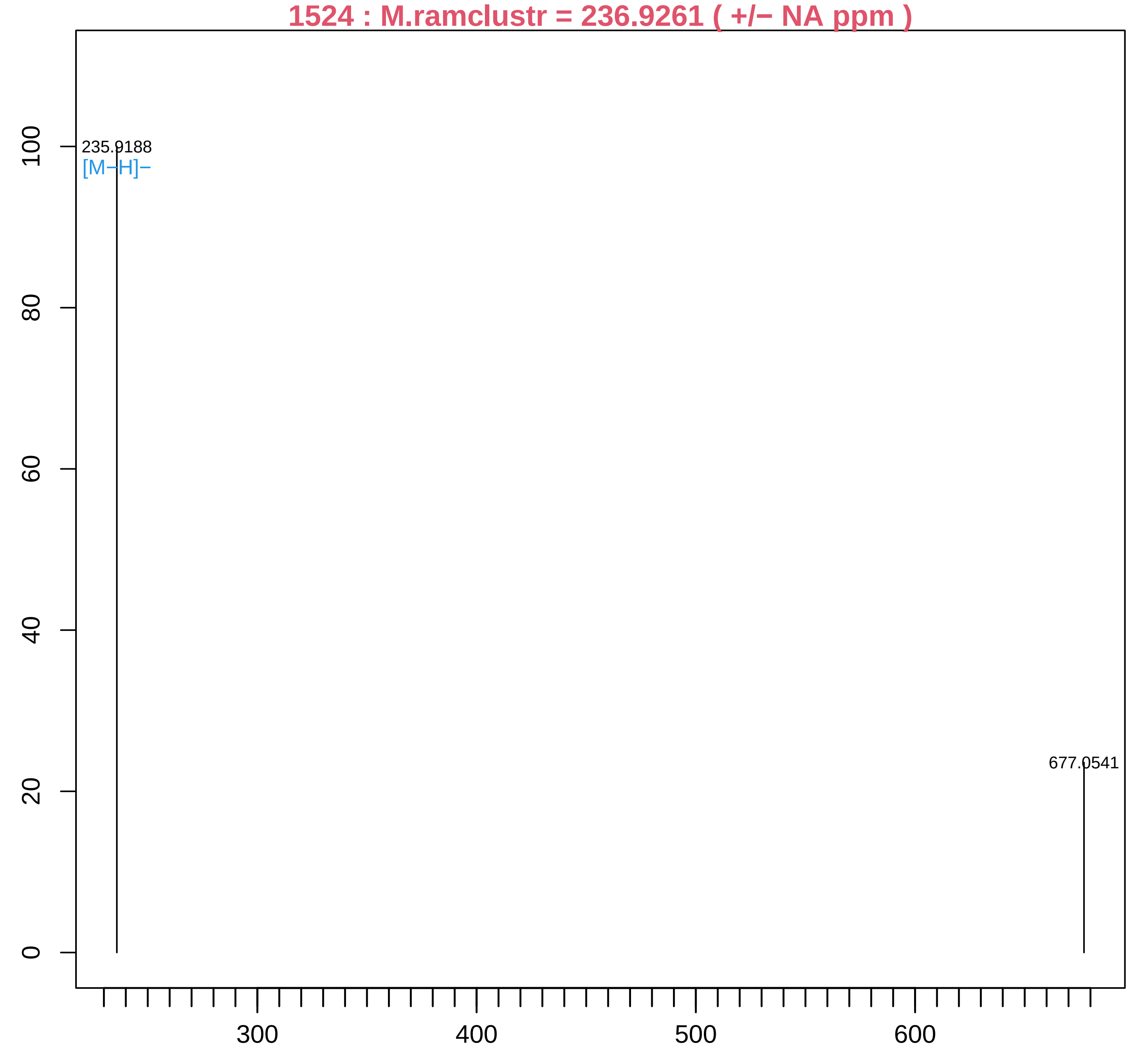
1524 : M.ramclustr = 236.9261 (+/− NA ppm)
235.9188
[M−H]−
677.0541
0
20
40
60
80
100
300
400
500
600

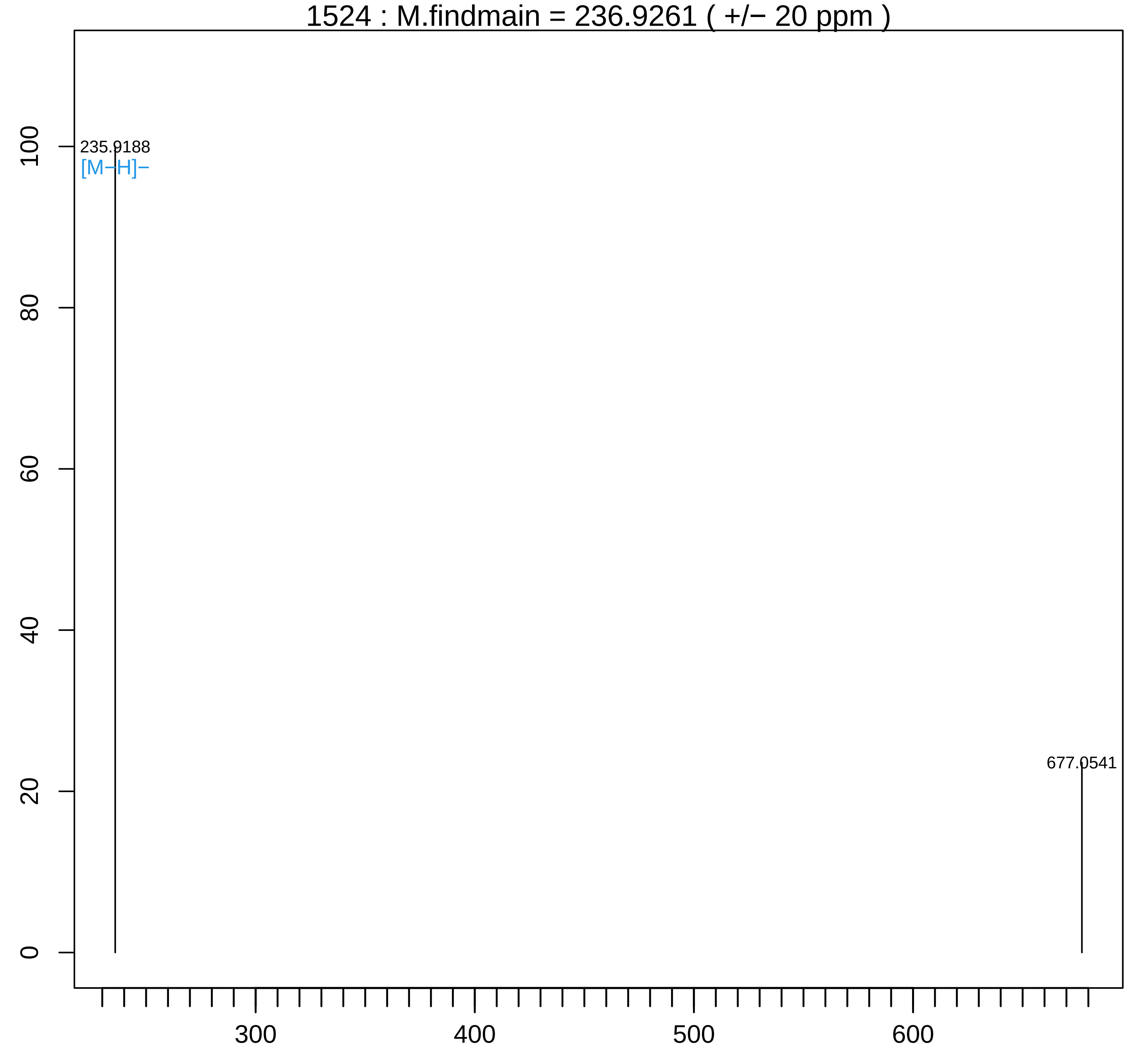
1524 : M.findmain = 236.9261 (+/− 20 ppm)
235.9188
[M−H]−
677.0541
0
20
40
60
80
100
300
400
500
600

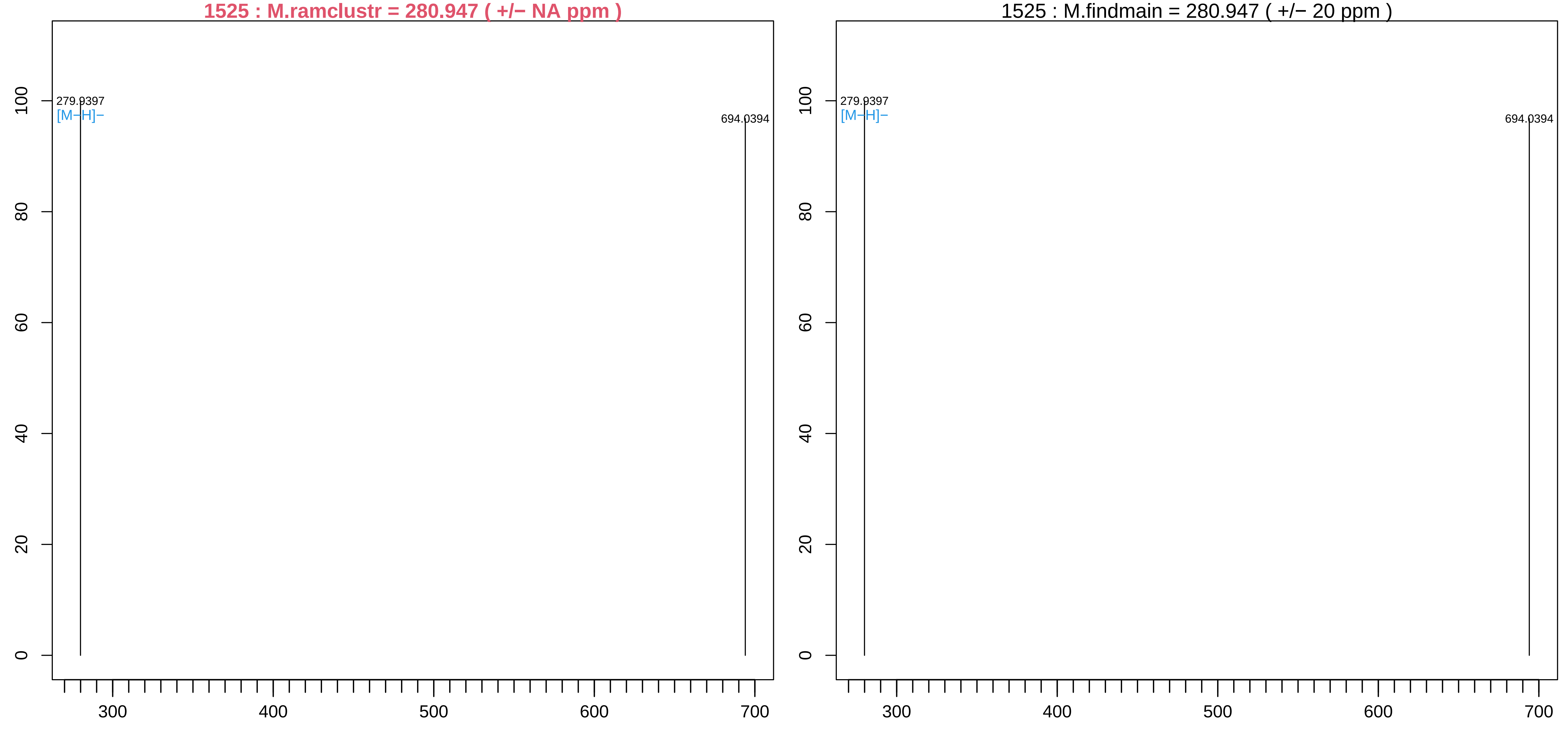

1525 : M.ramclustr = 280.947 (+/− NA ppm)
279.9397
[M−H]−
694.0394
1525 : M.findmain = 280.947 (+/− 20 ppm)
279.9397
[M−H]−
694.0394
0
20
40
60
80
100
300
400
500
600
700

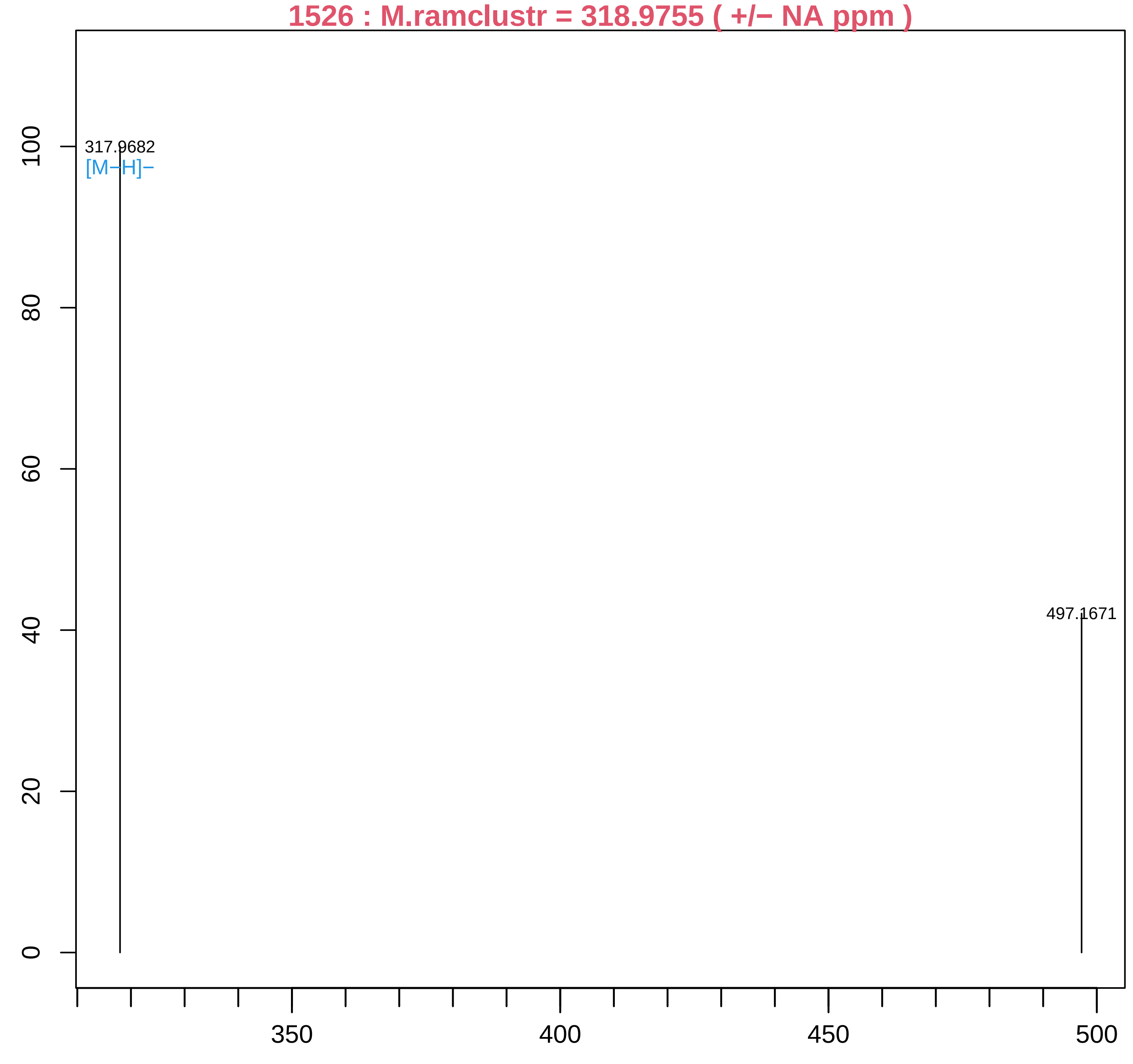
1526 : M.ramclustr = 318.9755 (+/− NA ppm)
317.9682
[M−H]−
497.1671
0
20
40
60
80
100
350
400
450
500

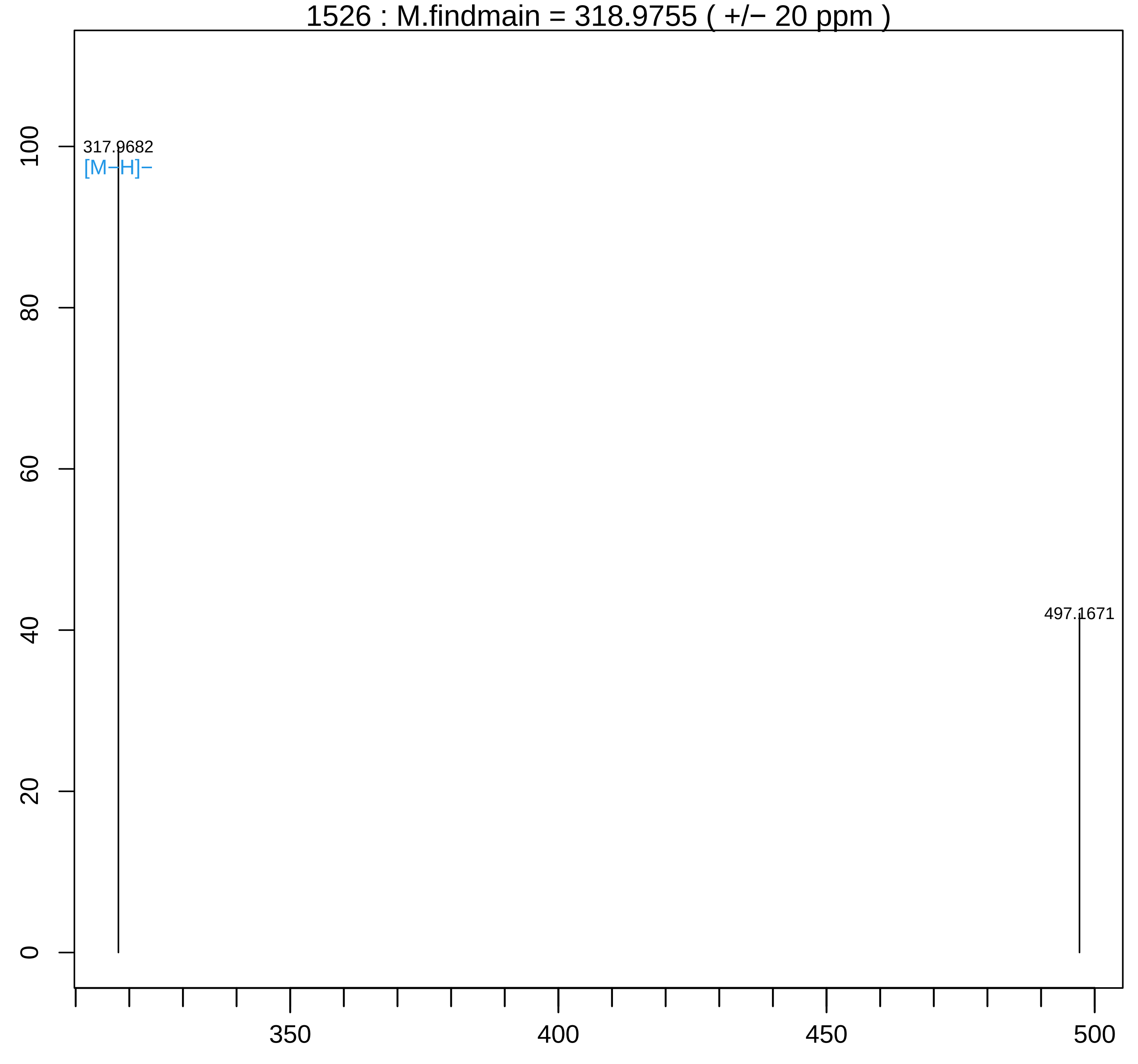
1526 : M.findmain = 318.9755 (+/− 20 ppm)
317.9682
[M−H]−
497.1671
0
20
40
60
80
100
350
400
450
500

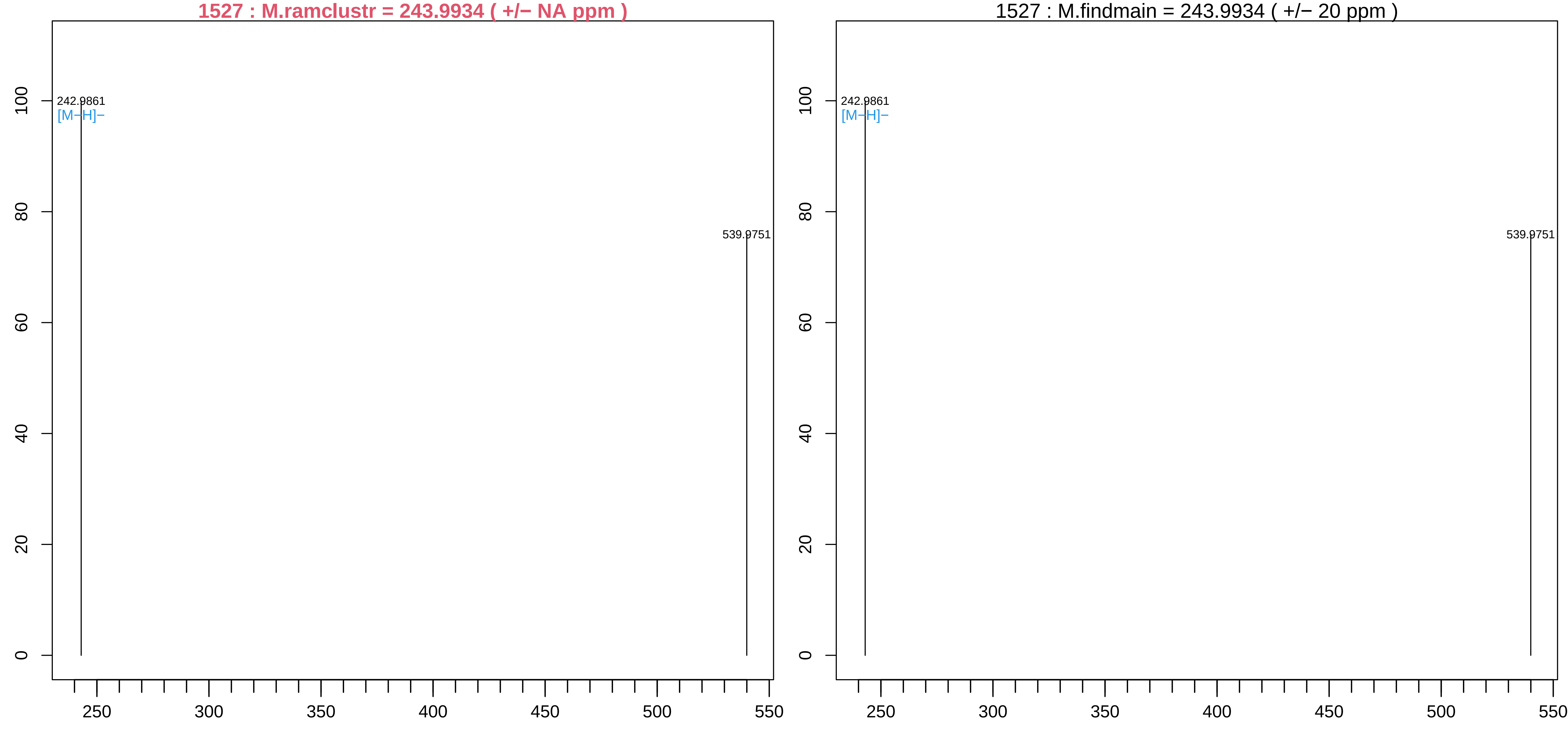
1527 : M.ramclustr = 243.9934 (+/− NA ppm)
242.9861
[M−H]−
539.9751
1527 : M.findmain = 243.9934 (+/− 20 ppm)
242.9861
[M−H]−
539.9751
0
20
40
60
80
100
250
300
350
400
450
500
550

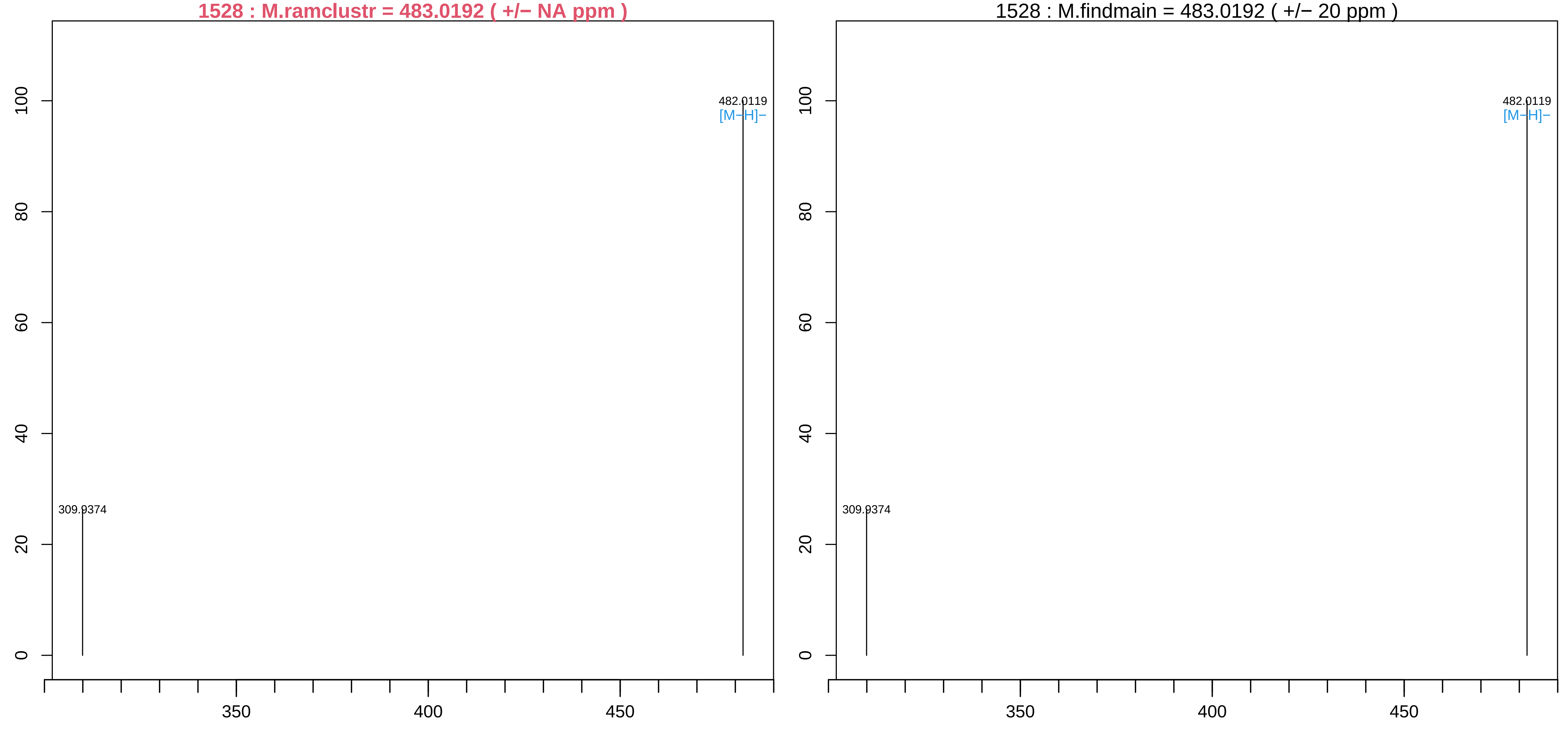
1528 : M.ramclustr = 483.0192 (+/− NA ppm)
1528 : M.findmain = 483.0192 (+/− 20 ppm)
482.0119
[M−H]−
309.9374
0
20
40
60
80
100
350
400
450

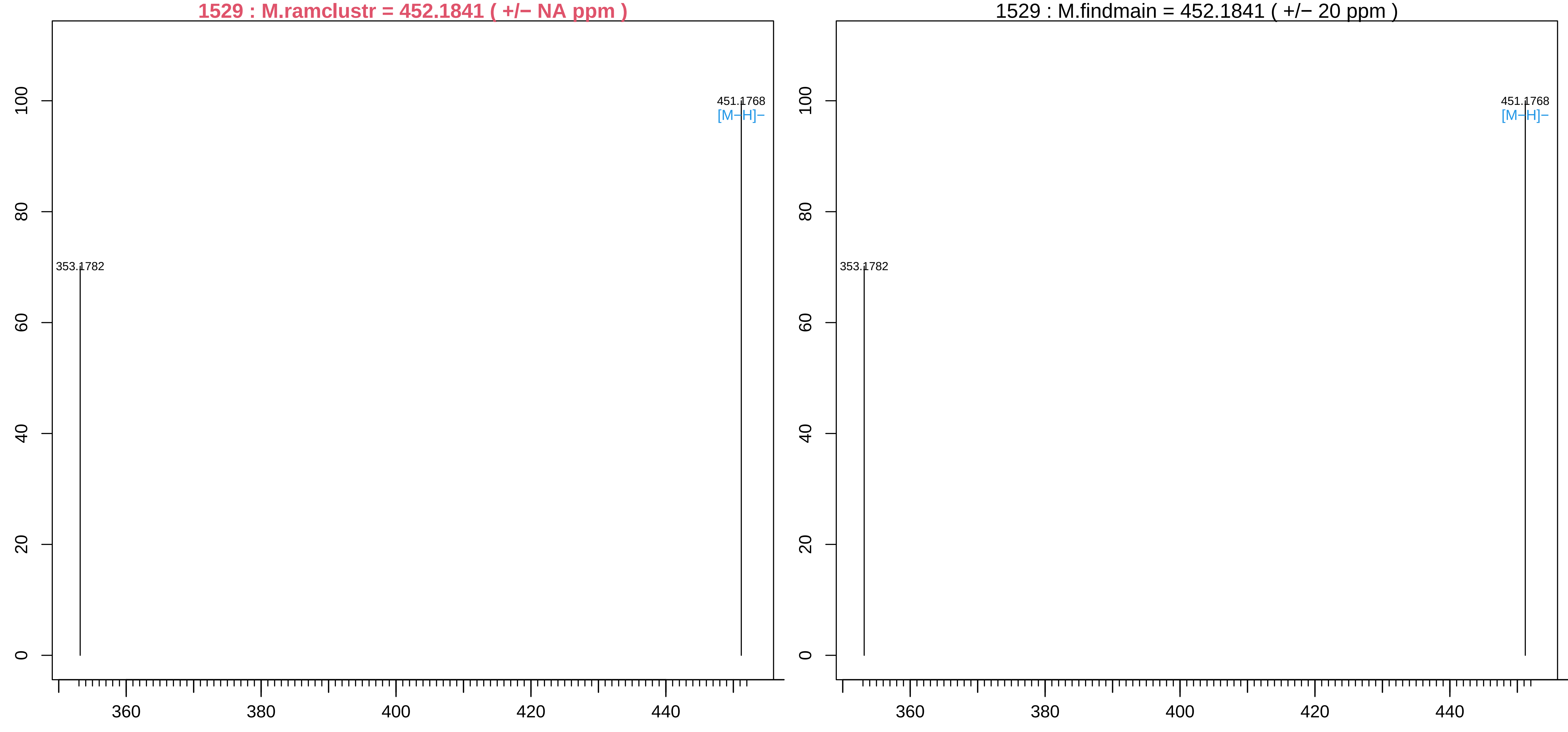

1529 : M.ramclustr = 452.1841 (+/− NA ppm)
1529 : M.findmain = 452.1841 (+/− 20 ppm)
451.1768
[M−H]−
353.1782
0
20
40
60
80
100
360
380
400
420
440

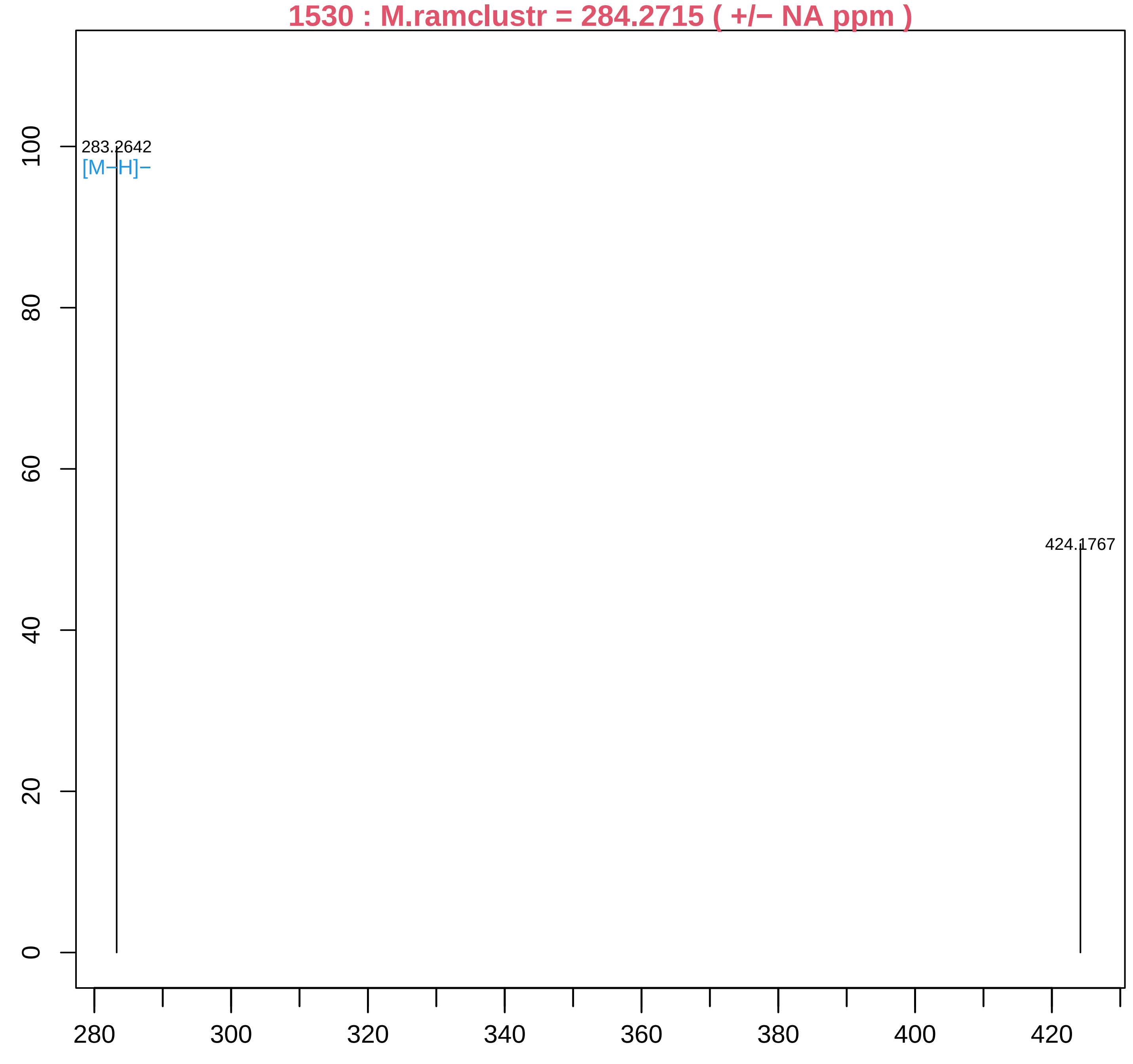

1530 : M.ramclustr = 284.2715 (+/− NA ppm)
283.2642
[M−H]−
424.1767
0
20
40
60
80
100
280
300
320
340
360
380
400
420

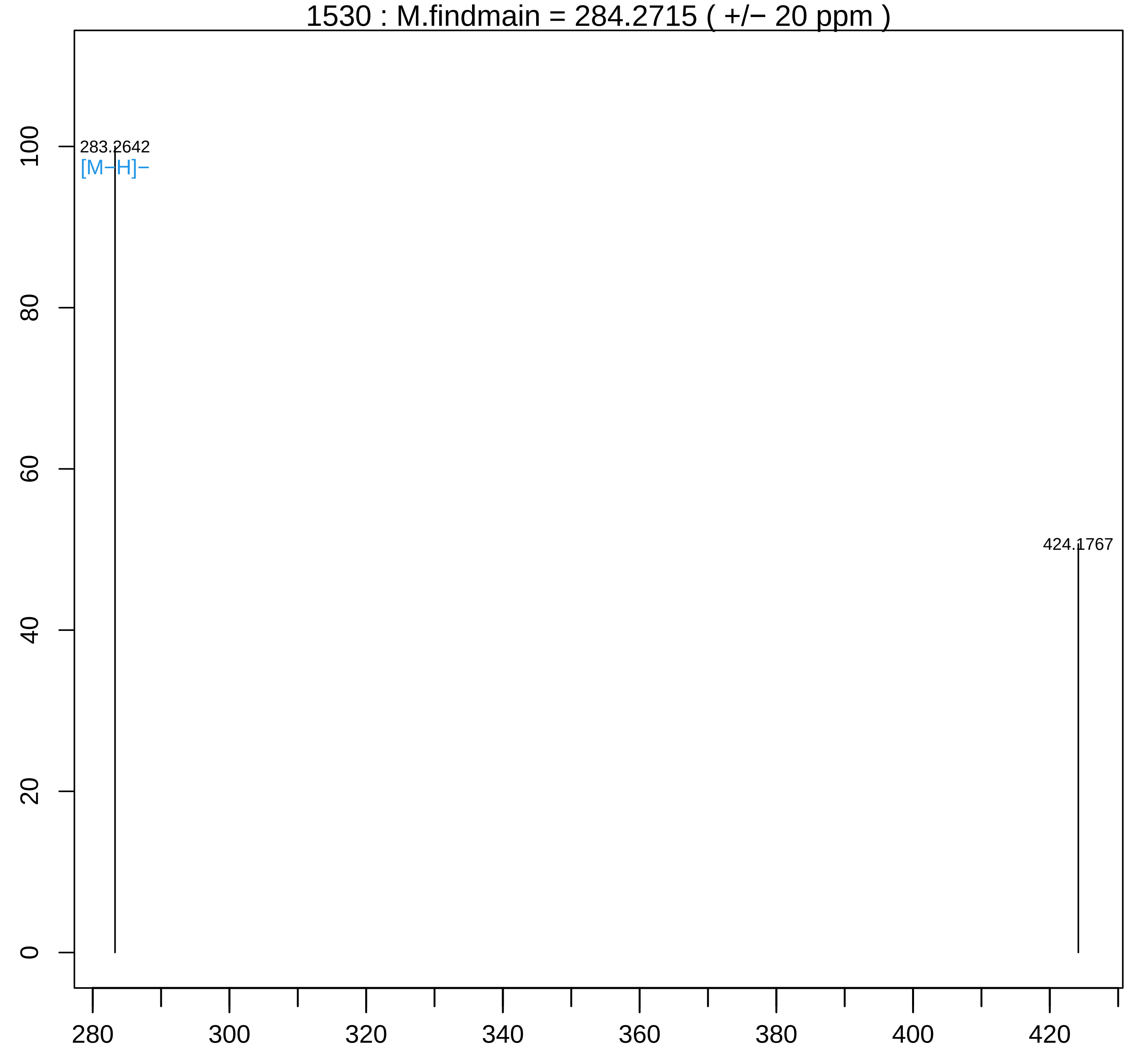

1530 : M.findmain = 284.2715 (+/− 20 ppm)
283.2642
[M−H]−
424.1767
0
20
40
60
80
100
280
300
320
340
360
380
400
420

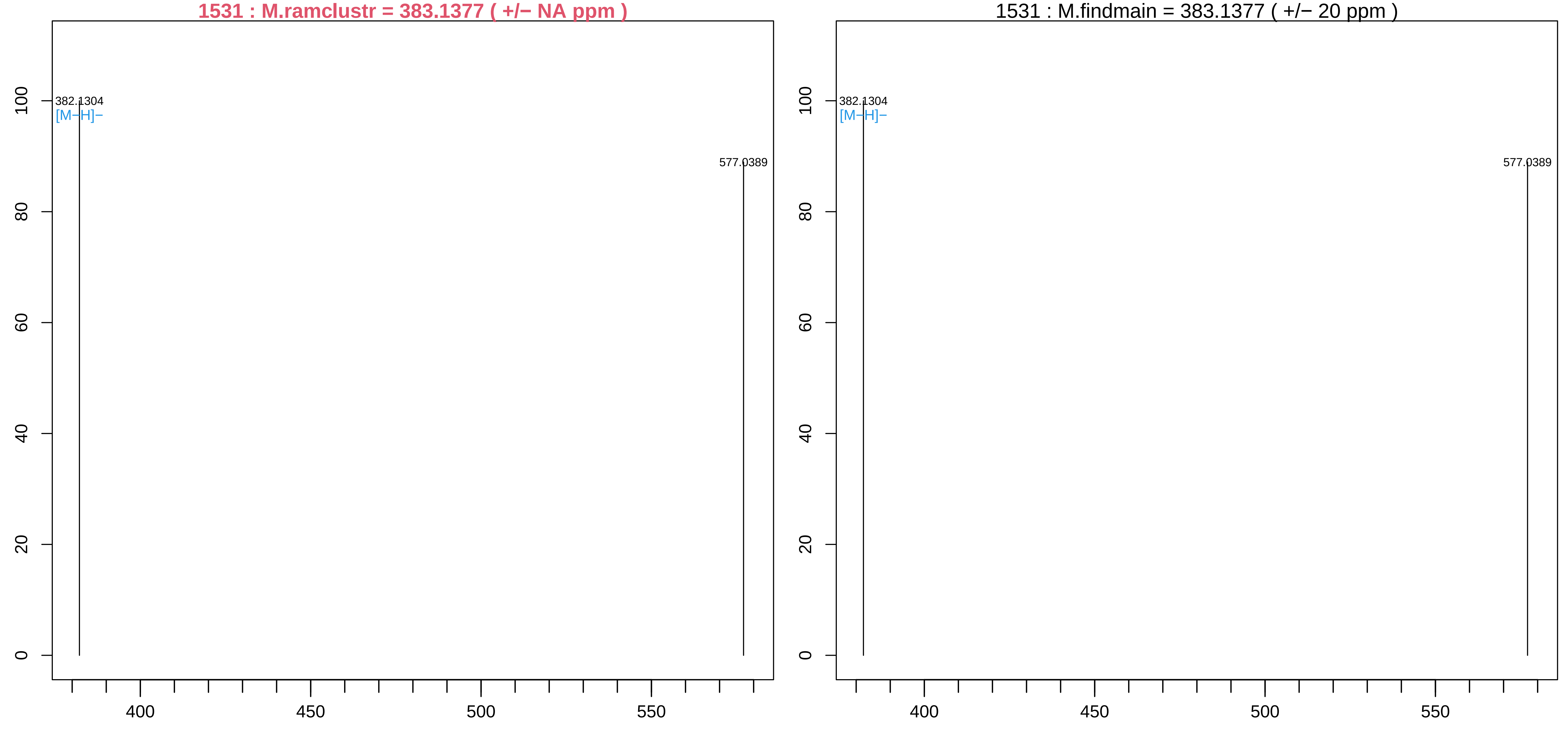
1531 : M.ramclustr = 383.1377 (+/− NA ppm)
382.1304
[M−H]−
577.0389
1531 : M.findmain = 383.1377 (+/− 20 ppm)
382.1304
[M−H]−
577.0389
0
20
40
60
80
100
400
450
500
550

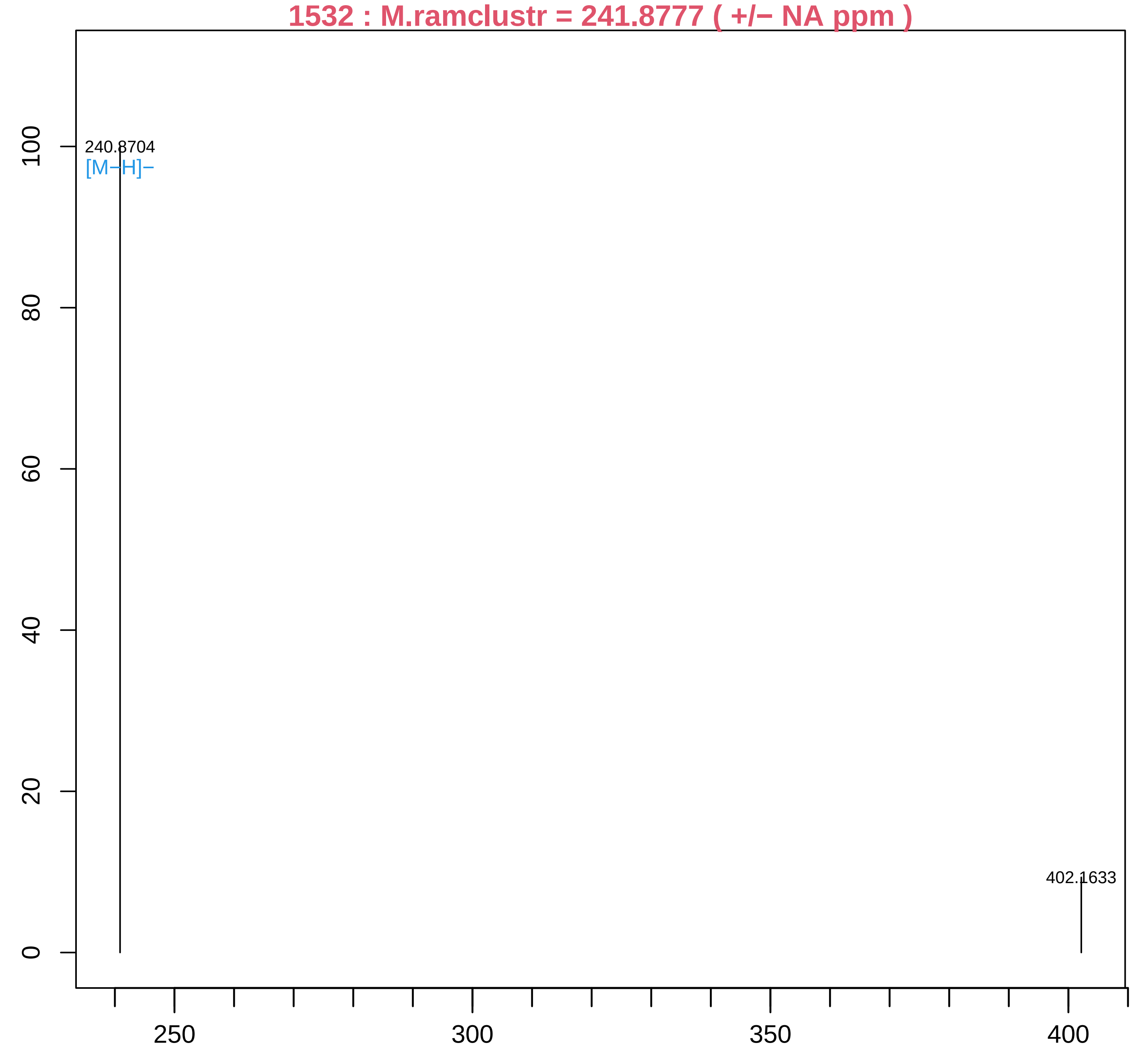
1532 : M.ramclustr = 241.8777 (+/− NA ppm)
240.8704
[M−H]−
402.1633
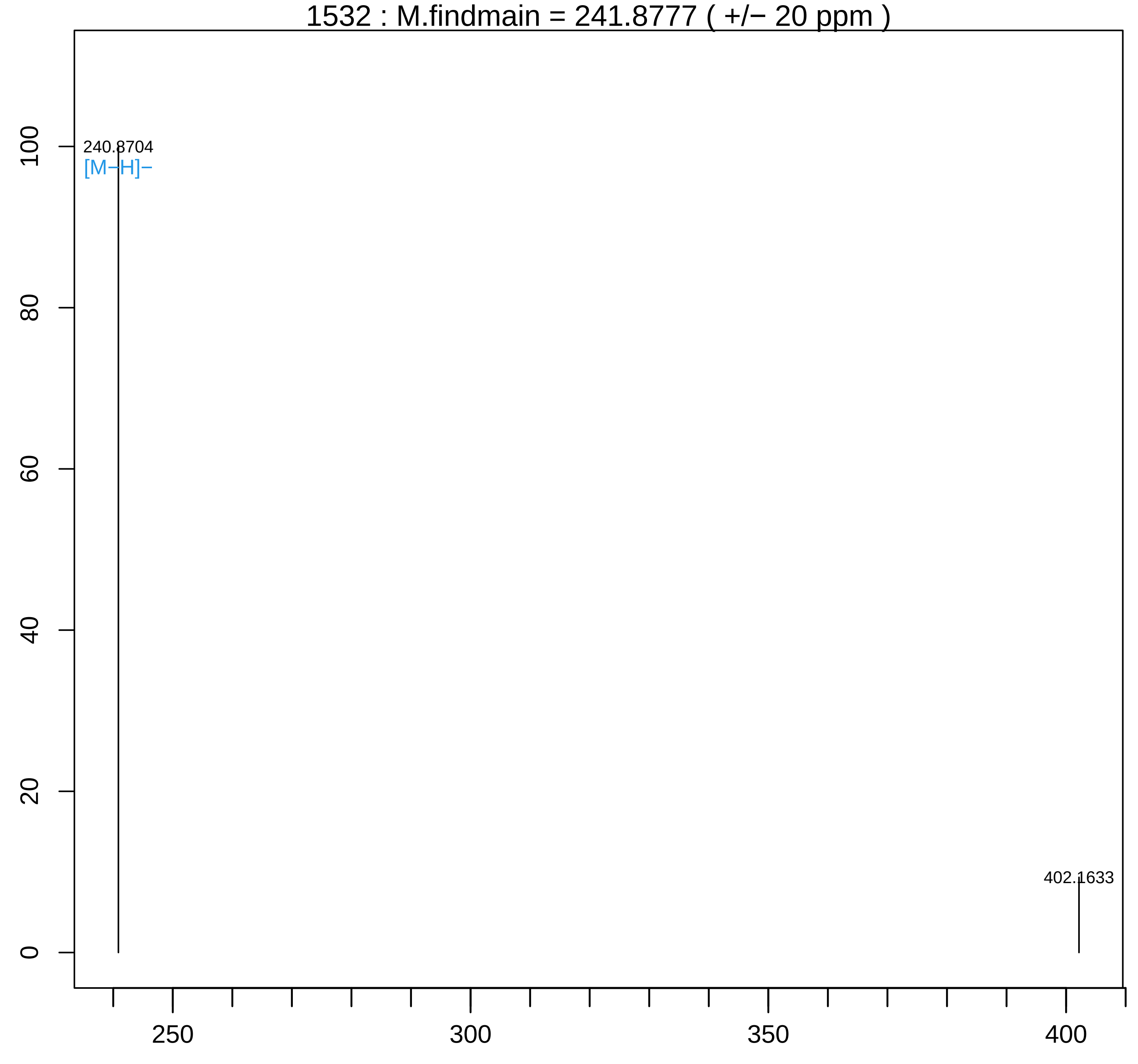
1532 : M.findmain = 241.8777 (+/− 20 ppm)
240.8704
[M−H]−
402.1633

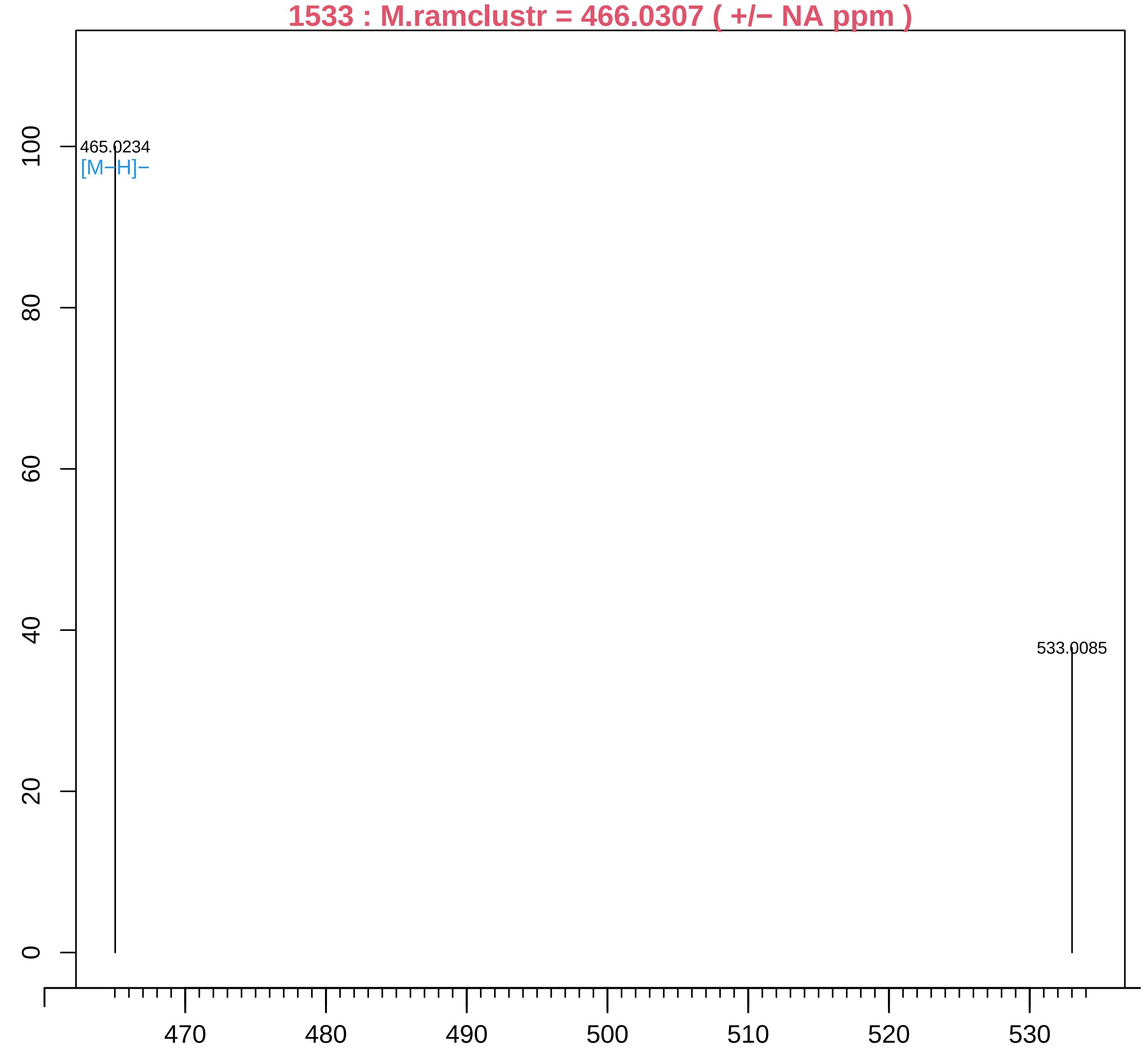

1533 : M.ramclustr = 466.0307 (+/− NA ppm)
465.0234
[M−H]−
533.0085
0
20
40
60
80
100
470
480
490
500
510
520
530

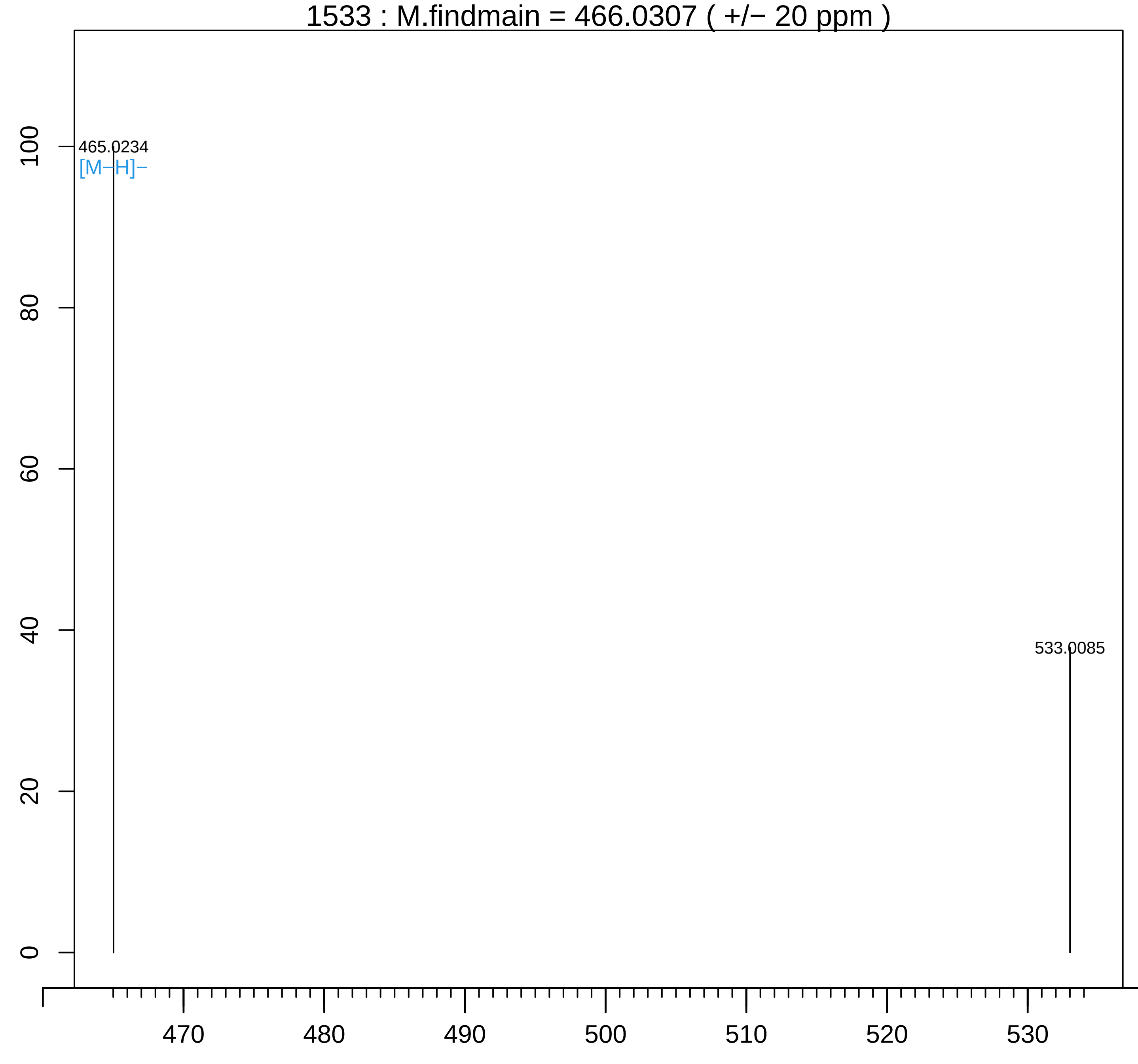

1533 : M.findmain = 466.0307 (+/− 20 ppm)
465.0234
[M−H]−
533.0085
0
20
40
60
80
100
470
480
490
500
510
520
530

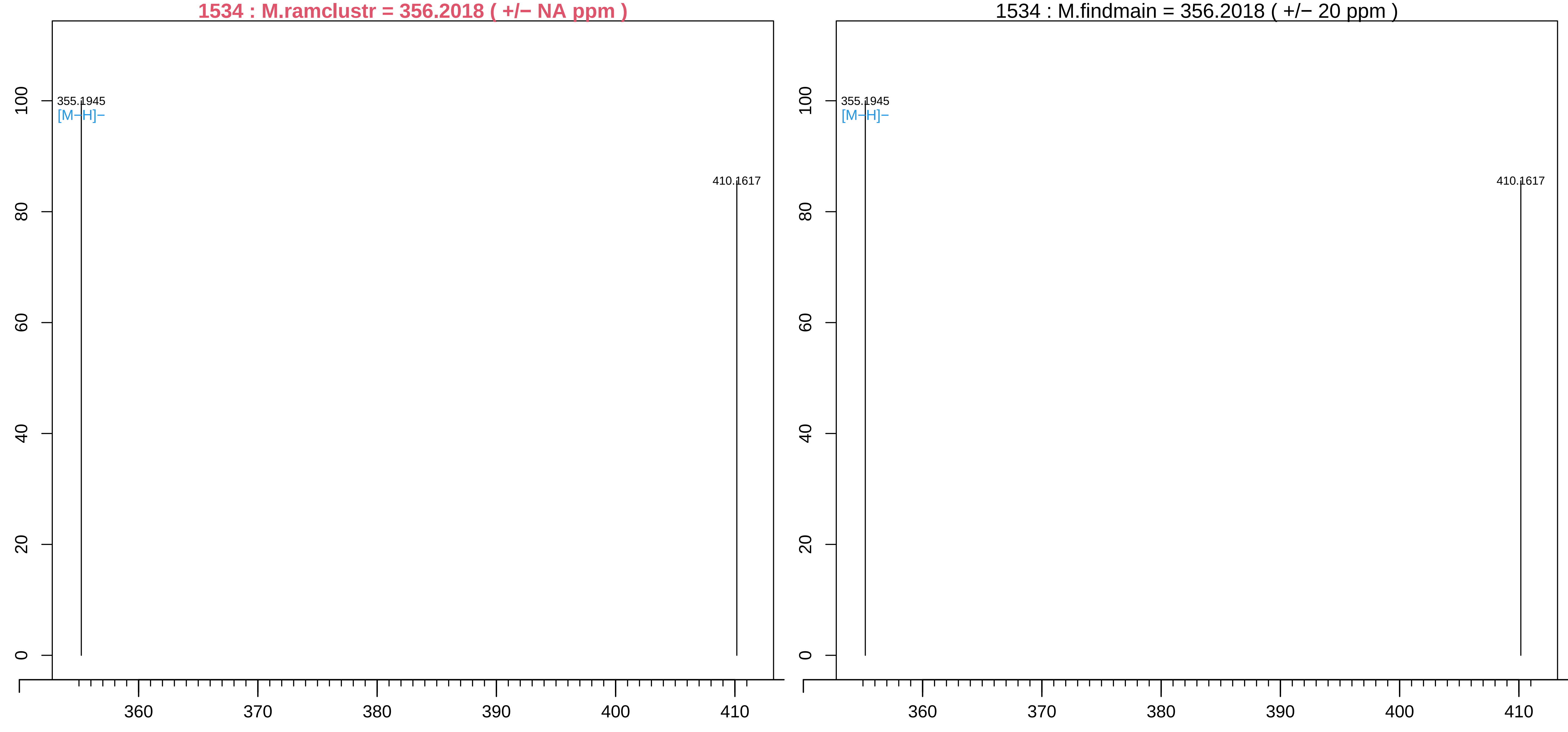
1534 : M.ramclustr = 356.2018 (+/− NA ppm)
355.1945
[M−H]−
410.1617
1534 : M.findmain = 356.2018 (+/− 20 ppm)
355.1945
[M−H]−
410.1617
0
20
40
60
80
100
360
370
380
390
400
410

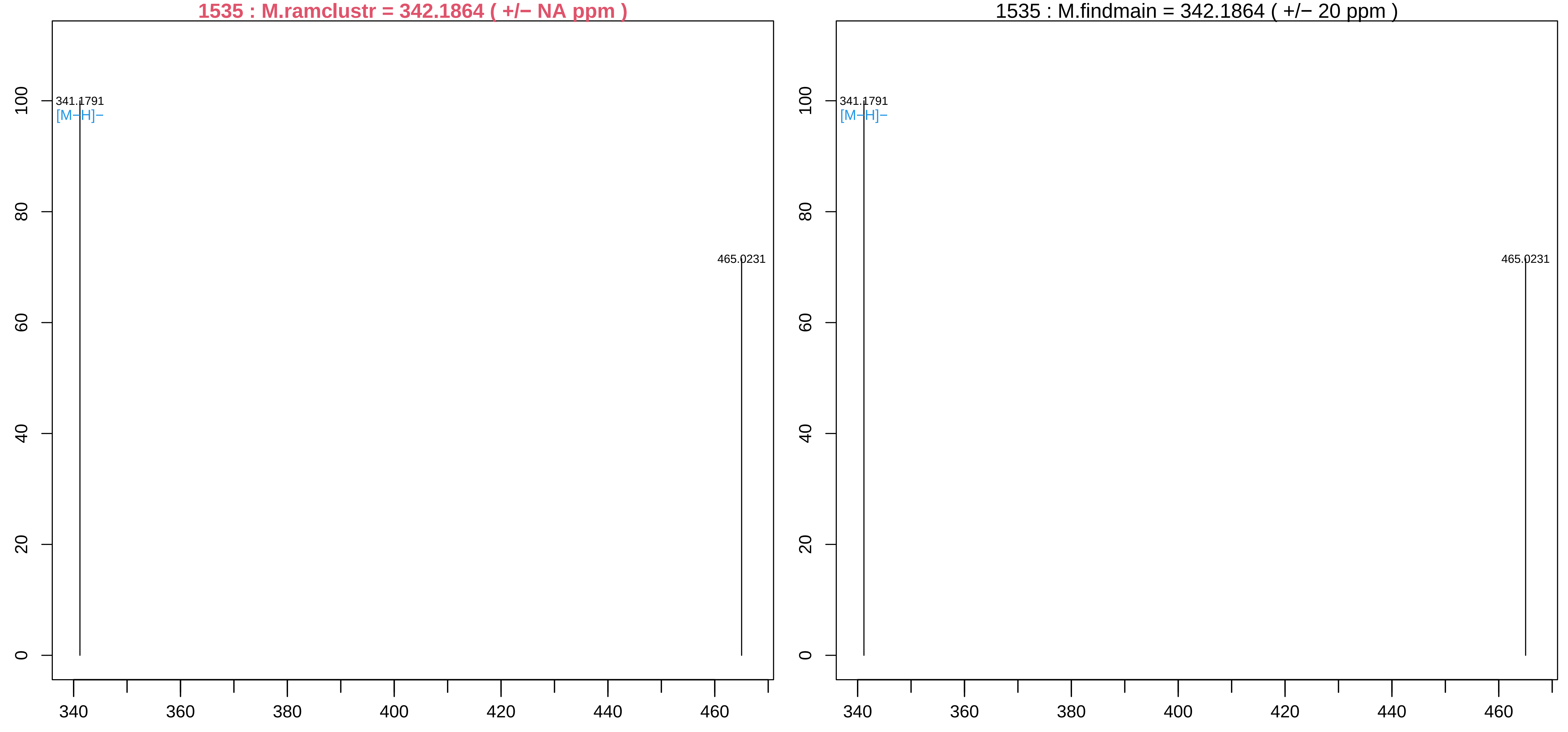
1535 : M.ramclustr = 342.1864 (+/− NA ppm)
341.1791
[M−H]−
465.0231
1535 : M.findmain = 342.1864 (+/− 20 ppm)
341.1791
[M−H]−
465.0231
0
20
40
60
80
100
340
360
380
400
420
440
460

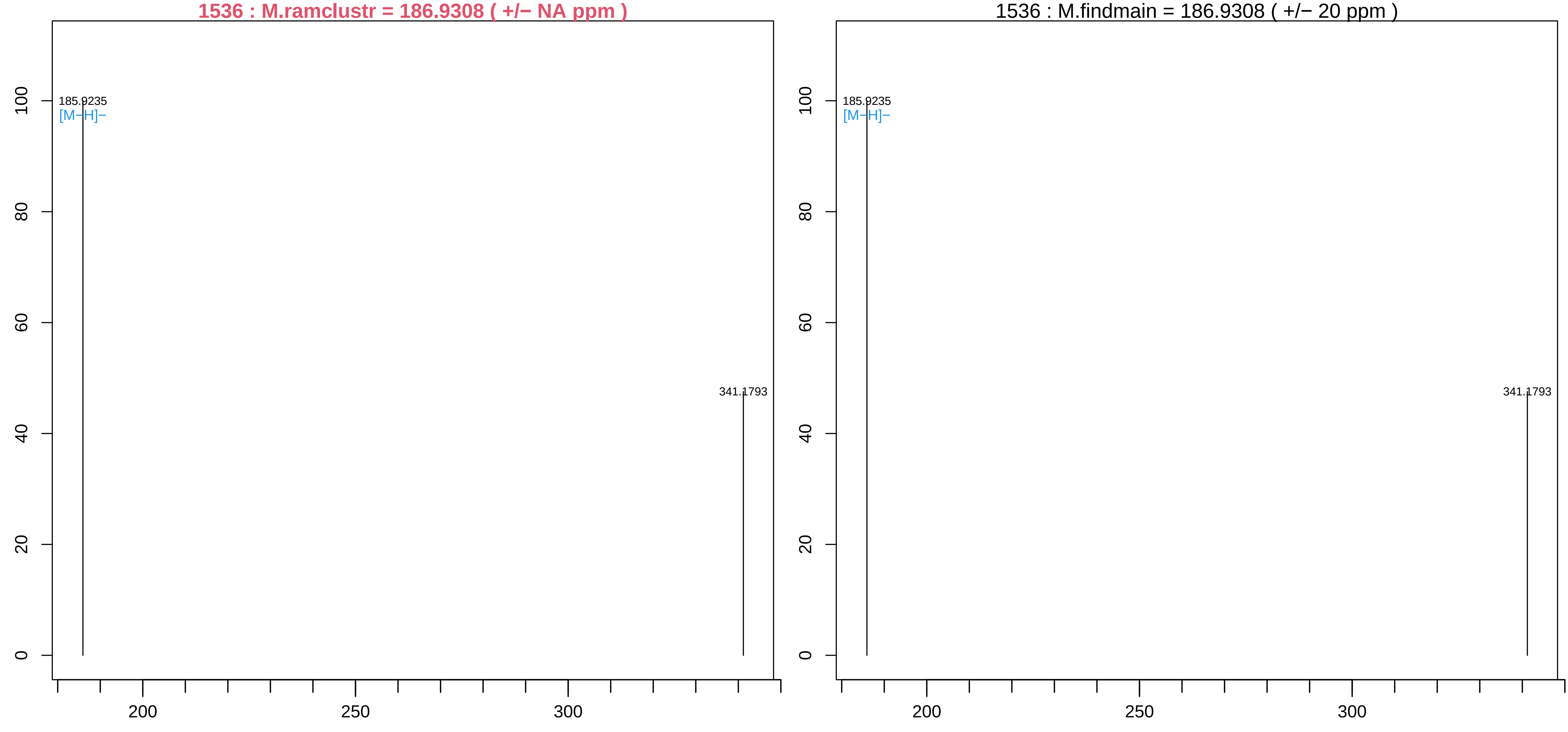
1536 : M.ramclustr = 186.9308 (+/− NA ppm)
1536 : M.findmain = 186.9308 (+/− 20 ppm)
185.9235
[M−H]−
341.1793
0
20
40
60
80
100
200
250
300

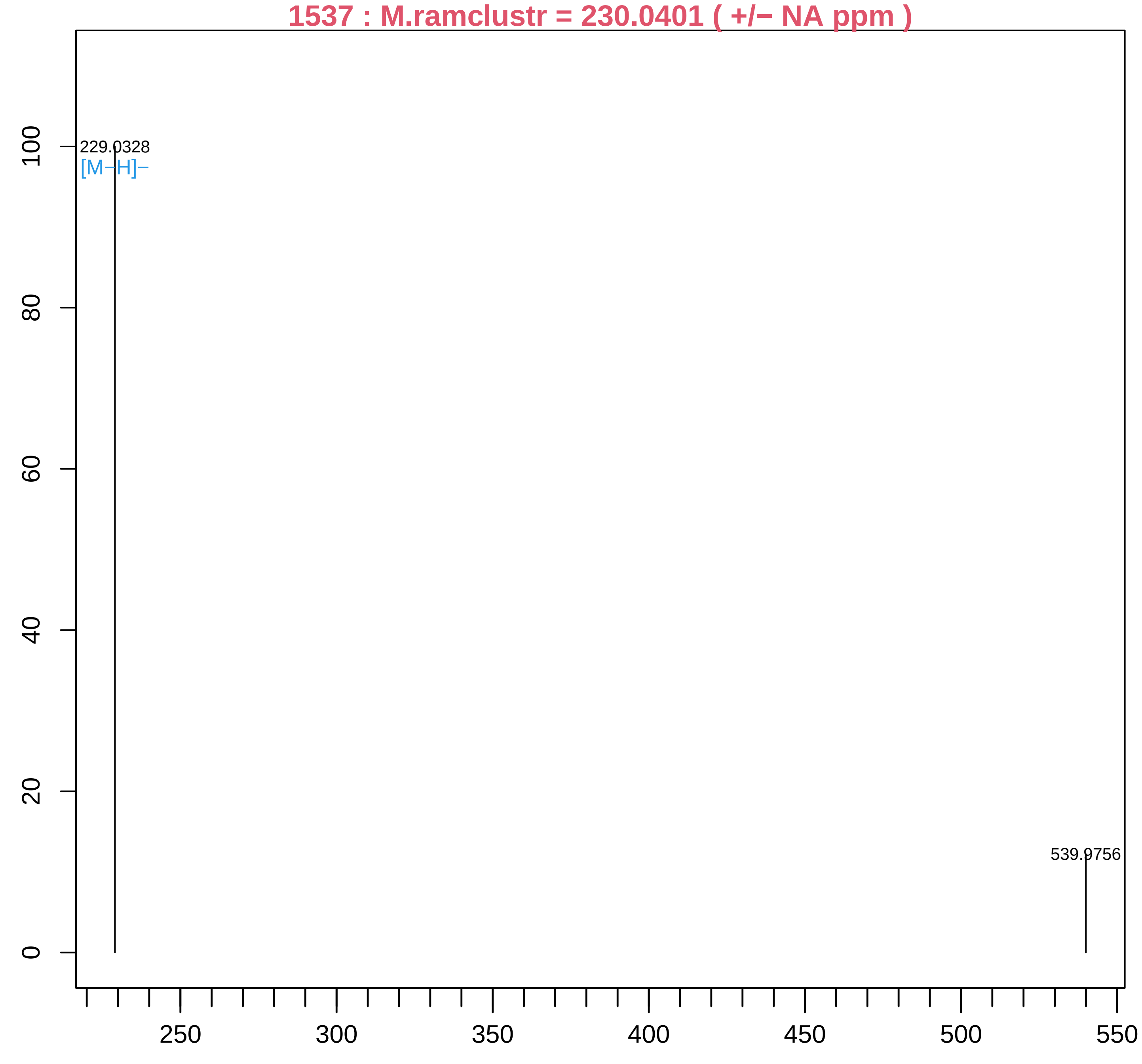
1537 : M.ramclustr = 230.0401 (+/− NA ppm)
229.0328
[M−H]−
539.9756
0
20
40
60
80
100
250
300
350
400
450
500
550

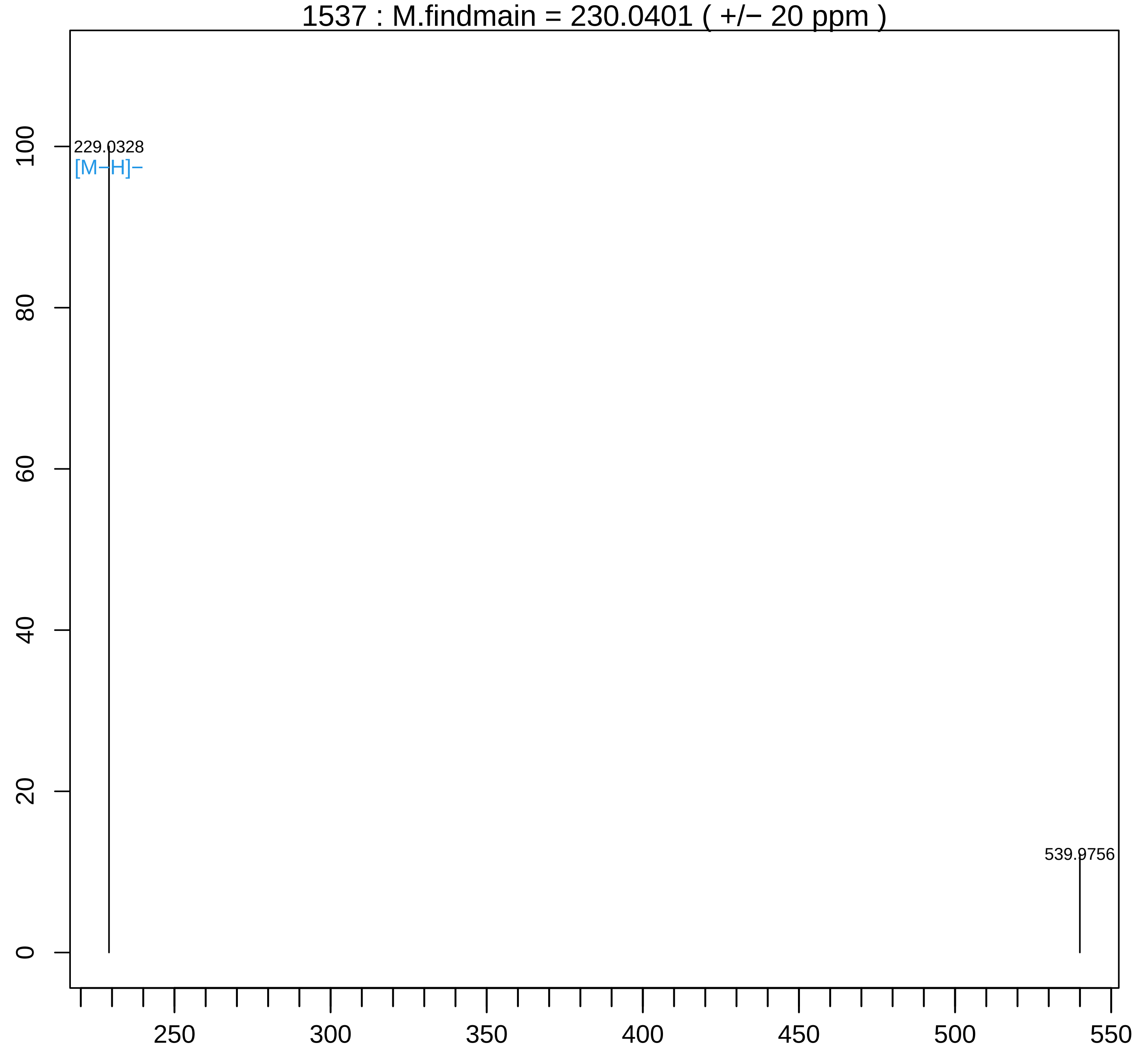
1537 : M.findmain = 230.0401 (+/− 20 ppm)
229.0328
[M−H]−
539.9756
0
20
40
60
80
100
250
300
350
400
450
500
550

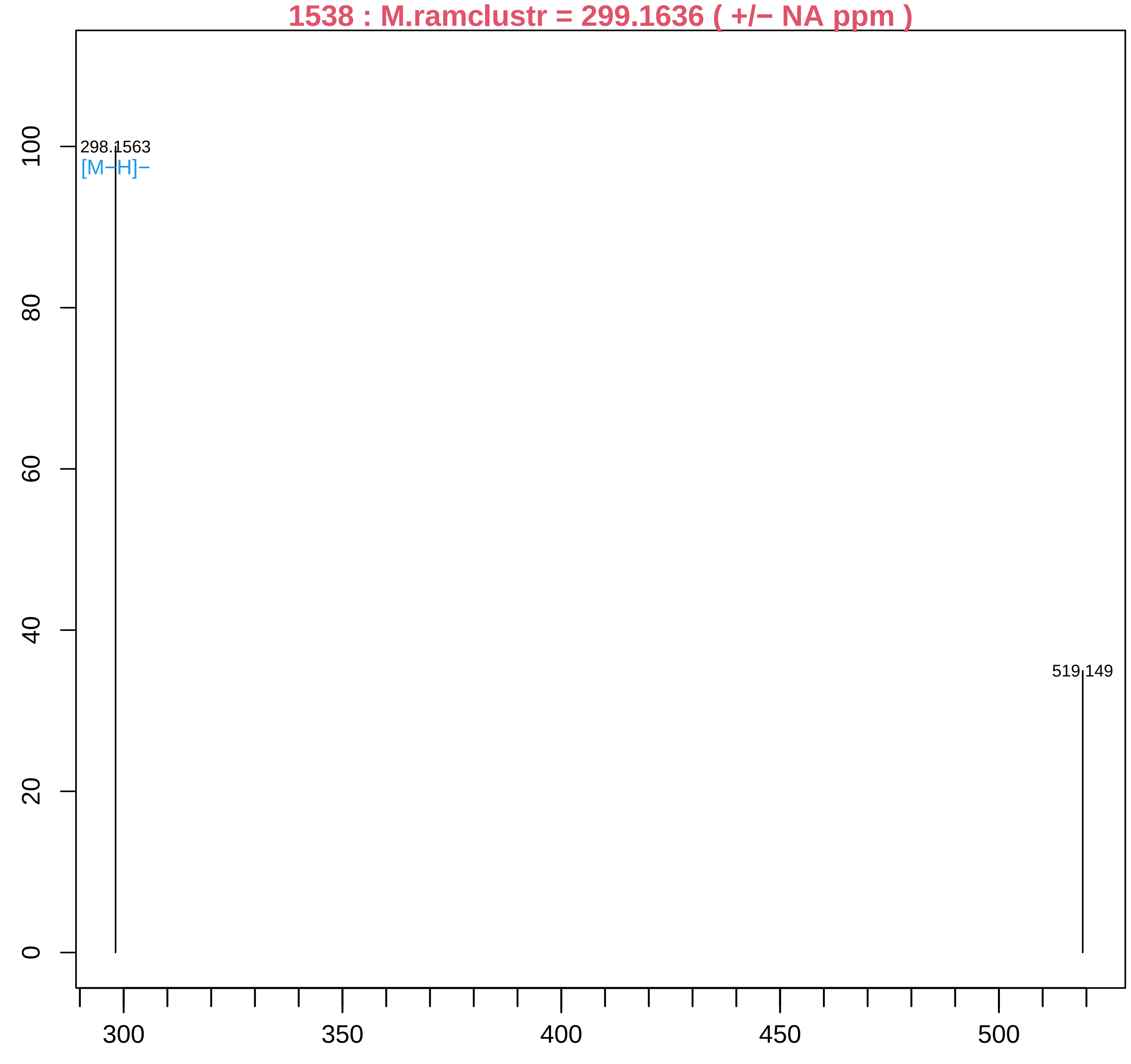

1538 : M.ramclustr = 299.1636 (+/− NA ppm)
298.1563
[M−H]−
519.149
0
20
40
60
80
100
300
350
400
450
500

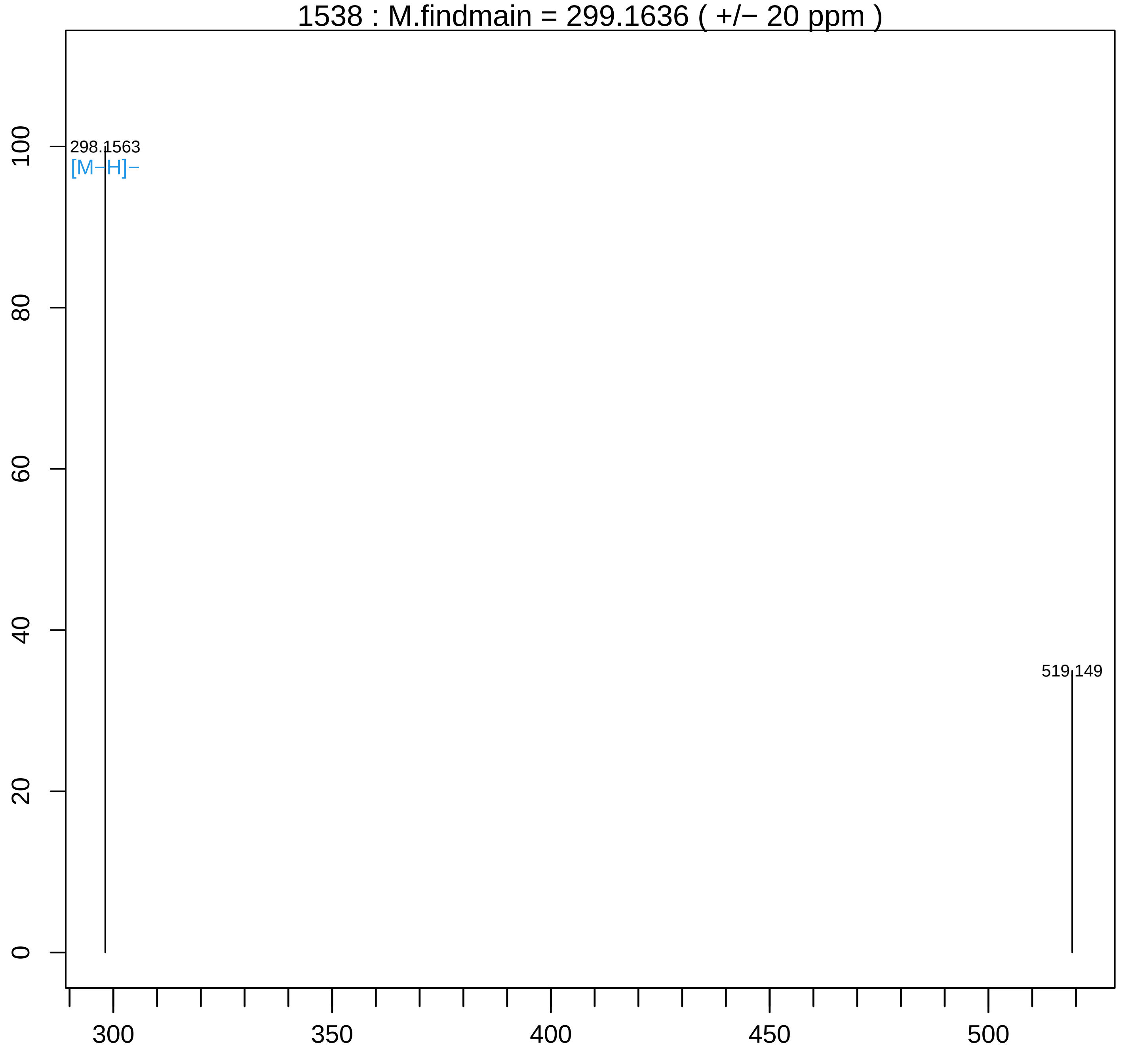

1538 : M.findmain = 299.1636 (+/− 20 ppm)
298.1563
[M−H]−
519.149
0
20
40
60
80
100
300
350
400
450
500

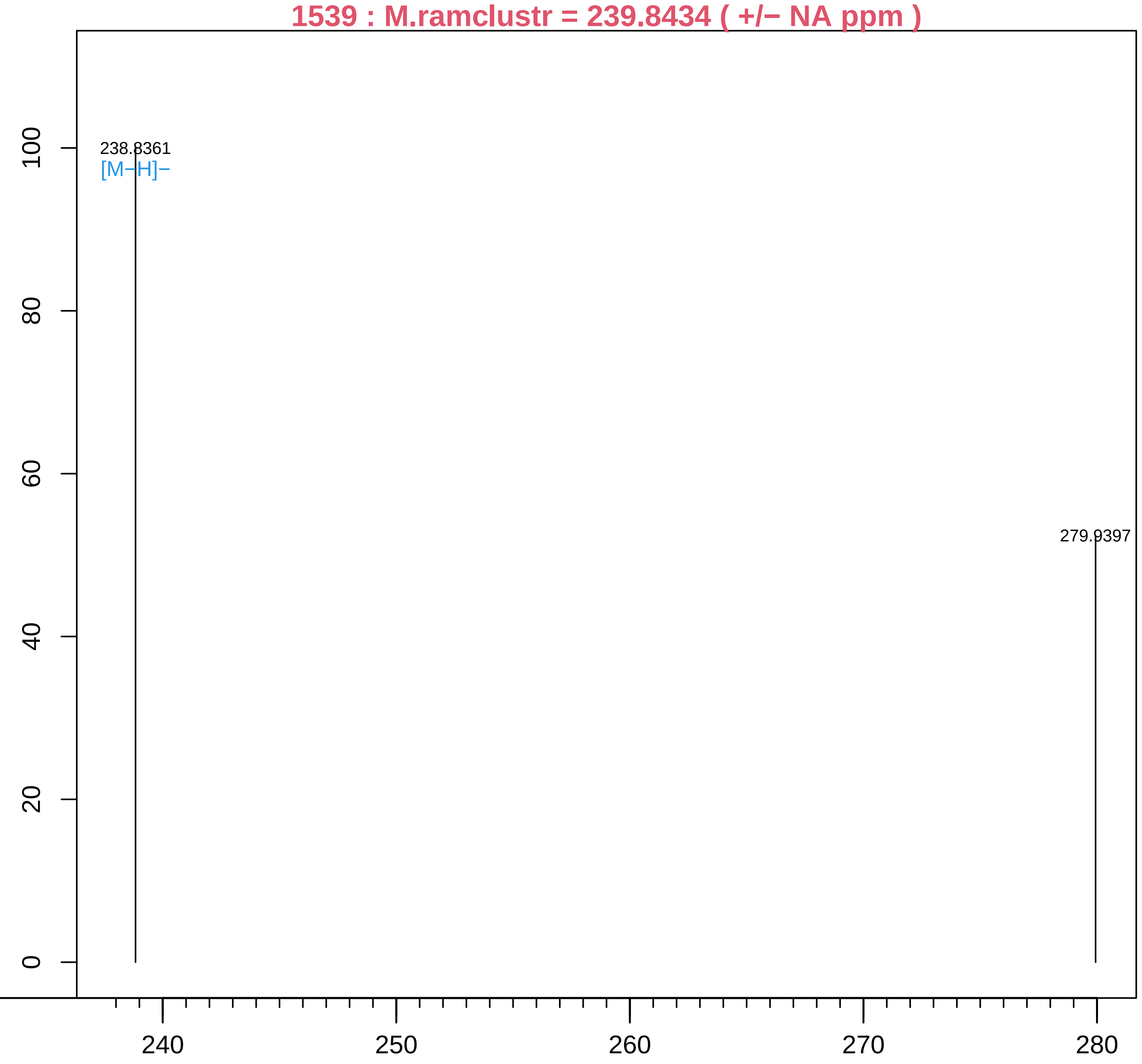
1539 : M.ramclustr = 239.8434 (+/− NA ppm)
238.8361
[M−H]−
279.9397
0
20
40
60
80
100
240
250
260
270
280

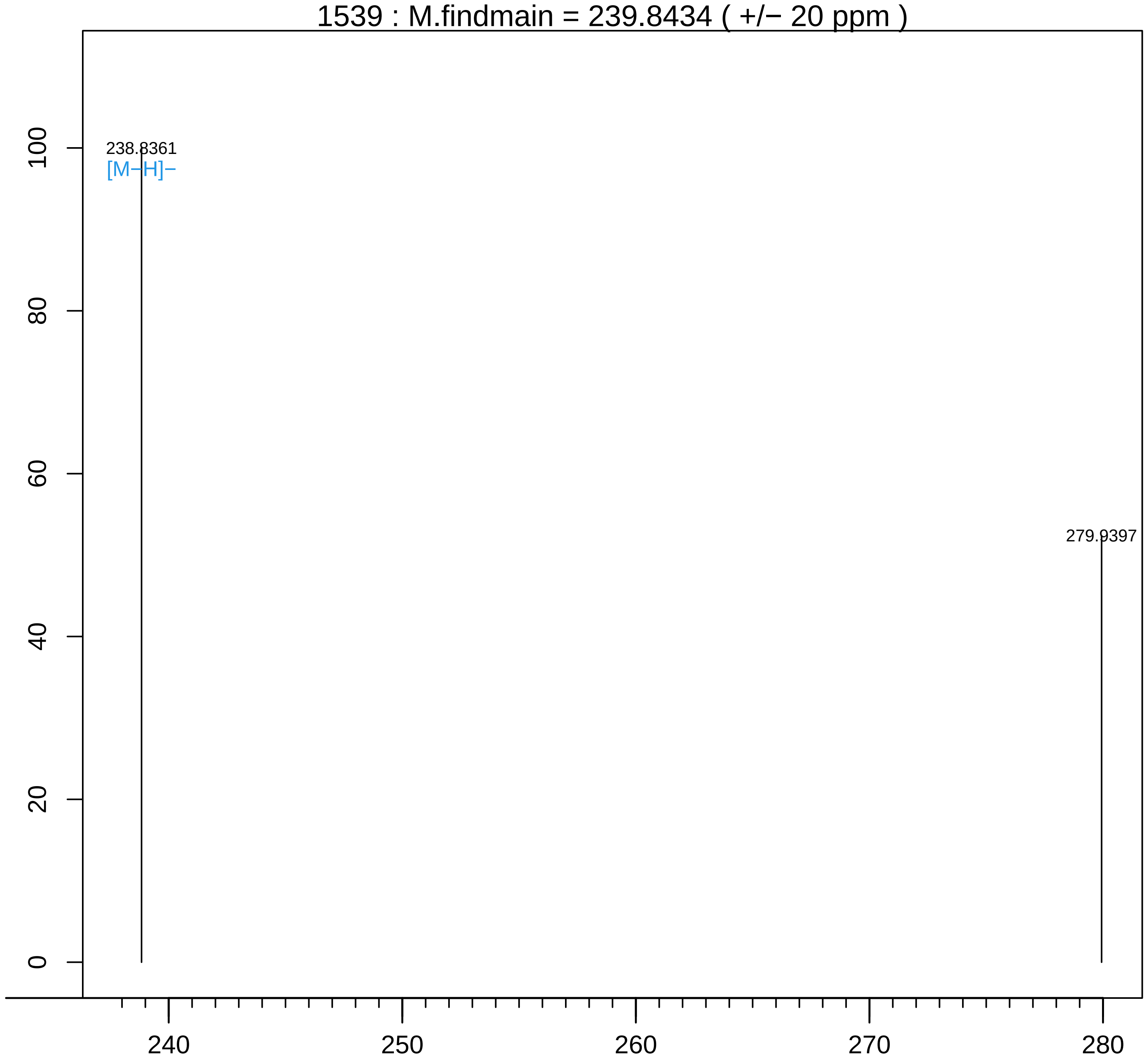
1539 : M.findmain = 239.8434 (+/− 20 ppm)
238.8361
[M−H]−
279.9397
0
20
40
60
80
100
240
250
260
270
280

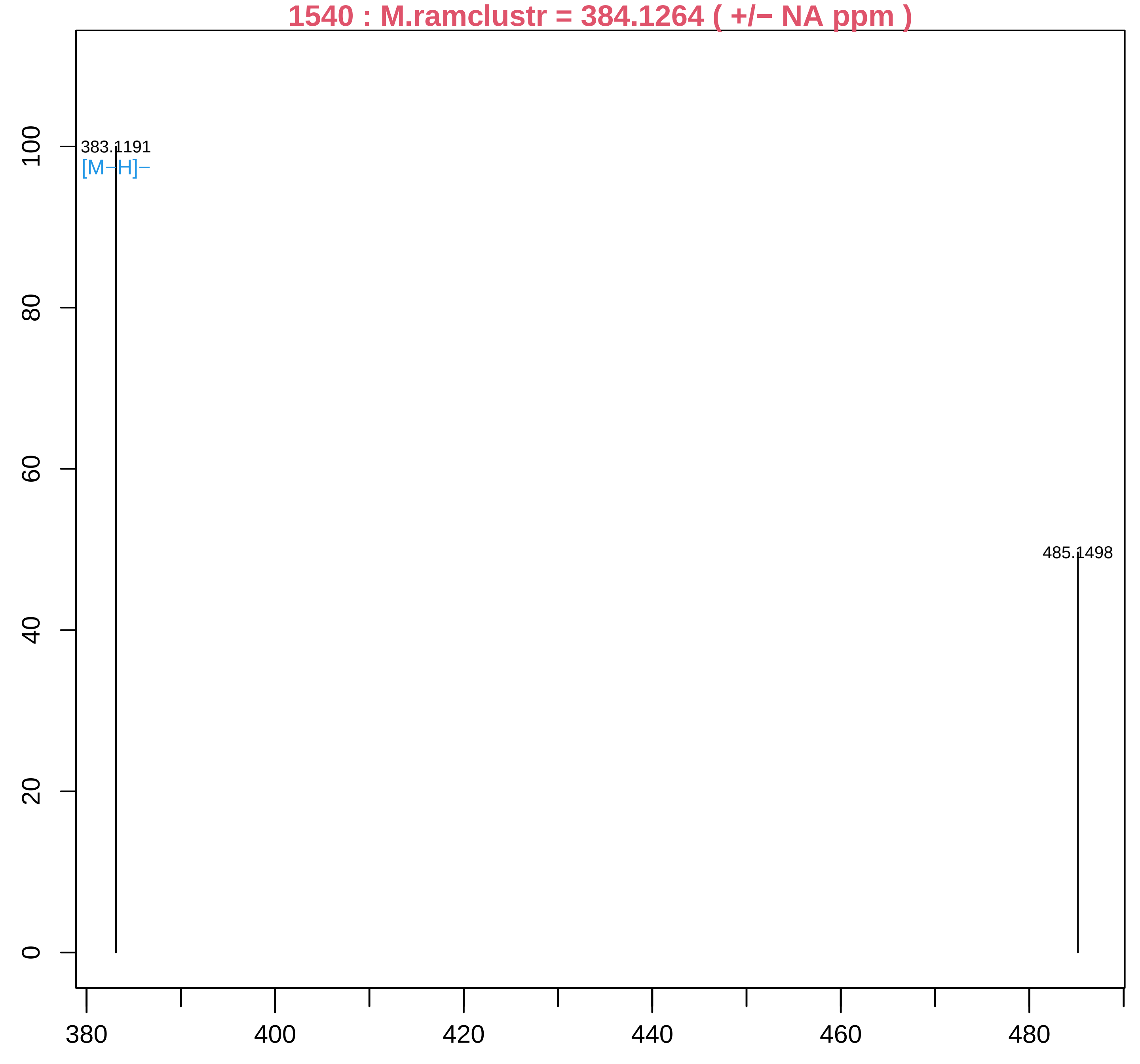

1540 : M.ramclustr = 384.1264 (+/− NA ppm)
383.1191
[M−H]−
485.1498
0
20
40
60
80
100
380
400
420
440
460
480

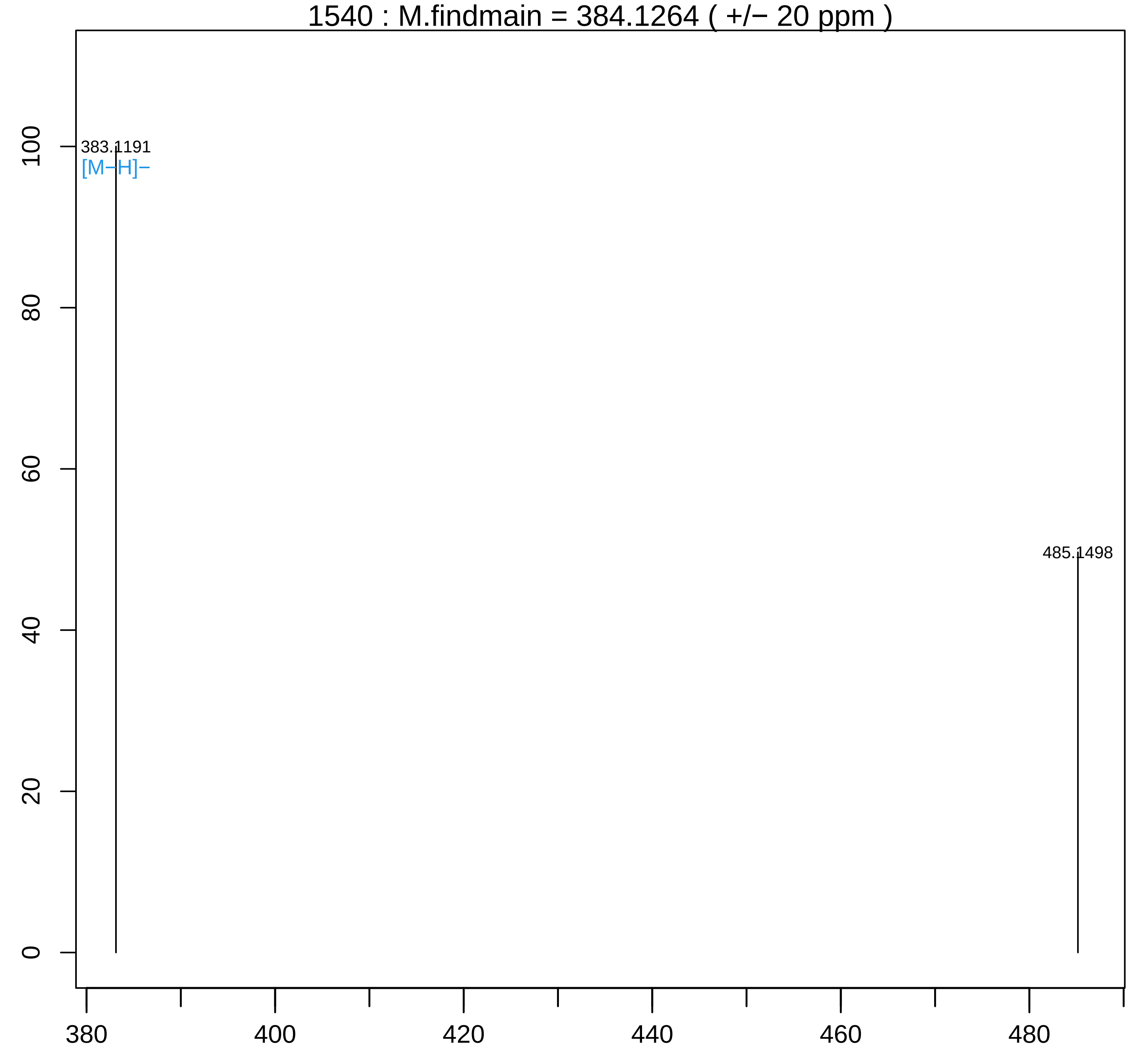

1540 : M.findmain = 384.1264 (+/− 20 ppm)
383.1191
[M−H]−
485.1498
0
20
40
60
80
100
380
400
420
440
460
480

1541 : M.ramclustr = 282.9247 (+/− NA ppm)

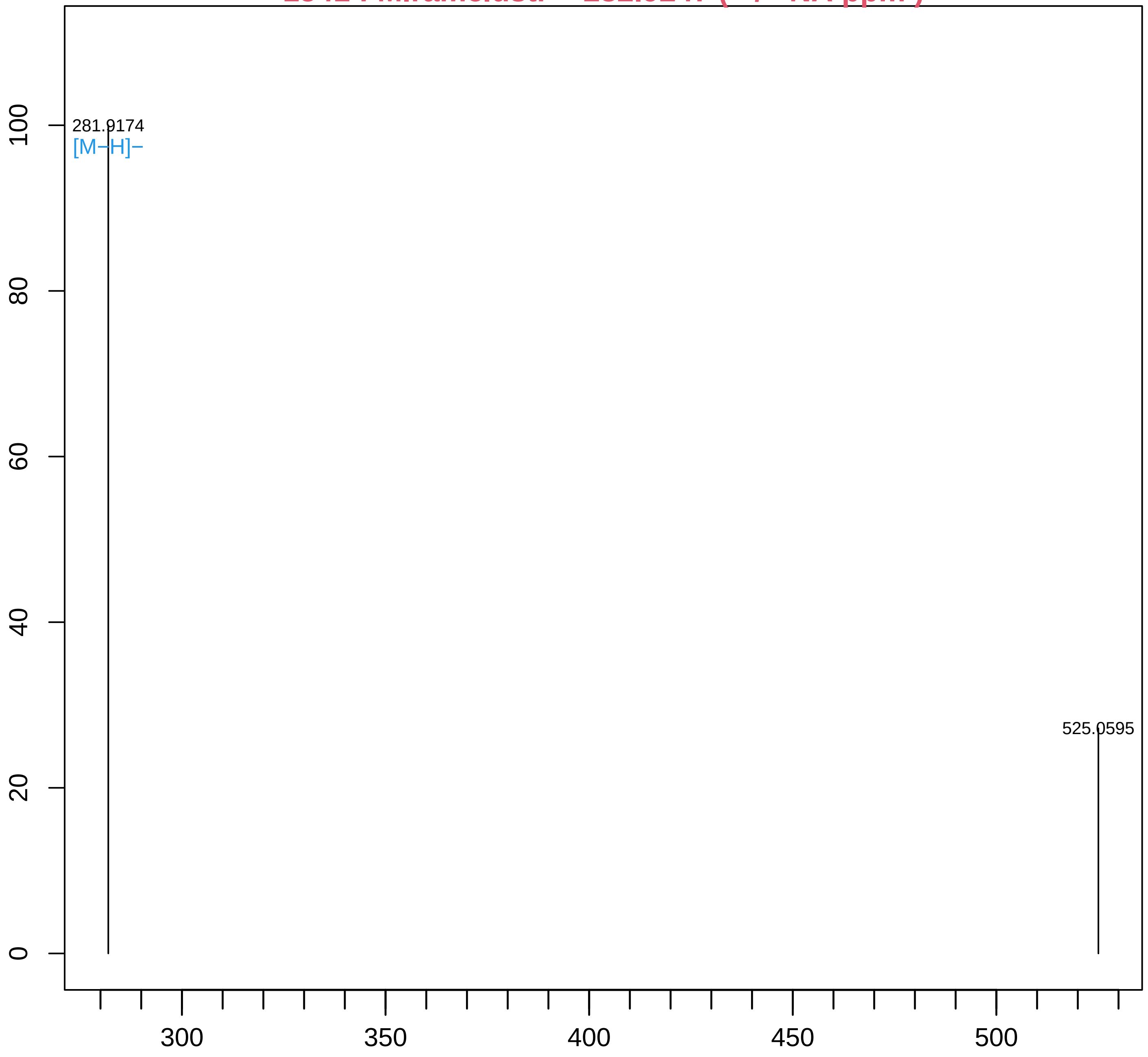

1541 : M.findmain = 282.9247 (+/− 20 ppm)

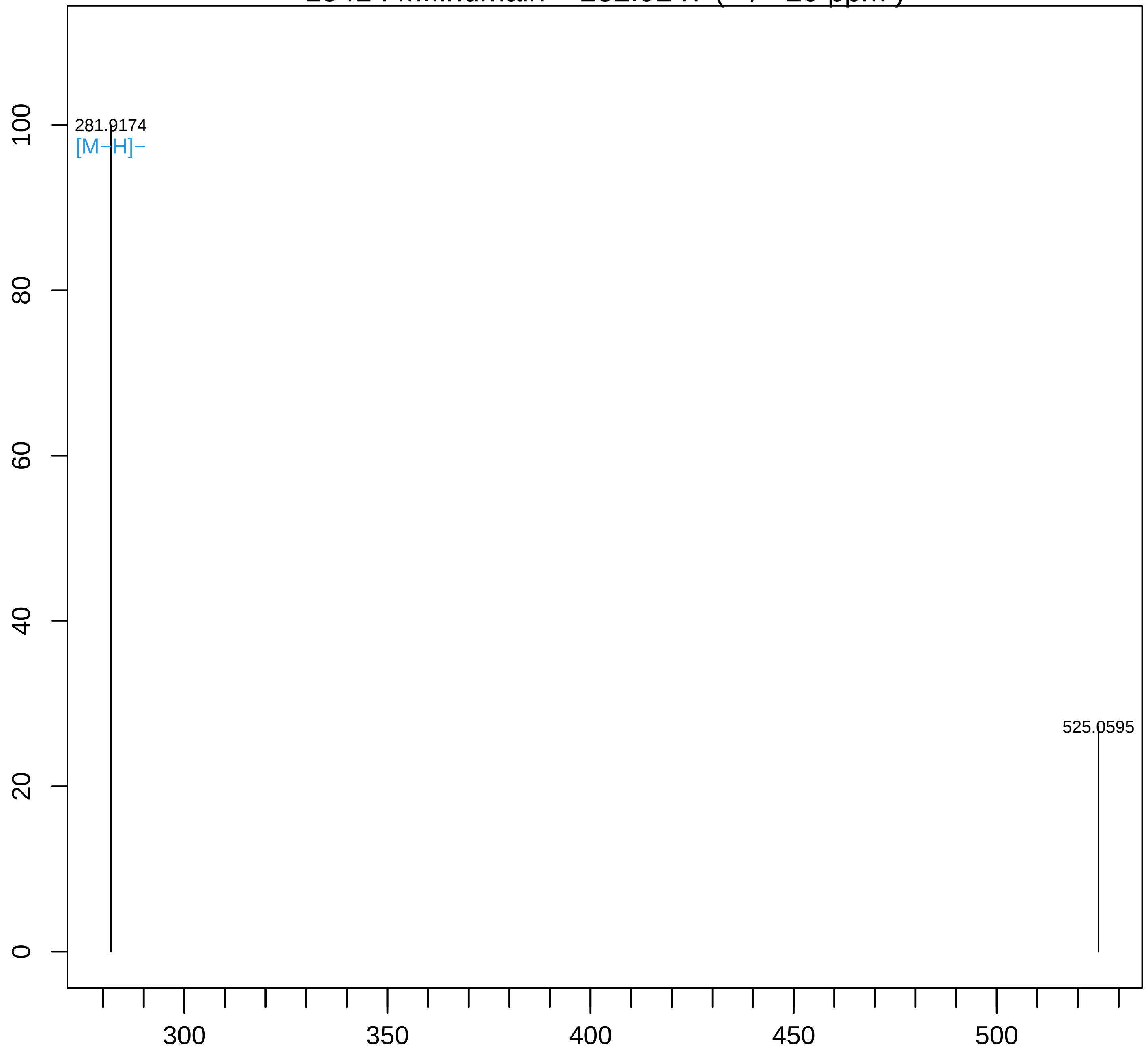

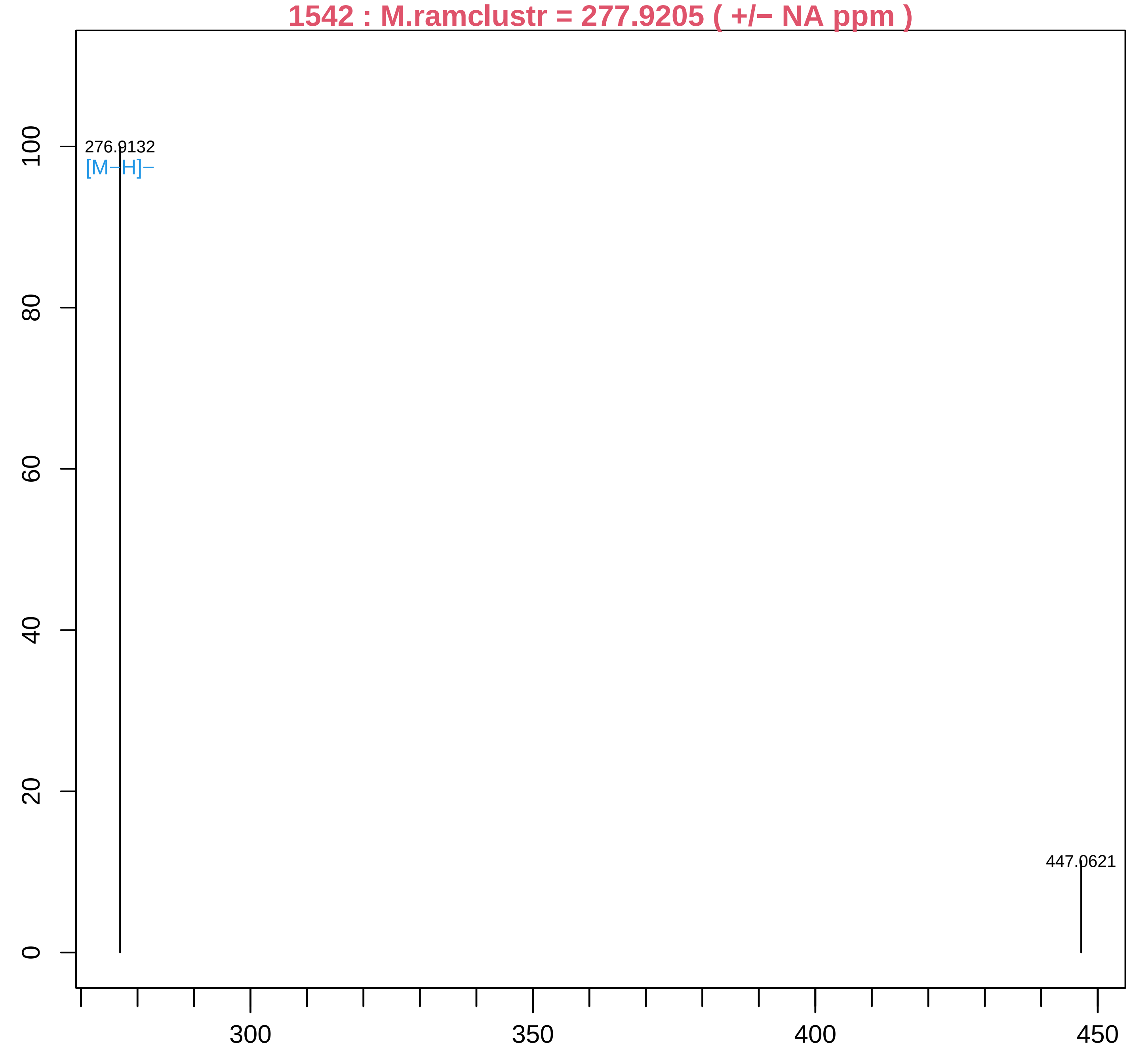
1542 : M.ramclustr = 277.9205 (+/− NA ppm)
276.9132
[M−H]−
447.0621
0
20
40
60
80
100
300
350
400
450

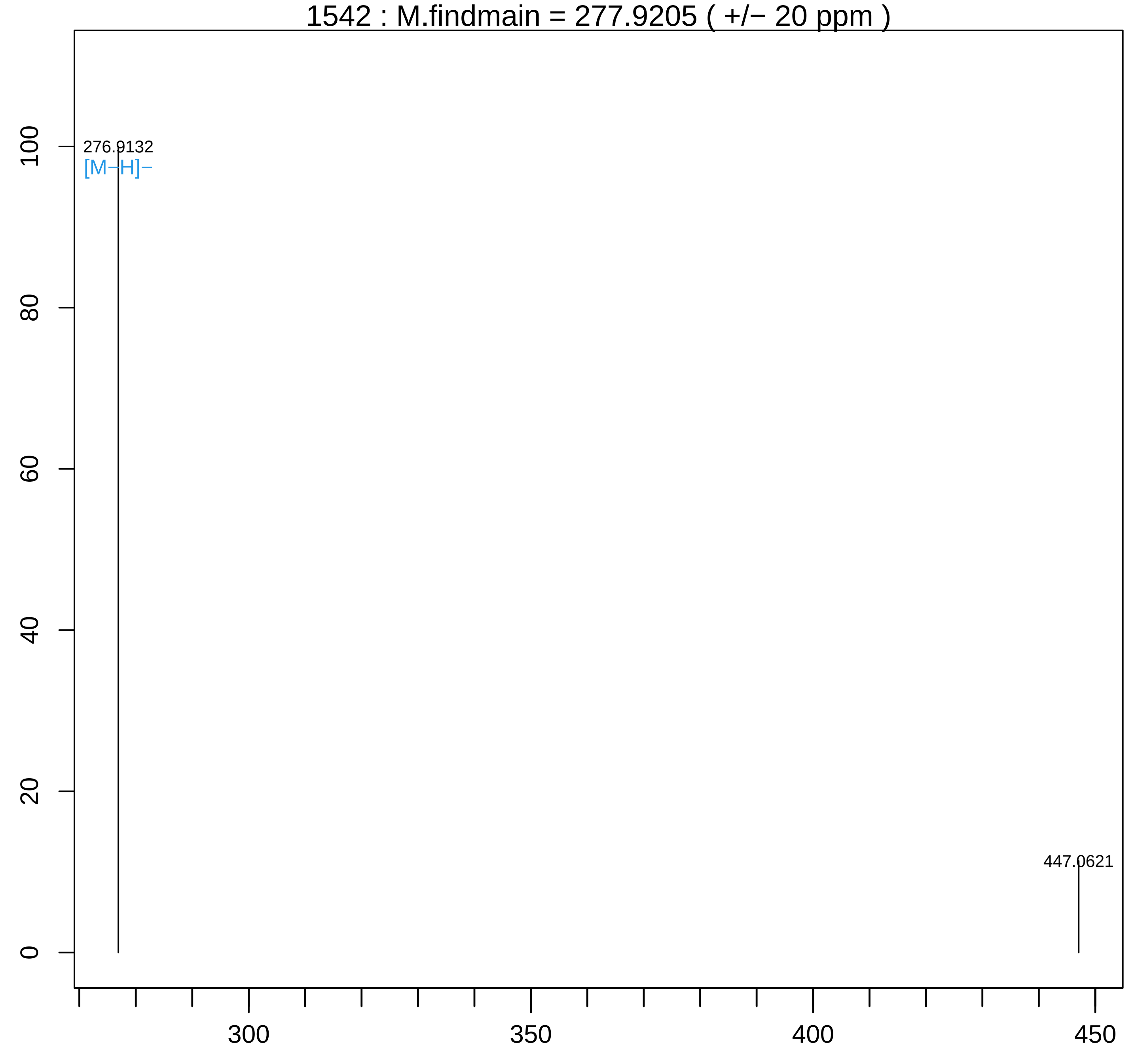
1542 : M.findmain = 277.9205 (+/− 20 ppm)
276.9132
[M−H]−
447.0621
0
20
40
60
80
100
300
350
400
450

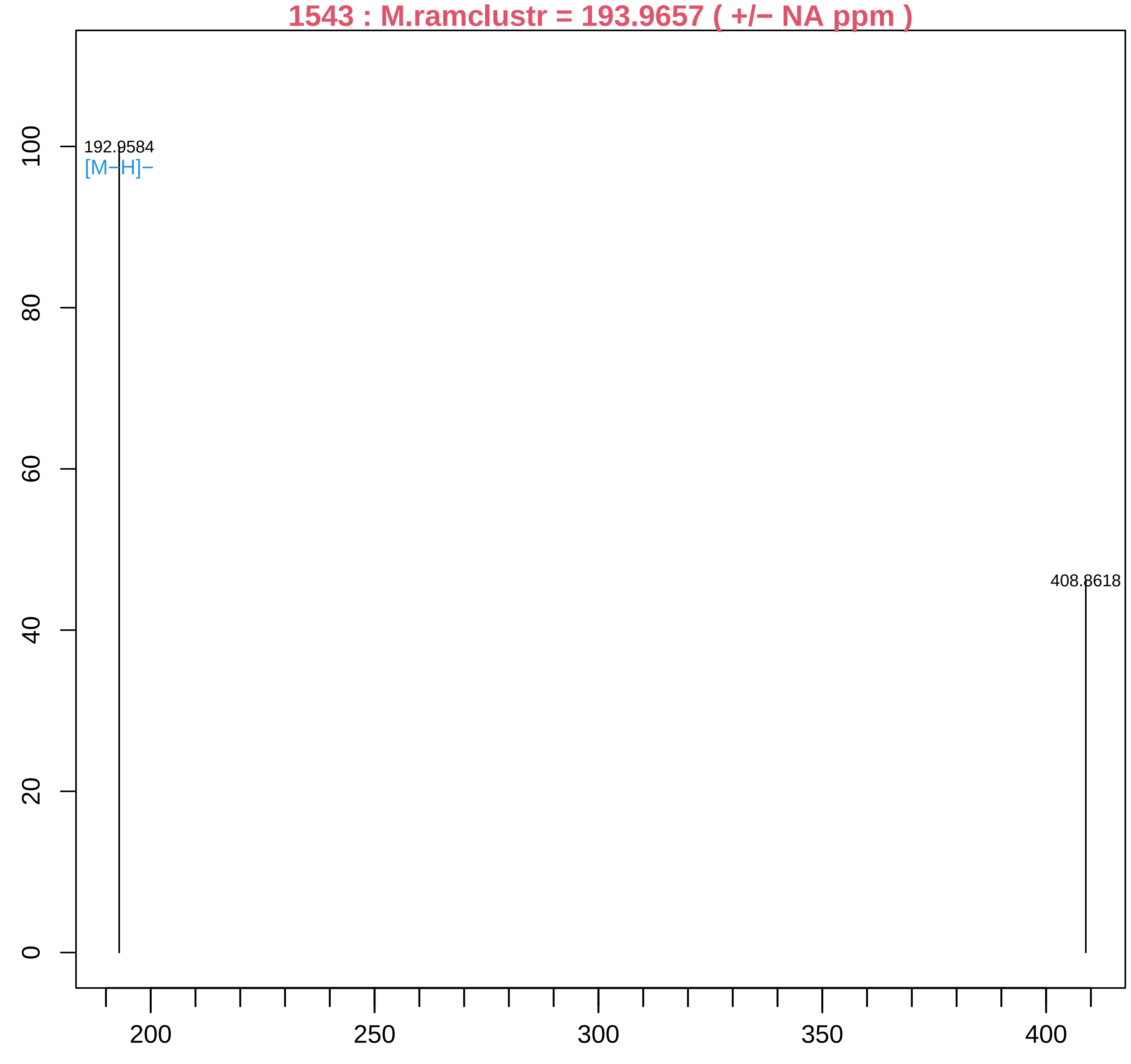
1543 : M.ramclustr = 193.9657 (+/− NA ppm)
192.9584
[M−H]−
408.8618
0
20
40
60
80
100
200
250
300
350
400

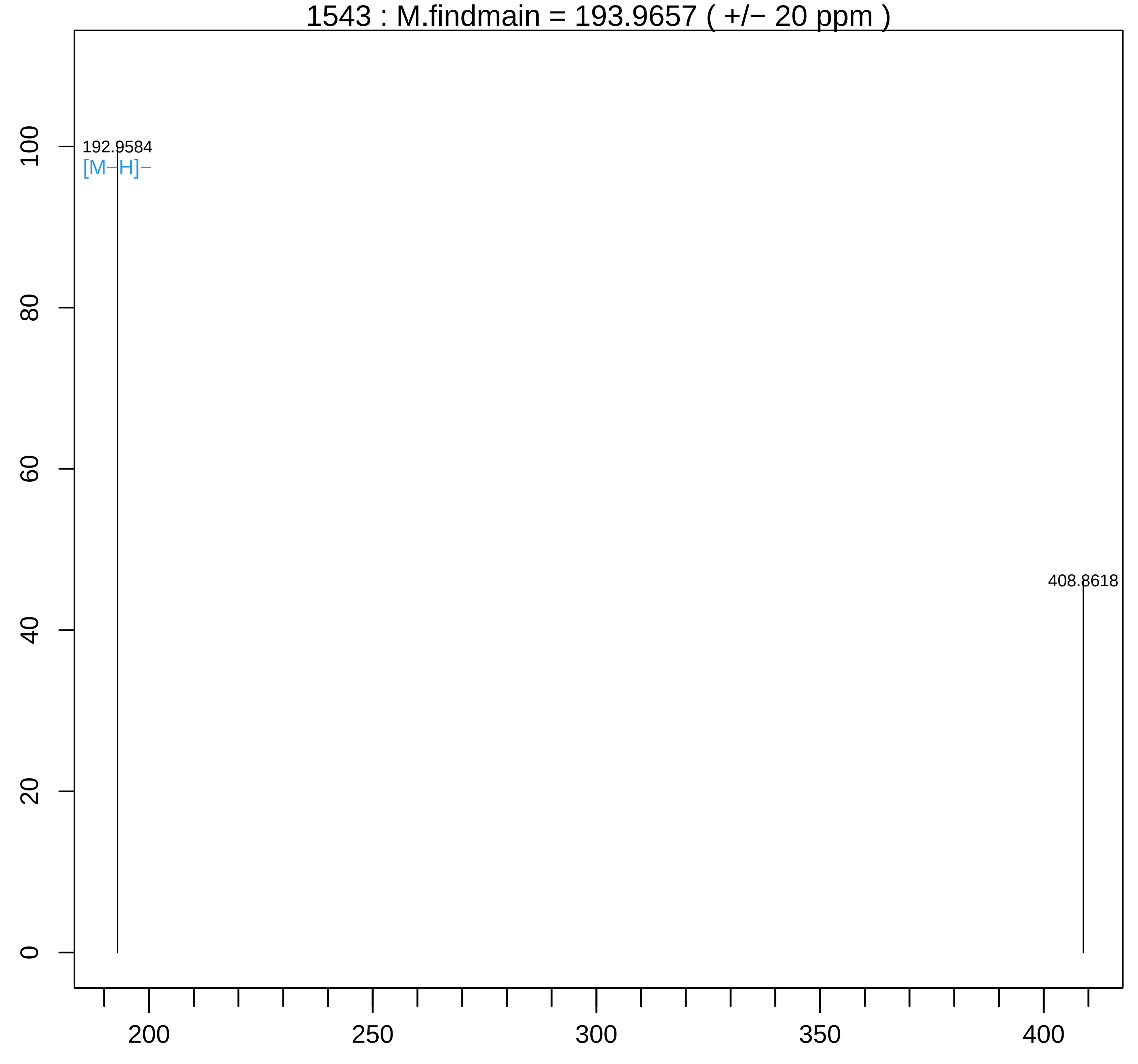
1543 : M.findmain = 193.9657 (+/− 20 ppm)
192.9584
[M−H]−
408.8618
0
20
40
60
80
100
200
250
300
350
400

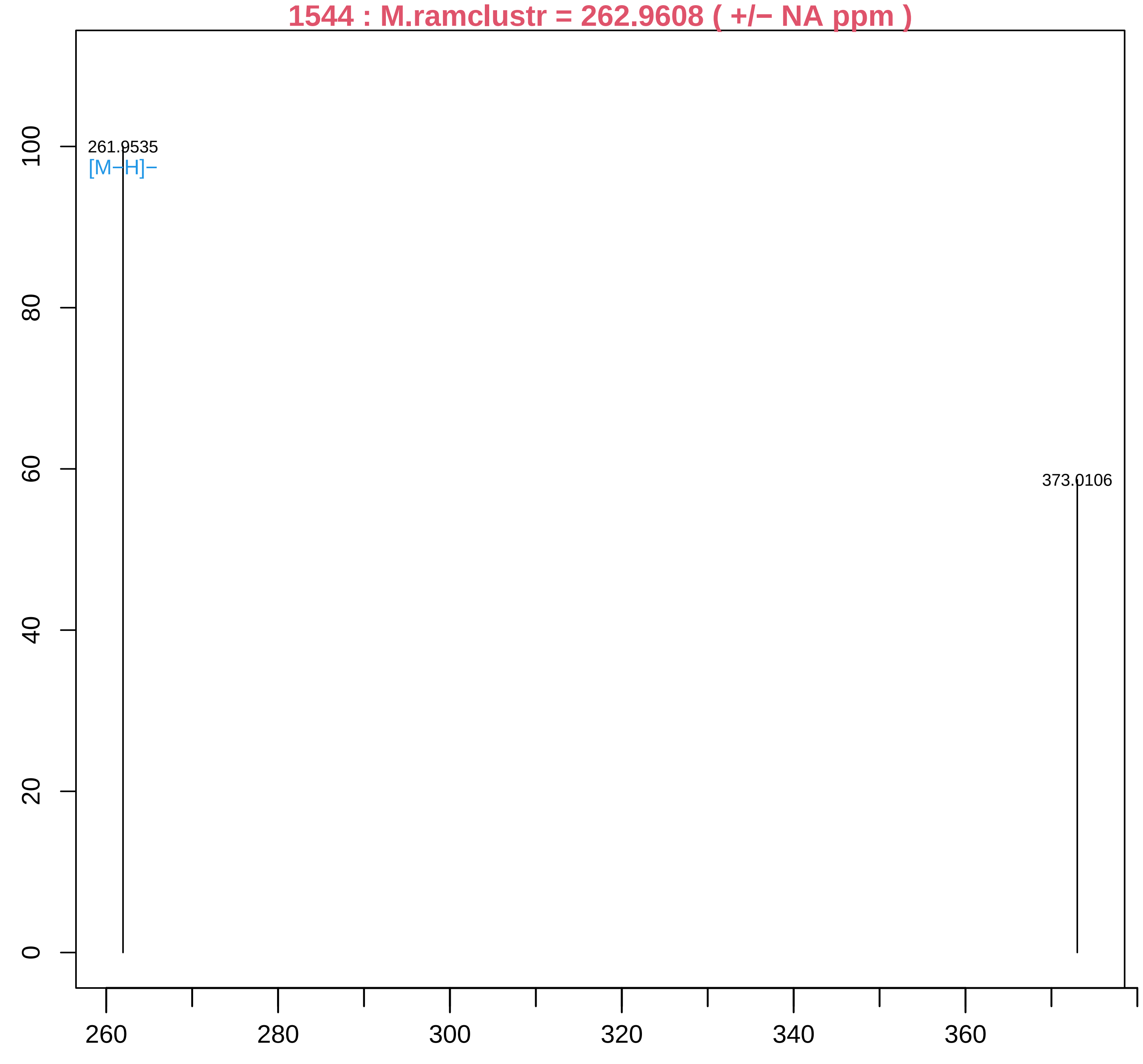
1544 : M.ramclustr = 262.9608 (+/− NA ppm)
261.9535
[M−H]−
373.0106
0
20
40
60
80
100
260
280
300
320
340
360

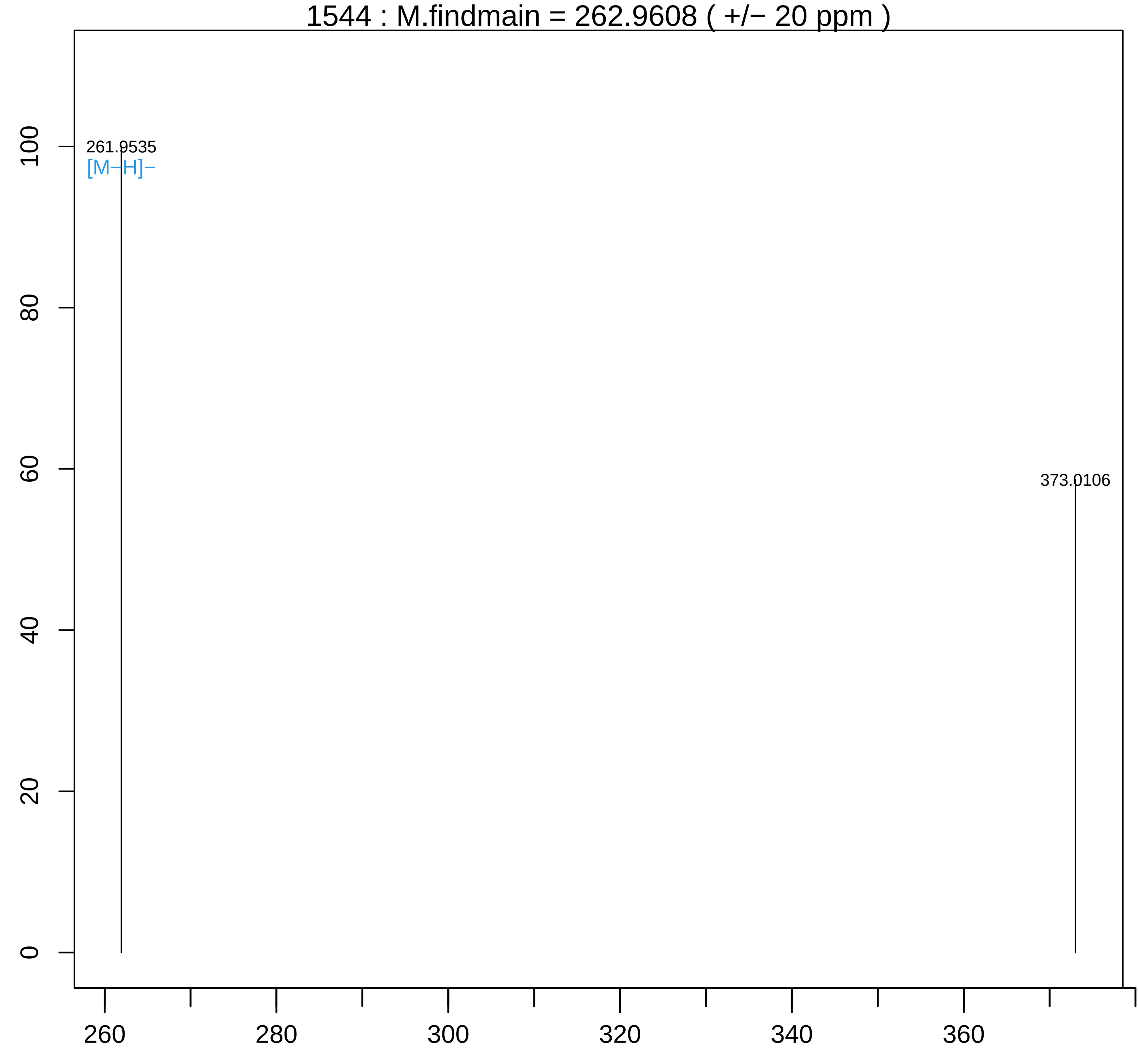
1544 : M.findmain = 262.9608 (+/− 20 ppm)
261.9535
[M−H]−
373.0106
0
20
40
60
80
100
260
280
300
320
340
360

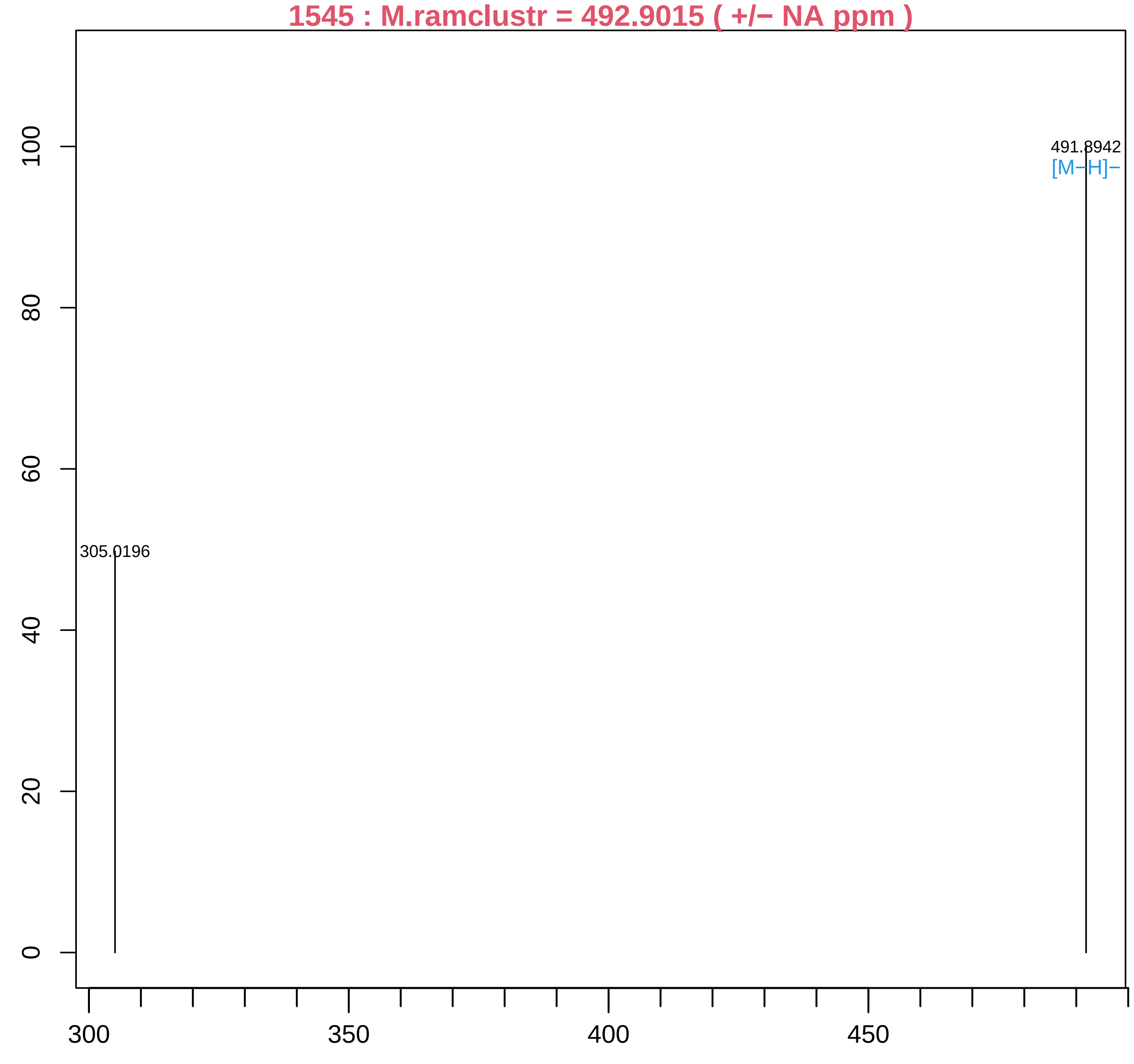
1545 : M.ramclustr = 492.9015 (+/− NA ppm)
491.8942
[M−H]−
305.0196
0
20
40
60
80
100
300
350
400
450

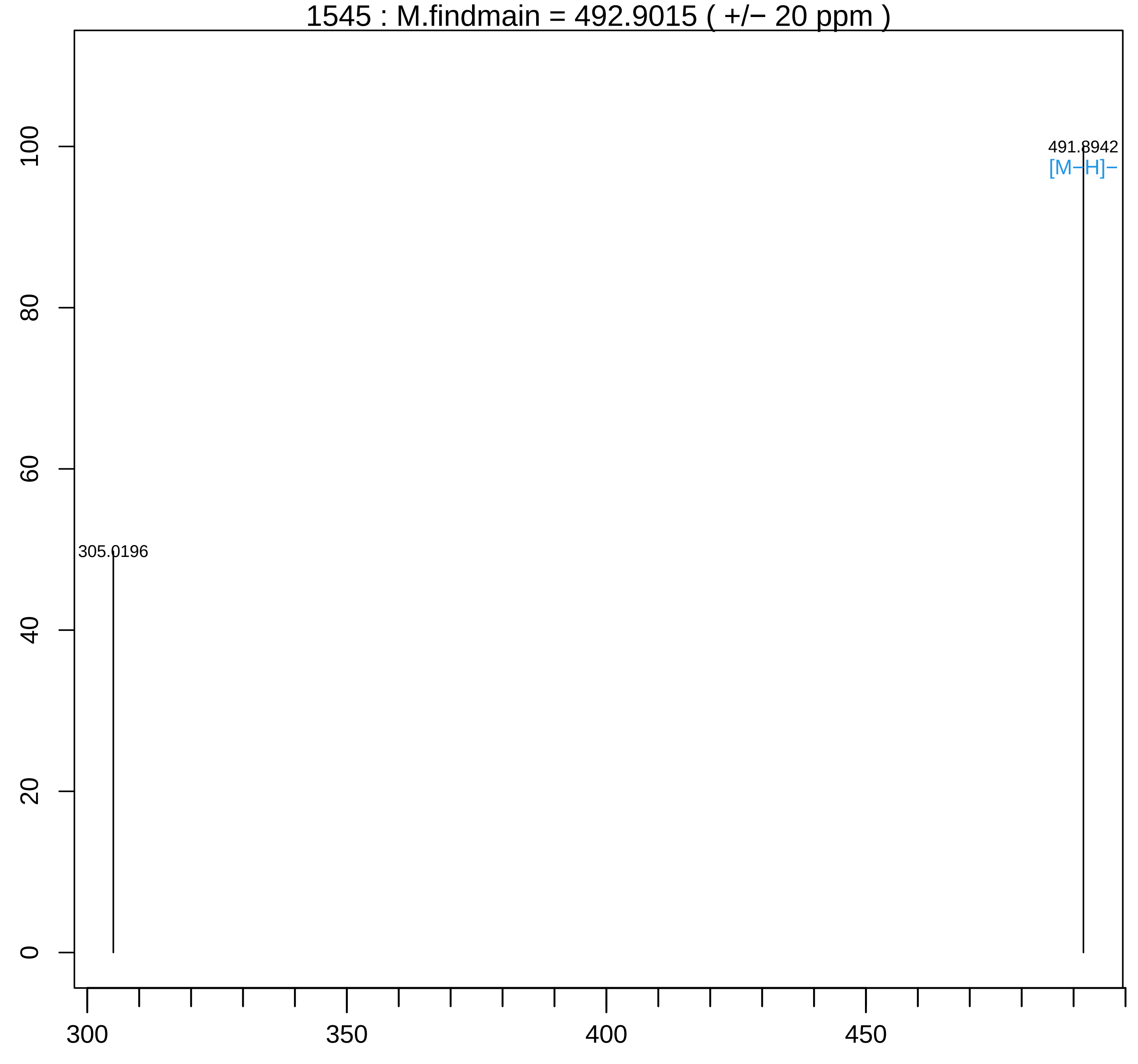
1545 : M.findmain = 492.9015 (+/− 20 ppm)
491.8942
[M−H]−
305.0196
0
20
40
60
80
100
300
350
400
450

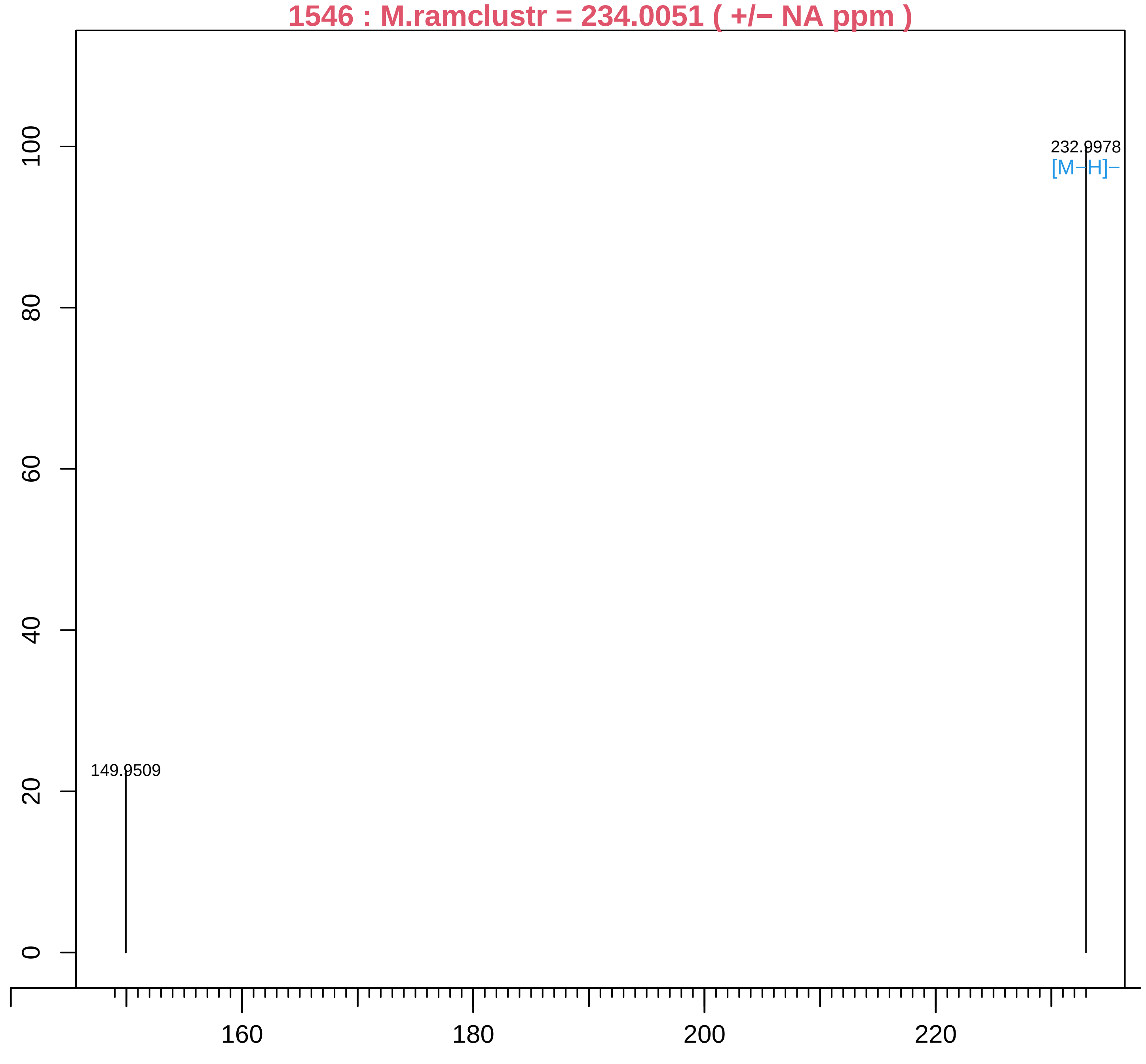
1546 : M.ramclustr = 234.0051 (+/− NA ppm)
232.9978
[M−H]−
149.9509
0
20
40
60
80
100
160
180
200
220

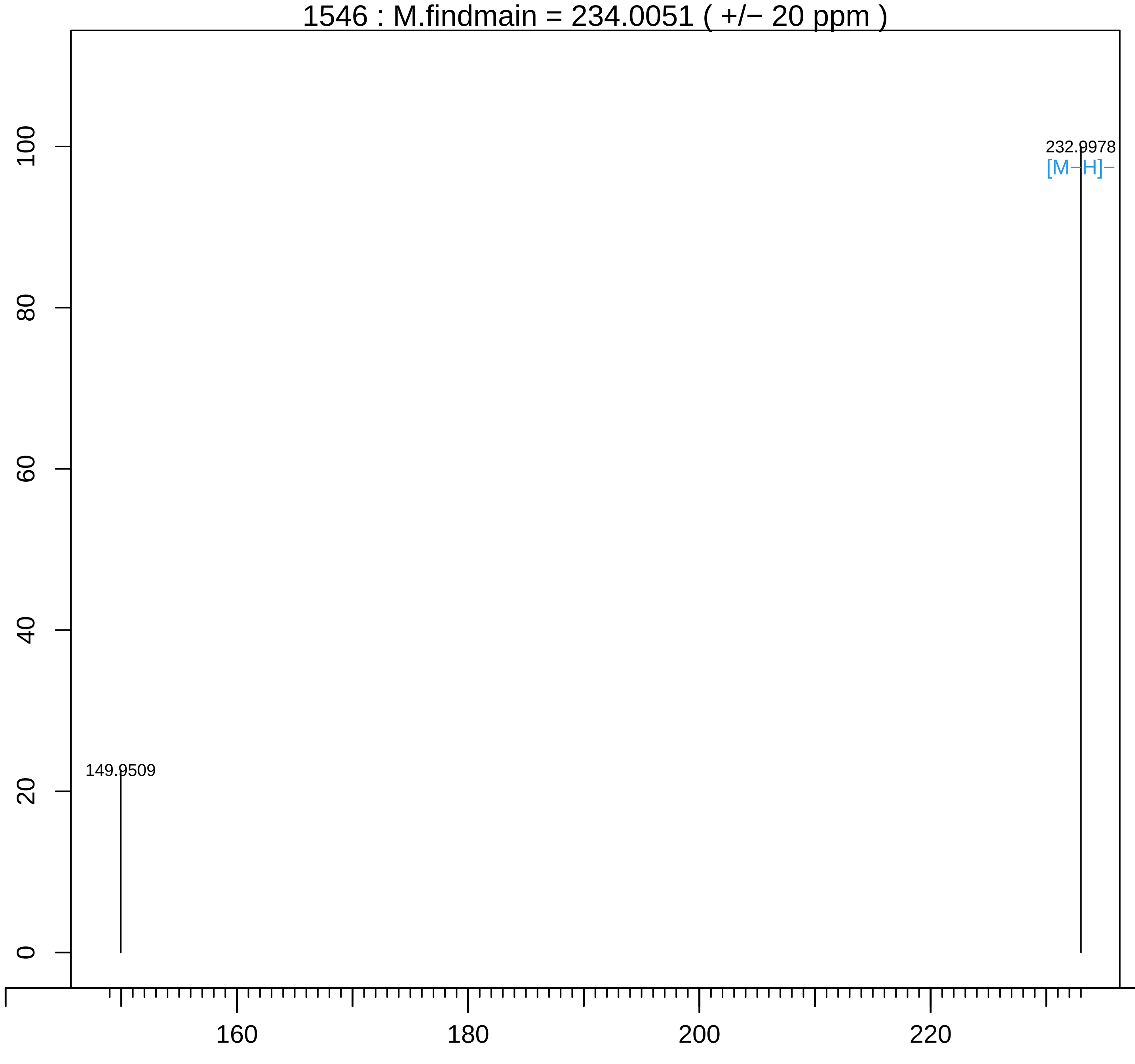
1546 : M.findmain = 234.0051 (+/− 20 ppm)
232.9978
[M−H]−
149.9509
0
20
40
60
80
100
160
180
200
220

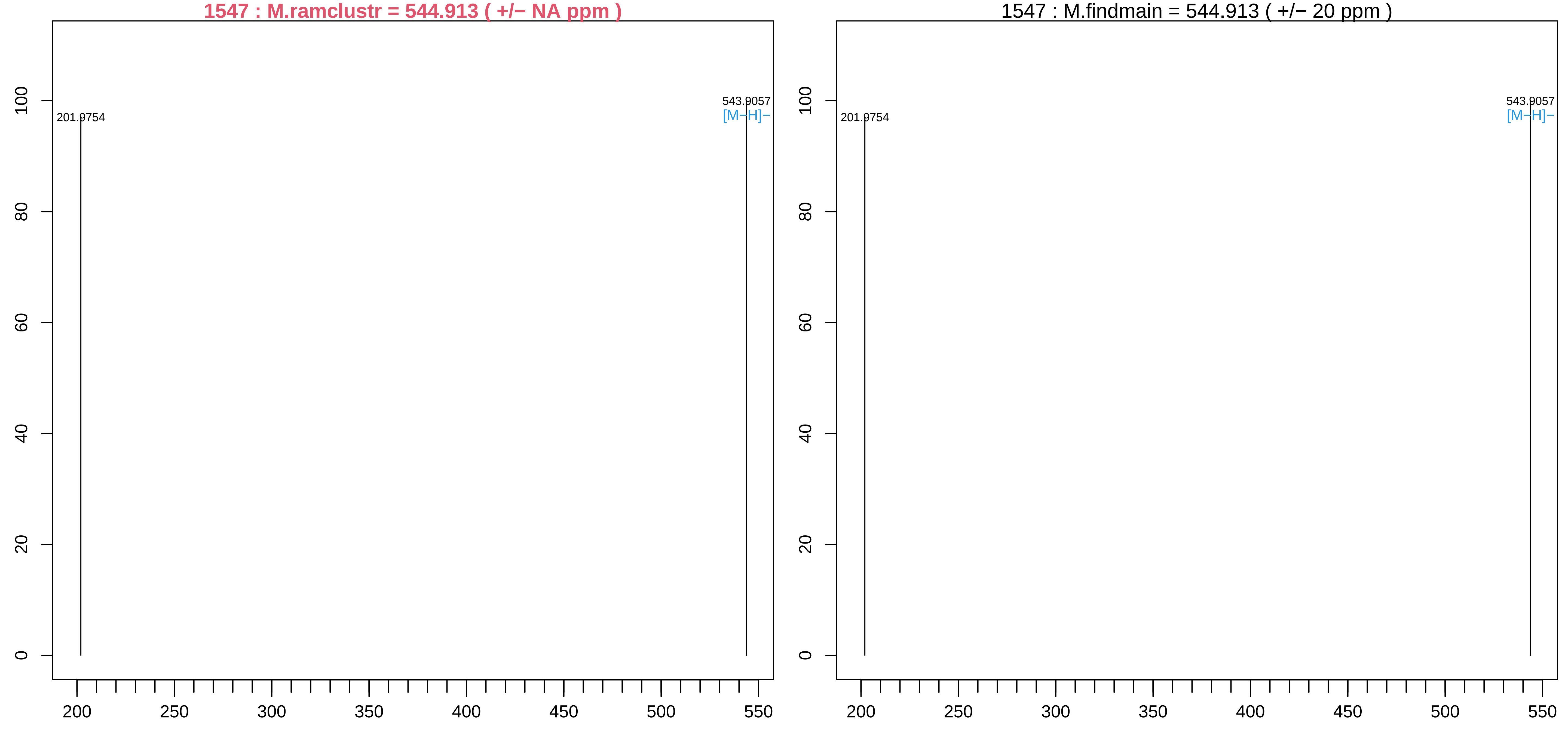
1547 : M.ramclustr = 544.913 (+/− NA ppm)
201.9754
543.9057
[M−H]−
1547 : M.findmain = 544.913 (+/− 20 ppm)
201.9754
543.9057
[M−H]−
0
20
40
60
80
100
200
250
300
350
400
450
500
550

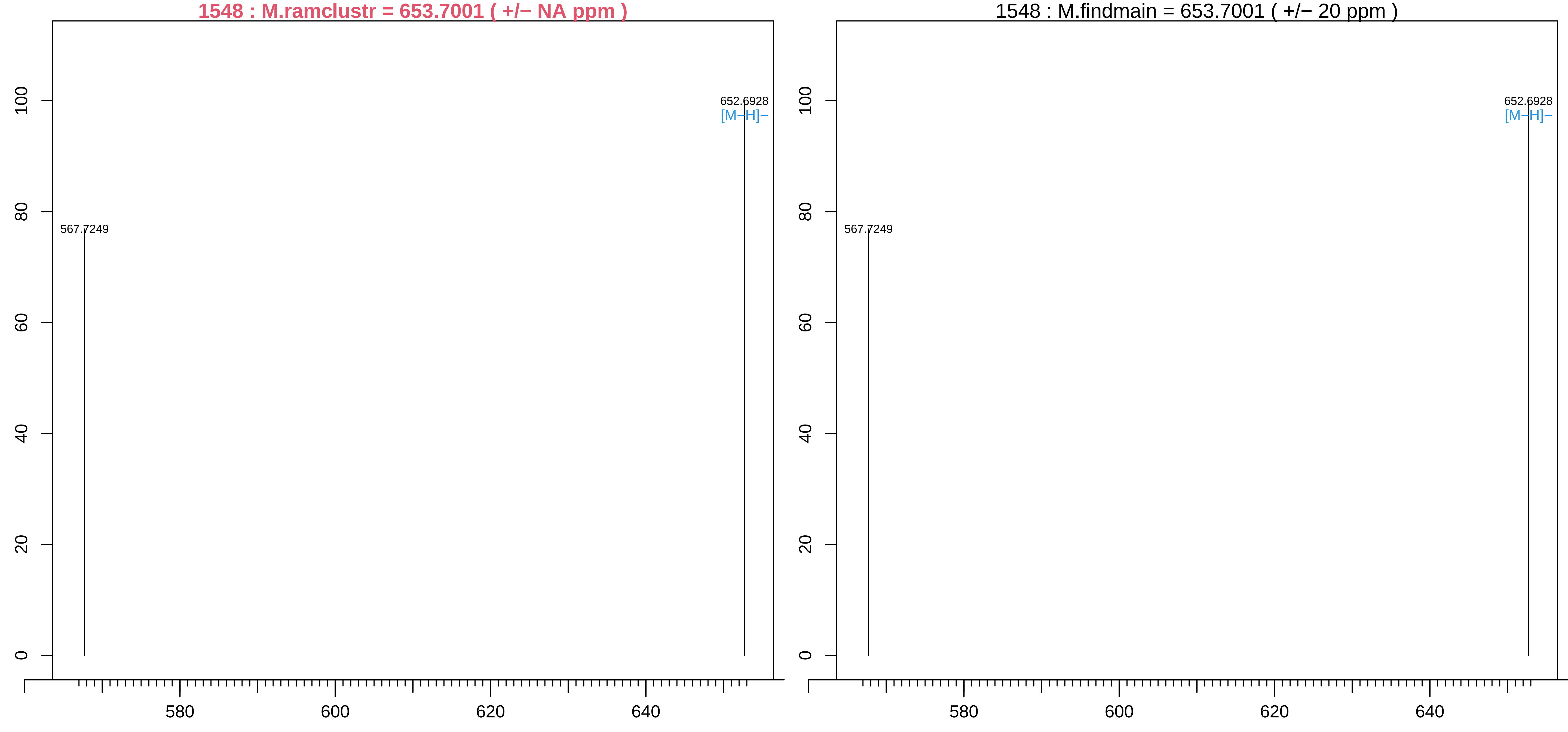

1548 : M.ramclustr = 653.7001 (+/− NA ppm)
567.7249
652.6928
[M−H]−
1548 : M.findmain = 653.7001 (+/− 20 ppm)
567.7249
652.6928
[M−H]−

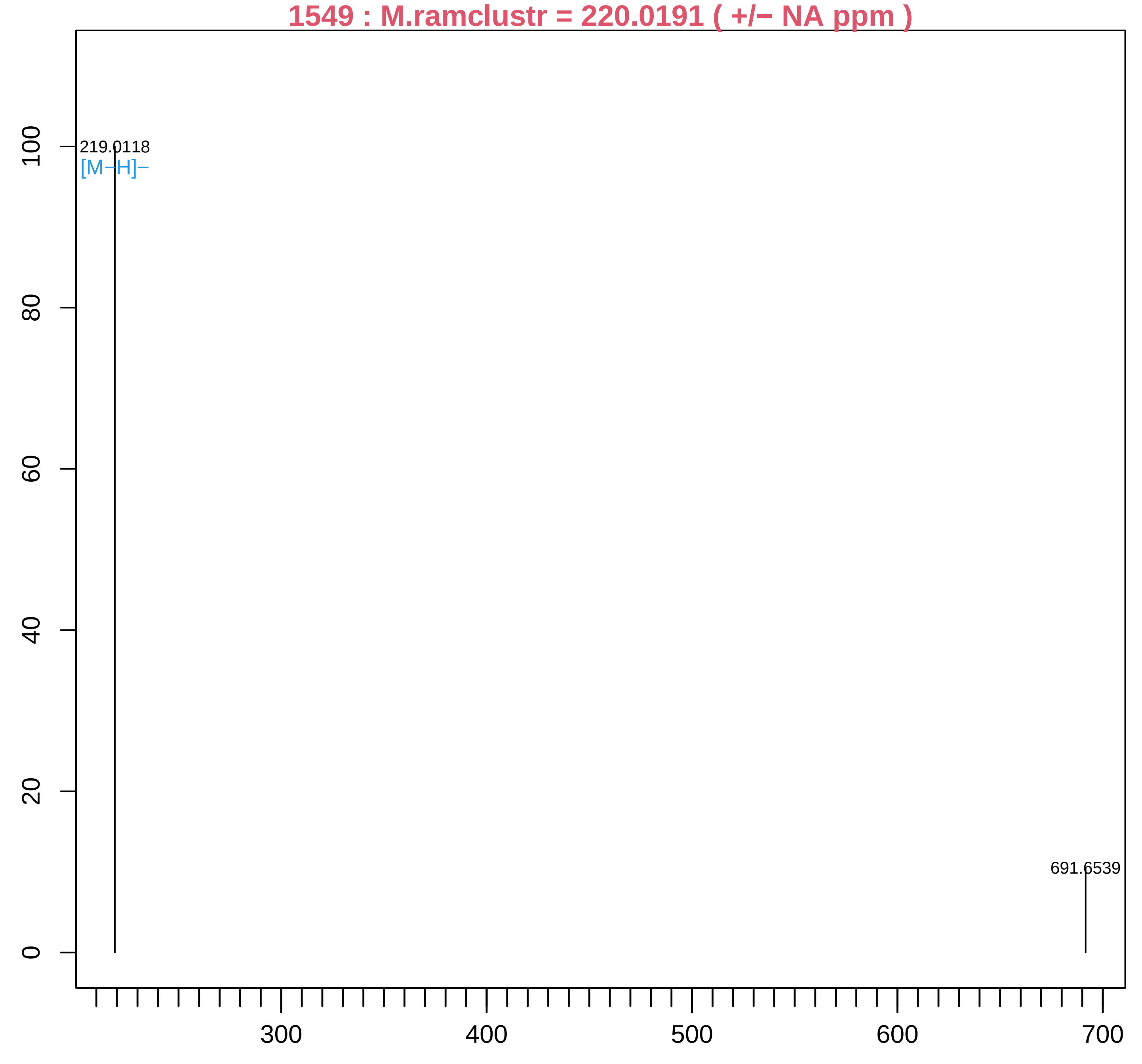
1549 : M.ramclustr = 220.0191 (+/− NA ppm)
219.0118
[M−H]−
691.6539
0
20
40
60
80
100
300
400
500
600
700

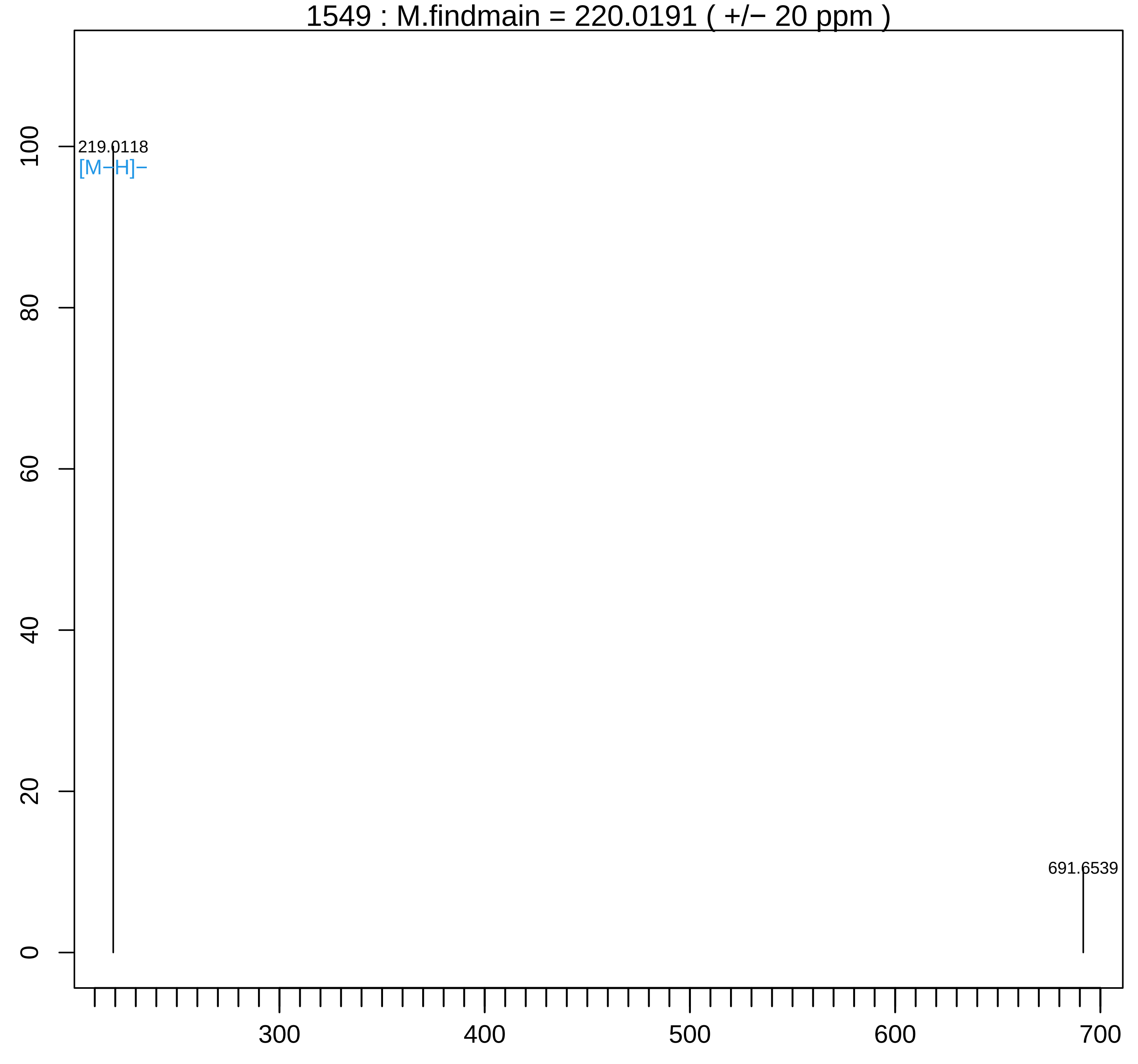
1549 : M.findmain = 220.0191 (+/− 20 ppm)
219.0118
[M−H]−
691.6539
0
20
40
60
80
100
300
400
500
600
700

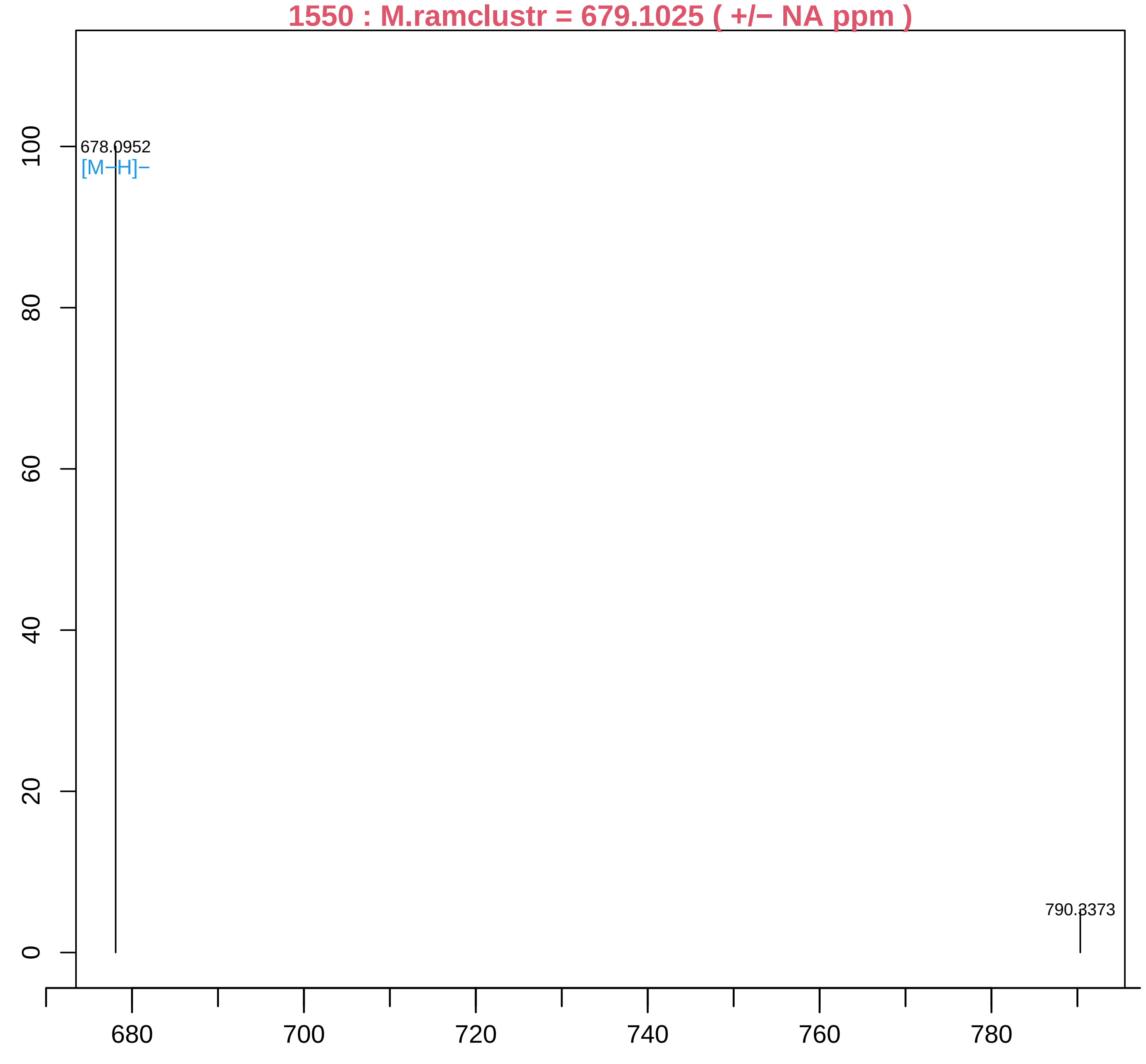

1550 : M.ramclustr = 679.1025 (+/− NA ppm)
678.0952
[M−H]−
790.3373
0
20
40
60
80
100
680
700
720
740
760
780

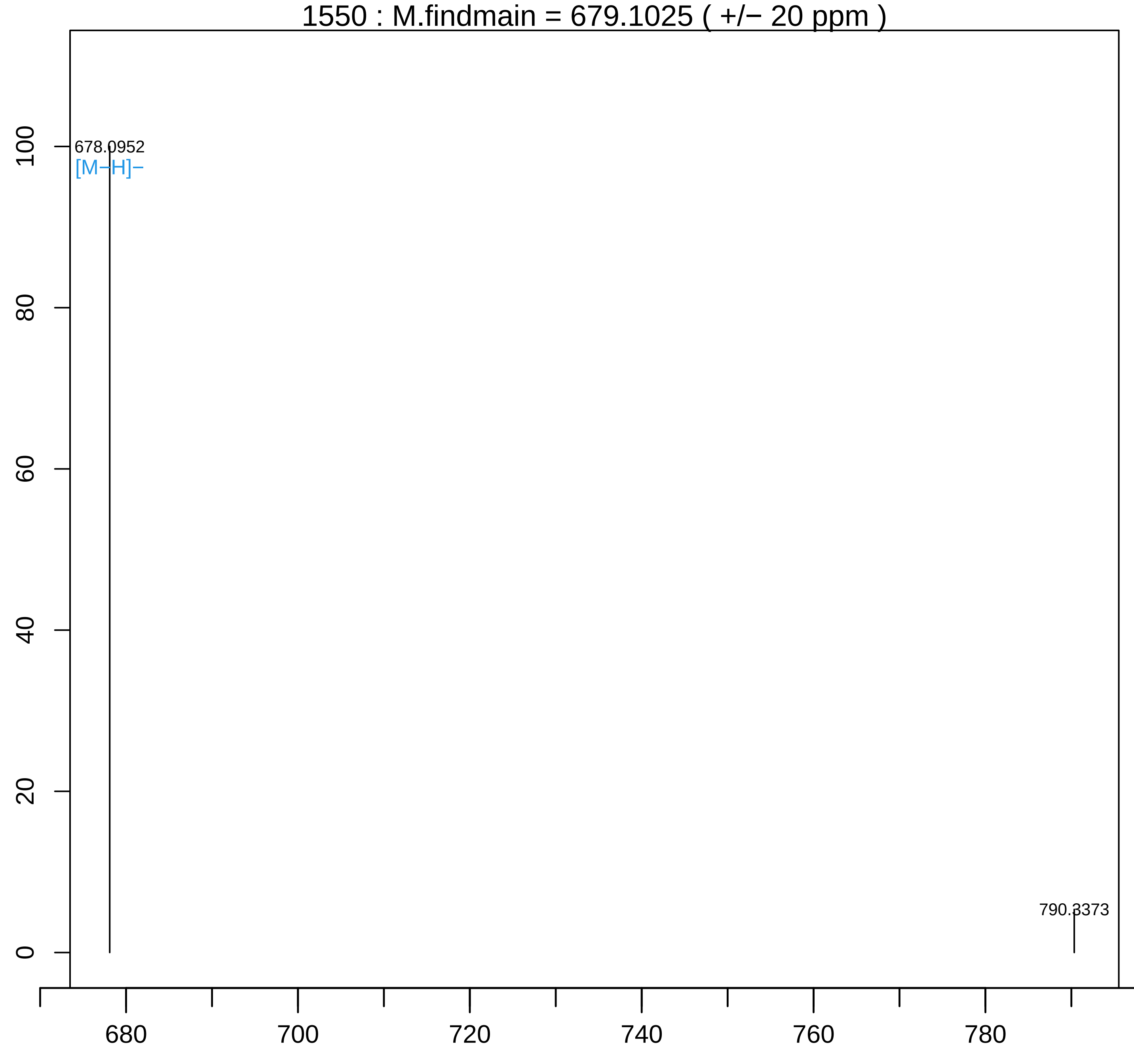

1550 : M.findmain = 679.1025 (+/− 20 ppm)
678.0952
[M−H]−
790.3373
0
20
40
60
80
100
680
700
720
740
760
780

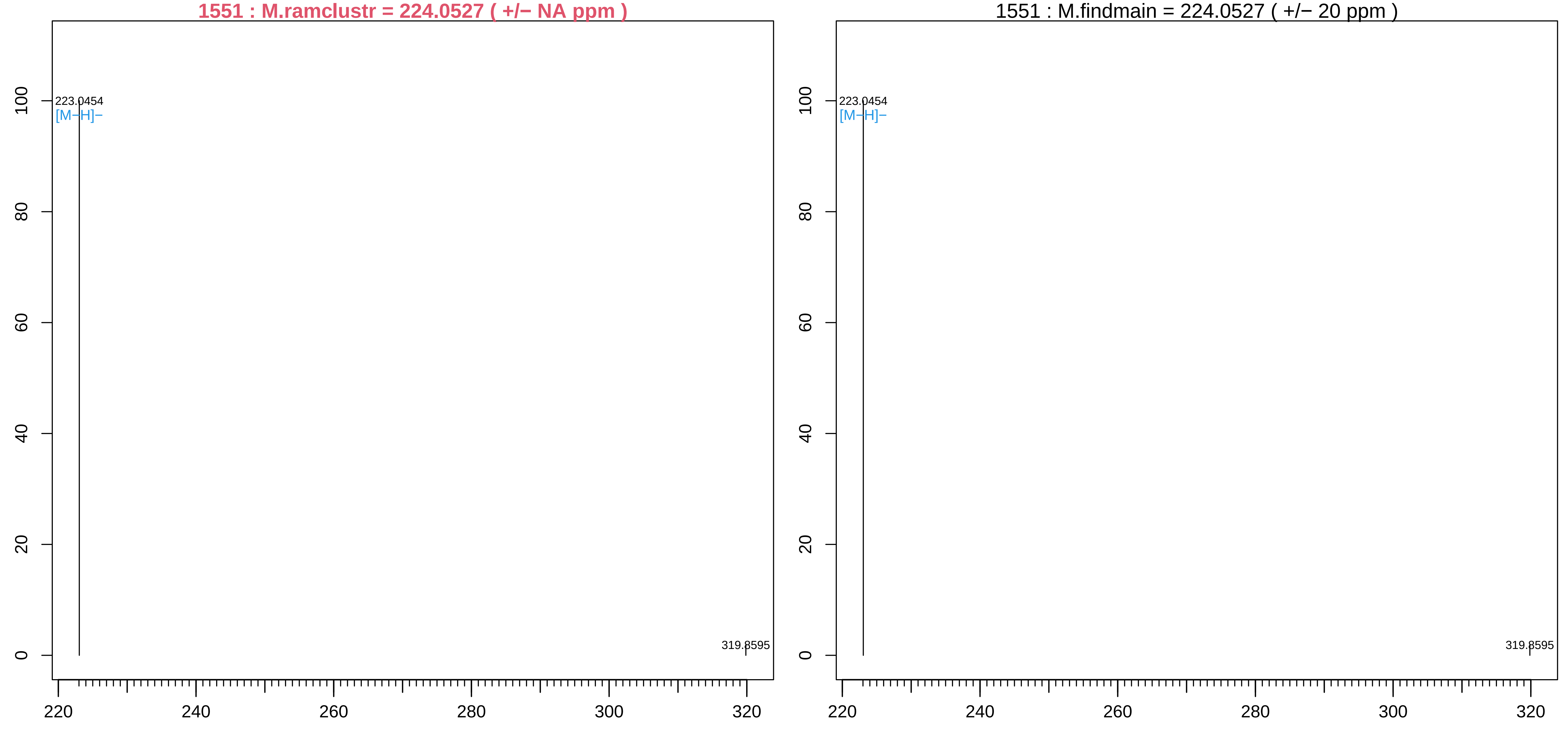
1551 : M.ramclustr = 224.0527 (+/− NA ppm)
223.0454
[M−H]−
319.8595
0
20
40
60
80
100
220
240
260
280
300
320
1551 : M.findmain = 224.0527 (+/− 20 ppm)
223.0454
[M−H]−
319.8595
0
20
40
60
80
100
220
240
260
280
300
320

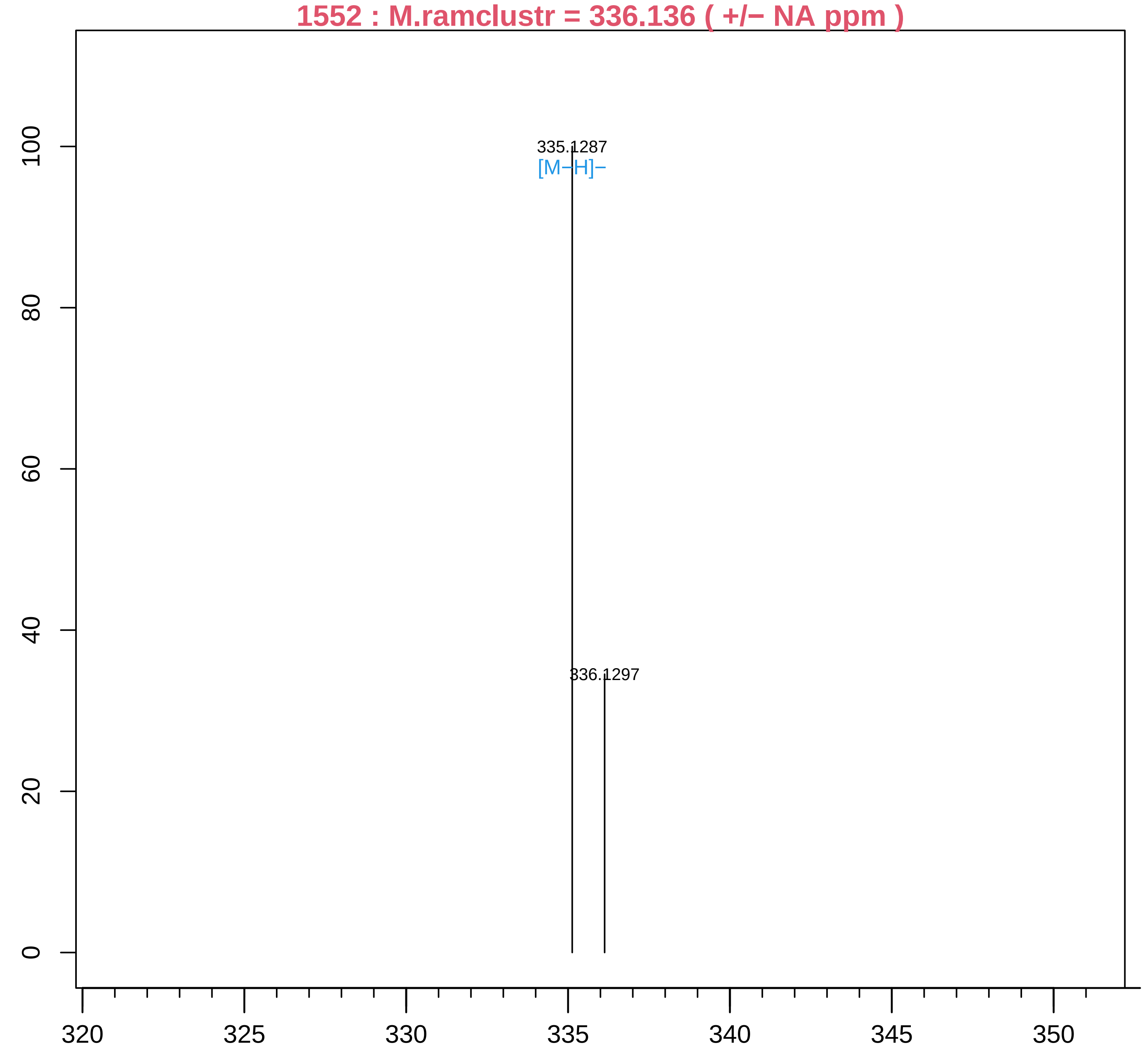

1552 : M.ramclustr = 336.136 (+/− NA ppm)
335.1287
[M−H]−
336.1297
0
20
40
60
80
100
320
325
330
335
340
345
350

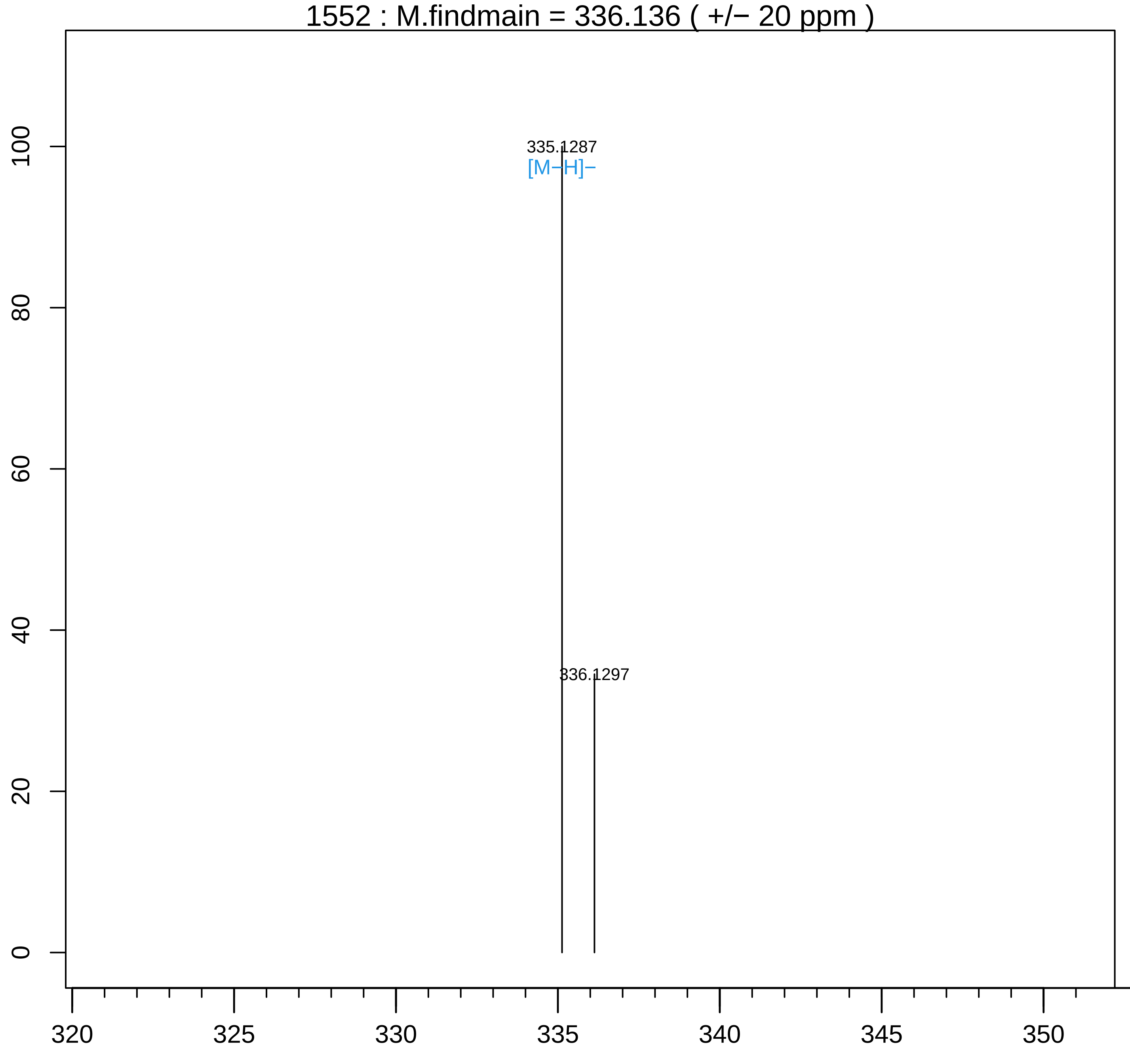

1552 : M.findmain = 336.136 (+/− 20 ppm)
335.1287
[M−H]−
336.1297
0
20
40
60
80
100
320
325
330
335
340
345
350

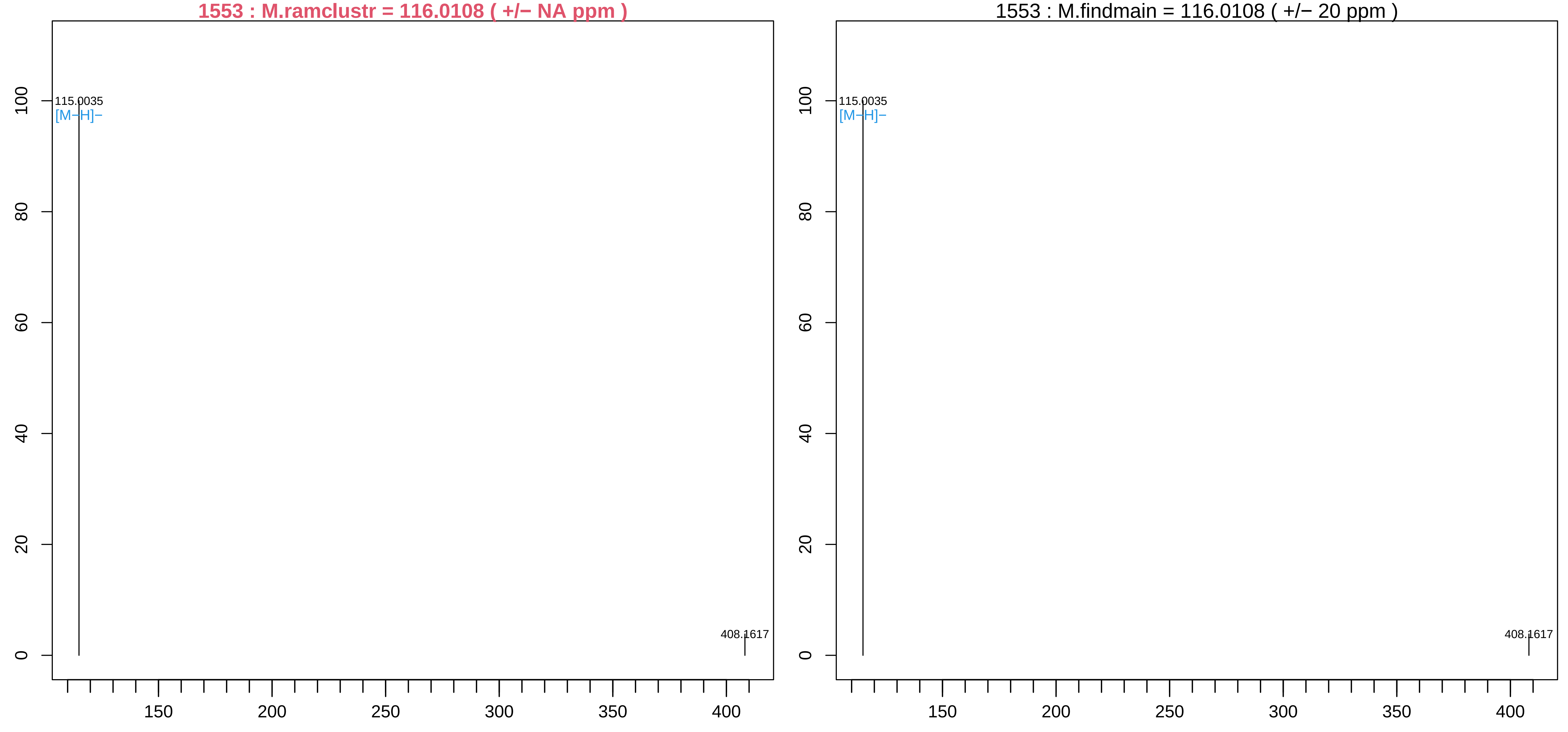
1553 : M.ramclustr = 116.0108 (+/− NA ppm)
115.0035
[M−H]−
408.1617
0
20
40
60
80
100
150
200
250
300
350
400
1553 : M.findmain = 116.0108 (+/− 20 ppm)
115.0035
[M−H]−
408.1617
0
20
40
60
80
100
150
200
250
300
350
400

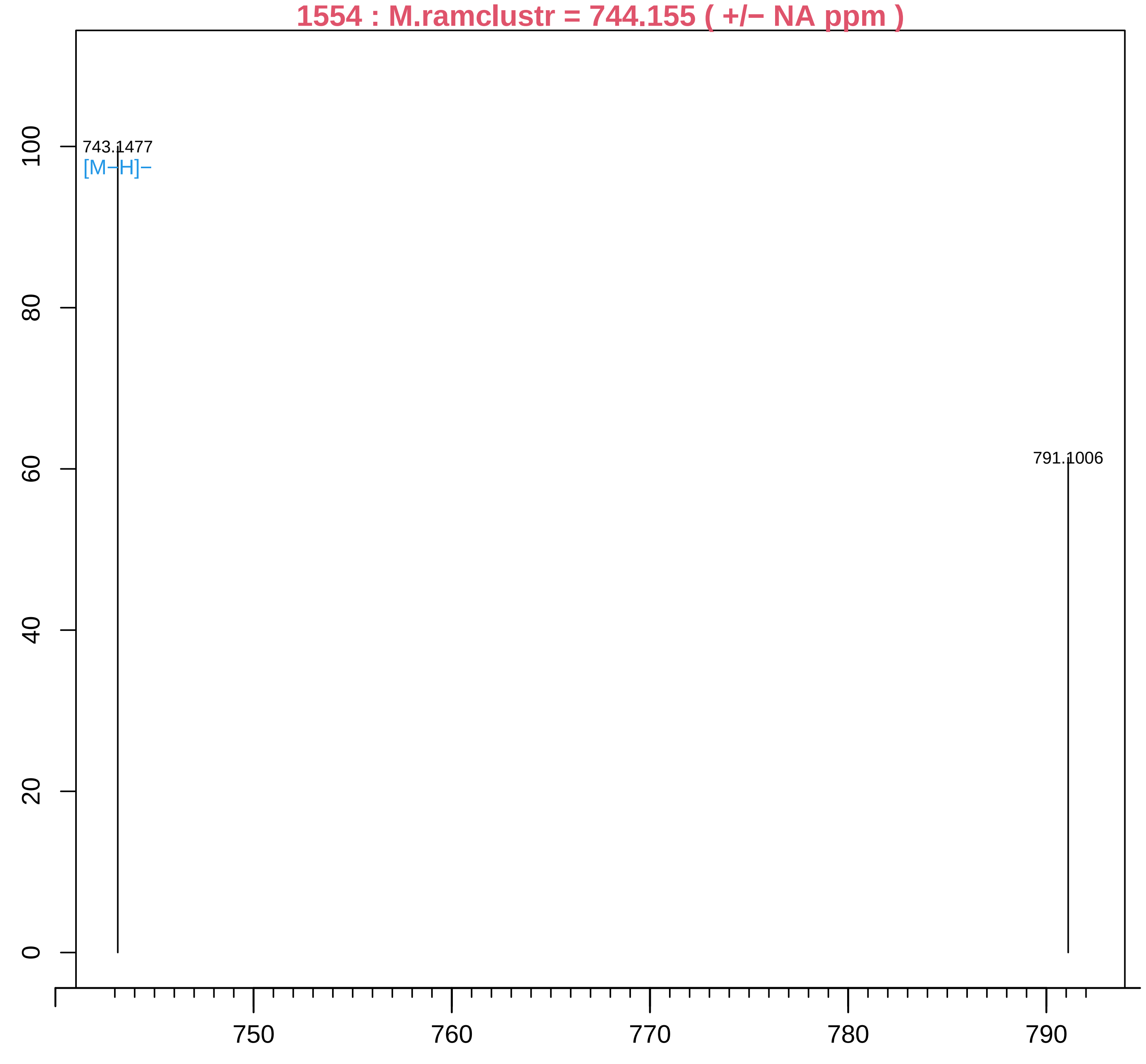

1554 : M.ramclustr = 744.155 (+/− NA ppm)
743.1477
[M−H]−
791.1006
0
20
40
60
80
100
750
760
770
780
790

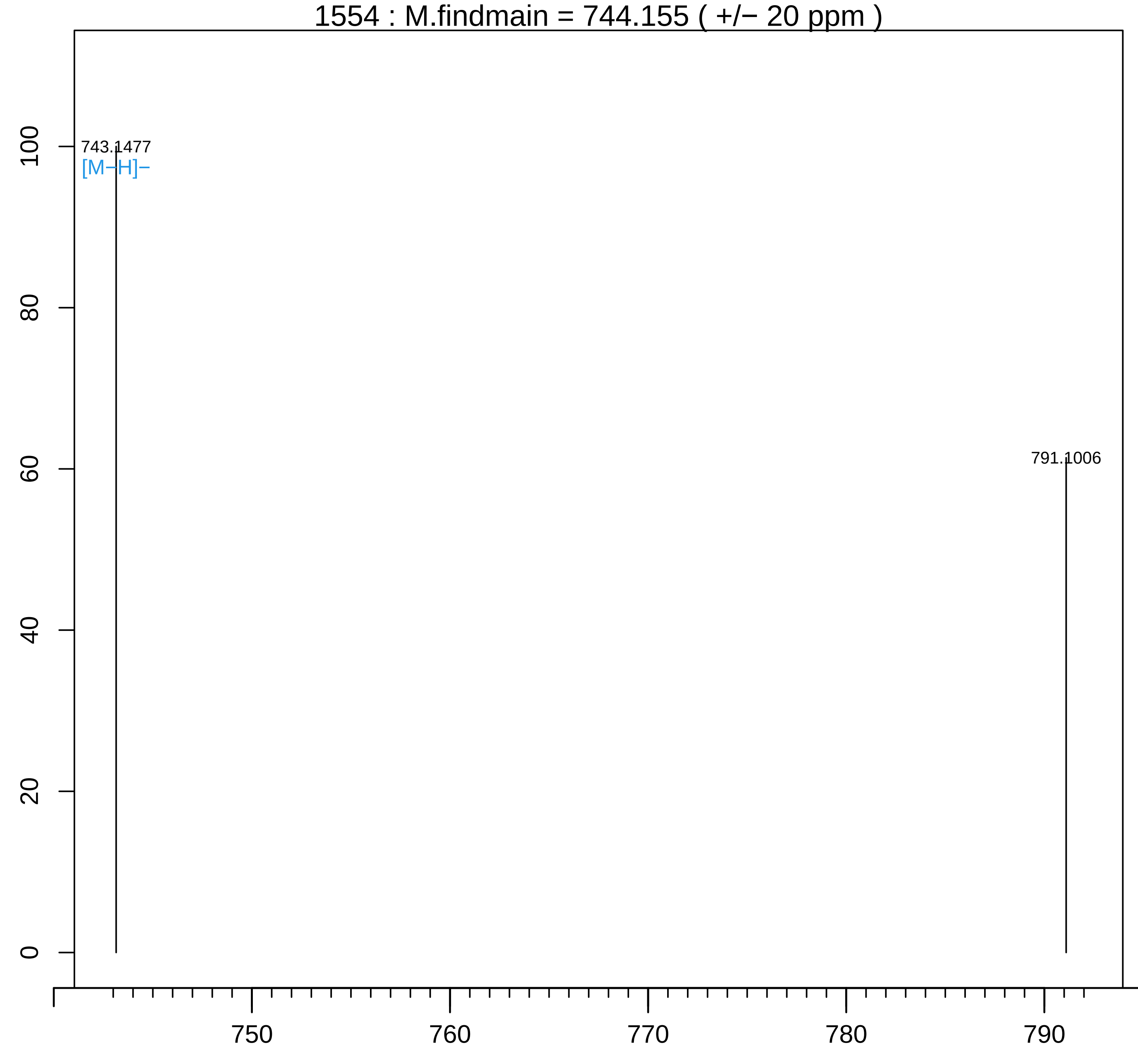

1554 : M.findmain = 744.155 (+/− 20 ppm)
743.1477
[M−H]−
791.1006
0
20
40
60
80
100
750
760
770
780
790

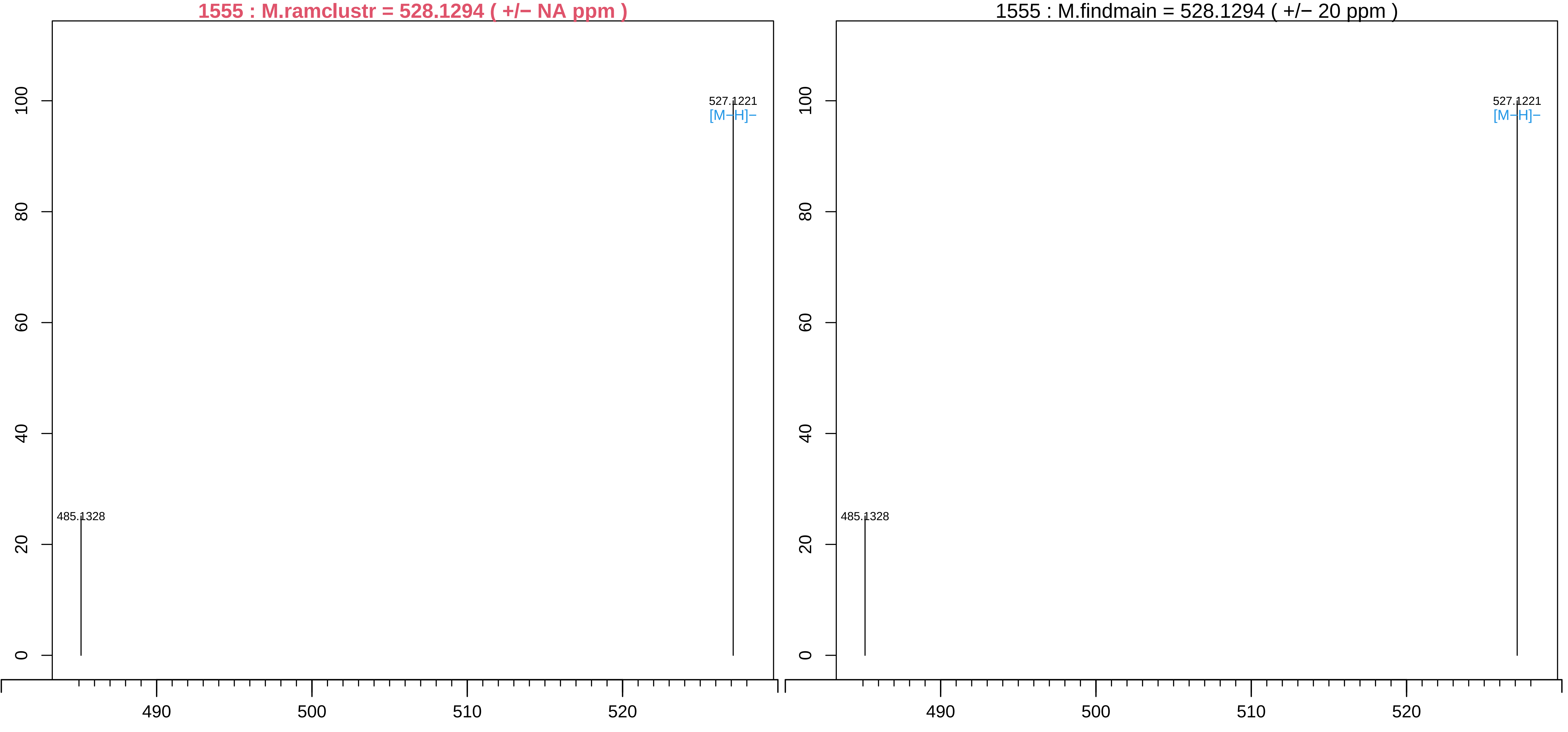

1555 : M.ramclustr = 528.1294 (+/− NA ppm)
527.1221
[M−H]−
485.1328
1555 : M.findmain = 528.1294 (+/− 20 ppm)
527.1221
[M−H]−
485.1328
0
20
40
60
80
100
490
500
510
520

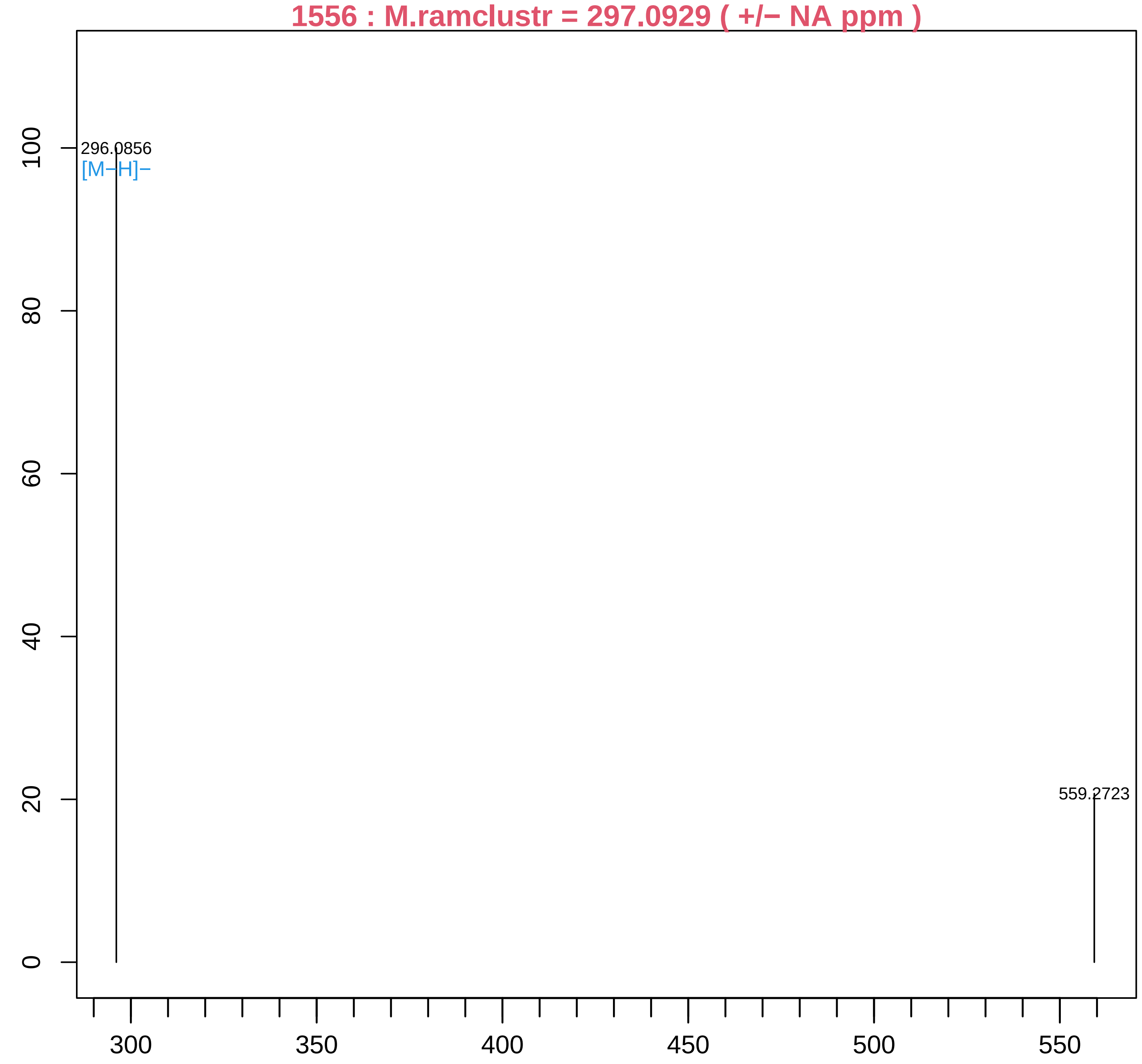
1556 : M.ramclustr = 297.0929 (+/− NA ppm)
296.0856
[M−H]−
559.2723
0
20
40
60
80
100
300
350
400
450
500
550

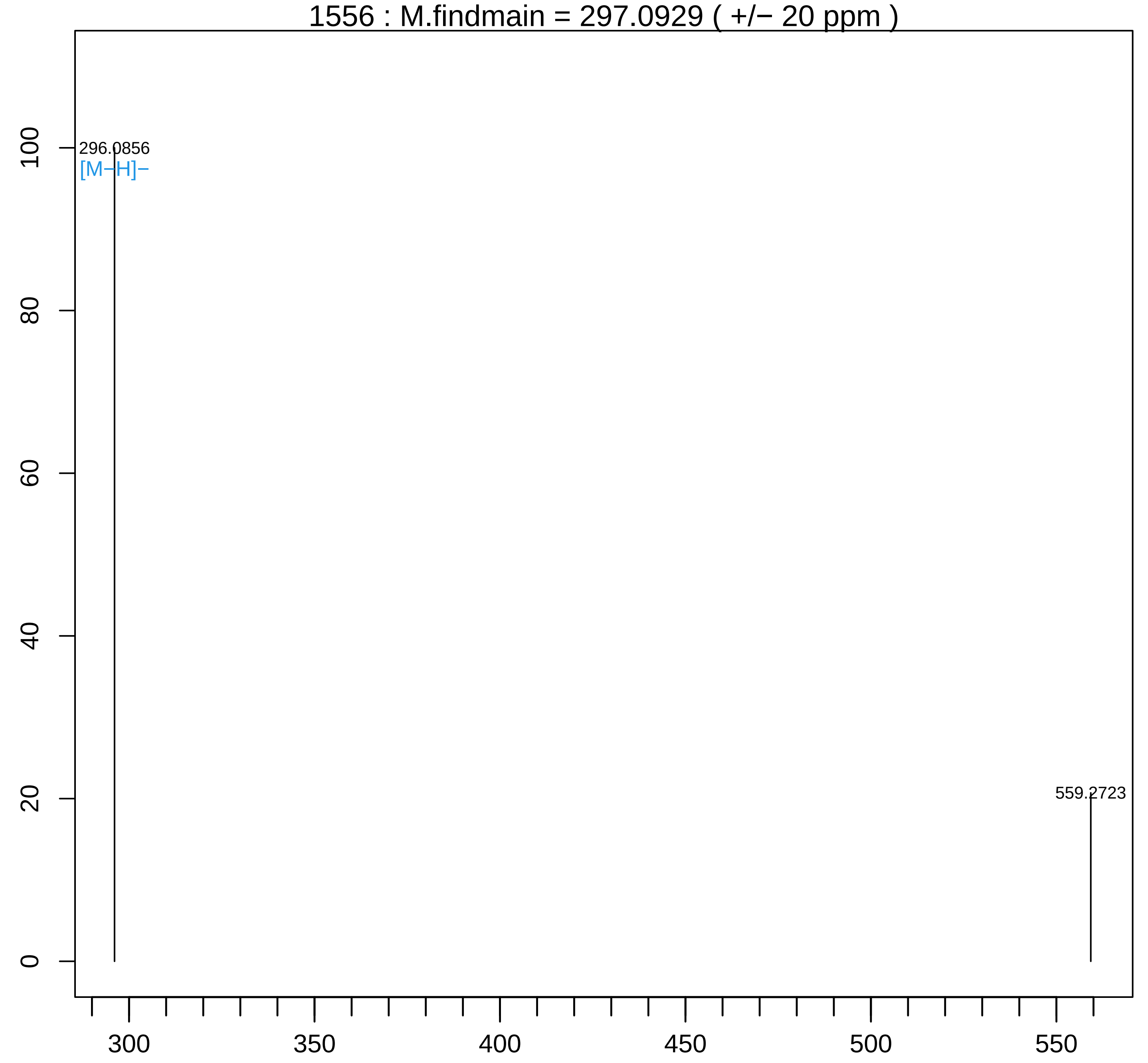
1556 : M.findmain = 297.0929 (+/− 20 ppm)
296.0856
[M−H]−
559.2723
0
20
40
60
80
100
300
350
400
450
500
550

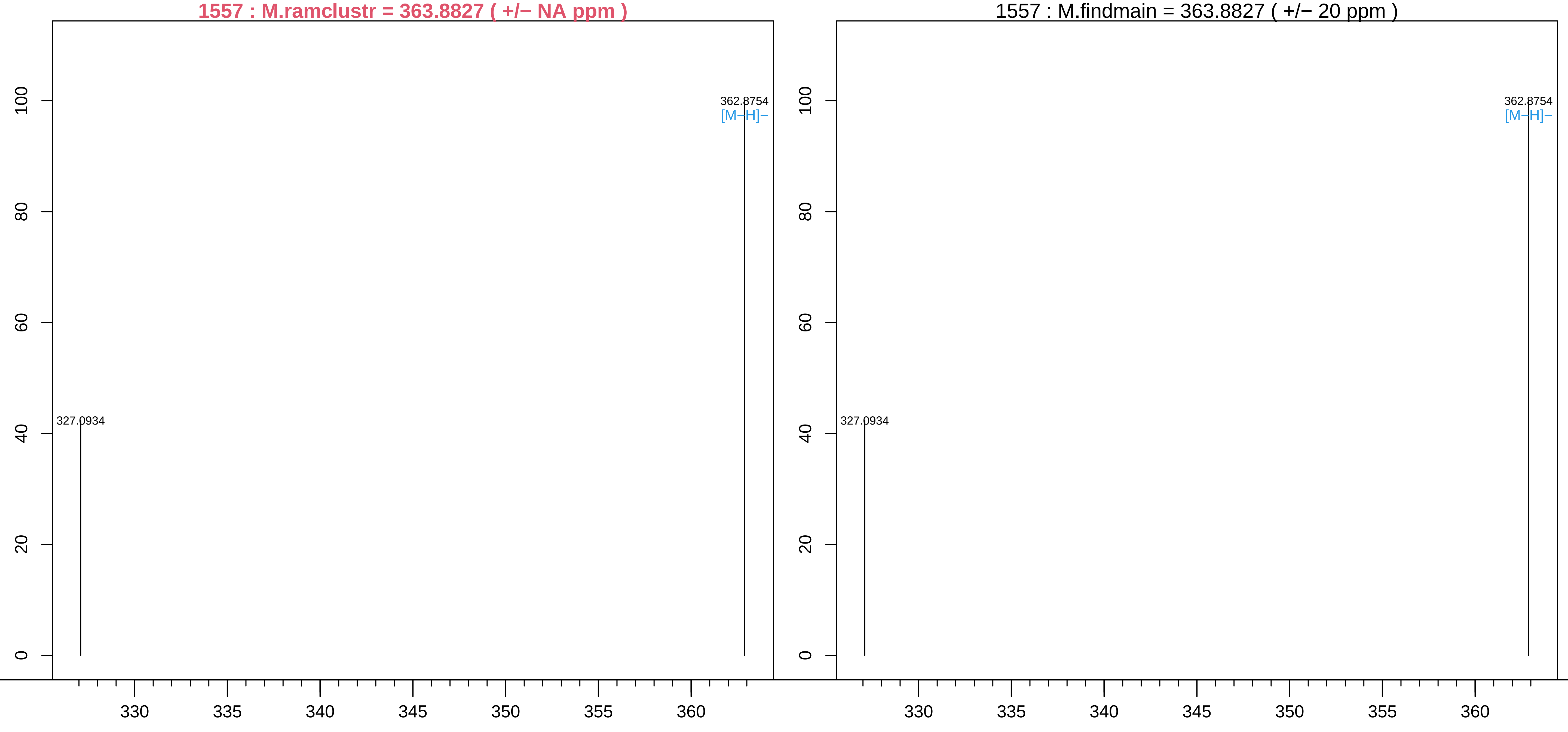
1557 : M.ramclustr = 363.8827 (+/− NA ppm)
1557 : M.findmain = 363.8827 (+/− 20 ppm)
362.8754
[M−H]−
327.0934
0
20
40
60
80
100
330
335
340
345
350
355
360

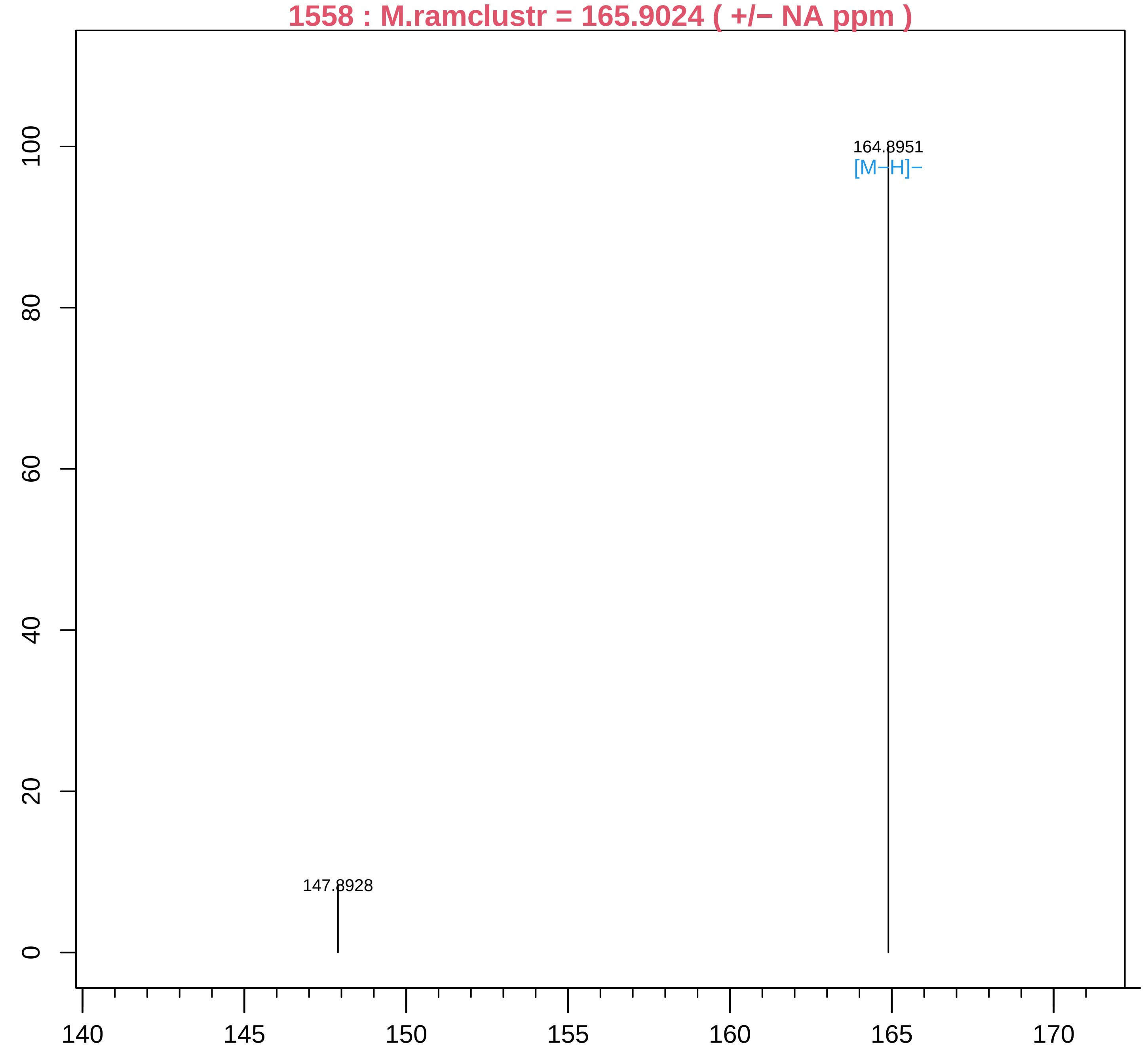
1558 : M.ramclustr = 165.9024 (+/− NA ppm)
164.8951
[M−H]−
147.8928
0
20
40
60
80
100
140
145
150
155
160
165
170

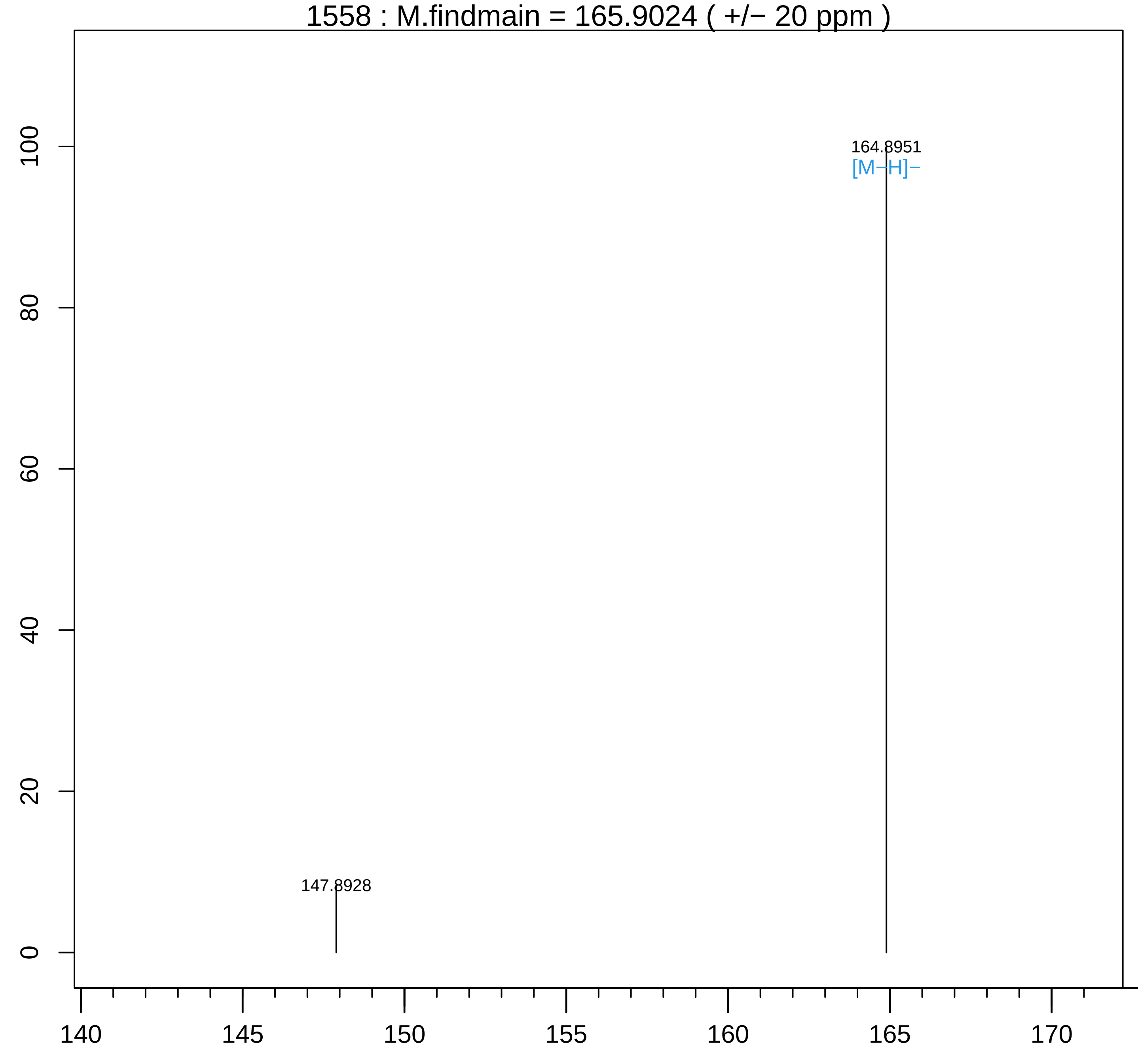
1558 : M.findmain = 165.9024 (+/− 20 ppm)
164.8951
[M−H]−
147.8928
0
20
40
60
80
100
140
145
150
155
160
165
170

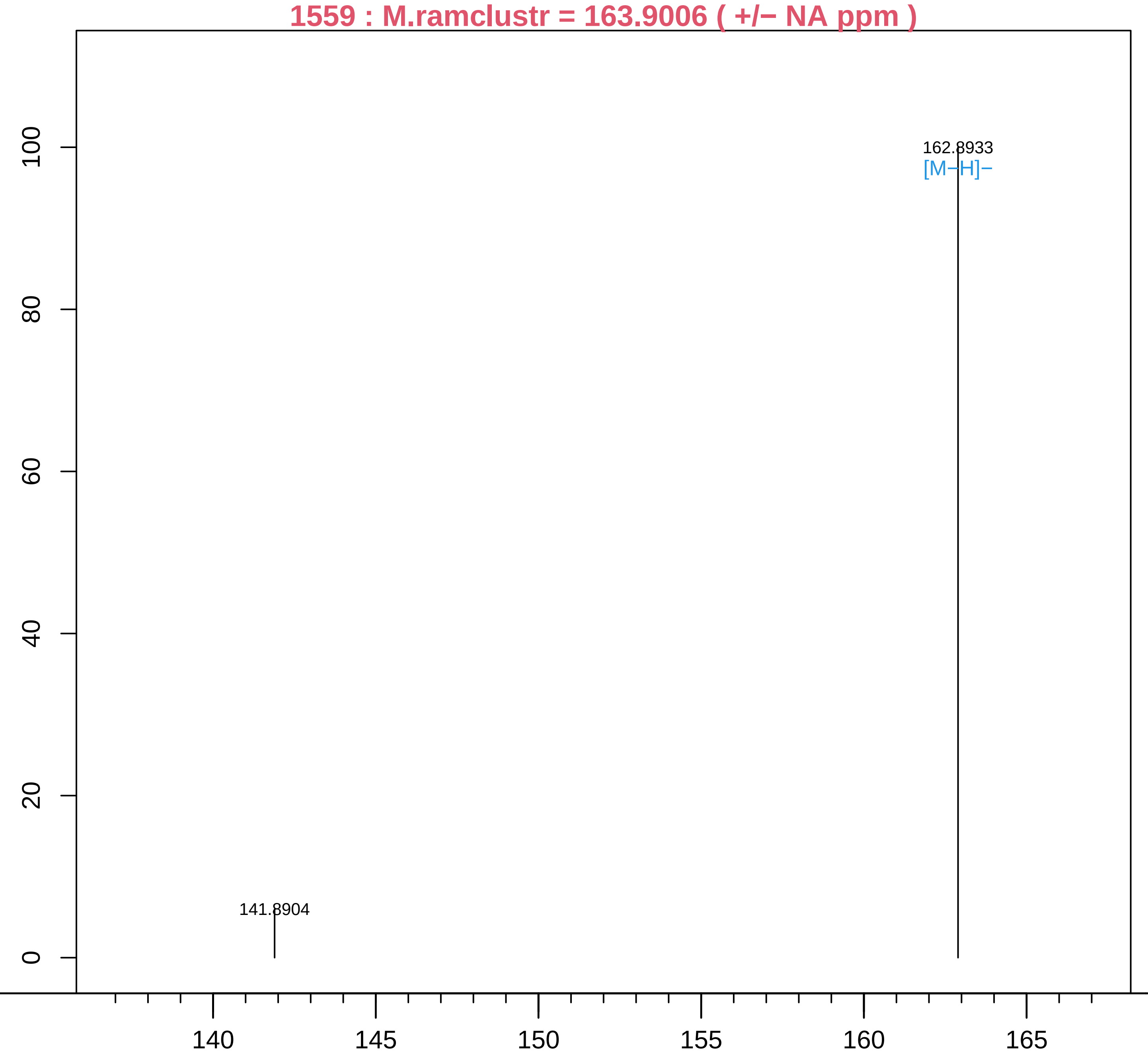

1559 : M.ramclustr = 163.9006 (+/− NA ppm)
162.8933
[M−H]−
141.8904
0
20
40
60
80
100
140
145
150
155
160
165

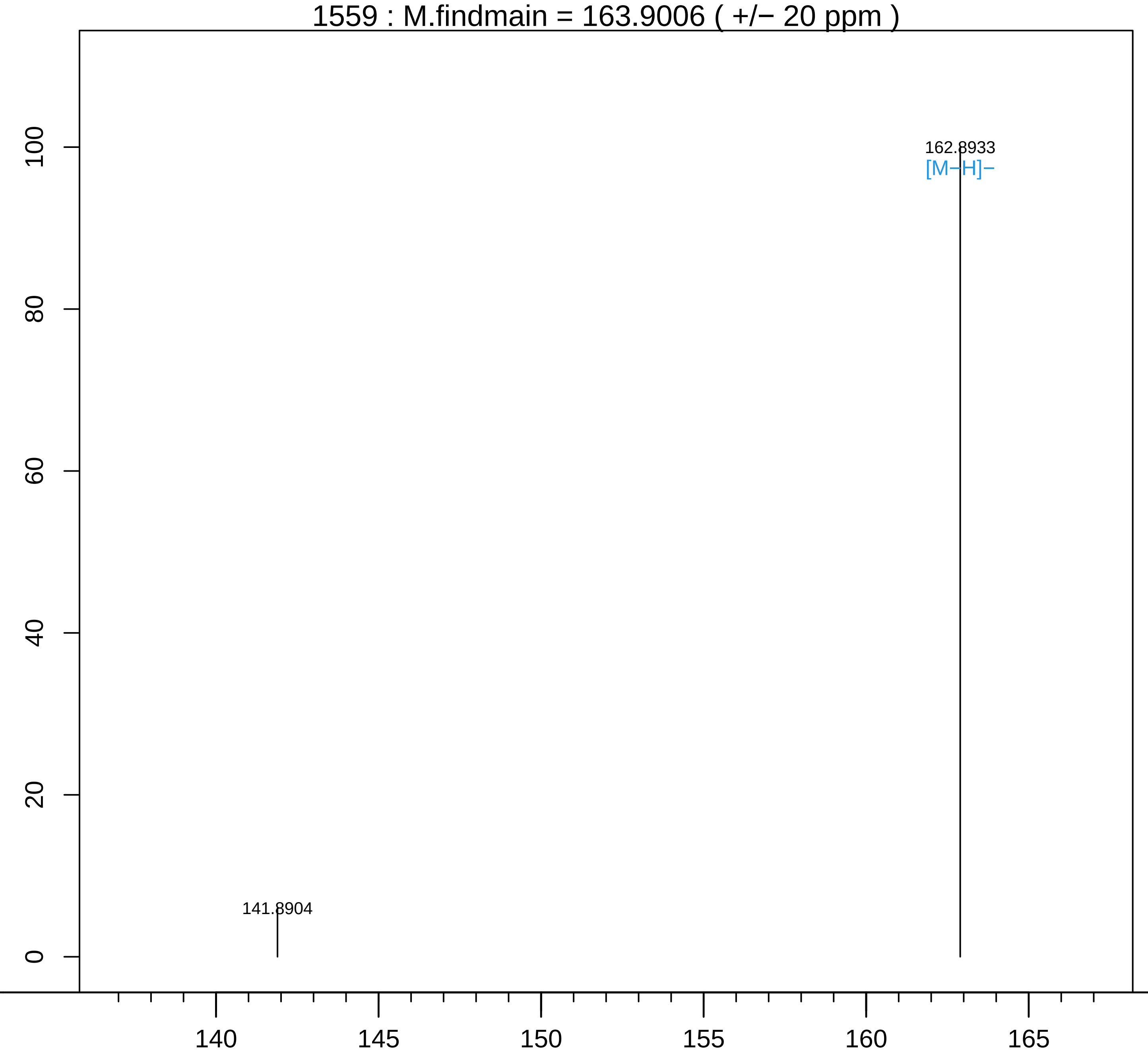

1559 : M.findmain = 163.9006 (+/− 20 ppm)
162.8933
[M−H]−
141.8904
0
20
40
60
80
100
140
145
150
155
160
165

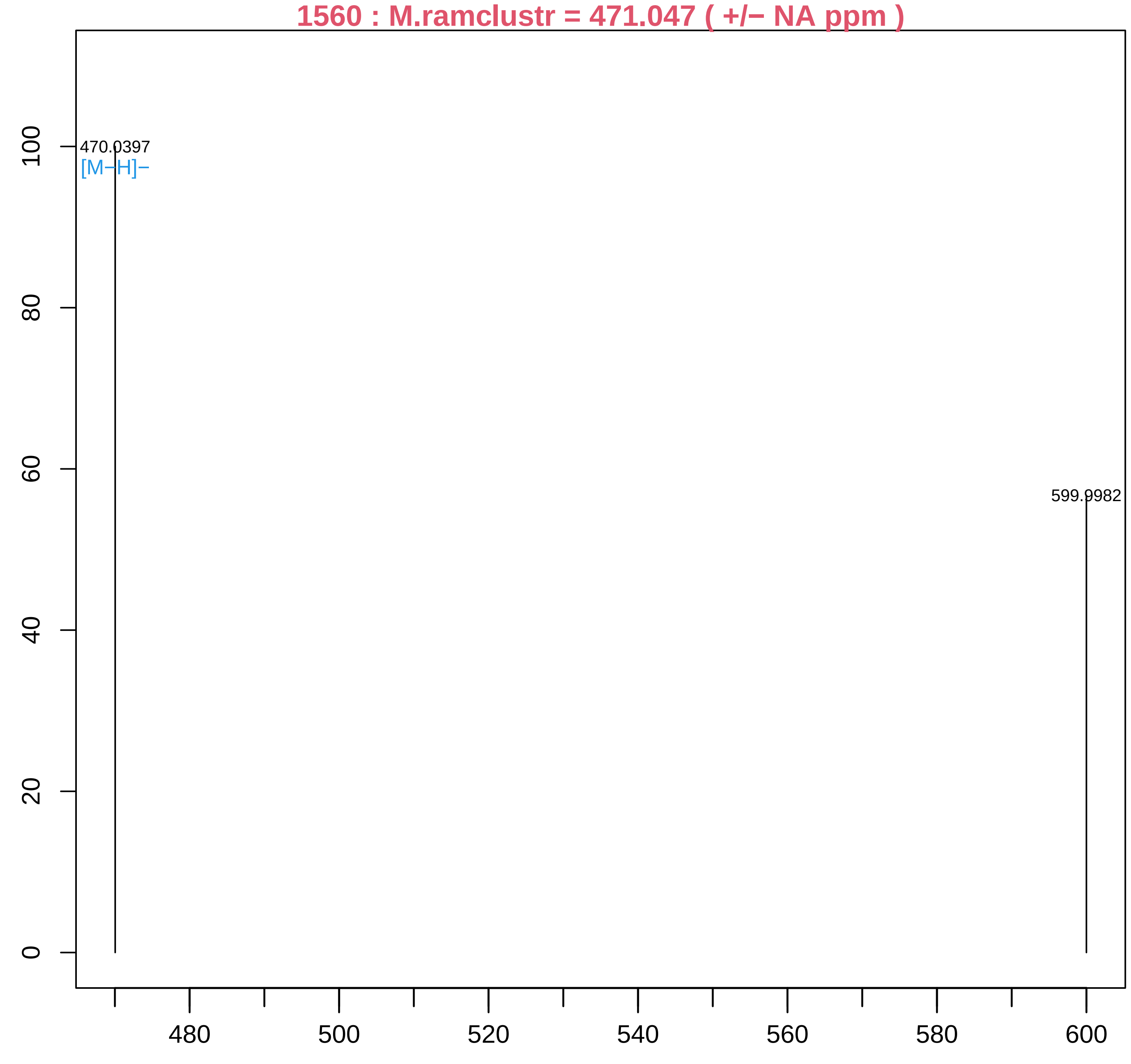
1560 : M.ramclustr = 471.047 (+/− NA ppm)
470.0397
[M−H]−
599.9982
0
20
40
60
80
100
480
500
520
540
560
580
600

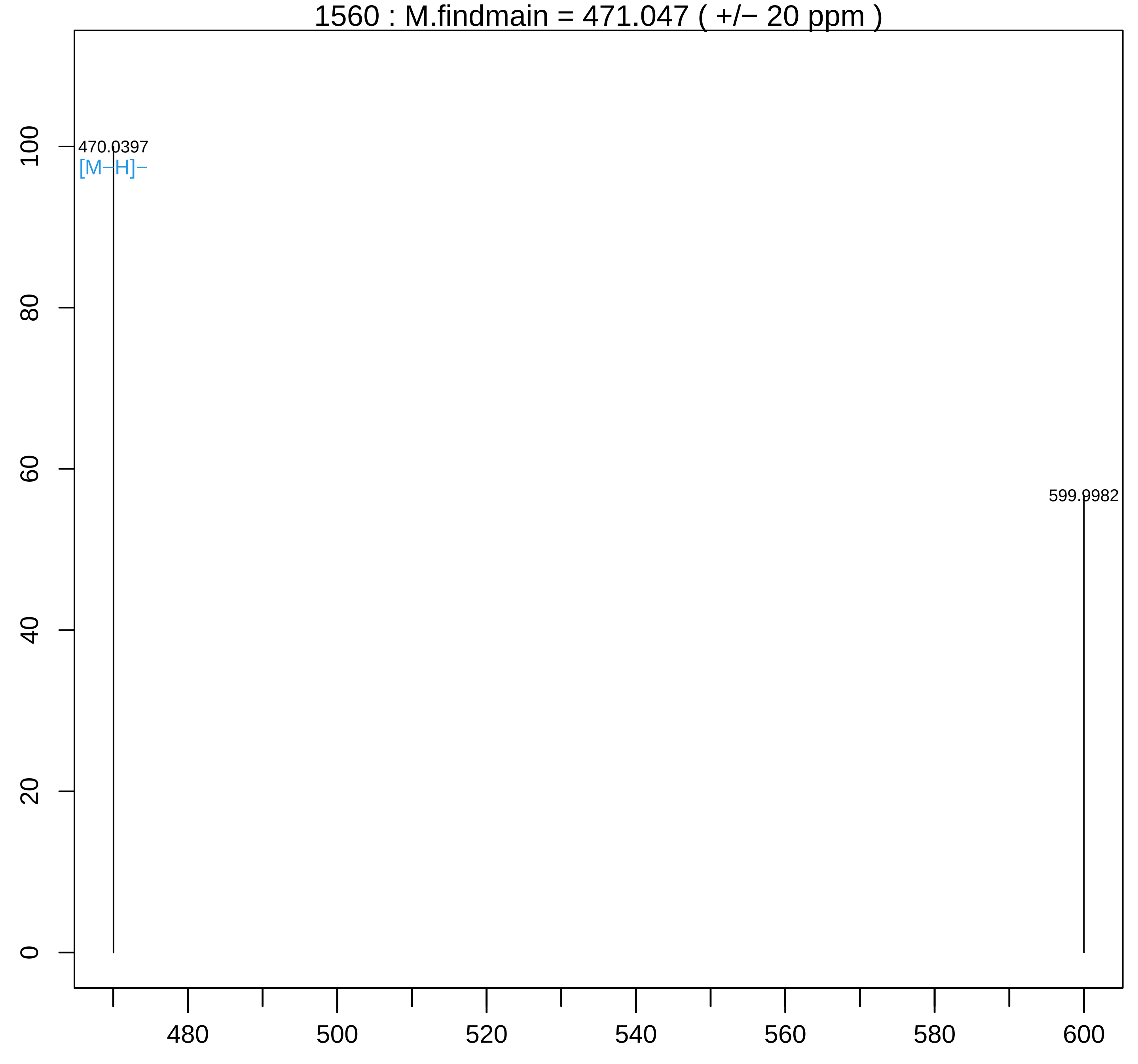
1560 : M.findmain = 471.047 (+/− 20 ppm)
470.0397
[M−H]−
599.9982
0
20
40
60
80
100
480
500
520
540
560
580
600

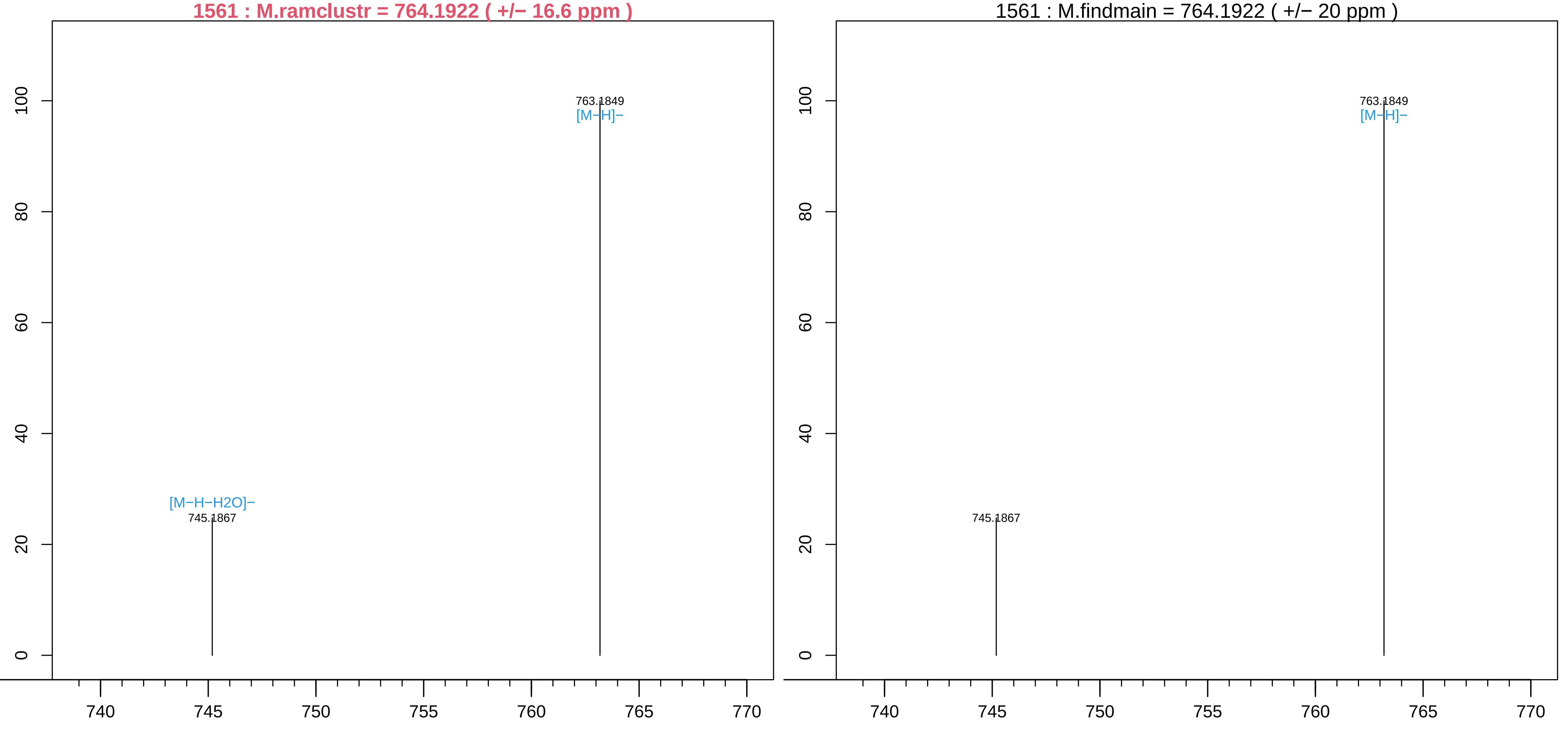
1561 : M.ramclustr = 764.1922 (+/− 16.6 ppm)
763.1849
[M−H]−
[M−H−H2O]−
745.1867
1561 : M.findmain = 764.1922 (+/− 20 ppm)
763.1849
[M−H]−
745.1867
0
20
40
60
80
100
740
745
750
755
760
765
770

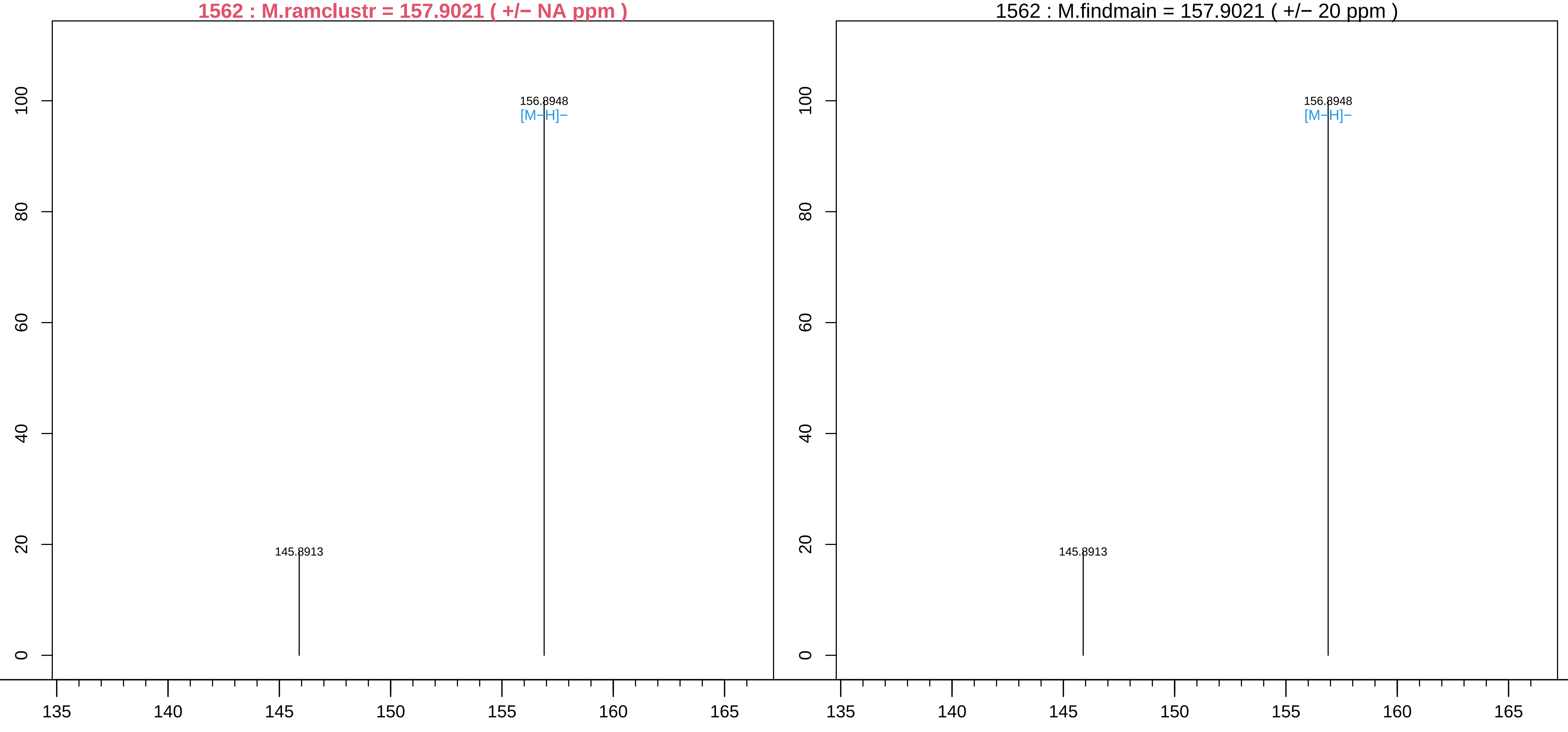

1562 : M.ramclustr = 157.9021 (+/− NA ppm)
156.8948
[M−H]−
145.8913
1562 : M.findmain = 157.9021 (+/− 20 ppm)
156.8948
[M−H]−
145.8913
0
20
40
60
80
100
135
140
145
150
155
160
165

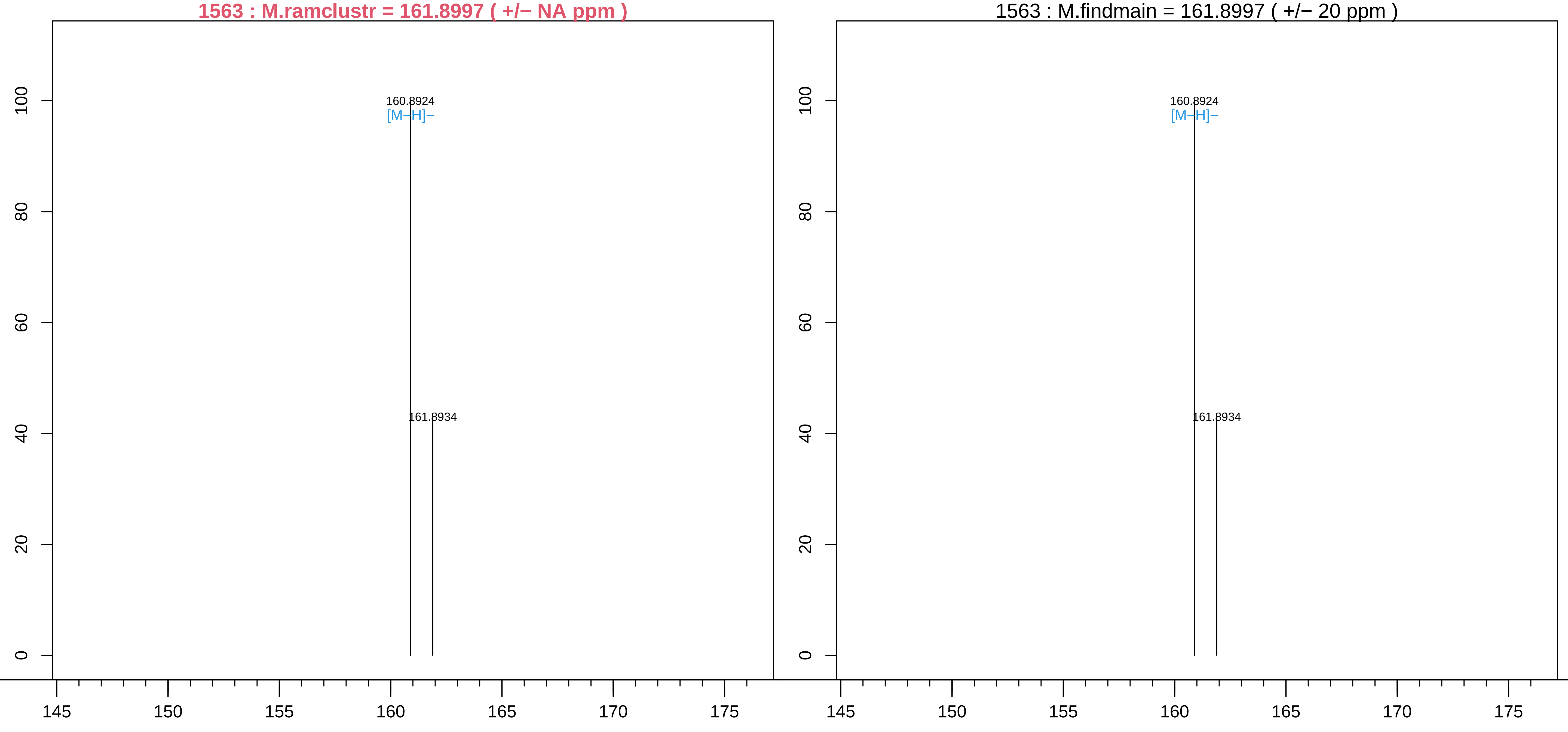
1563 : M.ramclustr = 161.8997 (+/− NA ppm)
160.8924
[M−H]−
161.8934
1563 : M.findmain = 161.8997 (+/− 20 ppm)
160.8924
[M−H]−
161.8934
0
20
40
60
80
100
145
150
155
160
165
170
175

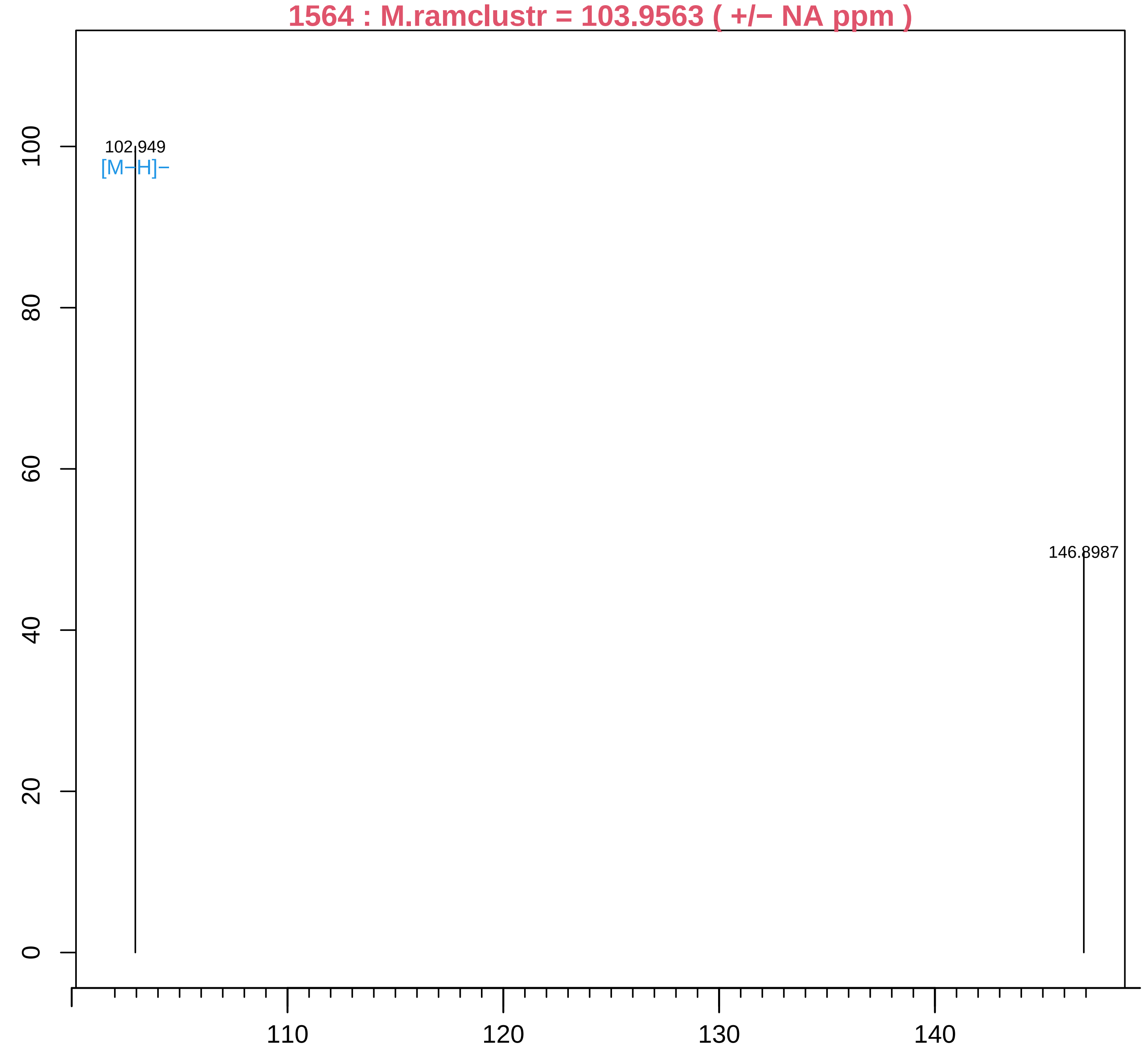
1564 : M.ramclustr = 103.9563 (+/− NA ppm)
102.949
[M−H]−
146.8987
0
20
40
60
80
100
110
120
130
140

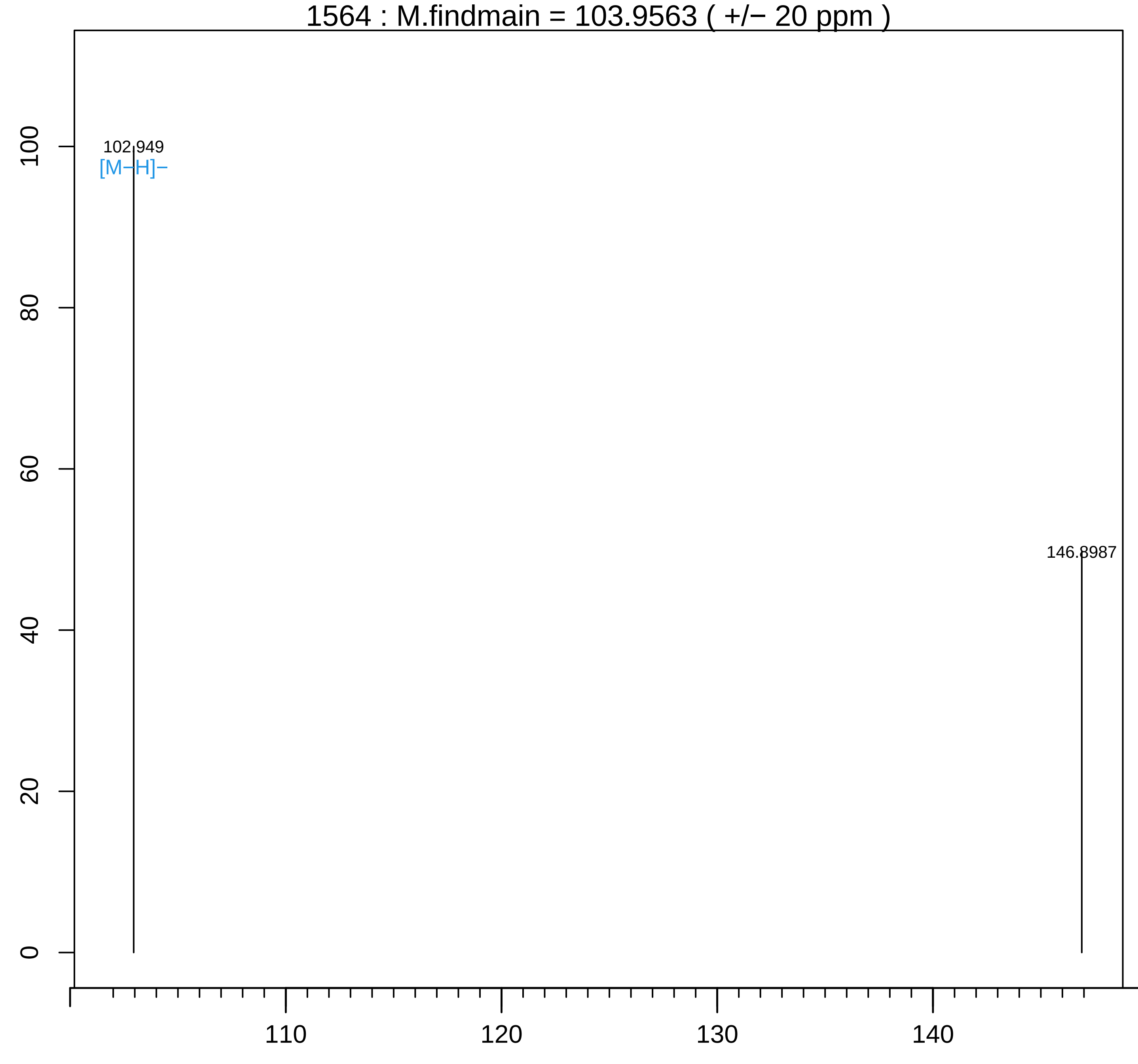
1564 : M.findmain = 103.9563 (+/− 20 ppm)
102.949
[M−H]−
146.8987
0
20
40
60
80
100
110
120
130
140

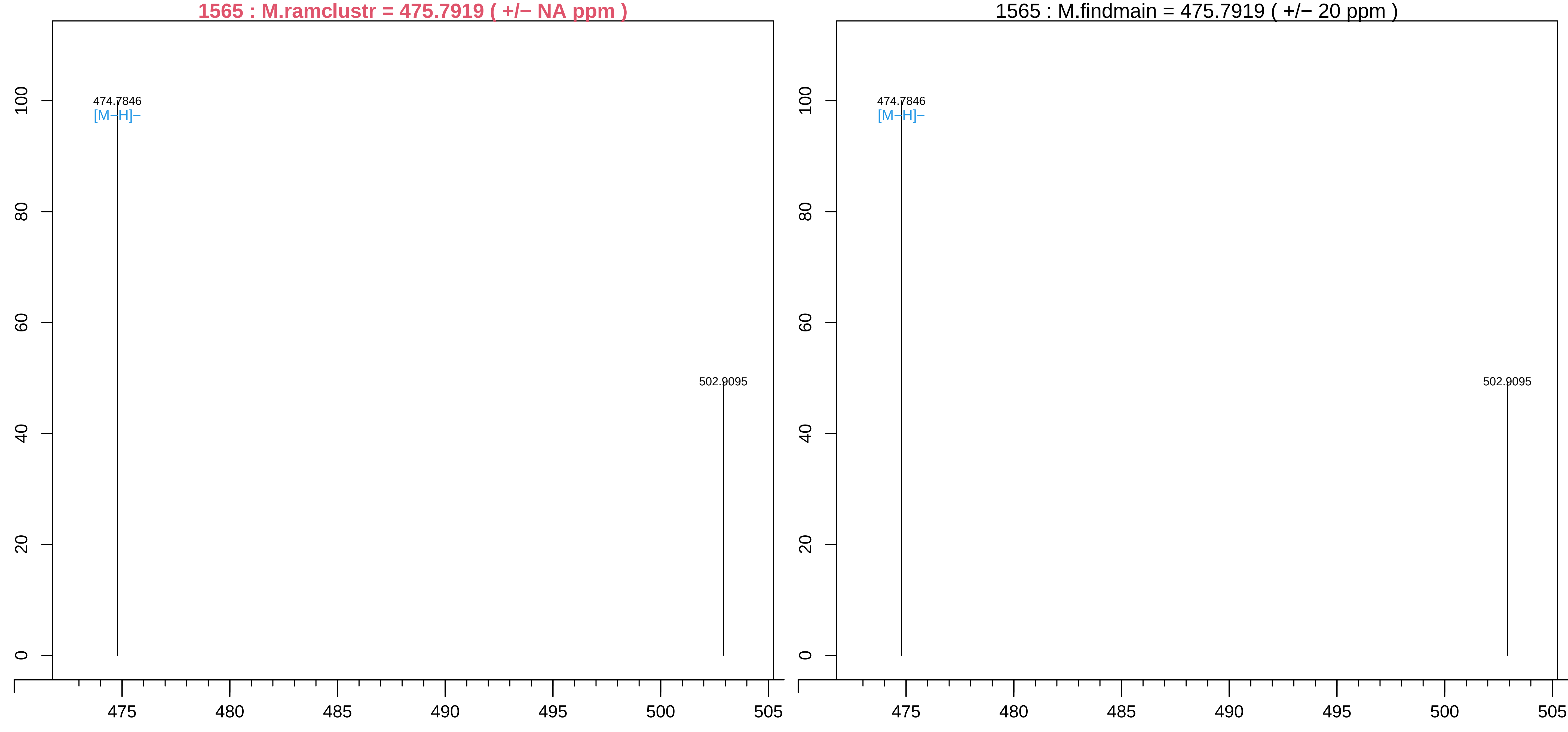
1565 : M.ramclustr = 475.7919 (+/− NA ppm)
474.7846
[M−H]−
502.9095
1565 : M.findmain = 475.7919 (+/− 20 ppm)
474.7846
[M−H]−
502.9095
0
20
40
60
80
100
475
480
485
490
495
500
505

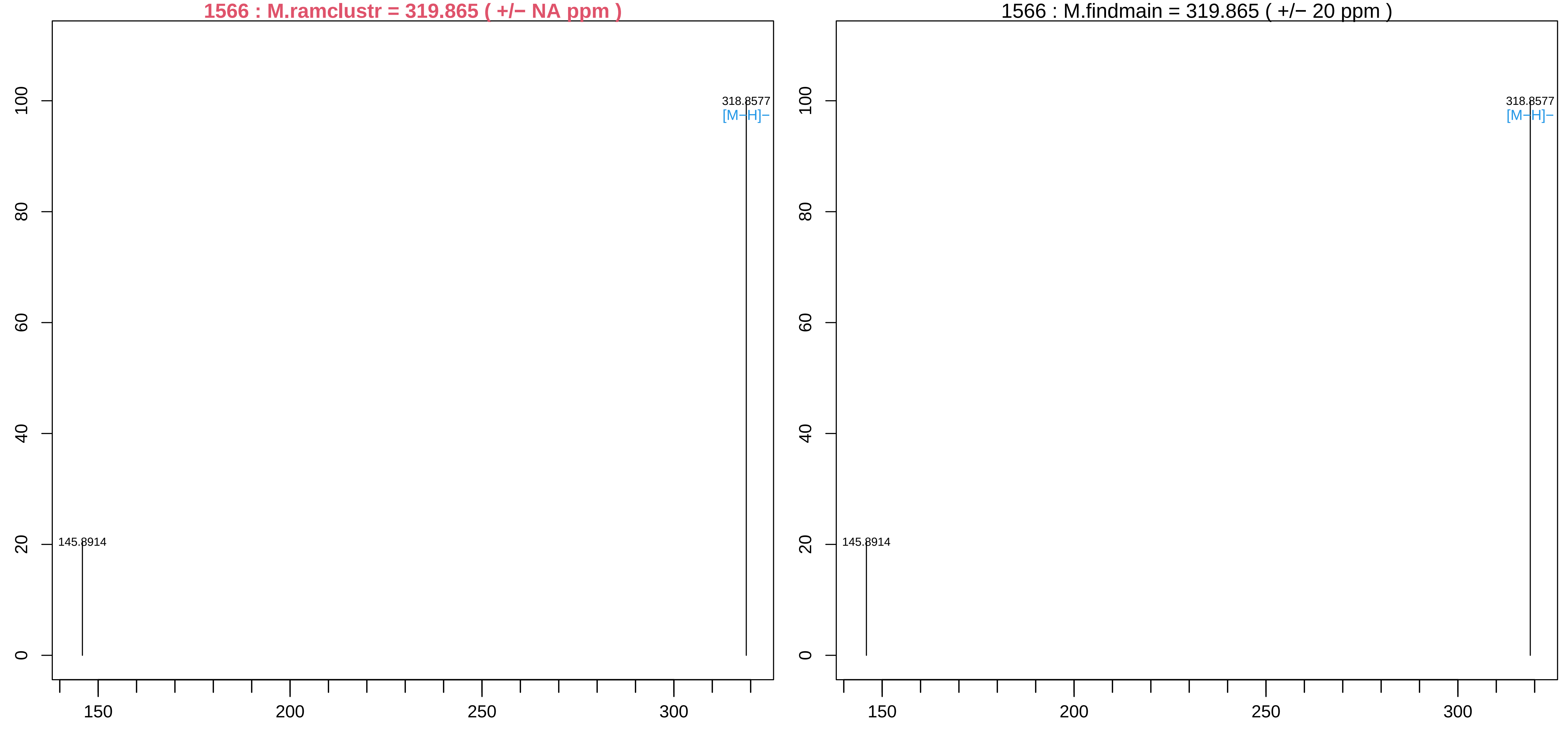
1566 : M.ramclustr = 319.865 (+/− NA ppm)
318.8577
[M−H]−
145.8914
1566 : M.findmain = 319.865 (+/− 20 ppm)
318.8577
[M−H]−
145.8914
0
20
40
60
80
100
150
200
250
300

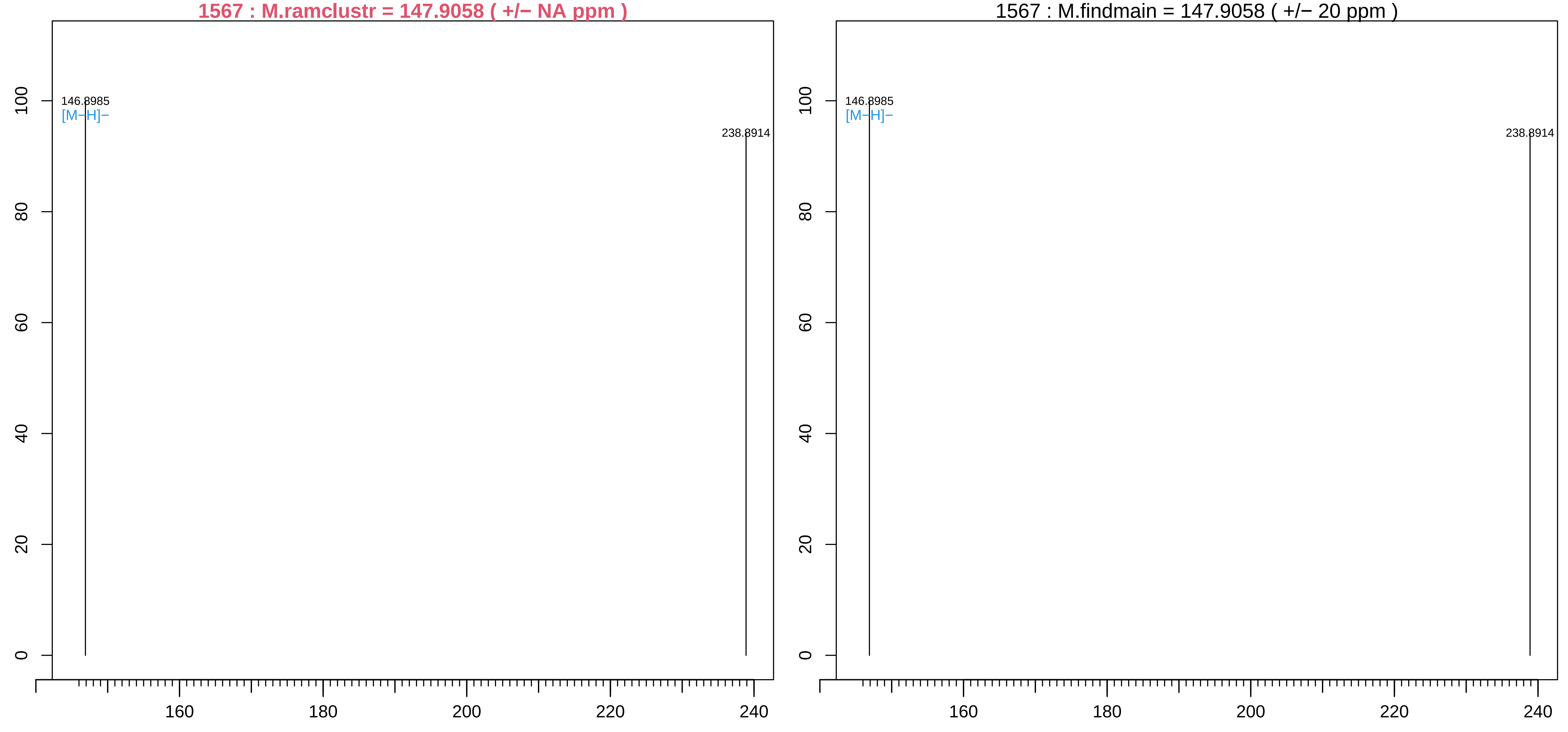

1567 : M.ramclustr = 147.9058 (+/− NA ppm)
146.8985
[M−H]−
238.8914
0
20
40
60
80
100
160
180
200
220
240
1567 : M.findmain = 147.9058 (+/− 20 ppm)
146.8985
[M−H]−
238.8914
0
20
40
60
80
100
160
180
200
220
240

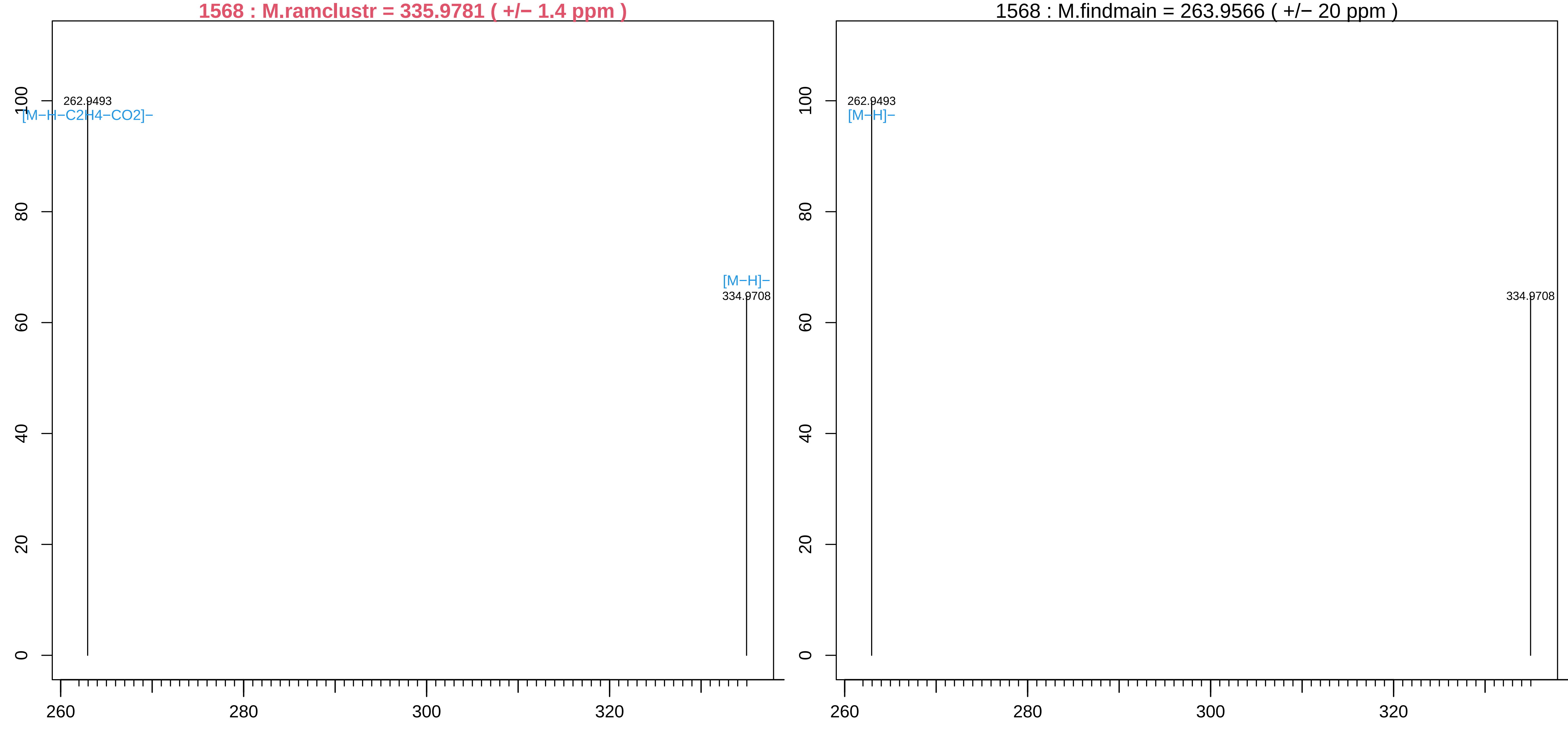
1568 : M.ramclustr = 335.9781 (+/− 1.4 ppm)
262.9493
[M−H−C2H4−CO2]−
[M−H]−
334.9708
1568 : M.findmain = 263.9566 (+/− 20 ppm)
262.9493
[M−H]−
334.9708
0
20
40
60
80
100
260
280
300
320

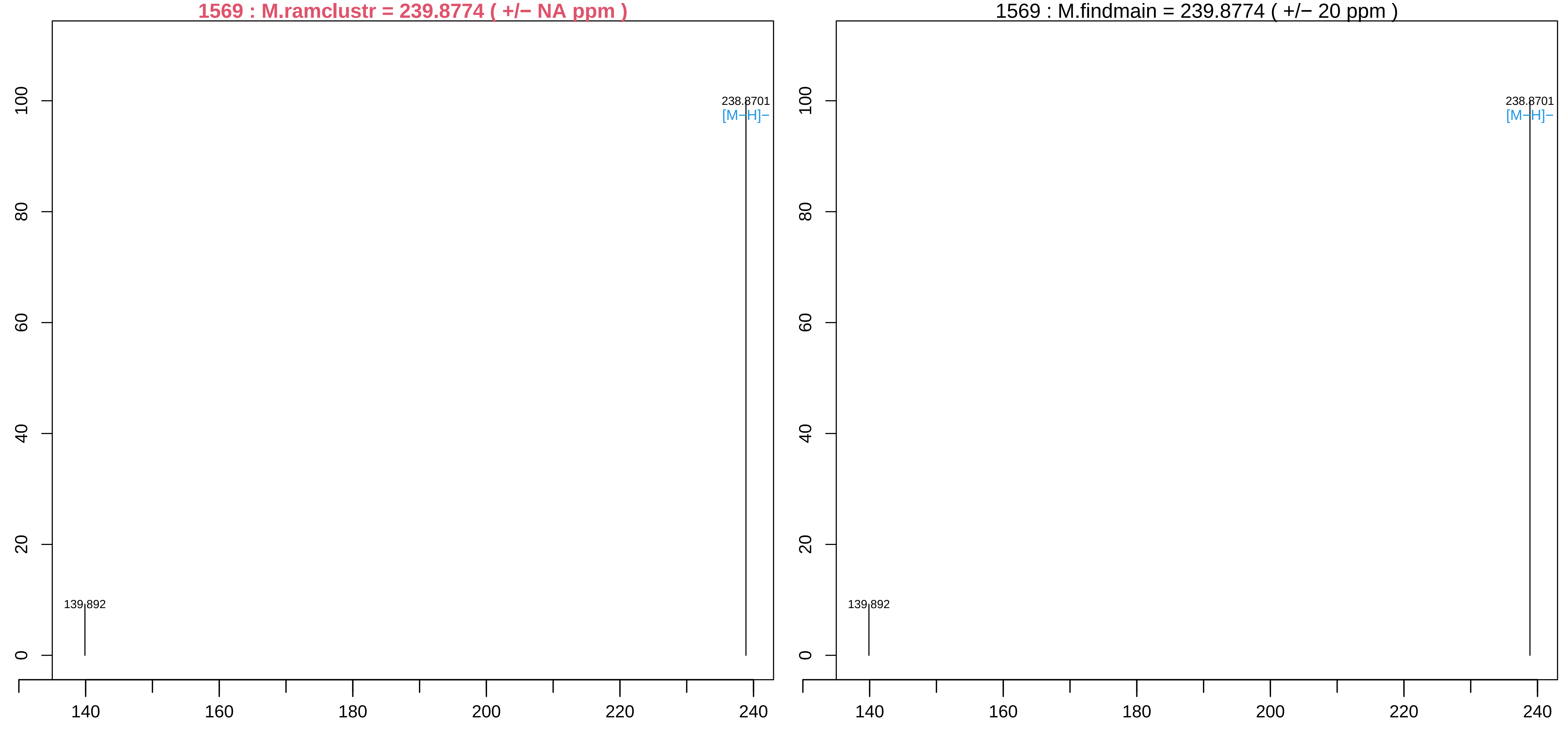

1569 : M.ramclustr = 239.8774 (+/− NA ppm)
238.8701
[M−H]−
139.892
0
20
40
60
80
100
140
160
180
200
220
240
1569 : M.findmain = 239.8774 (+/− 20 ppm)
238.8701
[M−H]−
139.892

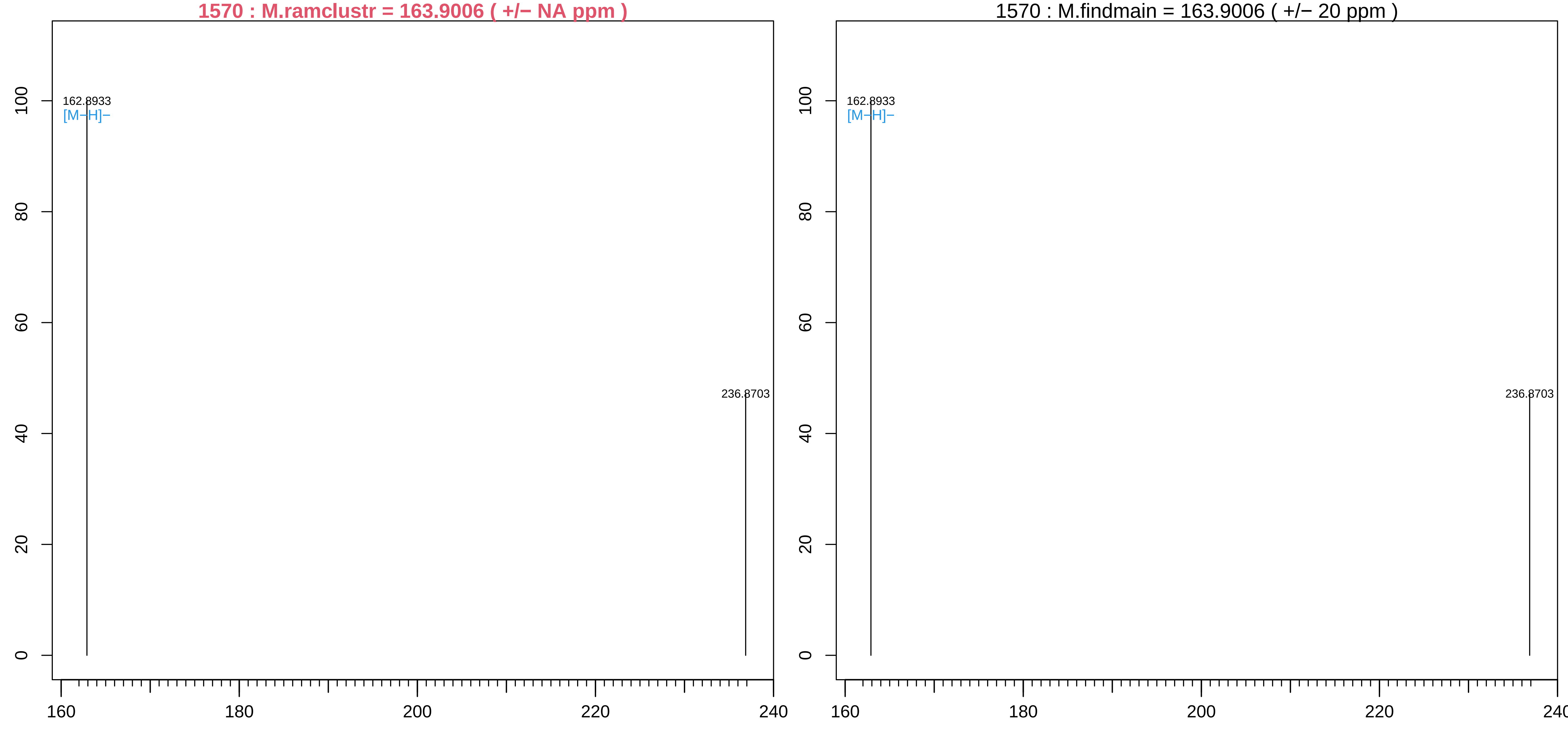
1570 : M.ramclustr = 163.9006 (+/− NA ppm)
162.8933
[M−H]−
236.8703
1570 : M.findmain = 163.9006 (+/− 20 ppm)
162.8933
[M−H]−
236.8703
0
20
40
60
80
100
160
180
200
220
240

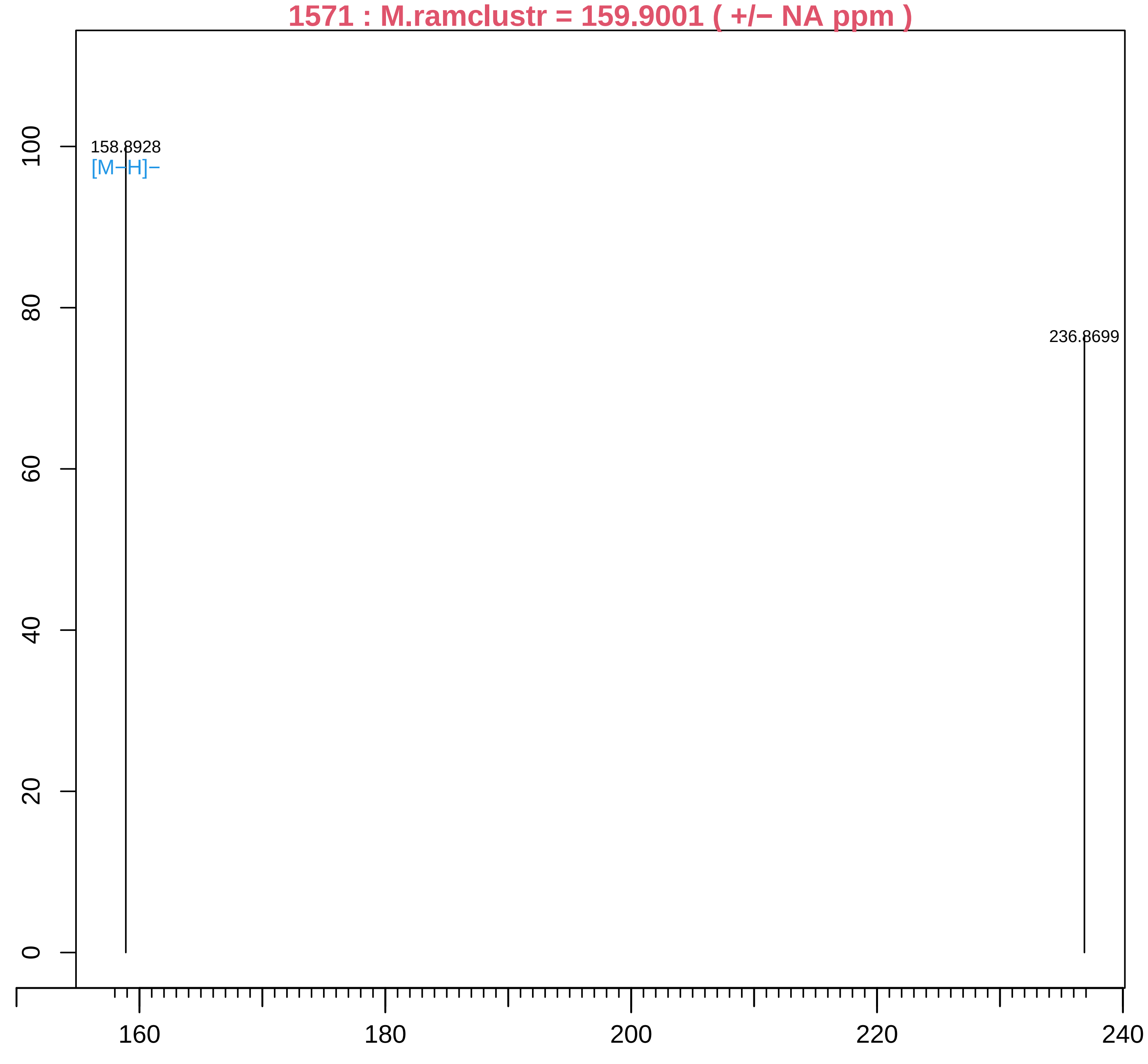
1571 : M.ramclustr = 159.9001 (+/− NA ppm)
158.8928
[M−H]−
236.8699
0
20
40
60
80
100
160
180
200
220
240

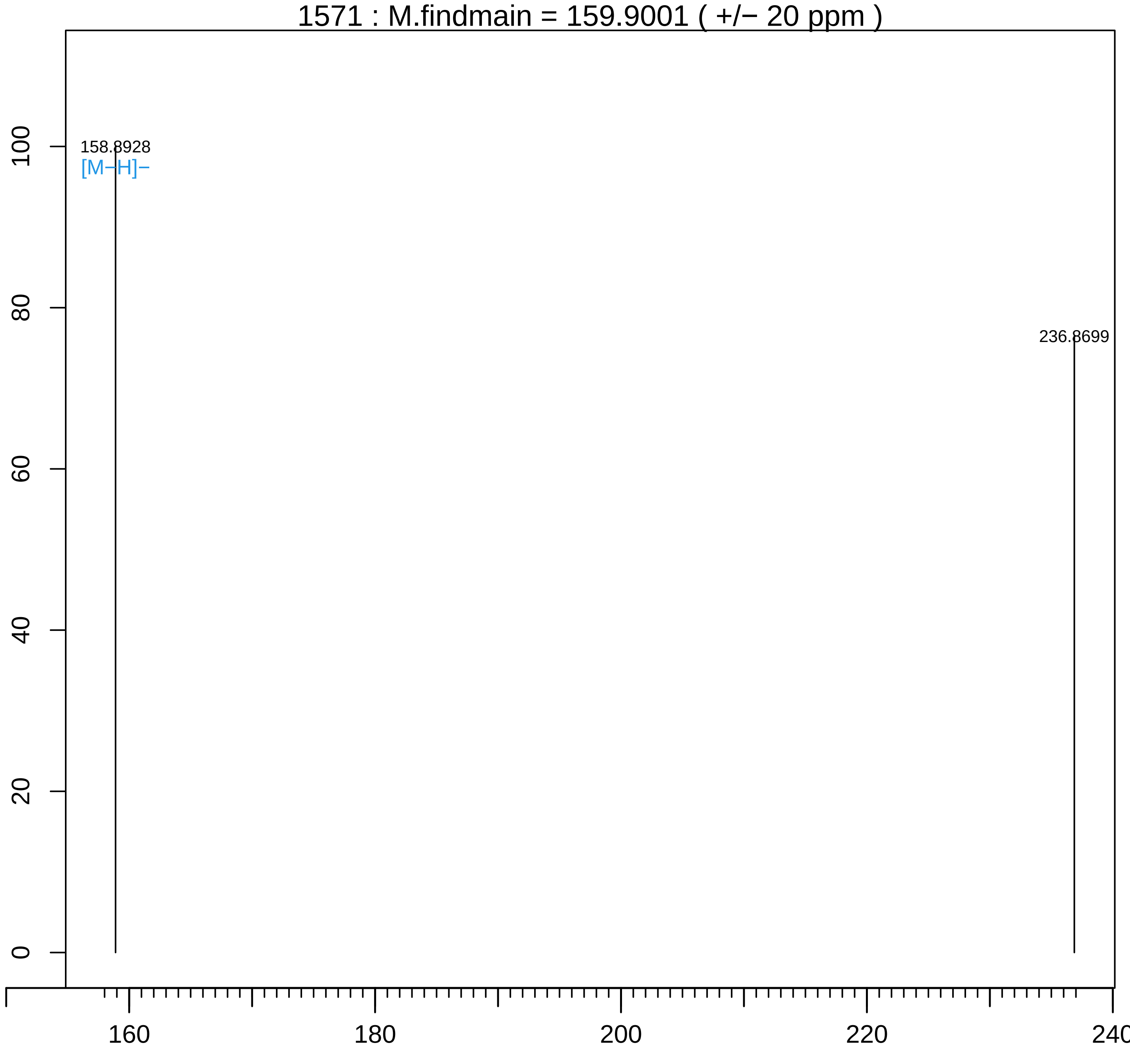
1571 : M.findmain = 159.9001 (+/− 20 ppm)
158.8928
[M−H]−
236.8699
0
20
40
60
80
100
160
180
200
220
240

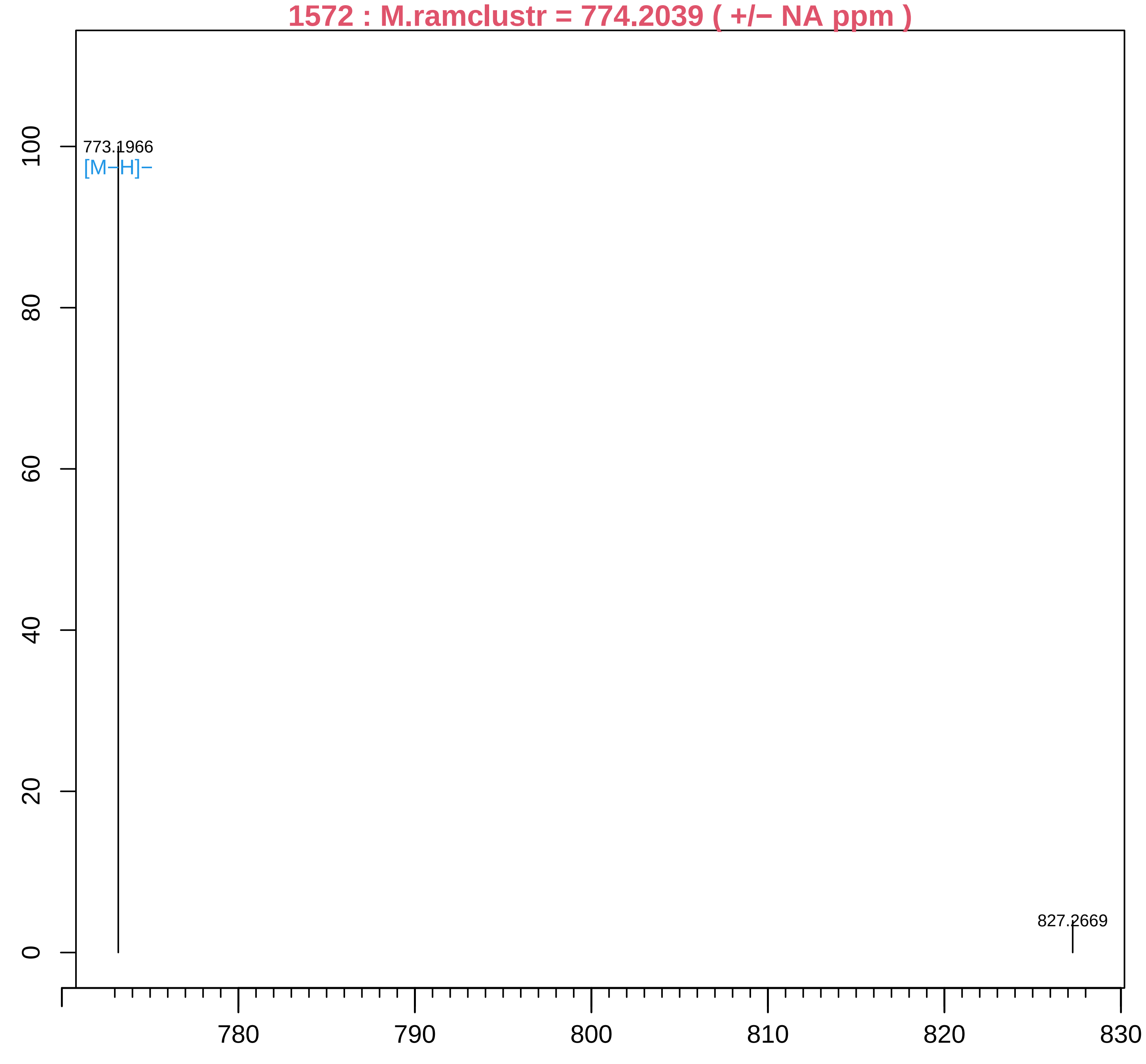

1572 : M.ramclustr = 774.2039 (+/− NA ppm)
773.1966
[M−H]−
827.2669
0
20
40
60
80
100
780
790
800
810
820
830

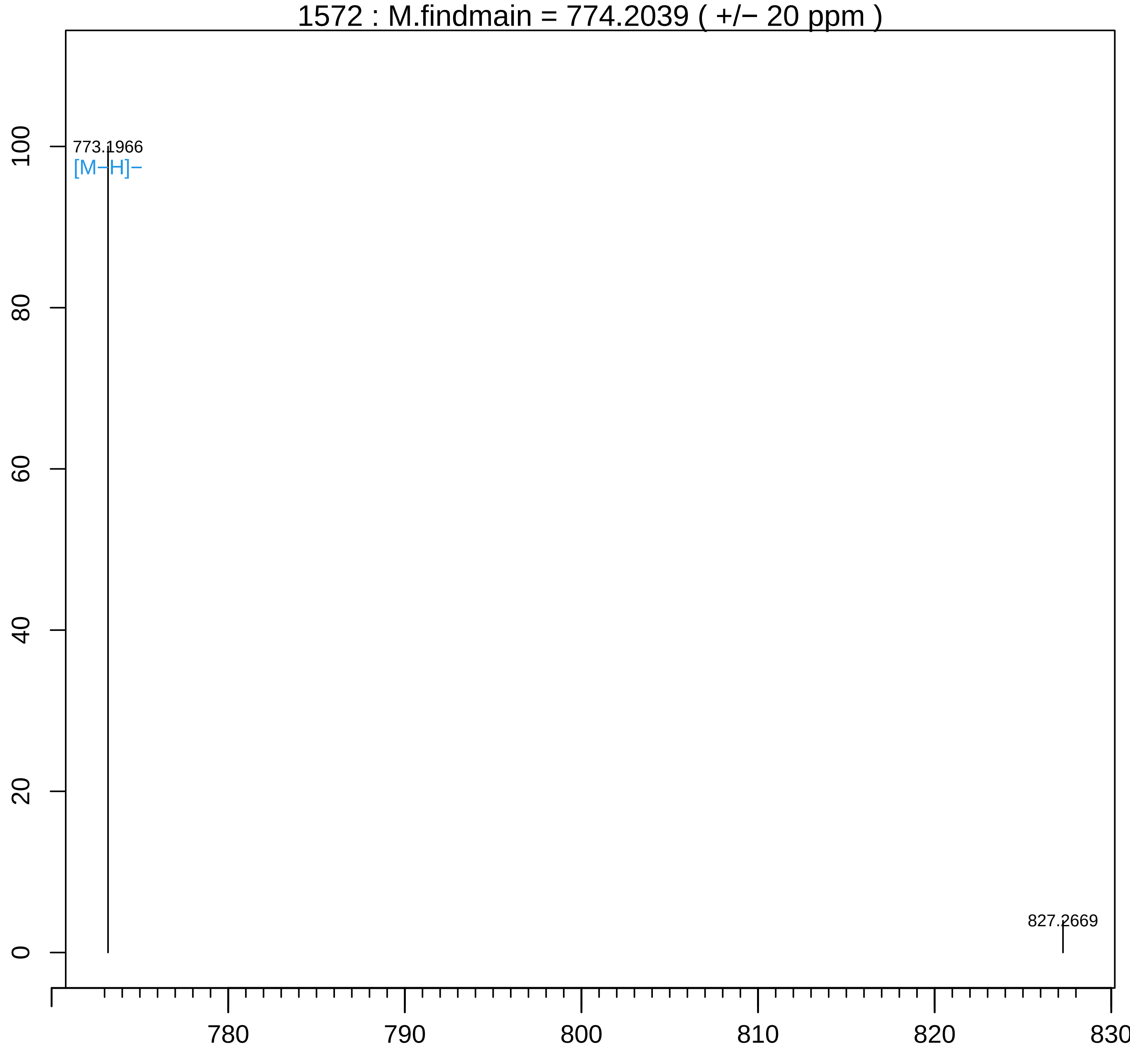

1572 : M.findmain = 774.2039 (+/− 20 ppm)
773.1966
[M−H]−
827.2669
0
20
40
60
80
100
780
790
800
810
820
830

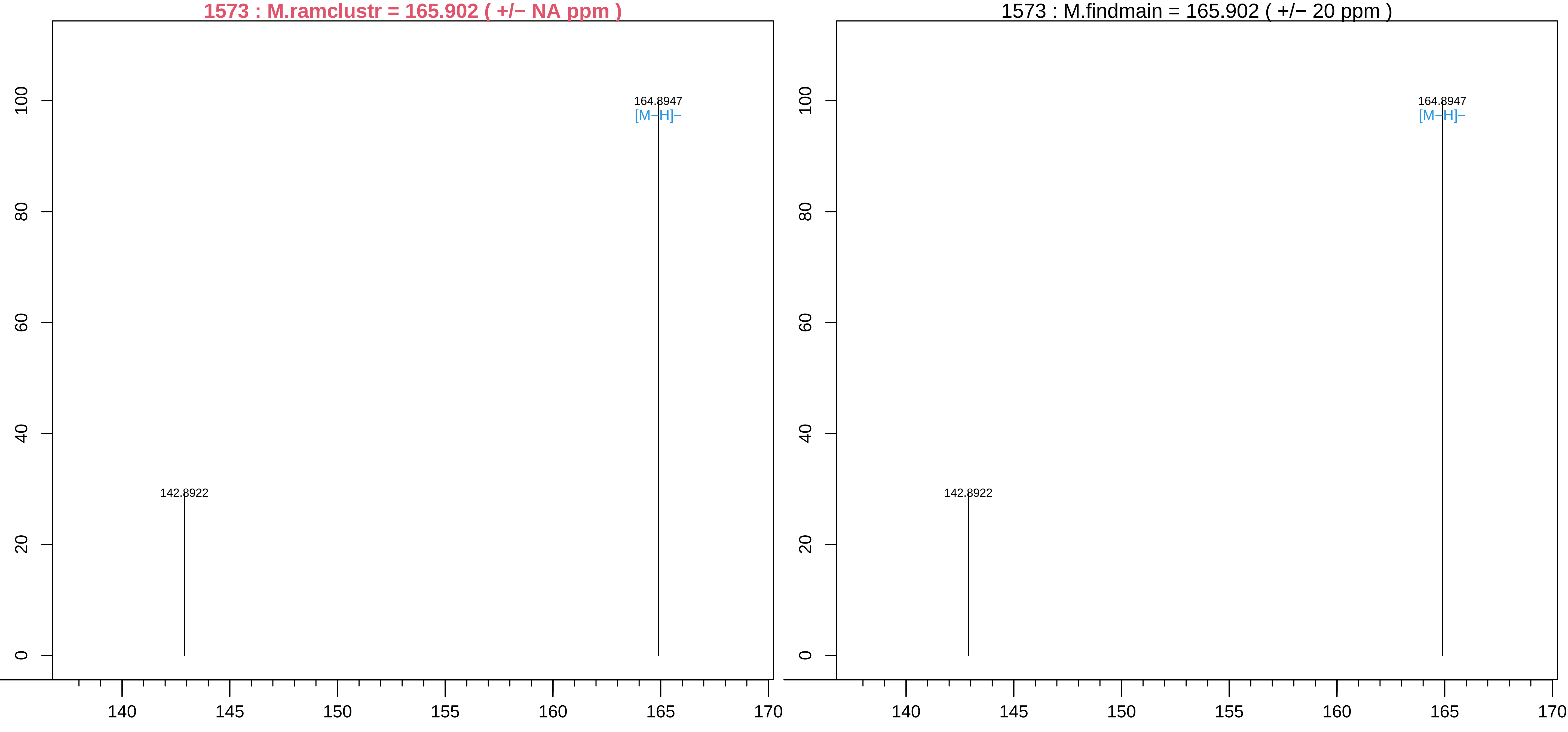
1573 : M.ramclustr = 165.902 (+/− NA ppm)
164.8947
[M−H]−
142.8922
0
20
40
60
80
100
140
145
150
155
160
165
170
1573 : M.findmain = 165.902 (+/− 20 ppm)
164.8947
[M−H]−
142.8922

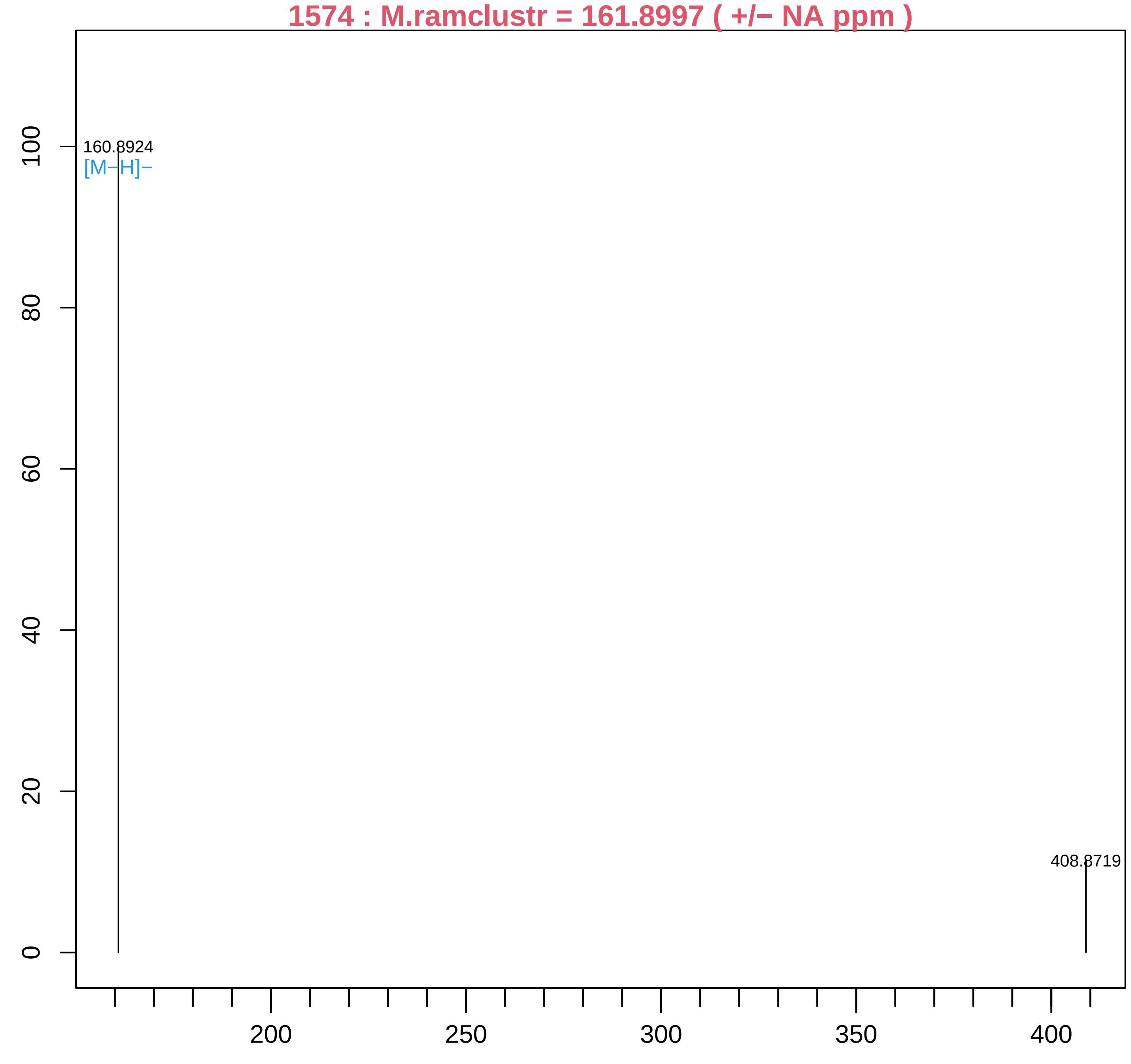

1574 : M.ramclustr = 161.8997 (+/− NA ppm)
160.8924
[M−H]−
408.8719
0
20
40
60
80
100
200
250
300
350
400

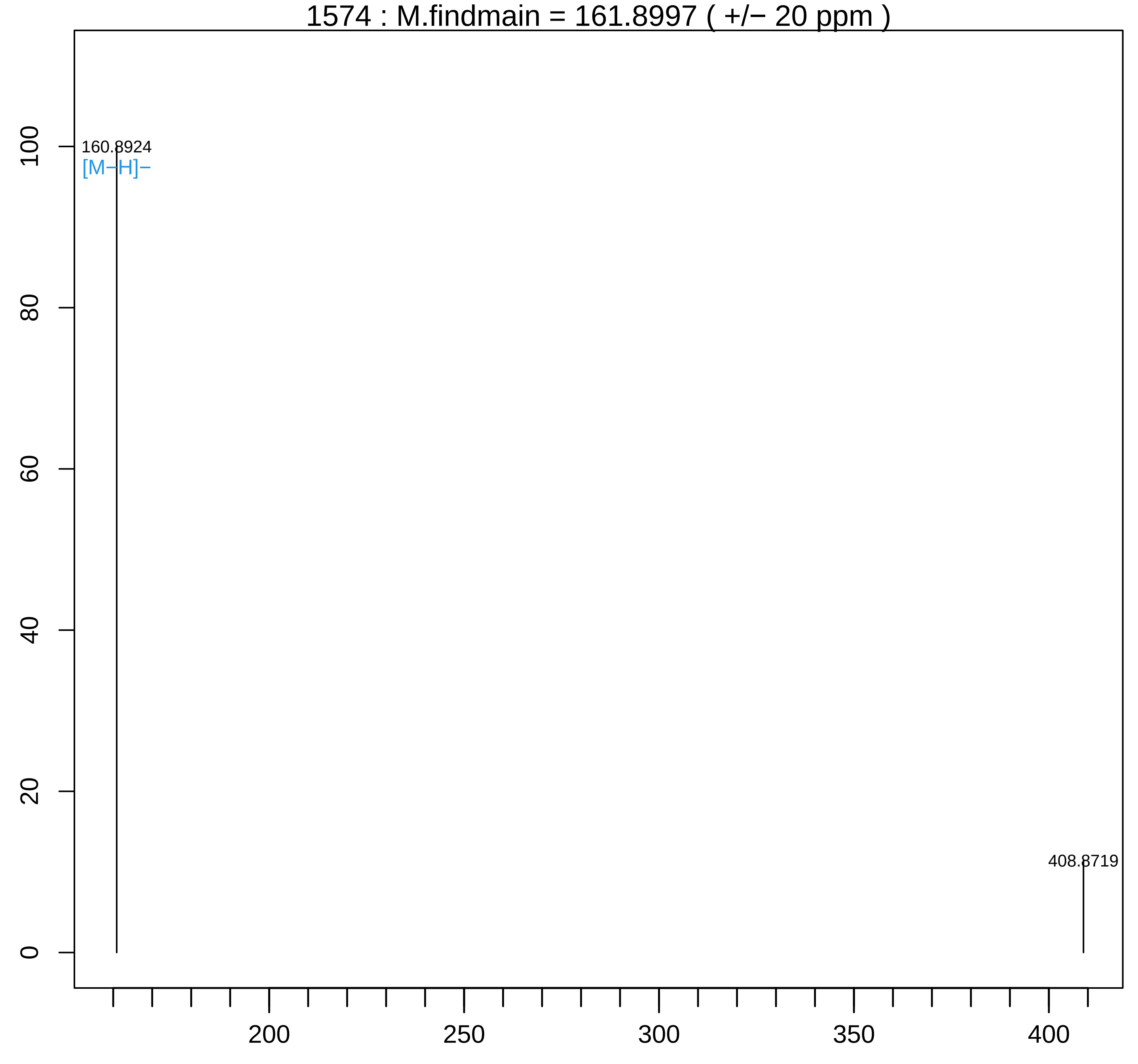

1574 : M.findmain = 161.8997 (+/− 20 ppm)
160.8924
[M−H]−
408.8719
0
20
40
60
80
100
200
250
300
350
400

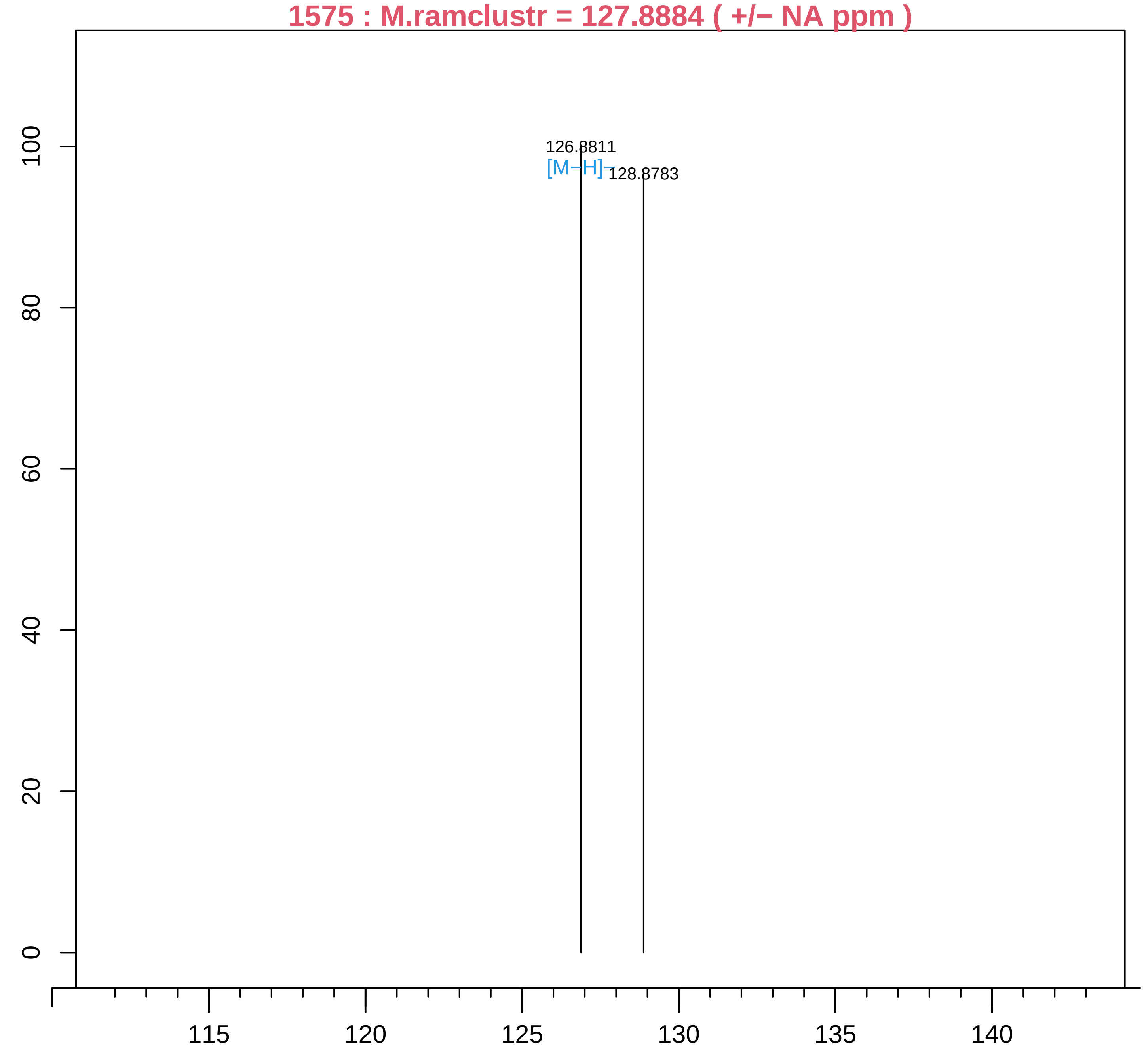
1575 : M.ramclustr = 127.8884 (+/− NA ppm)
126.8811
[M−H]−
128.8783
0
20
40
60
80
100
115
120
125
130
135
140

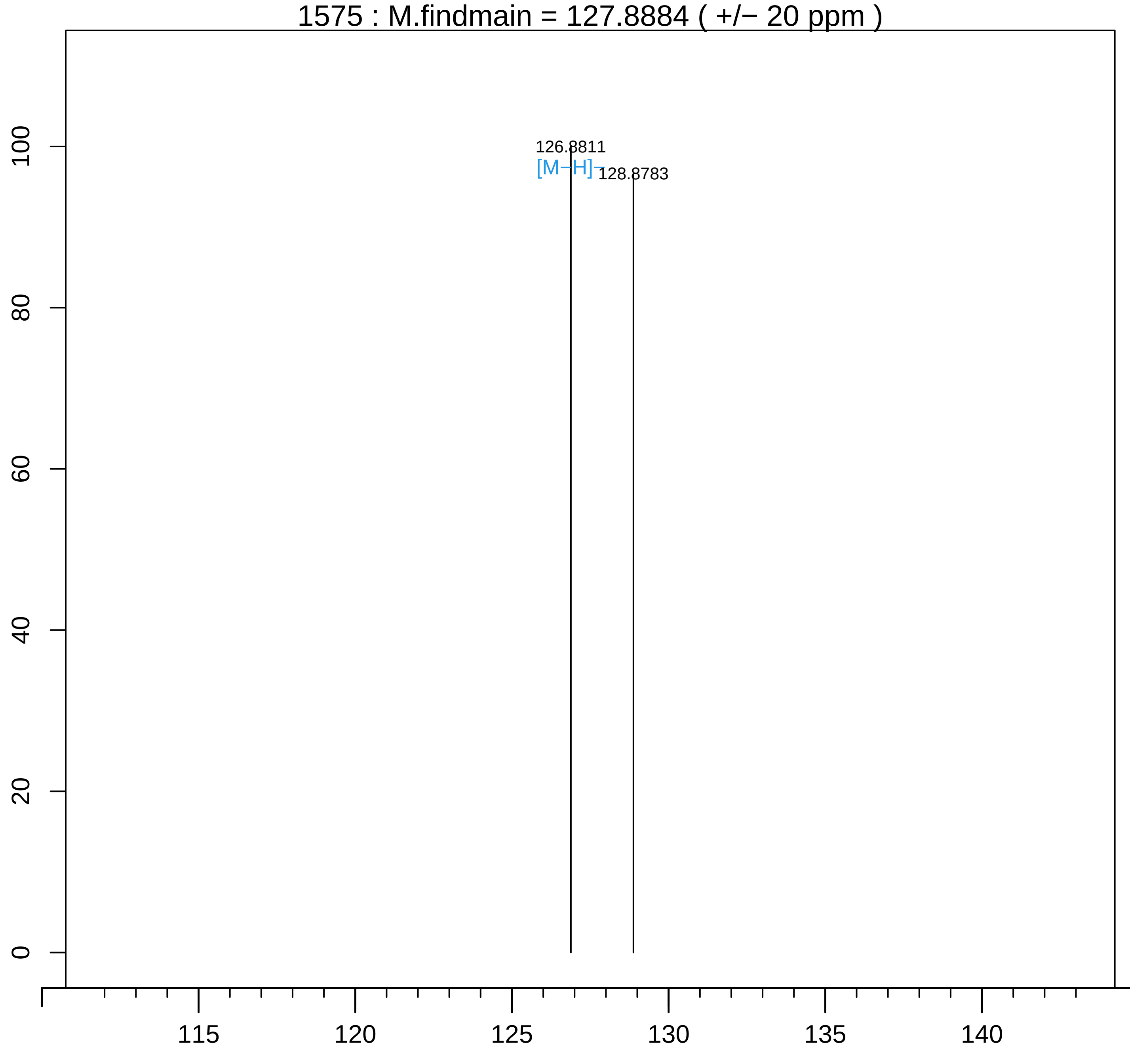
1575 : M.findmain = 127.8884 (+/− 20 ppm)
126.8811
[M−H]−
128.8783
0
20
40
60
80
100
115
120
125
130
135
140

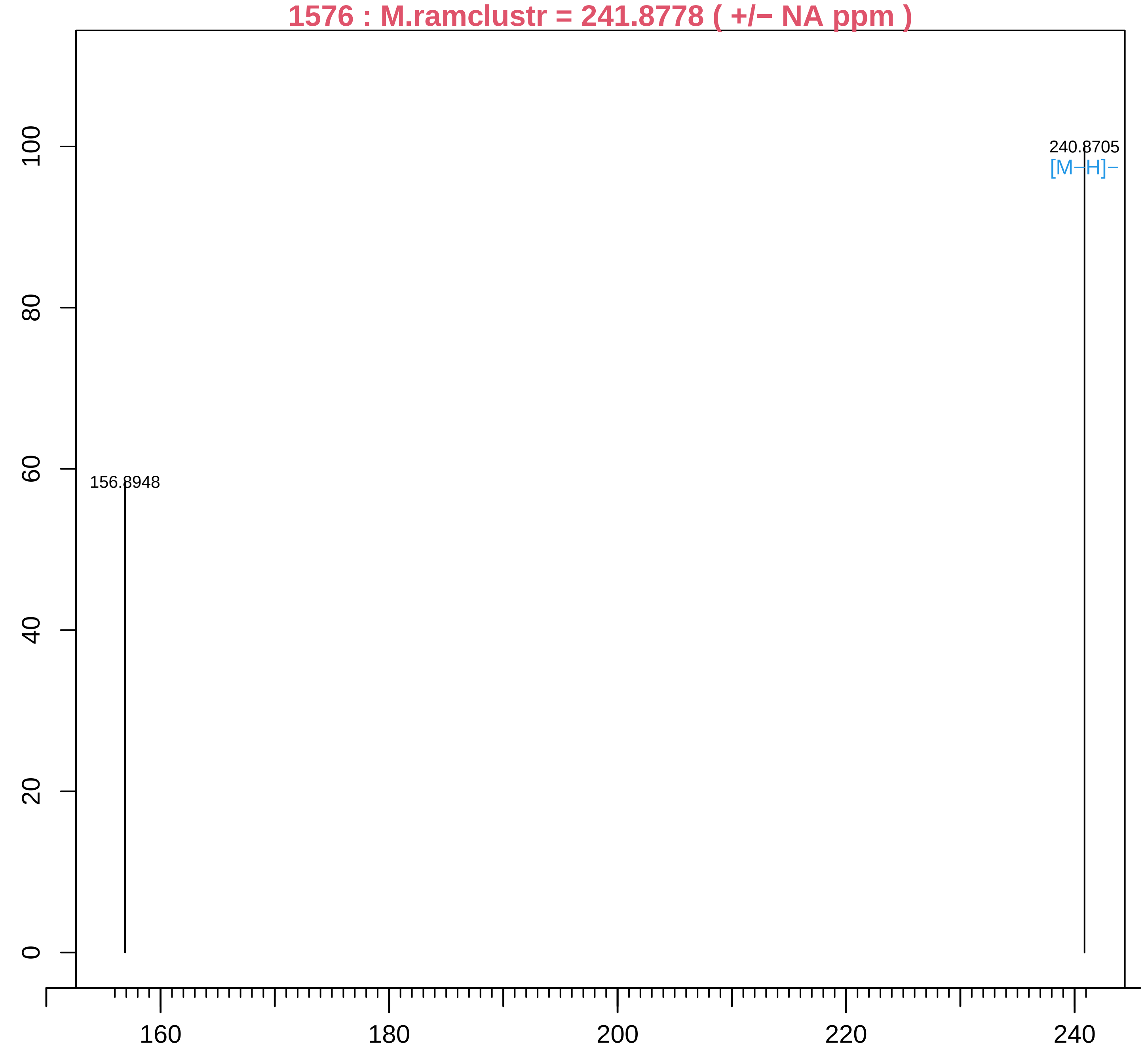
1576 : M.ramclustr = 241.8778 (+/− NA ppm)
240.8705
[M−H]−
156.8948
0
20
40
60
80
100
160
180
200
220
240

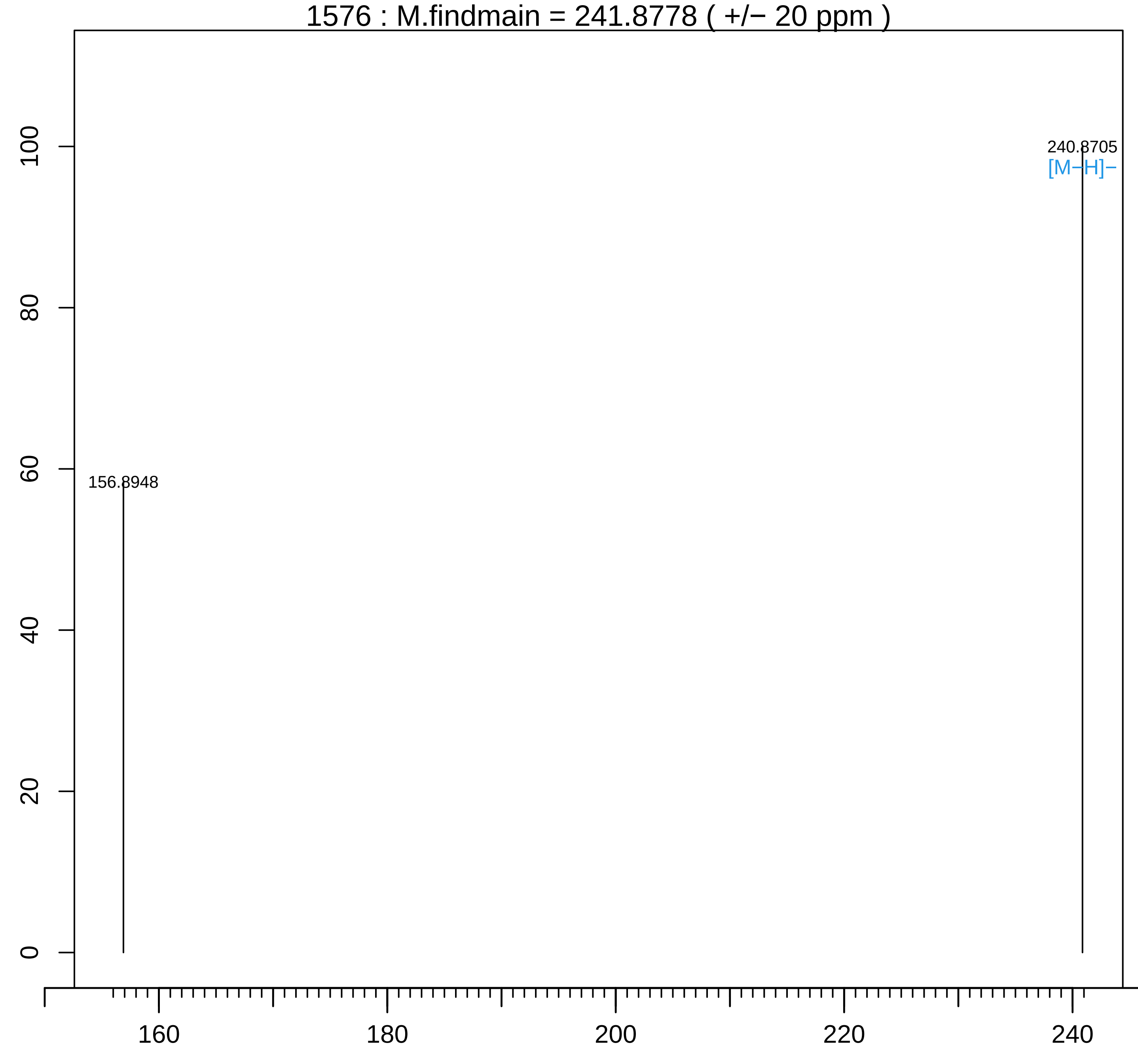
1576 : M.findmain = 241.8778 (+/− 20 ppm)
240.8705
[M−H]−
156.8948
0
20
40
60
80
100
160
180
200
220
240

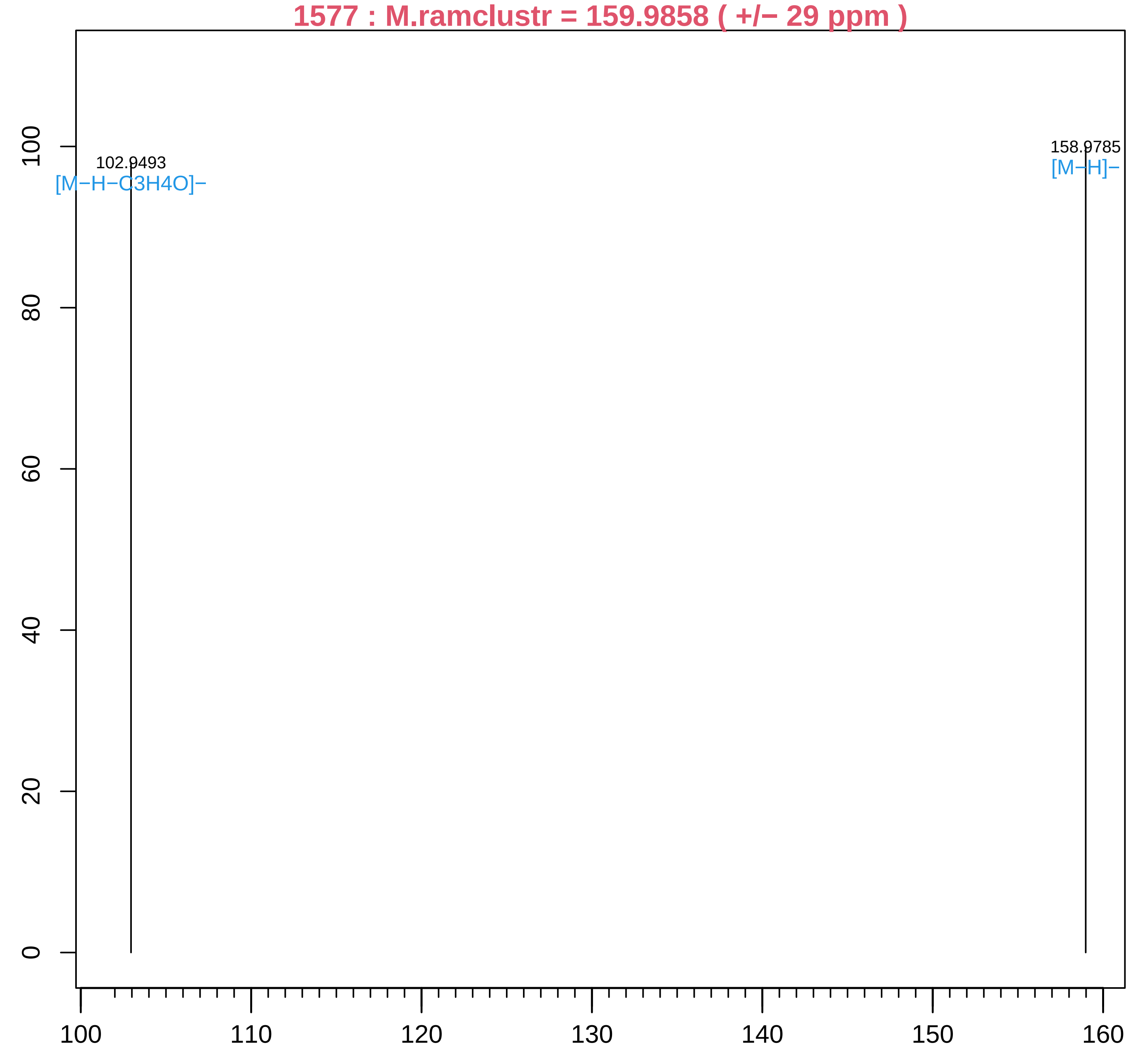
1577 : M.ramclustr = 159.9858 (+/− 29 ppm)
102.9493
[M−H−C3H4O]−
158.9785
[M−H]−
0
20
40
60
80
100
100
110
120
130
140
150
160

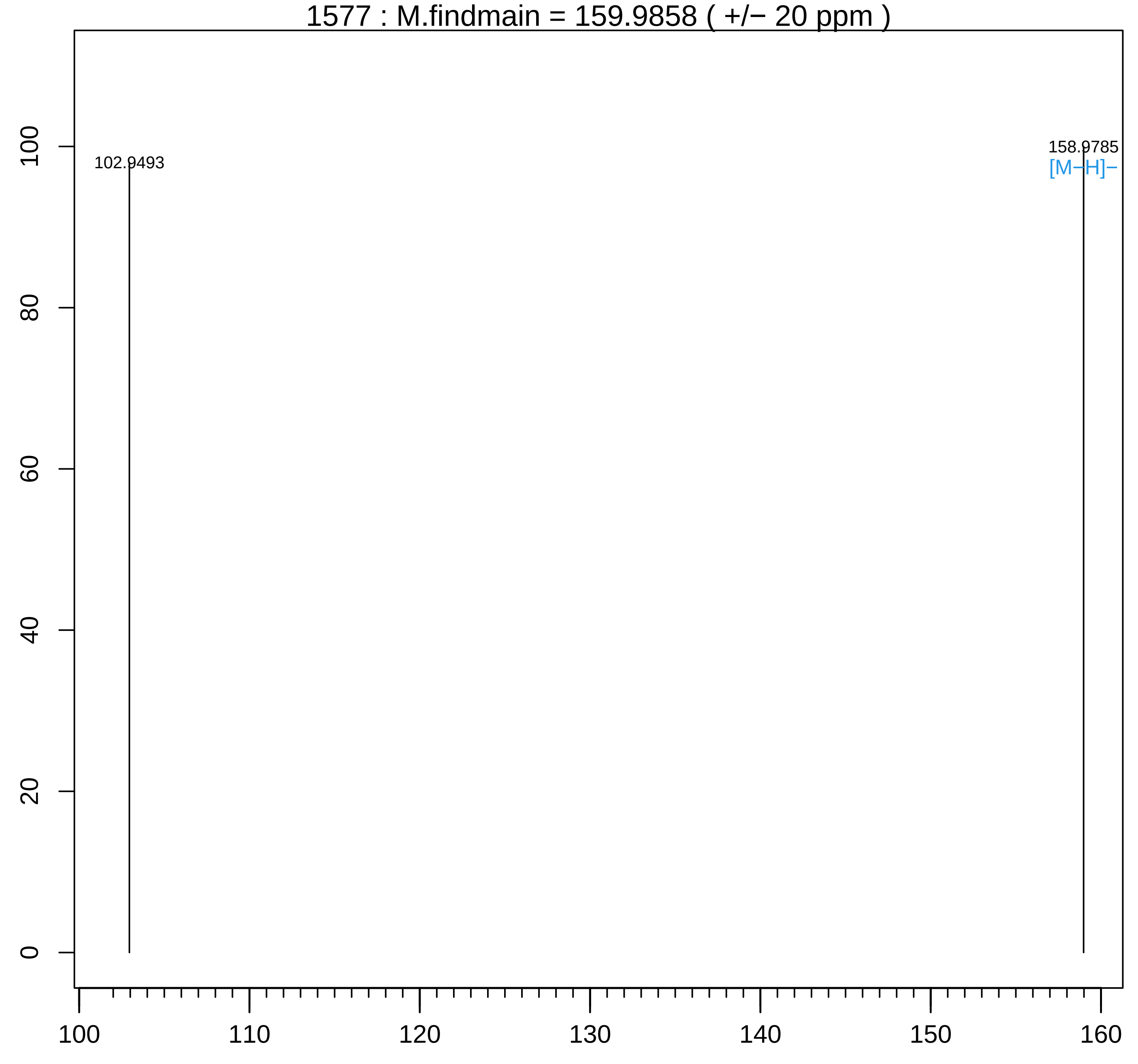
1577 : M.findmain = 159.9858 (+/− 20 ppm)
102.9493
158.9785
[M−H]−
0
20
40
60
80
100
100
110
120
130
140
150
160

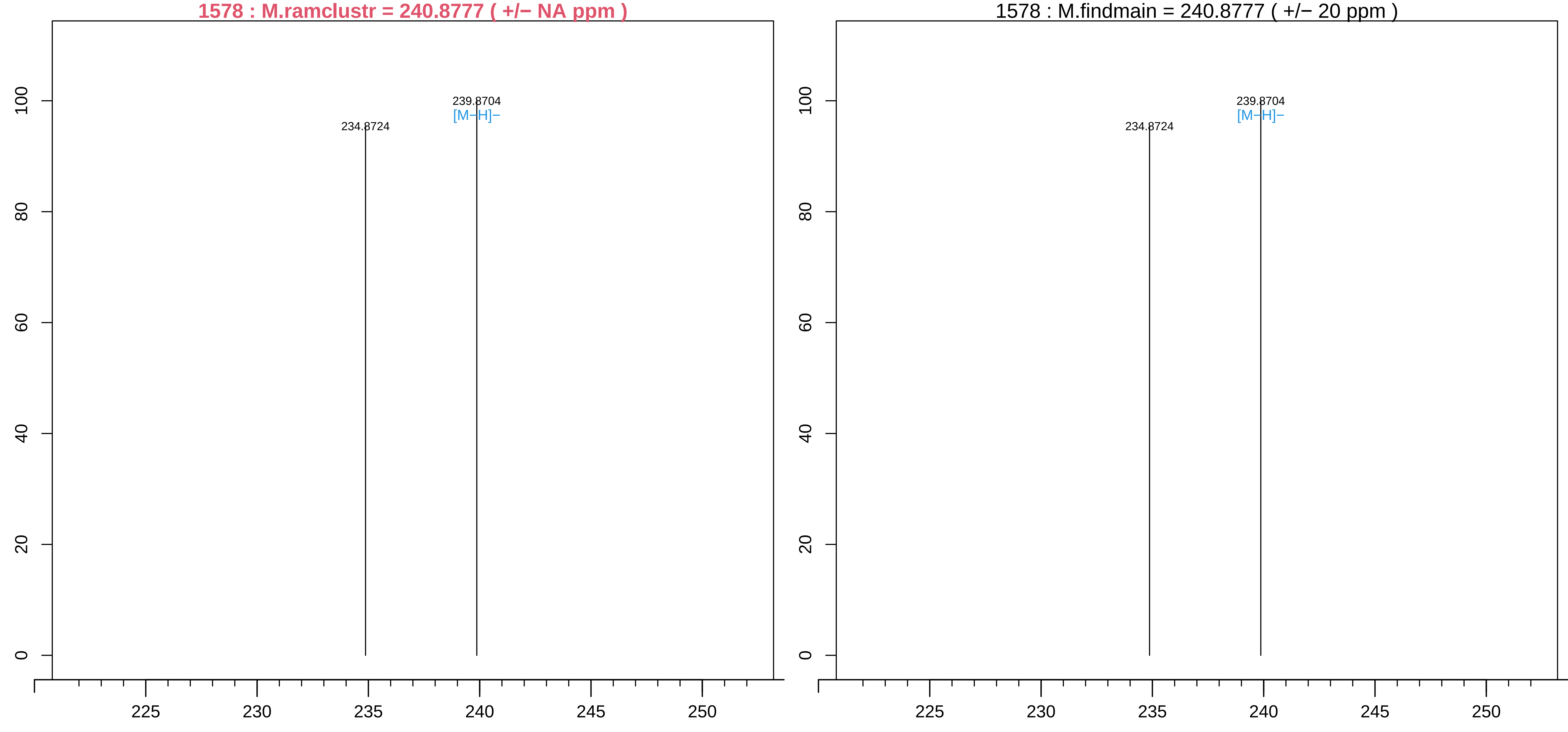
1578 : M.ramclustr = 240.8777 (+/− NA ppm)
1578 : M.findmain = 240.8777 (+/− 20 ppm)
234.8724
239.8704
[M−H]−
234.8724
239.8704
[M−H]−
0
20
40
60
80
100
225
230
235
240
245
250

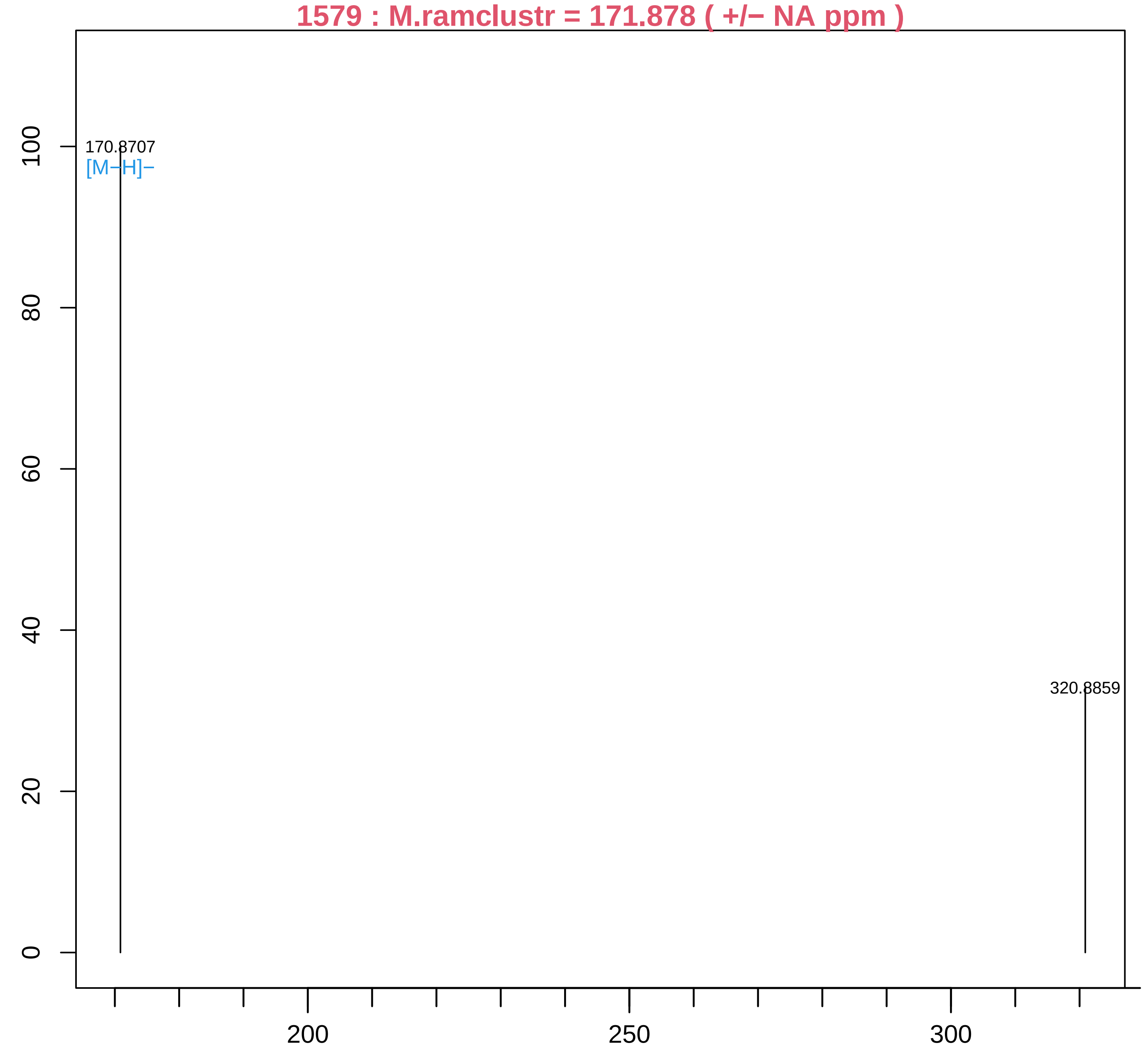
1579 : M.ramclustr = 171.878 (+/− NA ppm)
170.8707
[M−H]−
320.8859
0
20
40
60
80
100
200
250
300

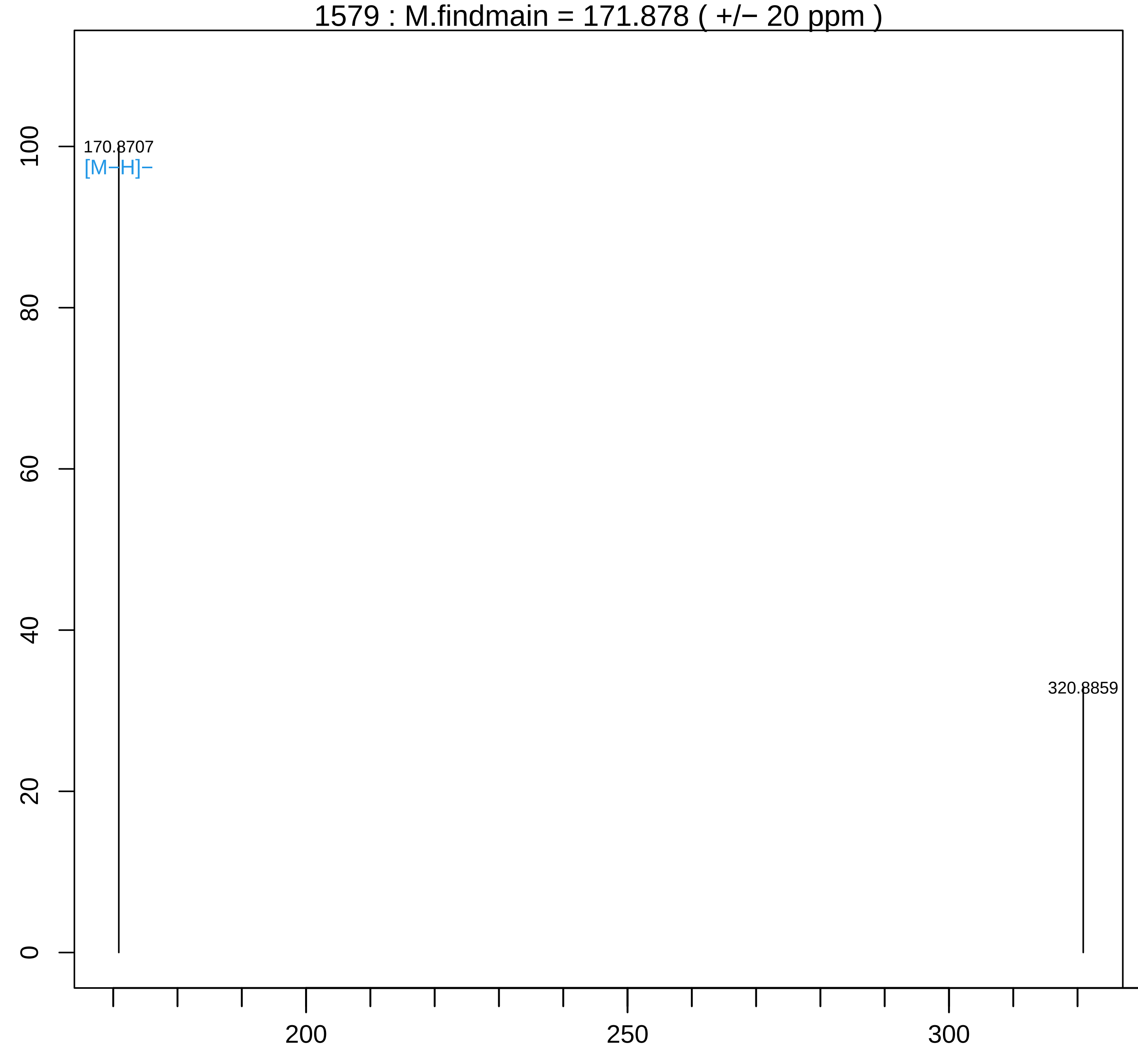
1579 : M.findmain = 171.878 (+/− 20 ppm)
170.8707
[M−H]−
320.8859
0
20
40
60
80
100
200
250
300

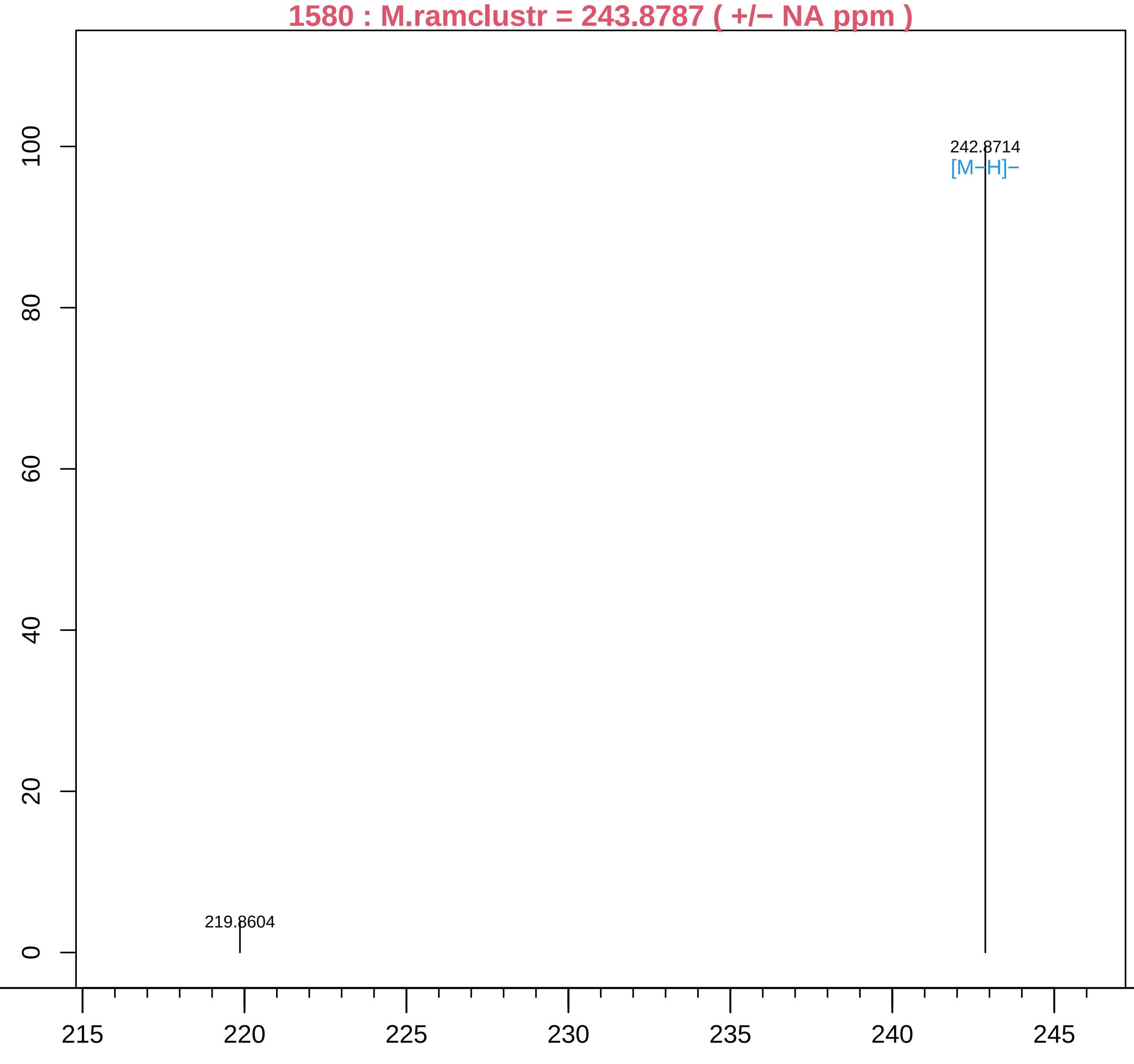

1580 : M.ramclustr = 243.8787 (+/− NA ppm)
242.8714
[M−H]−
219.8604

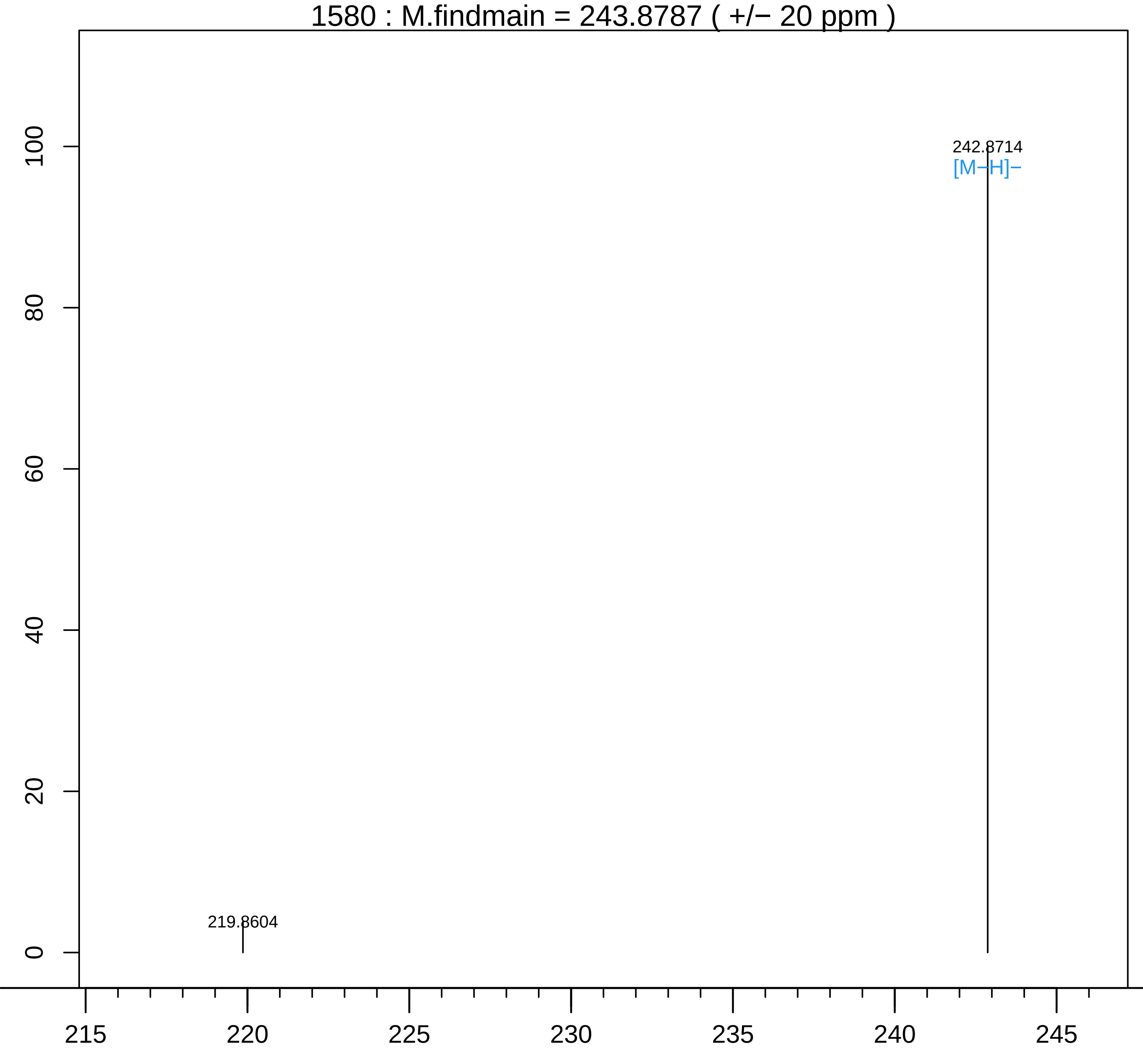

1580 : M.findmain = 243.8787 (+/− 20 ppm)
242.8714
[M−H]−
219.8604

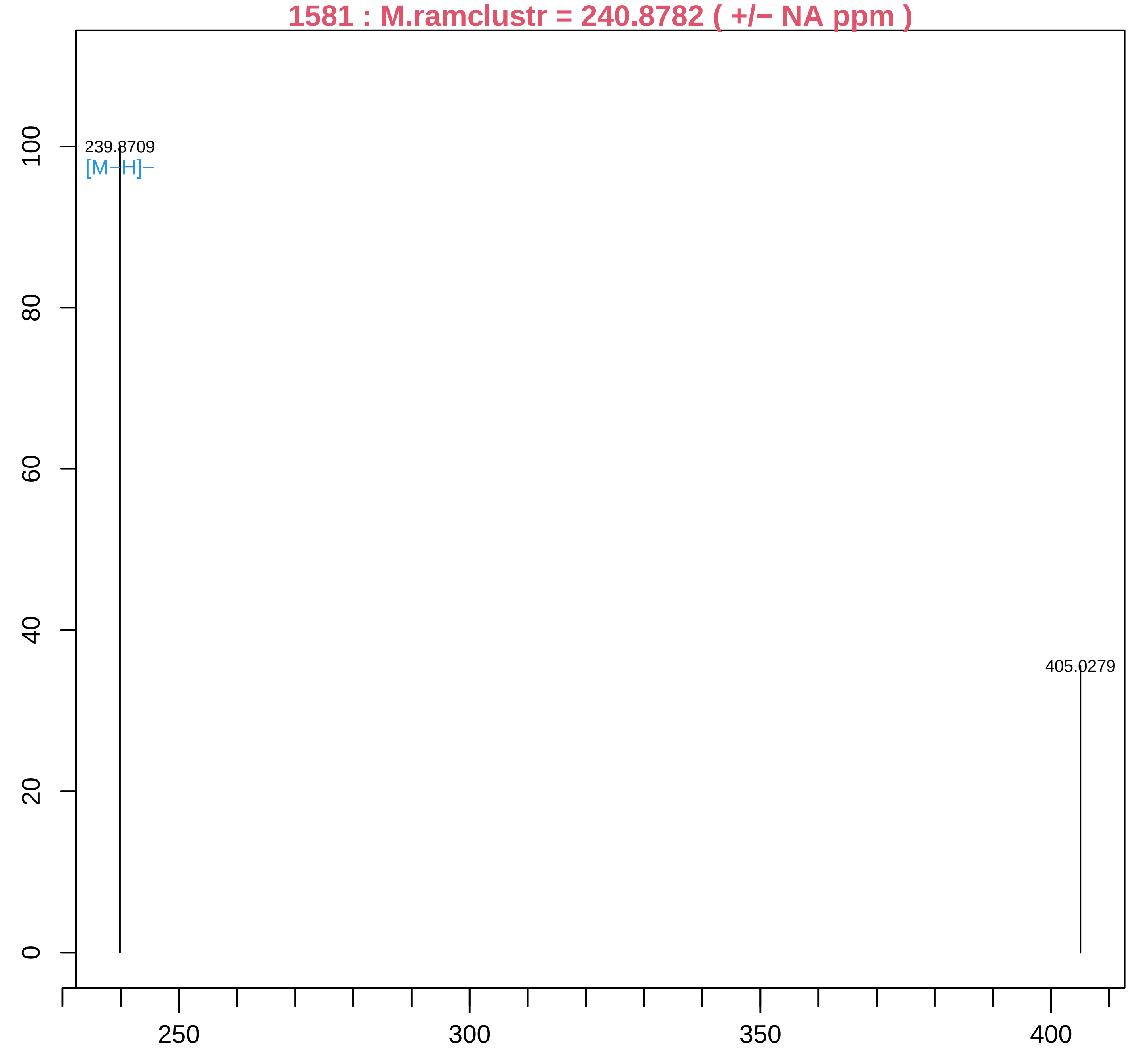
1581 : M.ramclustr = 240.8782 (+/− NA ppm)
239.8709
[M−H]−
405.0279
0
20
40
60
80
100
250
300
350
400

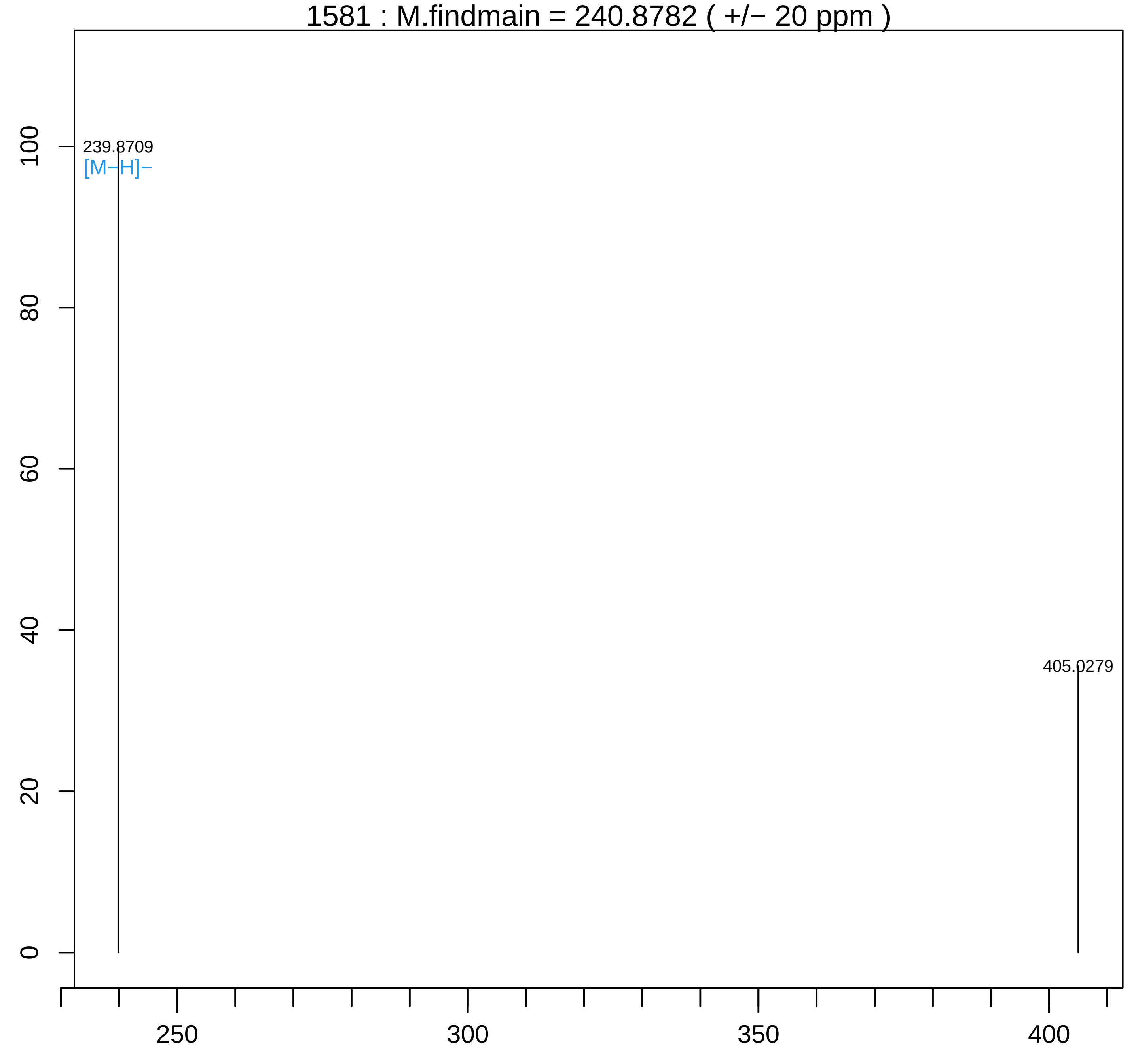
1581 : M.findmain = 240.8782 (+/− 20 ppm)
239.8709
[M−H]−
405.0279
0
20
40
60
80
100
250
300
350
400

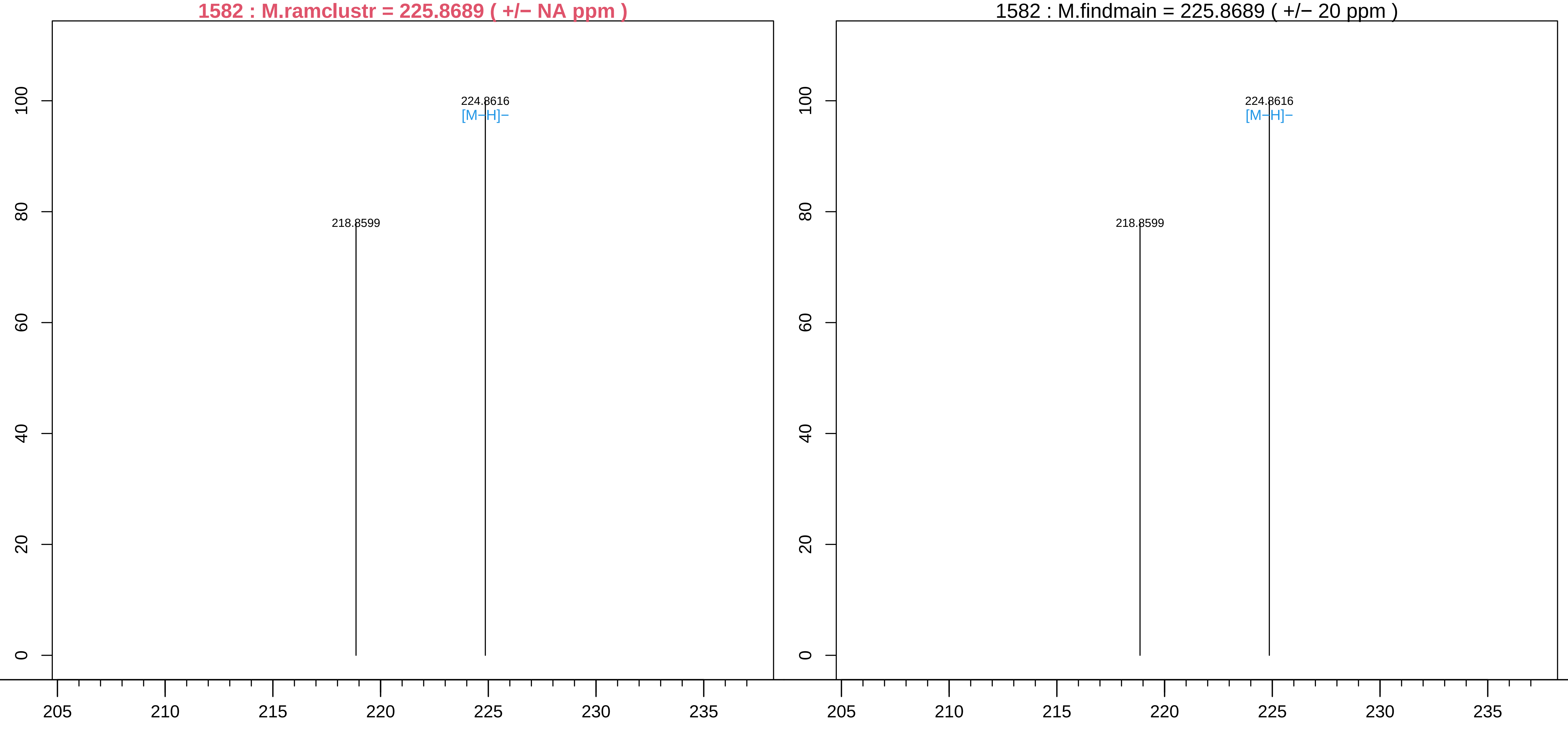

1582 : M.ramclustr = 225.8689 (+/− NA ppm)
1582 : M.findmain = 225.8689 (+/− 20 ppm)
224.8616
[M−H]−
218.8599
224.8616
[M−H]−
218.8599
0
20
40
60
80
100
205
210
215
220
225
230
235

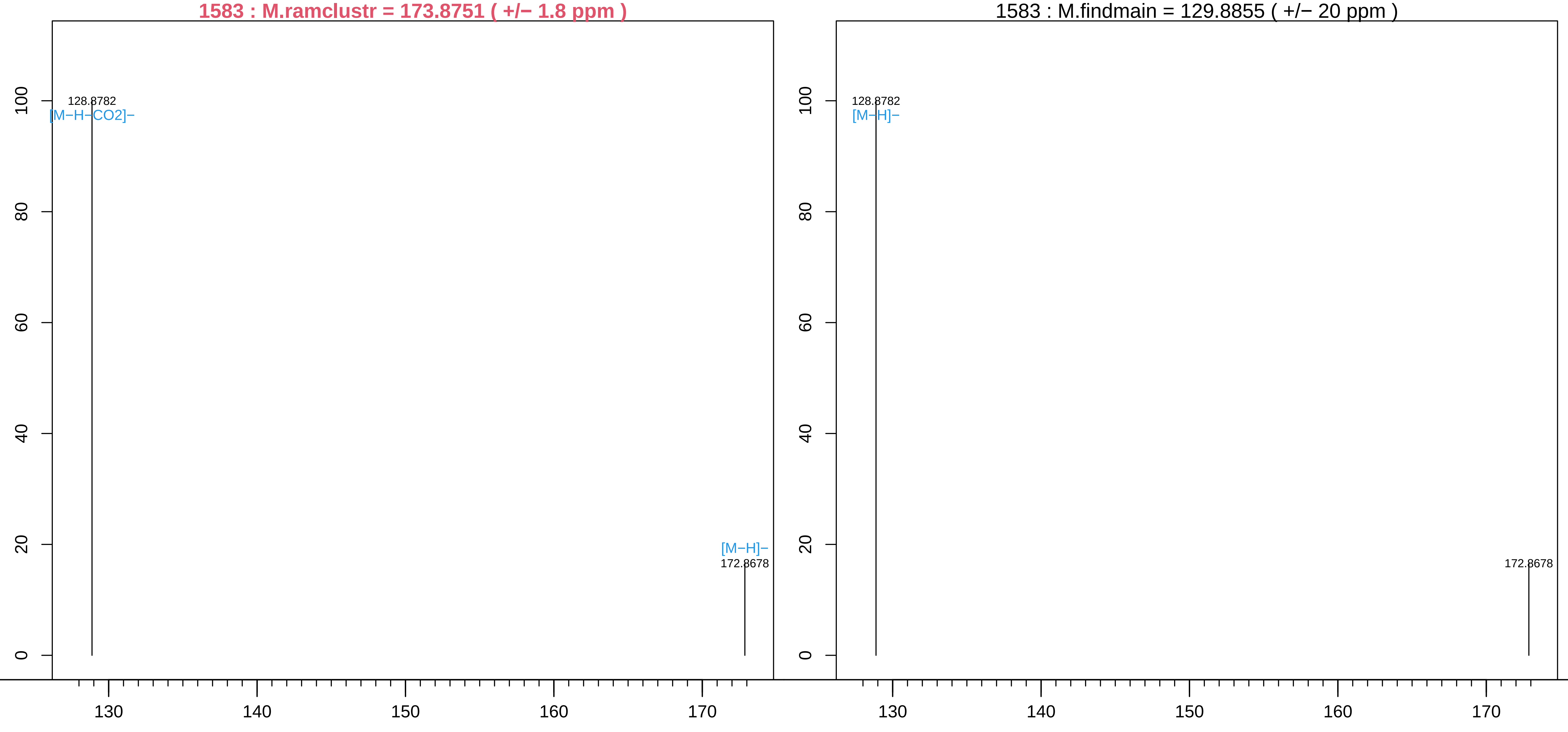
1583 : M.ramclustr = 173.8751 (+/− 1.8 ppm)
128.8782
[M−H−CO2]−
[M−H]−
172.8678
1583 : M.findmain = 129.8855 (+/− 20 ppm)
128.8782
[M−H]−
172.8678
0
20
40
60
80
100
130
140
150
160
170

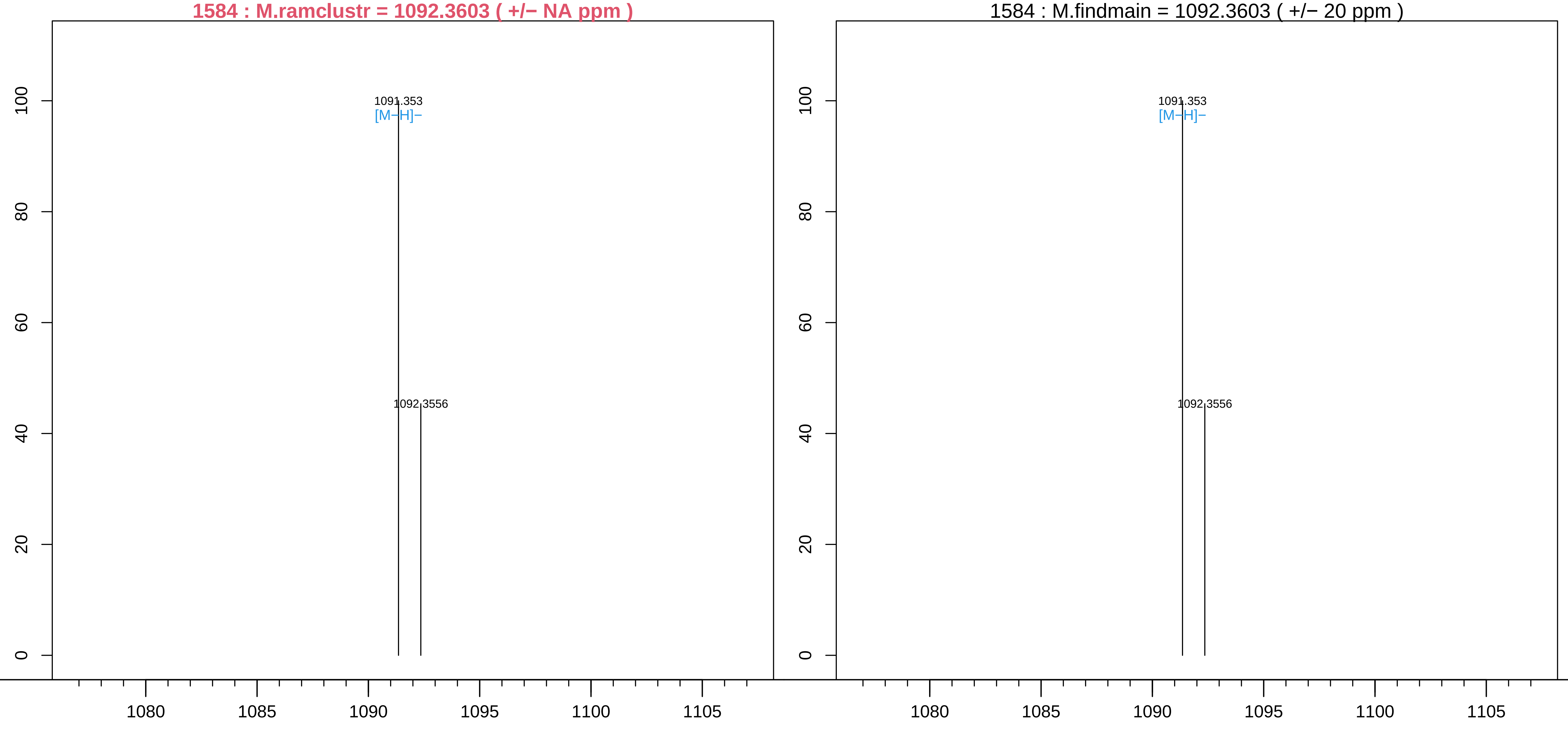
1584 : M.ramclustr = 1092.3603 (+/− NA ppm)
1091.353
[M−H]−
1092.3556
1584 : M.findmain = 1092.3603 (+/− 20 ppm)
1091.353
[M−H]−
1092.3556
0
20
40
60
80
100
1080
1085
1090
1095
1100
1105

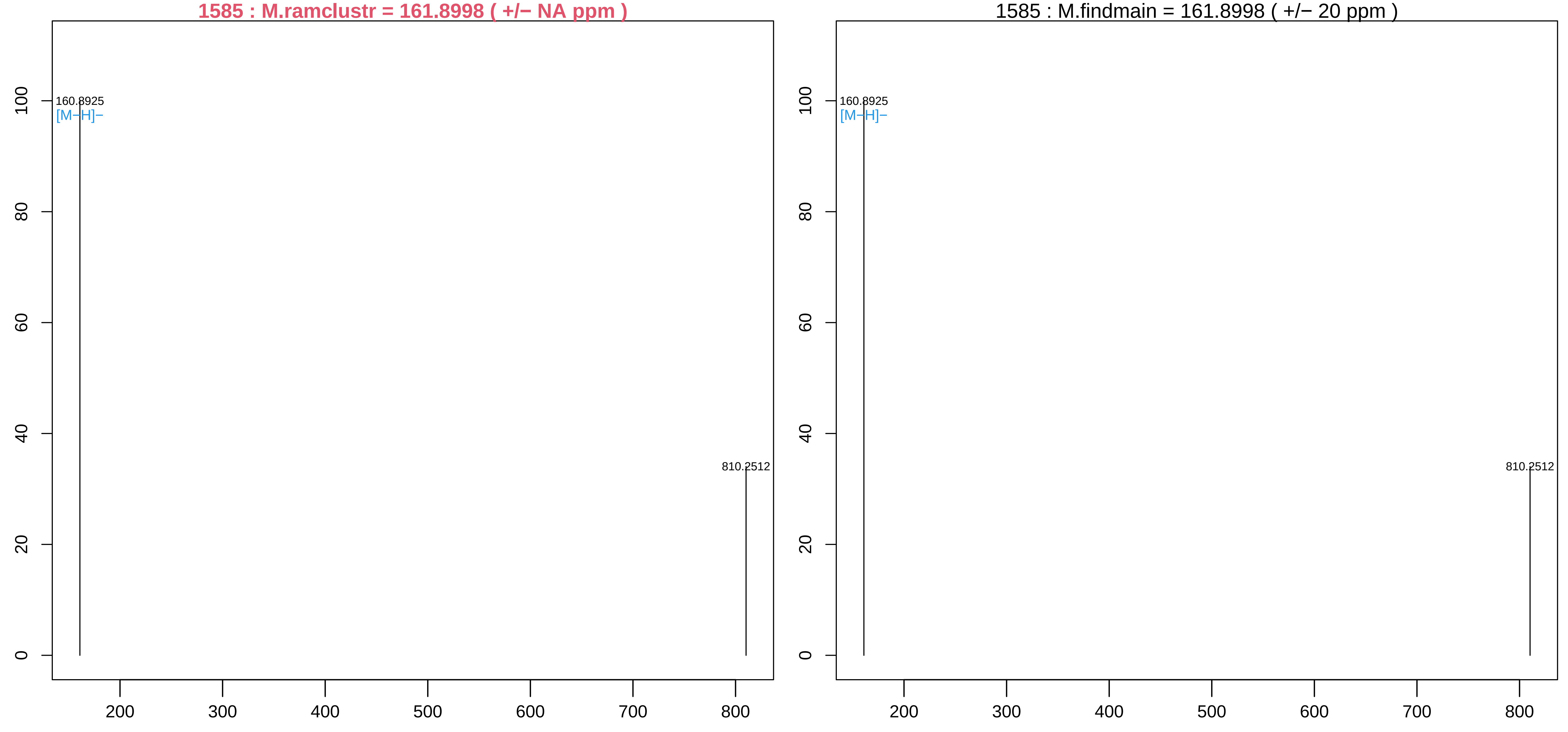
1585 : M.ramclustr = 161.8998 (+/− NA ppm)
160.8925
[M−H]−
810.2512
1585 : M.findmain = 161.8998 (+/− 20 ppm)
160.8925
[M−H]−
810.2512
0
20
40
60
80
100
200
300
400
500
600
700
800

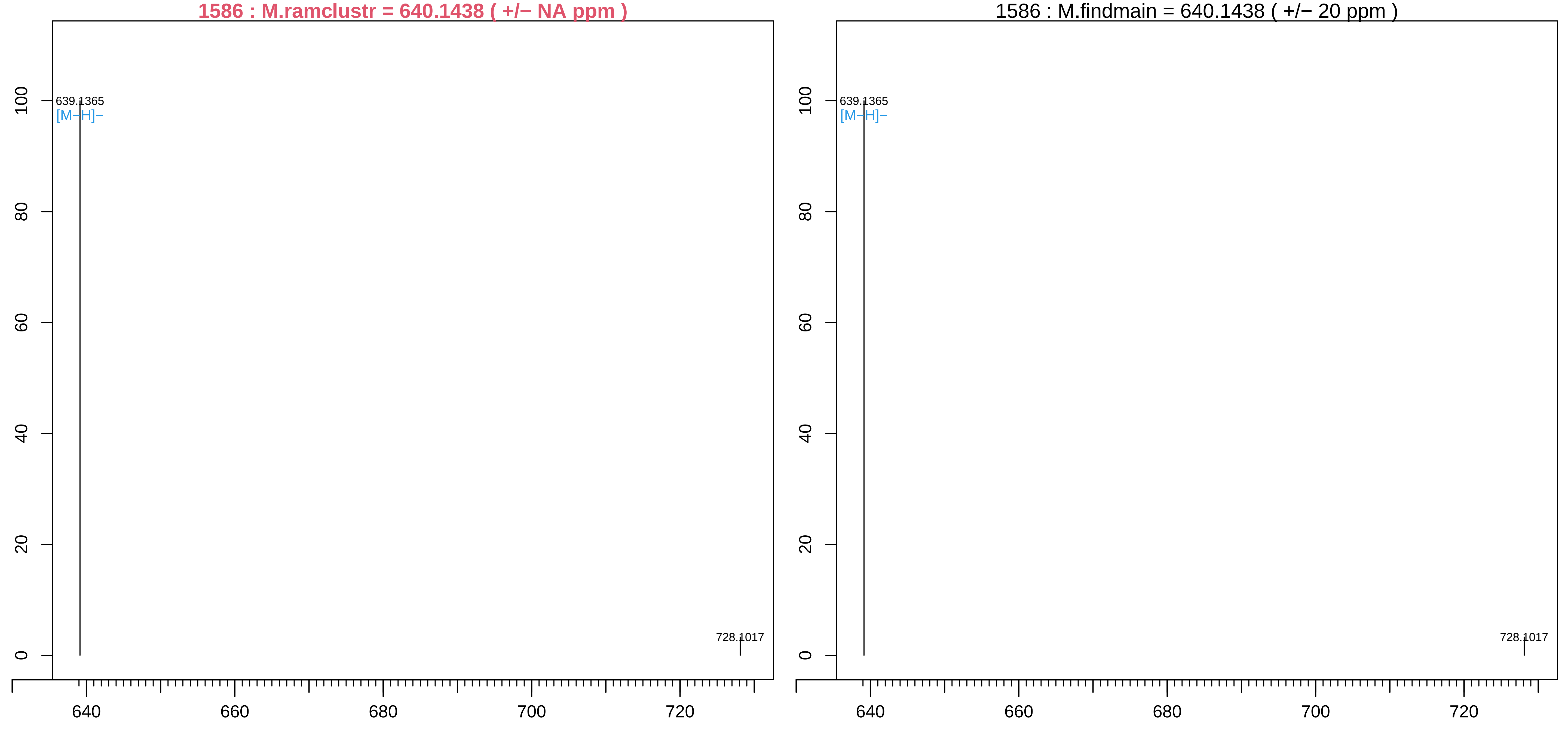
1586 : M.ramclustr = 640.1438 (+/− NA ppm)
639.1365
[M−H]−
728.1017
0
20
40
60
80
100
640
660
680
700
720
1586 : M.findmain = 640.1438 (+/− 20 ppm)
639.1365
[M−H]−
728.1017
0
20
40
60
80
100
640
660
680
700
720

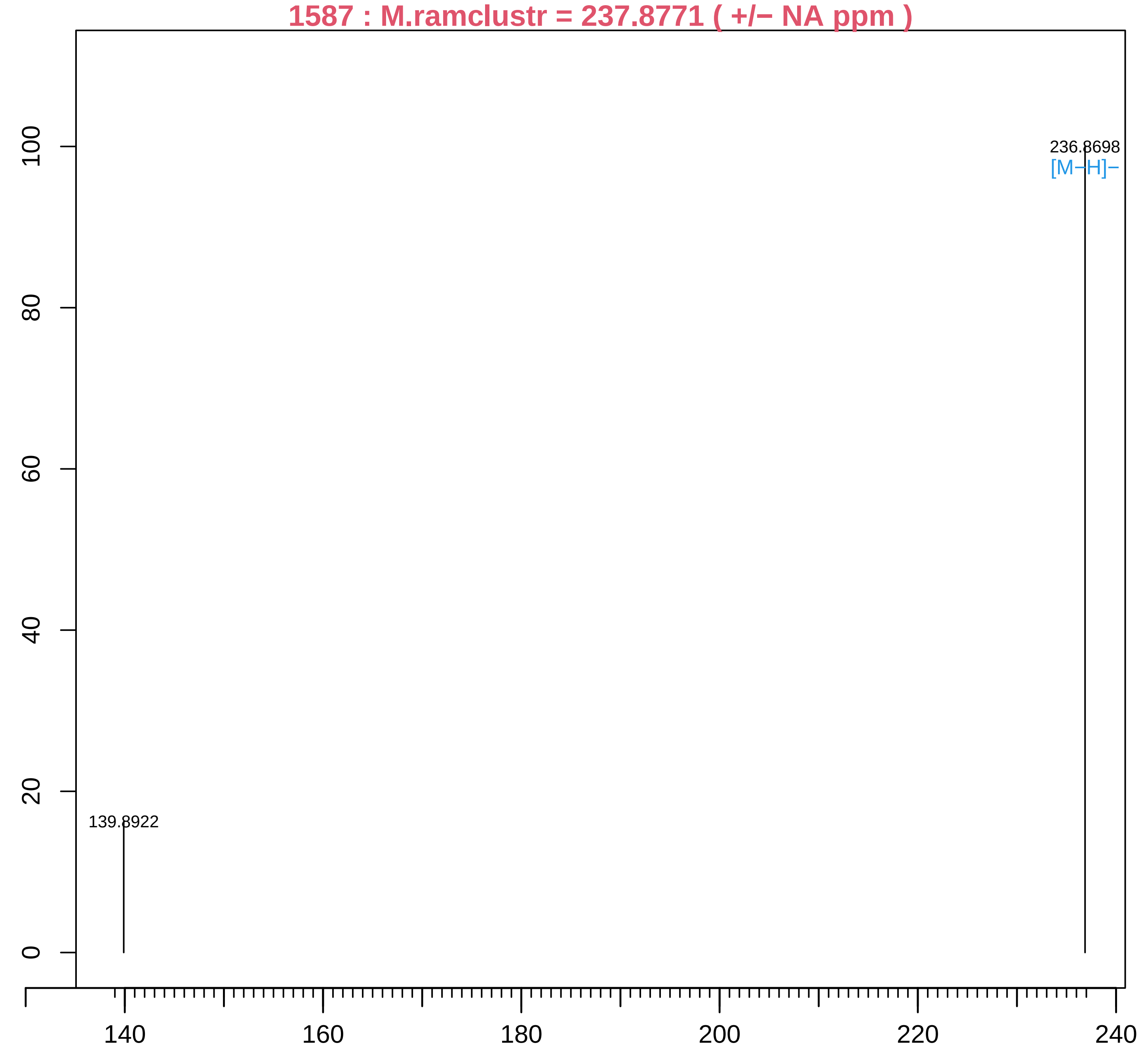
1587 : M.ramclustr = 237.8771 (+/− NA ppm)
236.8698
[M−H]−
139.8922
0
20
40
60
80
100
140
160
180
200
220
240

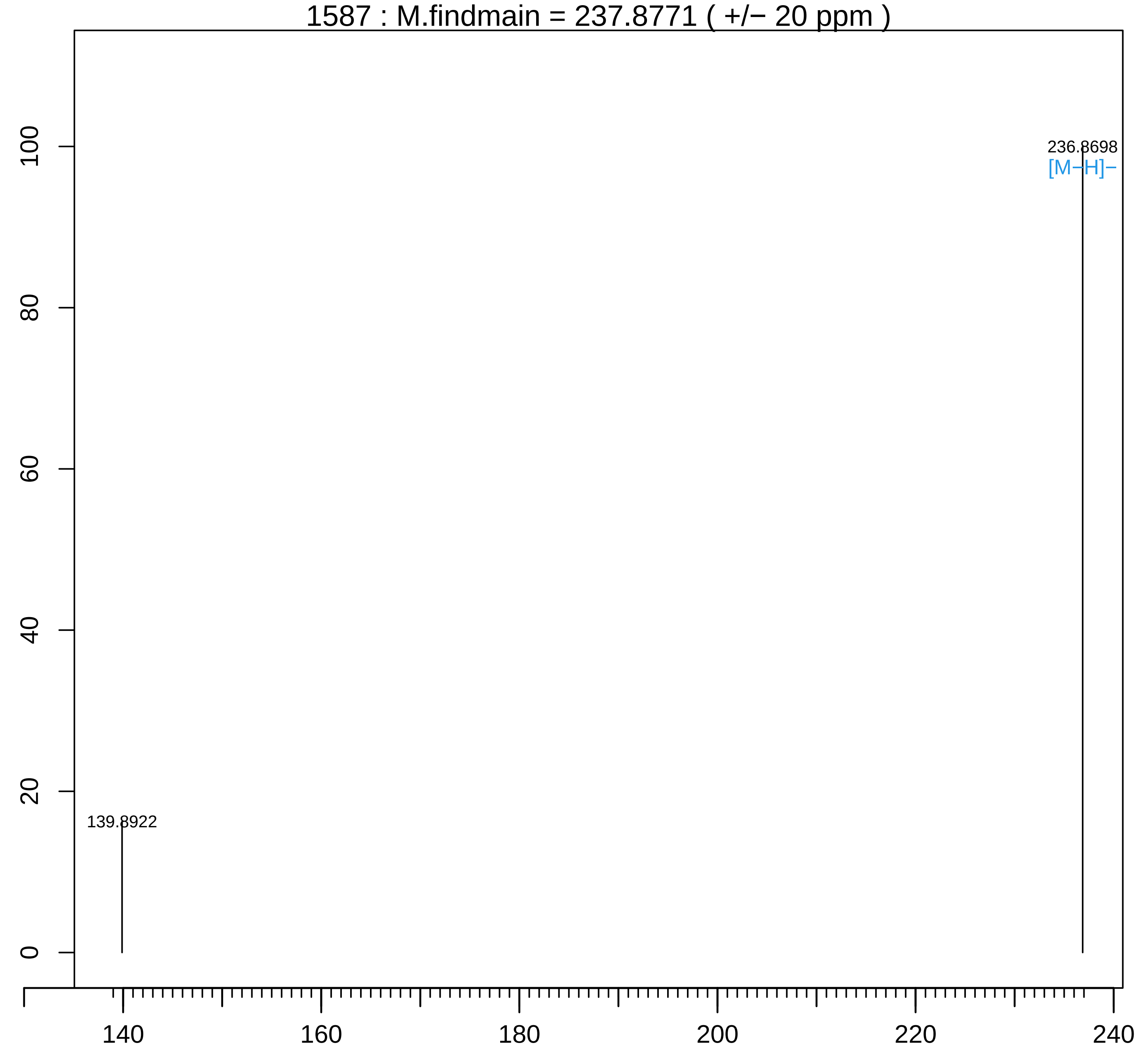
1587 : M.findmain = 237.8771 (+/− 20 ppm)
236.8698
[M−H]−
139.8922
0
20
40
60
80
100
140
160
180
200
220
240

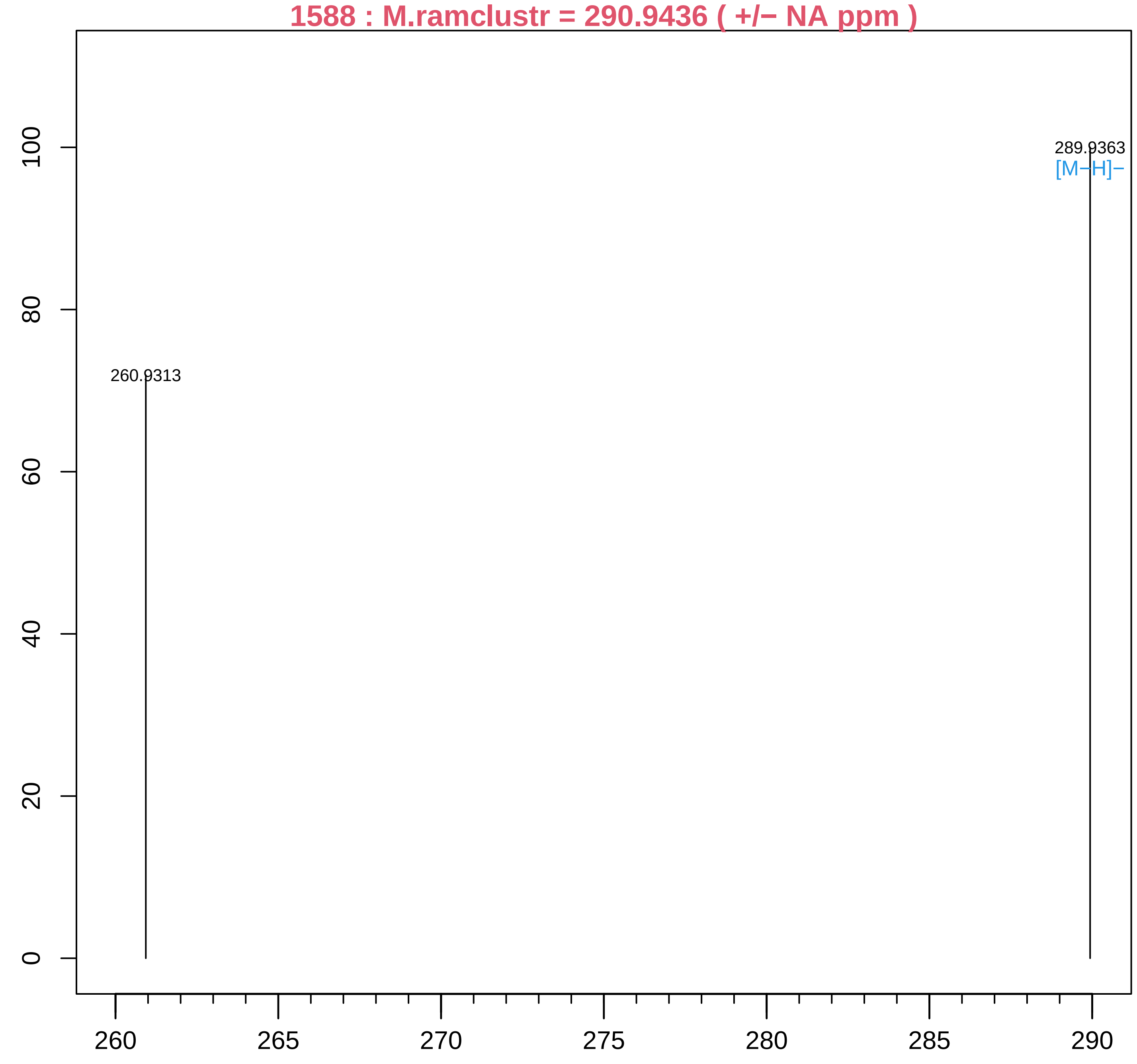
1588 : M.ramclustr = 290.9436 (+/− NA ppm)
289.9363
[M−H]−
260.9313
0
20
40
60
80
100
260
265
270
275
280
285
290

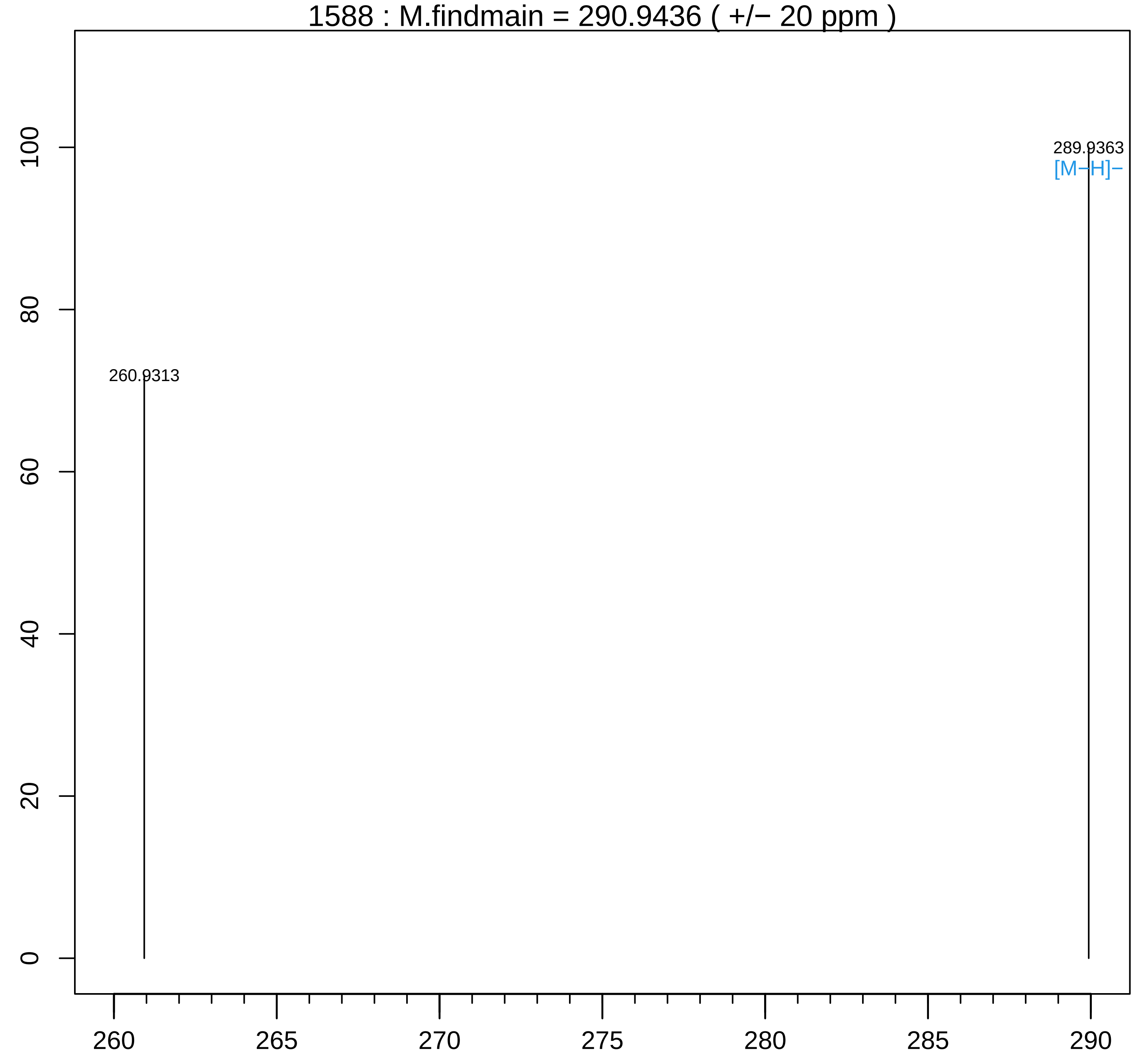
1588 : M.findmain = 290.9436 (+/− 20 ppm)
289.9363
[M−H]−
260.9313
0
20
40
60
80
100
260
265
270
275
280
285
290

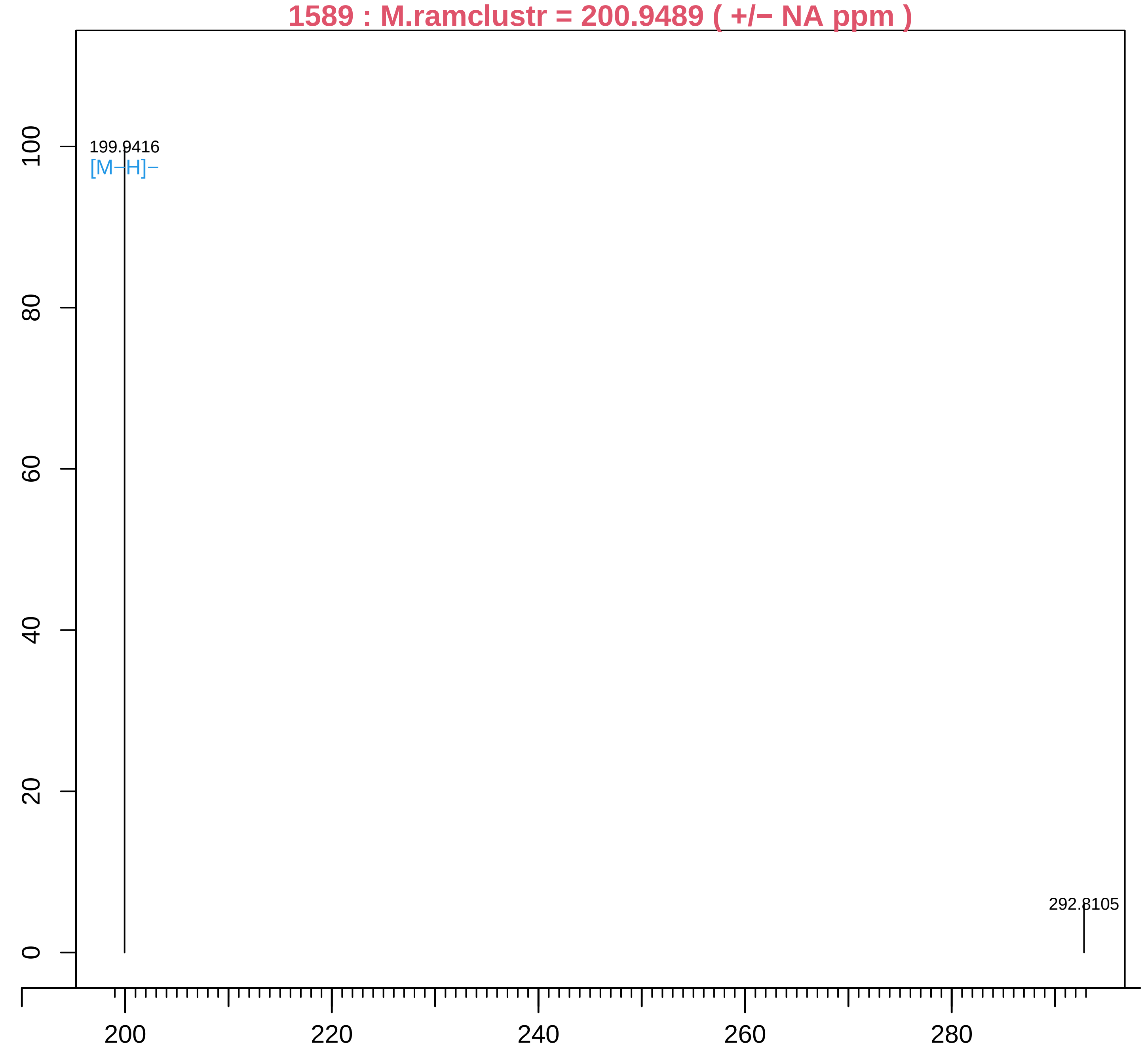
1589 : M.ramclustr = 200.9489 (+/− NA ppm)
199.9416
[M−H]−
292.8105
0
20
40
60
80
100
200
220
240
260
280

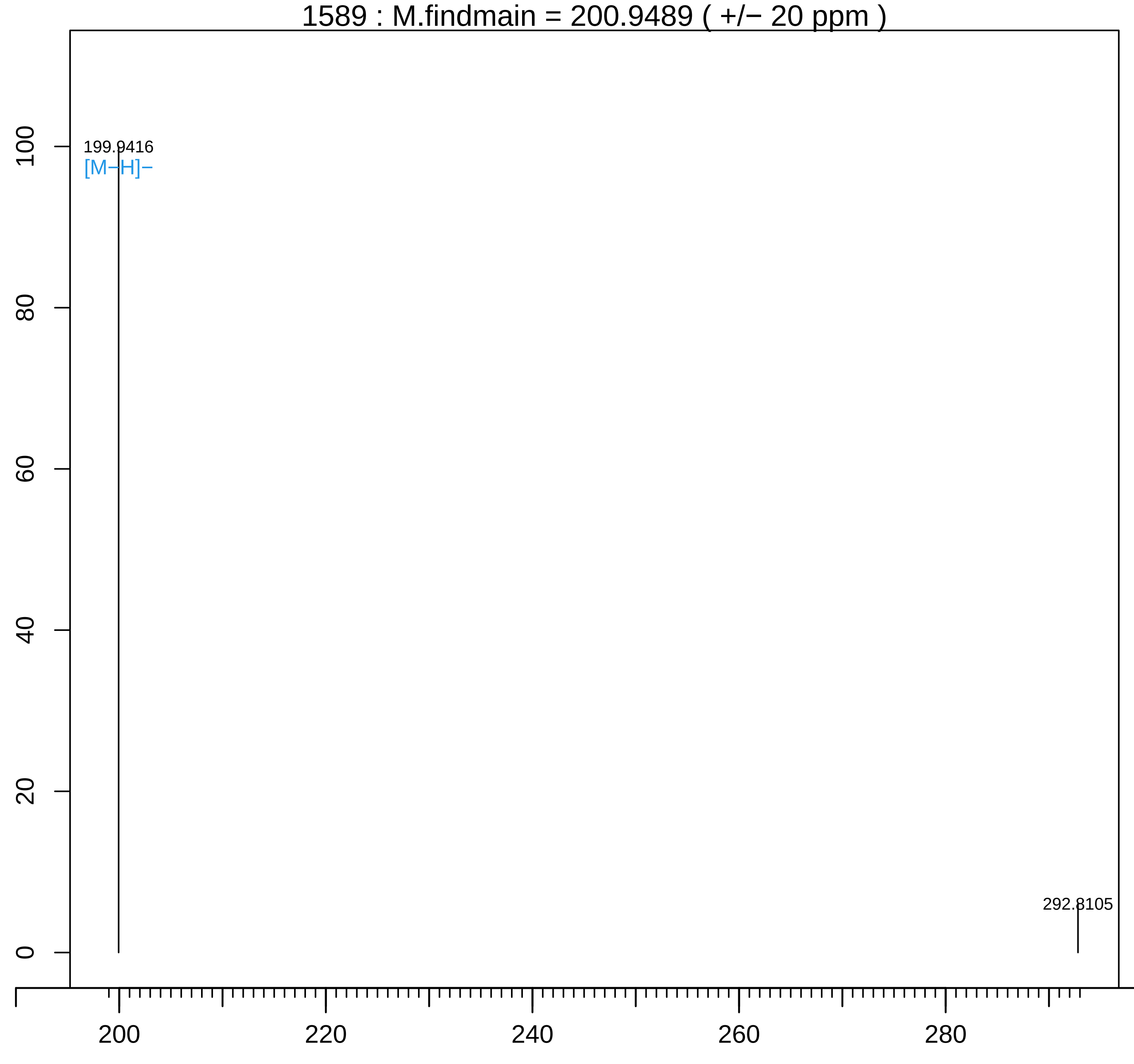
1589 : M.findmain = 200.9489 (+/− 20 ppm)
199.9416
[M−H]−
292.8105
0
20
40
60
80
100
200
220
240
260
280

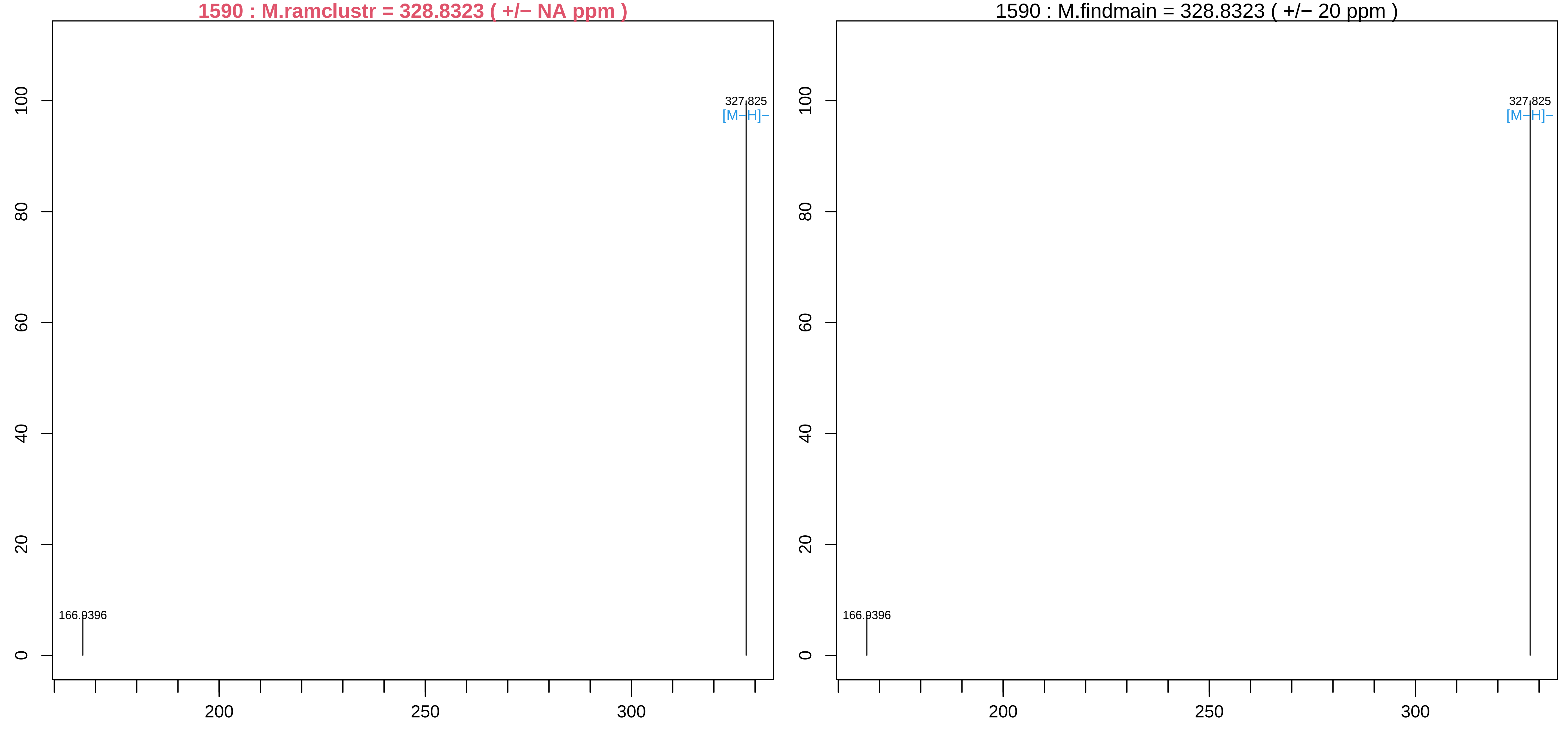

1590 : M.ramclustr = 328.8323 (+/− NA ppm)
327.825
[M−H]−
166.9396
0
20
40
60
80
100
200
250
300
1590 : M.findmain = 328.8323 (+/− 20 ppm)
327.825
[M−H]−
166.9396
0
20
40
60
80
100
200
250
300

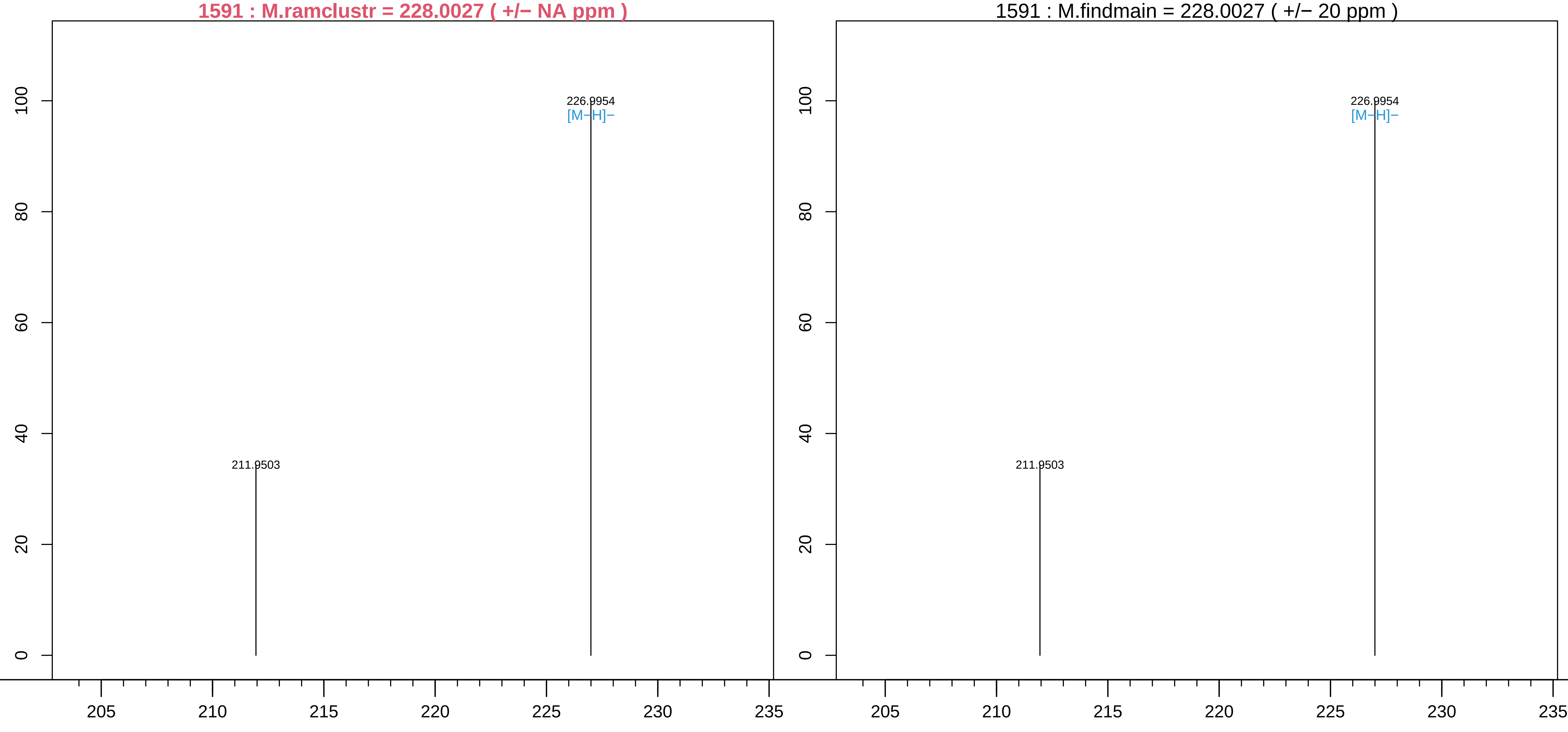
1591 : M.ramclustr = 228.0027 (+/− NA ppm)
226.9954
[M−H]−
211.9503
1591 : M.findmain = 228.0027 (+/− 20 ppm)
226.9954
[M−H]−
211.9503
0
20
40
60
80
100
205
210
215
220
225
230
235

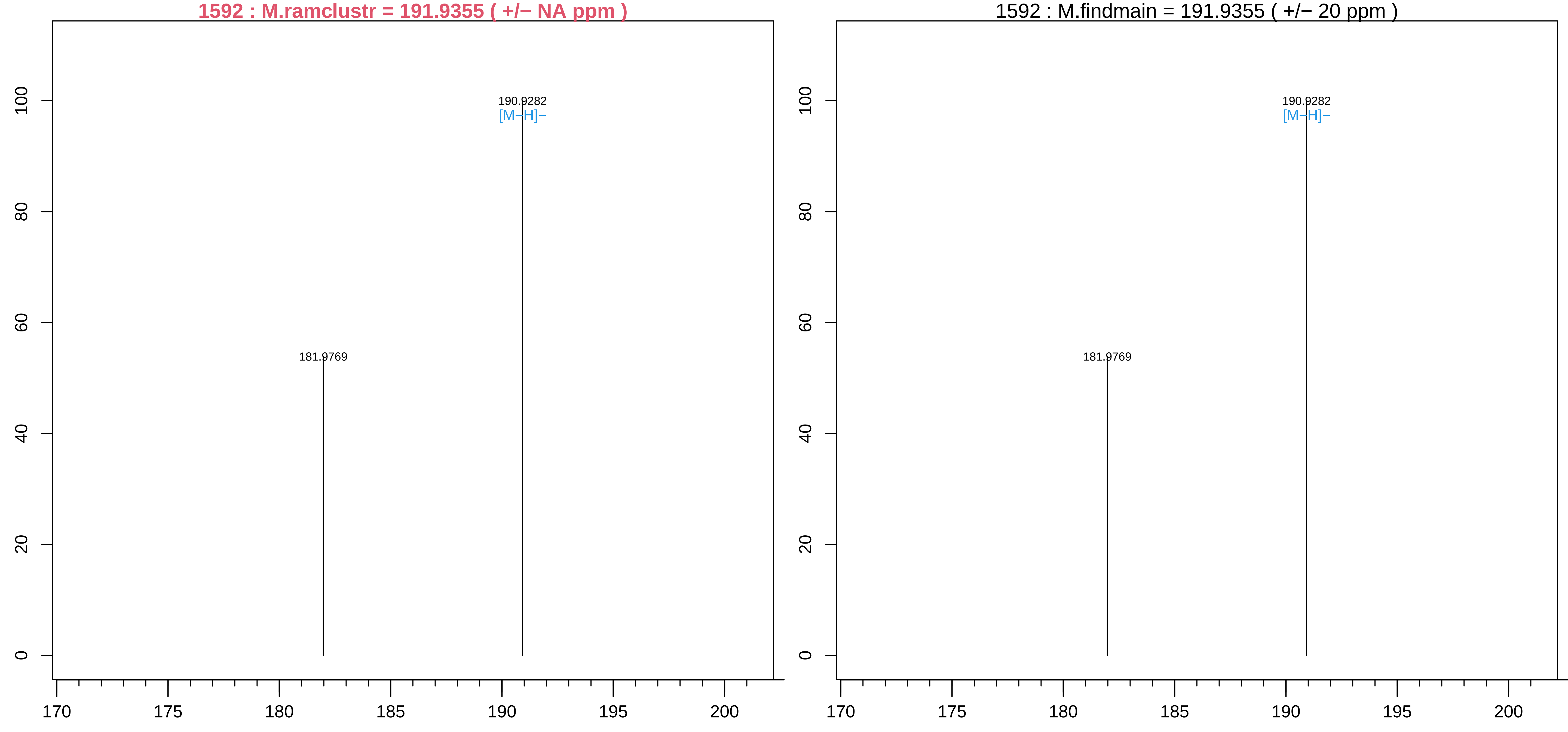
1592 : M.ramclustr = 191.9355 (+/− NA ppm)
190.9282
[M−H]−
181.9769
1592 : M.findmain = 191.9355 (+/− 20 ppm)
190.9282
[M−H]−
181.9769
0
20
40
60
80
100
170
175
180
185
190
195
200

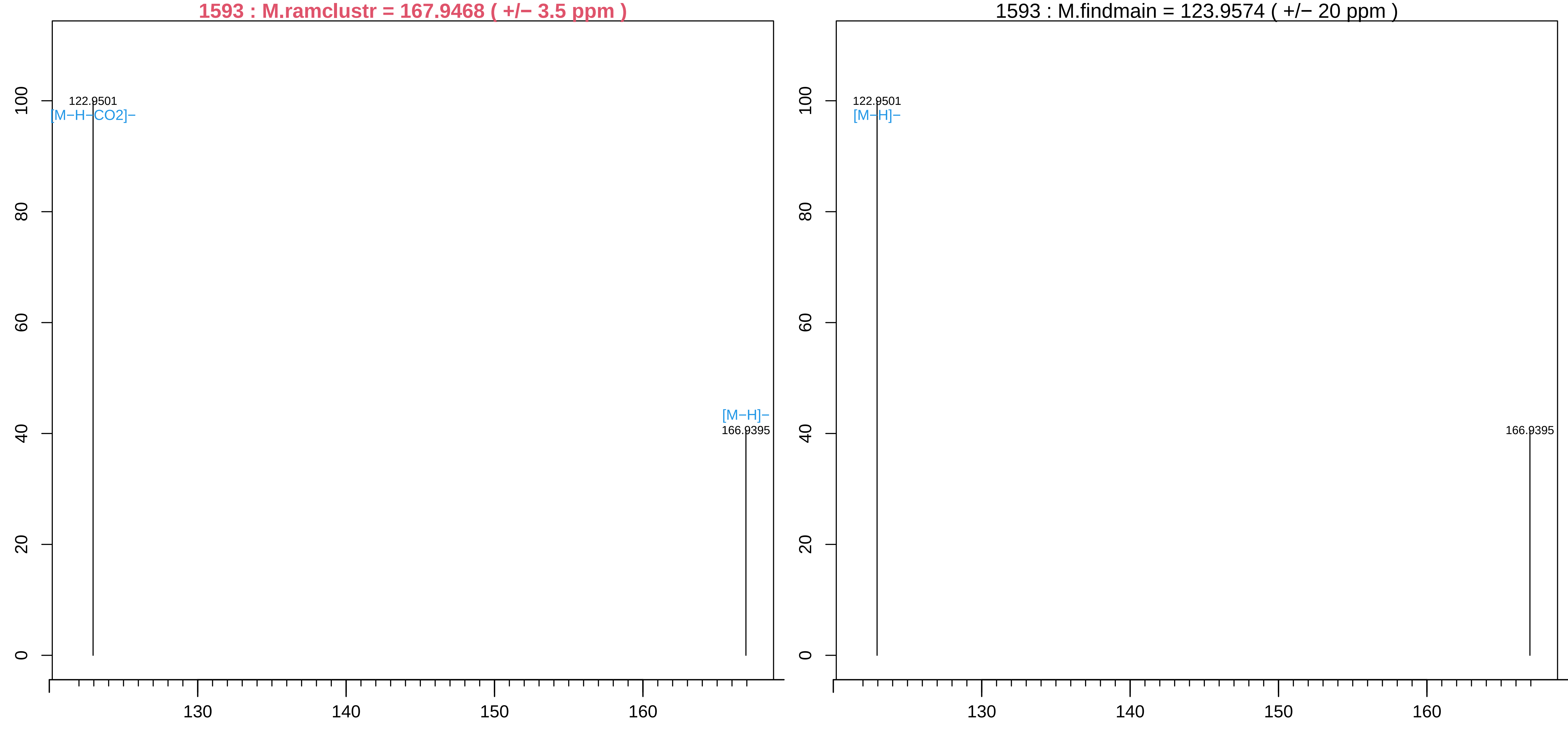
1593 : M.ramclustr = 167.9468 (+/− 3.5 ppm)
122.9501
[M−H−CO2]−
[M−H]−
166.9395
1593 : M.findmain = 123.9574 (+/− 20 ppm)
122.9501
[M−H]−
166.9395
0
20
40
60
80
100
130
140
150
160

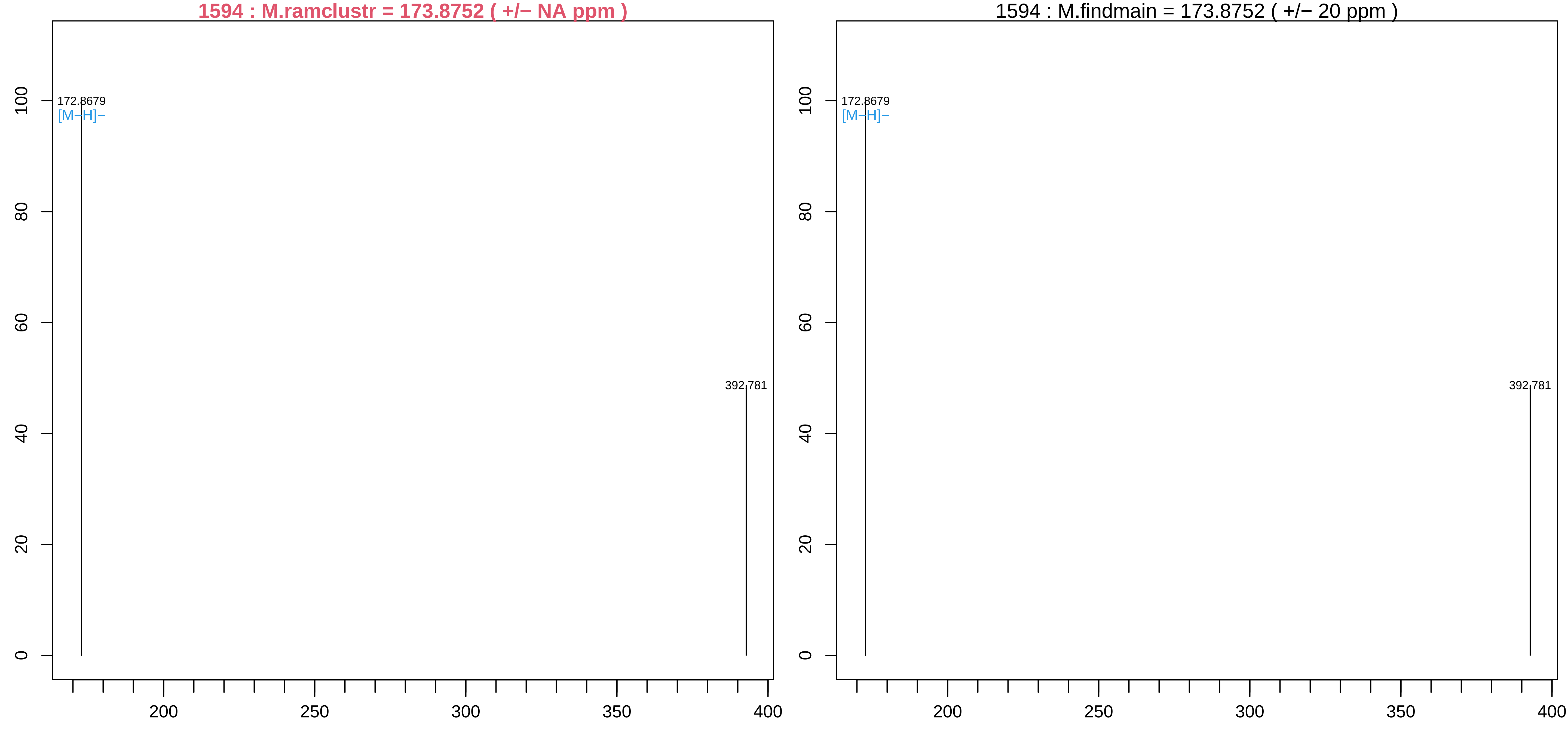
1594 : M.ramclustr = 173.8752 (+/− NA ppm)
172.8679
[M−H]−
392.781
1594 : M.findmain = 173.8752 (+/− 20 ppm)
172.8679
[M−H]−
392.781
0
20
40
60
80
100
200
250
300
350
400

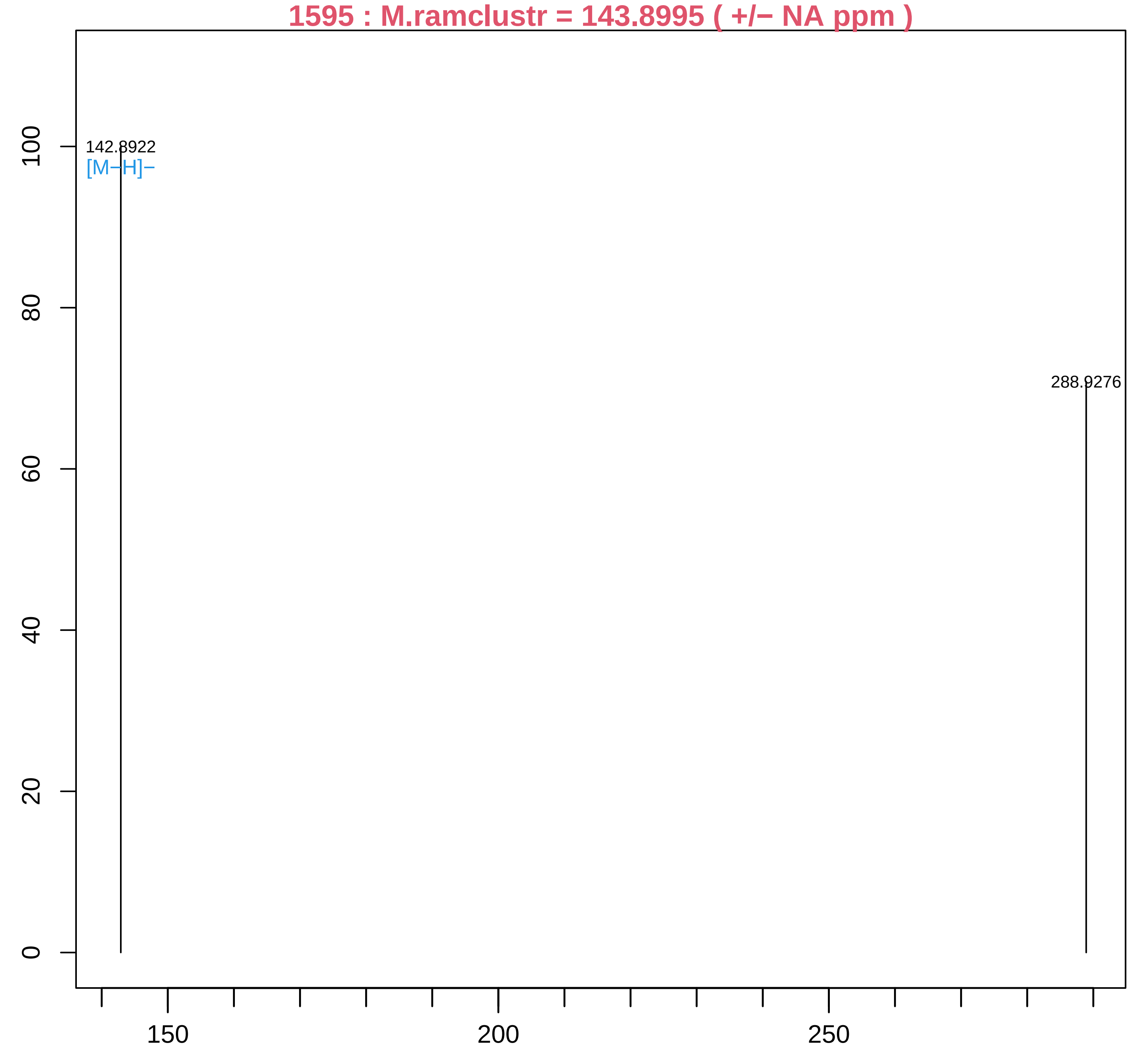
1595 : M.ramclustr = 143.8995 (+/− NA ppm)
142.8922
[M−H]−
288.9276

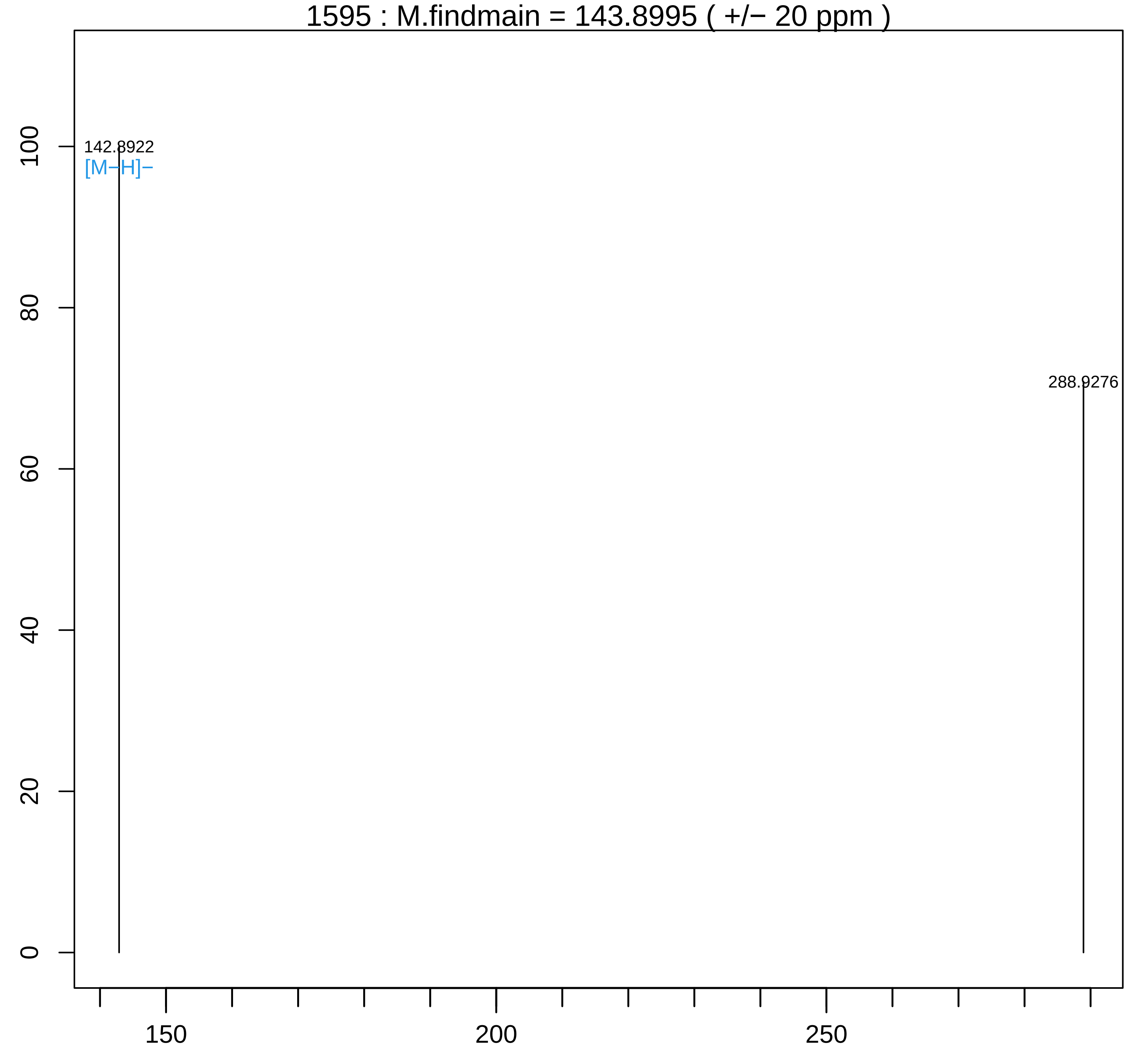
1595 : M.findmain = 143.8995 (+/− 20 ppm)
142.8922
[M−H]−
288.9276

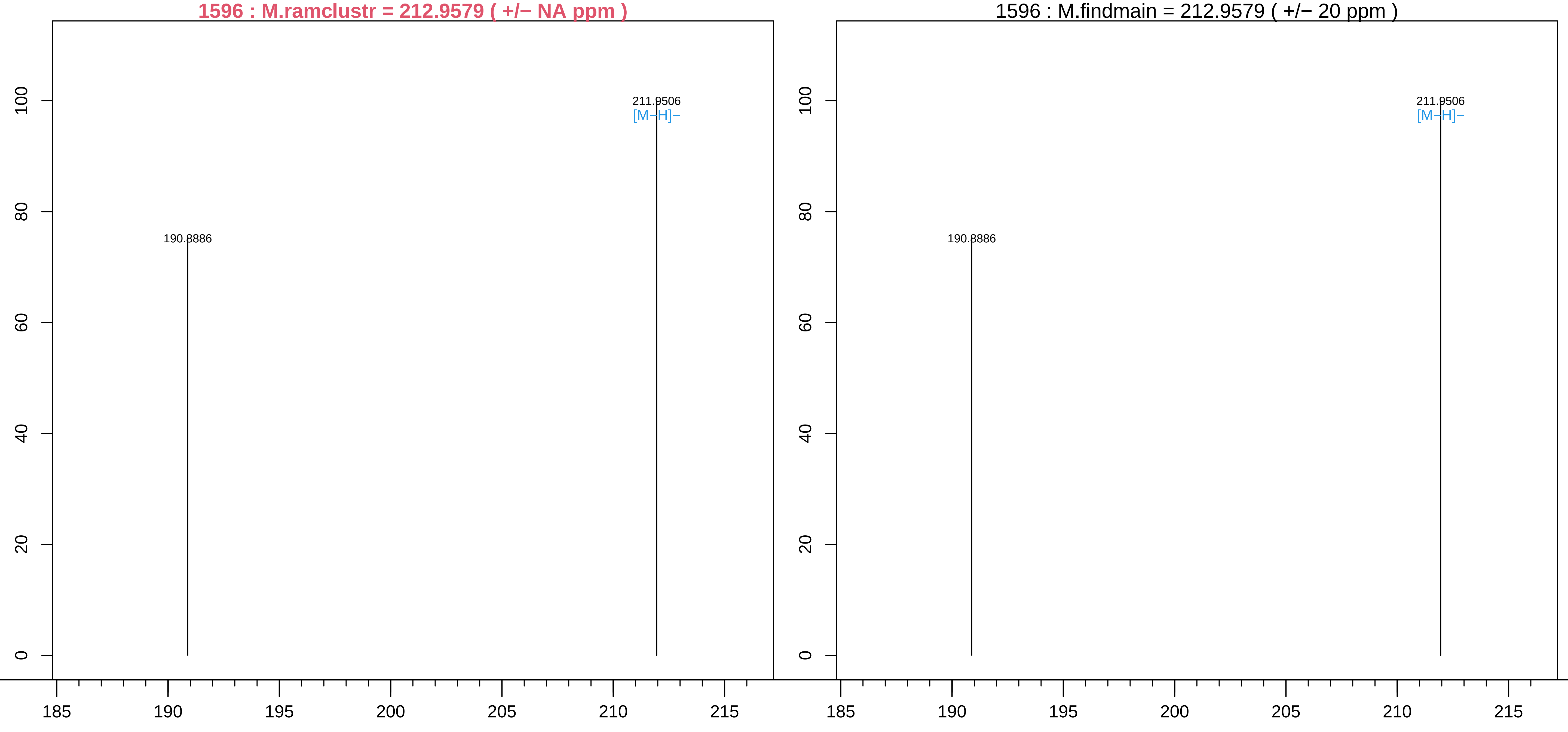
1596 : M.ramclustr = 212.9579 (+/− NA ppm)
1596 : M.findmain = 212.9579 (+/− 20 ppm)
190.8886
211.9506
[M−H]−
0
20
40
60
80
100
185
190
195
200
205
210
215

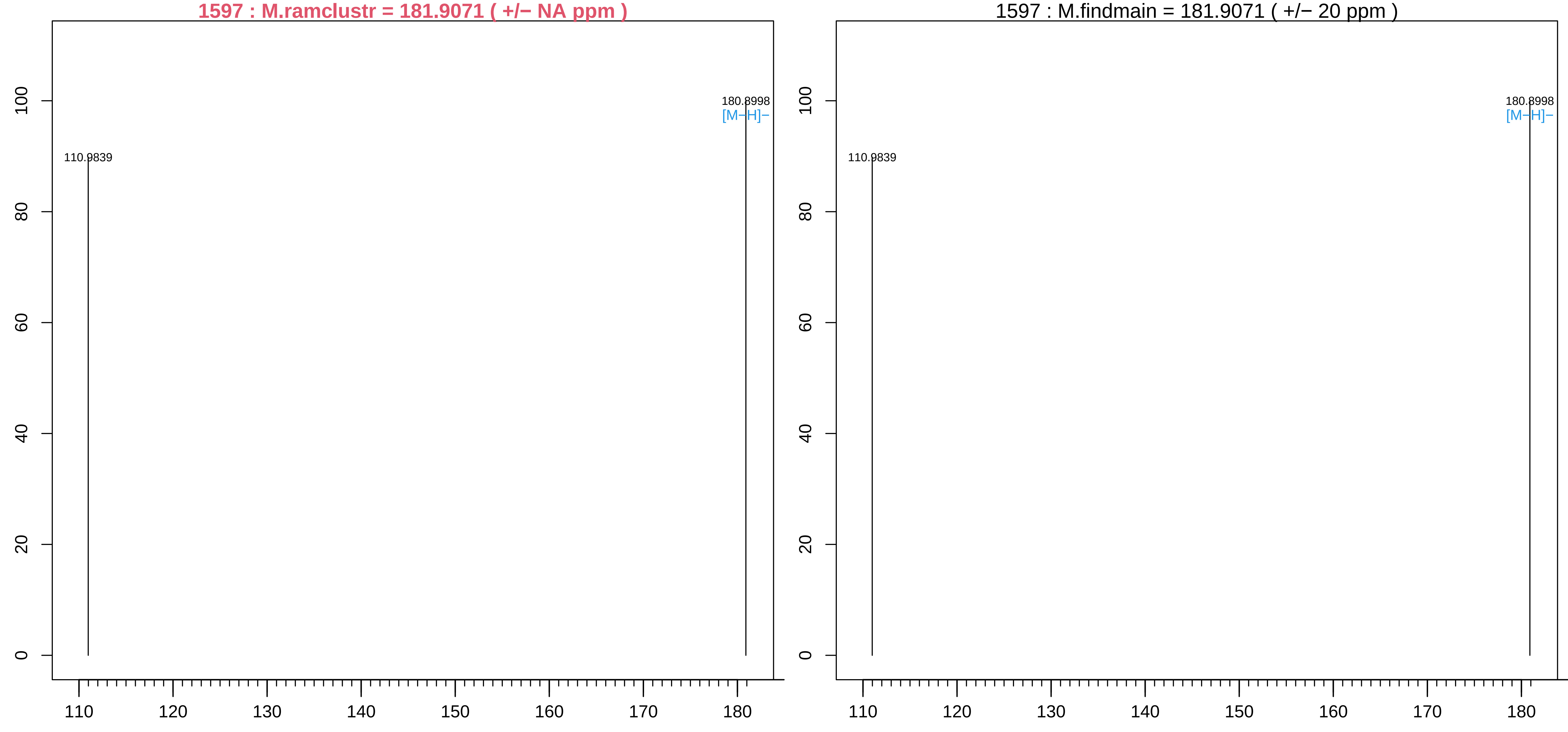
1597 : M.ramclustr = 181.9071 (+/− NA ppm)
110.9839
180.8998
[M−H]−
1597 : M.findmain = 181.9071 (+/− 20 ppm)
110.9839
180.8998
[M−H]−
0
20
40
60
80
100
110
120
130
140
150
160
170
180

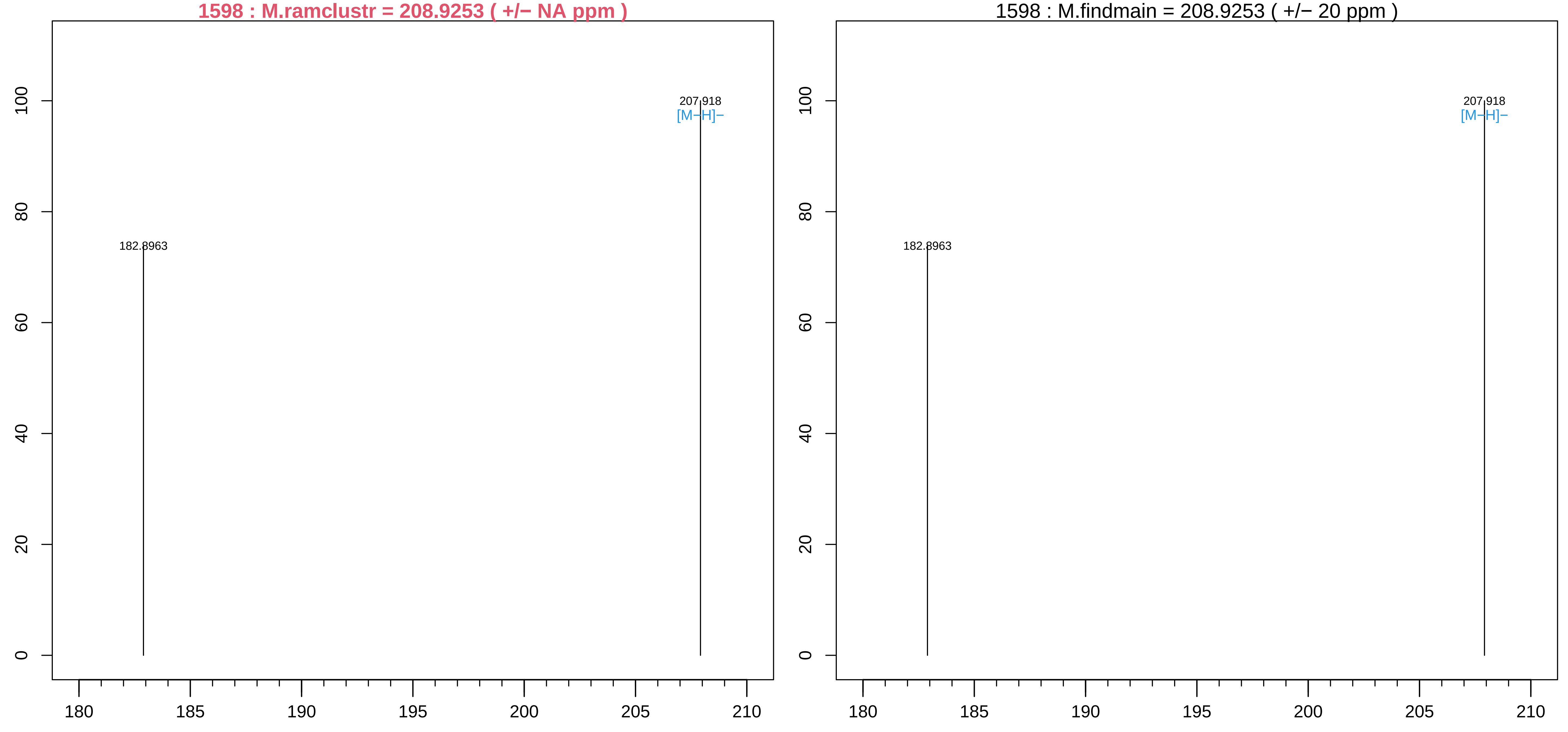
1598 : M.ramclustr = 208.9253 (+/− NA ppm)
207.918
[M−H]−
182.8963
1598 : M.findmain = 208.9253 (+/− 20 ppm)
207.918
[M−H]−
182.8963
0
20
40
60
80
100
180
185
190
195
200
205
210

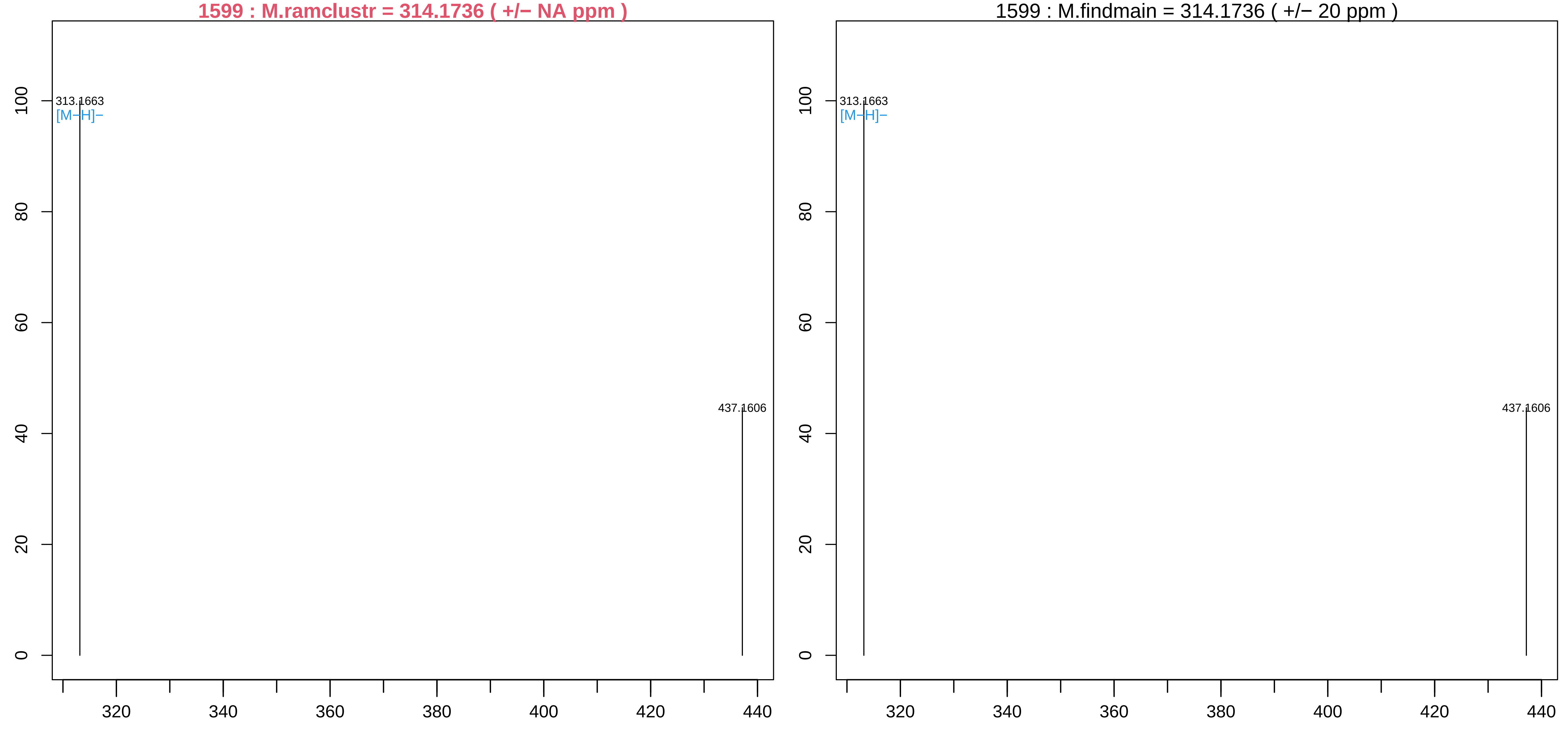
1599 : M.ramclustr = 314.1736 (+/− NA ppm)
313.1663
[M−H]−
437.1606
1599 : M.findmain = 314.1736 (+/− 20 ppm)
313.1663
[M−H]−
437.1606
0
20
40
60
80
100
320
340
360
380
400
420
440

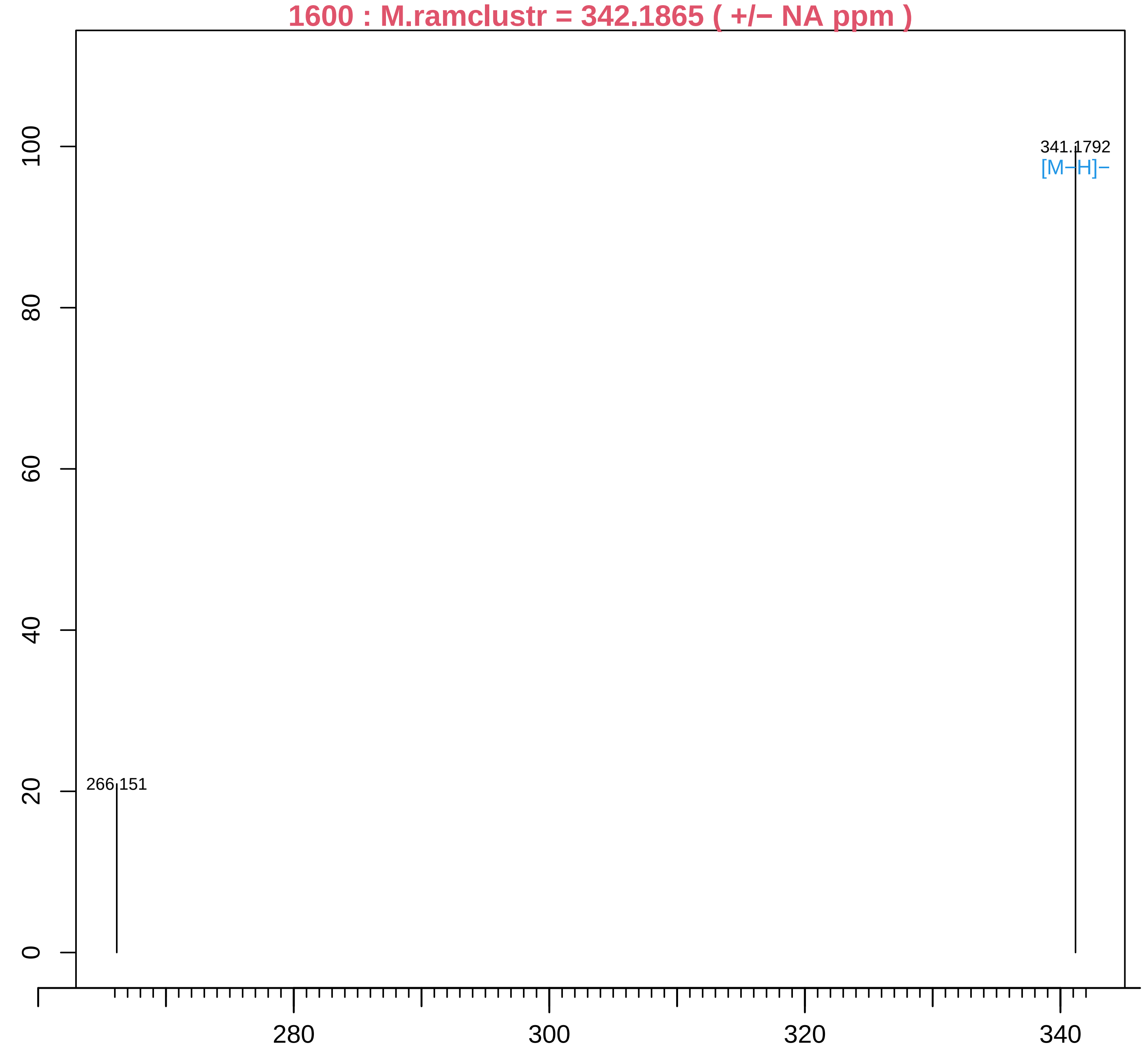
1600 : M.ramclustr = 342.1865 (+/− NA ppm)
341.1792
[M−H]−
266.151
0
20
40
60
80
100
280
300
320
340

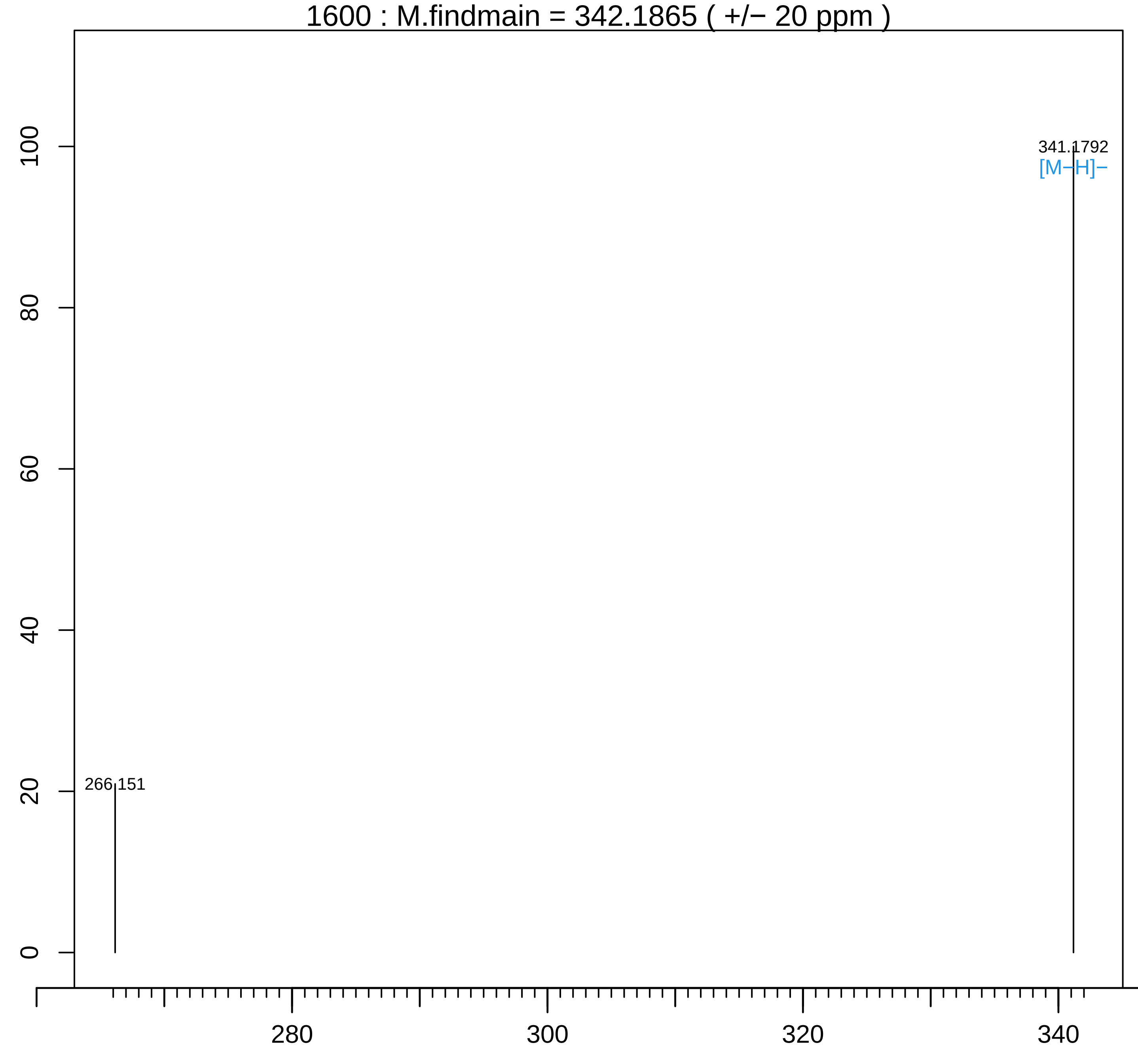
1600 : M.findmain = 342.1865 (+/− 20 ppm)
341.1792
[M−H]−
266.151
0
20
40
60
80
100
280
300
320
340

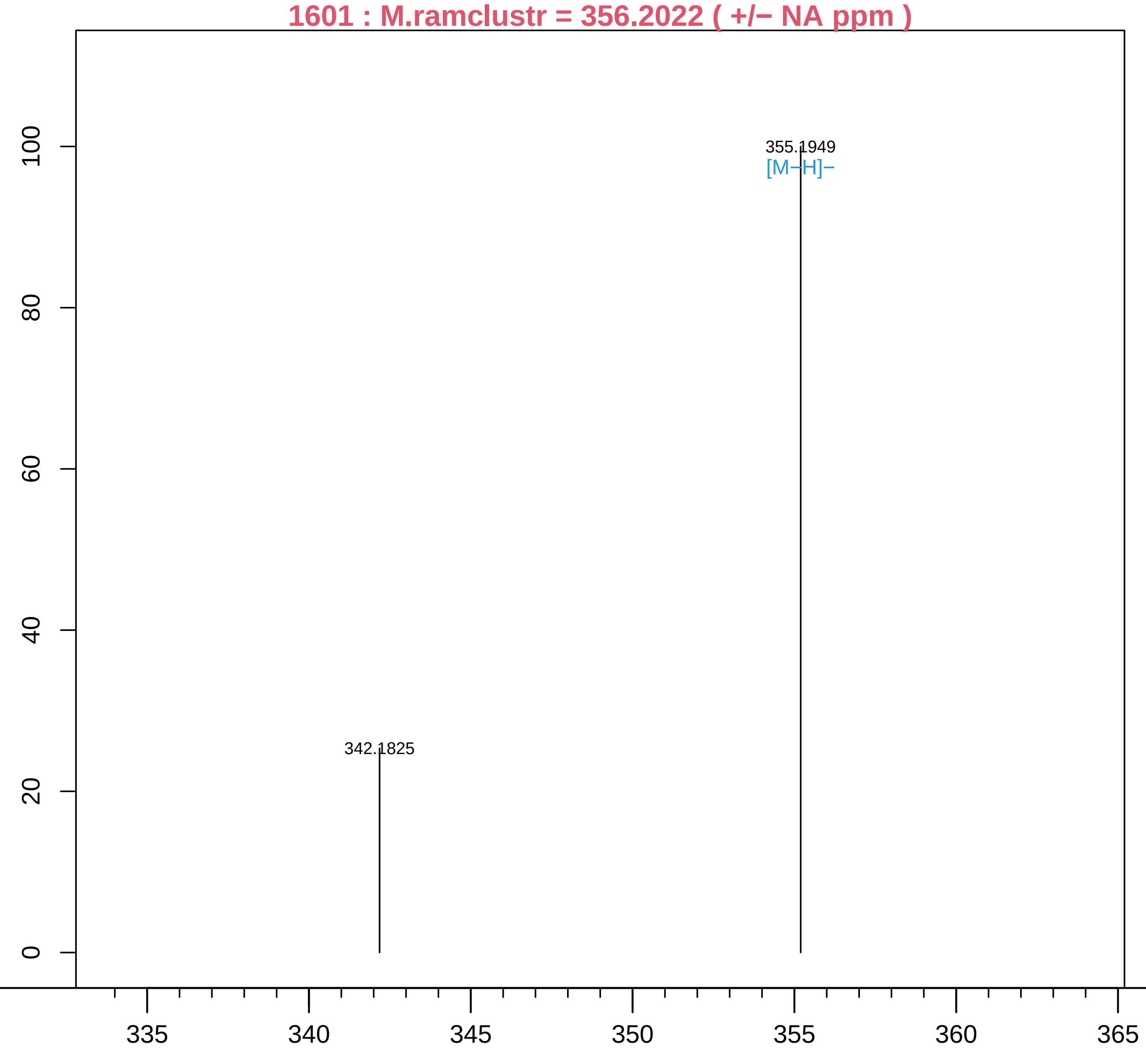
1601 : M.ramclustr = 356.2022 (+/− NA ppm)
355.1949
[M−H]−
342.1825
0
20
40
60
80
100
335
340
345
350
355
360
365

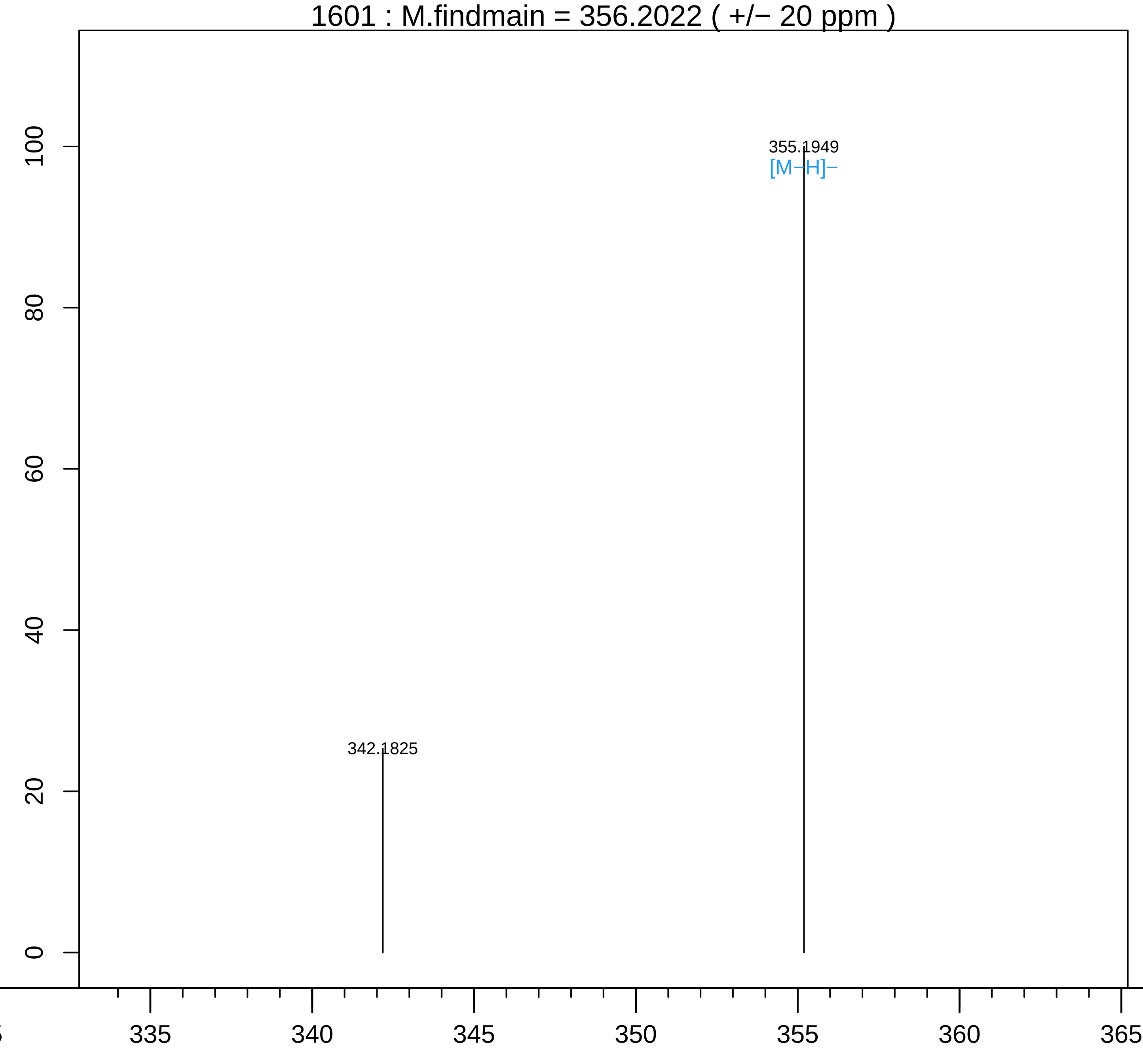
1601 : M.findmain = 356.2022 (+/− 20 ppm)
355.1949
[M−H]−
342.1825
0
20
40
60
80
100
335
340
345
350
355
360
365

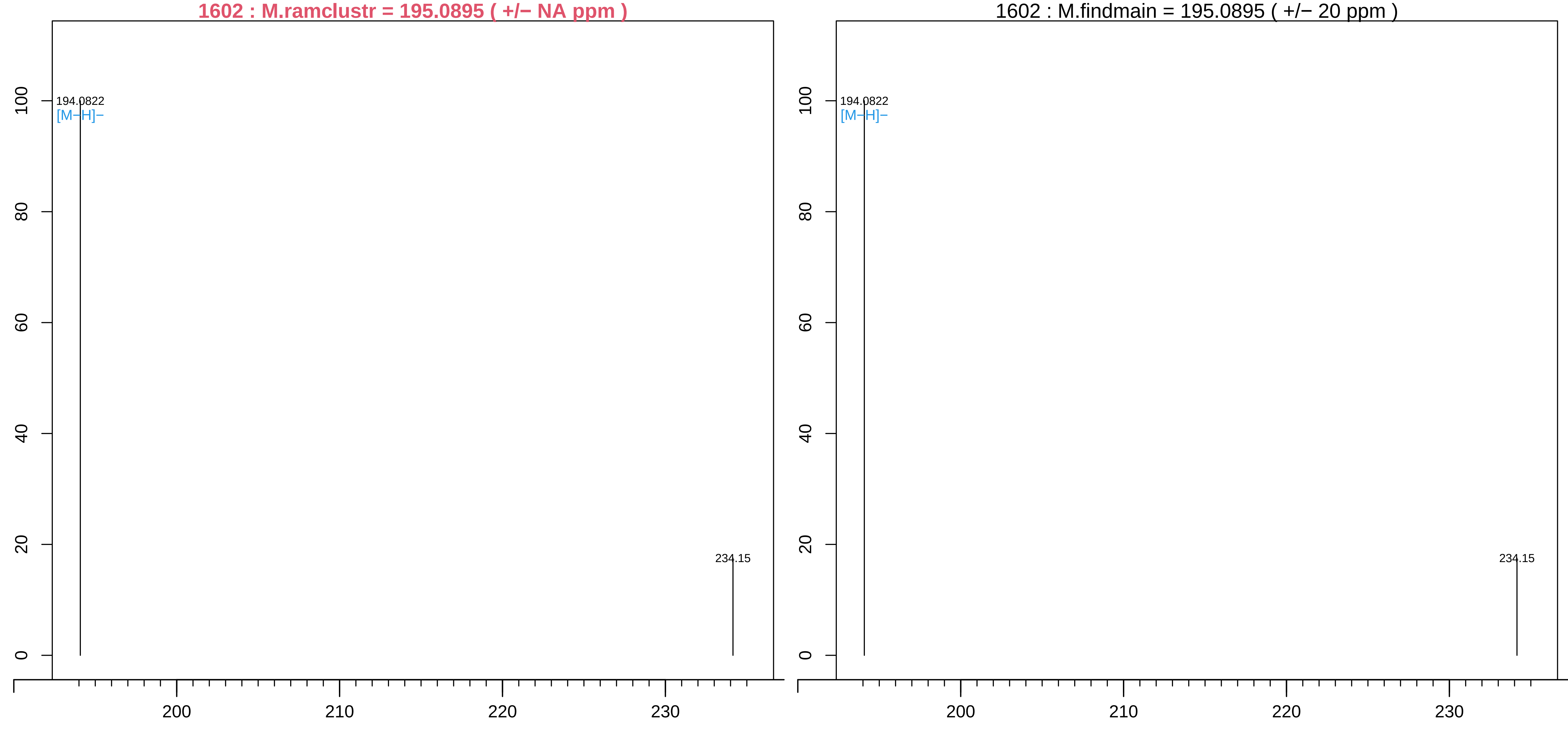
1602 : M.ramclustr = 195.0895 (+/− NA ppm)
194.0822
[M−H]−
234.15
1602 : M.findmain = 195.0895 (+/− 20 ppm)
194.0822
[M−H]−
234.15
0
20
40
60
80
100
200
210
220
230

1603 : M.ramclustr = 326.1916 (+/− NA ppm)

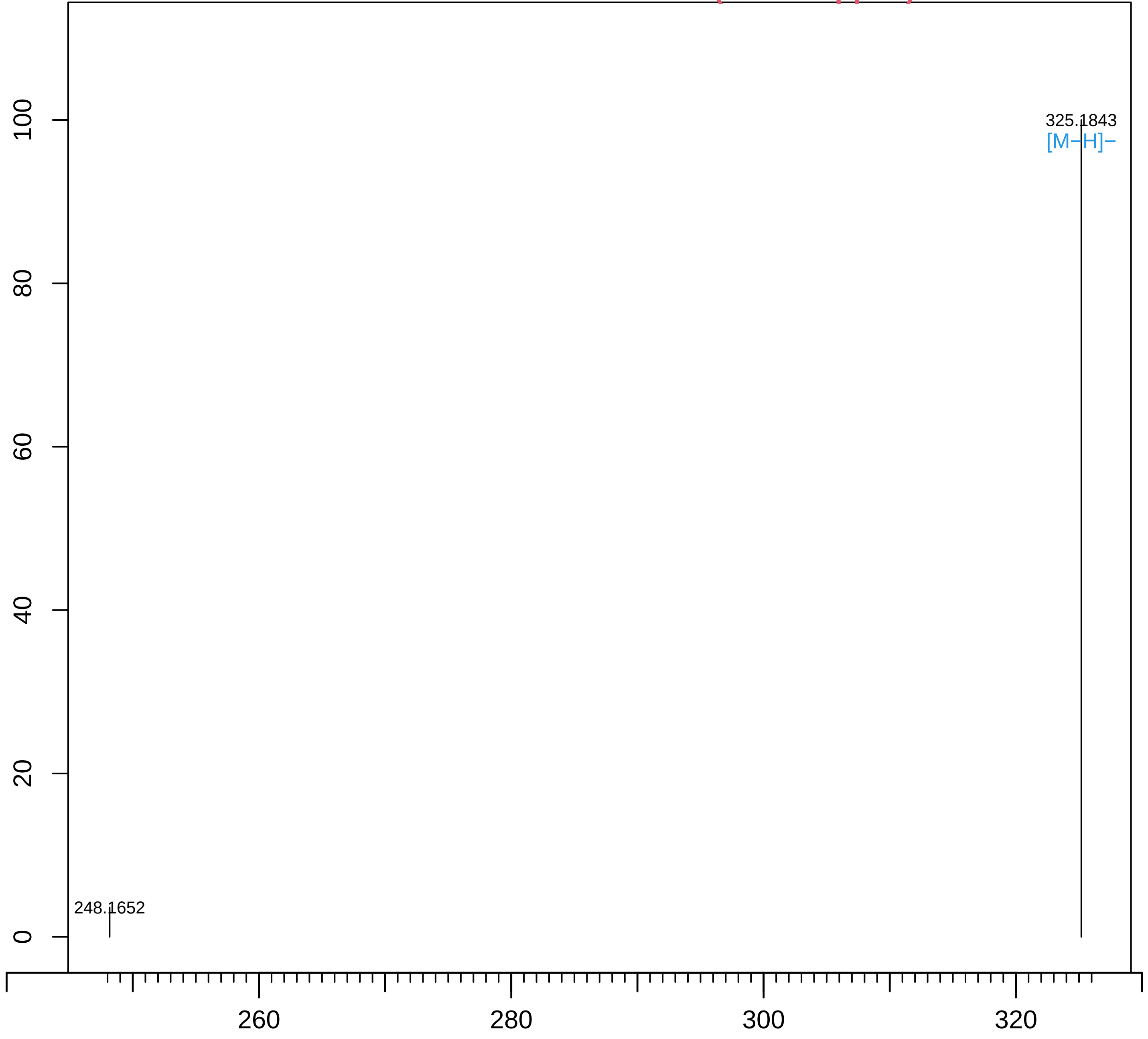

1603 : M.findmain = 326.1916 (+/− 20 ppm)

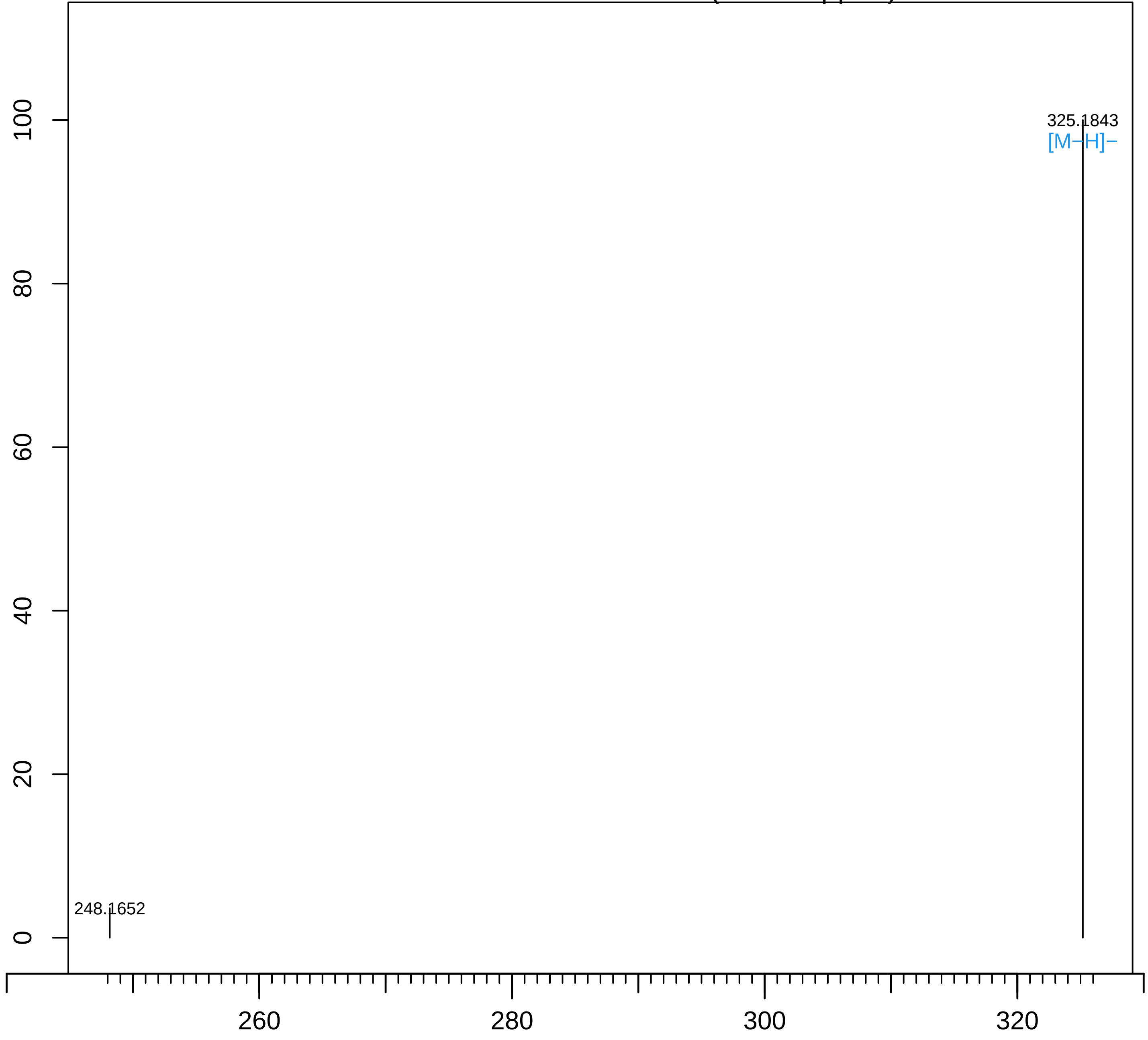

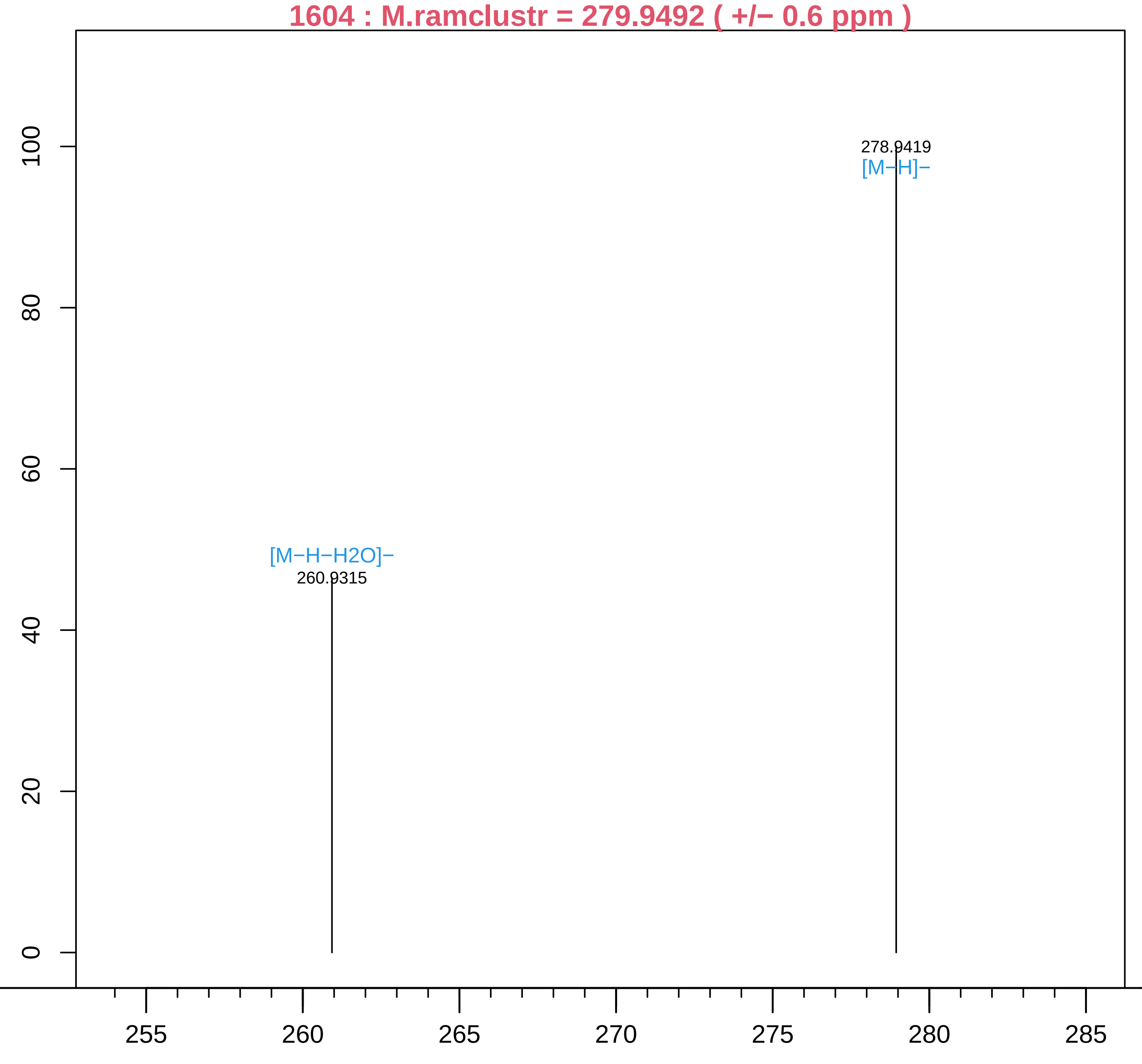
1604 : M.ramclustr = 279.9492 (+/− 0.6 ppm)
278.9419
[M−H]−
[M−H−H2O]−
260.9315
0
20
40
60
80
100
255
260
265
270
275
280
285

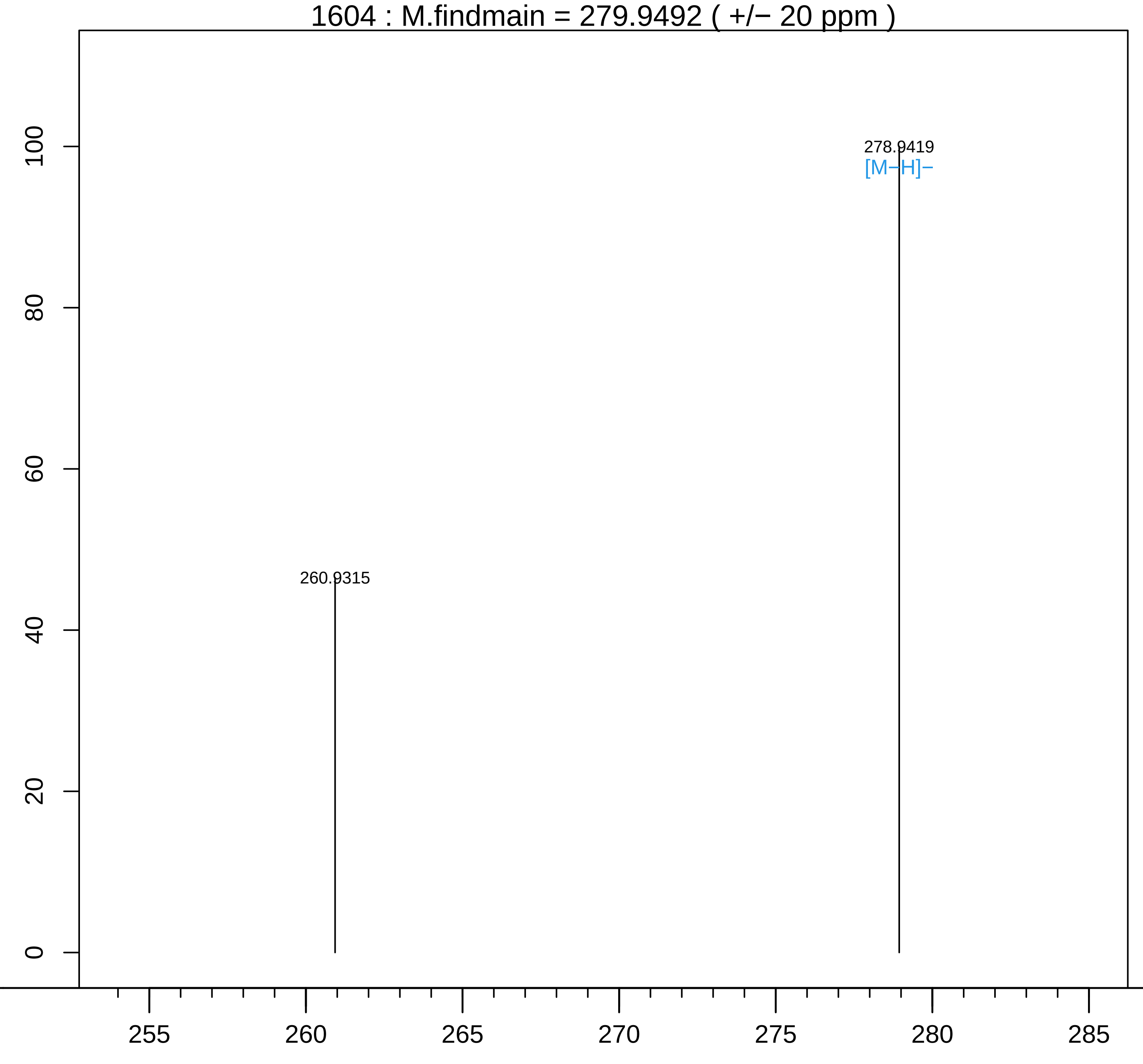
1604 : M.findmain = 279.9492 (+/− 20 ppm)
278.9419
[M−H]−
260.9315
0
20
40
60
80
100
255
260
265
270
275
280
285

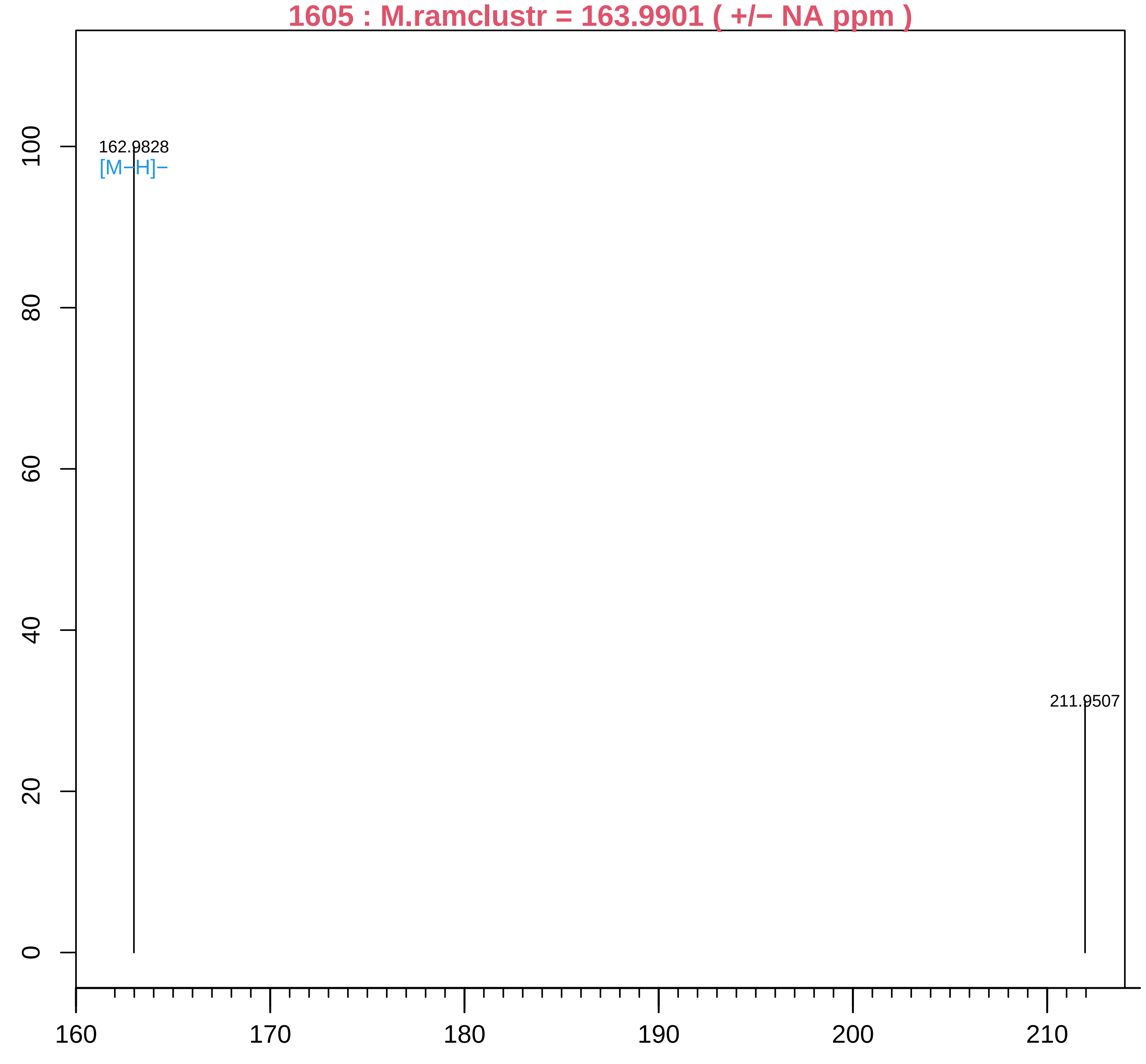
1605 : M.ramclustr = 163.9901 (+/− NA ppm)
162.9828
[M−H]−
211.9507
0
20
40
60
80
100
160
170
180
190
200
210

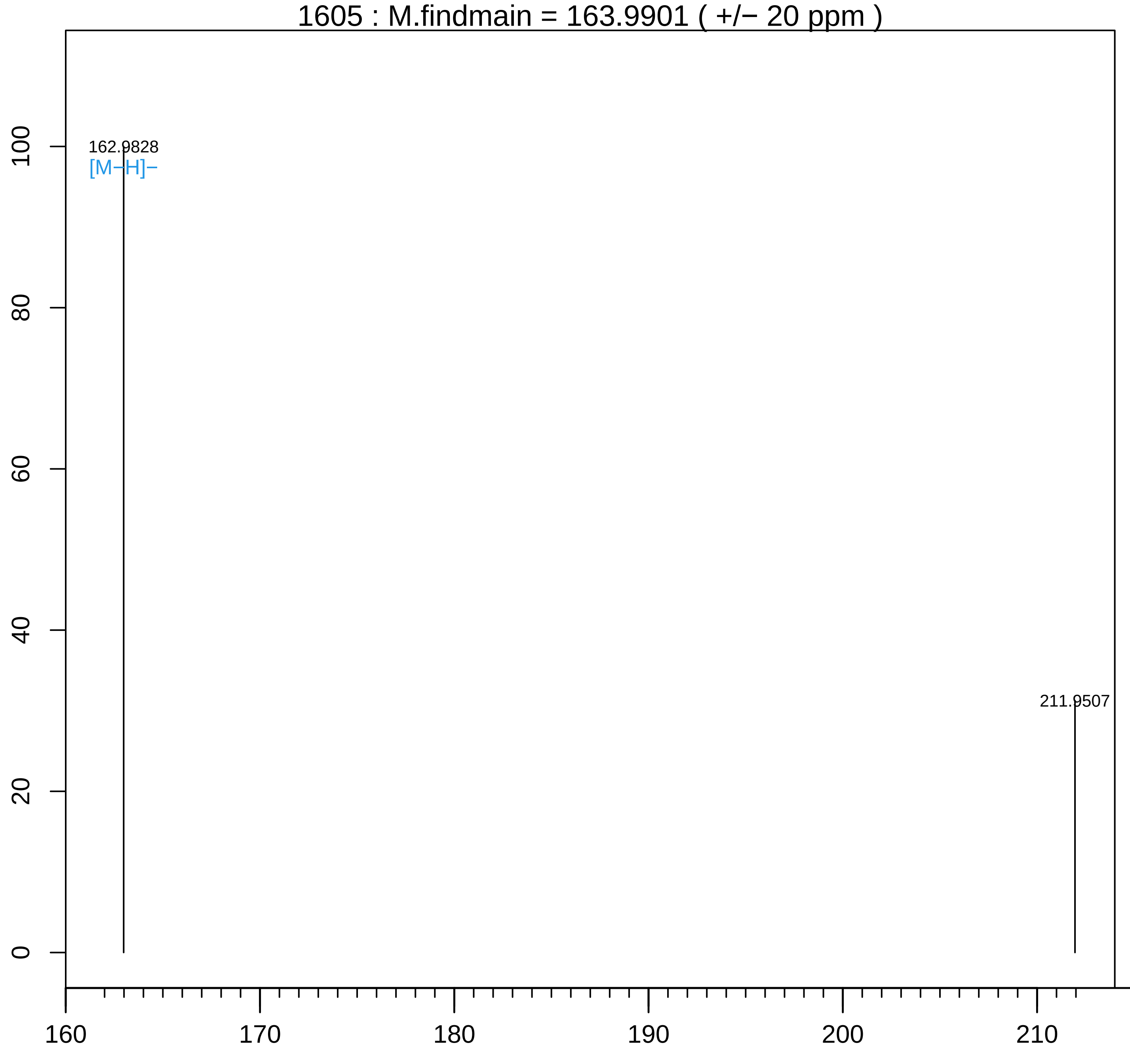
1605 : M.findmain = 163.9901 (+/− 20 ppm)
162.9828
[M−H]−
211.9507
0
20
40
60
80
100
160
170
180
190
200
210

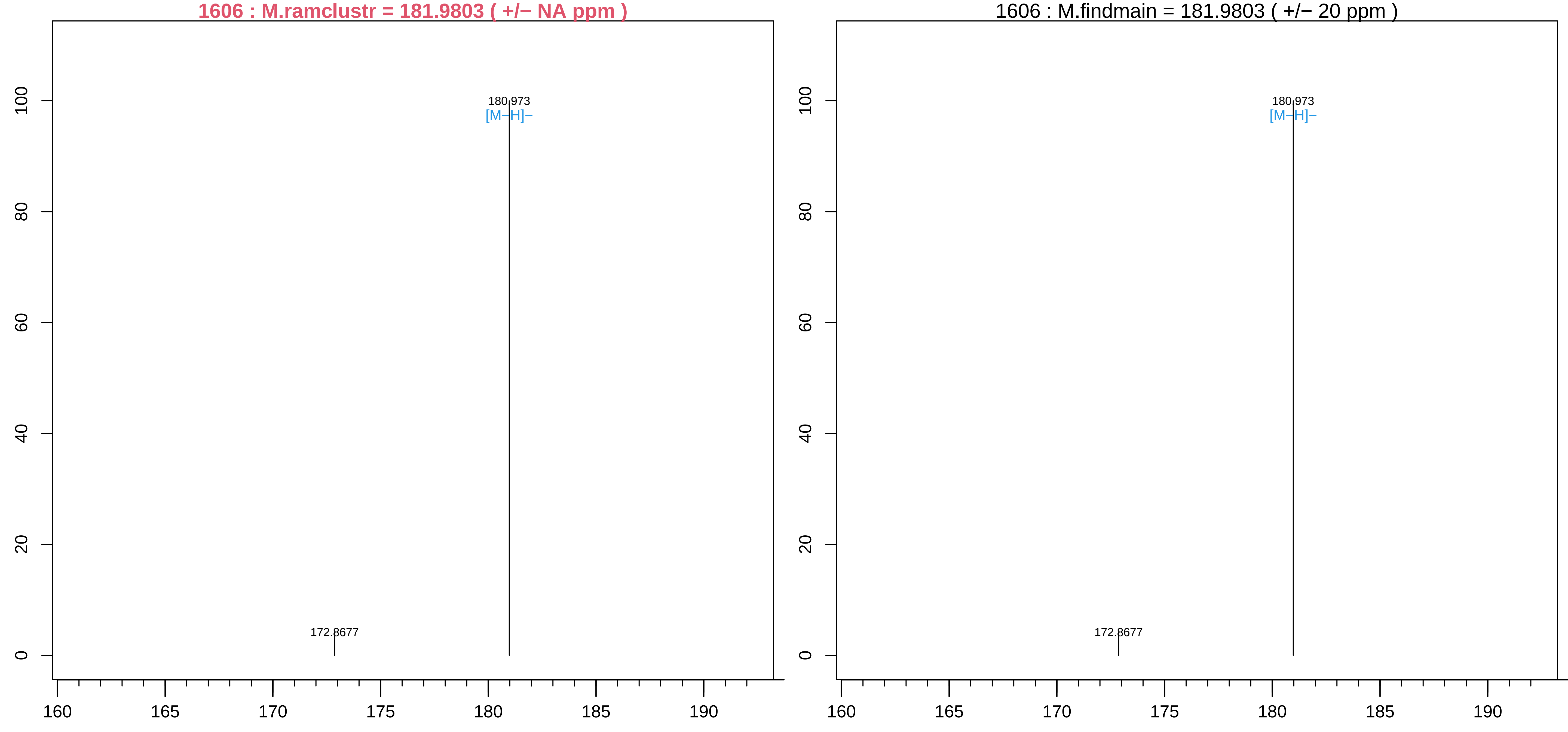
1606 : M.ramclustr = 181.9803 (+/− NA ppm)
1606 : M.findmain = 181.9803 (+/− 20 ppm)
180.973
[M−H]−
172.8677
0
20
40
60
80
100
160
165
170
175
180
185
190

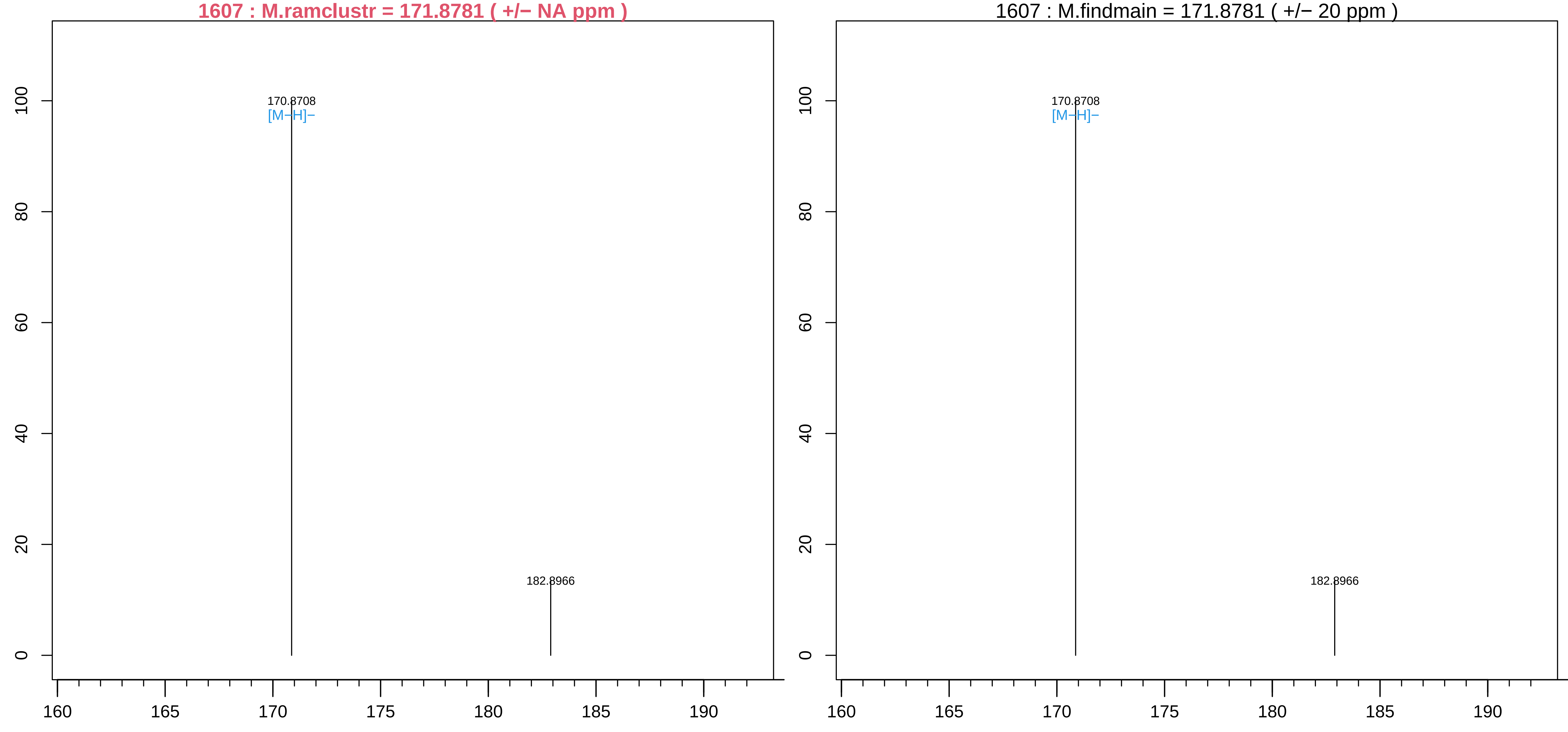

1607 : M.ramclustr = 171.8781 (+/− NA ppm)
170.8708
[M−H]−
182.8966
0
20
40
60
80
100
160
165
170
175
180
185
190
1607 : M.findmain = 171.8781 (+/− 20 ppm)
170.8708
[M−H]−
182.8966
0
20
40
60
80
100
160
165
170
175
180
185
190

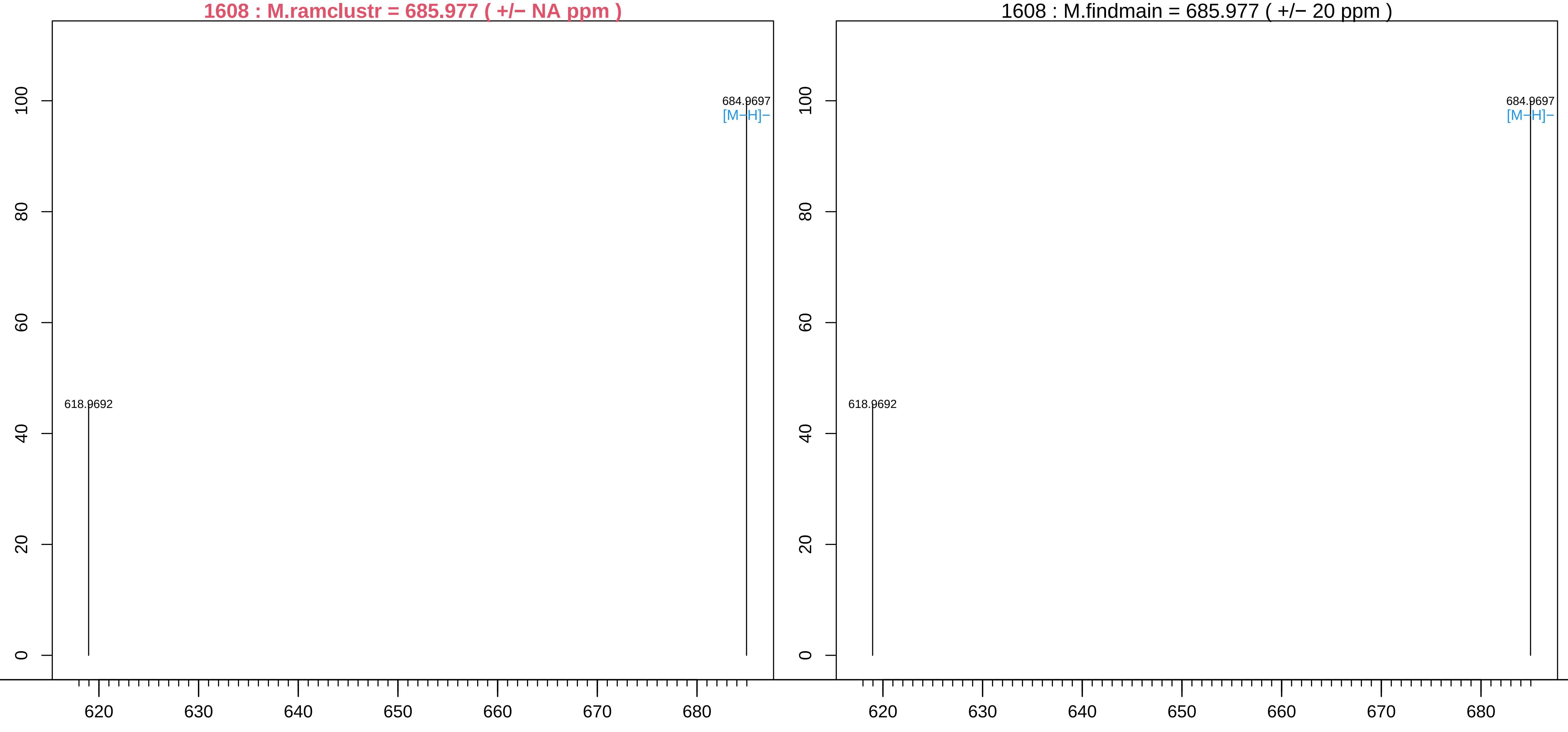

1608 : M.ramclustr = 685.977 (+/− NA ppm)
1608 : M.findmain = 685.977 (+/− 20 ppm)
684.9697
[M−H]−
618.9692
684.9697
[M−H]−
618.9692
0
20
40
60
80
100
620
630
640
650
660
670
680

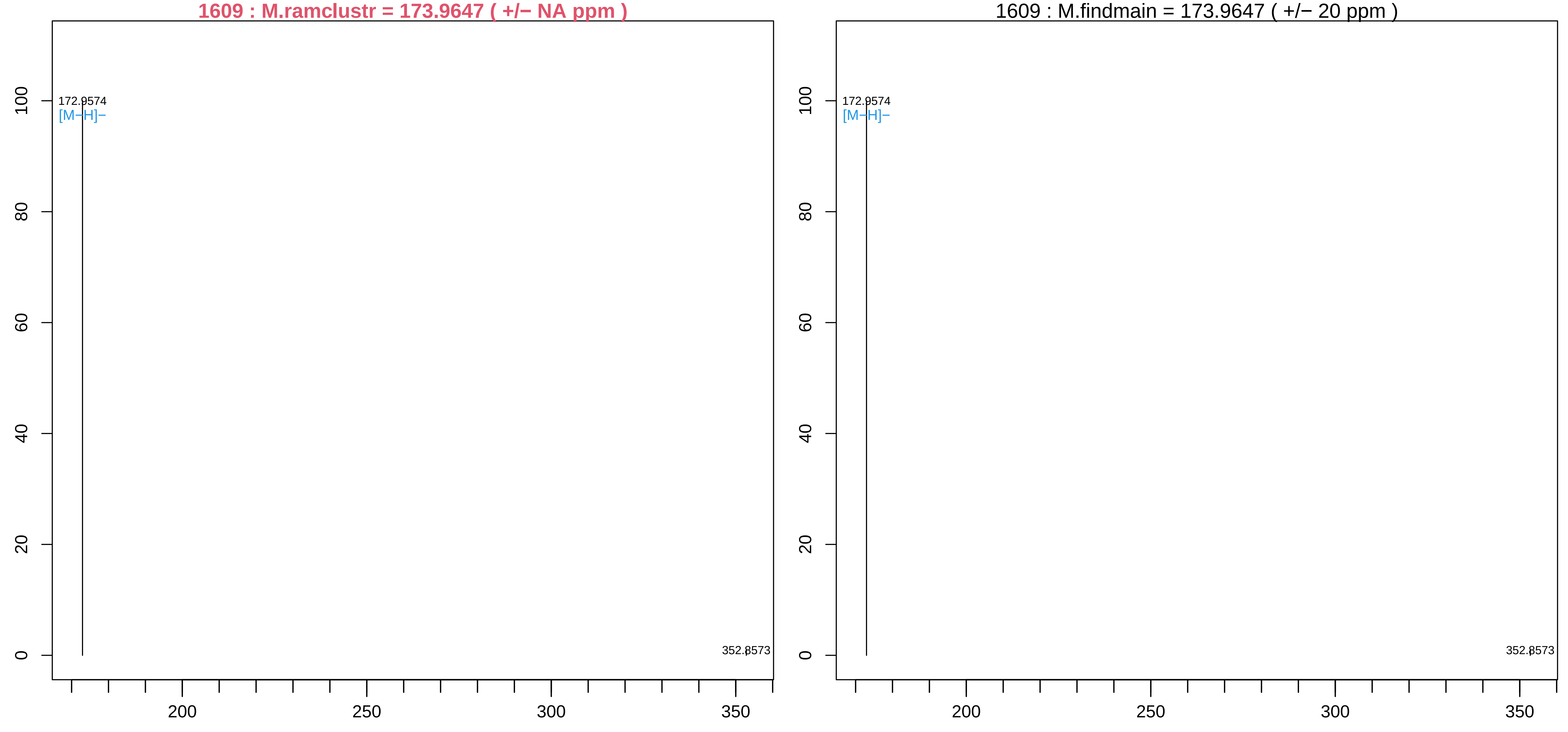
1609 : M.ramclustr = 173.9647 (+/− NA ppm)
172.9574
[M−H]−
352.8573
1609 : M.findmain = 173.9647 (+/− 20 ppm)
172.9574
[M−H]−
352.8573
0
20
40
60
80
100
200
250
300
350

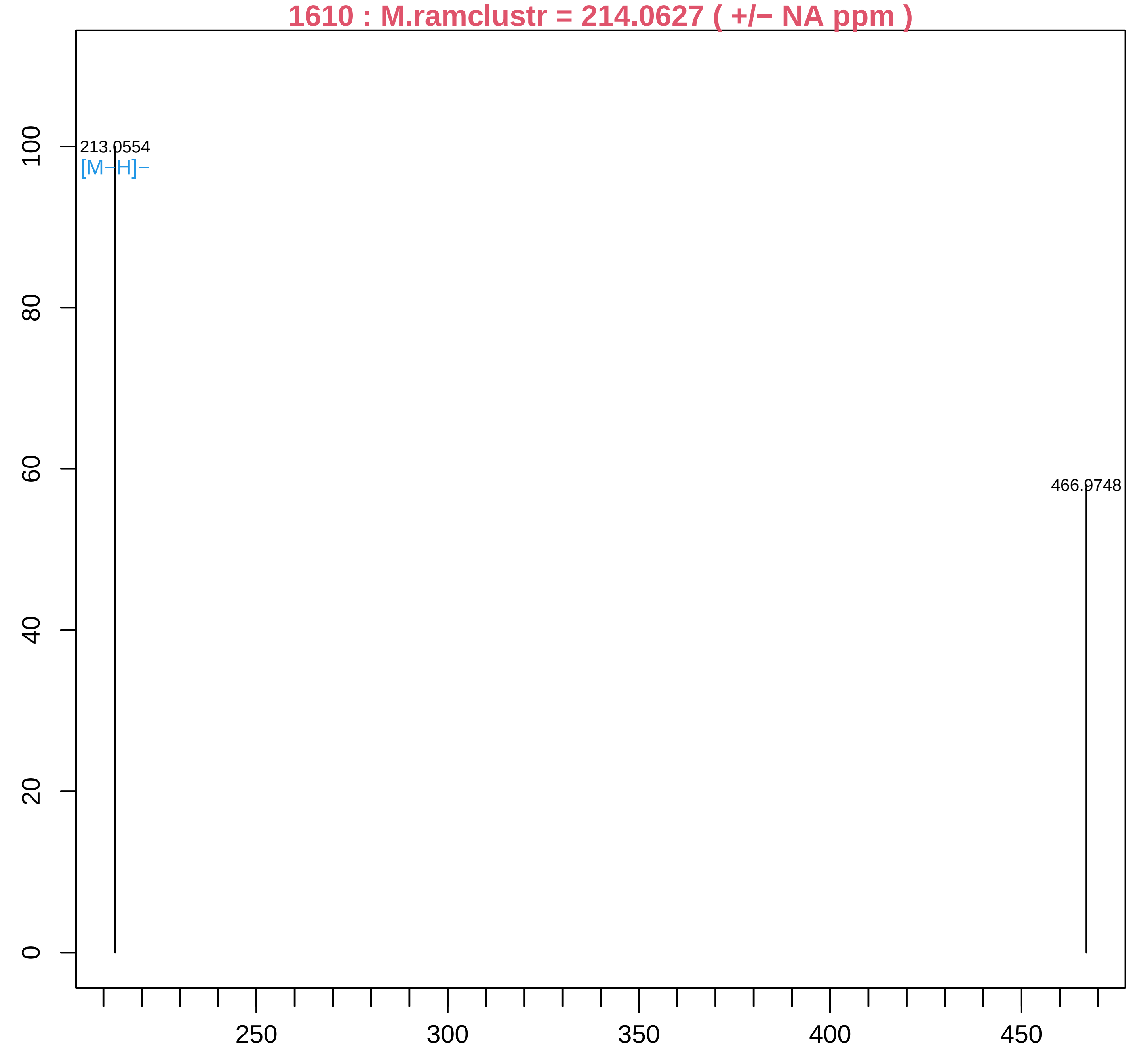

1610 : M.ramclustr = 214.0627 (+/− NA ppm)
213.0554
[M−H]−
466.9748
0
20
40
60
80
100
250
300
350
400
450

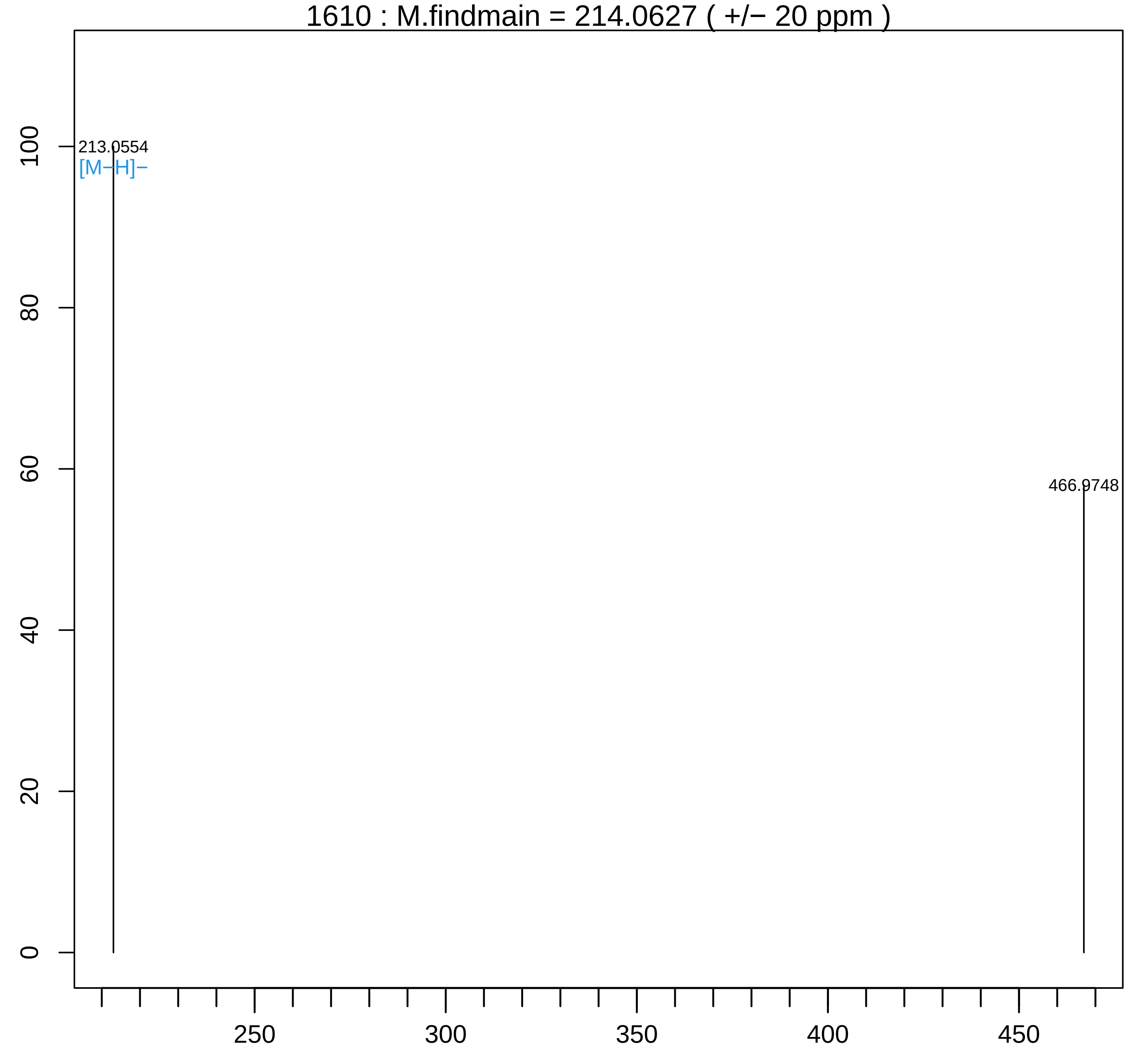

1610 : M.findmain = 214.0627 (+/− 20 ppm)
213.0554
[M−H]−
466.9748
0
20
40
60
80
100
250
300
350
400
450

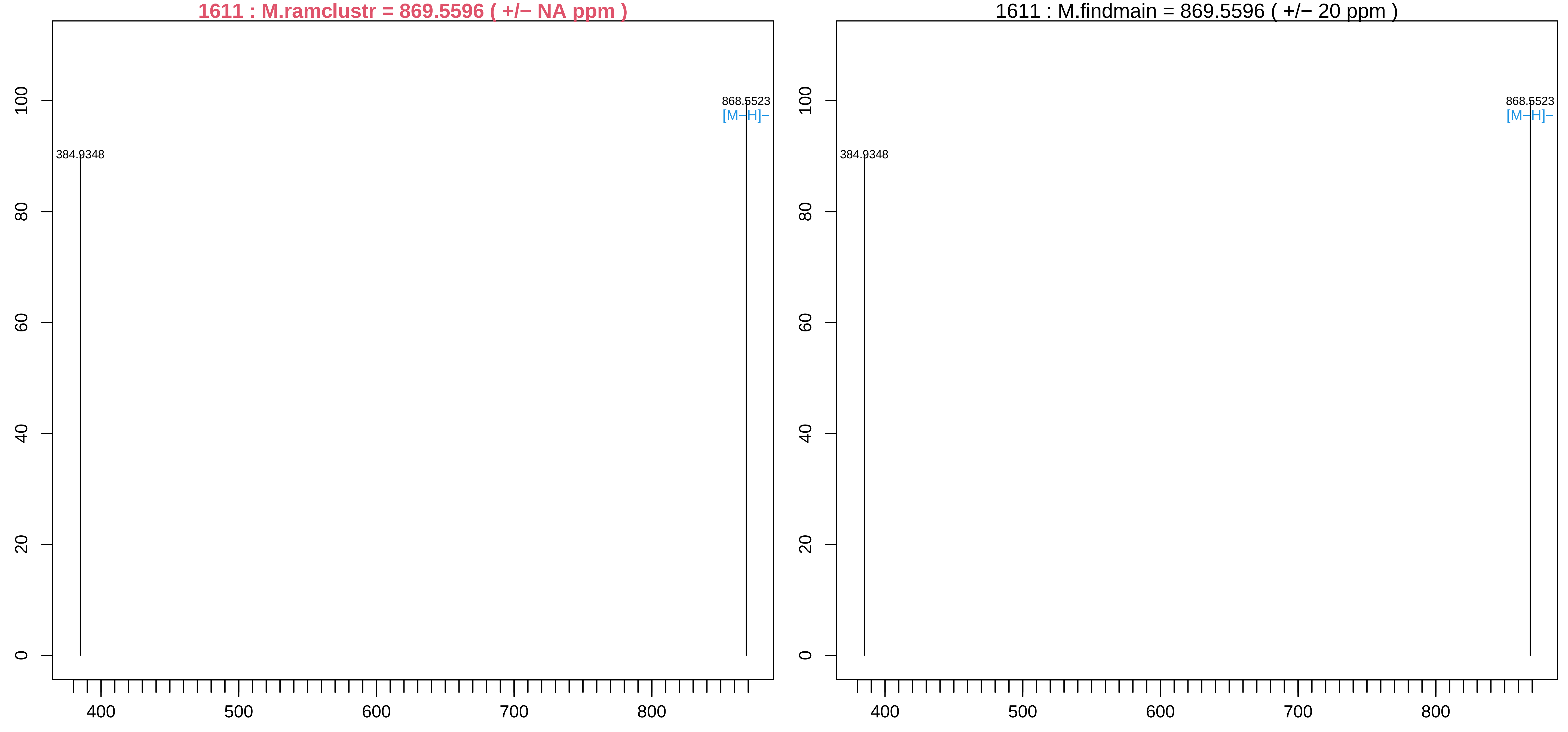
1611 : M.ramclustr = 869.5596 (+/− NA ppm)
384.9348
868.5523
[M−H]−
0
20
40
60
80
100
400
500
600
700
800
1611 : M.findmain = 869.5596 (+/− 20 ppm)
384.9348
868.5523
[M−H]−
0
20
40
60
80
100
400
500
600
700
800

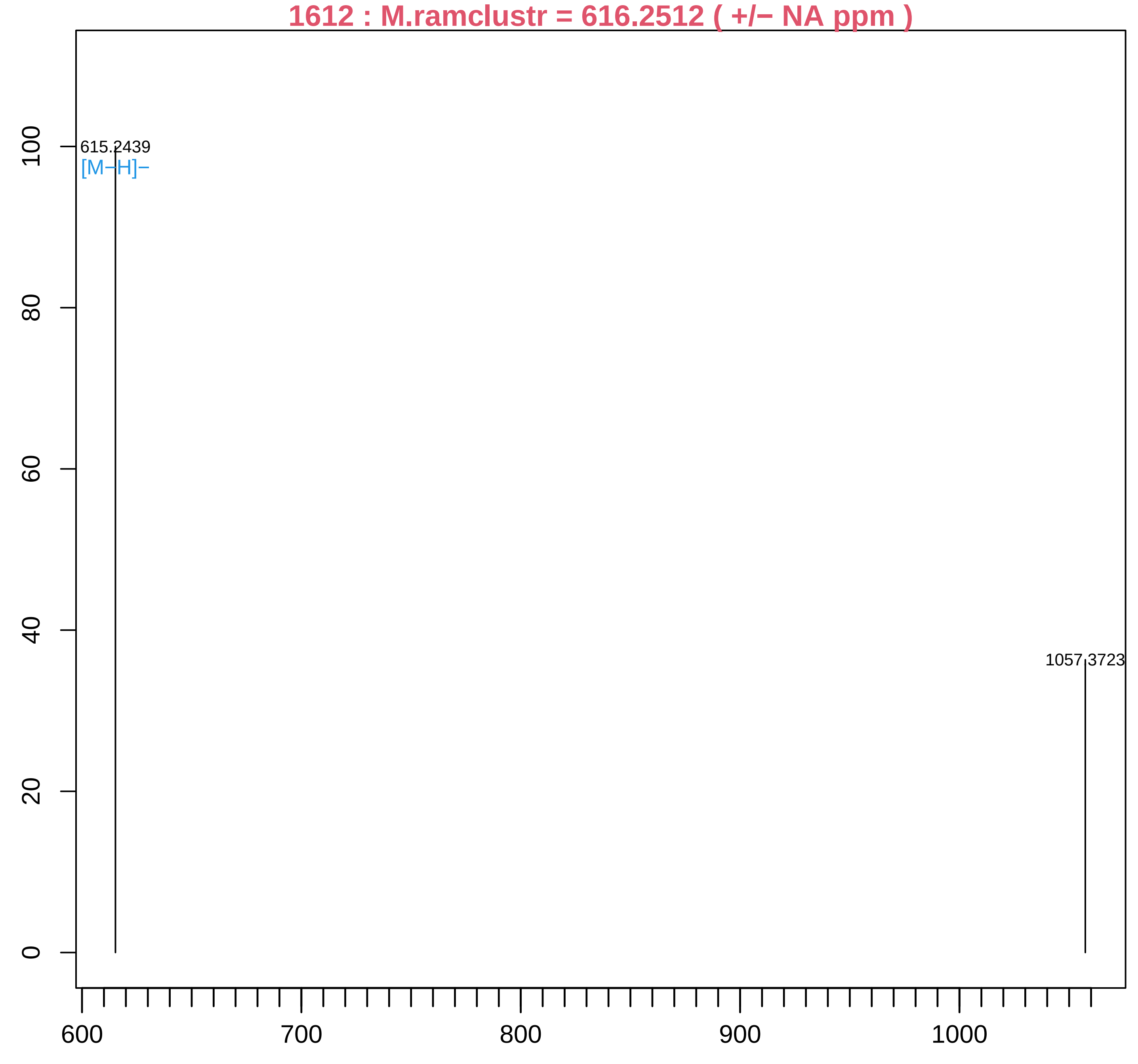

1612 : M.ramclustr = 616.2512 (+/− NA ppm)
615.2439
[M−H]−
1057.3723
0
20
40
60
80
100
600
700
800
900
1000

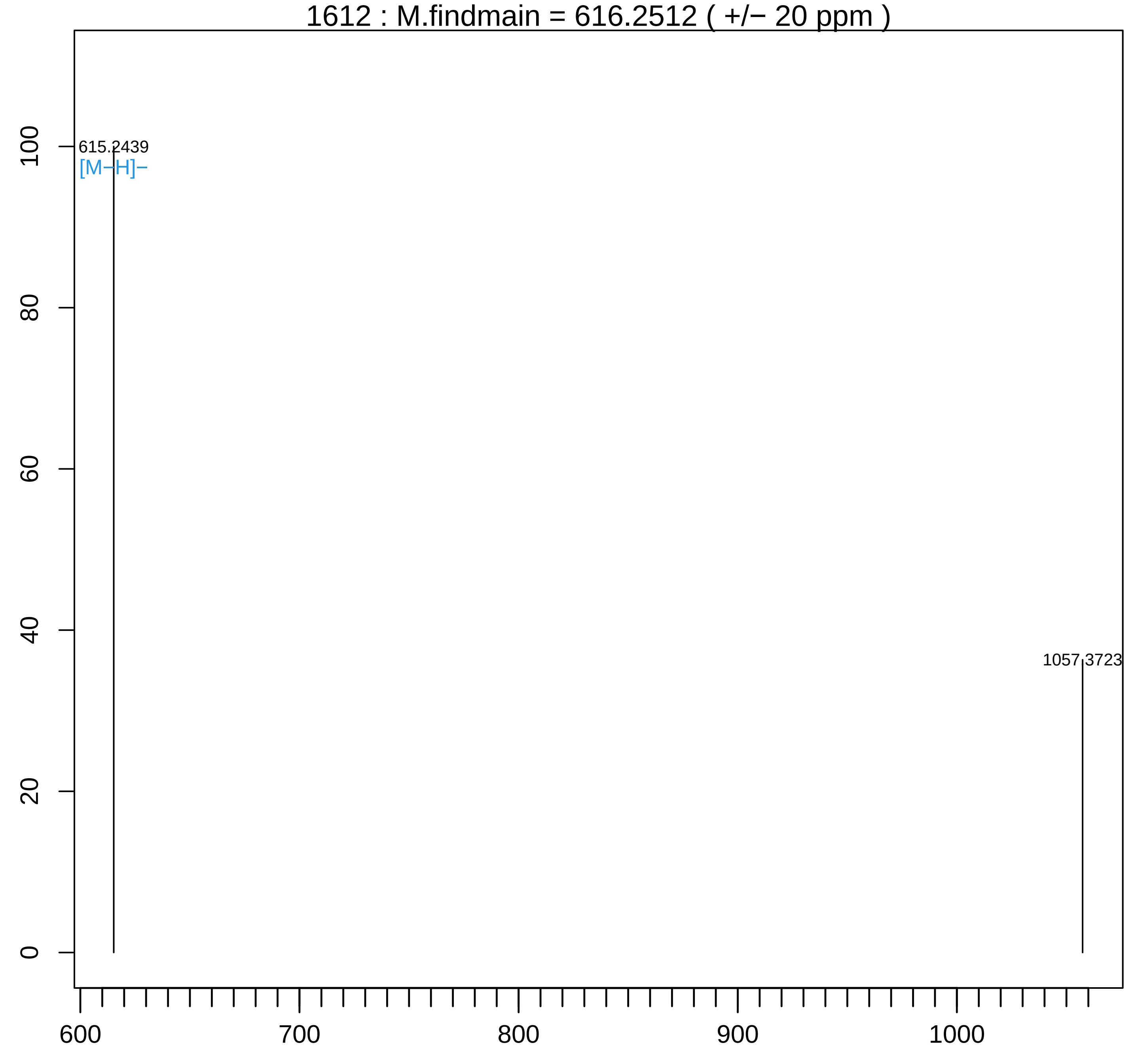

1612 : M.findmain = 616.2512 (+/− 20 ppm)
615.2439
[M−H]−
1057.3723
0
20
40
60
80
100
600
700
800
900
1000

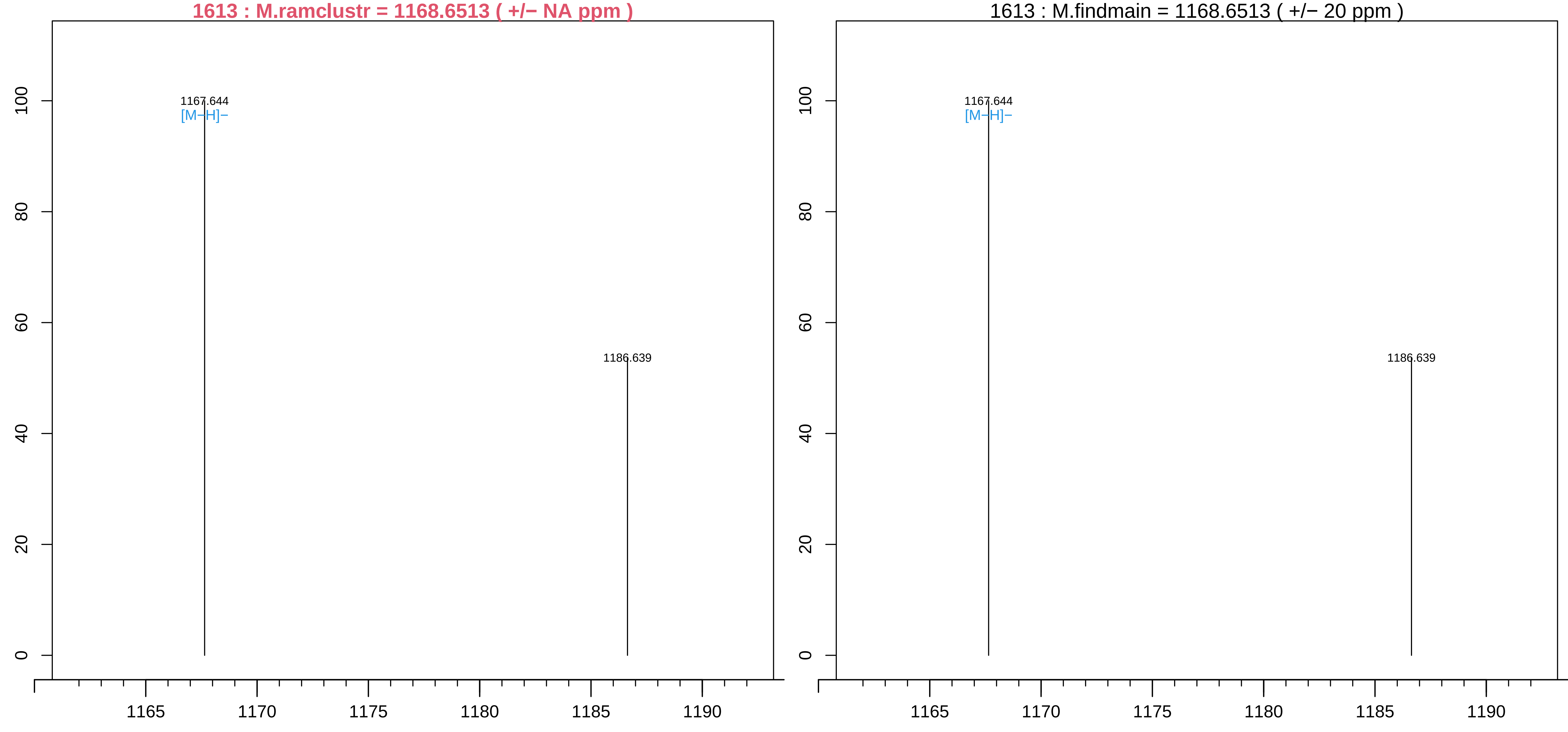
1613 : M.ramclustr = 1168.6513 (+/− NA ppm)
1167.644
[M−H]−
1186.639
1613 : M.findmain = 1168.6513 (+/− 20 ppm)
1167.644
[M−H]−
1186.639
0
20
40
60
80
100
1165
1170
1175
1180
1185
1190

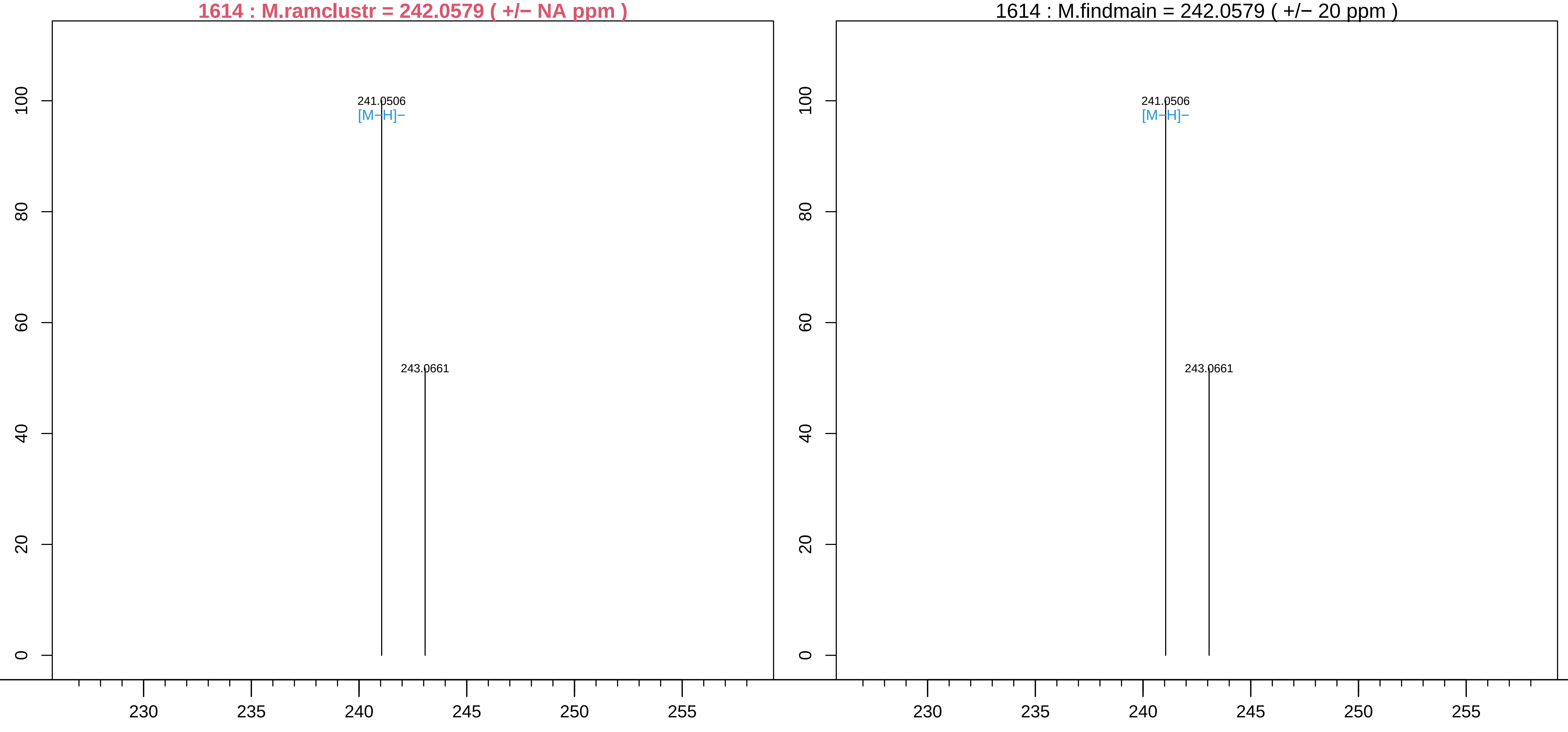

1614 : M.ramclustr = 242.0579 (+/− NA ppm)
241.0506
[M−H]−
243.0661
1614 : M.findmain = 242.0579 (+/− 20 ppm)
241.0506
[M−H]−
243.0661
0
20
40
60
80
100
230
235
240
245
250
255

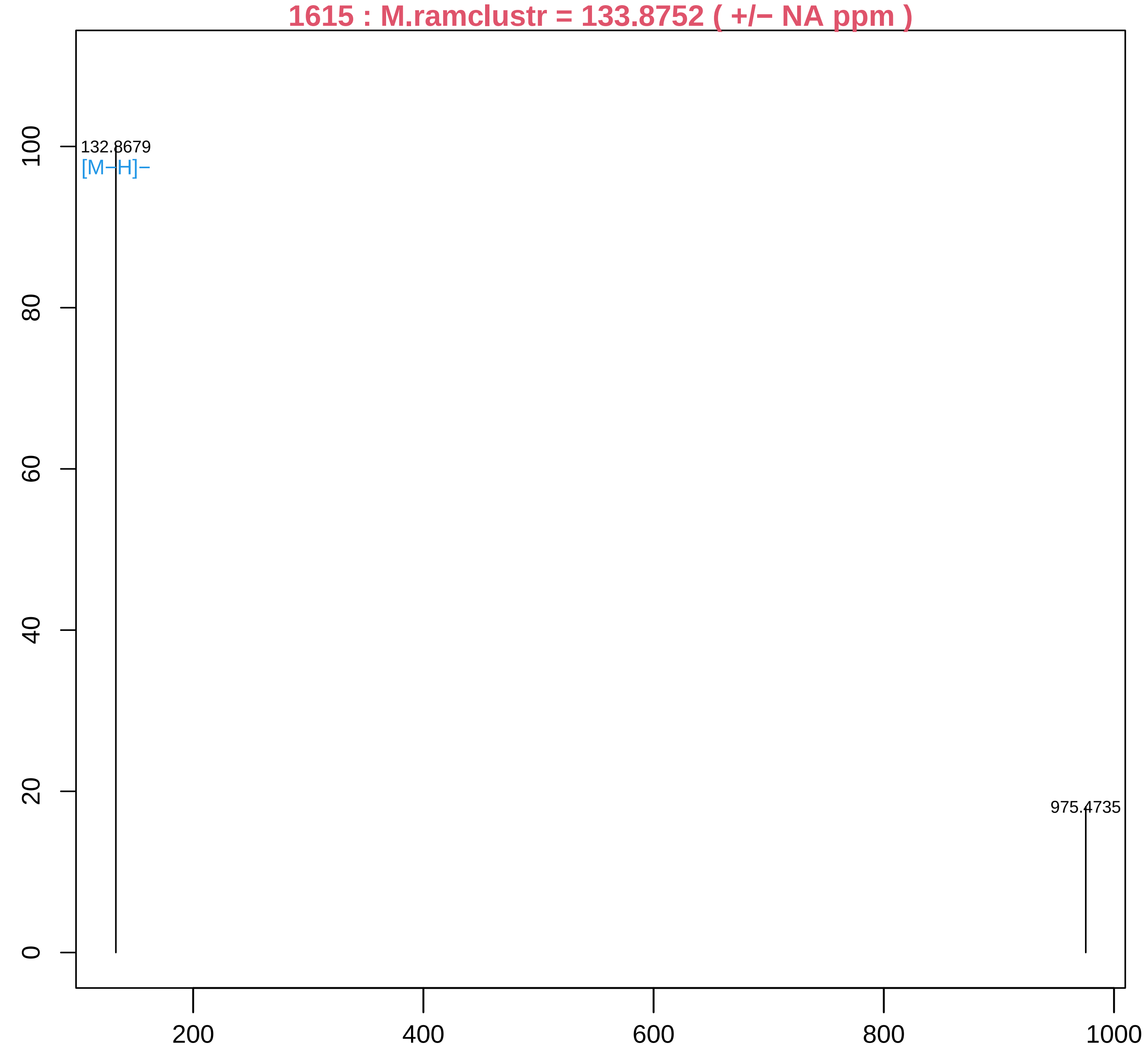
1615 : M.ramclustr = 133.8752 (+/− NA ppm)
132.8679
[M−H]−
975.4735
0
20
40
60
80
100
200
400
600
800
1000

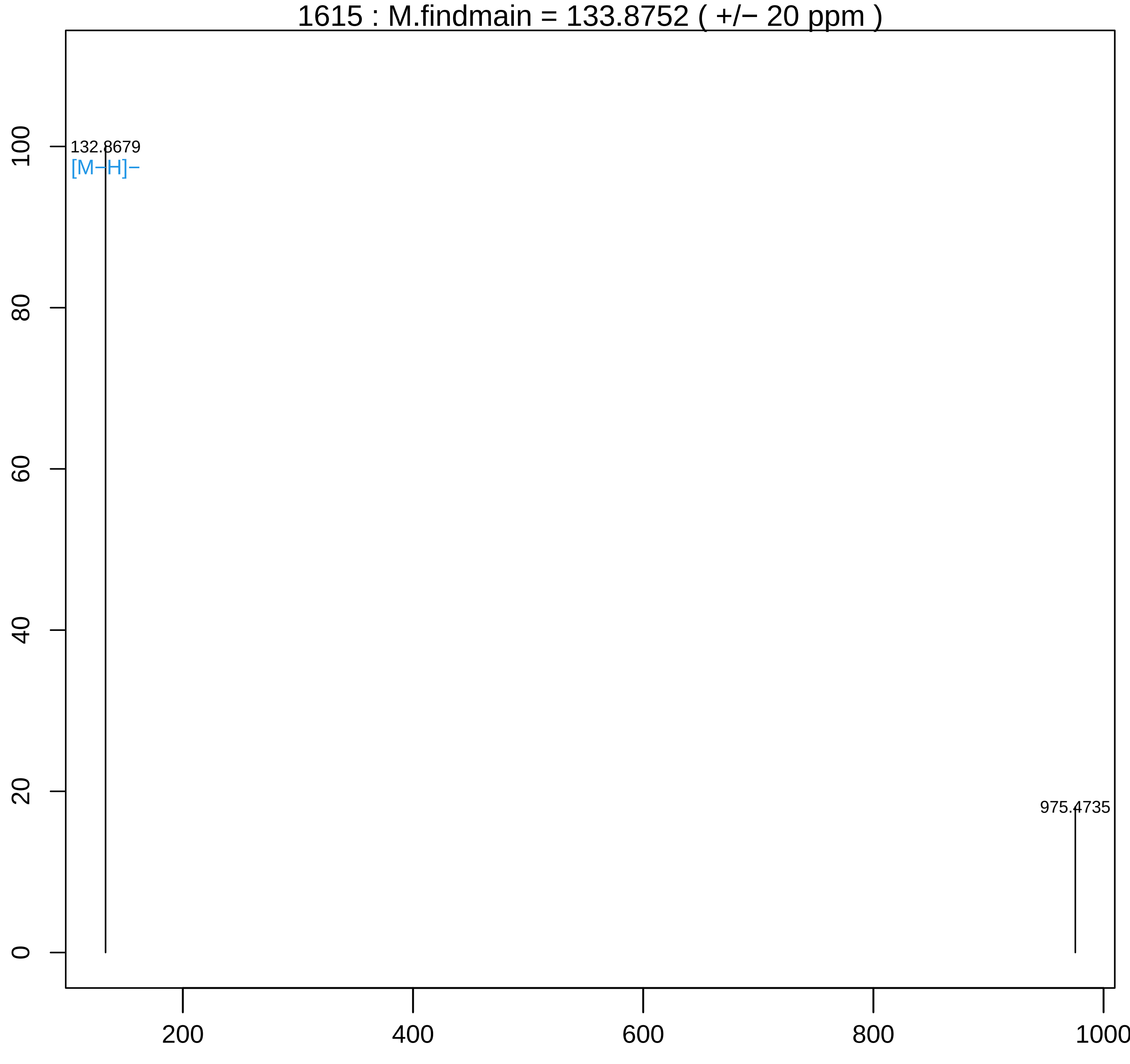
1615 : M.findmain = 133.8752 (+/− 20 ppm)
132.8679
[M−H]−
975.4735
0
20
40
60
80
100
200
400
600
800
1000

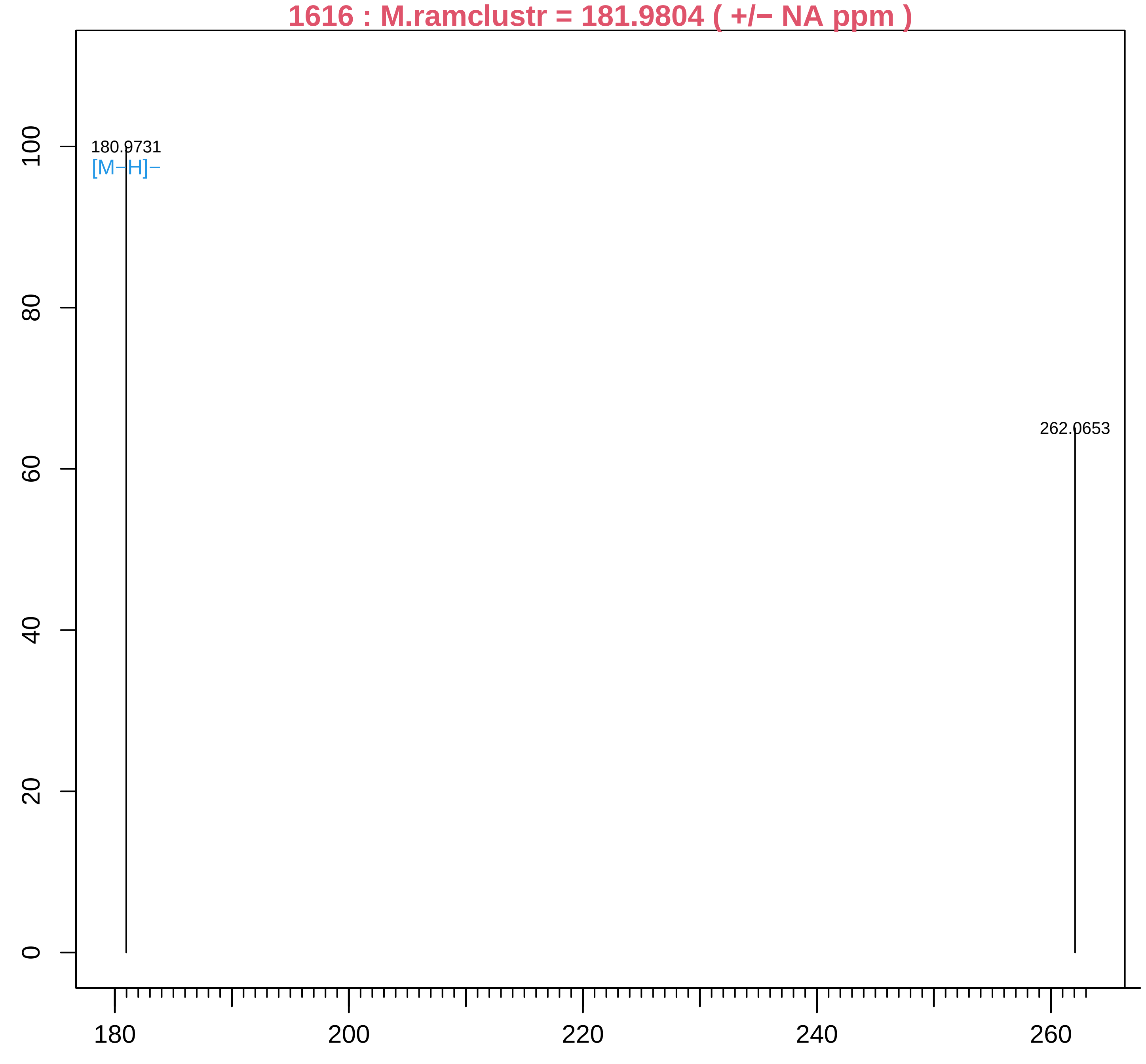
1616 : M.ramclustr = 181.9804 (+/− NA ppm)
180.9731
[M−H]−
262.0653

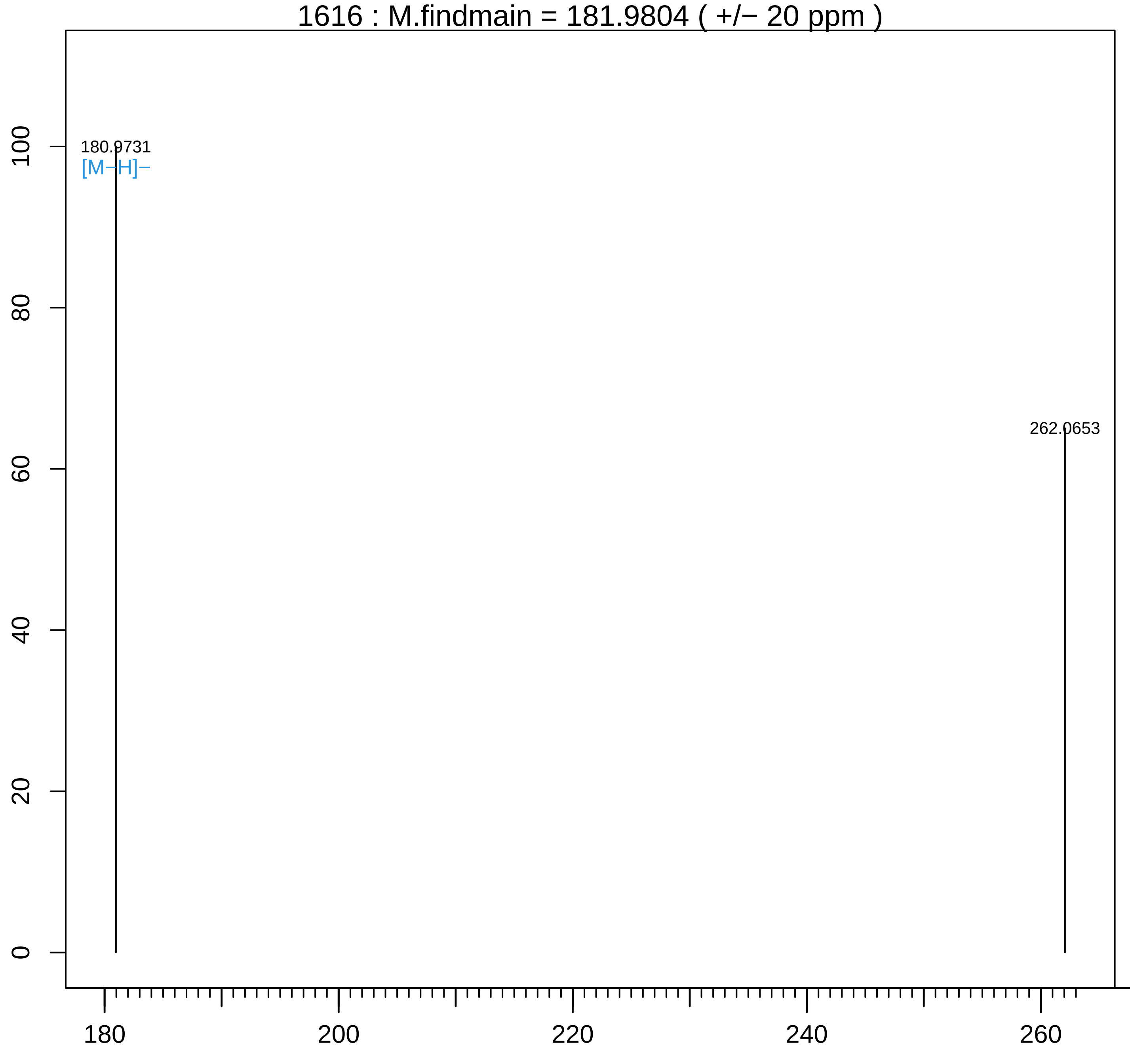
1616 : M.findmain = 181.9804 (+/− 20 ppm)
180.9731
[M−H]−
262.0653

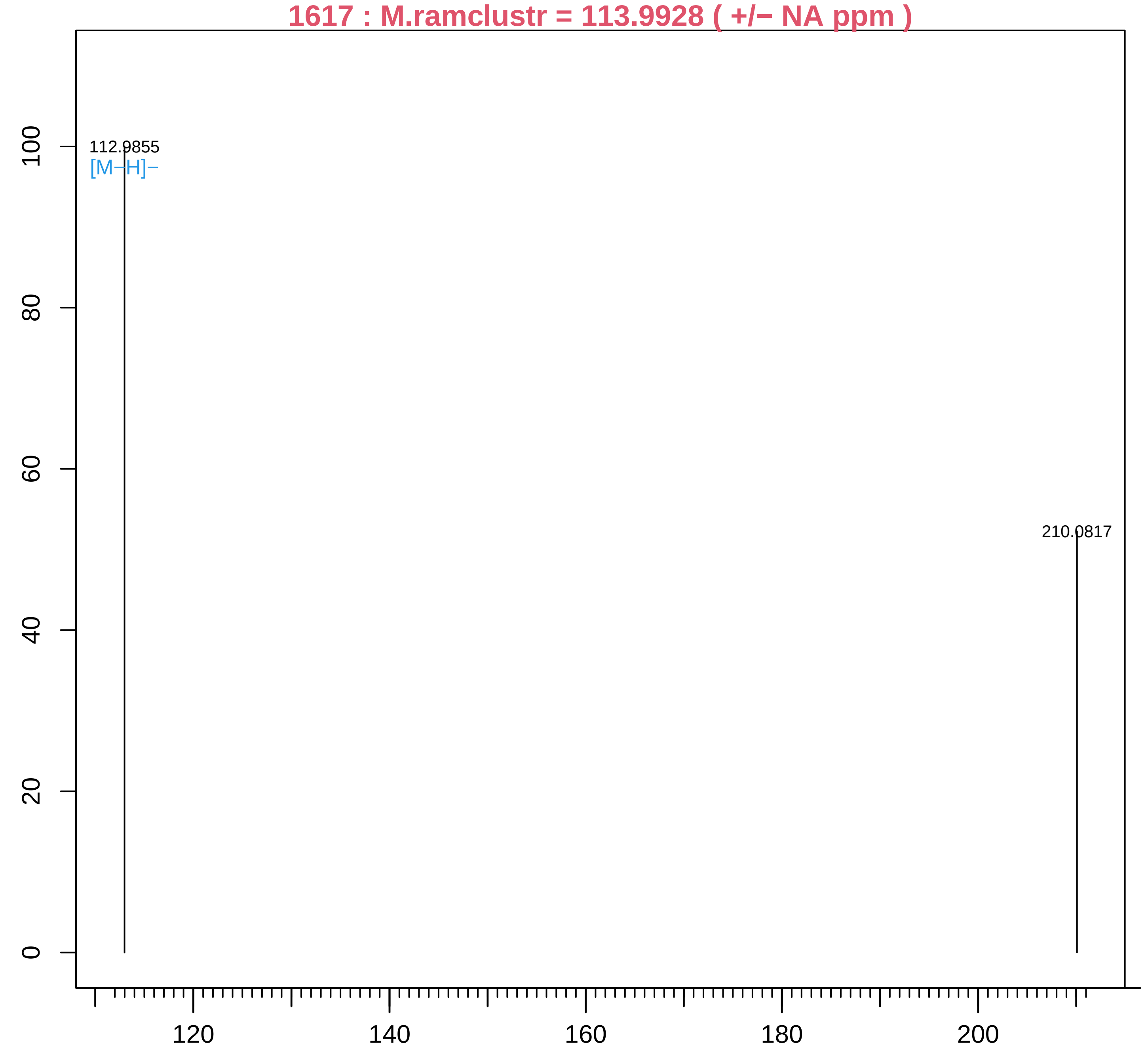
1617 : M.ramclustr = 113.9928 (+/− NA ppm)
112.9855
[M−H]−
210.0817
0
20
40
60
80
100
120
140
160
180
200

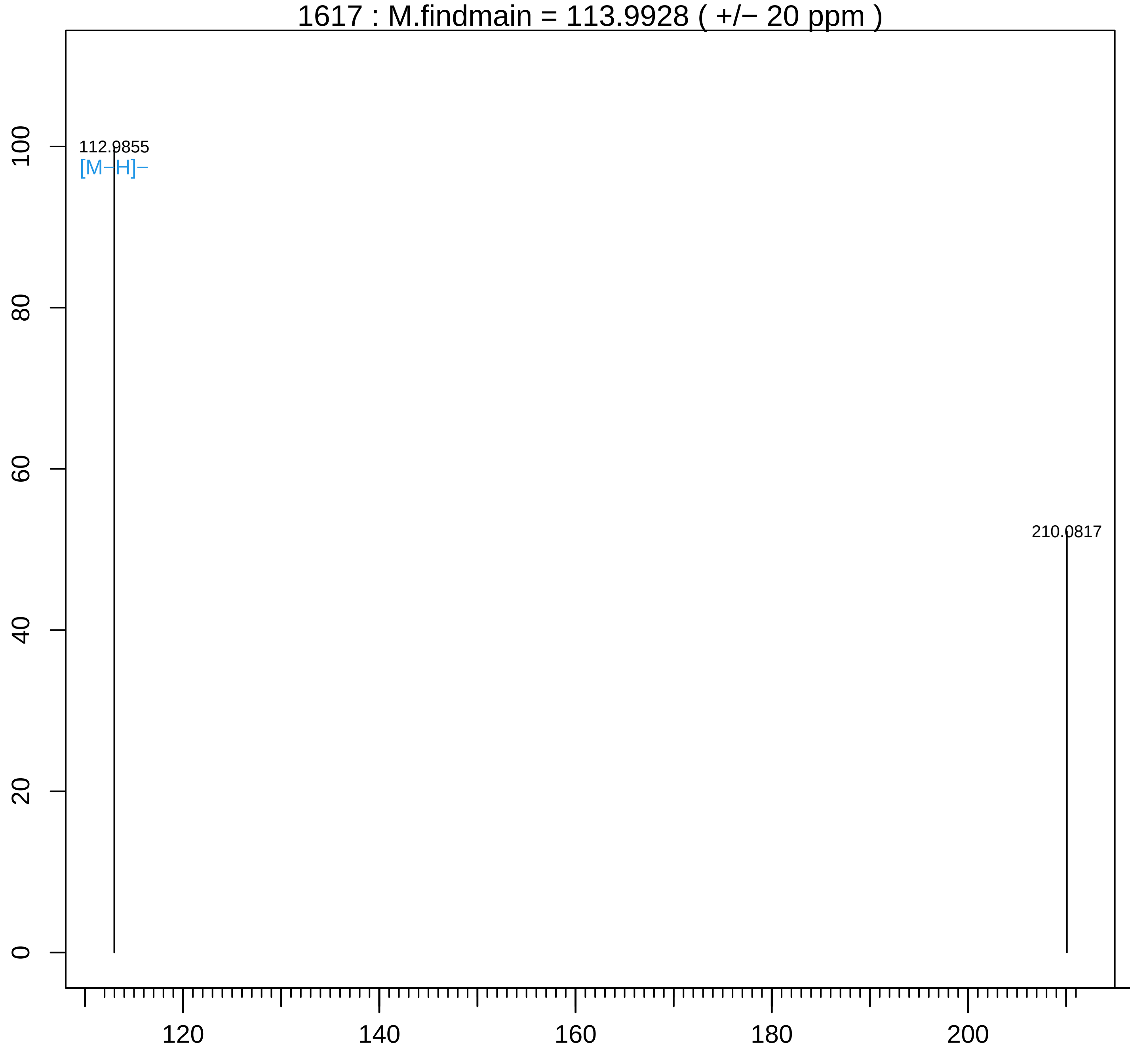
1617 : M.findmain = 113.9928 (+/− 20 ppm)
112.9855
[M−H]−
210.0817
0
20
40
60
80
100
120
140
160
180
200

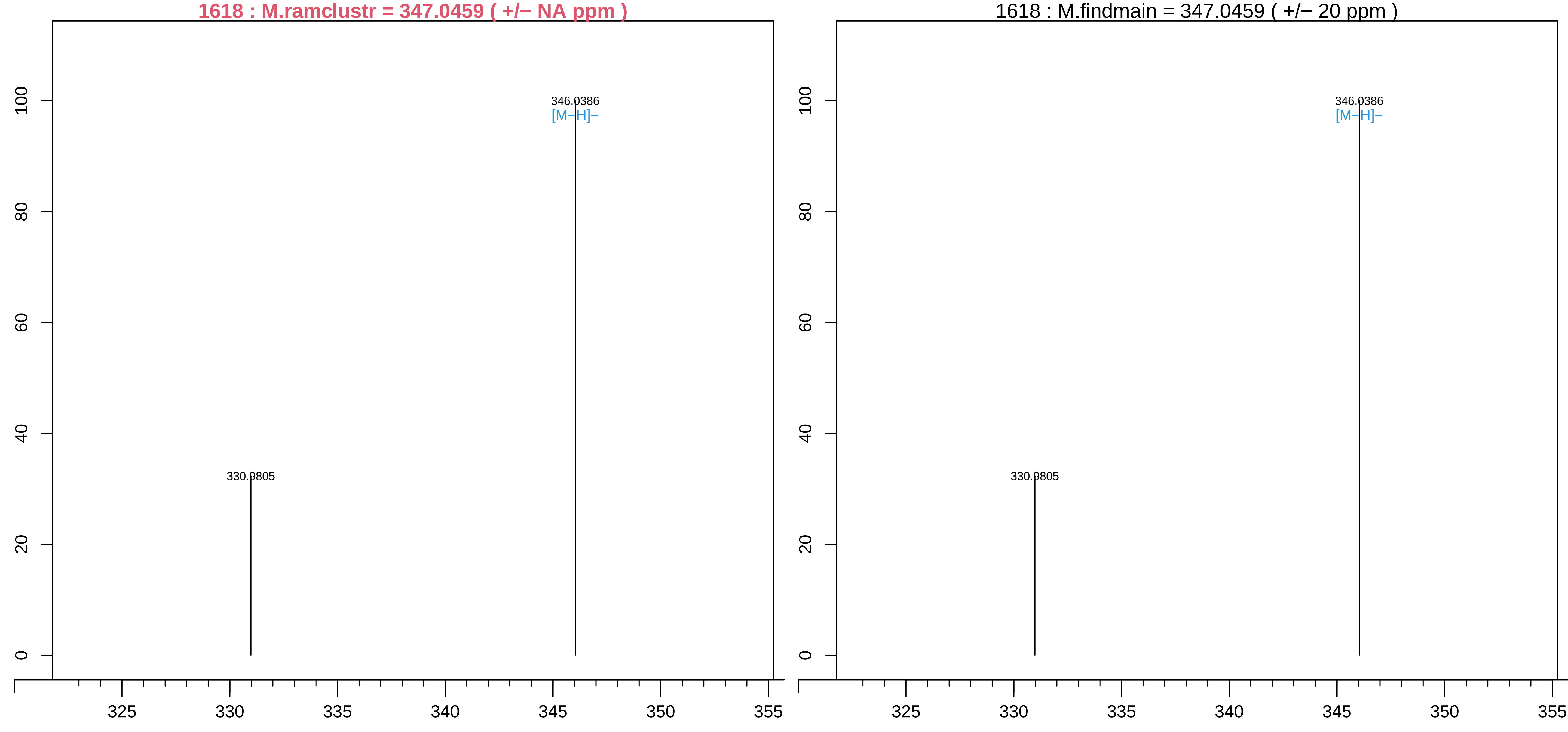

1618 : M.ramclustr = 347.0459 (+/− NA ppm)
346.0386
[M−H]−
330.9805
1618 : M.findmain = 347.0459 (+/− 20 ppm)
346.0386
[M−H]−
330.9805
0
20
40
60
80
100
325
330
335
340
345
350
355

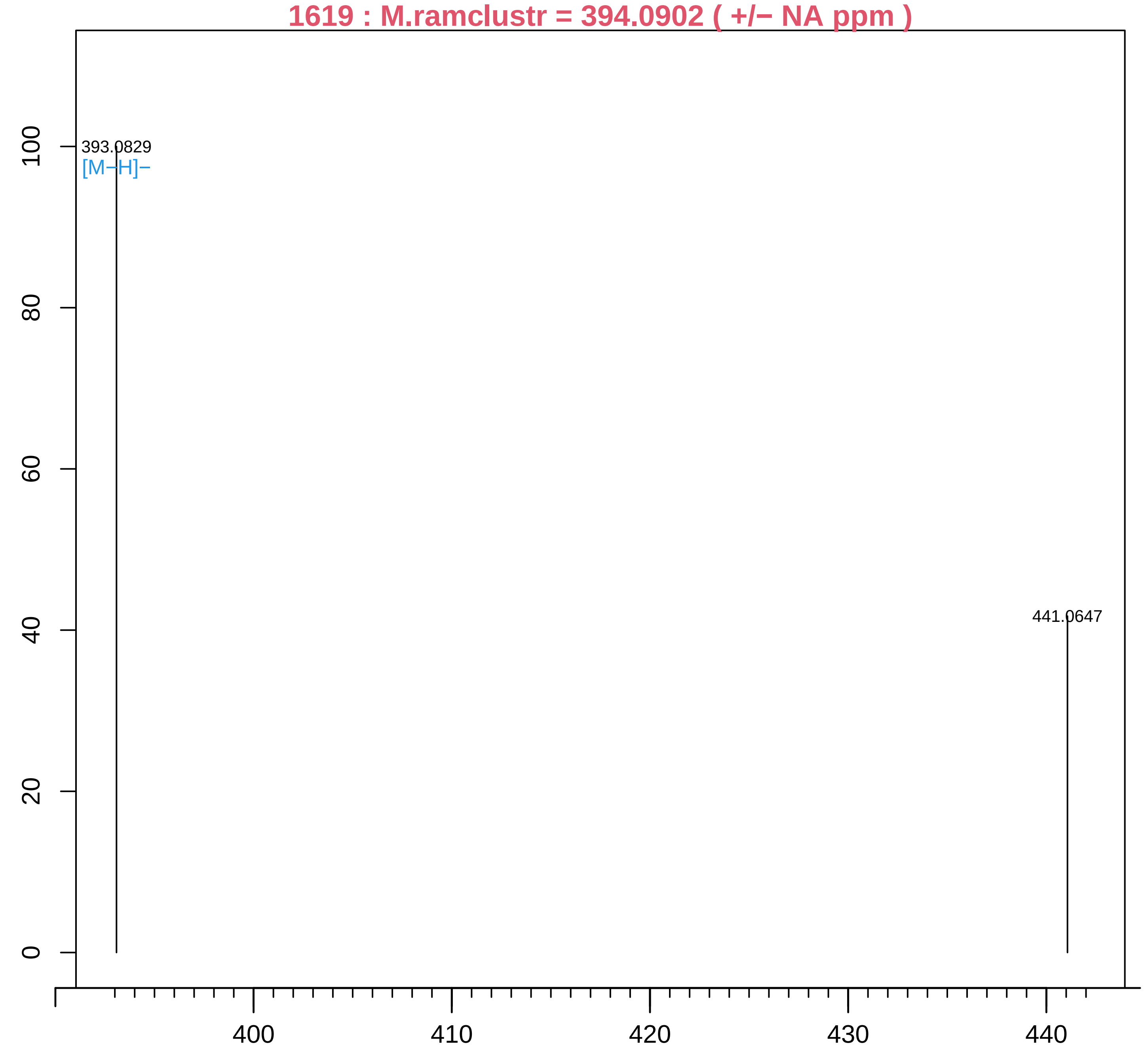
1619 : M.ramclustr = 394.0902 (+/− NA ppm)
393.0829
[M−H]−
441.0647
0
20
40
60
80
100
400
410
420
430
440

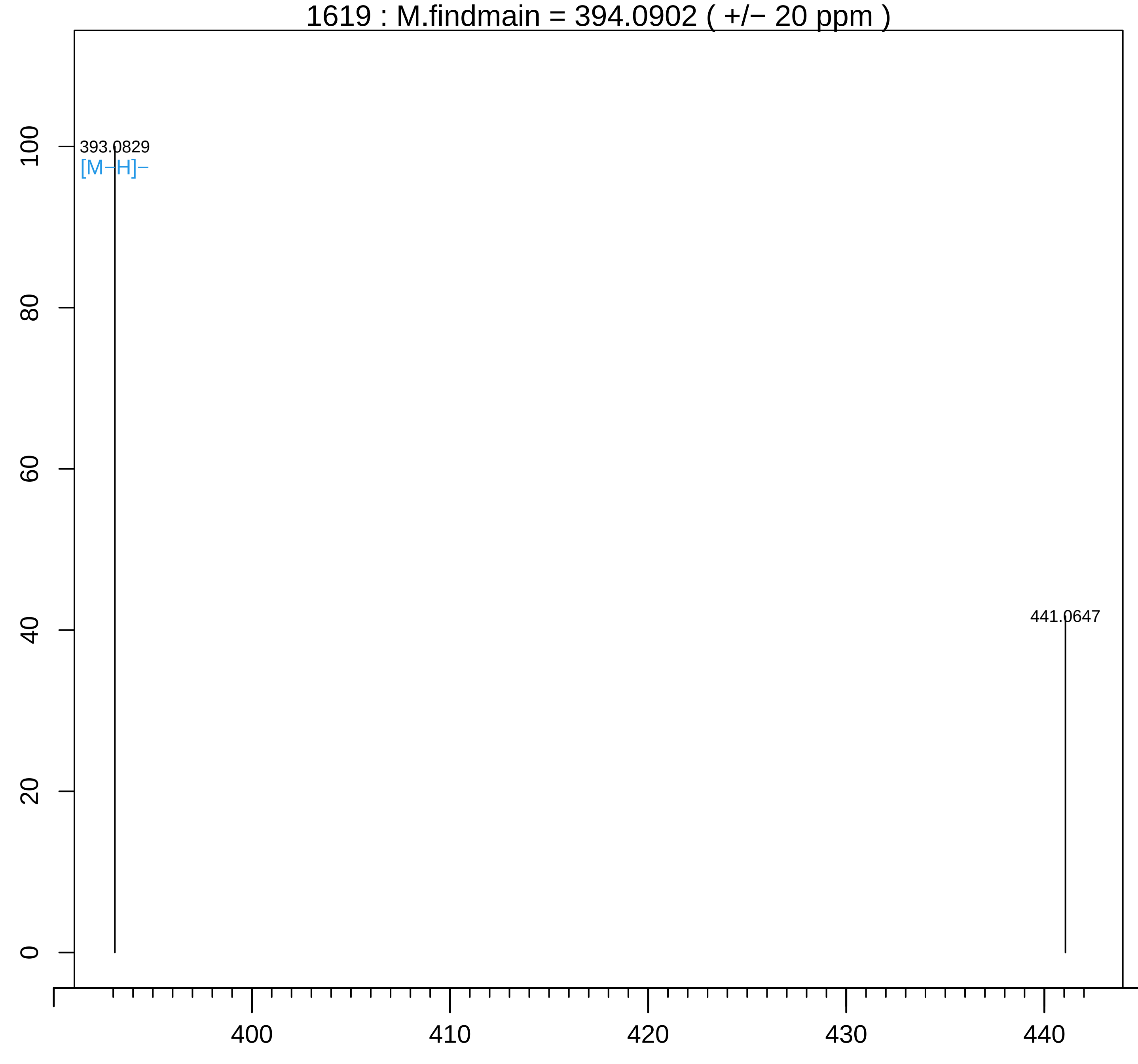
1619 : M.findmain = 394.0902 (+/− 20 ppm)
393.0829
[M−H]−
441.0647
0
20
40
60
80
100
400
410
420
430
440

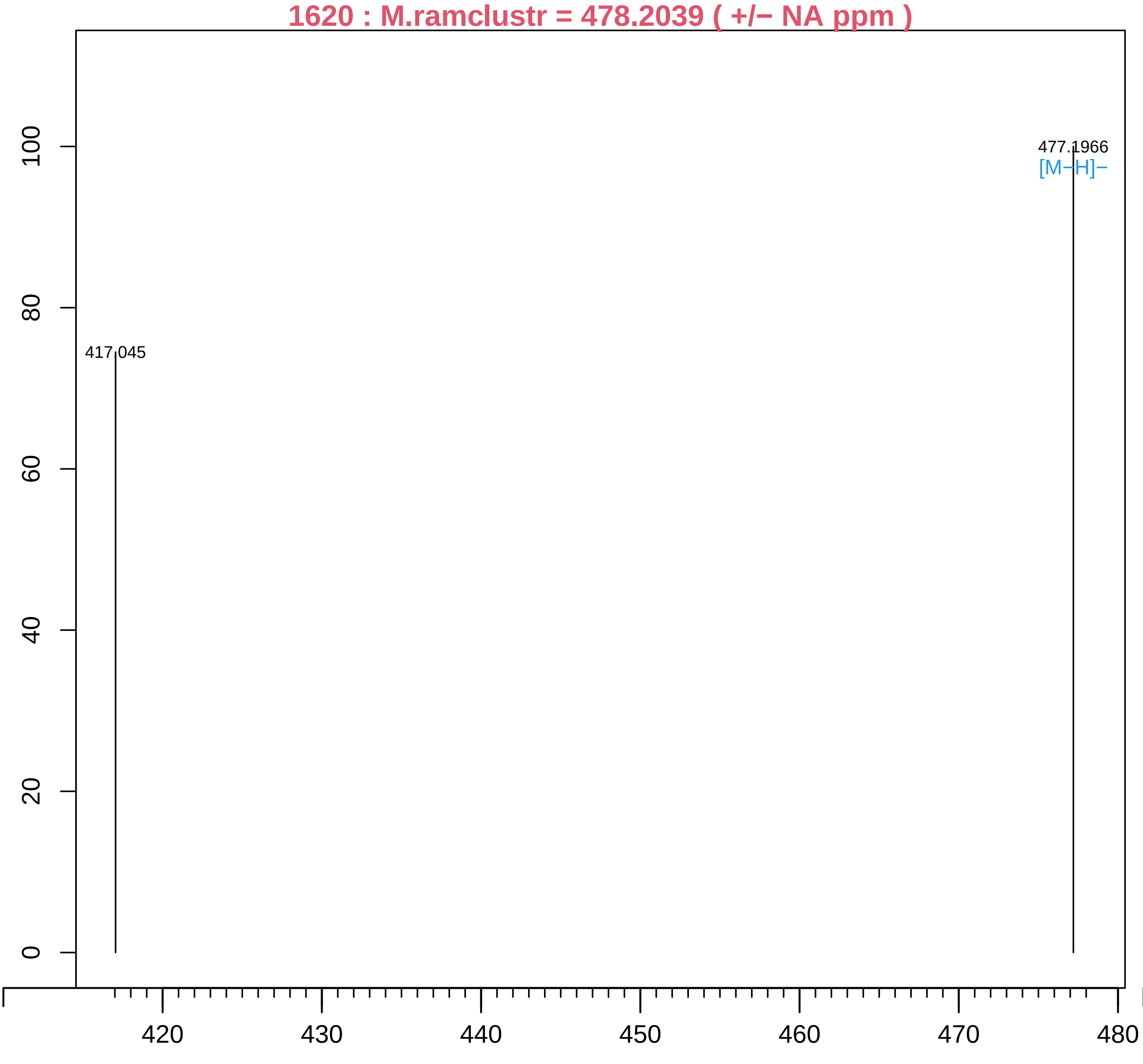
1620 : M.ramclustr = 478.2039 (+/− NA ppm)
477.1966
[M−H]−
417.045
0
20
40
60
80
100
420
430
440
450
460
470
480

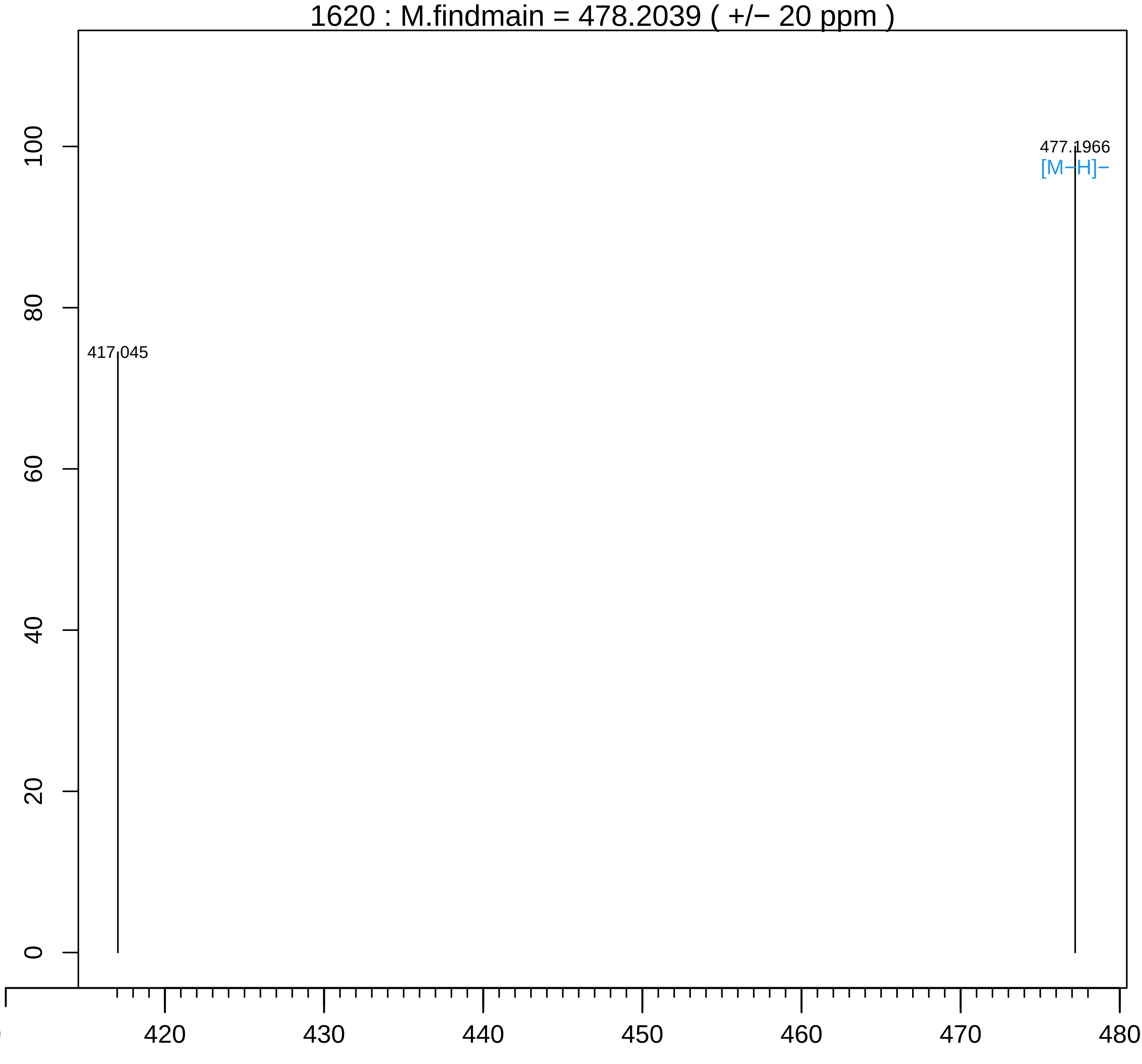
1620 : M.findmain = 478.2039 (+/− 20 ppm)
477.1966
[M−H]−
417.045
0
20
40
60
80
100
420
430
440
450
460
470
480

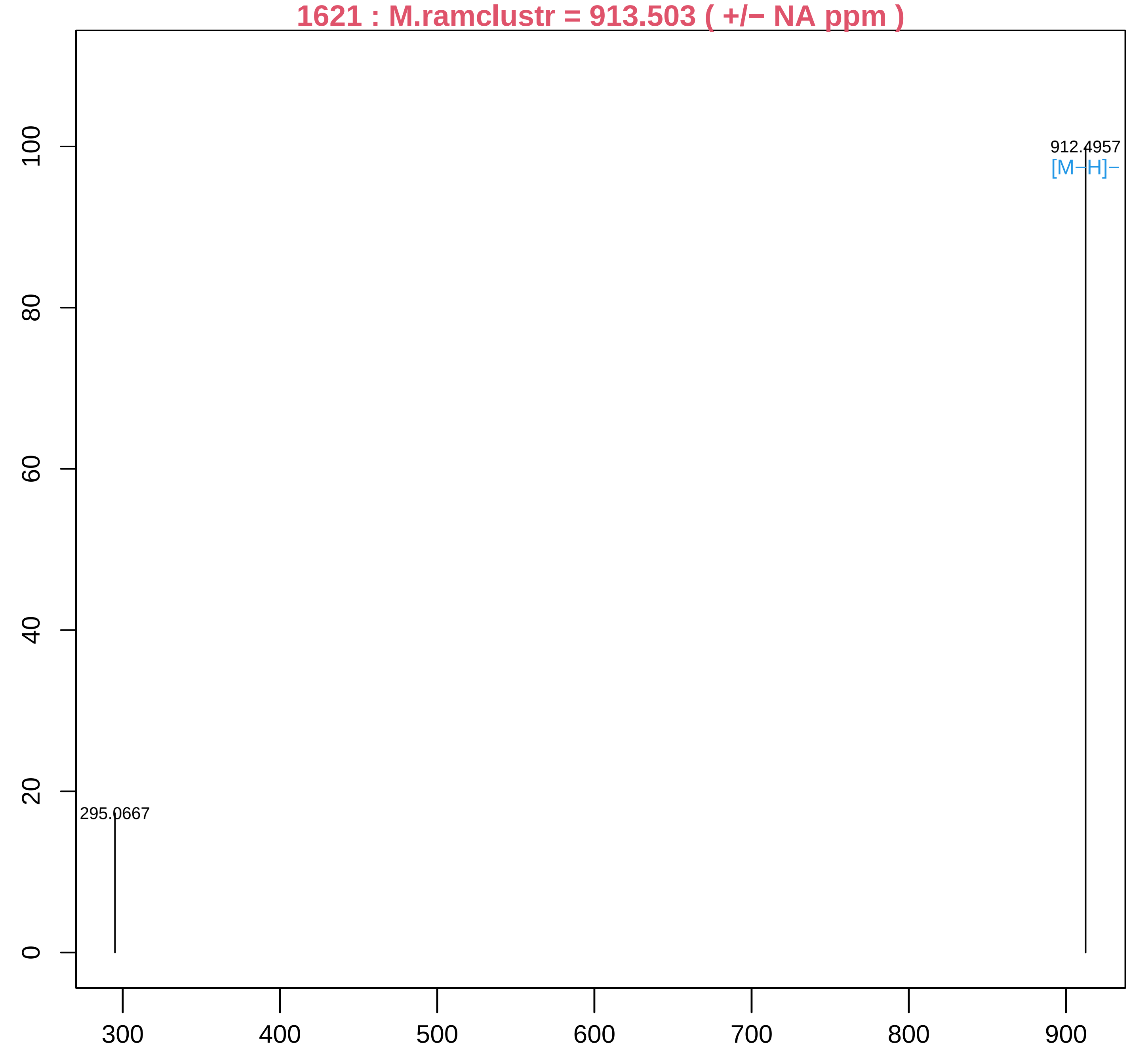
1621 : M.ramclustr = 913.503 (+/− NA ppm)
912.4957
[M−H]−
295.0667
0
20
40
60
80
100
300
400
500
600
700
800
900

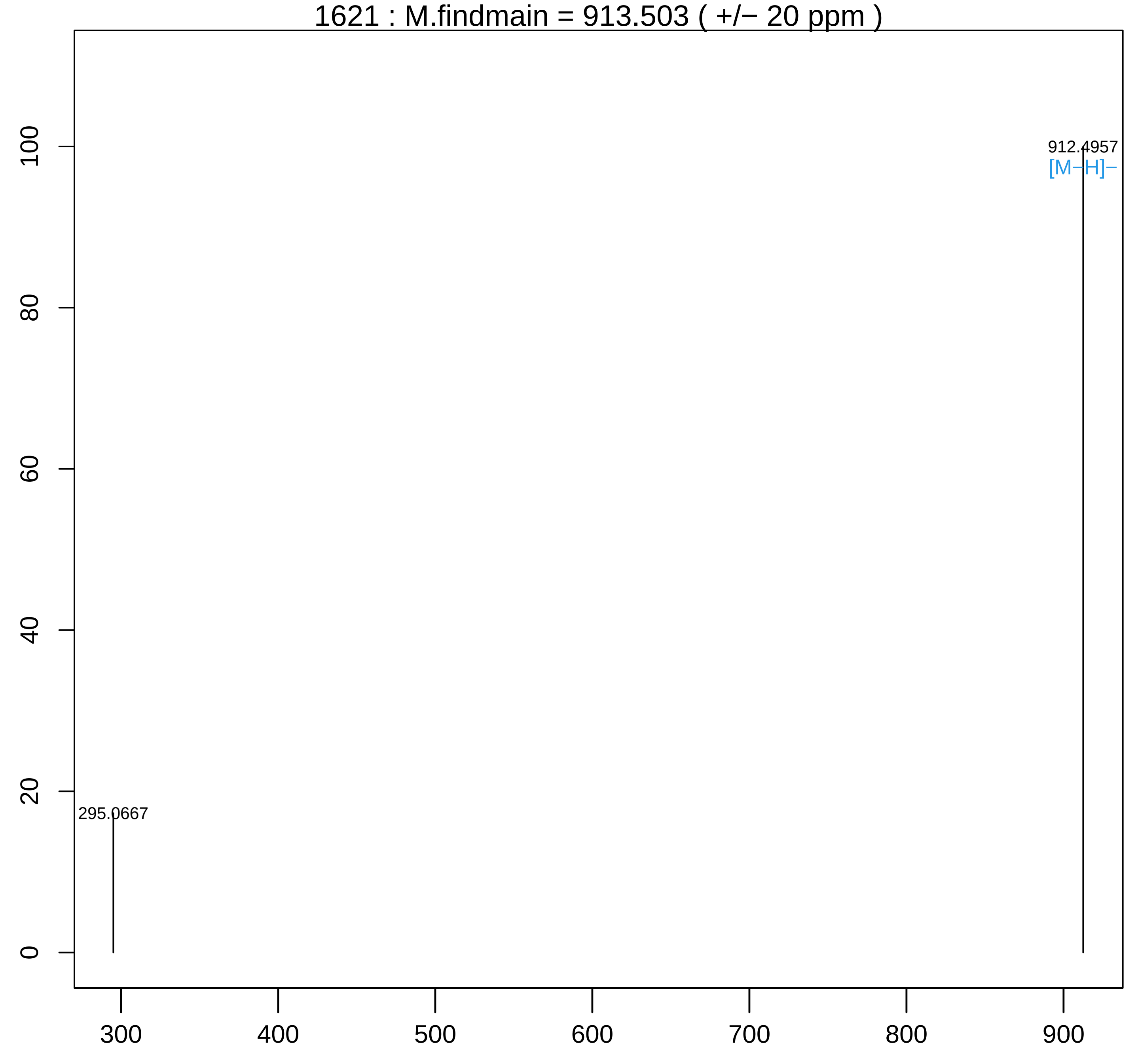
1621 : M.findmain = 913.503 (+/− 20 ppm)
912.4957
[M−H]−
295.0667
0
20
40
60
80
100
300
400
500
600
700
800
900

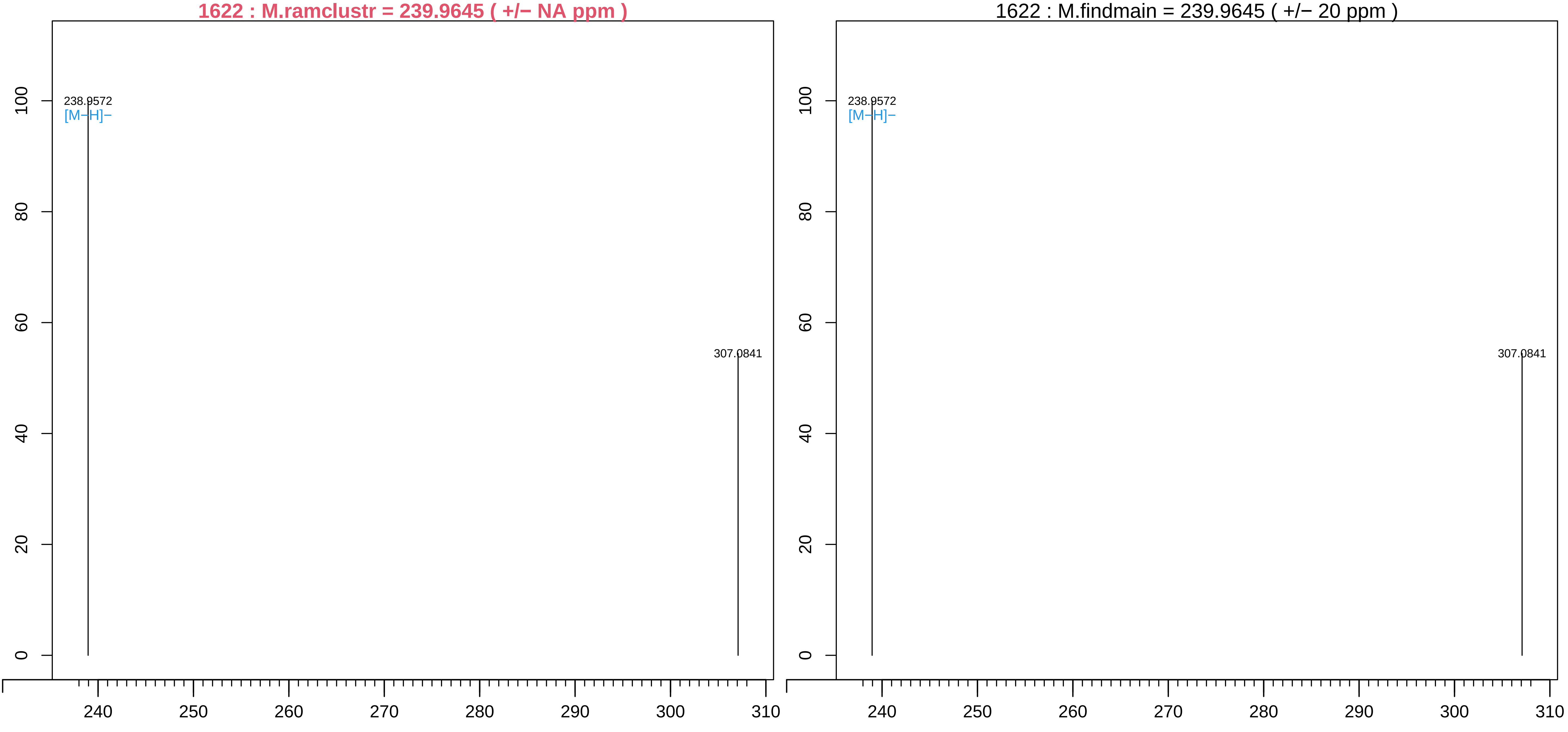
1622 : M.ramclustr = 239.9645 (+/− NA ppm)
238.9572
[M−H]−
307.0841
1622 : M.findmain = 239.9645 (+/− 20 ppm)
238.9572
[M−H]−
307.0841
0
20
40
60
80
100
240
250
260
270
280
290
300
310

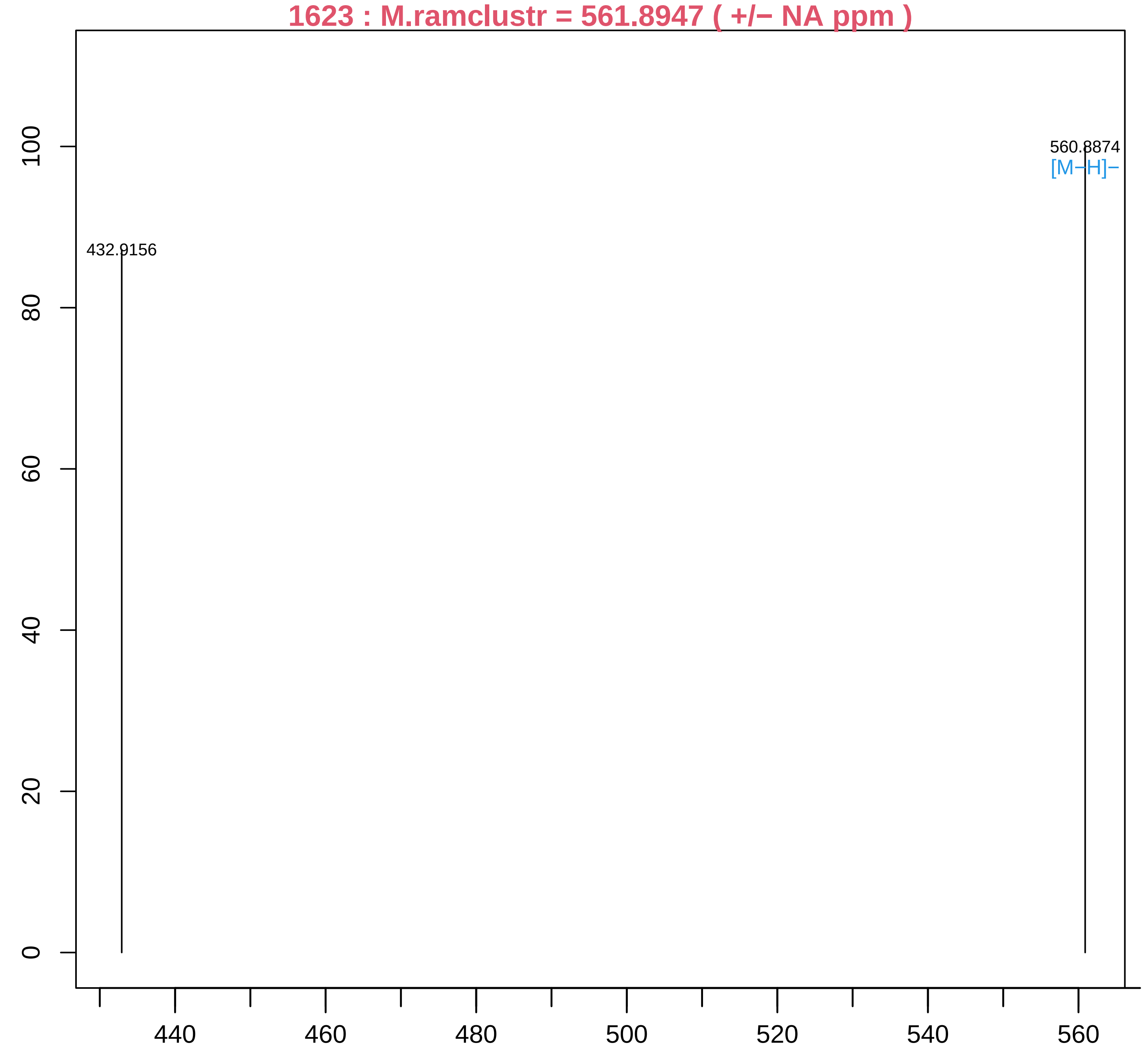
1623 : M.ramclustr = 561.8947 (+/− NA ppm)
560.8874
[M−H]−
432.9156
0
20
40
60
80
100
440
460
480
500
520
540
560

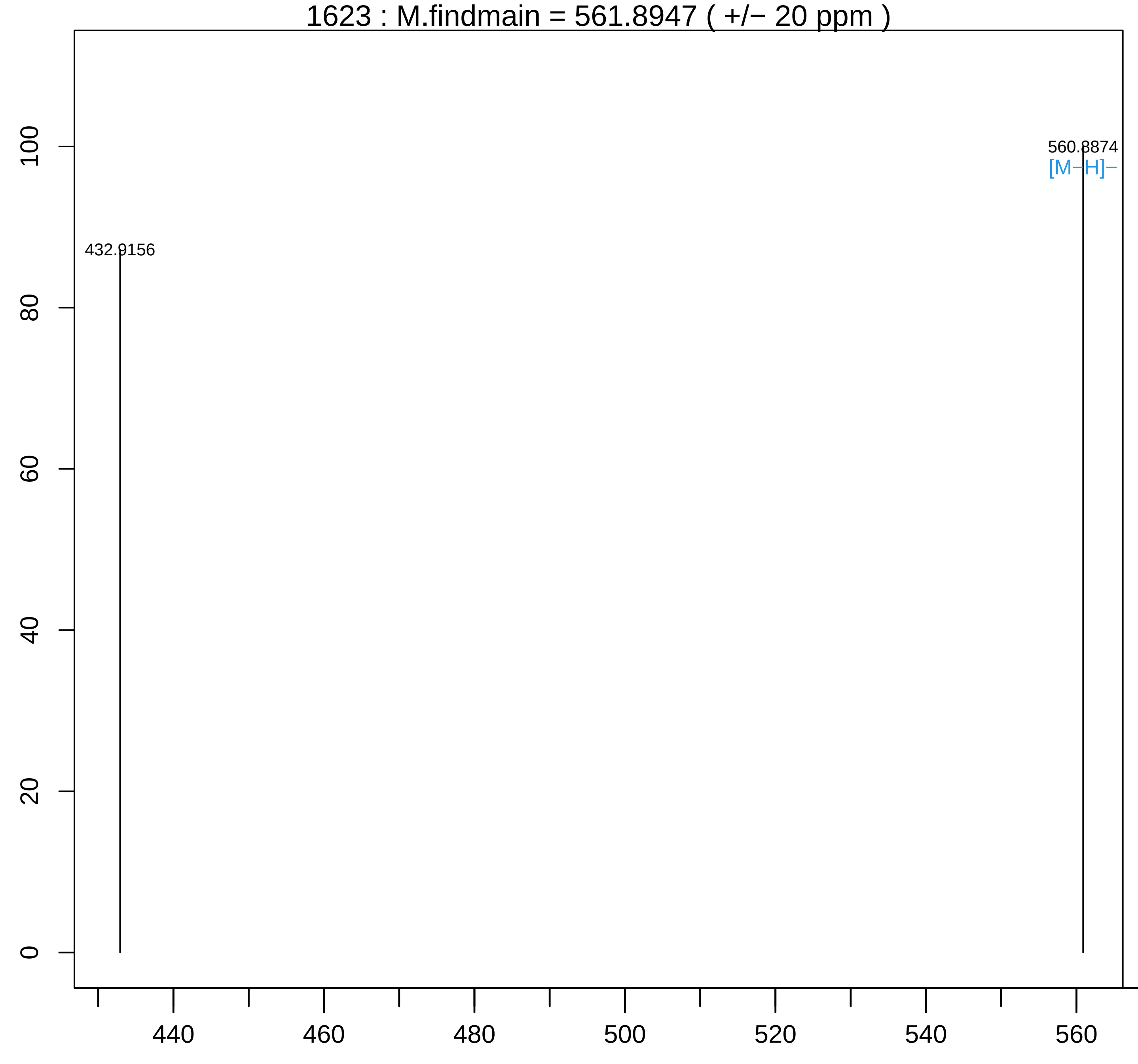
1623 : M.findmain = 561.8947 (+/− 20 ppm)
560.8874
[M−H]−
432.9156
0
20
40
60
80
100
440
460
480
500
520
540
560

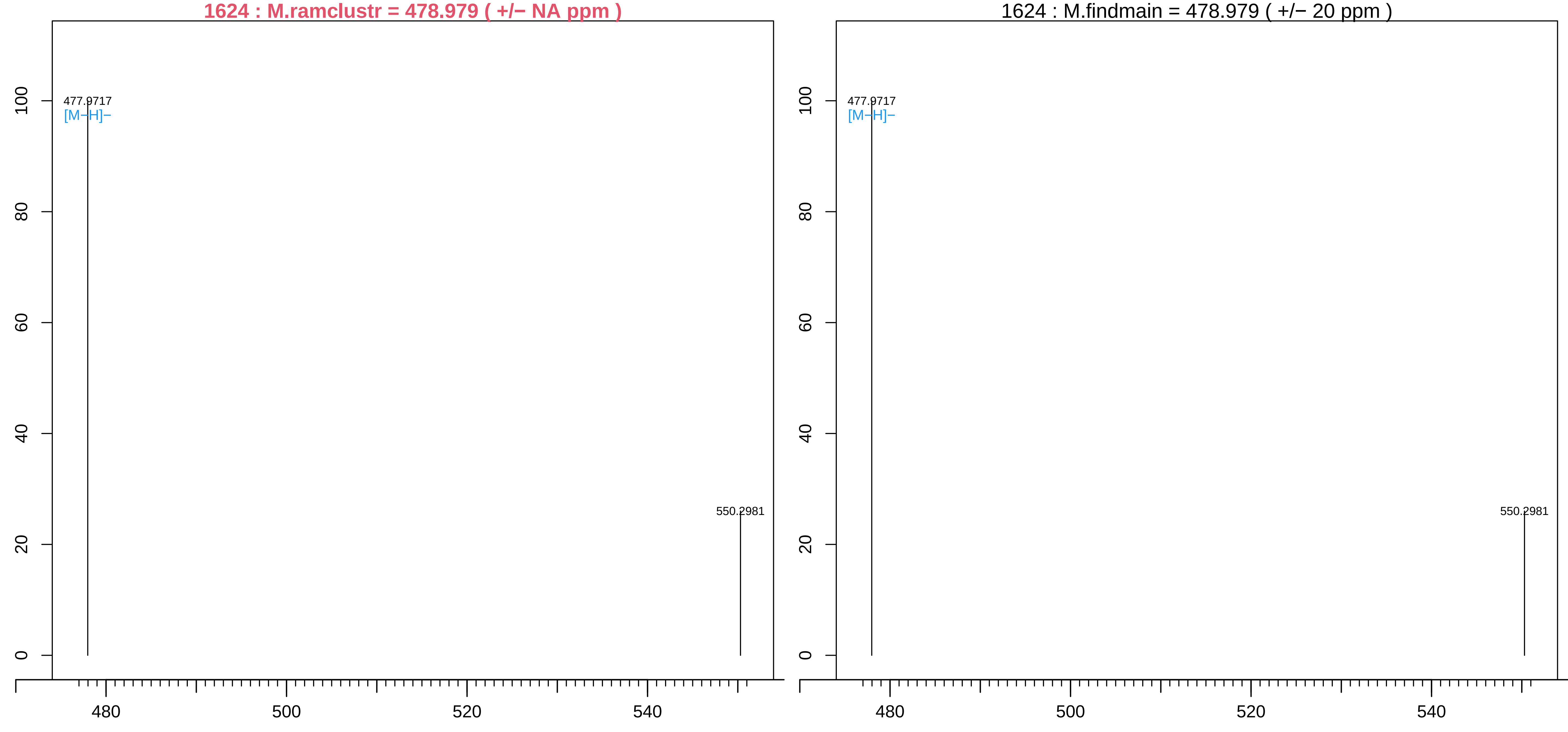
1624 : M.ramclustr = 478.979 (+/− NA ppm)
477.9717
[M−H]−
550.2981
1624 : M.findmain = 478.979 (+/− 20 ppm)
477.9717
[M−H]−
550.2981

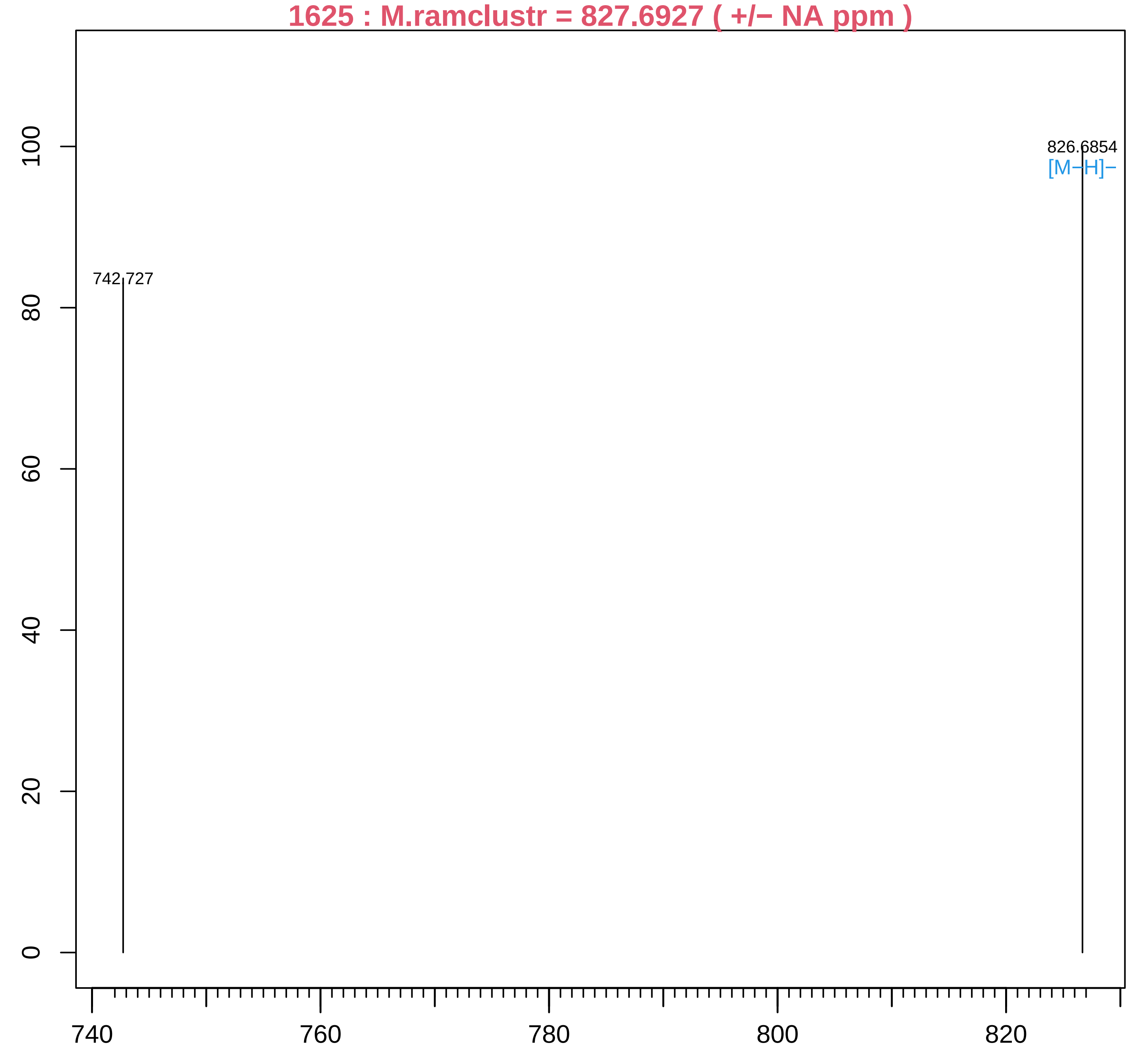
1625 : M.ramclustr = 827.6927 (+/− NA ppm)
826.6854
[M−H]−
742.727
0
20
40
60
80
100
740
760
780
800
820

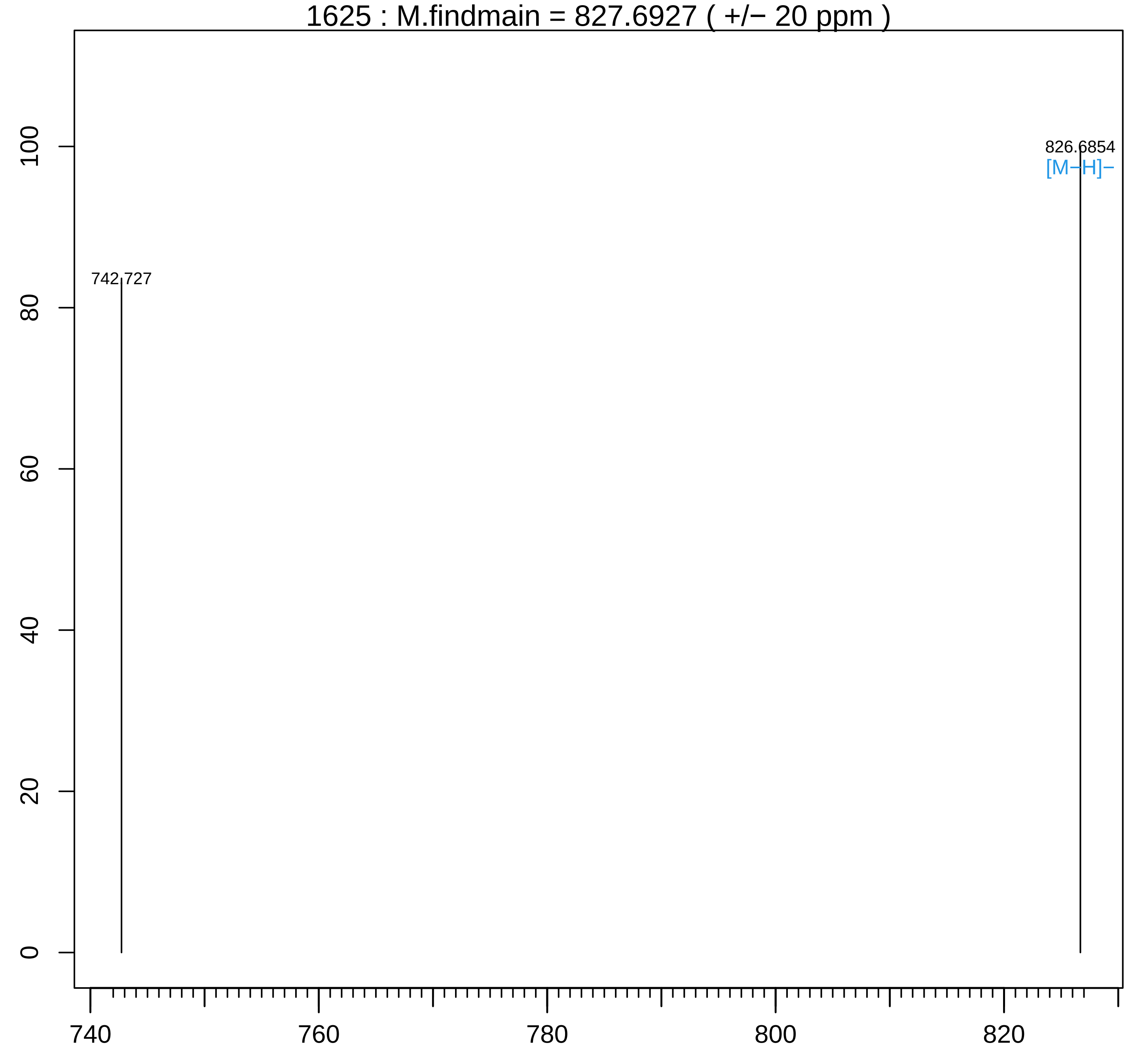
1625 : M.findmain = 827.6927 (+/− 20 ppm)
826.6854
[M−H]−
742.727
0
20
40
60
80
100
740
760
780
800
820

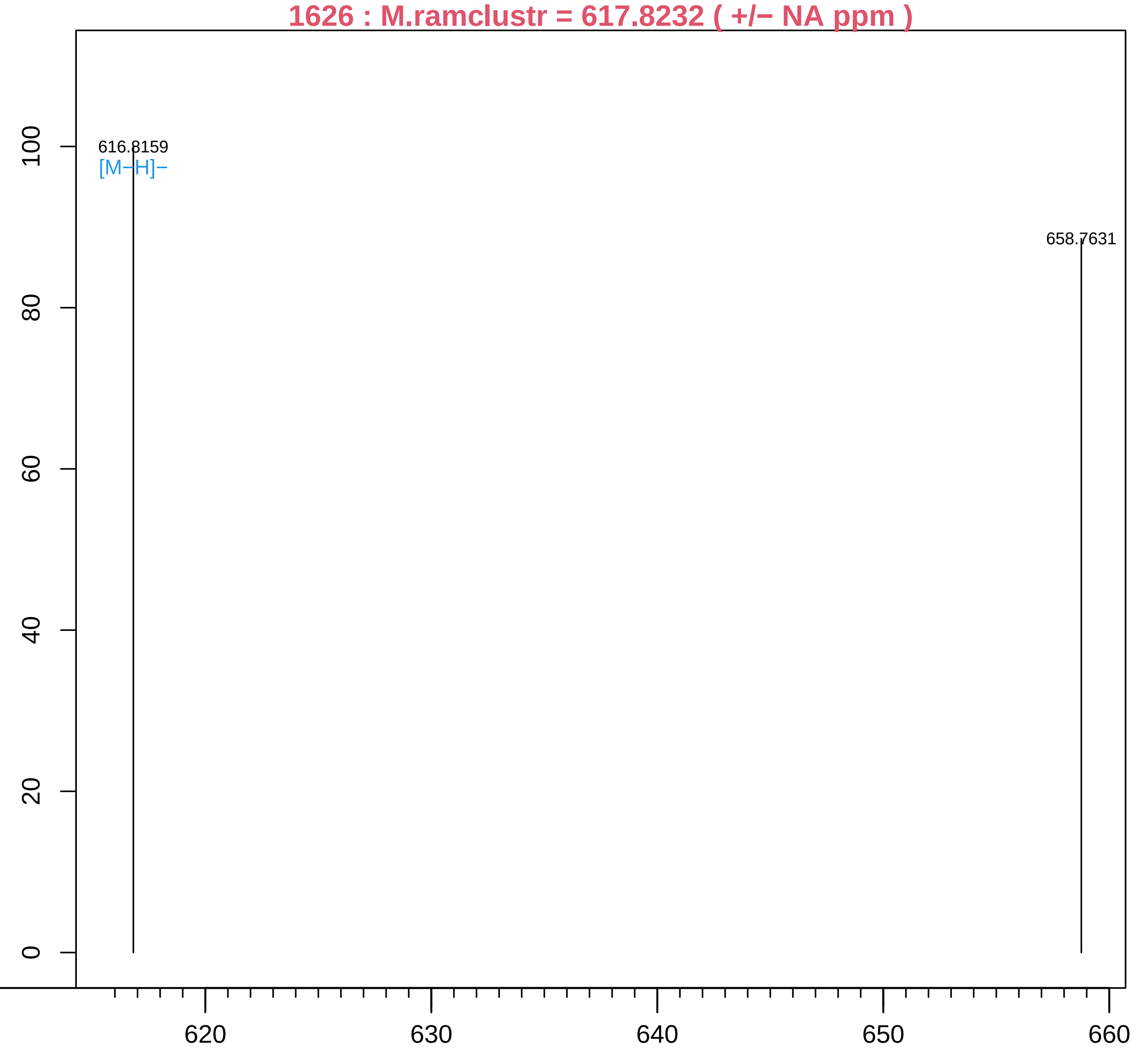
1626 : M.ramclustr = 617.8232 (+/− NA ppm)
616.8159
[M−H]−
658.7631
0
20
40
60
80
100
620
630
640
650
660

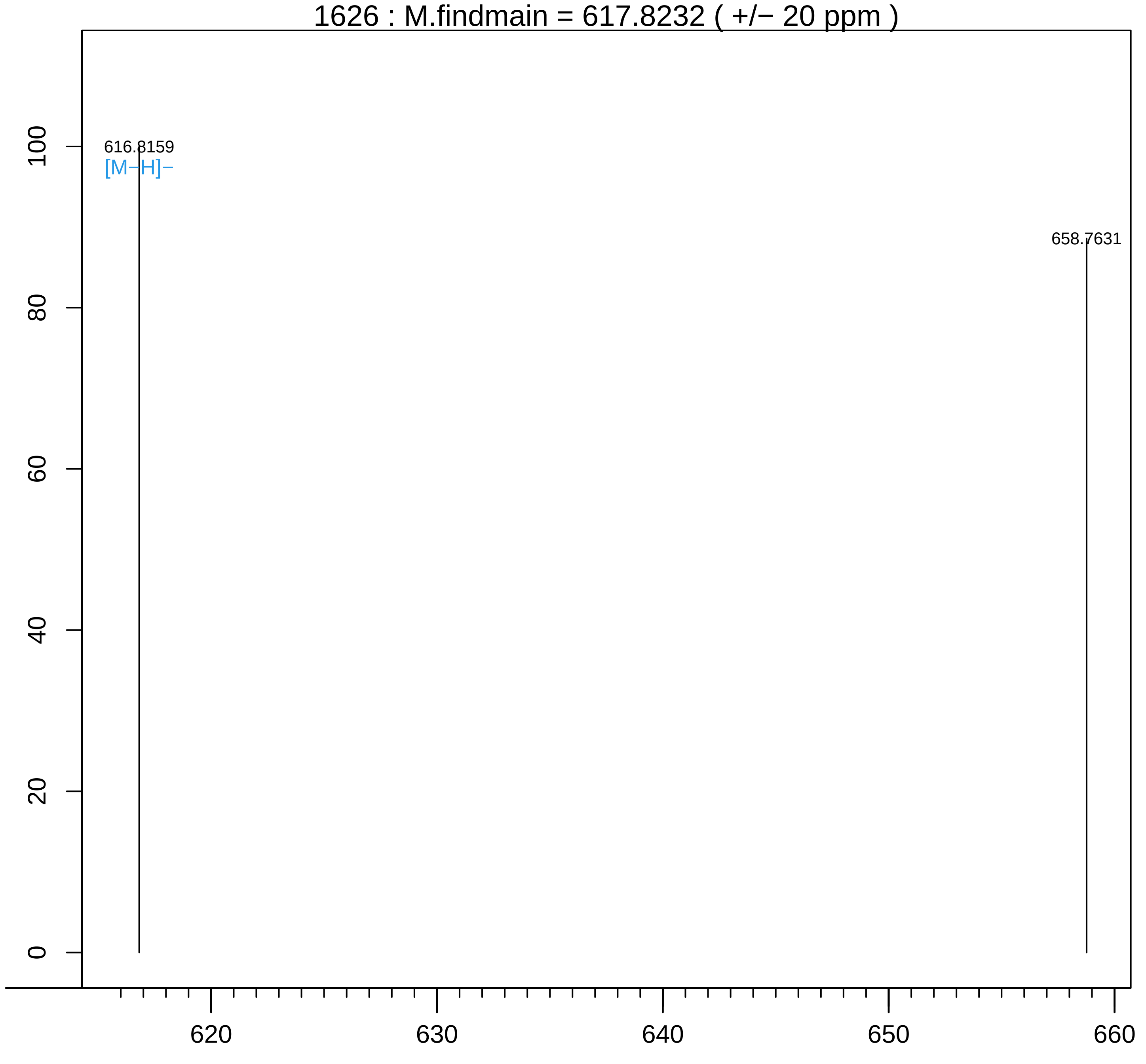
1626 : M.findmain = 617.8232 (+/− 20 ppm)
616.8159
[M−H]−
658.7631
0
20
40
60
80
100
620
630
640
650
660

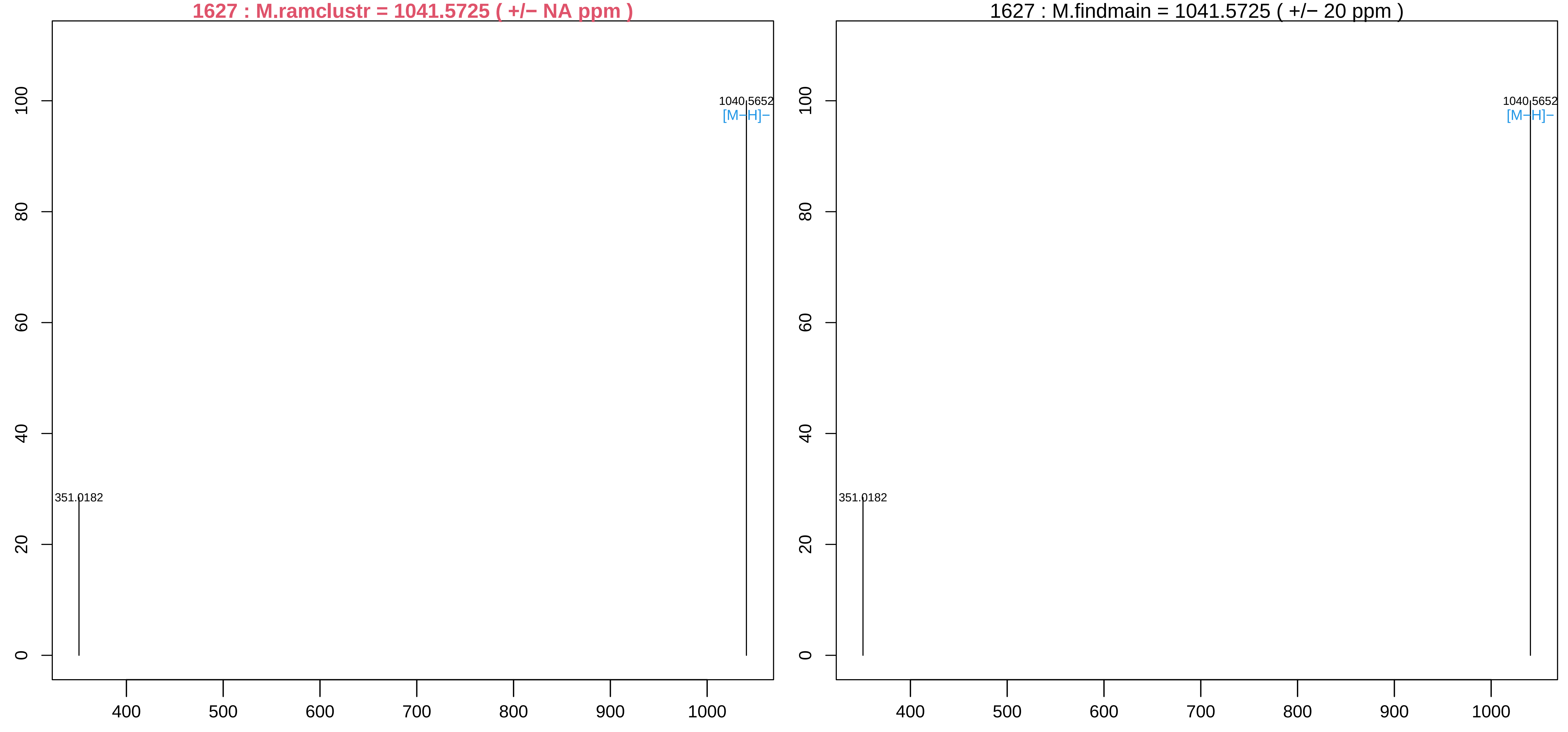
1627 : M.ramclustr = 1041.5725 (+/− NA ppm)
1040.5652
[M−H]−
351.0182
1627 : M.findmain = 1041.5725 (+/− 20 ppm)
1040.5652
[M−H]−
351.0182
0
20
40
60
80
100
400
500
600
700
800
900
1000

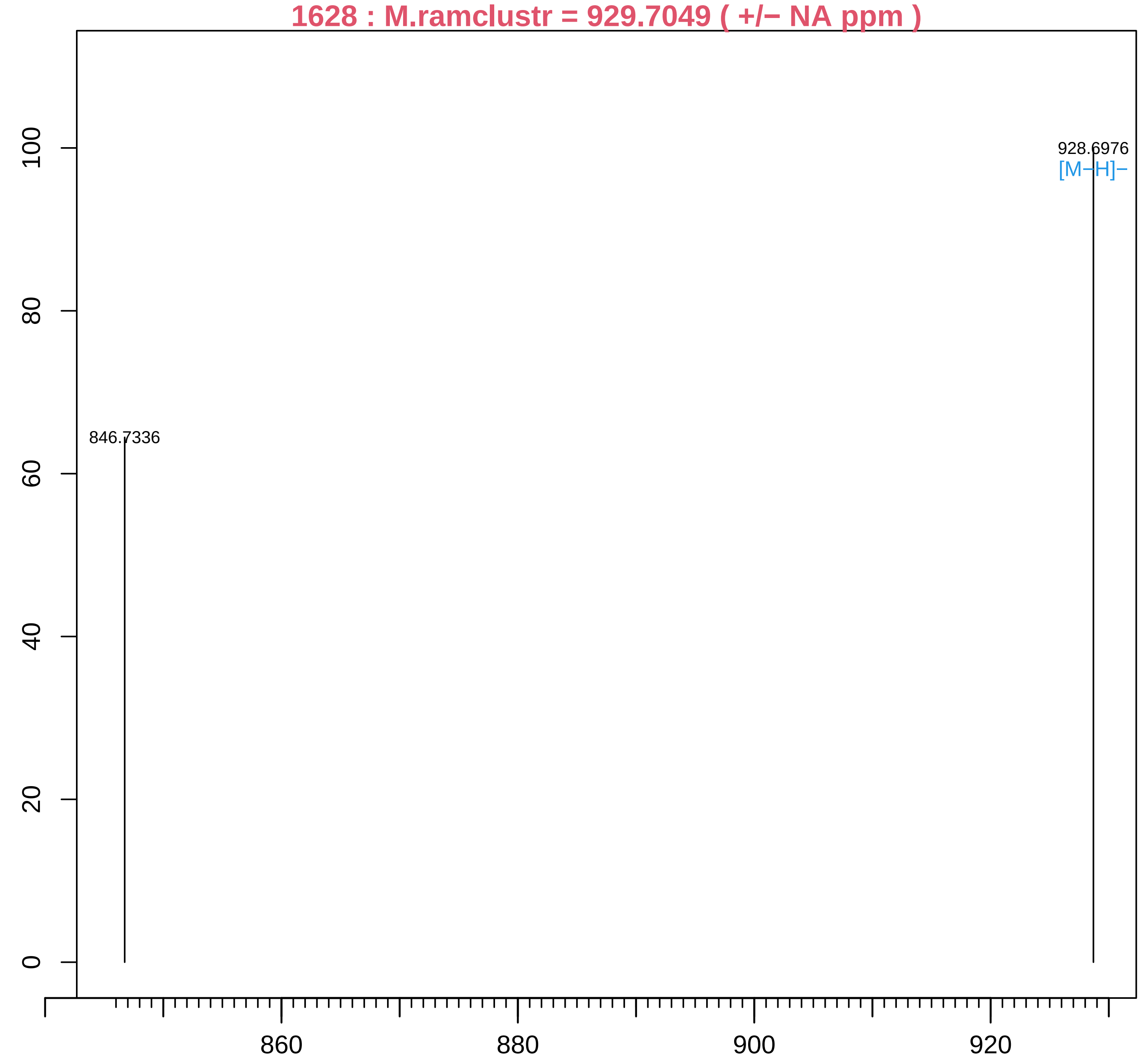
1628 : M.ramclustr = 929.7049 (+/− NA ppm)
928.6976
[M−H]−
846.7336
0
20
40
60
80
100
860
880
900
920

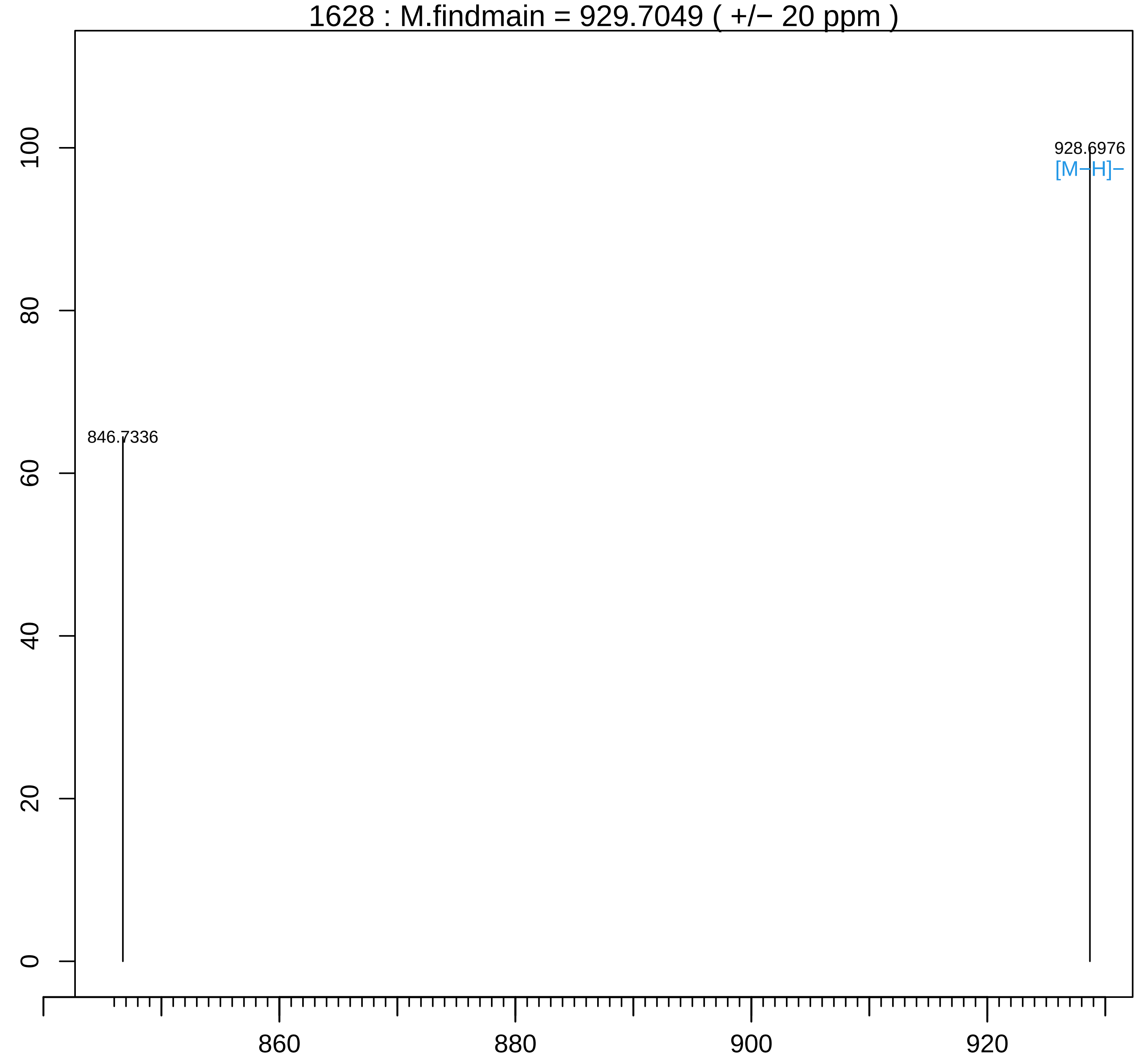
1628 : M.findmain = 929.7049 (+/− 20 ppm)
928.6976
[M−H]−
846.7336
0
20
40
60
80
100
860
880
900
920

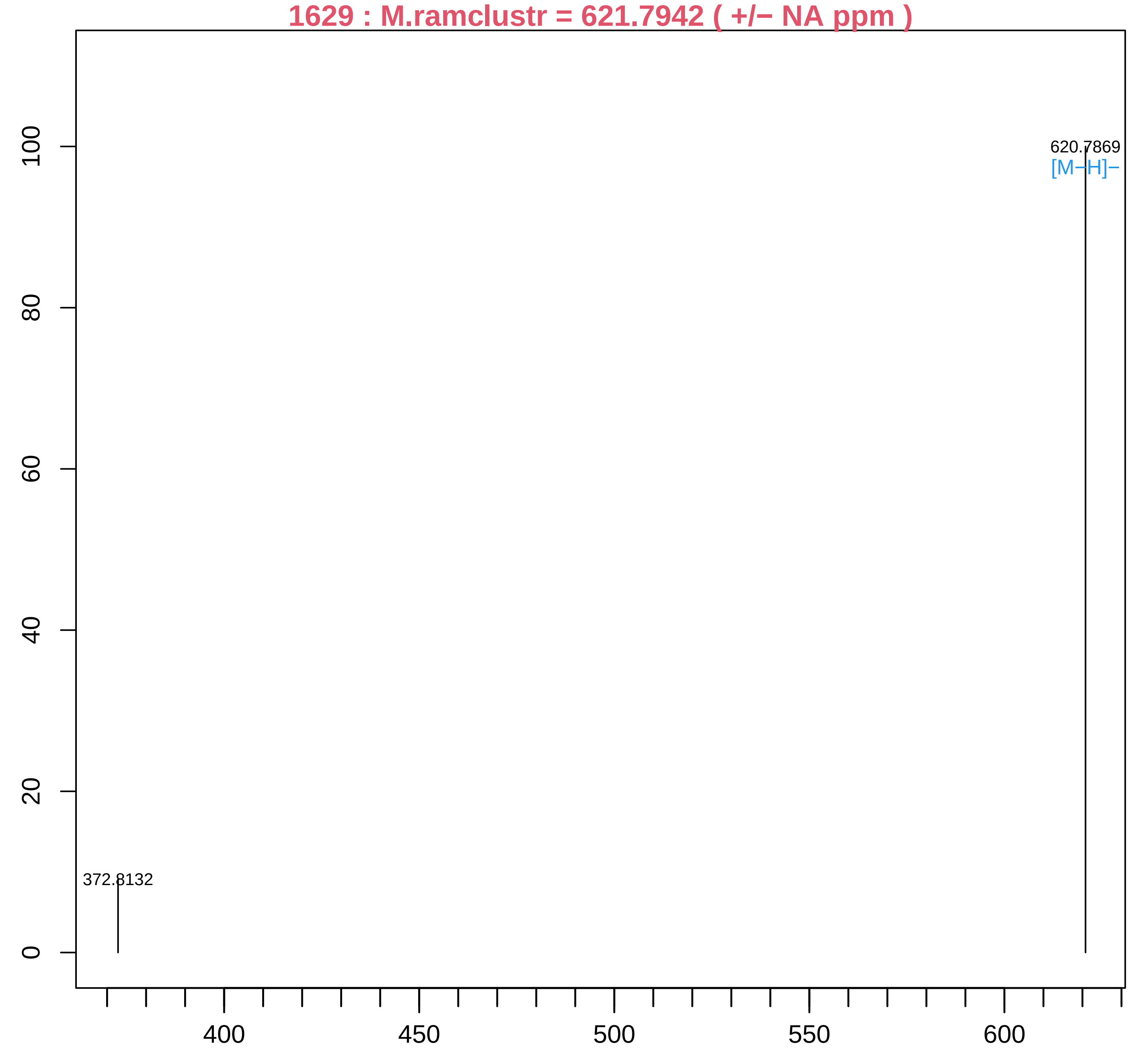
1629 : M.ramclustr = 621.7942 (+/− NA ppm)
620.7869
[M−H]−
372.8132
0
20
40
60
80
100
400
450
500
550
600

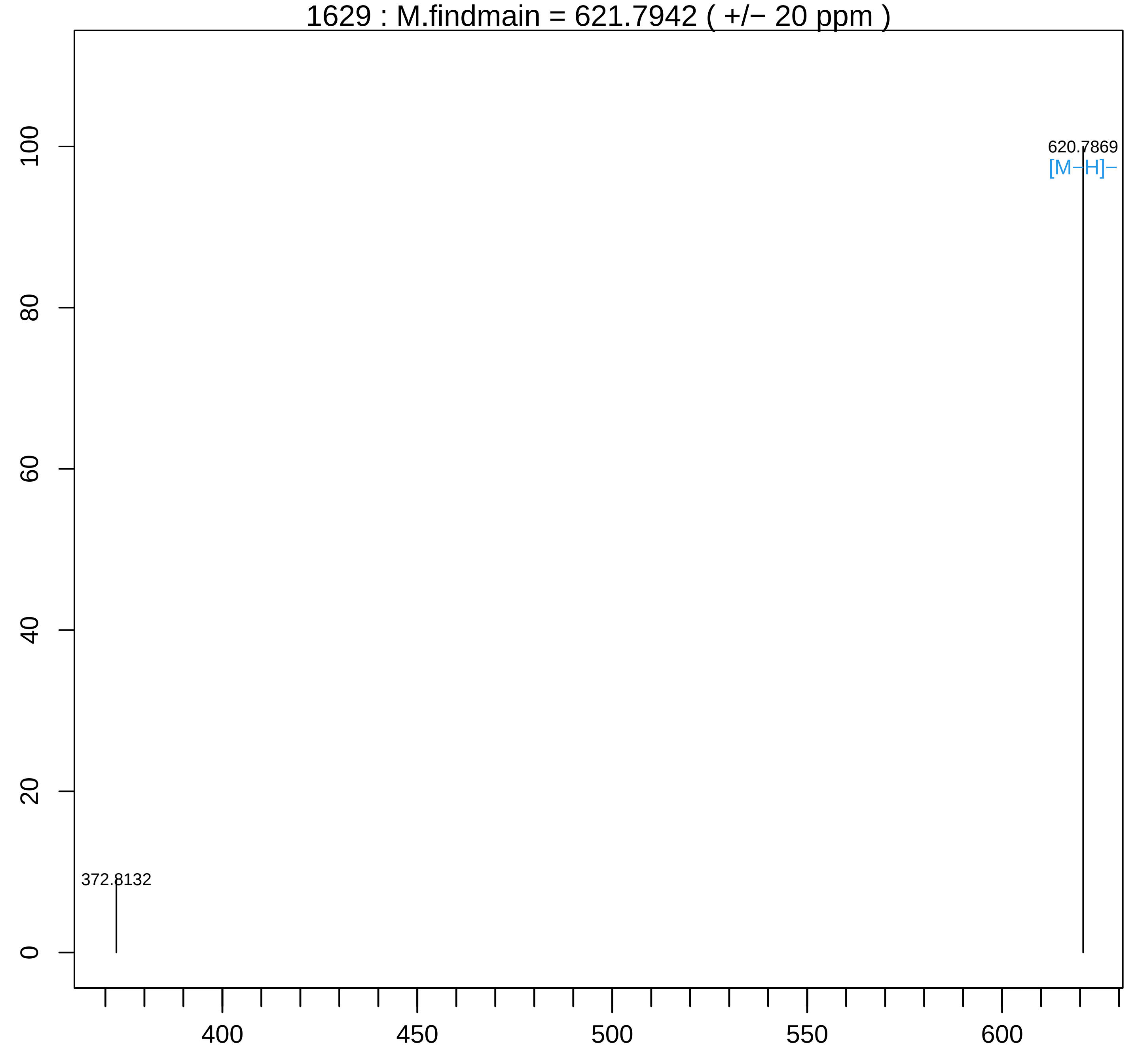
1629 : M.findmain = 621.7942 (+/− 20 ppm)
620.7869
[M−H]−
372.8132
0
20
40
60
80
100
400
450
500
550
600

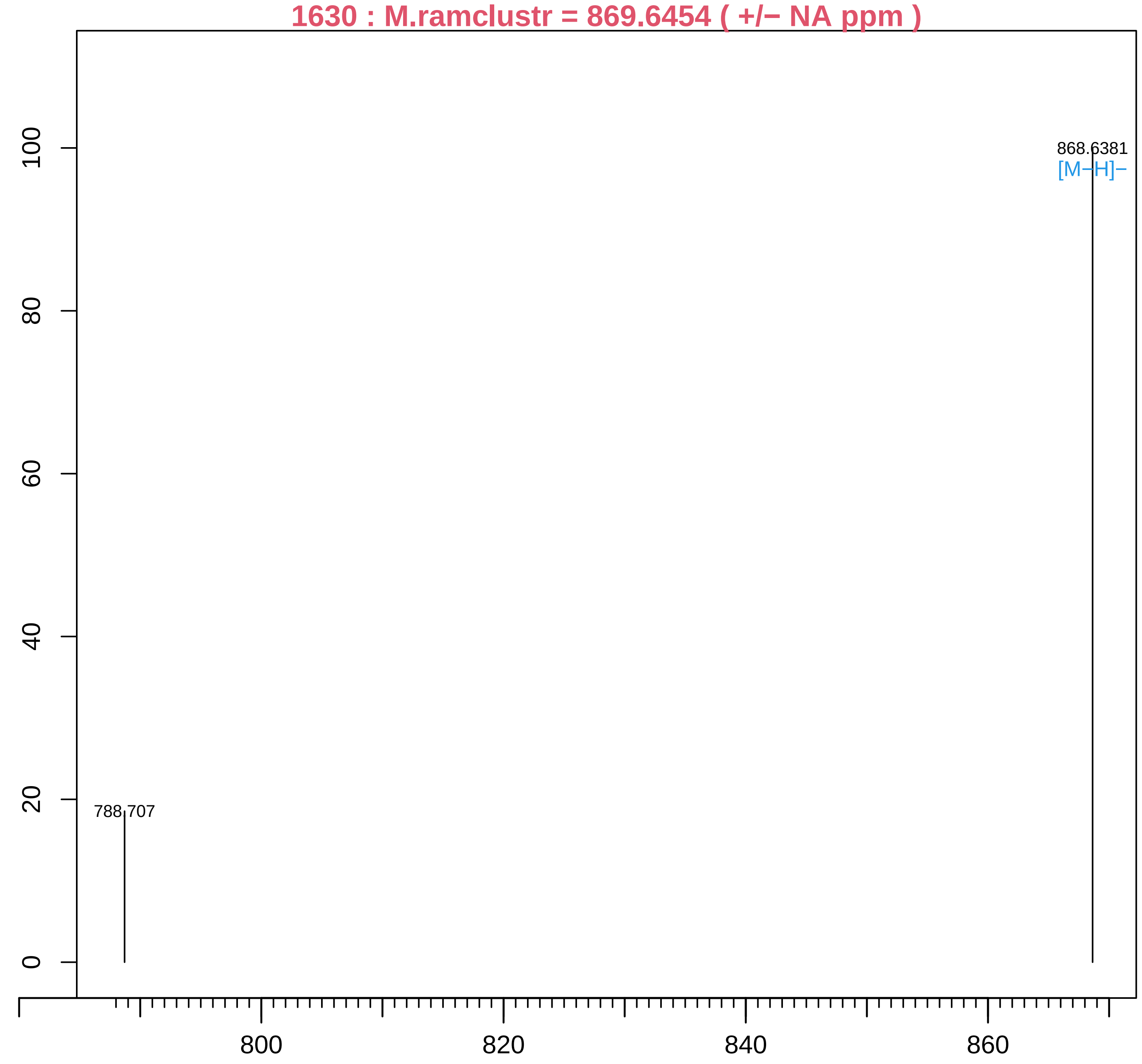

1630 : M.ramclustr = 869.6454 (+/− NA ppm)
868.6381
[M−H]−
788.707
0
20
40
60
80
100
800
820
840
860

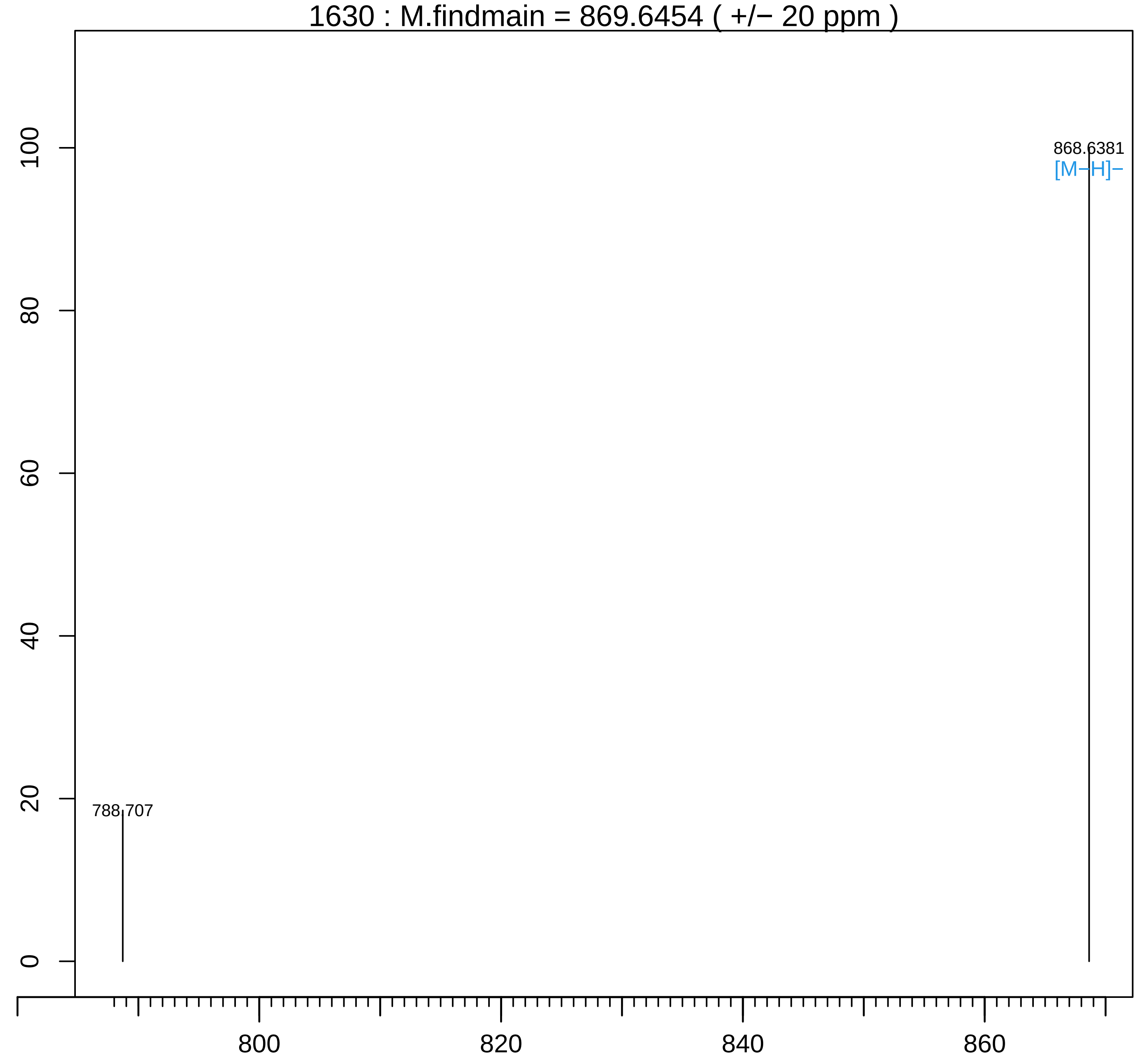

1630 : M.findmain = 869.6454 (+/− 20 ppm)
868.6381
[M−H]−
788.707
0
20
40
60
80
100
800
820
840
860

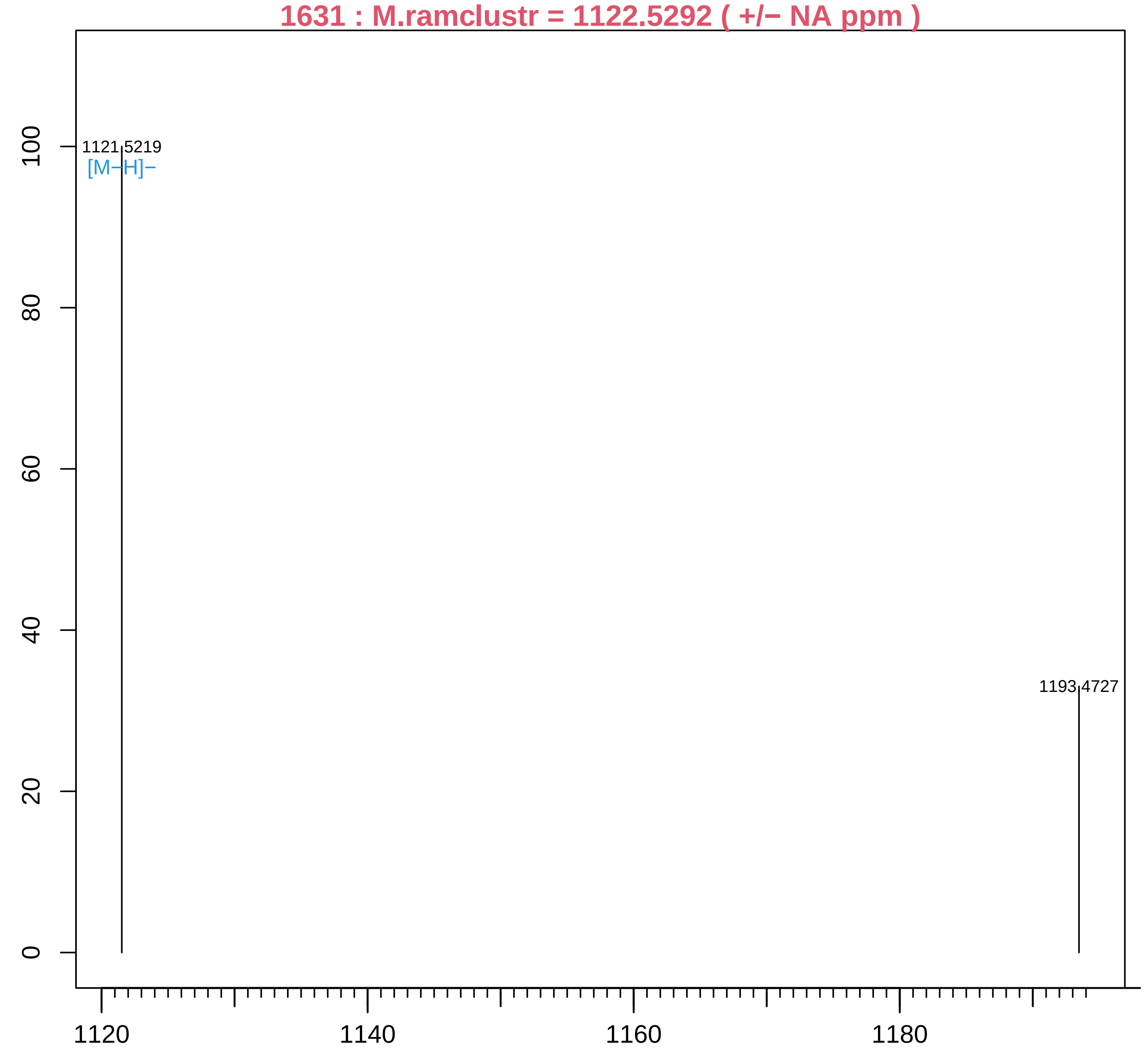
1631 : M.ramclustr = 1122.5292 (+/− NA ppm)
1121.5219
[M−H]−
1193.4727
0
20
40
60
80
100
1120
1140
1160
1180

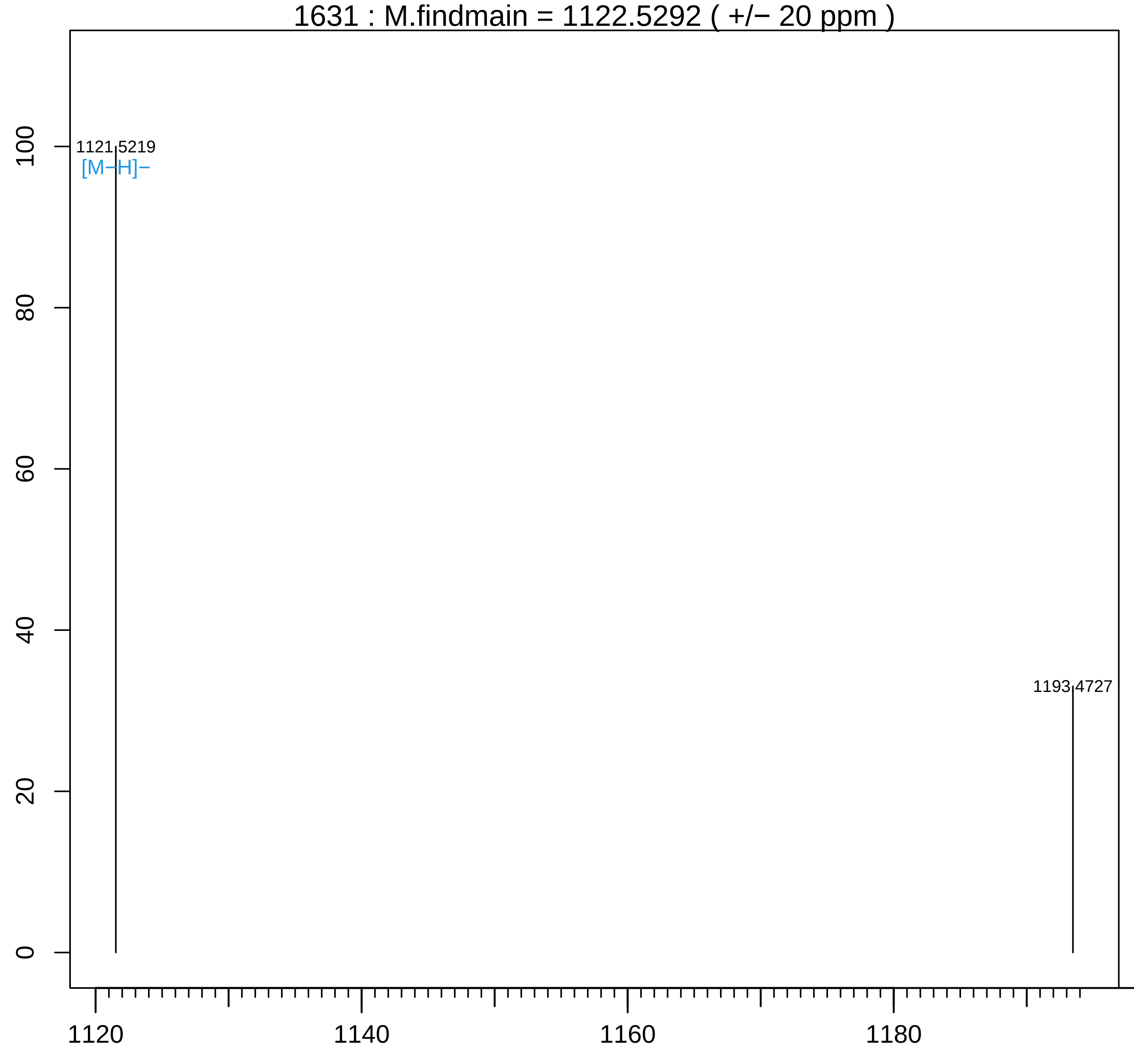
1631 : M.findmain = 1122.5292 (+/− 20 ppm)
1121.5219
[M−H]−
1193.4727
0
20
40
60
80
100
1120
1140
1160
1180

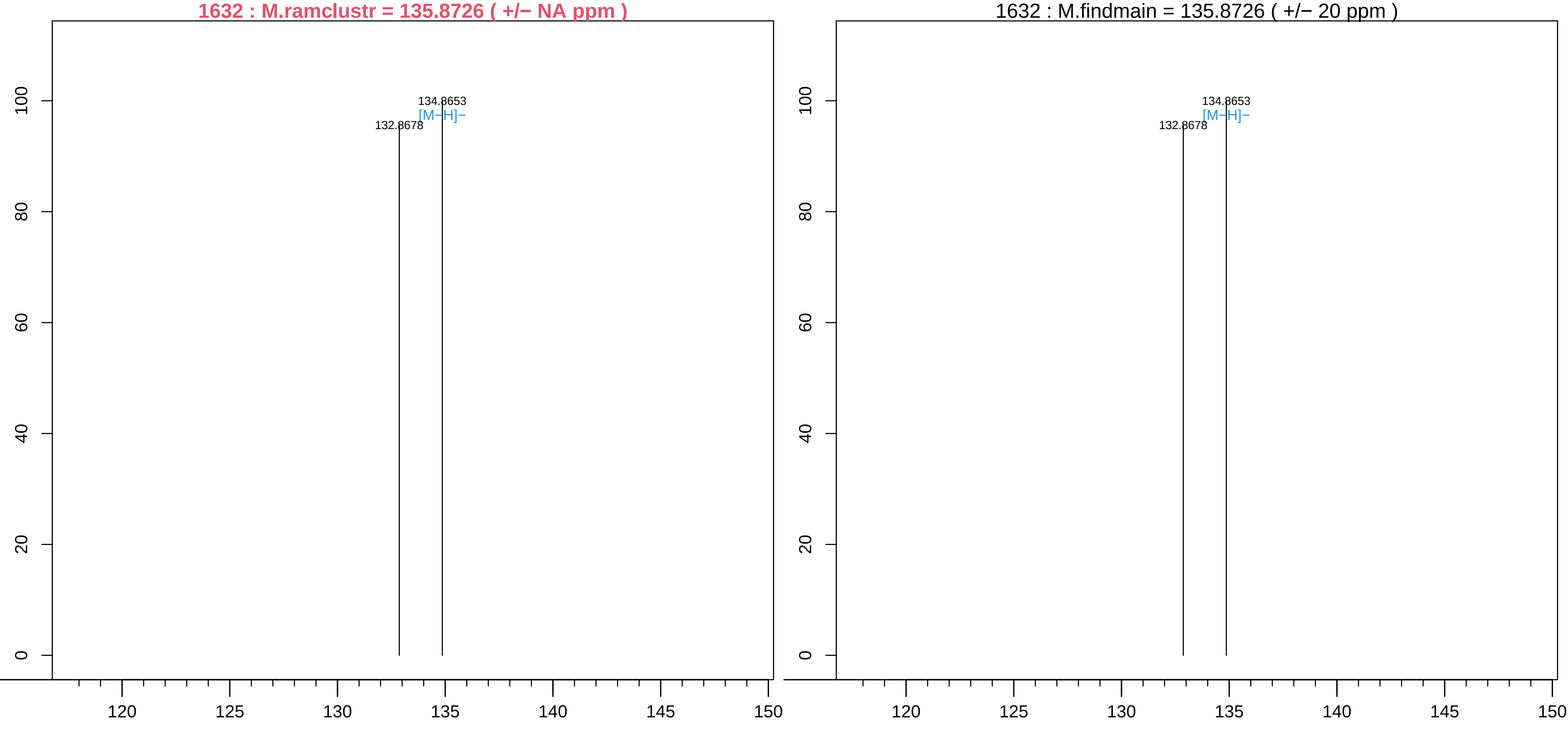
1632 : M.ramclustr = 135.8726 (+/− NA ppm)
134.8653
[M−H]−
132.8678
0
20
40
60
80
100
120
125
130
135
140
145
150
1632 : M.findmain = 135.8726 (+/− 20 ppm)
134.8653
[M−H]−
132.8678
0
20
40
60
80
100
120
125
130
135
140
145
150

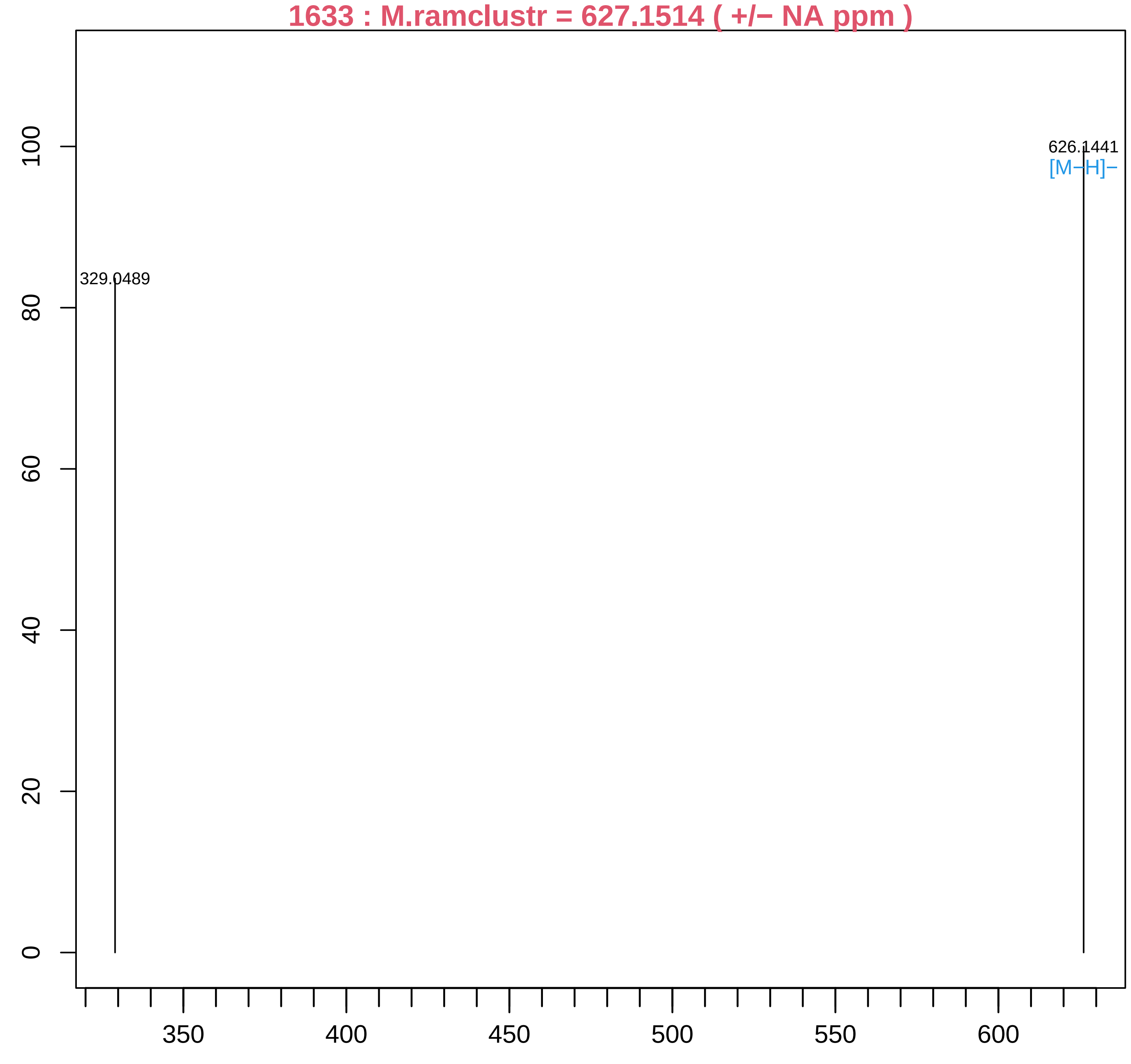
1633 : M.ramclustr = 627.1514 (+/− NA ppm)
626.1441
[M−H]−
329.0489
0
20
40
60
80
100
350
400
450
500
550
600

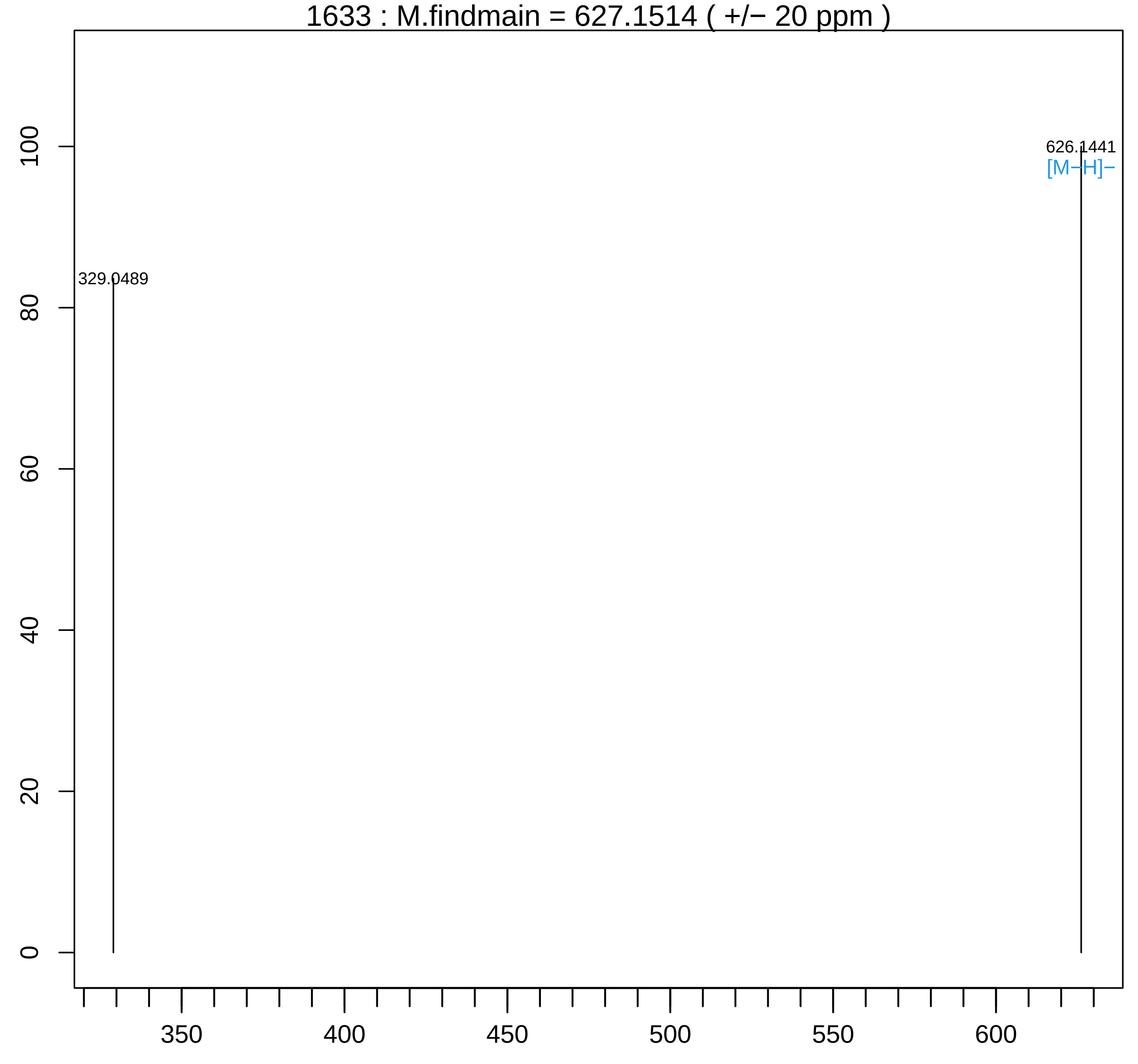
1633 : M.findmain = 627.1514 (+/− 20 ppm)
626.1441
[M−H]−
329.0489
0
20
40
60
80
100
350
400
450
500
550
600

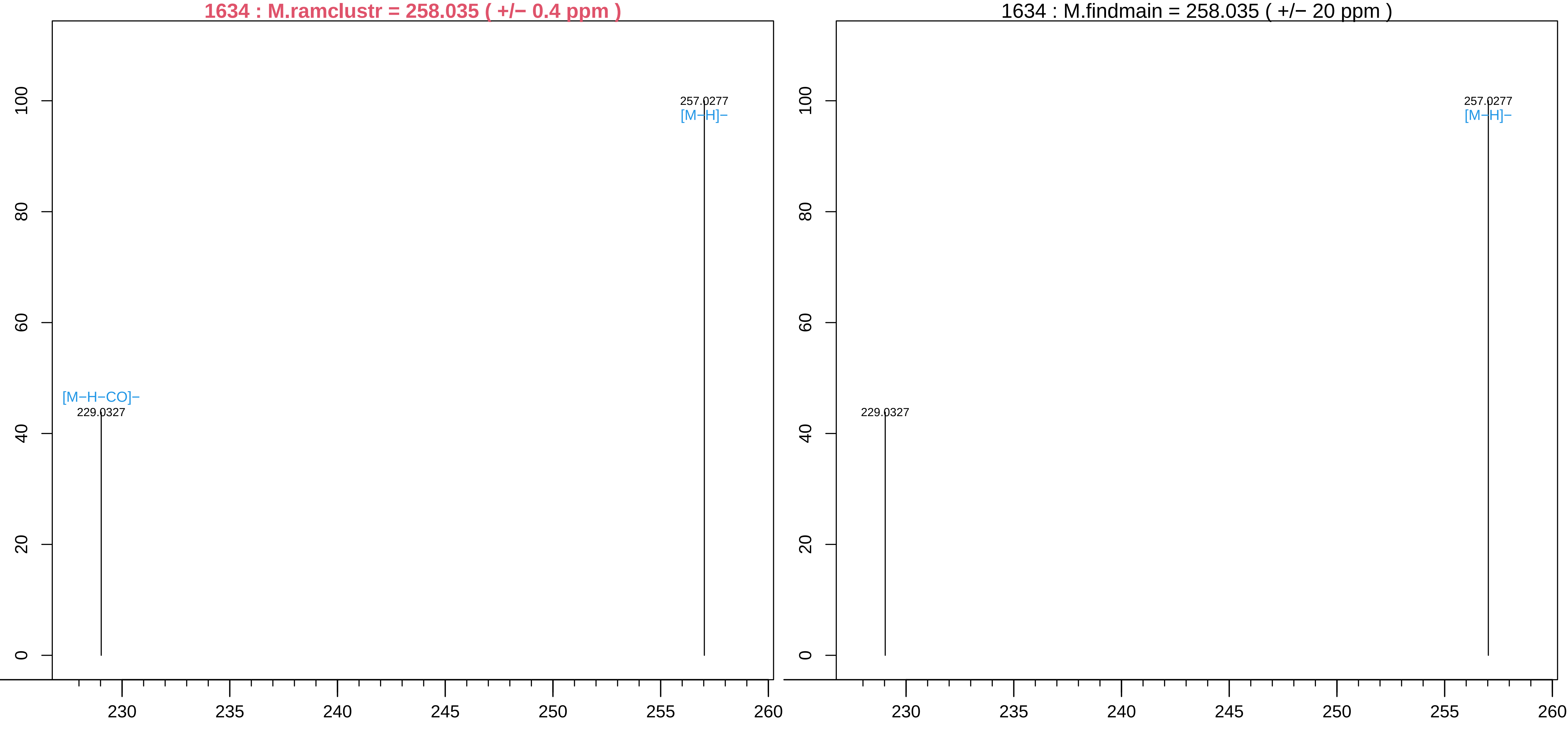
1634 : M.ramclustr = 258.035 (+/− 0.4 ppm)
257.0277
[M−H]−
[M−H−CO]−
229.0327
1634 : M.findmain = 258.035 (+/− 20 ppm)
257.0277
[M−H]−
229.0327
0
20
40
60
80
100
230
235
240
245
250
255
260

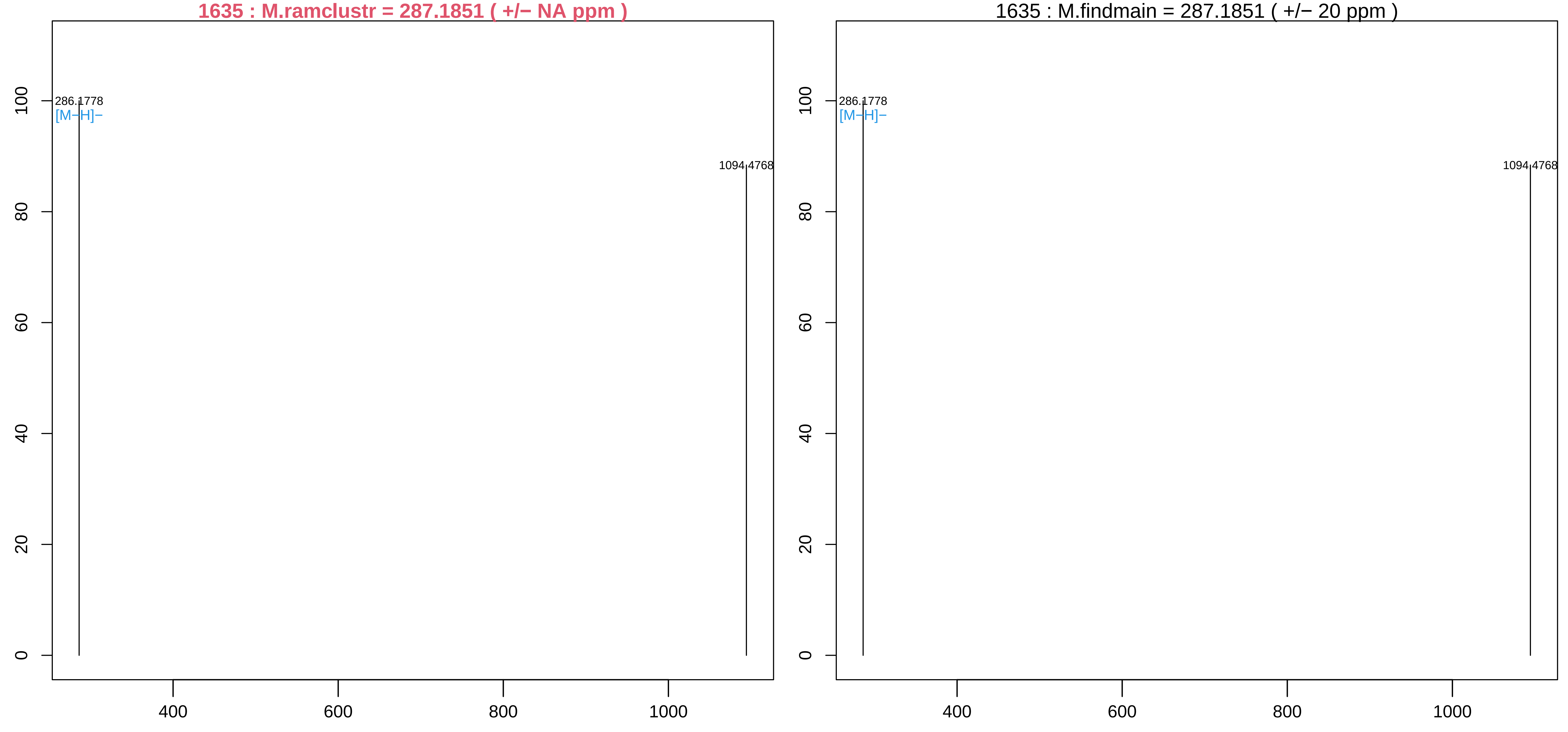
1635 : M.ramclustr = 287.1851 (+/− NA ppm)
286.1778
[M−H]−
1094.4768
0
20
40
60
80
100
400
600
800
1000
1635 : M.findmain = 287.1851 (+/− 20 ppm)
286.1778
[M−H]−
1094.4768
0
20
40
60
80
100
400
600
800
1000

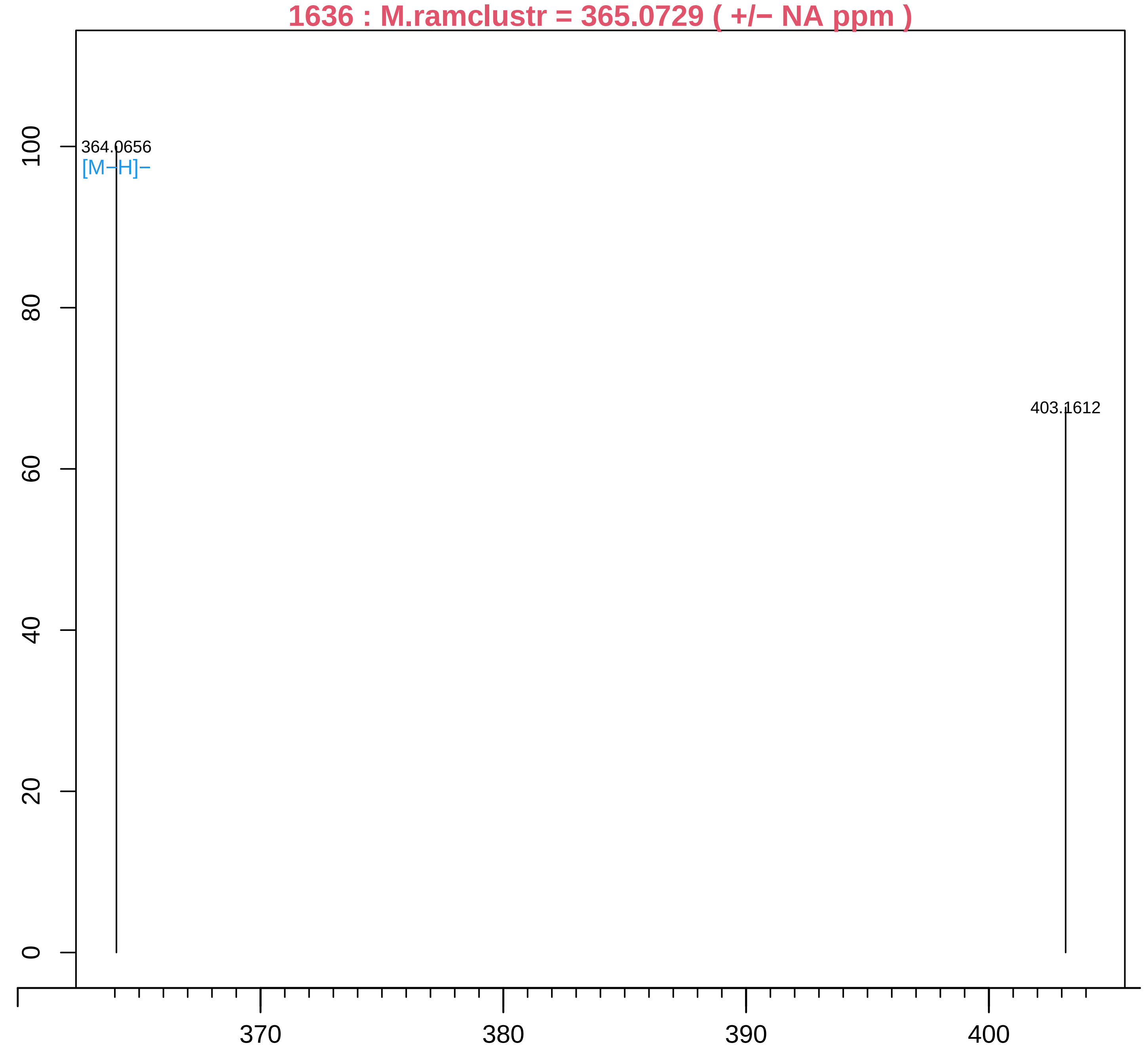

1636 : M.ramclustr = 365.0729 (+/− NA ppm)
364.0656
[M−H]−
403.1612
0
20
40
60
80
100
370
380
390
400

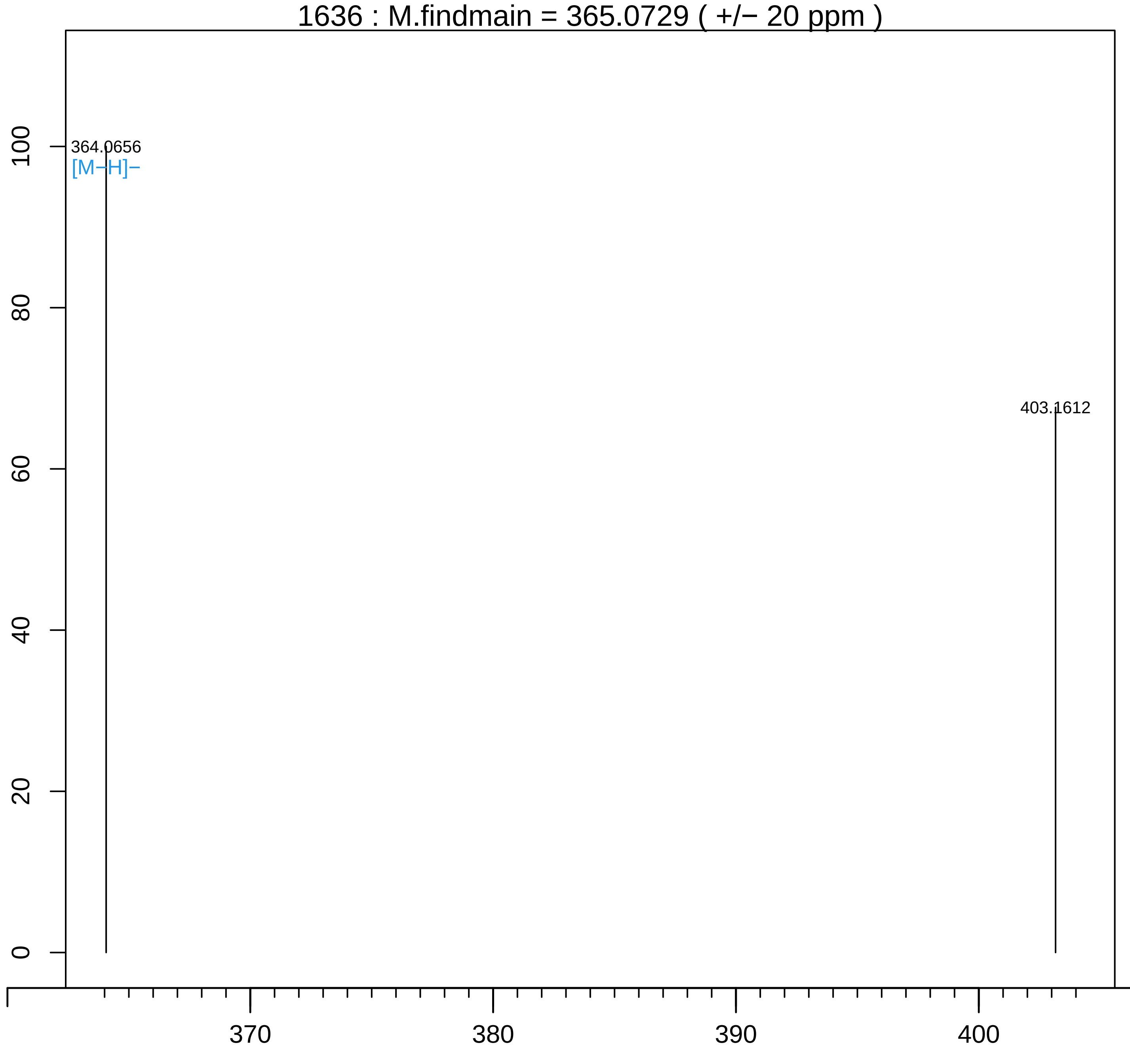

1636 : M.findmain = 365.0729 (+/− 20 ppm)
364.0656
[M−H]−
403.1612
0
20
40
60
80
100
370
380
390
400

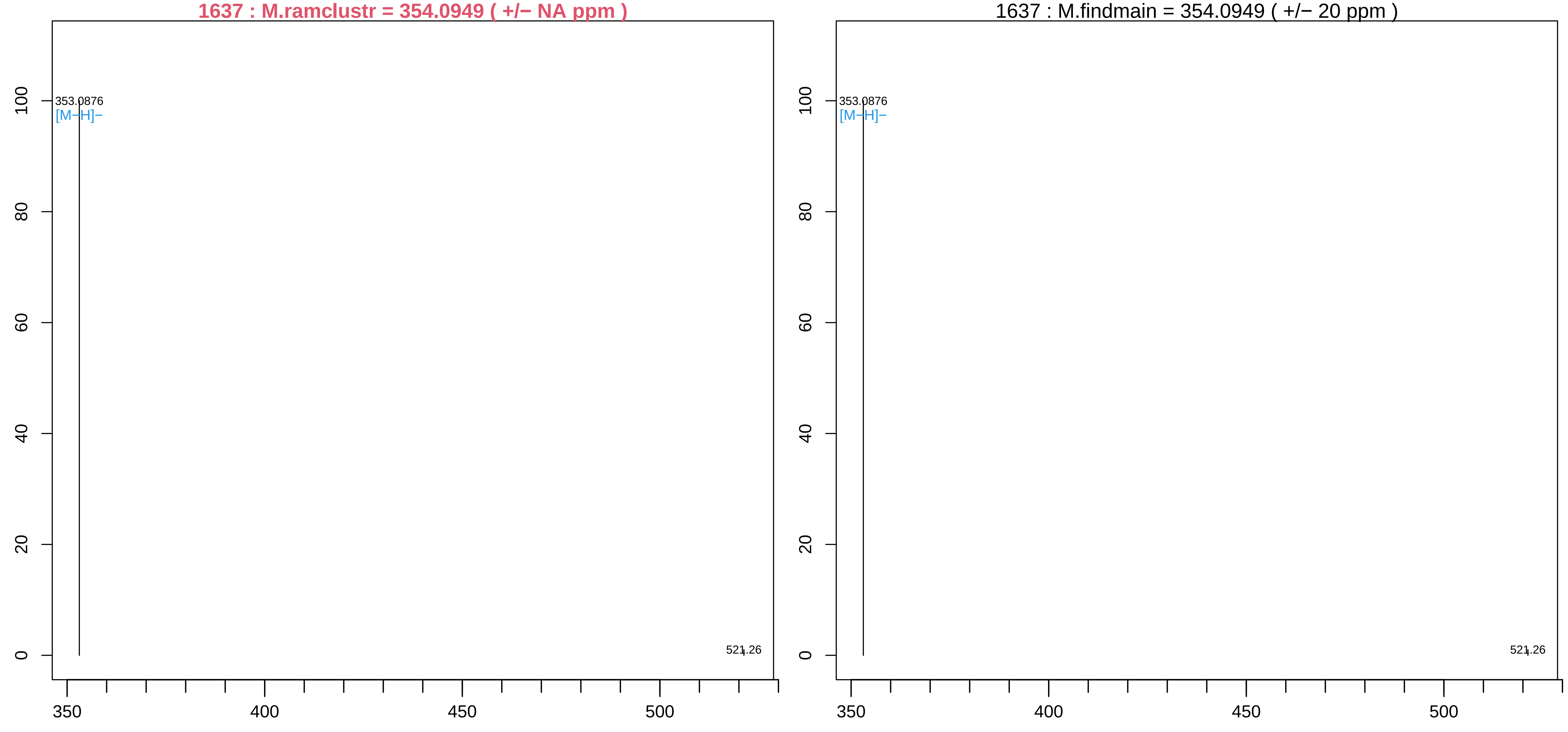
1637 : M.ramclustr = 354.0949 (+/− NA ppm)
353.0876
[M−H]−
521.26
1637 : M.findmain = 354.0949 (+/− 20 ppm)
353.0876
[M−H]−
521.26
0
20
40
60
80
100
350
400
450
500

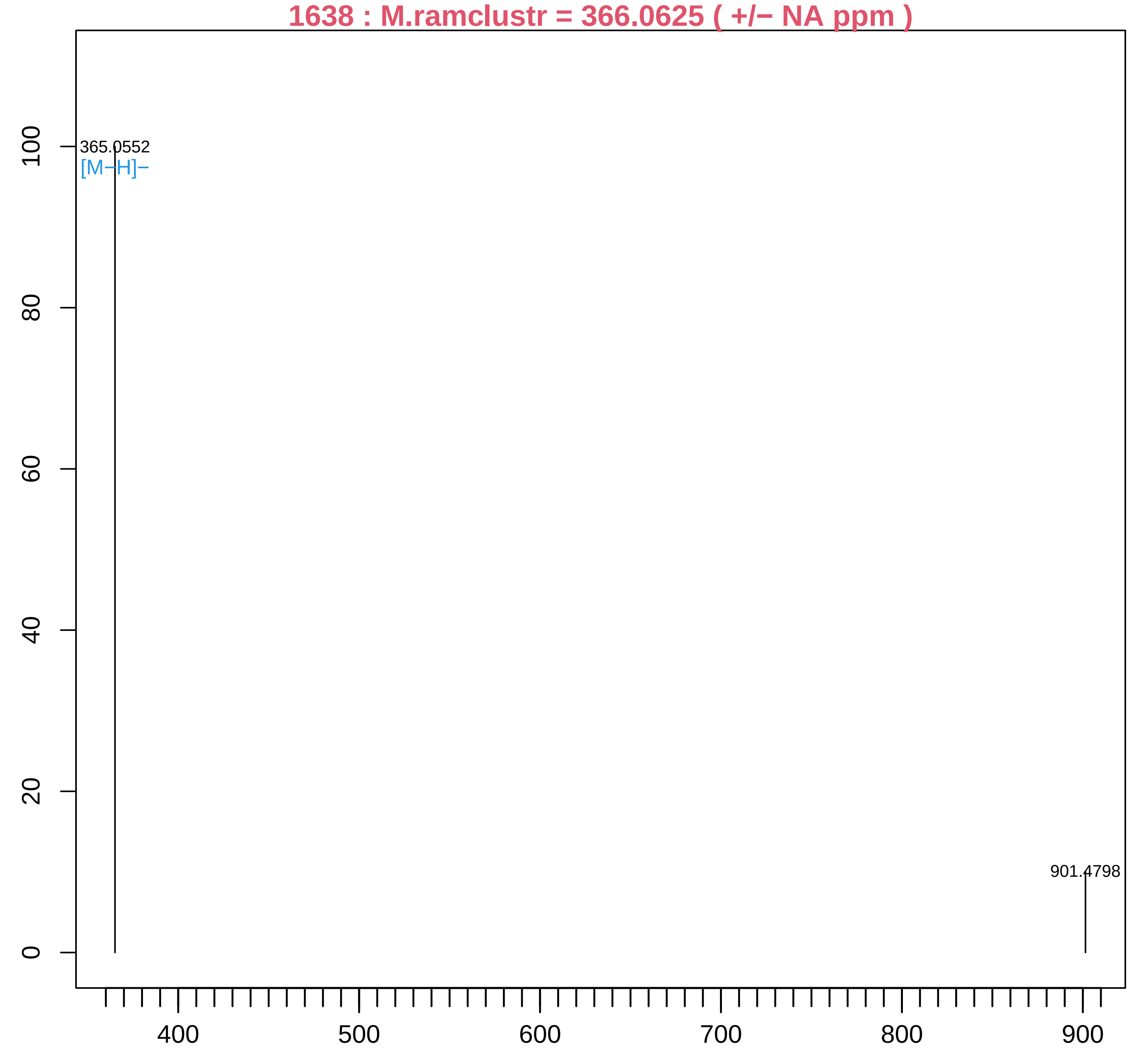
1638 : M.ramclustr = 366.0625 (+/− NA ppm)
365.0552
[M−H]−
901.4798
0
20
40
60
80
100
400
500
600
700
800
900

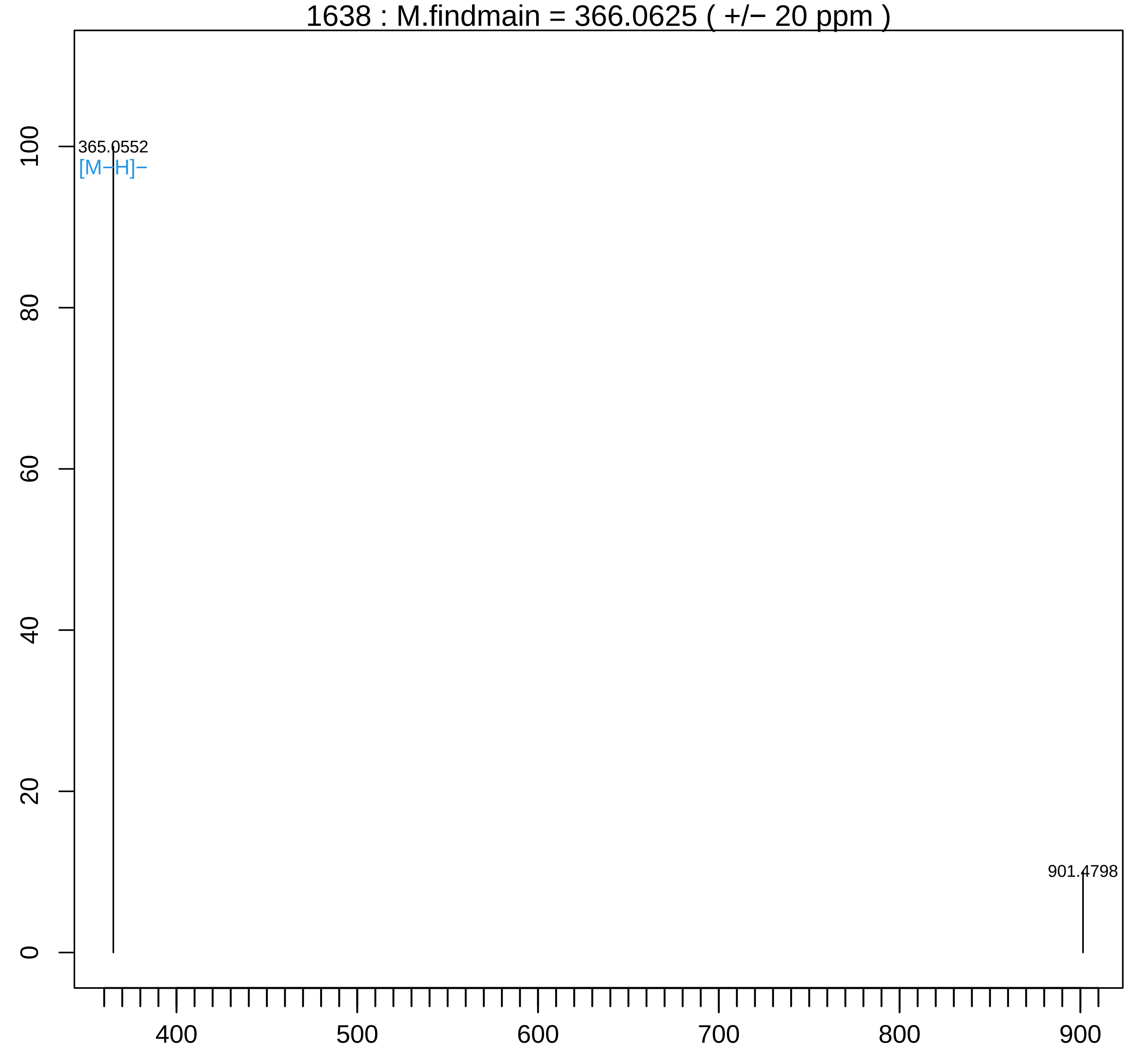
1638 : M.findmain = 366.0625 (+/− 20 ppm)
365.0552
[M−H]−
901.4798
0
20
40
60
80
100
400
500
600
700
800
900

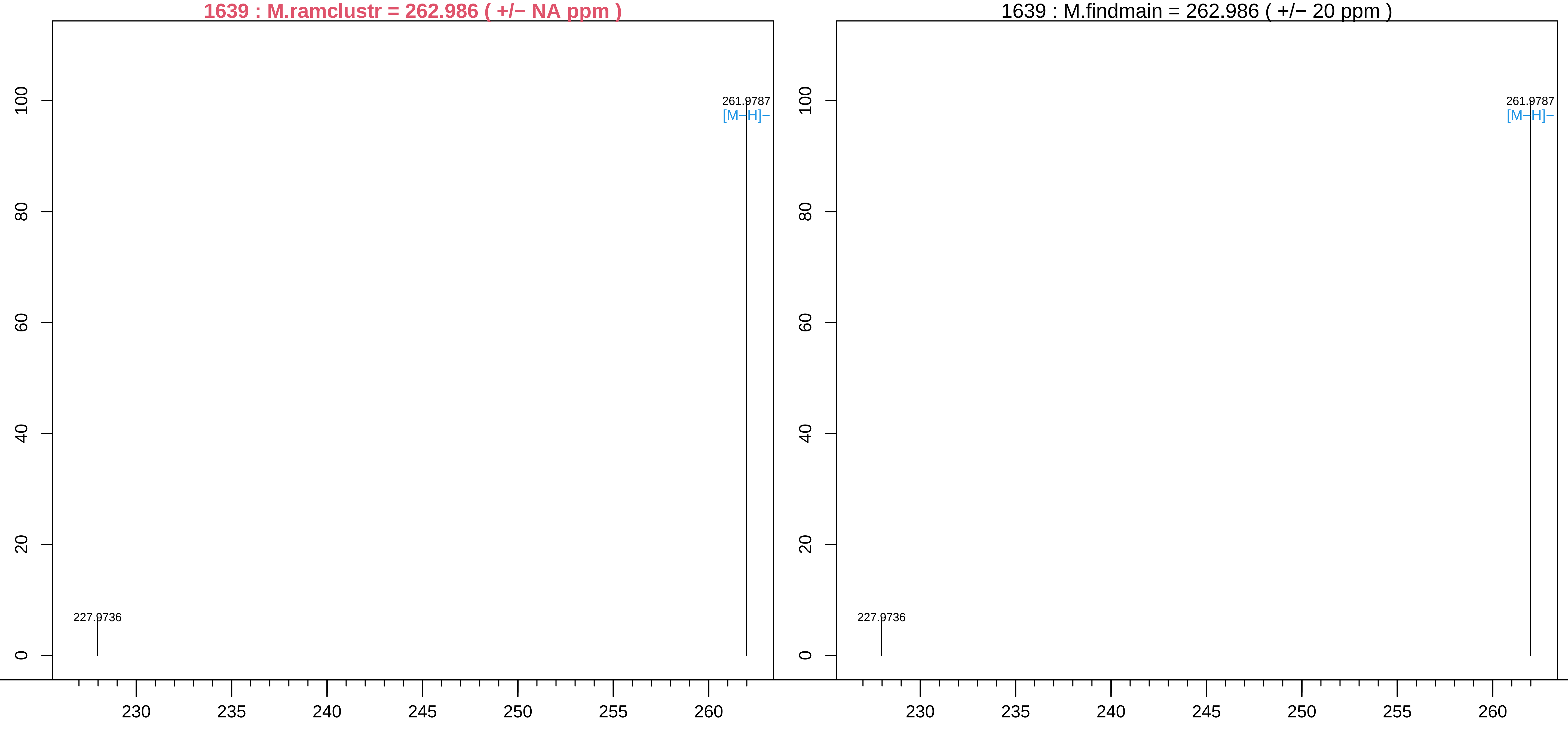

1639 : M.ramclustr = 262.986 (+/− NA ppm)
1639 : M.findmain = 262.986 (+/− 20 ppm)
261.9787
[M−H]−
227.9736
0
20
40
60
80
100
230
235
240
245
250
255
260

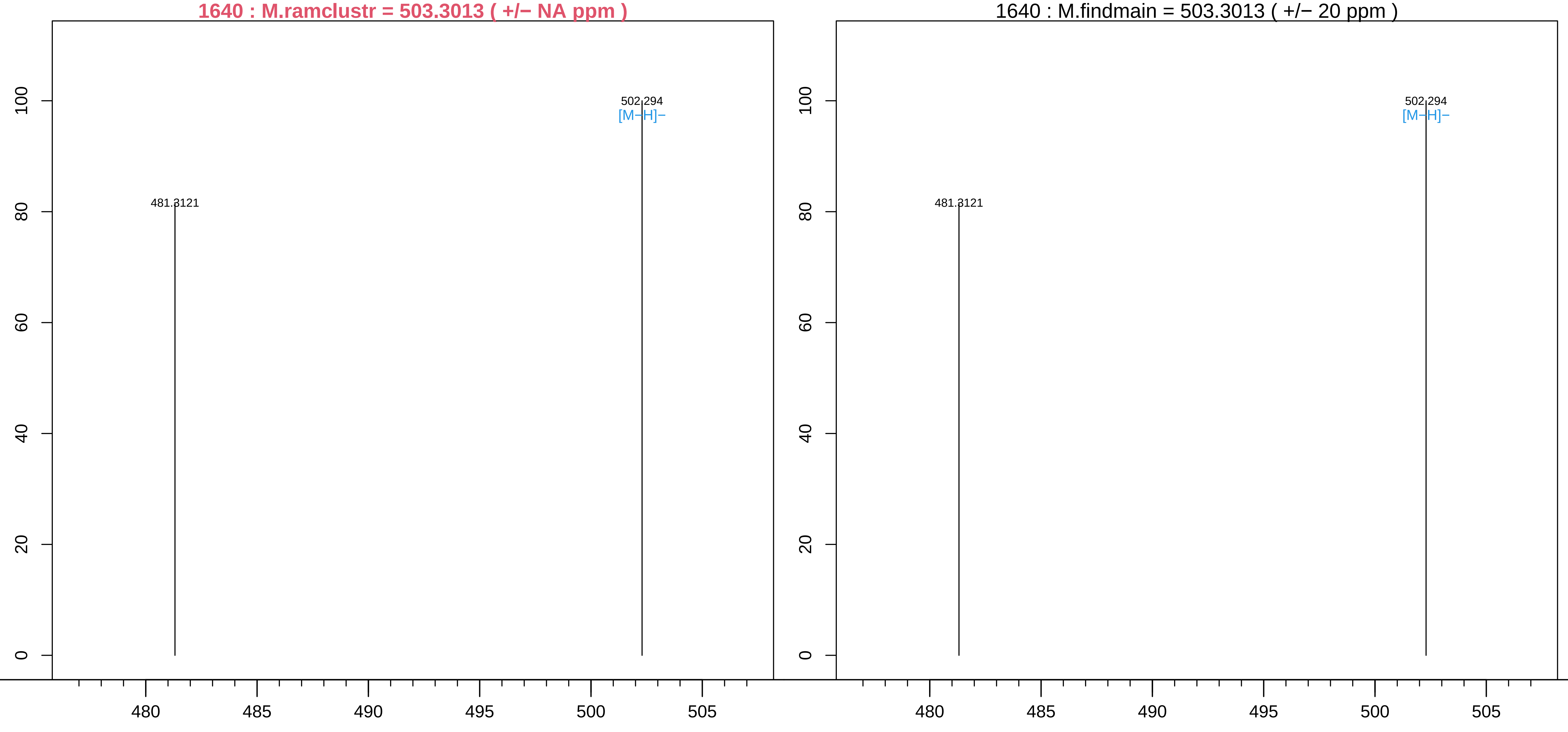
1640 : M.ramclustr = 503.3013 (+/− NA ppm)
1640 : M.findmain = 503.3013 (+/− 20 ppm)
502.294
[M−H]−
481.3121
502.294
[M−H]−
481.3121
0
20
40
60
80
100
480
485
490
495
500
505

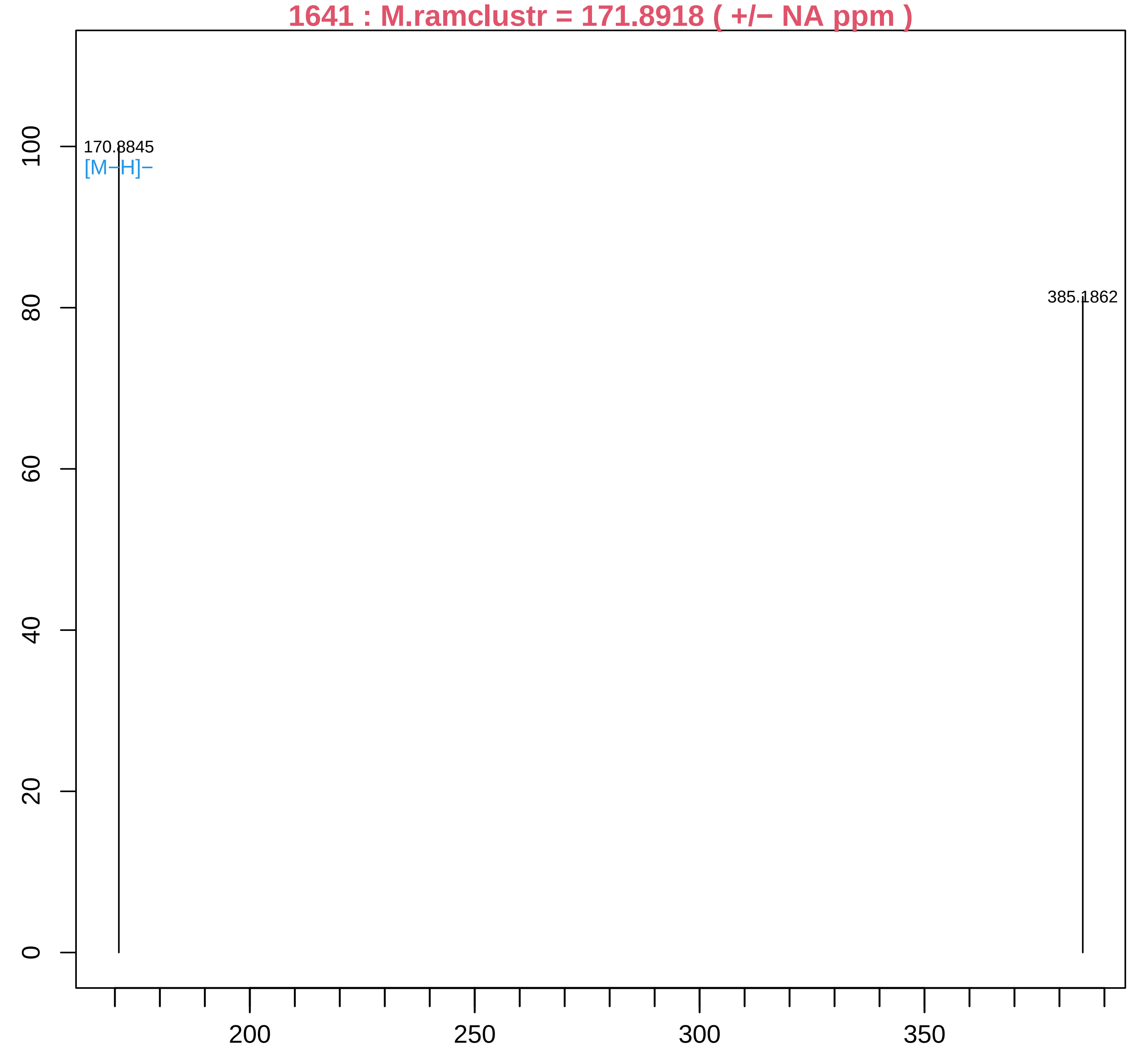
1641 : M.ramclustr = 171.8918 (+/− NA ppm)
170.8845
[M−H]−
385.1862
0
20
40
60
80
100
200
250
300
350

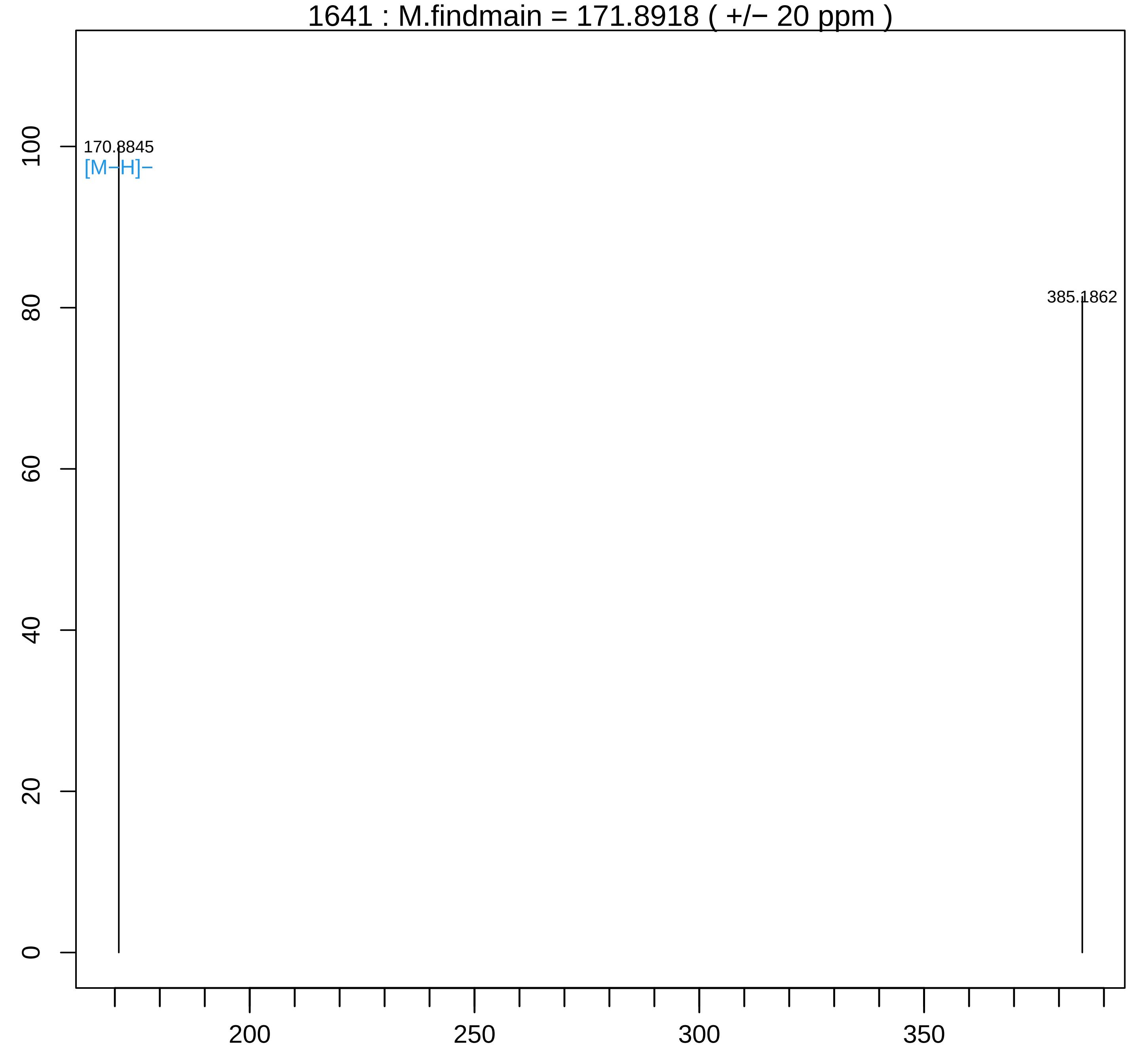
1641 : M.findmain = 171.8918 (+/− 20 ppm)
170.8845
[M−H]−
385.1862
0
20
40
60
80
100
200
250
300
350

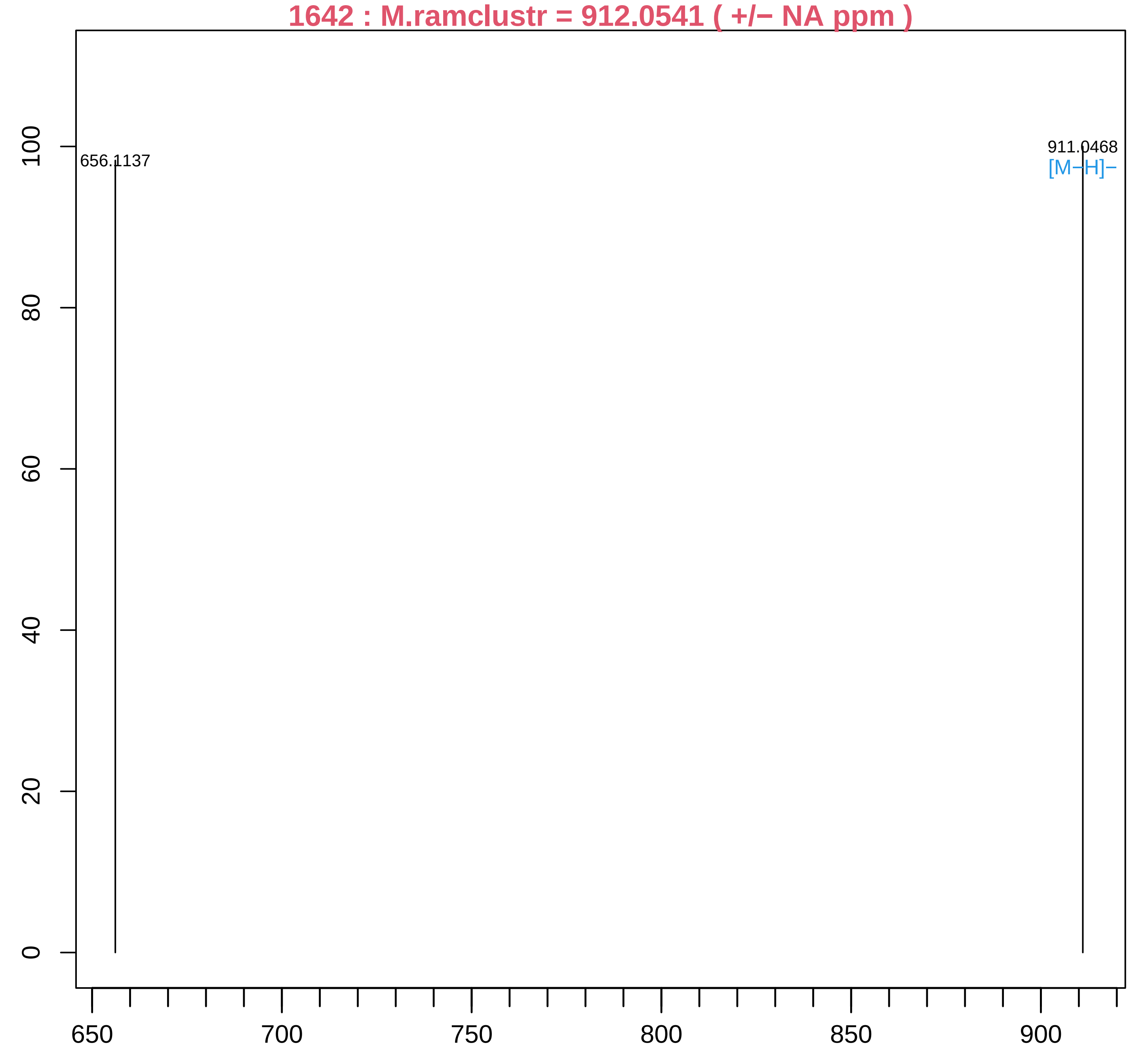
1642 : M.ramclustr = 912.0541 (+/− NA ppm)
656.1137
911.0468
[M−H]−
0
20
40
60
80
100
650
700
750
800
850
900

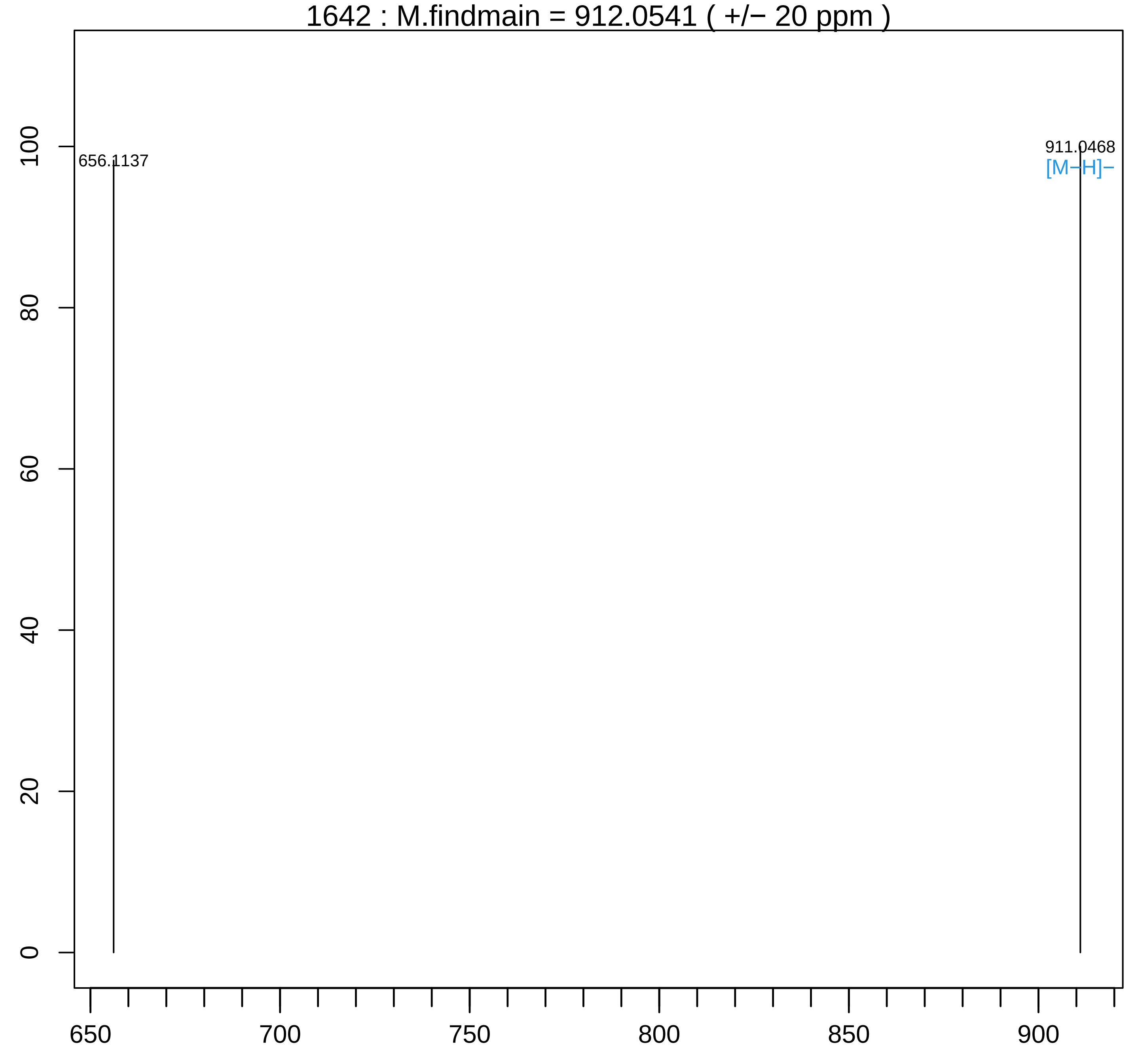
1642 : M.findmain = 912.0541 (+/− 20 ppm)
656.1137
911.0468
[M−H]−
0
20
40
60
80
100
650
700
750
800
850
900

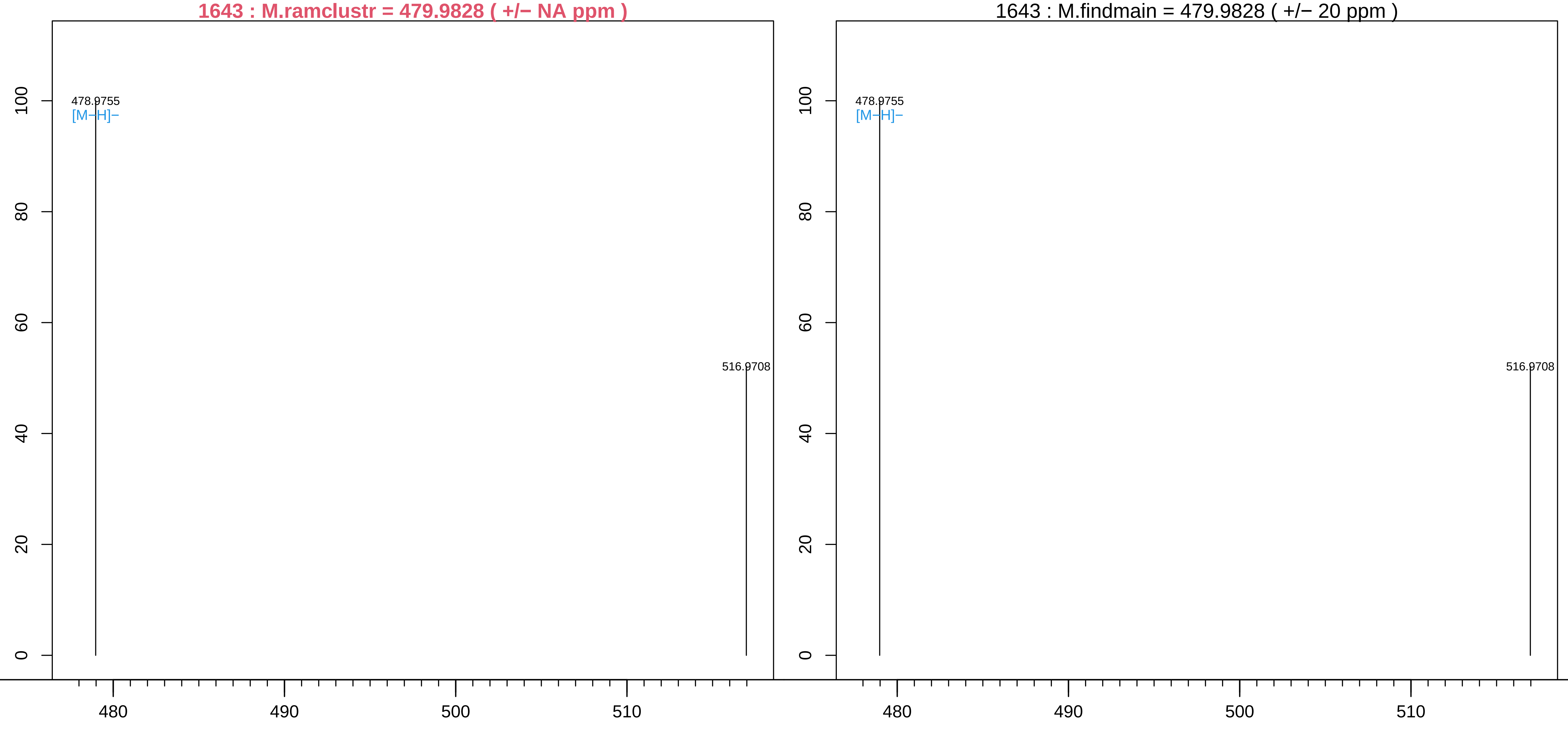
1643 : M.ramclustr = 479.9828 (+/− NA ppm)
478.9755
[M−H]−
516.9708
1643 : M.findmain = 479.9828 (+/− 20 ppm)
478.9755
[M−H]−
516.9708
0
20
40
60
80
100
480
490
500
510

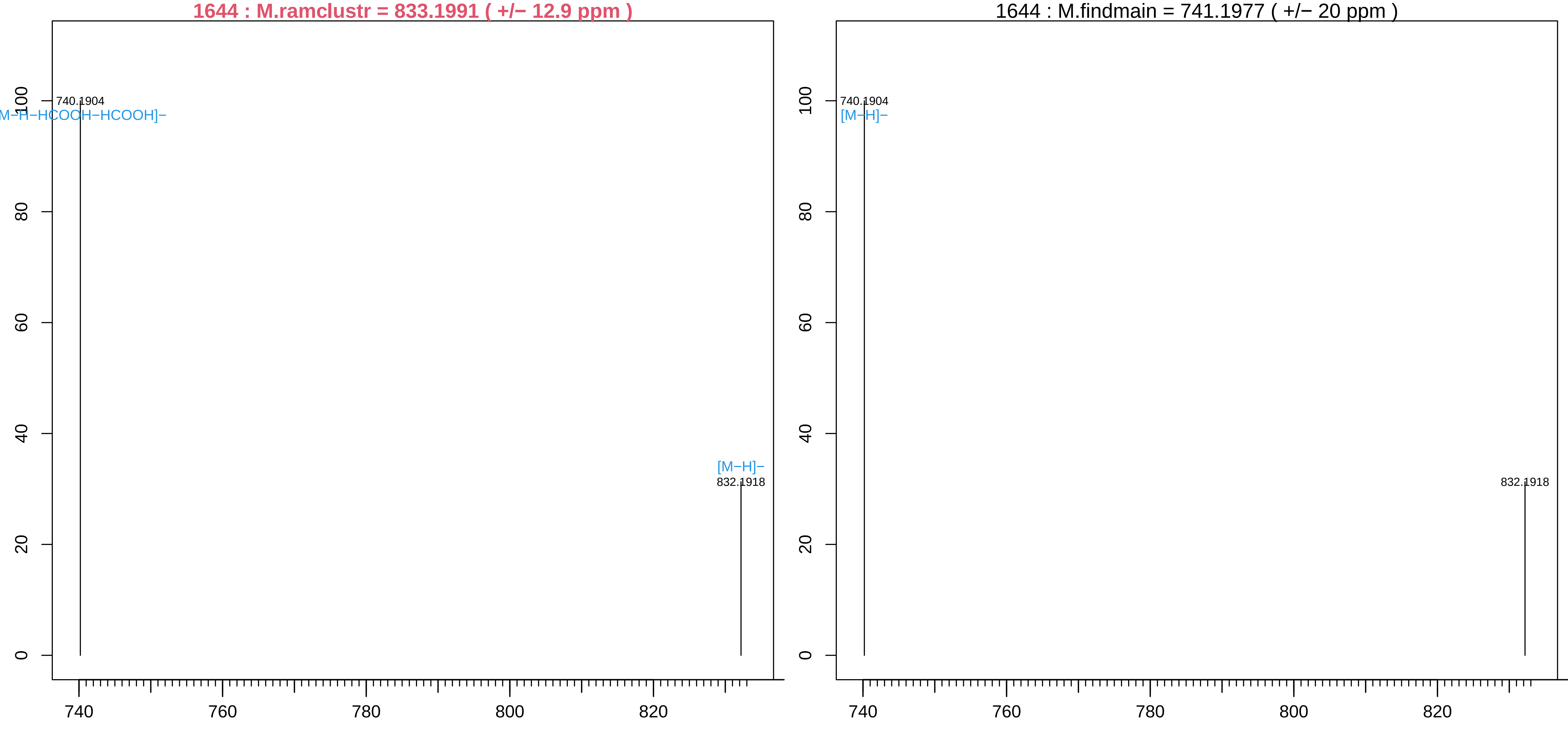
1644 : M.ramclustr = 833.1991 (+/− 12.9 ppm)
740.1904
[M−H−HCOOH−HCOOH]−
[M−H]−
832.1918
1644 : M.findmain = 741.1977 (+/− 20 ppm)
740.1904
[M−H]−
832.1918
0
20
40
60
80
100
740
760
780
800
820

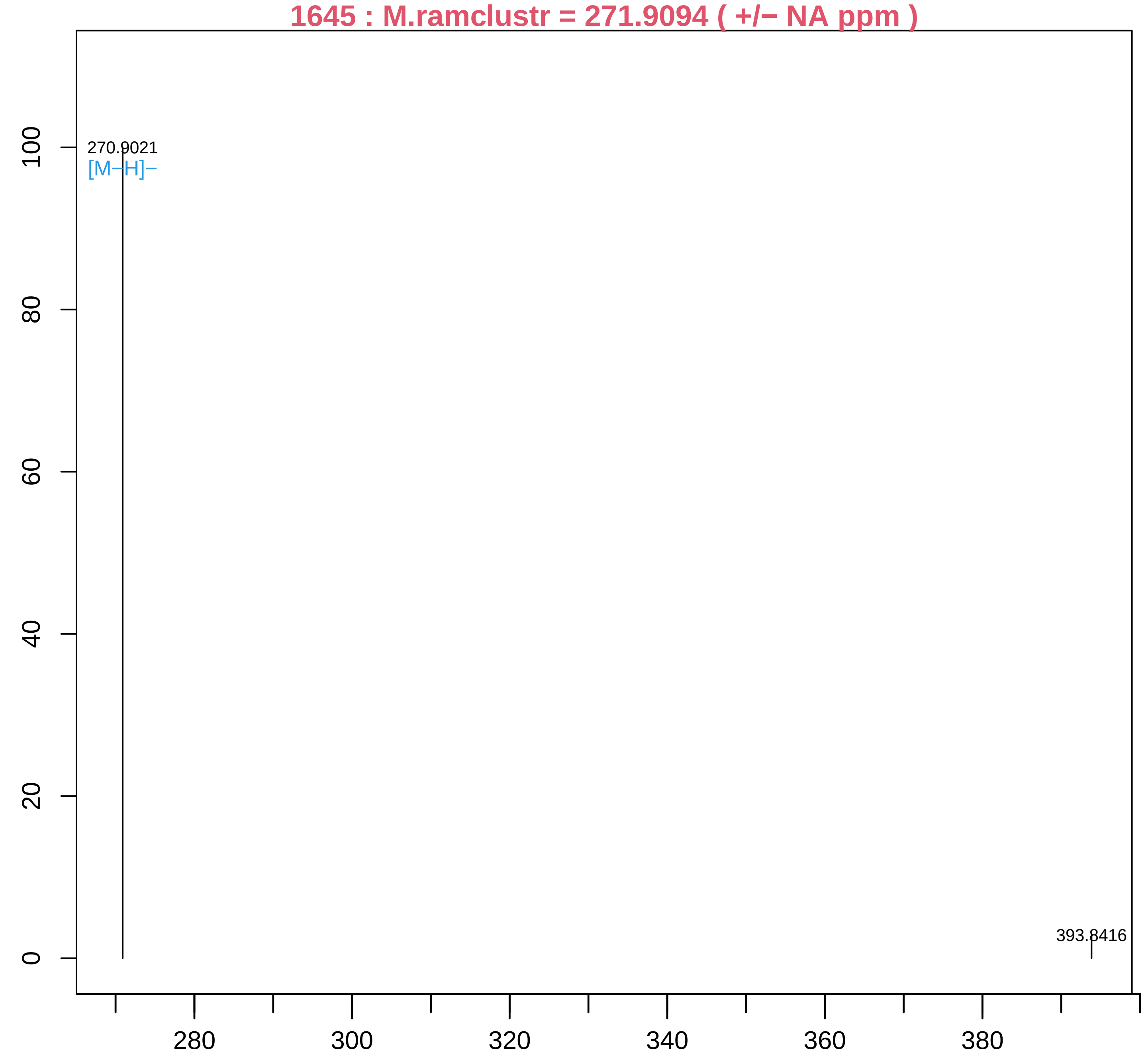
1645 : M.ramclustr = 271.9094 (+/− NA ppm)
270.9021
[M−H]−
393.8416
0
20
40
60
80
100
280
300
320
340
360
380

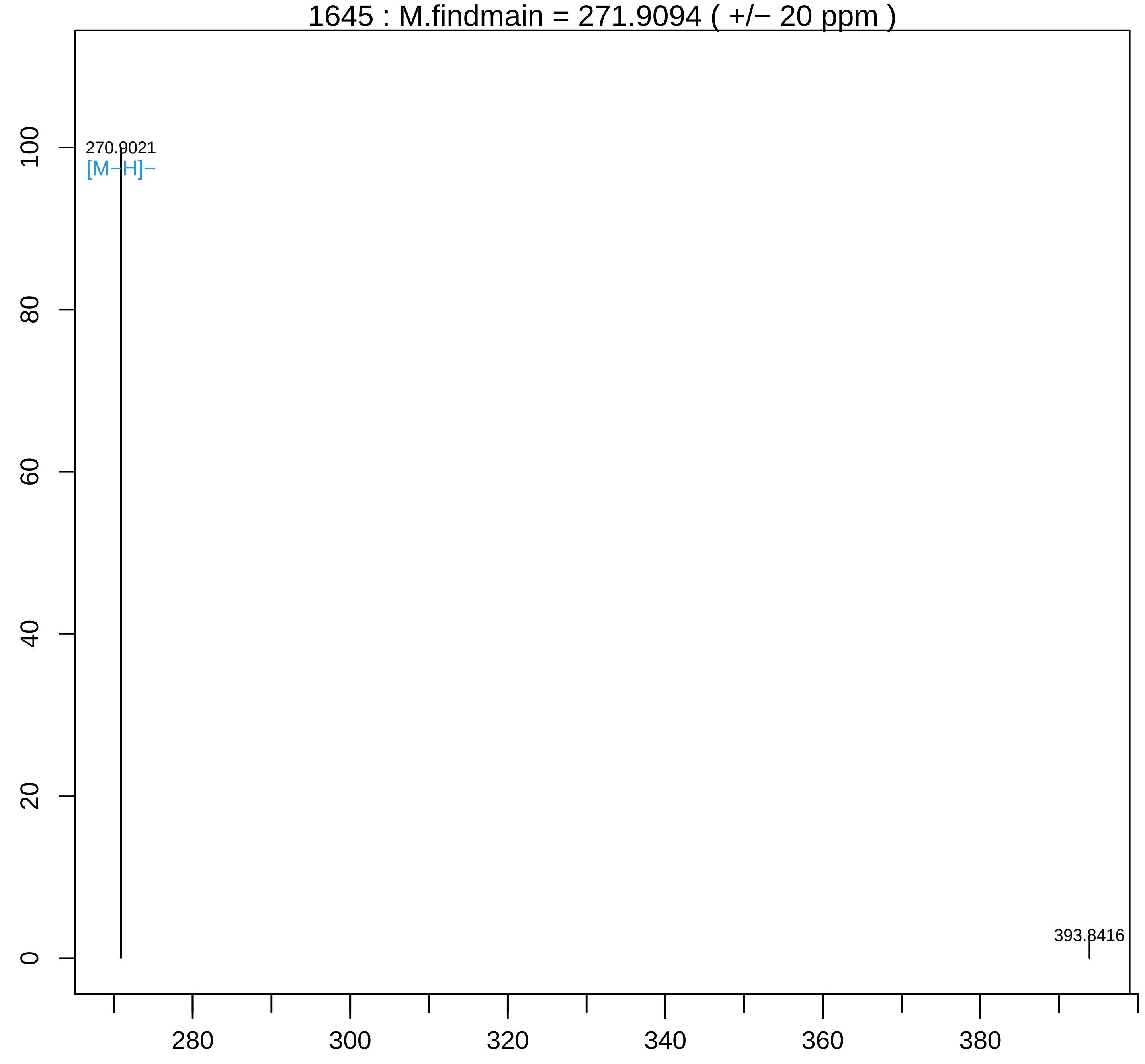
1645 : M.findmain = 271.9094 (+/− 20 ppm)
270.9021
[M−H]−
393.8416
0
20
40
60
80
100
280
300
320
340
360
380

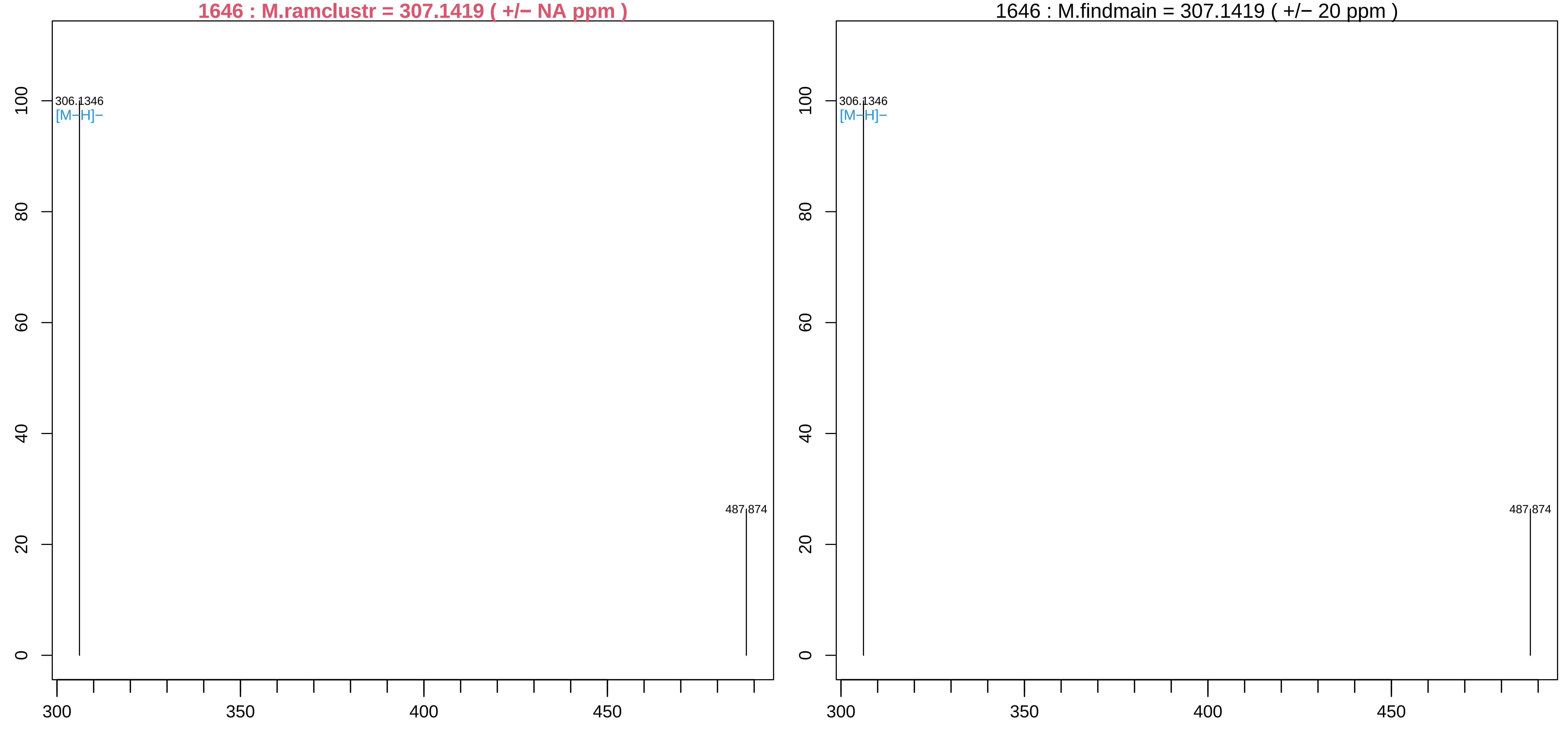

1646 : M.ramclustr = 307.1419 (+/− NA ppm)
306.1346
[M−H]−
487.874
1646 : M.findmain = 307.1419 (+/− 20 ppm)
306.1346
[M−H]−
487.874
0
20
40
60
80
100
300
350
400
450

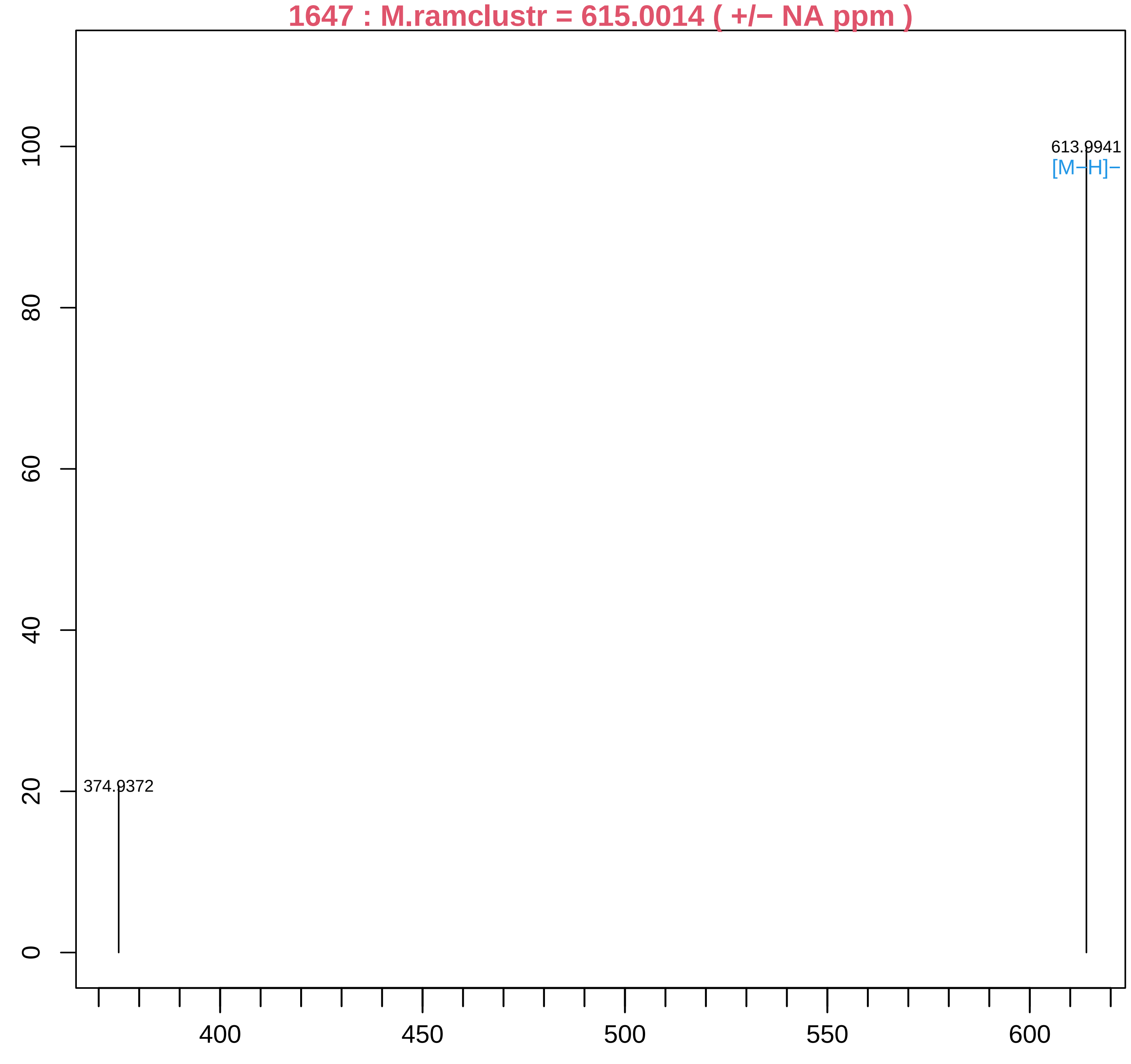
1647 : M.ramclustr = 615.0014 (+/− NA ppm)
613.9941
[M−H]−
374.9372
0
20
40
60
80
100
400
450
500
550
600

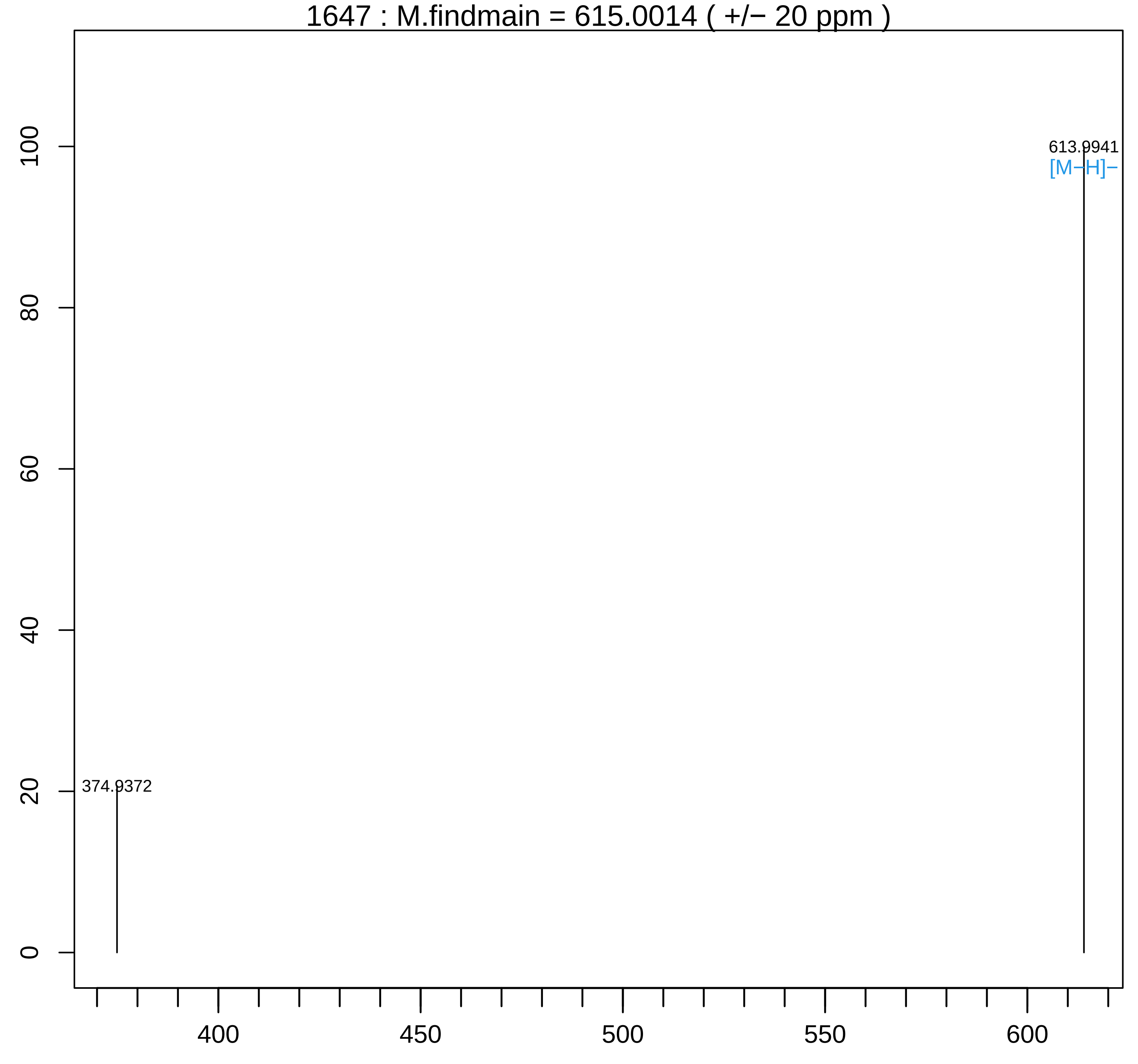
1647 : M.findmain = 615.0014 (+/− 20 ppm)
613.9941
[M−H]−
374.9372
0
20
40
60
80
100
400
450
500
550
600

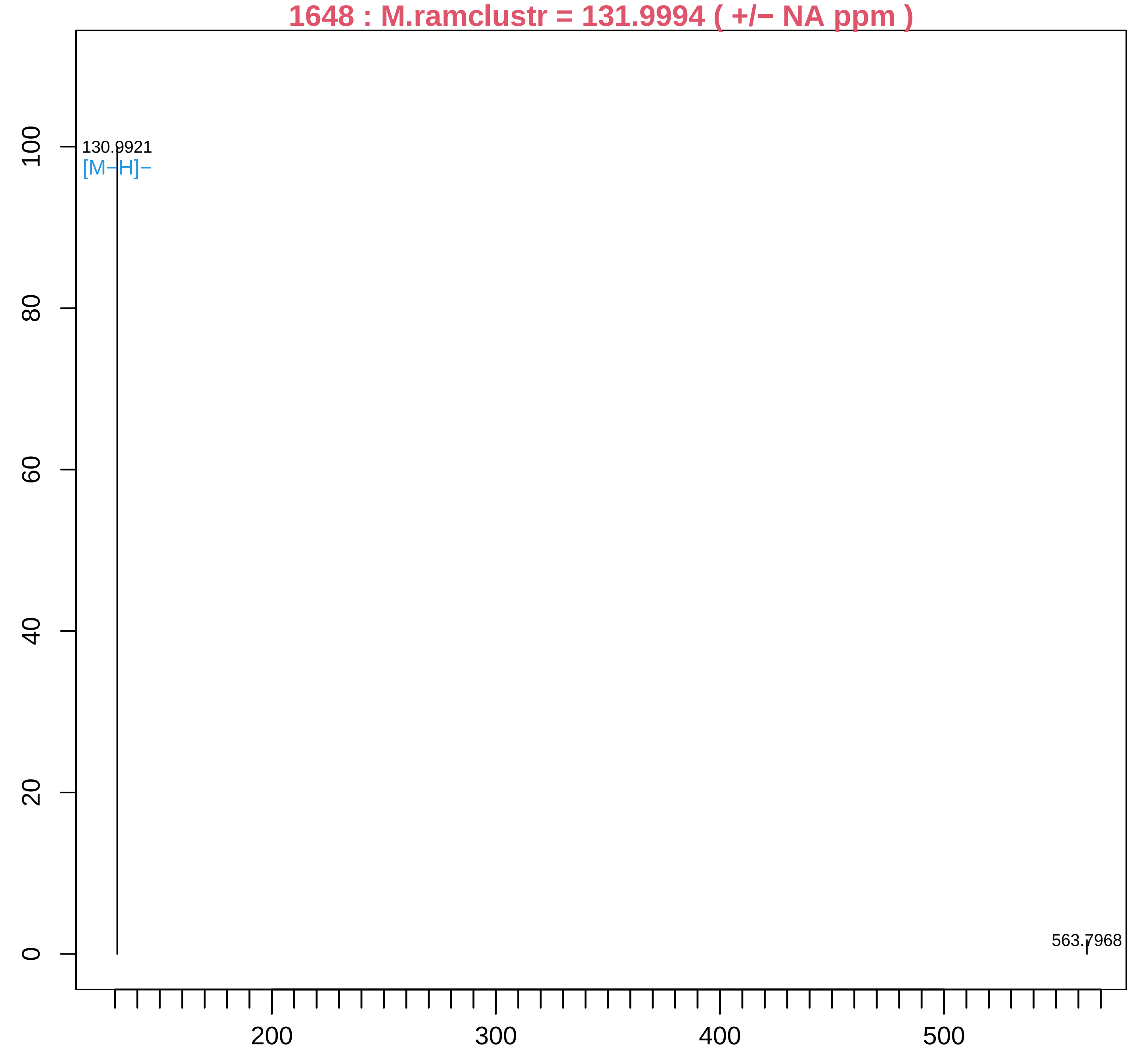
1648 : M.ramclustr = 131.9994 (+/− NA ppm)
130.9921
[M−H]−
563.7968
0
20
40
60
80
100
200
300
400
500

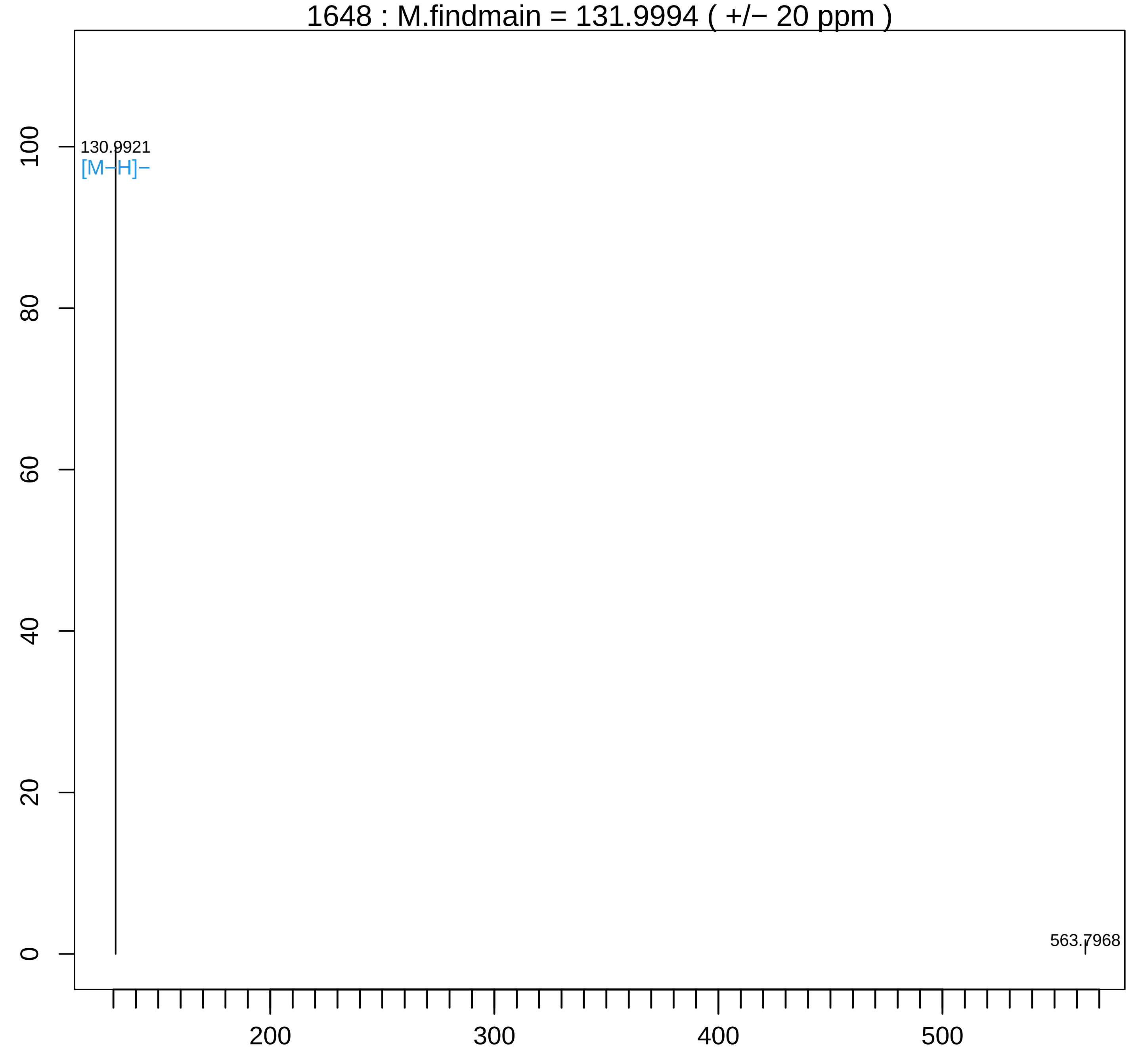
1648 : M.findmain = 131.9994 (+/− 20 ppm)
130.9921
[M−H]−
563.7968
0
20
40
60
80
100
200
300
400
500

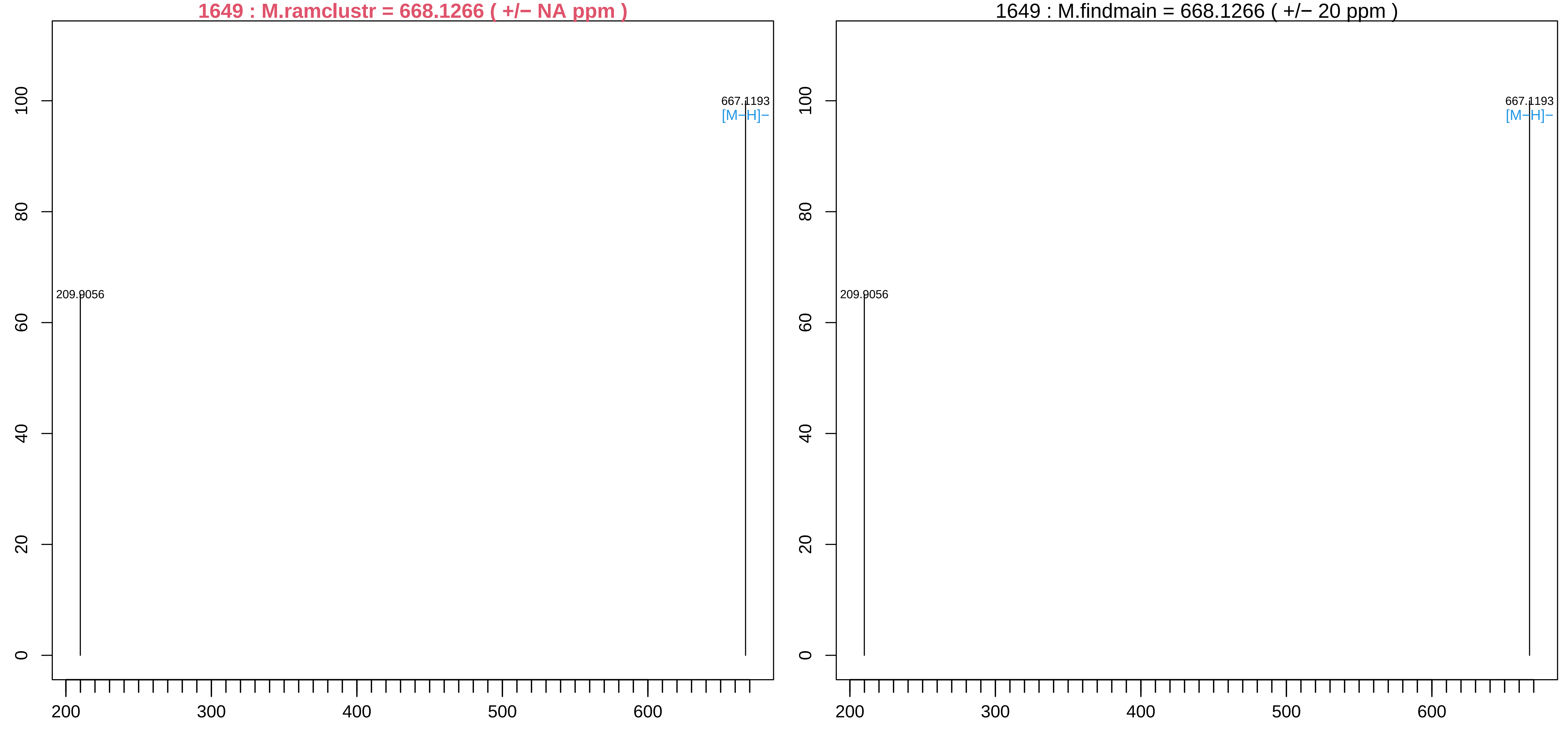
1649 : M.ramclustr = 668.1266 (+/− NA ppm)
667.1193
[M−H]−
209.9056
1649 : M.findmain = 668.1266 (+/− 20 ppm)
667.1193
[M−H]−
209.9056
0
20
40
60
80
100
200
300
400
500
600

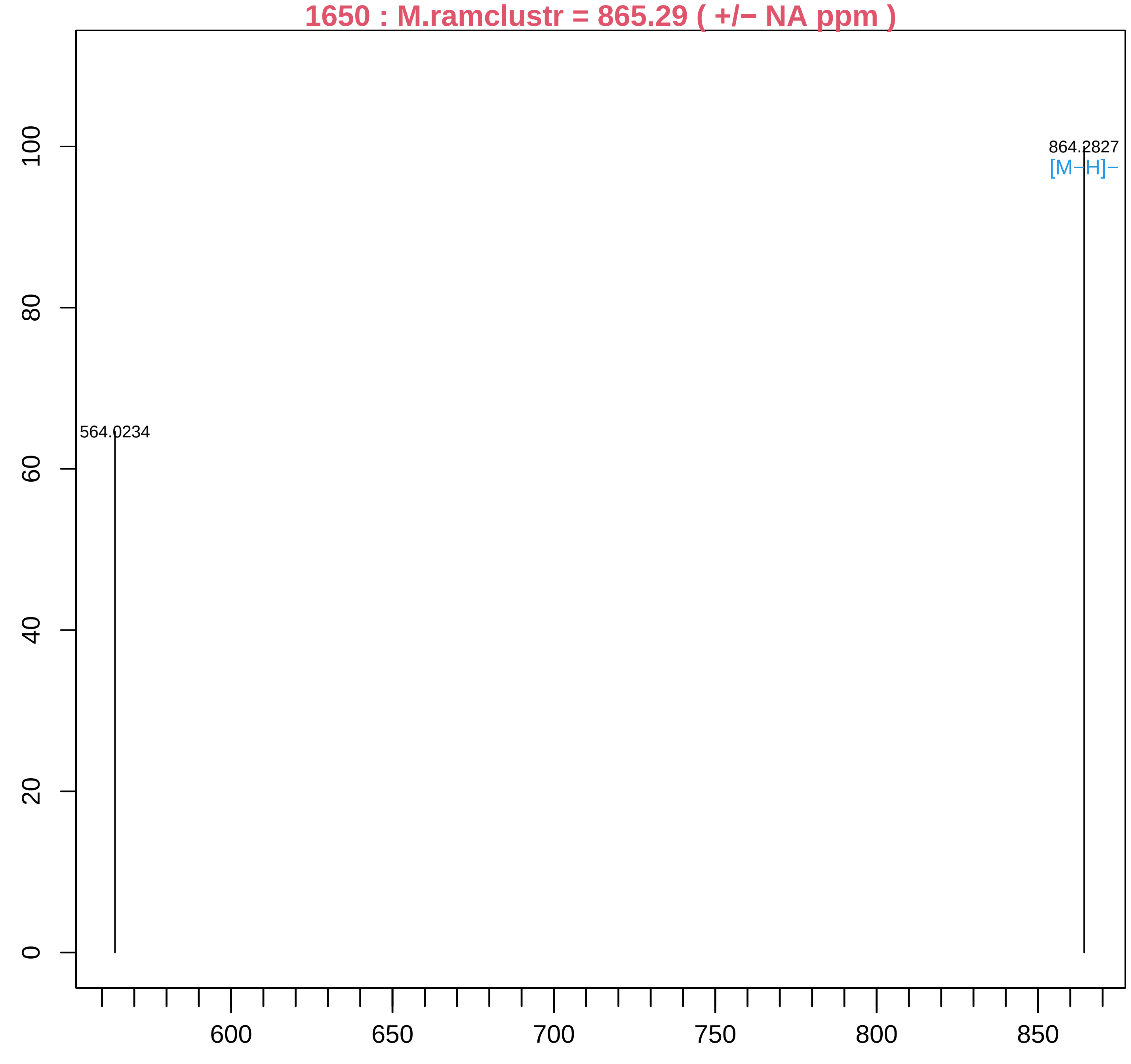
1650 : M.ramclustr = 865.29 (+/− NA ppm)
864.2827
[M−H]−
564.0234
0
20
40
60
80
100
600
650
700
750
800
850

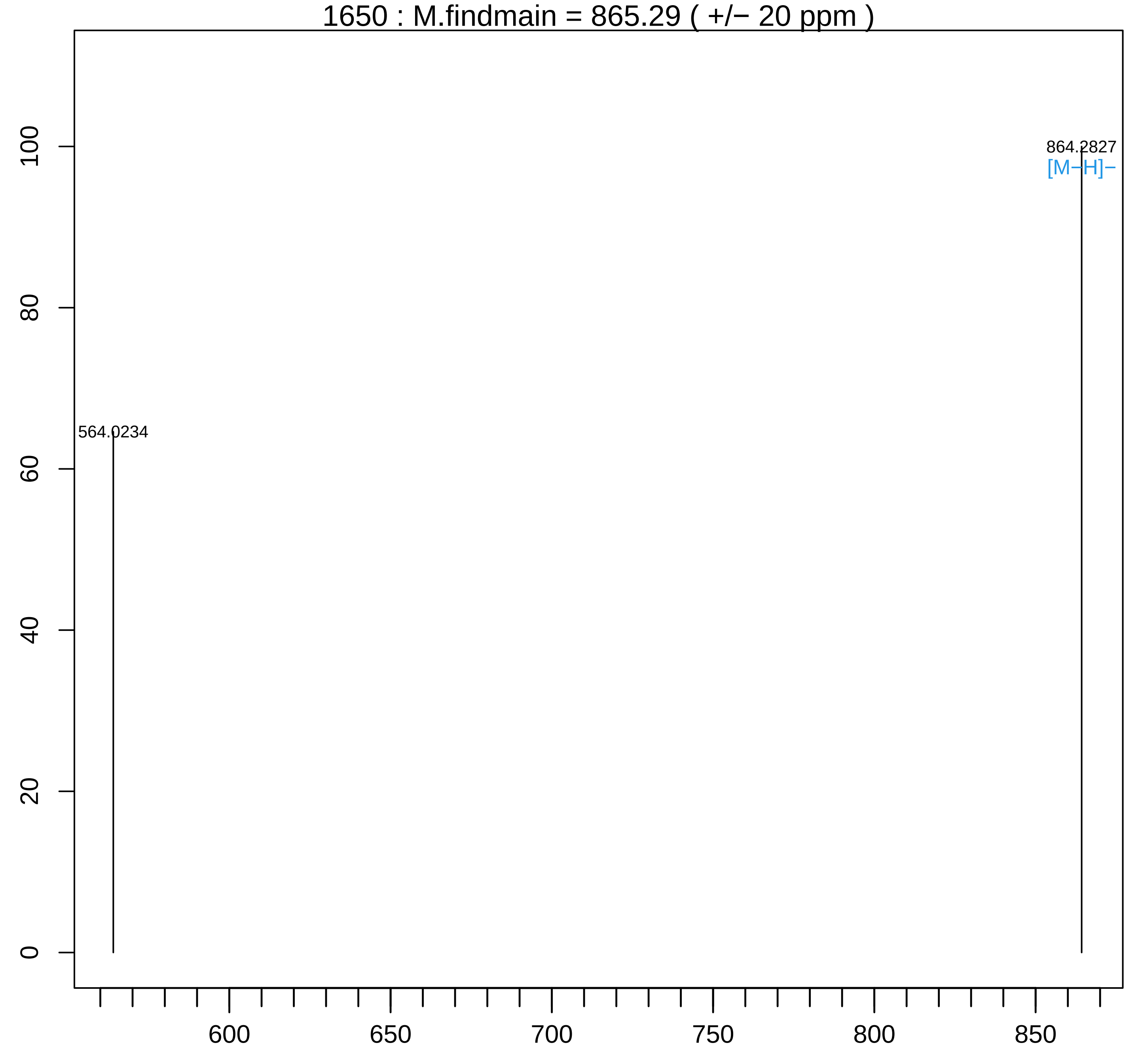
1650 : M.findmain = 865.29 (+/− 20 ppm)
864.2827
[M−H]−
564.0234
0
20
40
60
80
100
600
650
700
750
800
850

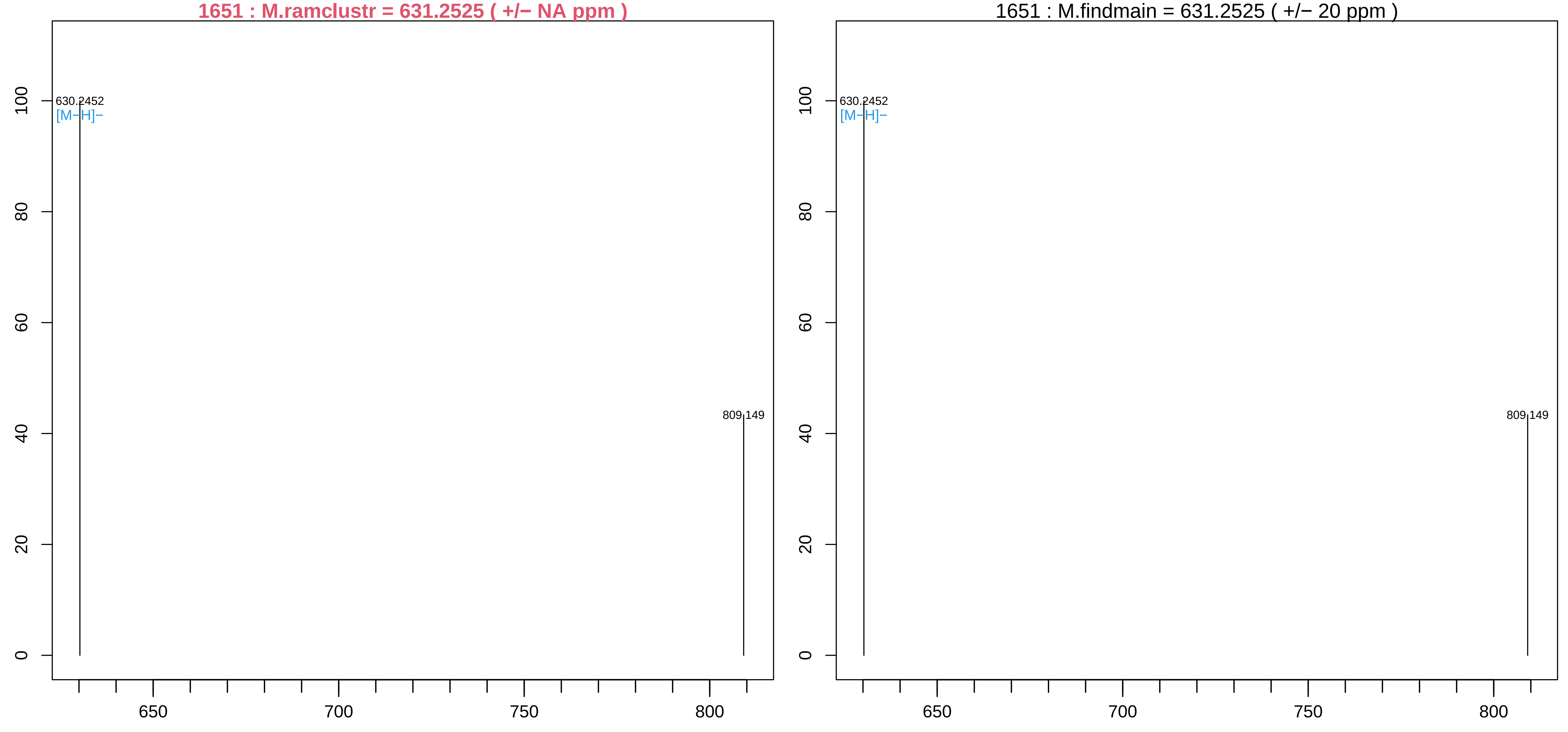
1651 : M.ramclustr = 631.2525 (+/− NA ppm)
630.2452
[M−H]−
809.149
1651 : M.findmain = 631.2525 (+/− 20 ppm)
630.2452
[M−H]−
809.149

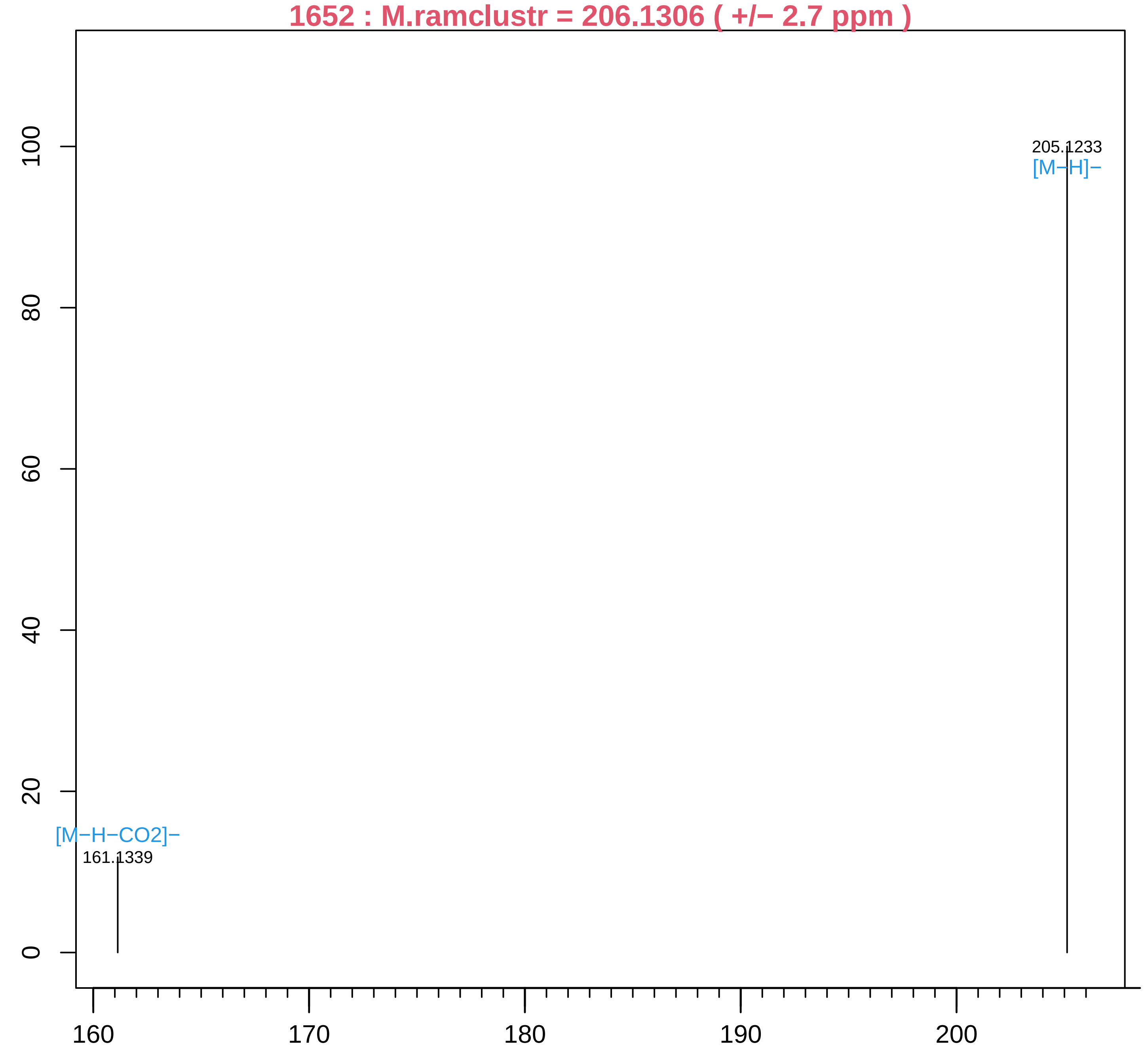
1652 : M.ramclustr = 206.1306 (+/− 2.7 ppm)
205.1233
[M−H]−
[M−H−CO2]−
161.1339
0
20
40
60
80
100
160
170
180
190
200

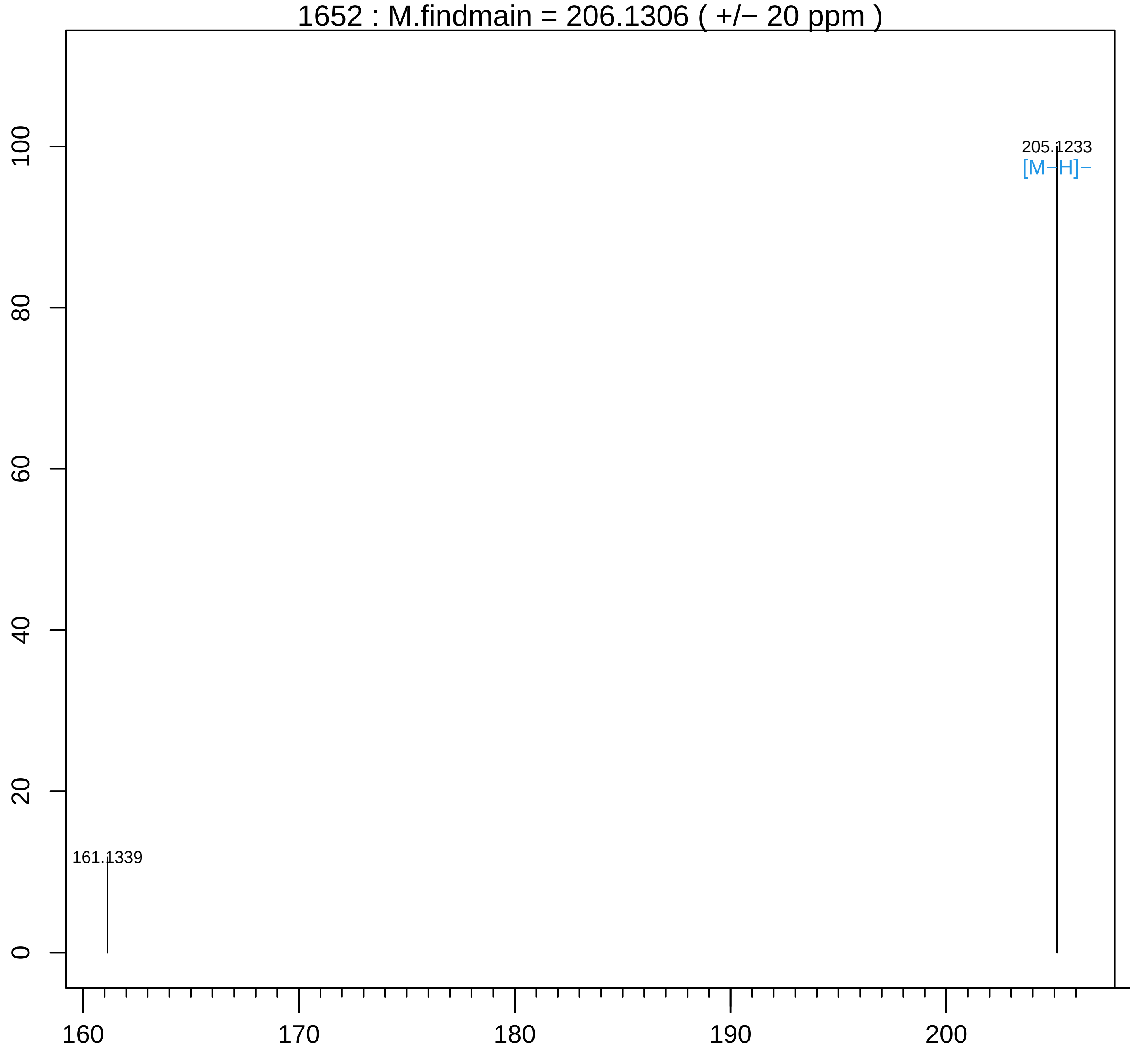
1652 : M.findmain = 206.1306 (+/− 20 ppm)
205.1233
[M−H]−
161.1339
0
20
40
60
80
100
160
170
180
190
200

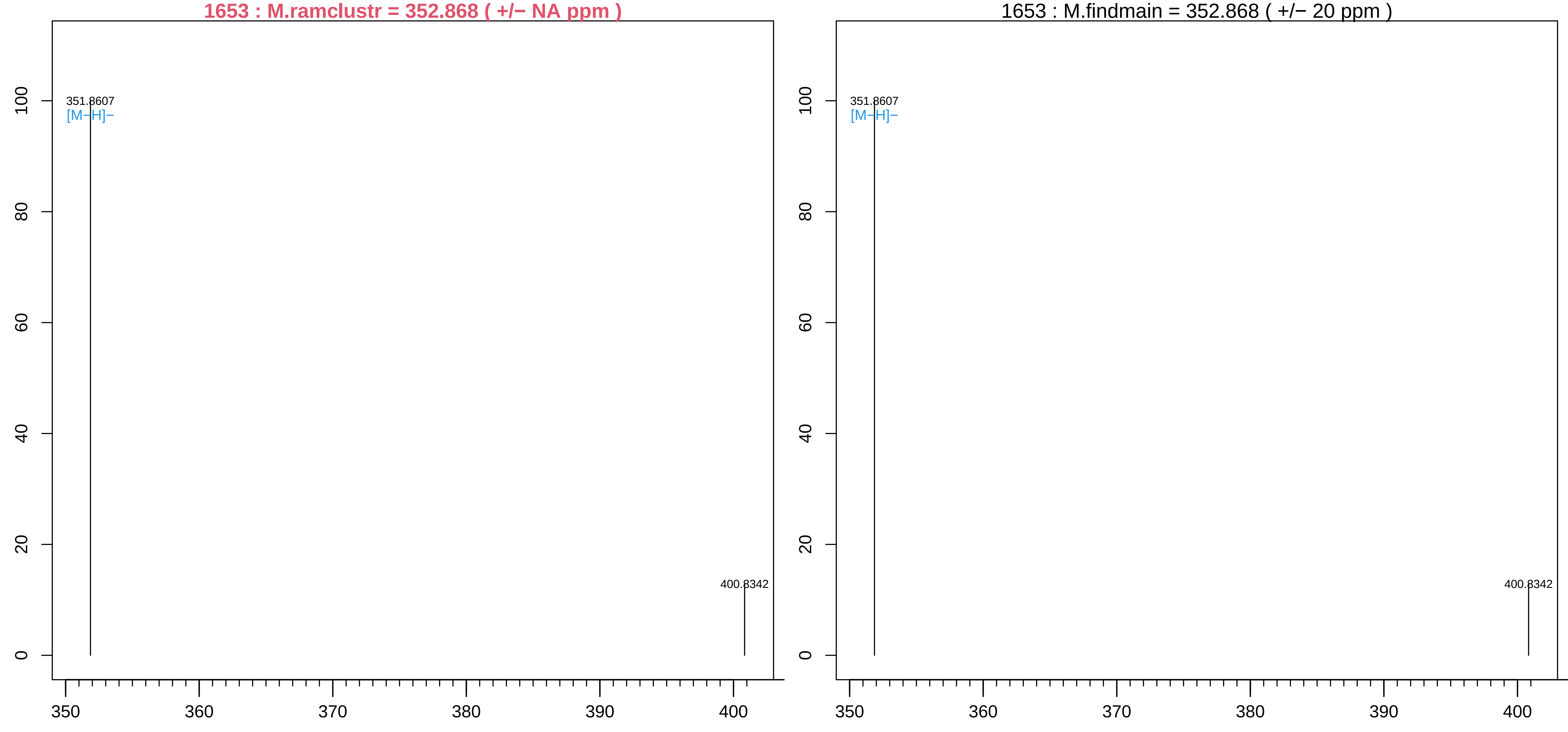
1653 : M.ramclustr = 352.868 (+/− NA ppm)
351.8607
[M−H]−
400.8342
1653 : M.findmain = 352.868 (+/− 20 ppm)
351.8607
[M−H]−
400.8342
0
20
40
60
80
100
350
360
370
380
390
400

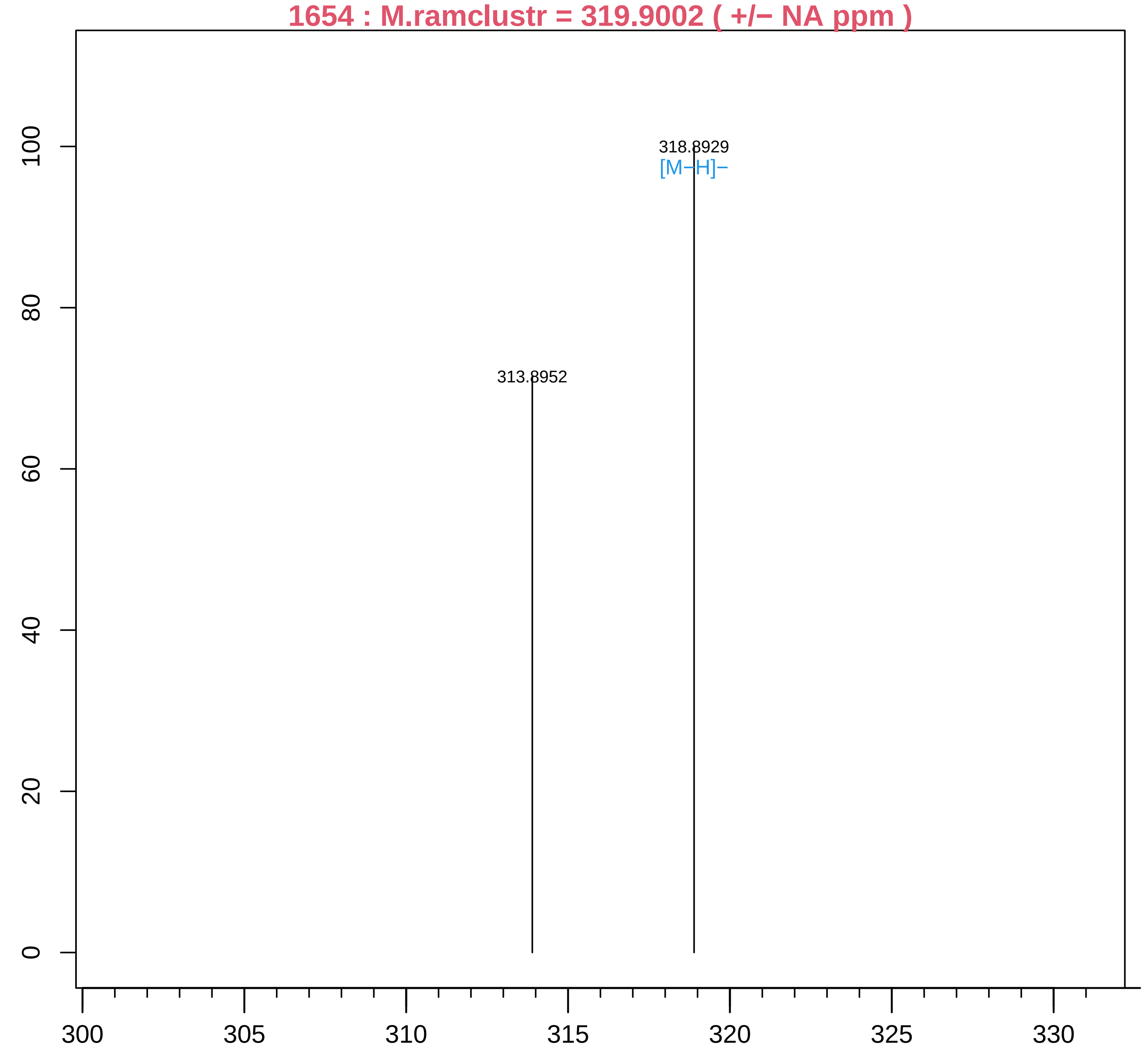
1654 : M.ramclustr = 319.9002 (+/− NA ppm)
318.8929
[M−H]−
313.8952
0
20
40
60
80
100
300
305
310
315
320
325
330

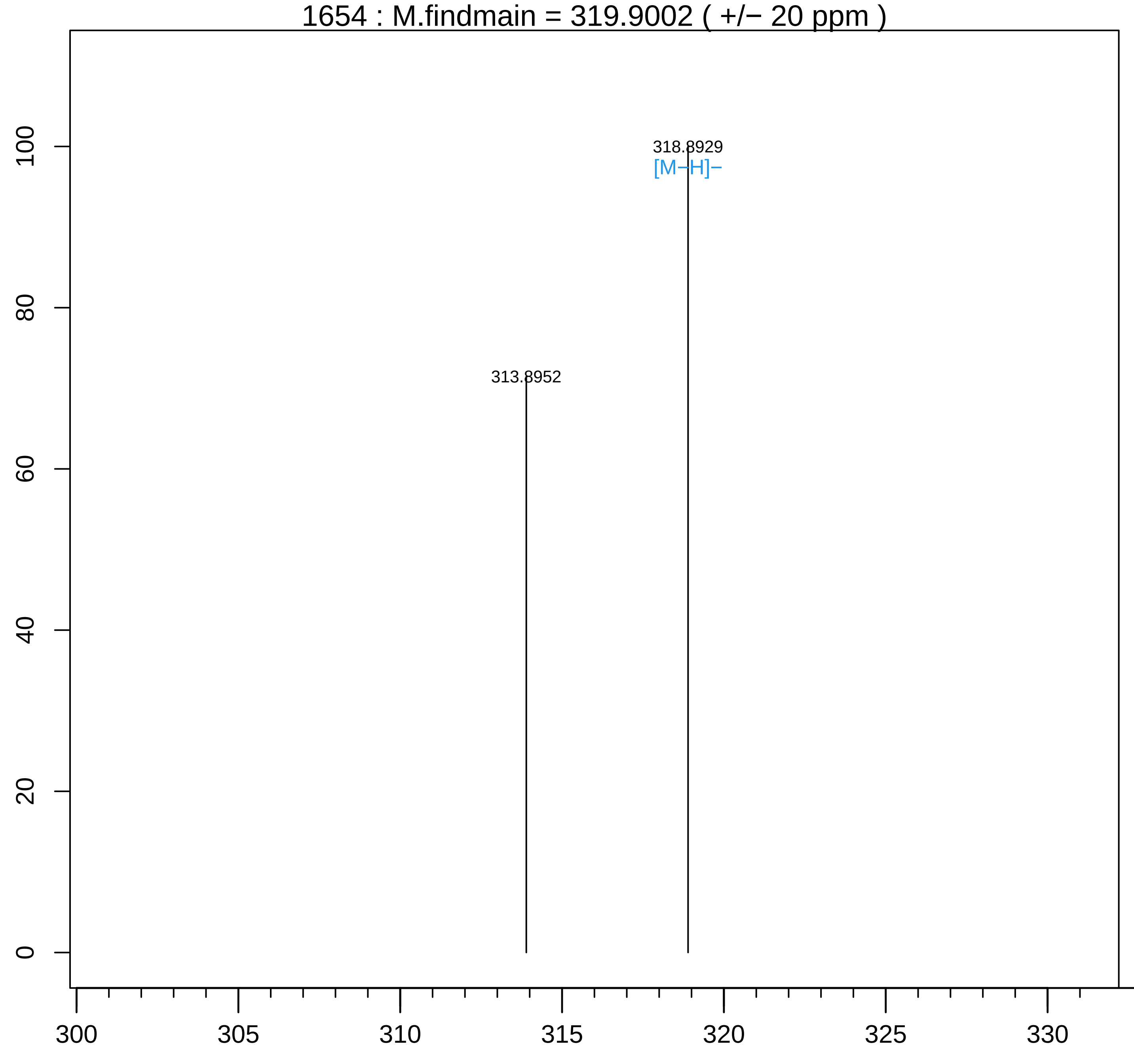
1654 : M.findmain = 319.9002 (+/− 20 ppm)
318.8929
[M−H]−
313.8952
0
20
40
60
80
100
300
305
310
315
320
325
330

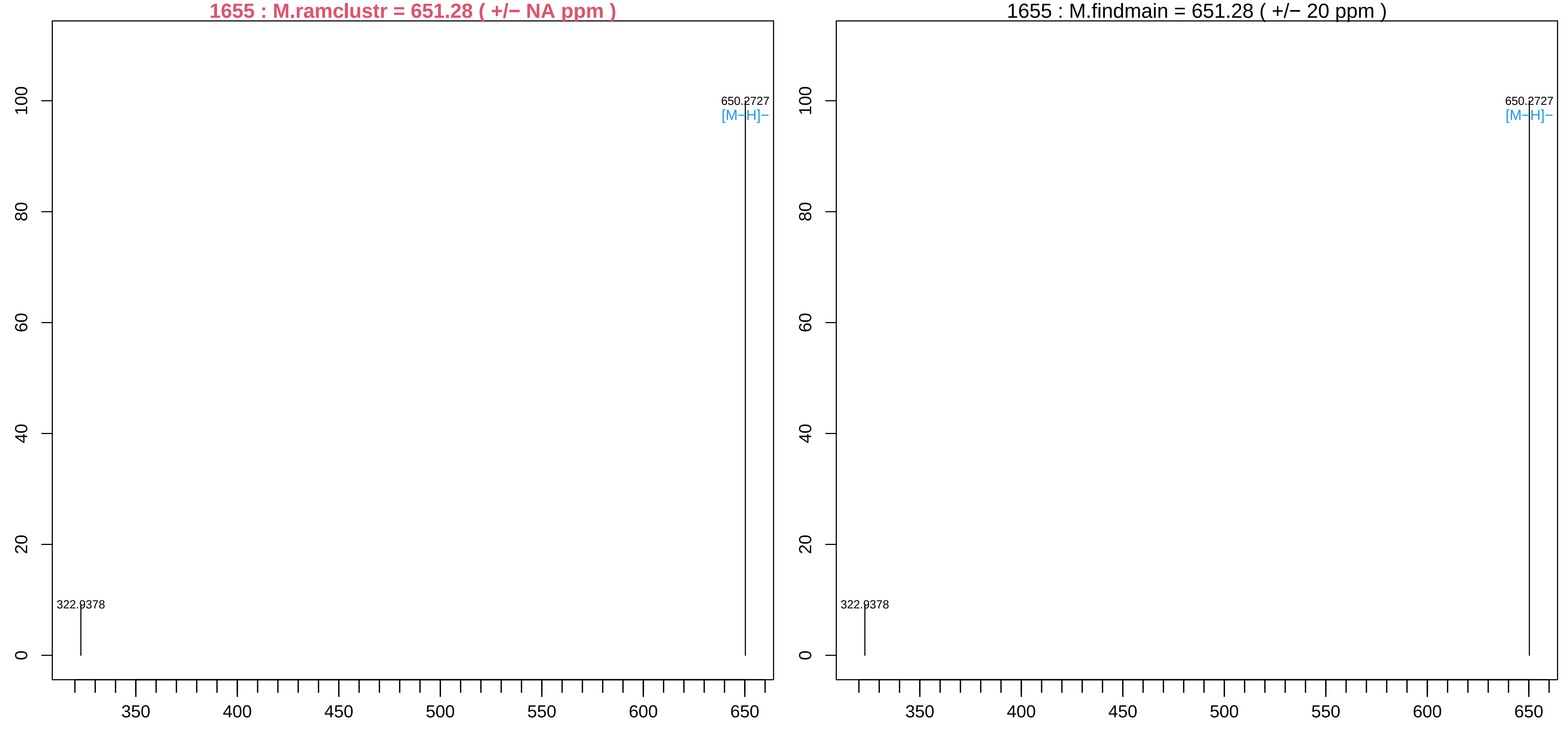

1655 : M.ramclustr = 651.28 (+/− NA ppm)
650.2727
[M−H]−
322.9378
1655 : M.findmain = 651.28 (+/− 20 ppm)
650.2727
[M−H]−
322.9378
0
20
40
60
80
100
350
400
450
500
550
600
650

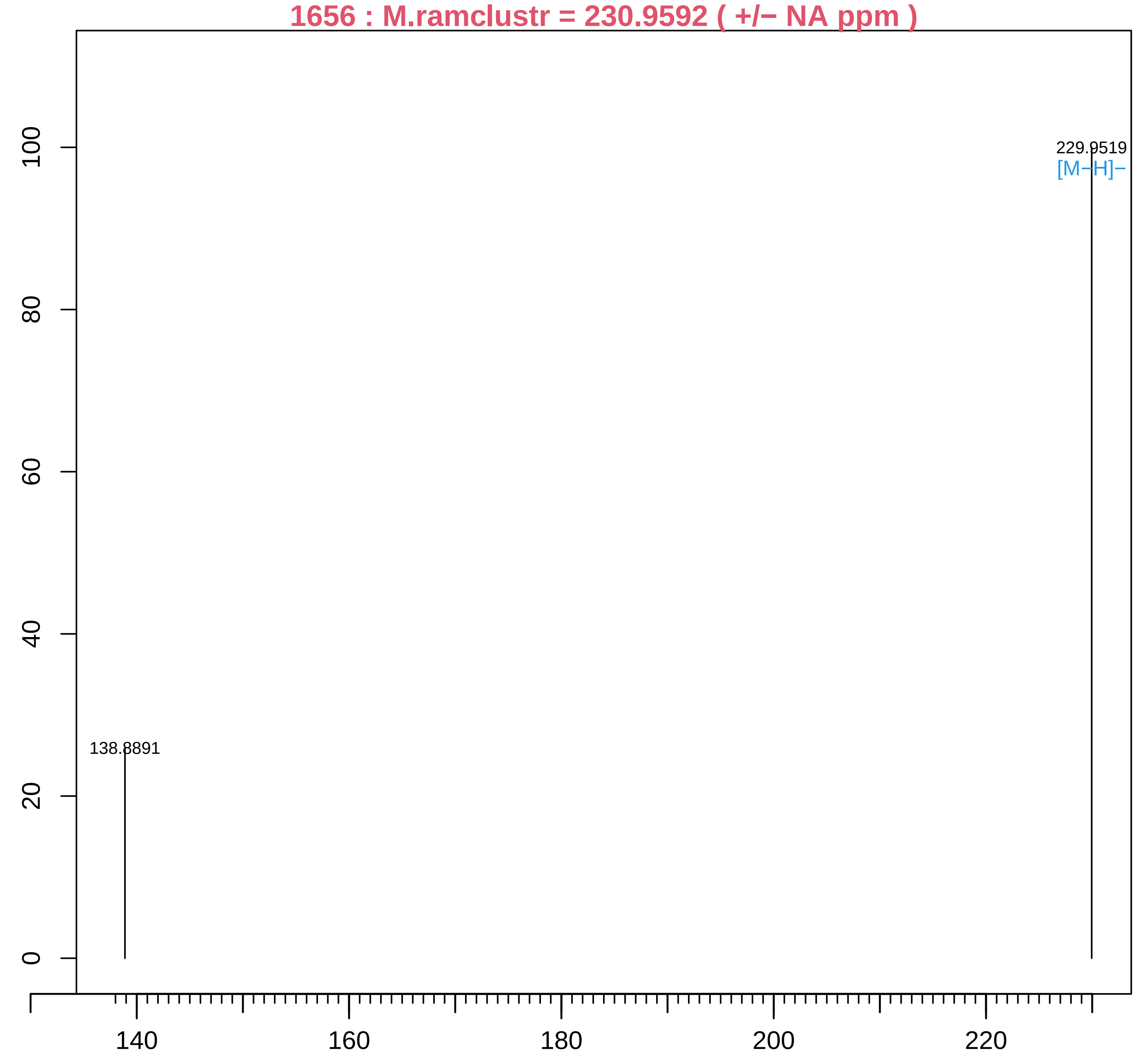
1656 : M.ramclustr = 230.9592 (+/− NA ppm)
229.9519
[M−H]−
138.8891
0
20
40
60
80
100
140
160
180
200
220

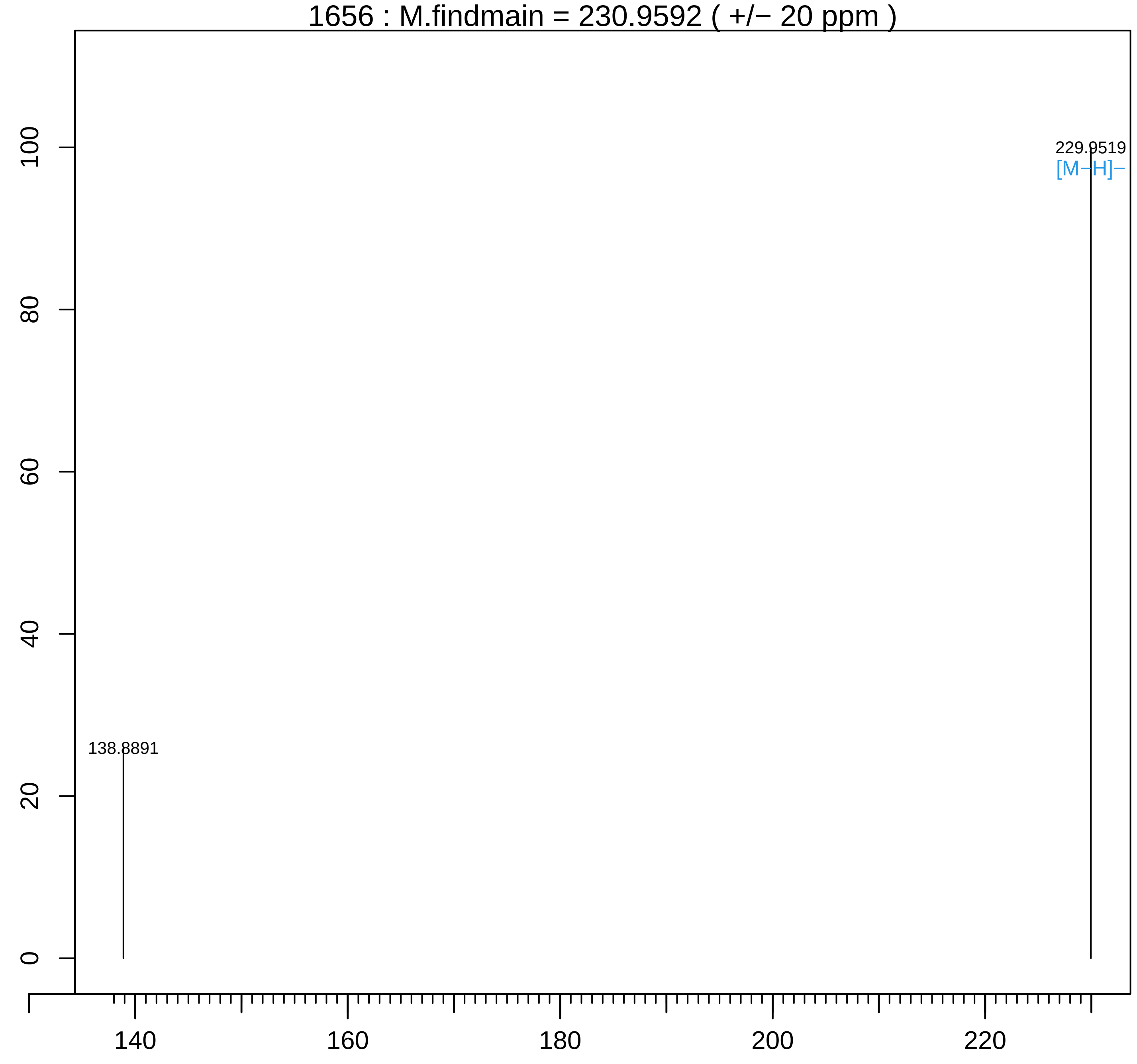
1656 : M.findmain = 230.9592 (+/− 20 ppm)
229.9519
[M−H]−
138.8891
0
20
40
60
80
100
140
160
180
200
220

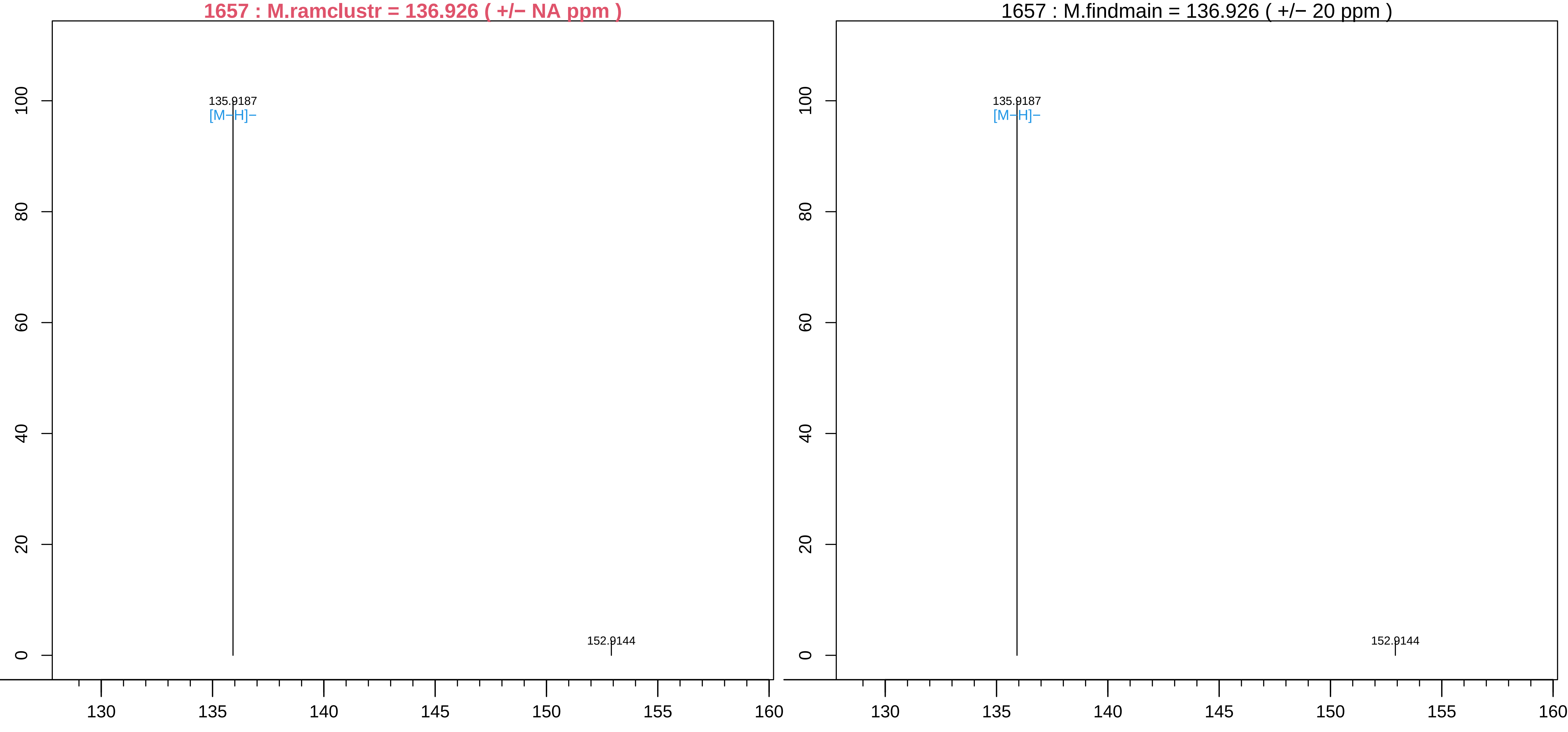

1657 : M.ramclustr = 136.926 (+/− NA ppm)
135.9187
[M−H]−
152.9144
1657 : M.findmain = 136.926 (+/− 20 ppm)
135.9187
[M−H]−
152.9144

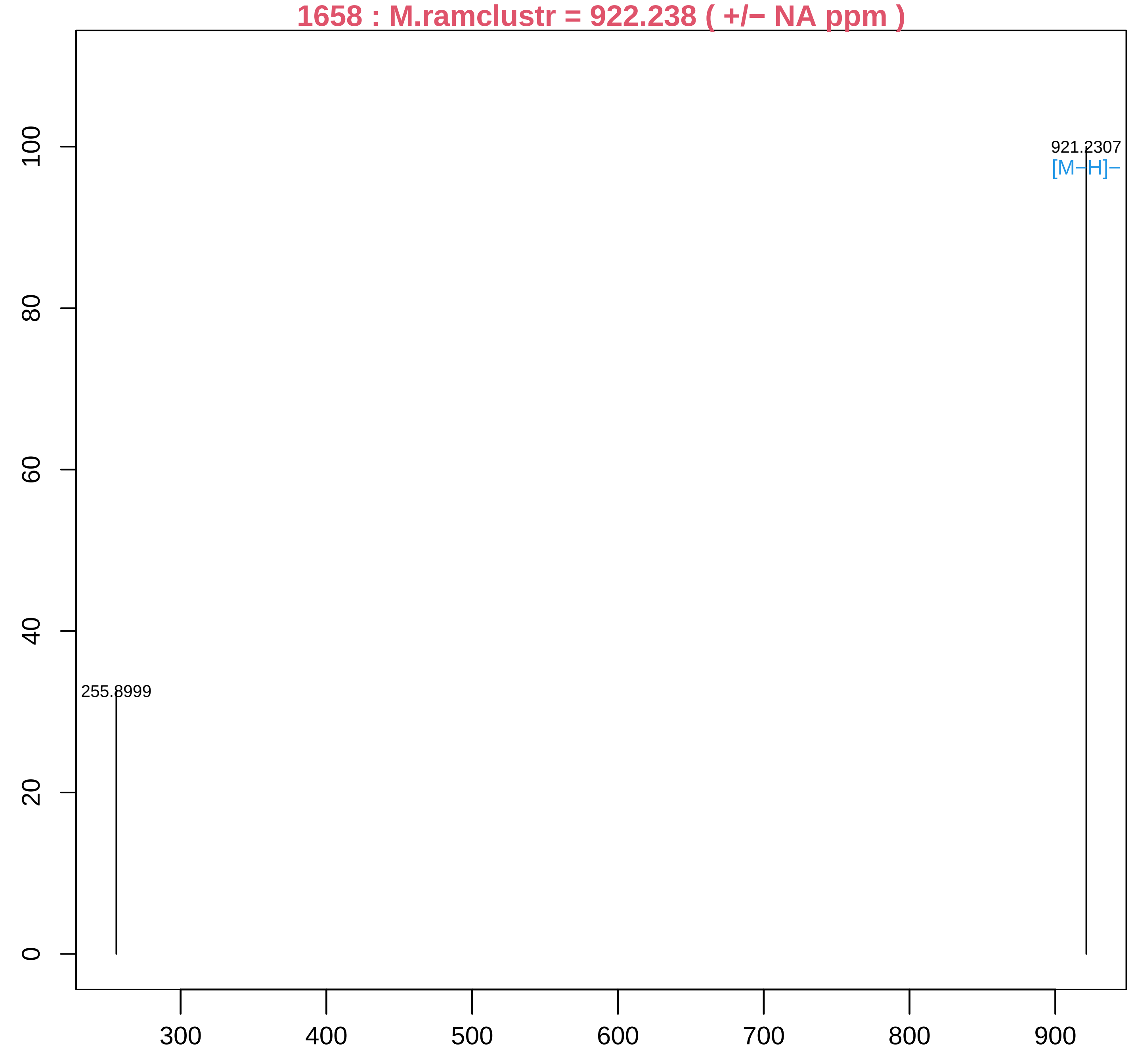
1658 : M.ramclustr = 922.238 (+/− NA ppm)
921.2307
[M−H]−
255.8999
0
20
40
60
80
100
300
400
500
600
700
800
900

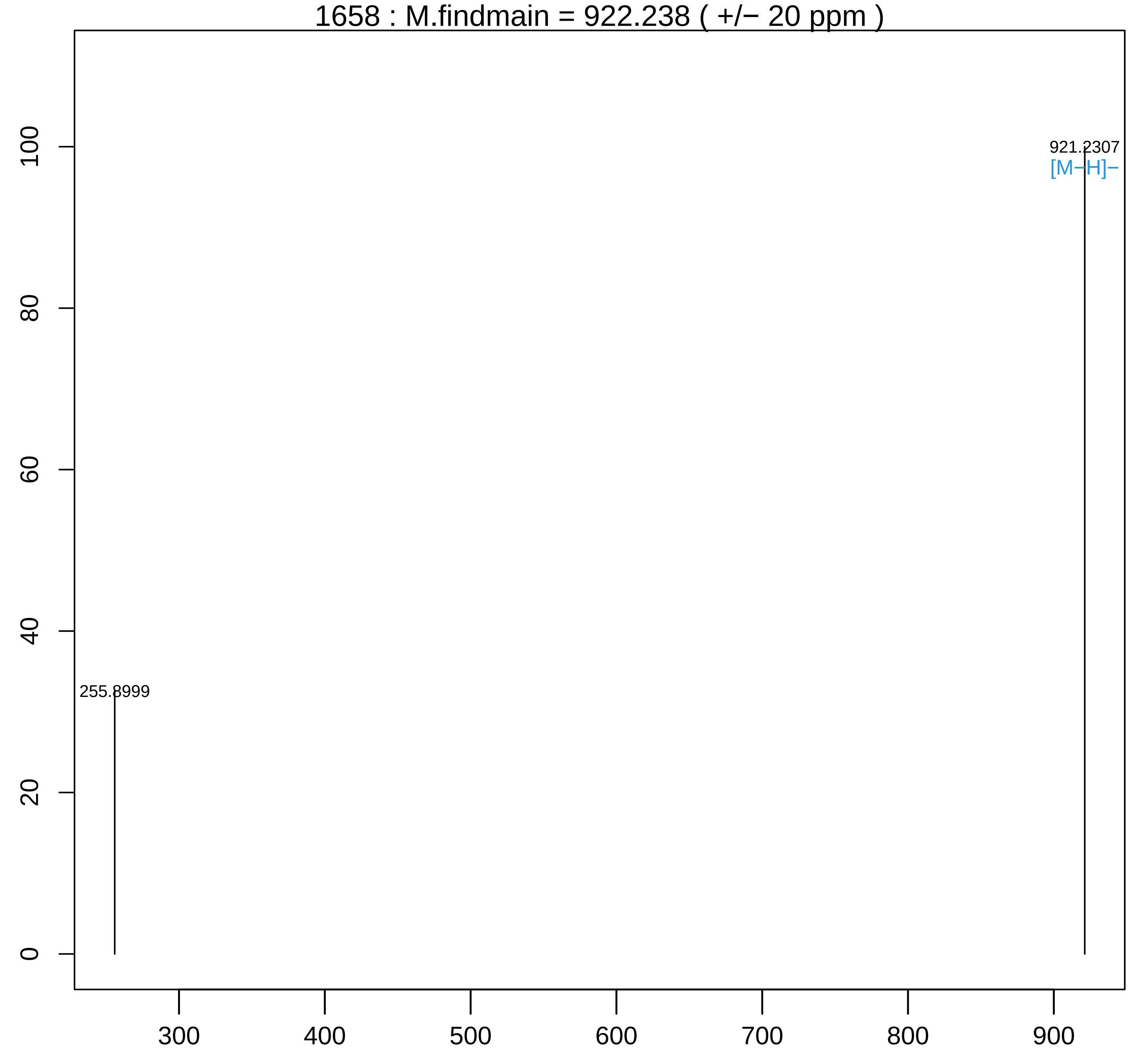
1658 : M.findmain = 922.238 (+/− 20 ppm)
921.2307
[M−H]−
255.8999
0
20
40
60
80
100
300
400
500
600
700
800
900

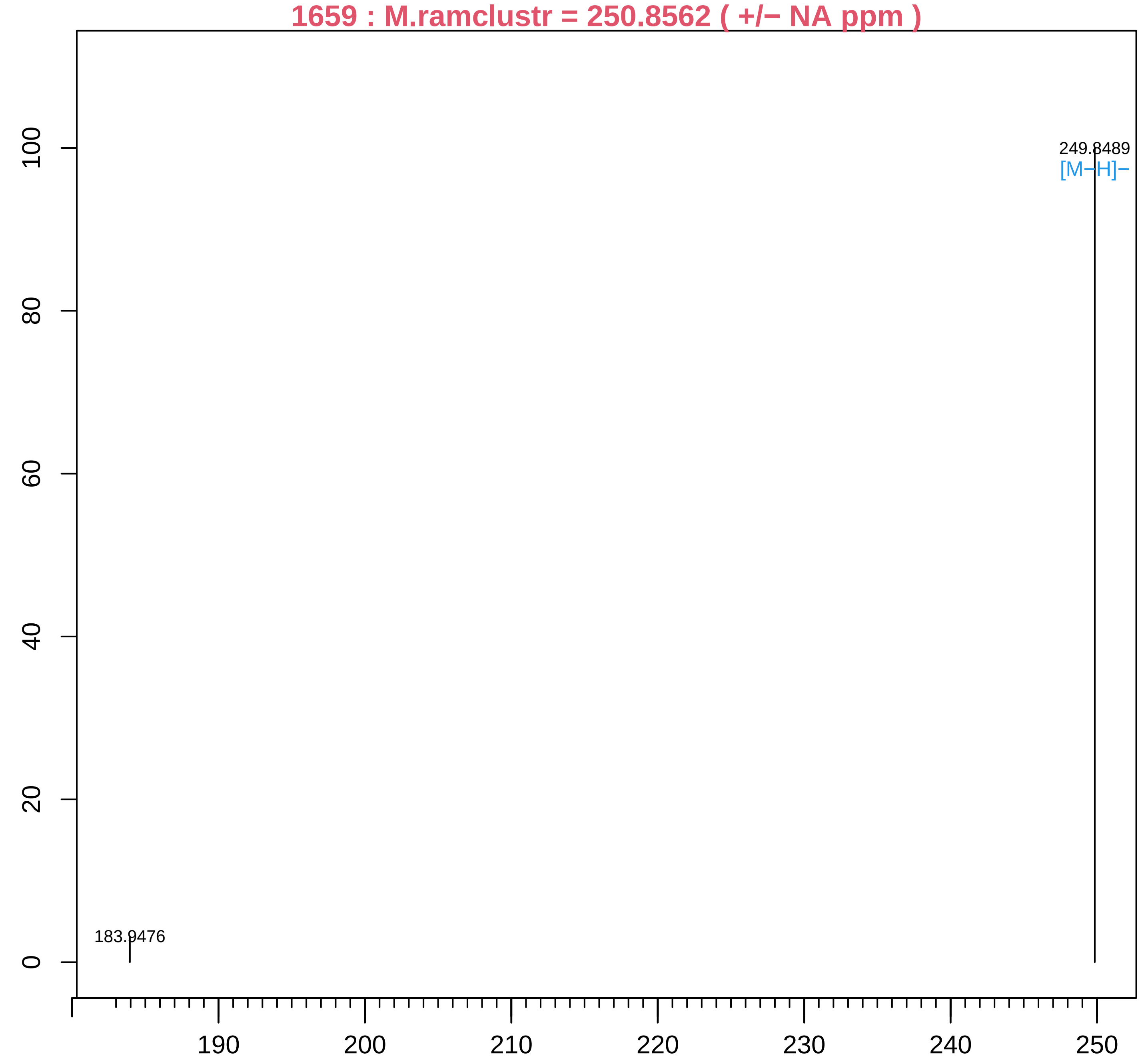
1659 : M.ramclustr = 250.8562 (+/− NA ppm)
249.8489
[M−H]−
183.9476
0
20
40
60
80
100
190
200
210
220
230
240
250

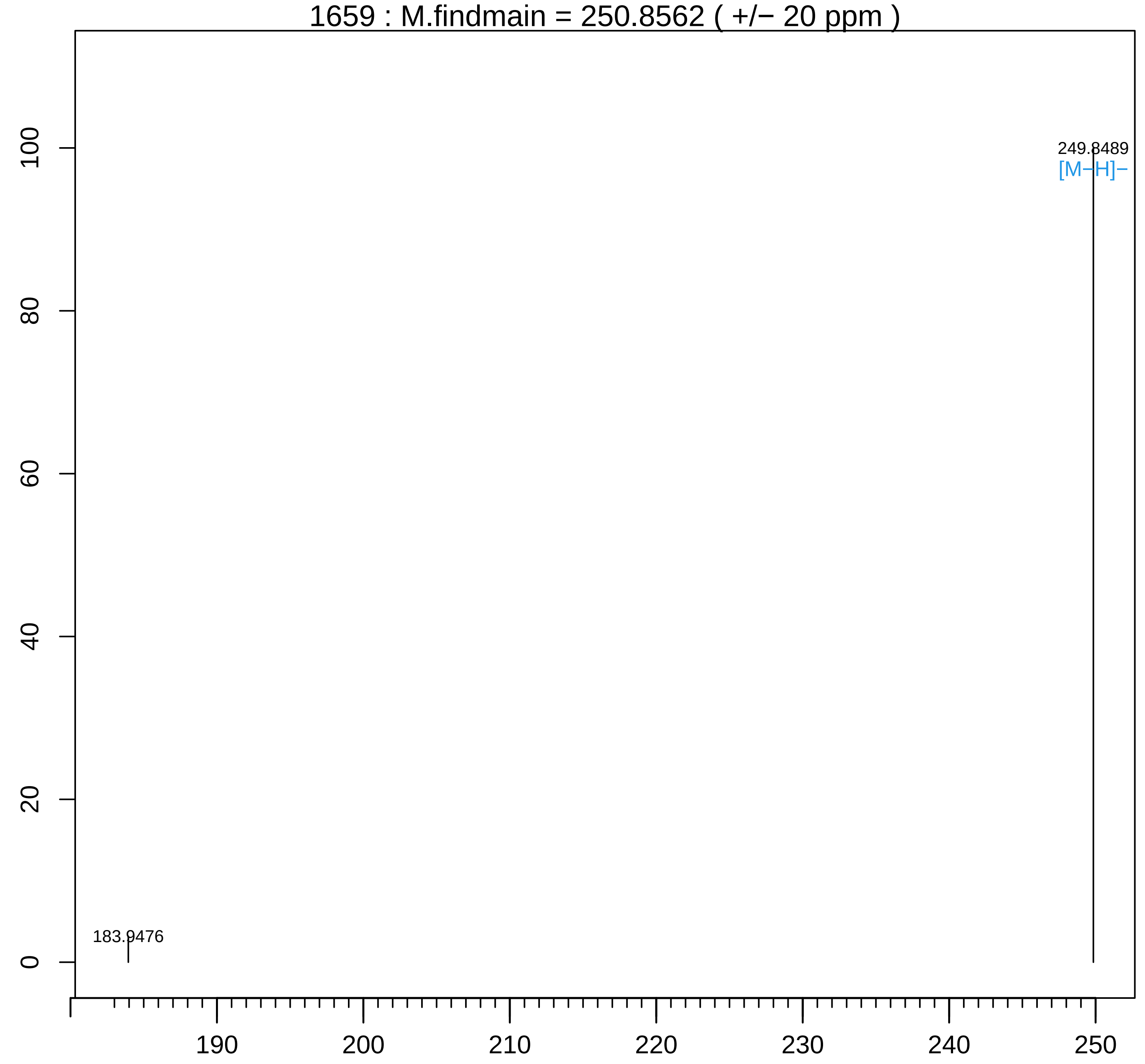
1659 : M.findmain = 250.8562 (+/− 20 ppm)
249.8489
[M−H]−
183.9476
0
20
40
60
80
100
190
200
210
220
230
240
250

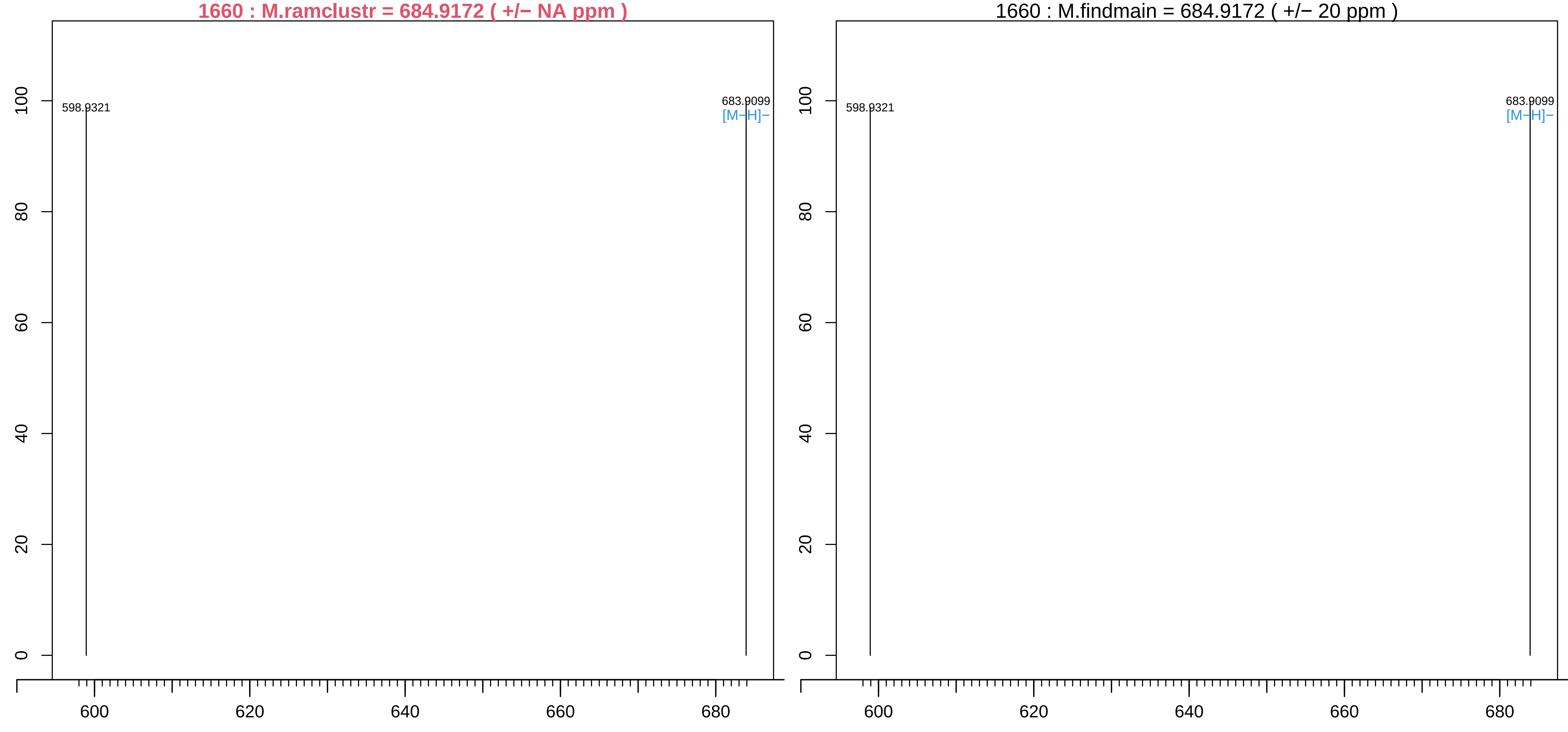
1660 : M.ramclustr = 684.9172 (+/− NA ppm)
598.9321
683.9099
[M−H]−
1660 : M.findmain = 684.9172 (+/− 20 ppm)
598.9321
683.9099
[M−H]−

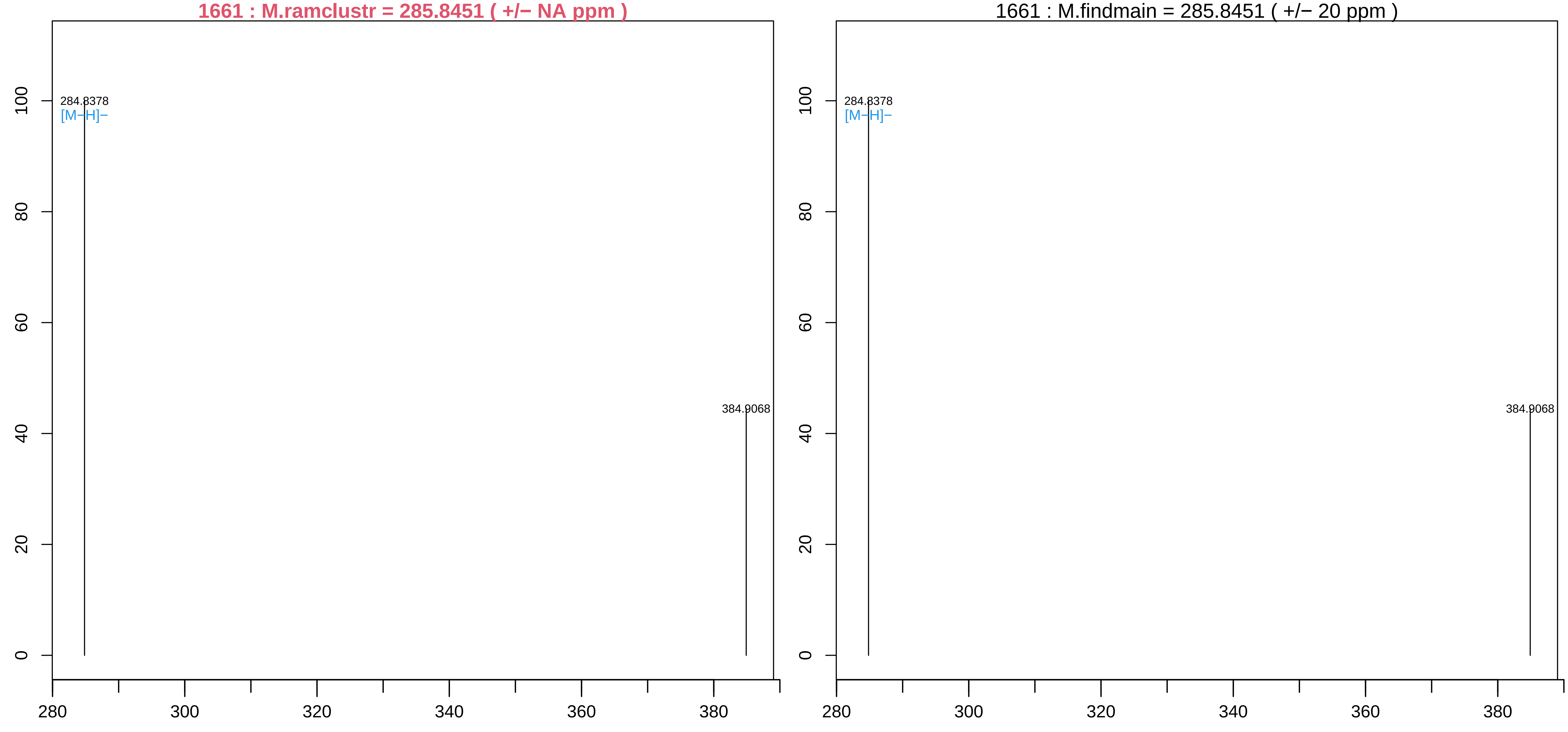

1661 : M.ramclustr = 285.8451 (+/− NA ppm)
284.8378
[M−H]−
384.9068
1661 : M.findmain = 285.8451 (+/− 20 ppm)
284.8378
[M−H]−
384.9068
0
20
40
60
80
100
280
300
320
340
360
380

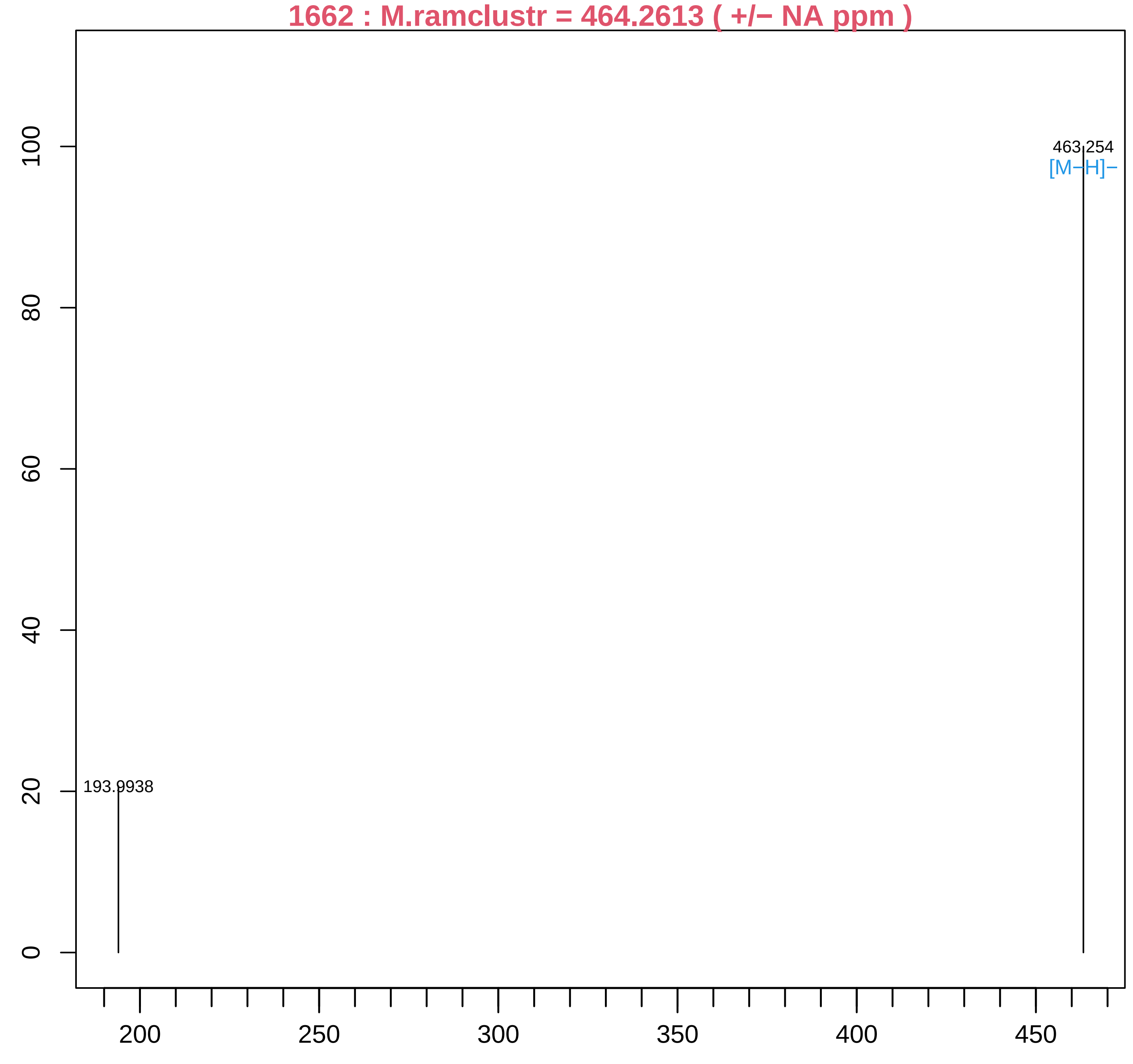
1662 : M.ramclustr = 464.2613 (+/− NA ppm)
463.254
[M−H]−
193.9938

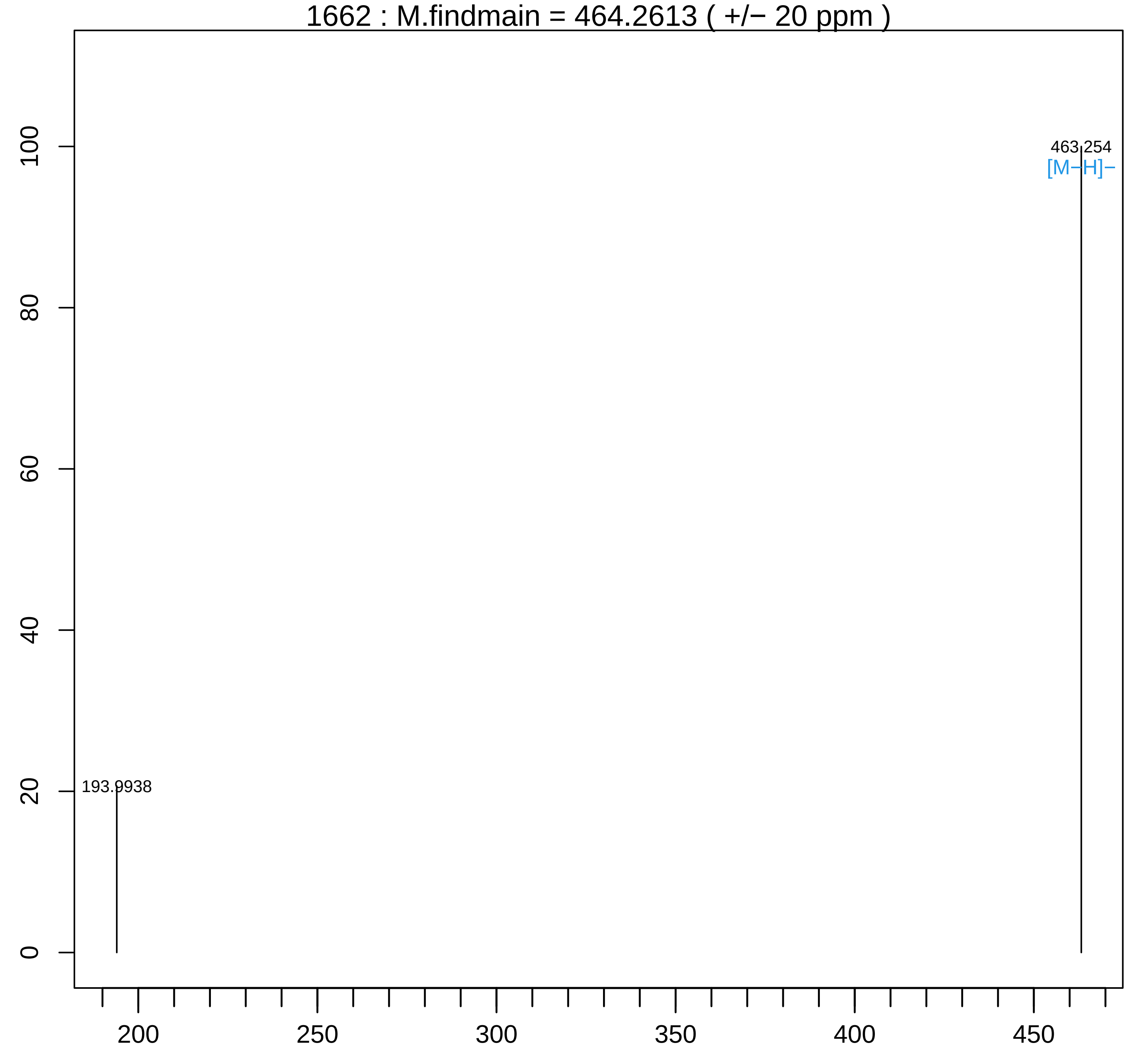
1662 : M.findmain = 464.2613 (+/− 20 ppm)
463.254
[M−H]−
193.9938

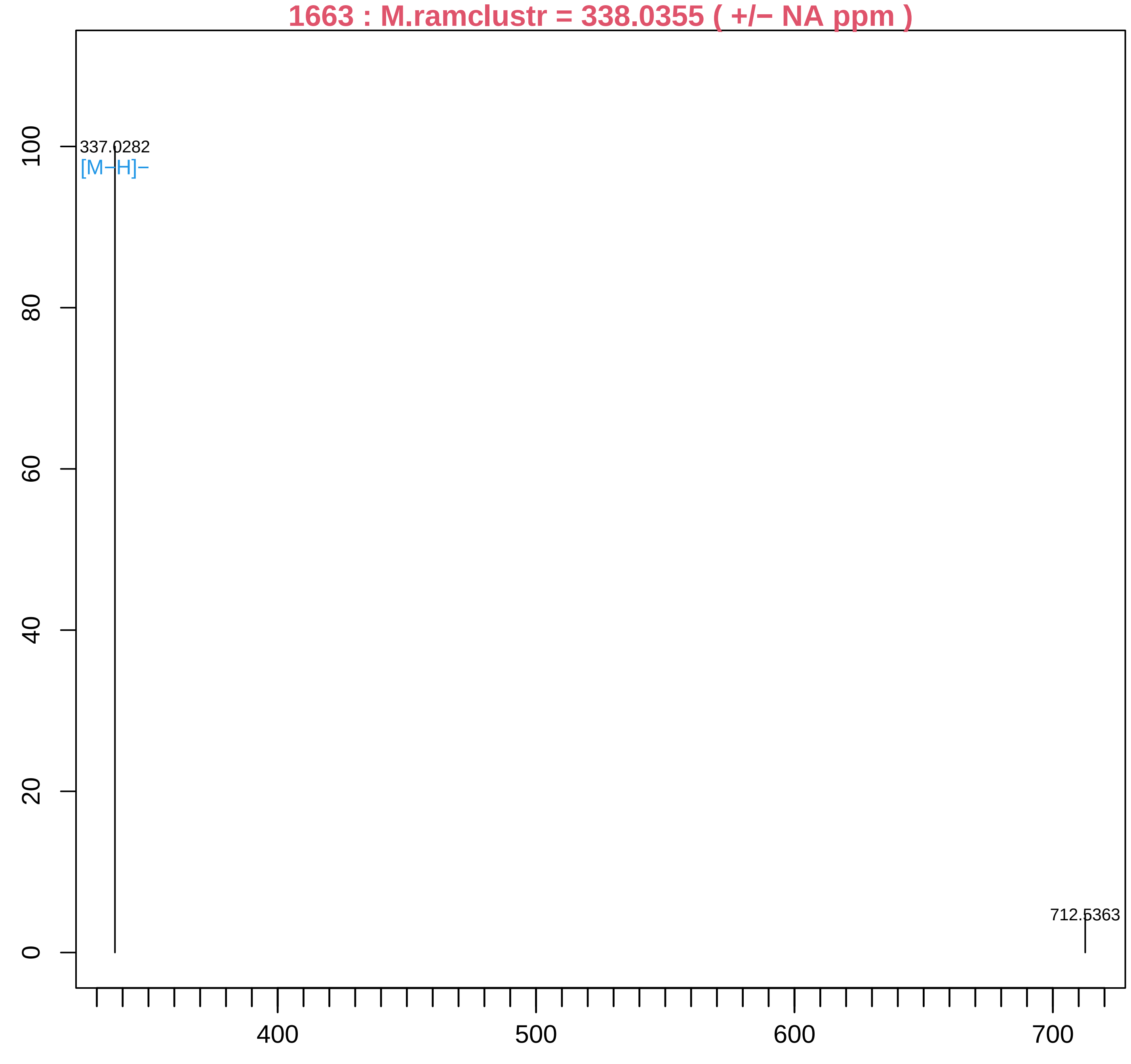

1663 : M.ramclustr = 338.0355 (+/− NA ppm)
337.0282
[M−H]−
712.5363
0
20
40
60
80
100
400
500
600
700

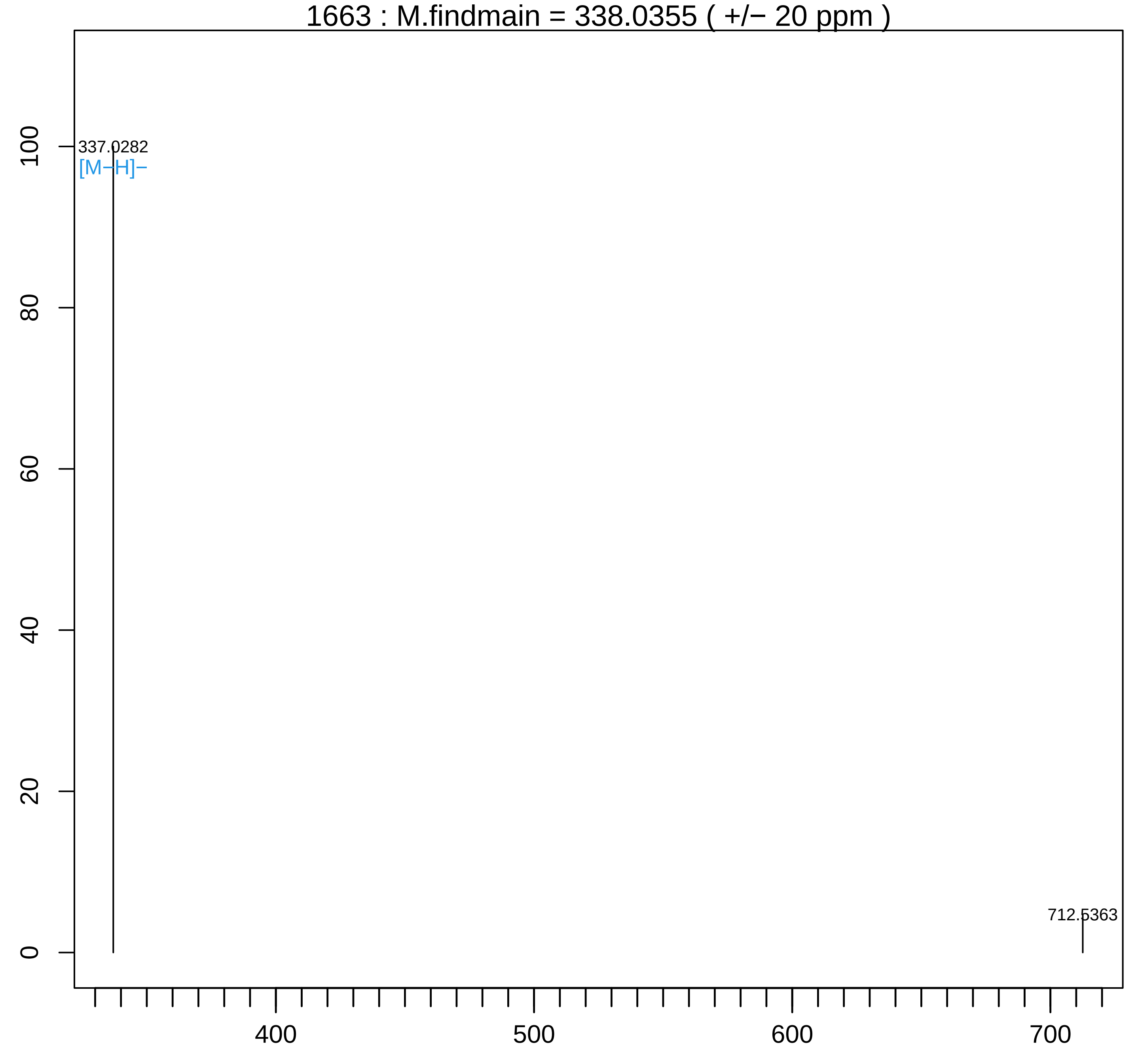

1663 : M.findmain = 338.0355 (+/− 20 ppm)
337.0282
[M−H]−
712.5363
0
20
40
60
80
100
400
500
600
700

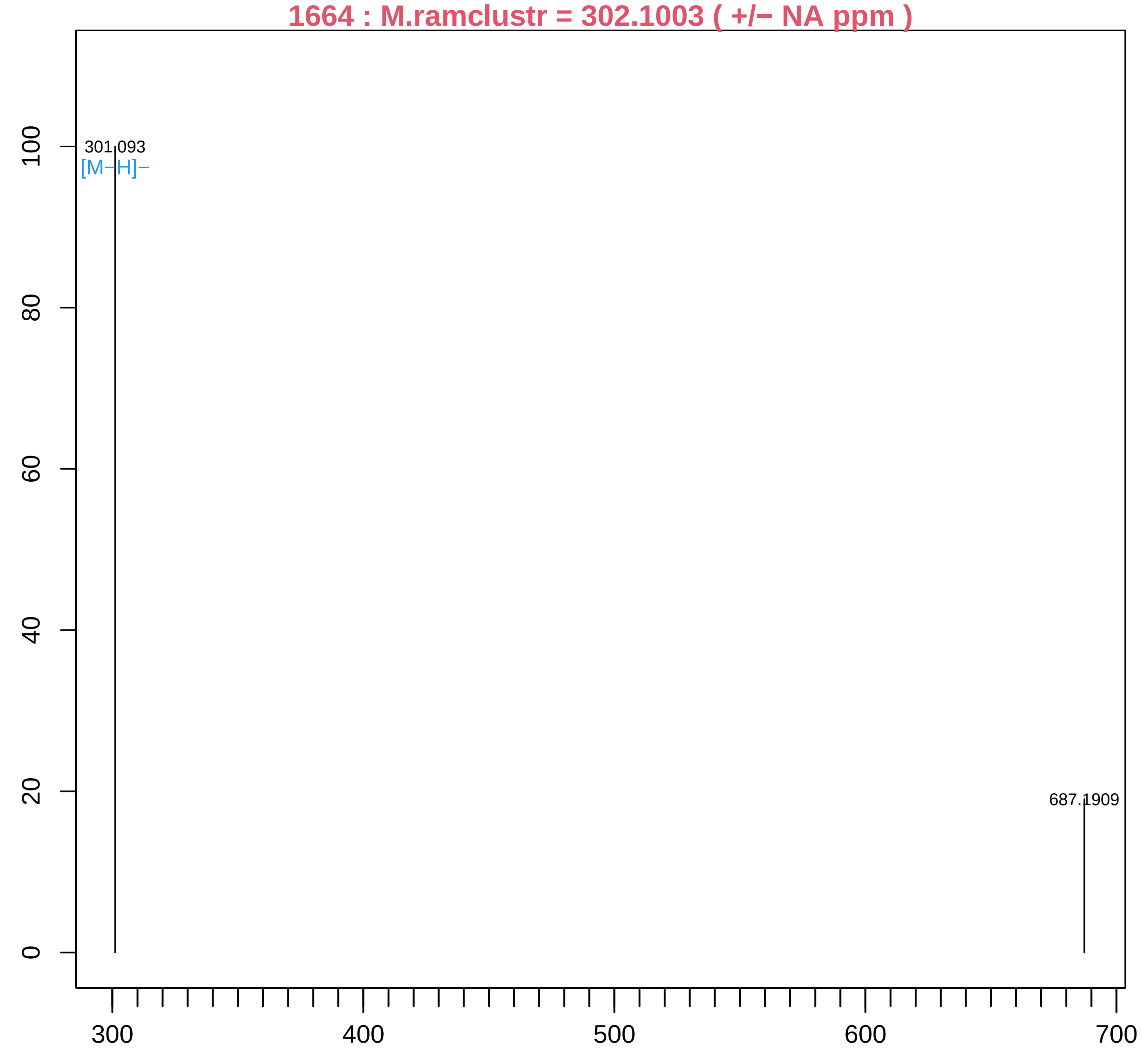
1664 : M.ramclustr = 302.1003 (+/− NA ppm)
301.093
[M−H]−
687.1909
0
20
40
60
80
100
300
400
500
600
700

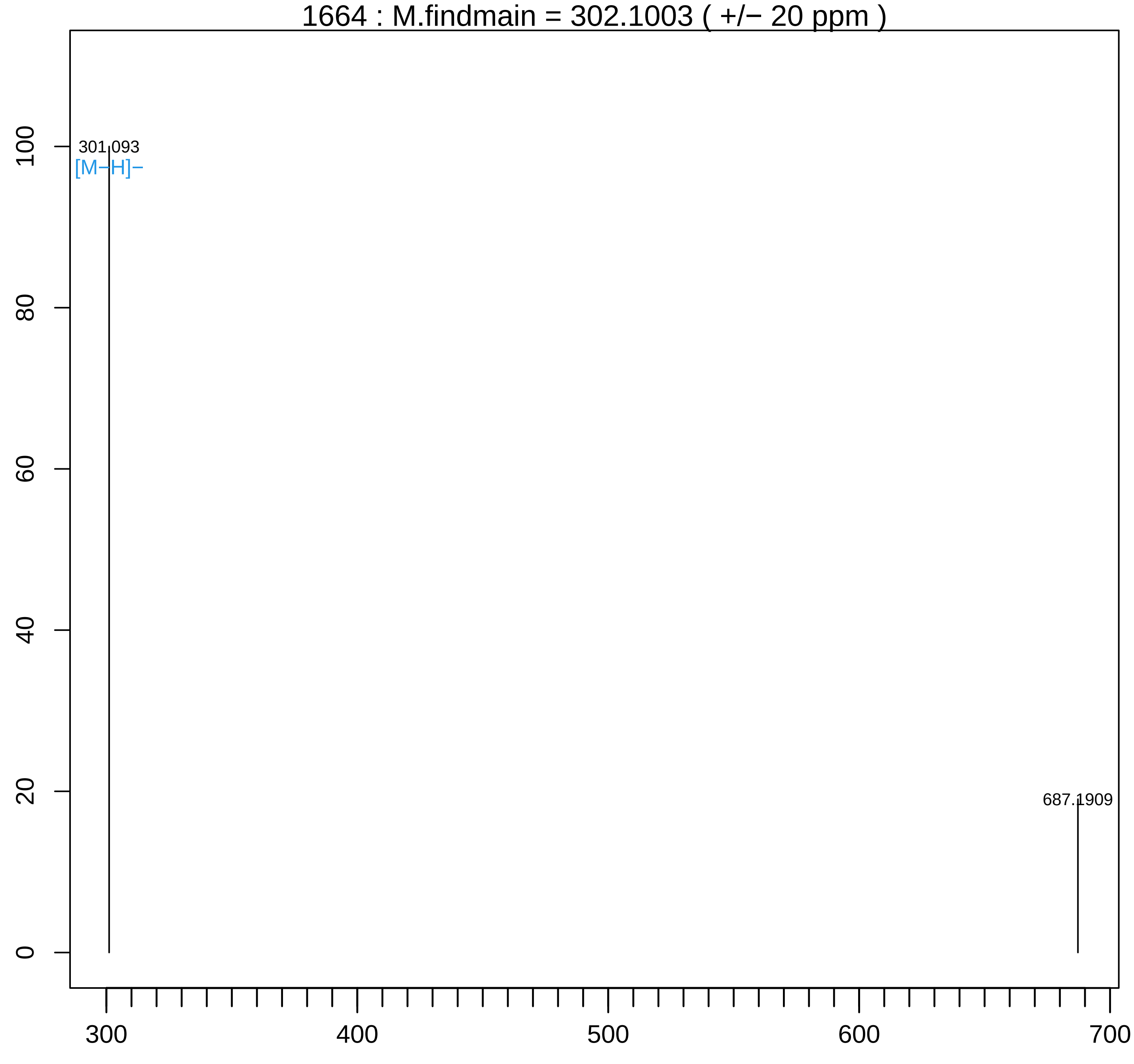
1664 : M.findmain = 302.1003 (+/− 20 ppm)
301.093
[M−H]−
687.1909
0
20
40
60
80
100
300
400
500
600
700

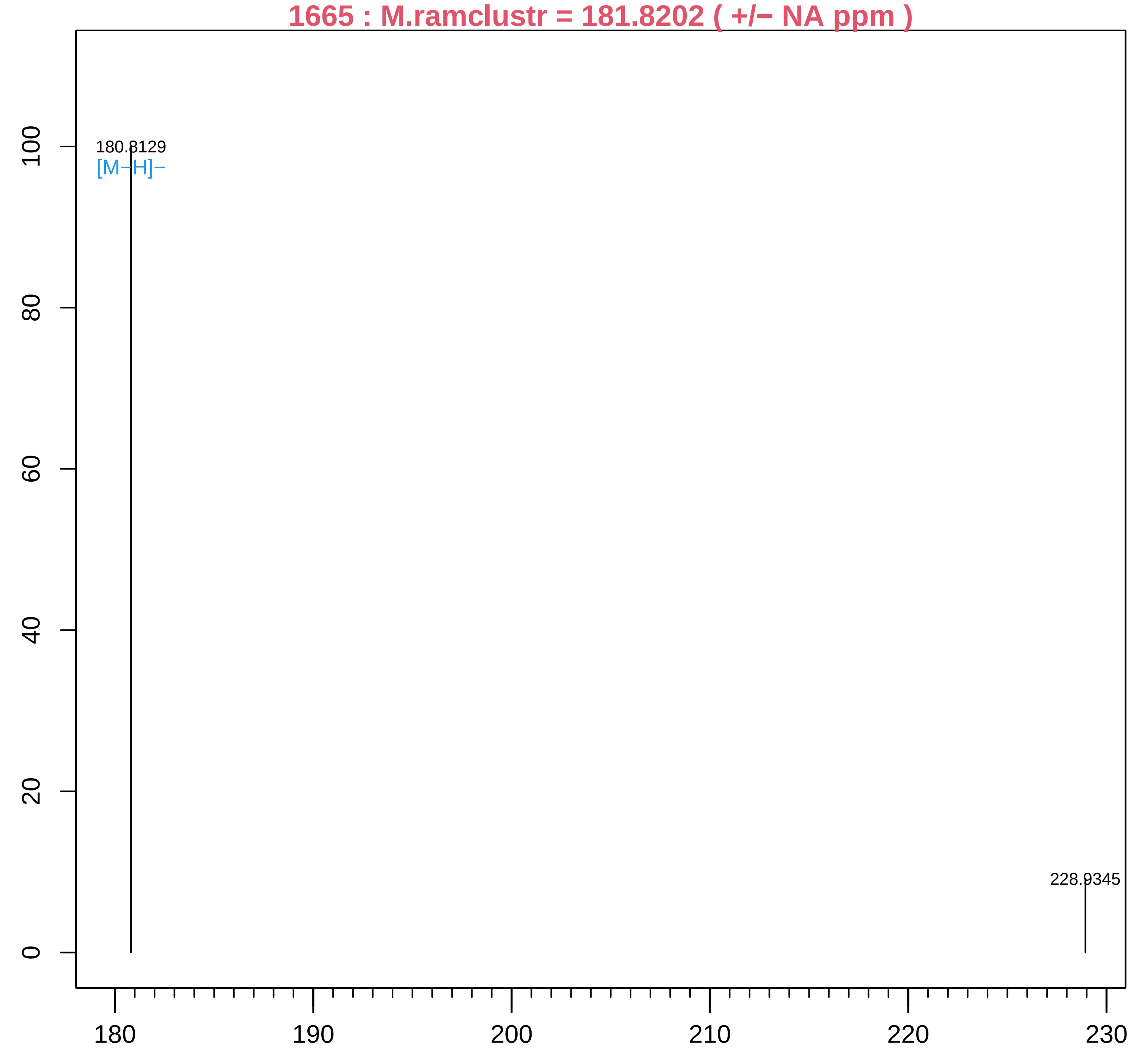
1665 : M.ramclustr = 181.8202 (+/− NA ppm)
180.8129
[M−H]−
228.9345
0
20
40
60
80
100
180
190
200
210
220
230

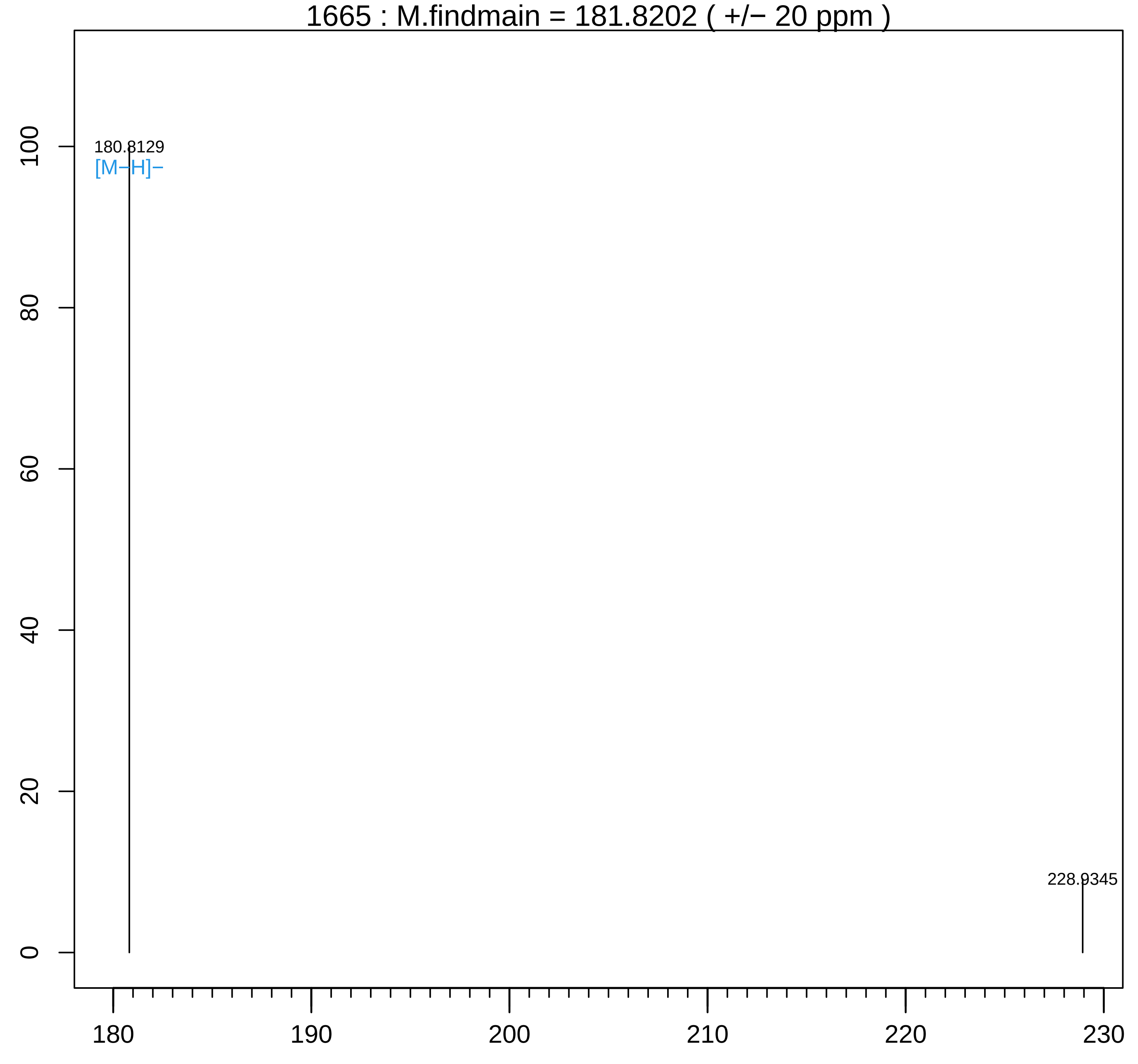
1665 : M.findmain = 181.8202 (+/− 20 ppm)
180.8129
[M−H]−
228.9345
0
20
40
60
80
100
180
190
200
210
220
230

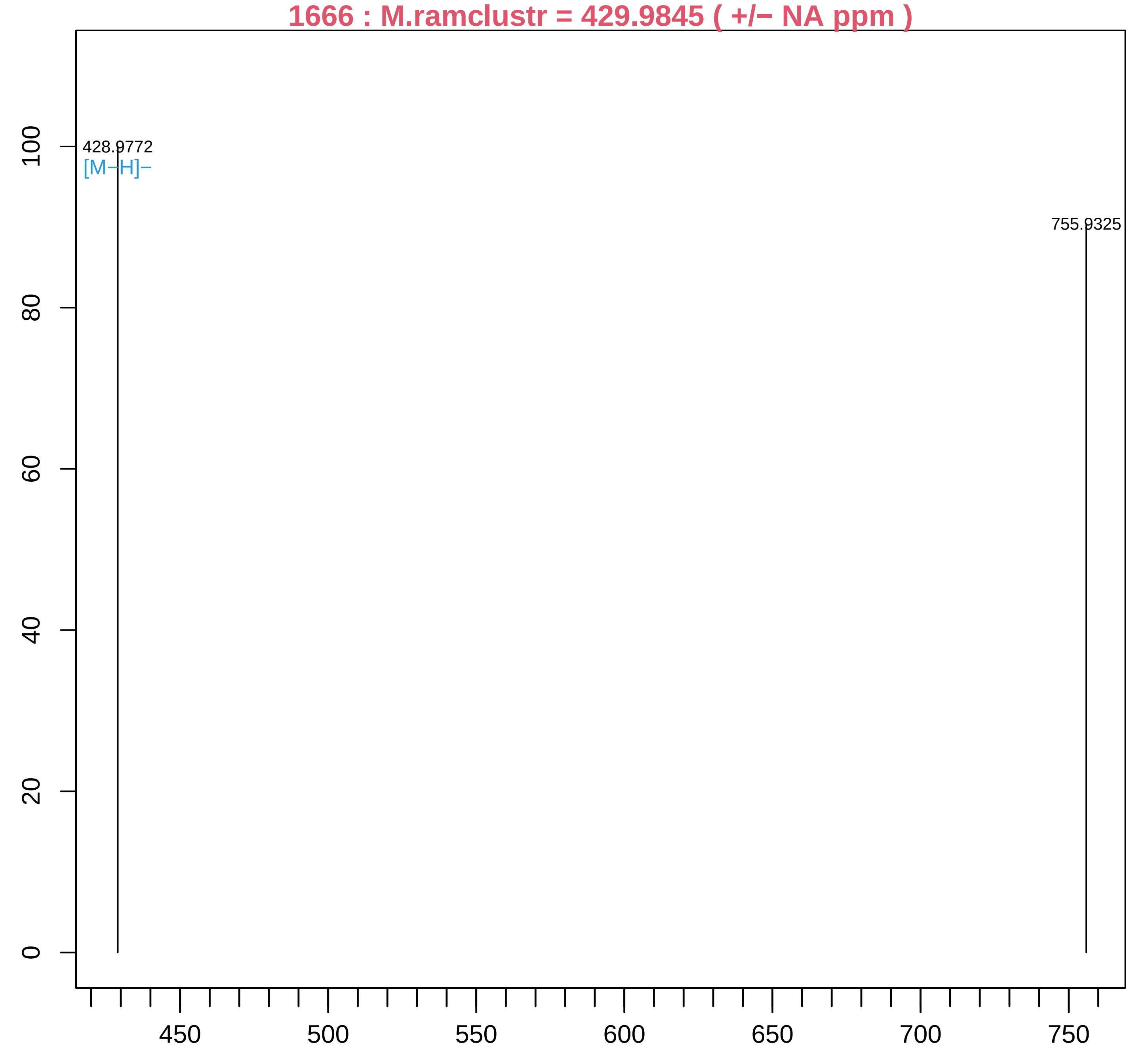
1666 : M.ramclustr = 429.9845 (+/− NA ppm)
428.9772
[M−H]−
755.9325
0
20
40
60
80
100
450
500
550
600
650
700
750

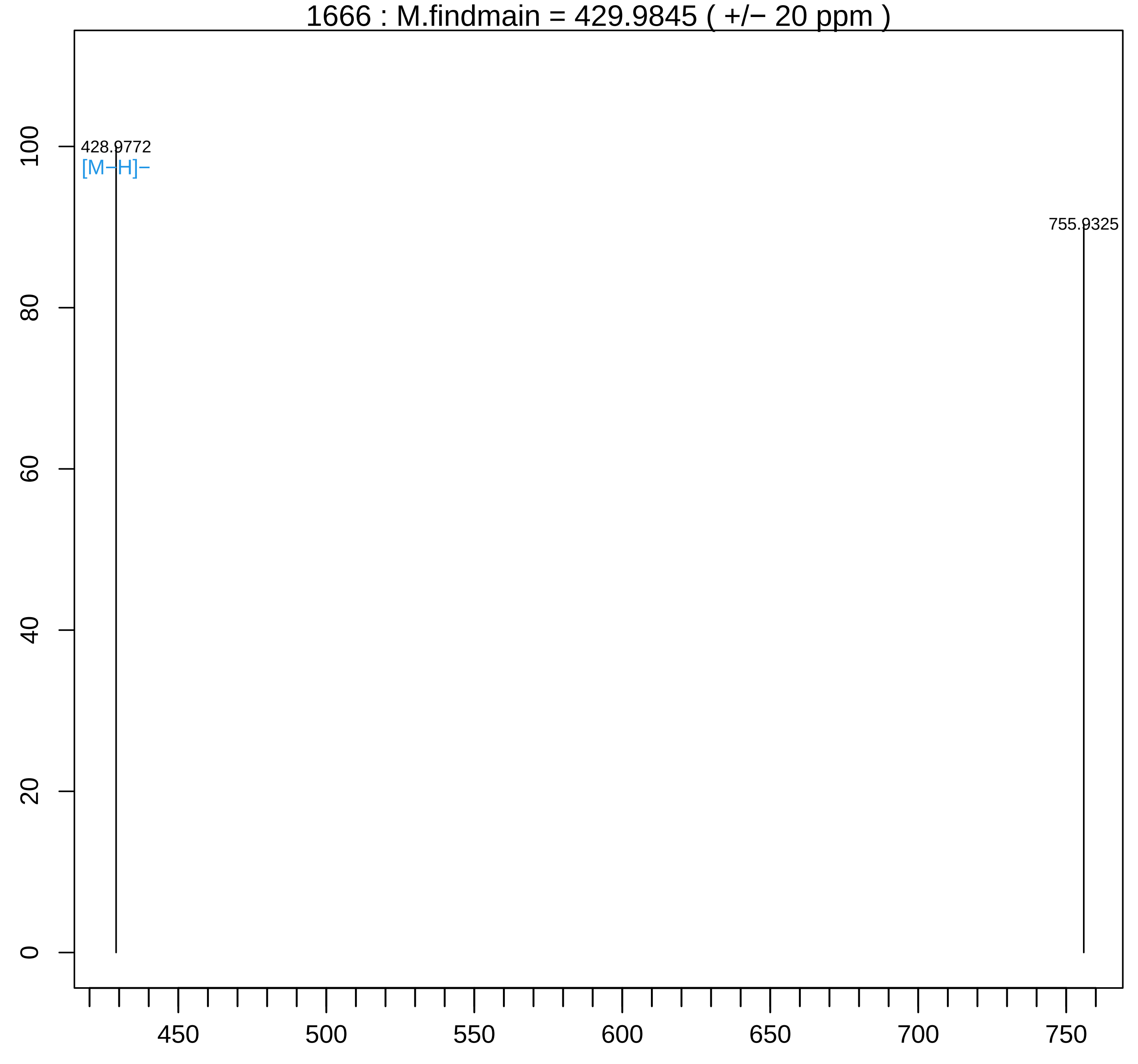
1666 : M.findmain = 429.9845 (+/− 20 ppm)
428.9772
[M−H]−
755.9325
0
20
40
60
80
100
450
500
550
600
650
700
750

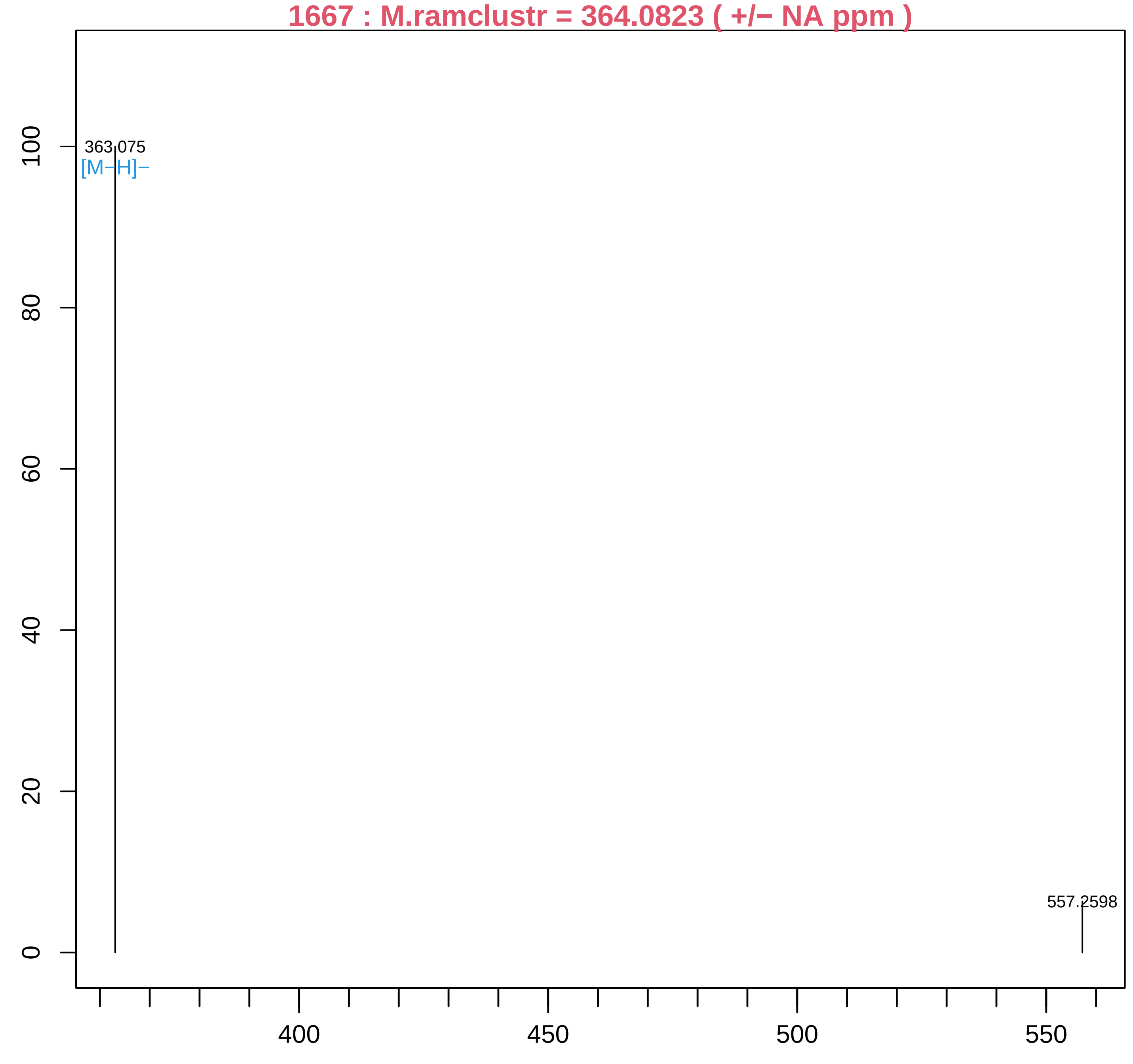
1667 : M.ramclustr = 364.0823 (+/− NA ppm)
363.075
[M−H]−
557.2598
0
20
40
60
80
100
400
450
500
550

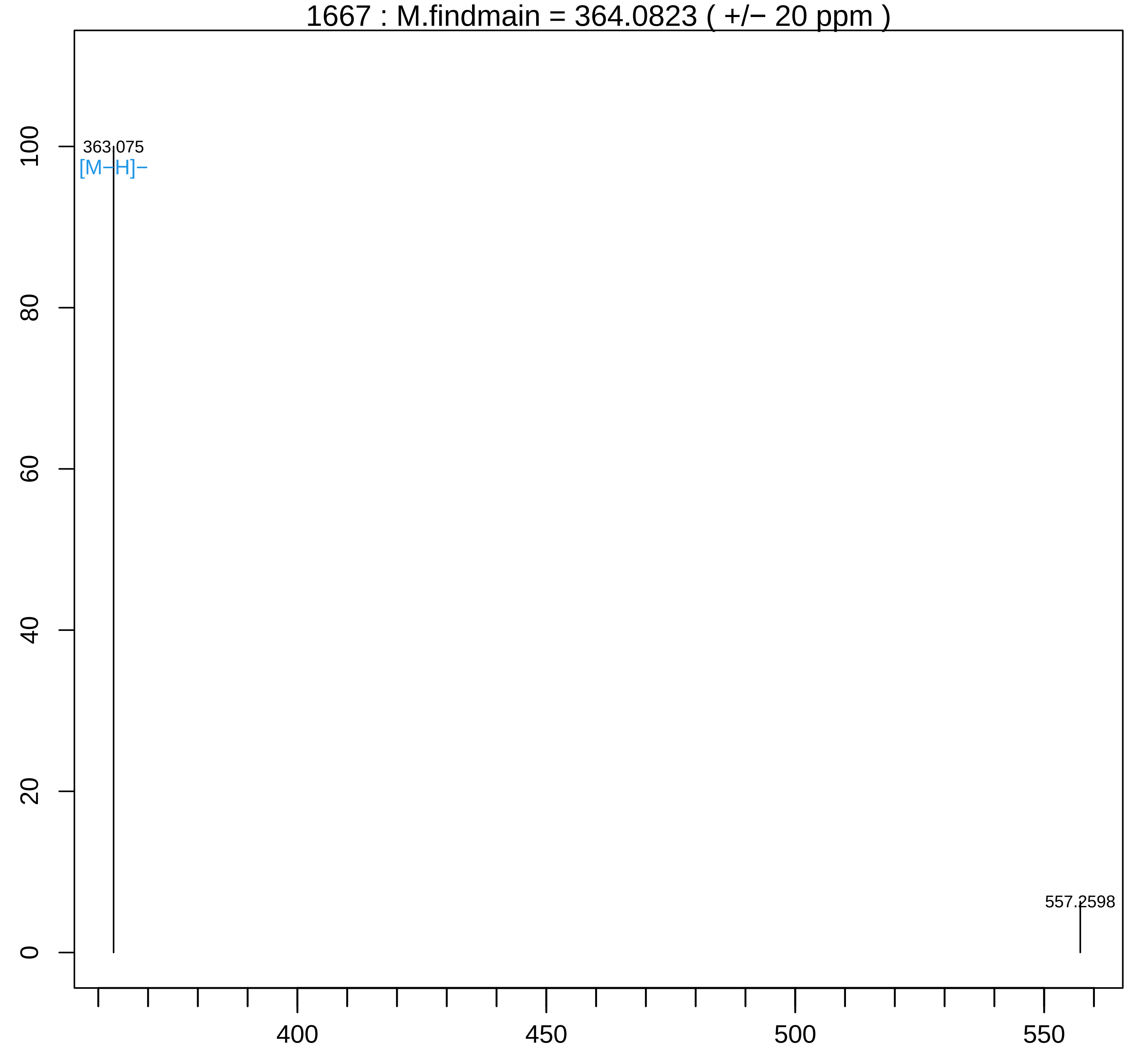
1667 : M.findmain = 364.0823 (+/− 20 ppm)
363.075
[M−H]−
557.2598
0
20
40
60
80
100
400
450
500
550

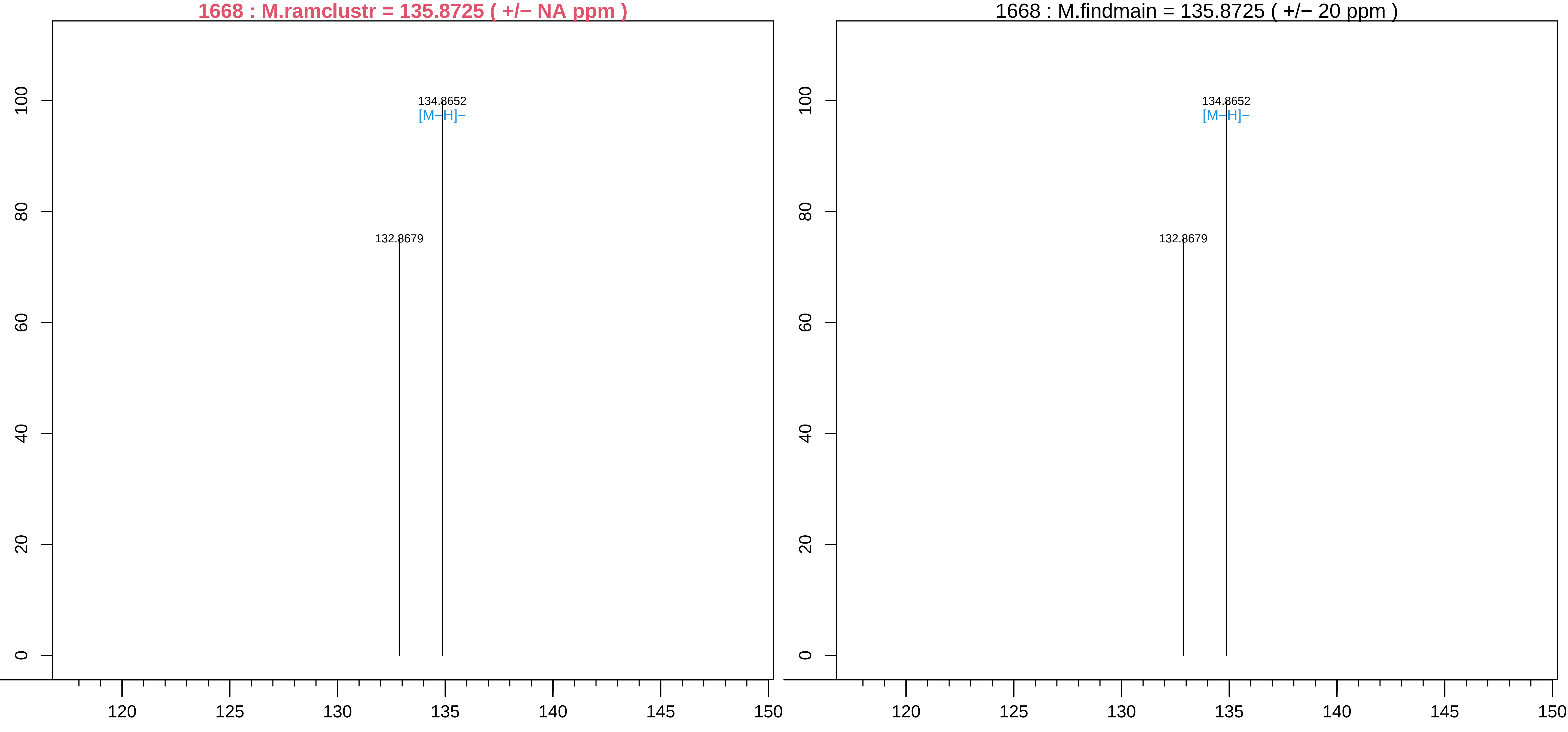
1668 : M.ramclustr = 135.8725 (+/− NA ppm)
134.8652
[M−H]−
132.8679
1668 : M.findmain = 135.8725 (+/− 20 ppm)
134.8652
[M−H]−
132.8679
0
20
40
60
80
100
120
125
130
135
140
145
150

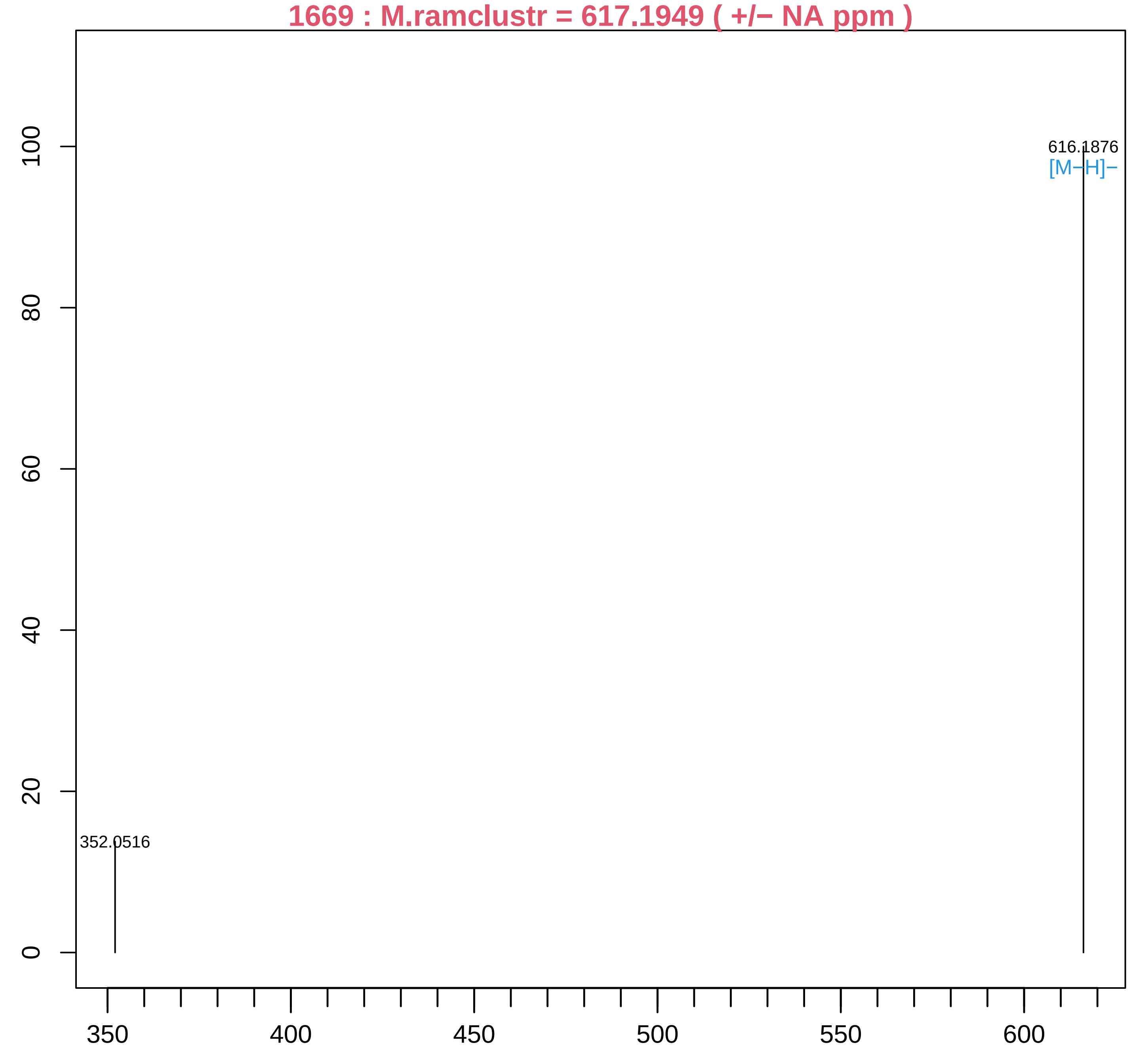
1669 : M.ramclustr = 617.1949 (+/− NA ppm)
616.1876
[M−H]−
352.0516
0
20
40
60
80
100
350
400
450
500
550
600

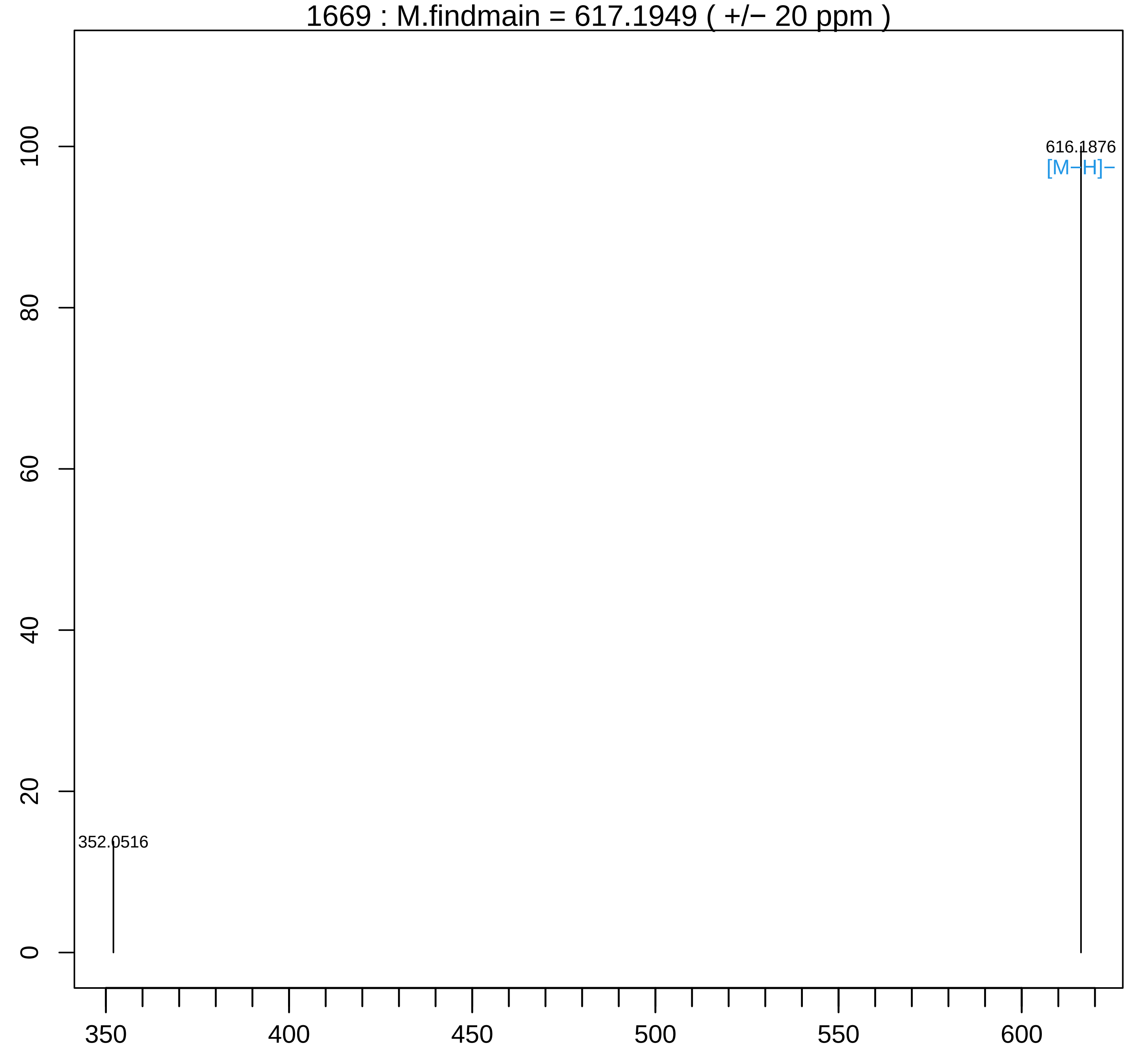
1669 : M.findmain = 617.1949 (+/− 20 ppm)
616.1876
[M−H]−
352.0516
0
20
40
60
80
100
350
400
450
500
550
600

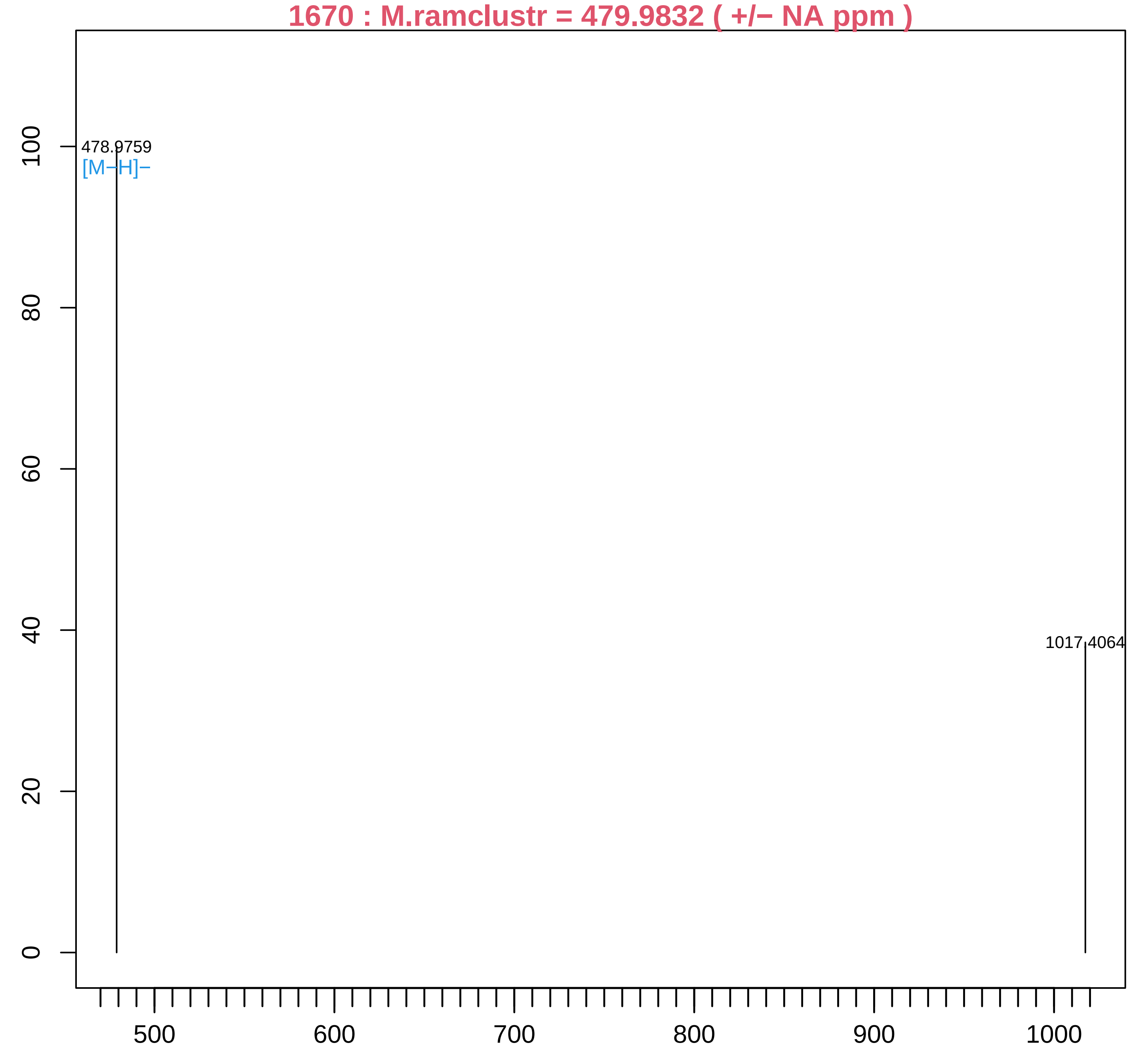
1670 : M.ramclustr = 479.9832 (+/− NA ppm)
478.9759
[M−H]−
1017.4064
0
20
40
60
80
100
500
600
700
800
900
1000

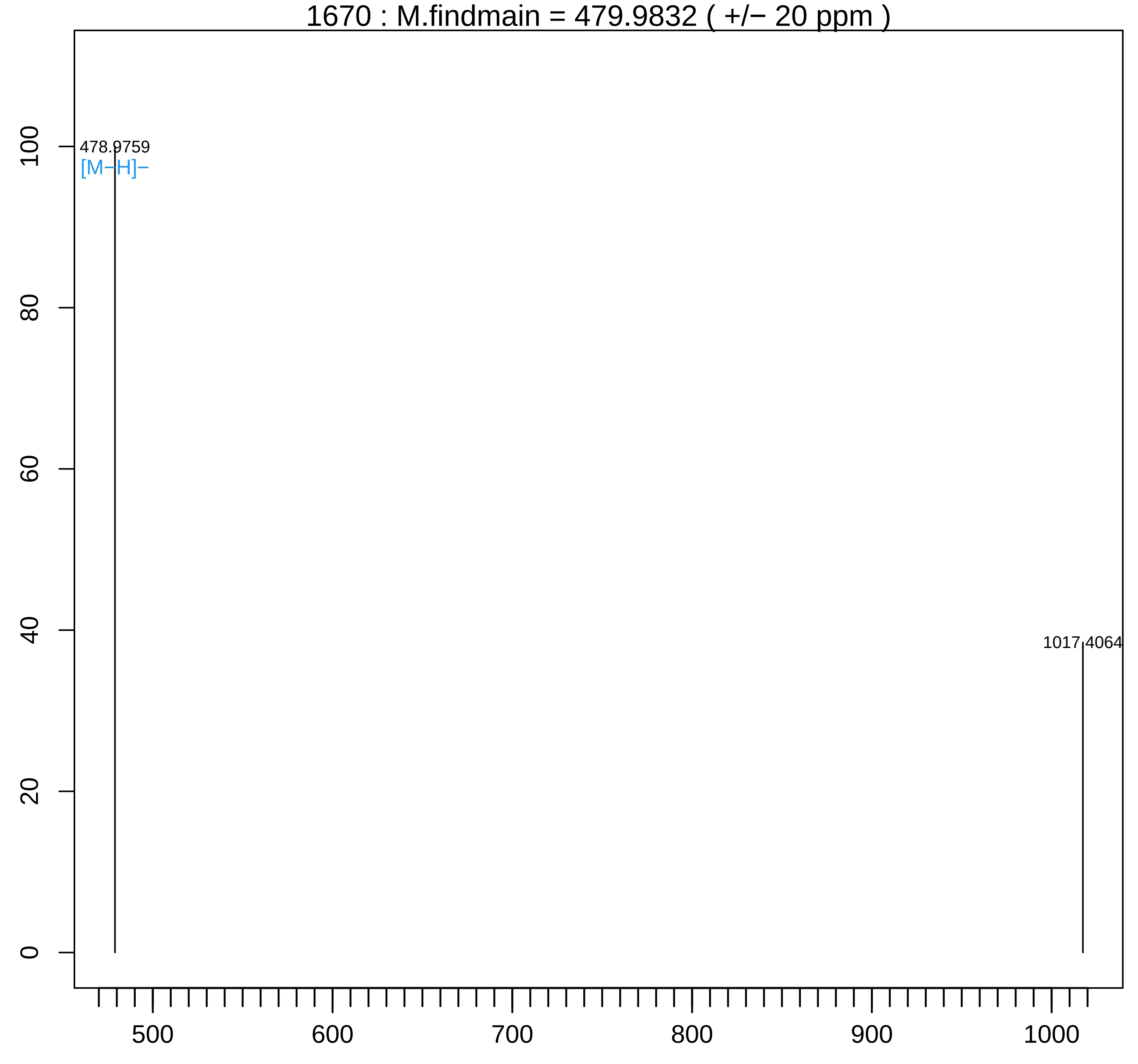
1670 : M.findmain = 479.9832 (+/− 20 ppm)
478.9759
[M−H]−
1017.4064
0
20
40
60
80
100
500
600
700
800
900
1000

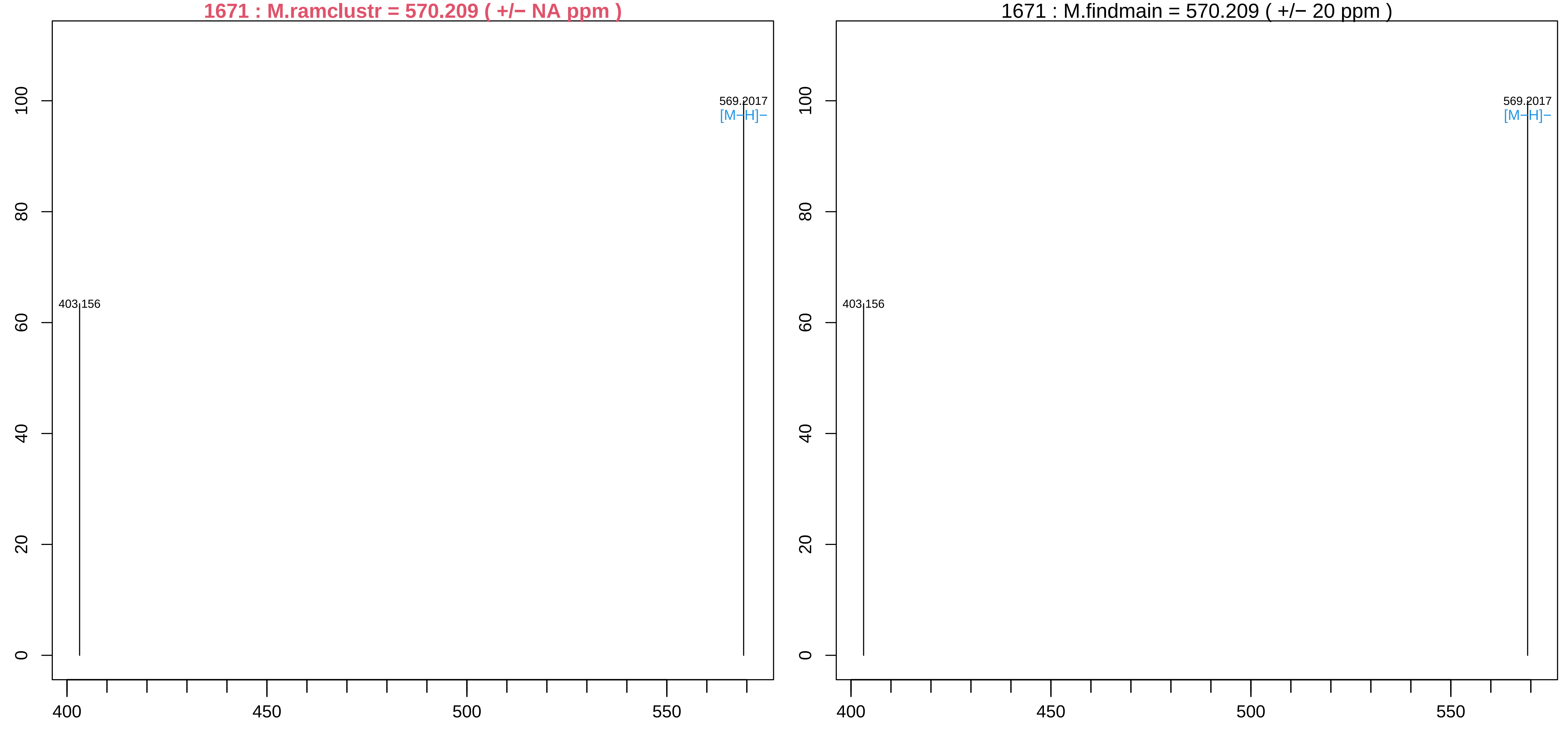
1671 : M.ramclustr = 570.209 (+/− NA ppm)
1671 : M.findmain = 570.209 (+/− 20 ppm)
569.2017
[M−H]−
403.156
0
20
40
60
80
100
400
450
500
550

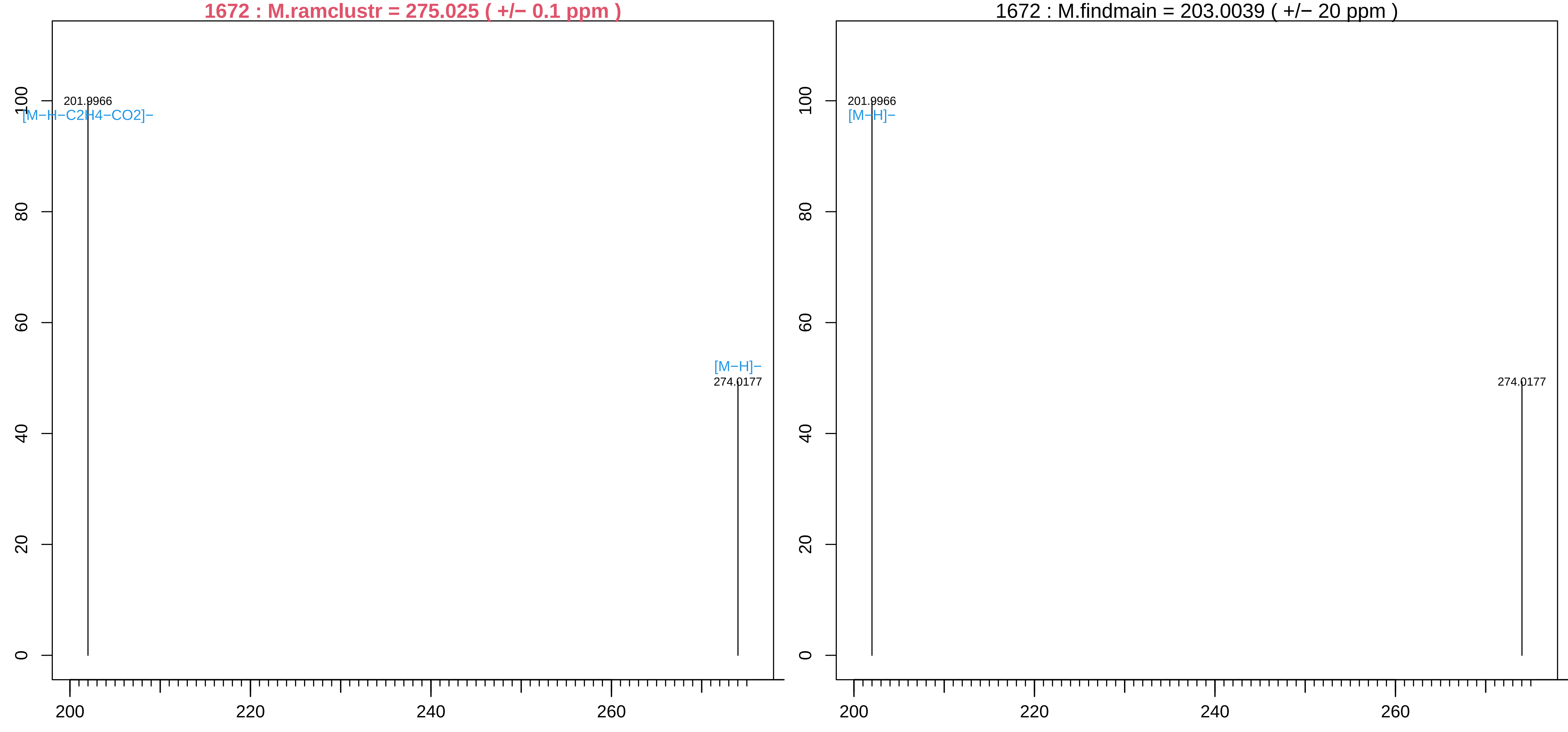
1672 : M.ramclustr = 275.025 (+/− 0.1 ppm)
201.9966
[M−H−C2H4−CO2]−
[M−H]−
274.0177
1672 : M.findmain = 203.0039 (+/− 20 ppm)
201.9966
[M−H]−
274.0177
0
20
40
60
80
100
200
220
240
260

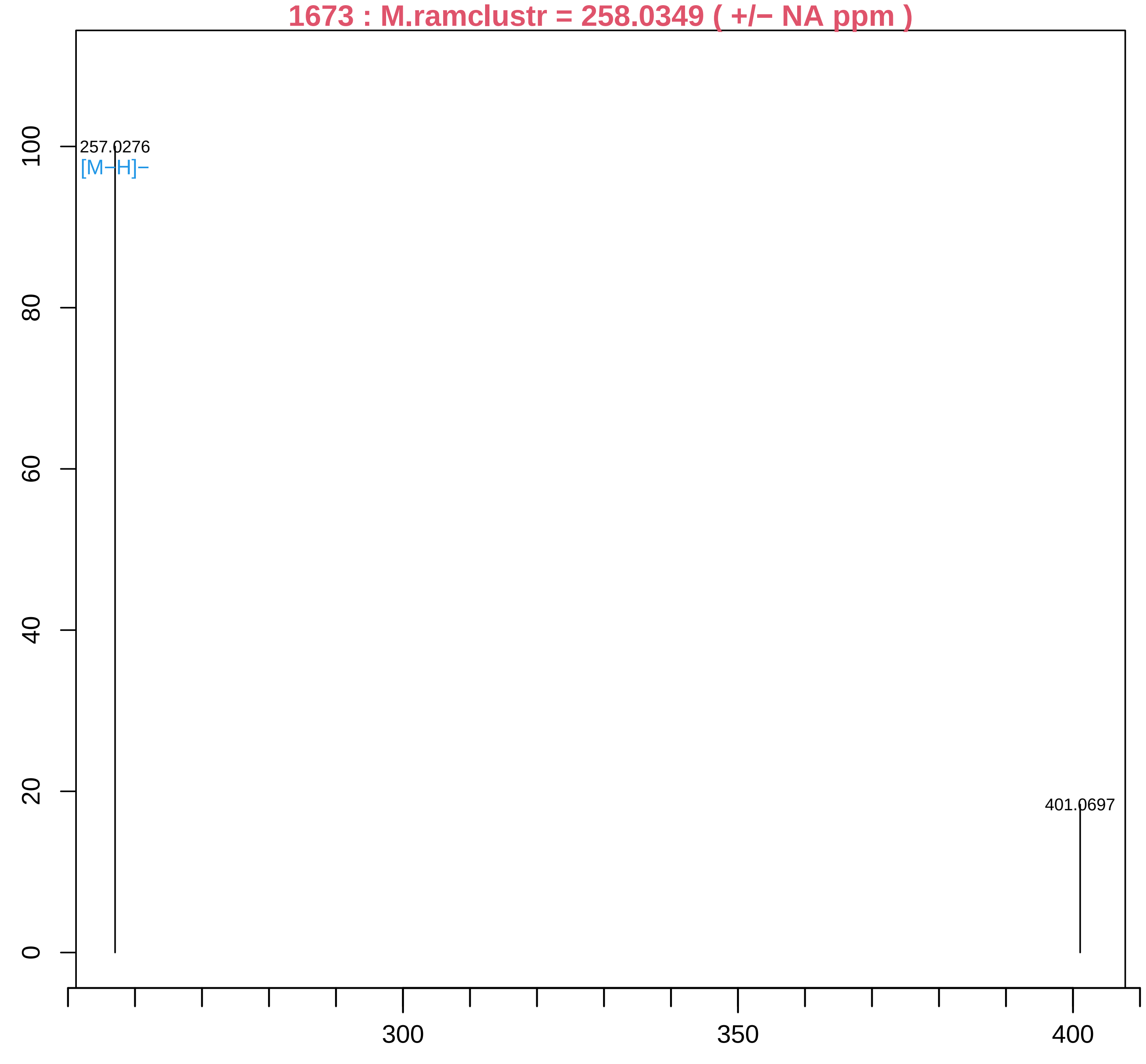

1673 : M.ramclustr = 258.0349 (+/− NA ppm)
257.0276
[M−H]−
401.0697
0
20
40
60
80
100
300
350
400

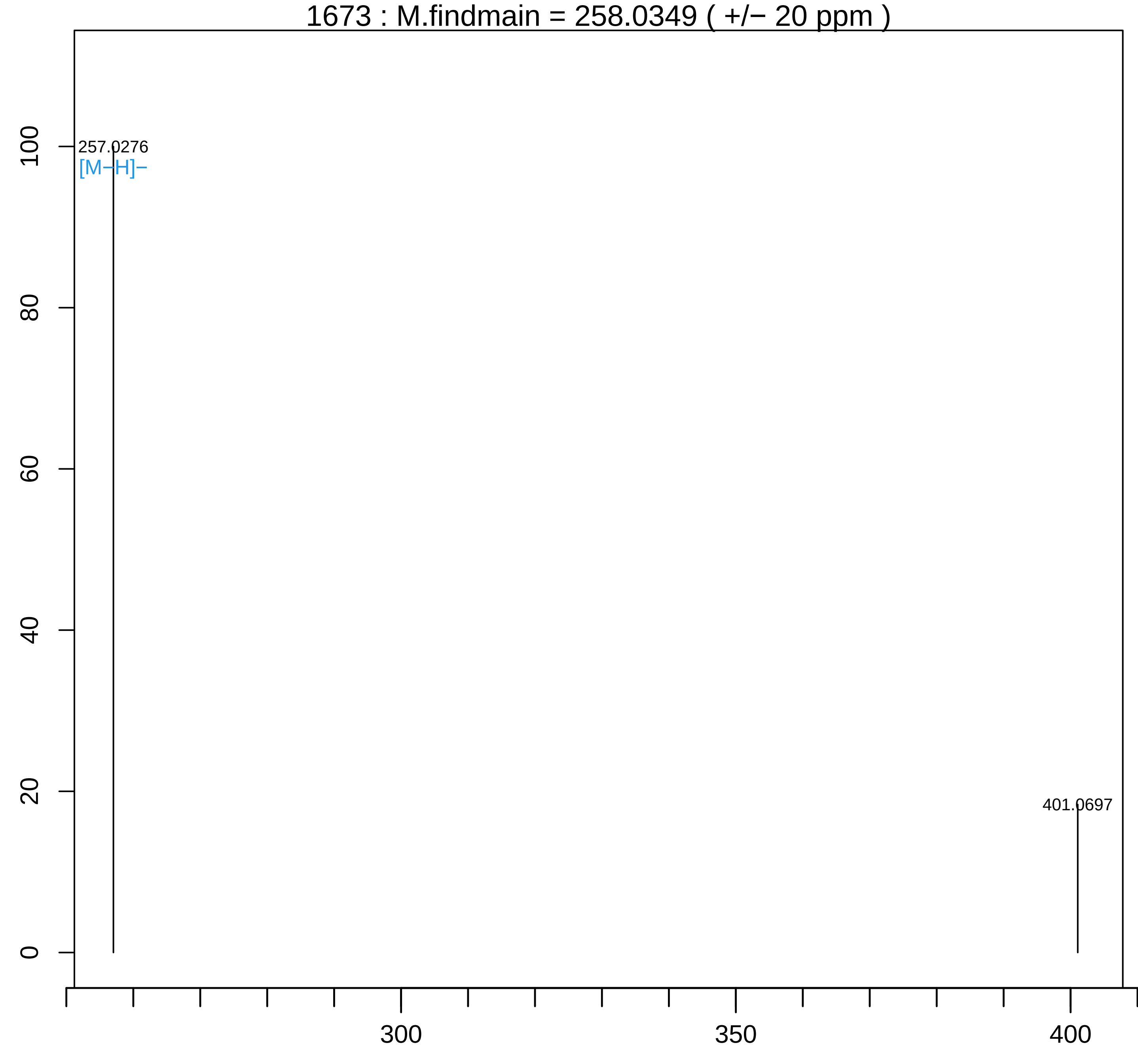

1673 : M.findmain = 258.0349 (+/− 20 ppm)
257.0276
[M−H]−
401.0697
0
20
40
60
80
100
300
350
400

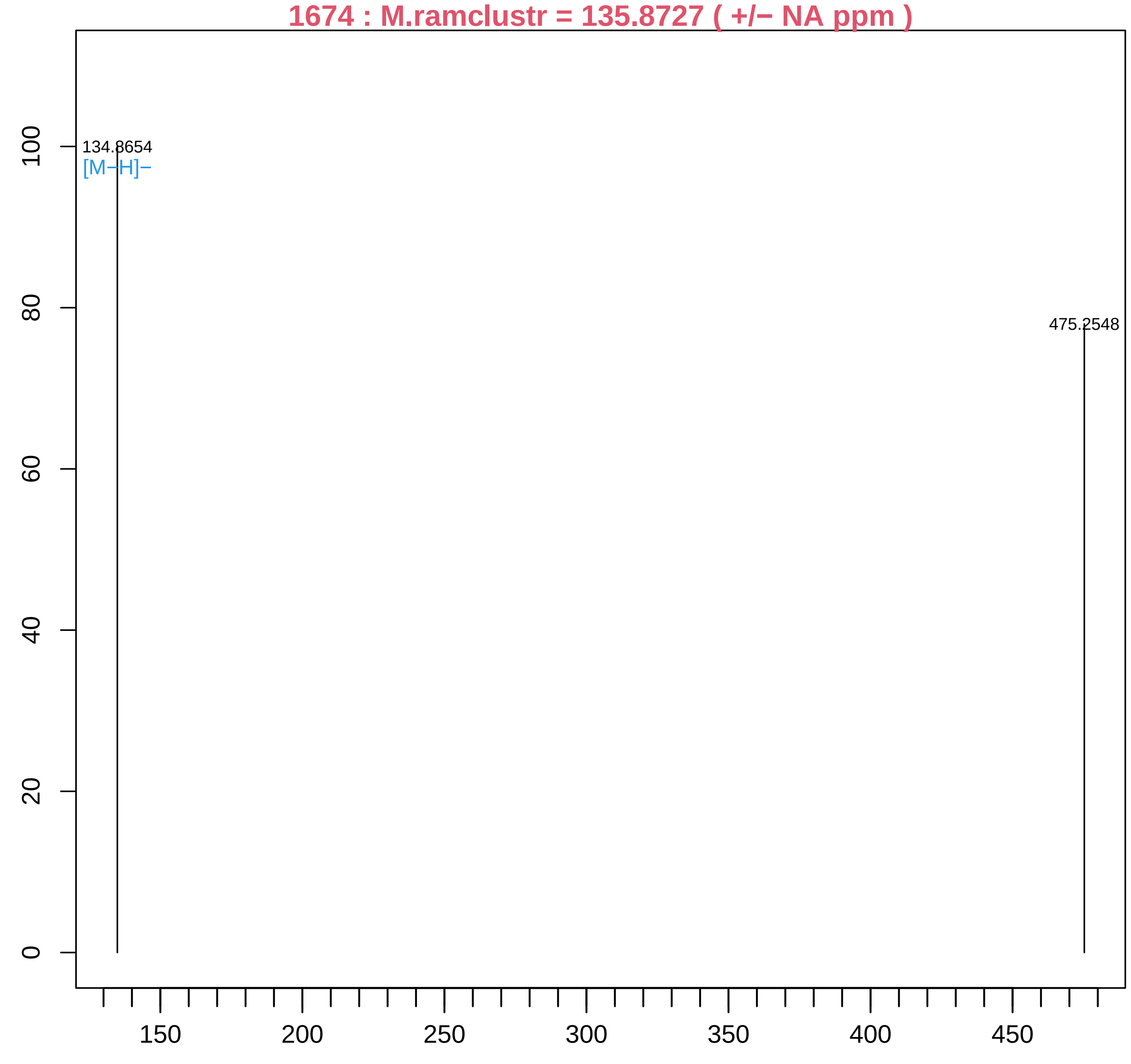

1674 : M.ramclustr = 135.8727 (+/− NA ppm)
134.8654
[M−H]−
475.2548
0
20
40
60
80
100
150
200
250
300
350
400
450

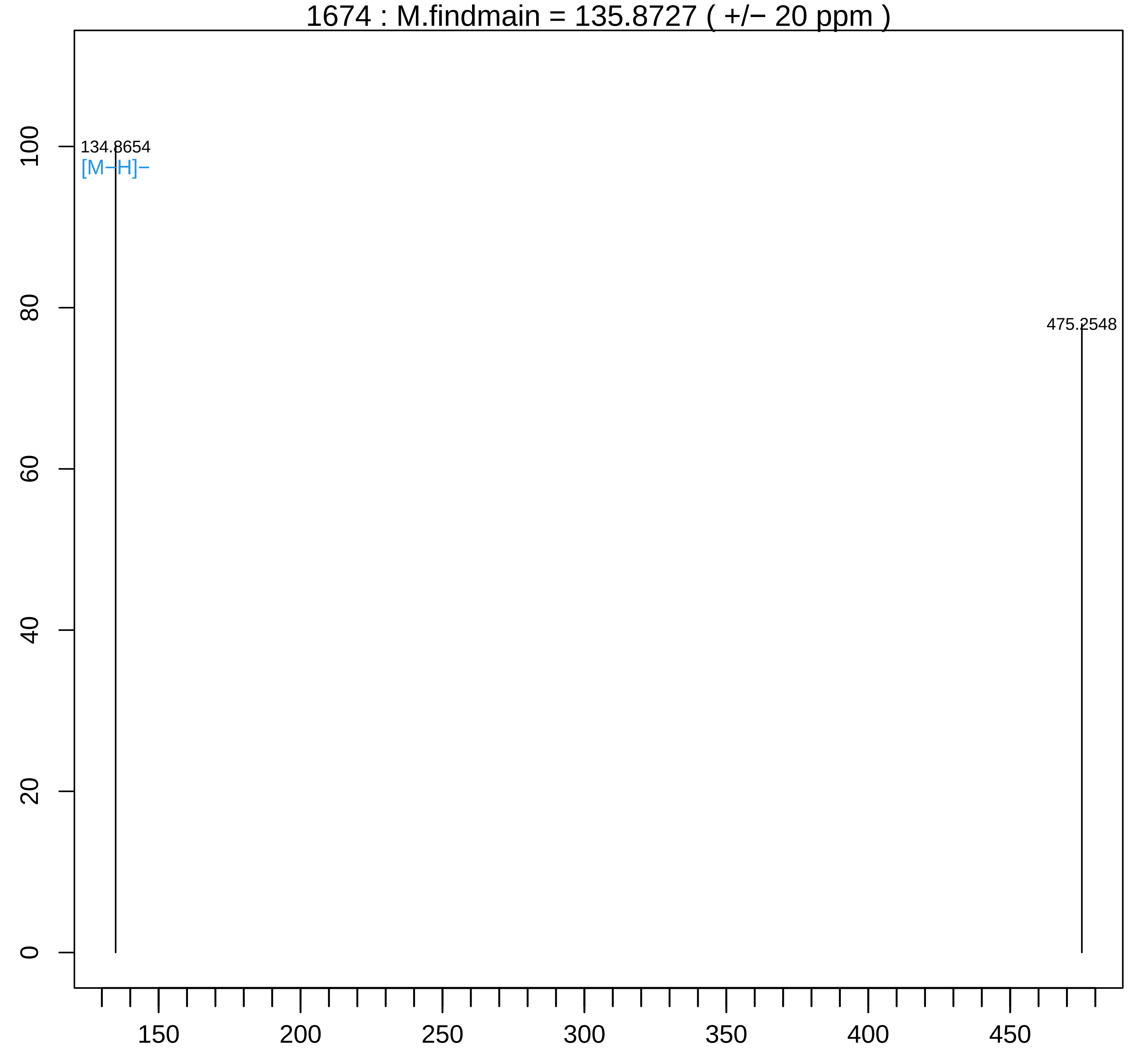

1674 : M.findmain = 135.8727 (+/− 20 ppm)
134.8654
[M−H]−
475.2548
0
20
40
60
80
100
150
200
250
300
350
400
450

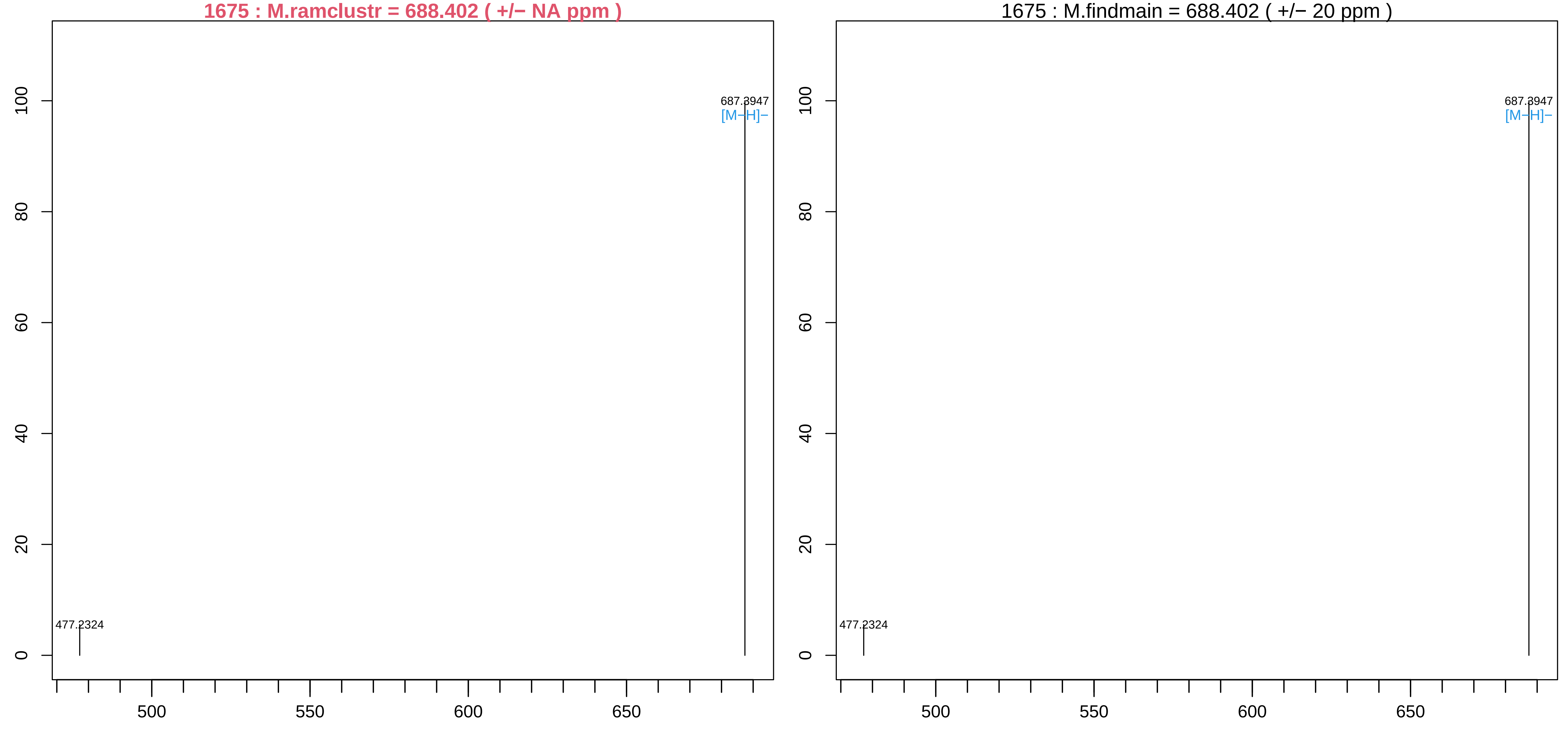
1675 : M.ramclustr = 688.402 (+/− NA ppm)
687.3947
[M−H]−
477.2324
1675 : M.findmain = 688.402 (+/− 20 ppm)
687.3947
[M−H]−
477.2324
0
20
40
60
80
100
500
550
600
650

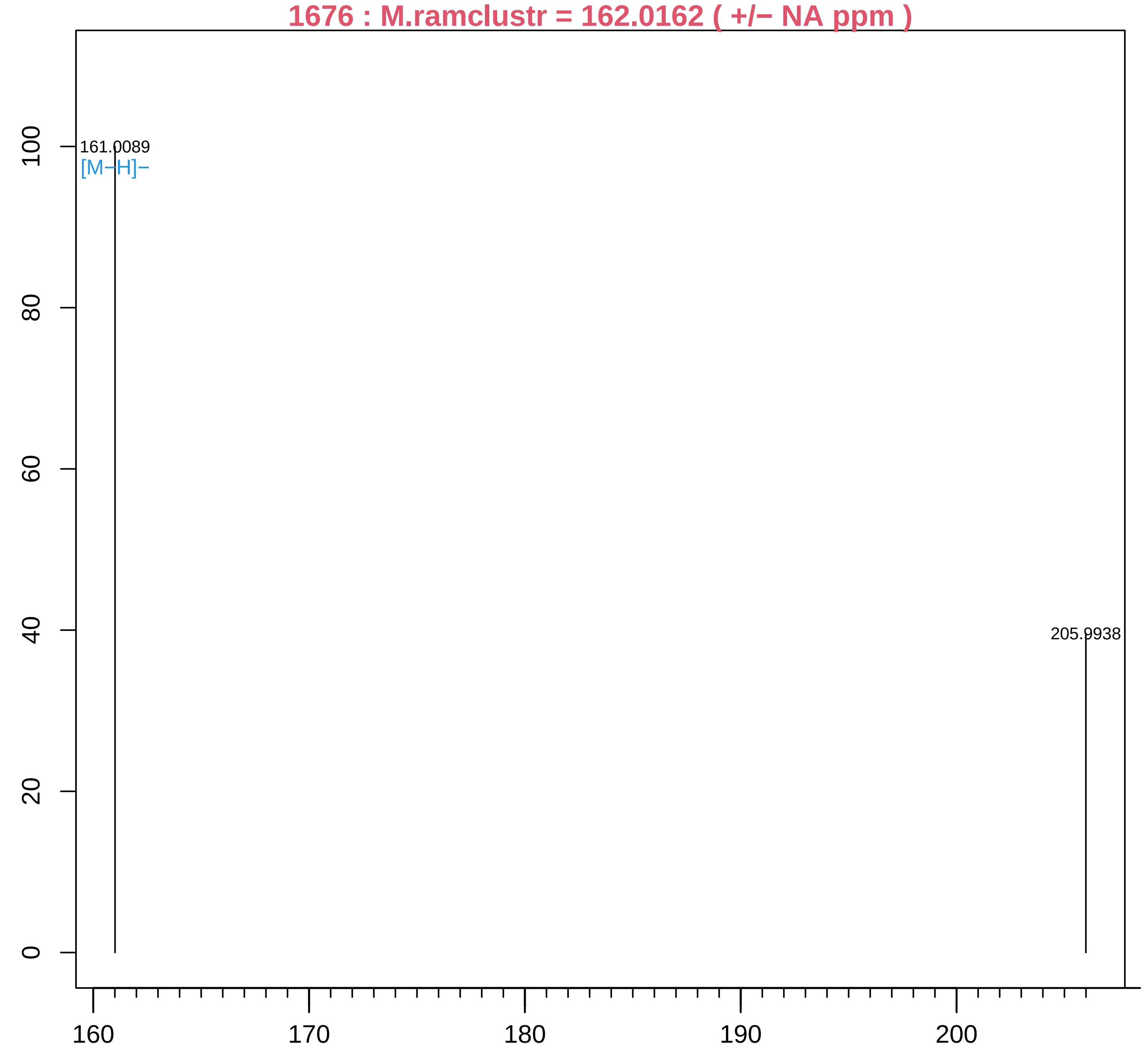
1676 : M.ramclustr = 162.0162 (+/− NA ppm)
161.0089
[M−H]−
205.9938
0
20
40
60
80
100
160
170
180
190
200

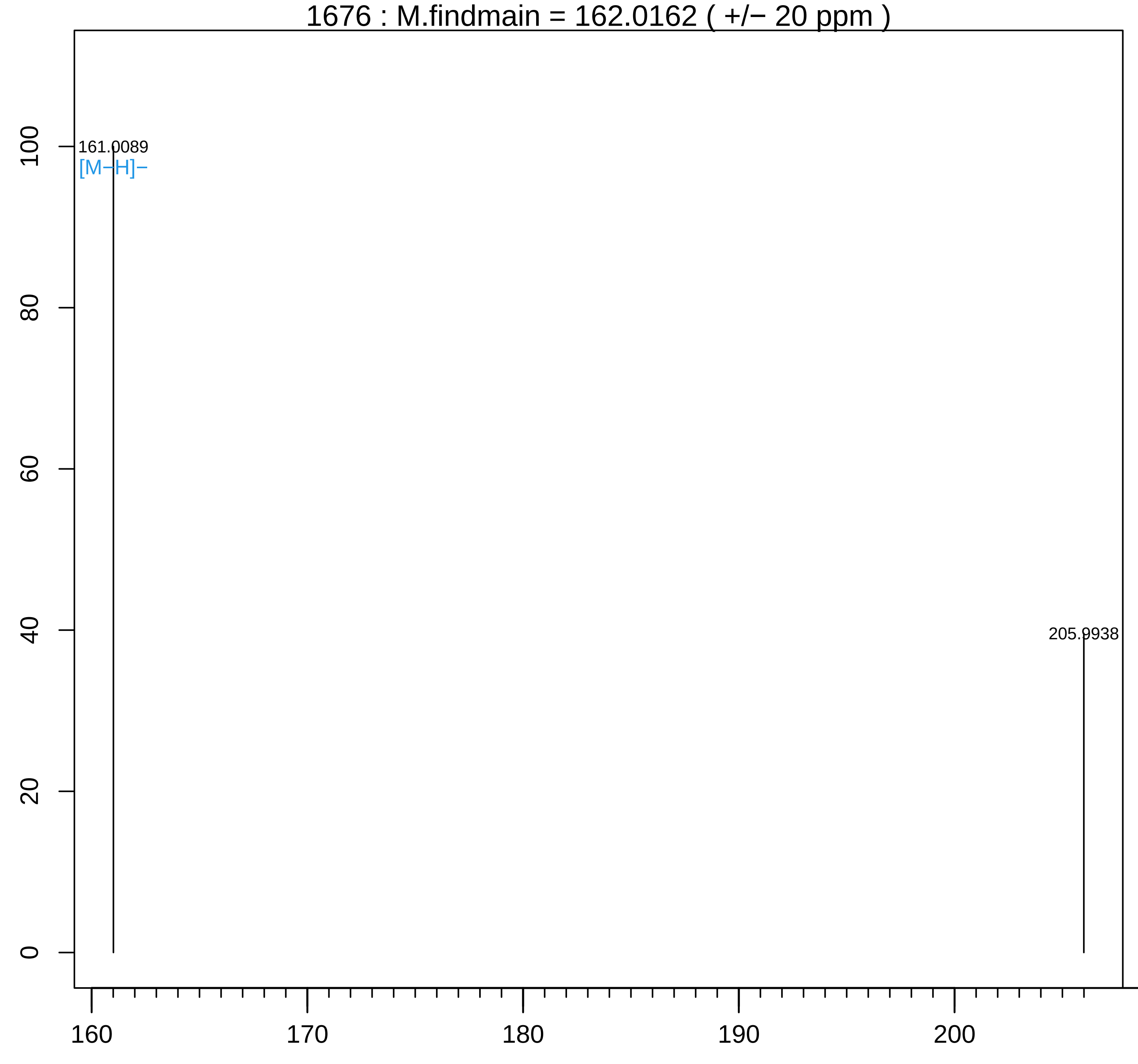
1676 : M.findmain = 162.0162 (+/− 20 ppm)
161.0089
[M−H]−
205.9938
0
20
40
60
80
100
160
170
180
190
200

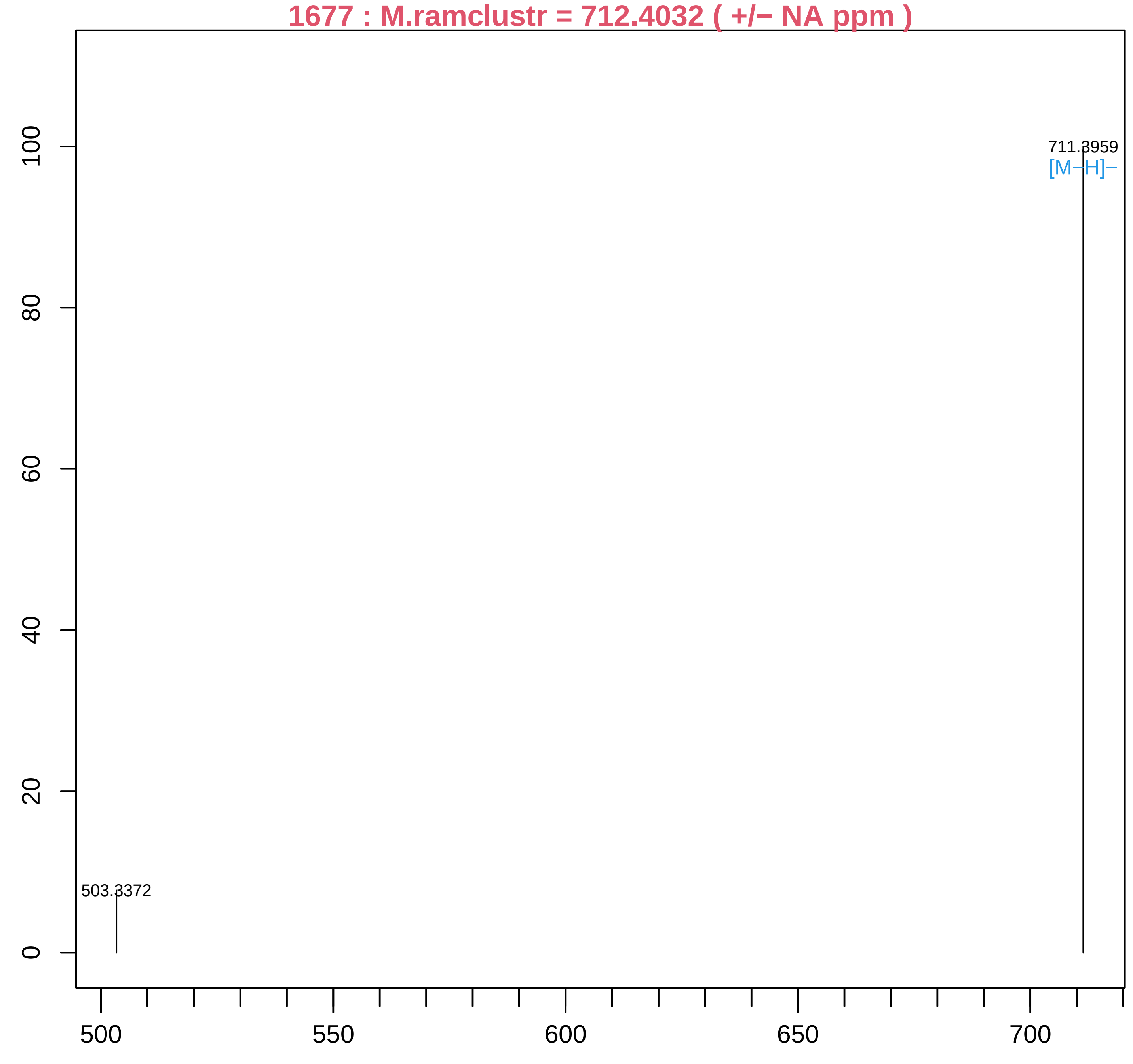
1677 : M.ramclustr = 712.4032 (+/− NA ppm)
711.3959
[M−H]−
503.3372
0
20
40
60
80
100
500
550
600
650
700

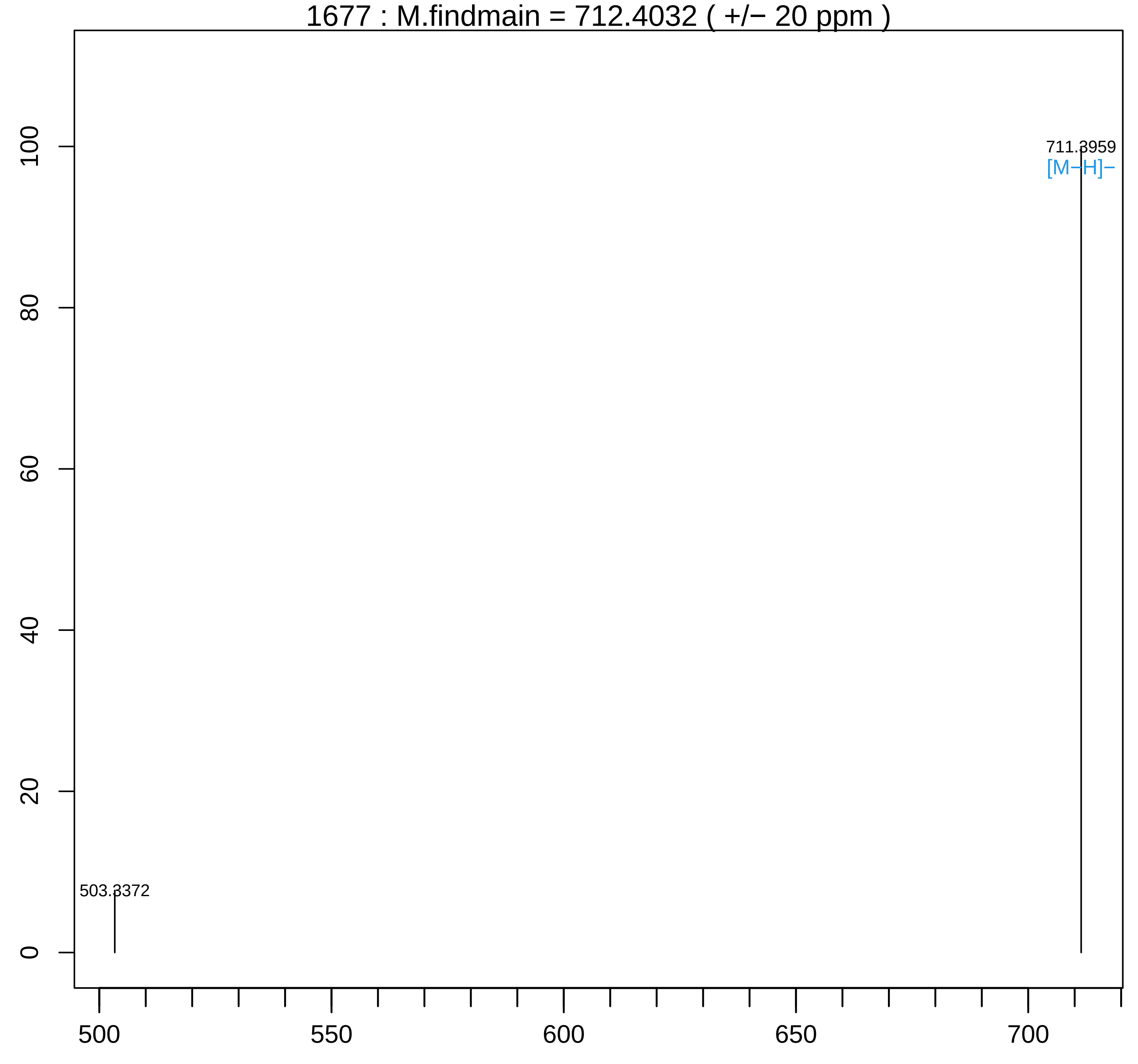
1677 : M.findmain = 712.4032 (+/− 20 ppm)
711.3959
[M−H]−
503.3372
0
20
40
60
80
100
500
550
600
650
700

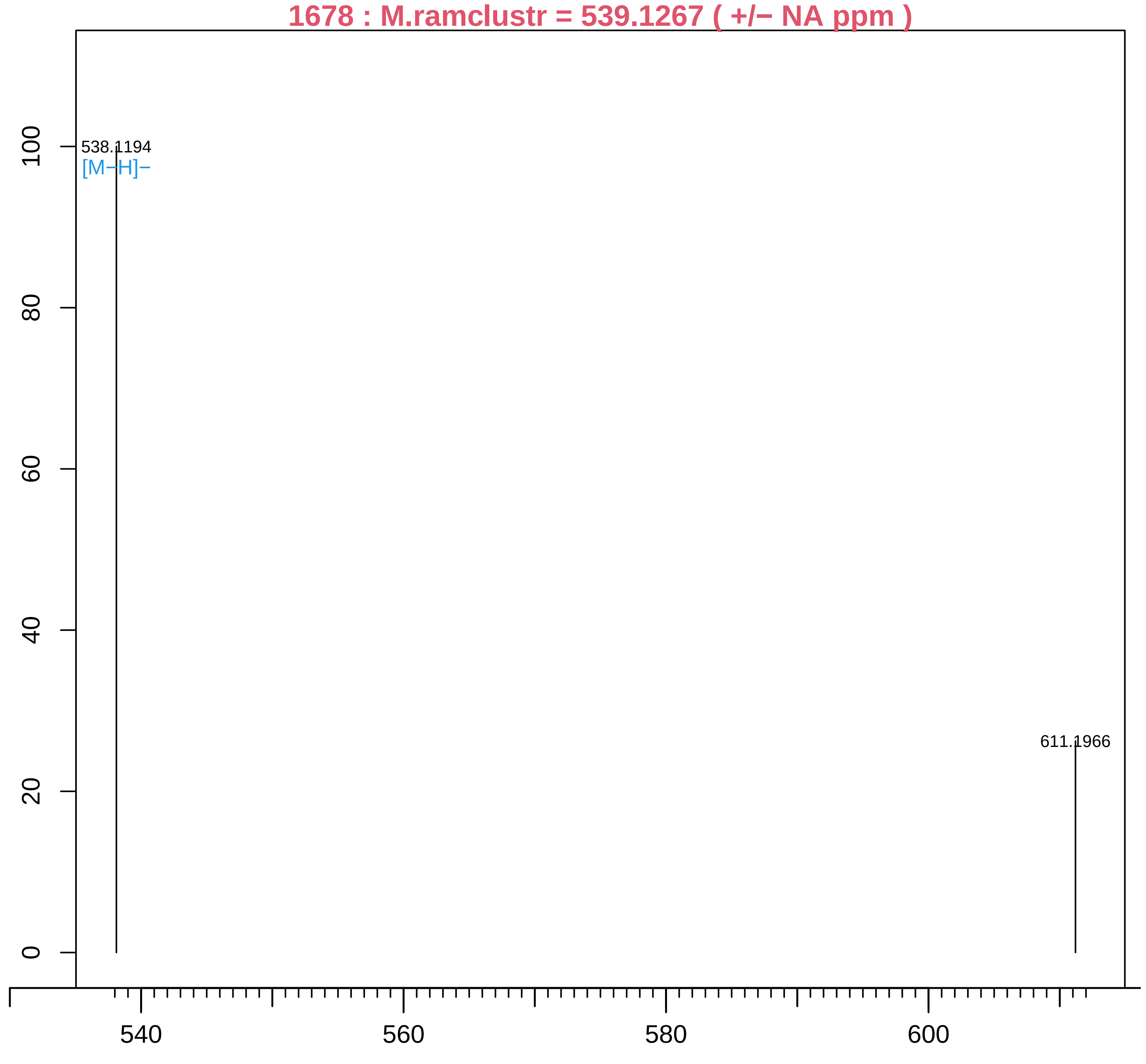

1678 : M.ramclustr = 539.1267 (+/− NA ppm)
538.1194
[M−H]−
611.1966
0
20
40
60
80
100
540
560
580
600

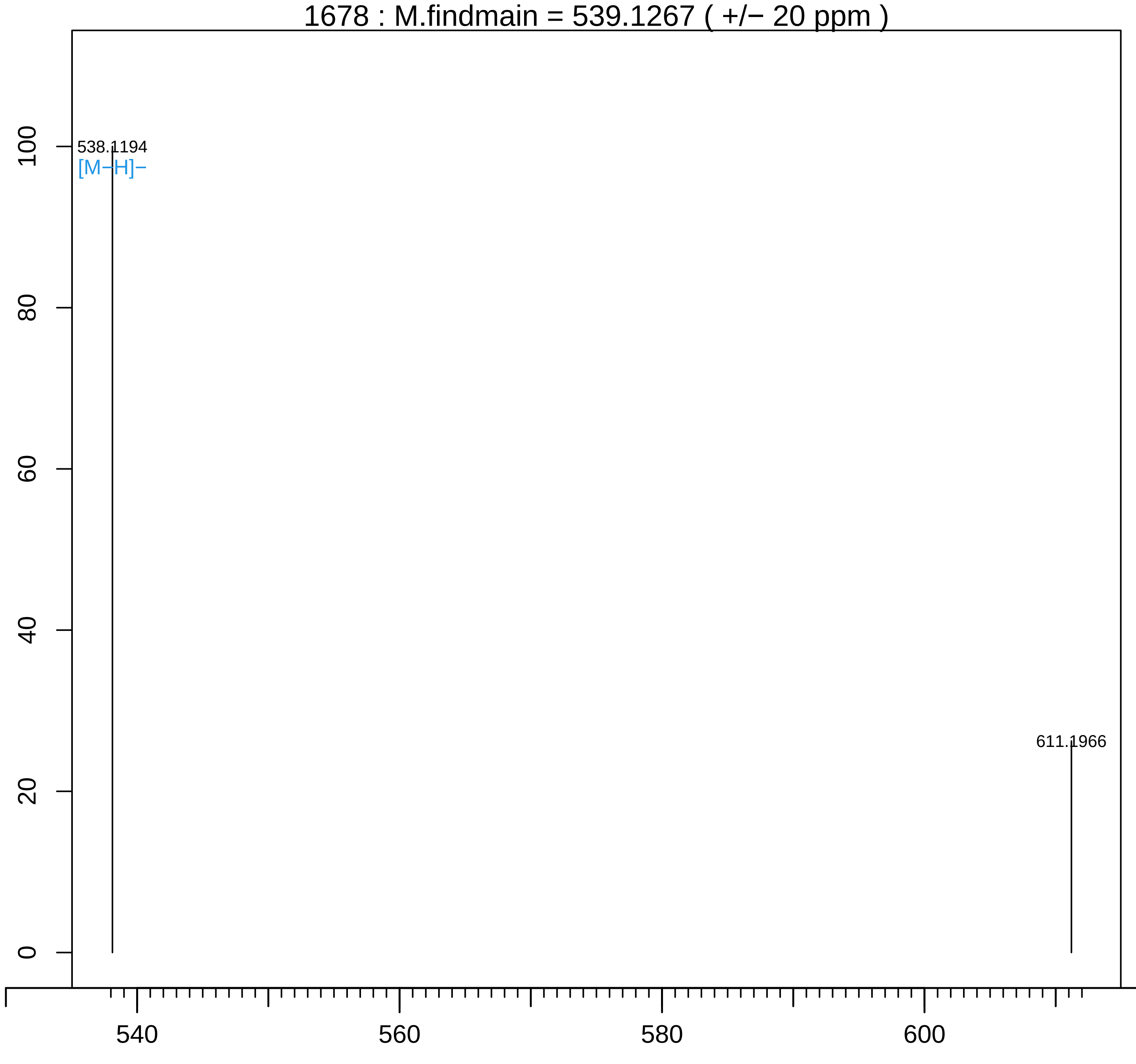

1678 : M.findmain = 539.1267 (+/− 20 ppm)
538.1194
[M−H]−
611.1966
0
20
40
60
80
100
540
560
580
600

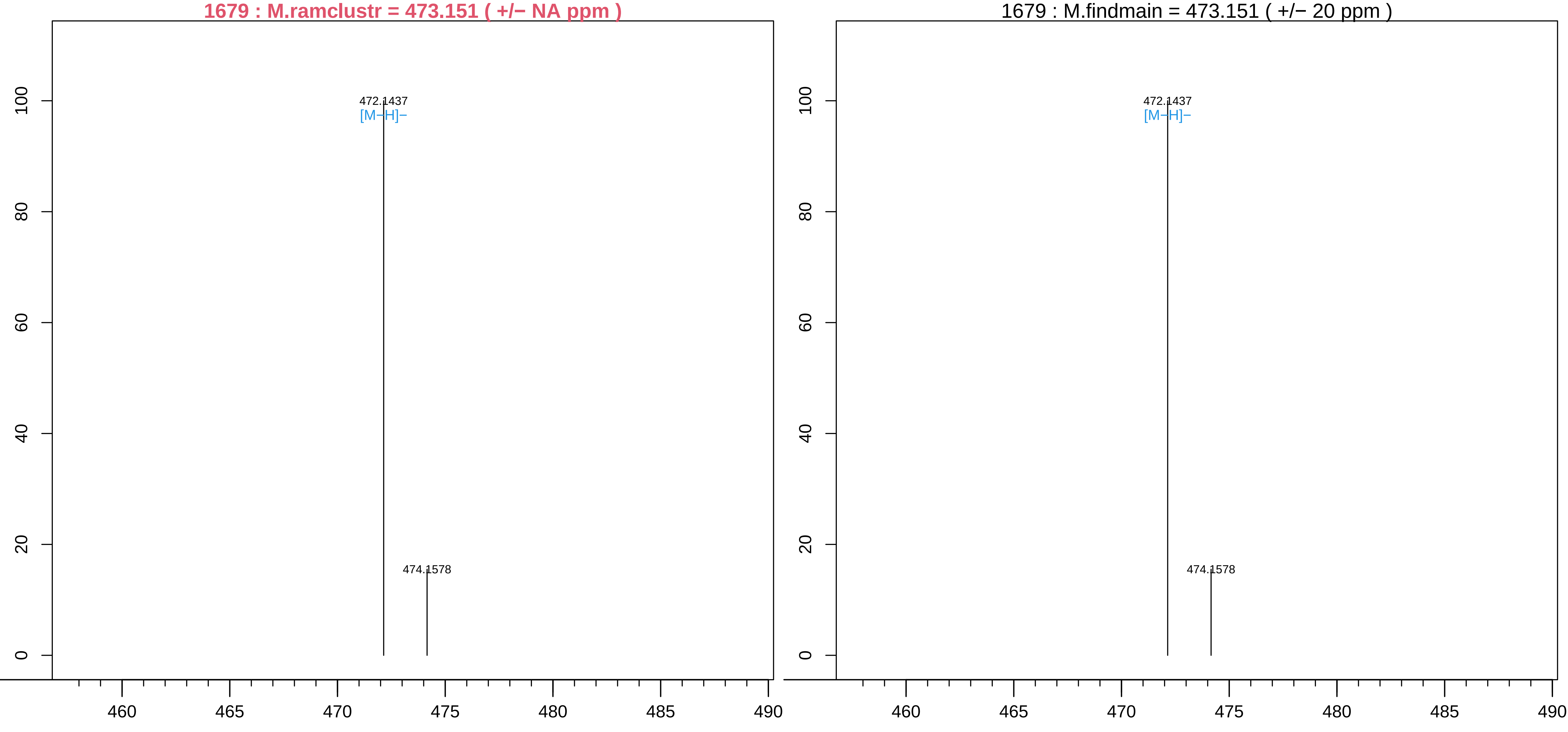
1679 : M.ramclustr = 473.151 (+/− NA ppm)
1679 : M.findmain = 473.151 (+/− 20 ppm)
472.1437
[M−H]−
474.1578
0
20
40
60
80
100
460
465
470
475
480
485
490

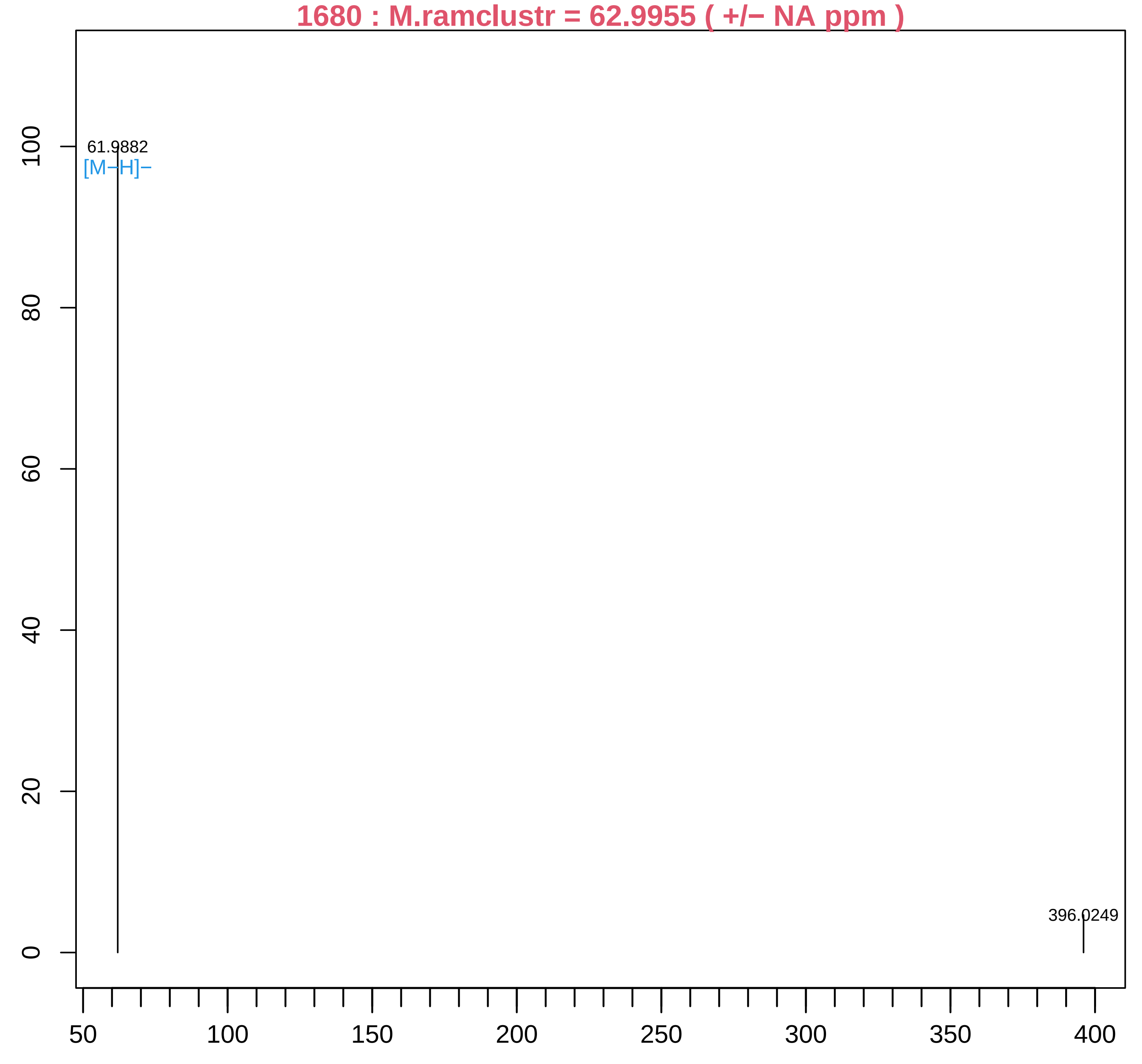
1680 : M.ramclustr = 62.9955 (+/− NA ppm)
61.9882
[M−H]−
396.0249
0
20
40
60
80
100
50
100
150
200
250
300
350
400

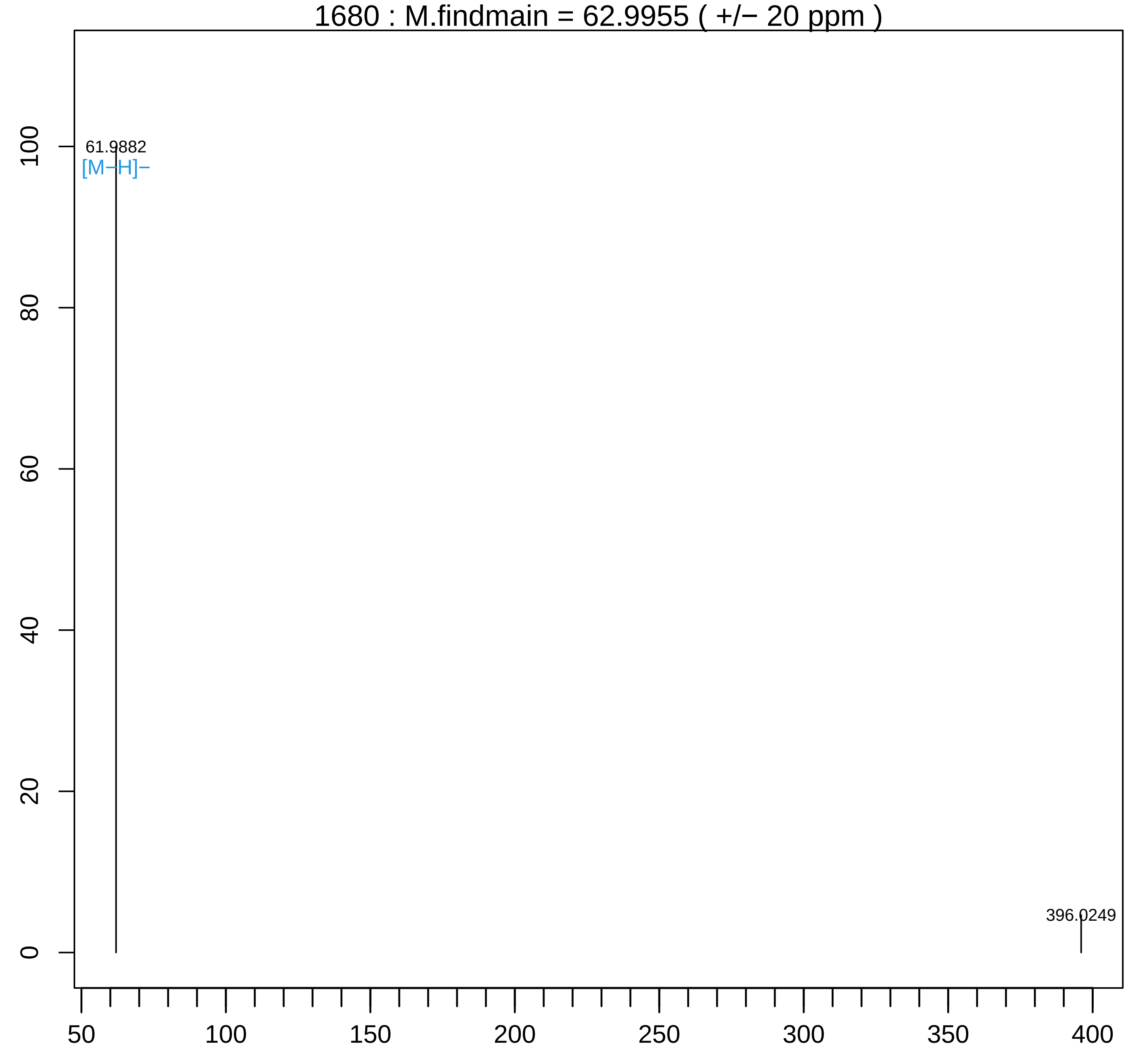
1680 : M.findmain = 62.9955 (+/− 20 ppm)
61.9882
[M−H]−
396.0249
0
20
40
60
80
100
50
100
150
200
250
300
350
400

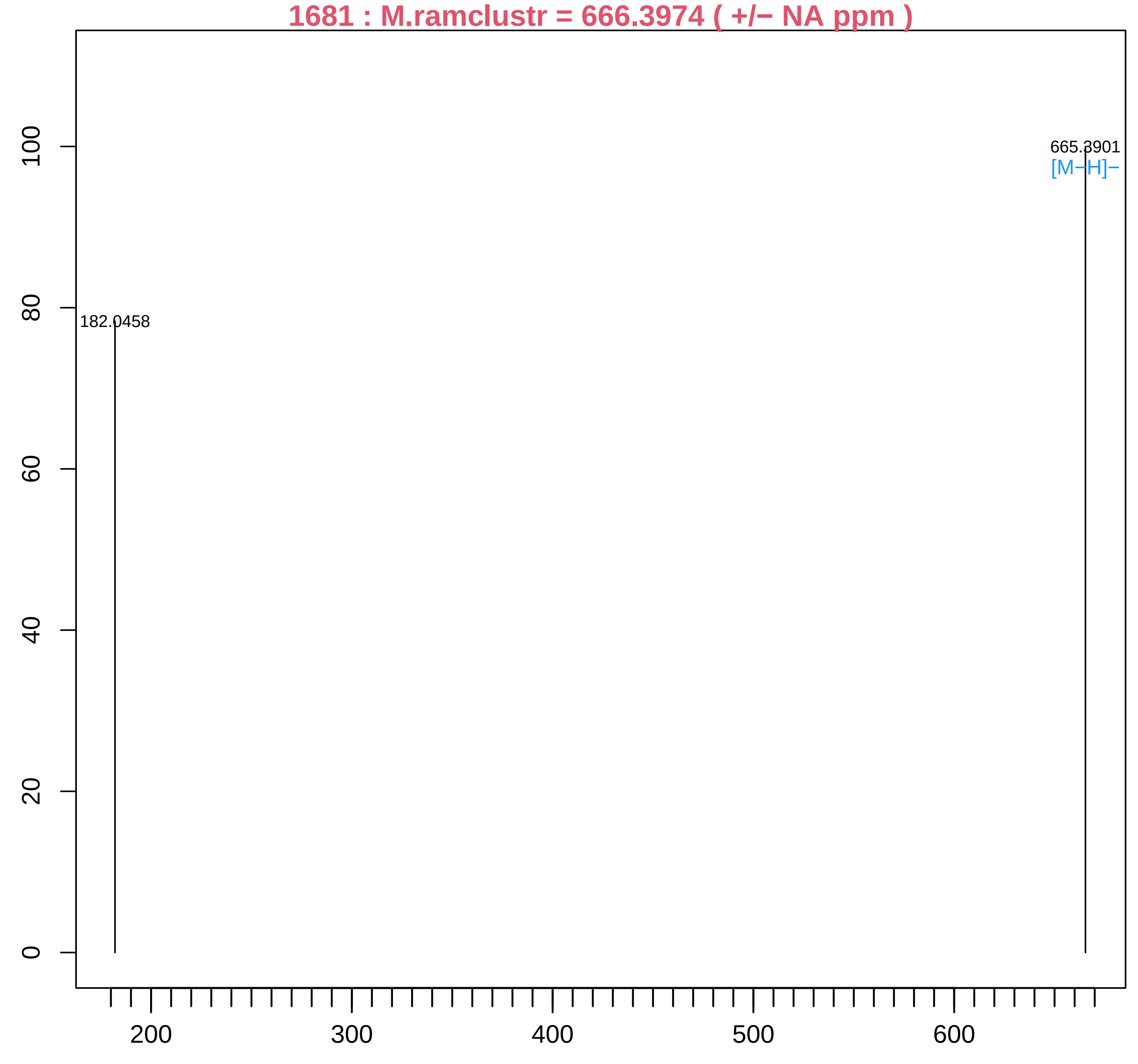
1681 : M.ramclustr = 666.3974 (+/− NA ppm)
665.3901
[M−H]−
182.0458
0
20
40
60
80
100
200
300
400
500
600

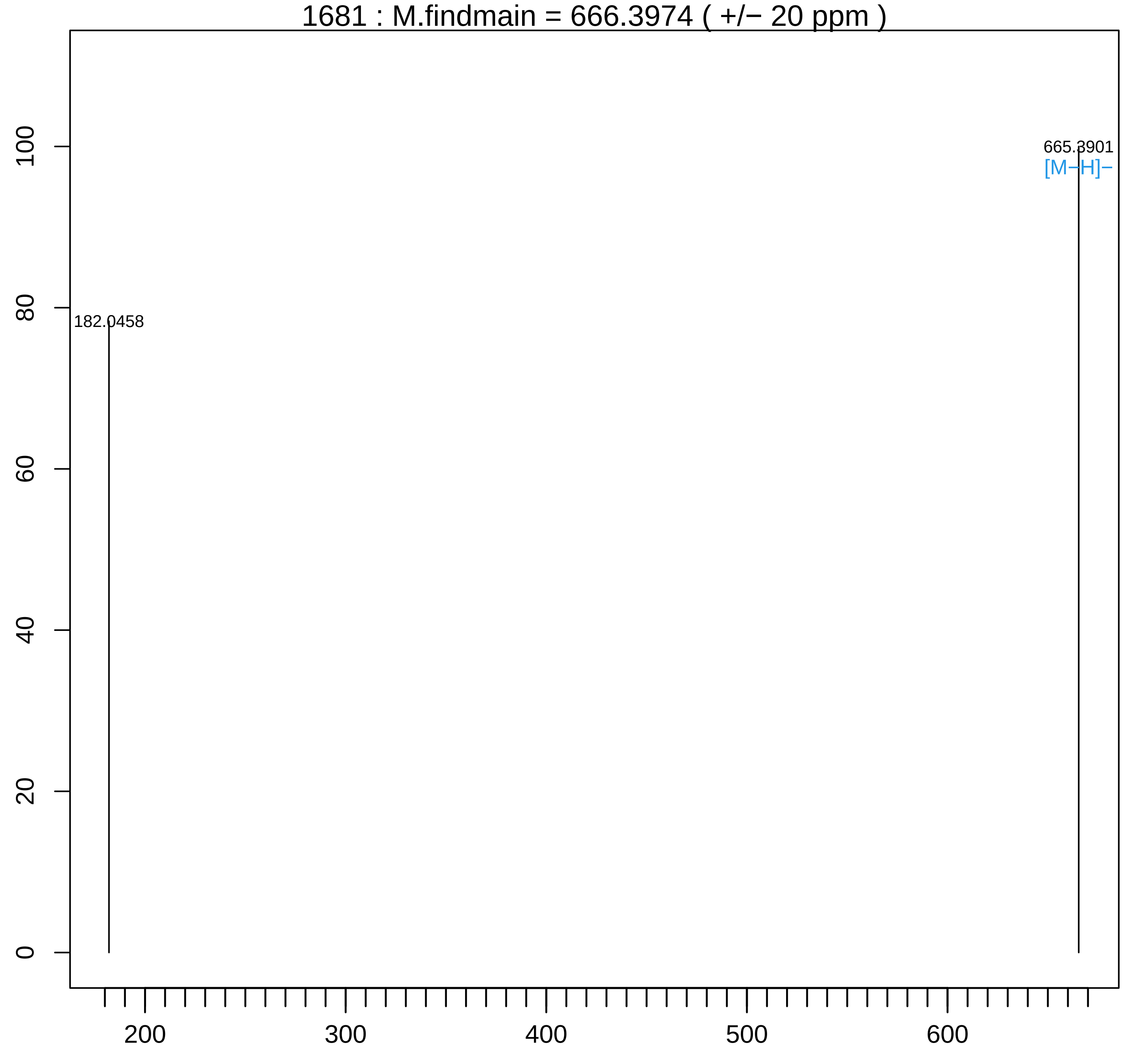
1681 : M.findmain = 666.3974 (+/− 20 ppm)
665.3901
[M−H]−
182.0458
0
20
40
60
80
100
200
300
400
500
600

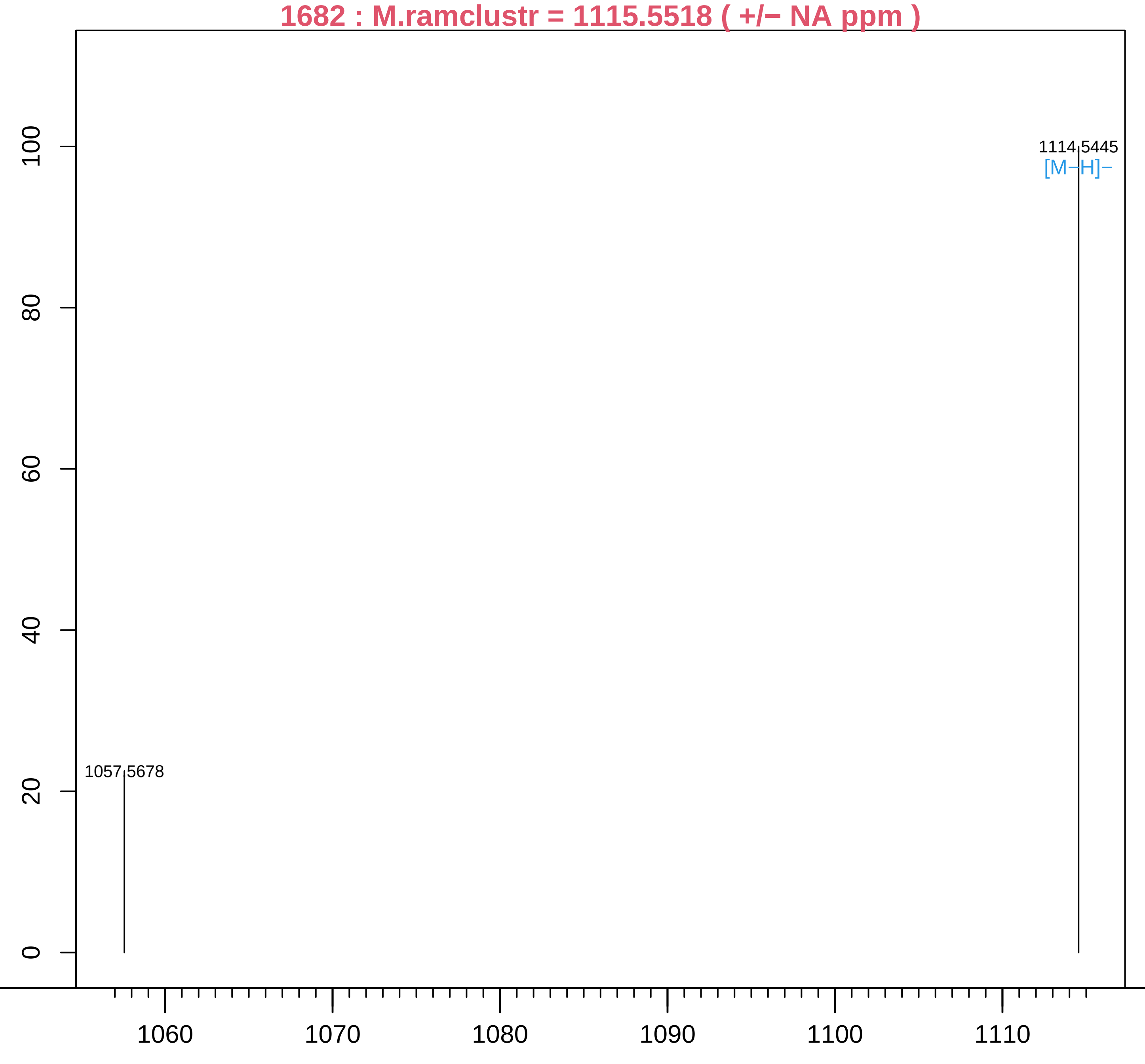
1682 : M.ramclustr = 1115.5518 (+/− NA ppm)
1114.5445
[M−H]−
1057.5678
0
20
40
60
80
100
1060
1070
1080
1090
1100
1110

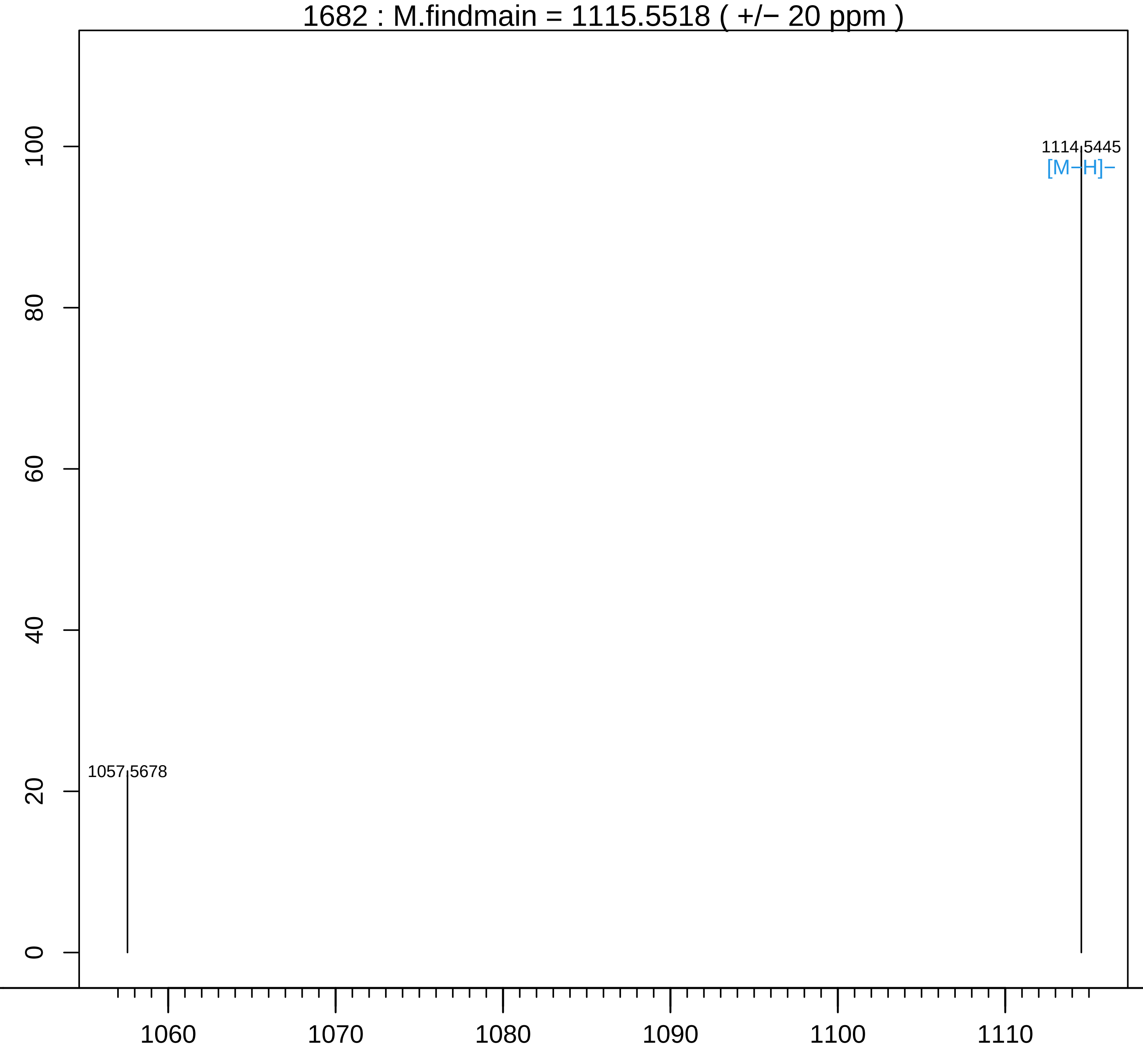
1682 : M.findmain = 1115.5518 (+/− 20 ppm)
1114.5445
[M−H]−
1057.5678
0
20
40
60
80
100
1060
1070
1080
1090
1100
1110

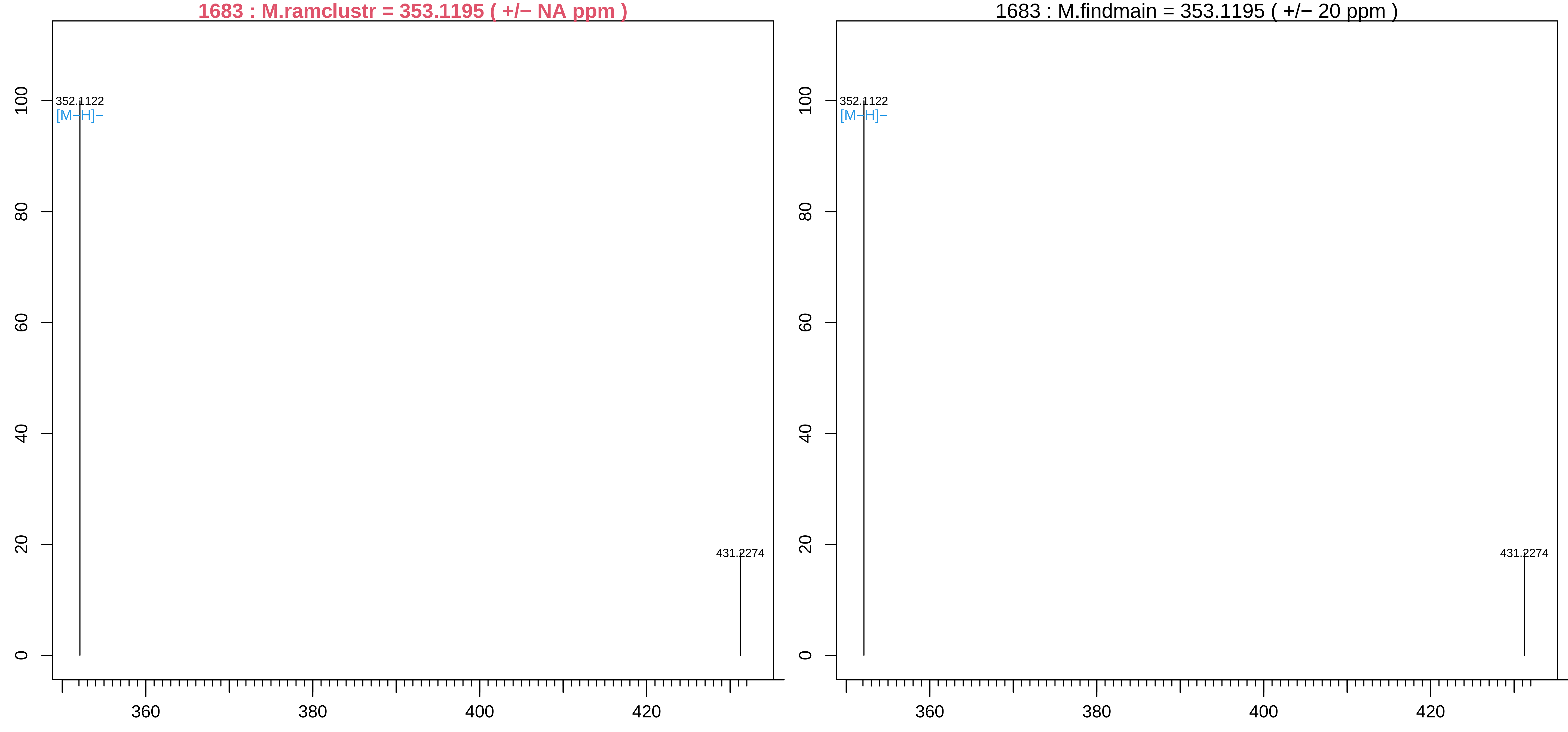
1683 : M.ramclustr = 353.1195 (+/− NA ppm)
352.1122
[M−H]−
431.2274
1683 : M.findmain = 353.1195 (+/− 20 ppm)
352.1122
[M−H]−
431.2274
0
20
40
60
80
100
360
380
400
420

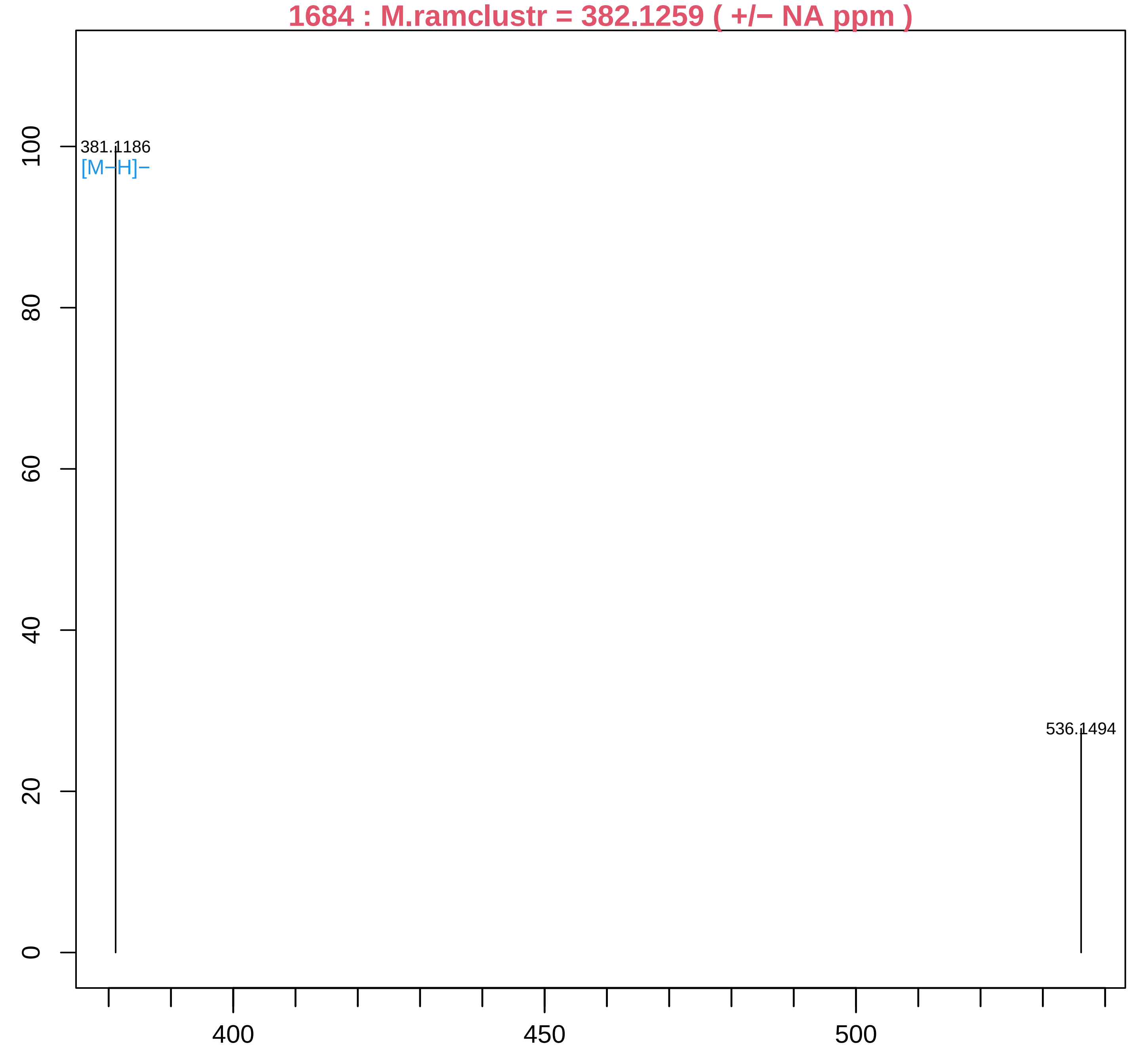
1684 : M.ramclustr = 382.1259 (+/− NA ppm)
381.1186
[M−H]−
536.1494
0
20
40
60
80
100
400
450
500

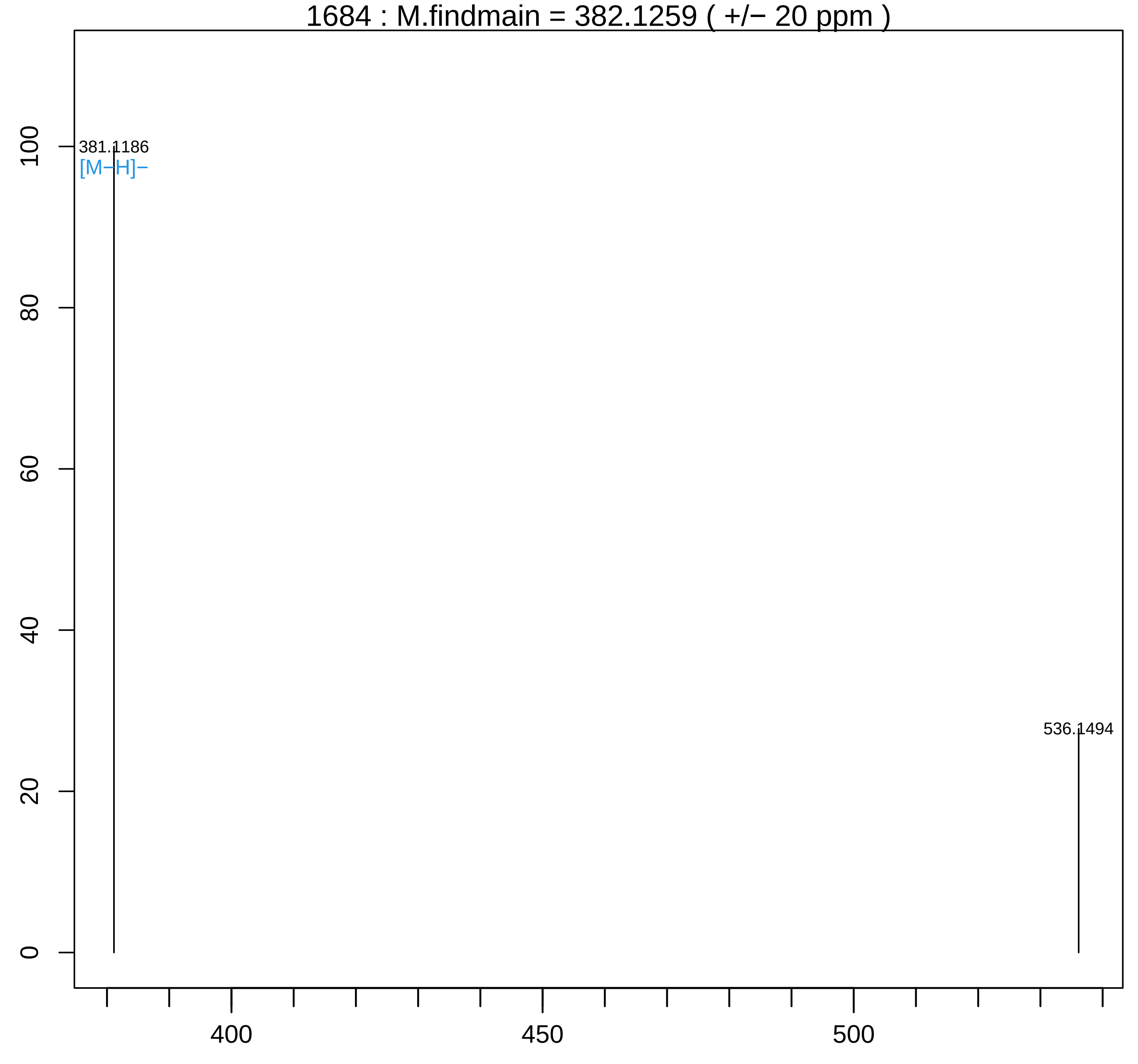
1684 : M.findmain = 382.1259 (+/− 20 ppm)
381.1186
[M−H]−
536.1494
0
20
40
60
80
100
400
450
500

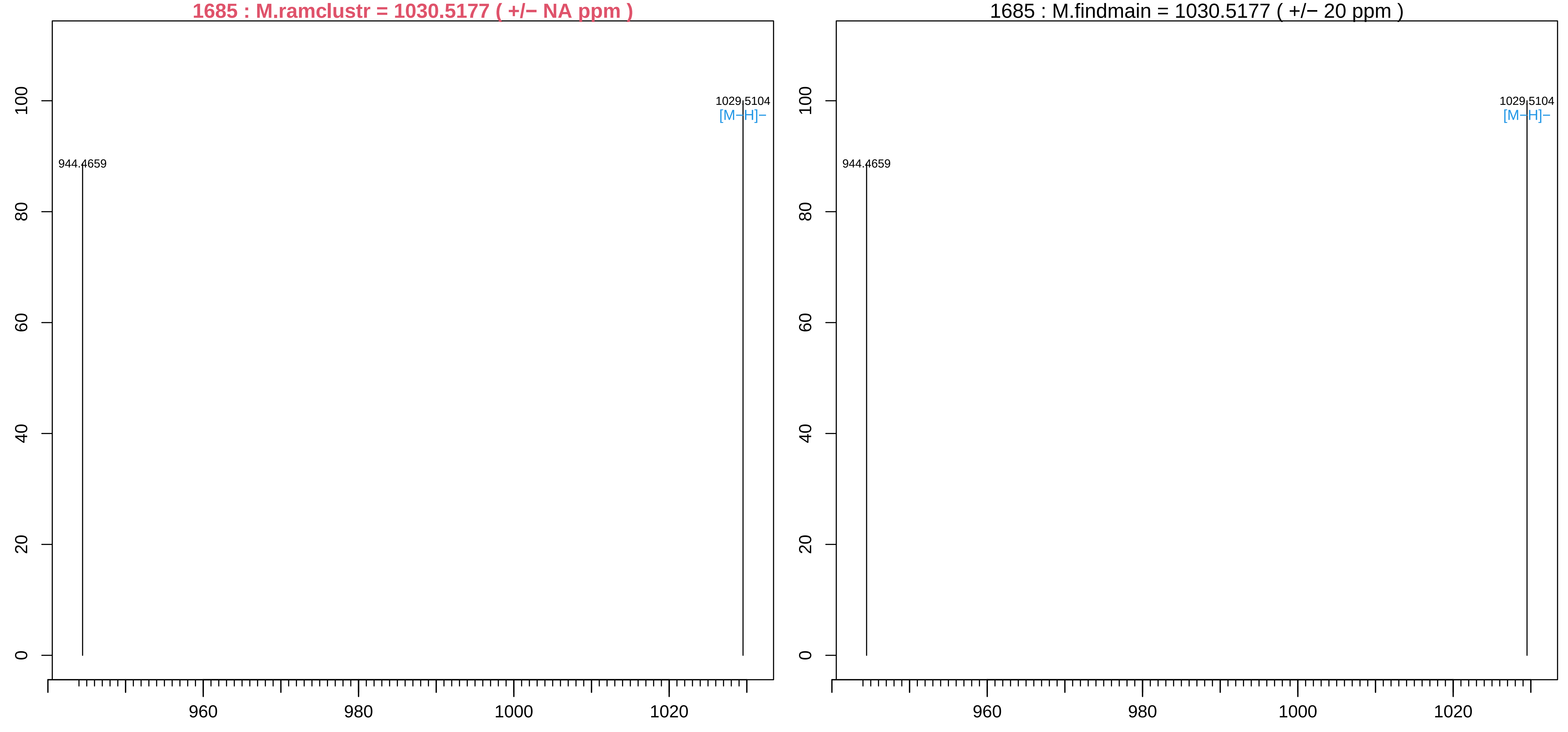
1685 : M.ramclustr = 1030.5177 (+/− NA ppm)
1029.5104
[M−H]−
944.4659
1685 : M.findmain = 1030.5177 (+/− 20 ppm)
1029.5104
[M−H]−
944.4659
0
20
40
60
80
100
960
980
1000
1020

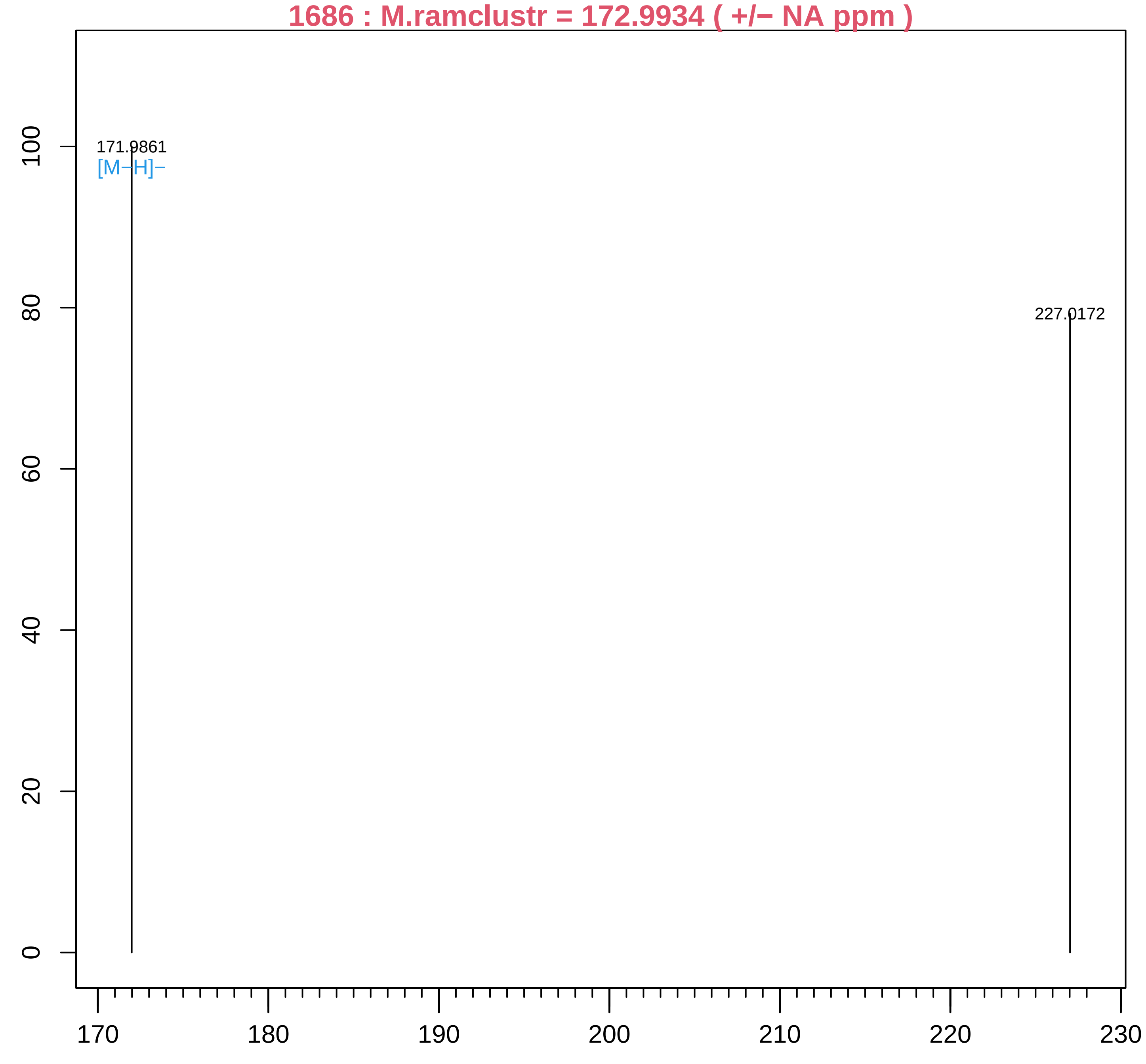
1686 : M.ramclustr = 172.9934 (+/− NA ppm)
171.9861
[M−H]−
227.0172
0
20
40
60
80
100
170
180
190
200
210
220
230
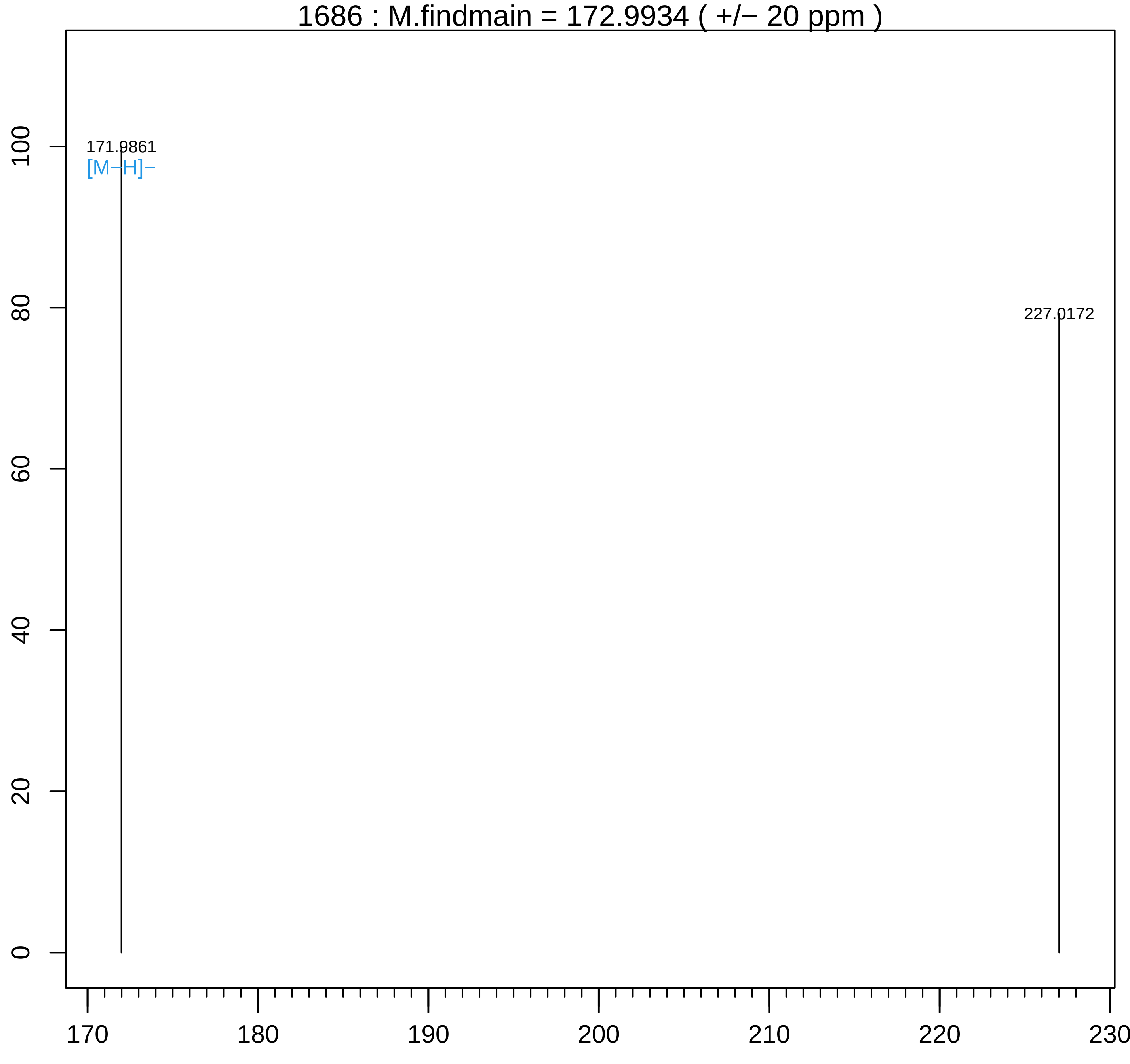
1686 : M.findmain = 172.9934 (+/− 20 ppm)
171.9861
[M−H]−
227.0172
0
20
40
60
80
100
170
180
190
200
210
220
230

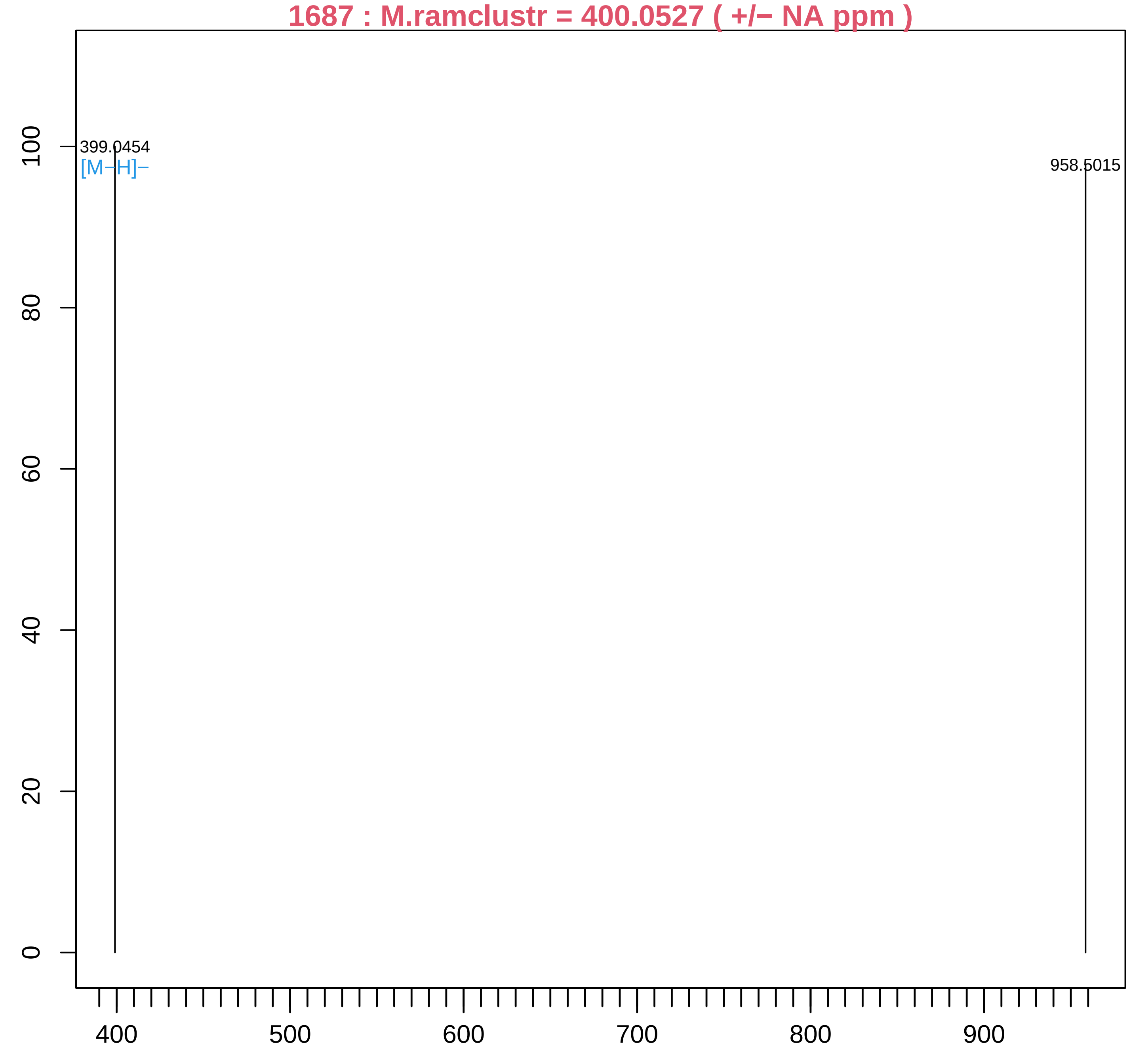

1687 : M.ramclustr = 400.0527 (+/− NA ppm)
399.0454
[M−H]−
958.5015
0
20
40
60
80
100
400
500
600
700
800
900

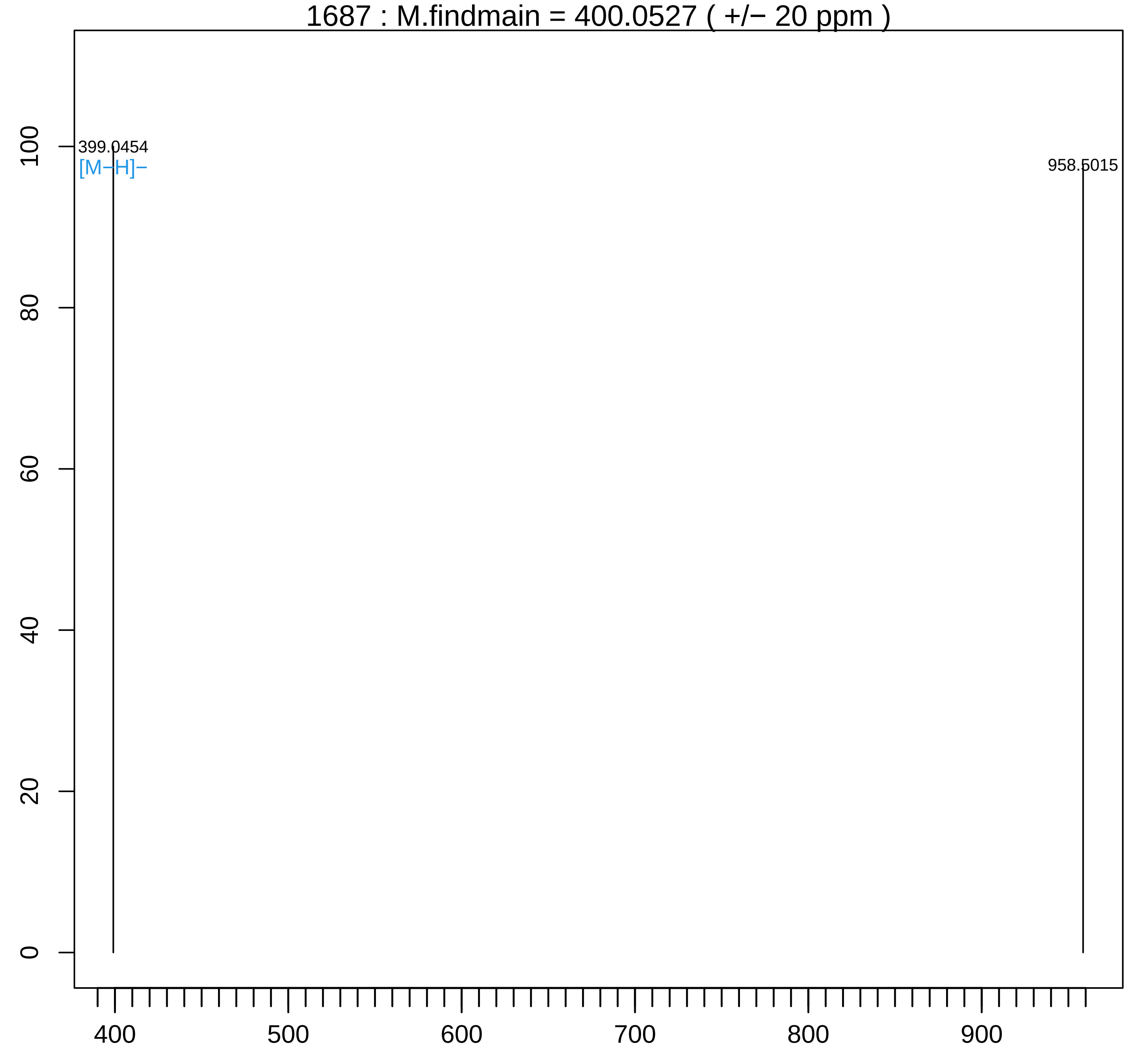

1687 : M.findmain = 400.0527 (+/− 20 ppm)
399.0454
[M−H]−
958.5015
0
20
40
60
80
100
400
500
600
700
800
900

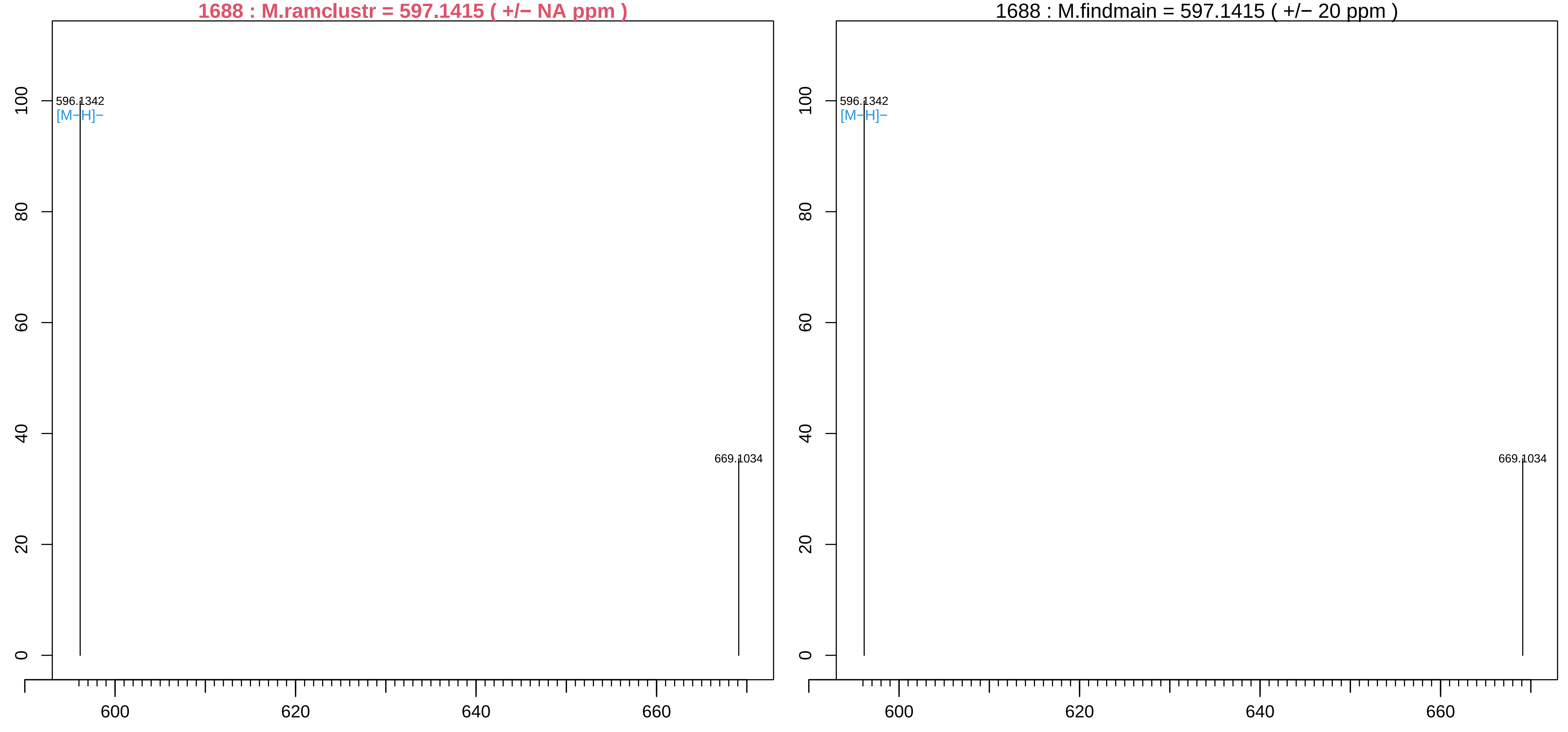
1688 : M.ramclustr = 597.1415 (+/− NA ppm)
596.1342
[M−H]−
669.1034
0
20
40
60
80
100
600
620
640
660
1688 : M.findmain = 597.1415 (+/− 20 ppm)
596.1342
[M−H]−
669.1034
0
20
40
60
80
100
600
620
640
660

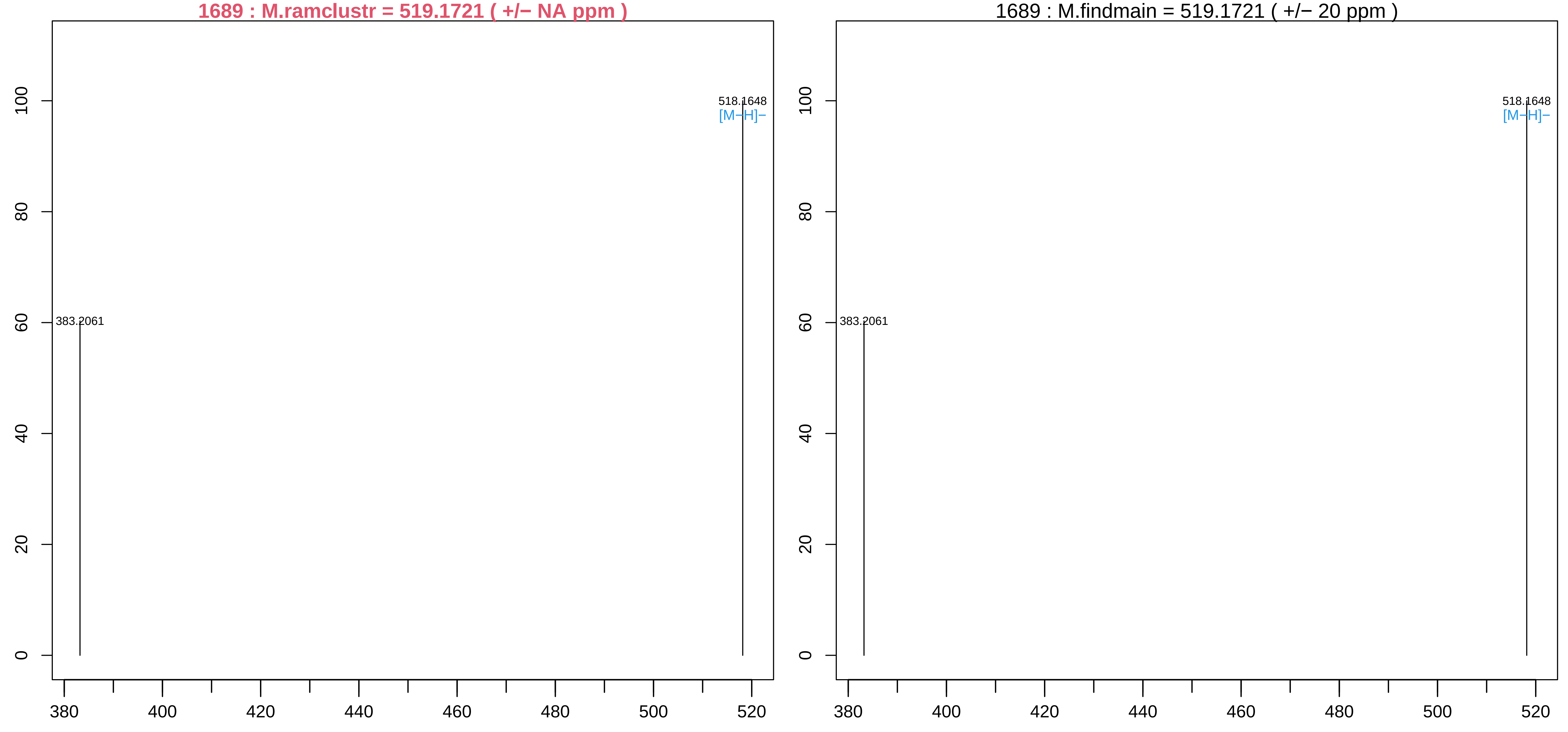
1689 : M.ramclustr = 519.1721 (+/− NA ppm)
518.1648
[M−H]−
383.2061
0
20
40
60
80
100
380
400
420
440
460
480
500
520
1689 : M.findmain = 519.1721 (+/− 20 ppm)
518.1648
[M−H]−
383.2061
0
20
40
60
80
100
380
400
420
440
460
480
500
520

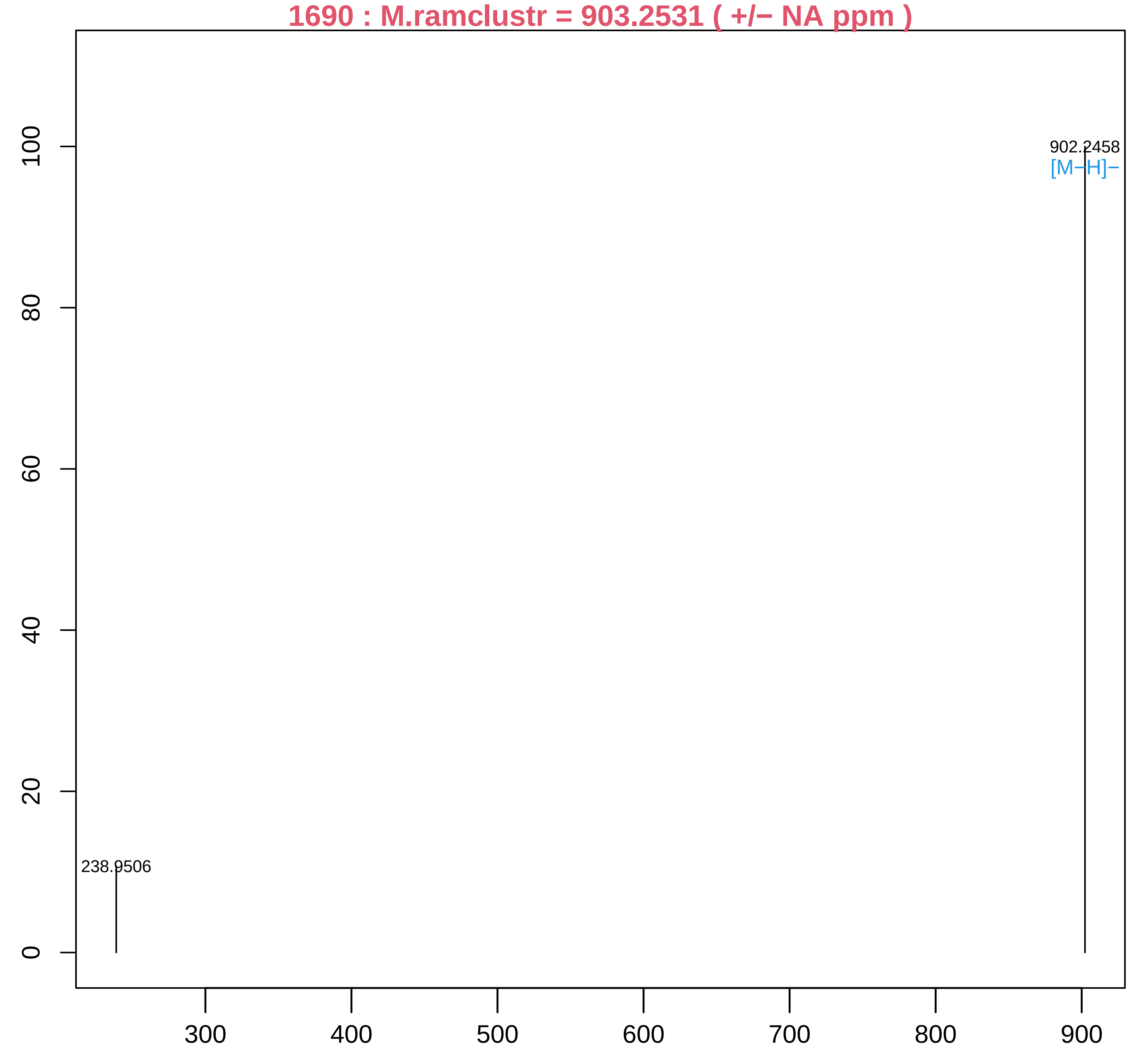
1690 : M.ramclustr = 903.2531 (+/− NA ppm)
902.2458
[M−H]−
238.9506
0
20
40
60
80
100
300
400
500
600
700
800
900

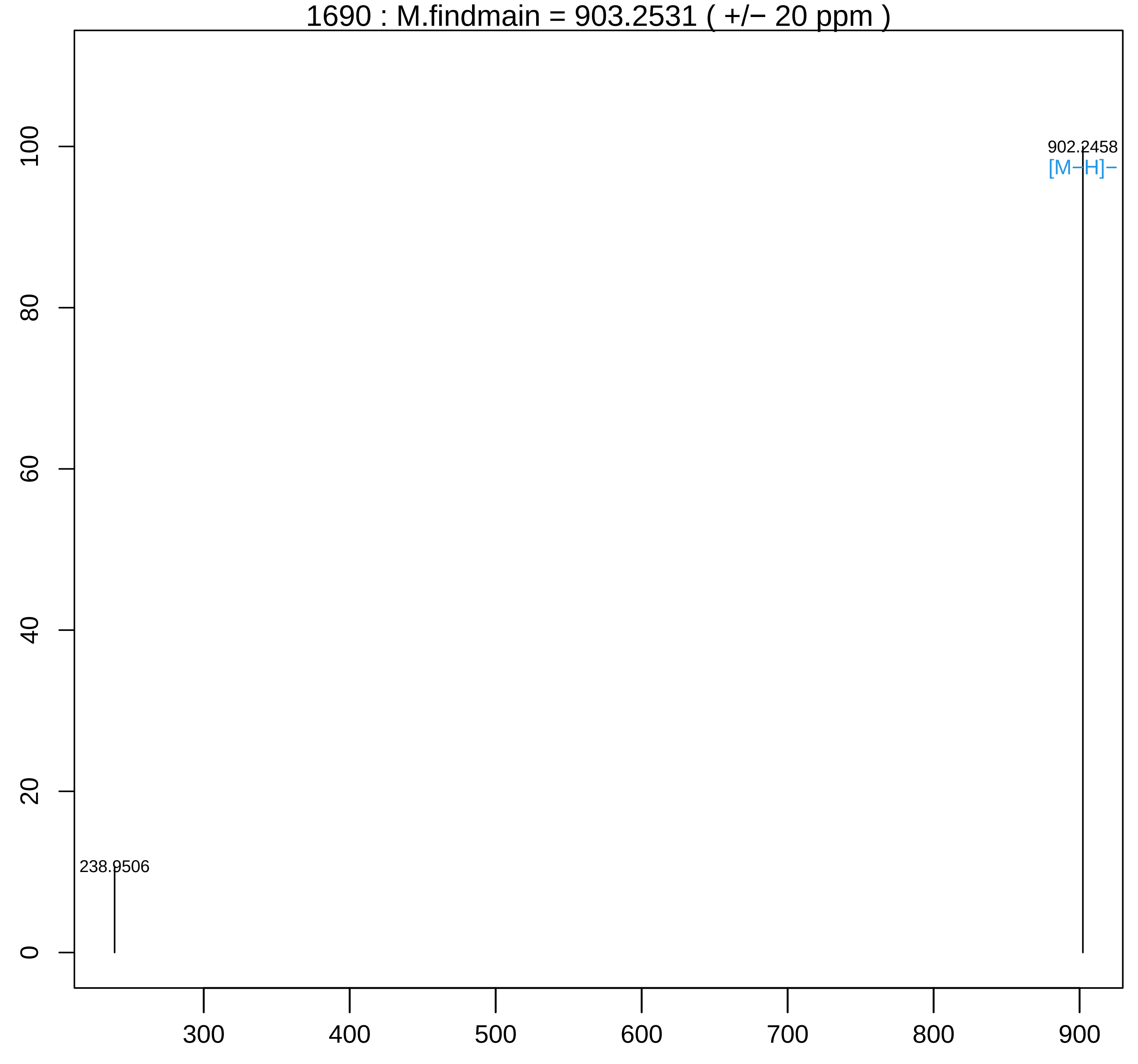
1690 : M.findmain = 903.2531 (+/− 20 ppm)
902.2458
[M−H]−
238.9506
0
20
40
60
80
100
300
400
500
600
700
800
900

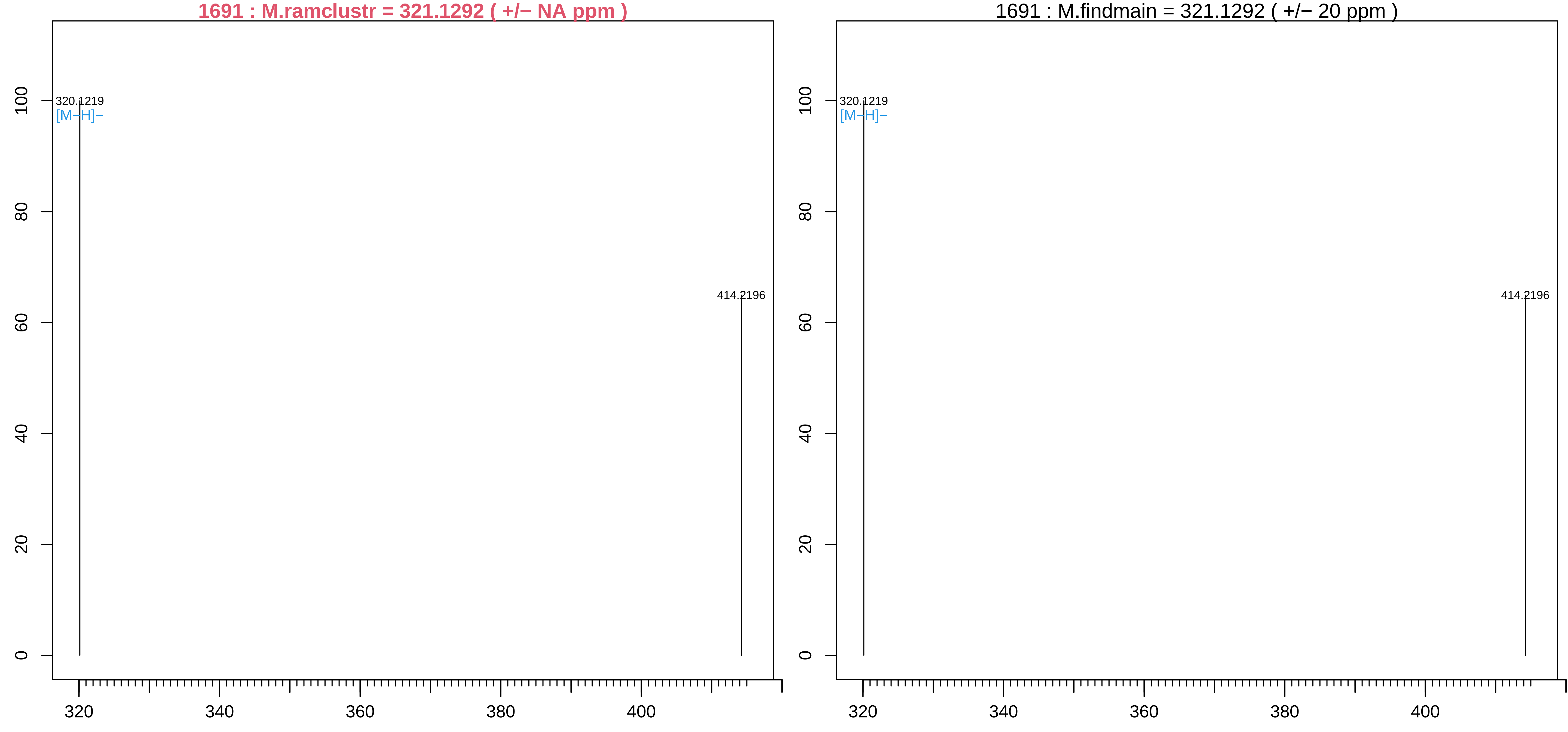
1691 : M.ramclustr = 321.1292 (+/− NA ppm)
320.1219
[M−H]−
414.2196
1691 : M.findmain = 321.1292 (+/− 20 ppm)
320.1219
[M−H]−
414.2196
0
20
40
60
80
100
320
340
360
380
400

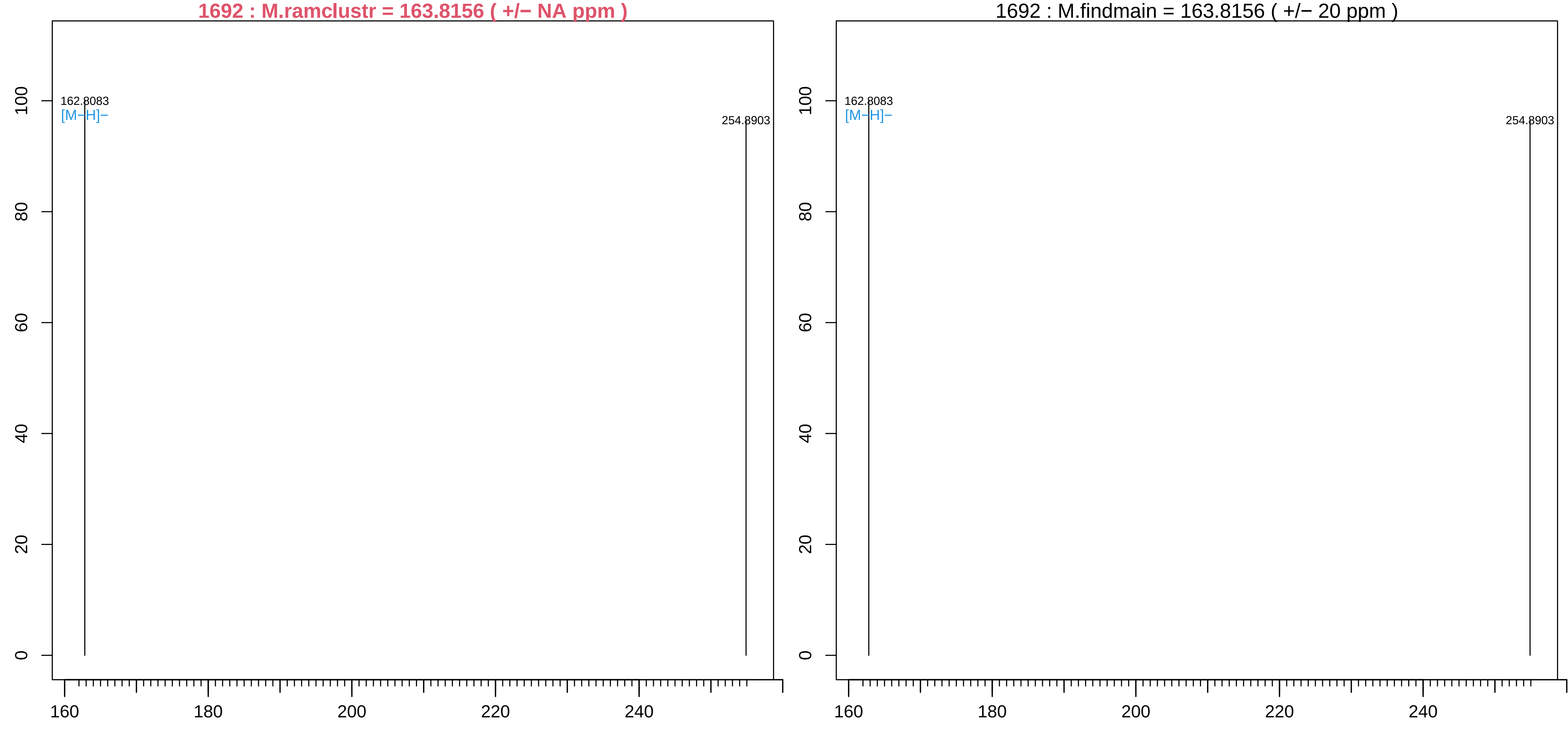
1692 : M.ramclustr = 163.8156 (+/− NA ppm)
162.8083
[M−H]−
254.8903
1692 : M.findmain = 163.8156 (+/− 20 ppm)
162.8083
[M−H]−
254.8903
0
20
40
60
80
100
160
180
200
220
240

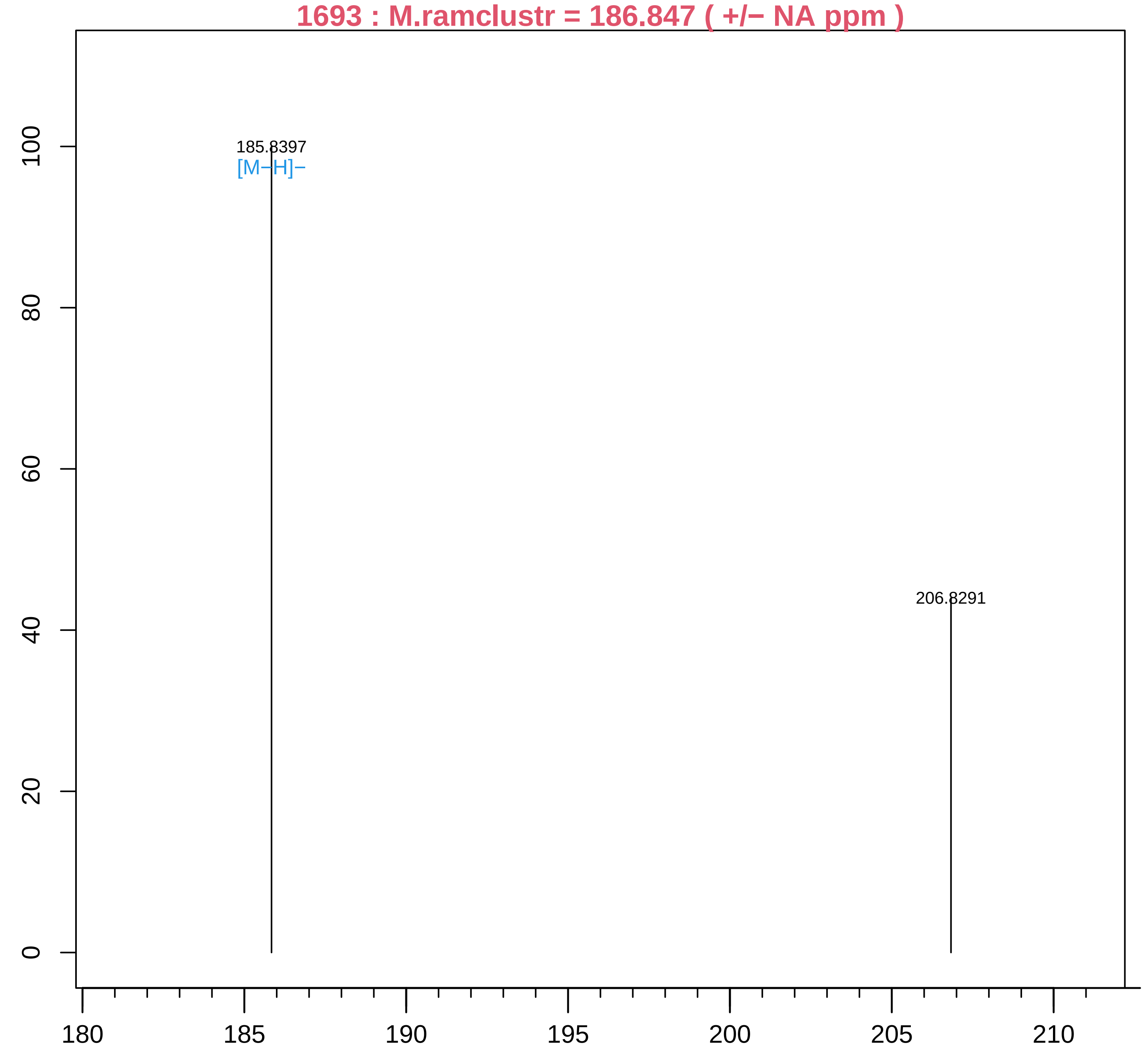
1693 : M.ramclustr = 186.847 (+/− NA ppm)
185.8397
[M−H]−
206.8291
0
20
40
60
80
100
180
185
190
195
200
205
210

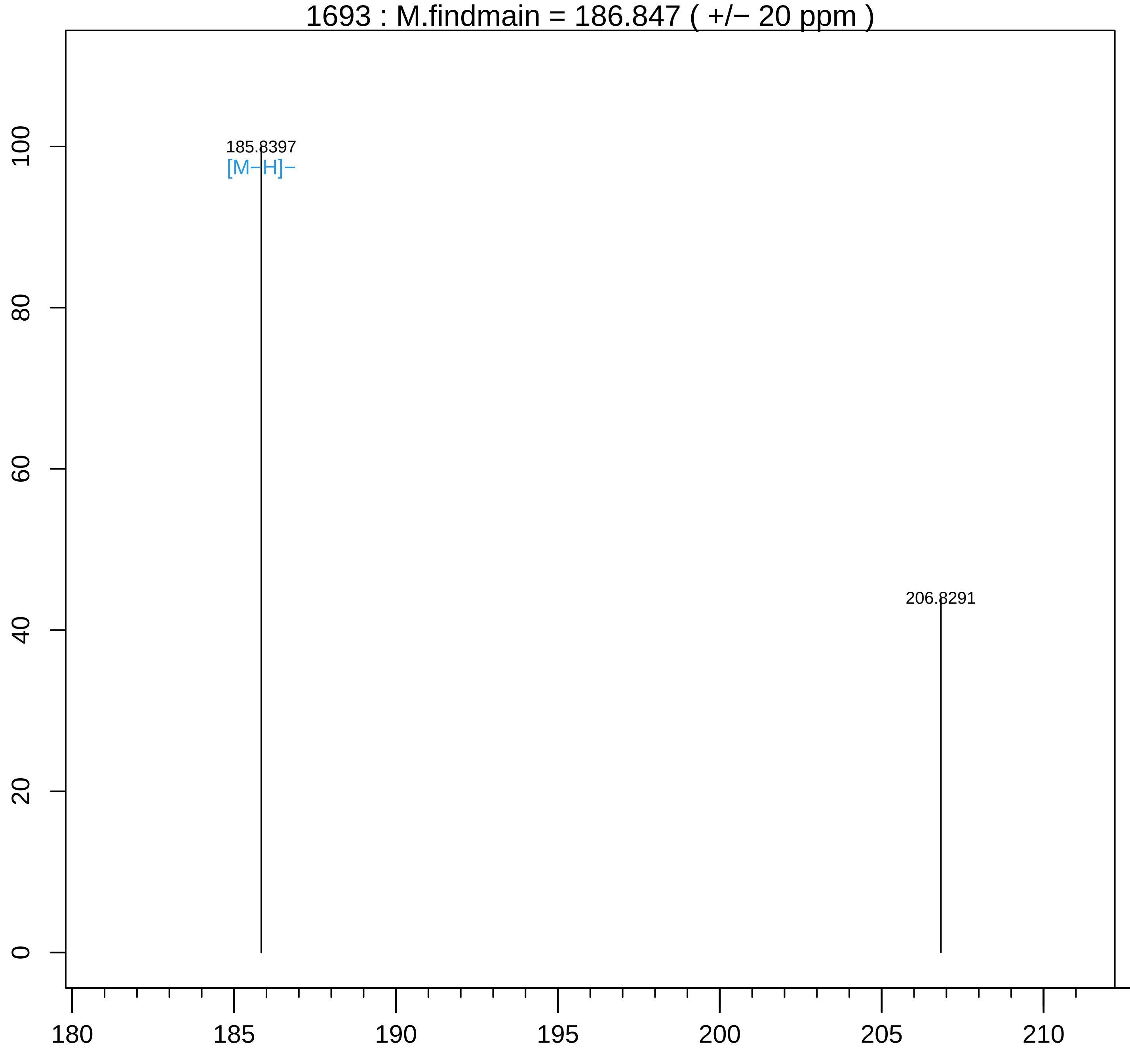
1693 : M.findmain = 186.847 (+/− 20 ppm)
185.8397
[M−H]−
206.8291
0
20
40
60
80
100
180
185
190
195
200
205
210

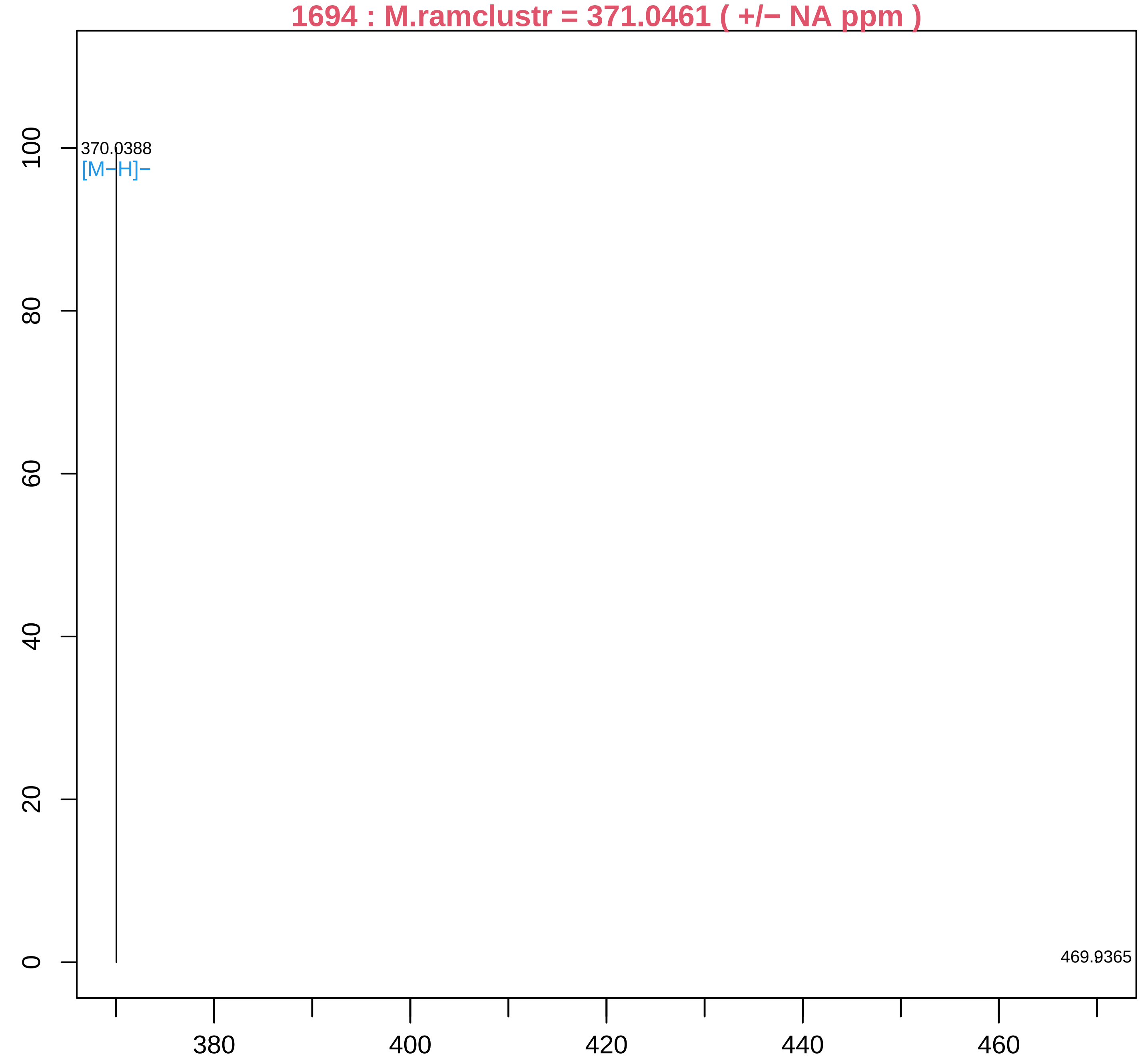
1694 : M.ramclustr = 371.0461 (+/− NA ppm)
370.0388
[M−H]−
469.9365
0
20
40
60
80
100
380
400
420
440
460

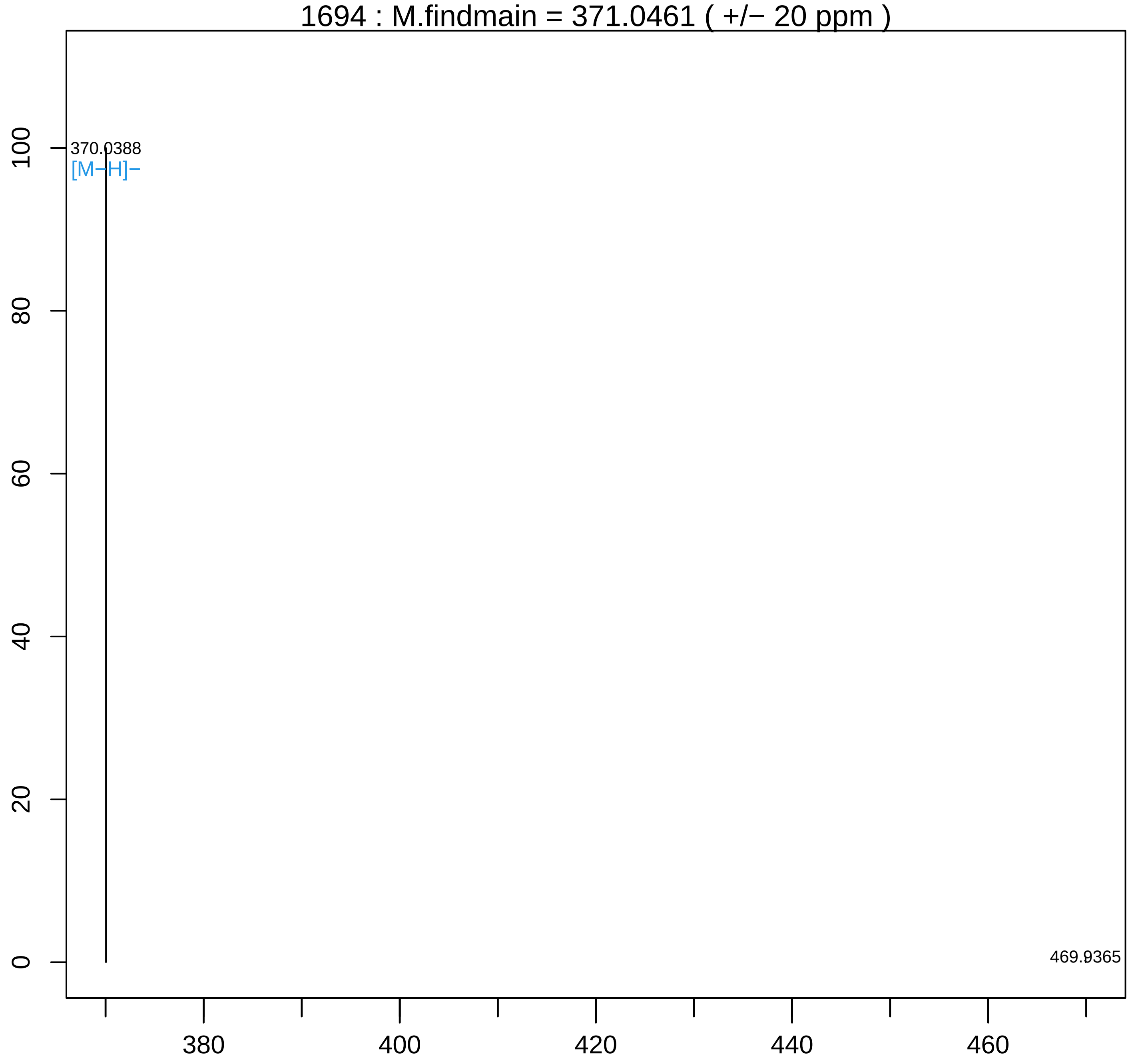
1694 : M.findmain = 371.0461 (+/− 20 ppm)
370.0388
[M−H]−
469.9365
0
20
40
60
80
100
380
400
420
440
460

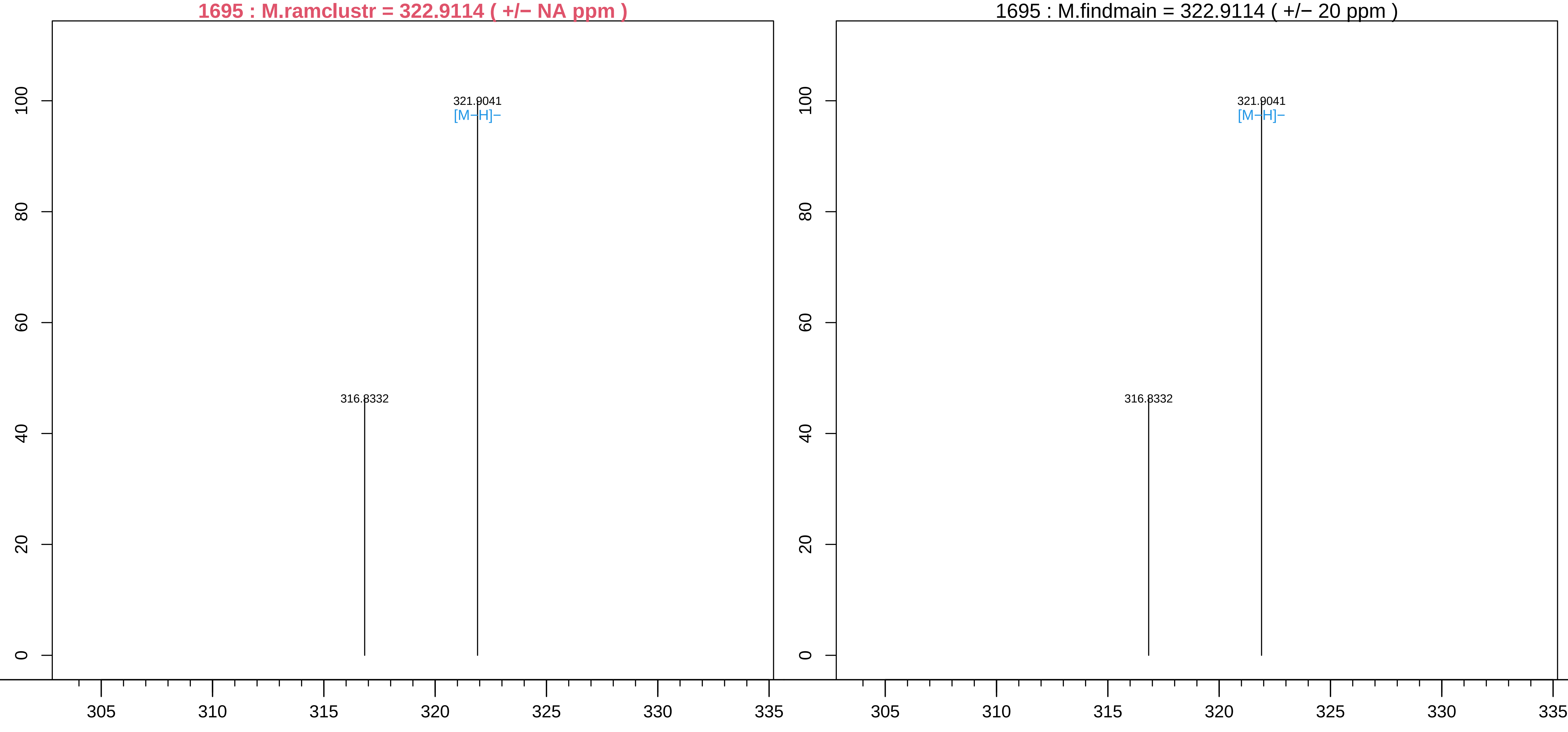

1695 : M.ramclustr = 322.9114 (+/− NA ppm)
321.9041
[M−H]−
316.8332
1695 : M.findmain = 322.9114 (+/− 20 ppm)
321.9041
[M−H]−
316.8332

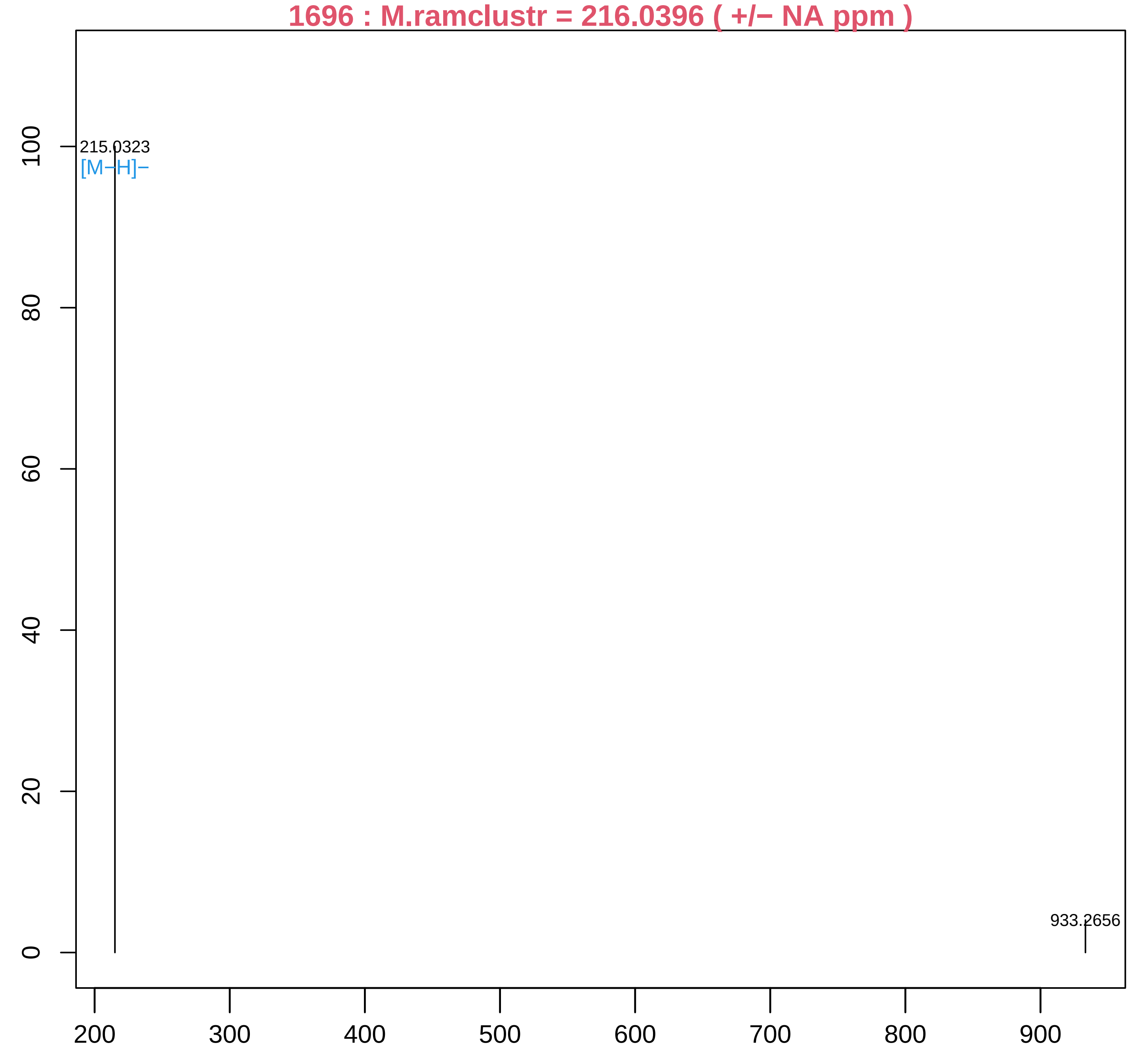
1696 : M.ramclustr = 216.0396 (+/− NA ppm)
215.0323
[M−H]−
933.2656
0
20
40
60
80
100
200
300
400
500
600
700
800
900

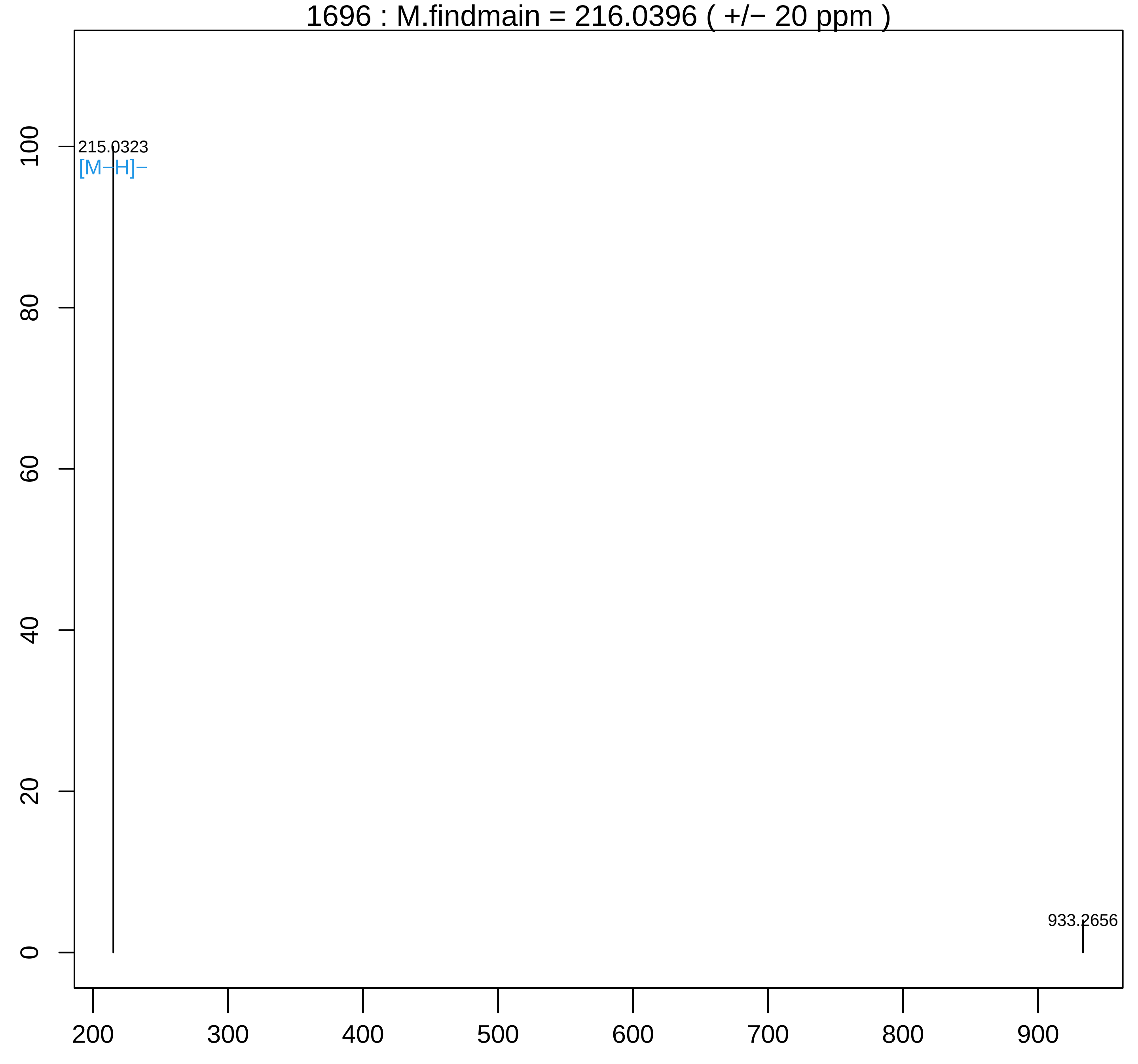
1696 : M.findmain = 216.0396 (+/− 20 ppm)
215.0323
[M−H]−
933.2656
0
20
40
60
80
100
200
300
400
500
600
700
800
900

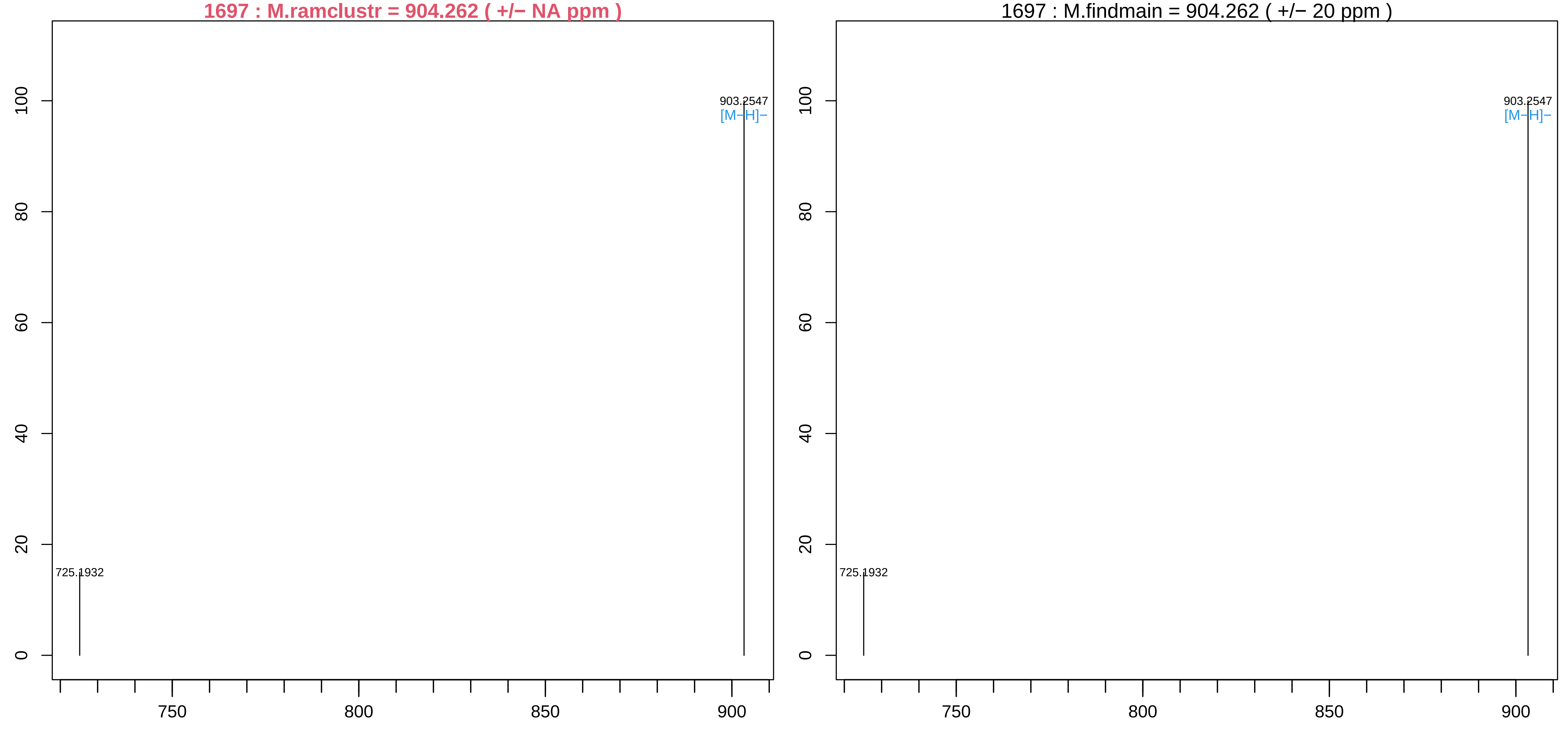
1697 : M.ramclustr = 904.262 (+/− NA ppm)
903.2547
[M−H]−
725.1932
0
20
40
60
80
100
750
800
850
900
1697 : M.findmain = 904.262 (+/− 20 ppm)
903.2547
[M−H]−
725.1932
0
20
40
60
80
100
750
800
850
900

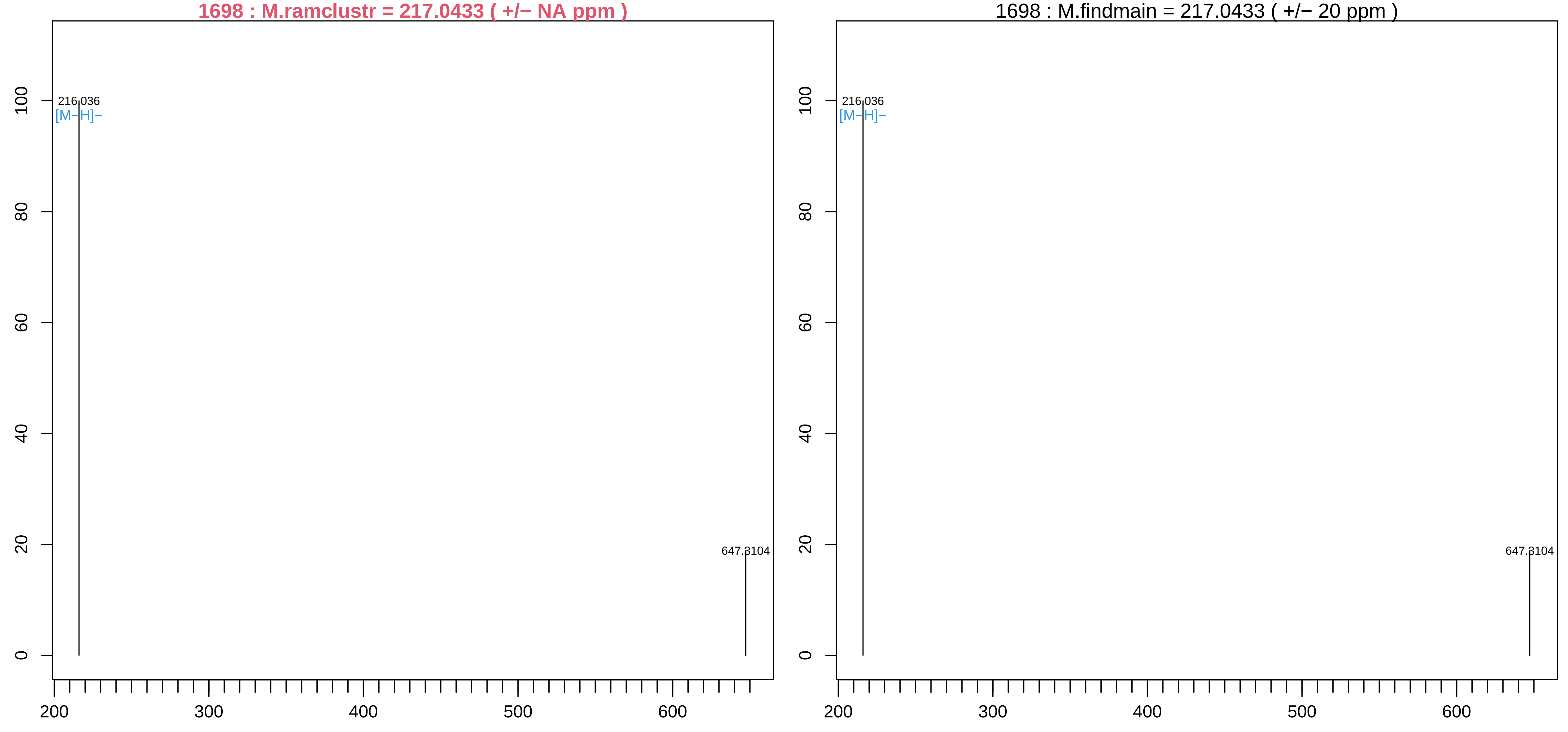
1698 : M.ramclustr = 217.0433 (+/− NA ppm)
216.036
[M−H]−
647.3104
1698 : M.findmain = 217.0433 (+/− 20 ppm)
216.036
[M−H]−
647.3104
0
20
40
60
80
100
200
300
400
500
600

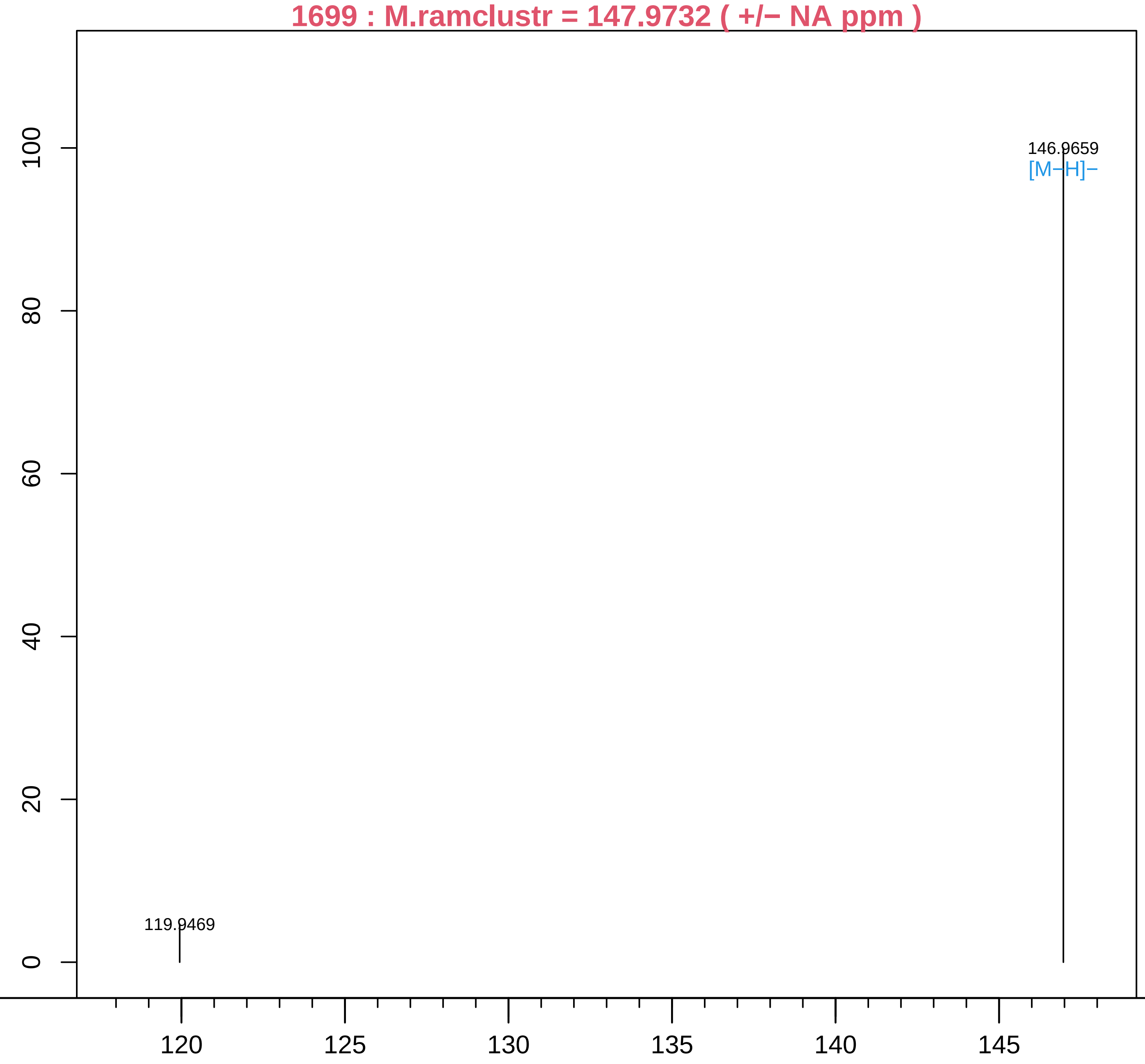

1699 : M.ramclustr = 147.9732 (+/− NA ppm)
146.9659
[M−H]−
119.9469
0
20
40
60
80
100
120
125
130
135
140
145

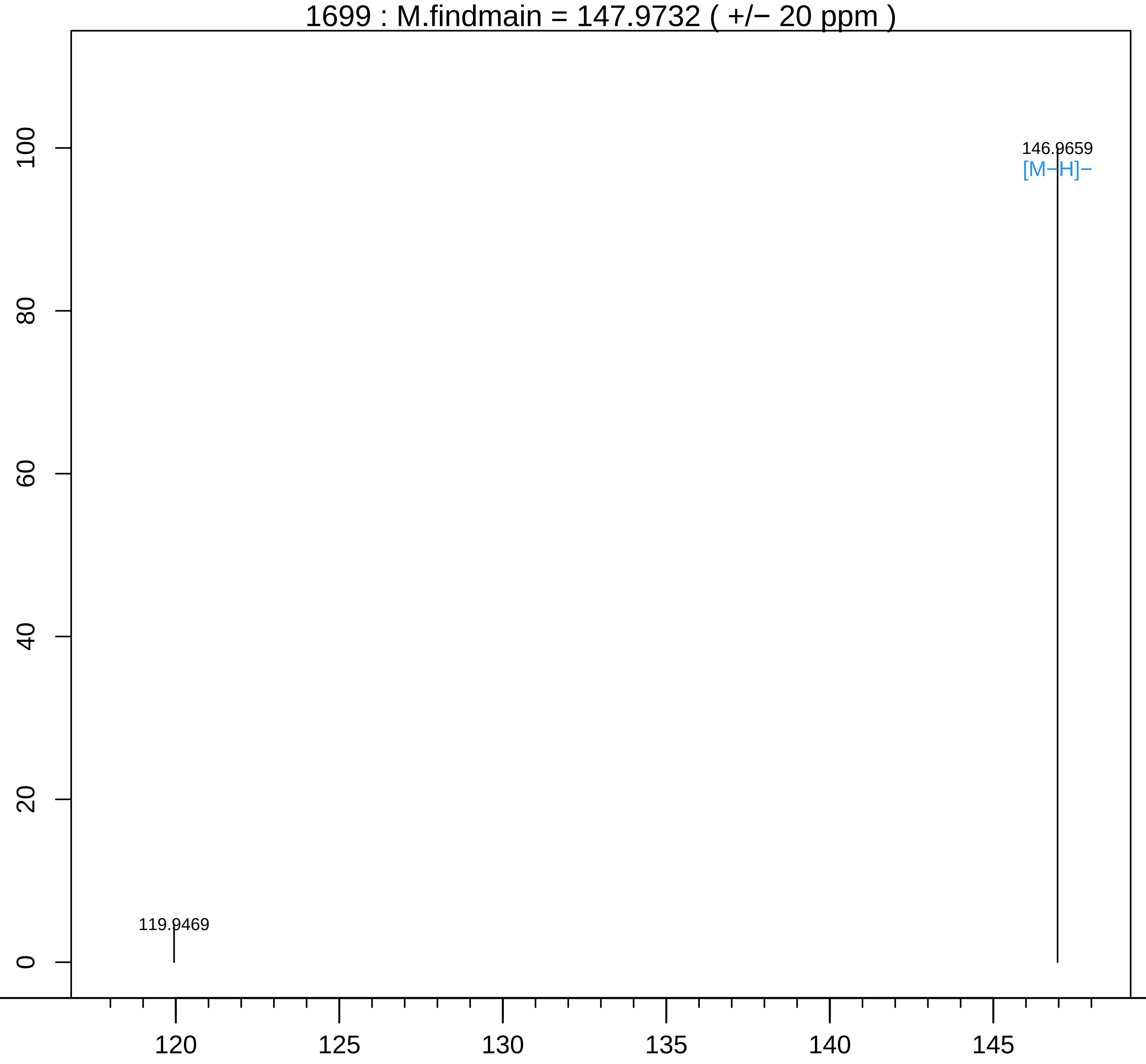

1699 : M.findmain = 147.9732 (+/− 20 ppm)
146.9659
[M−H]−
119.9469
0
20
40
60
80
100
120
125
130
135
140
145

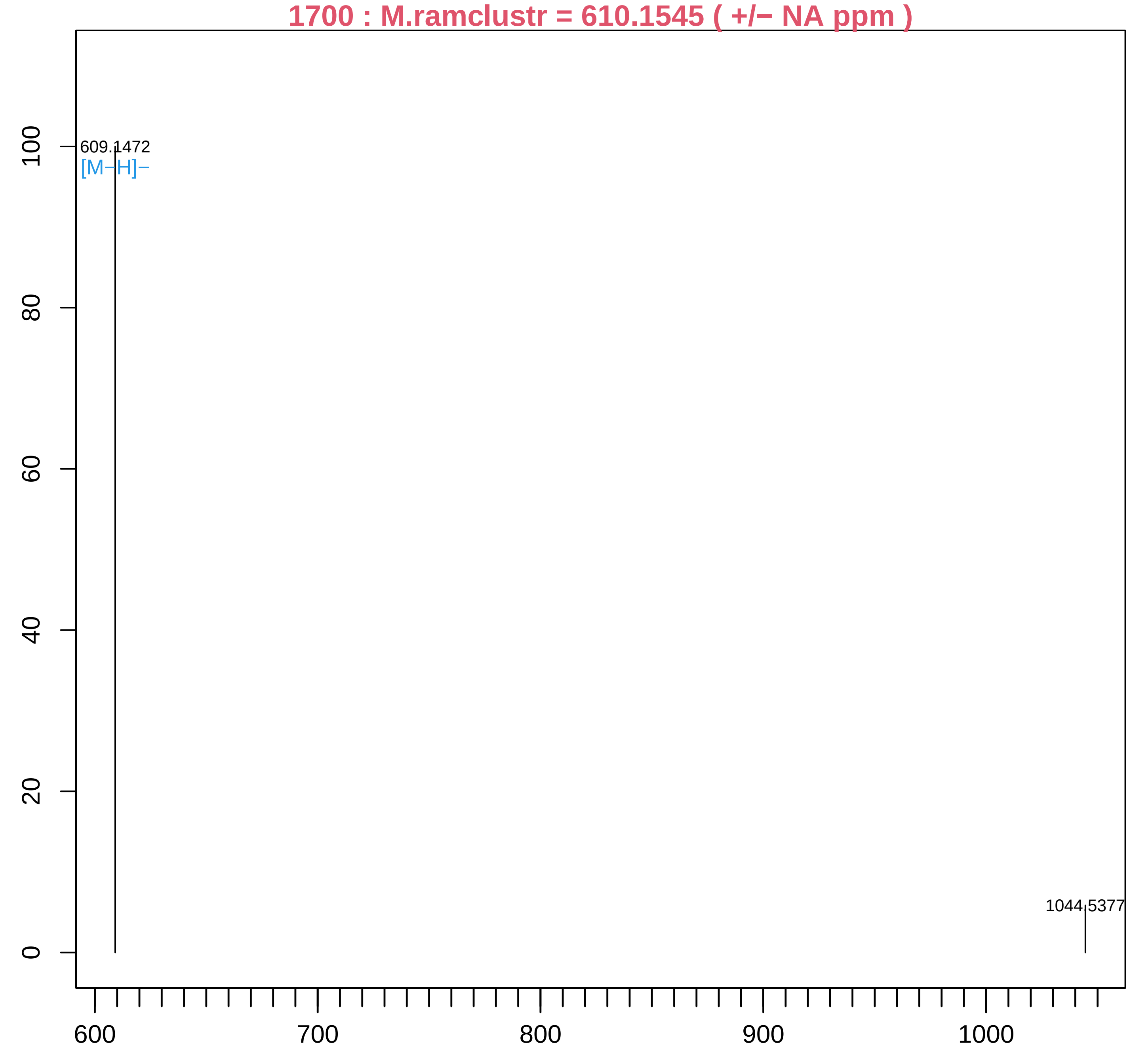
1700 : M.ramclustr = 610.1545 (+/− NA ppm)
609.1472
[M−H]−
1044.5377
0
20
40
60
80
100
600
700
800
900
1000

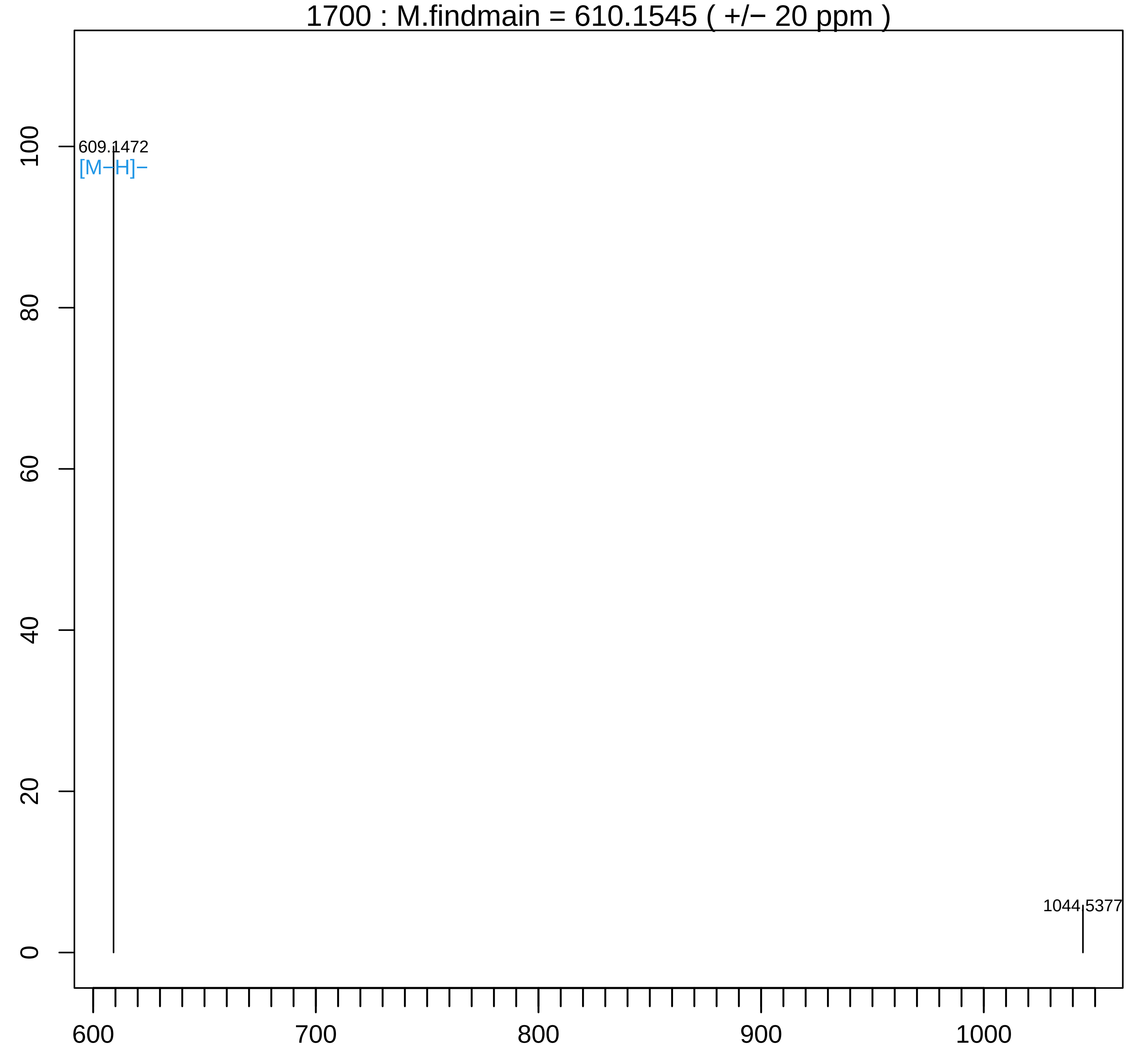
1700 : M.findmain = 610.1545 (+/− 20 ppm)
609.1472
[M−H]−
1044.5377
0
20
40
60
80
100
600
700
800
900
1000

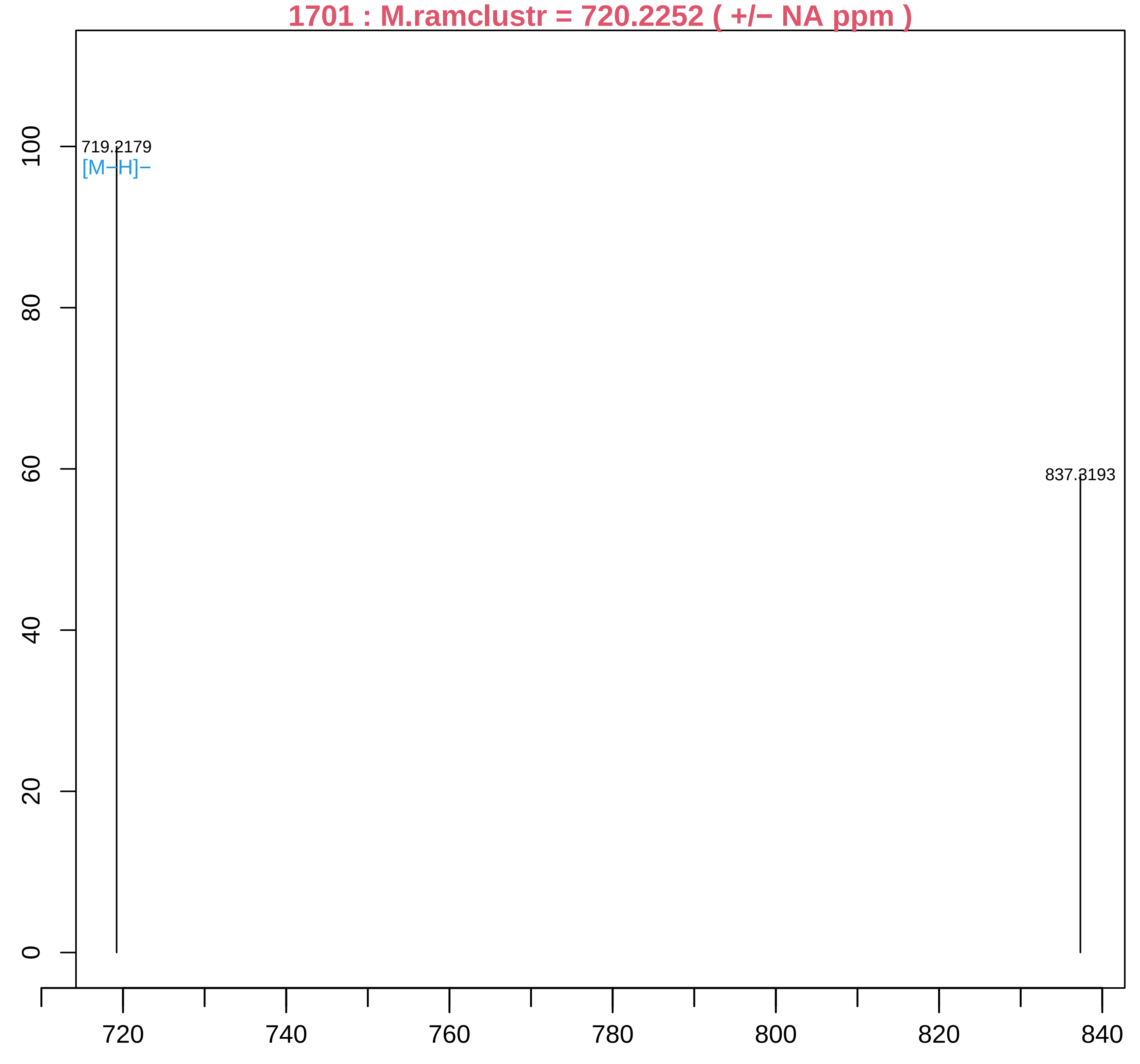
1701 : M.ramclustr = 720.2252 (+/− NA ppm)
719.2179
[M−H]−
837.3193
0
20
40
60
80
100
720
740
760
780
800
820
840

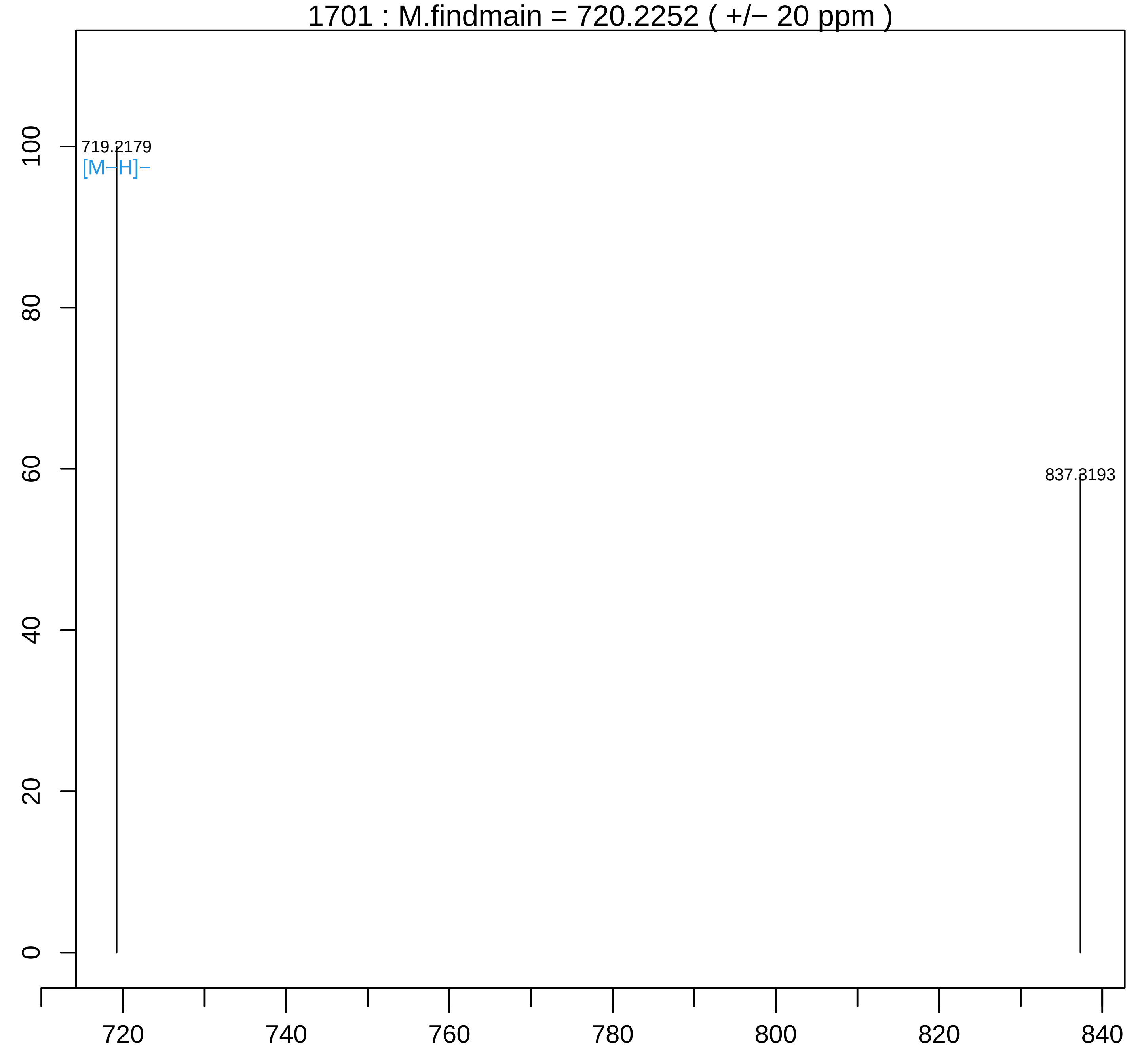
1701 : M.findmain = 720.2252 (+/− 20 ppm)
719.2179
[M−H]−
837.3193
0
20
40
60
80
100
720
740
760
780
800
820
840

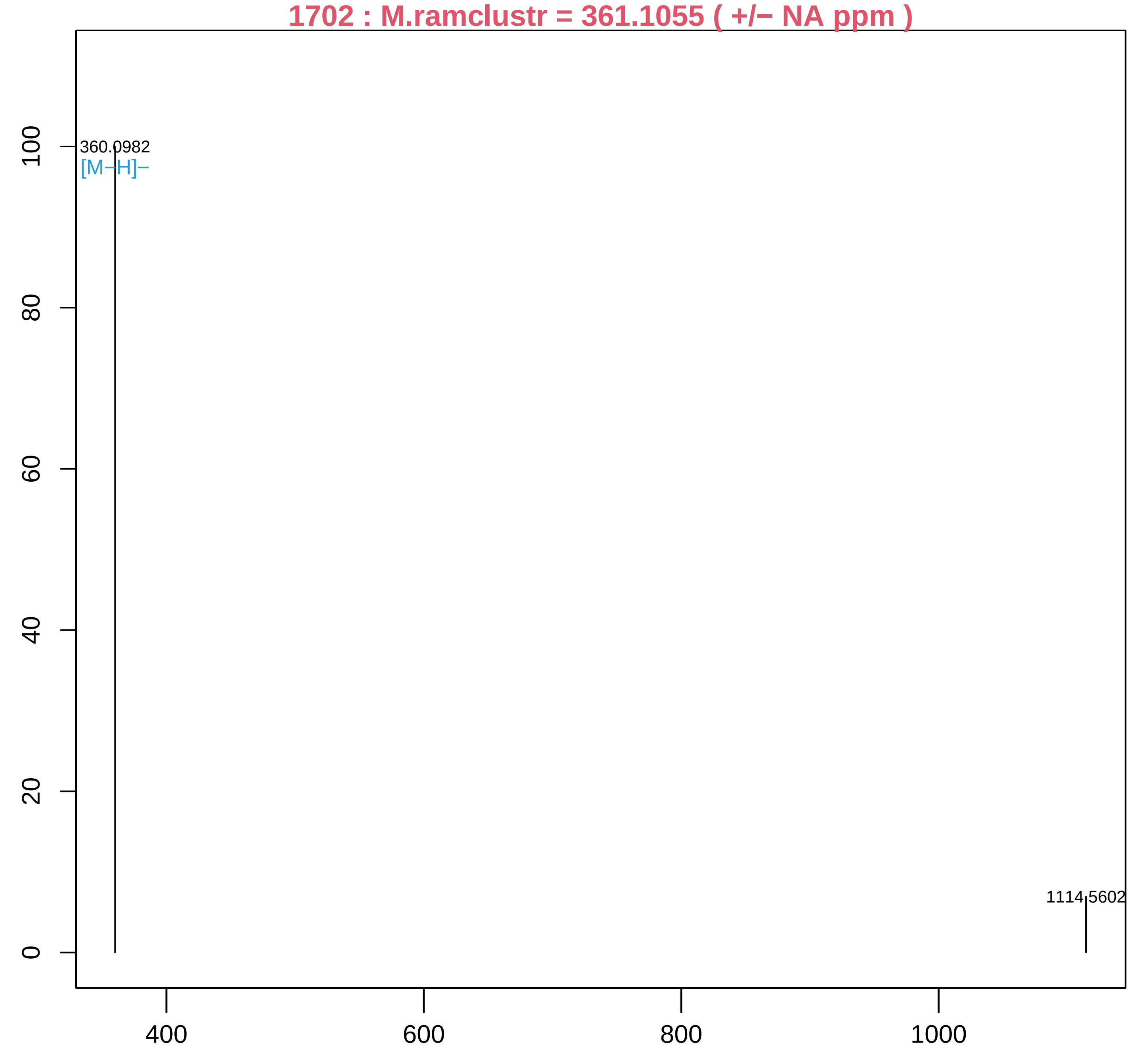
1702 : M.ramclustr = 361.1055 (+/− NA ppm)
360.0982
[M−H]−
1114.5602
0
20
40
60
80
100
400
600
800
1000

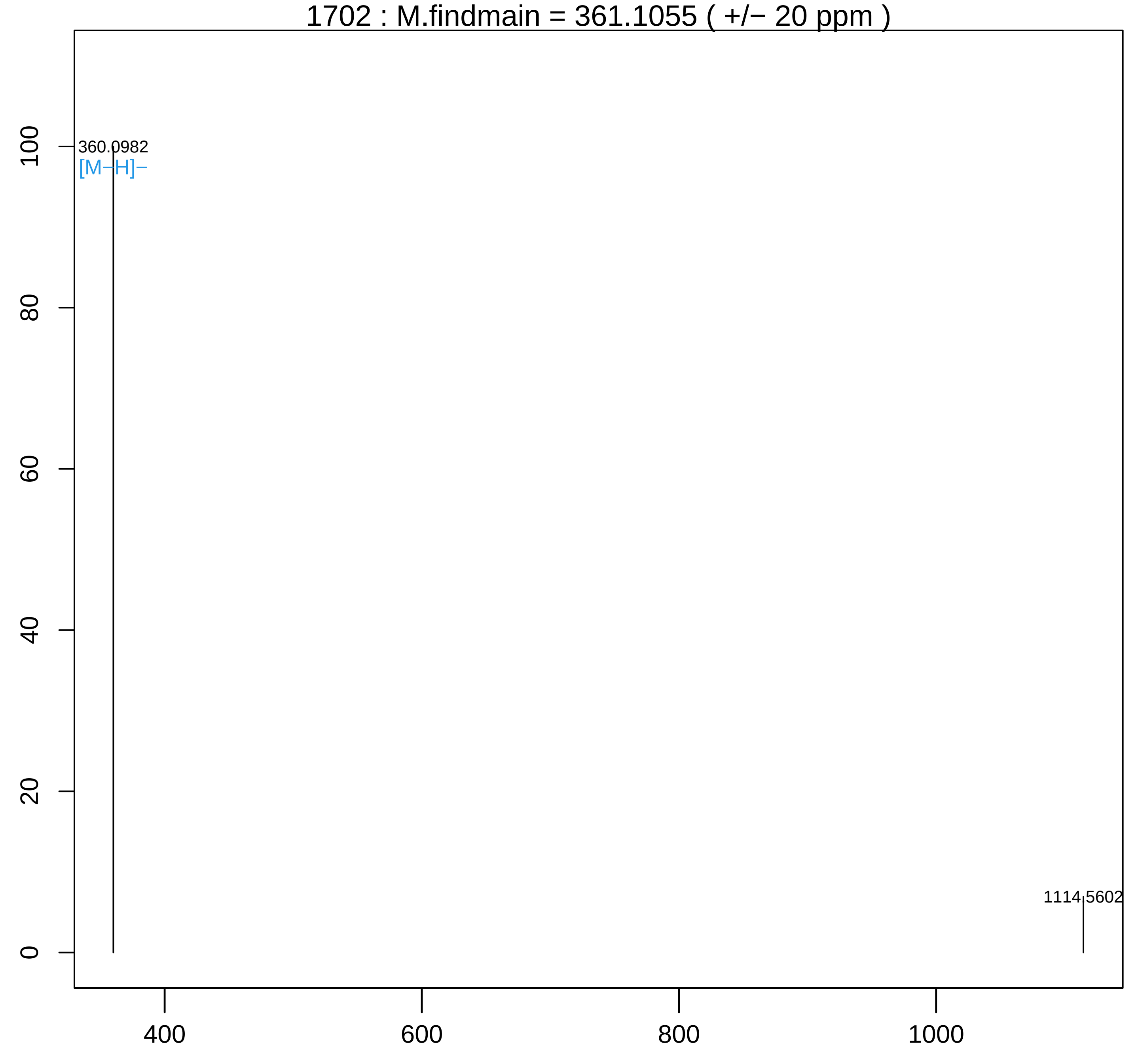
1702 : M.findmain = 361.1055 (+/− 20 ppm)
360.0982
[M−H]−
1114.5602
0
20
40
60
80
100
400
600
800
1000

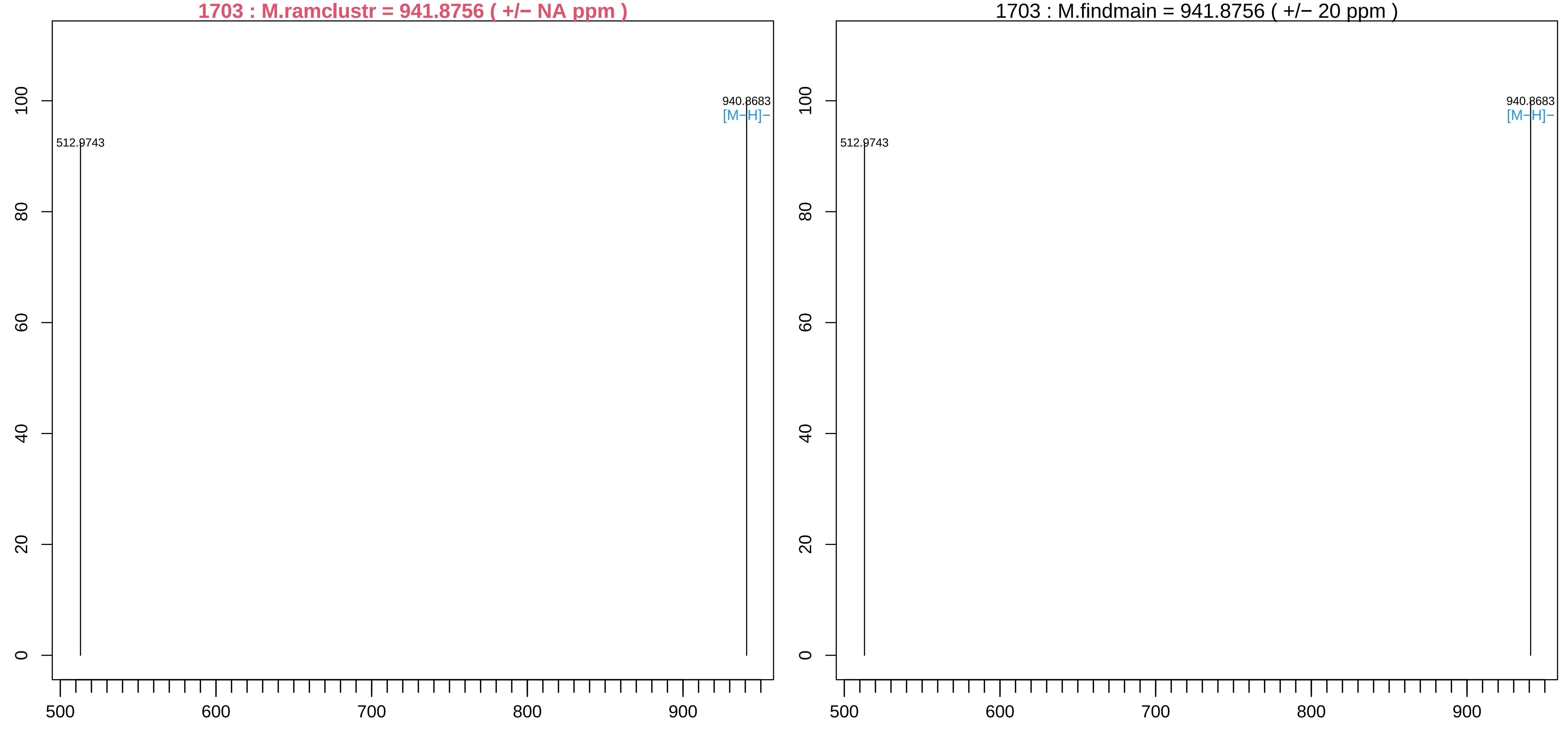
1703 : M.ramclustr = 941.8756 (+/− NA ppm)
512.9743
940.8683
[M−H]−
1703 : M.findmain = 941.8756 (+/− 20 ppm)
512.9743
940.8683
[M−H]−
0
20
40
60
80
100
500
600
700
800
900

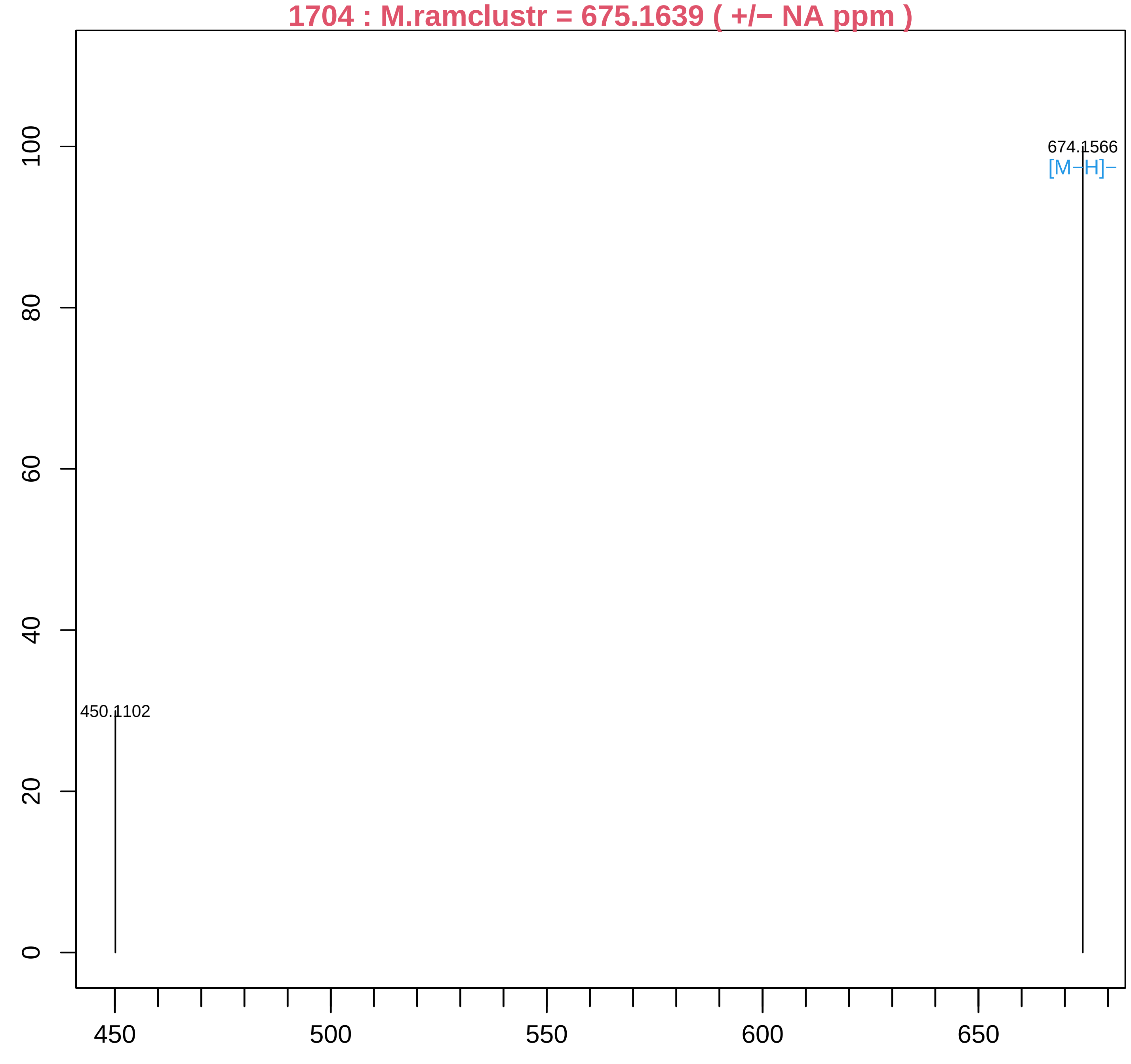

1704 : M.ramclustr = 675.1639 (+/− NA ppm)
674.1566
[M−H]−
450.1102
0
20
40
60
80
100
450
500
550
600
650

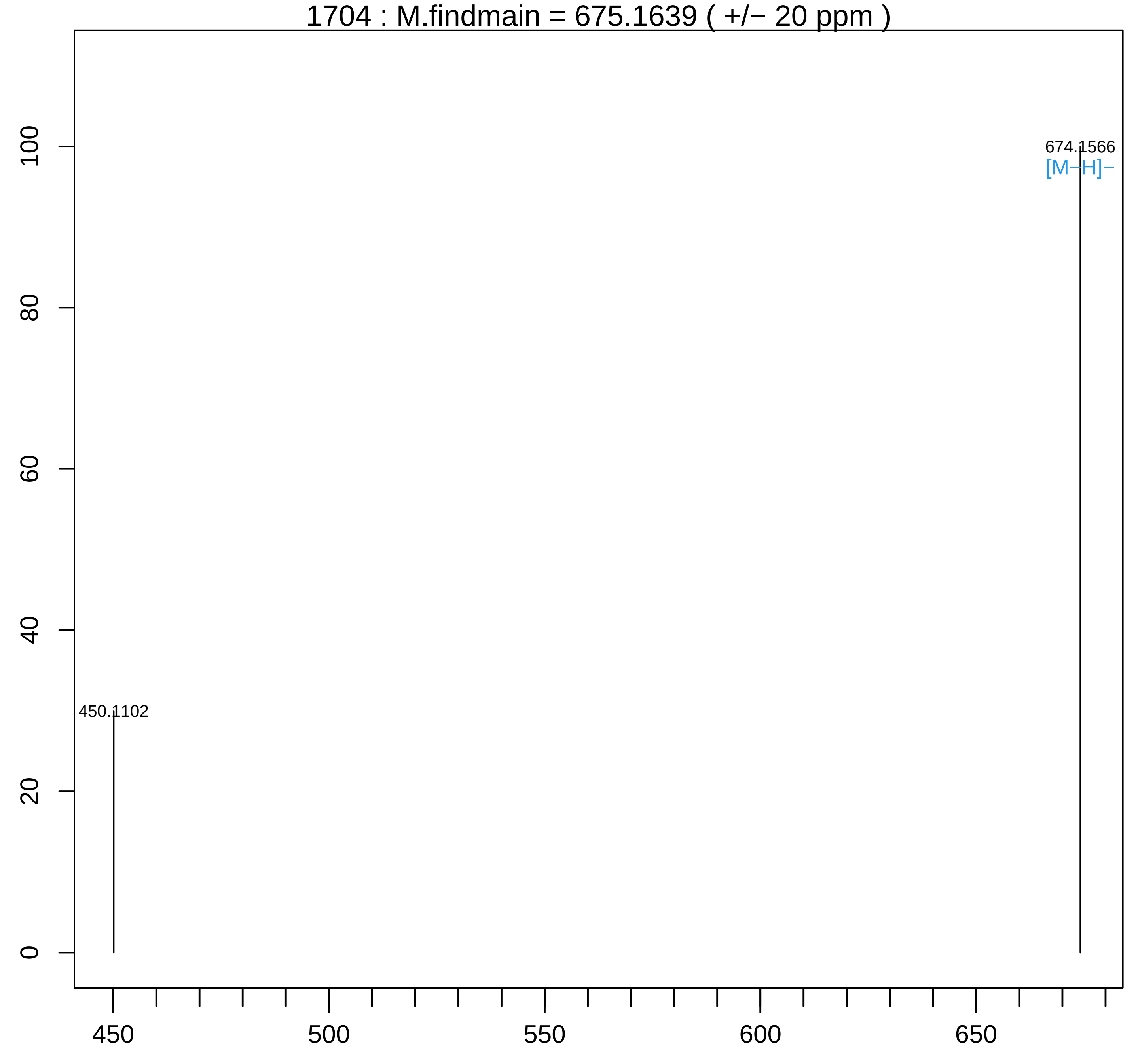

1704 : M.findmain = 675.1639 (+/− 20 ppm)
674.1566
[M−H]−
450.1102
0
20
40
60
80
100
450
500
550
600
650

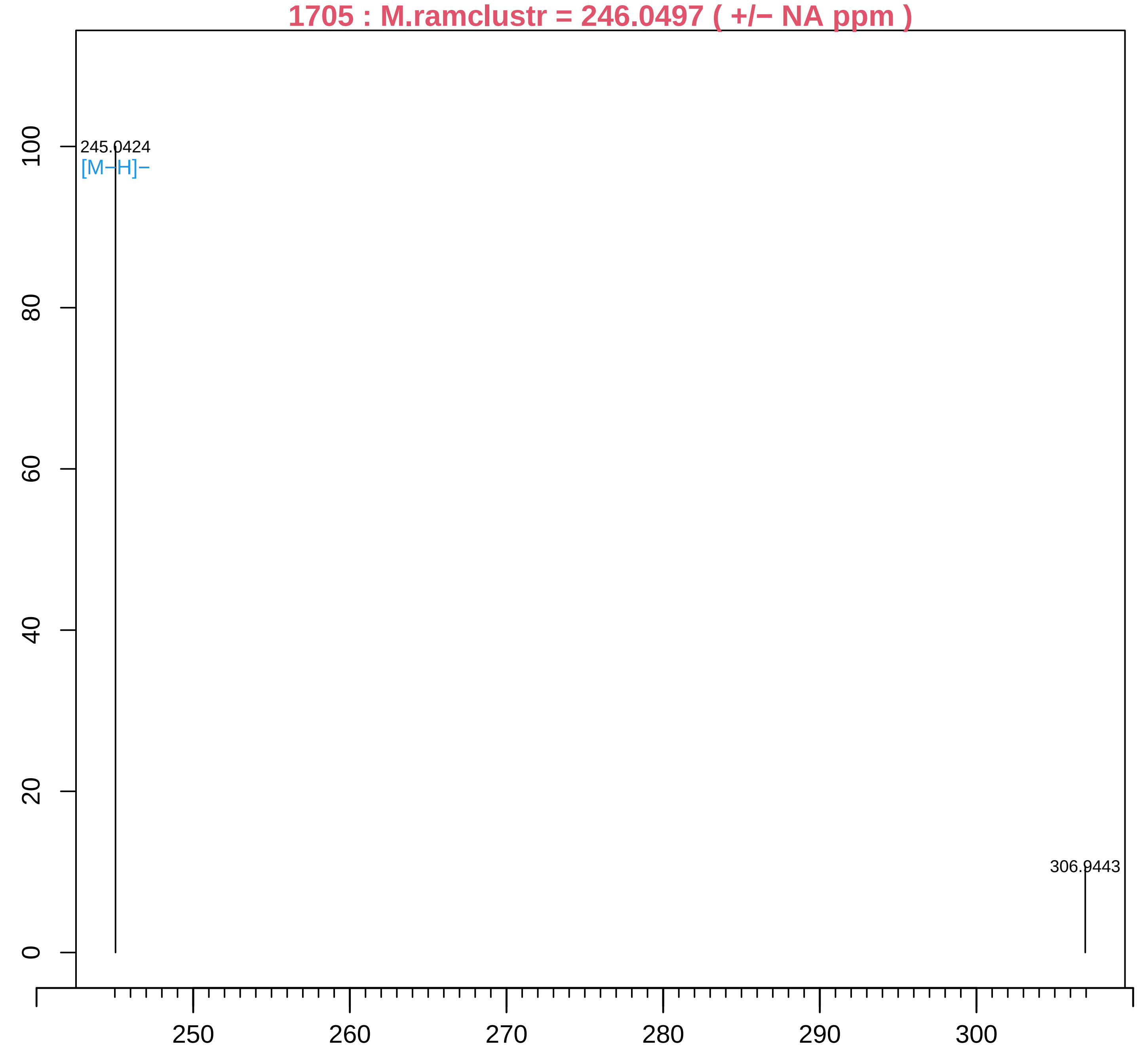
1705 : M.ramclustr = 246.0497 (+/− NA ppm)
245.0424
[M−H]−
306.9443
0
20
40
60
80
100
250
260
270
280
290
300

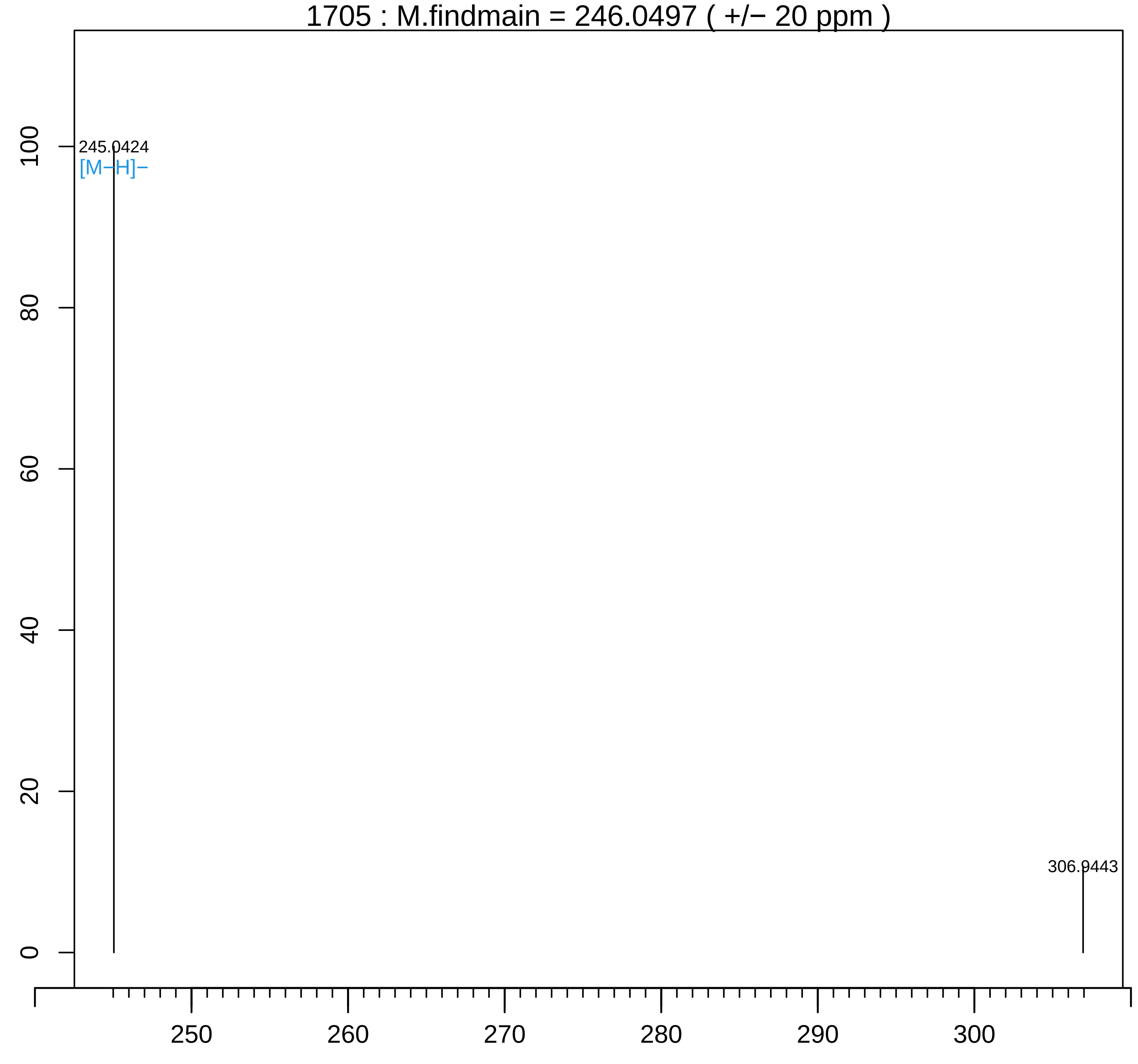
1705 : M.findmain = 246.0497 (+/− 20 ppm)
245.0424
[M−H]−
306.9443
0
20
40
60
80
100
250
260
270
280
290
300

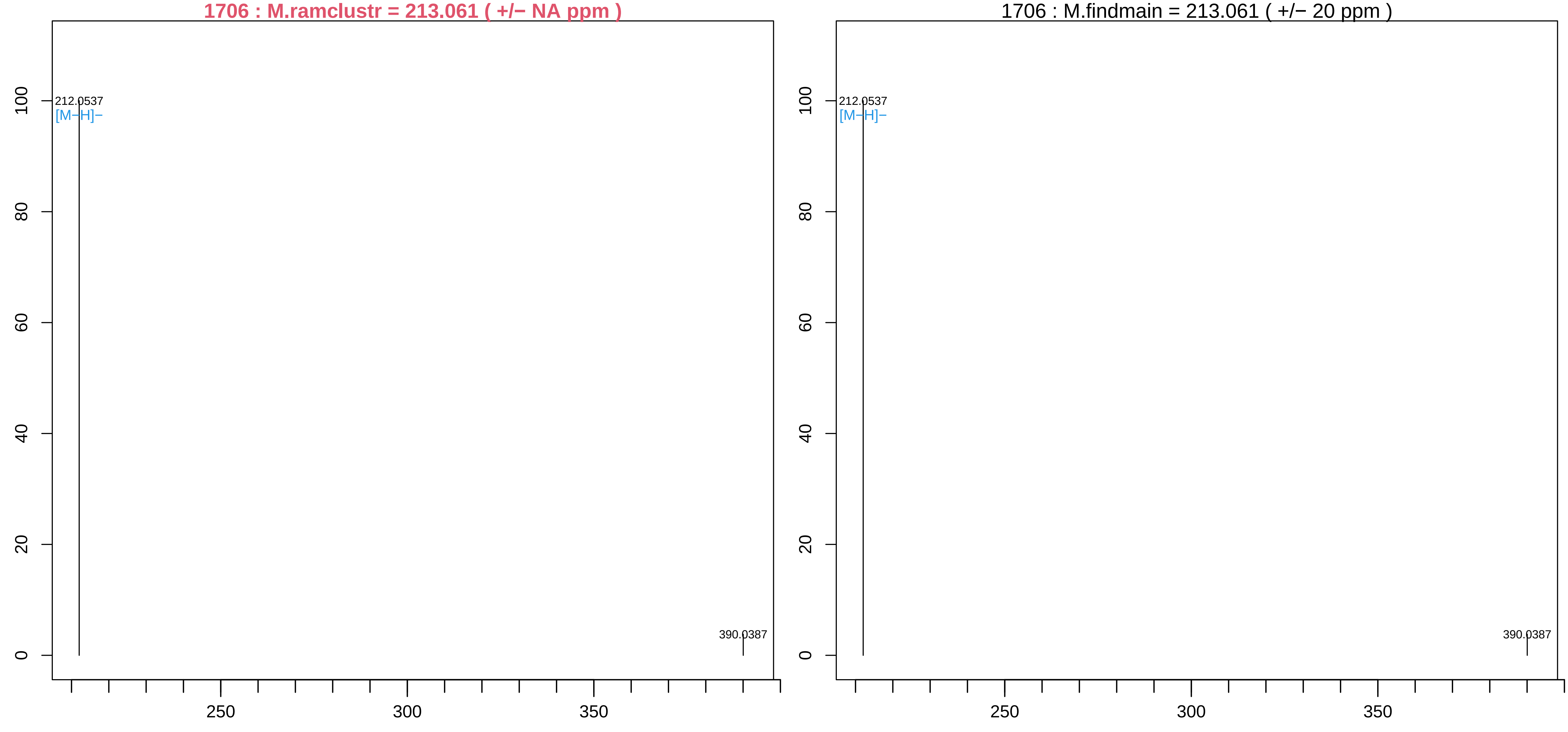
1706 : M.ramclustr = 213.061 (+/− NA ppm)
212.0537
[M−H]−
390.0387
1706 : M.findmain = 213.061 (+/− 20 ppm)
212.0537
[M−H]−
390.0387
0
20
40
60
80
100
250
300
350

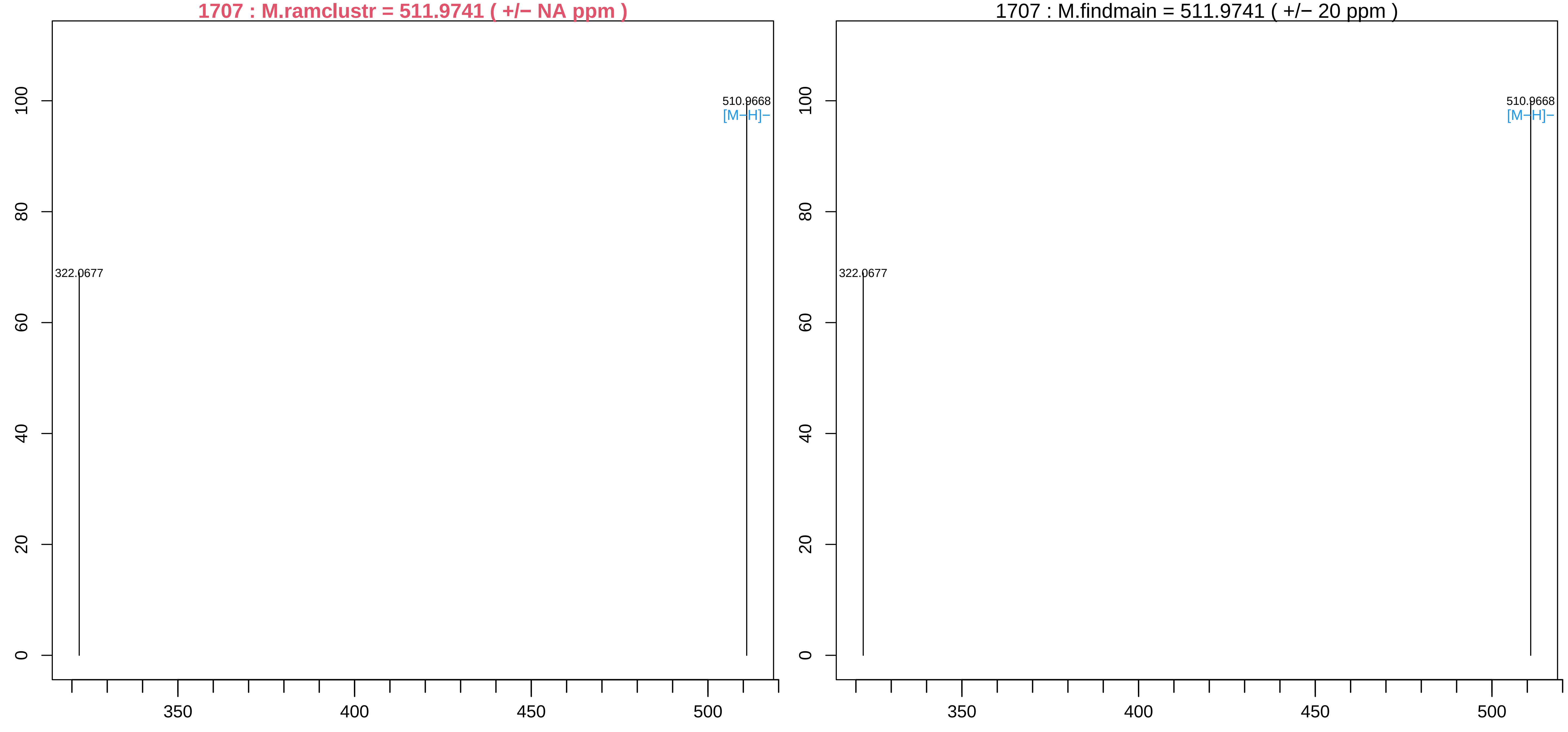
1707 : M.ramclustr = 511.9741 (+/− NA ppm)
1707 : M.findmain = 511.9741 (+/− 20 ppm)
510.9668
[M−H]−
322.0677
0
20
40
60
80
100
350
400
450
500

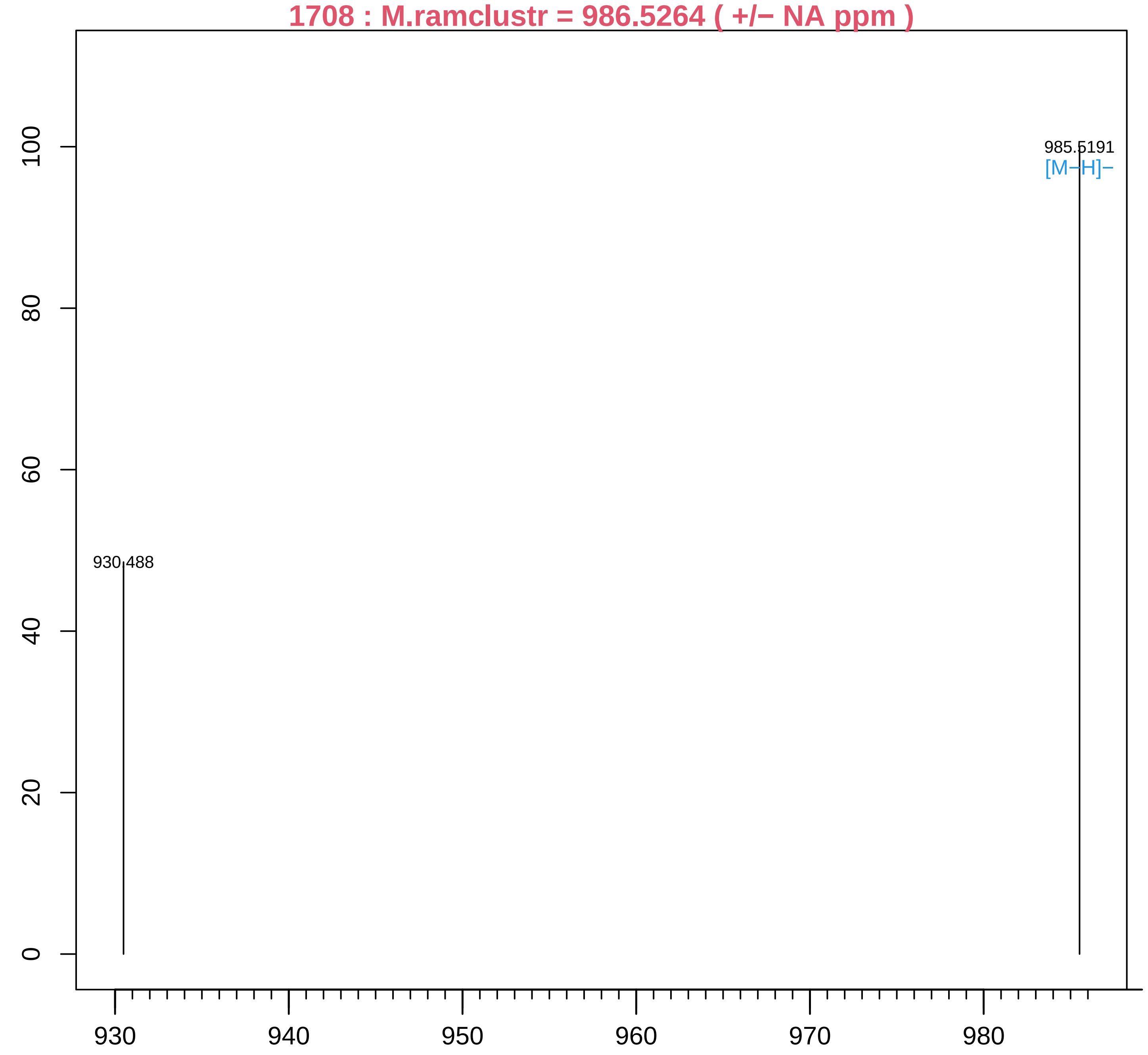
1708 : M.ramclustr = 986.5264 (+/− NA ppm)
985.5191
[M−H]−
930.488
0
20
40
60
80
100
930
940
950
960
970
980

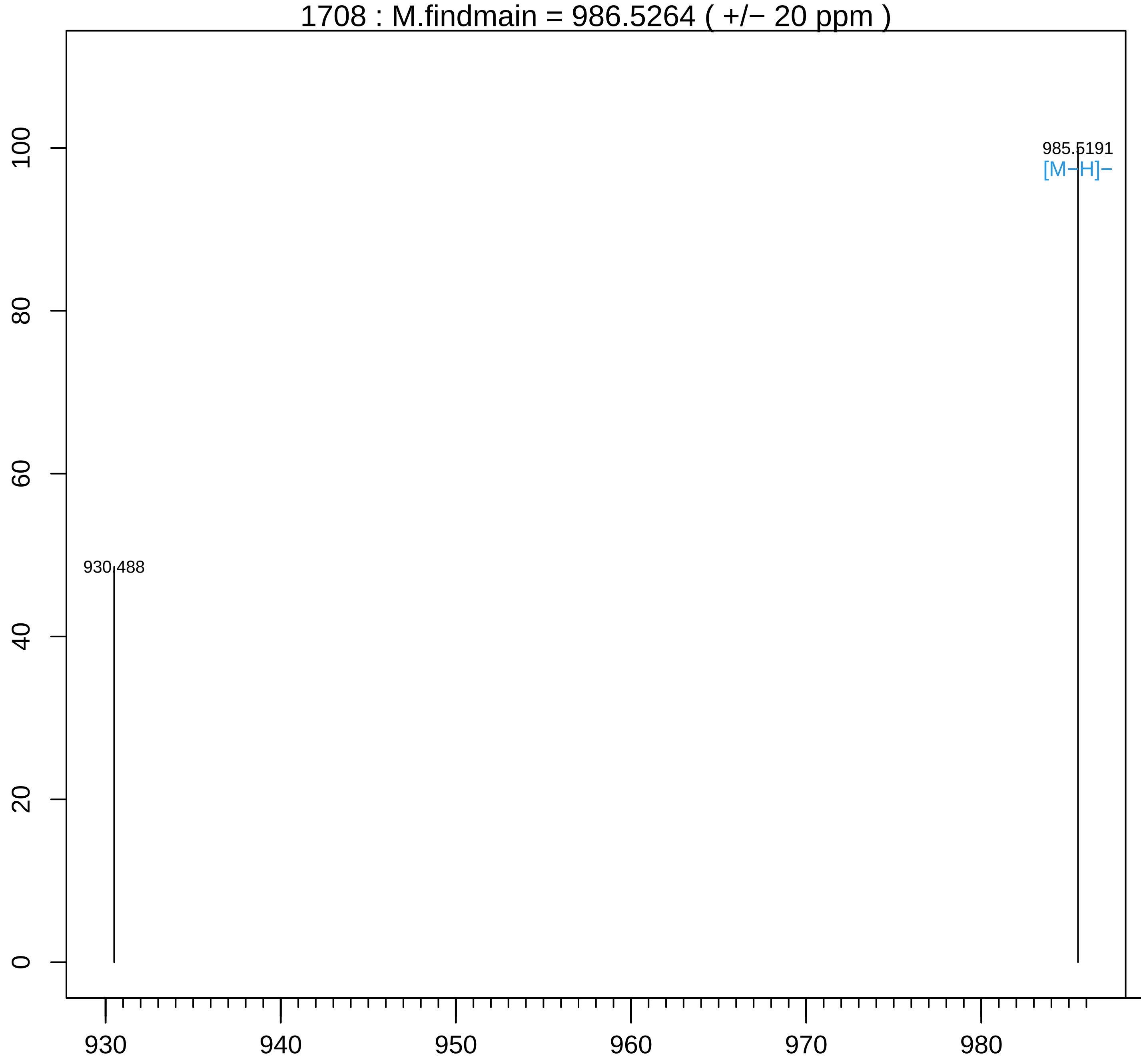
1708 : M.findmain = 986.5264 (+/− 20 ppm)
985.5191
[M−H]−
930.488
0
20
40
60
80
100
930
940
950
960
970
980

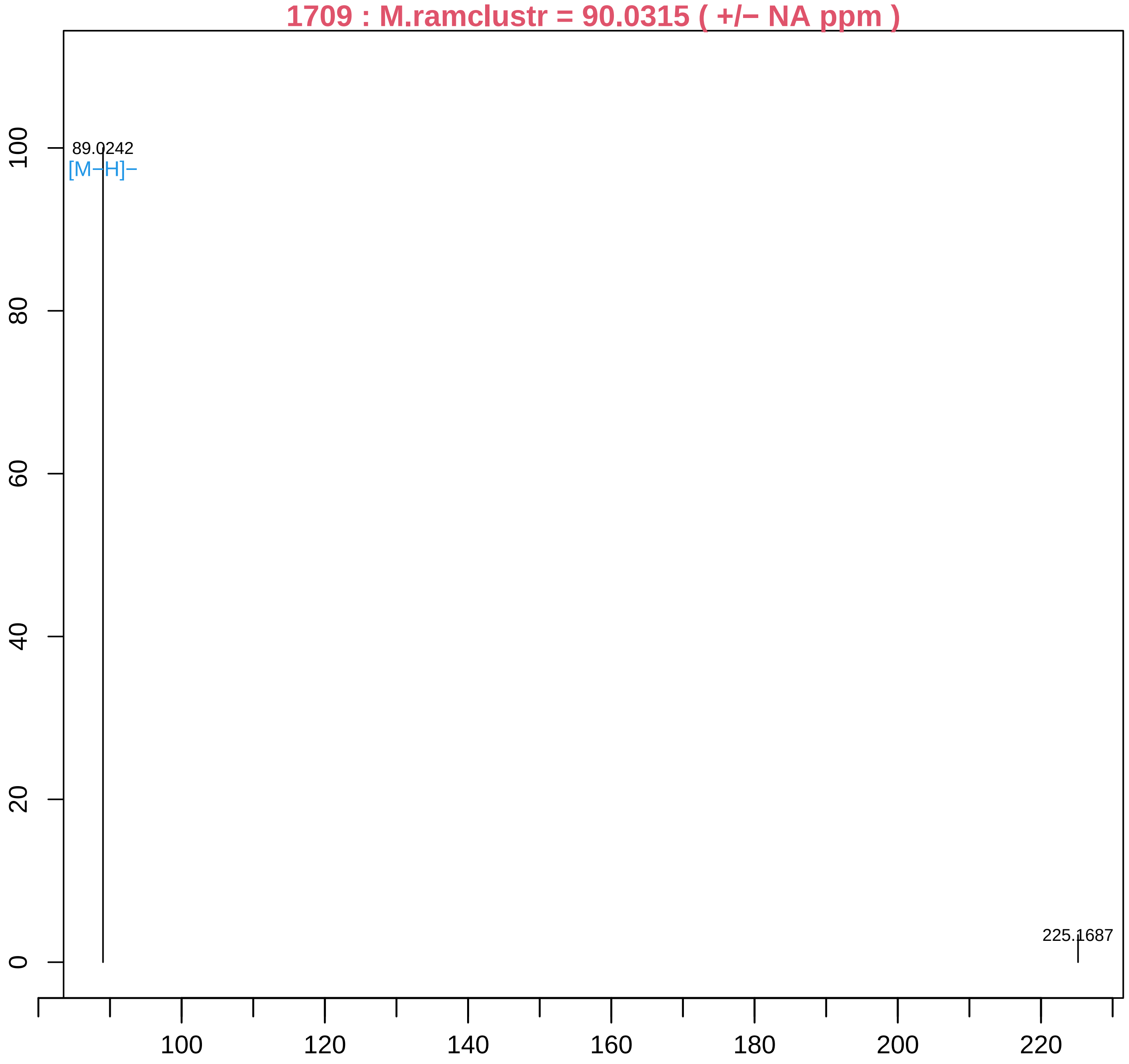
1709 : M.ramclustr = 90.0315 (+/− NA ppm)
89.0242
[M−H]−
225.1687
0
20
40
60
80
100
100
120
140
160
180
200
220

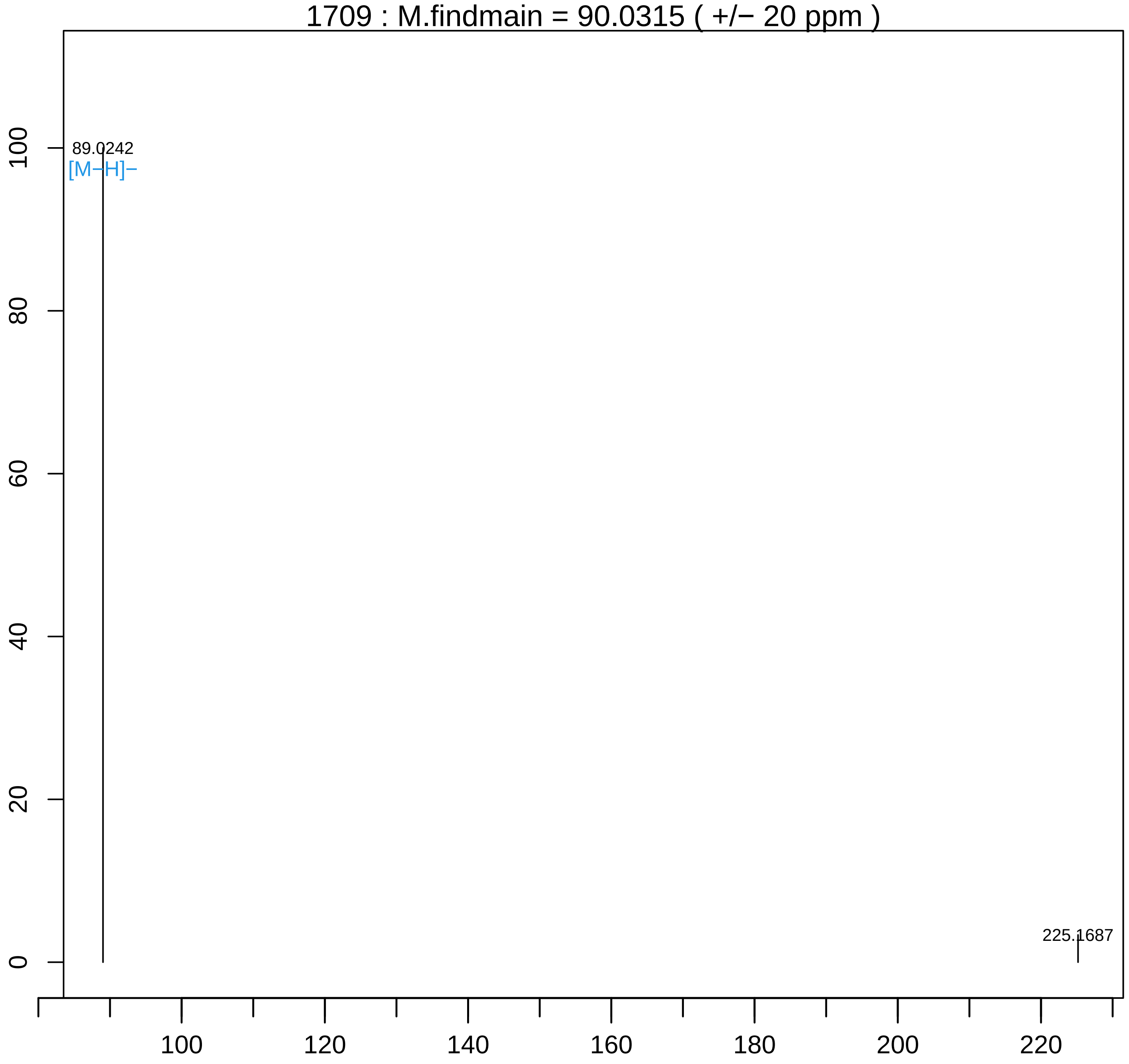
1709 : M.findmain = 90.0315 (+/− 20 ppm)
89.0242
[M−H]−
225.1687
0
20
40
60
80
100
100
120
140
160
180
200
220

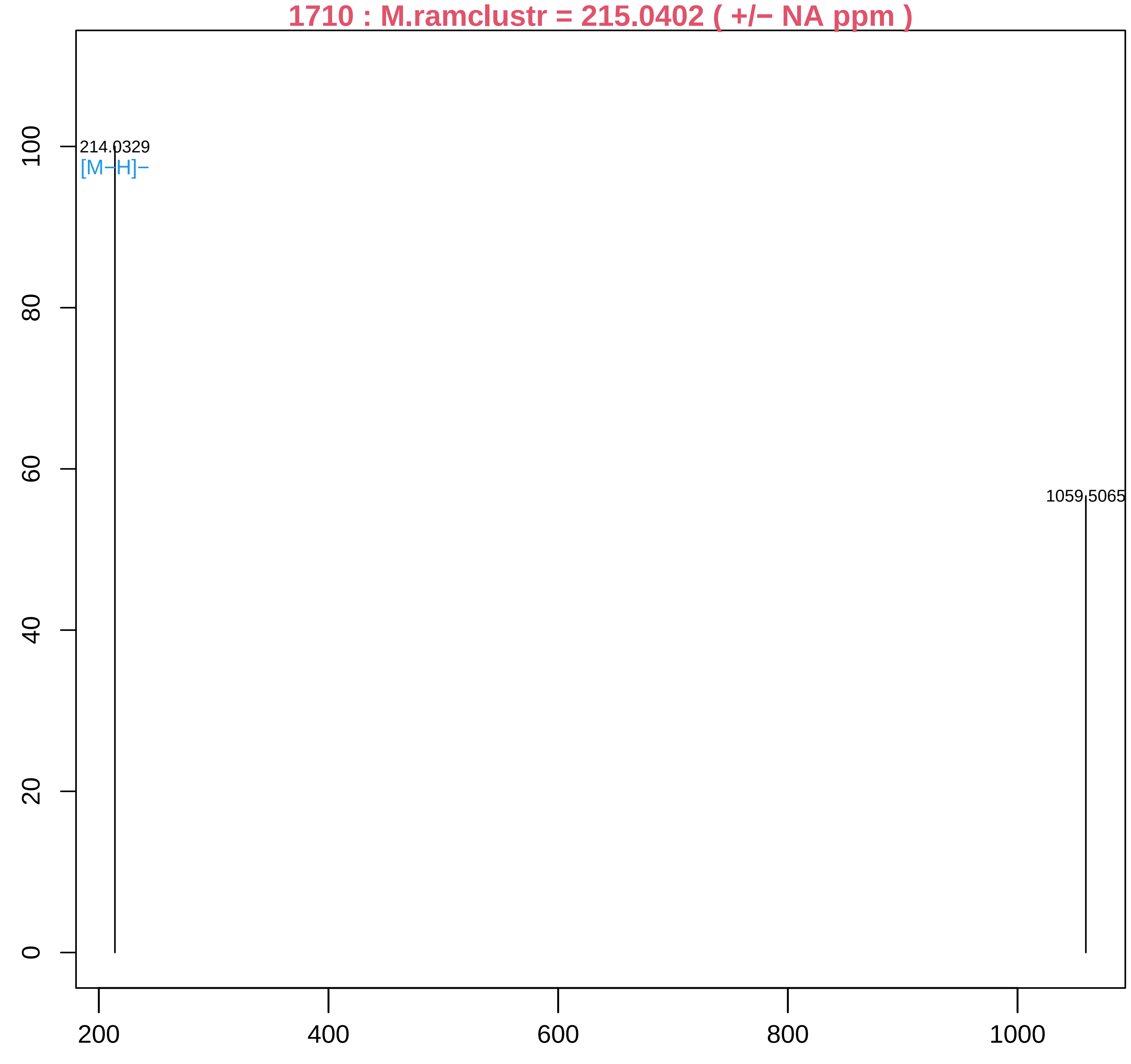
1710 : M.ramclustr = 215.0402 (+/− NA ppm)
214.0329
[M−H]−
1059.5065
0
20
40
60
80
100
200
400
600
800
1000

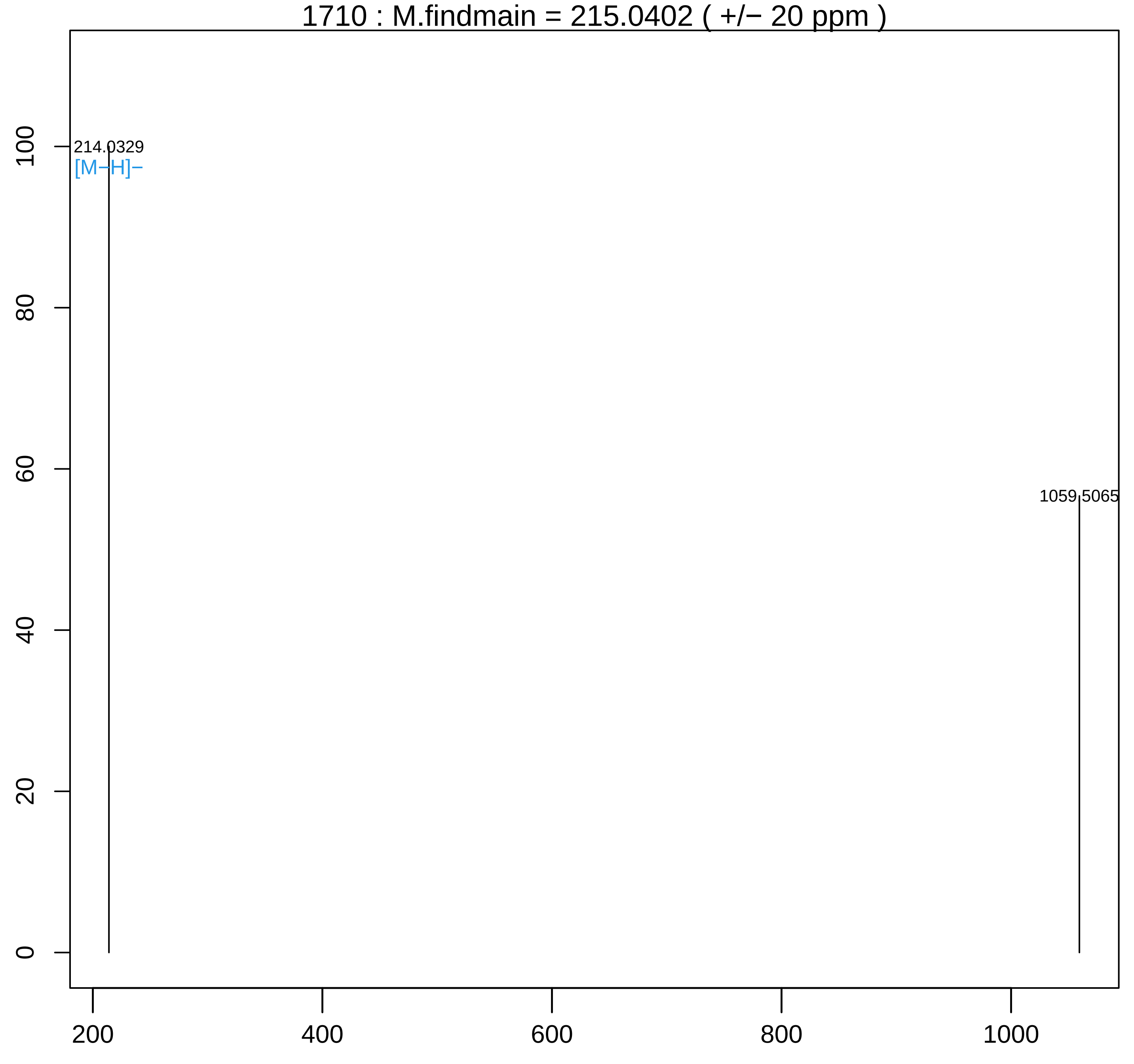
1710 : M.findmain = 215.0402 (+/− 20 ppm)
214.0329
[M−H]−
1059.5065
0
20
40
60
80
100
200
400
600
800
1000

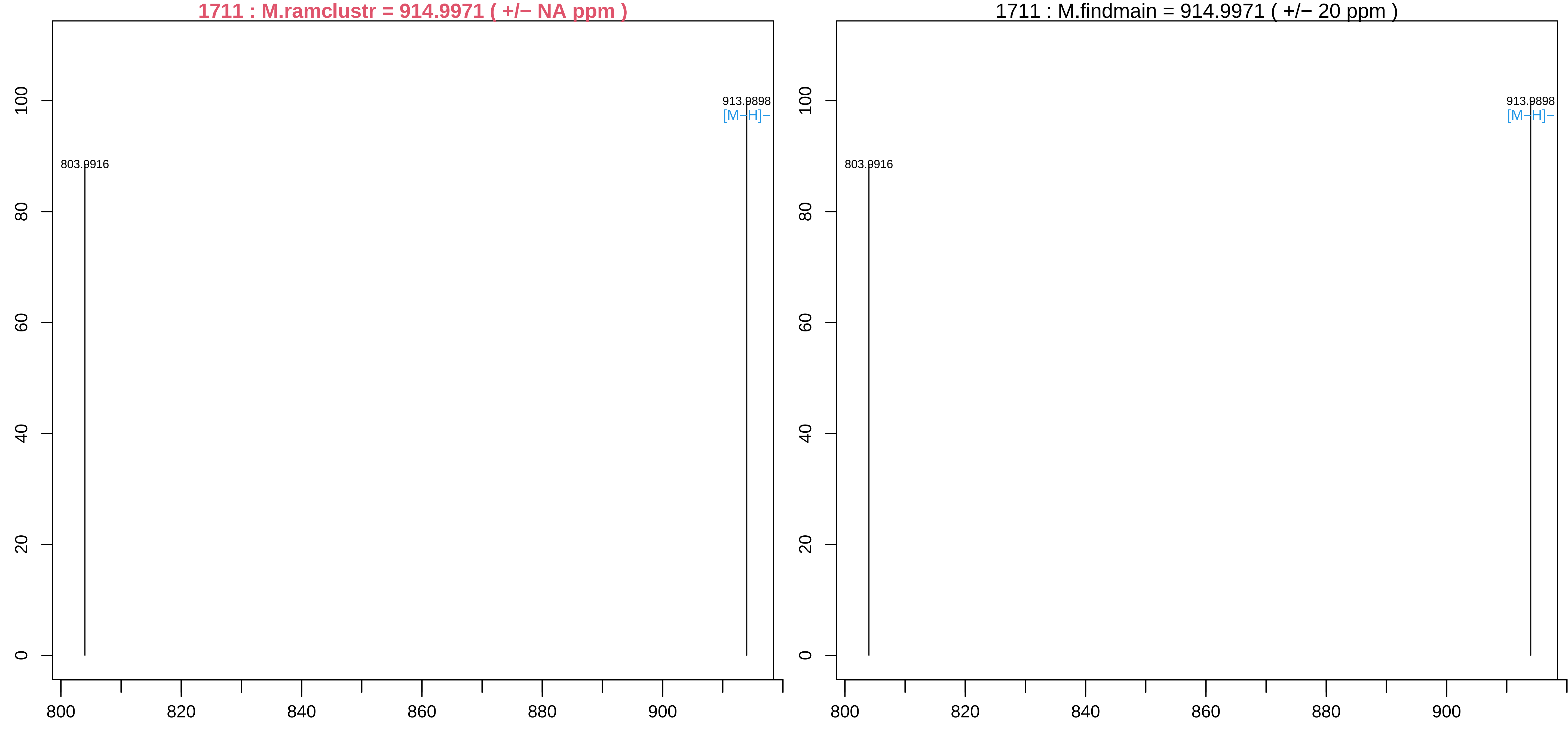
1711 : M.ramclustr = 914.9971 (+/− NA ppm)
1711 : M.findmain = 914.9971 (+/− 20 ppm)
913.9898
[M−H]−
803.9916
0
20
40
60
80
100
800
820
840
860
880
900

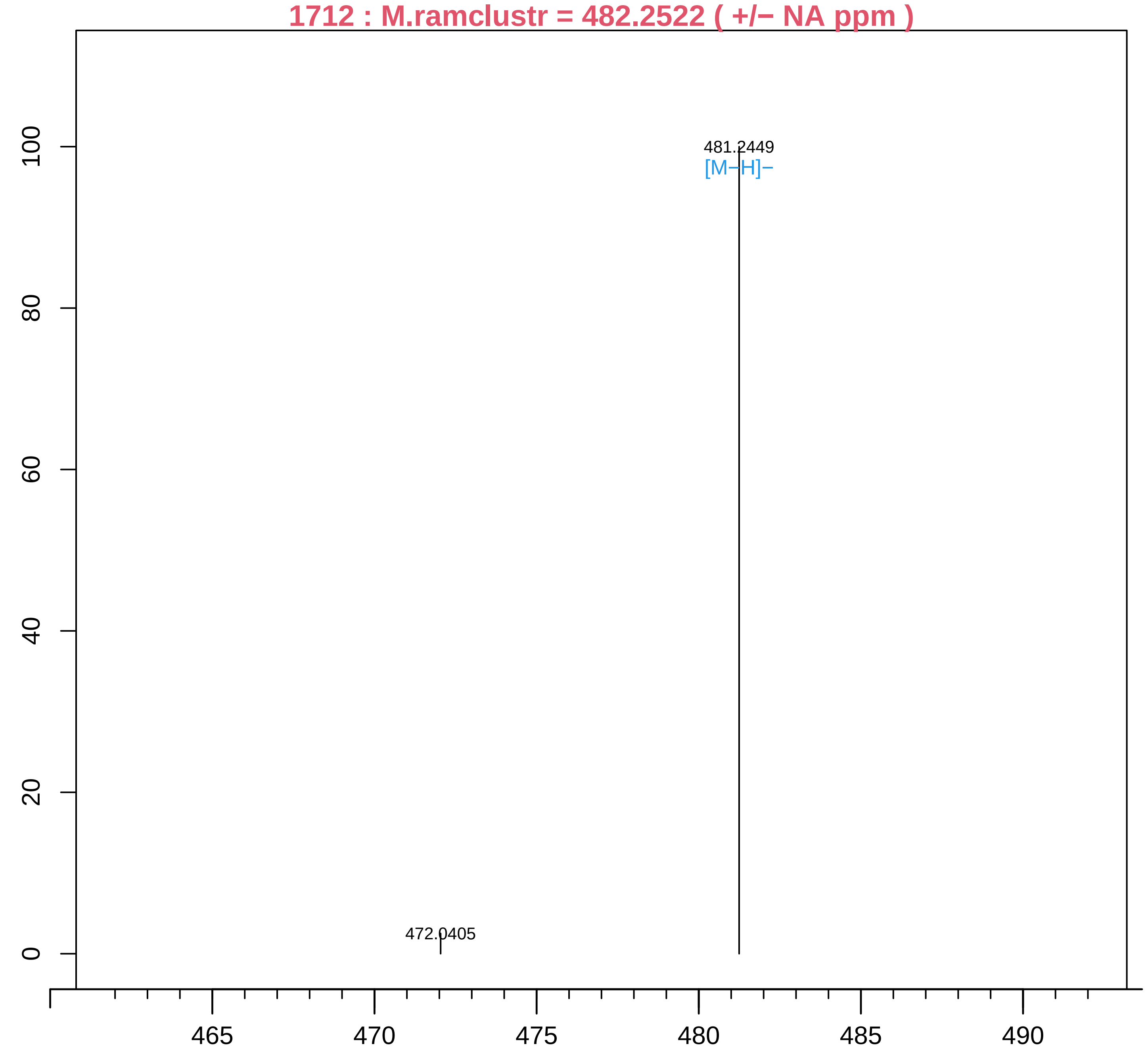

1712 : M.ramclustr = 482.2522 (+/− NA ppm)
481.2449
[M−H]−
472.0405
0
20
40
60
80
100
465
470
475
480
485
490

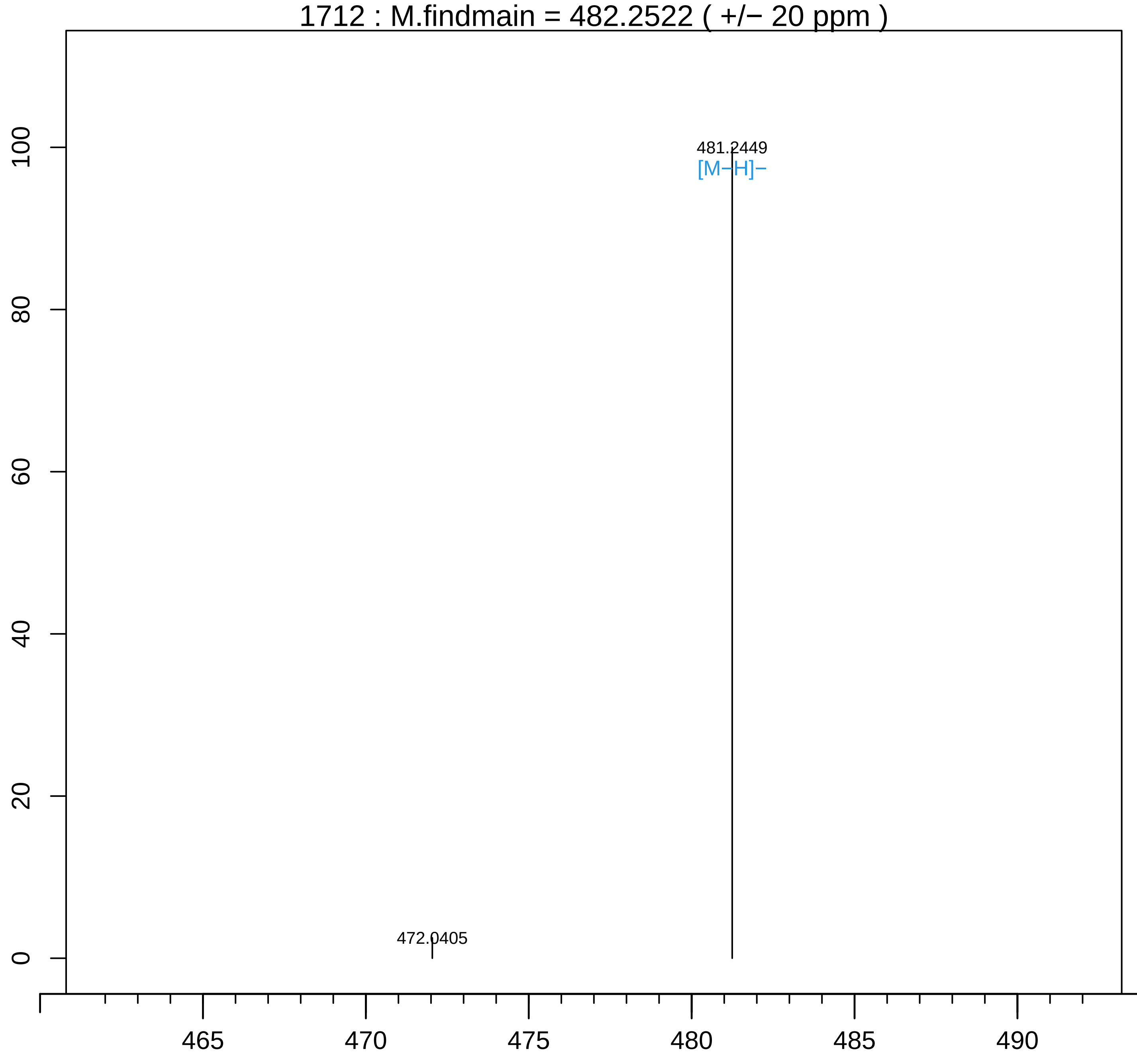

1712 : M.findmain = 482.2522 (+/− 20 ppm)
481.2449
[M−H]−
472.0405
0
20
40
60
80
100
465
470
475
480
485
490

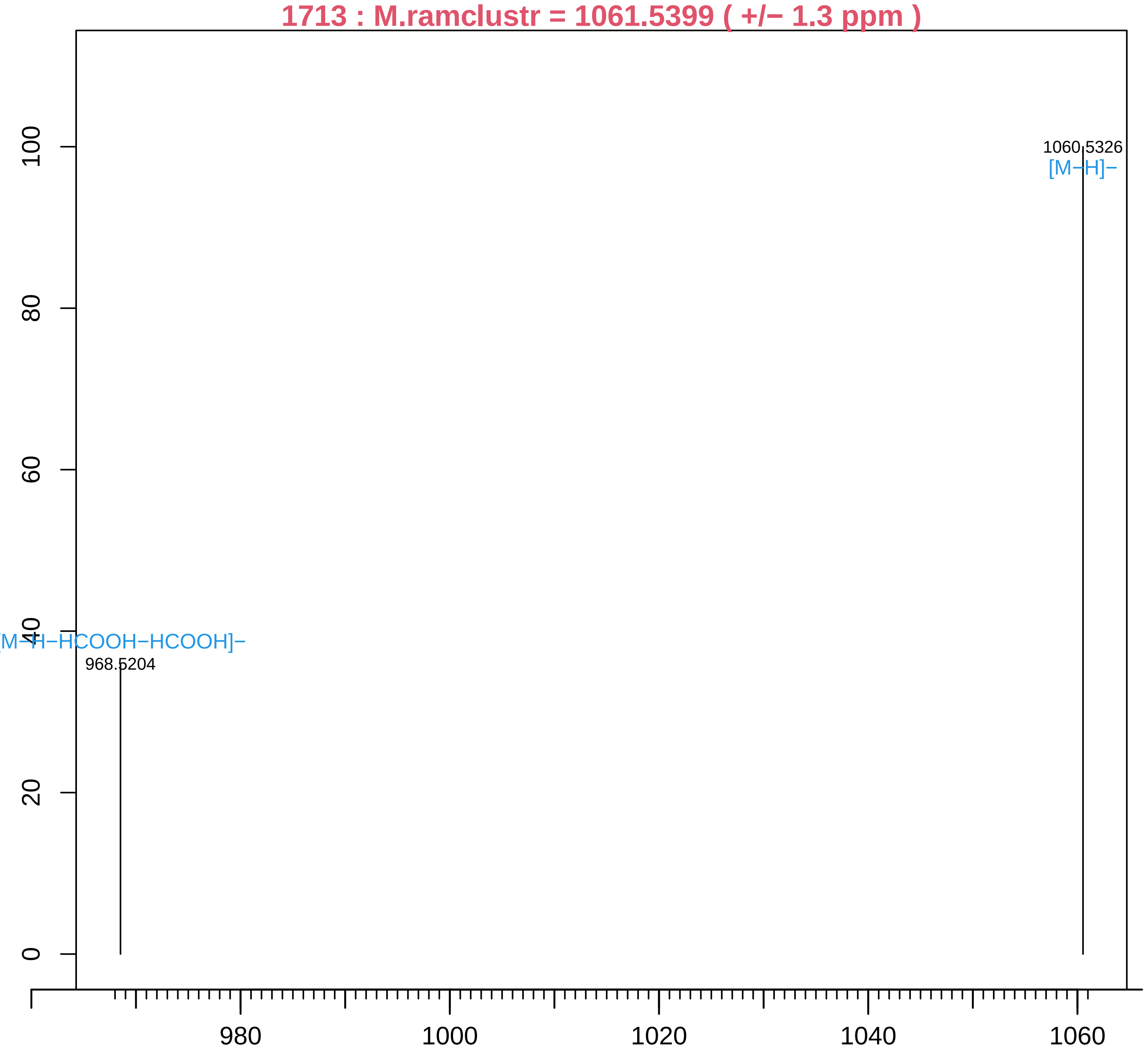
1713 : M.ramclustr = 1061.5399 (+/− 1.3 ppm)
1060.5326
[M−H]−
[M−H−HCOOH−HCOOH]−
968.5204
0
20
40
60
80
100
980
1000
1020
1040
1060

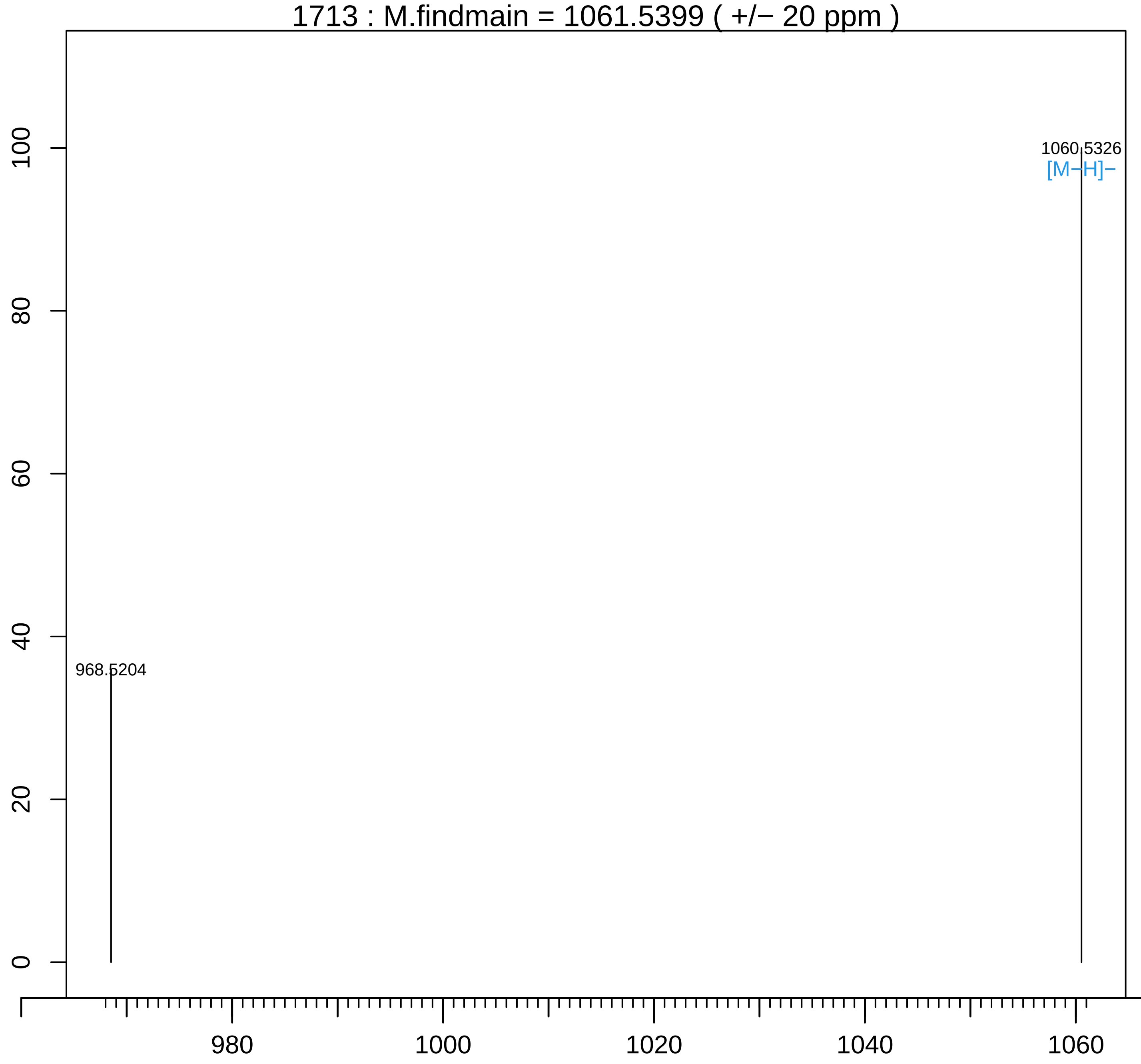
1713 : M.findmain = 1061.5399 (+/− 20 ppm)
1060.5326
[M−H]−
968.5204
0
20
40
60
80
100
980
1000
1020
1040
1060

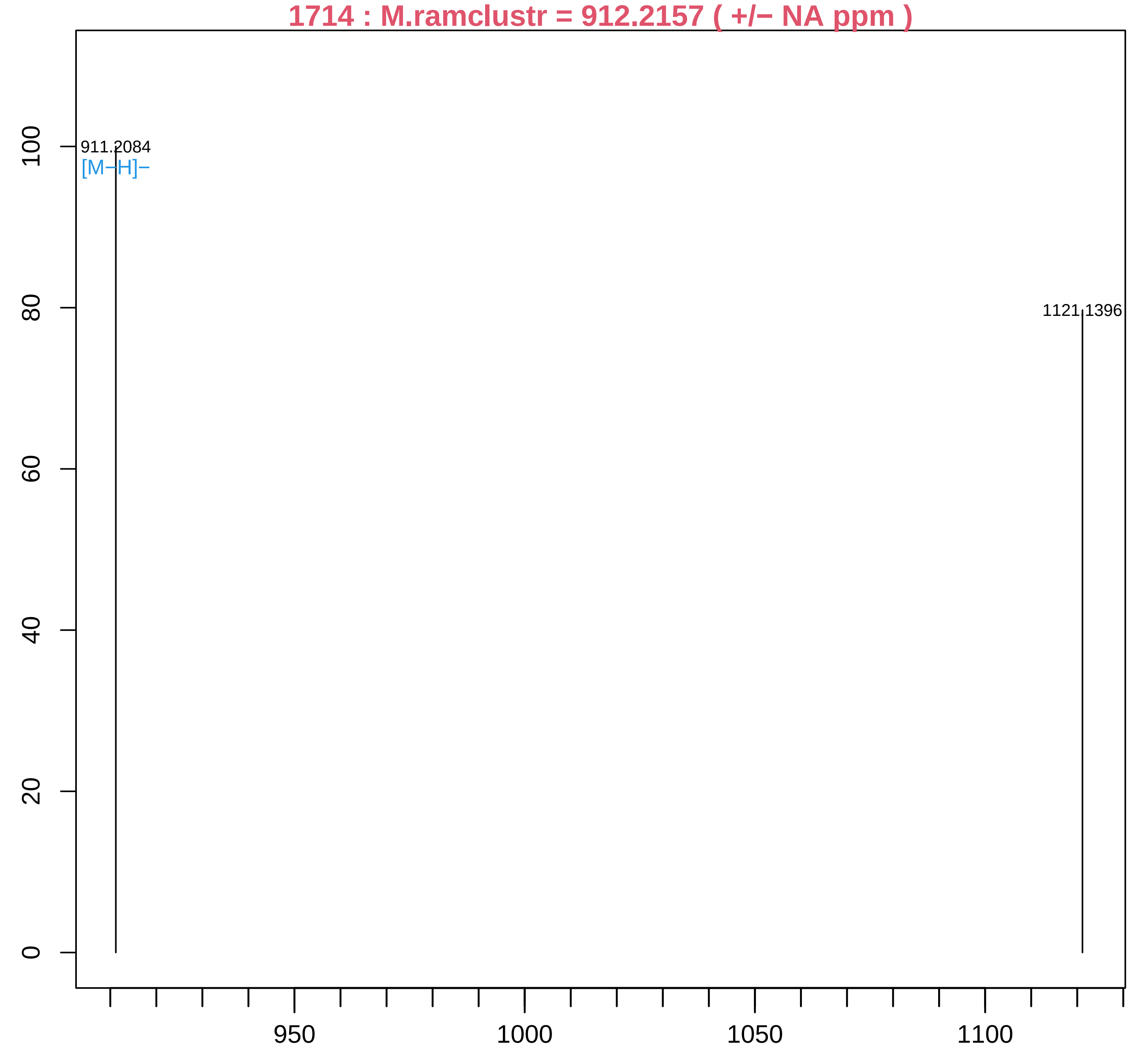

1714 : M.ramclustr = 912.2157 (+/− NA ppm)
911.2084
[M−H]−
1121.1396
0
20
40
60
80
100
950
1000
1050
1100

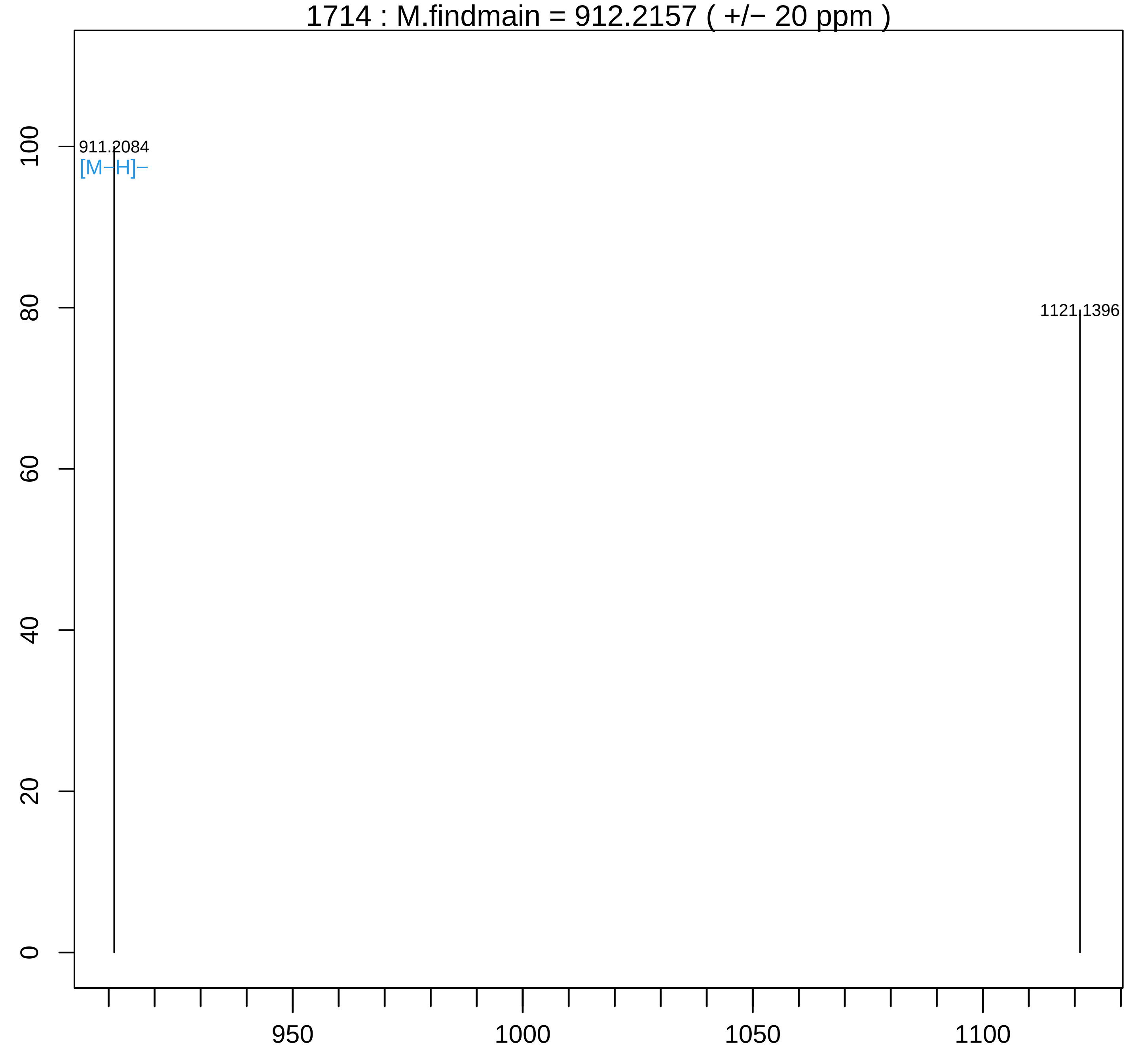

1714 : M.findmain = 912.2157 (+/− 20 ppm)
911.2084
[M−H]−
1121.1396
0
20
40
60
80
100
950
1000
1050
1100

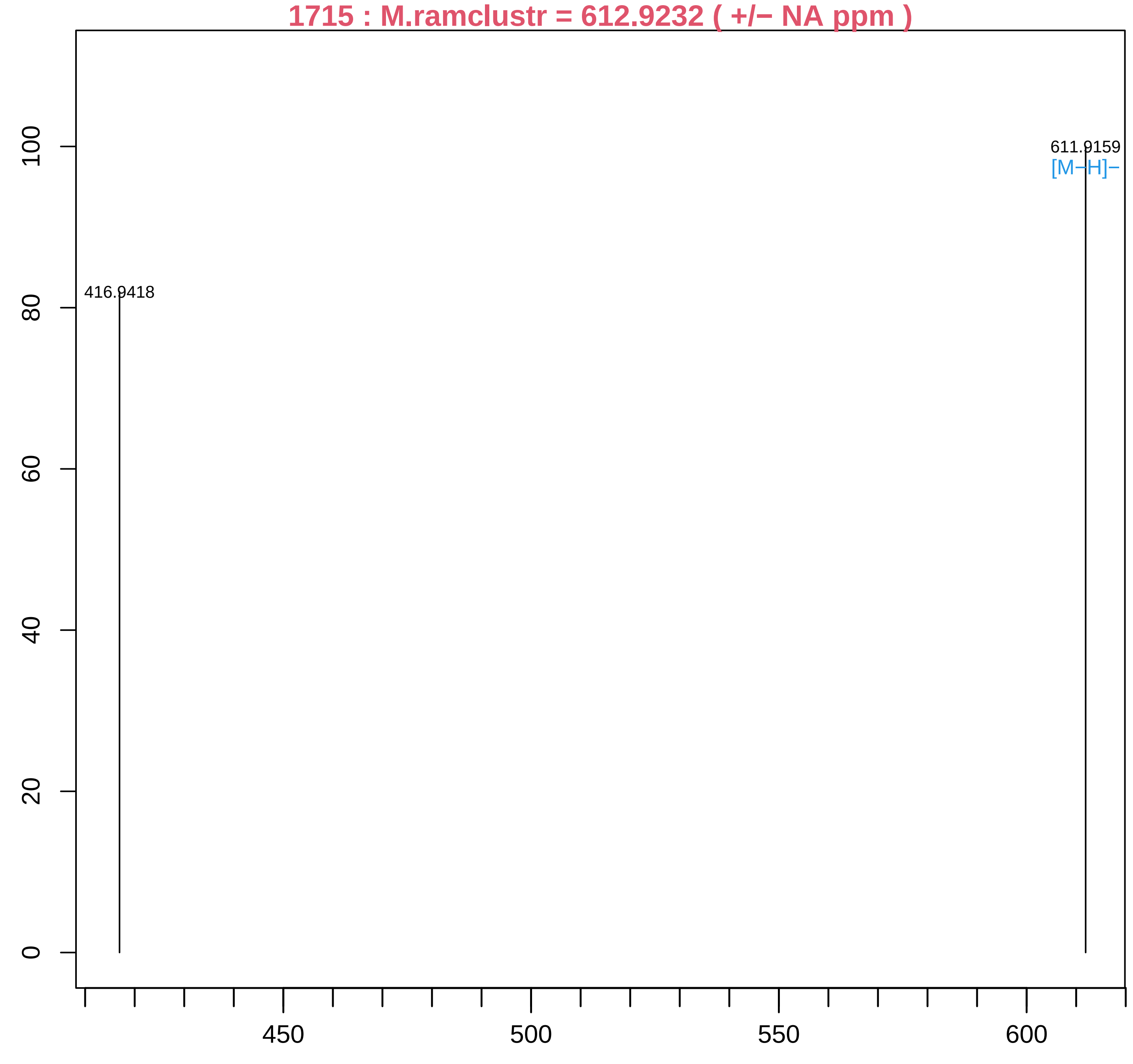

1715 : M.ramclustr = 612.9232 (+/− NA ppm)
611.9159
[M−H]−
416.9418
0
20
40
60
80
100
450
500
550
600

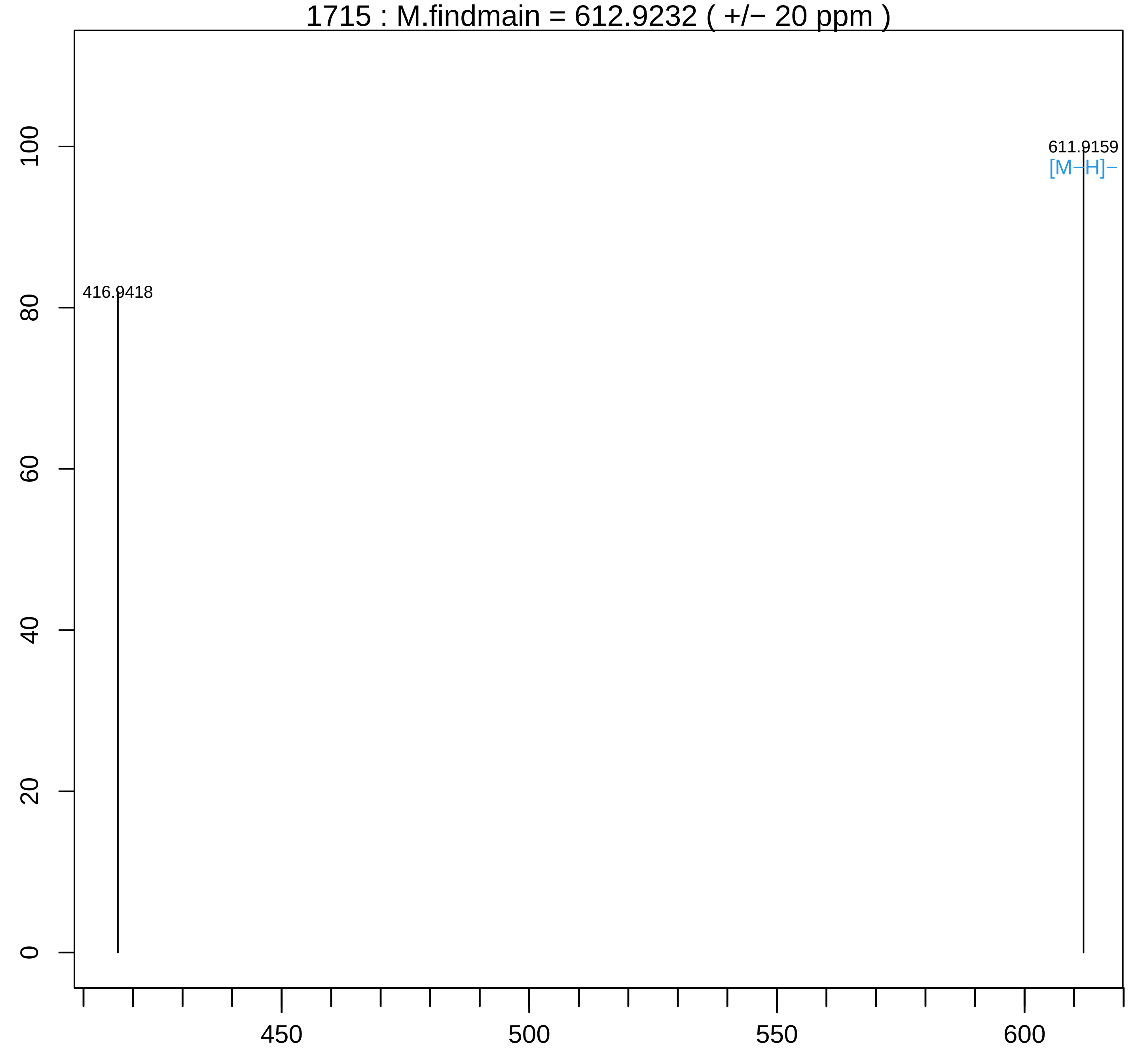

1715 : M.findmain = 612.9232 (+/− 20 ppm)
611.9159
[M−H]−
416.9418
0
20
40
60
80
100
450
500
550
600

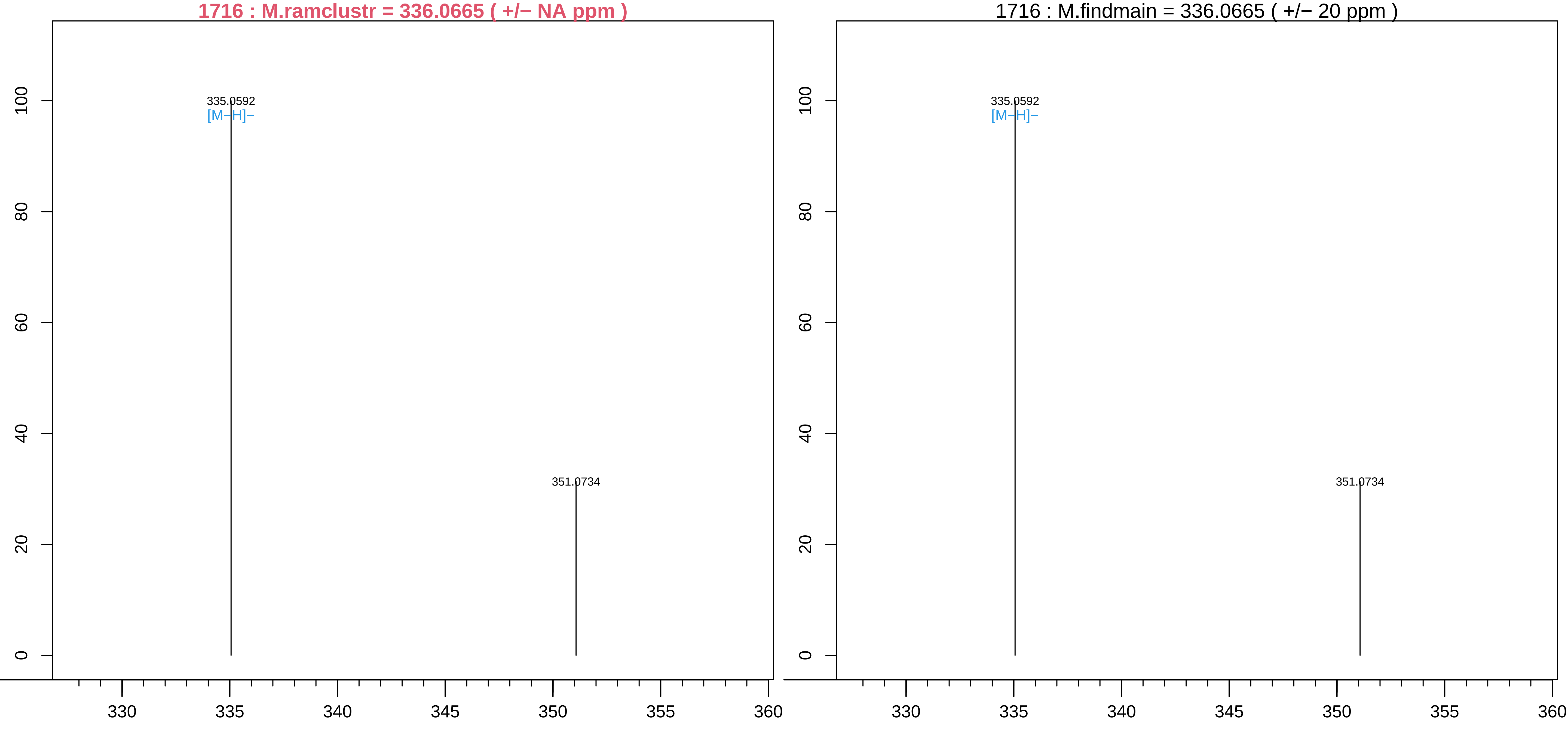
1716 : M.ramclustr = 336.0665 (+/− NA ppm)
335.0592
[M−H]−
351.0734
1716 : M.findmain = 336.0665 (+/− 20 ppm)
335.0592
[M−H]−
351.0734
0
20
40
60
80
100
330
335
340
345
350
355
360

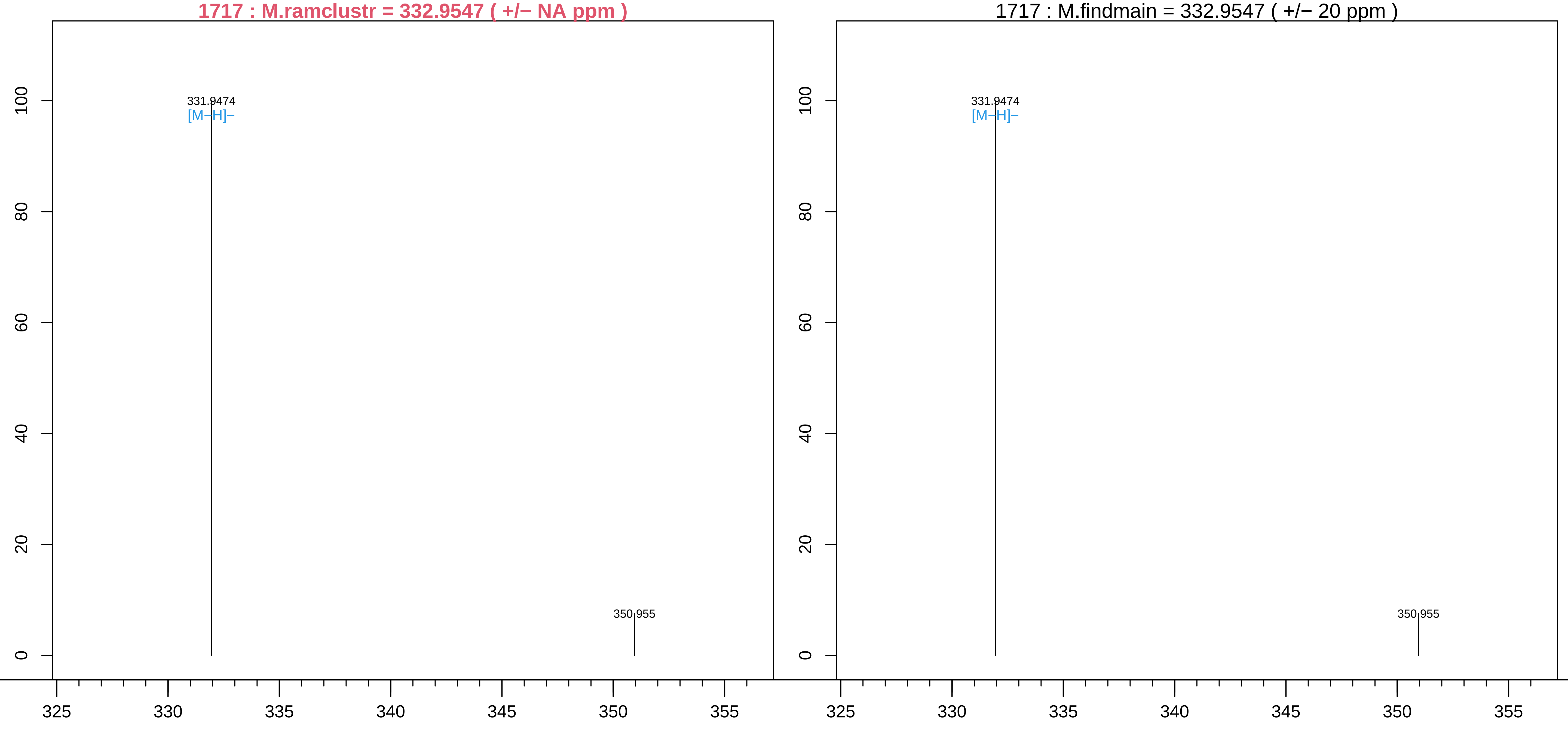
1717 : M.ramclustr = 332.9547 (+/− NA ppm)
331.9474
[M−H]−
350.955
1717 : M.findmain = 332.9547 (+/− 20 ppm)
331.9474
[M−H]−
350.955
0
20
40
60
80
100
325
330
335
340
345
350
355

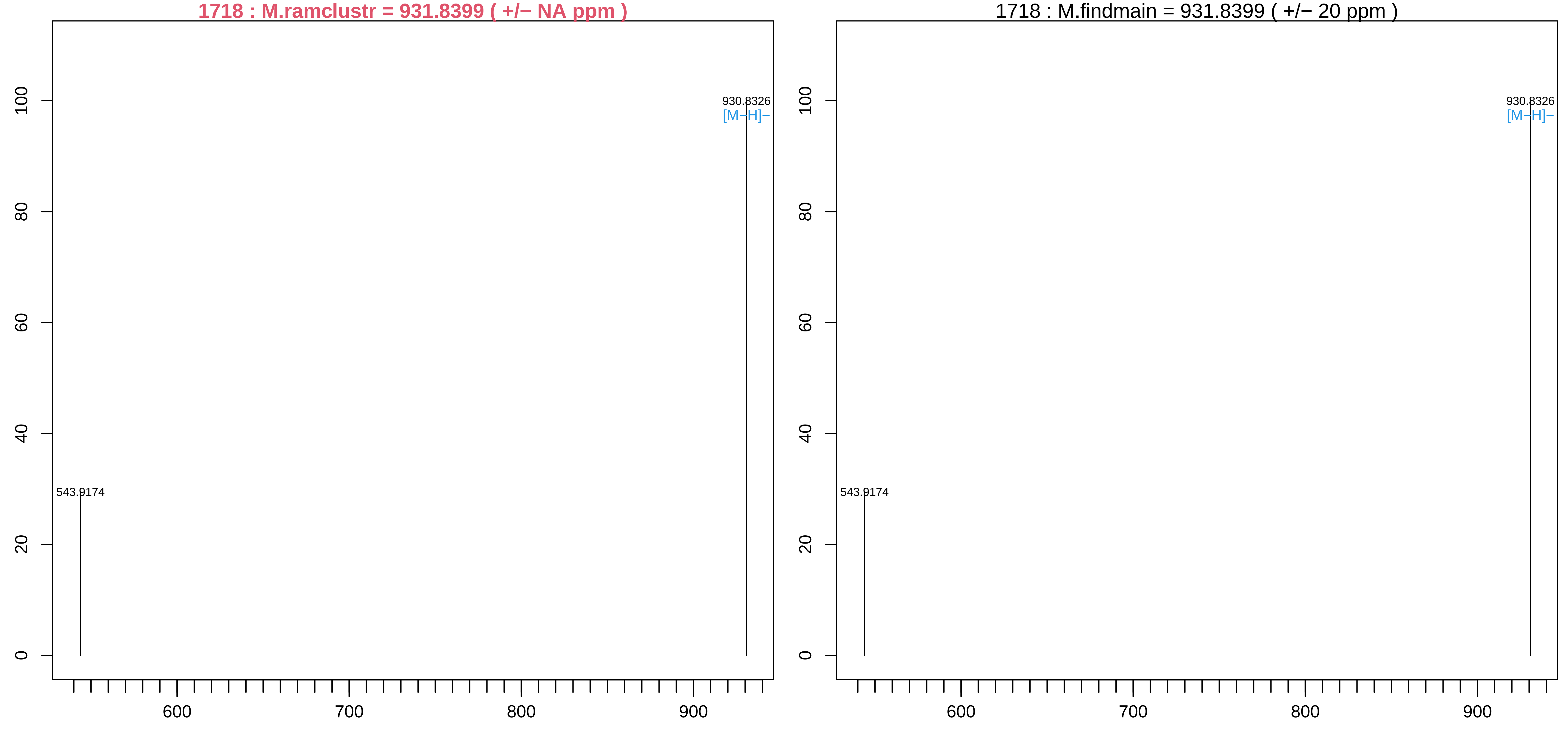
1718 : M.ramclustr = 931.8399 (+/− NA ppm)
930.8326
[M−H]−
543.9174
1718 : M.findmain = 931.8399 (+/− 20 ppm)
930.8326
[M−H]−
543.9174
0
20
40
60
80
100
600
700
800
900

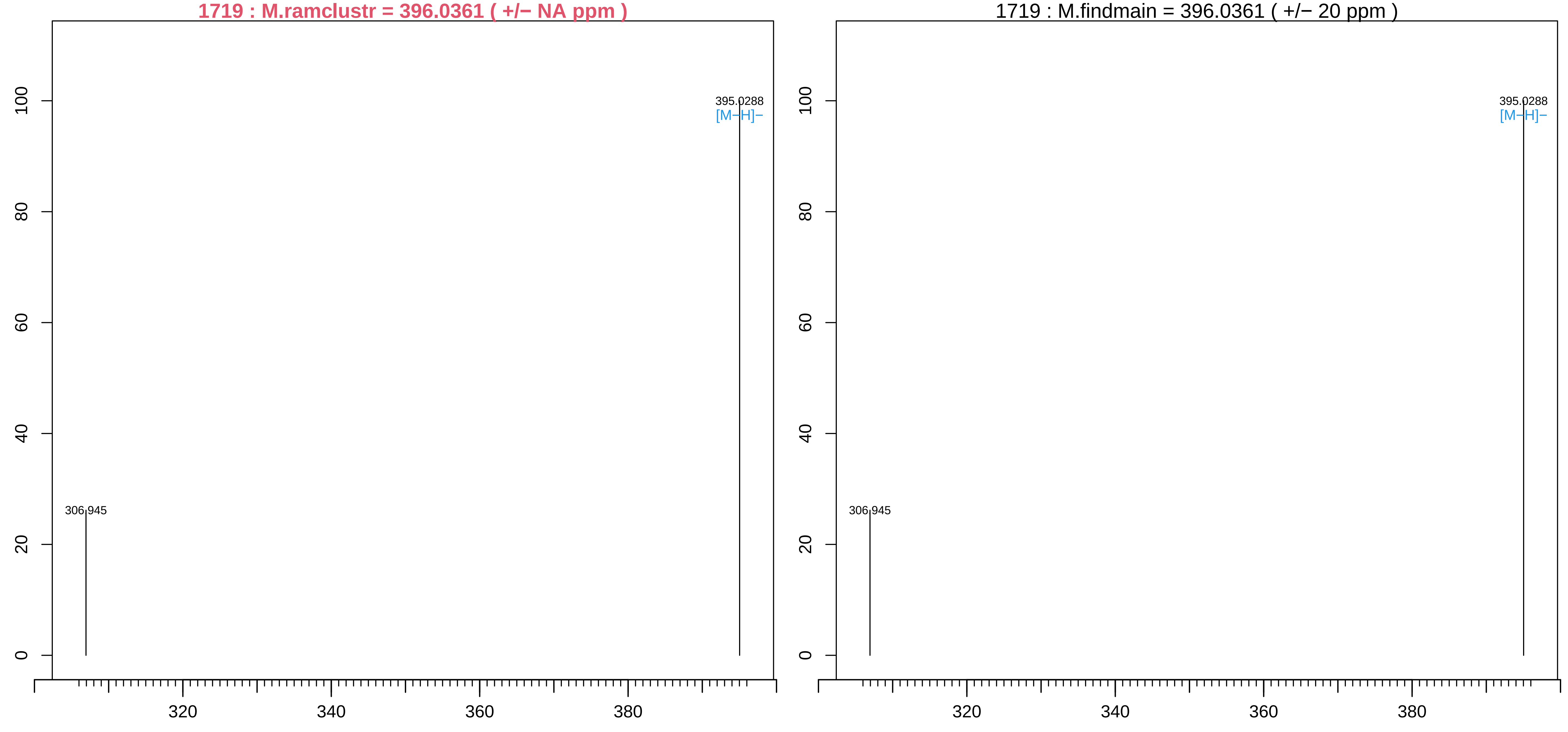
1719 : M.ramclustr = 396.0361 (+/− NA ppm)
1719 : M.findmain = 396.0361 (+/− 20 ppm)
395.0288
[M−H]−
306.945
395.0288
[M−H]−
306.945
0
20
40
60
80
100
320
340
360
380

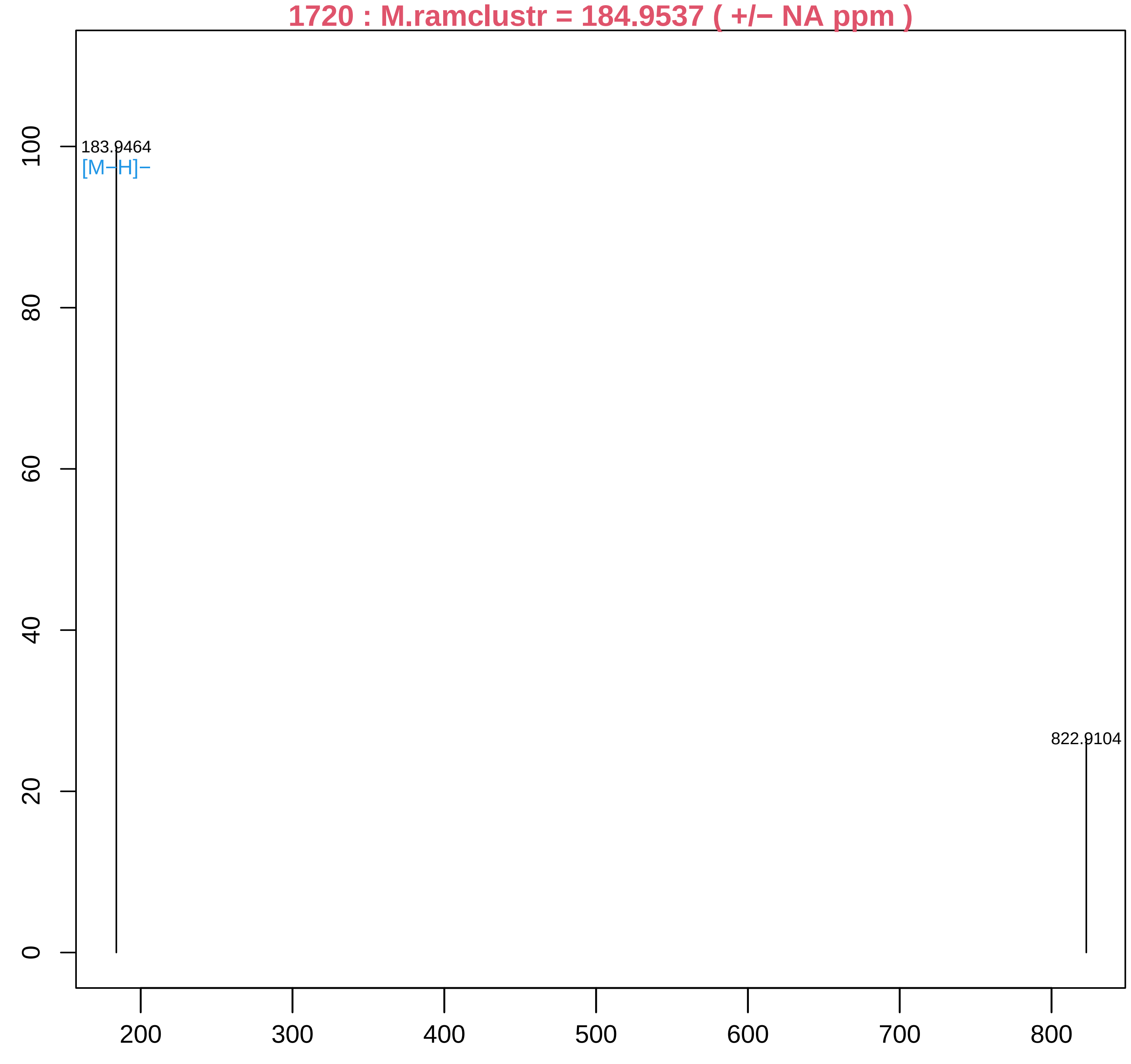

1720 : M.ramclustr = 184.9537 (+/− NA ppm)
183.9464
[M−H]−
822.9104
0
20
40
60
80
100
200
300
400
500
600
700
800

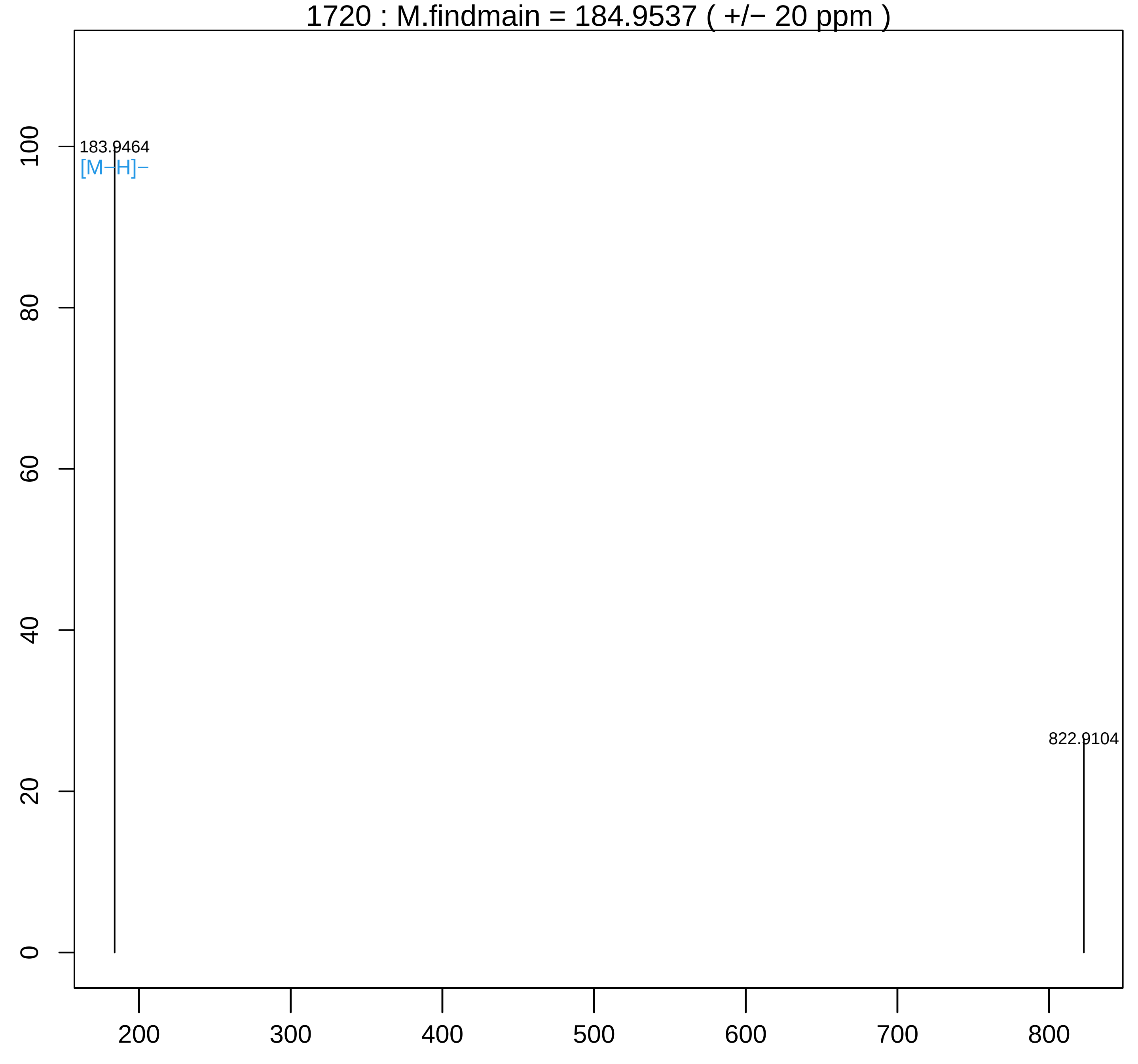

1720 : M.findmain = 184.9537 (+/− 20 ppm)
183.9464
[M−H]−
822.9104
0
20
40
60
80
100
200
300
400
500
600
700
800

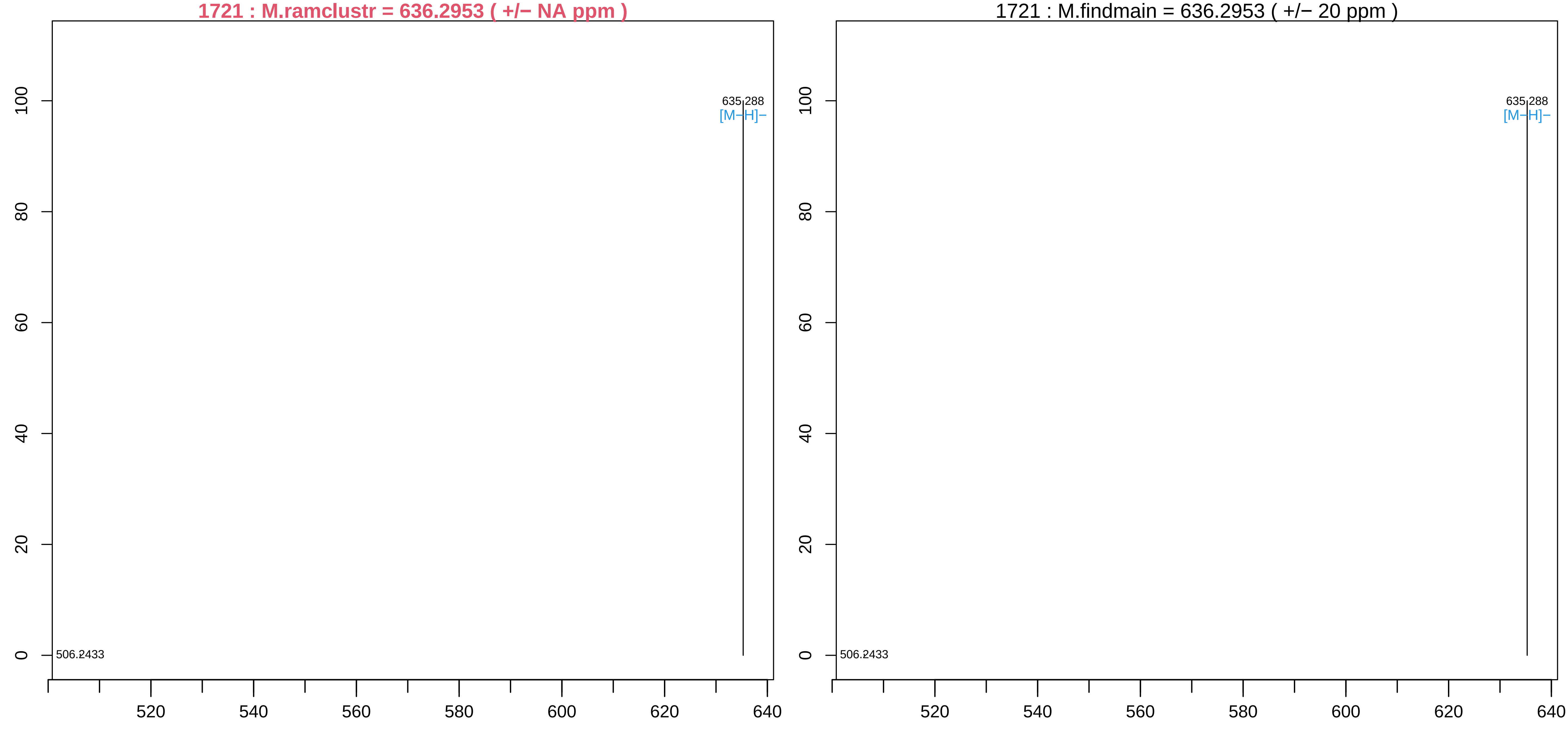
1721 : M.ramclustr = 636.2953 (+/− NA ppm)
635.288
[M−H]−
506.2433
1721 : M.findmain = 636.2953 (+/− 20 ppm)
635.288
[M−H]−
506.2433
0
20
40
60
80
100
520
540
560
580
600
620
640

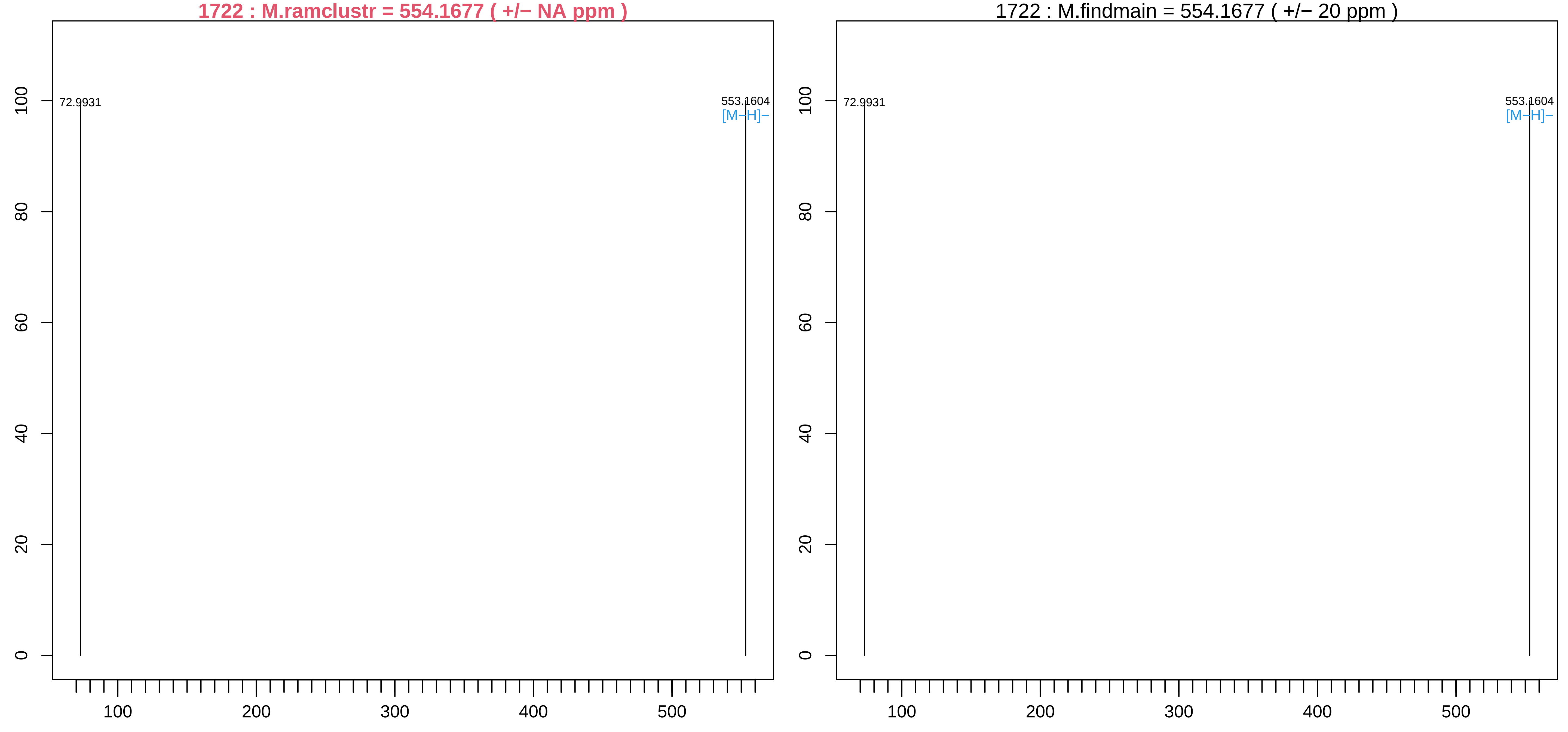
1722 : M.ramclustr = 554.1677 (+/− NA ppm)
72.9931
553.1604
[M−H]−
1722 : M.findmain = 554.1677 (+/− 20 ppm)
72.9931
553.1604
[M−H]−
0
20
40
60
80
100
100
200
300
400
500

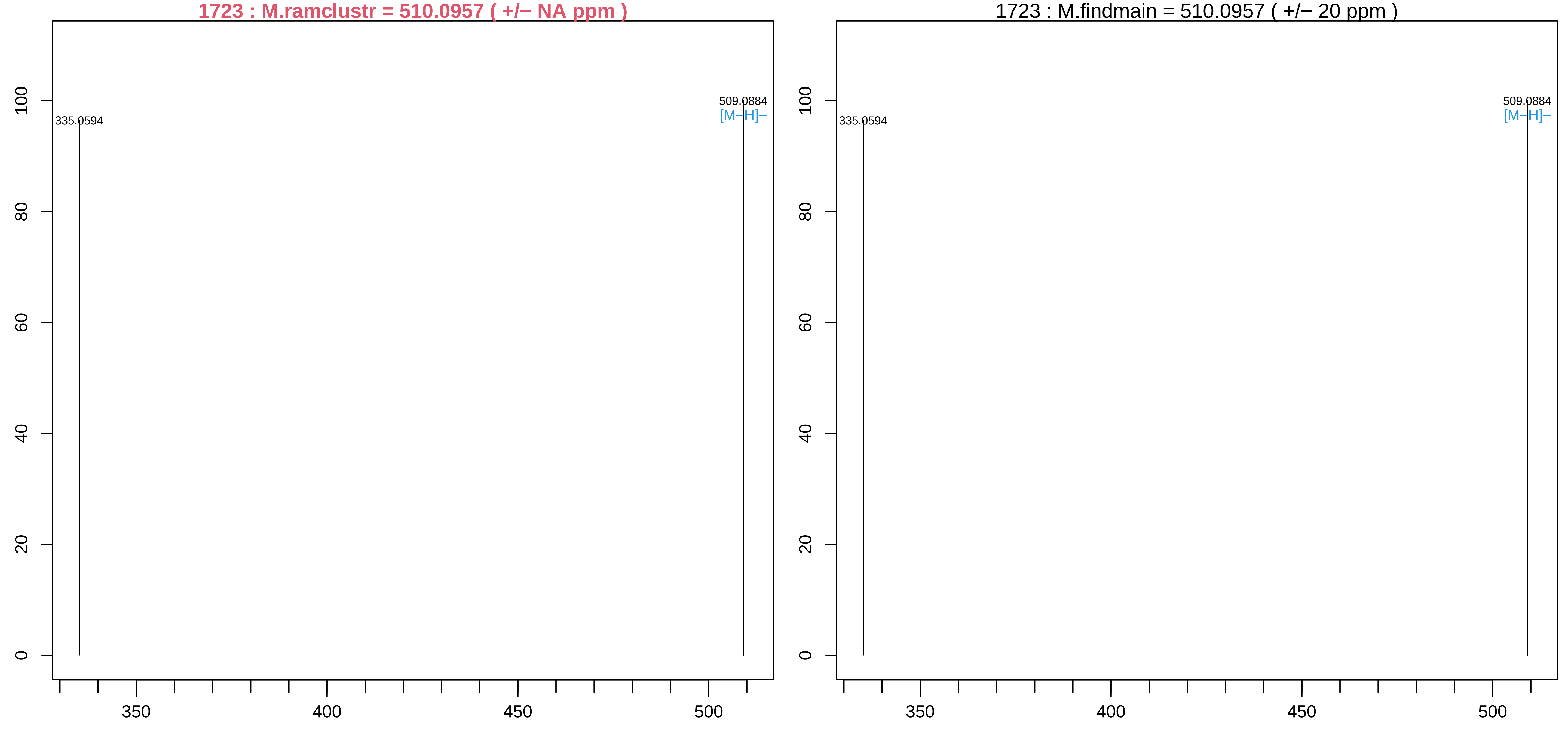
1723 : M.ramclustr = 510.0957 (+/− NA ppm)
335.0594
509.0884
[M−H]−
0
20
40
60
80
100
350
400
450
500
1723 : M.findmain = 510.0957 (+/− 20 ppm)
335.0594
509.0884
[M−H]−
0
20
40
60
80
100
350
400
450
500

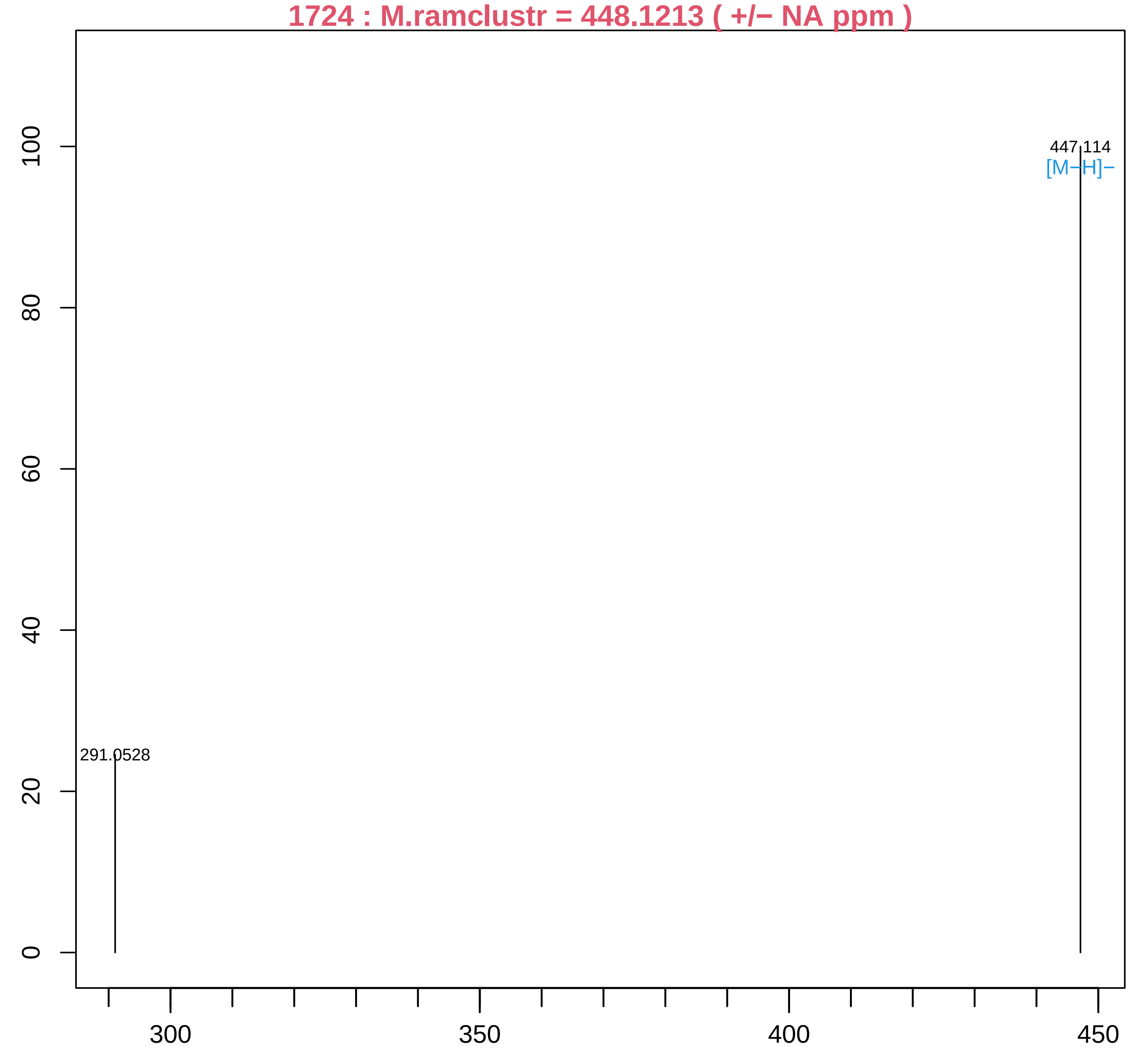
1724 : M.ramclustr = 448.1213 (+/− NA ppm)
447.114
[M−H]−
291.0528
0
20
40
60
80
100
300
350
400
450

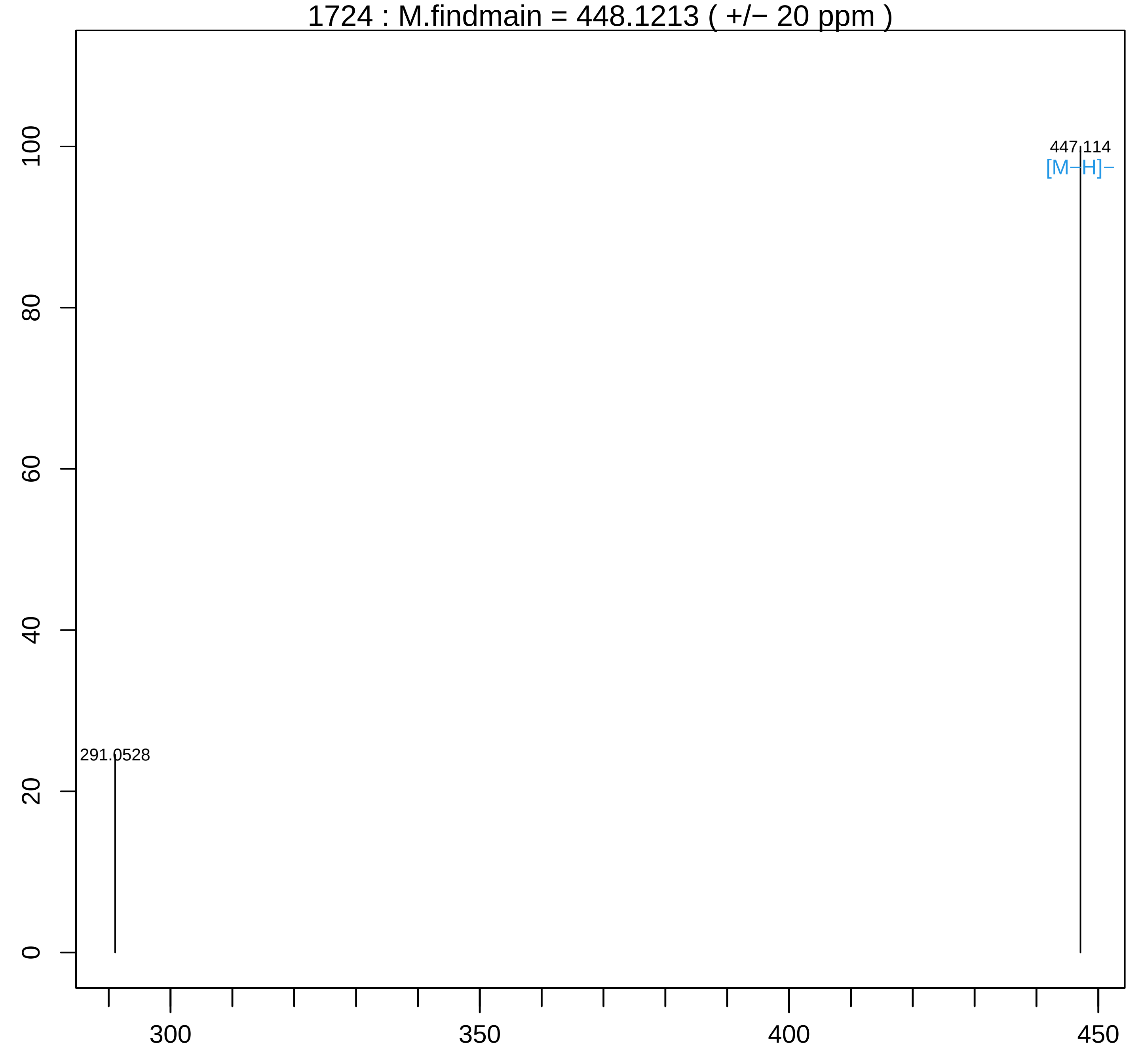
1724 : M.findmain = 448.1213 (+/− 20 ppm)
447.114
[M−H]−
291.0528
0
20
40
60
80
100
300
350
400
450

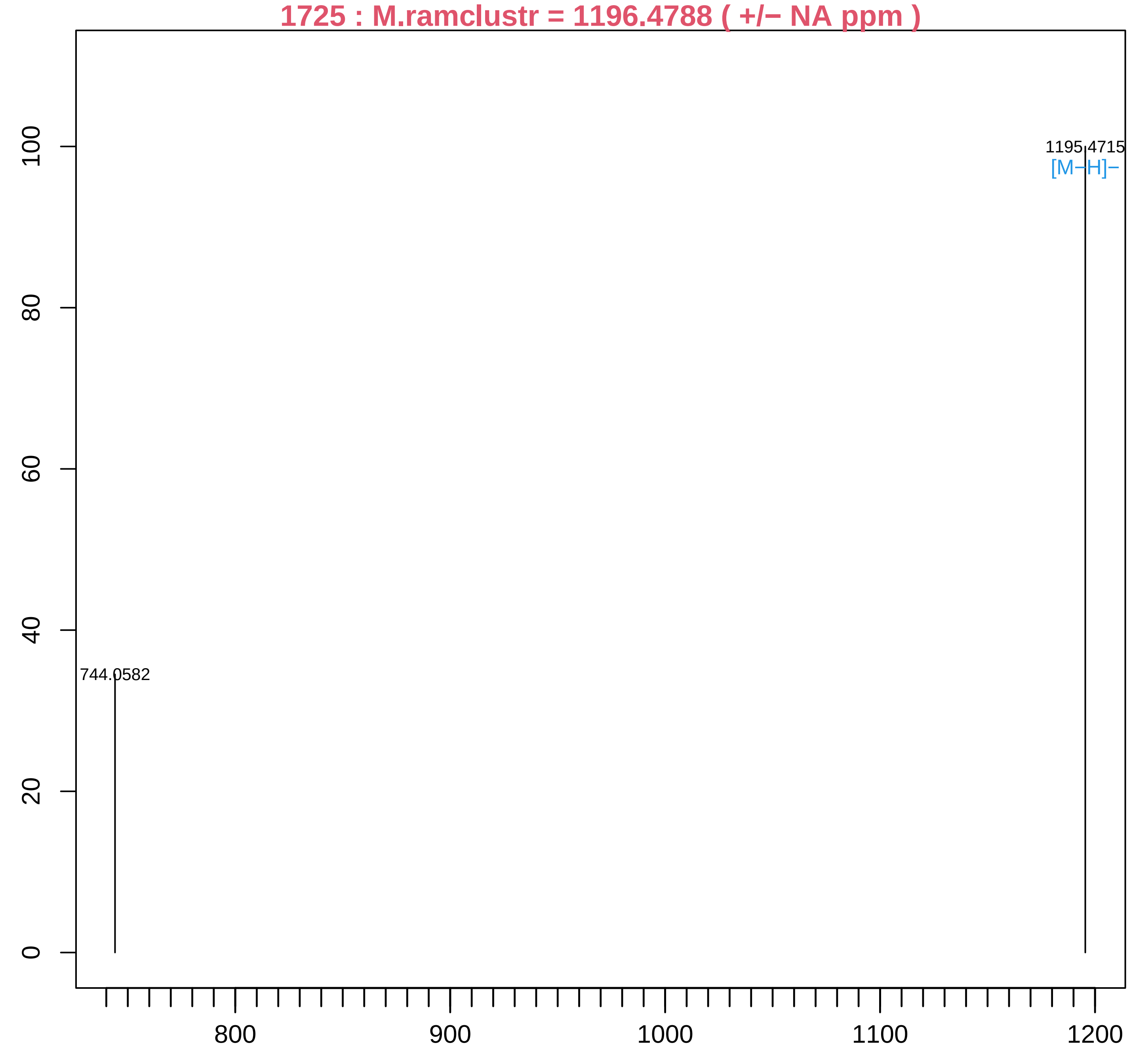
1725 : M.ramclustr = 1196.4788 (+/− NA ppm)
1195.4715
[M−H]−
744.0582
0
20
40
60
80
100
800
900
1000
1100
1200

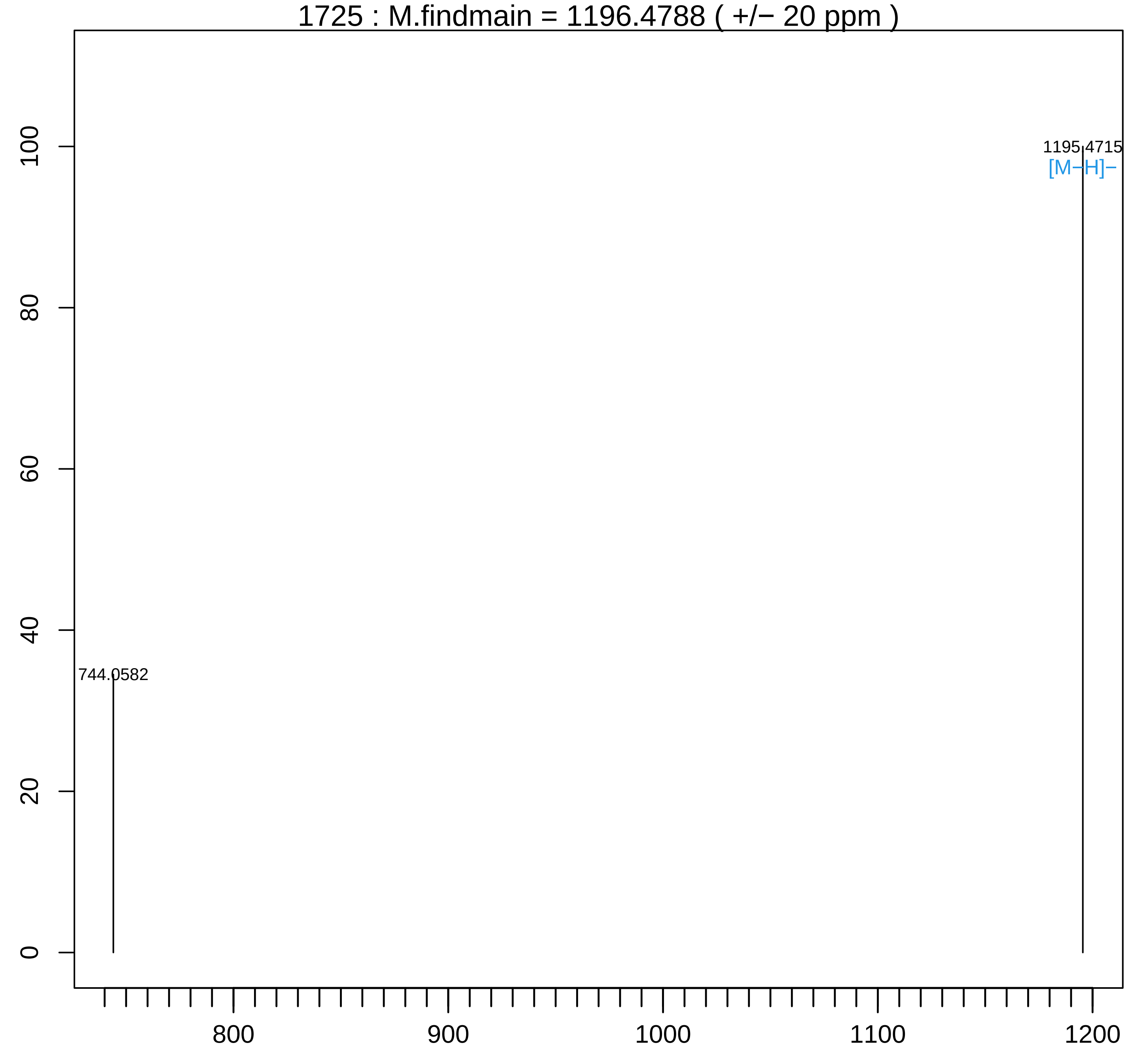
1725 : M.findmain = 1196.4788 (+/− 20 ppm)
1195.4715
[M−H]−
744.0582
0
20
40
60
80
100
800
900
1000
1100
1200

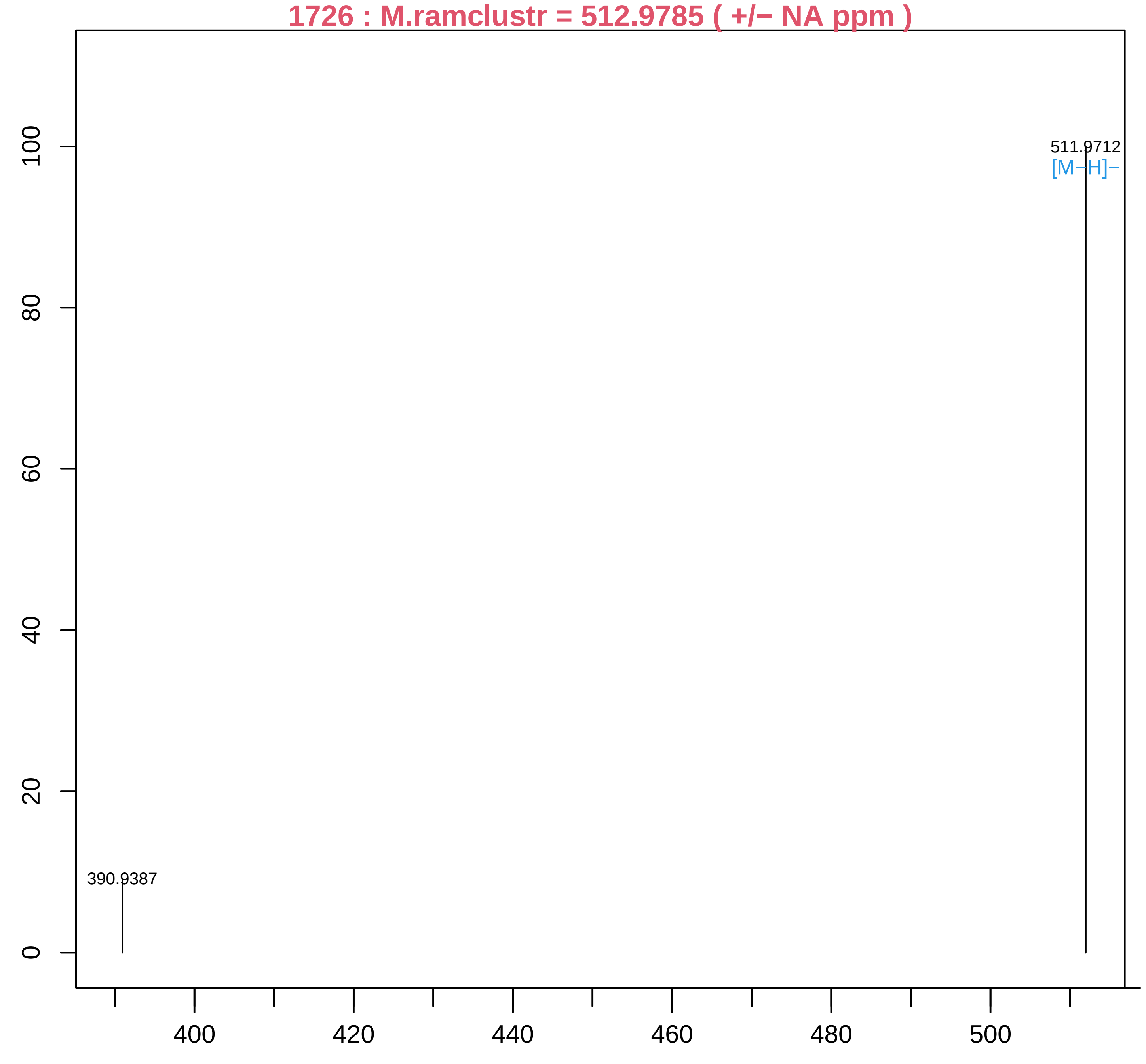

1726 : M.ramclustr = 512.9785 (+/− NA ppm)
511.9712
[M−H]−
390.9387
0
20
40
60
80
100
400
420
440
460
480
500

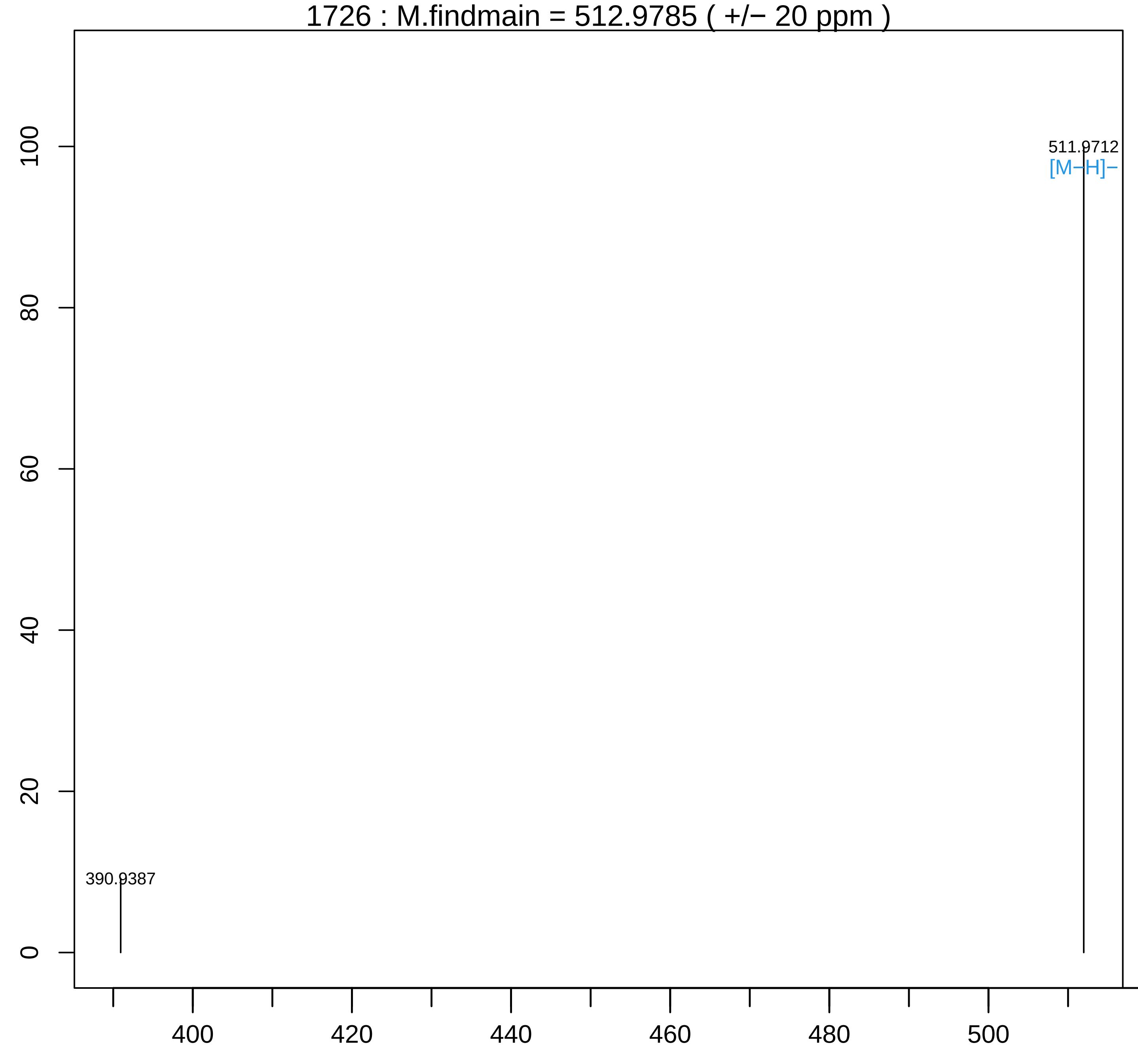

1726 : M.findmain = 512.9785 (+/− 20 ppm)
511.9712
[M−H]−
390.9387
0
20
40
60
80
100
400
420
440
460
480
500

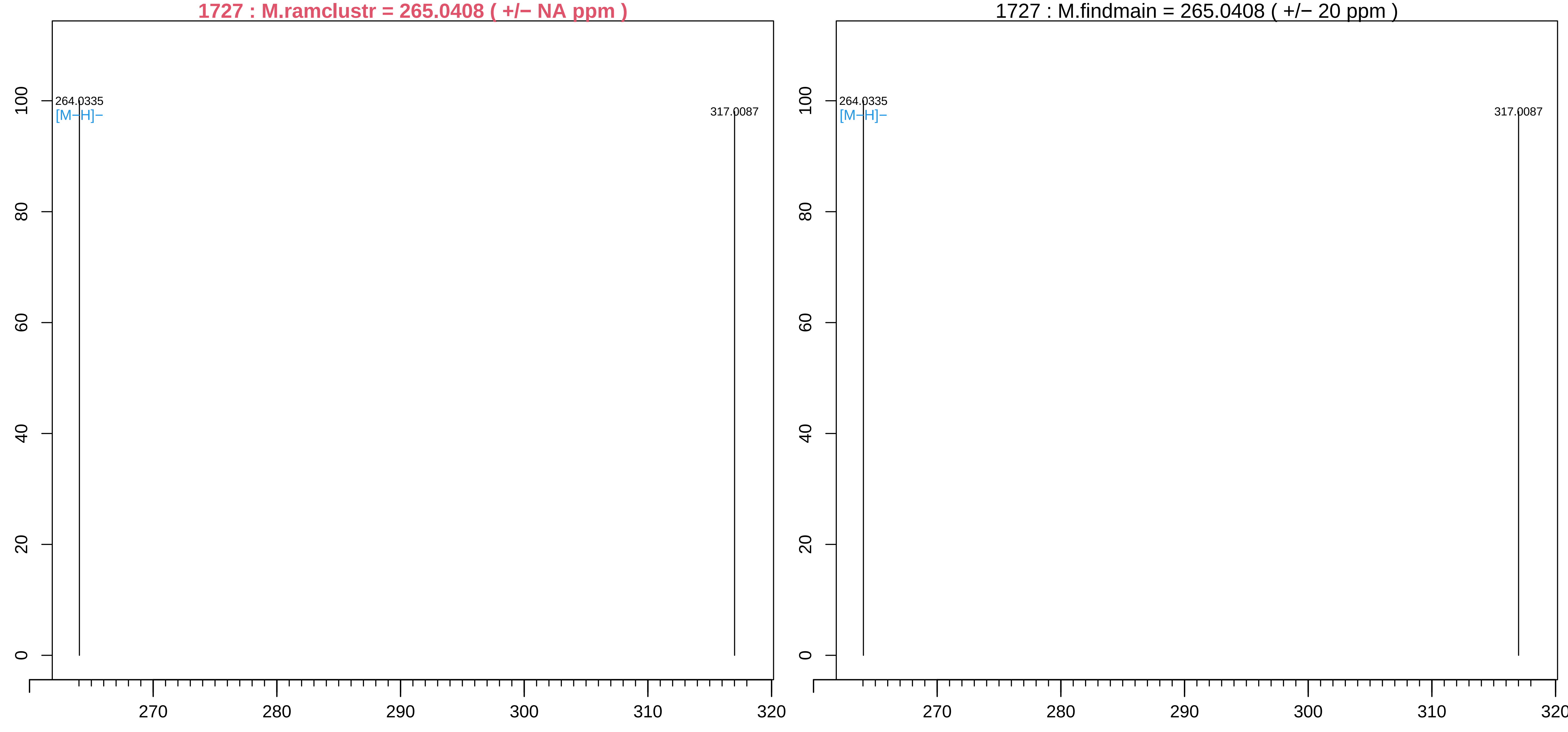
1727 : M.ramclustr = 265.0408 (+/− NA ppm)
264.0335
[M−H]−
317.0087
1727 : M.findmain = 265.0408 (+/− 20 ppm)
264.0335
[M−H]−
317.0087
0
20
40
60
80
100
270
280
290
300
310
320

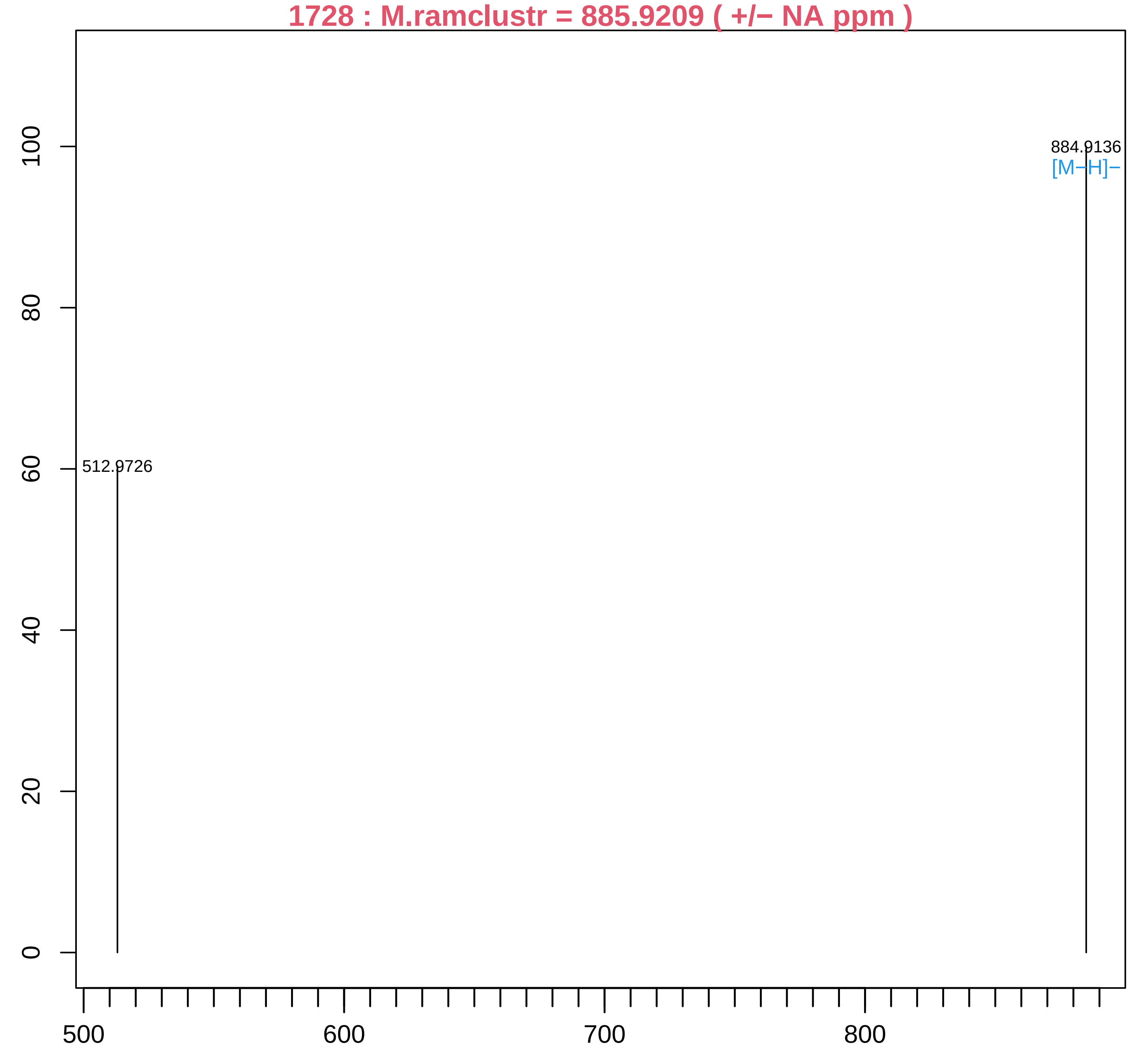
1728 : M.ramclustr = 885.9209 (+/− NA ppm)
884.9136
[M−H]−
512.9726
0
20
40
60
80
100
500
600
700
800

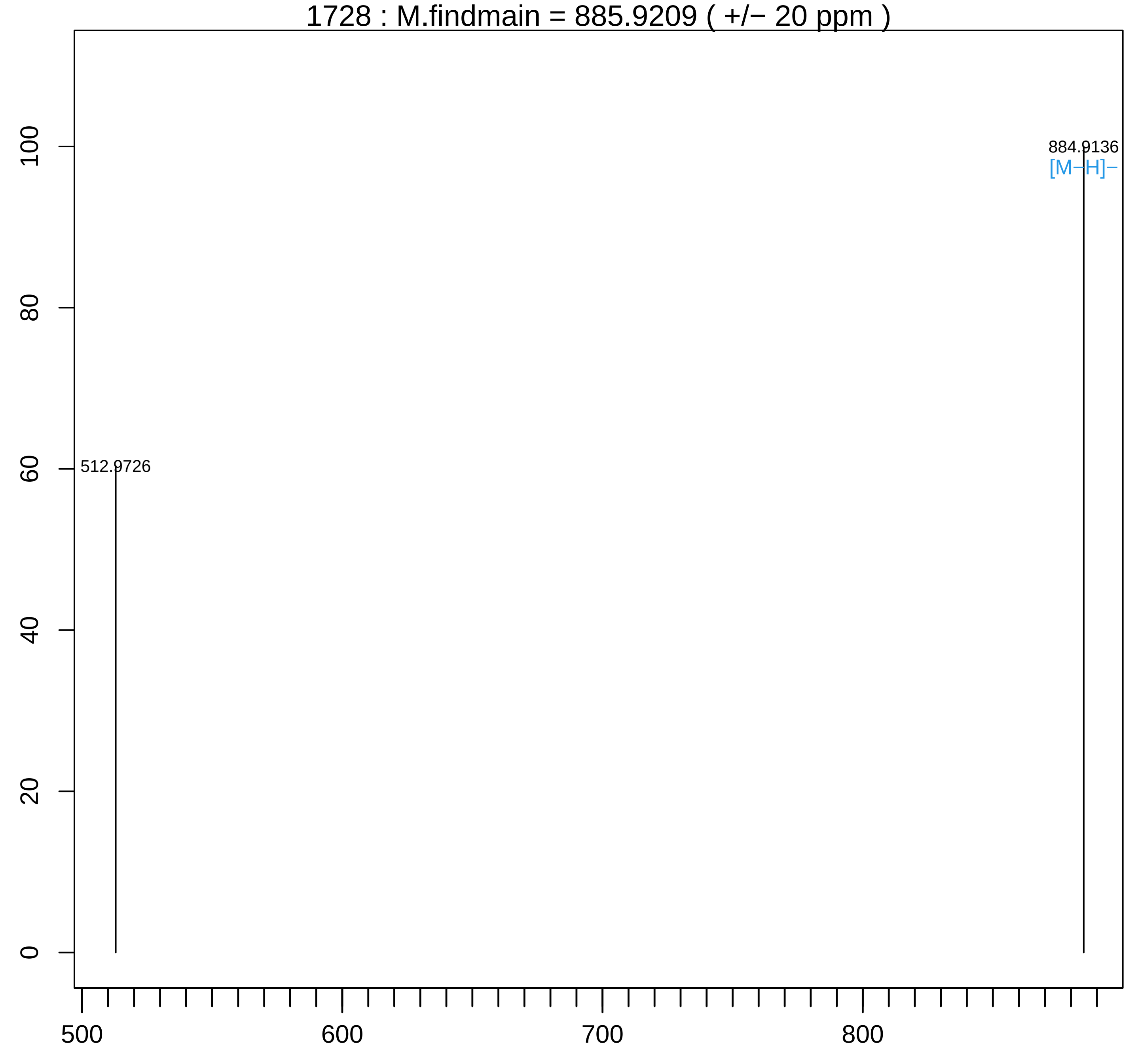
1728 : M.findmain = 885.9209 (+/− 20 ppm)
884.9136
[M−H]−
512.9726
0
20
40
60
80
100
500
600
700
800

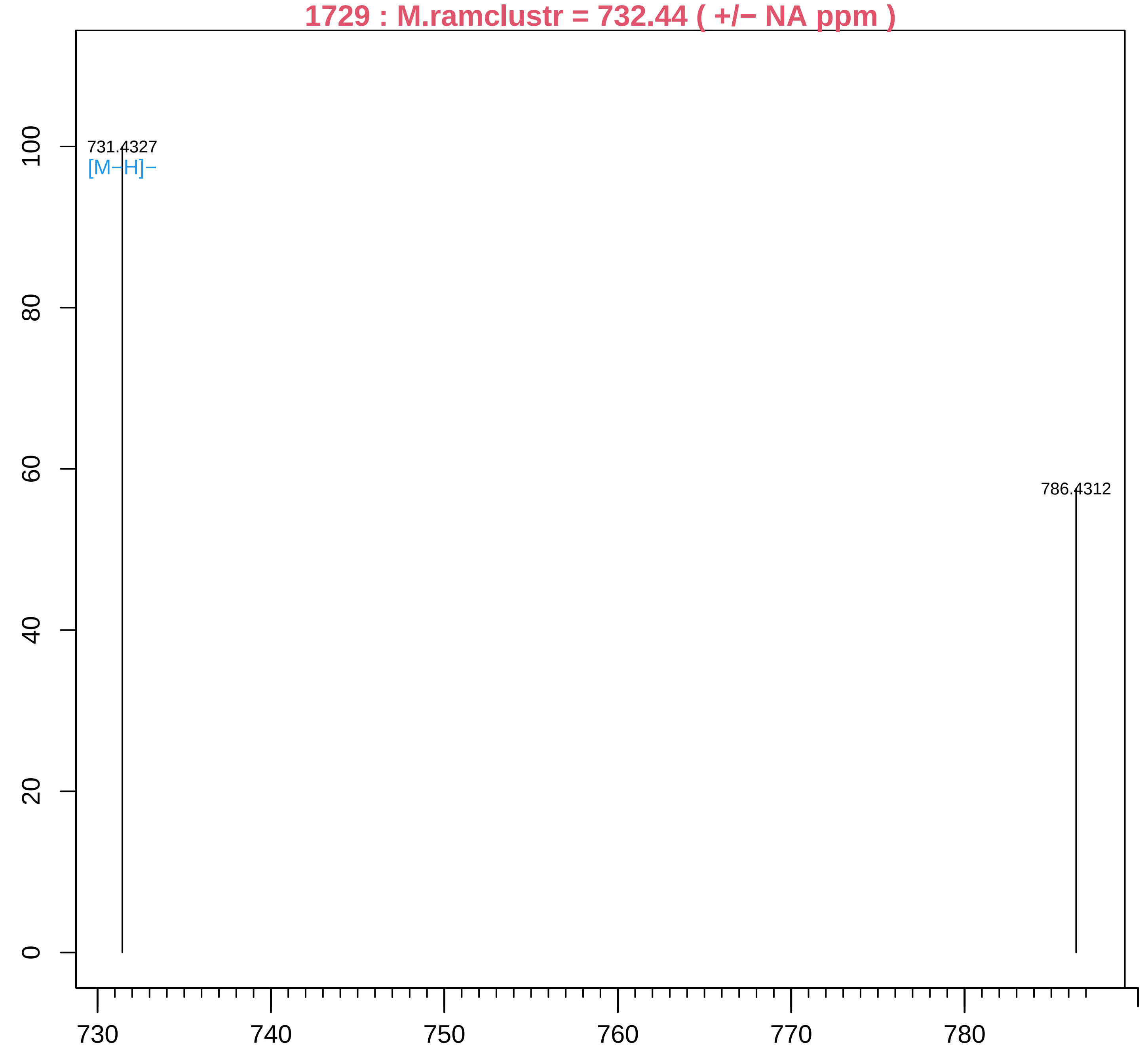
1729 : M.ramclustr = 732.44 (+/− NA ppm)
731.4327
[M−H]−
786.4312
0
20
40
60
80
100
730
740
750
760
770
780

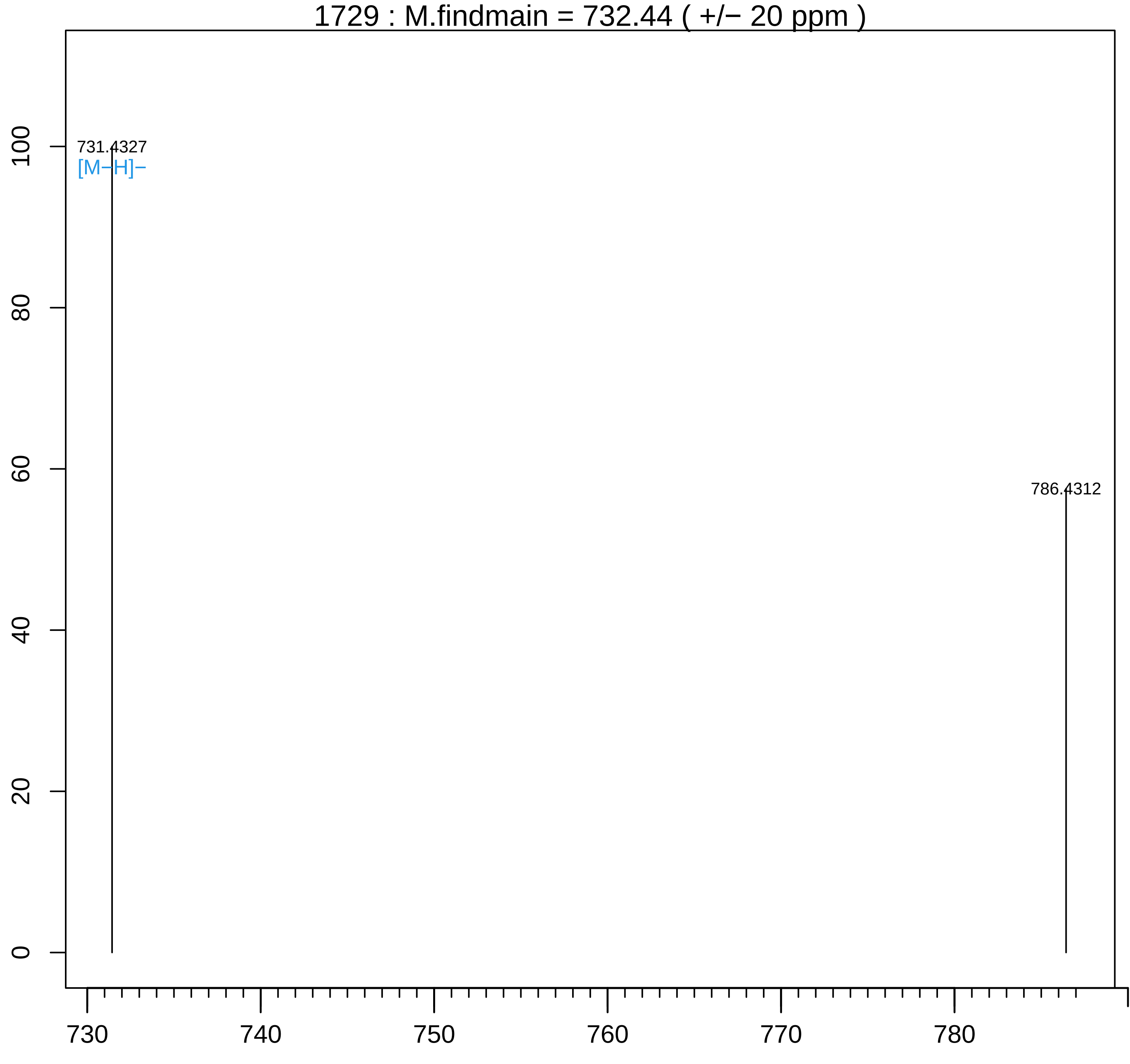
1729 : M.findmain = 732.44 (+/− 20 ppm)
731.4327
[M−H]−
786.4312
0
20
40
60
80
100
730
740
750
760
770
780

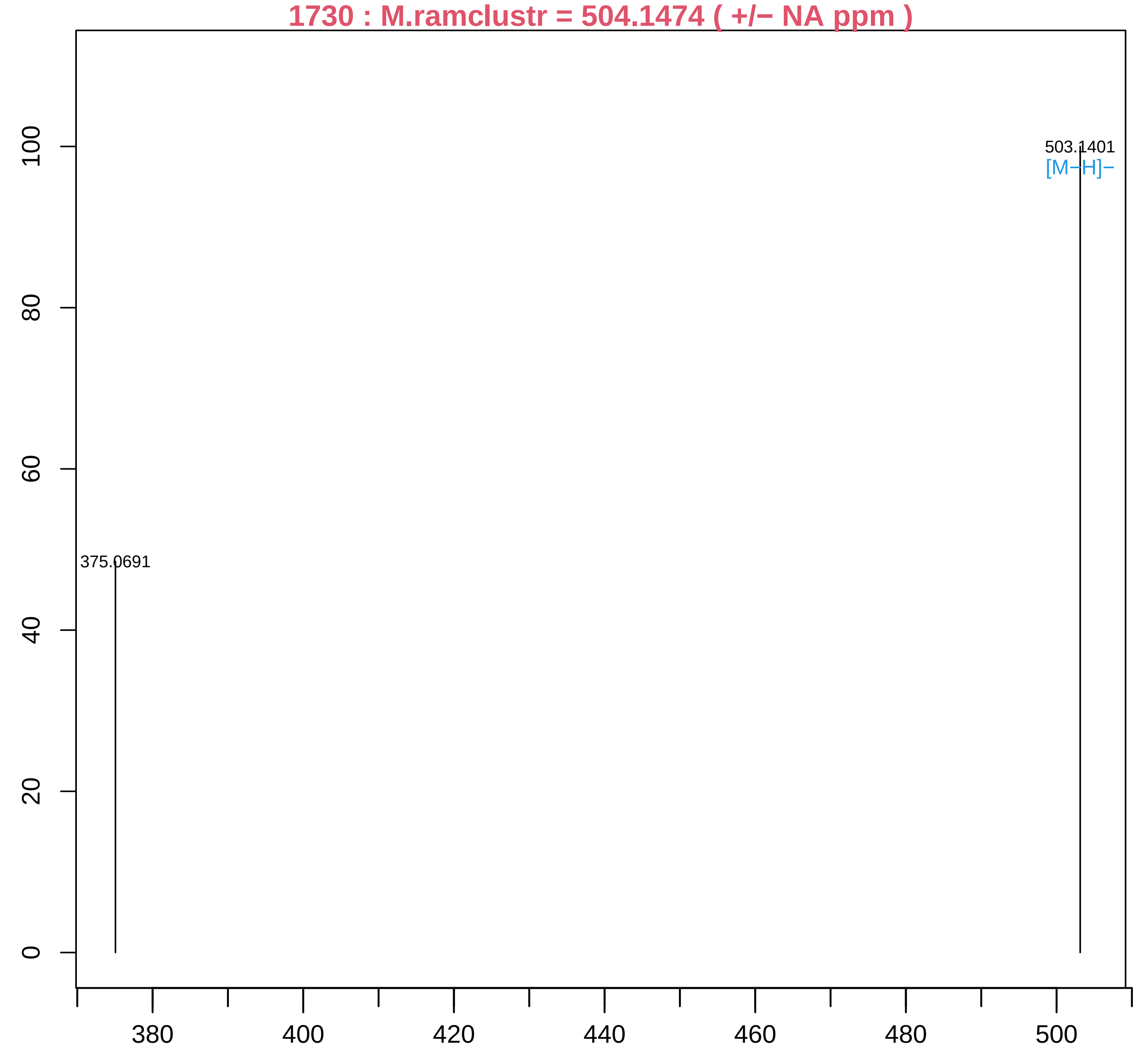

1730 : M.ramclustr = 504.1474 (+/− NA ppm)
503.1401
[M−H]−
375.0691
0
20
40
60
80
100
380
400
420
440
460
480
500

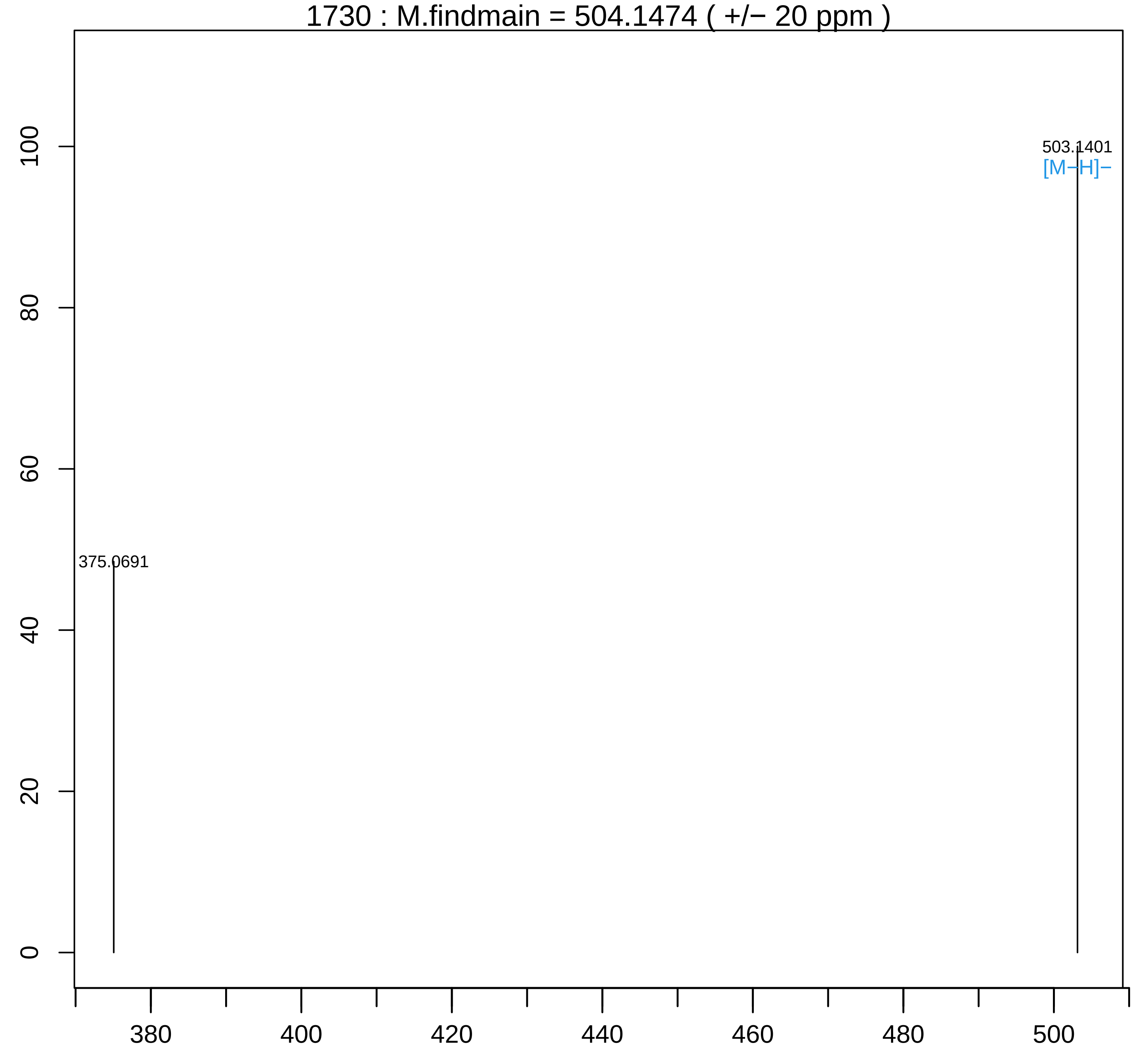

1730 : M.findmain = 504.1474 (+/− 20 ppm)
503.1401
[M−H]−
375.0691
0
20
40
60
80
100
380
400
420
440
460
480
500

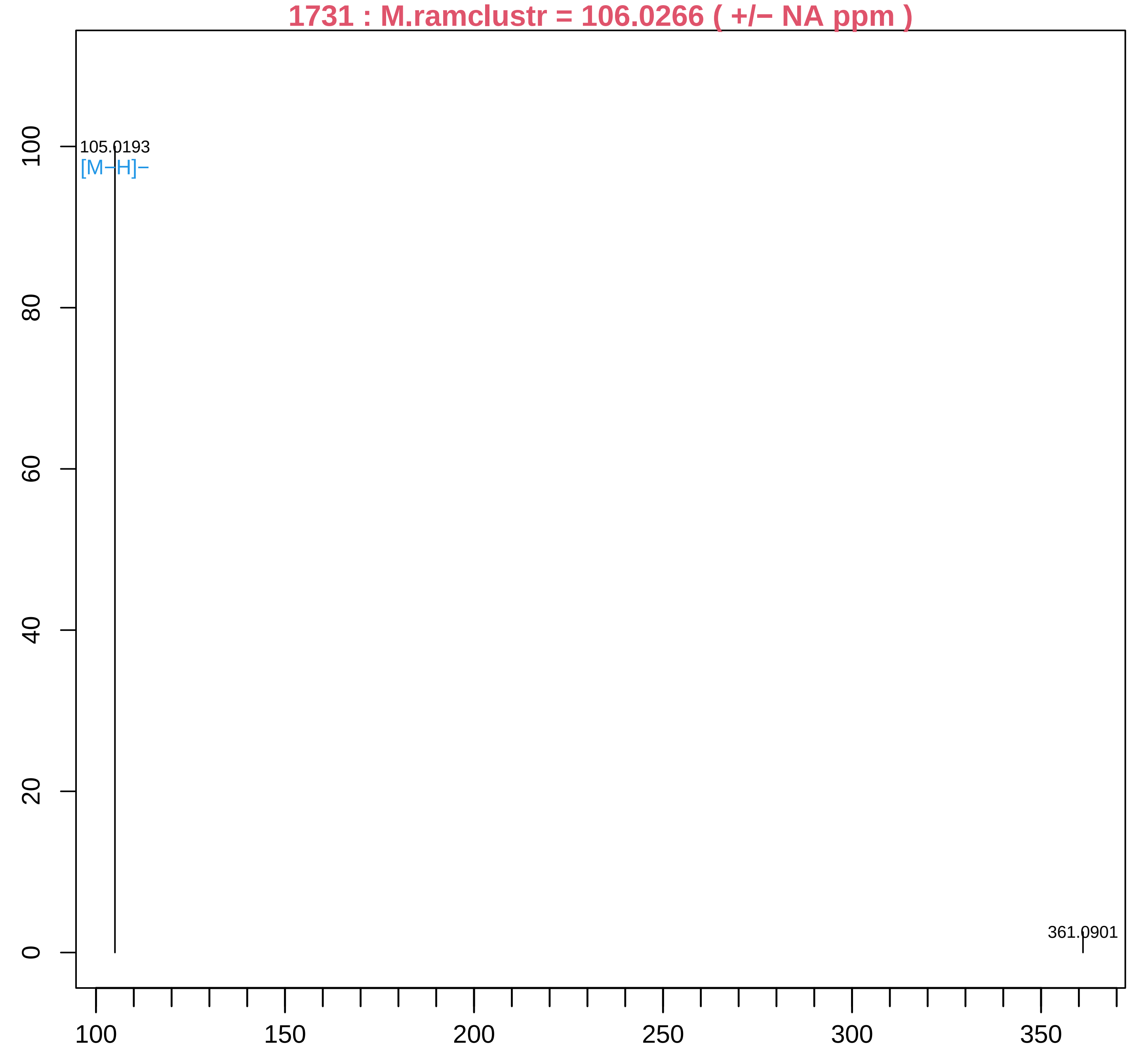
1731 : M.ramclustr = 106.0266 (+/− NA ppm)
105.0193
[M−H]−
361.0901
0
20
40
60
80
100
100
150
200
250
300
350

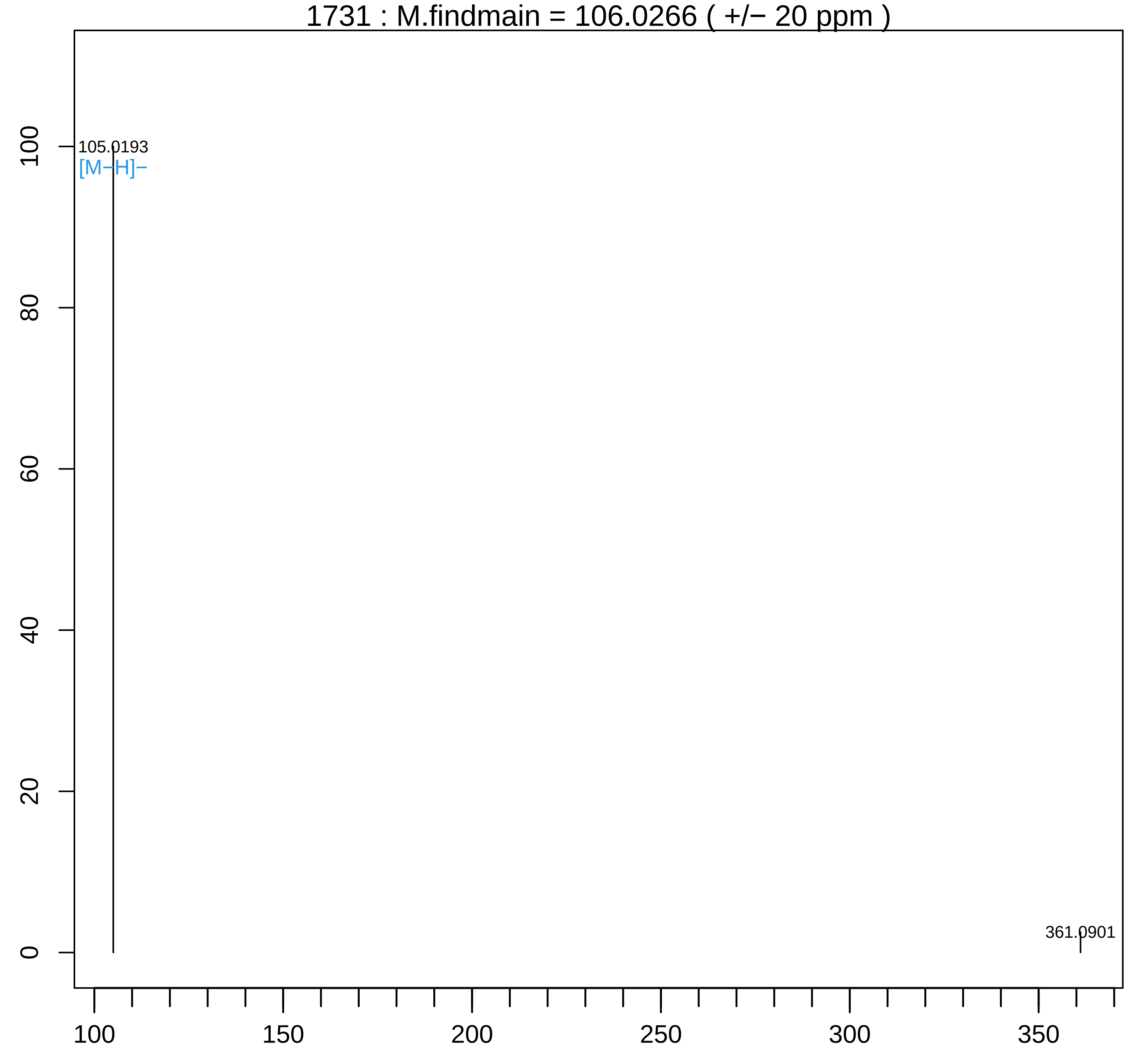
1731 : M.findmain = 106.0266 (+/− 20 ppm)
105.0193
[M−H]−
361.0901
0
20
40
60
80
100
100
150
200
250
300
350

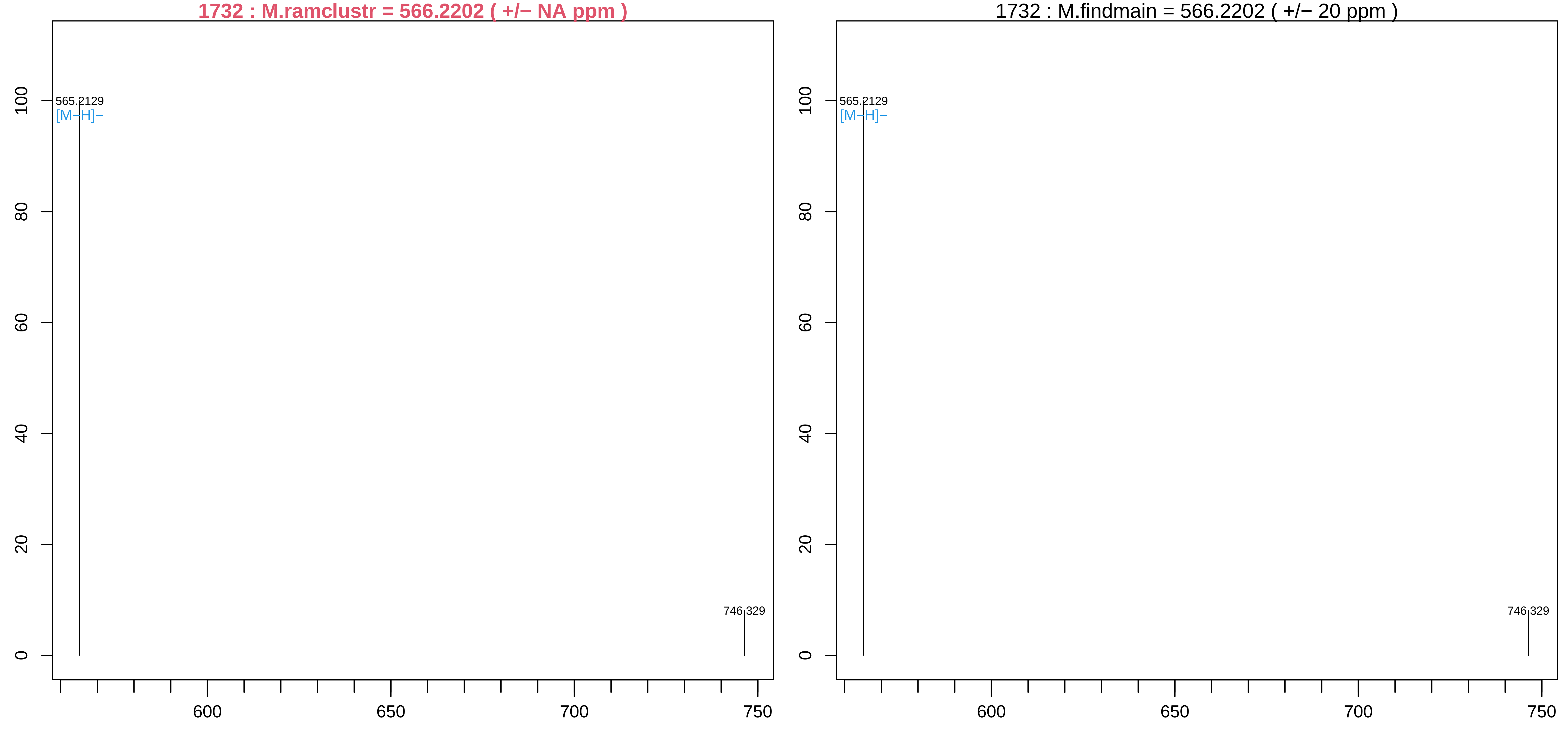
1732 : M.ramclustr = 566.2202 (+/− NA ppm)
565.2129
[M−H]−
746.329
1732 : M.findmain = 566.2202 (+/− 20 ppm)
565.2129
[M−H]−
746.329

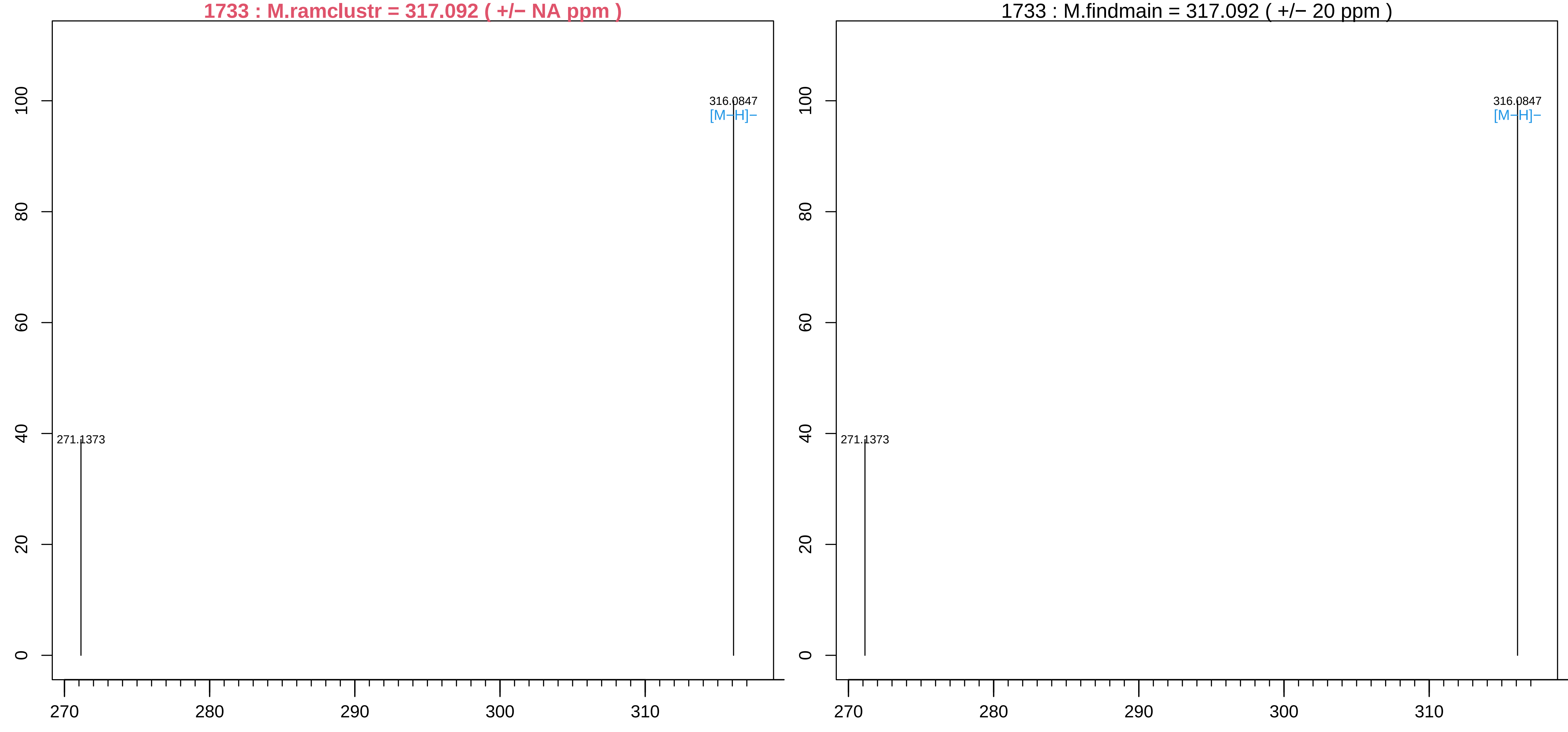
1733 : M.ramclustr = 317.092 (+/− NA ppm)
316.0847
[M−H]−
271.1373
1733 : M.findmain = 317.092 (+/− 20 ppm)
316.0847
[M−H]−
271.1373

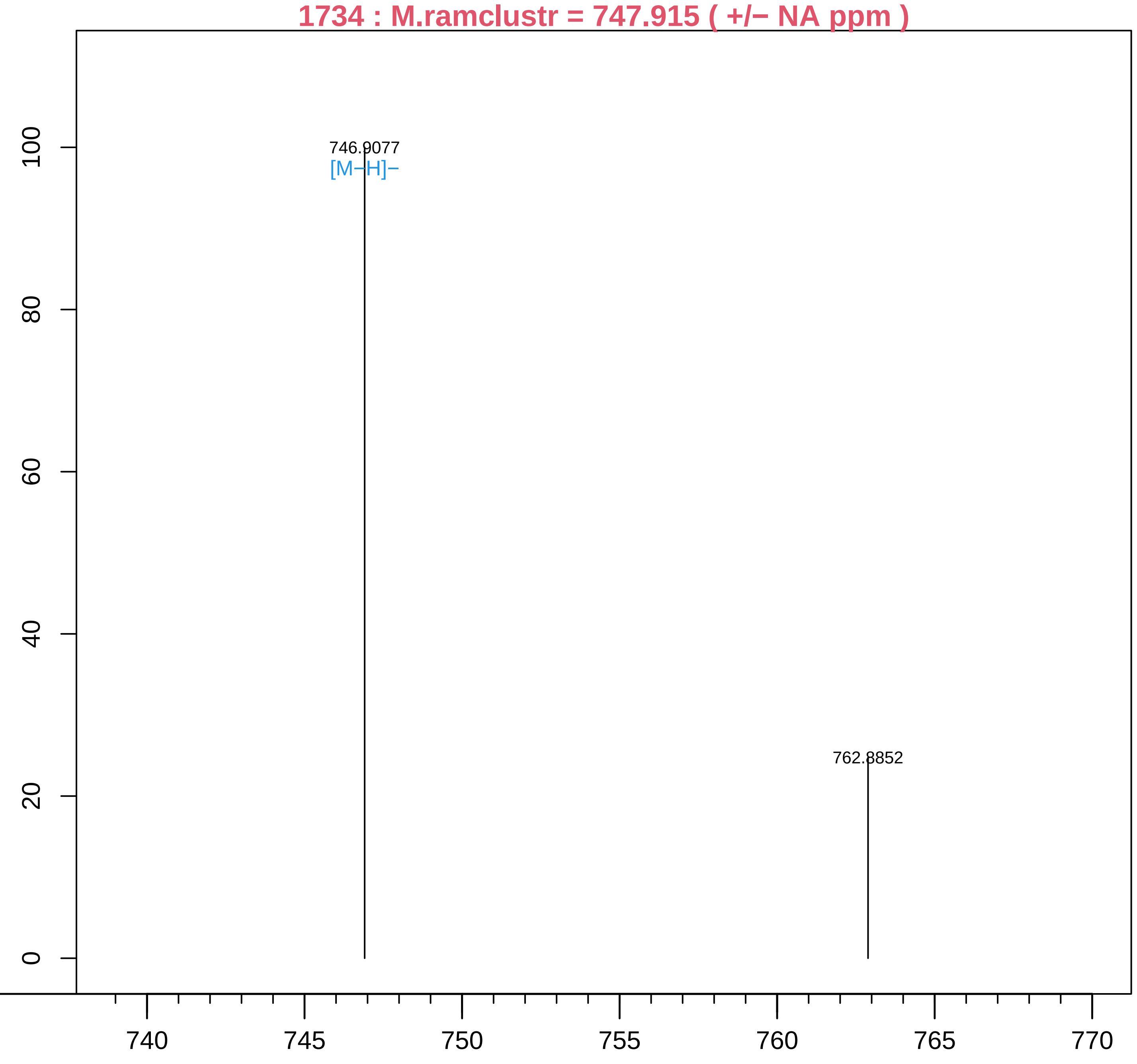
1734 : M.ramclustr = 747.915 (+/− NA ppm)
746.9077
[M−H]−
762.8852
0
20
40
60
80
100
740
745
750
755
760
765
770

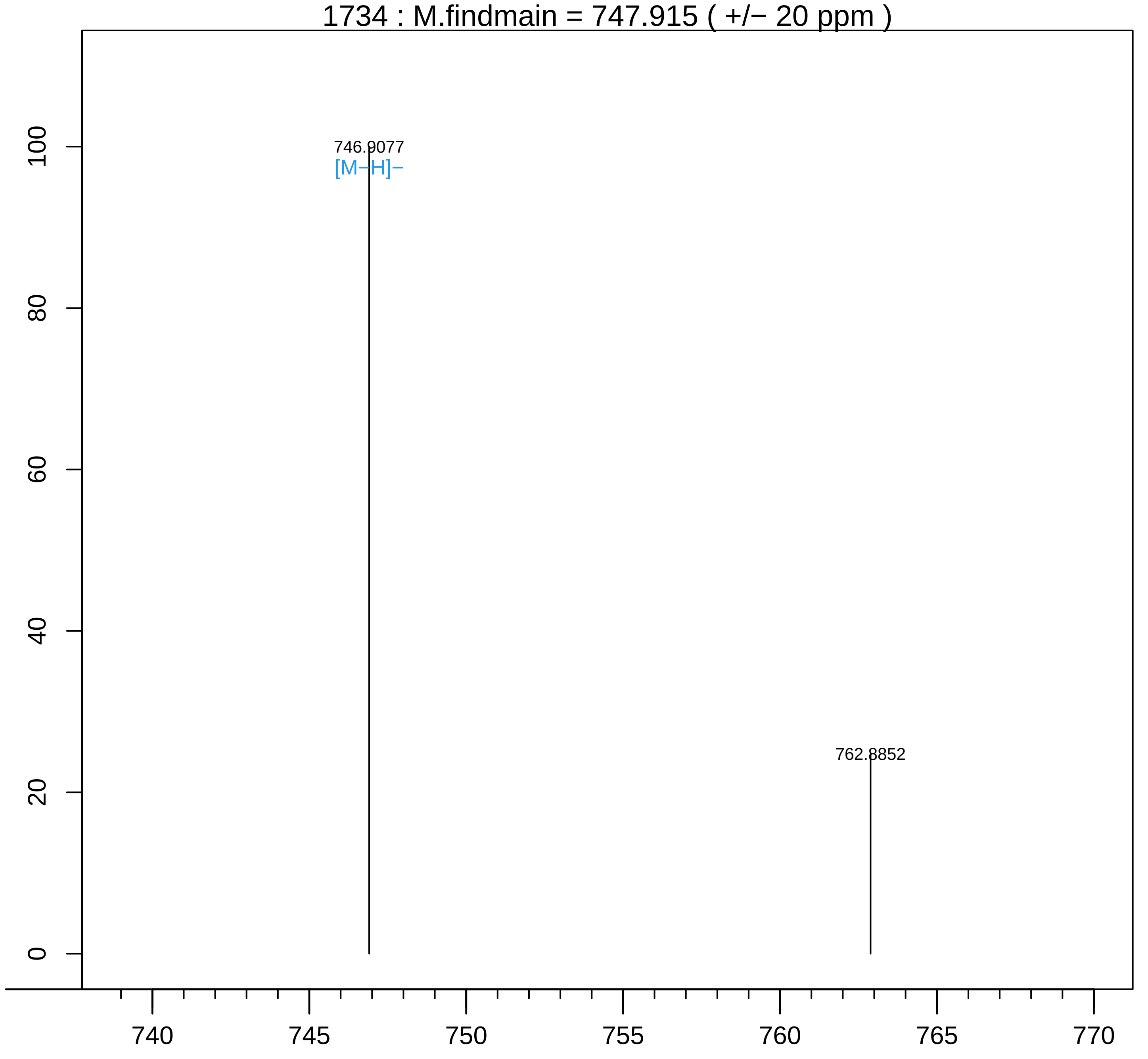
1734 : M.findmain = 747.915 (+/− 20 ppm)
746.9077
[M−H]−
762.8852
0
20
40
60
80
100
740
745
750
755
760
765
770

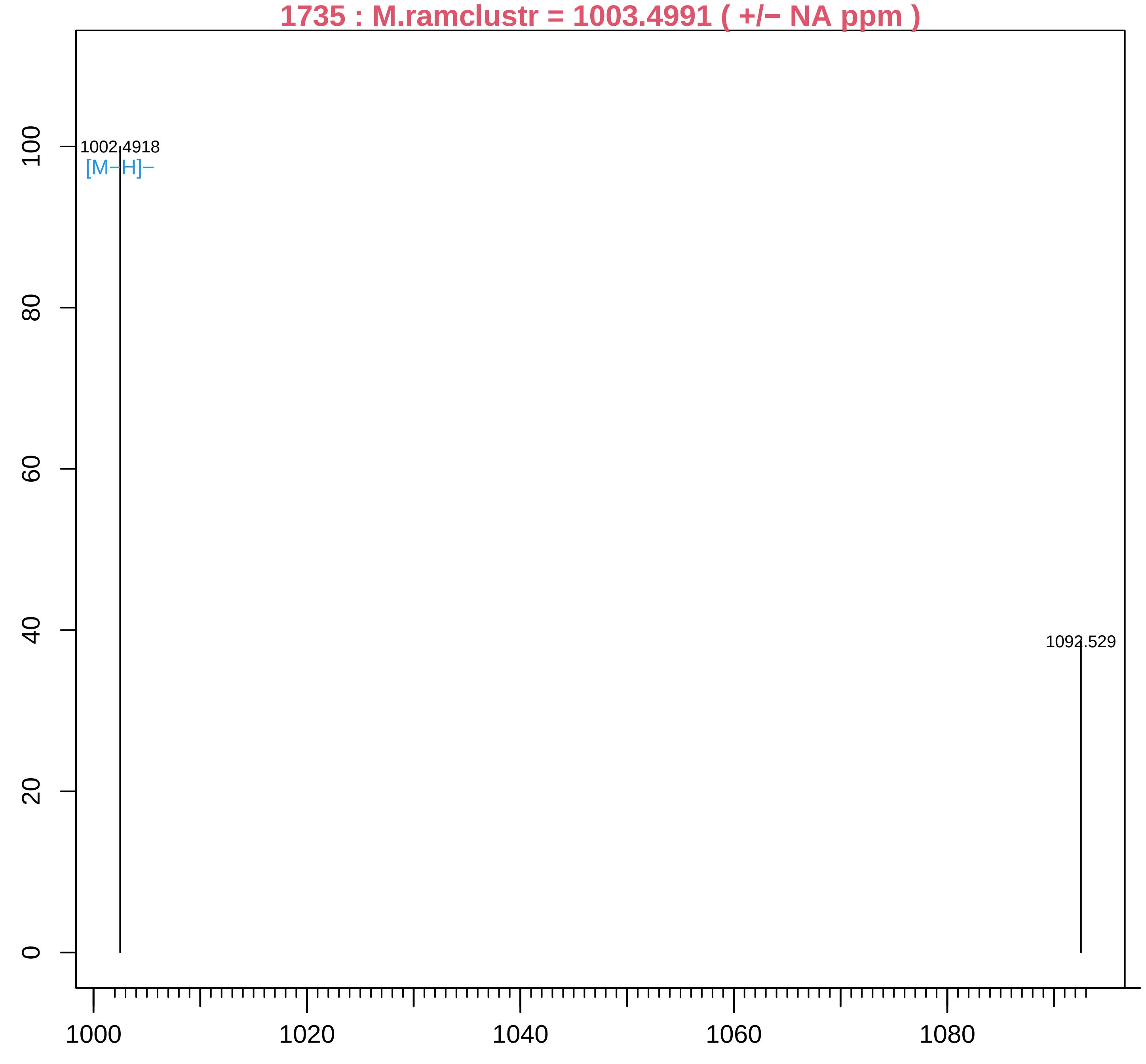

1735 : M.ramclustr = 1003.4991 (+/− NA ppm)
1002.4918
[M−H]−
1092.529
0
20
40
60
80
100
1000
1020
1040
1060
1080

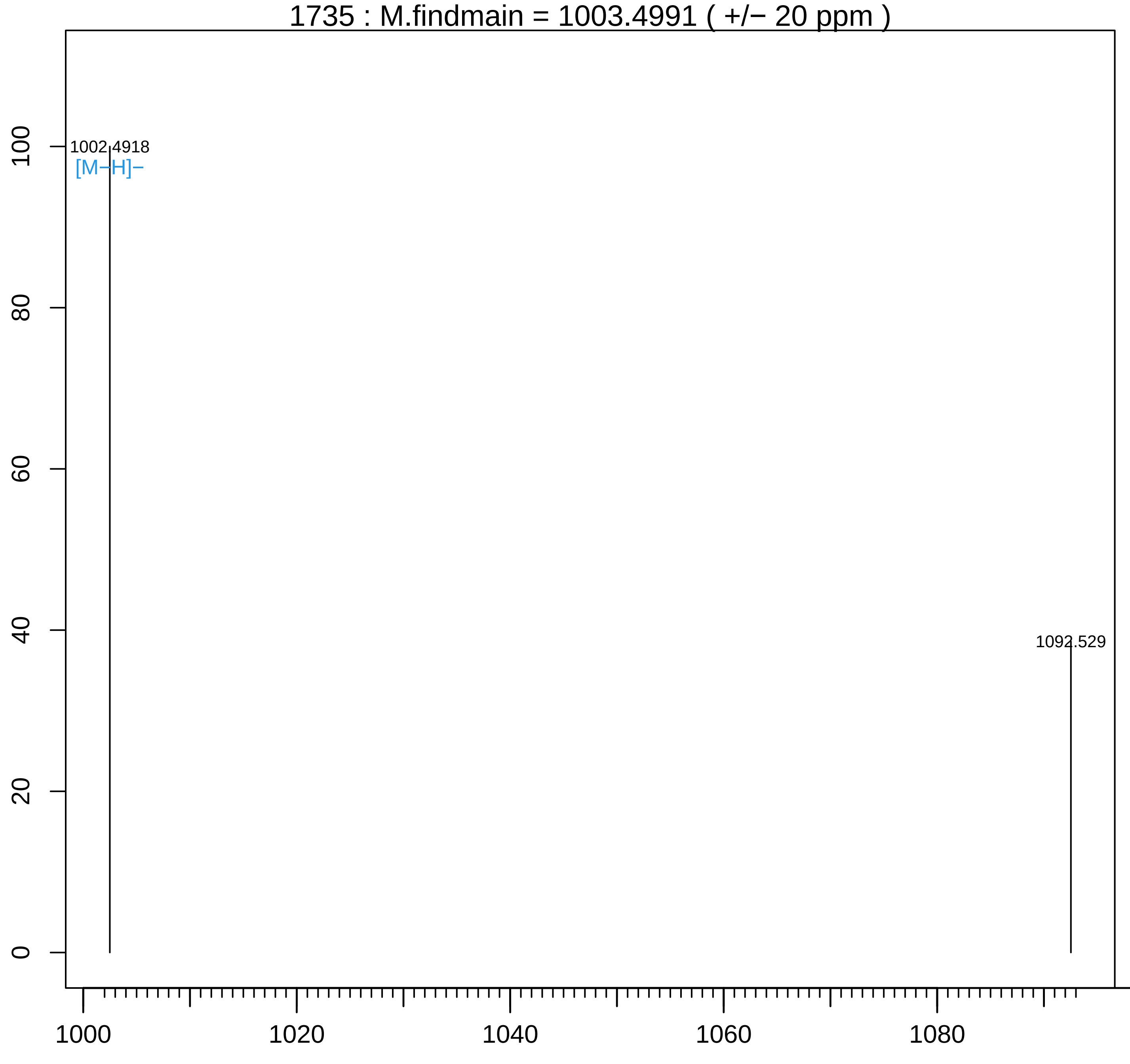

1735 : M.findmain = 1003.4991 (+/− 20 ppm)
1002.4918
[M−H]−
1092.529
0
20
40
60
80
100
1000
1020
1040
1060
1080

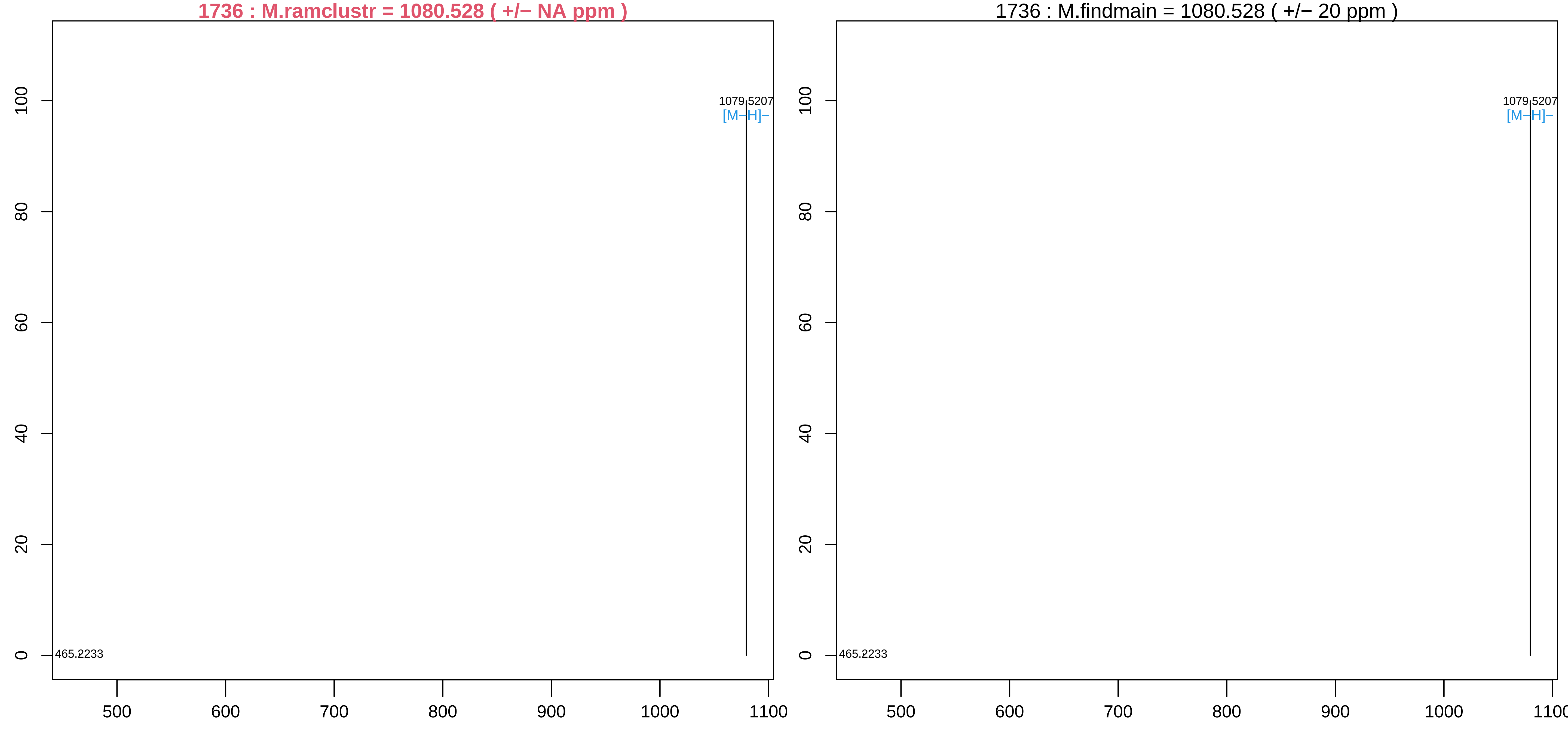

1736 : M.ramclustr = 1080.528 (+/− NA ppm)
1079.5207
[M−H]−
465.2233
1736 : M.findmain = 1080.528 (+/− 20 ppm)
1079.5207
[M−H]−
465.2233

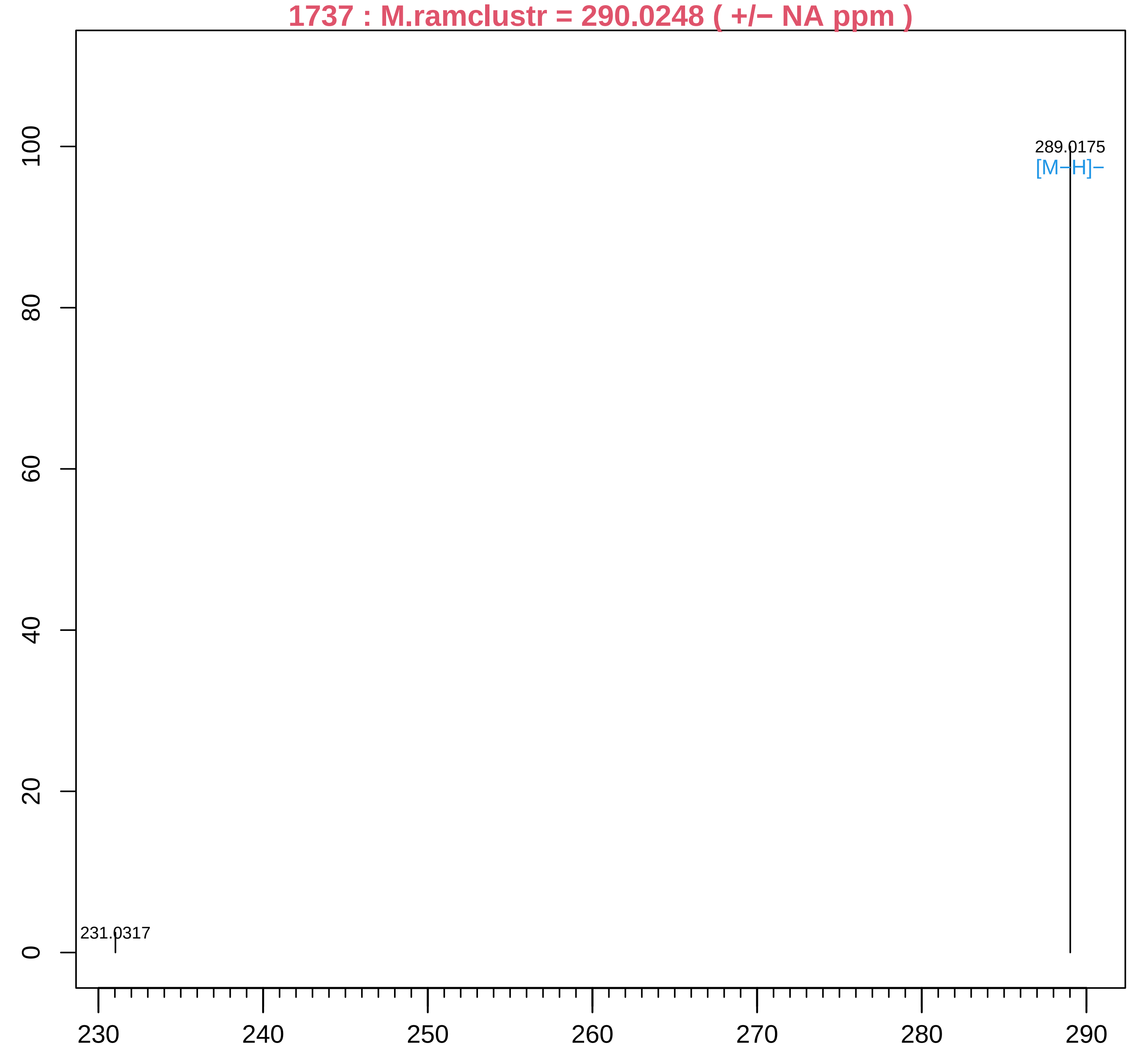

1737 : M.ramclustr = 290.0248 (+/− NA ppm)
289.0175
[M−H]−
231.0317

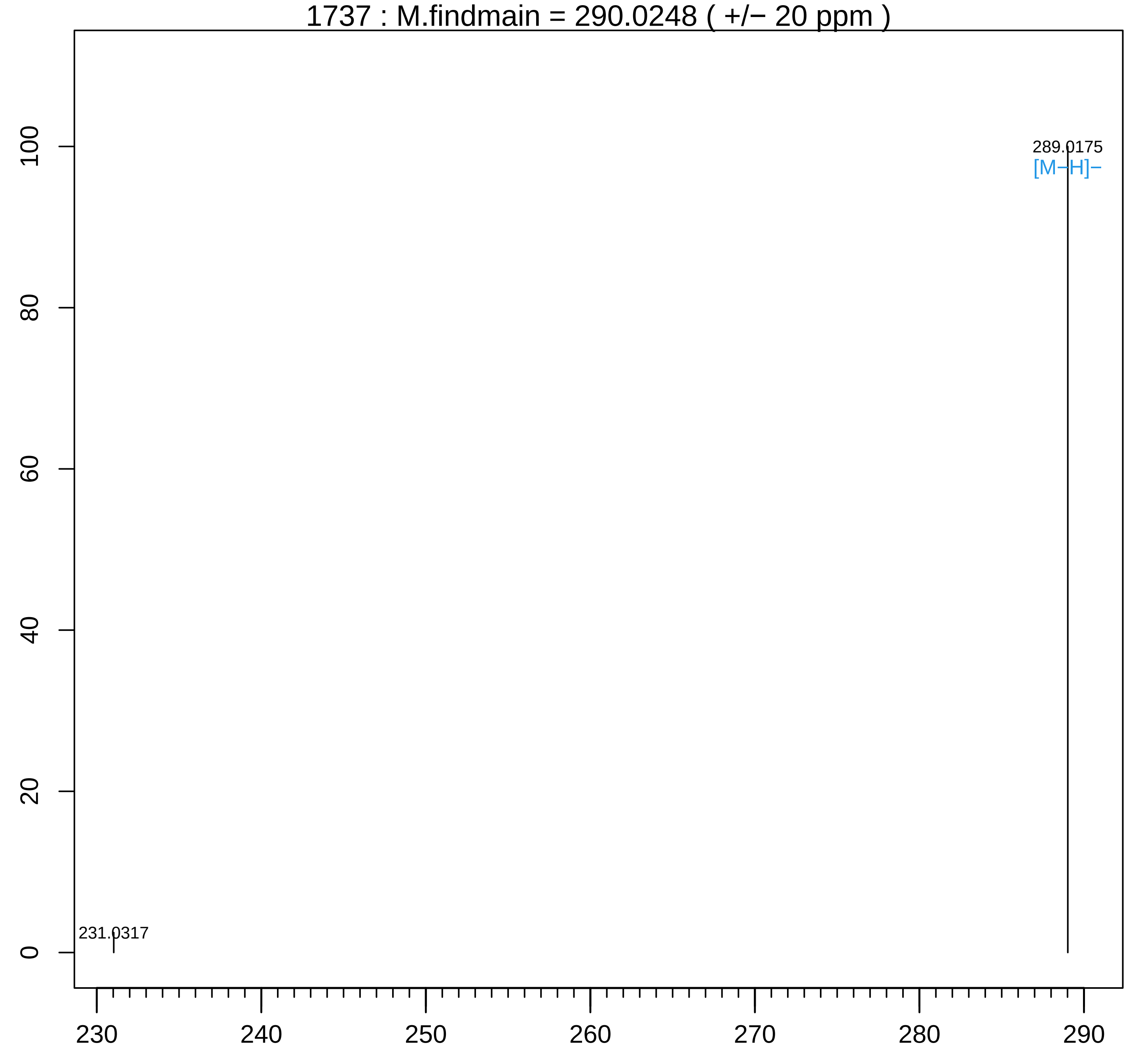

1737 : M.findmain = 290.0248 (+/− 20 ppm)
289.0175
[M−H]−
231.0317

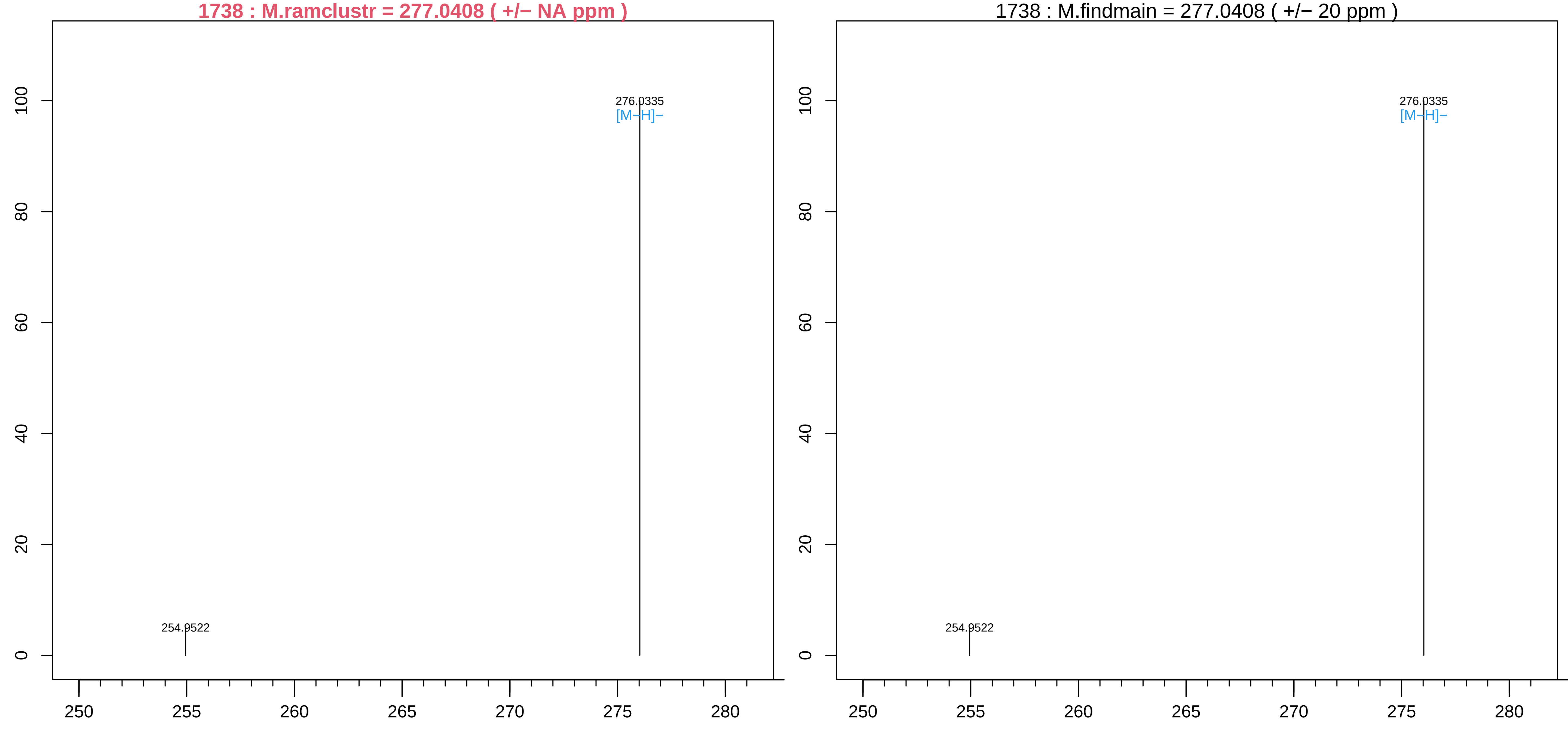

1738 : M.ramclustr = 277.0408 (+/− NA ppm)
276.0335
[M−H]−
254.9522
0
20
40
60
80
100
250
255
260
265
270
275
280
1738 : M.findmain = 277.0408 (+/− 20 ppm)
276.0335
[M−H]−
254.9522
0
20
40
60
80
100
250
255
260
265
270
275
280

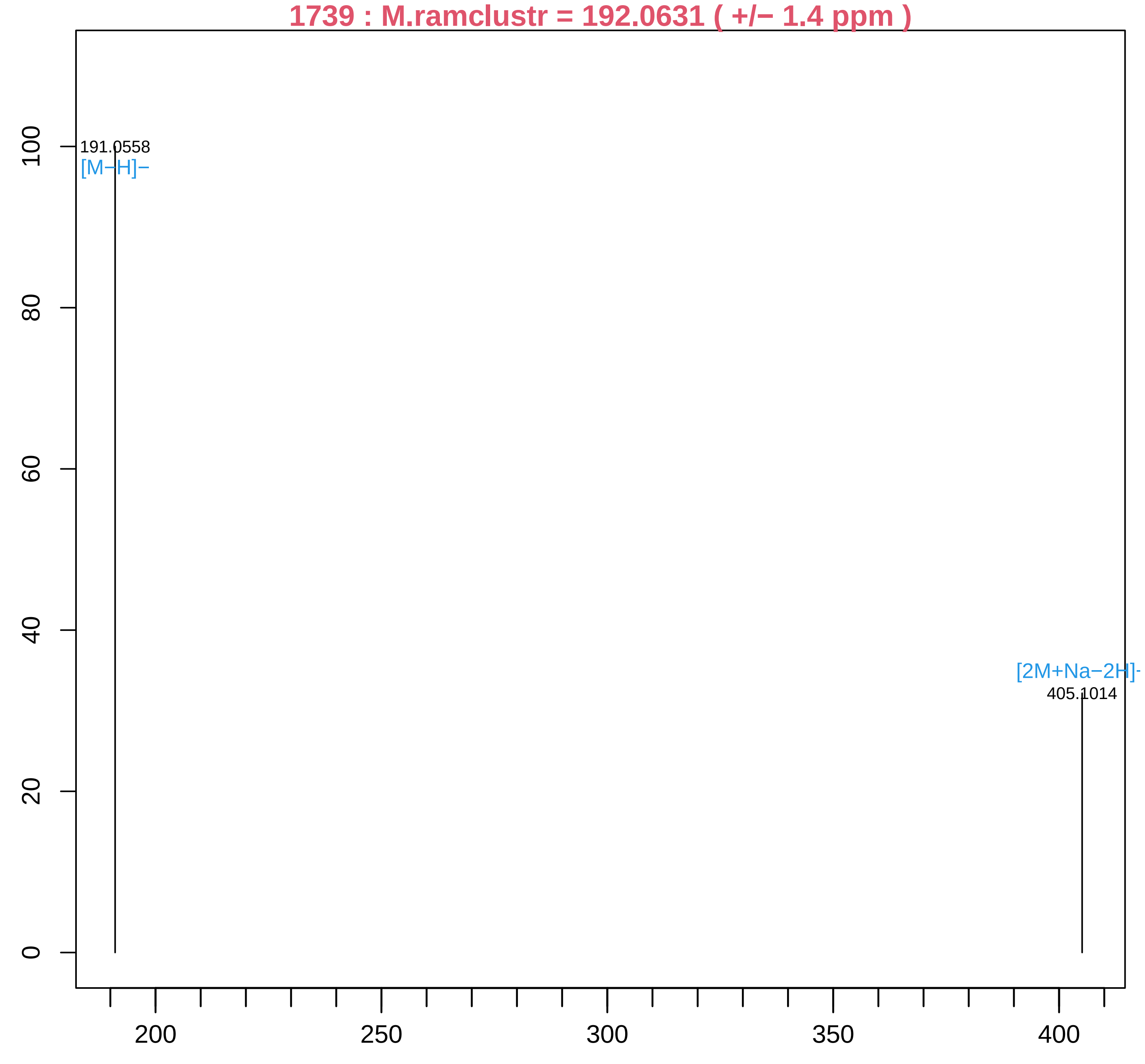

1739 : M.ramclustr = 192.0631 (+/− 1.4 ppm)
191.0558
[M−H]−
[2M+Na−2H]-
405.1014
0
20
40
60
80
100
200
250
300
350
400

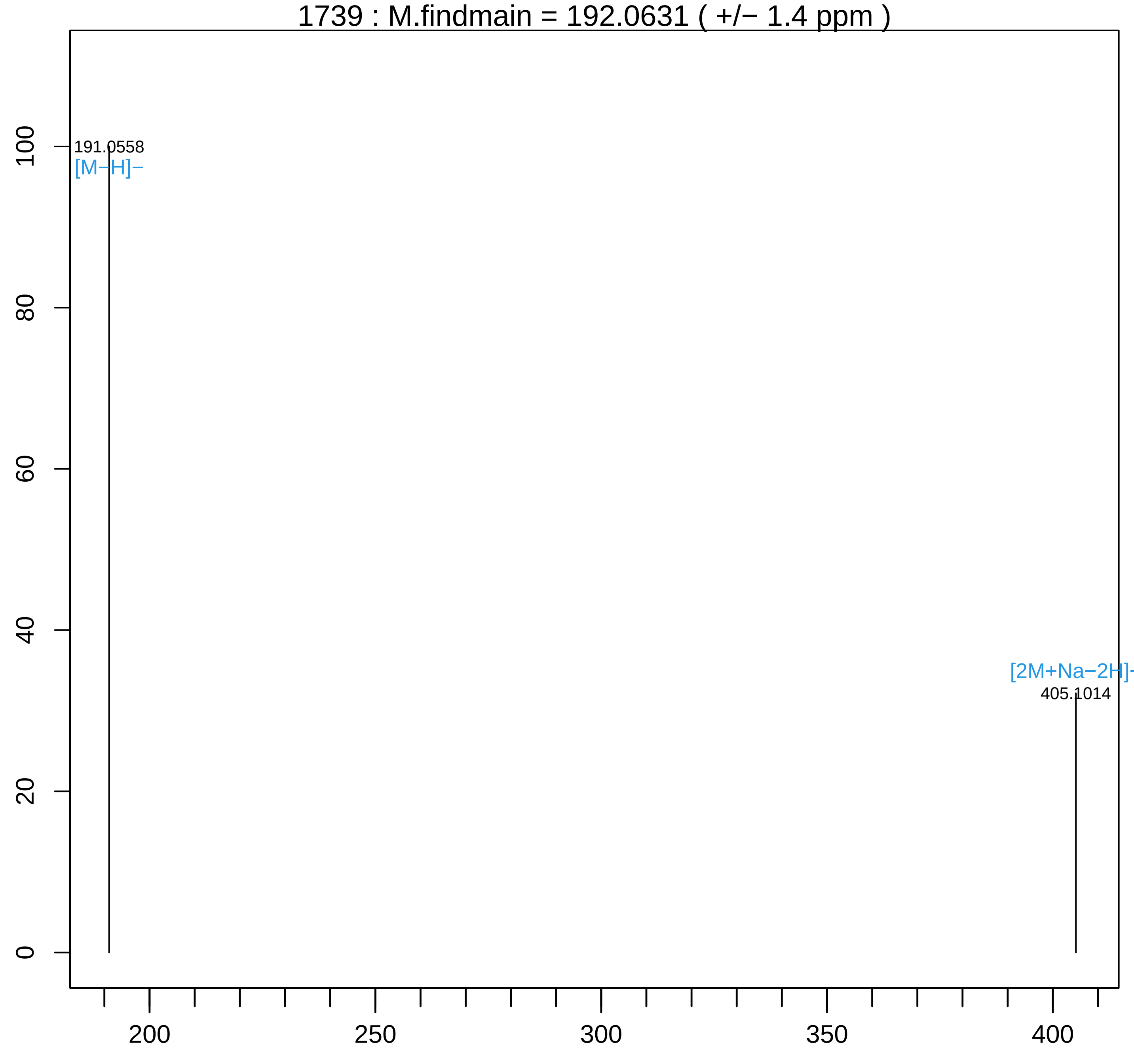

1739 : M.findmain = 192.0631 (+/− 1.4 ppm)
191.0558
[M−H]−
[2M+Na−2H]-
405.1014
0
20
40
60
80
100
200
250
300
350
400

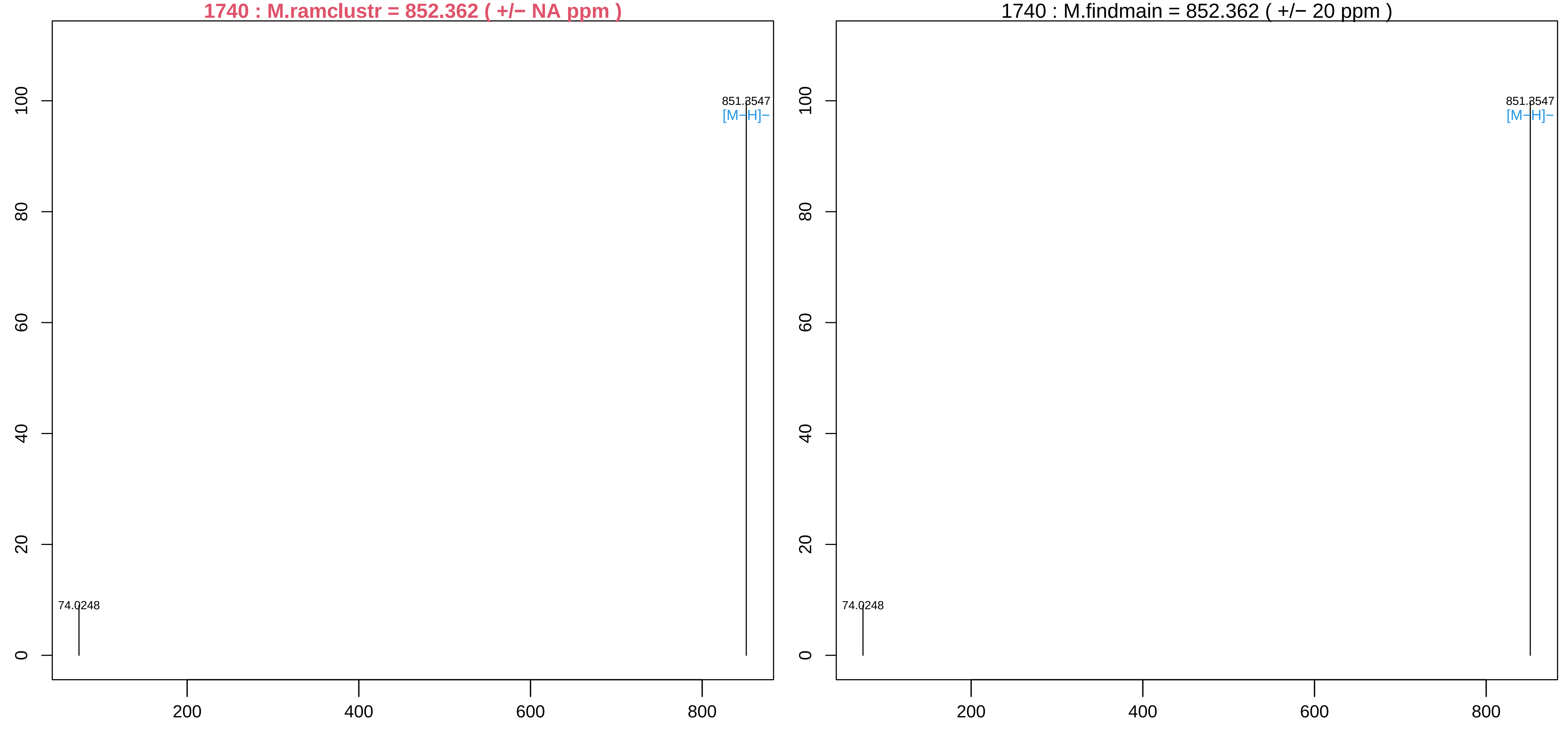
1740 : M.ramclustr = 852.362 (+/− NA ppm)
1740 : M.findmain = 852.362 (+/− 20 ppm)
851.3547
[M−H]−
74.0248
0
20
40
60
80
100
200
400
600
800

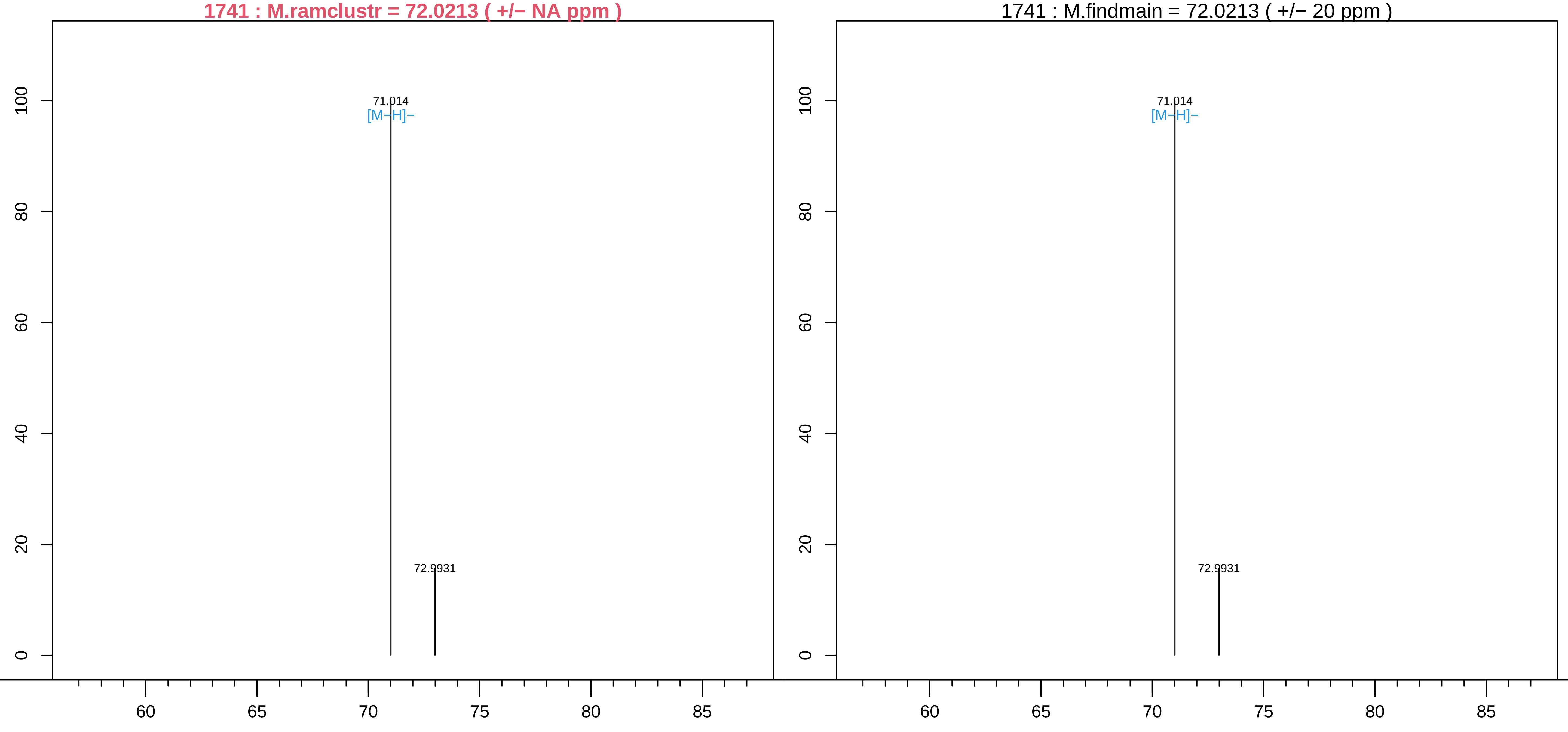
1741 : M.ramclustr = 72.0213 (+/− NA ppm)
1741 : M.findmain = 72.0213 (+/− 20 ppm)
71.014
[M−H]−
72.9931
0
20
40
60
80
100
60
65
70
75
80
85

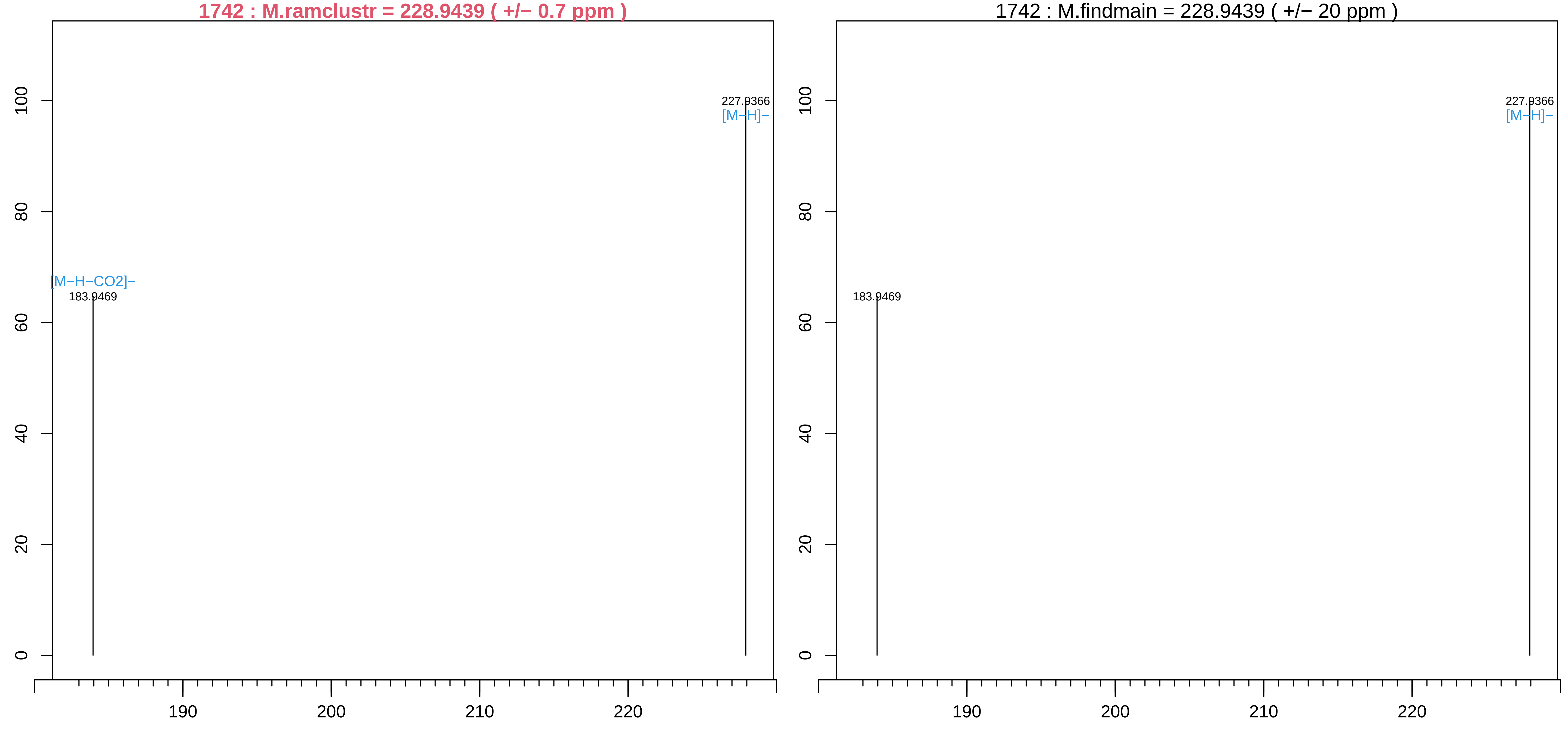
1742 : M.ramclustr = 228.9439 (+/− 0.7 ppm)
227.9366
[M−H]−
[M−H−CO2]−
183.9469
0
20
40
60
80
100
190
200
210
220
1742 : M.findmain = 228.9439 (+/− 20 ppm)
227.9366
[M−H]−
183.9469
0
20
40
60
80
100
190
200
210
220

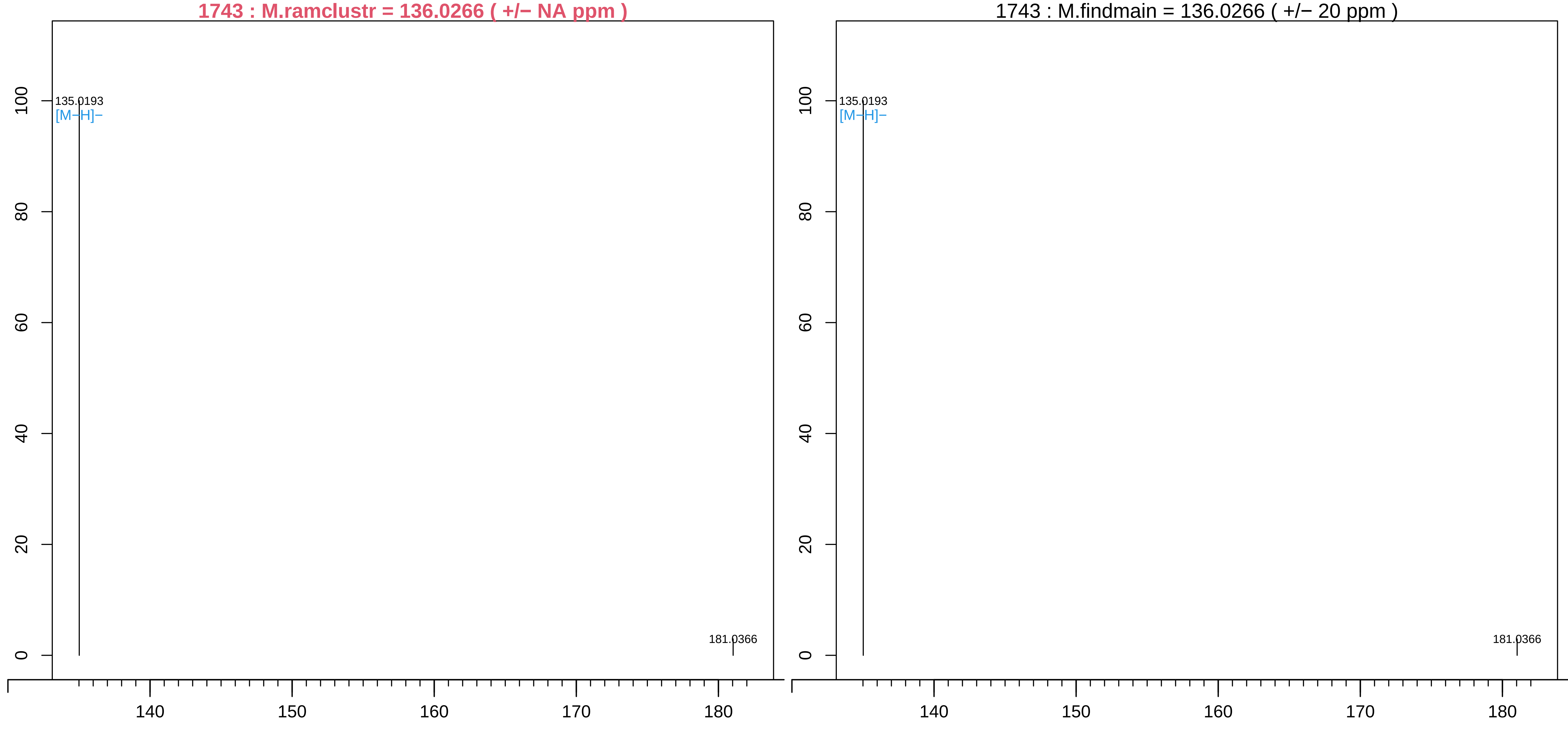

1743 : M.ramclustr = 136.0266 (+/− NA ppm)
135.0193
[M−H]−
181.0366
1743 : M.findmain = 136.0266 (+/− 20 ppm)
135.0193
[M−H]−
181.0366
0
20
40
60
80
100
140
150
160
170
180

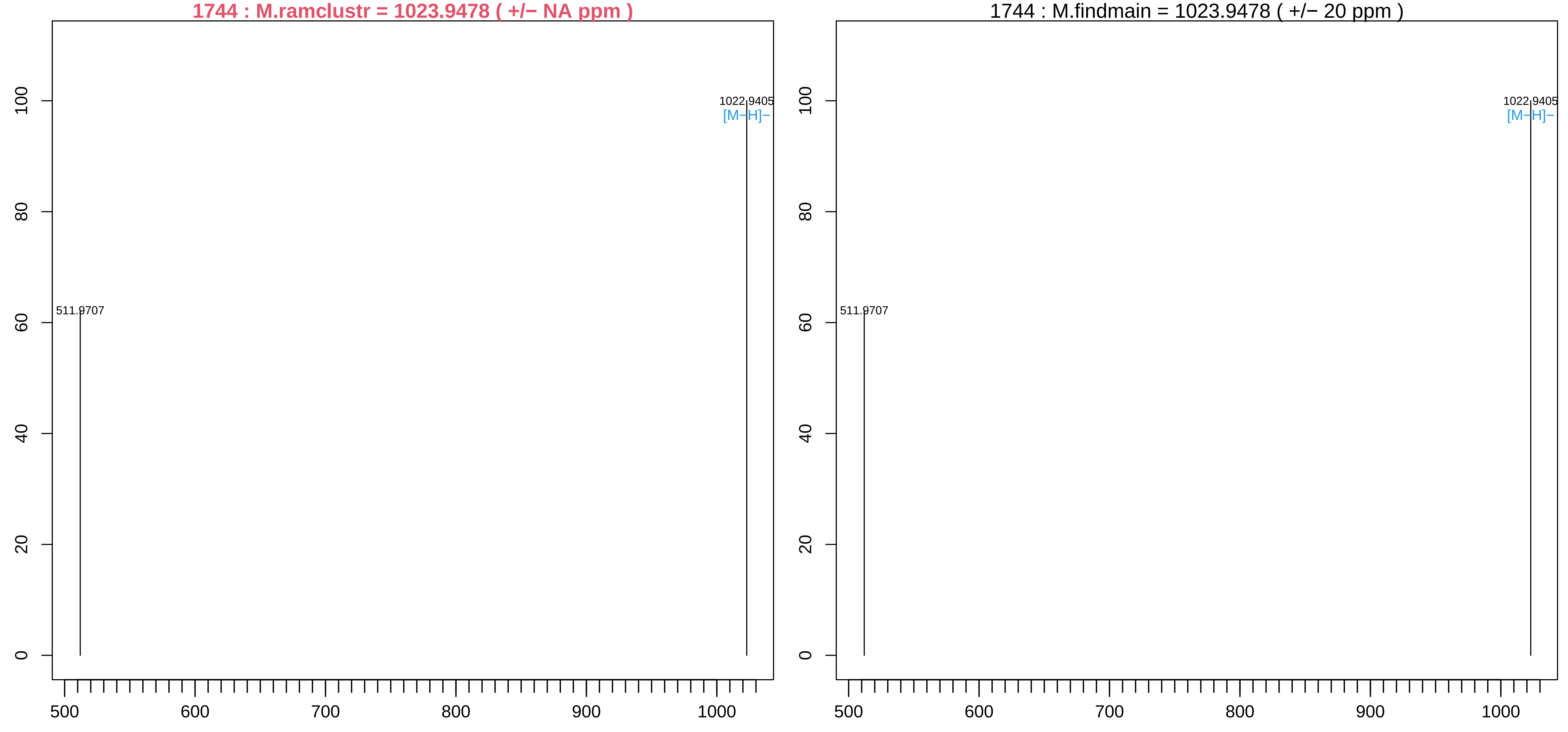

1744 : M.ramclustr = 1023.9478 (+/− NA ppm)
1022.9405
[M−H]−
511.9707
1744 : M.findmain = 1023.9478 (+/− 20 ppm)
1022.9405
[M−H]−
511.9707

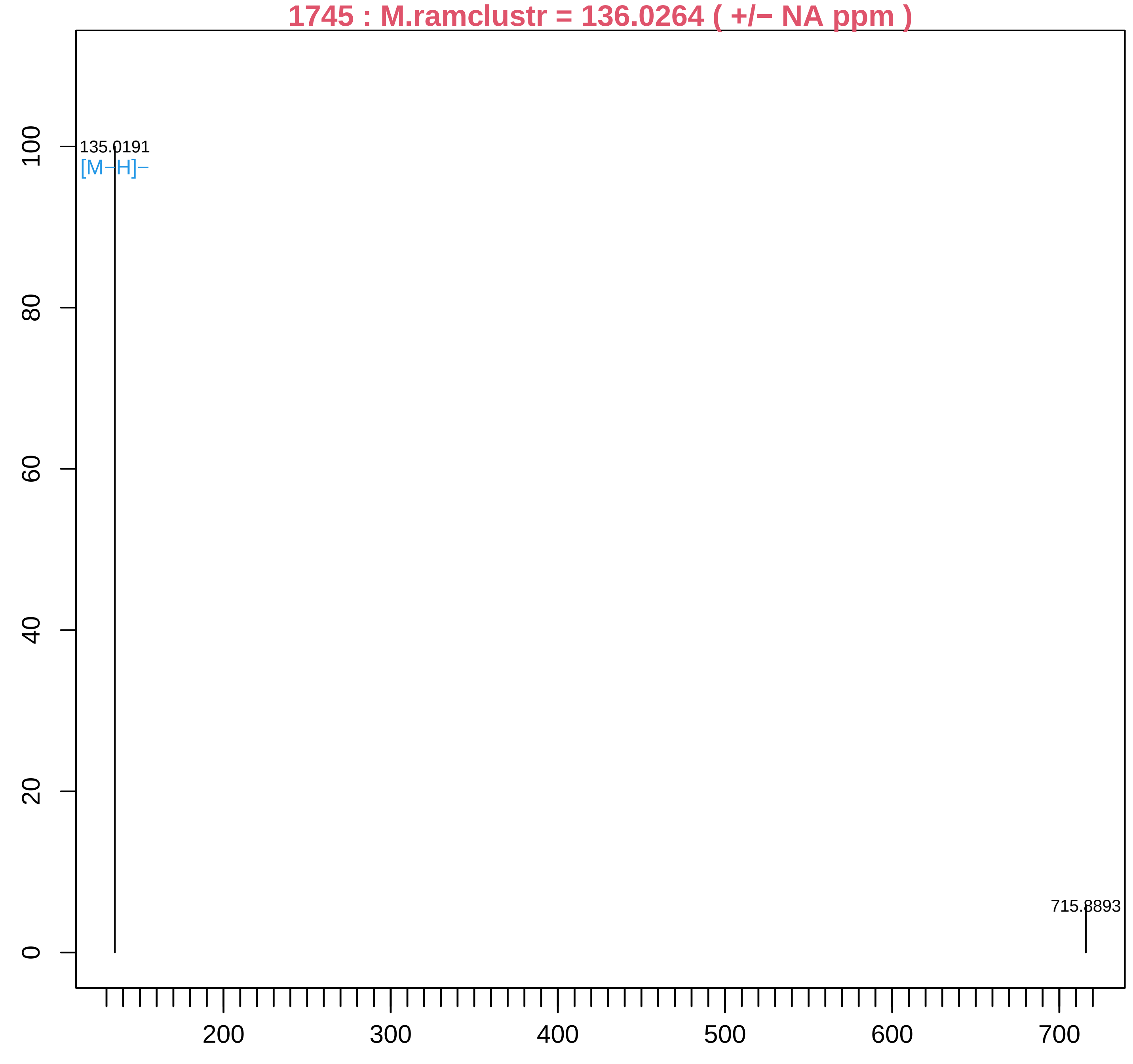

1745 : M.ramclustr = 136.0264 (+/− NA ppm)
135.0191
[M−H]−
715.8893
0
20
40
60
80
100
200
300
400
500
600
700

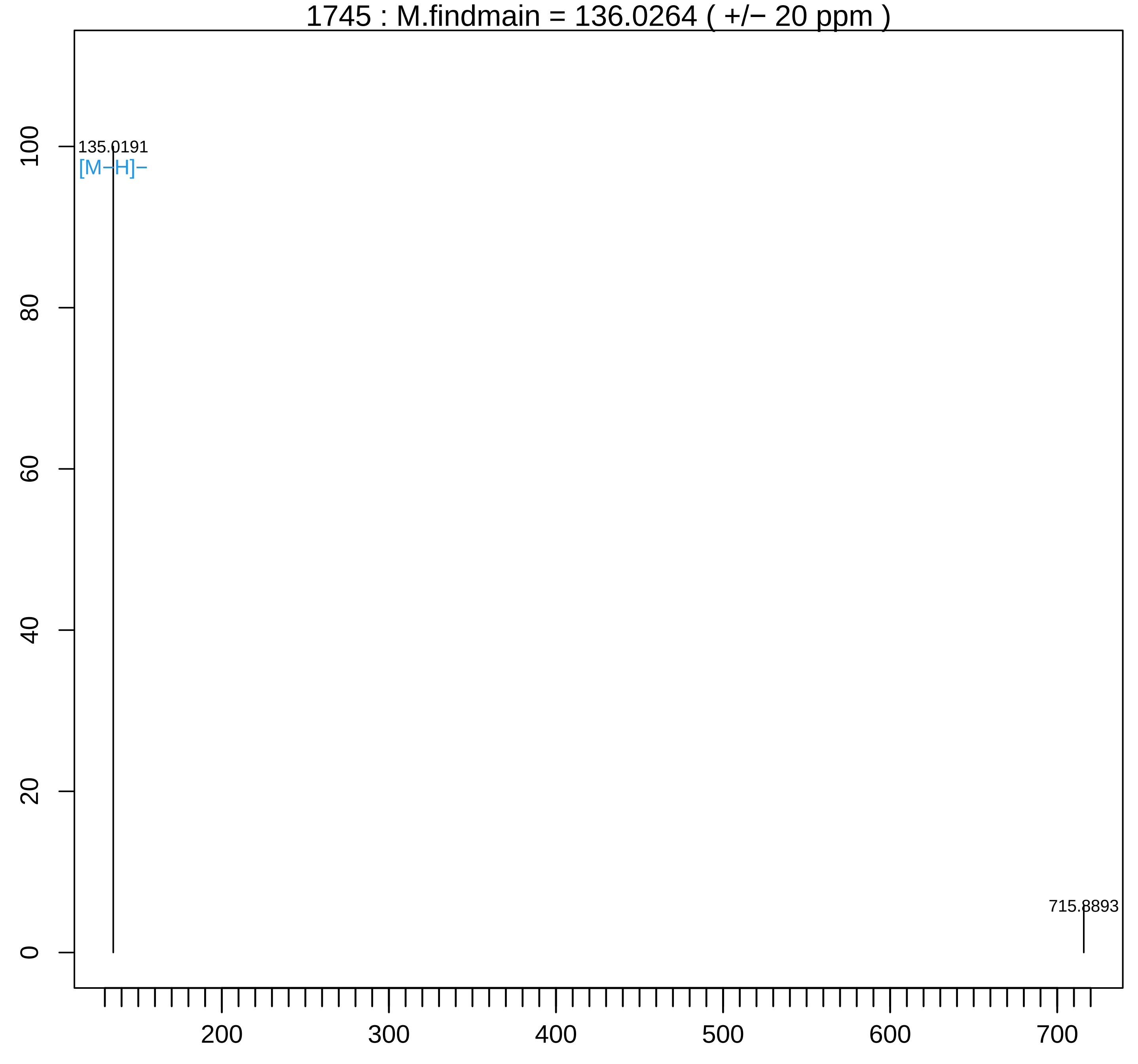

1745 : M.findmain = 136.0264 (+/− 20 ppm)
135.0191
[M−H]−
715.8893
0
20
40
60
80
100
200
300
400
500
600
700

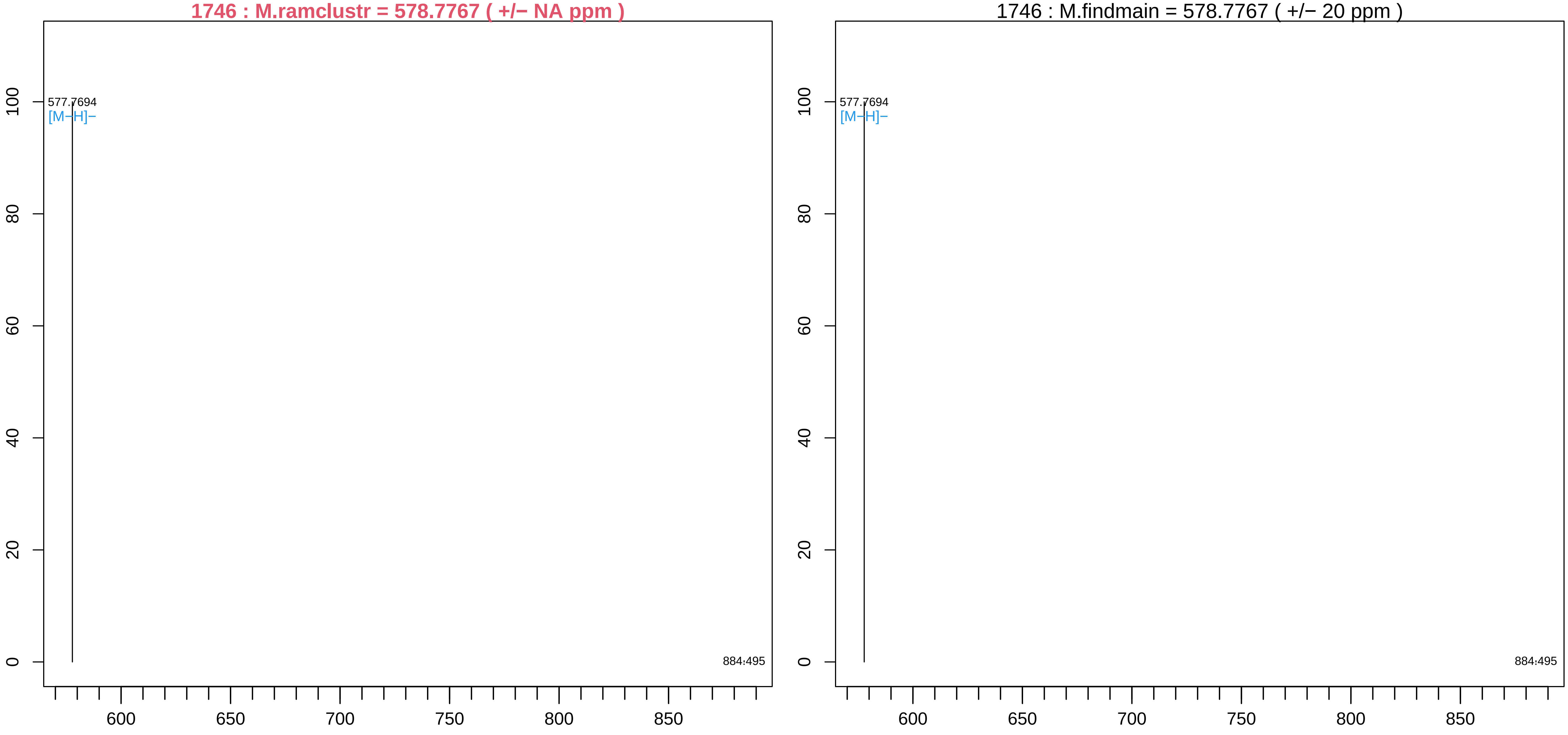
1746 : M.ramclustr = 578.7767 (+/− NA ppm)
577.7694
[M−H]−
884.495
0
20
40
60
80
100
600
650
700
750
800
850
1746 : M.findmain = 578.7767 (+/− 20 ppm)
577.7694
[M−H]−
884.495
0
20
40
60
80
100
600
650
700
750
800
850

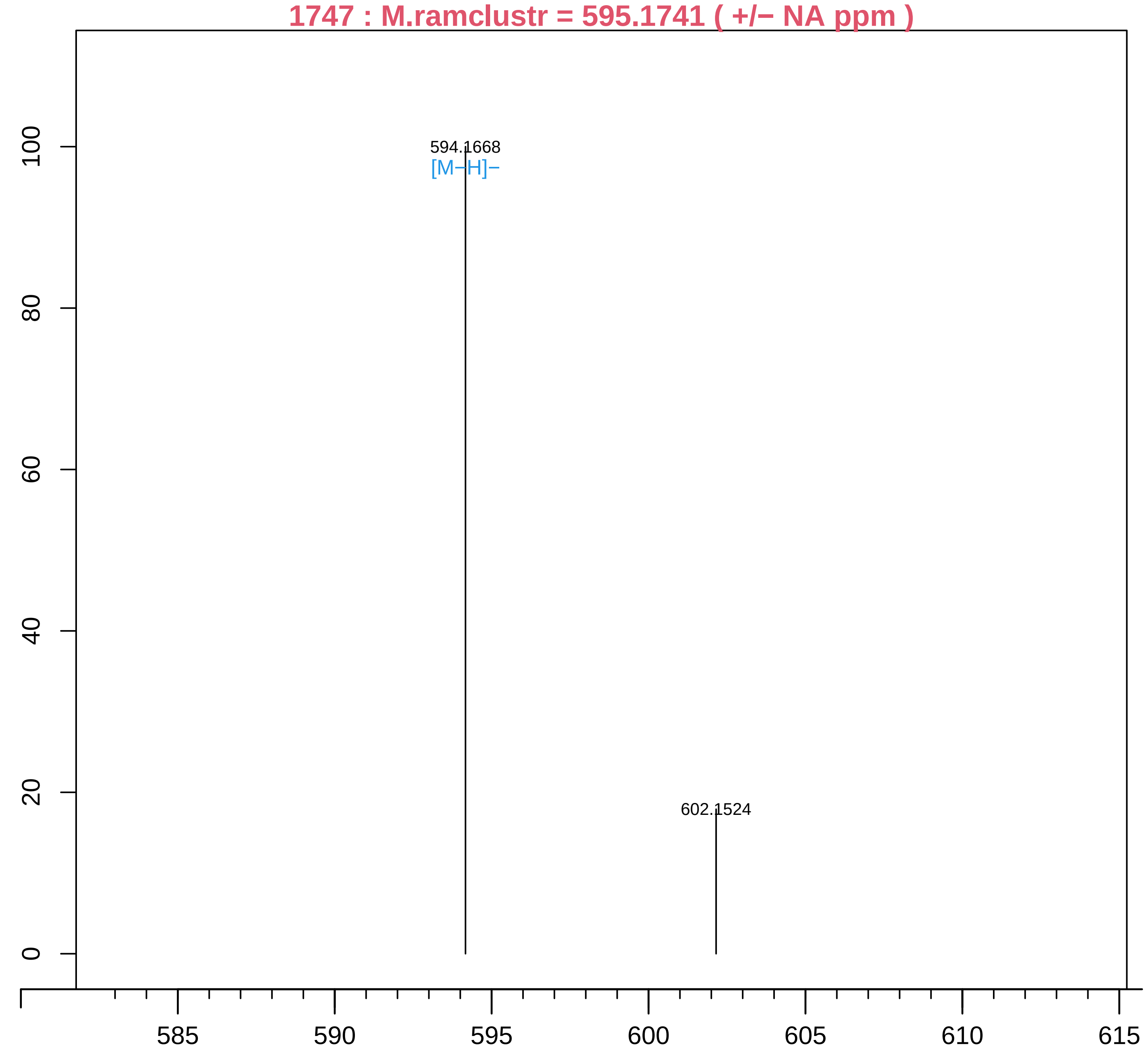
1747 : M.ramclustr = 595.1741 (+/− NA ppm)
594.1668
[M−H]−
602.1524
0
20
40
60
80
100
585
590
595
600
605
610
615

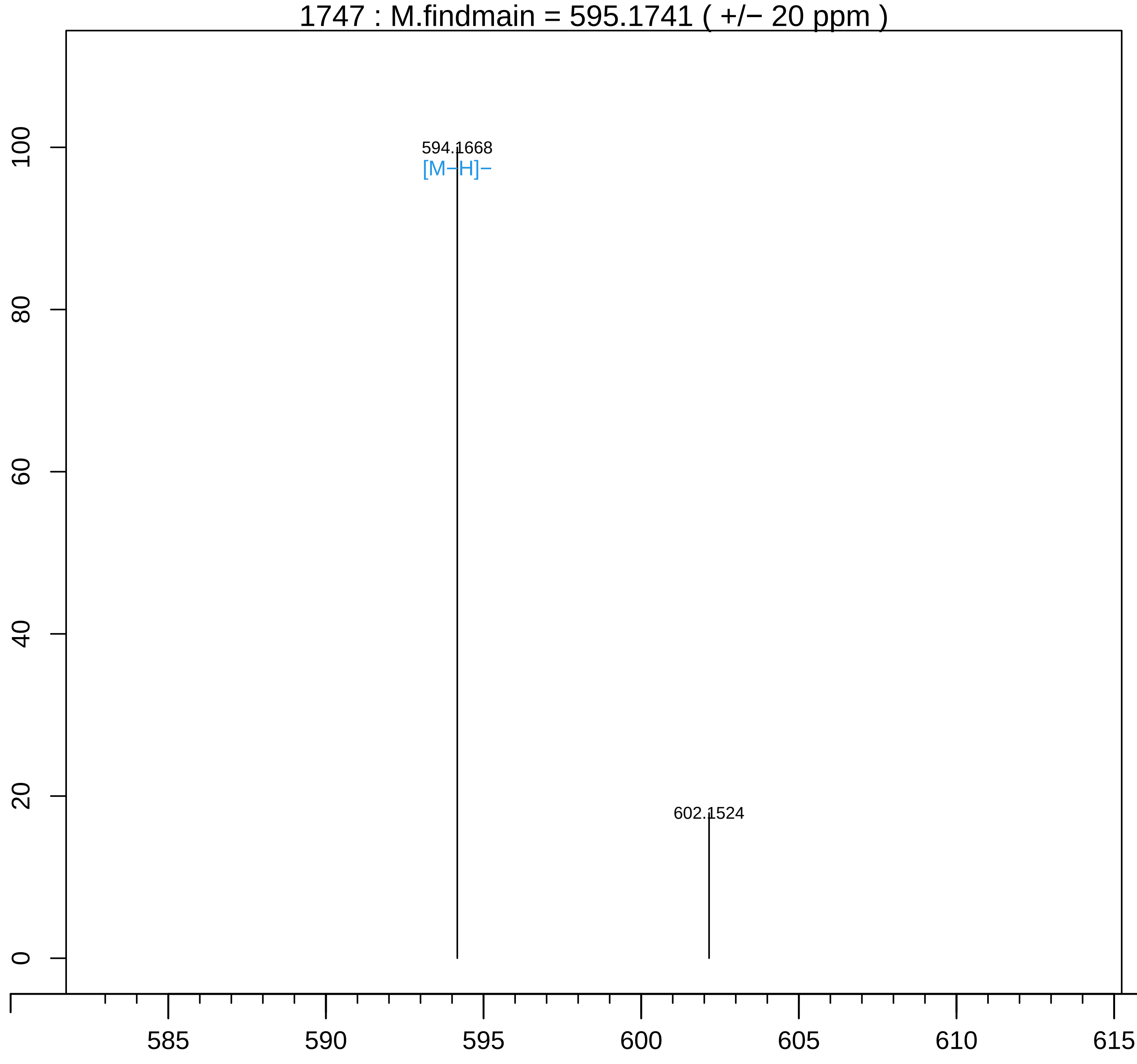
1747 : M.findmain = 595.1741 (+/− 20 ppm)
594.1668
[M−H]−
602.1524
0
20
40
60
80
100
585
590
595
600
605
610
615

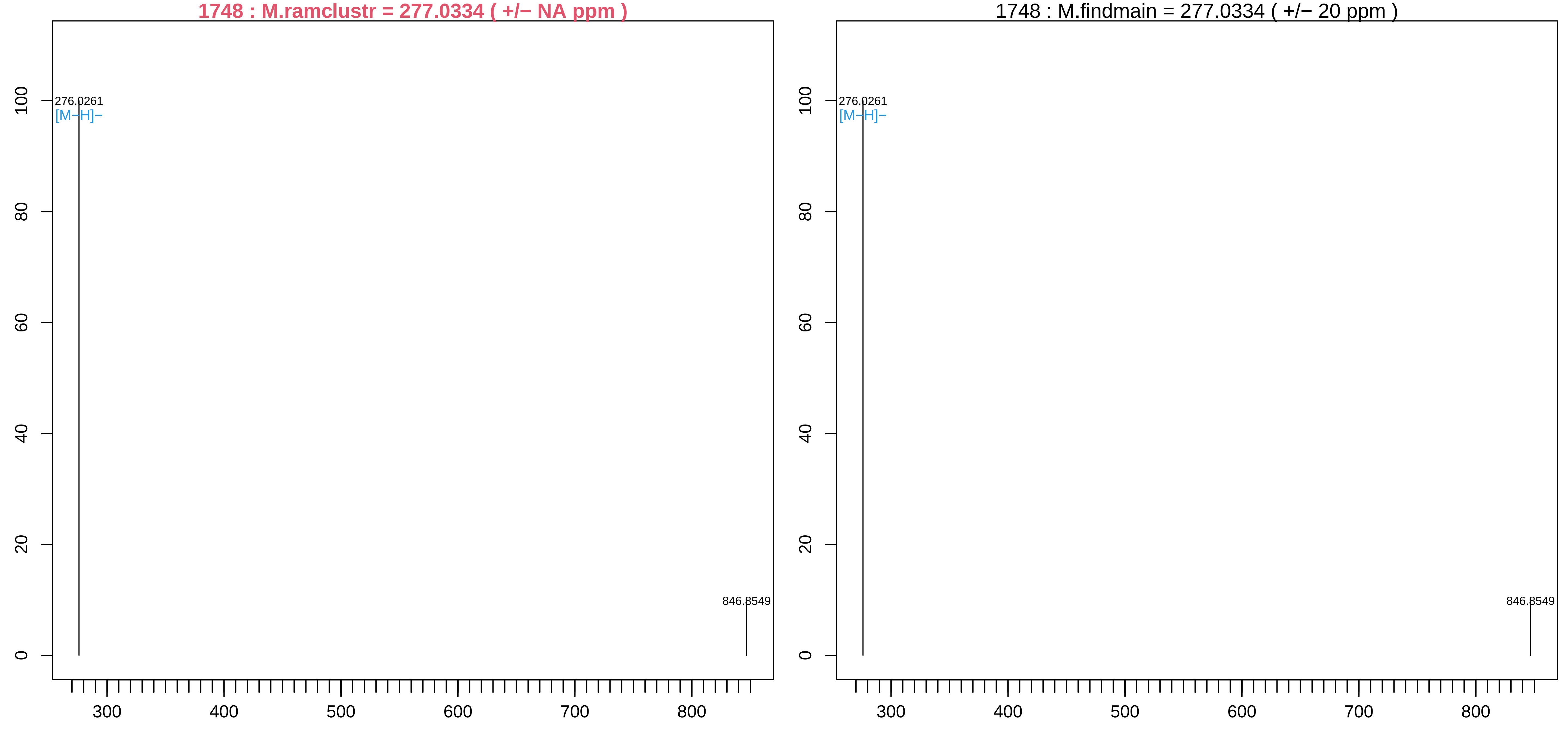

1748 : M.ramclustr = 277.0334 (+/− NA ppm)
276.0261
[M−H]−
846.8549
0
20
40
60
80
100
300
400
500
600
700
800
1748 : M.findmain = 277.0334 (+/− 20 ppm)
276.0261
[M−H]−
846.8549
0
20
40
60
80
100
300
400
500
600
700
800

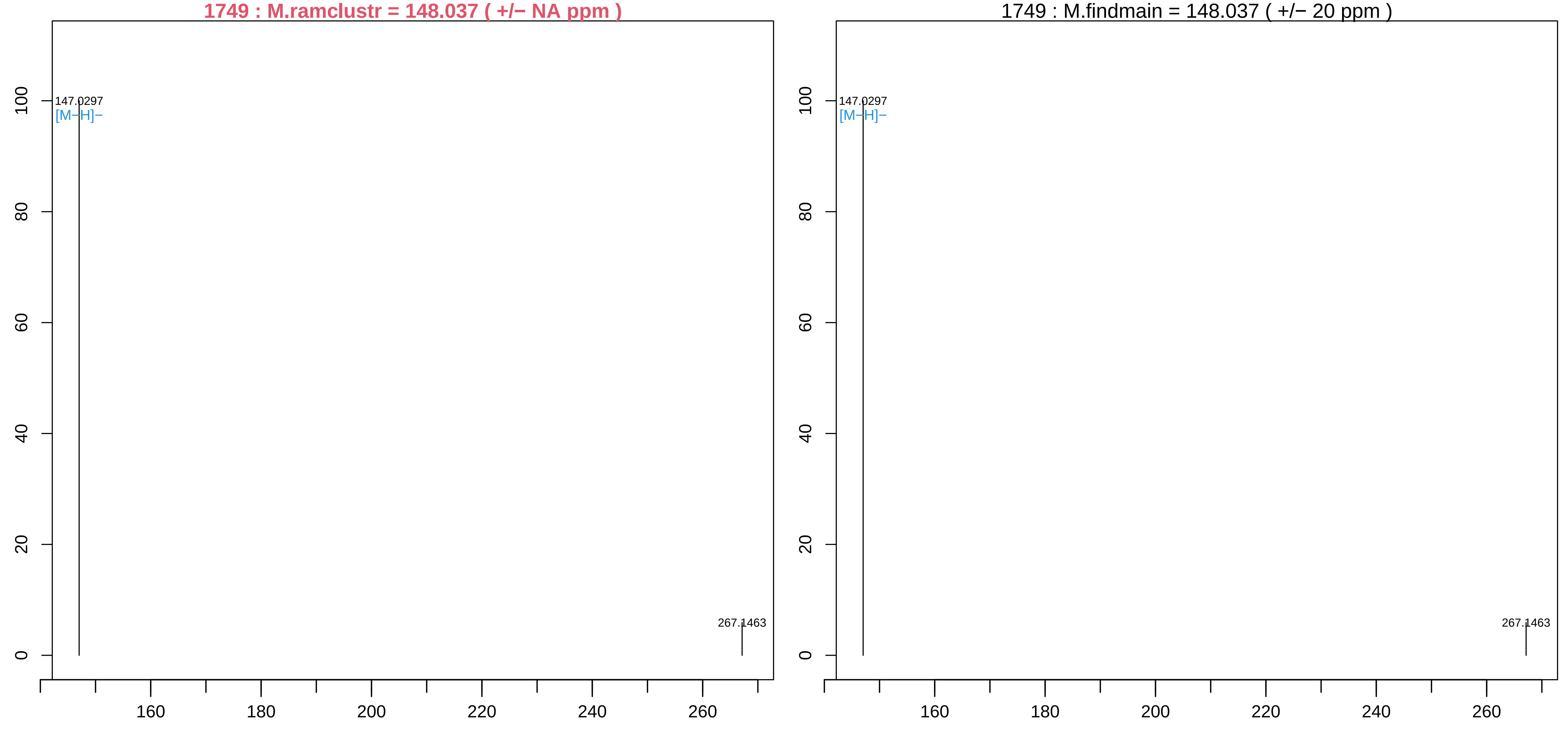
1749 : M.ramclustr = 148.037 (+/− NA ppm)
147.0297
[M−H]−
267.1463
0
20
40
60
80
100
160
180
200
220
240
260
1749 : M.findmain = 148.037 (+/− 20 ppm)
147.0297
[M−H]−
267.1463
0
20
40
60
80
100
160
180
200
220
240
260

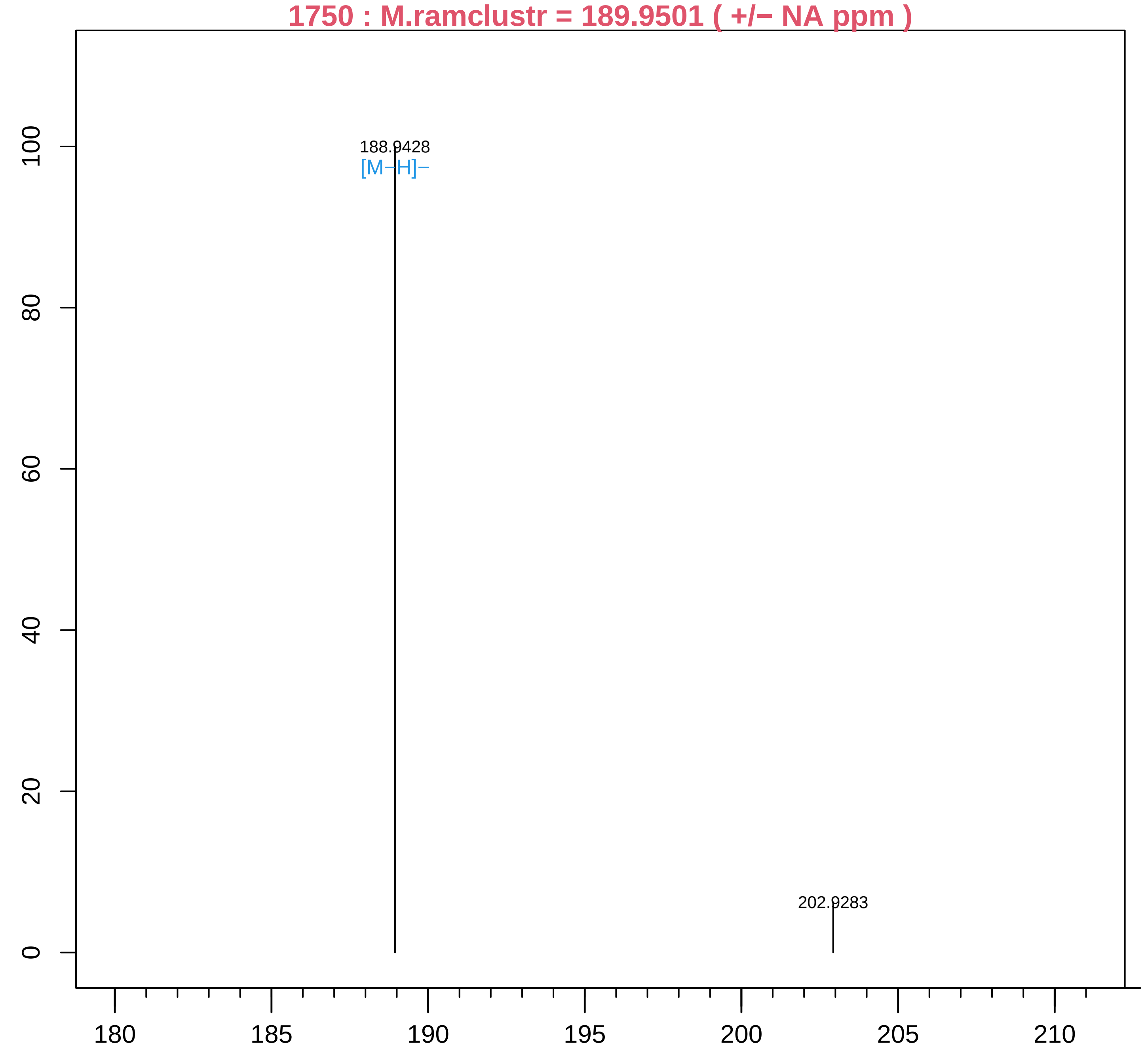
1750 : M.ramclustr = 189.9501 (+/− NA ppm)
188.9428
[M−H]−
202.9283
0
20
40
60
80
100
180
185
190
195
200
205
210

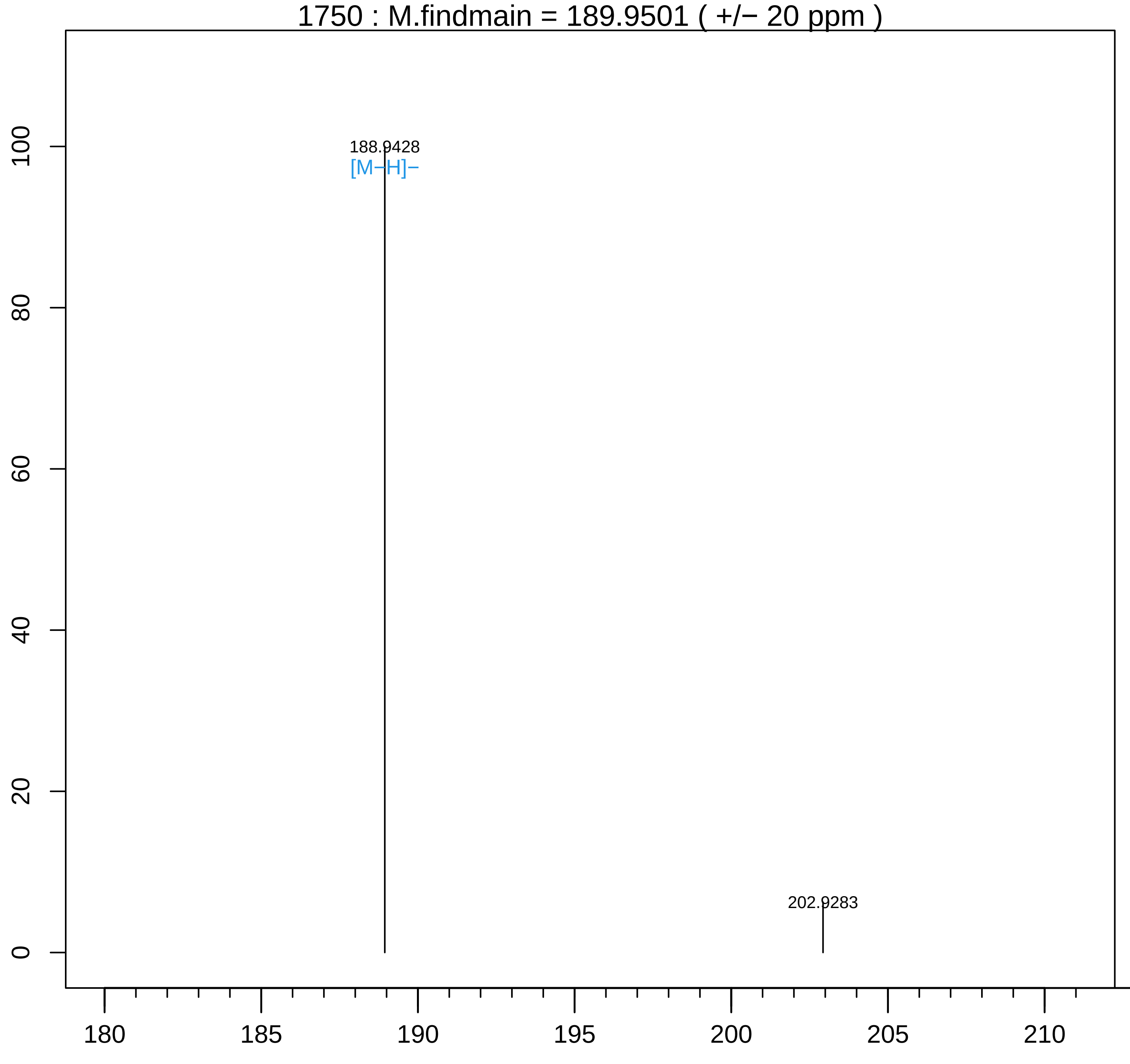
1750 : M.findmain = 189.9501 (+/− 20 ppm)
188.9428
[M−H]−
202.9283
0
20
40
60
80
100
180
185
190
195
200
205
210

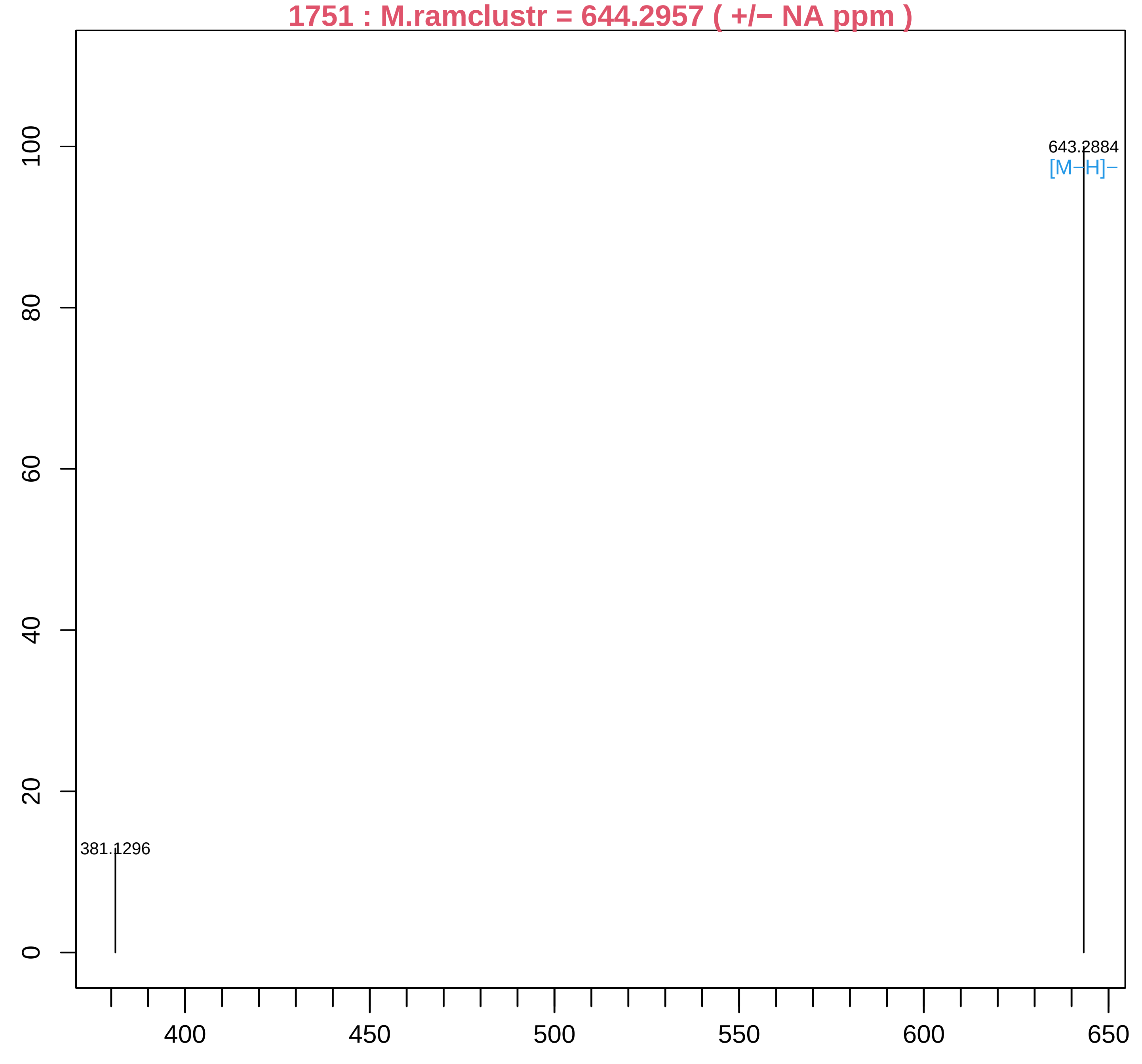
1751 : M.ramclustr = 644.2957 (+/− NA ppm)
643.2884
[M−H]−
381.1296
0
20
40
60
80
100
400
450
500
550
600
650

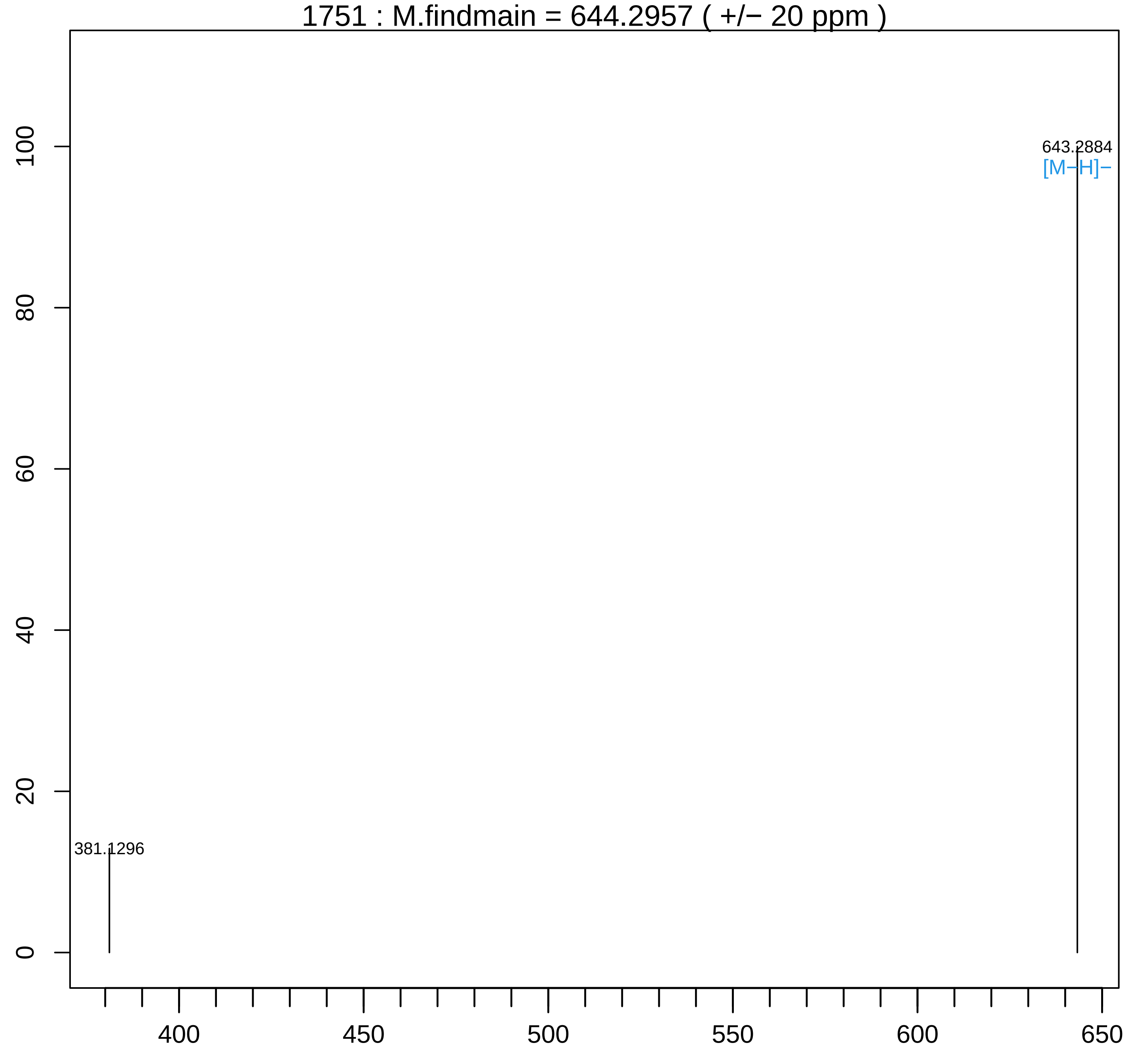
1751 : M.findmain = 644.2957 (+/− 20 ppm)
643.2884
[M−H]−
381.1296
0
20
40
60
80
100
400
450
500
550
600
650

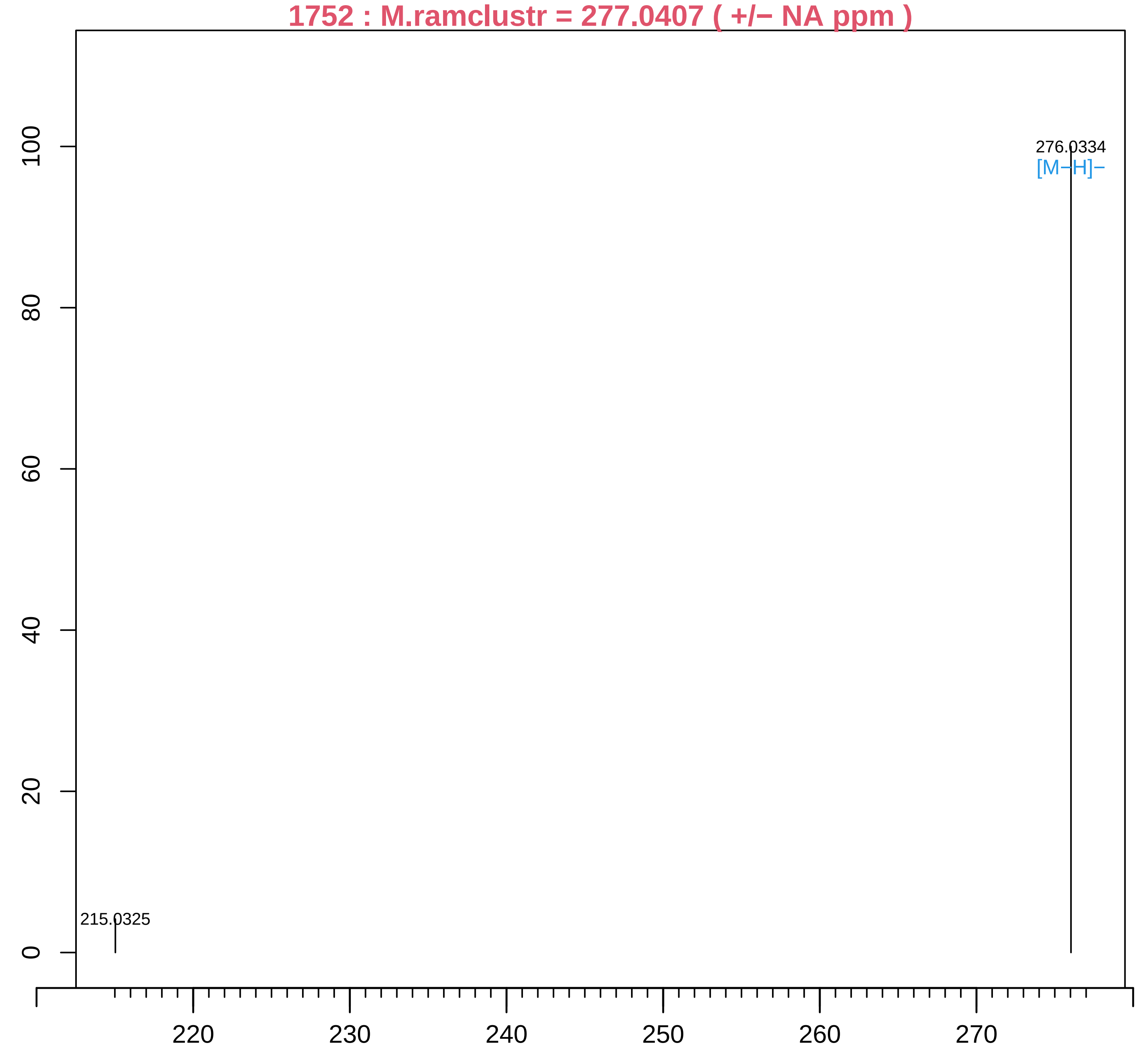

1752 : M.ramclustr = 277.0407 (+/− NA ppm)
276.0334
[M−H]−
215.0325
0
20
40
60
80
100
220
230
240
250
260
270

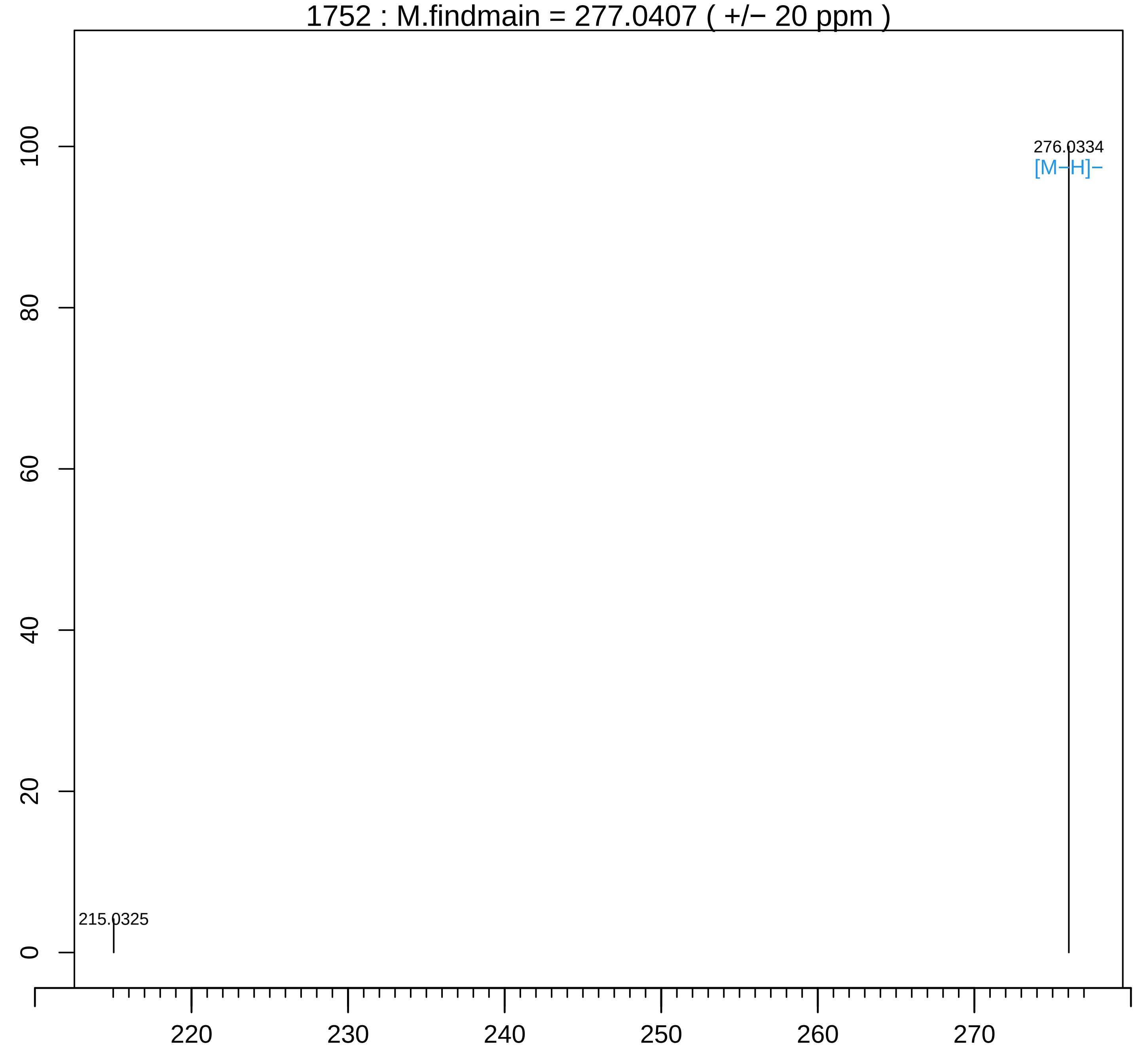

1752 : M.findmain = 277.0407 (+/− 20 ppm)
276.0334
[M−H]−
215.0325
0
20
40
60
80
100
220
230
240
250
260
270

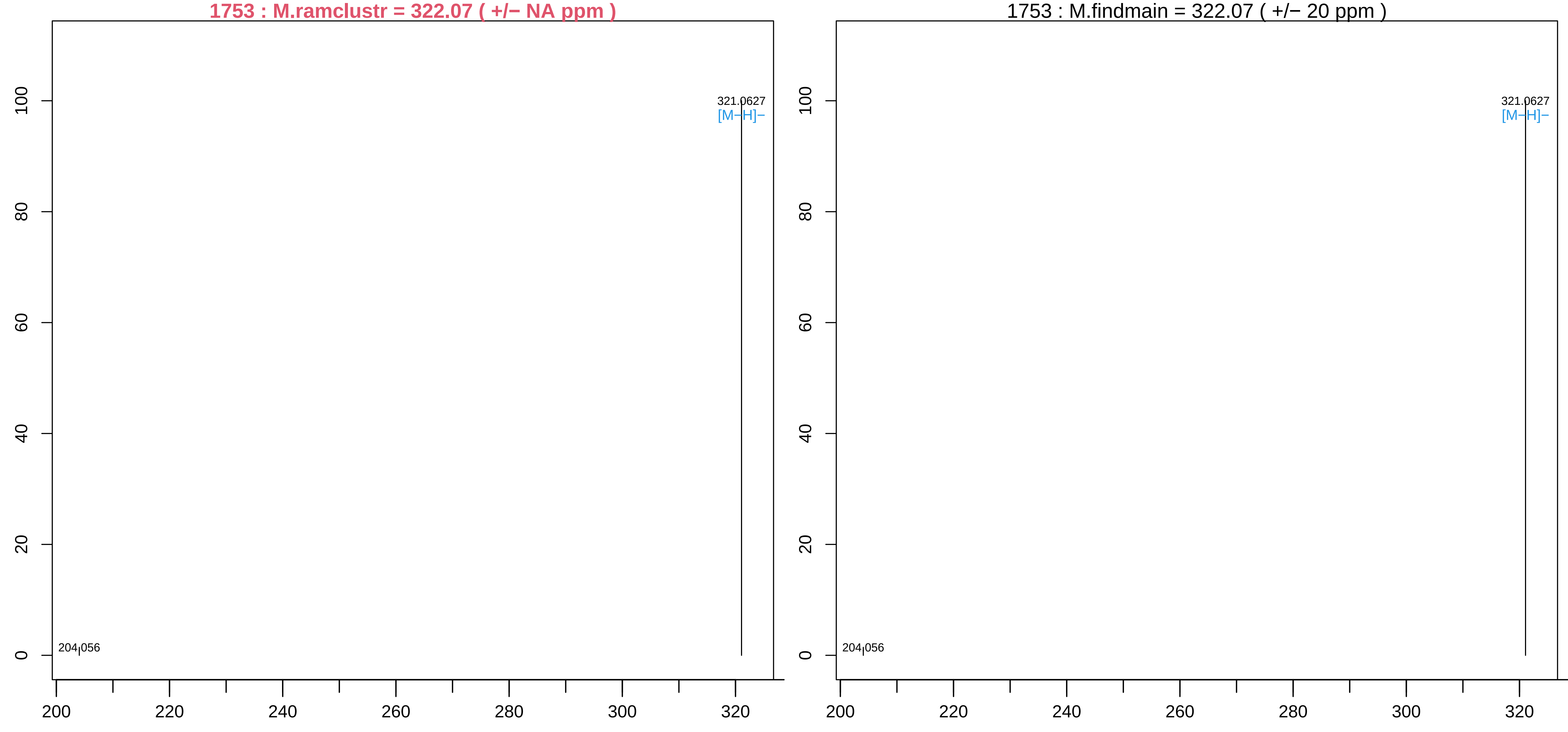
1753 : M.ramclustr = 322.07 (+/− NA ppm)
321.0627
[M−H]−
204.056
0
20
40
60
80
100
200
220
240
260
280
300
320
1753 : M.findmain = 322.07 (+/− 20 ppm)
321.0627
[M−H]−
204.056
0
20
40
60
80
100
200
220
240
260
280
300
320

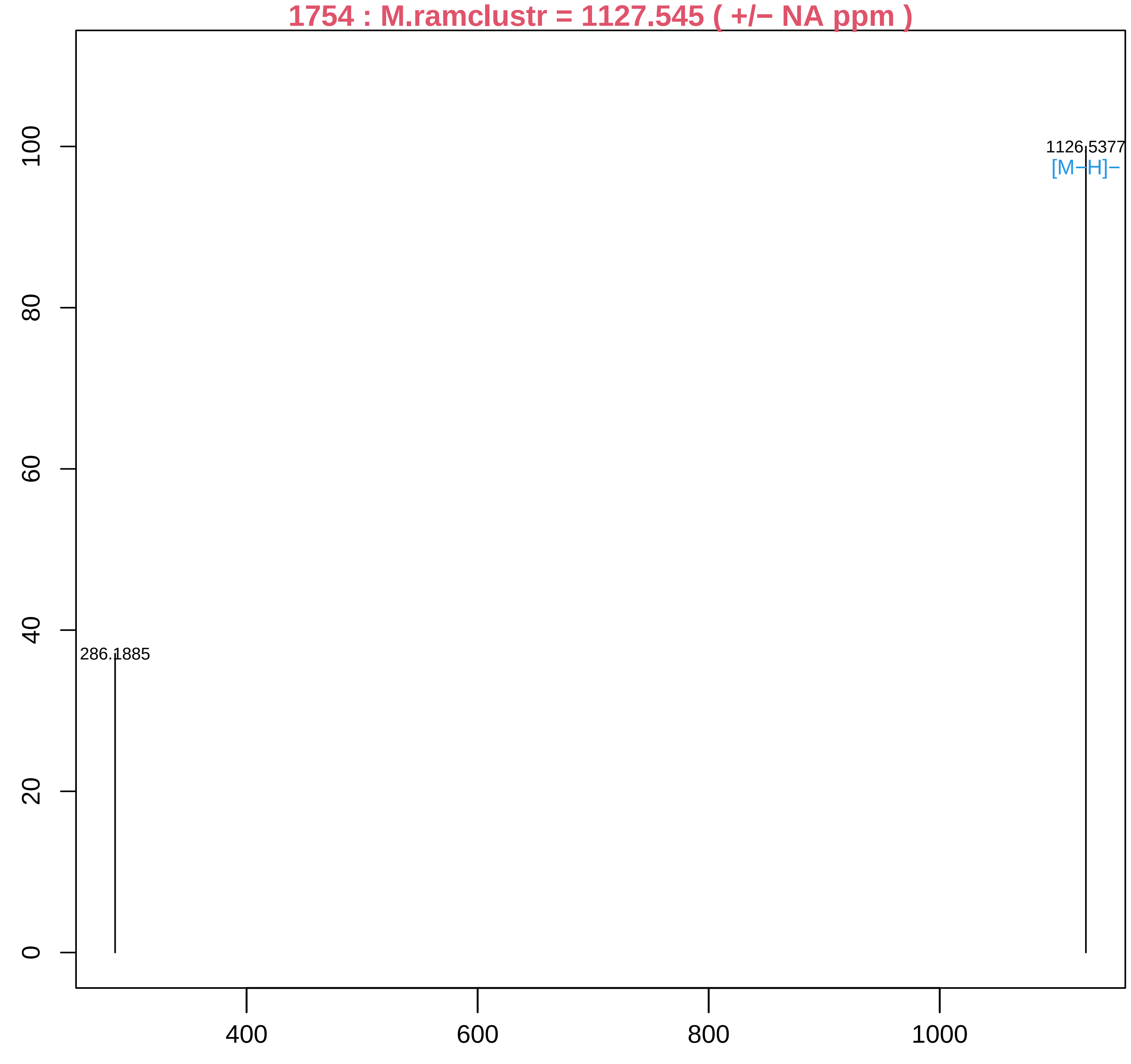
1754 : M.ramclustr = 1127.545 (+/− NA ppm)
1126.5377
[M−H]−
286.1885
0
20
40
60
80
100
400
600
800
1000

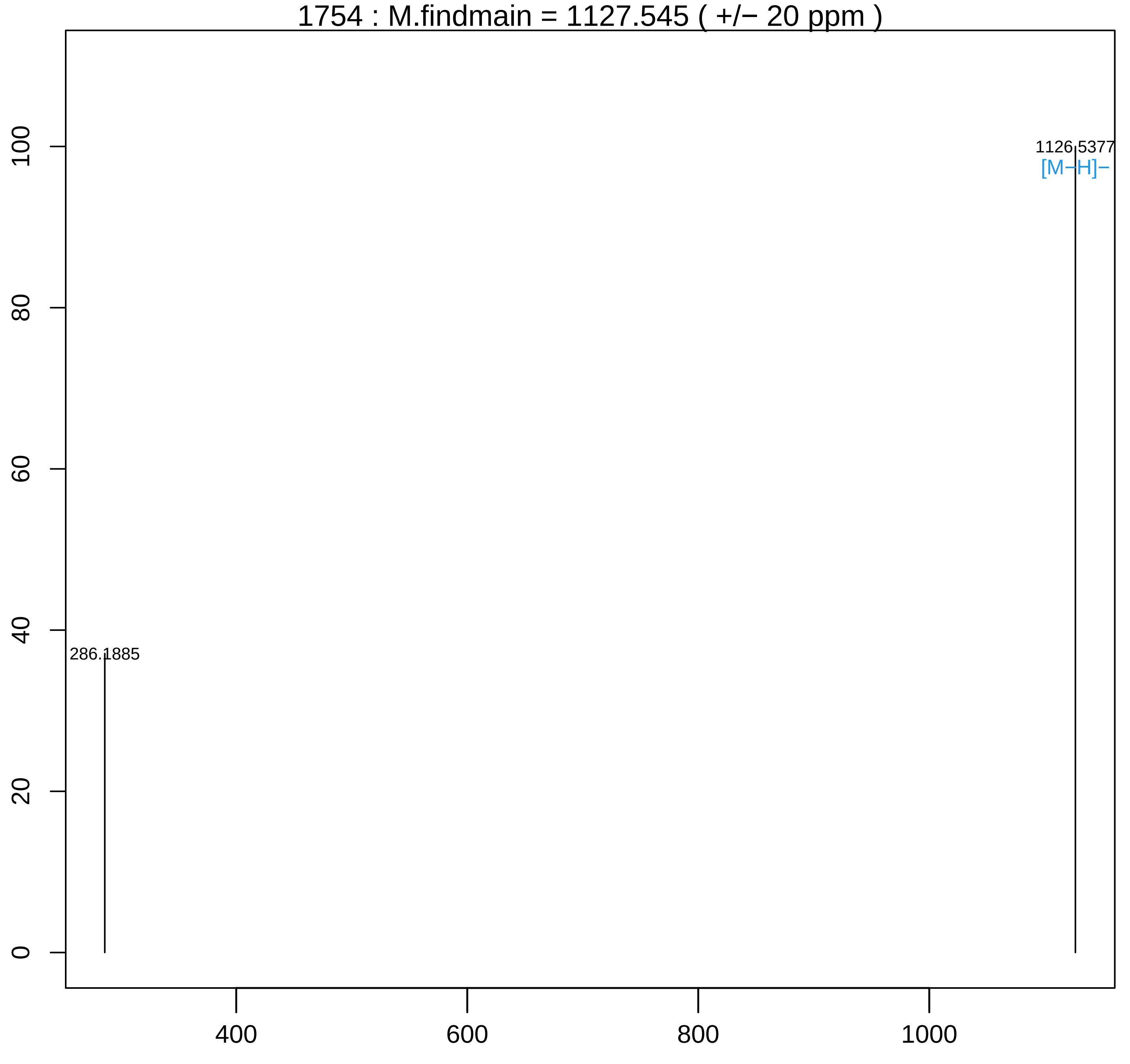
1754 : M.findmain = 1127.545 (+/− 20 ppm)
1126.5377
[M−H]−
286.1885
0
20
40
60
80
100
400
600
800
1000

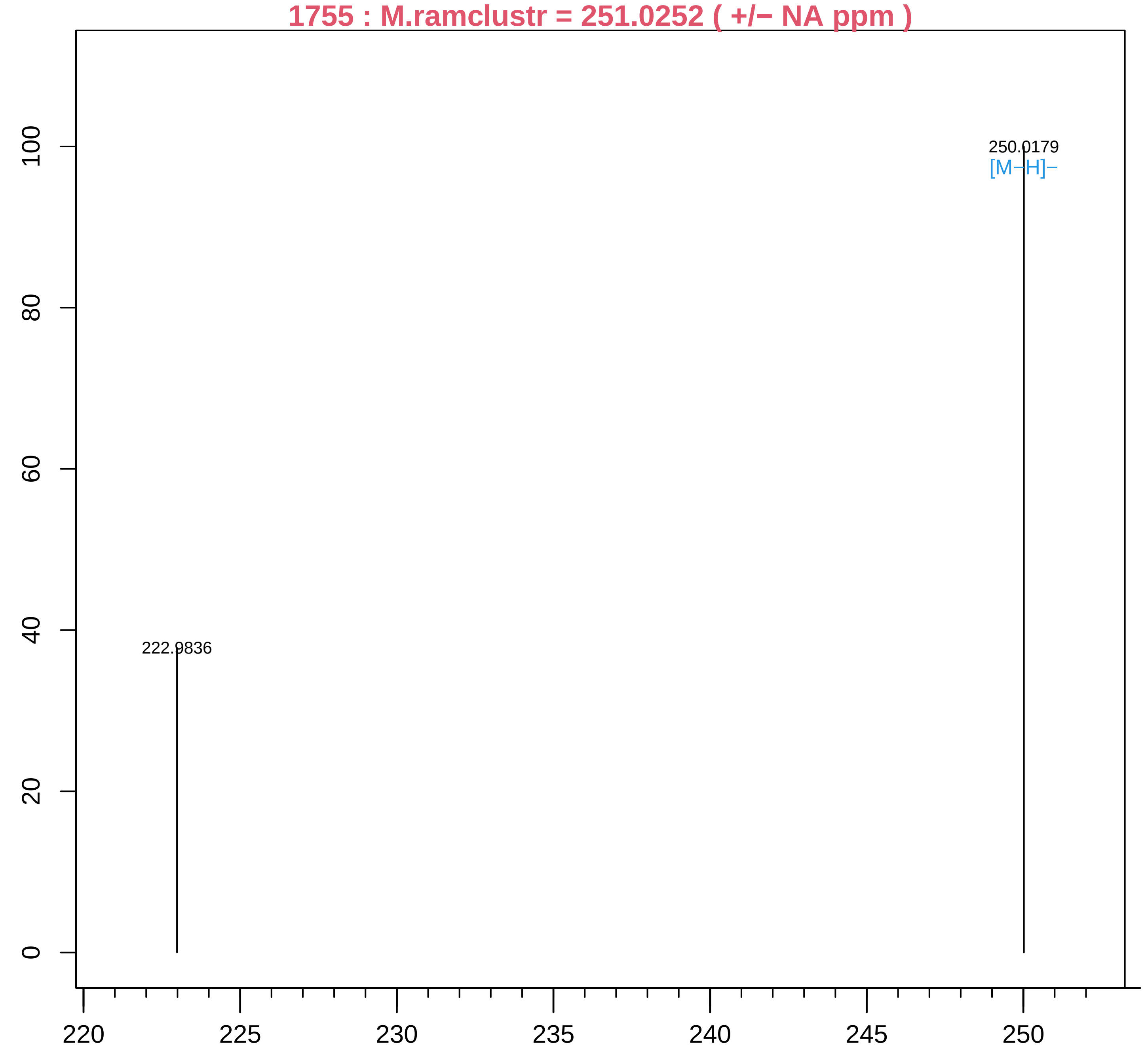
1755 : M.ramclustr = 251.0252 (+/− NA ppm)
250.0179
[M−H]−
222.9836
0
20
40
60
80
100
220
225
230
235
240
245
250

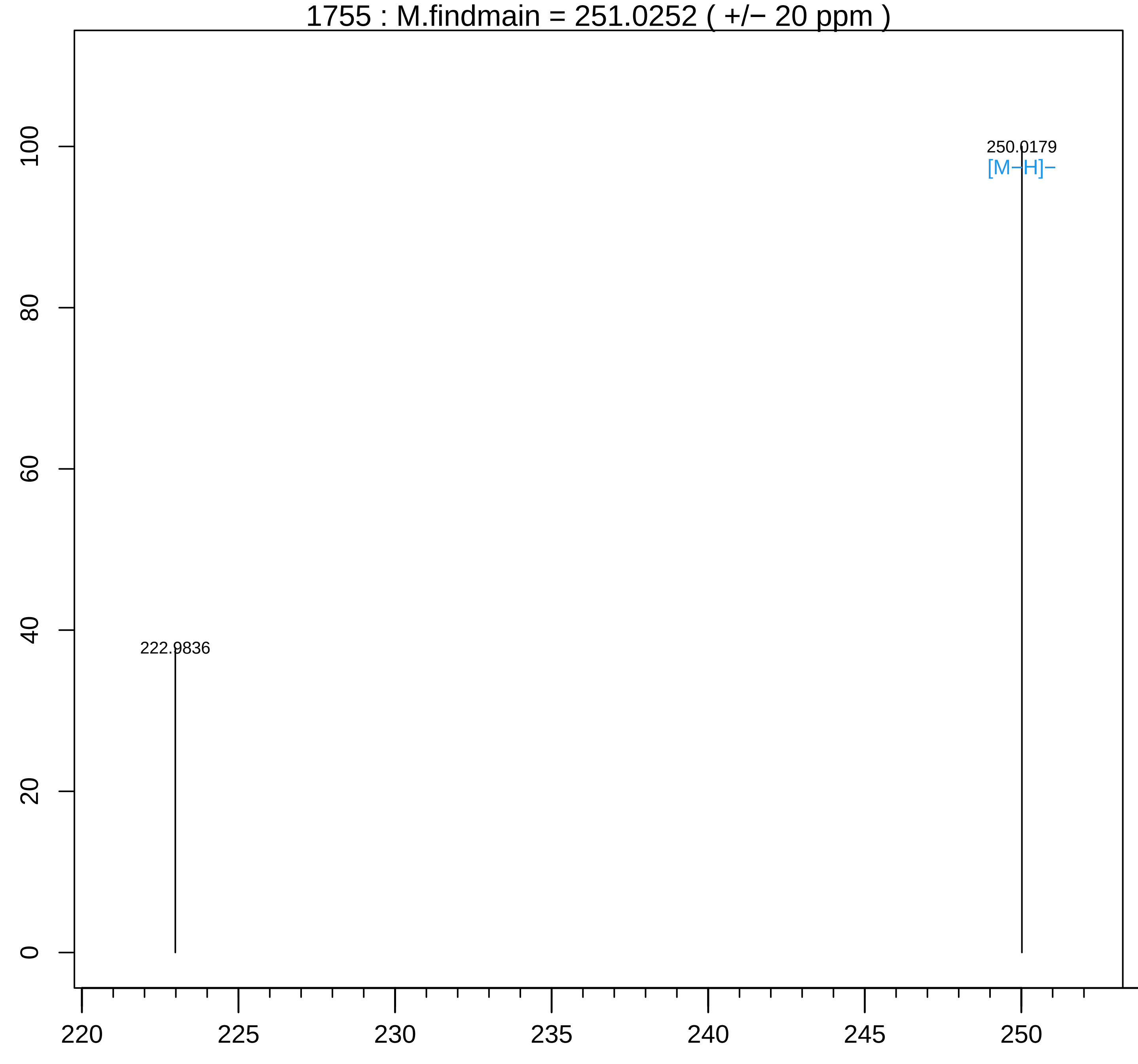
1755 : M.findmain = 251.0252 (+/− 20 ppm)
250.0179
[M−H]−
222.9836
0
20
40
60
80
100
220
225
230
235
240
245
250

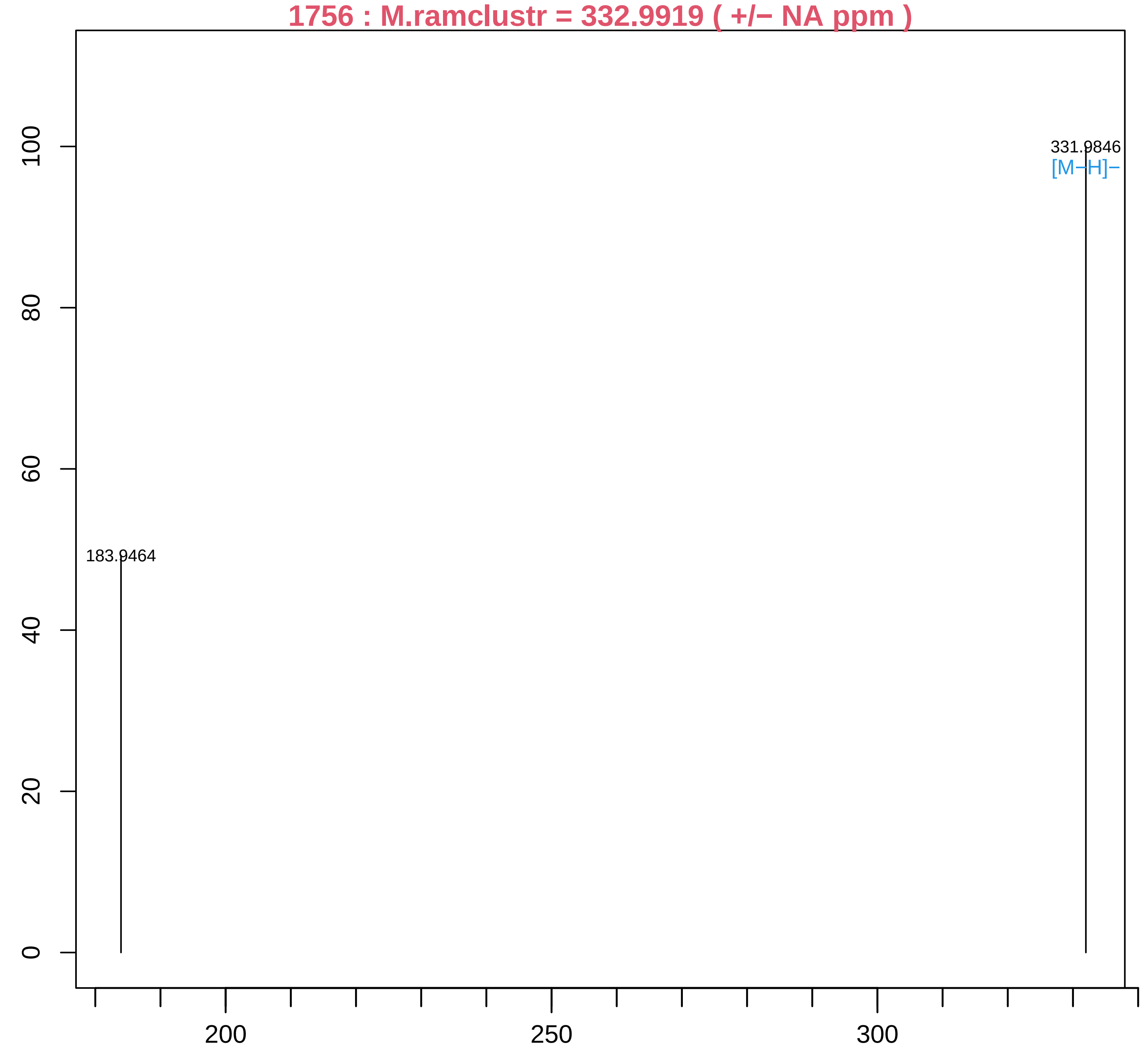
1756 : M.ramclustr = 332.9919 (+/− NA ppm)
331.9846
[M−H]−
183.9464
0
20
40
60
80
100
200
250
300

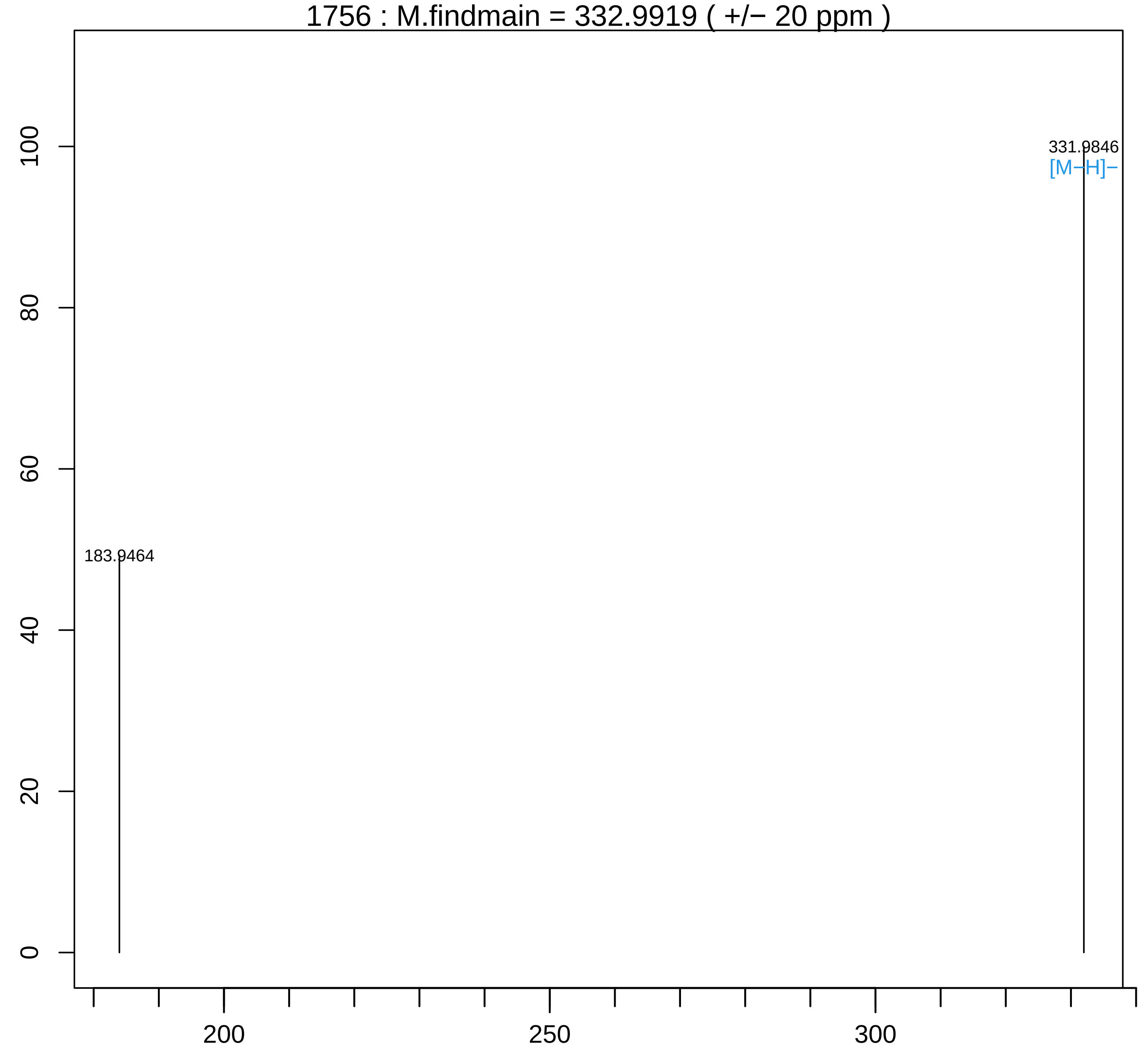
1756 : M.findmain = 332.9919 (+/− 20 ppm)
331.9846
[M−H]−
183.9464
0
20
40
60
80
100
200
250
300

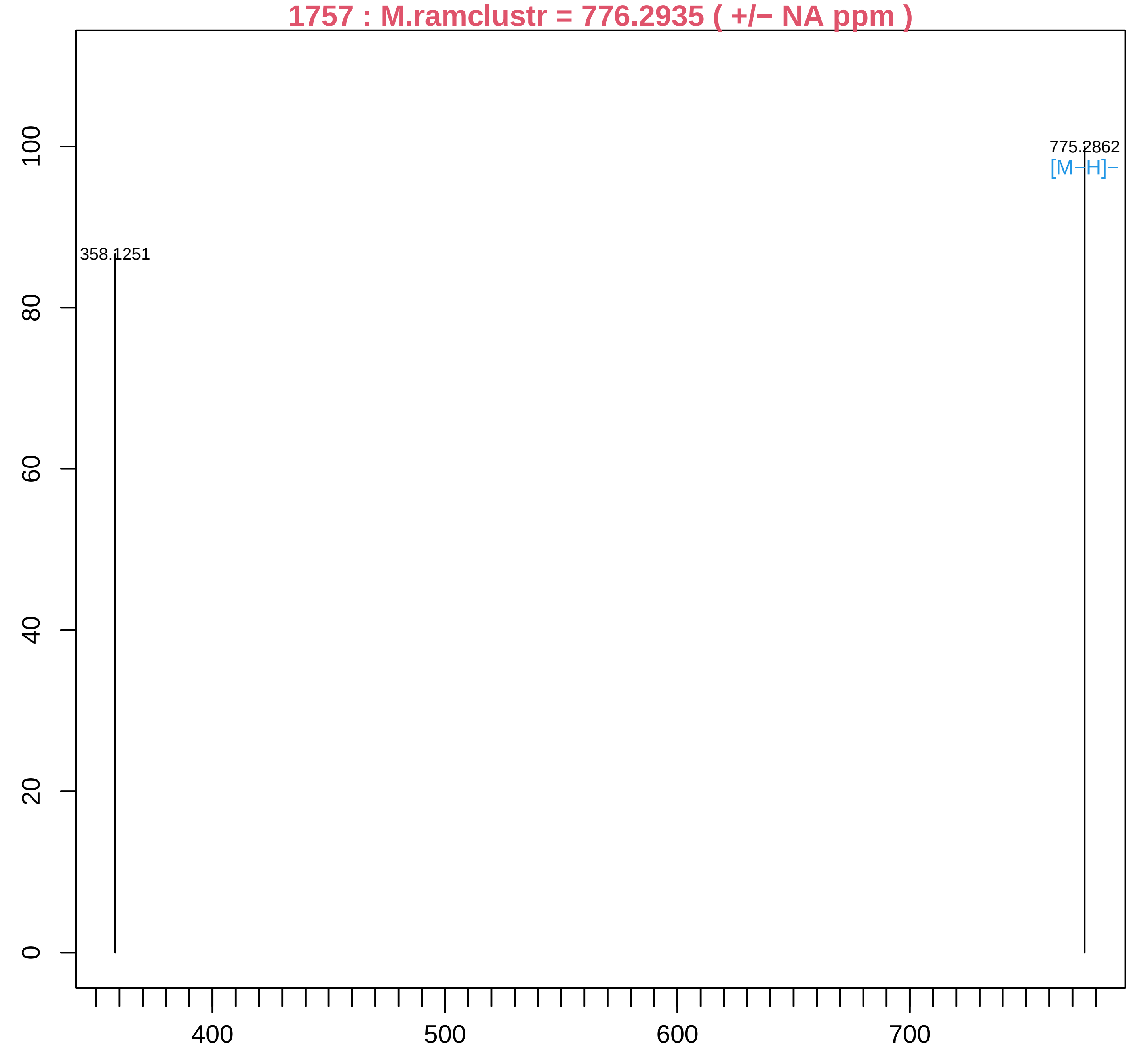
1757 : M.ramclustr = 776.2935 (+/− NA ppm)
358.1251
775.2862
[M−H]−
0
20
40
60
80
100
400
500
600
700

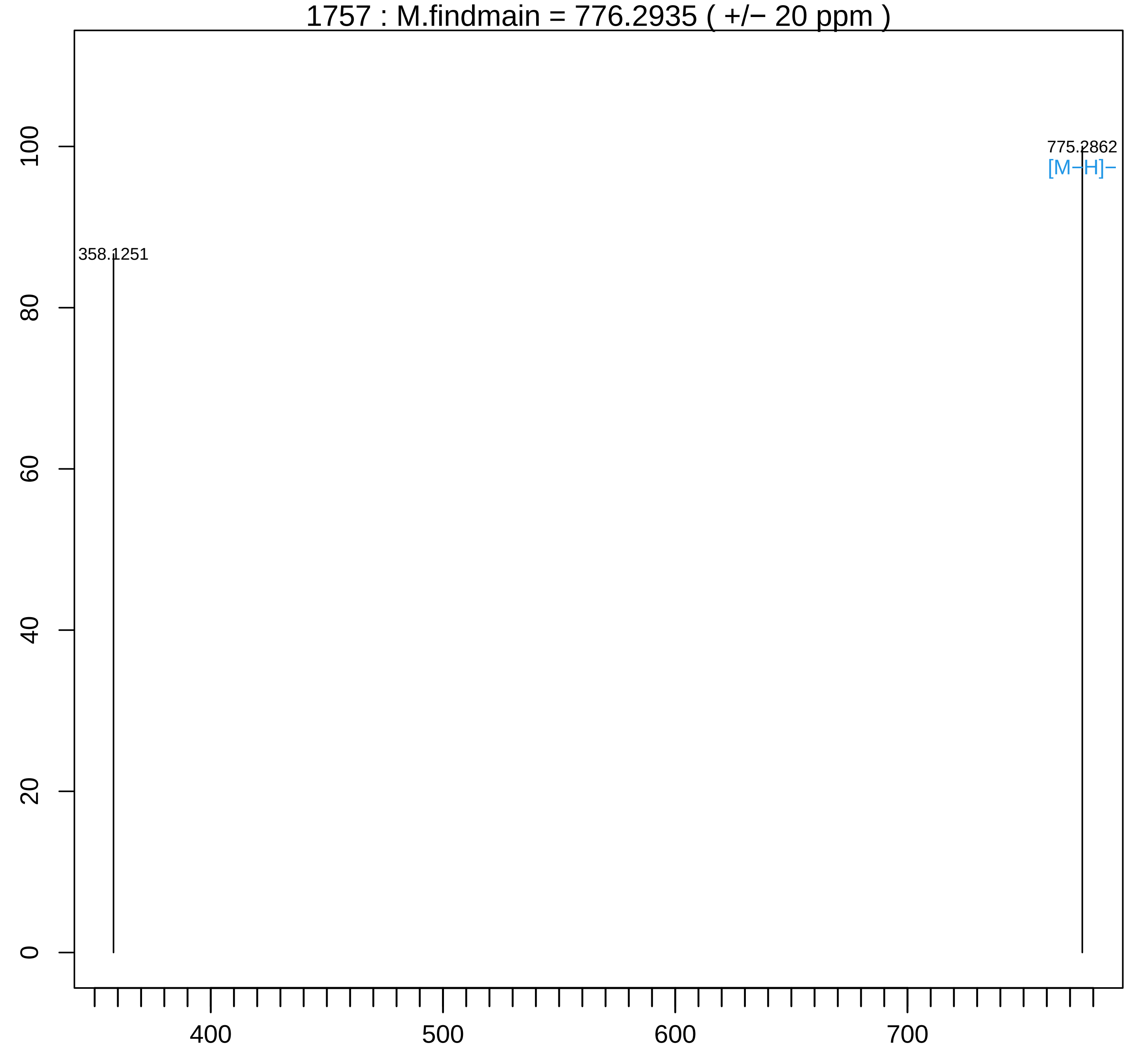
1757 : M.findmain = 776.2935 (+/− 20 ppm)
358.1251
775.2862
[M−H]−
0
20
40
60
80
100
400
500
600
700

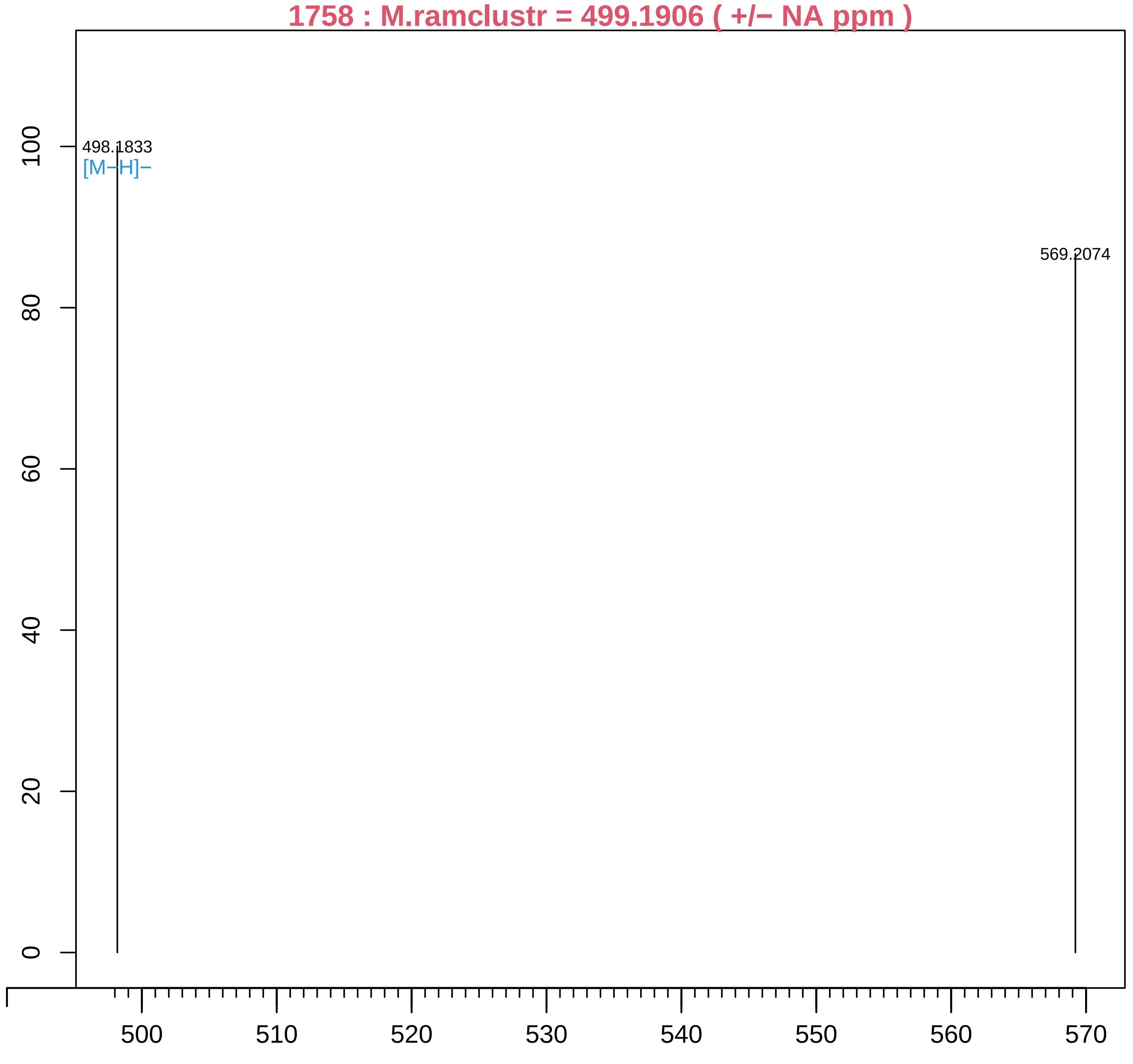
1758 : M.ramclustr = 499.1906 (+/− NA ppm)
498.1833
[M−H]−
569.2074
0
20
40
60
80
100
500
510
520
530
540
550
560
570

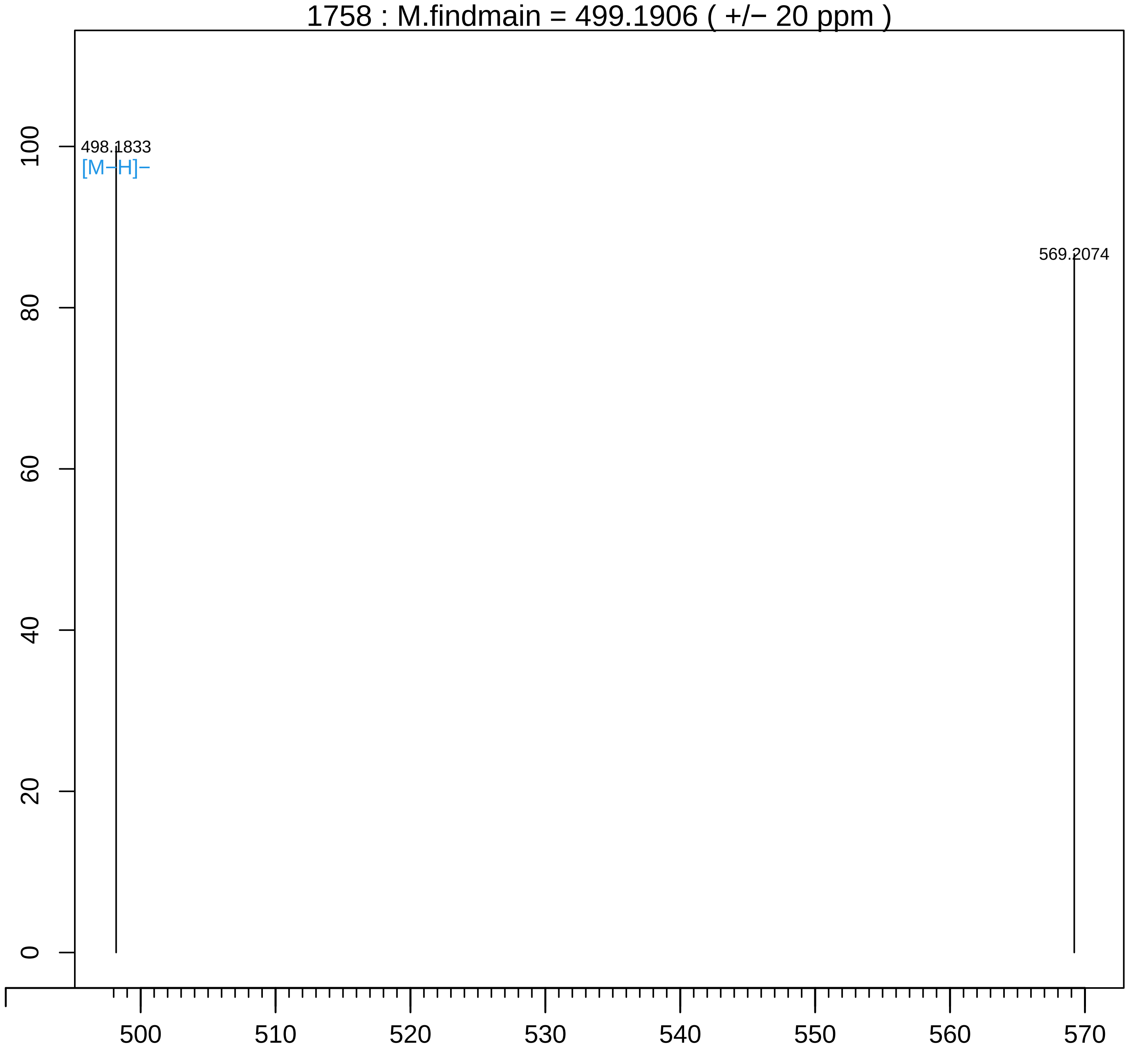
1758 : M.findmain = 499.1906 (+/− 20 ppm)
498.1833
[M−H]−
569.2074
0
20
40
60
80
100
500
510
520
530
540
550
560
570

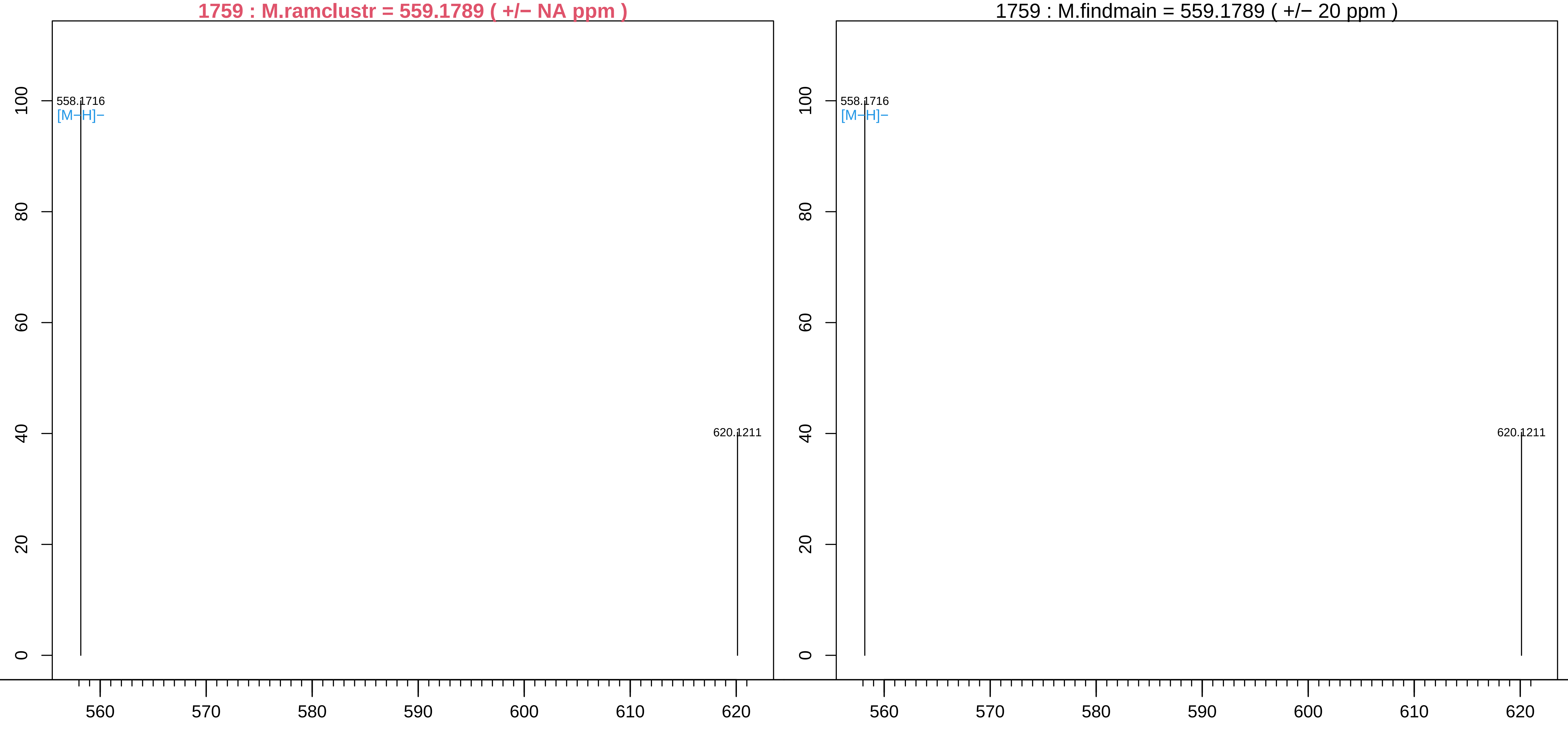
1759 : M.ramclustr = 559.1789 (+/− NA ppm)
558.1716
[M−H]−
620.1211
1759 : M.findmain = 559.1789 (+/− 20 ppm)
558.1716
[M−H]−
620.1211
0
20
40
60
80
100
560
570
580
590
600
610
620

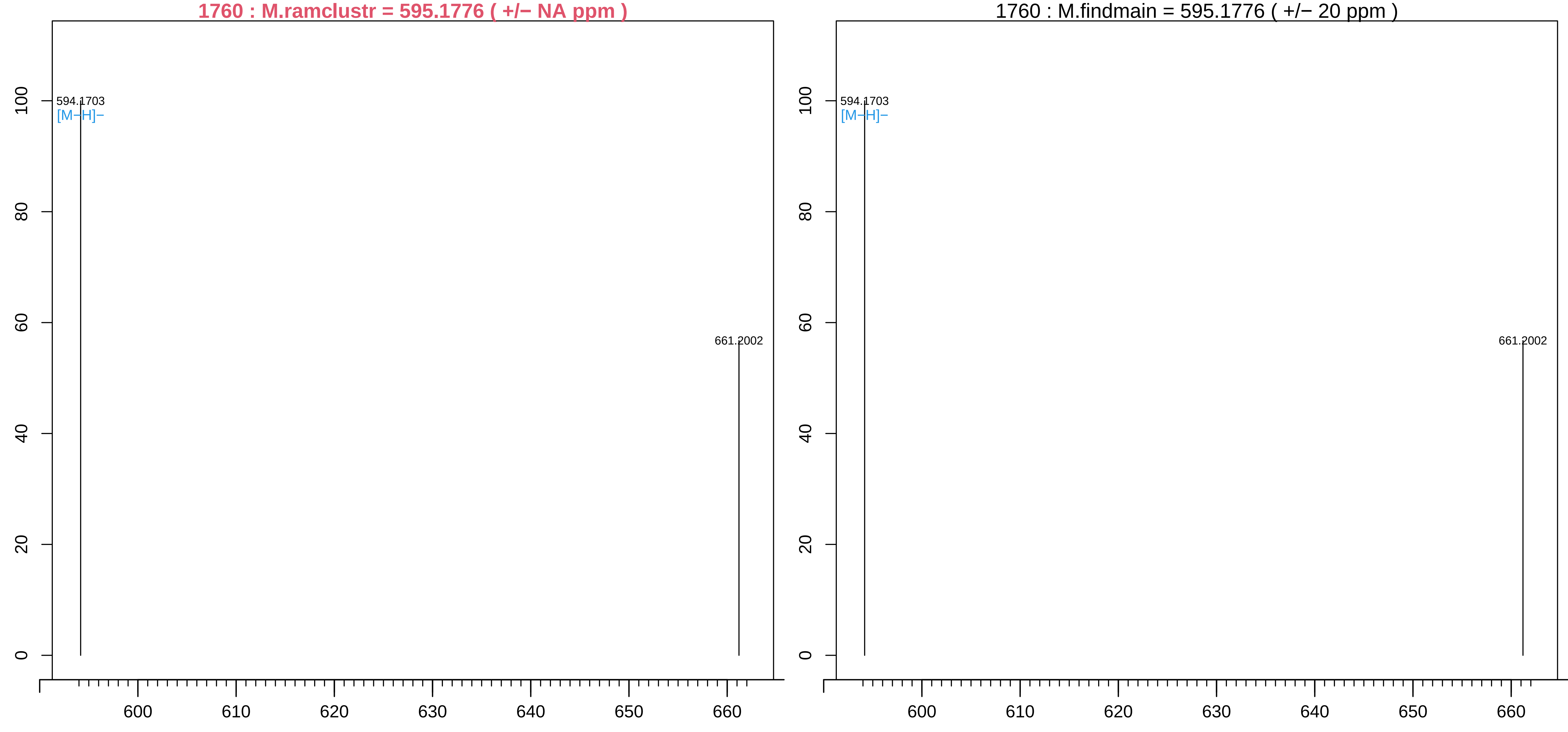
1760 : M.ramclustr = 595.1776 (+/− NA ppm)
594.1703
[M−H]−
661.2002
1760 : M.findmain = 595.1776 (+/− 20 ppm)
594.1703
[M−H]−
661.2002
0
20
40
60
80
100
600
610
620
630
640
650
660

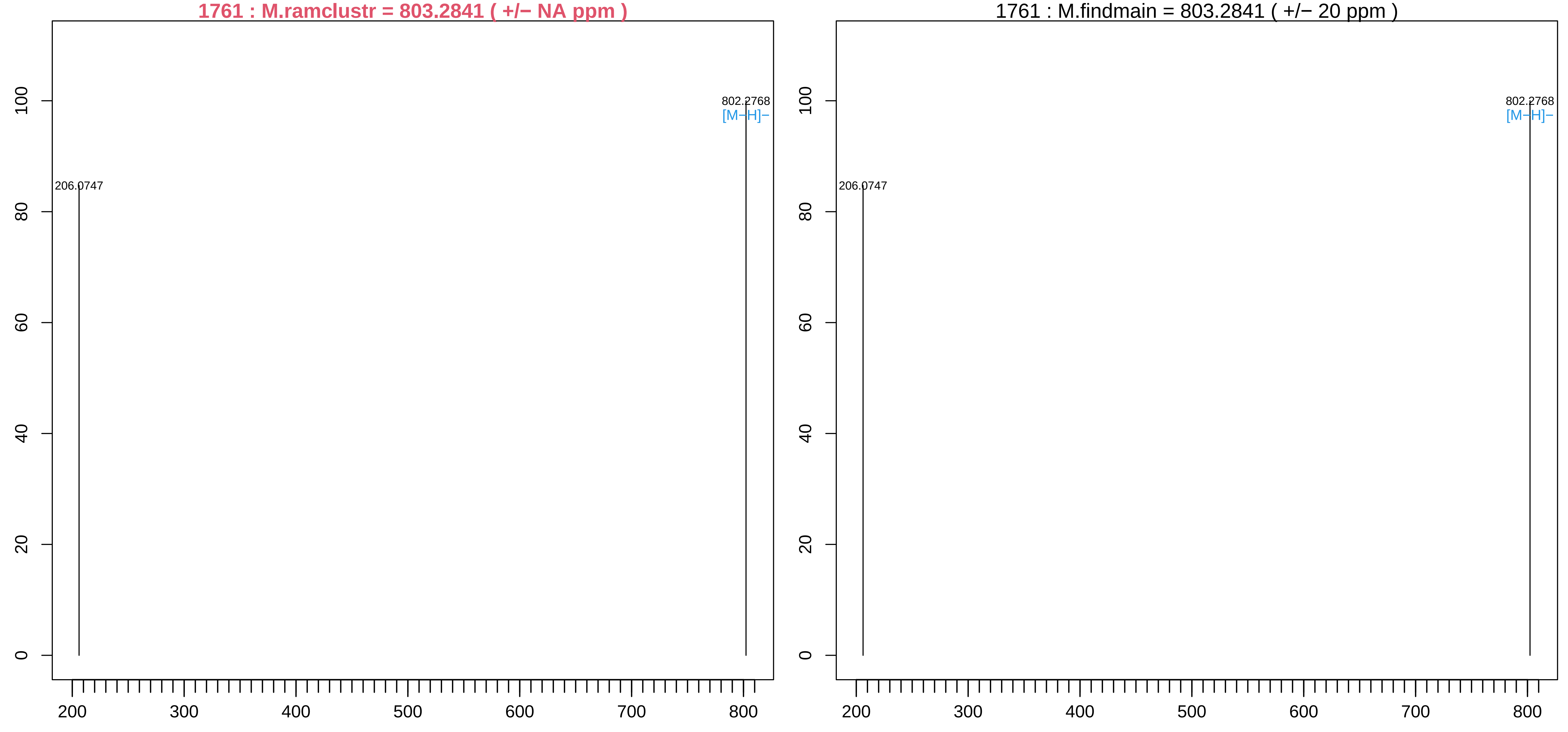
1761 : M.ramclustr = 803.2841 (+/− NA ppm)
1761 : M.findmain = 803.2841 (+/− 20 ppm)
802.2768
[M−H]−
206.0747
0
20
40
60
80
100
200
300
400
500
600
700
800

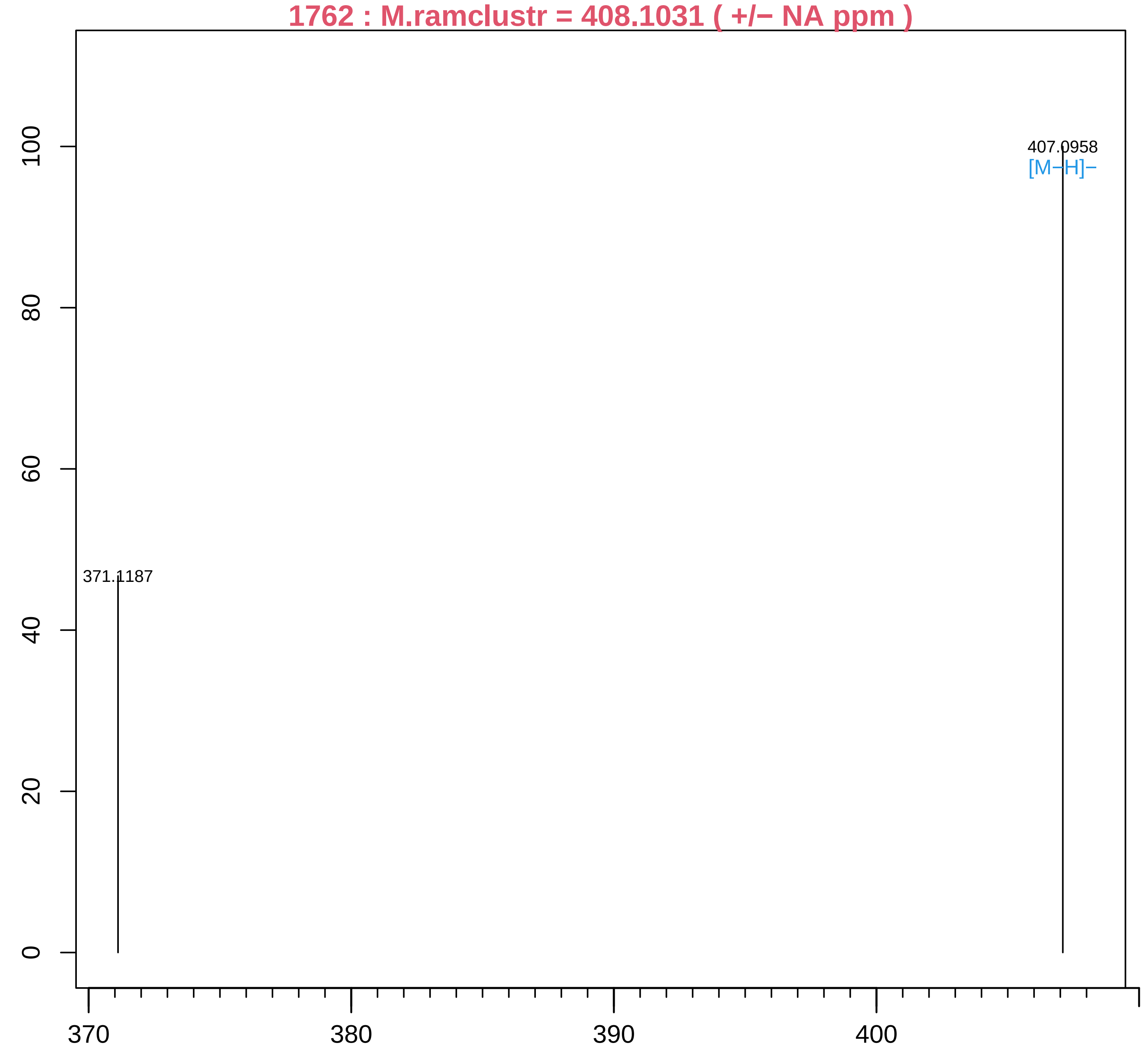

1762 : M.ramclustr = 408.1031 (+/− NA ppm)
407.0958
[M−H]−
371.1187
0
20
40
60
80
100
370
380
390
400

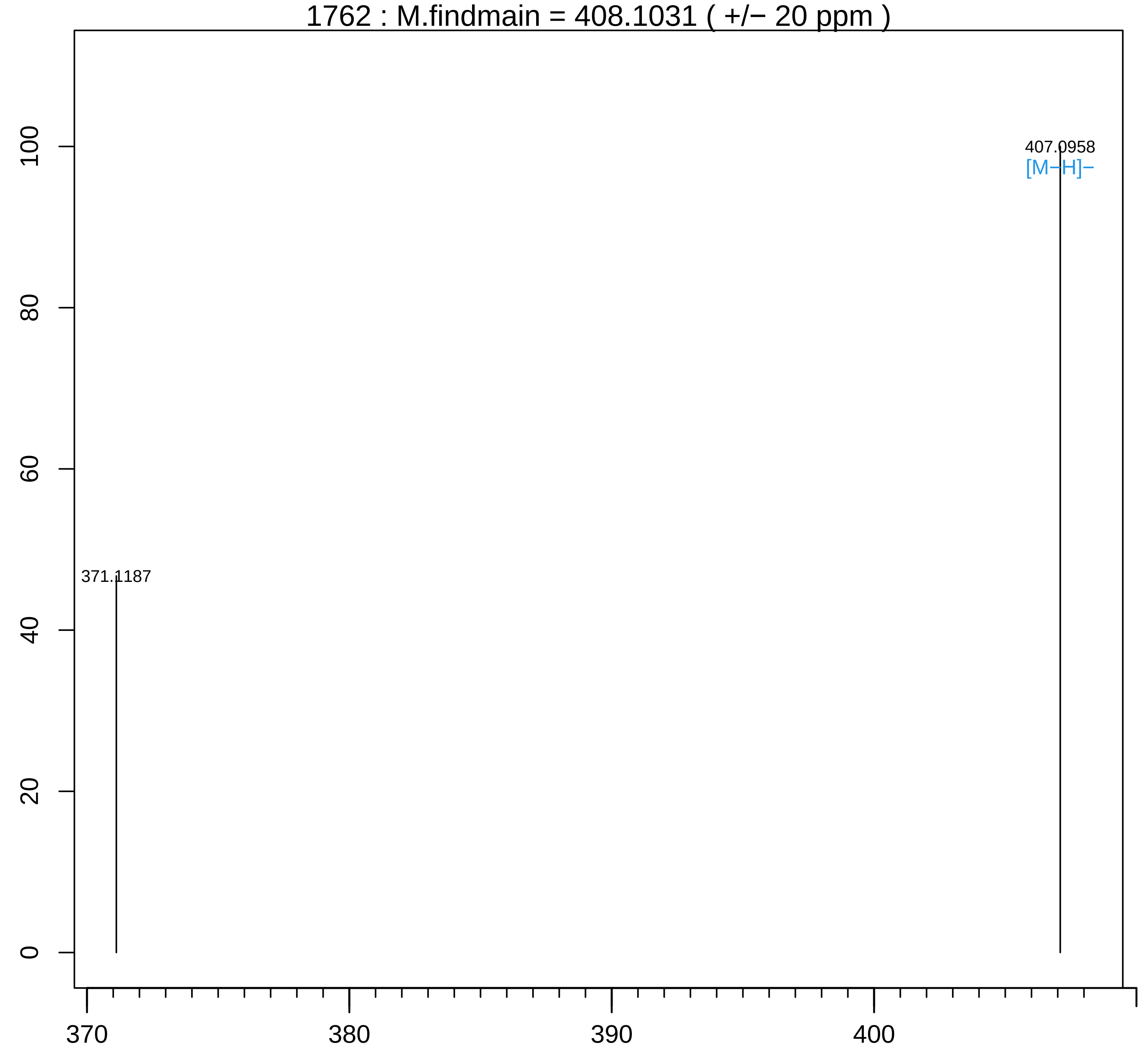

1762 : M.findmain = 408.1031 (+/− 20 ppm)
407.0958
[M−H]−
371.1187
0
20
40
60
80
100
370
380
390
400

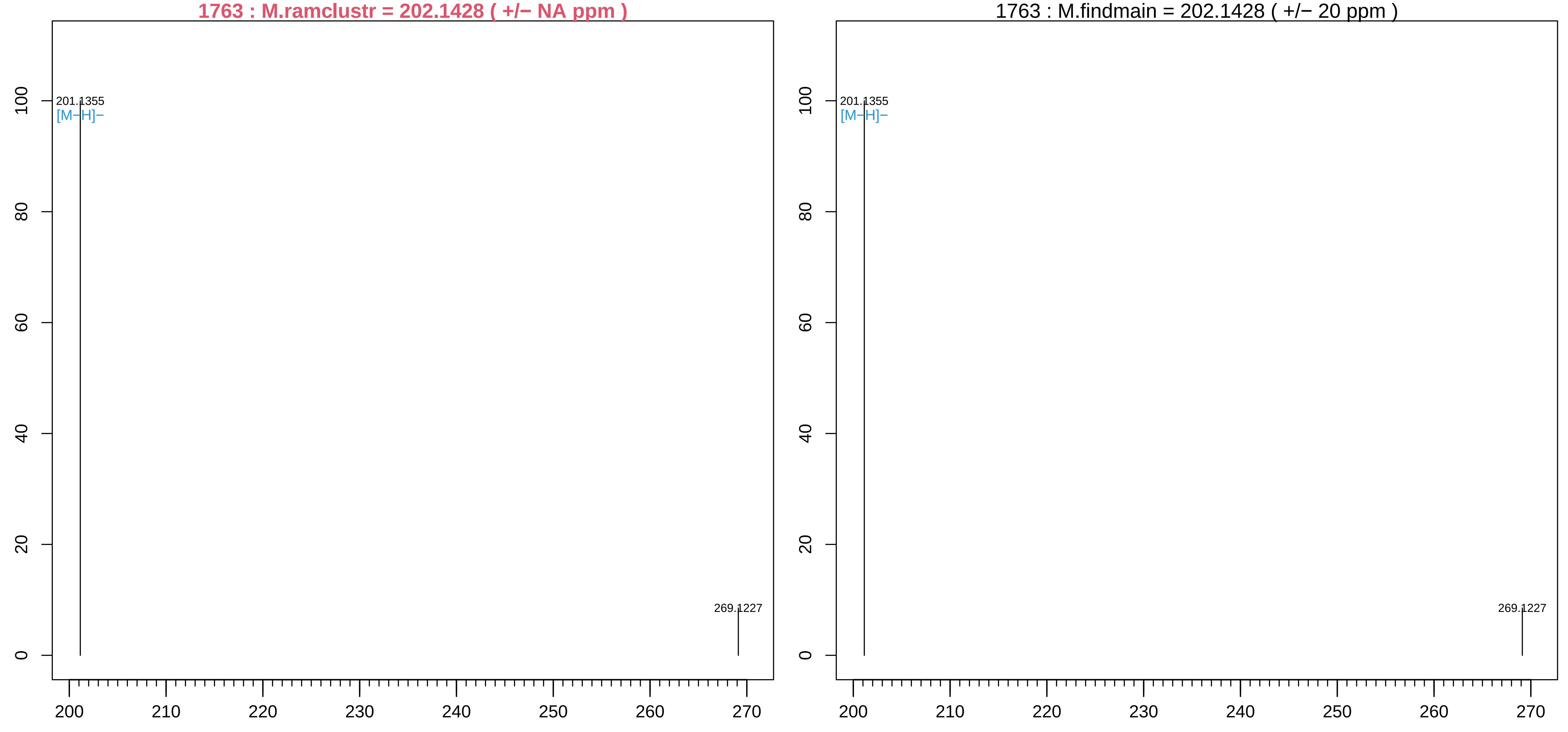
1763 : M.ramclustr = 202.1428 (+/− NA ppm)
201.1355
[M−H]−
269.1227
1763 : M.findmain = 202.1428 (+/− 20 ppm)
201.1355
[M−H]−
269.1227
0
20
40
60
80
100
200
210
220
230
240
250
260
270

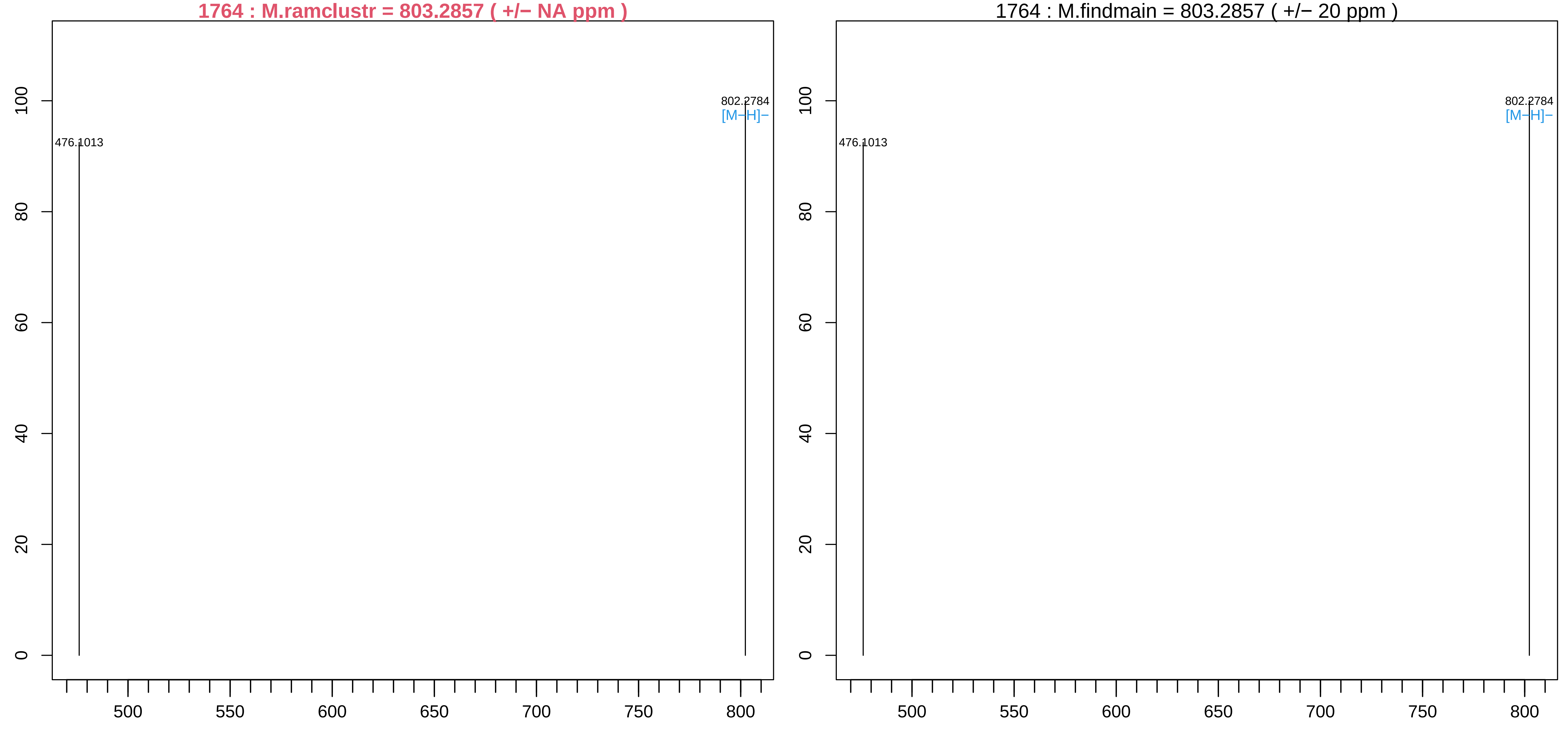
1764 : M.ramclustr = 803.2857 (+/− NA ppm)
1764 : M.findmain = 803.2857 (+/− 20 ppm)
802.2784
[M−H]−
476.1013
0
20
40
60
80
100
500
550
600
650
700
750
800

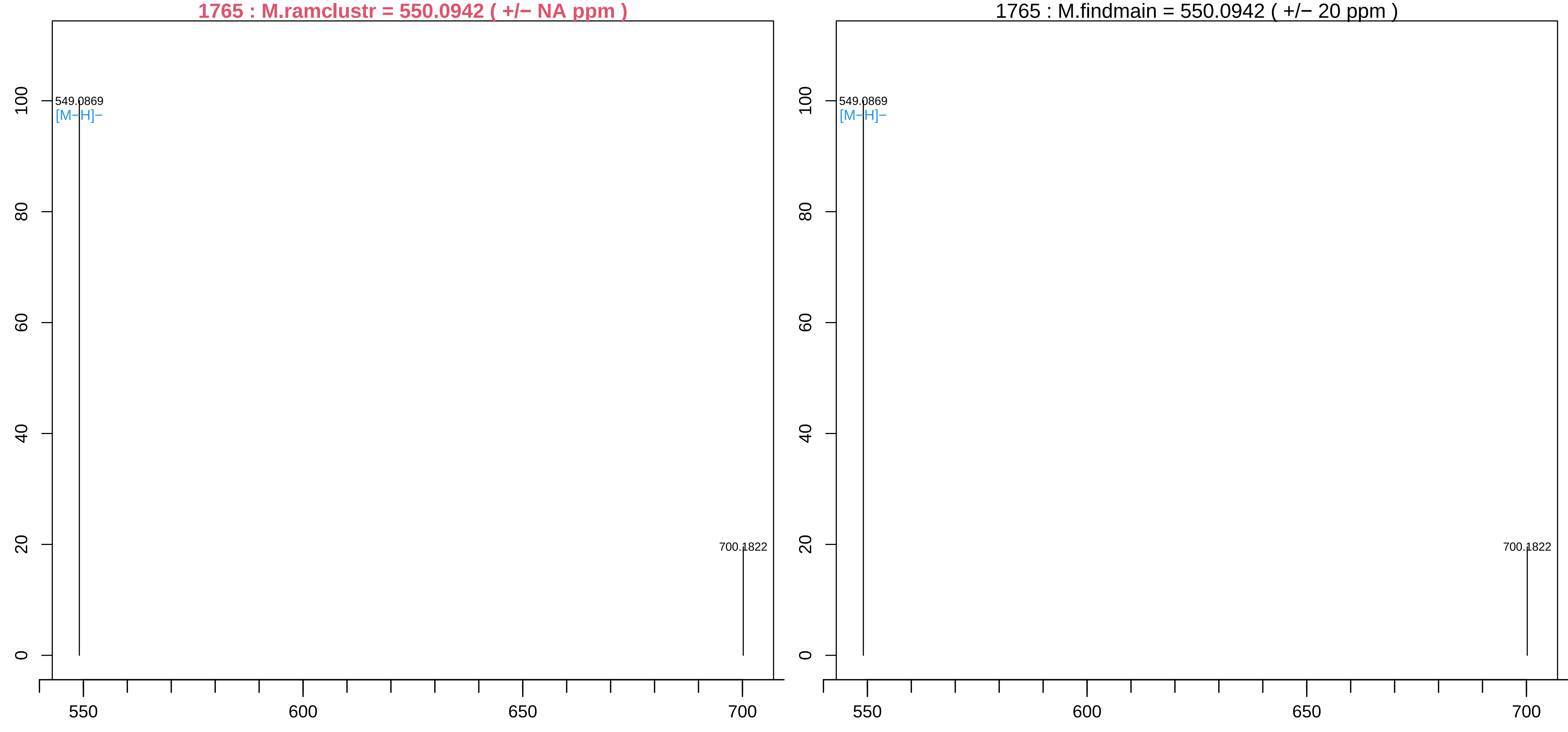

1765 : M.ramclustr = 550.0942 (+/− NA ppm)
549.0869
[M−H]−
700.1822
1765 : M.findmain = 550.0942 (+/− 20 ppm)
549.0869
[M−H]−
700.1822
0
20
40
60
80
100
550
600
650
700

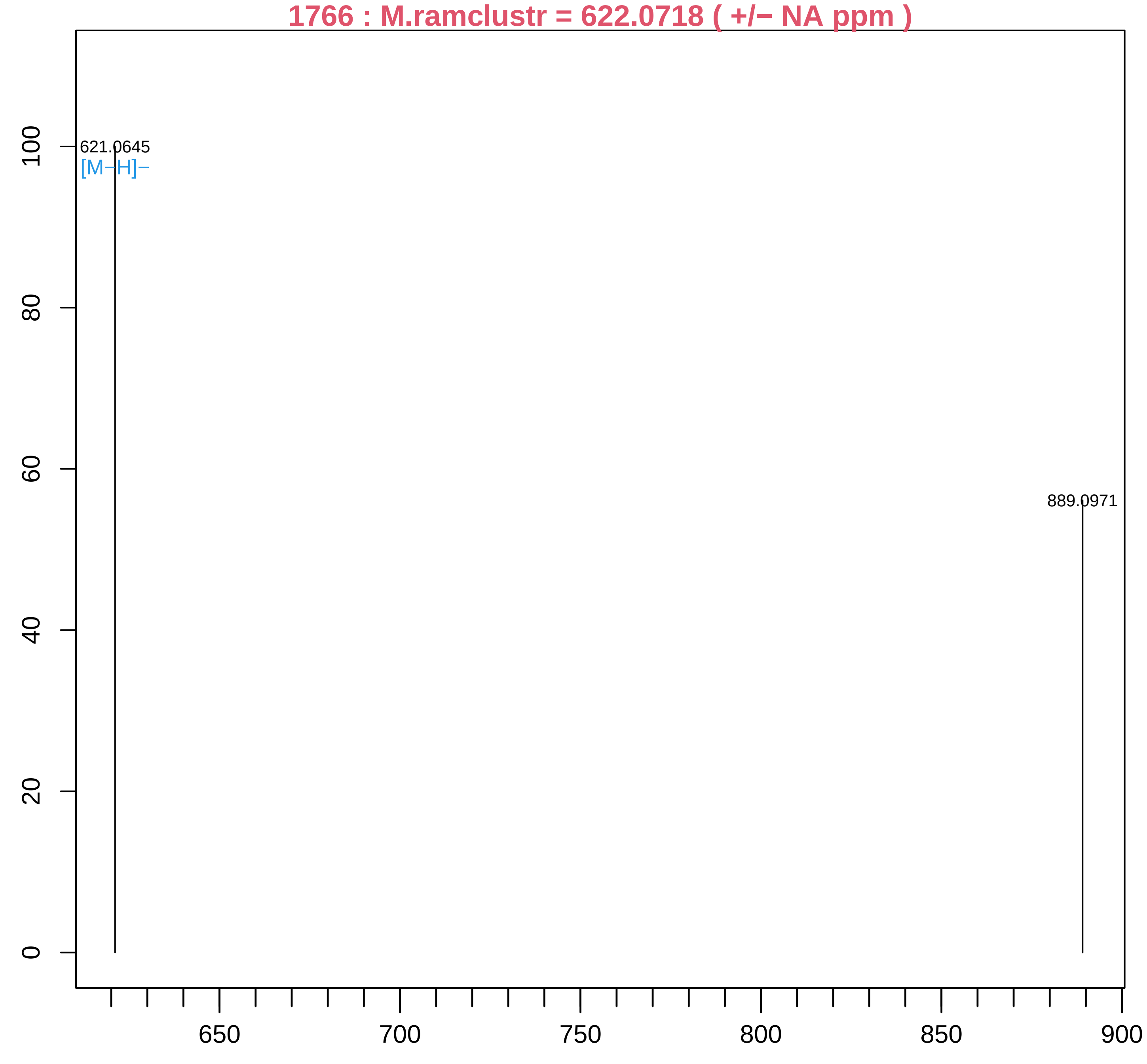

1766 : M.ramclustr = 622.0718 (+/− NA ppm)
621.0645
[M−H]−
889.0971
0
20
40
60
80
100
650
700
750
800
850
900

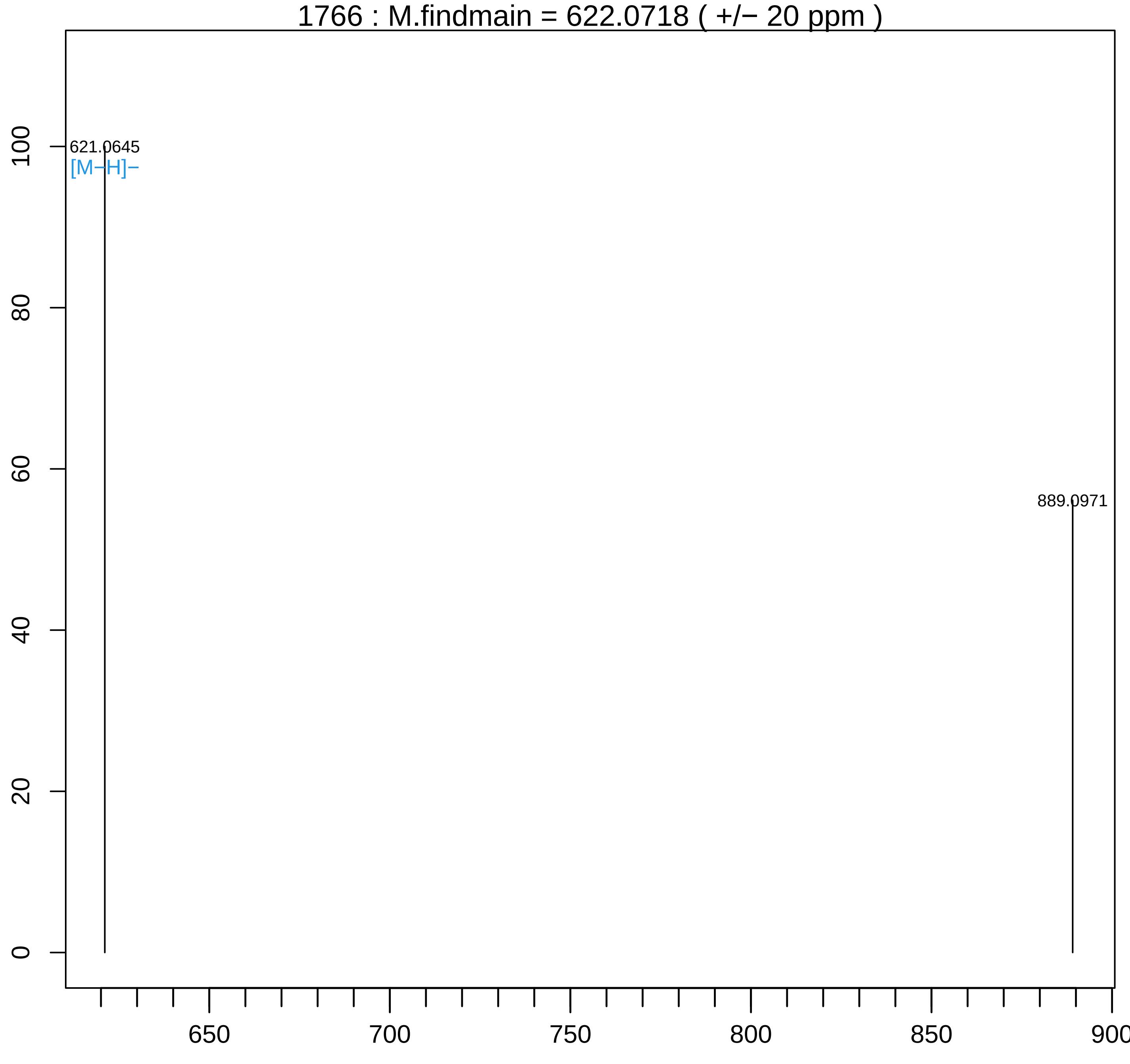

1766 : M.findmain = 622.0718 (+/− 20 ppm)
621.0645
[M−H]−
889.0971
0
20
40
60
80
100
650
700
750
800
850
900

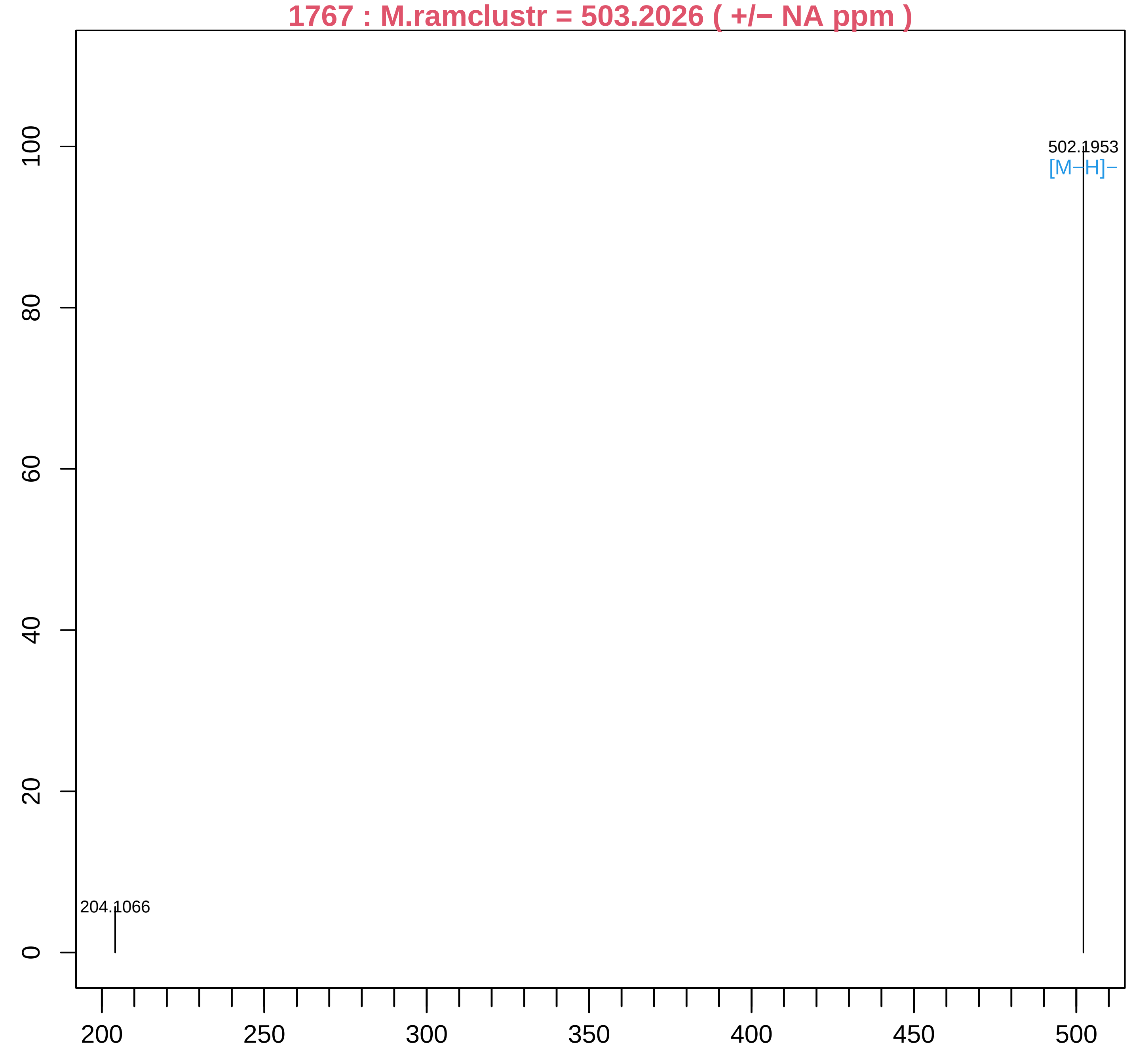
1767 : M.ramclustr = 503.2026 (+/− NA ppm)
502.1953
[M−H]−
204.1066
0
20
40
60
80
100
200
250
300
350
400
450
500

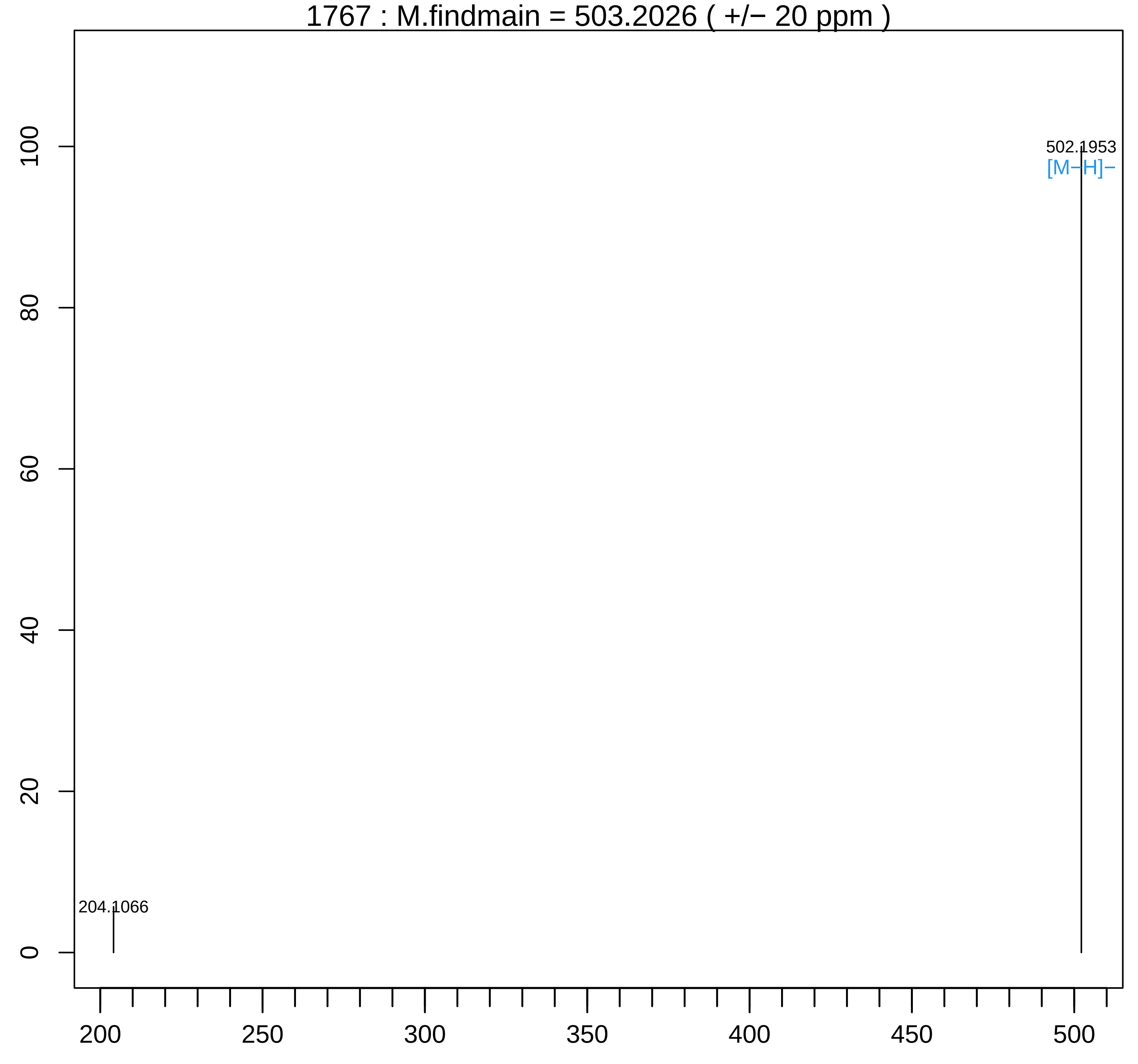
1767 : M.findmain = 503.2026 (+/− 20 ppm)
502.1953
[M−H]−
204.1066
0
20
40
60
80
100
200
250
300
350
400
450
500

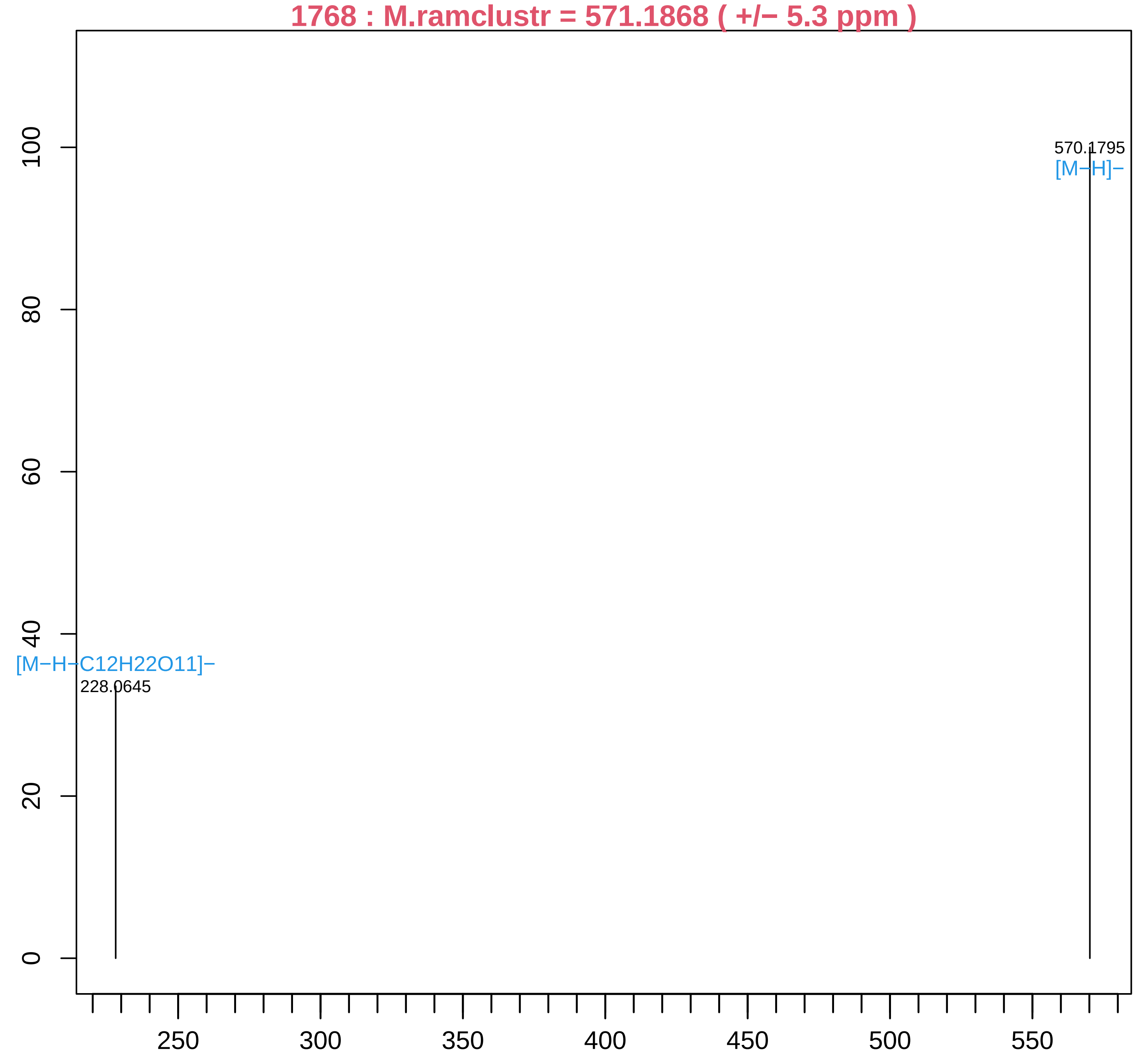
1768 : M.ramclustr = 571.1868 (+/− 5.3 ppm)
570.1795
[M−H]−
[M−H−C12H22O11]−
228.0645
0
20
40
60
80
100
250
300
350
400
450
500
550

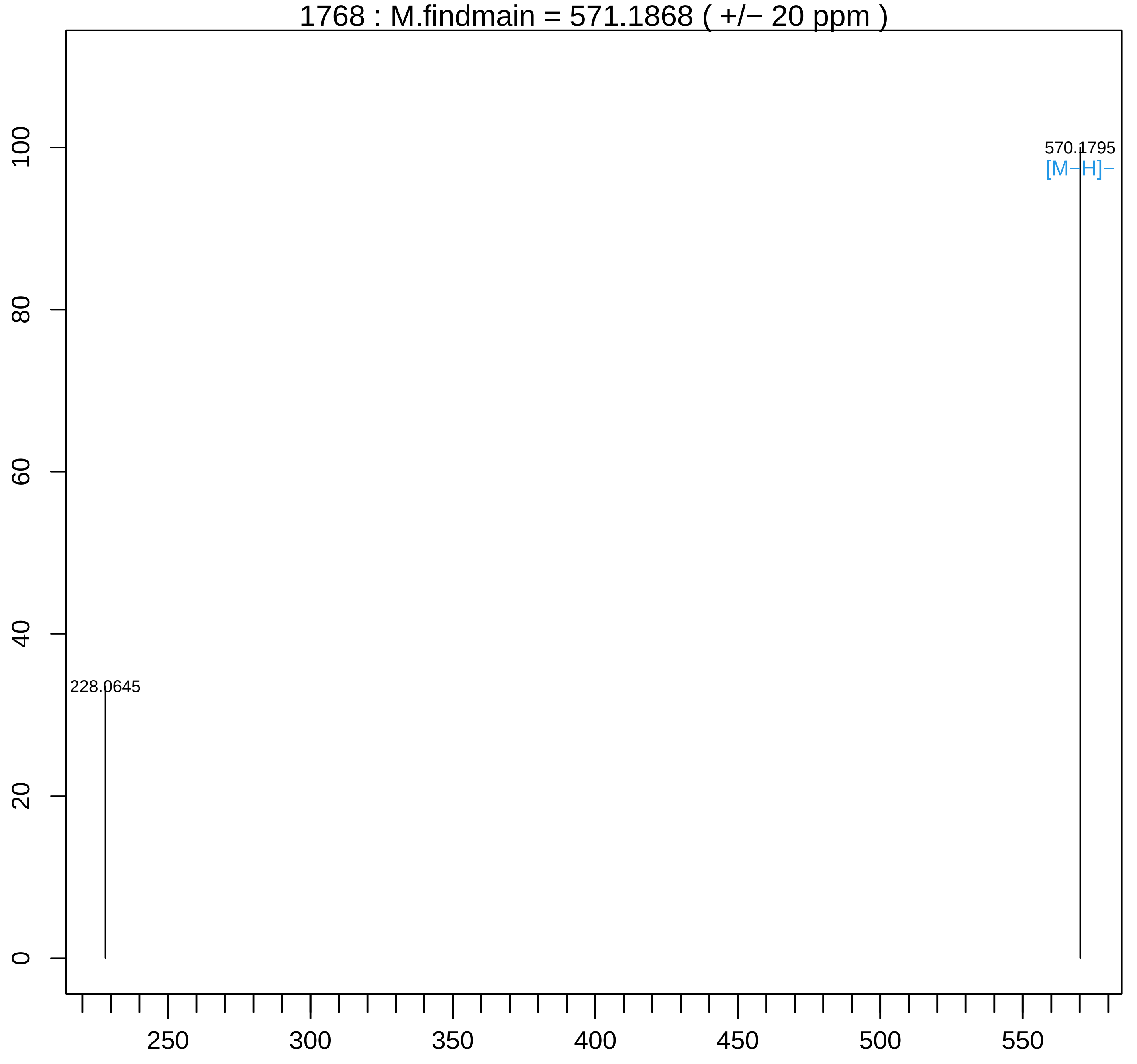
1768 : M.findmain = 571.1868 (+/− 20 ppm)
570.1795
[M−H]−
228.0645
0
20
40
60
80
100
250
300
350
400
450
500
550

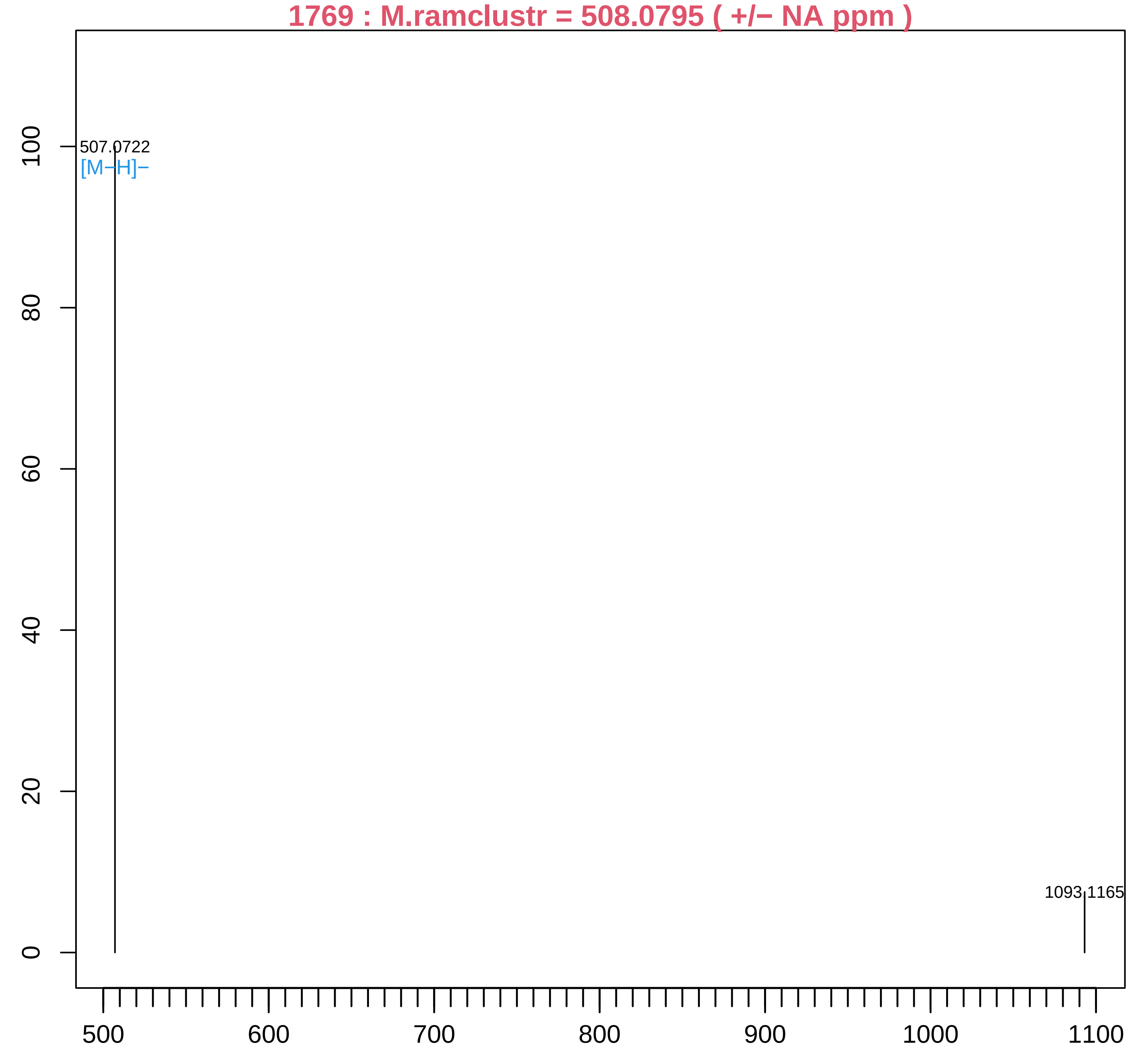
1769 : M.ramclustr = 508.0795 (+/− NA ppm)
507.0722
[M−H]−
1093.1165
0
20
40
60
80
100
500
600
700
800
900
1000
1100

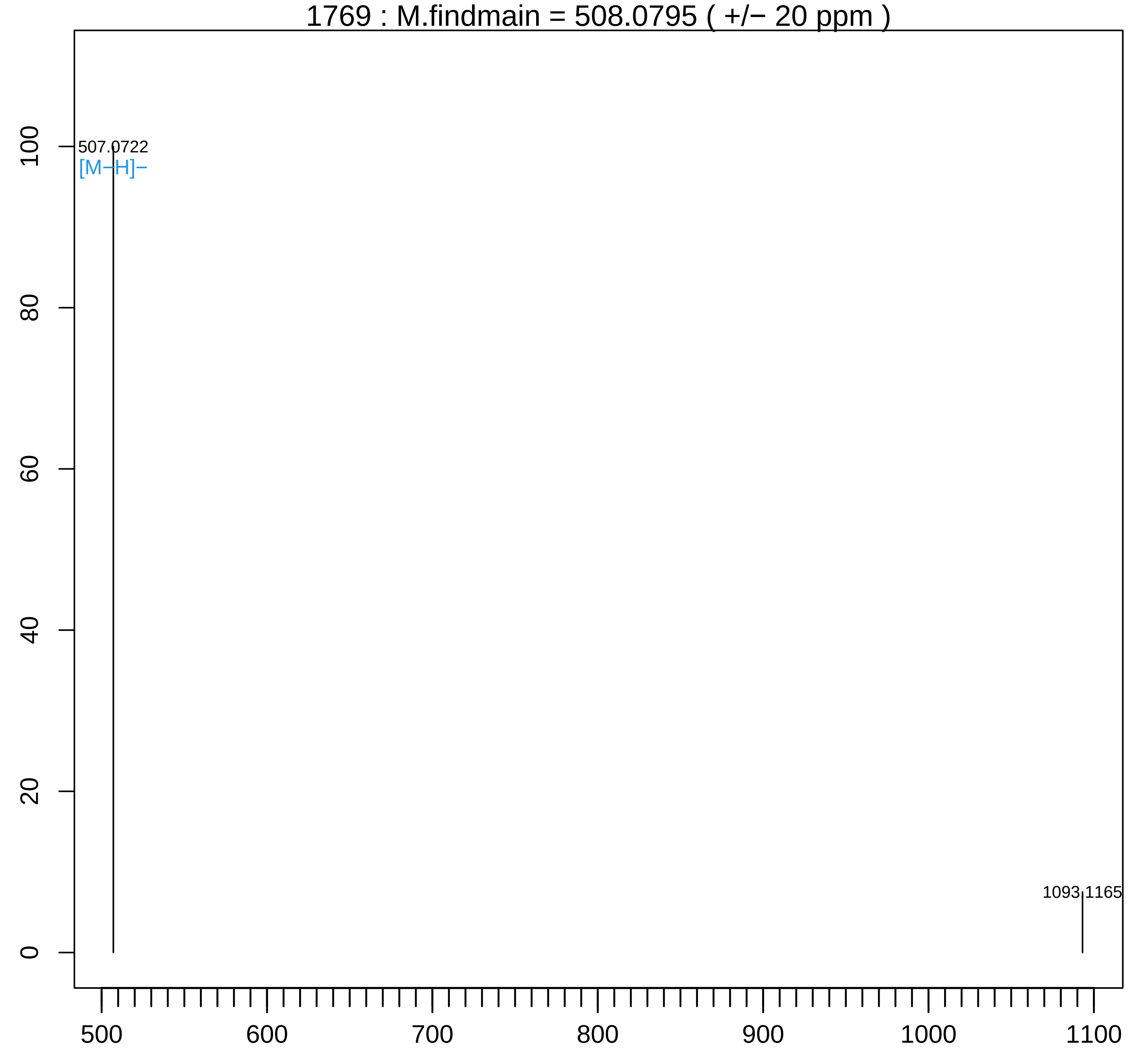
1769 : M.findmain = 508.0795 (+/− 20 ppm)
507.0722
[M−H]−
1093.1165
0
20
40
60
80
100
500
600
700
800
900
1000
1100

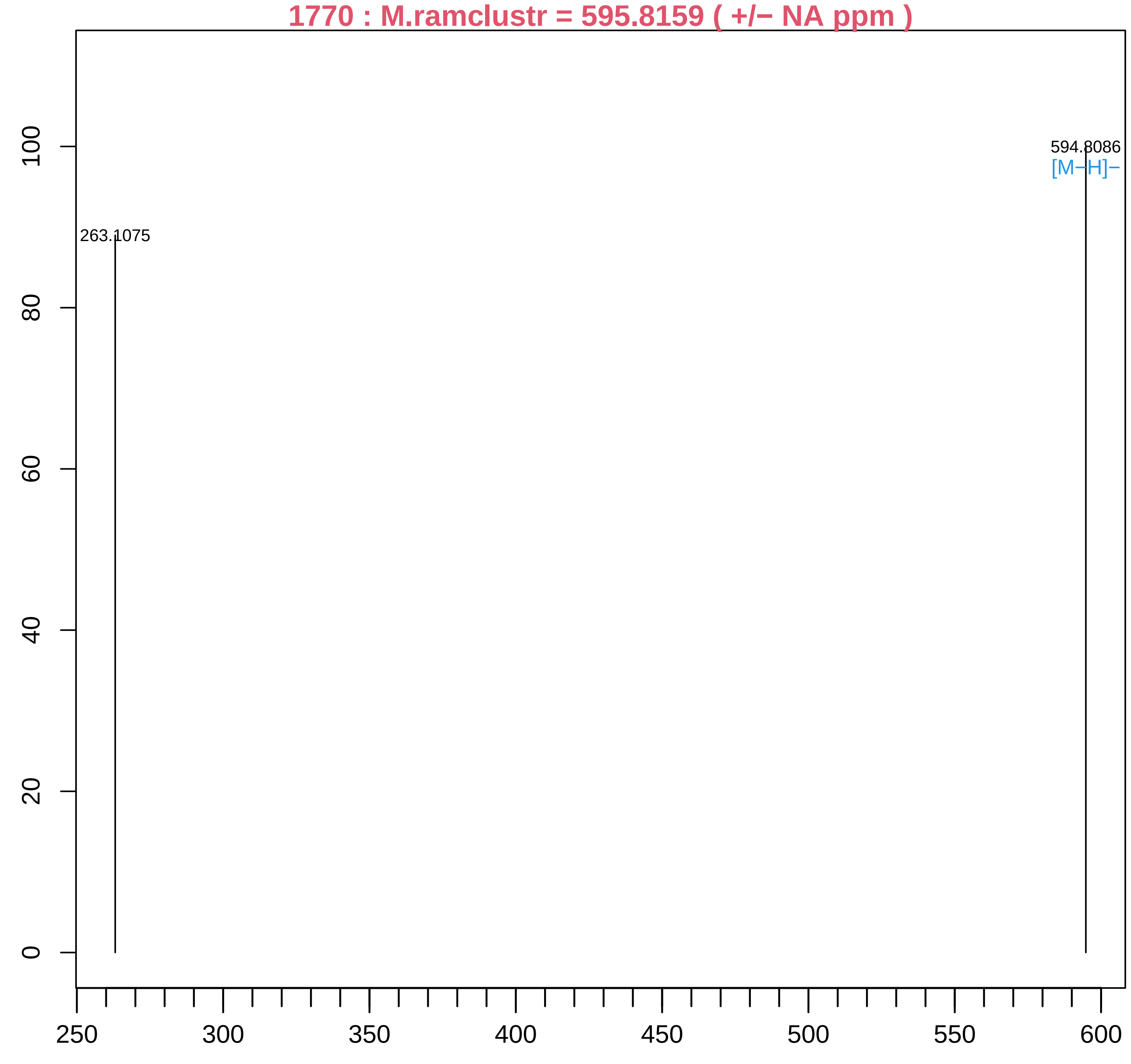
1770 : M.ramclustr = 595.8159 (+/− NA ppm)
594.8086
[M−H]−
263.1075
0
20
40
60
80
100
250
300
350
400
450
500
550
600

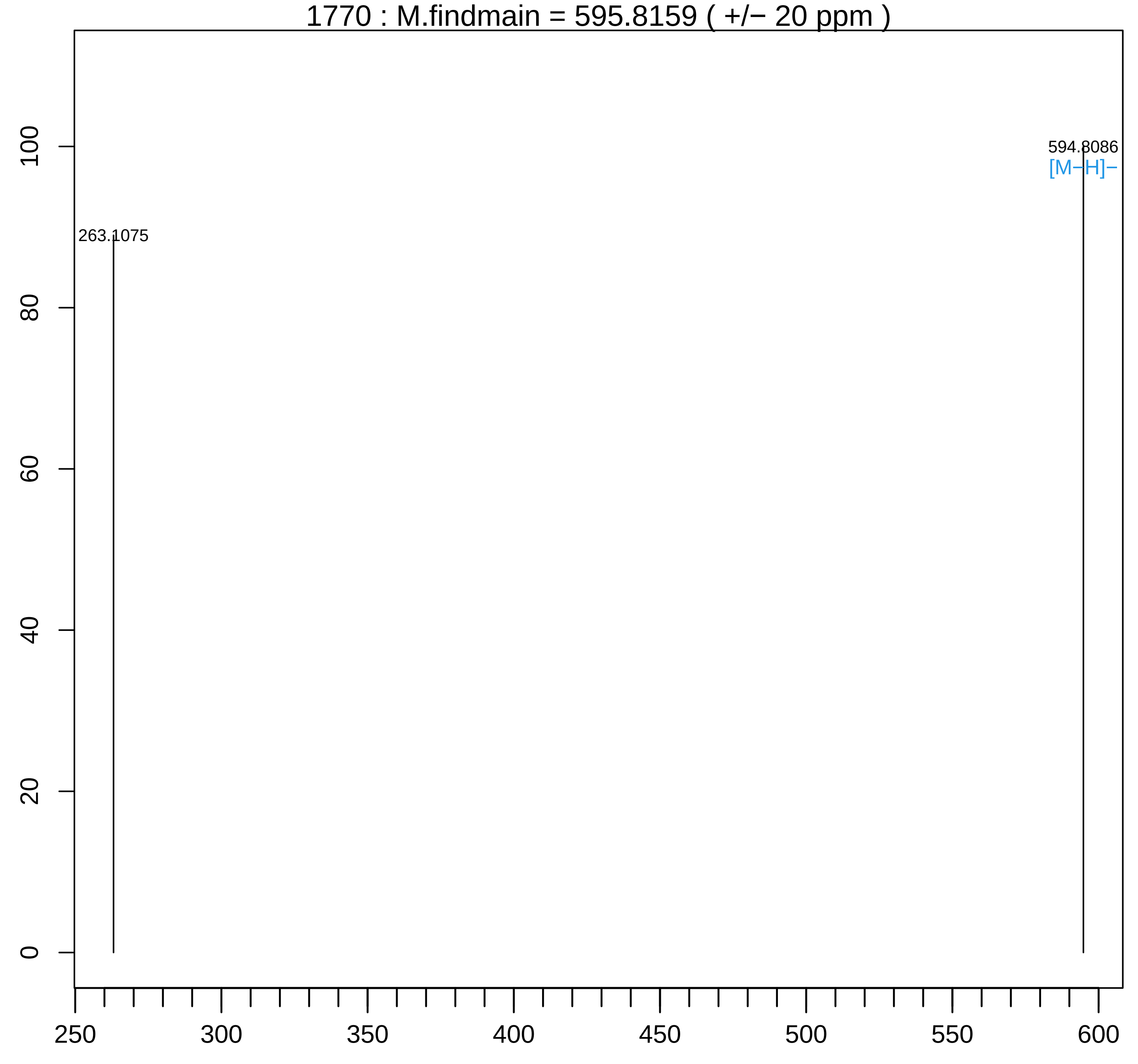
1770 : M.findmain = 595.8159 (+/− 20 ppm)
594.8086
[M−H]−
263.1075
0
20
40
60
80
100
250
300
350
400
450
500
550
600

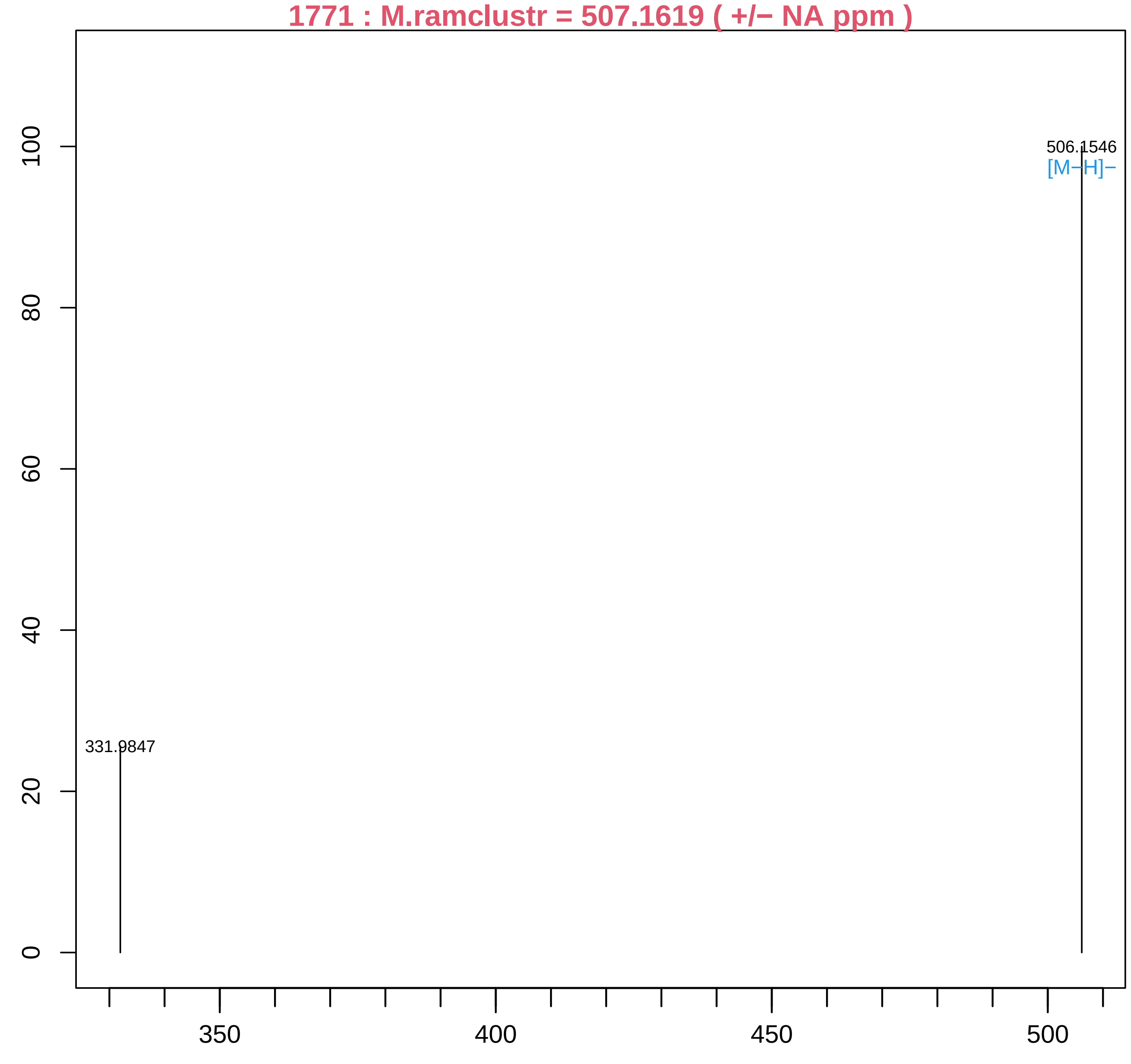
1771 : M.ramclustr = 507.1619 (+/− NA ppm)
506.1546
[M−H]−
331.9847
0
20
40
60
80
100
350
400
450
500

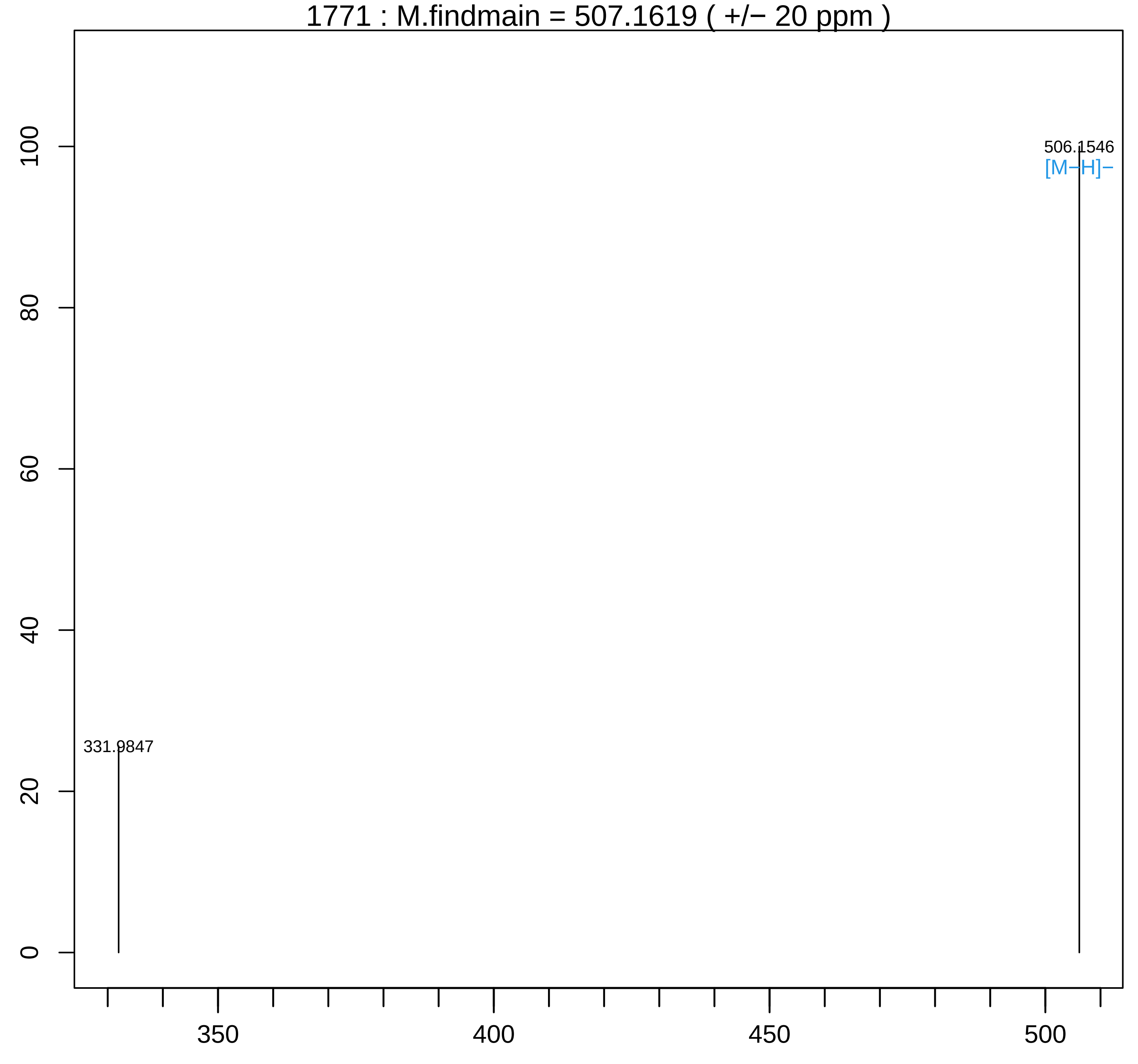
1771 : M.findmain = 507.1619 (+/− 20 ppm)
506.1546
[M−H]−
331.9847
0
20
40
60
80
100
350
400
450
500

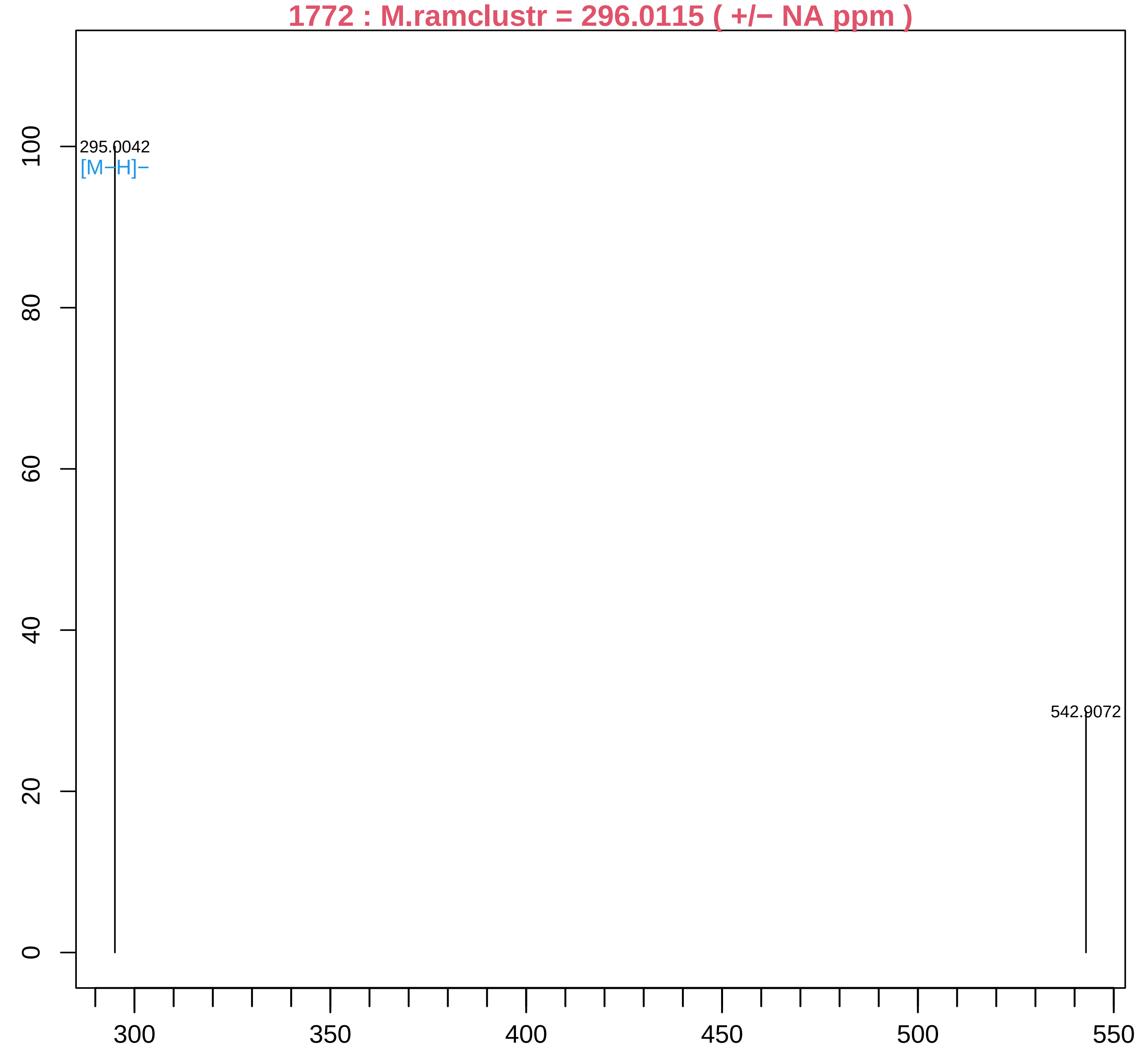
1772 : M.ramclustr = 296.0115 (+/− NA ppm)
295.0042
[M−H]−
542.9072
0
20
40
60
80
100
300
350
400
450
500
550

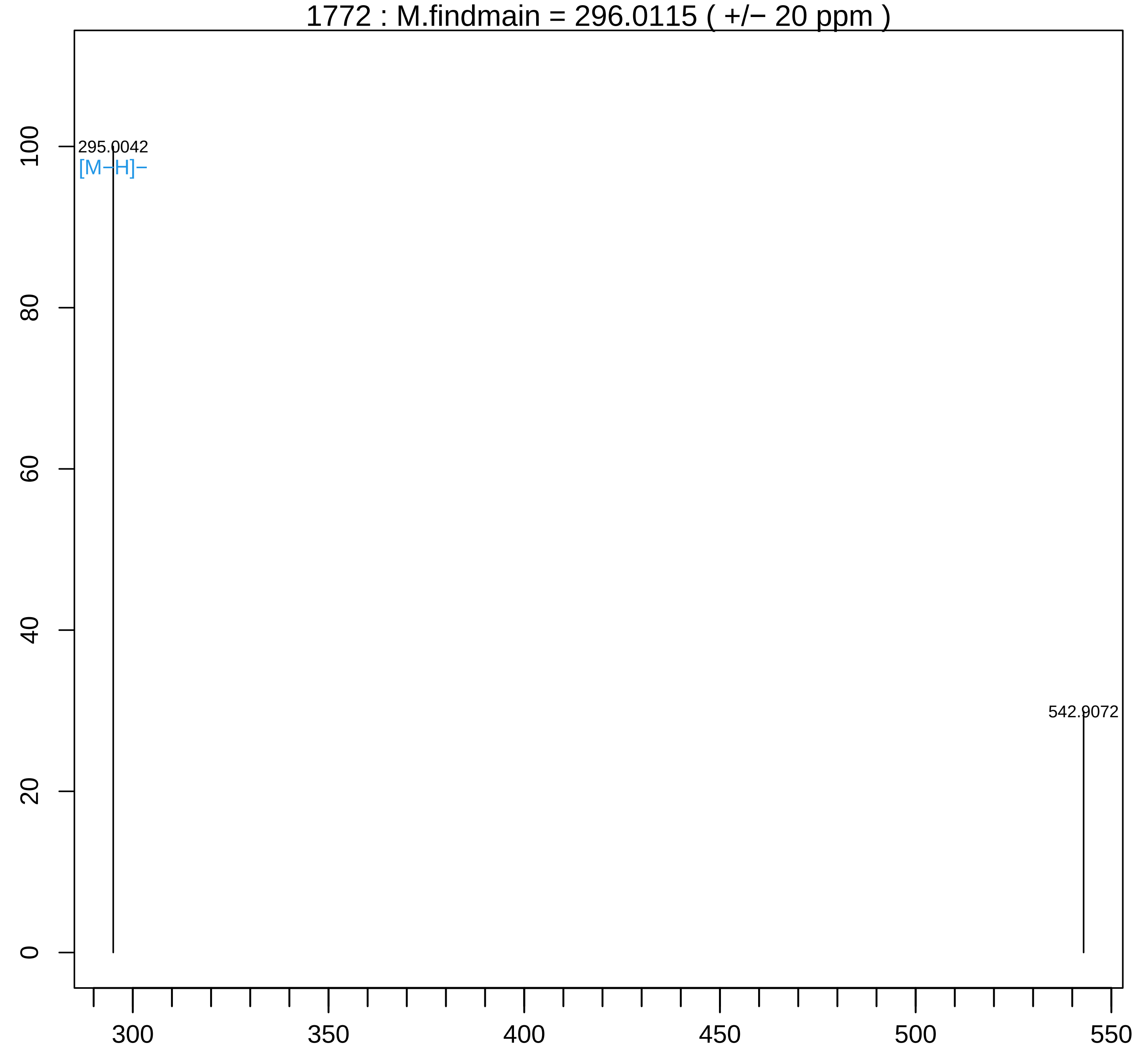
1772 : M.findmain = 296.0115 (+/− 20 ppm)
295.0042
[M−H]−
542.9072
0
20
40
60
80
100
300
350
400
450
500
550

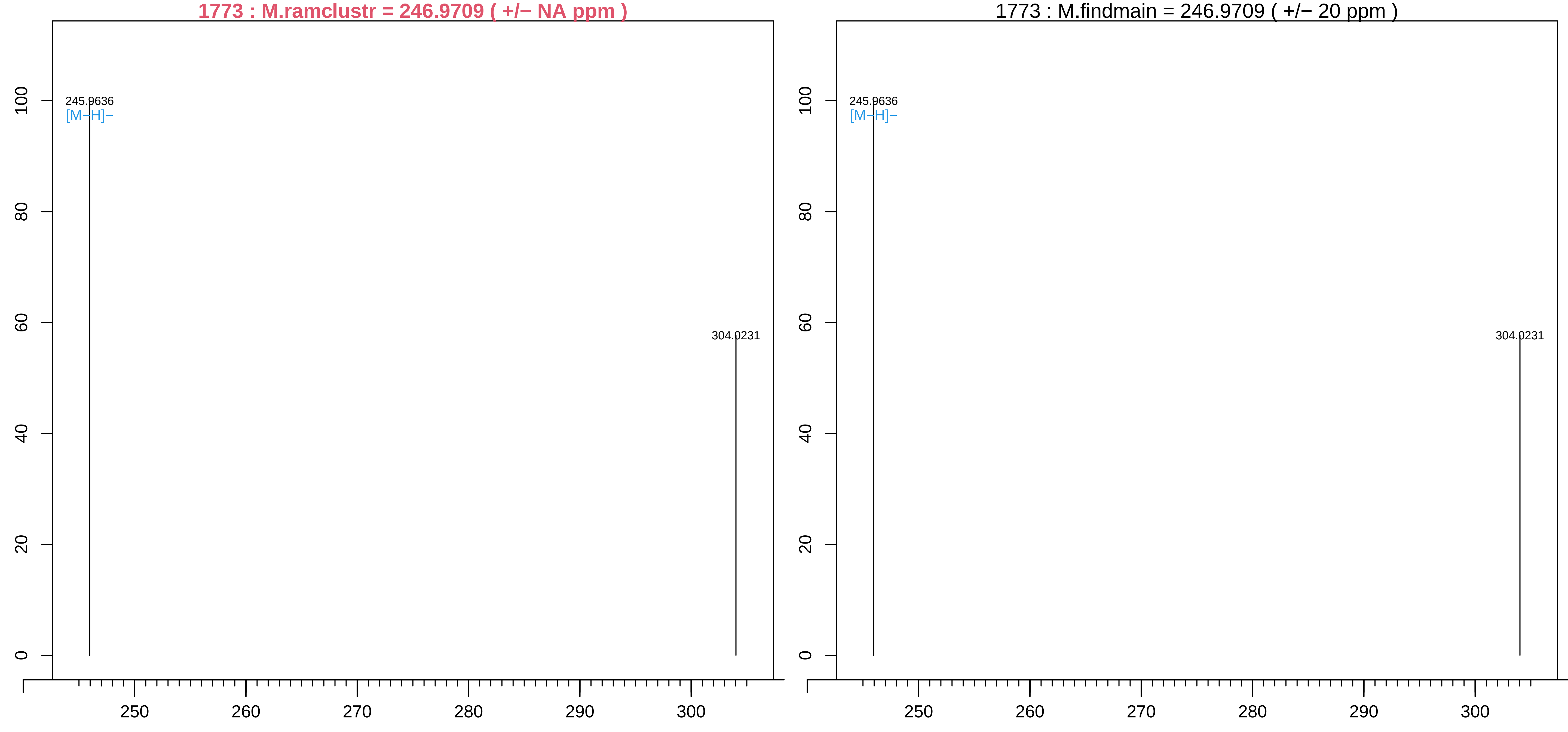
1773 : M.ramclustr = 246.9709 (+/− NA ppm)
245.9636
[M−H]−
304.0231
0
20
40
60
80
100
250
260
270
280
290
300
1773 : M.findmain = 246.9709 (+/− 20 ppm)
245.9636
[M−H]−
304.0231
0
20
40
60
80
100
250
260
270
280
290
300

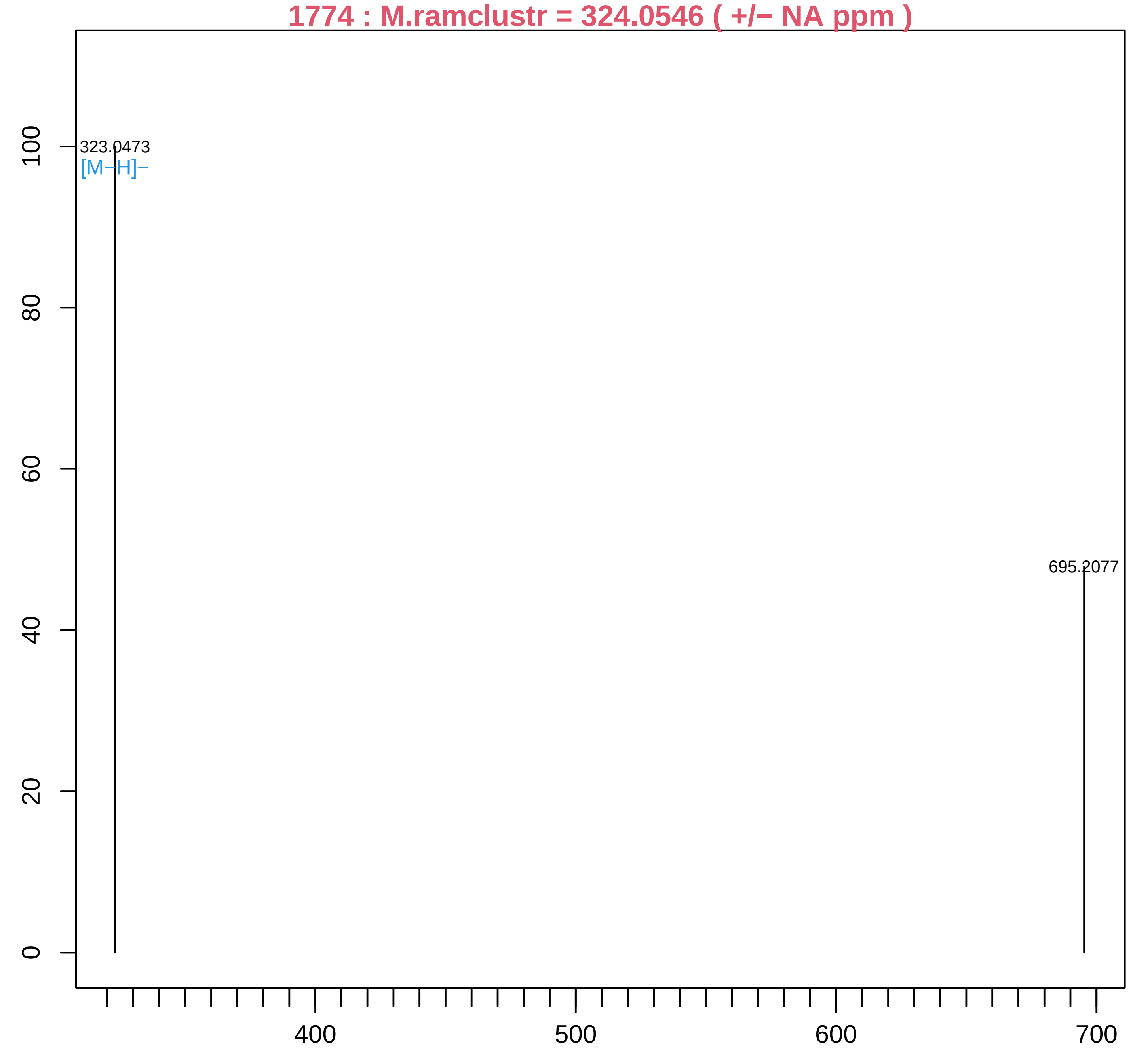
1774 : M.ramclustr = 324.0546 (+/− NA ppm)
323.0473
[M−H]−
695.2077
0
20
40
60
80
100
400
500
600
700

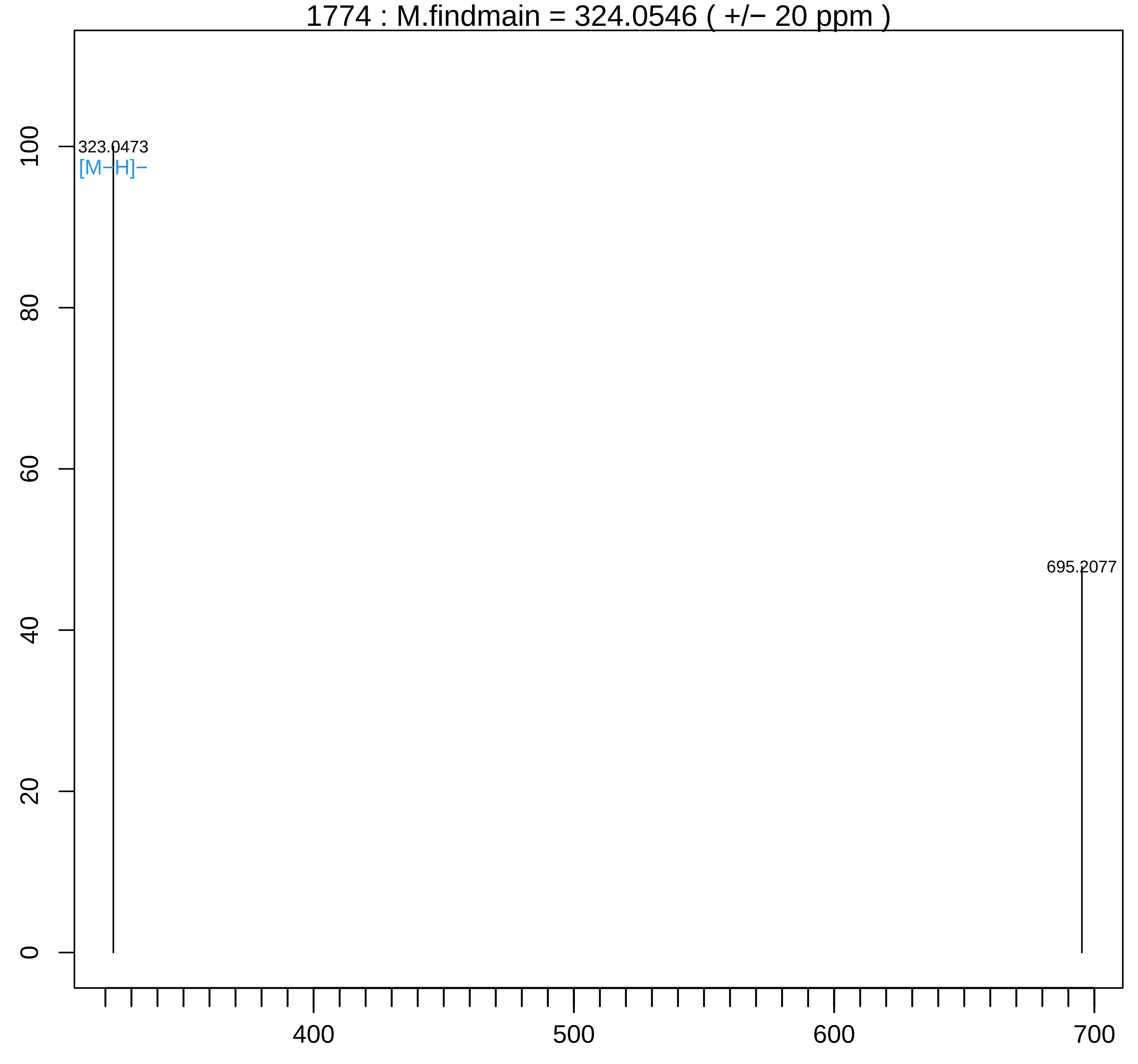
1774 : M.findmain = 324.0546 (+/− 20 ppm)
323.0473
[M−H]−
695.2077
0
20
40
60
80
100
400
500
600
700

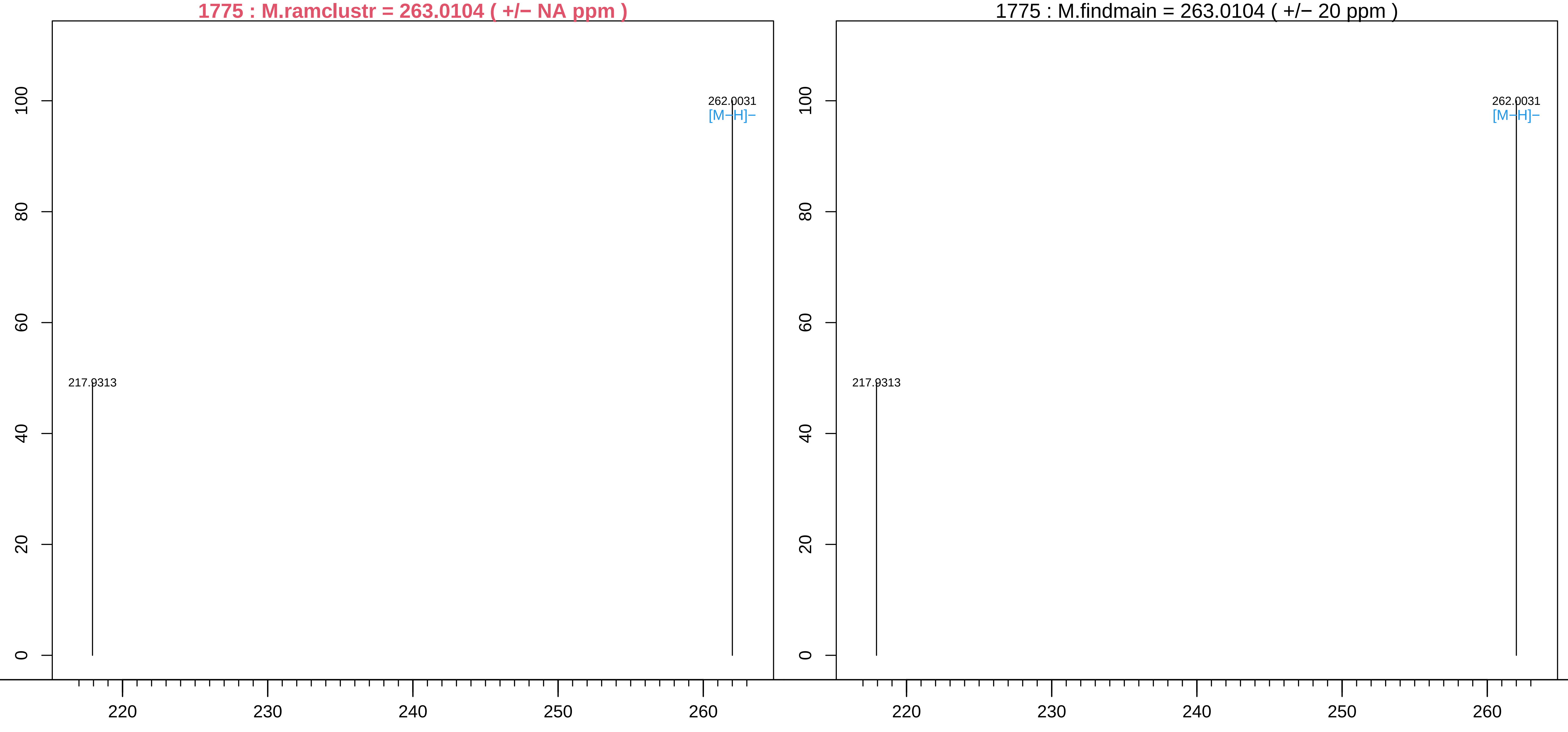
1775 : M.ramclustr = 263.0104 (+/− NA ppm)
262.0031
[M−H]−
217.9313
1775 : M.findmain = 263.0104 (+/− 20 ppm)
262.0031
[M−H]−
217.9313
0
20
40
60
80
100
220
230
240
250
260

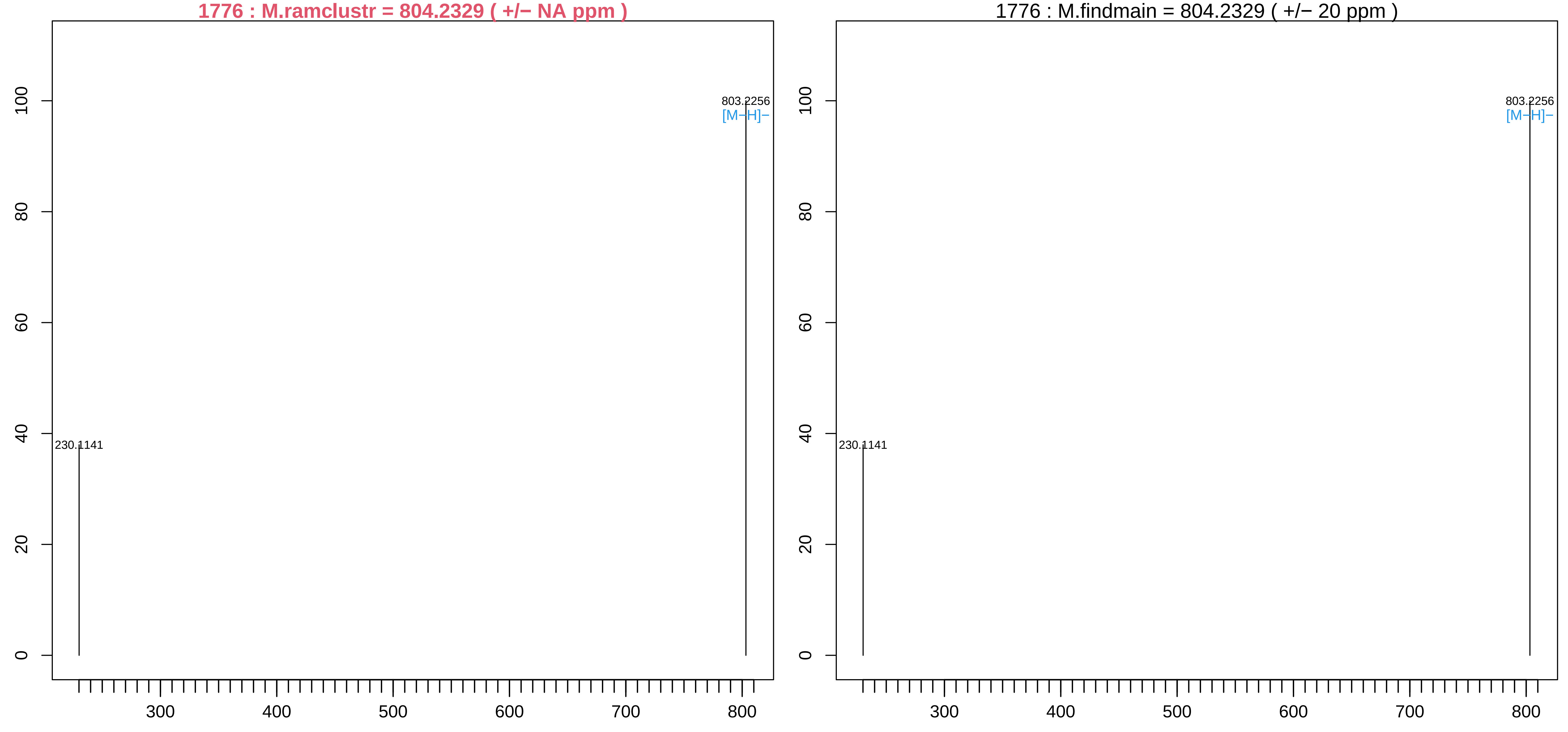
1776 : M.ramclustr = 804.2329 (+/− NA ppm)
803.2256
[M−H]−
230.1141
1776 : M.findmain = 804.2329 (+/− 20 ppm)
803.2256
[M−H]−
230.1141
0
20
40
60
80
100
300
400
500
600
700
800

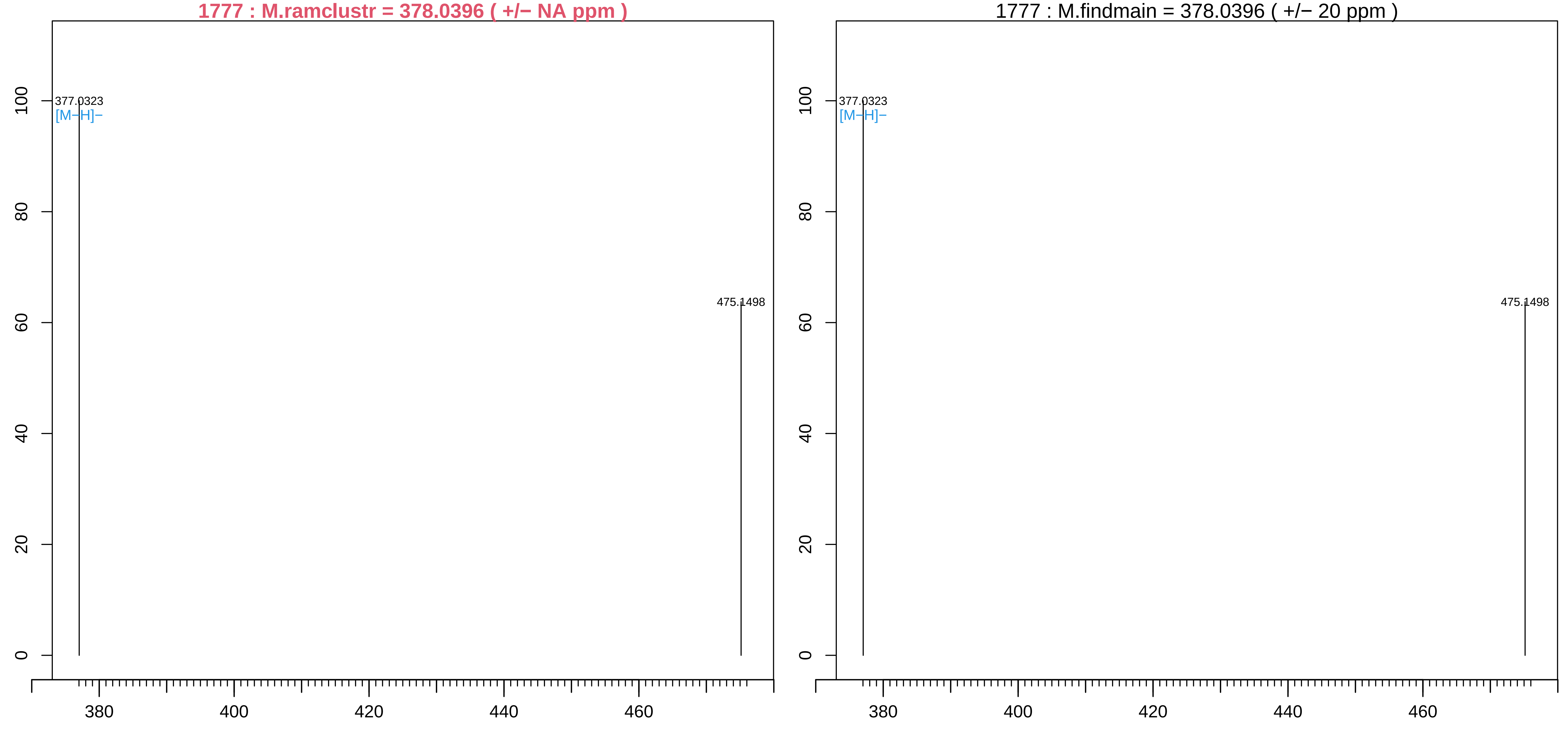
1777 : M.ramclustr = 378.0396 (+/− NA ppm)
377.0323
[M−H]−
475.1498
1777 : M.findmain = 378.0396 (+/− 20 ppm)
377.0323
[M−H]−
475.1498
0
20
40
60
80
100
380
400
420
440
460

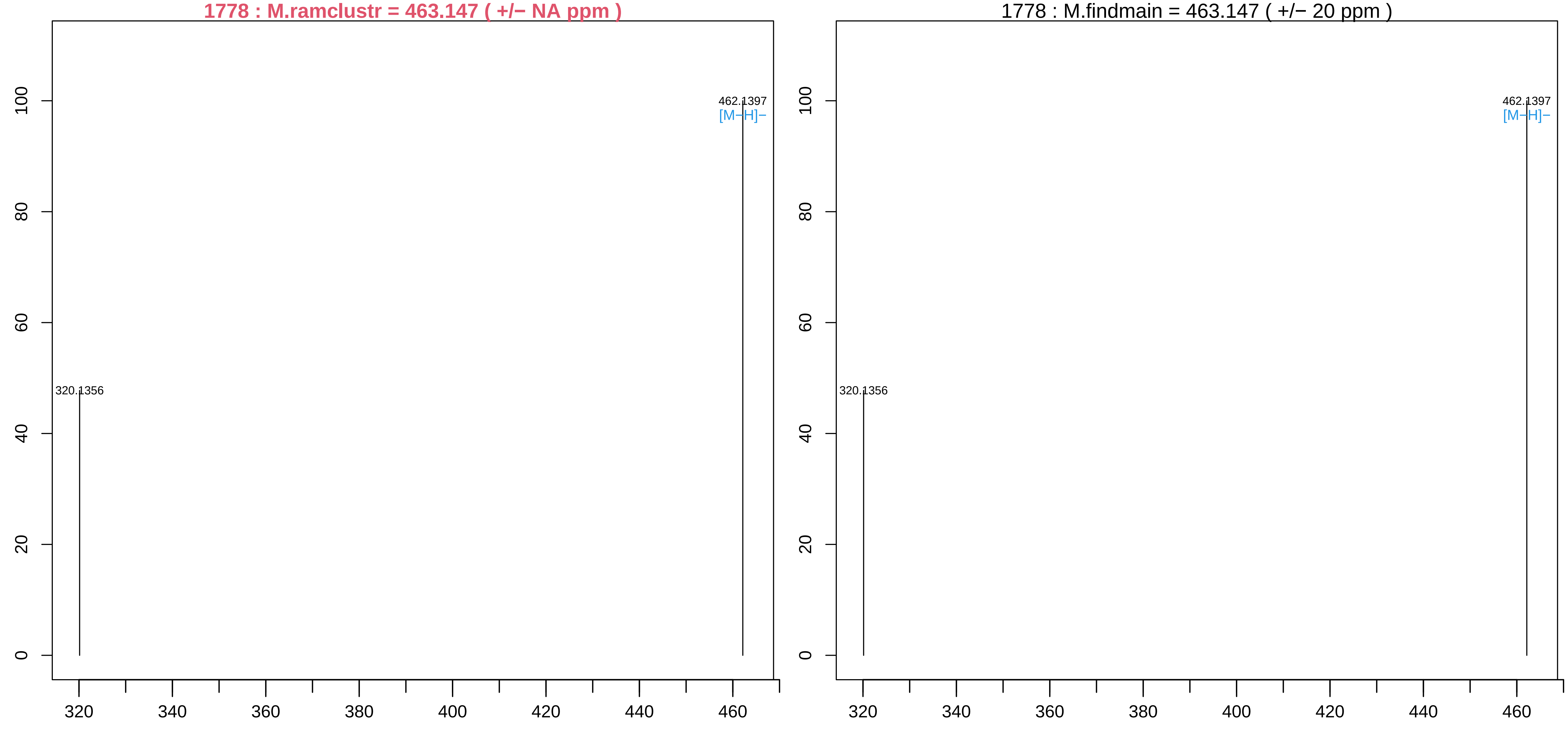
1778 : M.ramclustr = 463.147 (+/− NA ppm)
1778 : M.findmain = 463.147 (+/− 20 ppm)
462.1397
[M−H]−
320.1356
0
20
40
60
80
100
320
340
360
380
400
420
440
460

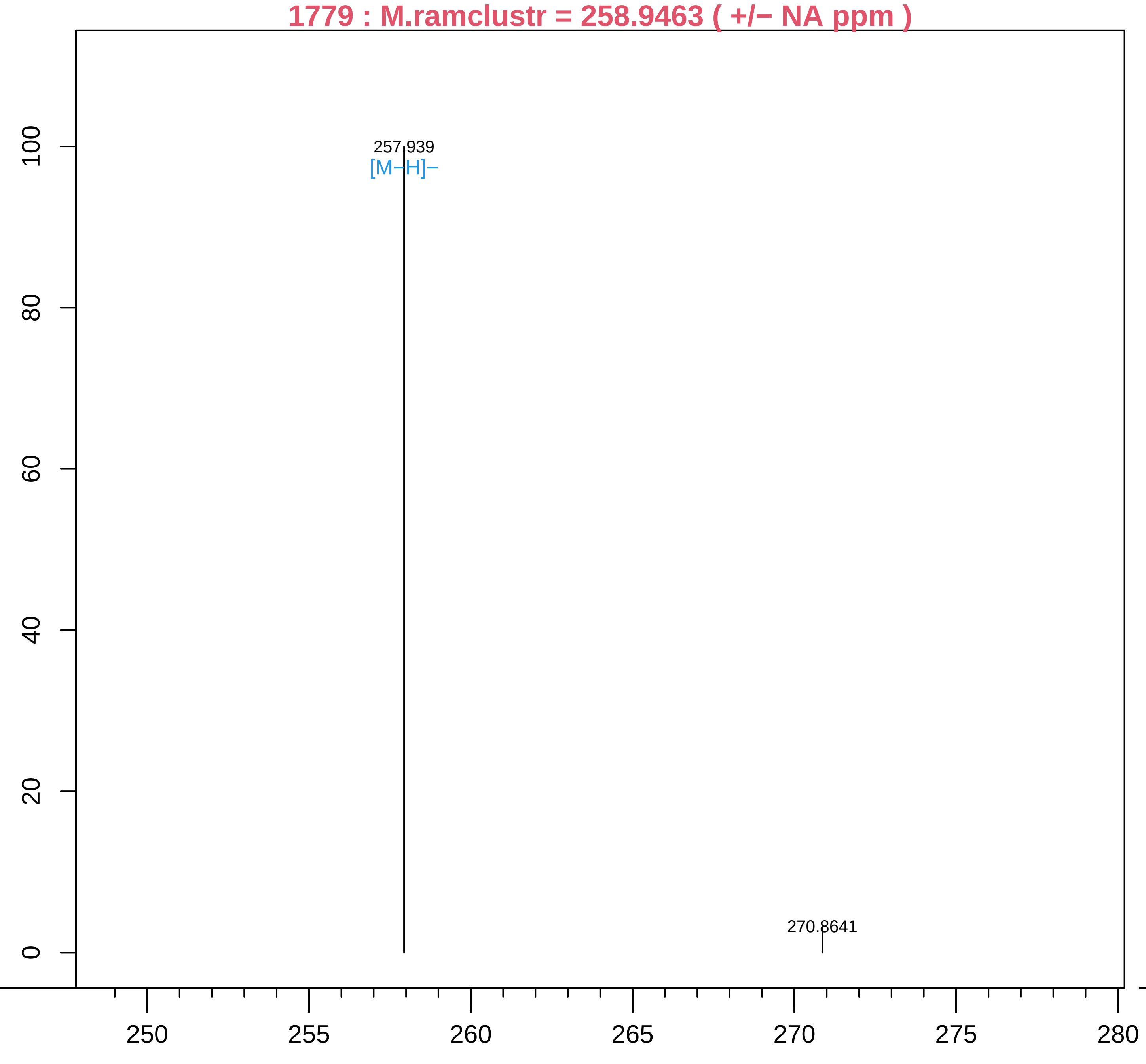

1779 : M.ramclustr = 258.9463 (+/− NA ppm)
257.939
[M−H]−
270.8641
0
20
40
60
80
100
250
255
260
265
270
275
280

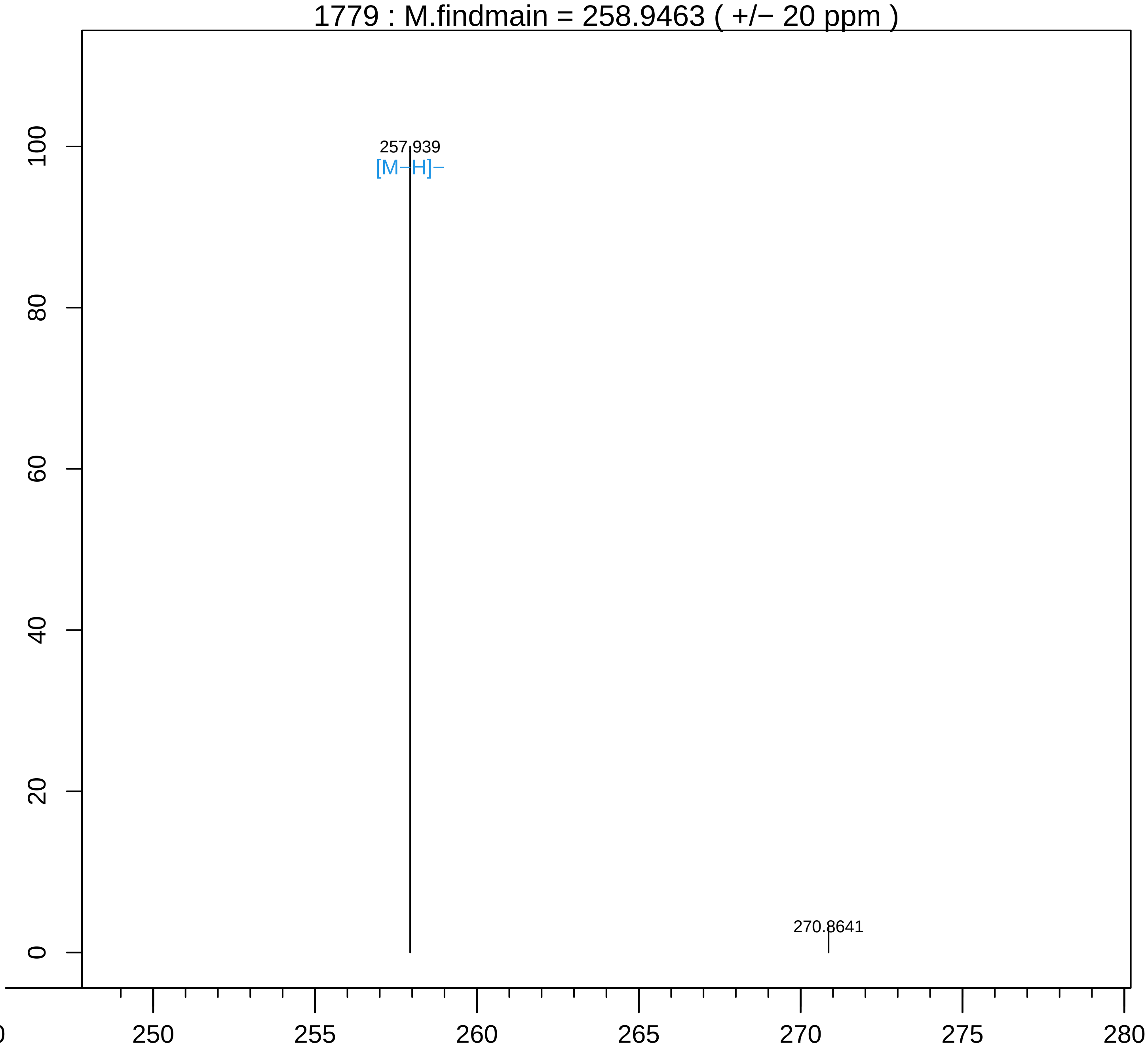

1779 : M.findmain = 258.9463 (+/− 20 ppm)
257.939
[M−H]−
270.8641
0
20
40
60
80
100
250
255
260
265
270
275
280

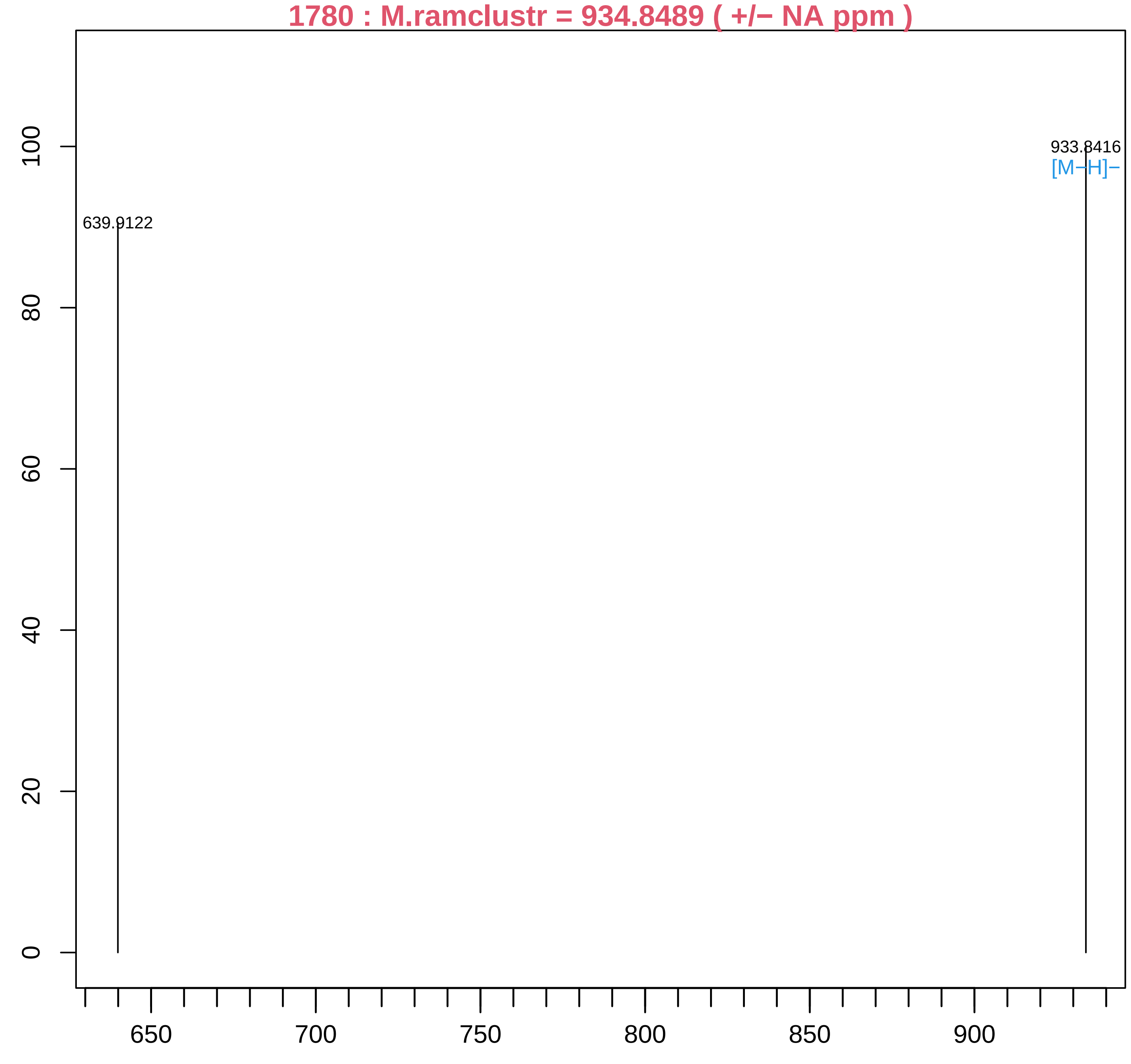
1780 : M.ramclustr = 934.8489 (+/− NA ppm)
933.8416
[M−H]−
639.9122
0
20
40
60
80
100
650
700
750
800
850
900

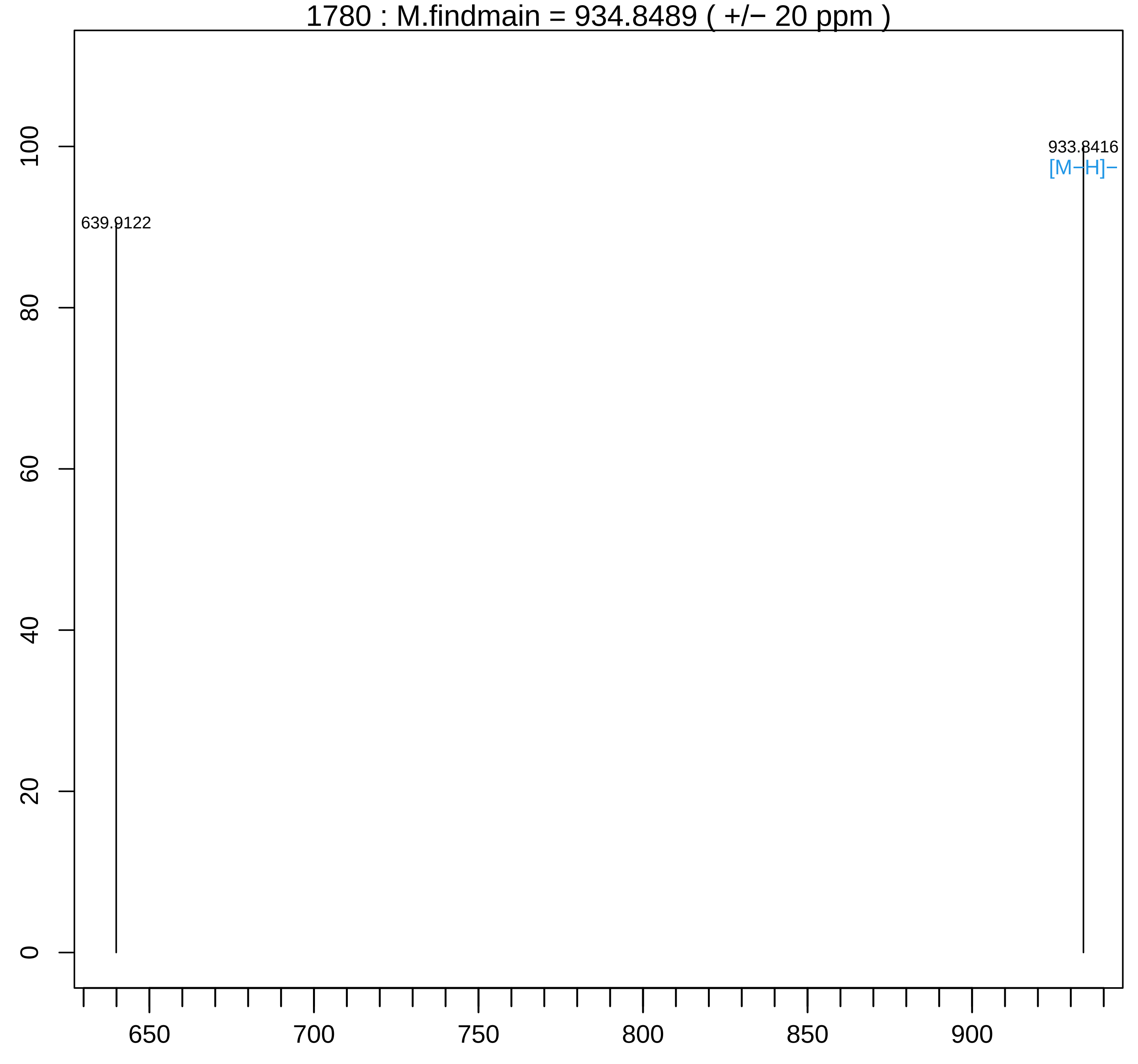
1780 : M.findmain = 934.8489 (+/− 20 ppm)
933.8416
[M−H]−
639.9122
0
20
40
60
80
100
650
700
750
800
850
900

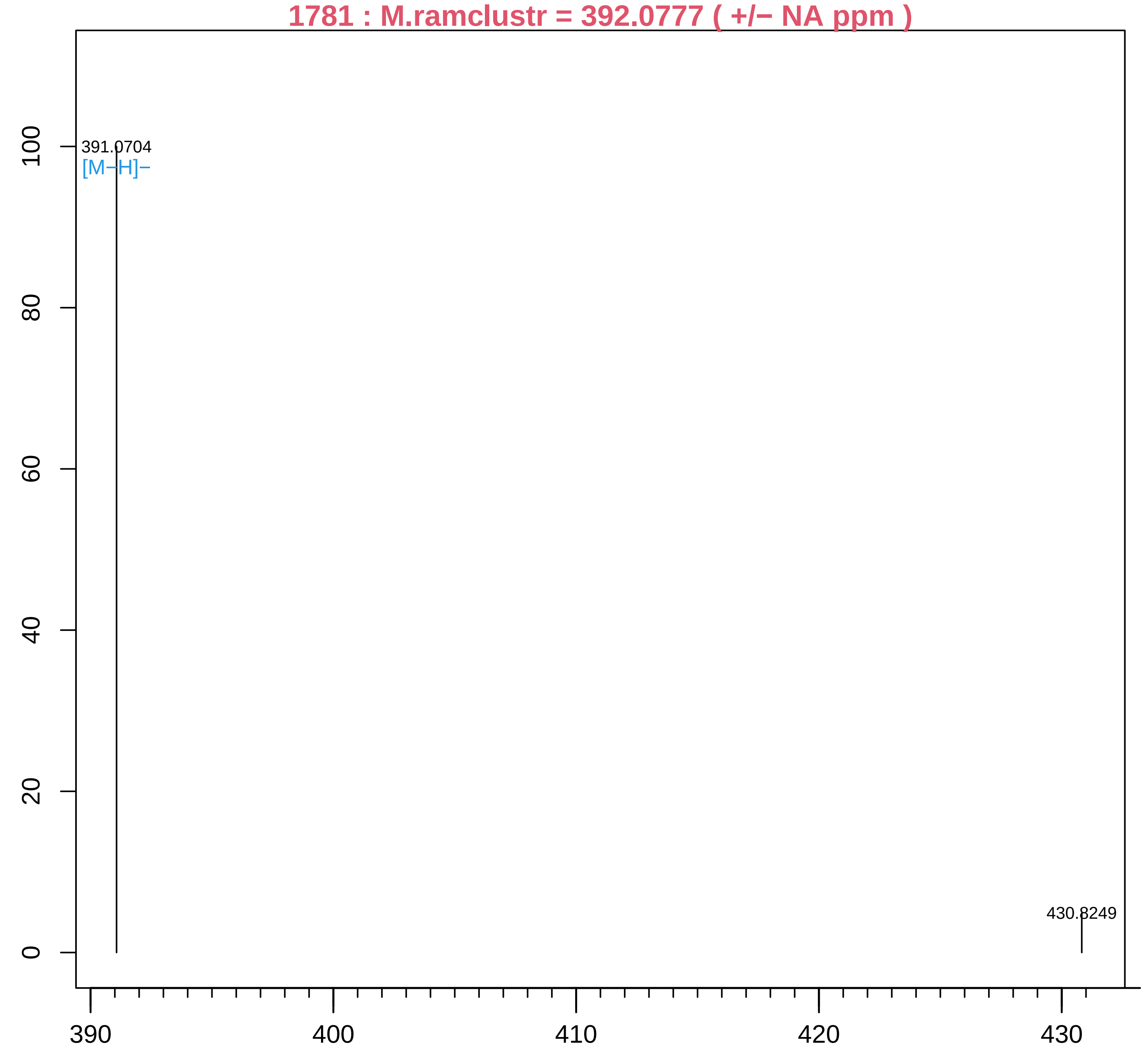

1781 : M.ramclustr = 392.0777 (+/− NA ppm)
391.0704
[M−H]−
430.8249
0
20
40
60
80
100
390
400
410
420
430

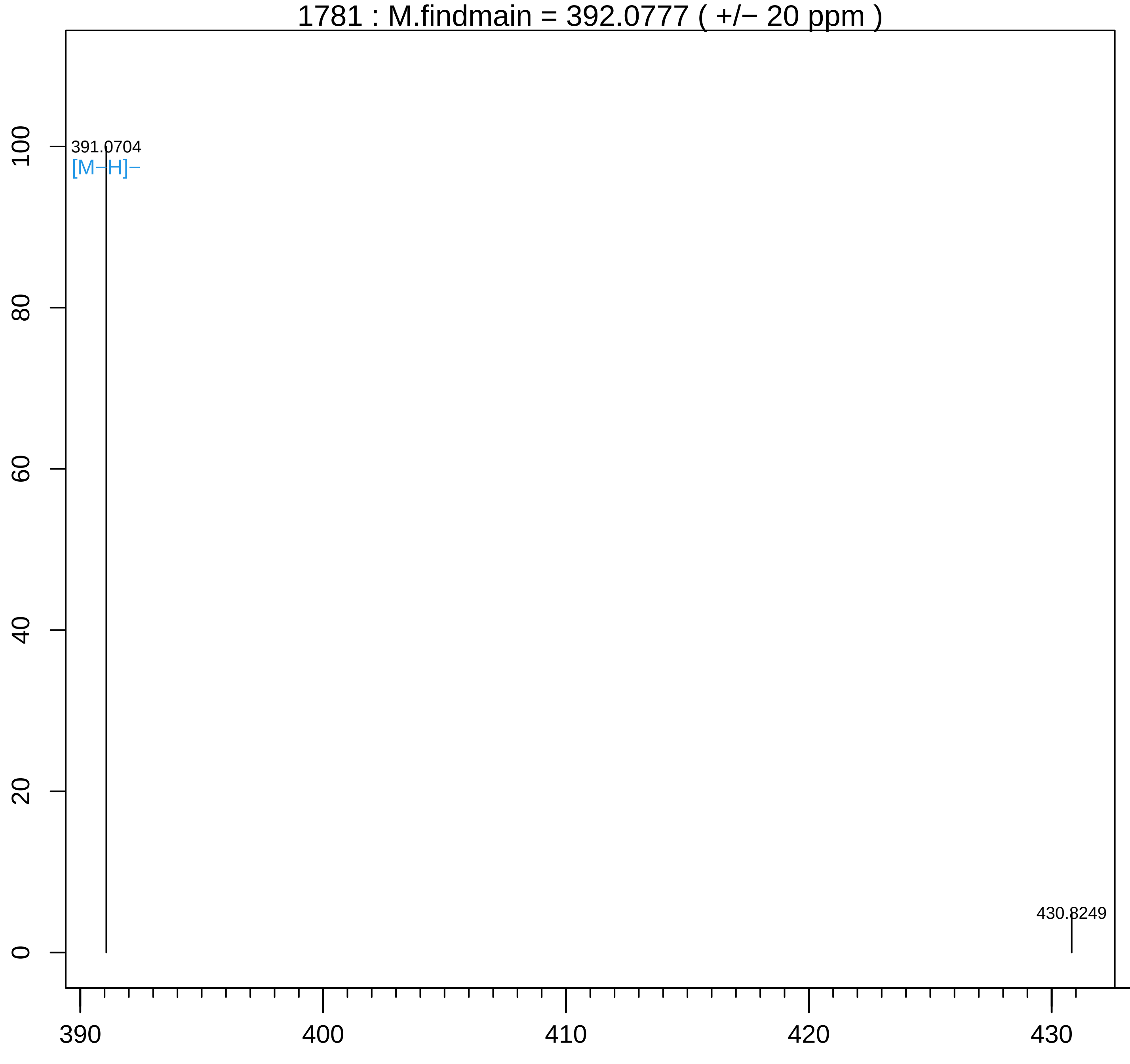

1781 : M.findmain = 392.0777 (+/− 20 ppm)
391.0704
[M−H]−
430.8249
0
20
40
60
80
100
390
400
410
420
430

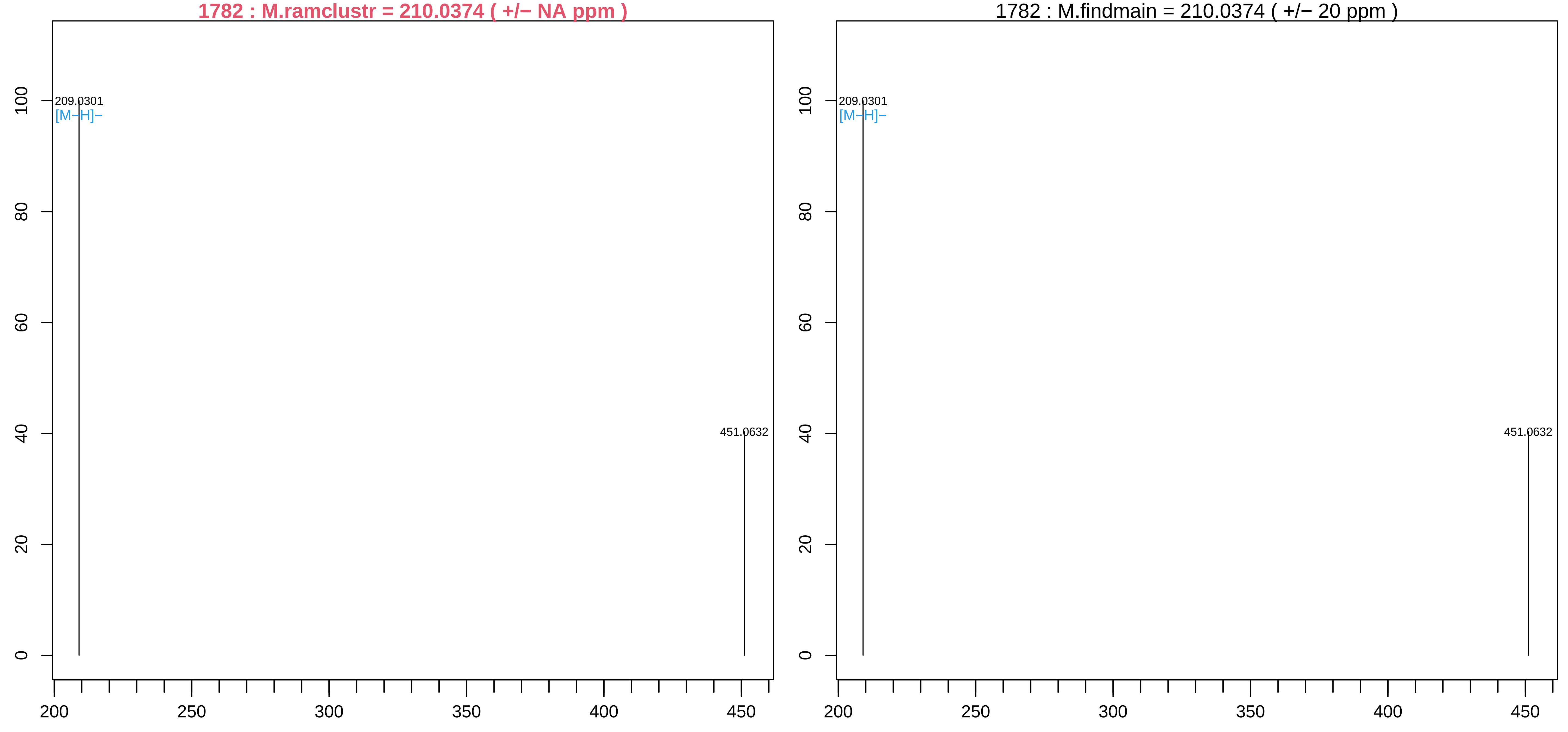
1782 : M.ramclustr = 210.0374 (+/− NA ppm)
209.0301
[M−H]−
451.0632
1782 : M.findmain = 210.0374 (+/− 20 ppm)
209.0301
[M−H]−
451.0632

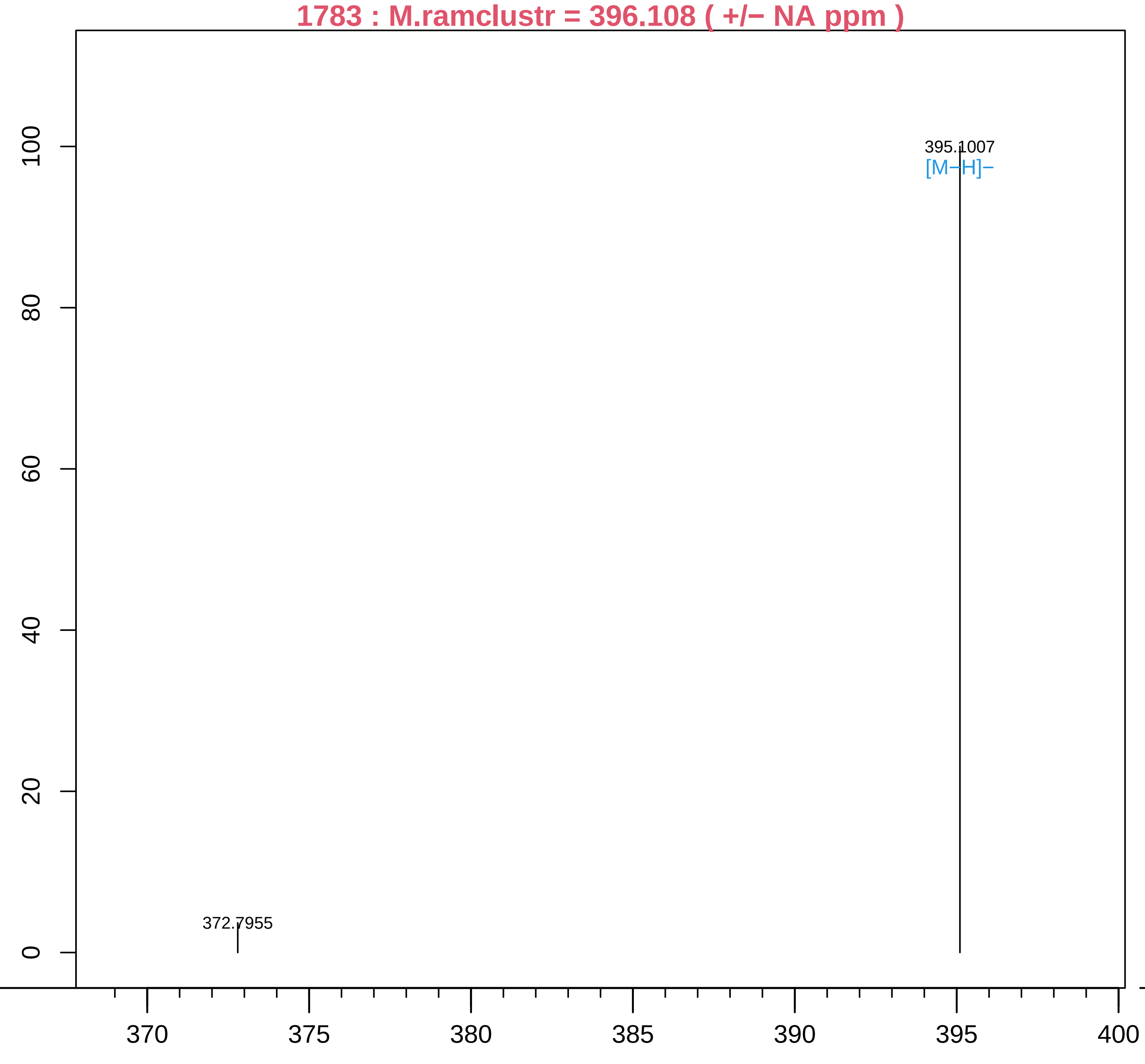

1783 : M.ramclustr = 396.108 (+/− NA ppm)
395.1007
[M−H]−
372.7955
0
20
40
60
80
100
370
375
380
385
390
395
400

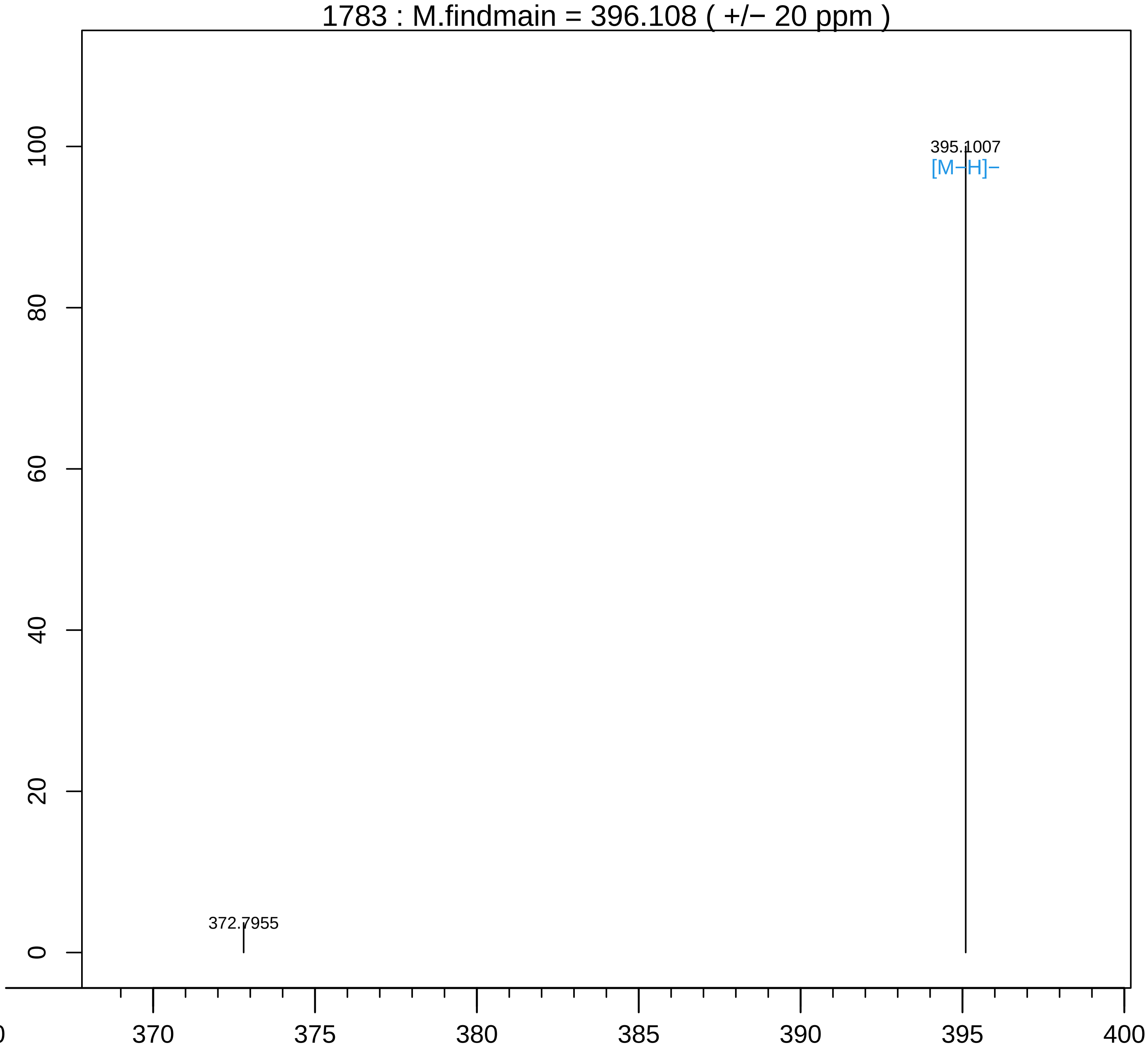

1783 : M.findmain = 396.108 (+/− 20 ppm)
395.1007
[M−H]−
372.7955
0
20
40
60
80
100
370
375
380
385
390
395
400

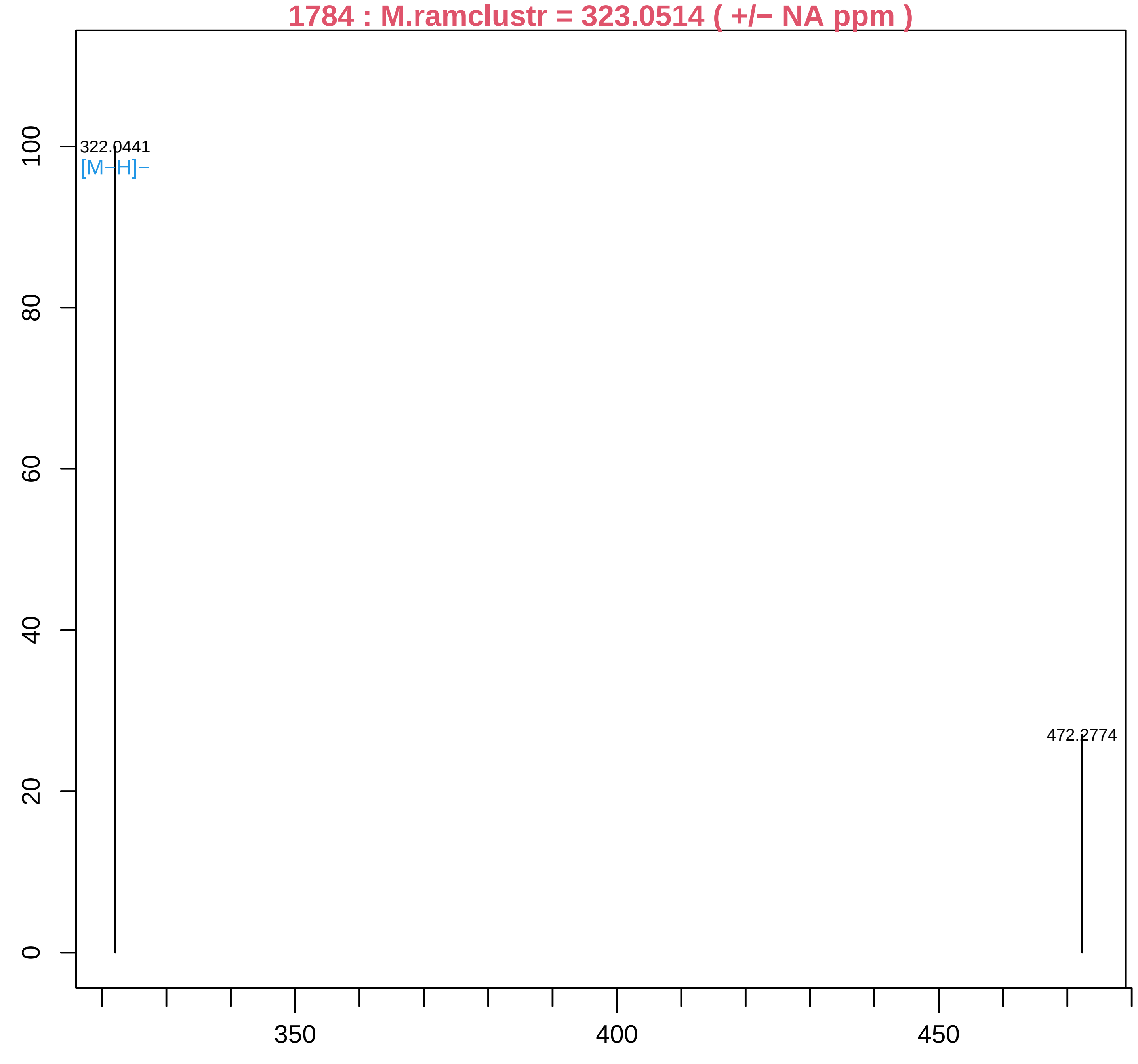
1784 : M.ramclustr = 323.0514 (+/− NA ppm)
322.0441
[M−H]−
472.2774
0
20
40
60
80
100
350
400
450

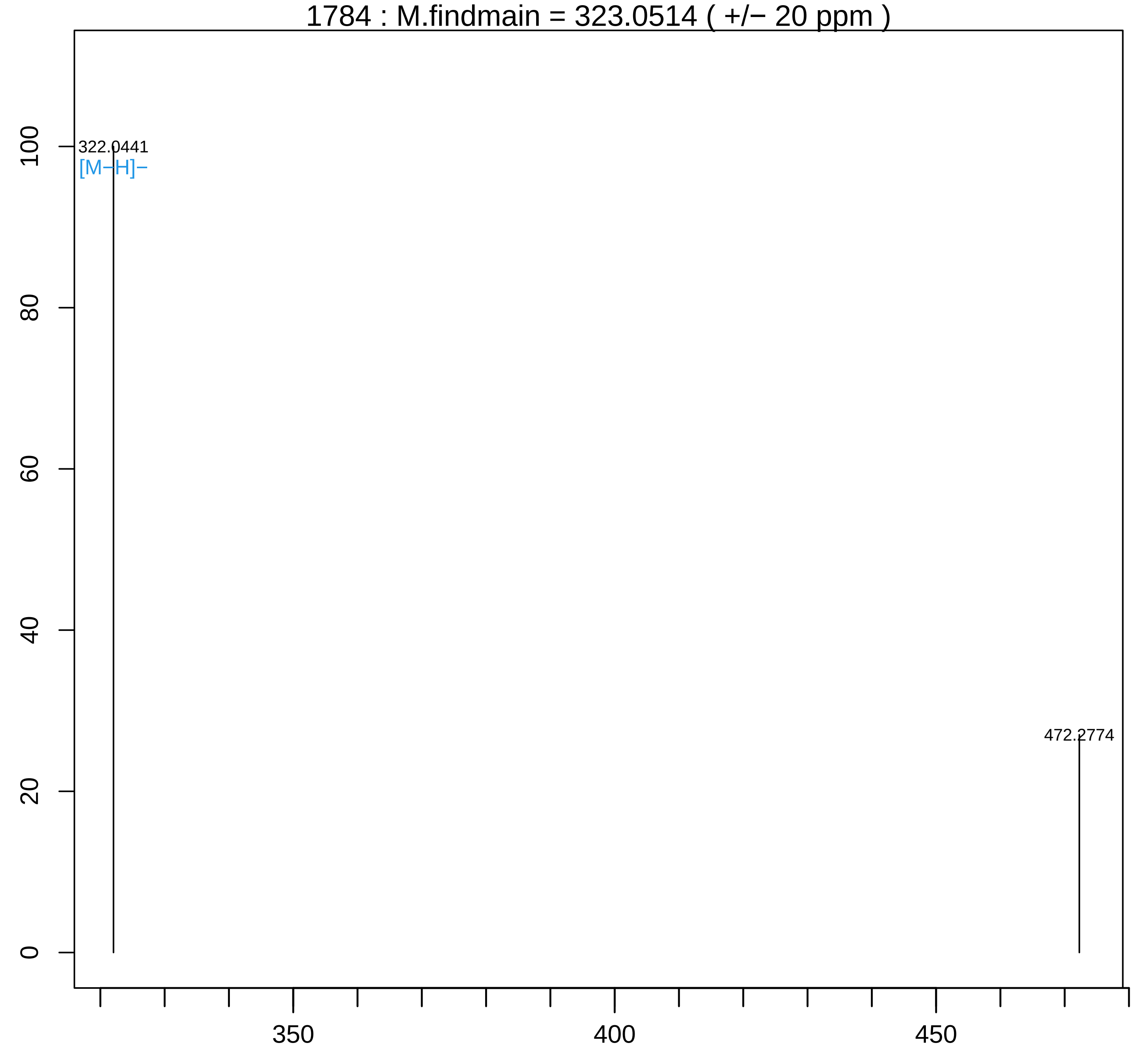
1784 : M.findmain = 323.0514 (+/− 20 ppm)
322.0441
[M−H]−
472.2774
0
20
40
60
80
100
350
400
450

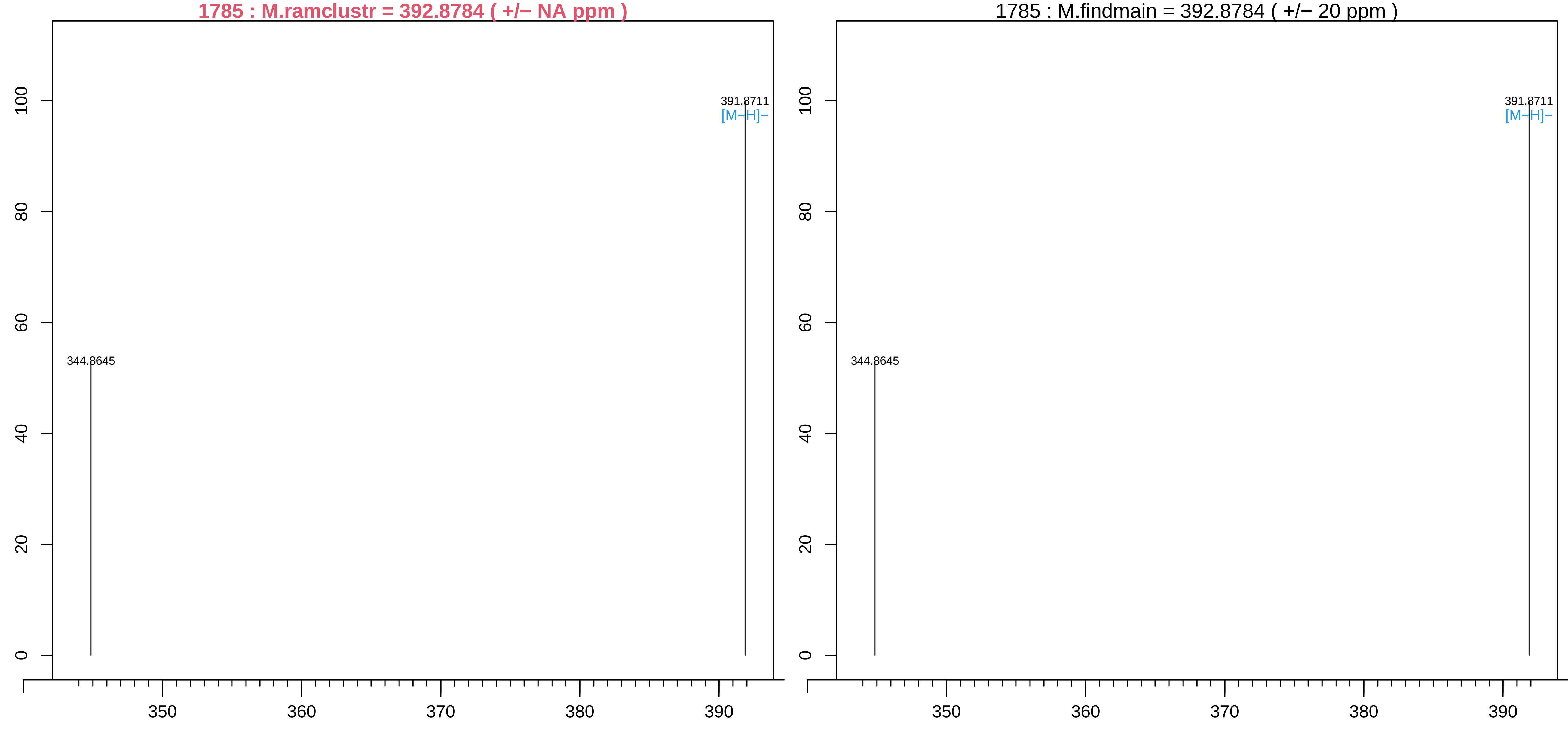
1785 : M.ramclustr = 392.8784 (+/− NA ppm)
344.8645
391.8711
[M−H]−
1785 : M.findmain = 392.8784 (+/− 20 ppm)
344.8645
391.8711
[M−H]−
0
20
40
60
80
100
350
360
370
380
390

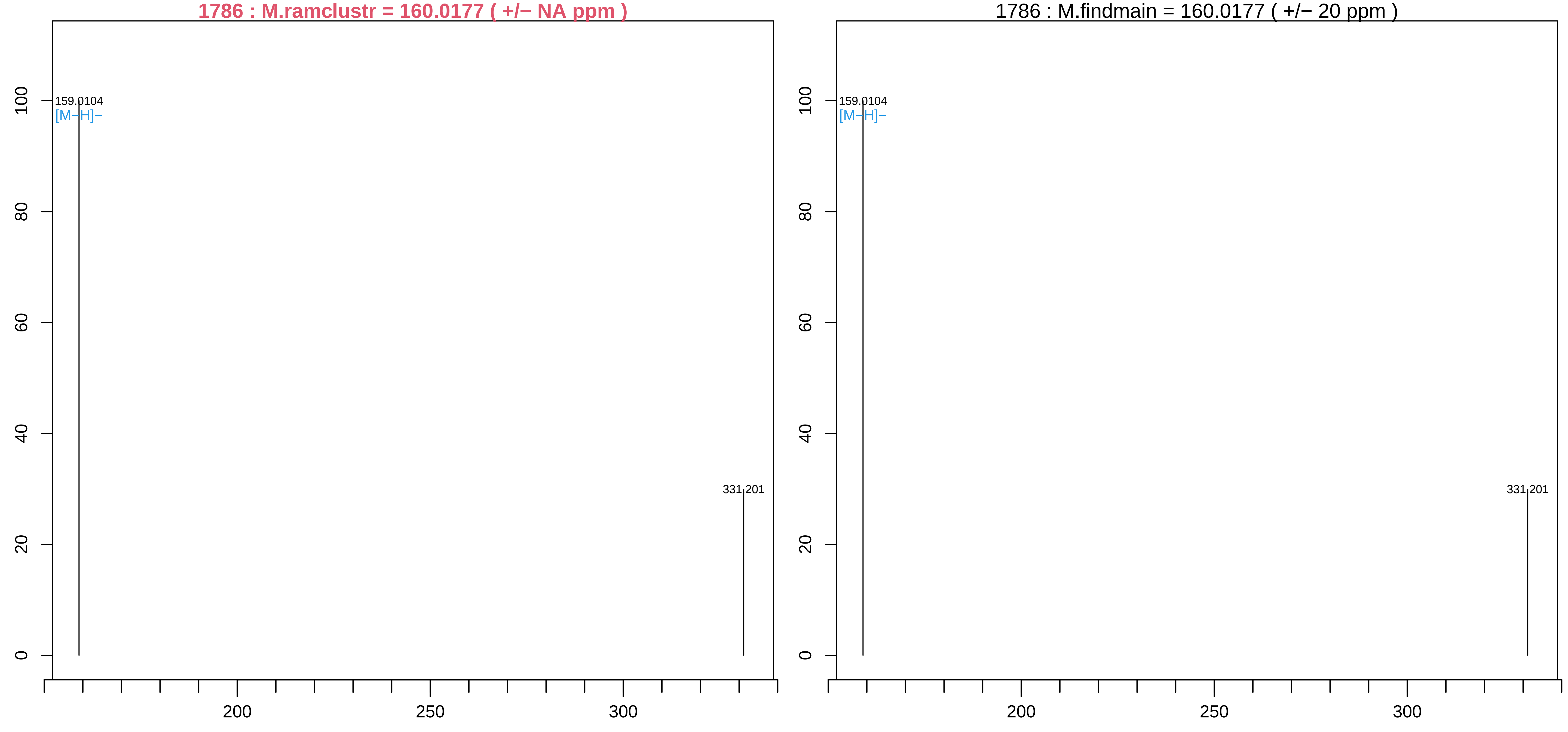
1786 : M.ramclustr = 160.0177 (+/− NA ppm)
159.0104
[M−H]−
331.201
0
20
40
60
80
100
200
250
300
1786 : M.findmain = 160.0177 (+/− 20 ppm)
159.0104
[M−H]−
331.201
0
20
40
60
80
100
200
250
300

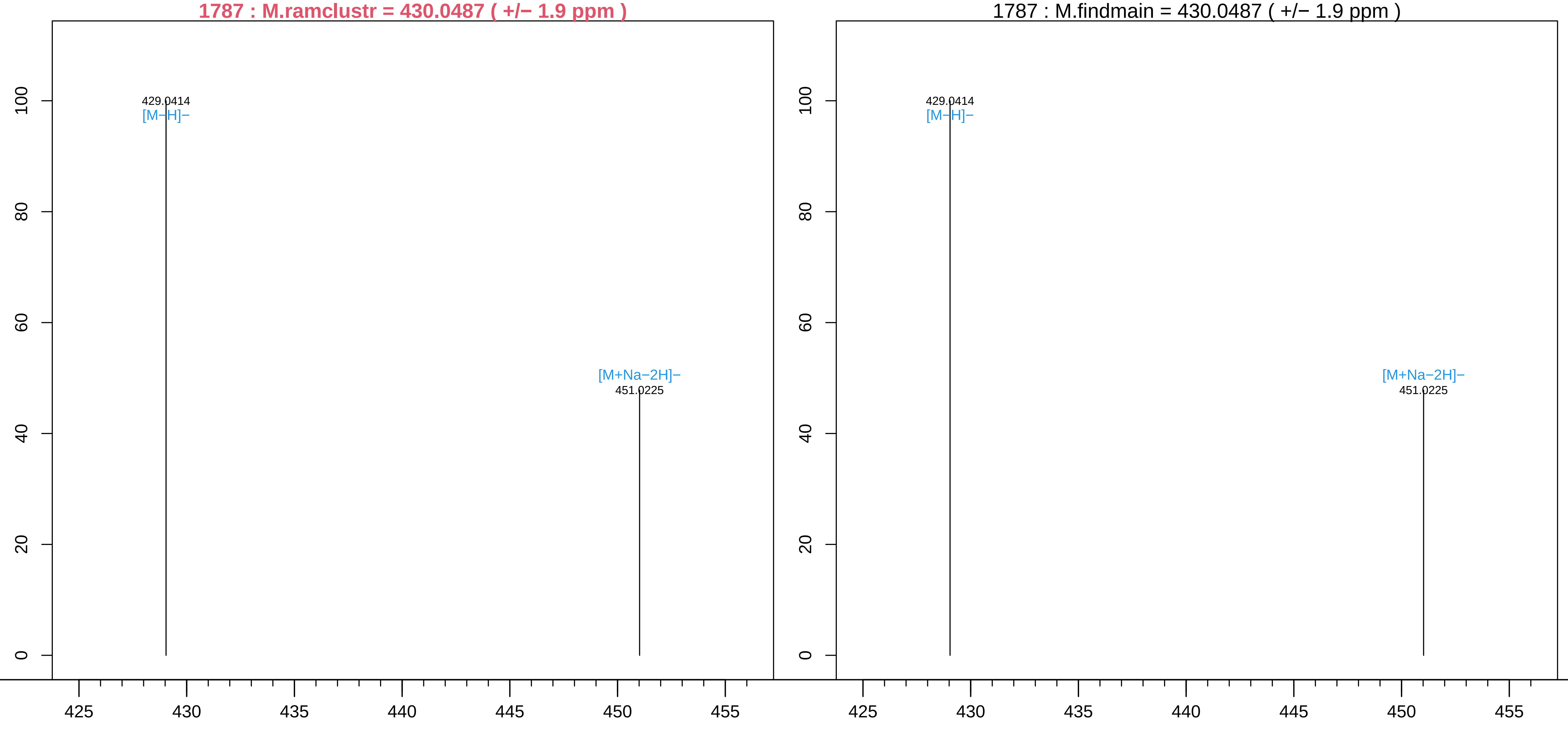
1787 : M.ramclustr = 430.0487 (+/− 1.9 ppm)
429.0414
[M−H]−
[M+Na−2H]−
451.0225
1787 : M.findmain = 430.0487 (+/− 1.9 ppm)
429.0414
[M−H]−
[M+Na−2H]−
451.0225
0
20
40
60
80
100
425
430
435
440
445
450
455

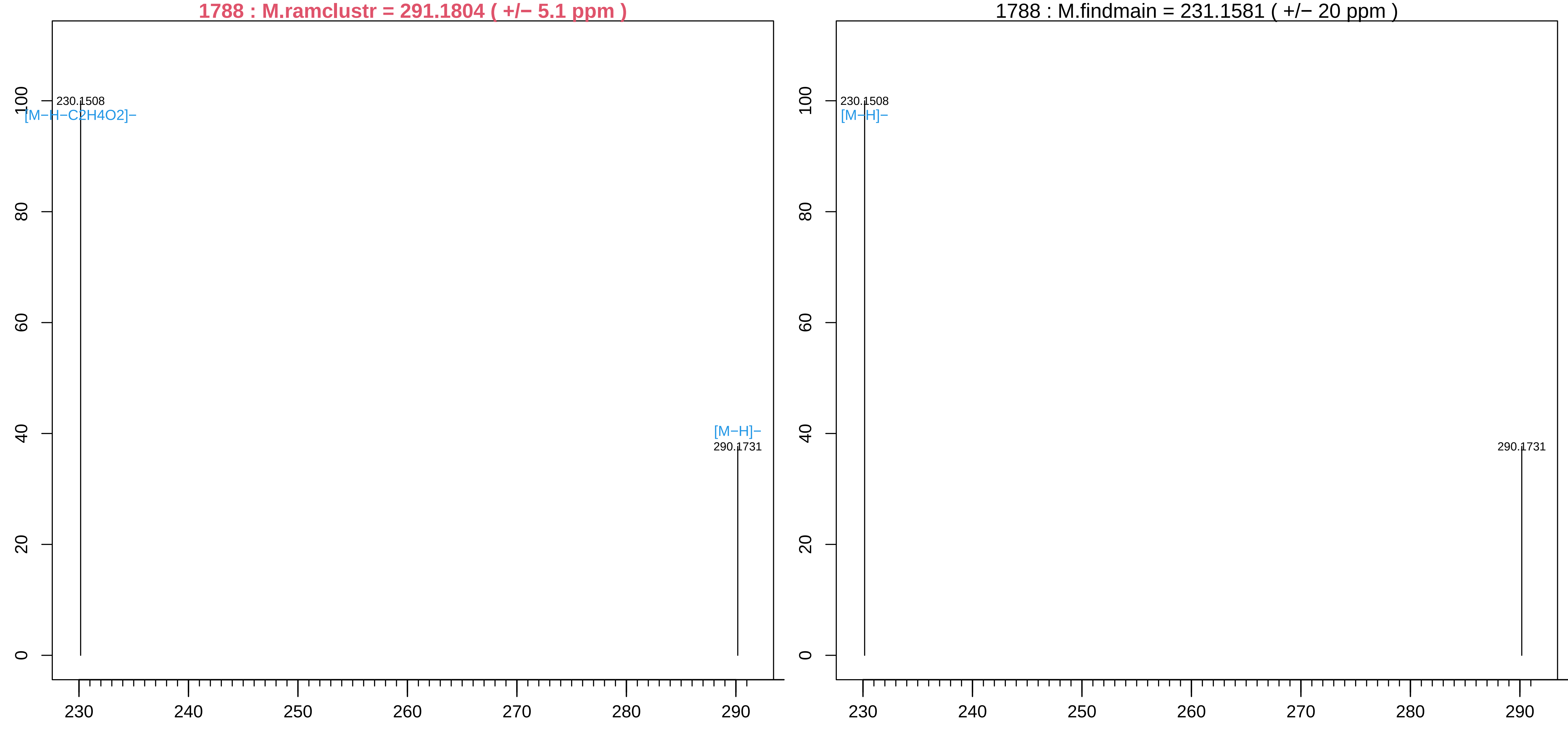
1788 : M.ramclustr = 291.1804 (+/− 5.1 ppm)
230.1508
[M−H−C2H4O2]−
[M−H]−
290.1731
1788 : M.findmain = 231.1581 (+/− 20 ppm)
230.1508
[M−H]−
290.1731
0
20
40
60
80
100
230
240
250
260
270
280
290

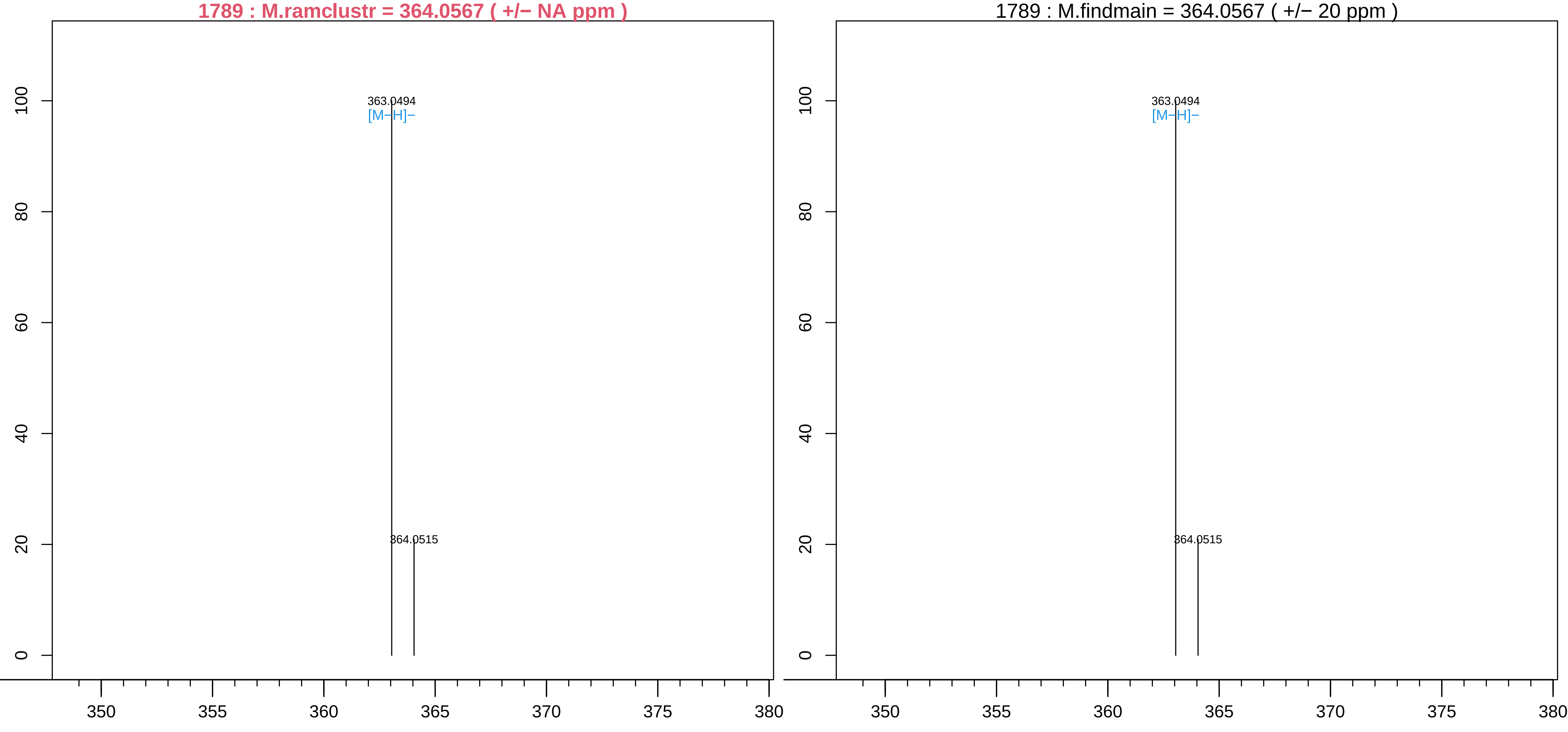

1789 : M.ramclustr = 364.0567 (+/− NA ppm)
363.0494
[M−H]−
364.0515
1789 : M.findmain = 364.0567 (+/− 20 ppm)
363.0494
[M−H]−
364.0515
0
20
40
60
80
100
350
355
360
365
370
375
380

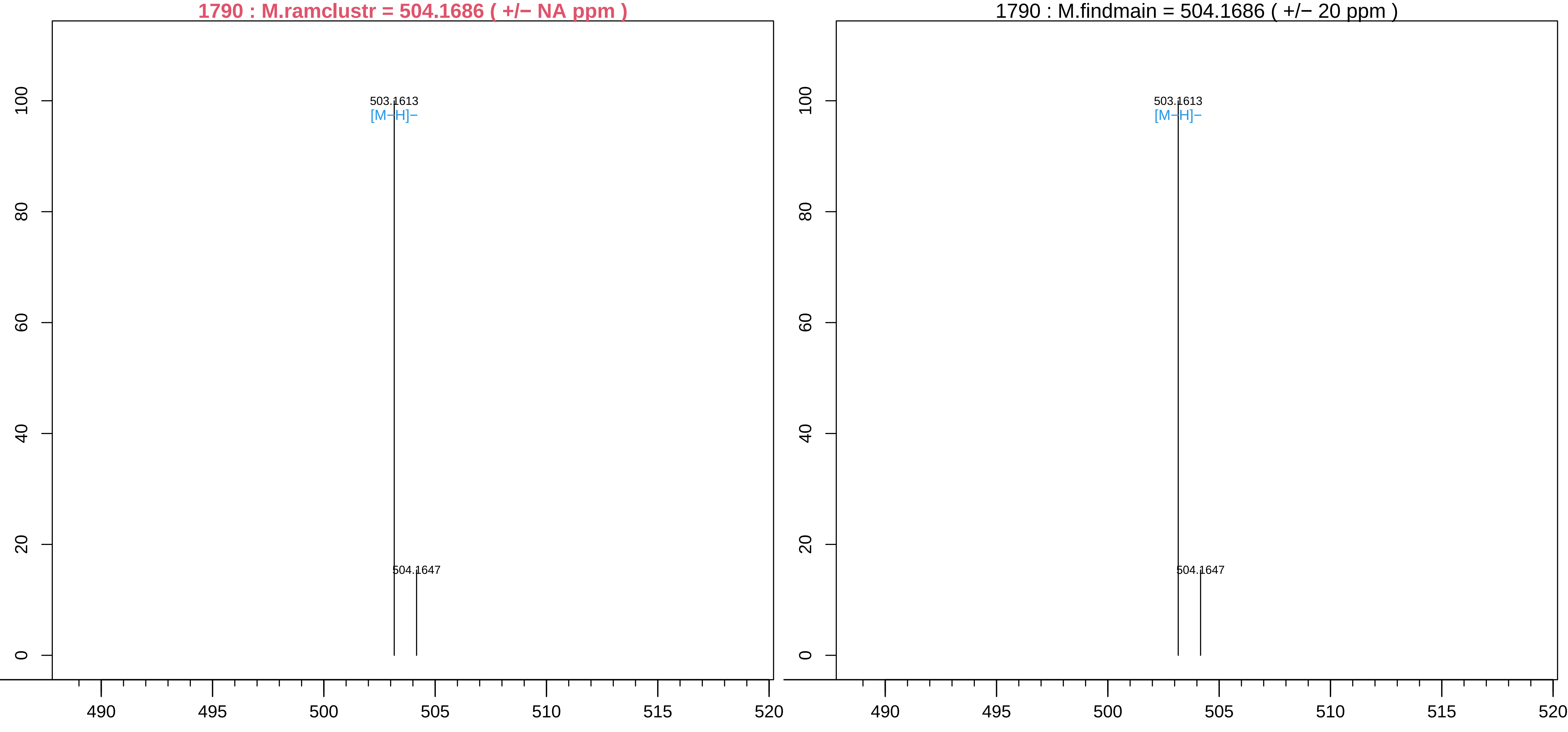
1790 : M.ramclustr = 504.1686 (+/− NA ppm)
503.1613
[M−H]−
504.1647
1790 : M.findmain = 504.1686 (+/− 20 ppm)
503.1613
[M−H]−
504.1647
0
20
40
60
80
100
490
495
500
505
510
515
520

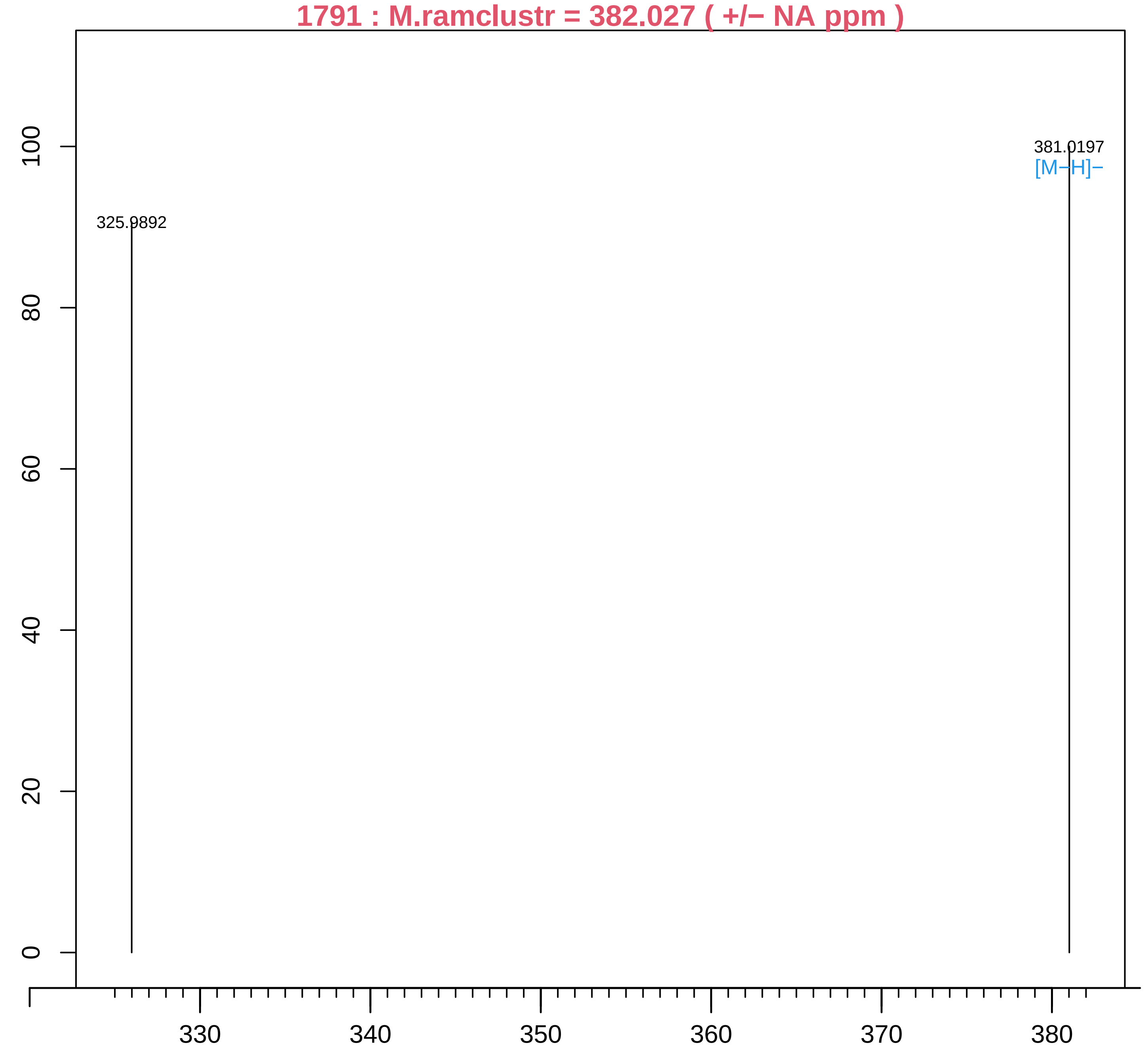
1791 : M.ramclustr = 382.027 (+/− NA ppm)
381.0197
[M−H]−
325.9892
0
20
40
60
80
100
330
340
350
360
370
380

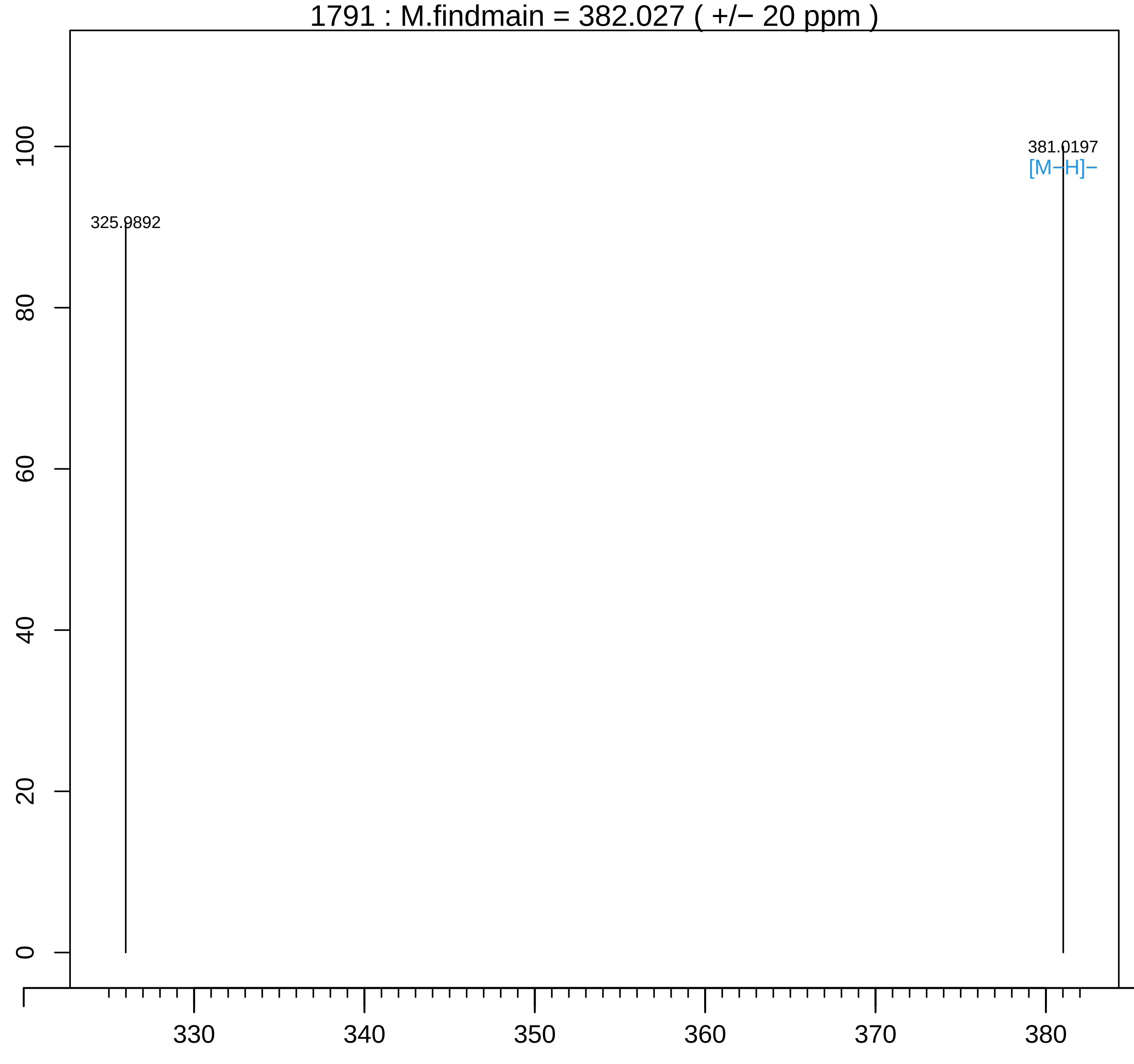
1791 : M.findmain = 382.027 (+/− 20 ppm)
381.0197
[M−H]−
325.9892
0
20
40
60
80
100
330
340
350
360
370
380

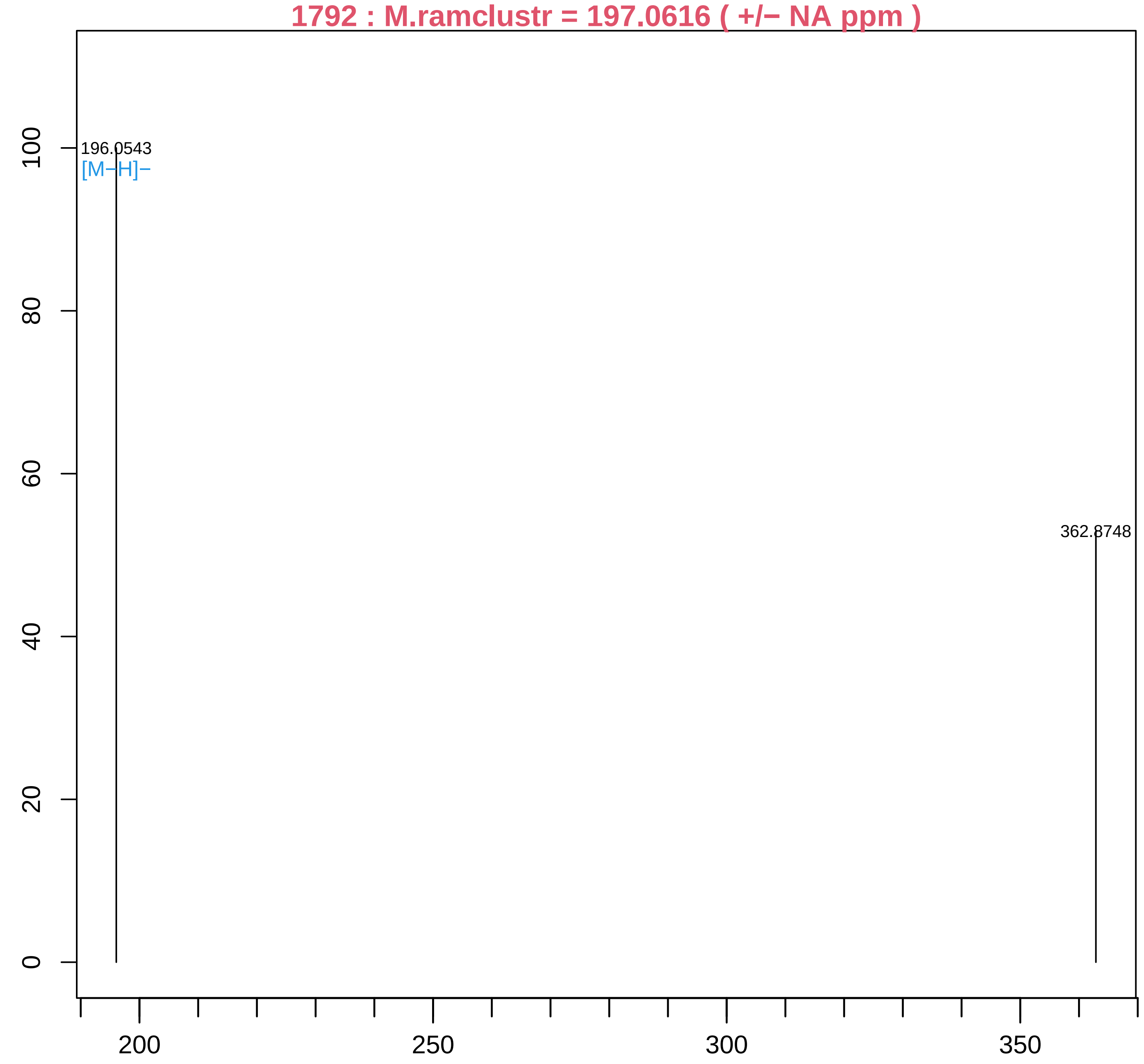
1792 : M.ramclustr = 197.0616 (+/− NA ppm)
196.0543
[M−H]−
362.8748
0
20
40
60
80
100
200
250
300
350

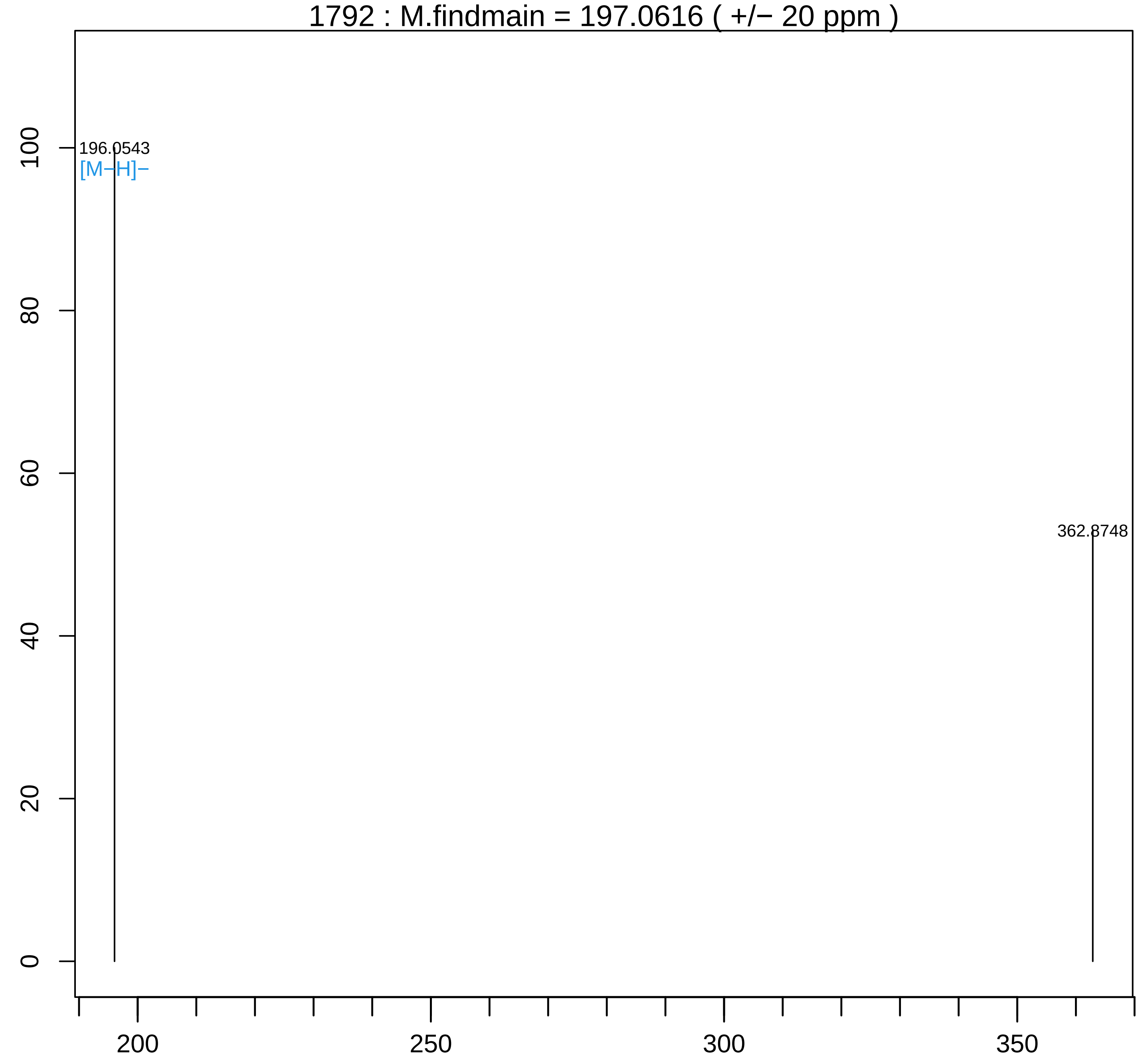
1792 : M.findmain = 197.0616 (+/− 20 ppm)
196.0543
[M−H]−
362.8748
0
20
40
60
80
100
200
250
300
350

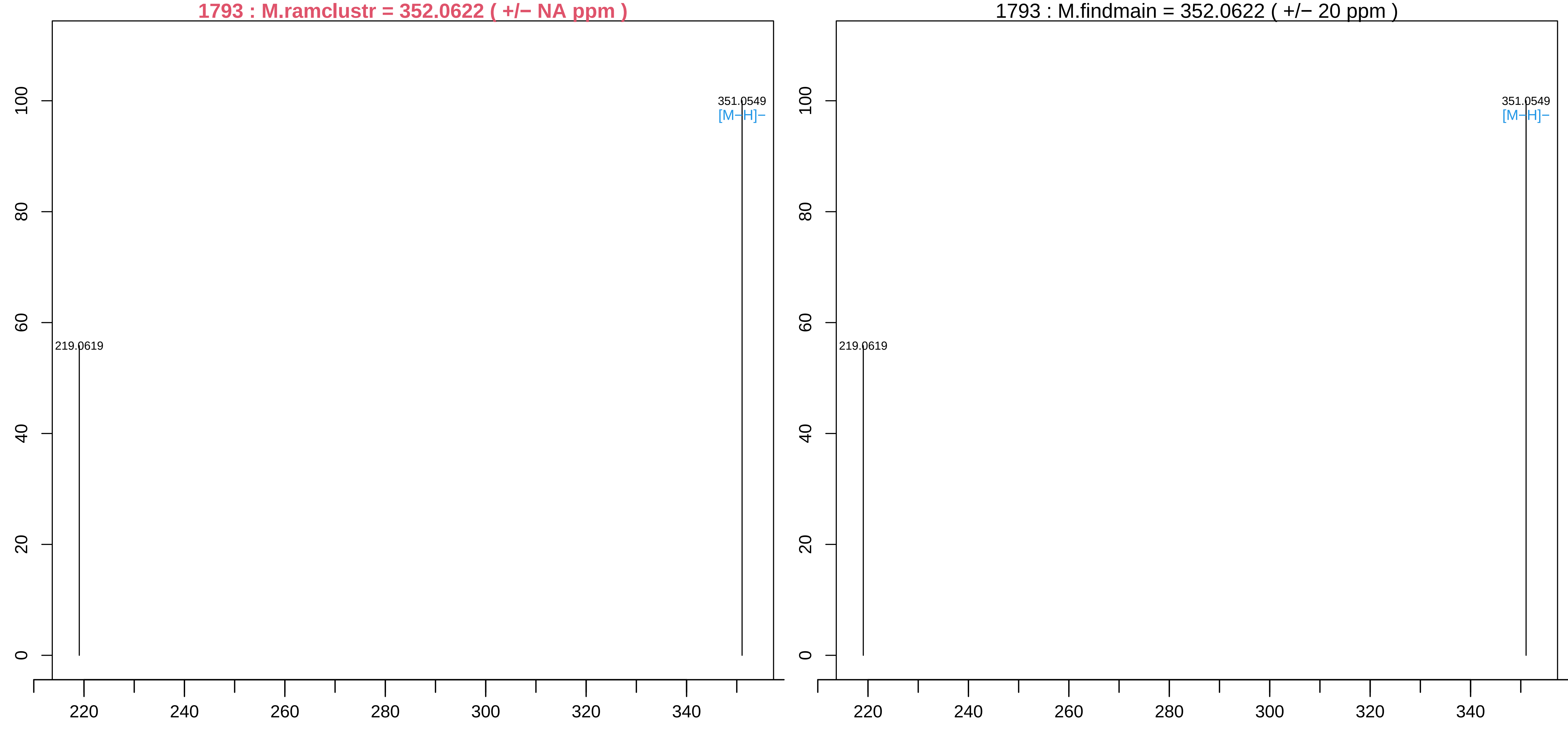
1793 : M.ramclustr = 352.0622 (+/− NA ppm)
1793 : M.findmain = 352.0622 (+/− 20 ppm)
351.0549
[M−H]−
219.0619
0
20
40
60
80
100
220
240
260
280
300
320
340
