# Supplementary material for: Plant necrotrophic bacterial disease resistance phenotypes, QTL, and metabolites identified through integrated genetic mapping and metabolomics in Solanum species
Source: Front Plant Sci. 2024 Mar 5;15:1336513. doi: 10.3389/fpls.2024.1336513 (PMC10949924; doi:10.3389/fpls.2024.1336513)
Supplement: Supplementary file 2 [file DataSheet_2.pdf]

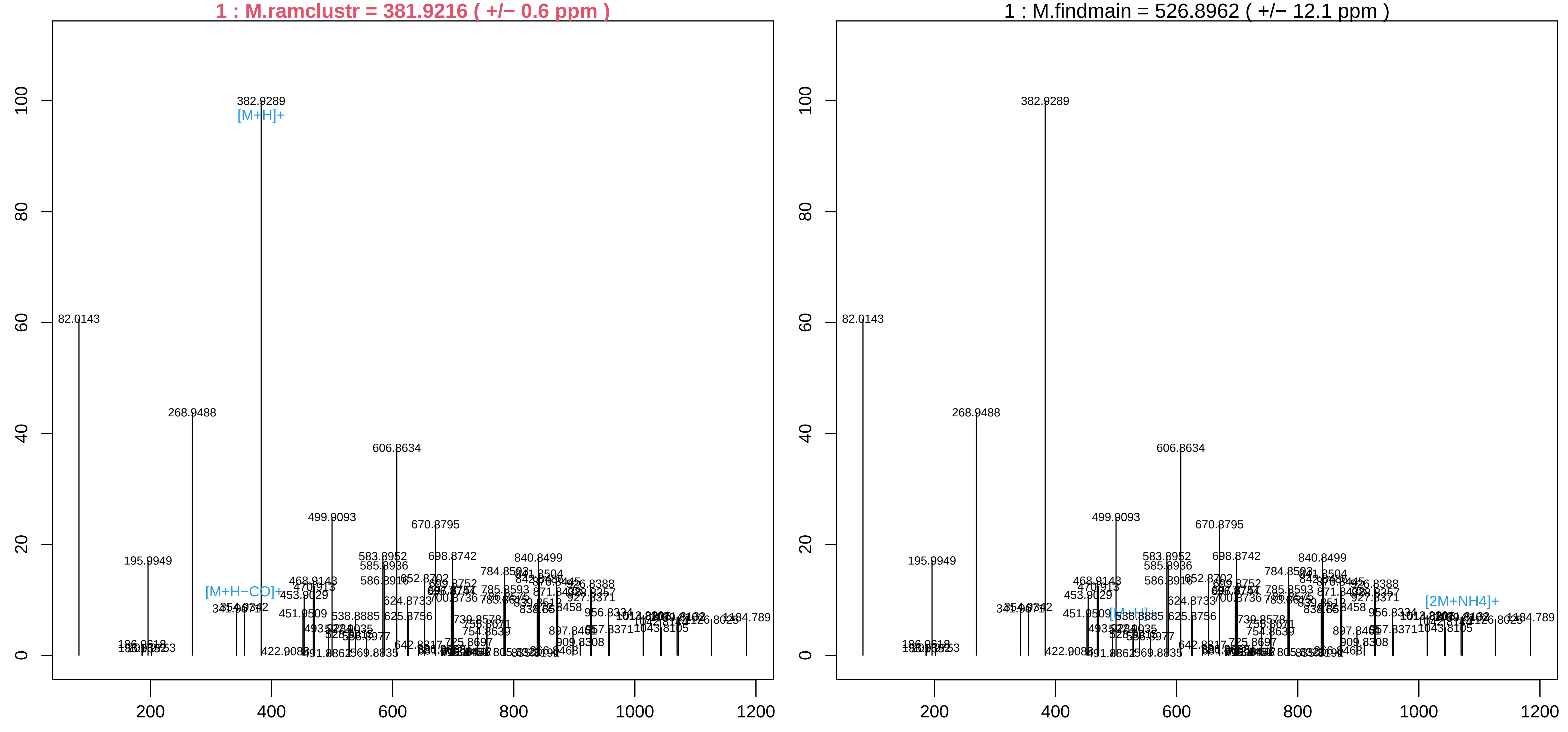

1 : M.ramclustr = 381.9216 (+/− 0.6 ppm)
1 : M.findmain = 526.8962 (+/− 12.1 ppm)
382.9289
[M+H]+
82.0143
268.9488
606.8634
499.9093
670.8795
195.9949
583.8952
585.8936
698.8742
840.8499
784.8593
[M+H−CO]+
453.9029
451.9509
538.8885
625.3756
624.8733
956.8334
897.8465
909.8308
1184.789
422.9088
569.8835
[2M+NH4]+
0
20
40
60
80
100
200
400
600
800
1000
1200

2 : M.ramclustr = 612.4755 (+/− 1.1 ppm)

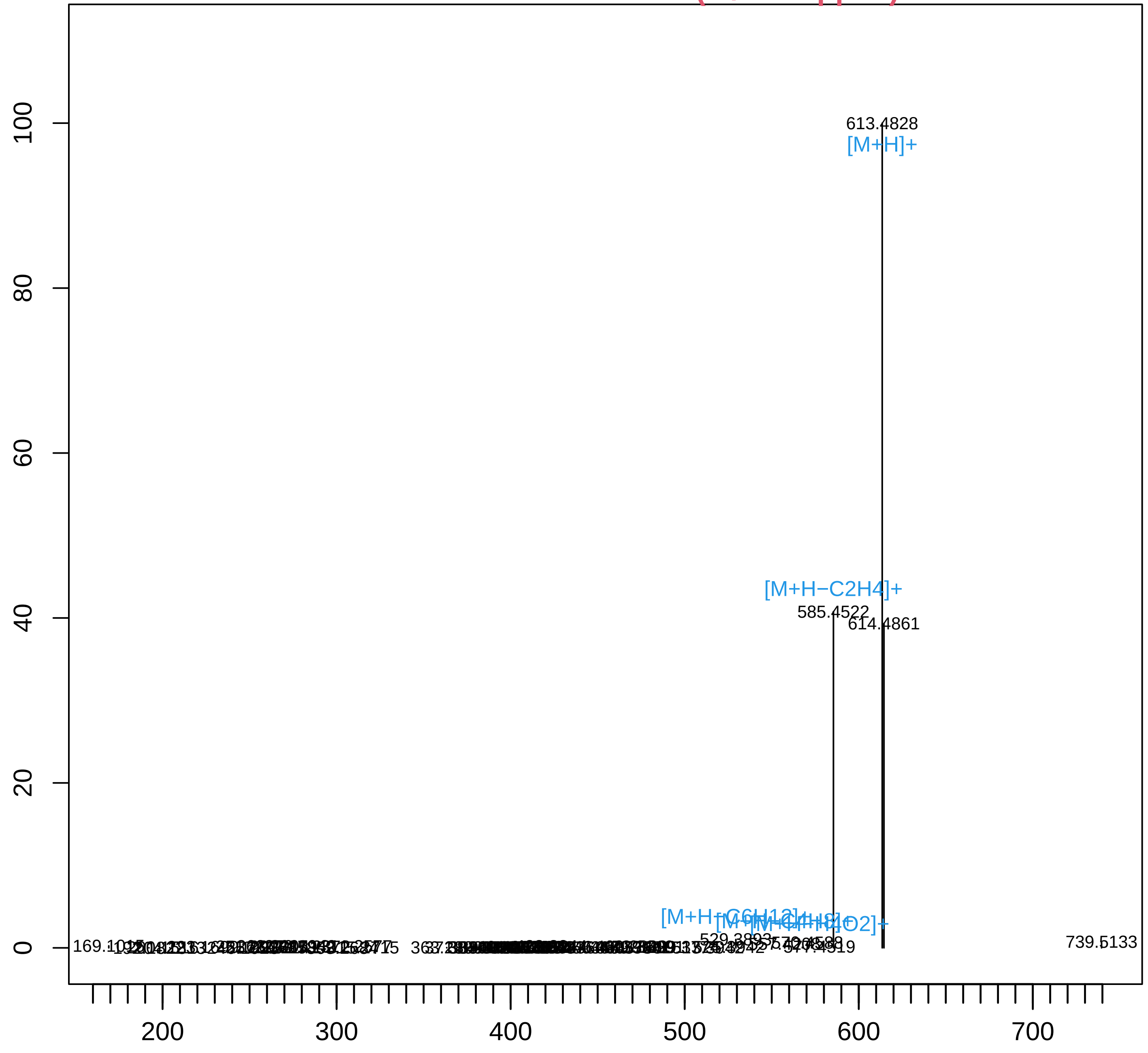

2 : M.findmain = 612.4755 (+/− 20 ppm)

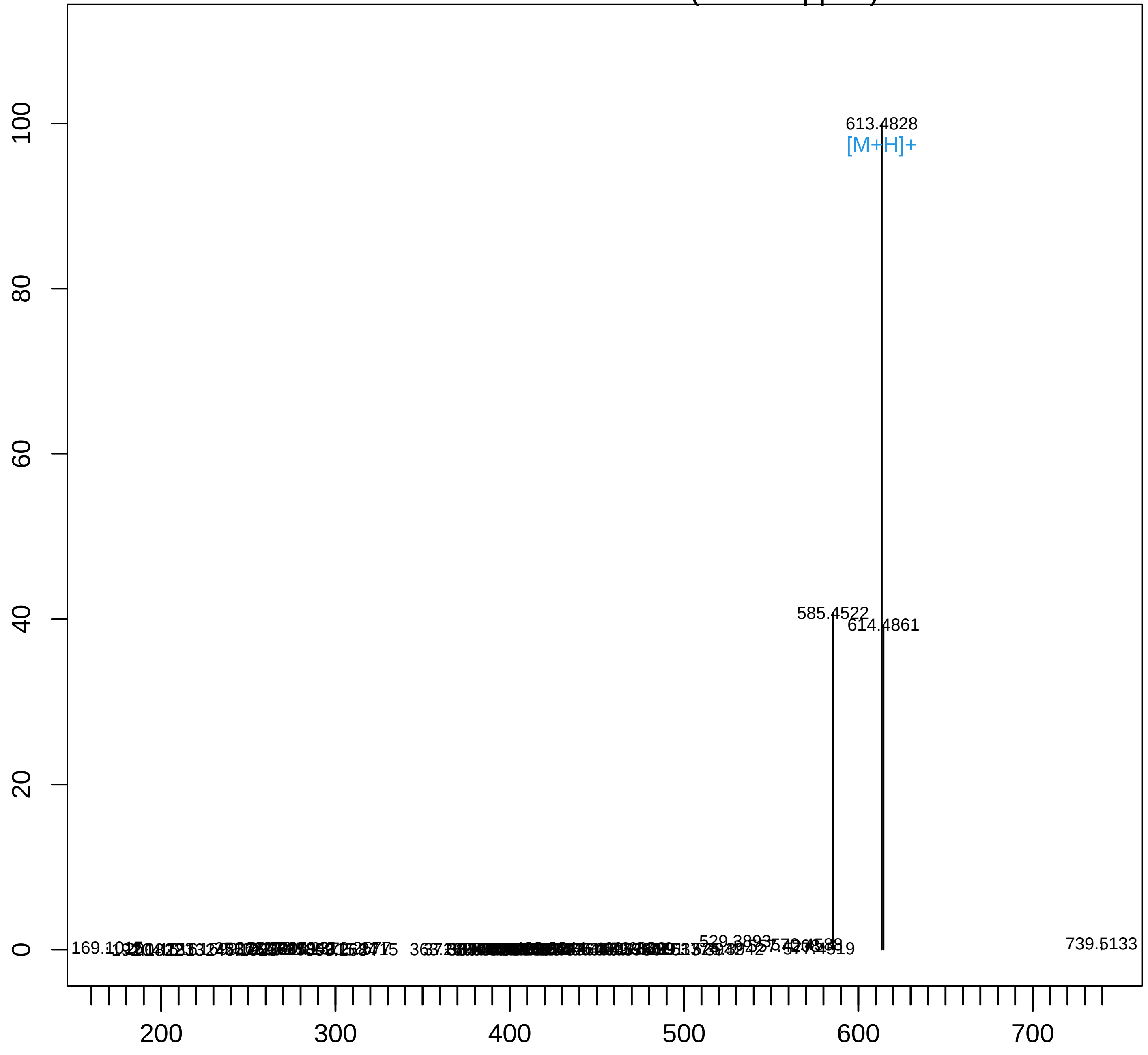

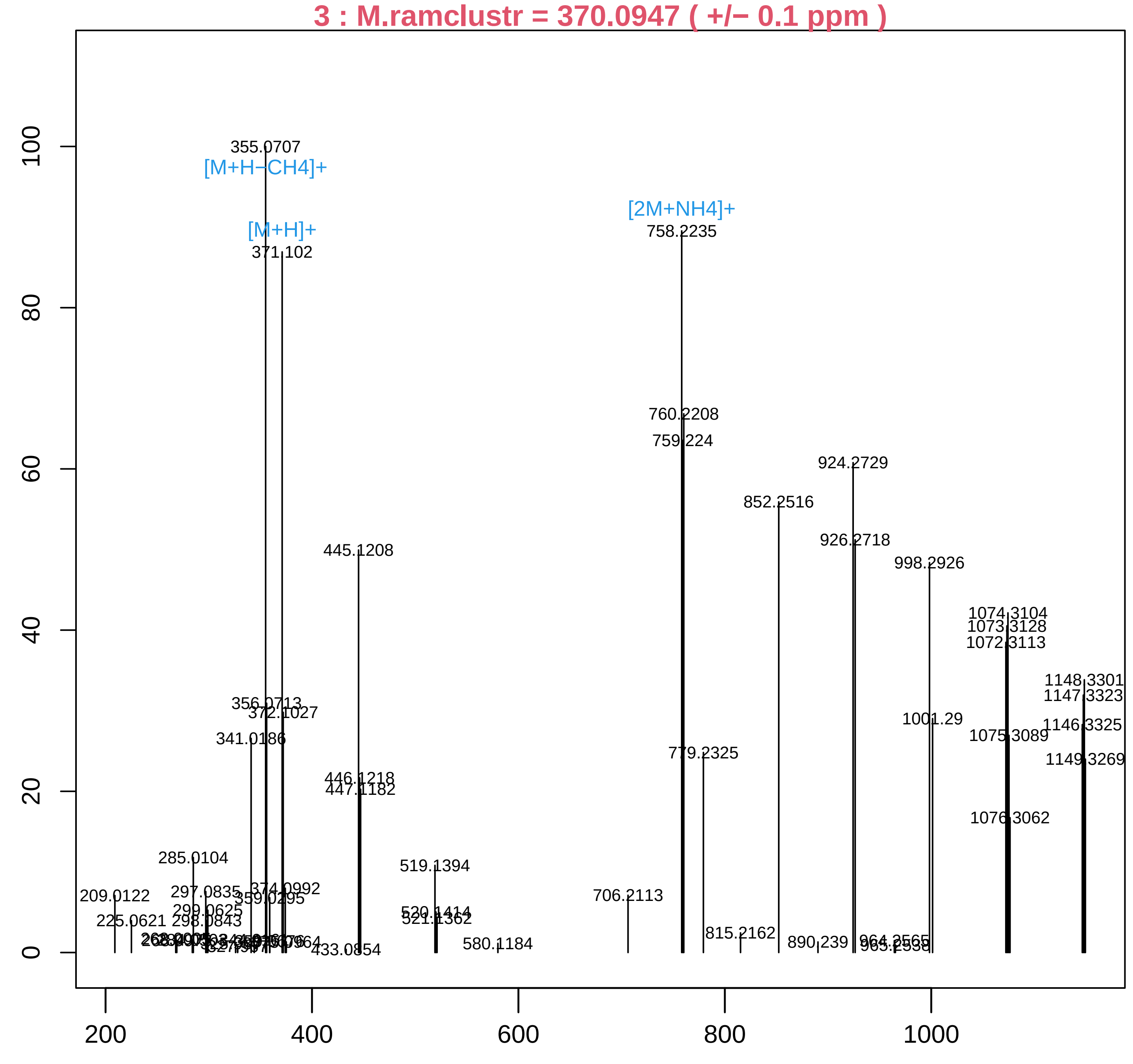
3 : M.ramclustr = 370.0947 (+/− 0.1 ppm)
355.0707
[M+H−CH4]+
[M+H]+
371.102
[2M+NH4]+
758.2235
760.2208
759.224
924.2729
852.2516
926.2718
445.1208
998.2926
1074.3104
1073.3128
1072.3113
1148.3301
1147.3323
356.0713
372.1027
1001.29
341.0186
1146.3325
1075.3089
779.2325
1149.3269
446.1218
447.1182
1076.3062
285.0104
519.1394
209.0122
297.0835
374.0992
706.2113
359.0295
299.0625
520.1414
521.1362
225.0621
298.0843
815.2162
890.239
964.2565
965.2538
580.1184
433.0854
0
20
40
60
80
100
200
400
600
800
1000
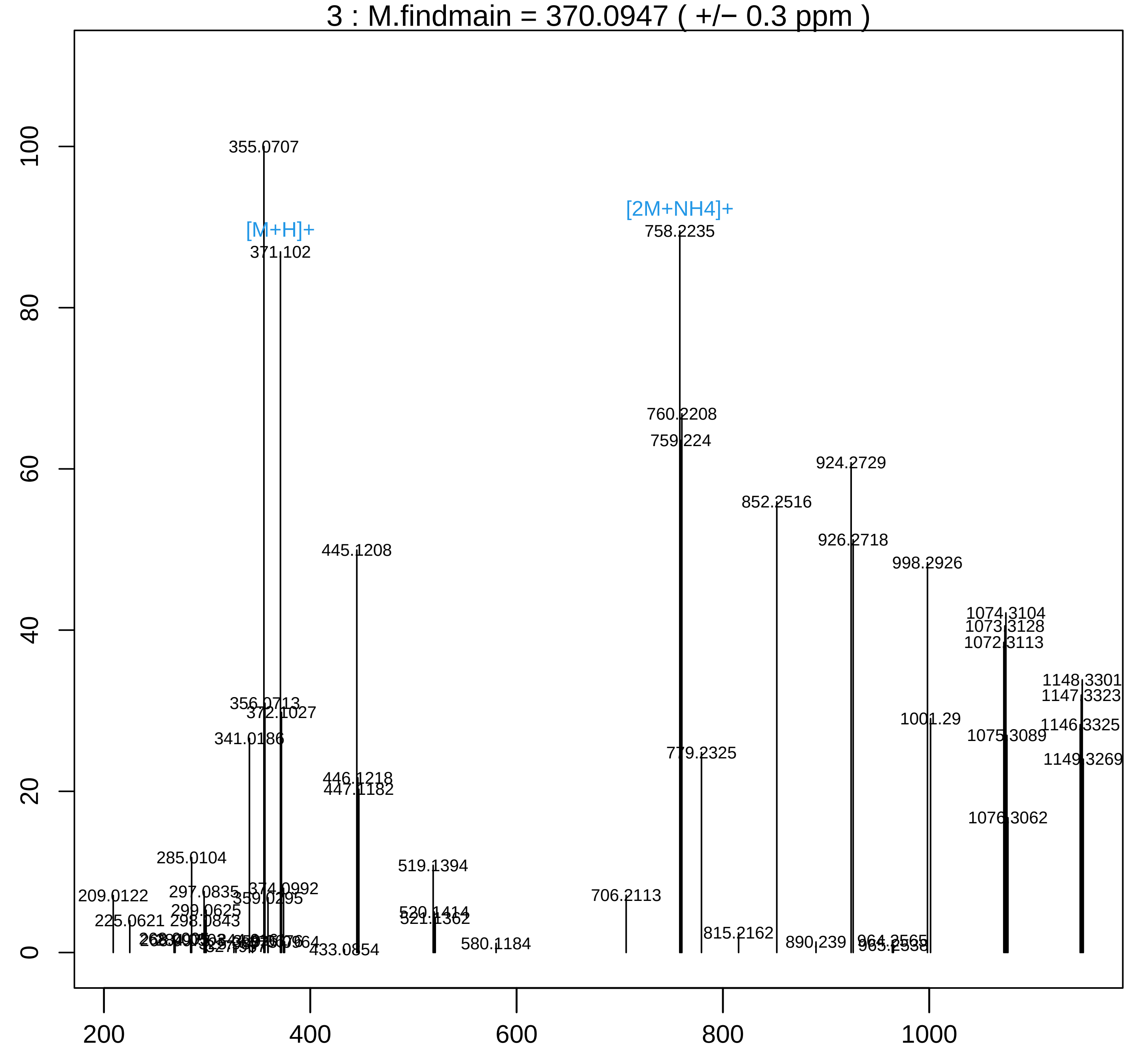
3 : M.findmain = 370.0947 (+/− 0.3 ppm)
355.0707
[M+H]+
371.102
[2M+NH4]+
758.2235
760.2208
759.224
924.2729
852.2516
926.2718
445.1208
998.2926
1074.3104
1073.3128
1072.3113
1148.3301
1147.3323
356.0713
372.1027
1001.29
341.0186
1146.3325
1075.3089
779.2325
1149.3269
446.1218
447.1182
1076.3062
285.0104
519.1394
209.0122
297.0835
374.0992
706.2113
359.0295
299.0625
520.1414
521.1362
225.0621
298.0843
815.2162
890.239
964.2565
965.2538
580.1184
433.0854
0
20
40
60
80
100
200
400
600
800
1000

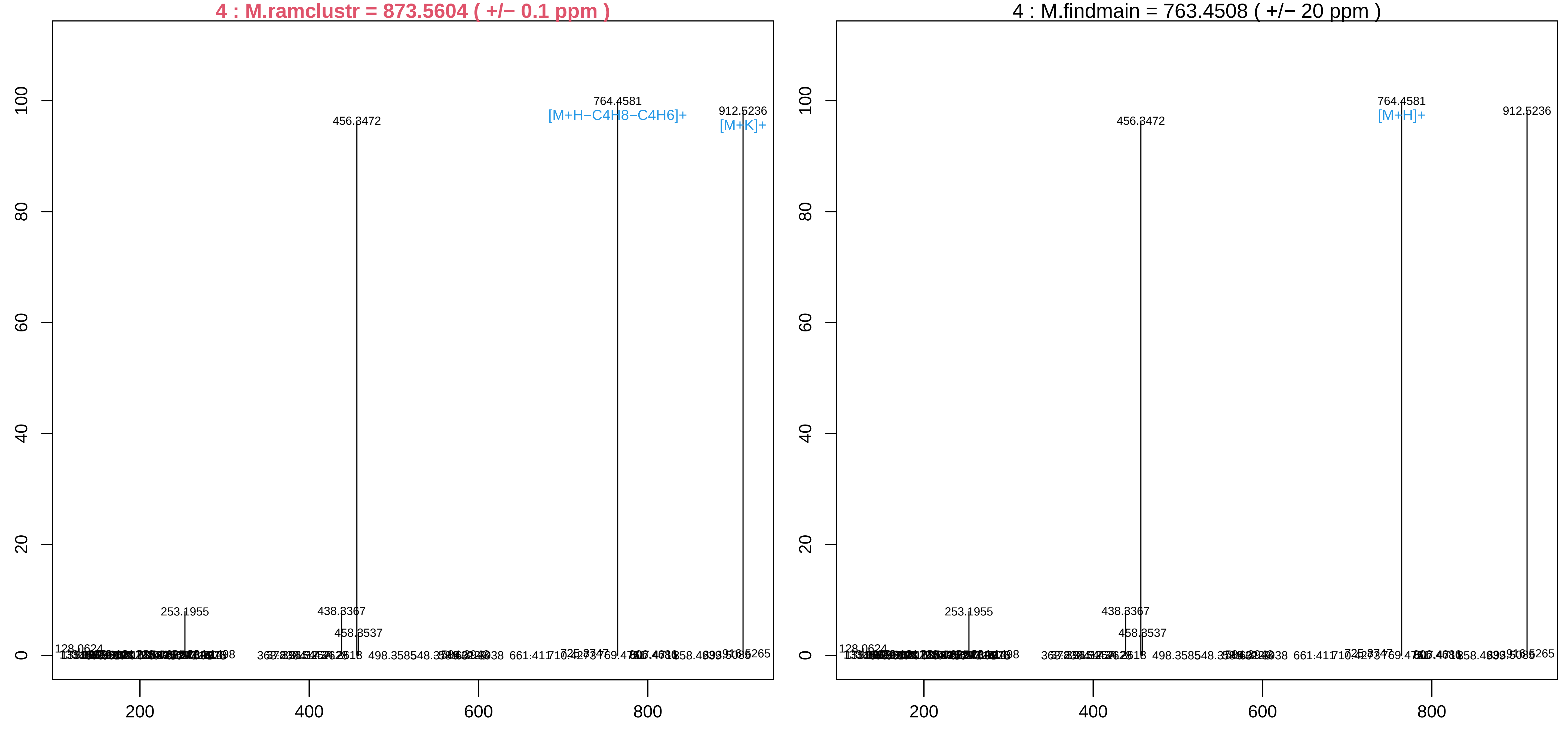
4 : M.ramclustr = 873.5604 (+/− 0.1 ppm)
764.4581
[M+H−C4H8−C4H6]+
912.5236
[M+K]+
456.3472
253.1955
438.3367
458.3537
128.0624
4 : M.findmain = 763.4508 (+/− 20 ppm)
764.4581
[M+H]+
912.5236
456.3472
253.1955
438.3367
458.3537
128.0624

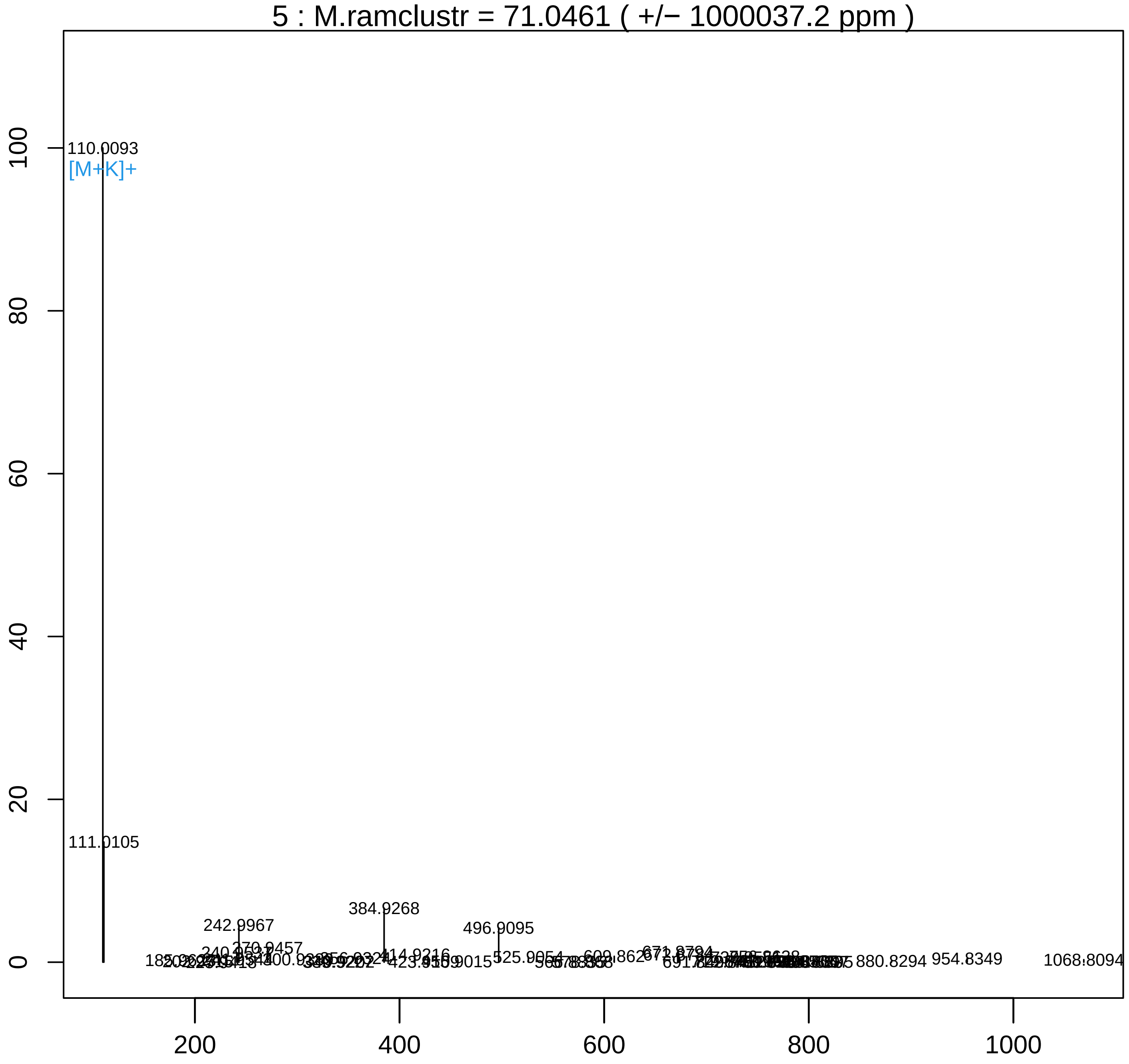
5 : M.ramclustr = 71.0461 (+/− 1000037.2 ppm)
110.0093
[M+K]+
111.0105
242.9967
270.9457
384.9268
414.9216
496.9095
525.9054
609.8626
671.8794
880.8294
954.8349
1068.8094
0
20
40
60
80
100
200
400
600
800
1000

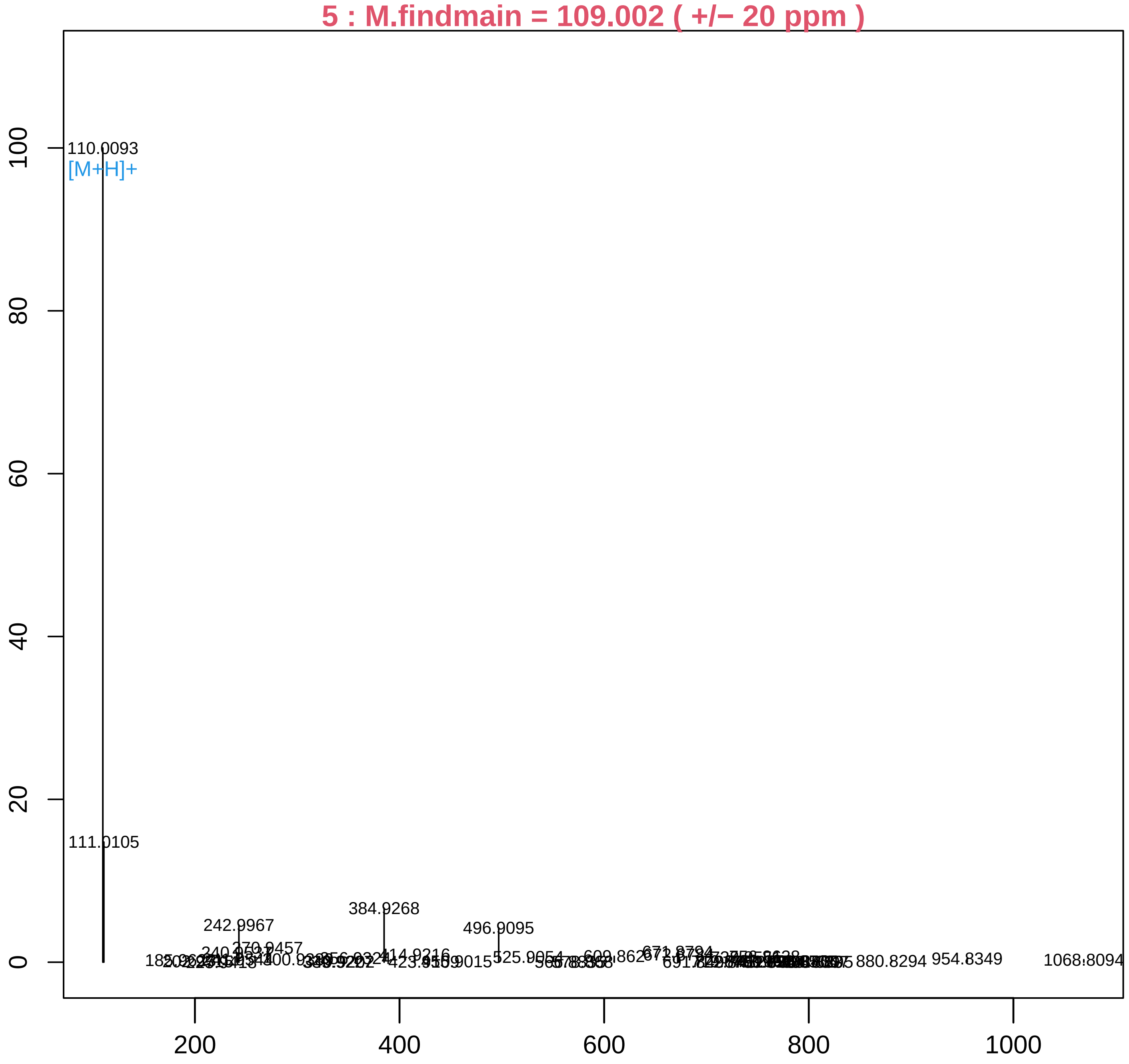
5 : M.findmain = 109.002 (+/− 20 ppm)
110.0093
[M+H]+
111.0105
242.9967
270.9457
384.9268
414.9216
496.9095
525.9054
609.8626
671.8794
880.8294
954.8349
1068.8094
0
20
40
60
80
100
200
400
600
800
1000

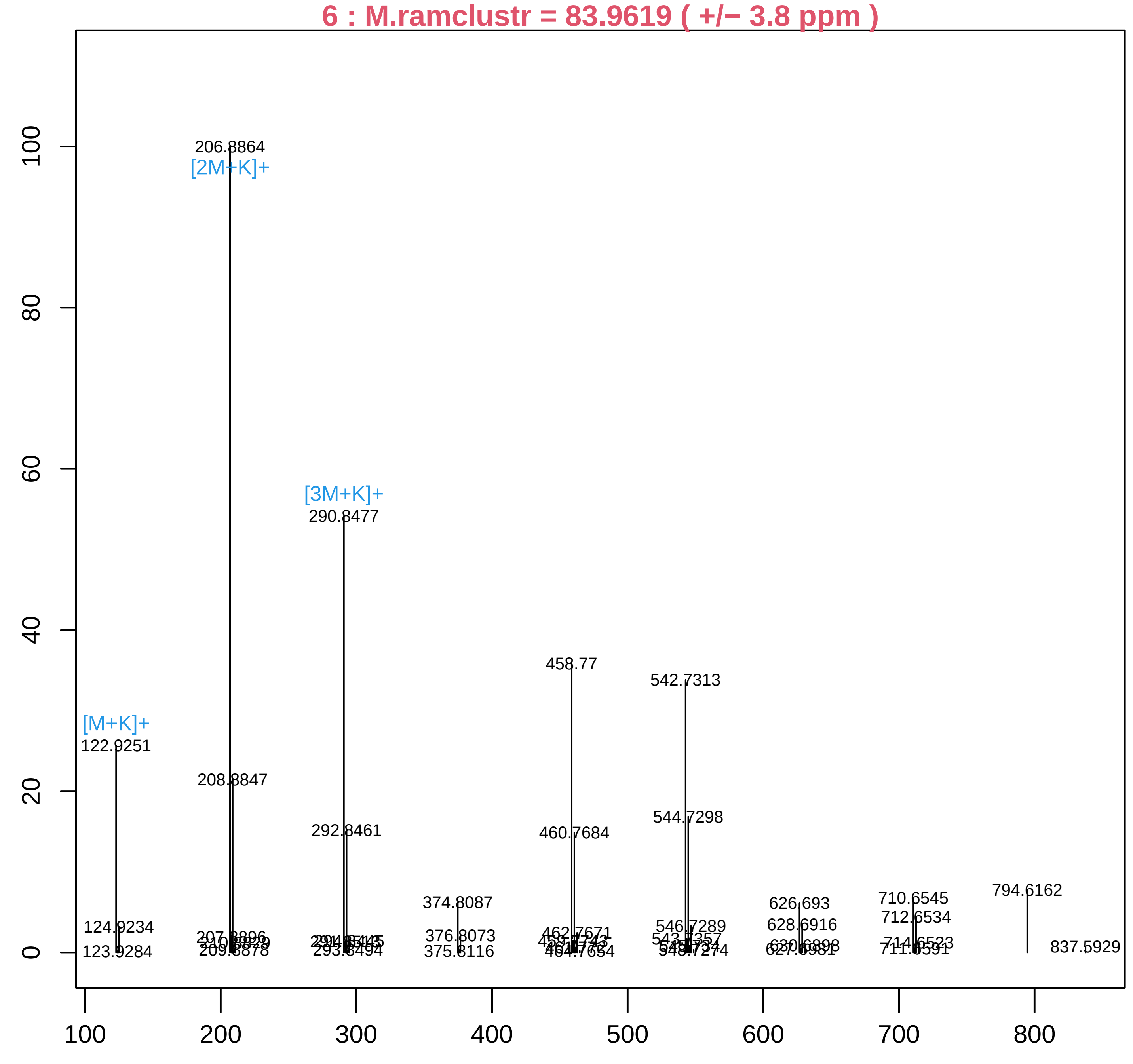
6 : M.ramclustr = 83.9619 (+/− 3.8 ppm)
206.8864
[2M+K]+
[3M+K]+
290.8477
[M+K]+
122.9251
208.8847
292.8461
460.7684
458.77
542.7313
544.7298
374.8087
626.693
628.6916
710.6545
712.6534
794.6162
837.5929
124.9234
123.9284
207.8896
209.8878
293.8494
376.8073
375.8116
462.7671
464.7654
546.7289
548.7274
627.6981
714.6523
711.6591
0
20
40
60
80
100
100
200
300
400
500
600
700
800

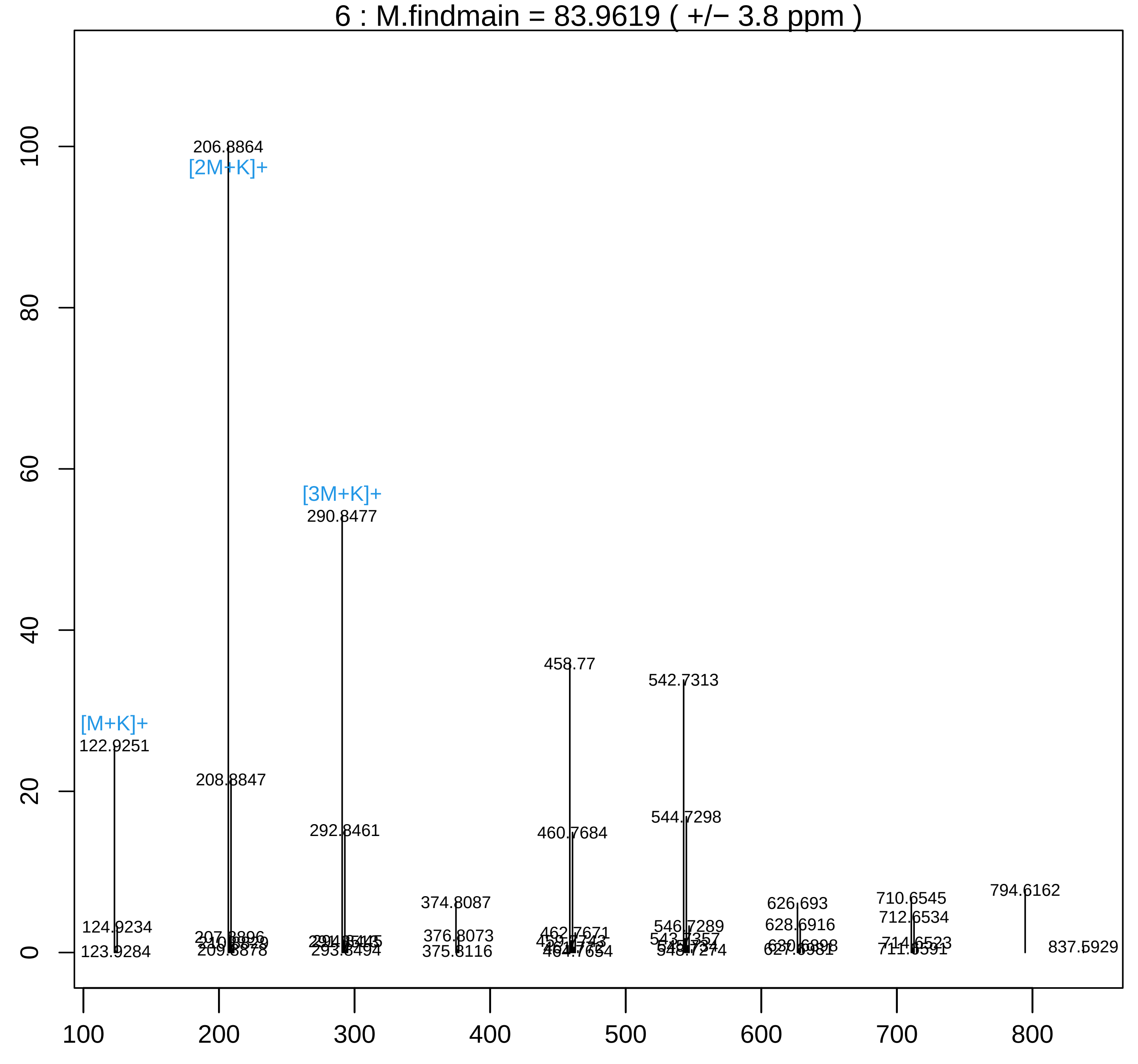
6 : M.findmain = 83.9619 (+/− 3.8 ppm)
206.8864
[2M+K]+
[3M+K]+
290.8477
[M+K]+
122.9251
208.8847
292.8461
460.7684
458.77
542.7313
544.7298
374.8087
626.693
628.6916
710.6545
712.6534
794.6162
837.5929
124.9234
123.9284
207.8896
209.8878
293.8494
376.8073
375.8116
462.7671
464.7654
546.7289
548.7274
627.6981
714.6523
711.6591
0
20
40
60
80
100
100
200
300
400
500
600
700
800

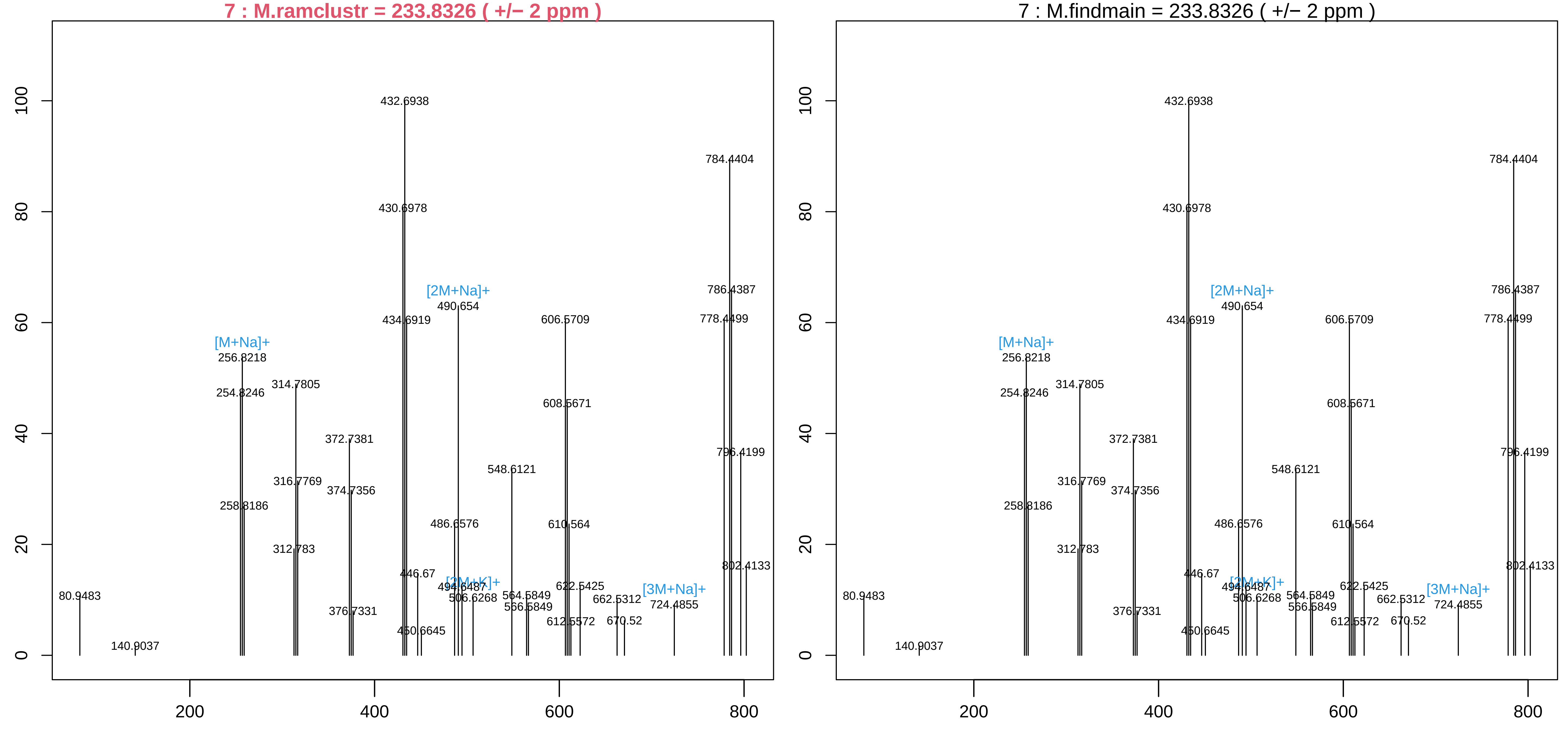
7 : M.ramclustr = 233.8326 (+/− 2 ppm)
7 : M.findmain = 233.8326 (+/− 2 ppm)
0
20
40
60
80
100
200
400
600
800
[M+Na]+
[2M+Na]+
[2M+K]+
[3M+Na]+
80.9483
140.9037
254.8246
256.8218
258.8186
312.783
314.7805
316.7769
372.7381
374.7356
376.7331
430.6978
432.6938
434.6919
446.67
450.6645
486.6576
490.654
494.6487
506.6268
548.6121
564.5849
566.5849
606.5709
608.5671
610.564
612.5572
622.5425
662.5312
670.52
724.4855
778.4499
784.4404
786.4387
796.4199
802.4133

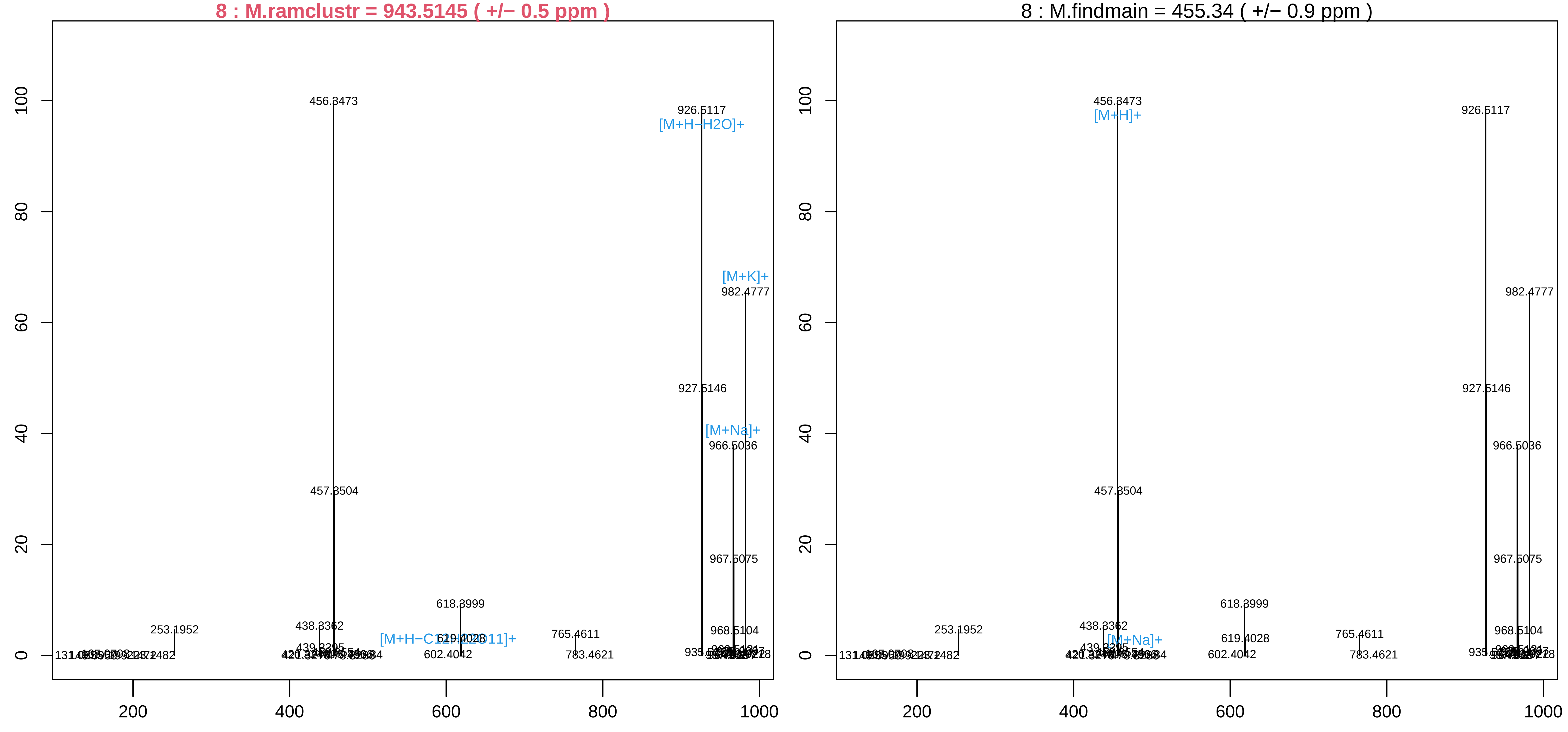
8 : M.ramclustr = 943.5145 (+/− 0.5 ppm)
8 : M.findmain = 455.34 (+/− 0.9 ppm)
456.3473
926.5117
[M+H−H2O]+
[M+H]+
[M+K]+
982.4777
927.5146
[M+Na]+
966.5036
457.3504
967.5075
618.3999
253.1952
438.3362
968.5104
765.4611
619.4028
602.4042
783.4621
0
20
40
60
80
100
200
400
600
800
1000

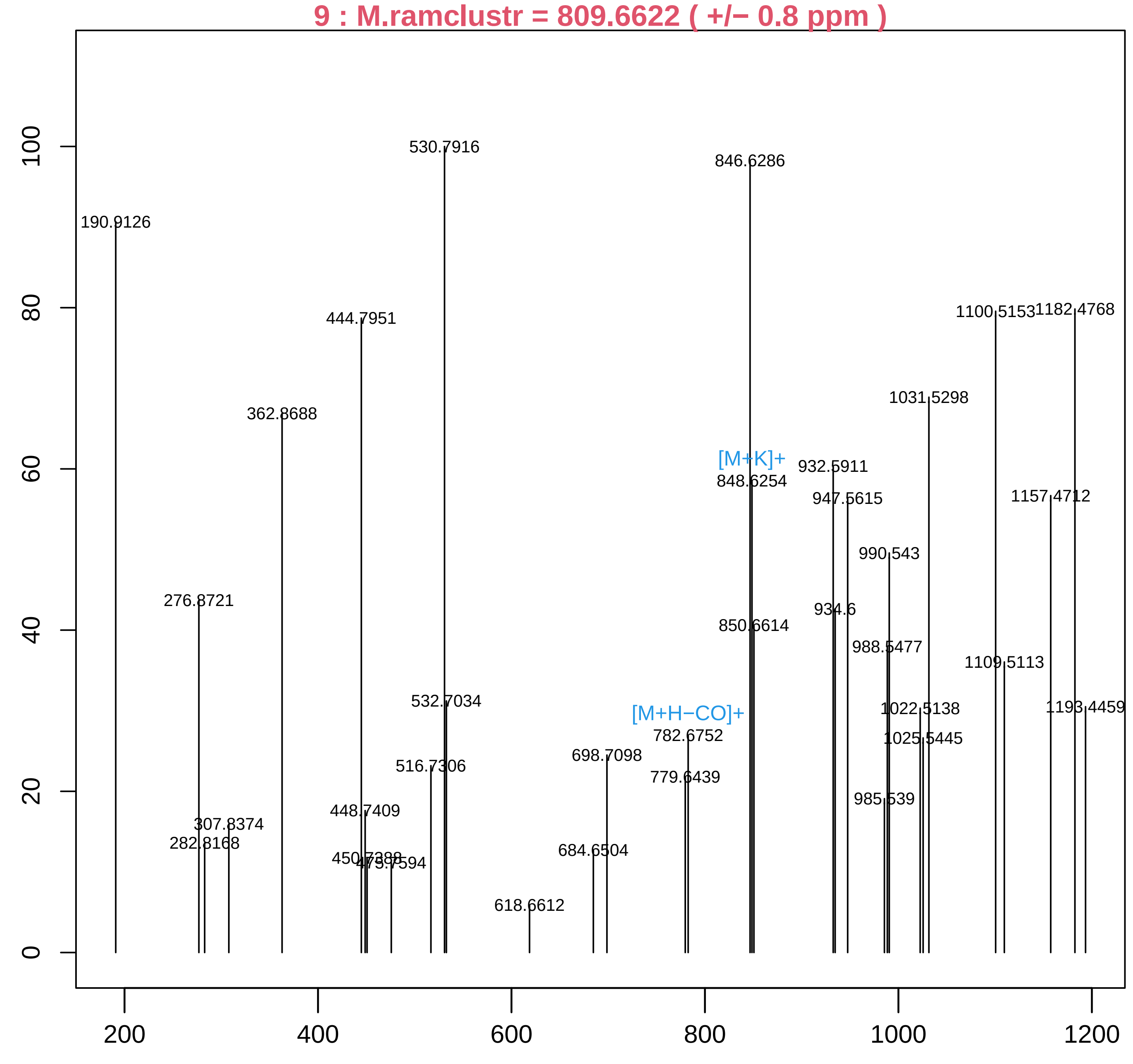
9 : M.ramclustr = 809.6622 (+/− 0.8 ppm)
[M+K]+
[M+H−CO]+
190.9126
276.8721
282.8168
307.8374
362.8688
444.7951
448.7409
450.7388
475.7594
516.7306
530.7916
532.7034
618.6612
684.6504
698.7098
779.6439
782.6752
846.6286
848.6254
850.6614
932.5911
934.6
947.5615
985.539
988.5477
990.543
1022.5138
1025.5445
1031.5298
1100.5153
1109.5113
1157.4712
1182.4768
1193.4459

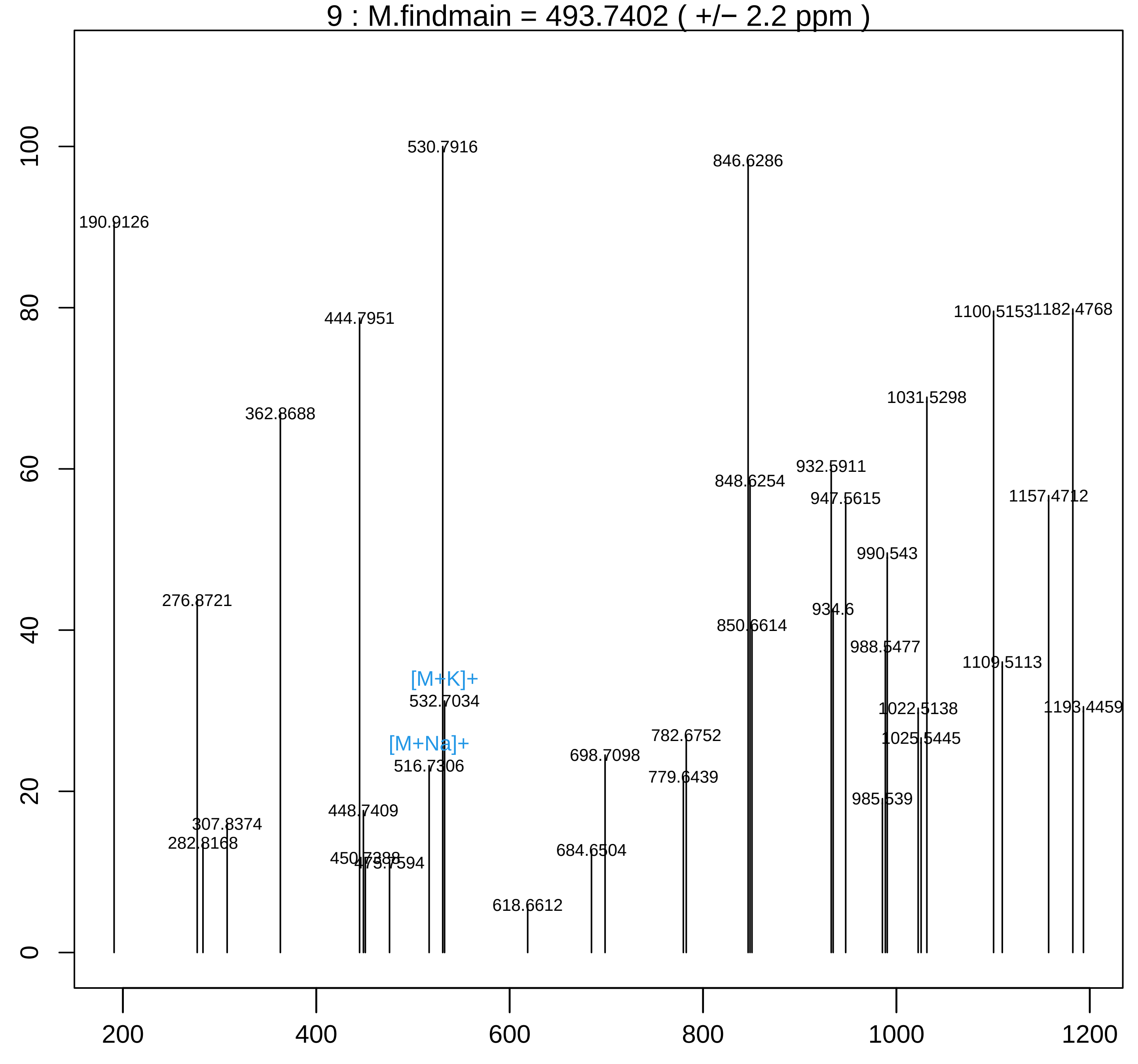
9 : M.findmain = 493.7402 (+/− 2.2 ppm)
[M+K]+
[M+Na]+
190.9126
276.8721
282.8168
307.8374
362.8688
444.7951
448.7409
450.7388
475.7594
516.7306
530.7916
532.7034
618.6612
684.6504
698.7098
779.6439
782.6752
846.6286
848.6254
850.6614
932.5911
934.6
947.5615
985.539
988.5477
990.543
1022.5138
1025.5445
1031.5298
1100.5153
1109.5113
1157.4712
1182.4768
1193.4459

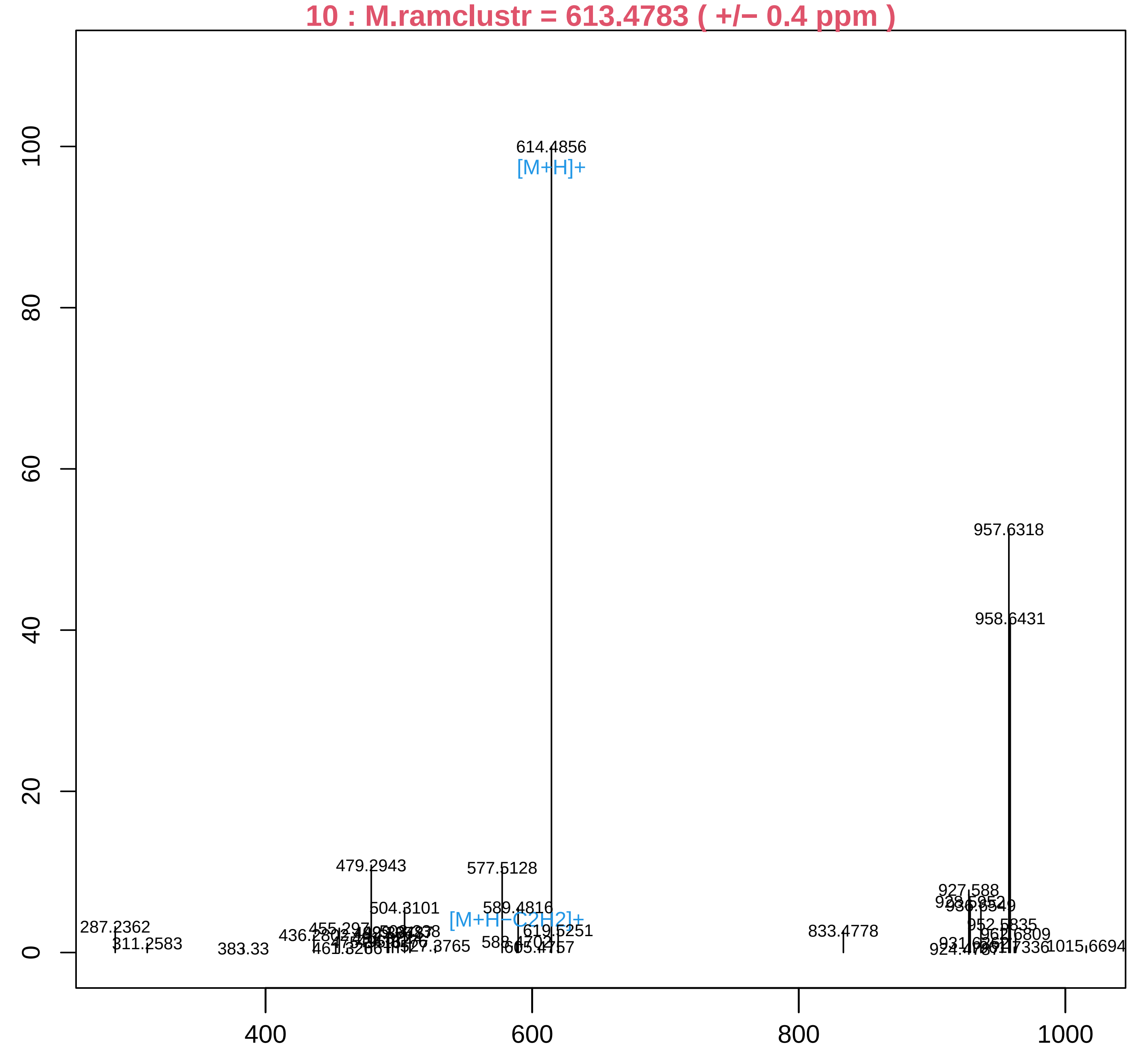
10 : M.ramclustr = 613.4783 (+/− 0.4 ppm)
614.4856
[M+H]+
957.6318
958.6431
479.2943
577.5128
504.3101
589.4816
619.5251
287.2362
311.2583
383.33
833.4778
927.588
952.5835
1015.6694
0
20
40
60
80
100
400
600
800
1000
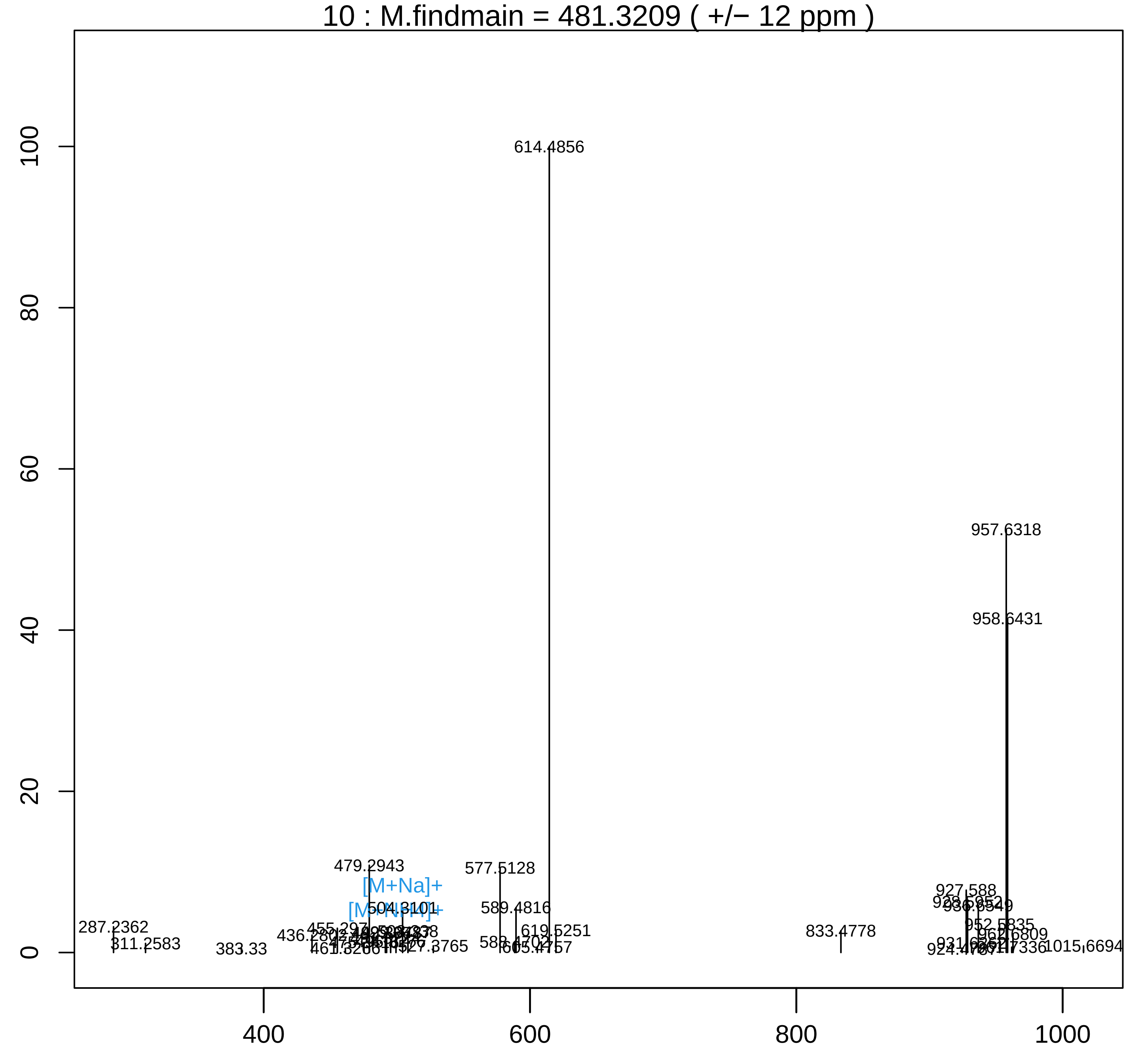
10 : M.findmain = 481.3209 (+/− 12 ppm)
614.4856
957.6318
958.6431
479.2943
[M+Na]+
577.5128
504.3101
589.4816
619.5251
287.2362
311.2583
383.33
833.4778
927.588
952.5835
1015.6694
0
20
40
60
80
100
400
600
800
1000

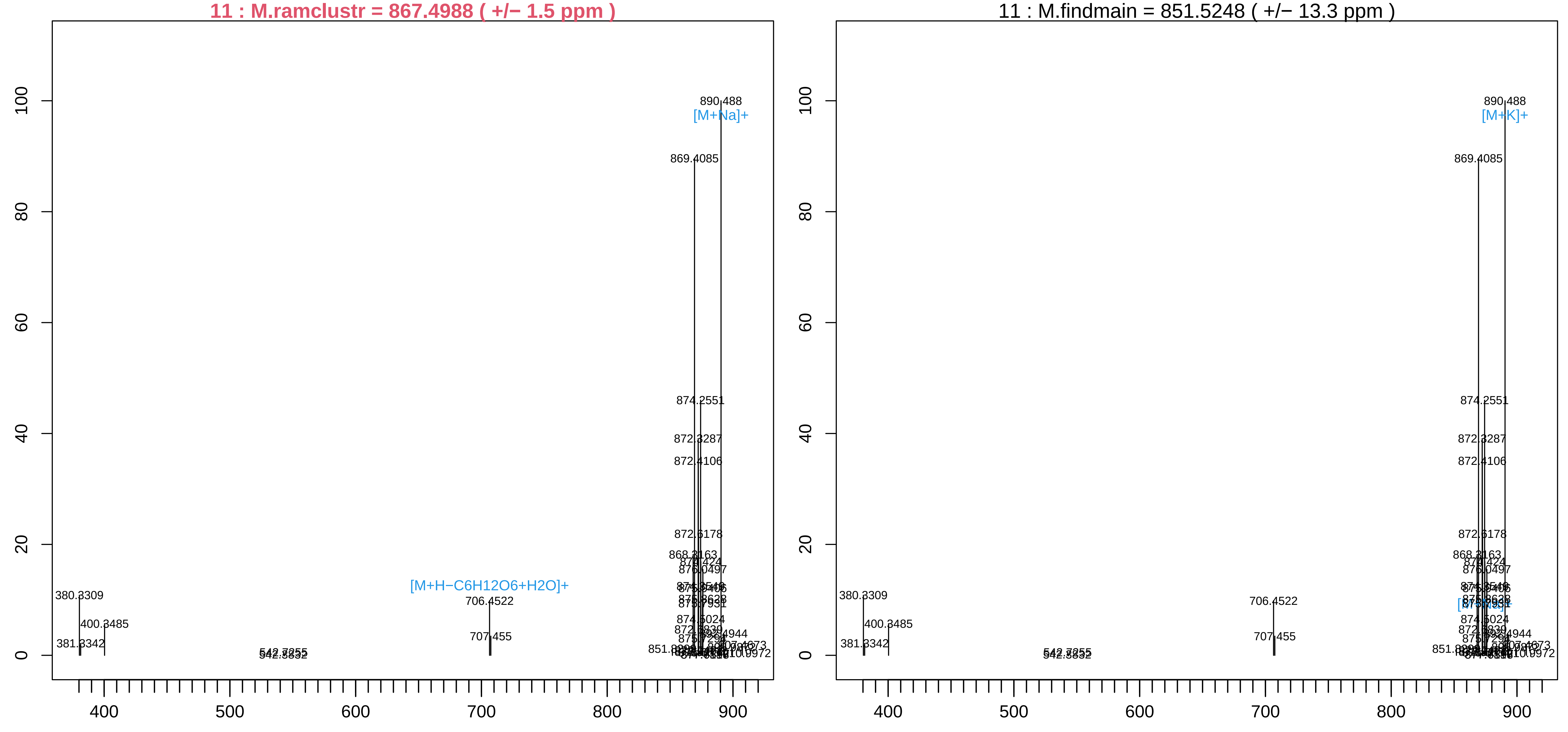

11 : M.ramclustr = 867.4988 (+/− 1.5 ppm)
890.488
[M+Na]+
869.4085
874.2551
872.3287
872.4106
872.5178
868.3163
[M+H−C6H12O6+H2O]+
706.4522
707.455
380.3309
400.3485
381.3342
11 : M.findmain = 851.5248 (+/− 13.3 ppm)
890.488
[M+K]+
869.4085
874.2551
872.3287
872.4106
872.5178
868.3163
706.4522
707.455
380.3309
400.3485
381.3342

12 : M.ramclustr = 435.8682 (+/− 4.8 ppm)

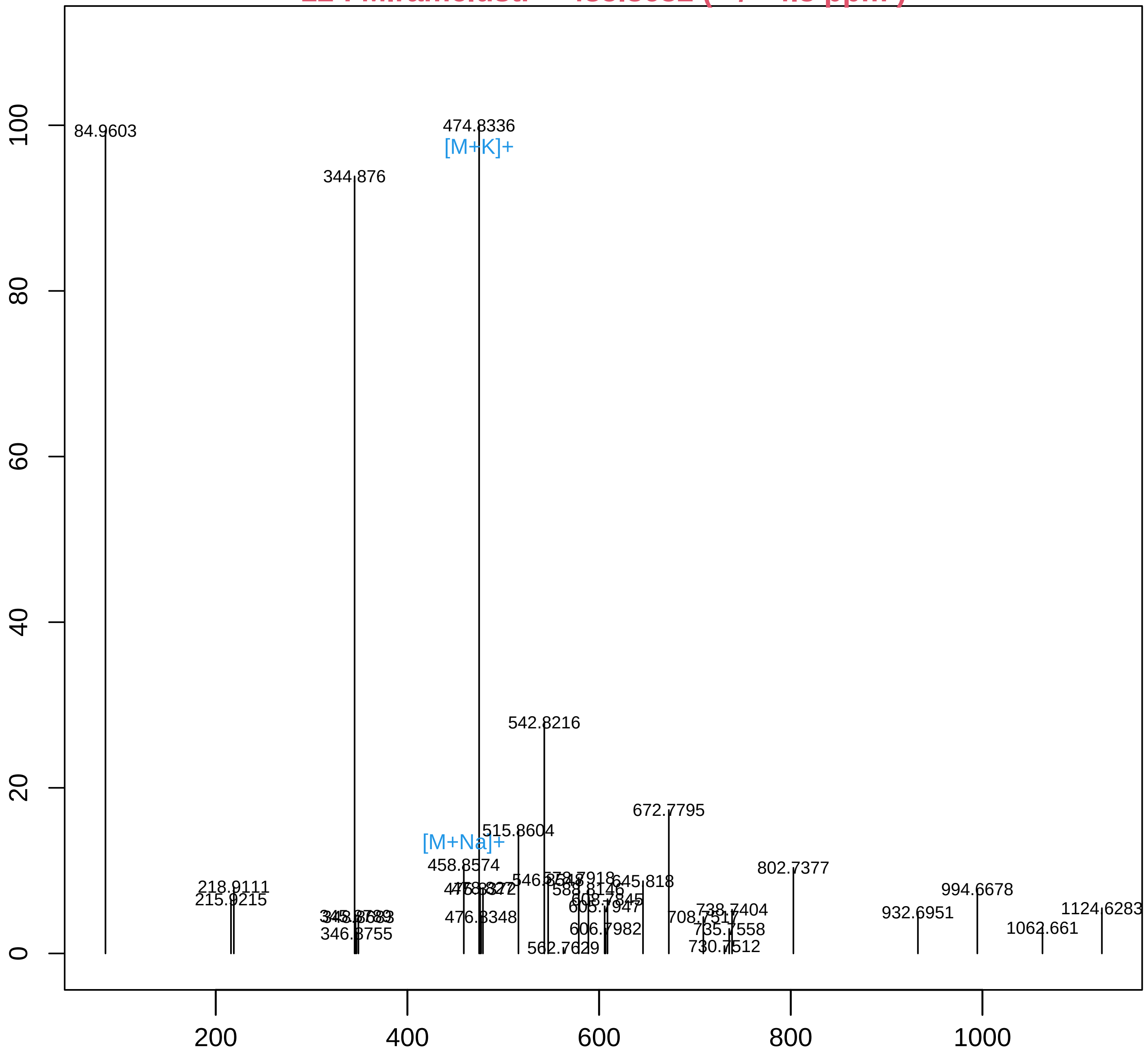

12 : M.findmain = 435.8704 (+/− 4.9 ppm)

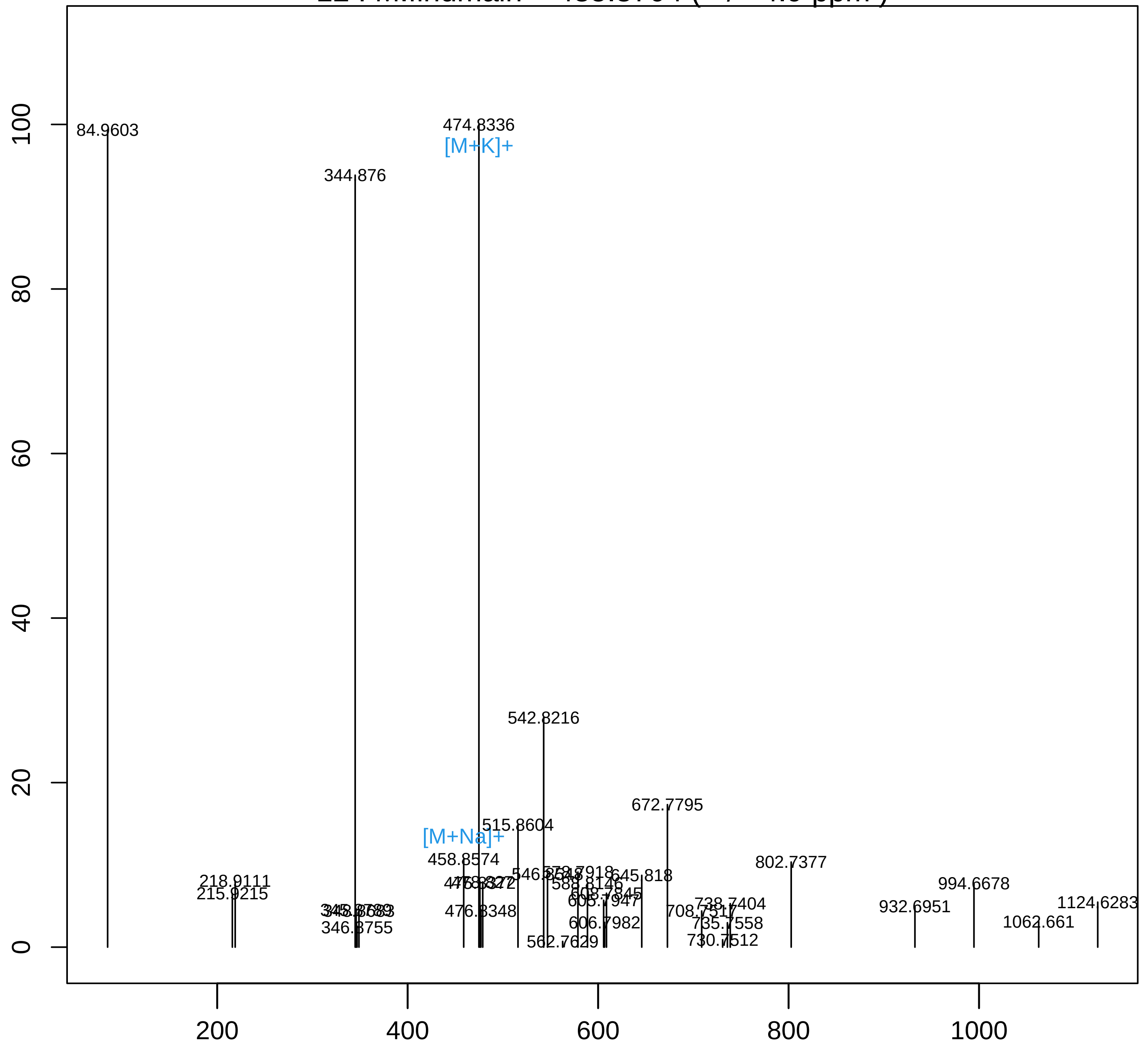

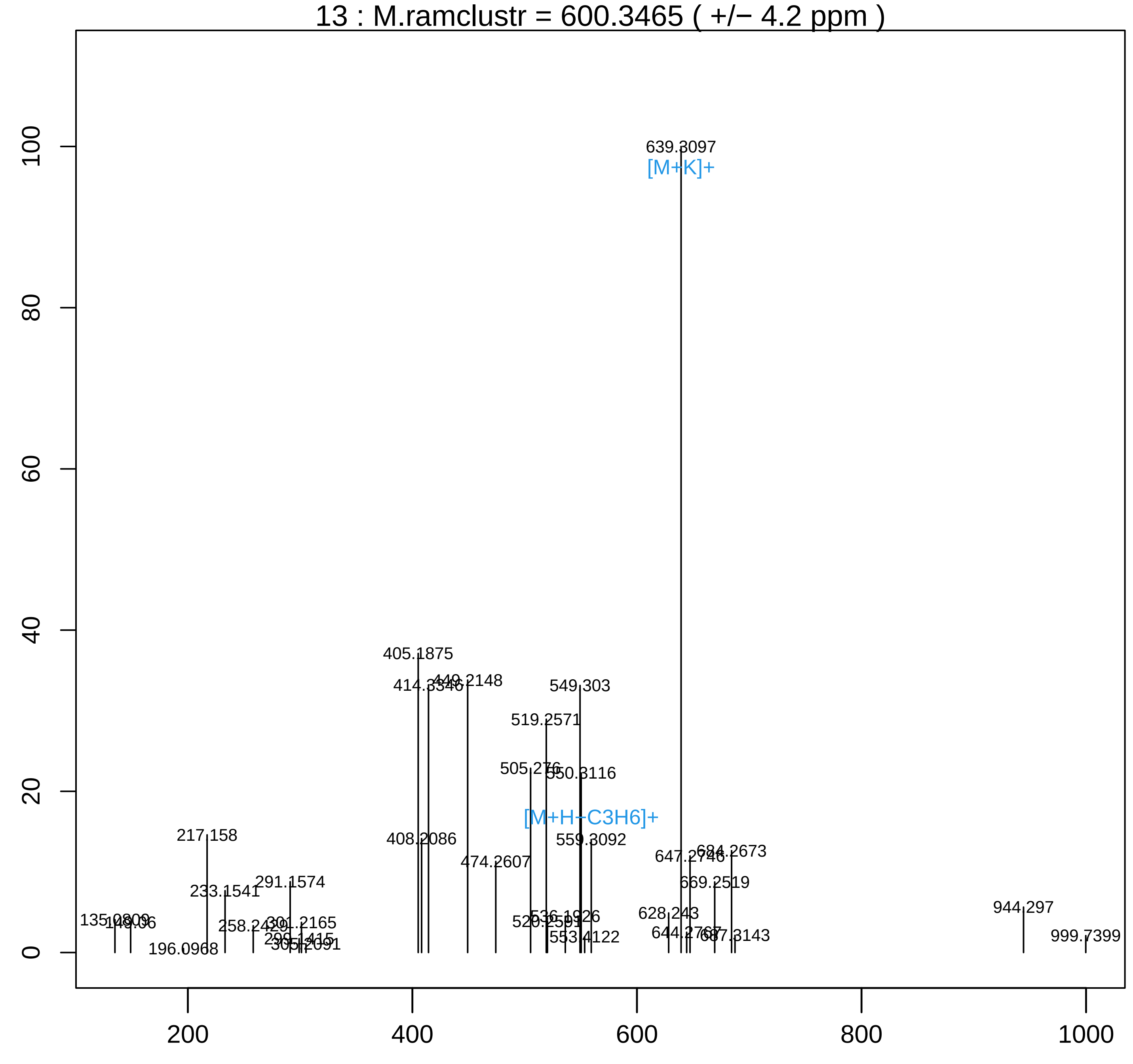
13 : M.ramclustr = 600.3465 (+/− 4.2 ppm)
639.3097
[M+K]+
405.1875
414.3546
449.2148
549.303
519.2571
505.276
550.3116
[M+H−C3H6]+
559.3092
217.158
408.2086
474.2607
647.2746
684.2673
669.2519
291.1574
233.1541
944.297
628.243
301.2165
258.2439
536.1926
520.2591
553.4122
644.2767
687.3143
999.7399
196.0968
0
20
40
60
80
100
200
400
600
800
1000

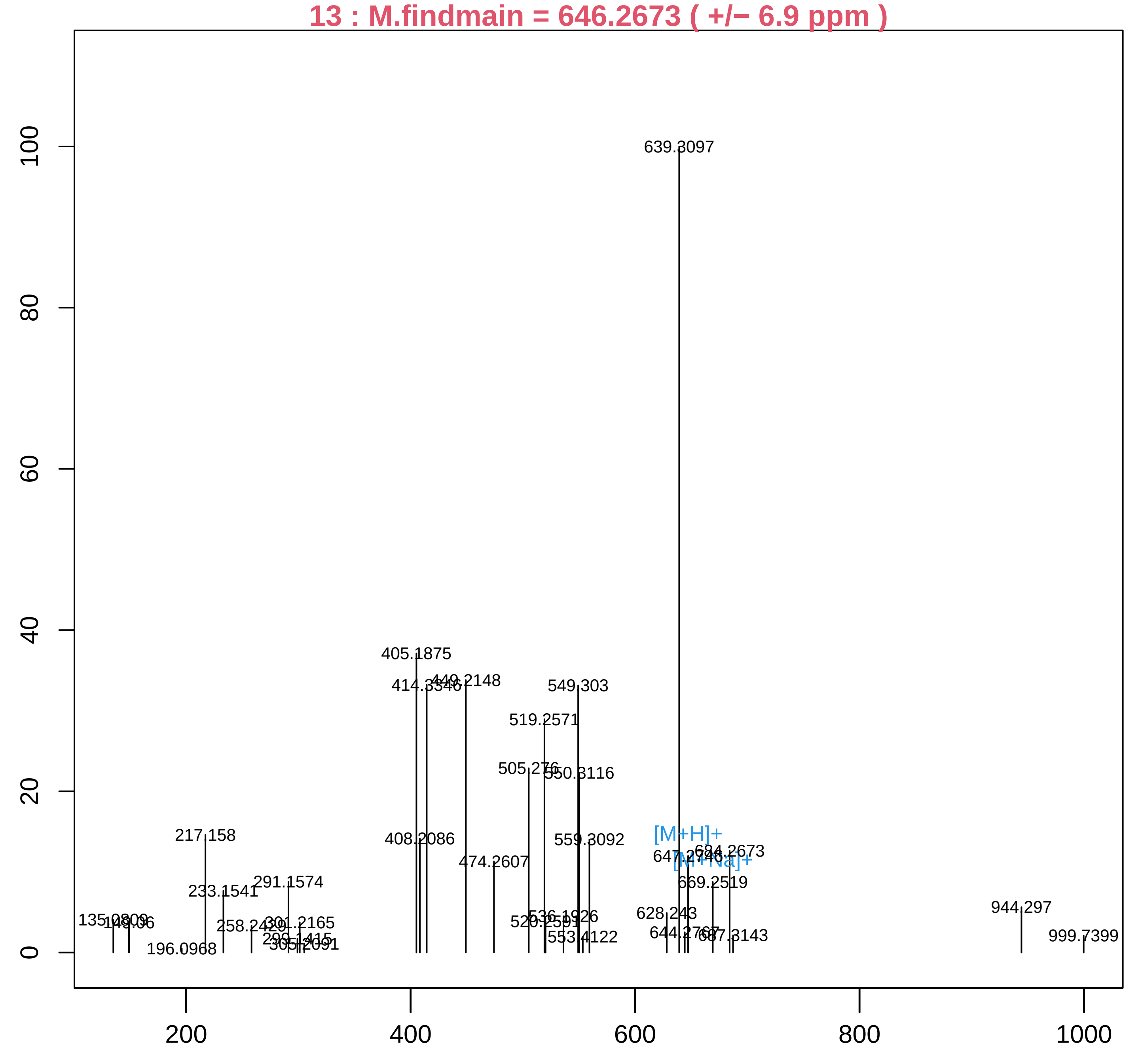
13 : M.findmain = 646.2673 (+/− 6.9 ppm)
639.3097
405.1875
414.3546
449.2148
549.303
519.2571
505.276
550.3116
[M+H]+
559.3092
217.158
408.2086
474.2607
684.2673
[M+Na]+
669.2519
291.1574
233.1541
944.297
628.243
301.2165
258.2439
536.1926
520.2591
553.4122
644.2767
687.3143
999.7399
196.0968
0
20
40
60
80
100
200
400
600
800
1000

14 : M.ramclustr = 919.531 (+/− 0.2 ppm)

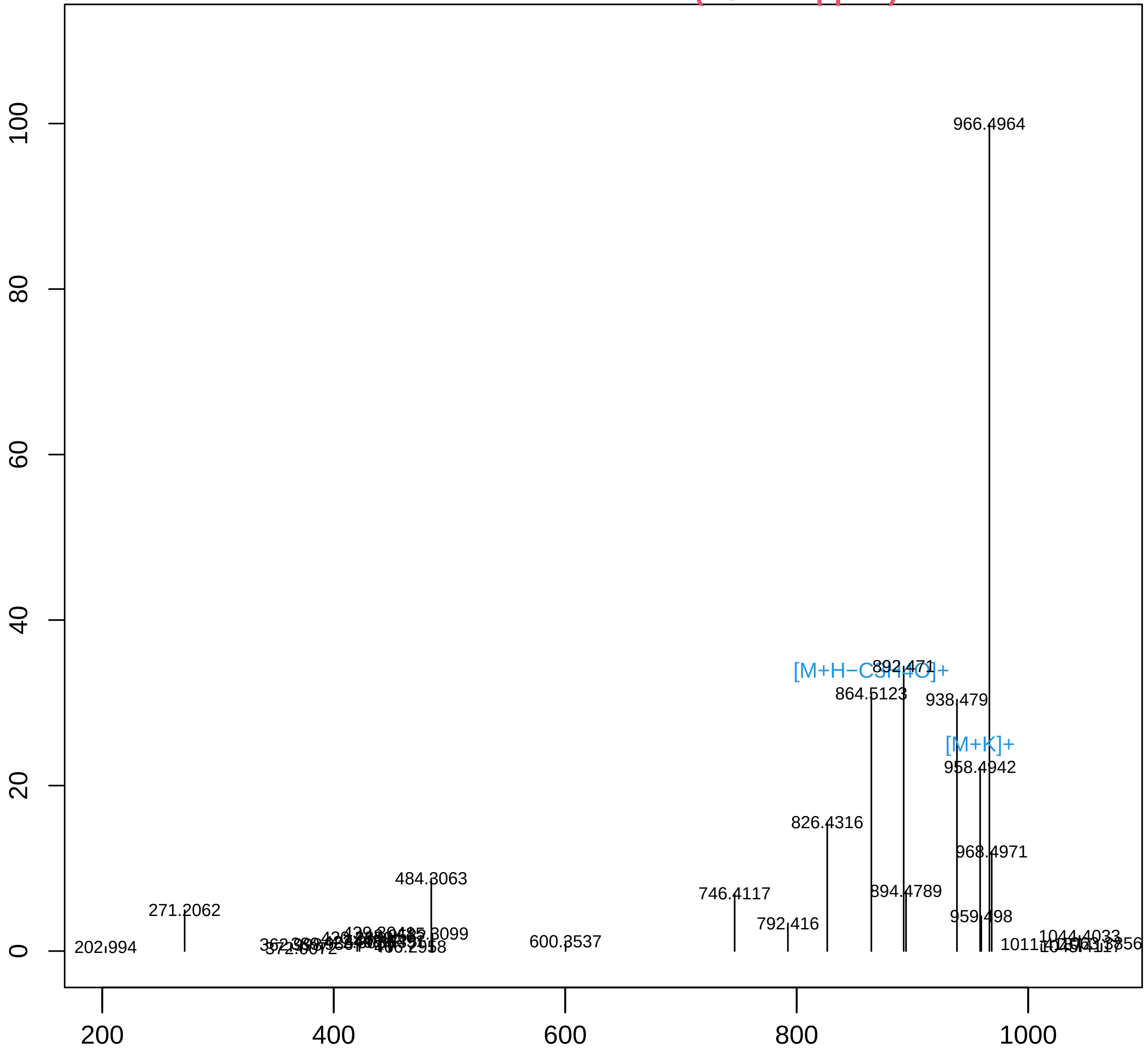

14 : M.findmain = 965.4891 (+/− 20 ppm)

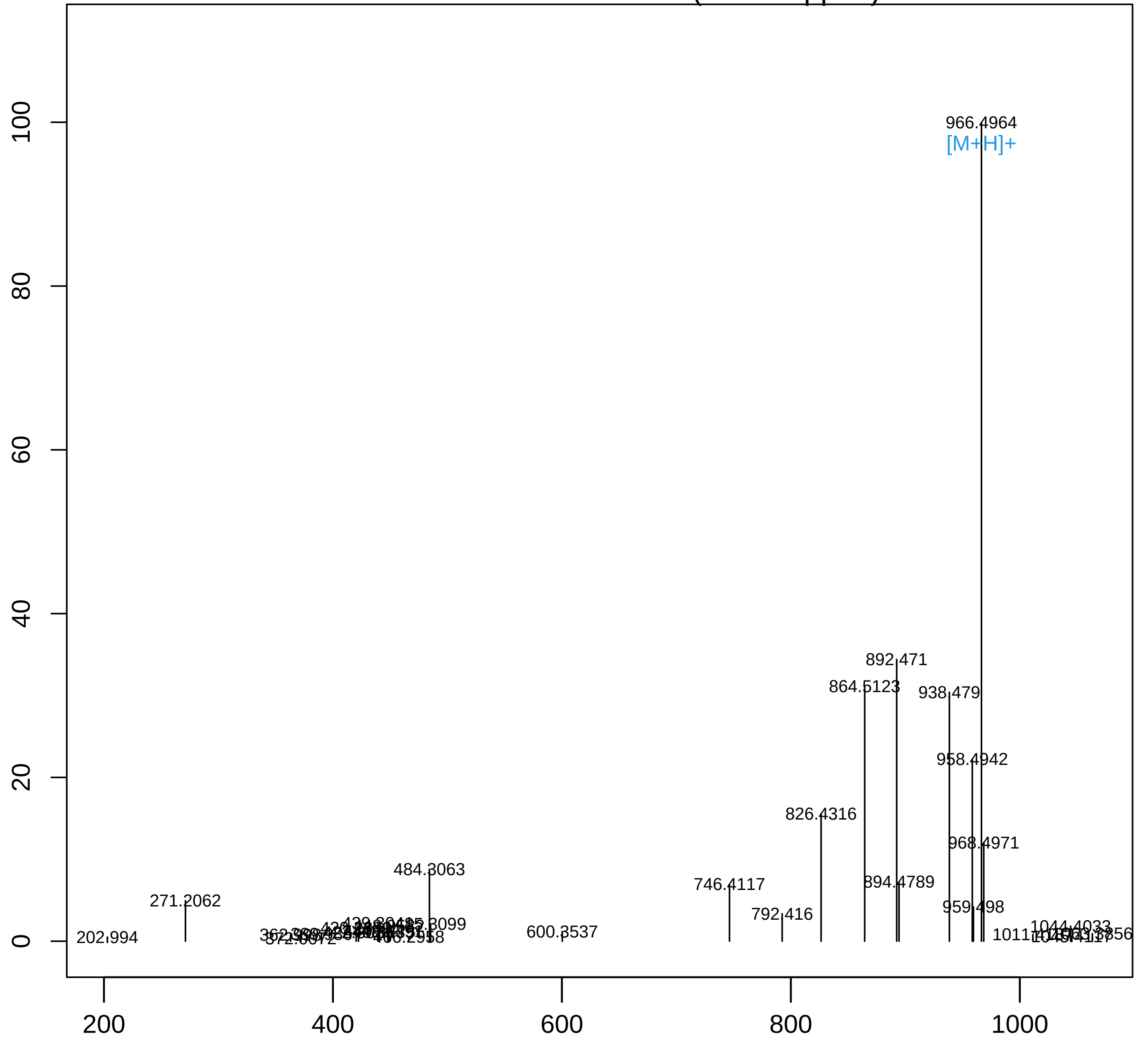

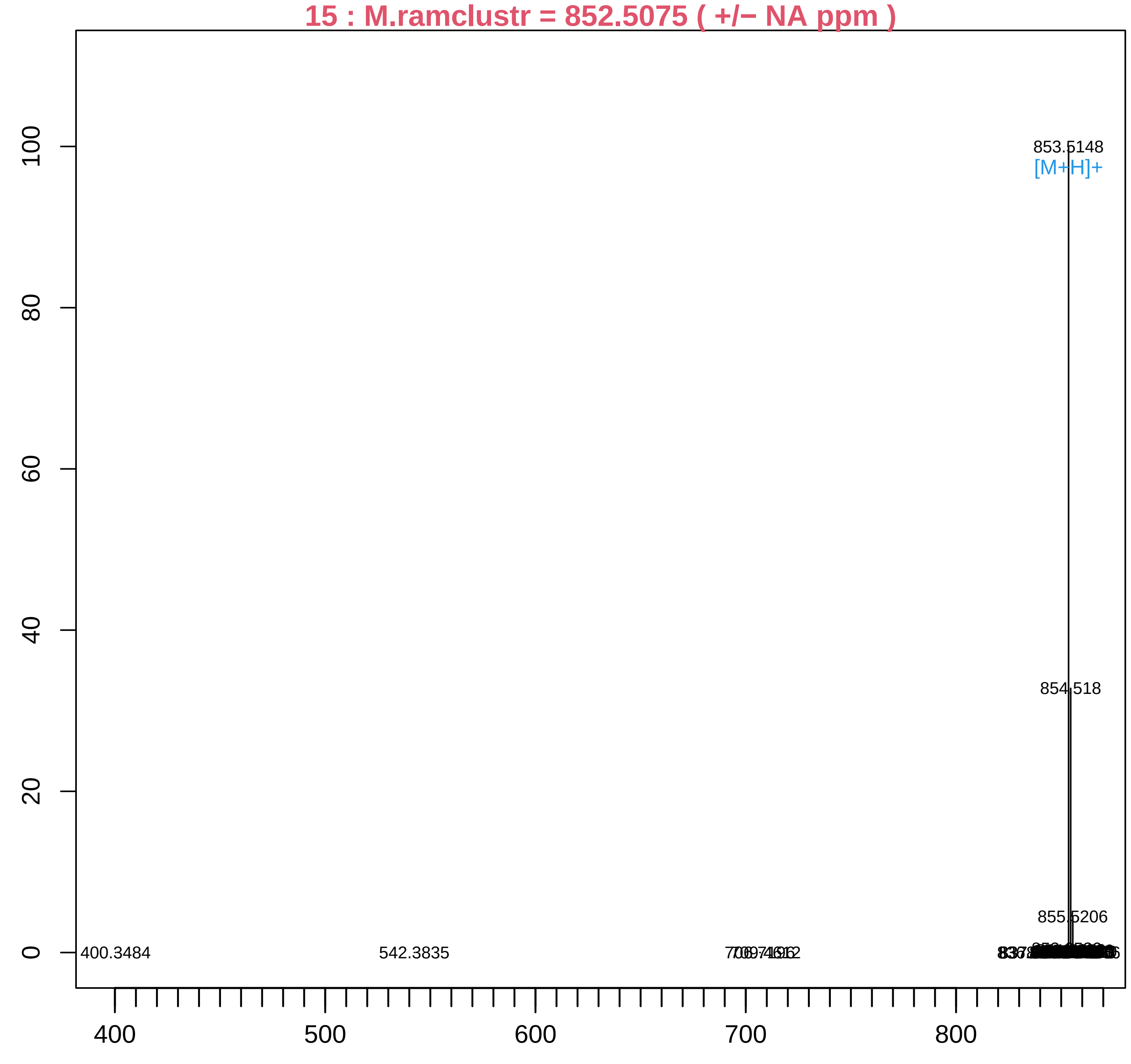
15 : M.ramclustr = 852.5075 (+/− NA ppm)
853.5148
[M+H]+
854.518
855.5206
400.3484
542.3835
0
20
40
60
80
100
400
500
600
700
800

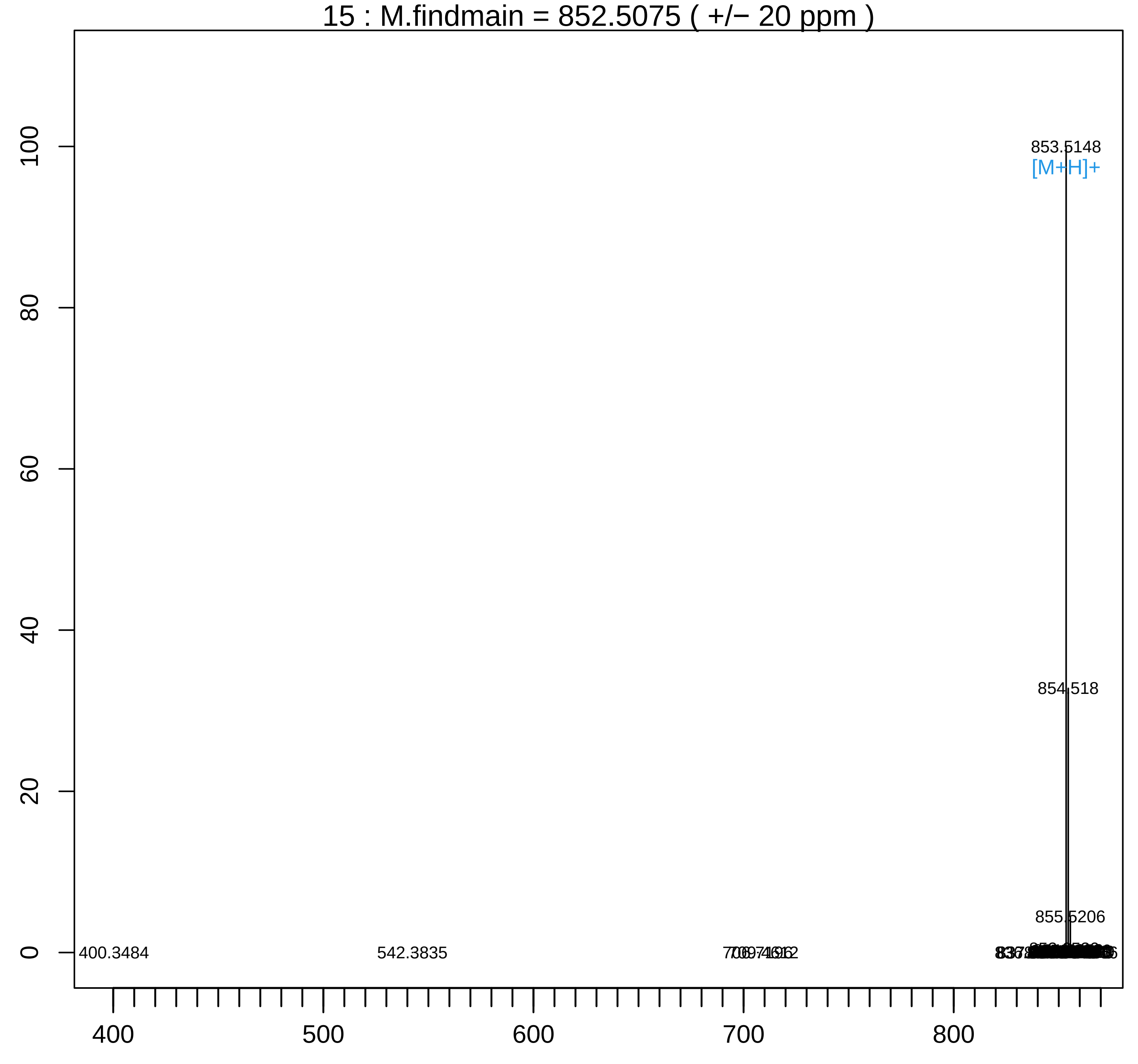
15 : M.findmain = 852.5075 (+/− 20 ppm)
853.5148
[M+H]+
854.518
855.5206
400.3484
542.3835
0
20
40
60
80
100
400
500
600
700
800

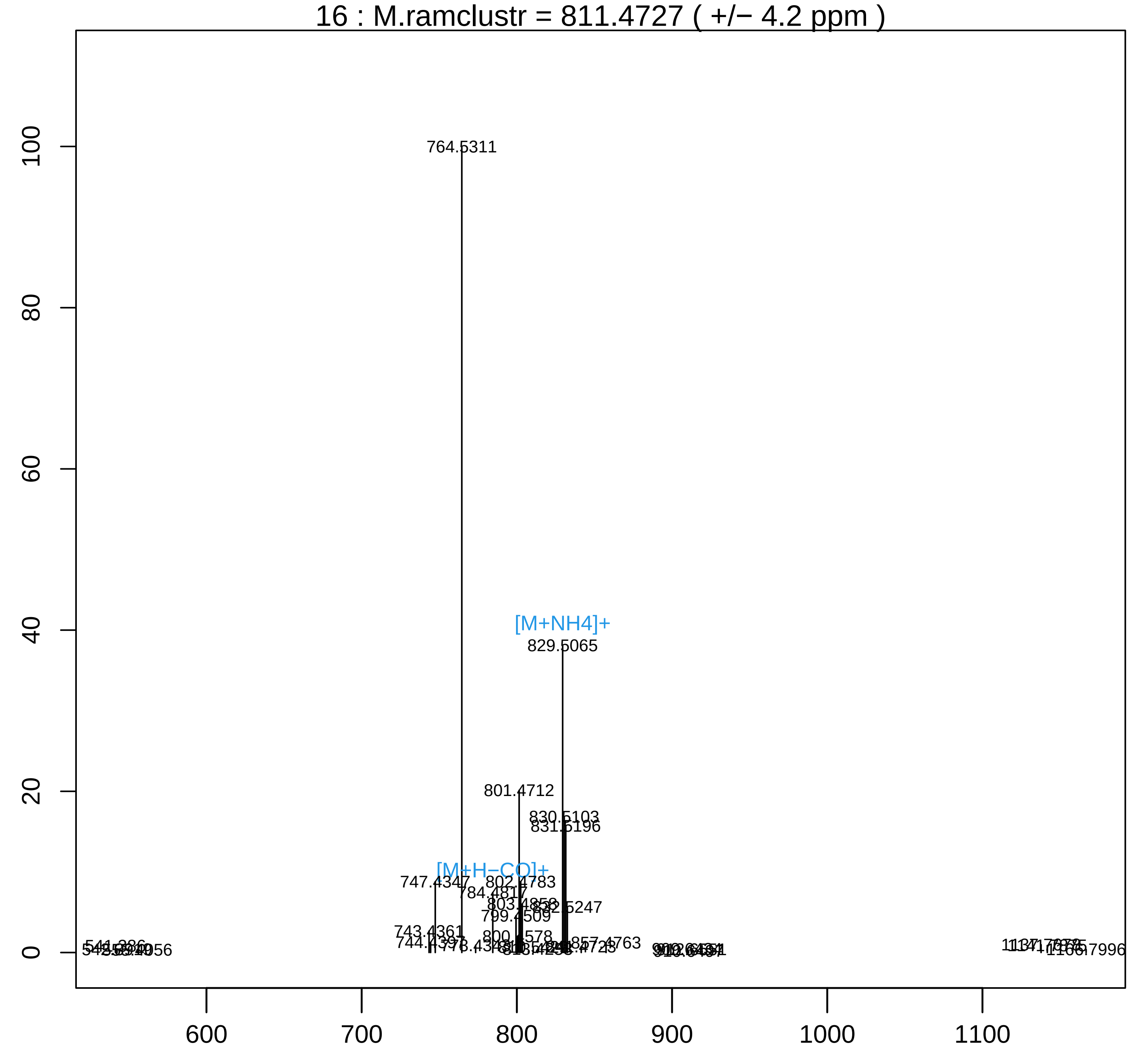

16 : M.ramclustr = 811.4727 (+/− 4.2 ppm)
764.5311
[M+NH4]+
829.5065
801.4712
830.5103
831.5196
[M+H−CO]+
747.4347
802.4783
743.4361
857.4763
1166.7996
0
20
40
60
80
100
600
700
800
900
1000
1100

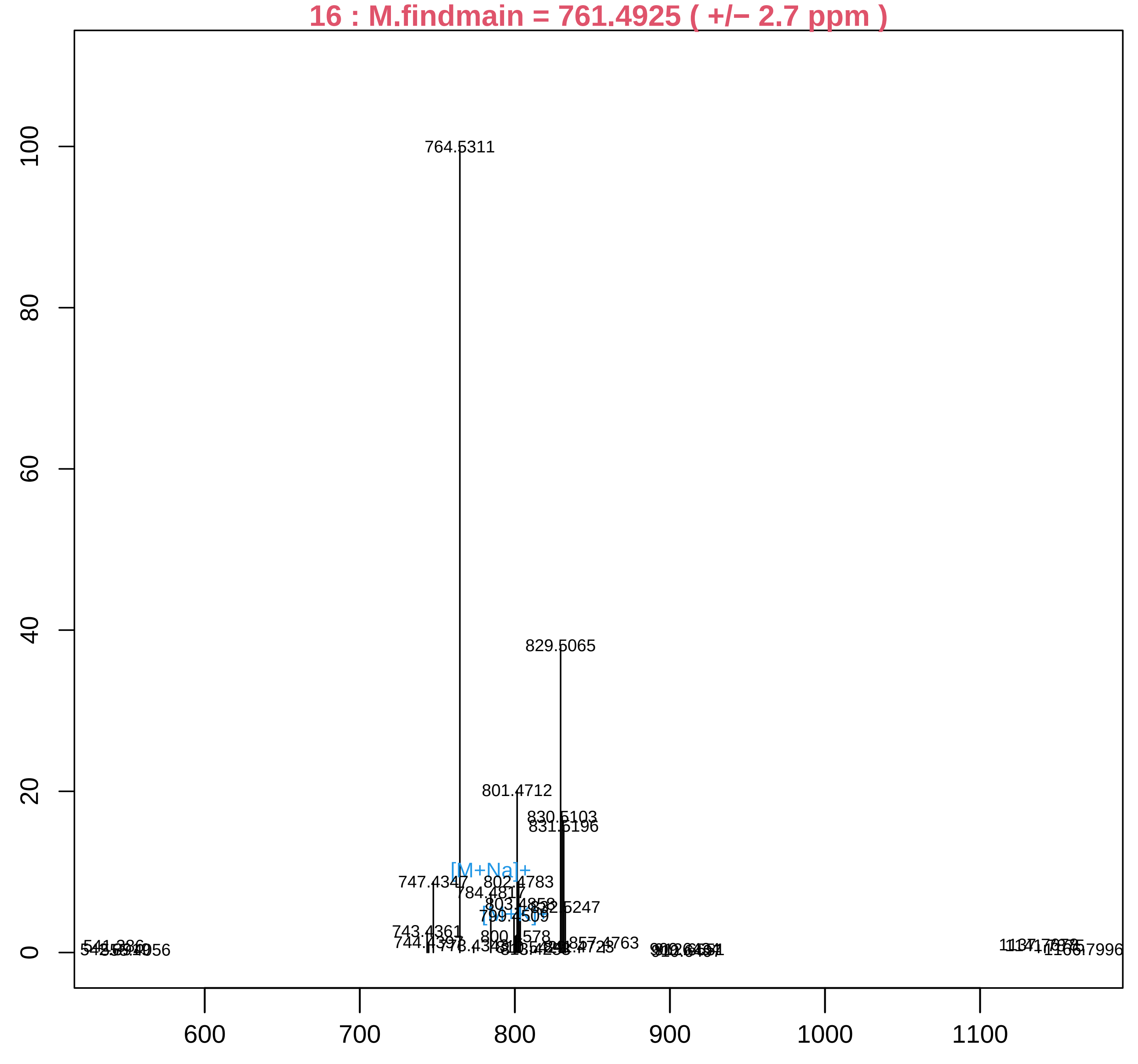

16 : M.findmain = 761.4925 (+/− 2.7 ppm)
764.5311
829.5065
801.4712
830.5103
831.5196
[M+Na]+
747.4347
802.4783
743.4361
857.4763
1166.7996
0
20
40
60
80
100
600
700
800
900
1000
1100

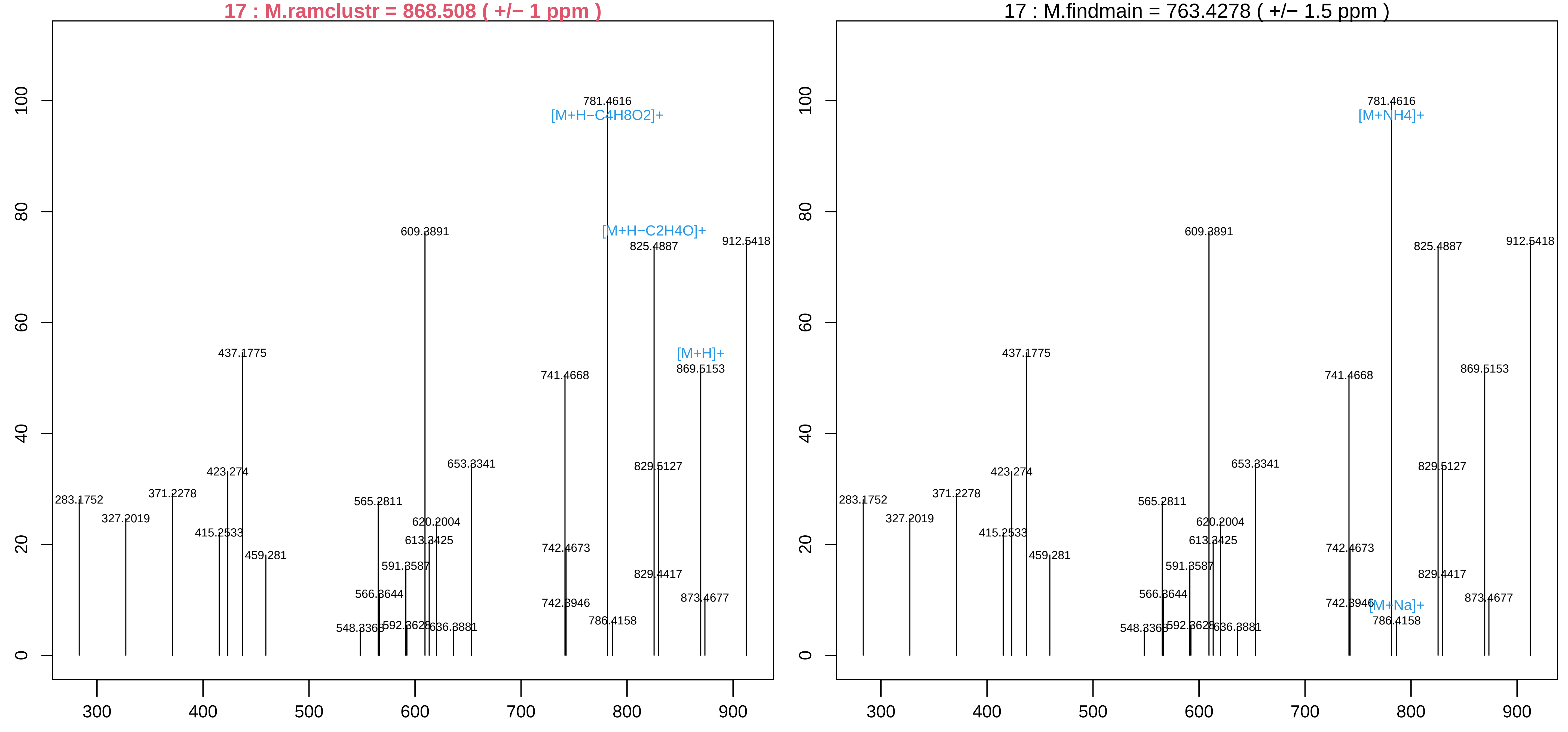
17 : M.ramclustr = 868.508 (+/− 1 ppm)
17 : M.findmain = 763.4278 (+/− 1.5 ppm)
781.4616
[M+H−C4H8O2]+
[M+H−C2H4O]+
825.4887
912.5418
609.3891
[M+H]+
869.5153
437.1775
741.4668
653.3341
829.5127
423.274
371.2278
283.1752
565.2811
327.2019
620.2004
415.2533
613.3425
459.281
742.4673
591.3587
829.4417
566.3644
873.4677
742.3946
786.4158
548.3368
592.3628
636.3881
[M+NH4]+
[M+Na]+
0
20
40
60
80
100
300
400
500
600
700
800
900

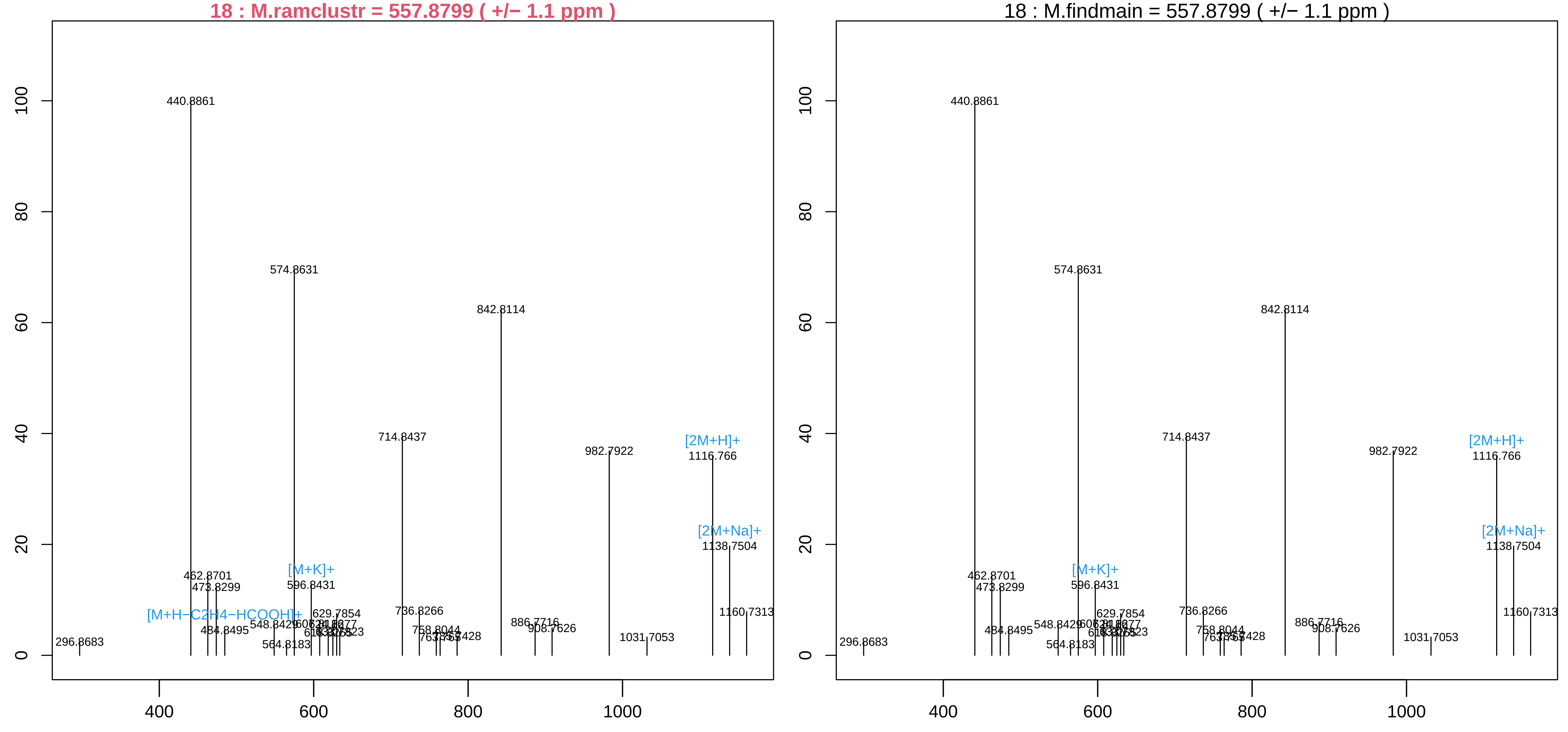
18 : M.ramclustr = 557.8799 (+/− 1.1 ppm)
440.8861
574.8631
842.8114
714.8437
982.7922
[2M+H]+
1116.766
[2M+Na]+
1138.7504
[M+K]+
596.8431
462.8701
473.8299
[M+H−C2H4−HCOOH]+
484.8495
548.8429
564.8183
629.7854
736.8266
758.8044
886.7716
908.7626
1031.7053
1160.7313
296.8683
18 : M.findmain = 557.8799 (+/− 1.1 ppm)
440.8861
574.8631
842.8114
714.8437
982.7922
[2M+H]+
1116.766
[2M+Na]+
1138.7504
[M+K]+
596.8431
462.8701
473.8299
484.8495
548.8429
564.8183
629.7854
736.8266
758.8044
886.7716
908.7626
1031.7053
1160.7313
296.8683

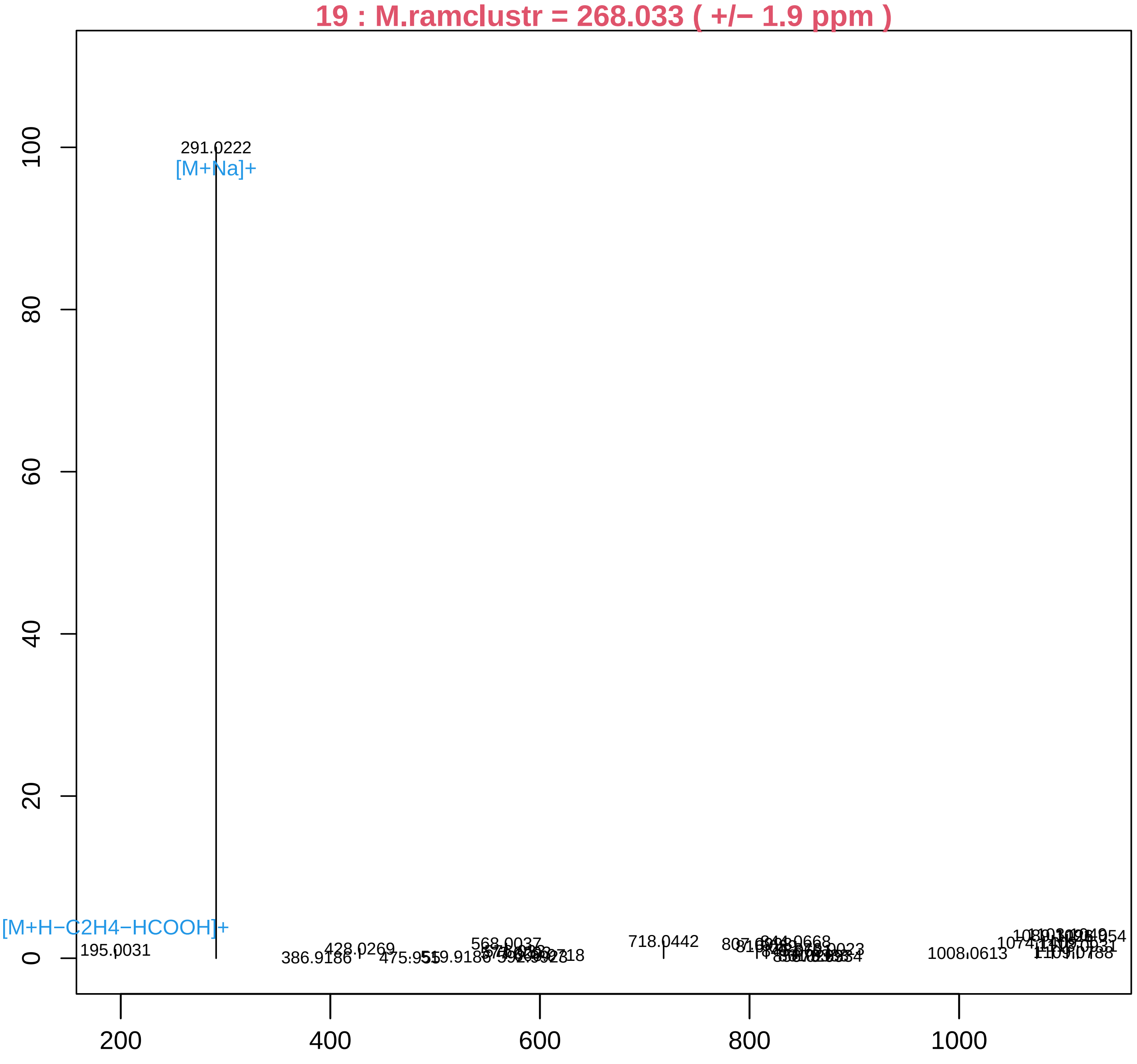

19 : M.ramclustr = 268.033 (+/− 1.9 ppm)
291.0222
[M+Na]+
[M+H−C2H4−HCOOH]+
195.0031
386.9186
428.0269
718.0442
1008.0613
0
20
40
60
80
100
200
400
600
800
1000

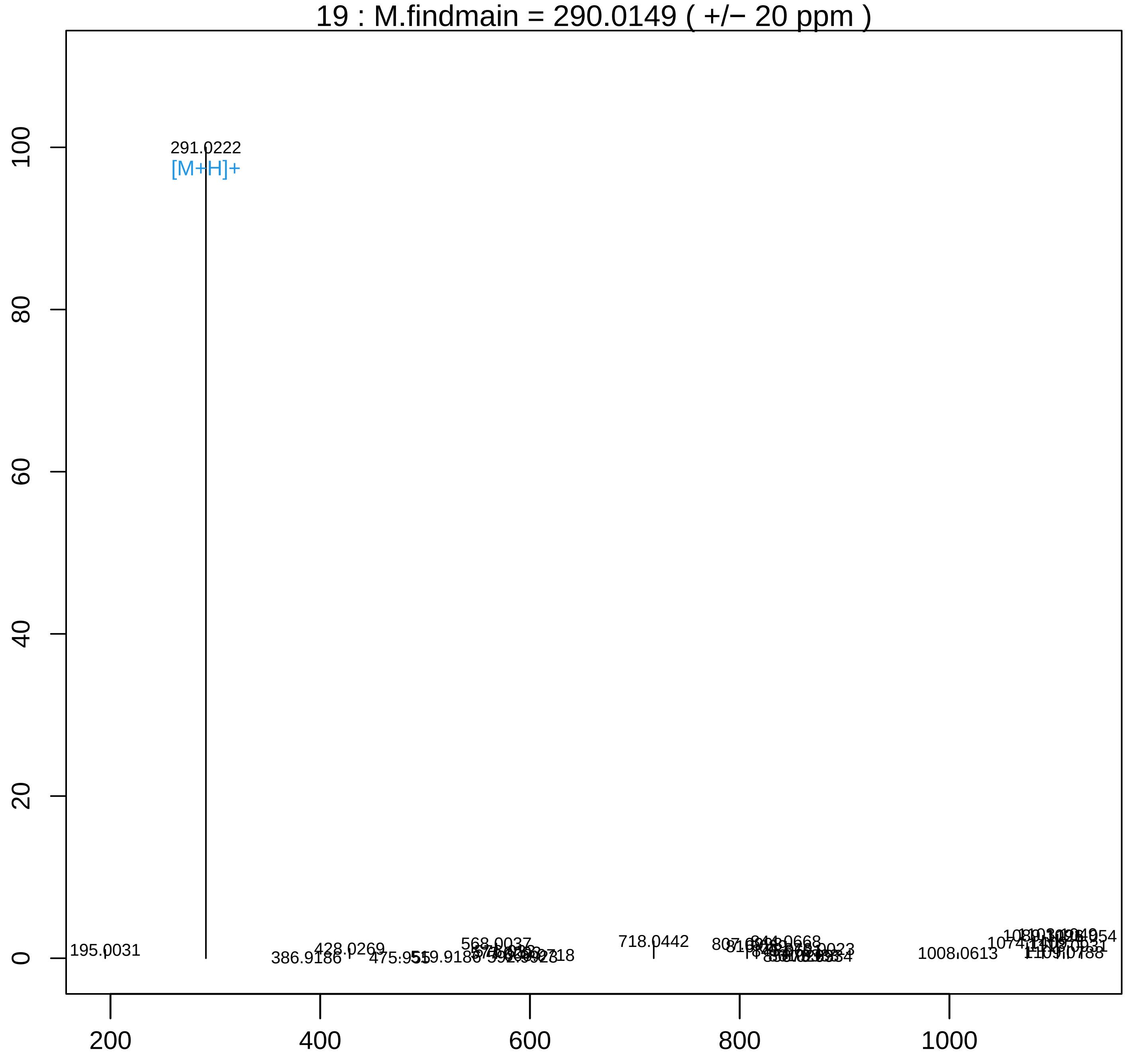

19 : M.findmain = 290.0149 (+/− 20 ppm)
291.0222
[M+H]+
195.0031
386.9186
428.0269
718.0442
1008.0613
0
20
40
60
80
100
200
400
600
800
1000

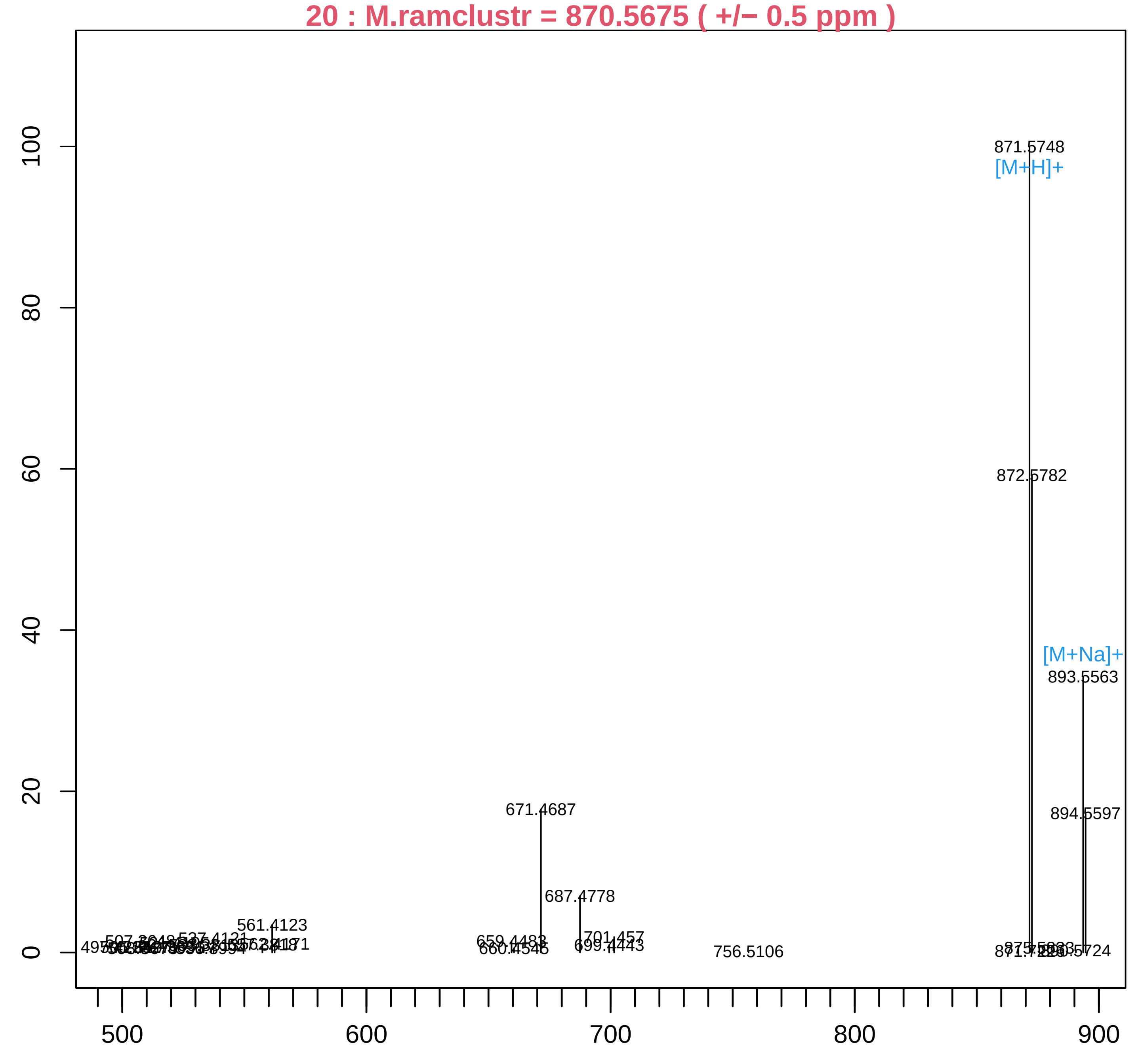
20 : M.ramclustr = 870.5675 (+/− 0.5 ppm)
871.5748
[M+H]+
872.5782
[M+Na]+
893.5563
894.5597
671.4687
687.4778
561.4123
659.4483
660.4546
701.457
699.4443
756.5106
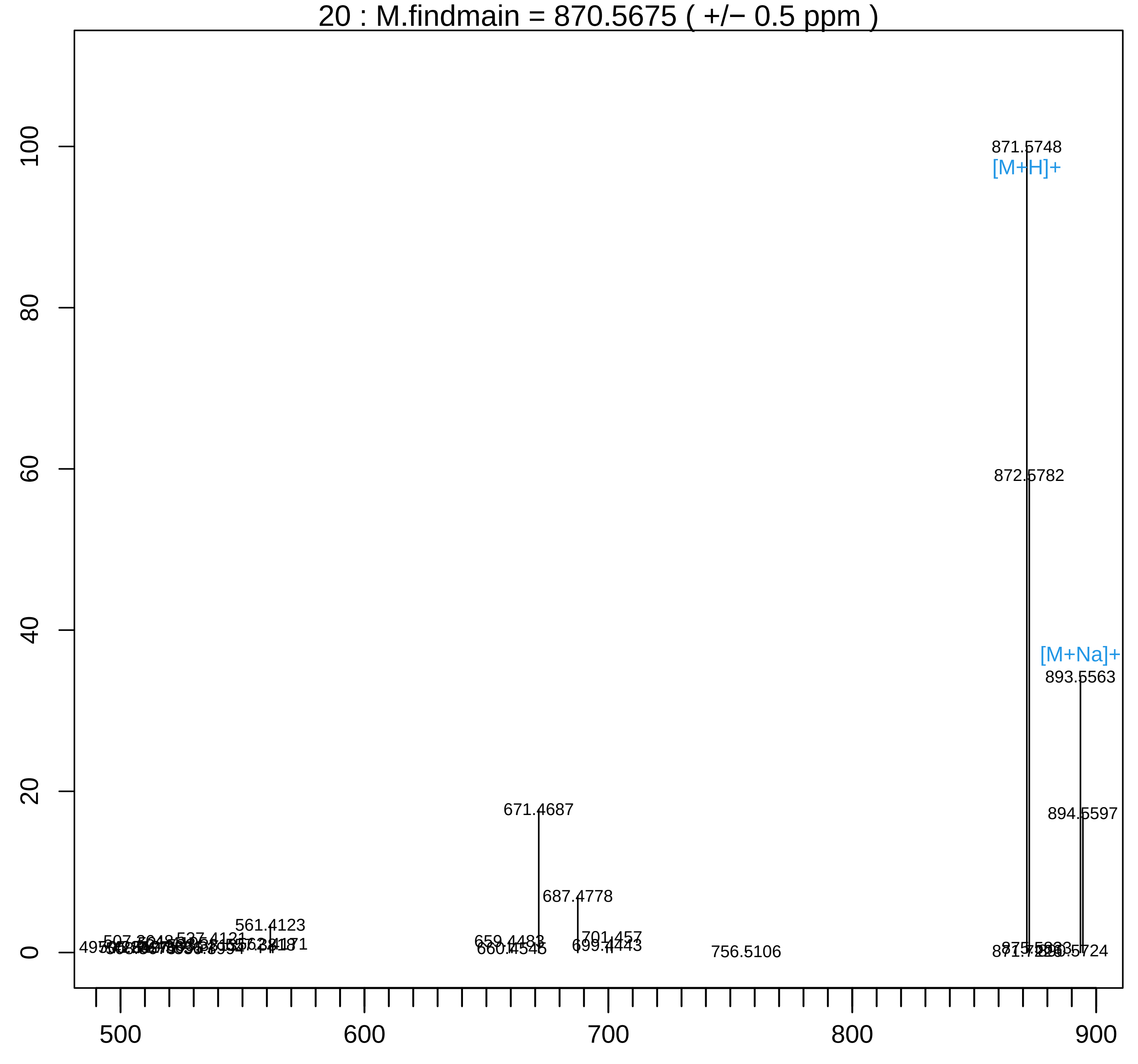
20 : M.findmain = 870.5675 (+/− 0.5 ppm)
871.5748
[M+H]+
872.5782
[M+Na]+
893.5563
894.5597
671.4687
687.4778
561.4123
659.4483
660.4546
701.457
699.4443
756.5106

21 : M.ramclustr = 614.4888 (+/− NA ppm)

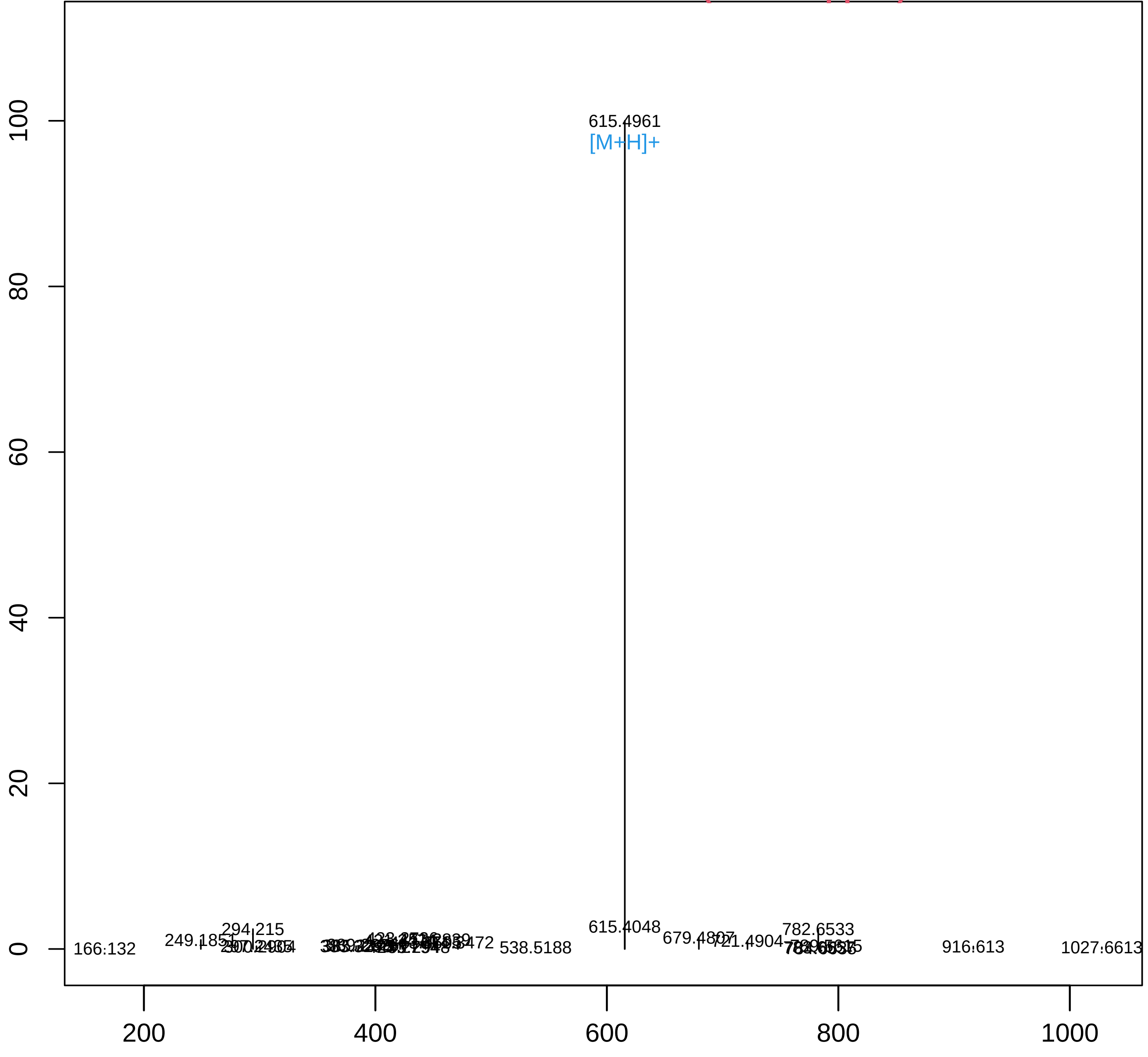

21 : M.findmain = 614.4888 (+/− 20 ppm)

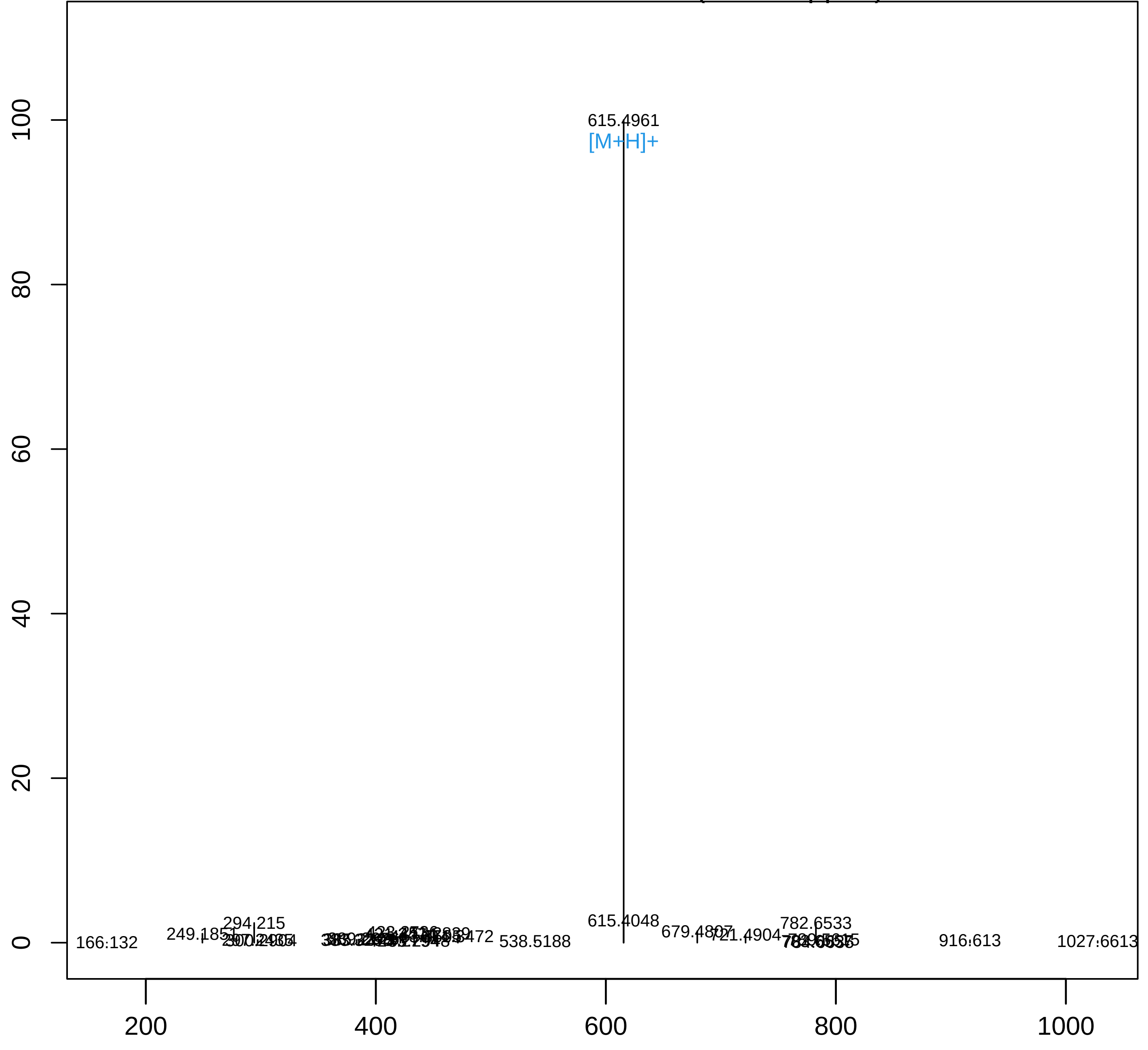

22 : M.ramclustr = 364.0985 (+/− NA ppm)

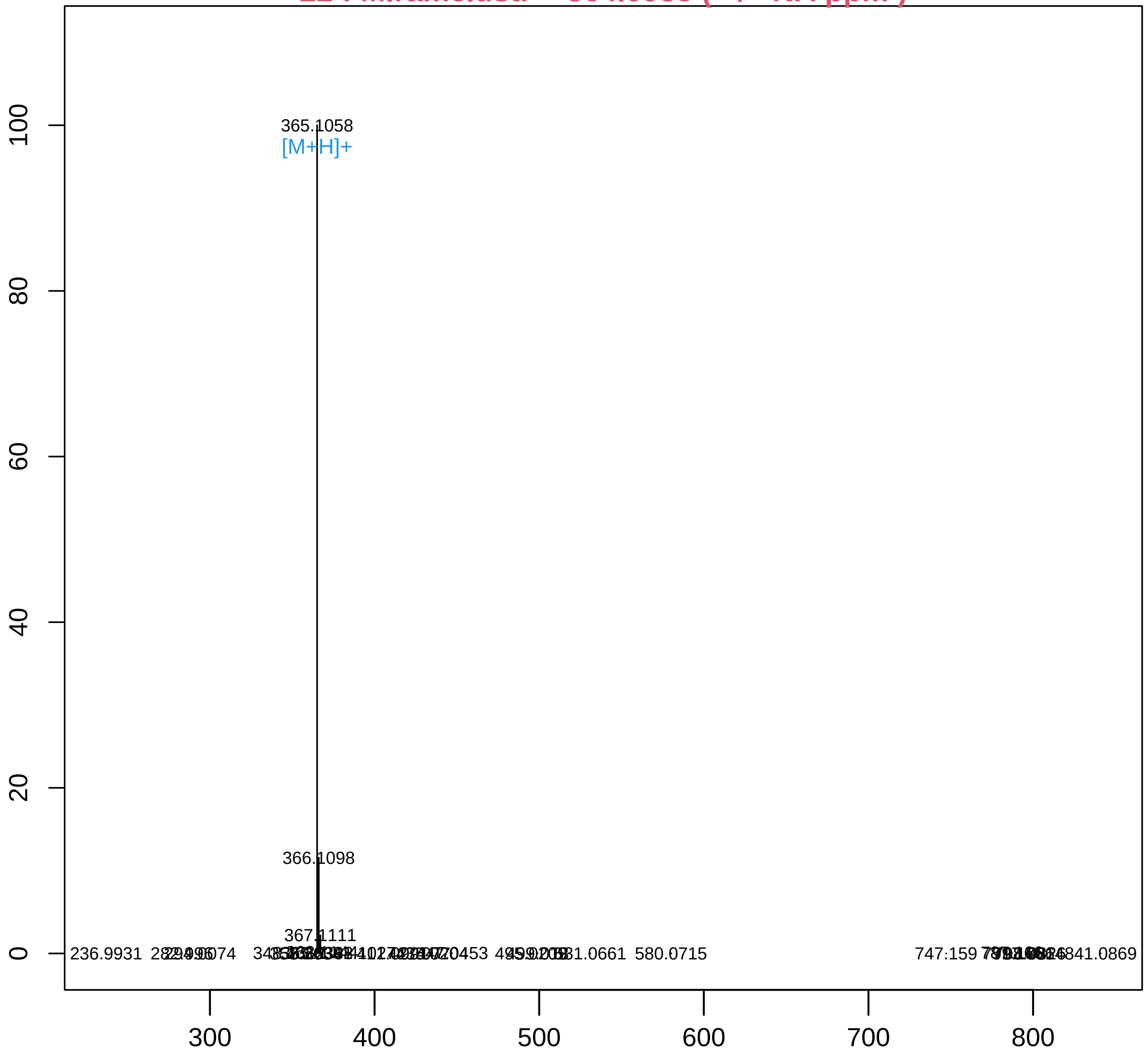

22 : M.findmain = 364.0985 (+/− 20 ppm)

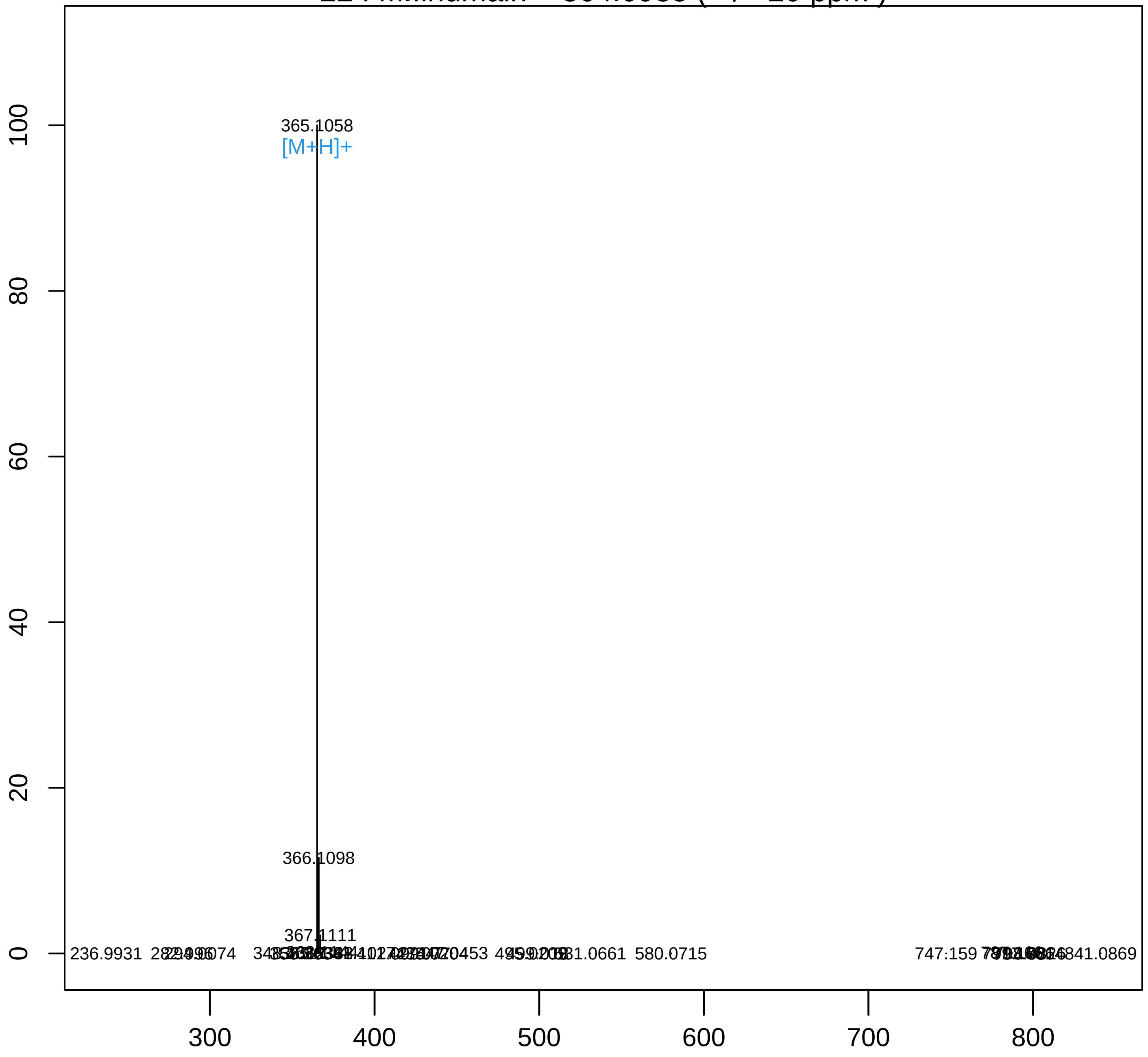

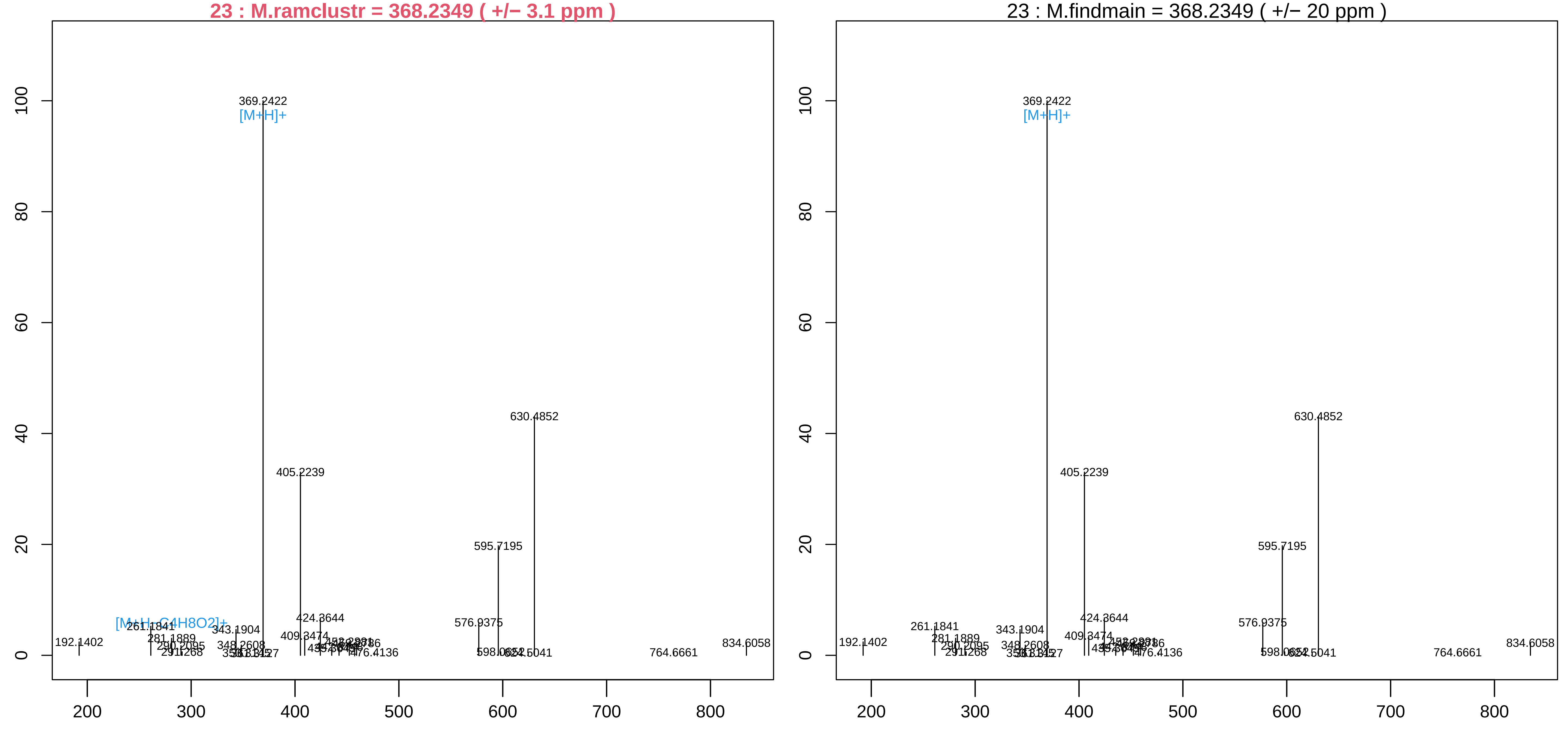
23 : M.ramclustr = 368.2349 (+/− 3.1 ppm)
369.2422
[M+H]+
630.4852
405.2239
595.7195
424.3644
576.9375
[M+H-C4H8O2]+
261.1841
343.1904
409.3474
192.1402
281.1889
290.2095
291.1268
348.2608
476.4136
598.0652
624.5041
764.6661
834.6058
23 : M.findmain = 368.2349 (+/− 20 ppm)
369.2422
[M+H]+
630.4852
405.2239
595.7195
424.3644
576.9375
261.1841
343.1904
409.3474
192.1402
281.1889
290.2095
291.1268
348.2608
476.4136
598.0652
624.5041
764.6661
834.6058

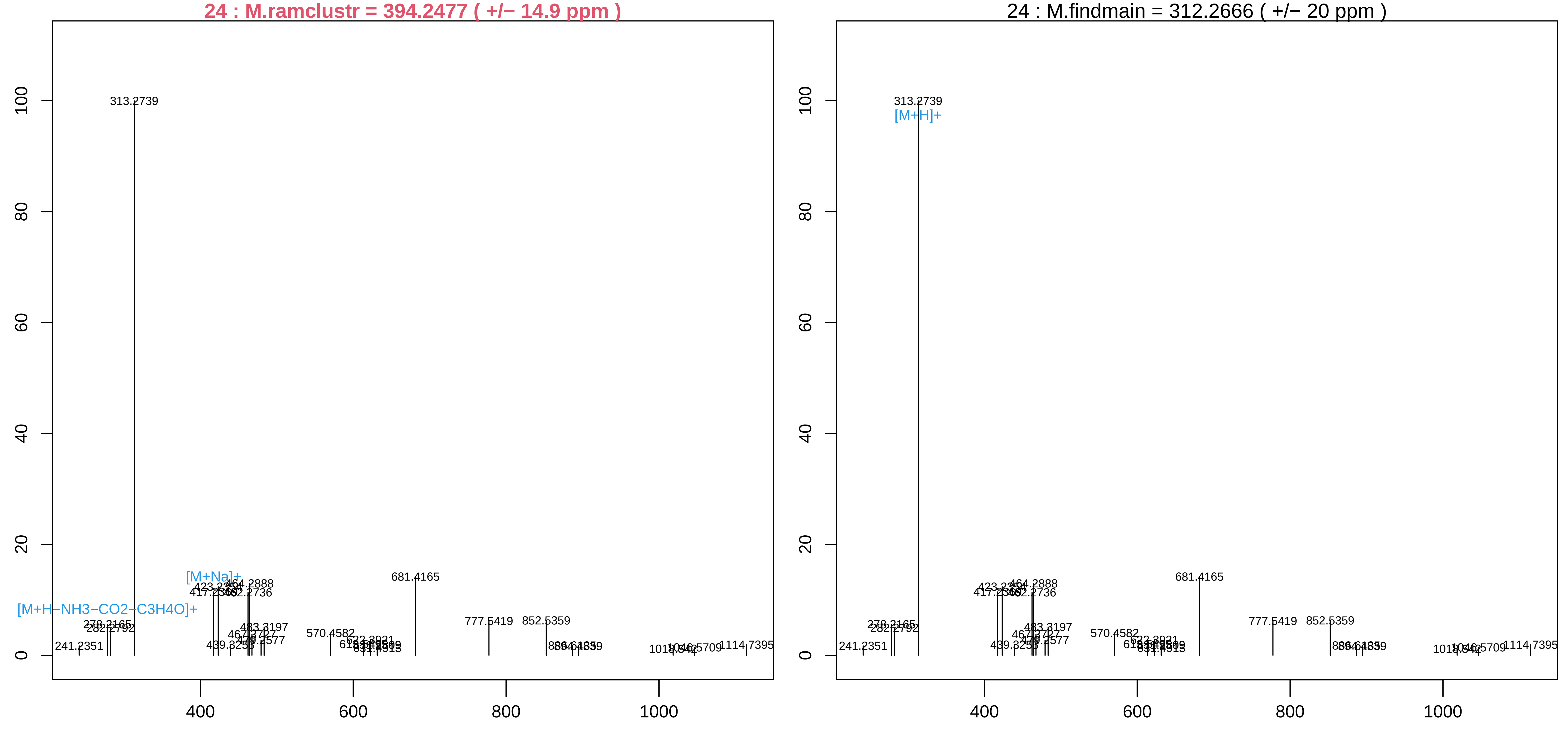
24 : M.ramclustr = 394.2477 (+/− 14.9 ppm)
313.2739
[M+Na]+
464.2888
681.4165
[M+H−NH3−CO2−C3H4O]+
777.5419
852.5359
570.4582
483.3197
241.2351
1114.7395
24 : M.findmain = 312.2666 (+/− 20 ppm)
313.2739
[M+H]+
464.2888
681.4165
777.5419
852.5359
570.4582
483.3197
241.2351
1114.7395
0
20
40
60
80
100
400
600
800
1000

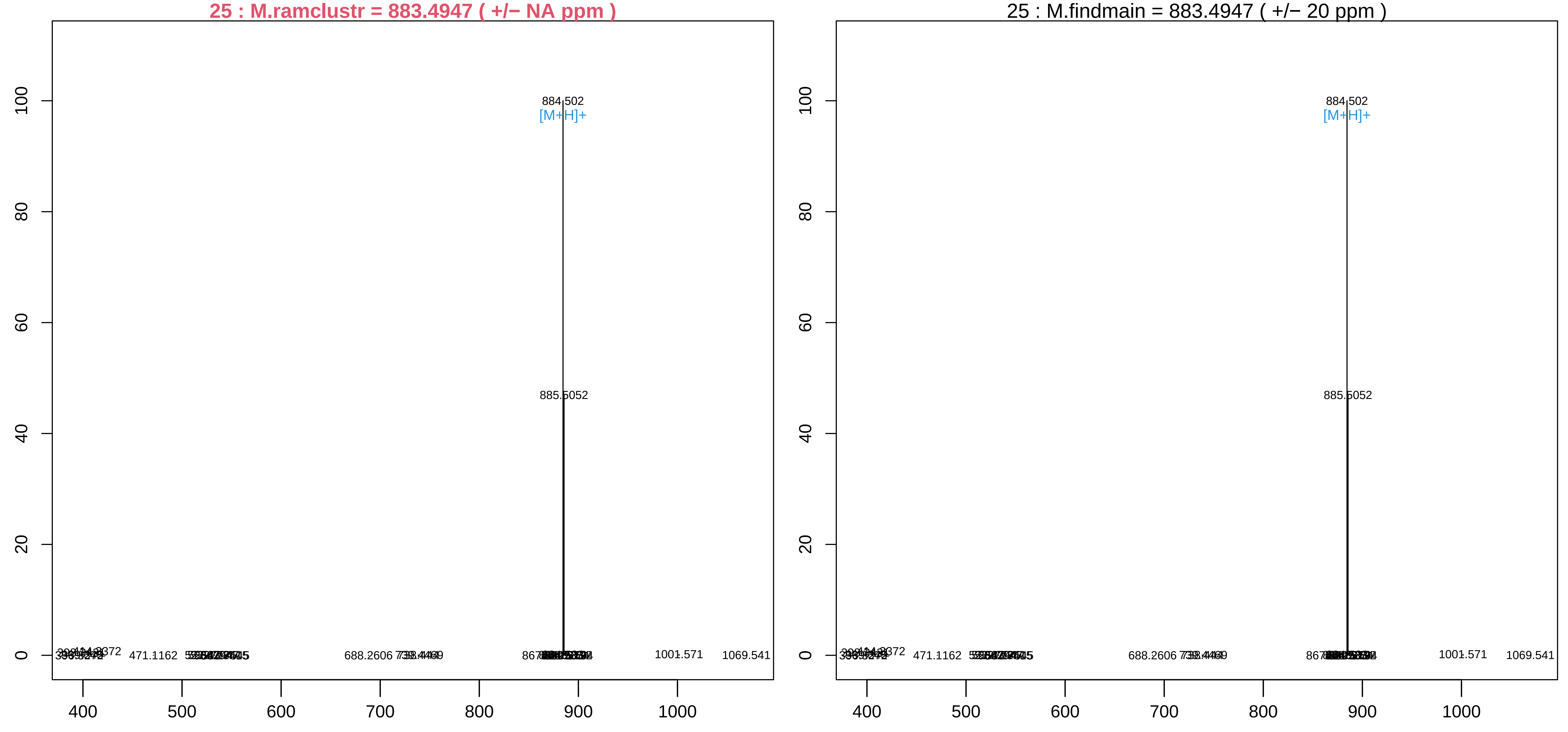
25 : M.ramclustr = 883.4947 (+/− NA ppm)
25 : M.findmain = 883.4947 (+/− 20 ppm)
884.502
[M+H]+
885.5052
471.1162
688.2606
1001.571
1069.541
0
20
40
60
80
100
400
500
600
700
800
900
1000

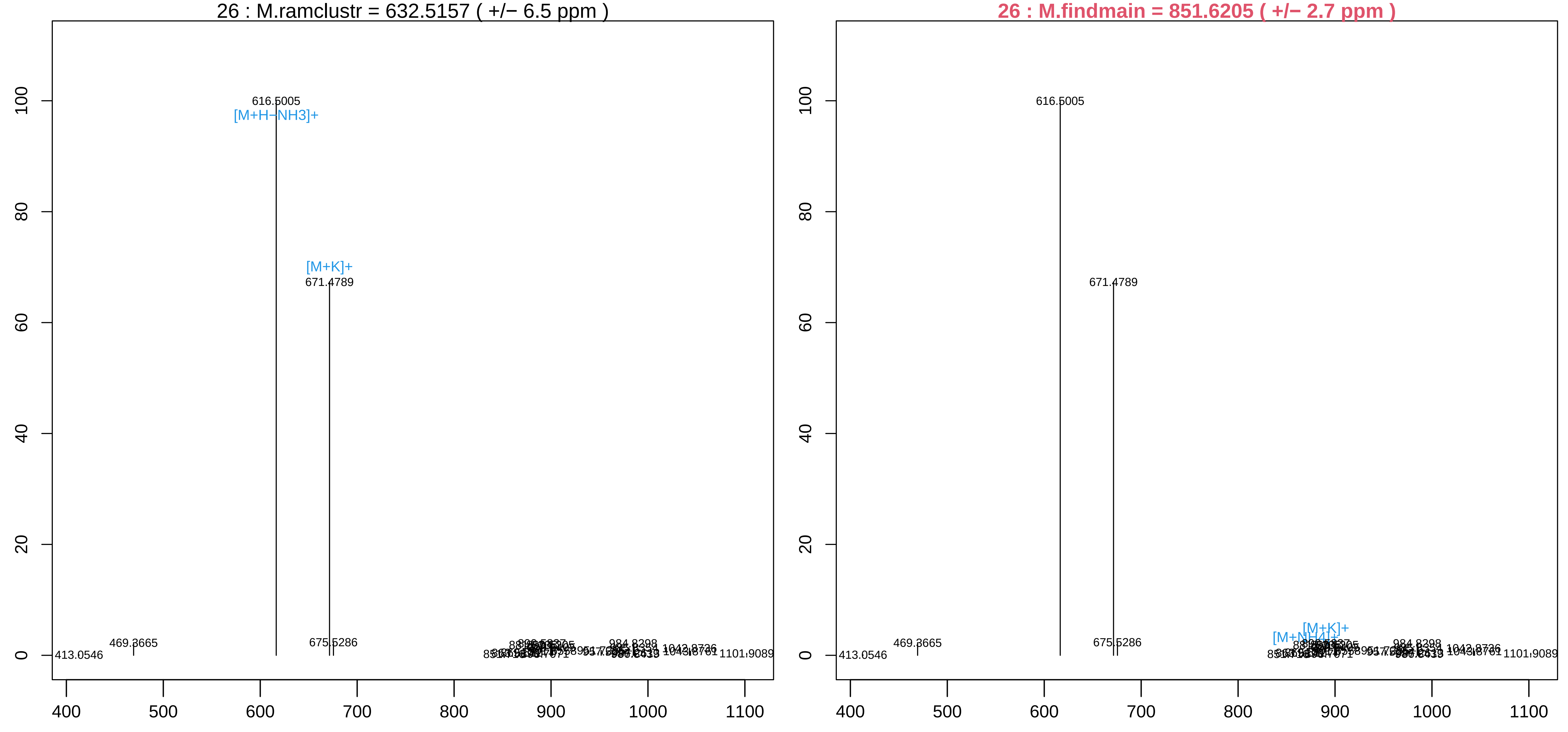
26 : M.ramclustr = 632.5157 (+/− 6.5 ppm)
26 : M.findmain = 851.6205 (+/− 2.7 ppm)
616.5005
[M+H−NH3]+
[M+K]+
671.4789
675.5286
469.3665
413.0546
984.8298
1042.8736
1101.9089
[M+K]+
0
20
40
60
80
100
400
500
600
700
800
900
1000
1100

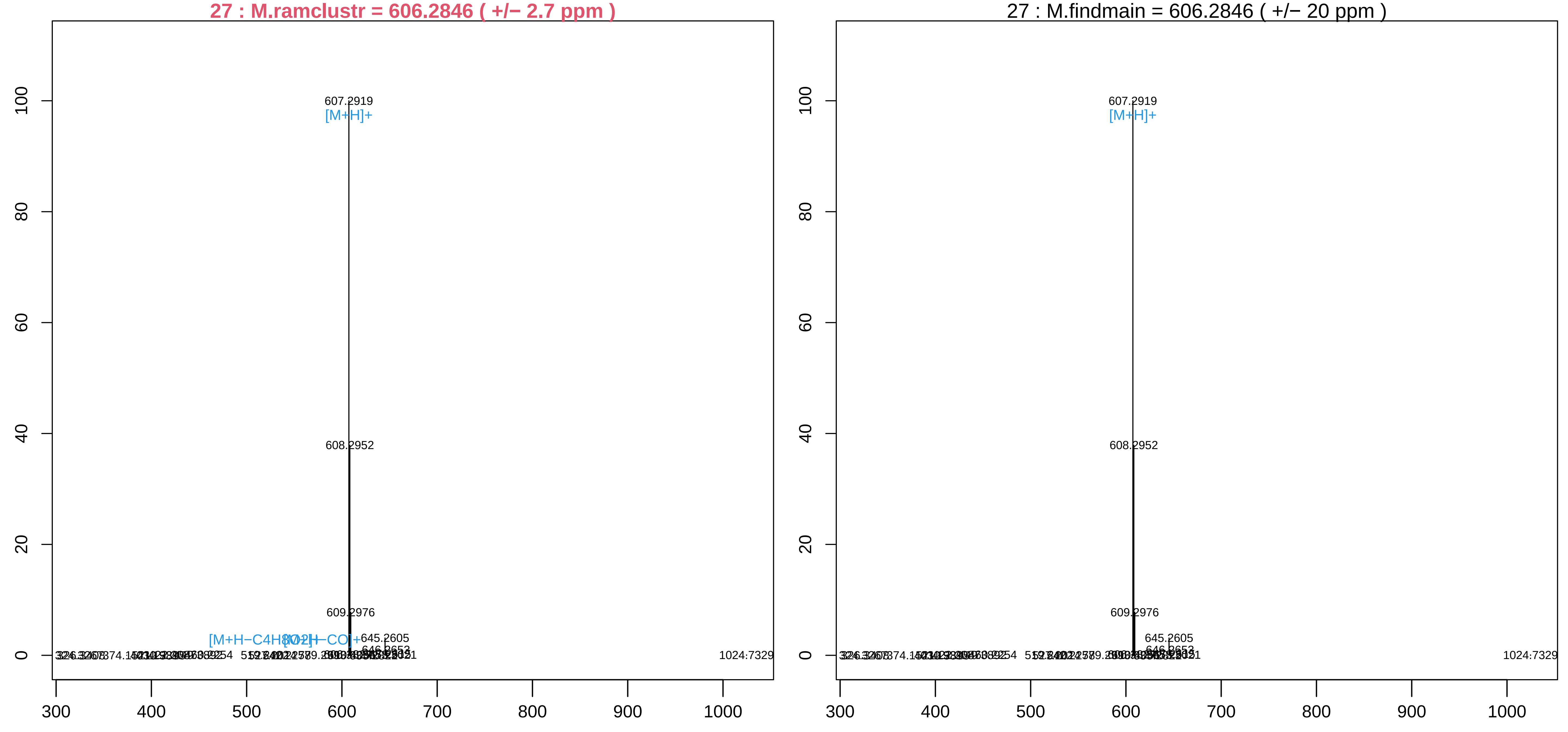
27 : M.ramclustr = 606.2846 (+/− 2.7 ppm)
607.2919
[M+H]+
608.2952
609.2976
645.2605
1024:7329
0
20
40
60
80
100
300
400
500
600
700
800
900
1000
27 : M.findmain = 606.2846 (+/− 20 ppm)
607.2919
[M+H]+
608.2952
609.2976
645.2605
1024:7329

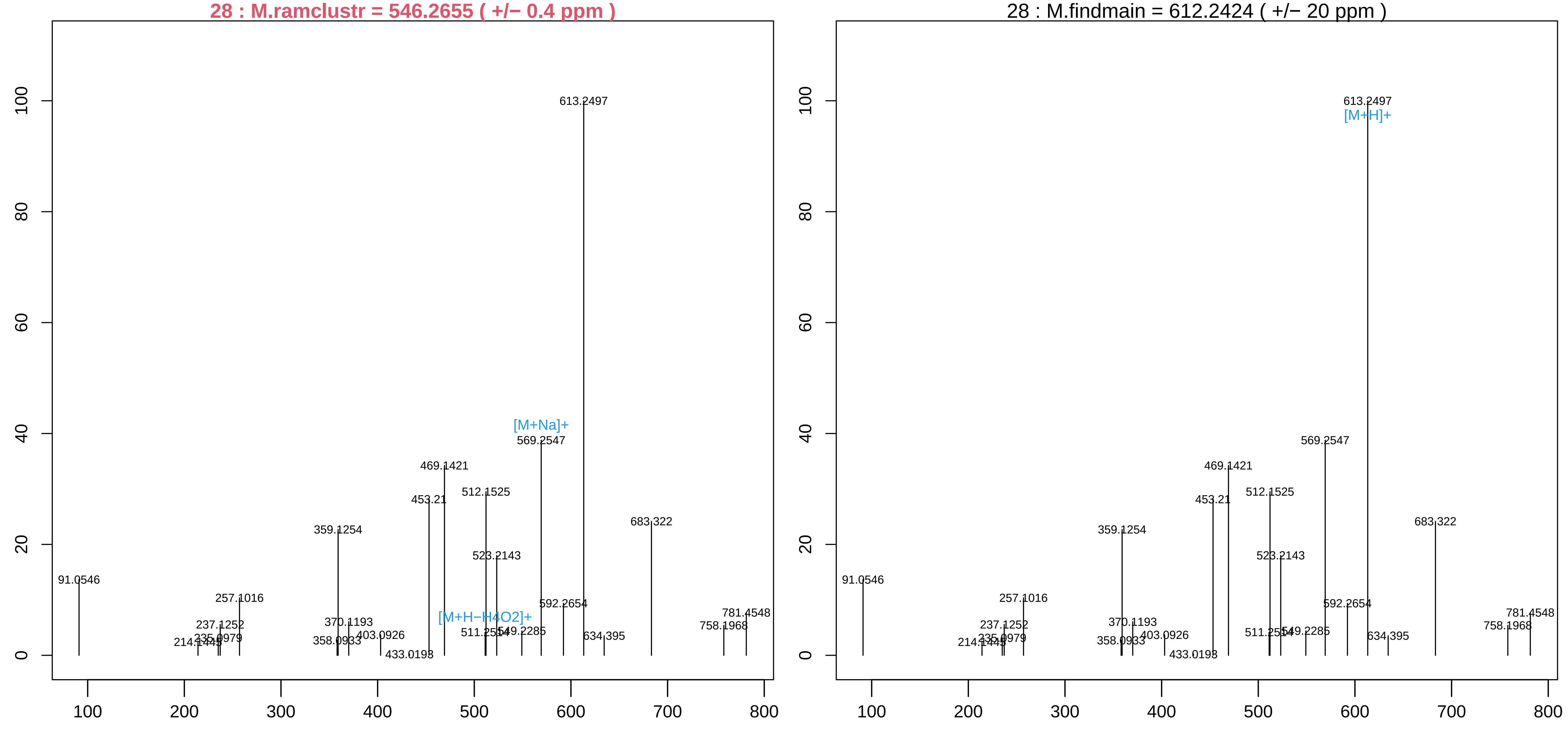

28 : M.ramclustr = 546.2655 (+/− 0.4 ppm)
613.2497
[M+Na]+
569.2547
469.1421
453.21
512.1525
359.1254
523.2143
683.322
91.0546
257.1016
592.2654
[M+H−H4O2]+
781.4548
237.1252
370.1193
758.1968
511.2554
549.2285
214.1445
235.0979
358.0933
403.0926
634.395
433.0198
28 : M.findmain = 612.2424 (+/− 20 ppm)
613.2497
[M+H]+
569.2547
469.1421
453.21
512.1525
359.1254
523.2143
683.322
91.0546
257.1016
592.2654
781.4548
237.1252
370.1193
758.1968
511.2554
549.2285
214.1445
235.0979
358.0933
403.0926
634.395
433.0198

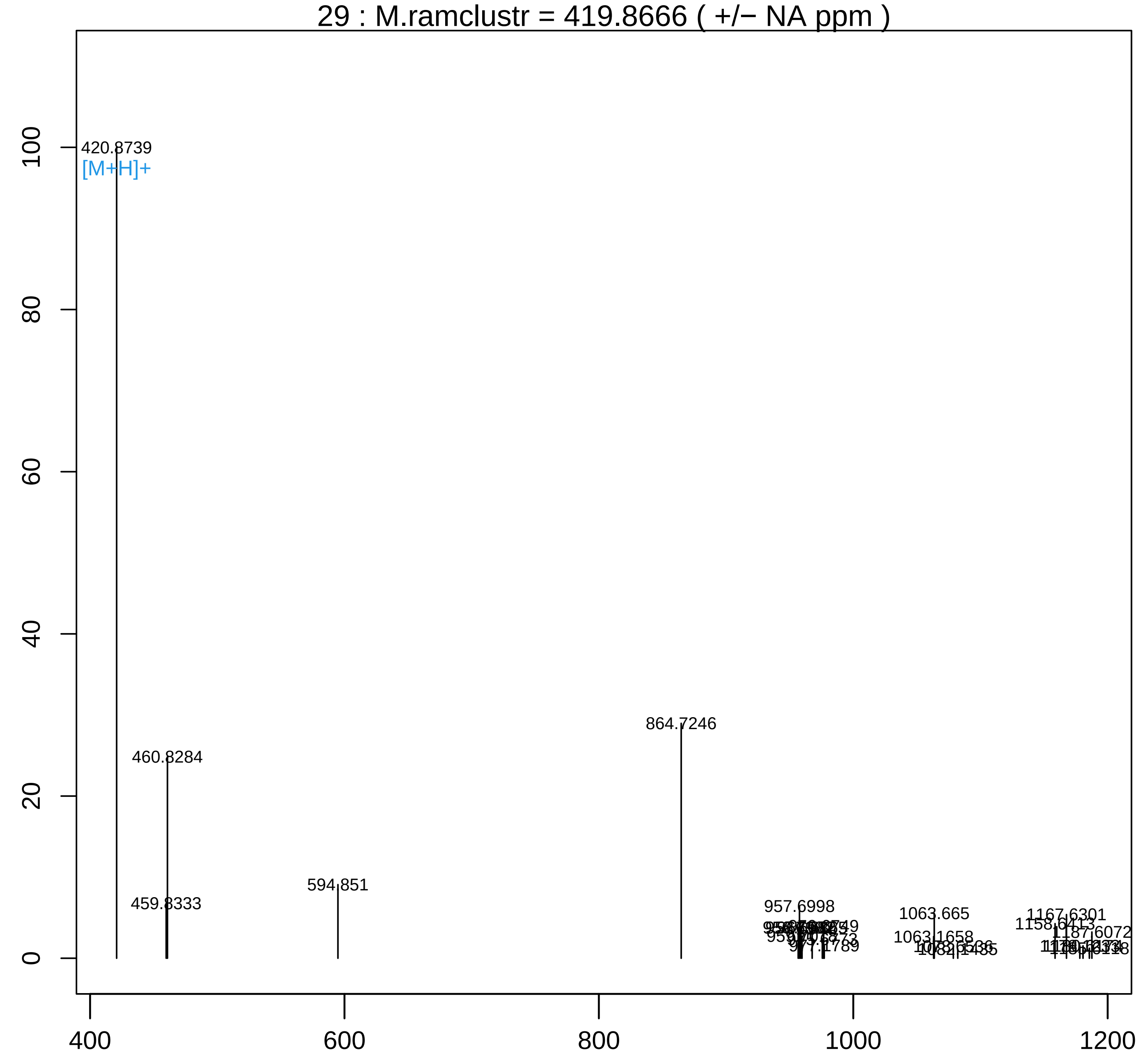
29 : M.ramclustr = 419.8666 (+/− NA ppm)
420.8739
[M+H]+
460.8284
459.8333
594.851
864.7246
957.6998
1063.665
1063.1658
1167.6301
1158.6413
1187.6072
977.1789
0
20
40
60
80
100
400
600
800
1000
1200
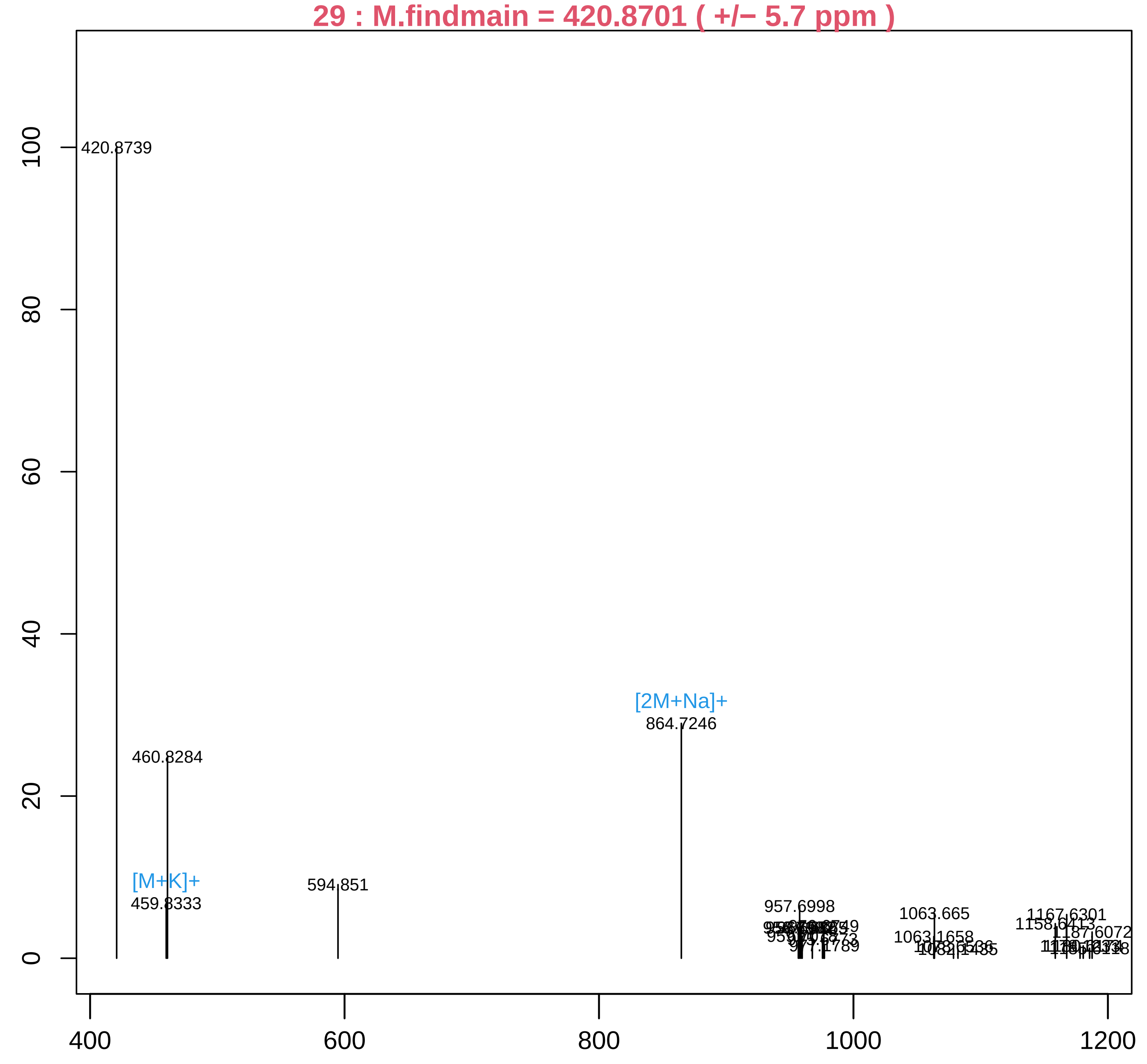
29 : M.findmain = 420.8701 (+/− 5.7 ppm)
420.8739
460.8284
[M+K]+
459.8333
594.851
[2M+Na]+
864.7246
957.6998
1063.665
1063.1658
1167.6301
1158.6413
1187.6072
977.1789
0
20
40
60
80
100
400
600
800
1000
1200

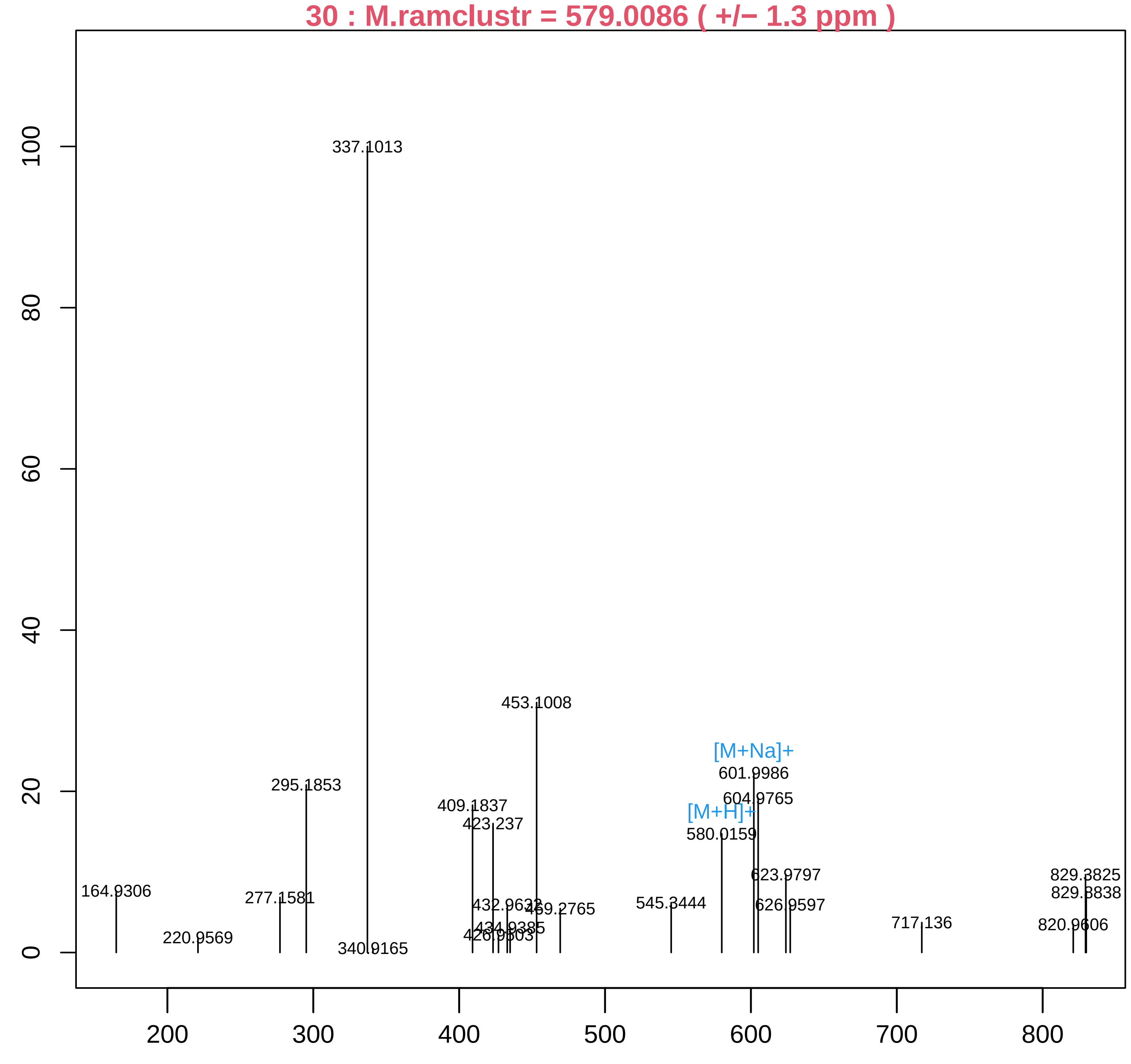

30 : M.ramclustr = 579.0086 (+/− 1.3 ppm)
337.1013
453.1008
[M+Na]+
601.9986
295.1853
409.1837
423.237
604.9765
[M+H]+
580.0159
623.9797
829.3825
829.3838
164.9306
277.1581
432.9632
469.2765
545.3444
626.9597
717.136
820.9606
434.9385
426.9503
220.9569
340.9165

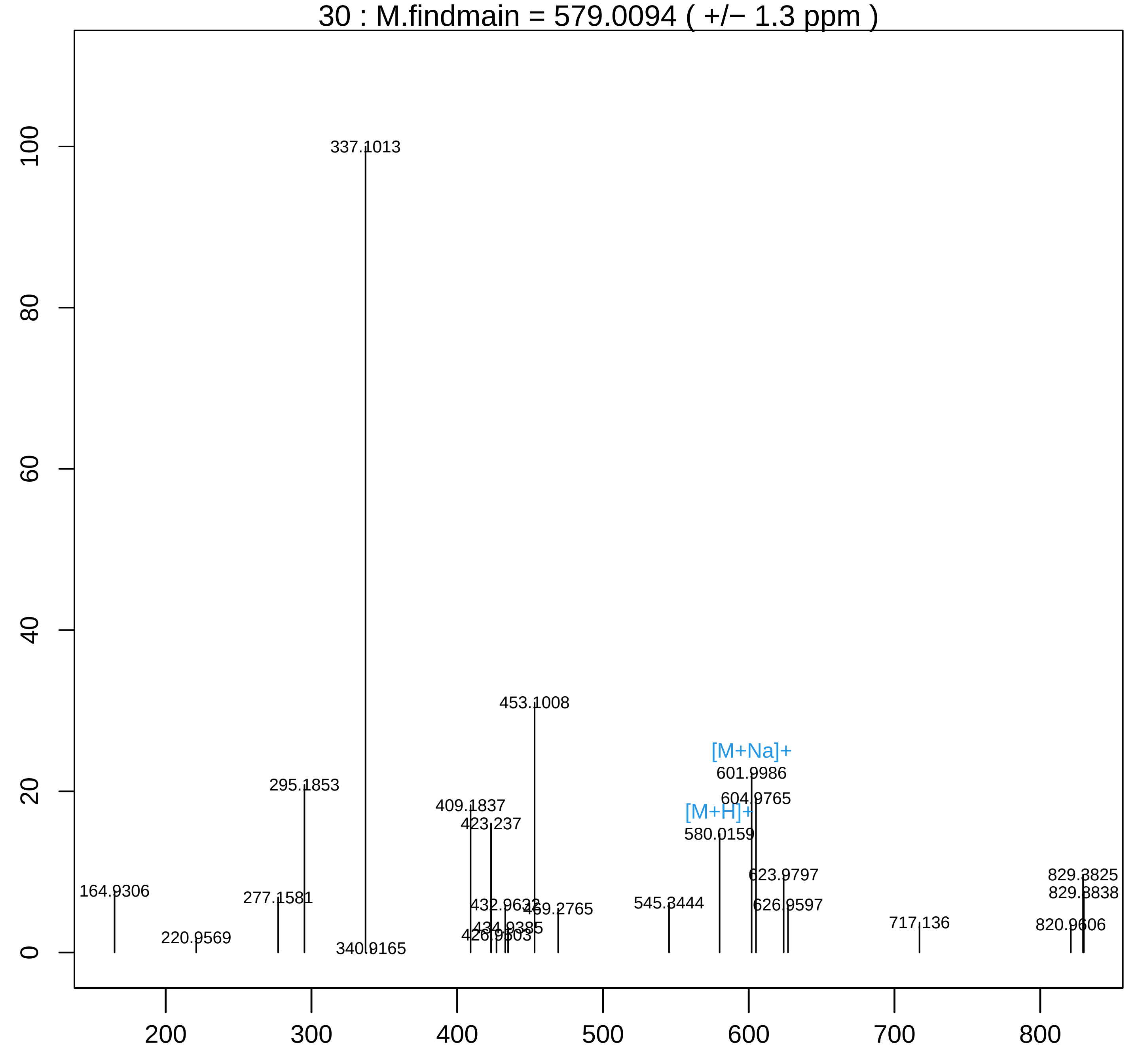

30 : M.findmain = 579.0094 (+/− 1.3 ppm)
337.1013
453.1008
[M+Na]+
601.9986
295.1853
409.1837
423.237
604.9765
[M+H]+
580.0159
623.9797
829.3825
829.3838
164.9306
277.1581
432.9632
469.2765
545.3444
626.9597
717.136
820.9606
434.9385
426.9503
220.9569
340.9165

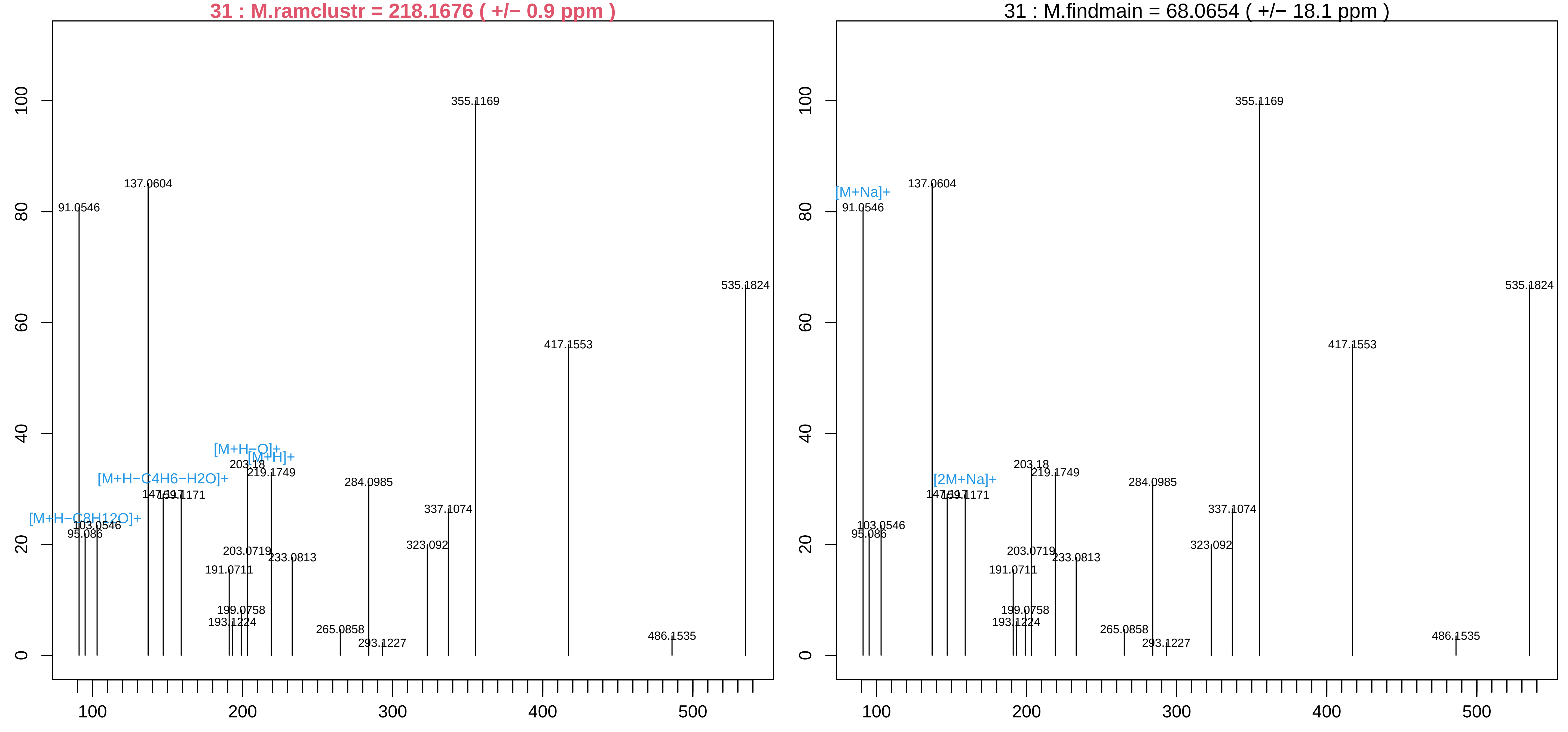

31 : M.ramclustr = 218.1676 (+/− 0.9 ppm)
31 : M.findmain = 68.0654 (+/− 18.1 ppm)
355.1169
137.0604
91.0546
535.1824
417.1553
[M+H−O]+
[M+H]+
203.18
219.1749
[M+H−C4H6−H2O]+
284.0985
147.1117
159.1171
337.1074
[M+H−C8H12O]+
103.0546
95.086
323.092
203.0719
233.0813
191.0711
199.0758
193.1224
265.0858
293.1227
486.1535
[M+Na]+
[2M+Na]+
0
20
40
60
80
100
200
300
400
500

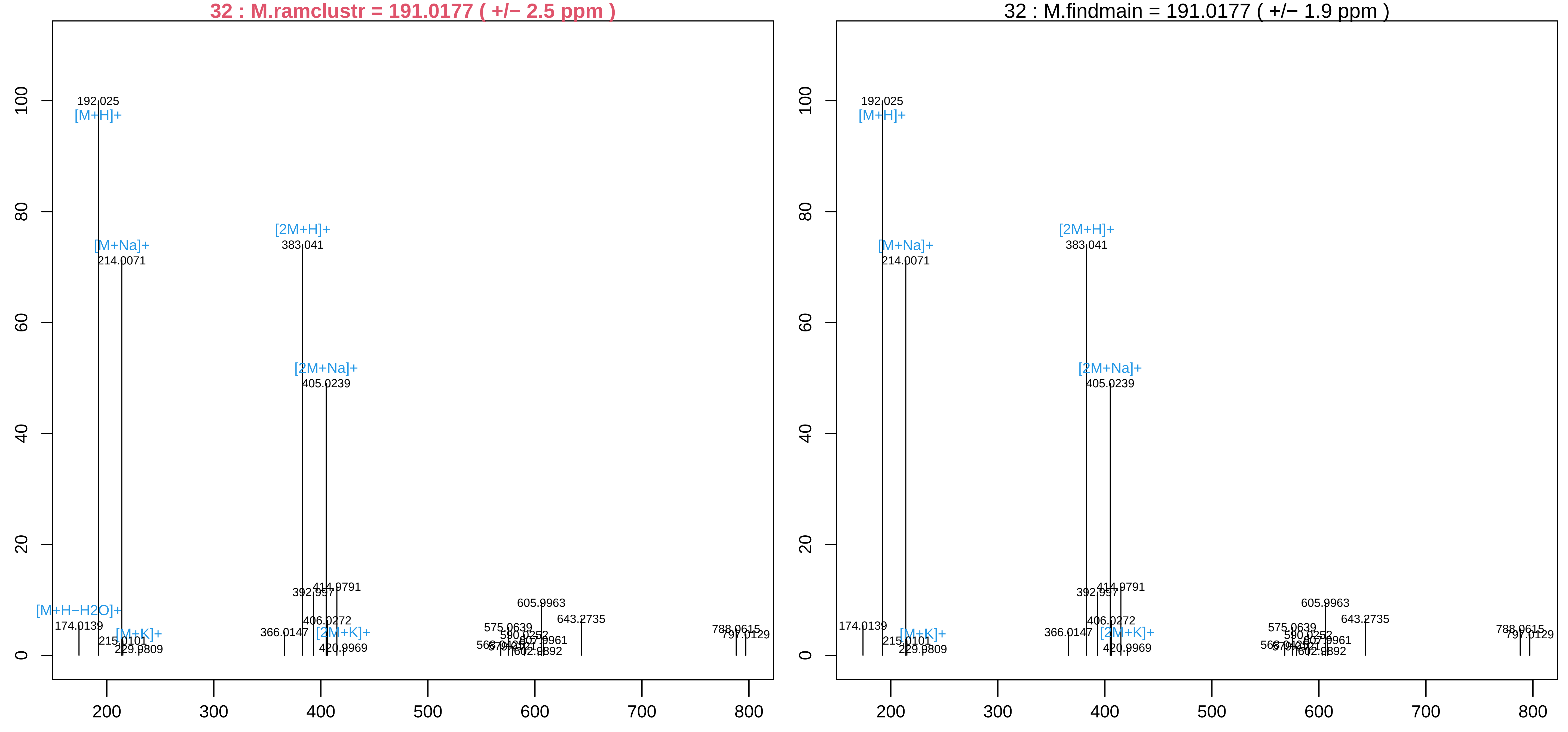
32 : M.ramclustr = 191.0177 (+/− 2.5 ppm)
32 : M.findmain = 191.0177 (+/− 1.9 ppm)
192.025
[M+H]+
[M+Na]+
214.0071
[2M+H]+
383.041
[2M+Na]+
405.0239
[M+H−H2O]+
174.0139
[M+K]+
215.0101
229.9809
366.0147
392.997
414.9791
406.0272
[2M+K]+
420.9969
605.9963
643.2735
575.0639
788.0615
797.0129
0
20
40
60
80
100
200
300
400
500
600
700
800

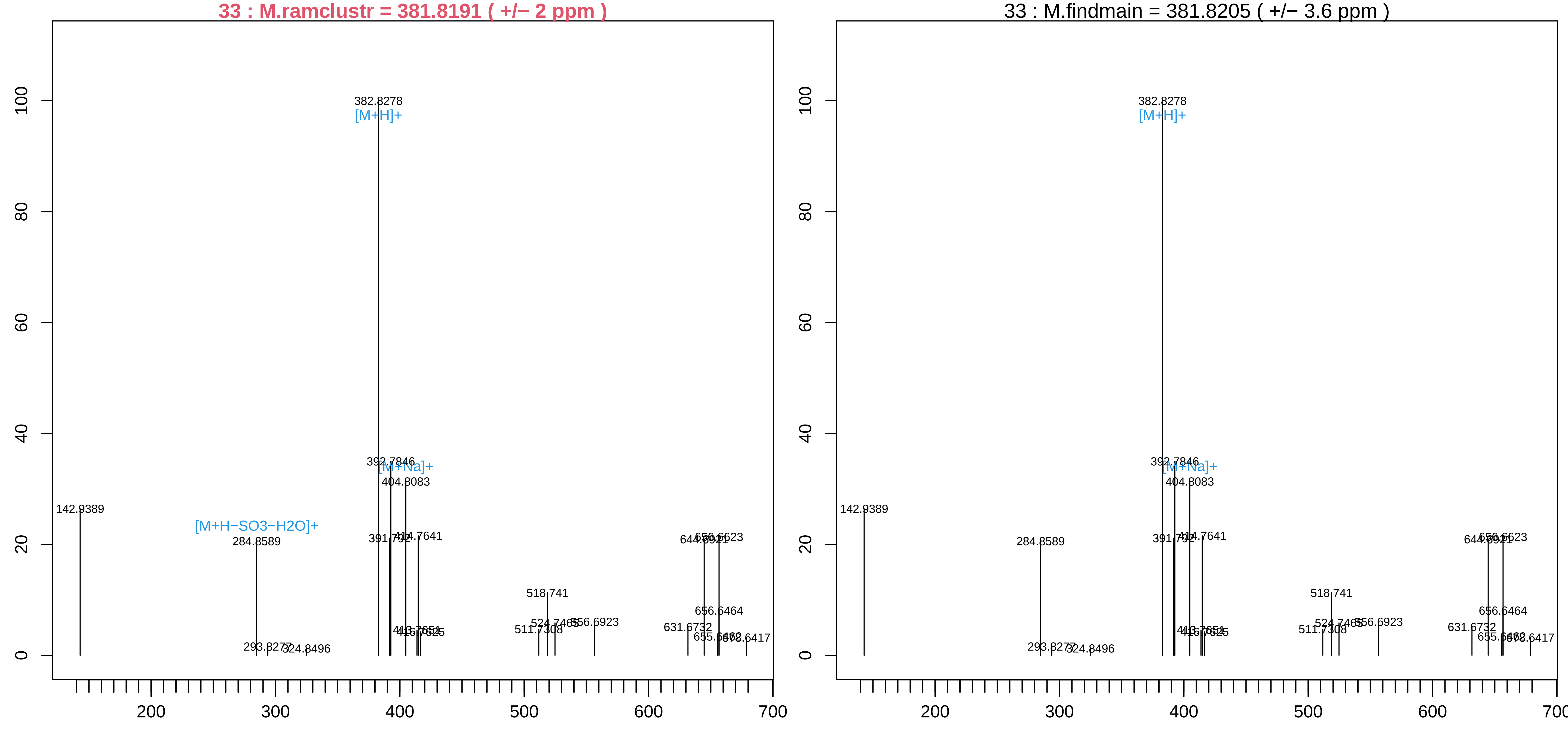

33 : M.ramclustr = 381.8191 (+/− 2 ppm)
33 : M.findmain = 381.8205 (+/− 3.6 ppm)
382.8278
[M+H]+
392.7846
[M+Na]+
404.8083
142.9389
[M+H−SO3−H2O]+
284.8589
391.792
414.7641
518.741
656.6623
644.6921
656.6464
631.6732
556.6923
524.7465
511.7308
413.7651
416.7625
655.6462
678.6417
293.8277
324.8496

34 : M.ramclustr = 730.5279 (+/− 8.1 ppm)

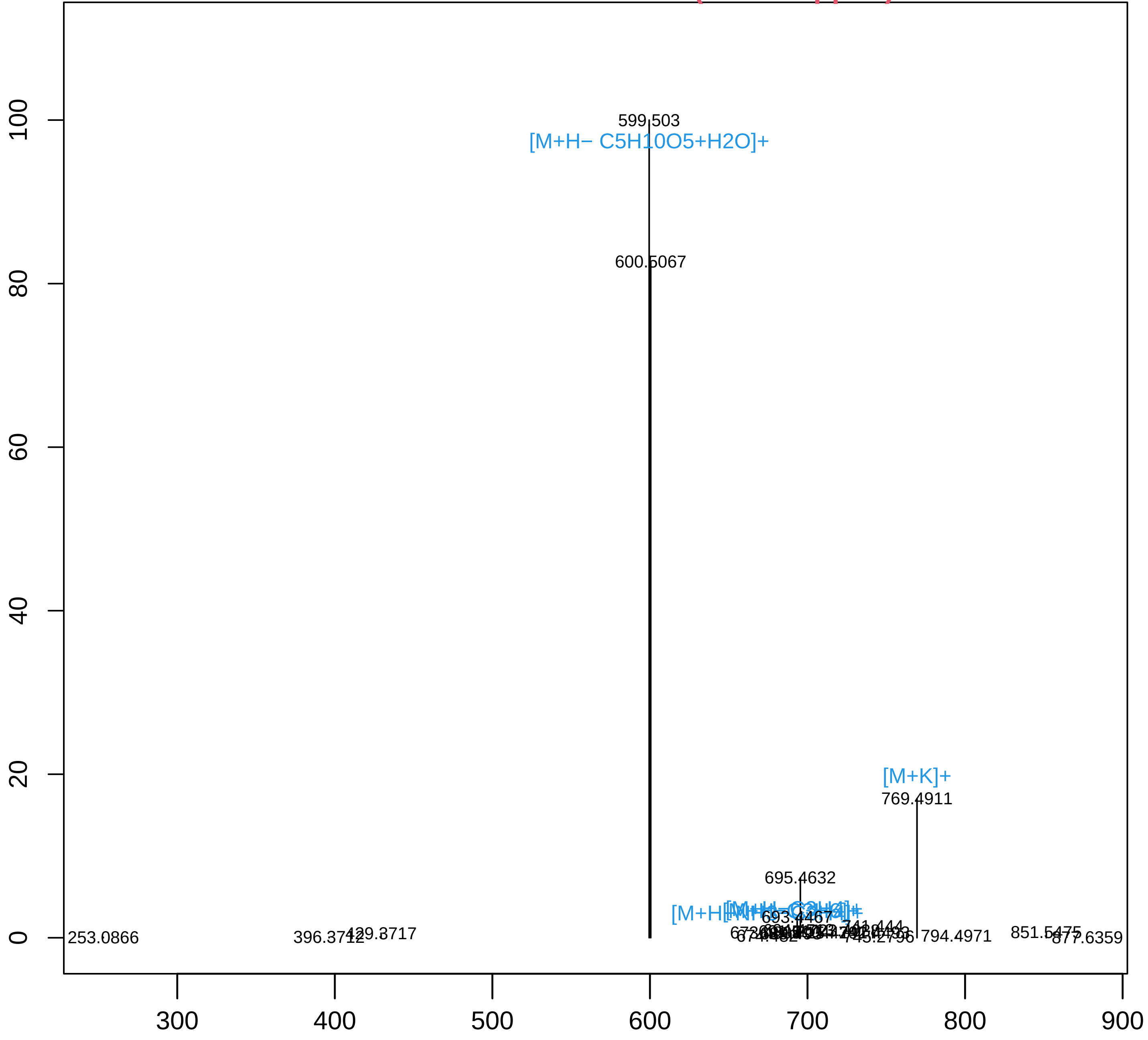

34 : M.findmain = 598.4957 (+/− 20 ppm)

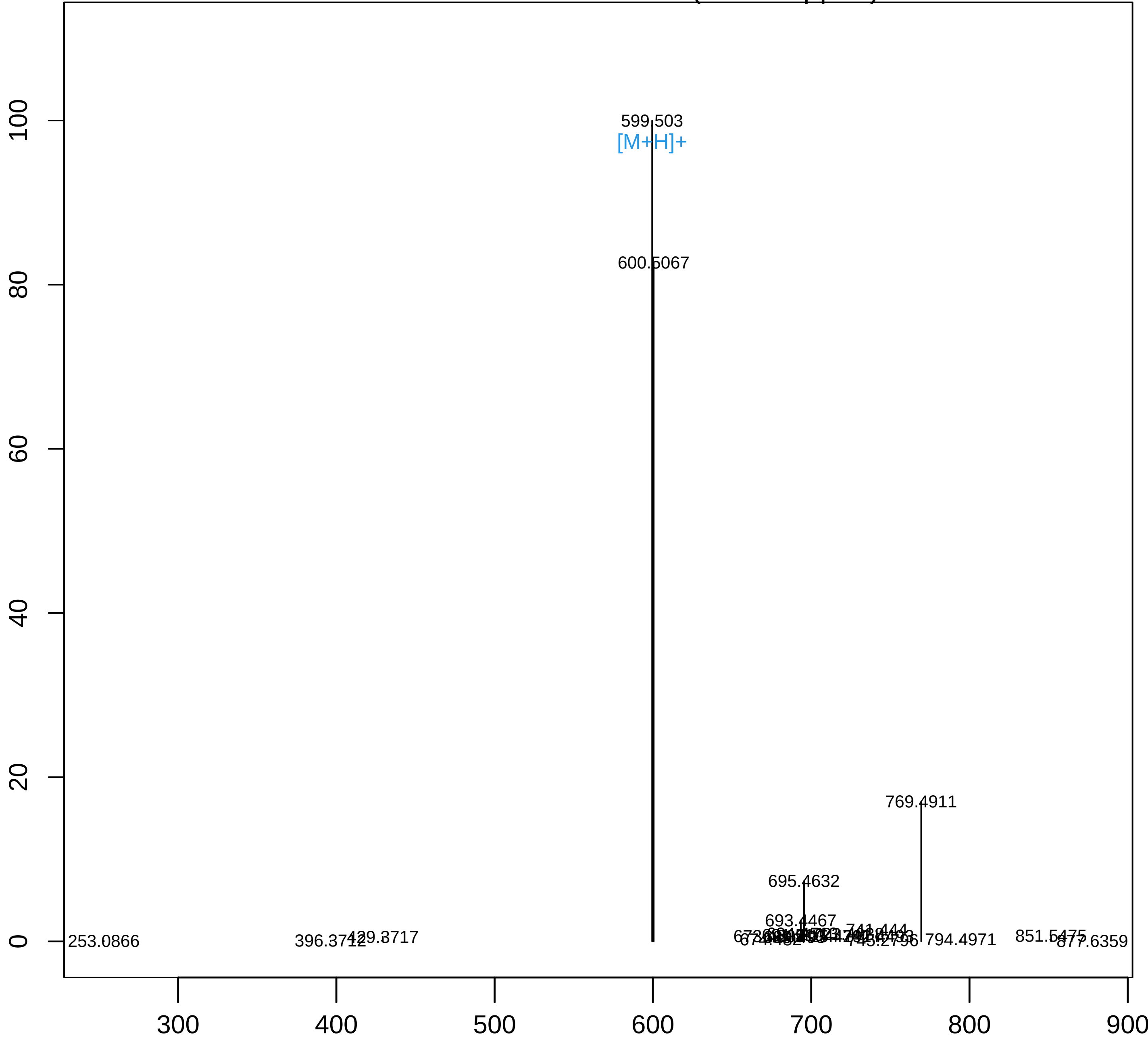

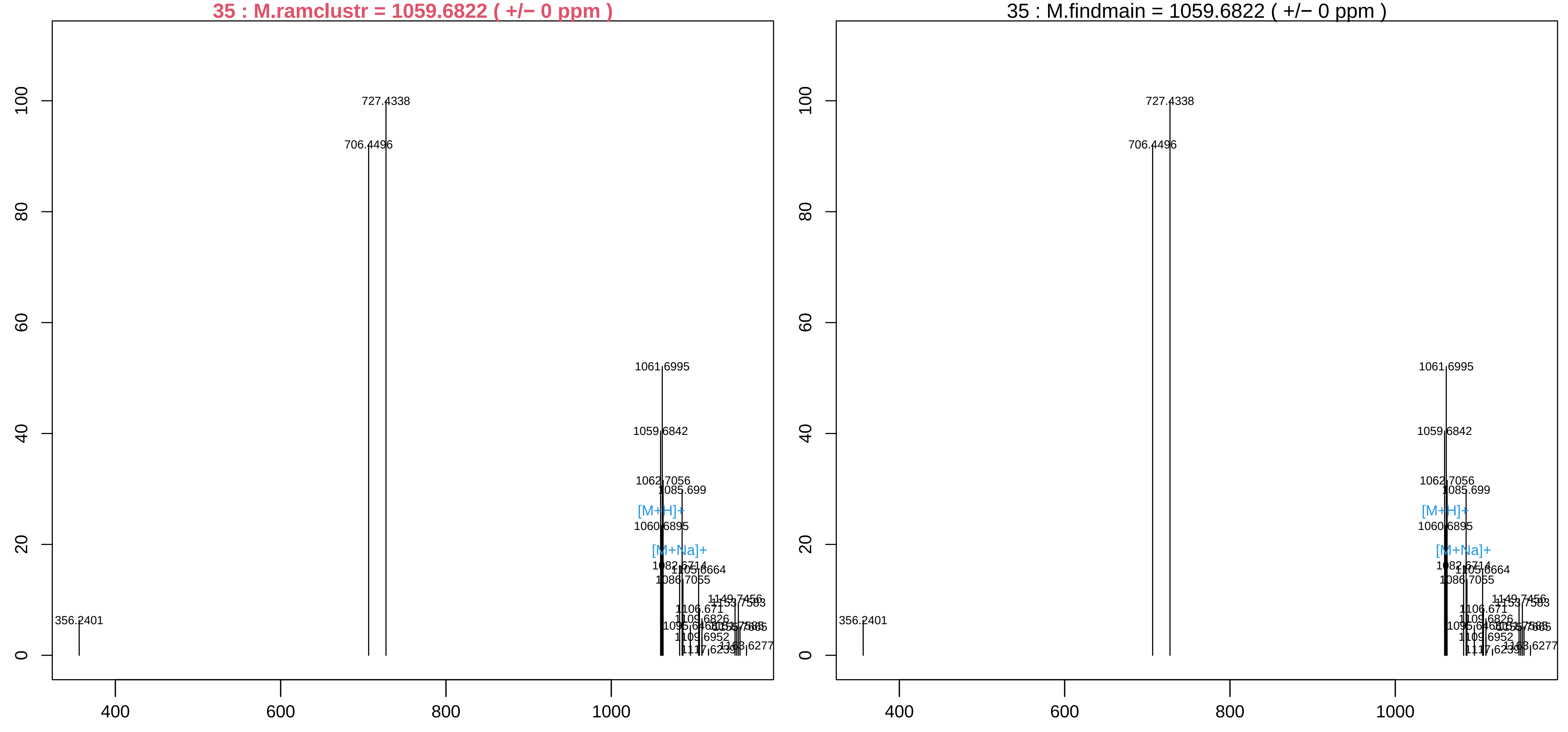
35 : M.ramclustr = 1059.6822 (+/− 0 ppm)
35 : M.findmain = 1059.6822 (+/− 0 ppm)
0
20
40
60
80
100
400
600
800
1000
727.4338
706.4496
1061.6995
1059.6842
1062.7056
1085.699
[M+H]+
1060.6895
[M+Na]+
1082.6714
1105.6664
1086.7055
1149.7456
1153.7583
1106.671
1109.6826
1109.6952
1163.6277
356.2401

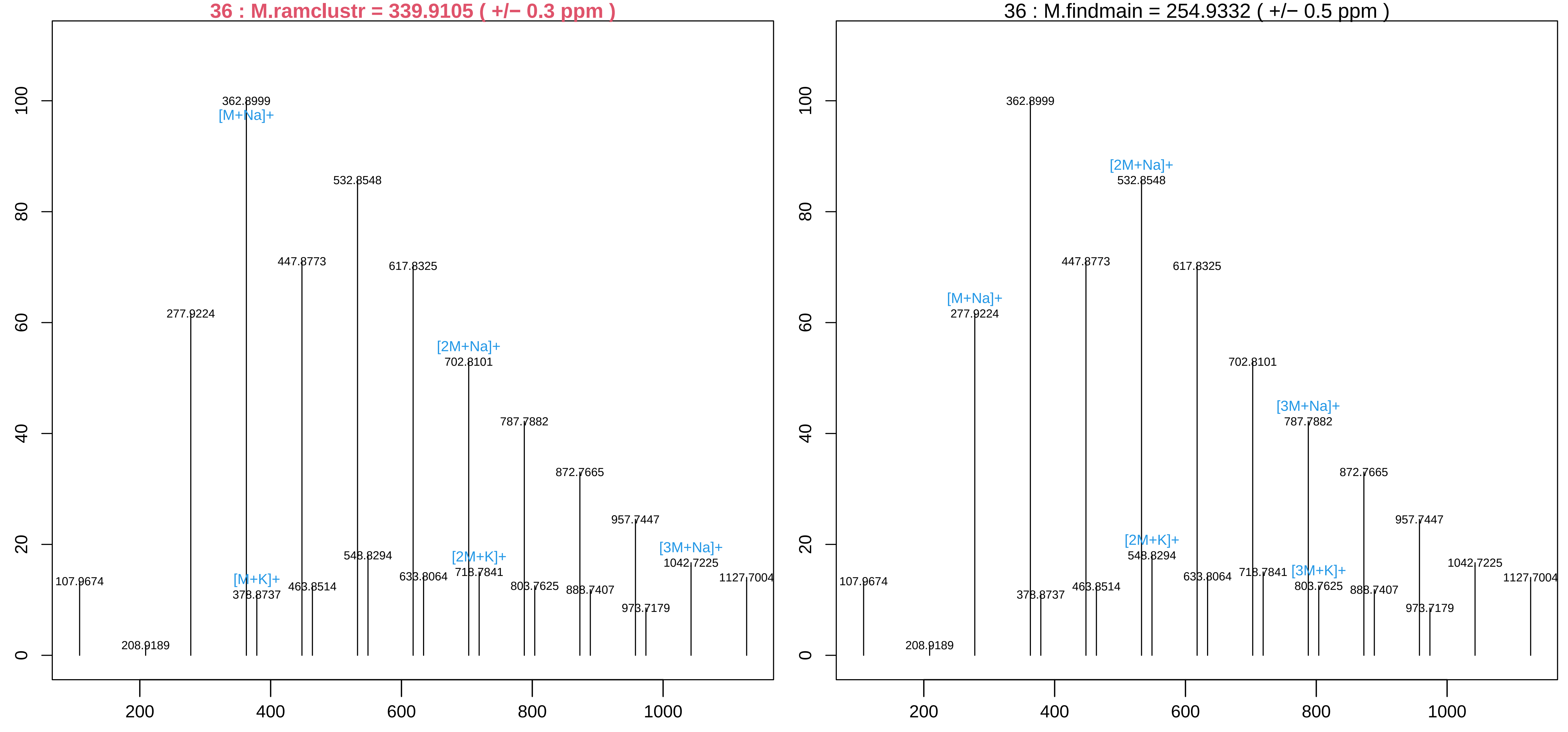
36 : M.ramclustr = 339.9105 (+/− 0.3 ppm)
362.8999
[M+Na]+
532.8548
447.8773
617.8325
277.9224
[2M+Na]+
702.8101
787.7882
872.7665
957.7447
[3M+Na]+
1042.7225
548.8294
[2M+K]+
718.7841
[M+K]+
378.8737
633.8064
463.8514
803.7625
888.7407
973.7179
1127.7004
107.9674
208.9189
0
20
40
60
80
100
200
400
600
800
1000
36 : M.findmain = 254.9332 (+/− 0.5 ppm)
362.8999
[2M+Na]+
532.8548
447.8773
617.8325
[M+Na]+
277.9224
702.8101
[3M+Na]+
787.7882
872.7665
957.7447
[2M+K]+
548.8294
1042.7225
1127.7004
107.9674
633.8064
718.7841
[3M+K]+
803.7625
888.7407
378.8737
463.8514
973.7179
208.9189
0
20
40
60
80
100
200
400
600
800
1000

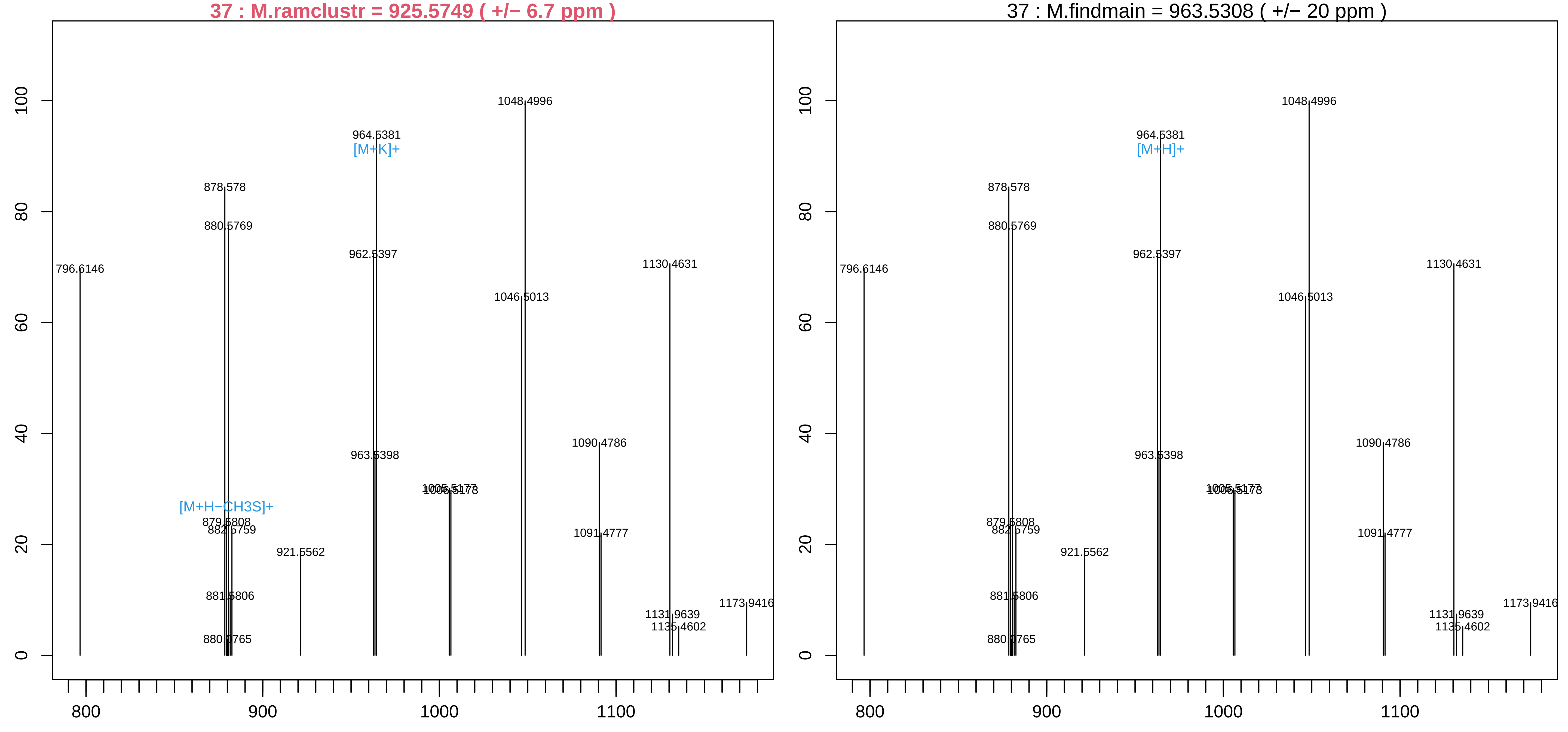
37 : M.ramclustr = 925.5749 (+/− 6.7 ppm)
37 : M.findmain = 963.5308 (+/− 20 ppm)
1048.4996
964.5381
[M+K]+
[M+H]+
878.578
880.5769
962.5397
796.6146
1130.4631
1046.5013
1090.4786
963.5398
1005.5177
1006.5178
[M+H−CH3S]+
879.5808
882.5759
1091.4777
921.5562
881.5806
1173.9416
1131.9639
1135.4602
880.0765
0
20
40
60
80
100
800
900
1000
1100

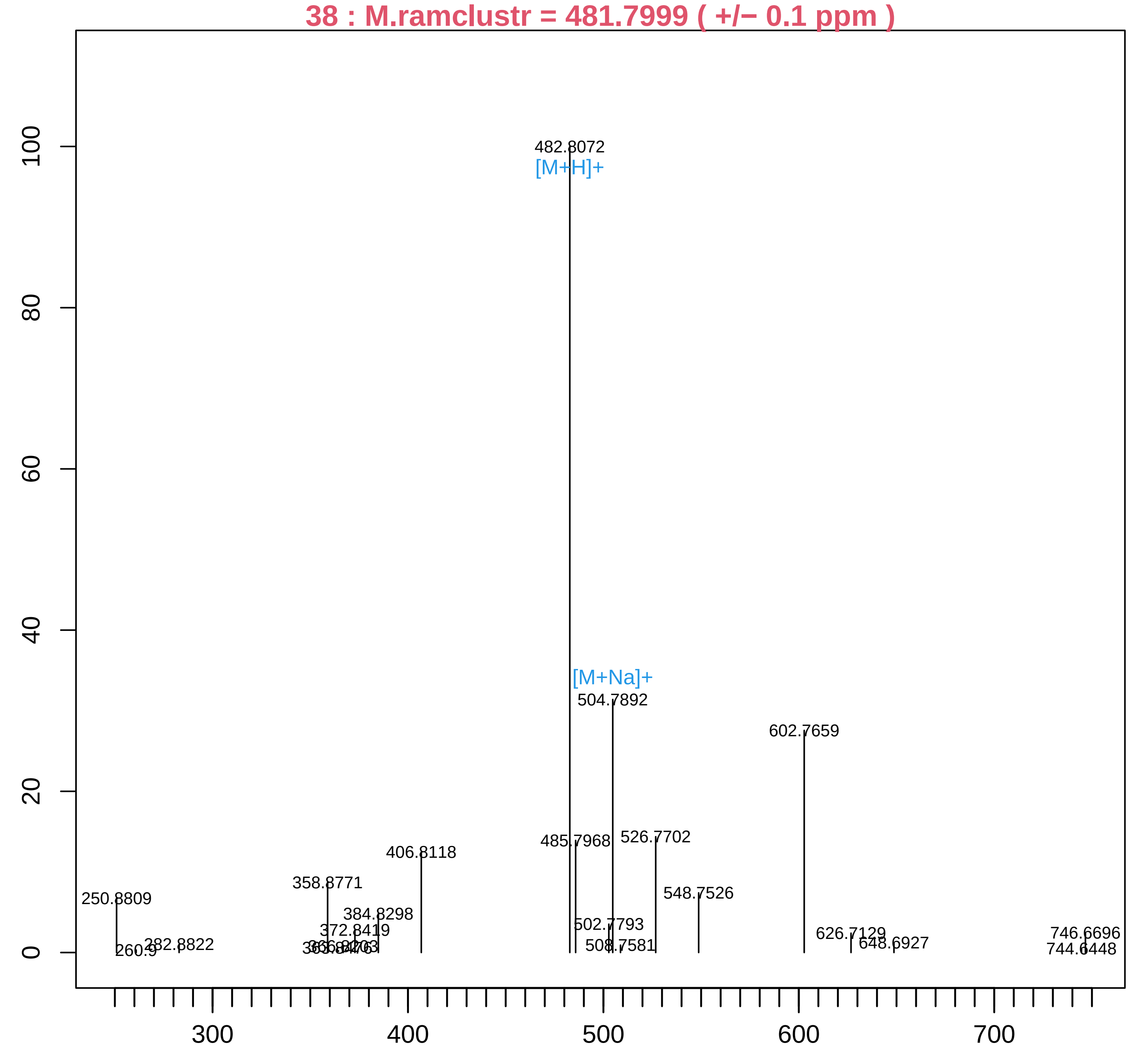
38 : M.ramclustr = 481.7999 (+/− 0.1 ppm)
482.8072
[M+H]+
[M+Na]+
504.7892
602.7659
485.7968
526.7702
406.8118
358.8771
548.7526
250.8809
384.8298
372.8419
502.7793
626.7129
746.6696
282.8822
508.7581
648.6927
744.6448
260.9
0
20
40
60
80
100
300
400
500
600
700
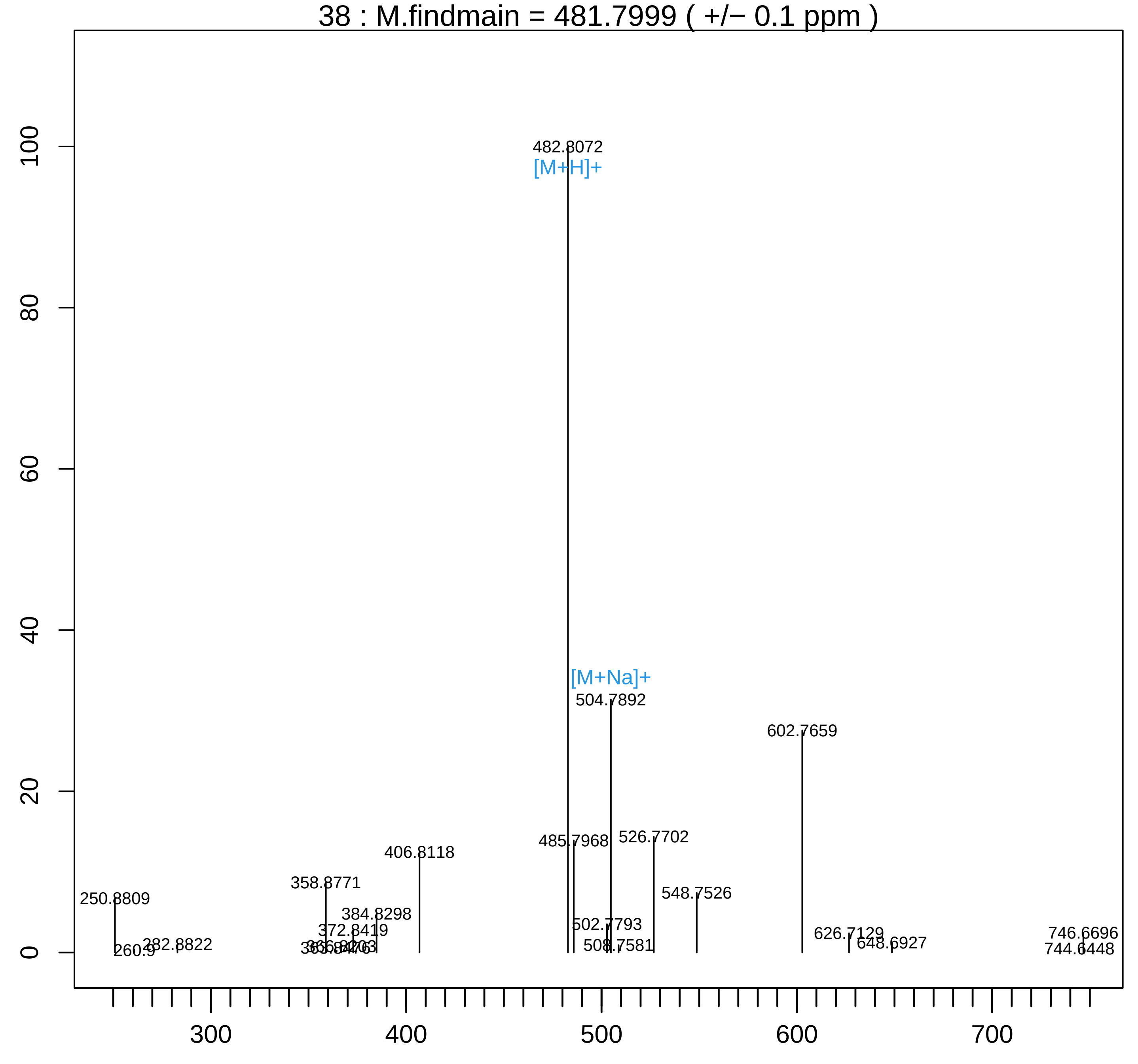
38 : M.findmain = 481.7999 (+/− 0.1 ppm)
482.8072
[M+H]+
[M+Na]+
504.7892
602.7659
485.7968
526.7702
406.8118
358.8771
548.7526
250.8809
384.8298
372.8419
502.7793
626.7129
746.6696
282.8822
508.7581
648.6927
744.6448
260.9
0
20
40
60
80
100
300
400
500
600
700

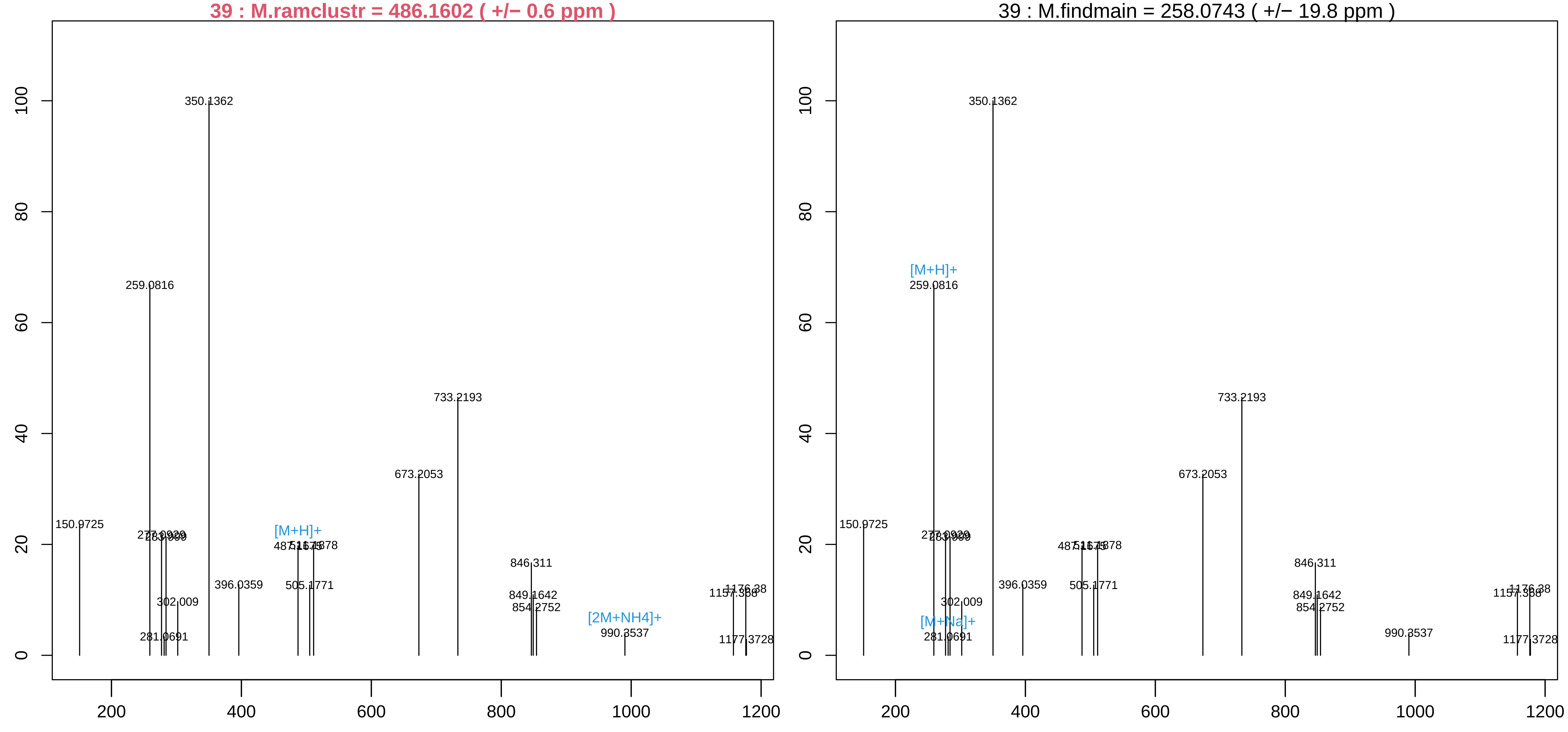
39 : M.ramclustr = 486.1602 (+/− 0.6 ppm)
39 : M.findmain = 258.0743 (+/− 19.8 ppm)
[M+H]+
[2M+NH4]+
[M+Na]+
150.9725
259.0816
350.1362
396.0359
302.009
281.0691
505.1771
673.2053
733.2193
846.311
849.1642
854.2752
990.3537
1177.3728
1176.38
0
20
40
60
80
100
200
400
600
800
1000
1200

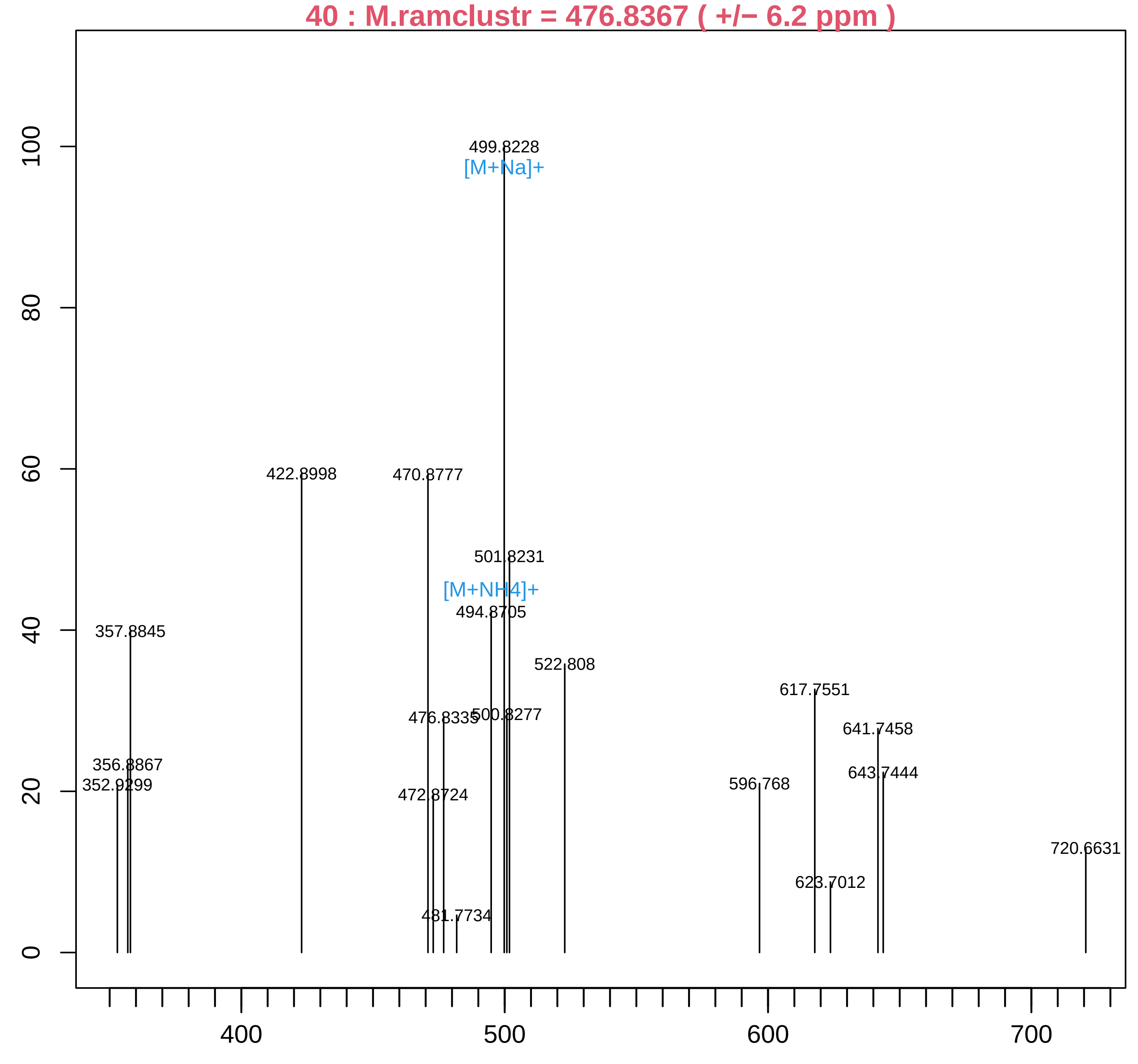
40 : M.ramclustr = 476.8367 (+/− 6.2 ppm)
499.8228
[M+Na]+
422.8998
470.8777
501.8231
[M+NH4]+
494.8705
357.8845
522.808
617.7551
476.8335
500.8277
641.7458
356.8867
352.9299
643.7444
472.8724
596.768
720.6631
623.7012
481.7734
0
20
40
60
80
100
400
500
600
700
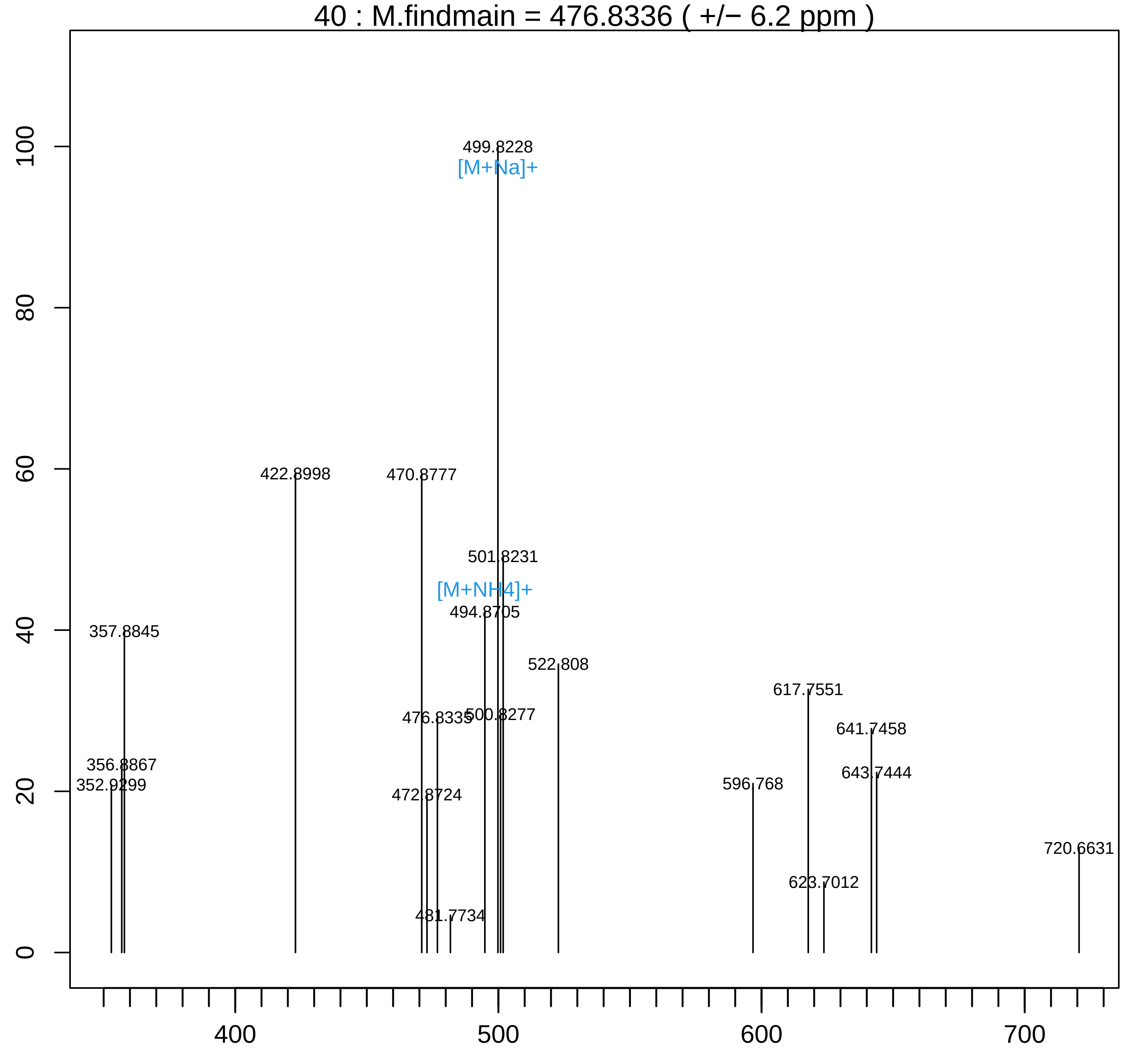
40 : M.findmain = 476.8336 (+/− 6.2 ppm)
499.8228
[M+Na]+
422.8998
470.8777
501.8231
[M+NH4]+
494.8705
357.8845
522.808
617.7551
476.8335
500.8277
641.7458
356.8867
352.9299
643.7444
472.8724
596.768
720.6631
623.7012
481.7734
0
20
40
60
80
100
400
500
600
700

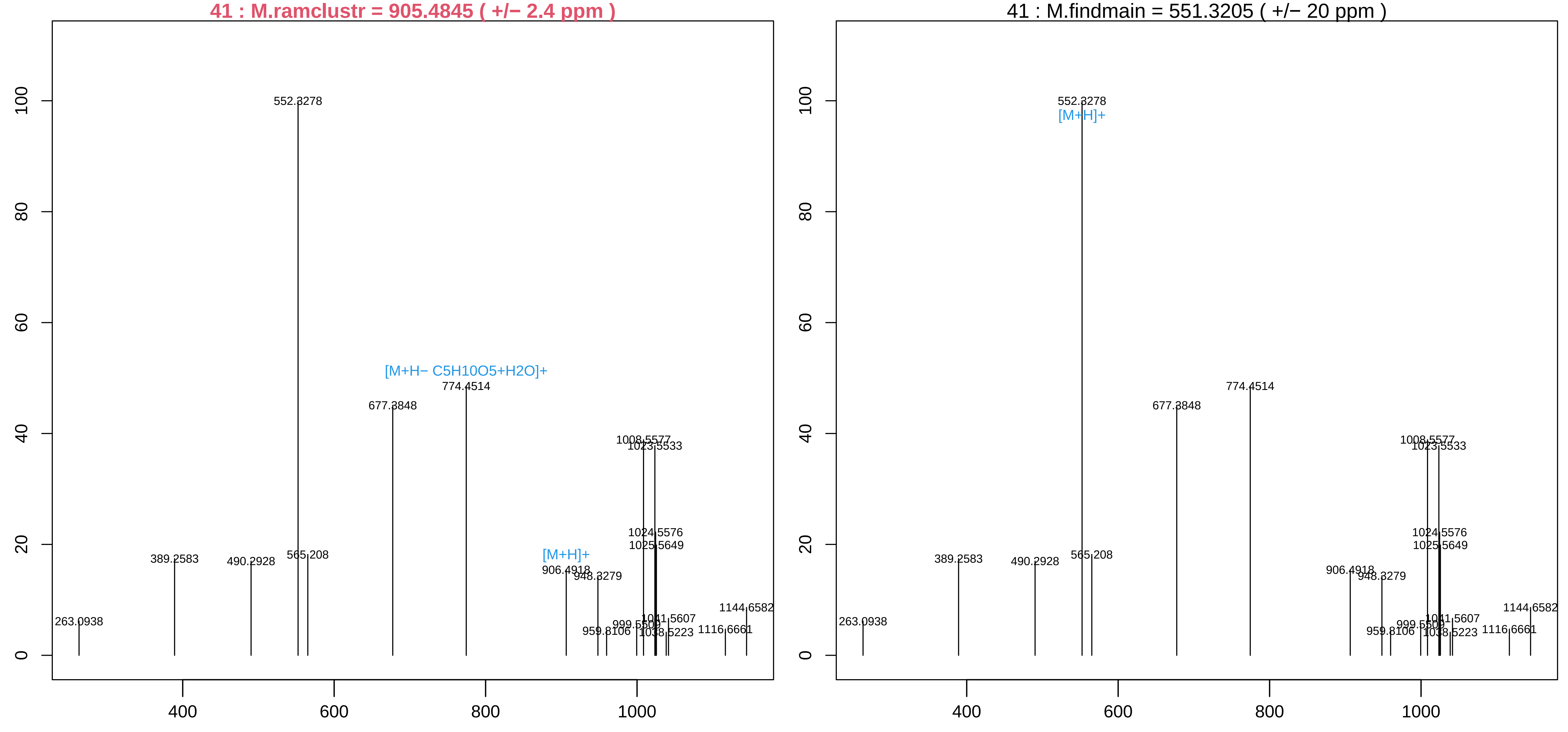

41 : M.ramclustr = 905.4845 (+/− 2.4 ppm)
552.3278
[M+H− C5H10O5+H2O]+
774.4514
677.3848
1008.5577
1023.5533
1024.5576
1025.5649
389.2583
490.2928
565.208
[M+H]+
906.4918
948.3279
263.0938
1144.6582
1041.5607
999.5509
959.8106
1038.5223
1116.6661
0
20
40
60
80
100
400
600
800
1000
41 : M.findmain = 551.3205 (+/− 20 ppm)
552.3278
[M+H]+
774.4514
677.3848
1008.5577
1023.5533
1024.5576
1025.5649
389.2583
490.2928
565.208
906.4918
948.3279
263.0938
1144.6582
1041.5607
999.5509
959.8106
1038.5223
1116.6661
0
20
40
60
80
100
400
600
800
1000

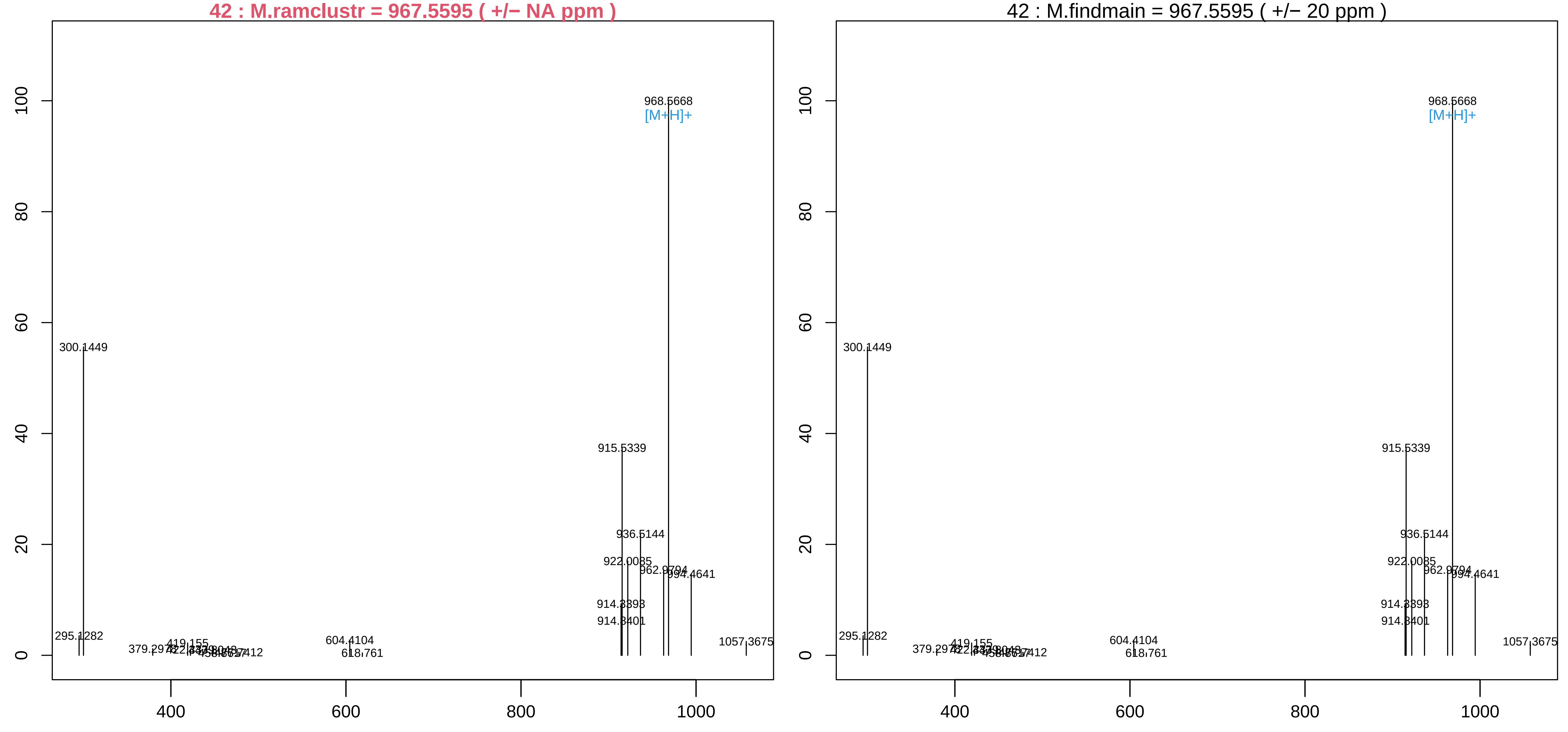
42 : M.ramclustr = 967.5595 (+/− NA ppm)
42 : M.findmain = 967.5595 (+/− 20 ppm)
968.5668
[M+H]+
300.1449
915.5339
936.5144
922.0035
962.9794
994.4641
914.3393
914.3401
295.1282
379.2978
419.155
604.4104
618.761
1057.3675
0
20
40
60
80
100
400
600
800
1000

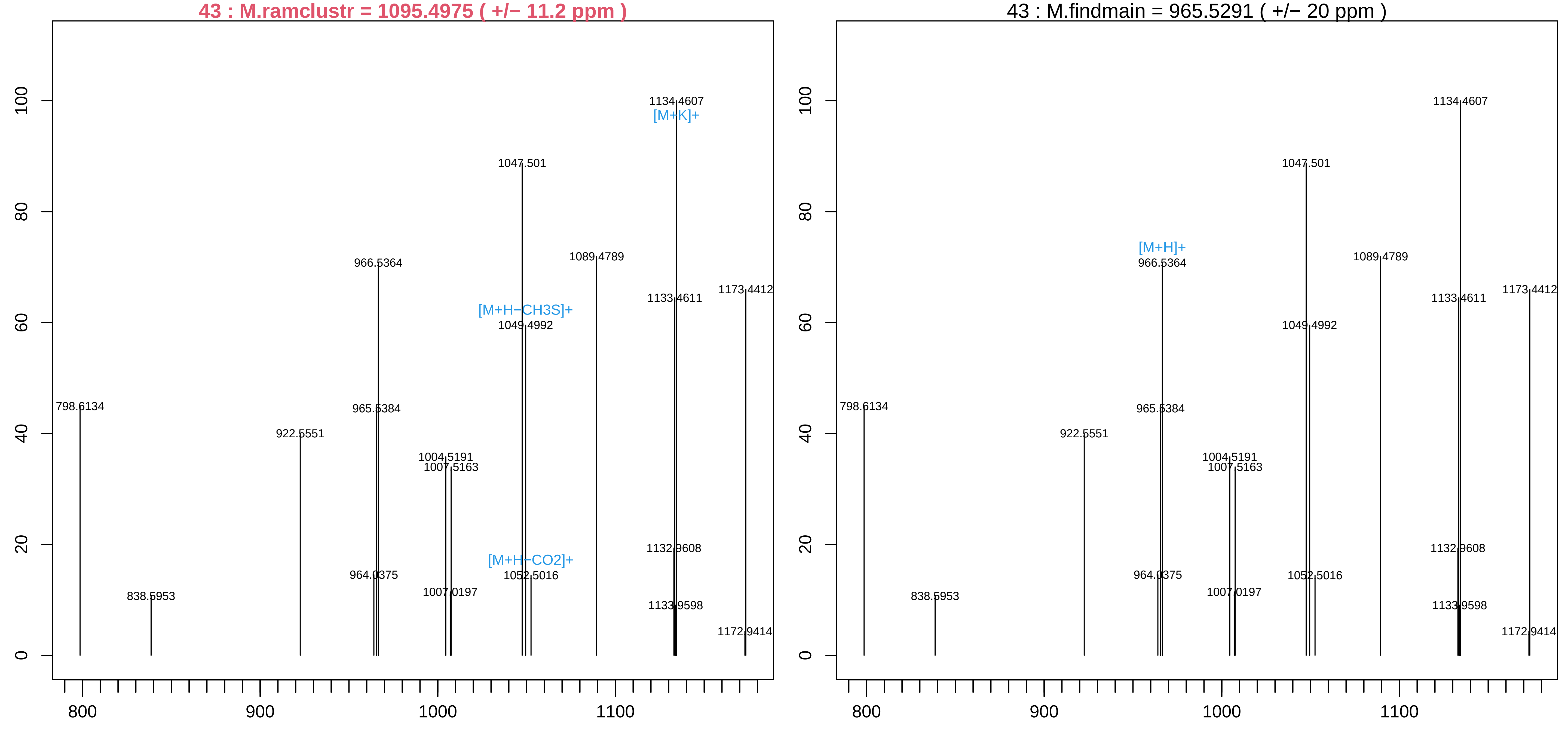
43 : M.ramclustr = 1095.4975 (+/− 11.2 ppm)
1134.4607
[M+K]+
1047.501
966.5364
1089.4789
1173.4412
1133.4611
[M+H−CH3S]+
1049.4992
798.6134
965.5384
922.5551
1004.5191
1007.5163
1132.9608
[M+H−CO2]+
964.0375
1052.5016
838.5953
1007.0197
1133.9598
1172.9414
43 : M.findmain = 965.5291 (+/− 20 ppm)
[M+H]+
0
20
40
60
80
100
800
900
1000
1100

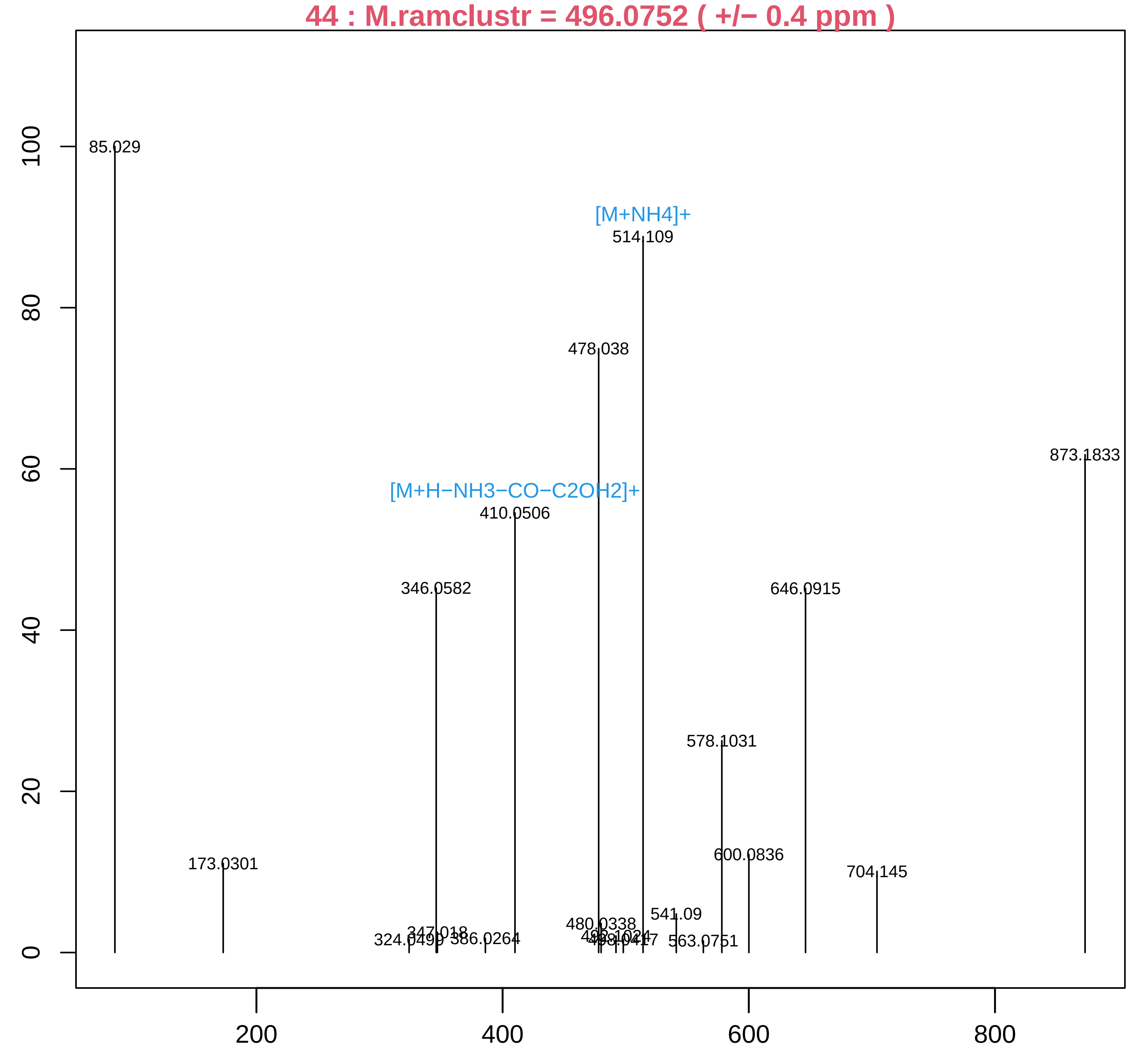
44 : M.ramclustr = 496.0752 (+/− 0.4 ppm)
[M+NH4]+
[M+H−NH3−CO−C2OH2]+
85.029
173.0301
324.0499
347.018
346.0582
386.0264
410.0506
478.038
480.0338
498.0417
514.109
541.09
563.0751
578.1031
600.0836
646.0915
704.145
873.1833
0
20
40
60
80
100
200
400
600
800

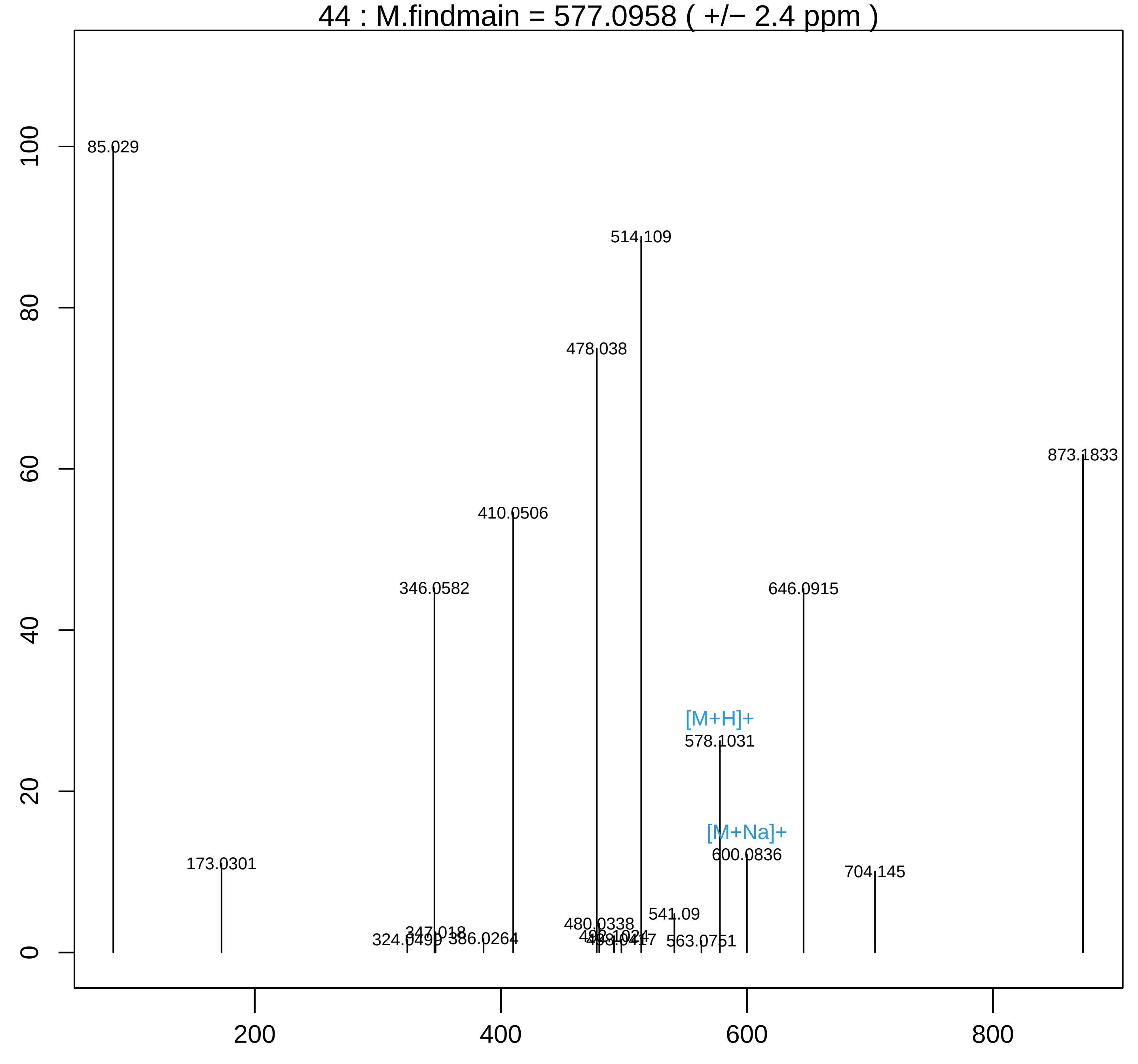
44 : M.findmain = 577.0958 (+/− 2.4 ppm)
[M+H]+
[M+Na]+
85.029
173.0301
324.0499
347.018
346.0582
386.0264
410.0506
478.038
480.0338
498.0417
514.109
541.09
563.0751
578.1031
600.0836
646.0915
704.145
873.1833
0
20
40
60
80
100
200
400
600
800

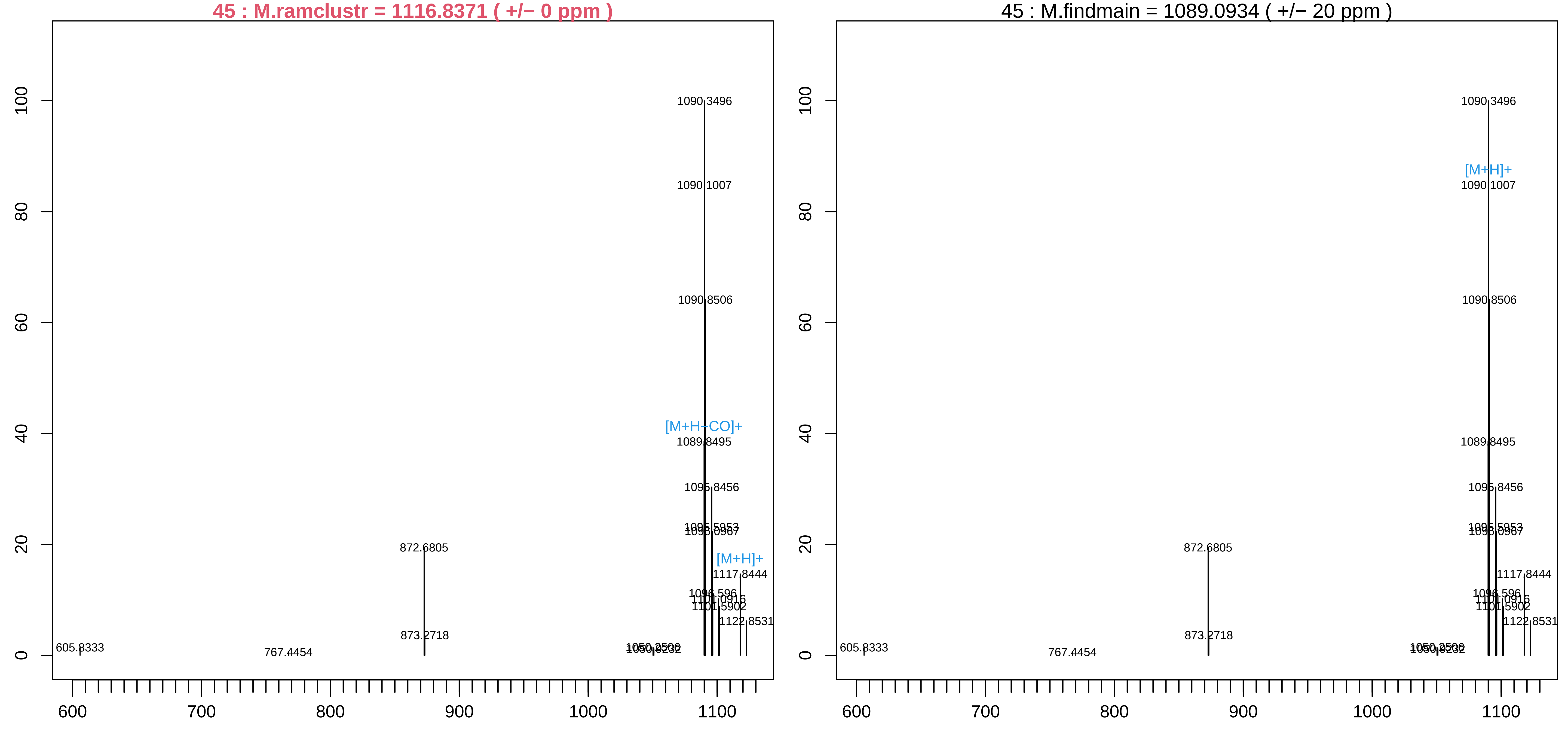
45 : M.ramclustr = 1116.8371 (+/− 0 ppm)
1090.3496
1090.1007
1090.8506
[M+H-CO]+
1089.8495
1095.8456
1095.5953
1093.0967
872.6805
[M+H]+
1117.8444
1096.596
1101.0916
1101.5902
1122.8531
873.2718
605.8333
767.4454
1050.2506
1050.6232
45 : M.findmain = 1089.0934 (+/− 20 ppm)
[M+H]+
0
20
40
60
80
100
600
700
800
900
1000
1100

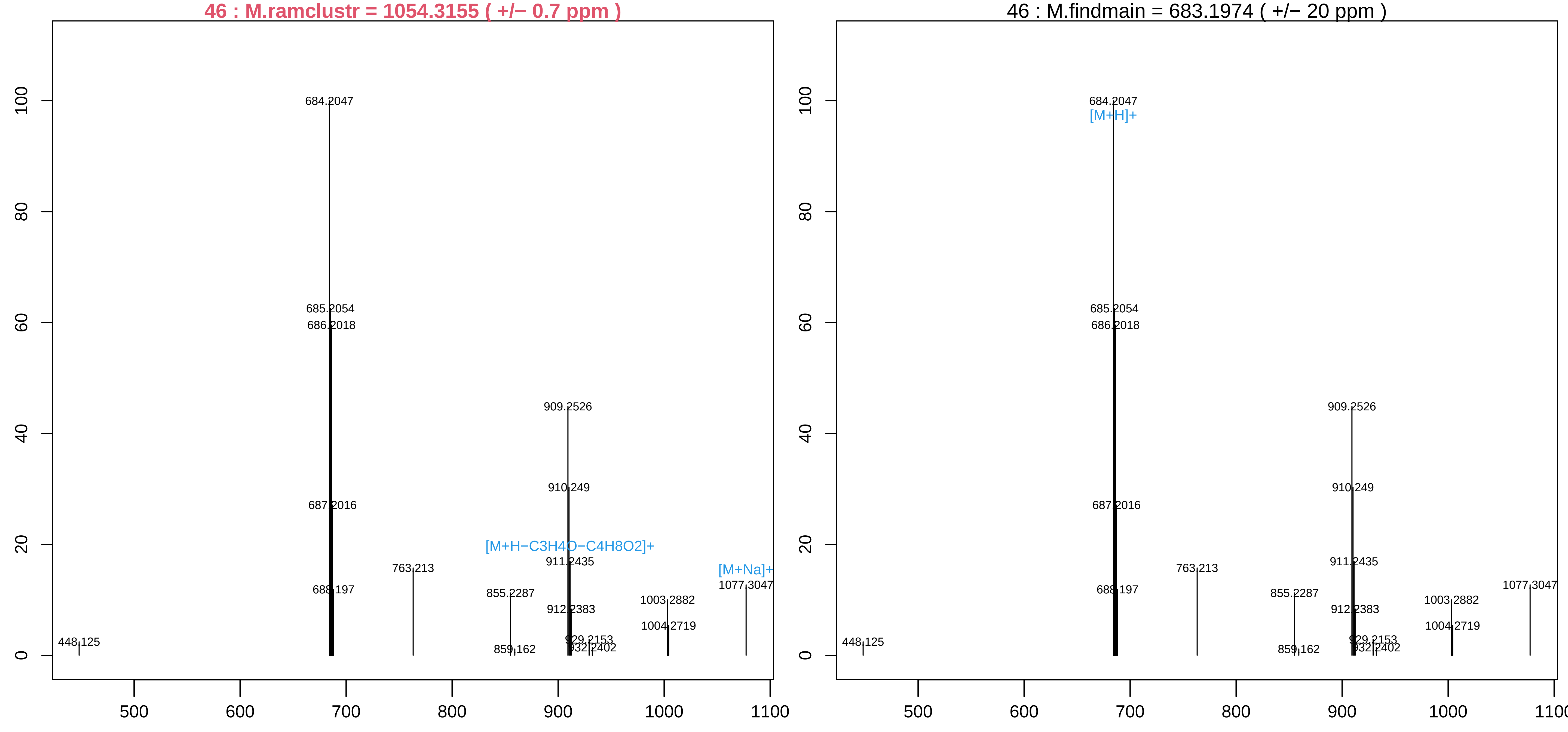
46 : M.ramclustr = 1054.3155 (+/− 0.7 ppm)
684.2047
685.2054
686.2018
687.2016
688.197
448.125
763.213
855.2287
859.162
909.2526
910.249
911.2435
912.2383
929.2153
932.2402
1003.2882
1004.2719
1077.3047
[M+H−C3H4O−C4H8O2]+
[M+Na]+
46 : M.findmain = 683.1974 (+/− 20 ppm)
[M+H]+
0
20
40
60
80
100
500
600
700
800
900
1000
1100

47 : M.ramclustr = 630.4839 (+/− 3.7 ppm)

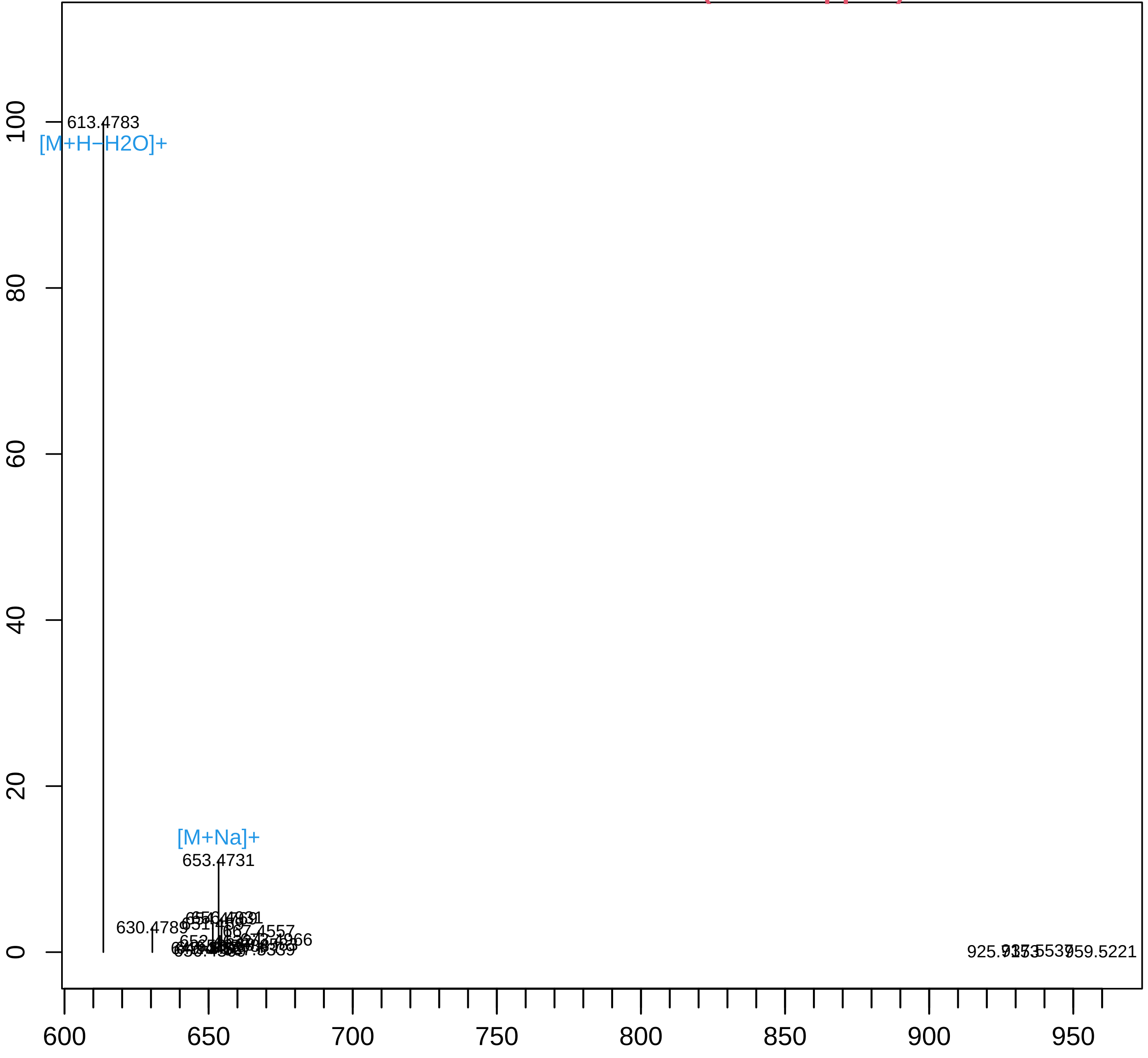

47 : M.findmain = 612.471 (+/− 20 ppm)

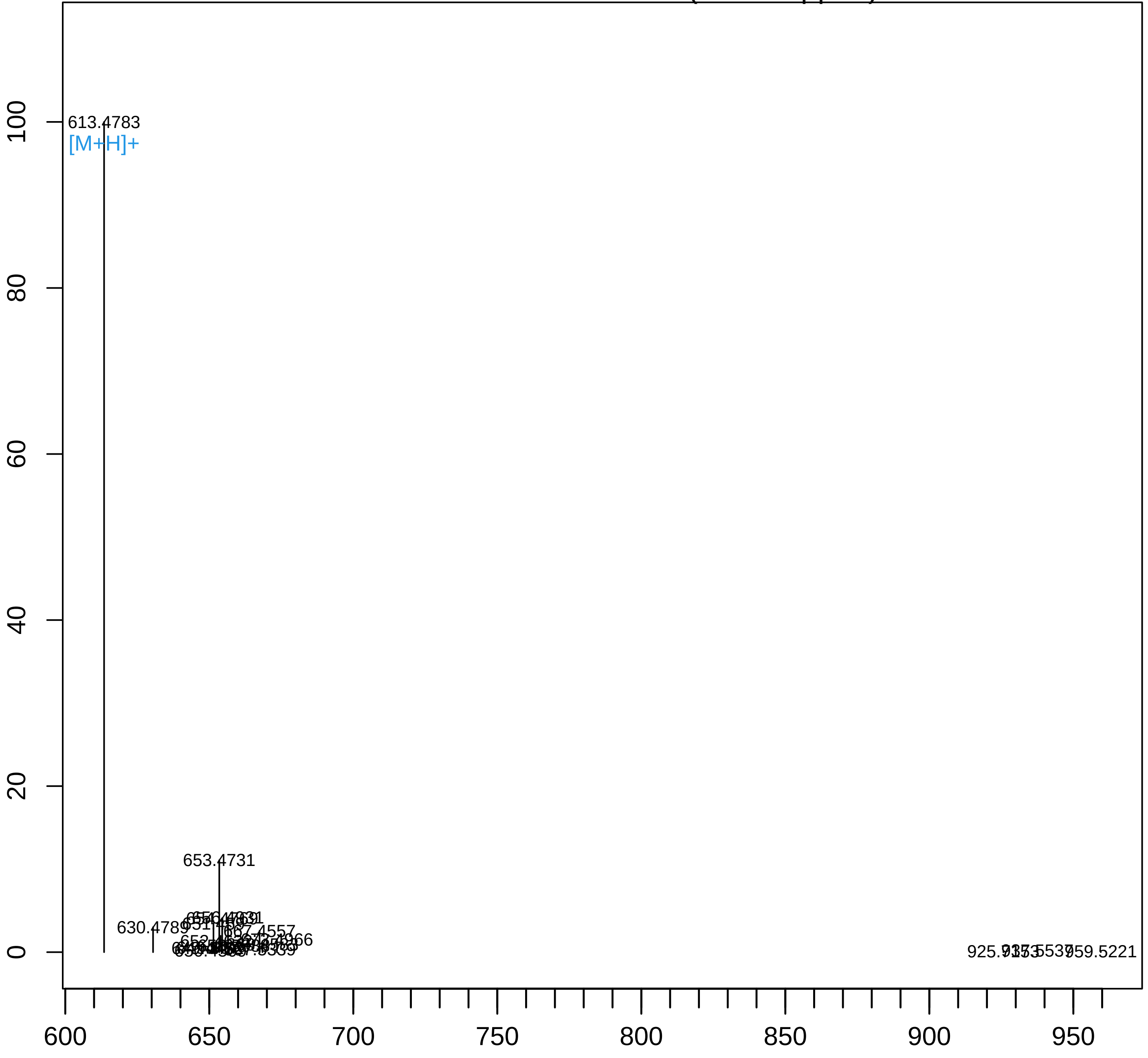

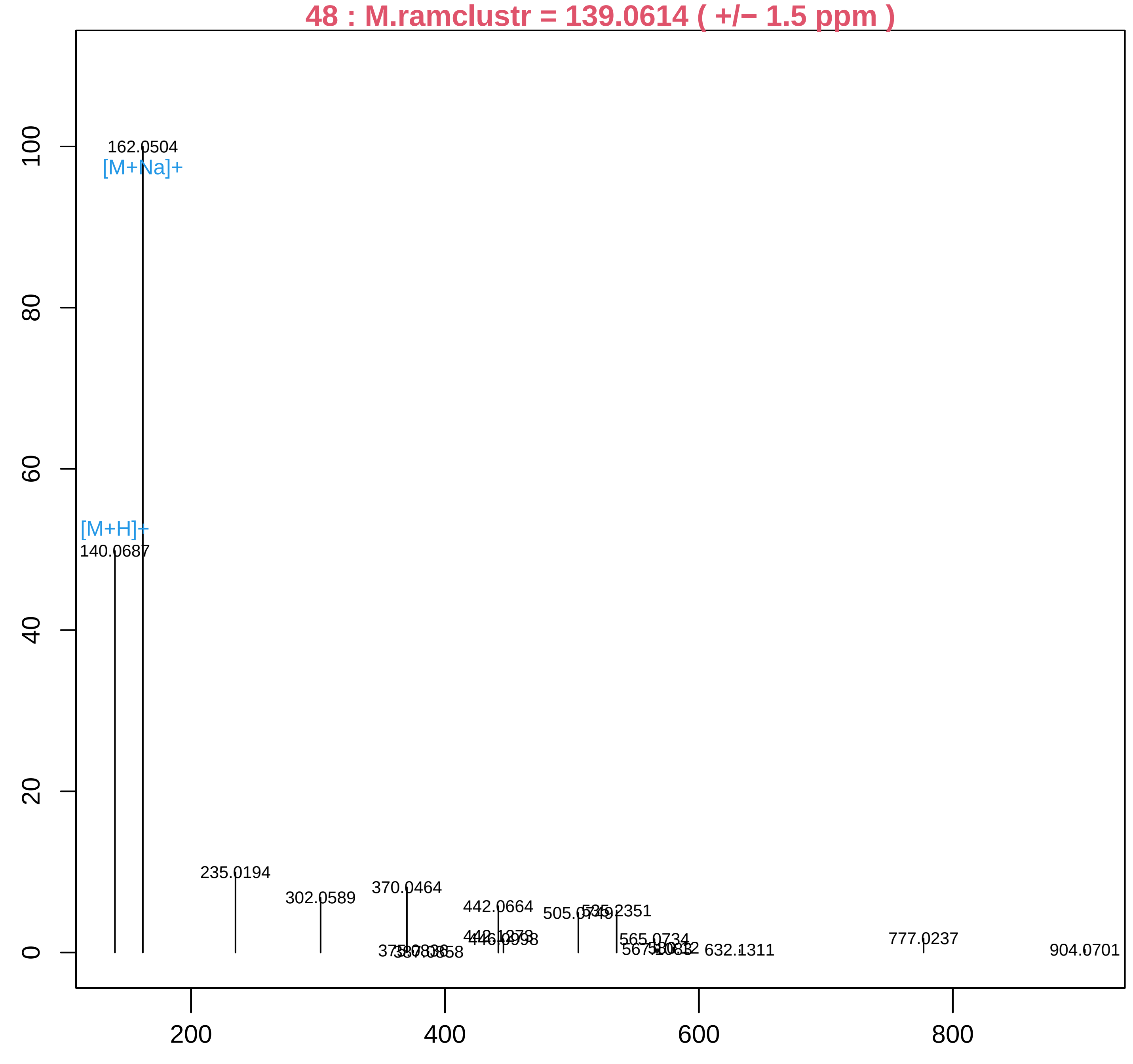
48 : M.ramclustr = 139.0614 (+/− 1.5 ppm)
162.0504
[M+Na]+
[M+H]+
140.0687
235.0194
302.0589
370.0464
442.0664
535.2351
565.0734
632.1311
777.0237
904.0701
0
20
40
60
80
100
200
400
600
800

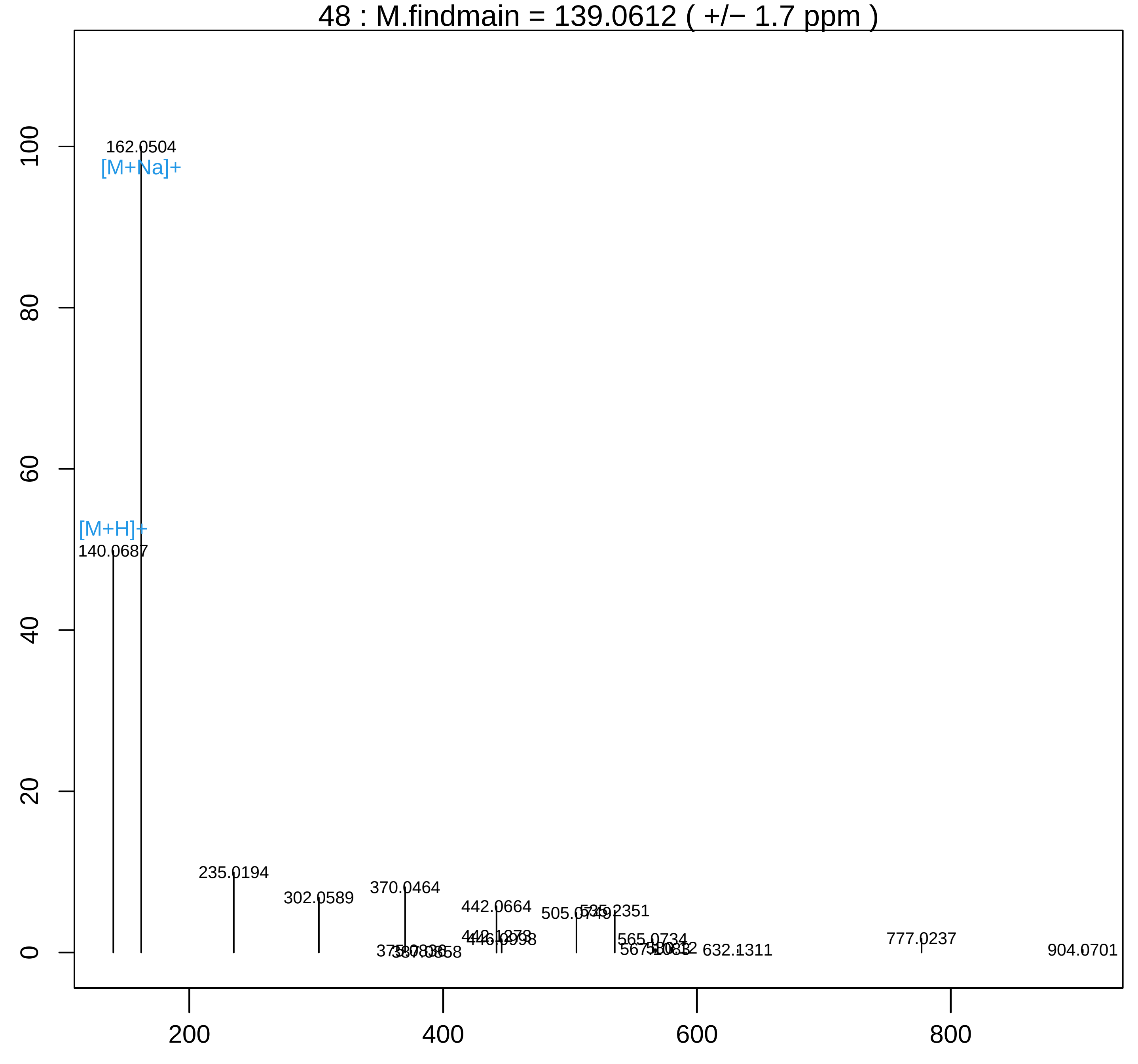
48 : M.findmain = 139.0612 (+/− 1.7 ppm)
162.0504
[M+Na]+
[M+H]+
140.0687
235.0194
302.0589
370.0464
442.0664
535.2351
565.0734
632.1311
777.0237
904.0701
0
20
40
60
80
100
200
400
600
800

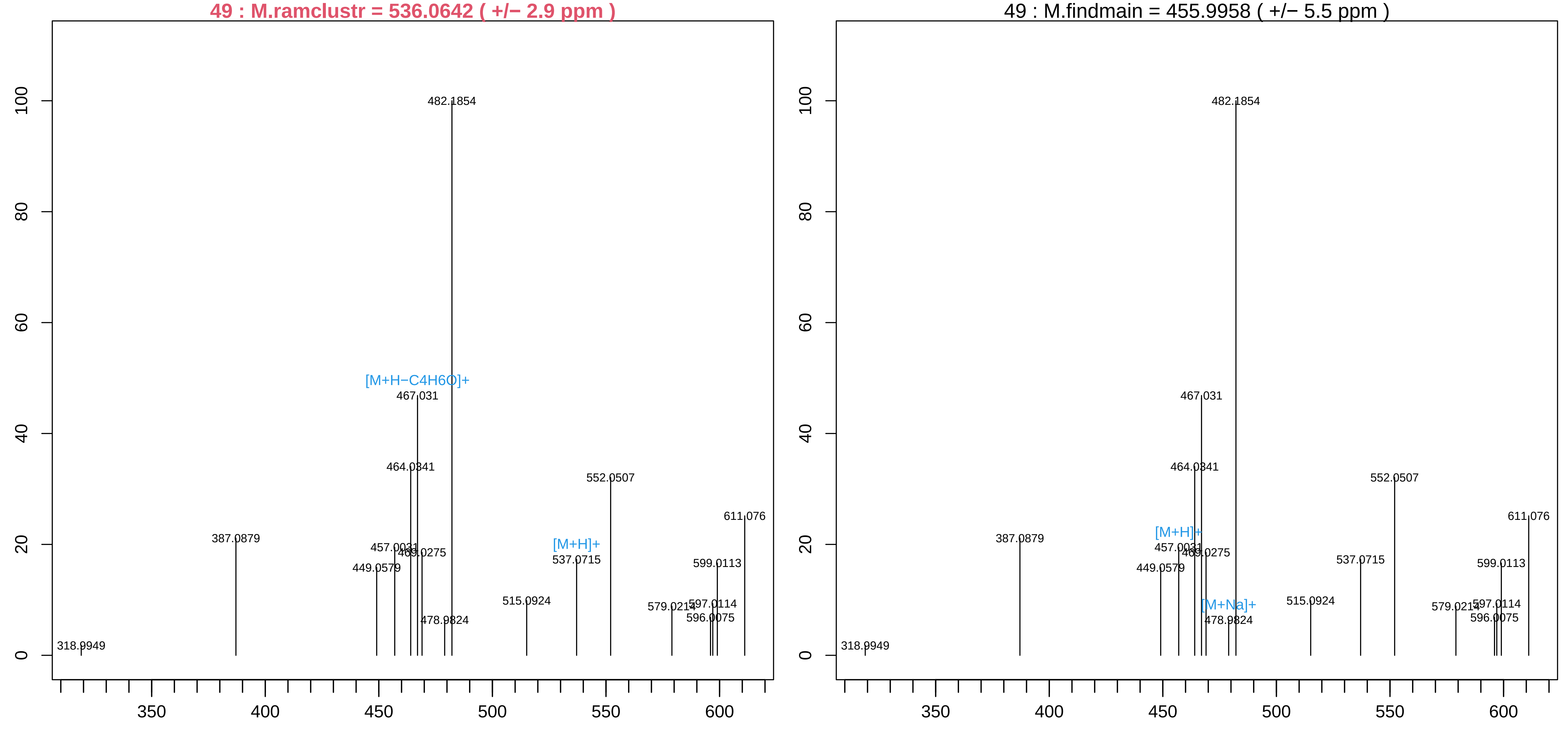

49 : M.ramclustr = 536.0642 (+/− 2.9 ppm)
[M+H−C4H6O]+
[M+H]+
482.1854
467.031
464.0341
552.0507
611.076
387.0879
457.0031
469.0275
449.0579
537.0715
599.0113
515.0924
579.0214
597.0114
596.0075
478.9824
318.9949
49 : M.findmain = 455.9958 (+/− 5.5 ppm)
[M+H]+
[M+Na]+
0
20
40
60
80
100
350
400
450
500
550
600

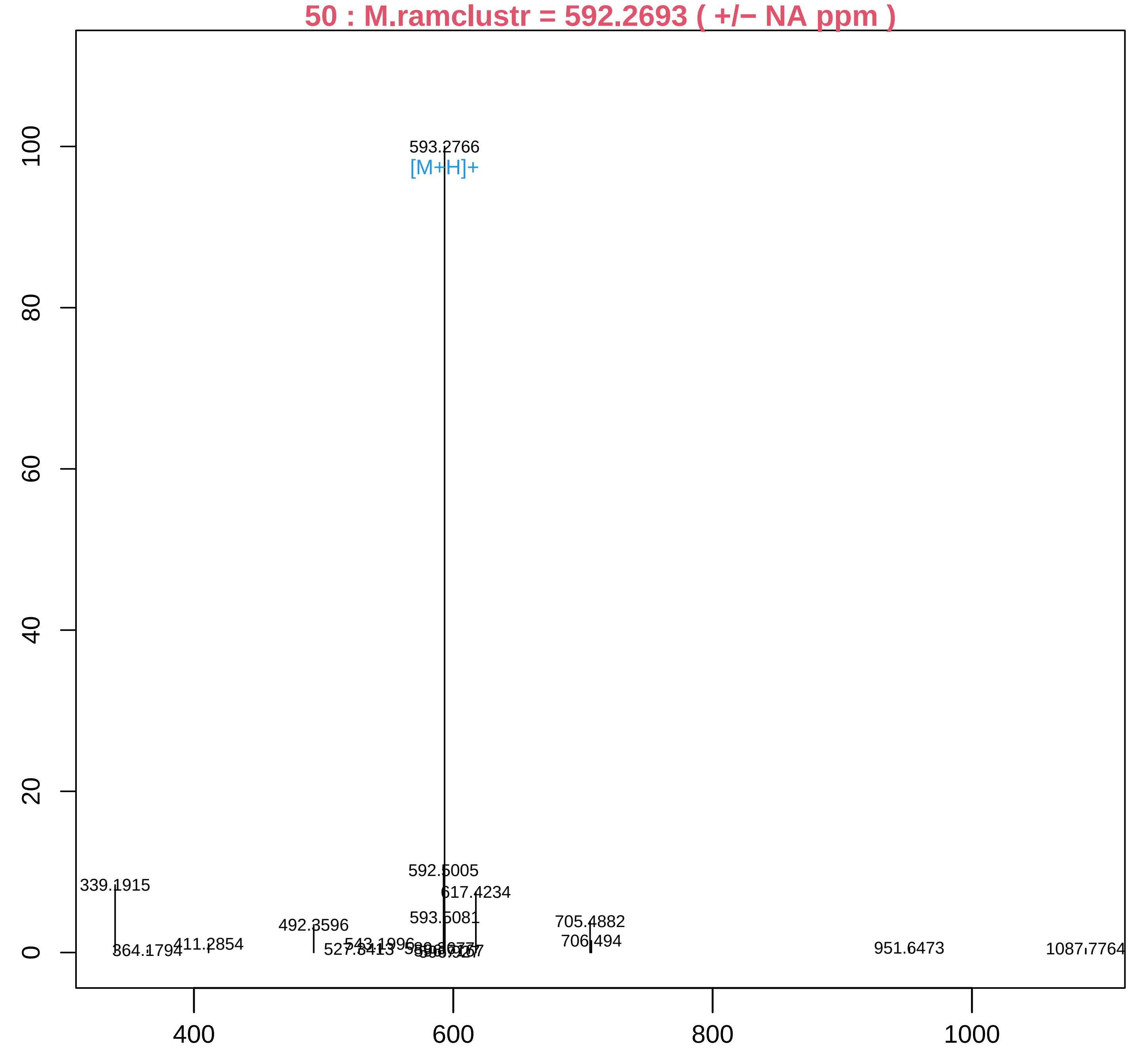
50 : M.ramclustr = 592.2693 (+/− NA ppm)
593.2766
[M+H]+
592.5005
339.1915
617.4234
593.5081
705.4882
492.3596
706.494
543.1996
411.2854
364.1794
527.5413
951.6473
1087.7764
0
20
40
60
80
100
400
600
800
1000

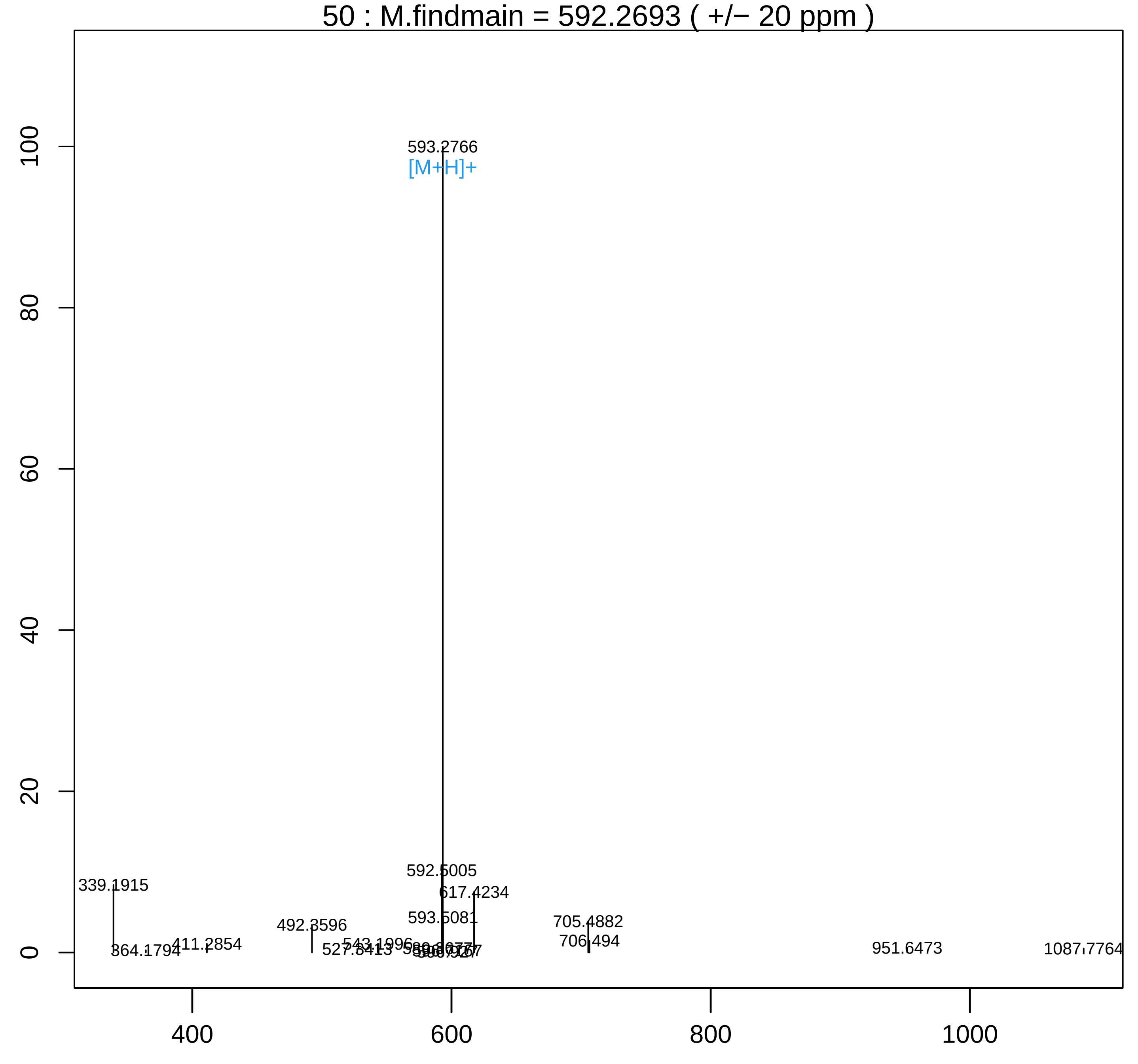
50 : M.findmain = 592.2693 (+/− 20 ppm)
593.2766
[M+H]+
592.5005
339.1915
617.4234
593.5081
705.4882
492.3596
706.494
543.1996
411.2854
364.1794
527.5413
951.6473
1087.7764
0
20
40
60
80
100
400
600
800
1000

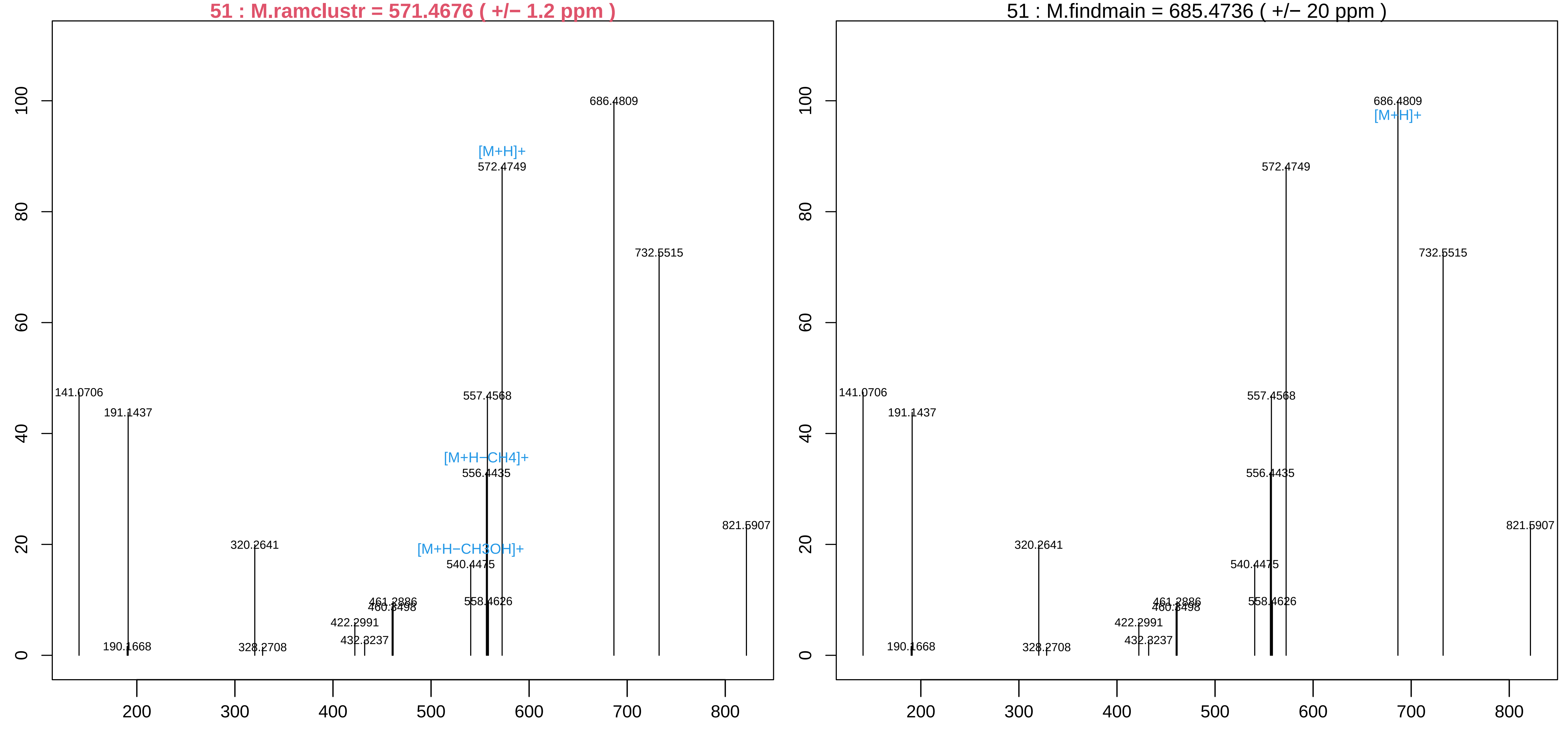

51 : M.ramclustr = 571.4676 (+/− 1.2 ppm)
686.4809
[M+H]+
572.4749
732.5515
141.0706
191.1437
557.4568
[M+H−CH4]+
556.4435
821.5907
320.2641
[M+H−CH3OH]+
540.4475
461.2886
460.3498
558.4626
422.2991
432.3237
190.1668
328.2708
0
20
40
60
80
100
200
300
400
500
600
700
800
51 : M.findmain = 685.4736 (+/− 20 ppm)
686.4809
[M+H]+
572.4749
732.5515
141.0706
191.1437
557.4568
556.4435
821.5907
320.2641
540.4475
461.2886
460.3498
558.4626
422.2991
432.3237
190.1668
328.2708

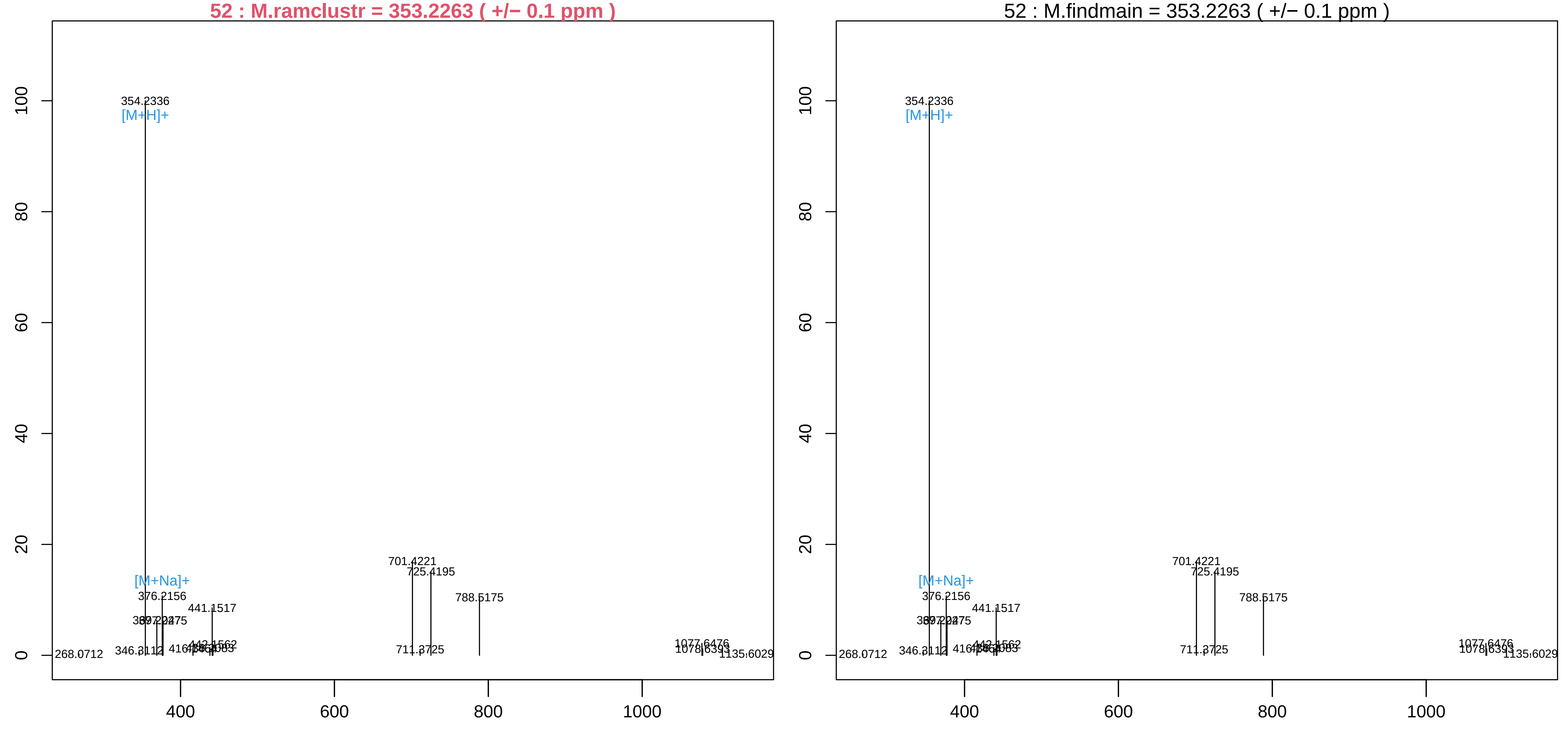
52 : M.ramclustr = 353.2263 (+/− 0.1 ppm)
354.2336
[M+H]+
[M+Na]+
376.2156
441.1517
442.1562
268.0712
346.3112
701.4221
725.4195
788.5175
711.3725
1077.6476
1078.6393
1135.6029
0
20
40
60
80
100
400
600
800
1000
52 : M.findmain = 353.2263 (+/− 0.1 ppm)
354.2336
[M+H]+
[M+Na]+
376.2156
441.1517
442.1562
268.0712
346.3112
701.4221
725.4195
788.5175
711.3725
1077.6476
1078.6393
1135.6029
0
20
40
60
80
100
400
600
800
1000

53 : M.ramclustr = 936.5797 (+/− 0.8 ppm)

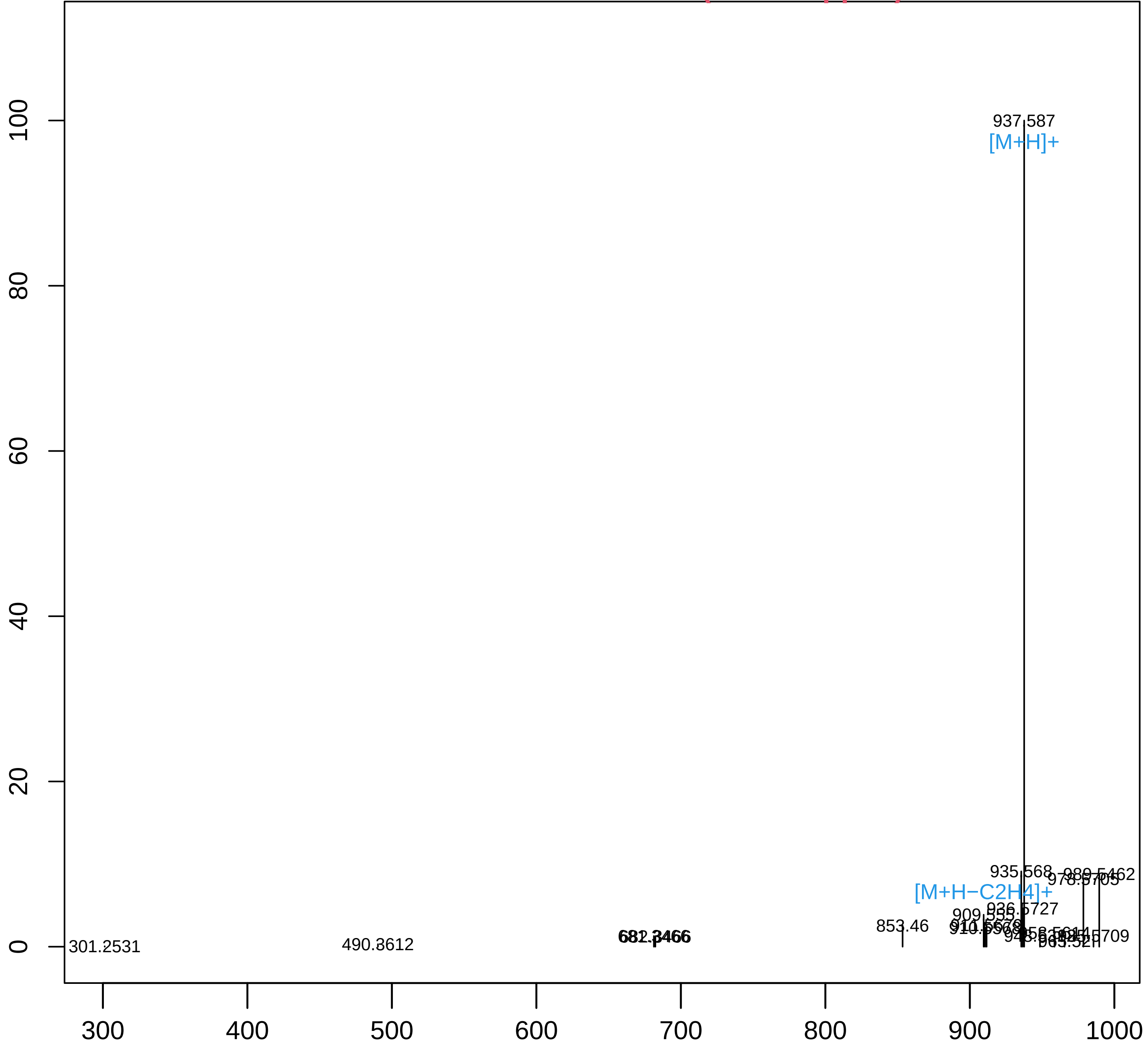

53 : M.findmain = 936.5797 (+/− 20 ppm)

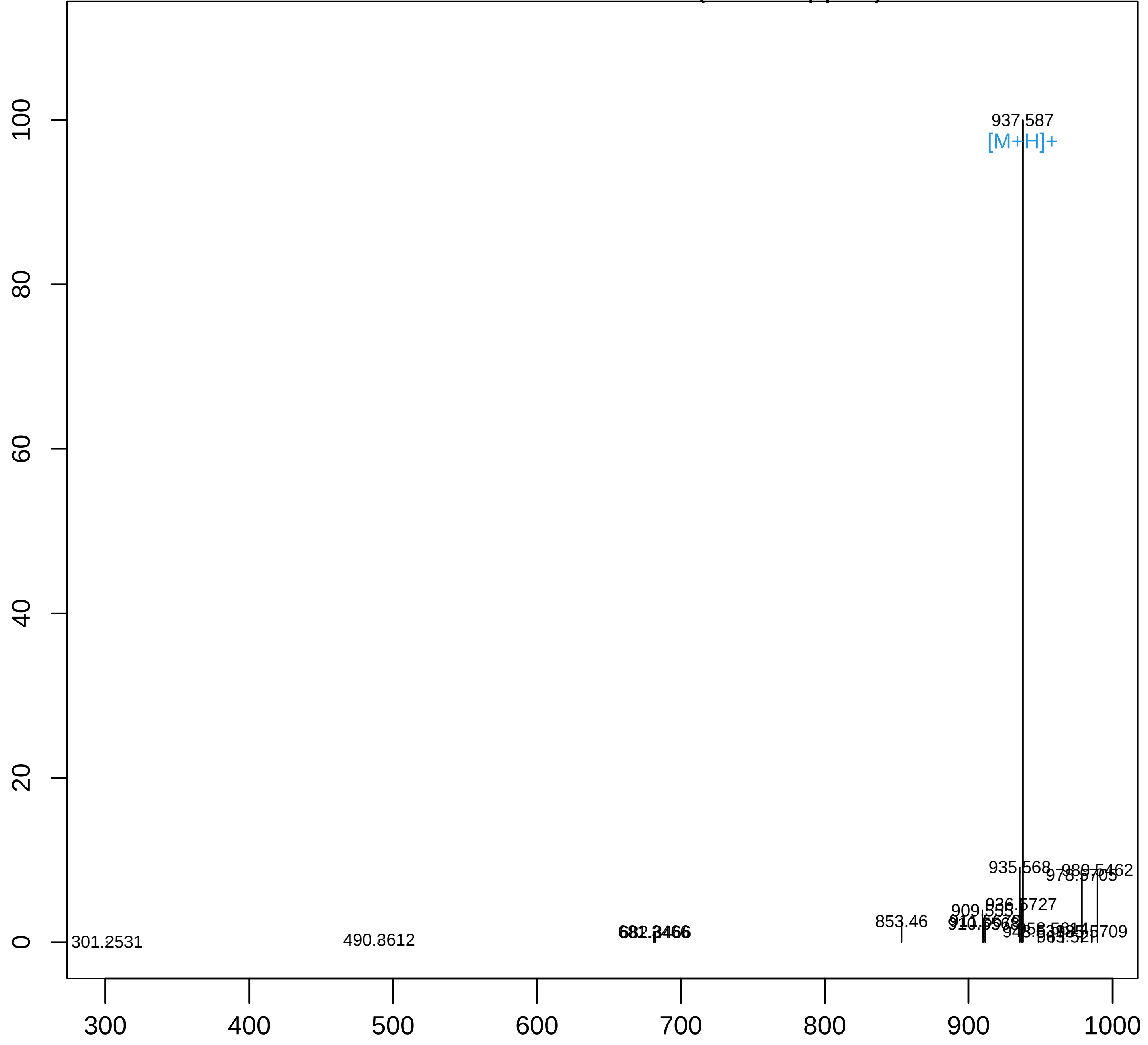

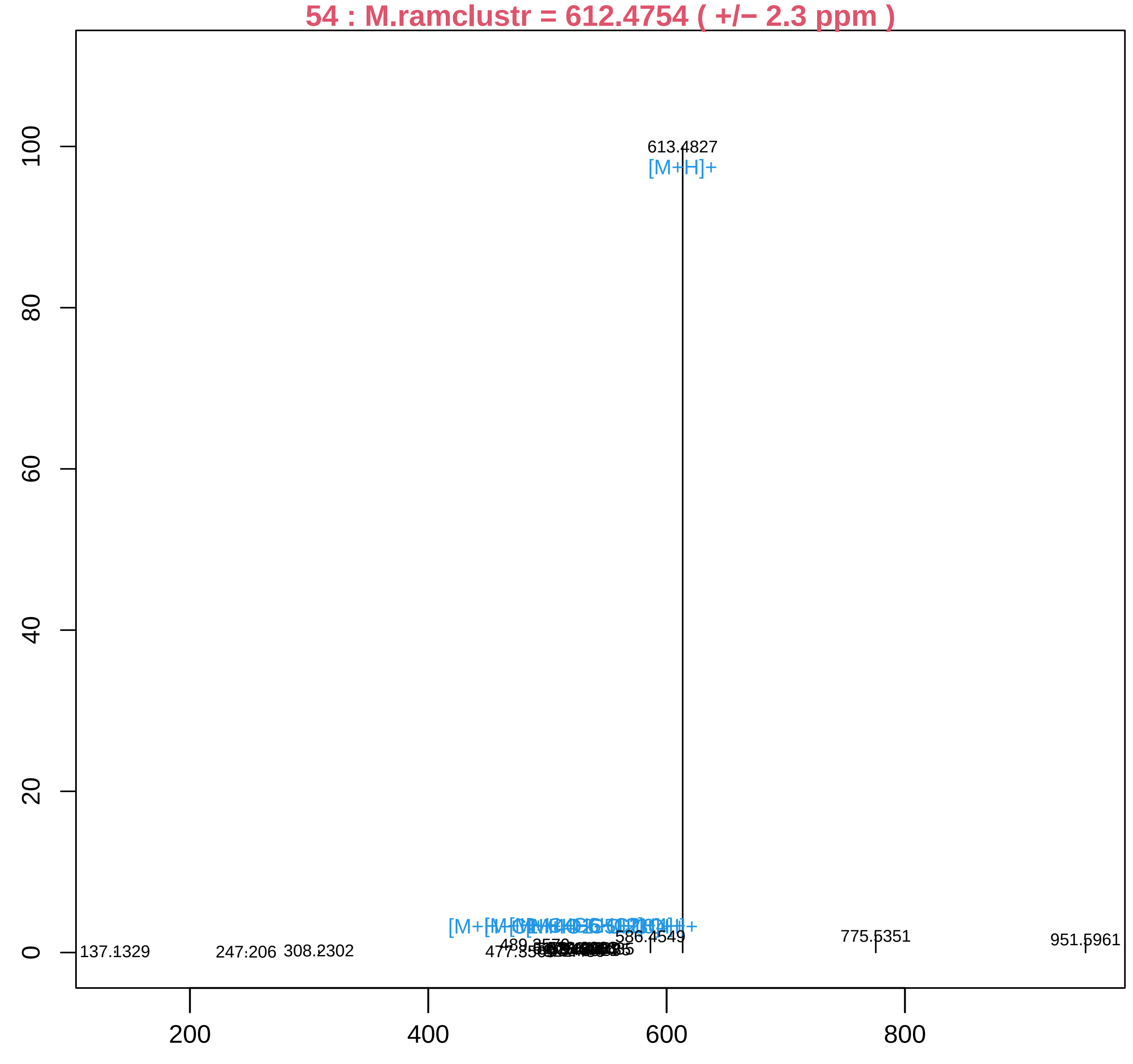
54 : M.ramclustr = 612.4754 (+/− 2.3 ppm)
613.4827
[M+H]+
586.4549
775.5351
951.5961
137.1329
247.206
308.2302
0
20
40
60
80
100
200
400
600
800

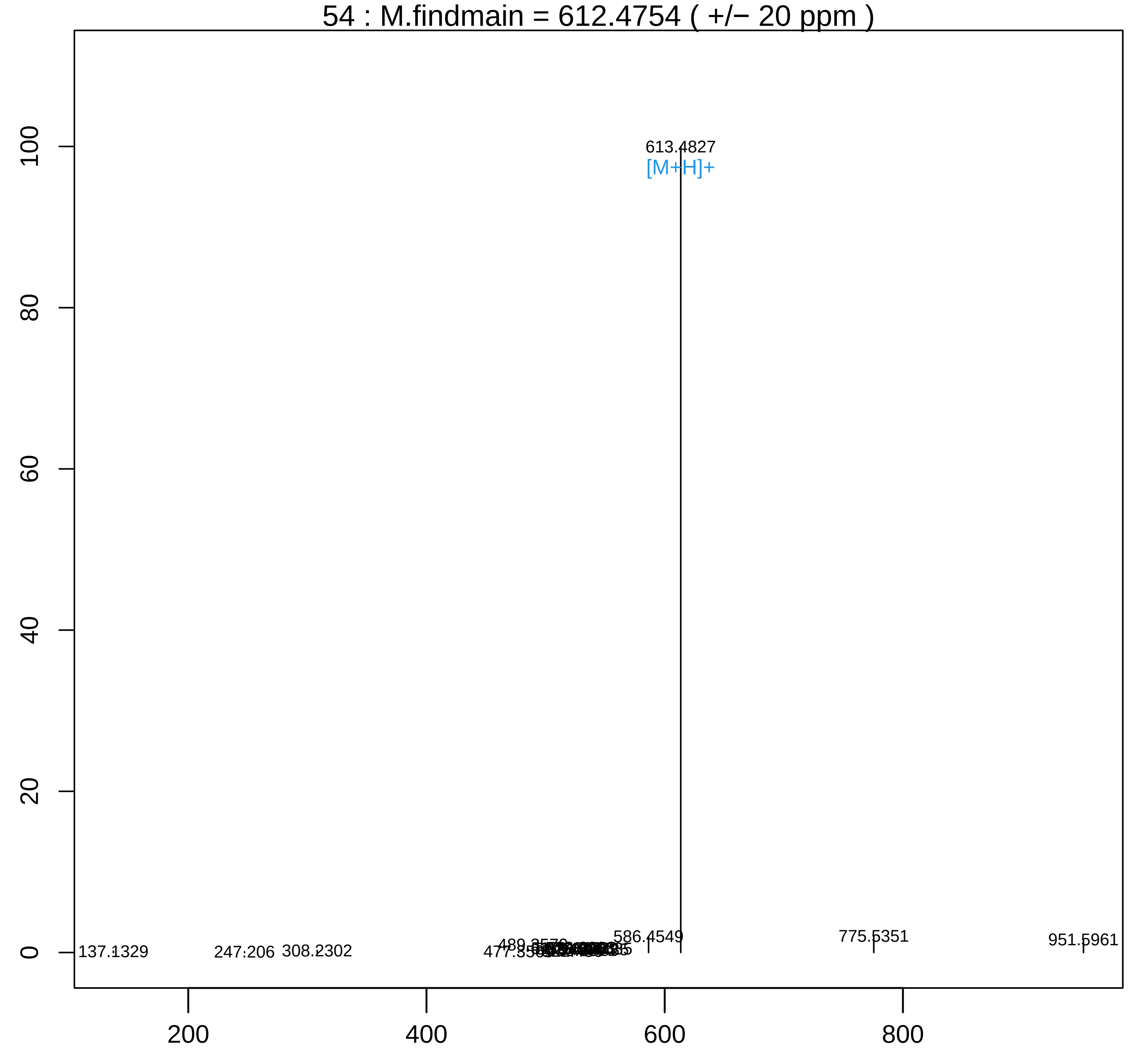
54 : M.findmain = 612.4754 (+/− 20 ppm)
613.4827
[M+H]+
586.4549
775.5351
951.5961
137.1329
247.206
308.2302
0
20
40
60
80
100
200
400
600
800

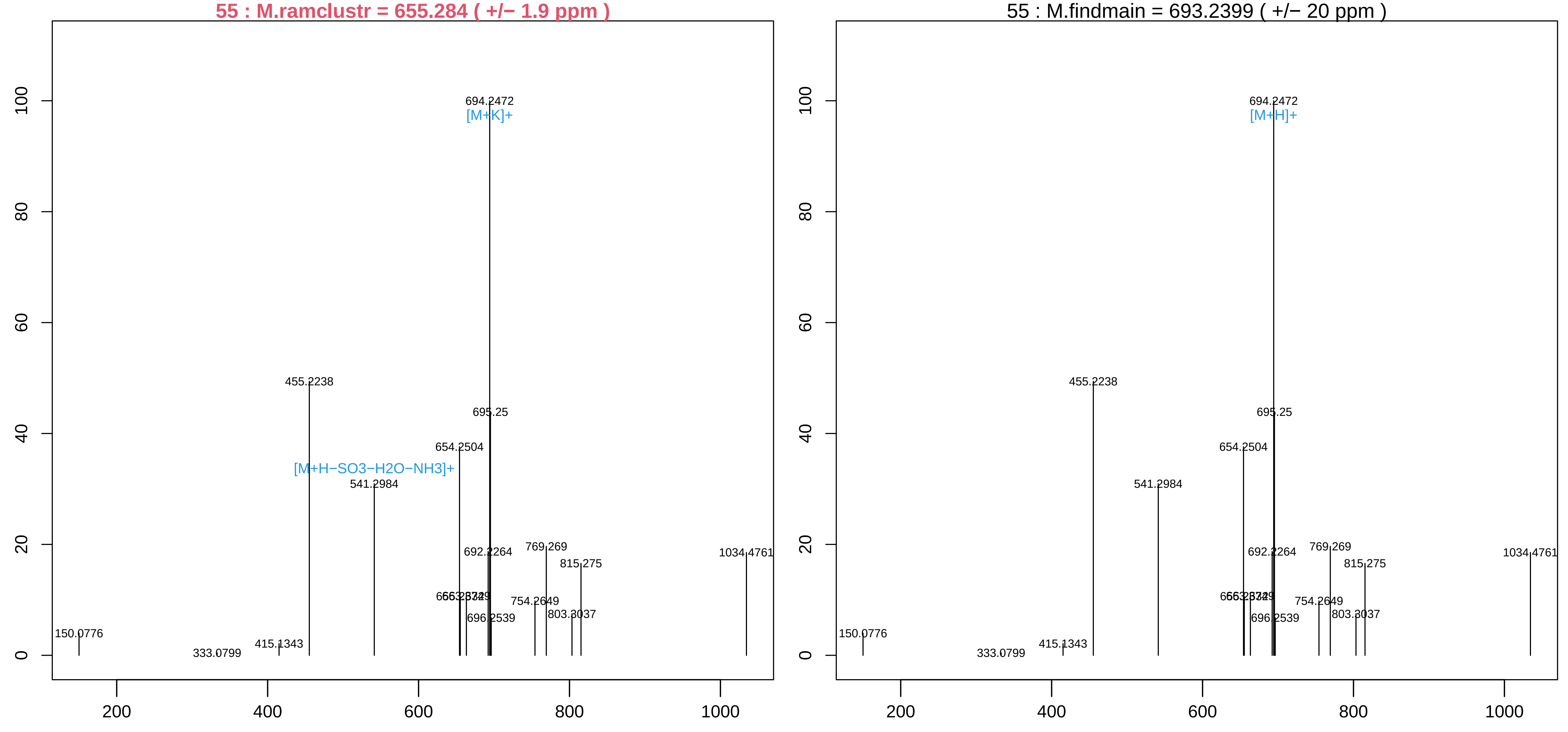

55 : M.ramclustr = 655.284 (+/− 1.9 ppm)
694.2472
[M+K]+
455.2238
695.25
654.2504
[M+H−SO3−H2O−NH3]+
541.2984
692.2264
769.269
815.275
1034.4761
754.2649
696.2539
803.3037
150.0776
415.1343
333.0799
0
20
40
60
80
100
200
400
600
800
1000
55 : M.findmain = 693.2399 (+/− 20 ppm)
694.2472
[M+H]+
455.2238
695.25
654.2504
541.2984
692.2264
769.269
815.275
1034.4761
754.2649
696.2539
803.3037
150.0776
415.1343
333.0799
0
20
40
60
80
100
200
400
600
800
1000

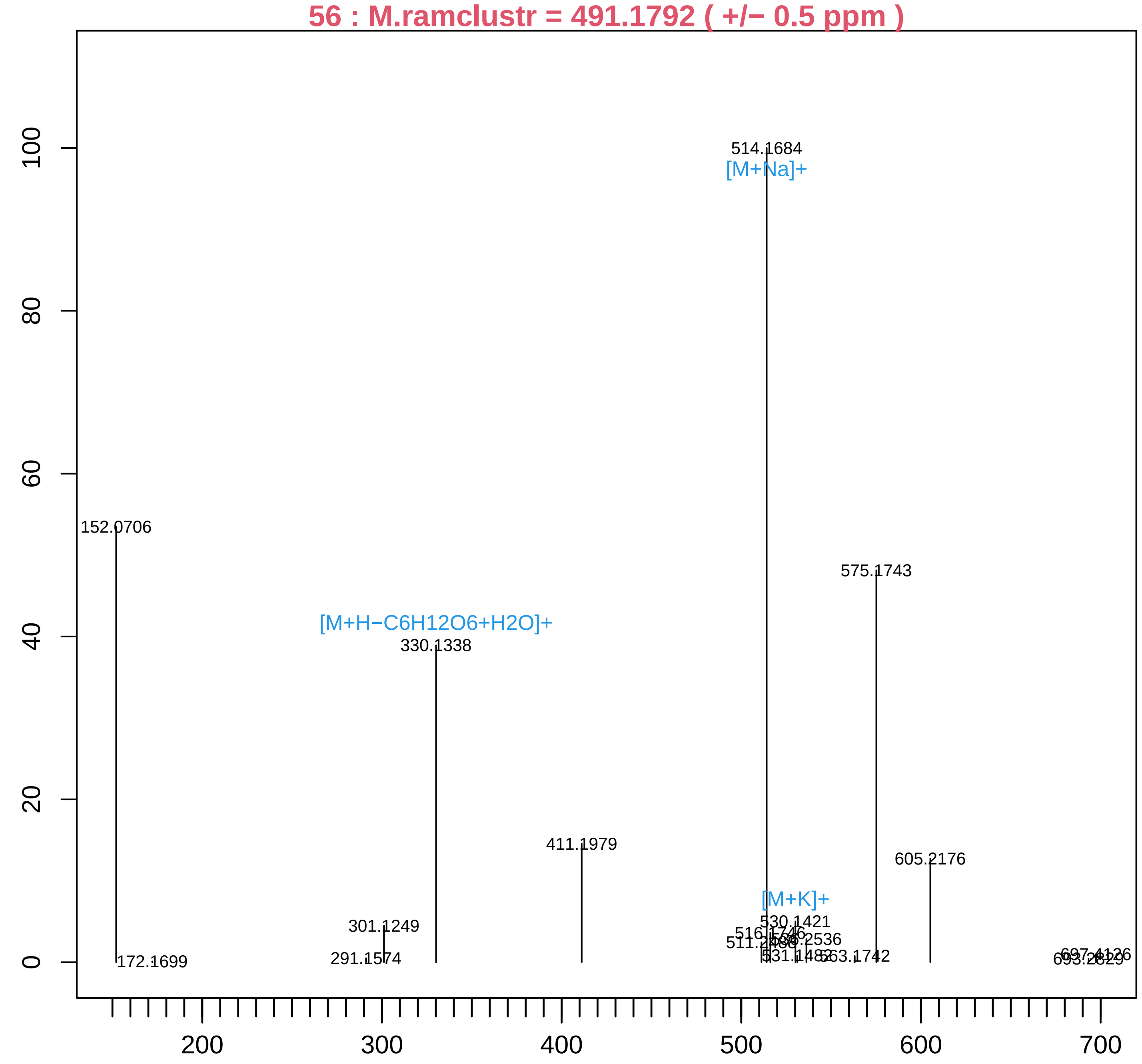

56 : M.ramclustr = 491.1792 (+/− 0.5 ppm)
514.1684
[M+Na]+
152.0706
575.1743
[M+H−C6H12O6+H2O]+
330.1338
411.1979
605.2176
[M+K]+
530.1421
301.1249
291.1574
172.1699
563.1742
697.4126
0
20
40
60
80
100
200
300
400
500
600
700

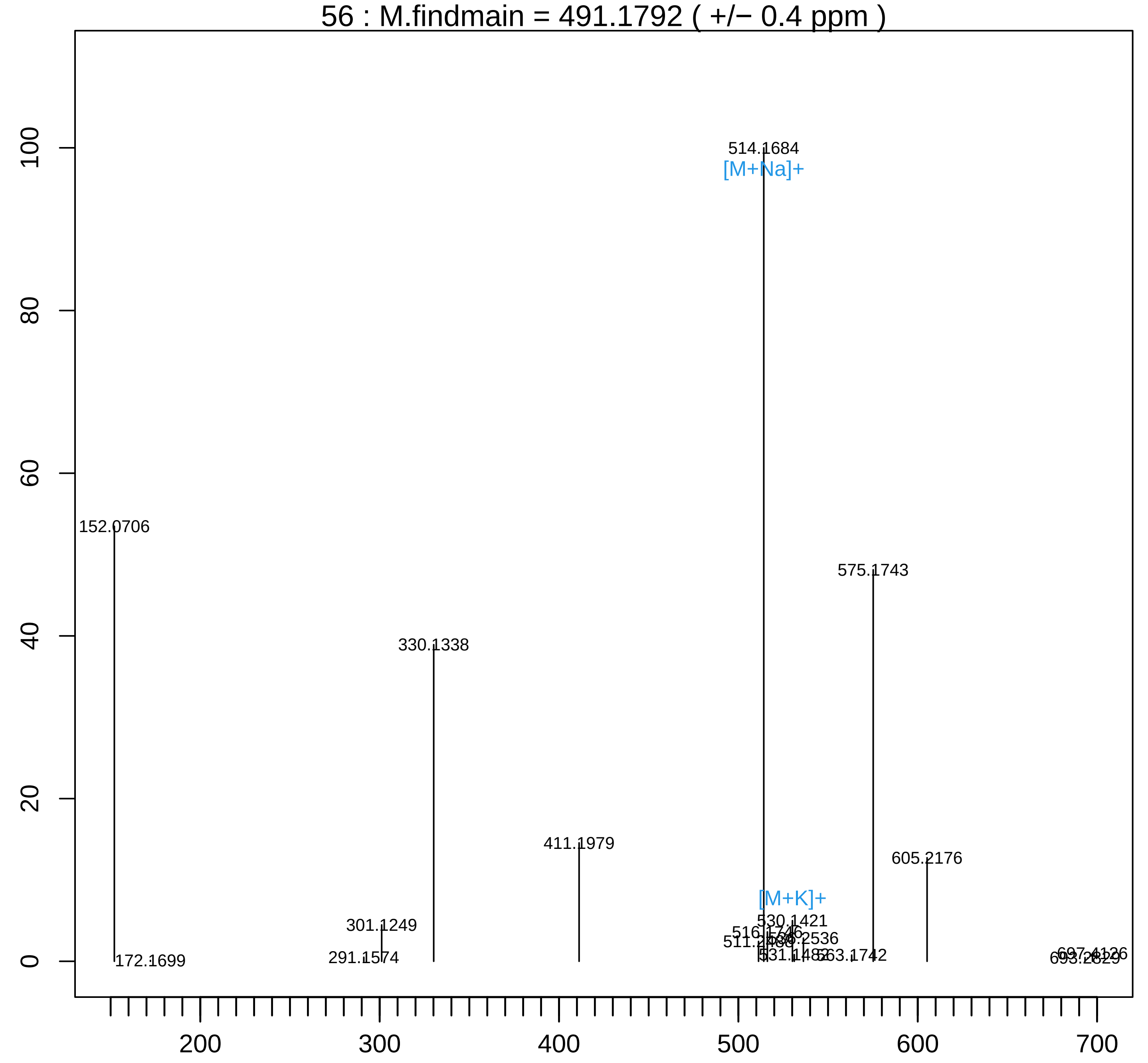

56 : M.findmain = 491.1792 (+/− 0.4 ppm)
514.1684
[M+Na]+
152.0706
575.1743
330.1338
411.1979
605.2176
[M+K]+
530.1421
301.1249
291.1574
172.1699
563.1742
697.4126
0
20
40
60
80
100
200
300
400
500
600
700

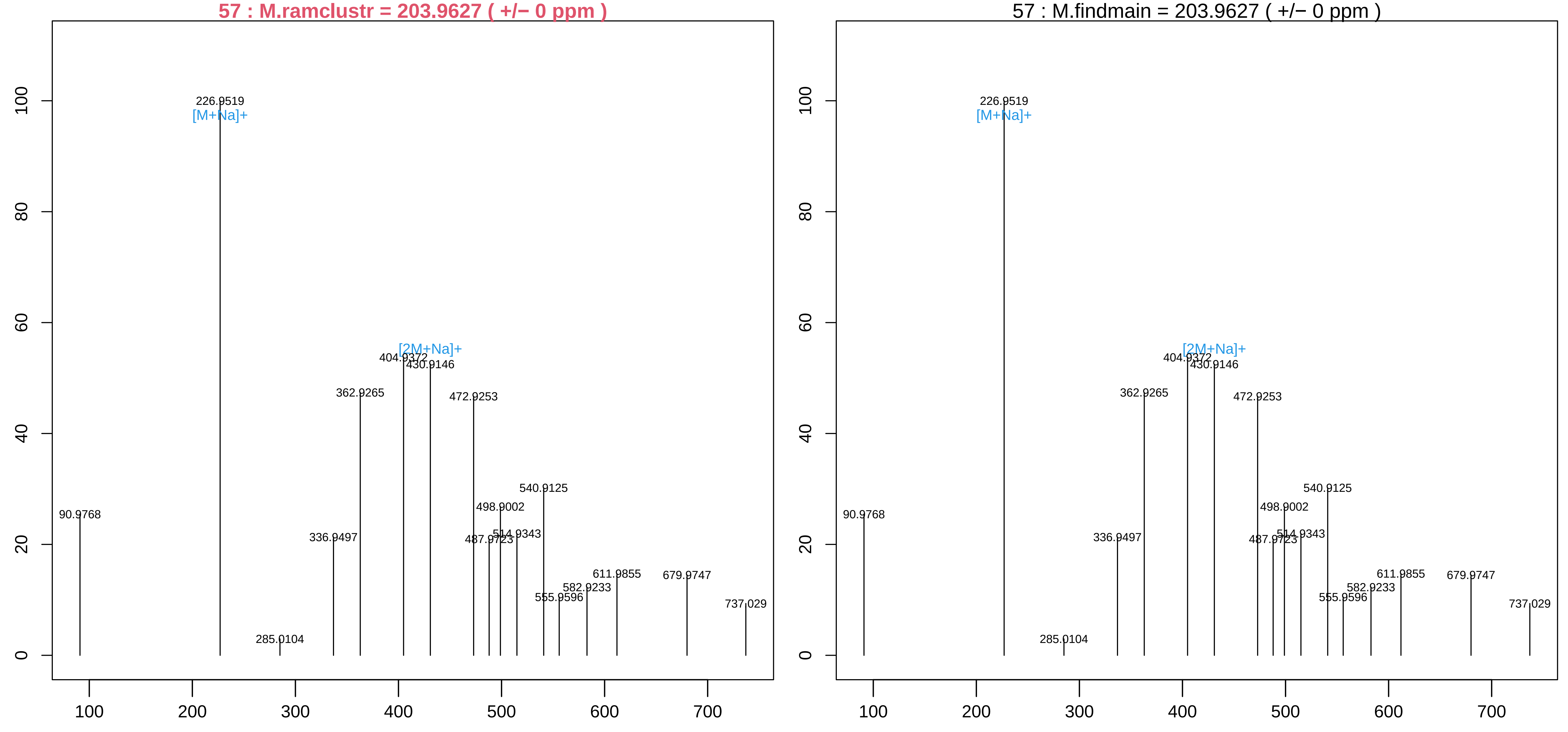
57 : M.ramclustr = 203.9627 (+/− 0 ppm)
57 : M.findmain = 203.9627 (+/− 0 ppm)
226.9519
[M+Na]+
[2M+Na]+
404.9372
430.9146
362.9265
472.9253
540.9125
498.9002
90.9768
336.9497
487.9723
514.9343
611.9855
679.9747
582.9233
555.9596
737.029
285.0104
0
20
40
60
80
100
100
200
300
400
500
600
700

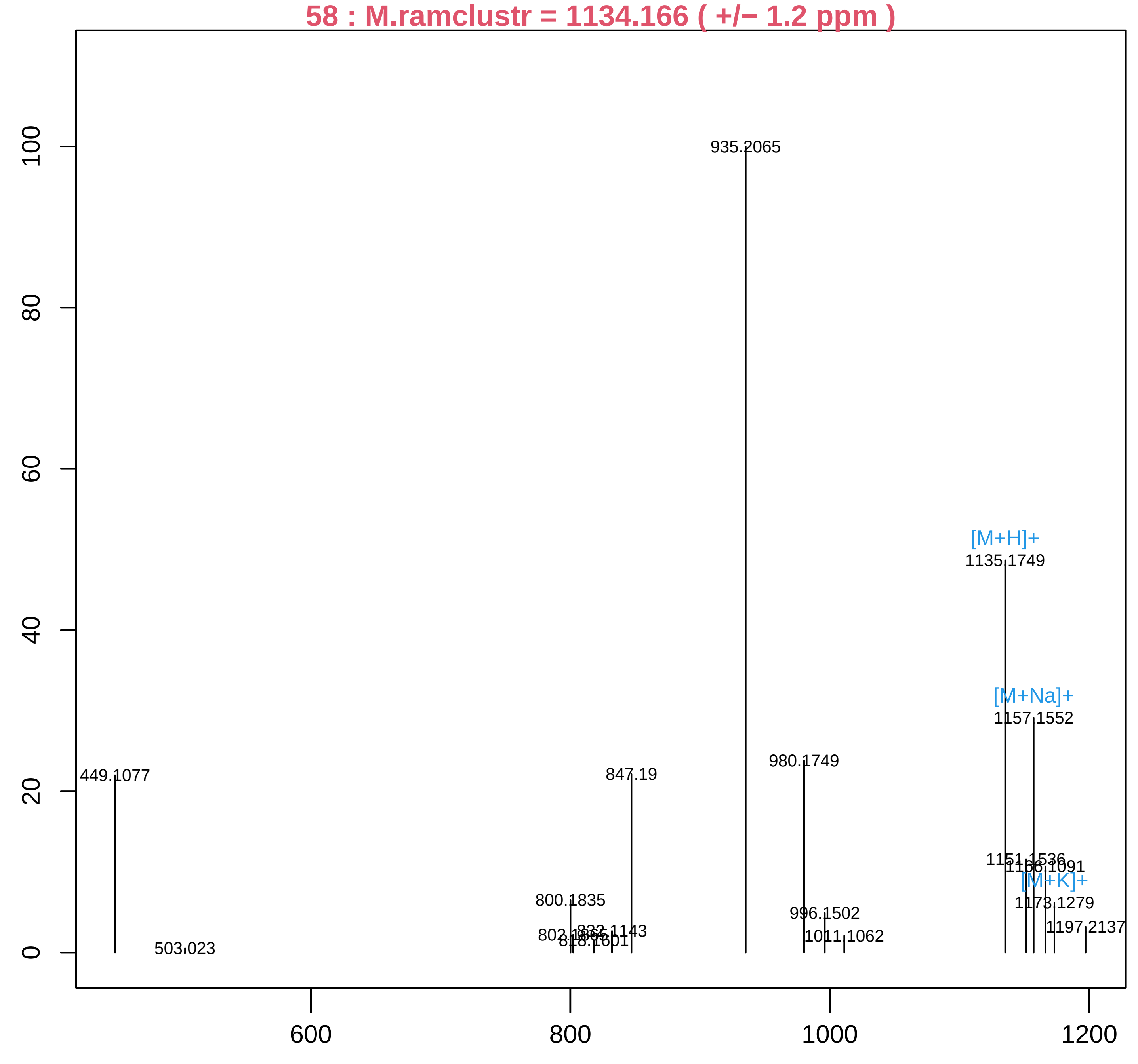
58 : M.ramclustr = 1134.166 (+/− 1.2 ppm)
0
20
40
60
80
100
600
800
1000
1200
935.2065
[M+H]+
1135.1749
[M+Na]+
1157.1552
980.1749
449.1077
847.19
1151.1536
1166.1091
[M+K]+
1173.1279
800.1835
996.1502
802.1866
832.1143
818.1601
1011.1062
1197.2137
503.023

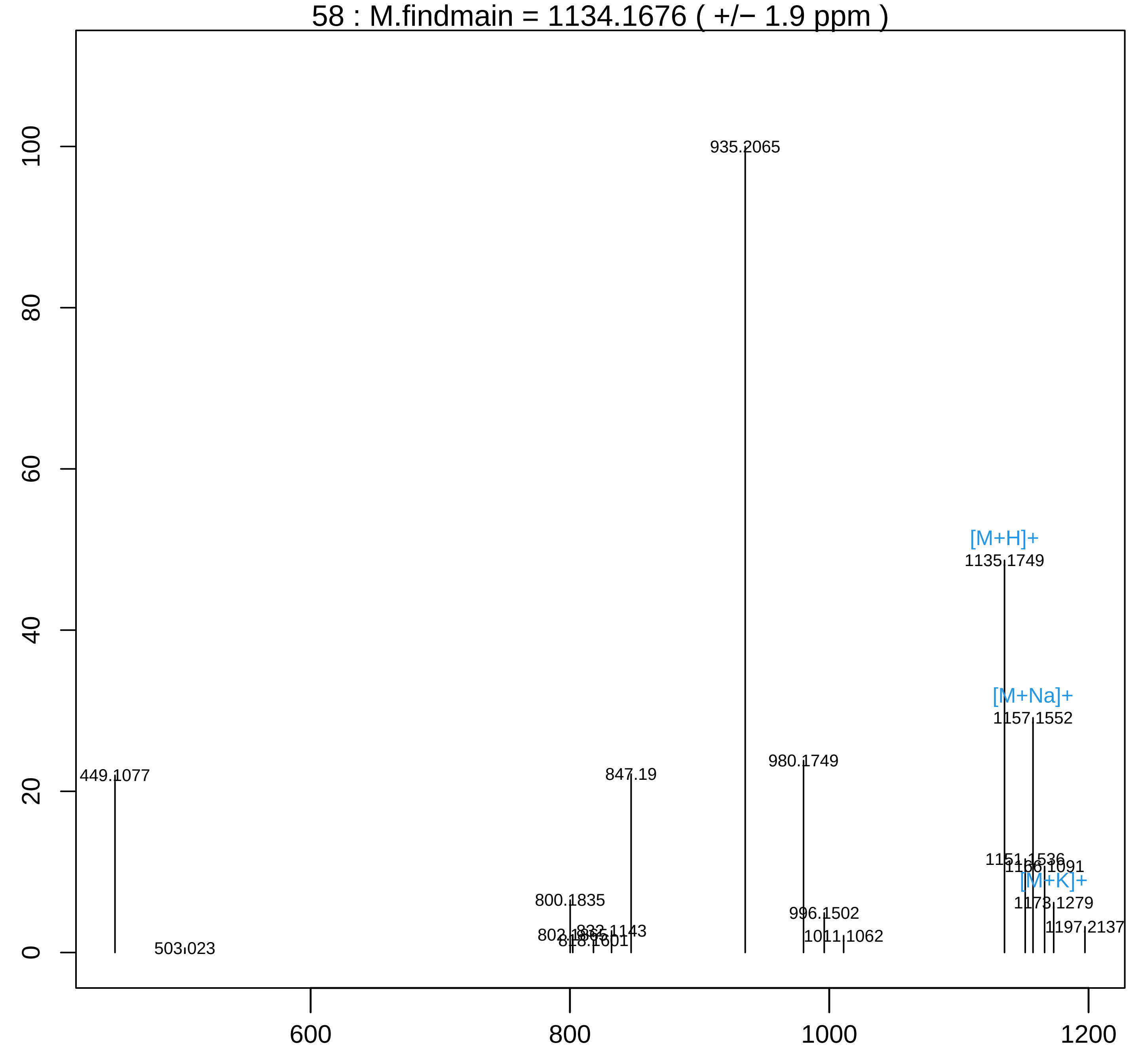
58 : M.findmain = 1134.1676 (+/− 1.9 ppm)
0
20
40
60
80
100
600
800
1000
1200
935.2065
[M+H]+
1135.1749
[M+Na]+
1157.1552
980.1749
449.1077
847.19
1151.1536
1166.1091
[M+K]+
1173.1279
800.1835
996.1502
802.1866
832.1143
818.1601
1011.1062
1197.2137
503.023

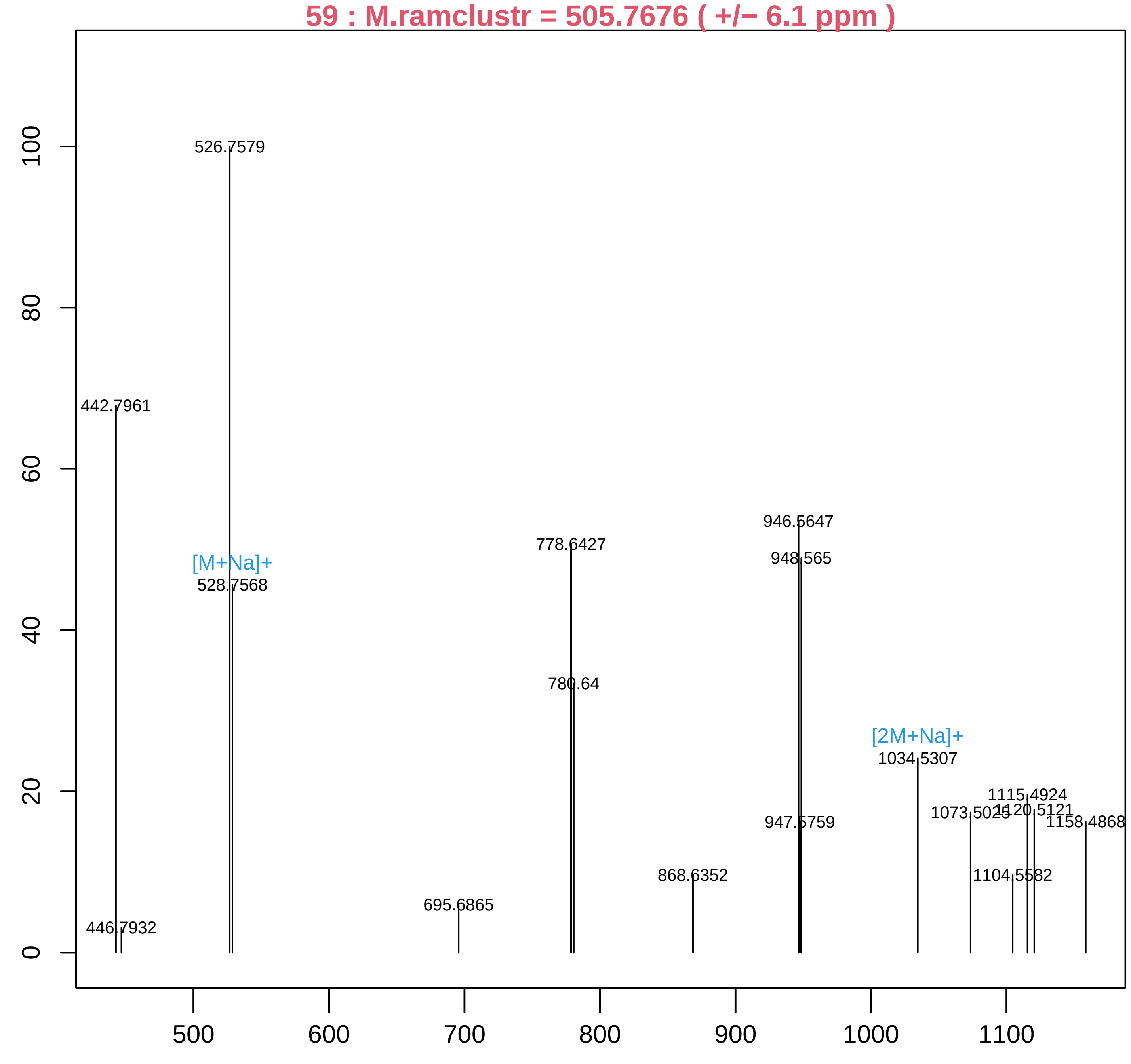
59 : M.ramclustr = 505.7676 (+/− 6.1 ppm)
526.7579
442.7961
[M+Na]+
528.7568
778.6427
780.64
946.5647
948.565
947.5759
[2M+Na]+
1034.5307
1073.5023
1115.4924
1120.5121
1158.4868
1104.5582
868.6352
695.6865
446.7932
0
20
40
60
80
100
500
600
700
800
900
1000
1100

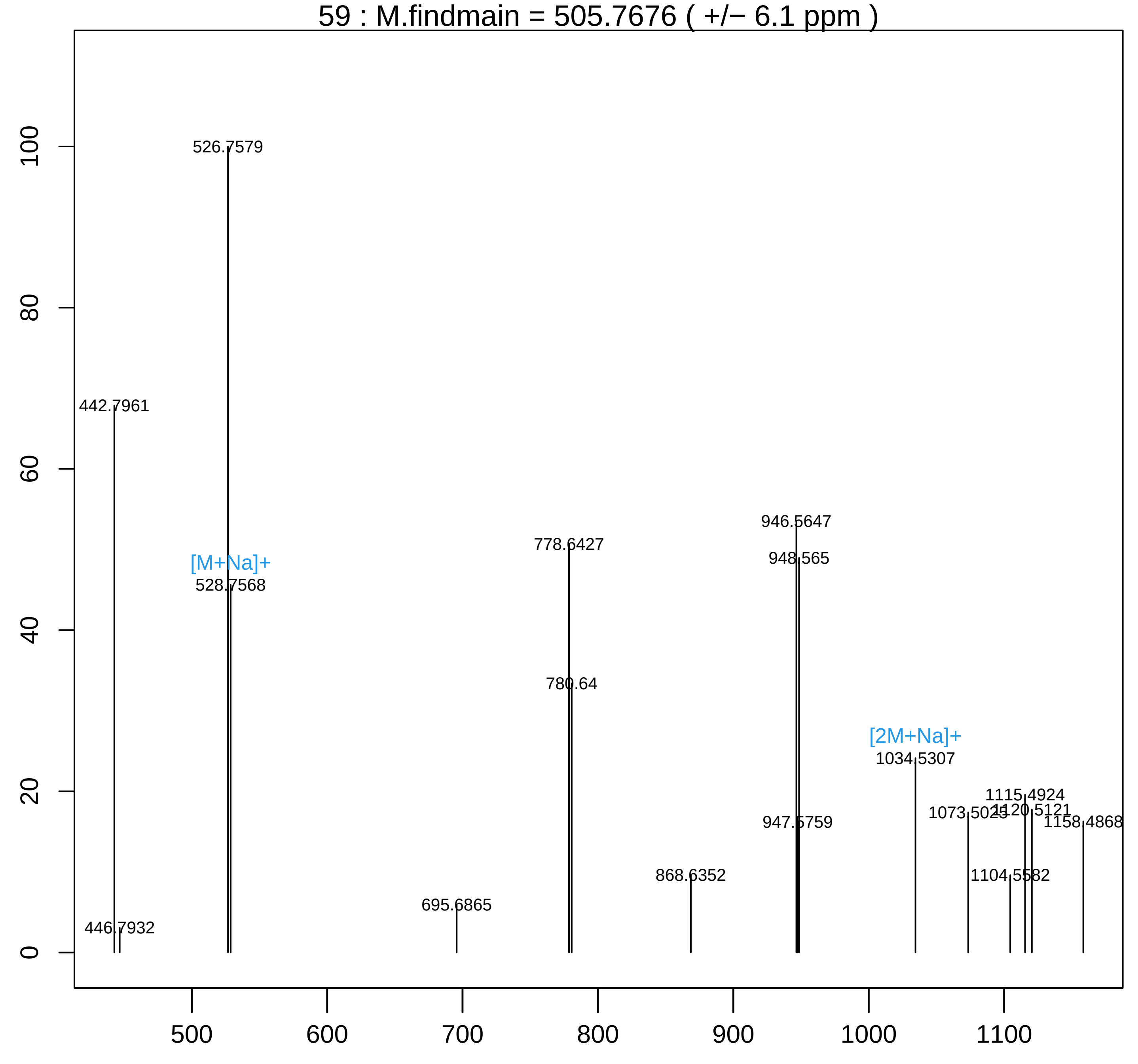
59 : M.findmain = 505.7676 (+/− 6.1 ppm)
526.7579
442.7961
[M+Na]+
528.7568
778.6427
780.64
946.5647
948.565
947.5759
[2M+Na]+
1034.5307
1073.5023
1115.4924
1120.5121
1158.4868
1104.5582
868.6352
695.6865
446.7932
0
20
40
60
80
100
500
600
700
800
900
1000
1100

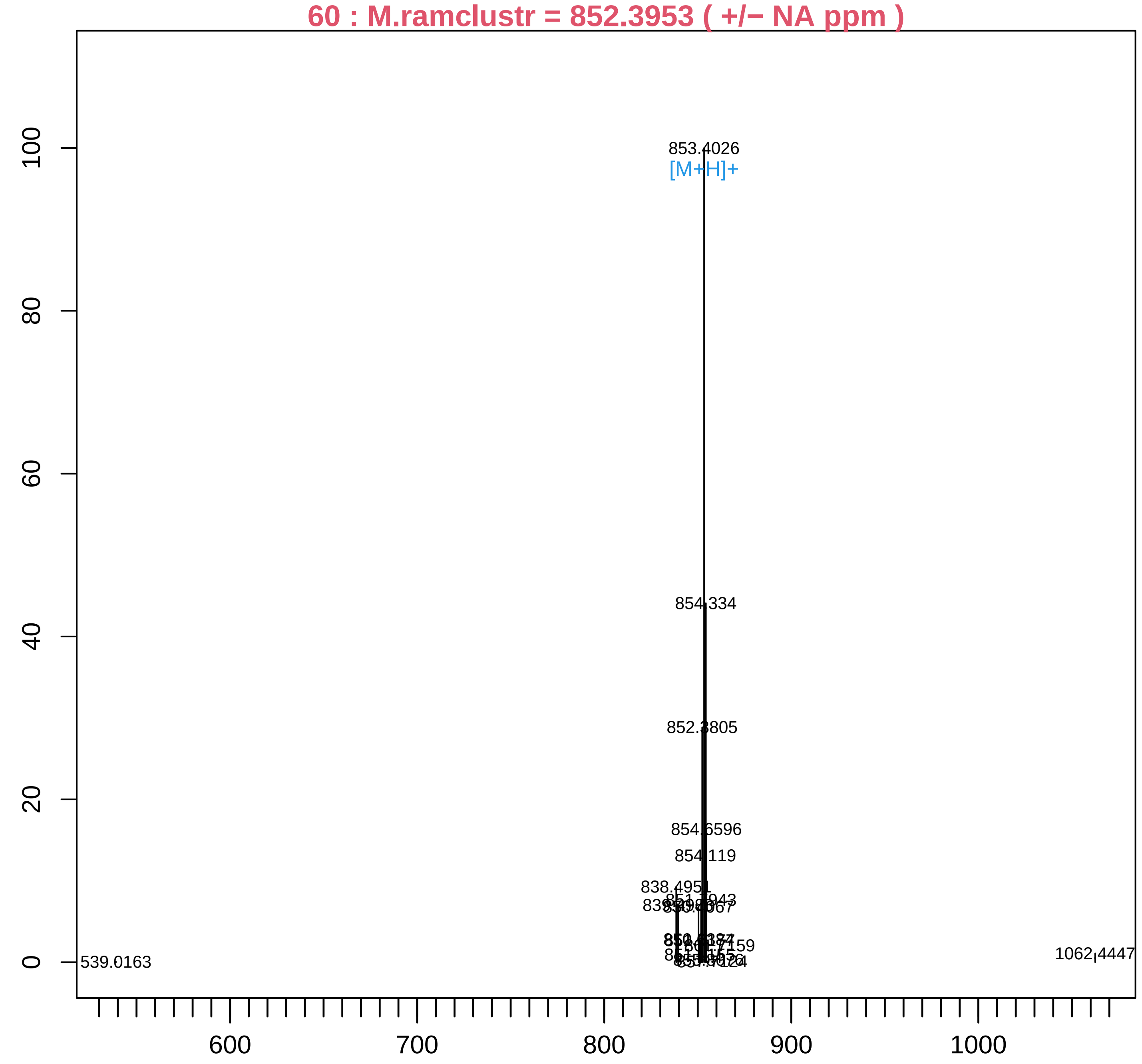
60 : M.ramclustr = 852.3953 (+/− NA ppm)
853.4026
[M+H]+
854.334
852.3805
854.6596
854.119
838.4951
539.0163
1062.4447
0
20
40
60
80
100
600
700
800
900
1000

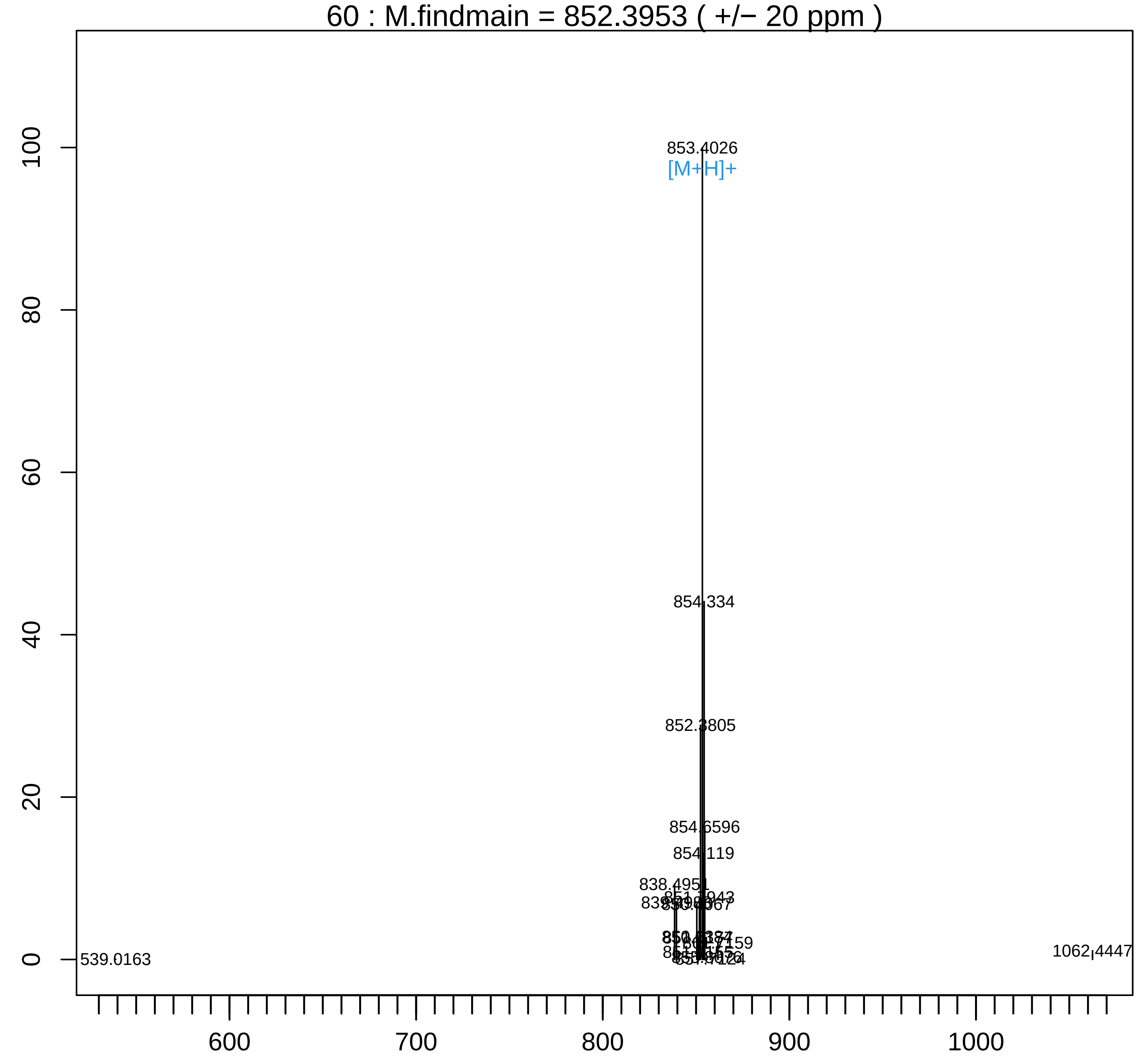
60 : M.findmain = 852.3953 (+/− 20 ppm)
853.4026
[M+H]+
854.334
852.3805
854.6596
854.119
838.4951
539.0163
1062.4447
0
20
40
60
80
100
600
700
800
900
1000

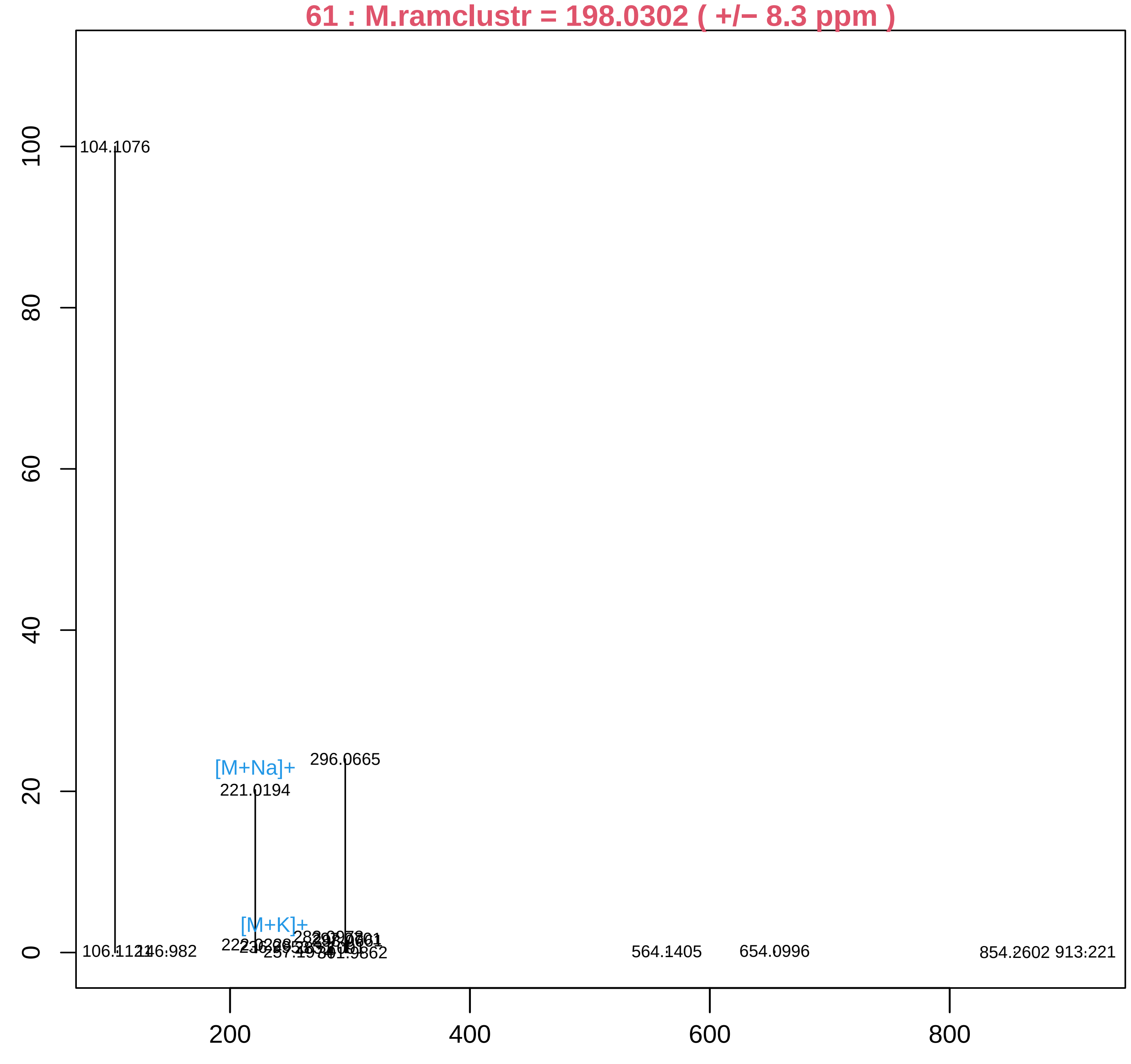
61 : M.ramclustr = 198.0302 (+/− 8.3 ppm)
104.1076
[M+Na]+
221.0194
296.0665
[M+K]+
106.1121
146.982
564.1405
654.0996
854.2602
913.221
0
20
40
60
80
100
200
400
600
800

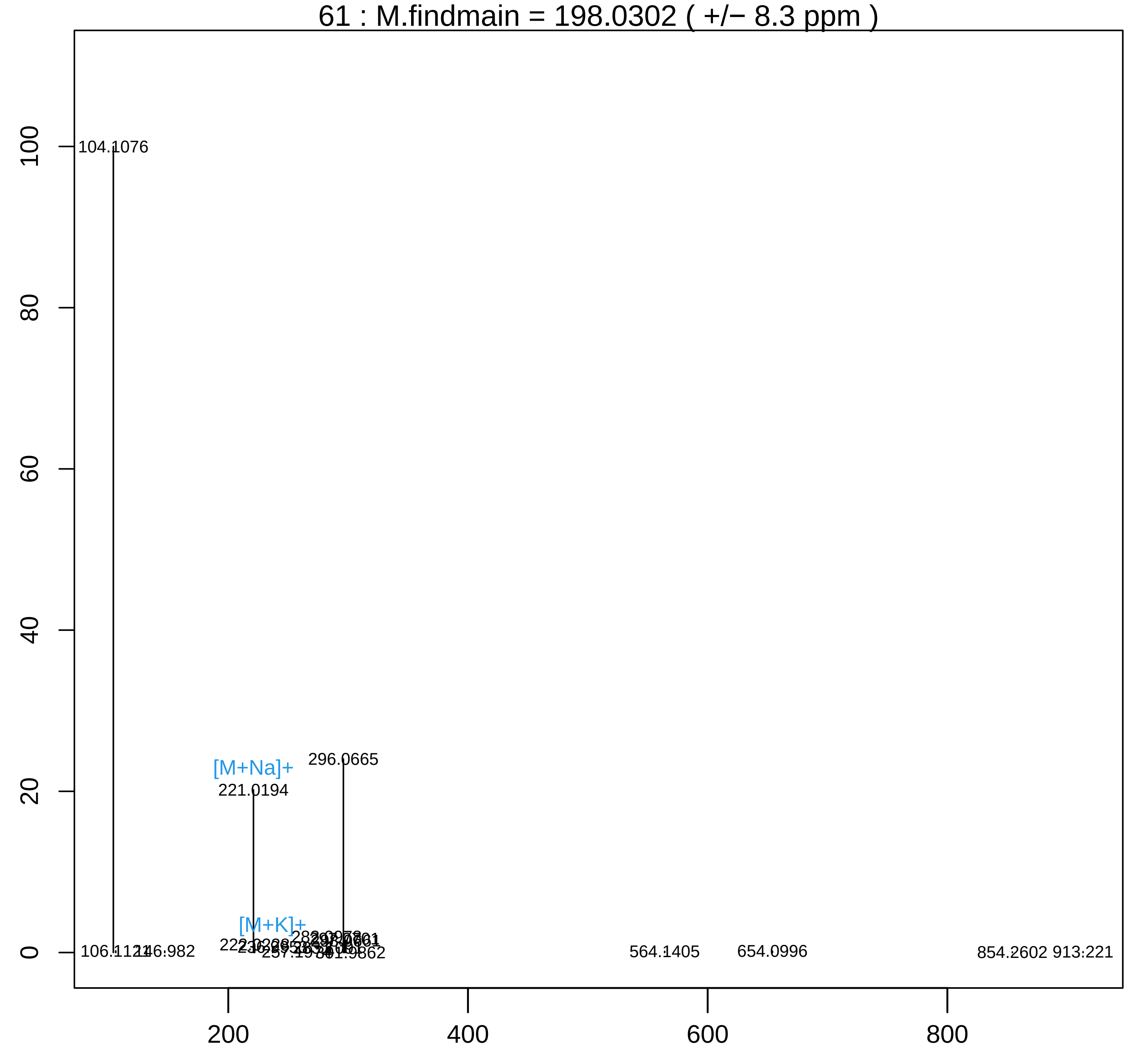
61 : M.findmain = 198.0302 (+/− 8.3 ppm)
104.1076
[M+Na]+
221.0194
296.0665
[M+K]+
106.1121
146.982
564.1405
654.0996
854.2602
913.221
0
20
40
60
80
100
200
400
600
800

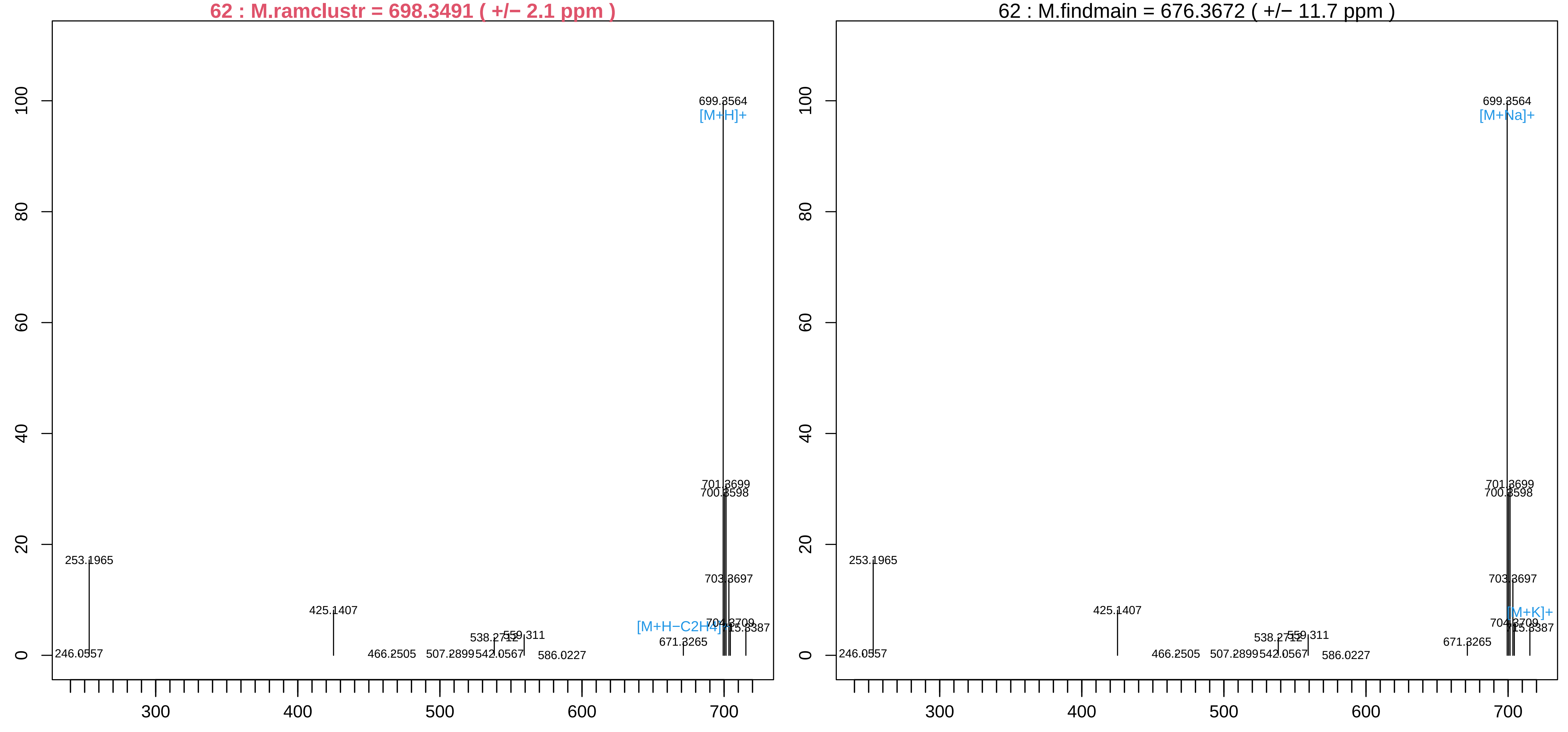
62 : M.ramclustr = 698.3491 (+/− 2.1 ppm)
699.3564
[M+H]+
701.3699
700.3598
253.1965
703.3697
425.1407
[M+H−C2H4]+
704.3709
715.3387
538.2712
559.311
671.3265
246.0557
466.2505
507.2899
542.0567
586.0227
62 : M.findmain = 676.3672 (+/− 11.7 ppm)
699.3564
[M+Na]+
701.3699
700.3598
253.1965
703.3697
425.1407
[M+K]+
704.3709
715.3387
538.2712
559.311
671.3265
246.0557
466.2505
507.2899
542.0567
586.0227
0
20
40
60
80
100
300
400
500
600
700

63 : M.ramclustr = 519.3325 (+/− 0.1 ppm)

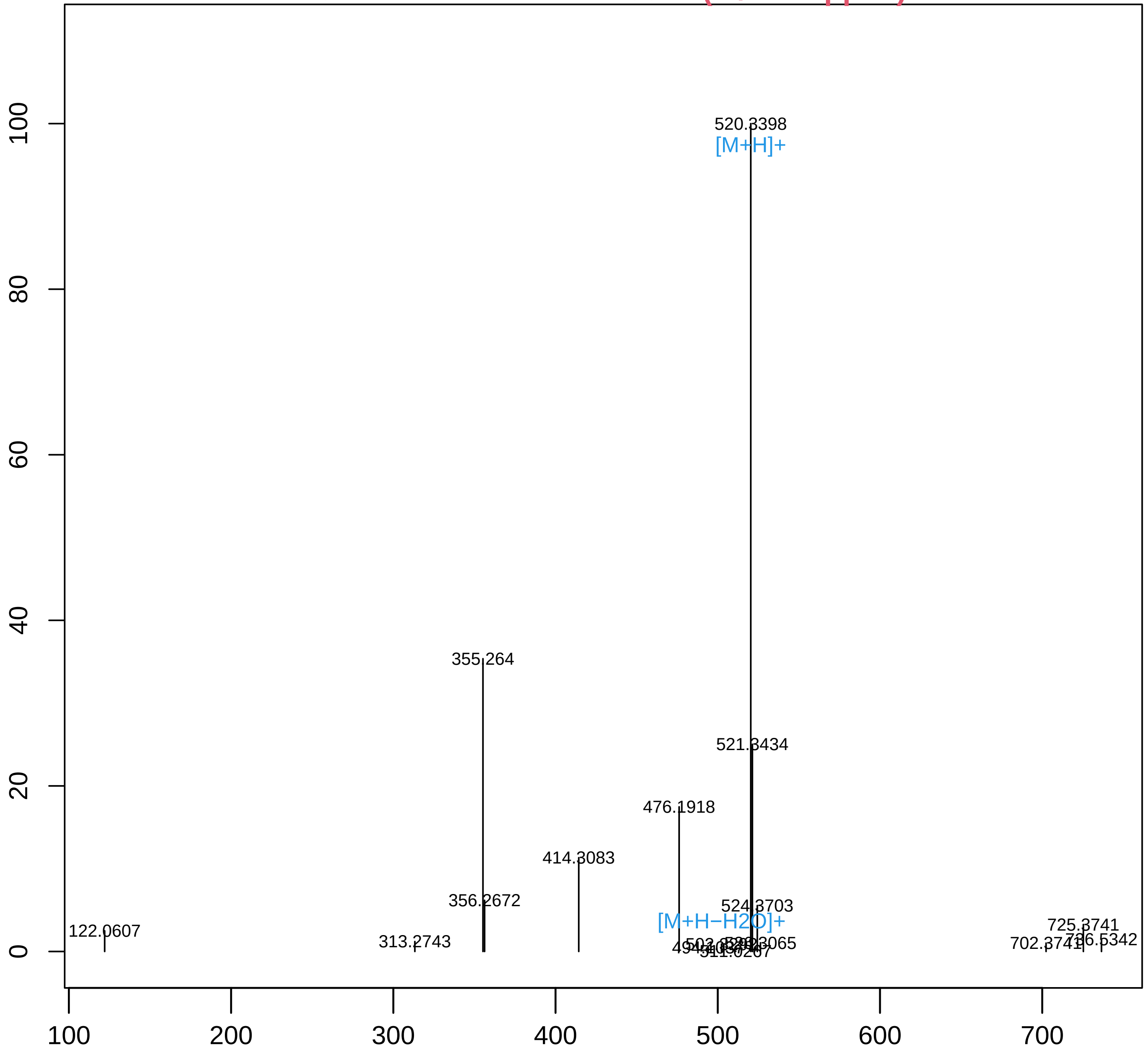

63 : M.findmain = 519.3325 (+/− 20 ppm)

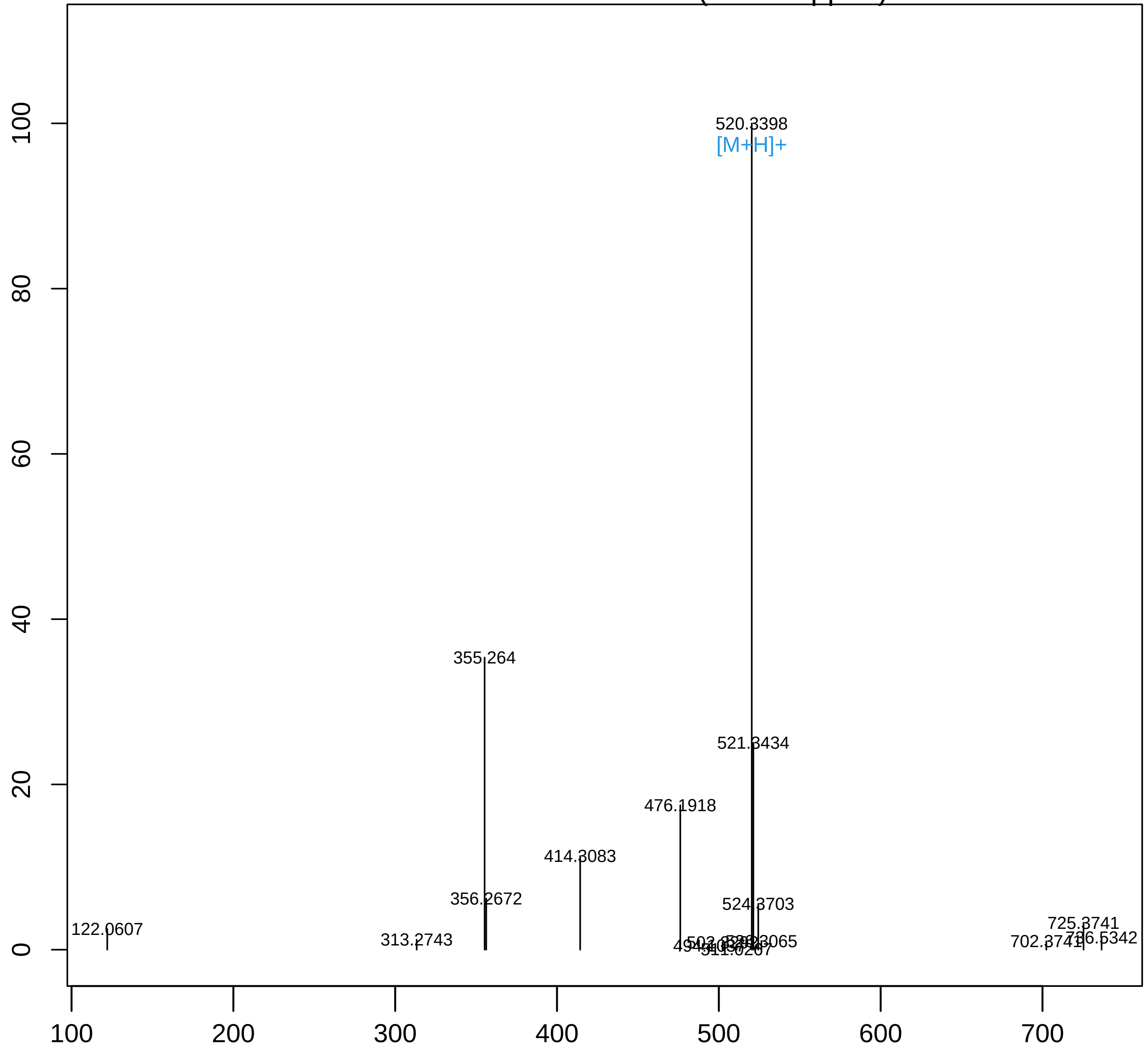

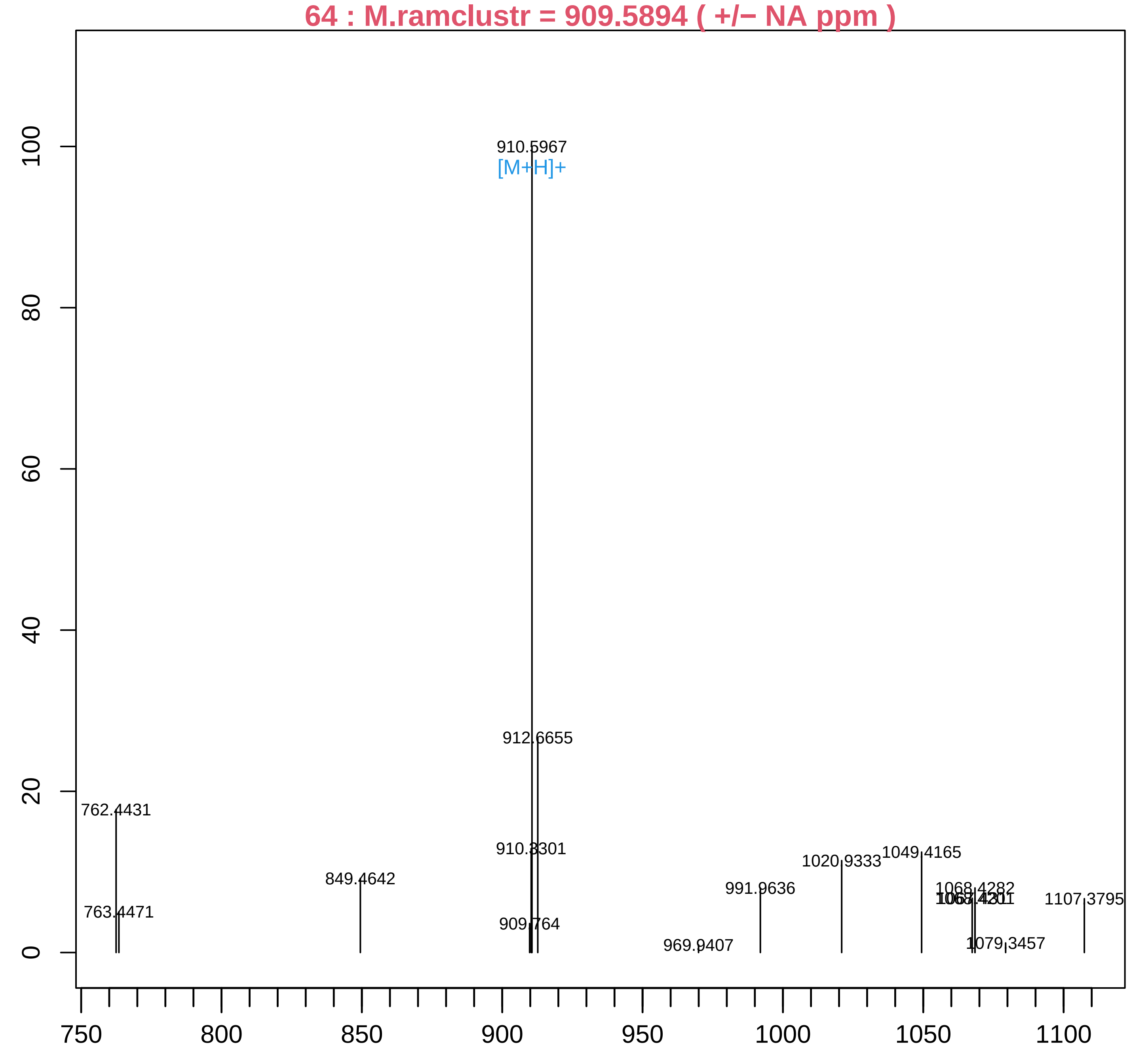
64 : M.ramclustr = 909.5894 (+/− NA ppm)
910.5967
[M+H]+
912.6655
762.4431
910.3301
1049.4165
1020.9333
849.4642
991.9636
1068.4282
1107.3795
763.4471
909.764
1079.3457
969.9407
0
20
40
60
80
100
750
800
850
900
950
1000
1050
1100
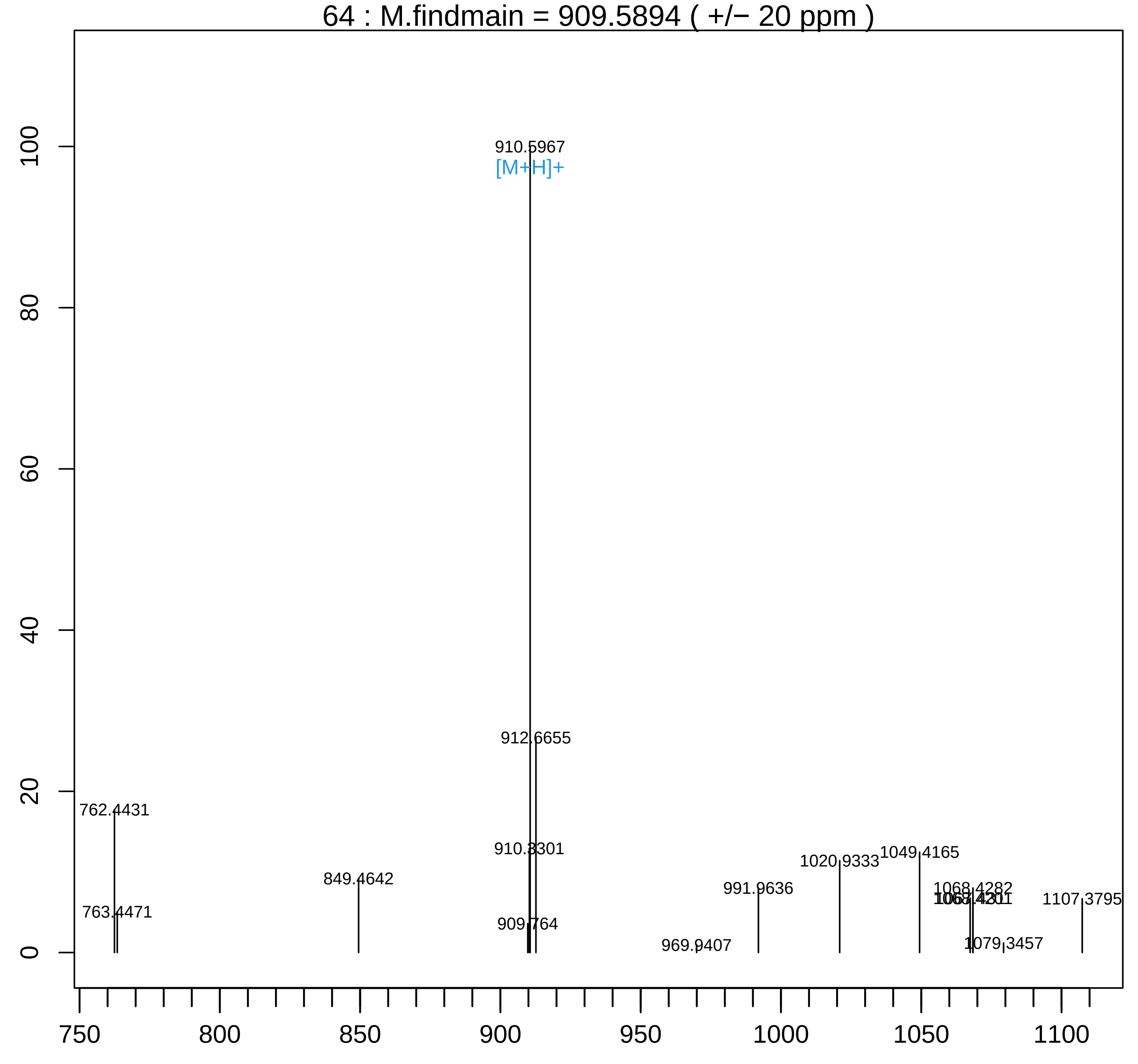
64 : M.findmain = 909.5894 (+/− 20 ppm)
910.5967
[M+H]+
912.6655
762.4431
910.3301
1049.4165
1020.9333
849.4642
991.9636
1068.4282
1107.3795
763.4471
909.764
1079.3457
969.9407
0
20
40
60
80
100
750
800
850
900
950
1000
1050
1100

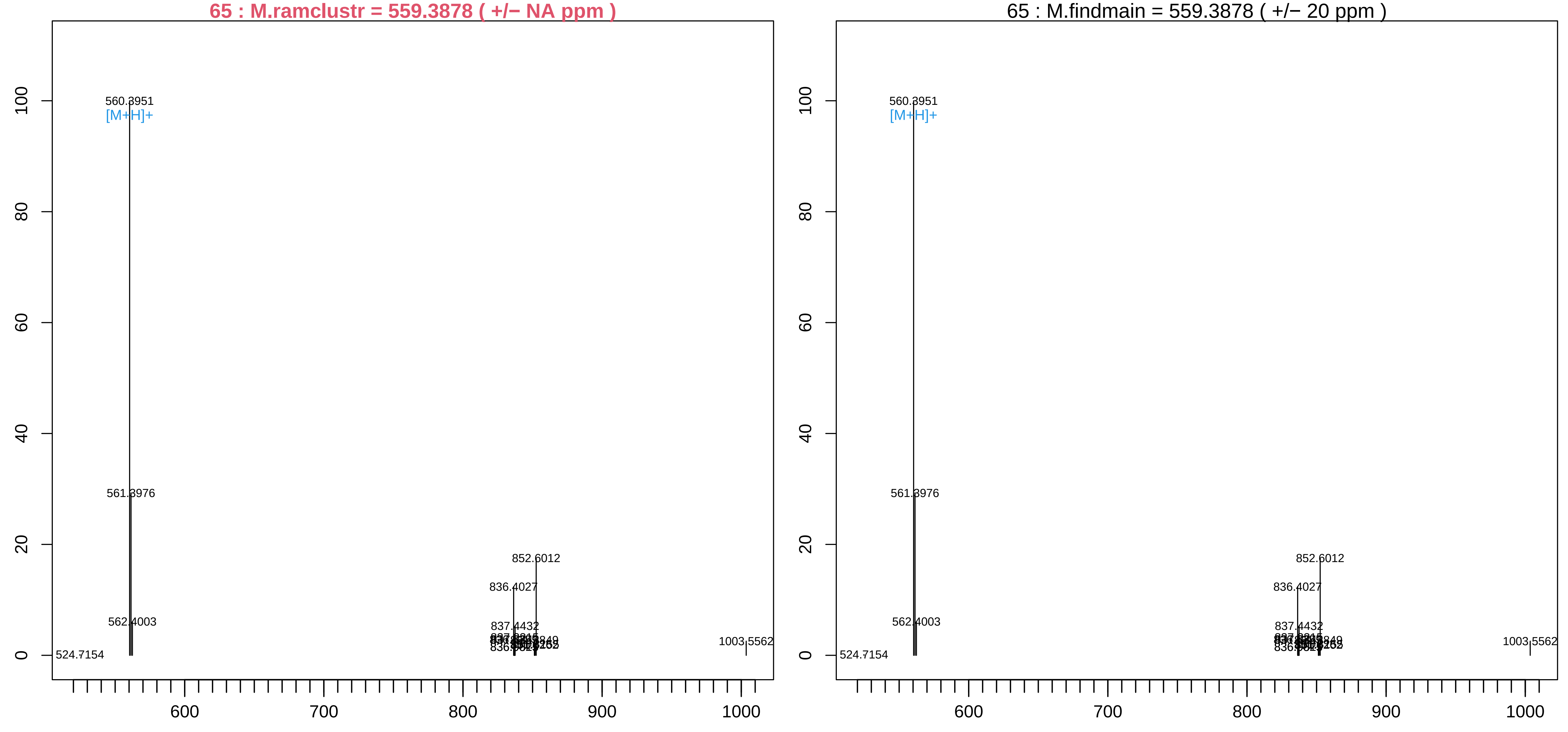
65 : M.ramclustr = 559.3878 (+/− NA ppm)
560.3951
[M+H]+
561.3976
562.4003
524.7154
852.6012
836.4027
837.4432
1003.5562
0
20
40
60
80
100
600
700
800
900
1000
65 : M.findmain = 559.3878 (+/− 20 ppm)
560.3951
[M+H]+
561.3976
562.4003
524.7154
852.6012
836.4027
837.4432
1003.5562
0
20
40
60
80
100
600
700
800
900
1000

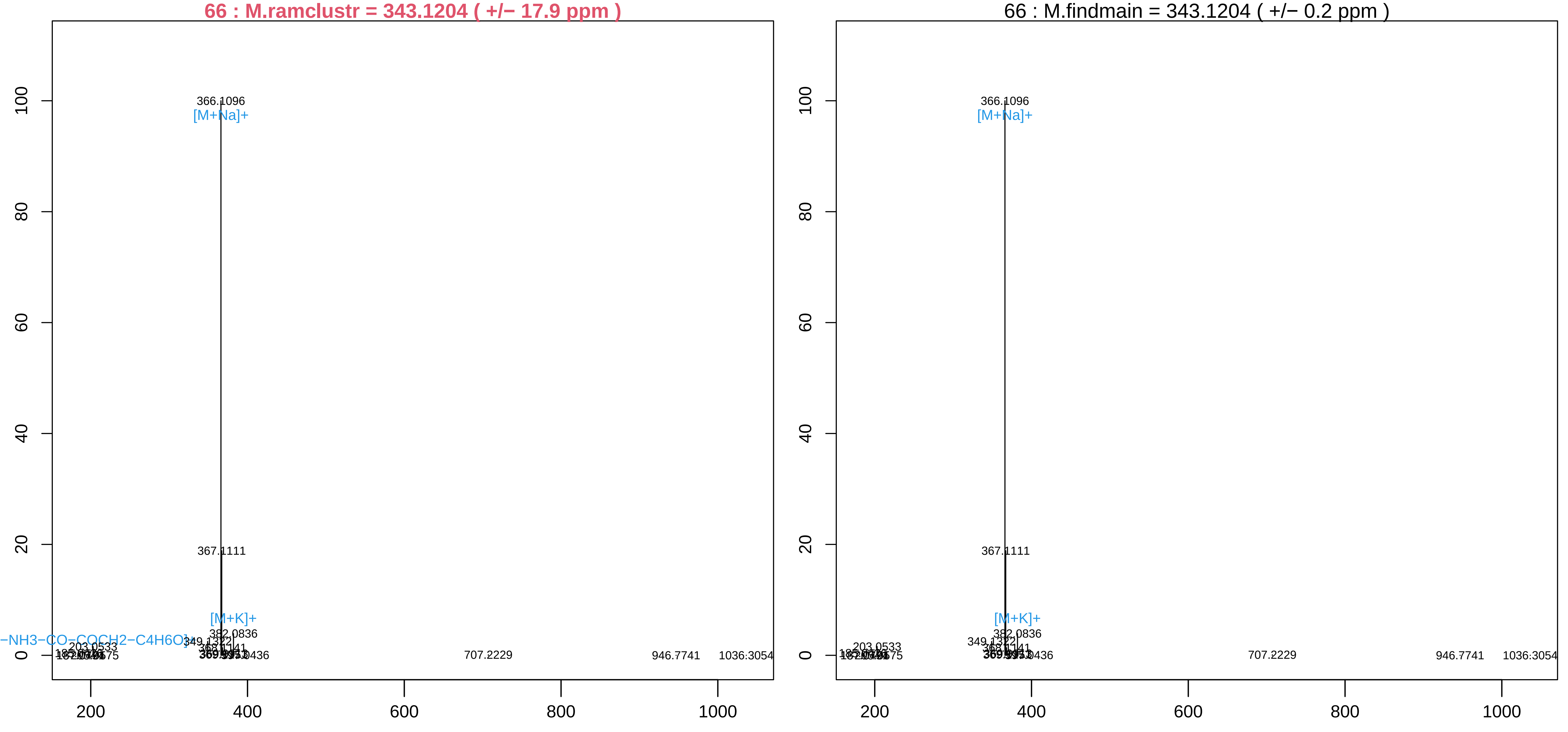

66 : M.ramclustr = 343.1204 (+/− 17.9 ppm)
366.1096
[M+Na]+
367.1111
[M+K]+
382.0836
349.1322
203.0533
707.2229
946.7741
1036:3054
66 : M.findmain = 343.1204 (+/− 0.2 ppm)
366.1096
[M+Na]+
367.1111
[M+K]+
382.0836
349.1322
203.0533
707.2229
946.7741
1036:3054

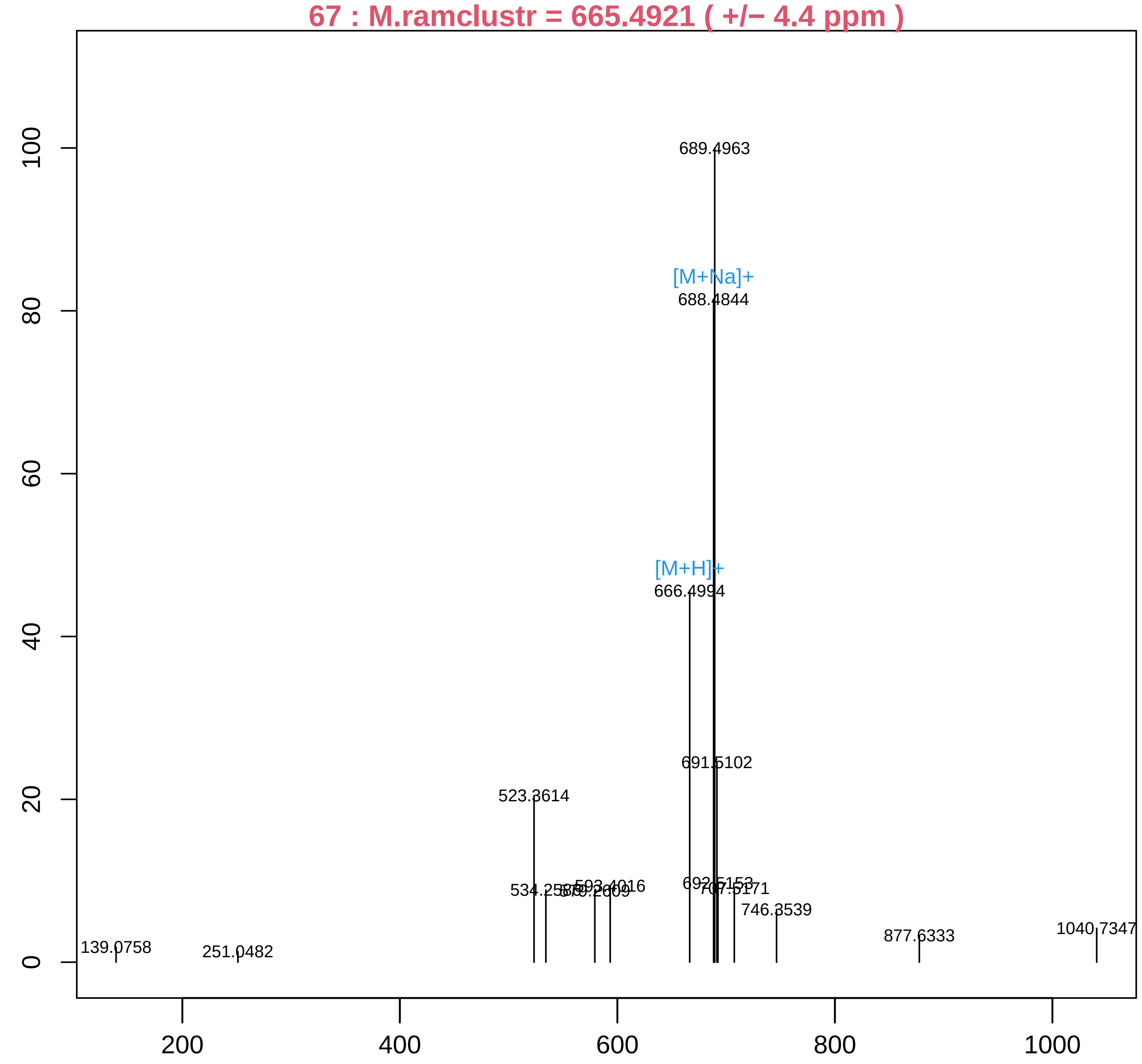
67 : M.ramclustr = 665.4921 (+/− 4.4 ppm)
689.4963
[M+Na]+
688.4844
[M+H]+
666.4994
691.5102
523.3614
593.4016
692.5153
707.5171
746.3539
877.6333
1040.7347
139.0758
251.0482
0
20
40
60
80
100
200
400
600
800
1000

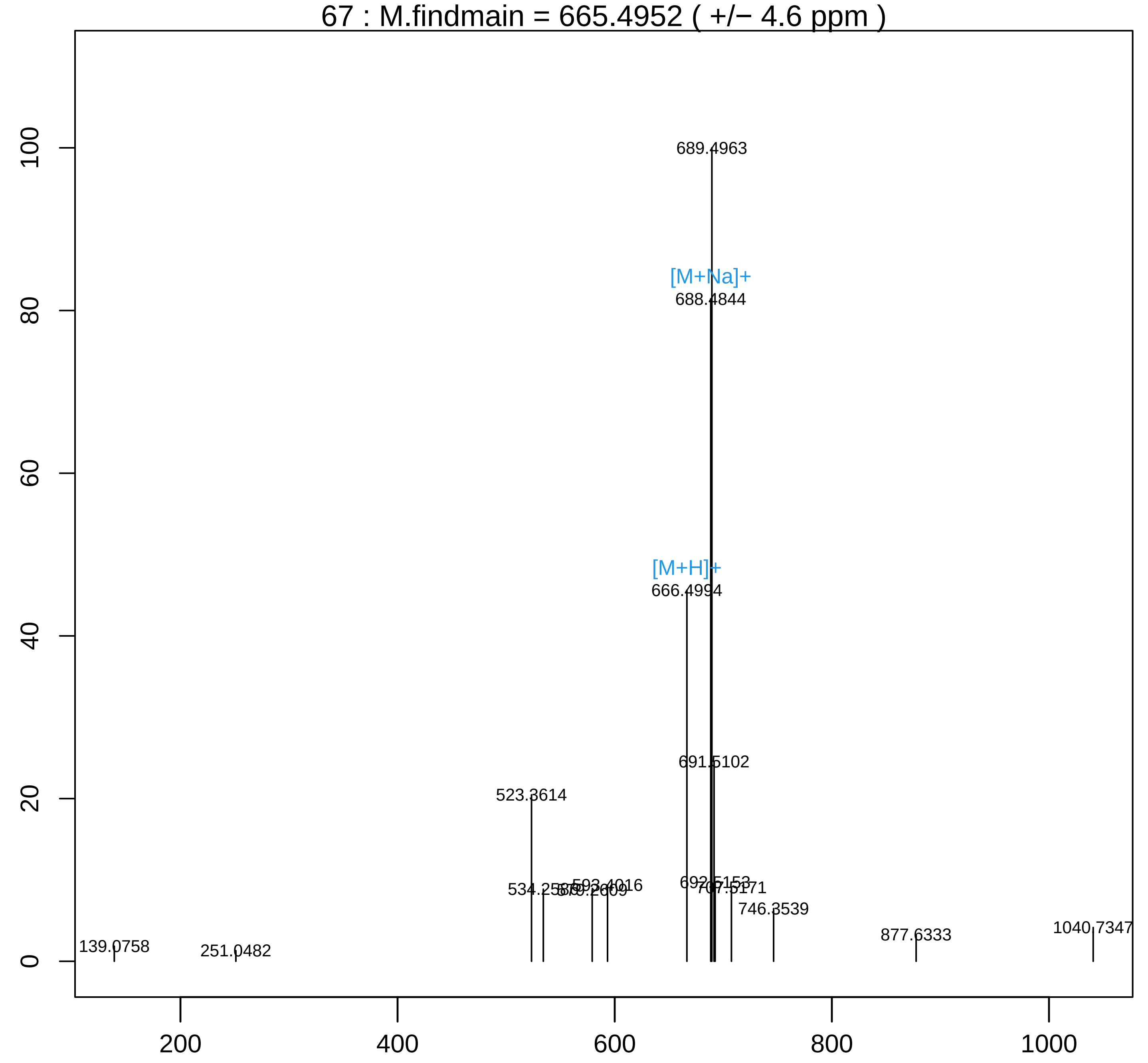
67 : M.findmain = 665.4952 (+/− 4.6 ppm)
689.4963
[M+Na]+
688.4844
[M+H]+
666.4994
691.5102
523.3614
593.4016
692.5153
707.5171
746.3539
877.6333
1040.7347
139.0758
251.0482
0
20
40
60
80
100
200
400
600
800
1000

68 : M.ramclustr = 104.0632 (+/− NA ppm)

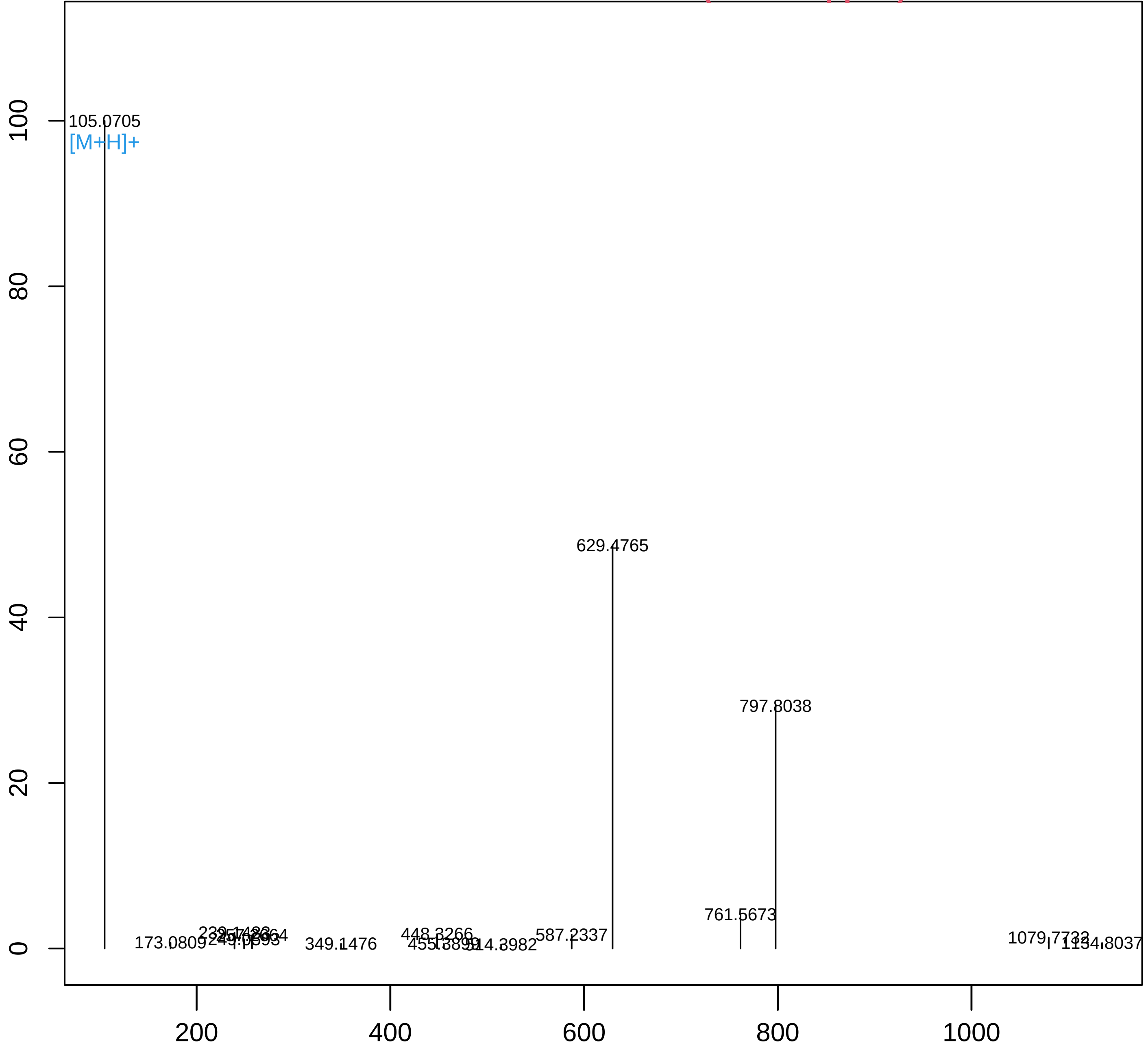

68 : M.findmain = 104.0632 (+/− 20 ppm)

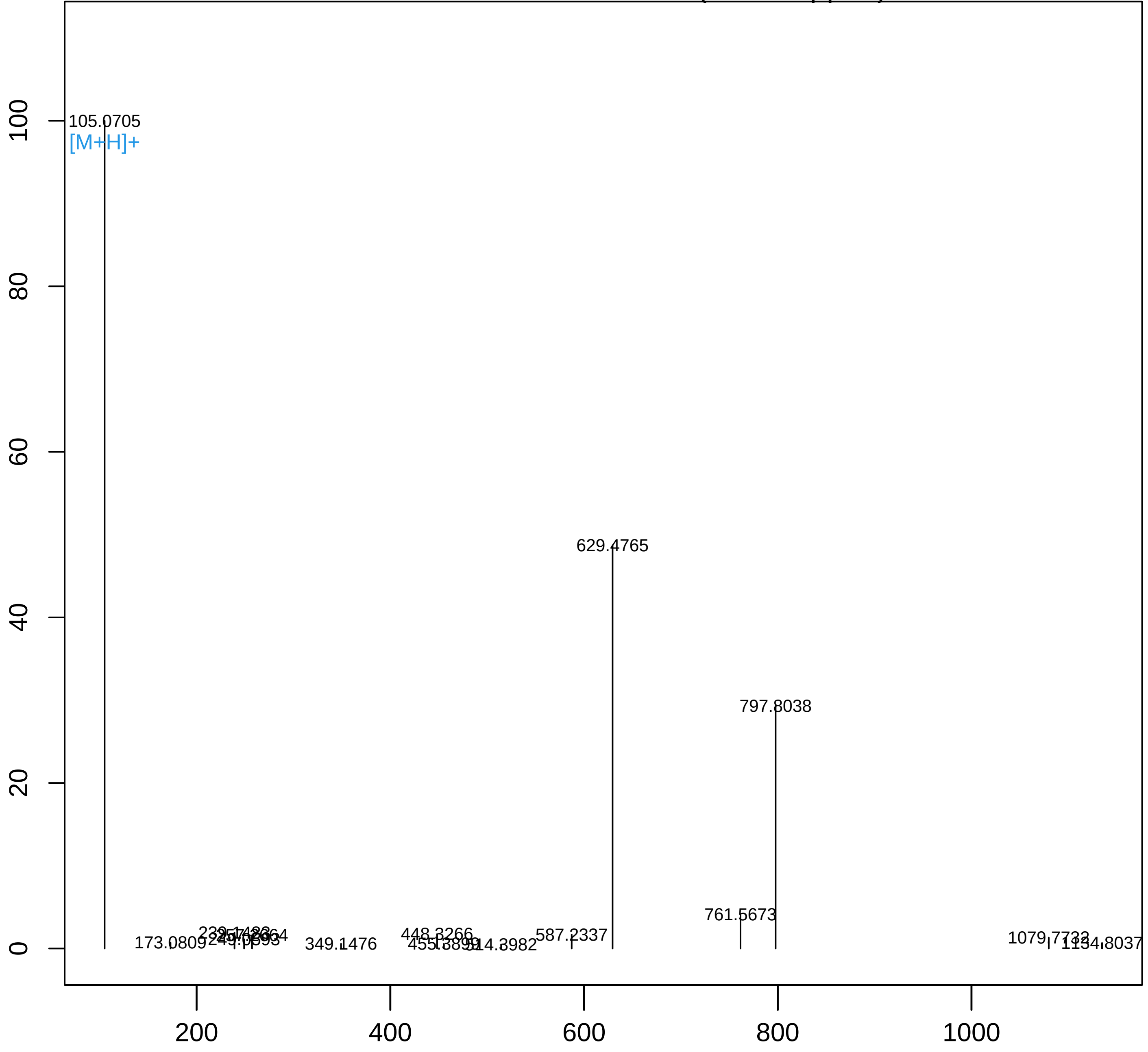

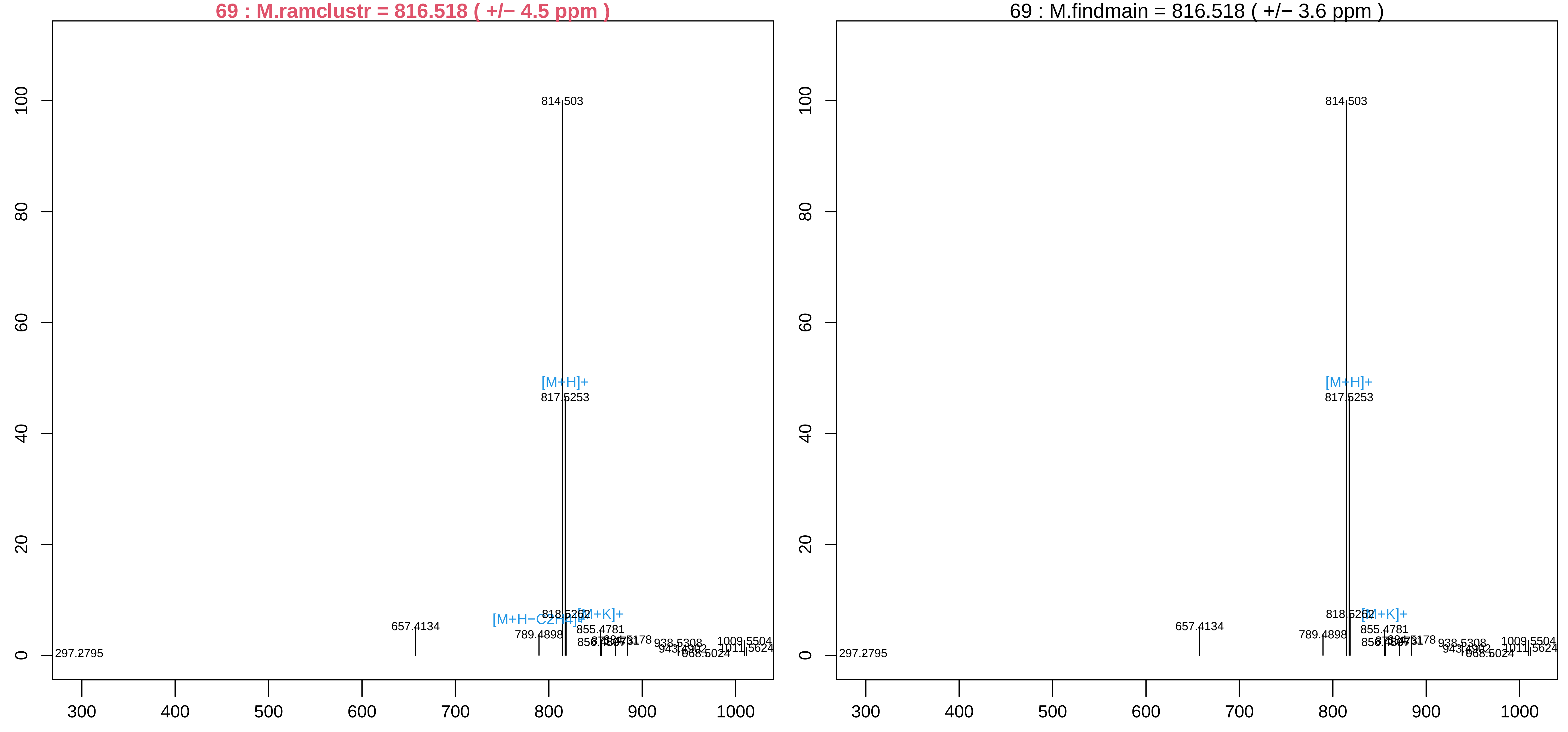
69 : M.ramclustr = 816.518 (+/− 4.5 ppm)
814.503
[M+H]+
817.5253
818.5262
[M+K]+
855.4781
657.4134
789.4898
938.5308
1009.5504
297.2795
69 : M.findmain = 816.518 (+/− 3.6 ppm)
814.503
[M+H]+
817.5253
818.5262
[M+K]+
855.4781
657.4134
789.4898
938.5308
1009.5504
297.2795

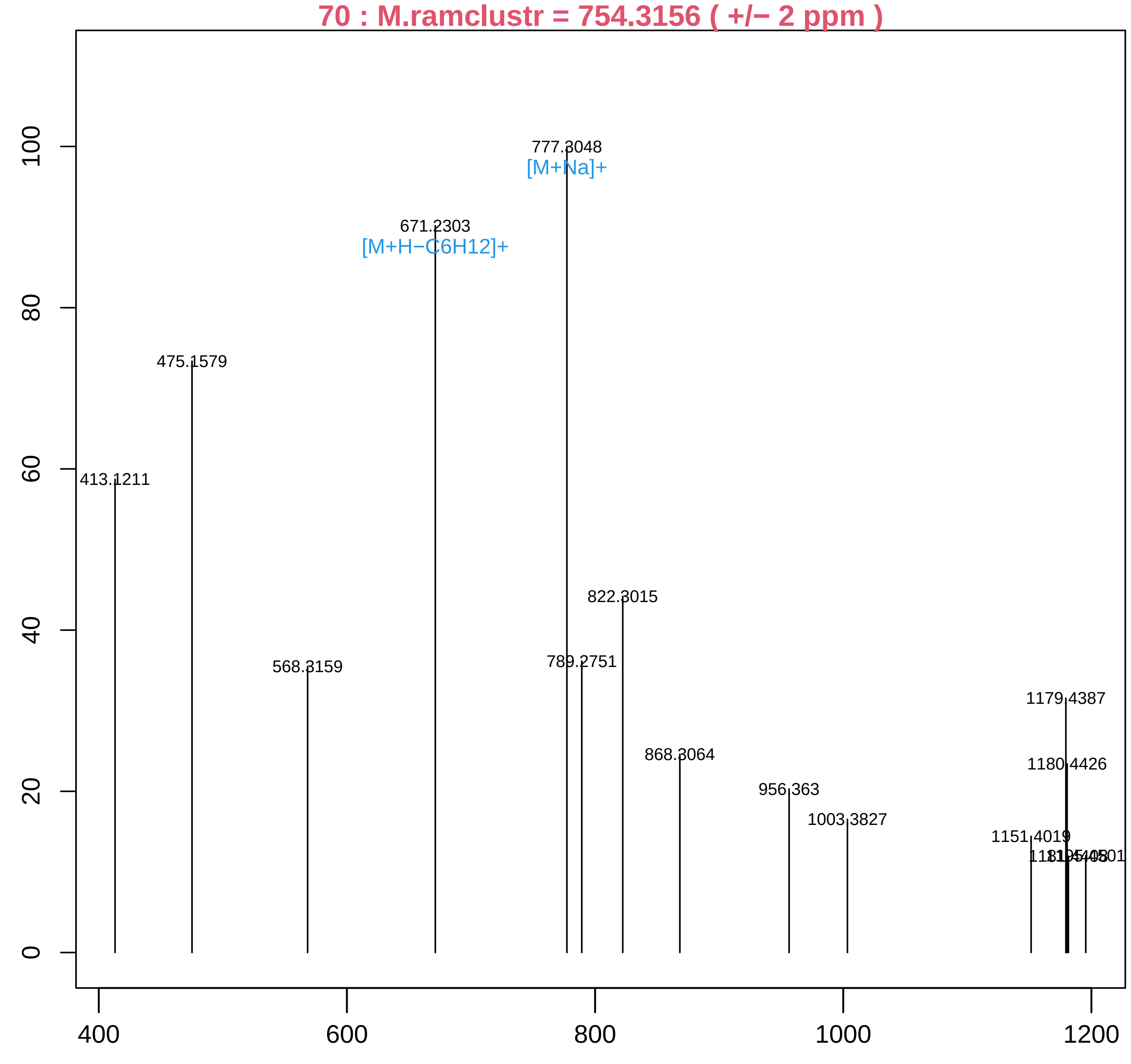
70 : M.ramclustr = 754.3156 (+/− 2 ppm)
777.3048
[M+Na]+
671.2303
[M+H−C6H12]+
475.1579
413.1211
568.3159
822.3015
789.2751
868.3064
956.363
1003.3827
1179.4387
1180.4426
1151.4019
0
20
40
60
80
100
400
600
800
1000
1200

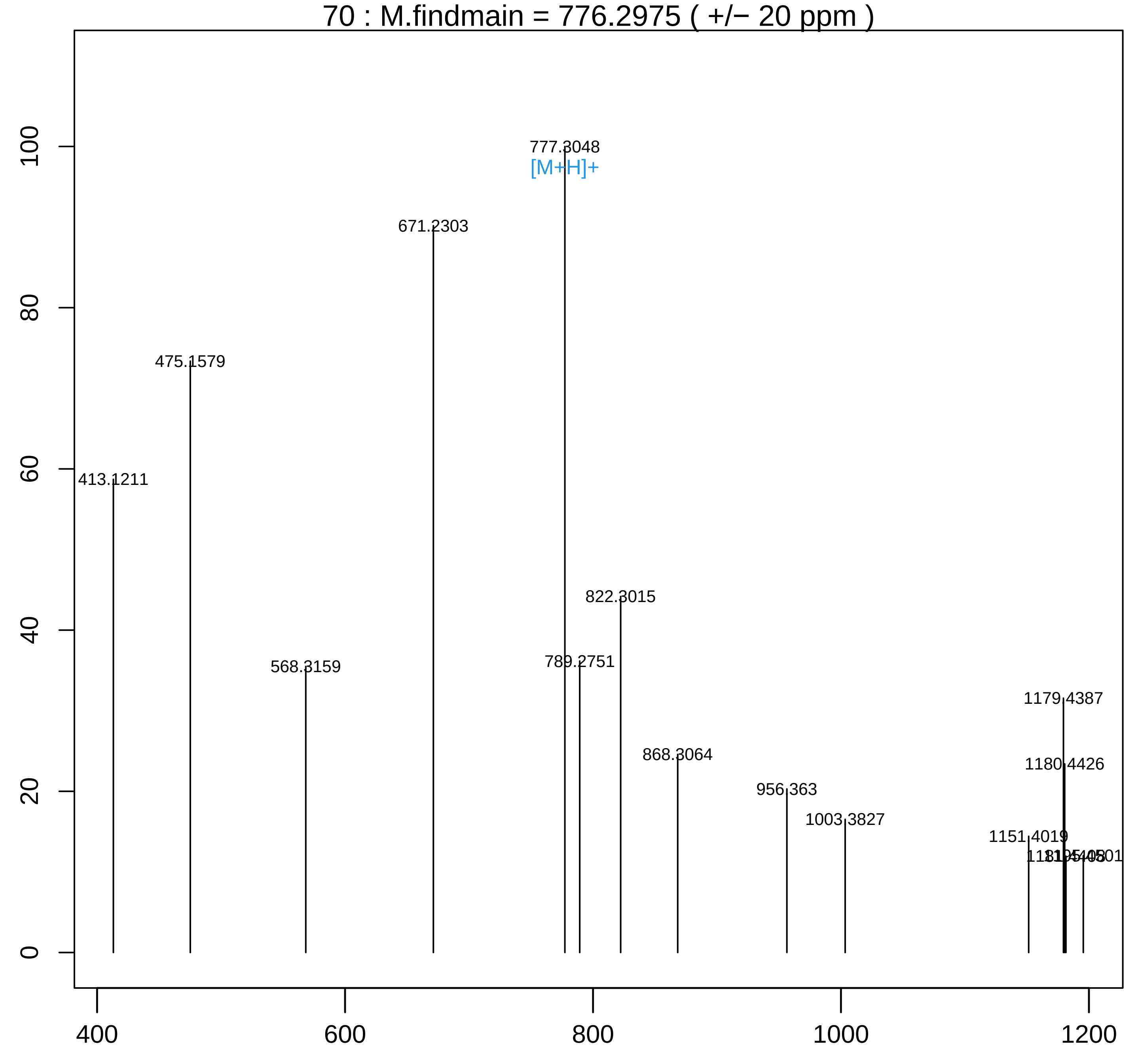
70 : M.findmain = 776.2975 (+/− 20 ppm)
777.3048
[M+H]+
671.2303
475.1579
413.1211
568.3159
822.3015
789.2751
868.3064
956.363
1003.3827
1179.4387
1180.4426
1151.4019
0
20
40
60
80
100
400
600
800
1000
1200

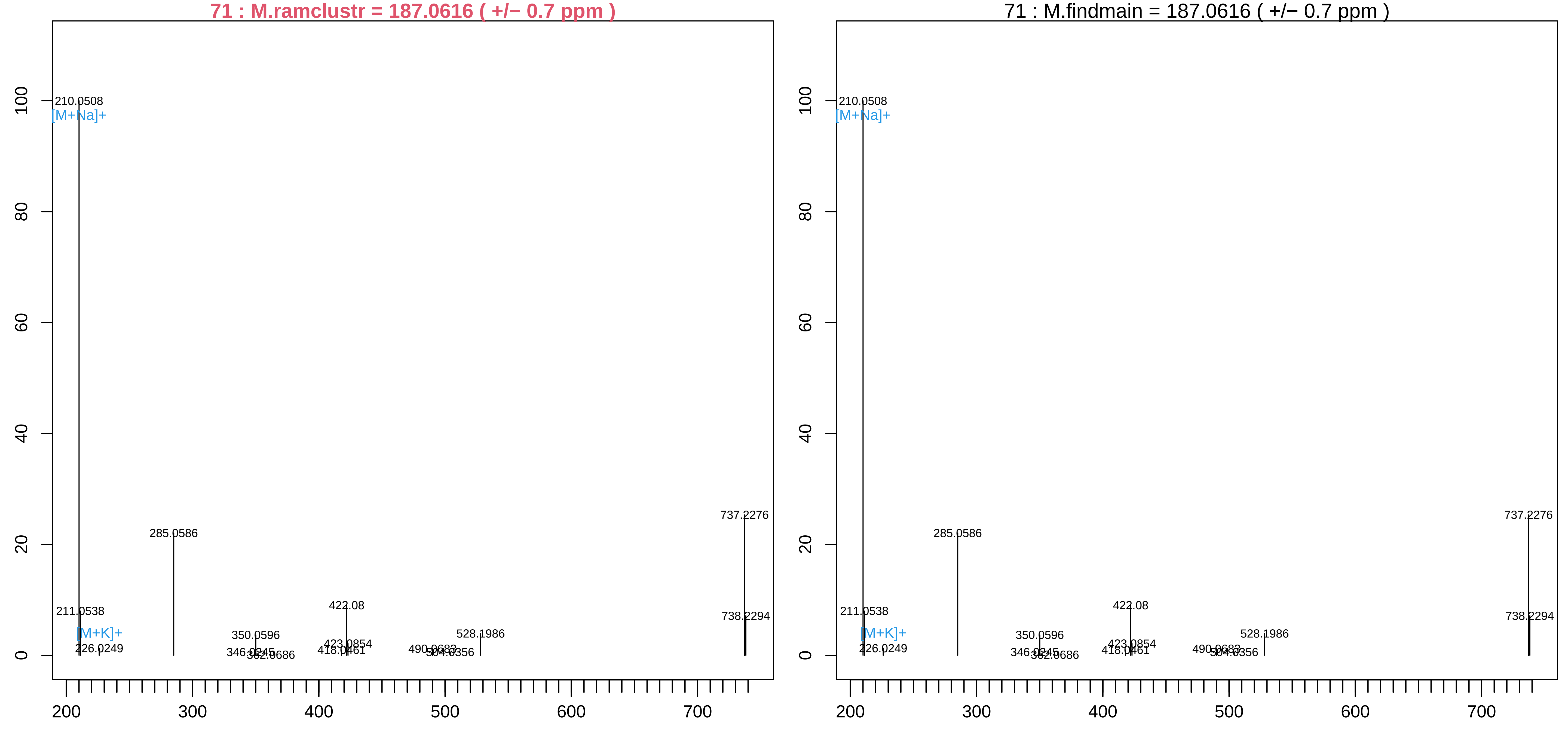

71 : M.ramclustr = 187.0616 (+/− 0.7 ppm)
210.0508
[M+Na]+
211.0538
[M+K]+
226.0249
285.0586
350.0596
346.0245
362.0686
422.08
423.0854
418.0461
490.0683
504.0356
528.1986
737.2276
738.2294
71 : M.findmain = 187.0616 (+/− 0.7 ppm)

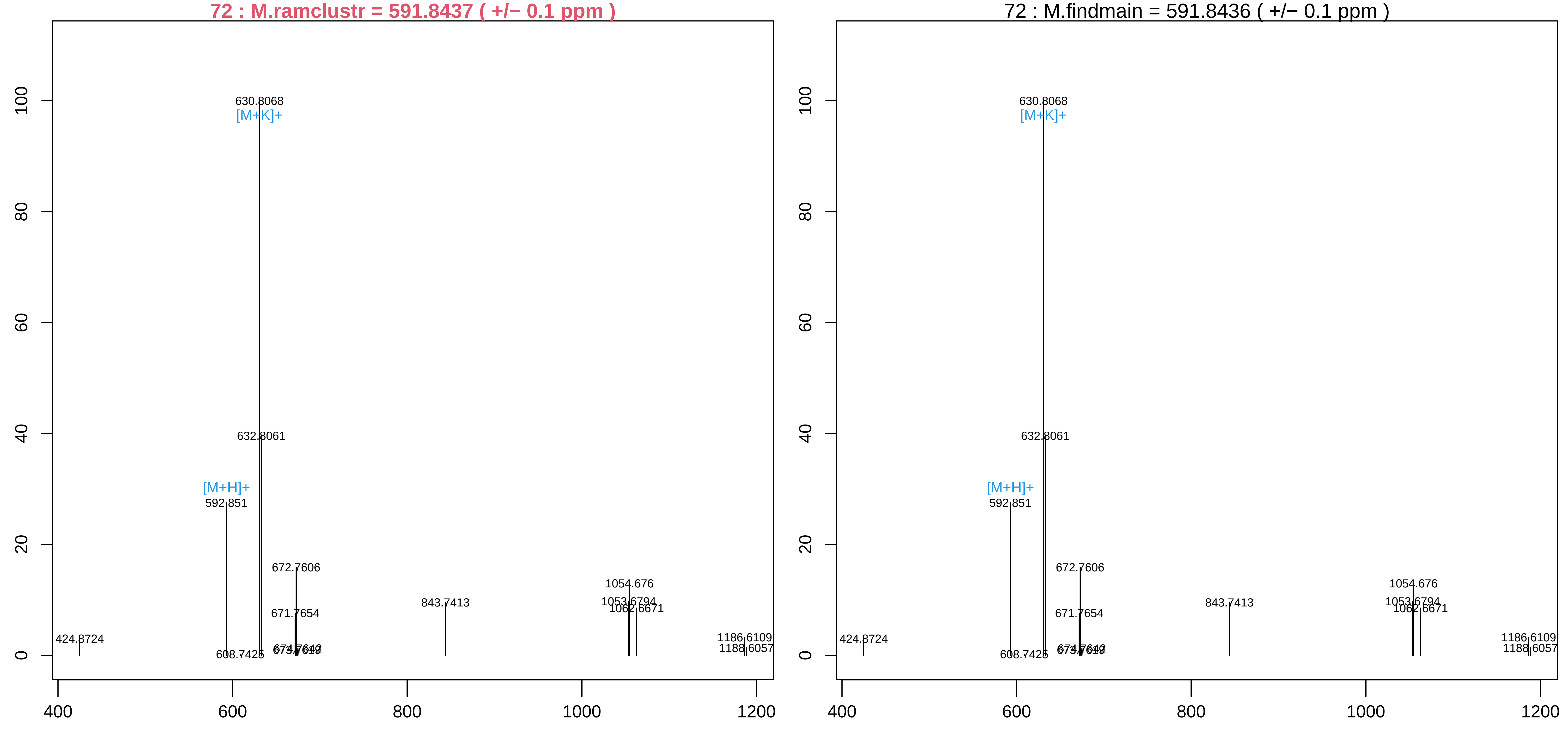
72 : M.ramclustr = 591.8437 (+/− 0.1 ppm)
630.8068
[M+K]+
632.8061
[M+H]+
592.851
672.7606
671.7654
843.7413
1054.676
1053.6794
1062.6671
424.8724
608.7425
1186.6109
1188.6057
72 : M.findmain = 591.8436 (+/− 0.1 ppm)
630.8068
[M+K]+
632.8061
[M+H]+
592.851
672.7606
671.7654
843.7413
1054.676
1053.6794
1062.6671
424.8724
608.7425
1186.6109
1188.6057

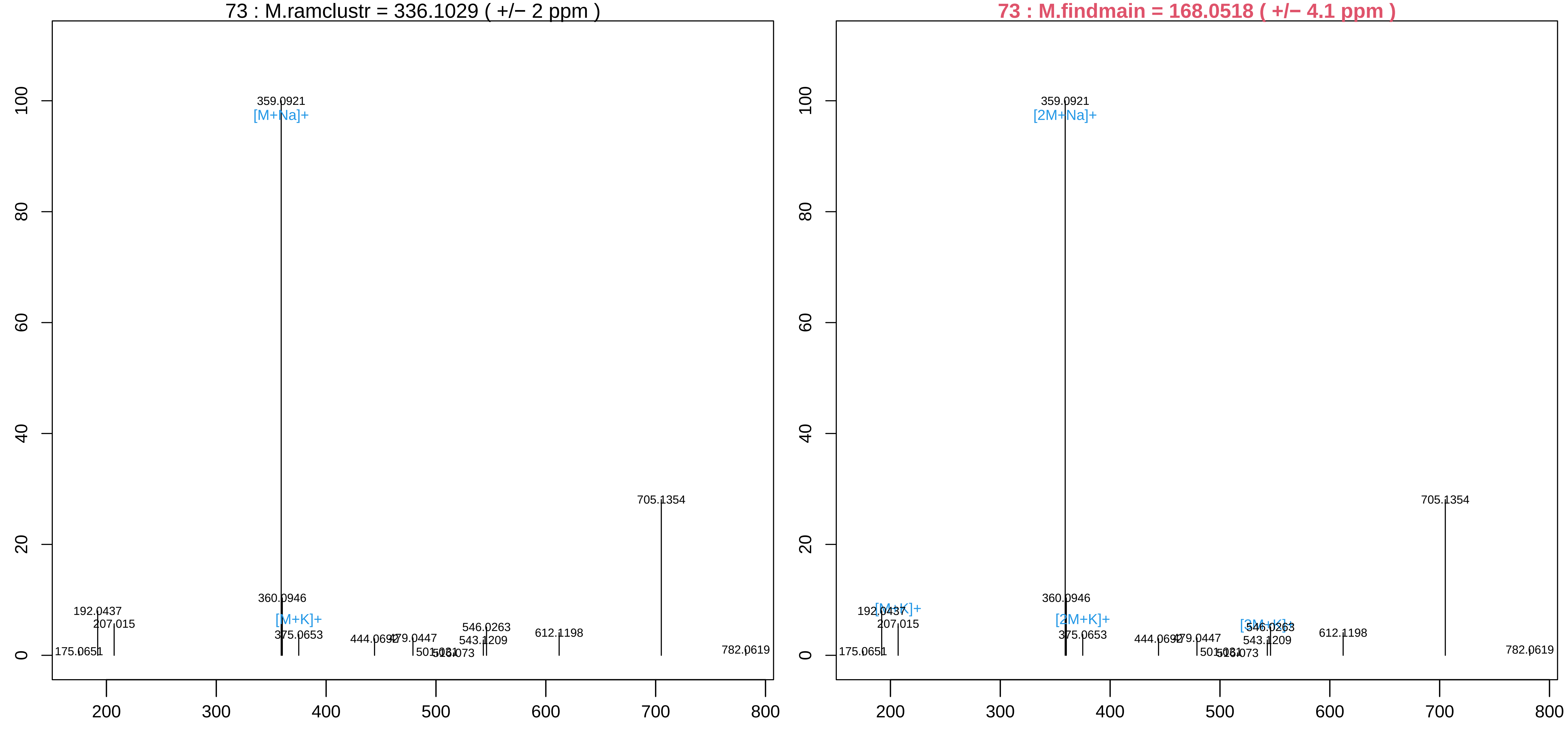
73 : M.ramclustr = 336.1029 (+/− 2 ppm)
359.0921
[M+Na]+
360.0946
[M+K]+
375.0653
192.0437
207.015
175.0651
444.0692
479.0447
501.0061
516.1073
546.0263
543.1209
612.1198
705.1354
782.0619
73 : M.findmain = 168.0518 (+/− 4.1 ppm)
[2M+Na]+
[M+K]+
[2M+K]+
[3M+K]+
0
20
40
60
80
100
200
300
400
500
600
700
800

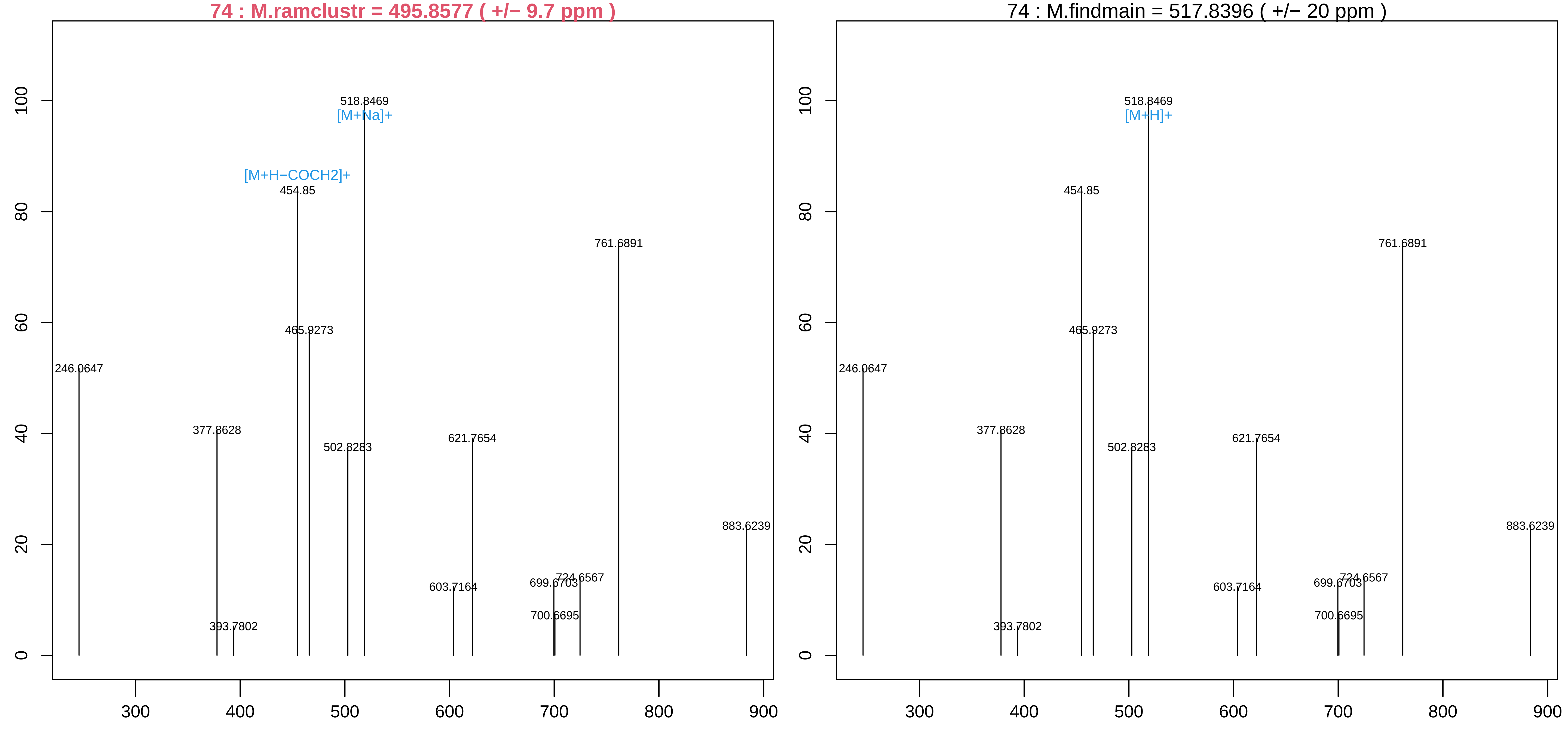

74 : M.ramclustr = 495.8577 (+/− 9.7 ppm)
518.8469
[M+Na]+
[M+H−COCH2]+
454.85
465.9273
246.0647
377.8628
502.8283
621.7654
761.6891
883.6239
603.7164
699.6703
724.6567
700.6695
393.7802
0
20
40
60
80
100
300
400
500
600
700
800
900
74 : M.findmain = 517.8396 (+/− 20 ppm)
518.8469
[M+H]+
454.85
465.9273
246.0647
377.8628
502.8283
621.7654
761.6891
883.6239
603.7164
699.6703
724.6567
700.6695
393.7802
0
20
40
60
80
100
300
400
500
600
700
800
900

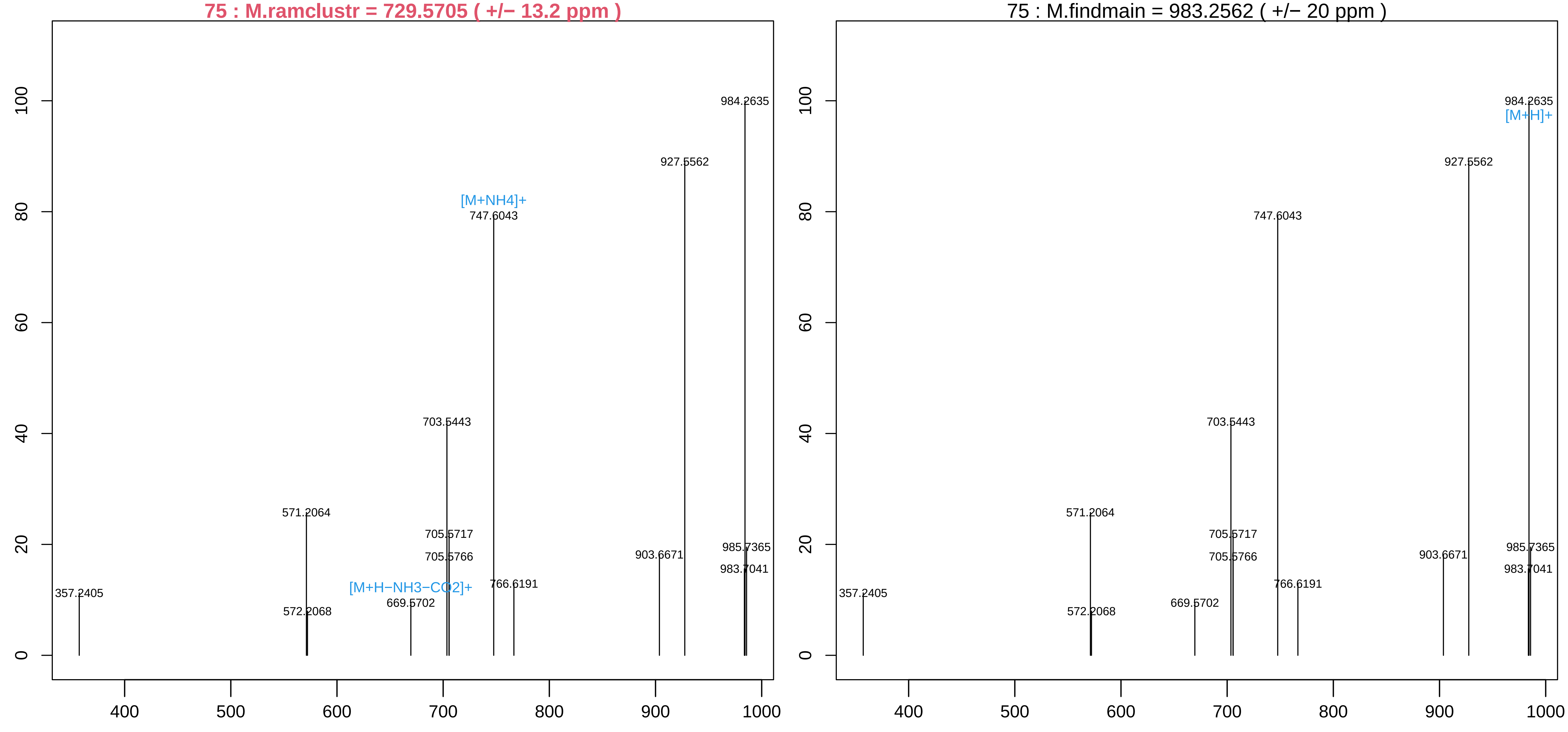

75 : M.ramclustr = 729.5705 (+/− 13.2 ppm)
984.2635
927.5562
[M+NH4]+
747.6043
703.5443
571.2064
705.5717
705.5766
903.6671
985.7365
983.7041
357.2405
[M+H−NH3−CO2]+
669.5702
572.2068
766.6191
0
20
40
60
80
100
400
500
600
700
800
900
1000
75 : M.findmain = 983.2562 (+/− 20 ppm)
984.2635
[M+H]+
927.5562
747.6043
703.5443
571.2064
705.5717
705.5766
903.6671
985.7365
983.7041
357.2405
669.5702
572.2068
766.6191
0
20
40
60
80
100
400
500
600
700
800
900
1000

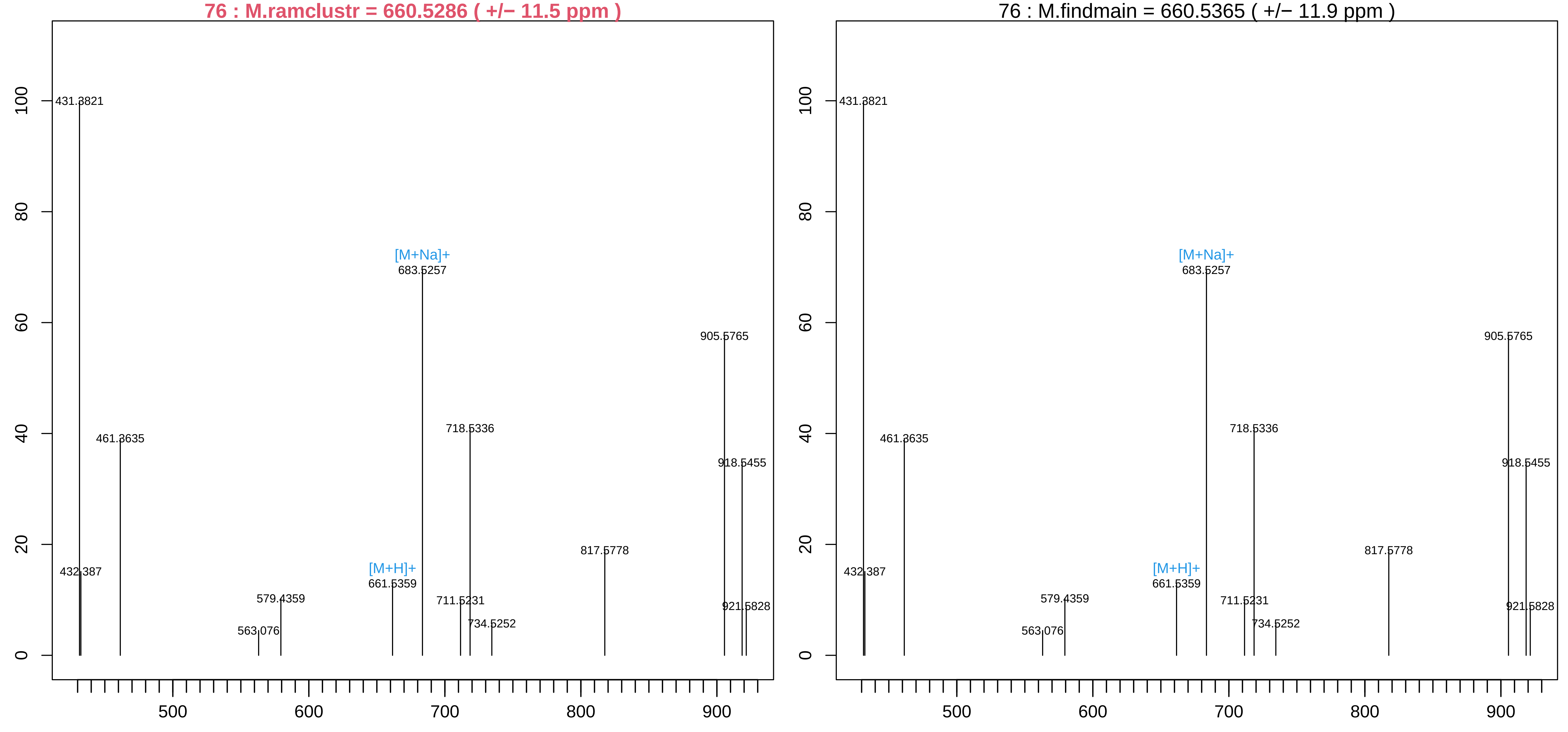

76 : M.ramclustr = 660.5286 (+/− 11.5 ppm)
76 : M.findmain = 660.5365 (+/− 11.9 ppm)
[M+Na]+
683.5257
[M+H]+
661.5359
431.3821
432.387
461.3635
563.076
579.4359
711.5231
718.5336
734.5252
817.5778
905.5765
918.5455
921.5828
0
20
40
60
80
100
500
600
700
800
900

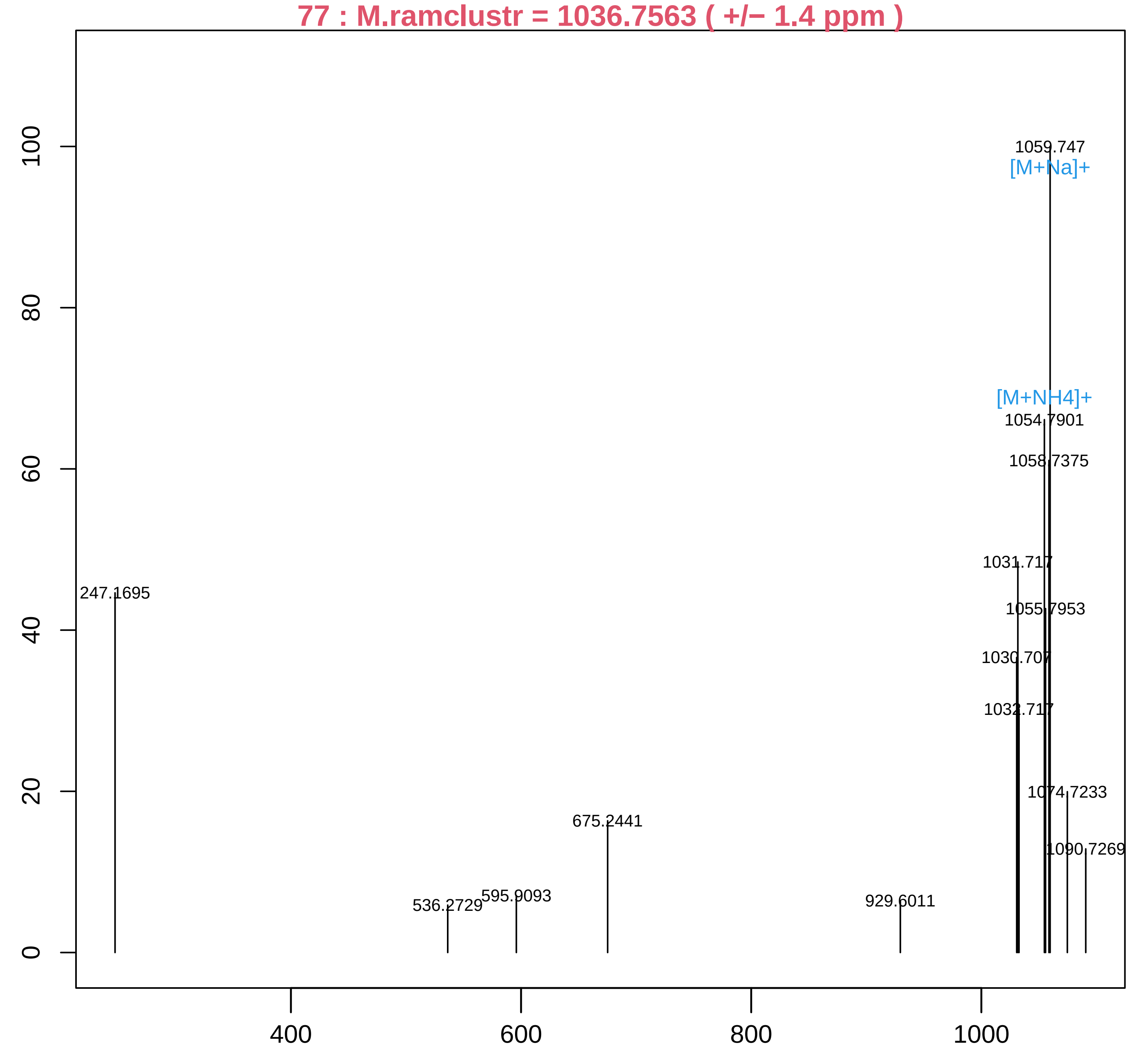
77 : M.ramclustr = 1036.7563 (+/− 1.4 ppm)
1059.747
[M+Na]+
[M+NH4]+
1054.7901
1058.7375
1031.717
247.1695
1055.7953
1030.707
1032.717
1074.7233
675.2441
1090.7269
595.9093
536.2729
929.6011
0
20
40
60
80
100
400
600
800
1000

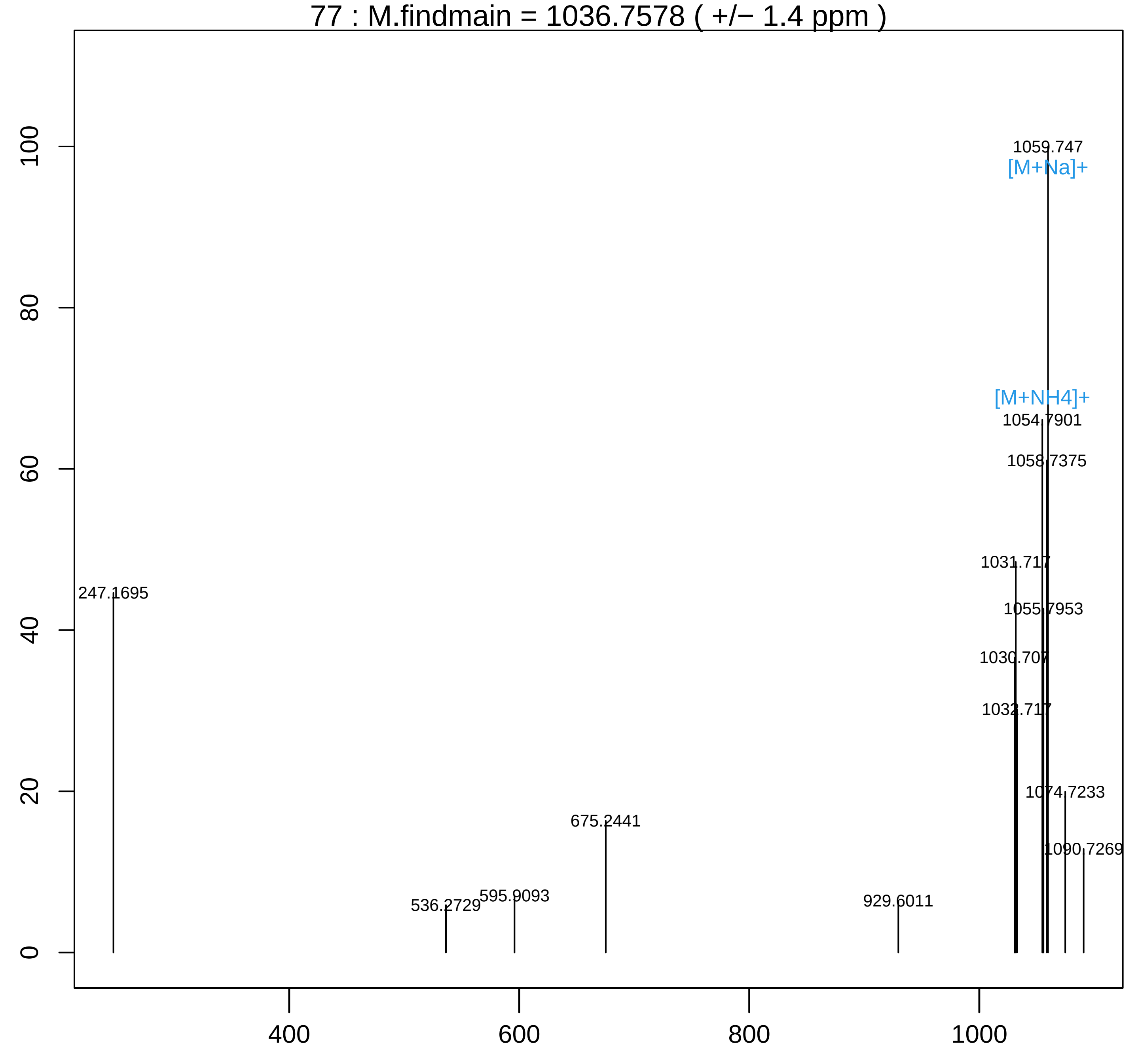
77 : M.findmain = 1036.7578 (+/− 1.4 ppm)
1059.747
[M+Na]+
[M+NH4]+
1054.7901
1058.7375
1031.717
247.1695
1055.7953
1030.707
1032.717
1074.7233
675.2441
1090.7269
595.9093
536.2729
929.6011
0
20
40
60
80
100
400
600
800
1000

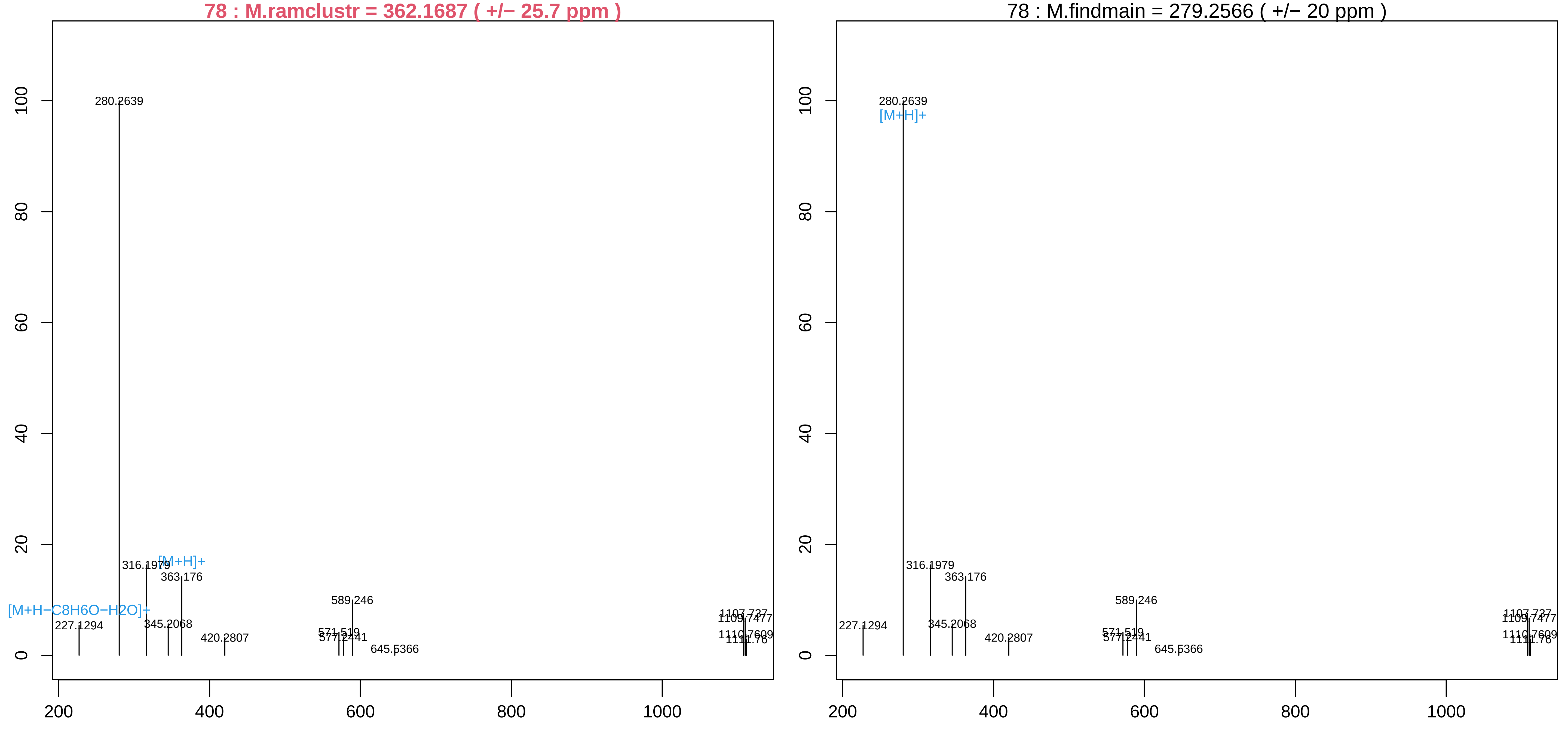

78 : M.ramclustr = 362.1687 (+/− 25.7 ppm)
280.2639
316.1979
[M+H]+
363.176
[M+H−C8H6O−H2O]+
227.1294
345.2068
420.2807
589.246
571.519
577.2441
645.5366
1107.737
1109.7477
1110.7609
1111.76
78 : M.findmain = 279.2566 (+/− 20 ppm)
280.2639
[M+H]+
316.1979
363.176
227.1294
345.2068
420.2807
589.246
571.519
577.2441
645.5366
1107.737
1109.7477
1110.7609
1111.76

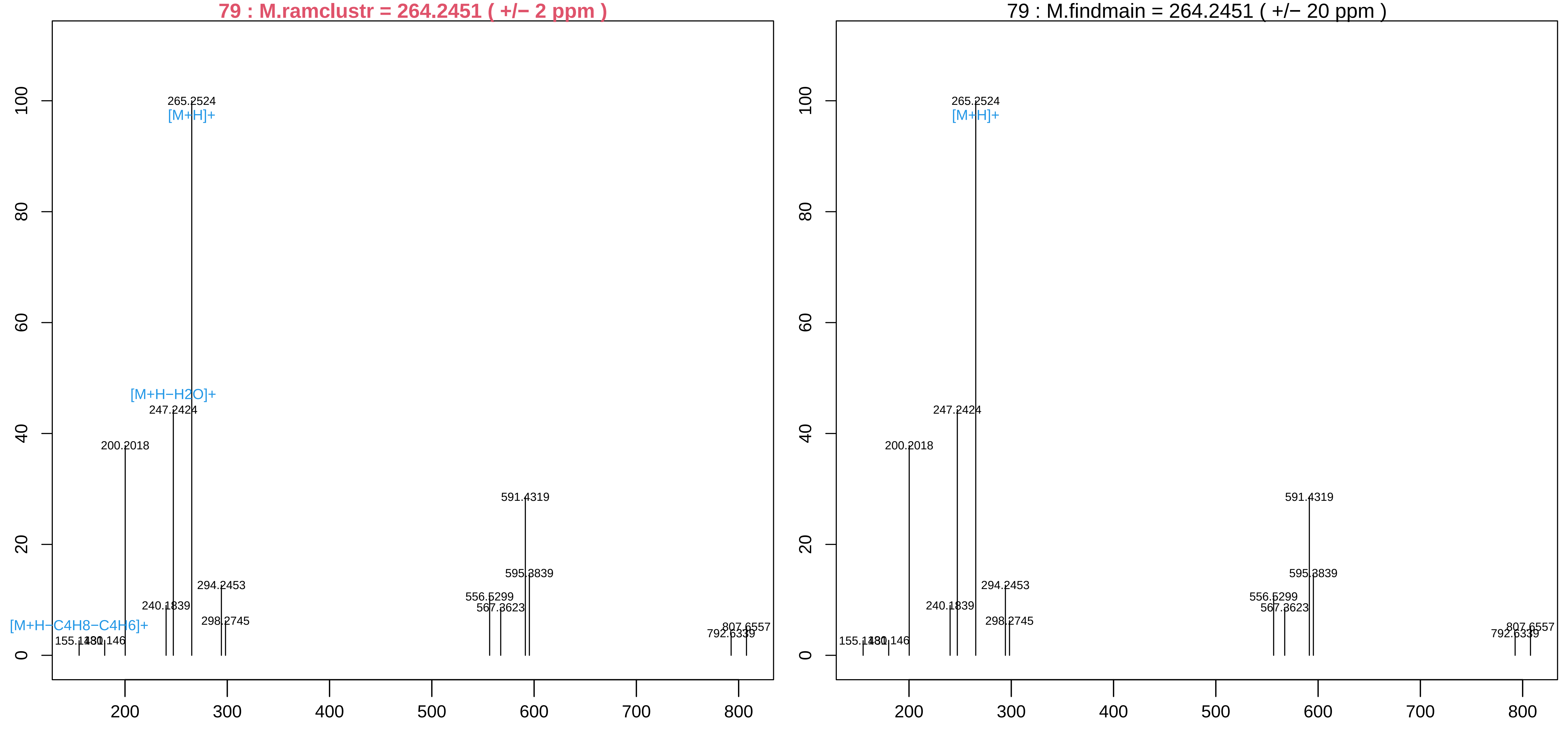
79 : M.ramclustr = 264.2451 (+/− 2 ppm)
79 : M.findmain = 264.2451 (+/− 20 ppm)
265.2524
[M+H]+
[M+H−H2O]+
247.2424
200.2018
591.4319
595.3839
294.2453
556.5299
567.3623
240.1839
298.2745
[M+H−C4H8−C4H6]+
807.6557
792.6339
155.1480.146
0
20
40
60
80
100
200
300
400
500
600
700
800

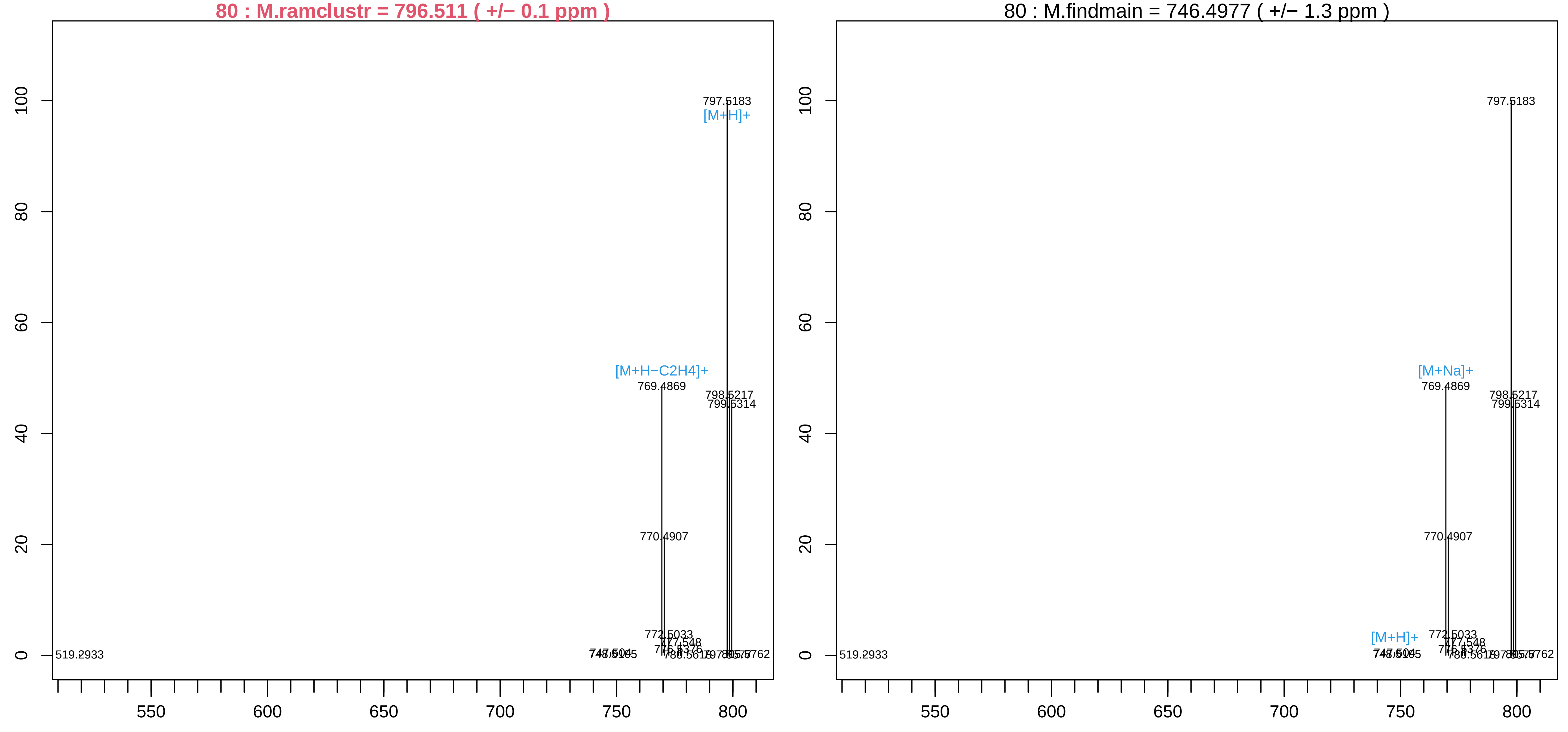
80 : M.ramclustr = 796.511 (+/− 0.1 ppm)
797.5183
[M+H]+
[M+H−C2H4]+
769.4869
798.5217
799.5314
770.4907
772.5033
777.548
519.2933
80 : M.findmain = 746.4977 (+/− 1.3 ppm)
797.5183
[M+Na]+
769.4869
798.5217
799.5314
770.4907
[M+H]+
772.5033
777.548
519.2933
0
20
40
60
80
100
550
600
650
700
750
800

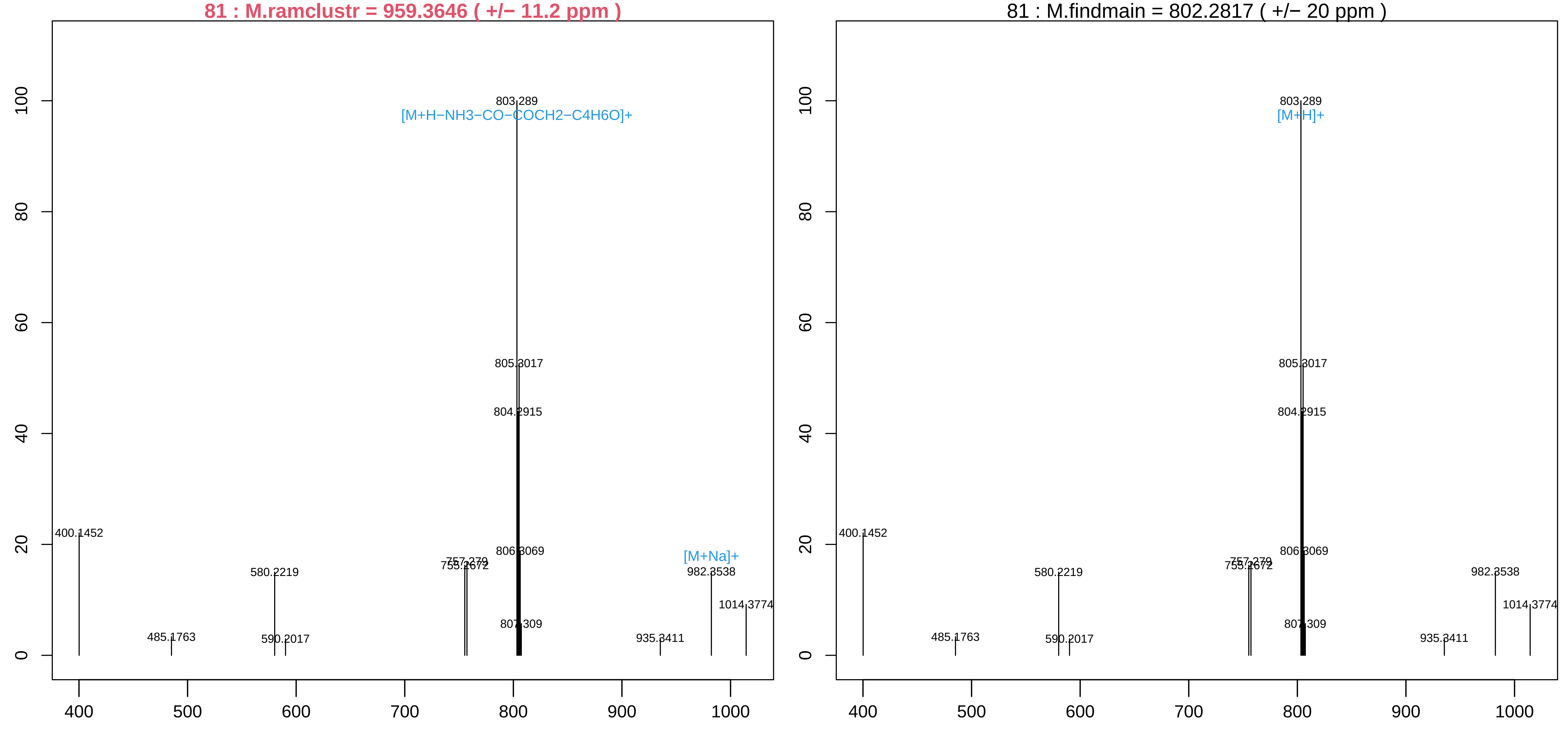
81 : M.ramclustr = 959.3646 (+/− 11.2 ppm)
[M+H−NH3−CO−COCH2−C4H6O]+
[M+Na]+
803.289
805.3017
804.2915
806.3069
807.309
757.279
755.2672
580.2219
590.2017
485.1763
400.1452
935.3411
982.3538
1014.3774
81 : M.findmain = 802.2817 (+/− 20 ppm)
[M+H]+
0
20
40
60
80
100
400
500
600
700
800
900
1000

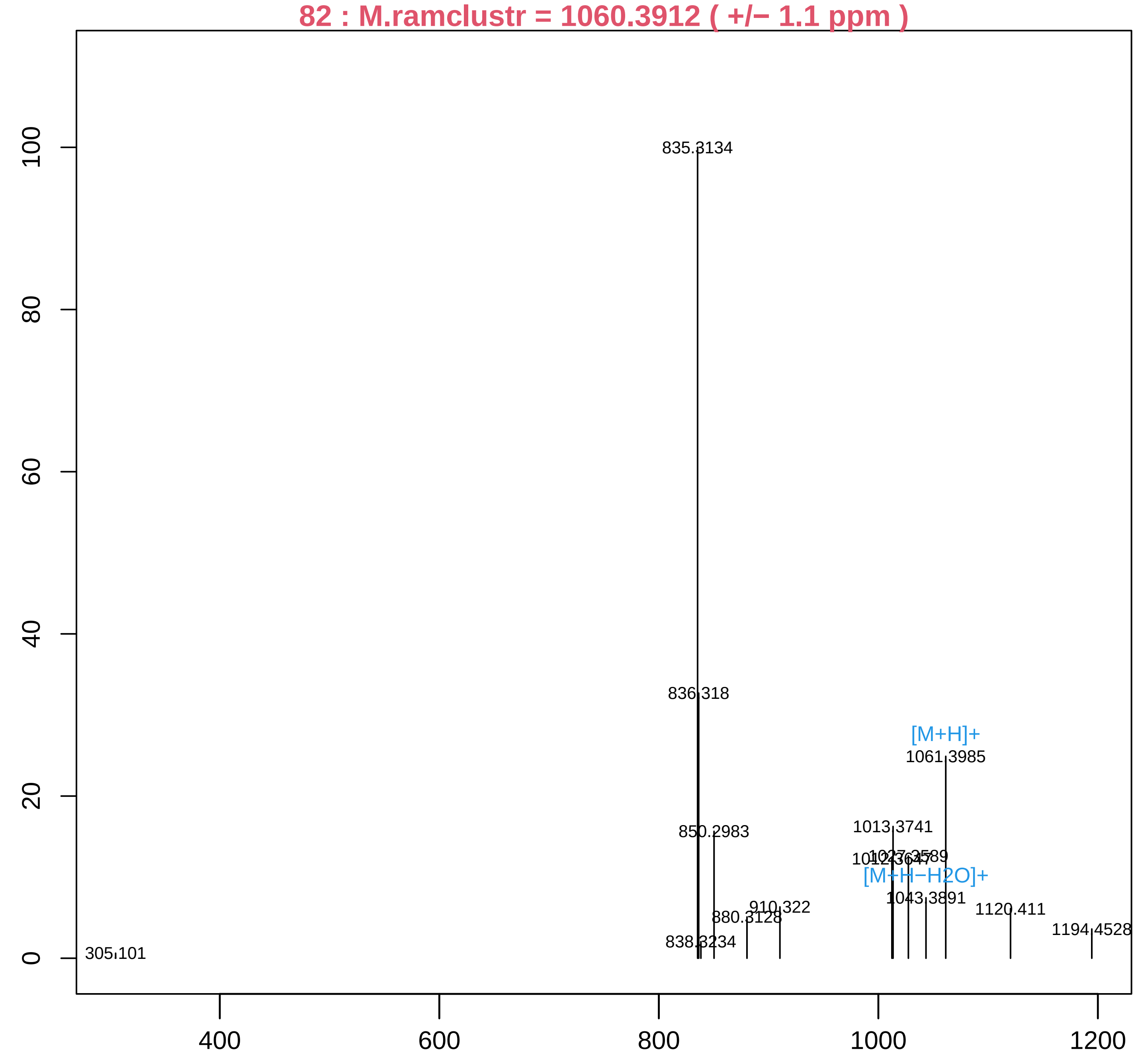
82 : M.ramclustr = 1060.3912 (+/− 1.1 ppm)
835.3134
836.318
850.2983
838.3234
880.3128
910.322
1013.3741
1012.3647
1027.3589
[M+H−H2O]+
1043.3891
[M+H]+
1061.3985
1120.411
1194.4528
305.101
0
20
40
60
80
100
400
600
800
1000
1200

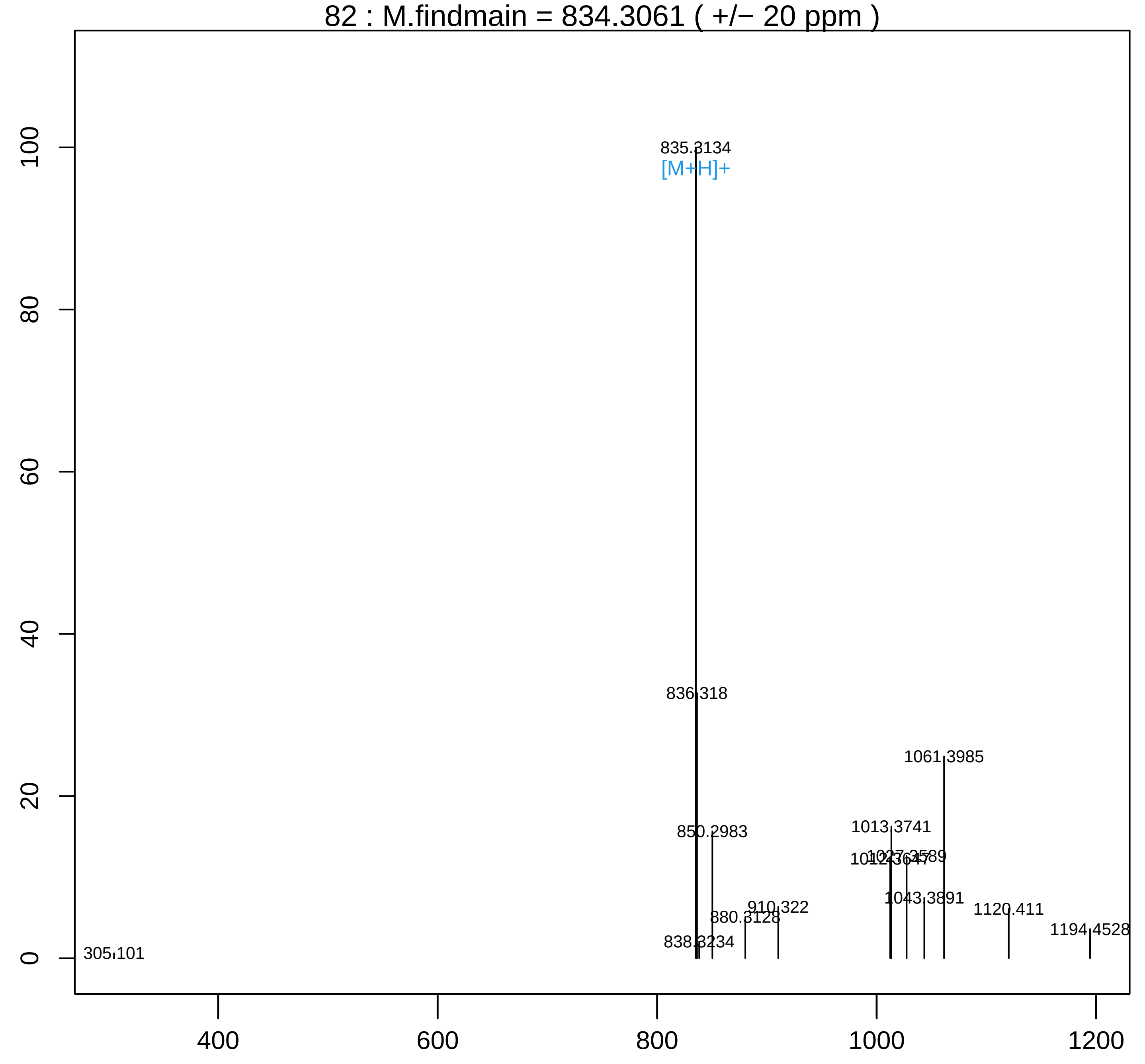
82 : M.findmain = 834.3061 (+/− 20 ppm)
835.3134
[M+H]+
836.318
850.2983
838.3234
880.3128
910.322
1013.3741
1012.3647
1027.3589
1043.3891
1061.3985
1120.411
1194.4528
305.101
0
20
40
60
80
100
400
600
800
1000
1200

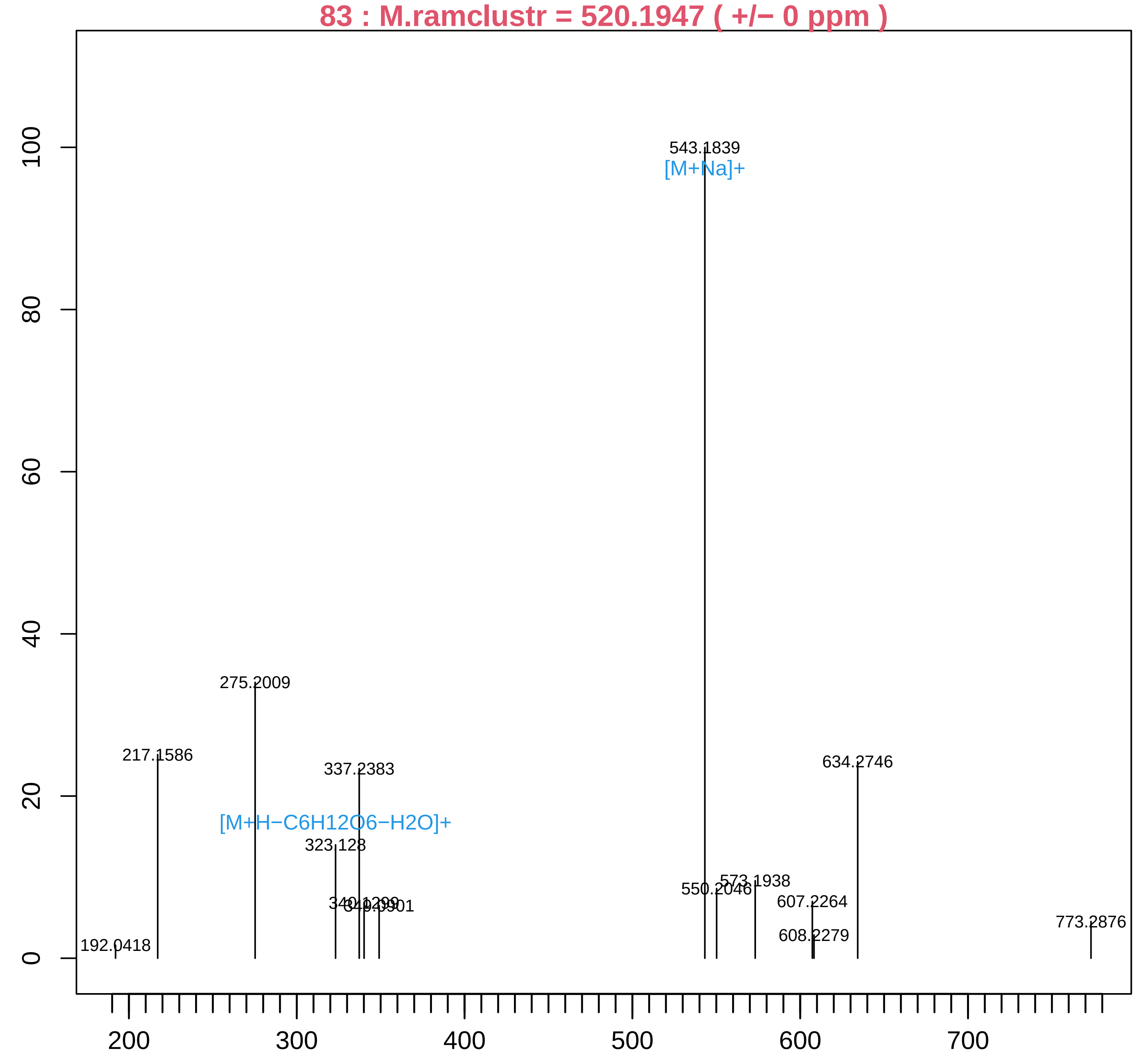
83 : M.ramclustr = 520.1947 (+/− 0 ppm)
543.1839
[M+Na]+
275.2009
217.1586
337.2383
634.2746
[M+H−C6H12O6−H2O]+
323.128
573.1938
550.2046
607.2264
340.1299
773.2876
608.2279
192.0418
0
20
40
60
80
100
200
300
400
500
600
700

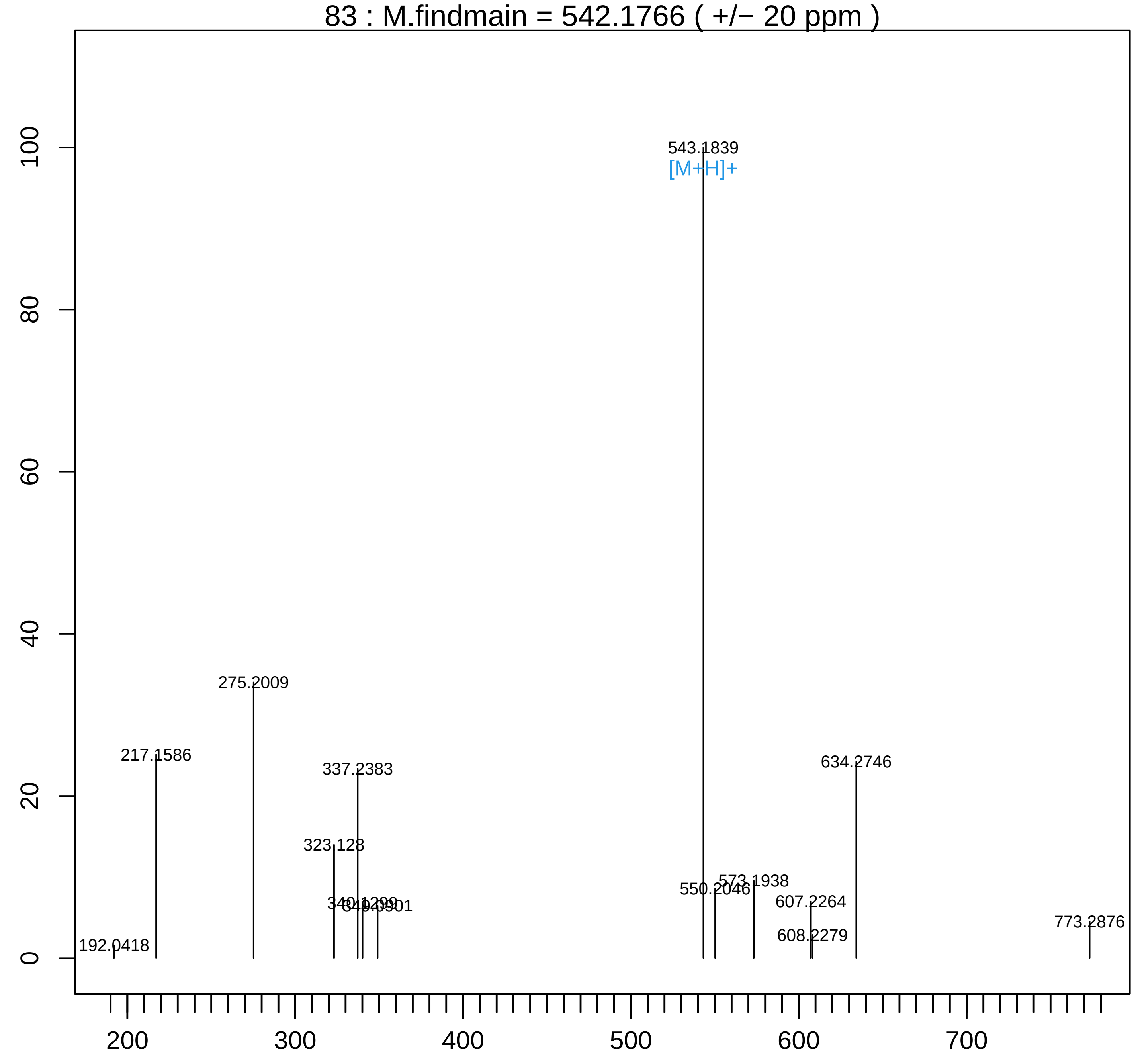
83 : M.findmain = 542.1766 (+/− 20 ppm)
543.1839
[M+H]+
275.2009
217.1586
337.2383
634.2746
323.128
573.1938
550.2046
607.2264
340.1299
773.2876
608.2279
192.0418
0
20
40
60
80
100
200
300
400
500
600
700

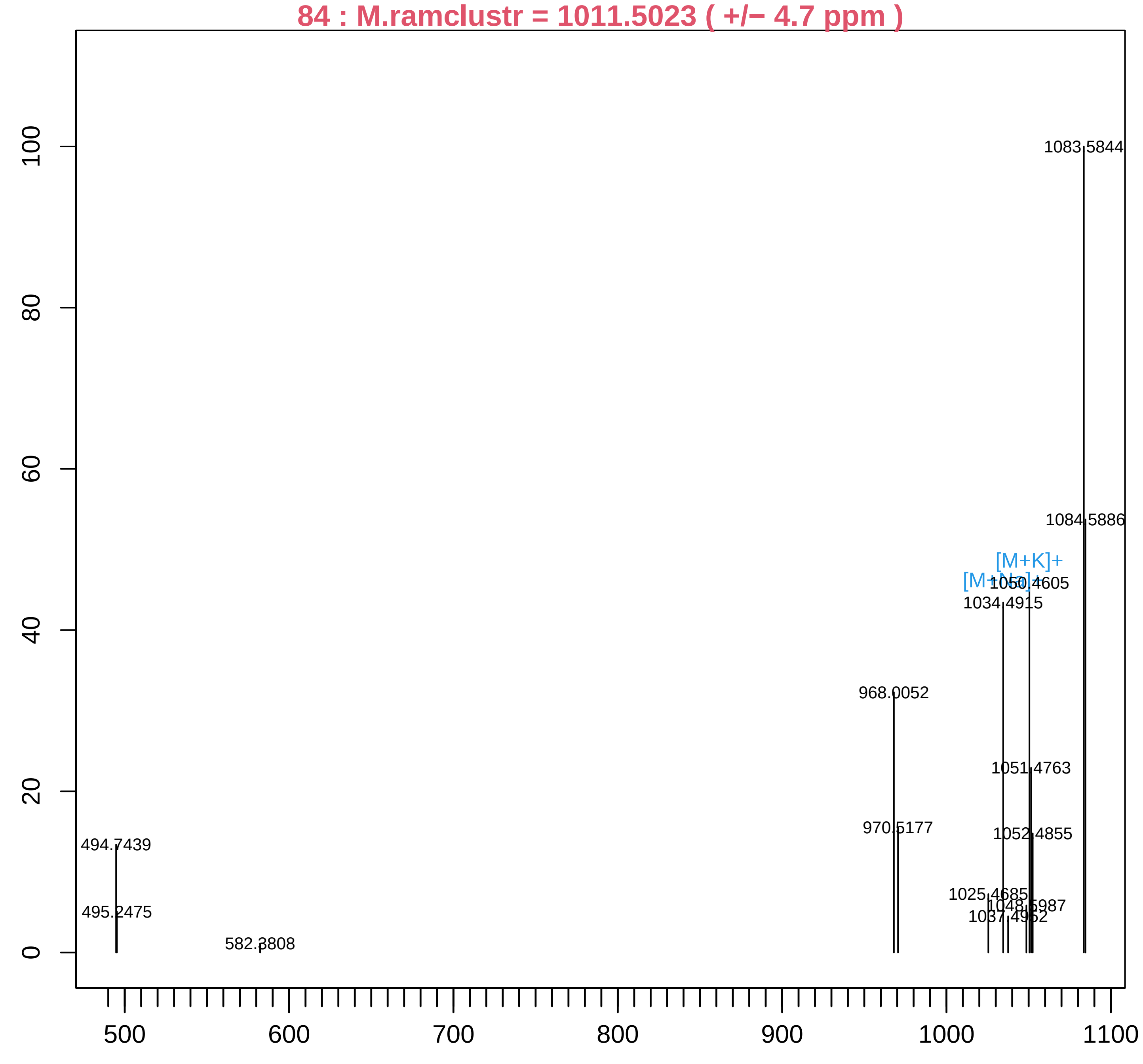
84 : M.ramclustr = 1011.5023 (+/− 4.7 ppm)
1083.5844
1084.5886
[M+K]+
[M+Na]+
1050.4605
1034.4915
968.0052
1051.4763
970.5177
1052.4855
494.7439
495.2475
582.3808
1025.4685
1048.5987
1037.4952
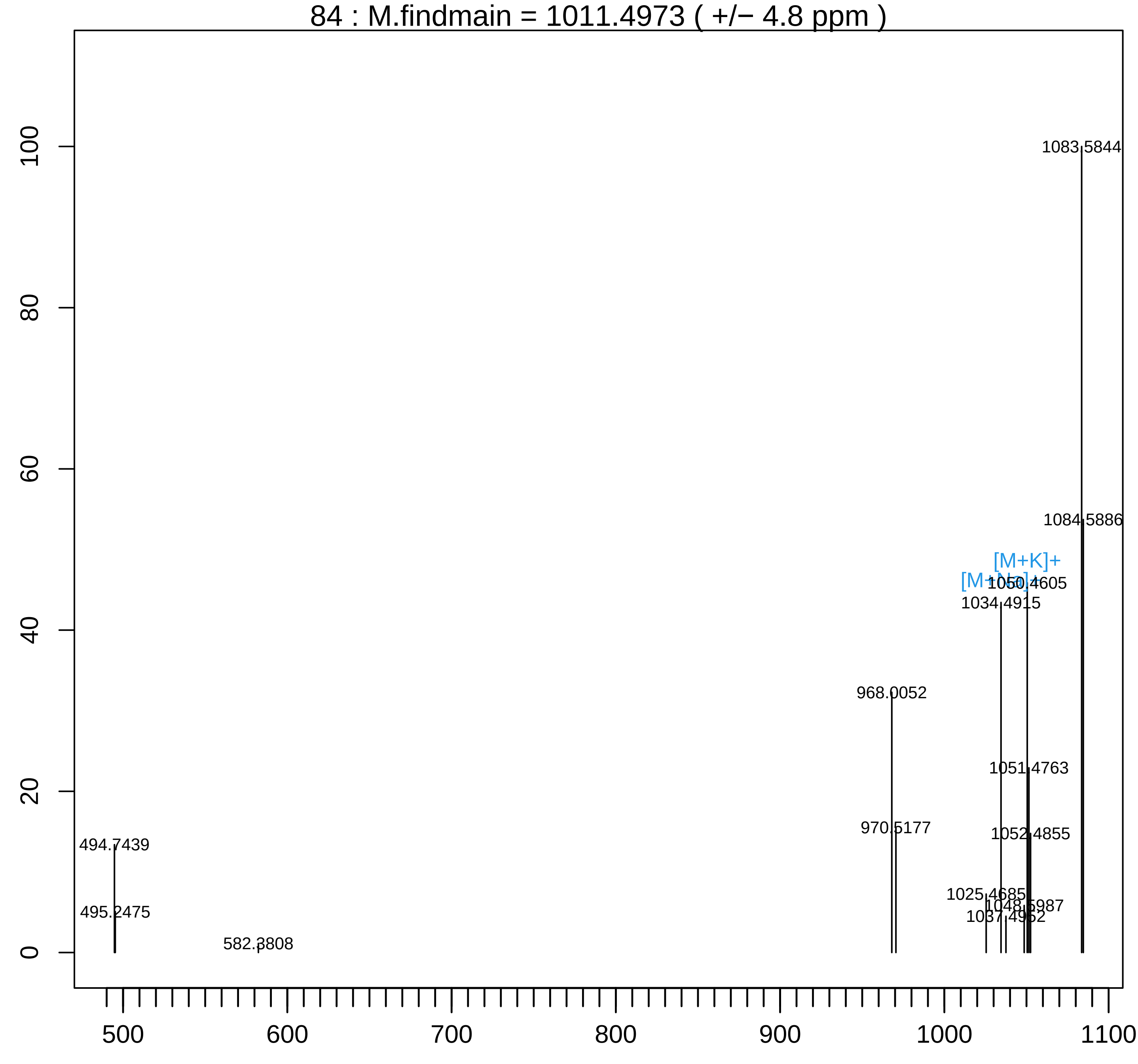
84 : M.findmain = 1011.4973 (+/− 4.8 ppm)
1083.5844
1084.5886
[M+K]+
[M+Na]+
1050.4605
1034.4915
968.0052
1051.4763
970.5177
1052.4855
494.7439
495.2475
582.3808
1025.4685
1048.5987
1037.4952

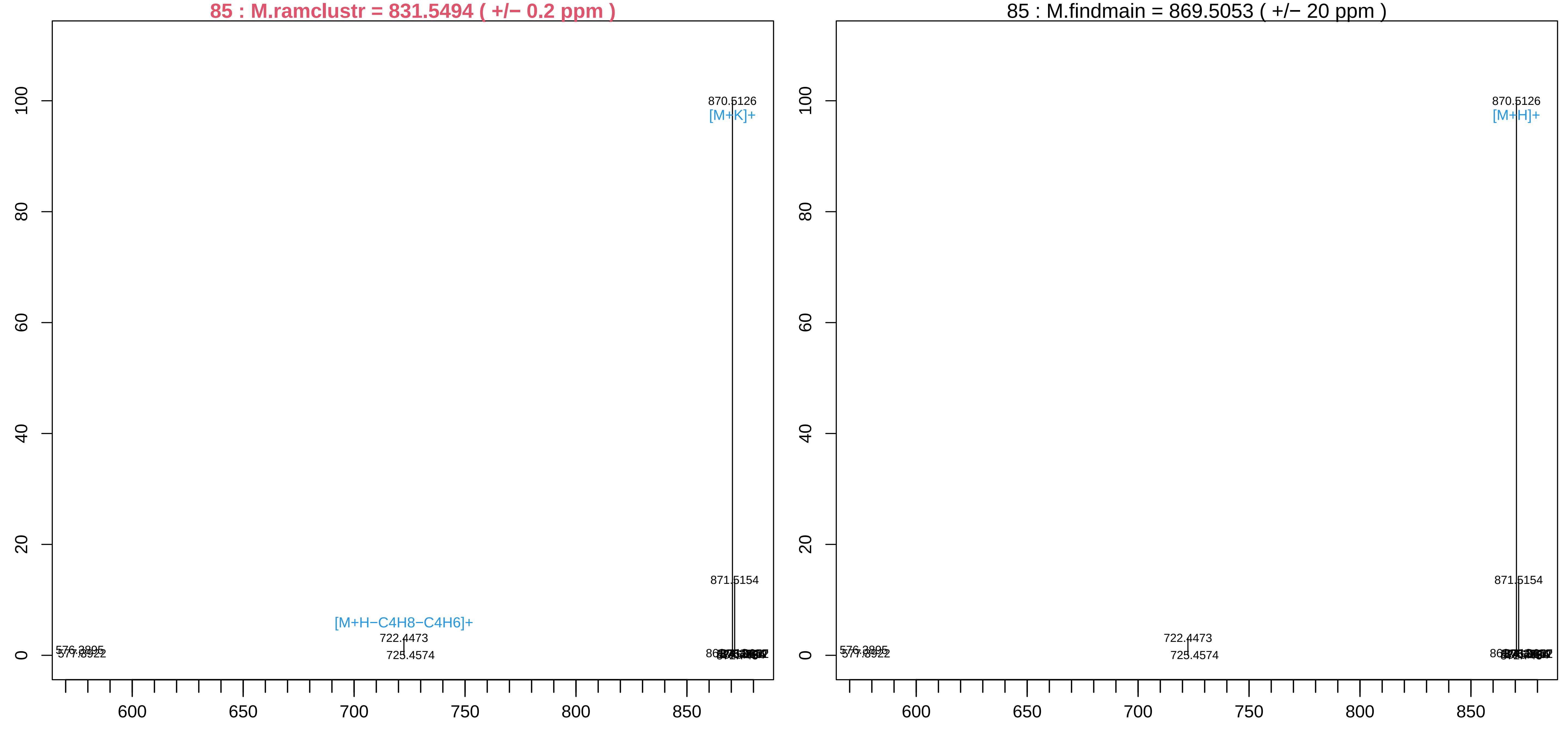
85 : M.ramclustr = 831.5494 (+/− 0.2 ppm)
870.5126
[M+K]+
871.5154
[M+H−C4H8−C4H6]+
722.4473
725.4574
576.3895
577.3922
85 : M.findmain = 869.5053 (+/− 20 ppm)
870.5126
[M+H]+
871.5154
722.4473
725.4574
576.3895
577.3922
0
20
40
60
80
100
600
650
700
750
800
850

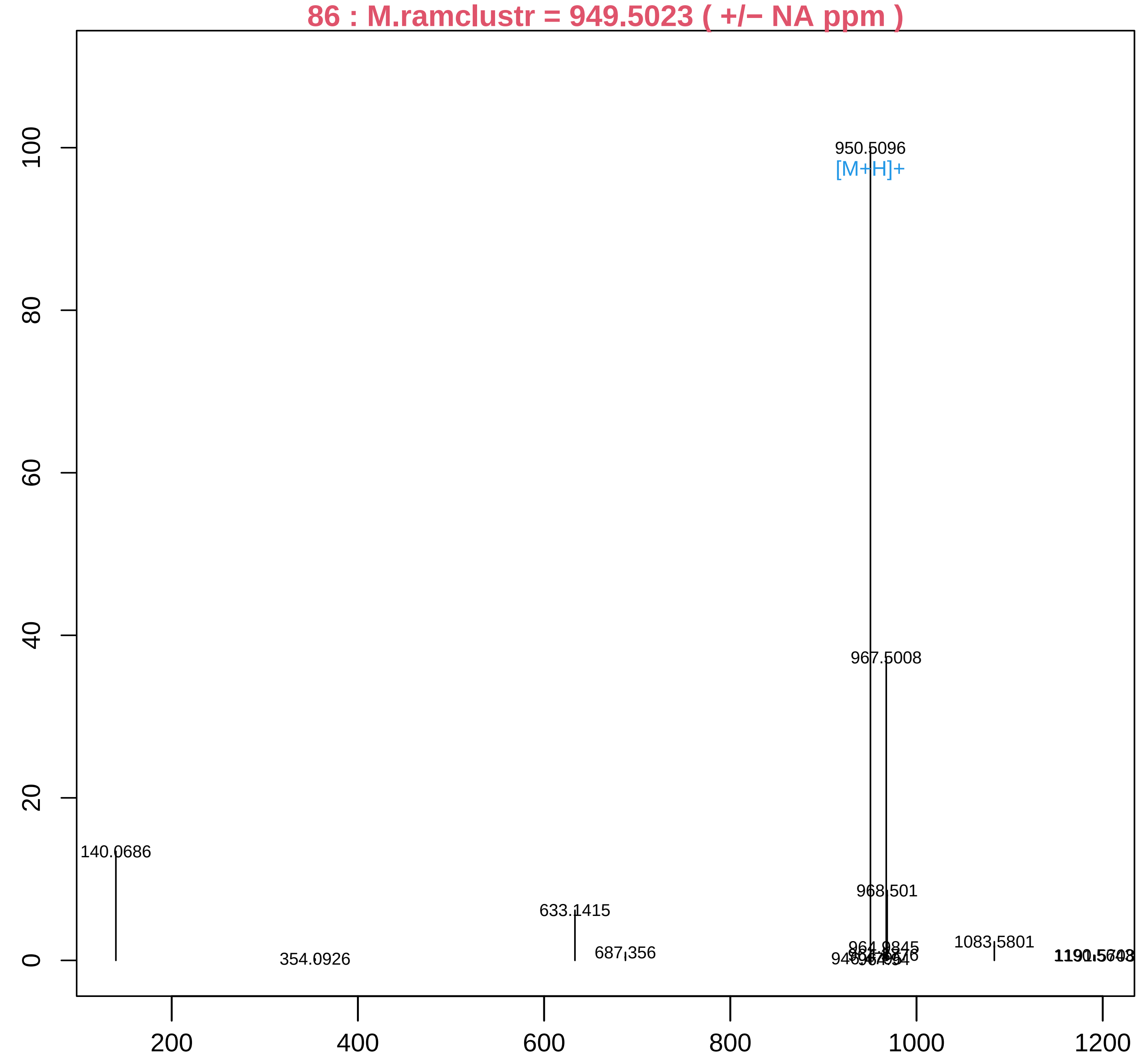
86 : M.ramclustr = 949.5023 (+/− NA ppm)
950.5096
[M+H]+
967.5008
968.501
140.0686
633.1415
687.356
964.9845
1083.5801
354.0926
0
20
40
60
80
100
200
400
600
800
1000
1200

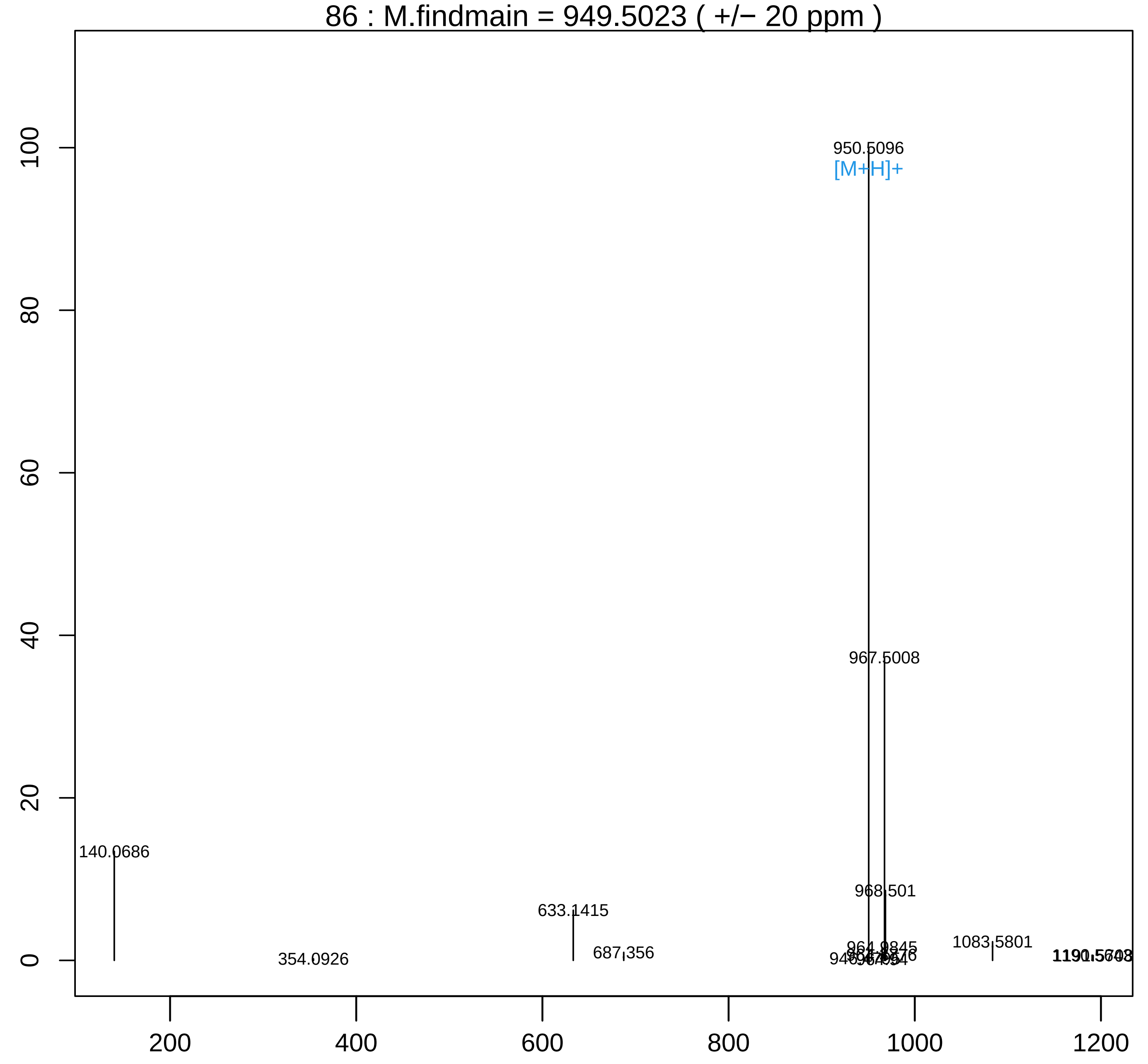
86 : M.findmain = 949.5023 (+/− 20 ppm)
950.5096
[M+H]+
967.5008
968.501
140.0686
633.1415
687.356
964.9845
1083.5801
354.0926
0
20
40
60
80
100
200
400
600
800
1000
1200

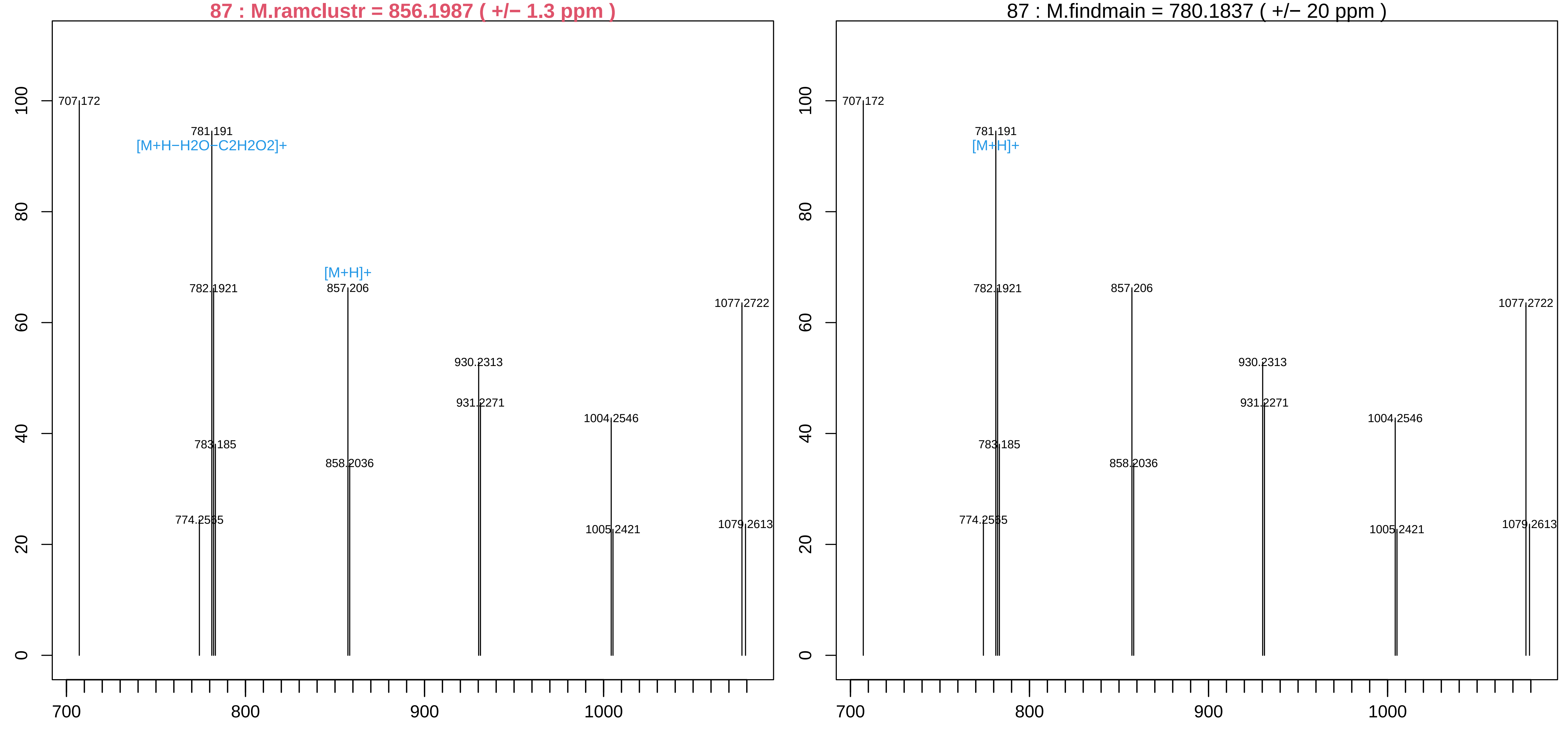

87 : M.ramclustr = 856.1987 (+/− 1.3 ppm)
707.172
781.191
[M+H−H2O−C2H2O2]+
782.1921
783.185
774.2555
[M+H]+
857.206
858.2036
930.2313
931.2271
1004.2546
1005.2421
1077.2722
1079.2613
0
20
40
60
80
100
700
800
900
1000
87 : M.findmain = 780.1837 (+/− 20 ppm)
707.172
781.191
[M+H]+
782.1921
783.185
774.2555
857.206
858.2036
930.2313
931.2271
1004.2546
1005.2421
1077.2722
1079.2613
0
20
40
60
80
100
700
800
900
1000

88 : M.ramclustr = 577.1944 (+/− NA ppm)

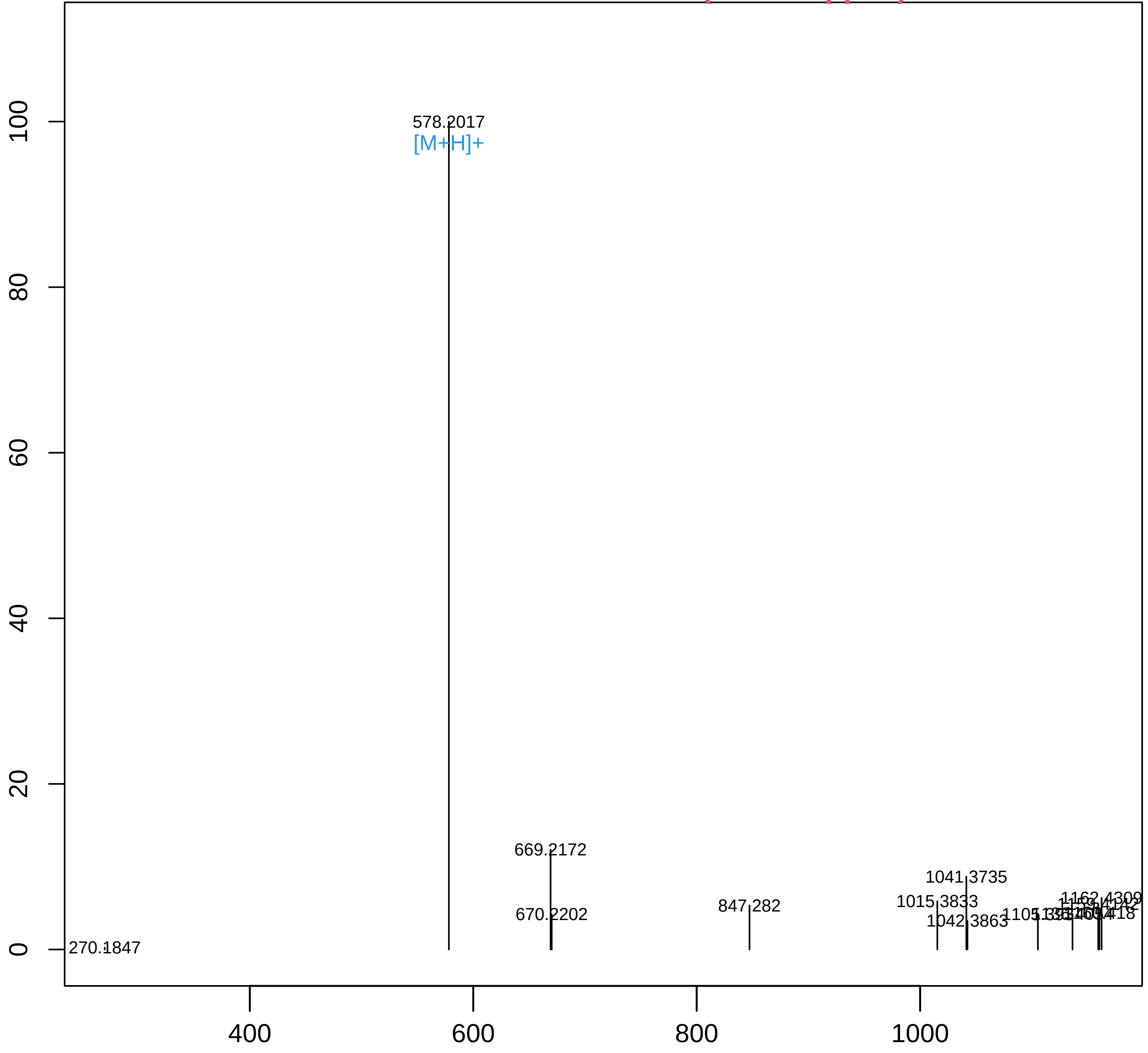

88 : M.findmain = 577.1944 (+/− 20 ppm)

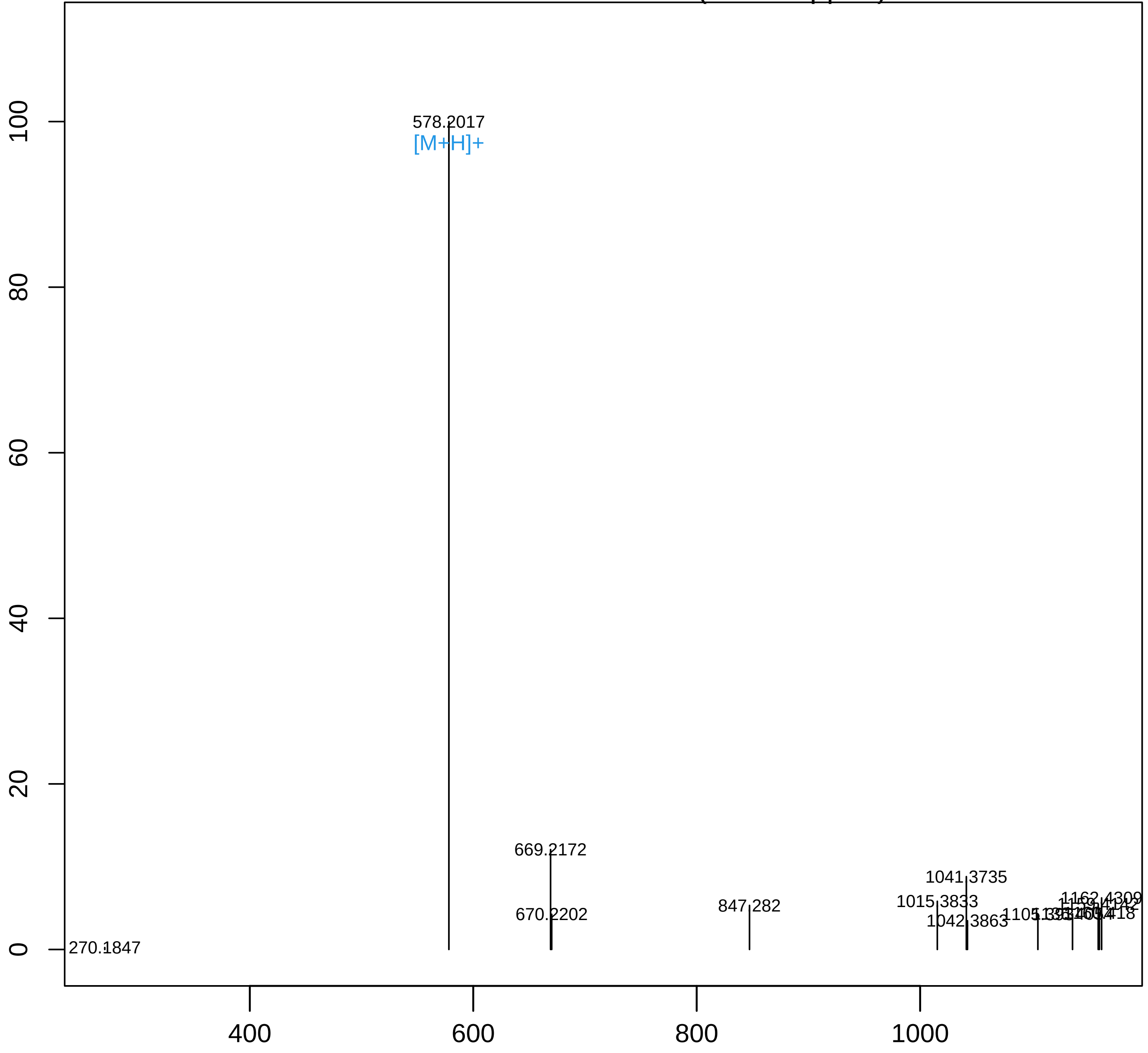

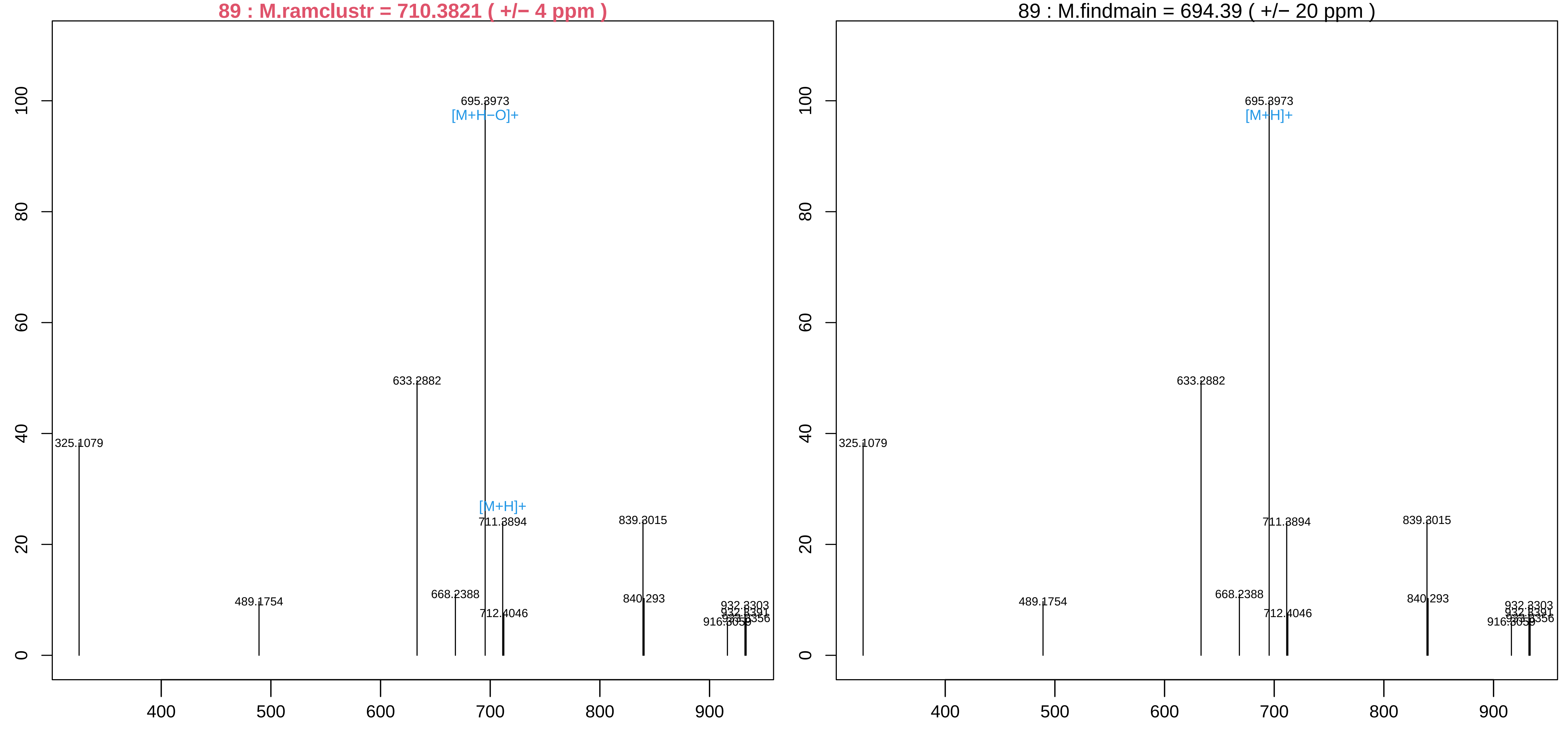
89 : M.ramclustr = 710.3821 (+/− 4 ppm)
695.3973
[M+H−O]+
633.2882
325.1079
[M+H]+
711.3894
839.3015
668.2388
489.1754
840.293
712.4046
932.3303
89 : M.findmain = 694.39 (+/− 20 ppm)
695.3973
[M+H]+
633.2882
325.1079
711.3894
839.3015
668.2388
489.1754
840.293
712.4046
932.3303
0
20
40
60
80
100
400
500
600
700
800
900

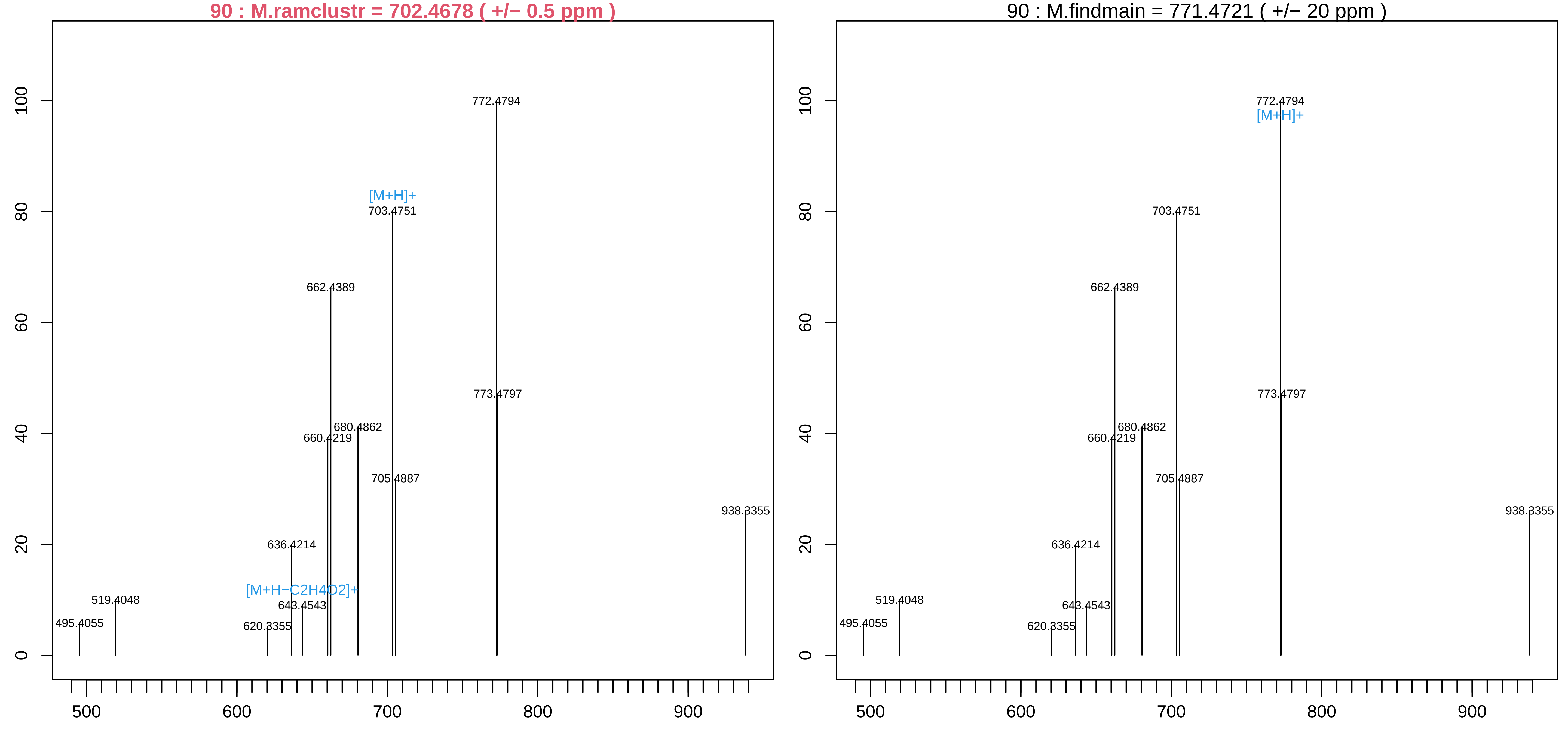
90 : M.ramclustr = 702.4678 (+/− 0.5 ppm)
772.4794
[M+H]+
703.4751
662.4389
773.4797
680.4862
660.4219
705.4887
938.3355
636.4214
[M+H−C2H4O2]+
519.4048
643.4543
495.4055
620.3355
0
20
40
60
80
100
500
600
700
800
900
90 : M.findmain = 771.4721 (+/− 20 ppm)
772.4794
[M+H]+
703.4751
662.4389
773.4797
680.4862
660.4219
705.4887
938.3355
636.4214
519.4048
643.4543
495.4055
620.3355
0
20
40
60
80
100
500
600
700
800
900

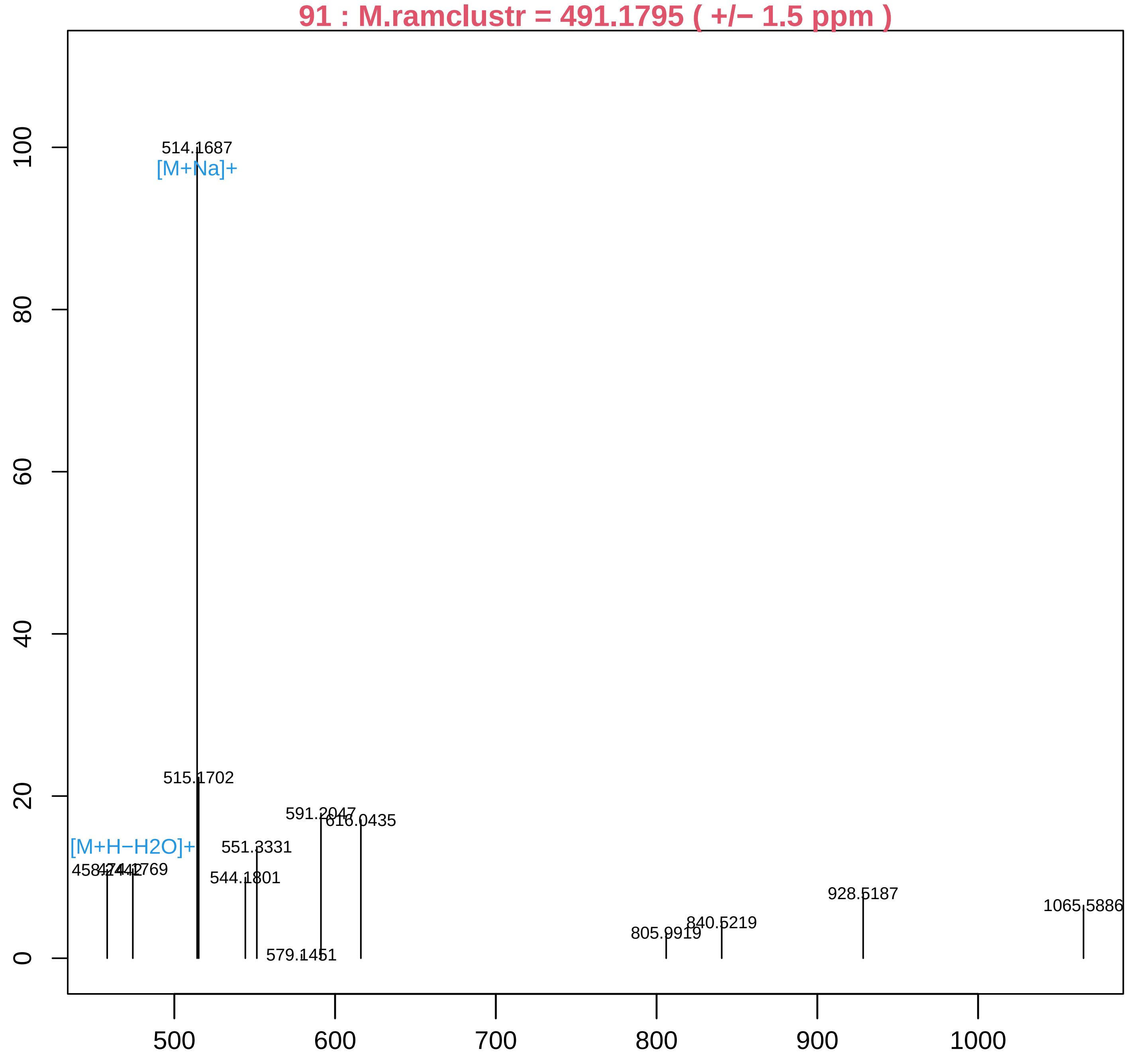

91 : M.ramclustr = 491.1795 (+/− 1.5 ppm)
514.1687
[M+Na]+
515.1702
[M+H−H2O]+
551.3331
544.1801
591.2047
616.0435
579.1451
805.9919
840.5219
928.5187
1065.5886
0
20
40
60
80
100
500
600
700
800
900
1000

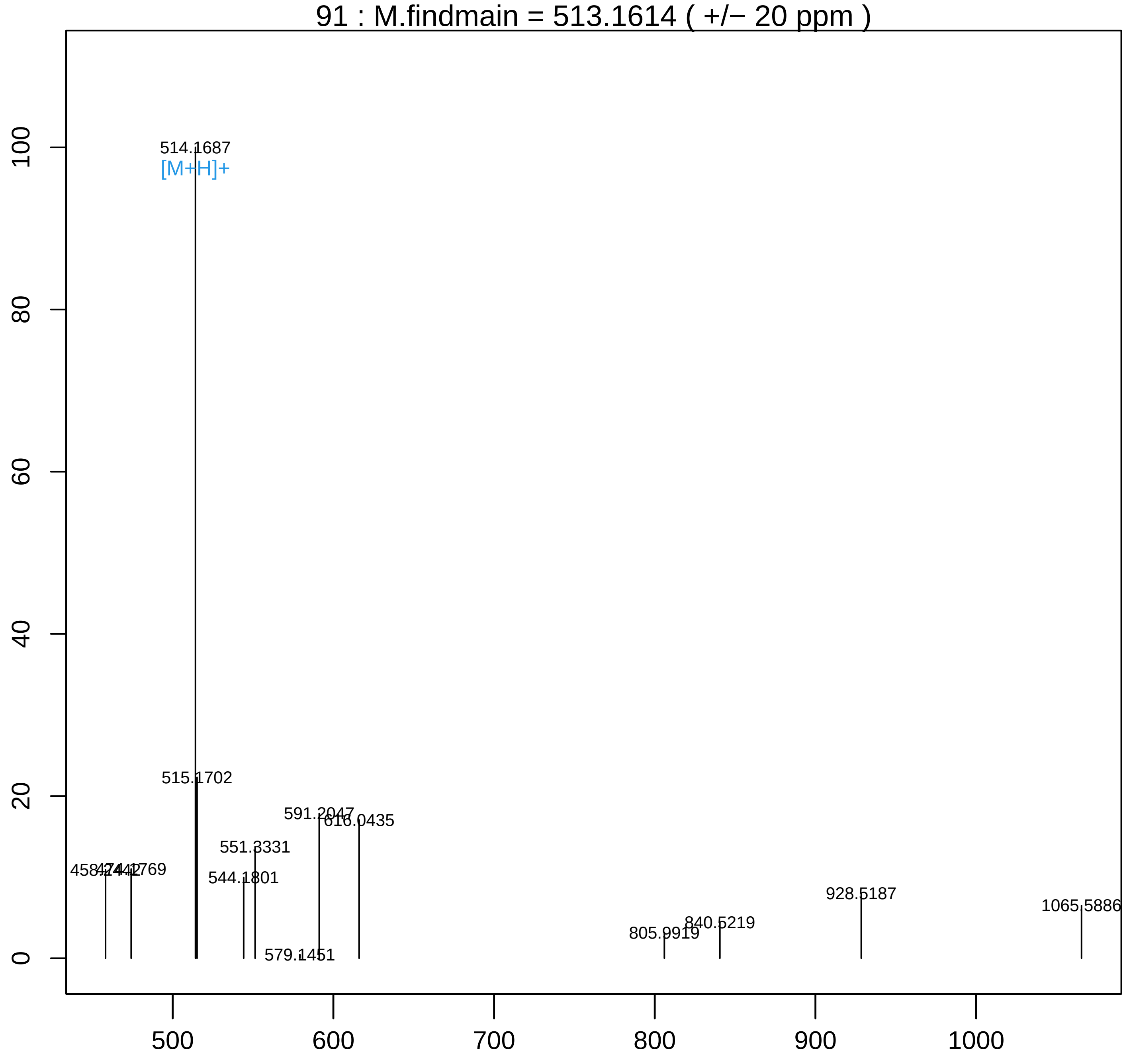

91 : M.findmain = 513.1614 (+/− 20 ppm)
514.1687
[M+H]+
515.1702
551.3331
544.1801
591.2047
616.0435
579.1451
805.9919
840.5219
928.5187
1065.5886
0
20
40
60
80
100
500
600
700
800
900
1000

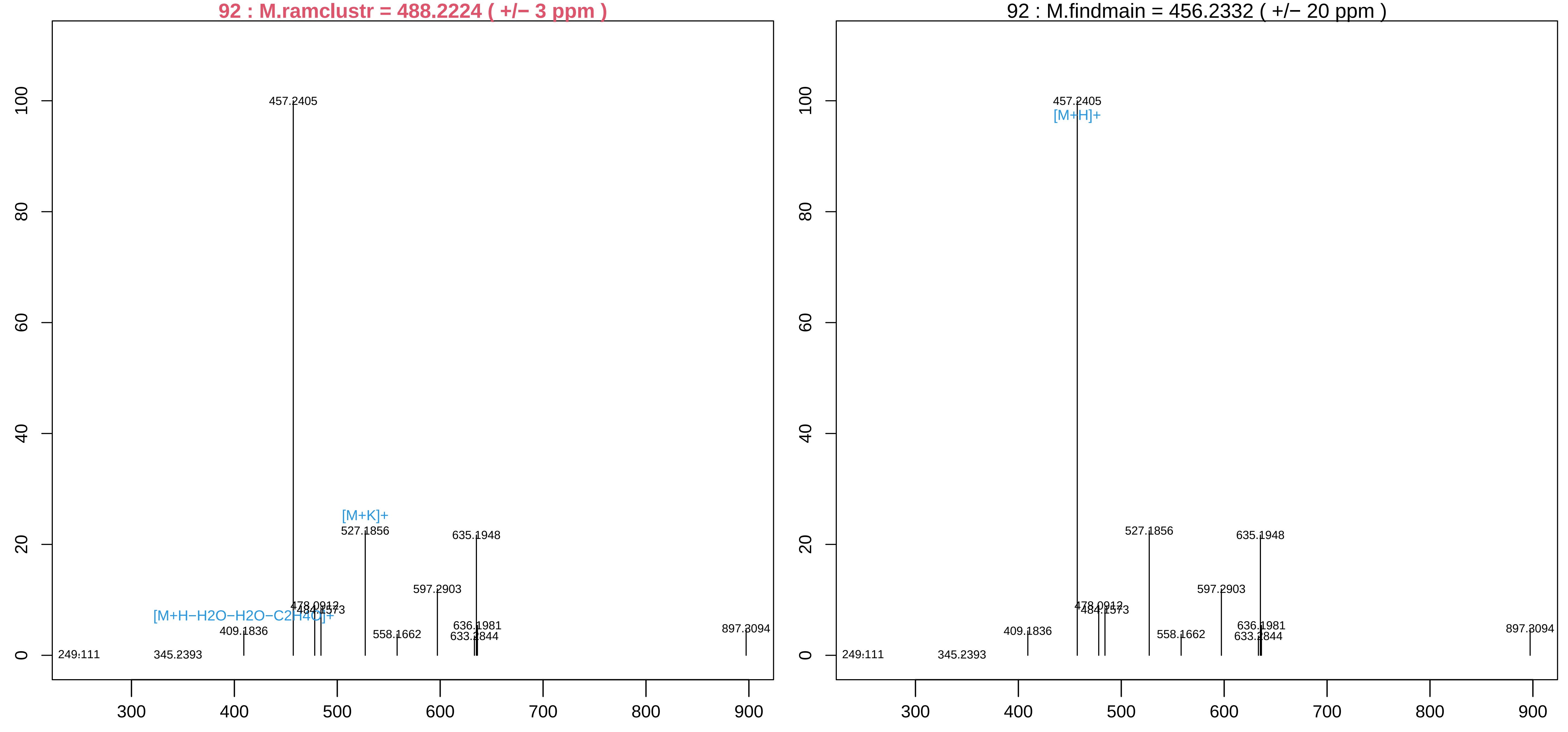
92 : M.ramclustr = 488.2224 (+/− 3 ppm)
457.2405
[M+K]+
527.1856
635.1948
597.2903
478.0912
484.1573
[M+H−H2O−H2O−C2H4O]+
409.1836
636.1981
633.2844
558.1662
897.3094
249.111
345.2393
92 : M.findmain = 456.2332 (+/− 20 ppm)
457.2405
[M+H]+
527.1856
635.1948
597.2903
478.0912
484.1573
409.1836
636.1981
633.2844
558.1662
897.3094
249.111
345.2393

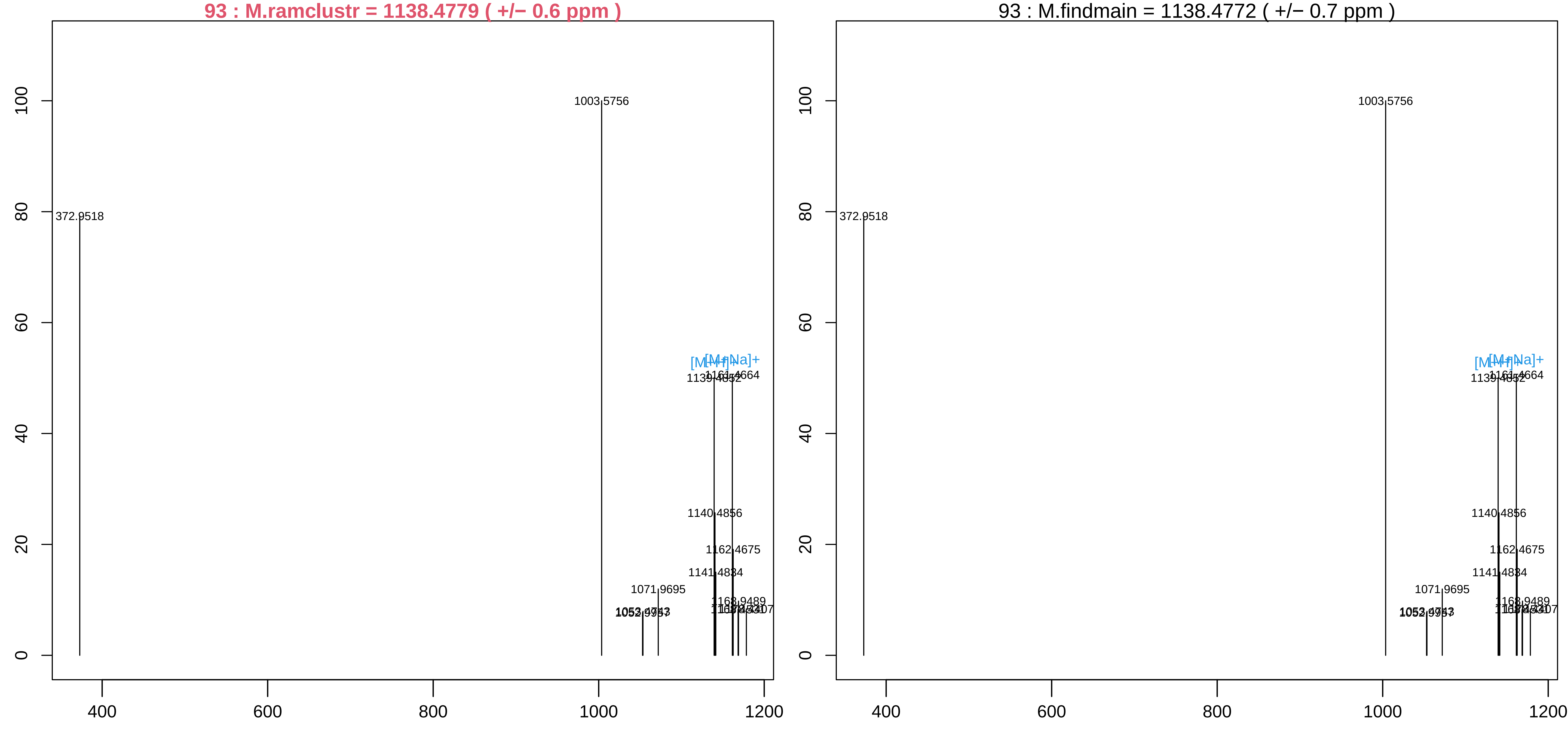
93 : M.ramclustr = 1138.4779 (+/− 0.6 ppm)
93 : M.findmain = 1138.4772 (+/− 0.7 ppm)
1003.5756
372.9518
[M+Na]+
[M+H]+
1161.4664
1140.4856
1162.4675
1141.4834
1071.9695
1168.9489
0
20
40
60
80
100
400
600
800
1000
1200

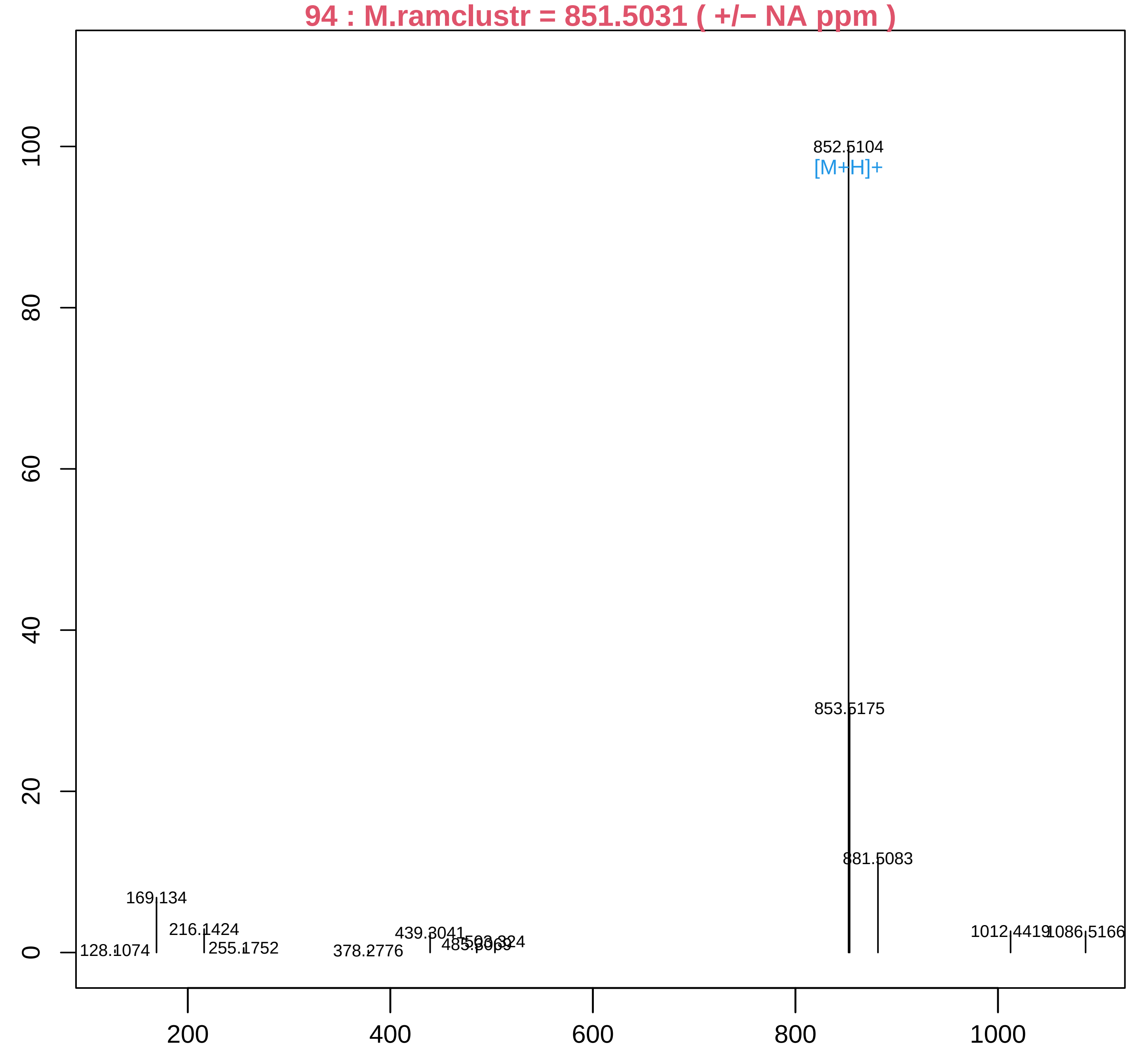
94 : M.ramclustr = 851.5031 (+/− NA ppm)
852.5104
[M+H]+
853.5175
881.5083
169.134
216.1424
128.1074
255.1752
378.2776
439.3041
1012.4419
1086.5166

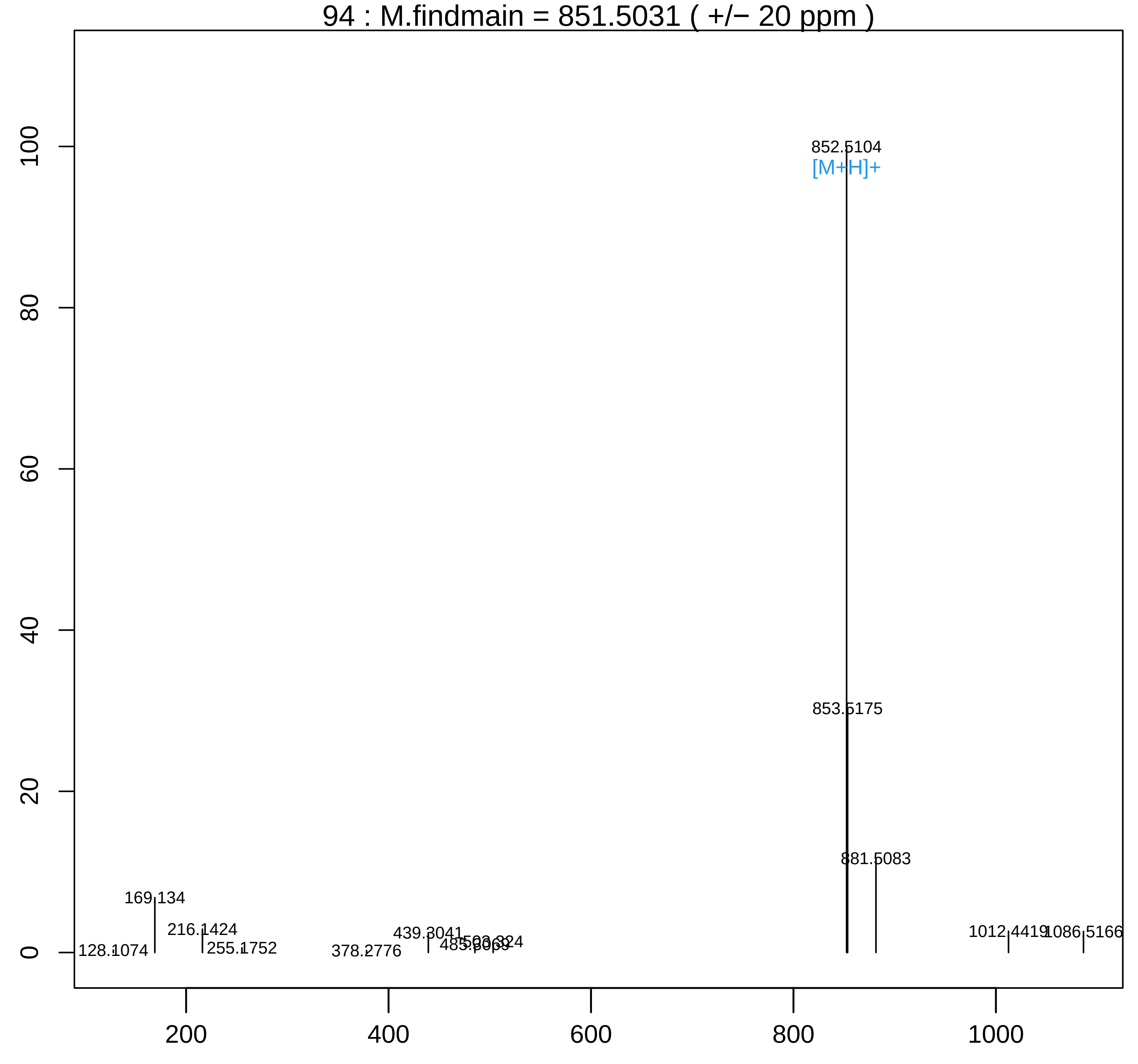
94 : M.findmain = 851.5031 (+/− 20 ppm)
852.5104
[M+H]+
853.5175
881.5083
169.134
216.1424
128.1074
255.1752
378.2776
439.3041
1012.4419
1086.5166

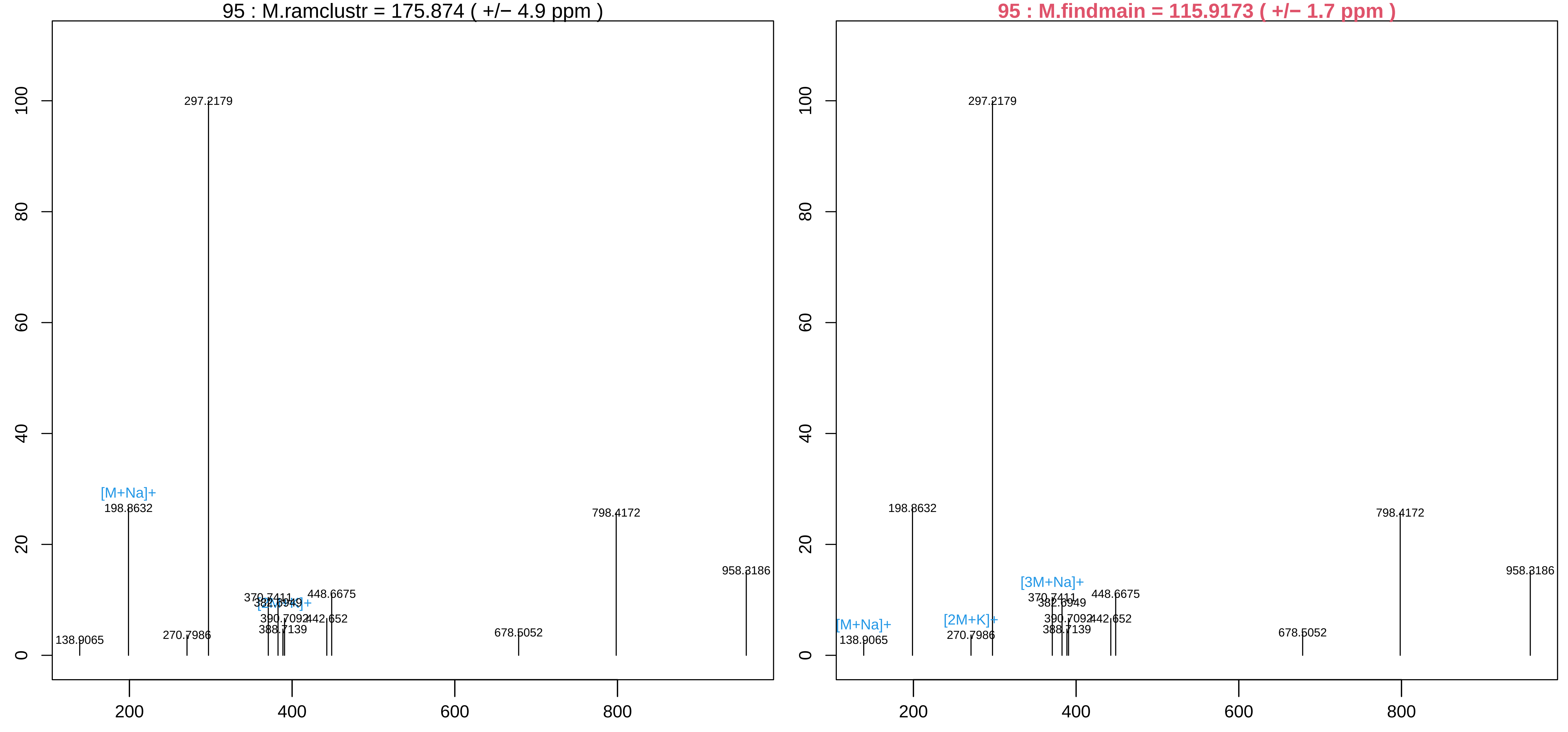
95 : M.ramclustr = 175.874 (+/− 4.9 ppm)
95 : M.findmain = 115.9173 (+/− 1.7 ppm)
0
20
40
60
80
100
200
400
600
800
297.2179
[M+Na]+
198.8632
798.4172
958.3186
448.6675
370.7411
382.6949
[3M+Na]+
[2M+K]+
390.7092
442.652
388.7139
138.9065
270.7986
678.5052

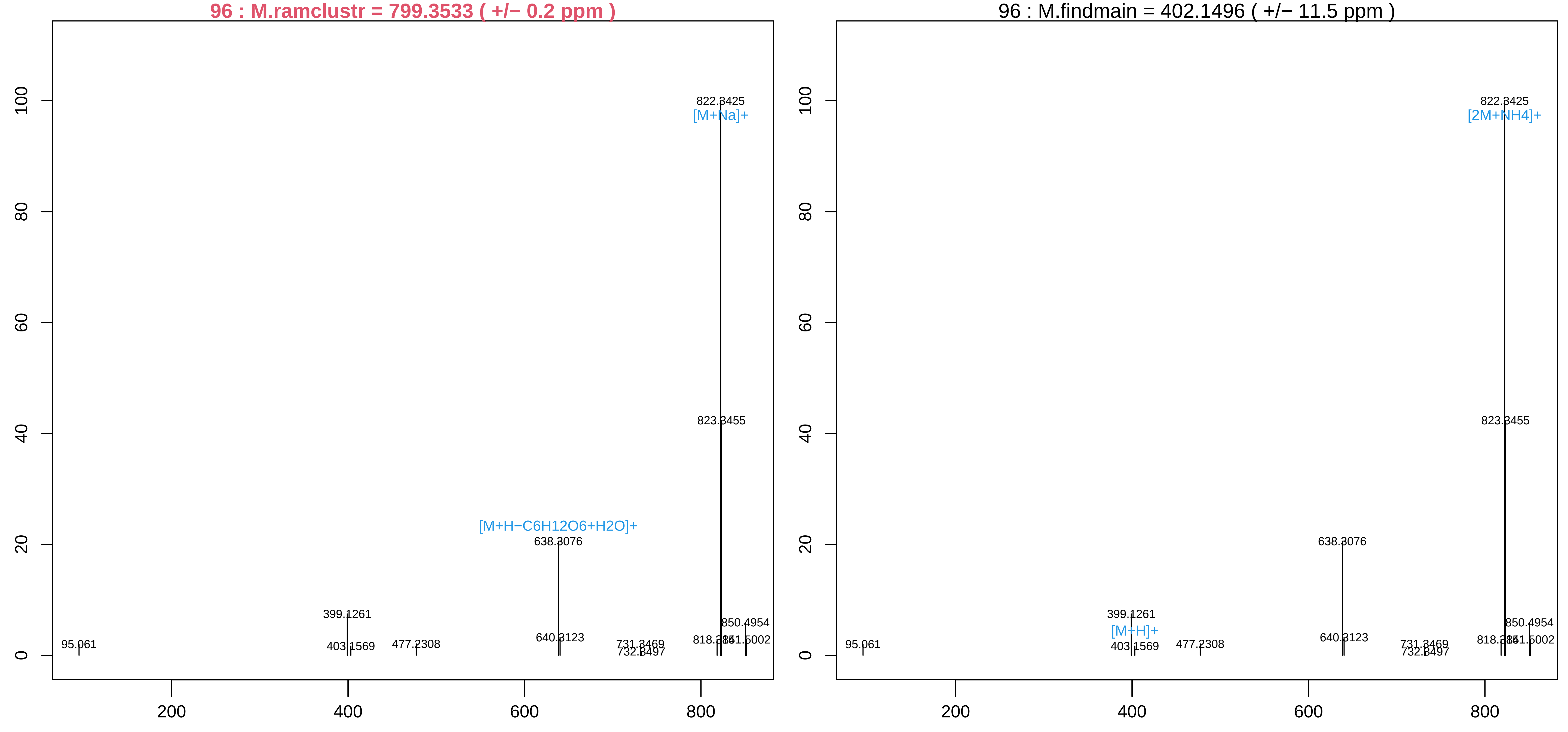

96 : M.ramclustr = 799.3533 (+/− 0.2 ppm)
822.3425
[M+Na]+
823.3455
[M+H−C6H12O6+H2O]+
638.3076
399.1261
850.4954
95.061
403.1569
477.2308
640.3123
731.3469
732.3497
818.3851
851.5002
96 : M.findmain = 402.1496 (+/− 11.5 ppm)
822.3425
[2M+NH4]+
823.3455
638.3076
399.1261
[M+H]+
403.1569
477.2308
640.3123
731.3469
732.3497
818.3851
850.4954
851.5002
95.061
0
20
40
60
80
100
200
400
600
800

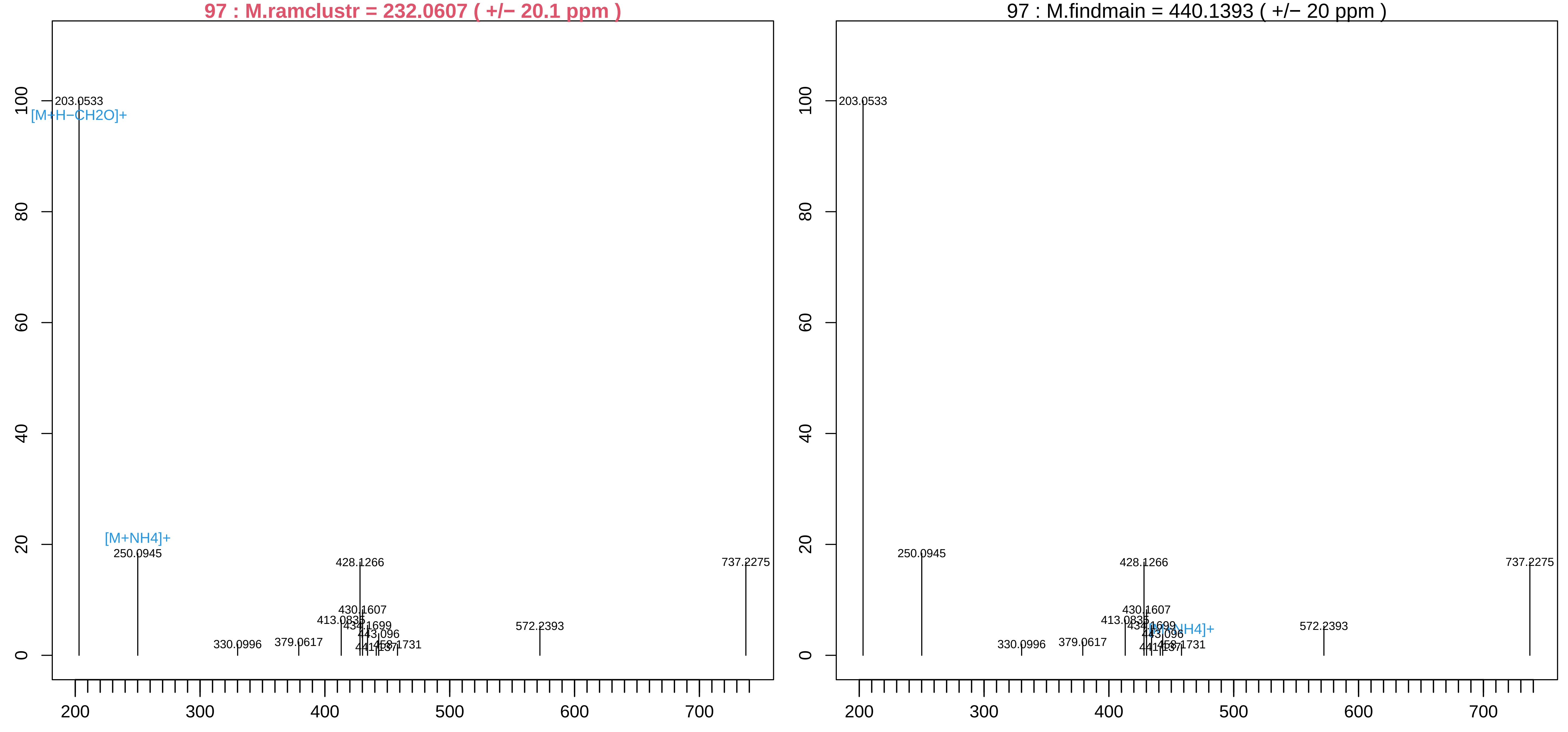
97 : M.ramclustr = 232.0607 (+/− 20.1 ppm)
203.0533
[M+H−CH2O]+
[M+NH4]+
250.0945
330.0996
379.0617
413.0835
428.1266
430.1607
434.1699
443.096
441.137
458.1731
572.2393
737.2275
97 : M.findmain = 440.1393 (+/− 20 ppm)
203.0533
250.0945
330.0996
379.0617
413.0835
428.1266
430.1607
434.1699
[M+NH4]+
443.096
441.137
458.1731
572.2393
737.2275

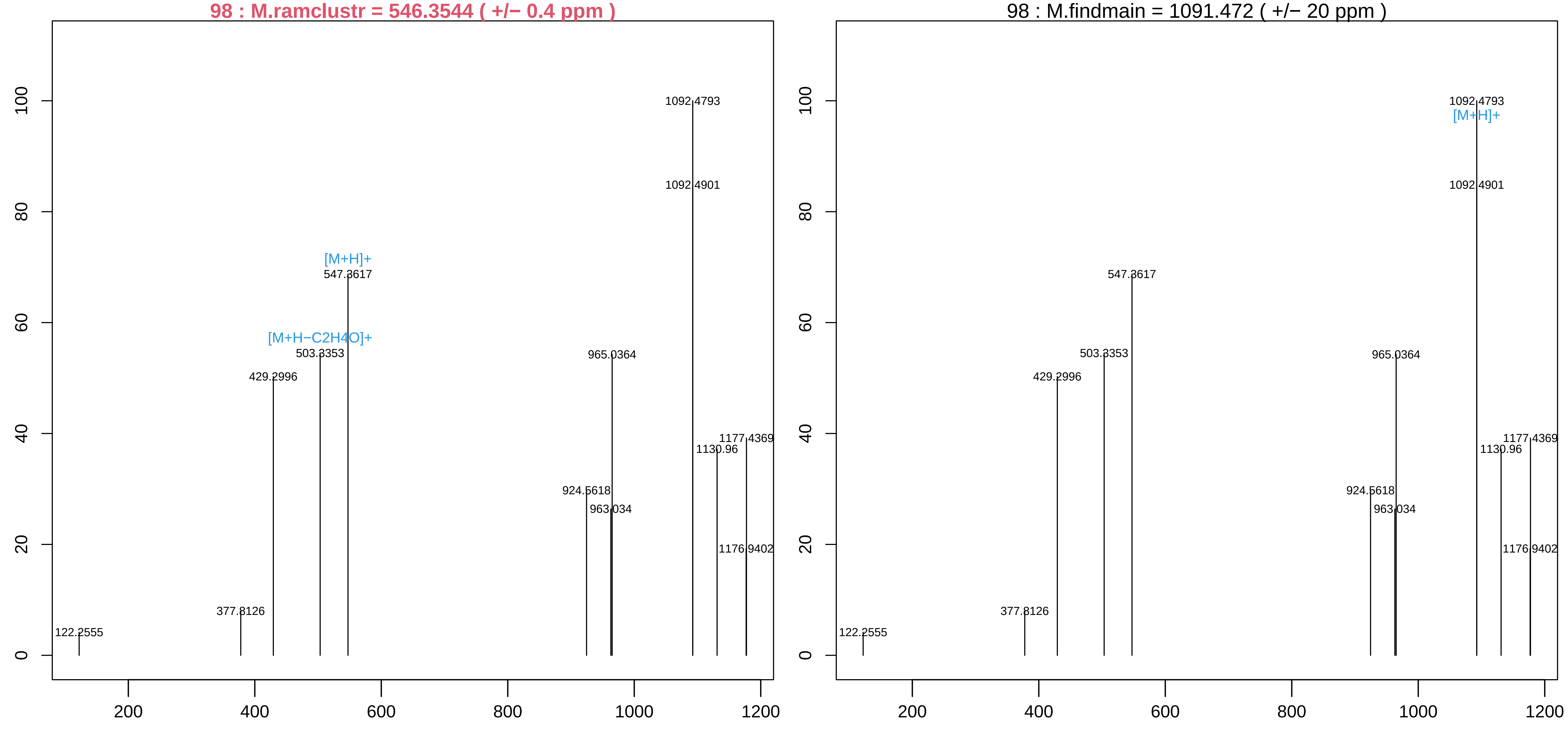
98 : M.ramclustr = 546.3544 (+/− 0.4 ppm)
[M+H]+
547.3617
[M+H−C2H4O]+
503.3353
429.2996
377.8126
122.2555
965.0364
924.5618
963.034
1092.4793
1092.4901
1130.96
1177.4369
1176.9402
98 : M.findmain = 1091.472 (+/− 20 ppm)
1092.4793
[M+H]+
1092.4901
547.3617
503.3353
429.2996
377.8126
122.2555
965.0364
924.5618
963.034
1130.96
1177.4369
1176.9402

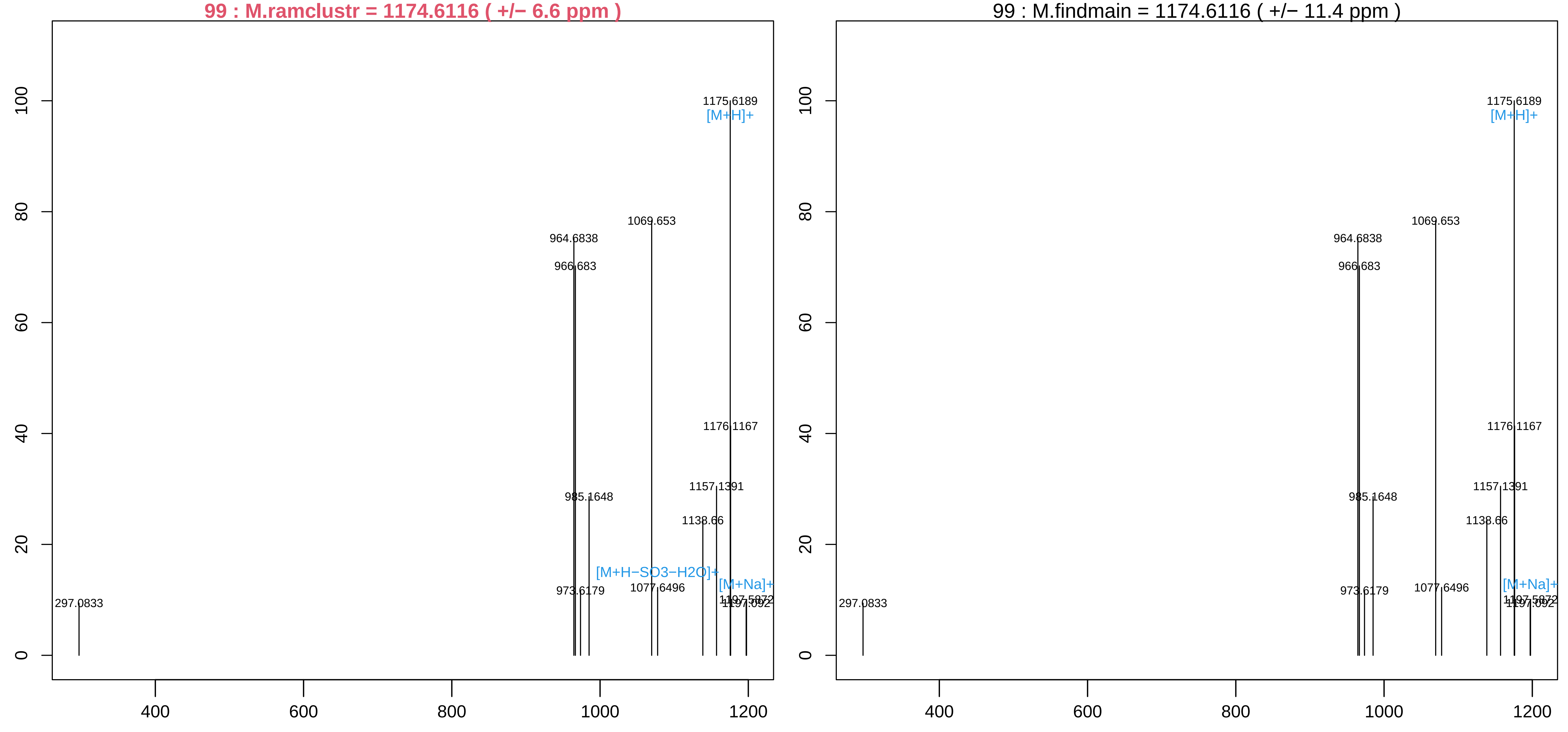

99 : M.ramclustr = 1174.6116 (+/− 6.6 ppm)
99 : M.findmain = 1174.6116 (+/− 11.4 ppm)
1175.6189
[M+H]+
1069.653
964.6838
966.683
1176.1167
1157.1391
985.1648
1138.66
[M+H−SO3−H2O]+
973.6179
1077.6496
[M+Na]+
1197.5872
1197.092
297.0833
0
20
40
60
80
100
400
600
800
1000
1200

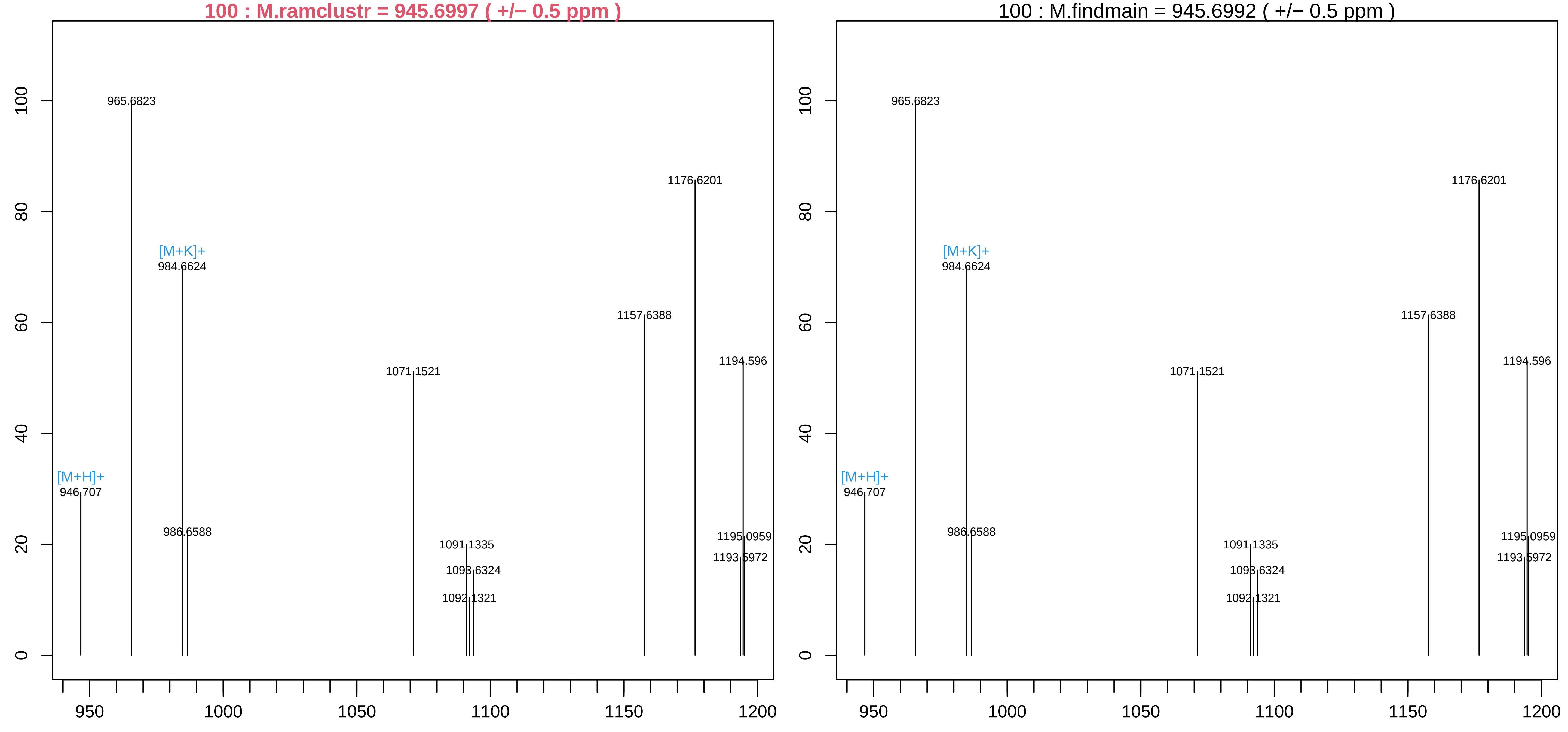

100 : M.ramclustr = 945.6997 (+/− 0.5 ppm)
965.6823
[M+K]+
984.6624
1176.6201
1157.6388
1194.596
1071.1521
[M+H]+
946.707
986.6588
1091.1335
1195.0959
1193.5972
1093.6324
1092.1321
0
20
40
60
80
100
950
1000
1050
1100
1150
1200
100 : M.findmain = 945.6992 (+/− 0.5 ppm)
965.6823
[M+K]+
984.6624
1176.6201
1157.6388
1194.596
1071.1521
[M+H]+
946.707
986.6588
1091.1335
1195.0959
1193.5972
1093.6324
1092.1321
0
20
40
60
80
100
950
1000
1050
1100
1150
1200

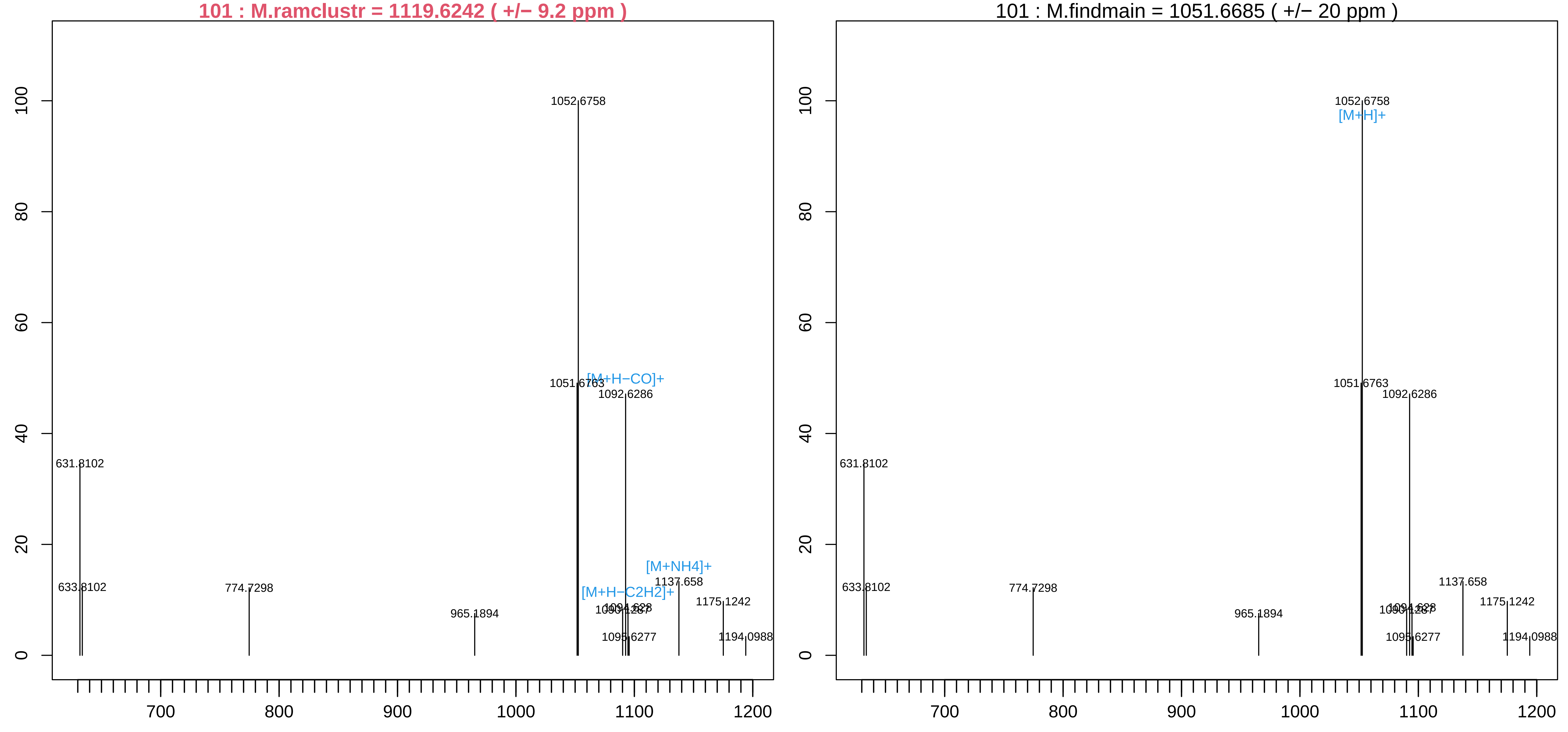
101 : M.ramclustr = 1119.6242 (+/− 9.2 ppm)
101 : M.findmain = 1051.6685 (+/− 20 ppm)
1052.6758
[M+H]+
1051.6763
[M+H−CO]+
1092.6286
631.8102
[M+NH4]+
1137.658
[M+H−C2H2]+
633.8102
774.7298
1175.1242
965.1894
1095.6277
1194.0988

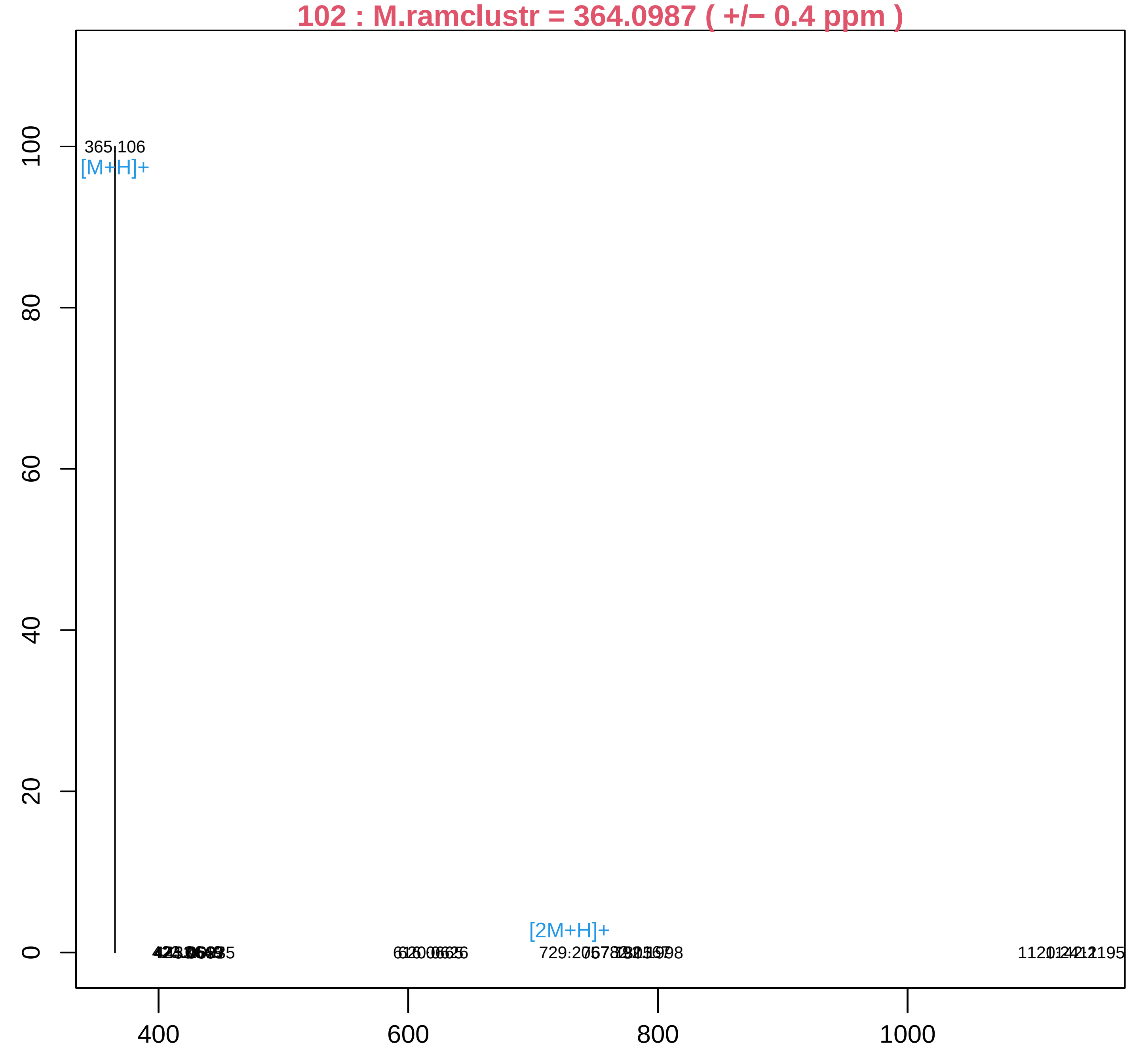
102 : M.ramclustr = 364.0987 (+/− 0.4 ppm)
365.106
[M+H]+
[2M+H]+
0
20
40
60
80
100
400
600
800
1000

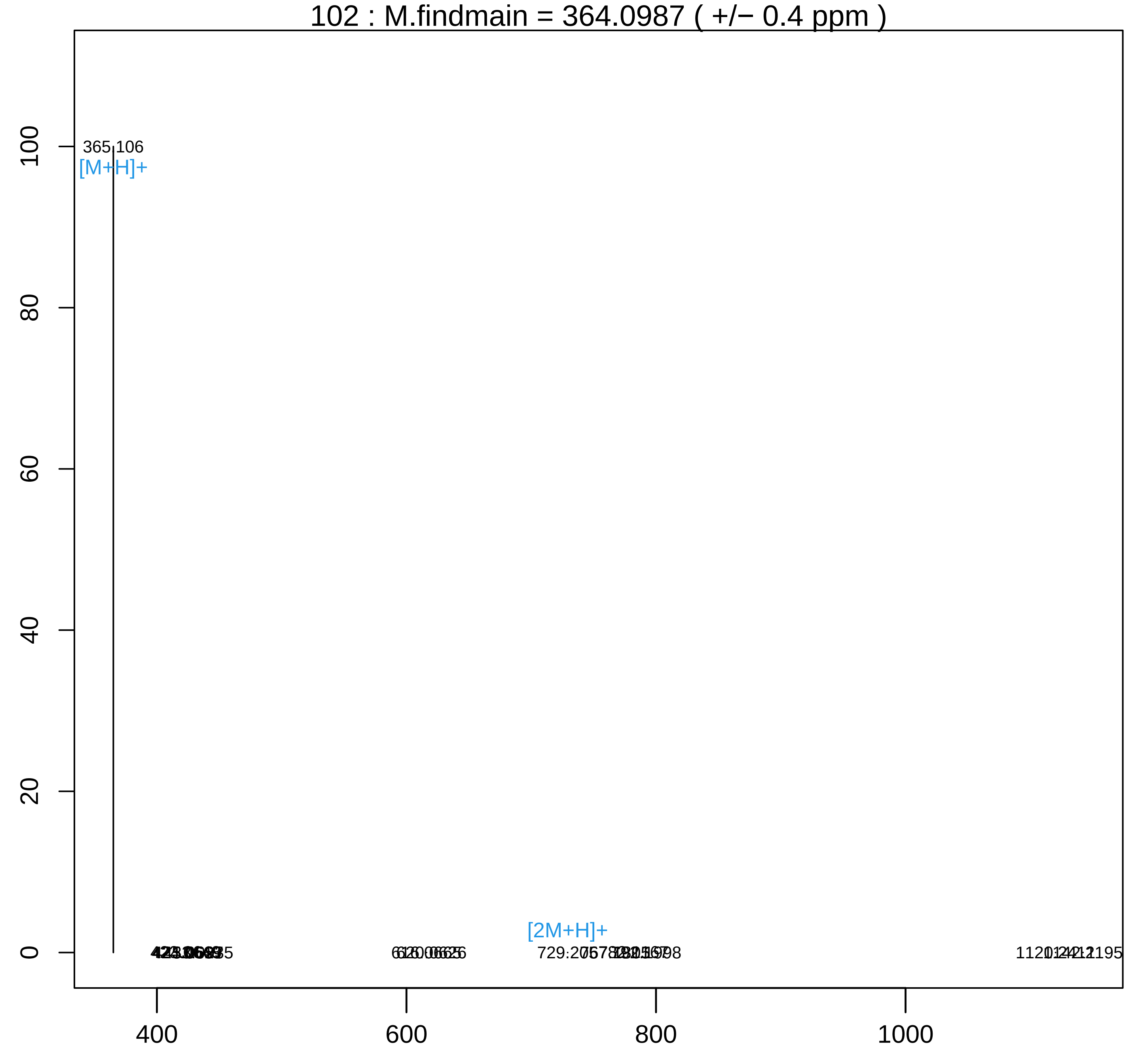
102 : M.findmain = 364.0987 (+/− 0.4 ppm)
365.106
[M+H]+
[2M+H]+
0
20
40
60
80
100
400
600
800
1000

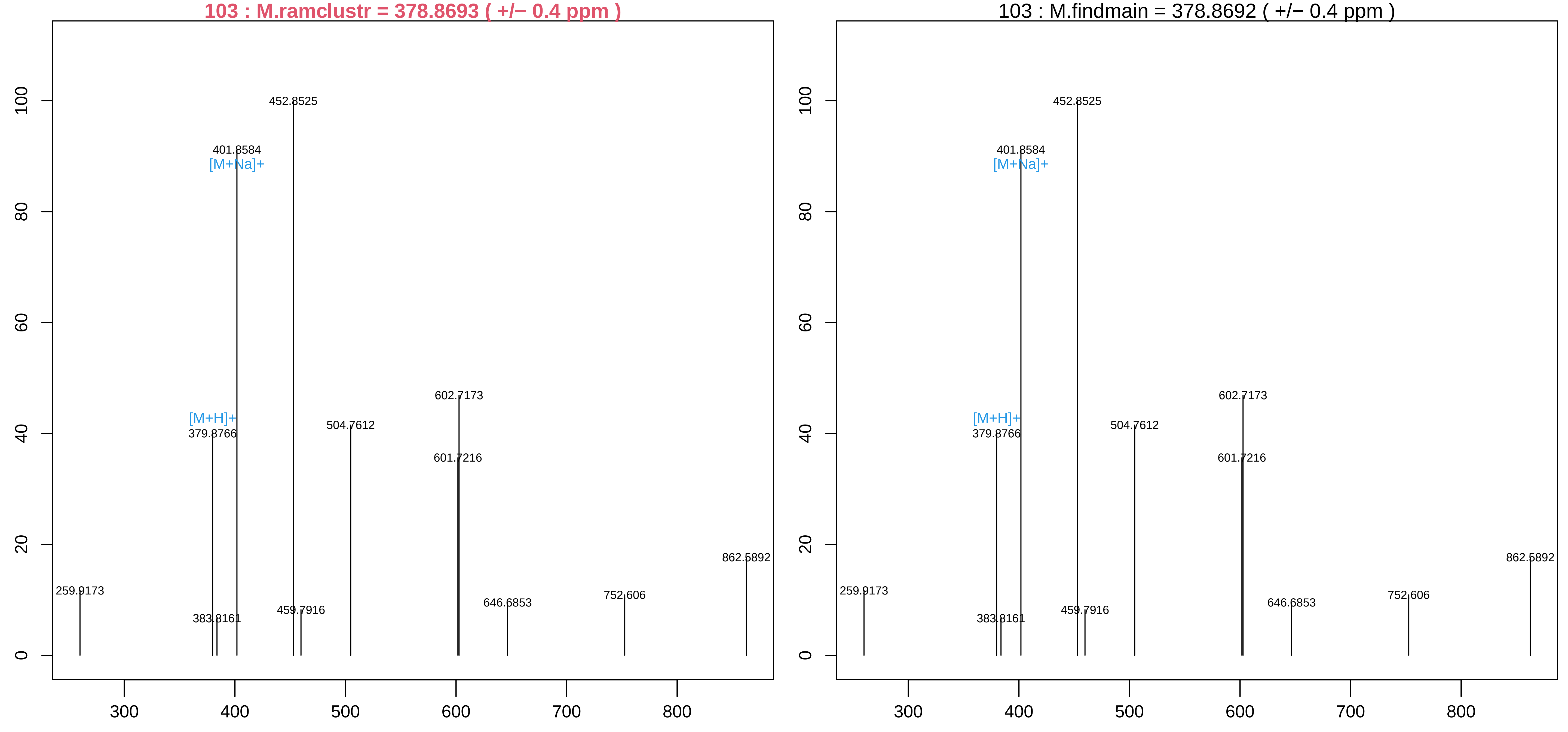

103 : M.ramclustr = 378.8693 (+/− 0.4 ppm)
452.8525
401.8584
[M+Na]+
[M+H]+
379.8766
504.7612
602.7173
601.7216
862.5892
259.9173
752.606
646.6853
459.7916
383.8161
103 : M.findmain = 378.8692 (+/− 0.4 ppm)

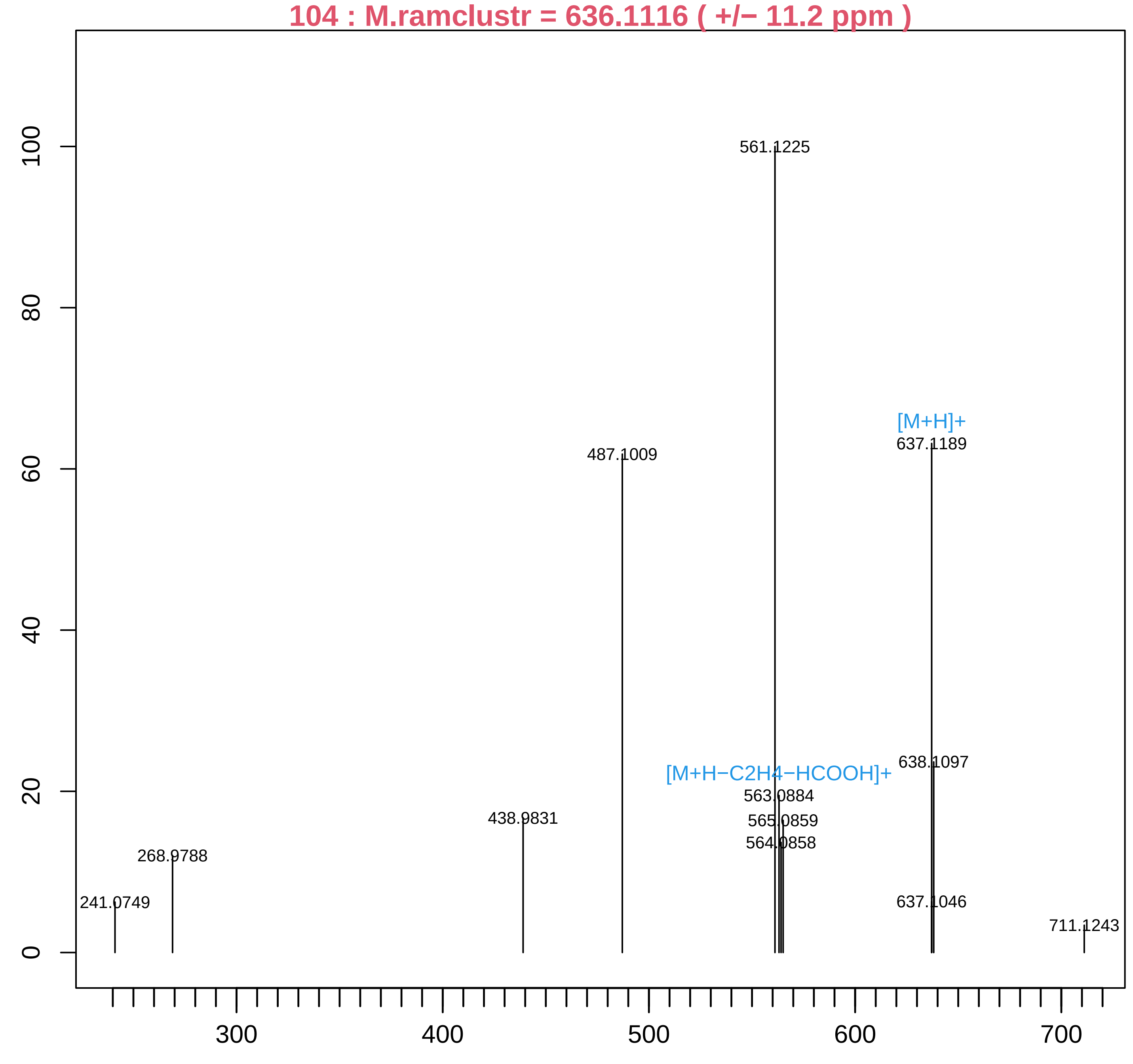
104 : M.ramclustr = 636.1116 (+/− 11.2 ppm)
561.1225
487.1009
[M+H]+
637.1189
[M+H−C2H4−HCOOH]+
563.0884
565.0859
564.0858
638.1097
438.9831
268.9788
241.0749
637.1046
711.1243
0
20
40
60
80
100
300
400
500
600
700

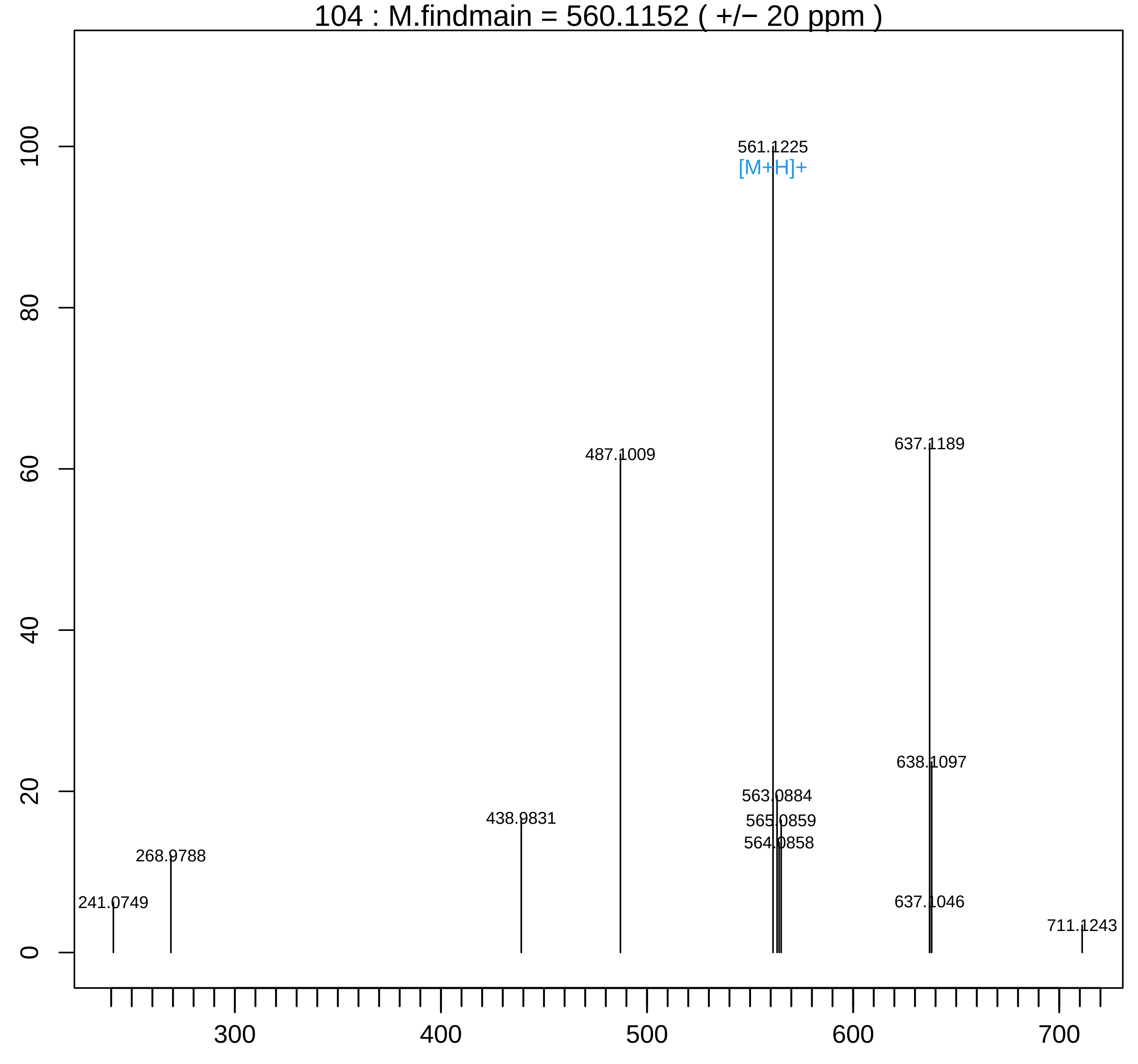
104 : M.findmain = 560.1152 (+/− 20 ppm)
561.1225
[M+H]+
487.1009
637.1189
563.0884
565.0859
564.0858
638.1097
438.9831
268.9788
241.0749
637.1046
711.1243
0
20
40
60
80
100
300
400
500
600
700

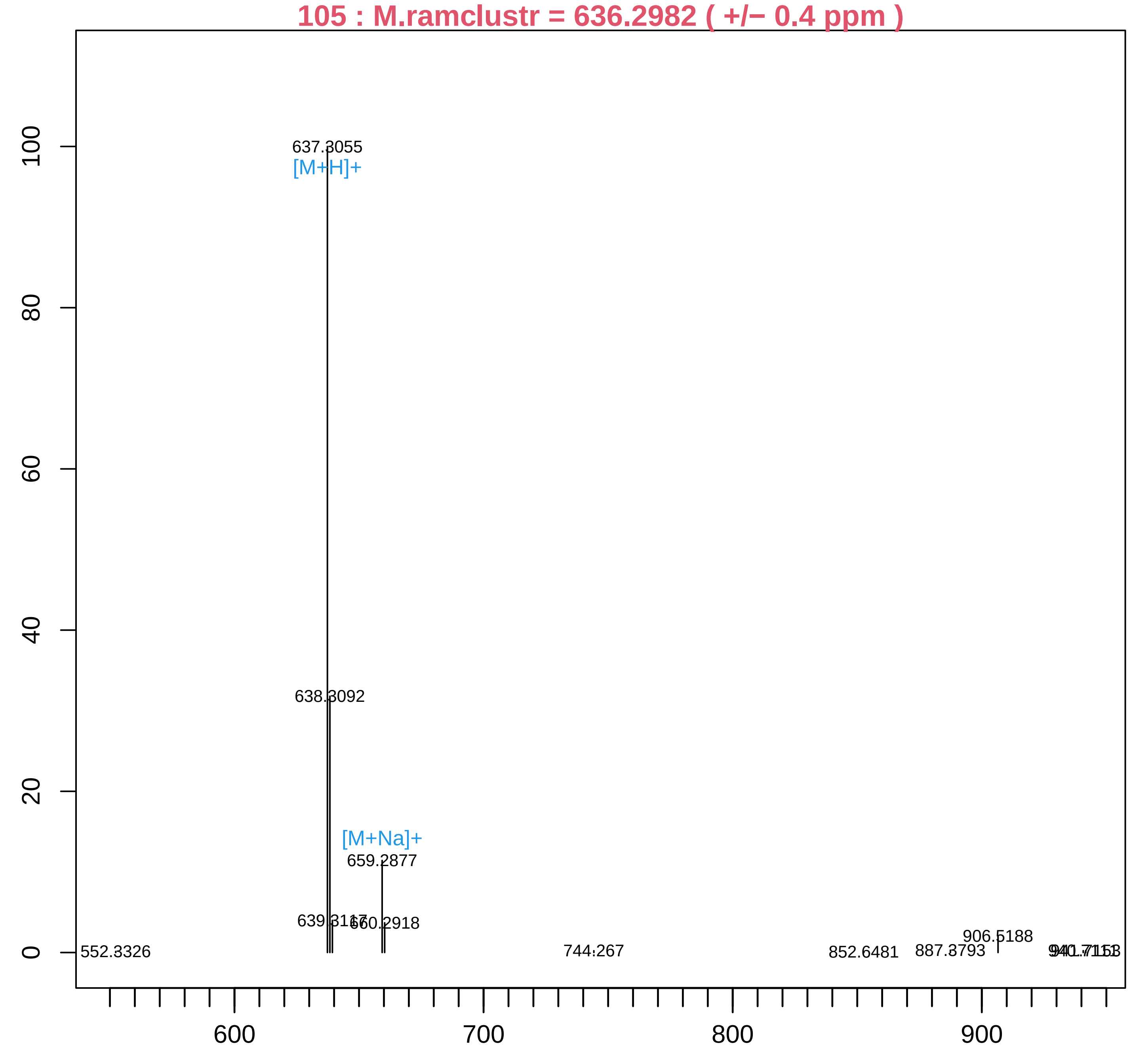
105 : M.ramclustr = 636.2982 (+/− 0.4 ppm)
637.3055
[M+H]+
638.3092
[M+Na]+
659.2877
639.3117
660.2918
552.3326
744.267
852.6481
887.8793
906.5188
941.7153
0
20
40
60
80
100
600
700
800
900

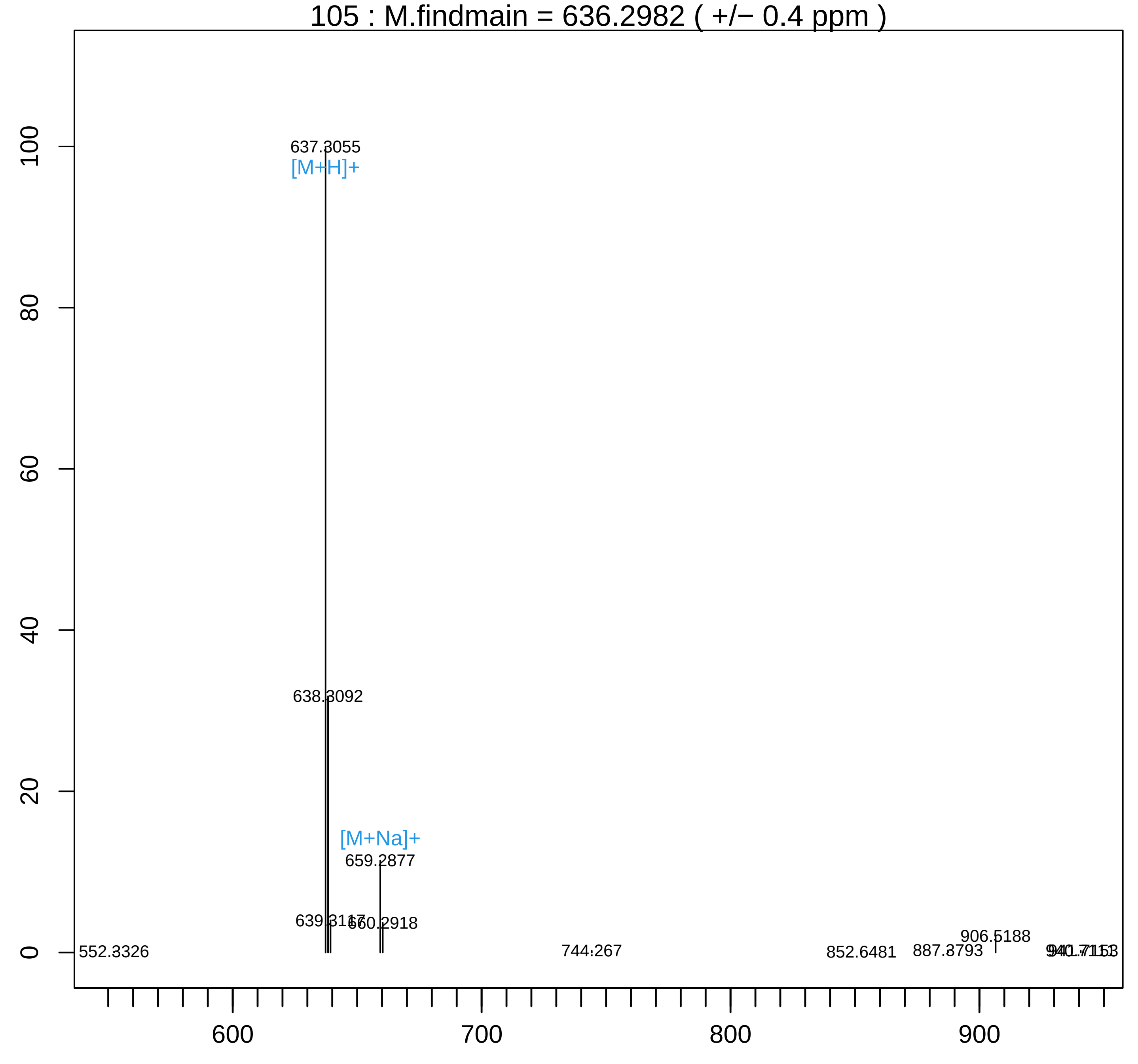
105 : M.findmain = 636.2982 (+/− 0.4 ppm)
637.3055
[M+H]+
638.3092
[M+Na]+
659.2877
639.3117
660.2918
552.3326
744.267
852.6481
887.8793
906.5188
941.7153
0
20
40
60
80
100
600
700
800
900

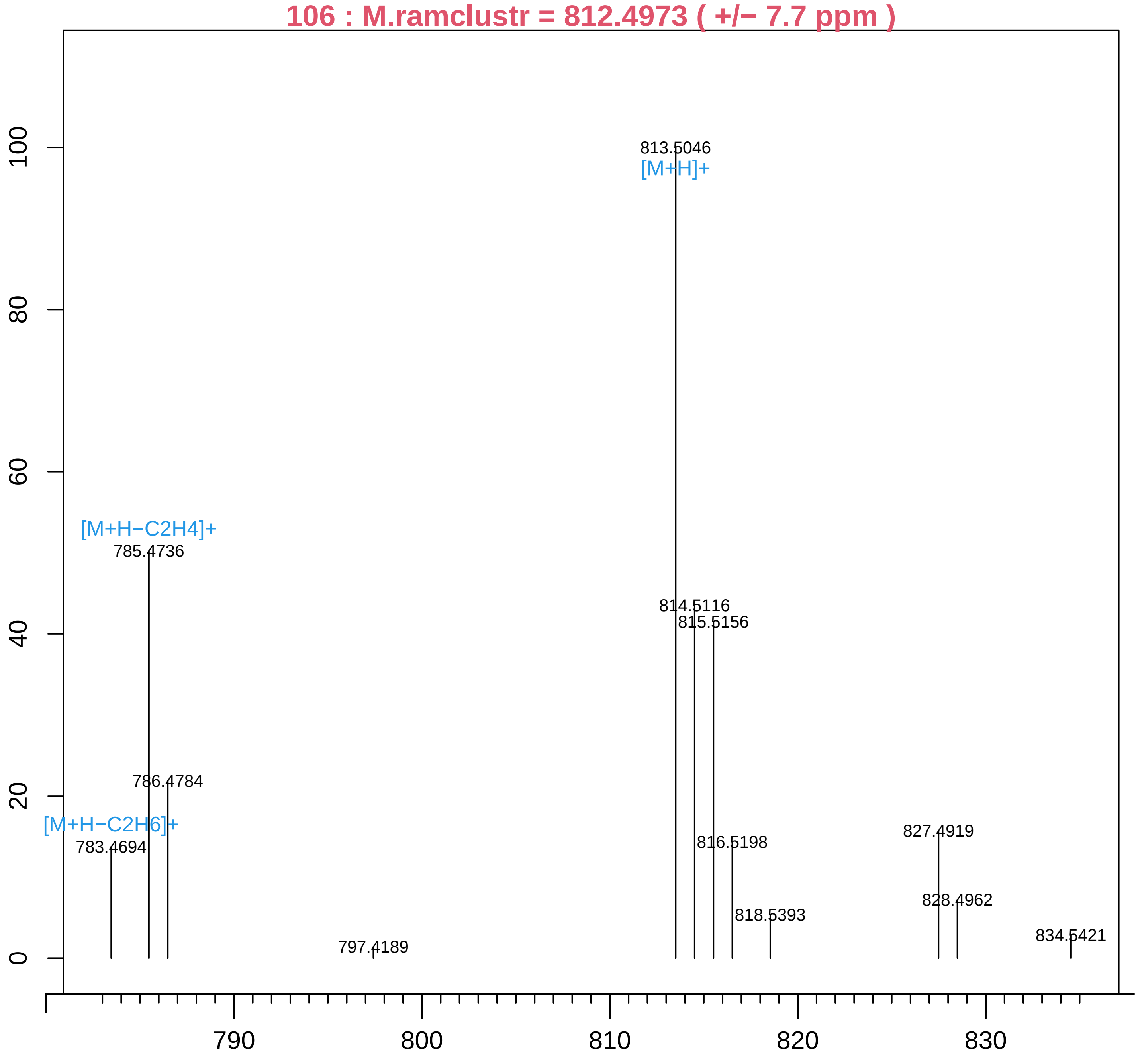
106 : M.ramclustr = 812.4973 (+/− 7.7 ppm)
813.5046
[M+H]+
[M+H−C2H4]+
785.4736
814.5116
815.5156
786.4784
[M+H−C2H6]+
783.4694
816.5198
827.4919
828.4962
818.5393
797.4189
834.5421
0
20
40
60
80
100
790
800
810
820
830
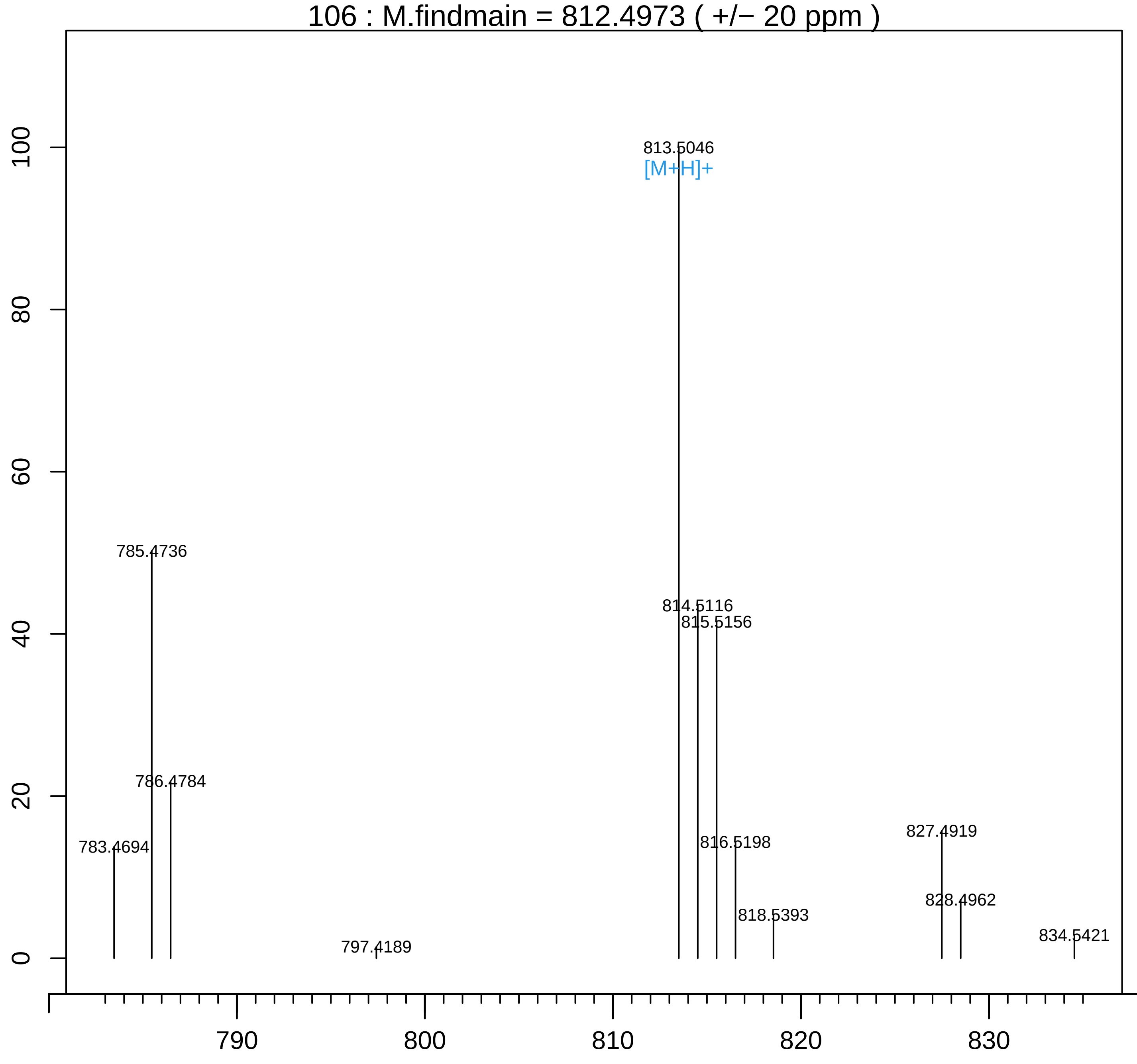
106 : M.findmain = 812.4973 (+/− 20 ppm)
813.5046
[M+H]+
785.4736
814.5116
815.5156
786.4784
783.4694
816.5198
827.4919
828.4962
818.5393
797.4189
834.5421
0
20
40
60
80
100
790
800
810
820
830

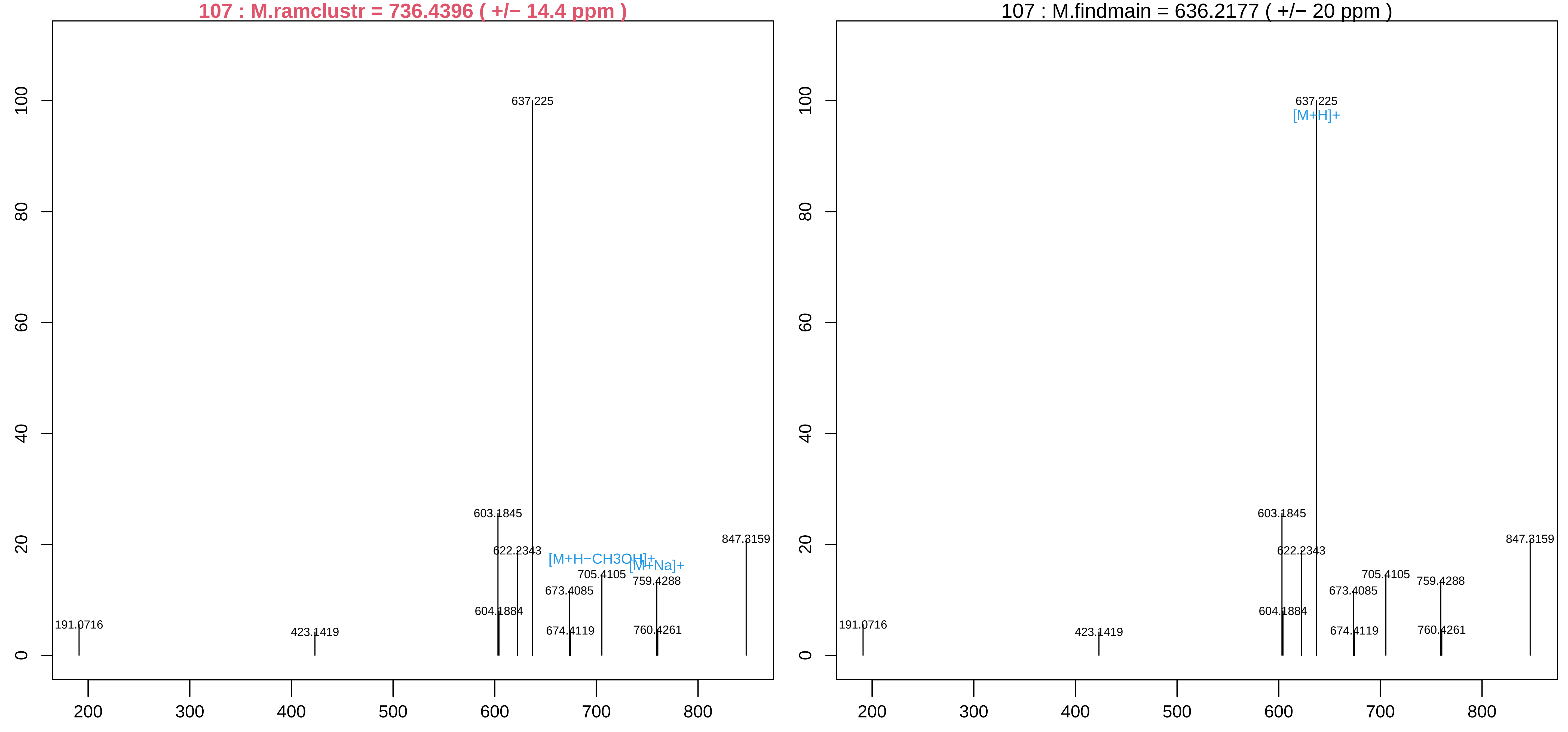
107 : M.ramclustr = 736.4396 (+/− 14.4 ppm)
637.225
603.1845
622.2343
[M+H−CH3OH]+
[M+Na]+
705.4105
673.4085
759.4288
847.3159
604.1884
191.0716
423.1419
674.4119
760.4261
107 : M.findmain = 636.2177 (+/− 20 ppm)
637.225
[M+H]+
603.1845
622.2343
705.4105
673.4085
759.4288
847.3159
604.1884
191.0716
423.1419
674.4119
760.4261
0
20
40
60
80
100
200
300
400
500
600
700
800

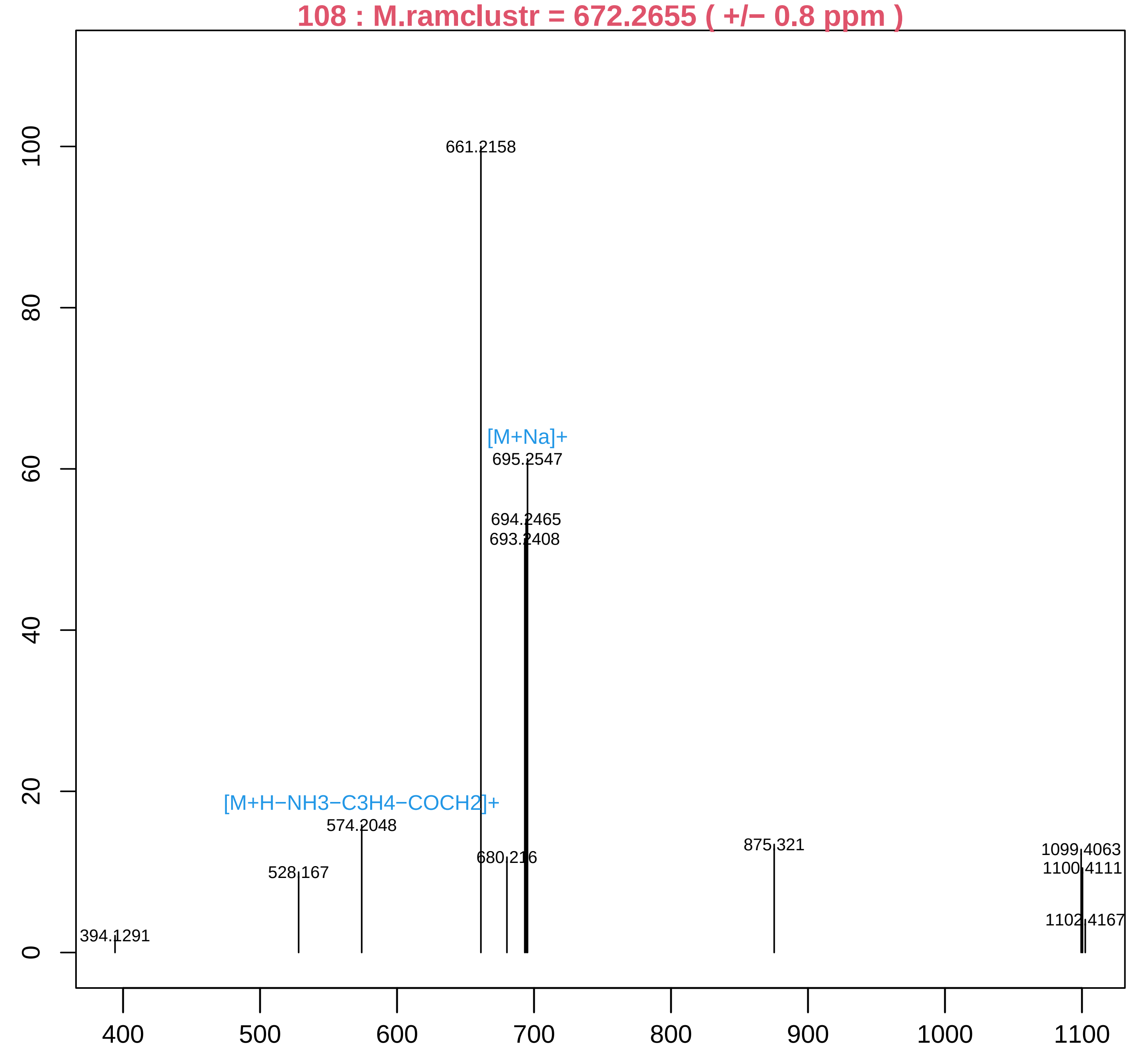
108 : M.ramclustr = 672.2655 (+/− 0.8 ppm)
661.2158
[M+Na]+
695.2547
694.2465
693.2408
[M+H−NH3−C3H4−COCH2]+
574.2048
680.216
875.321
528.167
1099.4063
1100.4111
1102.4167
394.1291
0
20
40
60
80
100
400
500
600
700
800
900
1000
1100

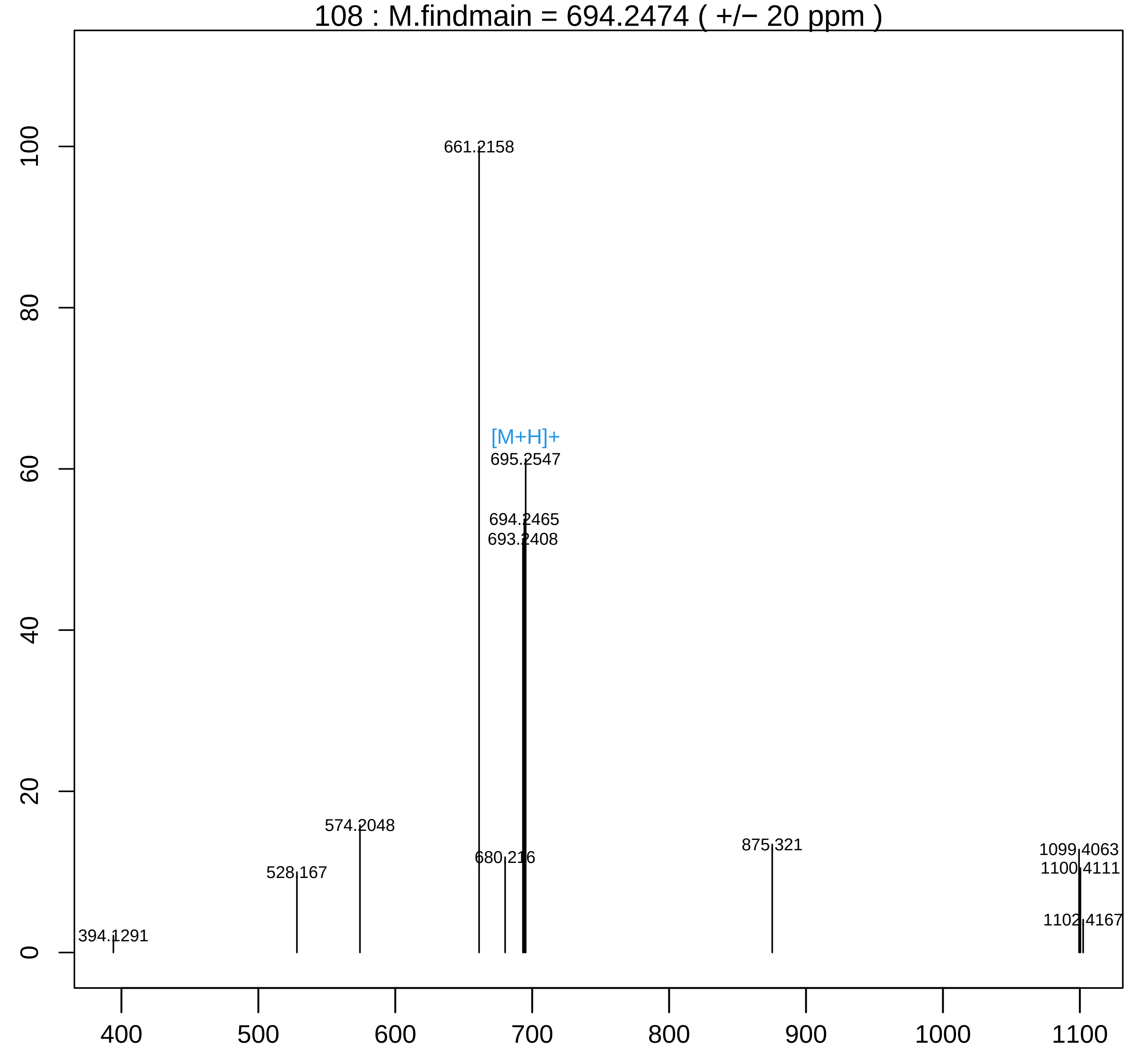
108 : M.findmain = 694.2474 (+/− 20 ppm)
661.2158
[M+H]+
695.2547
694.2465
693.2408
574.2048
680.216
875.321
528.167
1099.4063
1100.4111
1102.4167
394.1291
0
20
40
60
80
100
400
500
600
700
800
900
1000
1100

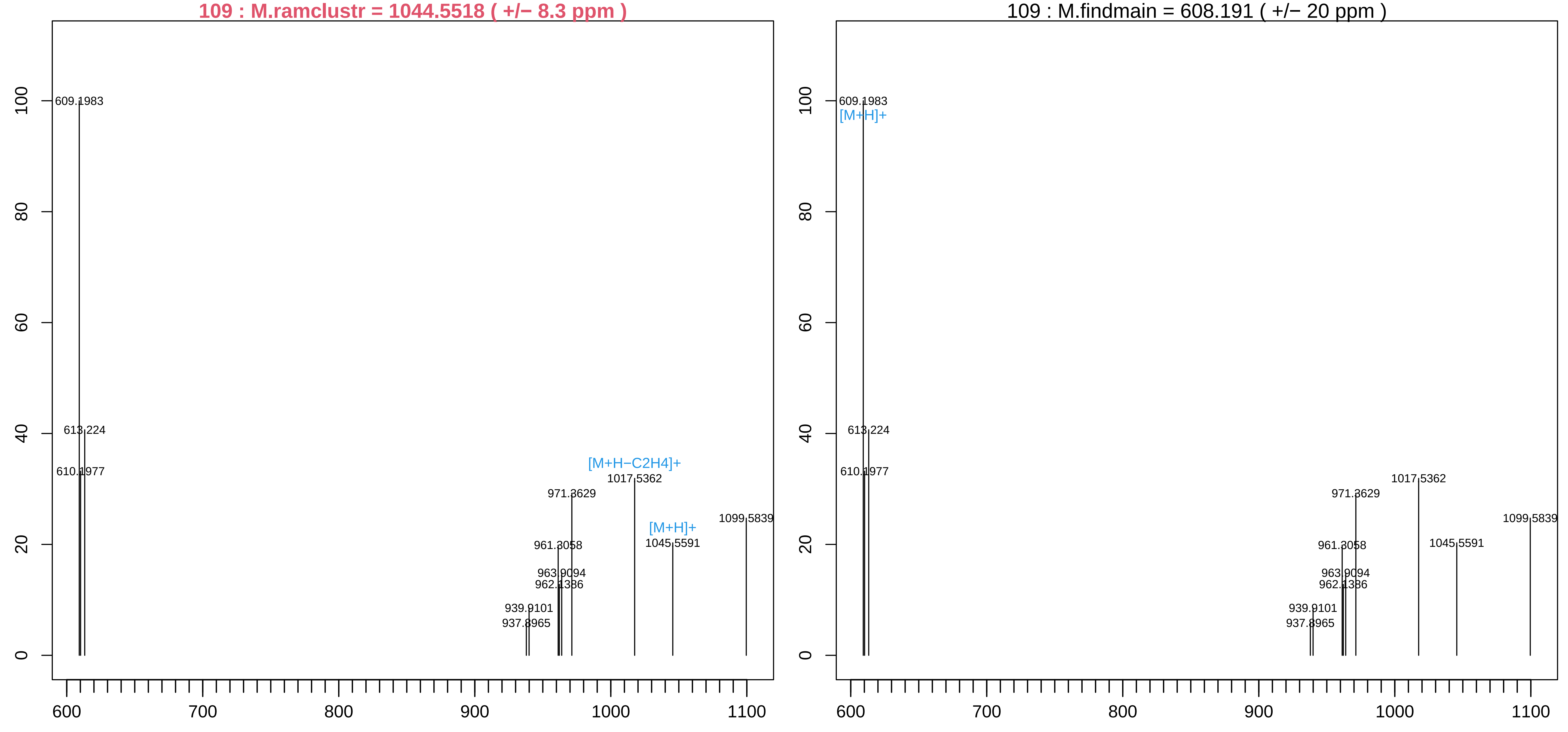
109 : M.ramclustr = 1044.5518 (+/− 8.3 ppm)
609.1983
613.224
610.1977
[M+H−C2H4]+
1017.5362
971.3629
[M+H]+
1045.5591
1099.5839
961.3058
963.9094
962.1386
939.9101
937.8965
109 : M.findmain = 608.191 (+/− 20 ppm)
609.1983
[M+H]+
613.224
610.1977
1017.5362
971.3629
1099.5839
961.3058
1045.5591
963.9094
962.1386
939.9101
937.8965
0
20
40
60
80
100
600
700
800
900
1000
1100

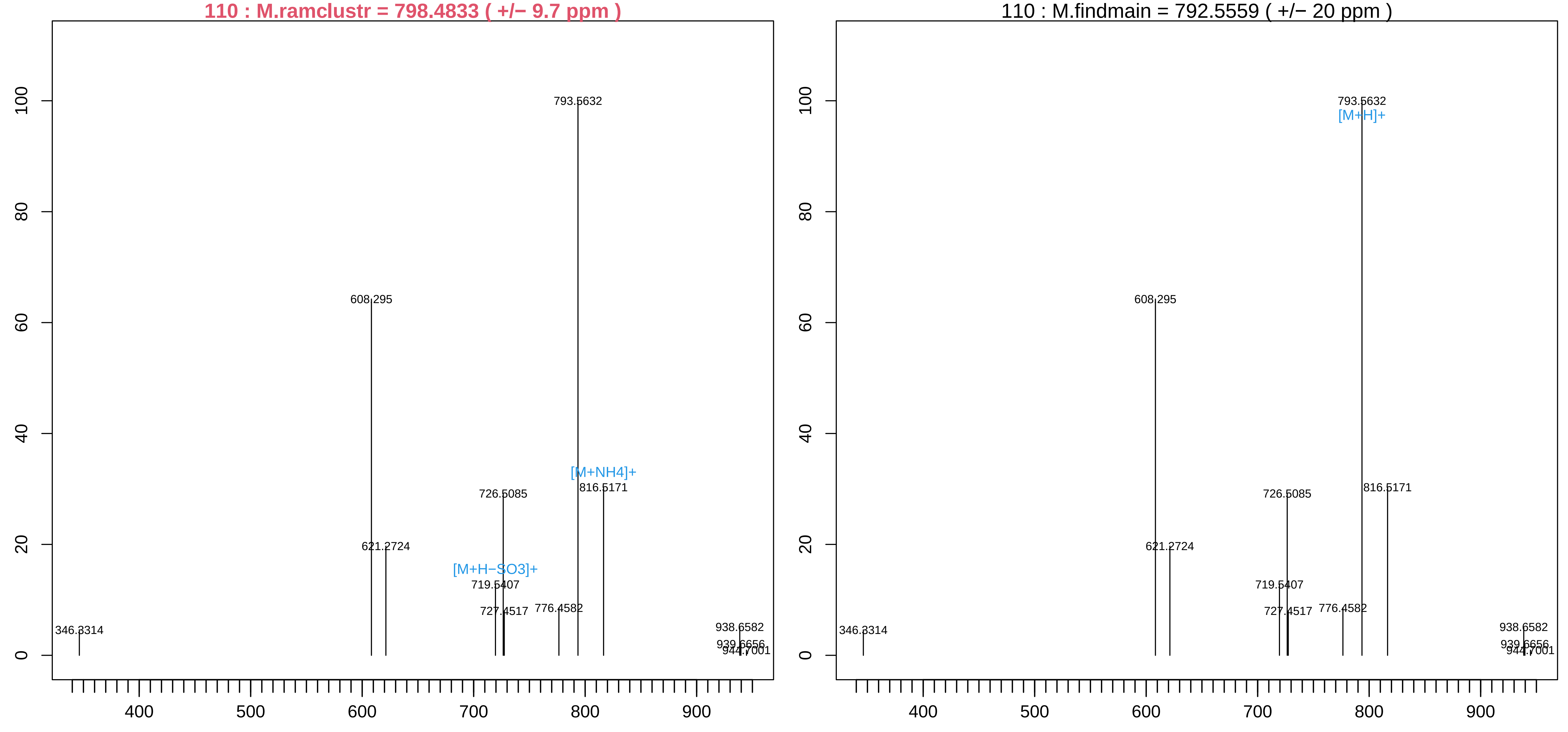

110 : M.ramclustr = 798.4833 (+/− 9.7 ppm)
110 : M.findmain = 792.5559 (+/− 20 ppm)
793.5632
[M+H]+
608.295
621.2724
726.5085
719.5407
727.4517
776.4582
816.5171
[M+NH4]+
[M+H−SO3]+
346.3314
938.6582
939.6656
944.7001
0
20
40
60
80
100
400
500
600
700
800
900

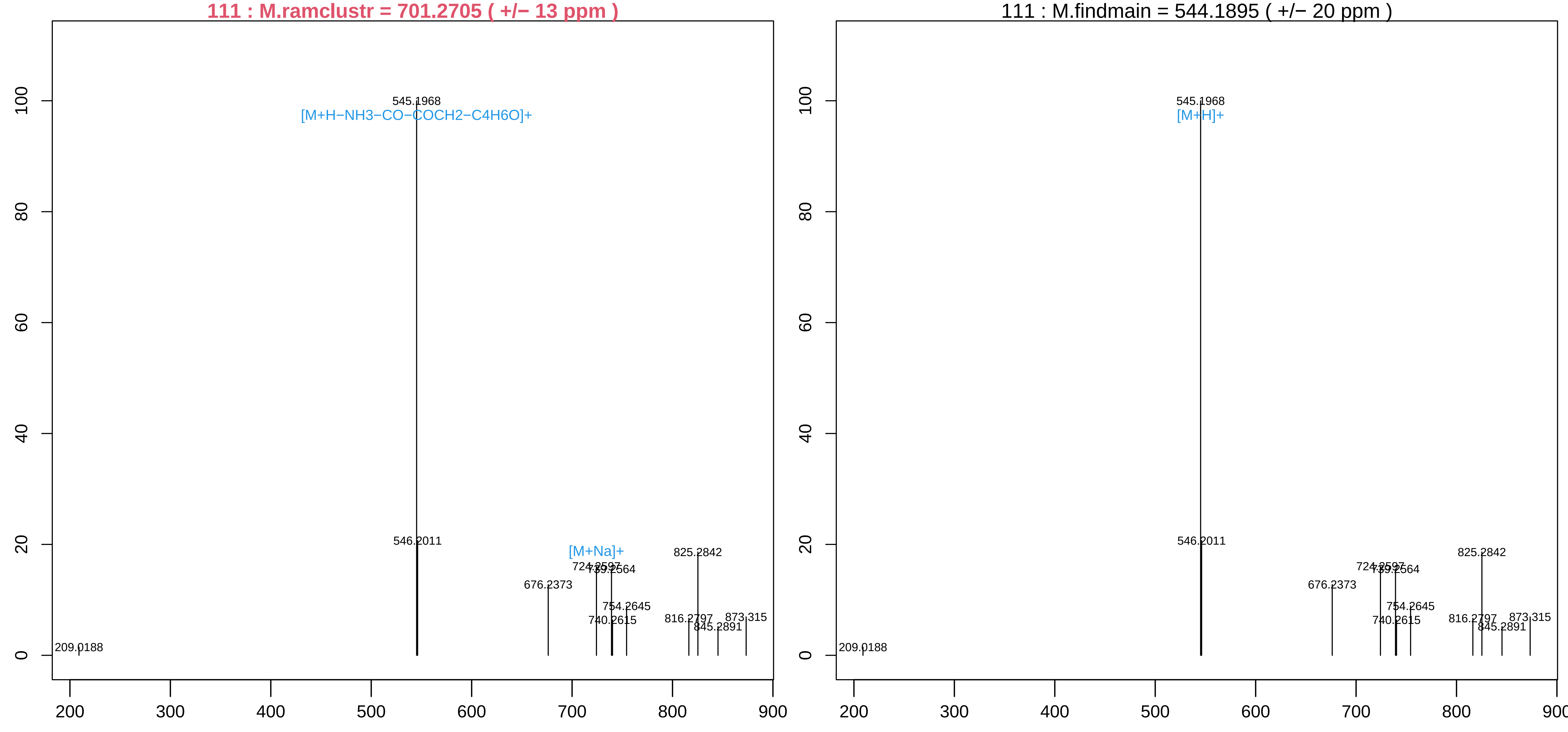
111 : M.ramclustr = 701.2705 (+/− 13 ppm)
545.1968
[M+H−NH3−CO−COCH2−C4H6O]+
546.2011
[M+Na]+
724.2597
739.2564
676.2373
754.2645
740.2615
825.2842
816.2797
845.2891
873.315
209.0188
111 : M.findmain = 544.1895 (+/− 20 ppm)
545.1968
[M+H]+
546.2011
724.2597
739.2564
676.2373
754.2645
740.2615
825.2842
816.2797
845.2891
873.315
209.0188
0
20
40
60
80
100
200
300
400
500
600
700
800
900

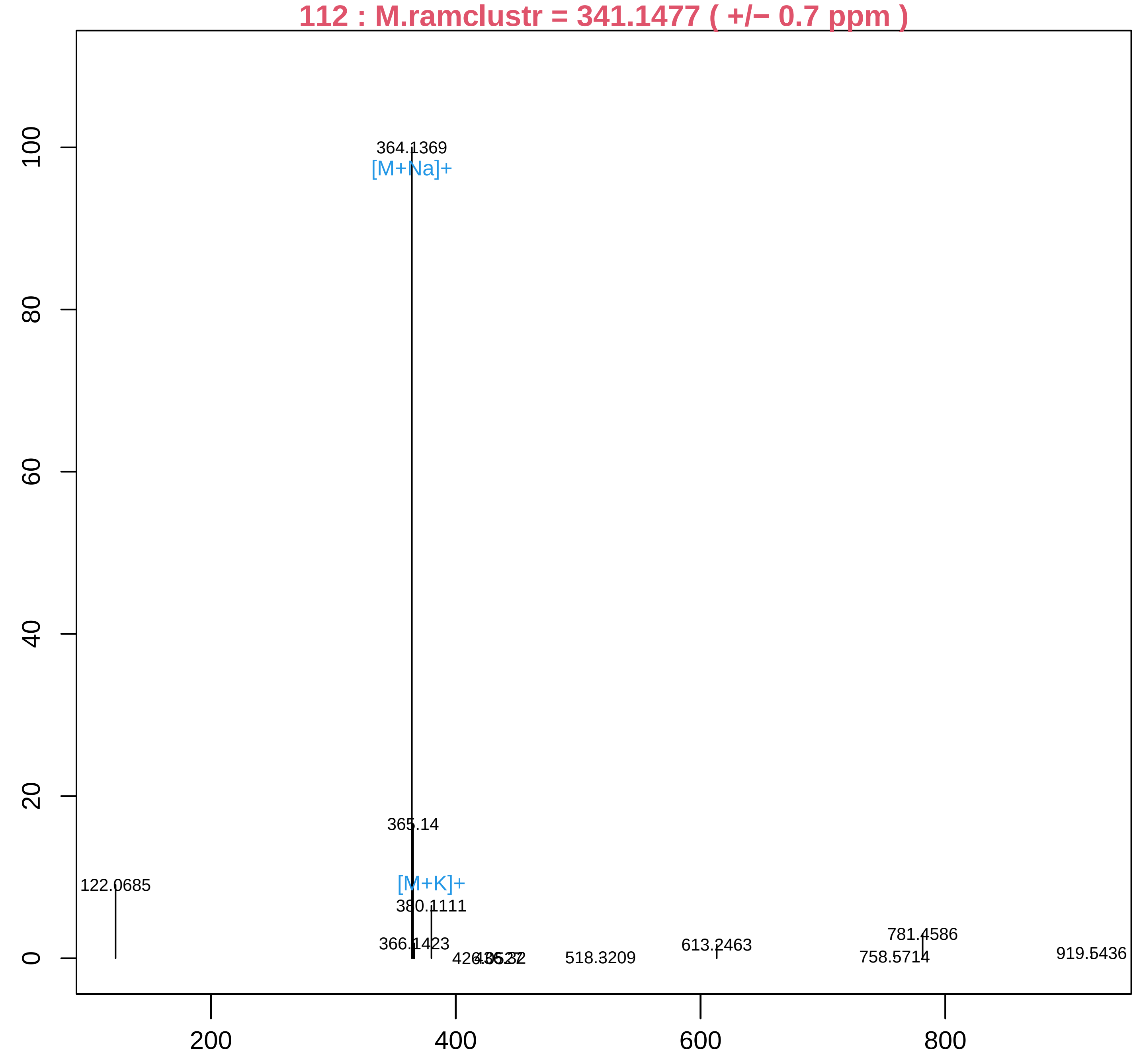
112 : M.ramclustr = 341.1477 (+/− 0.7 ppm)
364.1369
[M+Na]+
365.14
122.0685
[M+K]+
380.1111
366.1423
613.2463
781.4586
758.5714
919.5436
518.3209
0
20
40
60
80
100
200
400
600
800

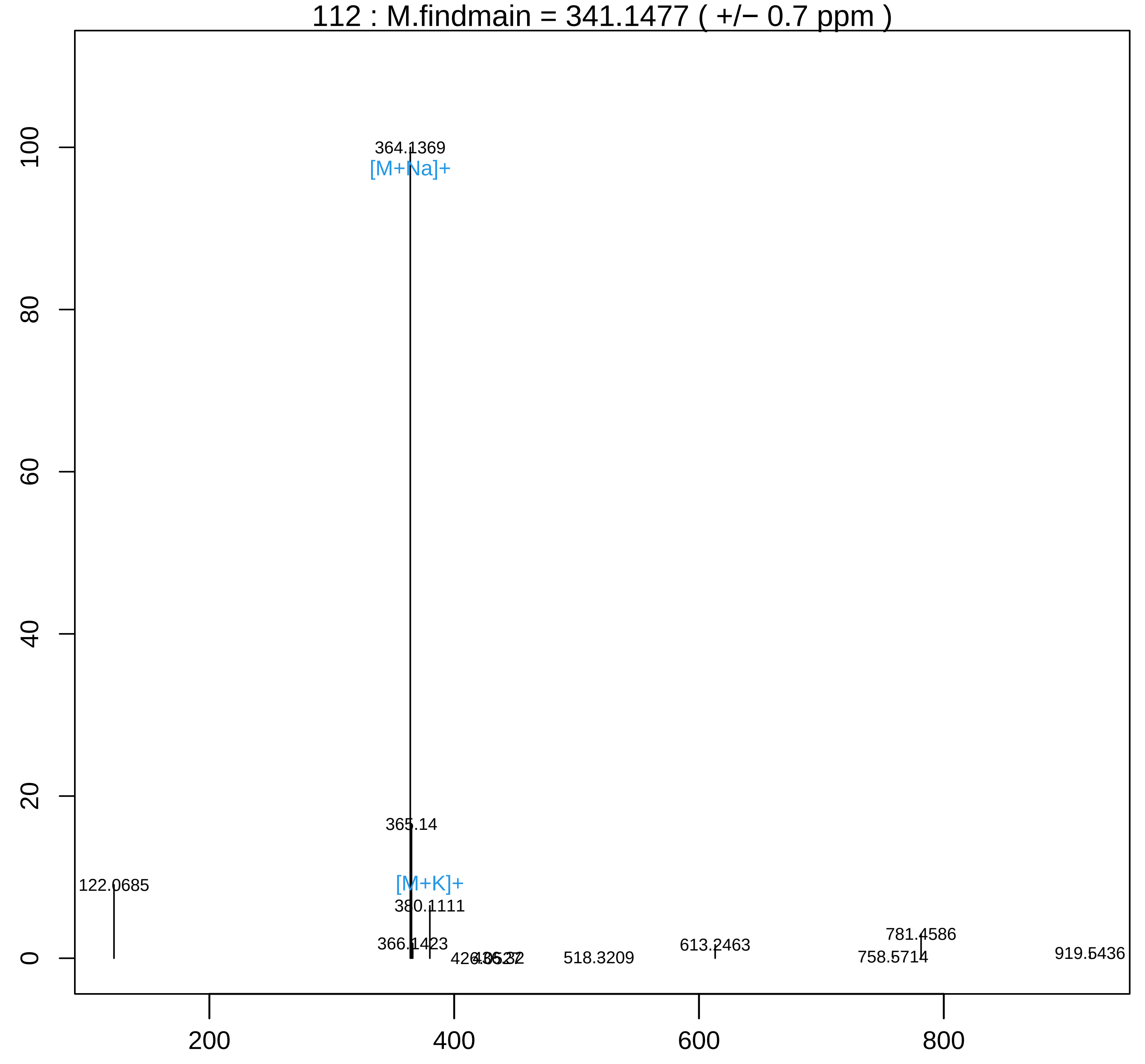
112 : M.findmain = 341.1477 (+/− 0.7 ppm)
364.1369
[M+Na]+
365.14
122.0685
[M+K]+
380.1111
366.1423
613.2463
781.4586
758.5714
919.5436
518.3209
0
20
40
60
80
100
200
400
600
800

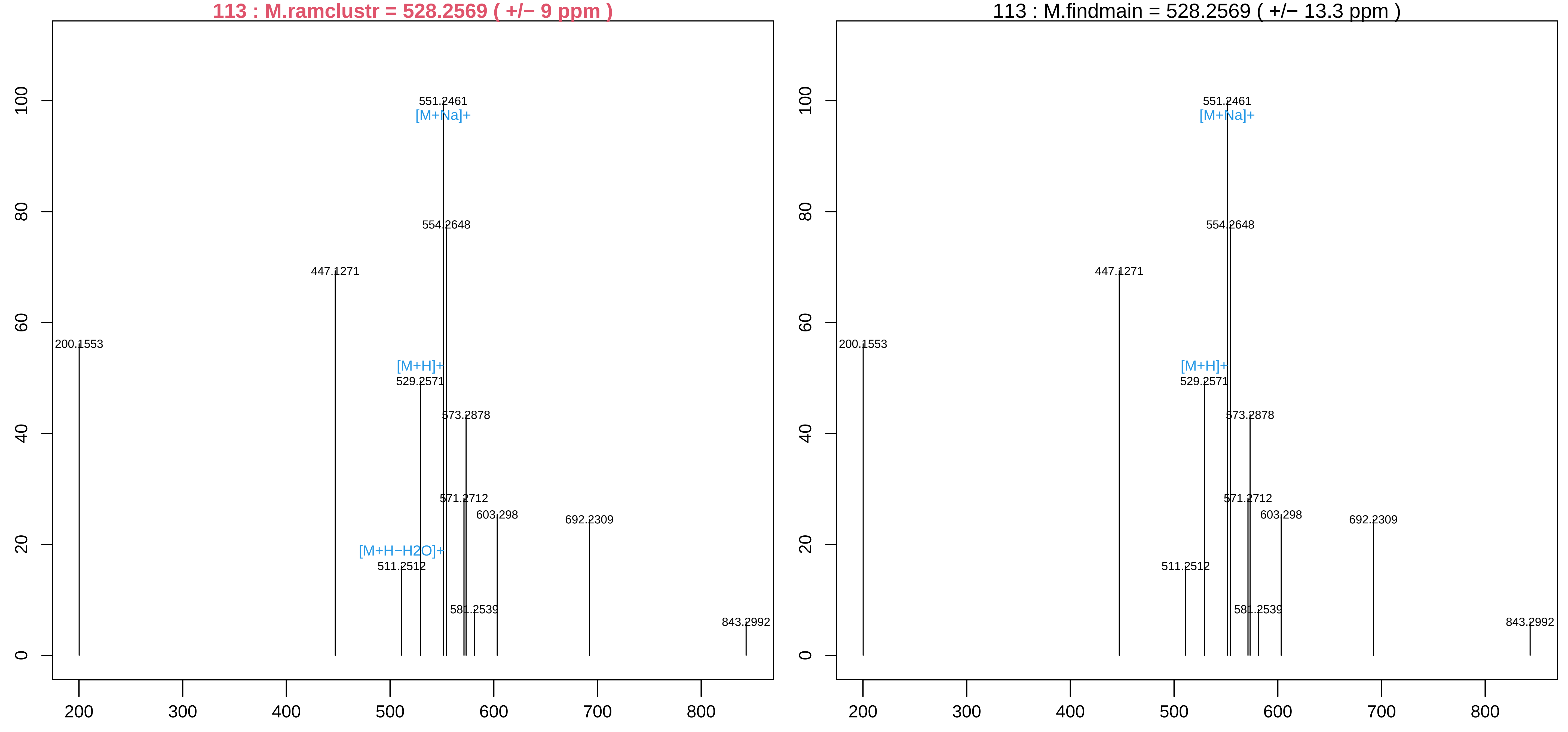

113 : M.ramclustr = 528.2569 (+/− 9 ppm)
551.2461
[M+Na]+
554.2648
447.1271
200.1553
[M+H]+
529.2571
573.2878
571.2712
603.298
692.2309
[M+H−H2O]+
511.2512
581.2539
843.2992
113 : M.findmain = 528.2569 (+/− 13.3 ppm)
0
20
40
60
80
100
200
300
400
500
600
700
800

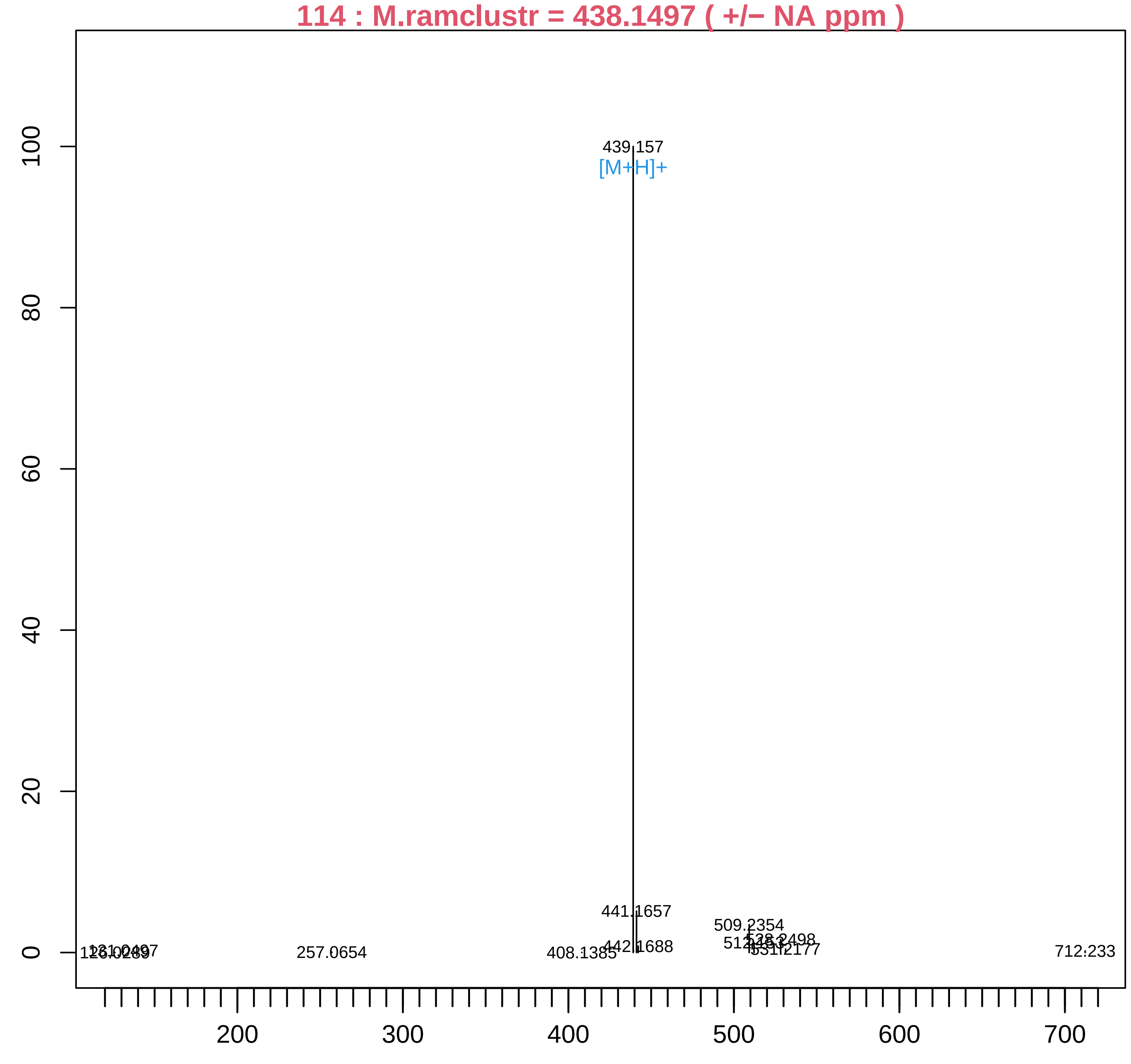
114 : M.ramclustr = 438.1497 (+/− NA ppm)
439.157
[M+H]+
441.1657
442.1688
509.2354
528.2498
531.2177
257.0654
408.1385
712.233
0
20
40
60
80
100
200
300
400
500
600
700

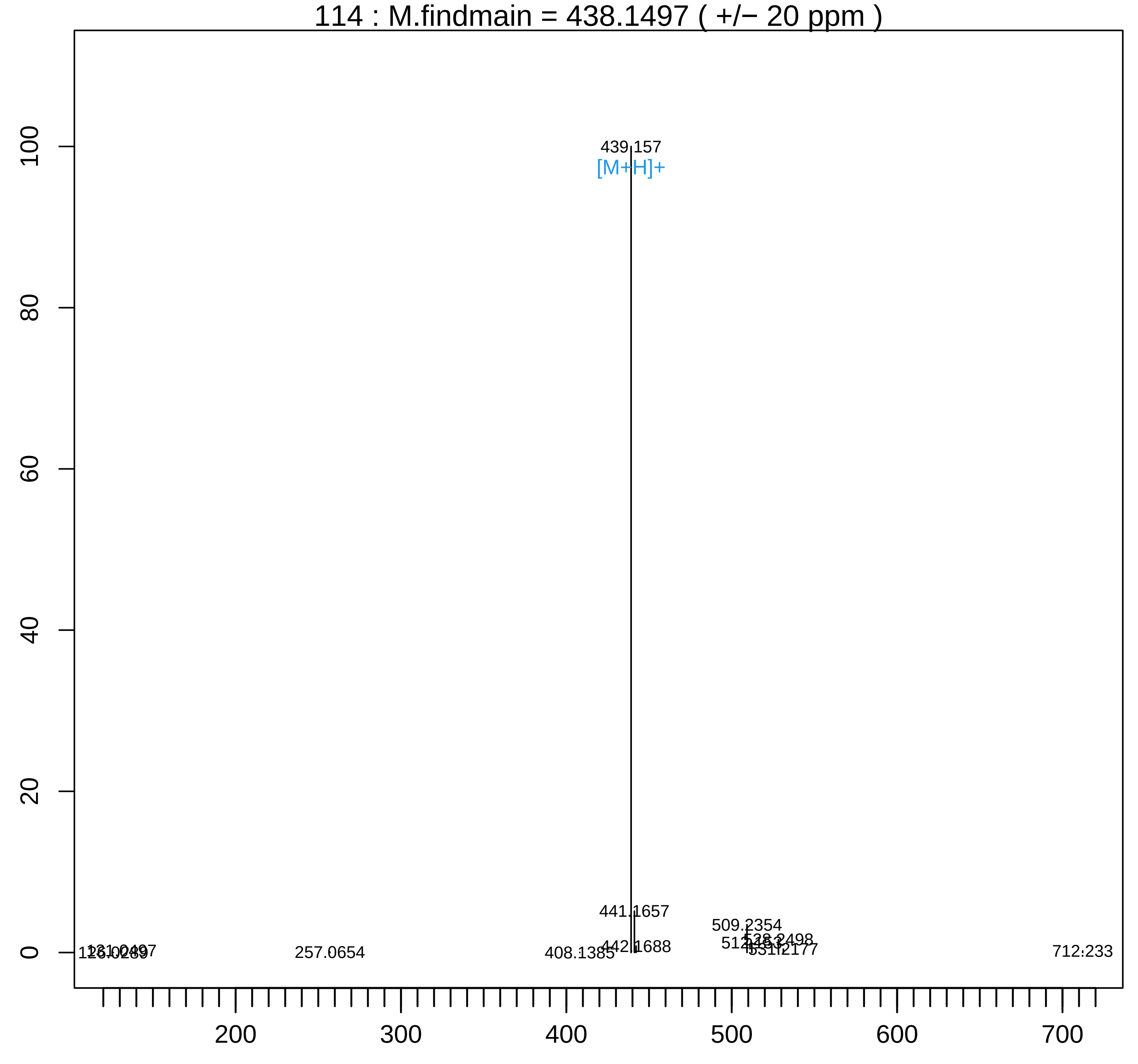
114 : M.findmain = 438.1497 (+/− 20 ppm)
439.157
[M+H]+
441.1657
442.1688
509.2354
528.2498
531.2177
257.0654
408.1385
712.233
0
20
40
60
80
100
200
300
400
500
600
700

115 : M.ramclustr = 182.1101 (+/− 2.3 ppm)

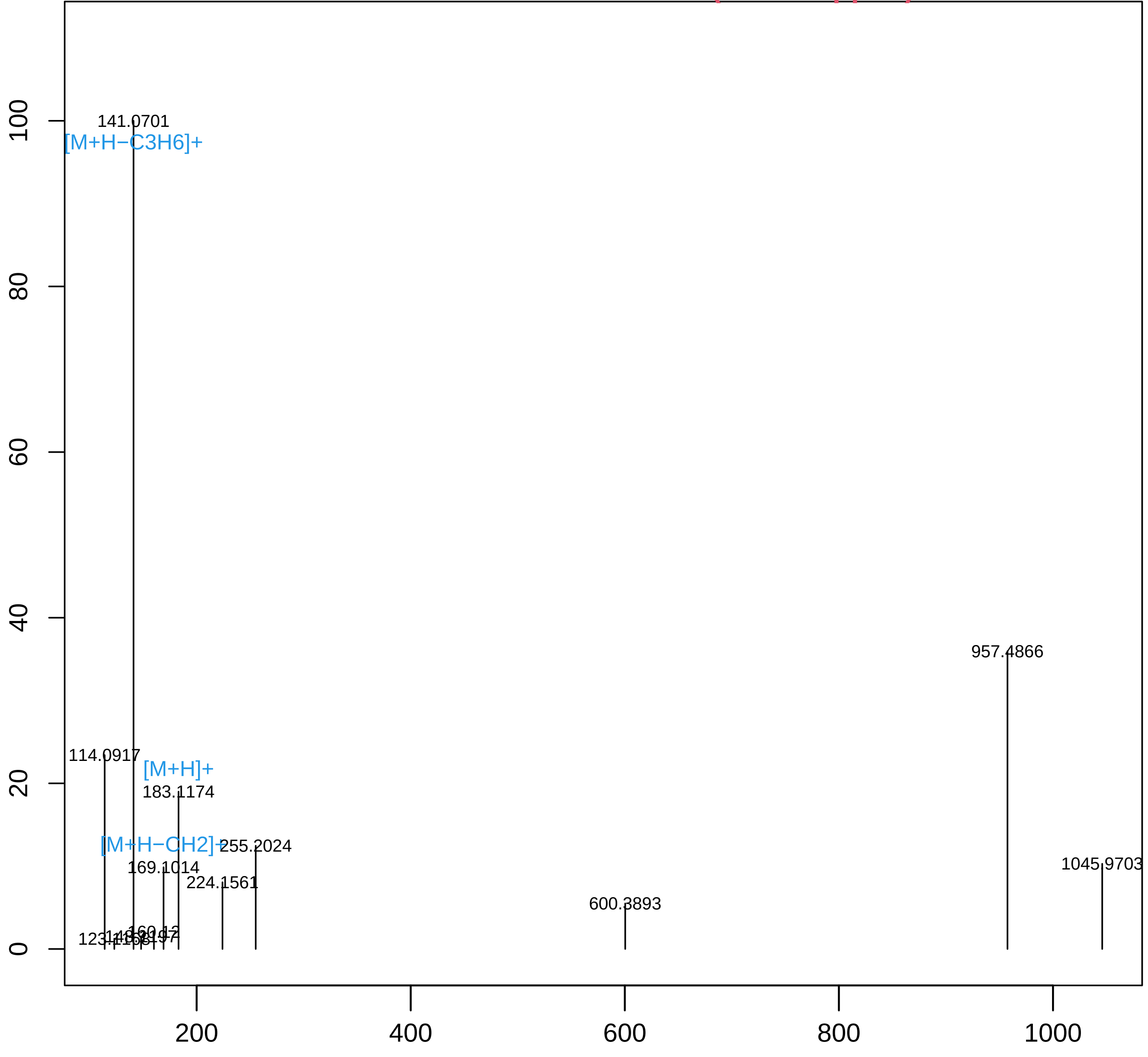

115 : M.findmain = 140.0628 (+/− 20 ppm)

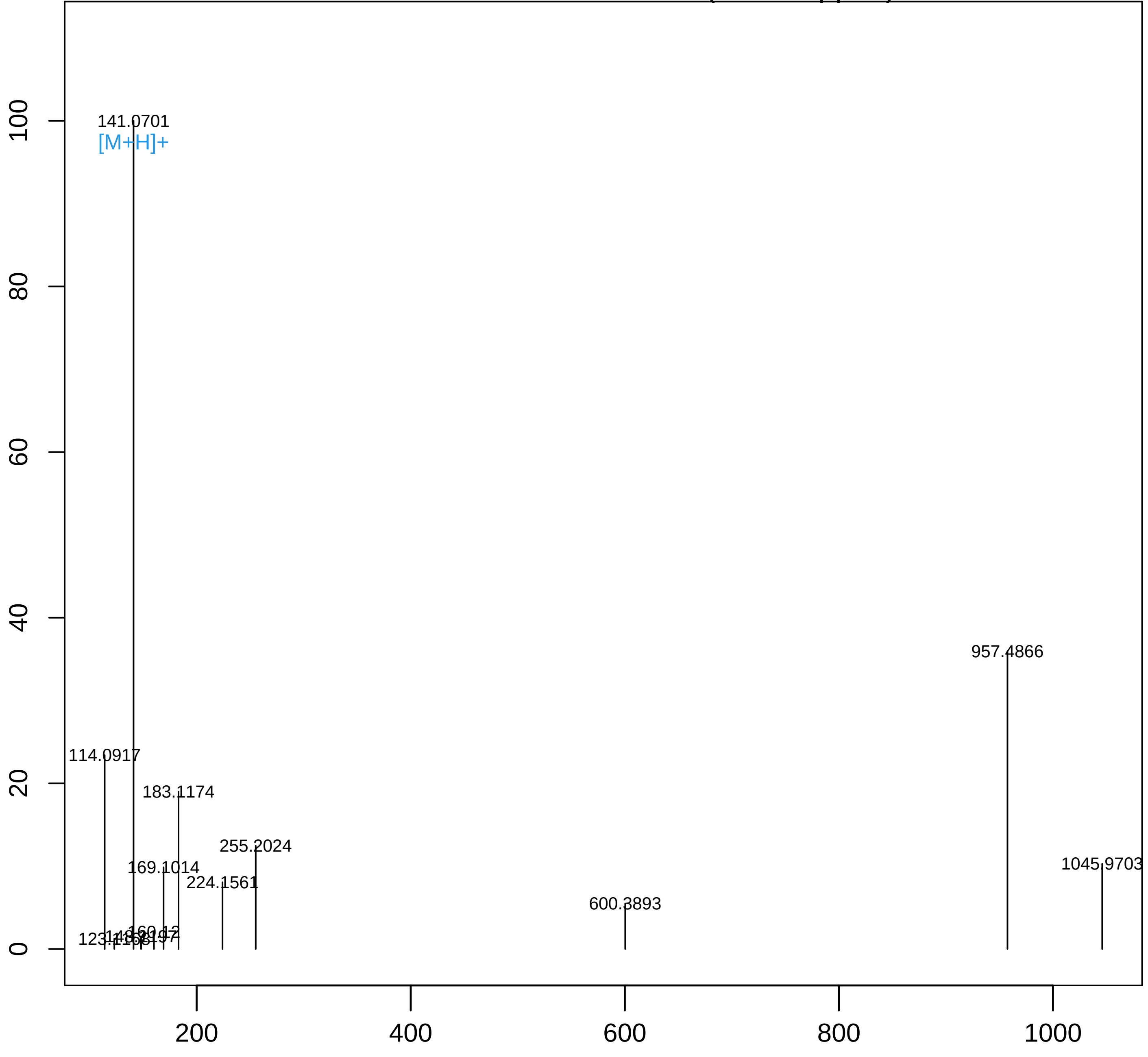

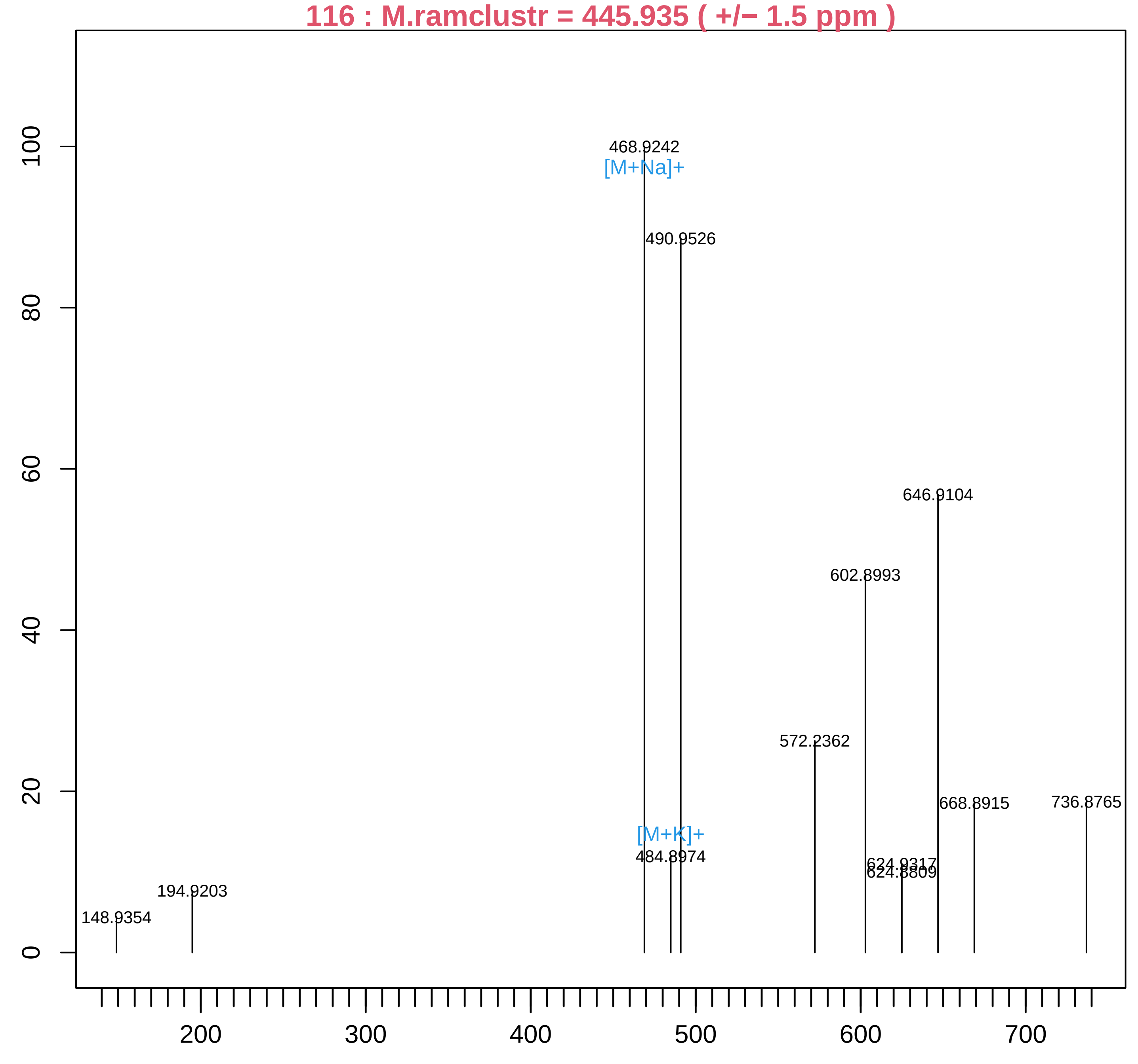
116 : M.ramclustr = 445.935 (+/− 1.5 ppm)
468.9242
[M+Na]+
490.9526
646.9104
602.8993
572.2362
668.8915
736.8765
[M+K]+
484.8974
624.9317
624.8809
194.9203
148.9354
0
20
40
60
80
100
200
300
400
500
600
700
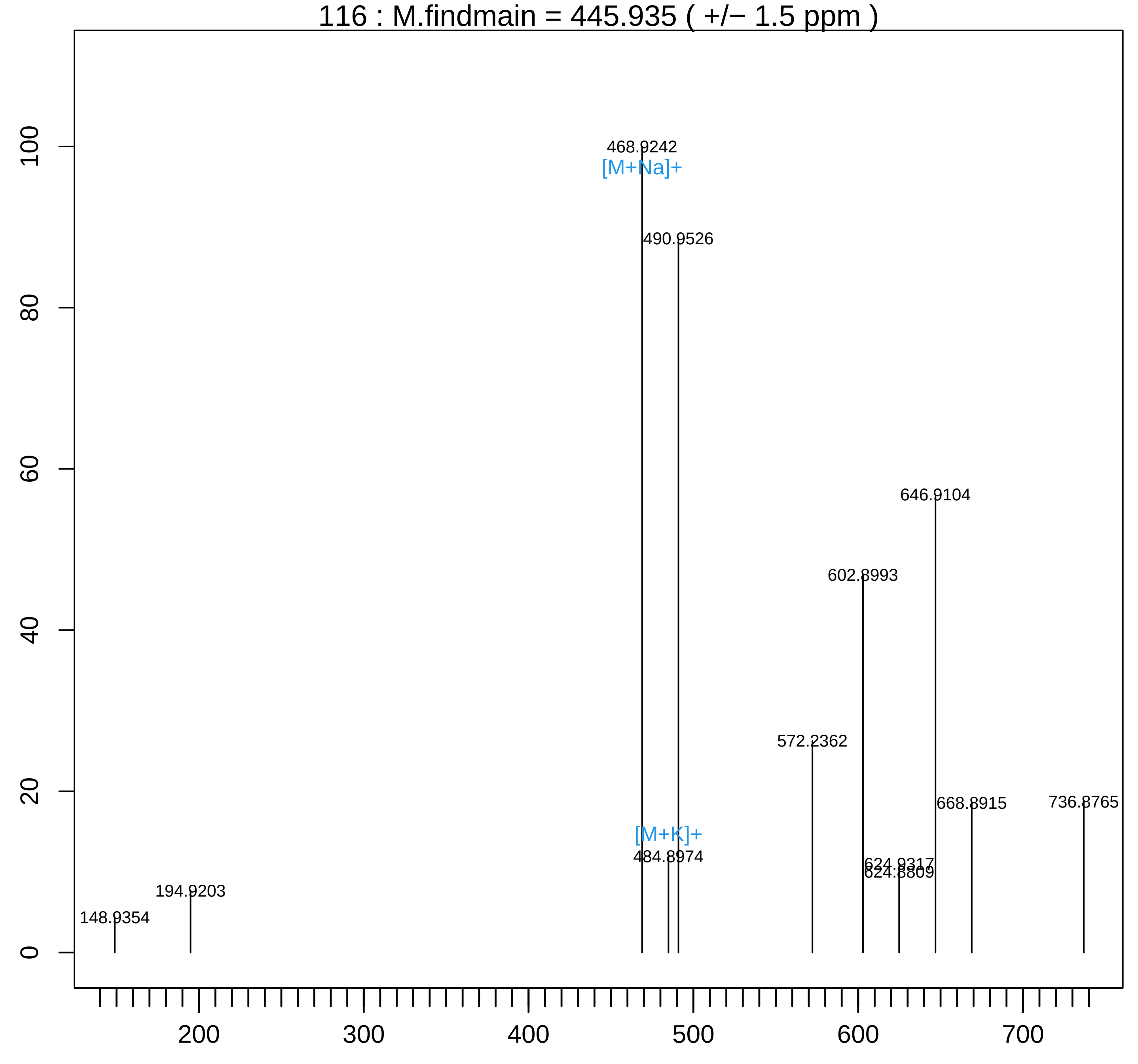
116 : M.findmain = 445.935 (+/− 1.5 ppm)
468.9242
[M+Na]+
490.9526
646.9104
602.8993
572.2362
668.8915
736.8765
[M+K]+
484.8974
624.9317
624.8809
194.9203
148.9354
0
20
40
60
80
100
200
300
400
500
600
700

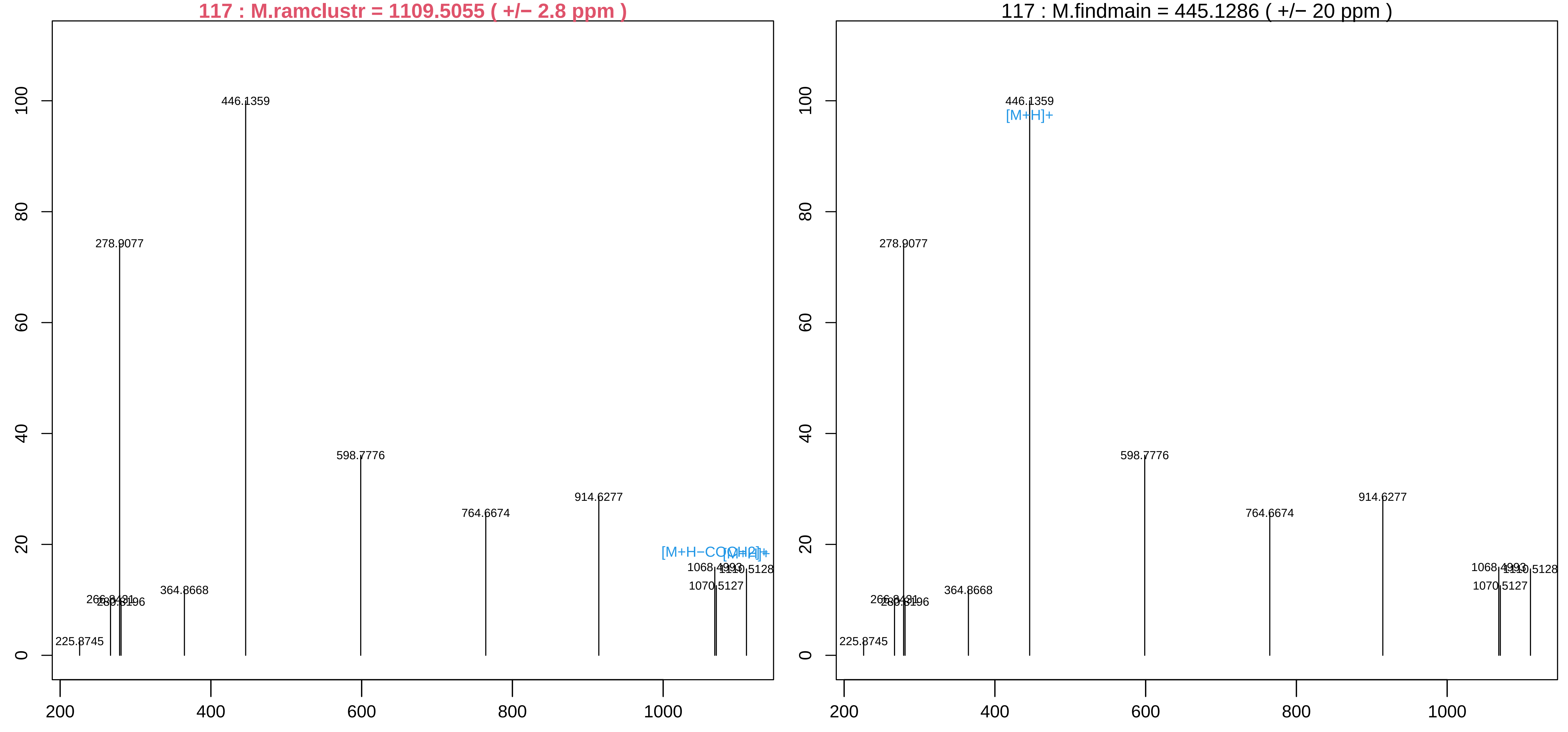
117 : M.ramclustr = 1109.5055 (+/− 2.8 ppm)
446.1359
278.9077
598.7776
914.6277
764.6674
[M+H−COOH2]+
[M+H]+
1068.4993
1110.5128
1070.5127
364.8668
266.8431
280.8196
225.8745
117 : M.findmain = 445.1286 (+/− 20 ppm)
446.1359
[M+H]+
278.9077
598.7776
914.6277
764.6674
1068.4993
1110.5128
1070.5127
364.8668
266.8431
280.8196
225.8745
0
20
40
60
80
100
200
400
600
800
1000

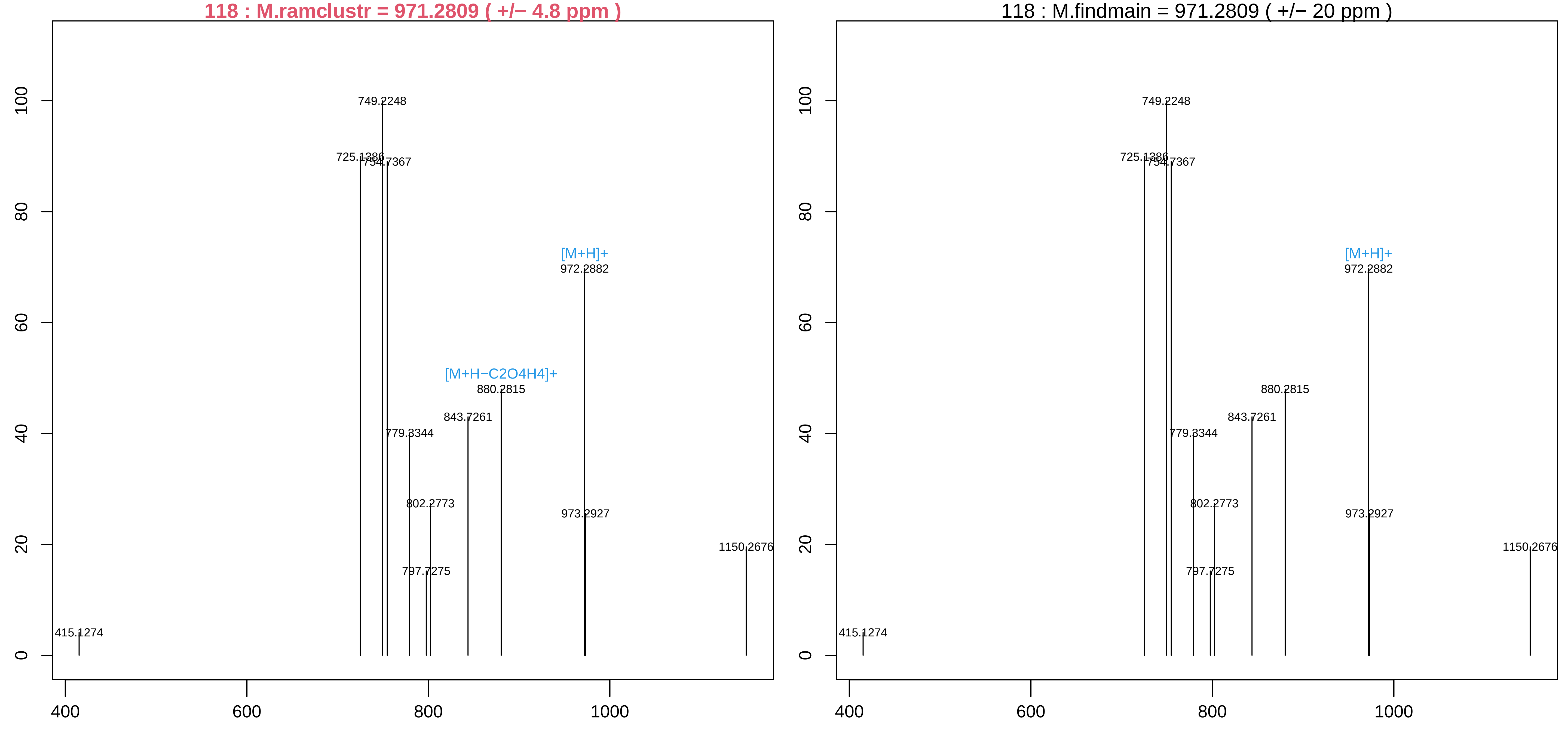
118 : M.ramclustr = 971.2809 (+/− 4.8 ppm)
749.2248
725.1386
754.7367
[M+H]+
972.2882
[M+H−C2O4H4]+
880.2815
843.7261
779.3344
802.2773
973.2927
1150.2676
797.7275
415.1274
118 : M.findmain = 971.2809 (+/− 20 ppm)
749.2248
725.1386
754.7367
[M+H]+
972.2882
880.2815
843.7261
779.3344
802.2773
973.2927
1150.2676
797.7275
415.1274

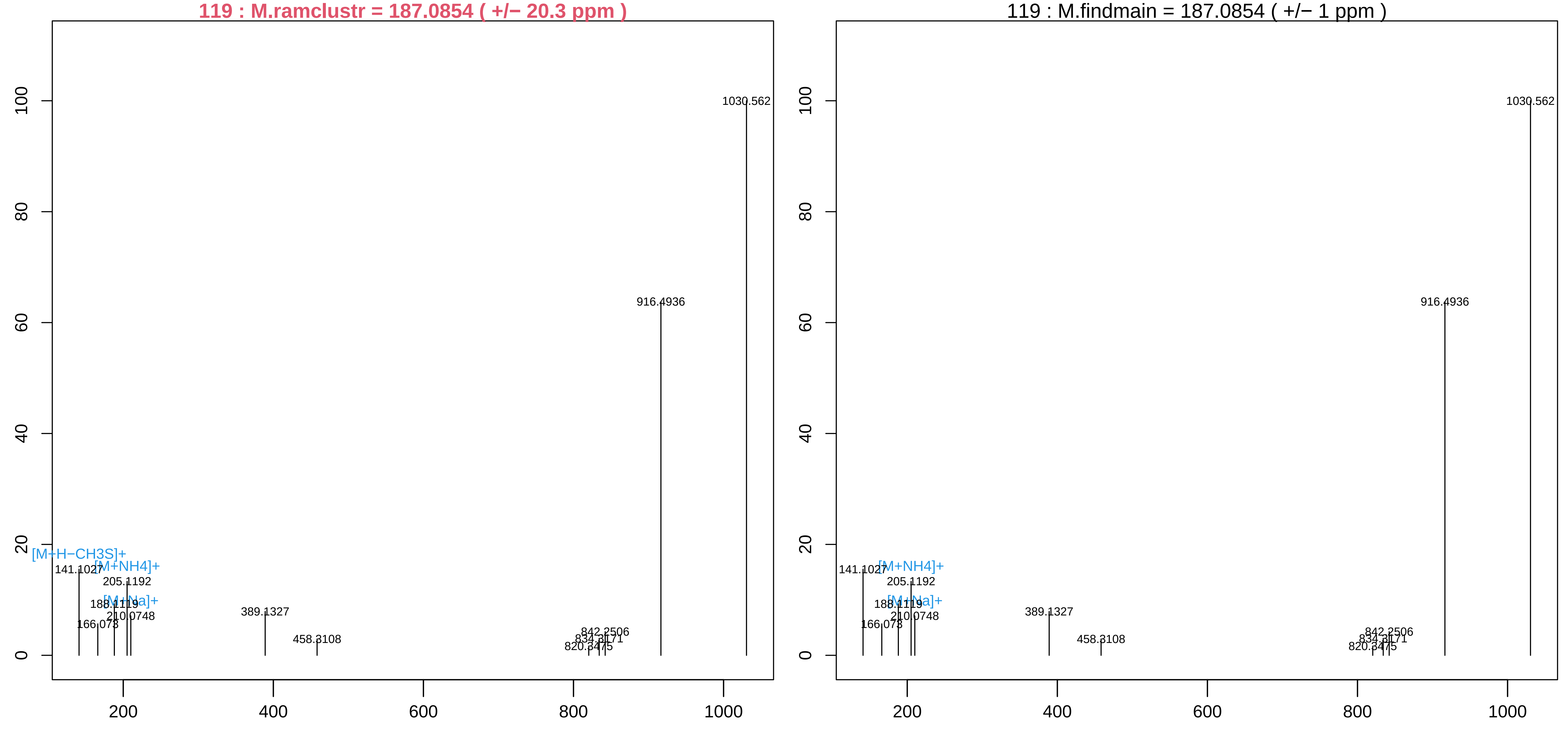
119 : M.ramclustr = 187.0854 (+/− 20.3 ppm)
119 : M.findmain = 187.0854 (+/− 1 ppm)
1030.562
916.4936
[M+H−CH3S]+
141.1027
[M+NH4]+
205.1192
[M+Na]+
188.1119
210.0748
166.073
389.1327
458.3108
842.2506
834.3171
820.3475
0
20
40
60
80
100
200
400
600
800
1000

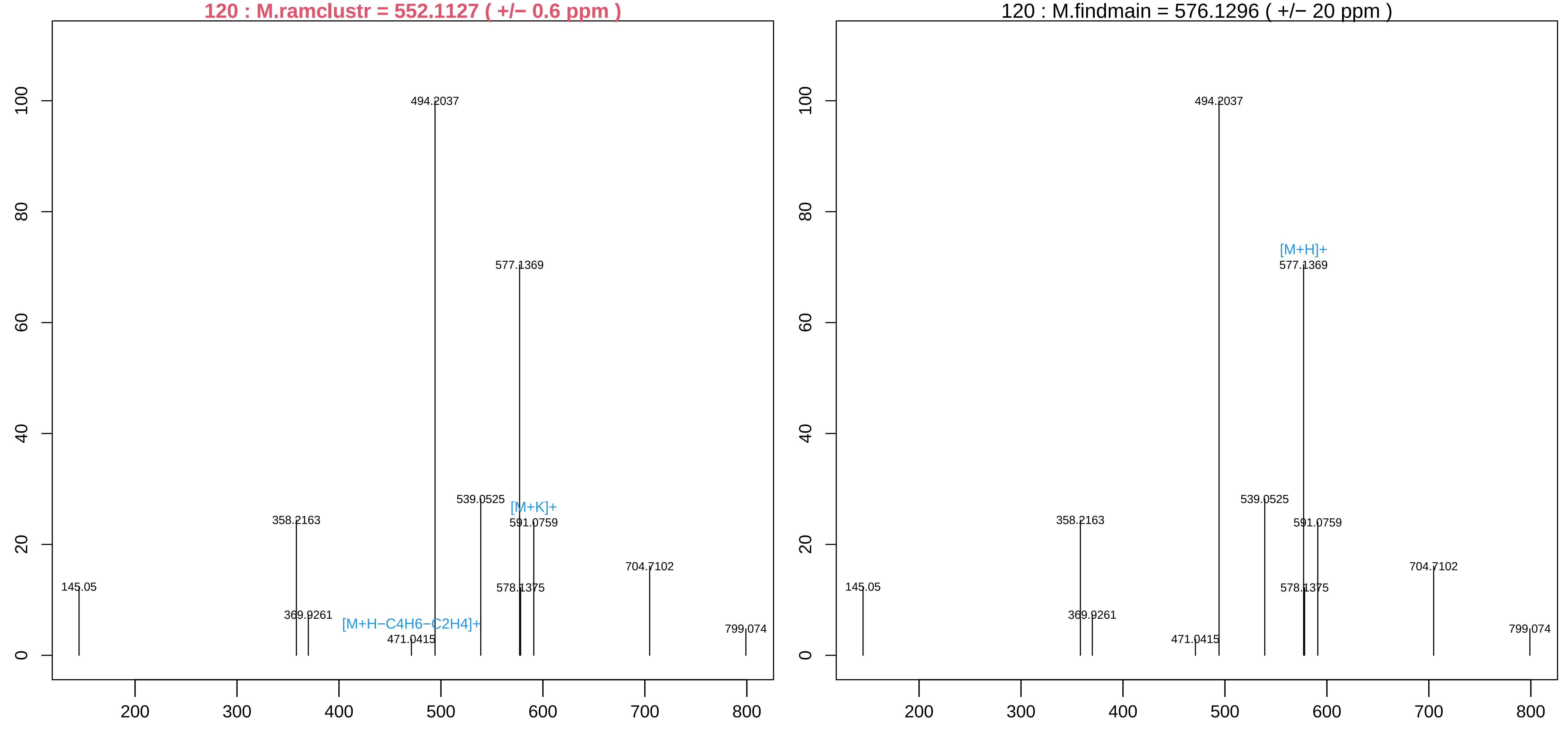

120 : M.ramclustr = 552.1127 (+/− 0.6 ppm)
494.2037
577.1369
539.0525
[M+K]+
591.0759
358.2163
704.7102
145.05
578.1375
369.9261
[M+H−C4H6−C2H4]+
471.0415
799.074
0
20
40
60
80
100
200
300
400
500
600
700
800
120 : M.findmain = 576.1296 (+/− 20 ppm)
494.2037
[M+H]+
577.1369
539.0525
591.0759
358.2163
704.7102
145.05
578.1375
369.9261
471.0415
799.074
0
20
40
60
80
100
200
300
400
500
600
700
800

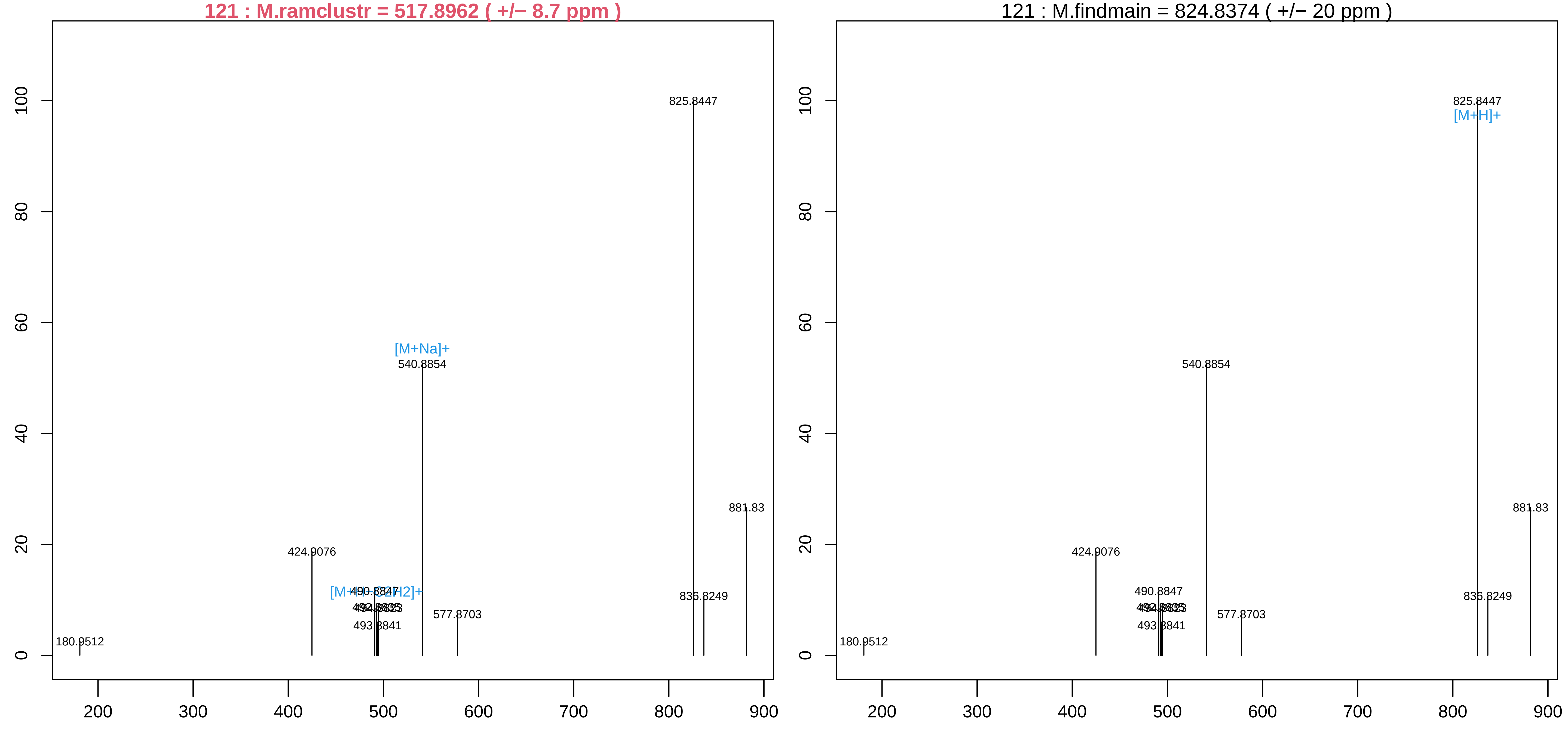
121 : M.ramclustr = 517.8962 (+/− 8.7 ppm)
121 : M.findmain = 824.8374 (+/− 20 ppm)
825.8447
[M+H]+
[M+Na]+
540.8854
881.83
424.9076
490.8847
493.8841
577.8703
836.8249
180.9512
0
20
40
60
80
100
200
300
400
500
600
700
800
900

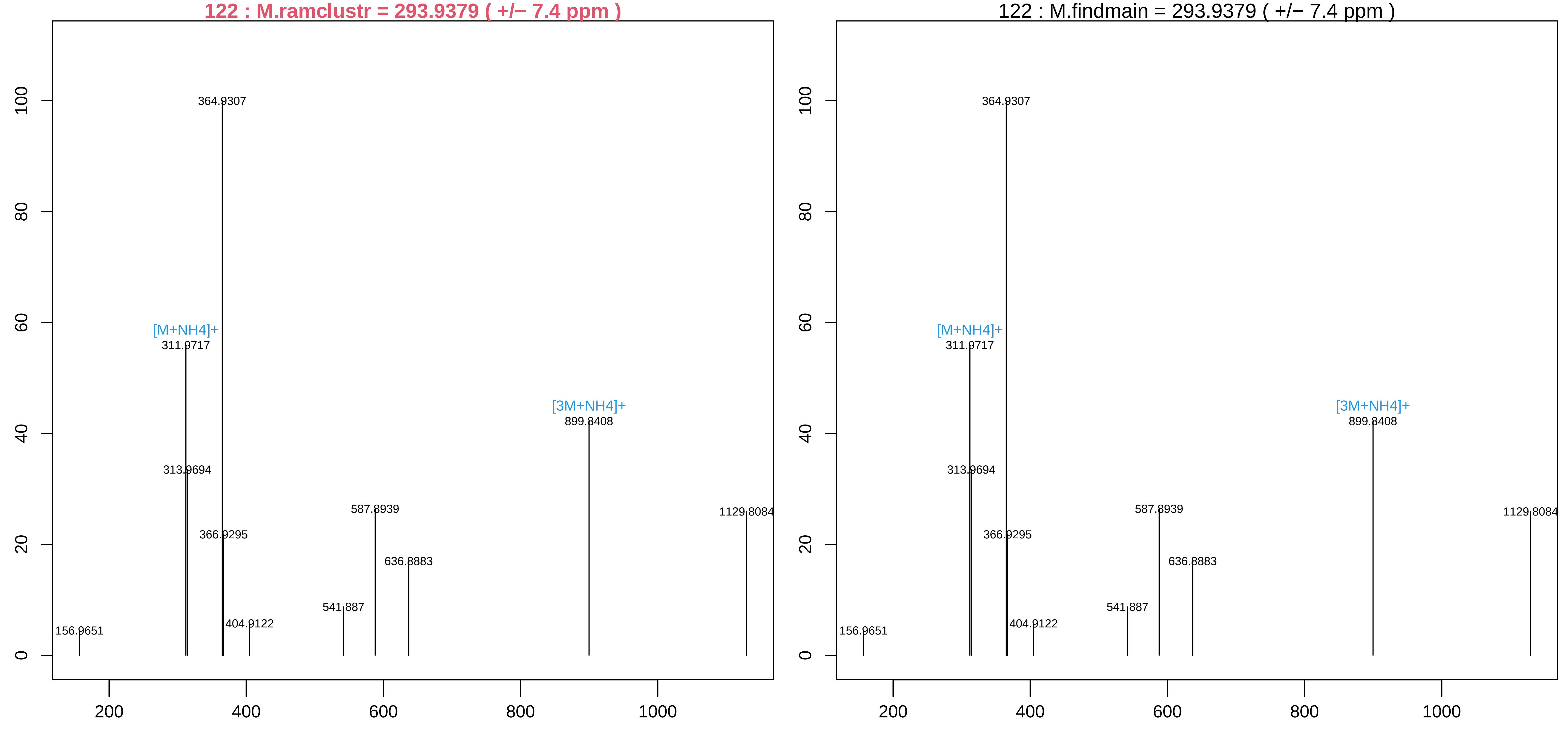

122 : M.ramclustr = 293.9379 (+/− 7.4 ppm)
122 : M.findmain = 293.9379 (+/− 7.4 ppm)
[M+NH4]+
[3M+NH4]+
364.9307
311.9717
899.8408
313.9694
587.8939
1129.8084
366.9295
636.8883
541.887
404.9122
156.9651
0
20
40
60
80
100
200
400
600
800
1000

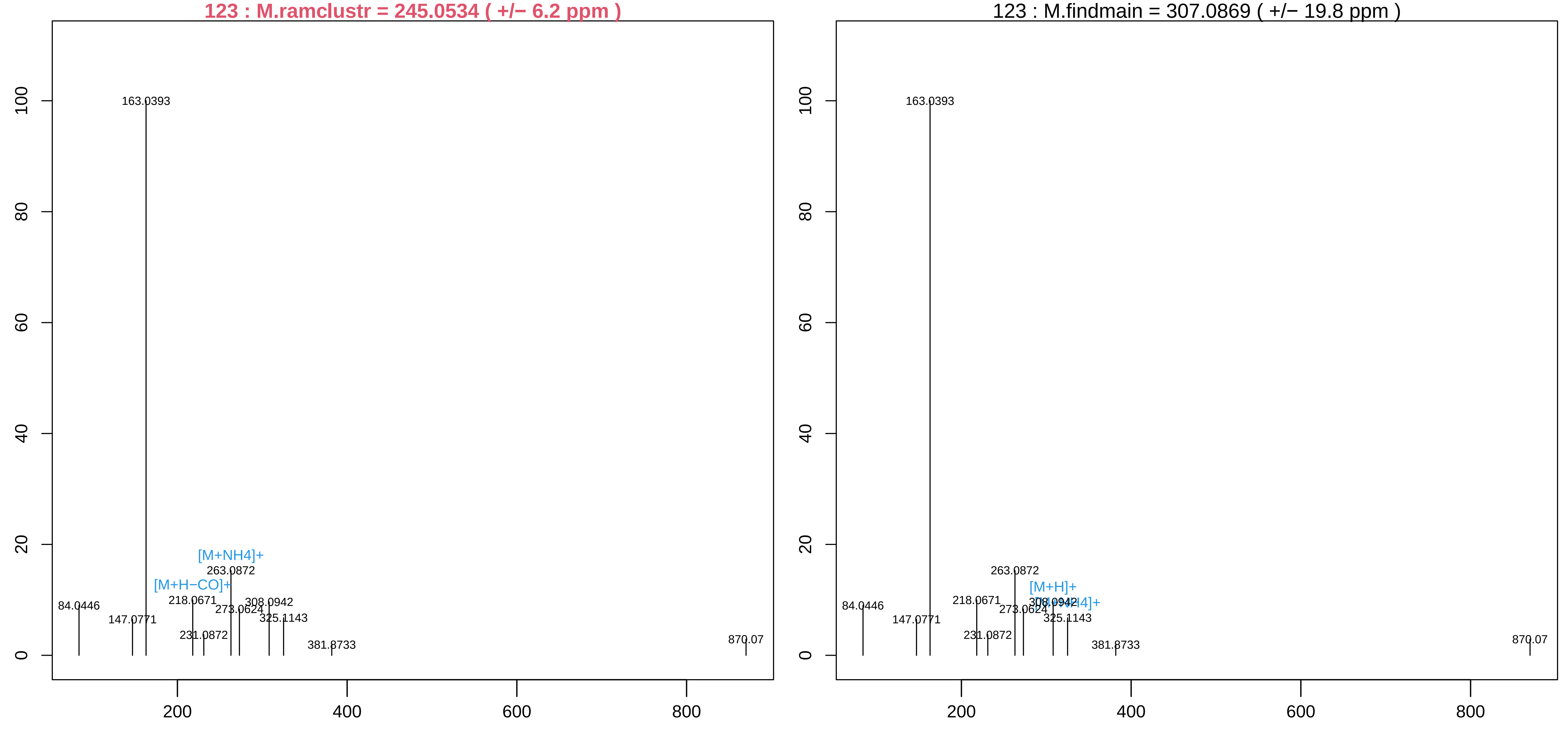

123 : M.ramclustr = 245.0534 (+/− 6.2 ppm)
163.0393
[M+NH4]+
263.0872
[M+H−CO]+
218.0671
84.0446
147.0771
273.0624
308.0942
325.1143
231.0872
381.8733
870.07
123 : M.findmain = 307.0869 (+/− 19.8 ppm)
163.0393
263.0872
[M+H]+
218.0671
84.0446
147.0771
273.0624
308.0942
325.1143
231.0872
381.8733
870.07

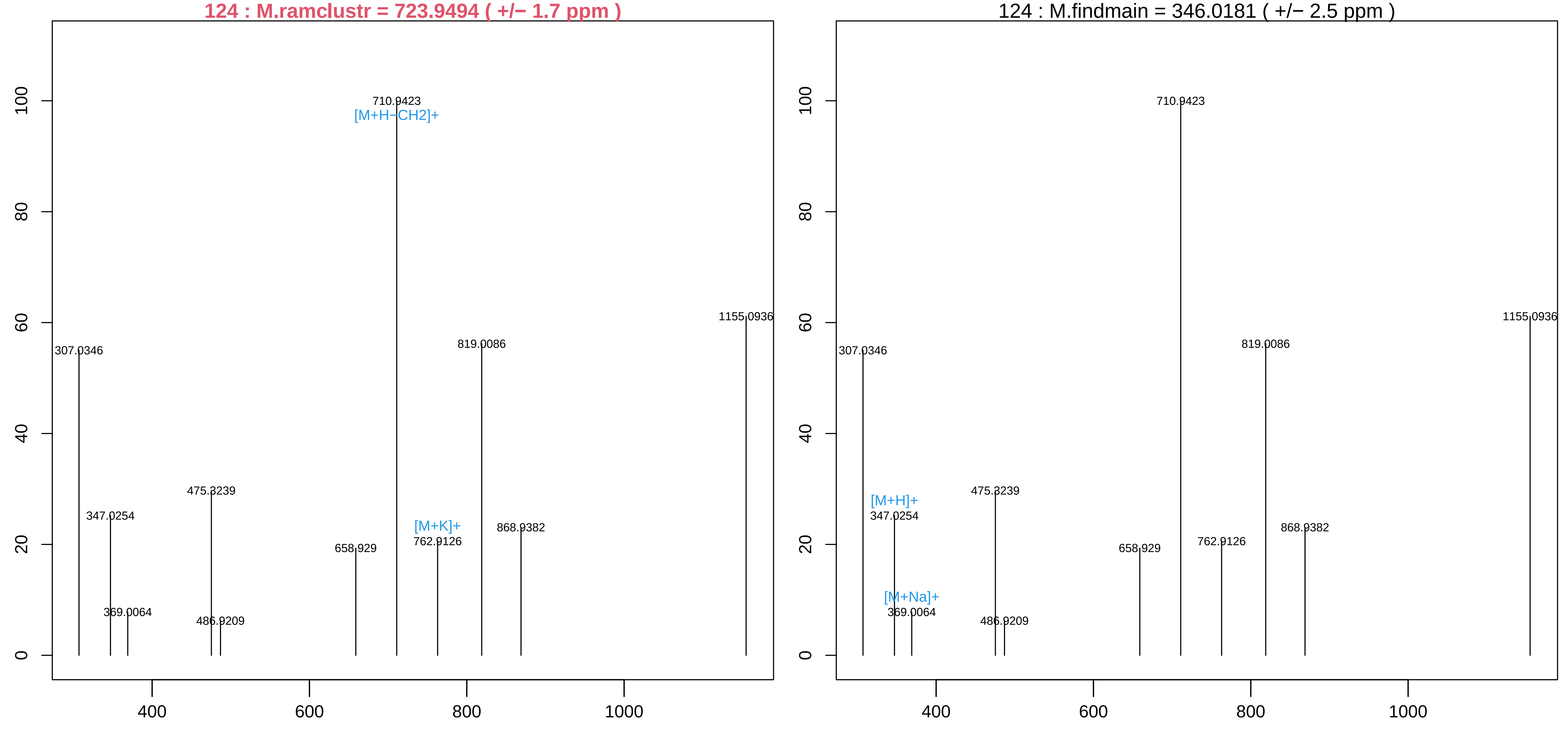
124 : M.ramclustr = 723.9494 (+/− 1.7 ppm)
710.9423
[M+H−CH2]+
1155.0936
307.0346
819.0086
475.3239
347.0254
[M+K]+
868.9382
762.9126
658.929
369.0064
486.9209
0
20
40
60
80
100
400
600
800
1000
124 : M.findmain = 346.0181 (+/− 2.5 ppm)
710.9423
1155.0936
307.0346
819.0086
475.3239
[M+H]+
347.0254
868.9382
762.9126
658.929
[M+Na]+
369.0064
486.9209

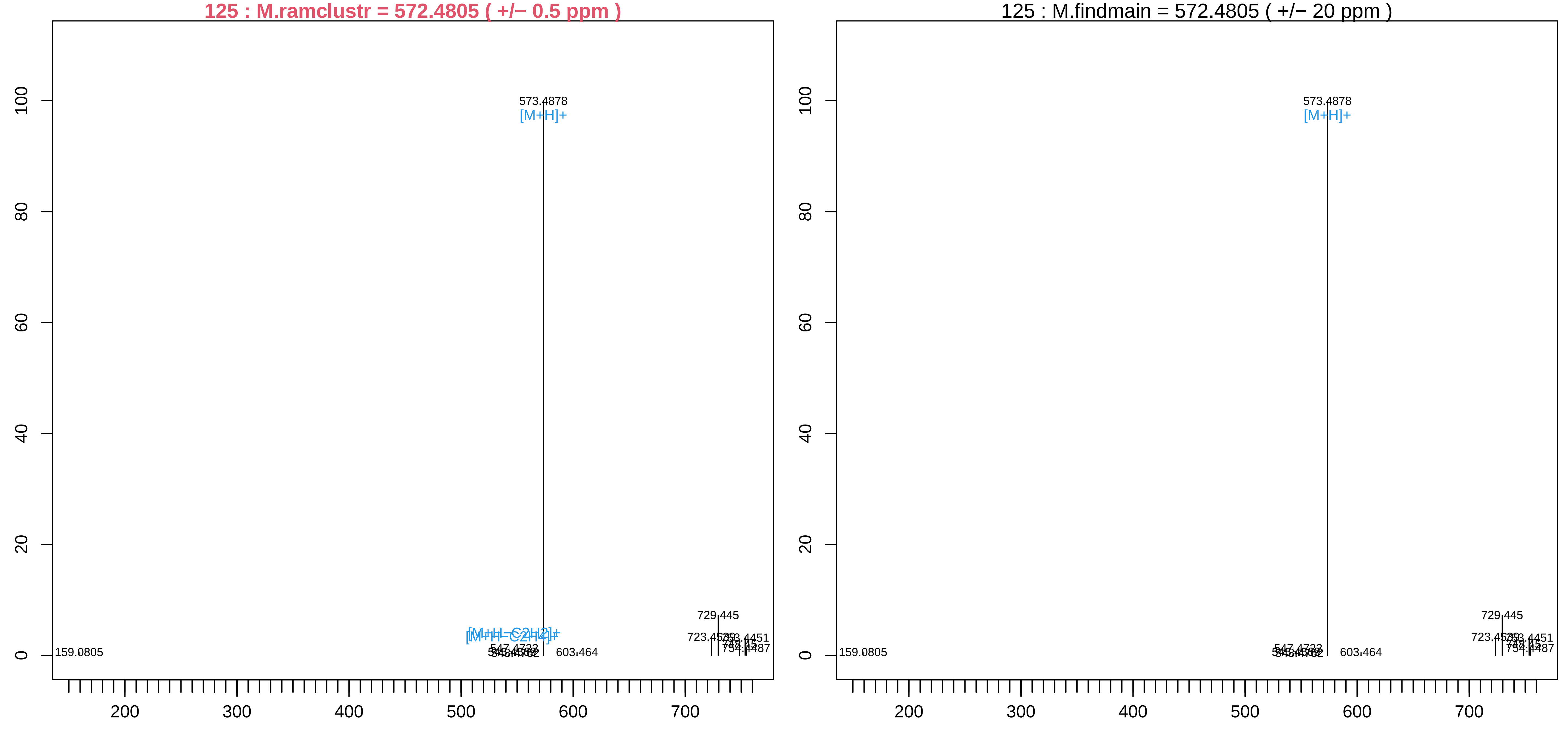
125 : M.ramclustr = 572.4805 (+/− 0.5 ppm)
125 : M.findmain = 572.4805 (+/− 20 ppm)
573.4878
[M+H]+
729.445
723.4539
753.4451
754.4487
603.464
159.0805
547.4723
[M+H−C2H2]+
[M+H−C2H4]+
0
20
40
60
80
100
200
300
400
500
600
700

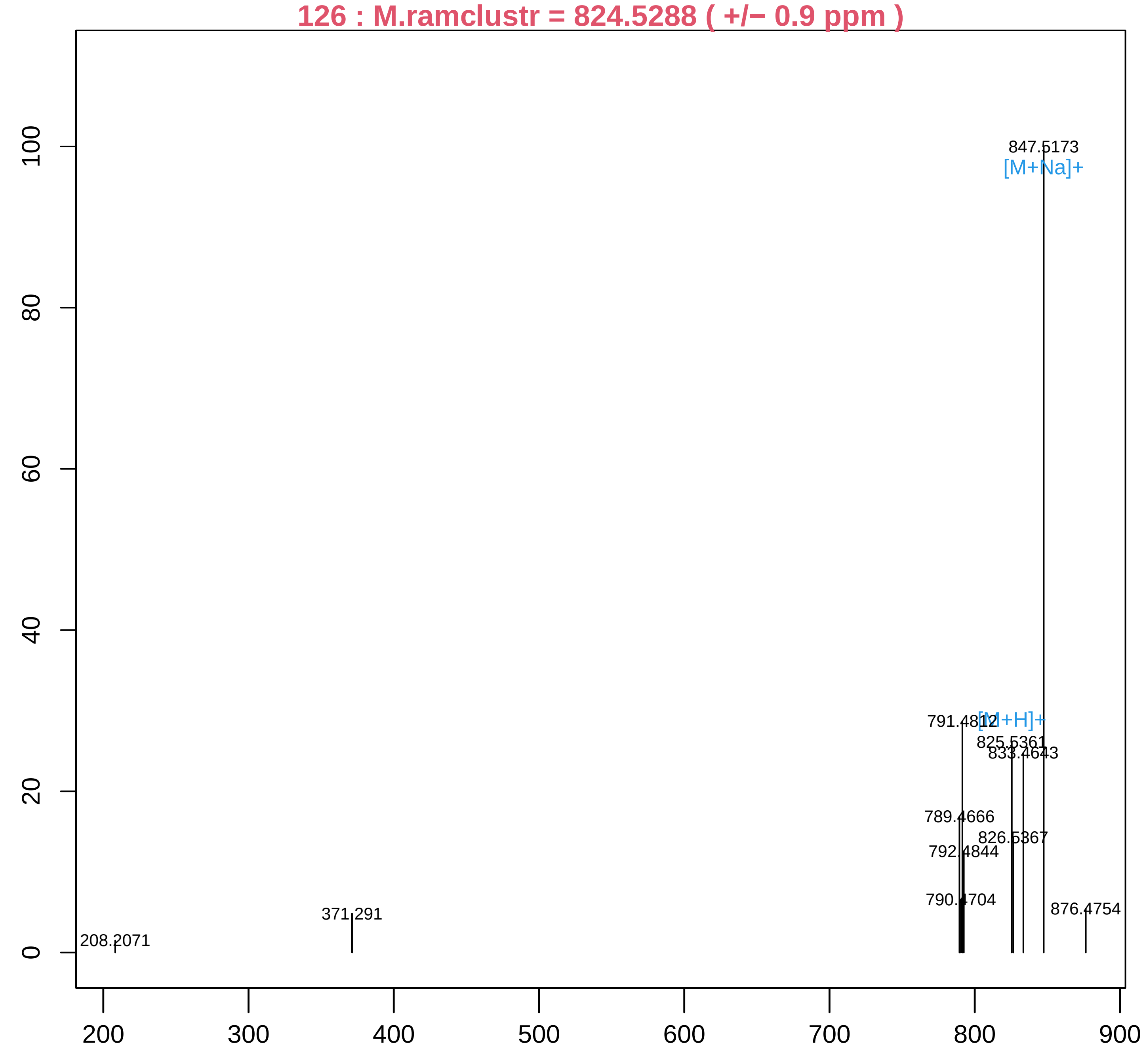
126 : M.ramclustr = 824.5288 (+/− 0.9 ppm)
847.5173
[M+Na]+
791.4812
[M+H]+
825.5361
833.4643
789.4666
826.5367
792.4844
790.4704
876.4754
371.291
208.2071
0
20
40
60
80
100
200
300
400
500
600
700
800
900

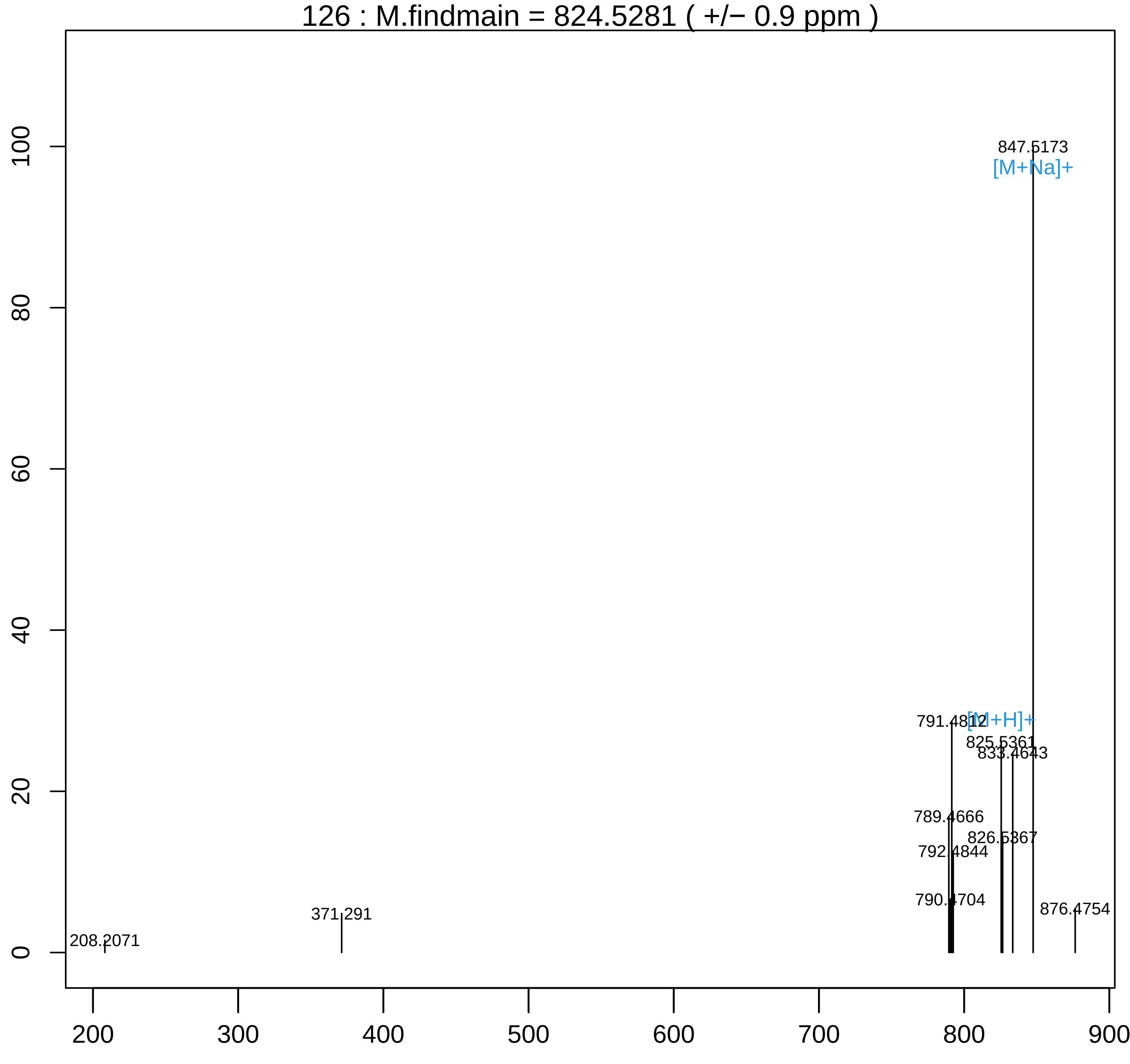
126 : M.findmain = 824.5281 (+/− 0.9 ppm)
847.5173
[M+Na]+
791.4812
[M+H]+
825.5361
833.4643
789.4666
826.5367
792.4844
790.4704
876.4754
371.291
208.2071
0
20
40
60
80
100
200
300
400
500
600
700
800
900

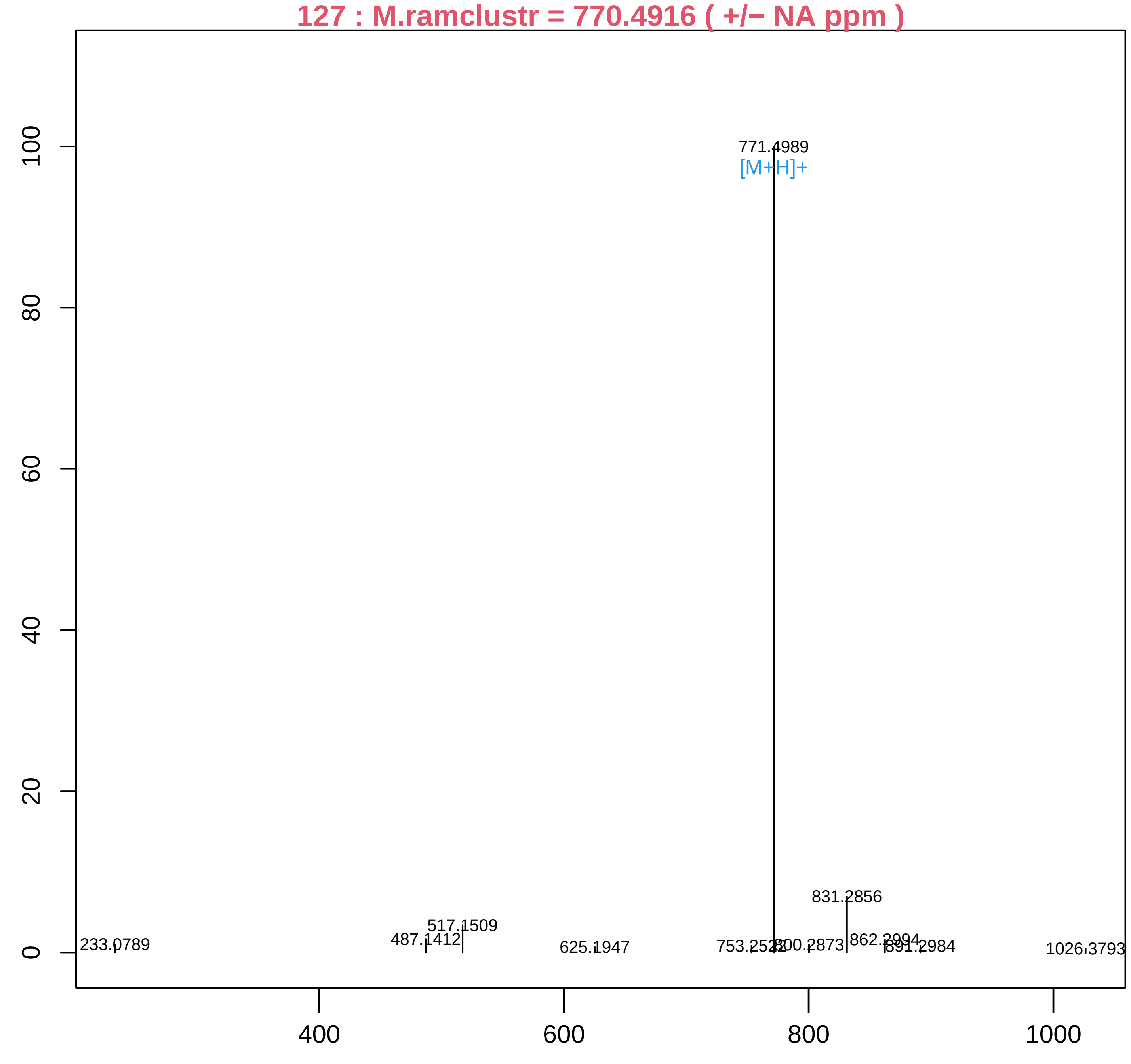
127 : M.ramclustr = 770.4916 (+/− NA ppm)
771.4989
[M+H]+
831.2856
517.1509
487.1412
233.0789
625.1947
753.2528
800.2873
862.2994
891.2984
1026.3793
0
20
40
60
80
100
400
600
800
1000
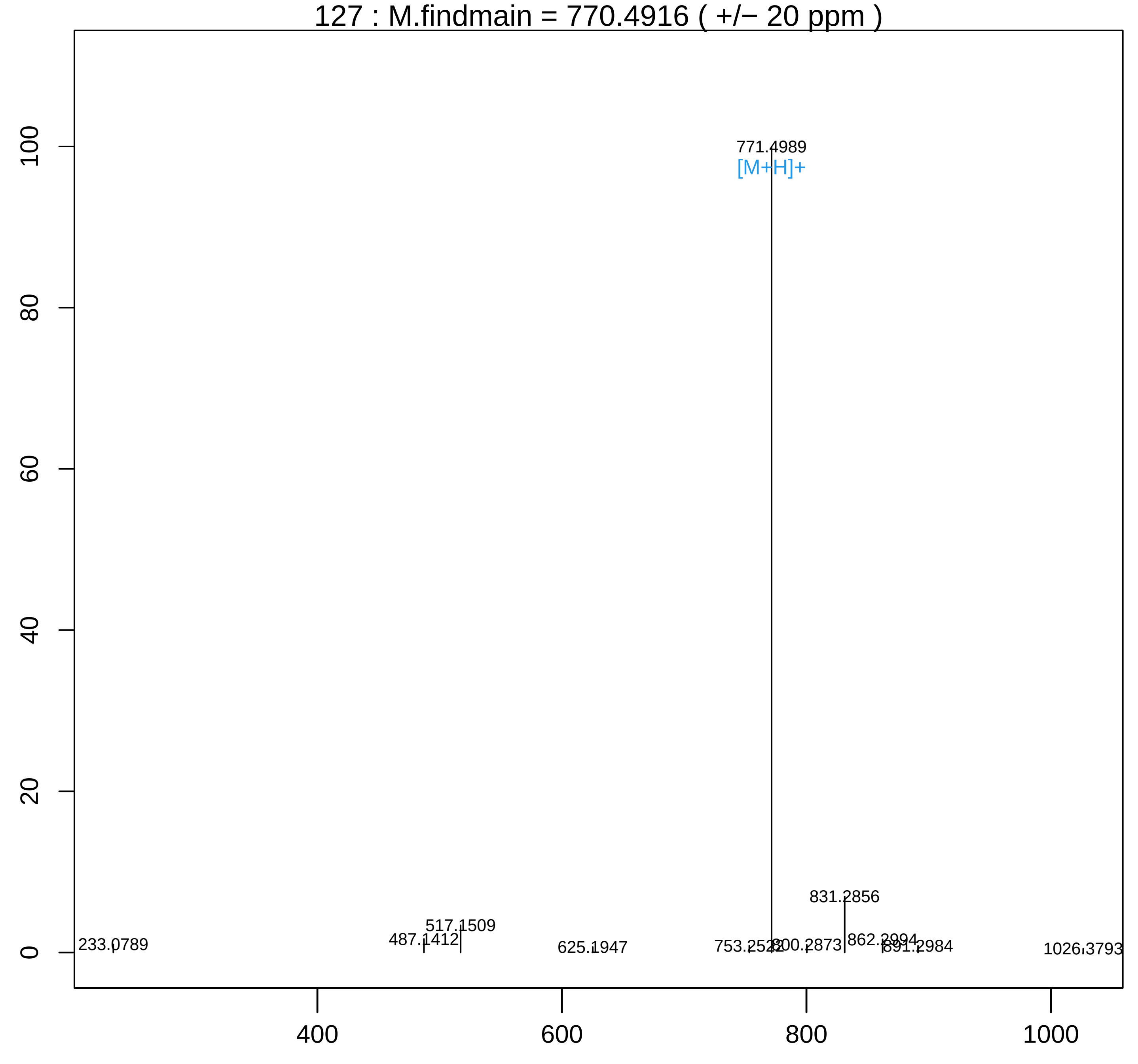
127 : M.findmain = 770.4916 (+/− 20 ppm)
771.4989
[M+H]+
831.2856
517.1509
487.1412
233.0789
625.1947
753.2528
800.2873
862.2994
891.2984
1026.3793
0
20
40
60
80
100
400
600
800
1000

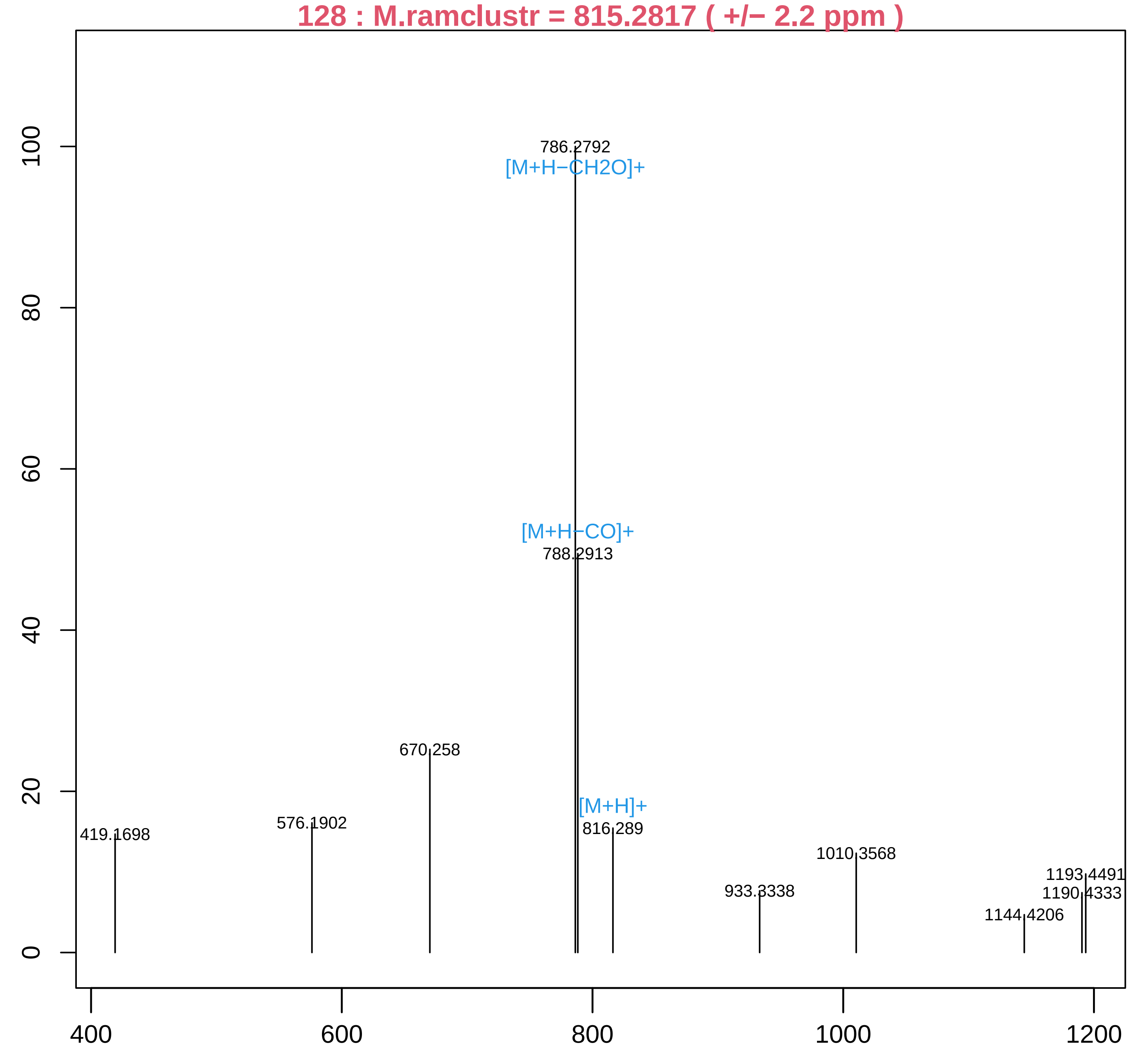
128 : M.ramclustr = 815.2817 (+/− 2.2 ppm)
786.2792
[M+H−CH2O]+
[M+H−CO]+
788.2913
[M+H]+
816.289
419.1698
576.1902
670.258
933.3338
1010.3568
1144.4206
1190.4333
1193.4491
0
20
40
60
80
100
400
600
800
1000
1200

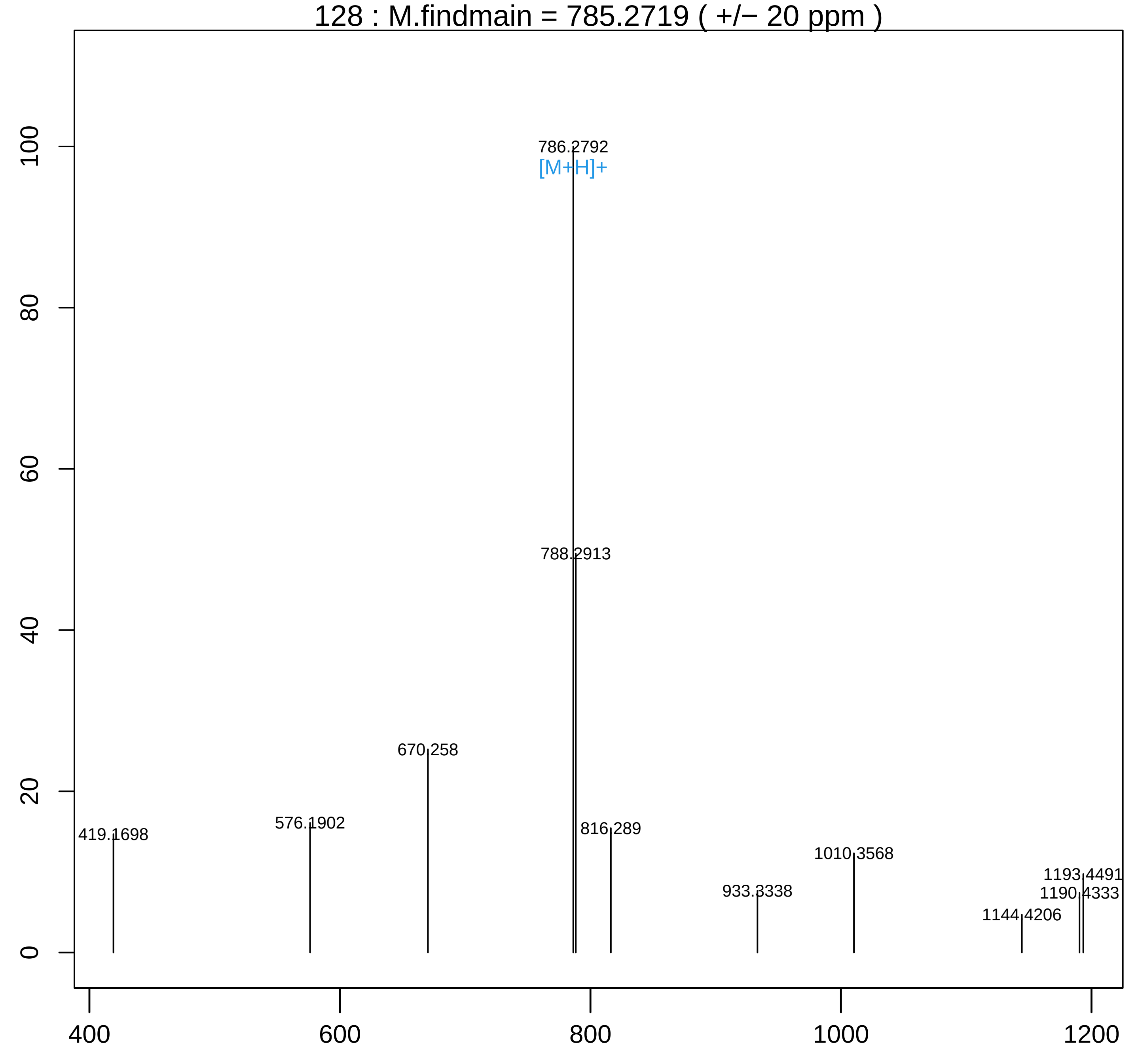
128 : M.findmain = 785.2719 (+/− 20 ppm)
786.2792
[M+H]+
788.2913
816.289
419.1698
576.1902
670.258
933.3338
1010.3568
1144.4206
1190.4333
1193.4491
0
20
40
60
80
100
400
600
800
1000
1200

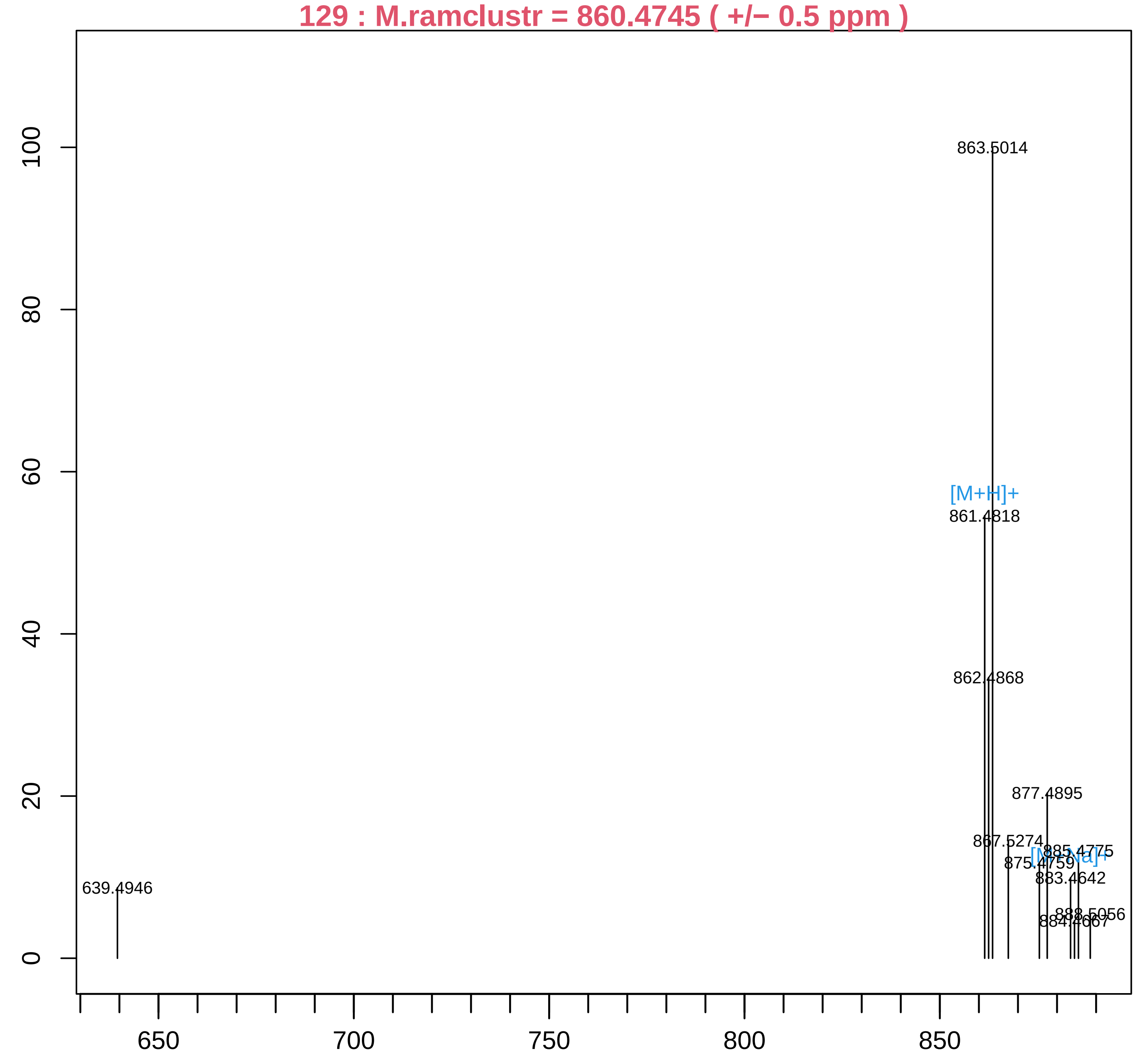
129 : M.ramclustr = 860.4745 (+/− 0.5 ppm)
863.5014
[M+H]+
861.4818
862.4868
877.4895
867.5274
885.4775
[M+Na]+
875.4759
883.4642
888.5056
884.4067
639.4946
0
20
40
60
80
100
650
700
750
800
850

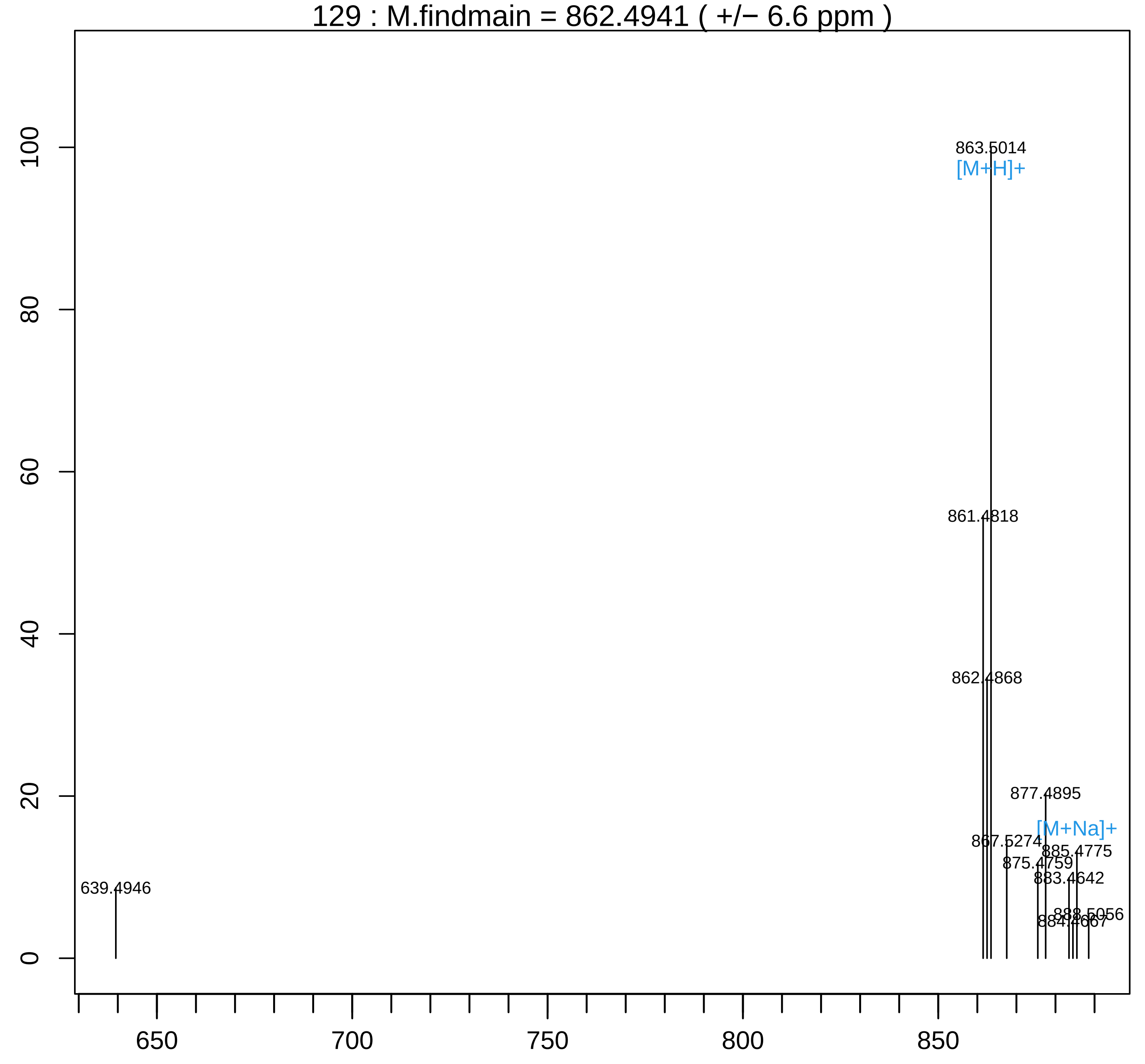
129 : M.findmain = 862.4941 (+/− 6.6 ppm)
863.5014
[M+H]+
861.4818
862.4868
877.4895
[M+Na]+
867.5274
885.4775
875.4759
883.4642
888.5056
884.4067
639.4946
0
20
40
60
80
100
650
700
750
800
850

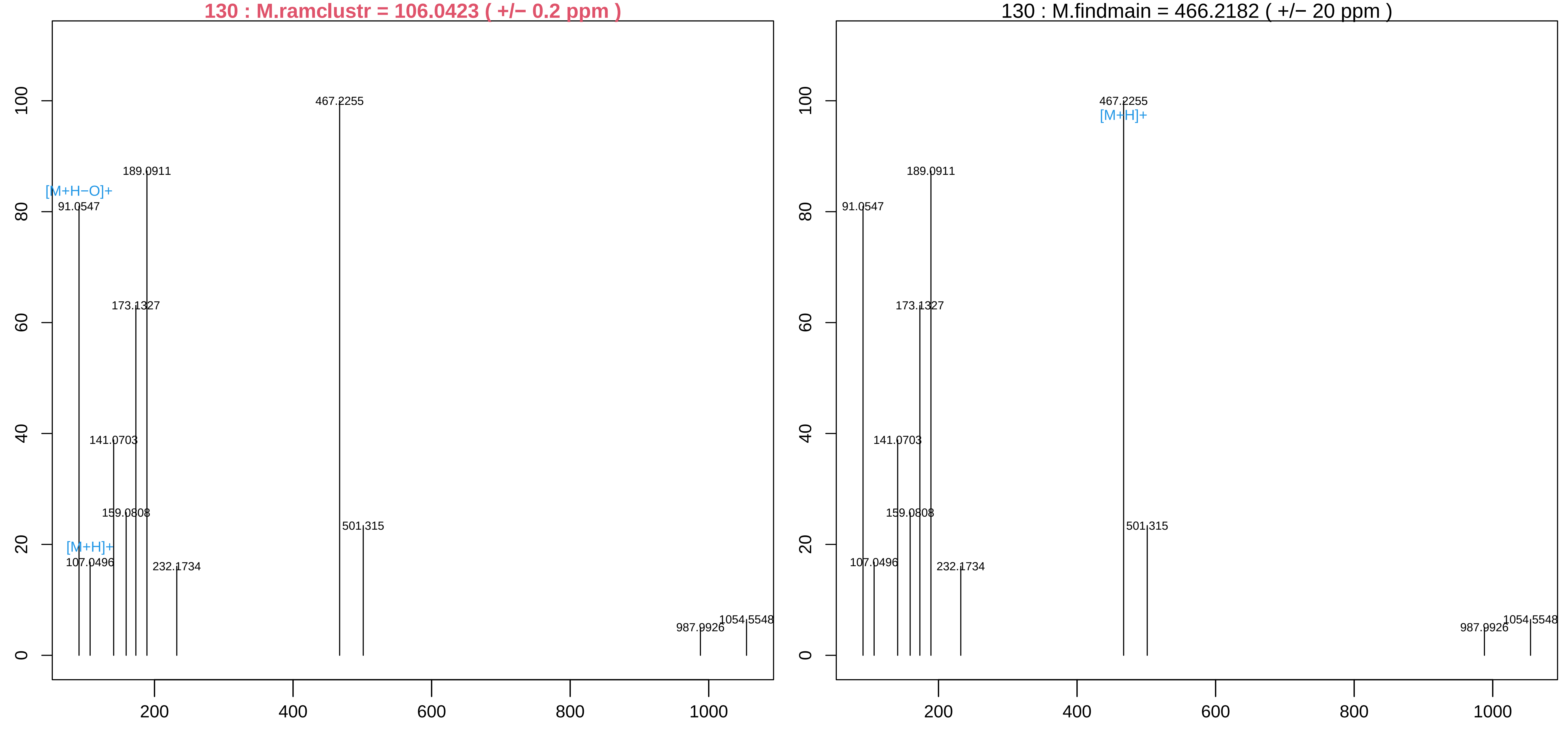

130 : M.ramclustr = 106.0423 (+/− 0.2 ppm)
[M+H−O]+
91.0547
189.0911
467.2255
173.1327
141.0703
159.0808
501.315
[M+H]+
107.0496
232.1734
987.9926
1054.5548
130 : M.findmain = 466.2182 (+/− 20 ppm)
467.2255
[M+H]+
189.0911
91.0547
173.1327
141.0703
159.0808
501.315
107.0496
232.1734
987.9926
1054.5548

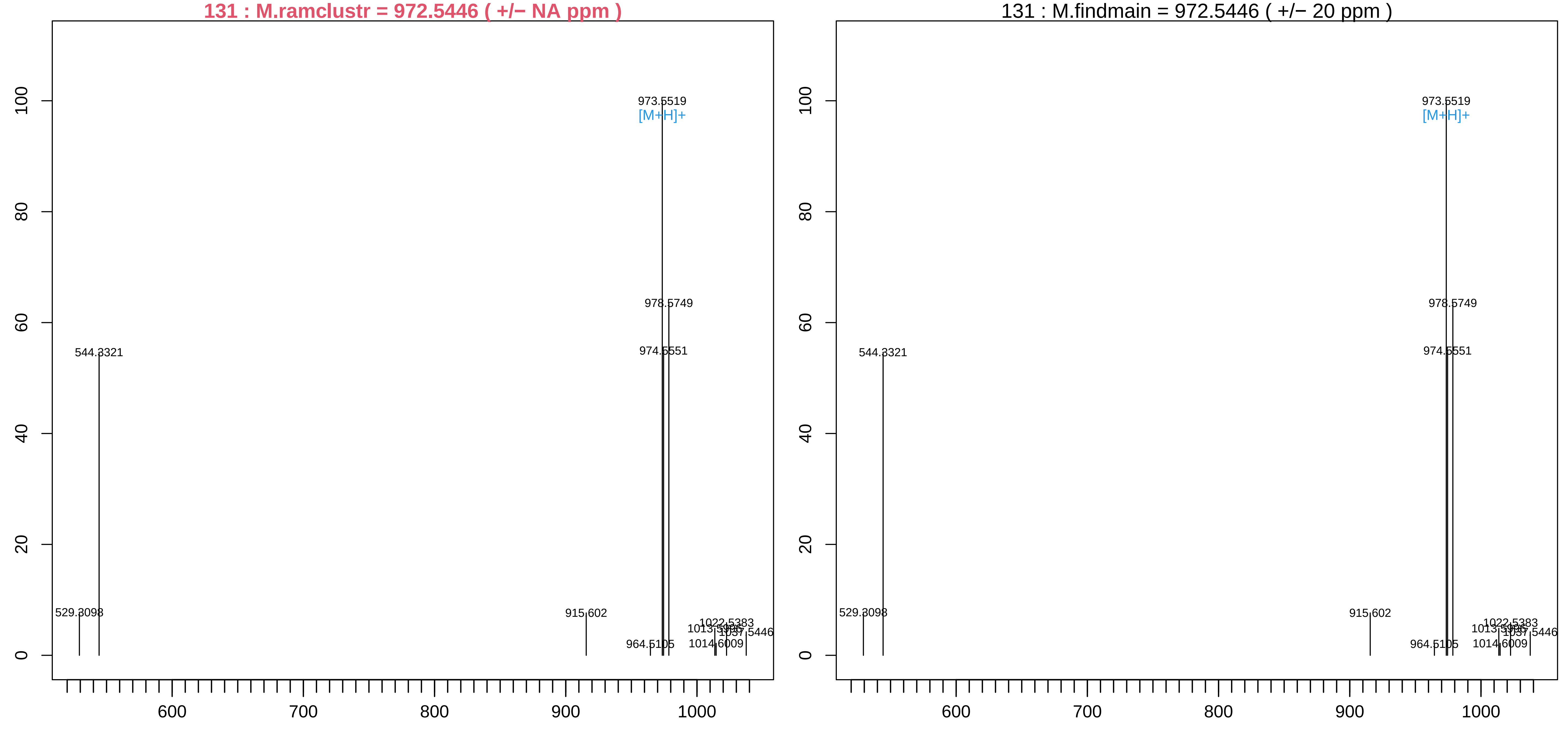
131 : M.ramclustr = 972.5446 (+/− NA ppm)
973.5519
[M+H]+
978.5749
974.5551
544.3321
529.3098
915.602
964.5105
1014.6009
1022.5383
1037.5446
131 : M.findmain = 972.5446 (+/− 20 ppm)
973.5519
[M+H]+
978.5749
974.5551
544.3321
529.3098
915.602
964.5105
1014.6009
1022.5383
1037.5446

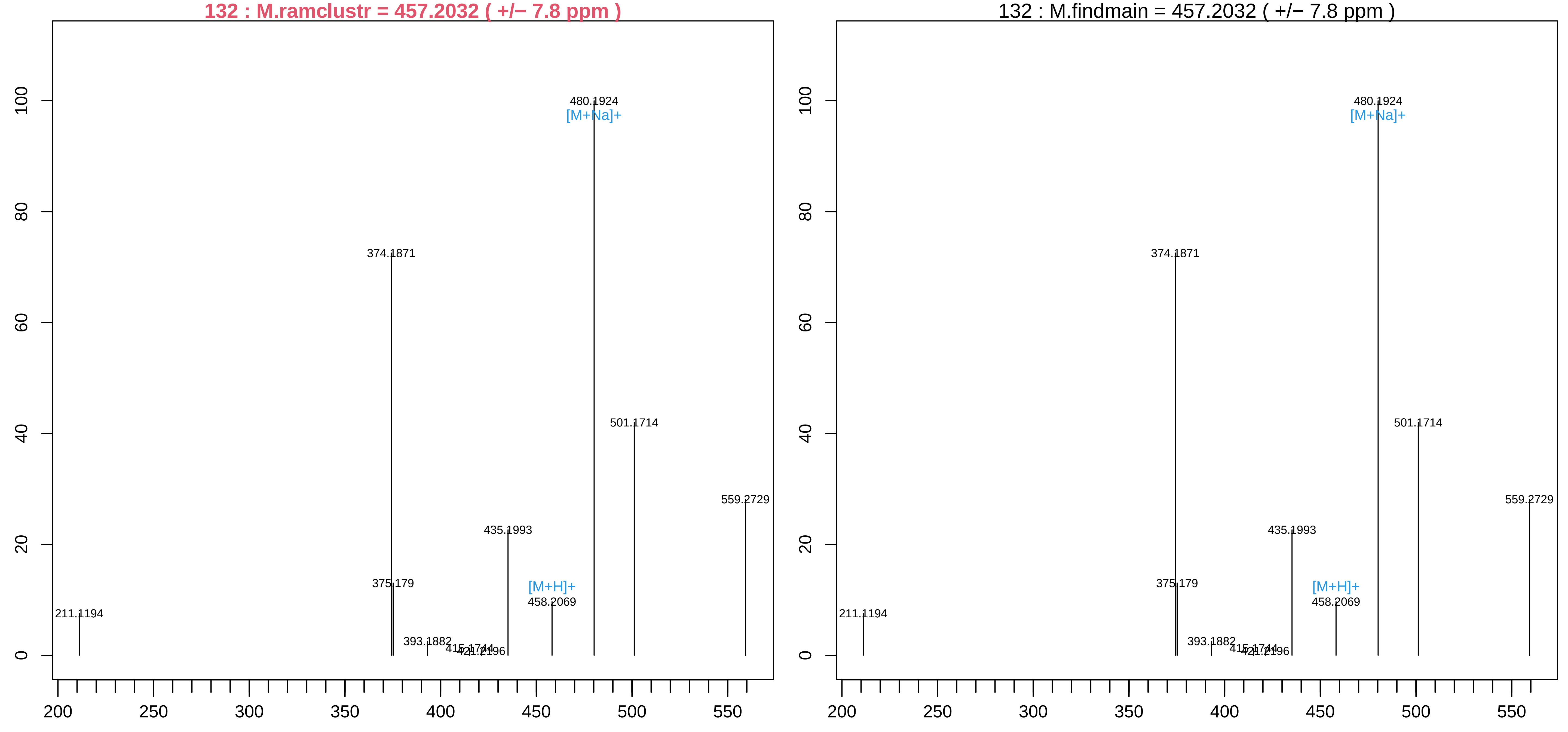
132 : M.ramclustr = 457.2032 (+/− 7.8 ppm)
132 : M.findmain = 457.2032 (+/− 7.8 ppm)
480.1924
[M+Na]+
374.1871
501.1714
559.2729
435.1993
375.179
[M+H]+
458.2069
211.1194
393.1882
415.1744
421.2196
0
20
40
60
80
100
200
250
300
350
400
450
500
550

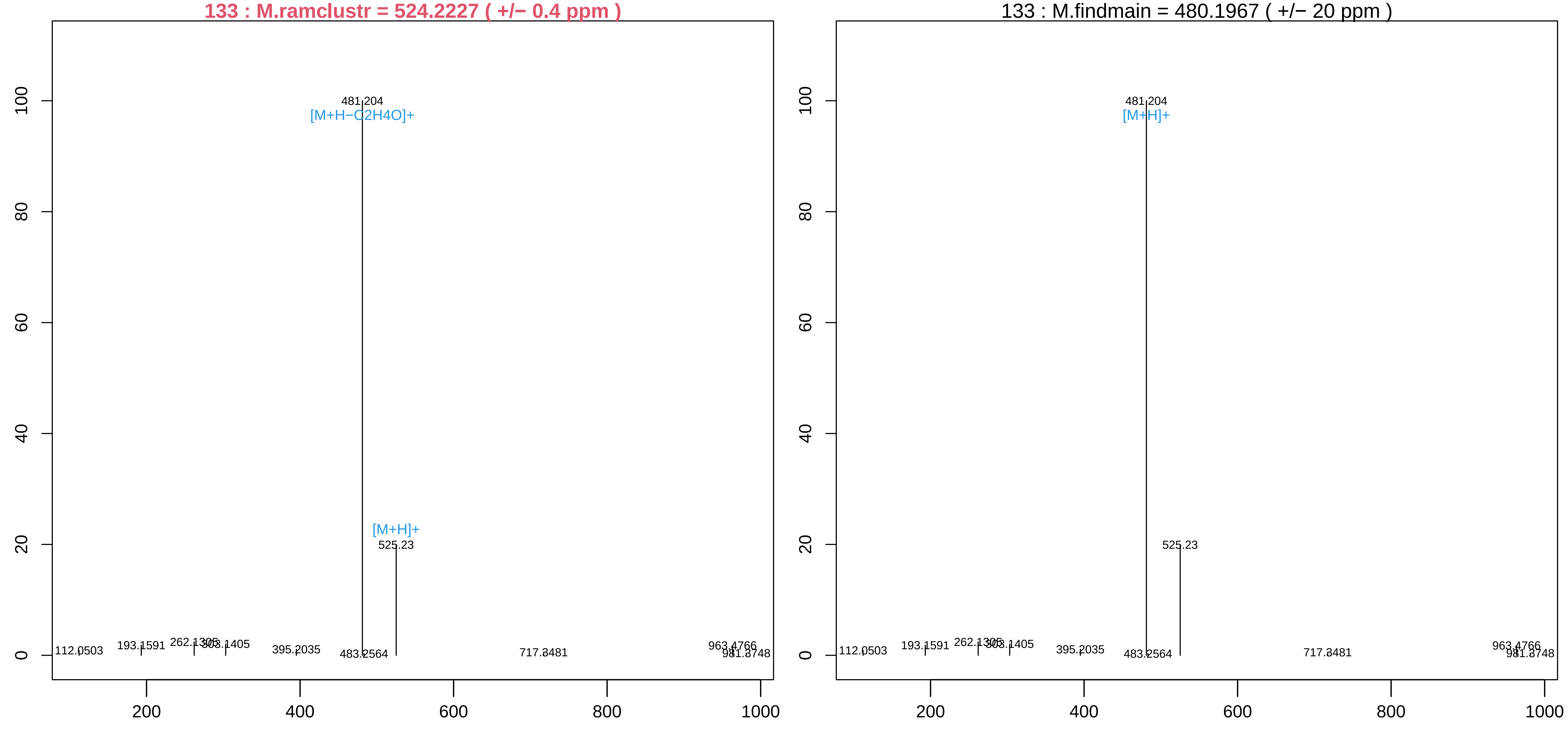
133 : M.ramclustr = 524.2227 (+/− 0.4 ppm)
481.204
[M+H−C2H4O]+
[M+H]+
525.23
112.0503
193.1591
262.1305
303.1405
395.2035
483.2564
717.8481
963.4766
981.8748
133 : M.findmain = 480.1967 (+/− 20 ppm)
481.204
[M+H]+
525.23
112.0503
193.1591
262.1305
303.1405
395.2035
483.2564
717.8481
963.4766
981.8748

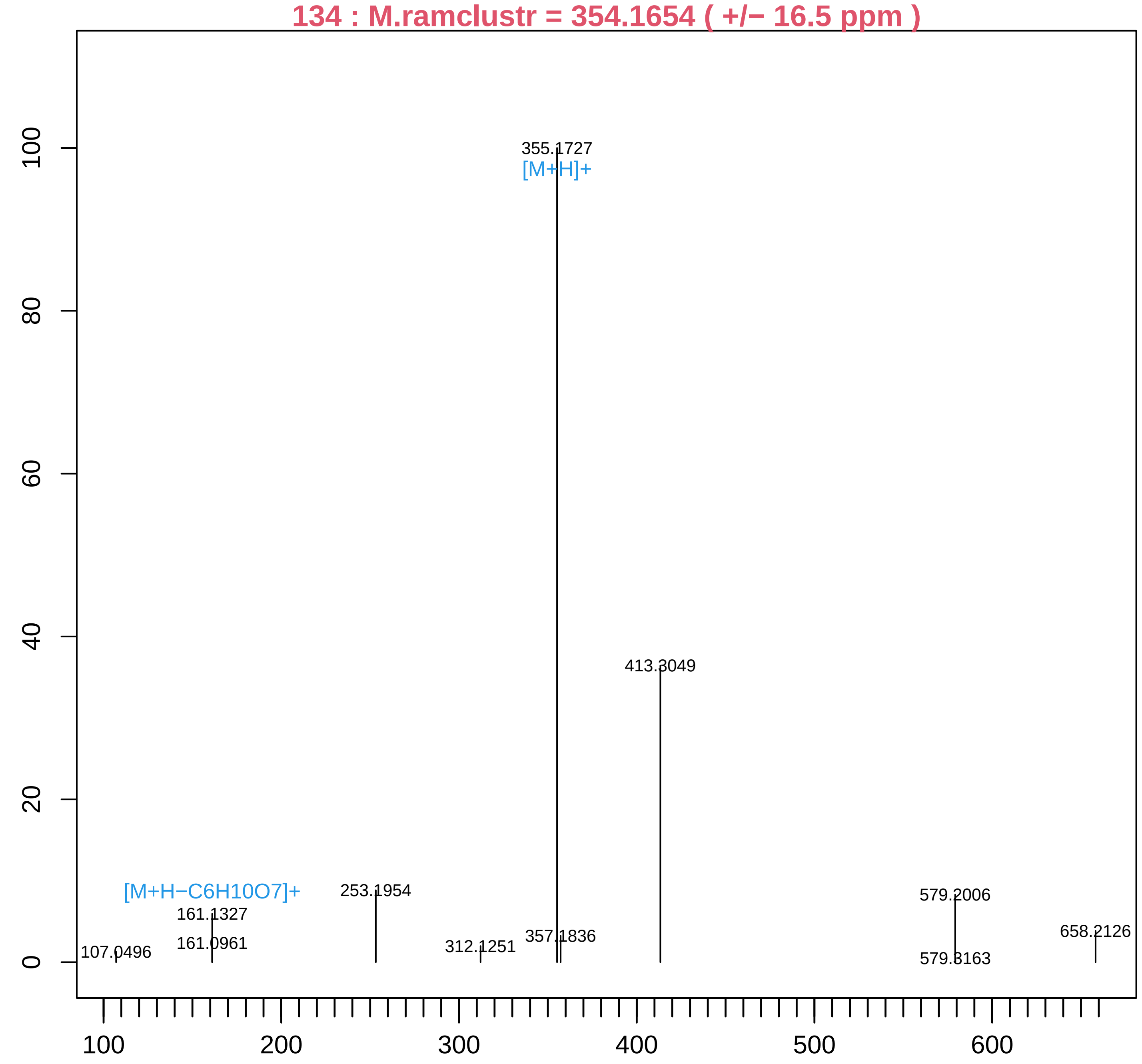
134 : M.ramclustr = 354.1654 (+/− 16.5 ppm)
355.1727
[M+H]+
413.3049
[M+H−C6H10O7]+
253.1954
161.1327
161.0961
107.0496
312.1251
357.1836
579.2006
579.3163
658.2126
0
20
40
60
80
100
200
300
400
500
600

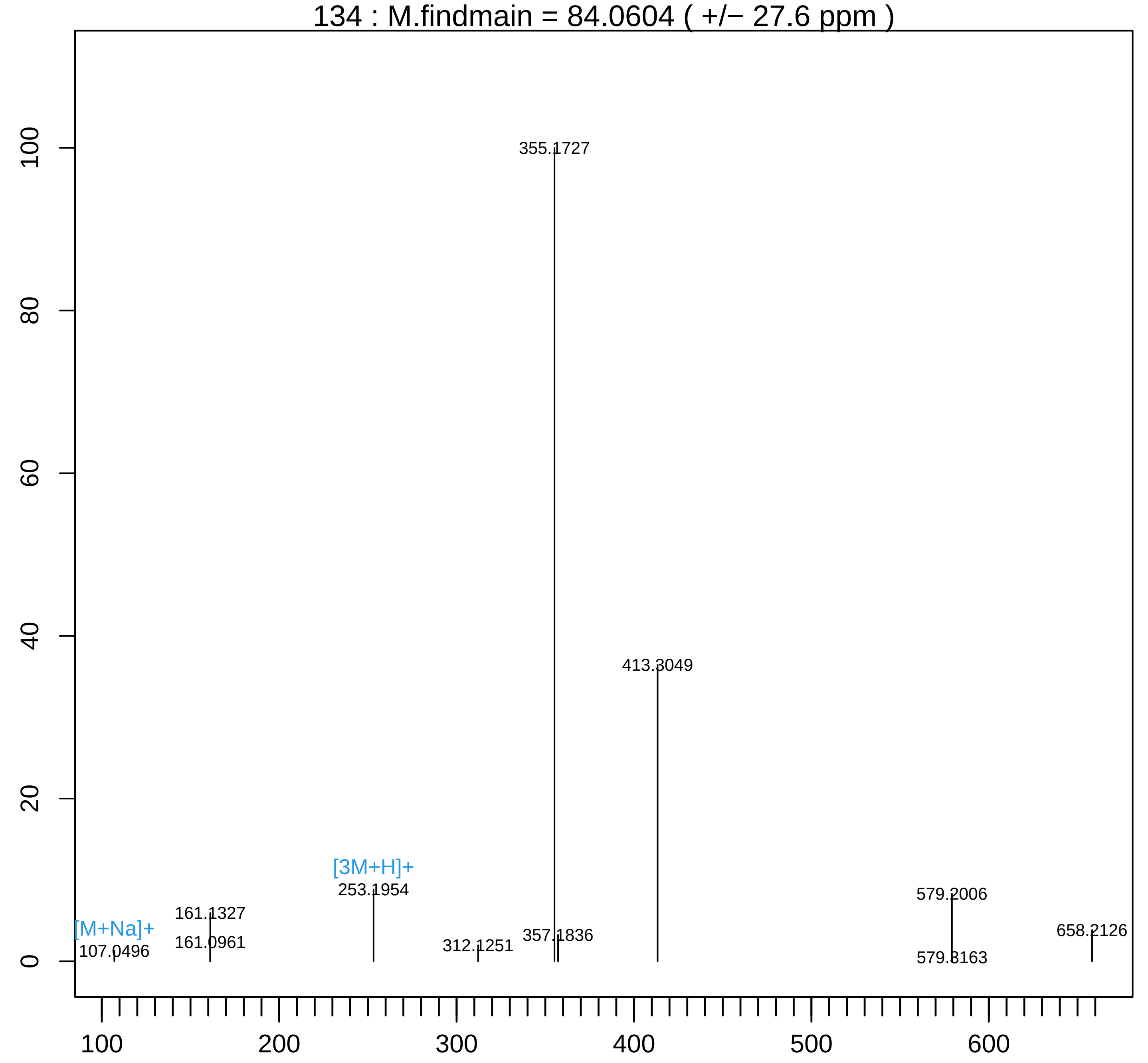
134 : M.findmain = 84.0604 (+/− 27.6 ppm)
355.1727
413.3049
[3M+H]+
253.1954
161.1327
161.0961
[M+Na]+
107.0496
312.1251
357.1836
579.2006
579.3163
658.2126
0
20
40
60
80
100
200
300
400
500
600

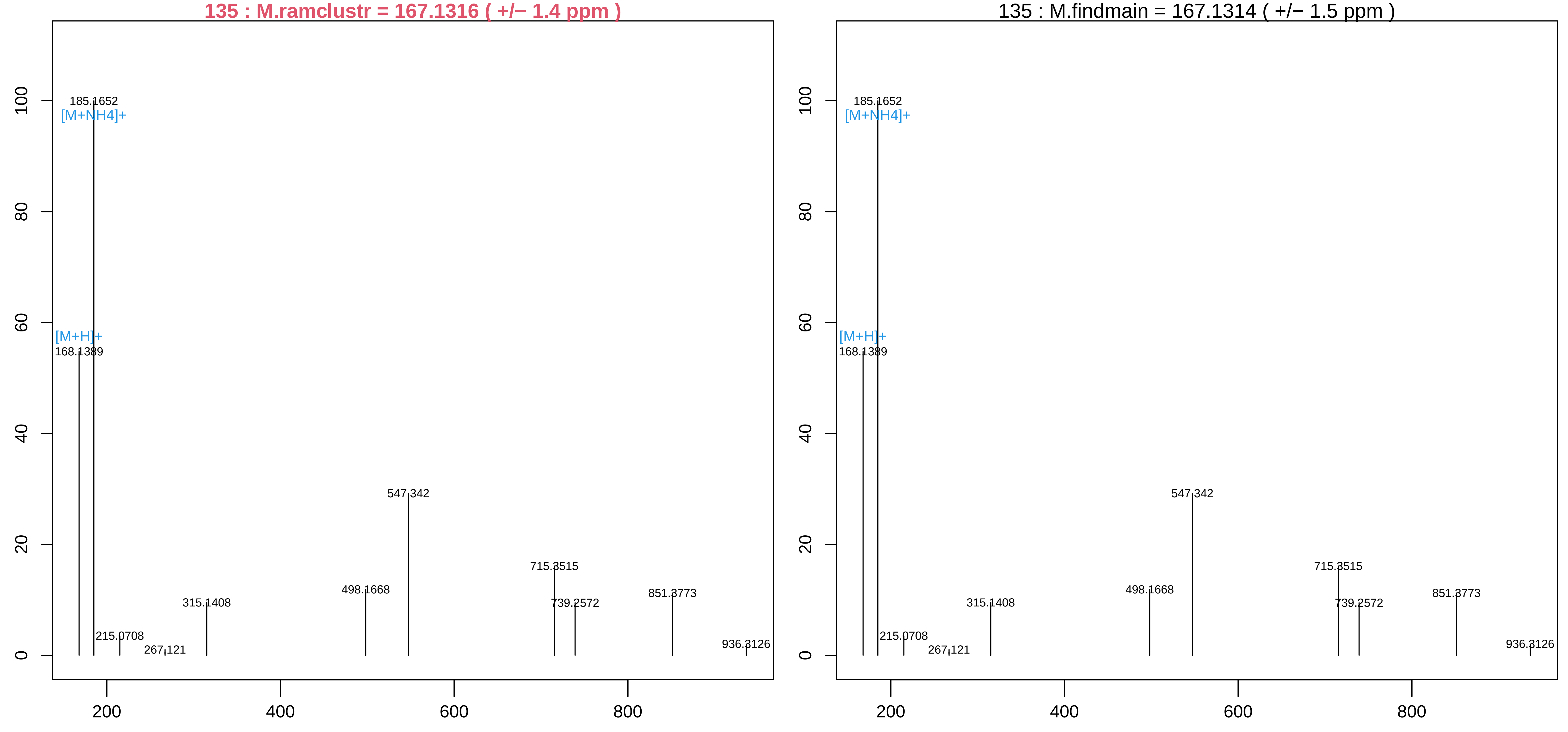

135 : M.ramclustr = 167.1316 (+/− 1.4 ppm)
185.1652
[M+NH4]+
[M+H]+
168.1389
215.0708
267.121
315.1408
498.1668
547.342
715.3515
739.2572
851.3773
936.3126
135 : M.findmain = 167.1314 (+/− 1.5 ppm)
185.1652
[M+NH4]+
[M+H]+
168.1389
215.0708
267.121
315.1408
498.1668
547.342
715.3515
739.2572
851.3773
936.3126

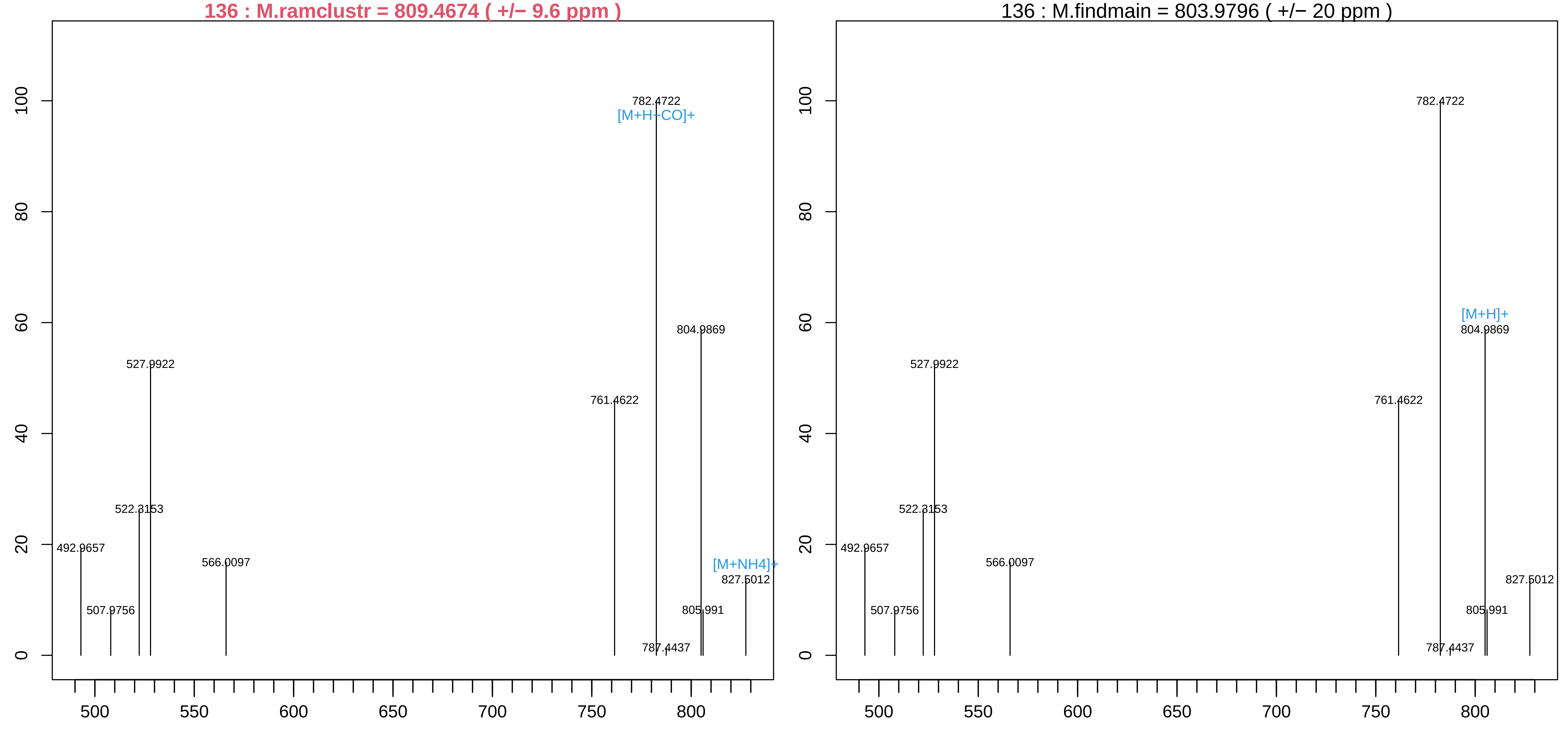
136 : M.ramclustr = 809.4674 (+/− 9.6 ppm)
782.4722
[M+H-CO]+
804.9869
527.9922
761.4622
522.3153
492.9657
566.0097
[M+NH4]+
827.5012
507.9756
805.991
787.4437
136 : M.findmain = 803.9796 (+/− 20 ppm)
782.4722
[M+H]+
804.9869
527.9922
761.4622
522.3153
492.9657
566.0097
827.5012
507.9756
805.991
787.4437

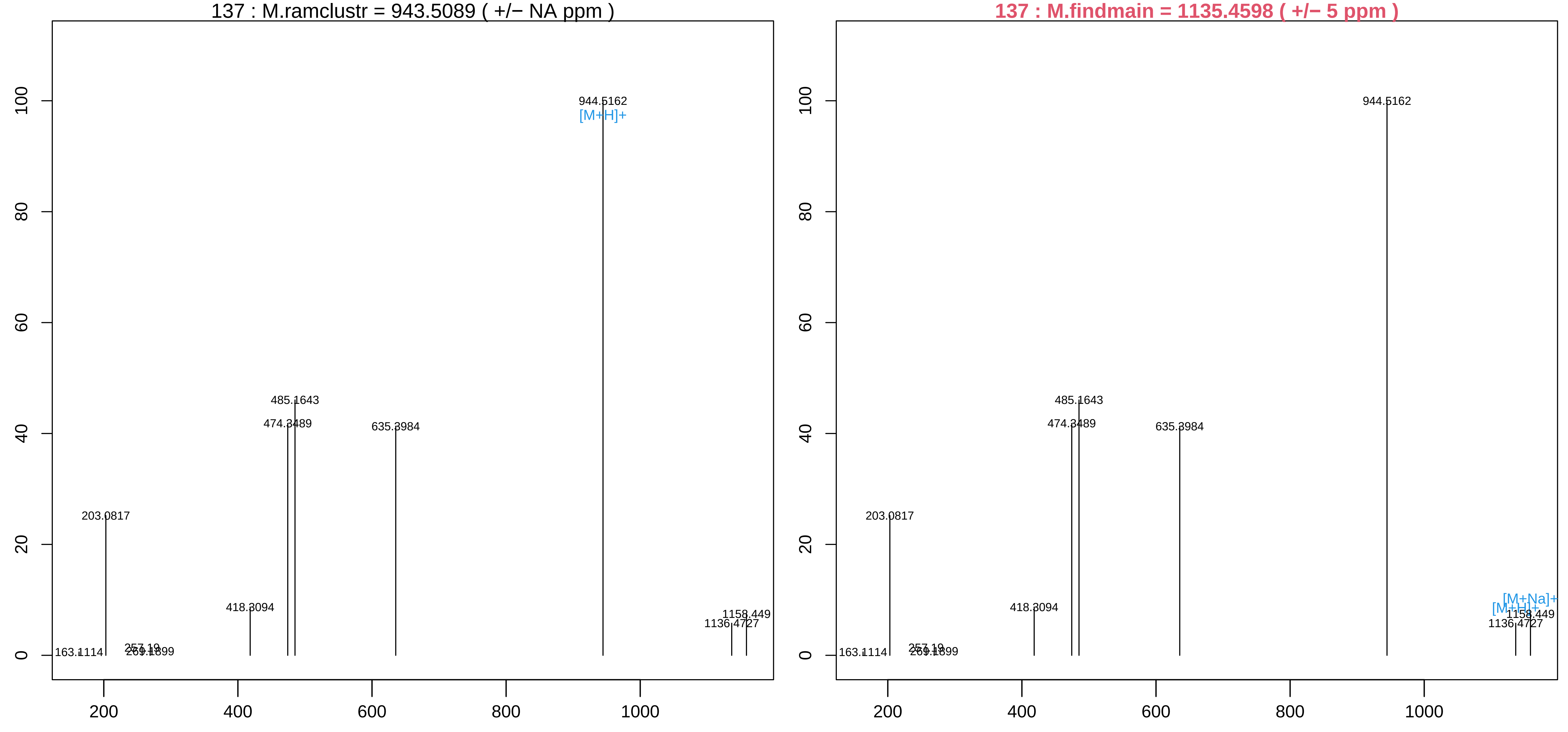

137 : M.ramclustr = 943.5089 (+/− NA ppm)
944.5162
[M+H]+
485.1643
474.3489
635.3984
203.0817
418.3094
1158.449
1136.4727
163.1114
257.19
269.1899
137 : M.findmain = 1135.4598 (+/− 5 ppm)
944.5162
485.1643
474.3489
635.3984
203.0817
418.3094
[M+Na]+
[M+H]+
1158.449
1136.4727
163.1114
257.19
269.1899
0
20
40
60
80
100
200
400
600
800
1000

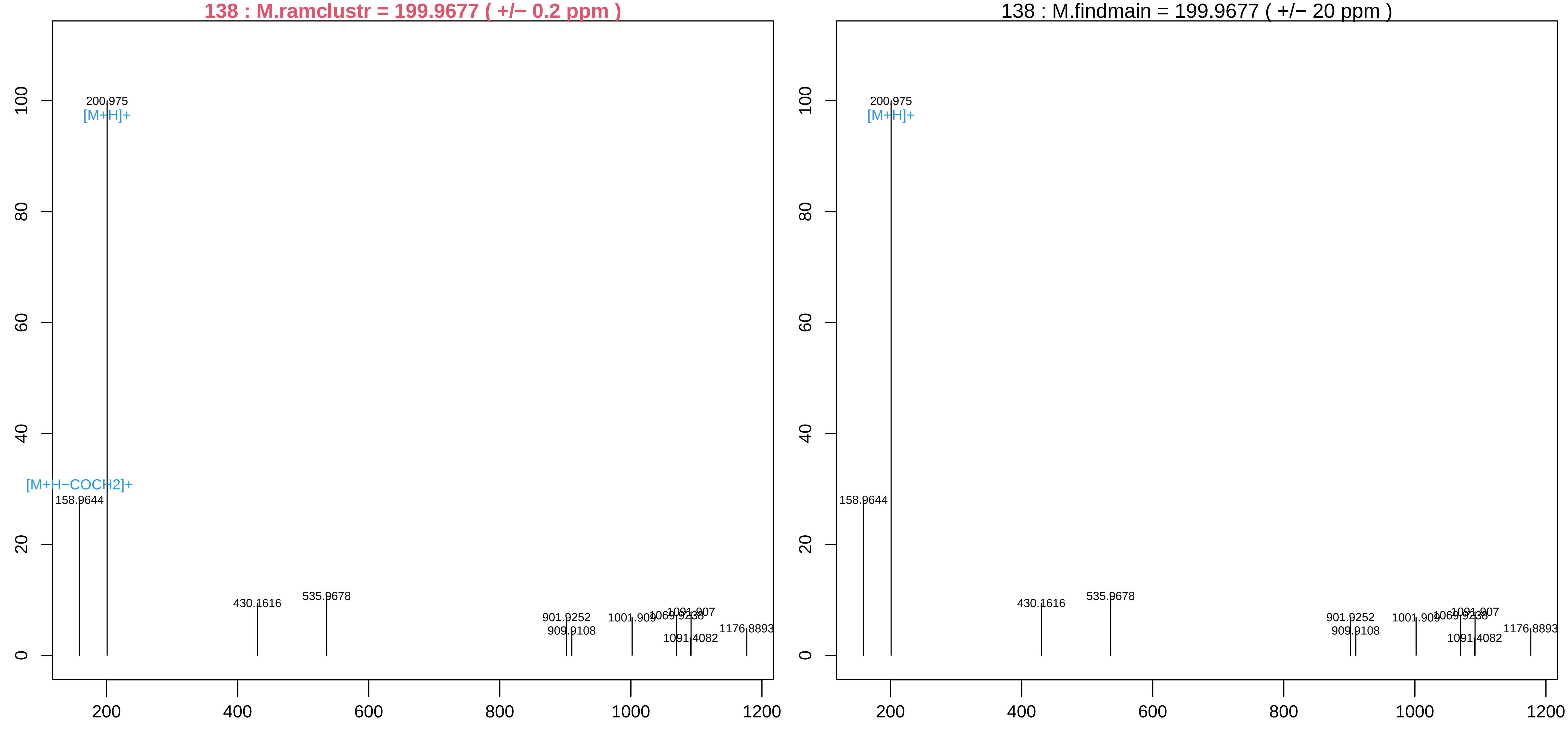
138 : M.ramclustr = 199.9677 (+/− 0.2 ppm)
138 : M.findmain = 199.9677 (+/− 20 ppm)
200.975
[M+H]+
[M+H−COCH2]+
158.9644
430.1616
535.9678
901.9252
909.9108
1001.909
1069.9238
1091.907
1091.4082
1176.8893
0
20
40
60
80
100
200
400
600
800
1000
1200

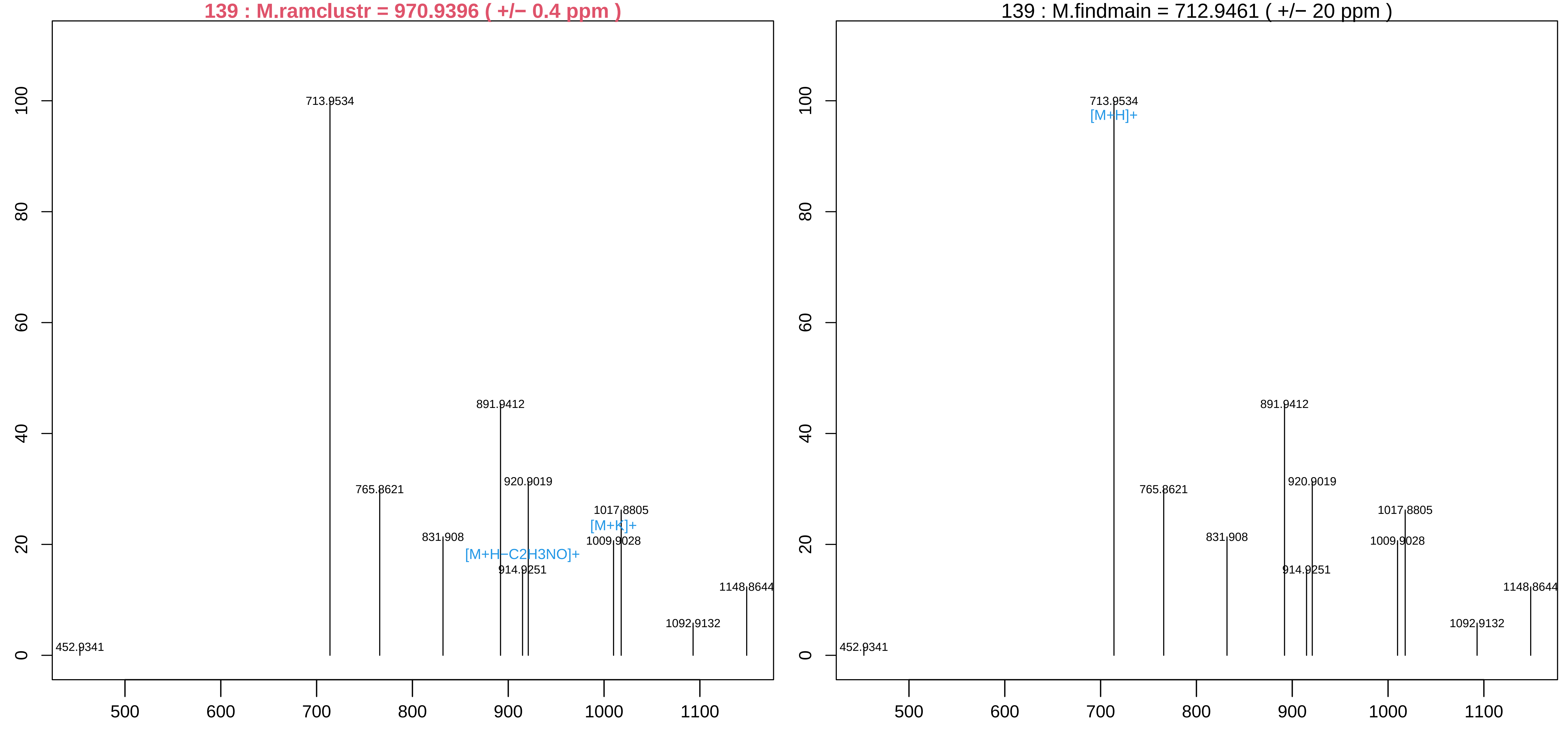
139 : M.ramclustr = 970.9396 (+/− 0.4 ppm)
713.9534
765.8621
831.908
891.9412
920.9019
914.9251
[M+H−C2H3NO]+
1017.8805
[M+K]+
1009.9028
1092.9132
1148.8644
452.9341
139 : M.findmain = 712.9461 (+/− 20 ppm)
713.9534
[M+H]+
765.8621
831.908
891.9412
920.9019
914.9251
1017.8805
1009.9028
1092.9132
1148.8644
452.9341

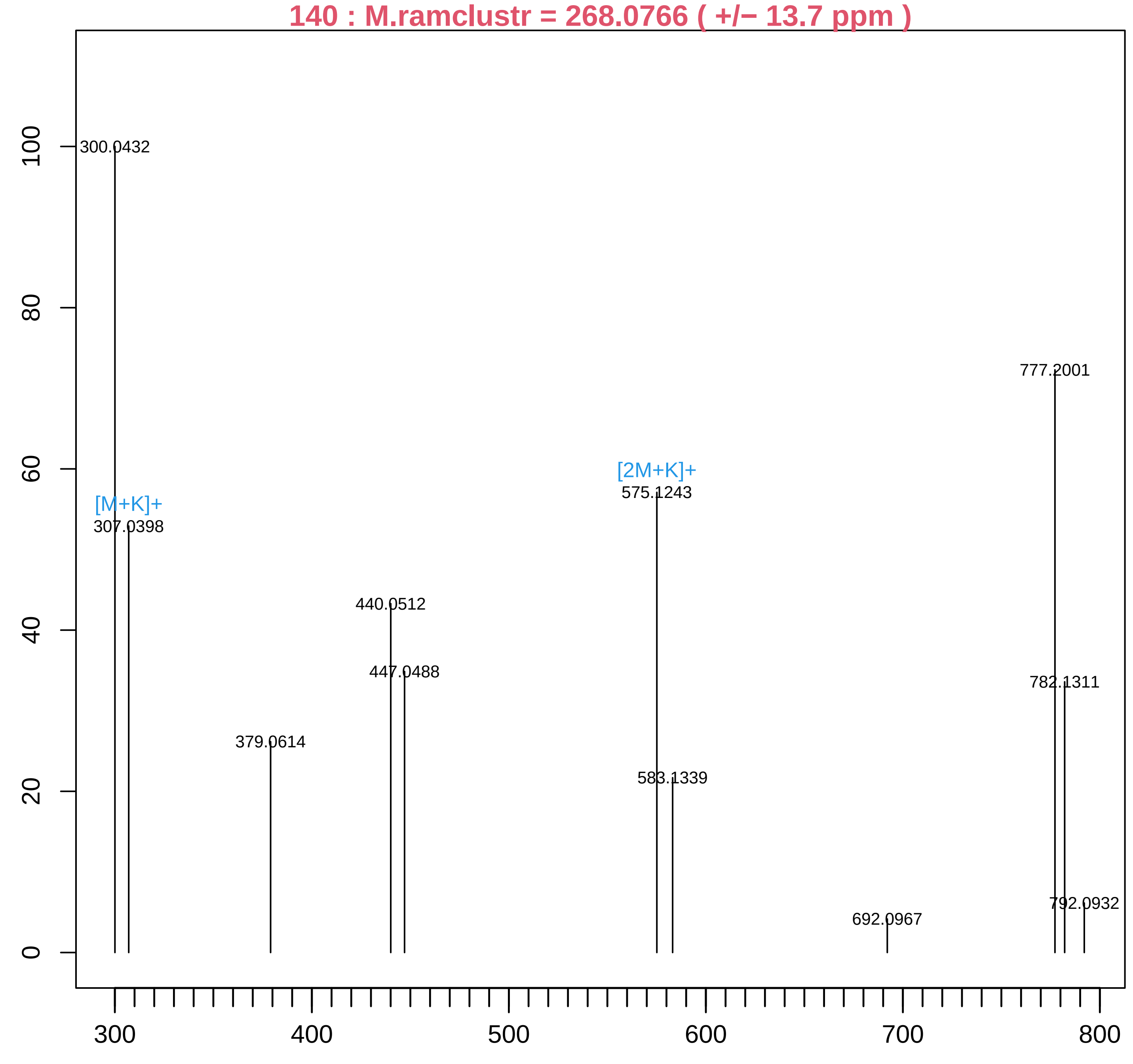
140 : M.ramclustr = 268.0766 (+/− 13.7 ppm)
300.0432
[M+K]+
307.0398
379.0614
440.0512
447.0488
[2M+K]+
575.1243
583.1339
692.0967
777.2001
782.1311
792.0932
0
20
40
60
80
100
300
400
500
600
700
800

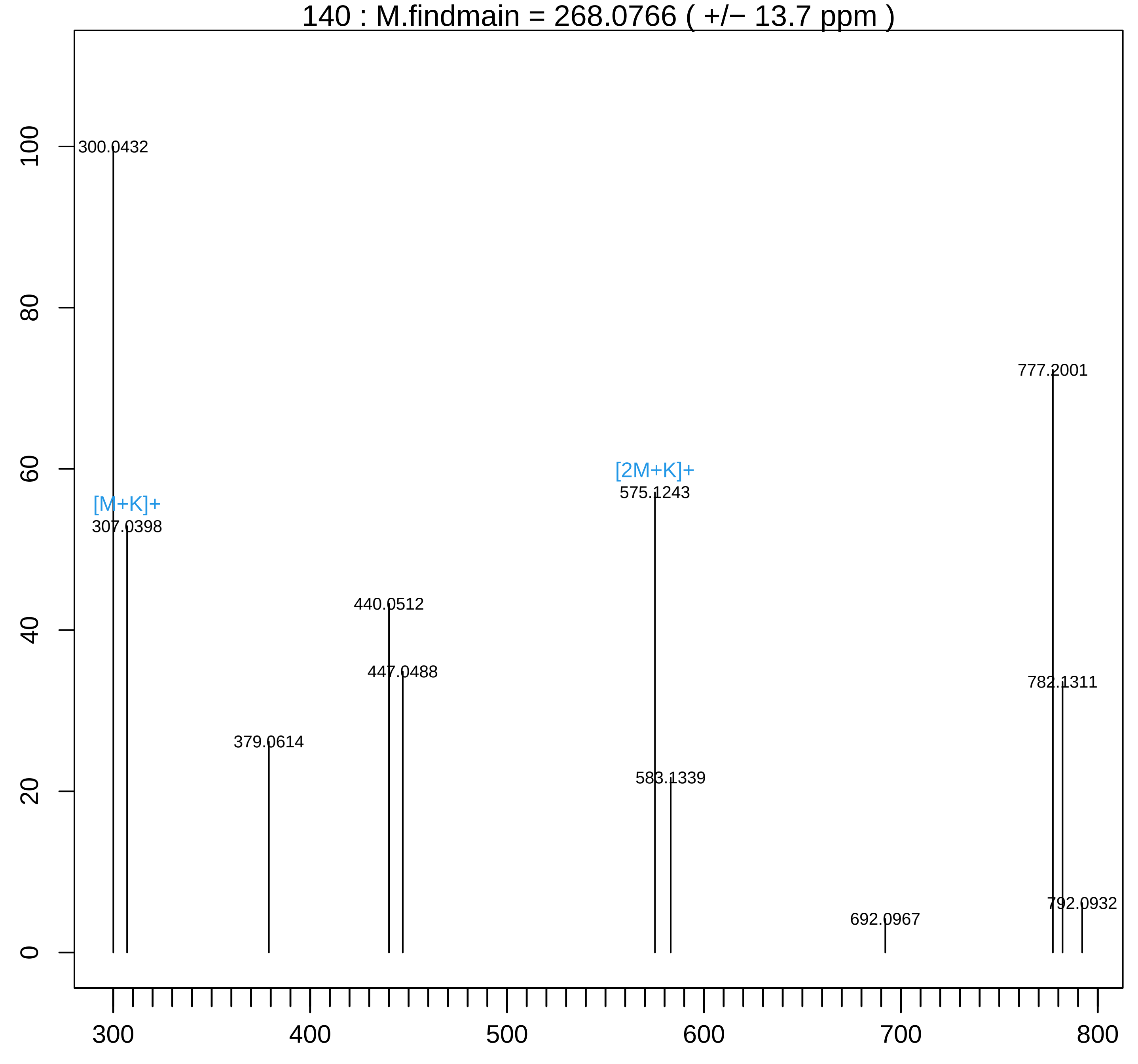
140 : M.findmain = 268.0766 (+/− 13.7 ppm)
300.0432
[M+K]+
307.0398
379.0614
440.0512
447.0488
[2M+K]+
575.1243
583.1339
692.0967
777.2001
782.1311
792.0932
0
20
40
60
80
100
300
400
500
600
700
800

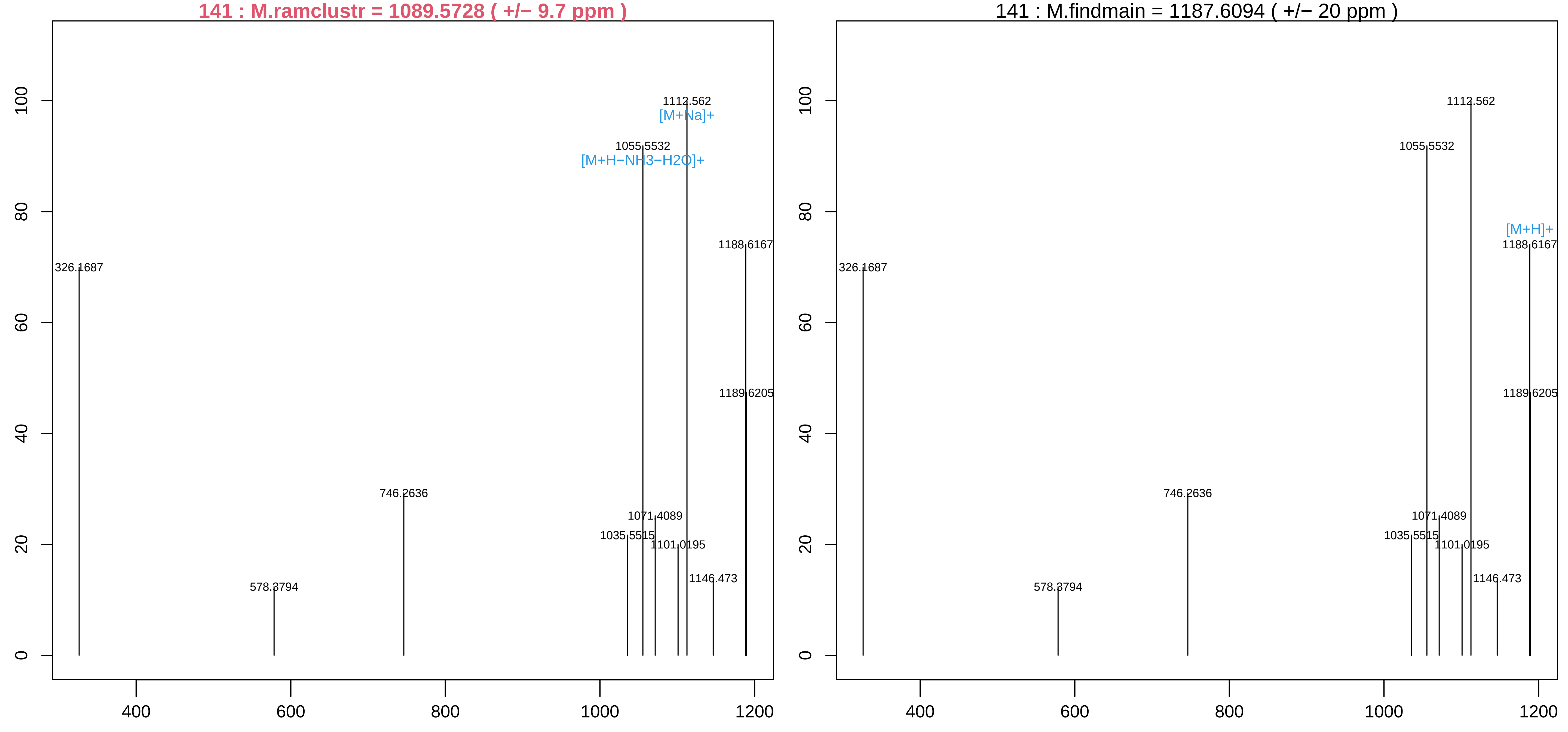

141 : M.ramclustr = 1089.5728 (+/− 9.7 ppm)
1112.562
[M+Na]+
1055.5532
[M+H−NH3−H2O]+
1188.6167
326.1687
1189.6205
746.2636
1071.4089
1035.5515
1101.0195
1146.473
578.3794
141 : M.findmain = 1187.6094 (+/− 20 ppm)
[M+H]+
0
20
40
60
80
100
400
600
800
1000
1200

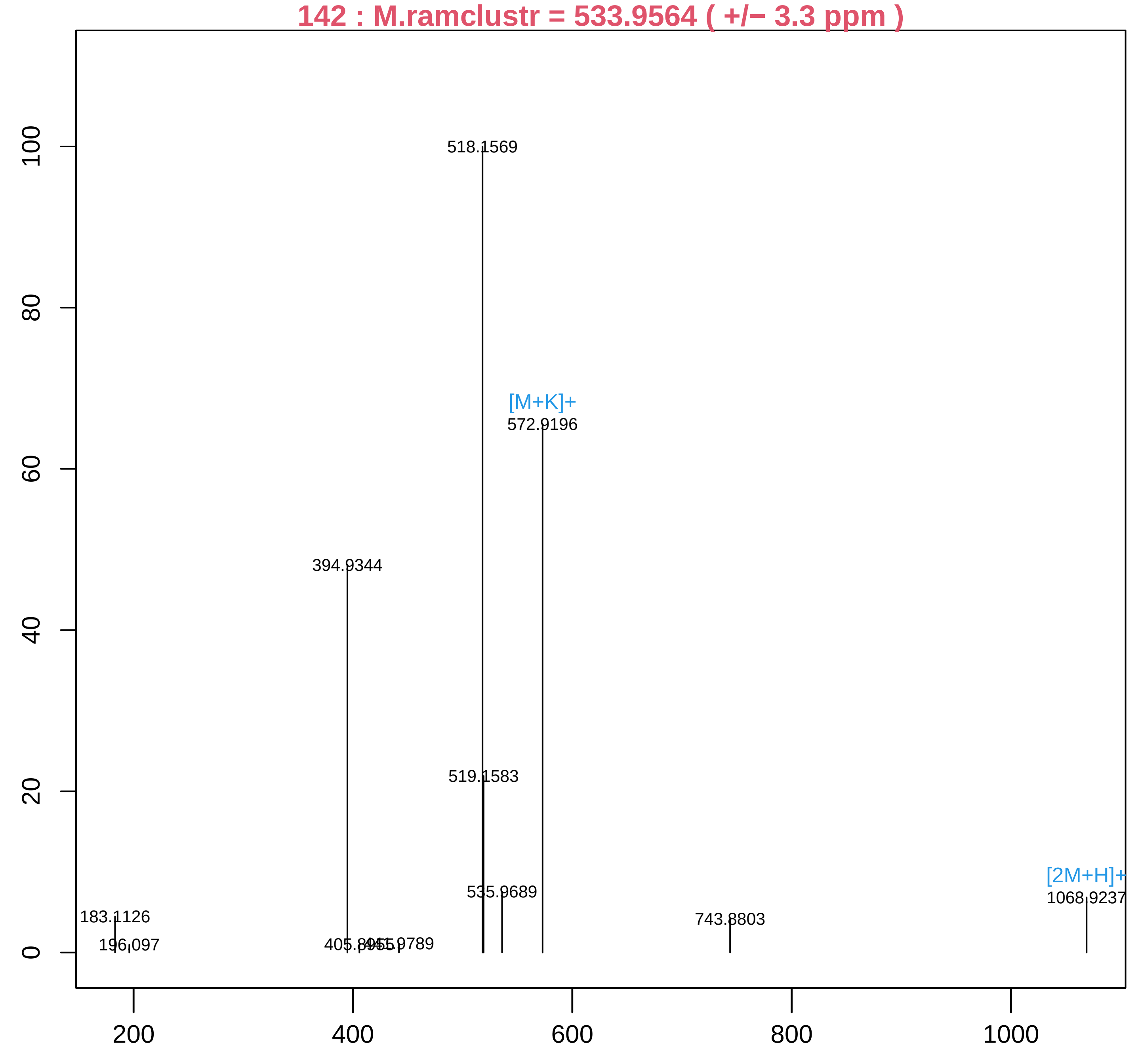
142 : M.ramclustr = 533.9564 (+/− 3.3 ppm)
518.1569
[M+K]+
572.9196
394.9344
519.1583
535.9689
743.8803
[2M+H]+
1068.9237
183.1126
196.097
0
20
40
60
80
100
200
400
600
800
1000

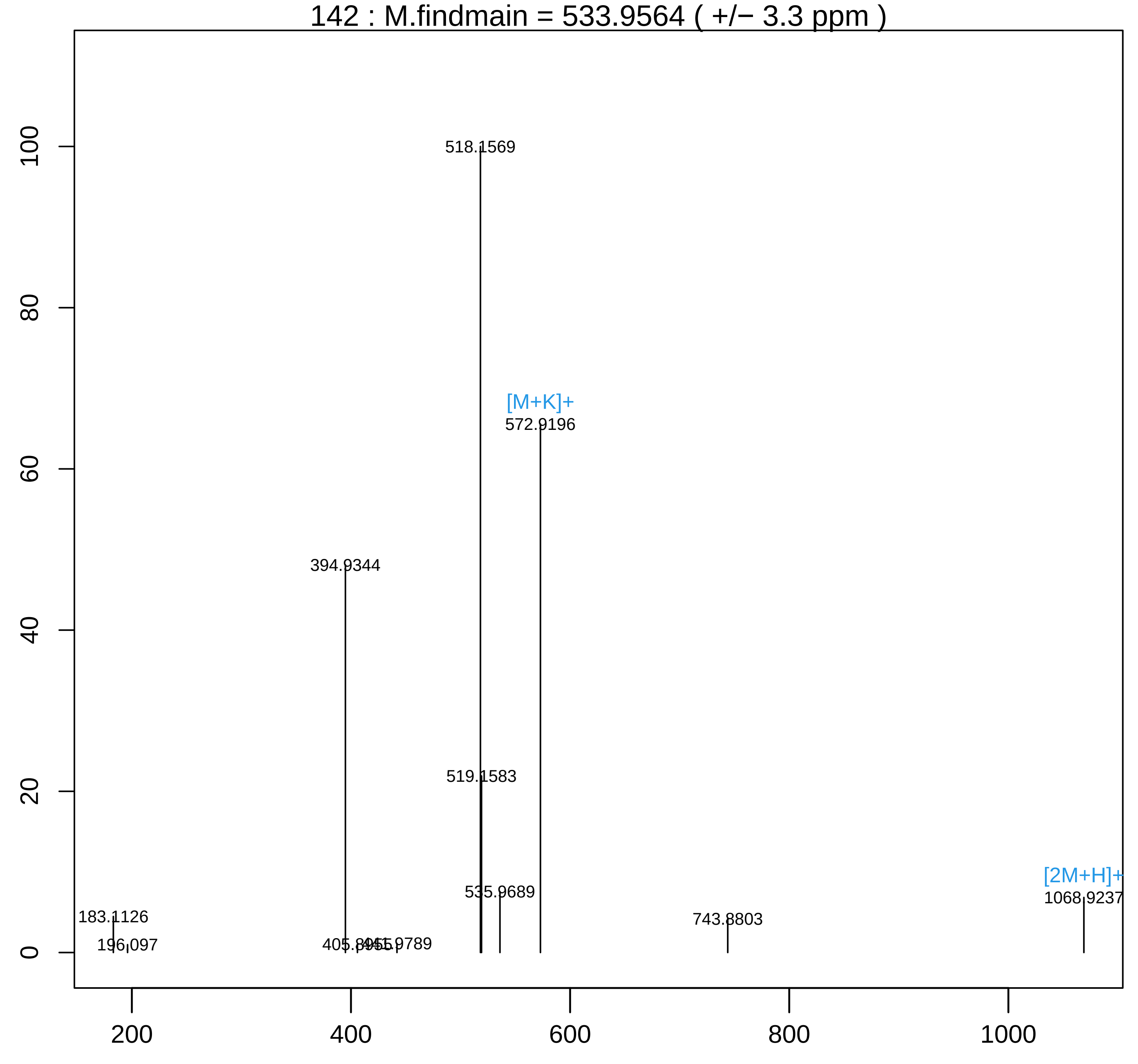
142 : M.findmain = 533.9564 (+/− 3.3 ppm)
518.1569
[M+K]+
572.9196
394.9344
519.1583
535.9689
743.8803
[2M+H]+
1068.9237
183.1126
196.097
0
20
40
60
80
100
200
400
600
800
1000

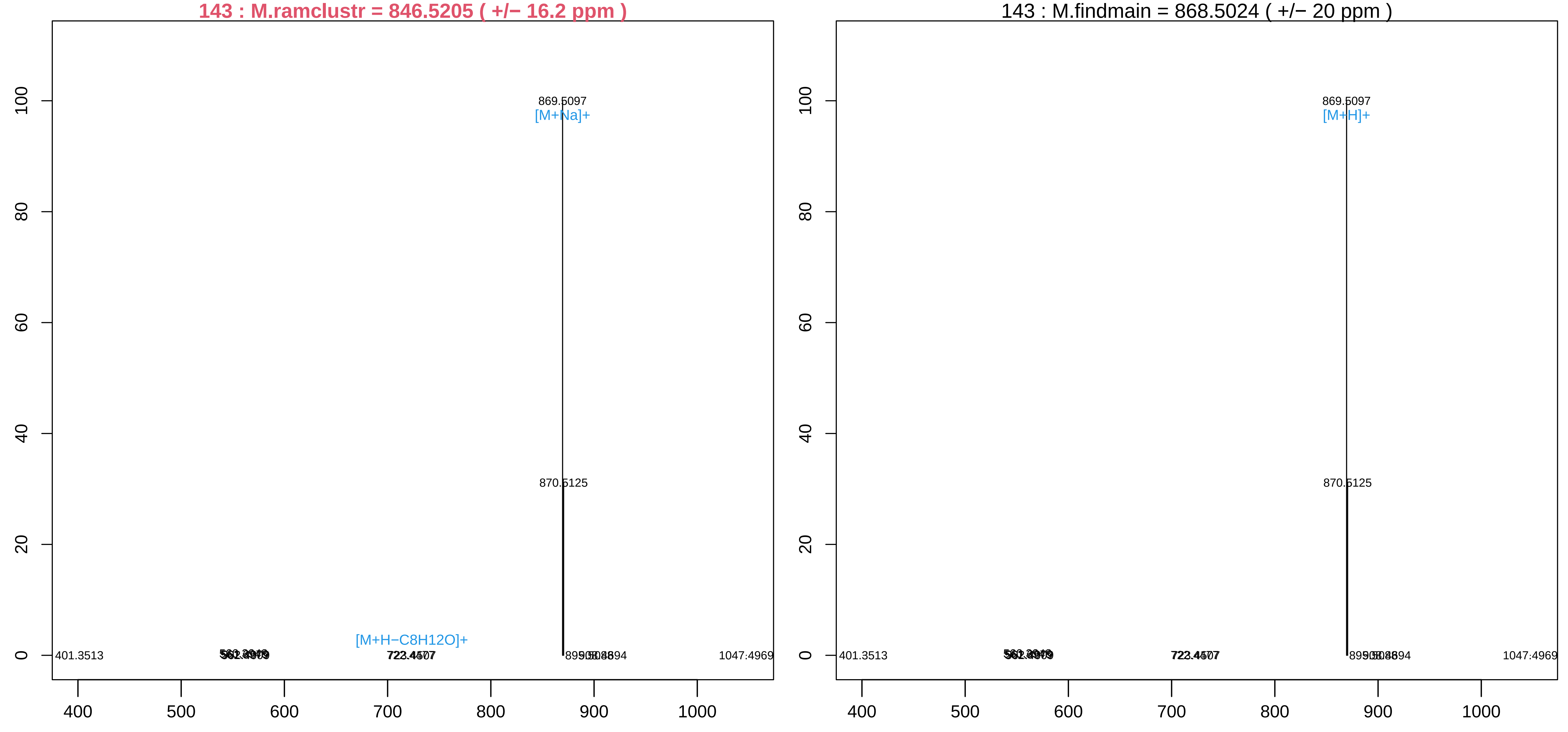
143 : M.ramclustr = 846.5205 (+/− 16.2 ppm)
143 : M.findmain = 868.5024 (+/− 20 ppm)
869.5097
[M+Na]+
[M+H]+
870.5125
401.3513
[M+H−C8H12O]+
1047.4969
0
20
40
60
80
100
400
500
600
700
800
900
1000

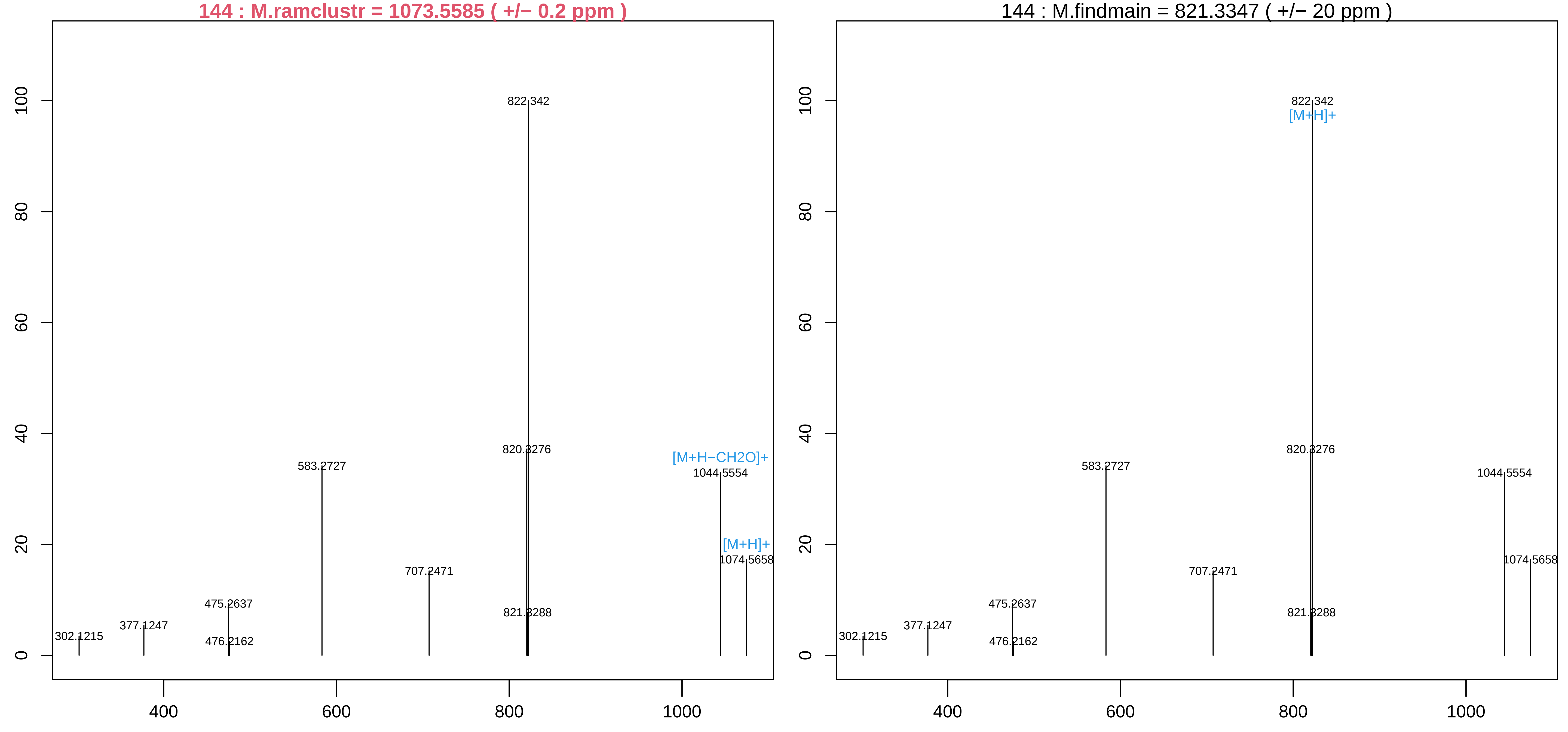
144 : M.ramclustr = 1073.5585 (+/− 0.2 ppm)
822.342
820.3276
583.2727
[M+H−CH2O]+
1044.5554
[M+H]+
1074.5658
707.2471
475.2637
821.3288
377.1247
302.1215
476.2162
144 : M.findmain = 821.3347 (+/− 20 ppm)
822.342
[M+H]+
820.3276
583.2727
1044.5554
1074.5658
707.2471
475.2637
821.3288
377.1247
302.1215
476.2162
0
20
40
60
80
100
400
600
800
1000

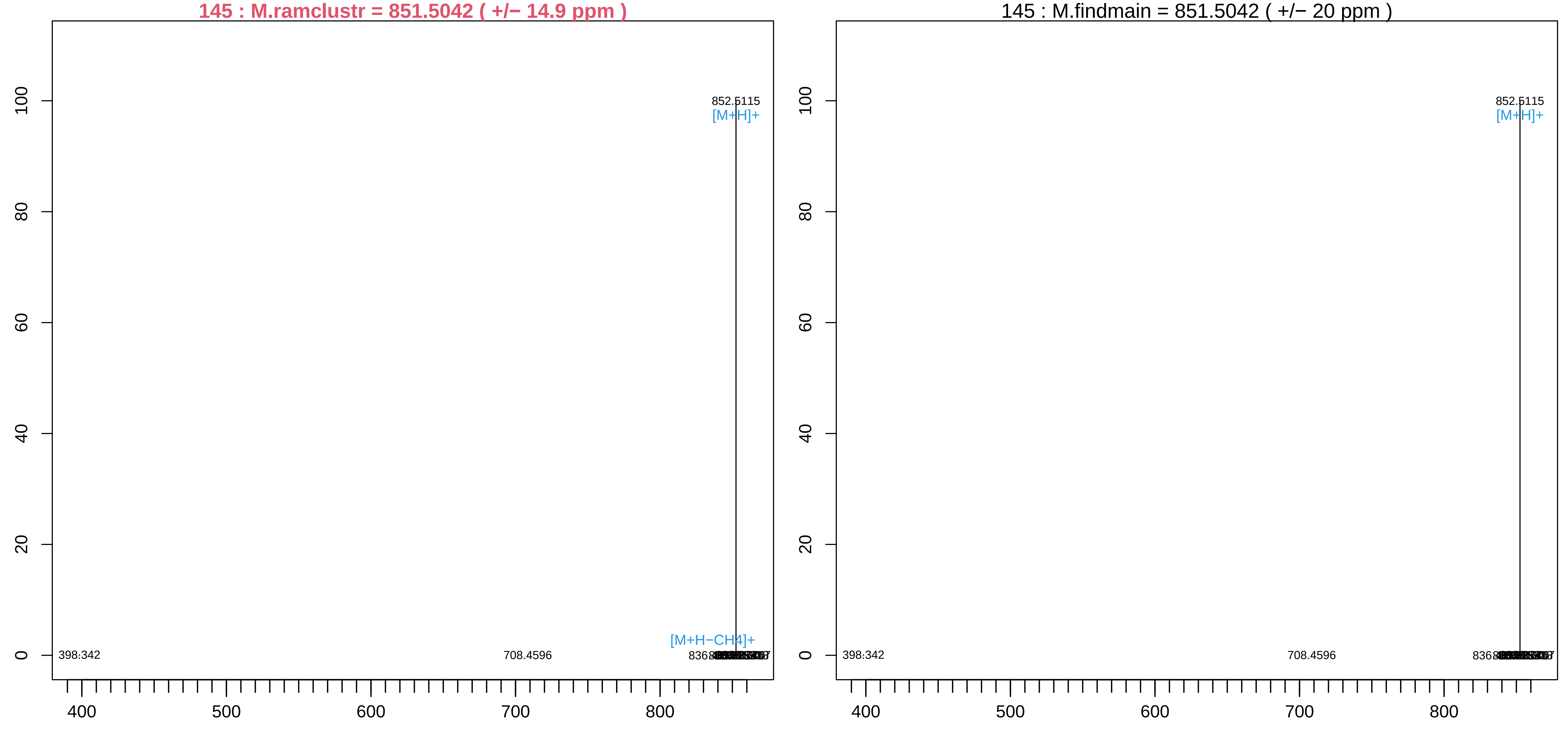
145 : M.ramclustr = 851.5042 (+/− 14.9 ppm)
852.5115
[M+H]+
[M+H−CH4]+
398:342
708.4596
145 : M.findmain = 851.5042 (+/− 20 ppm)
852.5115
[M+H]+
398:342
708.4596

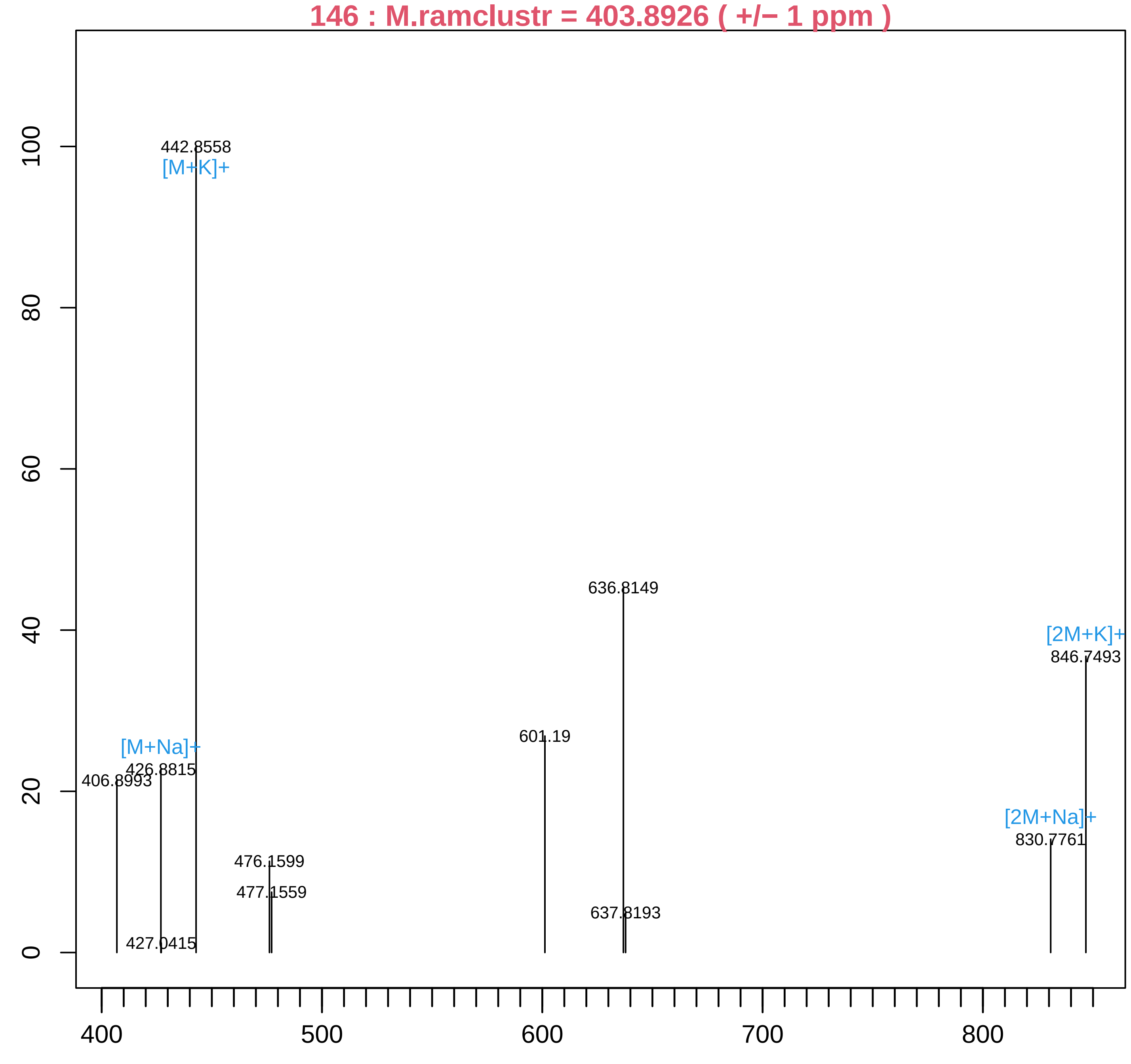
146 : M.ramclustr = 403.8926 (+/− 1 ppm)
442.8558
[M+K]+
636.8149
[2M+K]+
846.7493
[M+Na]+
426.8815
406.8993
601.19
[2M+Na]+
830.7761
476.1599
477.1559
637.8193
427.0415
0
20
40
60
80
100
400
500
600
700
800
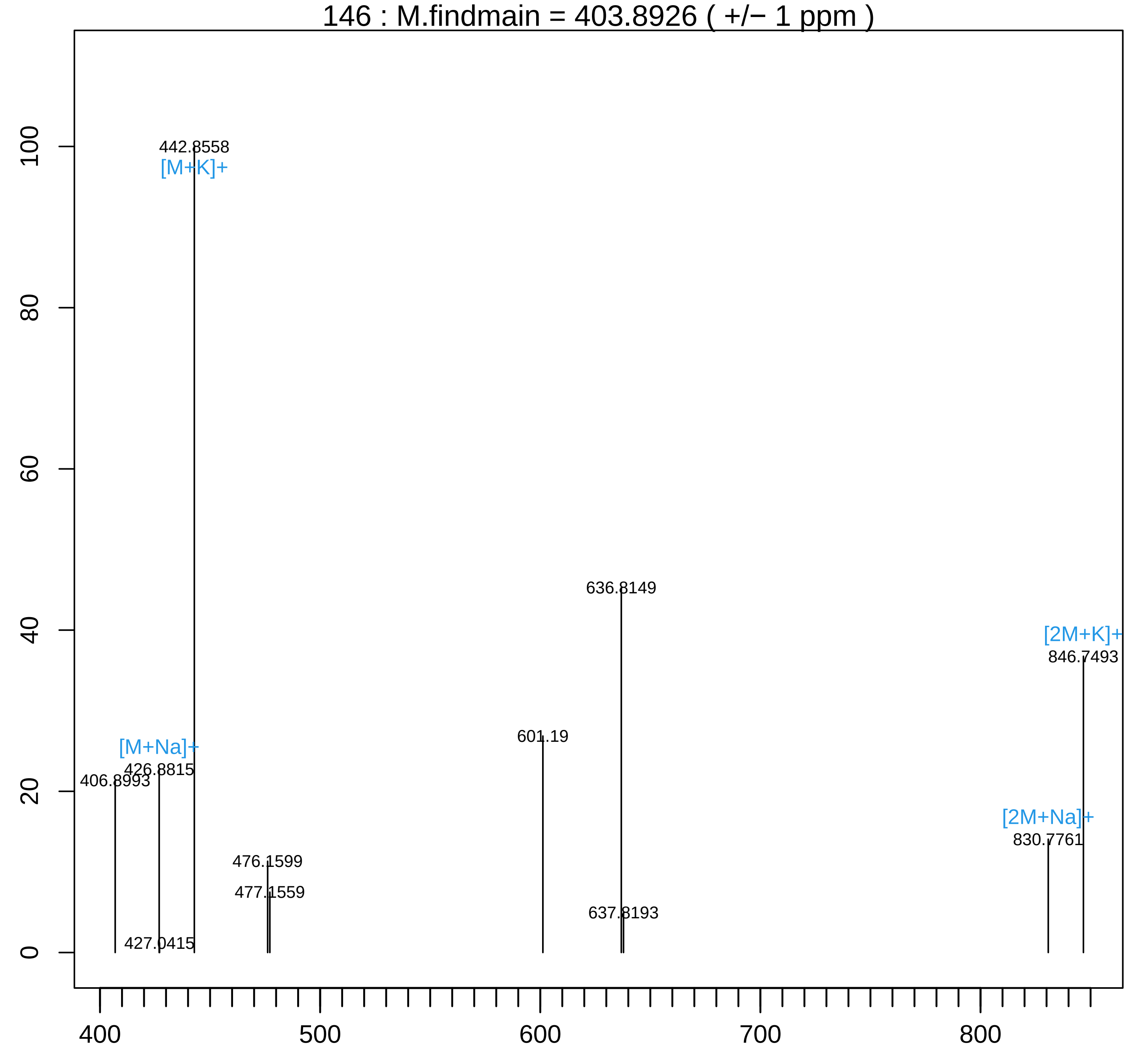
146 : M.findmain = 403.8926 (+/− 1 ppm)
442.8558
[M+K]+
636.8149
[2M+K]+
846.7493
[M+Na]+
426.8815
406.8993
601.19
[2M+Na]+
830.7761
476.1599
477.1559
637.8193
427.0415
0
20
40
60
80
100
400
500
600
700
800

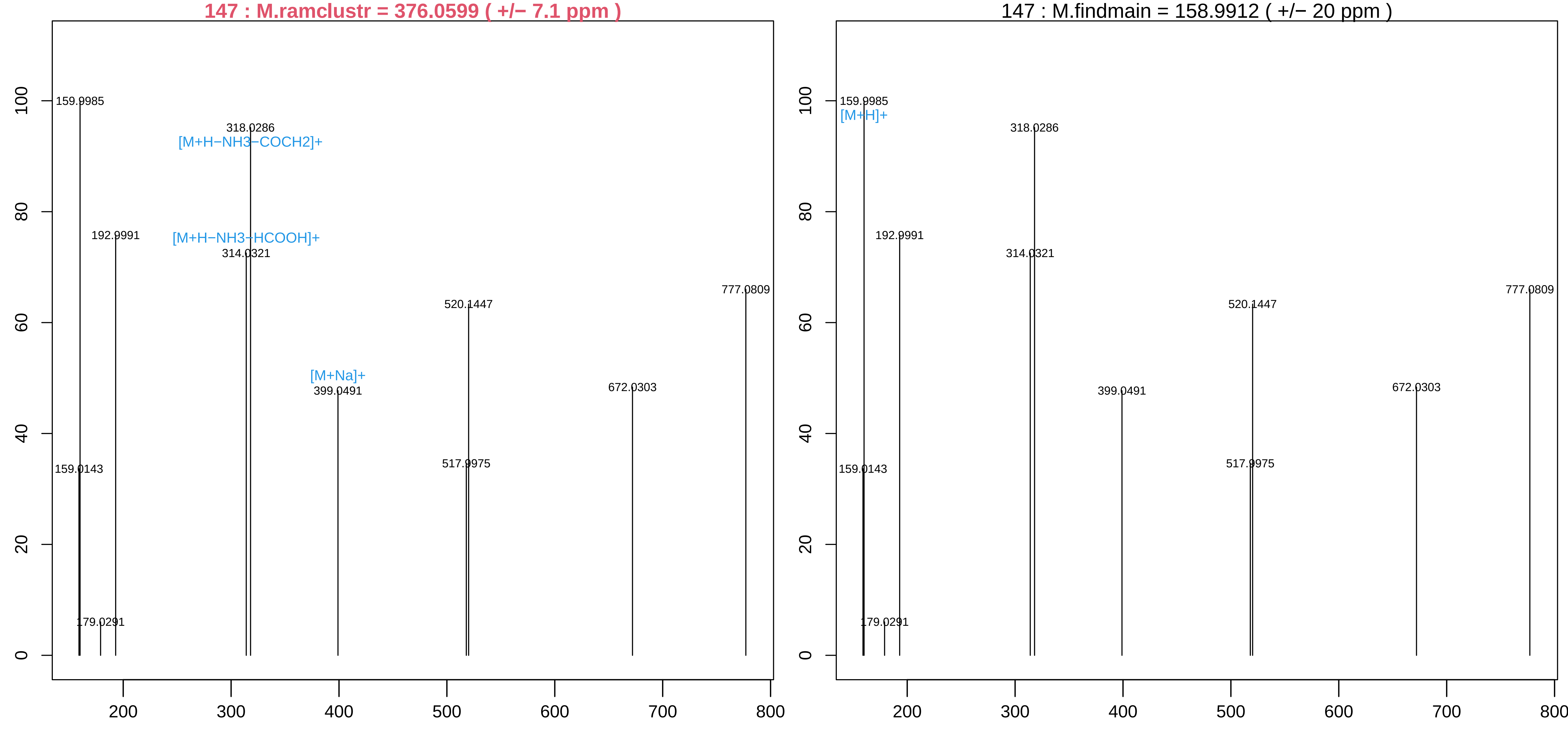

147 : M.ramclustr = 376.0599 (+/− 7.1 ppm)
159.9985
318.0286
[M+H−NH3−COCH2]+
192.9991
[M+H−NH3−HCOOH]+
314.0321
777.0809
520.1447
[M+Na]+
399.0491
672.0303
159.0143
517.9975
179.0291
0
20
40
60
80
100
200
300
400
500
600
700
800
147 : M.findmain = 158.9912 (+/− 20 ppm)
159.9985
[M+H]+
318.0286
192.9991
314.0321
777.0809
520.1447
399.0491
672.0303
159.0143
517.9975
179.0291
0
20
40
60
80
100
200
300
400
500
600
700
800

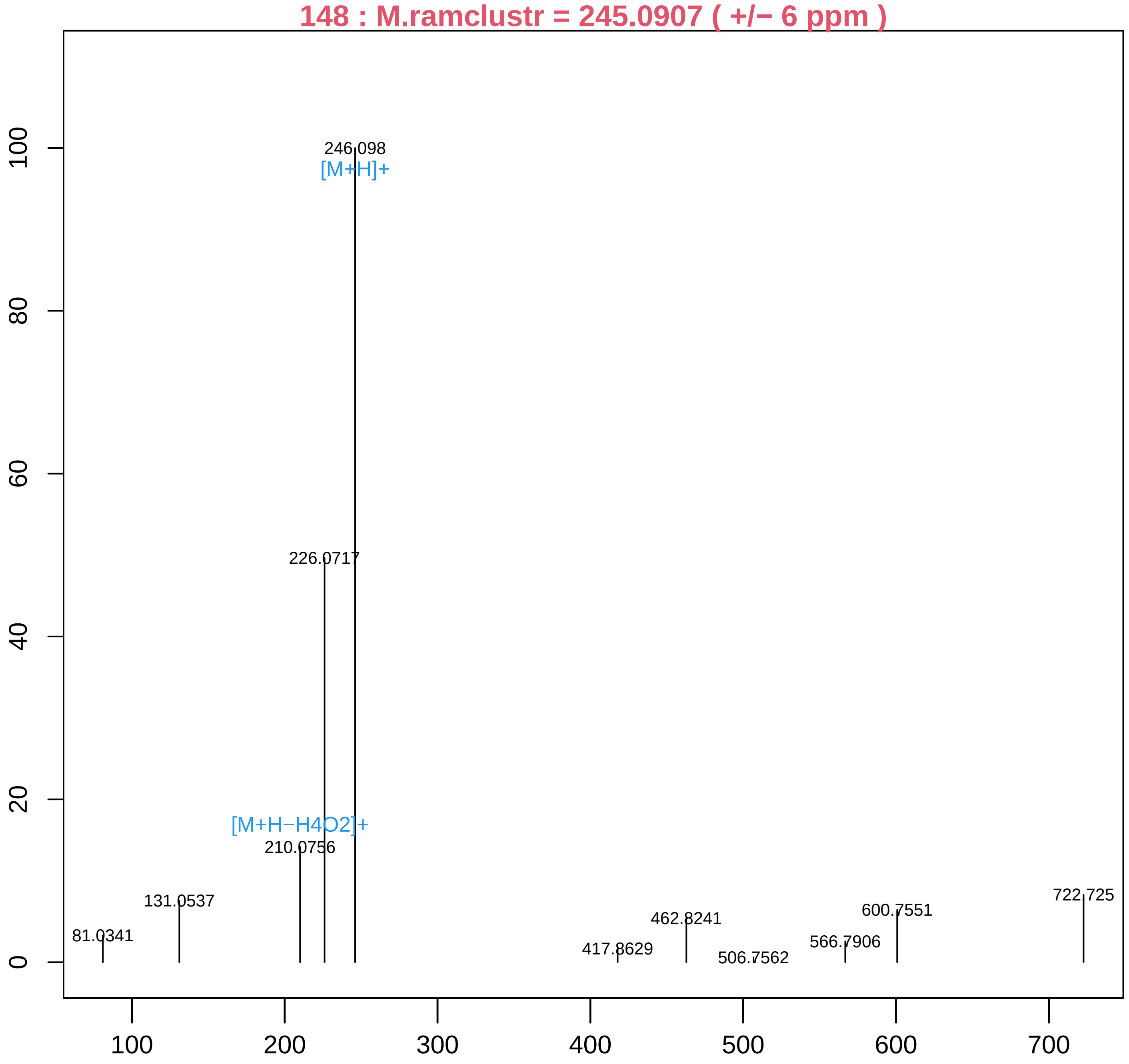
148 : M.ramclustr = 245.0907 (+/− 6 ppm)
246.098
[M+H]+
226.0717
[M+H−H4O2]+
210.0756
131.0537
81.0341
417.8629
462.8241
506.7562
566.7906
600.7551
722.725
0
20
40
60
80
100
100
200
300
400
500
600
700

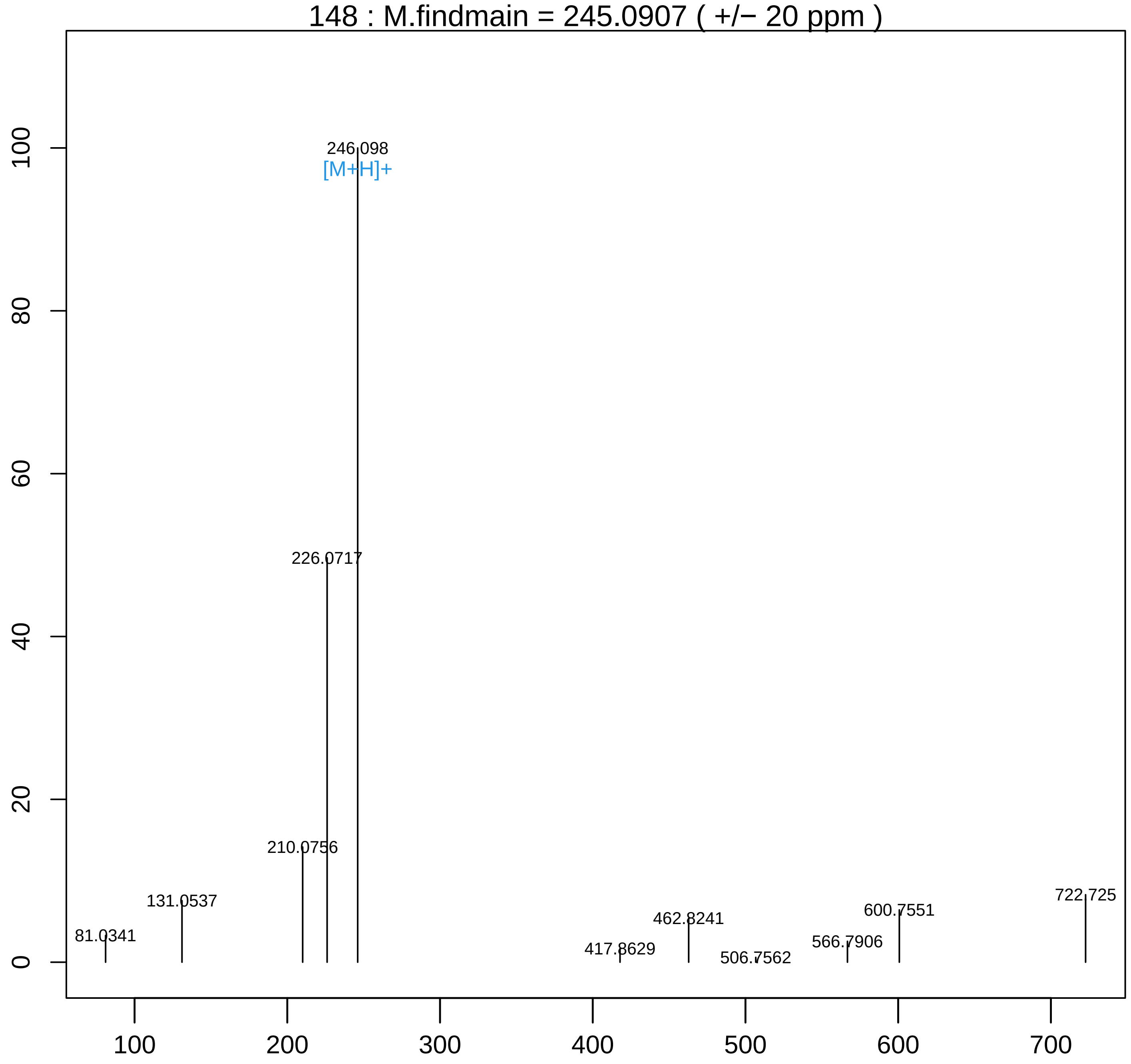
148 : M.findmain = 245.0907 (+/− 20 ppm)
246.098
[M+H]+
226.0717
210.0756
131.0537
81.0341
417.8629
462.8241
506.7562
566.7906
600.7551
722.725
0
20
40
60
80
100
100
200
300
400
500
600
700

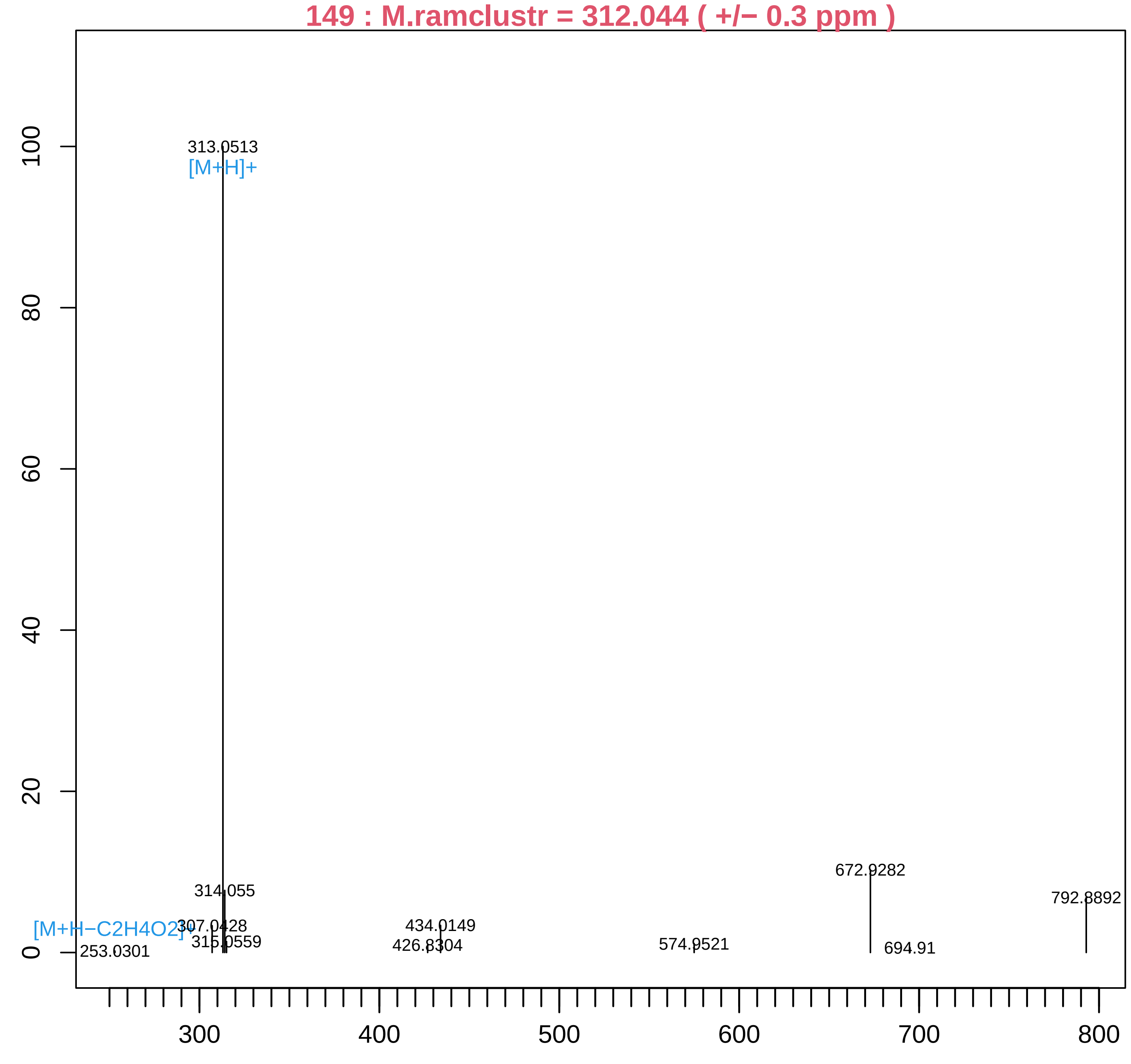
149 : M.ramclustr = 312.044 (+/− 0.3 ppm)
313.0513
[M+H]+
314.055
[M+H−C2H4O2]+
307.0428
315.0559
253.0301
434.0149
426.8304
574.9521
672.9282
694.91
792.8892

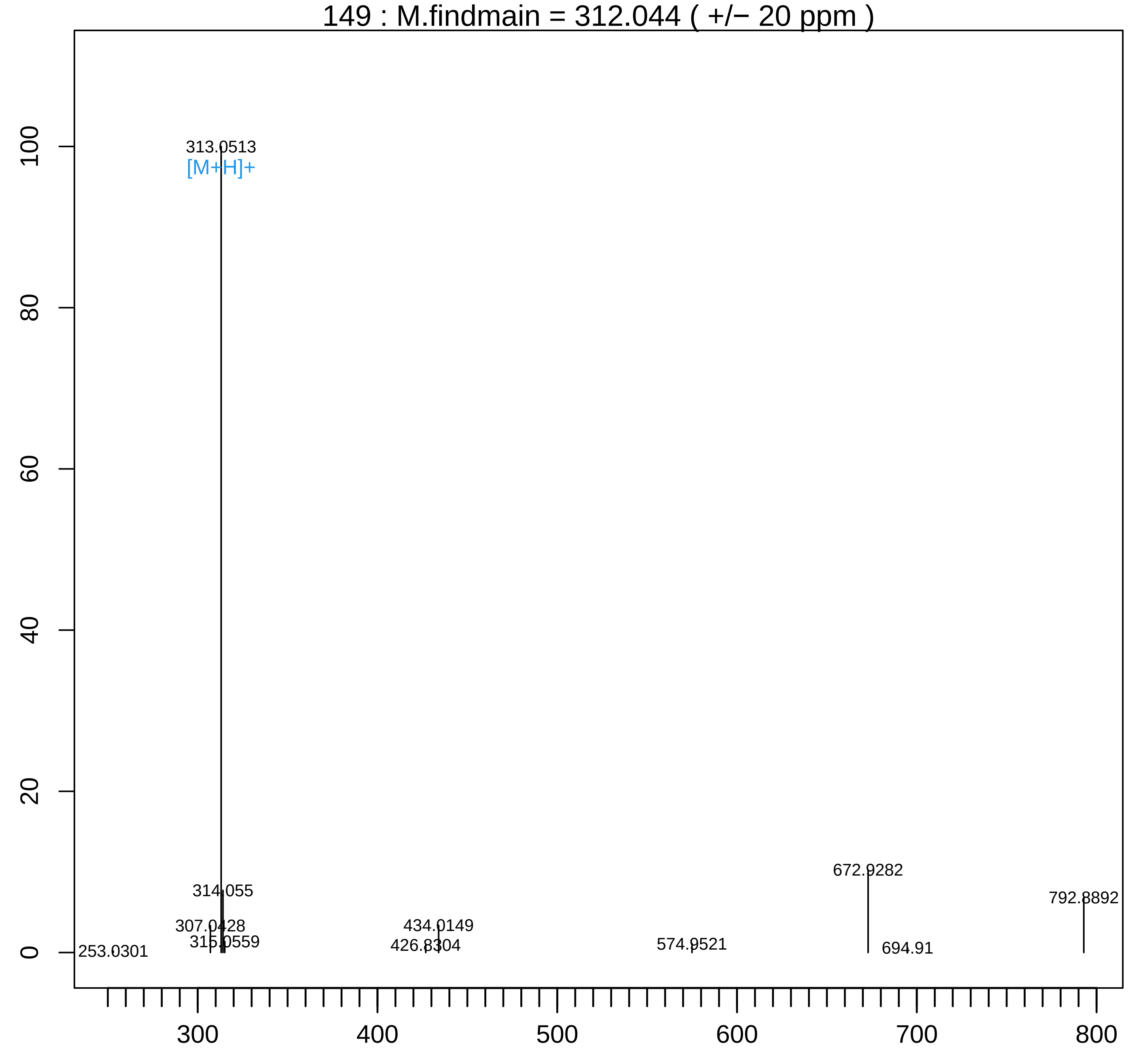
149 : M.findmain = 312.044 (+/− 20 ppm)
313.0513
[M+H]+
314.055
307.0428
315.0559
253.0301
434.0149
426.8304
574.9521
672.9282
694.91
792.8892

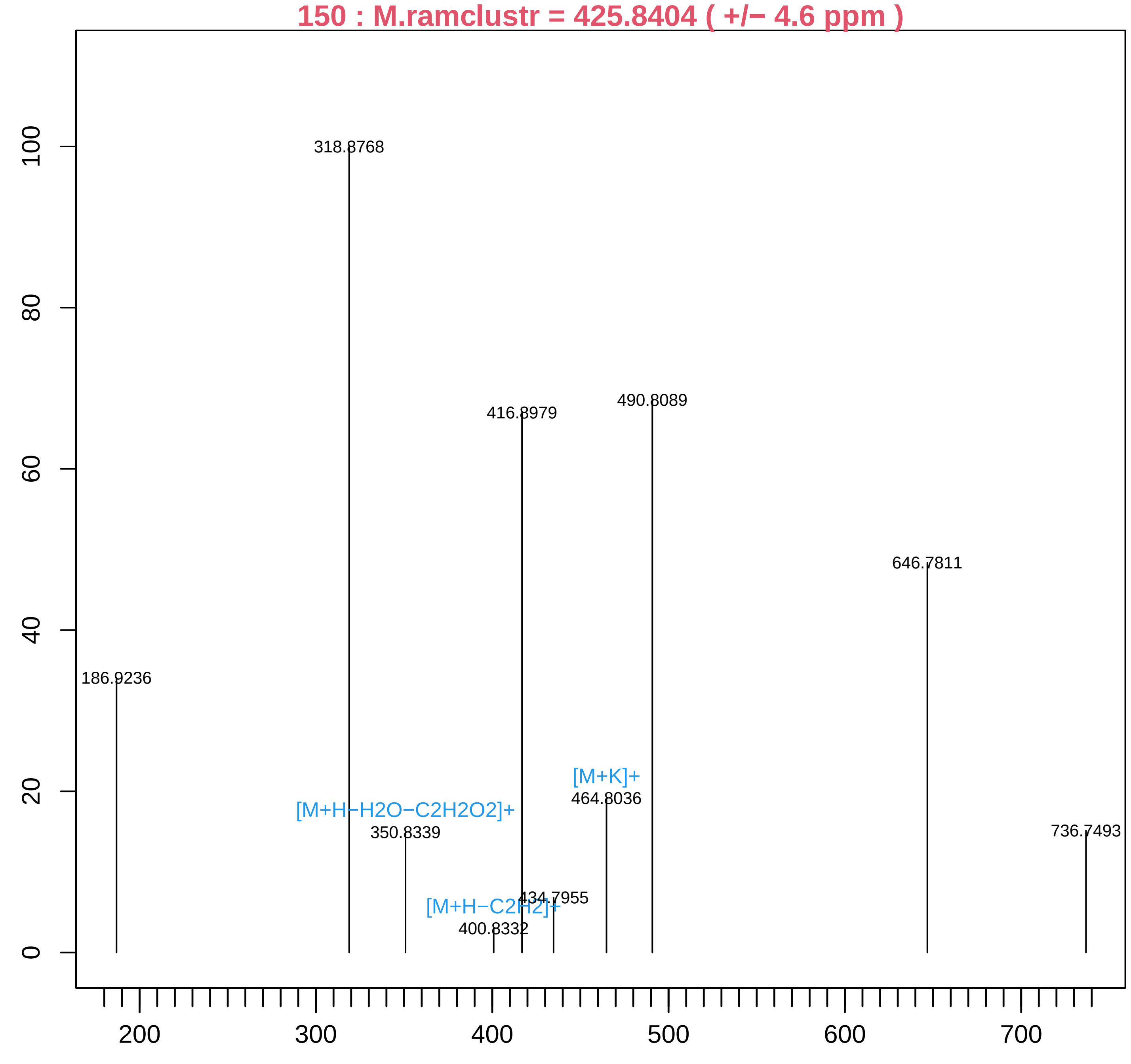
150 : M.ramclustr = 425.8404 (+/− 4.6 ppm)
318.8768
416.8979
490.8089
646.7811
186.9236
[M+K]+
464.8036
[M+H−H2O−C2H2O2]+
350.8339
736.7493
434.7955
[M+H−C2H2]+
400.8332
0
20
40
60
80
100
200
300
400
500
600
700

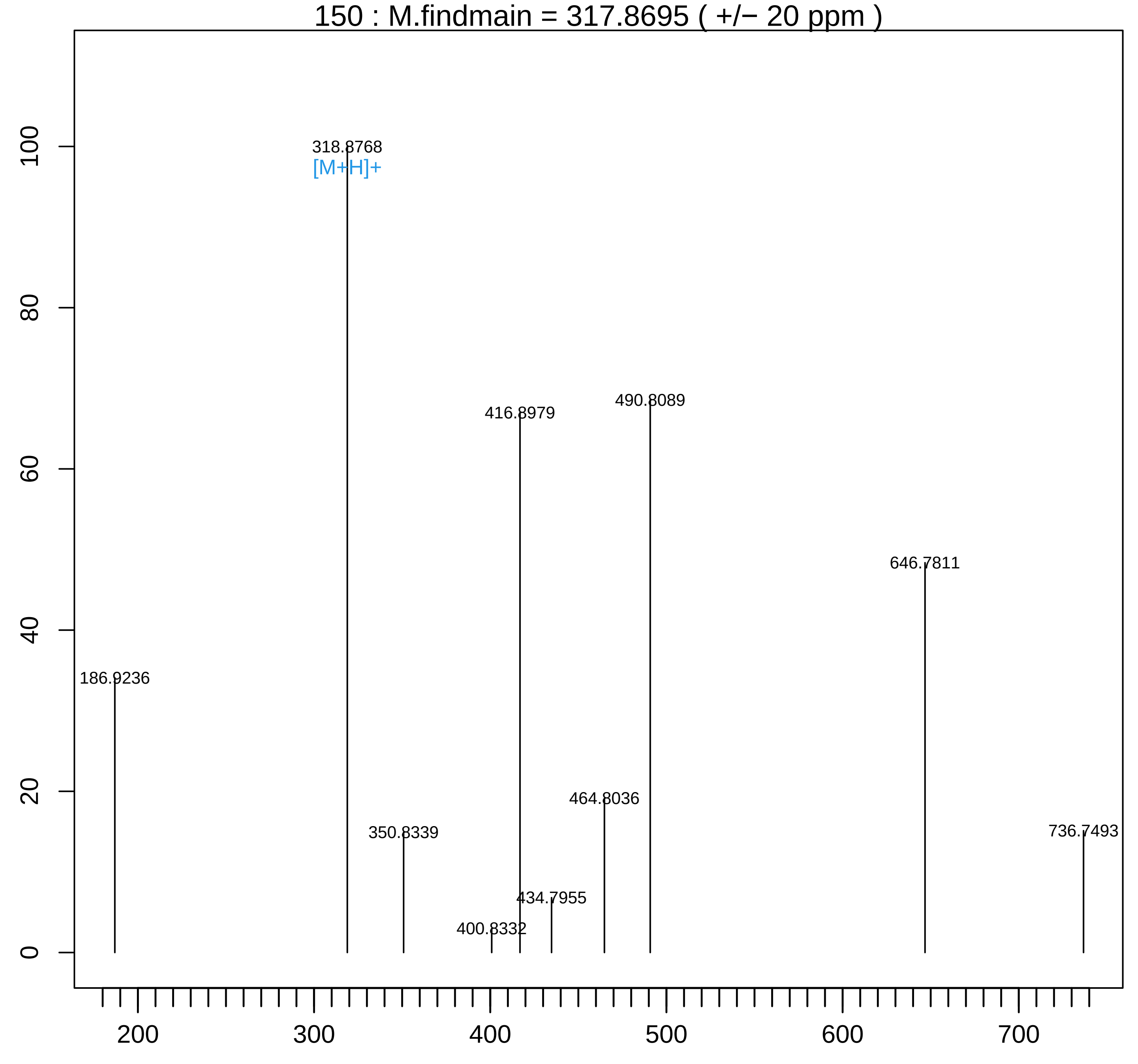
150 : M.findmain = 317.8695 (+/− 20 ppm)
318.8768
[M+H]+
416.8979
490.8089
646.7811
186.9236
464.8036
350.8339
736.7493
434.7955
400.8332
0
20
40
60
80
100
200
300
400
500
600
700

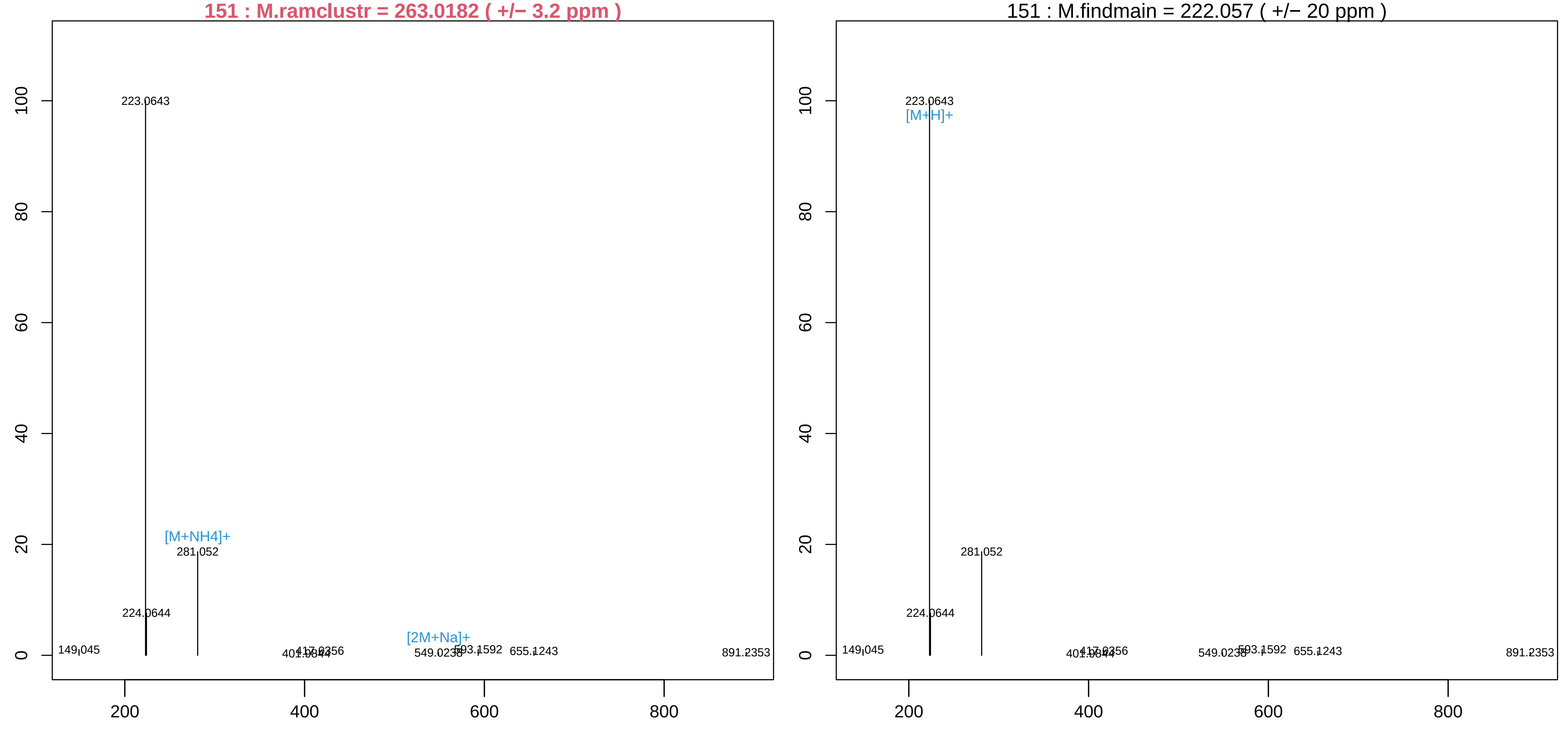
151 : M.ramclustr = 263.0182 (+/− 3.2 ppm)
223.0643
[M+NH4]+
281.052
224.0644
149.045
401.9844
417.0356
[2M+Na]+
549.0238
593.1592
655.1243
891.2353
151 : M.findmain = 222.057 (+/− 20 ppm)
223.0643
[M+H]+
281.052
224.0644
149.045
401.9844
417.0356
549.0238
593.1592
655.1243
891.2353
0
20
40
60
80
100
200
400
600
800

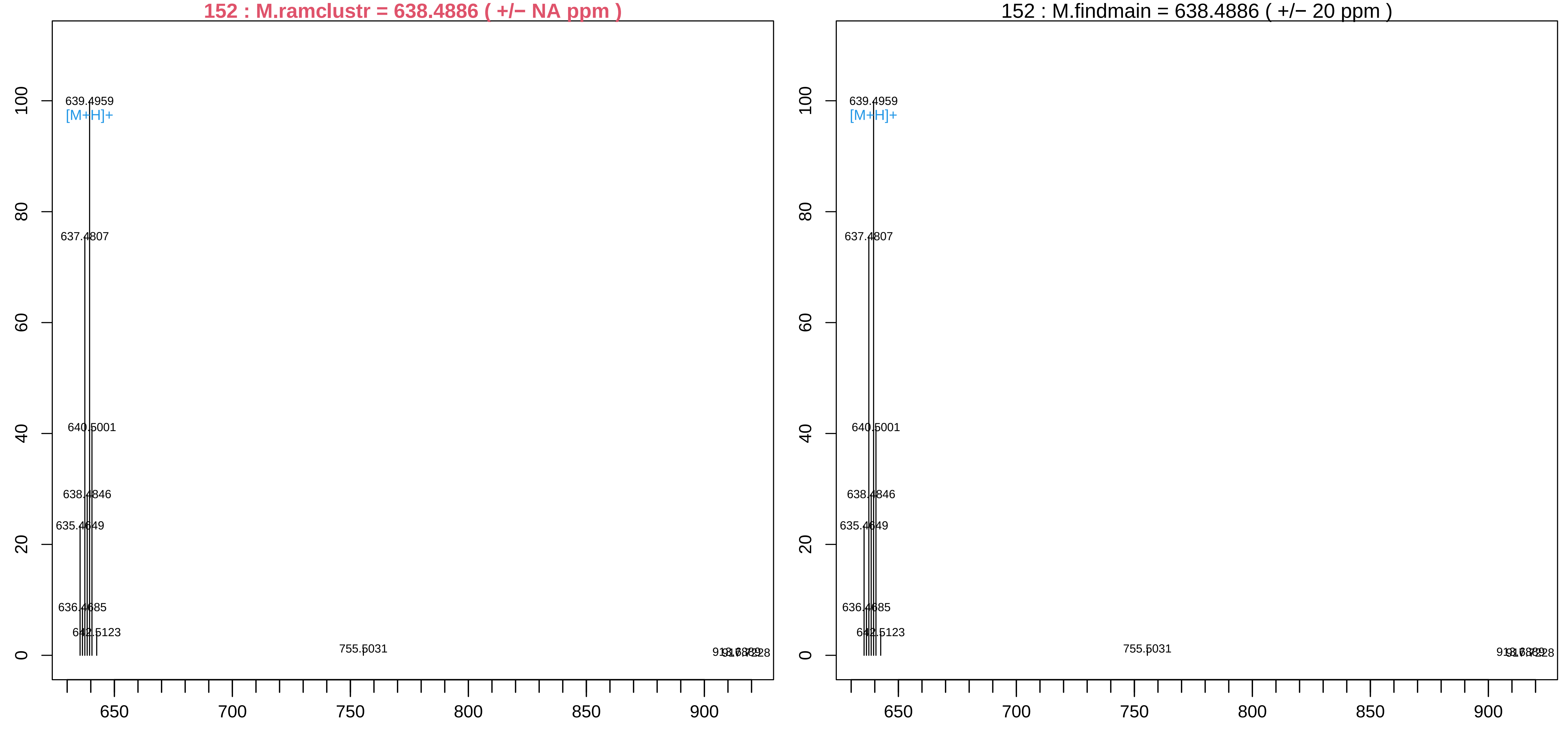
152 : M.ramclustr = 638.4886 (+/− NA ppm)
639.4959
[M+H]+
637.4807
640.5001
638.4846
635.4649
636.4685
642.5123
755.5031
0
20
40
60
80
100
650
700
750
800
850
900
152 : M.findmain = 638.4886 (+/− 20 ppm)
639.4959
[M+H]+
637.4807
640.5001
638.4846
635.4649
636.4685
642.5123
755.5031
0
20
40
60
80
100
650
700
750
800
850
900

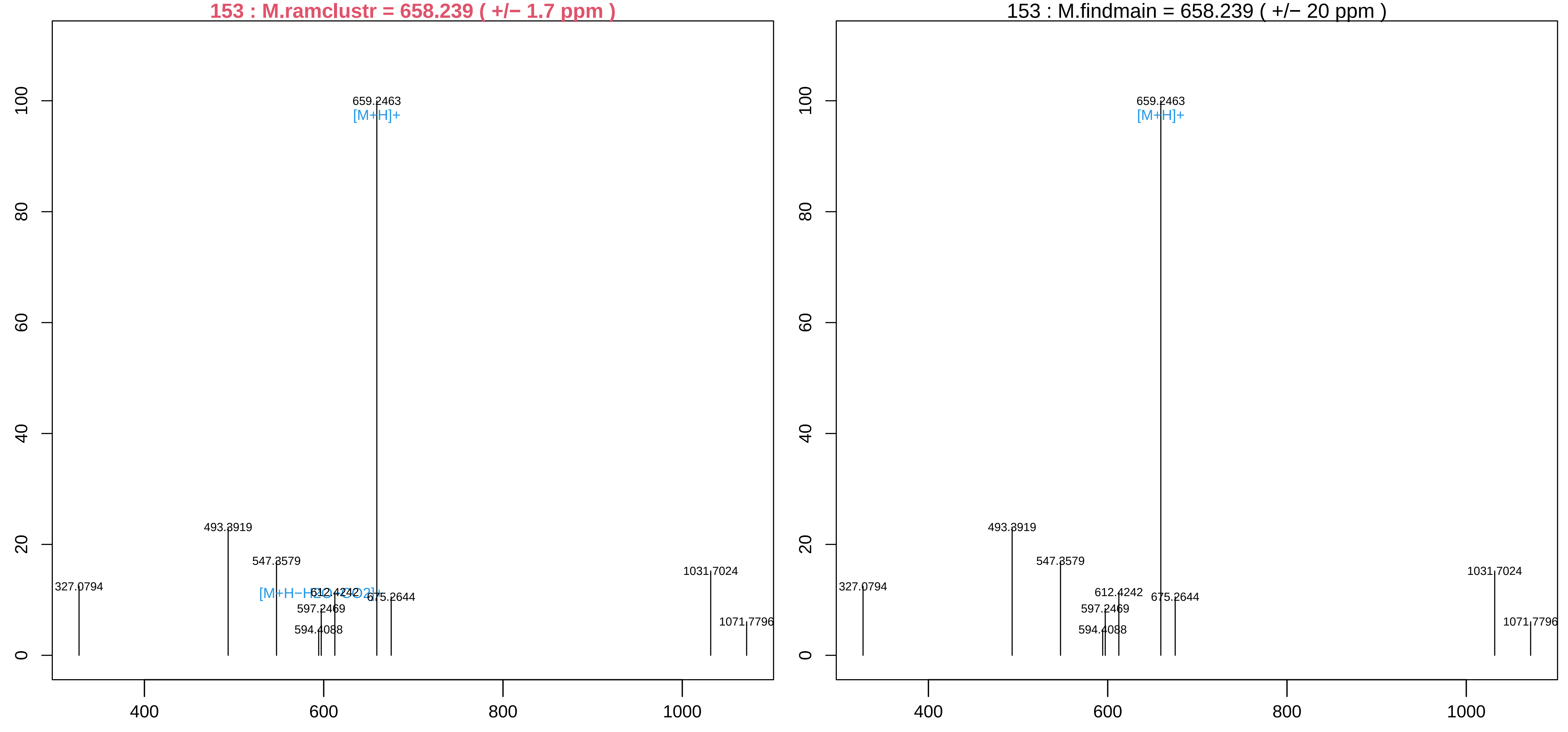
153 : M.ramclustr = 658.239 (+/− 1.7 ppm)
659.2463
[M+H]+
493.3919
547.3579
327.0794
[M+H−H2O−CO2]+
612.4242
597.2469
594.4088
675.2644
1031.7024
1071.7796
0
20
40
60
80
100
400
600
800
1000
153 : M.findmain = 658.239 (+/− 20 ppm)
659.2463
[M+H]+
493.3919
547.3579
327.0794
612.4242
597.2469
594.4088
675.2644
1031.7024
1071.7796
0
20
40
60
80
100
400
600
800
1000

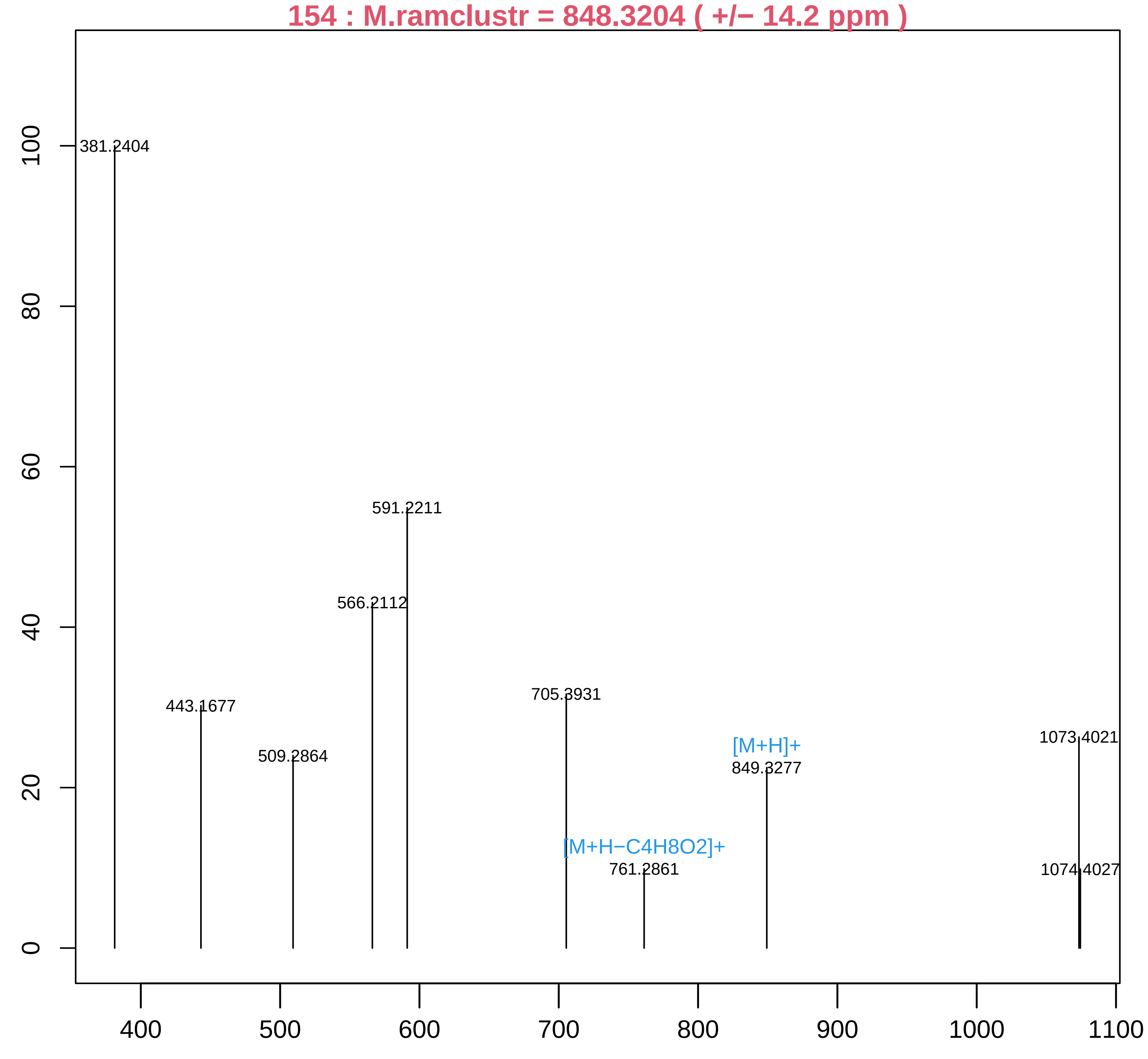

154 : M.ramclustr = 848.3204 (+/− 14.2 ppm)
381.2404
443.1677
509.2864
566.2112
591.2211
705.3931
[M+H−C4H8O2]+
761.2861
[M+H]+
849.3277
1073.4021
1074.4027
0
20
40
60
80
100
400
500
600
700
800
900
1000
1100

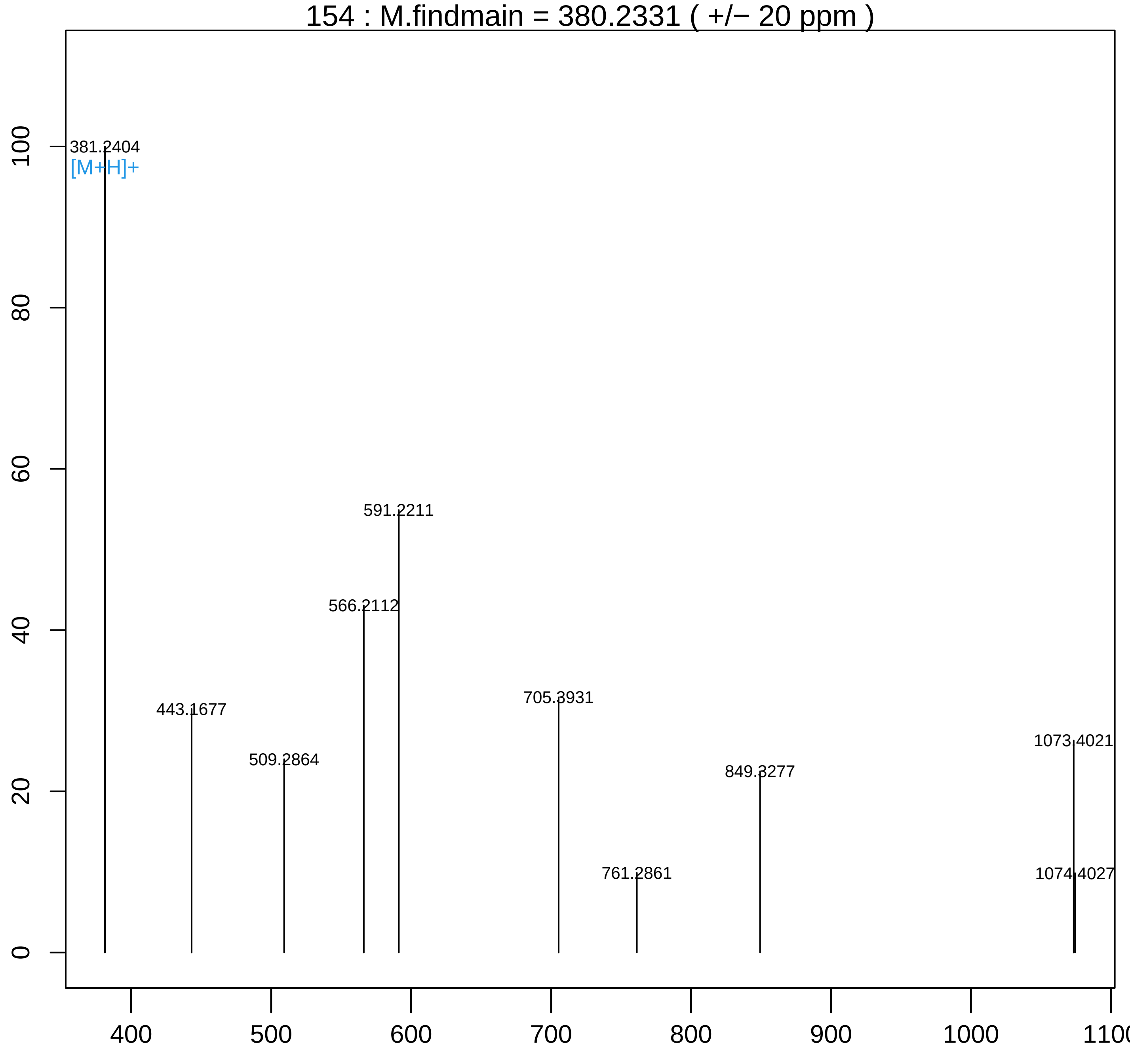

154 : M.findmain = 380.2331 (+/− 20 ppm)
381.2404
[M+H]+
443.1677
509.2864
566.2112
591.2211
705.3931
761.2861
849.3277
1073.4021
1074.4027
0
20
40
60
80
100
400
500
600
700
800
900
1000
1100

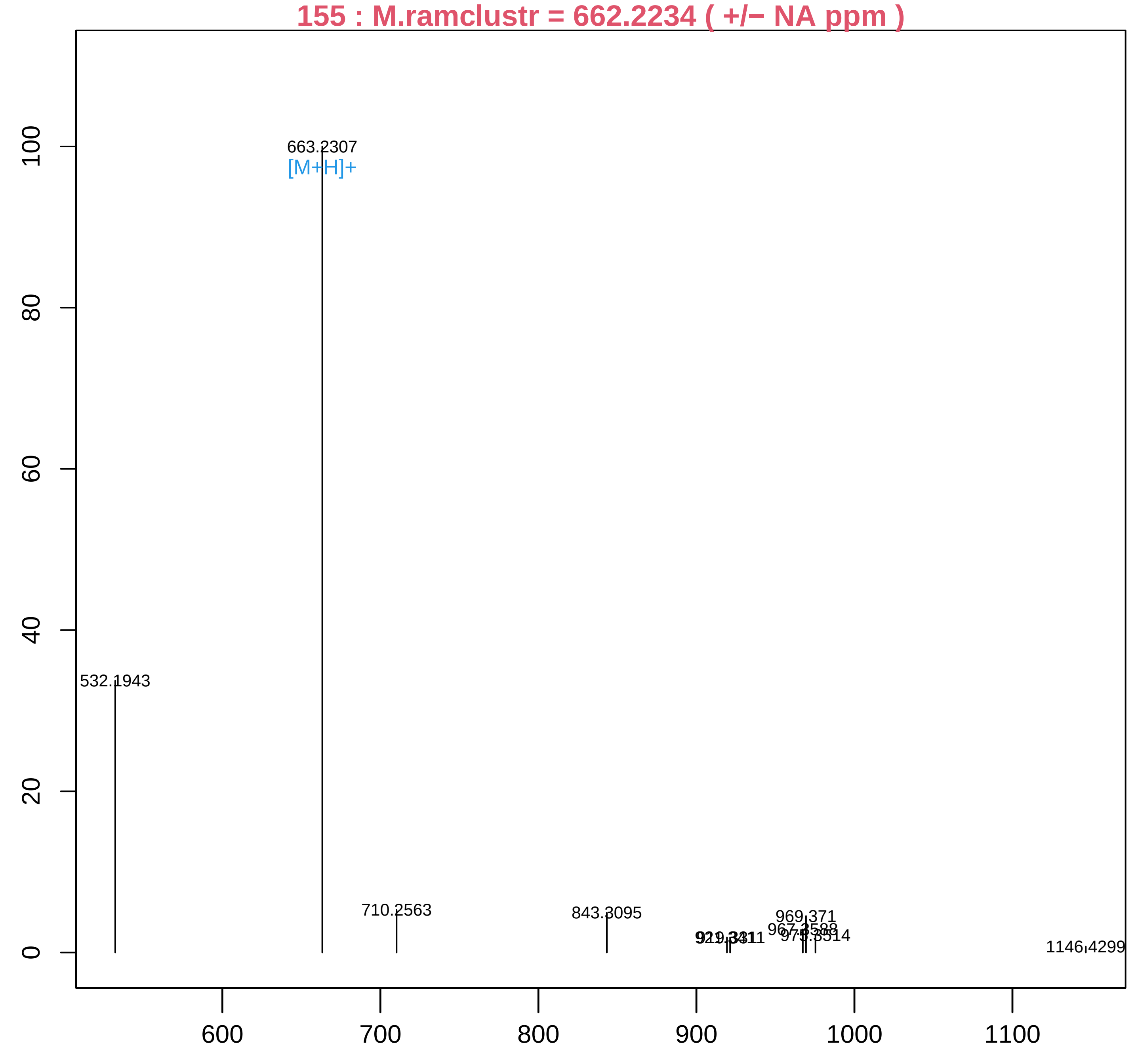

155 : M.ramclustr = 662.2234 (+/− NA ppm)
663.2307
[M+H]+
532.1943
710.2563
843.3095
969.371
1146.4299
0
20
40
60
80
100
600
700
800
900
1000
1100

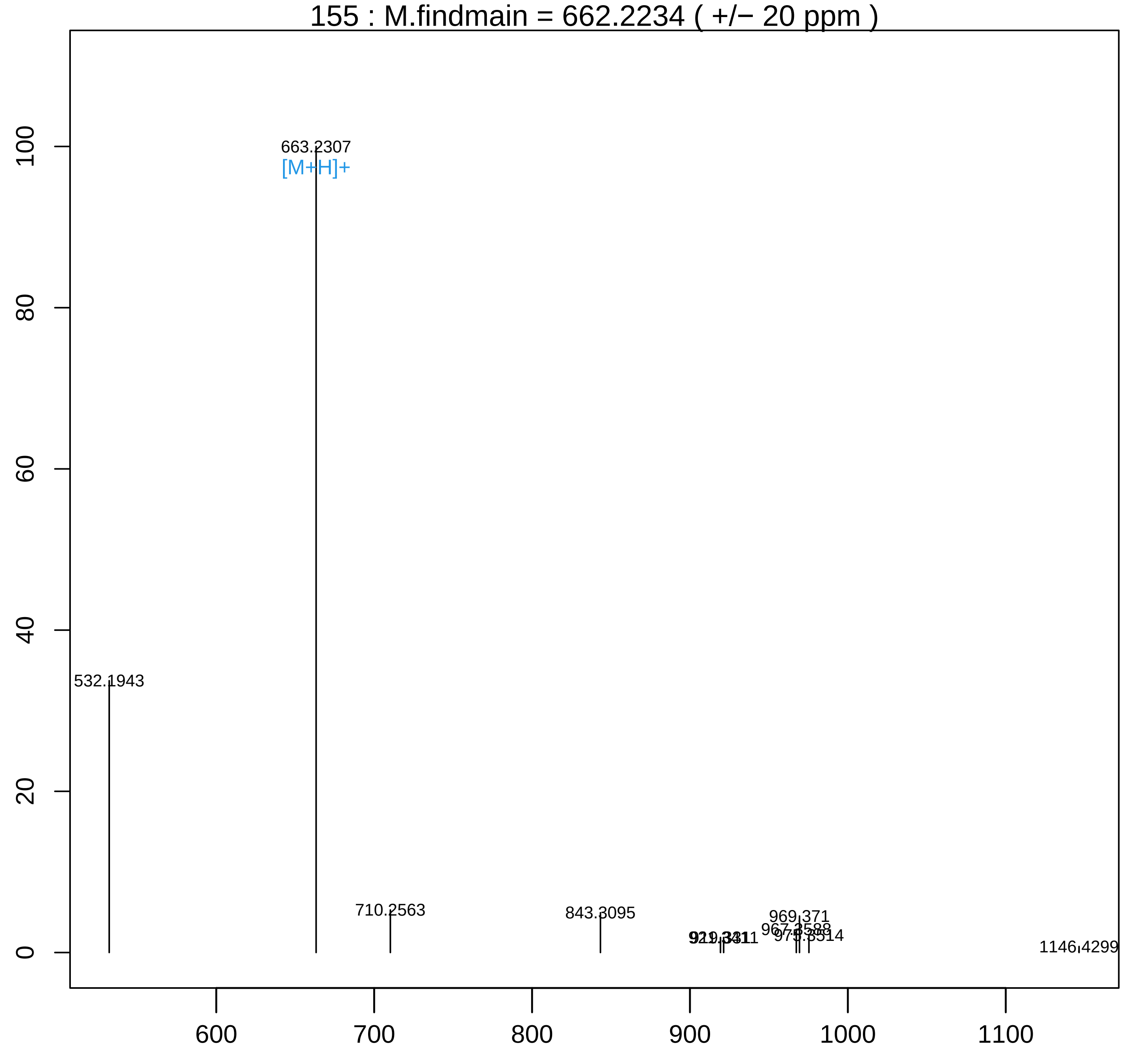

155 : M.findmain = 662.2234 (+/− 20 ppm)
663.2307
[M+H]+
532.1943
710.2563
843.3095
969.371
1146.4299
0
20
40
60
80
100
600
700
800
900
1000
1100

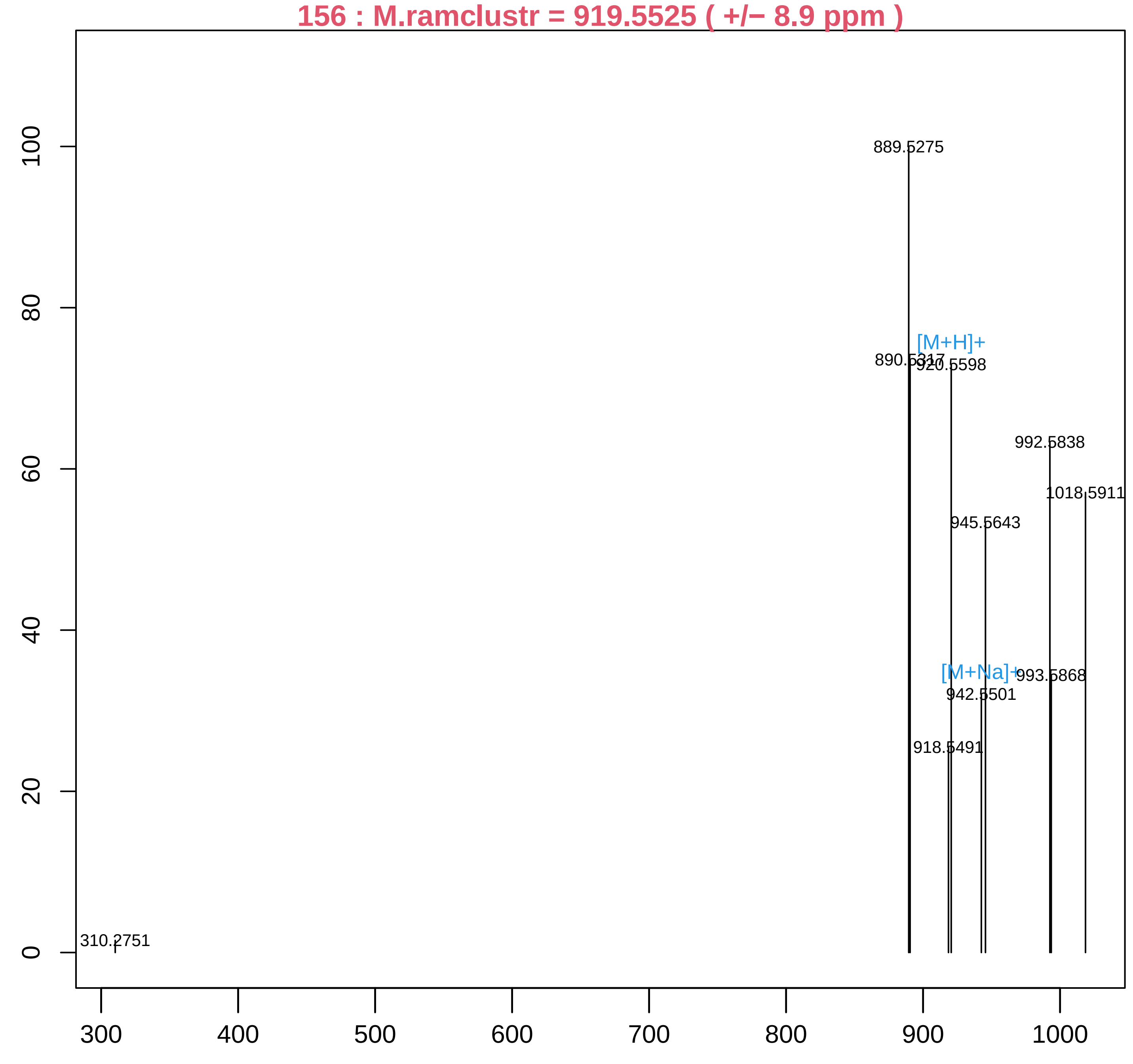
156 : M.ramclustr = 919.5525 (+/− 8.9 ppm)
889.5275
890.5317
920.5598
[M+H]+
992.5838
1018.5911
945.5643
[M+Na]+
993.5868
942.5501
918.5491
310.2751
0
20
40
60
80
100
300
400
500
600
700
800
900
1000
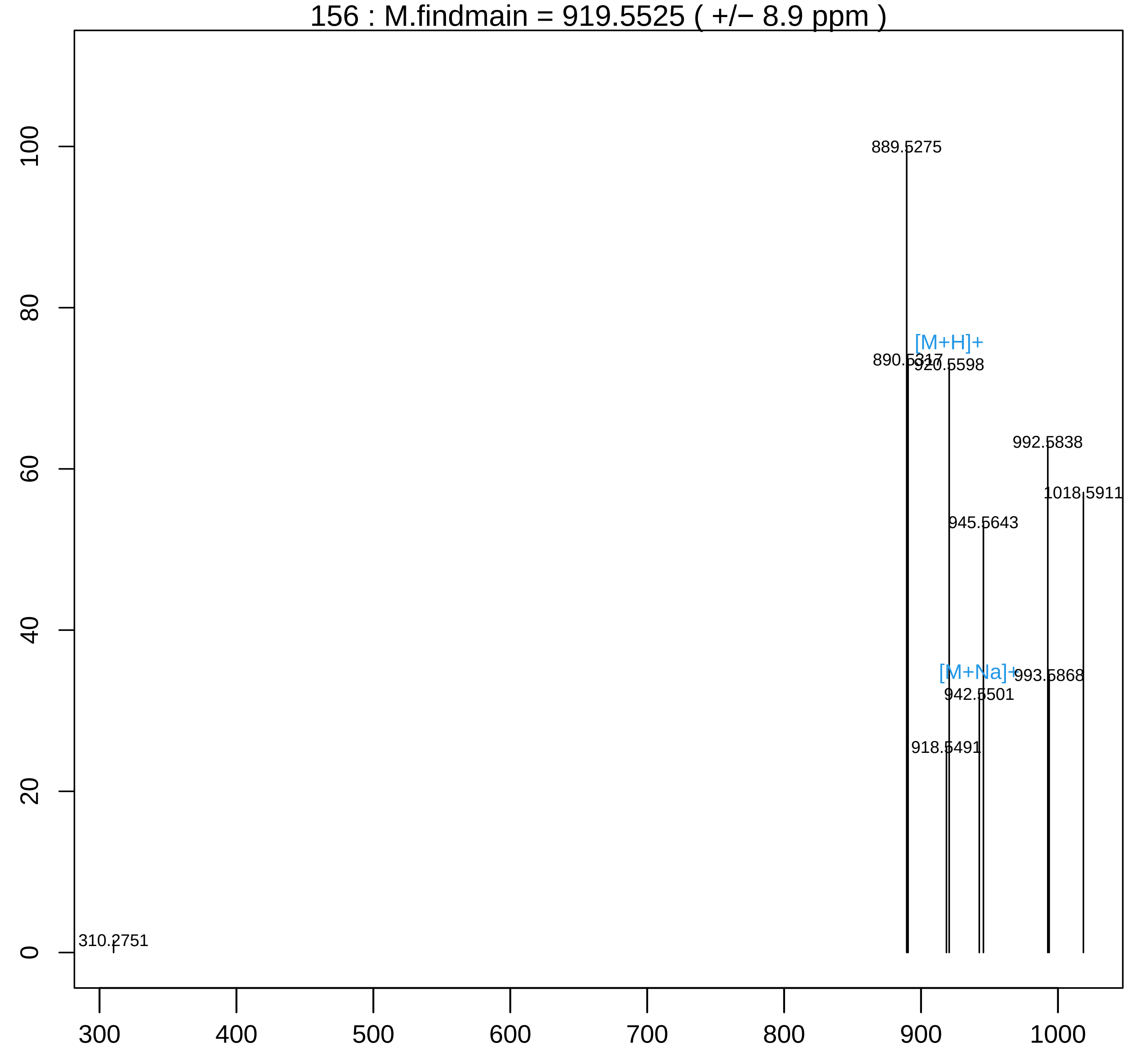
156 : M.findmain = 919.5525 (+/− 8.9 ppm)
889.5275
890.5317
920.5598
[M+H]+
992.5838
1018.5911
945.5643
[M+Na]+
993.5868
942.5501
918.5491
310.2751
0
20
40
60
80
100
300
400
500
600
700
800
900
1000

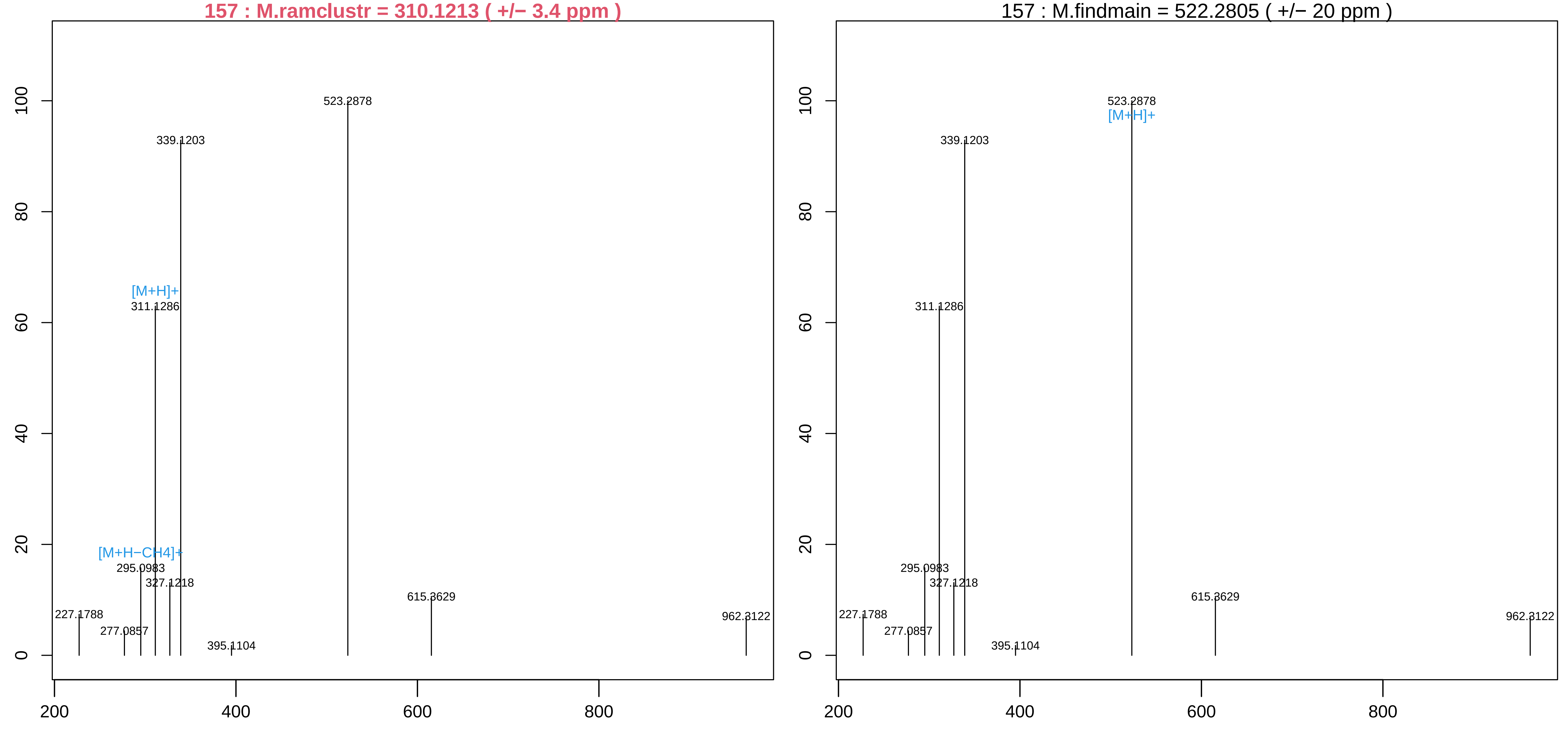
157 : M.ramclustr = 310.1213 (+/− 3.4 ppm)
523.2878
339.1203
[M+H]+
311.1286
[M+H−CH4]+
295.0983
327.1218
615.3629
227.1788
962.3122
277.0857
395.1104
157 : M.findmain = 522.2805 (+/− 20 ppm)
523.2878
[M+H]+
339.1203
311.1286
295.0983
327.1218
615.3629
227.1788
962.3122
277.0857
395.1104
0
20
40
60
80
100
200
400
600
800

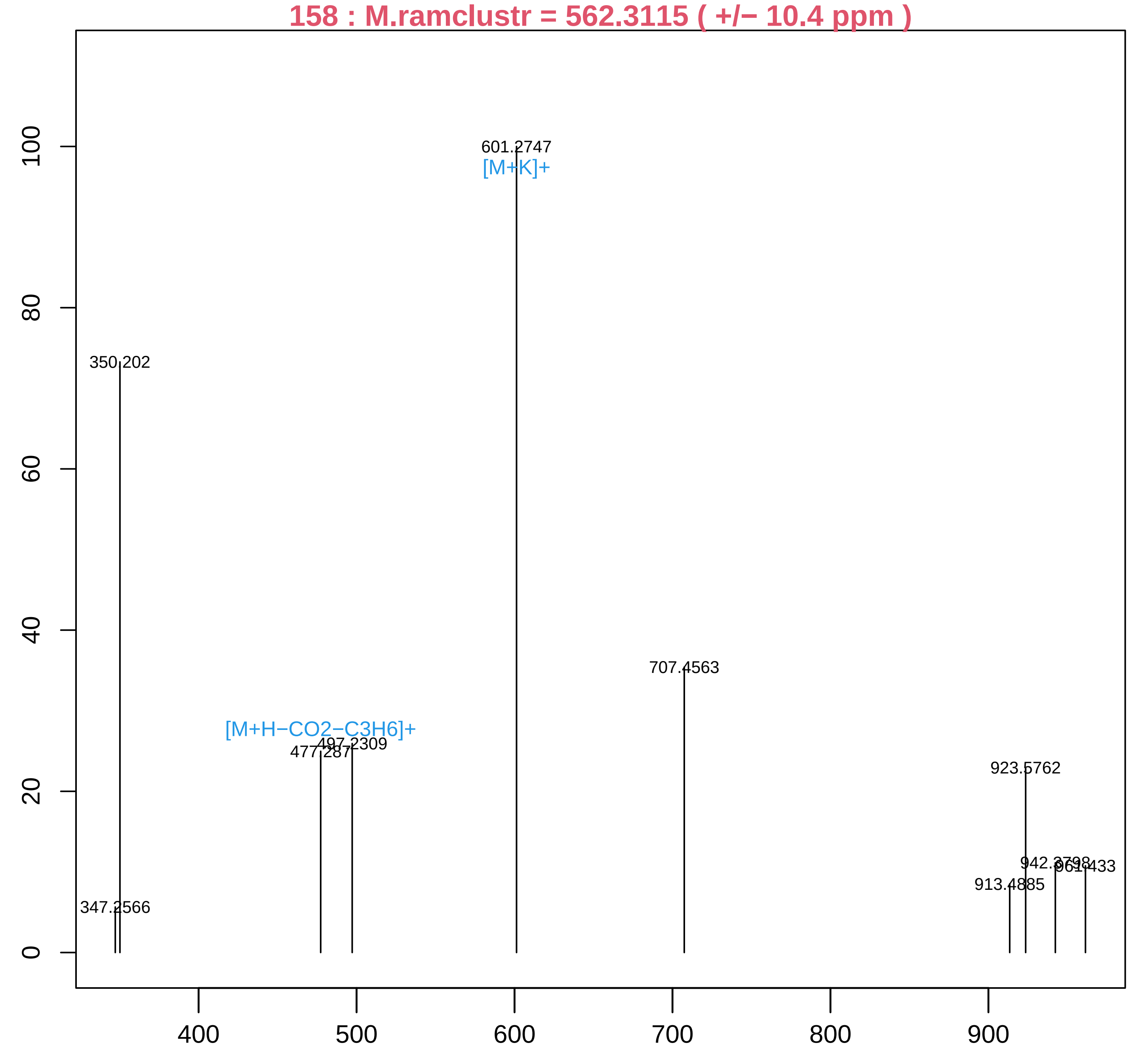

158 : M.ramclustr = 562.3115 (+/− 10.4 ppm)
601.2747
[M+K]+
350.202
707.4563
[M+H−CO2−C3H6]+
497.2309
477.287
923.5762
942.3798
961.433
913.4885
347.2566
0
20
40
60
80
100
400
500
600
700
800
900

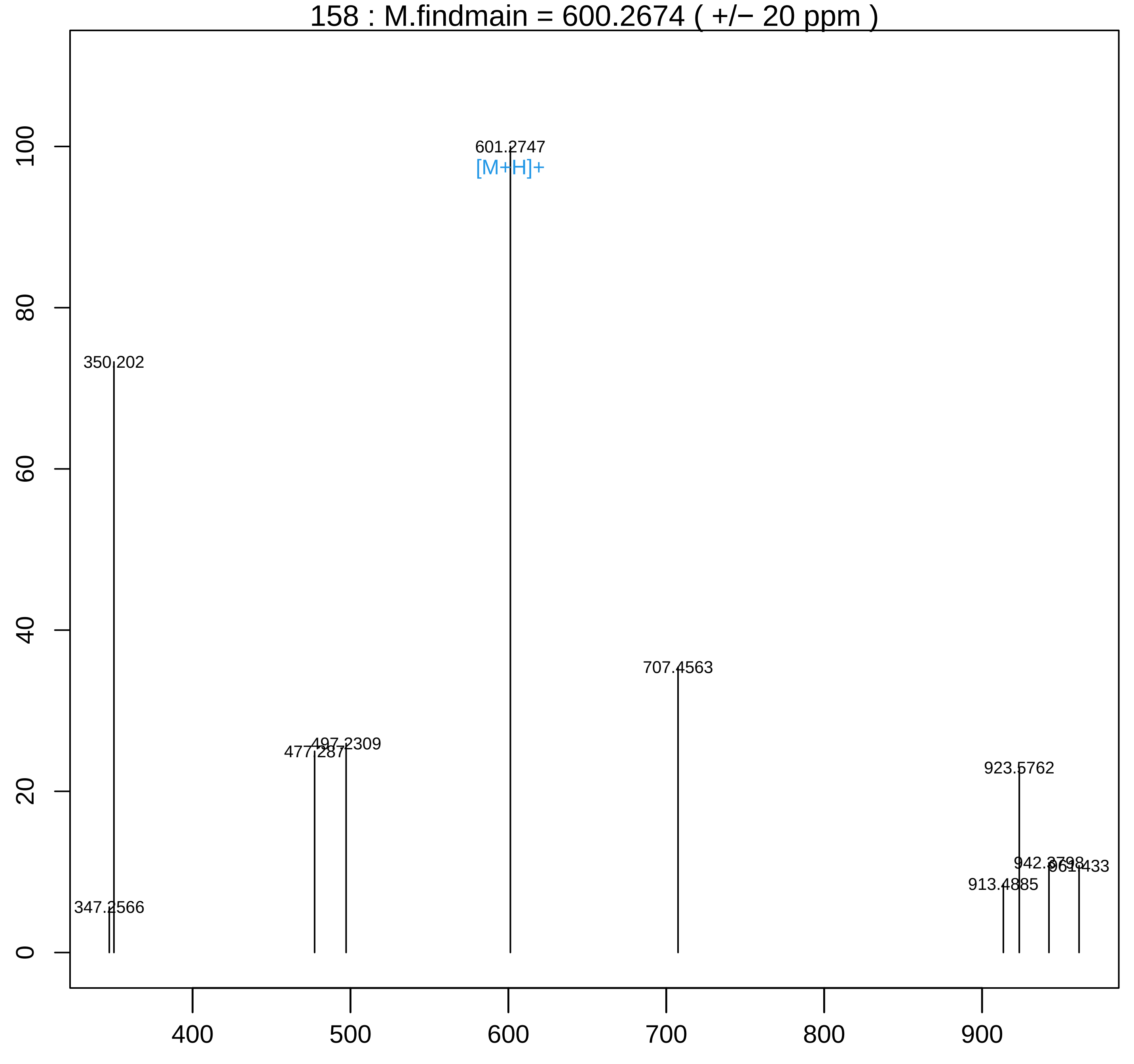

158 : M.findmain = 600.2674 (+/− 20 ppm)
601.2747
[M+H]+
350.202
707.4563
497.2309
477.287
923.5762
942.3798
961.433
913.4885
347.2566
0
20
40
60
80
100
400
500
600
700
800
900

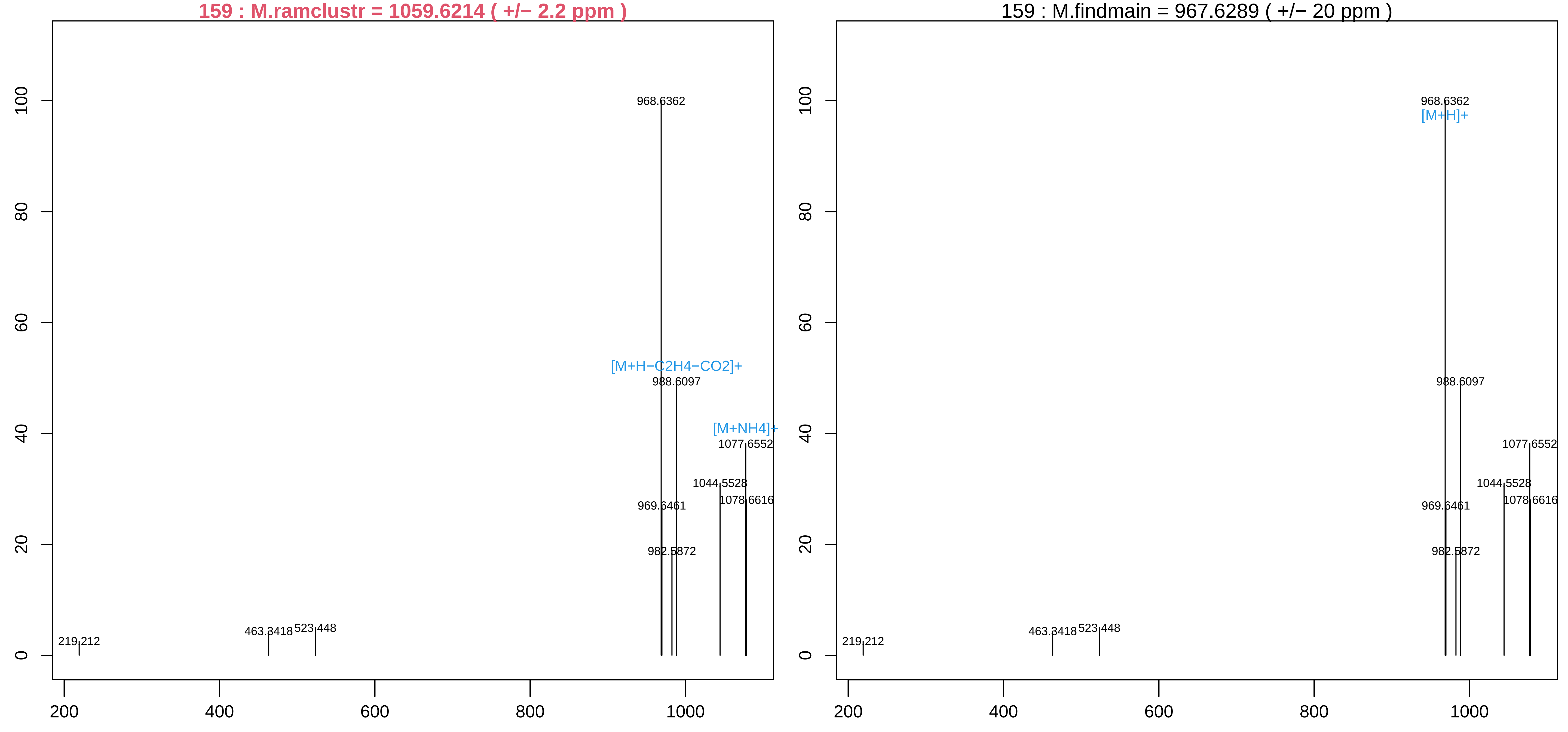
159 : M.ramclustr = 1059.6214 (+/− 2.2 ppm)
968.6362
[M+H−C2H4−CO2]+
988.6097
[M+NH4]+
1077.6552
1044.5528
1078.6616
969.6461
982.5872
219.212
463.3418
523.448
159 : M.findmain = 967.6289 (+/− 20 ppm)
968.6362
[M+H]+
988.6097
1077.6552
1044.5528
1078.6616
969.6461
982.5872
219.212
463.3418
523.448
0
20
40
60
80
100
200
400
600
800
1000

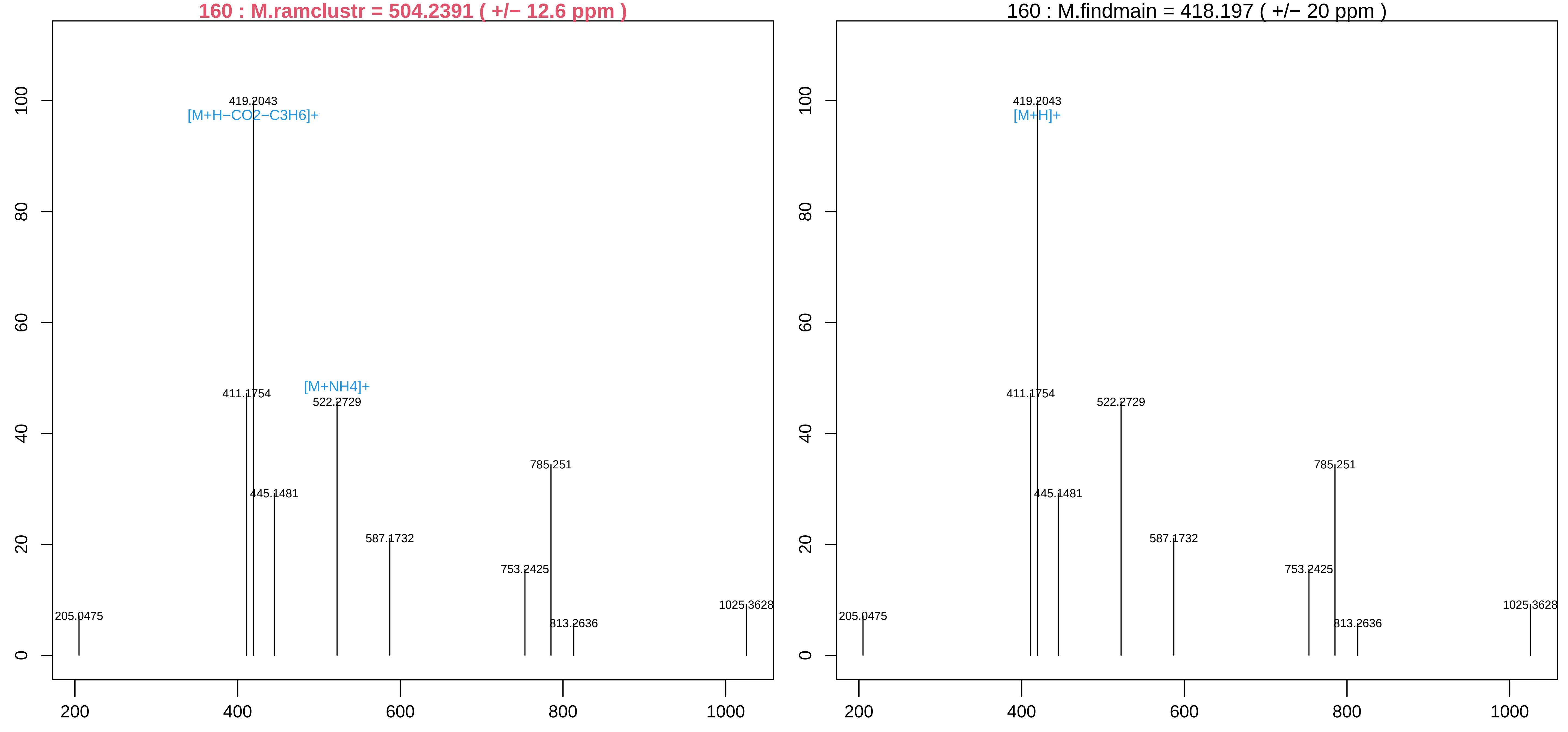

160 : M.ramclustr = 504.2391 (+/− 12.6 ppm)
419.2043
[M+H−CO2−C3H6]+
411.1754
[M+NH4]+
522.2729
445.1481
587.1732
785.251
753.2425
813.2636
1025.3628
205.0475
160 : M.findmain = 418.197 (+/− 20 ppm)
419.2043
[M+H]+
411.1754
522.2729
445.1481
587.1732
785.251
753.2425
813.2636
1025.3628
205.0475
0
20
40
60
80
100
200
400
600
800
1000

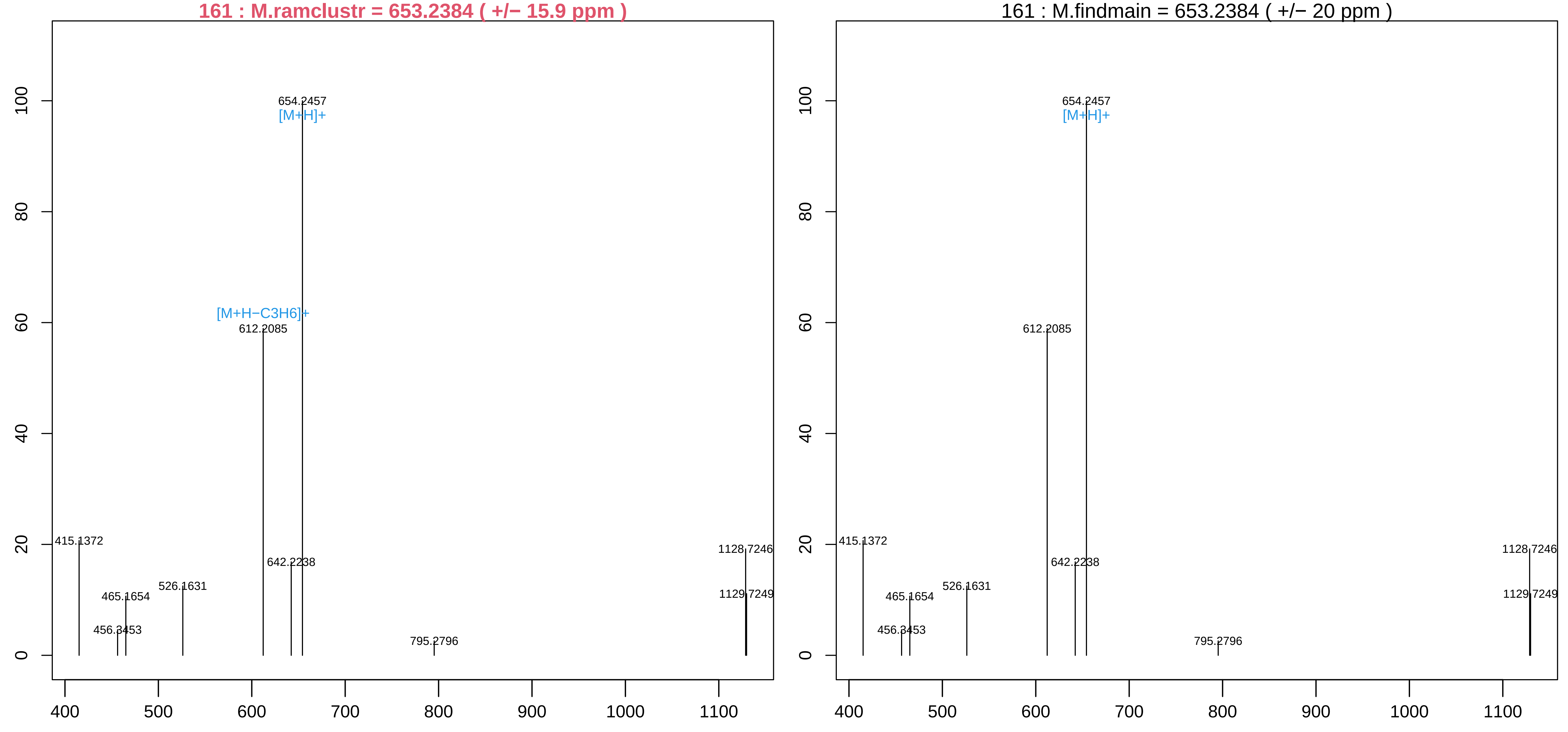
161 : M.ramclustr = 653.2384 (+/− 15.9 ppm)
654.2457
[M+H]+
[M+H−C3H6]+
612.2085
415.1372
642.2238
526.1631
465.1654
456.3453
795.2796
1128.7246
1129.7249
161 : M.findmain = 653.2384 (+/− 20 ppm)
0
20
40
60
80
100
400
500
600
700
800
900
1000
1100

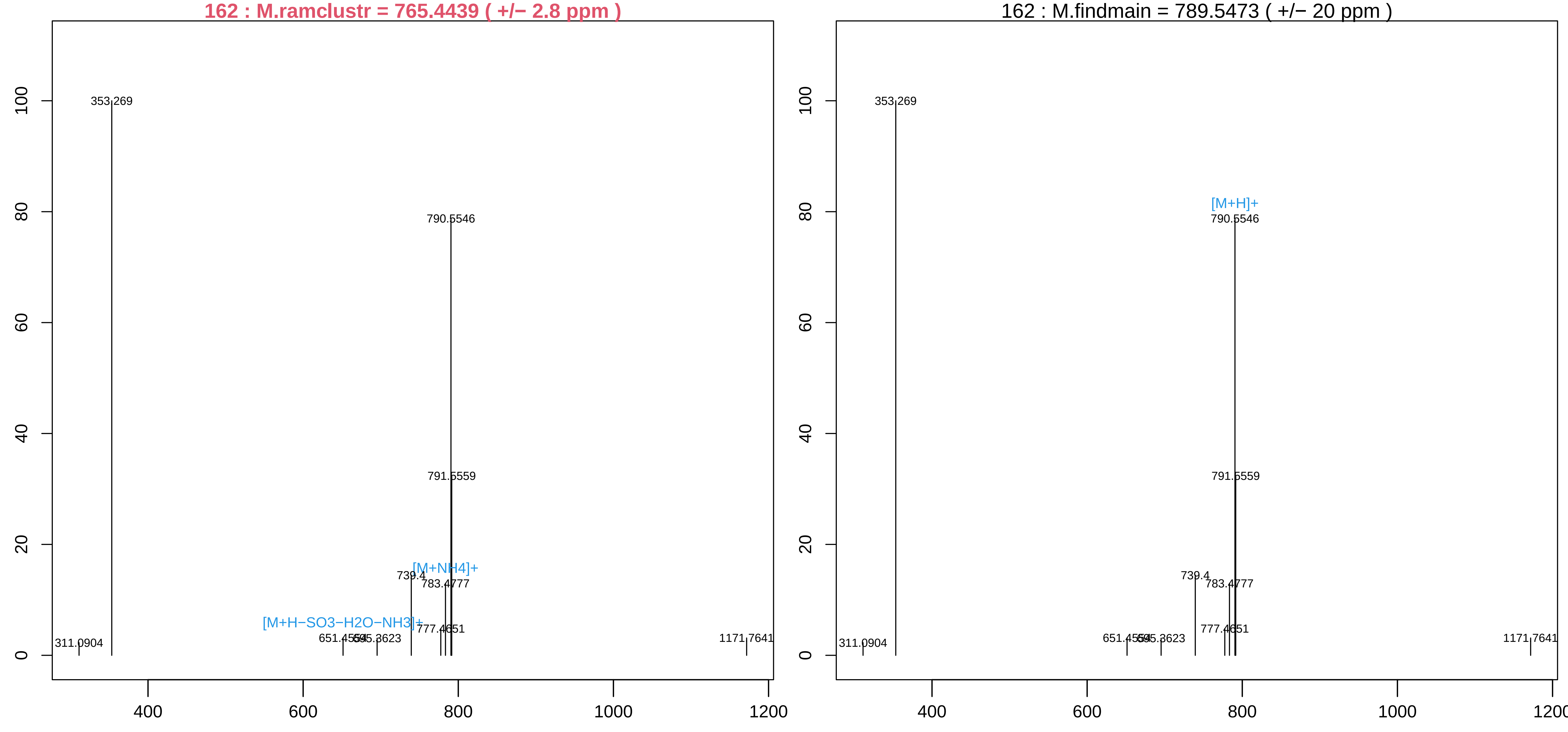
162 : M.ramclustr = 765.4439 (+/− 2.8 ppm)
353.269
790.5546
791.5559
[M+NH4]+
739.4
783.4777
[M+H−SO3−H2O−NH3]+
777.4651
311.0904
651.4569
695.3623
1171.7641
0
20
40
60
80
100
400
600
800
1000
1200
162 : M.findmain = 789.5473 (+/− 20 ppm)
353.269
[M+H]+
790.5546
791.5559
739.4
783.4777
777.4651
311.0904
651.4569
695.3623
1171.7641

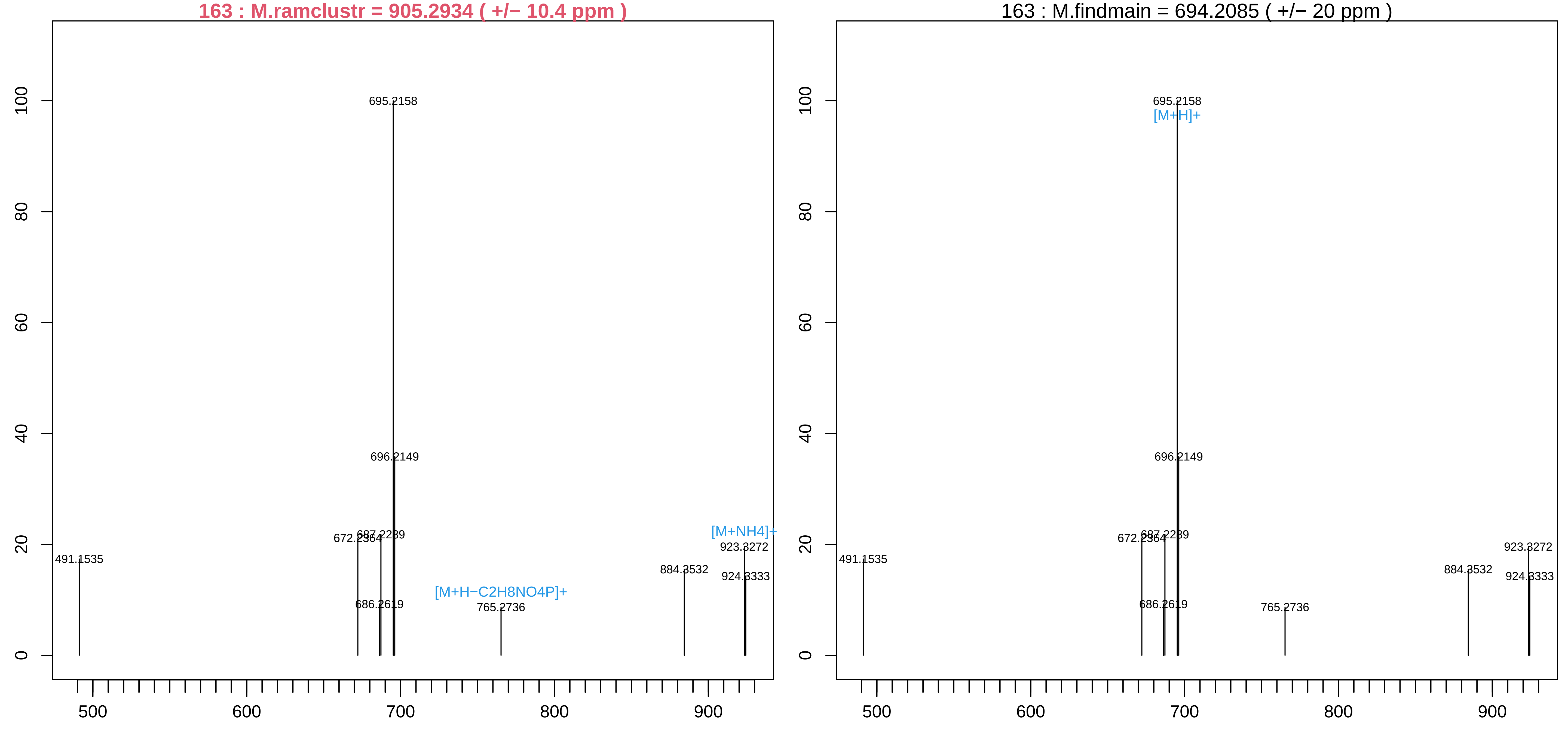
163 : M.ramclustr = 905.2934 (+/− 10.4 ppm)
695.2158
696.2149
687.2239
672.2364
491.1535
[M+NH4]+
923.3272
884.3532
924.3333
[M+H−C2H8NO4P]+
686.2619
765.2736
163 : M.findmain = 694.2085 (+/− 20 ppm)
695.2158
[M+H]+
696.2149
687.2239
672.2364
491.1535
923.3272
884.3532
924.3333
686.2619
765.2736

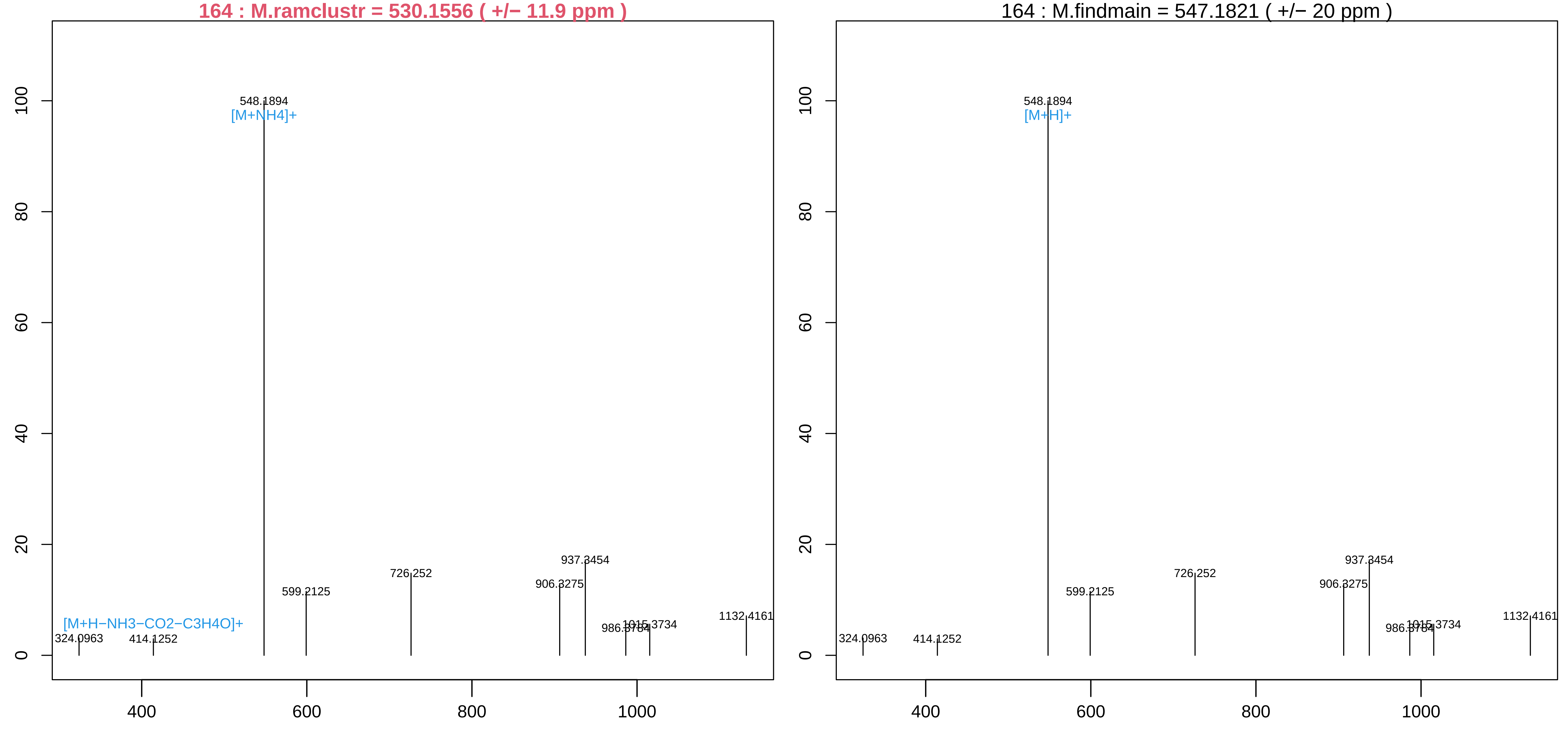
164 : M.ramclustr = 530.1556 (+/− 11.9 ppm)
548.1894
[M+NH4]+
[M+H−NH3−CO2−C3H4O]+
324.0963
414.1252
599.2125
726.252
906.3275
937.3454
986.3784
1015.3734
1132.4161
164 : M.findmain = 547.1821 (+/− 20 ppm)
548.1894
[M+H]+
324.0963
414.1252
599.2125
726.252
906.3275
937.3454
986.3784
1015.3734
1132.4161

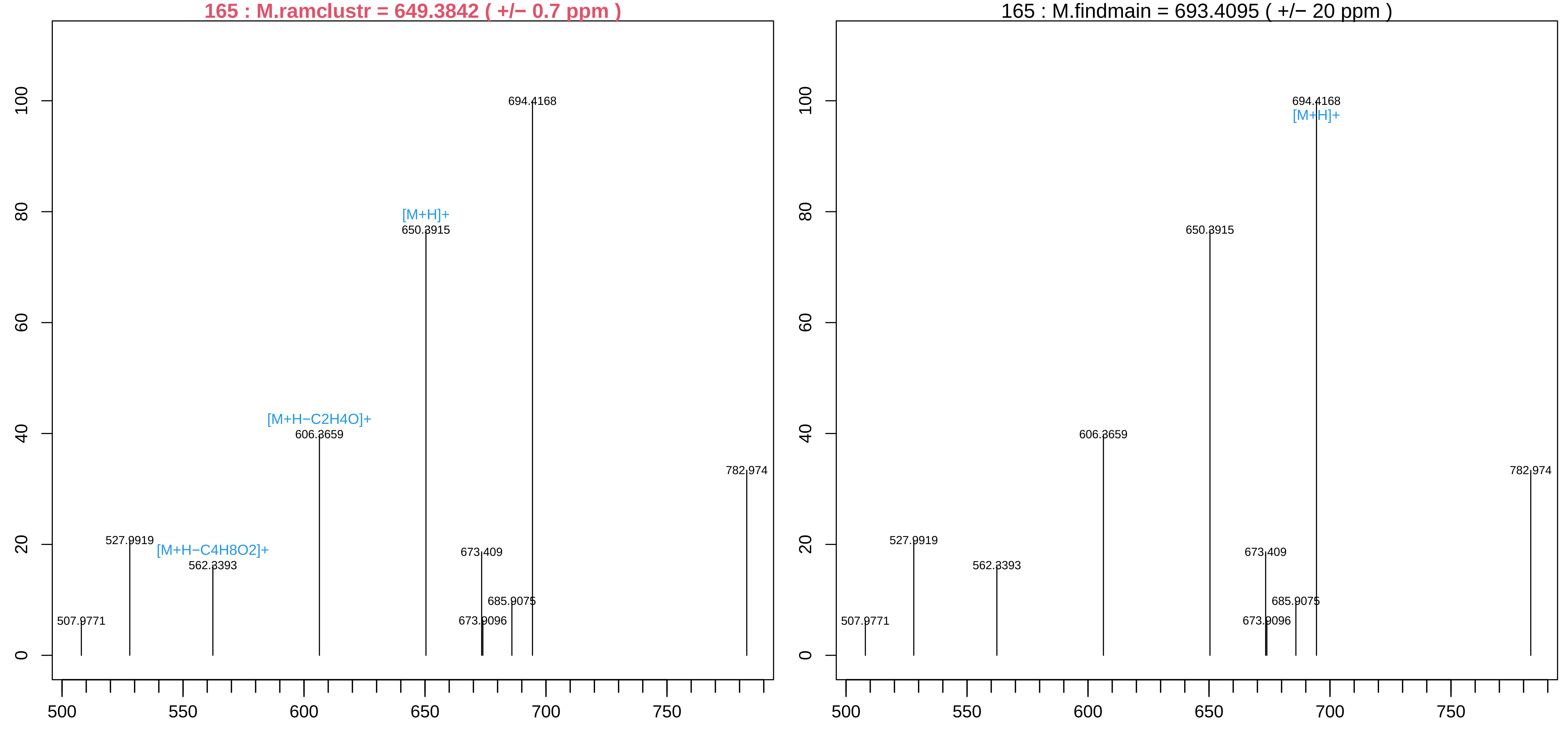
165 : M.ramclustr = 649.3842 (+/− 0.7 ppm)
[M+H]+
650.3915
694.4168
[M+H−C2H4O]+
606.3659
782.974
527.9919
[M+H−C4H8O2]+
562.3393
673.409
685.9075
673.9096
507.9771
165 : M.findmain = 693.4095 (+/− 20 ppm)
694.4168
[M+H]+
650.3915
606.3659
782.974
527.9919
562.3393
673.409
685.9075
673.9096
507.9771
0
20
40
60
80
100
500
550
600
650
700
750

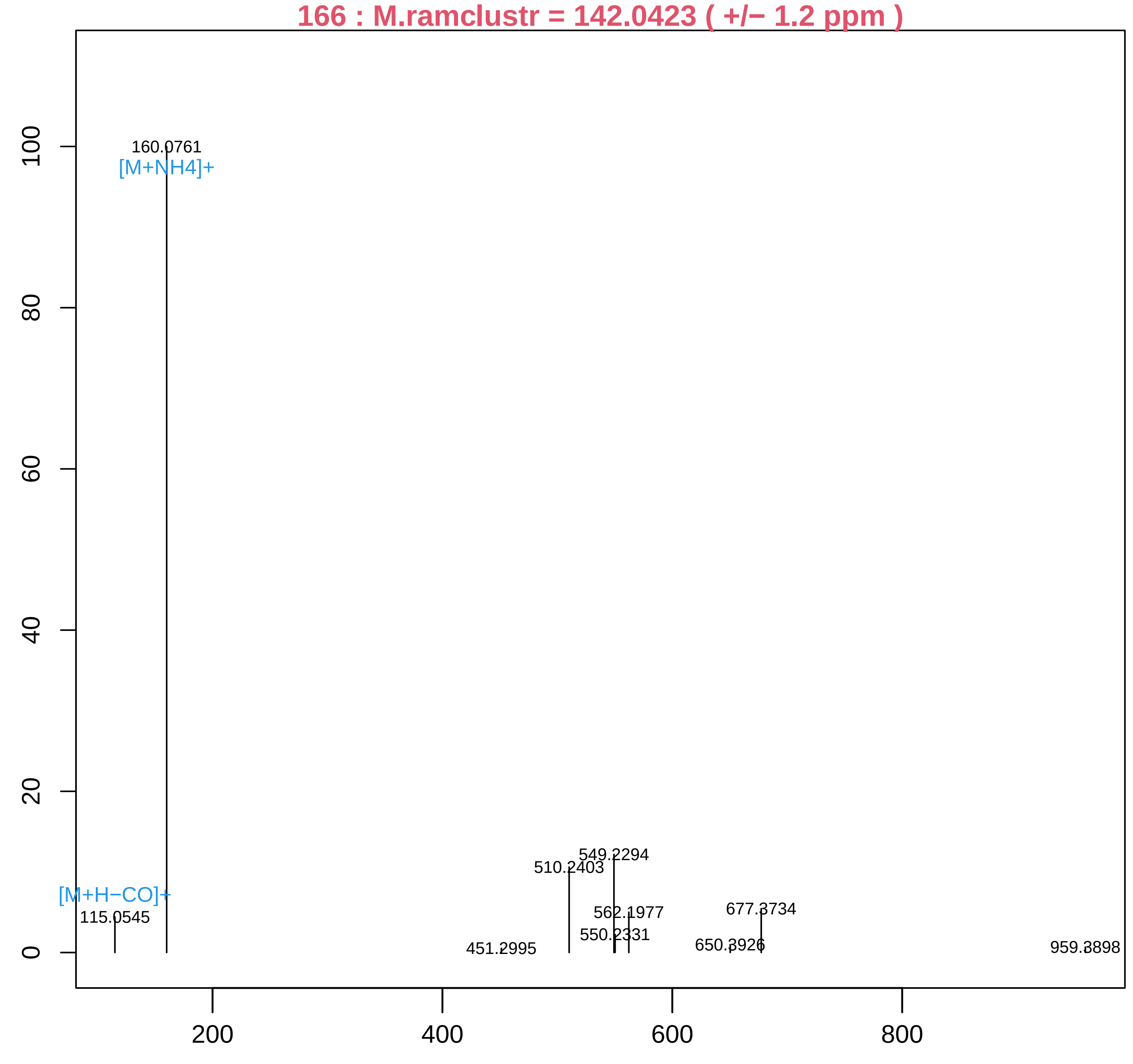

166 : M.ramclustr = 142.0423 (+/− 1.2 ppm)
160.0761
[M+NH4]+
[M+H−CO]+
115.0545
549.2294
510.2403
562.1977
550.2331
451.2995
677.3734
650.3926
959.3898
0
20
40
60
80
100
200
400
600
800

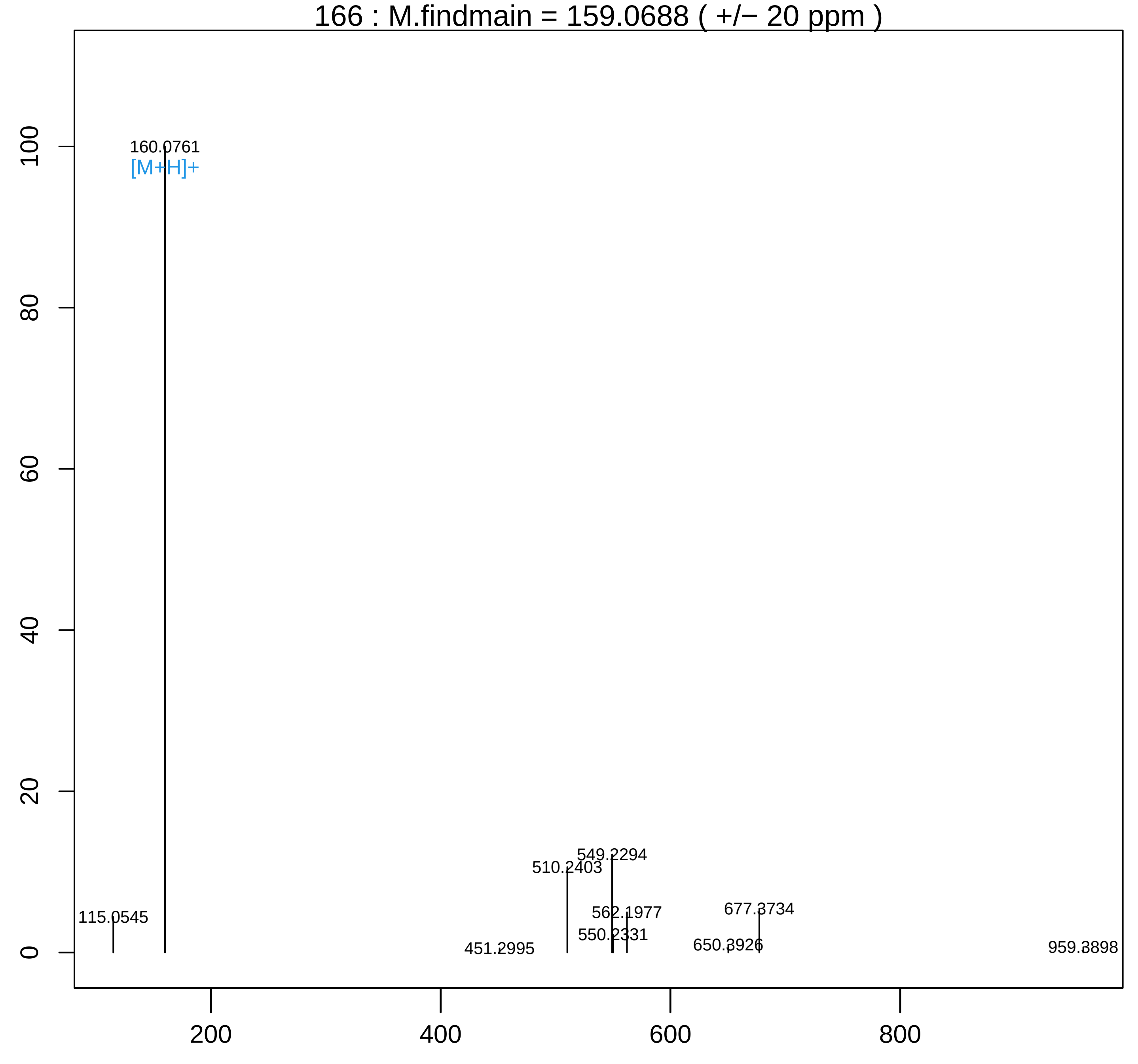

166 : M.findmain = 159.0688 (+/− 20 ppm)
160.0761
[M+H]+
115.0545
549.2294
510.2403
562.1977
550.2331
451.2995
677.3734
650.3926
959.3898
0
20
40
60
80
100
200
400
600
800

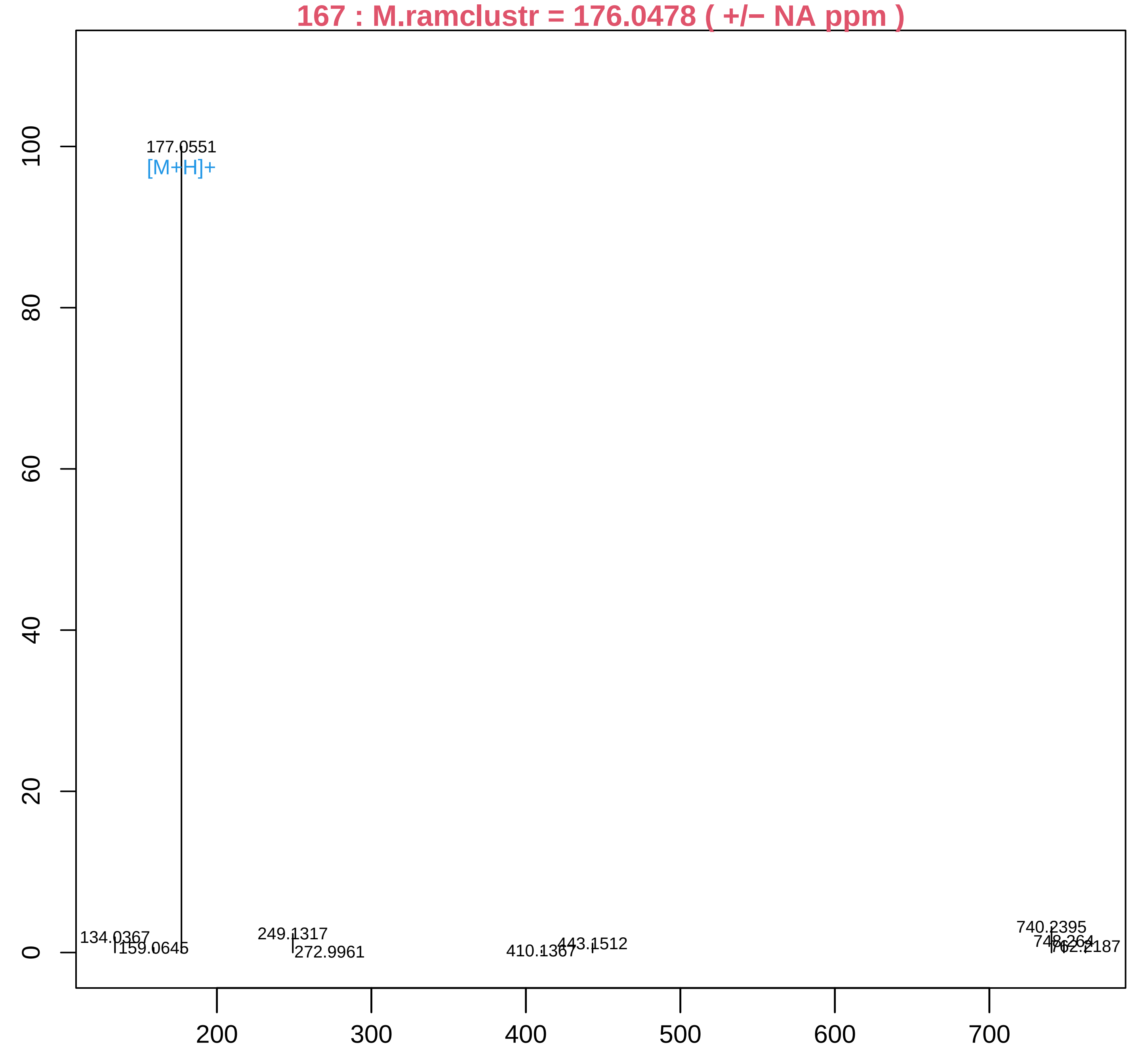
167 : M.ramclustr = 176.0478 (+/− NA ppm)
177.0551
[M+H]+
134.0367
159.0645
249.1317
272.9961
410.1367
443.1512
740.2395
0
20
40
60
80
100
200
300
400
500
600
700

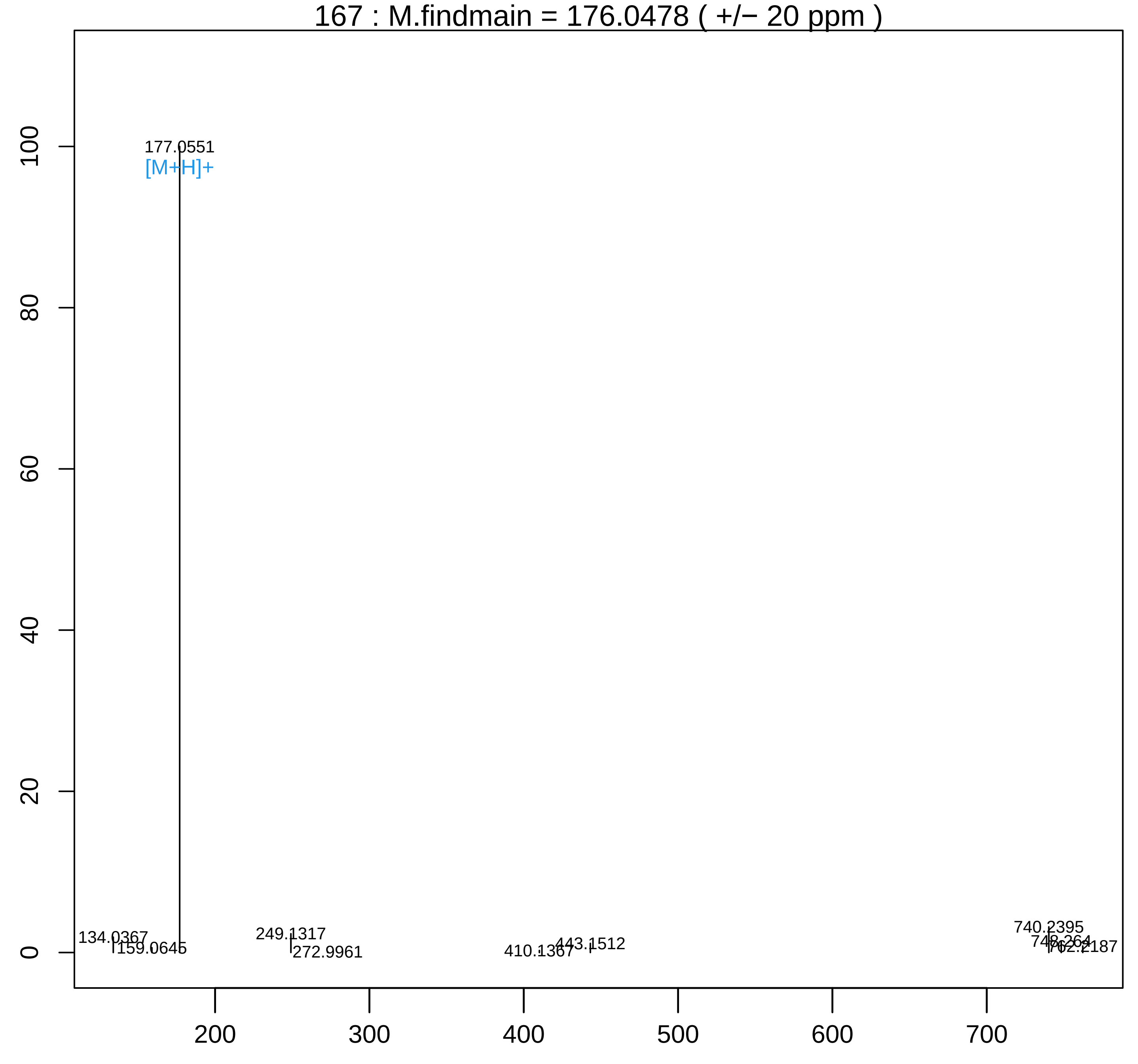
167 : M.findmain = 176.0478 (+/− 20 ppm)
177.0551
[M+H]+
134.0367
159.0645
249.1317
272.9961
410.1367
443.1512
740.2395
0
20
40
60
80
100
200
300
400
500
600
700

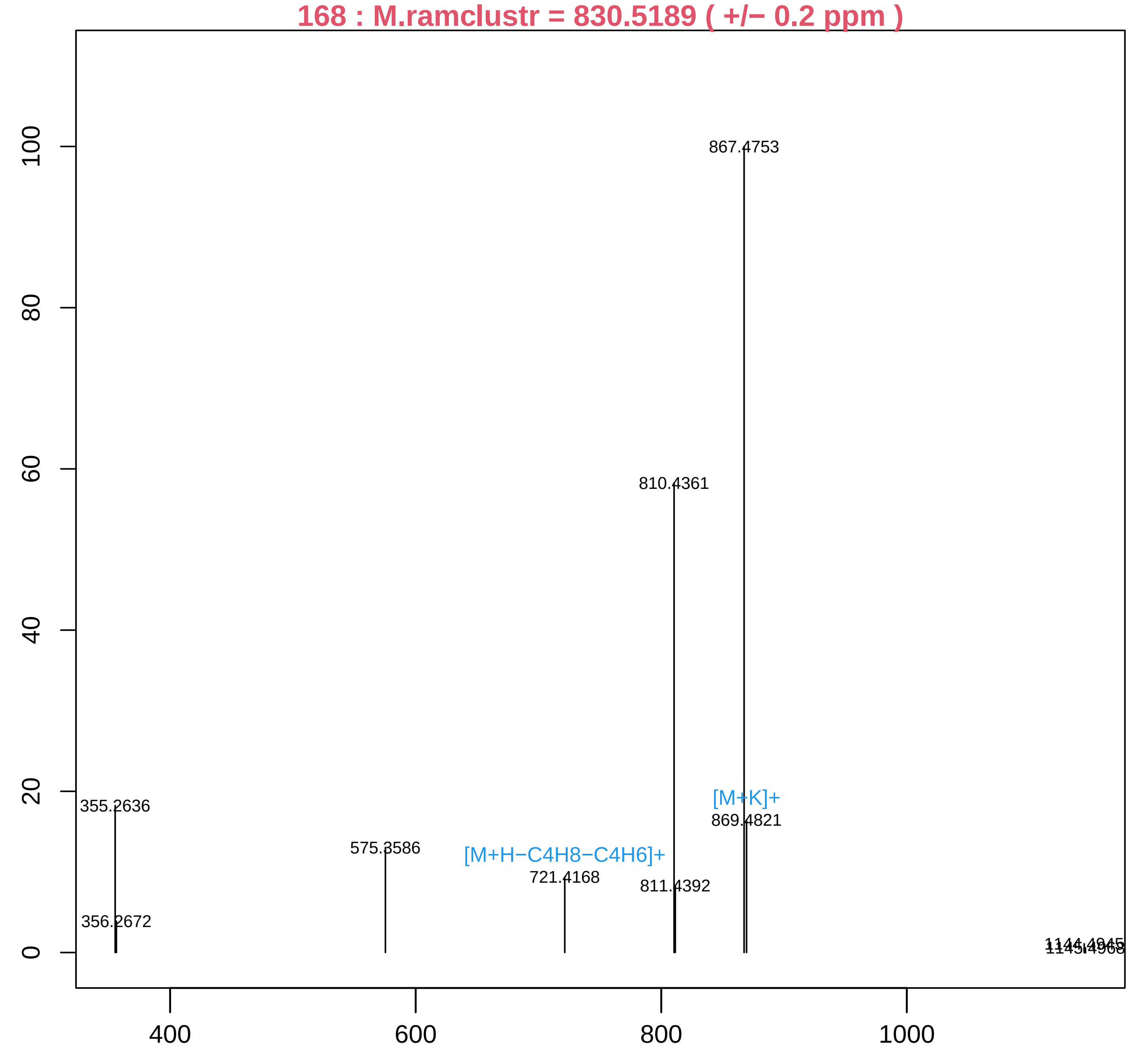
168 : M.ramclustr = 830.5189 (+/− 0.2 ppm)
867.4753
810.4361
355.2636
575.3586
[M+H−C4H8−C4H6]+
721.4168
811.4392
[M+K]+
869.4821
356.2672
1144.4945
1145.4968
0
20
40
60
80
100
400
600
800
1000

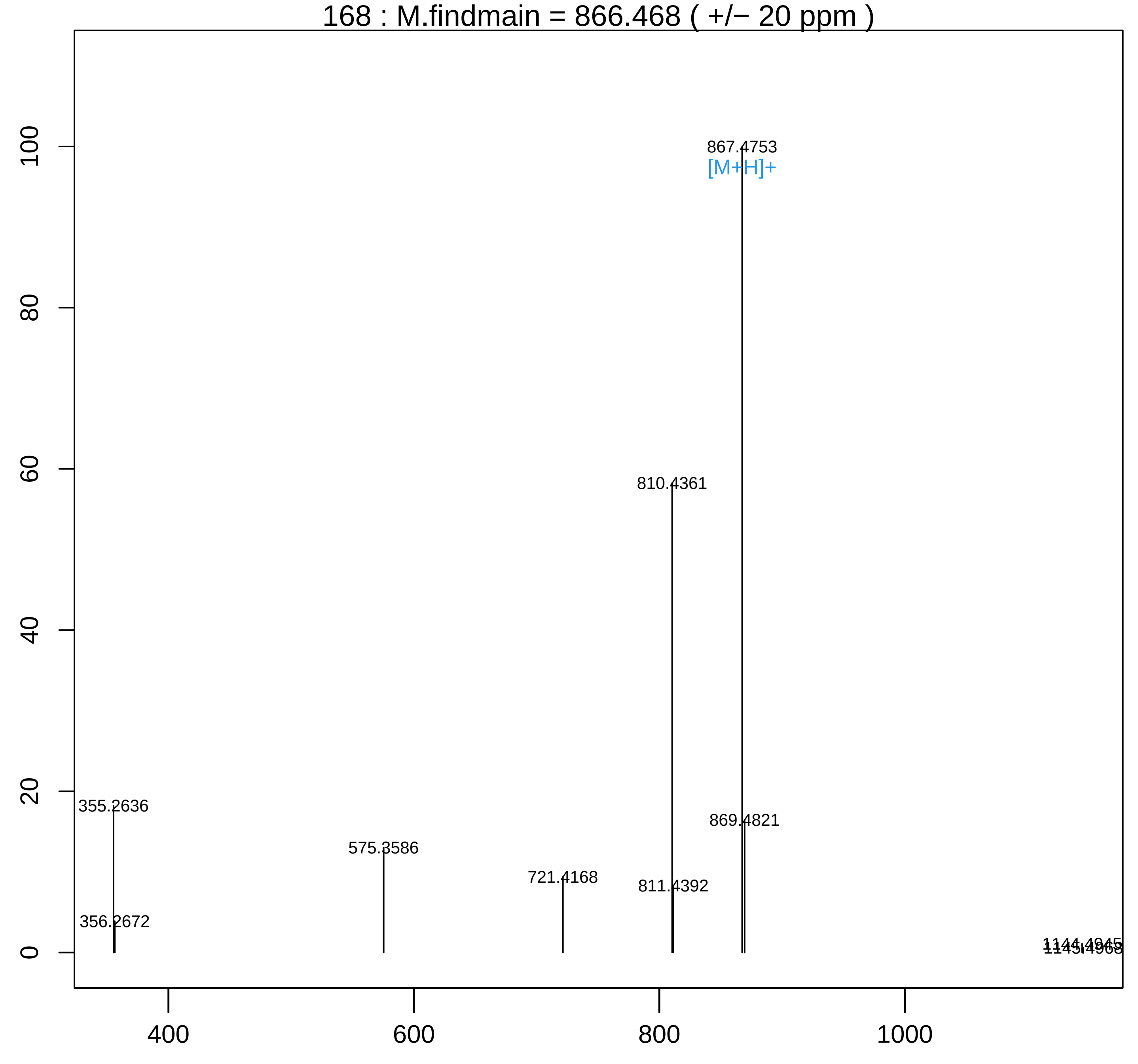
168 : M.findmain = 866.468 (+/− 20 ppm)
867.4753
[M+H]+
810.4361
355.2636
869.4821
575.3586
721.4168
811.4392
356.2672
1144.4945
1145.4968
0
20
40
60
80
100
400
600
800
1000

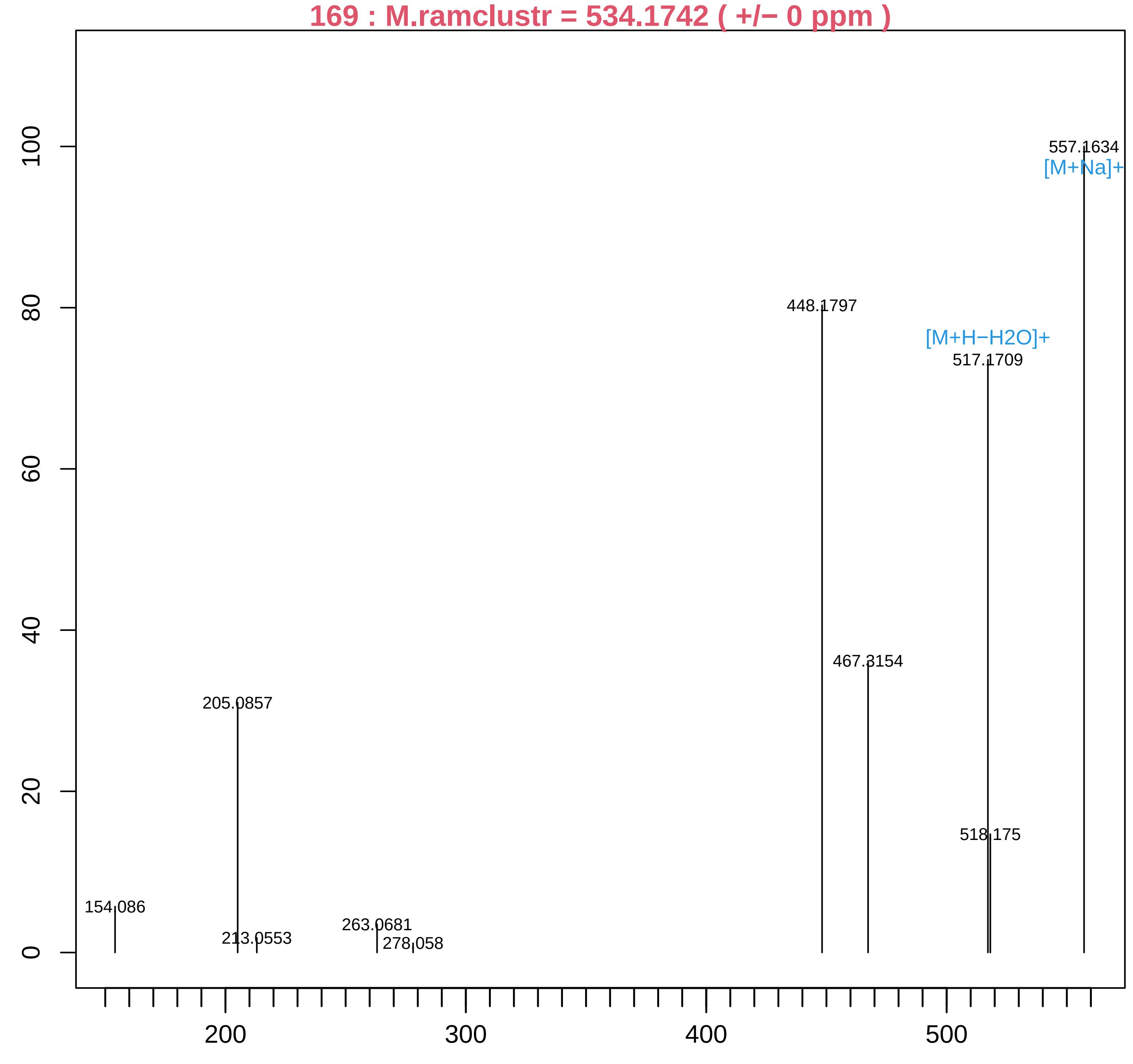
169 : M.ramclustr = 534.1742 (+/− 0 ppm)
557.1634
[M+Na]+
448.1797
[M+H−H2O]+
517.1709
467.3154
205.0857
518.175
154.086
213.0553
263.0681
278.058
0
20
40
60
80
100
200
300
400
500

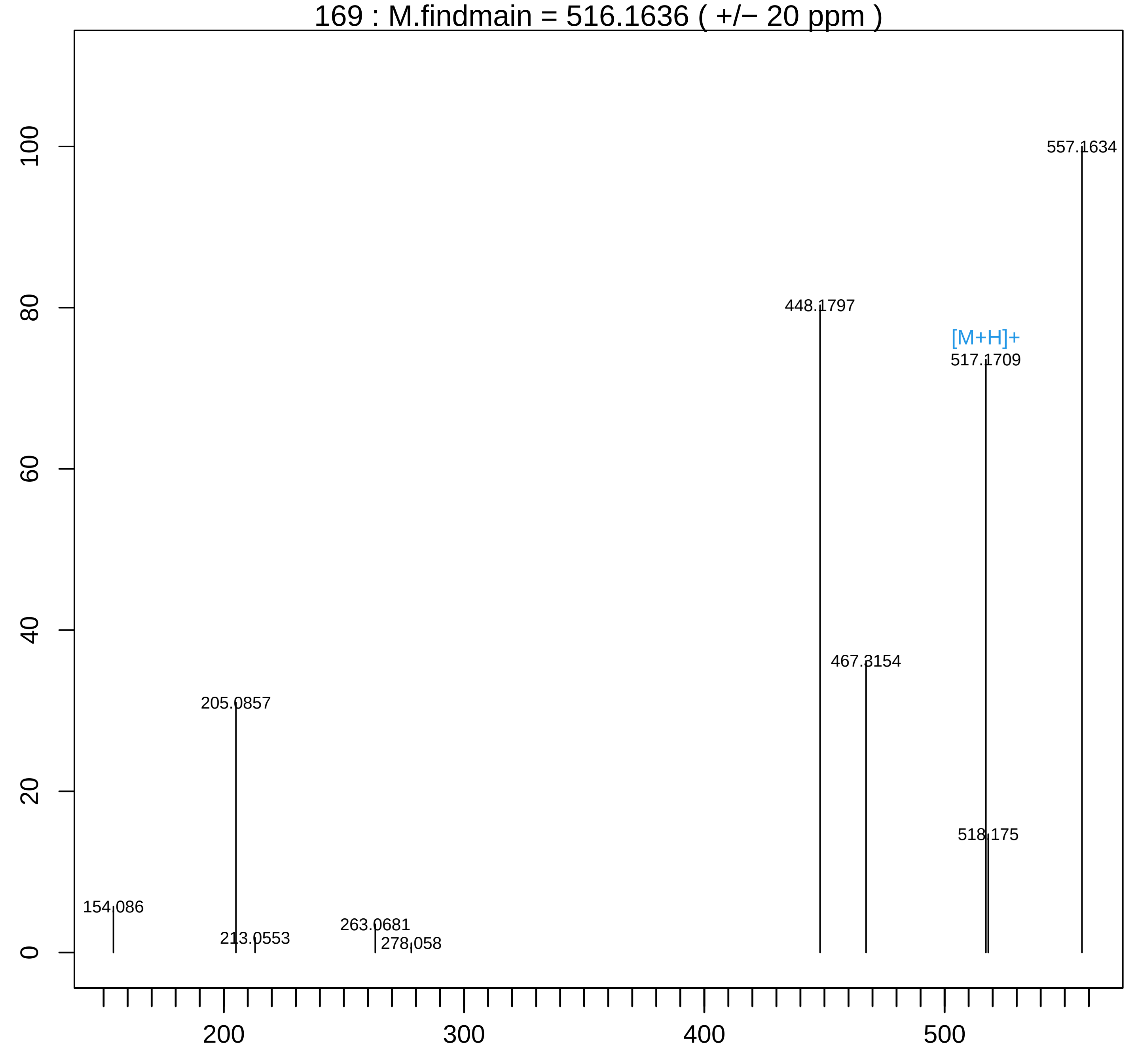
169 : M.findmain = 516.1636 (+/− 20 ppm)
557.1634
448.1797
[M+H]+
517.1709
467.3154
205.0857
518.175
154.086
213.0553
263.0681
278.058
0
20
40
60
80
100
200
300
400
500

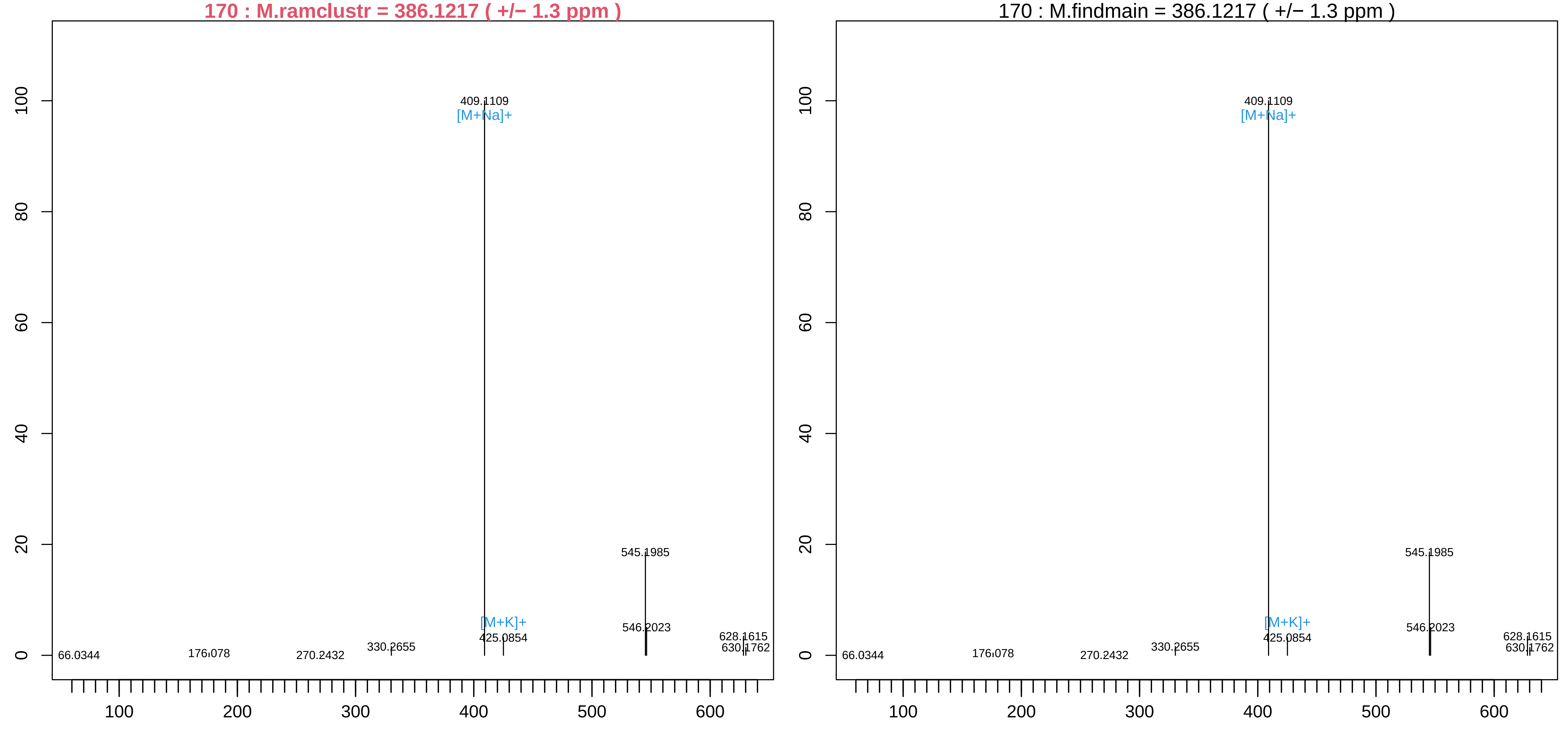
170 : M.ramclustr = 386.1217 (+/− 1.3 ppm)
409.1109
[M+Na]+
545.1985
[M+K]+
425.0854
546.2023
628.1615
630.1762
330.2655
66.0344
176.078
270.2432
170 : M.findmain = 386.1217 (+/− 1.3 ppm)
409.1109
[M+Na]+
545.1985
[M+K]+
425.0854
546.2023
628.1615
630.1762
330.2655
66.0344
176.078
270.2432

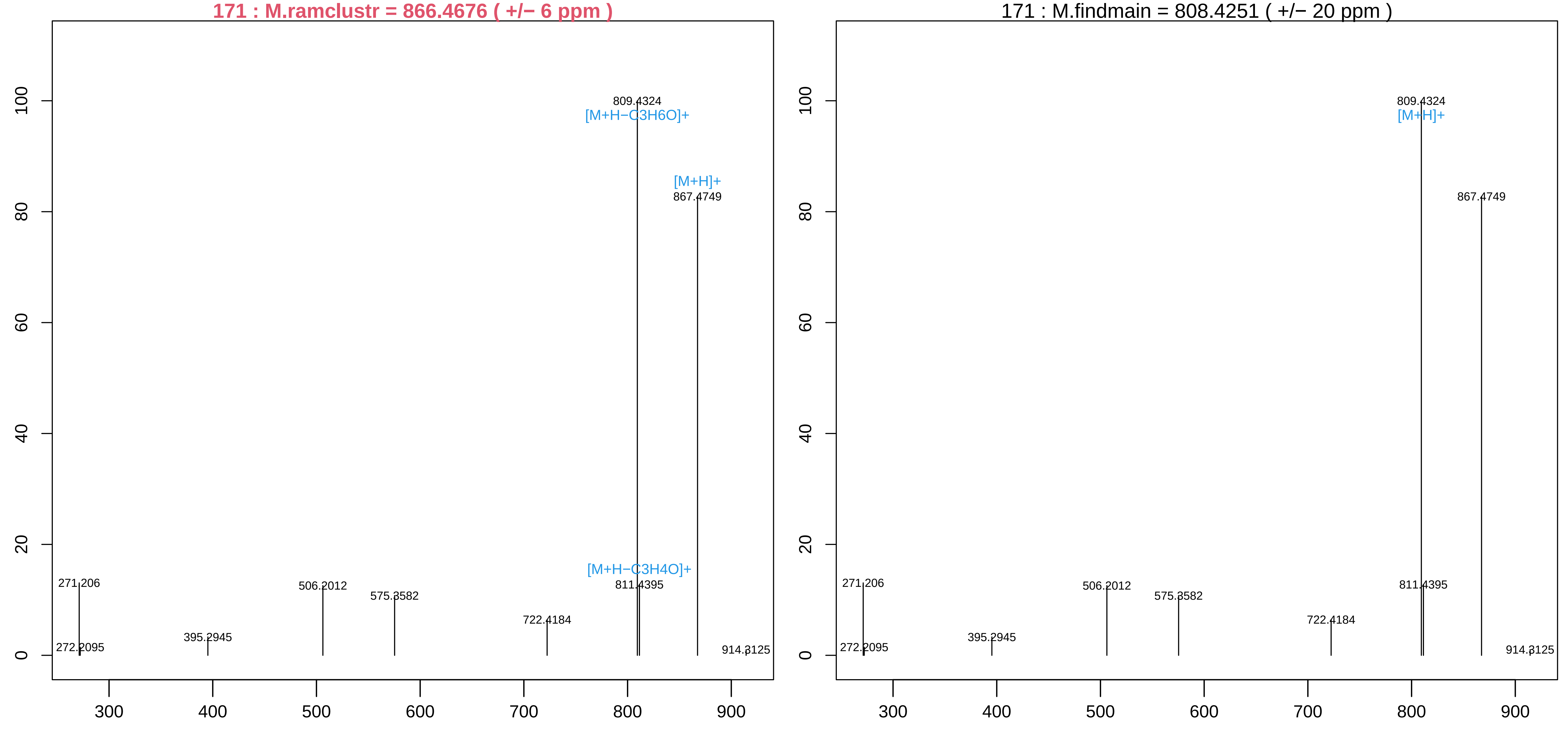
171 : M.ramclustr = 866.4676 (+/− 6 ppm)
809.4324
[M+H−C3H6O]+
[M+H]+
867.4749
[M+H−C3H4O]+
811.4395
271.206
272.2095
395.2945
506.2012
575.3582
722.4184
914.3125
171 : M.findmain = 808.4251 (+/− 20 ppm)
809.4324
[M+H]+
867.4749
271.206
272.2095
395.2945
506.2012
575.3582
722.4184
811.4395
914.3125

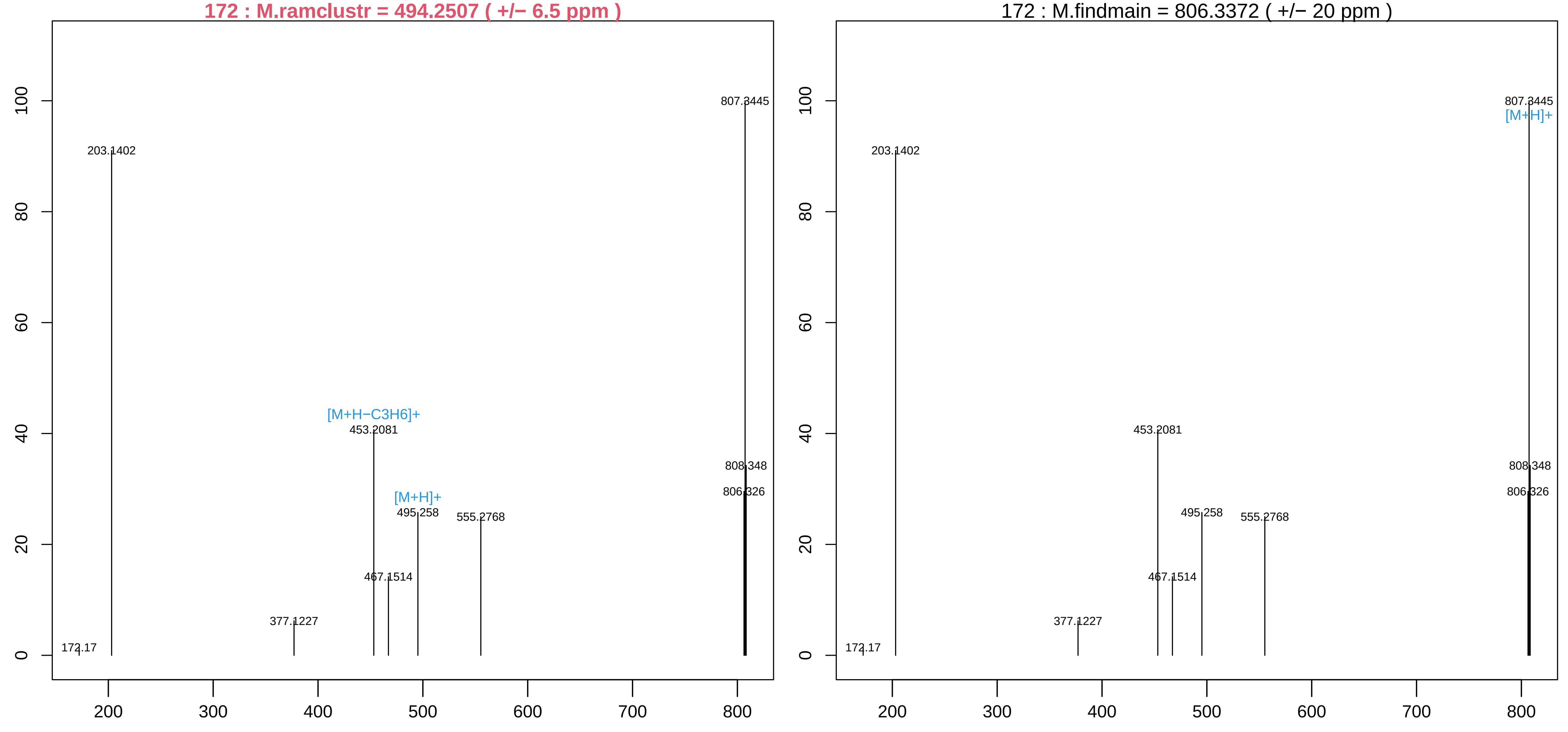

172 : M.ramclustr = 494.2507 (+/− 6.5 ppm)
807.3445
203.1402
[M+H−C3H6]+
453.2081
808.348
806.326
[M+H]+
495.258
555.2768
467.1514
377.1227
172.17
172 : M.findmain = 806.3372 (+/− 20 ppm)
807.3445
[M+H]+
203.1402
453.2081
808.348
806.326
495.258
555.2768
467.1514
377.1227
172.17

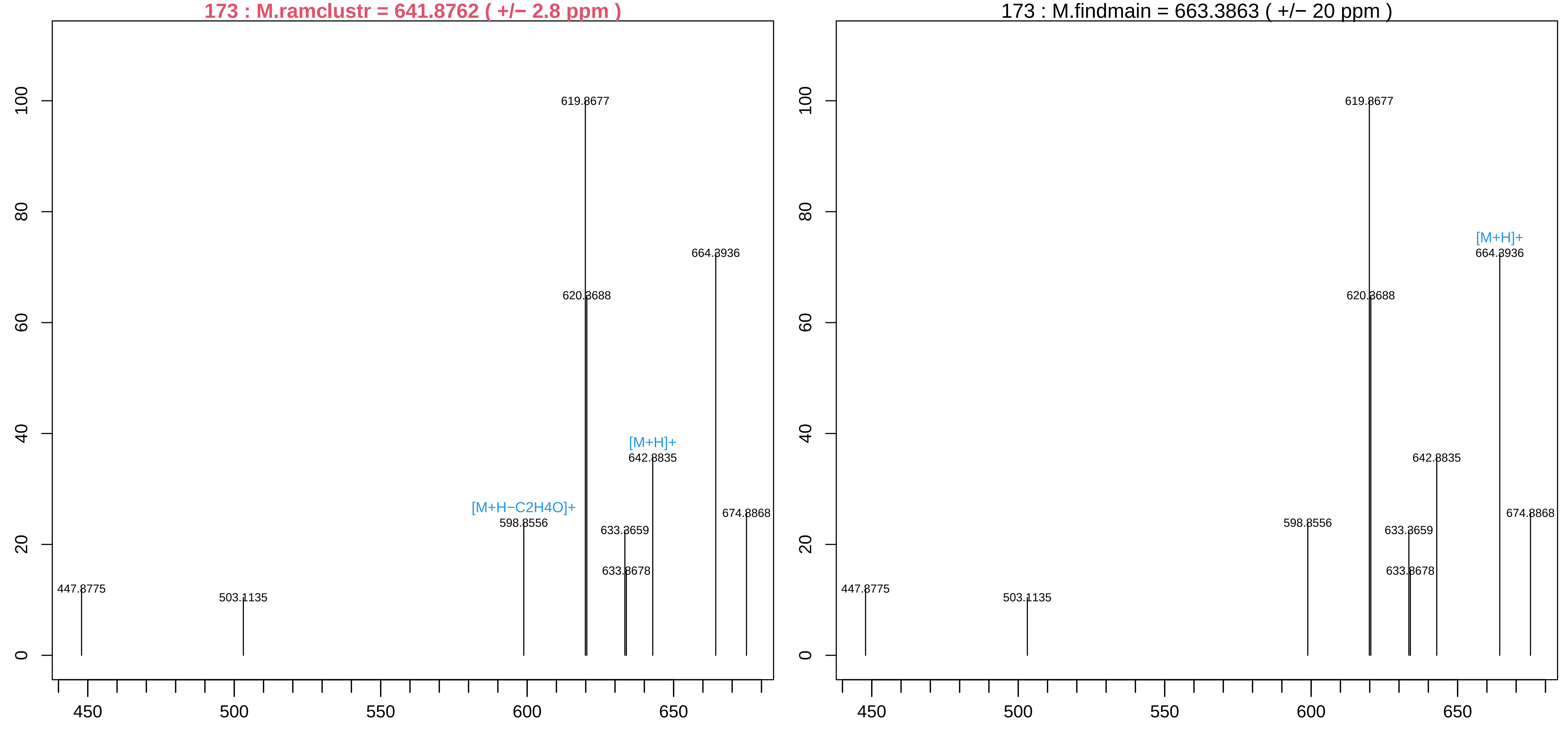
173 : M.ramclustr = 641.8762 (+/− 2.8 ppm)
619.8677
620.8688
664.3936
[M+H]+
642.8835
[M+H−C2H4O]+
598.8556
633.3659
633.8678
674.8868
447.8775
503.1135
173 : M.findmain = 663.3863 (+/− 20 ppm)
619.8677
620.8688
[M+H]+
664.3936
642.8835
598.8556
633.3659
633.8678
674.8868
447.8775
503.1135

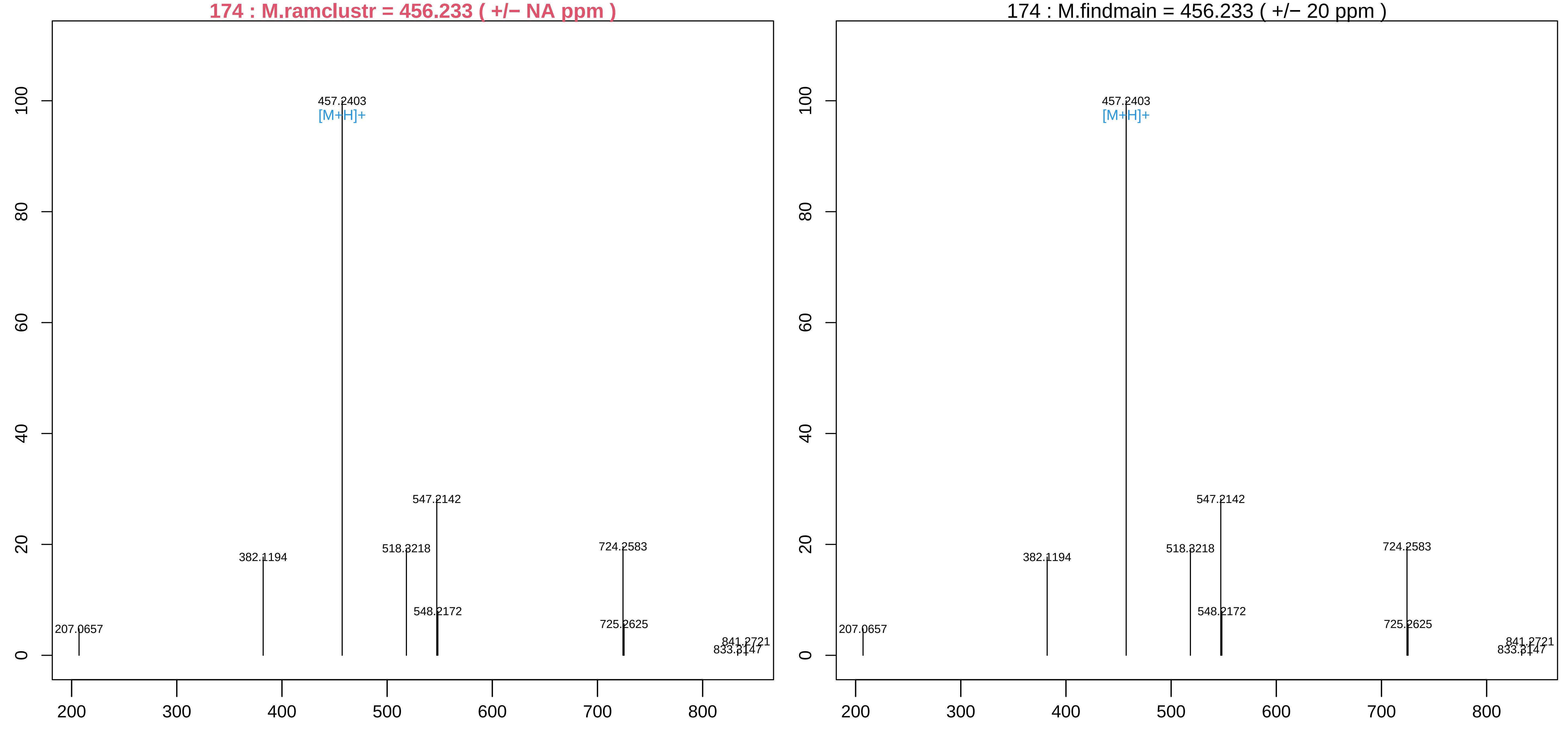

174 : M.ramclustr = 456.233 (+/− NA ppm)
457.2403
[M+H]+
547.2142
518.3218
382.1194
724.2583
548.2172
725.2625
207.0657
841.2721
833.3147
174 : M.findmain = 456.233 (+/− 20 ppm)
457.2403
[M+H]+
547.2142
518.3218
382.1194
724.2583
548.2172
725.2625
207.0657
841.2721
833.3147

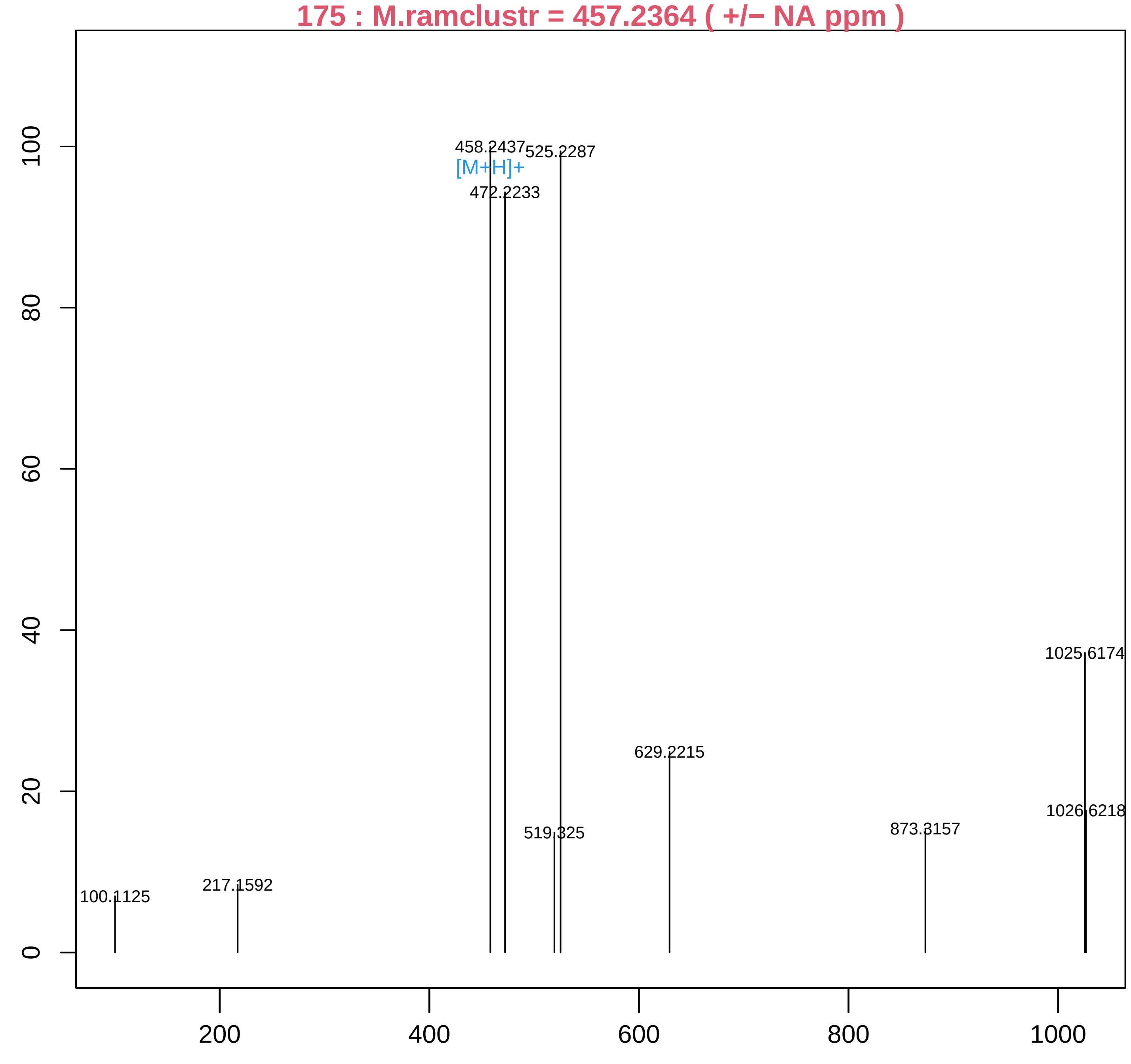
175 : M.ramclustr = 457.2364 (+/− NA ppm)
458.2437
[M+H]+
525.2287
472.2233
1025.6174
629.2215
1026.6218
519.325
873.3157
217.1592
100.1125
0
20
40
60
80
100
200
400
600
800
1000

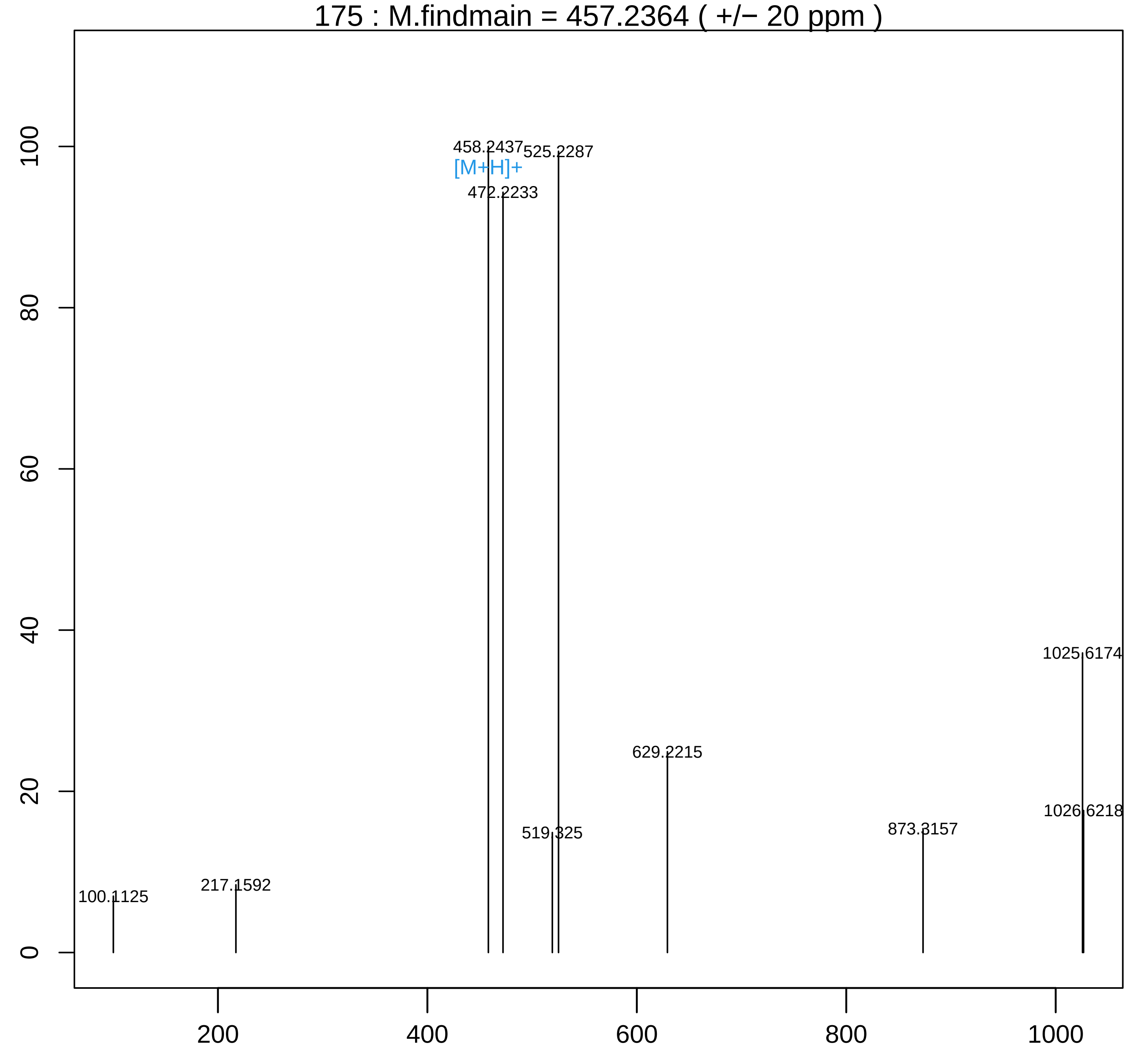
175 : M.findmain = 457.2364 (+/− 20 ppm)
458.2437
[M+H]+
525.2287
472.2233
1025.6174
629.2215
1026.6218
519.325
873.3157
217.1592
100.1125
0
20
40
60
80
100
200
400
600
800
1000

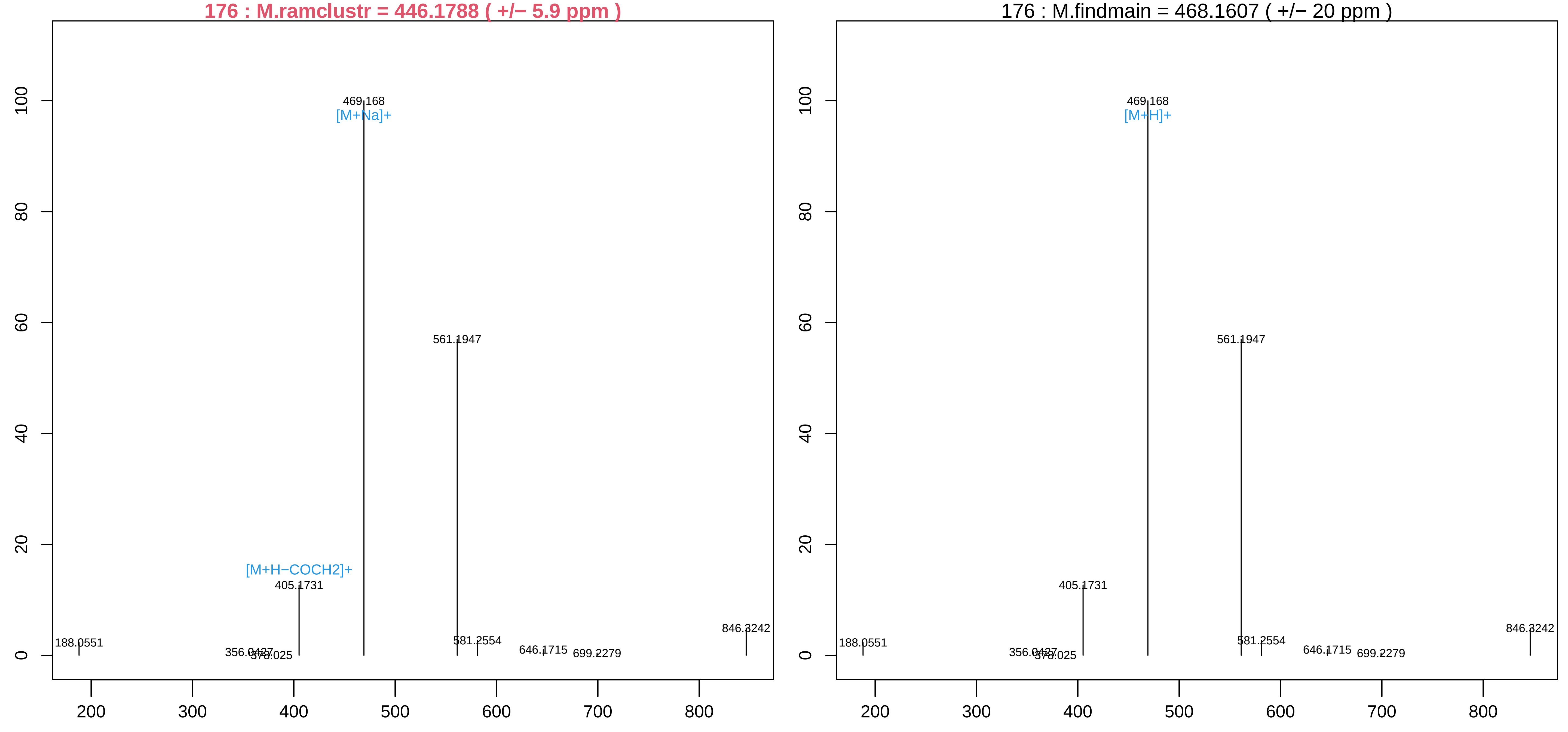

176 : M.ramclustr = 446.1788 (+/− 5.9 ppm)
469.168
[M+Na]+
561.1947
[M+H−COCH2]+
405.1731
846.3242
188.0551
581.2554
356.0427
378.025
646.1715
699.2279
176 : M.findmain = 468.1607 (+/− 20 ppm)
469.168
[M+H]+
561.1947
405.1731
846.3242
188.0551
581.2554
356.0427
378.025
646.1715
699.2279

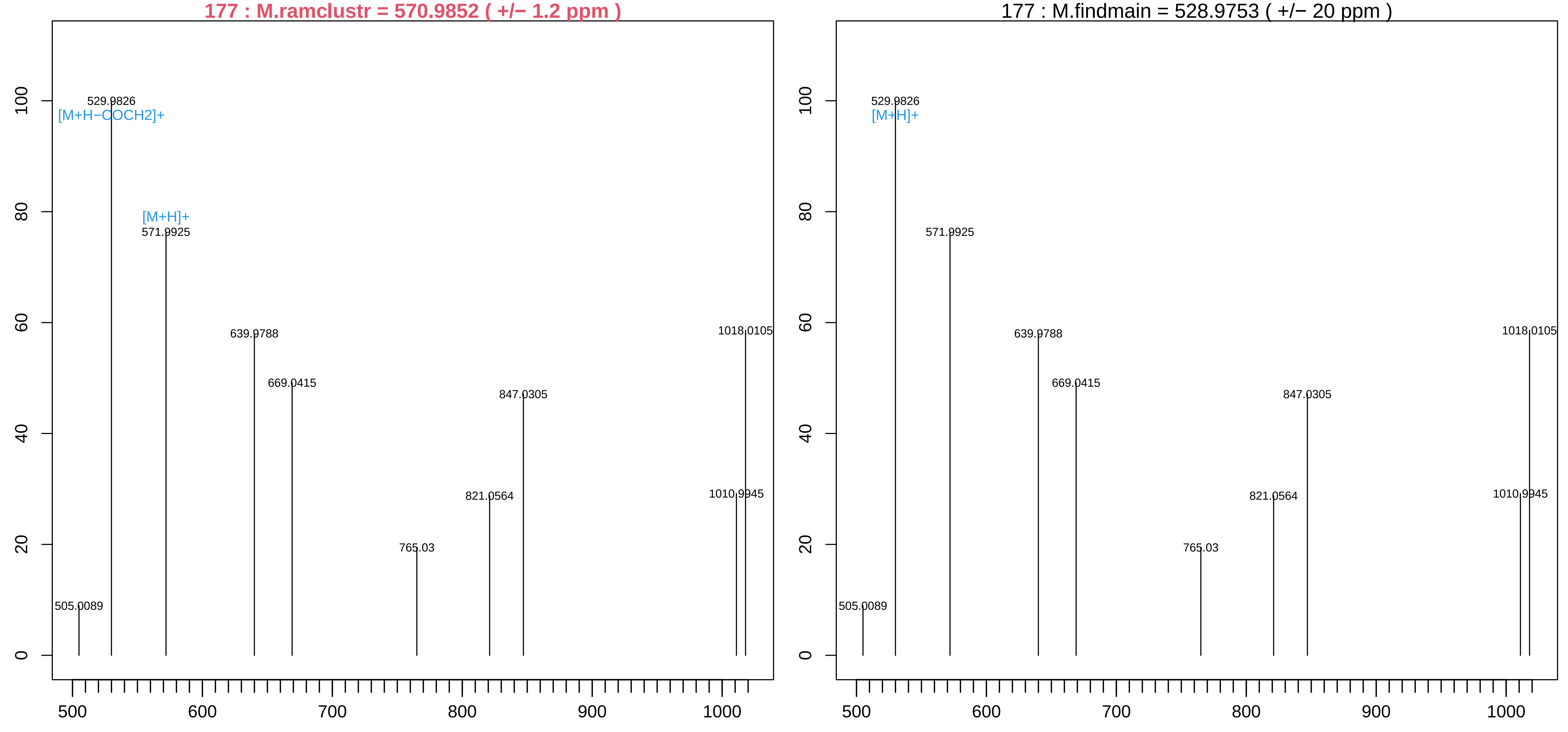

177 : M.ramclustr = 570.9852 (+/− 1.2 ppm)
529.9826
[M+H−COCH2]+
[M+H]+
571.9925
639.9788
669.0415
847.0305
1018.0105
821.0564
1010.9945
765.03
505.0089
177 : M.findmain = 528.9753 (+/− 20 ppm)
529.9826
[M+H]+
571.9925
639.9788
669.0415
847.0305
1018.0105
821.0564
1010.9945
765.03
505.0089

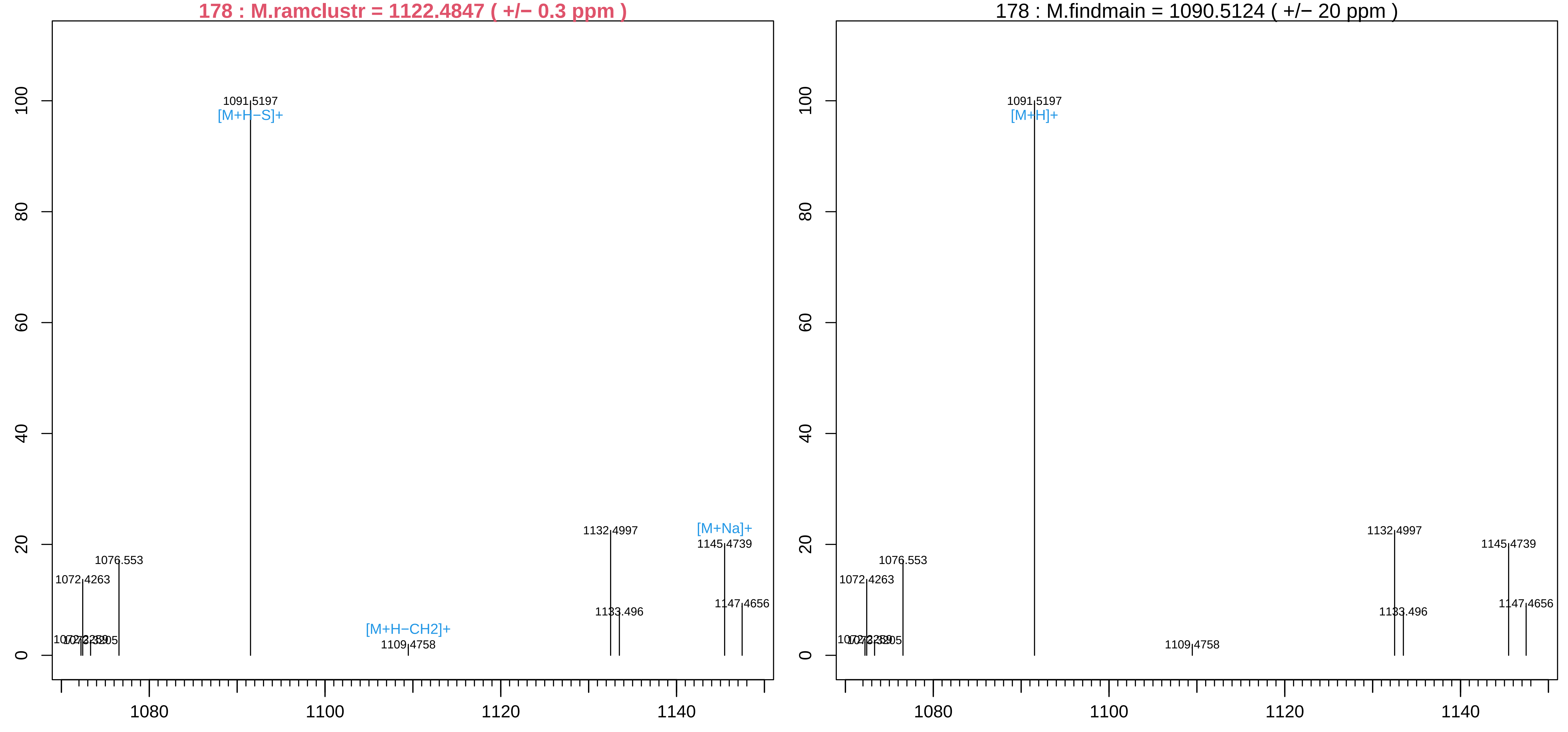
178 : M.ramclustr = 1122.4847 (+/− 0.3 ppm)
1091.5197
[M+H−S]+
1076.553
1072.4263
1132.4997
1133.496
[M+Na]+
1145.4739
1147.4656
[M+H−CH2]+
1109.4758
178 : M.findmain = 1090.5124 (+/− 20 ppm)
1091.5197
[M+H]+
1076.553
1072.4263
1132.4997
1133.496
1145.4739
1147.4656
1109.4758
0
20
40
60
80
100
1080
1100
1120
1140

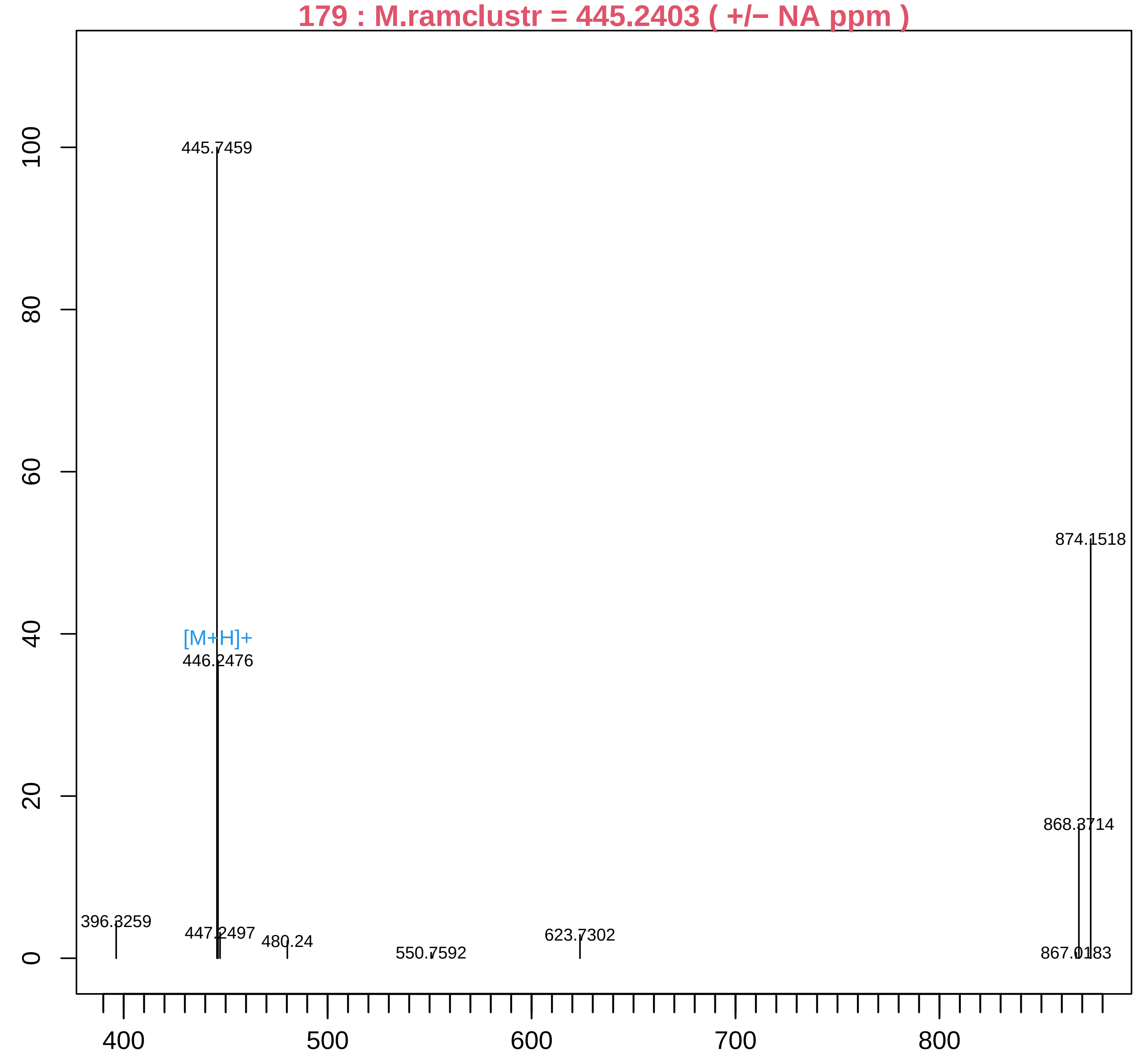
179 : M.ramclustr = 445.2403 (+/− NA ppm)
445.7459
[M+H]+
446.2476
874.1518
868.3714
396.3259
447.2497
480.24
550.7592
623.7302
867.0183
0
20
40
60
80
100
400
500
600
700
800

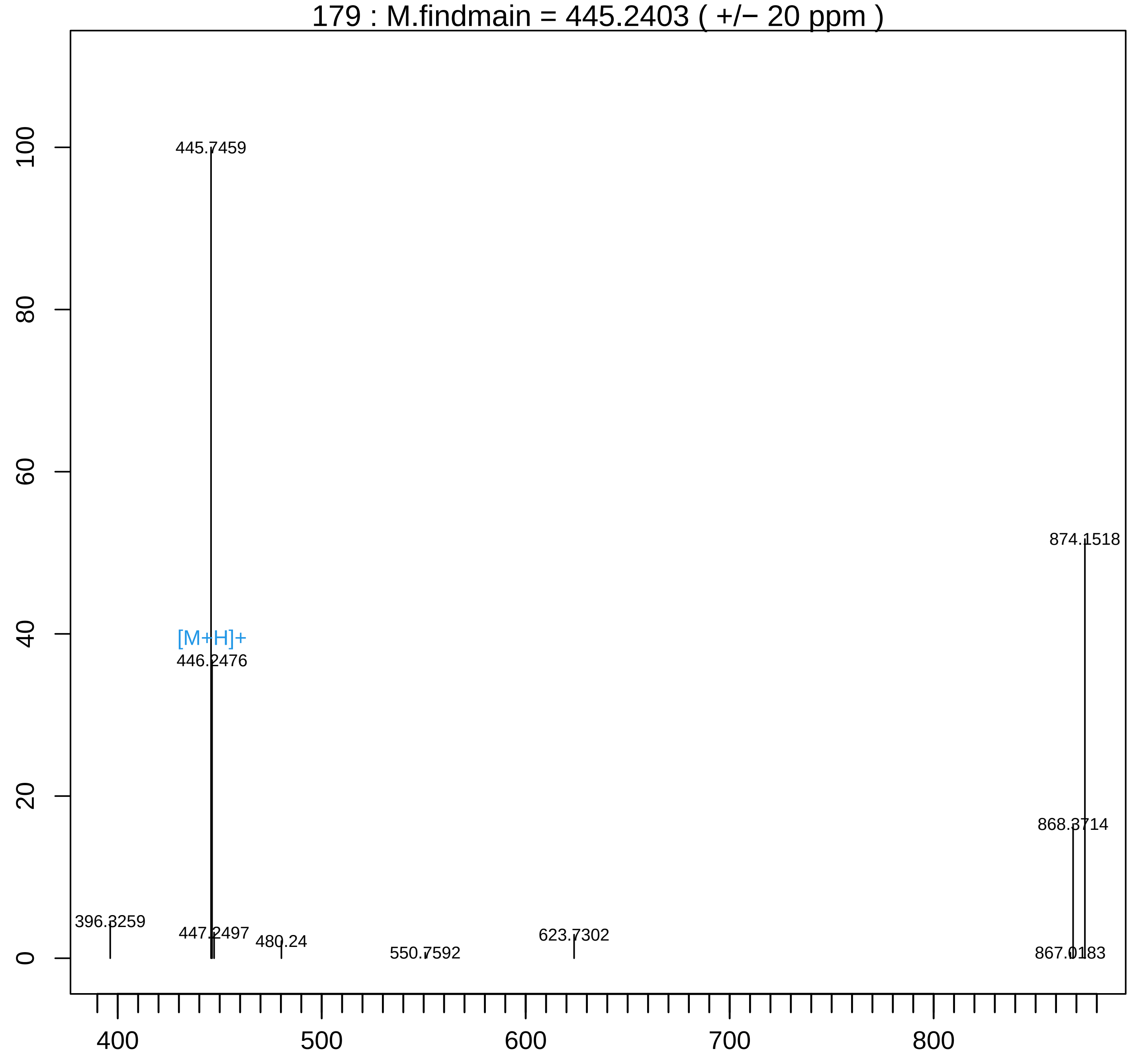
179 : M.findmain = 445.2403 (+/− 20 ppm)
445.7459
[M+H]+
446.2476
874.1518
868.3714
396.3259
447.2497
480.24
550.7592
623.7302
867.0183
0
20
40
60
80
100
400
500
600
700
800

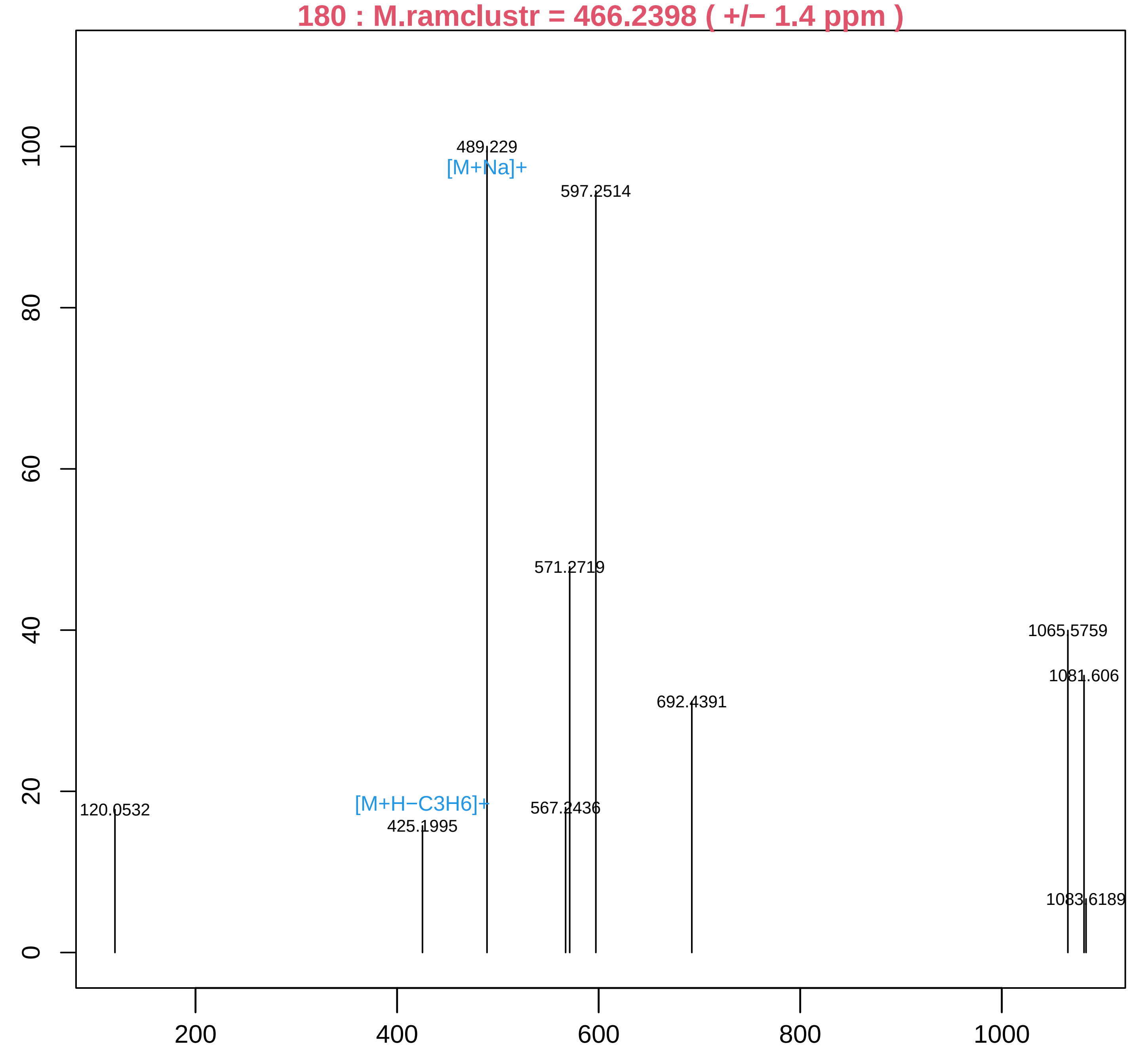

180 : M.ramclustr = 466.2398 (+/− 1.4 ppm)
489.229
[M+Na]+
597.2514
571.2719
1065.5759
1081.606
692.4391
[M+H−C3H6]+
120.0532
567.2436
425.1995
1083.6189
0
20
40
60
80
100
200
400
600
800
1000

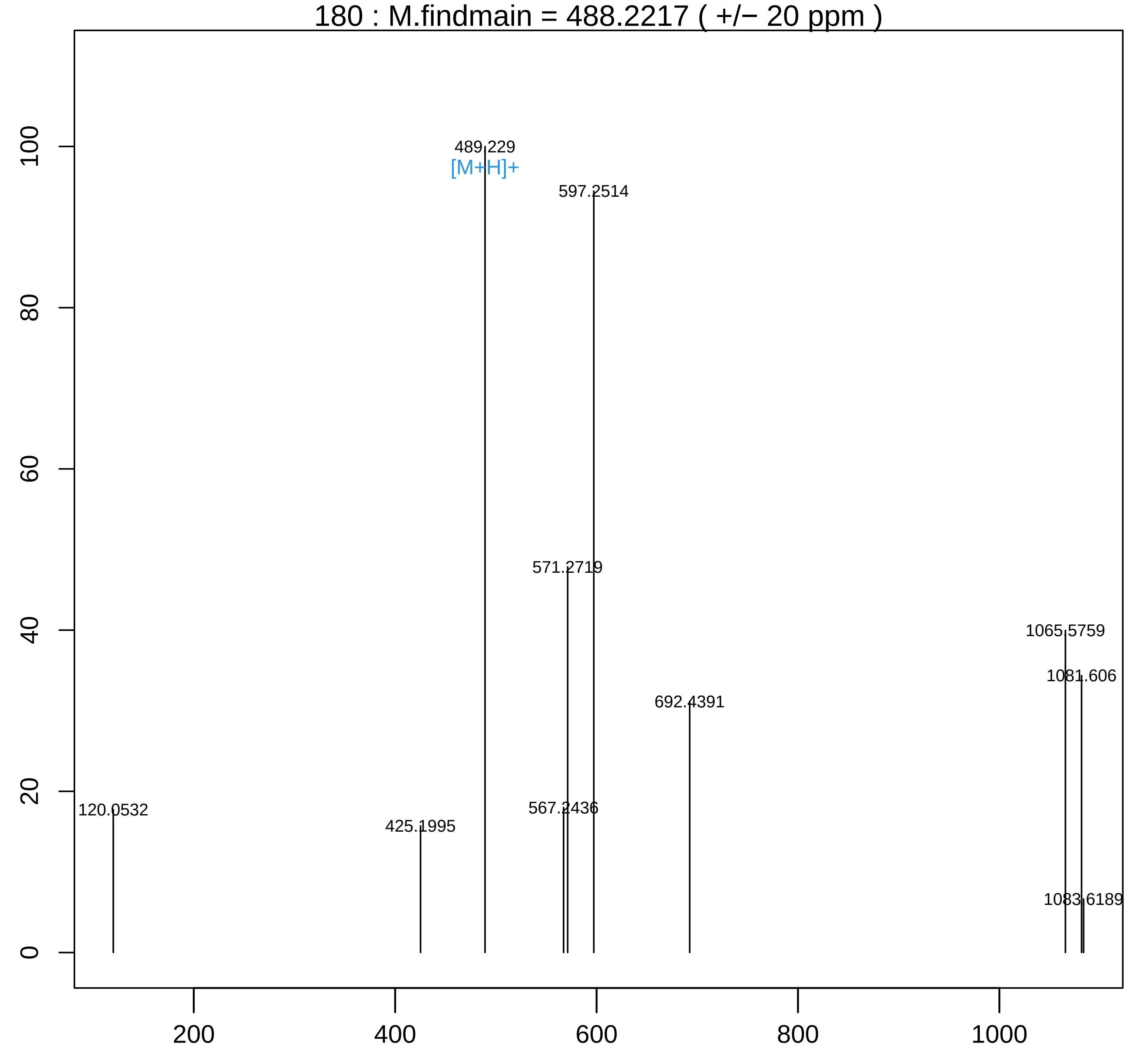

180 : M.findmain = 488.2217 (+/− 20 ppm)
489.229
[M+H]+
597.2514
571.2719
1065.5759
1081.606
692.4391
120.0532
567.2436
425.1995
1083.6189
0
20
40
60
80
100
200
400
600
800
1000

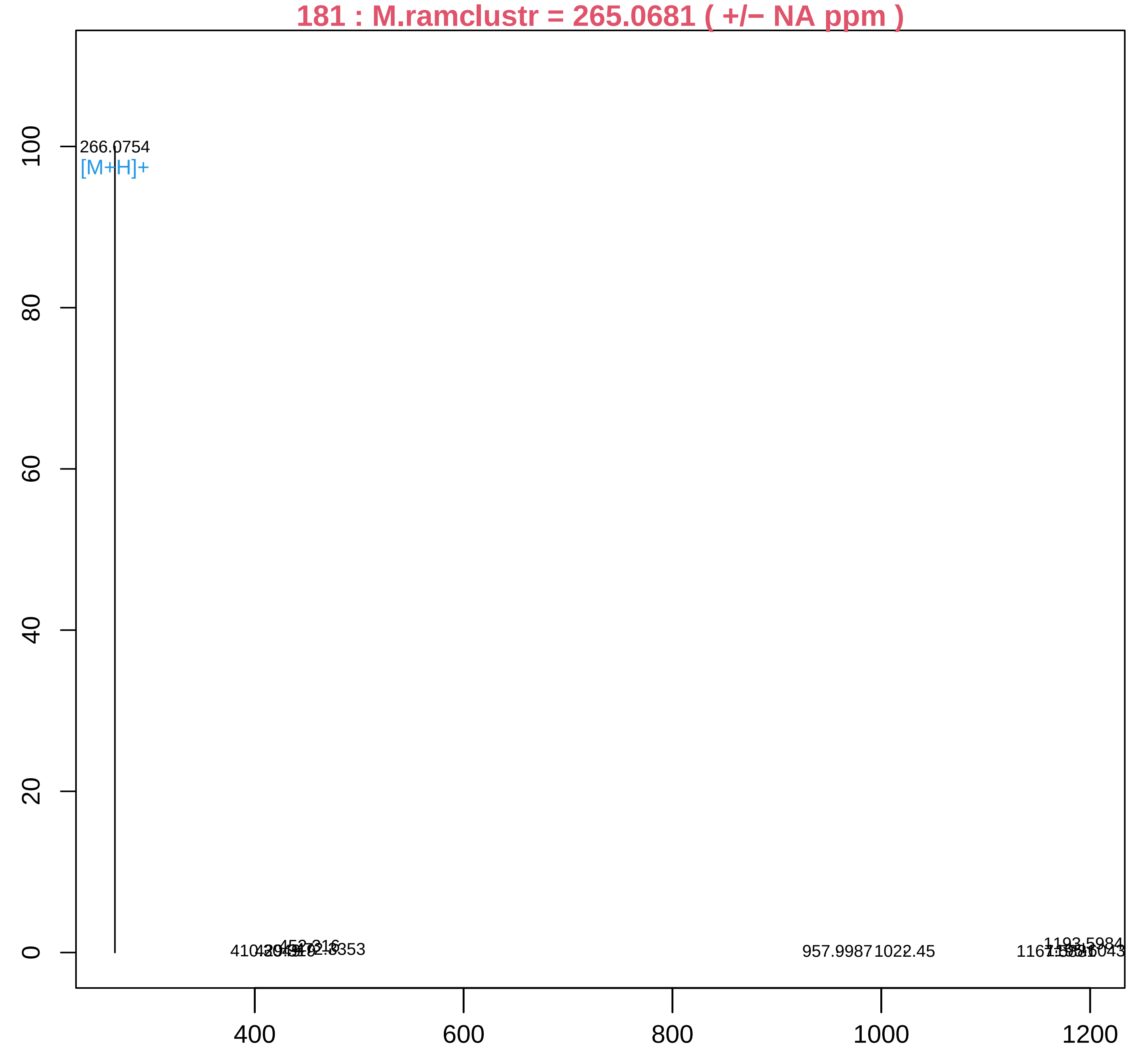
181 : M.ramclustr = 265.0681 (+/− NA ppm)
266.0754
[M+H]+
957.9987
1022.45
1193.5984
0
20
40
60
80
100
400
600
800
1000
1200

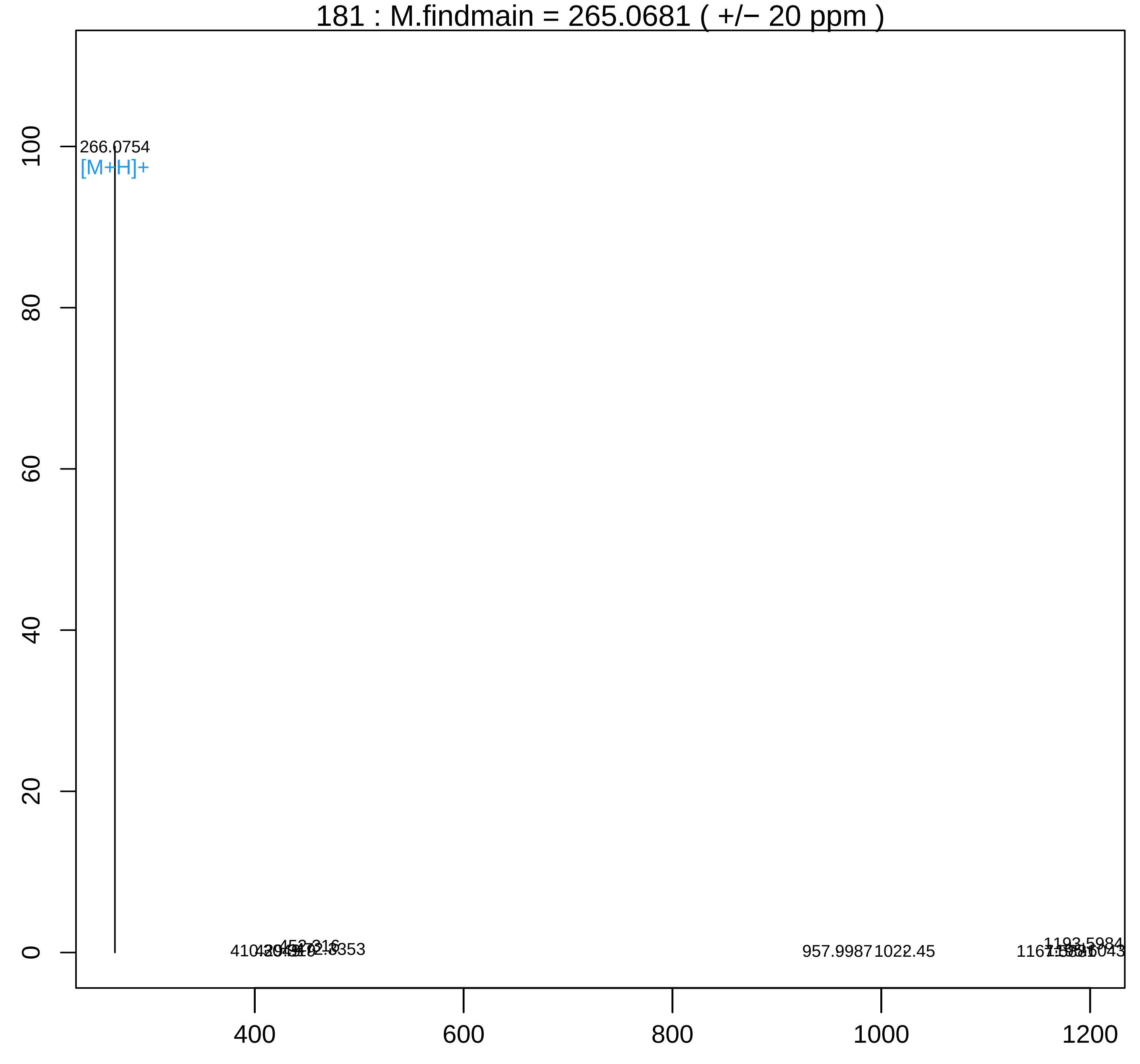
181 : M.findmain = 265.0681 (+/− 20 ppm)
266.0754
[M+H]+
957.9987
1022.45
1193.5984
0
20
40
60
80
100
400
600
800
1000
1200

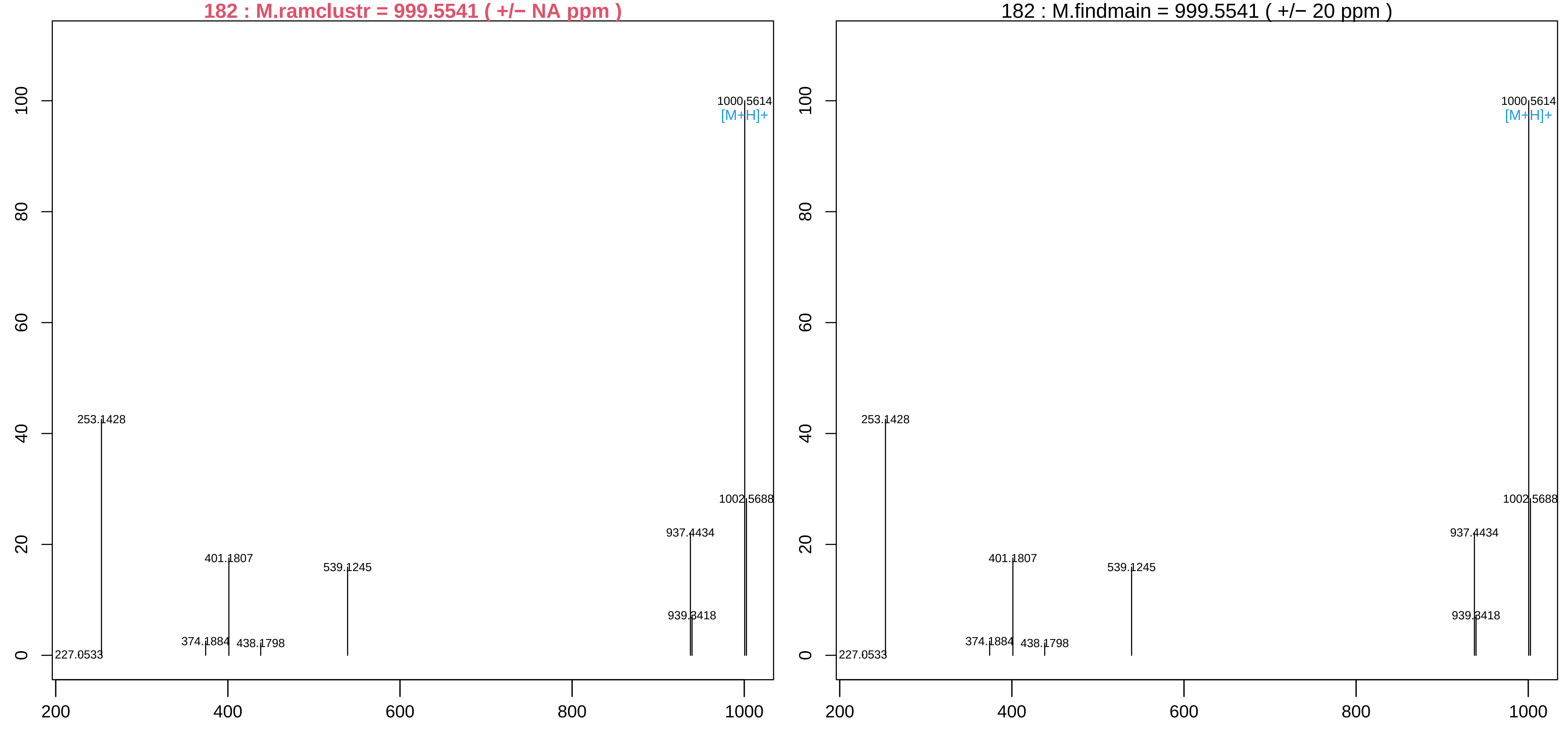

182 : M.ramclustr = 999.5541 (+/− NA ppm)
1000.5614
[M+H]+
253.1428
1002.5688
937.4434
401.1807
539.1245
939.3418
374.1884
438.1798
227.0533
0
20
40
60
80
100
200
400
600
800
1000
182 : M.findmain = 999.5541 (+/− 20 ppm)
1000.5614
[M+H]+
253.1428
1002.5688
937.4434
401.1807
539.1245
939.3418
374.1884
438.1798
227.0533
0
20
40
60
80
100
200
400
600
800
1000

183 : M.ramclustr = 1131.454 (+/− 7.4 ppm)

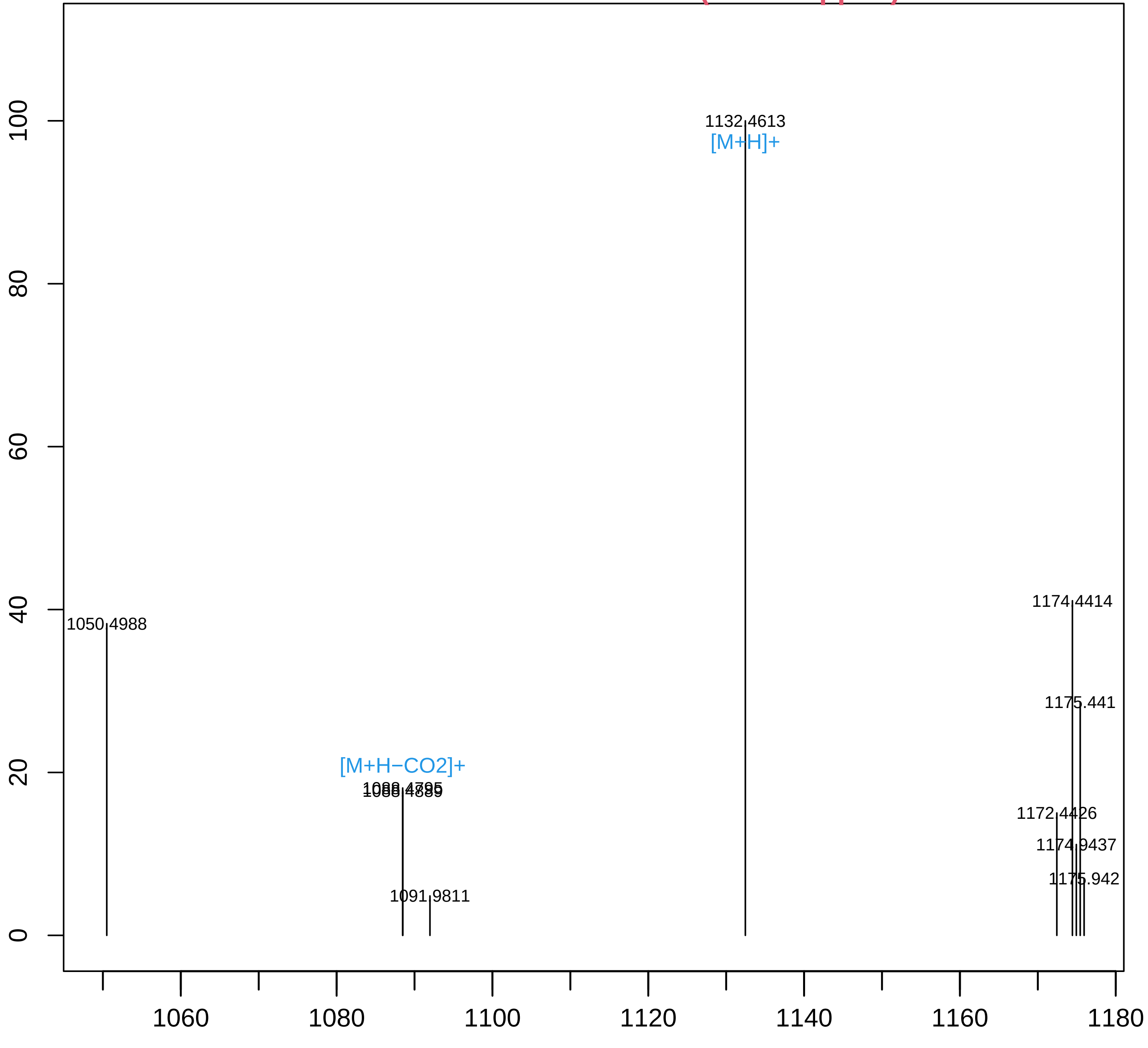

183 : M.findmain = 1131.454 (+/− 20 ppm)

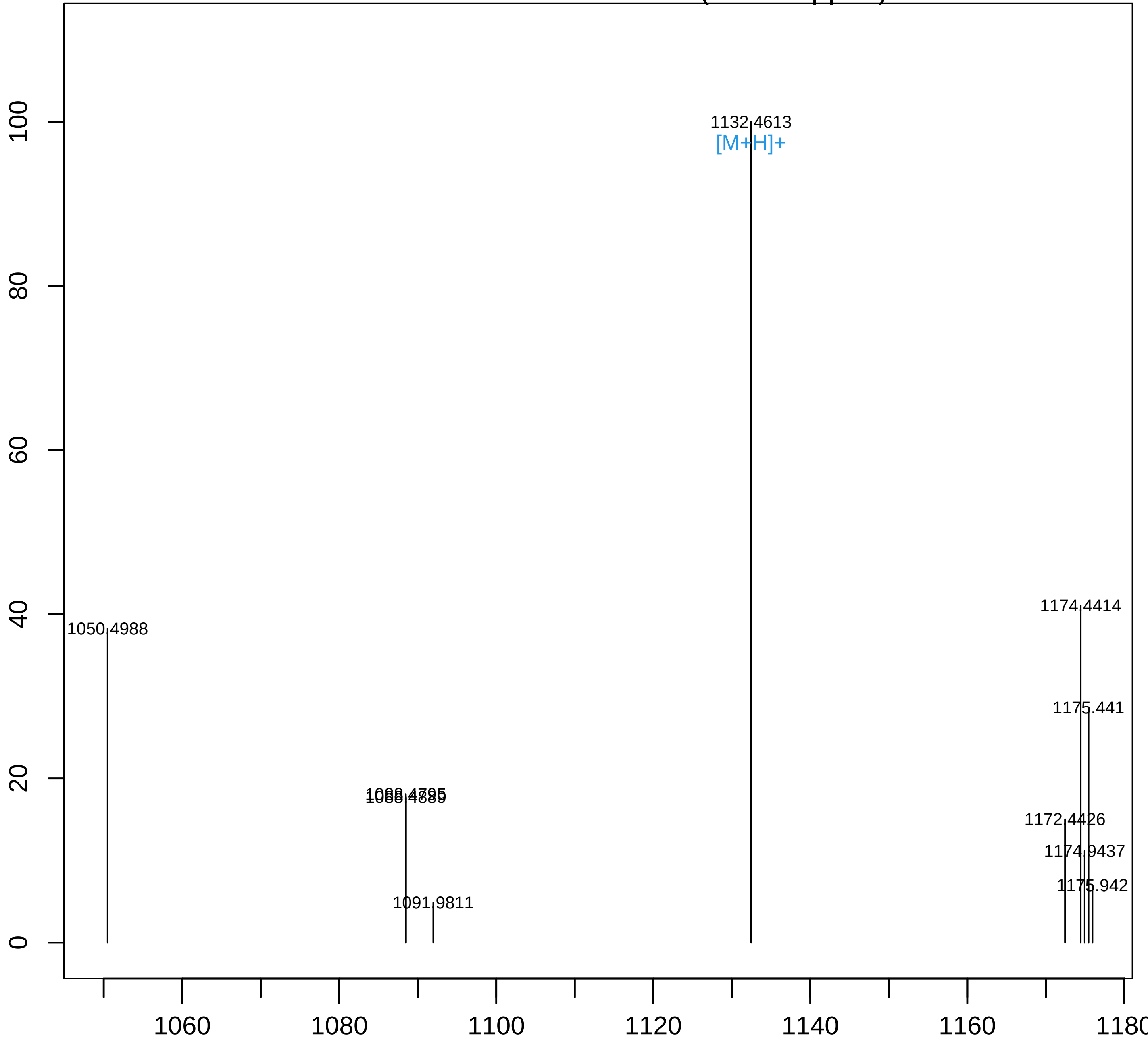

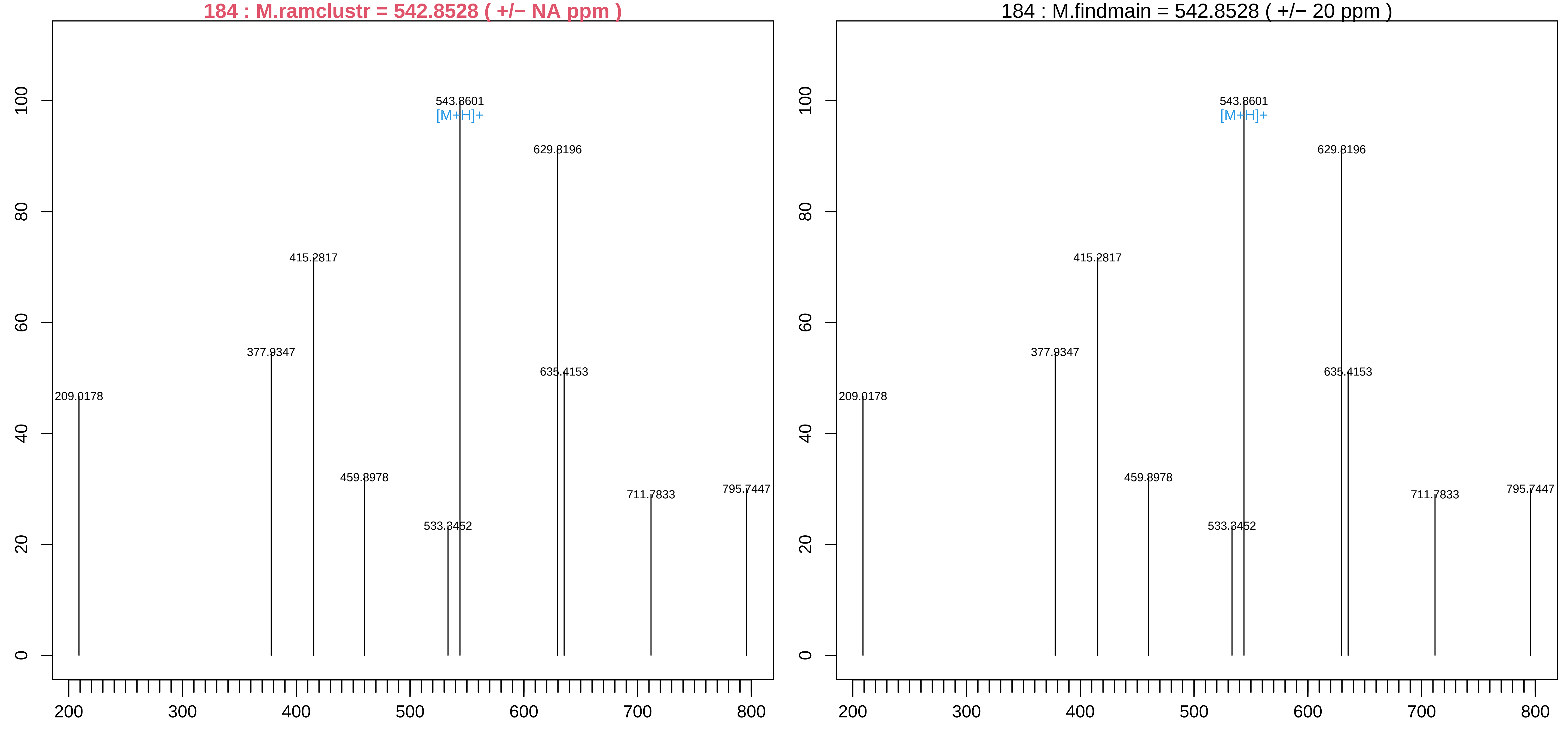
184 : M.ramclustr = 542.8528 (+/− NA ppm)
543.8601
[M+H]+
629.8196
415.2817
377.9347
635.4153
209.0178
459.8978
711.7833
795.7447
533.3452
184 : M.findmain = 542.8528 (+/− 20 ppm)
543.8601
[M+H]+
629.8196
415.2817
377.9347
635.4153
209.0178
459.8978
711.7833
795.7447
533.3452
0
20
40
60
80
100
200
300
400
500
600
700
800

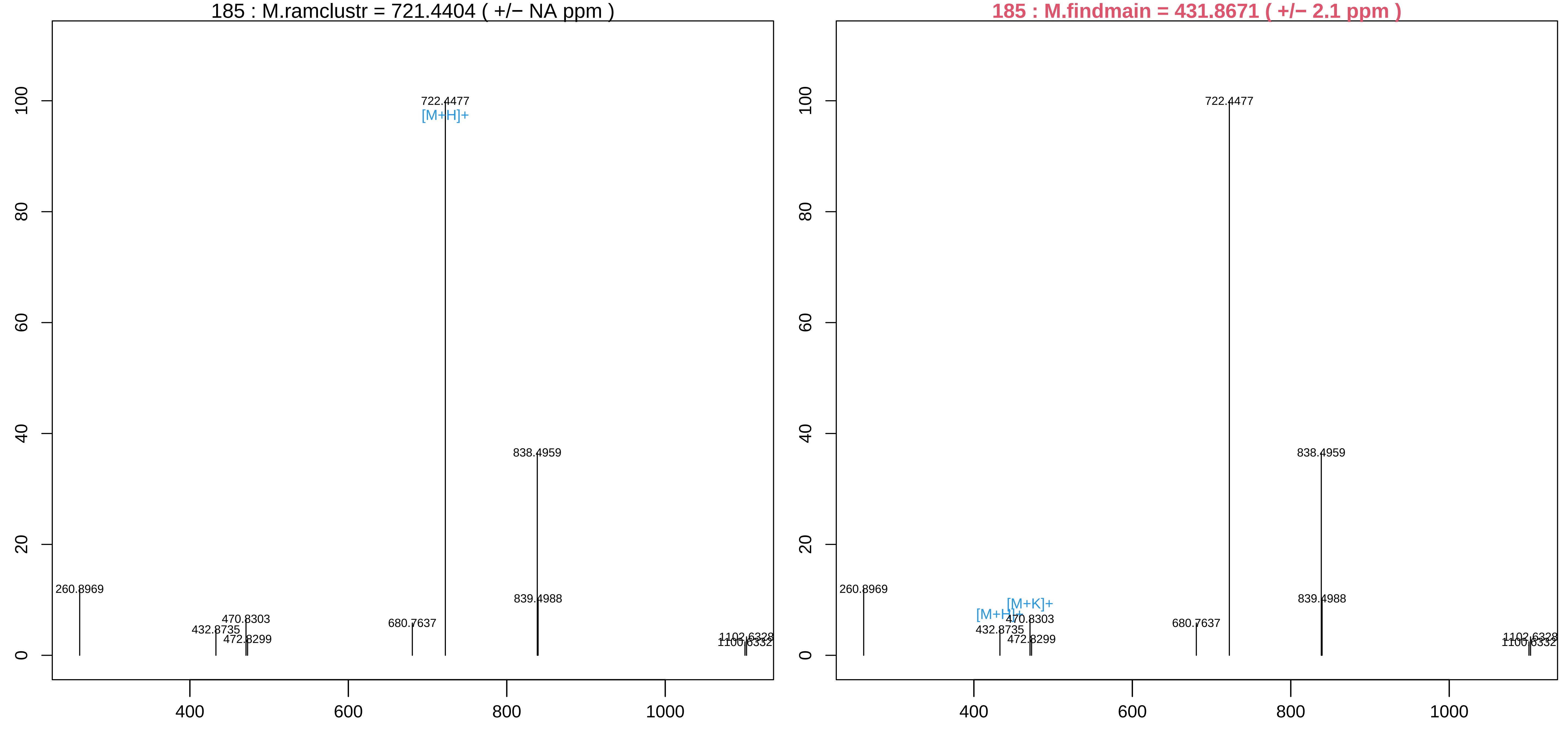
185 : M.ramclustr = 721.4404 (+/− NA ppm)
722.4477
[M+H]+
838.4959
839.4988
260.8969
470.8303
432.8735
472.8299
680.7637
1102.6328
1100.6332
185 : M.findmain = 431.8671 (+/− 2.1 ppm)
722.4477
838.4959
839.4988
260.8969
[M+K]+
[M+H]+
470.8303
432.8735
472.8299
680.7637
1102.6328
1100.6332
0
20
40
60
80
100
400
600
800
1000

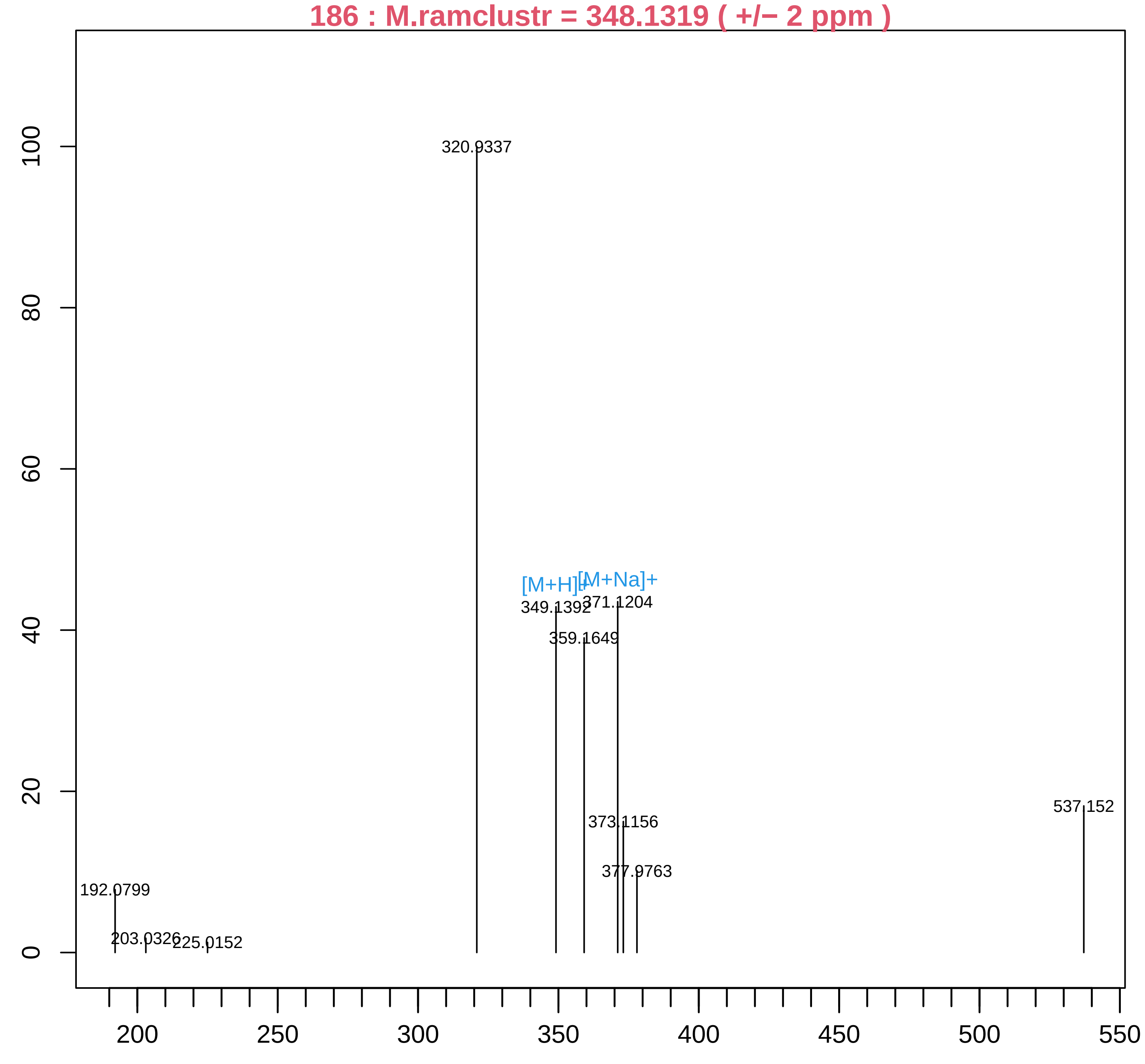
186 : M.ramclustr = 348.1319 (+/− 2 ppm)
320.9337
[M+H]+
349.1392
[M+Na]+
371.1204
359.1649
373.1156
377.9763
537.152
192.0799
203.0326
225.0152
0
20
40
60
80
100
200
250
300
350
400
450
500
550
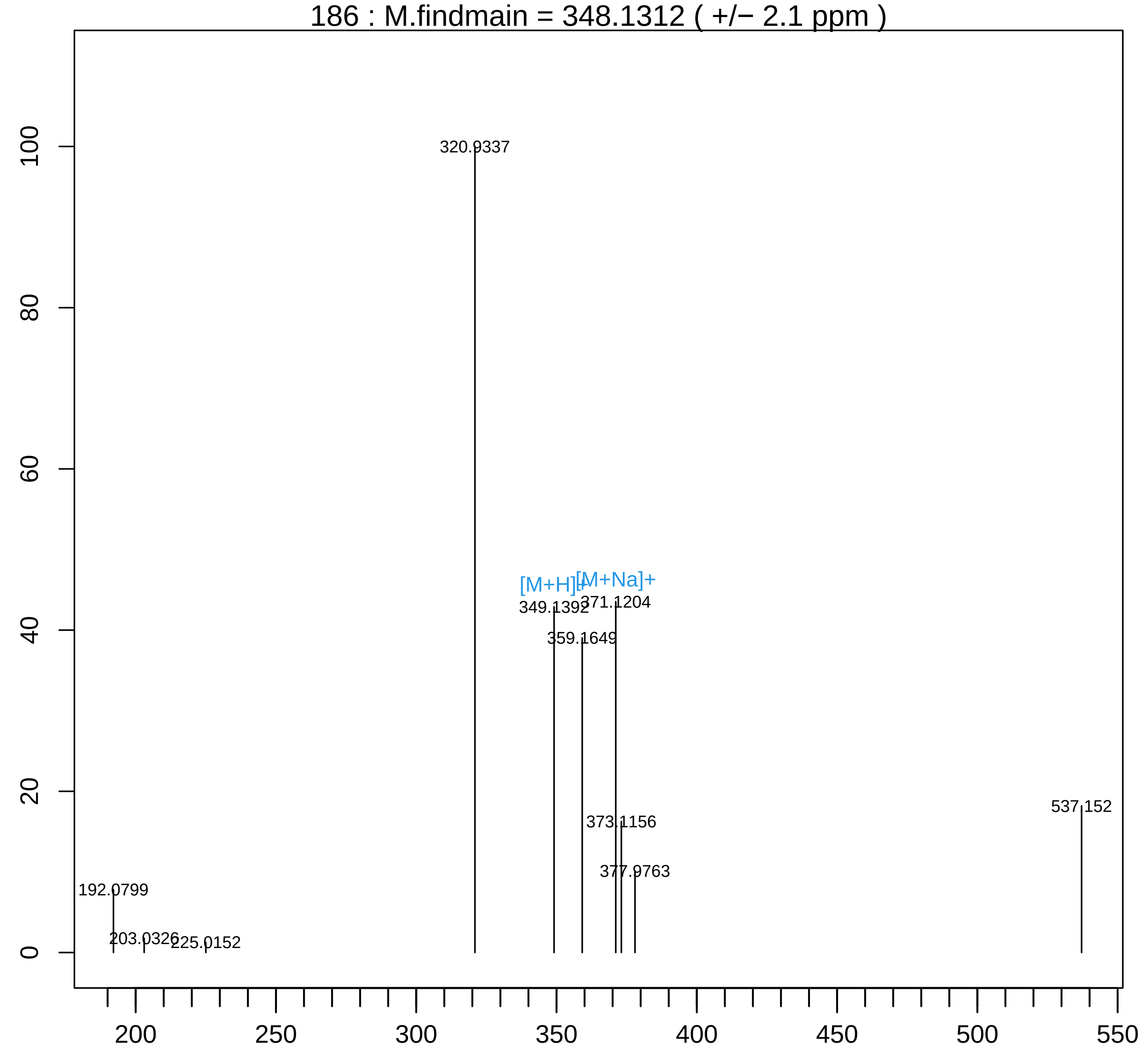
186 : M.findmain = 348.1312 (+/− 2.1 ppm)
320.9337
[M+H]+
349.1392
[M+Na]+
371.1204
359.1649
373.1156
377.9763
537.152
192.0799
203.0326
225.0152
0
20
40
60
80
100
200
250
300
350
400
450
500
550

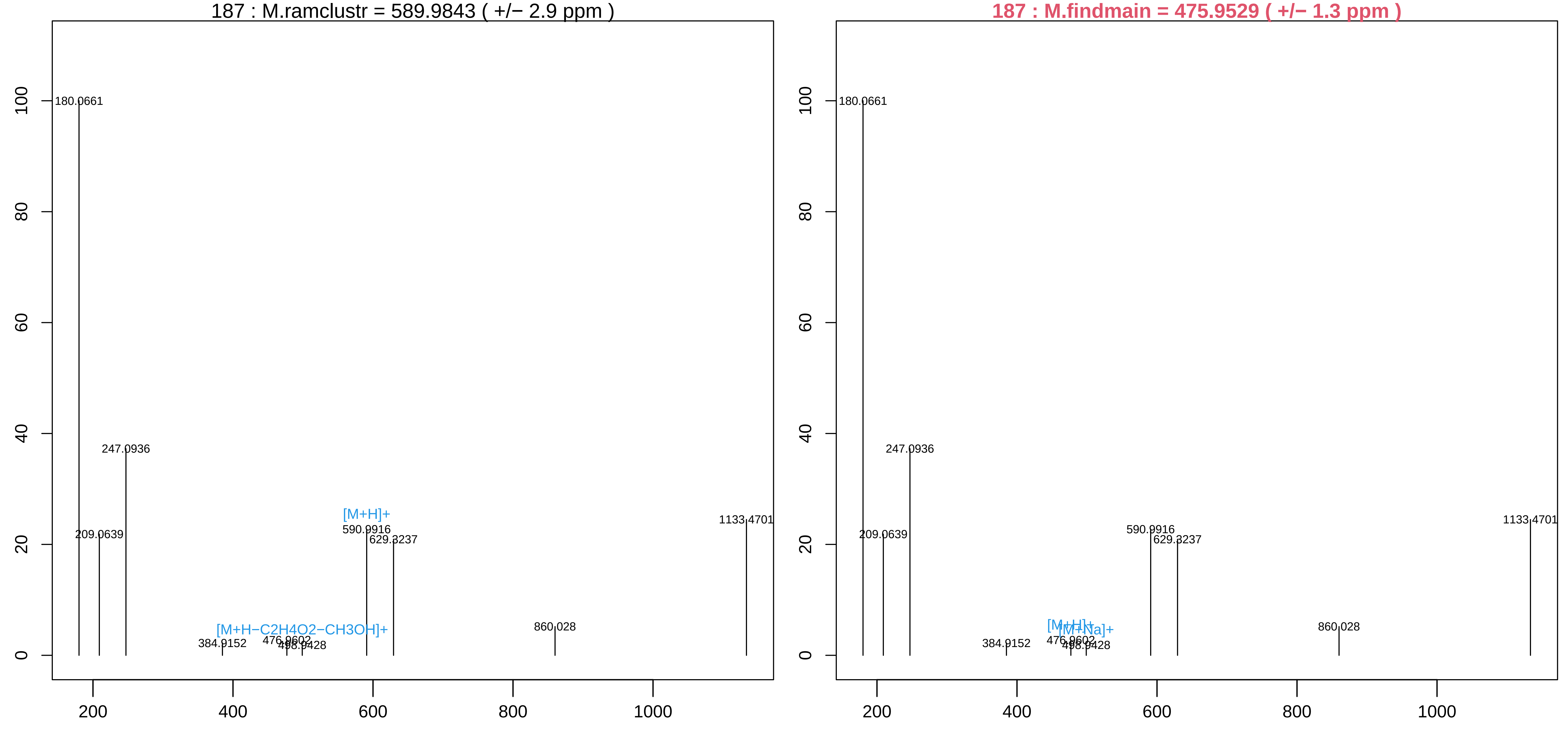
187 : M.ramclustr = 589.9843 (+/− 2.9 ppm)
180.0661
247.0936
209.0639
[M+H]+
590.9916
629.3237
1133.4701
860.028
[M+H−C2H4O2−CH3OH]+
384.9152
476.9602
498.9428
0
20
40
60
80
100
200
400
600
800
1000
187 : M.findmain = 475.9529 (+/− 1.3 ppm)
180.0661
247.0936
209.0639
590.9916
629.3237
1133.4701
860.028
[M+H]+
[M+Na]+
384.9152
476.9602
498.9428
0
20
40
60
80
100
200
400
600
800
1000

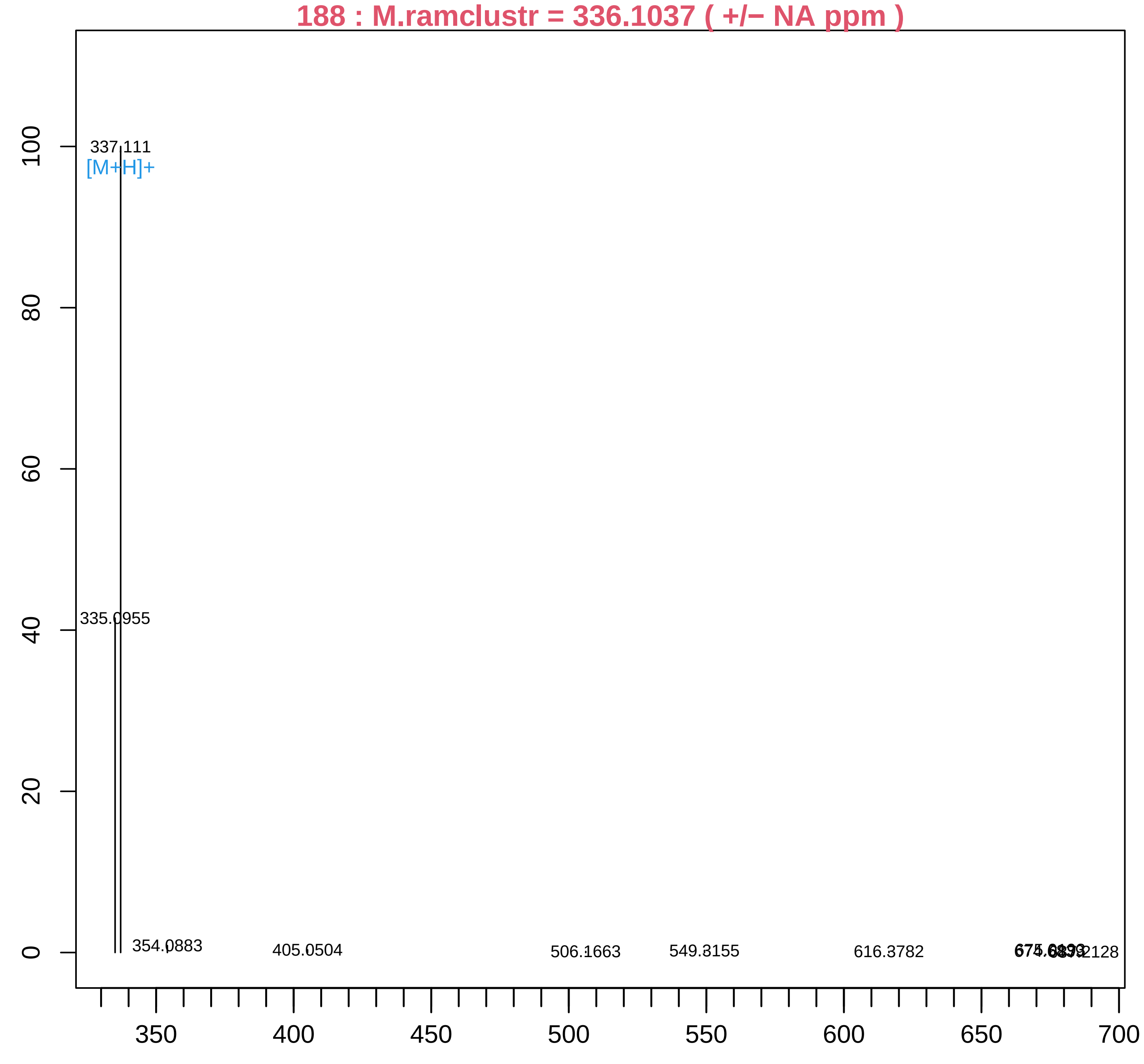
188 : M.ramclustr = 336.1037 (+/− NA ppm)
337.111
[M+H]+
335.0955
354.0883
405.0504
506.1663
549.8155
616.3782
675.6893
0
20
40
60
80
100
350
400
450
500
550
600
650
700

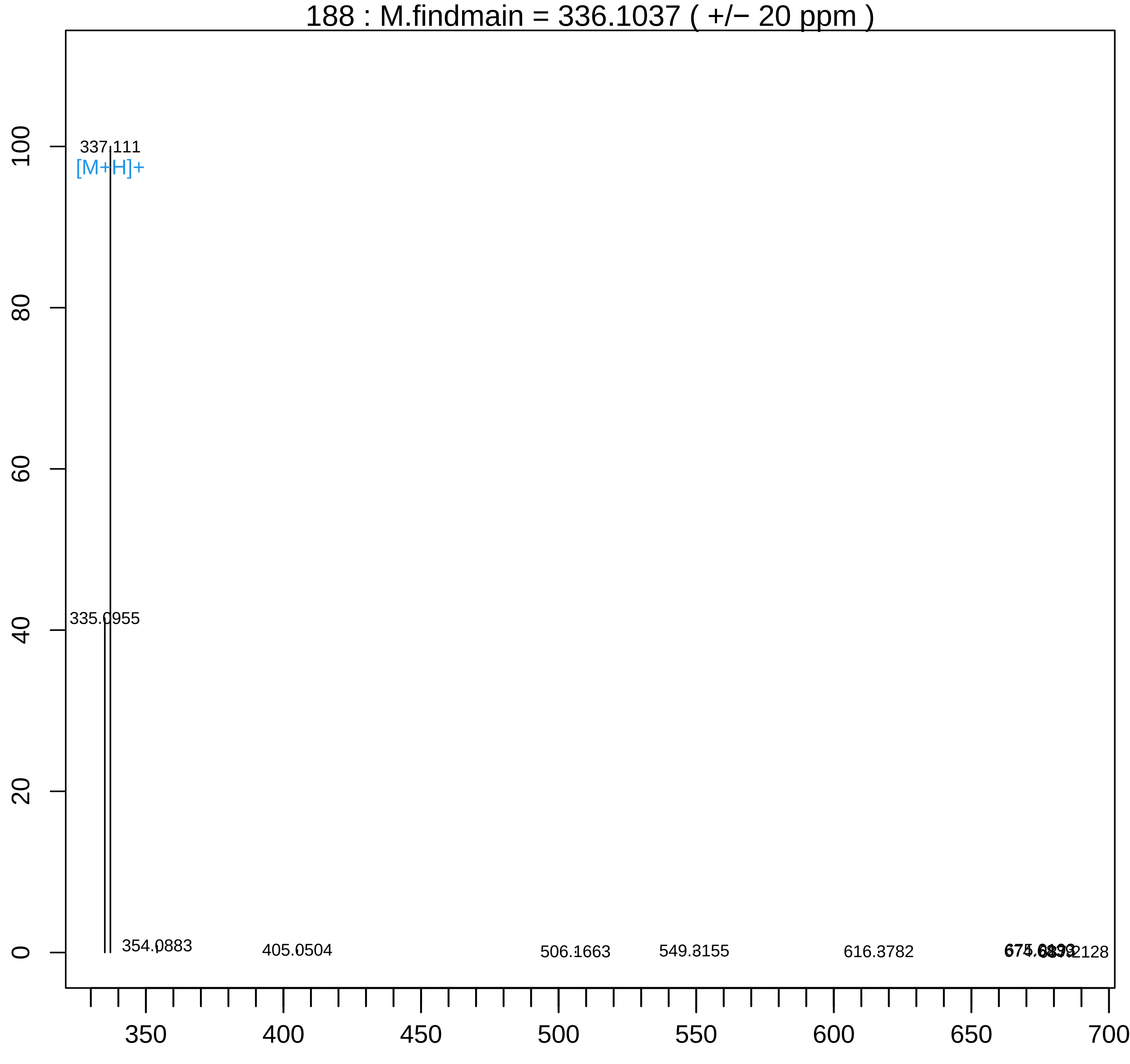
188 : M.findmain = 336.1037 (+/− 20 ppm)
337.111
[M+H]+
335.0955
354.0883
405.0504
506.1663
549.8155
616.3782
675.6893
0
20
40
60
80
100
350
400
450
500
550
600
650
700

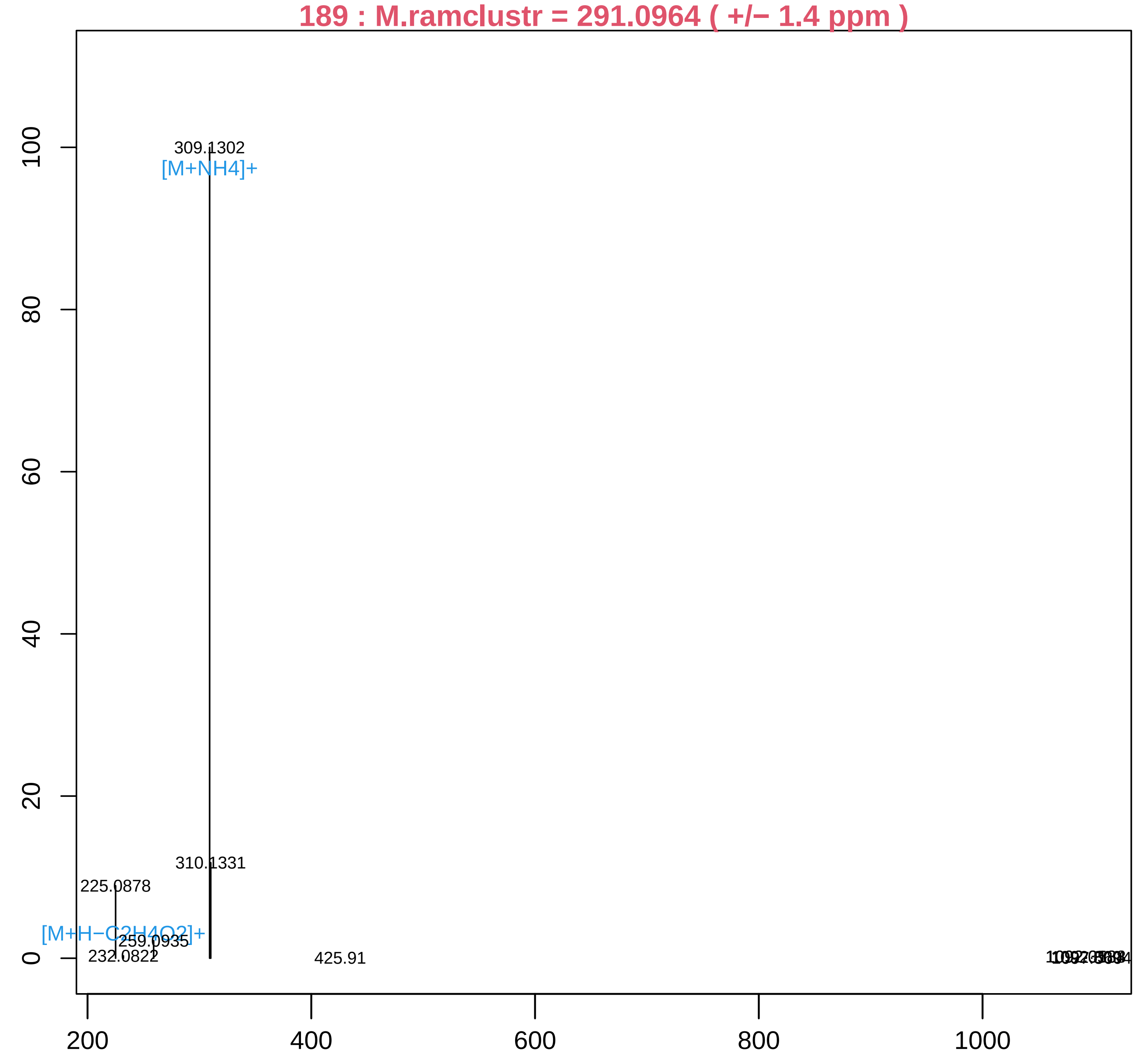
189 : M.ramclustr = 291.0964 (+/− 1.4 ppm)
309.1302
[M+NH4]+
310.1331
225.0878
[M+H−C2H4O2]+
259.0935
232.0822
425.91
0
20
40
60
80
100
200
400
600
800
1000

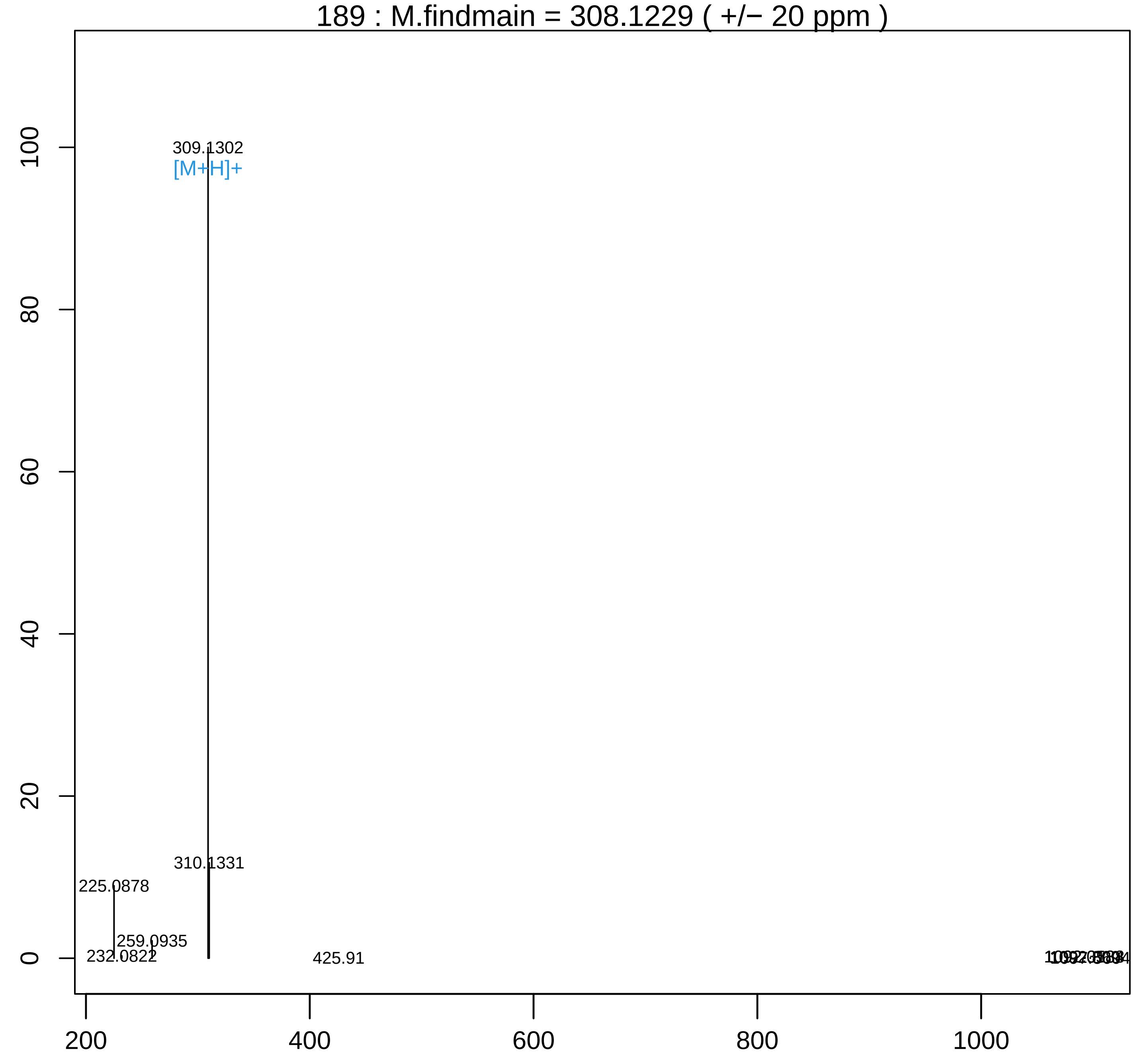
189 : M.findmain = 308.1229 (+/− 20 ppm)
309.1302
[M+H]+
310.1331
225.0878
259.0935
232.0822
425.91
0
20
40
60
80
100
200
400
600
800
1000

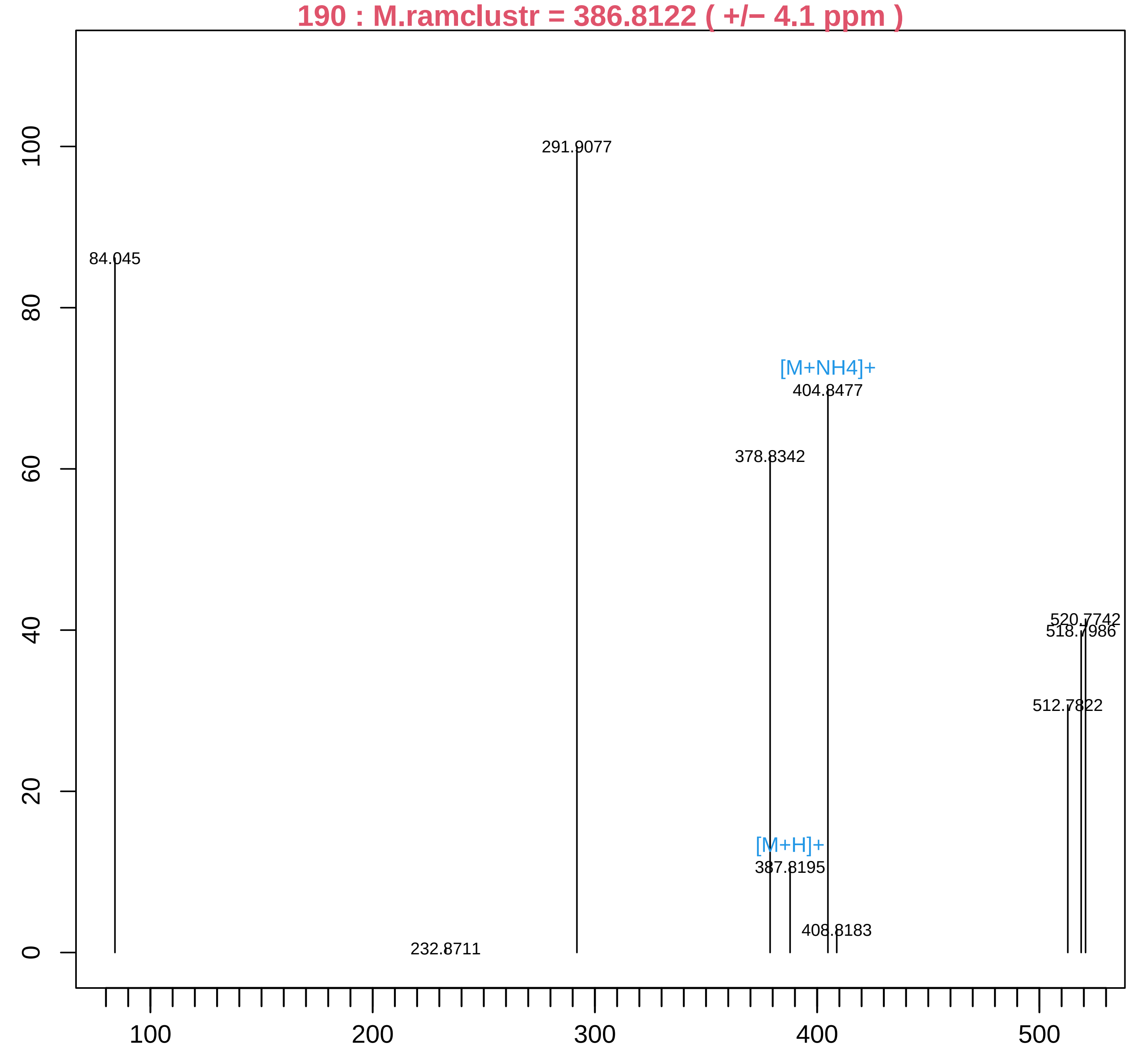

190 : M.ramclustr = 386.8122 (+/− 4.1 ppm)
84.045
291.9077
[M+NH4]+
404.8477
378.8342
520.7742
518.7986
512.7822
[M+H]+
387.8195
408.8183
232.8711
0
20
40
60
80
100
100
200
300
400
500

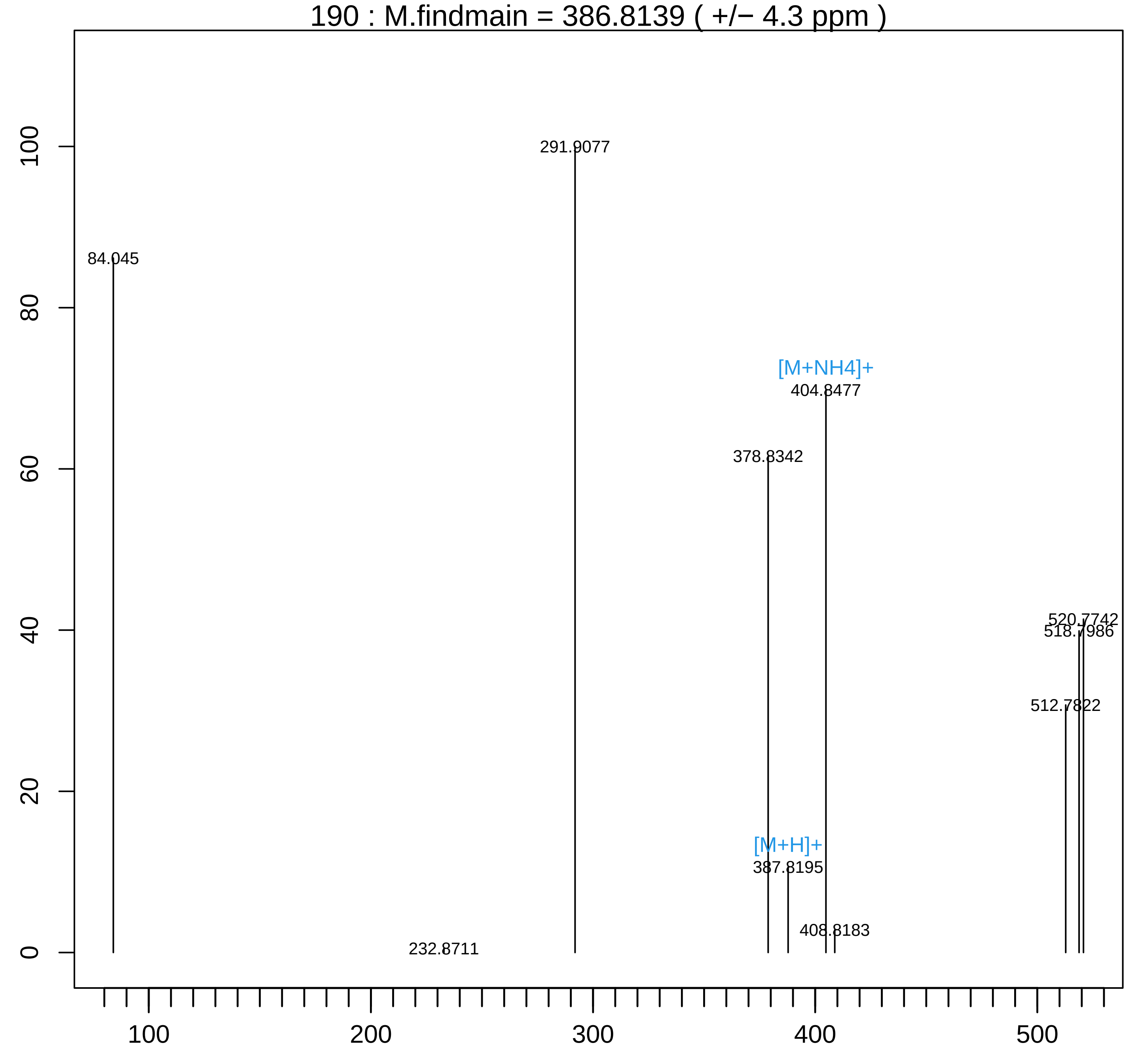

190 : M.findmain = 386.8139 (+/− 4.3 ppm)
84.045
291.9077
[M+NH4]+
404.8477
378.8342
520.7742
518.7986
512.7822
[M+H]+
387.8195
408.8183
232.8711
0
20
40
60
80
100
100
200
300
400
500

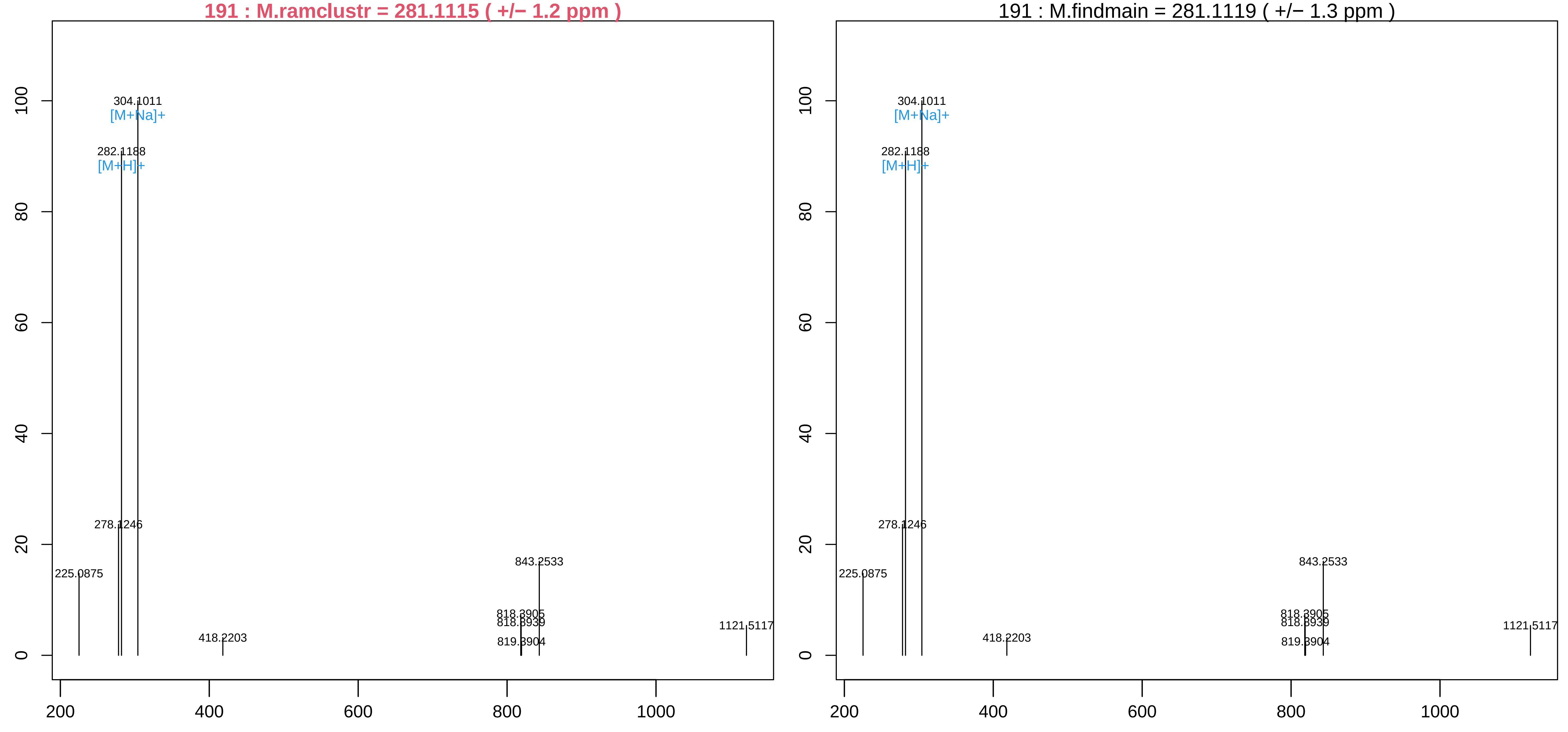
191 : M.ramclustr = 281.1115 (+/− 1.2 ppm)
304.1011
[M+Na]+
282.1188
[M+H]+
278.1246
225.0875
418.2203
843.2533
818.3905
818.3939
819.3904
1121.5117
191 : M.findmain = 281.1119 (+/− 1.3 ppm)
304.1011
[M+Na]+
282.1188
[M+H]+
278.1246
225.0875
418.2203
843.2533
818.3905
818.3939
819.3904
1121.5117

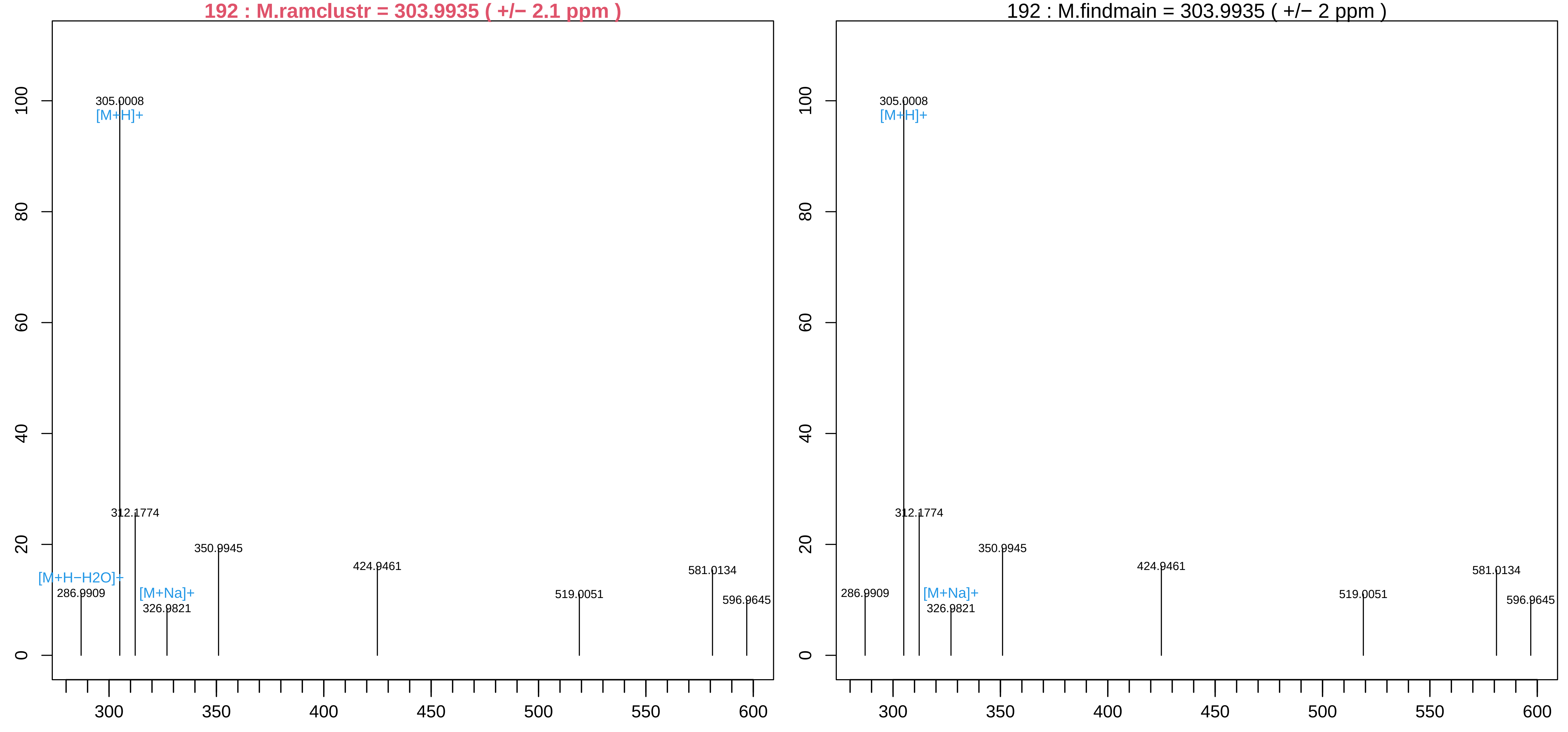

192 : M.ramclustr = 303.9935 (+/− 2.1 ppm)
305.0008
[M+H]+
312.1774
350.9945
[M+H−H2O]+
286.9909
[M+Na]+
326.9821
424.9461
519.0051
581.0134
596.9645
192 : M.findmain = 303.9935 (+/− 2 ppm)
305.0008
[M+H]+
312.1774
350.9945
286.9909
[M+Na]+
326.9821
424.9461
519.0051
581.0134
596.9645
0
20
40
60
80
100
300
350
400
450
500
550
600

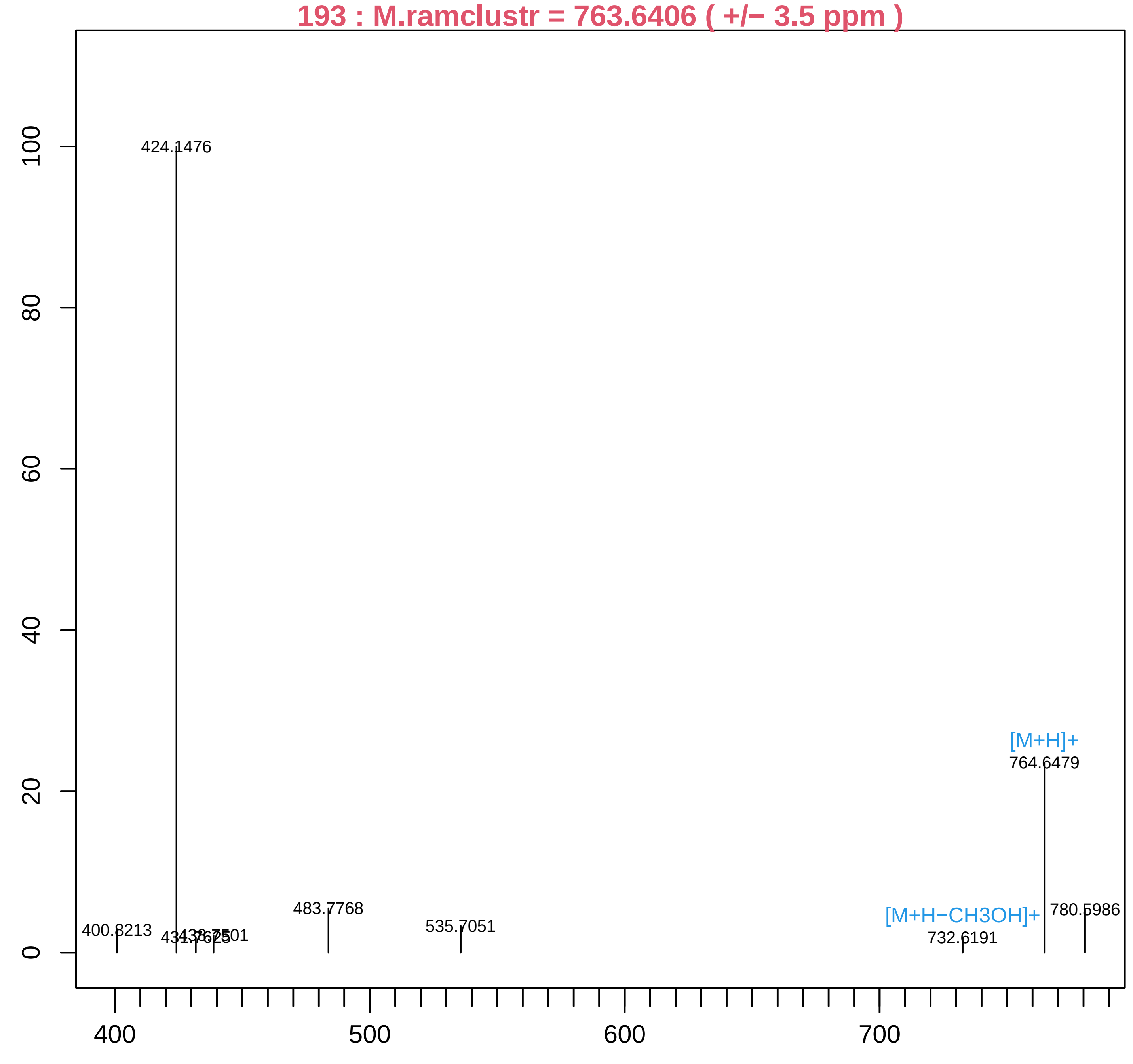

193 : M.ramclustr = 763.6406 (+/− 3.5 ppm)
424.1476
[M+H]+
764.6479
[M+H−CH3OH]+
732.6191
780.5986
400.8213
483.7768
535.7051
434.3625
438.7501
0
20
40
60
80
100
400
500
600
700

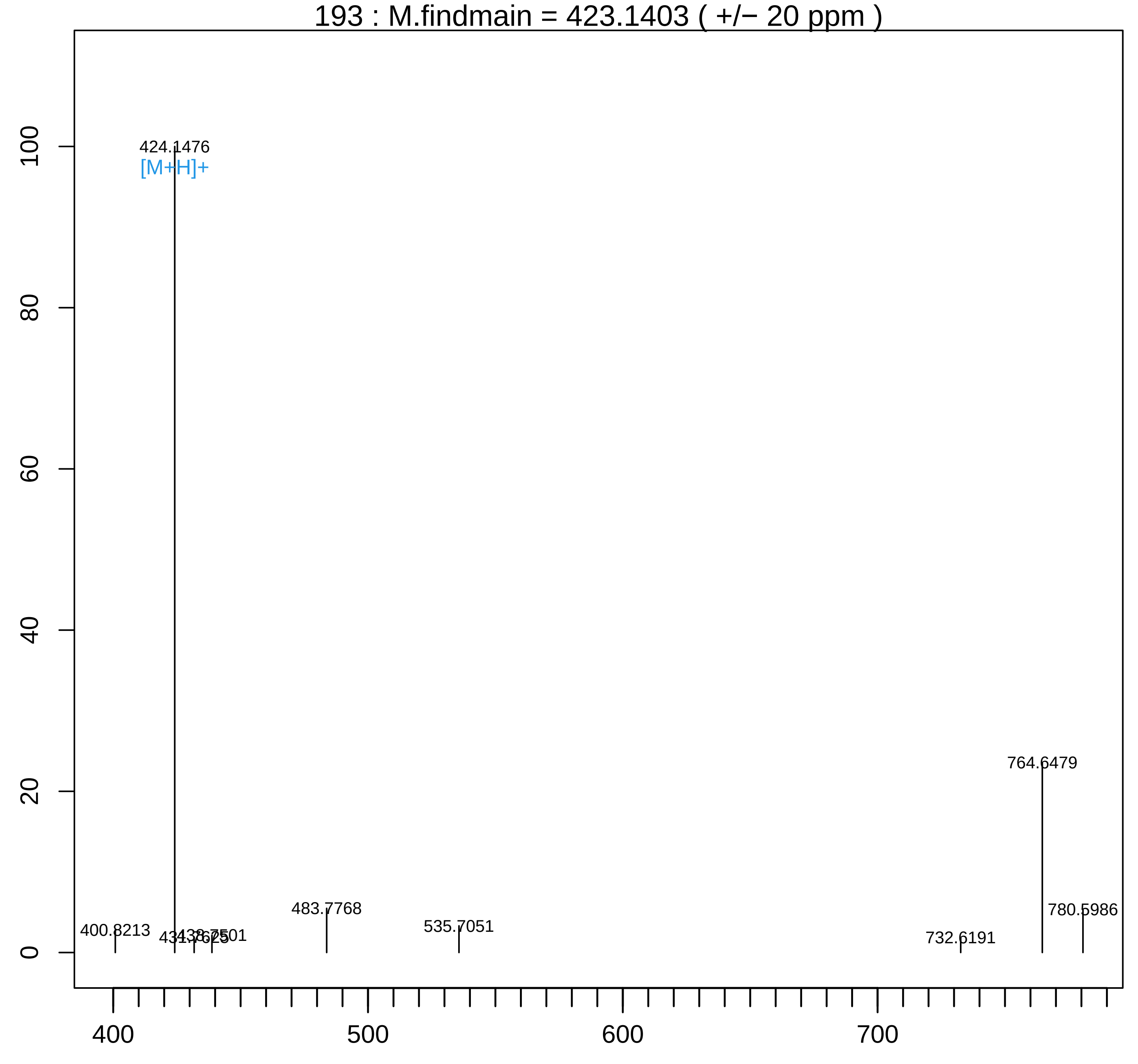

193 : M.findmain = 423.1403 (+/− 20 ppm)
424.1476
[M+H]+
764.6479
732.6191
780.5986
400.8213
483.7768
535.7051
434.3625
438.7501
0
20
40
60
80
100
400
500
600
700

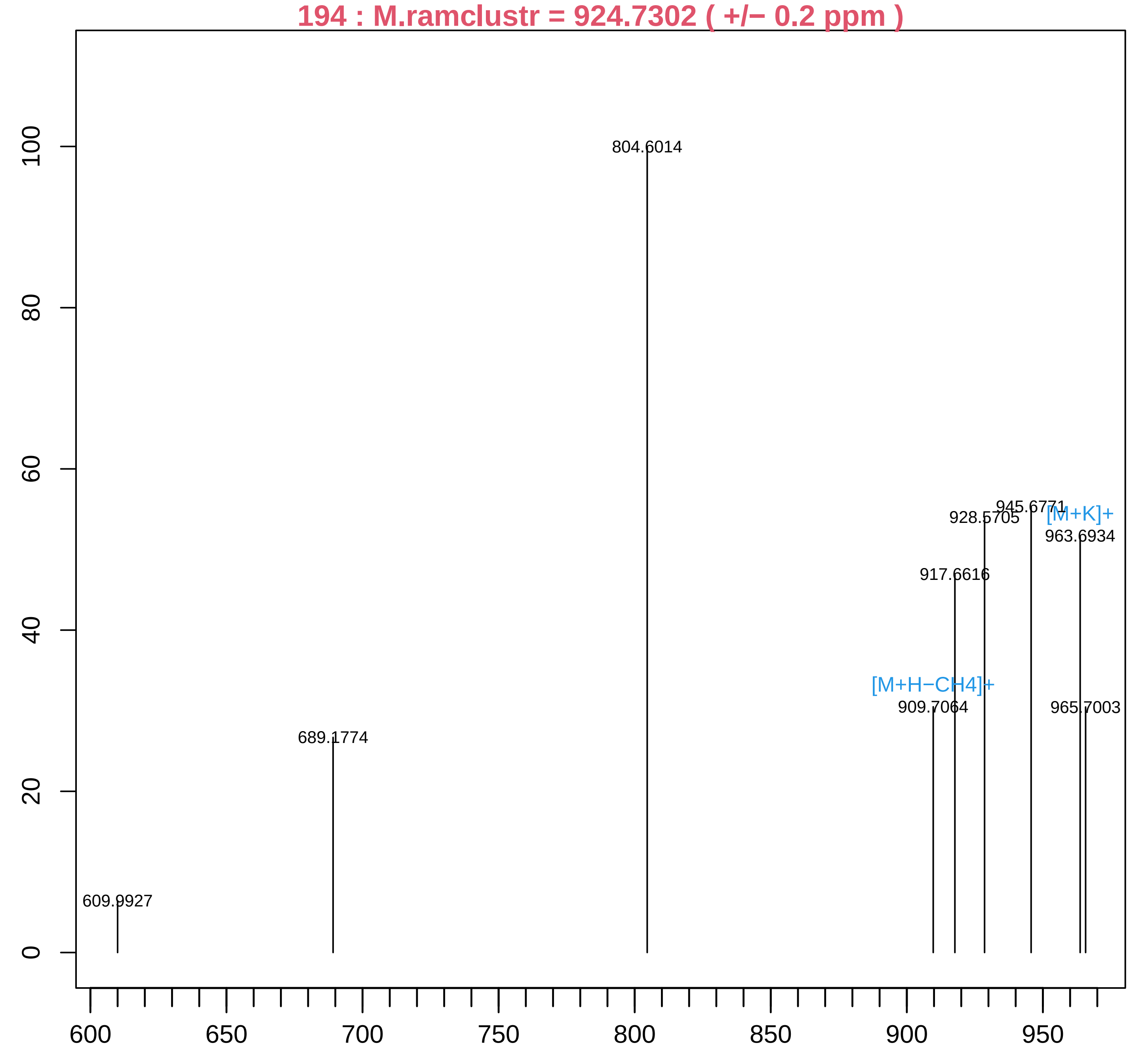
194 : M.ramclustr = 924.7302 (+/− 0.2 ppm)
804.6014
945.6771
[M+K]+
928.5705
963.6934
917.6616
[M+H−CH4]+
909.7064
965.7003
689.1774
609.9927
0
20
40
60
80
100
600
650
700
750
800
850
900
950

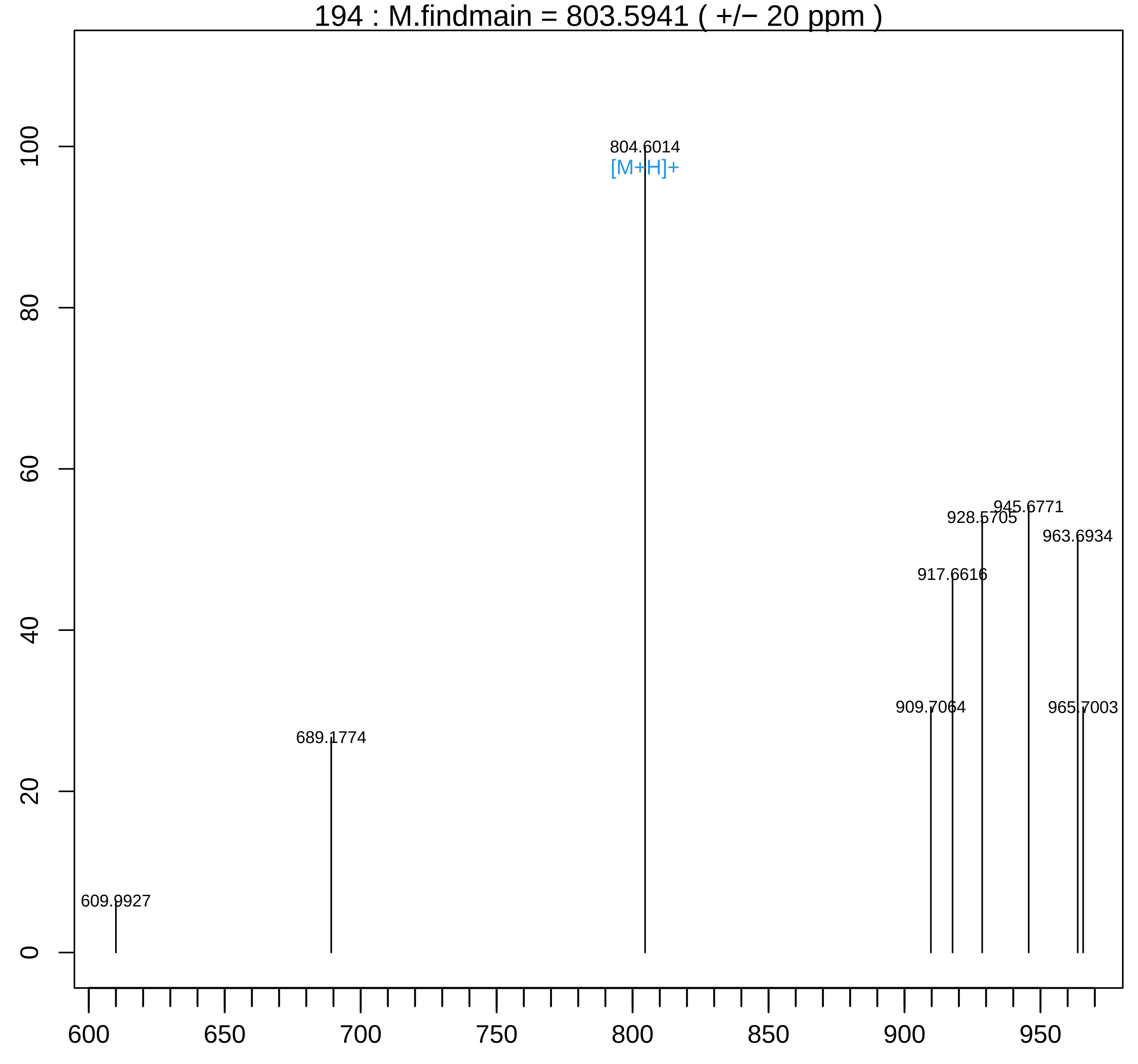
194 : M.findmain = 803.5941 (+/− 20 ppm)
804.6014
[M+H]+
945.6771
928.5705
963.6934
917.6616
909.7064
965.7003
689.1774
609.9927
0
20
40
60
80
100
600
650
700
750
800
850
900
950

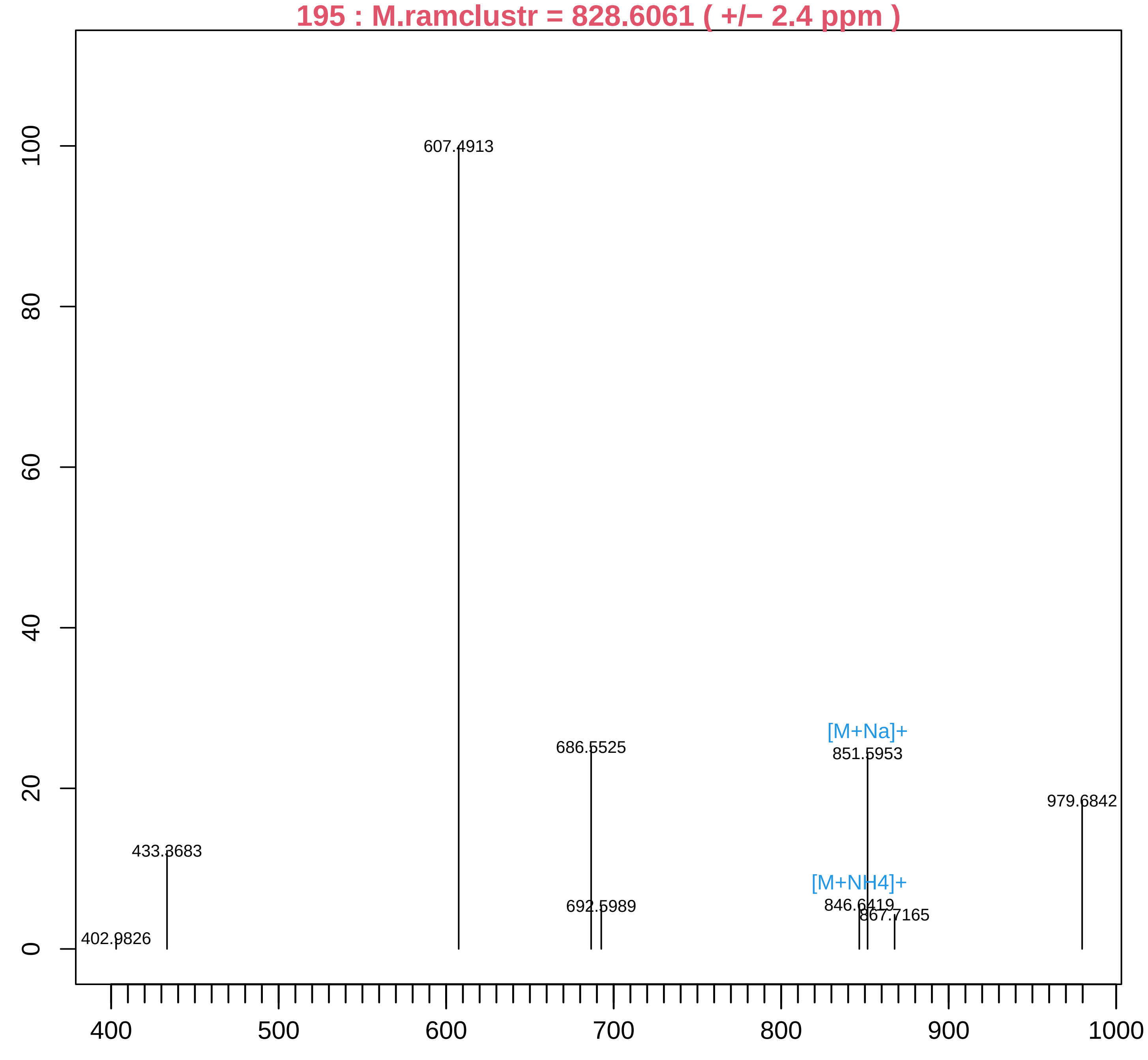

195 : M.ramclustr = 828.6061 (+/− 2.4 ppm)
0
20
40
60
80
100
400
500
600
700
800
900
1000
607.4913
686.5525
[M+Na]+
851.5953
979.6842
433.3683
[M+NH4]+
692.5989
846.6419
867.7165
402.9826

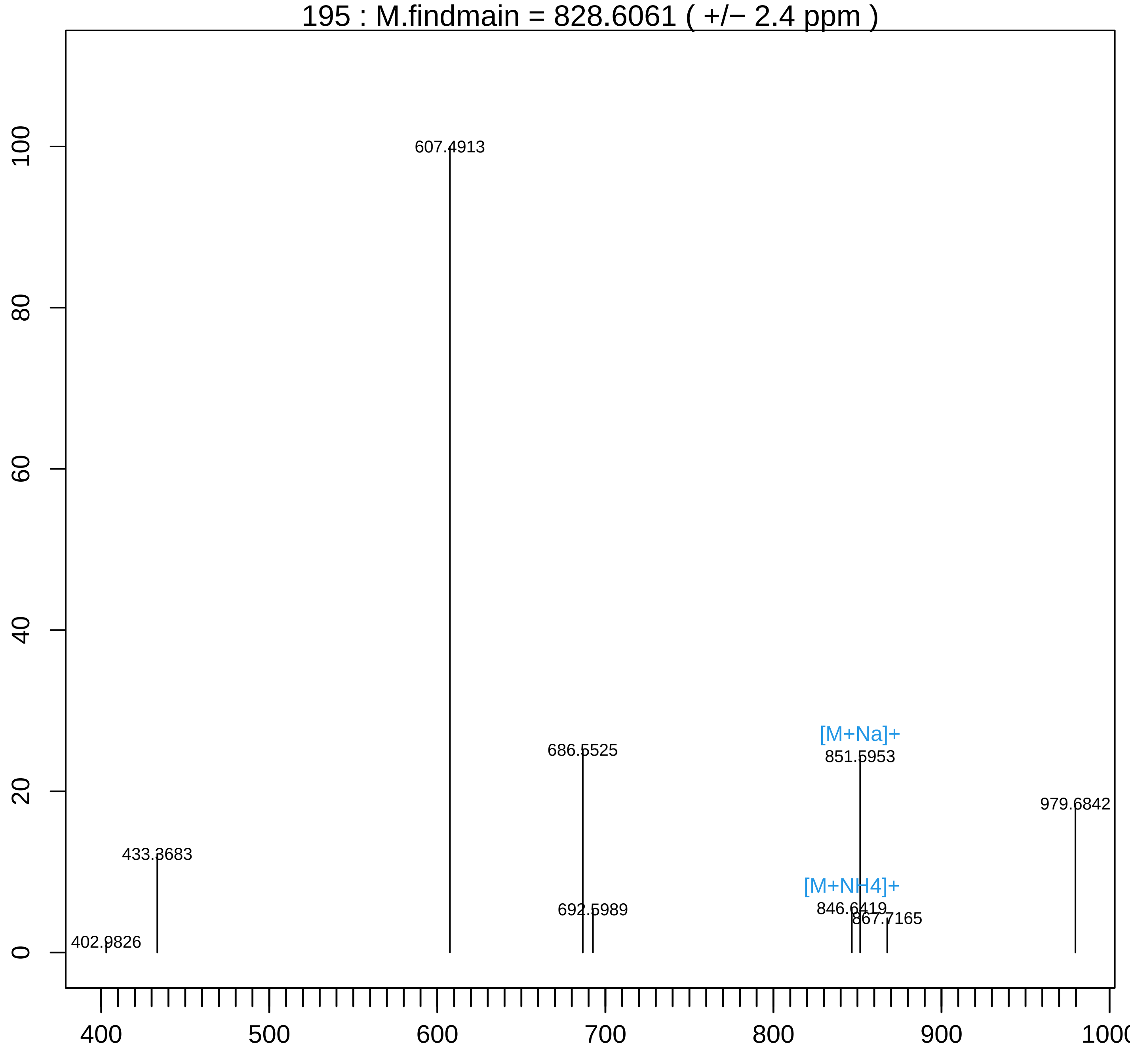

195 : M.findmain = 828.6061 (+/− 2.4 ppm)
0
20
40
60
80
100
400
500
600
700
800
900
607.4913
686.5525
[M+Na]+
851.5953
979.6842
433.3683
[M+NH4]+
692.5989
846.6419
867.7165
402.9826

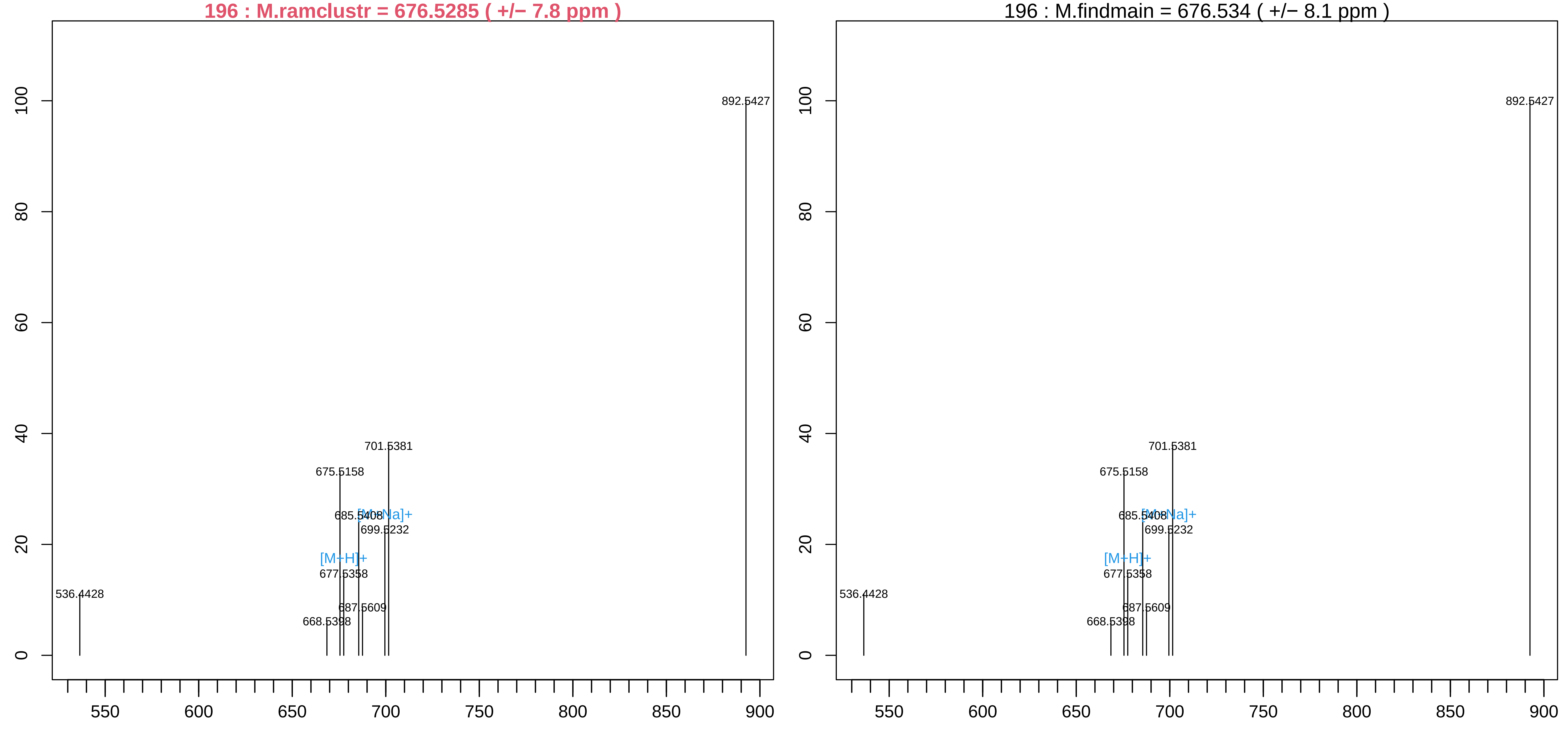
196 : M.ramclustr = 676.5285 (+/− 7.8 ppm)
892.5427
701.5381
675.5158
685.5408
[M+Na]+
699.5232
[M+H]+
677.5358
536.4428
687.5609
668.5398
196 : M.findmain = 676.534 (+/− 8.1 ppm)
892.5427
701.5381
675.5158
685.5408
[M+Na]+
699.5232
[M+H]+
677.5358
536.4428
687.5609
668.5398

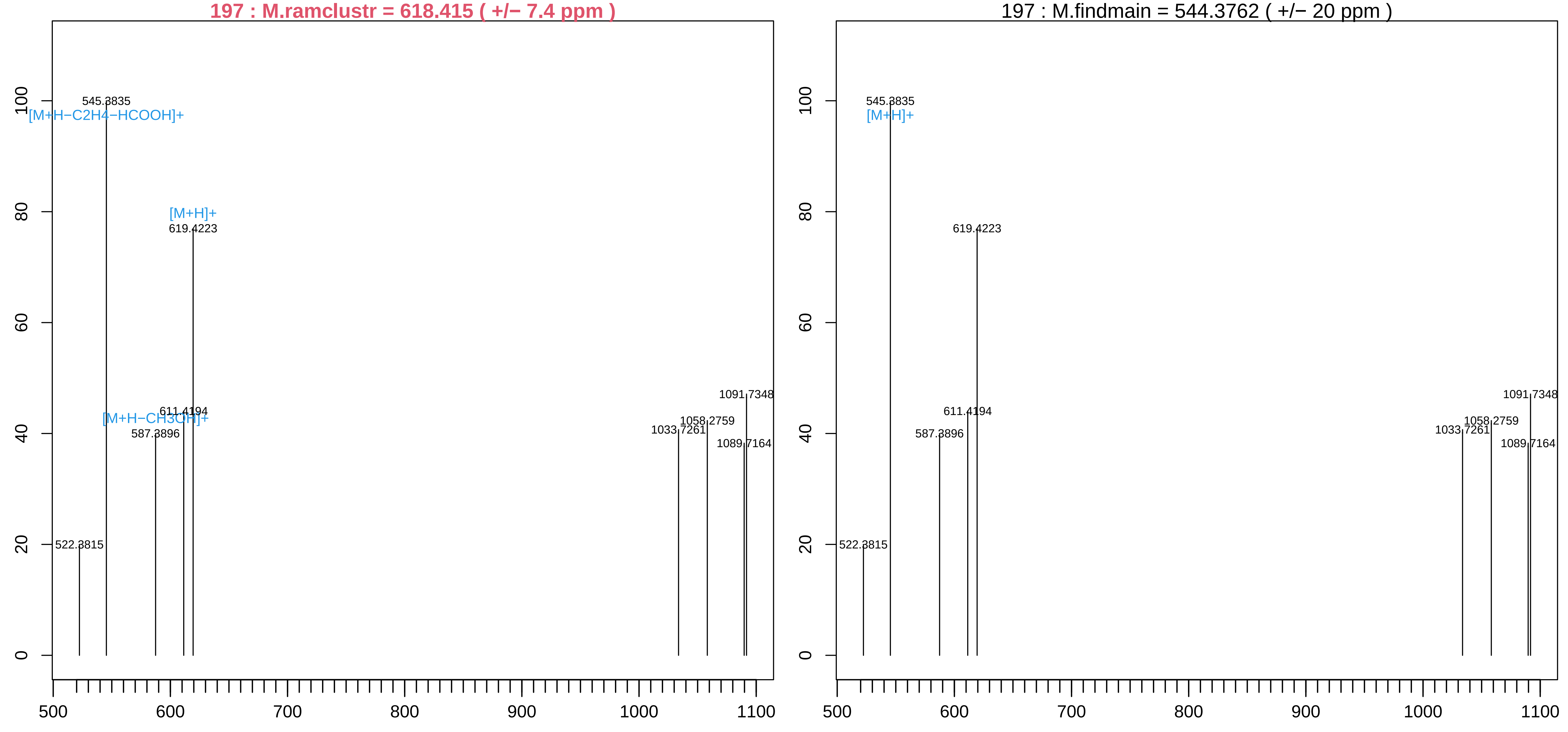

197 : M.ramclustr = 618.415 (+/− 7.4 ppm)
545.3835
[M+H−C2H4−HCOOH]+
[M+H]+
619.4223
611.4194
[M+H−CH3OH]+
587.3896
522.3815
1033.7261
1058.2759
1091.7348
1089.7164
197 : M.findmain = 544.3762 (+/− 20 ppm)
545.3835
[M+H]+
619.4223
611.4194
587.3896
522.3815
1033.7261
1058.2759
1091.7348
1089.7164

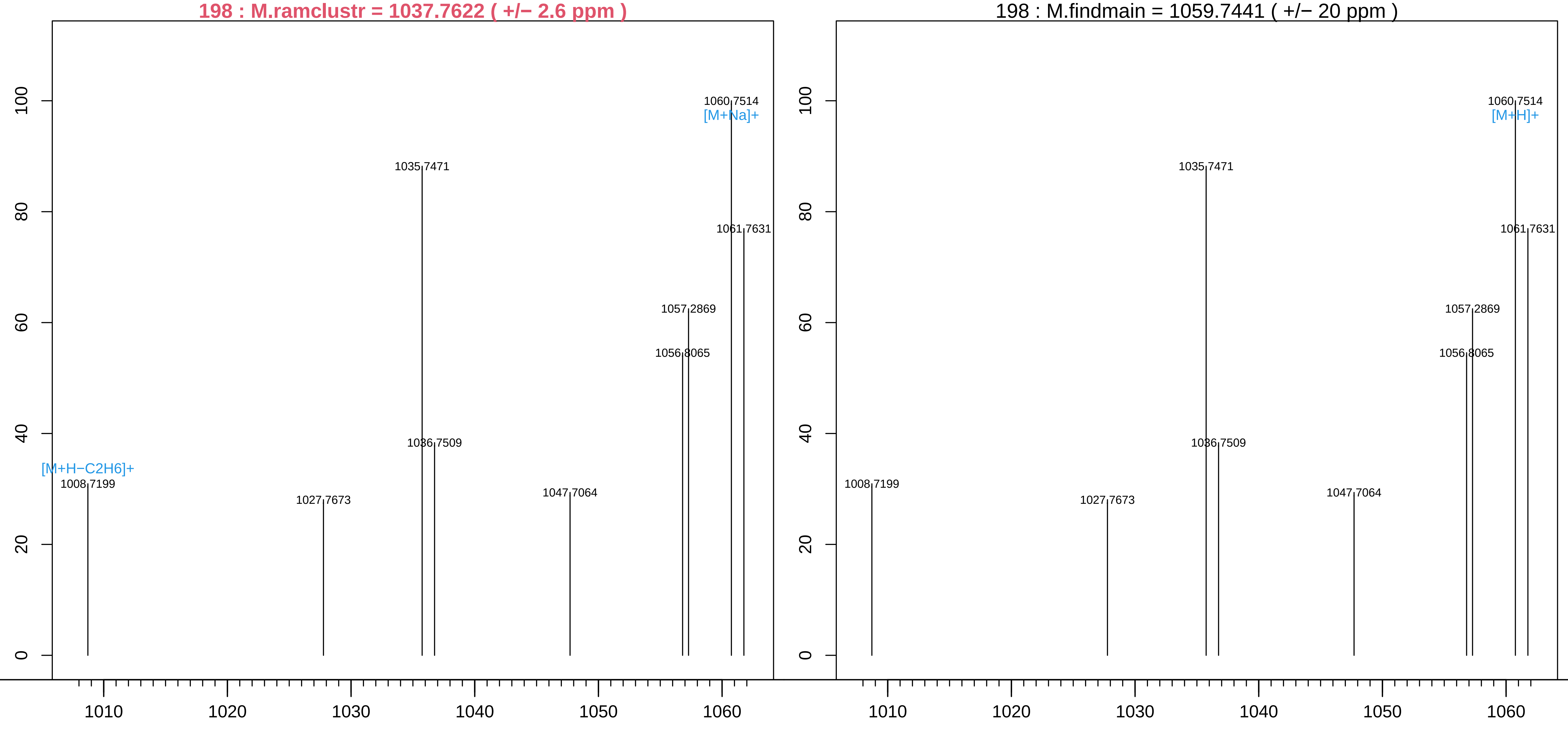

198 : M.ramclustr = 1037.7622 (+/− 2.6 ppm)
1060.7514
[M+Na]+
1035.7471
1061.7631
1057.2869
1056.8065
1036.7509
[M+H−C2H6]+
1008.7199
1027.7673
1047.7064
0
20
40
60
80
100
1010
1020
1030
1040
1050
1060
198 : M.findmain = 1059.7441 (+/− 20 ppm)
1060.7514
[M+H]+
1035.7471
1061.7631
1057.2869
1056.8065
1036.7509
1008.7199
1027.7673
1047.7064
0
20
40
60
80
100
1010
1020
1030
1040
1050
1060

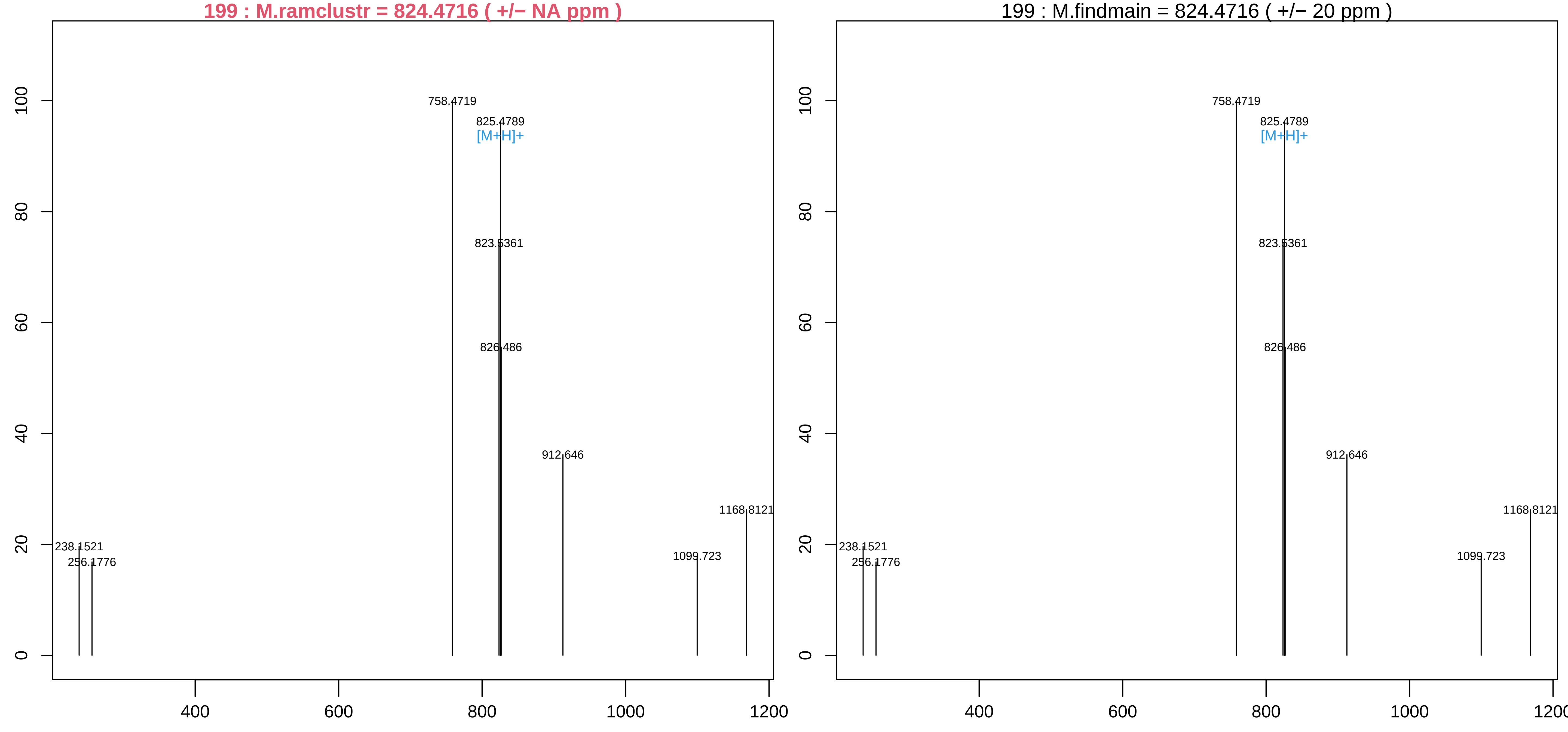
199 : M.ramclustr = 824.4716 (+/− NA ppm)
199 : M.findmain = 824.4716 (+/− 20 ppm)
758.4719
825.4789
[M+H]+
823.5361
826.486
912.646
1168.8121
1099.723
238.1521
256.1776
0
20
40
60
80
100
400
600
800
1000
1200

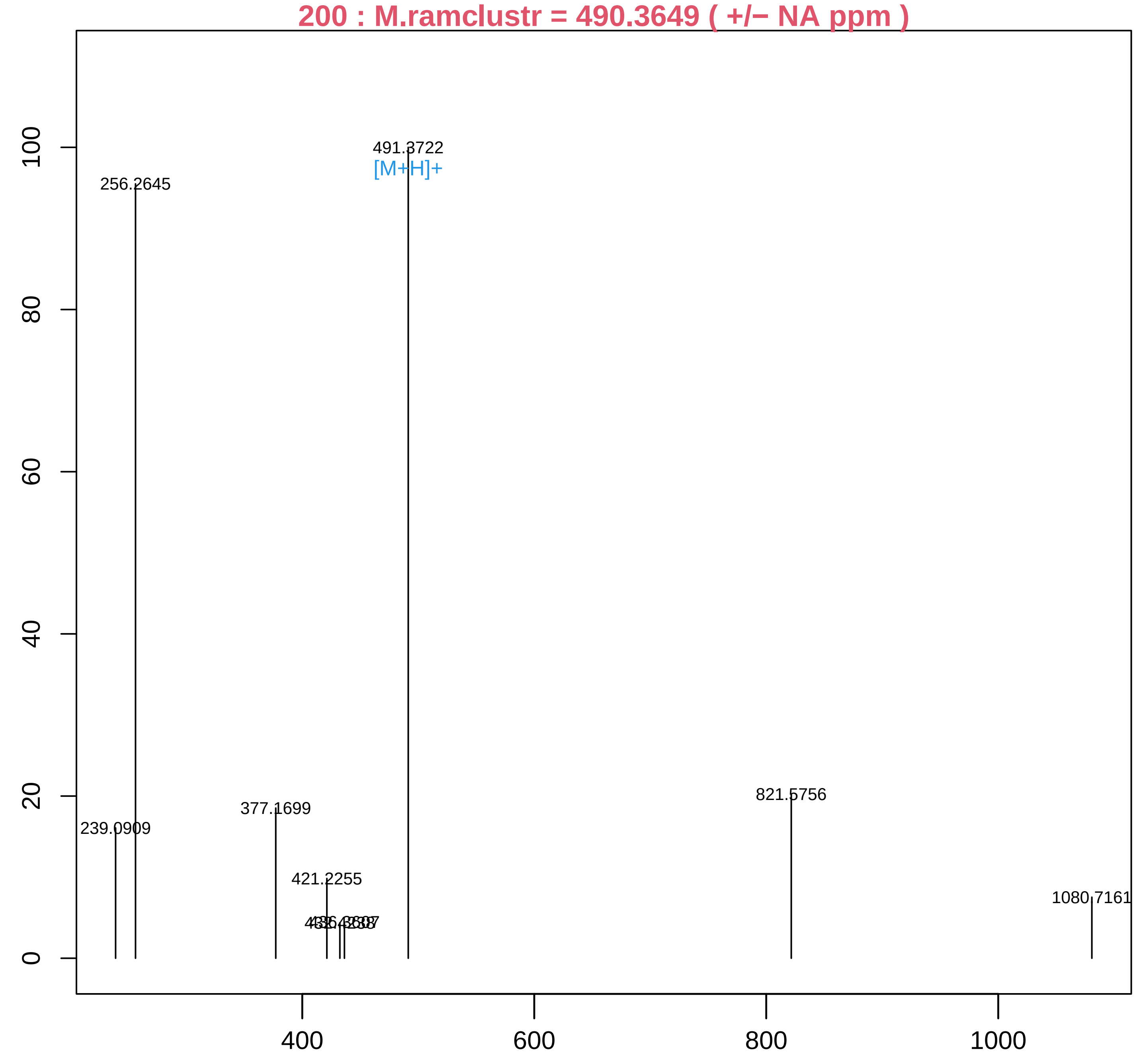
200 : M.ramclustr = 490.3649 (+/− NA ppm)
491.3722
[M+H]+
256.2645
239.0909
377.1699
421.2255
821.5756
1080.7161
0
20
40
60
80
100
400
600
800
1000

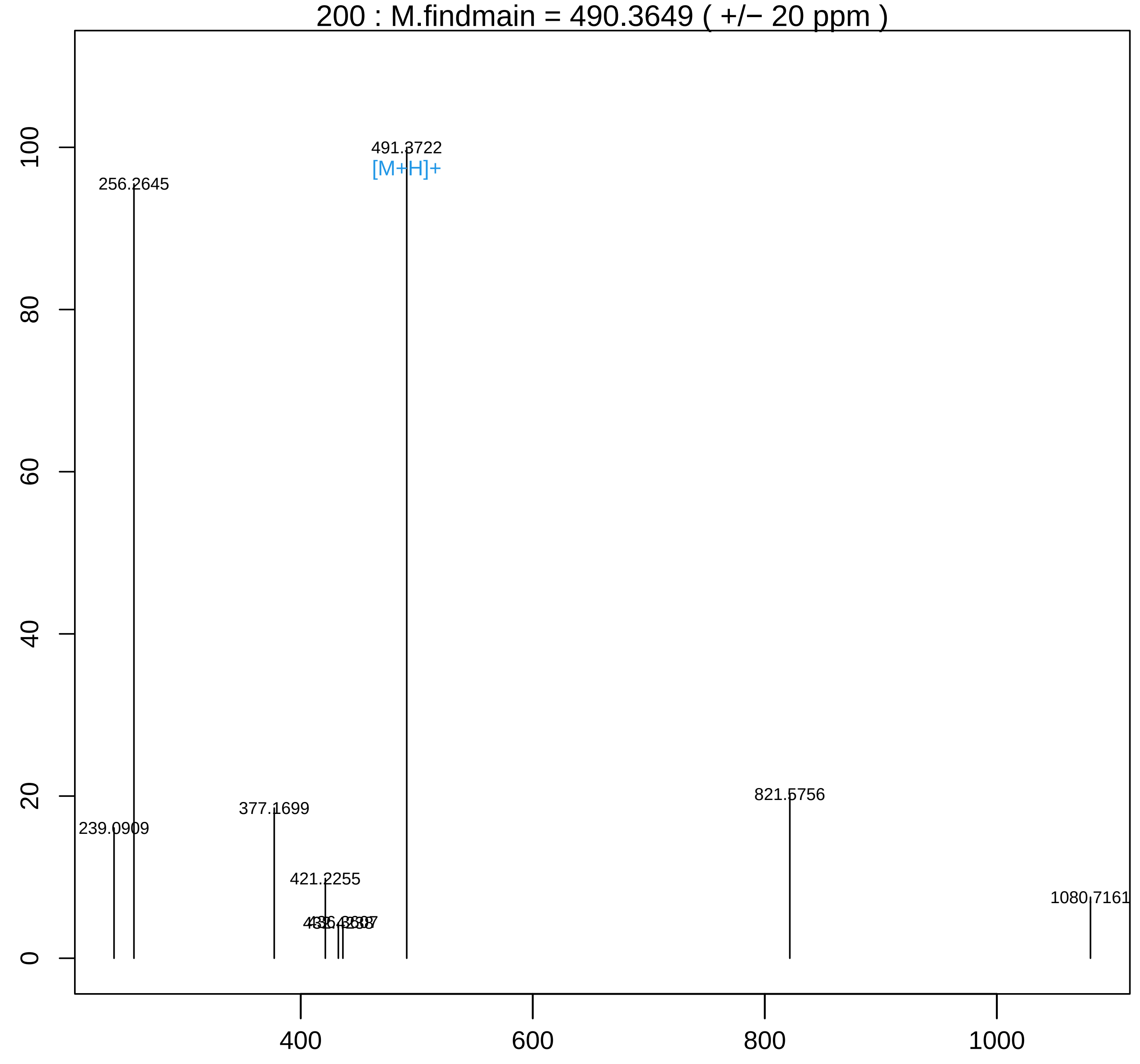
200 : M.findmain = 490.3649 (+/− 20 ppm)
491.3722
[M+H]+
256.2645
239.0909
377.1699
421.2255
821.5756
1080.7161
0
20
40
60
80
100
400
600
800
1000

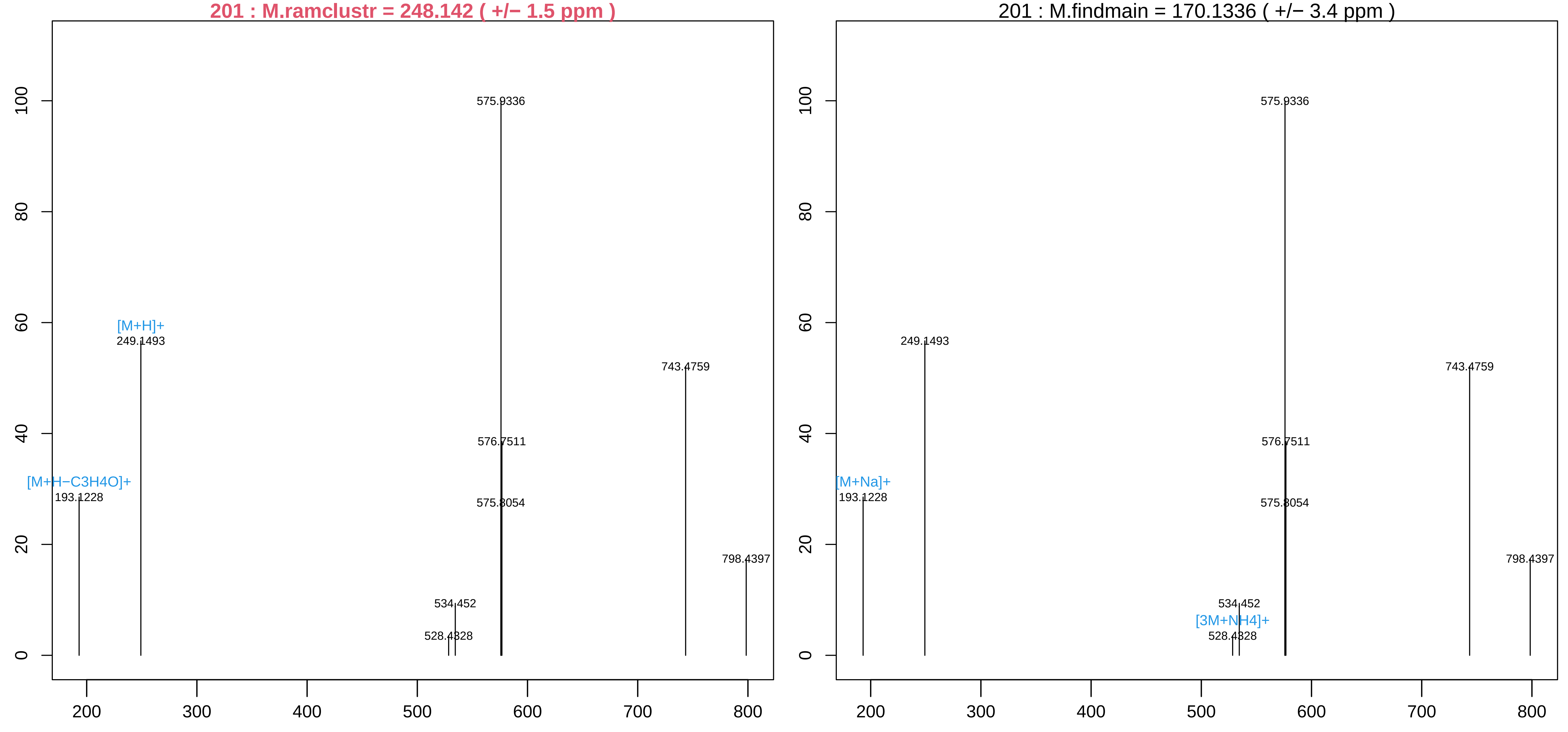
201 : M.ramclustr = 248.142 (+/− 1.5 ppm)
575.9336
[M+H]+
249.1493
743.4759
576.7511
[M+H−C3H4O]+
193.1228
575.8054
798.4397
534.452
528.4328
201 : M.findmain = 170.1336 (+/− 3.4 ppm)
575.9336
249.1493
743.4759
576.7511
[M+Na]+
193.1228
575.8054
798.4397
534.452
[3M+NH4]+
528.4328
0
20
40
60
80
100
200
300
400
500
600
700
800

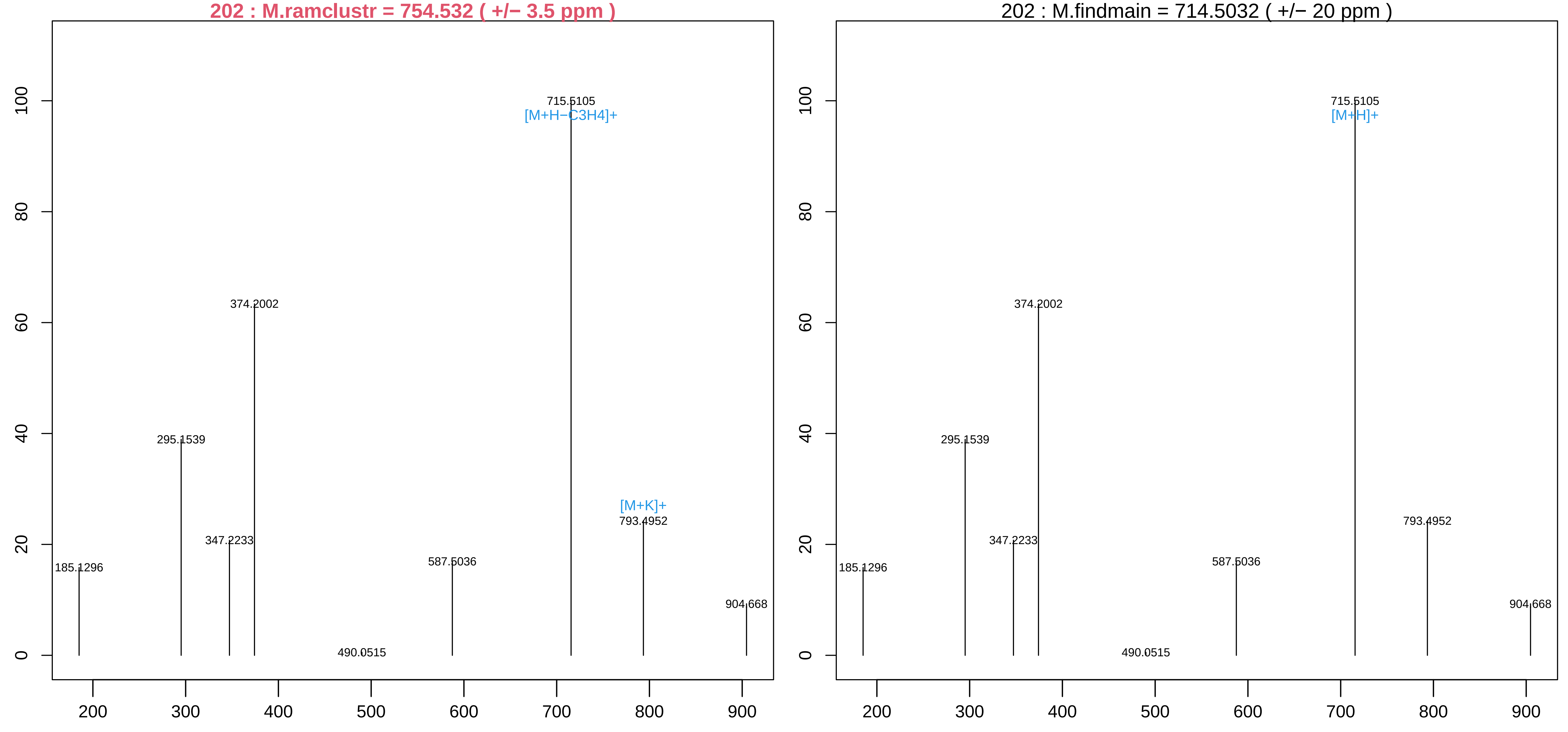

202 : M.ramclustr = 754.532 (+/− 3.5 ppm)
715.5105
[M+H−C3H4]+
374.2002
295.1539
347.2233
185.1296
587.5036
[M+K]+
793.4952
904.668
490.0515
202 : M.findmain = 714.5032 (+/− 20 ppm)
715.5105
[M+H]+
374.2002
295.1539
347.2233
185.1296
587.5036
793.4952
904.668
490.0515

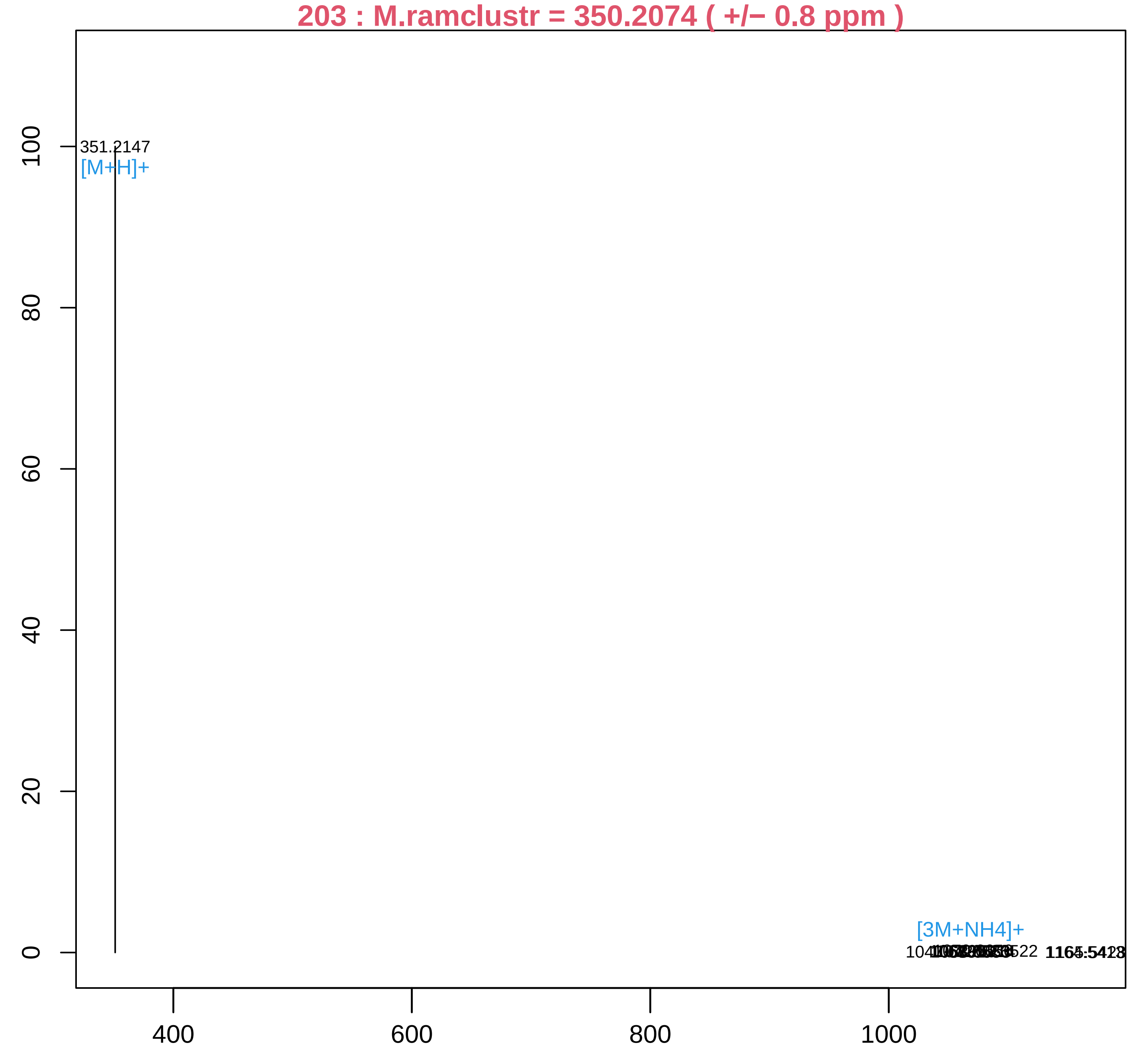

203 : M.ramclustr = 350.2074 (+/− 0.8 ppm)
351.2147
[M+H]+
[3M+NH4]+
1165.5428
0
20
40
60
80
100
400
600
800
1000

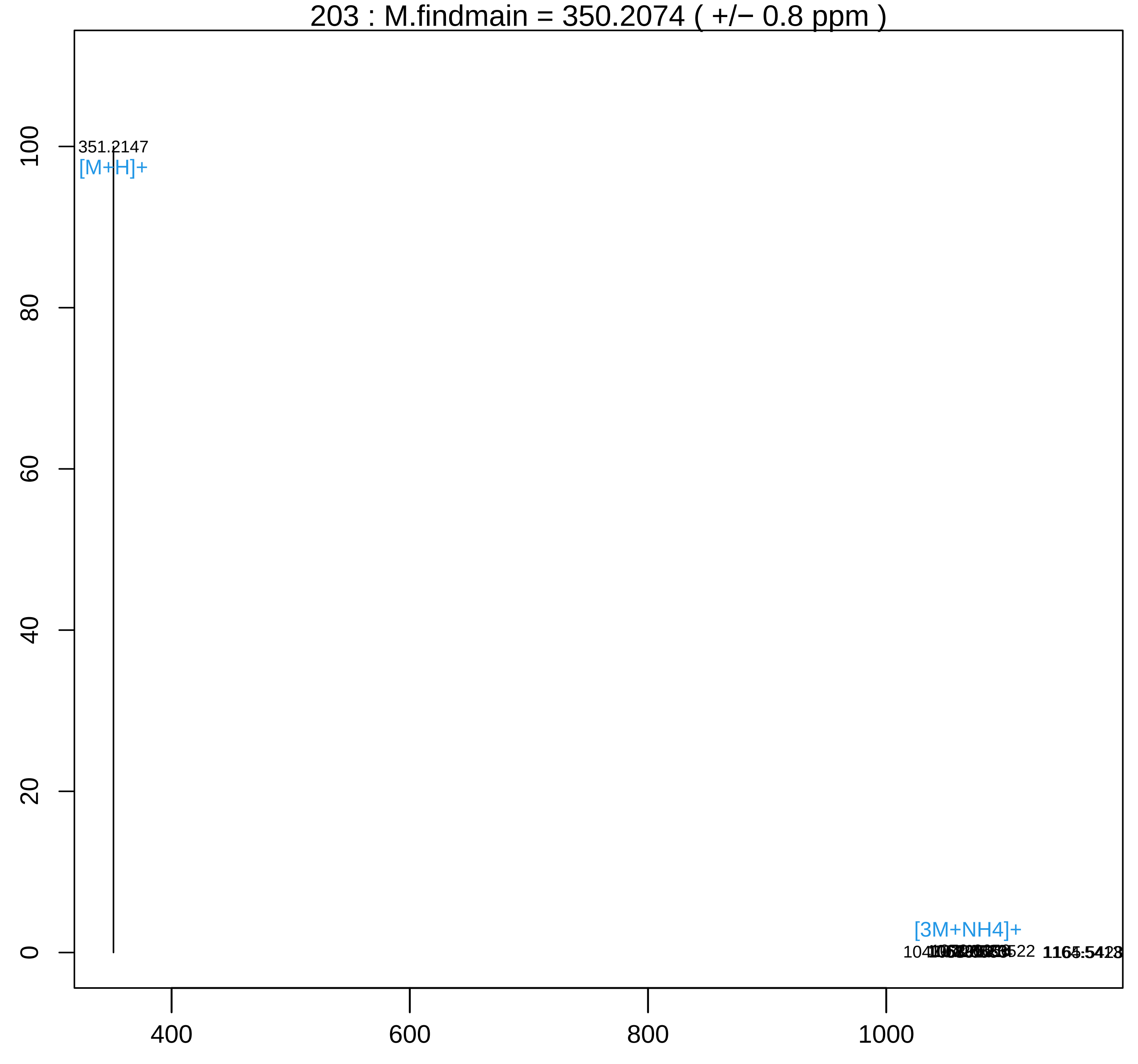

203 : M.findmain = 350.2074 (+/− 0.8 ppm)
351.2147
[M+H]+
[3M+NH4]+
1165.5428
0
20
40
60
80
100
400
600
800
1000

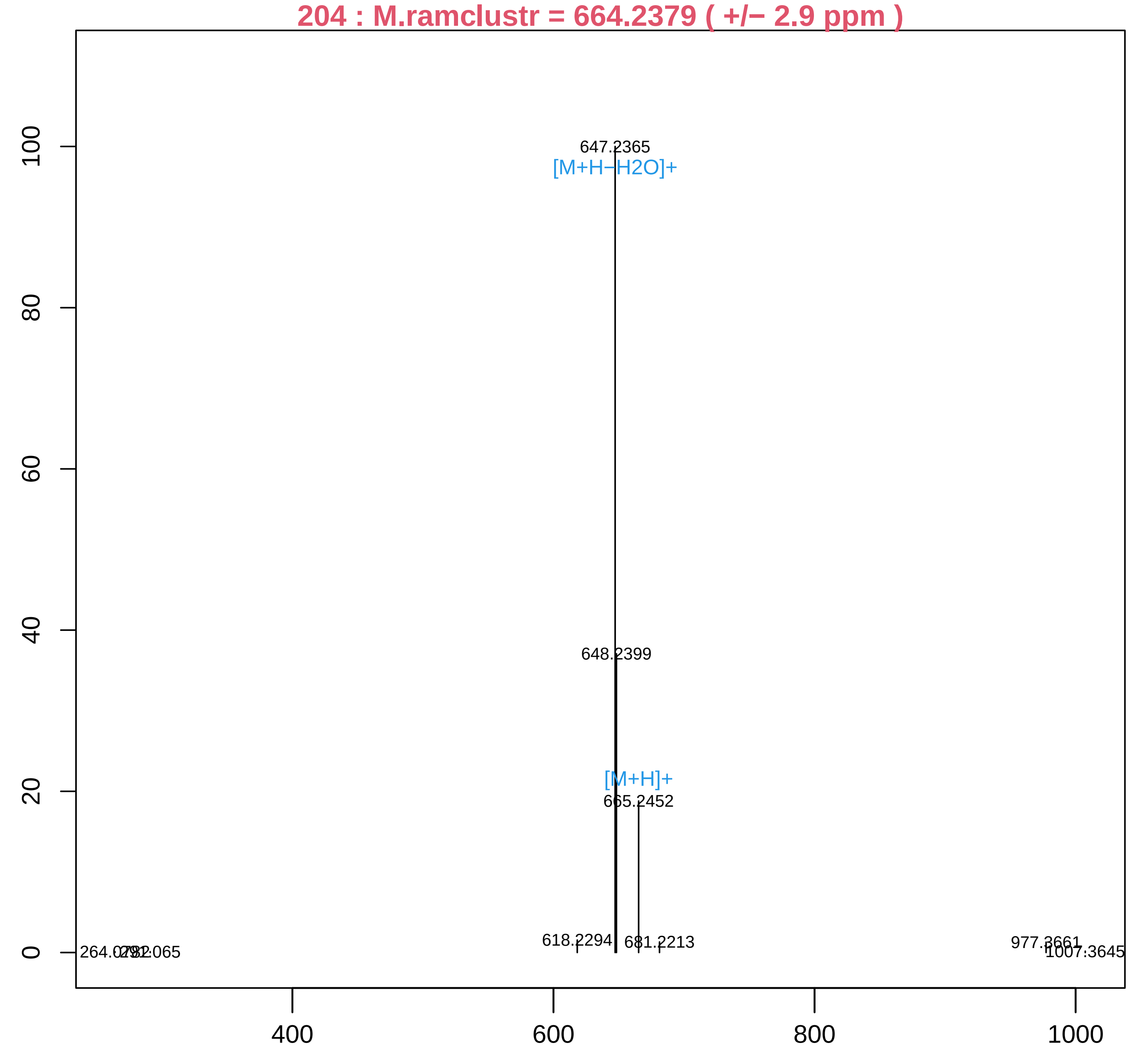
204 : M.ramclustr = 664.2379 (+/− 2.9 ppm)
647.2365
[M+H−H2O]+
648.2399
[M+H]+
665.2452
618.2294
681.2213
264.0782
282.065
977.3661
1007.3645
0
20
40
60
80
100
400
600
800
1000

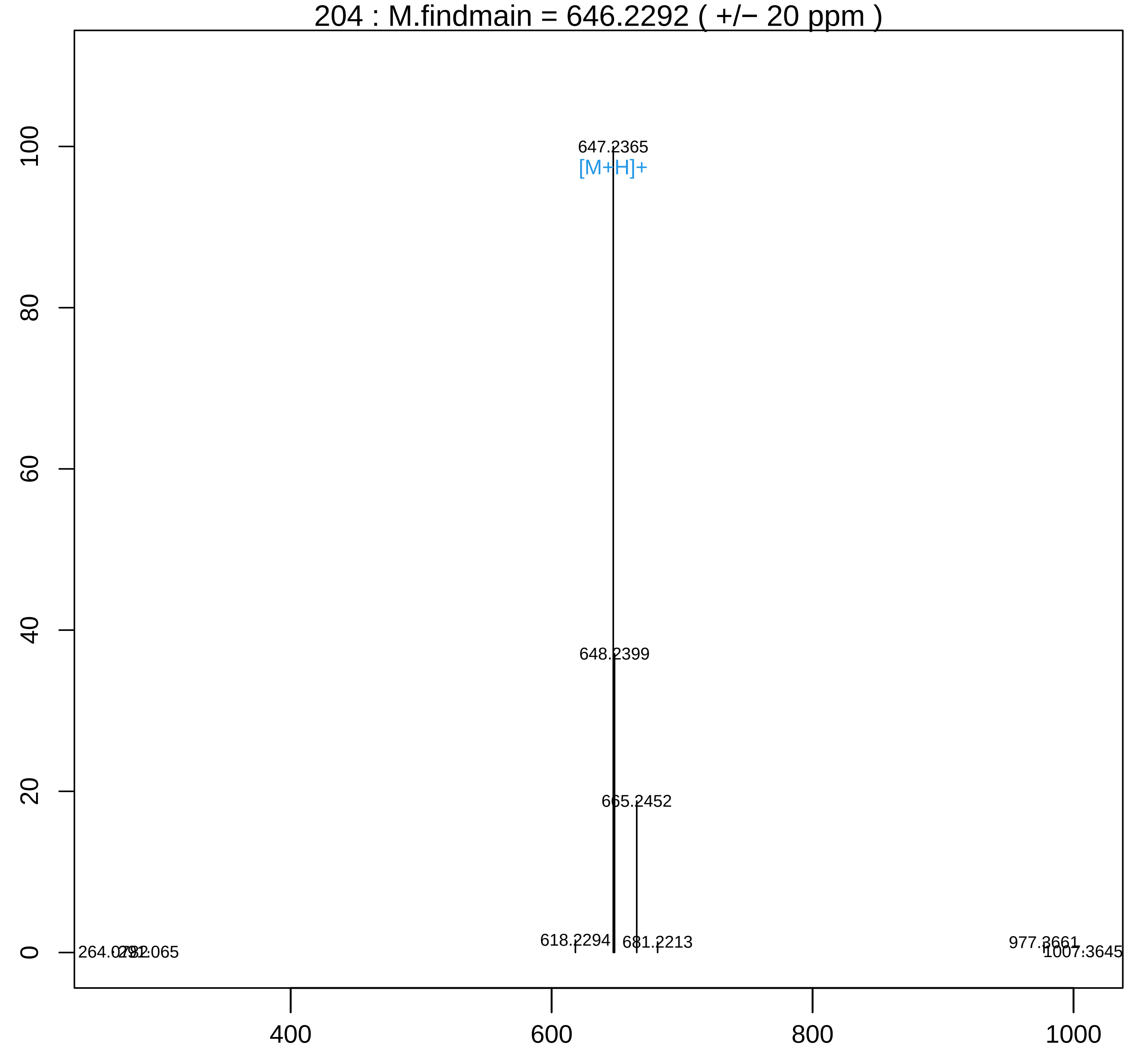
204 : M.findmain = 646.2292 (+/− 20 ppm)
647.2365
[M+H]+
648.2399
665.2452
618.2294
681.2213
264.0782
282.065
977.3661
1007.3645
0
20
40
60
80
100
400
600
800
1000

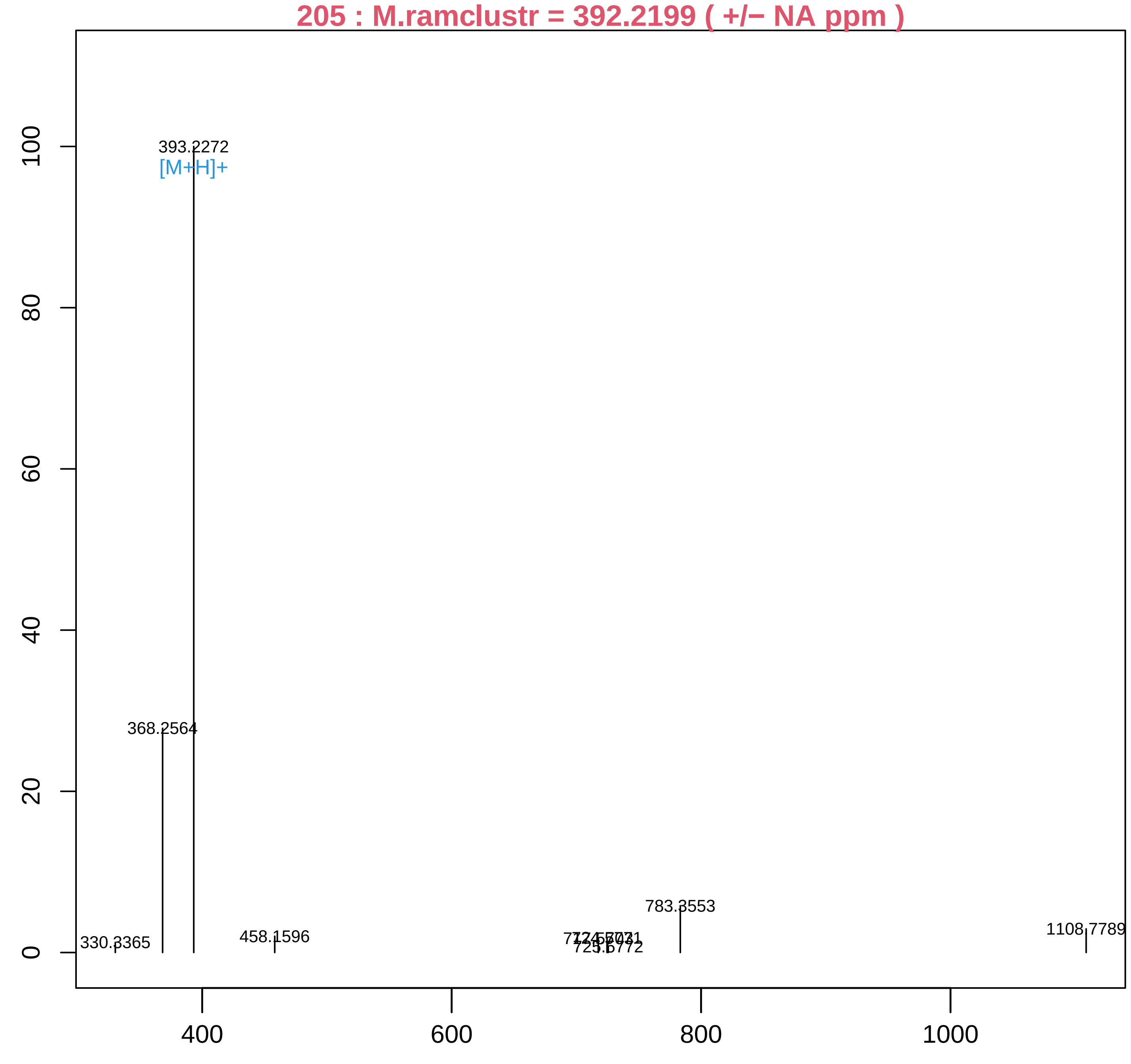
205 : M.ramclustr = 392.2199 (+/− NA ppm)
393.2272
[M+H]+
368.2564
330.3365
458.1596
783.3553
1108.7789
0
20
40
60
80
100
400
600
800
1000

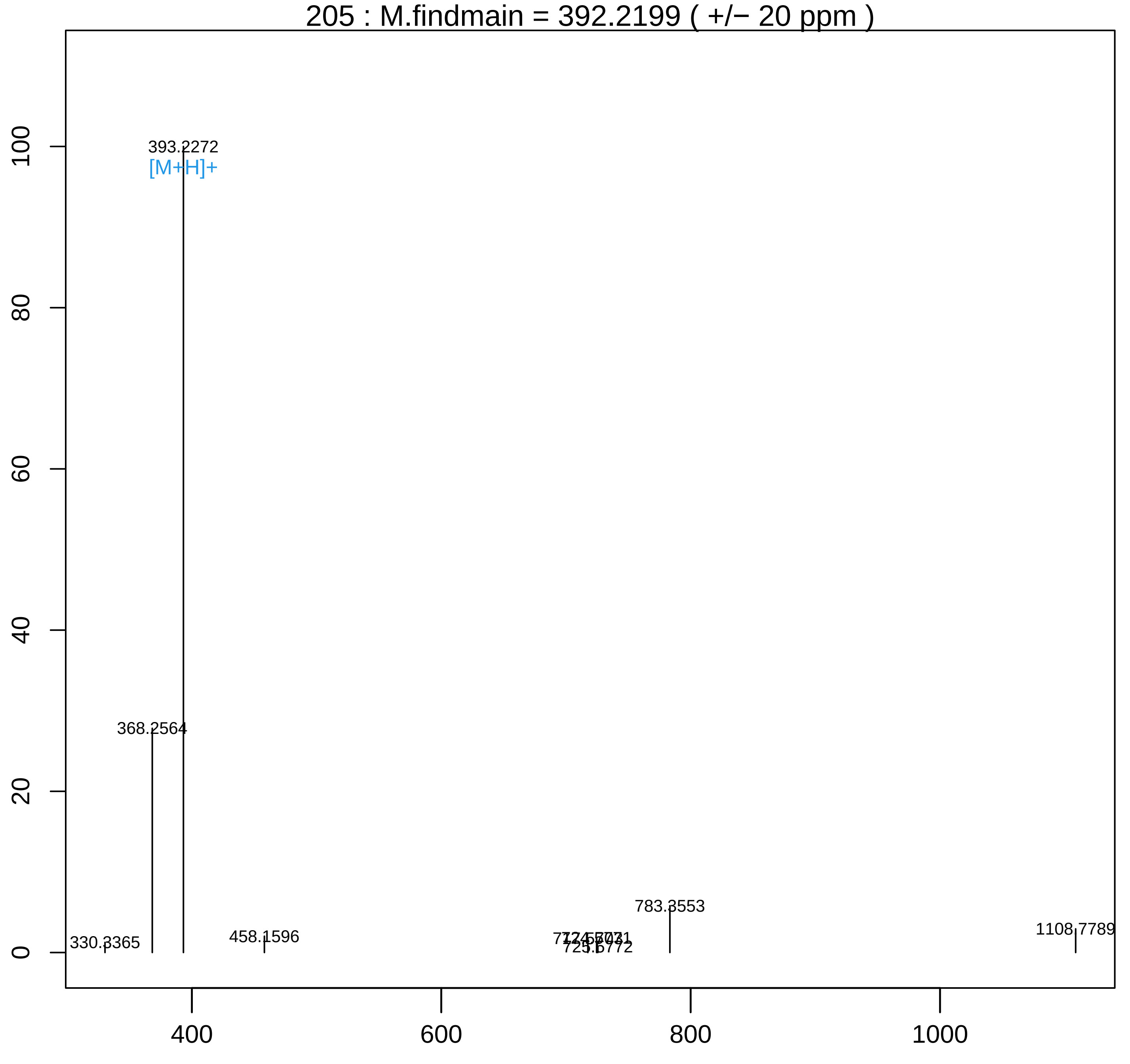
205 : M.findmain = 392.2199 (+/− 20 ppm)
393.2272
[M+H]+
368.2564
330.3365
458.1596
783.3553
1108.7789
0
20
40
60
80
100
400
600
800
1000

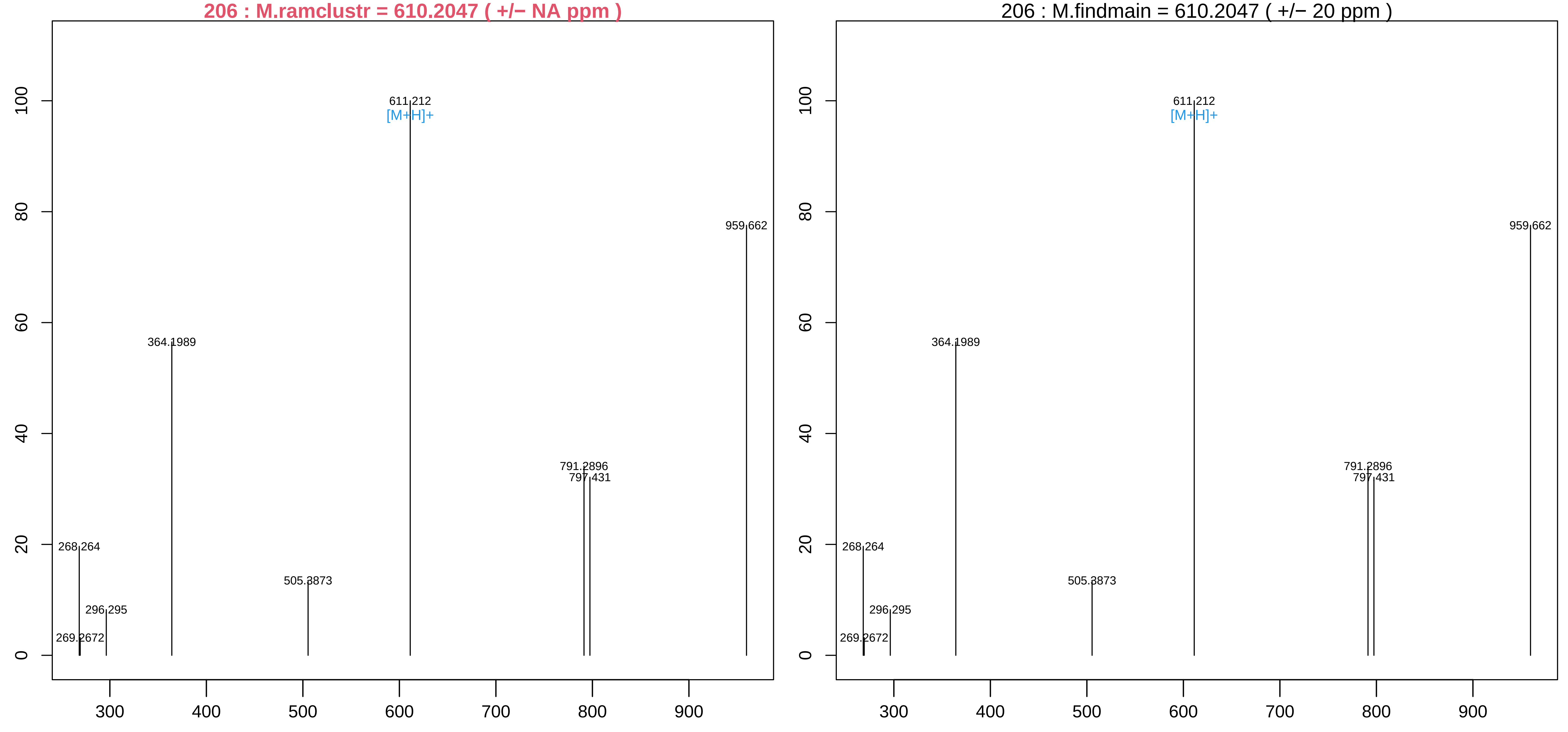
206 : M.ramclustr = 610.2047 (+/− NA ppm)
206 : M.findmain = 610.2047 (+/− 20 ppm)
611.212
[M+H]+
959.662
364.1989
791.2896
797.431
268.264
505.3873
296.295
269.2672
0
20
40
60
80
100
300
400
500
600
700
800
900

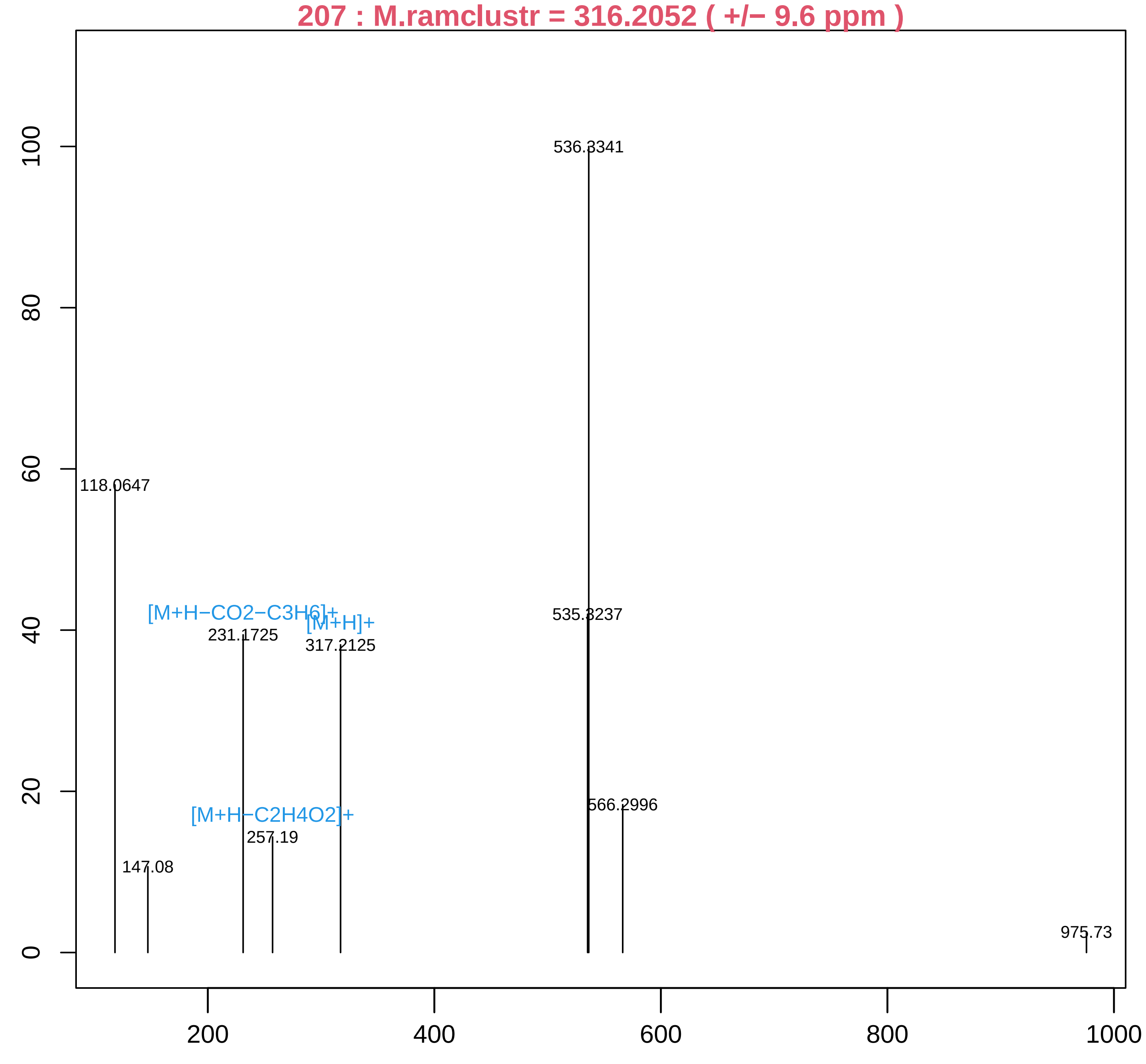
207 : M.ramclustr = 316.2052 (+/− 9.6 ppm)
536.3341
118.0647
[M+H−CO2−C3H6]+
231.1725
[M+H]+
317.2125
535.3237
[M+H−C2H4O2]+
257.19
566.2996
147.08
975.73
0
20
40
60
80
100
200
400
600
800
1000

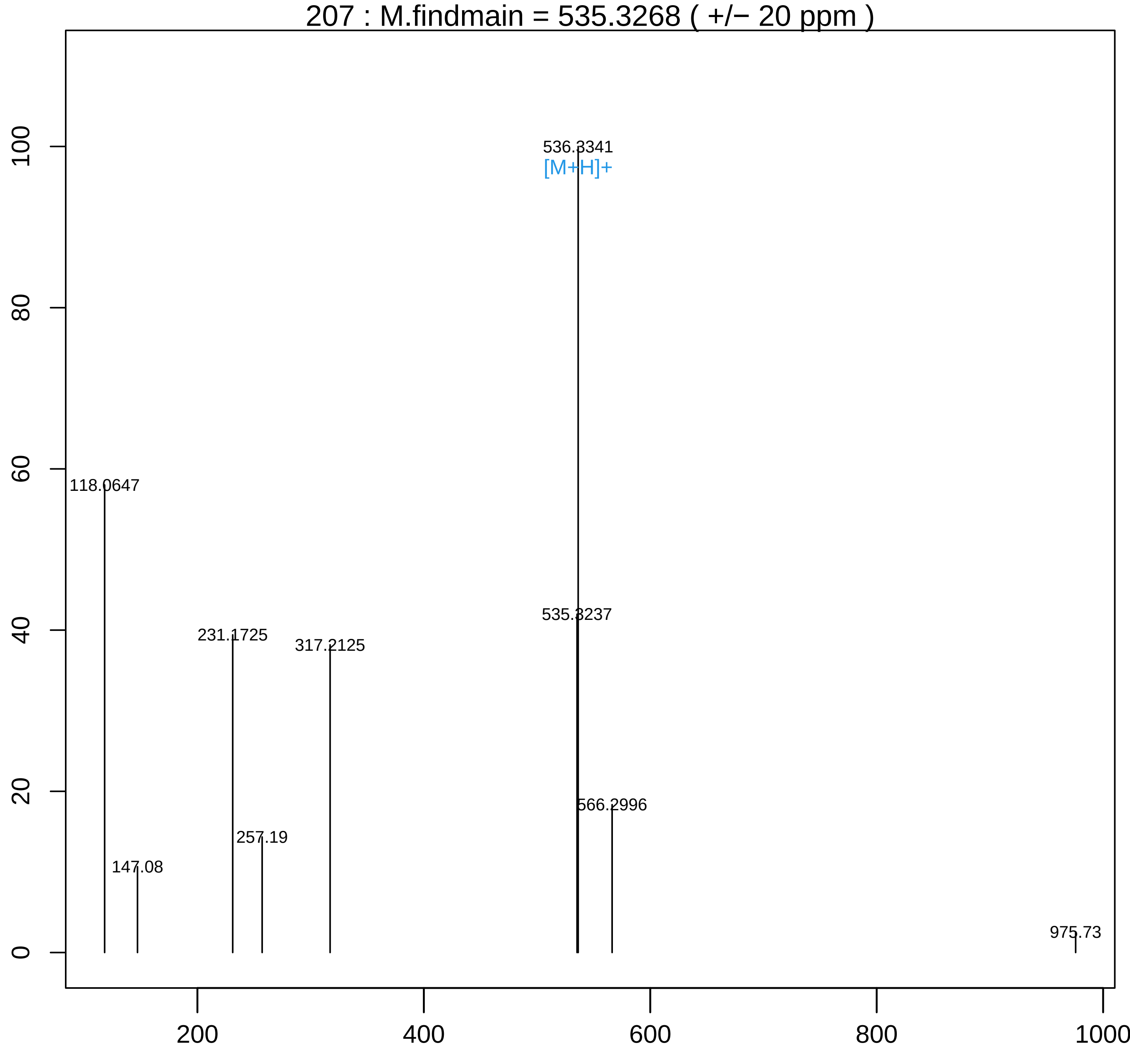
207 : M.findmain = 535.3268 (+/− 20 ppm)
536.3341
[M+H]+
118.0647
231.1725
317.2125
535.3237
566.2996
257.19
147.08
975.73
0
20
40
60
80
100
200
400
600
800
1000

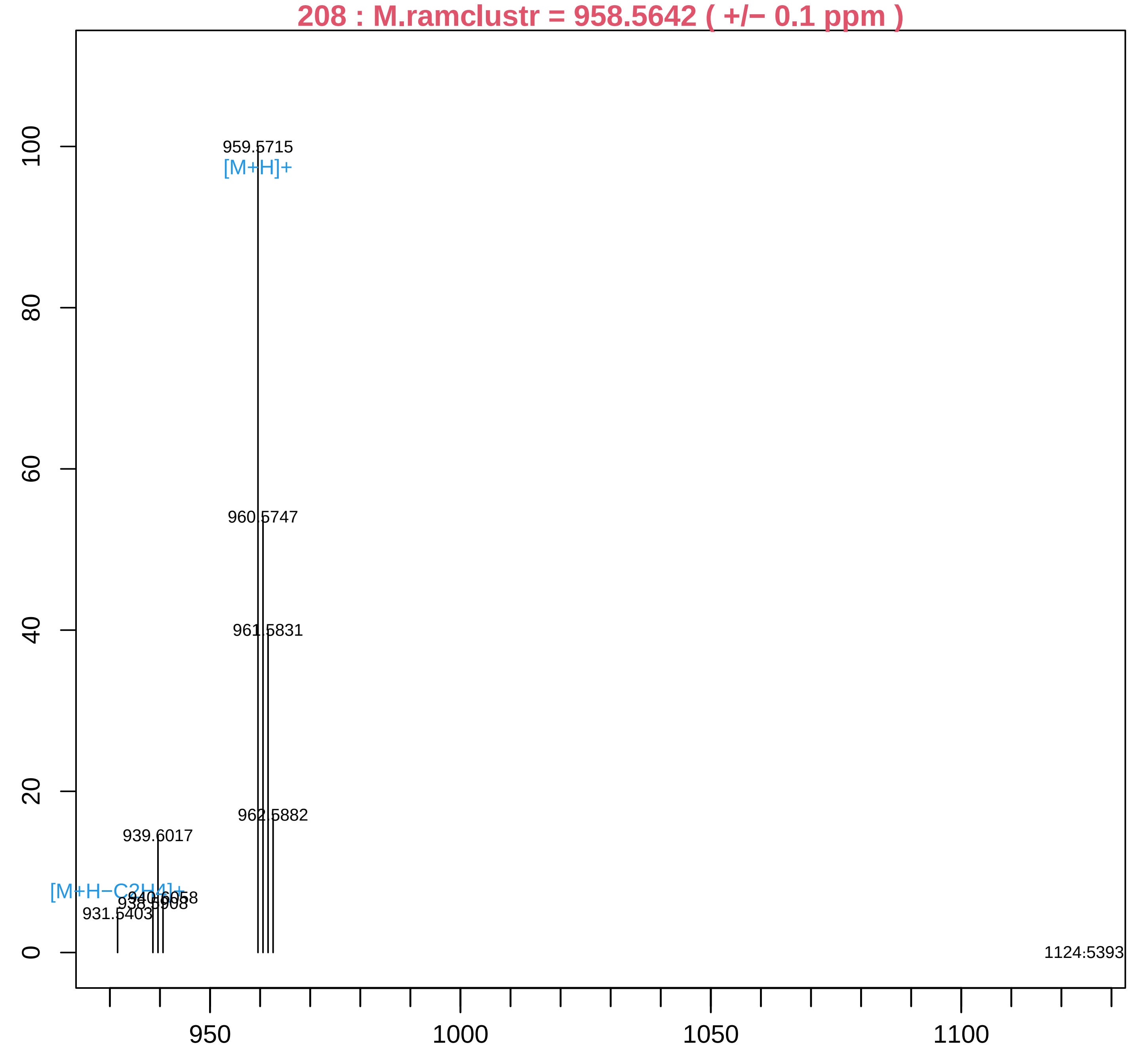
208 : M.ramclustr = 958.5642 (+/− 0.1 ppm)
959.5715
[M+H]+
960.5747
961.5831
962.5882
939.6017
[M+H−C2H4]+
940.6058
938.5908
931.5403
1124.5393
0
20
40
60
80
100
950
1000
1050
1100
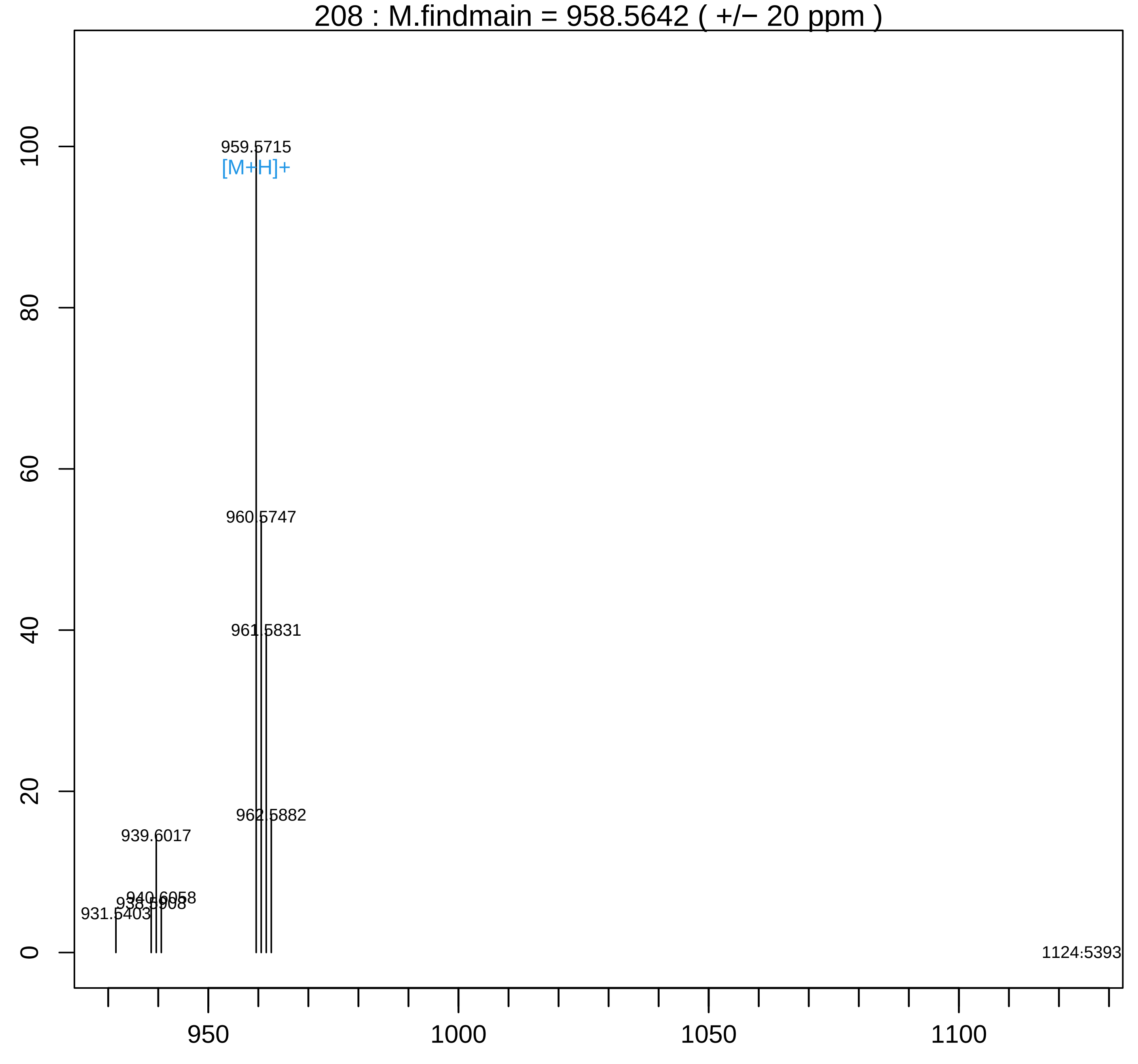
208 : M.findmain = 958.5642 (+/− 20 ppm)
959.5715
[M+H]+
960.5747
961.5831
962.5882
939.6017
940.6058
938.5908
931.5403
1124.5393
0
20
40
60
80
100
950
1000
1050
1100

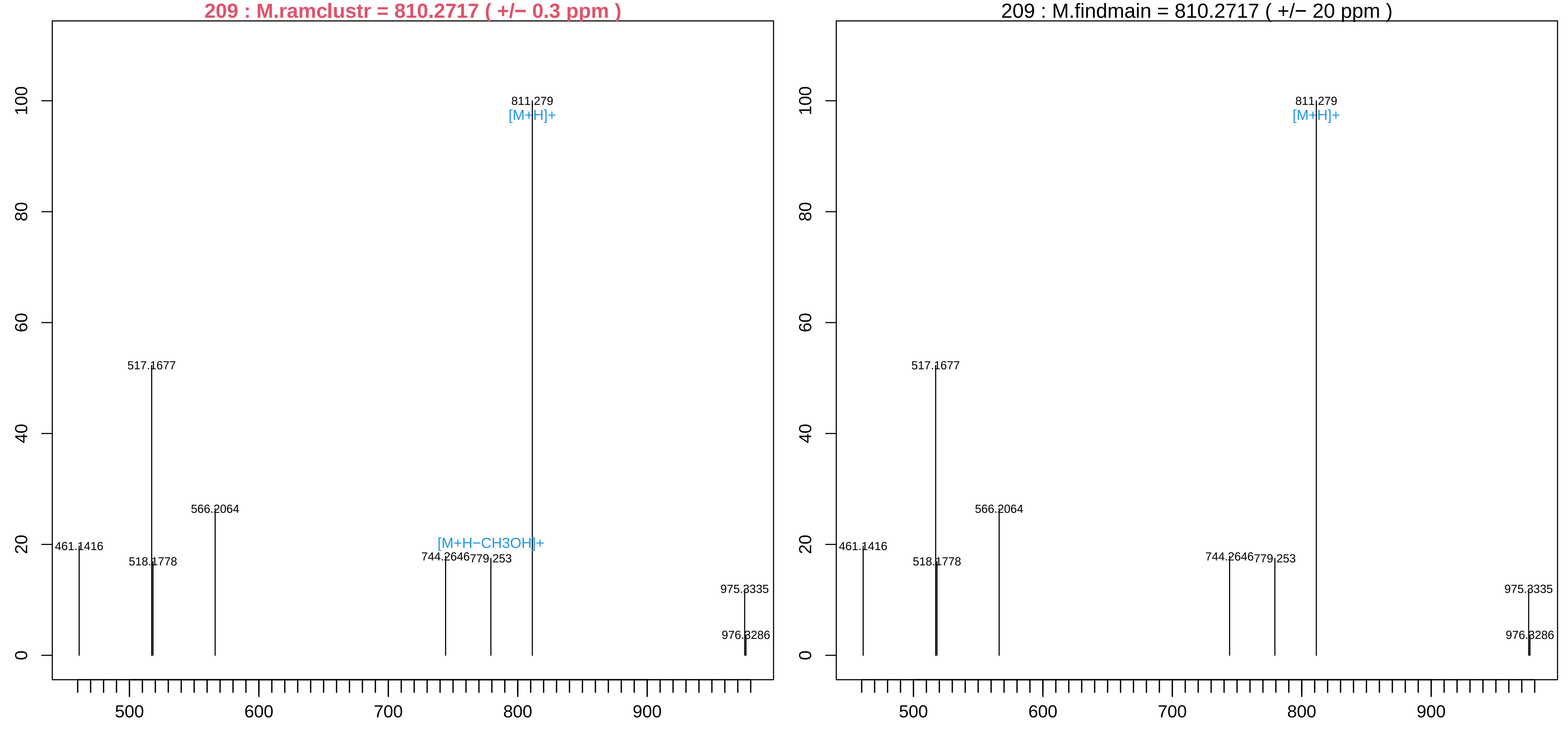
209 : M.ramclustr = 810.2717 (+/− 0.3 ppm)
811.279
[M+H]+
517.1677
566.2064
461.1416
518.1778
[M+H−CH3OH]+
744.2646
779.253
975.3335
976.3286
209 : M.findmain = 810.2717 (+/− 20 ppm)
811.279
[M+H]+
517.1677
566.2064
461.1416
518.1778
744.2646
779.253
975.3335
976.3286
0
20
40
60
80
100
500
600
700
800
900

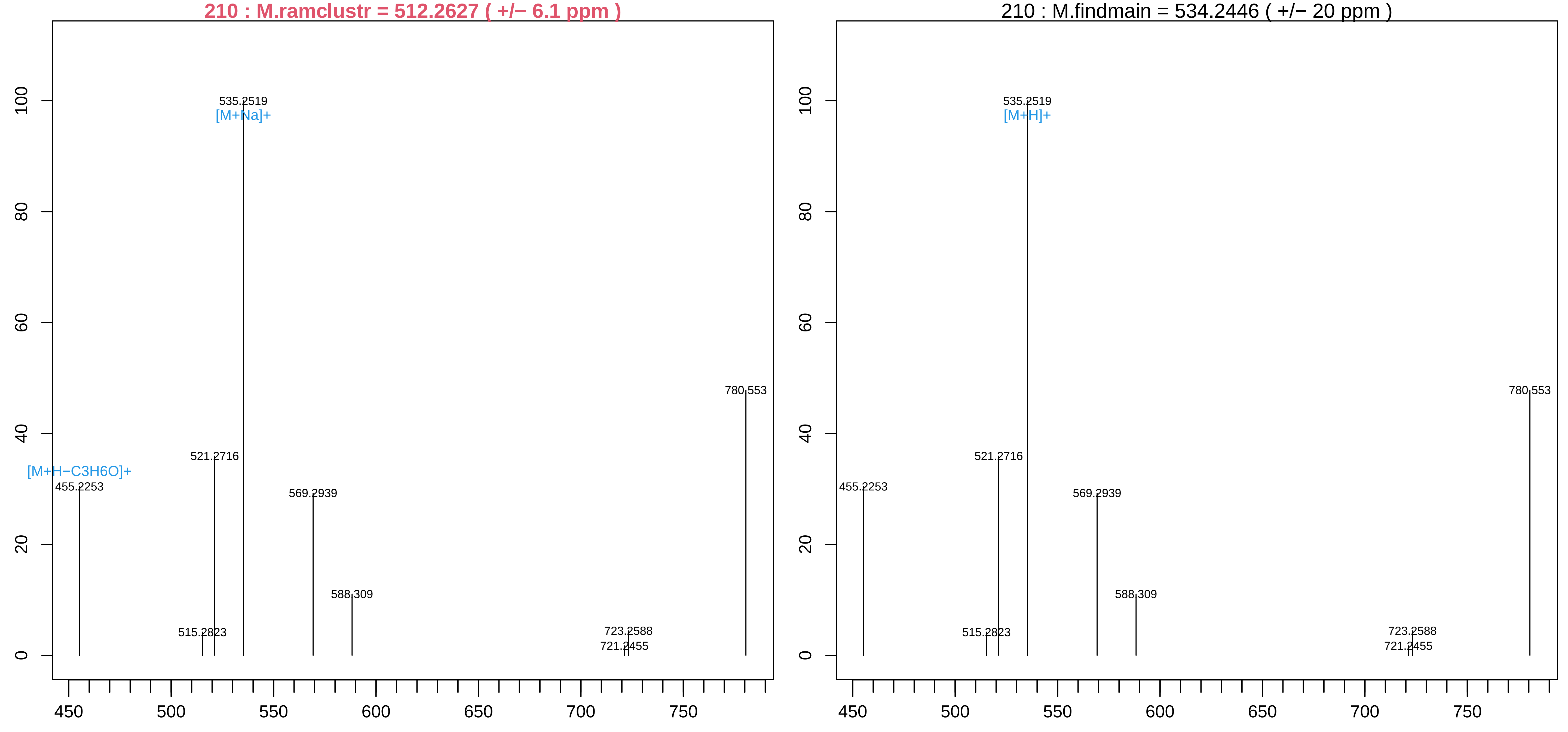

210 : M.ramclustr = 512.2627 (+/− 6.1 ppm)
535.2519
[M+Na]+
780.553
521.2716
[M+H−C3H6O]+
455.2253
569.2939
588.309
515.2823
723.2588
721.2455
210 : M.findmain = 534.2446 (+/− 20 ppm)
535.2519
[M+H]+
780.553
521.2716
455.2253
569.2939
588.309
515.2823
723.2588
721.2455

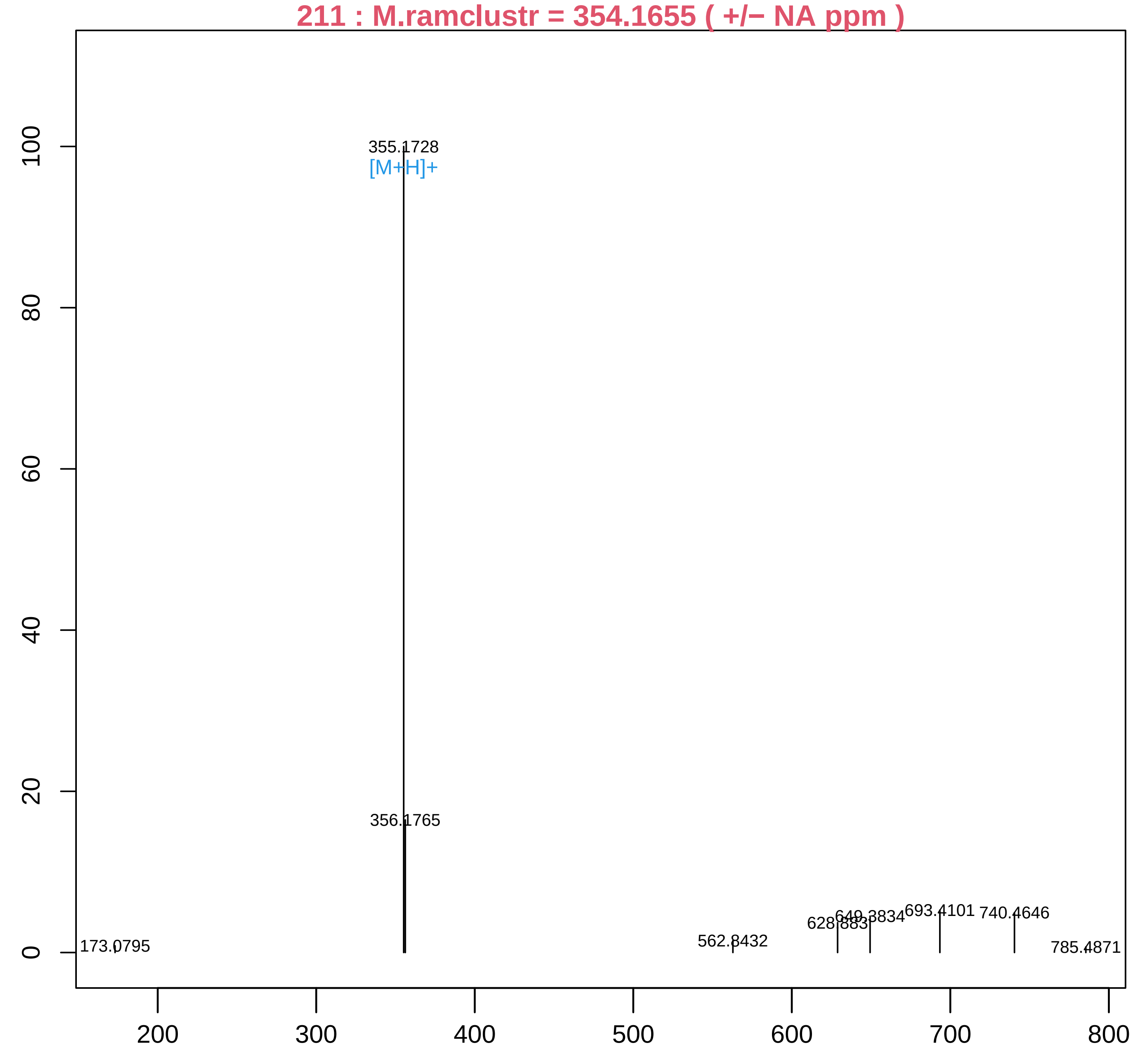
211 : M.ramclustr = 354.1655 (+/− NA ppm)
355.1728
[M+H]+
356.1765
173.0795
562.8432
628.883
649.3834
693.4101
740.4646
785.4871
0
20
40
60
80
100
200
300
400
500
600
700
800

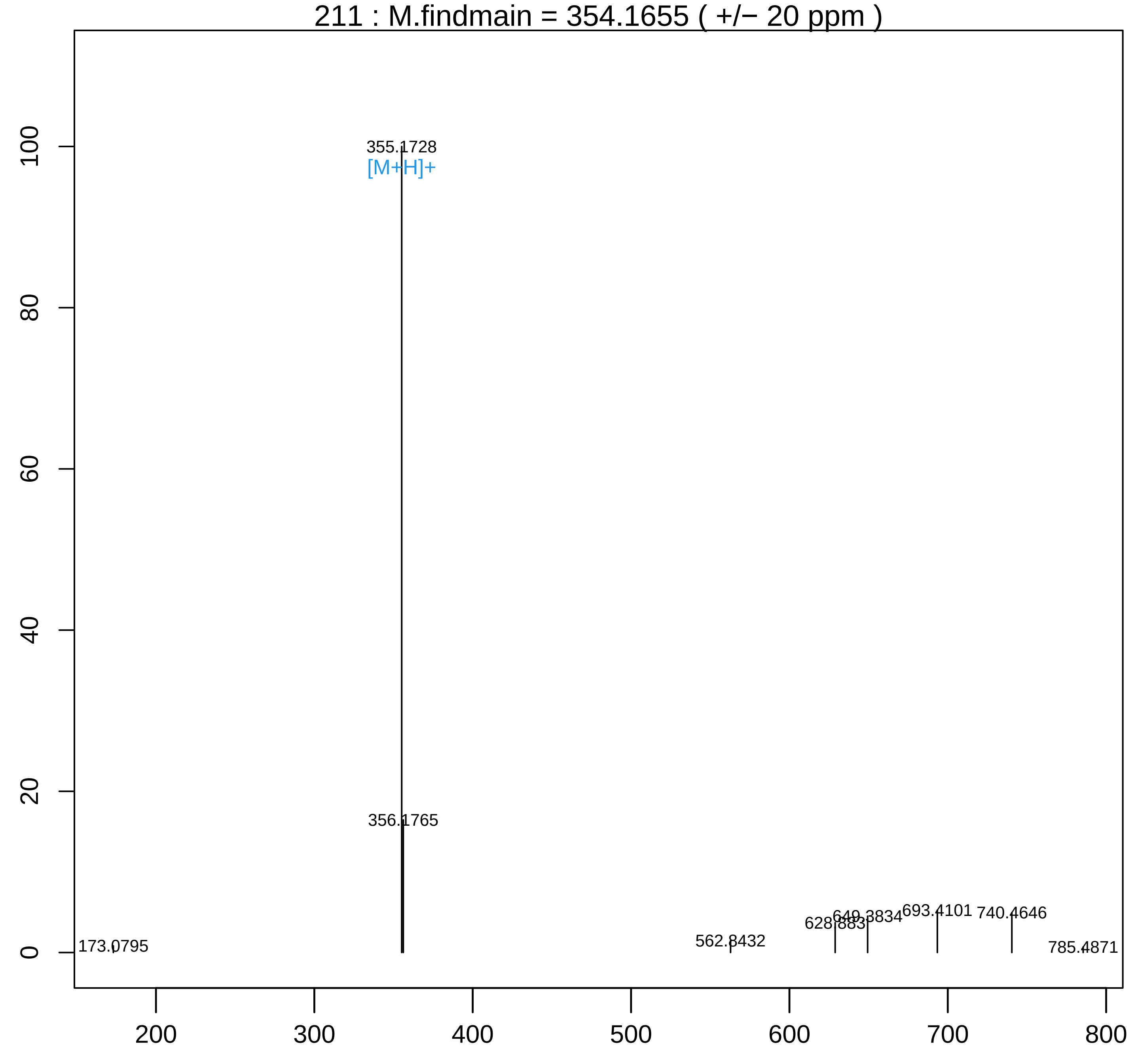
211 : M.findmain = 354.1655 (+/− 20 ppm)
355.1728
[M+H]+
356.1765
173.0795
562.8432
628.883
649.3834
693.4101
740.4646
785.4871
0
20
40
60
80
100
200
300
400
500
600
700
800

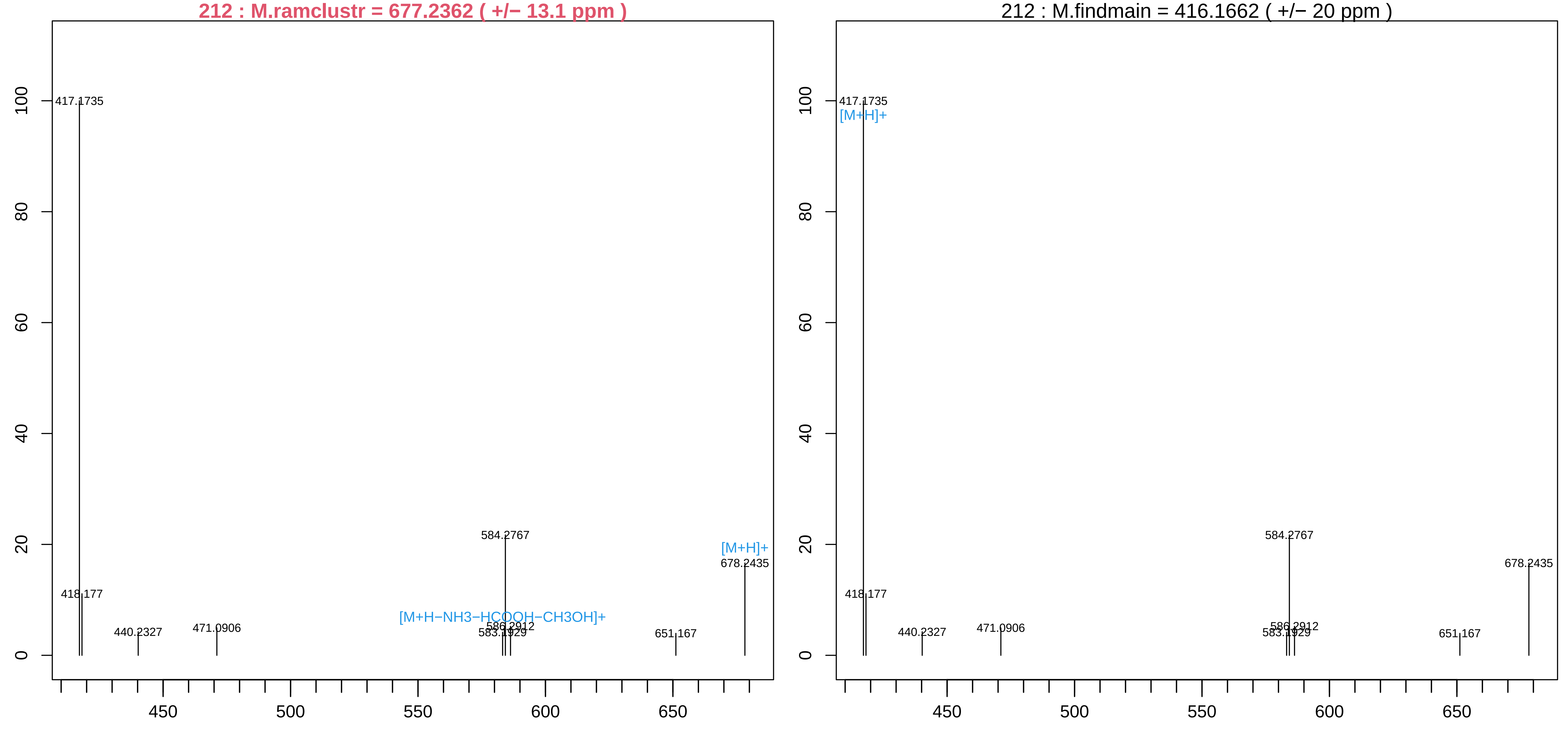
212 : M.ramclustr = 677.2362 (+/− 13.1 ppm)
417.1735
418.177
440.2327
471.0906
584.2767
[M+H−NH3−HCOOH−CH3OH]+
586.2912
583.1929
651.167
[M+H]+
678.2435
212 : M.findmain = 416.1662 (+/− 20 ppm)
417.1735
[M+H]+
418.177
440.2327
471.0906
584.2767
586.2912
583.1929
651.167
678.2435

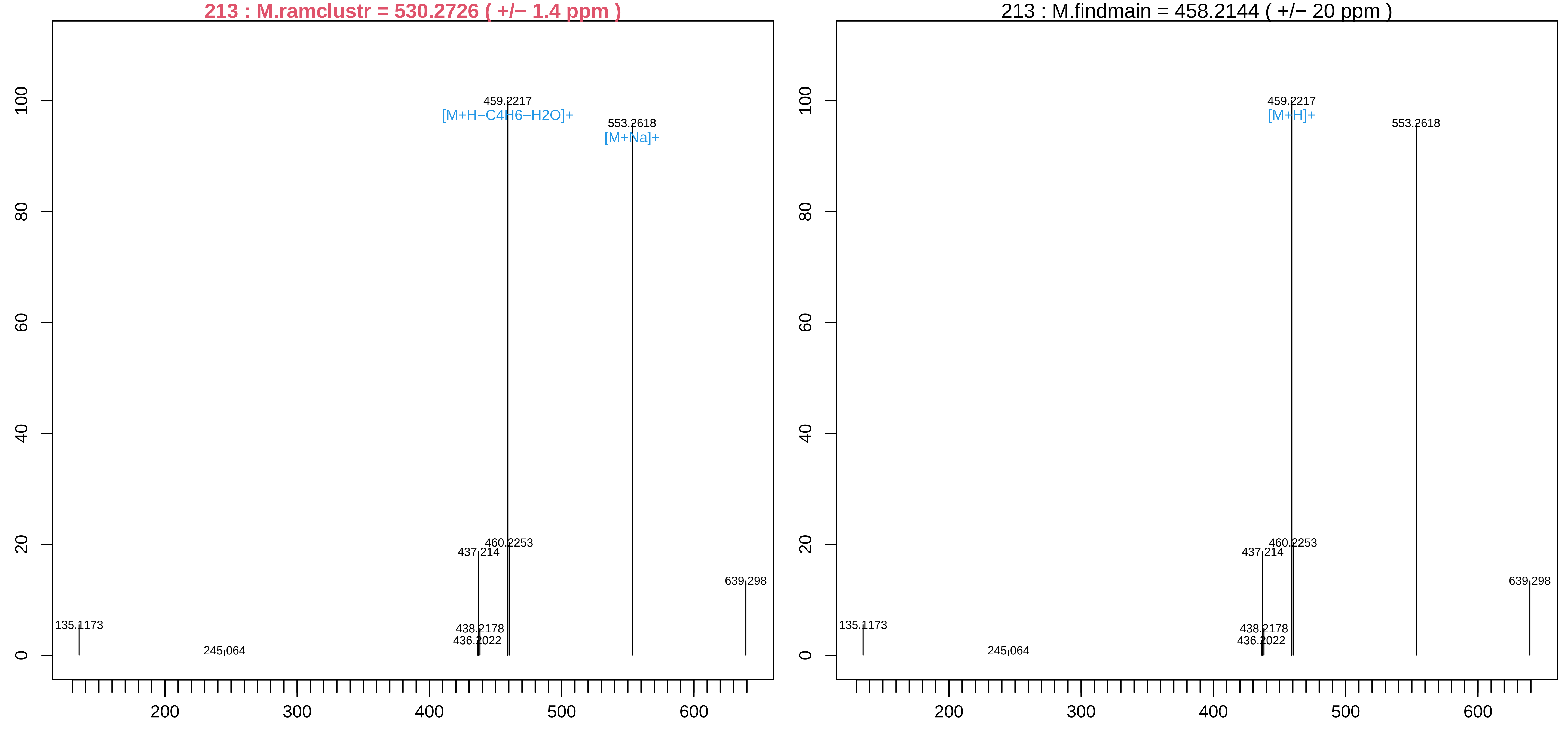

213 : M.ramclustr = 530.2726 (+/− 1.4 ppm)
459.2217
[M+H−C4H6−H2O]+
553.2618
[M+Na]+
460.2253
437.214
639.298
135.1173
438.2178
436.2022
245.064
213 : M.findmain = 458.2144 (+/− 20 ppm)
459.2217
[M+H]+
553.2618
460.2253
437.214
639.298
135.1173
438.2178
436.2022
245.064
0
20
40
60
80
100
200
300
400
500
600

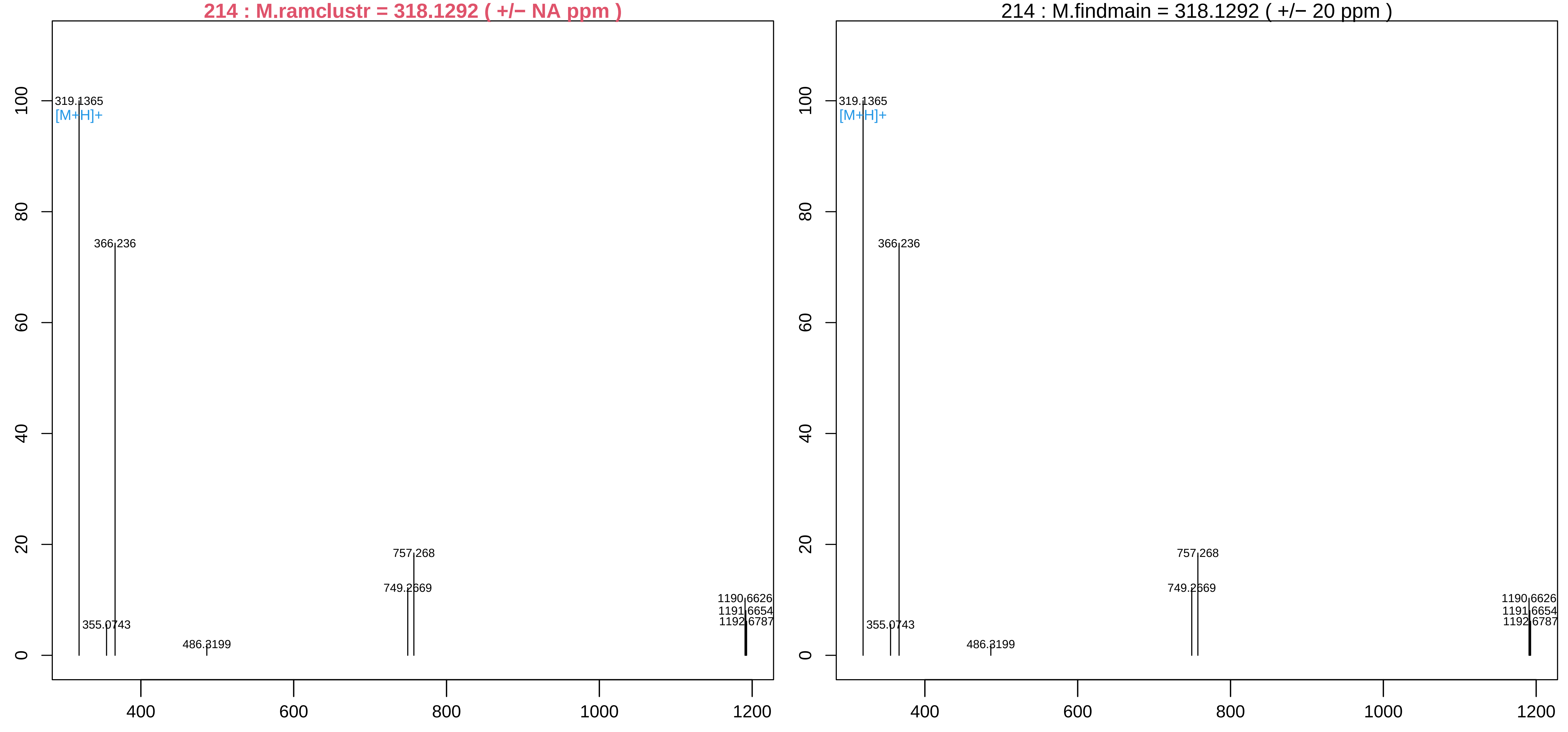

214 : M.ramclustr = 318.1292 (+/− NA ppm)
319.1365
[M+H]+
366.236
757.268
749.2669
1190.6626
1191.6654
1192.6787
355.0743
486.3199
0
20
40
60
80
100
400
600
800
1000
1200
214 : M.findmain = 318.1292 (+/− 20 ppm)
319.1365
[M+H]+
366.236
757.268
749.2669
1190.6626
1191.6654
1192.6787
355.0743
486.3199
0
20
40
60
80
100
400
600
800
1000
1200

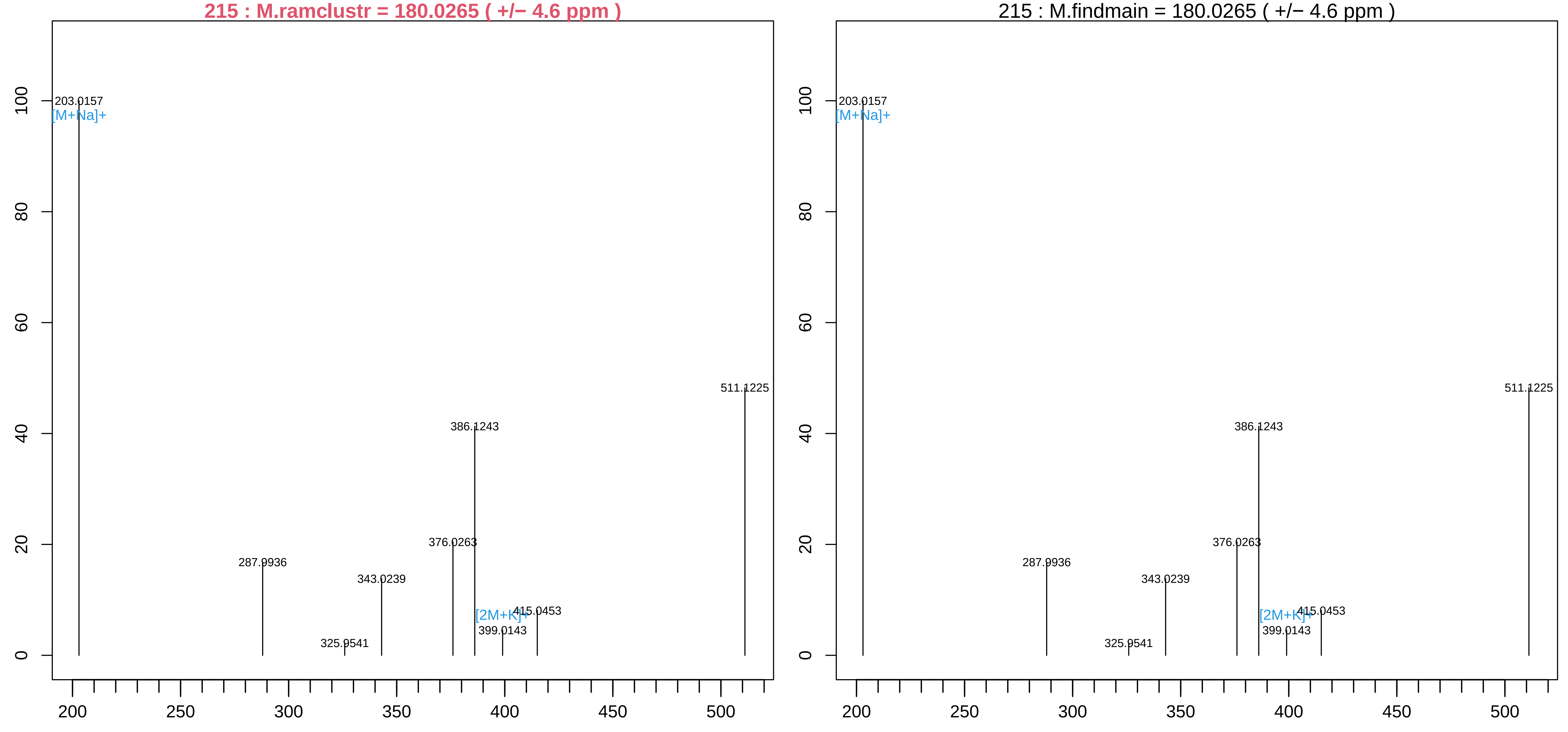
215 : M.ramclustr = 180.0265 (+/− 4.6 ppm)
203.0157
[M+Na]+
287.9936
325.9541
343.0239
376.0263
386.1243
[2M+K]+
399.0143
415.0453
511.1225
215 : M.findmain = 180.0265 (+/− 4.6 ppm)
203.0157
[M+Na]+
287.9936
325.9541
343.0239
376.0263
386.1243
[2M+K]+
399.0143
415.0453
511.1225
0
20
40
60
80
100
200
250
300
350
400
450
500

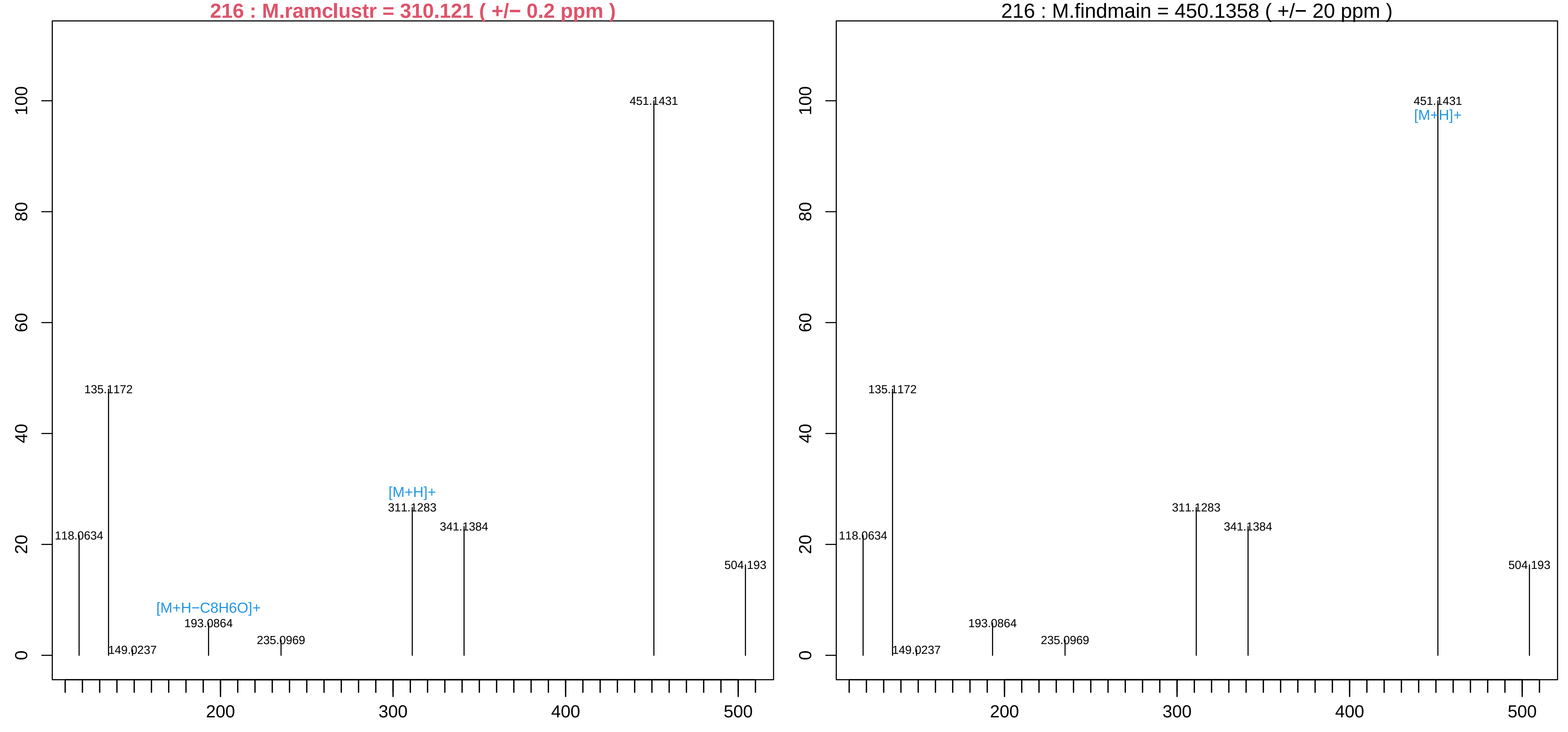
216 : M.ramclustr = 310.121 (+/− 0.2 ppm)
451.1431
135.1172
[M+H]+
311.1283
341.1384
118.0634
504.193
[M+H−C8H6O]+
193.0864
235.0969
149.0237
0
20
40
60
80
100
200
300
400
500
216 : M.findmain = 450.1358 (+/− 20 ppm)
451.1431
[M+H]+
135.1172
311.1283
341.1384
118.0634
504.193
193.0864
235.0969
149.0237
0
20
40
60
80
100
200
300
400
500

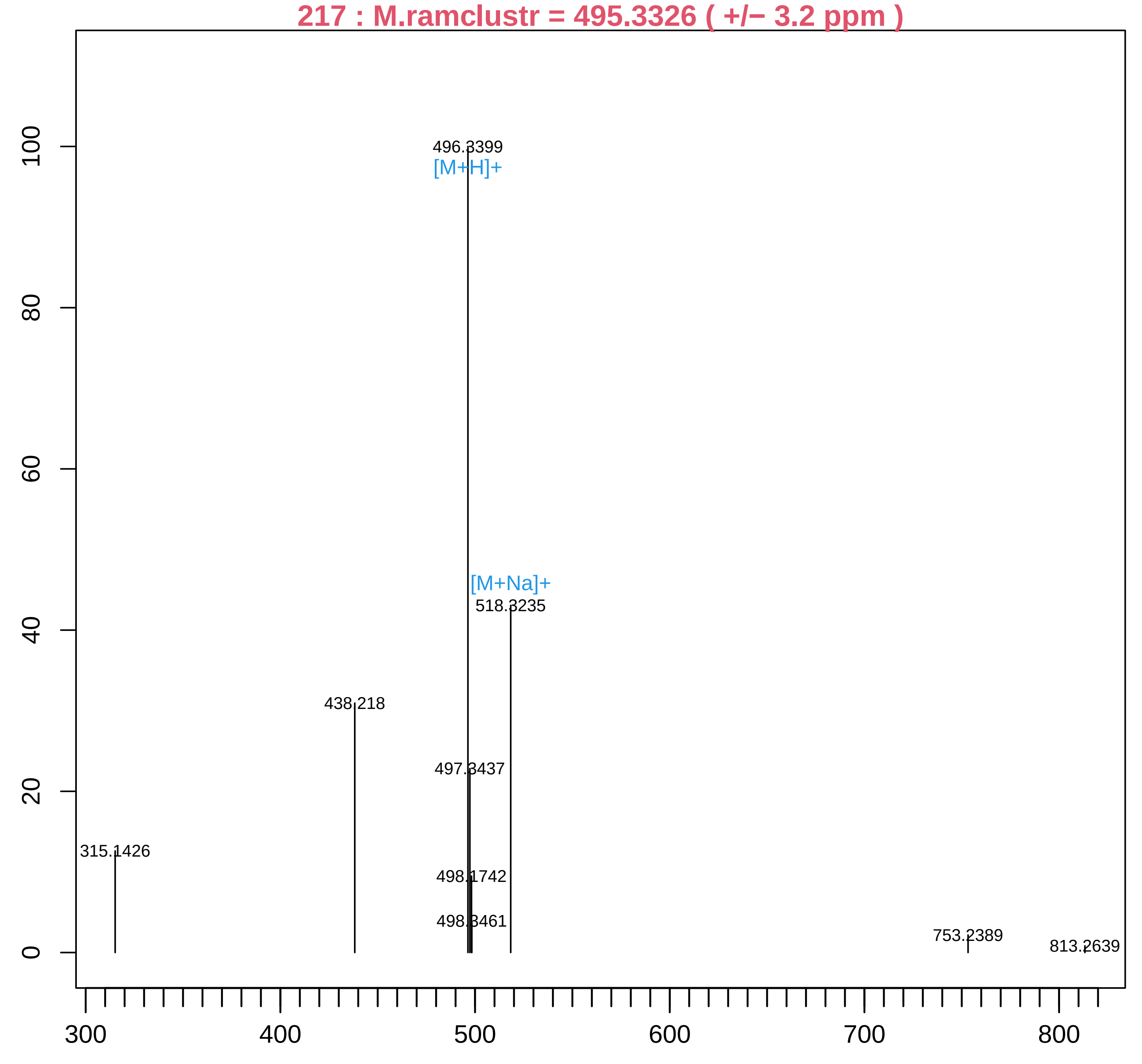

217 : M.ramclustr = 495.3326 (+/− 3.2 ppm)
496.3399
[M+H]+
[M+Na]+
518.3235
438.218
497.3437
315.1426
498.1742
498.3461
753.2389
813.2639

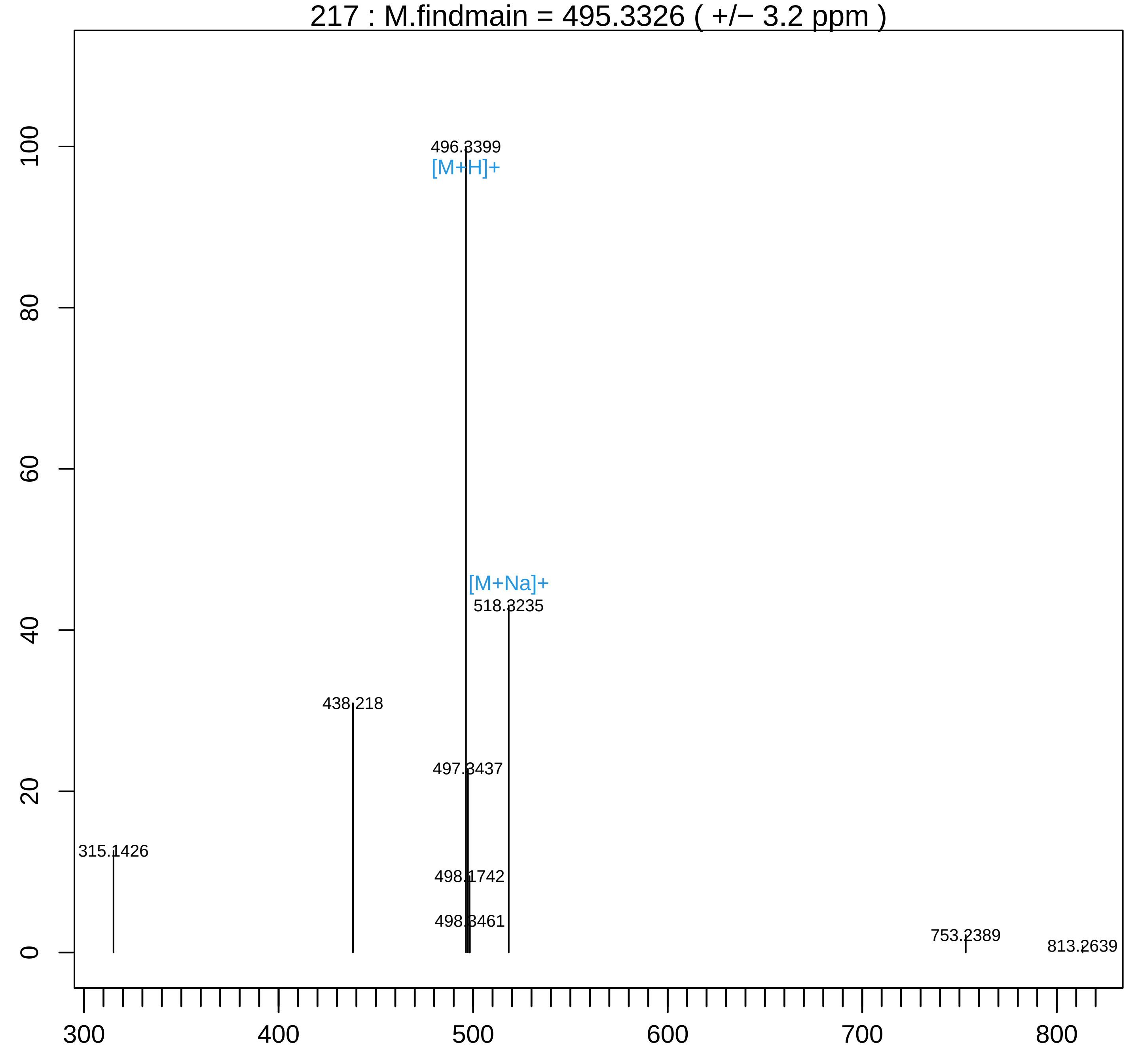

217 : M.findmain = 495.3326 (+/− 3.2 ppm)
496.3399
[M+H]+
[M+Na]+
518.3235
438.218
497.3437
315.1426
498.1742
498.3461
753.2389
813.2639

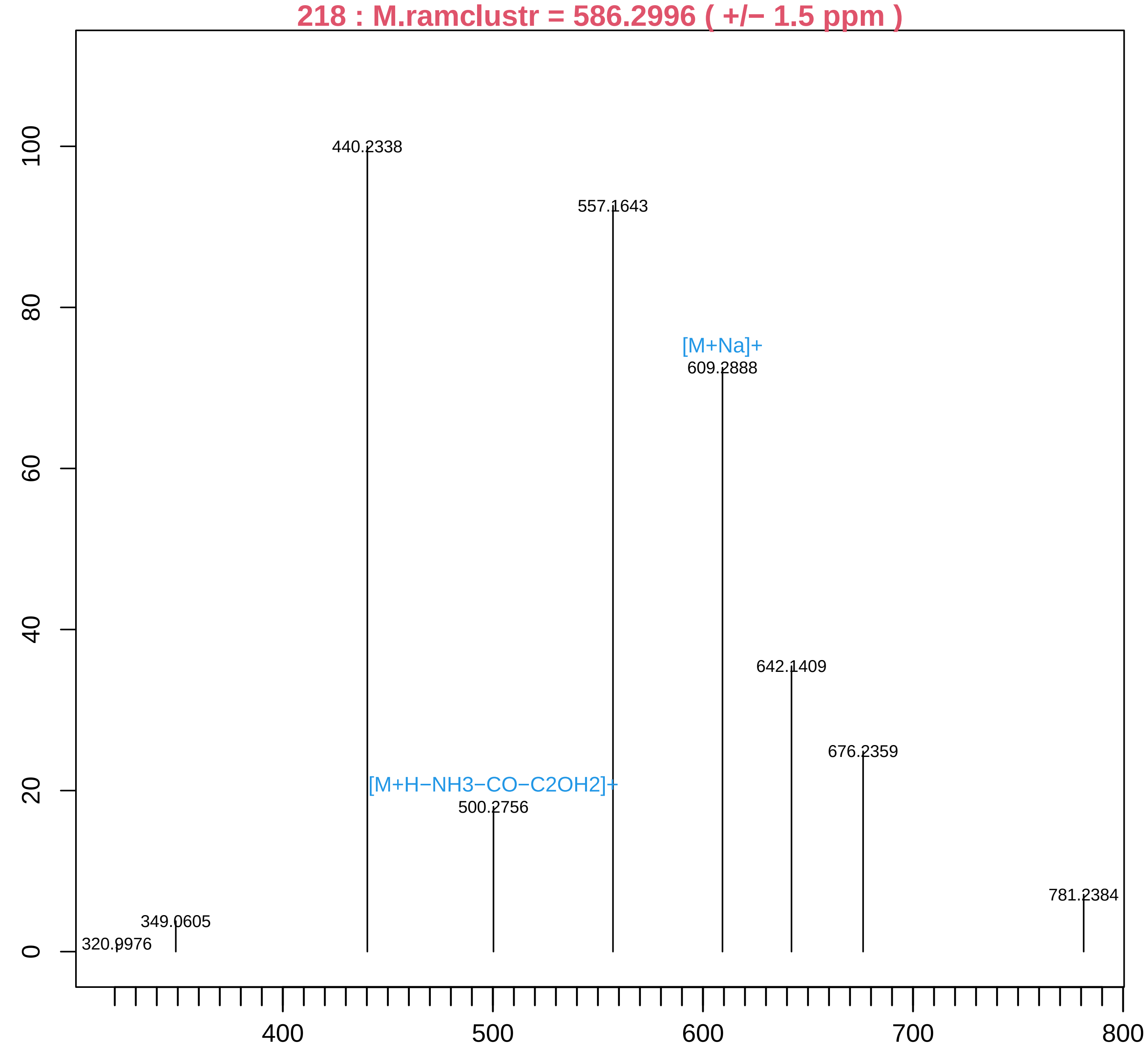
218 : M.ramclustr = 586.2996 (+/− 1.5 ppm)
0
20
40
60
80
100
400
500
600
700
800
440.2338
557.1643
[M+Na]+
609.2888
642.1409
676.2359
[M+H−NH3−CO−C2OH2]+
500.2756
781.2384
349.0605
320.9976

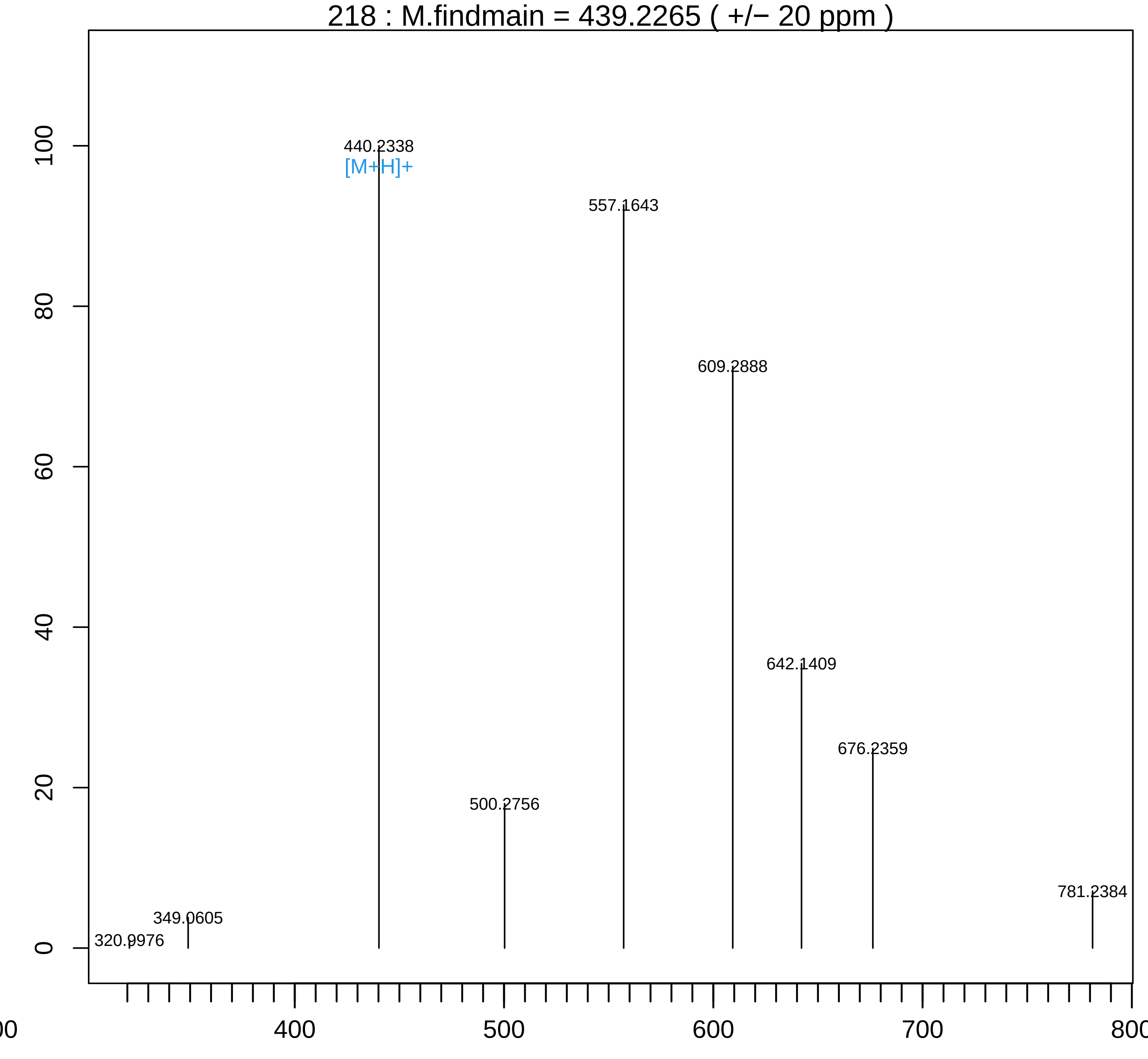
218 : M.findmain = 439.2265 (+/− 20 ppm)
0
20
40
60
80
100
400
500
600
700
800
440.2338
[M+H]+
557.1643
609.2888
642.1409
676.2359
500.2756
781.2384
349.0605
320.9976

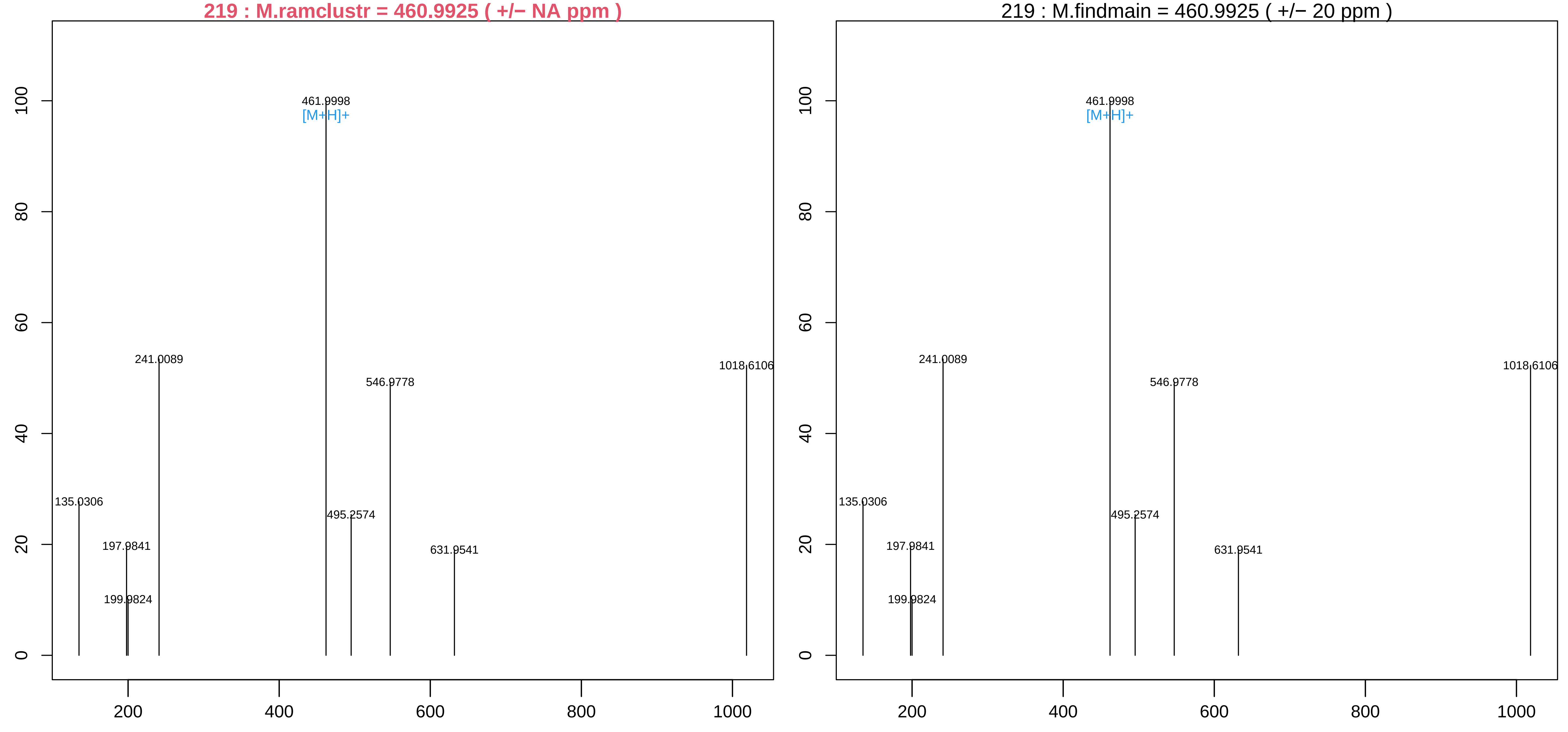
219 : M.ramclustr = 460.9925 (+/− NA ppm)
219 : M.findmain = 460.9925 (+/− 20 ppm)
461.9998
[M+H]+
241.0089
1018.6106
546.9778
135.0306
495.2574
197.9841
631.9541
199.9824
0
20
40
60
80
100
200
400
600
800
1000

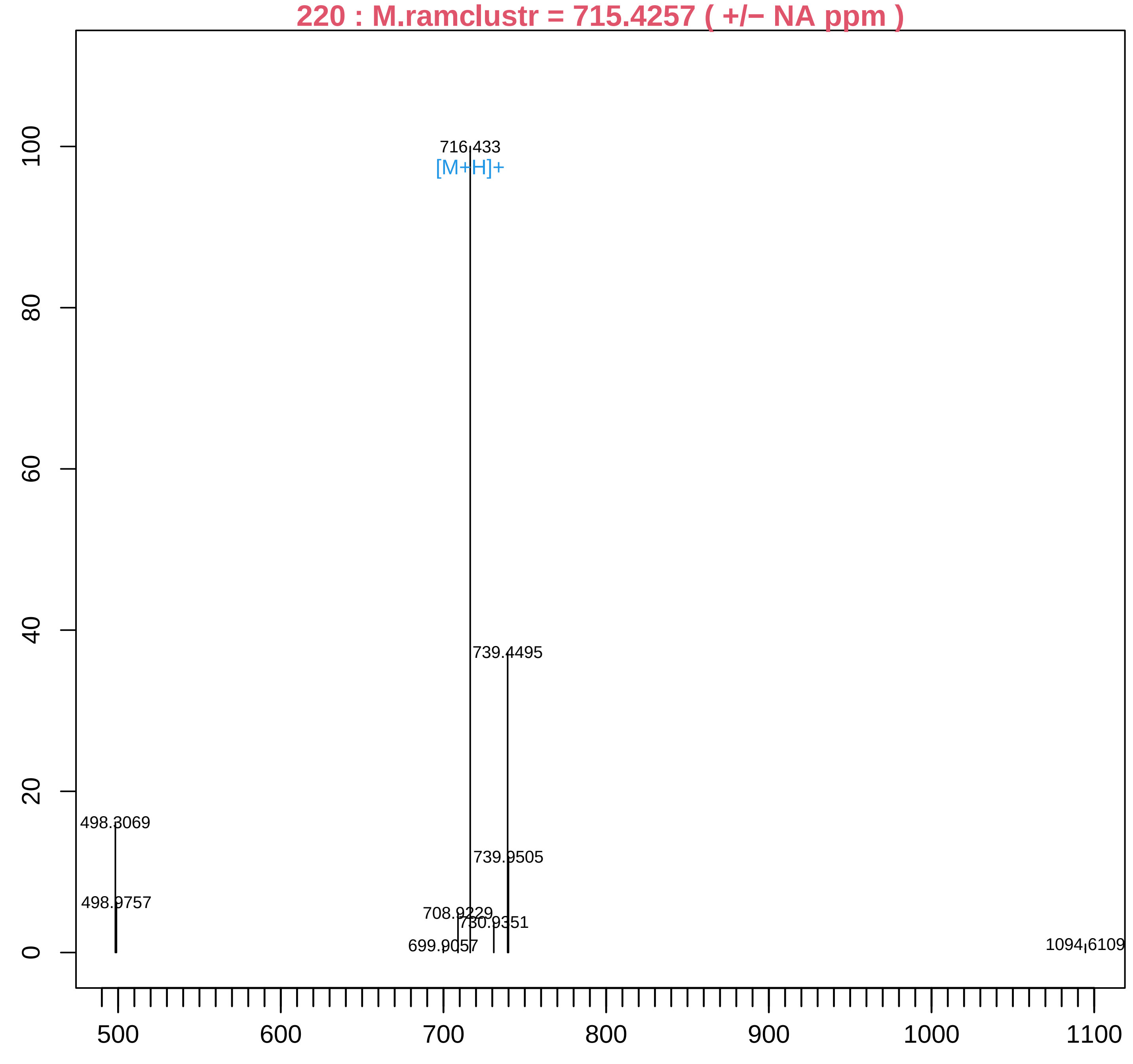
220 : M.ramclustr = 715.4257 (+/− NA ppm)
716.433
[M+H]+
739.4495
498.3069
739.9505
498.9757
708.9229
730.9351
699.9057
1094.6109
0
20
40
60
80
100
500
600
700
800
900
1000
1100
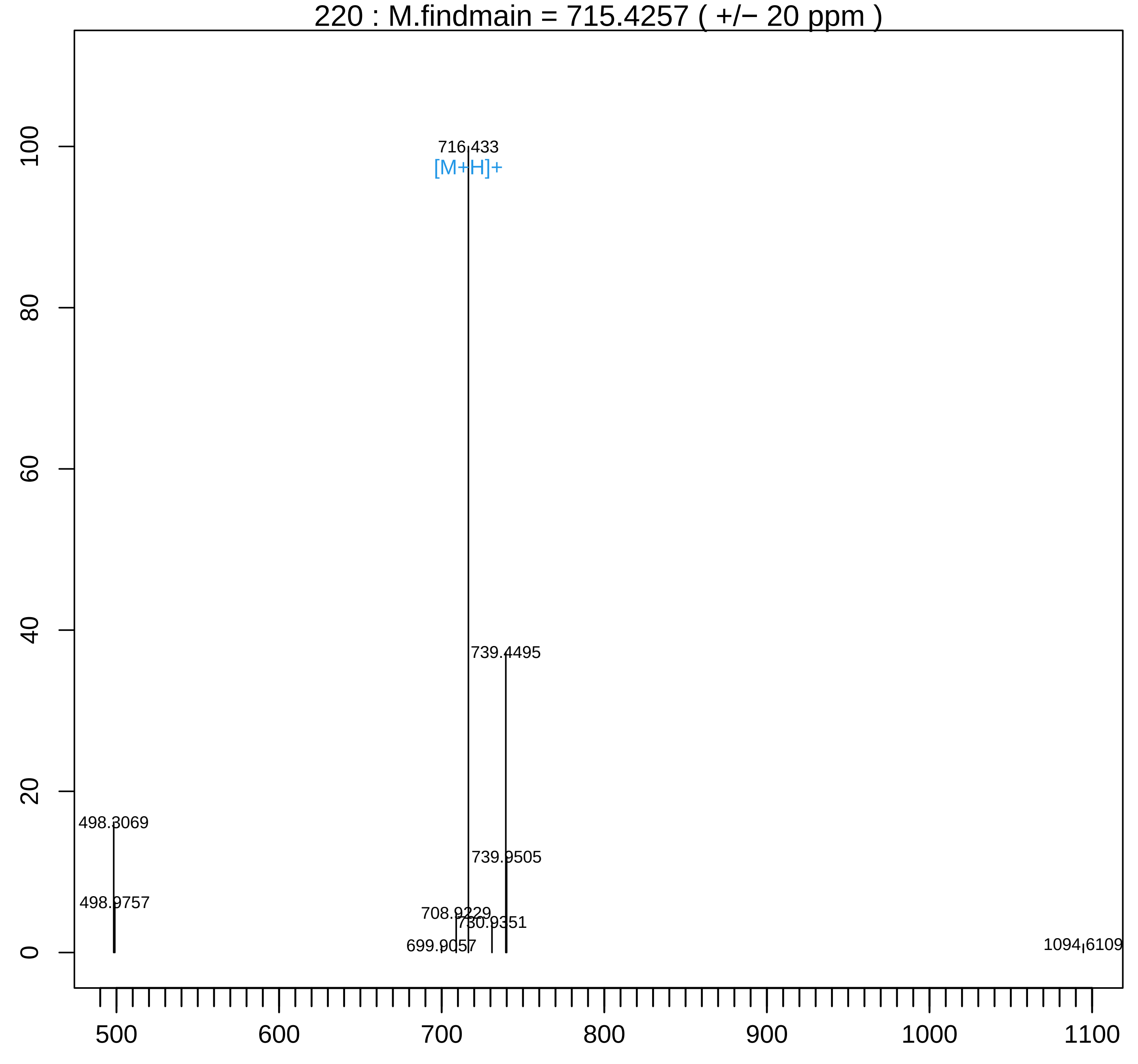
220 : M.findmain = 715.4257 (+/− 20 ppm)
716.433
[M+H]+
739.4495
498.3069
739.9505
498.9757
708.9229
730.9351
699.9057
1094.6109
0
20
40
60
80
100
500
600
700
800
900
1000
1100

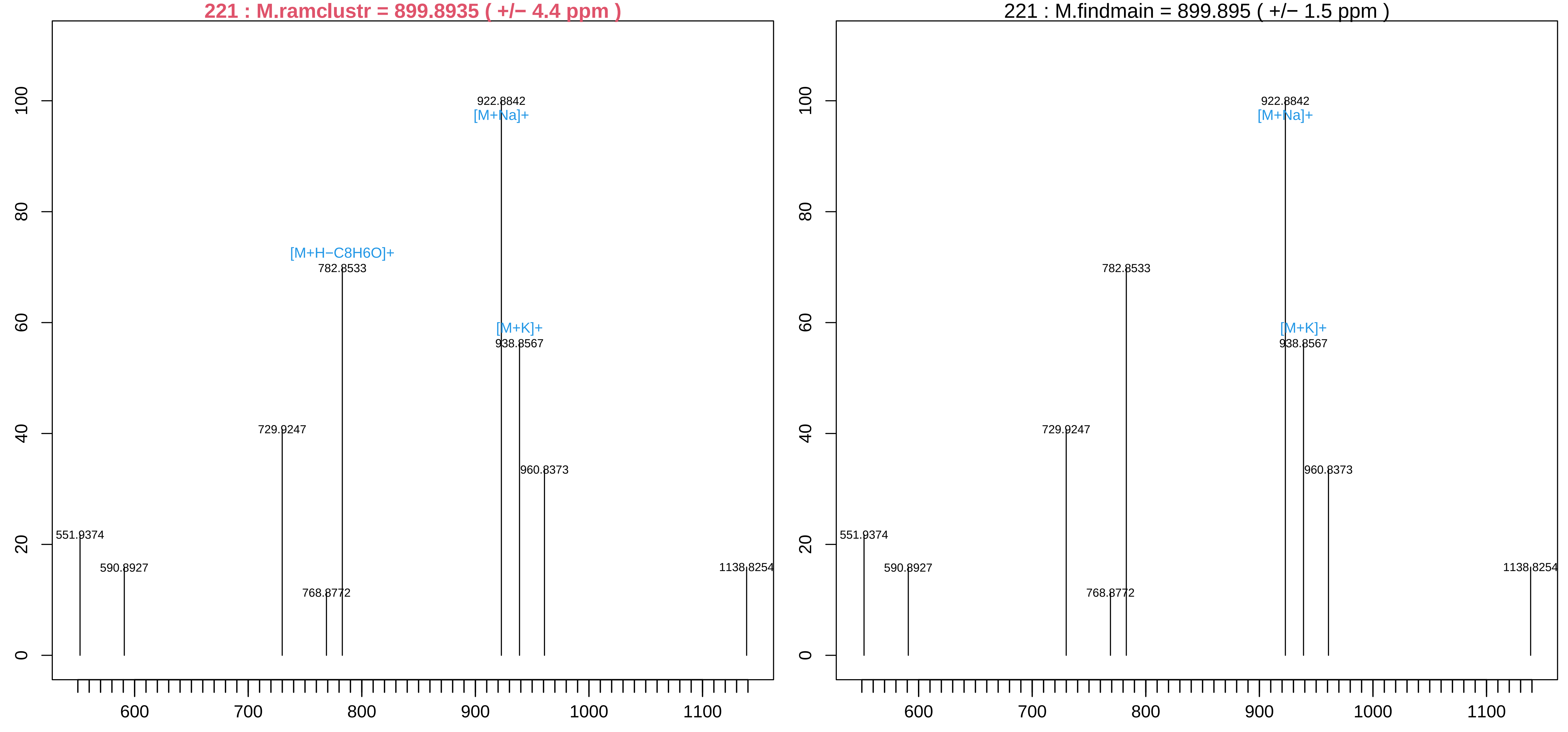

221 : M.ramclustr = 899.8935 (+/− 4.4 ppm)
922.8842
[M+Na]+
[M+H−C8H6O]+
782.8533
[M+K]+
938.8567
729.9247
960.8373
551.9374
590.8927
768.8772
1138.8254
221 : M.findmain = 899.895 (+/− 1.5 ppm)
922.8842
[M+Na]+
782.8533
[M+K]+
938.8567
729.9247
960.8373
551.9374
590.8927
768.8772
1138.8254

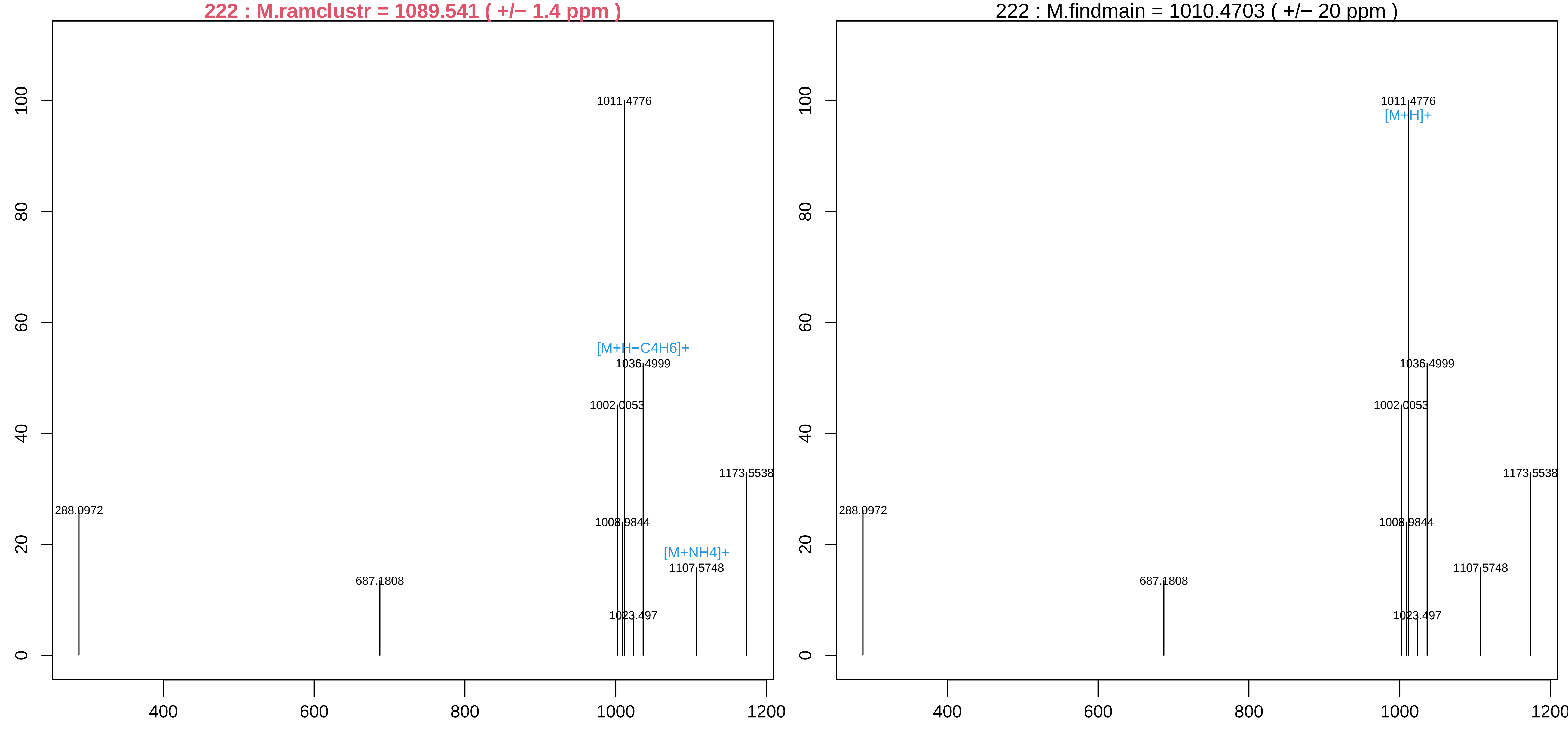
222 : M.ramclustr = 1089.541 (+/− 1.4 ppm)
1011.4776
[M+H−C4H6]+
1036.4999
1002.0053
1173.5538
288.0972
1008.9844
[M+NH4]+
1107.5748
687.1808
1023.497
0
20
40
60
80
100
400
600
800
1000
1200
222 : M.findmain = 1010.4703 (+/− 20 ppm)
1011.4776
[M+H]+
1036.4999
1002.0053
1173.5538
288.0972
1008.9844
1107.5748
687.1808
1023.497
0
20
40
60
80
100
400
600
800
1000
1200

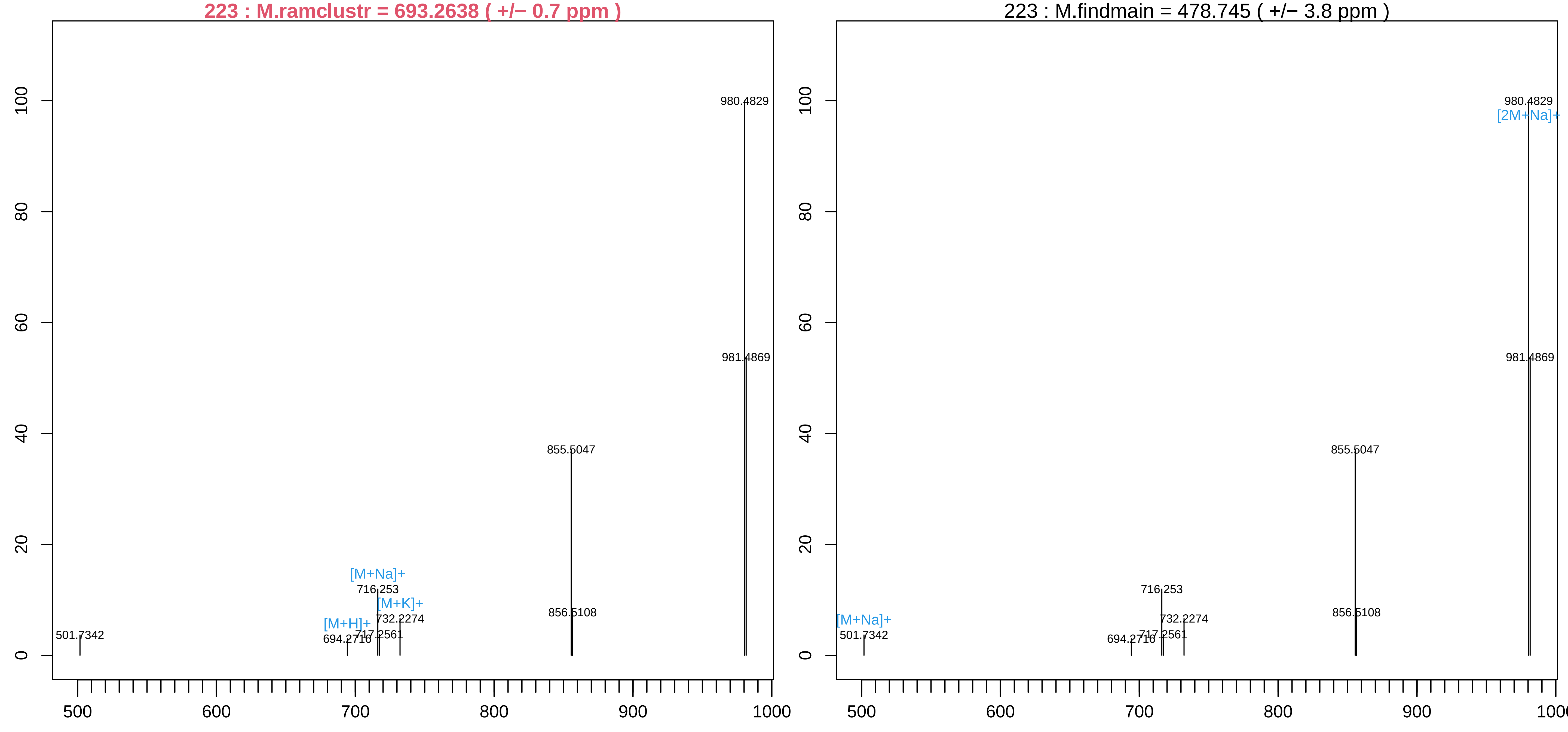
223 : M.ramclustr = 693.2638 (+/− 0.7 ppm)
980.4829
981.4869
855.5047
856.5108
[M+Na]+
716.253
[M+K]+
732.2274
[M+H]+
694.2716
717.2561
501.7342
223 : M.findmain = 478.745 (+/− 3.8 ppm)
980.4829
[2M+Na]+
981.4869
855.5047
856.5108
716.253
732.2274
694.2716
717.2561
[M+Na]+
501.7342
0
20
40
60
80
100
500
600
700
800
900
1000

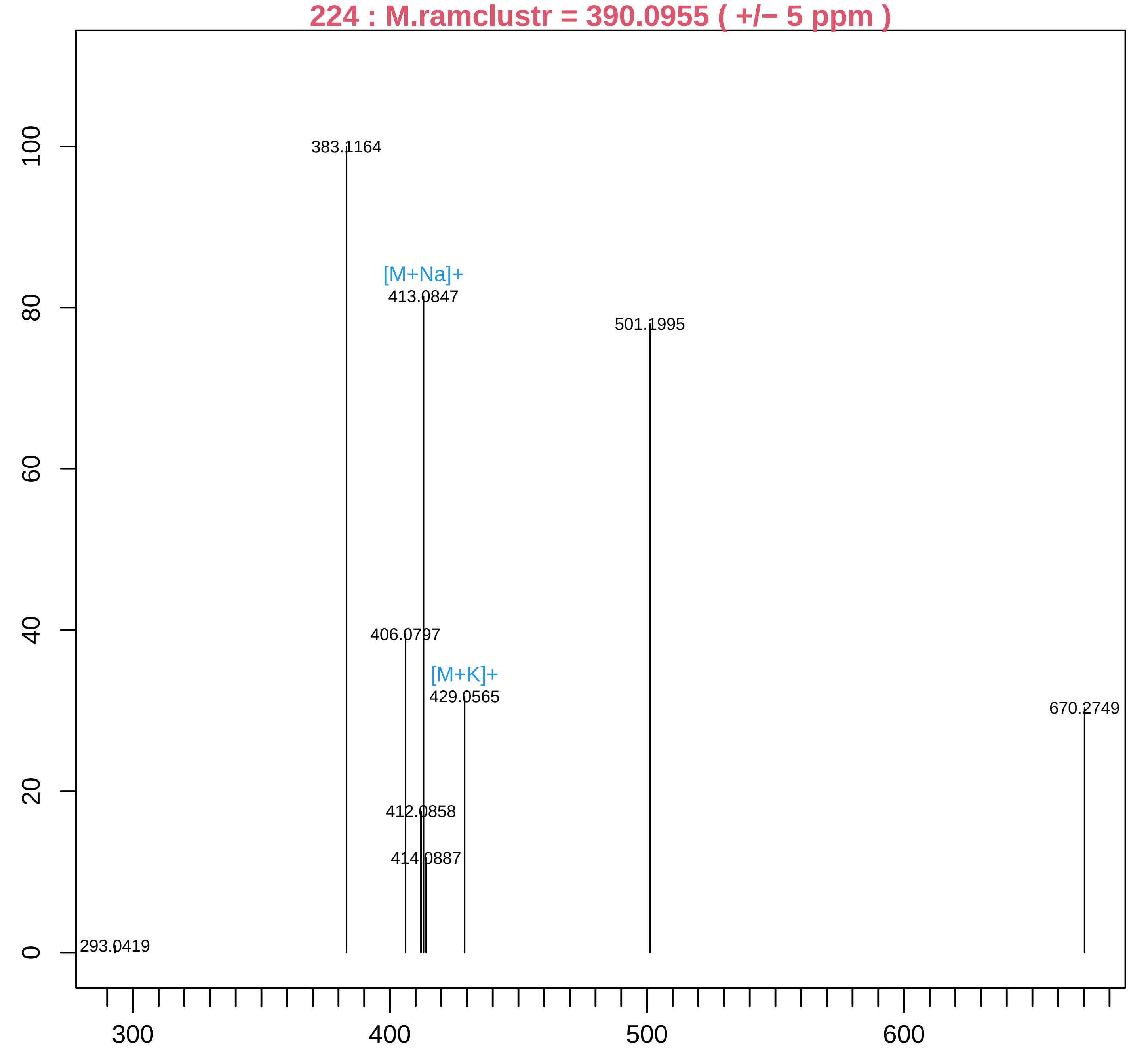

224 : M.ramclustr = 390.0955 (+/− 5 ppm)
383.1164
[M+Na]+
413.0847
501.1995
406.0797
[M+K]+
429.0565
670.2749
412.0858
414.0887
293.0419
0
20
40
60
80
100
300
400
500
600

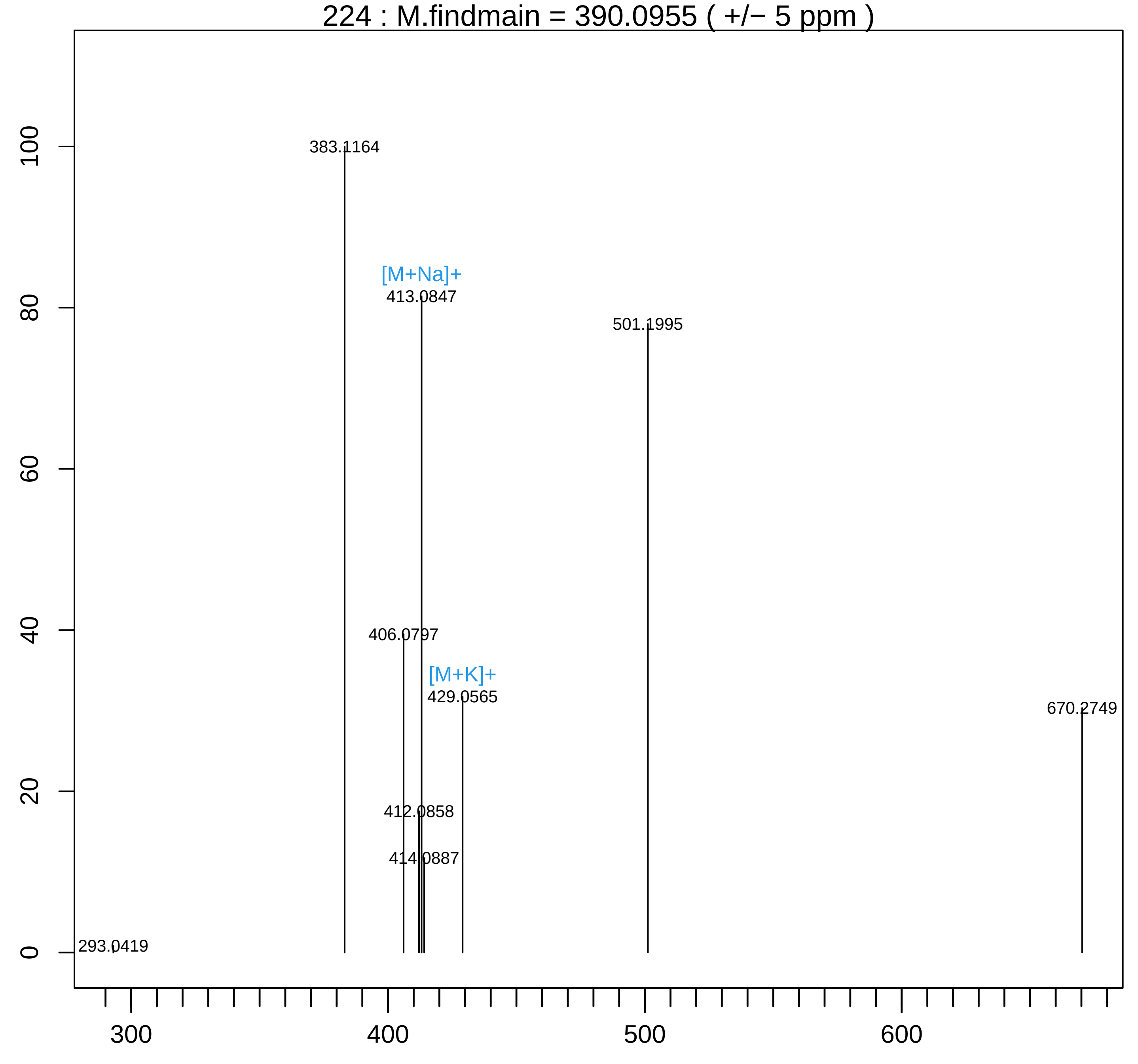

224 : M.findmain = 390.0955 (+/− 5 ppm)
383.1164
[M+Na]+
413.0847
501.1995
406.0797
[M+K]+
429.0565
670.2749
412.0858
414.0887
293.0419
0
20
40
60
80
100
300
400
500
600

225 : M.ramclustr = 711.9423 (+/− 0.2 ppm)

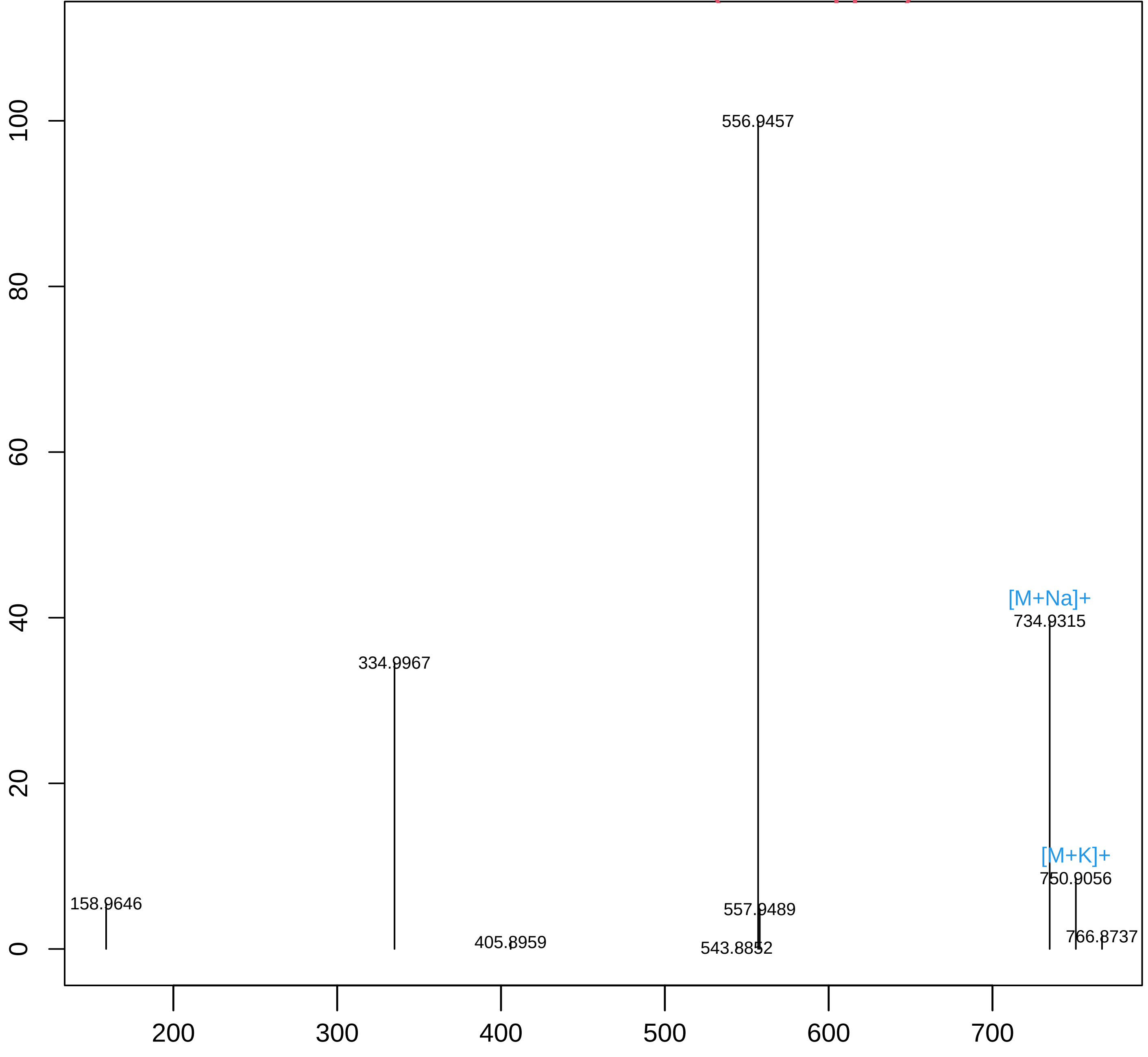

225 : M.findmain = 711.9423 (+/− 0.2 ppm)

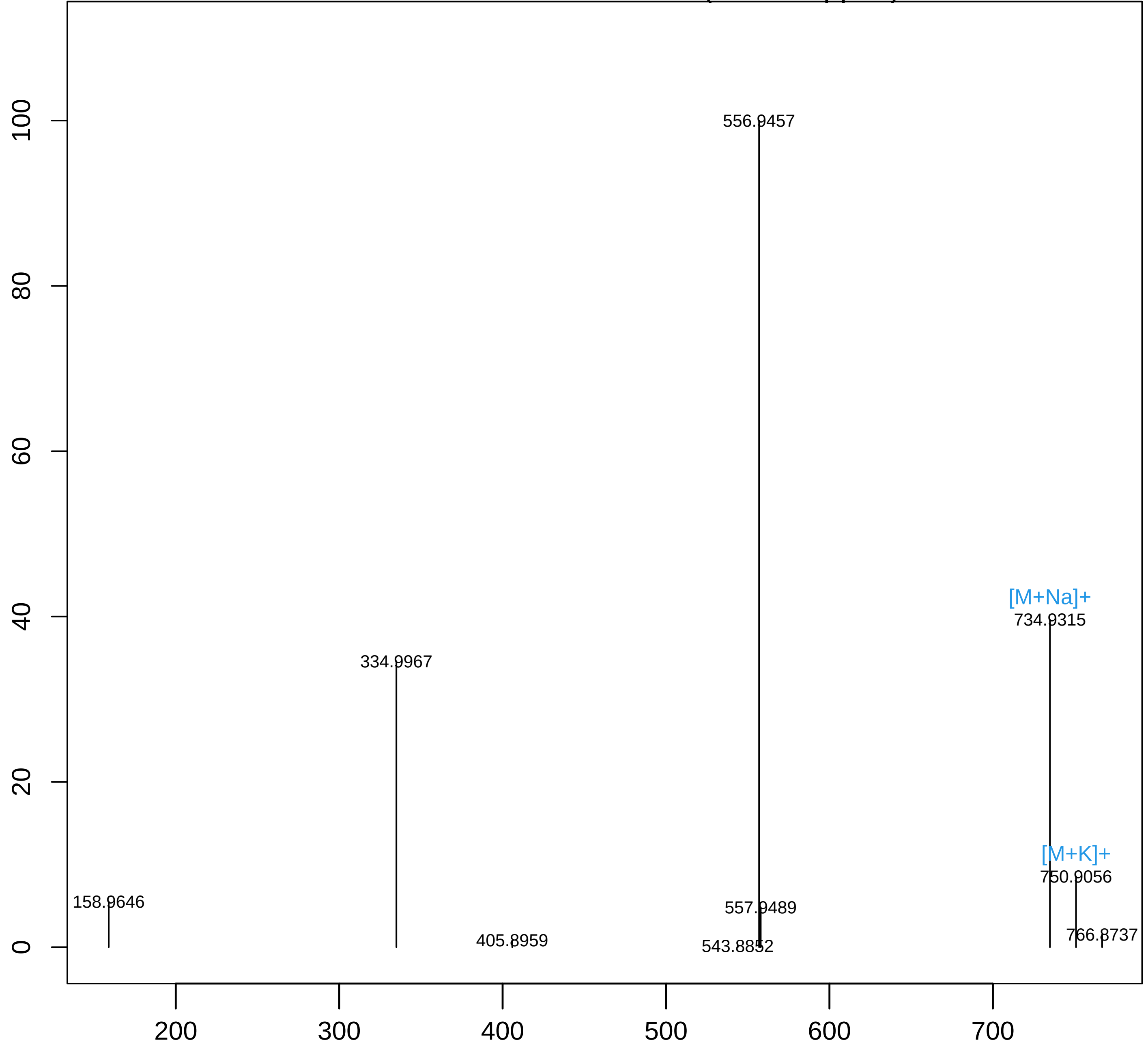

226 : M.ramclustr = 705.4453 (+/− NA ppm)

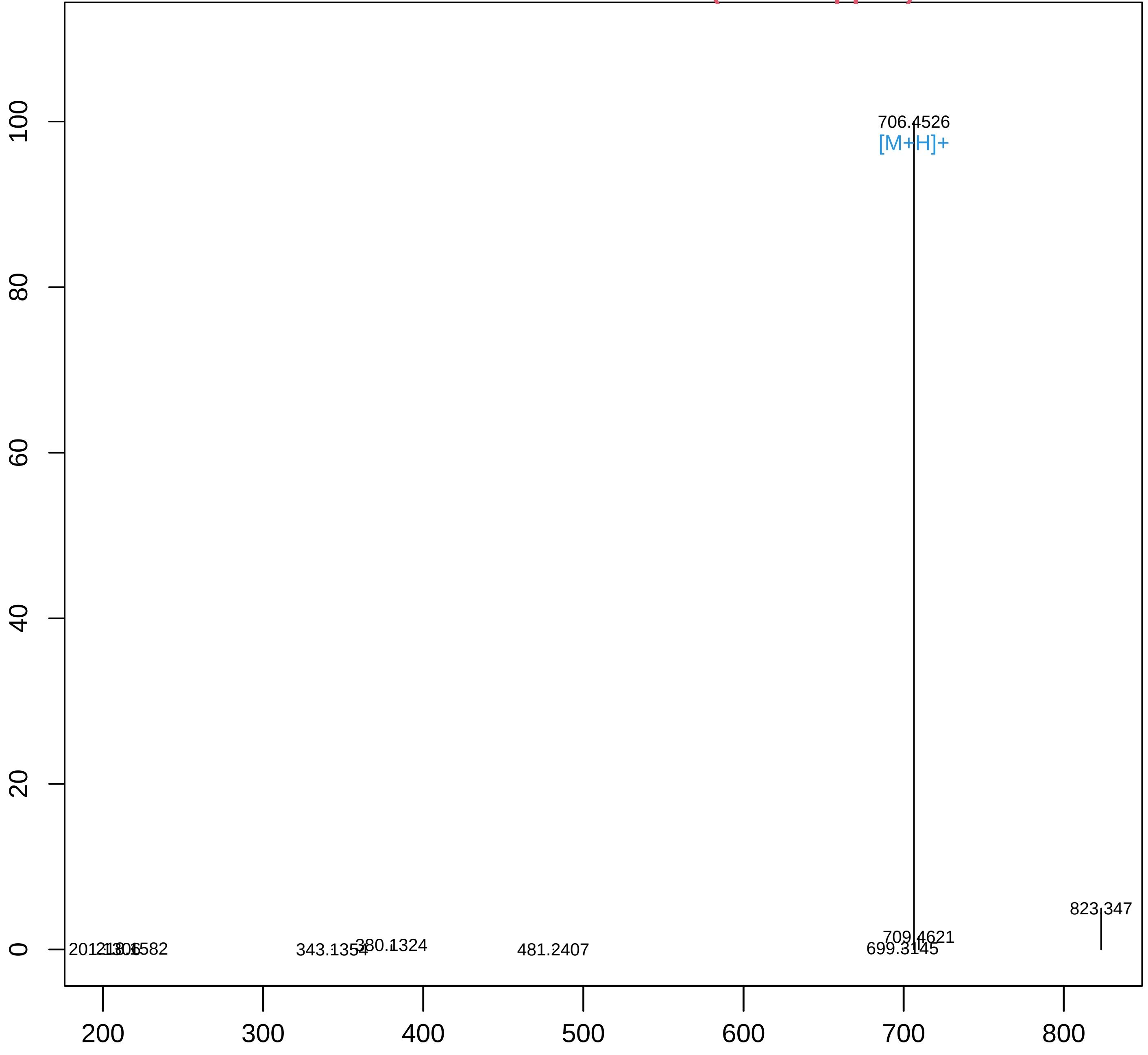

226 : M.findmain = 705.4453 (+/− 20 ppm)

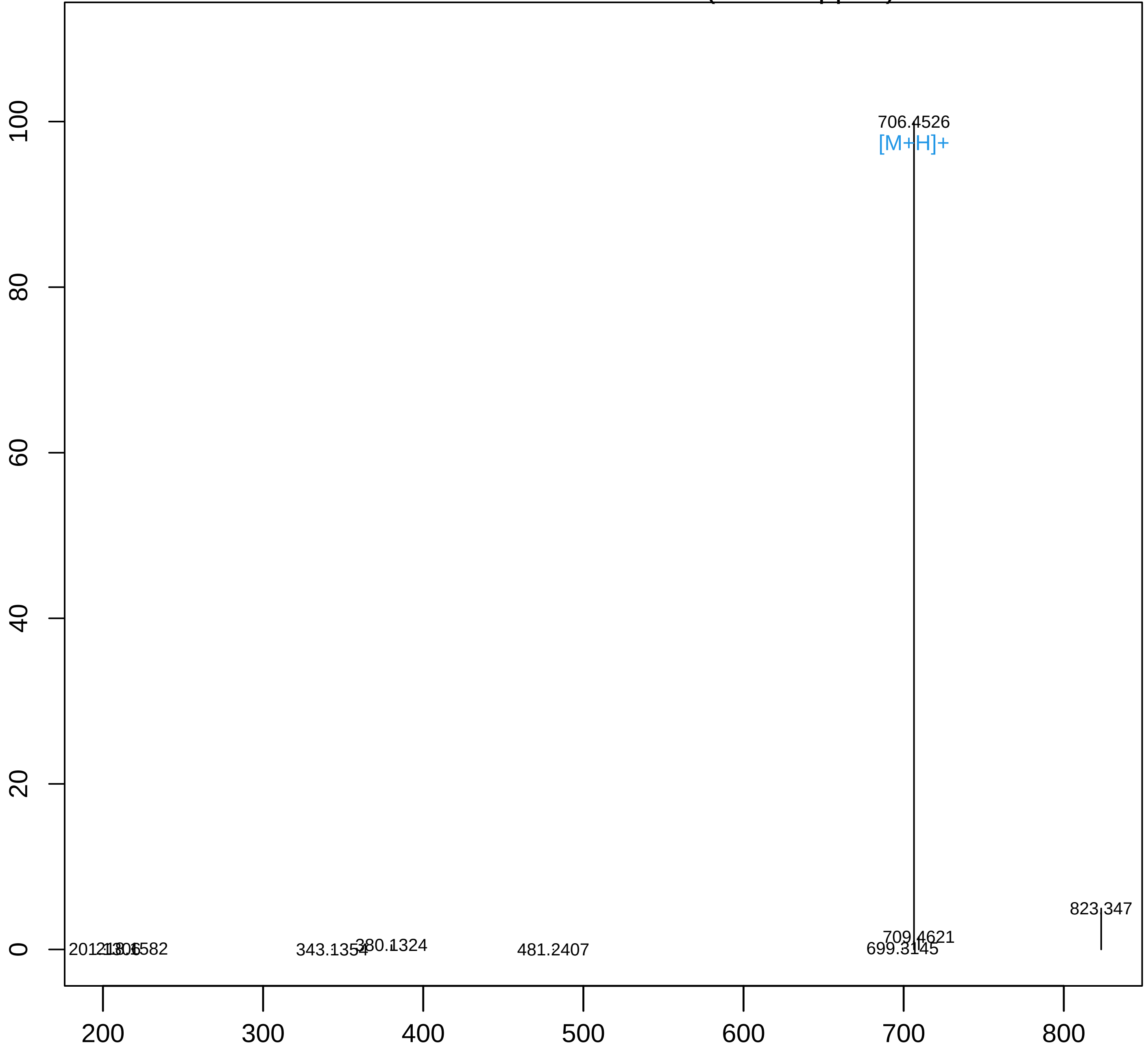

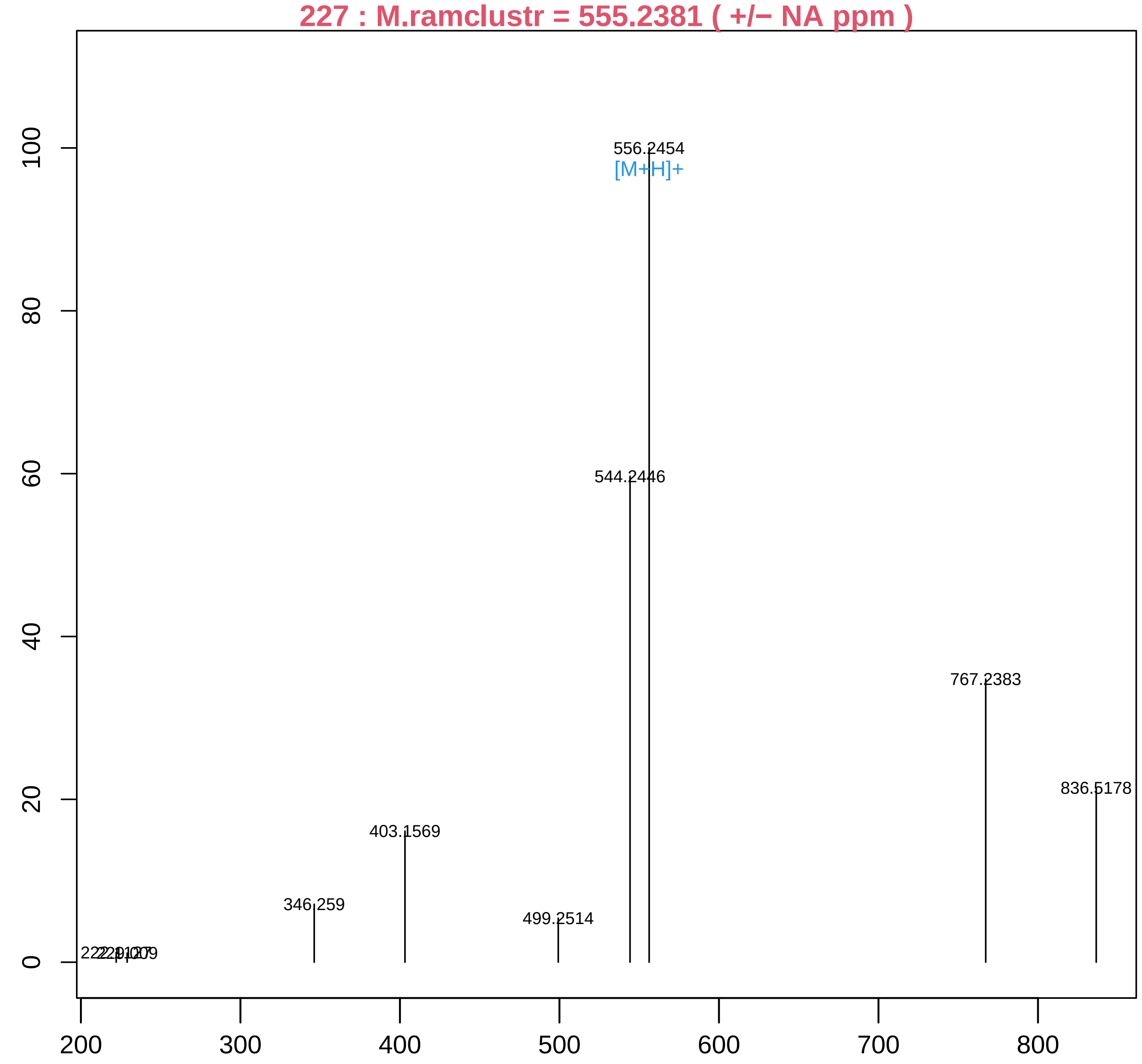
227 : M.ramclustr = 555.2381 (+/− NA ppm)
556.2454
[M+H]+
544.2446
767.2383
836.5178
403.1569
346.259
499.2514
0
20
40
60
80
100
200
300
400
500
600
700
800

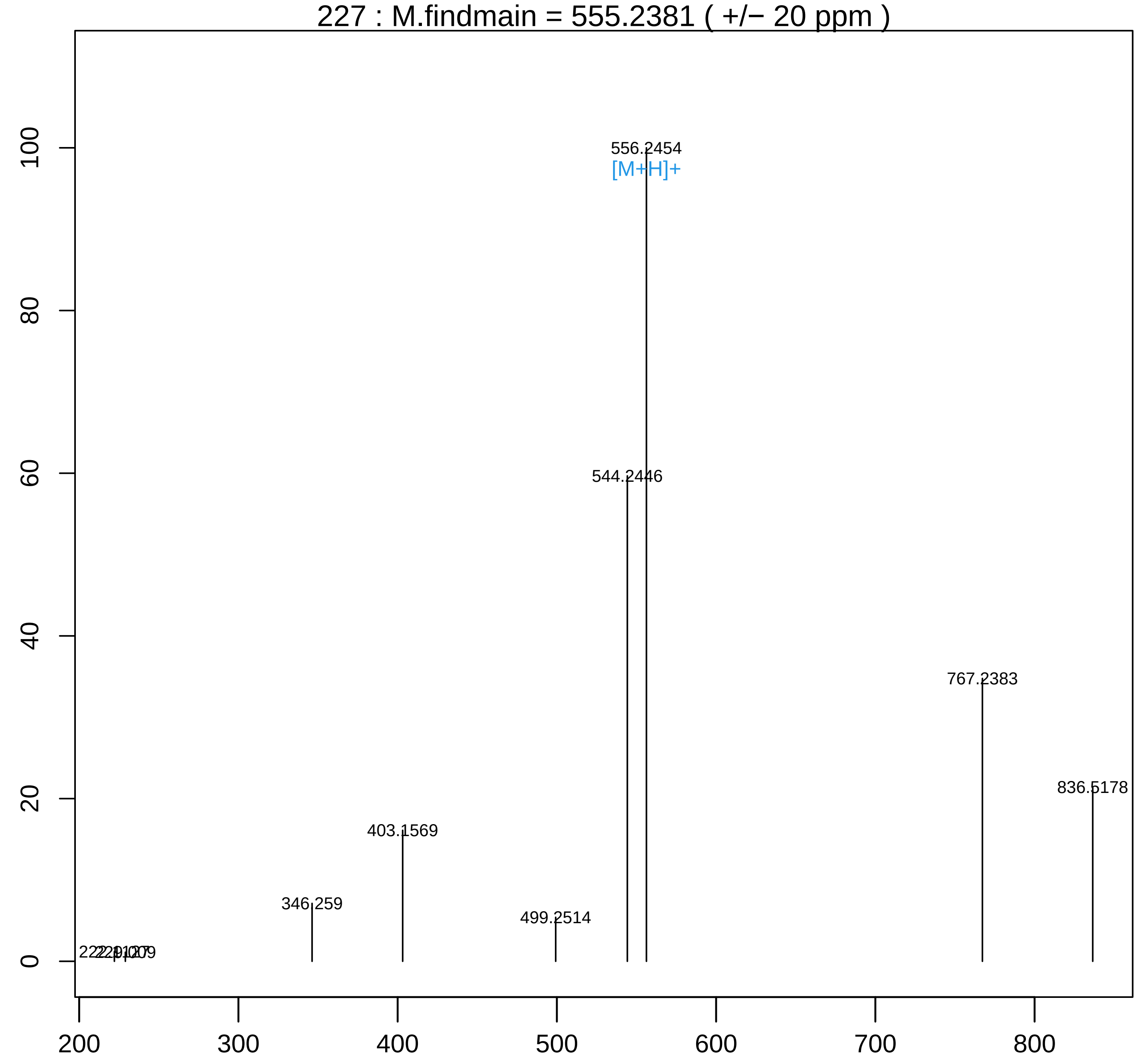
227 : M.findmain = 555.2381 (+/− 20 ppm)
556.2454
[M+H]+
544.2446
767.2383
836.5178
403.1569
346.259
499.2514
0
20
40
60
80
100
200
300
400
500
600
700
800

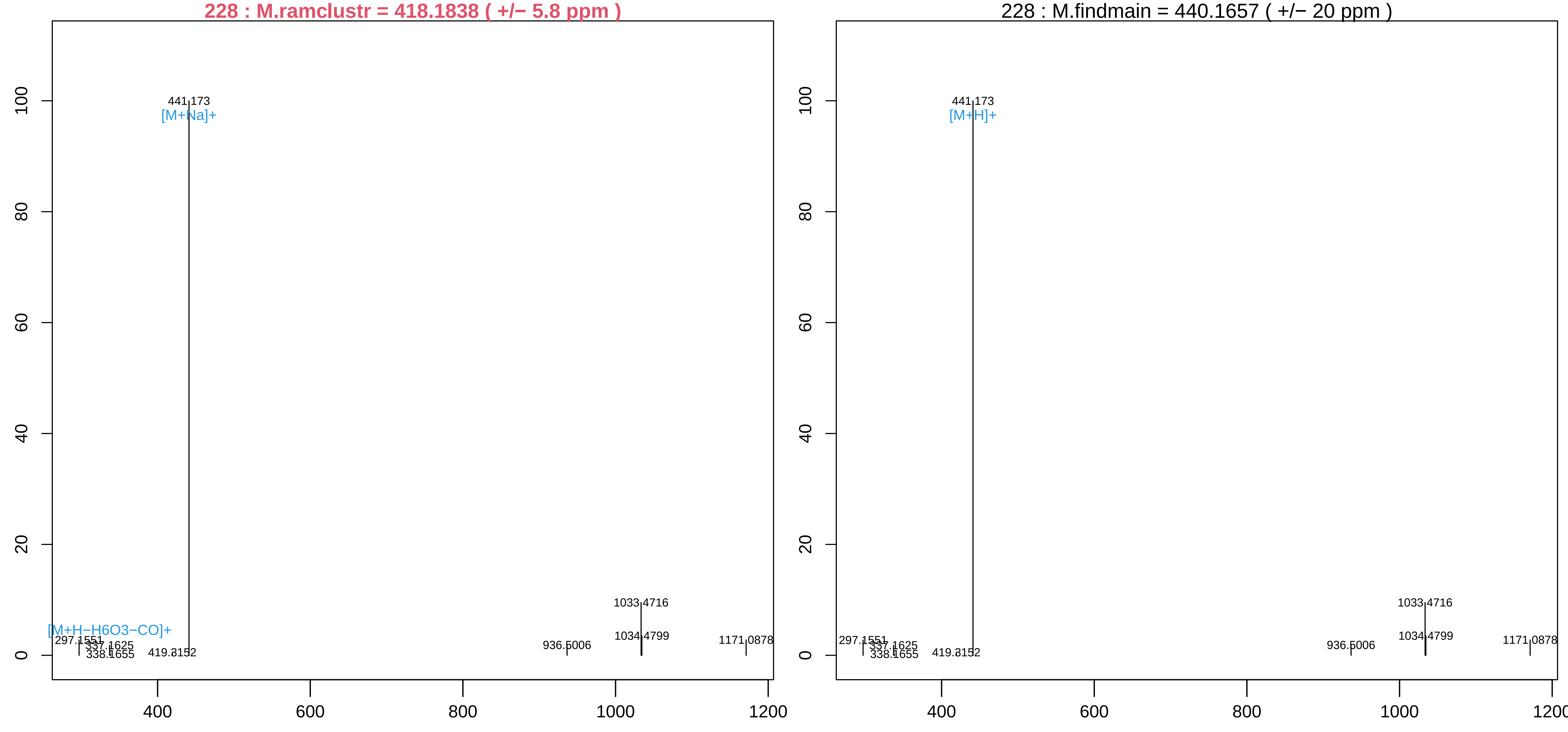
228 : M.ramclustr = 418.1838 (+/− 5.8 ppm)
441.173
[M+Na]+
[M+H−H6O3−CO]+
297.1551
337.1625
338.1655
419.8152
936.5006
1033.4716
1034.4799
1171.0878
0
20
40
60
80
100
400
600
800
1000
1200
228 : M.findmain = 440.1657 (+/− 20 ppm)
441.173
[M+H]+
297.1551
337.1625
338.1655
419.8152
936.5006
1033.4716
1034.4799
1171.0878
0
20
40
60
80
100
400
600
800
1000
1200

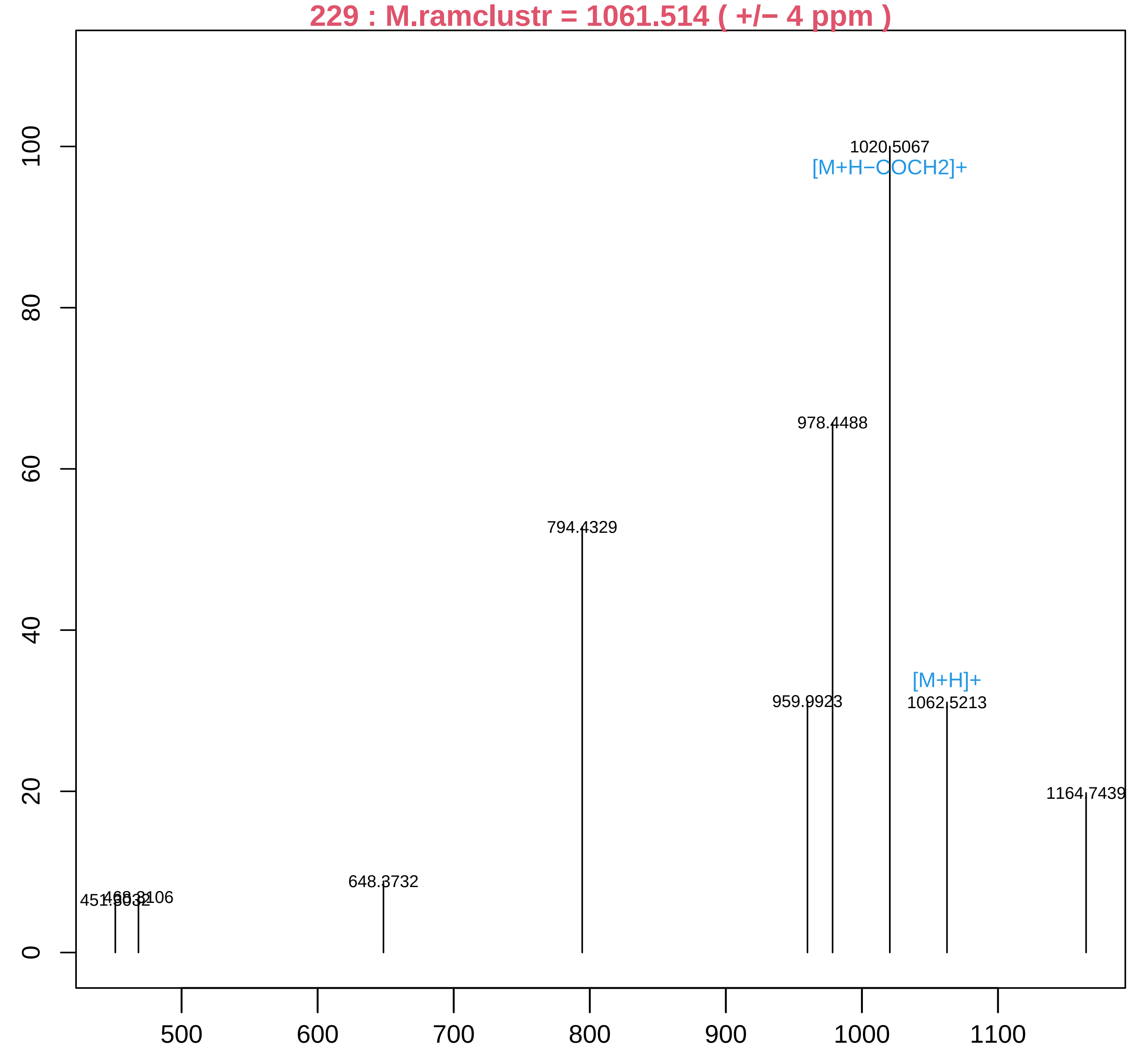
229 : M.ramclustr = 1061.514 (+/− 4 ppm)
1020.5067
[M+H−COCH2]+
978.4488
794.4329
959.9923
[M+H]+
1062.5213
1164.7439
648.3732
451.9032
468.3106
0
20
40
60
80
100
500
600
700
800
900
1000
1100

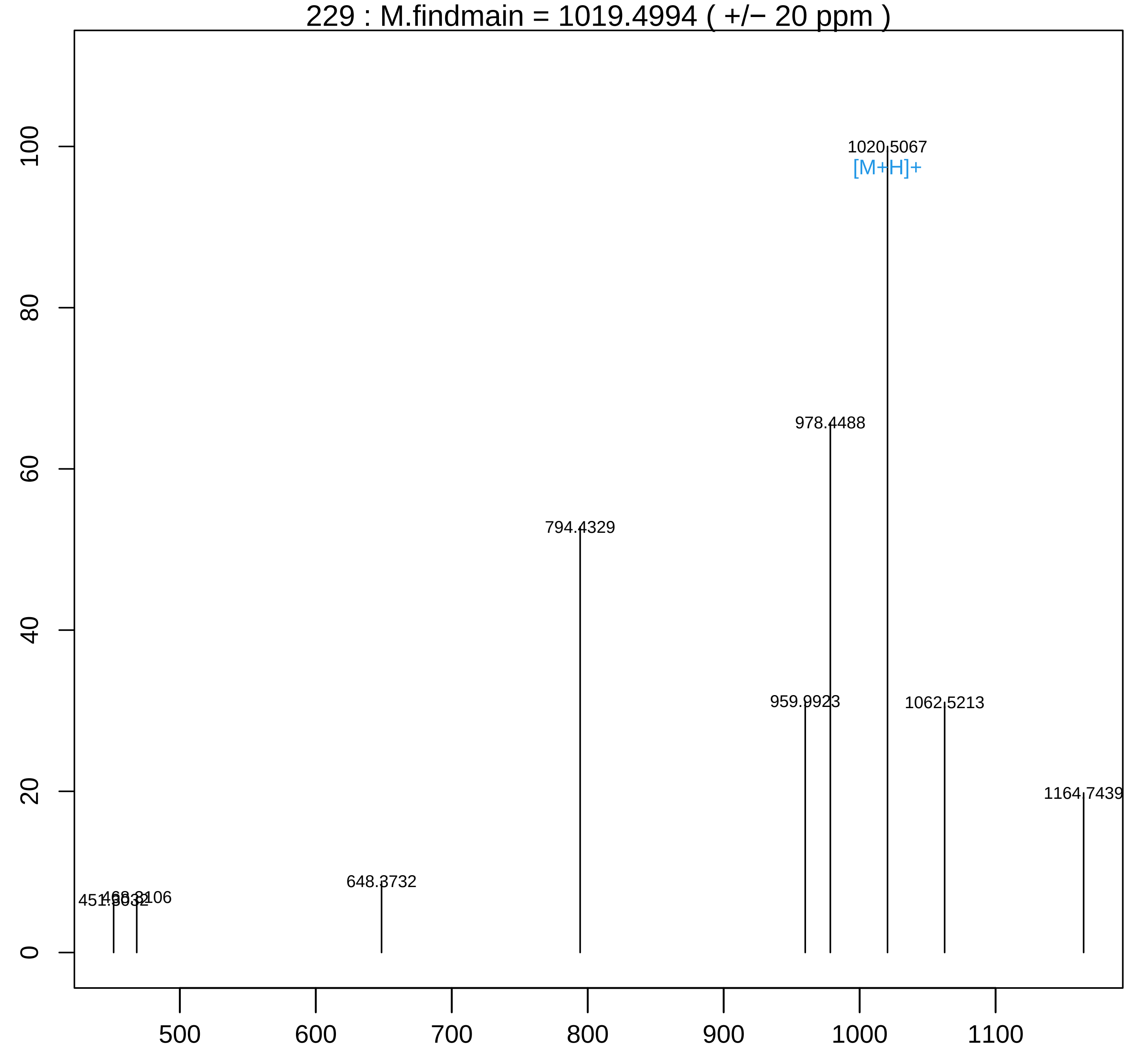
229 : M.findmain = 1019.4994 (+/− 20 ppm)
1020.5067
[M+H]+
978.4488
794.4329
959.9923
1062.5213
1164.7439
648.3732
451.9032
468.3106
0
20
40
60
80
100
500
600
700
800
900
1000
1100

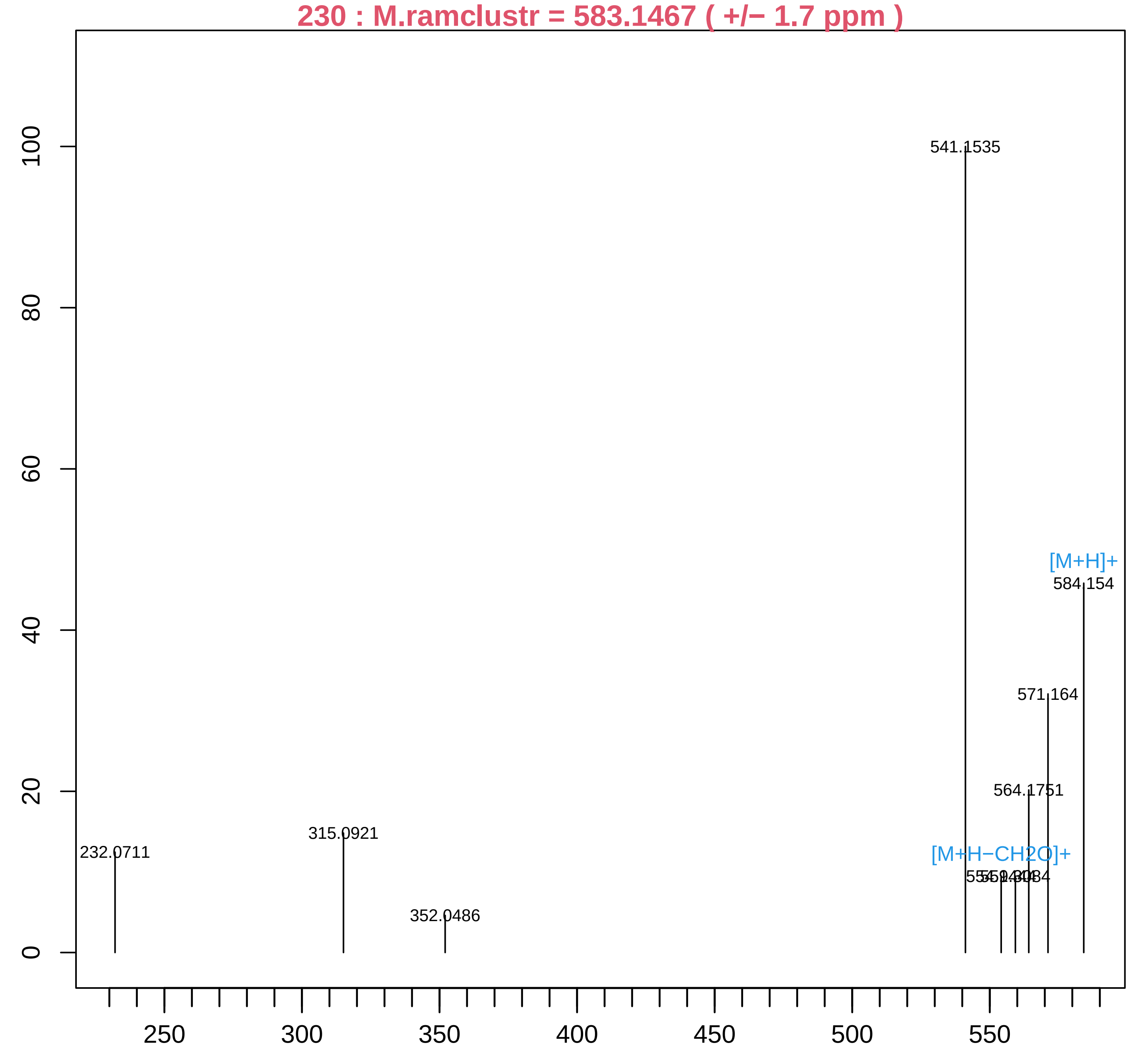
230 : M.ramclustr = 583.1467 (+/− 1.7 ppm)
541.1535
[M+H]+
584.154
571.164
564.1751
315.0921
232.0711
[M+H−CH2O]+
352.0486
0
20
40
60
80
100
250
300
350
400
450
500
550

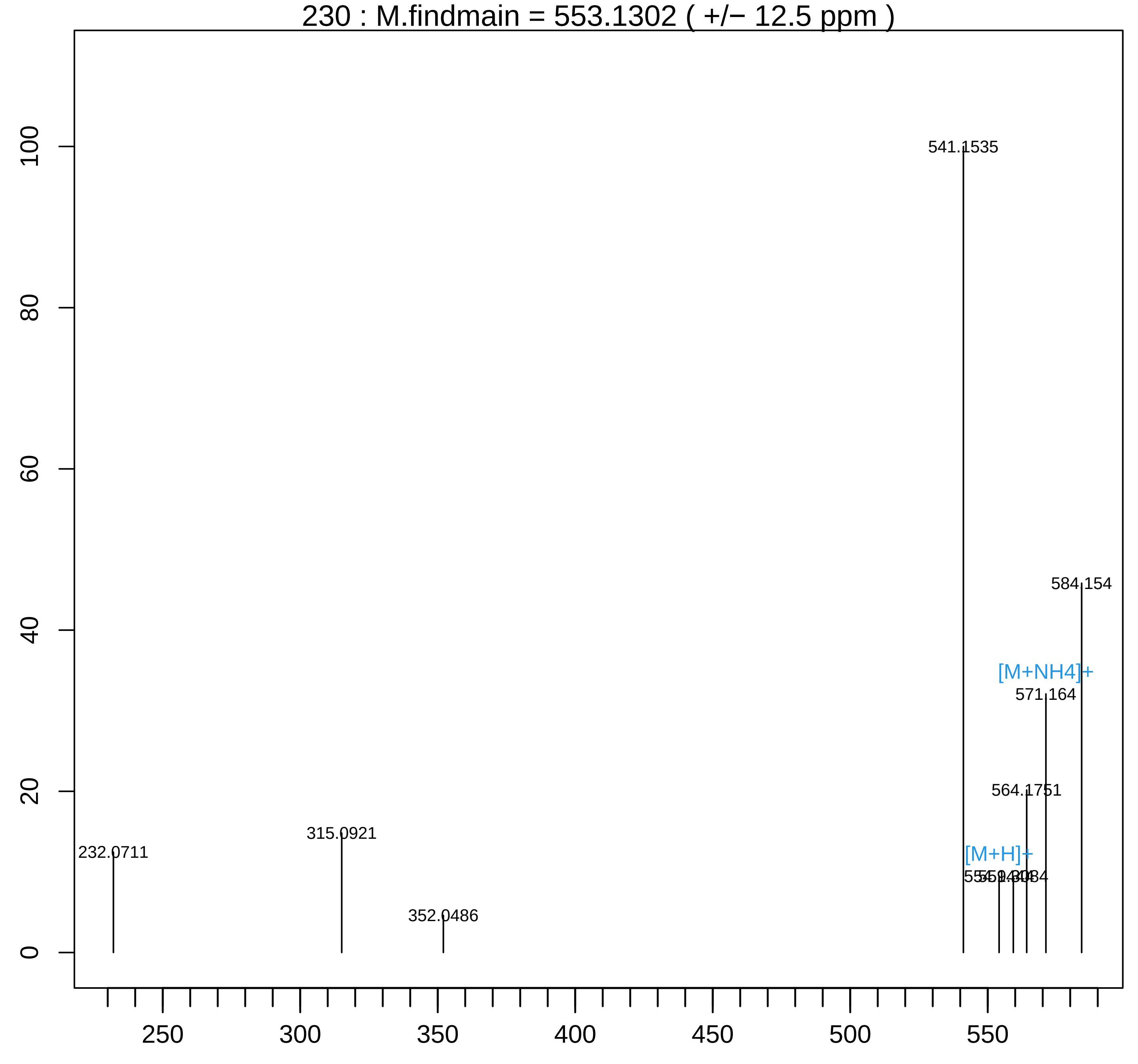
230 : M.findmain = 553.1302 (+/− 12.5 ppm)
541.1535
584.154
[M+NH4]+
571.164
564.1751
315.0921
232.0711
[M+H]+
352.0486
0
20
40
60
80
100
250
300
350
400
450
500
550

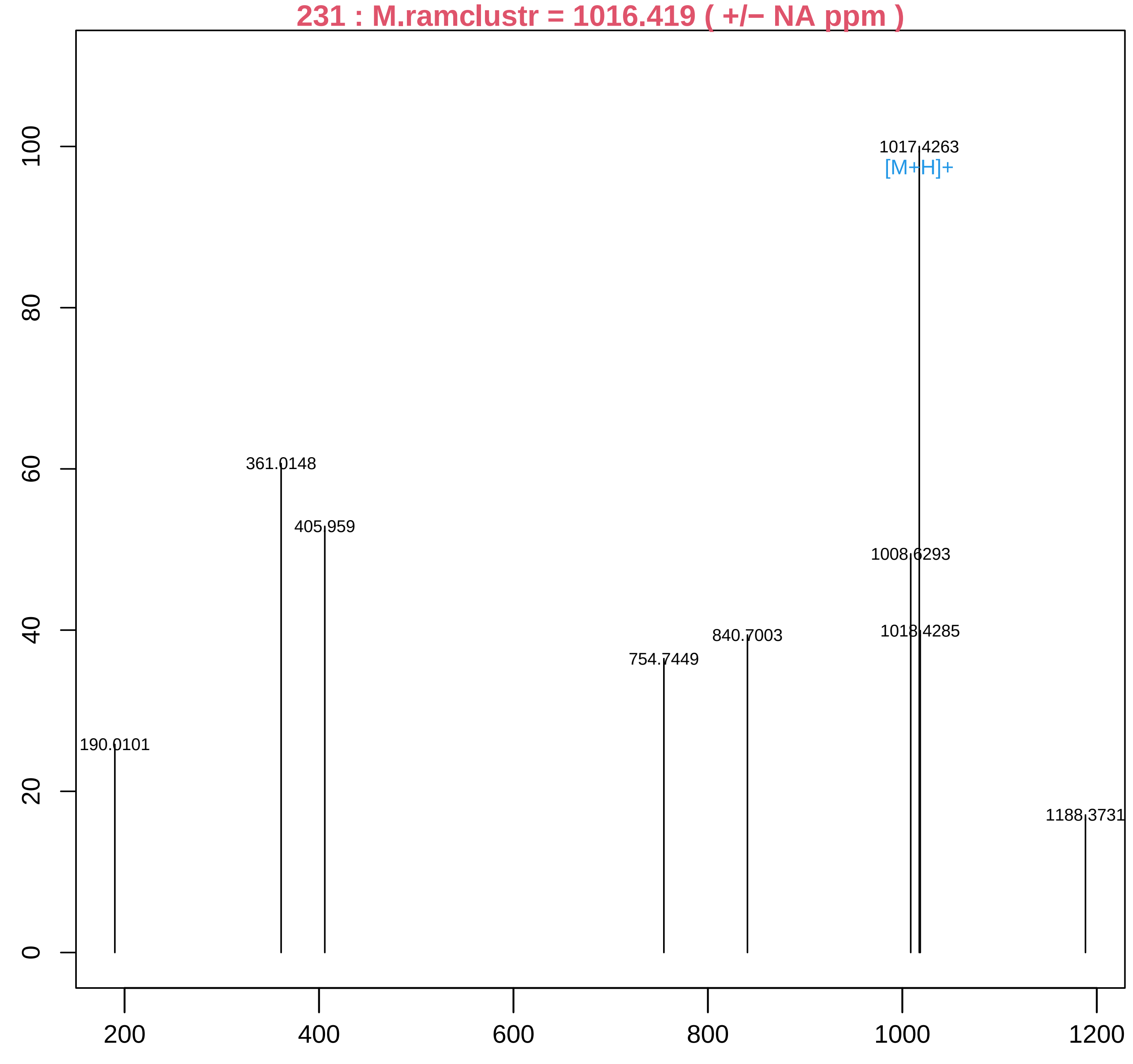
231 : M.ramclustr = 1016.419 (+/− NA ppm)
1017.4263
[M+H]+
361.0148
405.959
1008.6293
840.7003
1018.4285
754.7449
190.0101
1188.3731
0
20
40
60
80
100
200
400
600
800
1000
1200
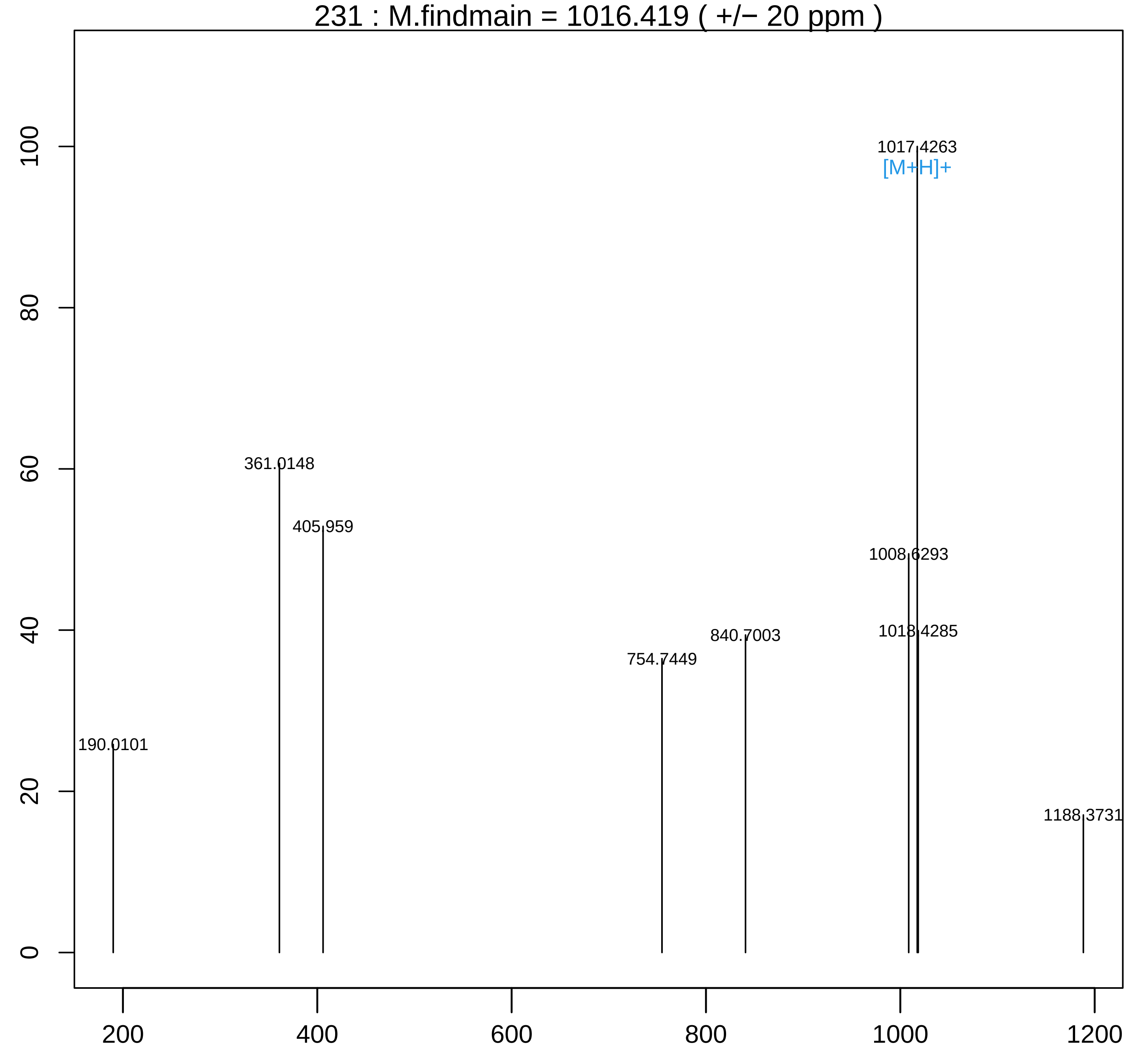
231 : M.findmain = 1016.419 (+/− 20 ppm)
1017.4263
[M+H]+
361.0148
405.959
1008.6293
840.7003
1018.4285
754.7449
190.0101
1188.3731
0
20
40
60
80
100
200
400
600
800
1000
1200

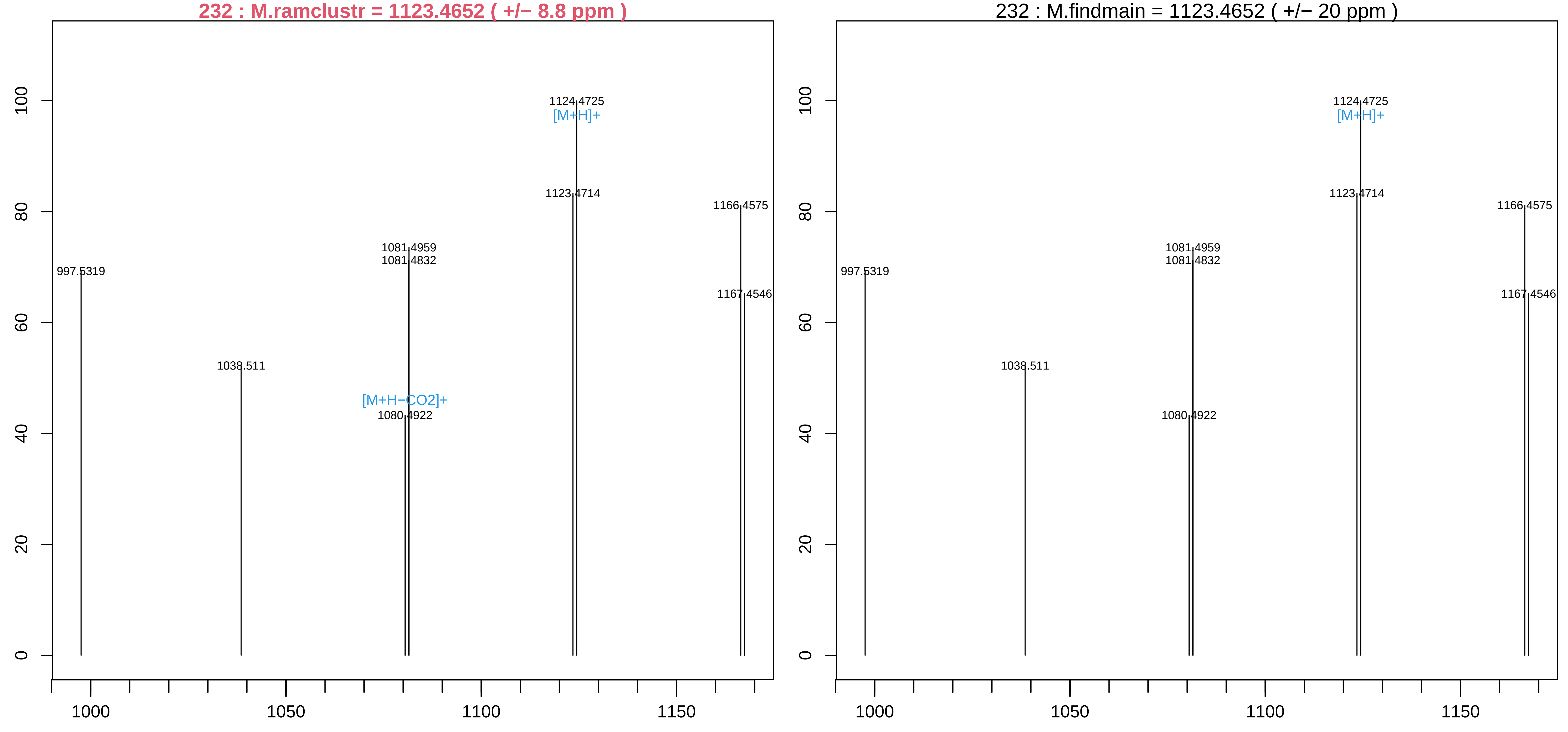
232 : M.ramclustr = 1123.4652 (+/− 8.8 ppm)
997.5319
1038.511
1081.4959
1081.4832
[M+H−CO2]+
1080.4922
1124.4725
[M+H]+
1123.4714
1166.4575
1167.4546
232 : M.findmain = 1123.4652 (+/− 20 ppm)
997.5319
1038.511
1081.4959
1081.4832
1080.4922
1124.4725
[M+H]+
1123.4714
1166.4575
1167.4546
0
20
40
60
80
100
1000
1050
1100
1150

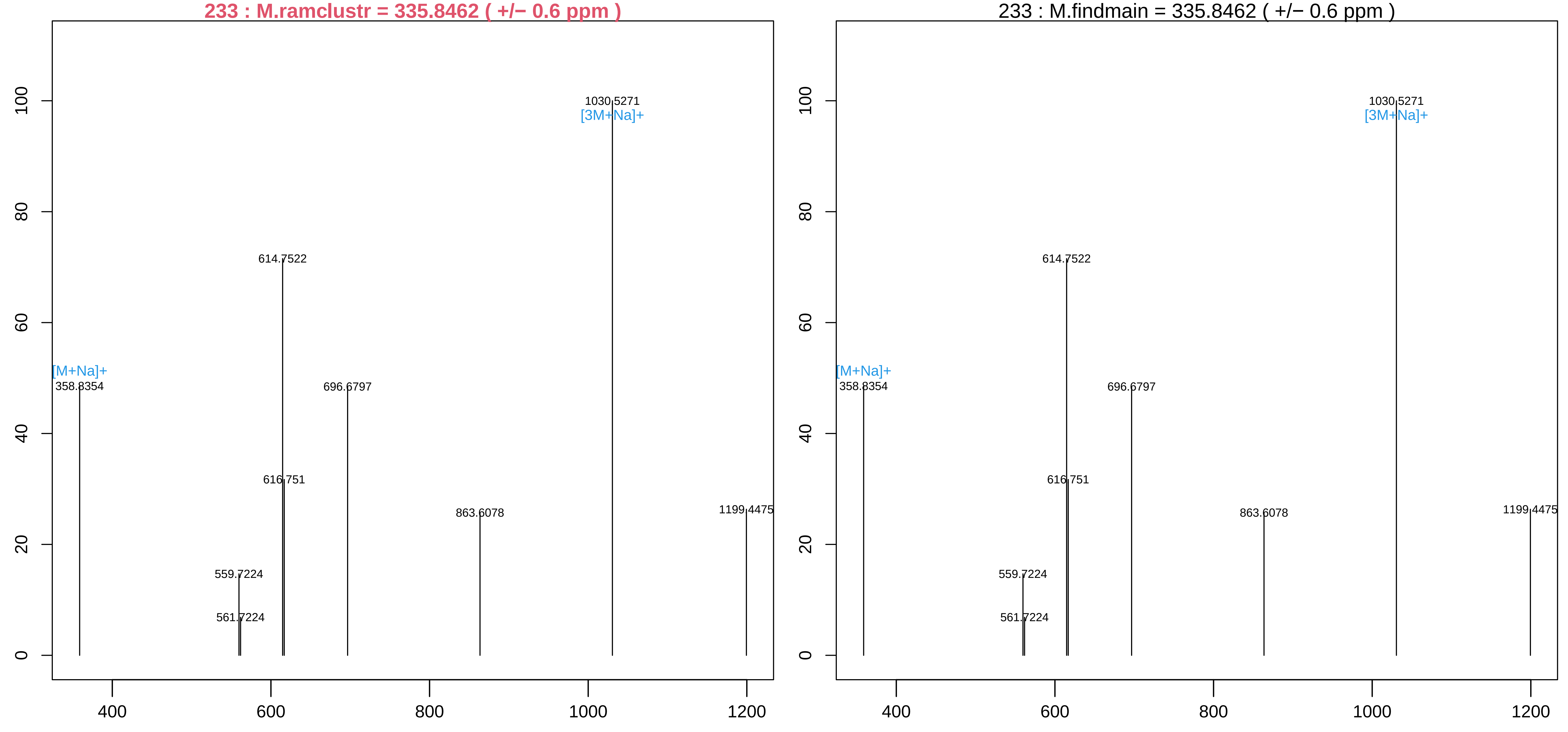

233 : M.ramclustr = 335.8462 (+/− 0.6 ppm)
233 : M.findmain = 335.8462 (+/− 0.6 ppm)
1030.5271
[3M+Na]+
614.7522
[M+Na]+
358.8354
696.6797
616.751
863.6078
1199.4475
559.7224
561.7224
0
20
40
60
80
100
400
600
800
1000
1200

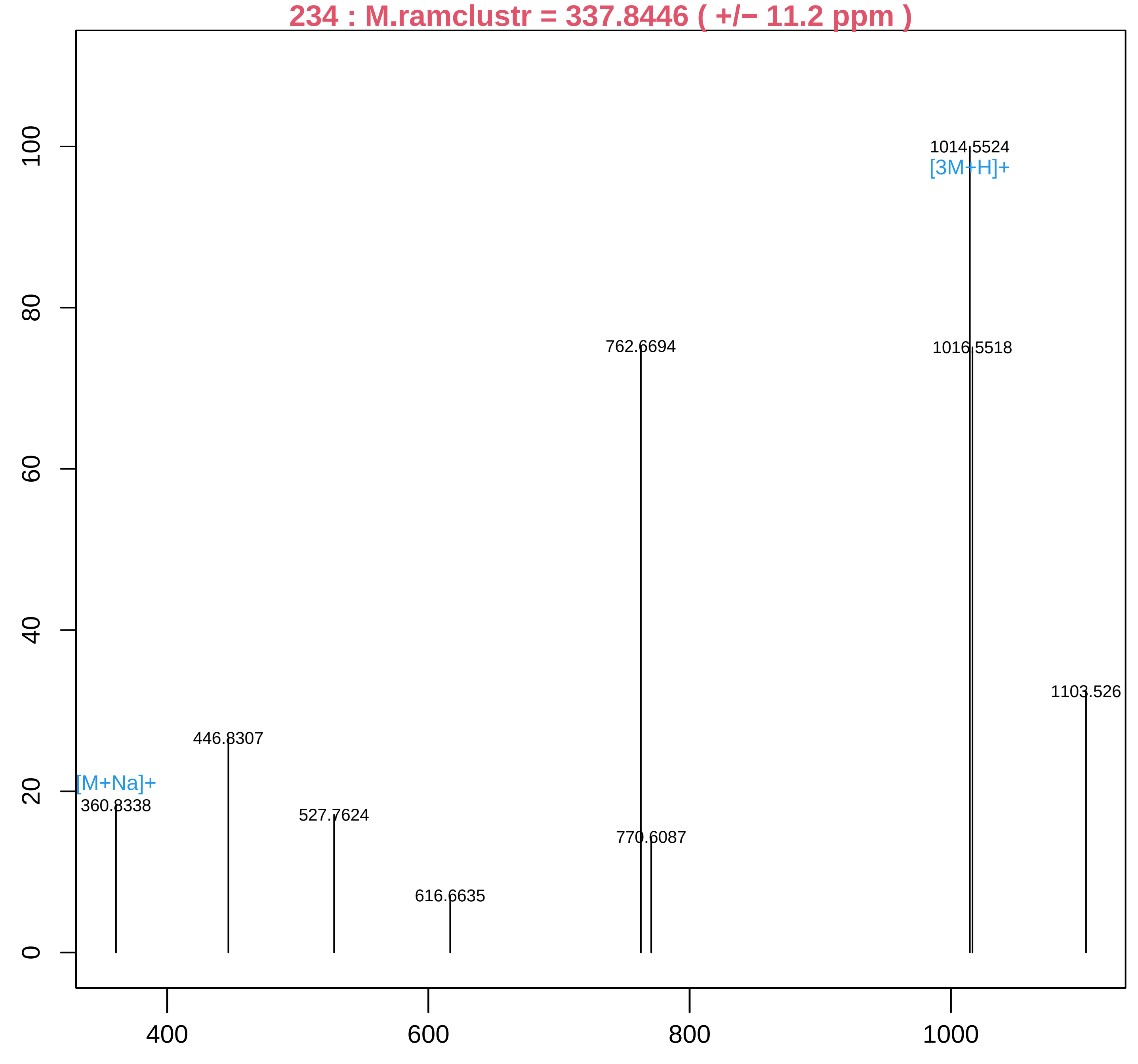
234 : M.ramclustr = 337.8446 (+/− 11.2 ppm)
1014.5524
[3M+H]+
1016.5518
762.6694
1103.526
446.8307
[M+Na]+
360.8338
527.7624
770.6087
616.6635
0
20
40
60
80
100
400
600
800
1000

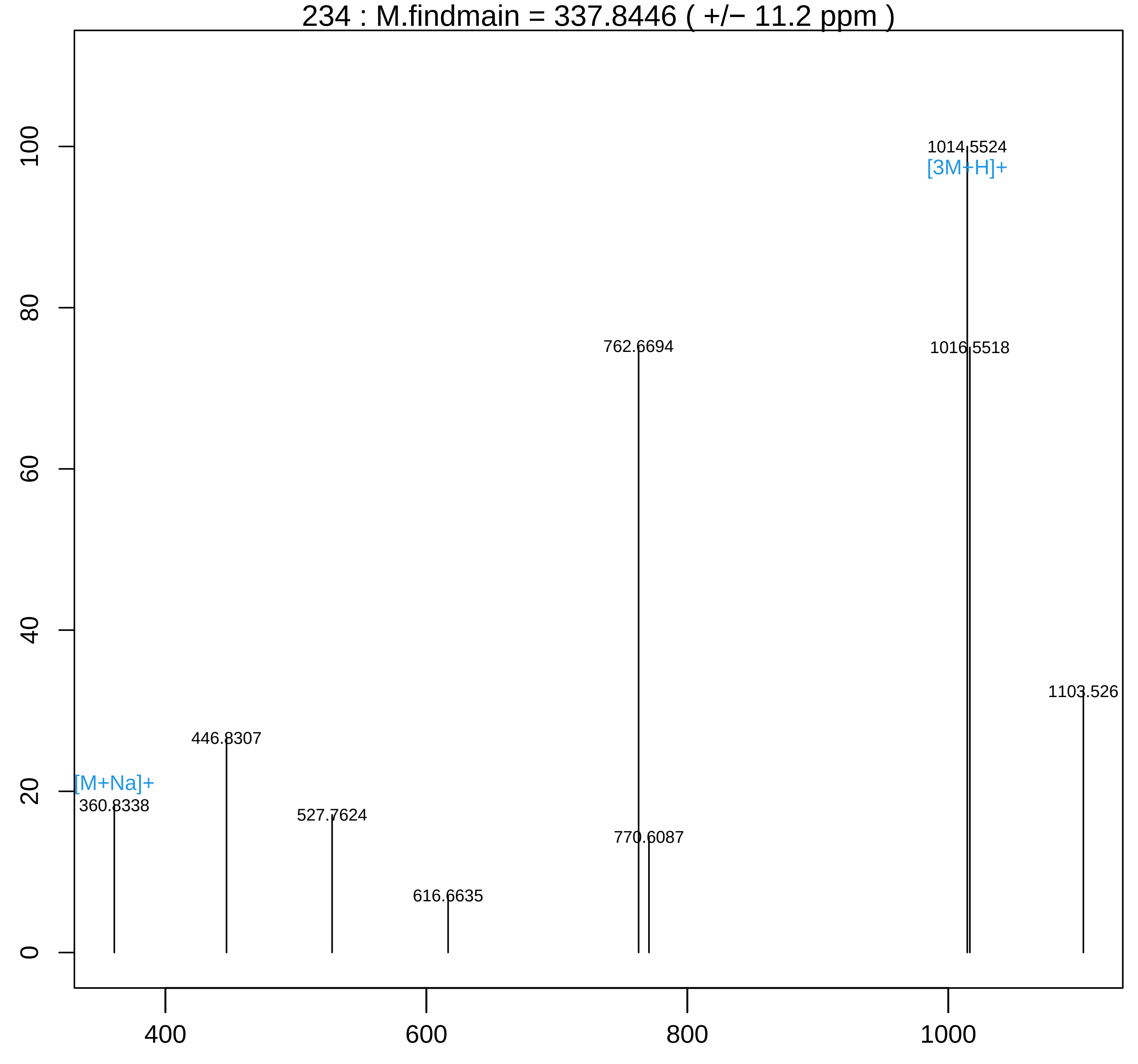
234 : M.findmain = 337.8446 (+/− 11.2 ppm)
1014.5524
[3M+H]+
1016.5518
762.6694
1103.526
446.8307
[M+Na]+
360.8338
527.7624
770.6087
616.6635
0
20
40
60
80
100
400
600
800
1000

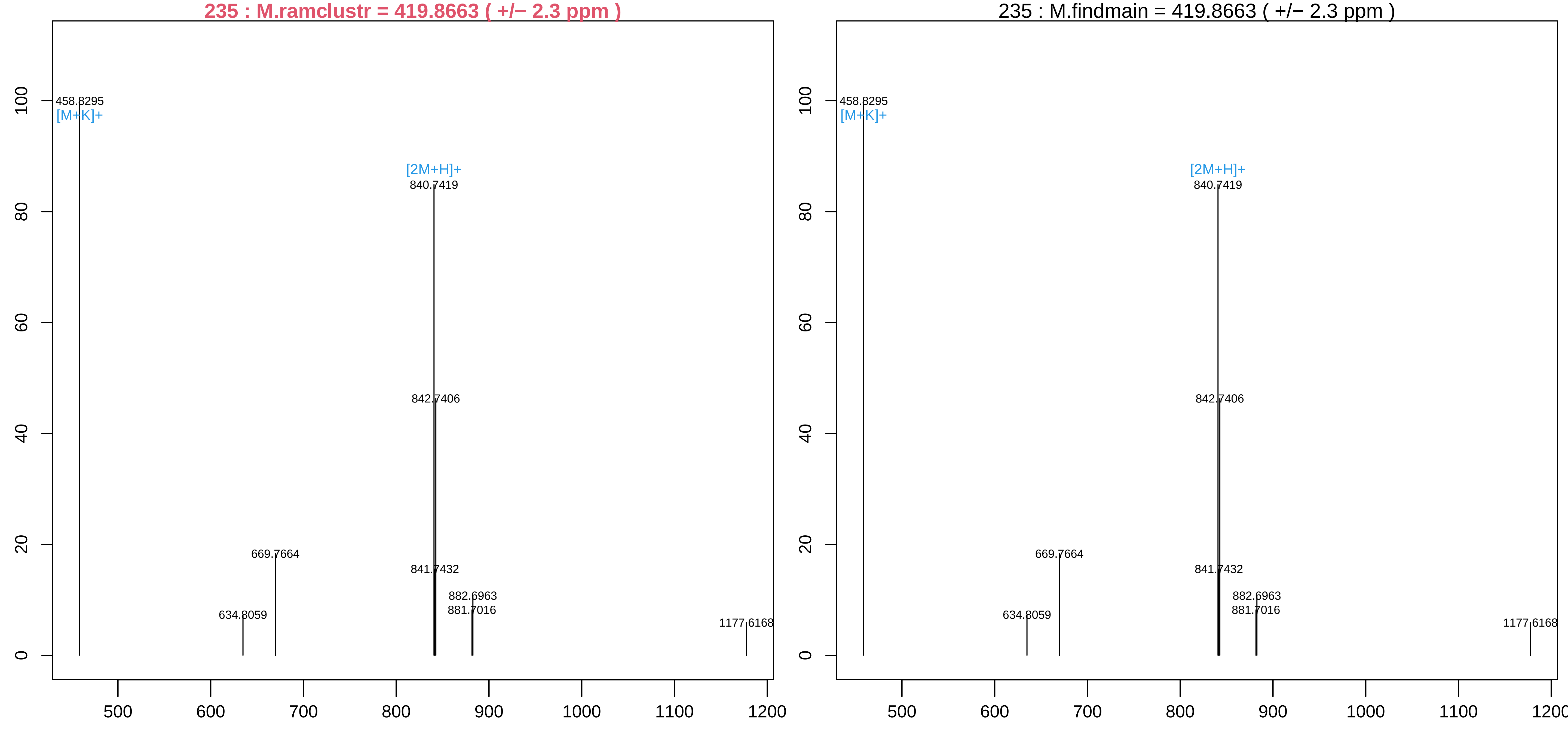

235 : M.ramclustr = 419.8663 (+/− 2.3 ppm)
458.8295
[M+K]+
[2M+H]+
840.7419
842.7406
669.7664
841.7432
882.6963
881.7016
634.8059
1177.6168
235 : M.findmain = 419.8663 (+/− 2.3 ppm)
458.8295
[M+K]+
[2M+H]+
840.7419
842.7406
669.7664
841.7432
882.6963
881.7016
634.8059
1177.6168

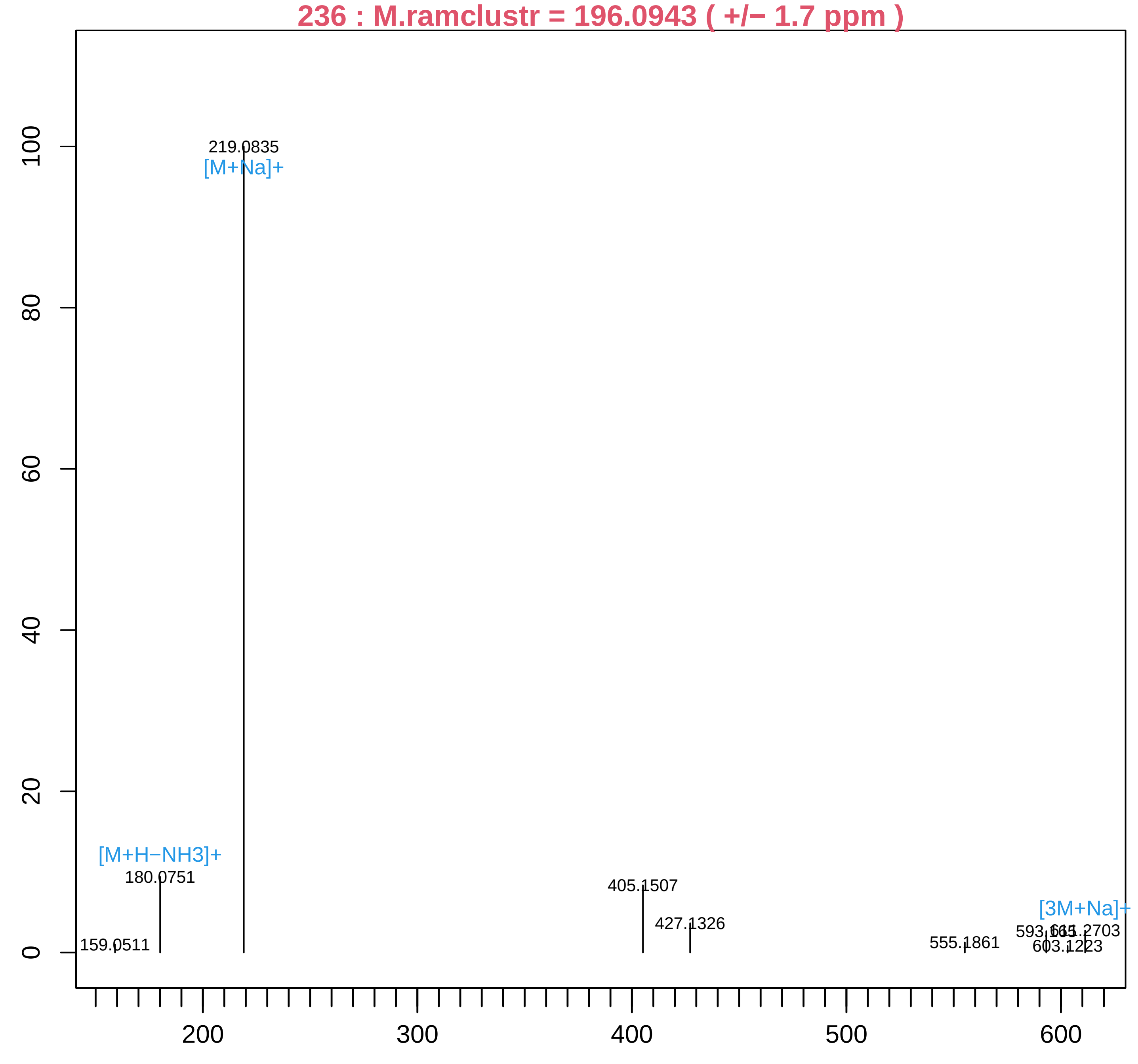
236 : M.ramclustr = 196.0943 (+/− 1.7 ppm)
219.0835
[M+Na]+
[M+H−NH3]+
180.0751
159.0511
405.1507
427.1326
555.1861
593.165
611.2703
603.1223
[3M+Na]+
0
20
40
60
80
100
200
300
400
500
600

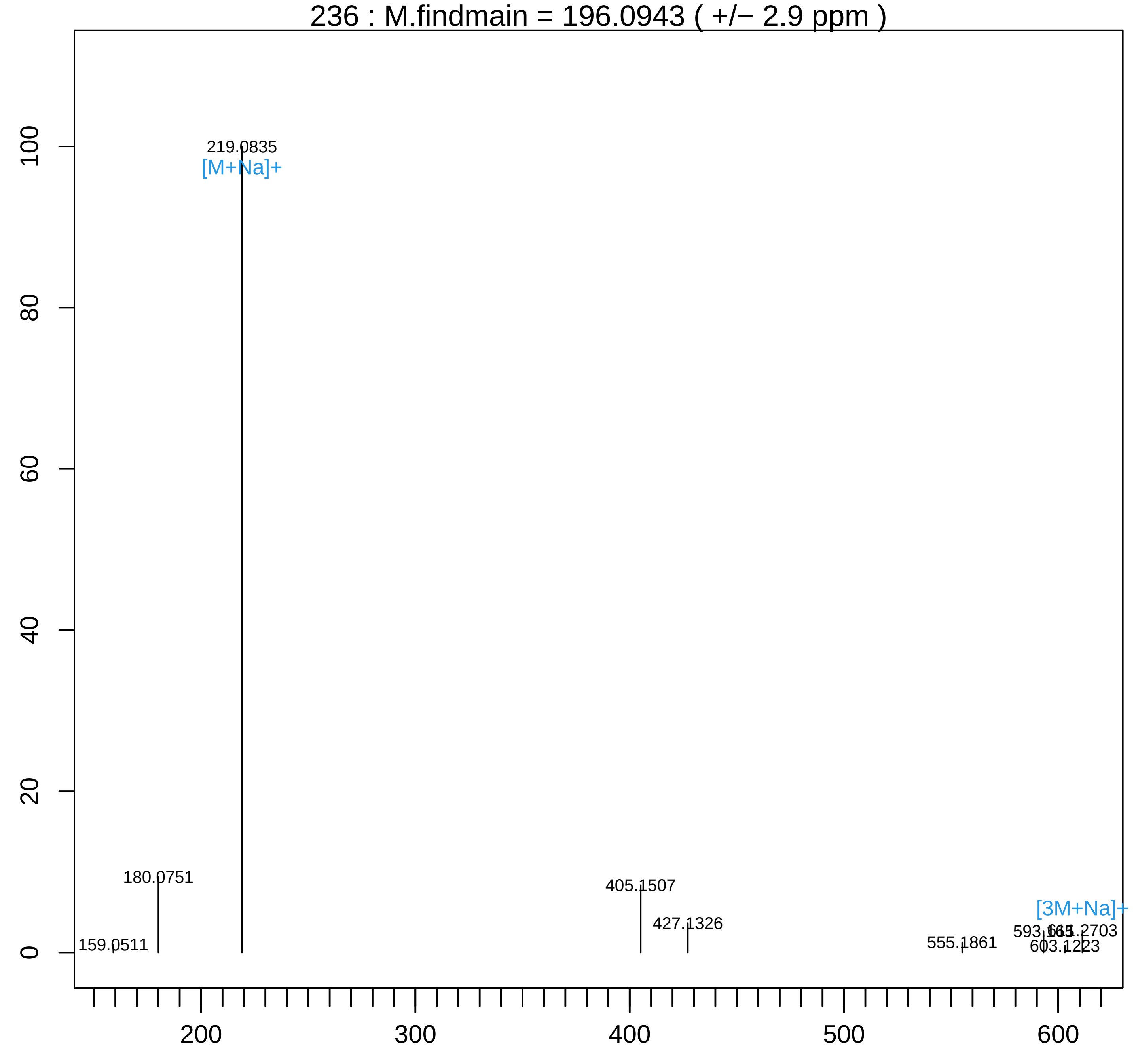
236 : M.findmain = 196.0943 (+/− 2.9 ppm)
219.0835
[M+Na]+
180.0751
159.0511
405.1507
427.1326
555.1861
593.165
611.2703
603.1223
[3M+Na]+
0
20
40
60
80
100
200
300
400
500
600

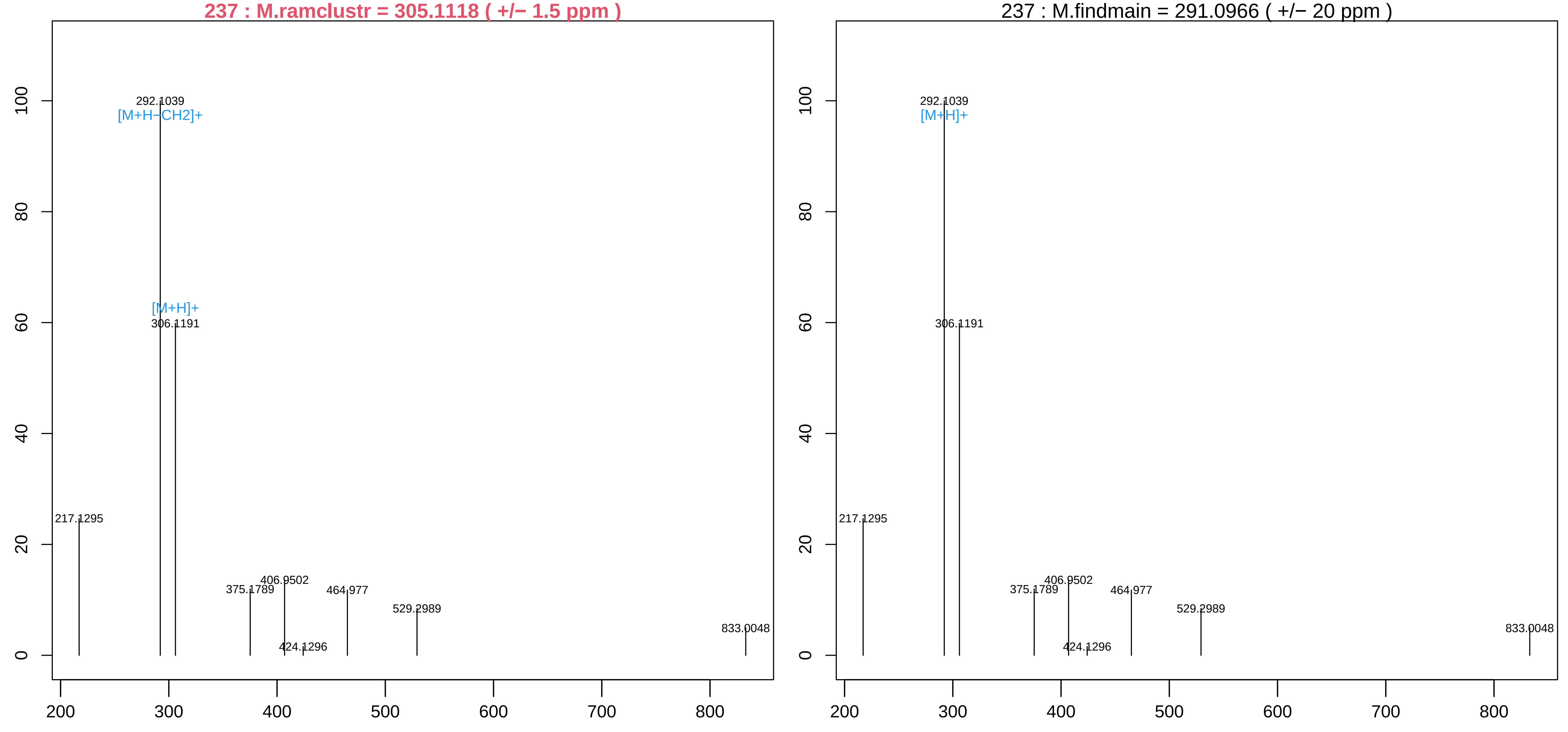

237 : M.ramclustr = 305.1118 (+/− 1.5 ppm)
292.1039
[M+H−CH2]+
[M+H]+
306.1191
217.1295
375.1789
406.9502
424.1296
464.977
529.2989
833.0048
237 : M.findmain = 291.0966 (+/− 20 ppm)
292.1039
[M+H]+
306.1191
217.1295
375.1789
406.9502
424.1296
464.977
529.2989
833.0048

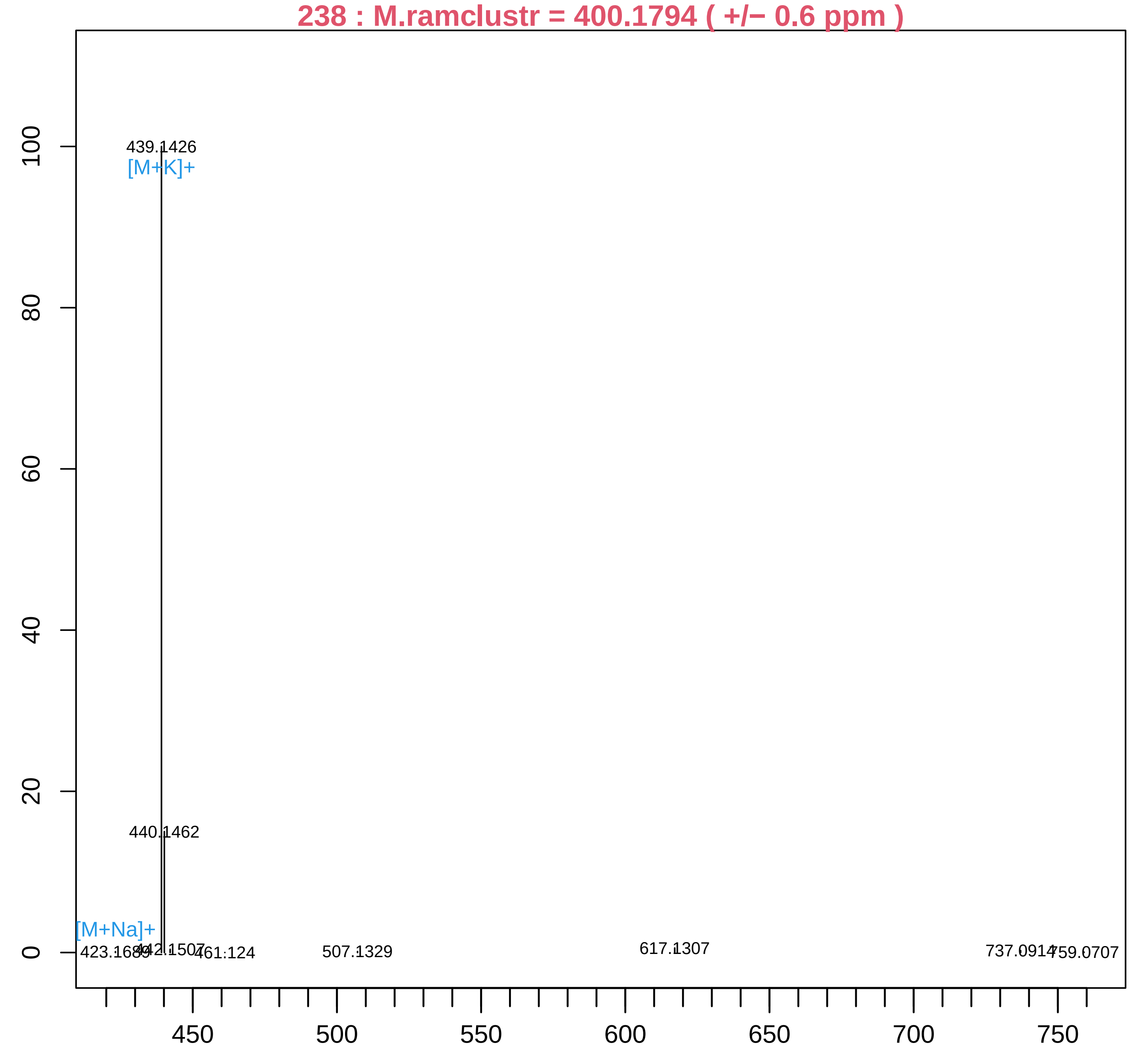

238 : M.ramclustr = 400.1794 (+/− 0.6 ppm)
439.1426
[M+K]+
440.1462
[M+Na]+
423.1689
442.1507
461.124
507.1329
617.1307
737.0914
759.0707
0
20
40
60
80
100
450
500
550
600
650
700
750

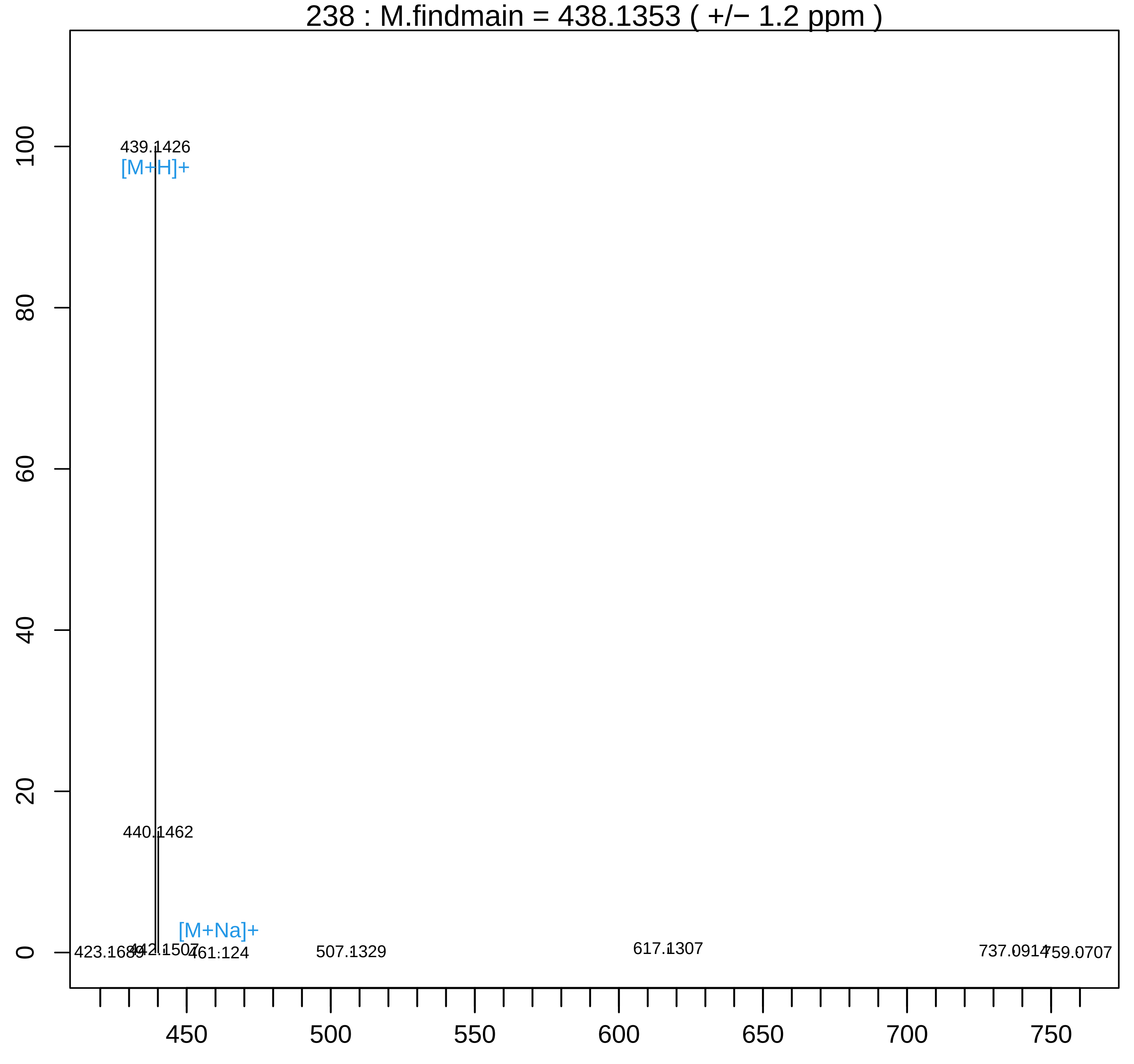

238 : M.findmain = 438.1353 (+/− 1.2 ppm)
439.1426
[M+H]+
440.1462
[M+Na]+
423.1689
442.1507
461.124
507.1329
617.1307
737.0914
759.0707
0
20
40
60
80
100
450
500
550
600
650
700
750

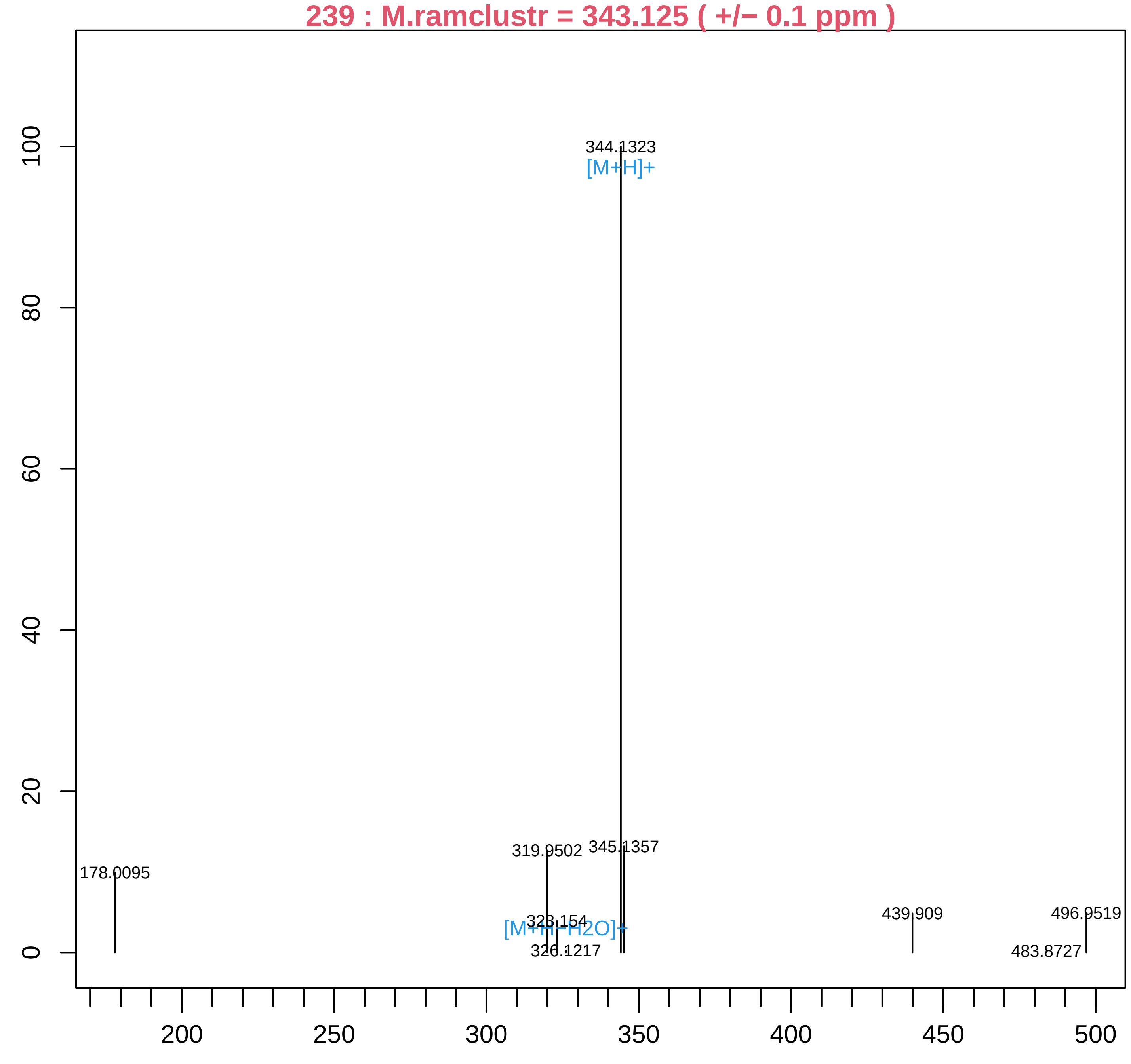
239 : M.ramclustr = 343.125 (+/− 0.1 ppm)
344.1323
[M+H]+
319.9502
345.1357
178.0095
323.154
[M+H−H2O]+
326.1217
439.909
496.9519
483.8727
0
20
40
60
80
100
200
250
300
350
400
450
500

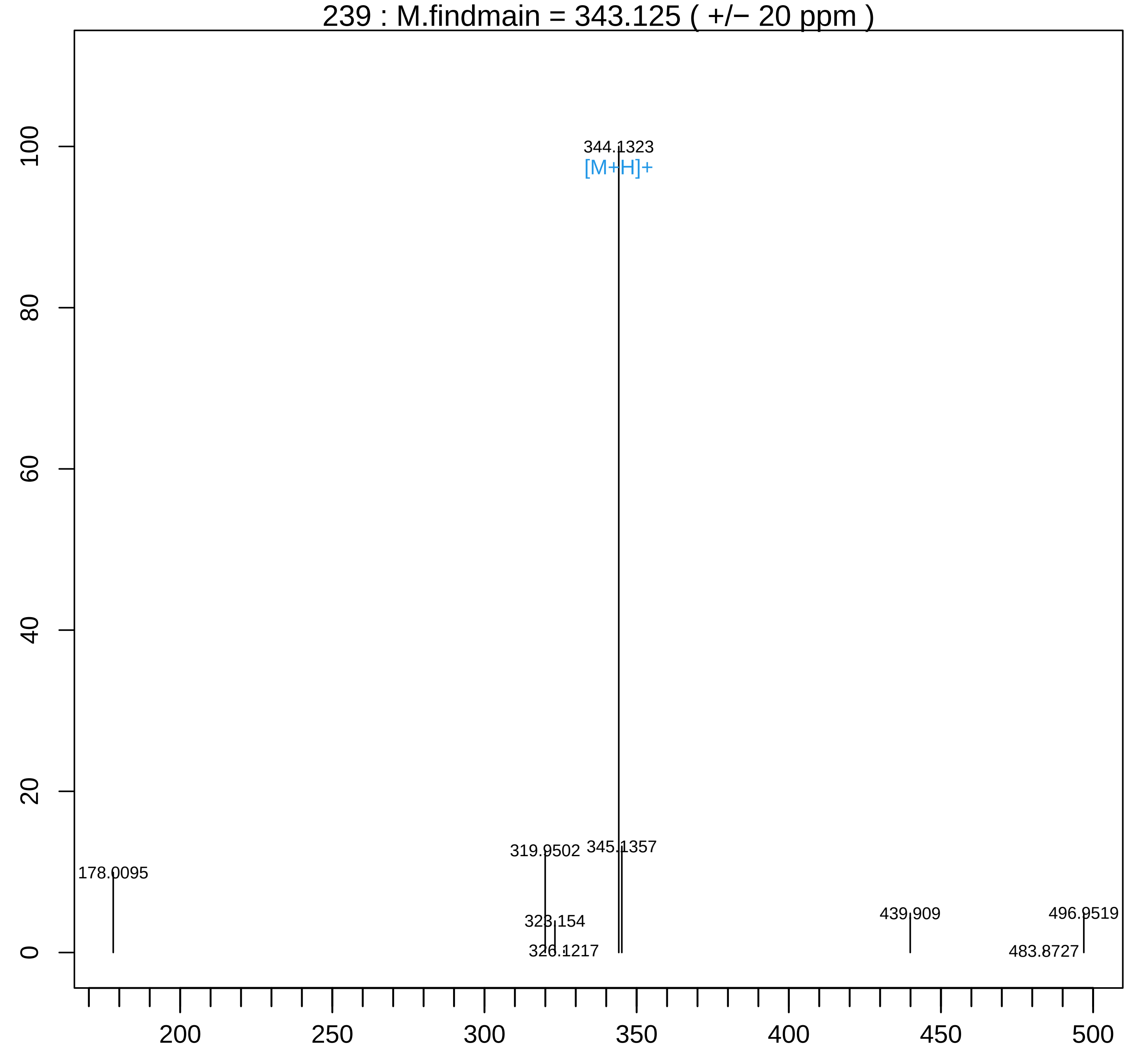
239 : M.findmain = 343.125 (+/− 20 ppm)
344.1323
[M+H]+
319.9502
345.1357
178.0095
323.154
326.1217
439.909
496.9519
483.8727
0
20
40
60
80
100
200
250
300
350
400
450
500

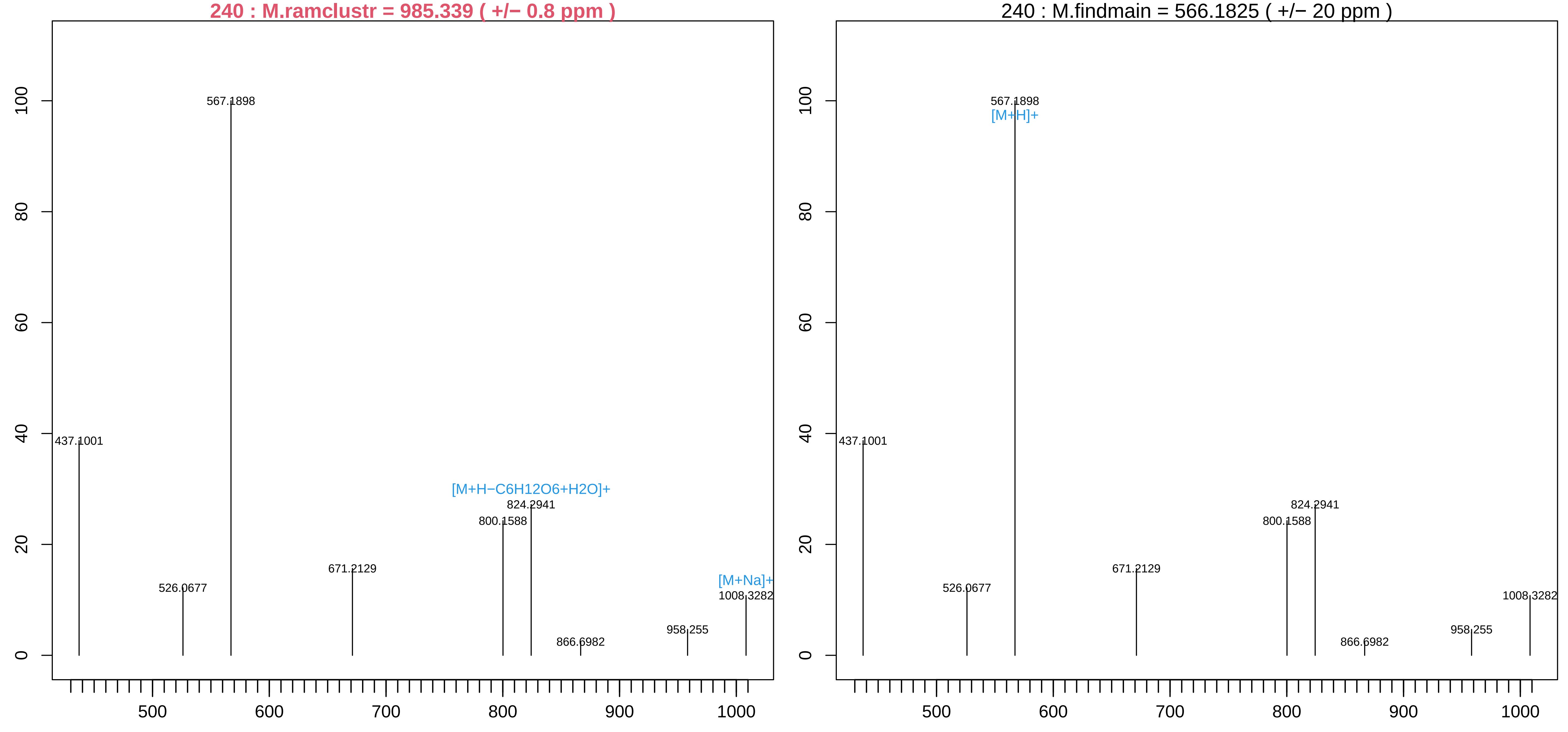

240 : M.ramclustr = 985.339 (+/− 0.8 ppm)
567.1898
437.1001
526.0677
671.2129
800.1588
824.2941
[M+H−C6H12O6+H2O]+
866.6982
958.255
1008.3282
[M+Na]+
240 : M.findmain = 566.1825 (+/− 20 ppm)
567.1898
[M+H]+
437.1001
526.0677
671.2129
800.1588
824.2941
866.6982
958.255
1008.3282

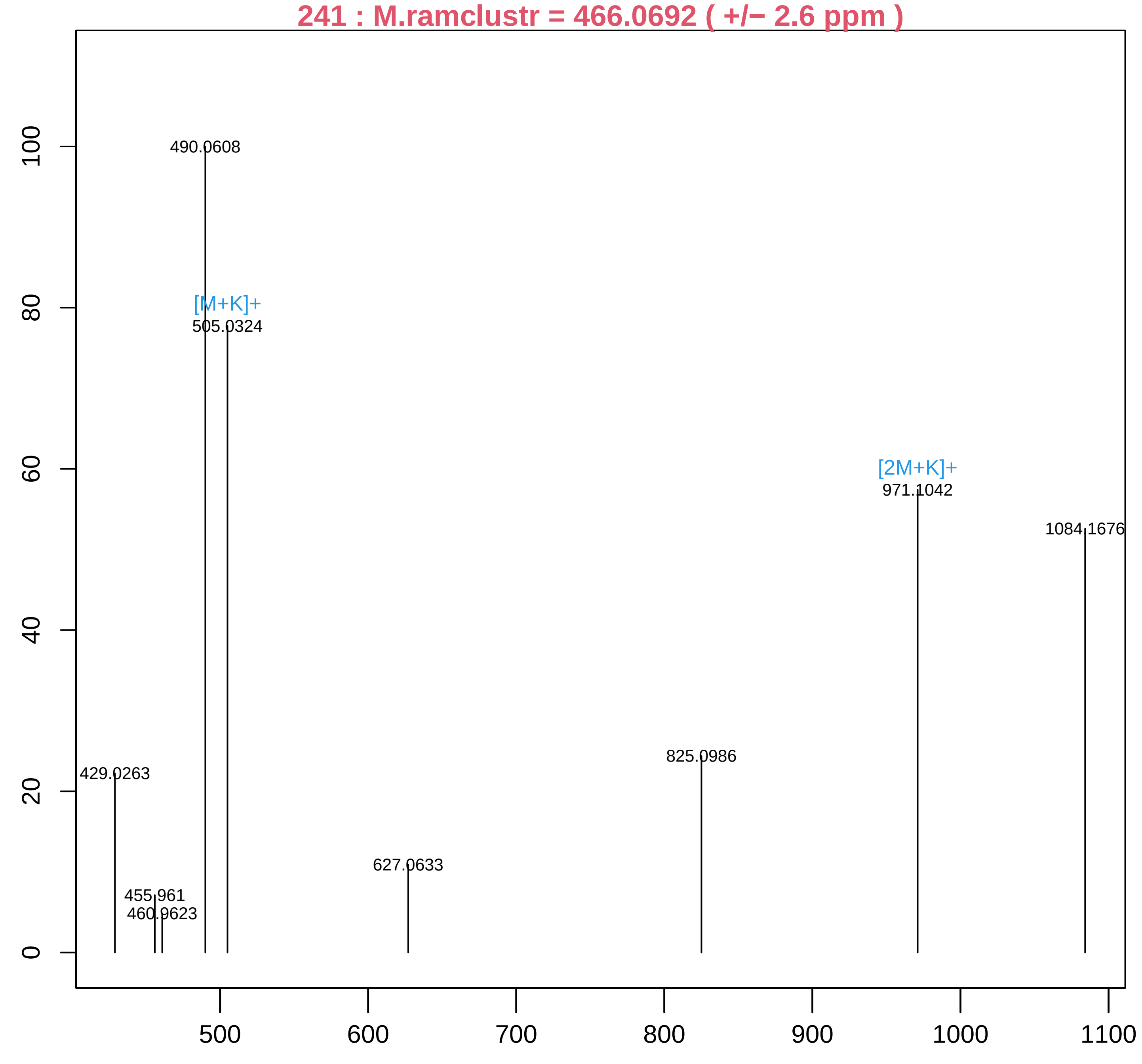

241 : M.ramclustr = 466.0692 (+/− 2.6 ppm)
490.0608
[M+K]+
505.0324
[2M+K]+
971.1042
1084.1676
825.0986
429.0263
627.0633
455.961
460.9623
0
20
40
60
80
100
500
600
700
800
900
1000
1100

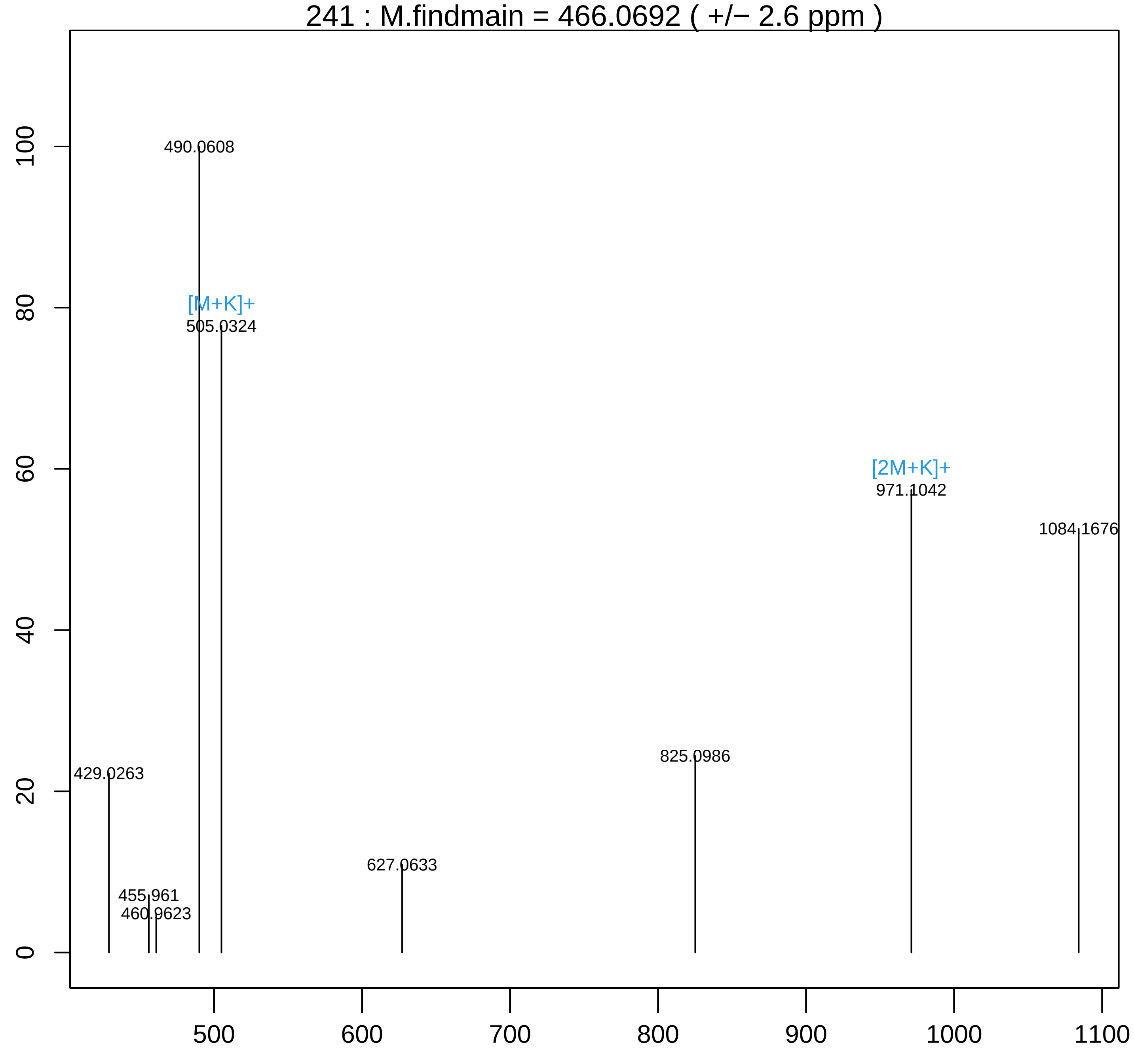

241 : M.findmain = 466.0692 (+/− 2.6 ppm)
490.0608
[M+K]+
505.0324
[2M+K]+
971.1042
1084.1676
825.0986
429.0263
627.0633
455.961
460.9623
0
20
40
60
80
100
500
600
700
800
900
1000
1100

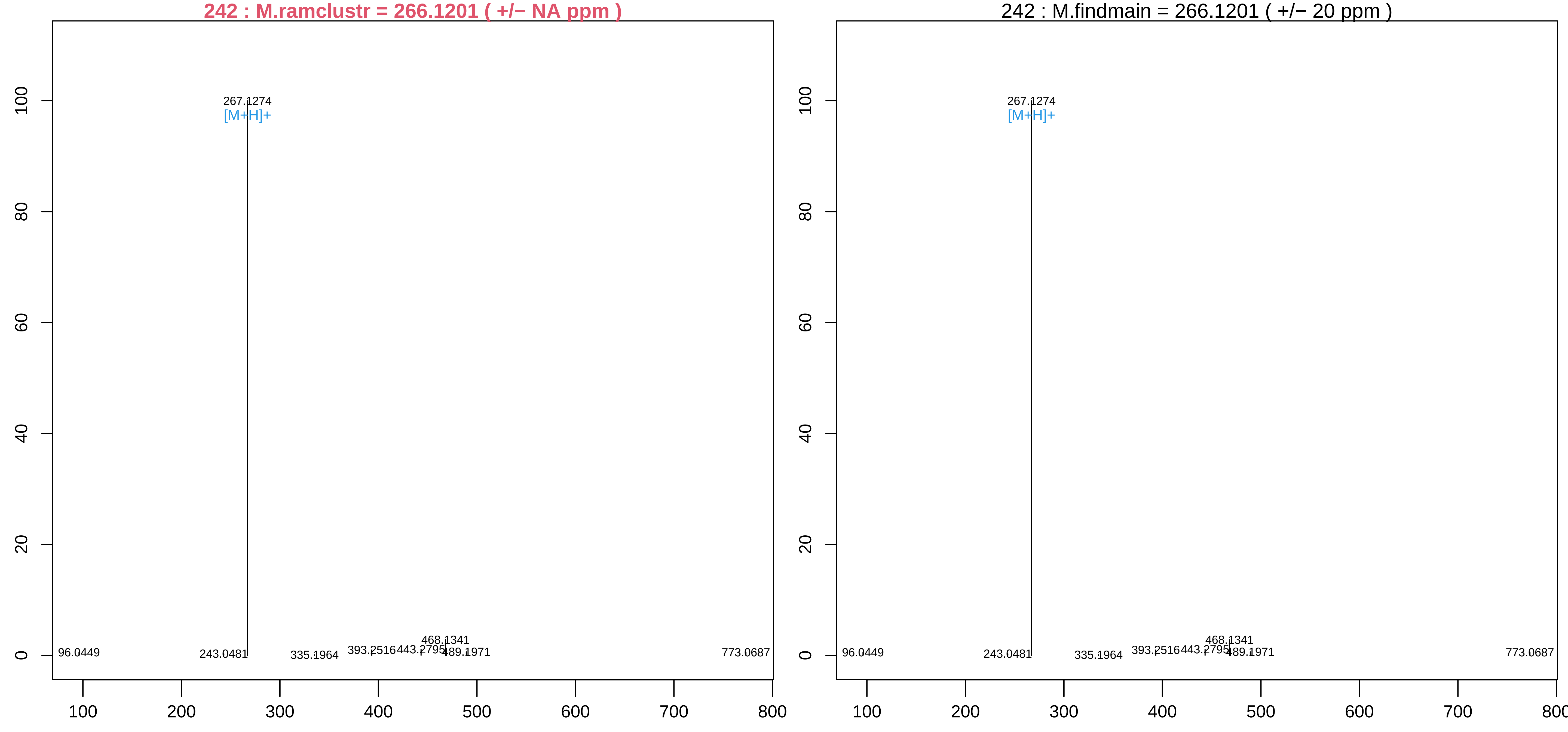
242 : M.ramclustr = 266.1201 (+/− NA ppm)
267.1274
[M+H]+
96.0449
243.0481
335.1964
393.2516
443.2795
468.1341
489.1971
773.0687
242 : M.findmain = 266.1201 (+/− 20 ppm)
267.1274
[M+H]+
96.0449
243.0481
335.1964
393.2516
443.2795
468.1341
489.1971
773.0687

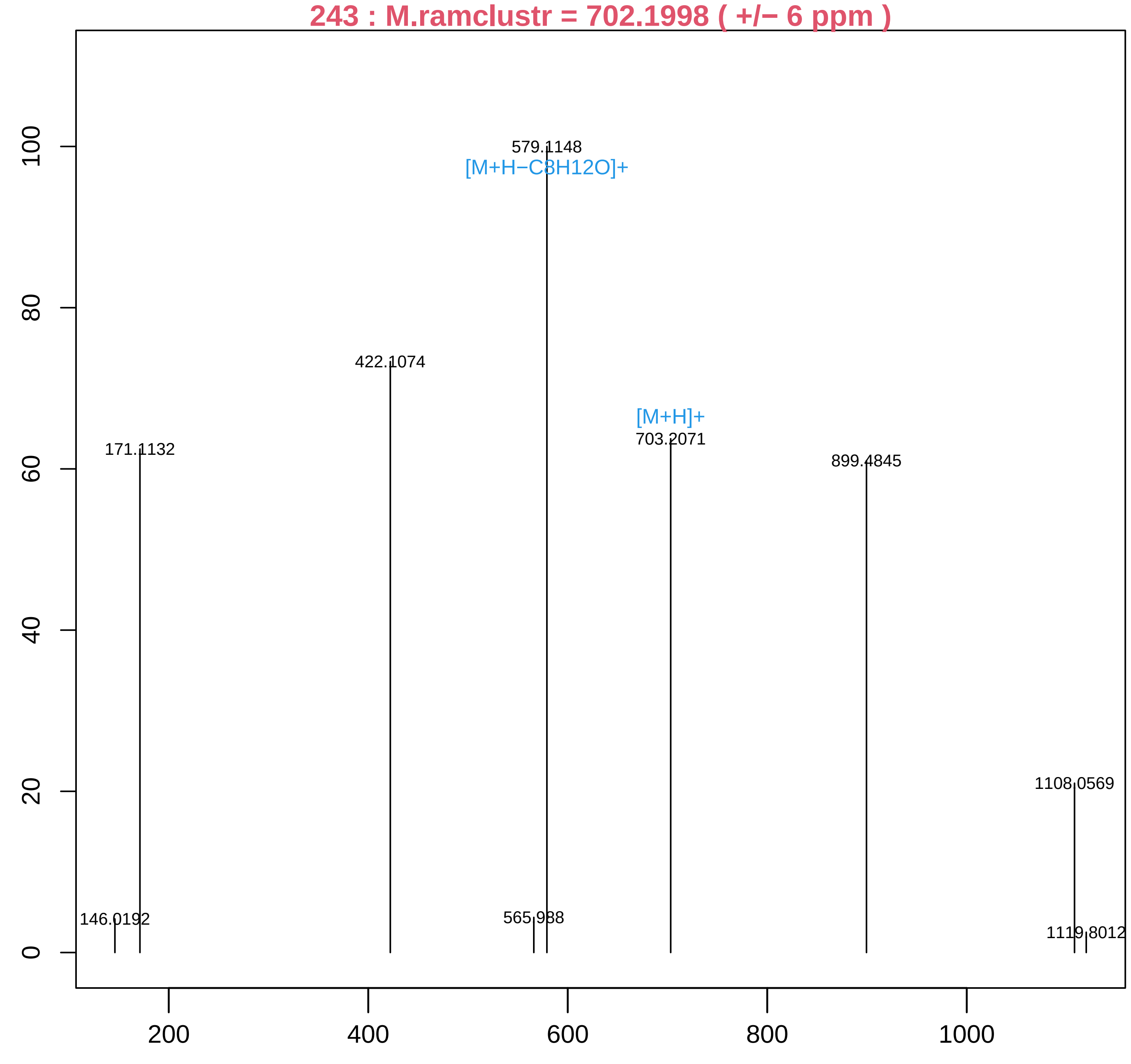
243 : M.ramclustr = 702.1998 (+/− 6 ppm)
579.1148
[M+H−C8H12O]+
422.1074
[M+H]+
703.2071
171.1132
899.4845
1108.0569
146.0192
565.988
1119.8012
0
20
40
60
80
100
200
400
600
800
1000
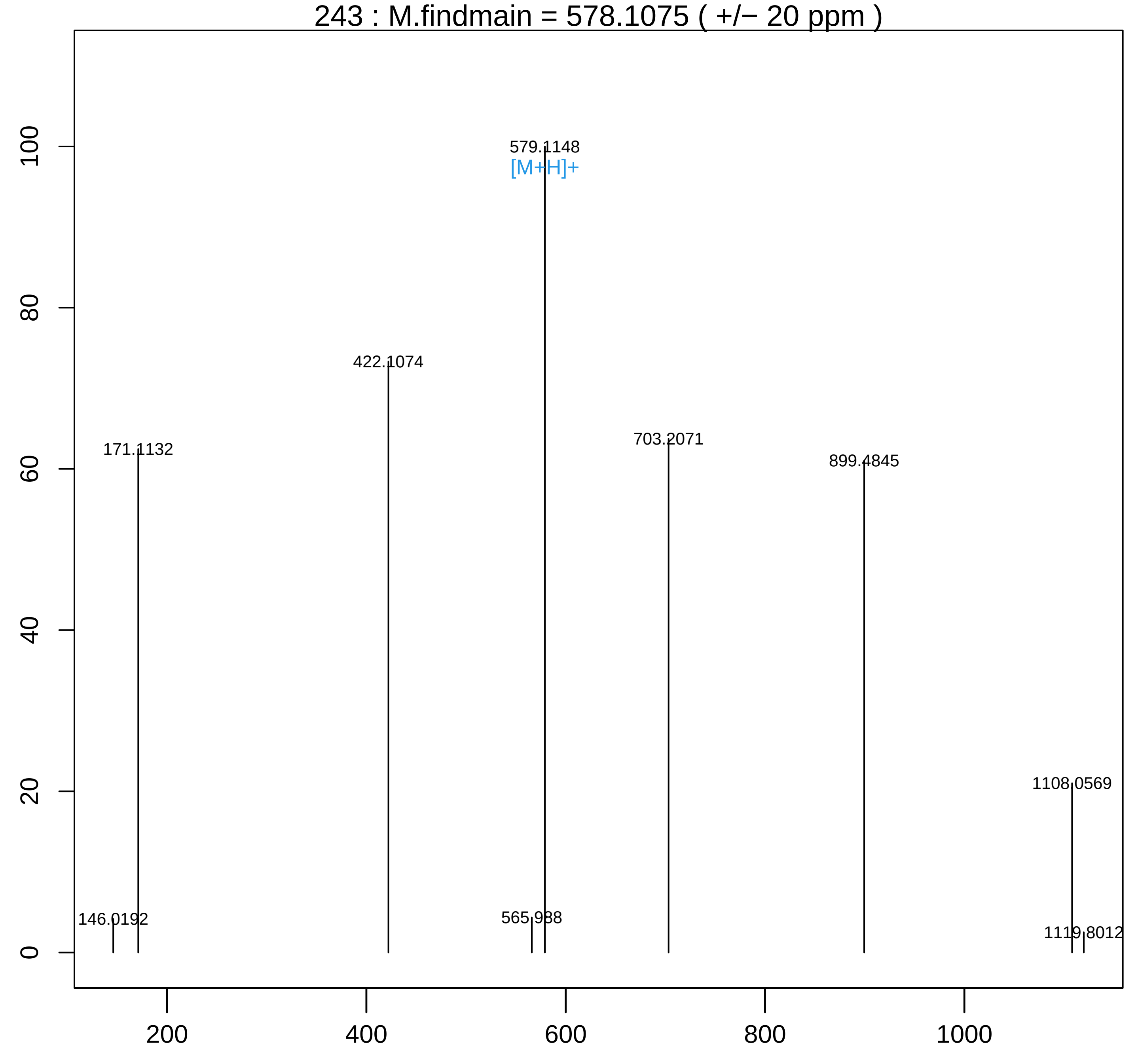
243 : M.findmain = 578.1075 (+/− 20 ppm)
579.1148
[M+H]+
422.1074
703.2071
171.1132
899.4845
1108.0569
146.0192
565.988
1119.8012
0
20
40
60
80
100
200
400
600
800
1000

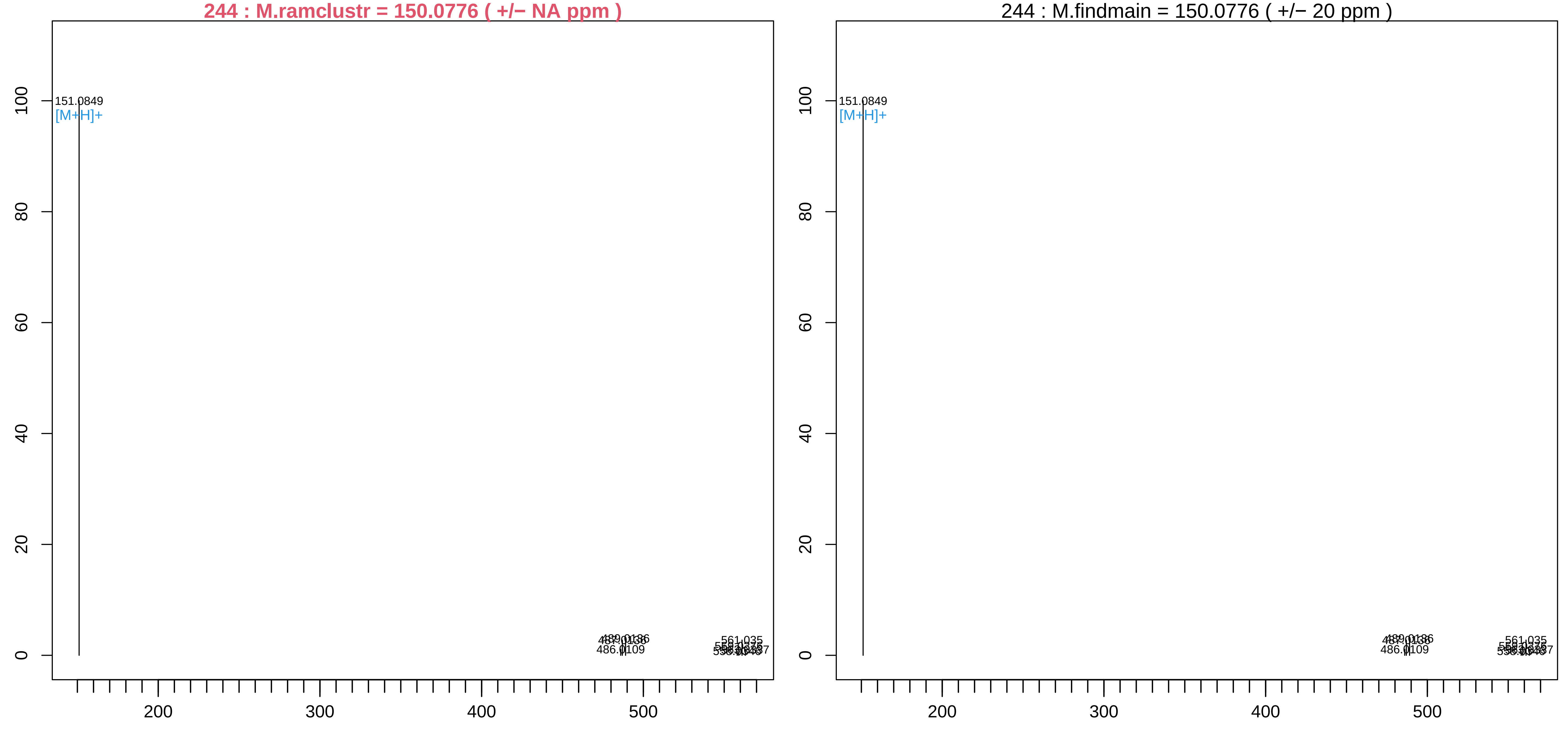
244 : M.ramclustr = 150.0776 (+/− NA ppm)
151.0849
[M+H]+
489.0186
486.0109
561.035
100
80
60
40
20
0
200
300
400
500
244 : M.findmain = 150.0776 (+/− 20 ppm)
151.0849
[M+H]+
489.0186
486.0109
561.035
100
80
60
40
20
0
200
300
400
500

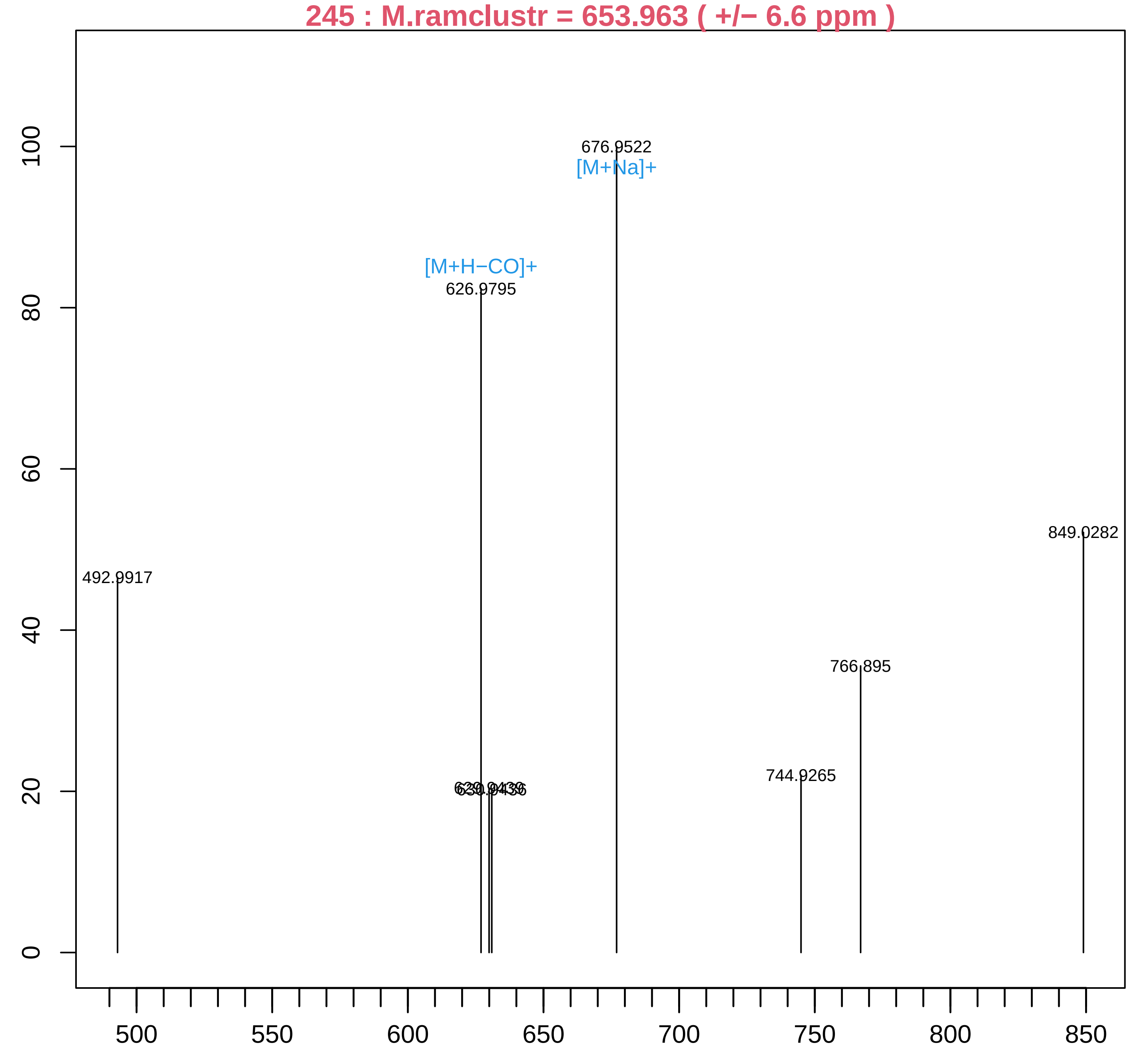
245 : M.ramclustr = 653.963 (+/− 6.6 ppm)
676.9522
[M+Na]+
[M+H−CO]+
626.9795
492.9917
849.0282
766.895
744.9265
0
20
40
60
80
100
500
550
600
650
700
750
800
850

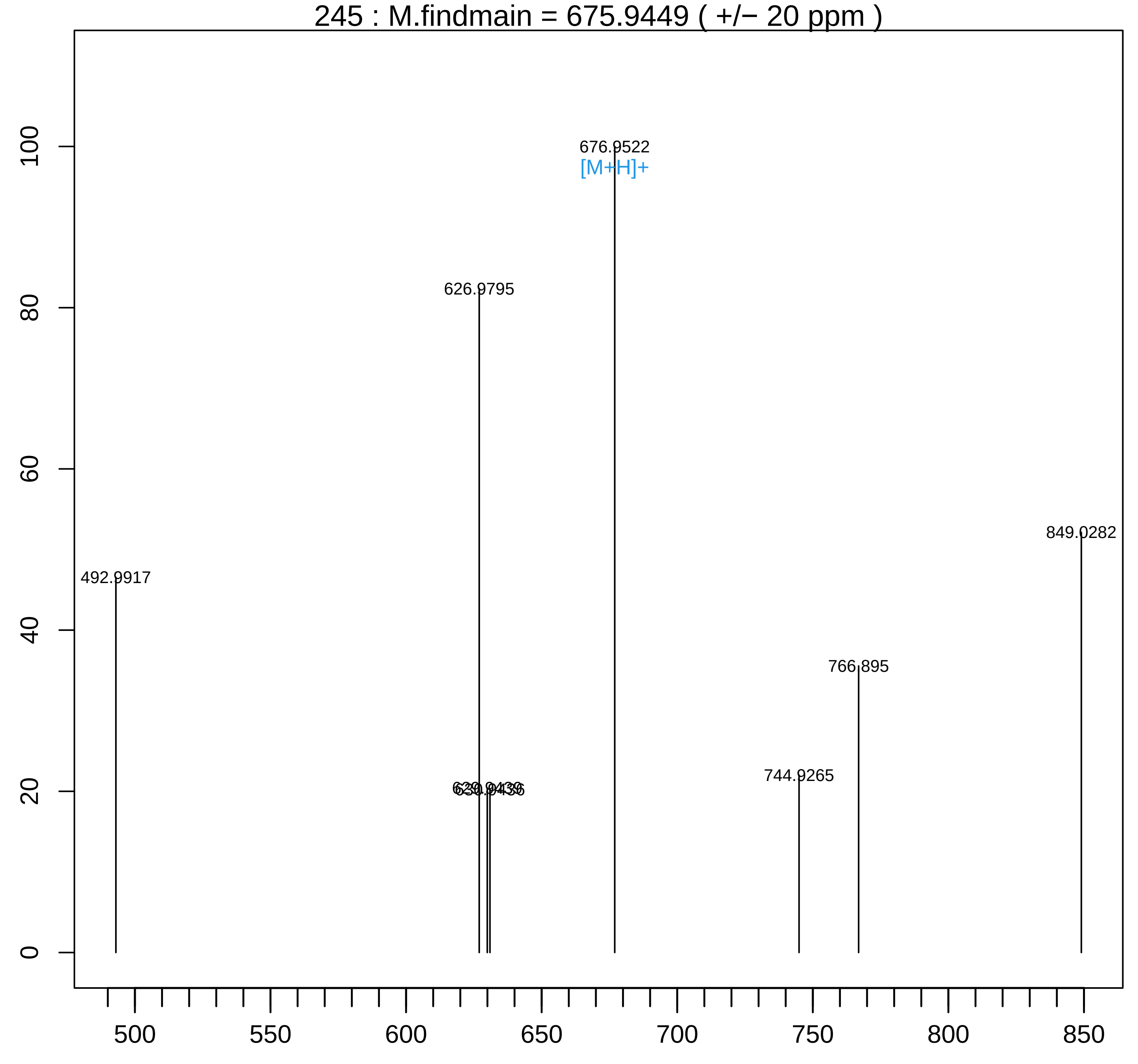
245 : M.findmain = 675.9449 (+/− 20 ppm)
676.9522
[M+H]+
626.9795
492.9917
849.0282
766.895
744.9265
0
20
40
60
80
100
500
550
600
650
700
750
800
850

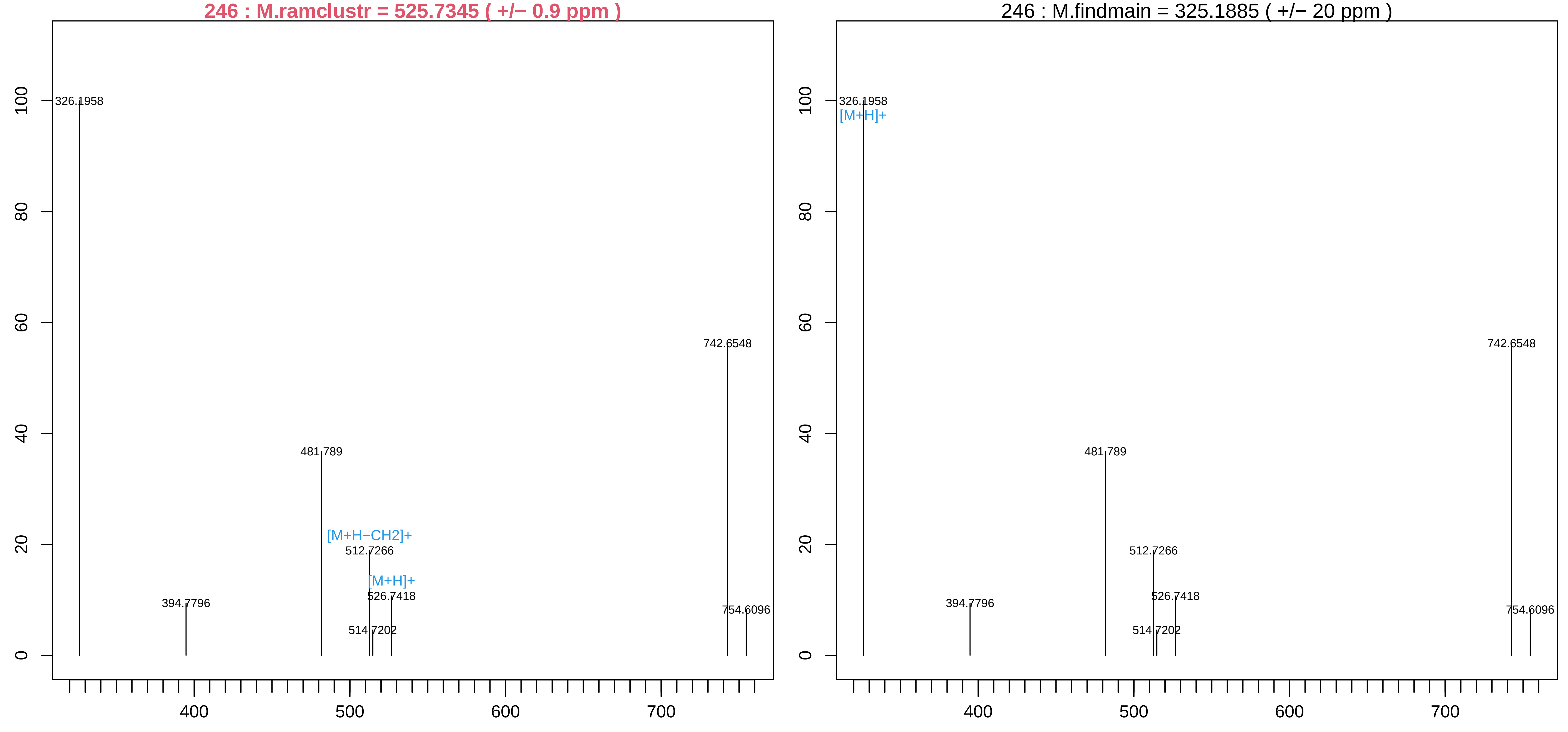
246 : M.ramclustr = 525.7345 (+/− 0.9 ppm)
326.1958
394.7796
481.789
[M+H−CH2]+
512.7266
[M+H]+
526.7418
514.7202
742.6548
754.6096
0
20
40
60
80
100
400
500
600
700
246 : M.findmain = 325.1885 (+/− 20 ppm)
326.1958
[M+H]+
394.7796
481.789
512.7266
526.7418
514.7202
742.6548
754.6096

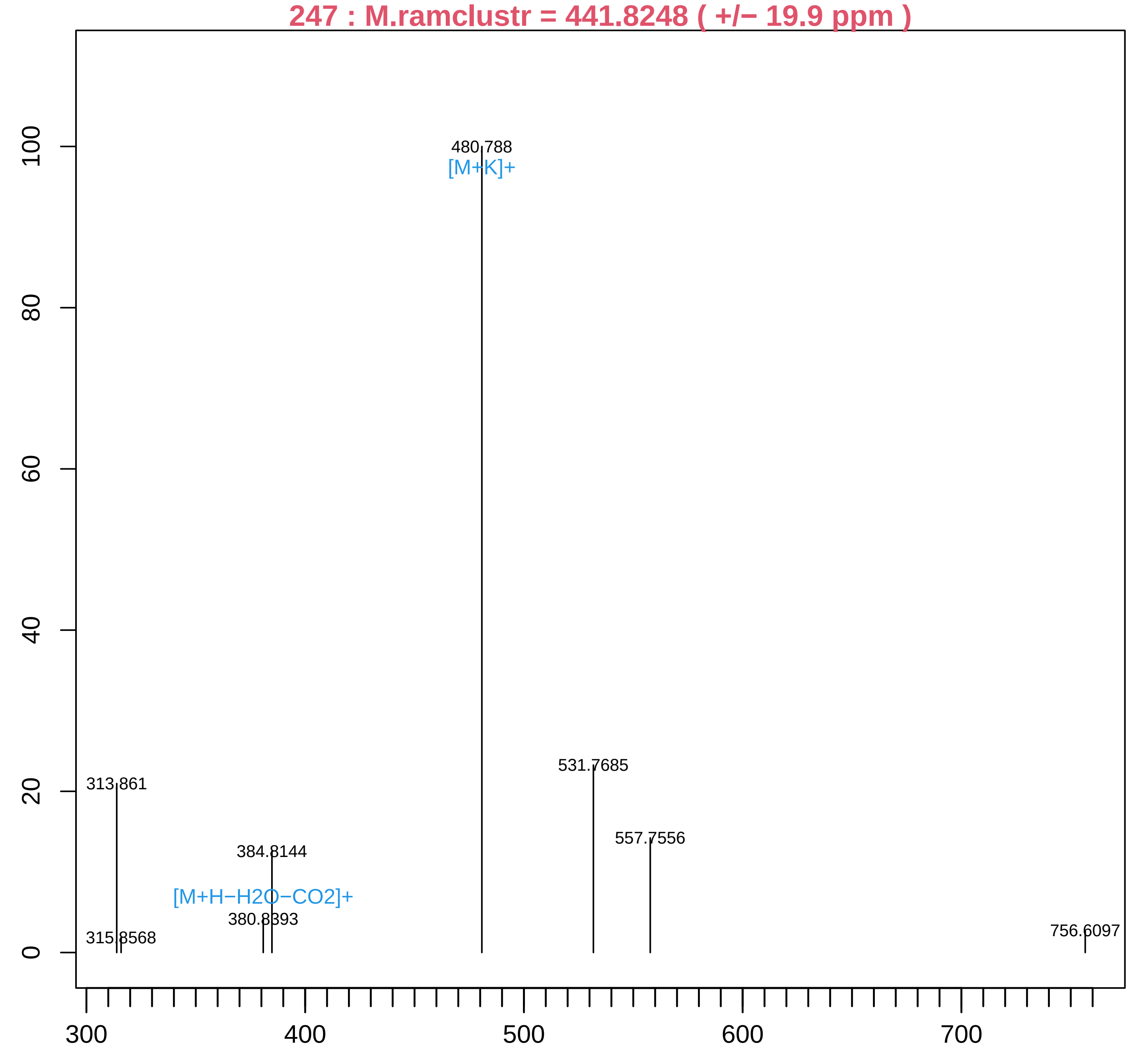

247 : M.ramclustr = 441.8248 (+/− 19.9 ppm)
480.788
[M+K]+
531.7685
557.7556
313.861
384.8144
[M+H−H2O−CO2]+
380.8393
315.8568
756.6097
0
20
40
60
80
100
300
400
500
600
700

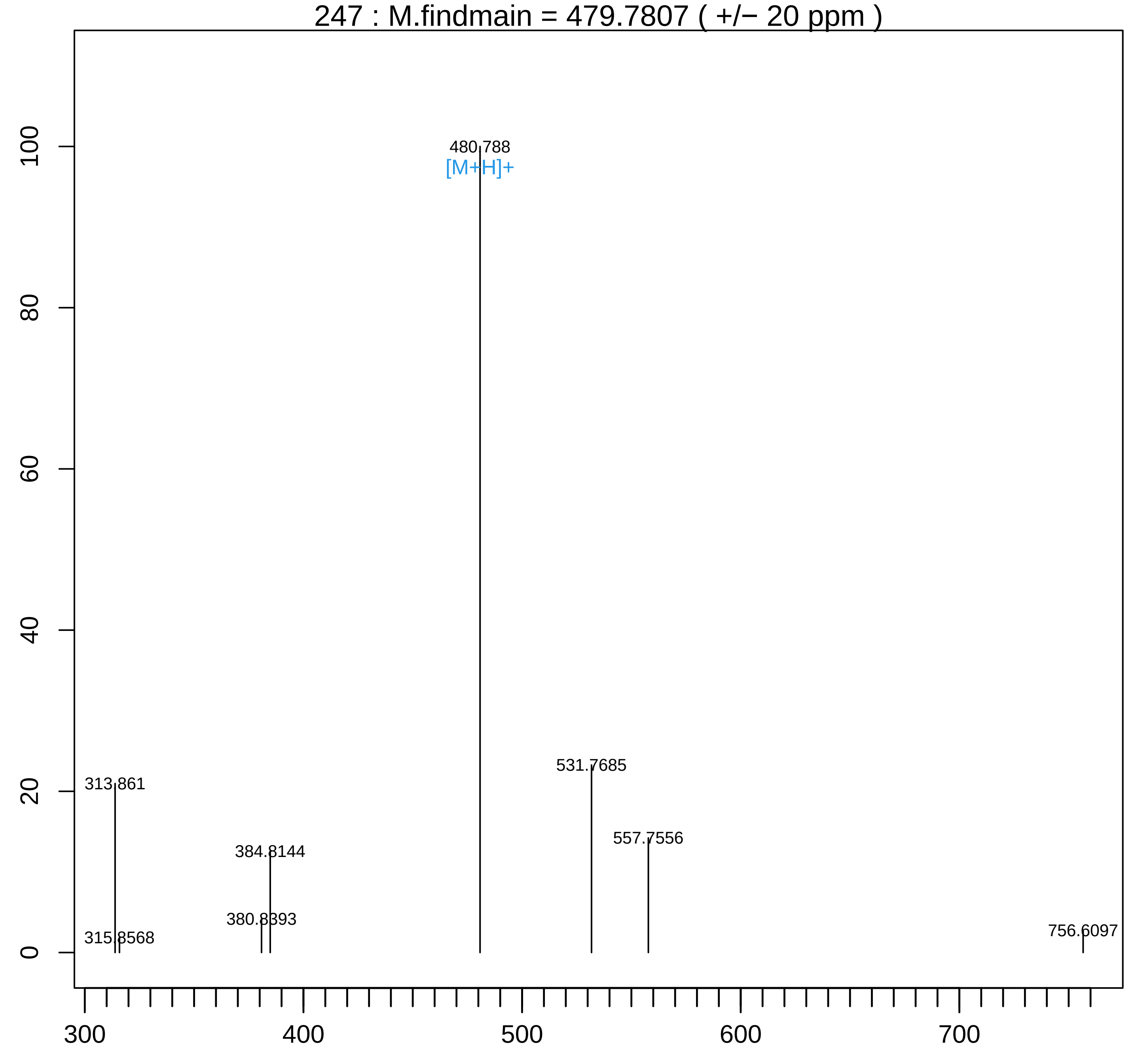

247 : M.findmain = 479.7807 (+/− 20 ppm)
480.788
[M+H]+
531.7685
557.7556
313.861
384.8144
380.8393
315.8568
756.6097
0
20
40
60
80
100
300
400
500
600
700

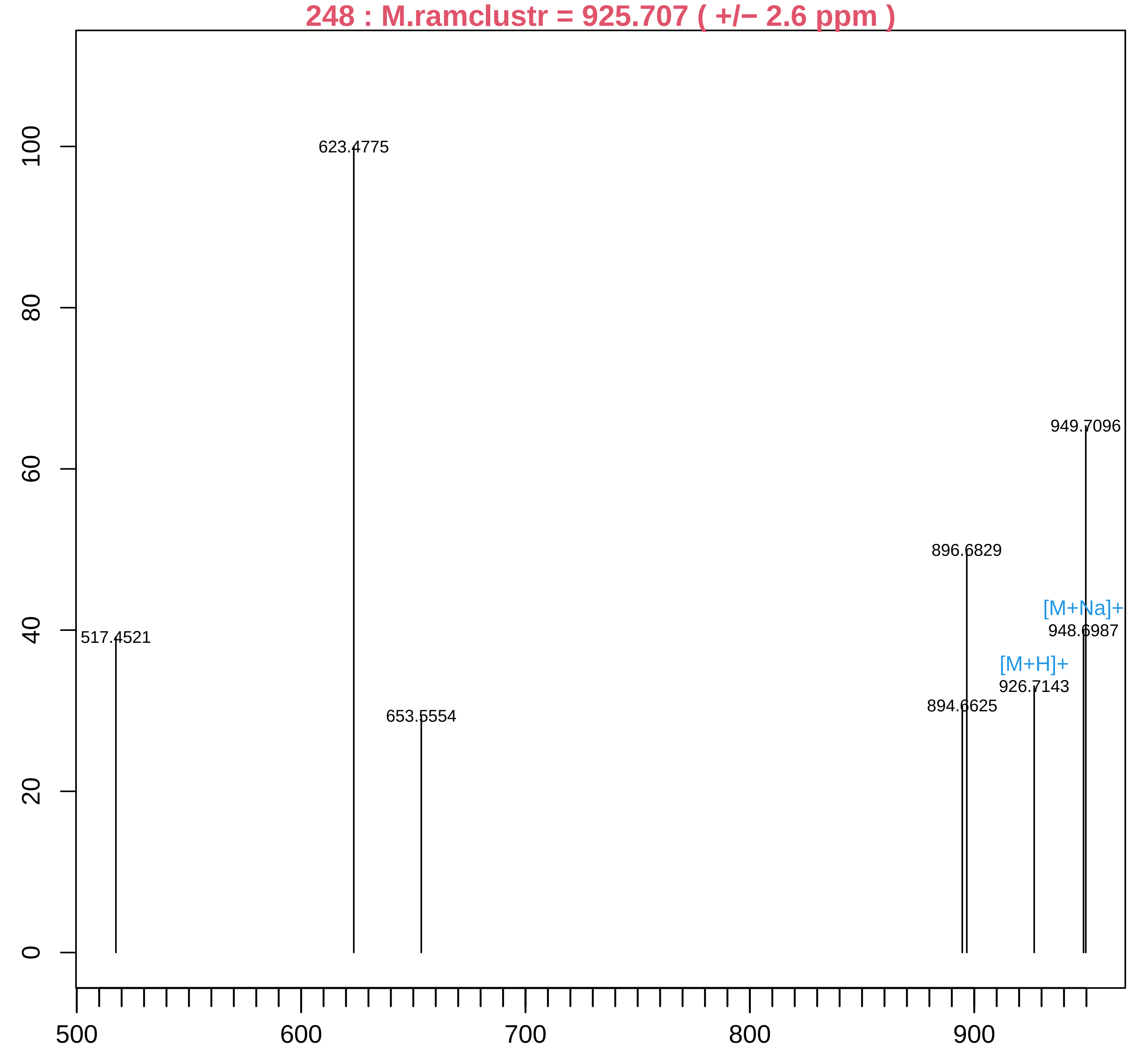

248 : M.ramclustr = 925.707 (+/− 2.6 ppm)
623.4775
949.7096
896.6829
[M+Na]+
948.6987
517.4521
[M+H]+
926.7143
894.6625
653.5554
0
20
40
60
80
100
500
600
700
800
900

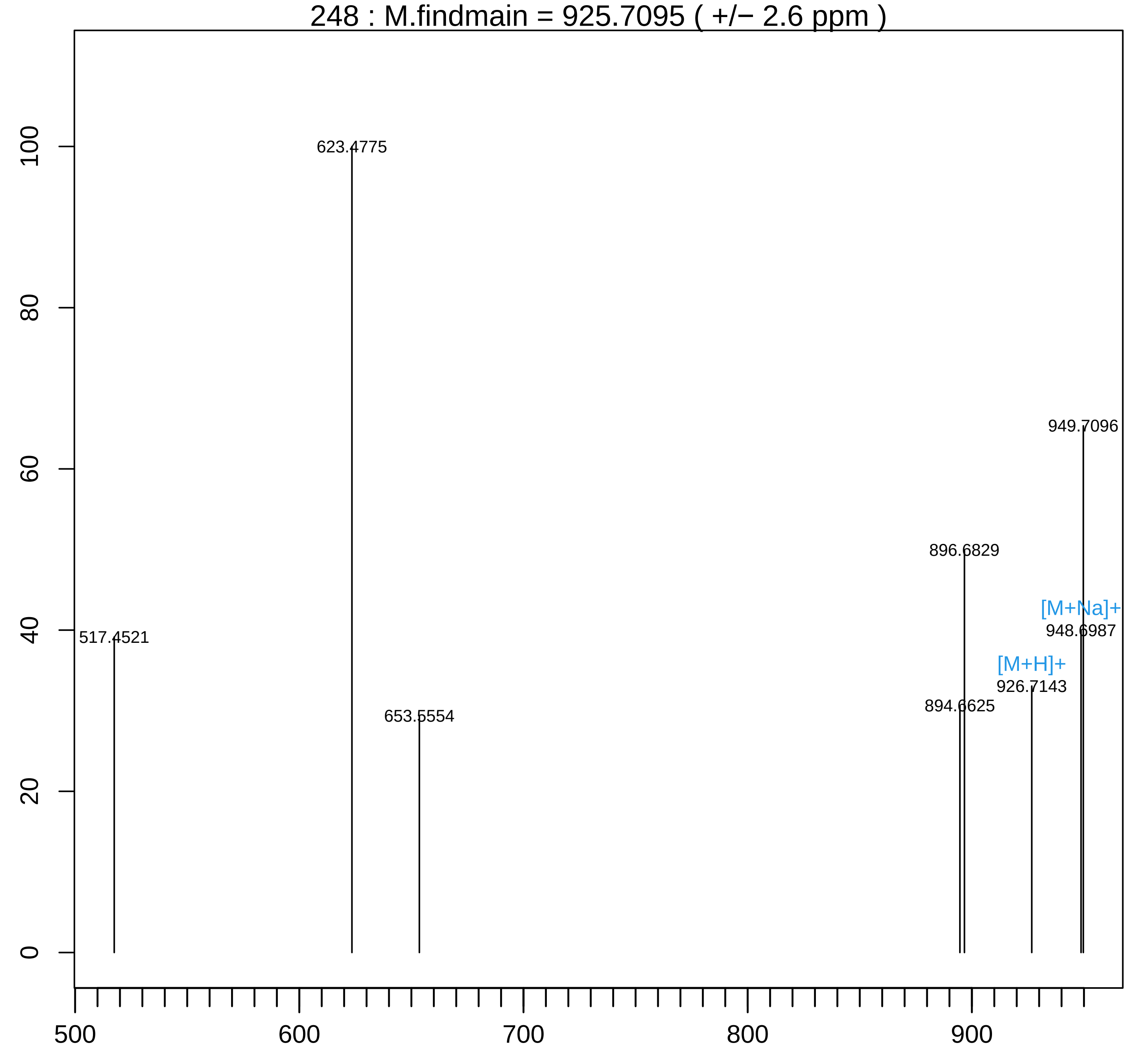

248 : M.findmain = 925.7095 (+/− 2.6 ppm)
623.4775
949.7096
896.6829
[M+Na]+
948.6987
517.4521
[M+H]+
926.7143
894.6625
653.5554
0
20
40
60
80
100
500
600
700
800
900

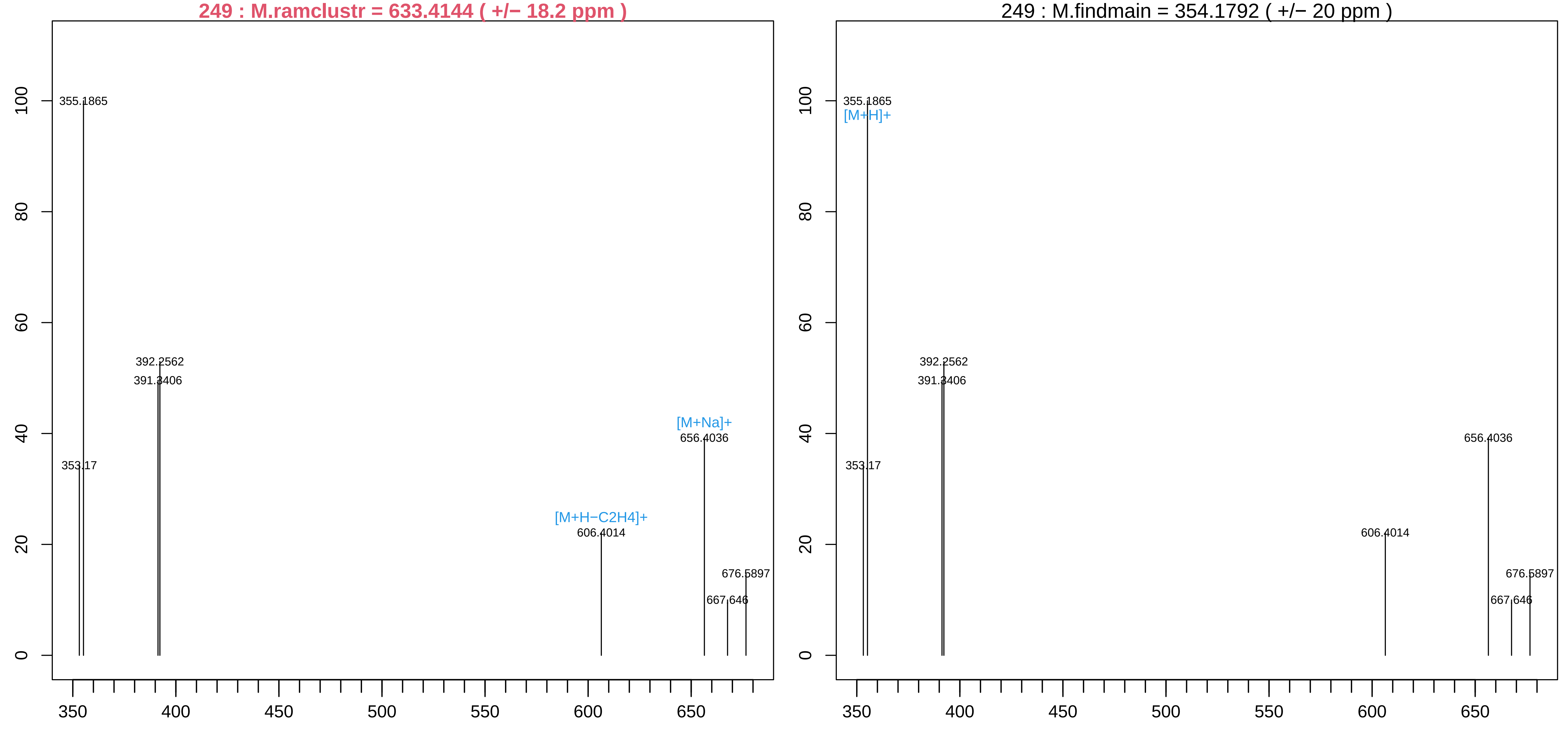
249 : M.ramclustr = 633.4144 (+/− 18.2 ppm)
355.1865
392.2562
391.3406
353.17
[M+Na]+
656.4036
[M+H−C2H4]+
606.4014
676.5897
667.646
0
20
40
60
80
100
350
400
450
500
550
600
650
249 : M.findmain = 354.1792 (+/− 20 ppm)
355.1865
[M+H]+
392.2562
391.3406
353.17
656.4036
606.4014
676.5897
667.646
0
20
40
60
80
100
350
400
450
500
550
600
650

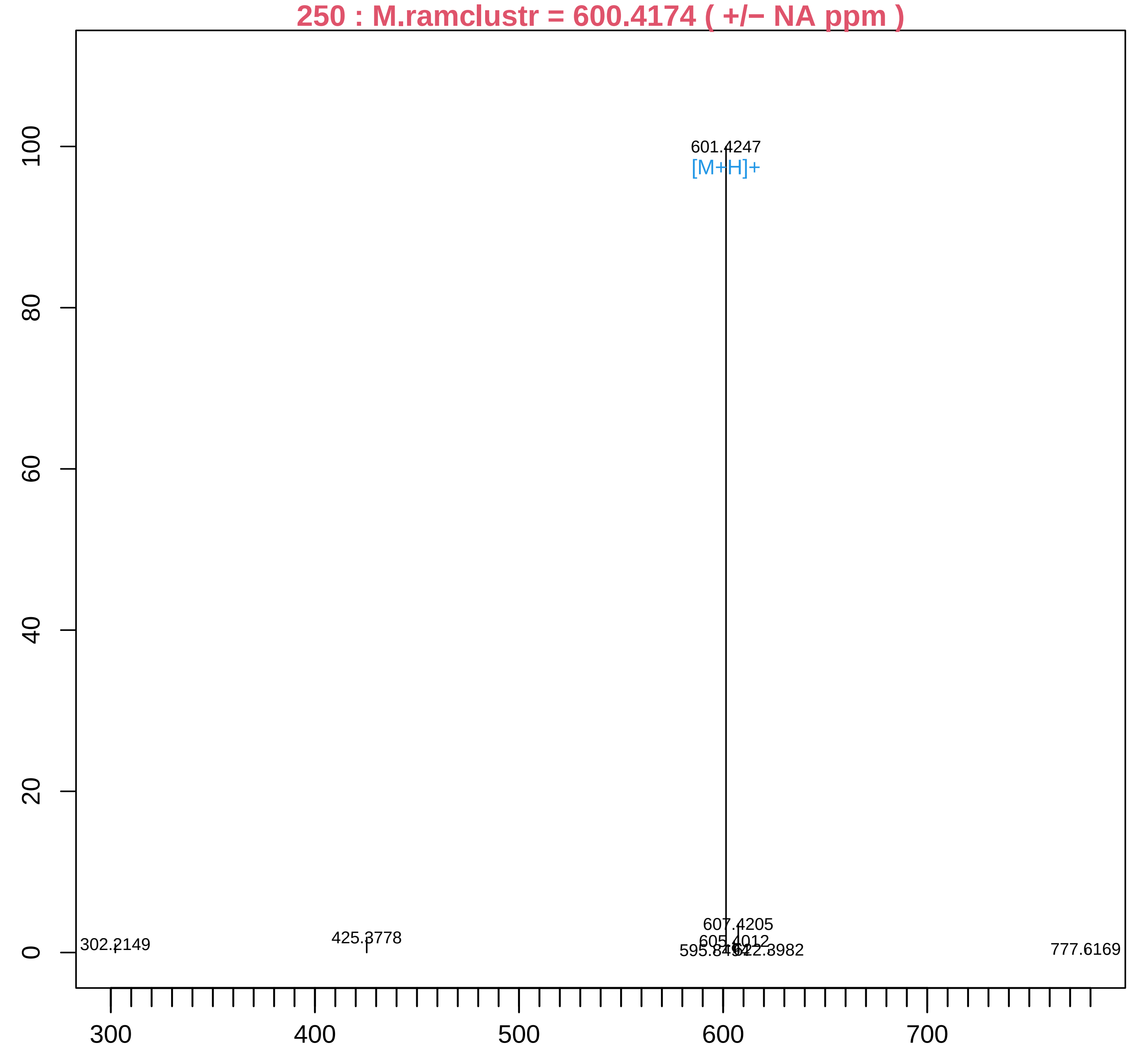
250 : M.ramclustr = 600.4174 (+/− NA ppm)
601.4247
[M+H]+
607.4205
605.4012
595.8451
612.3982
302.2149
425.3778
777.6169
0
20
40
60
80
100
300
400
500
600
700

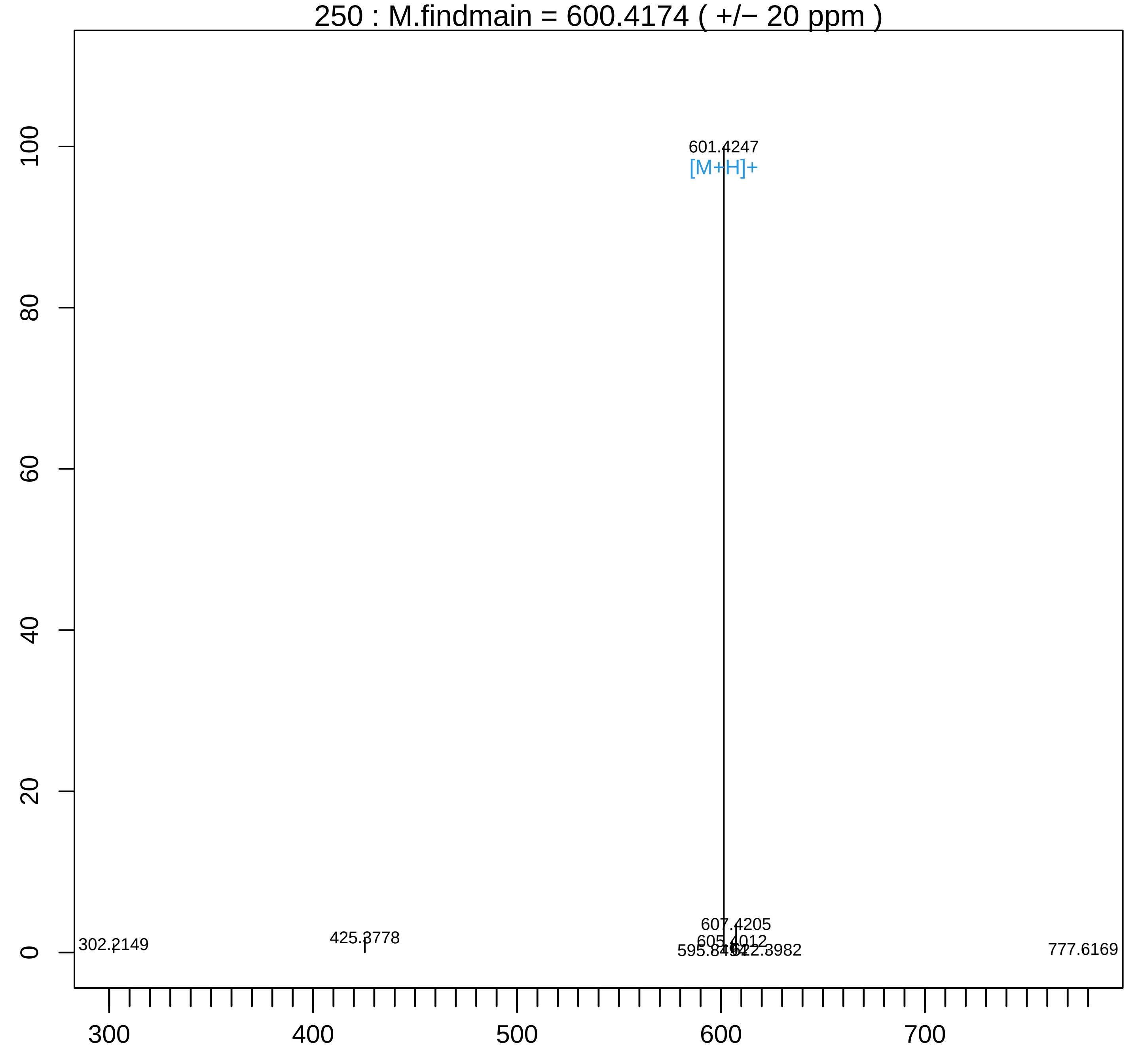
250 : M.findmain = 600.4174 (+/− 20 ppm)
601.4247
[M+H]+
607.4205
605.4012
595.8451
612.3982
302.2149
425.3778
777.6169
0
20
40
60
80
100
300
400
500
600
700

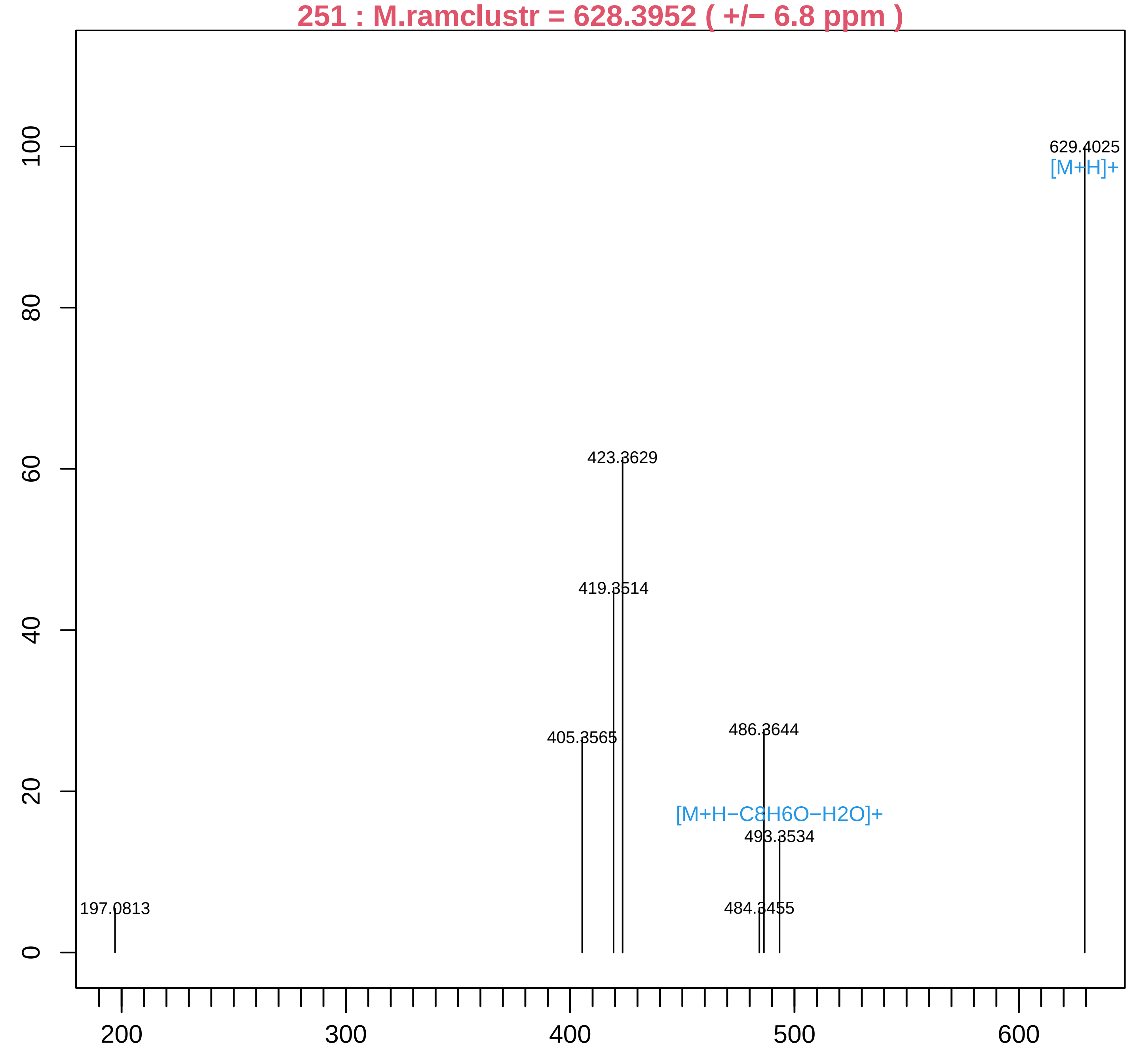
251 : M.ramclustr = 628.3952 (+/− 6.8 ppm)
629.4025
[M+H]+
423.3629
419.3514
405.3565
486.3644
[M+H−C8H6O−H2O]+
493.3534
484.3455
197.0813
0
20
40
60
80
100
200
300
400
500
600

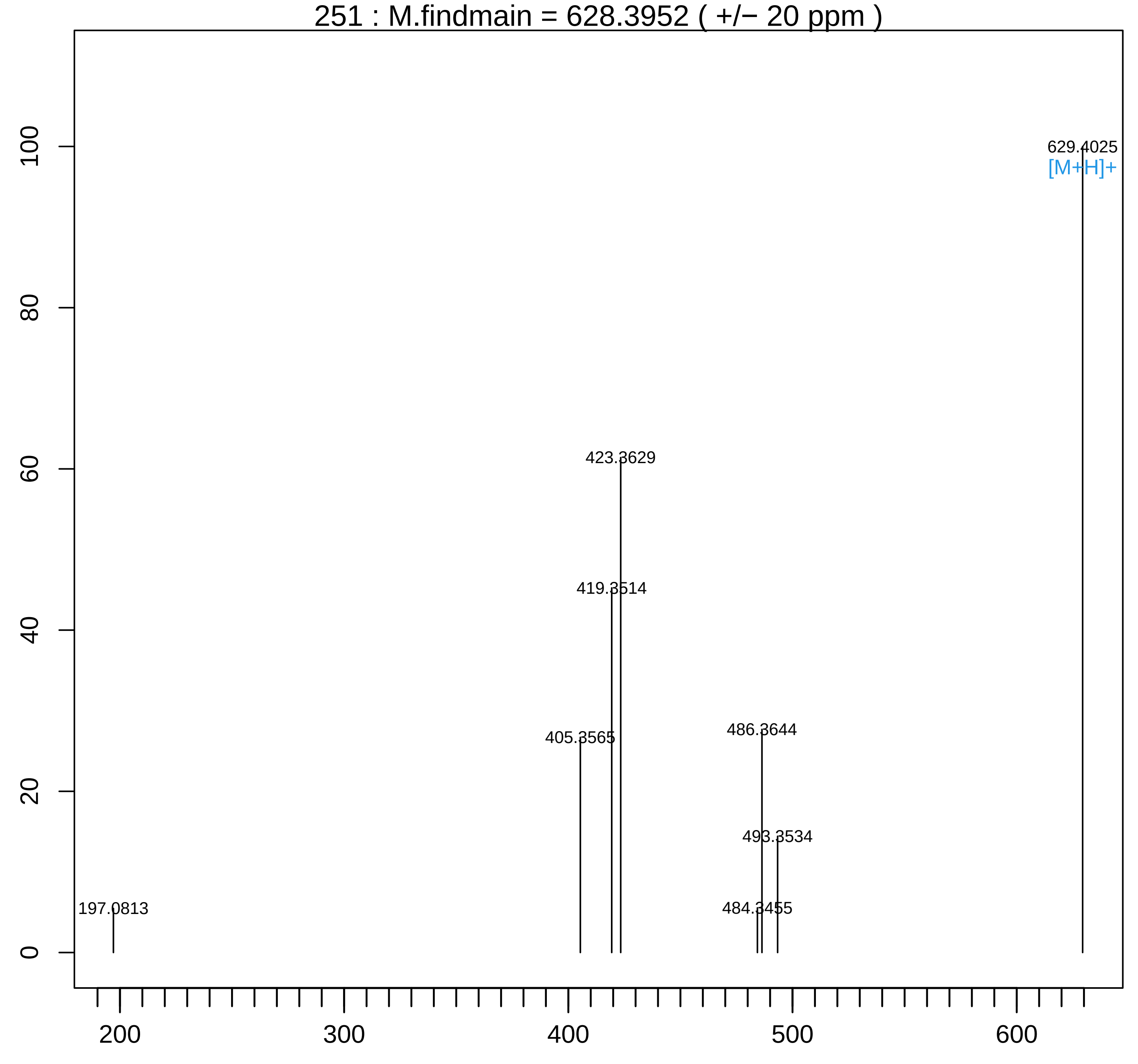
251 : M.findmain = 628.3952 (+/− 20 ppm)
629.4025
[M+H]+
423.3629
419.3514
405.3565
486.3644
493.3534
484.3455
197.0813
0
20
40
60
80
100
200
300
400
500
600

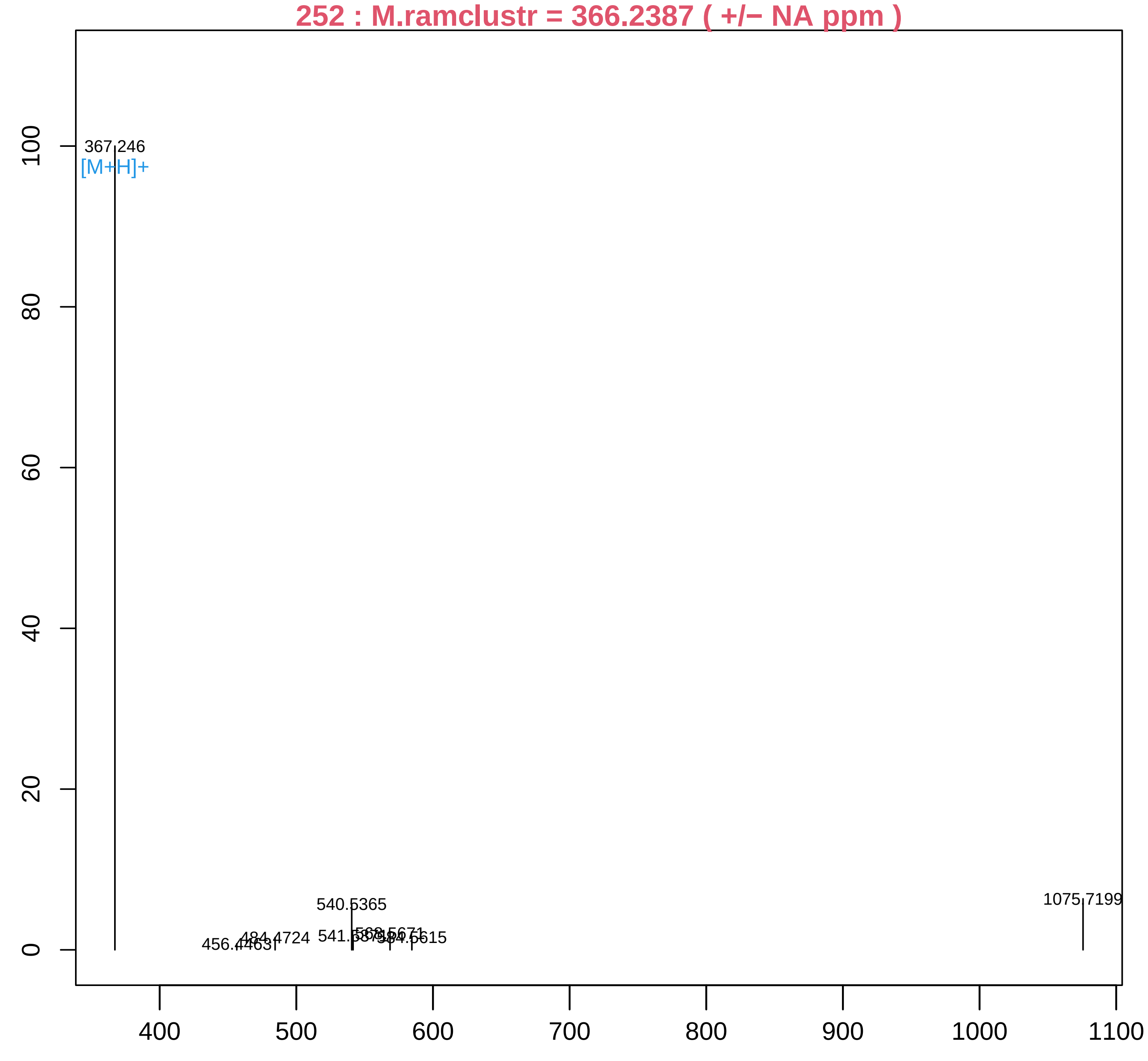

252 : M.ramclustr = 366.2387 (+/− NA ppm)
367.246
[M+H]+
540.5365
1075.7199
484.4724
456.4463

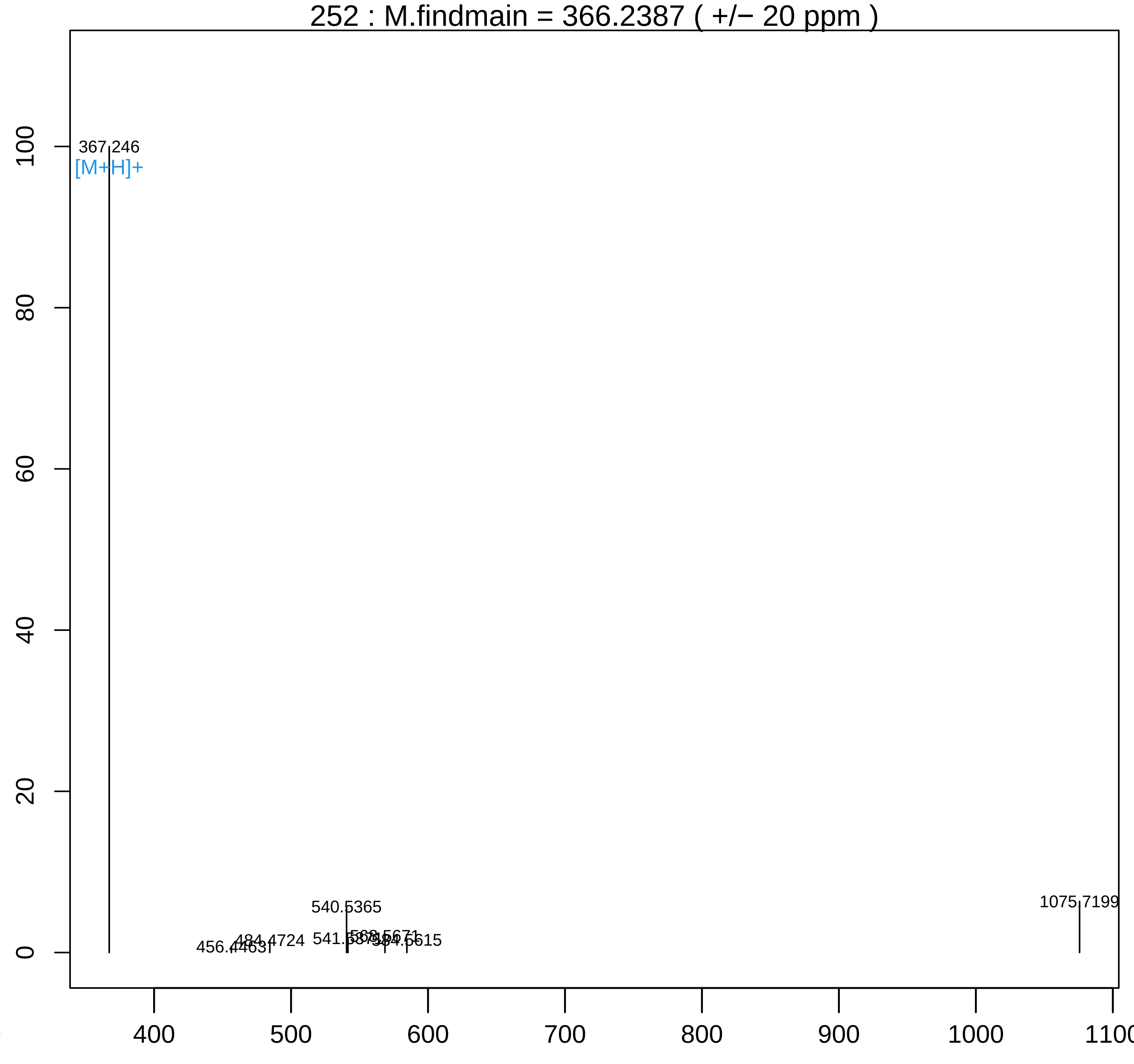

252 : M.findmain = 366.2387 (+/− 20 ppm)
367.246
[M+H]+
540.5365
1075.7199
484.4724
456.4463

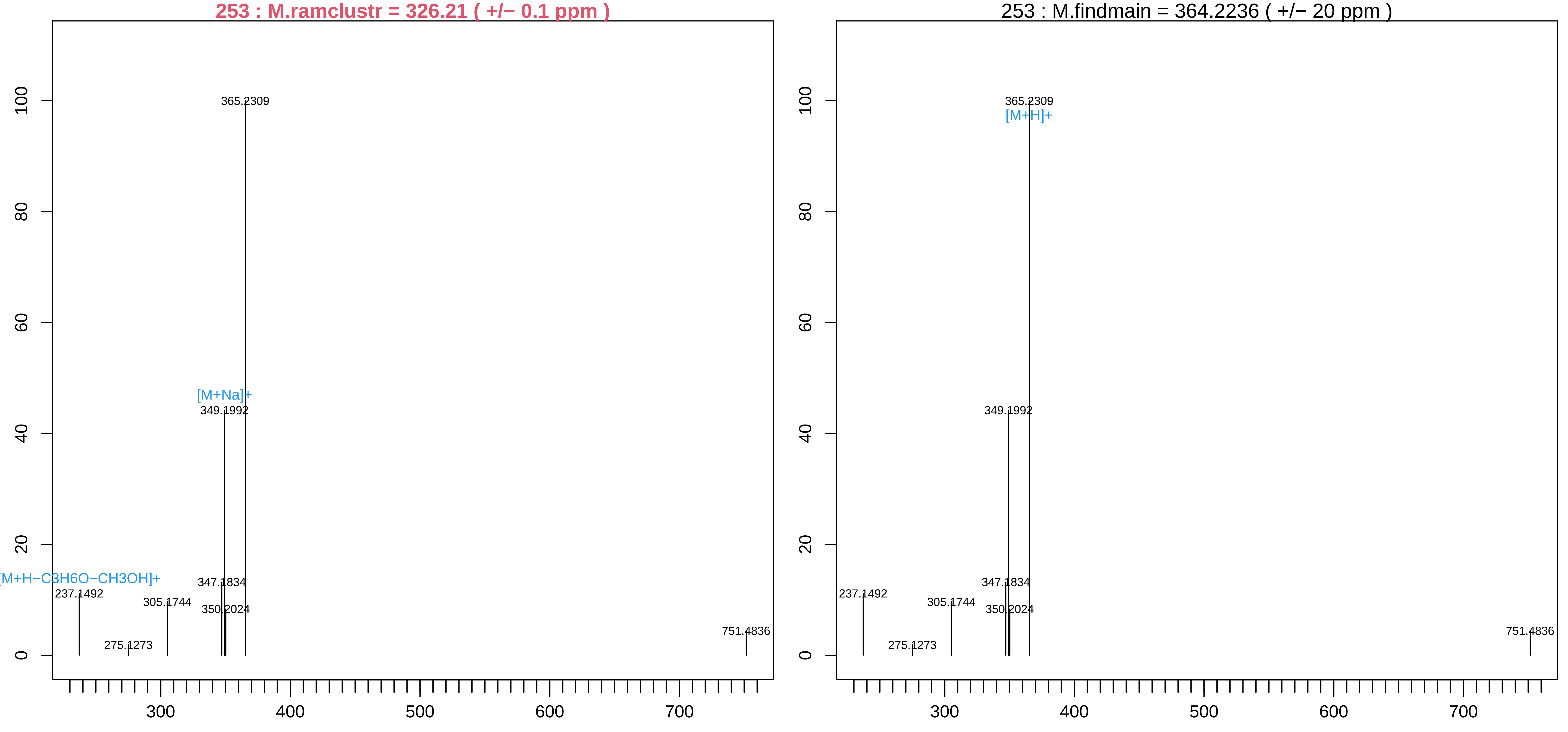
253 : M.ramclustr = 326.21 (+/− 0.1 ppm)
365.2309
[M+Na]+
349.1992
[M+H−C3H6O−CH3OH]+
237.1492
347.1834
305.1744
350.2024
275.1273
751.4836
253 : M.findmain = 364.2236 (+/− 20 ppm)
365.2309
[M+H]+
349.1992
237.1492
347.1834
305.1744
350.2024
275.1273
751.4836

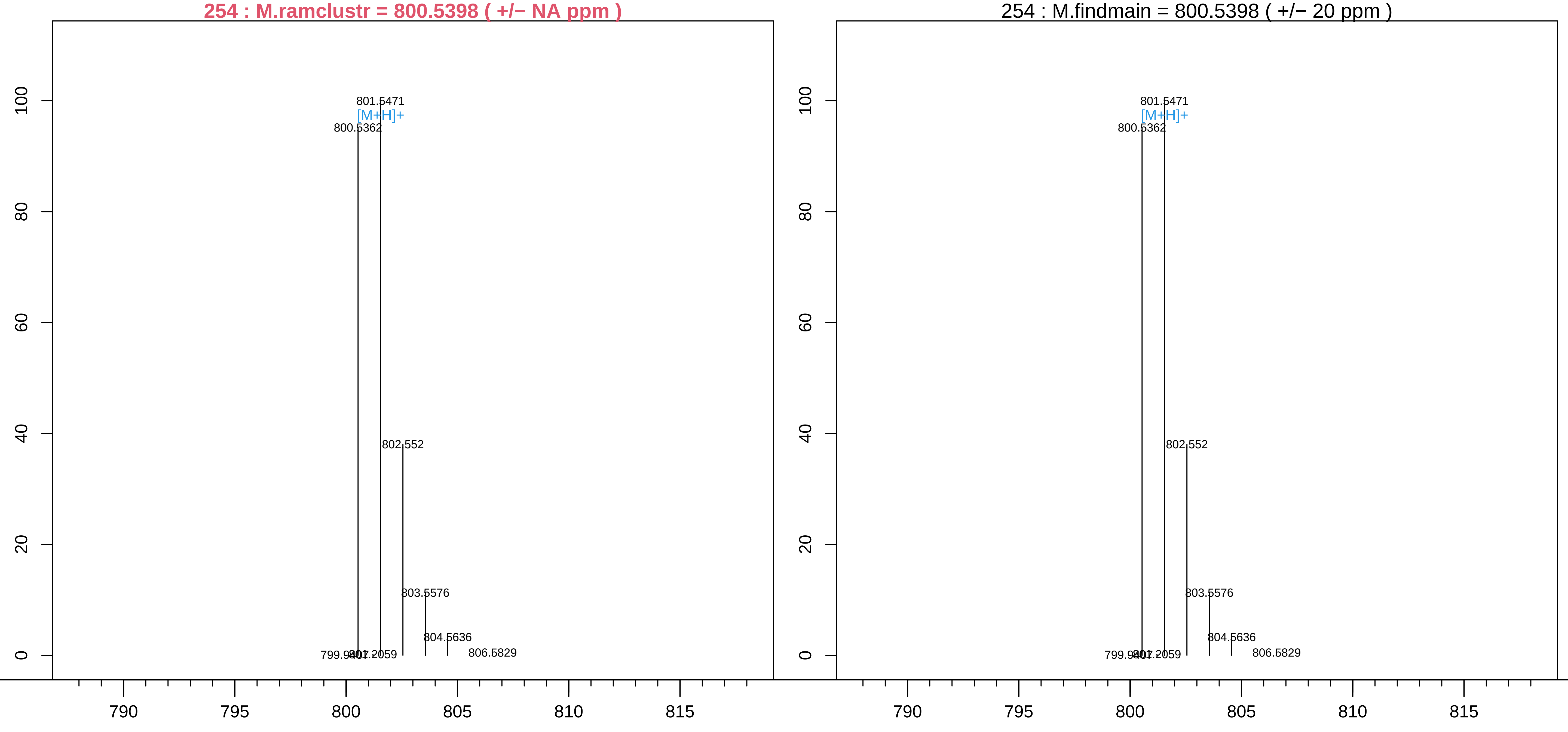
254 : M.ramclustr = 800.5398 (+/− NA ppm)
801.5471
[M+H]+
800.5362
802.552
803.5576
804.5636
806.5829
801.2059
0
20
40
60
80
100
790
795
800
805
810
815
254 : M.findmain = 800.5398 (+/− 20 ppm)
801.5471
[M+H]+
800.5362
802.552
803.5576
804.5636
806.5829
801.2059
0
20
40
60
80
100
790
795
800
805
810
815

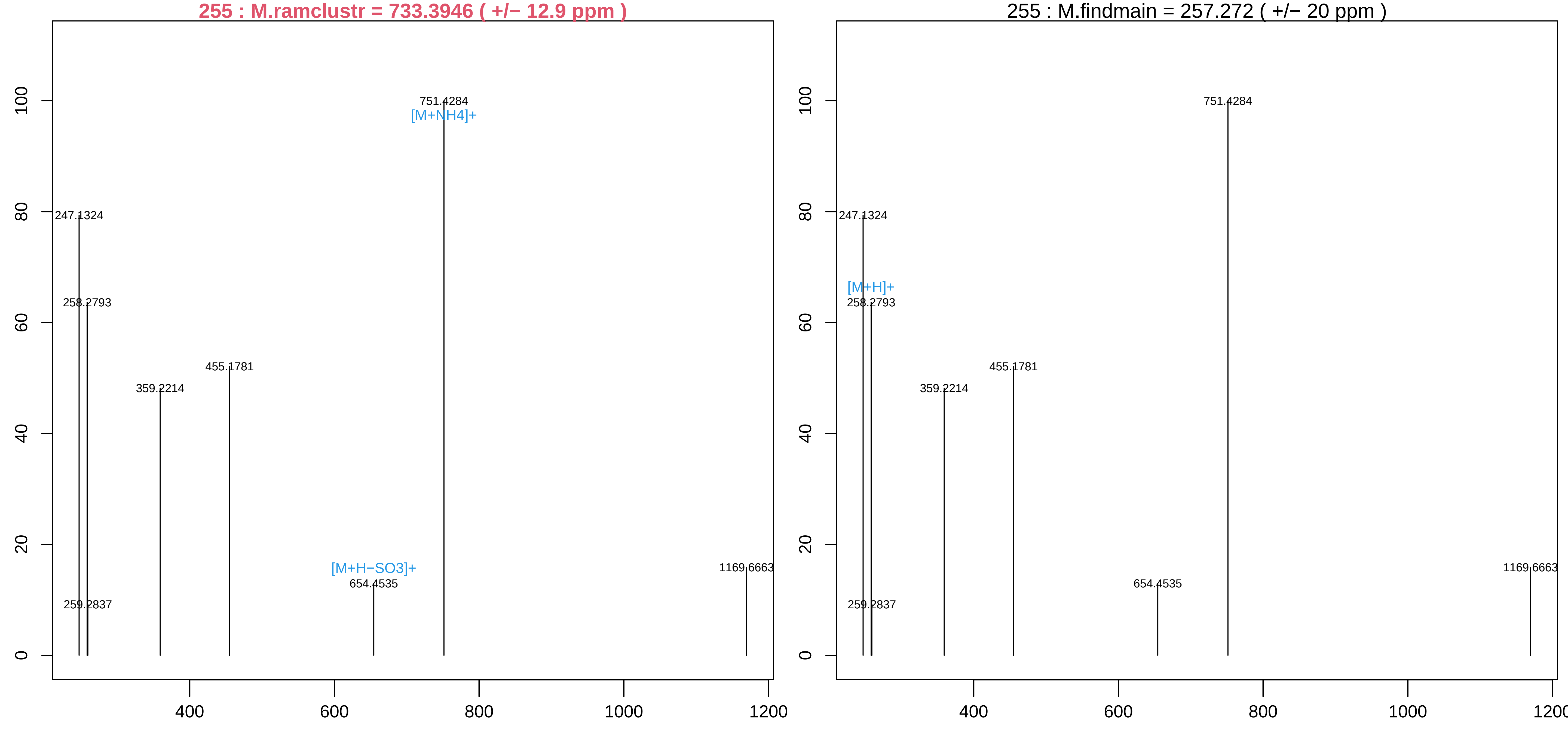
255 : M.ramclustr = 733.3946 (+/− 12.9 ppm)
751.4284
[M+NH4]+
247.1324
258.2793
359.2214
455.1781
[M+H−SO3]+
654.4535
259.2837
1169.6663
255 : M.findmain = 257.272 (+/− 20 ppm)
751.4284
247.1324
[M+H]+
258.2793
455.1781
359.2214
654.4535
259.2837
1169.6663
0
20
40
60
80
100
400
600
800
1000
1200

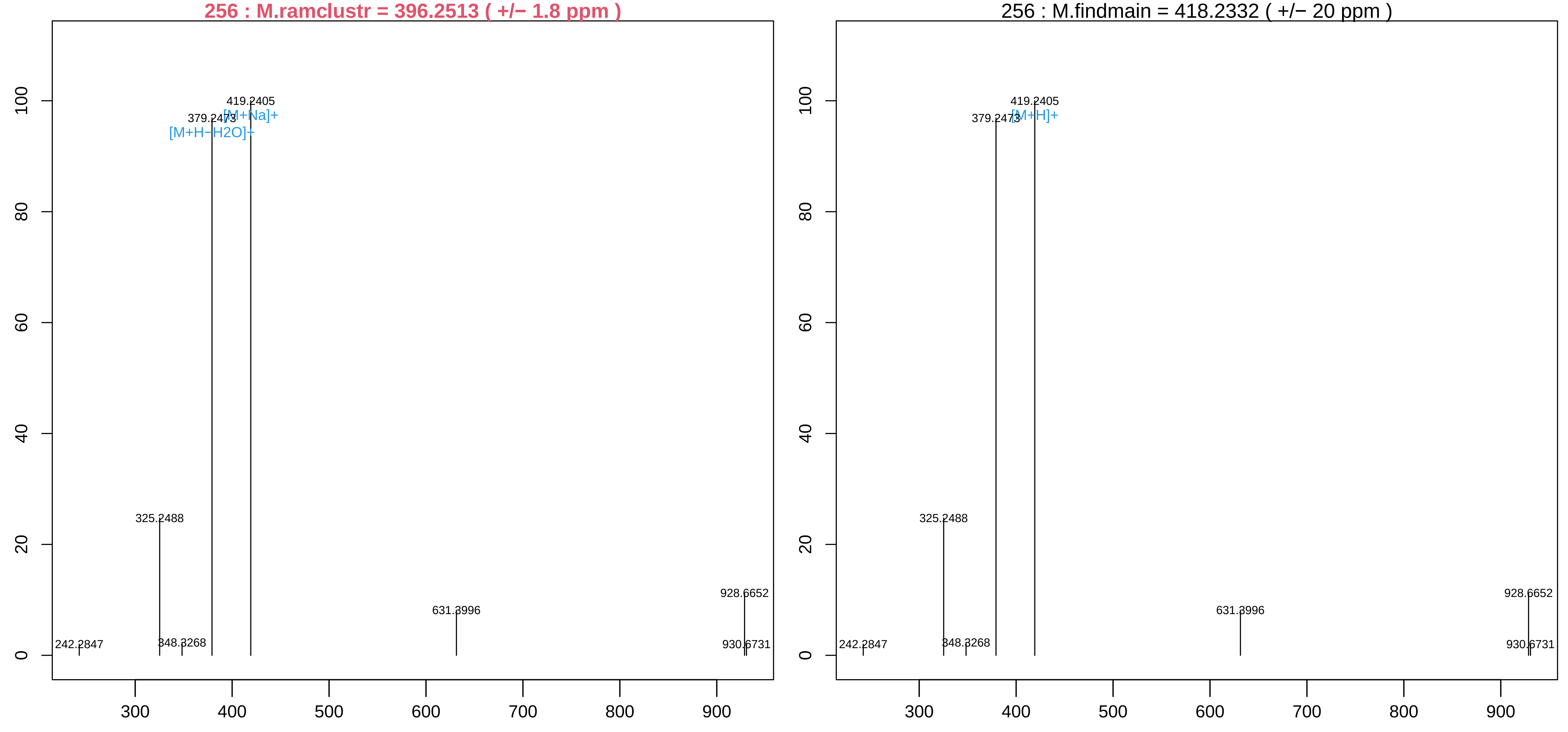

256 : M.ramclustr = 396.2513 (+/− 1.8 ppm)
419.2405
[M+Na]+
379.2473
[M+H−H2O]+
325.2488
242.2847
348.3268
631.3996
928.6652
930.6731
256 : M.findmain = 418.2332 (+/− 20 ppm)
419.2405
[M+H]+
379.2473
325.2488
242.2847
348.3268
631.3996
928.6652
930.6731
0
20
40
60
80
100
300
400
500
600
700
800
900

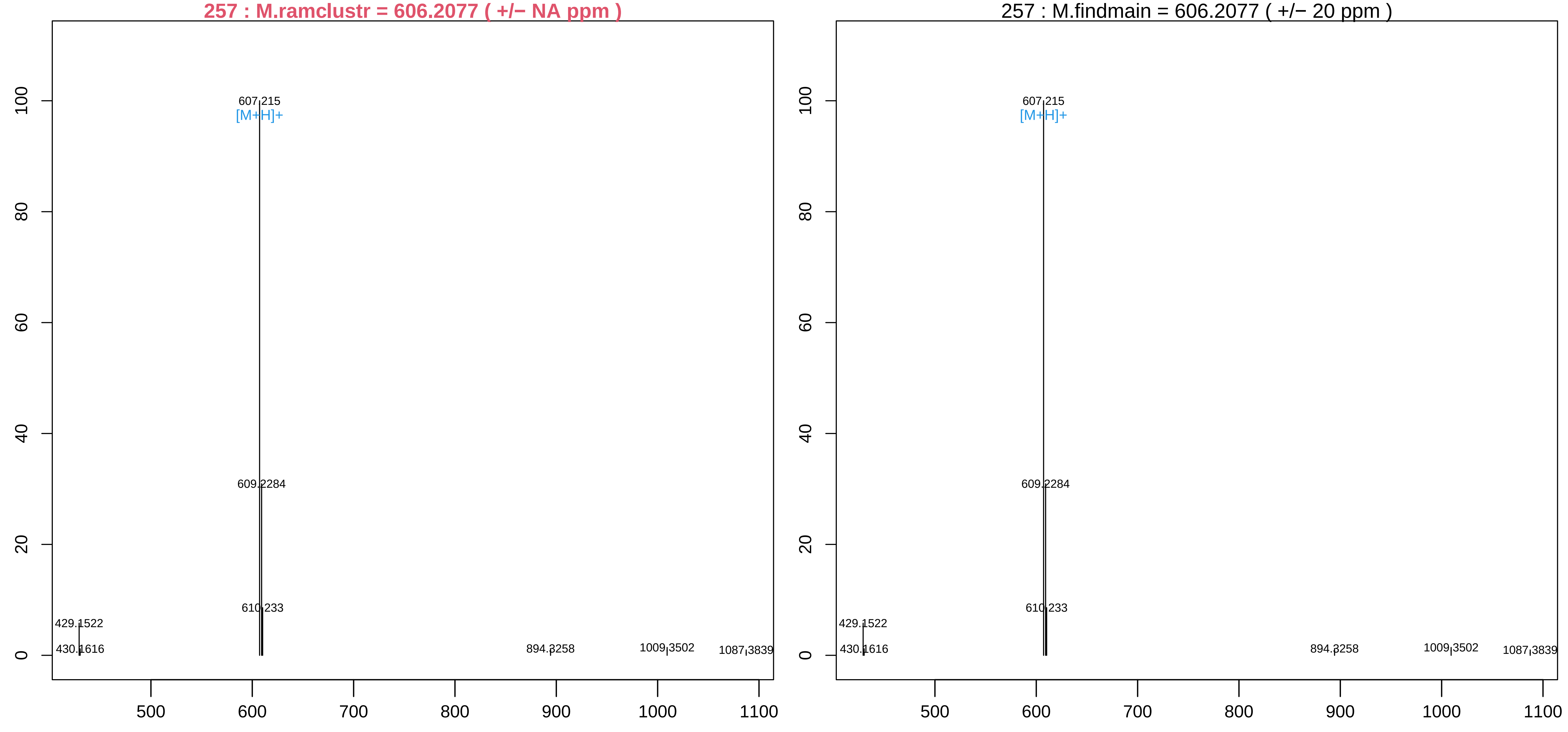

257 : M.ramclustr = 606.2077 (+/− NA ppm)
257 : M.findmain = 606.2077 (+/− 20 ppm)
607.215
[M+H]+
609.2284
610.233
429.1522
430.1616
894.3258
1009.3502
1087.3839
0
20
40
60
80
100
500
600
700
800
900
1000
1100

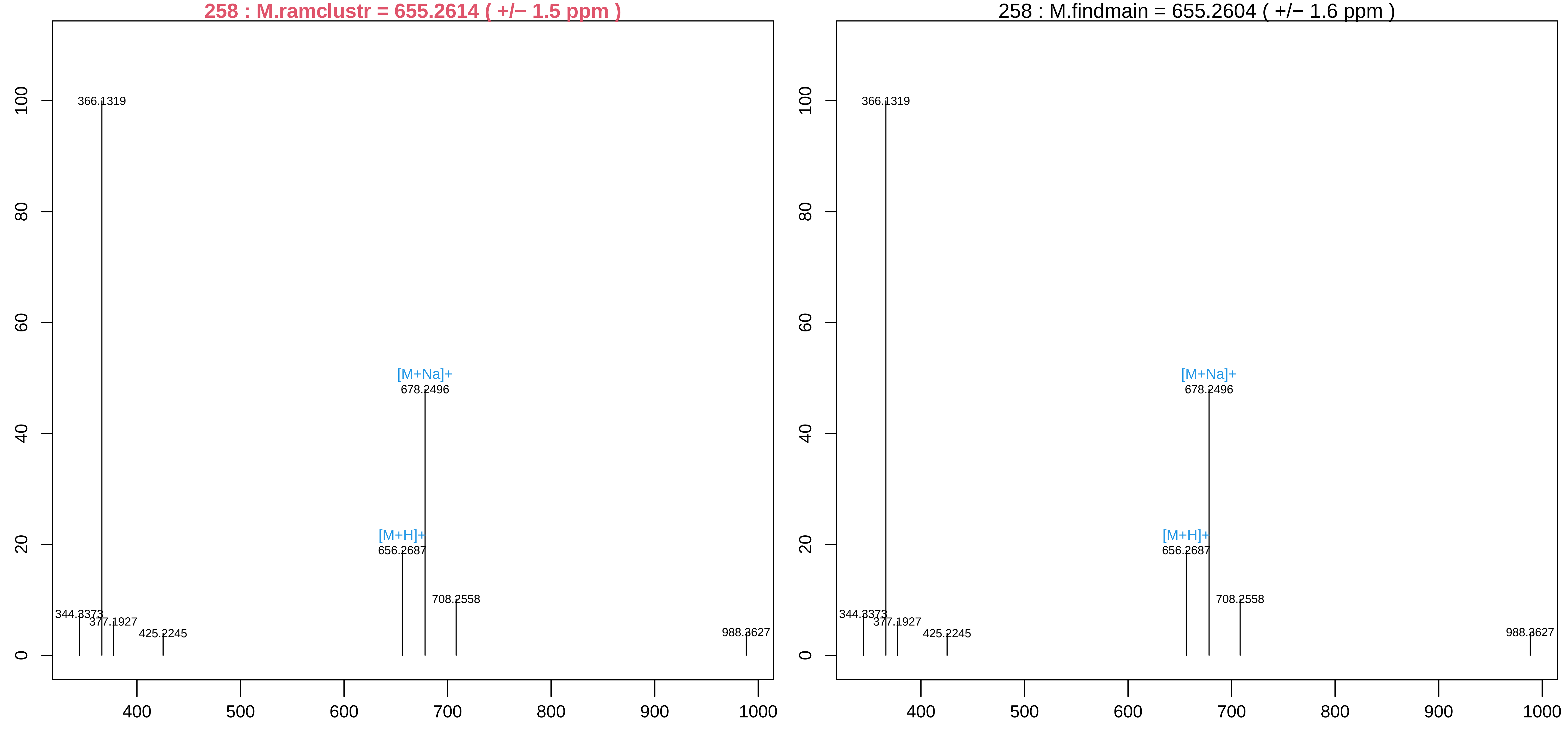
258 : M.ramclustr = 655.2614 (+/− 1.5 ppm)
366.1319
[M+Na]+
678.2496
[M+H]+
656.2687
708.2558
344.3373
377.1927
425.2245
988.3627
0
20
40
60
80
100
400
500
600
700
800
900
1000
258 : M.findmain = 655.2604 (+/− 1.6 ppm)
366.1319
[M+Na]+
678.2496
[M+H]+
656.2687
708.2558
344.3373
377.1927
425.2245
988.3627
0
20
40
60
80
100
400
500
600
700
800
900
1000

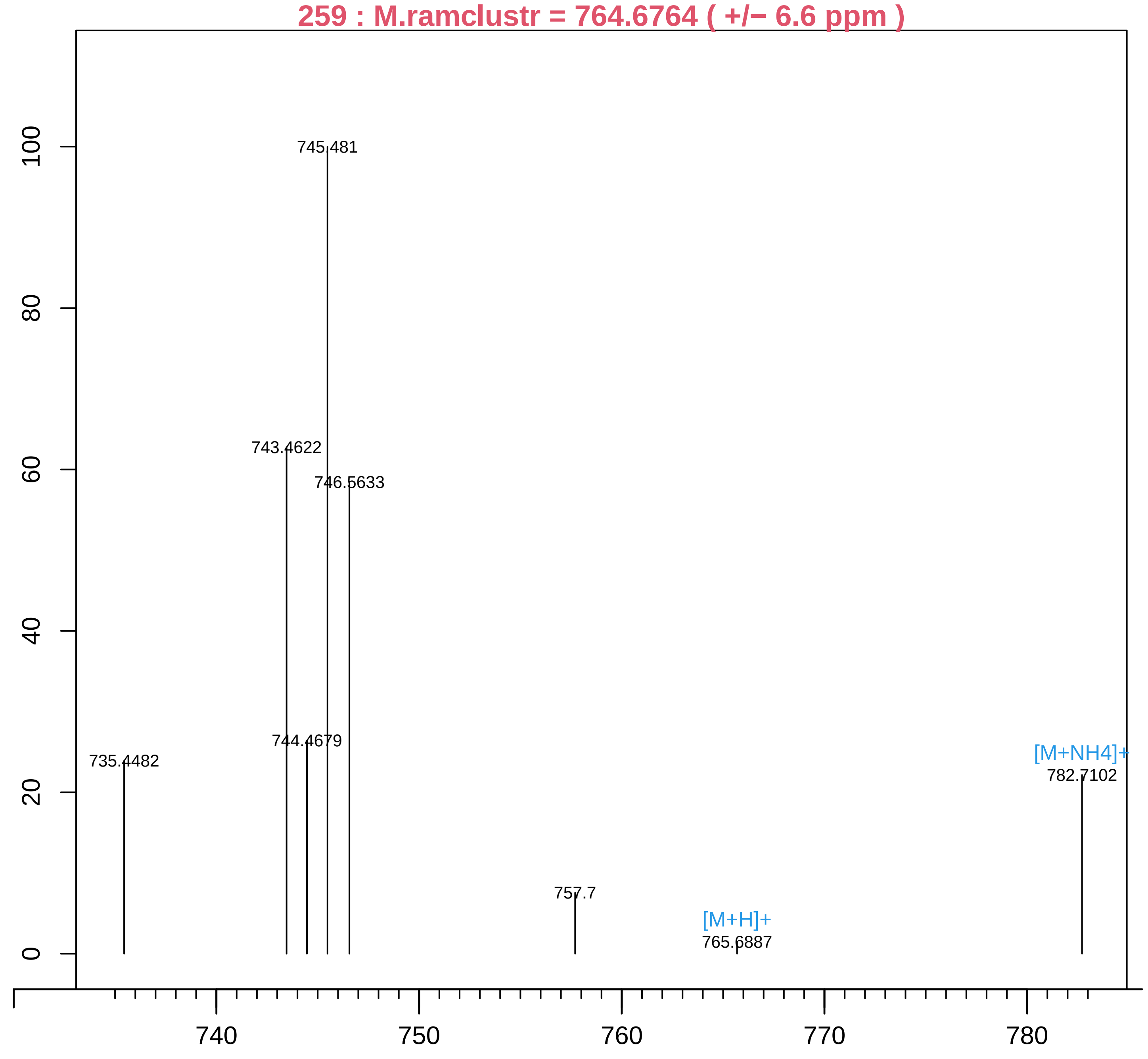
259 : M.ramclustr = 764.6764 (+/− 6.6 ppm)
745.481
743.4622
746.5633
744.4679
735.4482
[M+NH4]+
782.7102
757.7
[M+H]+
765.6887

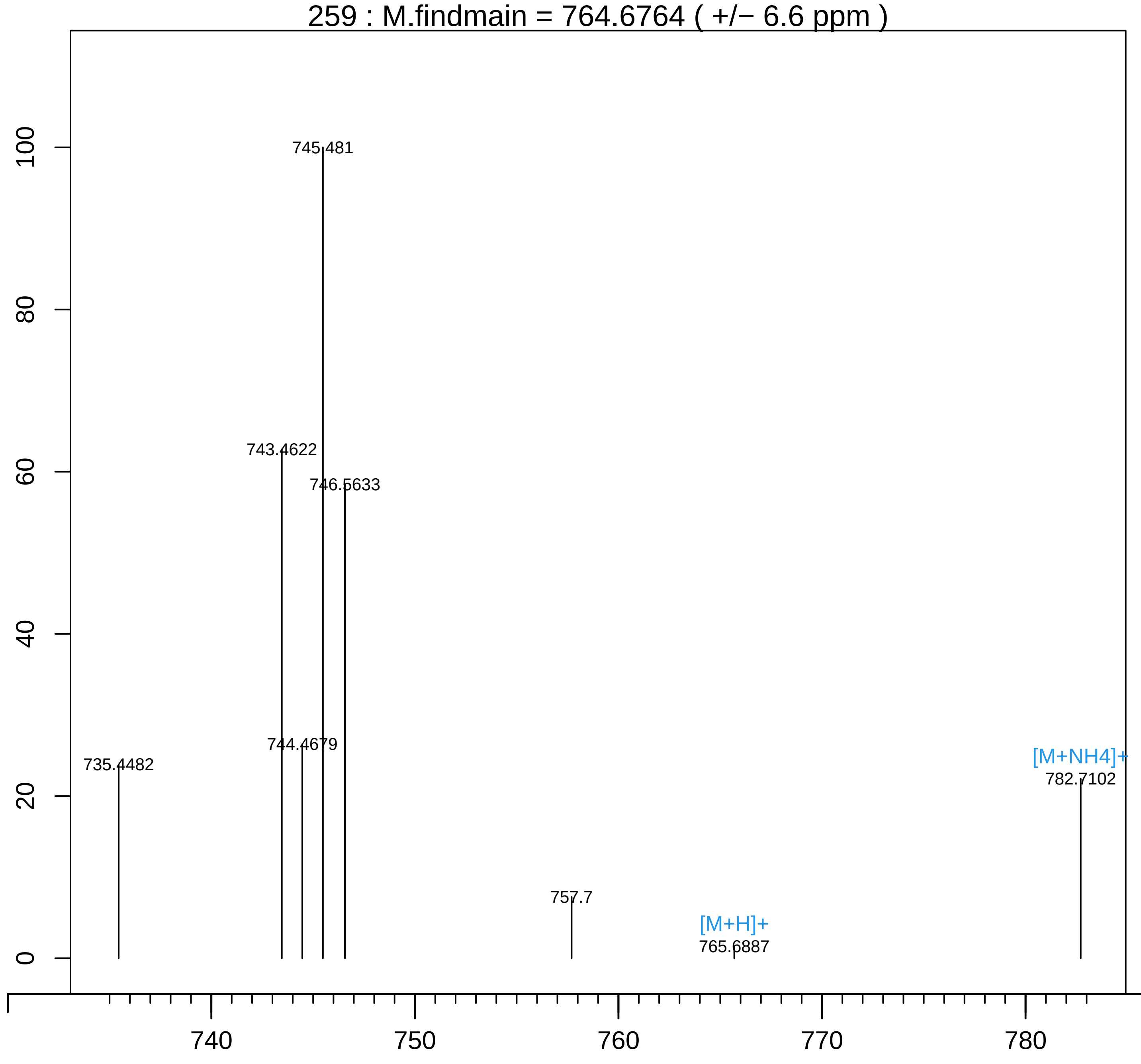
259 : M.findmain = 764.6764 (+/− 6.6 ppm)
745.481
743.4622
746.5633
744.4679
735.4482
[M+NH4]+
782.7102
757.7
[M+H]+
765.6887

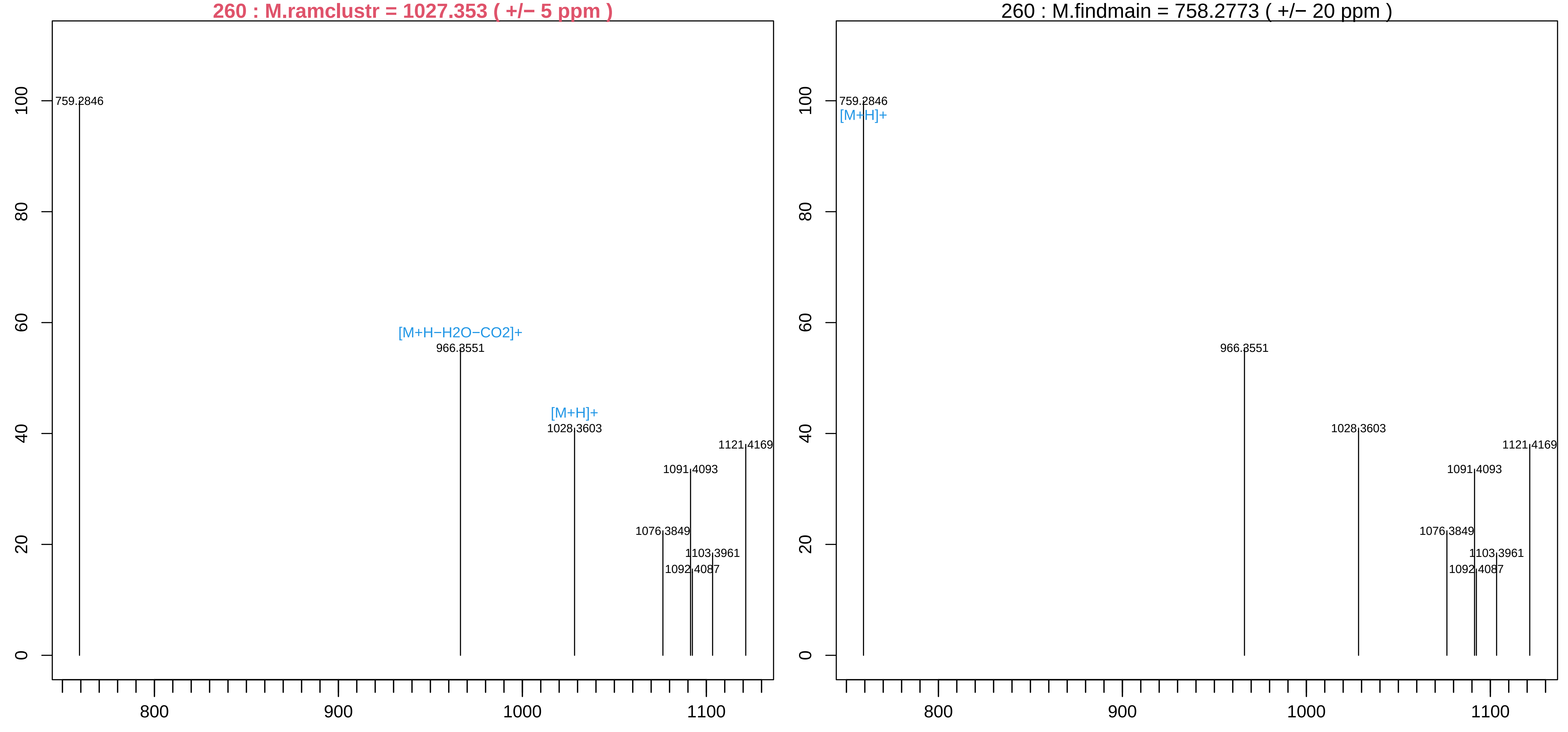

260 : M.ramclustr = 1027.353 (+/− 5 ppm)
759.2846
[M+H−H2O−CO2]+
966.3551
[M+H]+
1028.3603
1121.4169
1091.4093
1076.3849
1103.3961
1092.4087
0
20
40
60
80
100
800
900
1000
1100
260 : M.findmain = 758.2773 (+/− 20 ppm)
759.2846
[M+H]+
966.3551
1028.3603
1121.4169
1091.4093
1076.3849
1103.3961
1092.4087
0
20
40
60
80
100
800
900
1000
1100

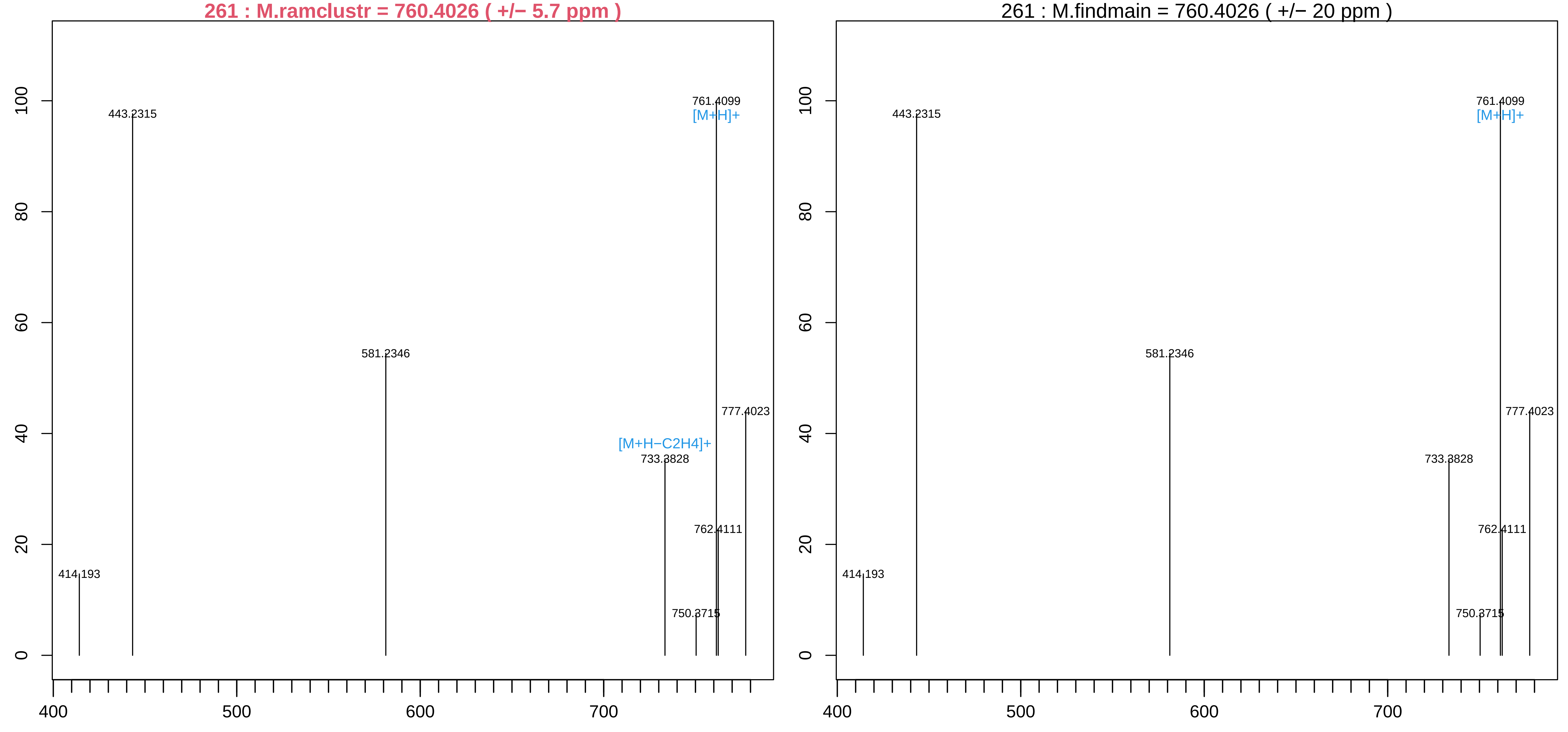

261 : M.ramclustr = 760.4026 (+/− 5.7 ppm)
443.2315
761.4099
[M+H]+
581.2346
777.4023
[M+H−C2H4]+
733.3828
762.4111
414.193
750.3715
261 : M.findmain = 760.4026 (+/− 20 ppm)
443.2315
761.4099
[M+H]+
581.2346
777.4023
733.3828
762.4111
414.193
750.3715
0
20
40
60
80
100
400
500
600
700

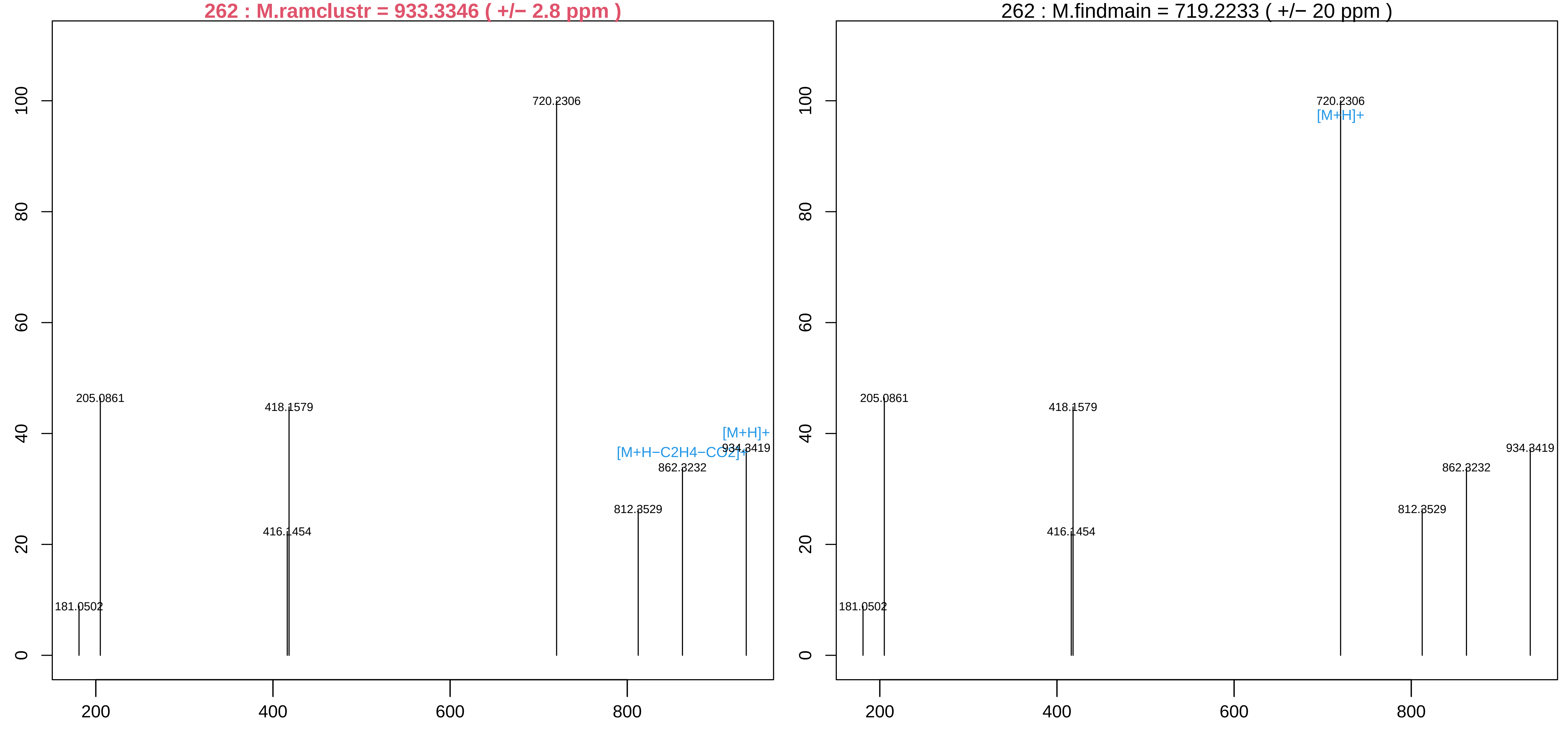

262 : M.ramclustr = 933.3346 (+/− 2.8 ppm)
720.2306
205.0861
418.1579
416.1454
181.0502
812.3529
862.3232
934.3419
[M+H]+
[M+H−C2H4−CO2]+
262 : M.findmain = 719.2233 (+/− 20 ppm)
720.2306
[M+H]+
205.0861
418.1579
416.1454
181.0502
812.3529
862.3232
934.3419

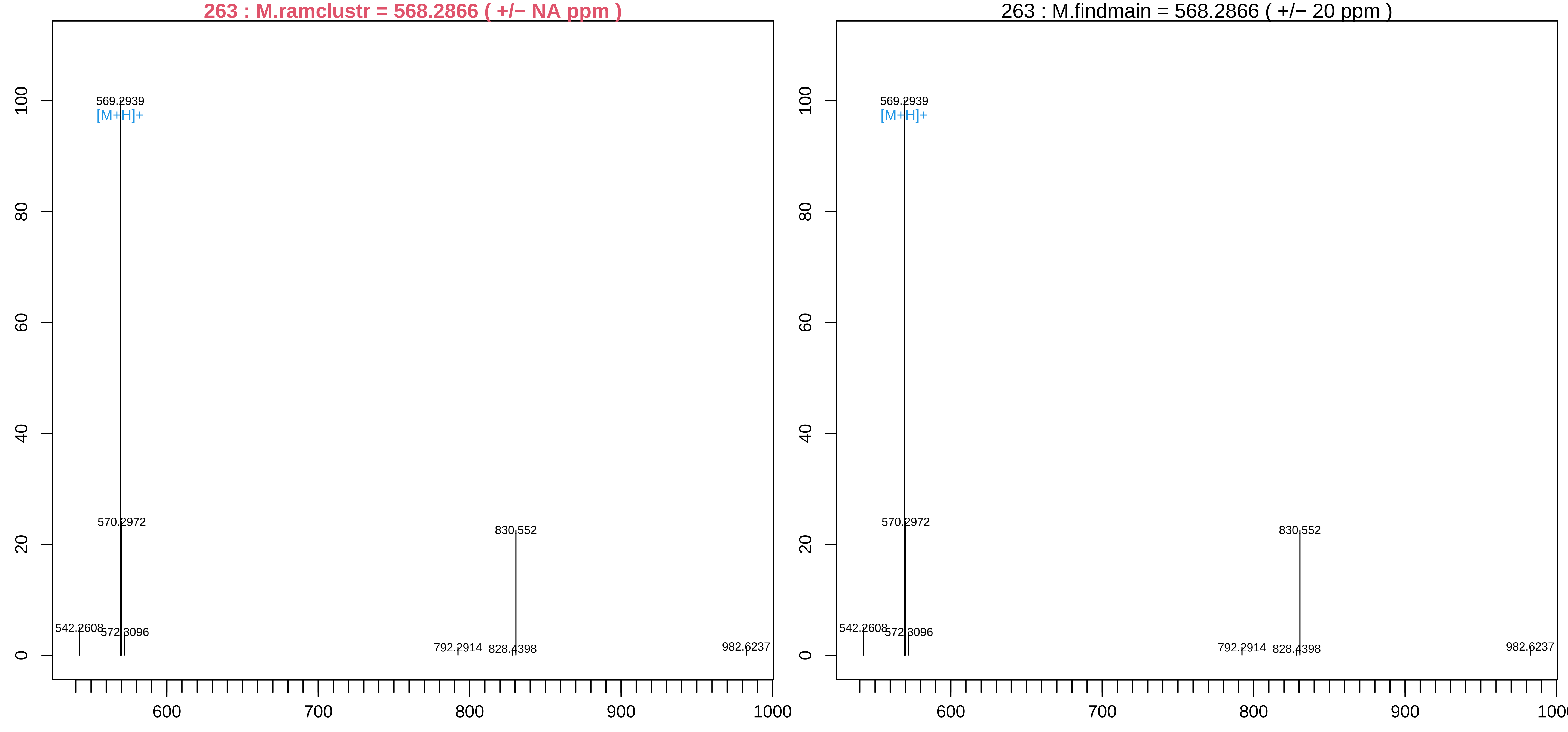
263 : M.ramclustr = 568.2866 (+/− NA ppm)
569.2939
[M+H]+
570.2972
830.552
542.2608
572.3096
792.2914
828.4398
982.6237
0
20
40
60
80
100
600
700
800
900
1000
263 : M.findmain = 568.2866 (+/− 20 ppm)
569.2939
[M+H]+
570.2972
830.552
542.2608
572.3096
792.2914
828.4398
982.6237
0
20
40
60
80
100
600
700
800
900
1000

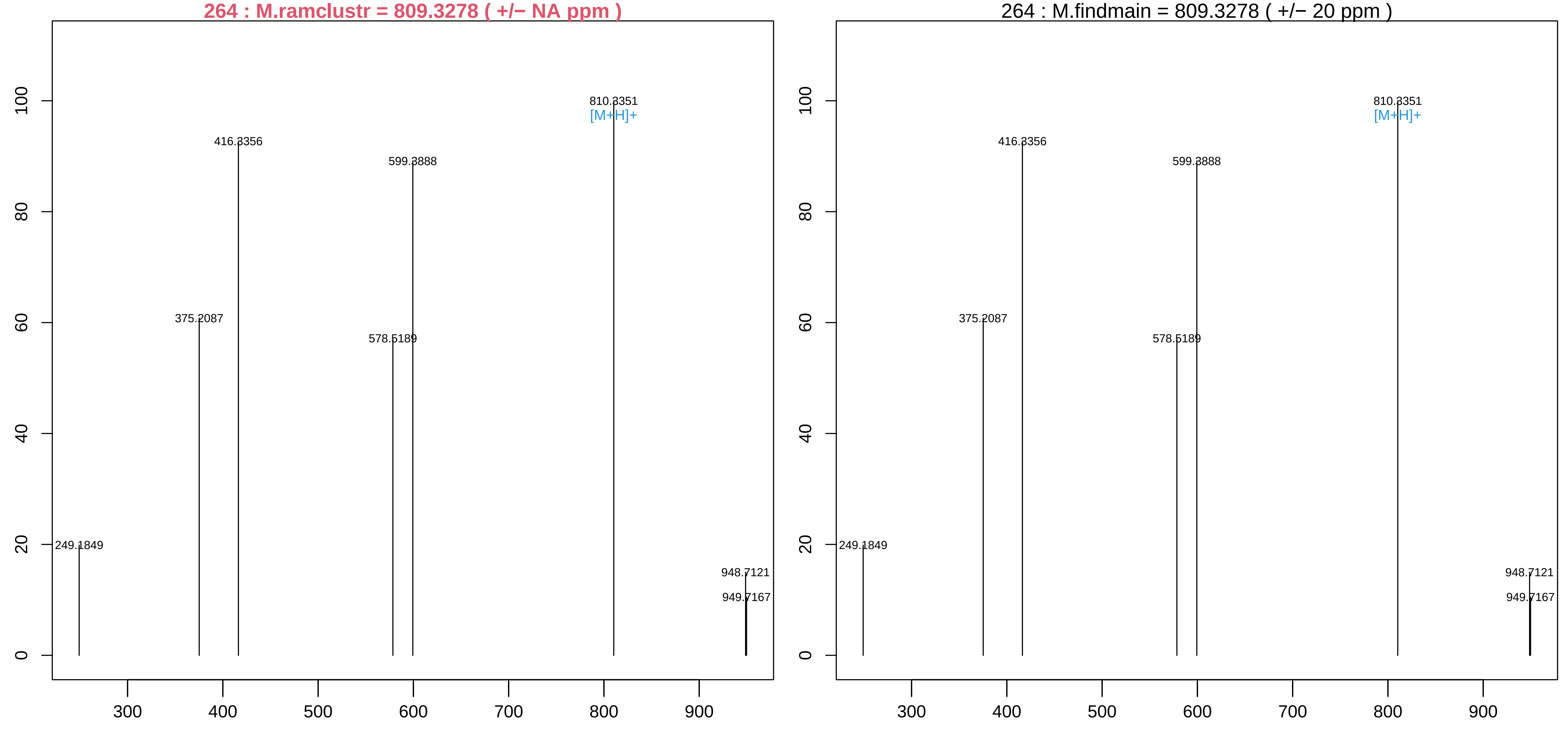
264 : M.ramclustr = 809.3278 (+/− NA ppm)
810.3351
[M+H]+
416.3356
599.3888
375.2087
578.5189
249.1849
948.7121
949.7167
264 : M.findmain = 809.3278 (+/− 20 ppm)
810.3351
[M+H]+
416.3356
599.3888
375.2087
578.5189
249.1849
948.7121
949.7167

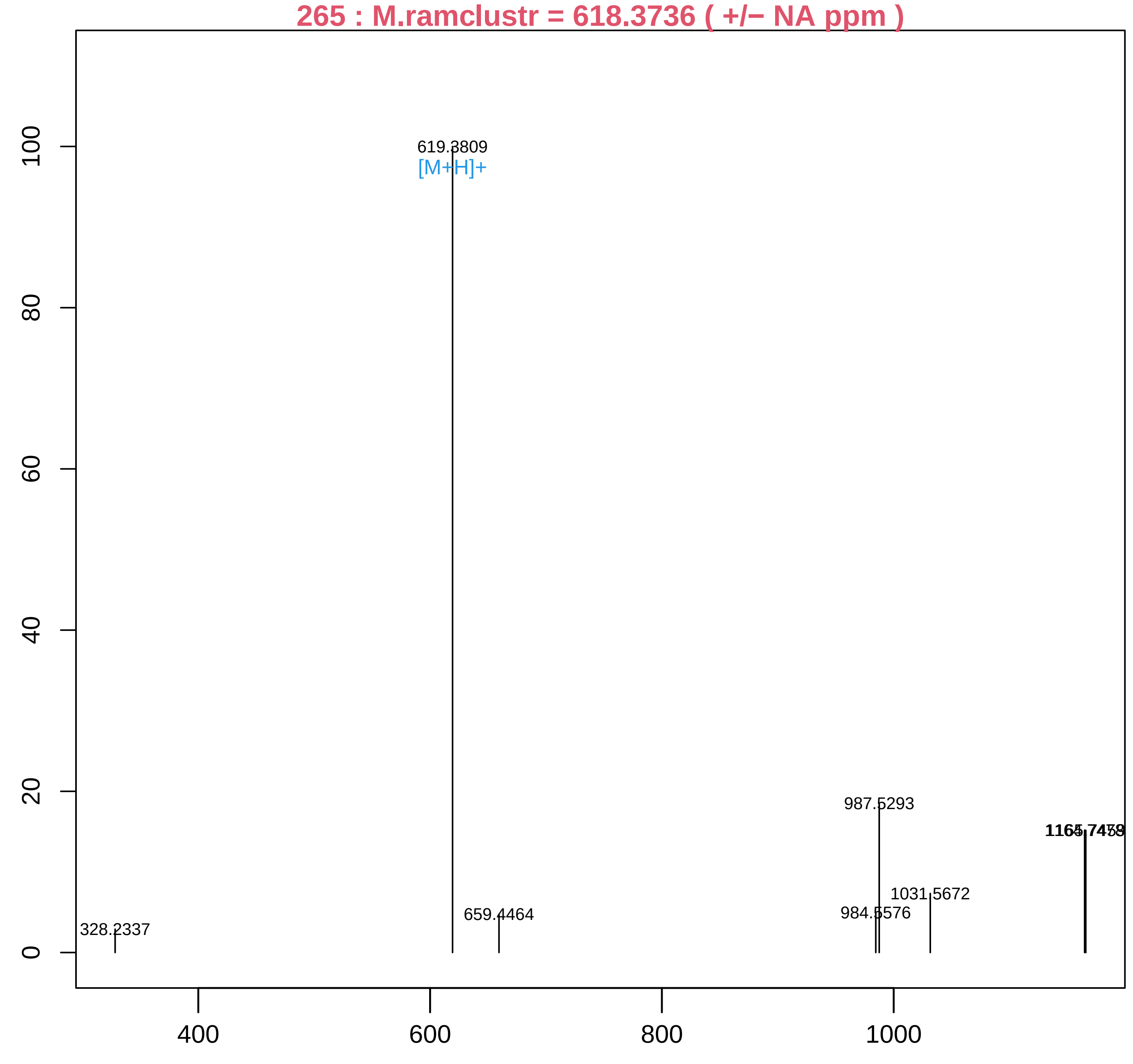
265 : M.ramclustr = 618.3736 (+/− NA ppm)
619.3809
[M+H]+
987.5293
1031.5672
984.5576
659.4464
328.2337
0
20
40
60
80
100
400
600
800
1000

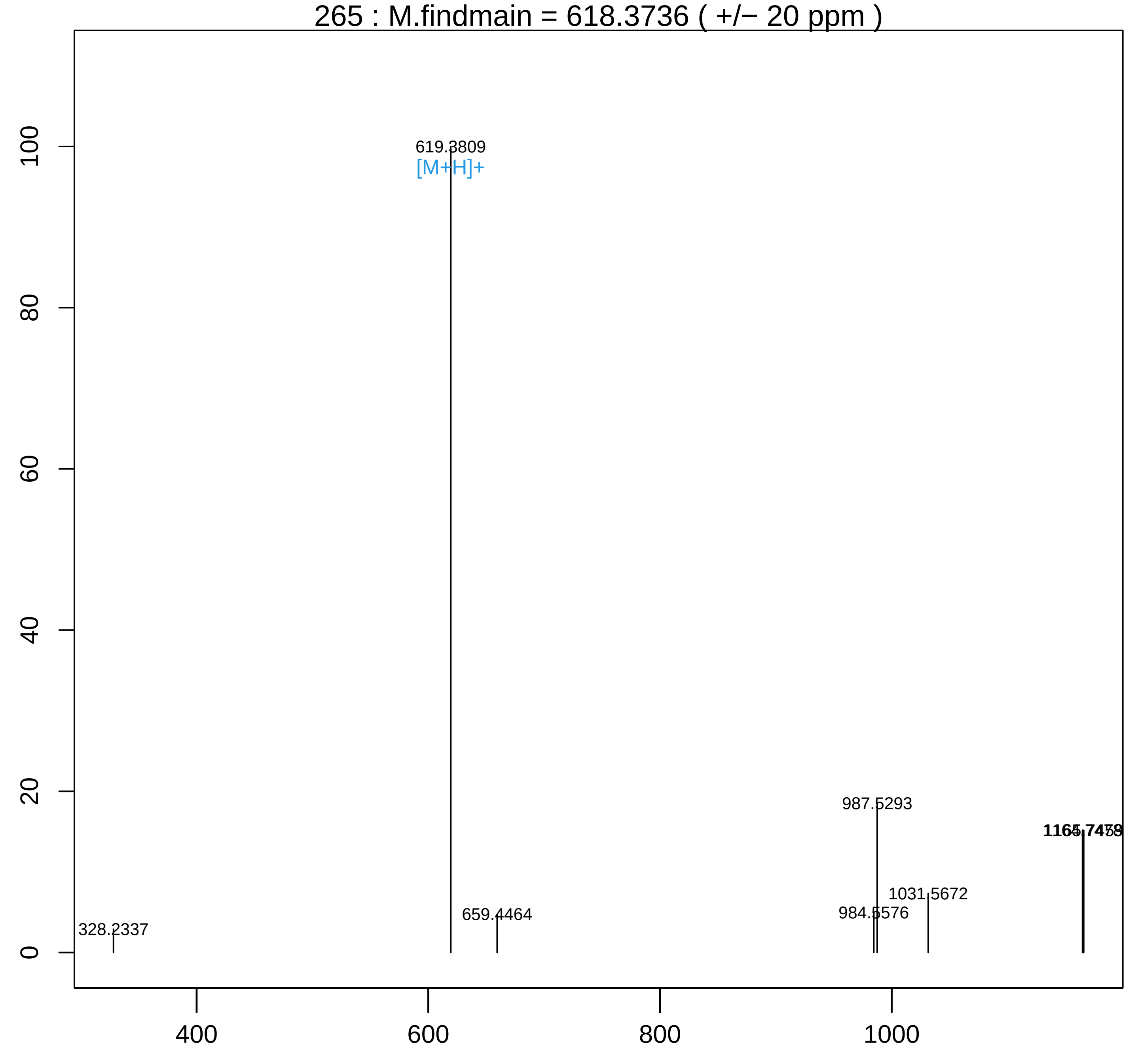
265 : M.findmain = 618.3736 (+/− 20 ppm)
619.3809
[M+H]+
987.5293
1031.5672
984.5576
659.4464
328.2337
0
20
40
60
80
100
400
600
800
1000

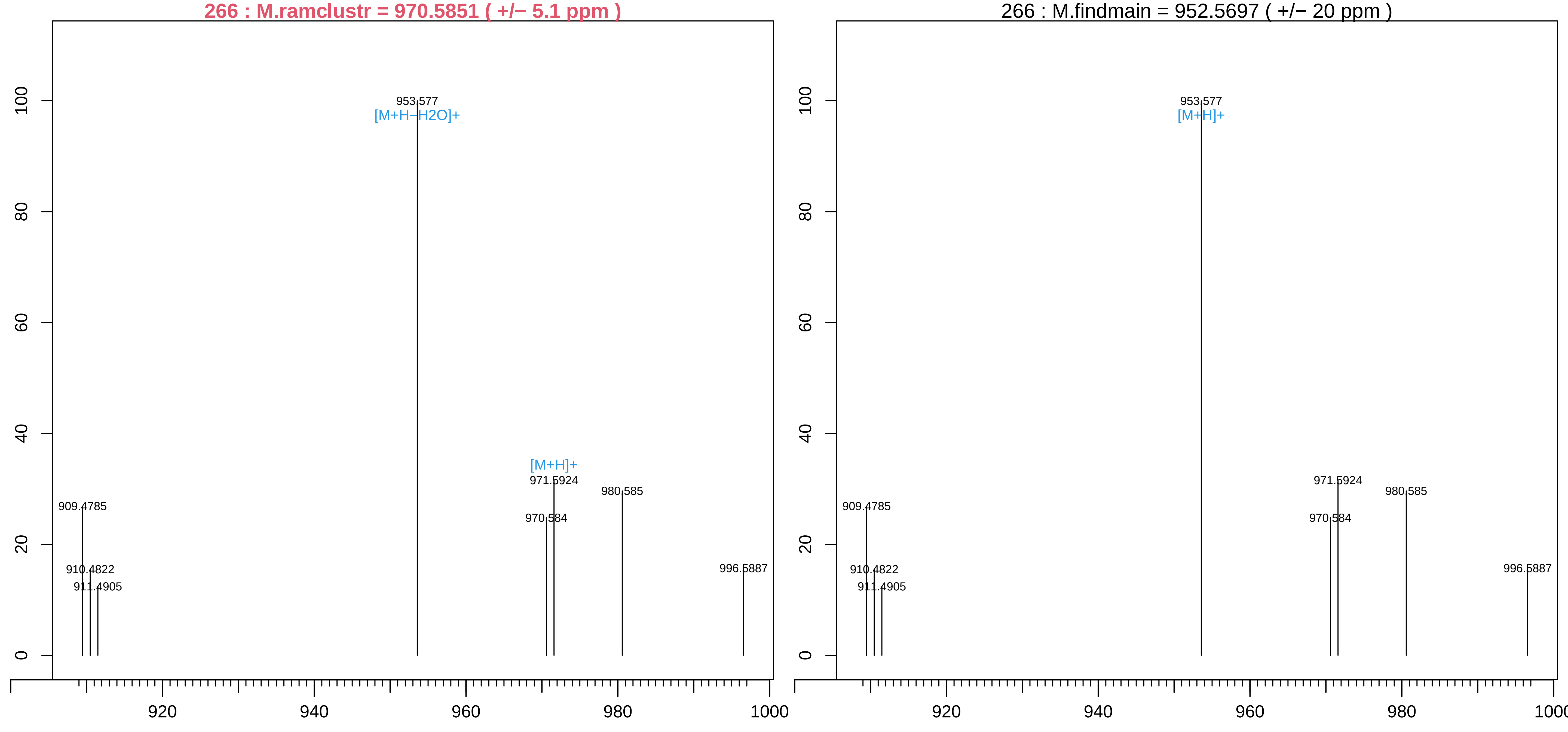
266 : M.ramclustr = 970.5851 (+/− 5.1 ppm)
953.577
[M+H−H2O]+
[M+H]+
971.5924
980.585
970.584
909.4785
910.4822
911.4905
996.5887
266 : M.findmain = 952.5697 (+/− 20 ppm)
953.577
[M+H]+
971.5924
980.585
970.584
909.4785
910.4822
911.4905
996.5887
0
20
40
60
80
100
920
940
960
980
1000

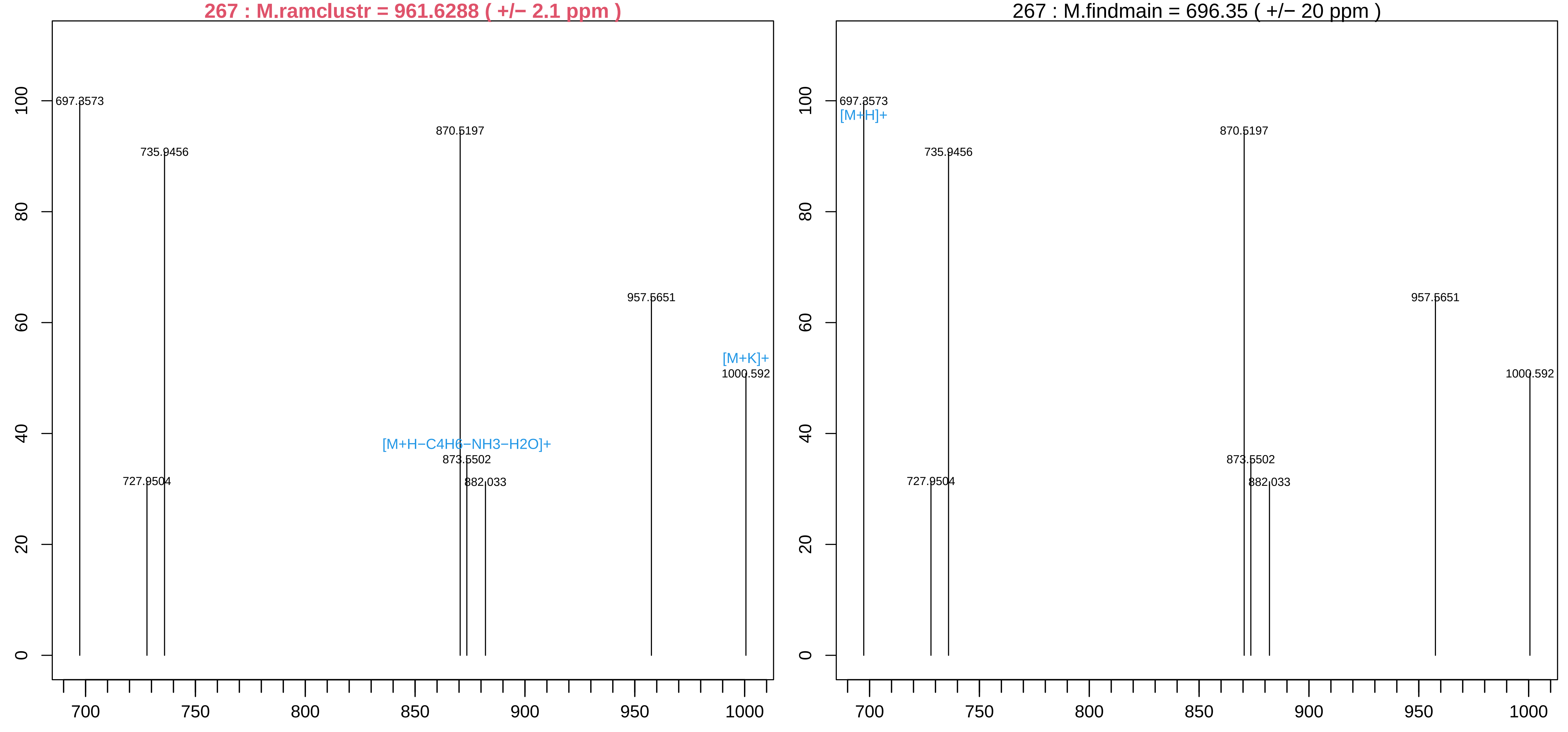
267 : M.ramclustr = 961.6288 (+/− 2.1 ppm)
697.3573
735.9456
727.9504
870.5197
[M+H−C4H6−NH3−H2O]+
873.5502
882.033
957.5651
[M+K]+
1000.592
267 : M.findmain = 696.35 (+/− 20 ppm)
697.3573
[M+H]+
735.9456
727.9504
870.5197
873.5502
882.033
957.5651
1000.592

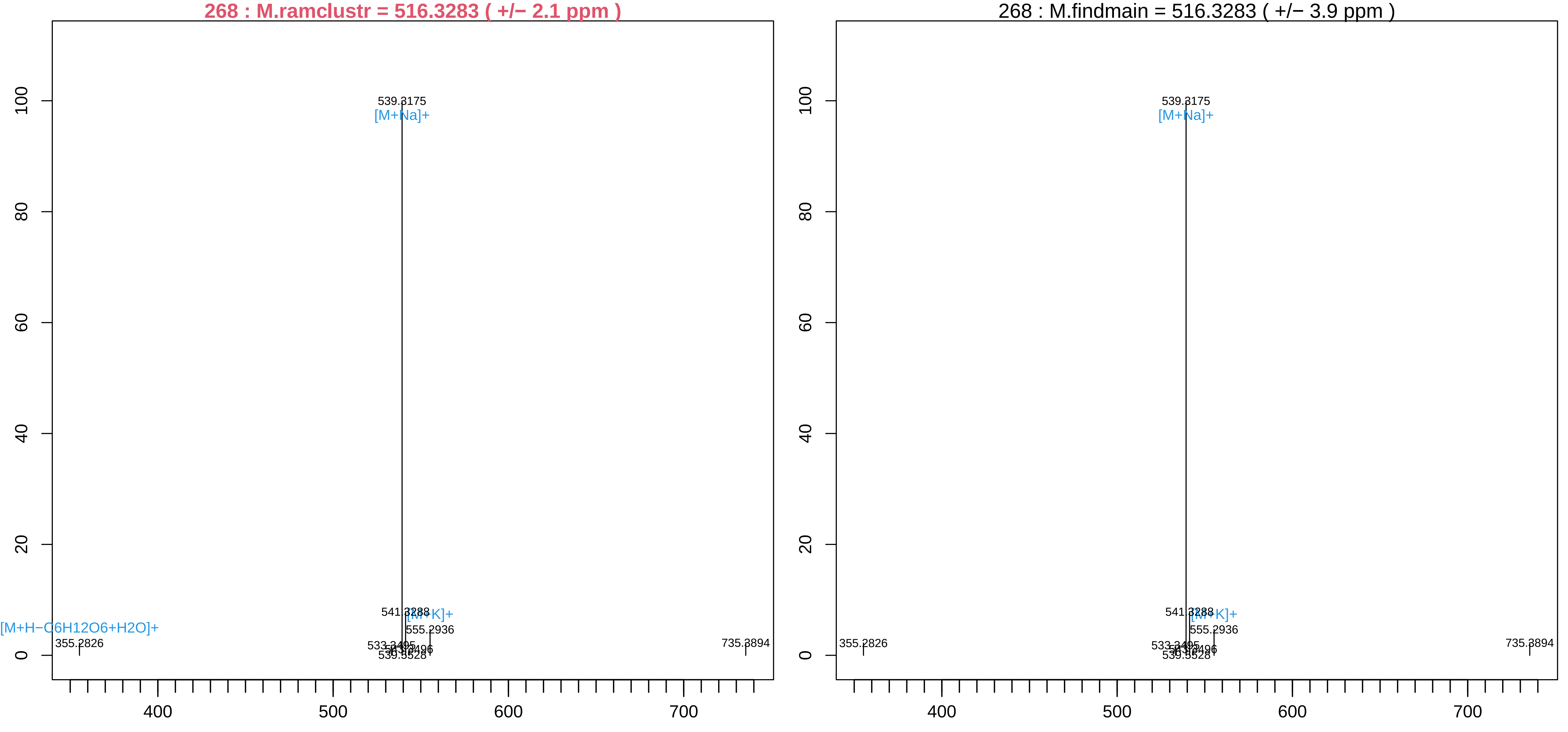
268 : M.ramclustr = 516.3283 (+/− 2.1 ppm)
539.3175
[M+Na]+
541.3288
[M+K]+
555.2936
533.3495
[M+H−C6H12O6+H2O]+
355.2826
735.3894
100
80
60
40
20
0
400
500
600
700
268 : M.findmain = 516.3283 (+/− 3.9 ppm)
539.3175
[M+Na]+
541.3288
[M+K]+
555.2936
533.3495
355.2826
735.3894
100
80
60
40
20
0
400
500
600
700

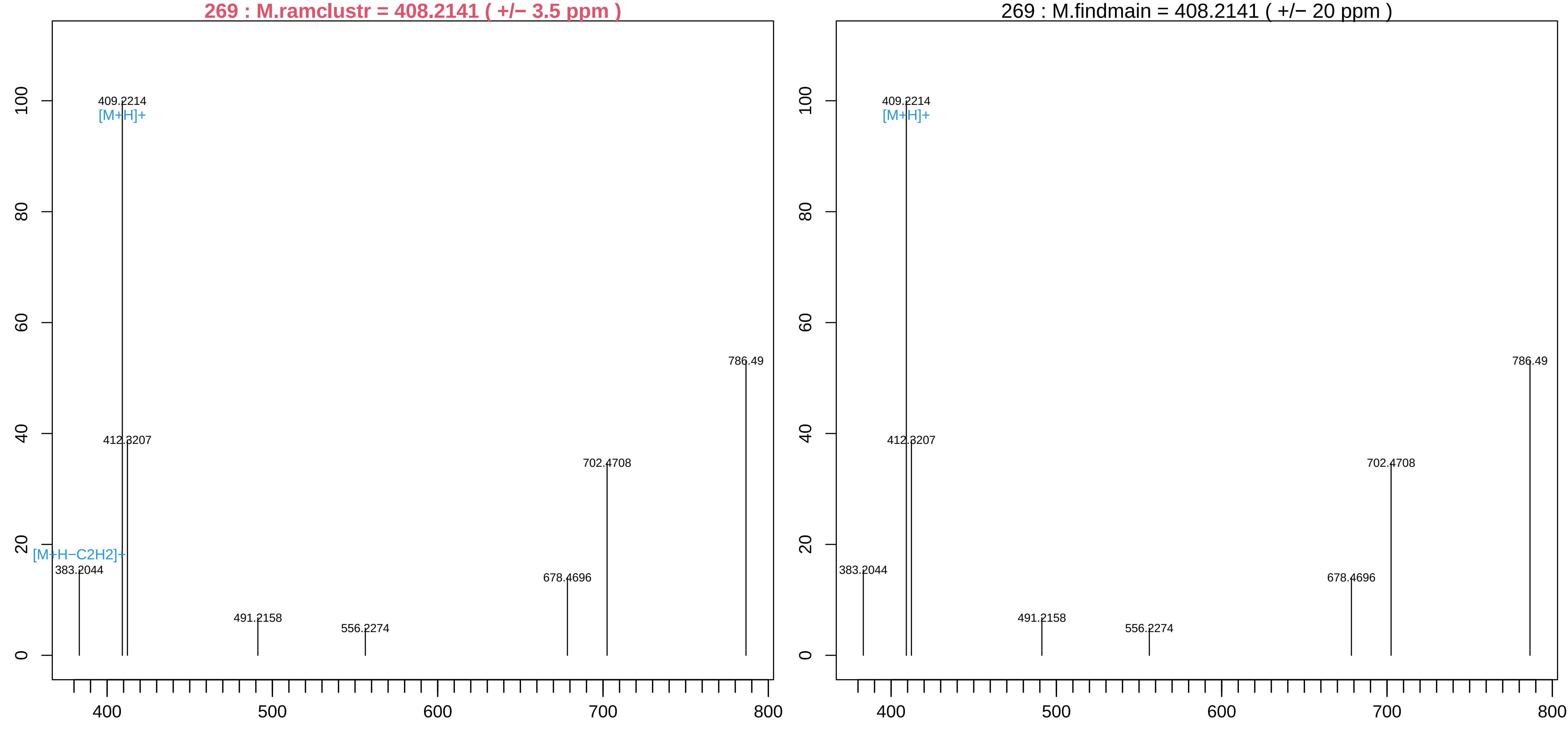

269 : M.ramclustr = 408.2141 (+/− 3.5 ppm)
409.2214
[M+H]+
412.3207
[M+H−C2H2]+
383.2044
491.2158
556.2274
678.4696
702.4708
786.49
269 : M.findmain = 408.2141 (+/− 20 ppm)
409.2214
[M+H]+
412.3207
383.2044
491.2158
556.2274
678.4696
702.4708
786.49

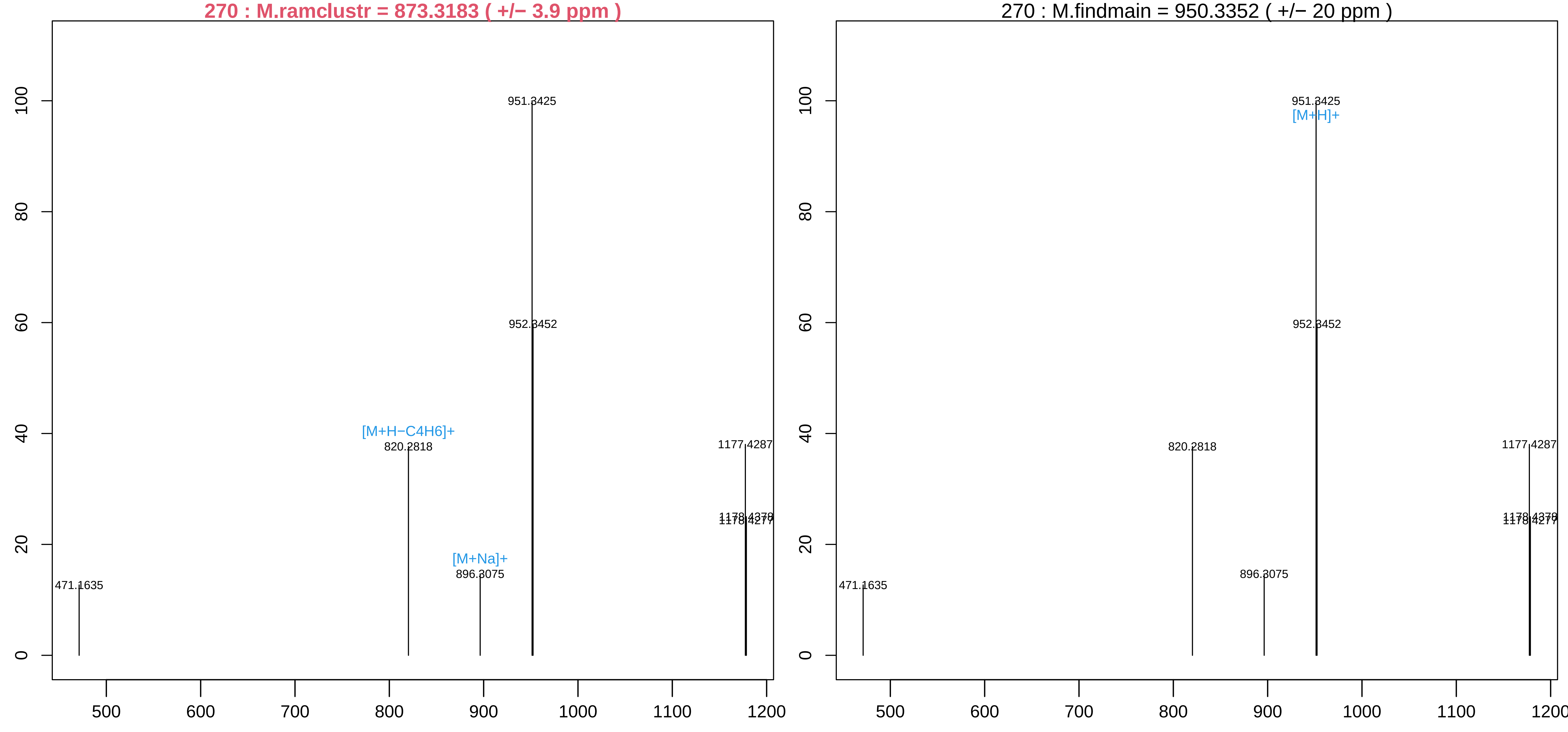
270 : M.ramclustr = 873.3183 (+/− 3.9 ppm)
951.3425
952.3452
[M+H−C4H6]+
820.2818
1177.4287
1178.4379
1178.4277
[M+Na]+
896.3075
471.1635
270 : M.findmain = 950.3352 (+/− 20 ppm)
951.3425
[M+H]+
952.3452
820.2818
1177.4287
1178.4379
1178.4277
896.3075
471.1635

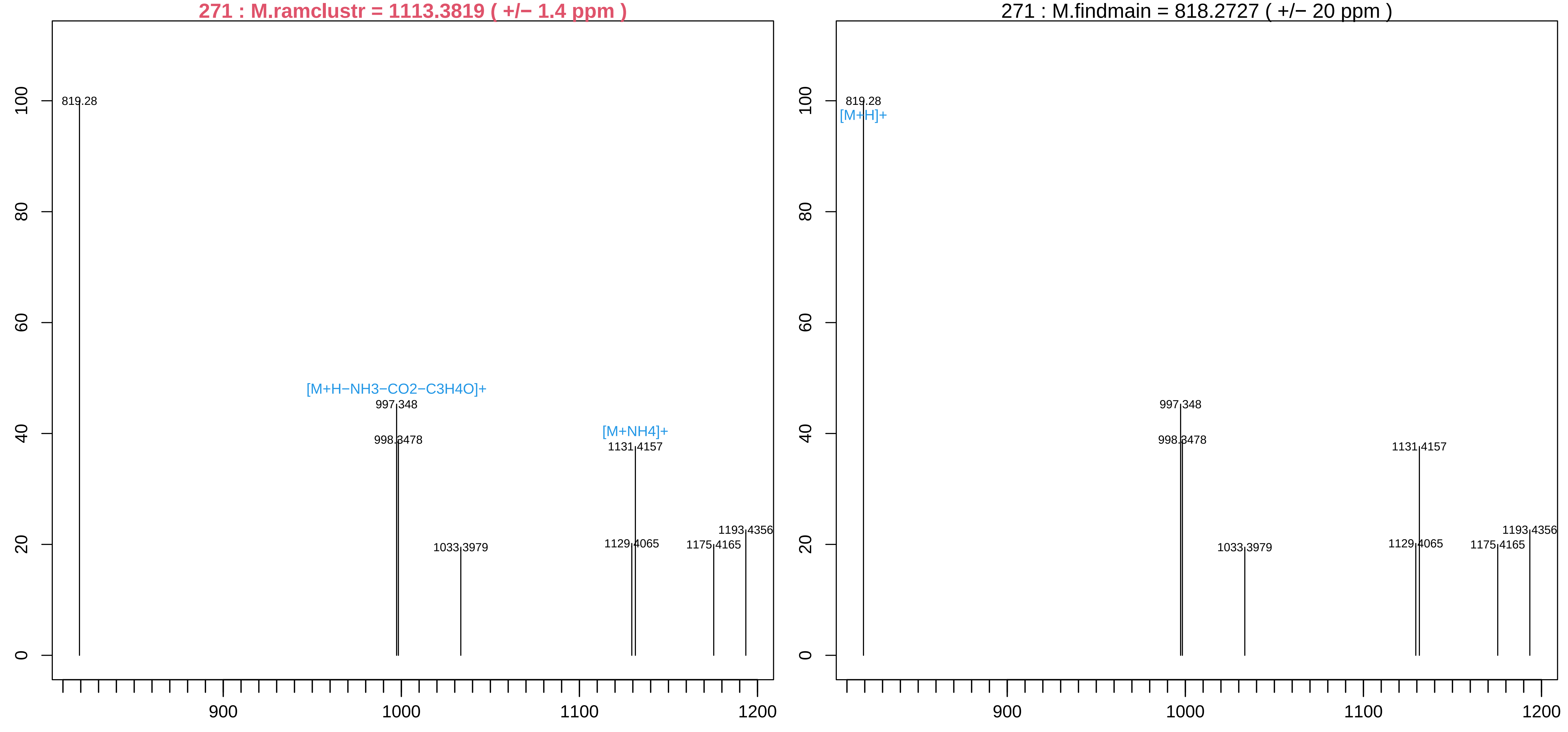
271 : M.ramclustr = 1113.3819 (+/− 1.4 ppm)
819.28
[M+H−NH3−CO2−C3H4O]+
997.348
998.3478
1033.3979
[M+NH4]+
1131.4157
1129.4065
1175.4165
1193.4356
271 : M.findmain = 818.2727 (+/− 20 ppm)
819.28
[M+H]+
997.348
998.3478
1033.3979
1131.4157
1129.4065
1175.4165
1193.4356
0
20
40
60
80
100
900
1000
1100
1200

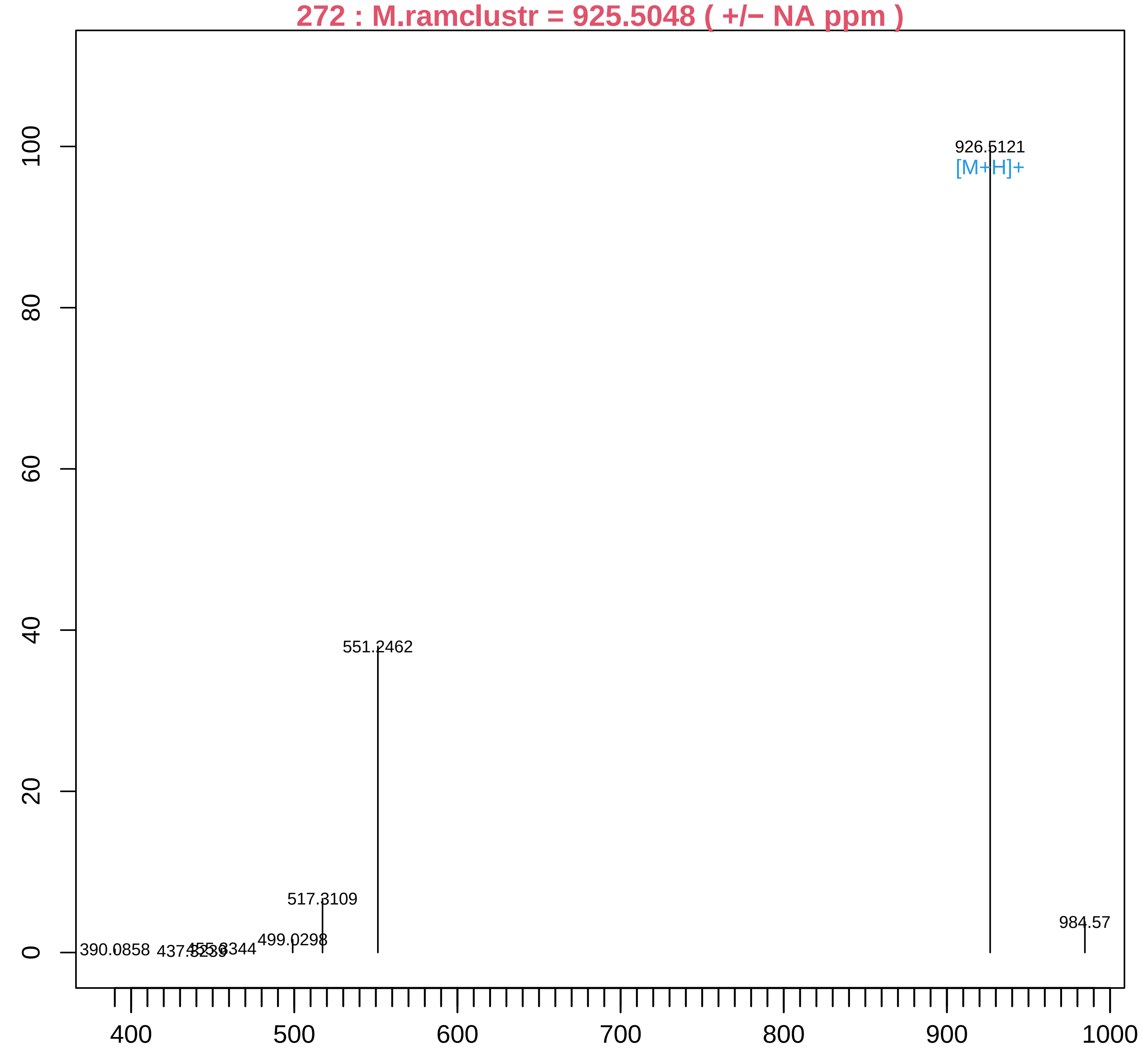
272 : M.ramclustr = 925.5048 (+/− NA ppm)
926.5121
[M+H]+
551.2462
517.3109
499.0298
390.0858
984.57
0
20
40
60
80
100
400
500
600
700
800
900
1000

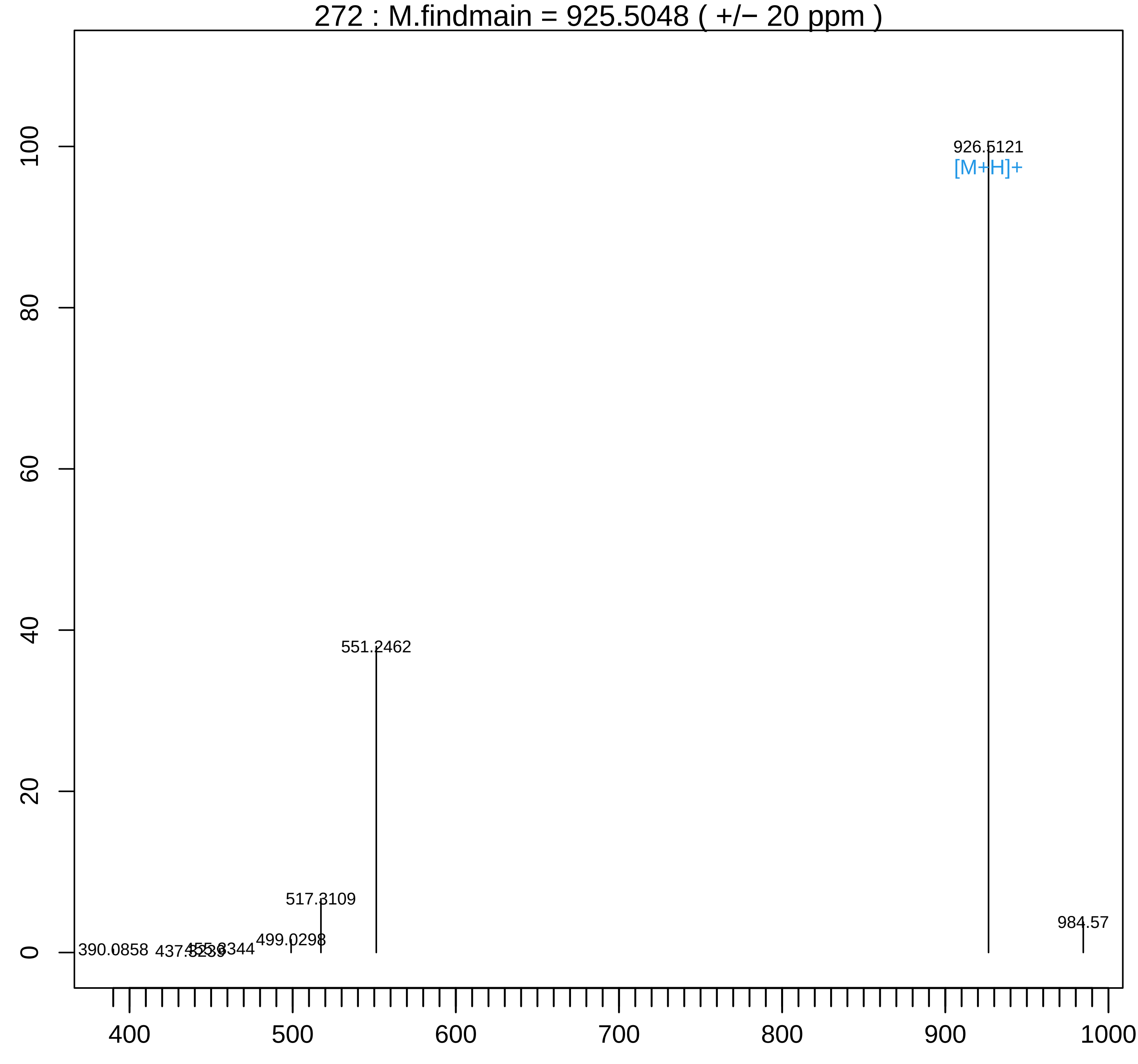
272 : M.findmain = 925.5048 (+/− 20 ppm)
926.5121
[M+H]+
551.2462
517.3109
499.0298
390.0858
984.57
0
20
40
60
80
100
400
500
600
700
800
900
1000

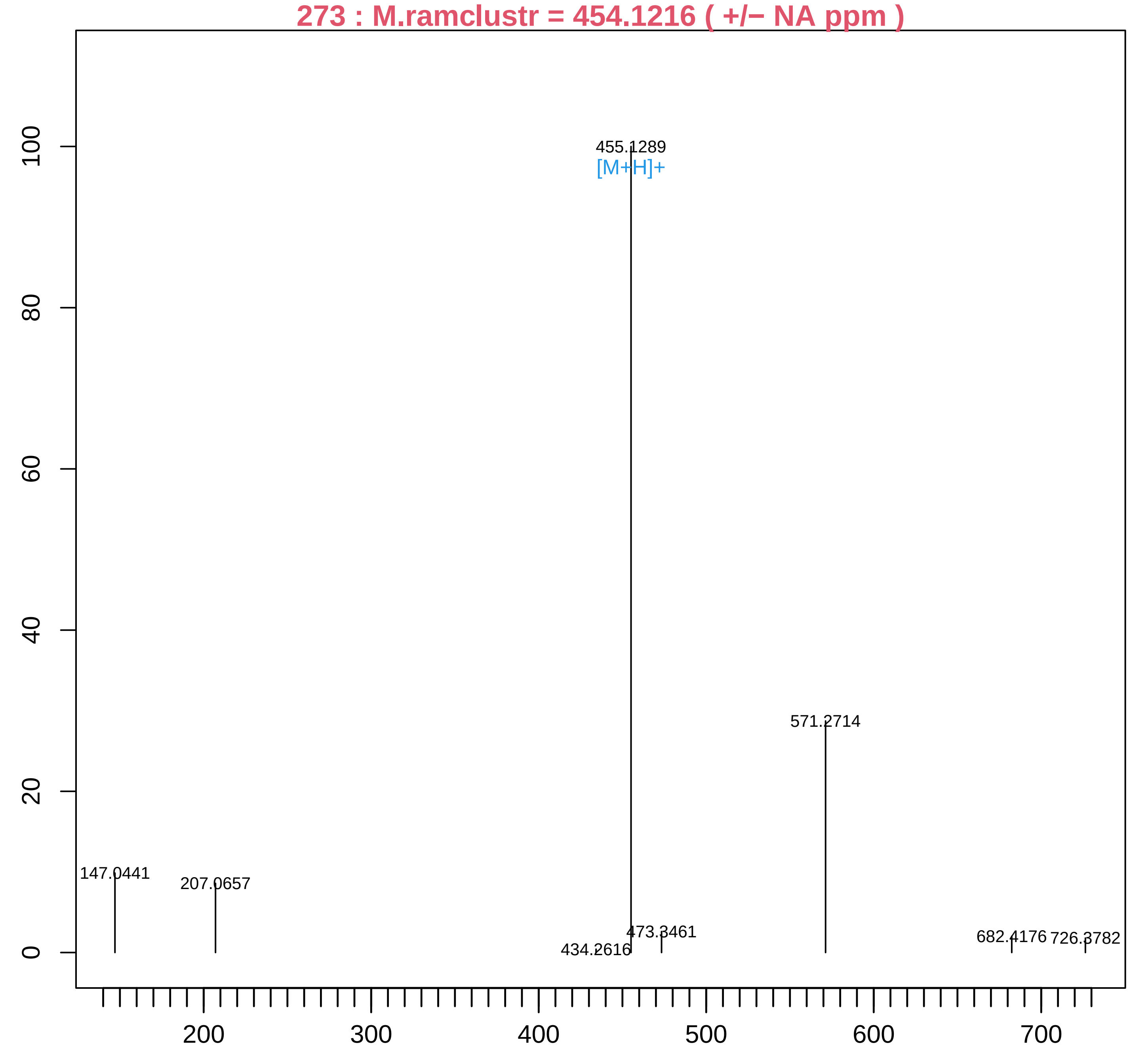
273 : M.ramclustr = 454.1216 (+/− NA ppm)
455.1289
[M+H]+
571.2714
147.0441
207.0657
434.2616
473.3461
682.4176
726.3782
0
20
40
60
80
100
200
300
400
500
600
700

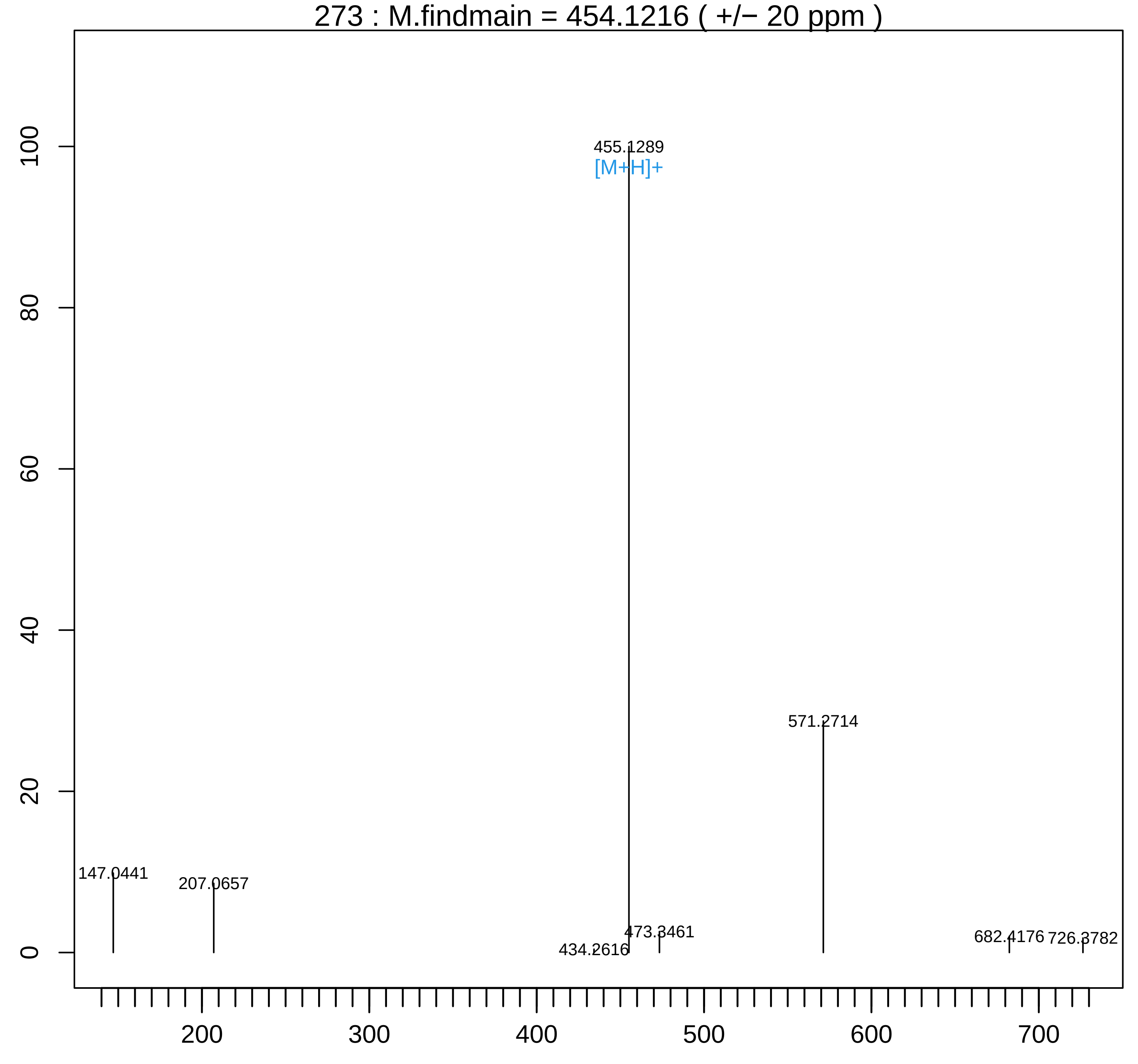
273 : M.findmain = 454.1216 (+/− 20 ppm)
455.1289
[M+H]+
571.2714
147.0441
207.0657
434.2616
473.3461
682.4176
726.3782
0
20
40
60
80
100
200
300
400
500
600
700

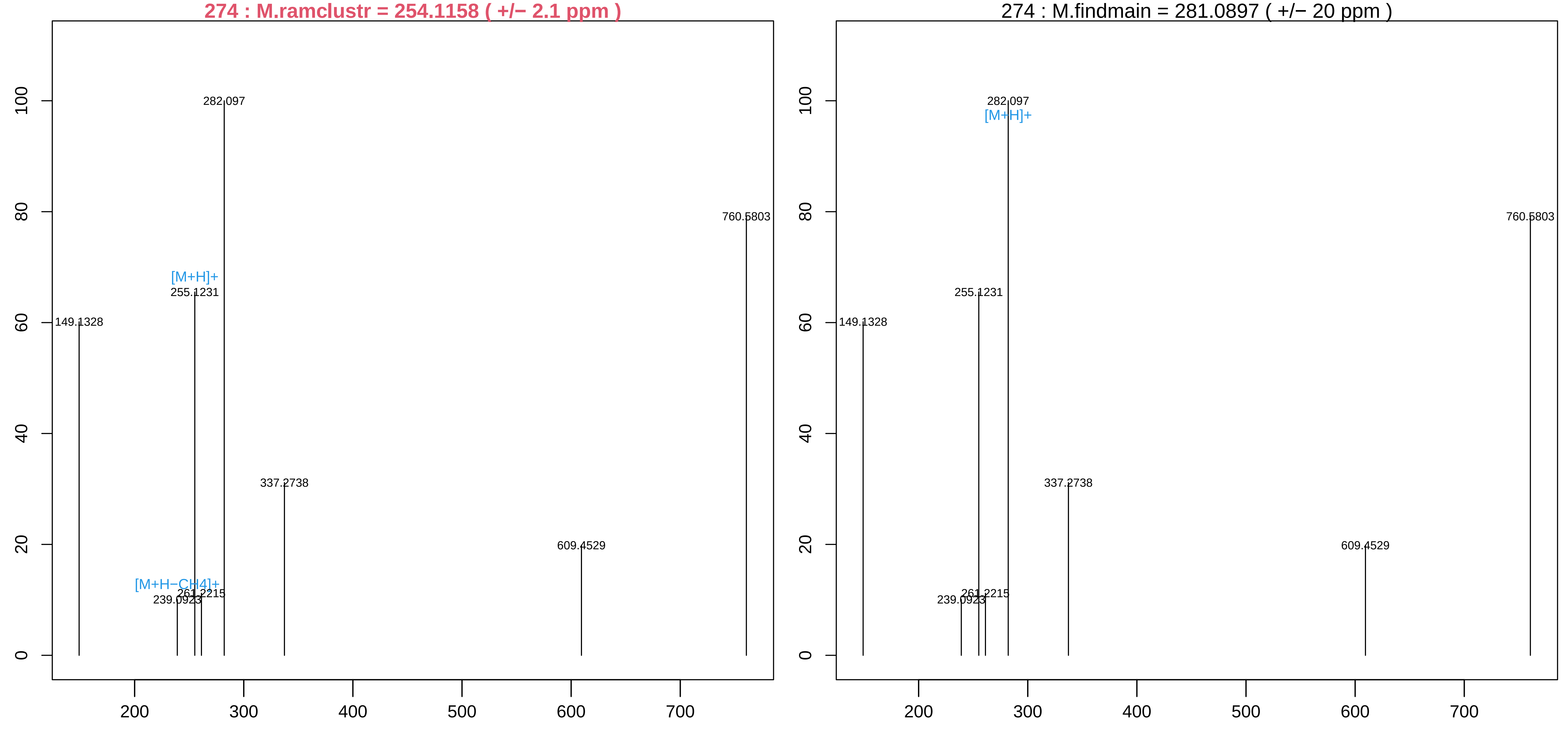
274 : M.ramclustr = 254.1158 (+/− 2.1 ppm)
282.097
760.5803
[M+H]+
255.1231
149.1328
337.2738
609.4529
[M+H−CH4]+
261.2215
239.0923
274 : M.findmain = 281.0897 (+/− 20 ppm)
282.097
[M+H]+
760.5803
255.1231
149.1328
337.2738
609.4529
261.2215
239.0923

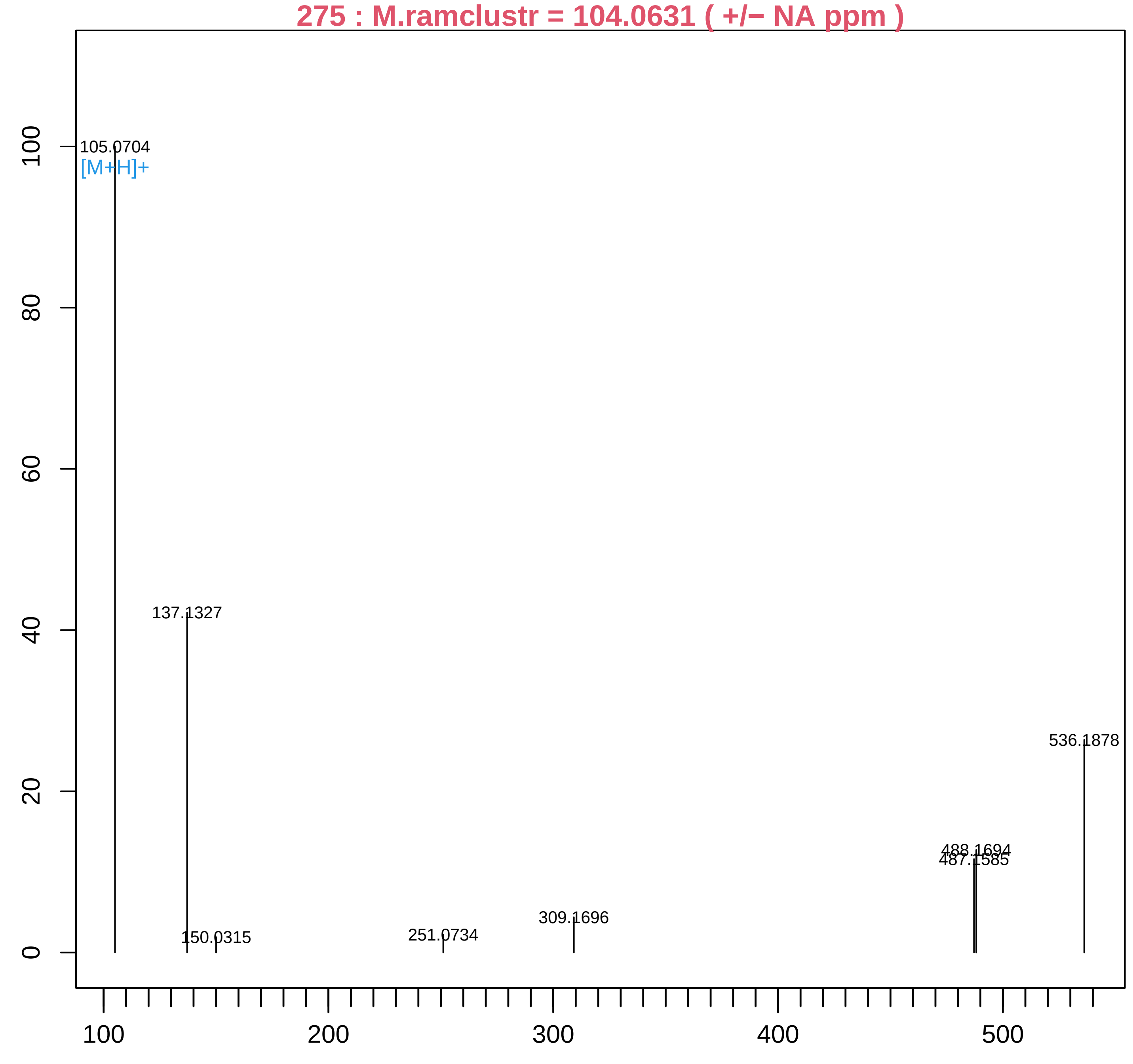
275 : M.ramclustr = 104.0631 (+/− NA ppm)
105.0704
[M+H]+
137.1327
150.0315
251.0734
309.1696
488.1694
487.1585
536.1878
0
20
40
60
80
100
100
200
300
400
500

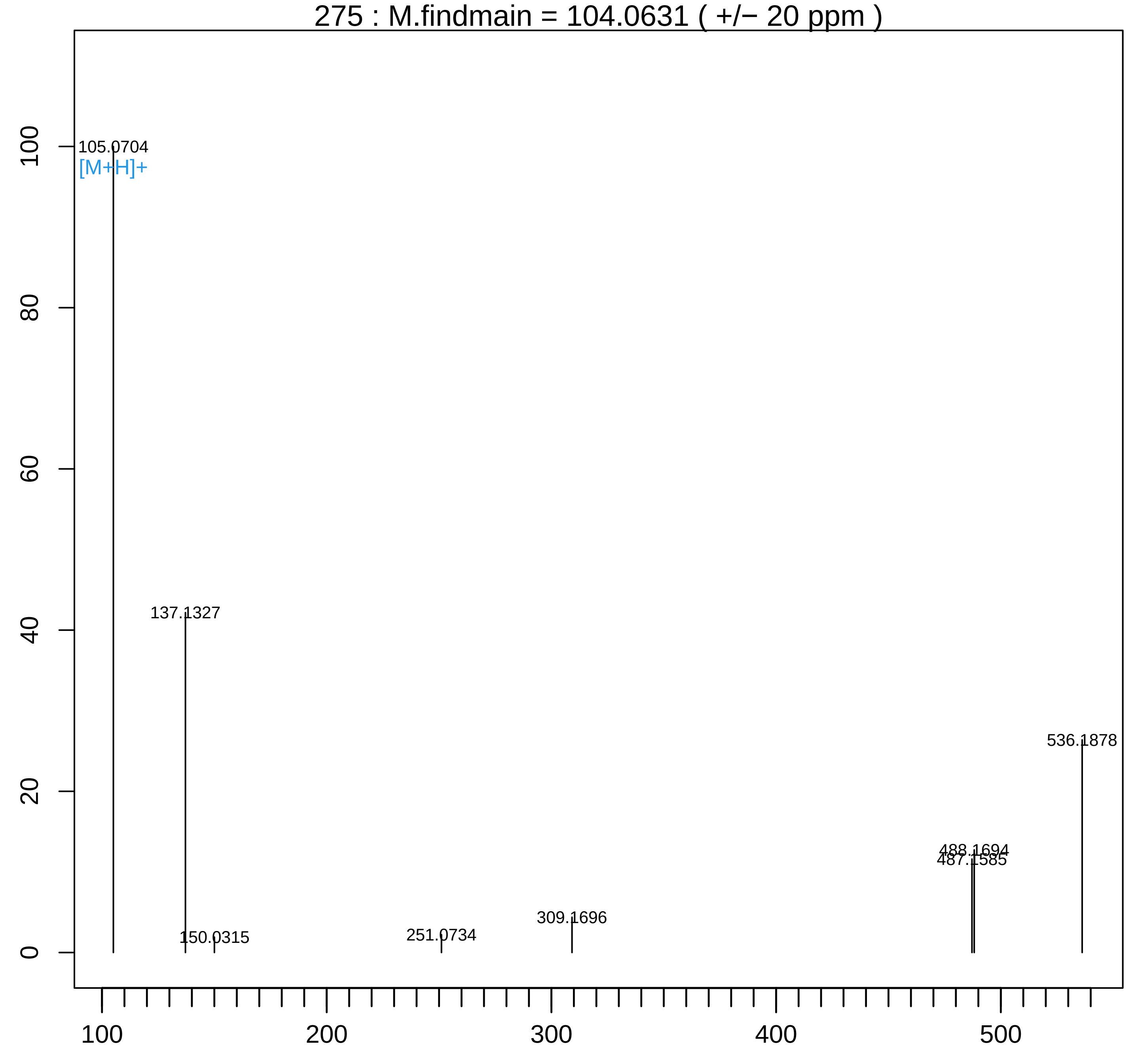
275 : M.findmain = 104.0631 (+/− 20 ppm)
105.0704
[M+H]+
137.1327
150.0315
251.0734
309.1696
488.1694
487.1585
536.1878
0
20
40
60
80
100
100
200
300
400
500

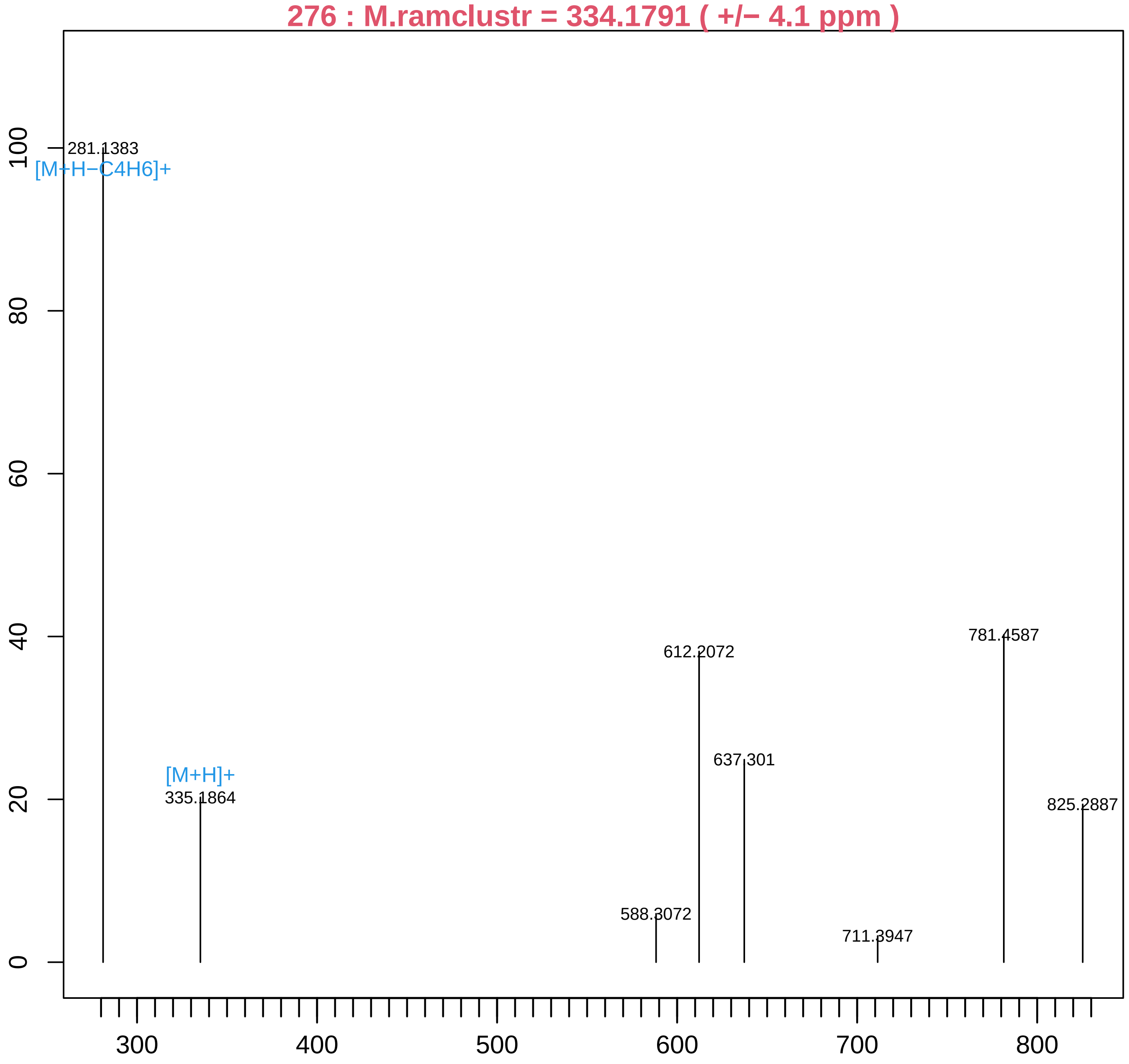
276 : M.ramclustr = 334.1791 (+/− 4.1 ppm)
281.1383
[M+H−C4H6]+
[M+H]+
335.1864
612.2072
637.301
781.4587
825.2887
588.3072
711.3947
0
20
40
60
80
100
300
400
500
600
700
800

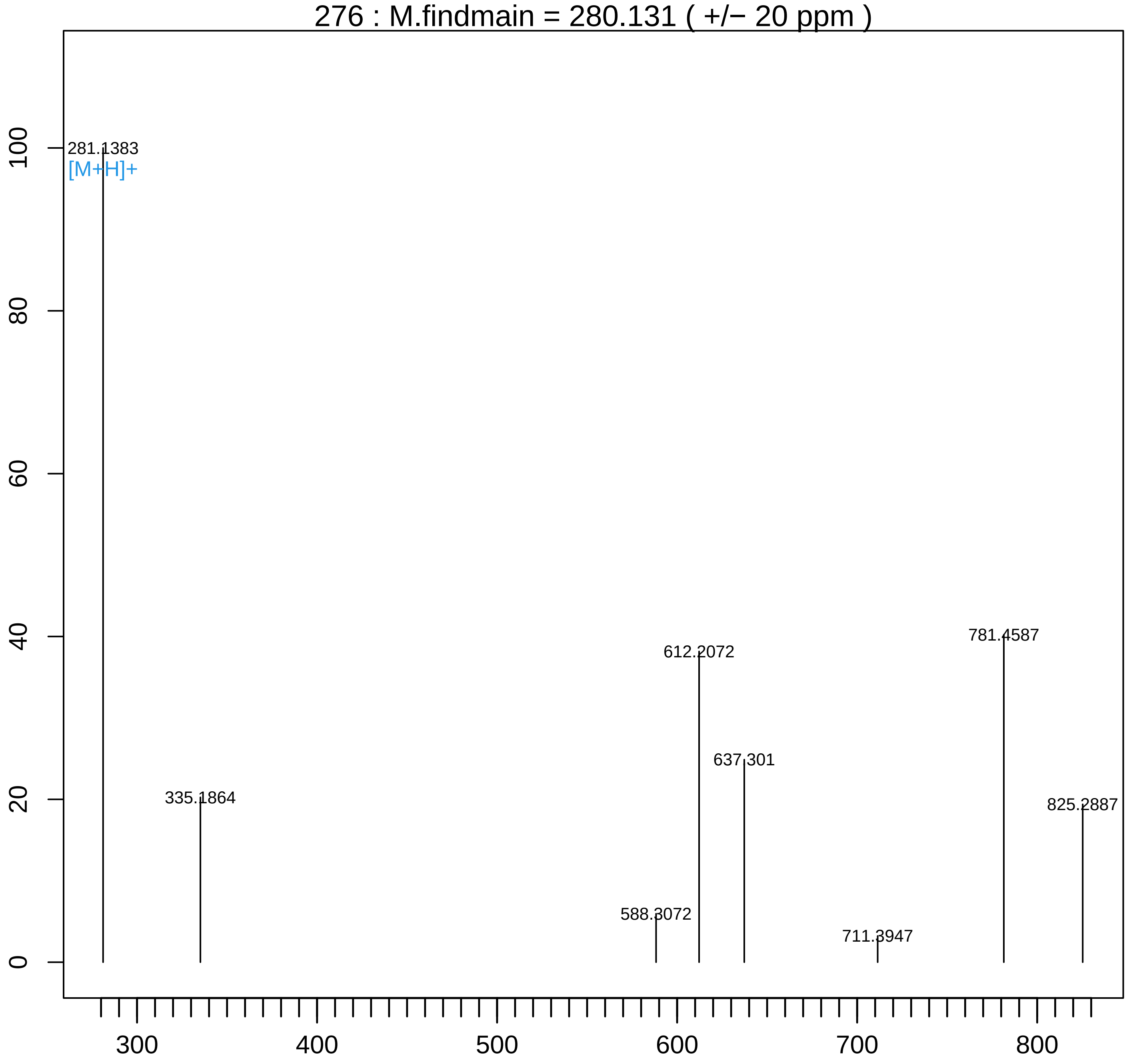
276 : M.findmain = 280.131 (+/− 20 ppm)
281.1383
[M+H]+
335.1864
612.2072
637.301
781.4587
825.2887
588.3072
711.3947
0
20
40
60
80
100
300
400
500
600
700
800

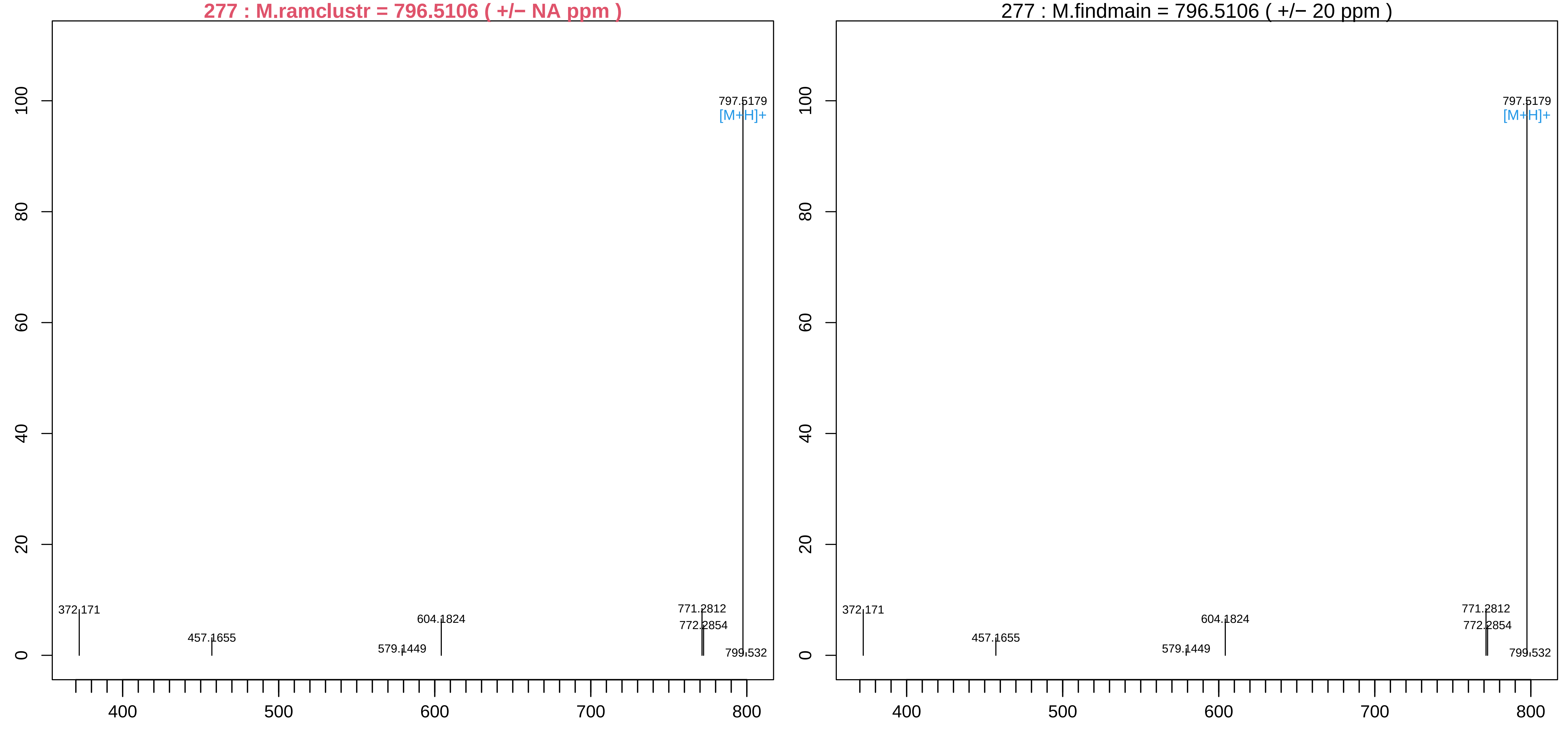
277 : M.ramclustr = 796.5106 (+/− NA ppm)
797.5179
[M+H]+
372.171
457.1655
579.1449
604.1824
771.2812
772.2854
799.532
0
20
40
60
80
100
400
500
600
700
800
277 : M.findmain = 796.5106 (+/− 20 ppm)
797.5179
[M+H]+
372.171
457.1655
579.1449
604.1824
771.2812
772.2854
799.532
0
20
40
60
80
100
400
500
600
700
800

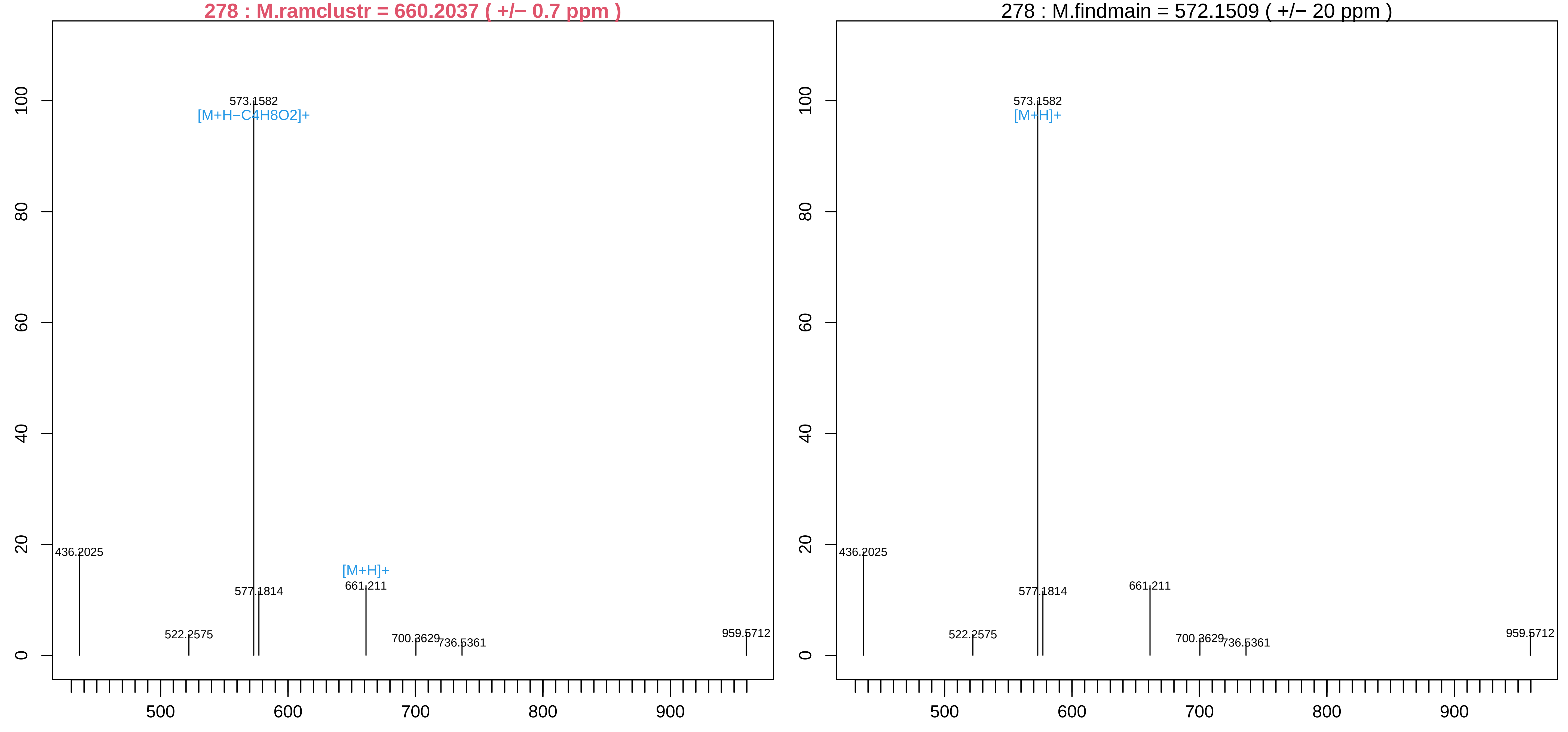

278 : M.ramclustr = 660.2037 (+/− 0.7 ppm)
573.1582
[M+H−C4H8O2]+
436.2025
522.2575
577.1814
[M+H]+
661.211
700.3629
736.5361
959.5712
0
20
40
60
80
100
500
600
700
800
900
278 : M.findmain = 572.1509 (+/− 20 ppm)
573.1582
[M+H]+
436.2025
522.2575
577.1814
661.211
700.3629
736.5361
959.5712
0
20
40
60
80
100
500
600
700
800
900

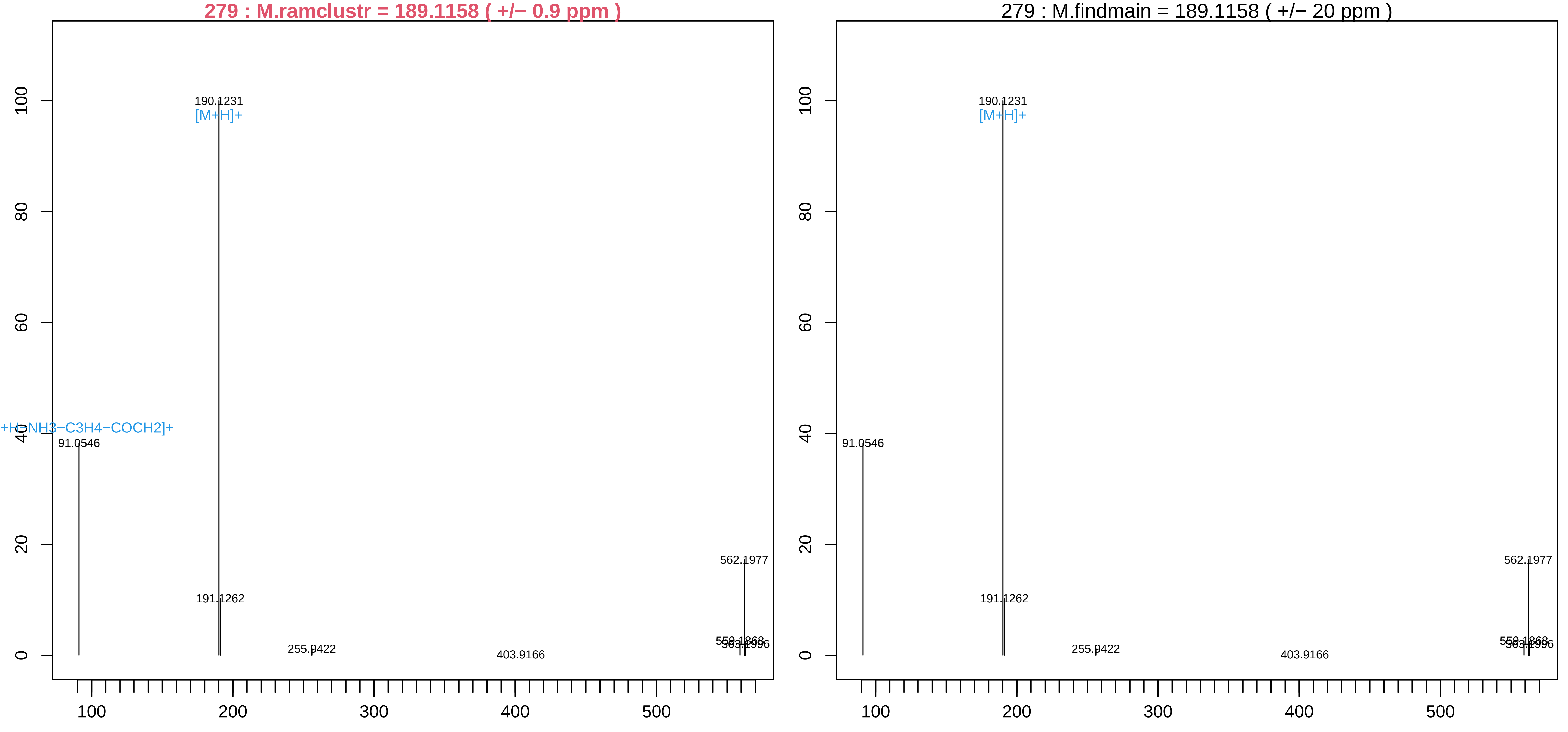

279 : M.ramclustr = 189.1158 (+/− 0.9 ppm)
190.1231
[M+H]+
+H−NH3−C3H4−COCH2]+
91.0546
191.1262
255.9422
403.9166
562.1977
559.1868
563.1996
0
20
40
60
80
100
100
200
300
400
500
279 : M.findmain = 189.1158 (+/− 20 ppm)
190.1231
[M+H]+
91.0546
191.1262
255.9422
403.9166
562.1977
559.1868
563.1996

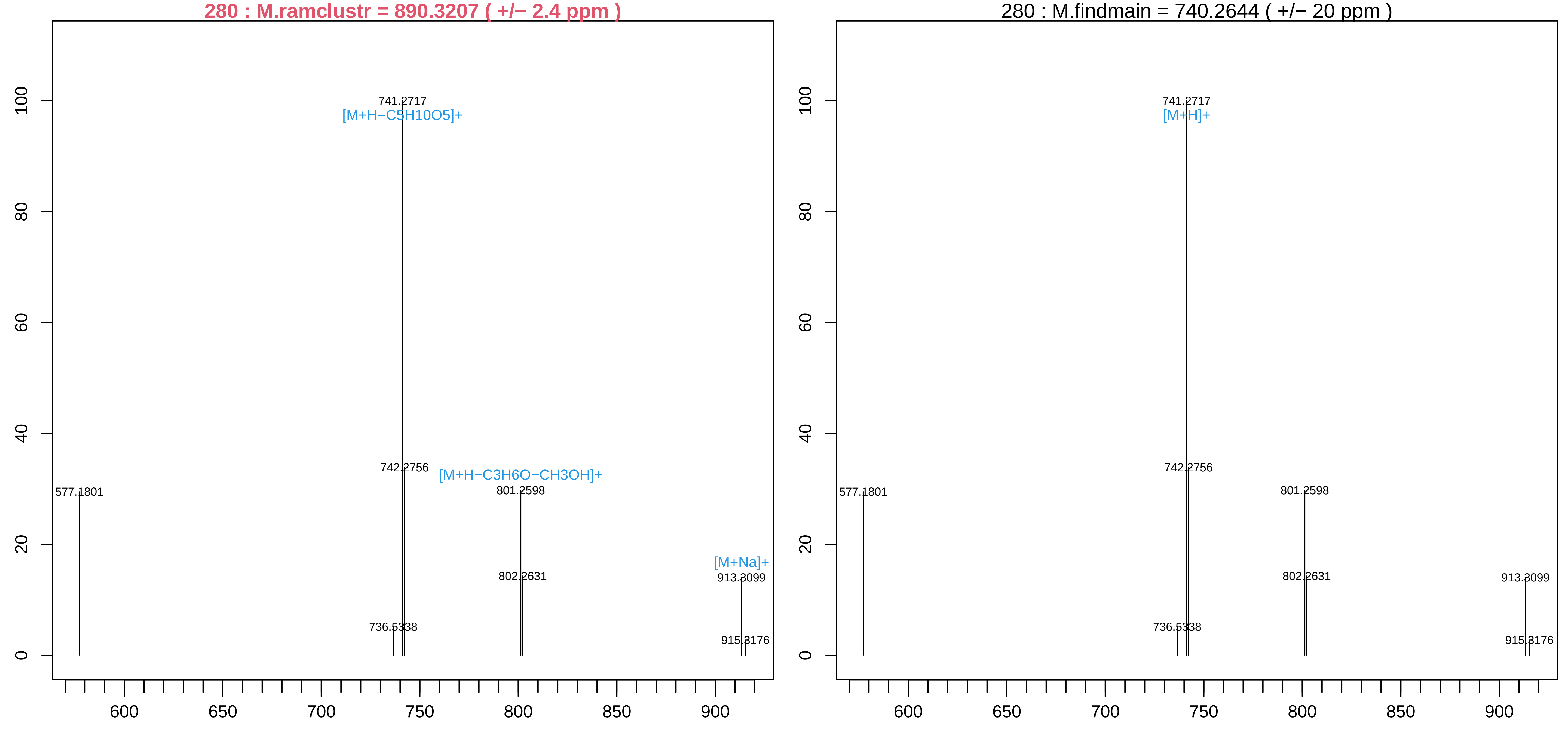
280 : M.ramclustr = 890.3207 (+/− 2.4 ppm)
741.2717
[M+H−C5H10O5]+
742.2756
[M+H−C3H6O−CH3OH]+
577.1801
801.2598
802.2631
[M+Na]+
913.3099
736.5338
915.3176
280 : M.findmain = 740.2644 (+/− 20 ppm)
741.2717
[M+H]+
742.2756
577.1801
801.2598
802.2631
913.3099
736.5338
915.3176

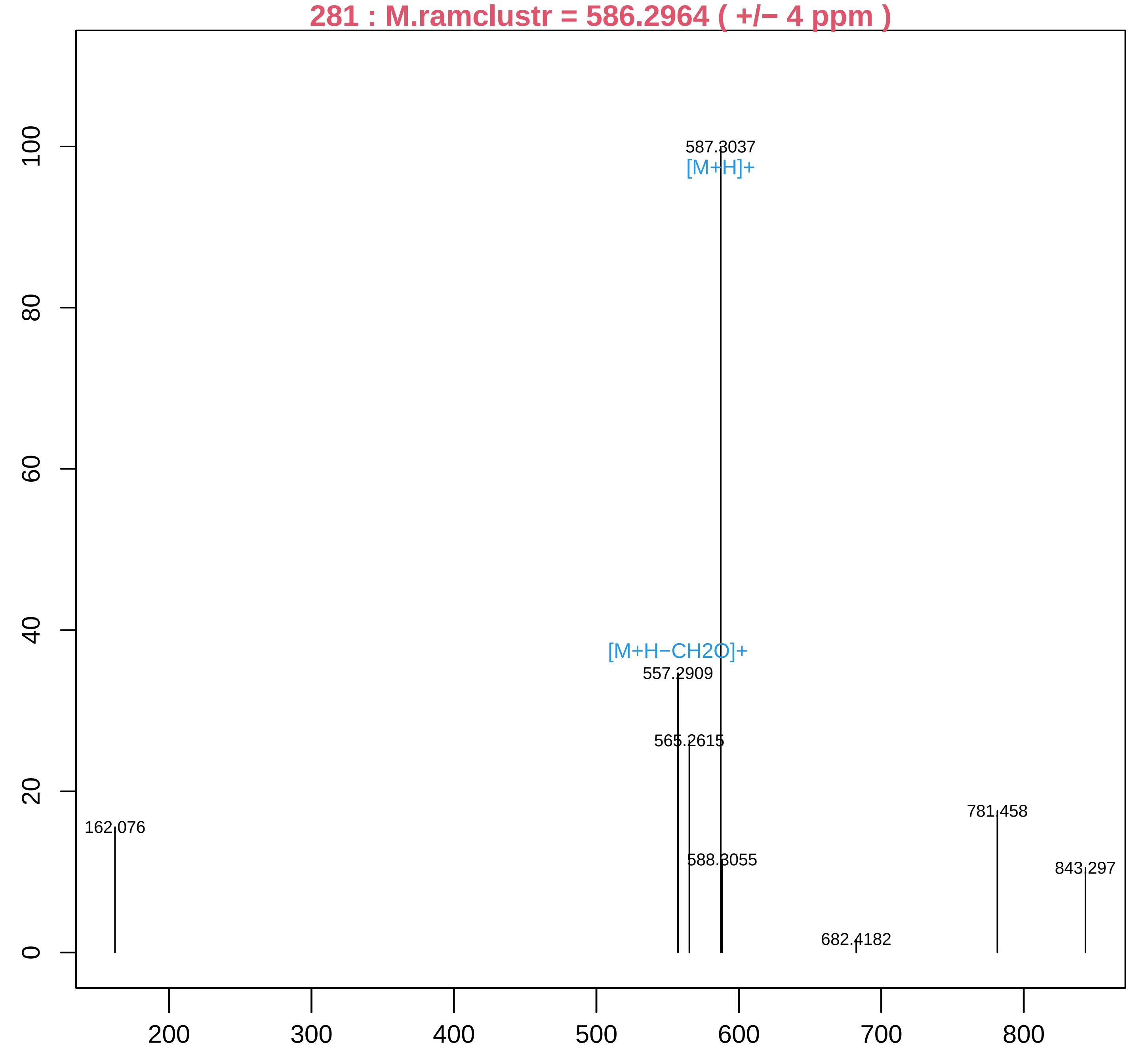
281 : M.ramclustr = 586.2964 (+/− 4 ppm)
587.3037
[M+H]+
[M+H−CH2O]+
557.2909
565.2615
162.076
588.3055
781.458
843.297
682.4182

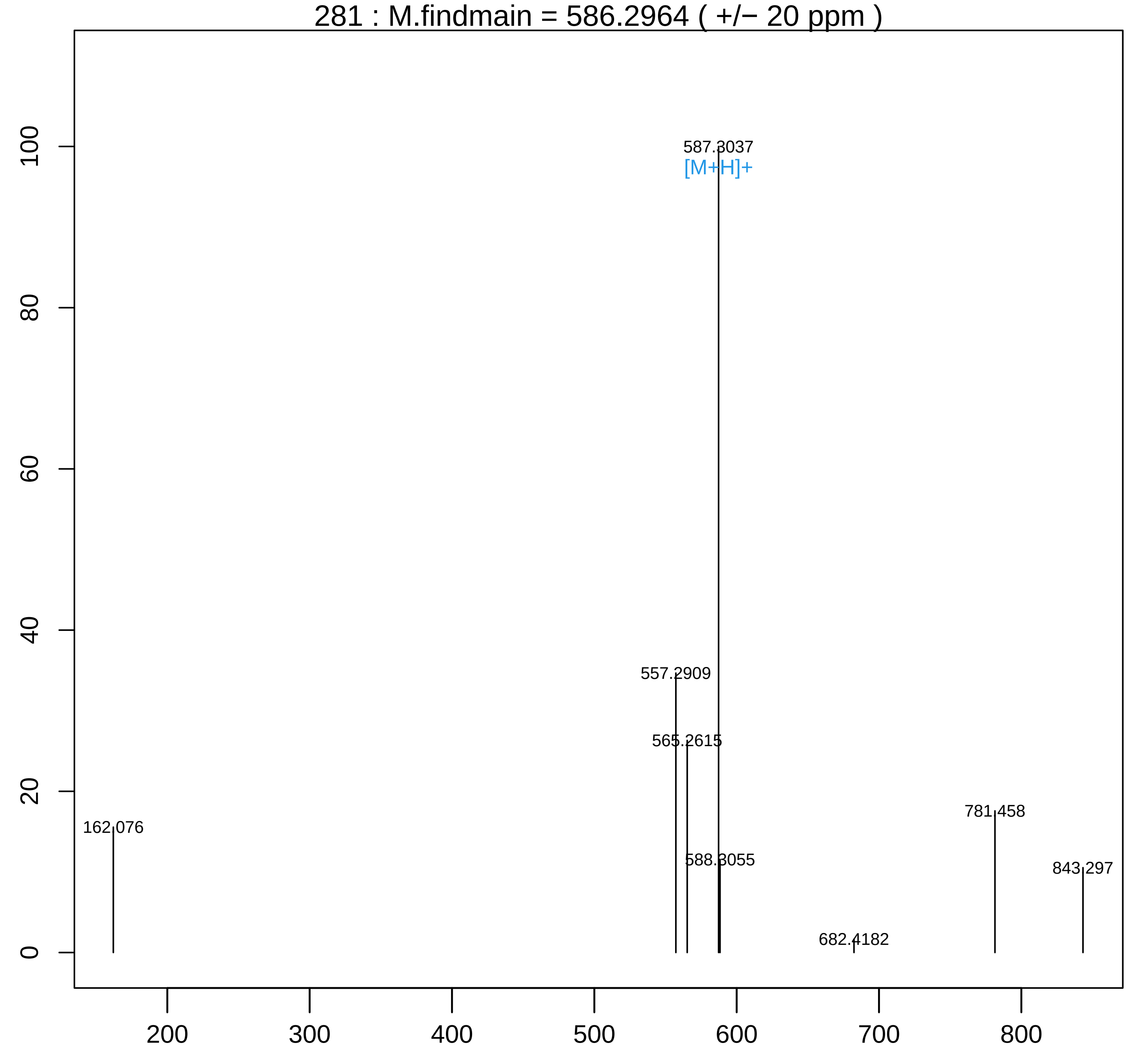
281 : M.findmain = 586.2964 (+/− 20 ppm)
587.3037
[M+H]+
557.2909
565.2615
162.076
588.3055
781.458
843.297
682.4182

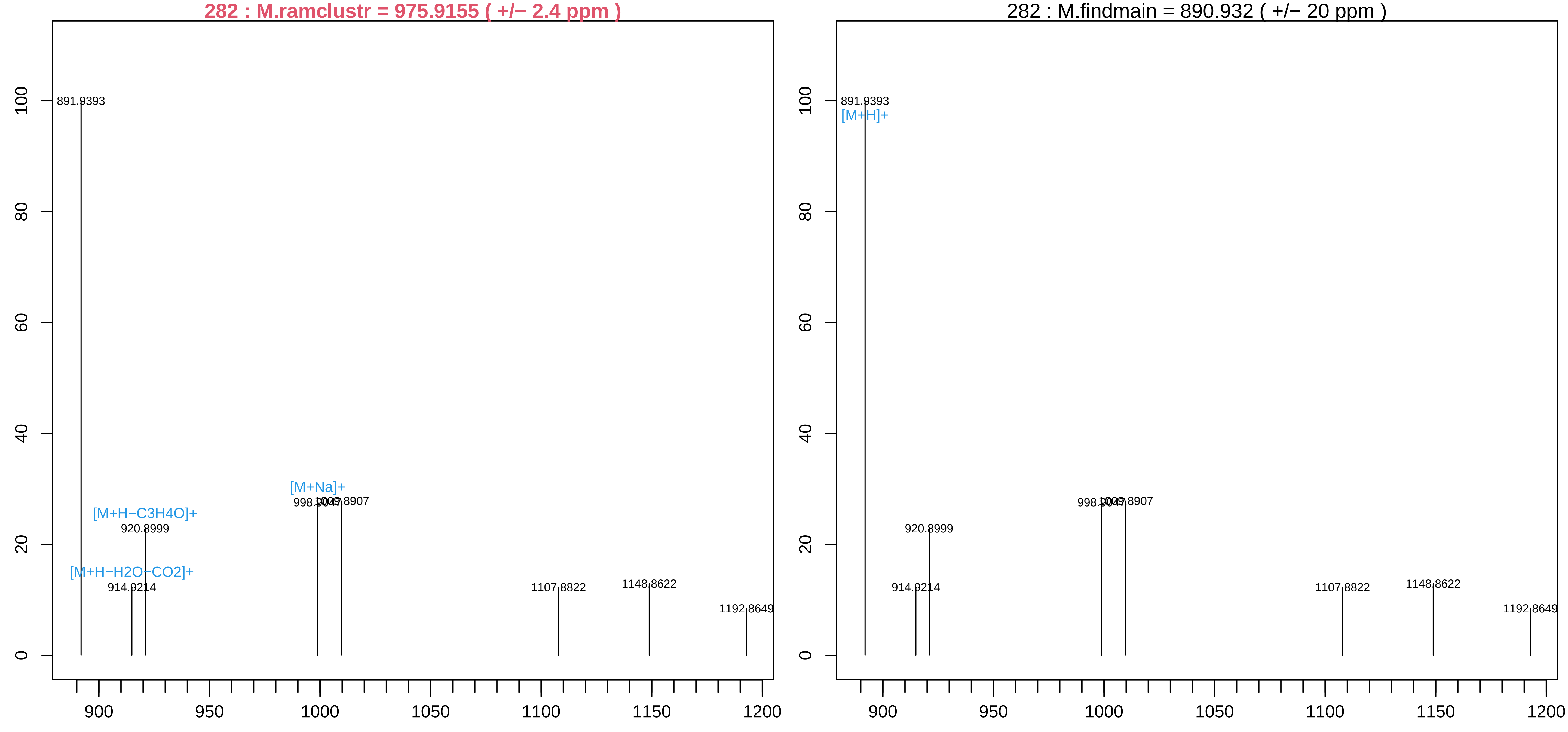

282 : M.ramclustr = 975.9155 (+/− 2.4 ppm)
891.9393
[M+H−C3H4O]+
920.8999
[M+H−H2O−CO2]+
914.9214
[M+Na]+
998.9047
1009.8907
1107.8822
1148.8622
1192.8649
282 : M.findmain = 890.932 (+/− 20 ppm)
891.9393
[M+H]+
920.8999
914.9214
998.9047
1009.8907
1107.8822
1148.8622
1192.8649
0
20
40
60
80
100
900
950
1000
1050
1100
1150
1200

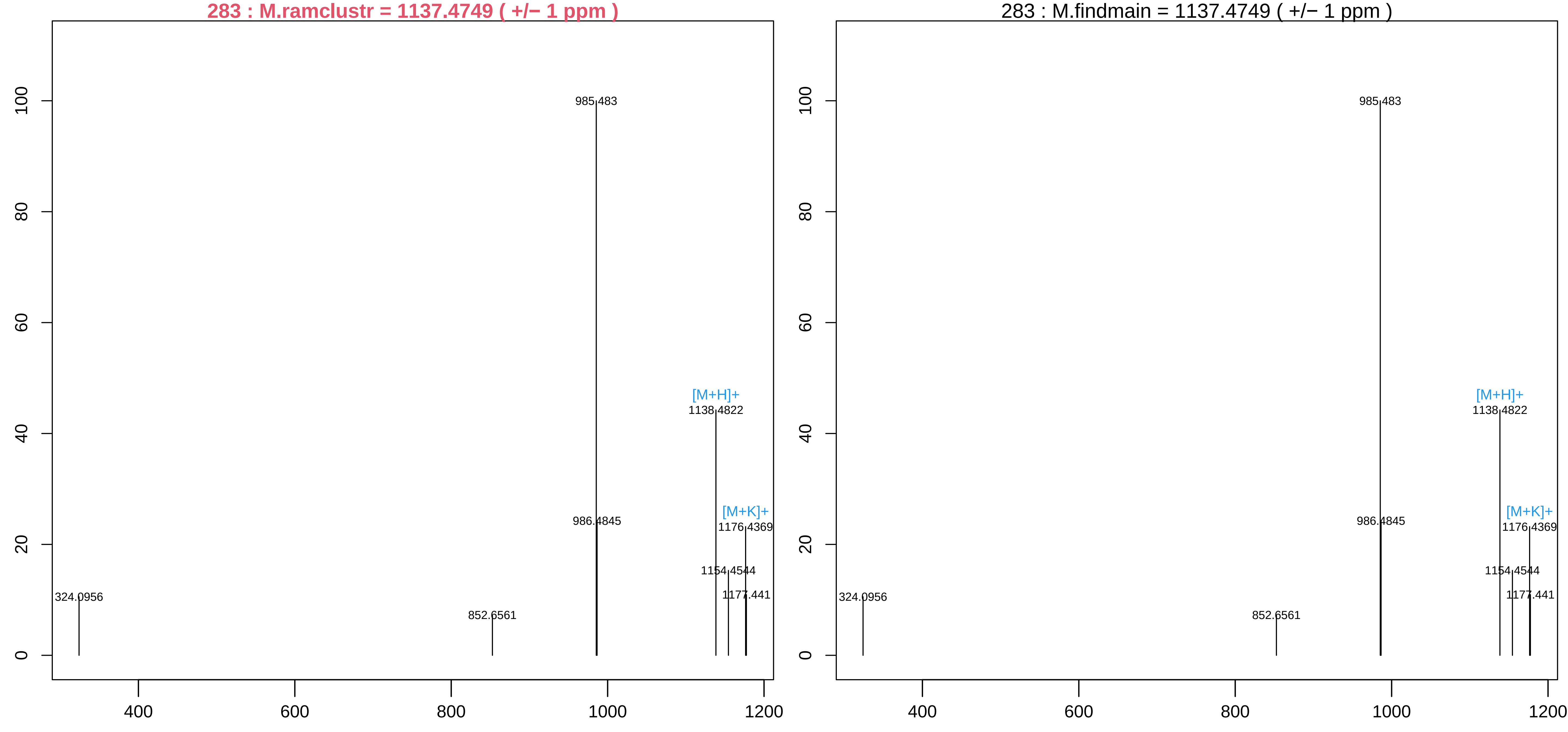
283 : M.ramclustr = 1137.4749 (+/− 1 ppm)
985.483
986.4845
[M+H]+
1138.4822
[M+K]+
1176.4369
1154.4544
1177.441
324.0956
852.6561
283 : M.findmain = 1137.4749 (+/− 1 ppm)
985.483
986.4845
[M+H]+
1138.4822
[M+K]+
1176.4369
1154.4544
1177.441
324.0956
852.6561

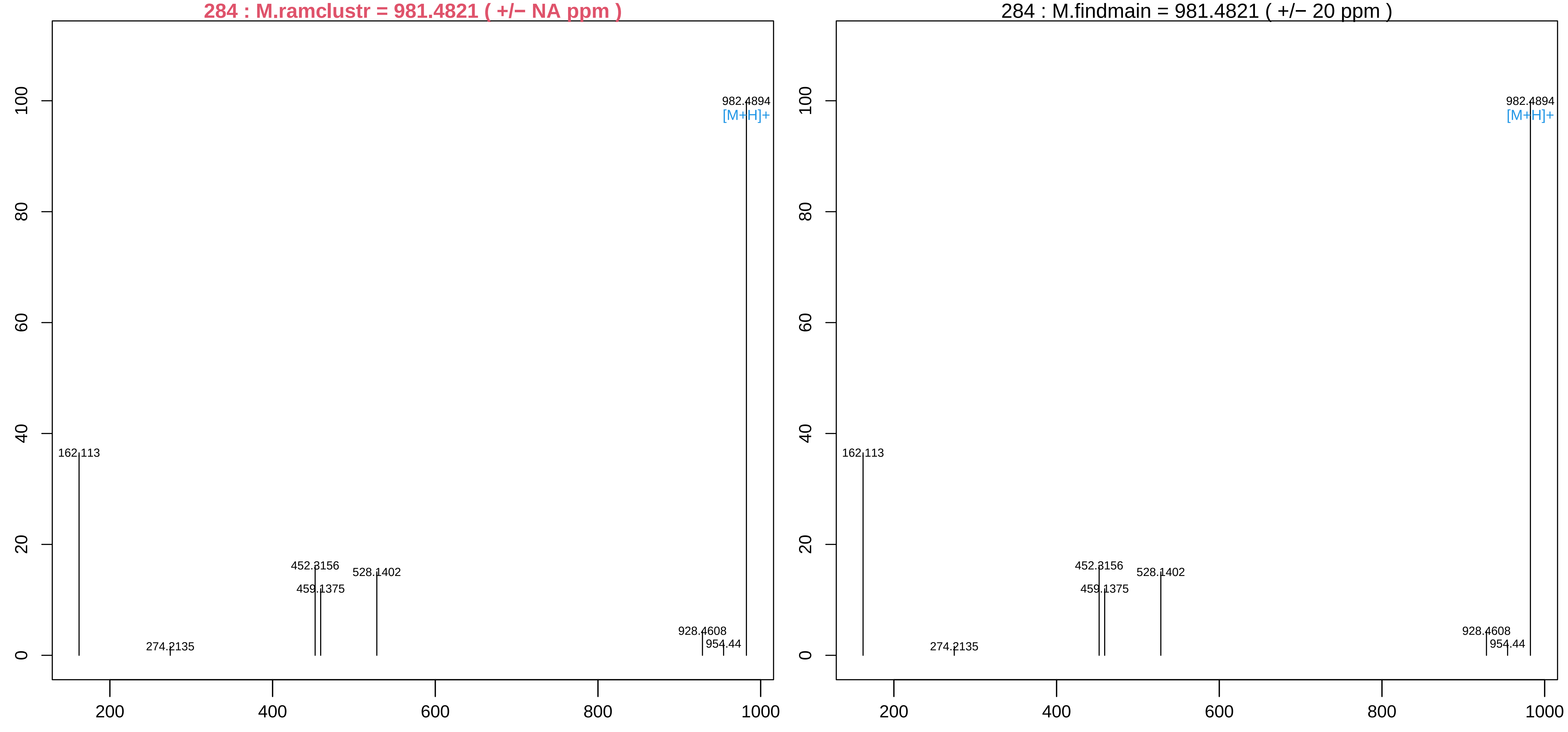
284 : M.ramclustr = 981.4821 (+/− NA ppm)
982.4894
[M+H]+
162.113
274.2135
452.3156
459.1375
528.1402
928.4608
954.44
0
20
40
60
80
100
200
400
600
800
1000
284 : M.findmain = 981.4821 (+/− 20 ppm)
982.4894
[M+H]+
162.113
274.2135
452.3156
459.1375
528.1402
928.4608
954.44
0
20
40
60
80
100
200
400
600
800
1000

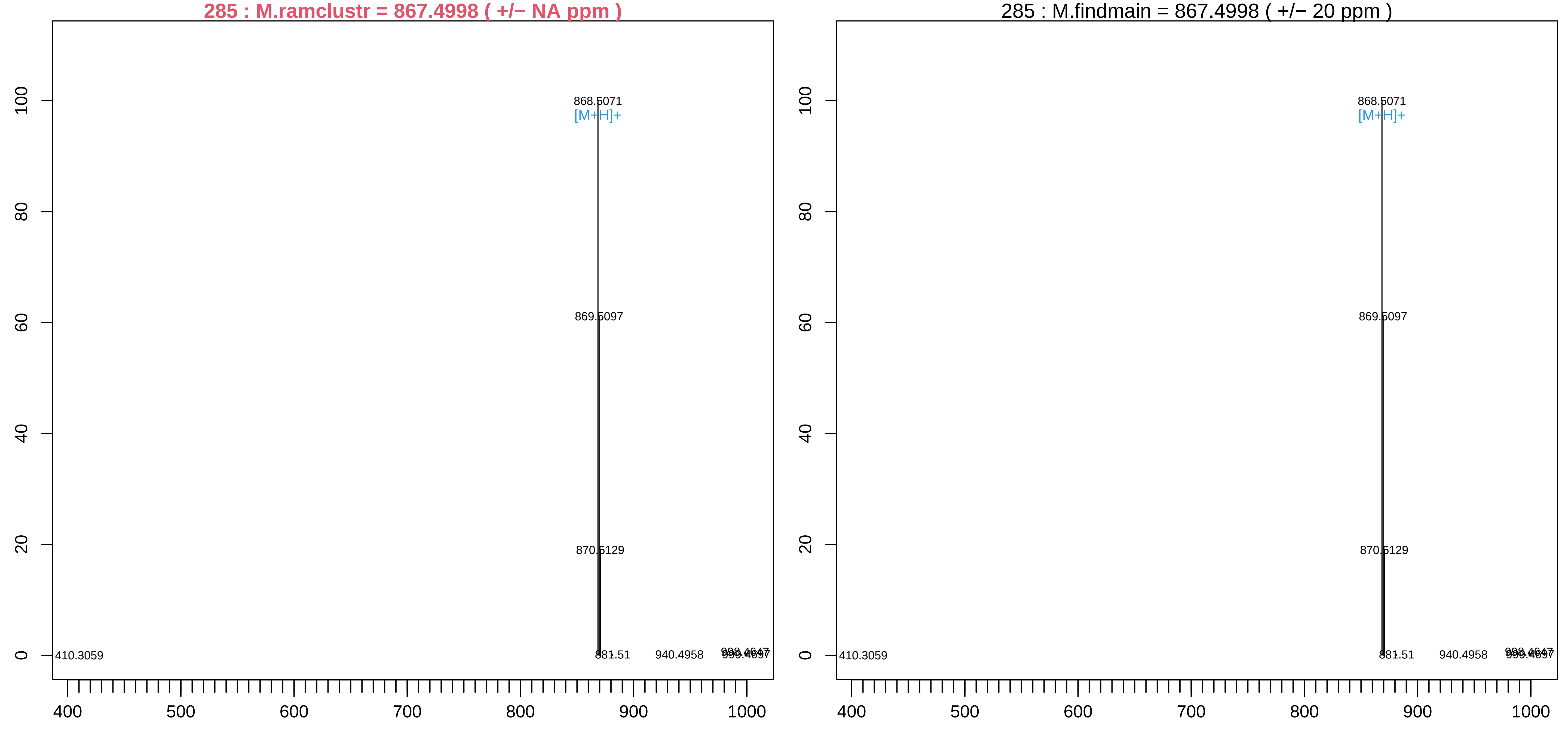
285 : M.ramclustr = 867.4998 (+/− NA ppm)
285 : M.findmain = 867.4998 (+/− 20 ppm)
868.5071
[M+H]+
869.5097
870.5129
410.3059
881.51
940.4958
0
20
40
60
80
100
400
500
600
700
800
900
1000

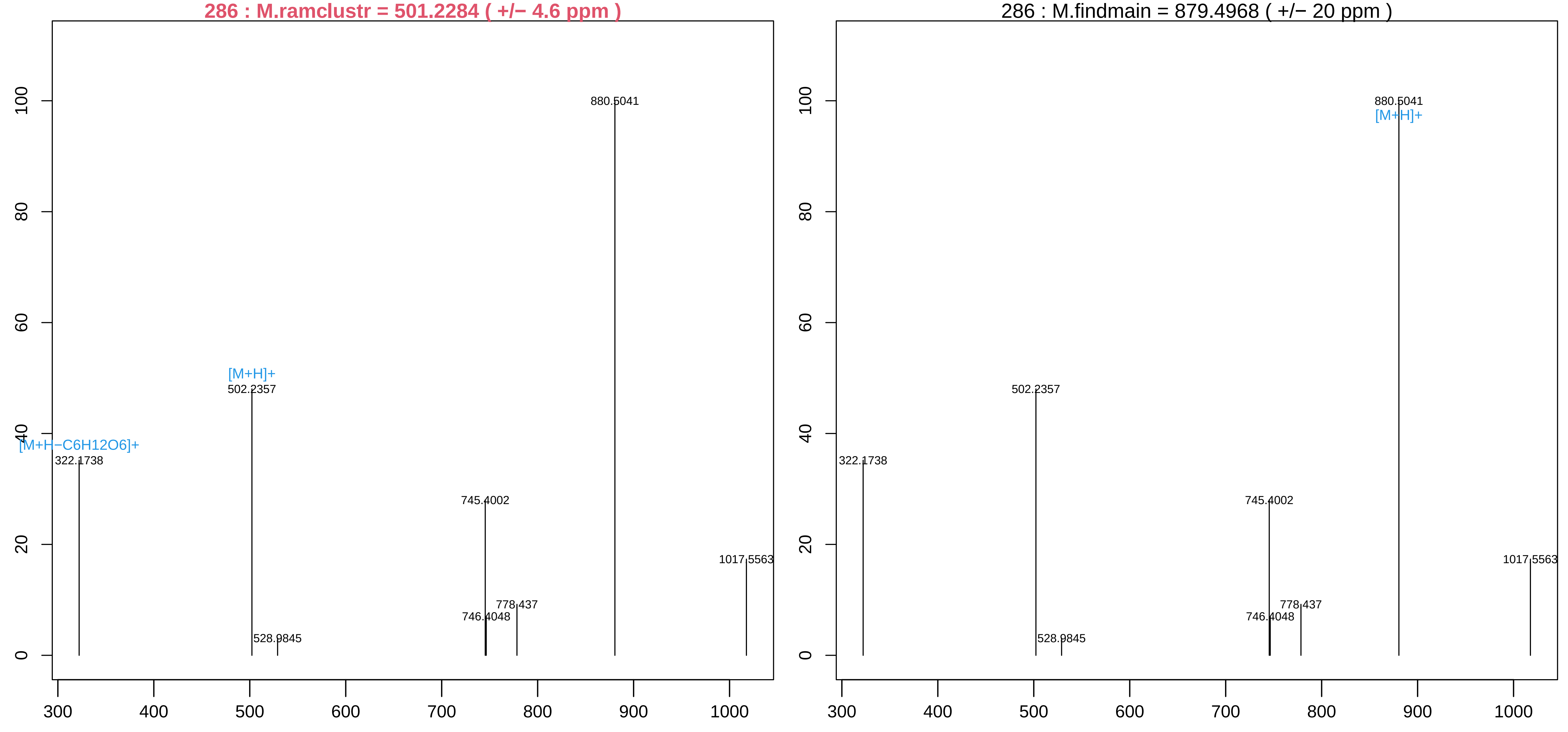
286 : M.ramclustr = 501.2284 (+/− 4.6 ppm)
[M+H−C6H12O6]+
322.1738
[M+H]+
502.2357
528.9845
745.4002
746.4048
778.437
880.5041
1017.5563
286 : M.findmain = 879.4968 (+/− 20 ppm)
322.1738
502.2357
528.9845
745.4002
746.4048
778.437
880.5041
[M+H]+
1017.5563

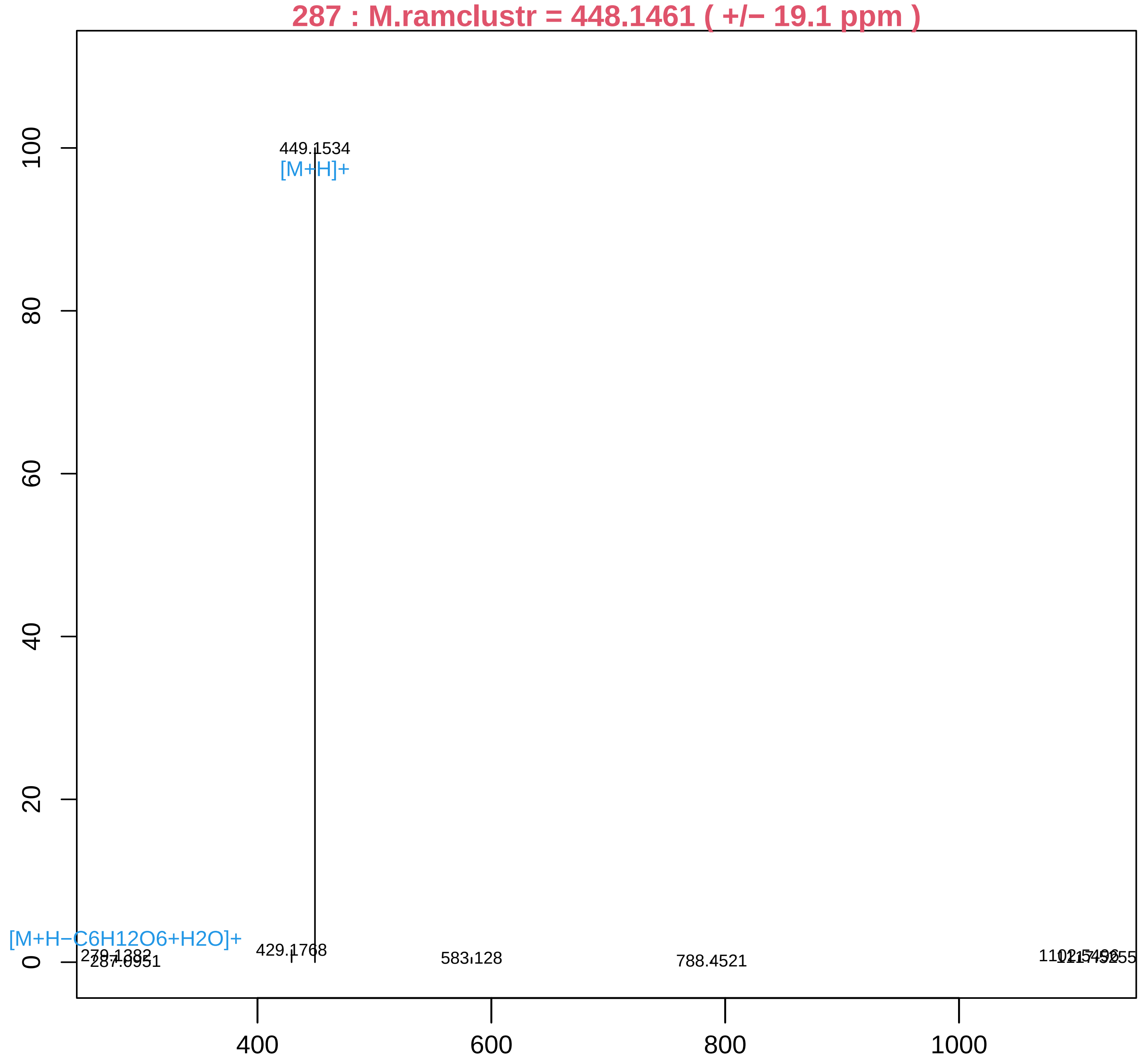
287 : M.ramclustr = 448.1461 (+/− 19.1 ppm)
449.1534
[M+H]+
[M+H−C6H12O6+H2O]+
429.1768
583.128
788.4521
0
20
40
60
80
100
400
600
800
1000

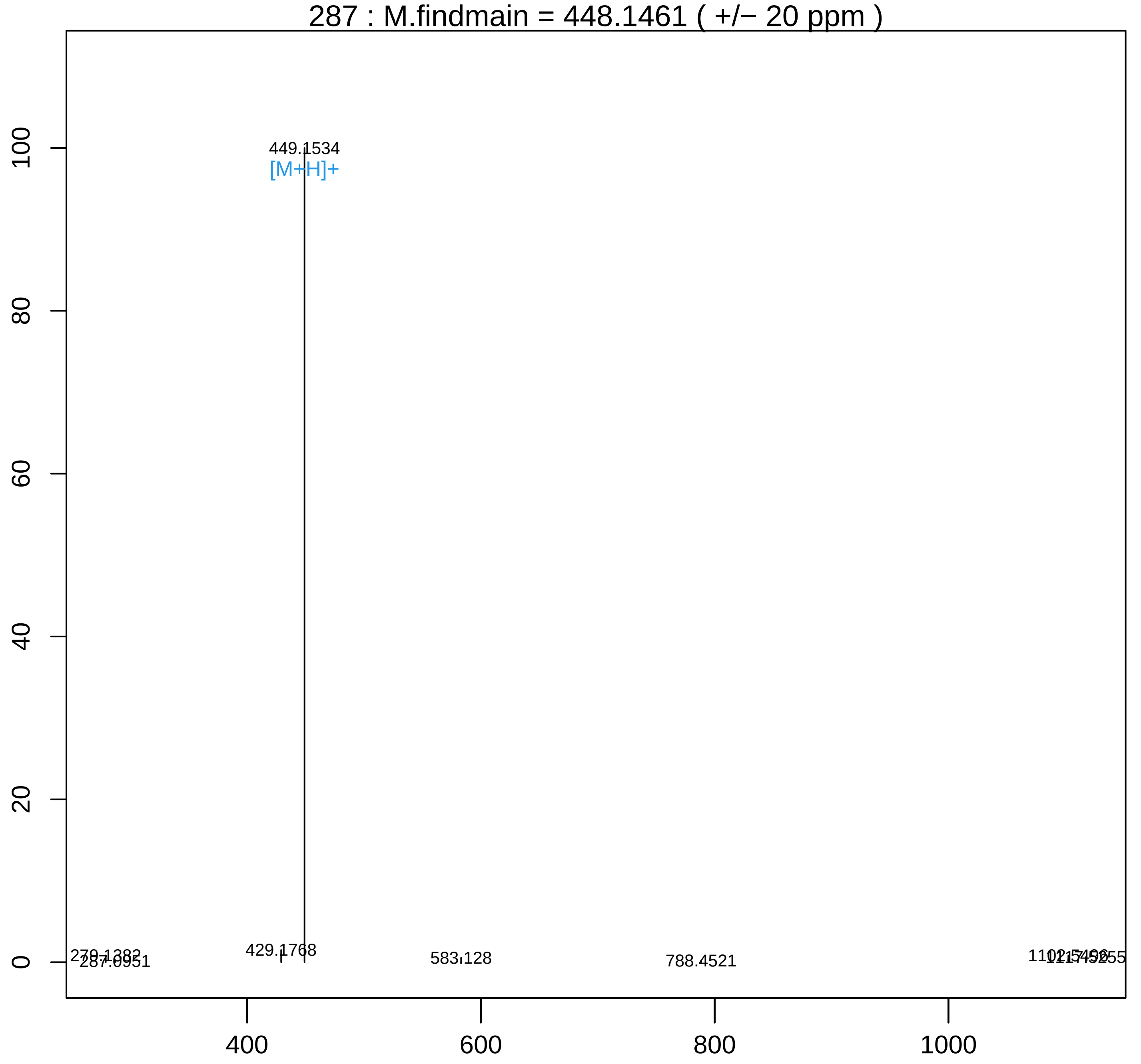
287 : M.findmain = 448.1461 (+/− 20 ppm)
449.1534
[M+H]+
429.1768
583.128
788.4521
0
20
40
60
80
100
400
600
800
1000

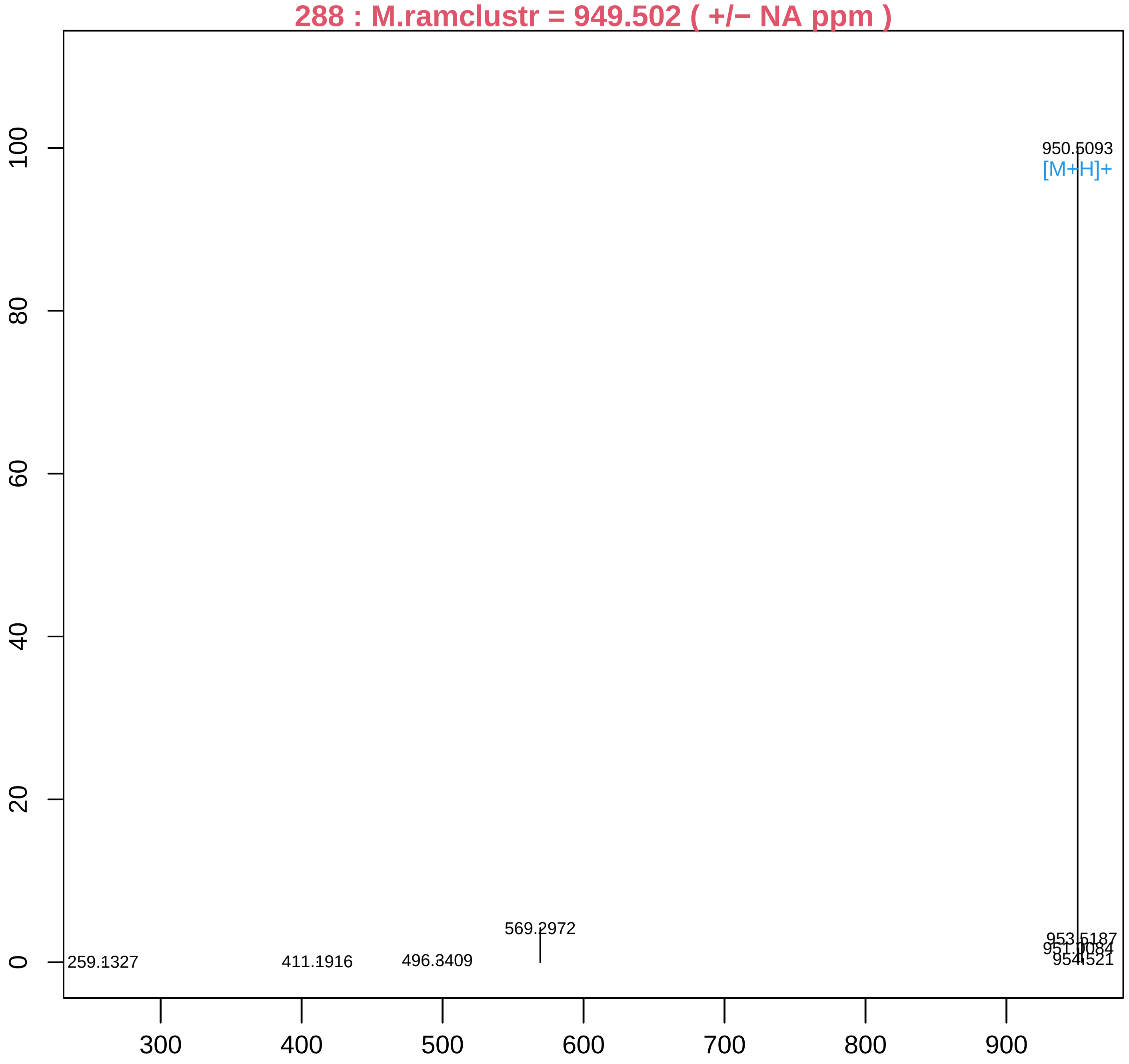
288 : M.ramclustr = 949.502 (+/− NA ppm)
950.5093
[M+H]+
569.2972
953.5187
951.0084
954.521
259.1327
411.1916
496.3409
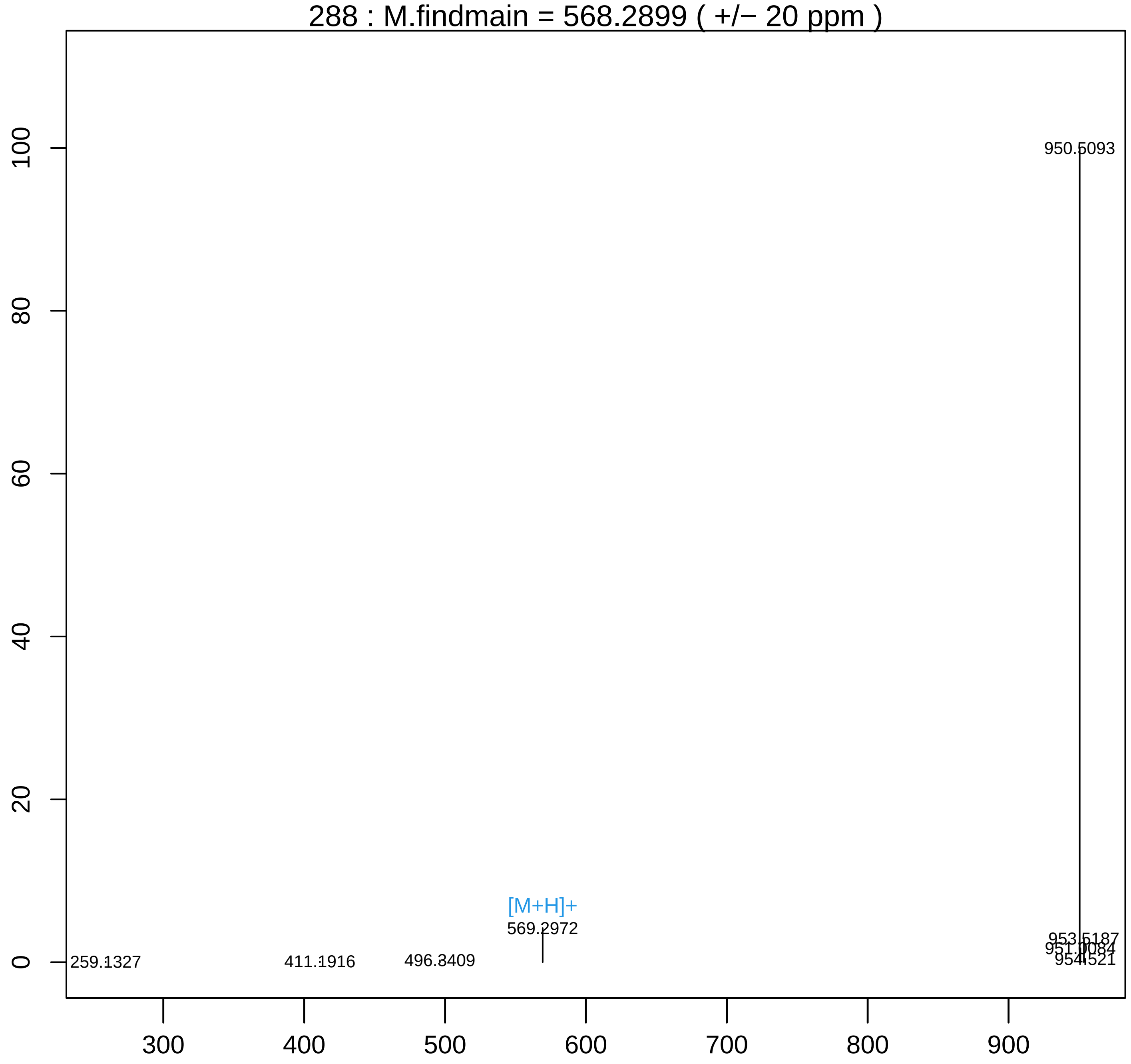
288 : M.findmain = 568.2899 (+/− 20 ppm)
950.5093
[M+H]+
569.2972
953.5187
951.0084
954.521
259.1327
411.1916
496.3409

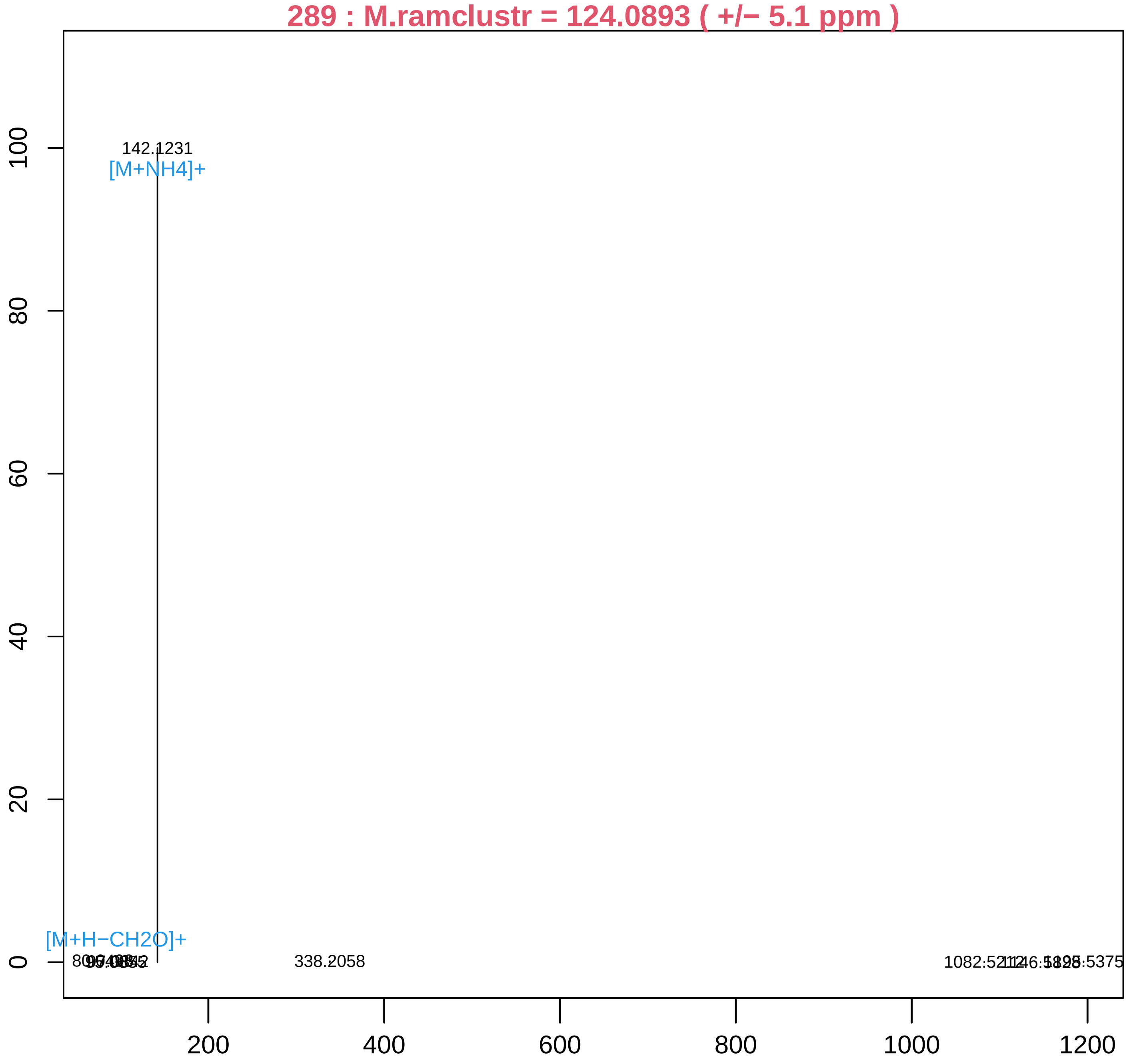
289 : M.ramclustr = 124.0893 (+/− 5.1 ppm)
142.1231
[M+NH4]+
[M+H−CH2O]+
338.2058
0
20
40
60
80
100
200
400
600
800
1000
1200
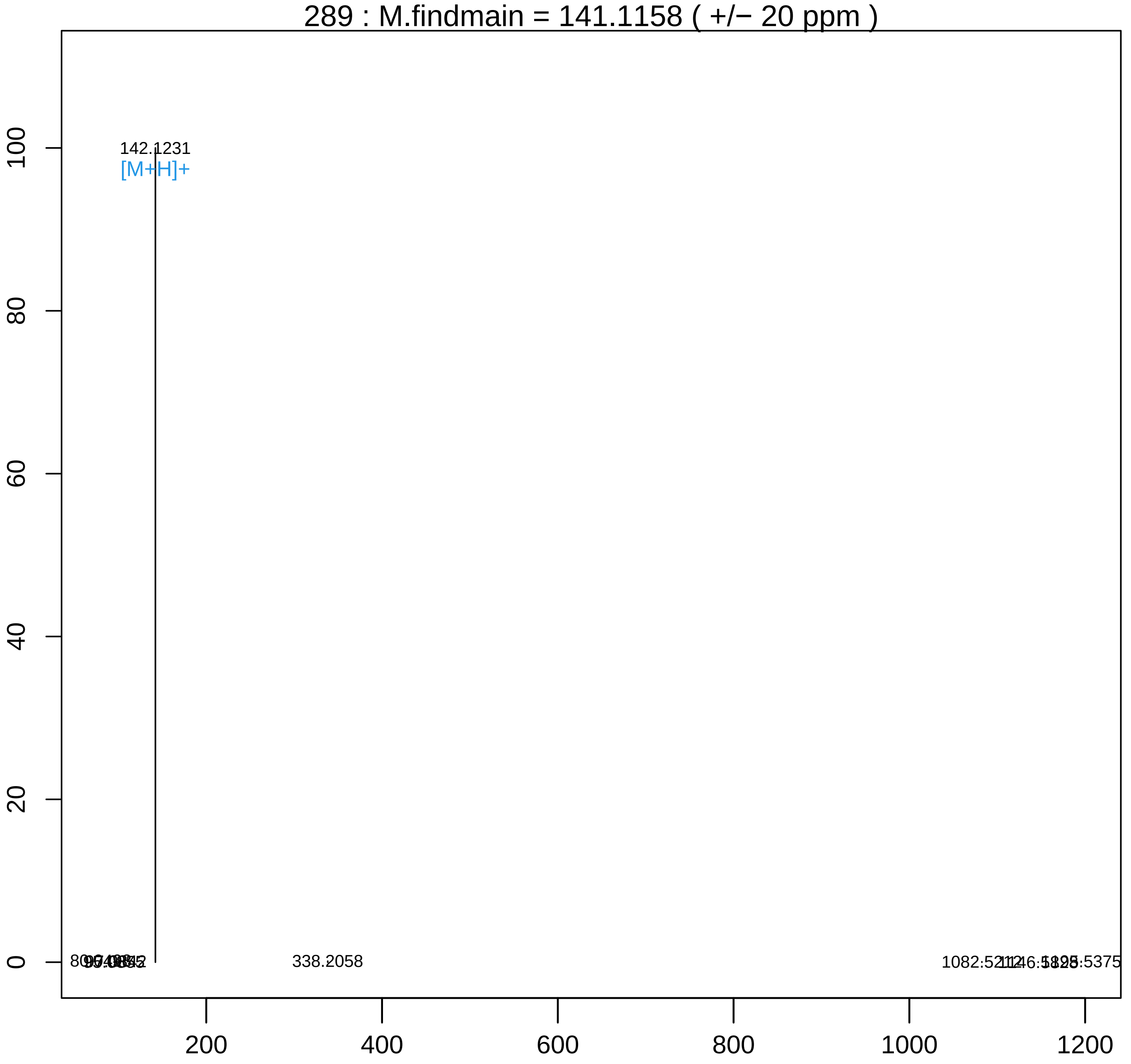
289 : M.findmain = 141.1158 (+/− 20 ppm)
142.1231
[M+H]+
338.2058
0
20
40
60
80
100
200
400
600
800
1000
1200

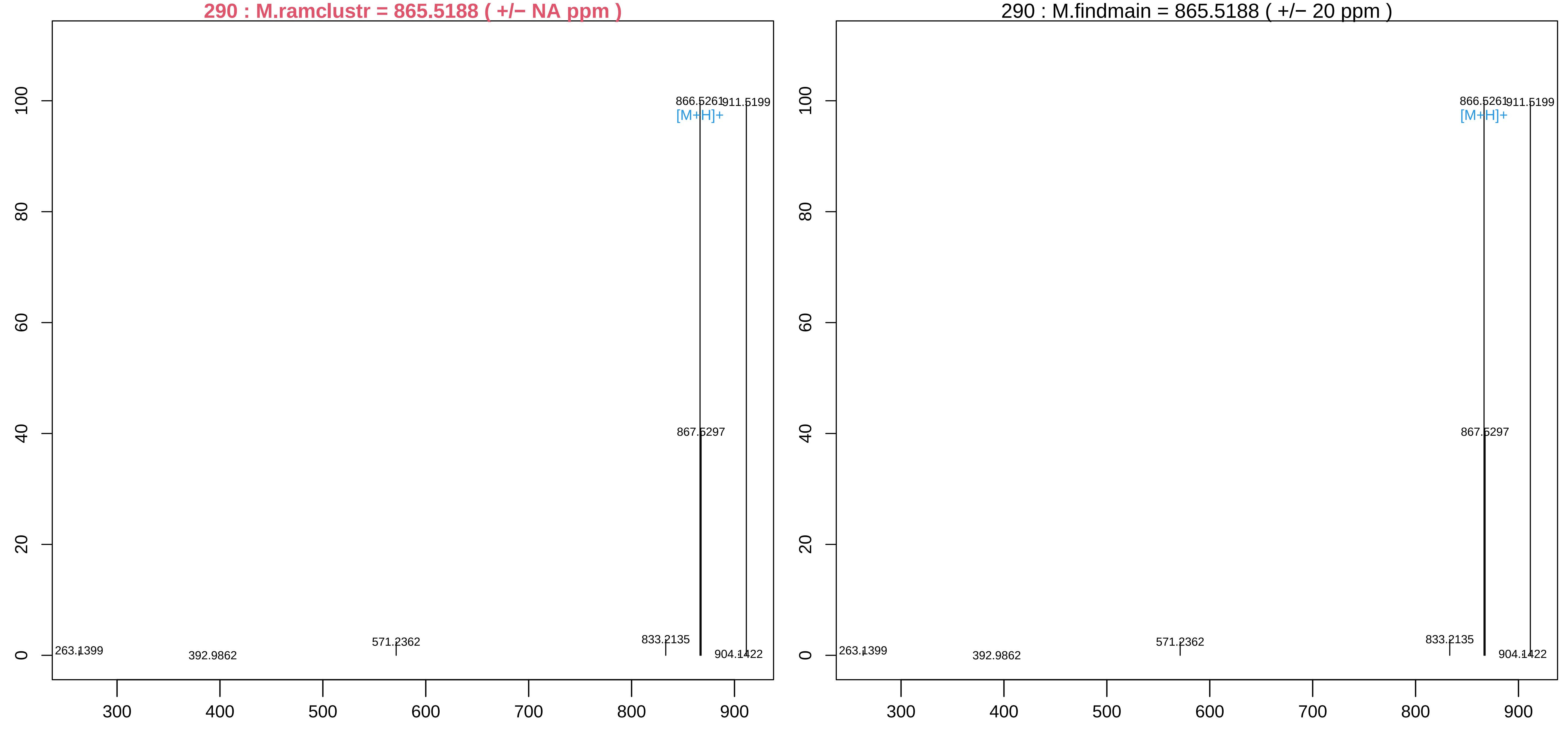

290 : M.ramclustr = 865.5188 (+/− NA ppm)
866.5261
[M+H]+
911.5199
867.5297
263.1399
392.9862
571.2362
833.2135
904.1422
0
20
40
60
80
100
300
400
500
600
700
800
900
290 : M.findmain = 865.5188 (+/− 20 ppm)
866.5261
[M+H]+
911.5199
867.5297
263.1399
392.9862
571.2362
833.2135
904.1422
0
20
40
60
80
100
300
400
500
600
700
800
900

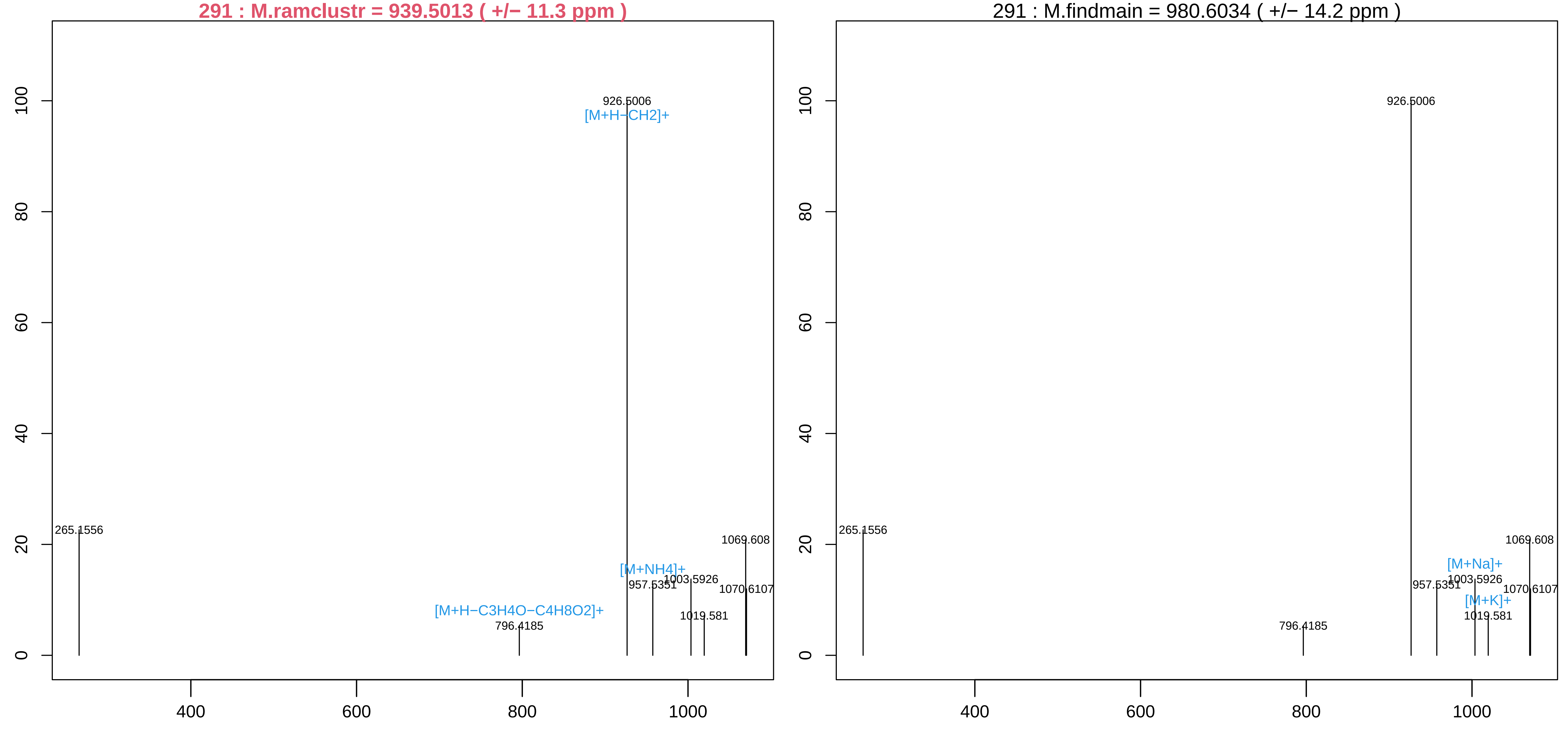
291 : M.ramclustr = 939.5013 (+/− 11.3 ppm)
926.5006
[M+H−CH2]+
265.1556
1069.608
[M+NH4]+
1003.5926
957.5351
1070.6107
[M+H−C3H4O−C4H8O2]+
796.4185
1019.581
291 : M.findmain = 980.6034 (+/− 14.2 ppm)
926.5006
265.1556
1069.608
[M+Na]+
1003.5926
957.5351
1070.6107
[M+K]+
1019.581
796.4185
0
20
40
60
80
100
400
600
800
1000

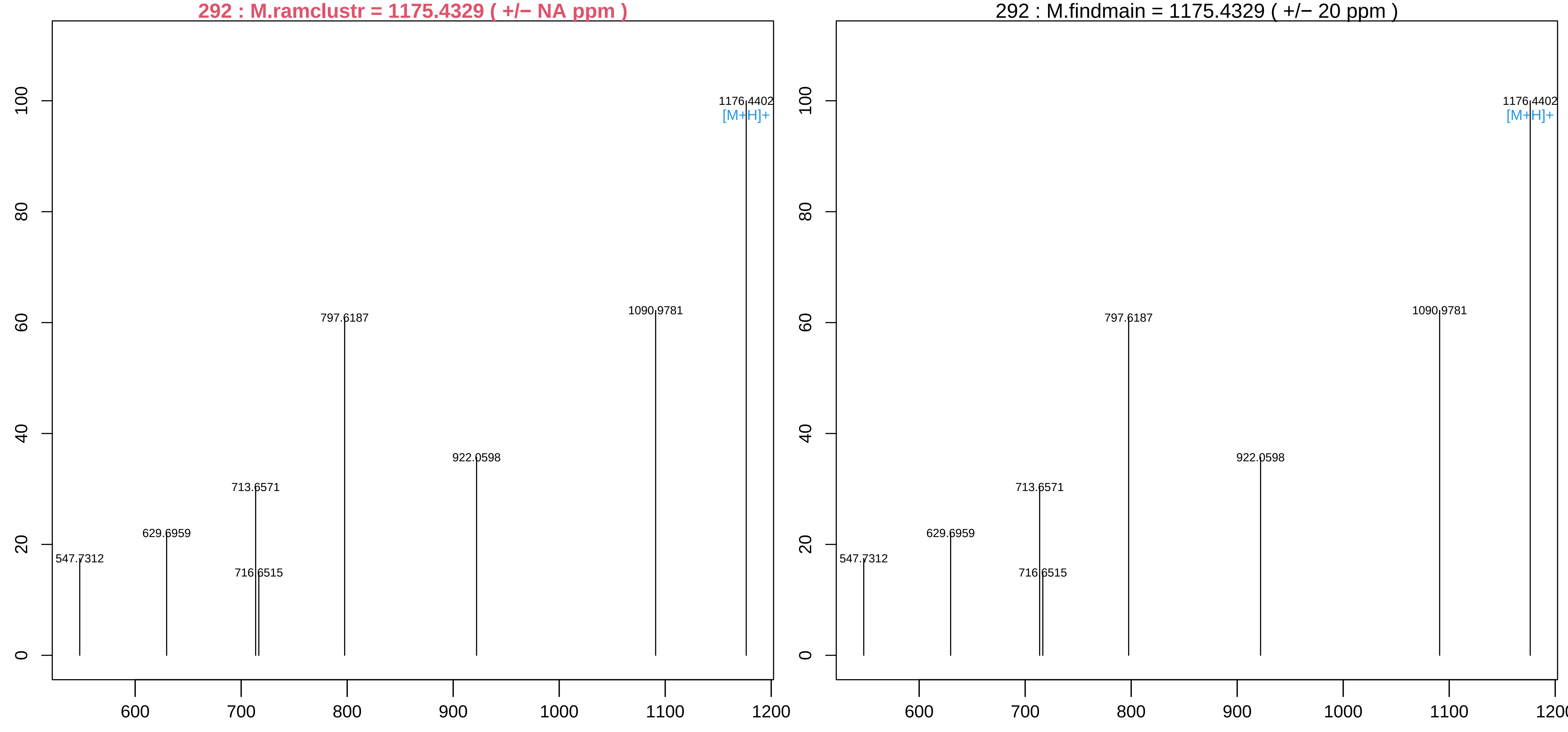
292 : M.ramclustr = 1175.4329 (+/− NA ppm)
1176.4402
[M+H]+
1090.9781
797.6187
922.0598
713.6571
629.6959
547.7312
716.6515
292 : M.findmain = 1175.4329 (+/− 20 ppm)
1176.4402
[M+H]+
1090.9781
797.6187
922.0598
713.6571
629.6959
547.7312
716.6515

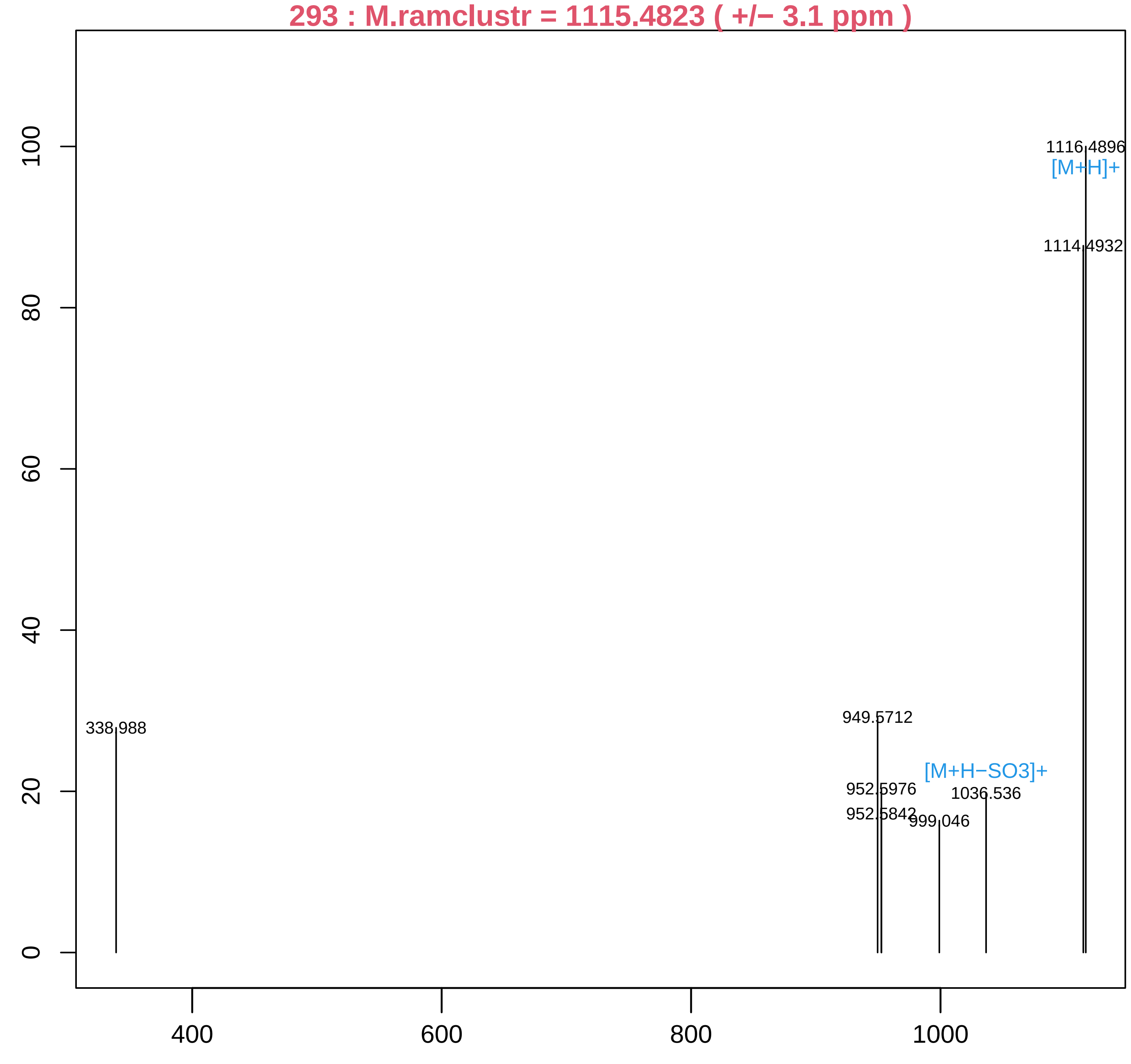
293 : M.ramclustr = 1115.4823 (+/− 3.1 ppm)
1116.4896
[M+H]+
1114.4932
338.988
949.5712
952.5976
952.5842
999.046
1036.536
[M+H−SO3]+
0
20
40
60
80
100
400
600
800
1000

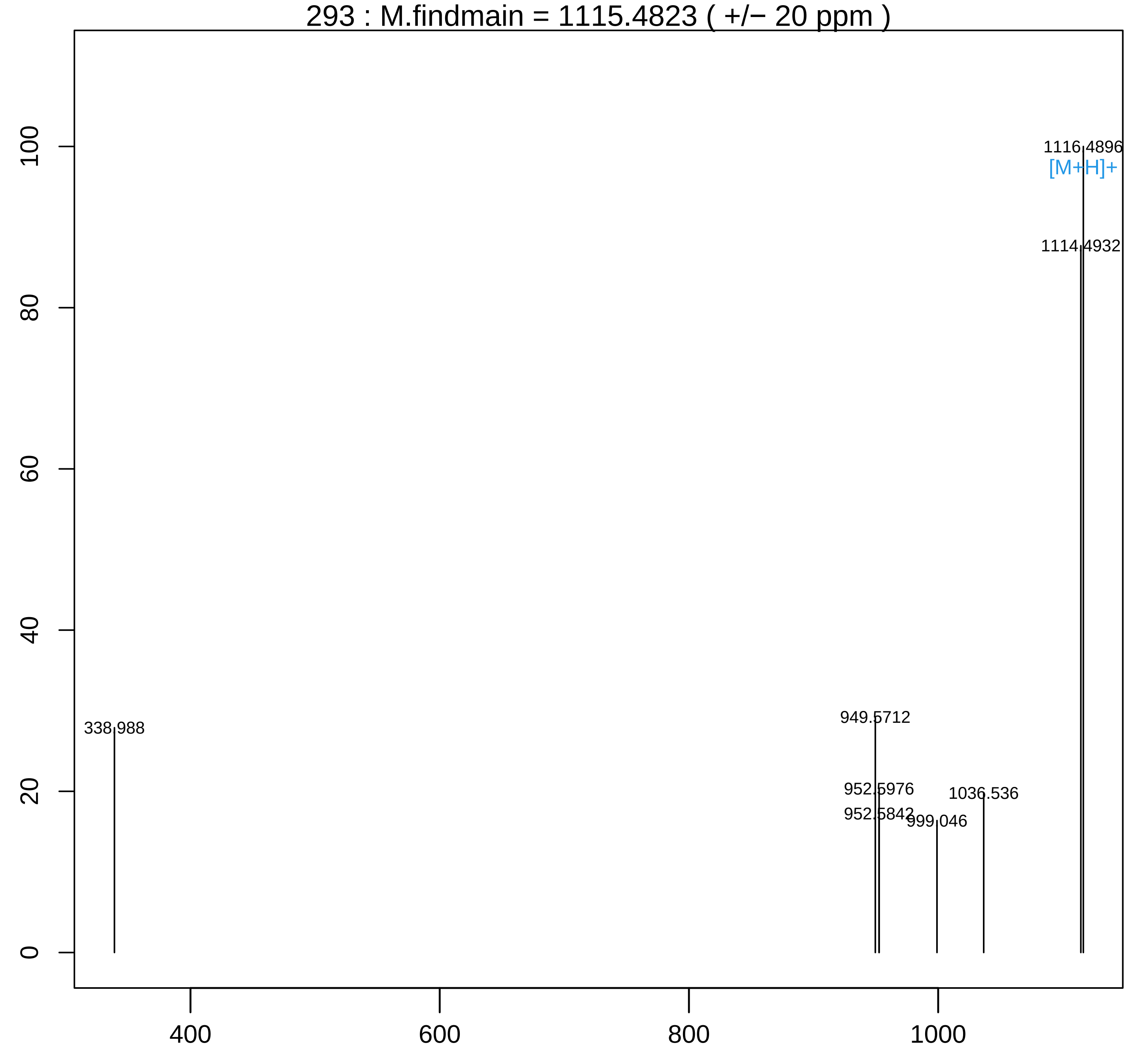
293 : M.findmain = 1115.4823 (+/− 20 ppm)
1116.4896
[M+H]+
1114.4932
338.988
949.5712
952.5976
952.5842
999.046
1036.536
0
20
40
60
80
100
400
600
800
1000

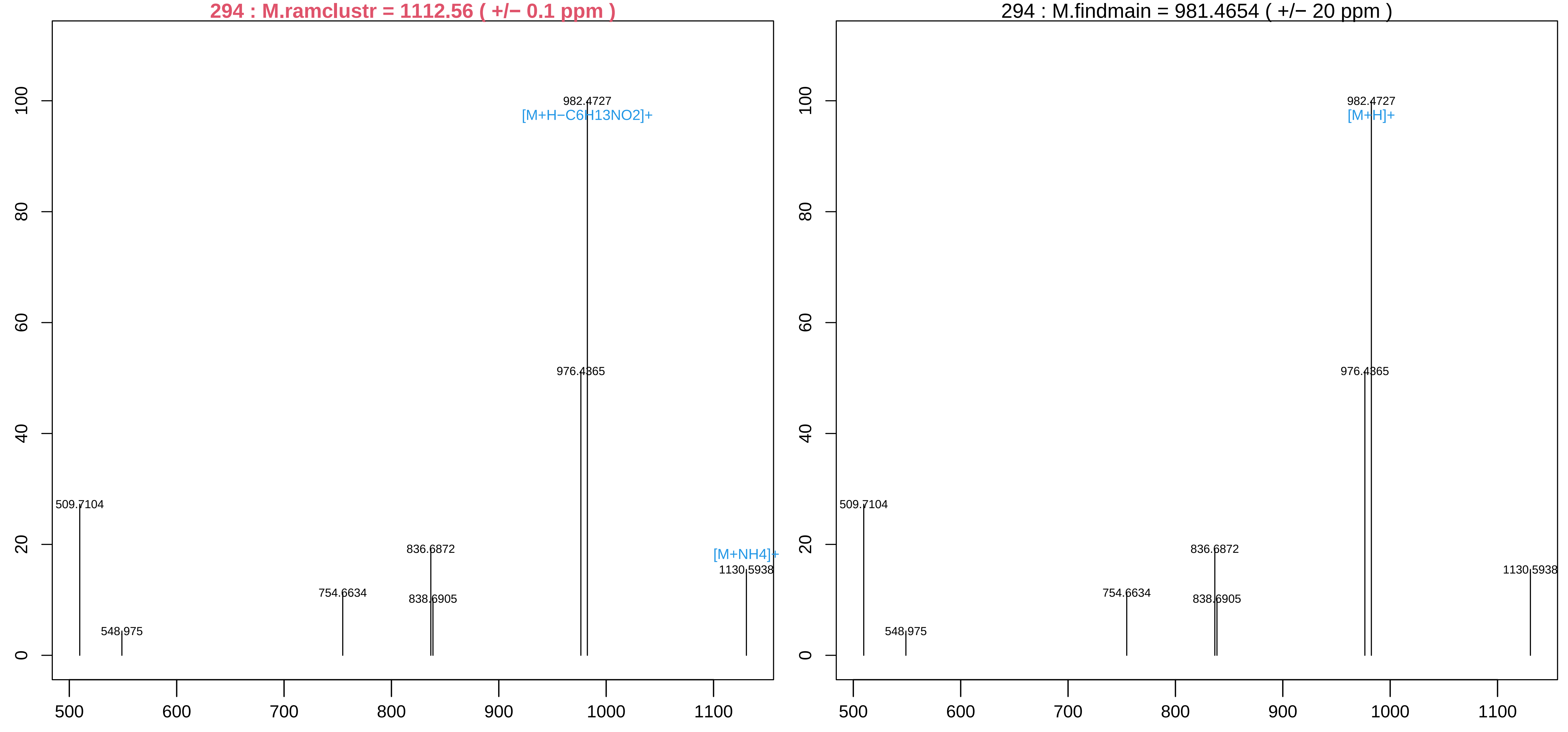

294 : M.ramclustr = 1112.56 (+/− 0.1 ppm)
982.4727
[M+H−C6H13NO2]+
976.4865
509.7104
836.6872
[M+NH4]+
1130.5938
754.6634
838.6905
548.975
294 : M.findmain = 981.4654 (+/− 20 ppm)
982.4727
[M+H]+
976.4865
509.7104
836.6872
1130.5938
754.6634
838.6905
548.975

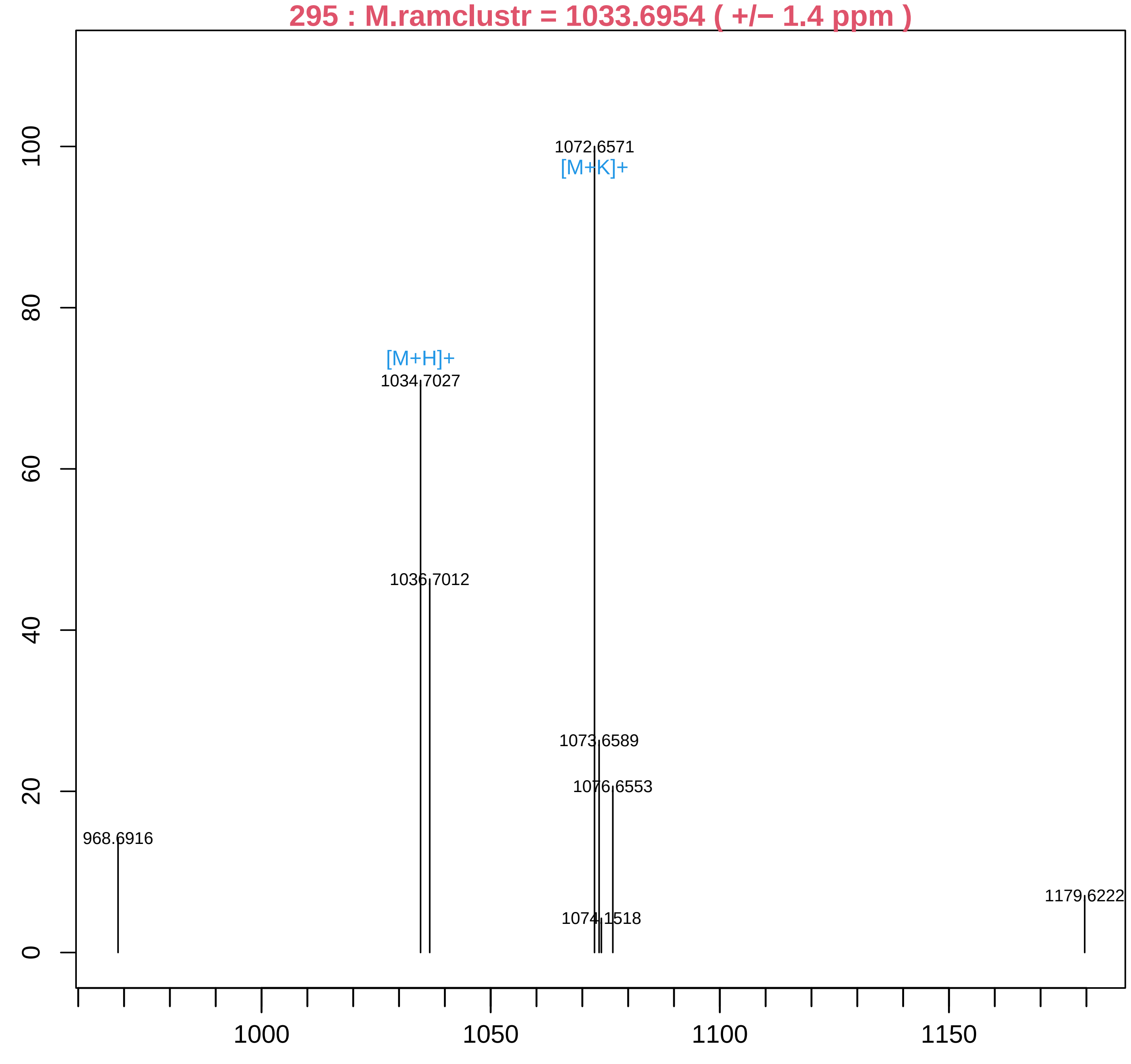
295 : M.ramclustr = 1033.6954 (+/− 1.4 ppm)
1072.6571
[M+K]+
[M+H]+
1034.7027
1036.7012
1073.6589
1076.6553
968.6916
1179.6222
1074.1518
0
20
40
60
80
100
1000
1050
1100
1150

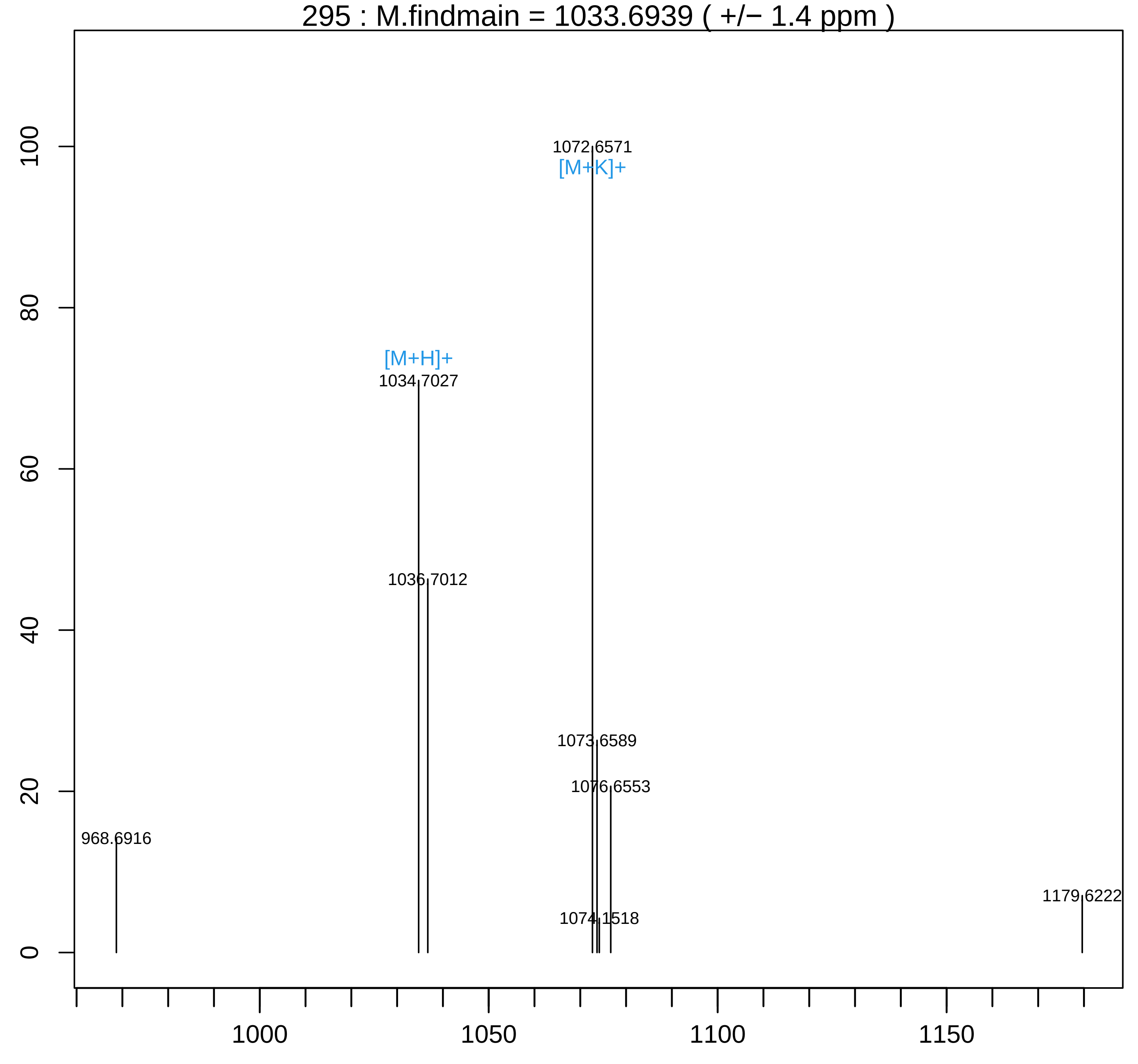
295 : M.findmain = 1033.6939 (+/− 1.4 ppm)
1072.6571
[M+K]+
[M+H]+
1034.7027
1036.7012
1073.6589
1076.6553
968.6916
1179.6222
1074.1518
0
20
40
60
80
100
1000
1050
1100
1150

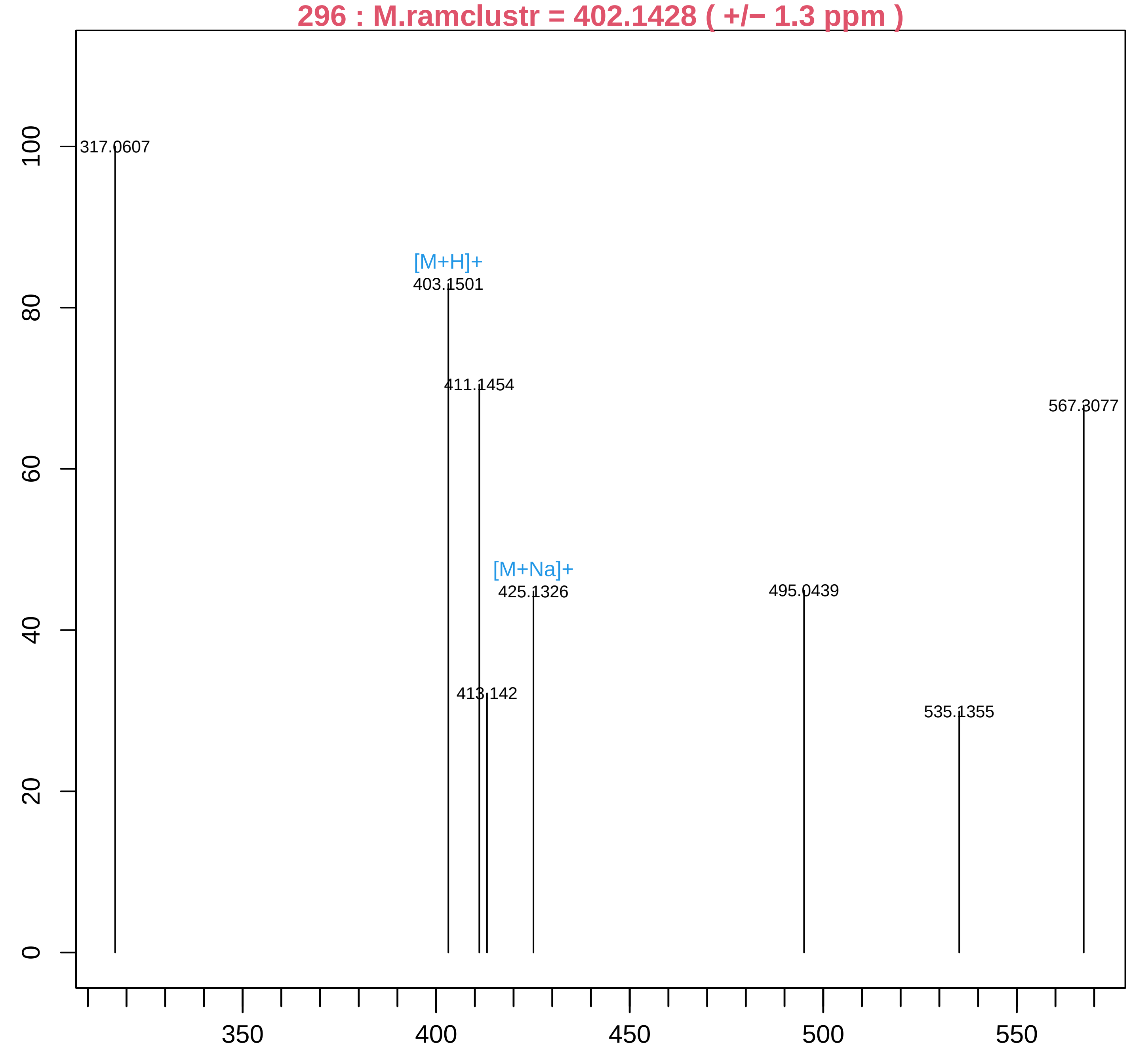
296 : M.ramclustr = 402.1428 (+/− 1.3 ppm)
317.0607
[M+H]+
403.1501
411.1454
[M+Na]+
425.1326
413.142
495.0439
535.1355
567.3077
0
20
40
60
80
100
350
400
450
500
550

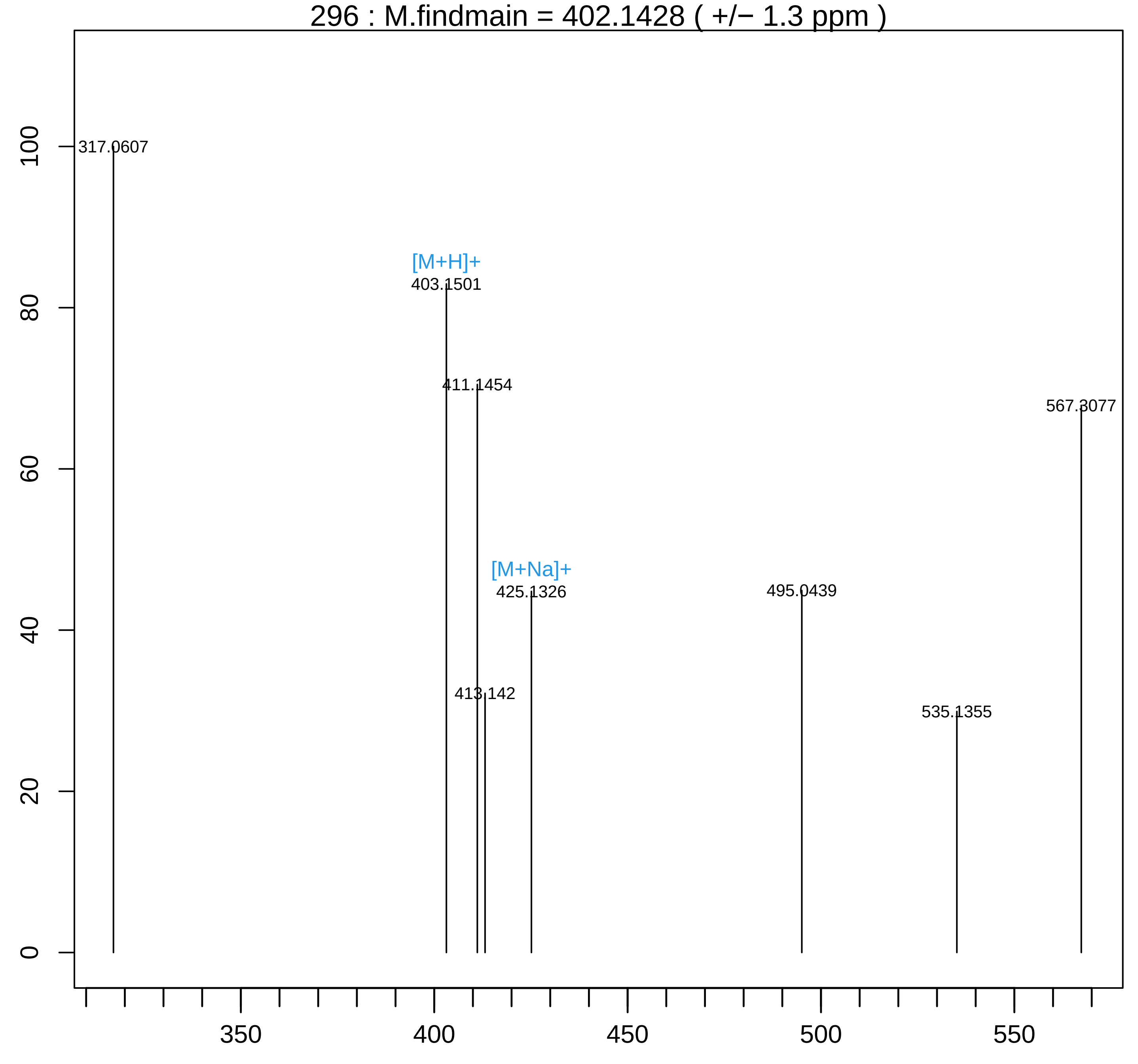
296 : M.findmain = 402.1428 (+/− 1.3 ppm)
317.0607
[M+H]+
403.1501
411.1454
[M+Na]+
425.1326
413.142
495.0439
535.1355
567.3077
0
20
40
60
80
100
350
400
450
500
550

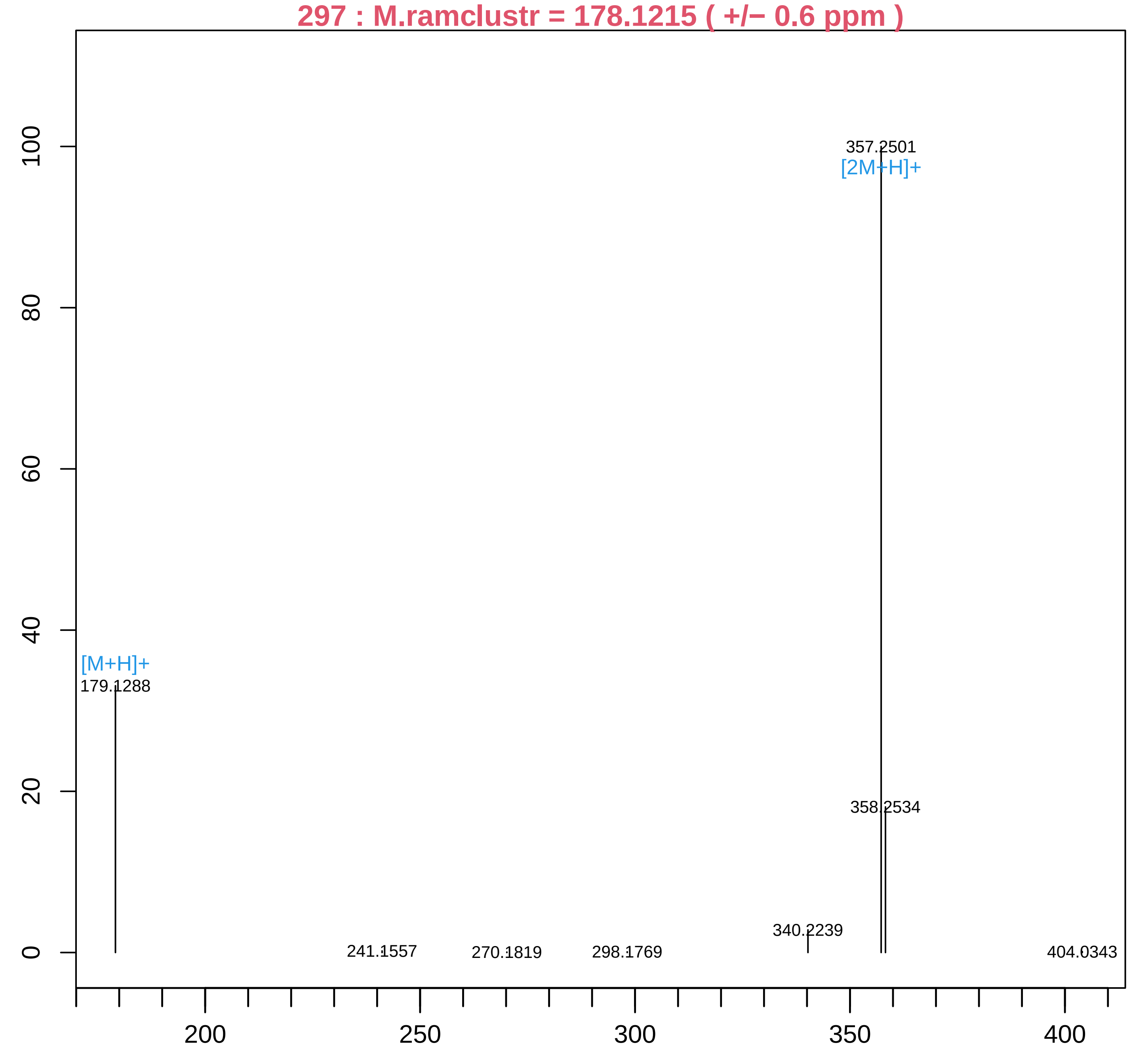
297 : M.ramclustr = 178.1215 (+/− 0.6 ppm)
357.2501
[2M+H]+
[M+H]+
179.1288
358.2534
340.2239
241.1557
270.1819
298.1769
404.0343
0
20
40
60
80
100
200
250
300
350
400

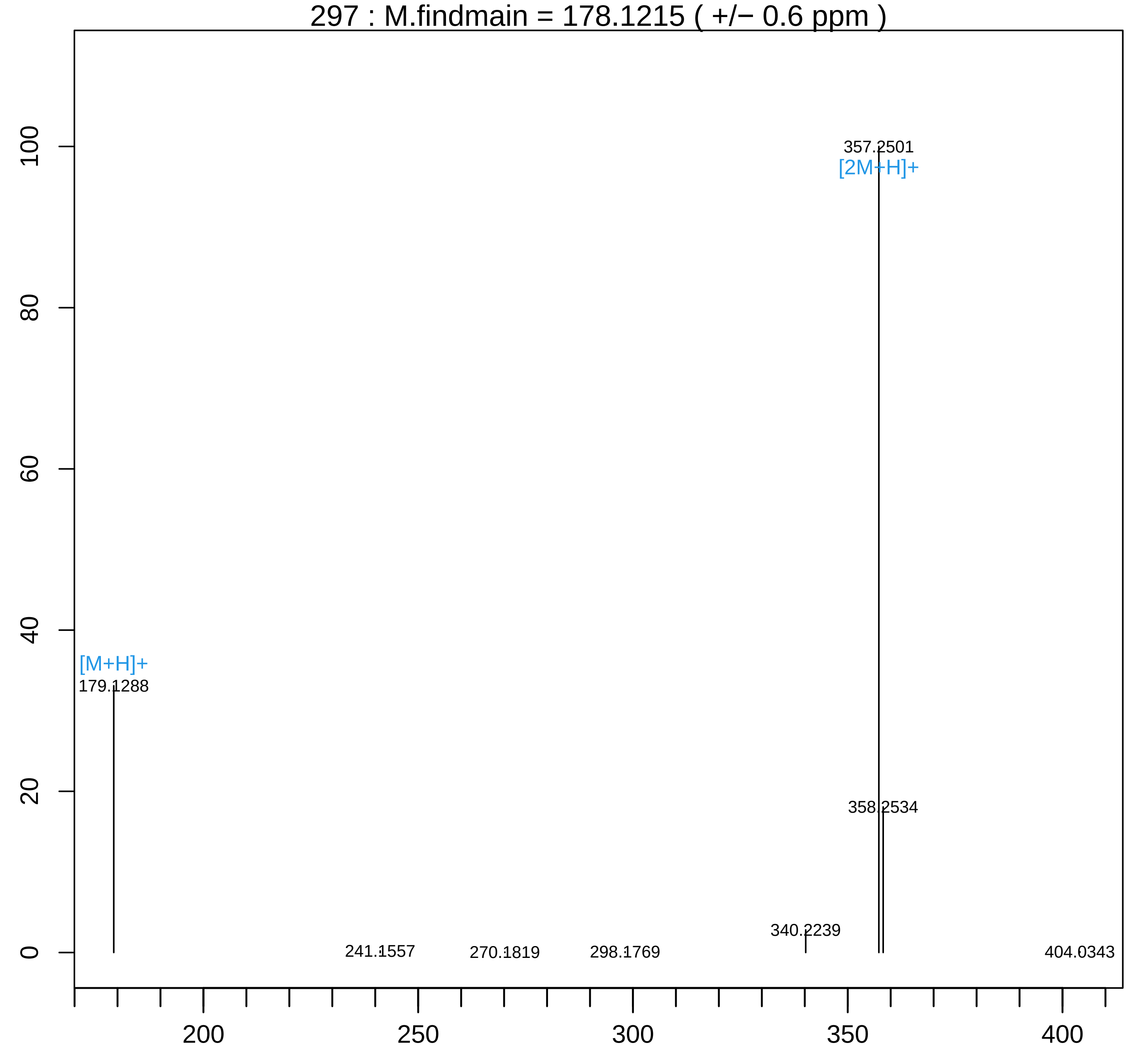
297 : M.findmain = 178.1215 (+/− 0.6 ppm)
357.2501
[2M+H]+
[M+H]+
179.1288
358.2534
340.2239
241.1557
270.1819
298.1769
404.0343
0
20
40
60
80
100
200
250
300
350
400

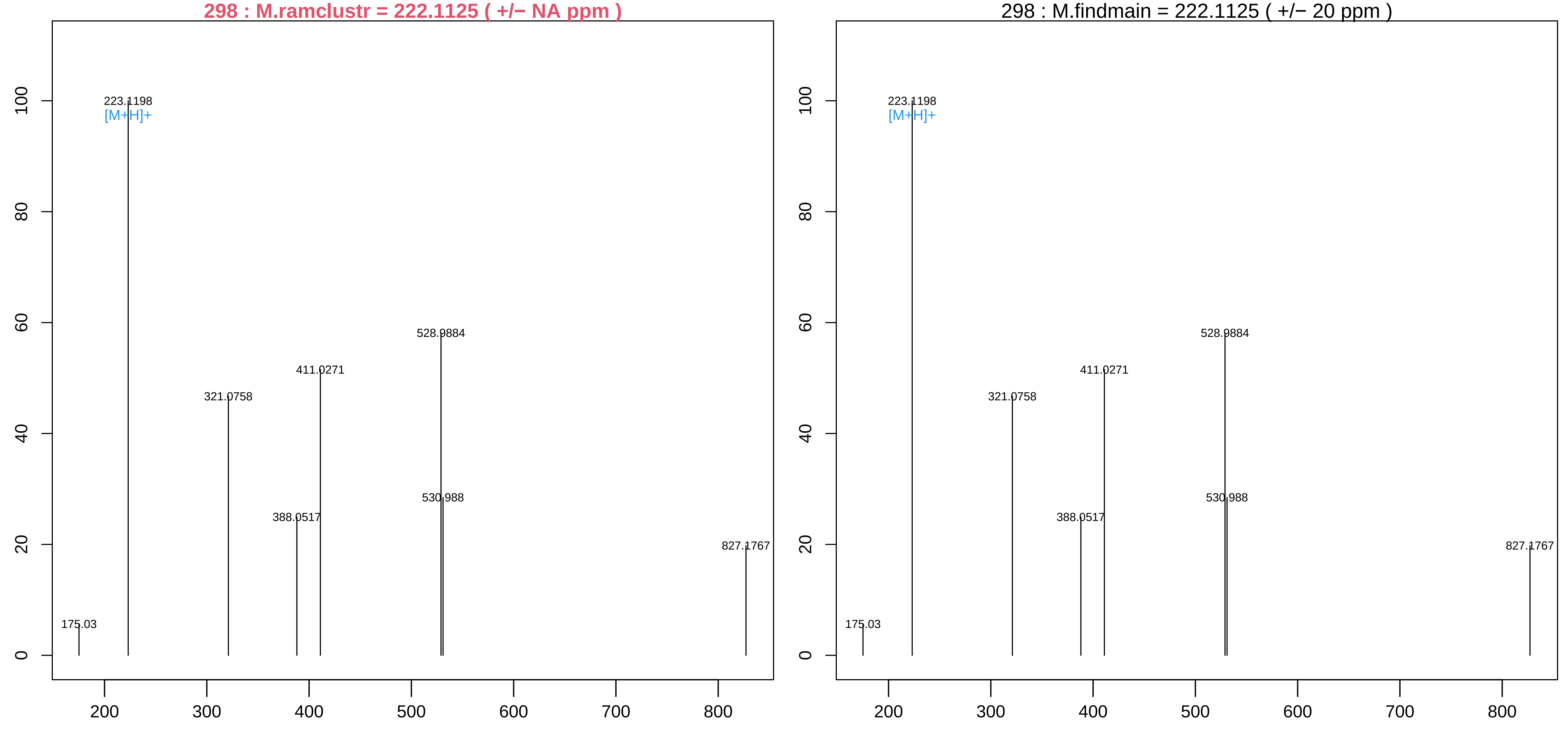
298 : M.ramclustr = 222.1125 (+/− NA ppm)
298 : M.findmain = 222.1125 (+/− 20 ppm)
223.1198
[M+H]+
528.9884
411.0271
321.0758
530.988
388.0517
827.1767
175.03
0
20
40
60
80
100
200
300
400
500
600
700
800

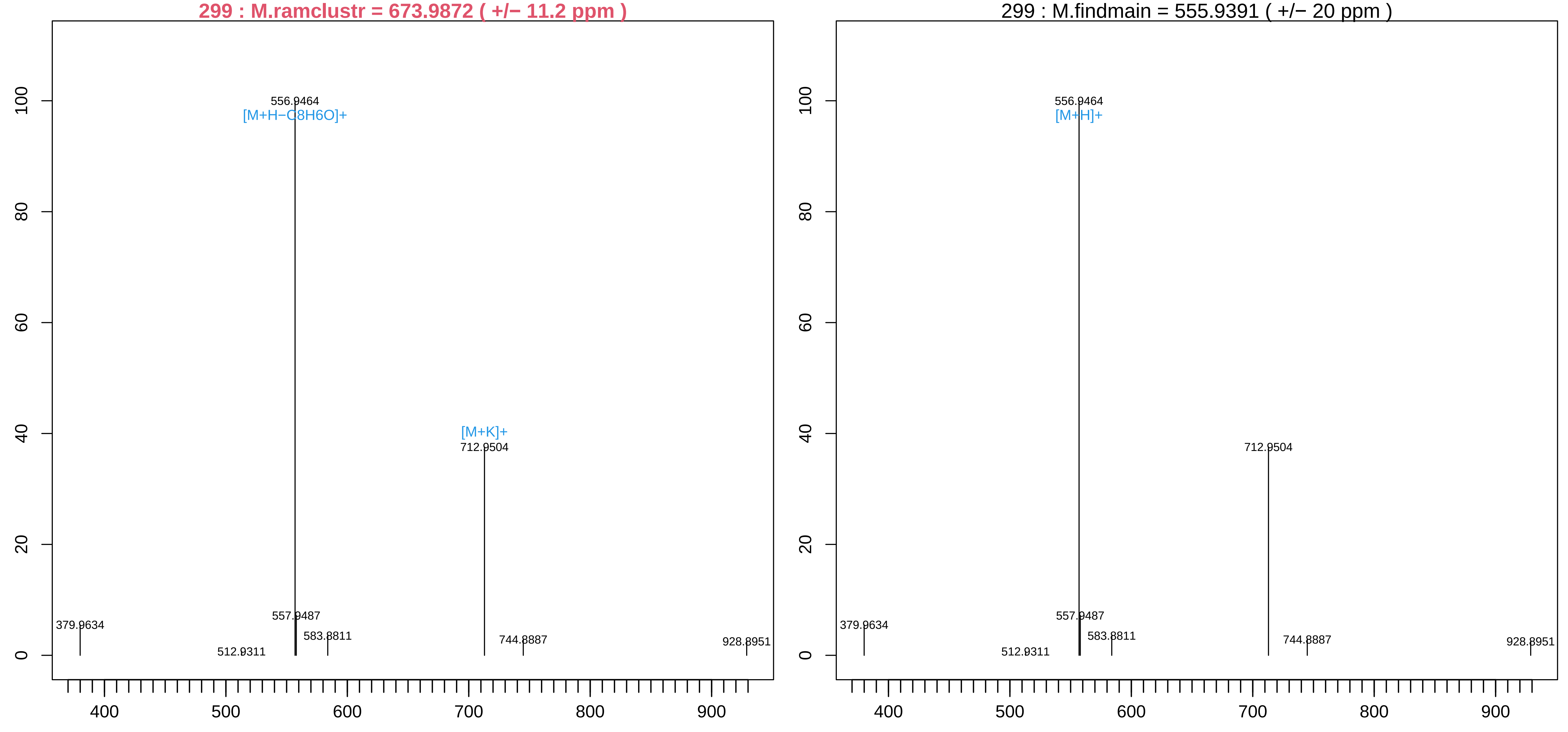

299 : M.ramclustr = 673.9872 (+/− 11.2 ppm)
556.9464
[M+H−C8H6O]+
[M+K]+
712.9504
379.9634
512.9311
557.9487
583.8811
744.8887
928.8951
299 : M.findmain = 555.9391 (+/− 20 ppm)
556.9464
[M+H]+
712.9504
379.9634
512.9311
557.9487
583.8811
744.8887
928.8951

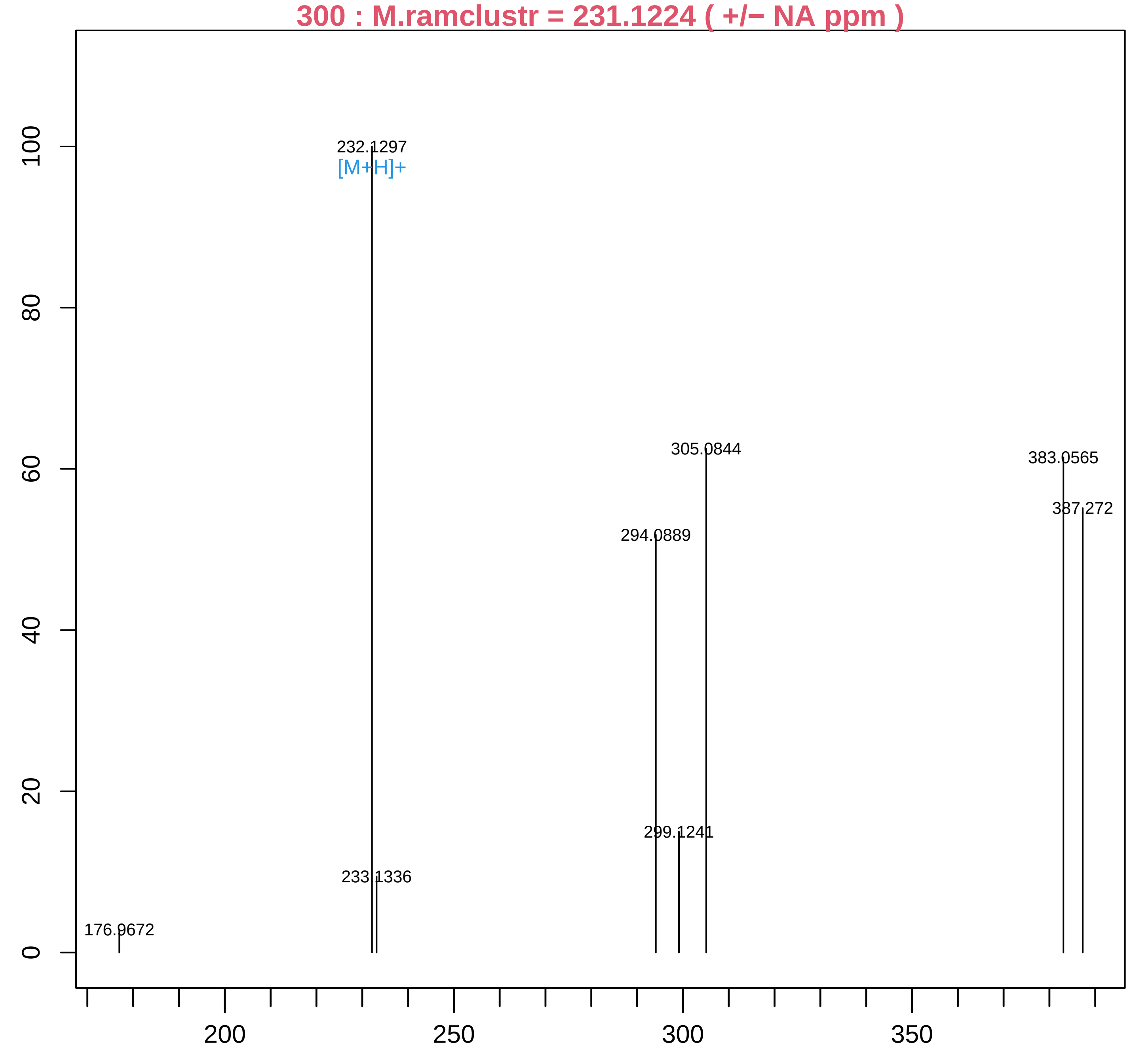
300 : M.ramclustr = 231.1224 (+/− NA ppm)
232.1297
[M+H]+
305.0844
383.0565
387.272
294.0889
299.1241
233.1336
176.9672
0
20
40
60
80
100
200
250
300
350

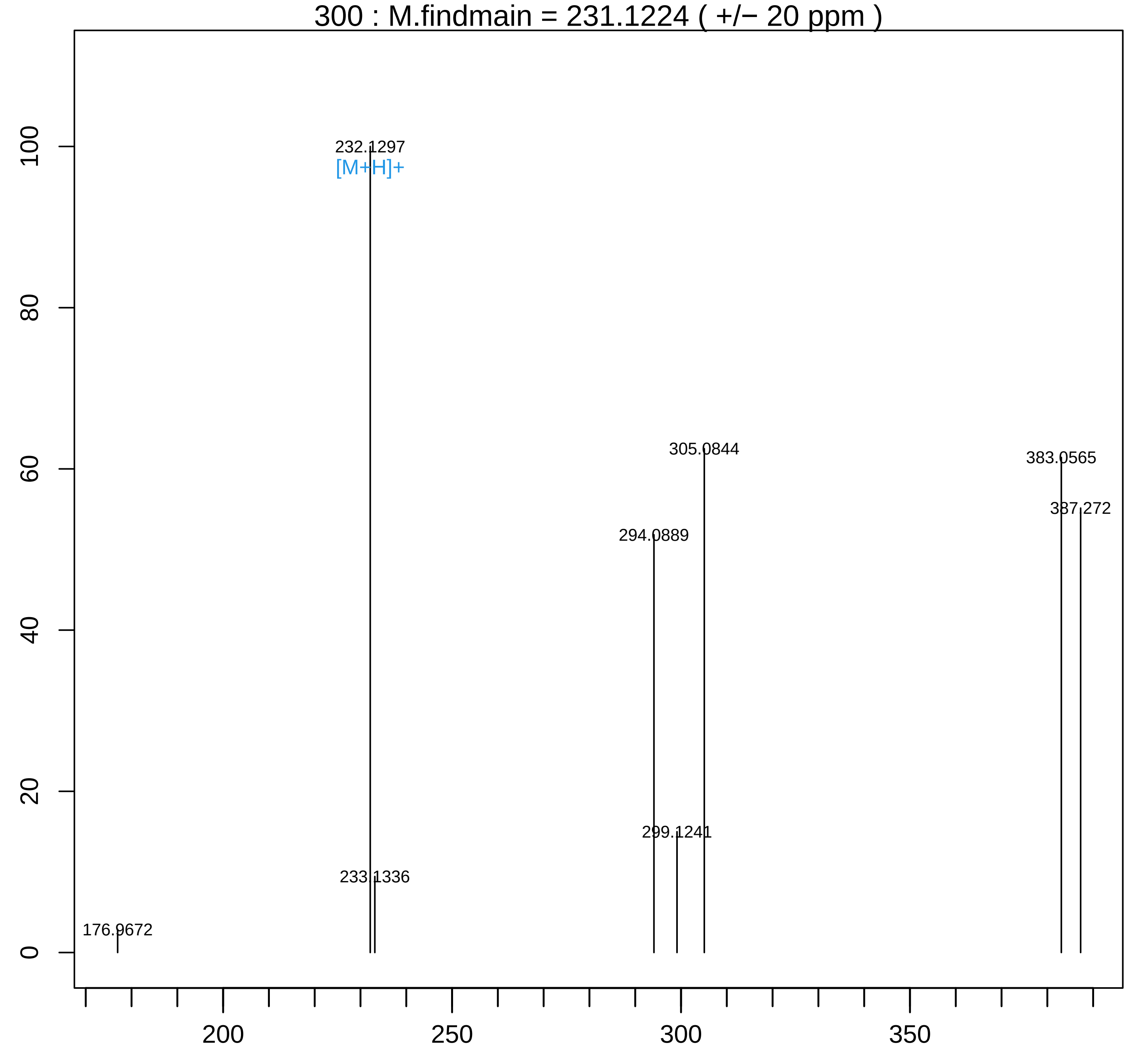
300 : M.findmain = 231.1224 (+/− 20 ppm)
232.1297
[M+H]+
305.0844
383.0565
387.272
294.0889
299.1241
233.1336
176.9672
0
20
40
60
80
100
200
250
300
350

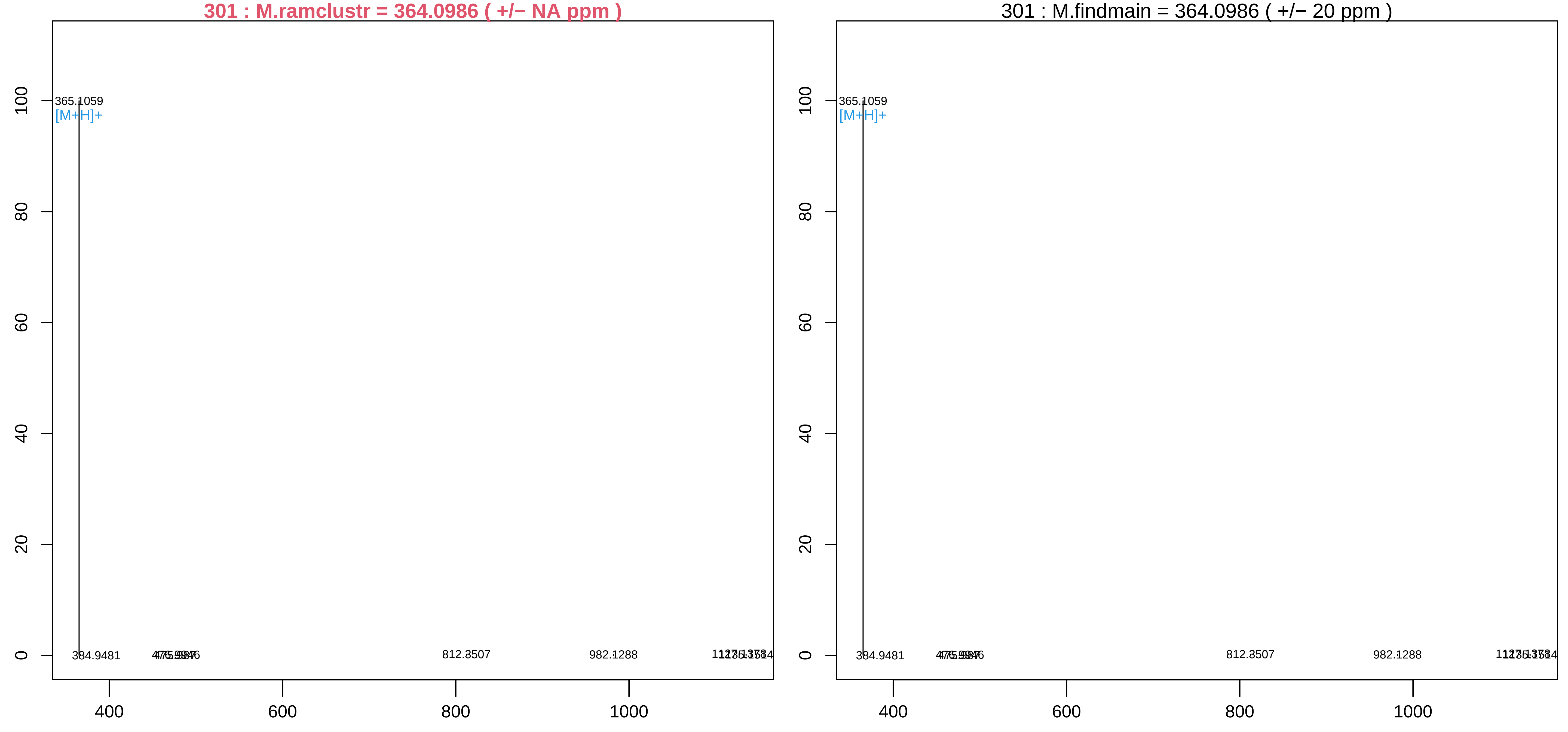
301 : M.ramclustr = 364.0986 (+/− NA ppm)
301 : M.findmain = 364.0986 (+/− 20 ppm)
365.1059
[M+H]+
384.9481
812.3507
982.1288
0
20
40
60
80
100
400
600
800
1000

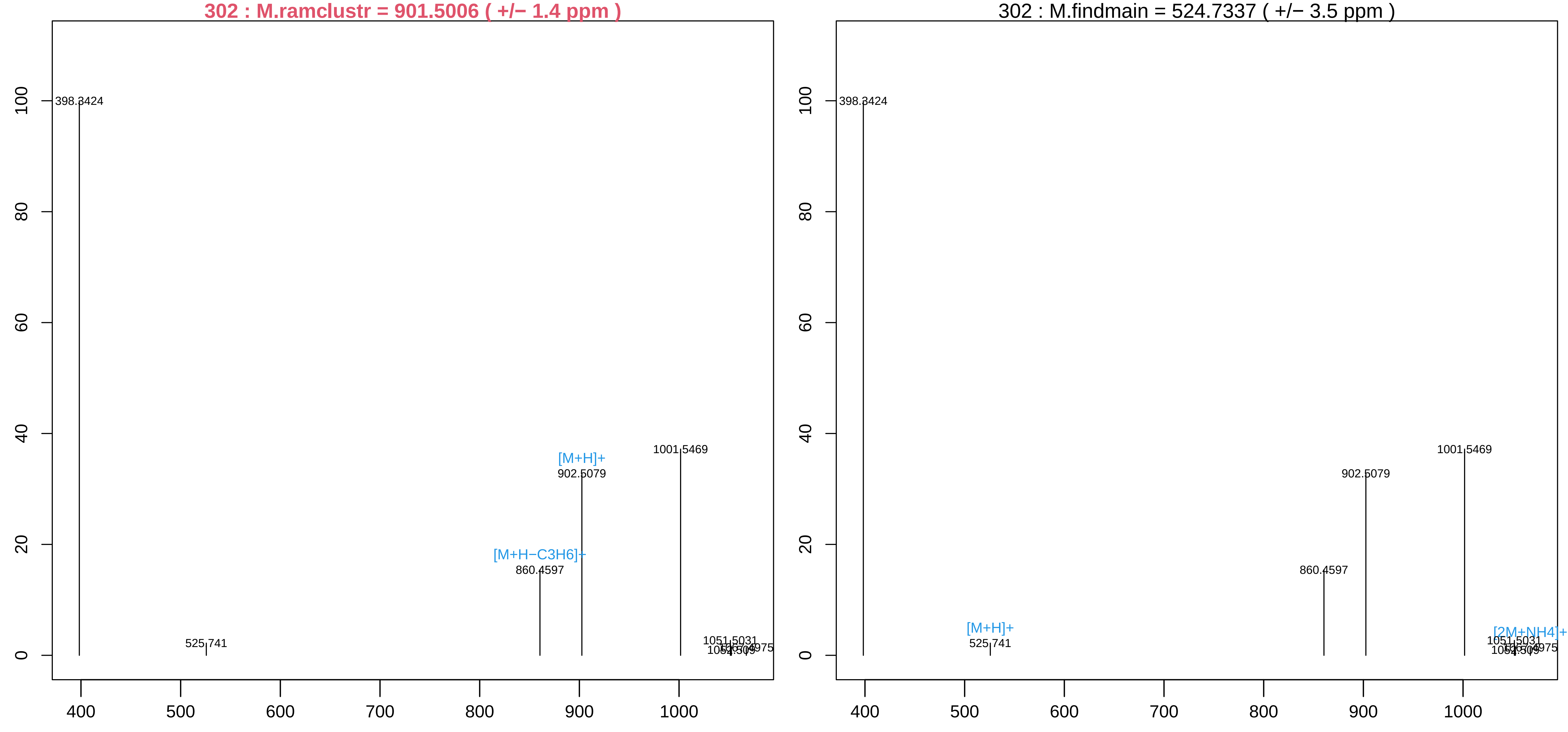
302 : M.ramclustr = 901.5006 (+/− 1.4 ppm)
302 : M.findmain = 524.7337 (+/− 3.5 ppm)
398.3424
[M+H]+
902.5079
1001.5469
[M+H−C3H6]+
860.4597
525.741
1051.5031
[2M+NH4]+
0
20
40
60
80
100
400
500
600
700
800
900
1000

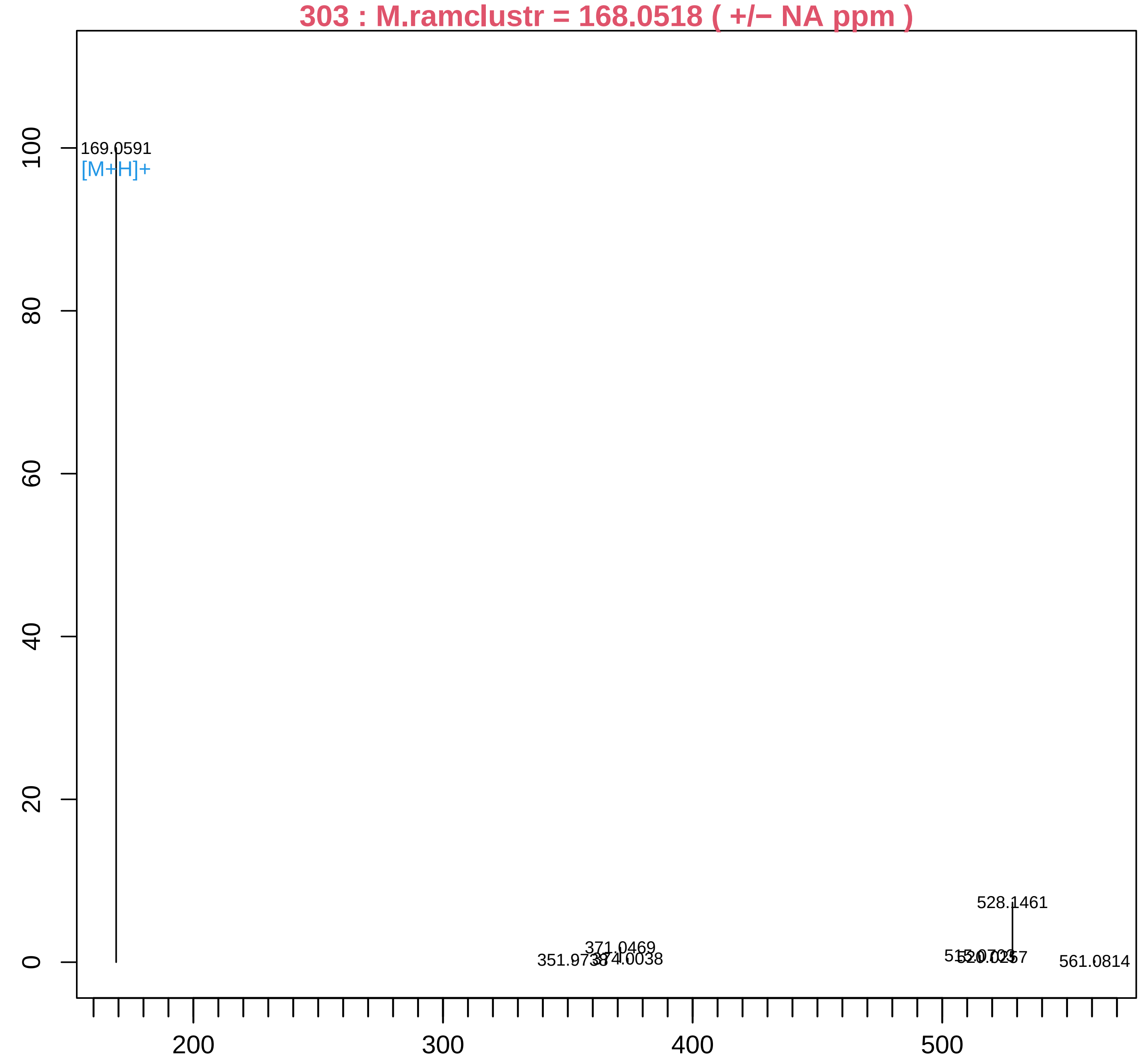
303 : M.ramclustr = 168.0518 (+/− NA ppm)
169.0591
[M+H]+
528.1461
371.0469
351.9738
374.0038
515.0703
520.0257
561.0814
0
20
40
60
80
100
200
300
400
500

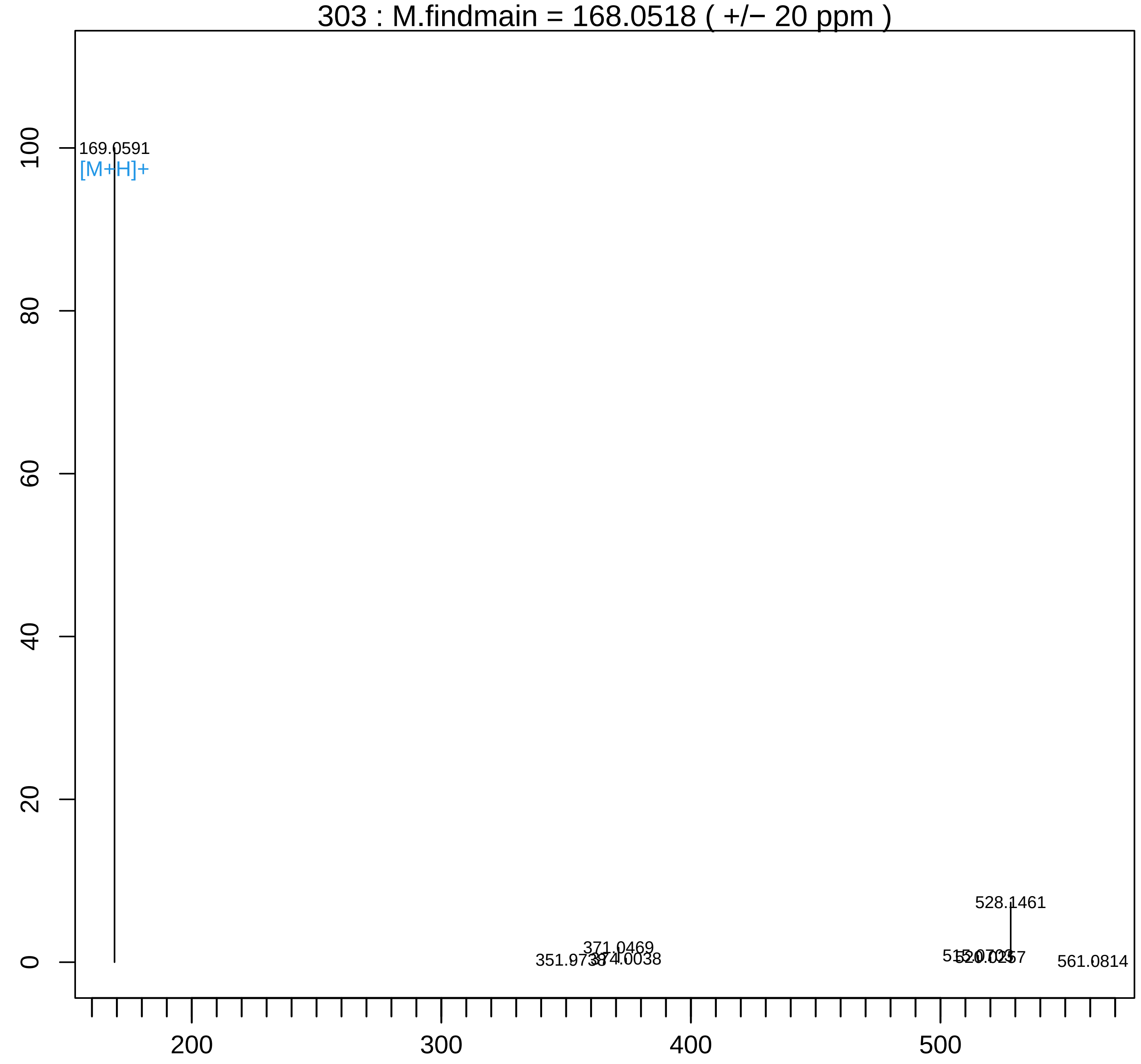
303 : M.findmain = 168.0518 (+/− 20 ppm)
169.0591
[M+H]+
528.1461
371.0469
351.9738
374.0038
515.0703
520.0257
561.0814
0
20
40
60
80
100
200
300
400
500

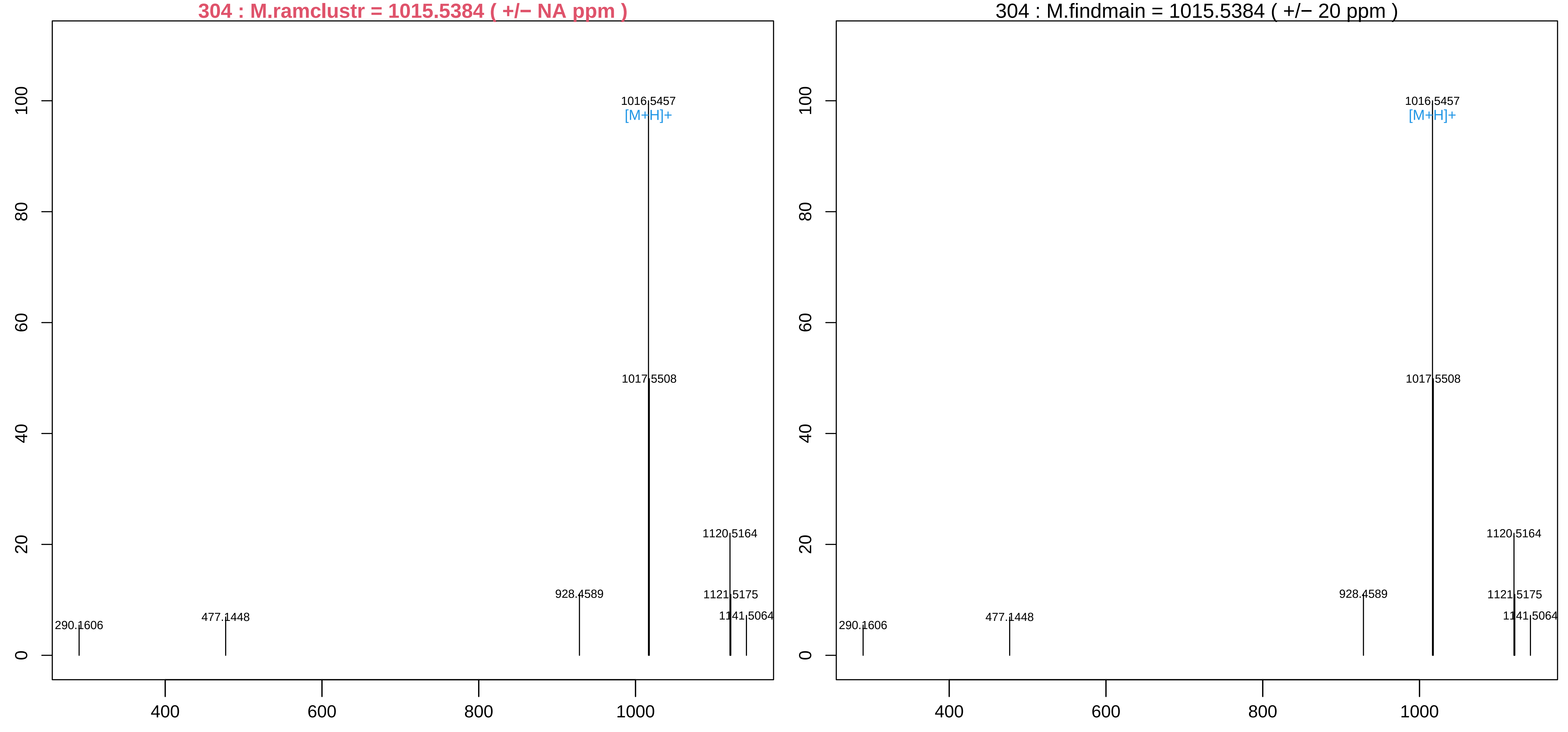

304 : M.ramclustr = 1015.5384 (+/− NA ppm)
304 : M.findmain = 1015.5384 (+/− 20 ppm)
1016.5457
[M+H]+
1017.5508
1120.5164
1121.5175
1141.5064
928.4589
477.1448
290.1606
0
20
40
60
80
100
400
600
800
1000

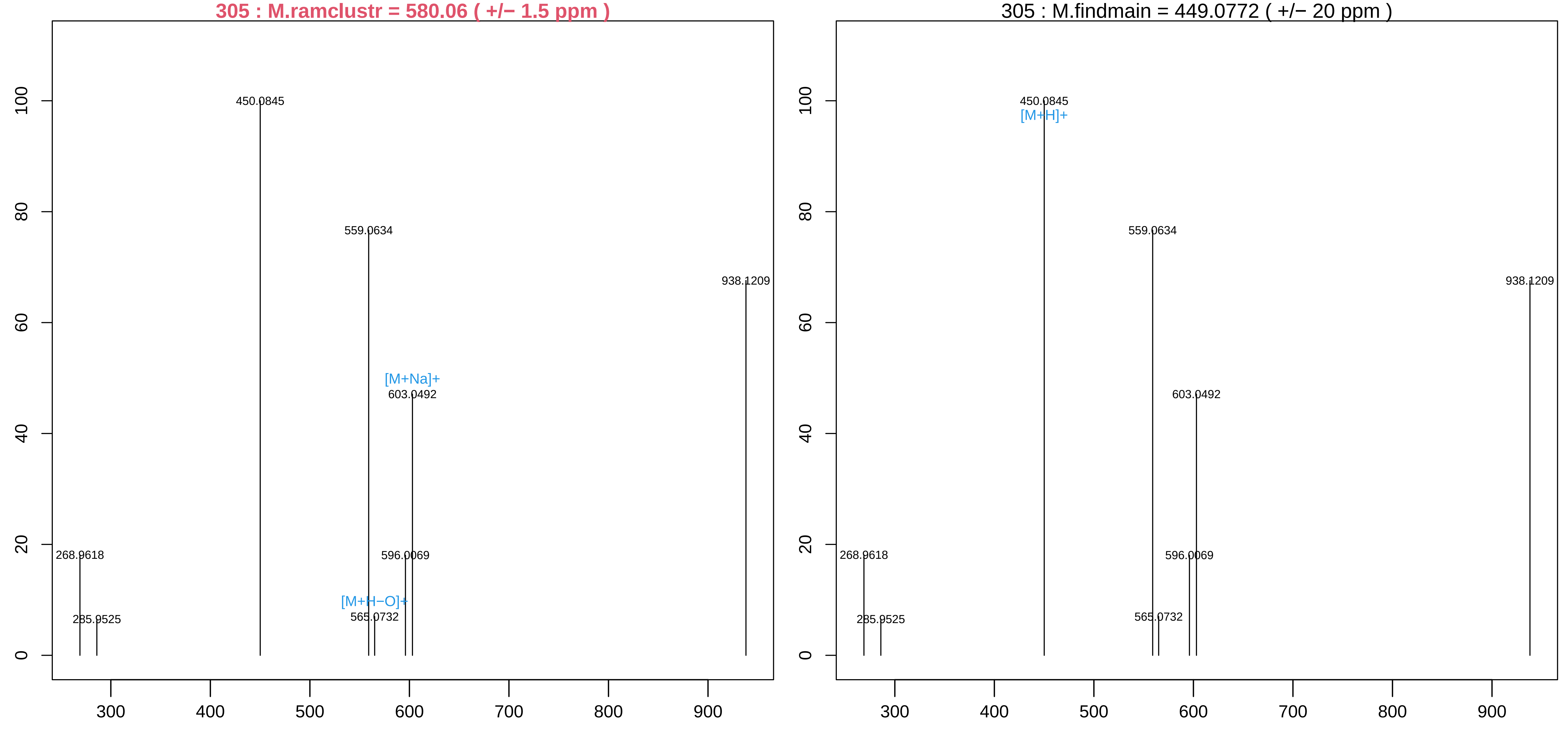
305 : M.ramclustr = 580.06 (+/− 1.5 ppm)
450.0845
559.0634
938.1209
[M+Na]+
603.0492
268.9618
596.0069
[M+H−O]+
285.9525
565.0732
305 : M.findmain = 449.0772 (+/− 20 ppm)
450.0845
[M+H]+
559.0634
938.1209
603.0492
268.9618
596.0069
565.0732
285.9525

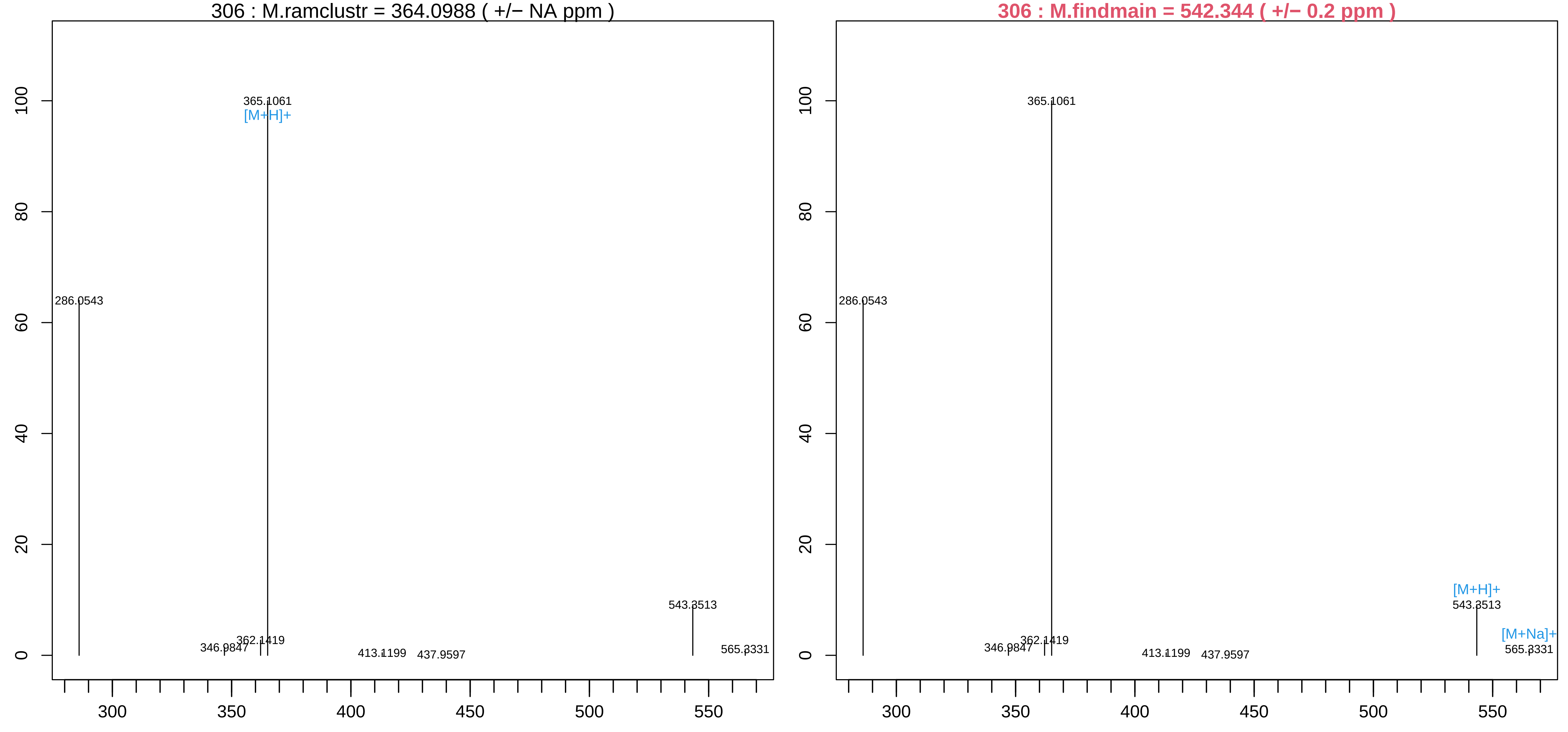

306 : M.ramclustr = 364.0988 (+/− NA ppm)
365.1061
[M+H]+
286.0543
543.3513
346.9847
362.1419
413.1199
437.9597
565.3331
0
20
40
60
80
100
300
350
400
450
500
550
306 : M.findmain = 542.344 (+/− 0.2 ppm)
365.1061
286.0543
[M+H]+
543.3513
[M+Na]+
565.3331
346.9847
362.1419
413.1199
437.9597

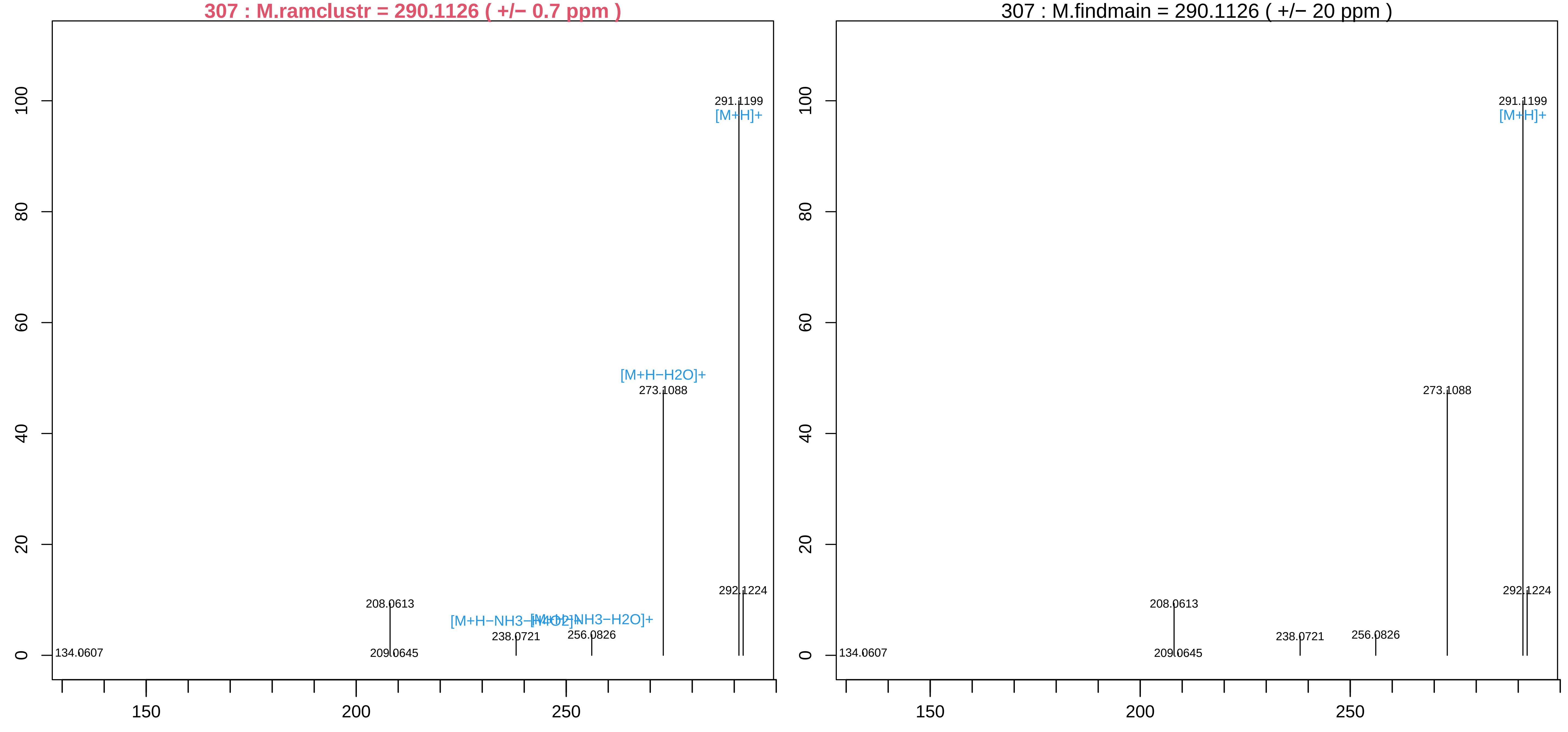
307 : M.ramclustr = 290.1126 (+/− 0.7 ppm)
291.1199
[M+H]+
[M+H−H2O]+
273.1088
292.1224
208.0613
[M+H−NH3−H4O2]+
[M+H−NH3−H2O]+
238.0721
256.0826
134.0607
209.0645
307 : M.findmain = 290.1126 (+/− 20 ppm)
291.1199
[M+H]+
273.1088
292.1224
208.0613
238.0721
256.0826
134.0607
209.0645
0
20
40
60
80
100
150
200
250

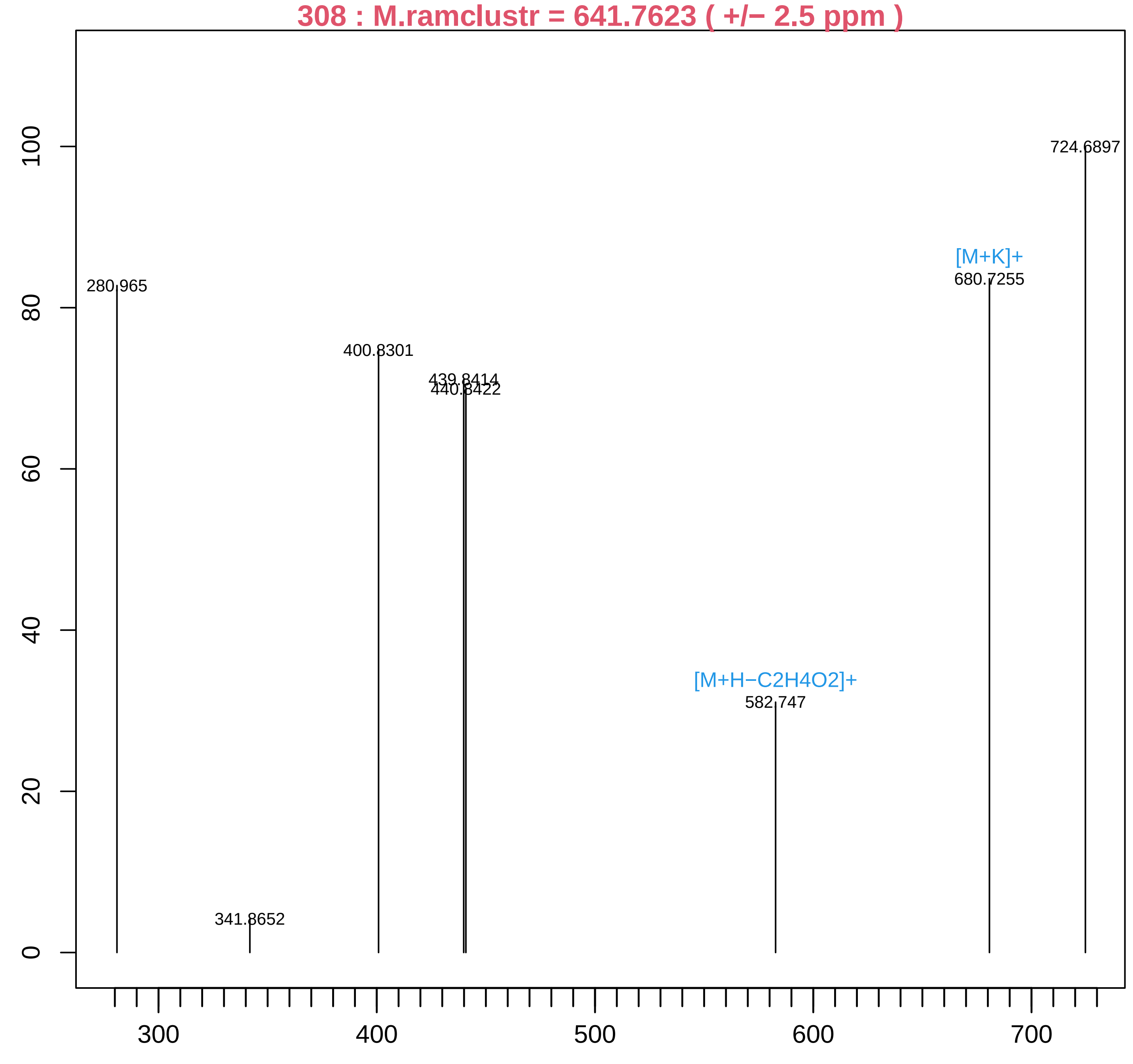
308 : M.ramclustr = 641.7623 (+/− 2.5 ppm)
280.965
341.8652
400.8301
439.8414
440.8422
[M+H−C2H4O2]+
582.747
[M+K]+
680.7255
724.6897
0
20
40
60
80
100
300
400
500
600
700

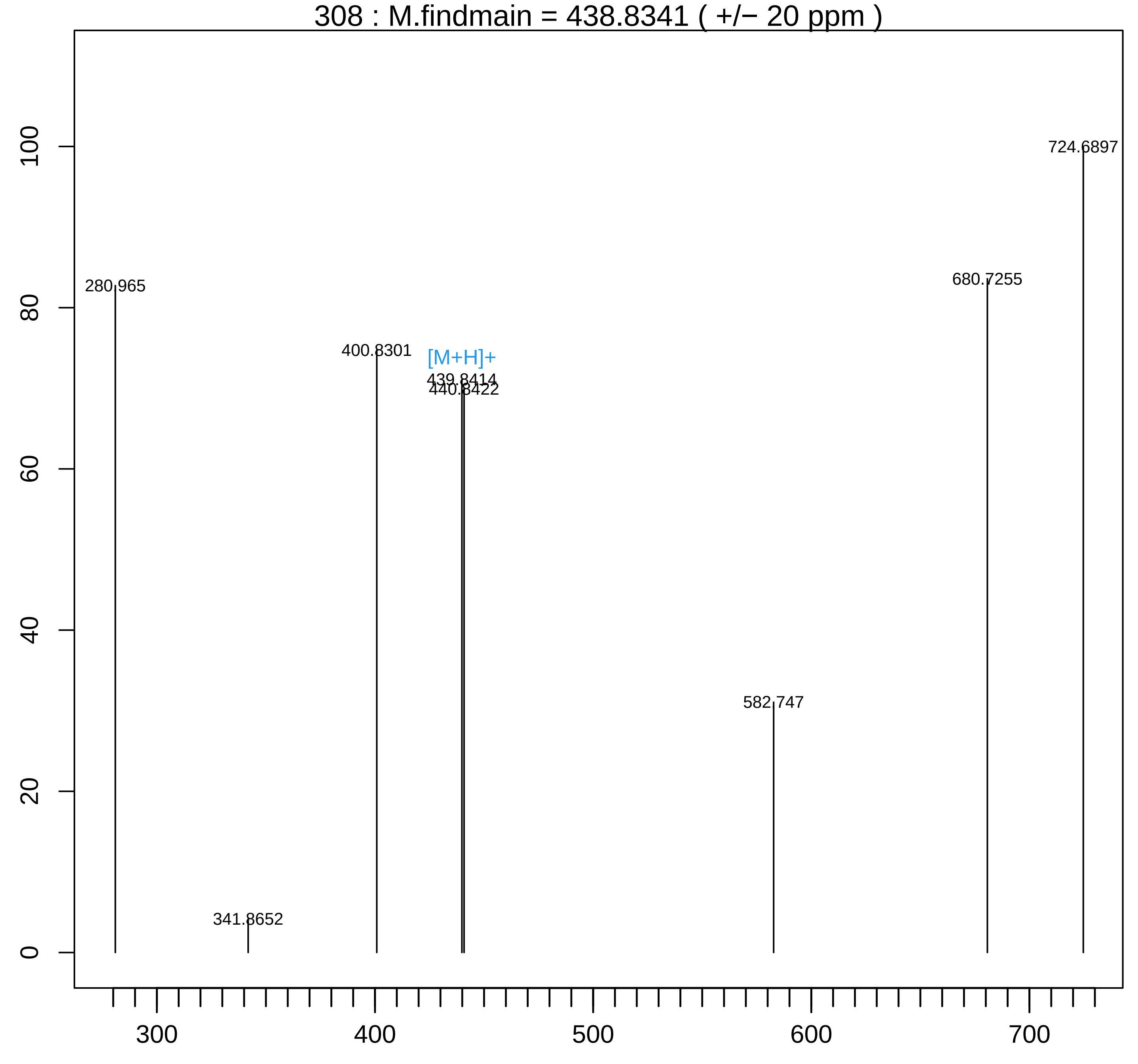
308 : M.findmain = 438.8341 (+/− 20 ppm)
280.965
341.8652
400.8301
[M+H]+
439.8414
440.8422
582.747
680.7255
724.6897
0
20
40
60
80
100
300
400
500
600
700

309 : M.ramclustr = 544.7874 (+/− 11.8 ppm)

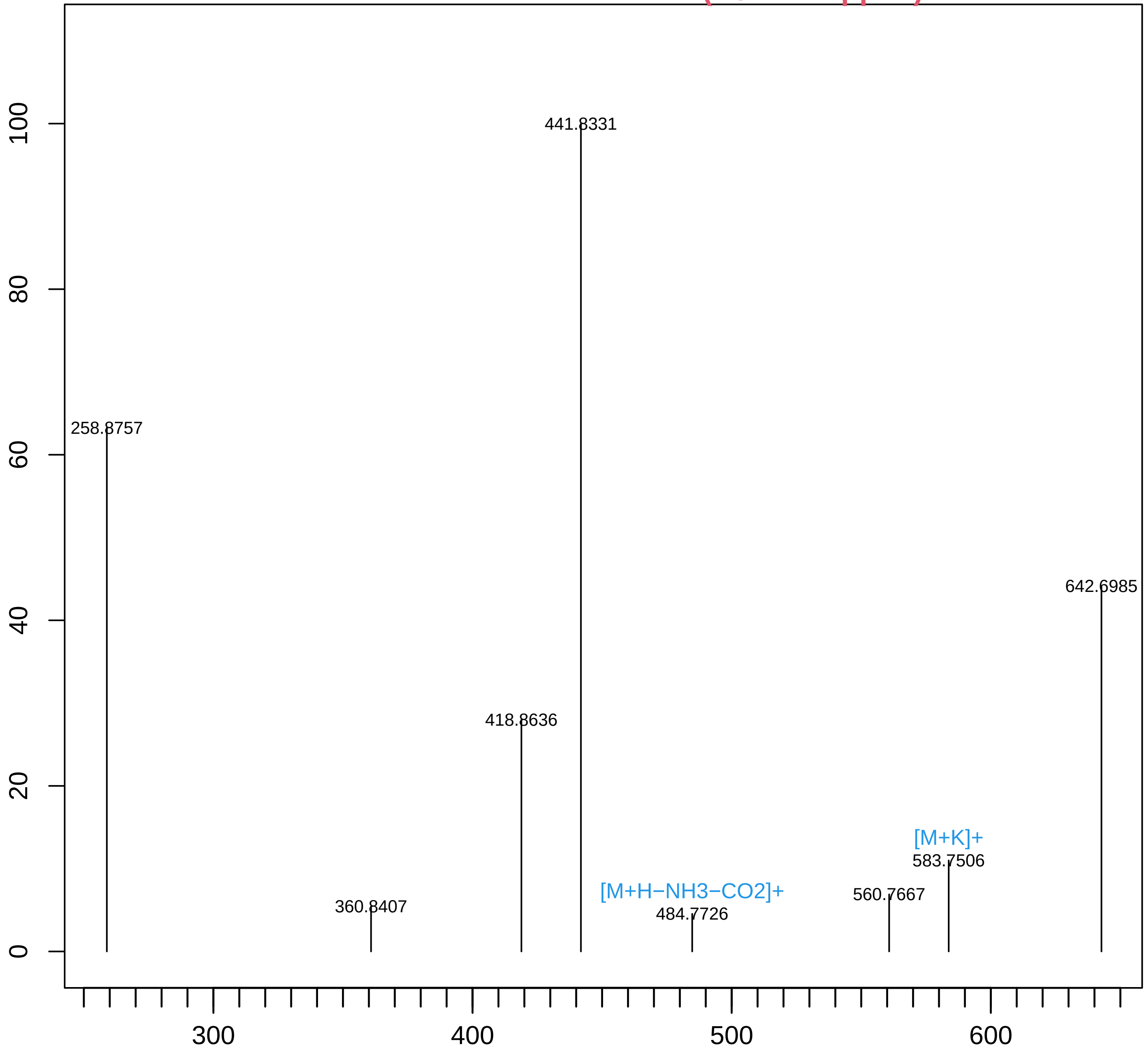

309 : M.findmain = 440.8258 (+/− 20 ppm)

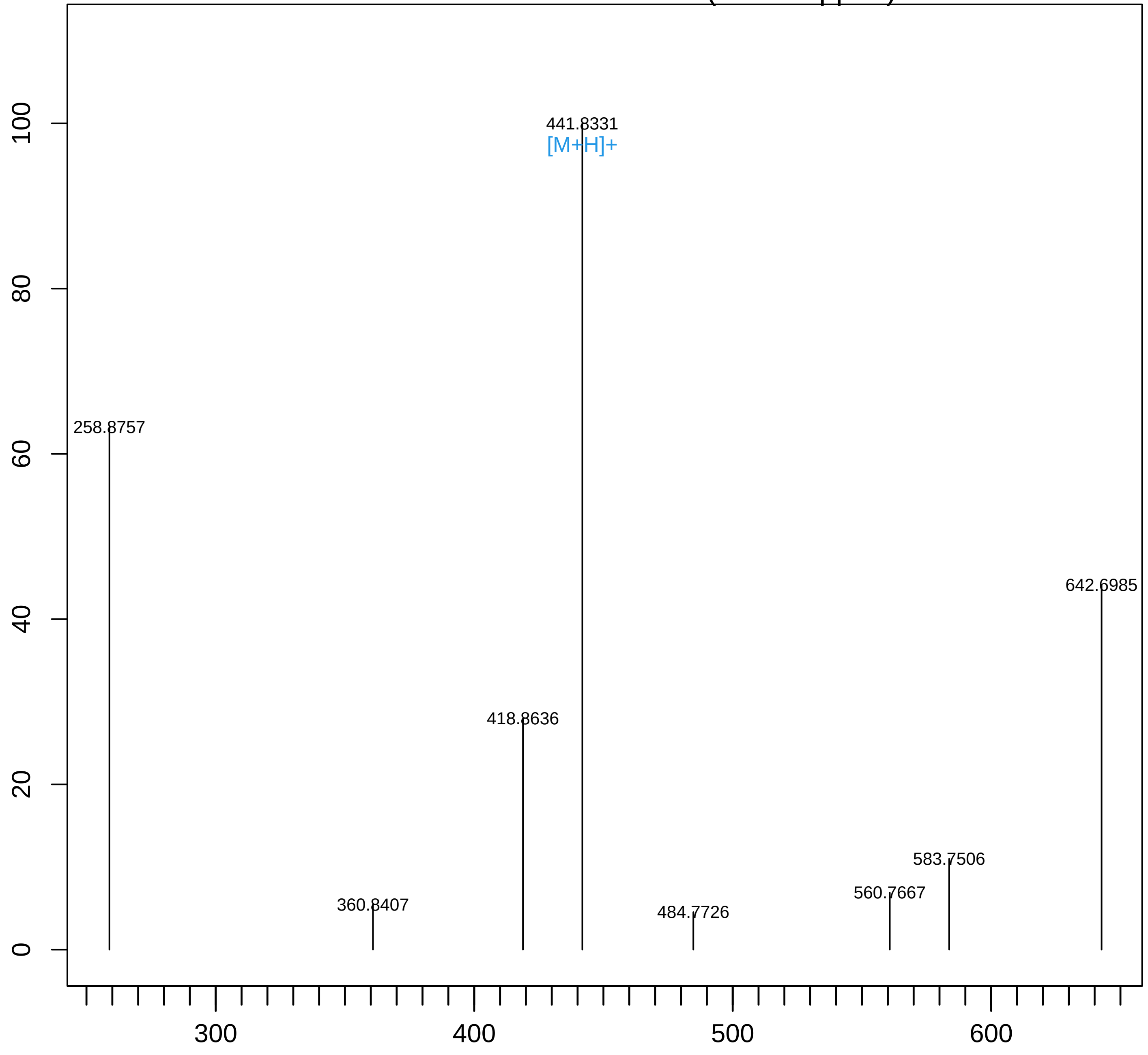

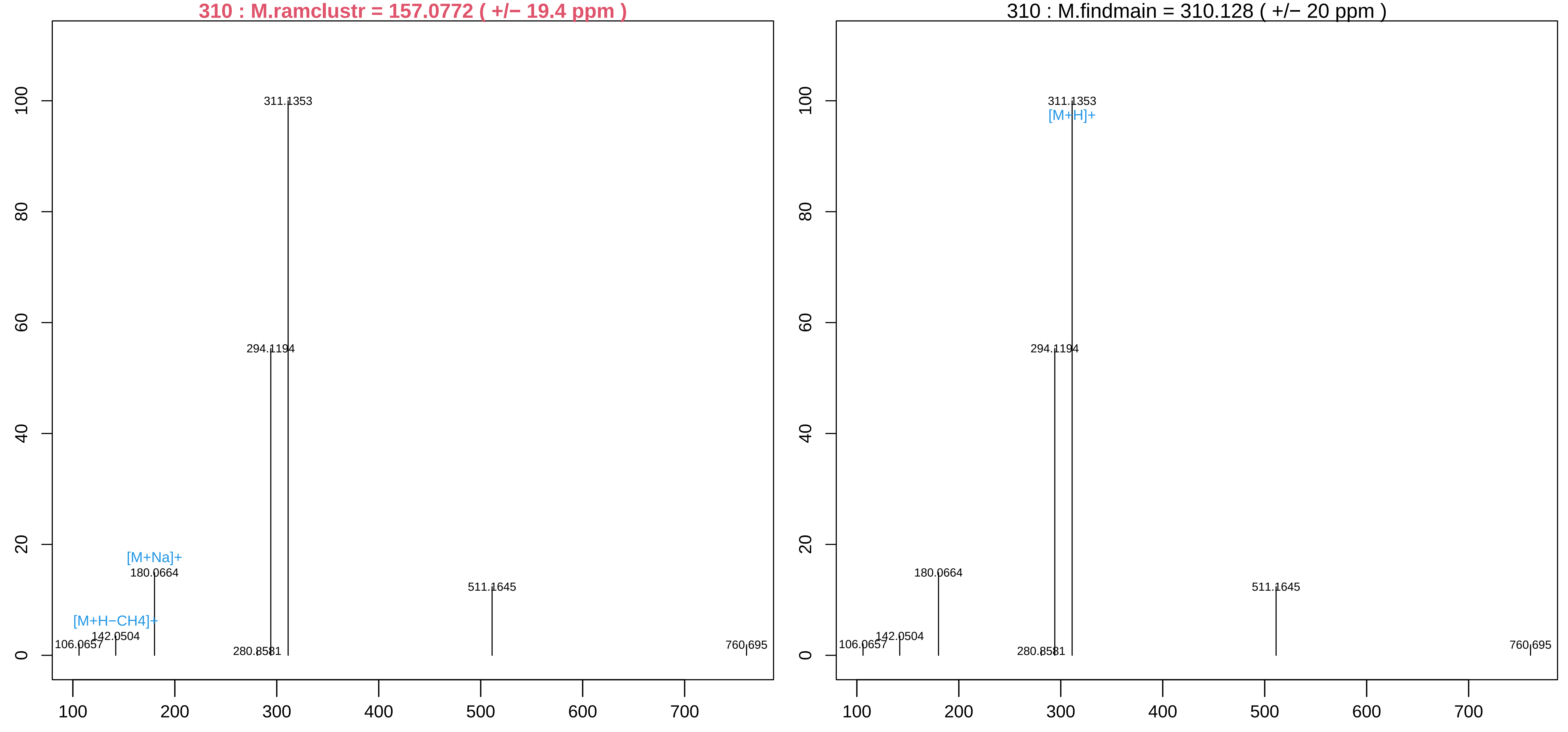

310 : M.ramclustr = 157.0772 (+/− 19.4 ppm)
311.1353
294.1194
[M+Na]+
180.0664
511.1645
[M+H−CH4]+
142.0504
106.0657
280.8581
760.695
310 : M.findmain = 310.128 (+/− 20 ppm)
311.1353
[M+H]+
294.1194
180.0664
511.1645
142.0504
106.0657
280.8581
760.695

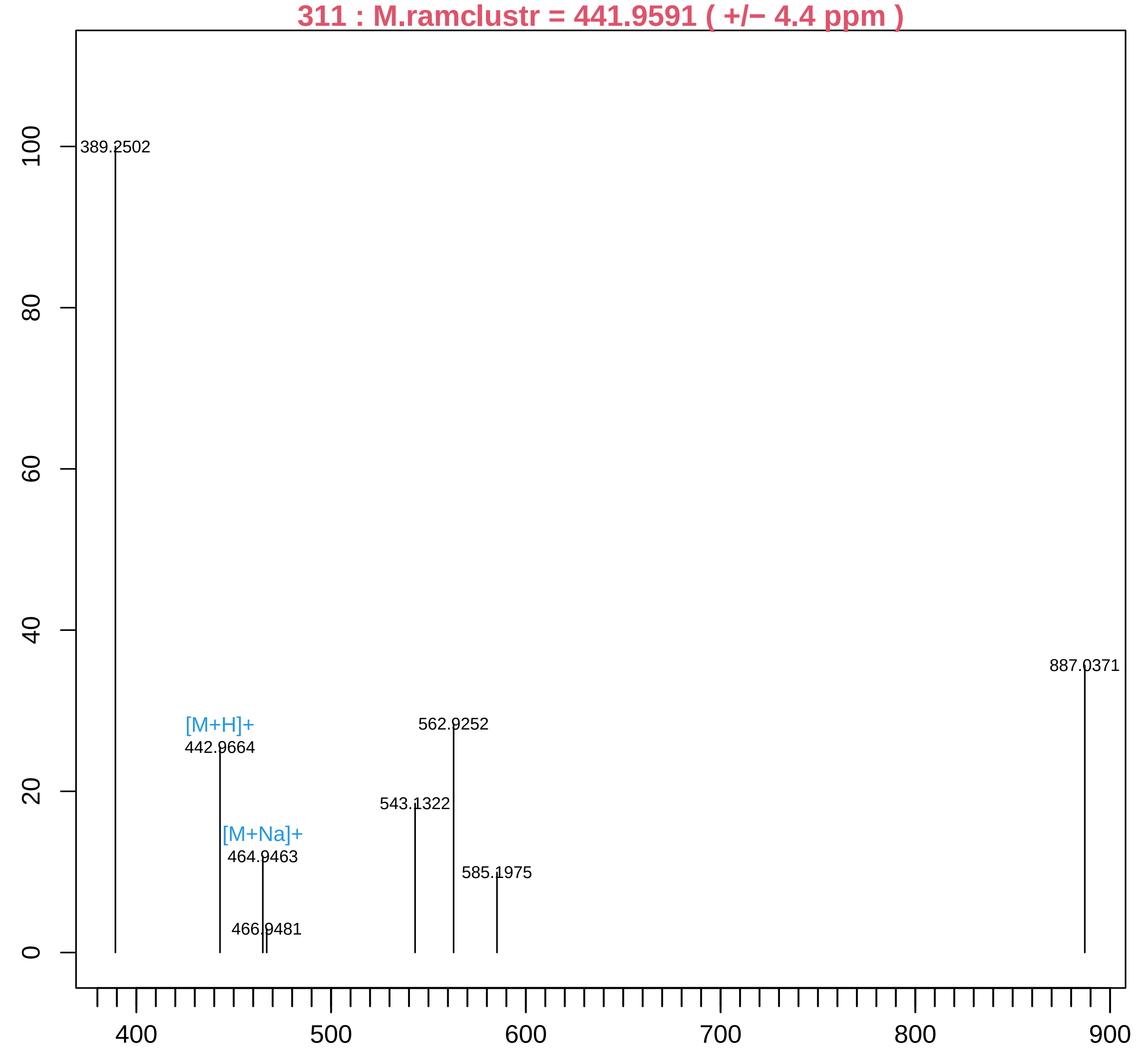
311 : M.ramclustr = 441.9591 (+/− 4.4 ppm)
389.2502
[M+H]+
442.9664
[M+Na]+
464.9463
466.9481
543.1322
562.9252
585.1975
887.0371
0
20
40
60
80
100
400
500
600
700
800
900

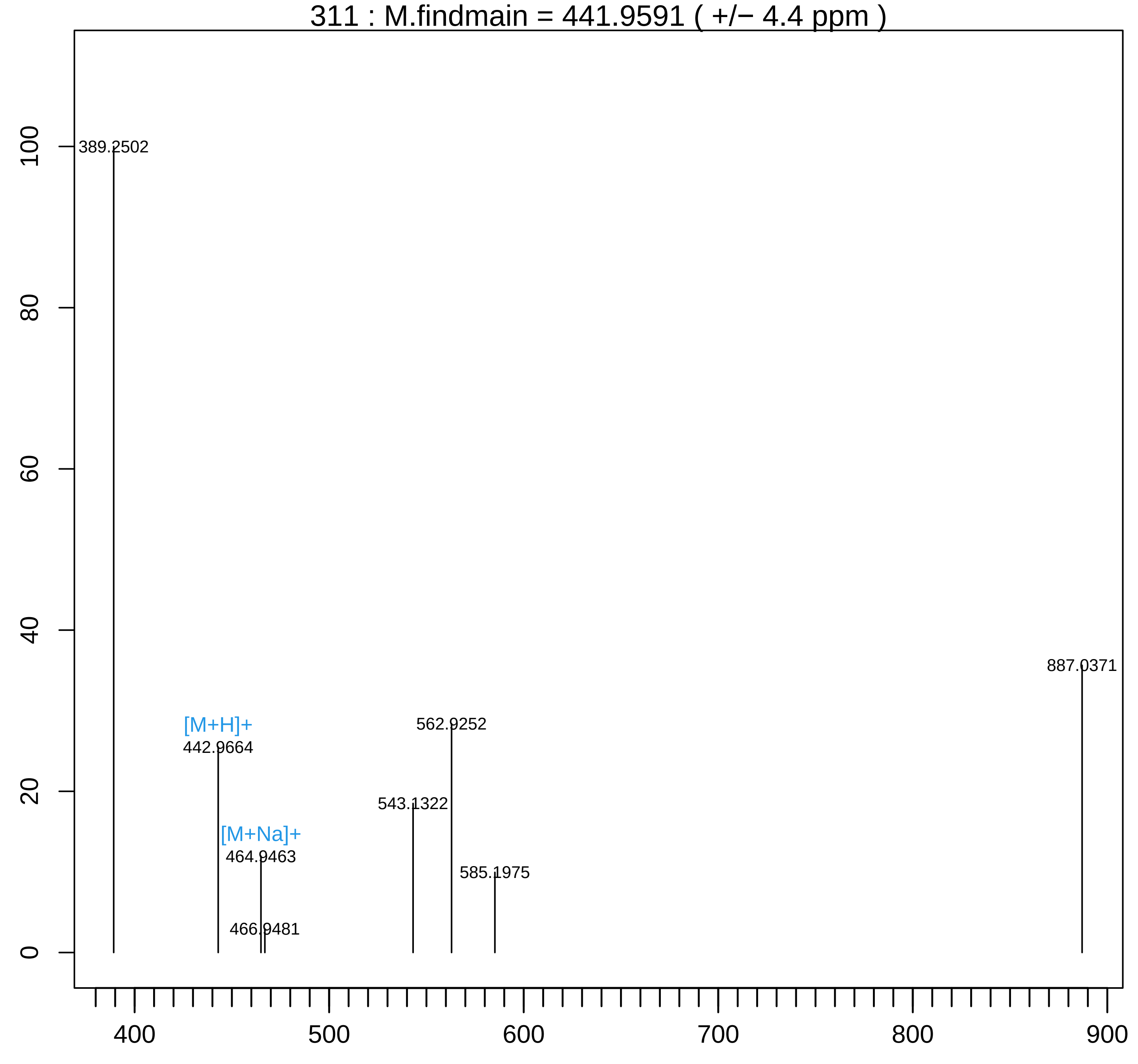
311 : M.findmain = 441.9591 (+/− 4.4 ppm)
389.2502
[M+H]+
442.9664
[M+Na]+
464.9463
466.9481
543.1322
562.9252
585.1975
887.0371
0
20
40
60
80
100
400
500
600
700
800
900

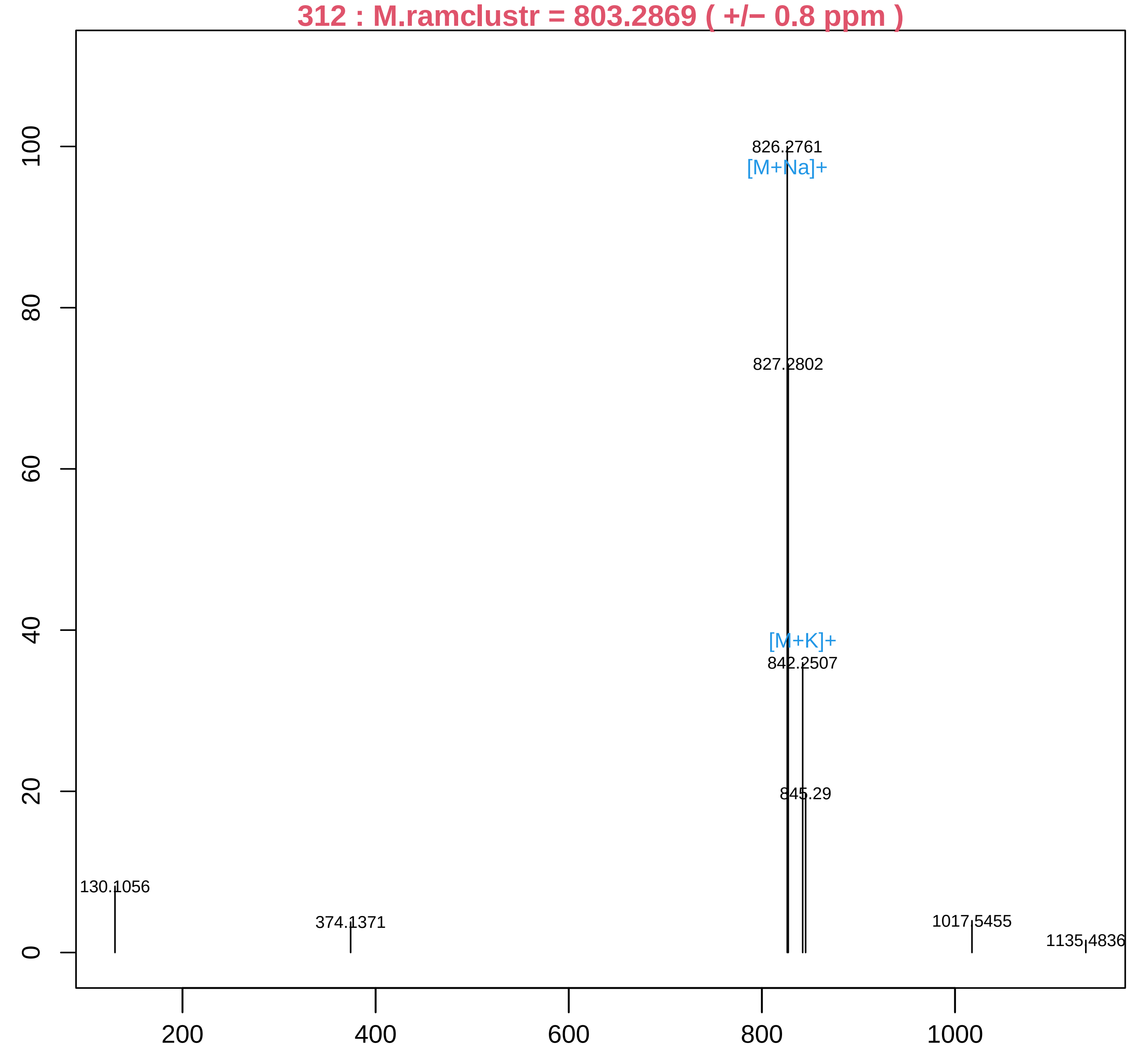

312 : M.ramclustr = 803.2869 (+/− 0.8 ppm)
826.2761
[M+Na]+
827.2802
[M+K]+
842.2507
845.29
130.1056
374.1371
1017.5455
1135.4836
0
20
40
60
80
100
200
400
600
800
1000

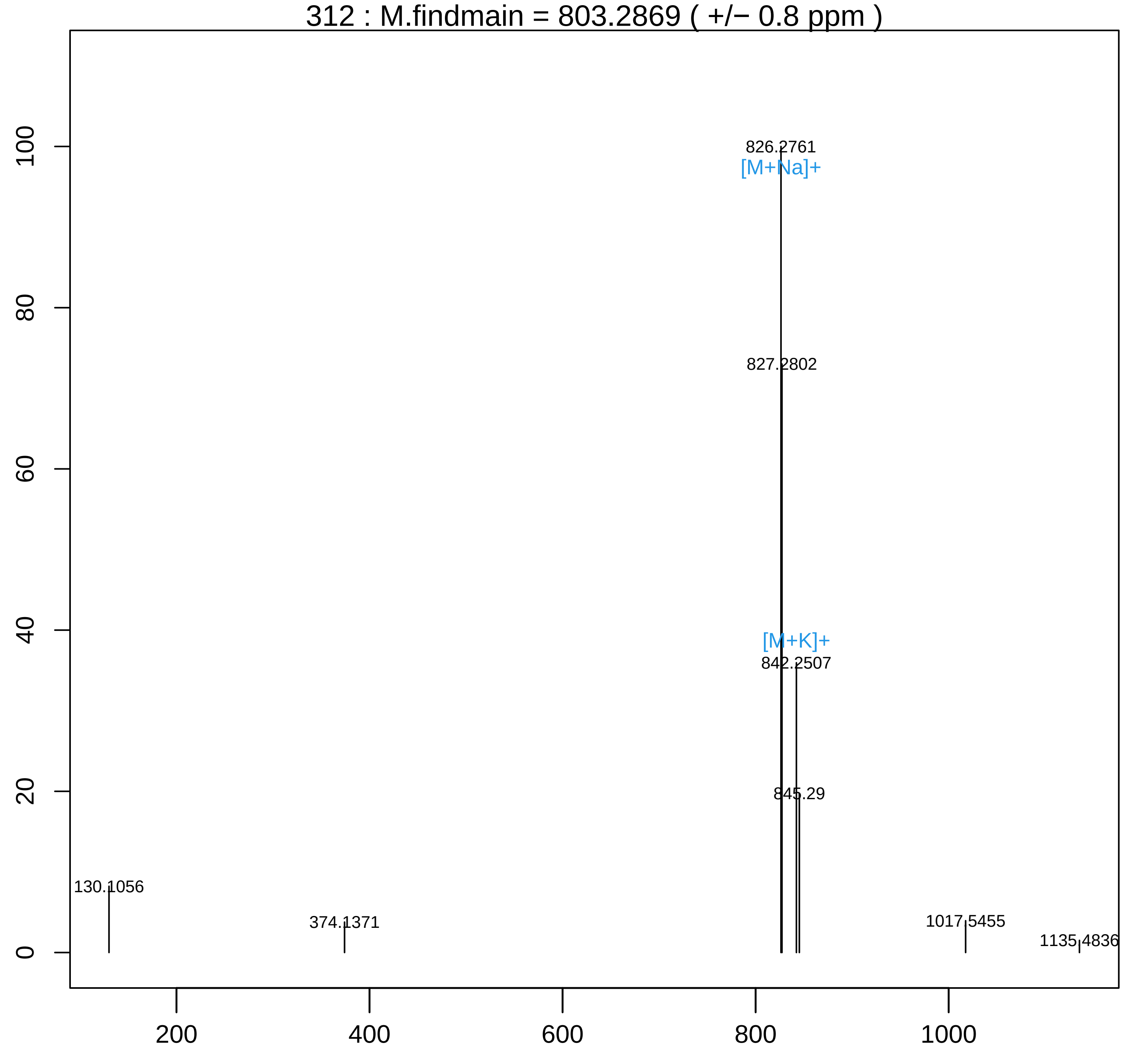

312 : M.findmain = 803.2869 (+/− 0.8 ppm)
826.2761
[M+Na]+
827.2802
[M+K]+
842.2507
845.29
130.1056
374.1371
1017.5455
1135.4836
0
20
40
60
80
100
200
400
600
800
1000

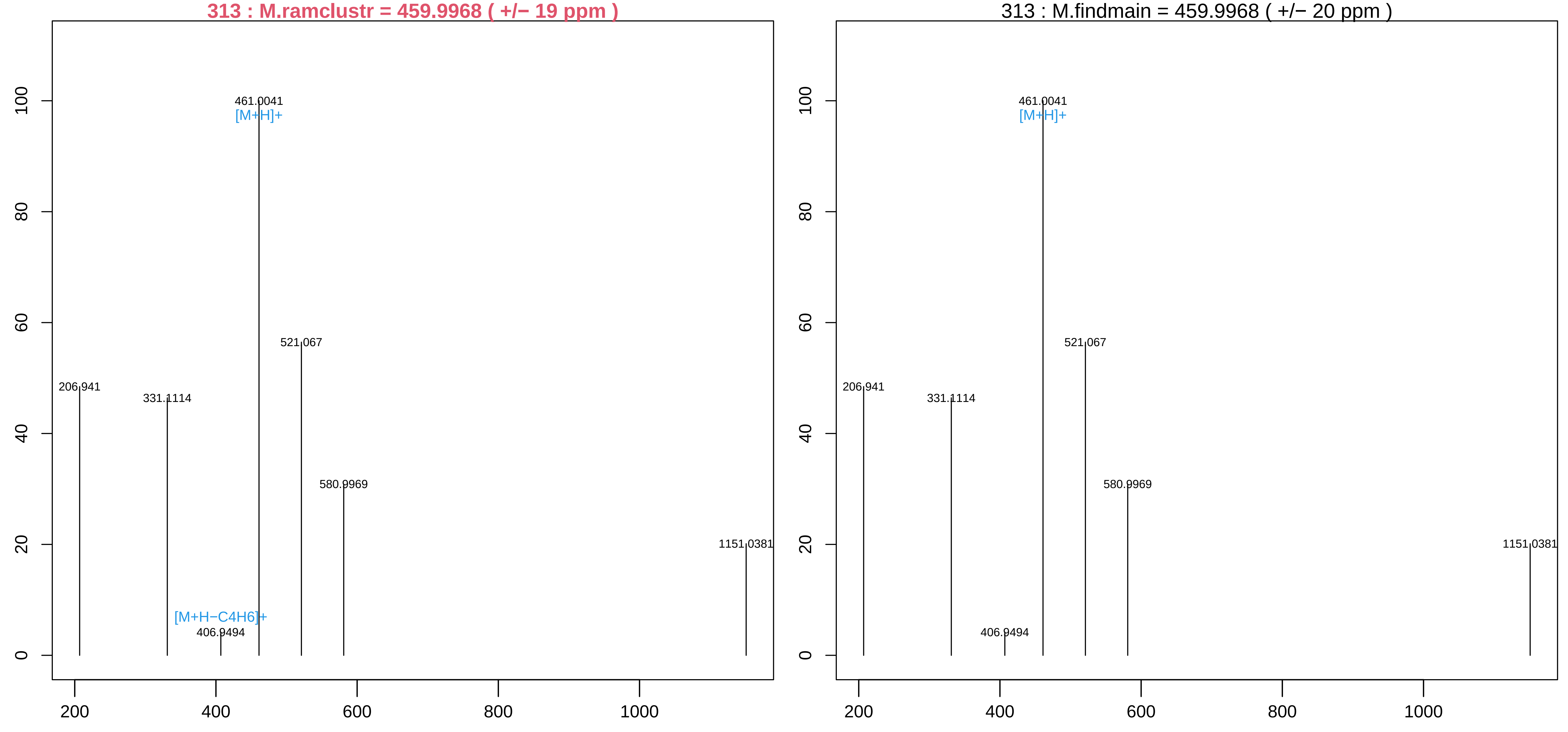
313 : M.ramclustr = 459.9968 (+/− 19 ppm)
461.0041
[M+H]+
521.067
206.941
331.1114
580.9969
1151.0381
[M+H−C4H6]+
406.9494
313 : M.findmain = 459.9968 (+/− 20 ppm)
461.0041
[M+H]+
521.067
206.941
331.1114
580.9969
1151.0381
406.9494

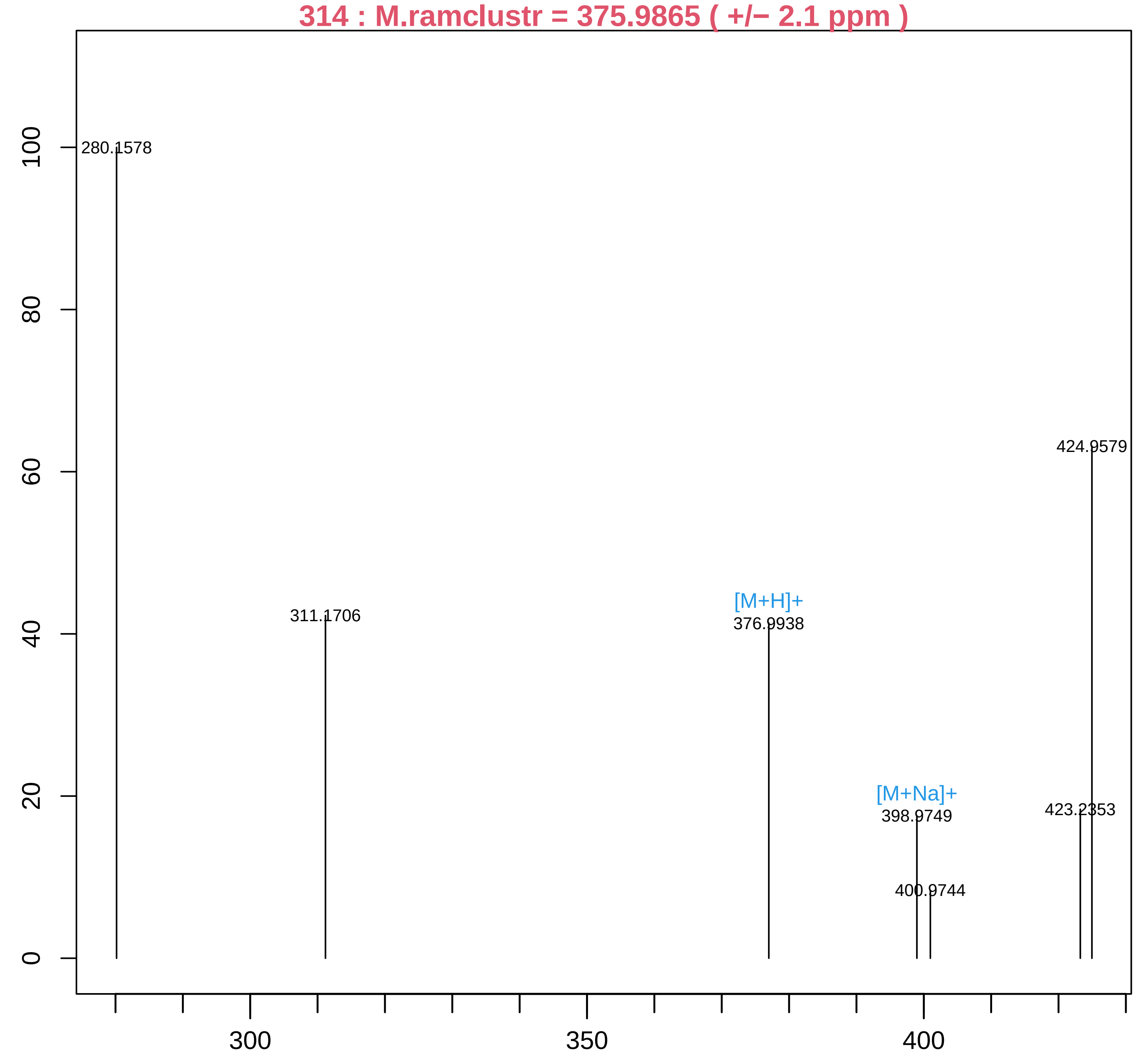

314 : M.ramclustr = 375.9865 (+/− 2.1 ppm)
280.1578
311.1706
[M+H]+
376.9938
[M+Na]+
398.9749
400.9744
423.2353
424.9579
0
20
40
60
80
100
300
350
400

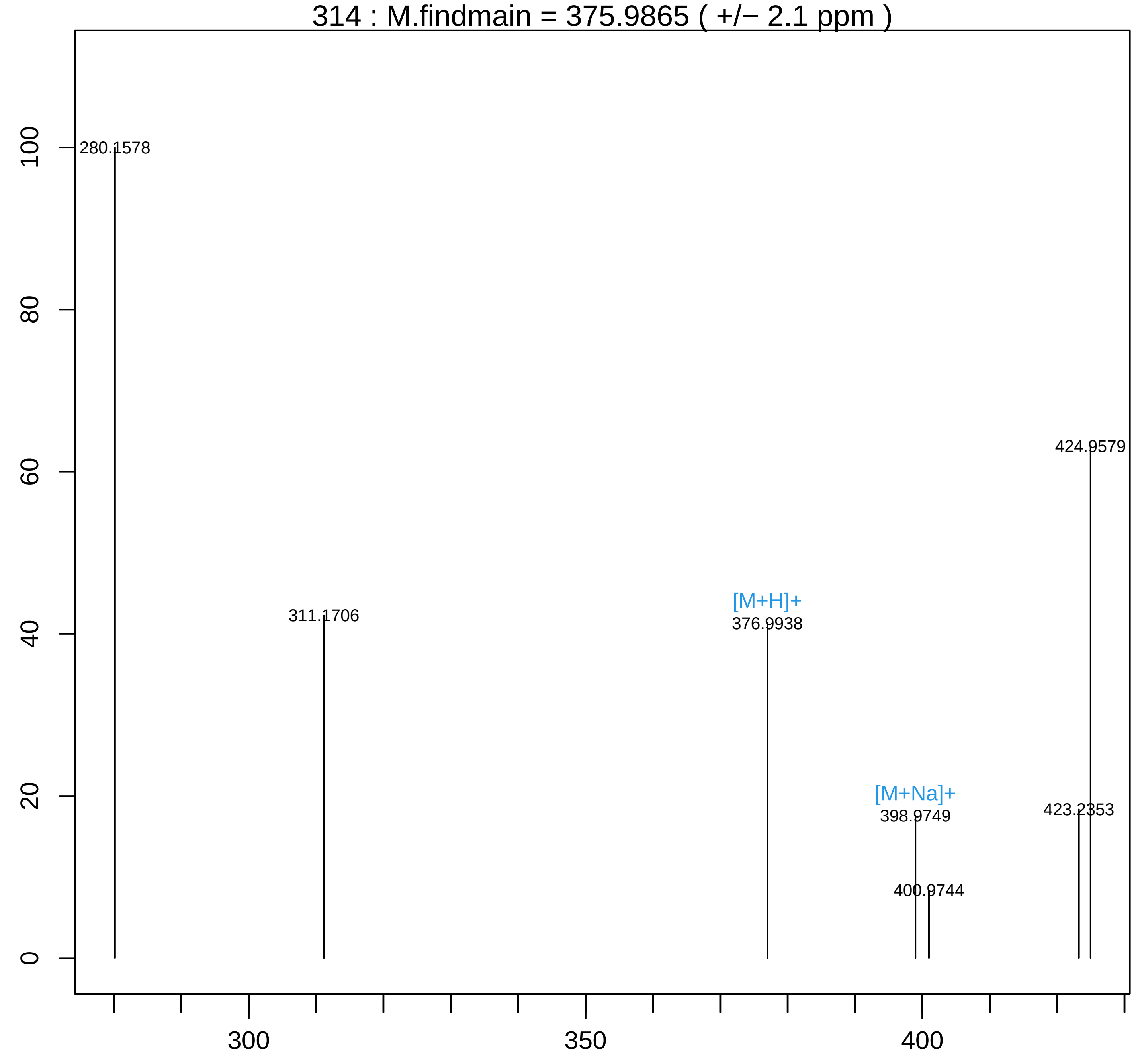

314 : M.findmain = 375.9865 (+/− 2.1 ppm)
280.1578
311.1706
[M+H]+
376.9938
[M+Na]+
398.9749
400.9744
423.2353
424.9579
0
20
40
60
80
100
300
350
400

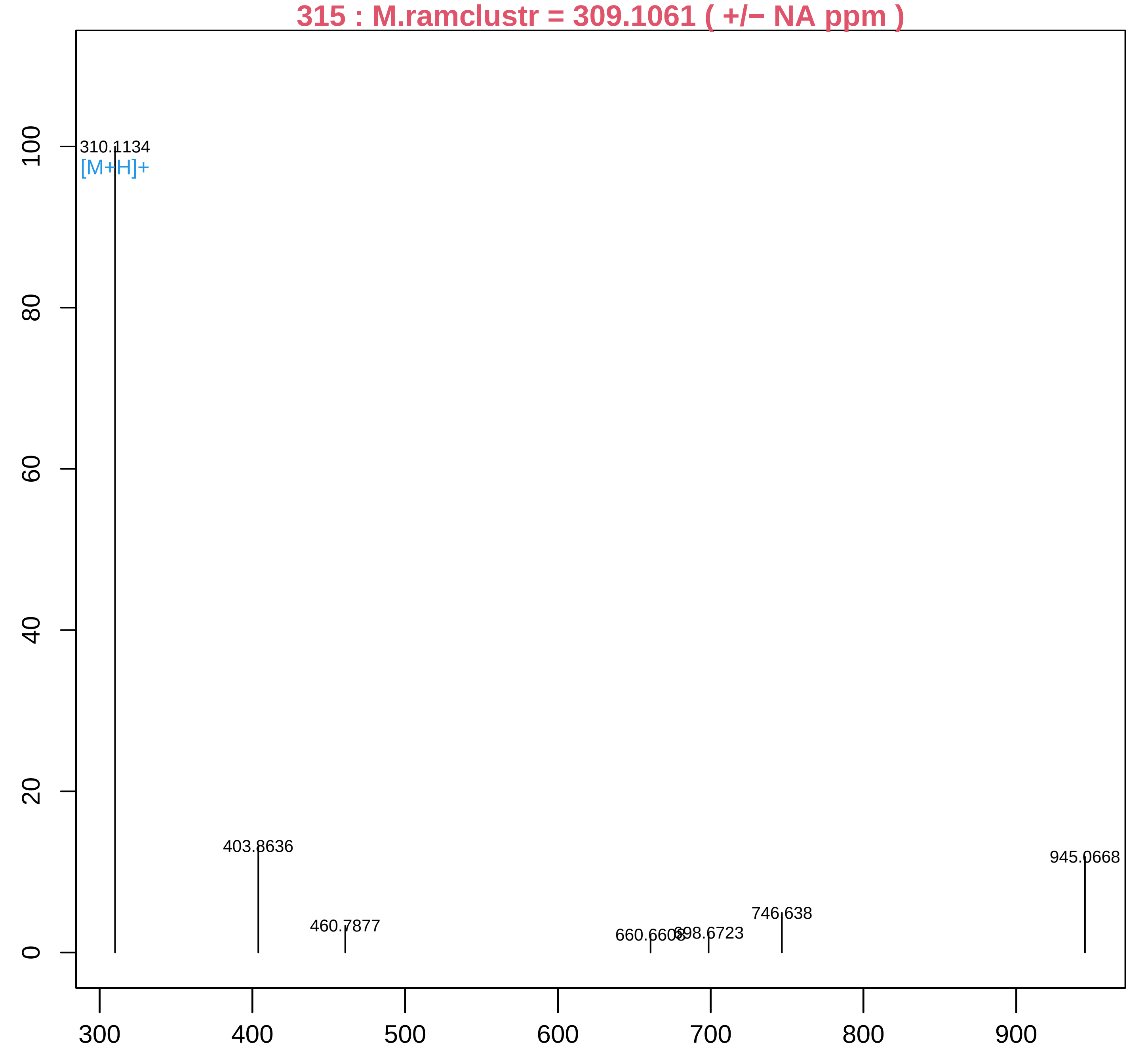
315 : M.ramclustr = 309.1061 (+/− NA ppm)
0
20
40
60
80
100
300
400
500
600
700
800
900
310.1134
[M+H]+
403.8636
460.7877
660.6608
698.6723
746.638
945.0668

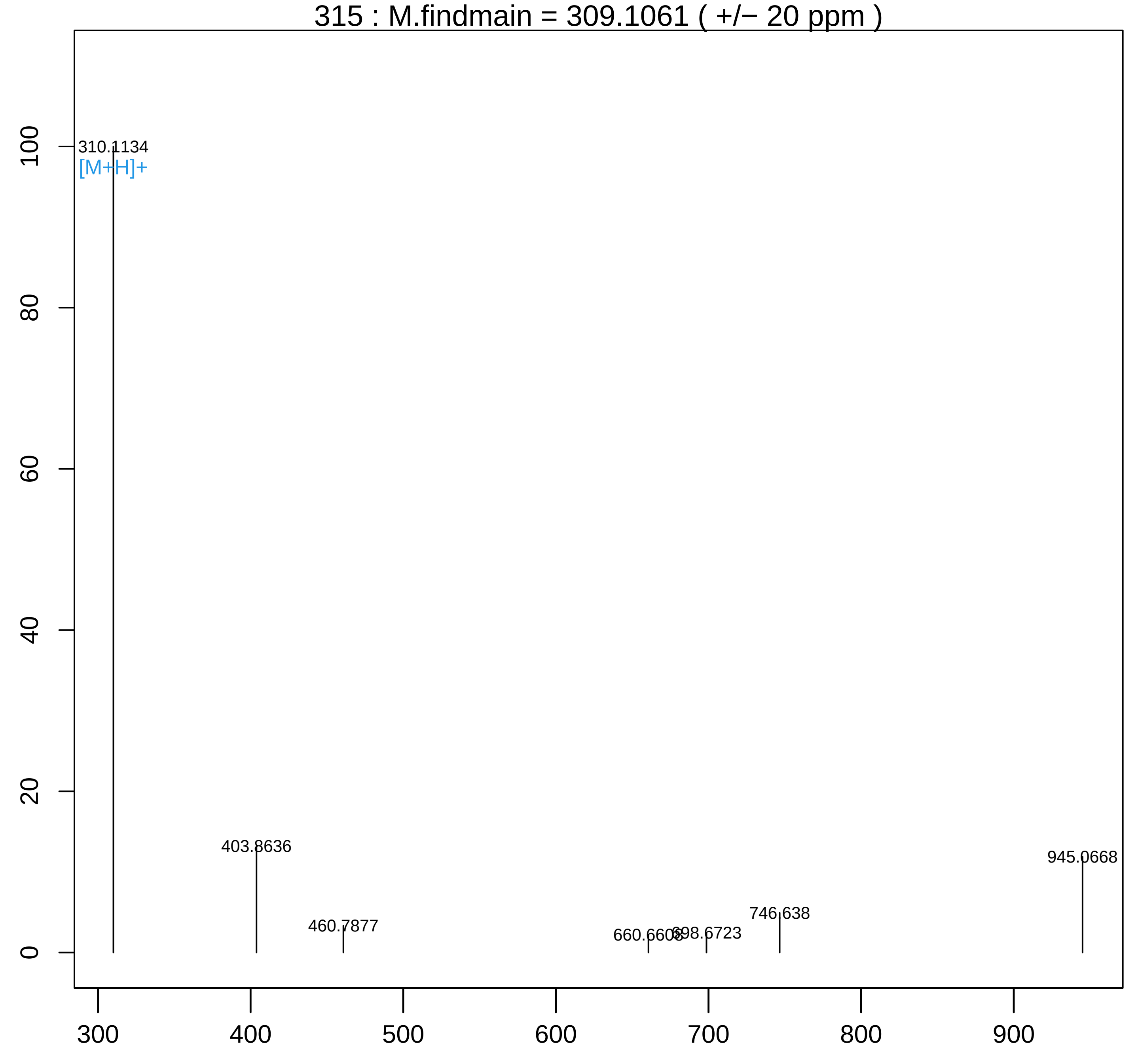
315 : M.findmain = 309.1061 (+/− 20 ppm)
0
20
40
60
80
100
300
400
500
600
700
800
900
310.1134
[M+H]+
403.8636
460.7877
660.6608
698.6723
746.638
945.0668

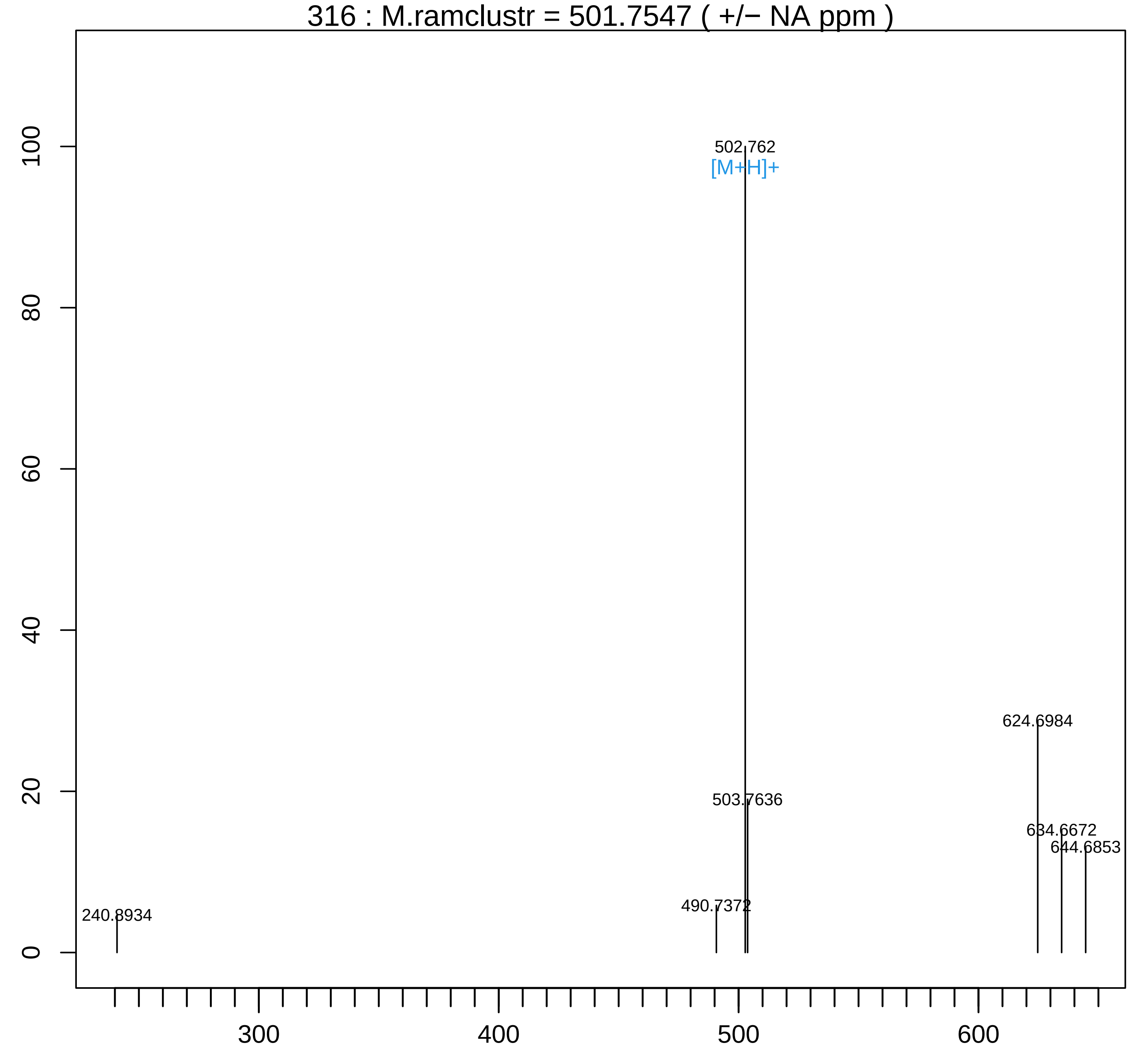
316 : M.ramclustr = 501.7547 (+/− NA ppm)
502.762
[M+H]+
503.7636
240.8934
490.7372
624.6984
634.6672
644.6853
0
20
40
60
80
100
300
400
500
600

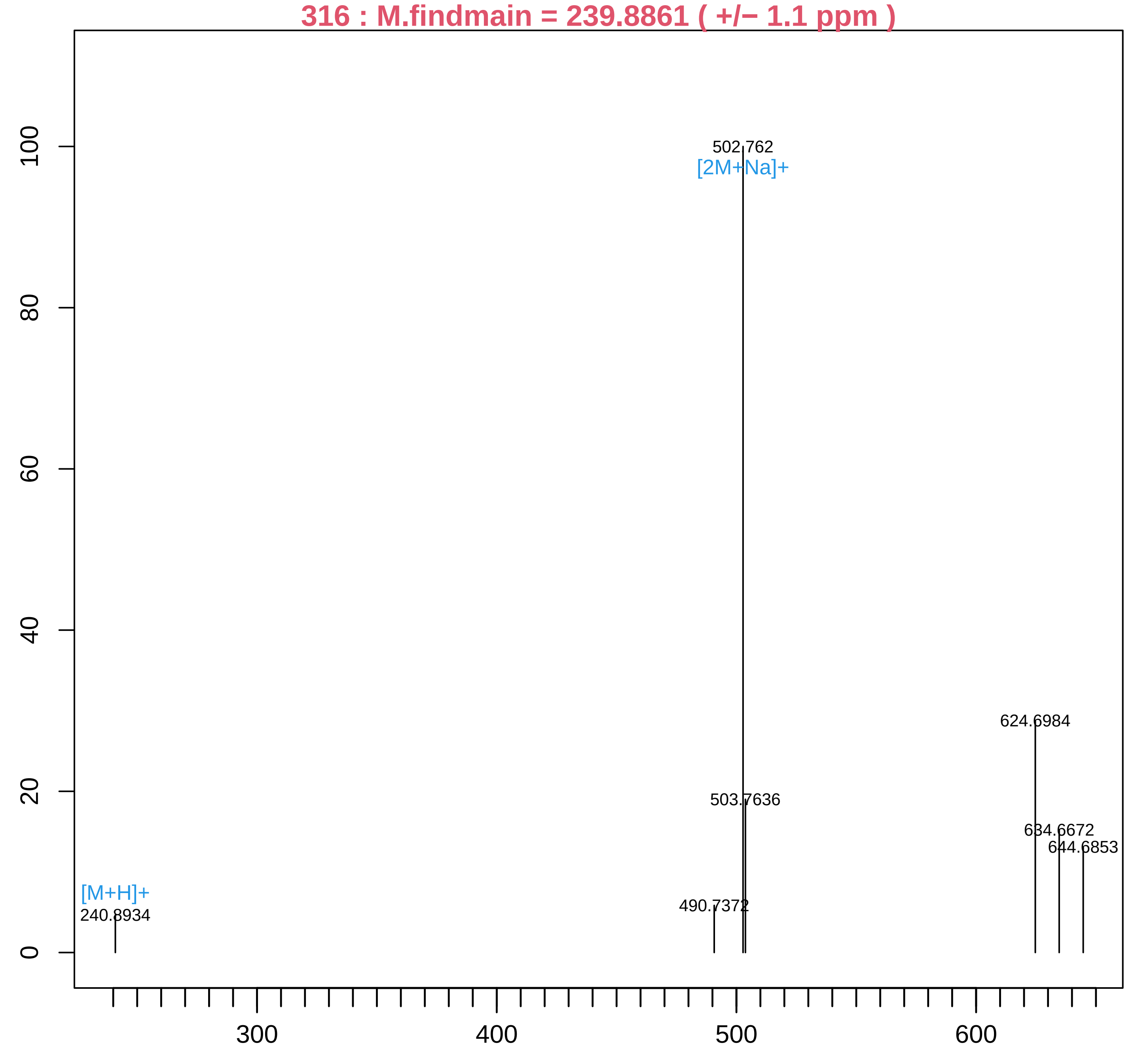
316 : M.findmain = 239.8861 (+/− 1.1 ppm)
502.762
[2M+Na]+
503.7636
[M+H]+
240.8934
490.7372
624.6984
634.6672
644.6853
0
20
40
60
80
100
300
400
500
600

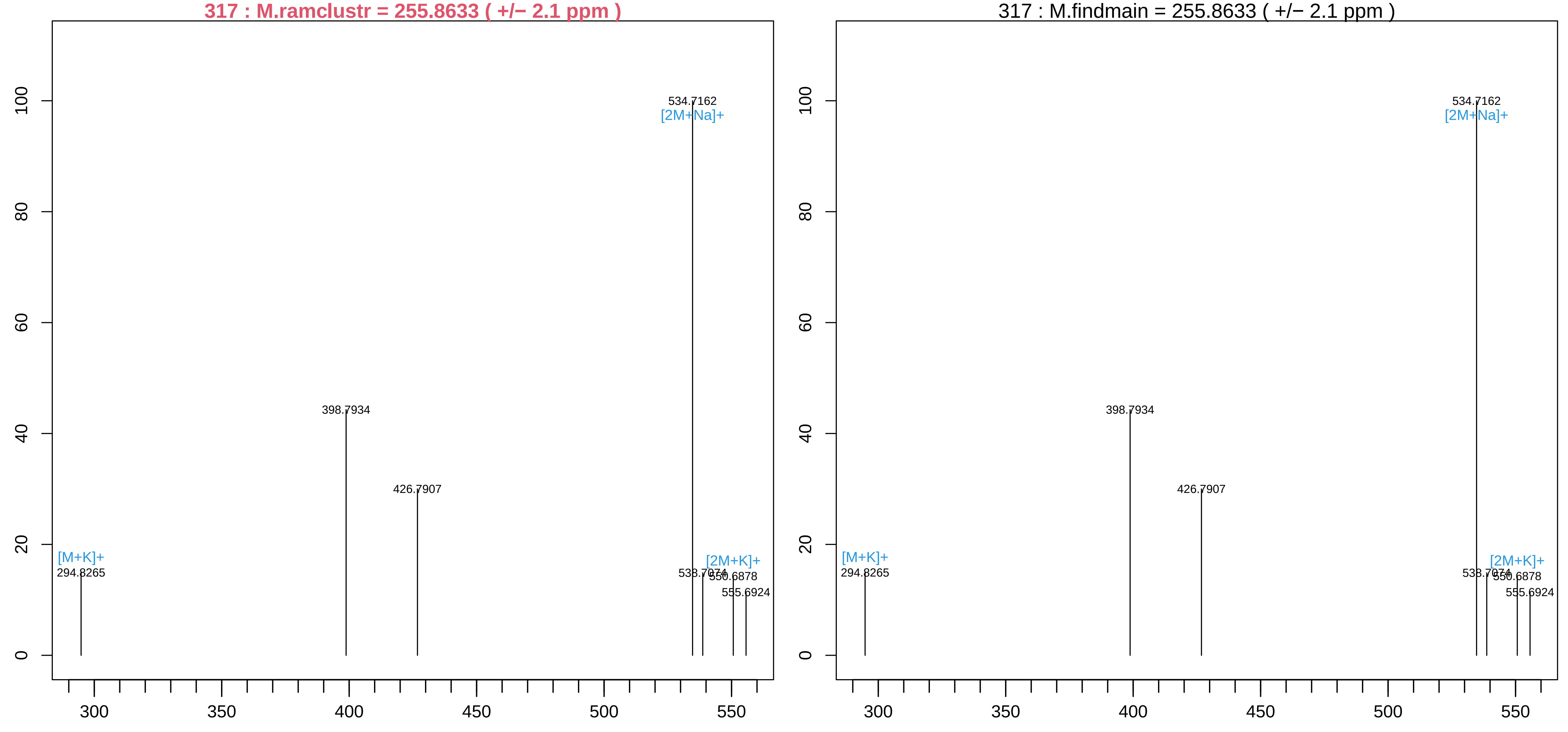
317 : M.ramclustr = 255.8633 (+/− 2.1 ppm)
317 : M.findmain = 255.8633 (+/− 2.1 ppm)
534.7162
[2M+Na]+
398.7934
426.7907
[M+K]+
294.8265
538.7074
550.6878
[2M+K]+
555.6924
0
20
40
60
80
100
300
350
400
450
500
550

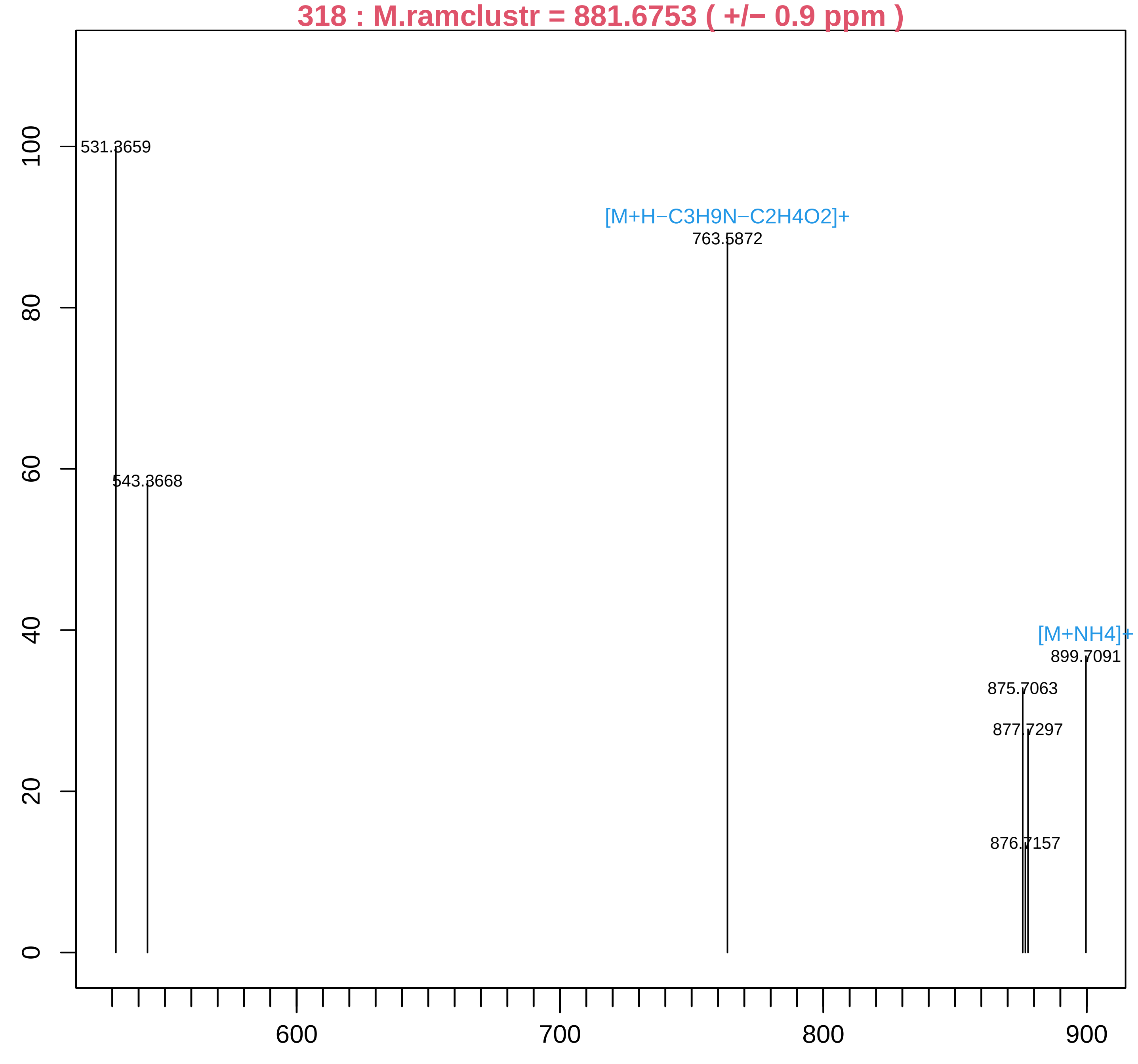
318 : M.ramclustr = 881.6753 (+/− 0.9 ppm)
531.3659
543.3668
[M+H−C3H9N−C2H4O2]+
763.5872
[M+NH4]+
899.7091
875.7063
877.7297
876.7157
0
20
40
60
80
100
600
700
800
900

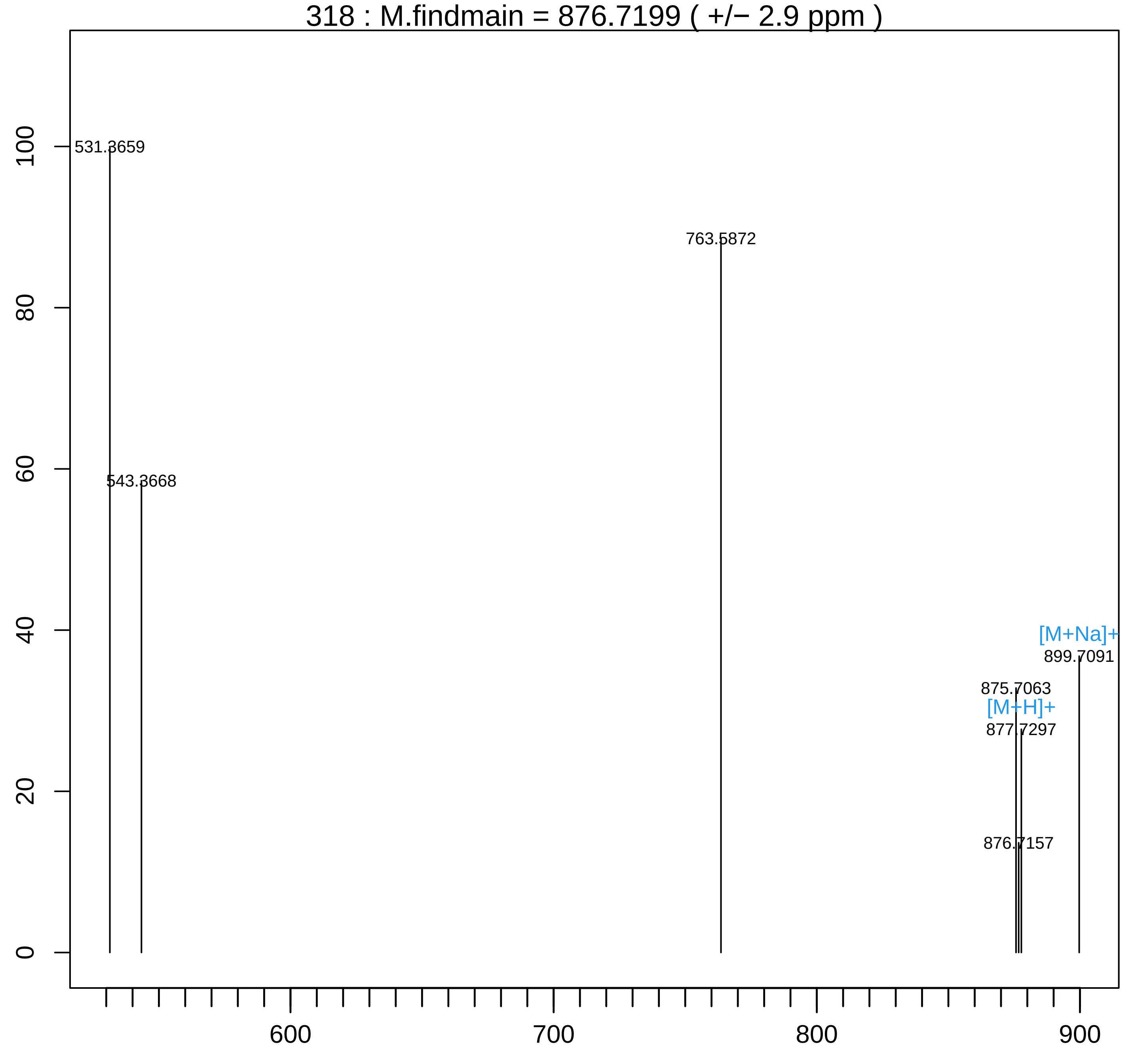
318 : M.findmain = 876.7199 (+/− 2.9 ppm)
531.3659
543.3668
763.5872
[M+Na]+
899.7091
875.7063
[M+H]+
877.7297
876.7157
0
20
40
60
80
100
600
700
800
900

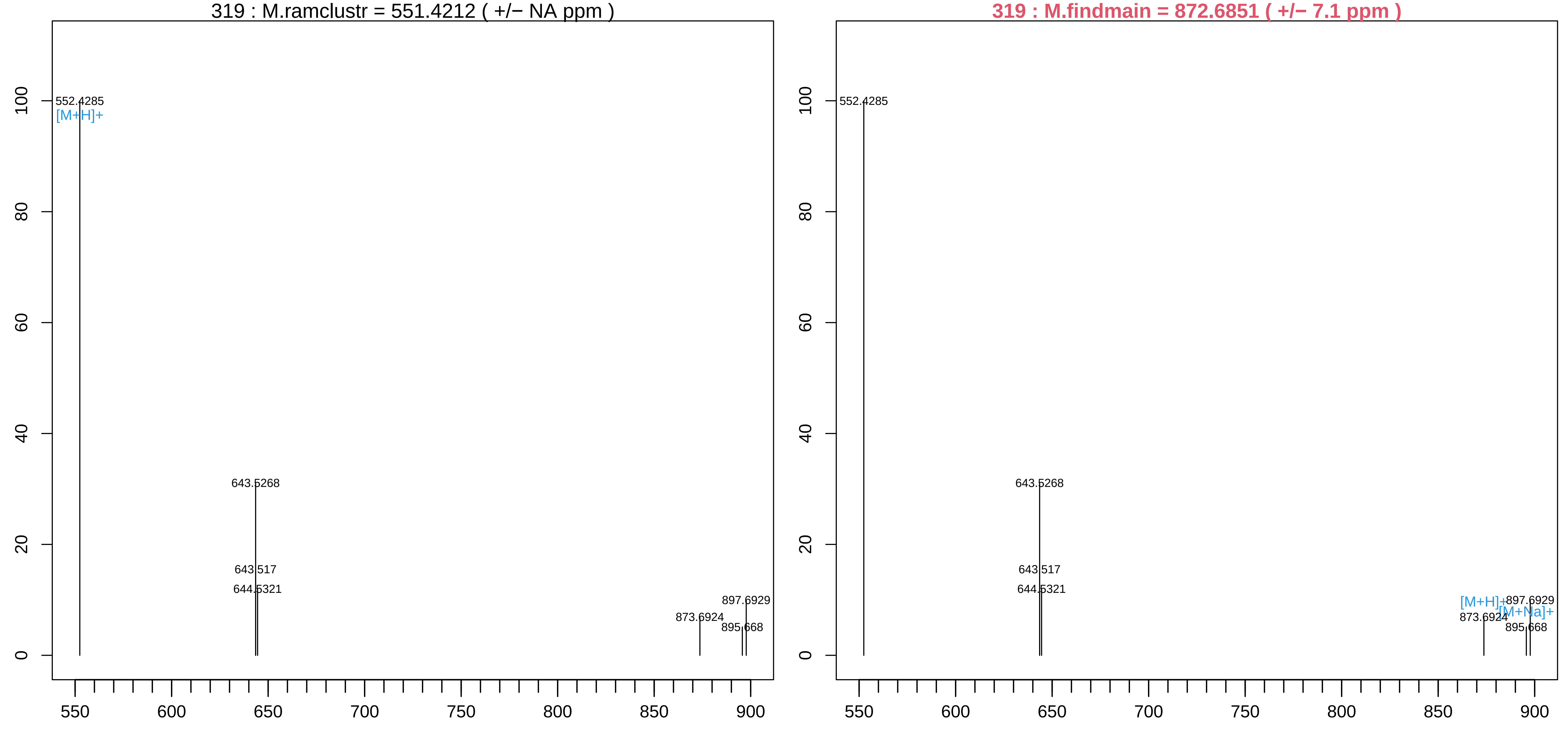
319 : M.ramclustr = 551.4212 (+/− NA ppm)
552.4285
[M+H]+
643.5268
643.517
644.5321
873.6924
897.6929
895.668
319 : M.findmain = 872.6851 (+/− 7.1 ppm)
552.4285
643.5268
643.517
644.5321
[M+H]+
873.6924
897.6929
[M+Na]+
895.668

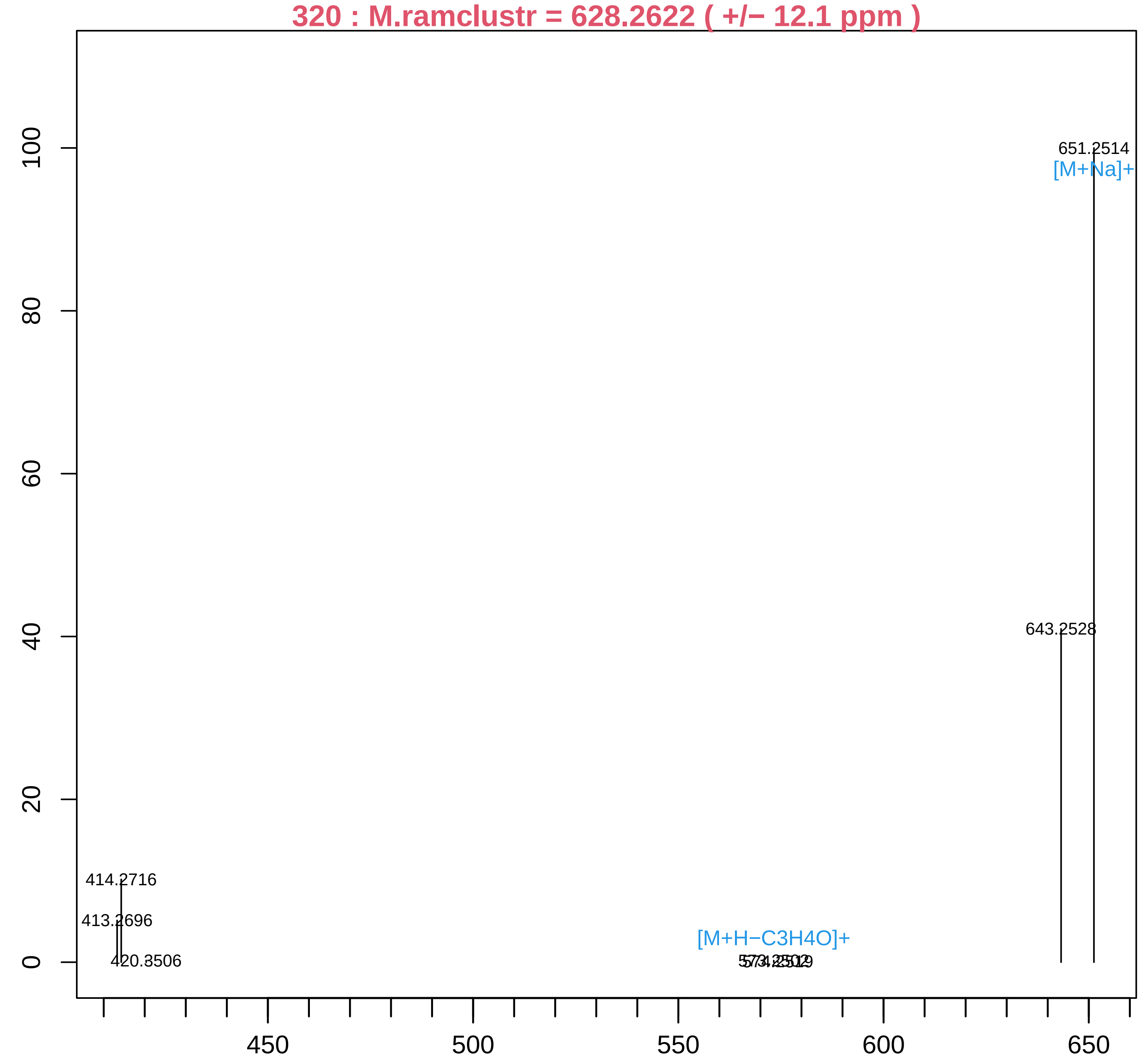
320 : M.ramclustr = 628.2622 (+/− 12.1 ppm)
651.2514
[M+Na]+
643.2528
414.2716
413.2696
420.3506
[M+H−C3H4O]+
0
20
40
60
80
100
450
500
550
600
650

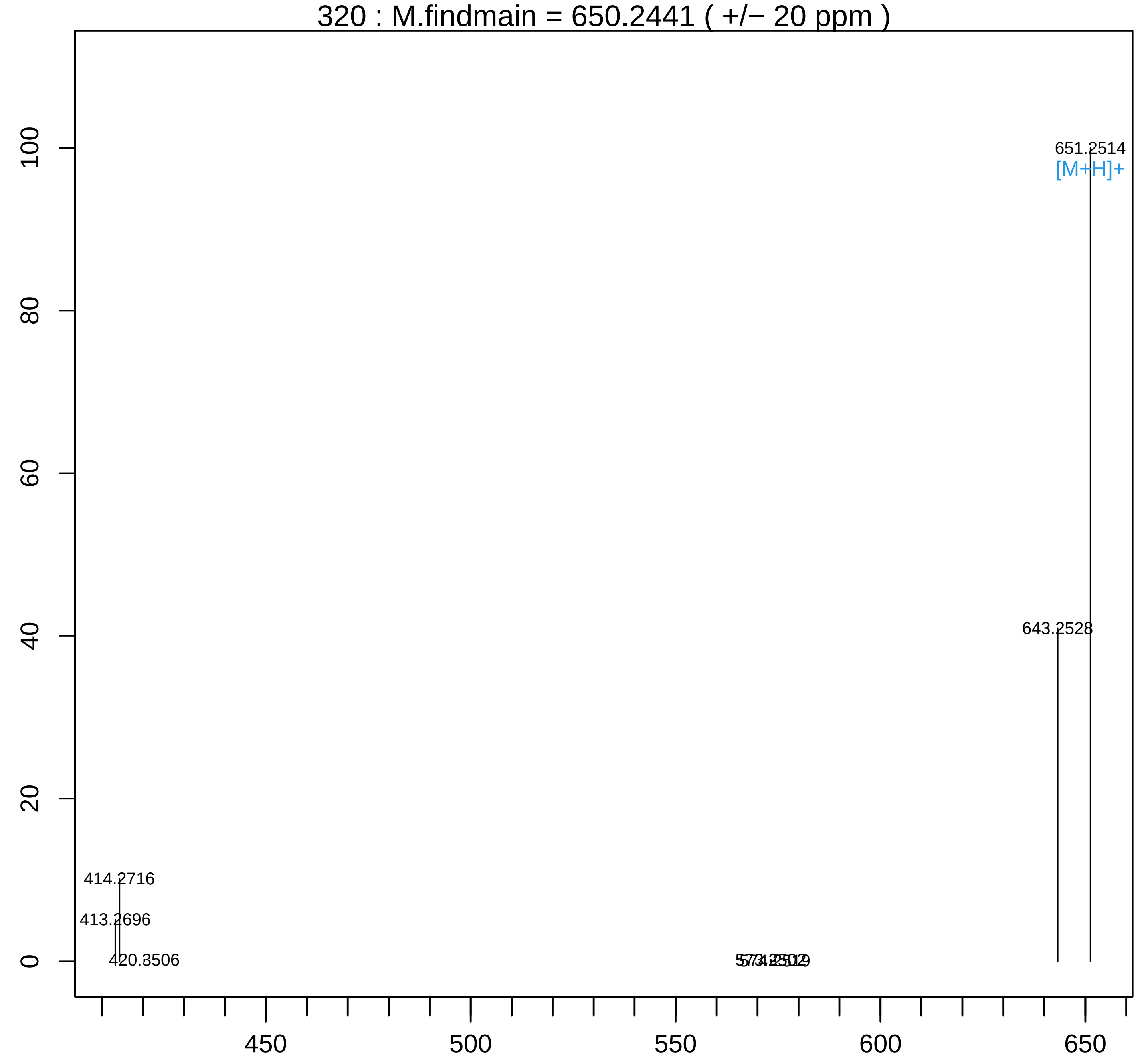
320 : M.findmain = 650.2441 (+/− 20 ppm)
651.2514
[M+H]+
643.2528
414.2716
413.2696
420.3506
0
20
40
60
80
100
450
500
550
600
650

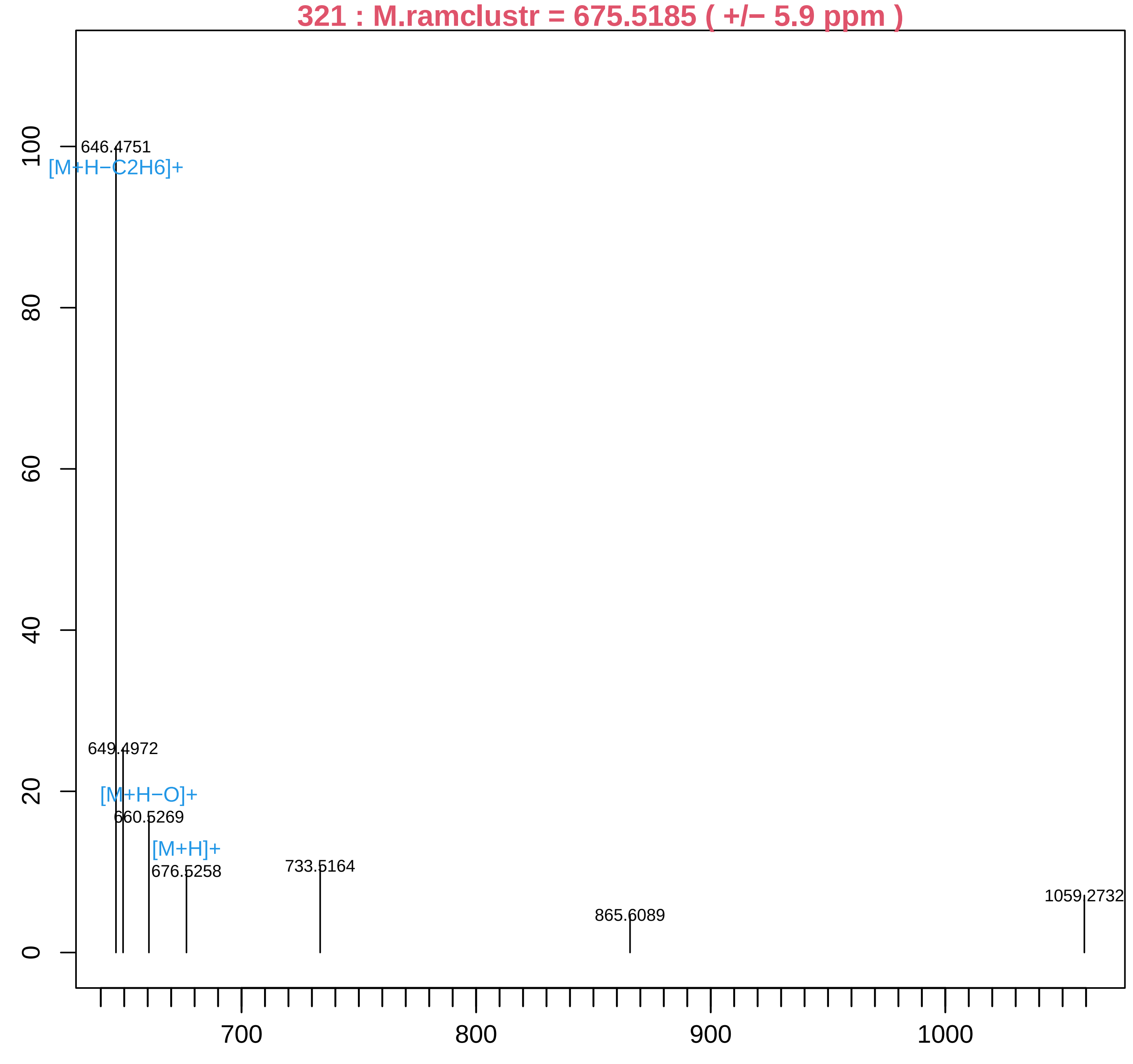
321 : M.ramclustr = 675.5185 (+/− 5.9 ppm)
646.4751
[M+H−C2H6]+
649.4972
[M+H−O]+
660.5269
[M+H]+
676.5258
733.5164
865.6089
1059.2732
0
20
40
60
80
100
700
800
900
1000
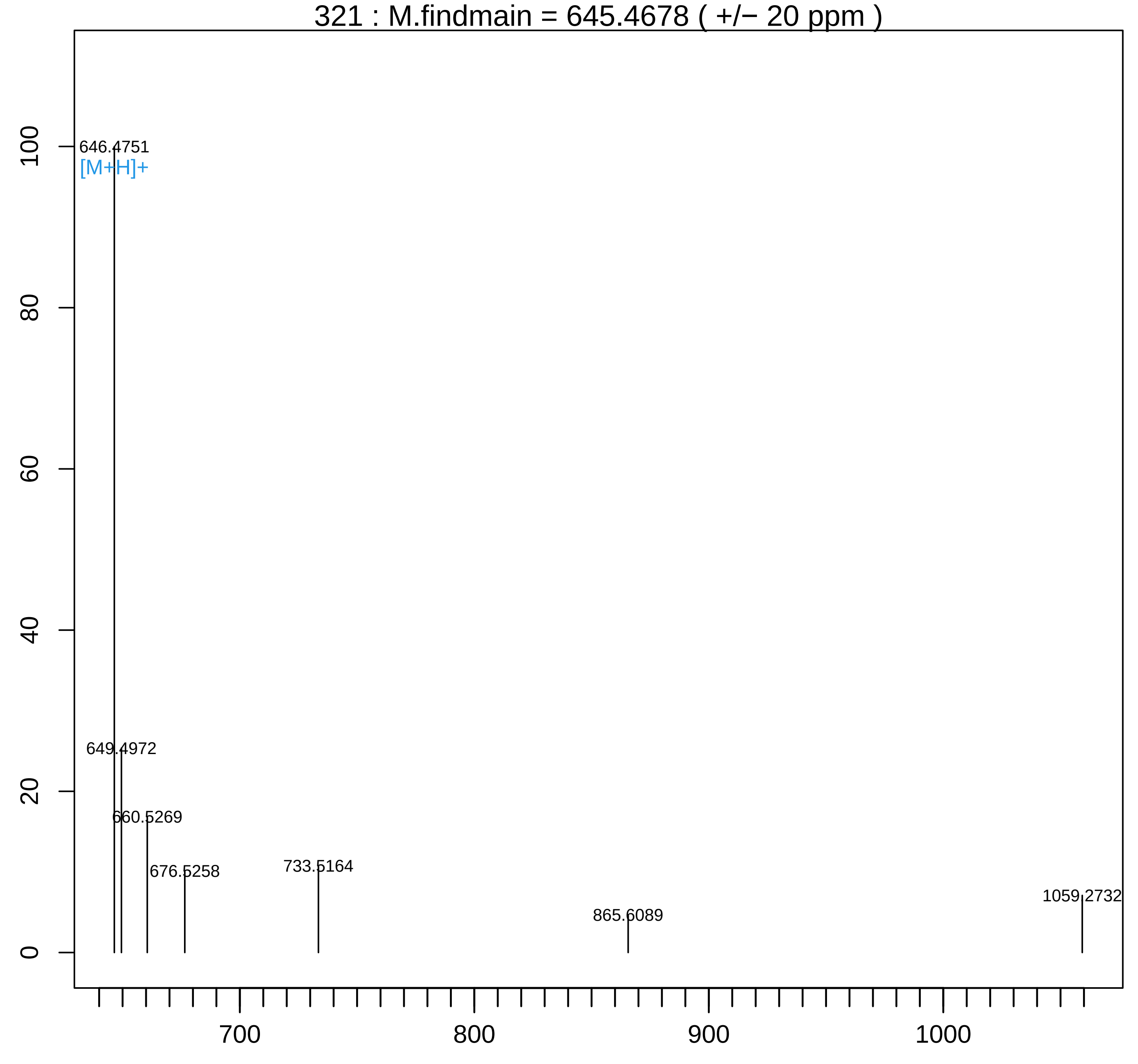
321 : M.findmain = 645.4678 (+/− 20 ppm)
646.4751
[M+H]+
649.4972
660.5269
676.5258
733.5164
865.6089
1059.2732
0
20
40
60
80
100
700
800
900
1000

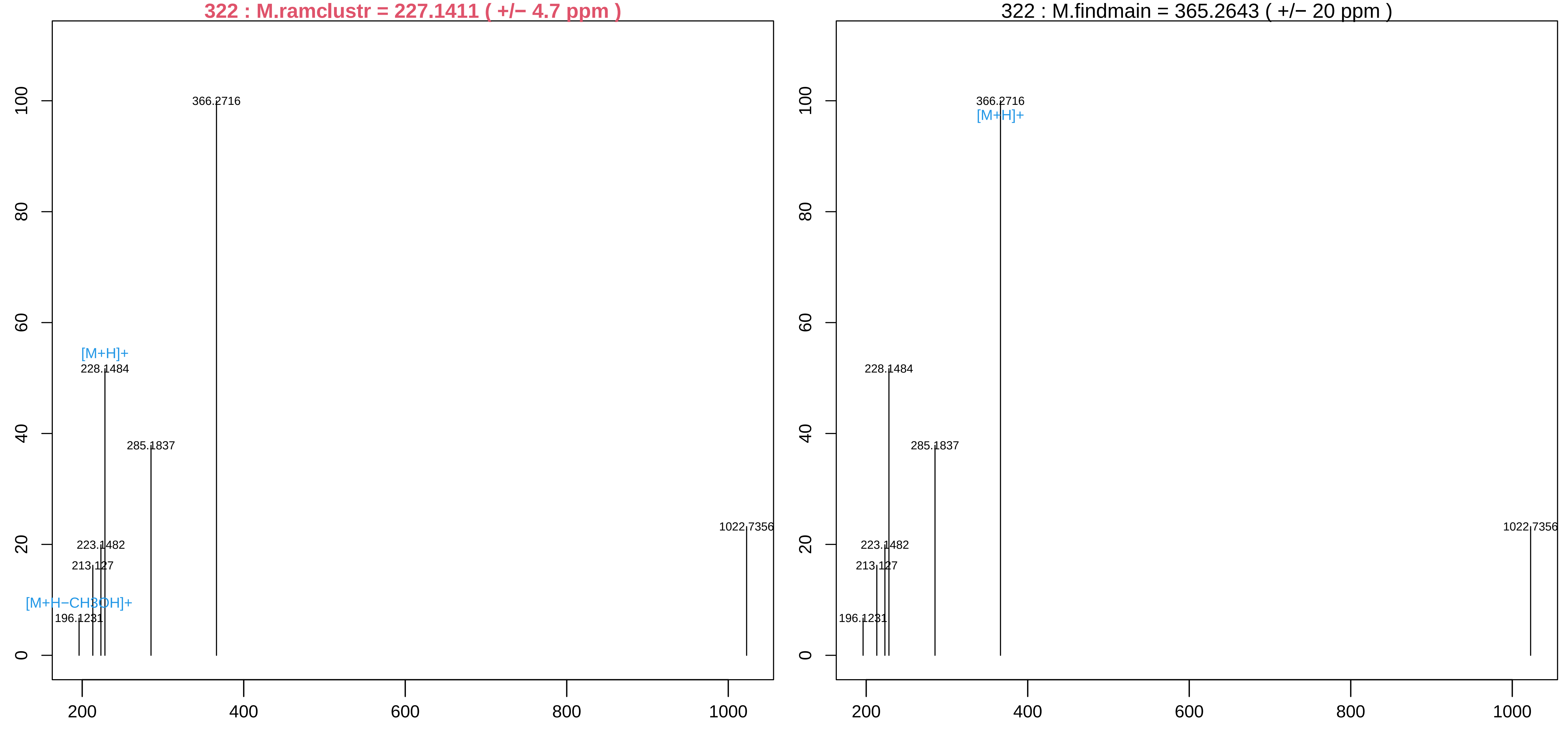

322 : M.ramclustr = 227.1411 (+/− 4.7 ppm)
366.2716
[M+H]+
228.1484
285.1837
223.1482
213.127
[M+H−CH3OH]+
196.1231
1022.7356
322 : M.findmain = 365.2643 (+/− 20 ppm)
366.2716
[M+H]+
228.1484
285.1837
223.1482
213.127
196.1231
1022.7356
0
20
40
60
80
100
200
400
600
800
1000

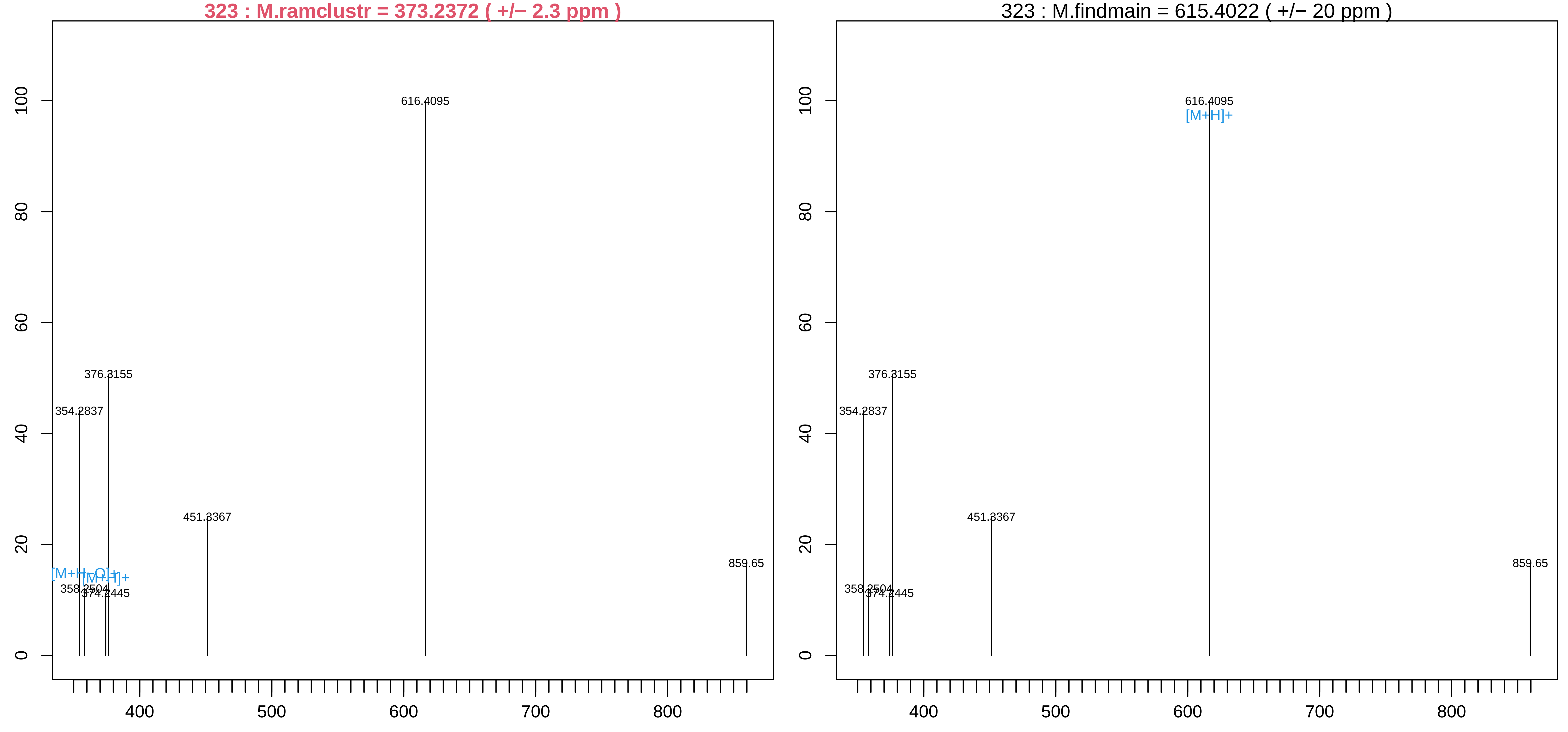

323 : M.ramclustr = 373.2372 (+/− 2.3 ppm)
616.4095
376.3155
354.2837
451.3367
859.65
[M+H−O]+
[M+H]+
358.2504
374.2445
0
20
40
60
80
100
400
500
600
700
800
323 : M.findmain = 615.4022 (+/− 20 ppm)
616.4095
[M+H]+
376.3155
354.2837
451.3367
859.65
358.2504
374.2445

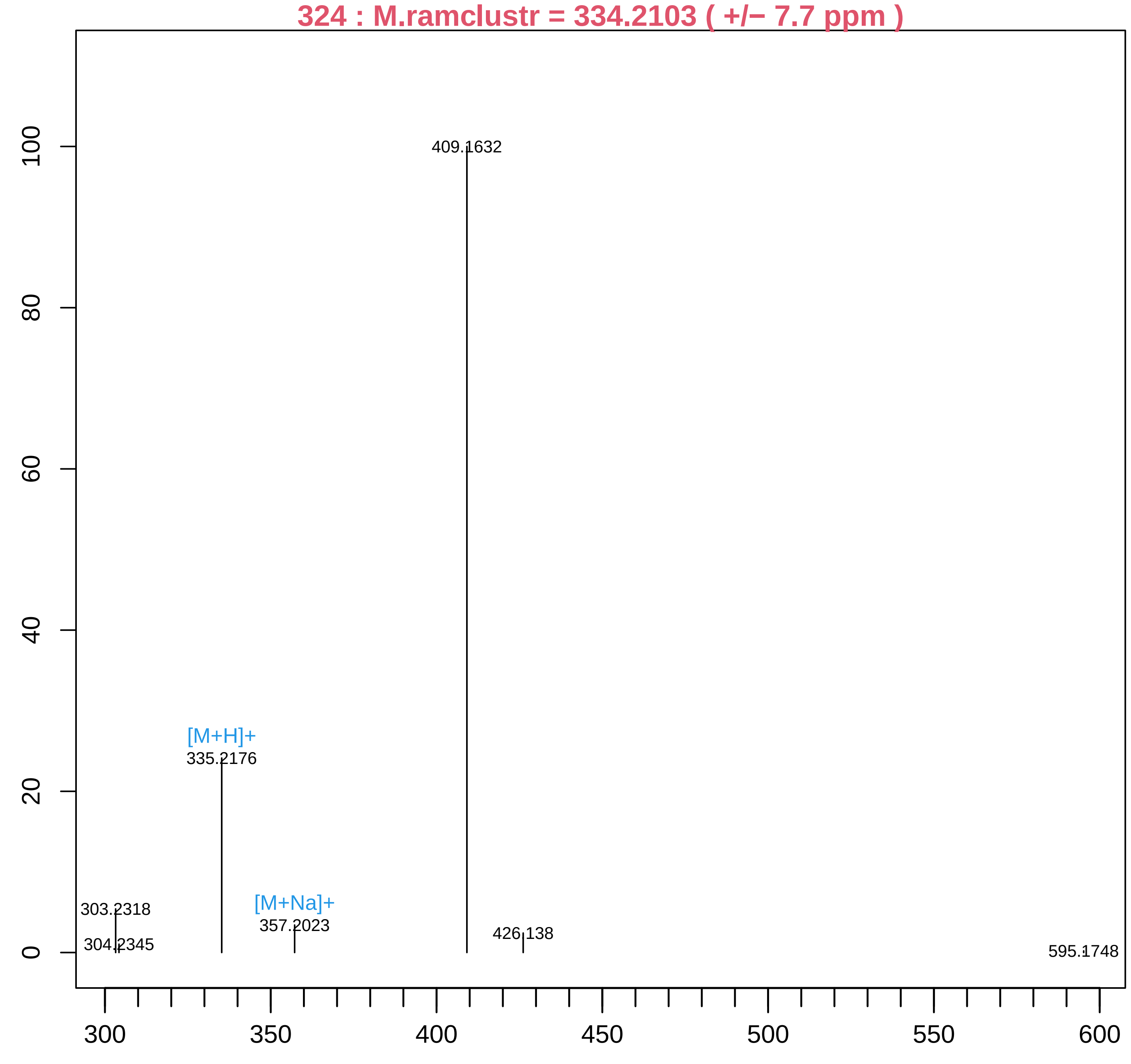
324 : M.ramclustr = 334.2103 (+/− 7.7 ppm)
409.1632
[M+H]+
335.2176
303.2318
304.2345
[M+Na]+
357.2023
426.138
595.1748
0
20
40
60
80
100
300
350
400
450
500
550
600

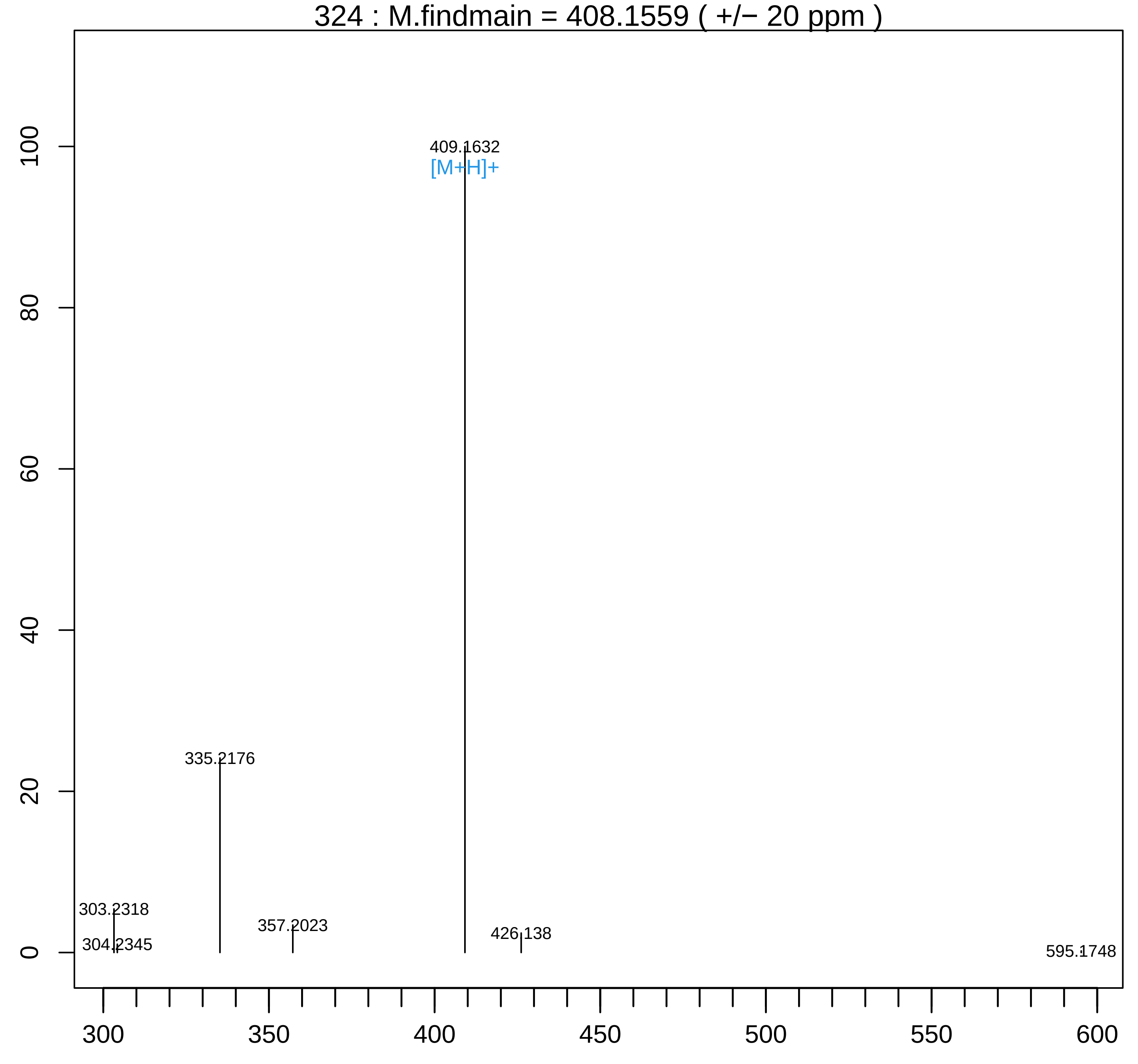
324 : M.findmain = 408.1559 (+/− 20 ppm)
409.1632
[M+H]+
335.2176
303.2318
304.2345
357.2023
426.138
595.1748
0
20
40
60
80
100
300
350
400
450
500
550
600

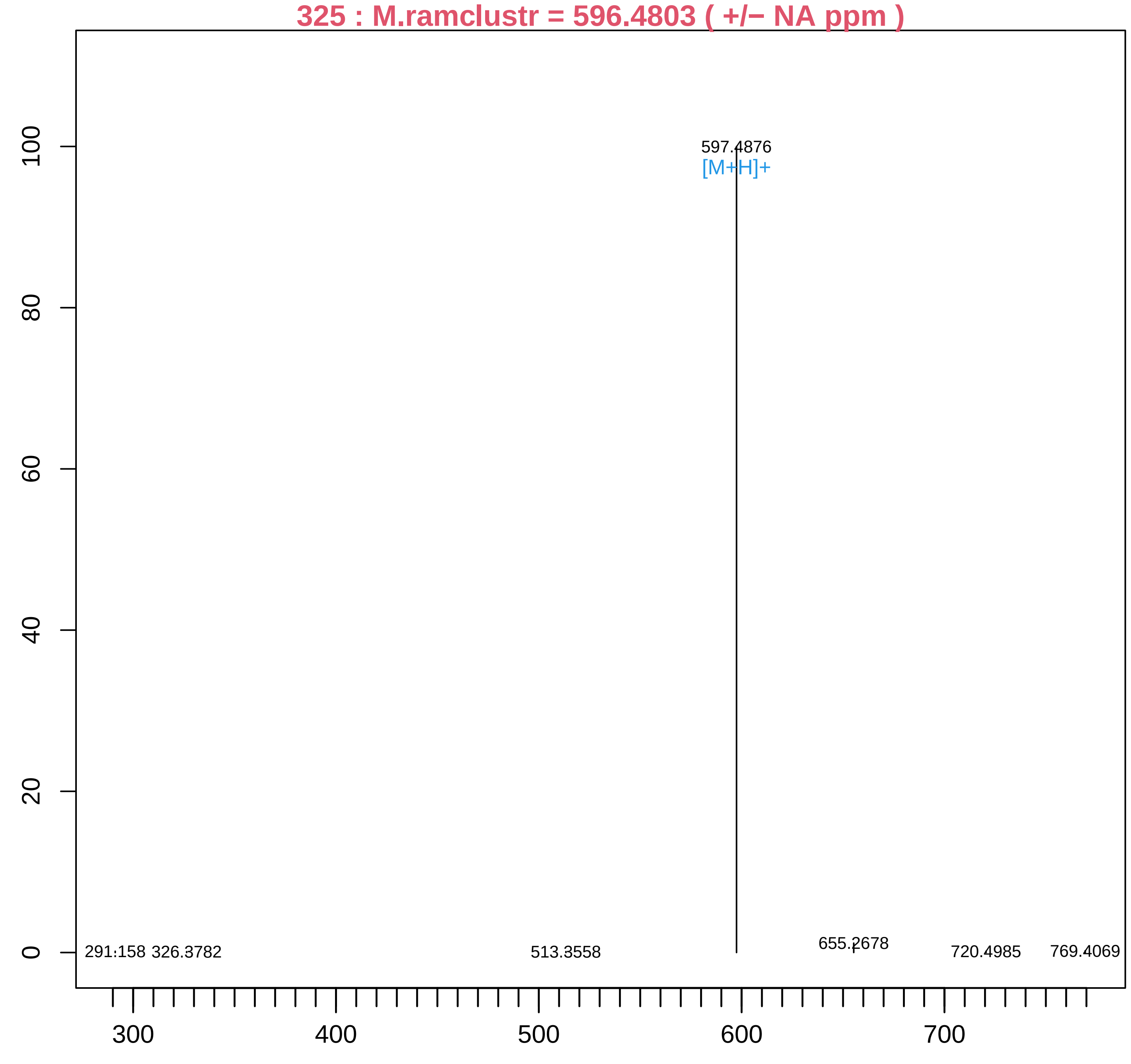
325 : M.ramclustr = 596.4803 (+/− NA ppm)
597.4876
[M+H]+
291:158 326.3782
513.3558
655.2678
720.4985
769.4069
0
20
40
60
80
100
300
400
500
600
700

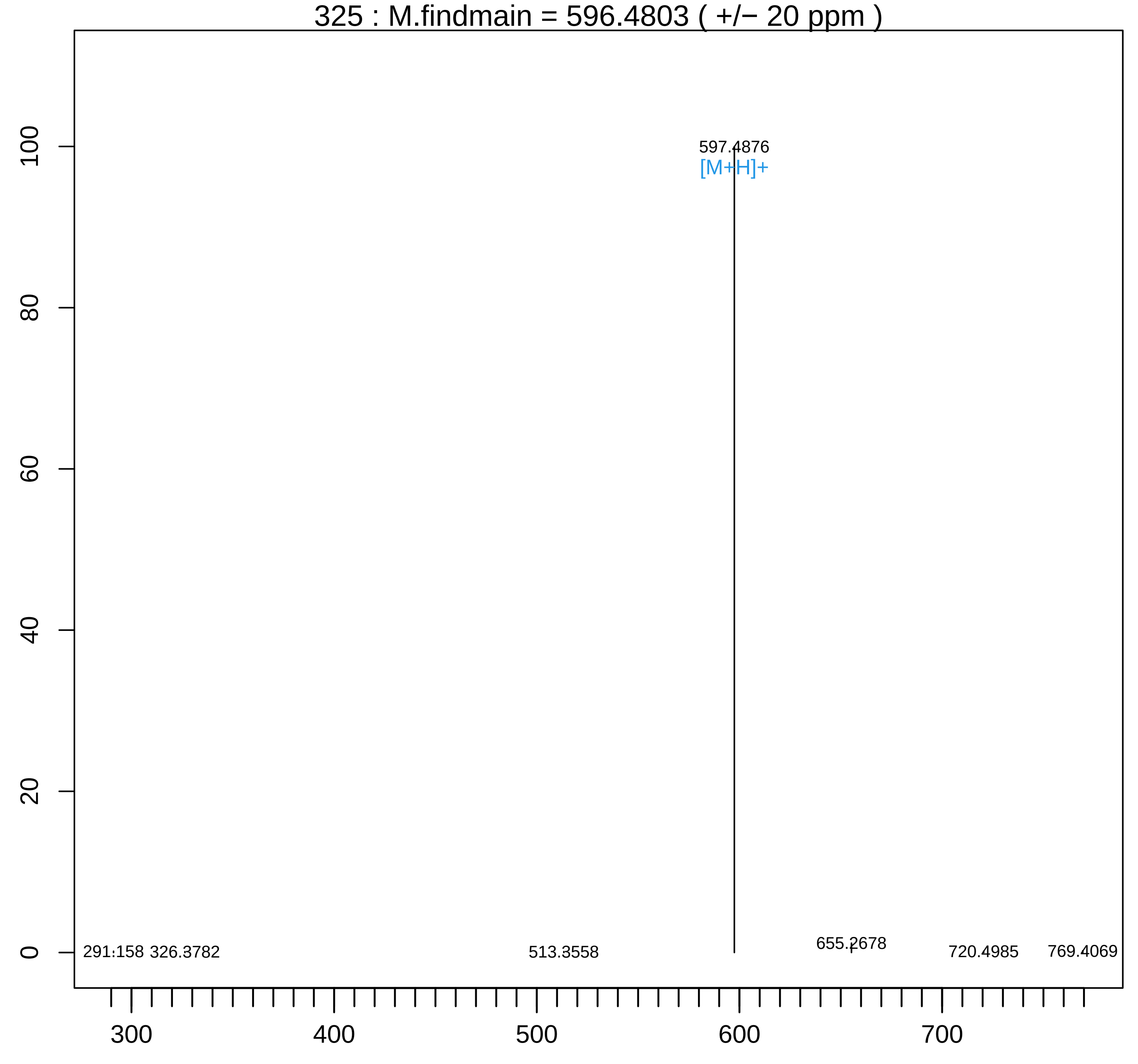
325 : M.findmain = 596.4803 (+/− 20 ppm)
597.4876
[M+H]+
291:158 326.3782
513.3558
655.2678
720.4985
769.4069
0
20
40
60
80
100
300
400
500
600
700

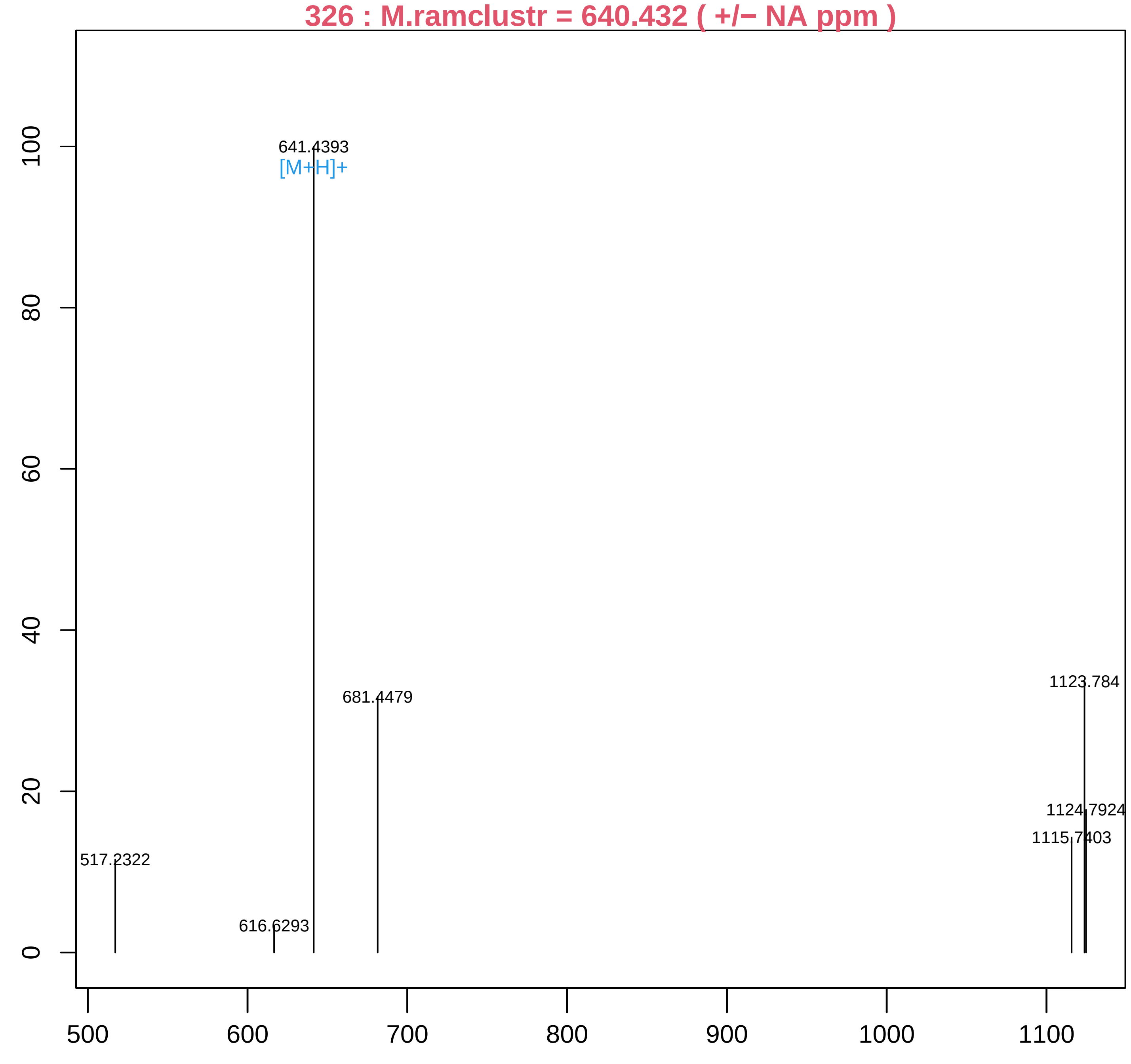

326 : M.ramclustr = 640.432 (+/− NA ppm)
641.4393
[M+H]+
681.4479
1123.784
1124.7924
1115.7403
517.2322
616.6293
0
20
40
60
80
100
500
600
700
800
900
1000
1100

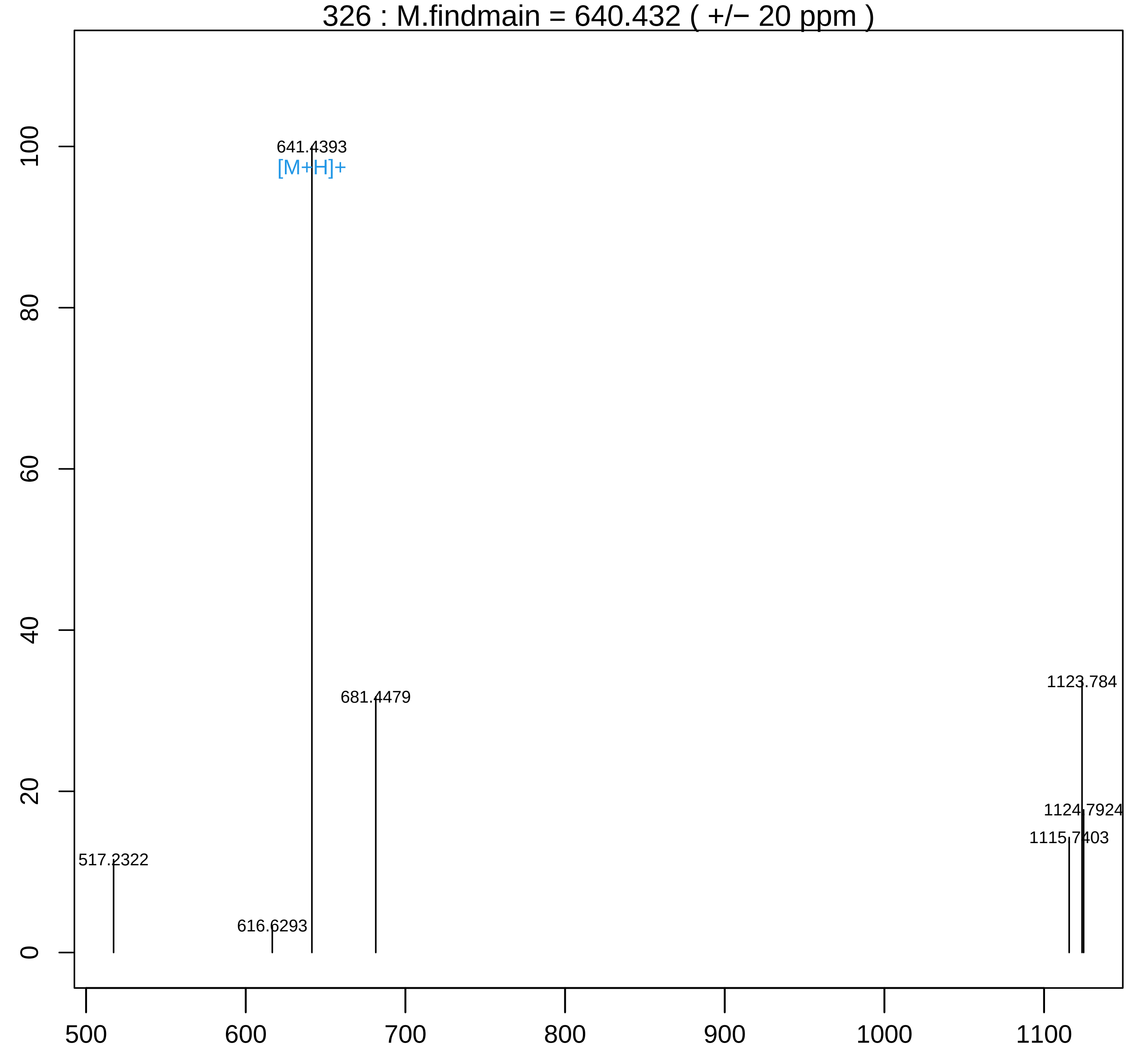

326 : M.findmain = 640.432 (+/− 20 ppm)
641.4393
[M+H]+
681.4479
1123.784
1124.7924
1115.7403
517.2322
616.6293
0
20
40
60
80
100
500
600
700
800
900
1000
1100

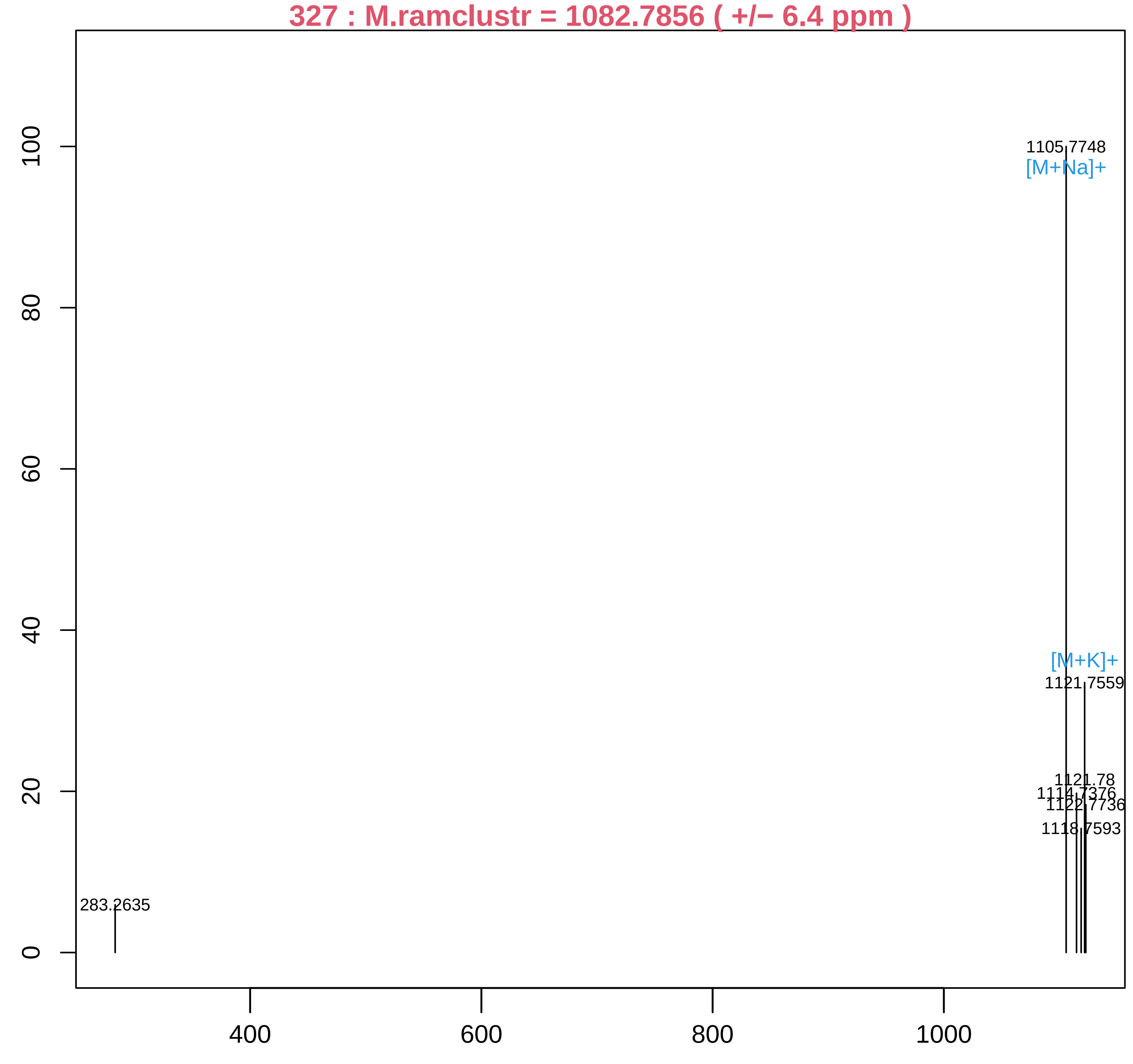
327 : M.ramclustr = 1082.7856 (+/− 6.4 ppm)
1105.7748
[M+Na]+
[M+K]+
1121.7559
1121.78
1114.7376
1122.7736
1118.7593
283.2635
0
20
40
60
80
100
400
600
800
1000
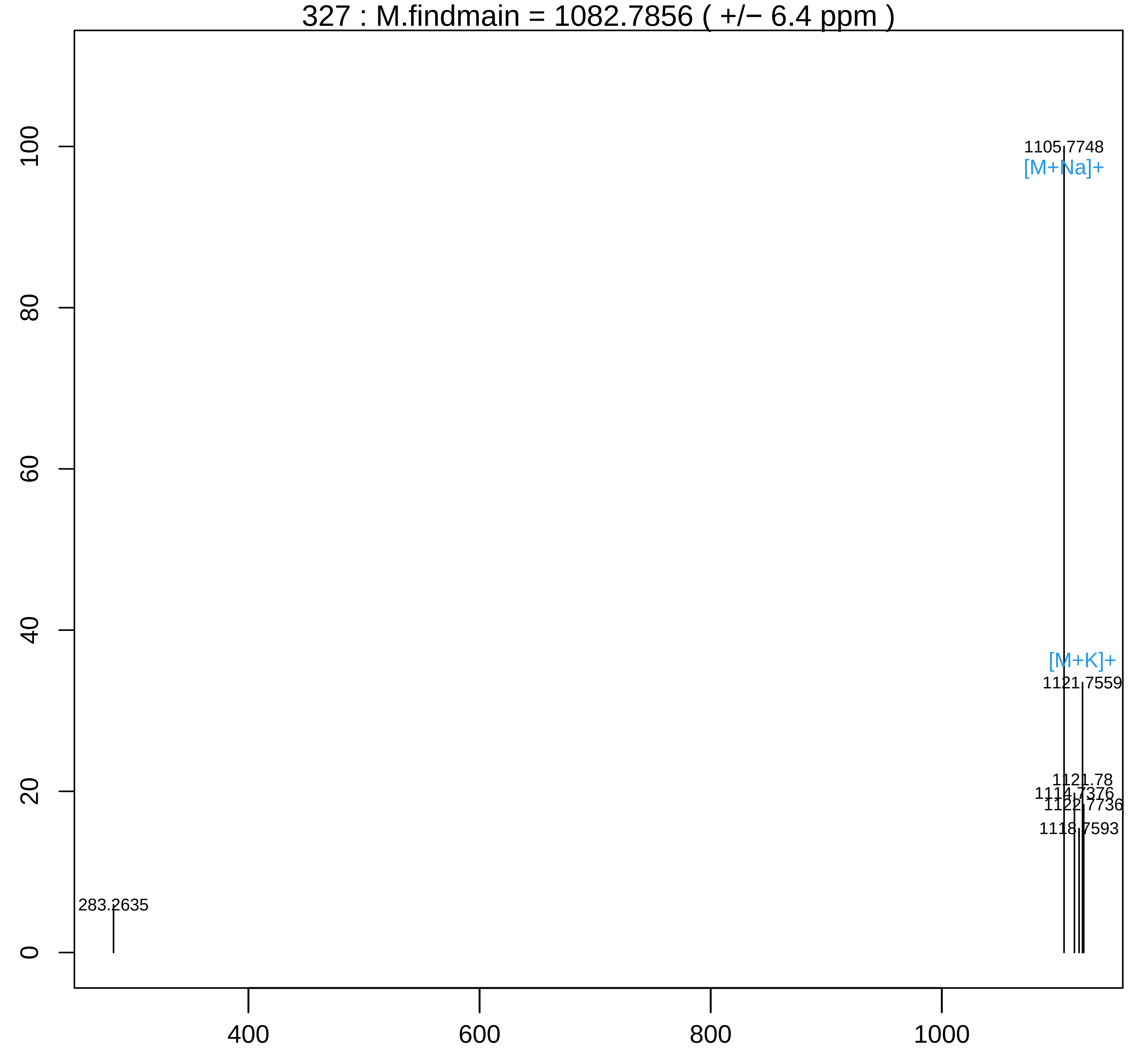
327 : M.findmain = 1082.7856 (+/− 6.4 ppm)
1105.7748
[M+Na]+
[M+K]+
1121.7559
1121.78
1114.7376
1122.7736
1118.7593
283.2635
0
20
40
60
80
100
400
600
800
1000

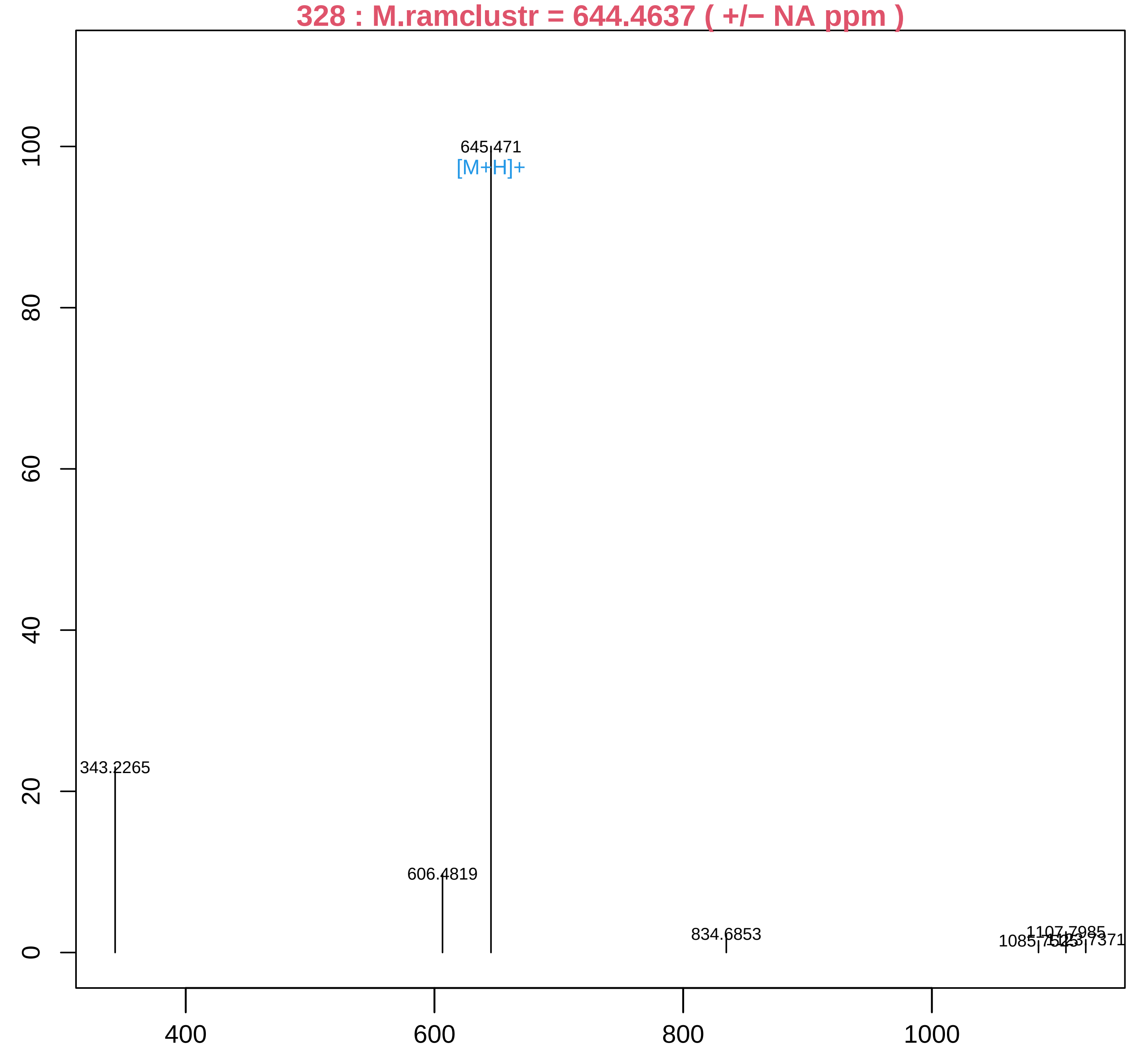
328 : M.ramclustr = 644.4637 (+/− NA ppm)
645.471
[M+H]+
343.2265
606.4819
834.6853
1107.7985
0
20
40
60
80
100
400
600
800
1000

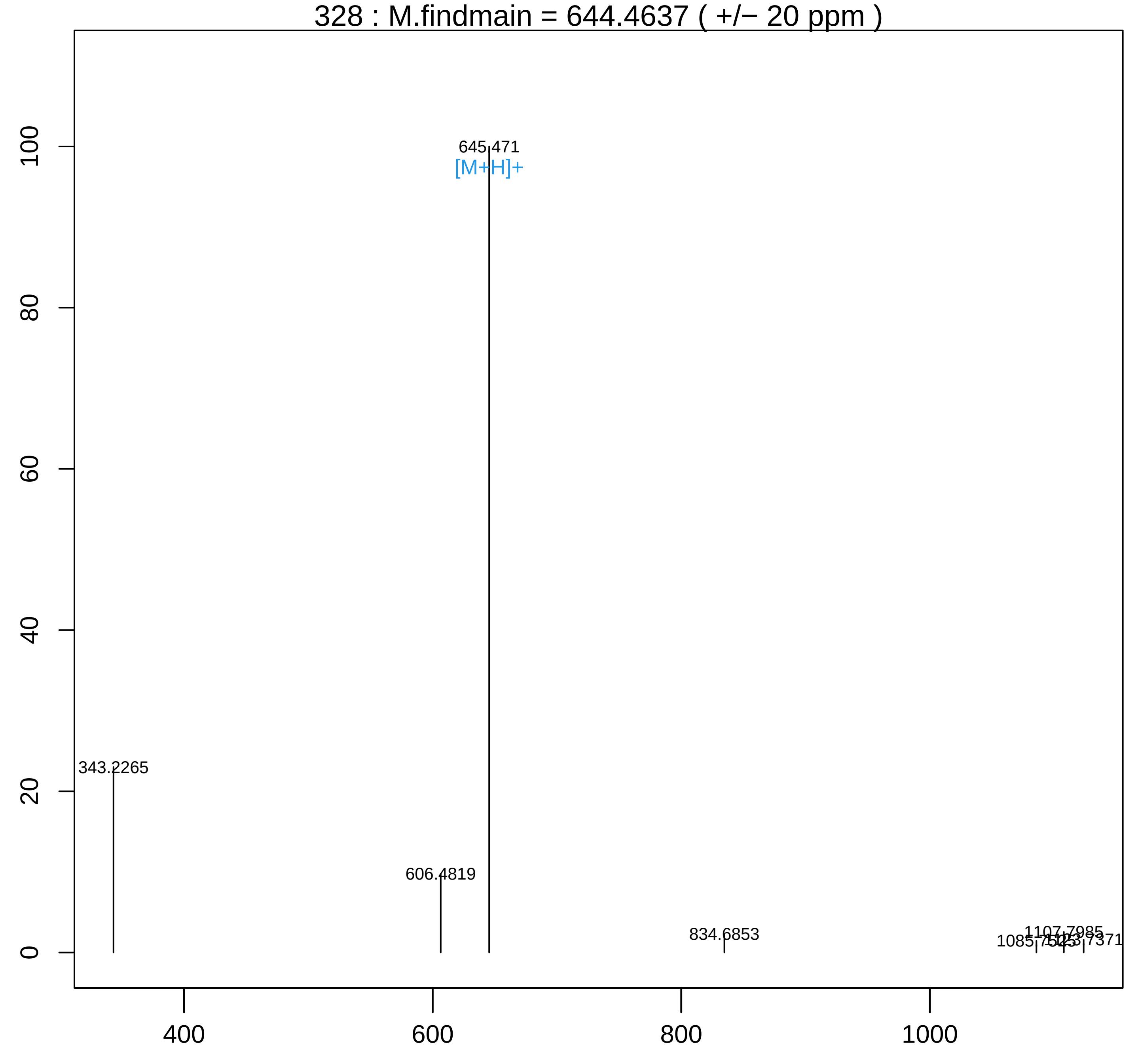
328 : M.findmain = 644.4637 (+/− 20 ppm)
645.471
[M+H]+
343.2265
606.4819
834.6853
1107.7985
0
20
40
60
80
100
400
600
800
1000

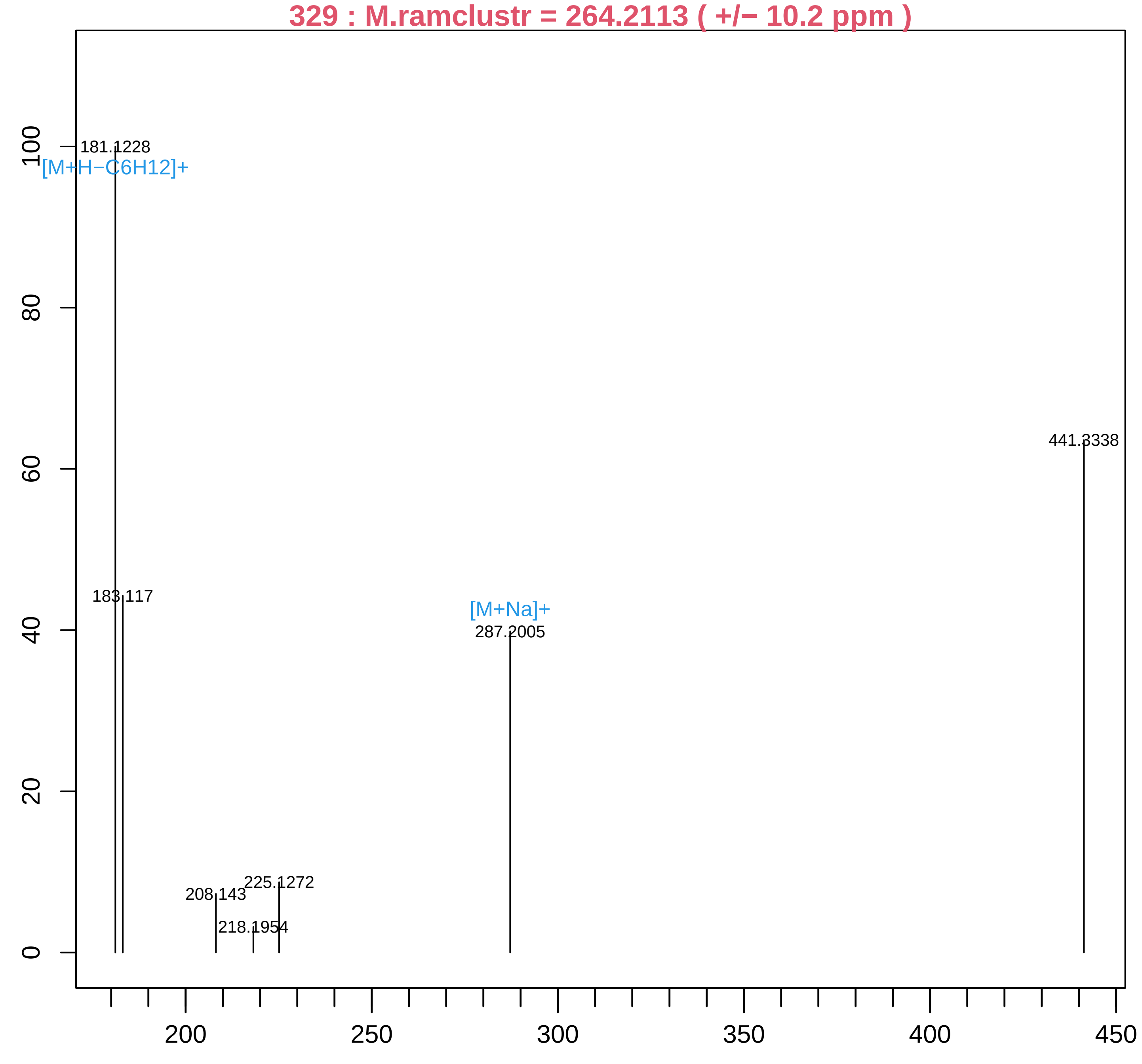

329 : M.ramclustr = 264.2113 (+/− 10.2 ppm)
181.1228
[M+H−C6H12]+
183.117
[M+Na]+
287.2005
441.3338
208.143
225.1272
218.1954

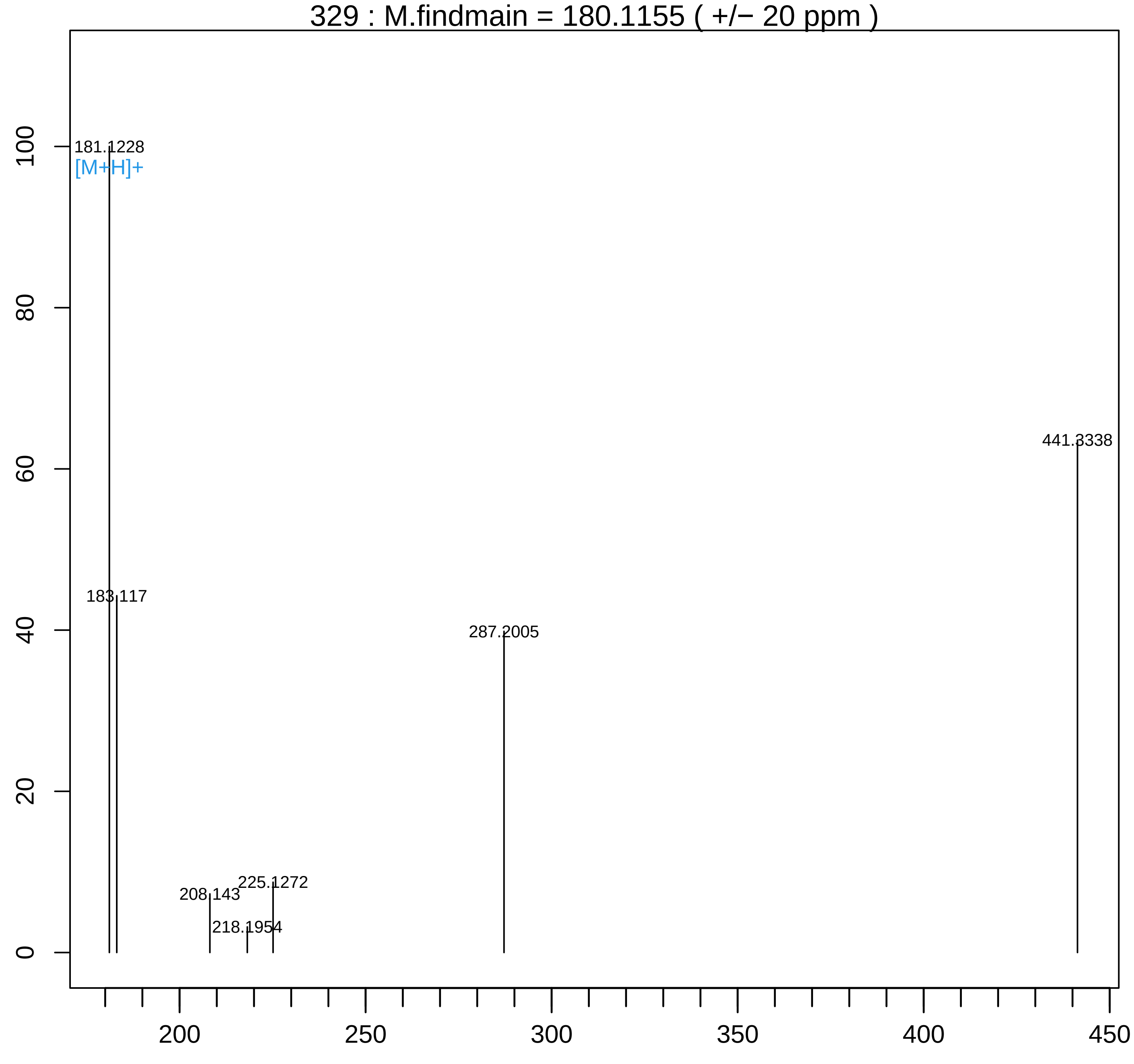

329 : M.findmain = 180.1155 (+/− 20 ppm)
181.1228
[M+H]+
183.117
287.2005
441.3338
208.143
225.1272
218.1954

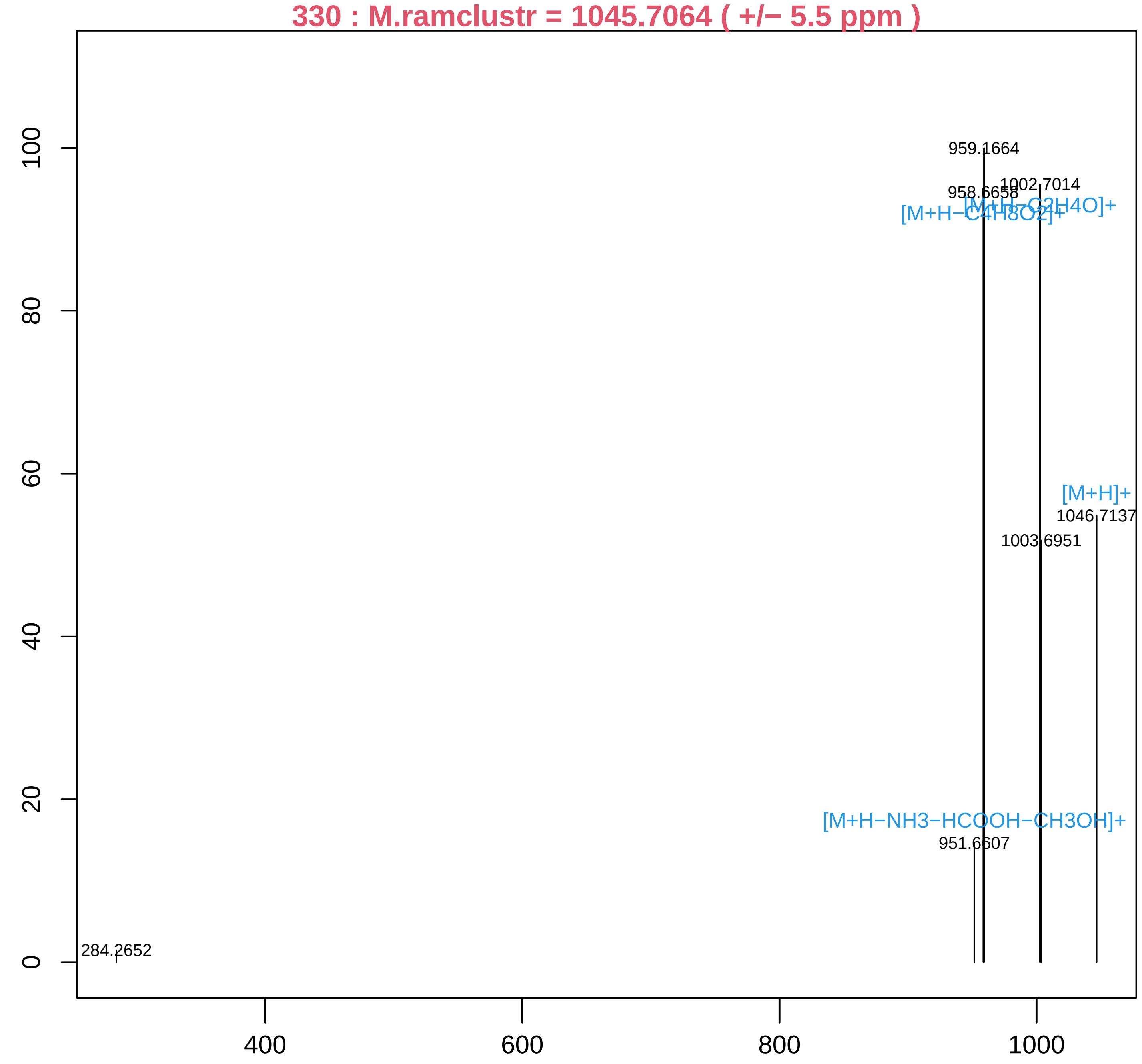
330 : M.ramclustr = 1045.7064 (+/− 5.5 ppm)
959.1664
958.6658
1002.7014
[M+H−C2H4O]+
[M+H−C4H8O2]+
[M+H]+
1046.7137
1003.6951
[M+H−NH3−HCOOH−CH3OH]+
951.6607
284.2652
0
20
40
60
80
100
400
600
800
1000

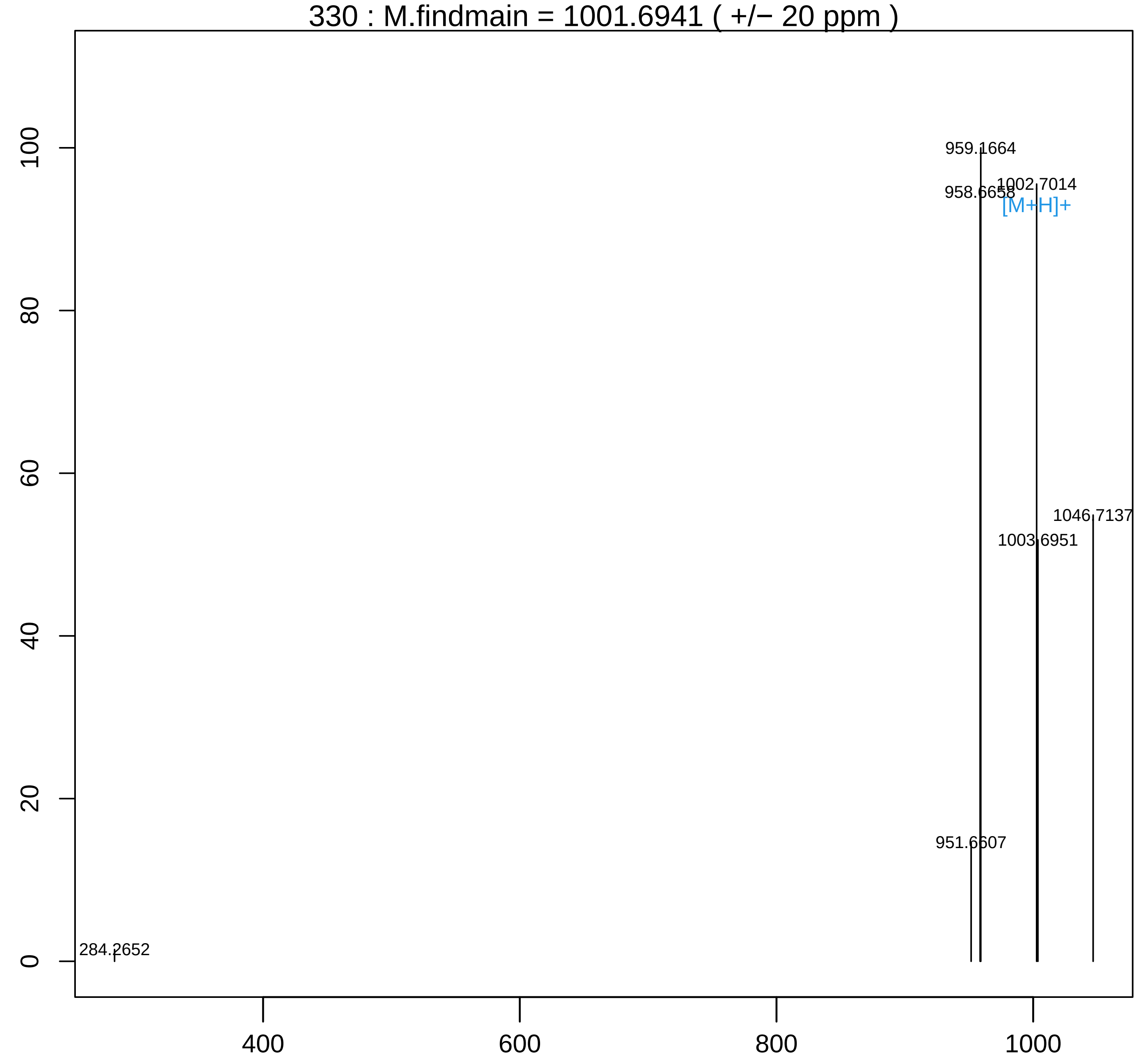
330 : M.findmain = 1001.6941 (+/− 20 ppm)
959.1664
958.6658
1002.7014
[M+H]+
1046.7137
1003.6951
951.6607
284.2652
0
20
40
60
80
100
400
600
800
1000

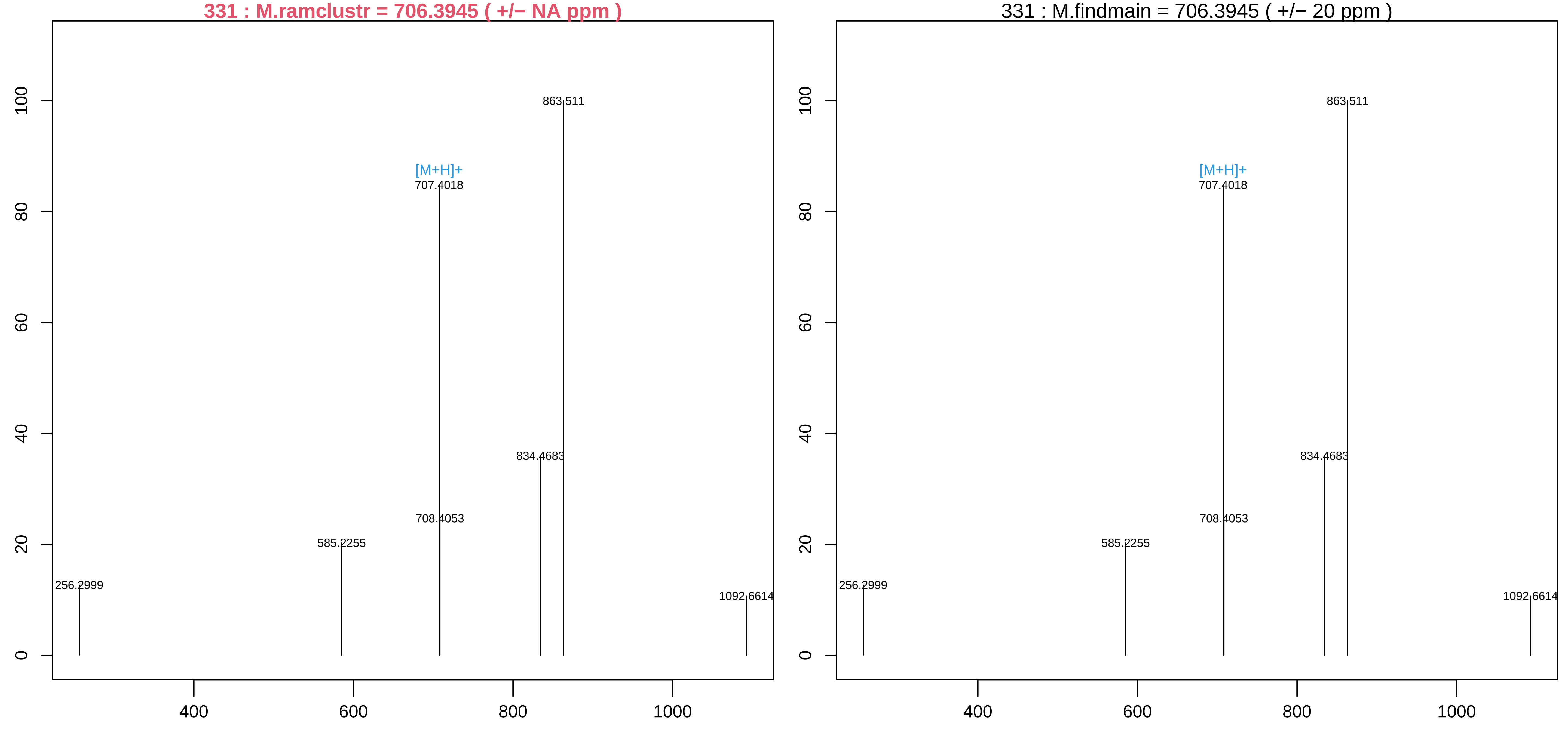
331 : M.ramclustr = 706.3945 (+/− NA ppm)
[M+H]+
707.4018
863.511
834.4683
708.4053
585.2255
256.2999
1092.6614
331 : M.findmain = 706.3945 (+/− 20 ppm)
[M+H]+
707.4018
863.511
834.4683
708.4053
585.2255
256.2999
1092.6614

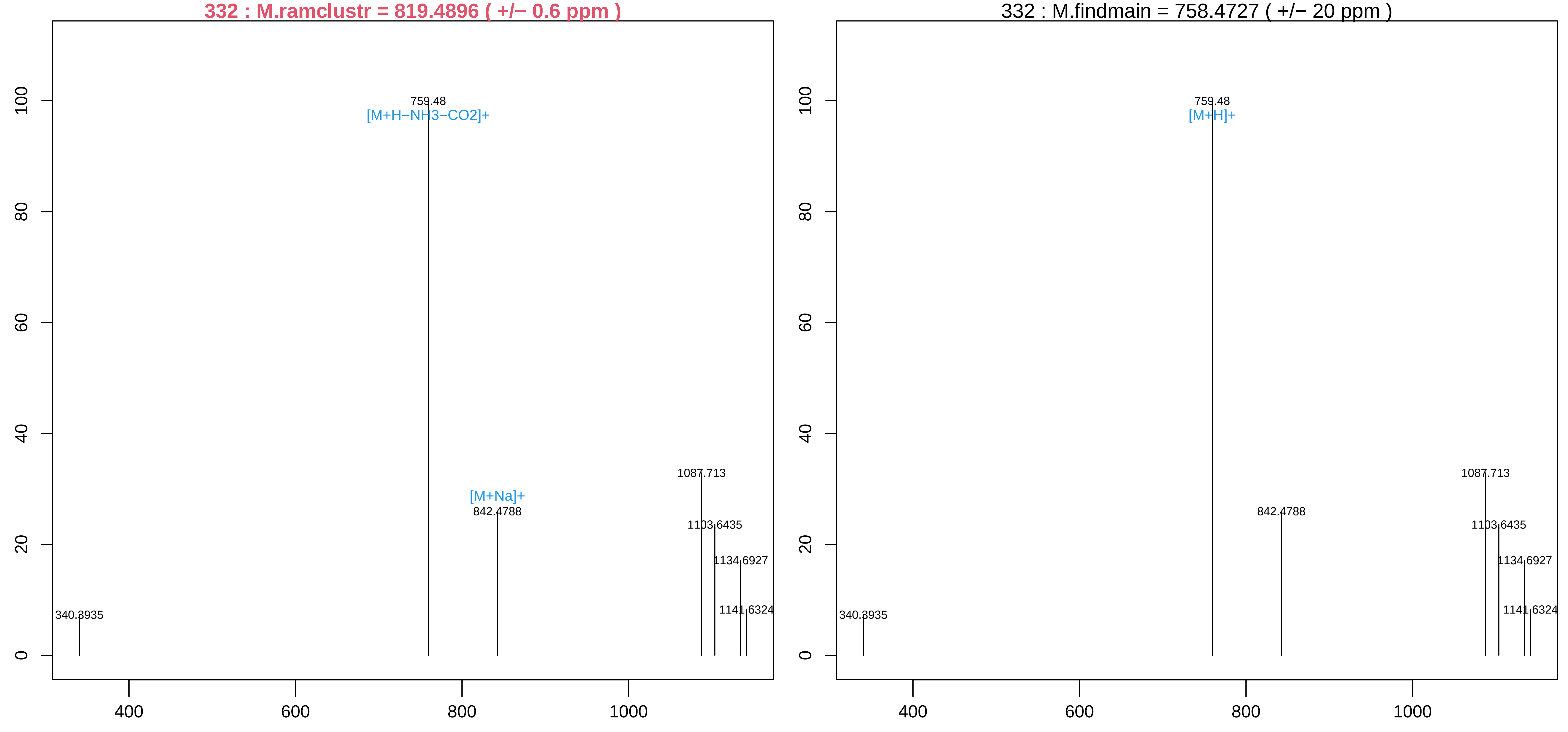
332 : M.ramclustr = 819.4896 (+/− 0.6 ppm)
759.48
[M+H−NH3−CO2]+
[M+Na]+
842.4788
1087.713
1103.6435
1134.6927
1141.6324
340.3935
0
20
40
60
80
100
400
600
800
1000
332 : M.findmain = 758.4727 (+/− 20 ppm)
759.48
[M+H]+
842.4788
1087.713
1103.6435
1134.6927
1141.6324
340.3935

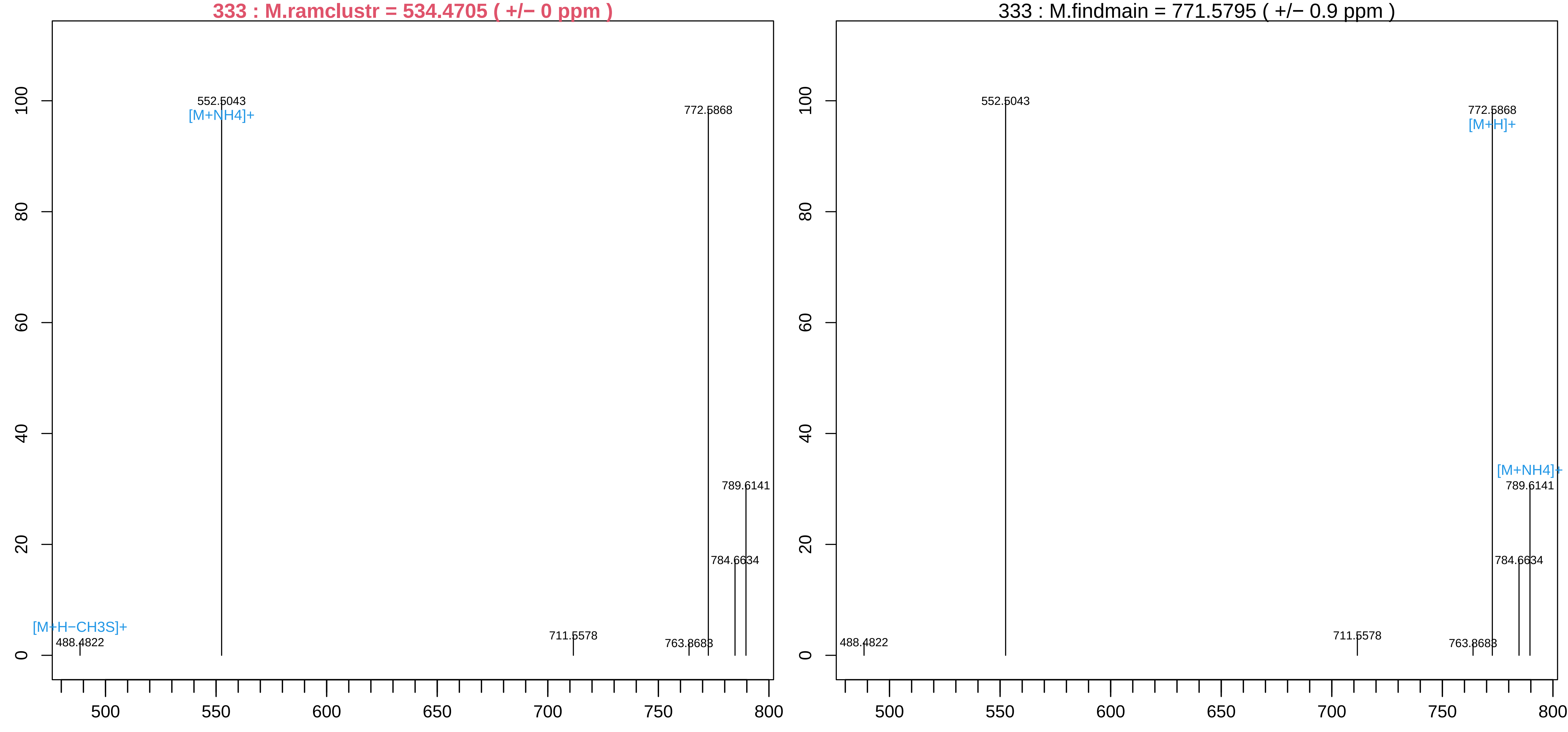
333 : M.ramclustr = 534.4705 (+/− 0 ppm)
552.5043
[M+NH4]+
772.5868
789.6141
784.6634
[M+H−CH3S]+
488.4822
711.5578
763.8683
333 : M.findmain = 771.5795 (+/− 0.9 ppm)
552.5043
772.5868
[M+H]+
[M+NH4]+
789.6141
784.6634
488.4822
711.5578
763.8683
0
20
40
60
80
100
500
550
600
650
700
750
800

334 : M.ramclustr = 696.5366 (+/− NA ppm)

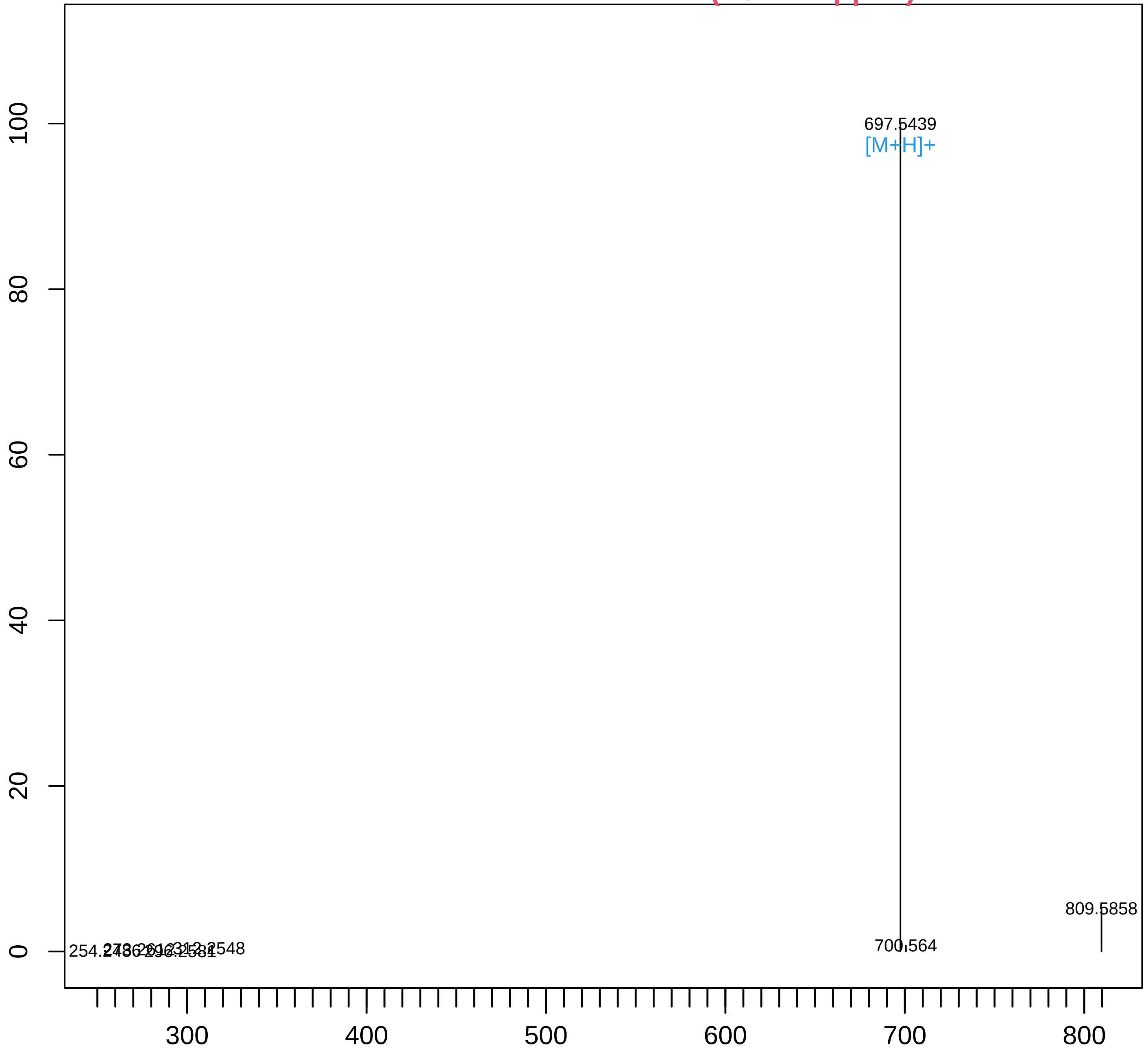

334 : M.findmain = 696.5366 (+/− 20 ppm)

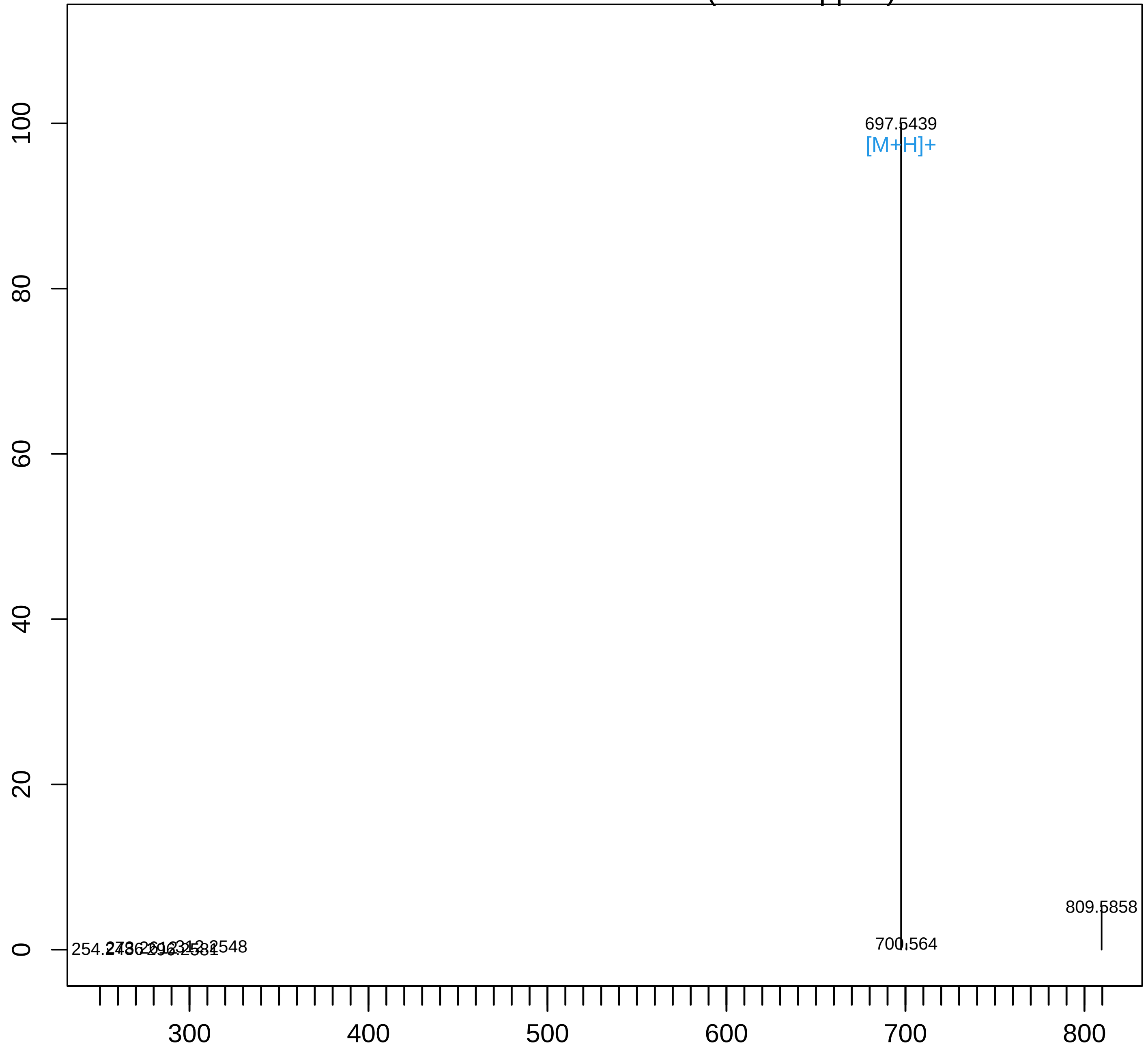

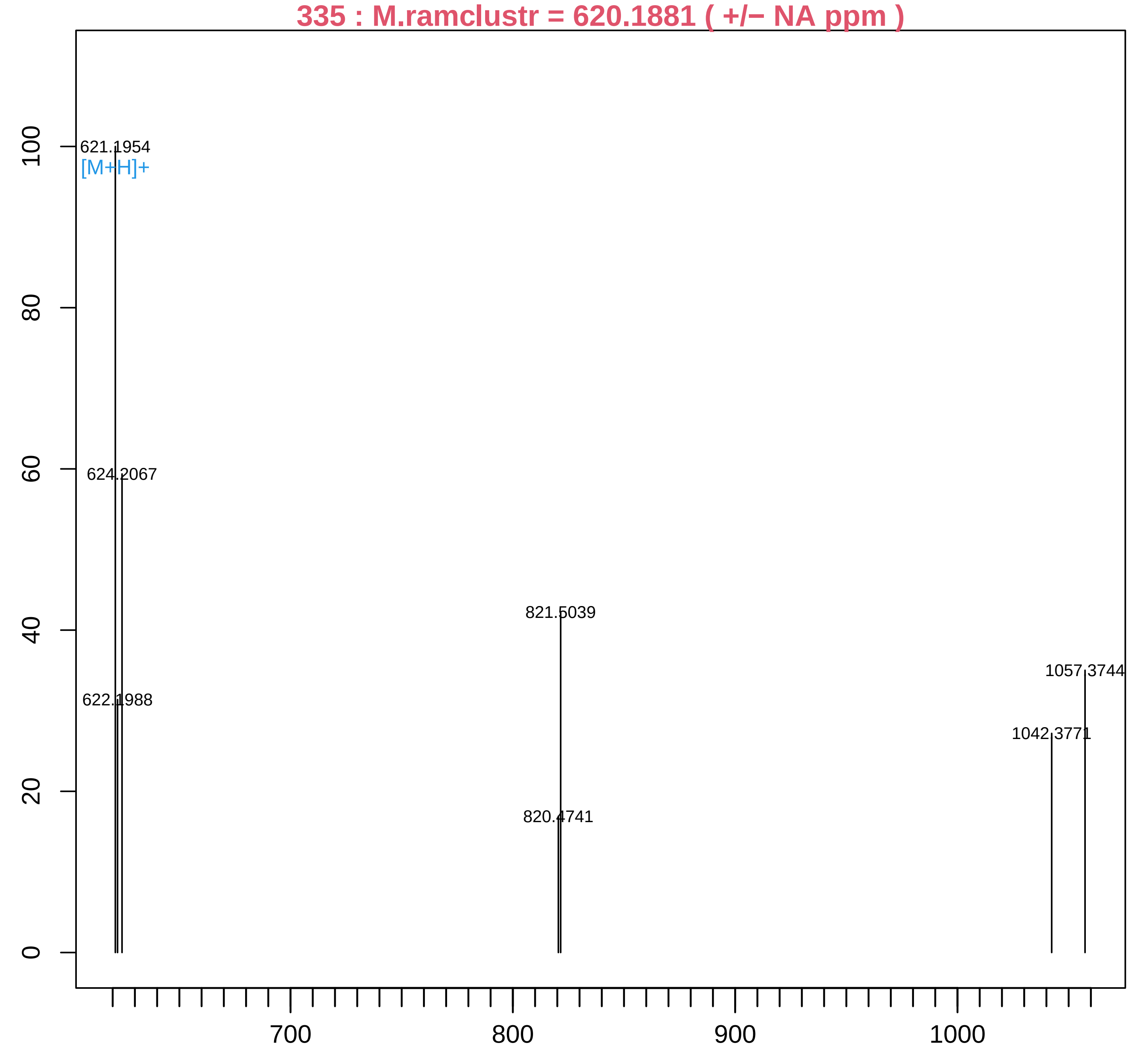
335 : M.ramclustr = 620.1881 (+/− NA ppm)
621.1954
[M+H]+
624.2067
622.1988
821.5039
820.4741
1057.3744
1042.3771
0
20
40
60
80
100
700
800
900
1000

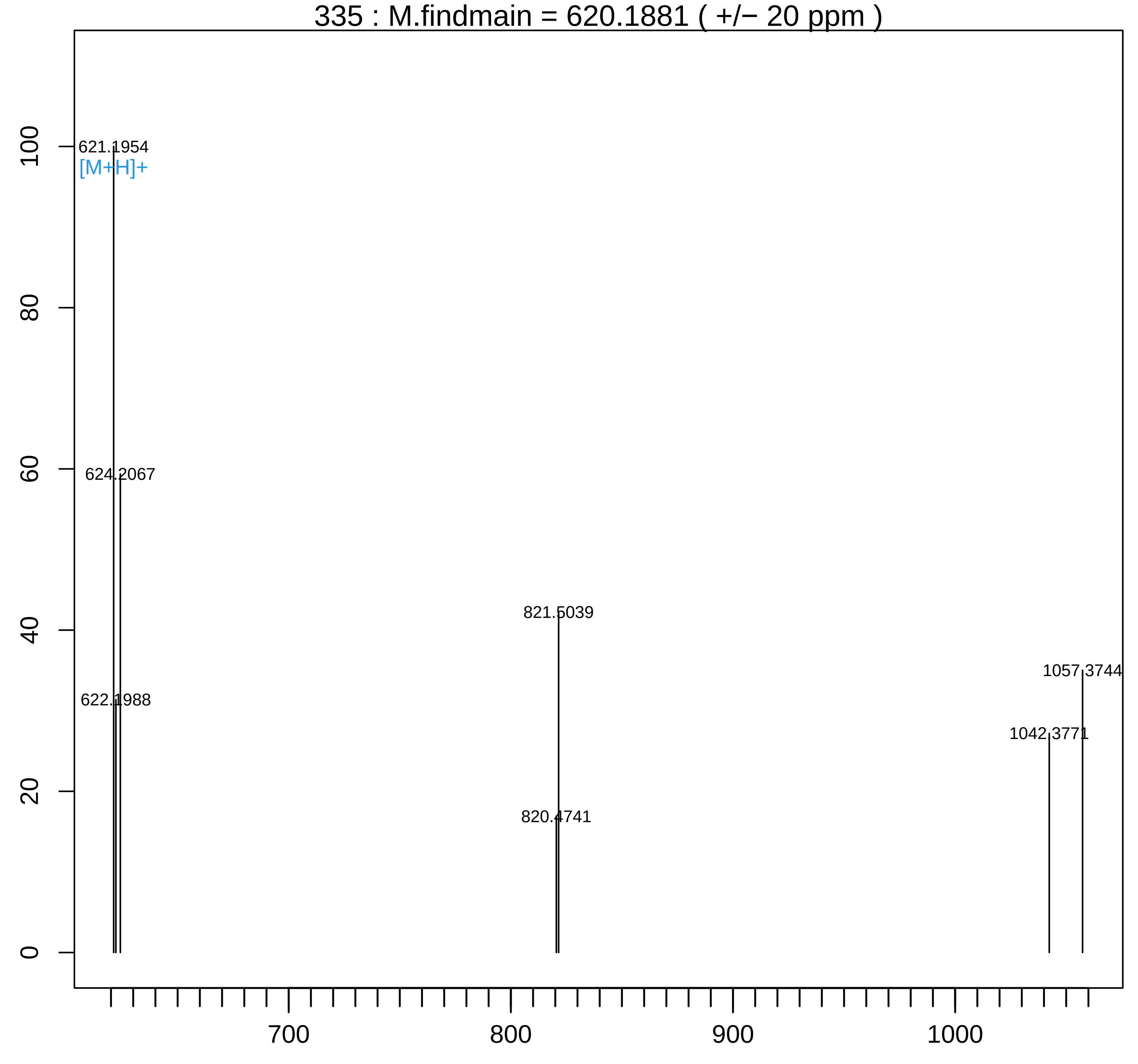
335 : M.findmain = 620.1881 (+/− 20 ppm)
621.1954
[M+H]+
624.2067
622.1988
821.5039
820.4741
1057.3744
1042.3771
0
20
40
60
80
100
700
800
900
1000

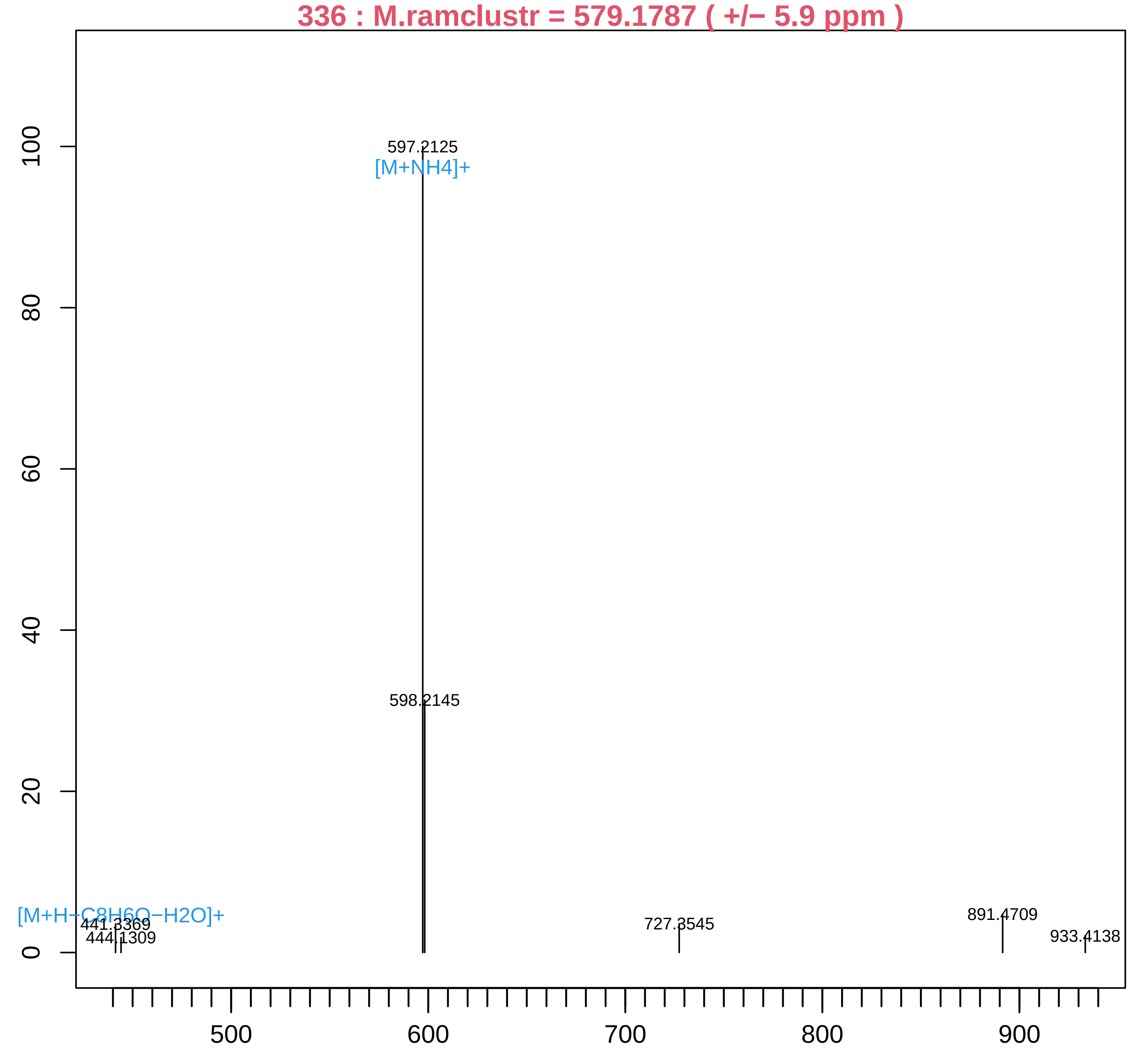

336 : M.ramclustr = 579.1787 (+/− 5.9 ppm)
597.2125
[M+NH4]+
598.2145
[M+H−C8H6O−H2O]+
441.3369
444.1309
727.3545
891.4709
933.4138
0
20
40
60
80
100
500
600
700
800
900

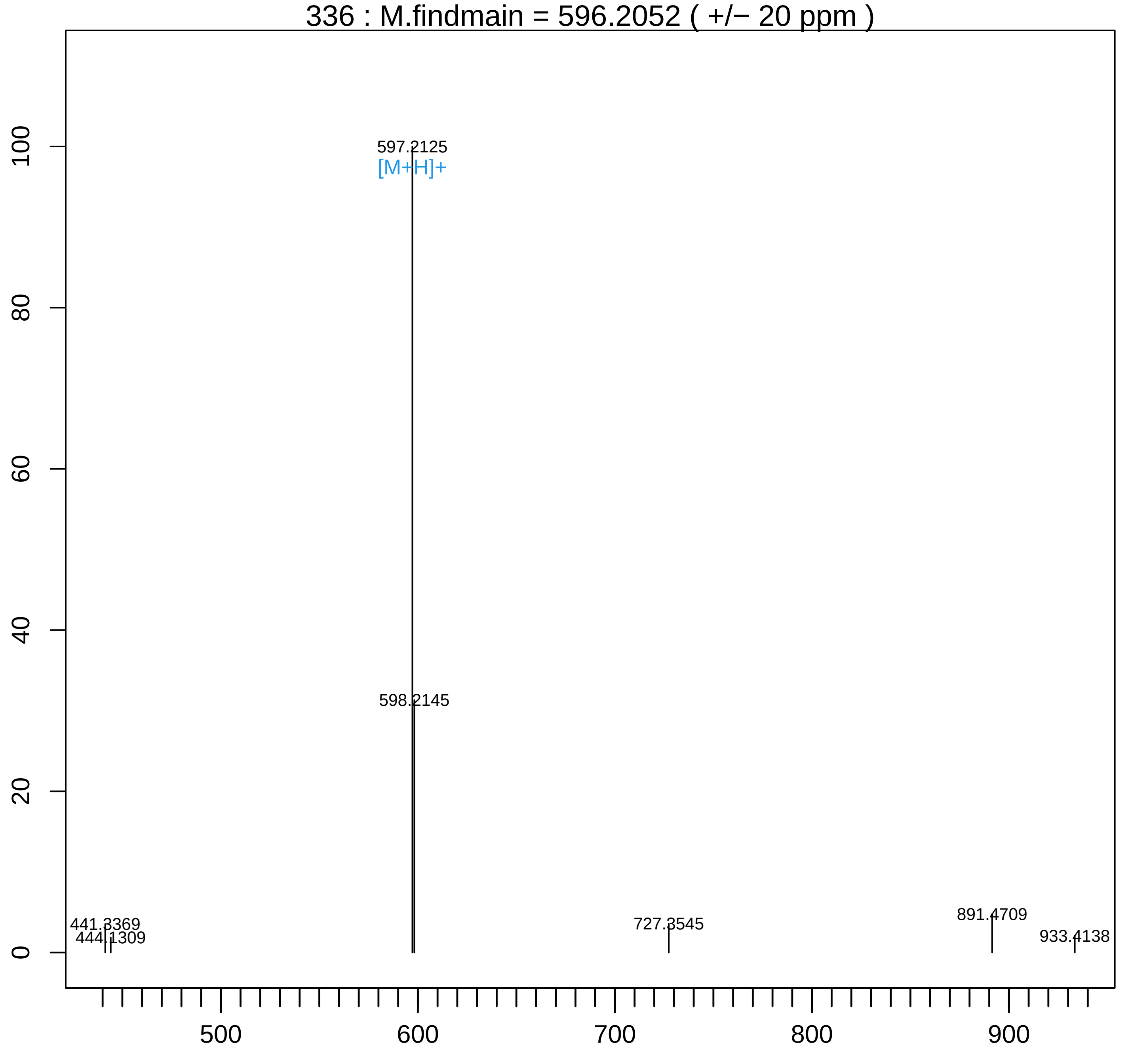

336 : M.findmain = 596.2052 (+/− 20 ppm)
597.2125
[M+H]+
598.2145
441.3369
444.1309
727.3545
891.4709
933.4138
0
20
40
60
80
100
500
600
700
800
900

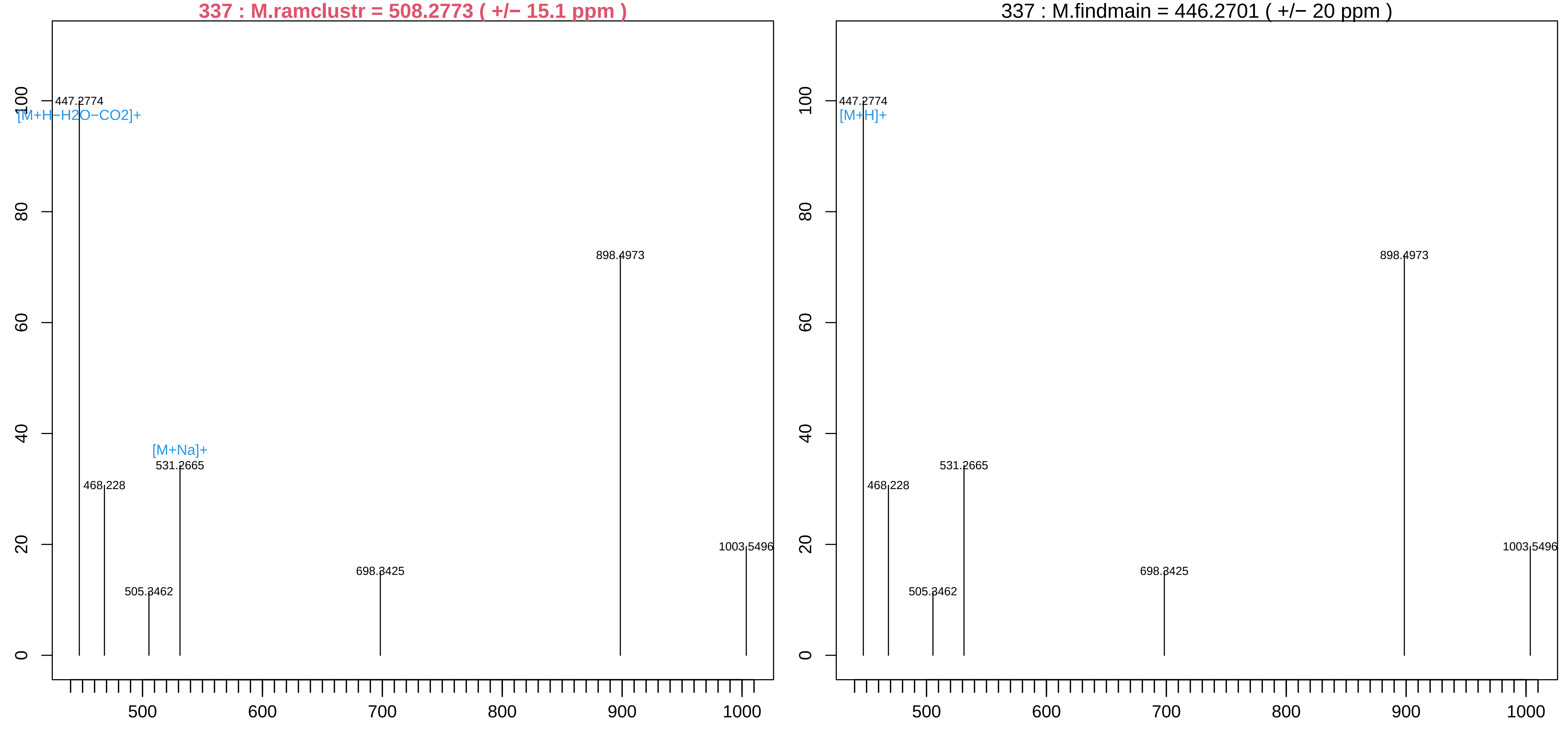

337 : M.ramclustr = 508.2773 (+/− 15.1 ppm)
447.2774
[M+H−H2O−CO2]+
468.228
505.3462
[M+Na]+
531.2665
698.3425
898.4973
1003.5496
337 : M.findmain = 446.2701 (+/− 20 ppm)
447.2774
[M+H]+
468.228
505.3462
531.2665
698.3425
898.4973
1003.5496

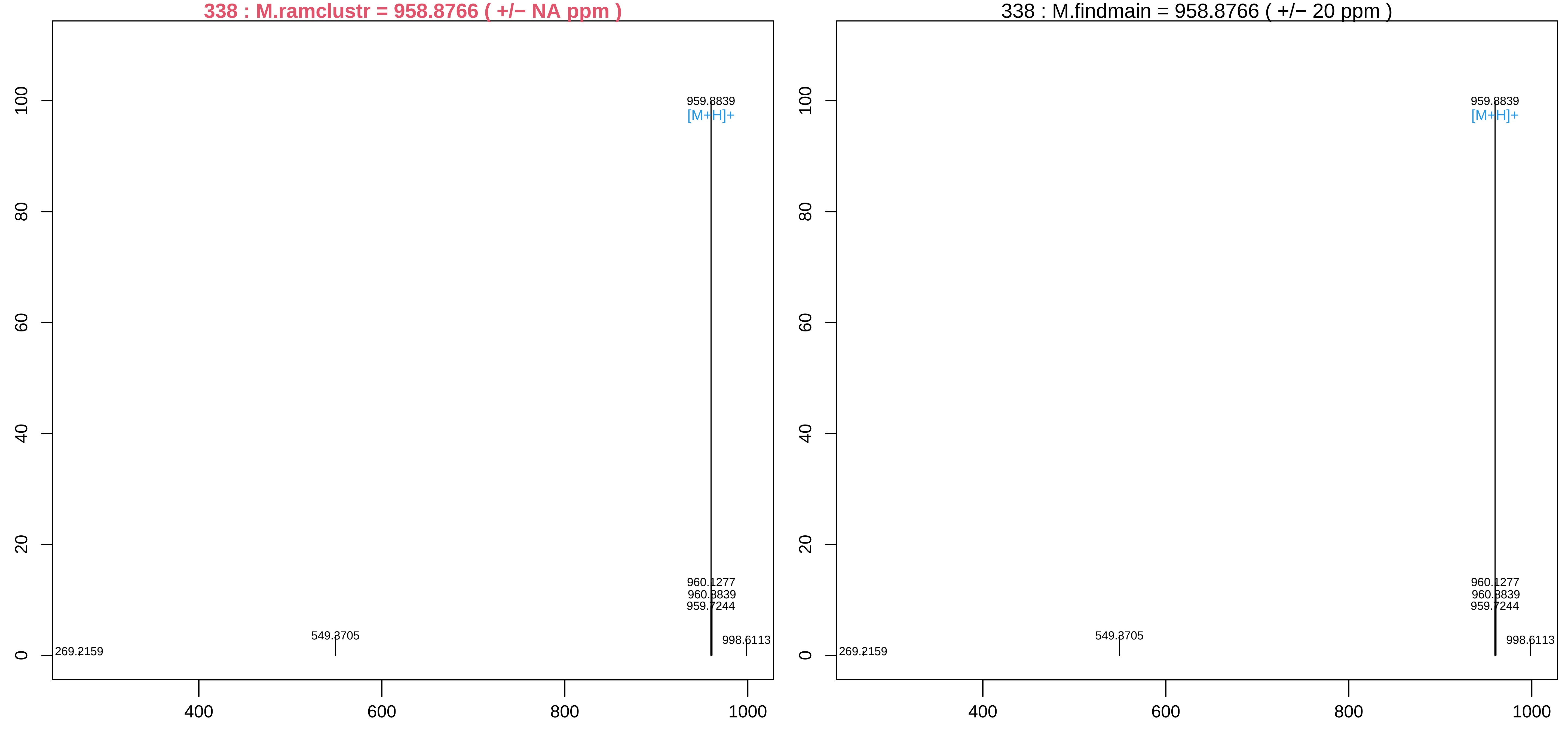
338 : M.ramclustr = 958.8766 (+/− NA ppm)
338 : M.findmain = 958.8766 (+/− 20 ppm)
959.8839
[M+H]+
960.1277
960.8839
959.7244
549.3705
998.6113
269.2159
0
20
40
60
80
100
400
600
800
1000

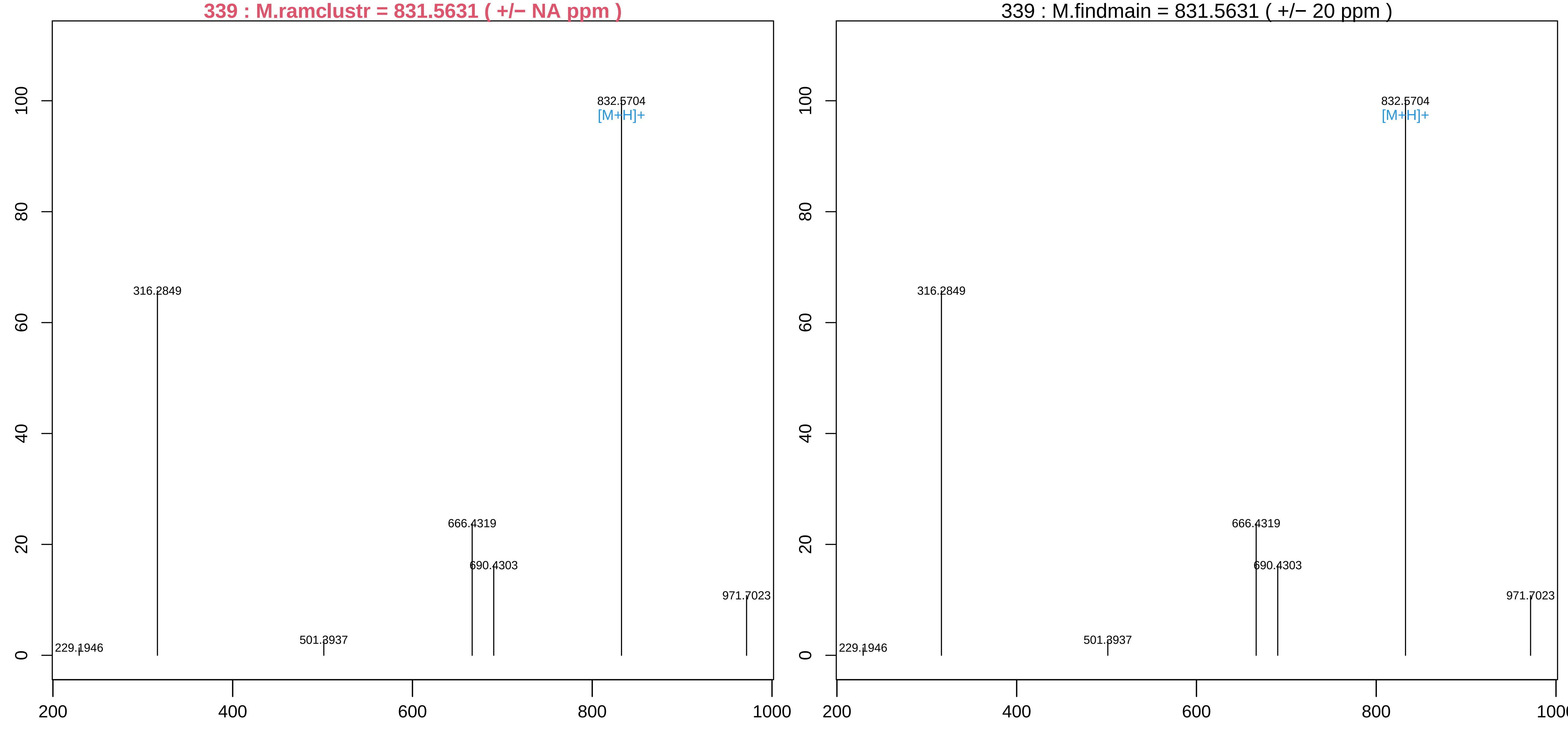
339 : M.ramclustr = 831.5631 (+/− NA ppm)
832.5704
[M+H]+
316.2849
666.4319
690.4303
971.7023
501.3937
229.1946
339 : M.findmain = 831.5631 (+/− 20 ppm)
832.5704
[M+H]+
316.2849
666.4319
690.4303
971.7023
501.3937
229.1946

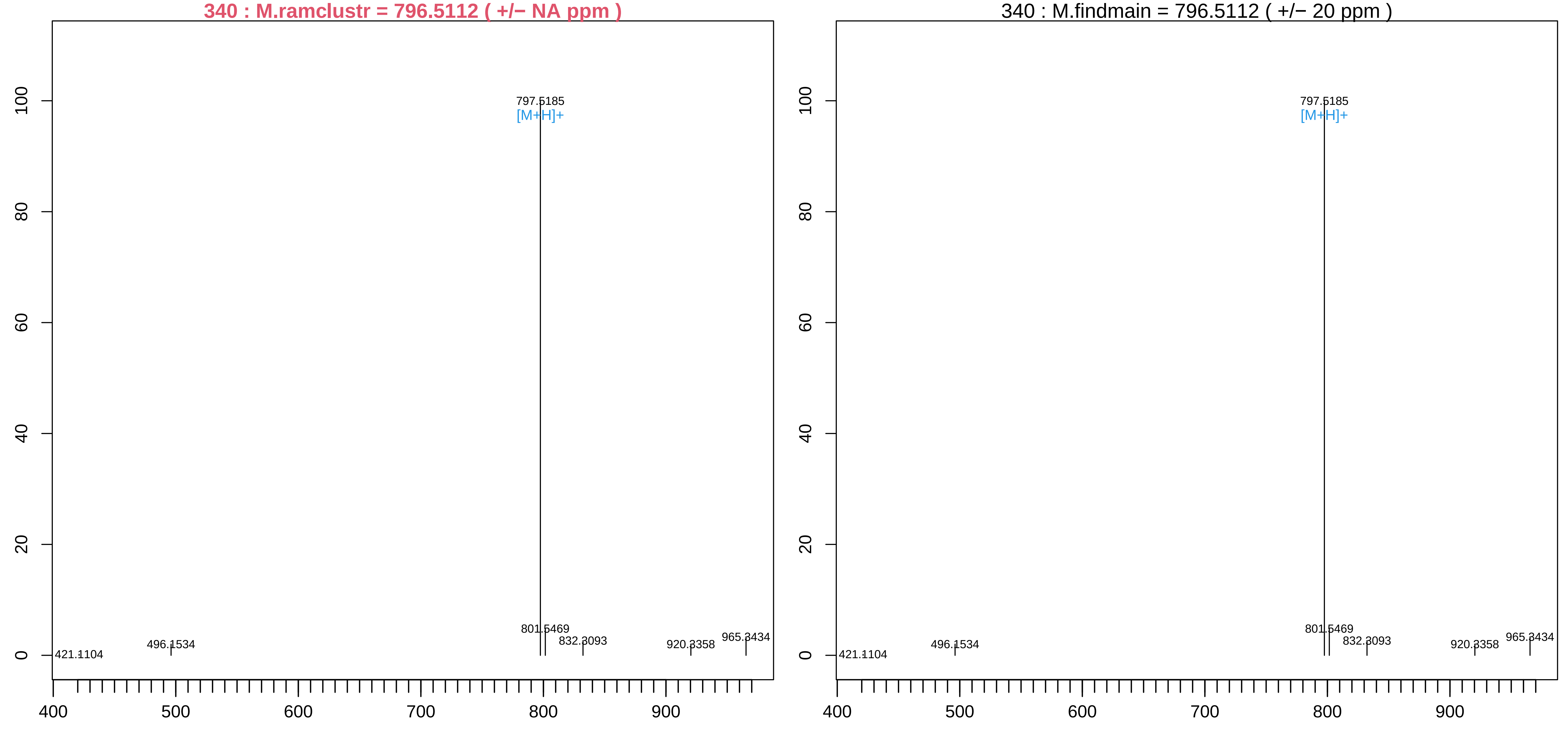

340 : M.ramclustr = 796.5112 (+/− NA ppm)
340 : M.findmain = 796.5112 (+/− 20 ppm)
797.5185
[M+H]+
801.5469
832.3093
920.3358
965.3434
496.1534
421.1104
0
20
40
60
80
100
400
500
600
700
800
900

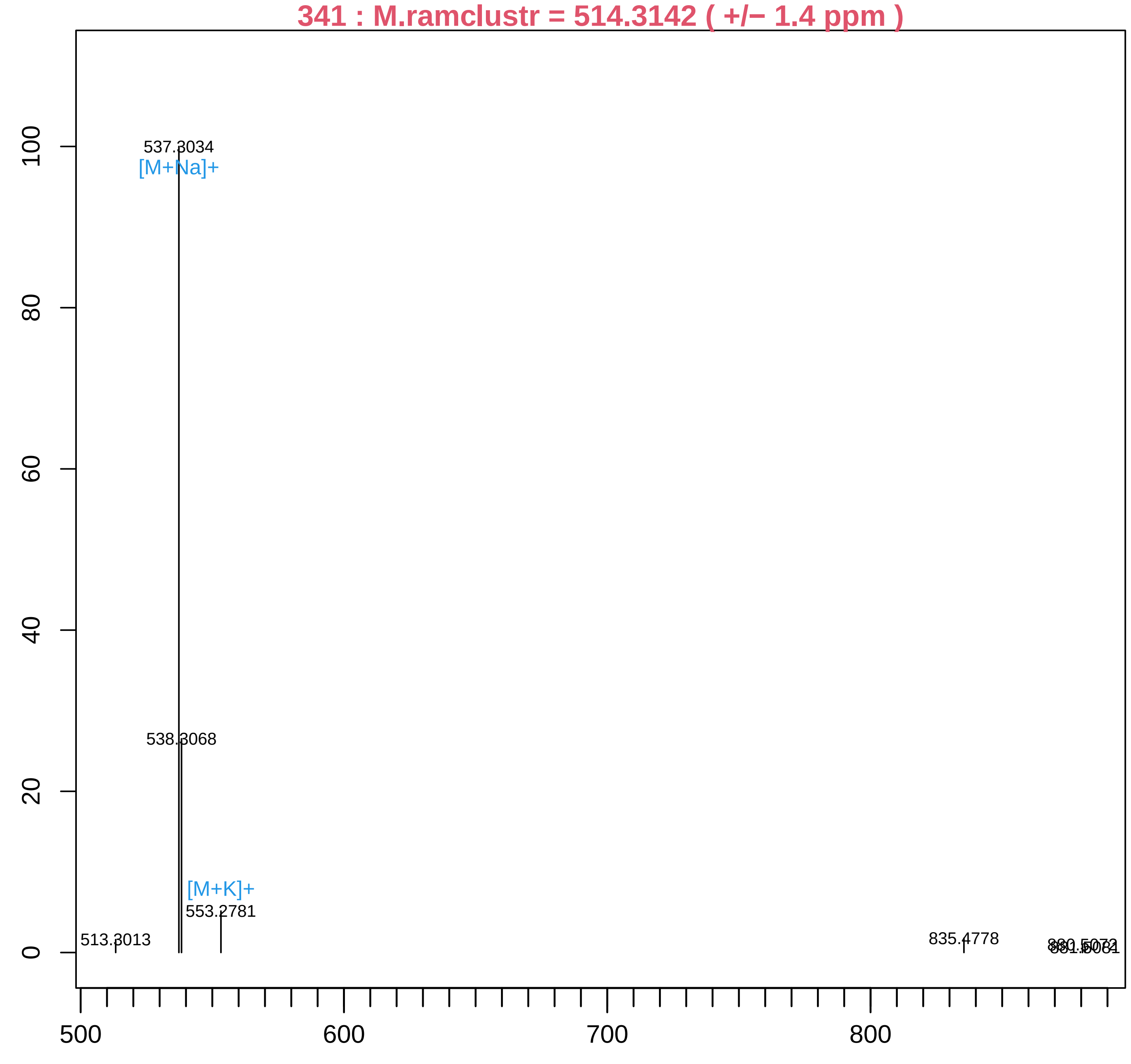
341 : M.ramclustr = 514.3142 (+/− 1.4 ppm)
537.3034
[M+Na]+
538.3068
[M+K]+
553.2781
513.3013
835.4778
0
20
40
60
80
100
500
600
700
800

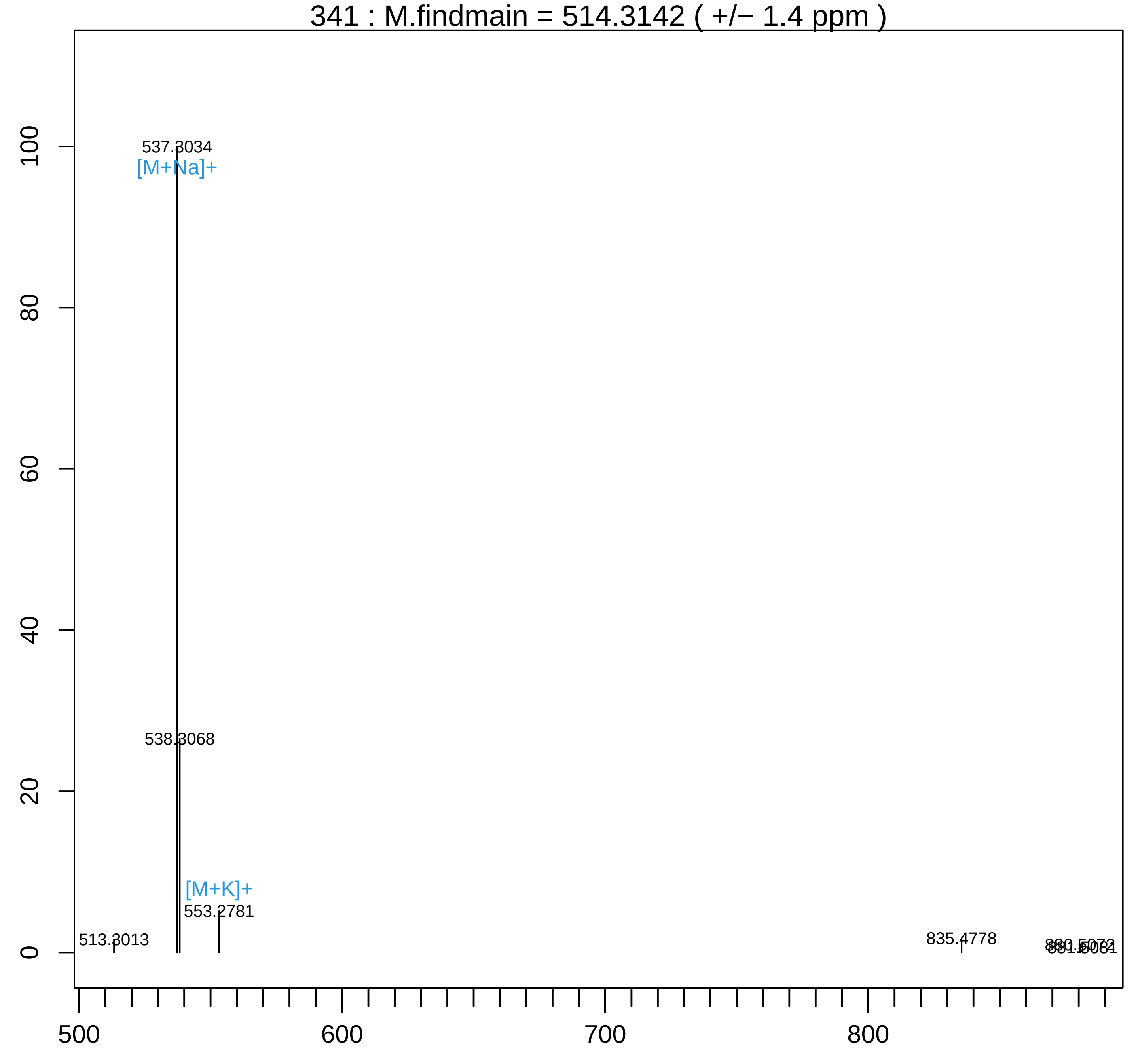
341 : M.findmain = 514.3142 (+/− 1.4 ppm)
537.3034
[M+Na]+
538.3068
[M+K]+
553.2781
513.3013
835.4778
0
20
40
60
80
100
500
600
700
800

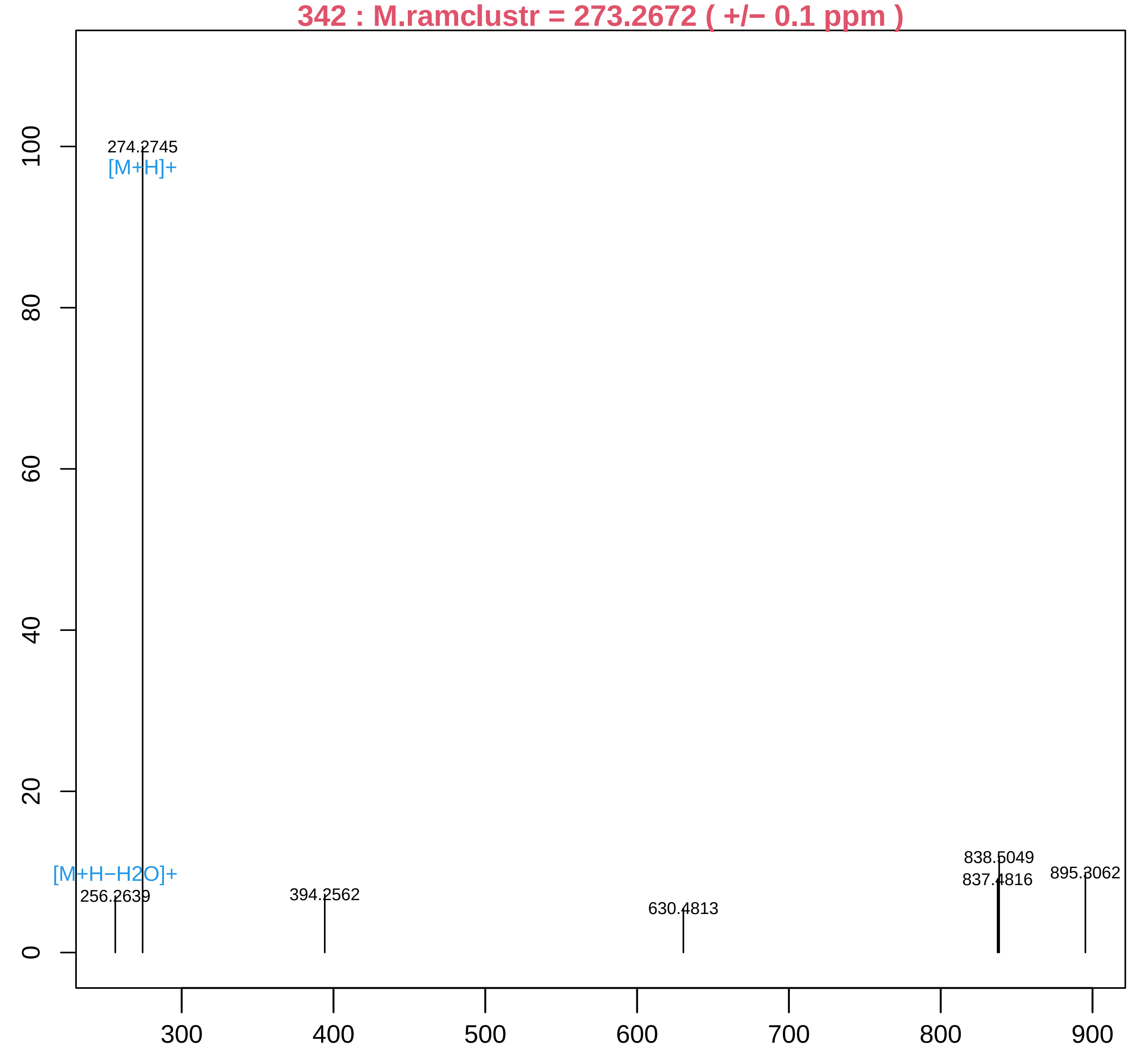

342 : M.ramclustr = 273.2672 (+/− 0.1 ppm)
274.2745
[M+H]+
[M+H−H2O]+
256.2639
394.2562
630.4813
838.5049
837.4816
895.3062
0
20
40
60
80
100
300
400
500
600
700
800
900

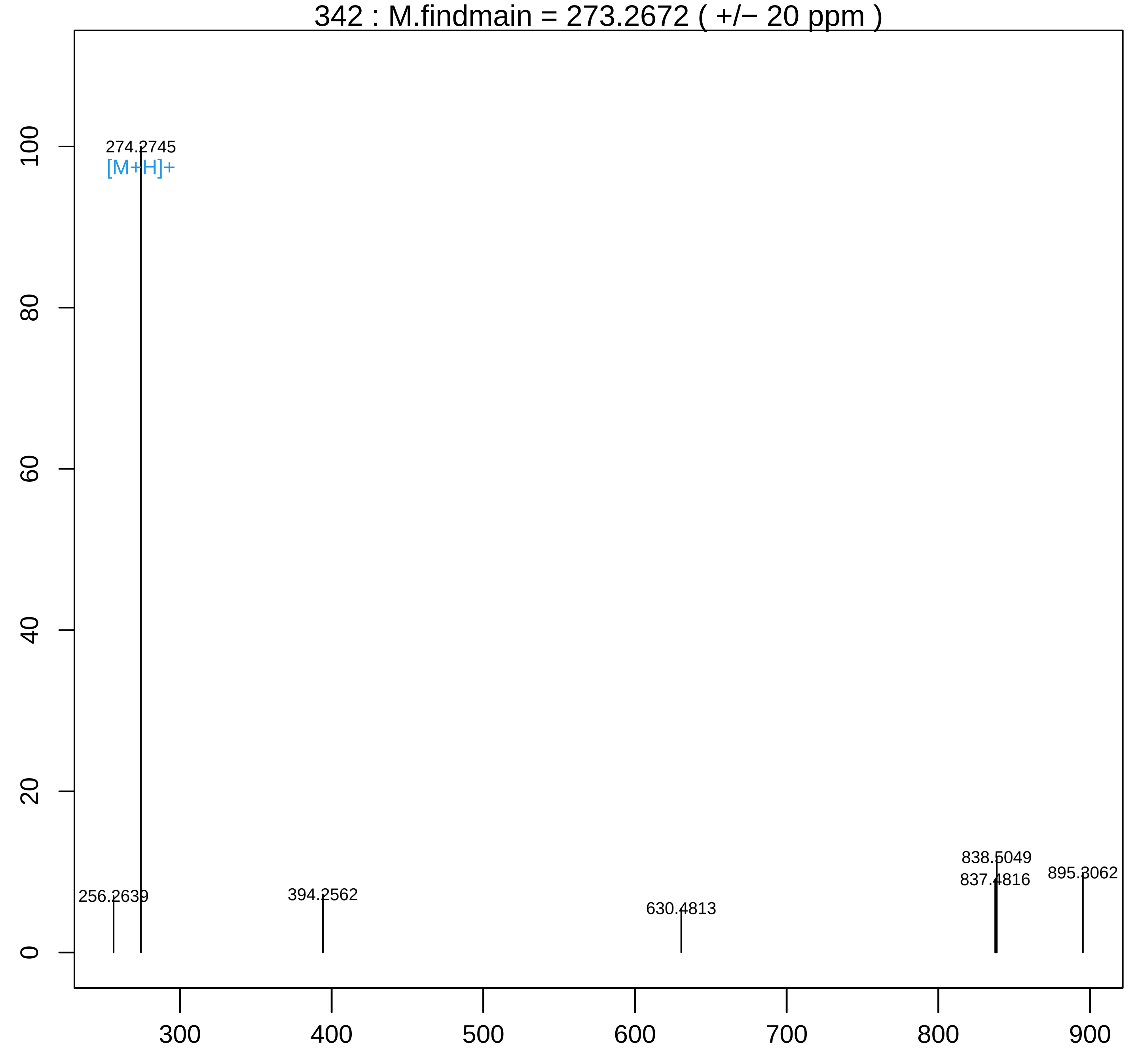

342 : M.findmain = 273.2672 (+/− 20 ppm)
274.2745
[M+H]+
256.2639
394.2562
630.4813
838.5049
837.4816
895.3062
0
20
40
60
80
100
300
400
500
600
700
800
900

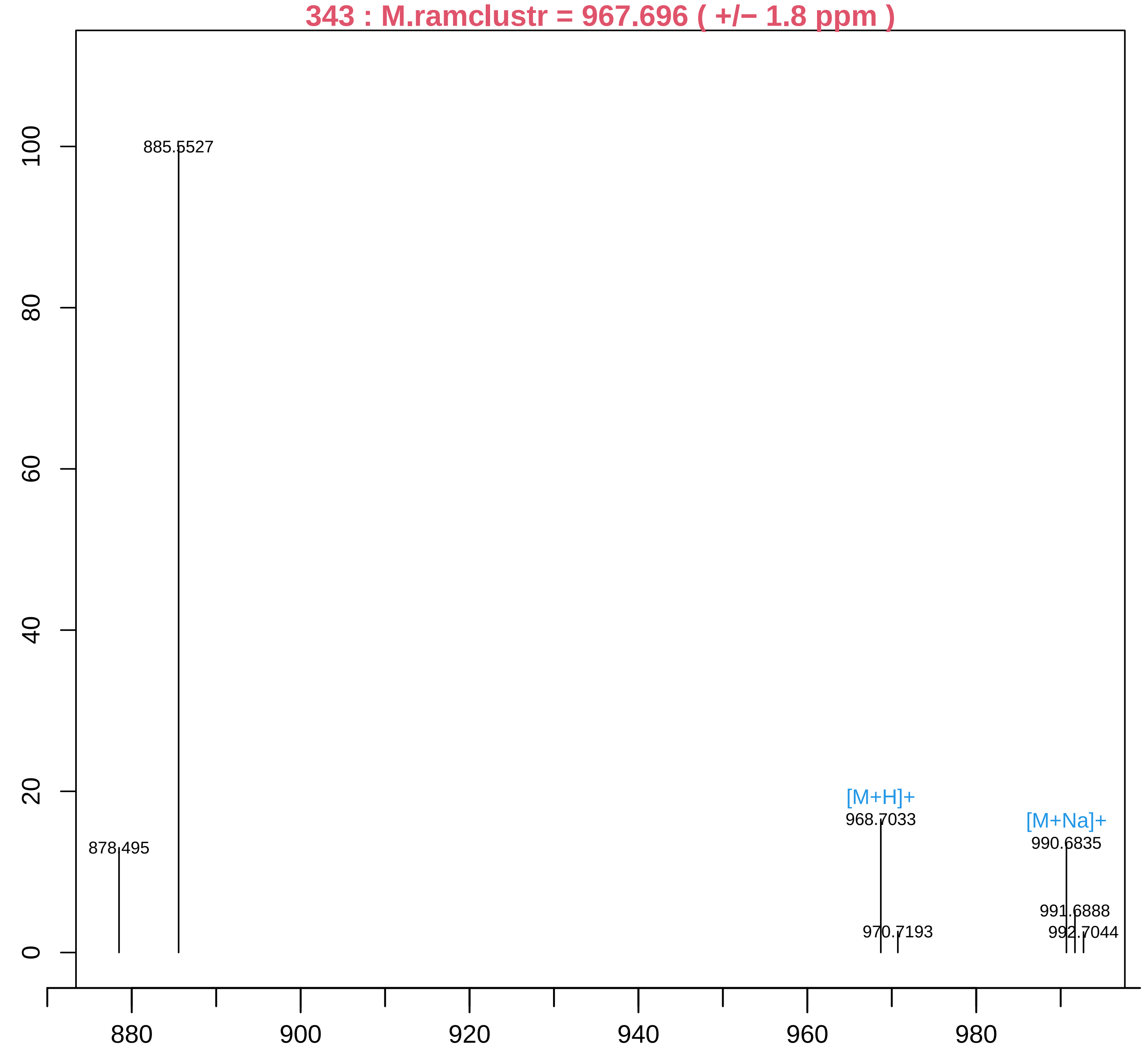
343 : M.ramclustr = 967.696 (+/− 1.8 ppm)
885.5527
878.495
[M+H]+
968.7033
970.7193
[M+Na]+
990.6835
991.6888
992.7044
0
20
40
60
80
100
880
900
920
940
960
980

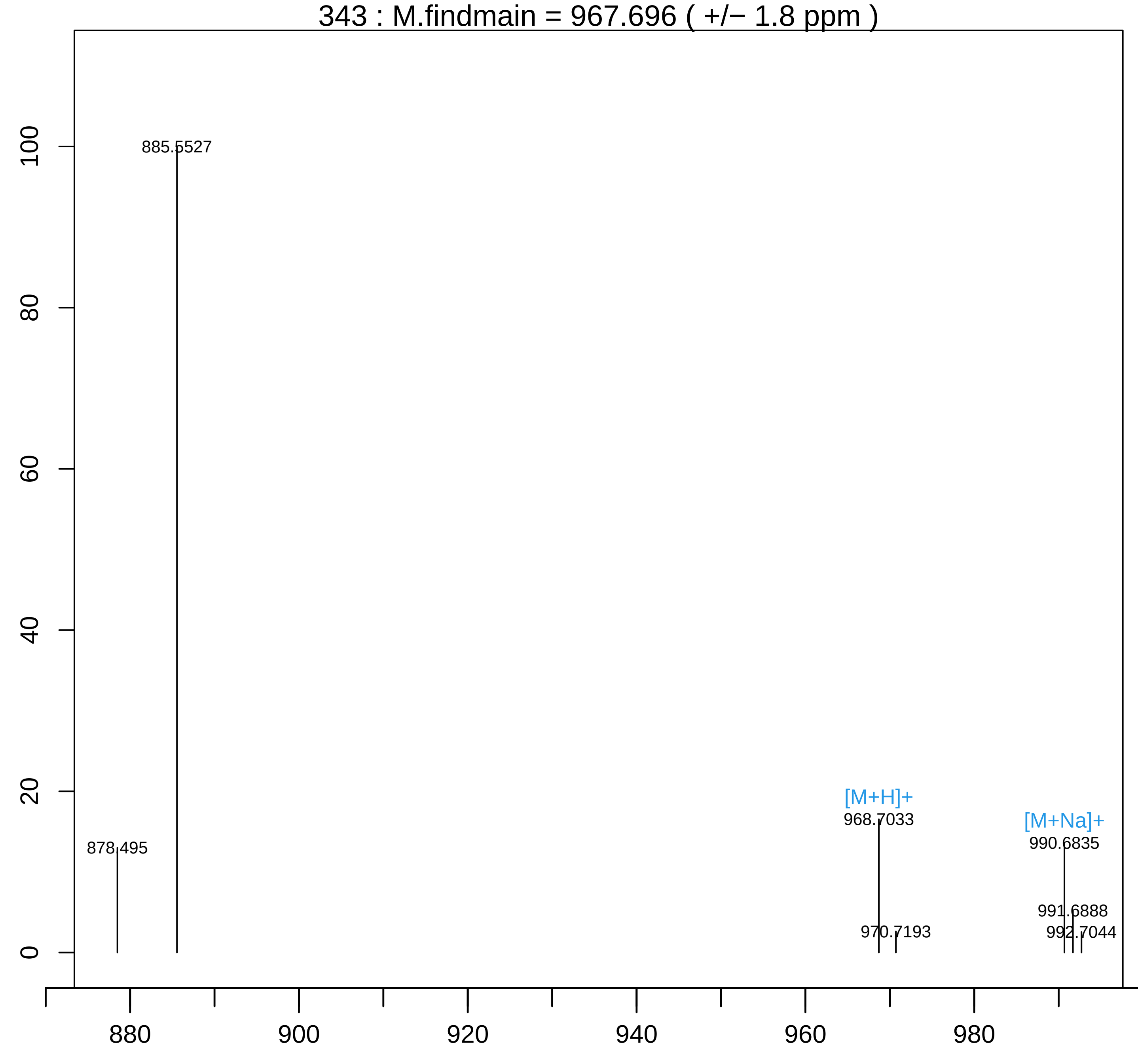
343 : M.findmain = 967.696 (+/− 1.8 ppm)
885.5527
878.495
[M+H]+
968.7033
970.7193
[M+Na]+
990.6835
991.6888
992.7044
0
20
40
60
80
100
880
900
920
940
960
980

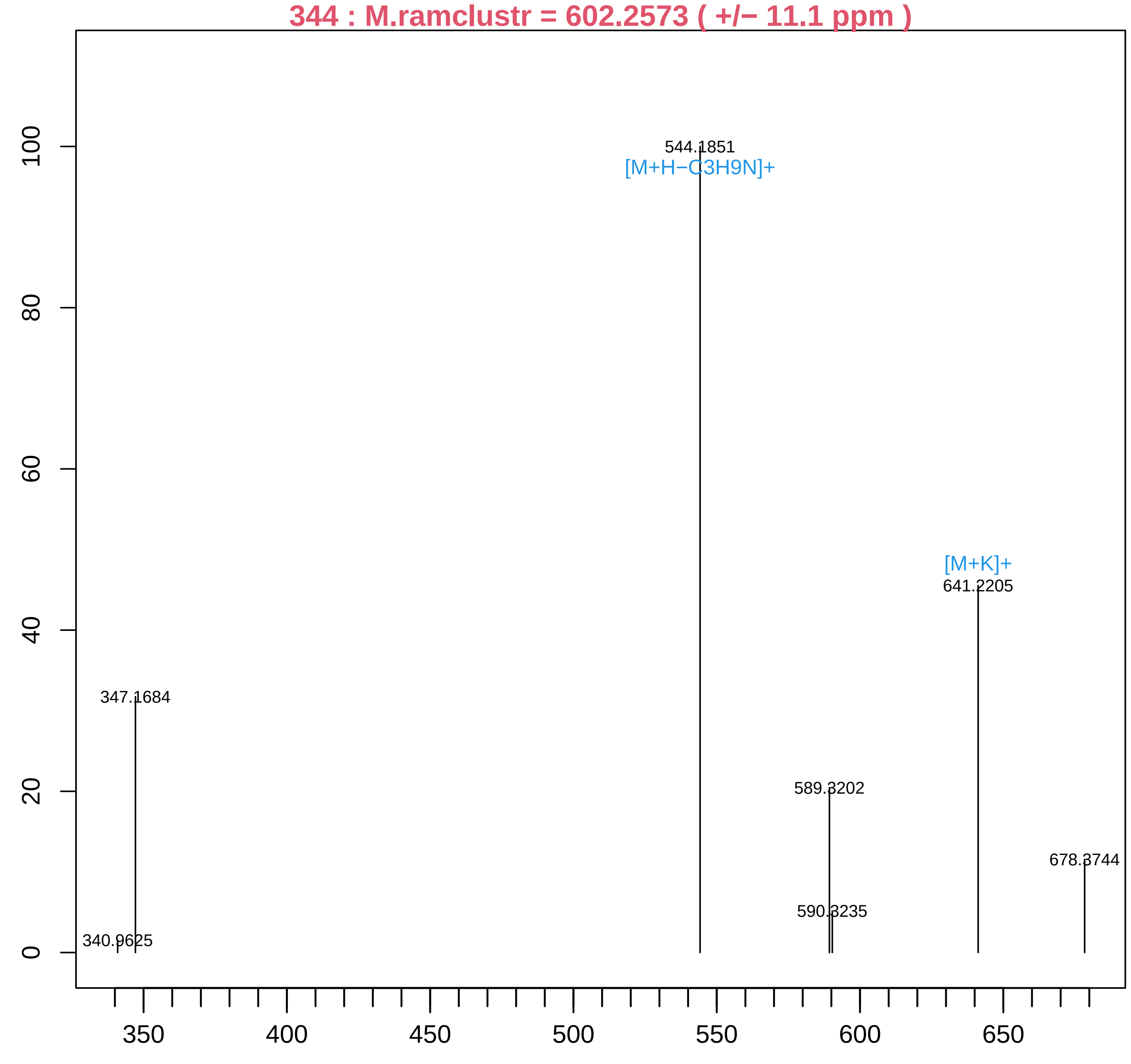
344 : M.ramclustr = 602.2573 (+/− 11.1 ppm)
544.1851
[M+H−C3H9N]+
[M+K]+
641.2205
347.1684
589.3202
678.3744
590.3235
340.9625
0
20
40
60
80
100
350
400
450
500
550
600
650

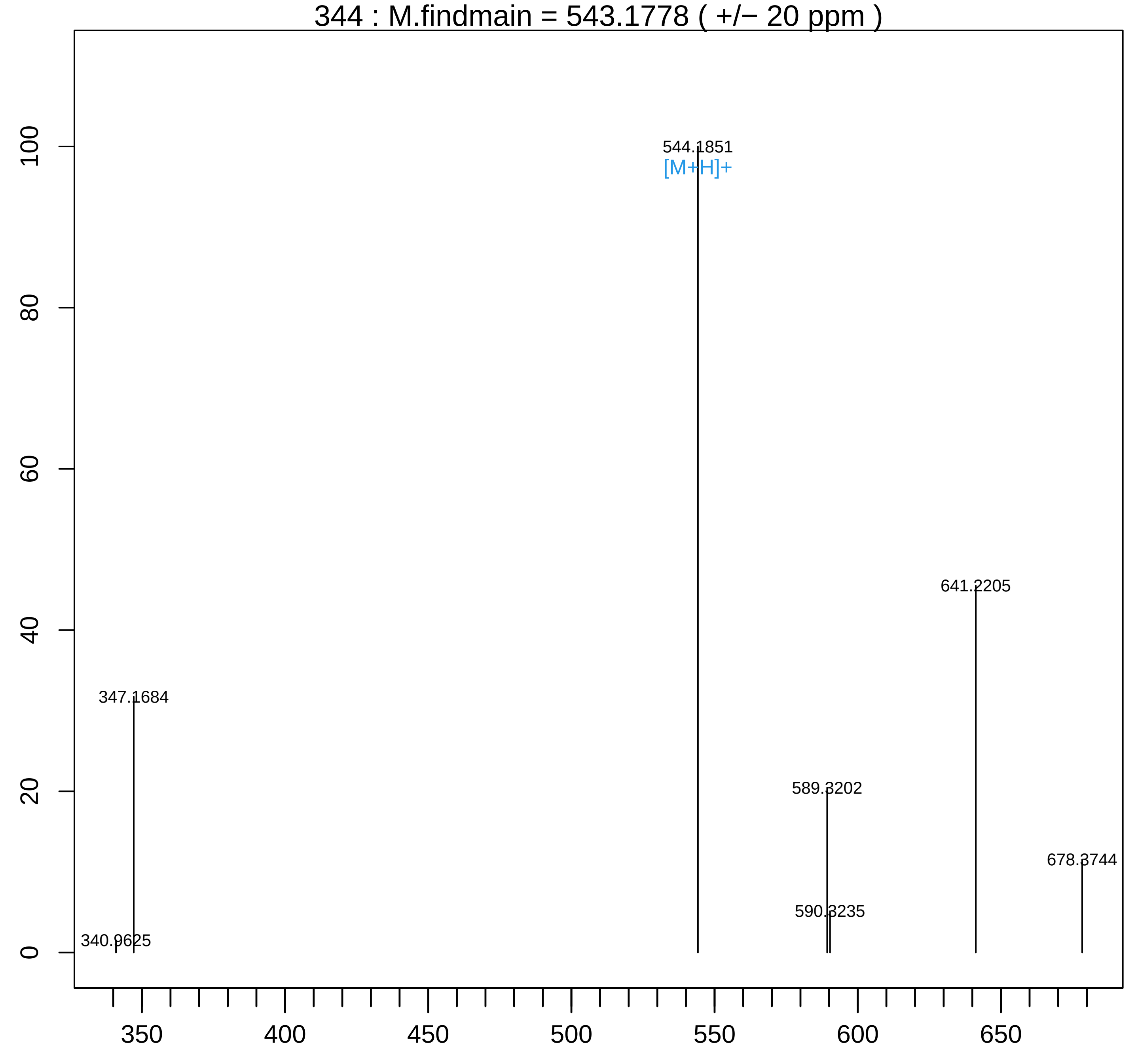
344 : M.findmain = 543.1778 (+/− 20 ppm)
544.1851
[M+H]+
641.2205
347.1684
589.3202
678.3744
590.3235
340.9625
0
20
40
60
80
100
350
400
450
500
550
600
650

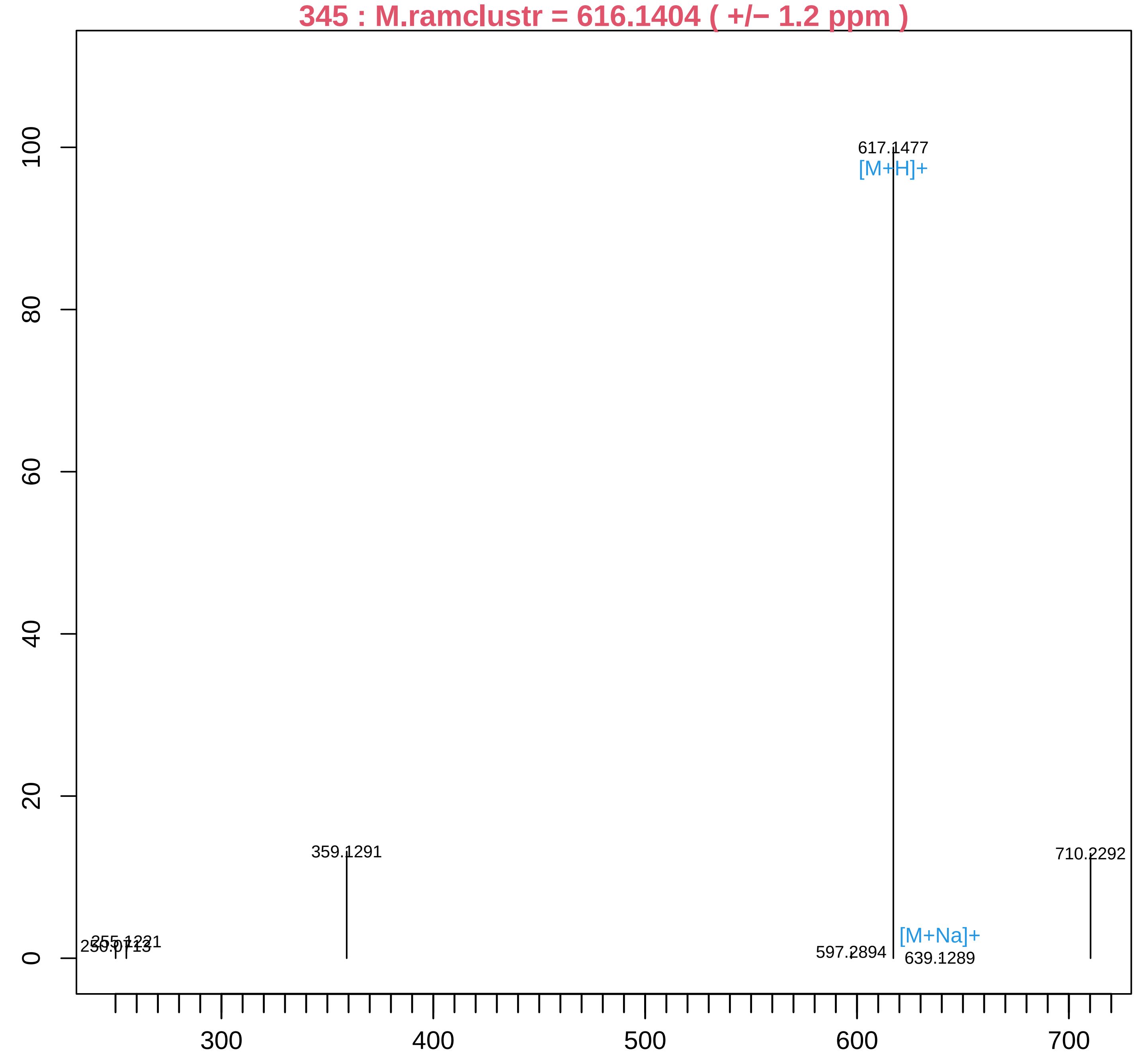
345 : M.ramclustr = 616.1404 (+/− 1.2 ppm)
617.1477
[M+H]+
359.1291
710.2292
255.1221
250.0113
597.2894
[M+Na]+
639.1289
0
20
40
60
80
100
300
400
500
600
700

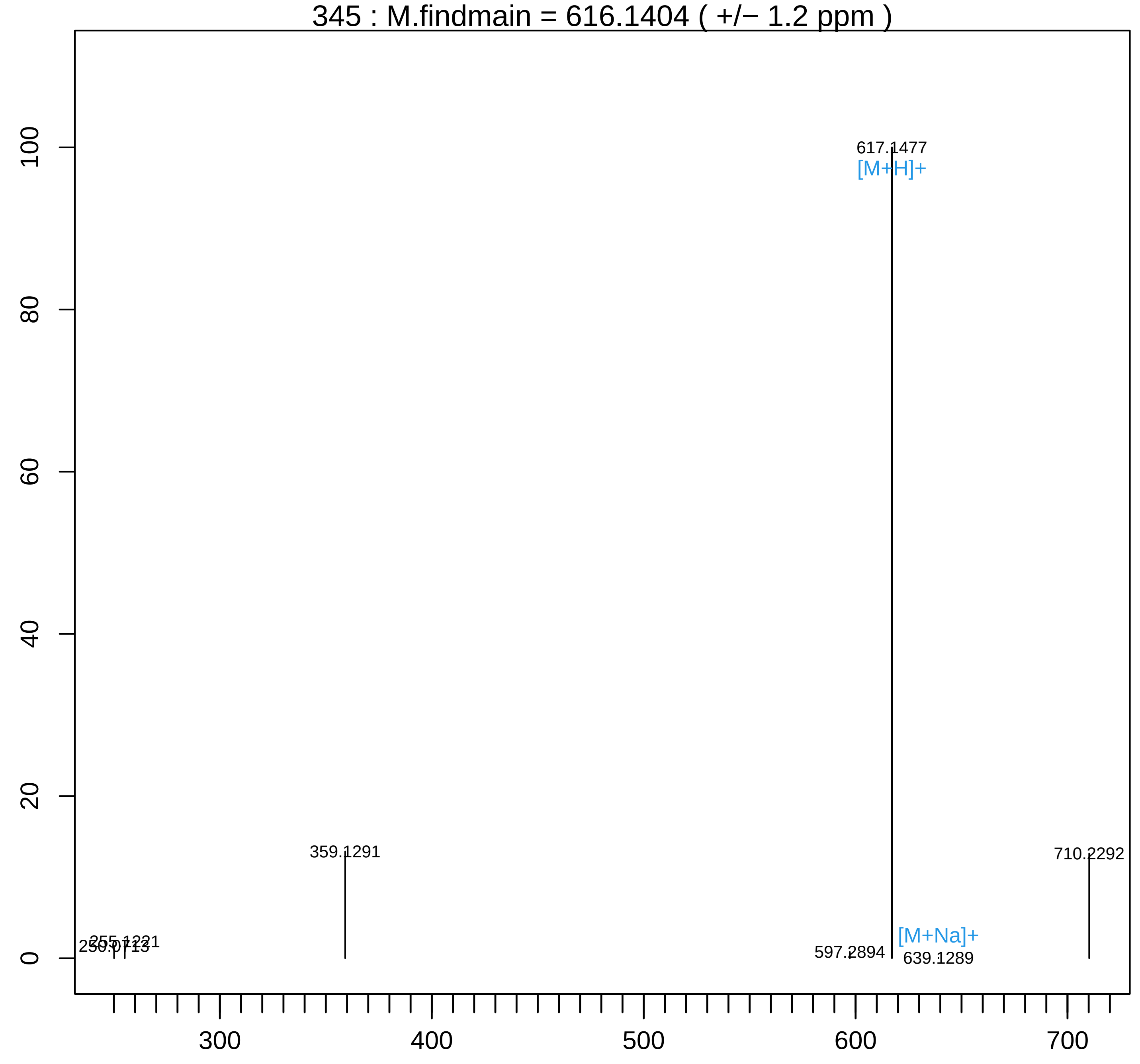
345 : M.findmain = 616.1404 (+/− 1.2 ppm)
617.1477
[M+H]+
359.1291
710.2292
255.1221
250.0113
597.2894
[M+Na]+
639.1289
0
20
40
60
80
100
300
400
500
600
700

346 : M.ramclustr = 528.1948 (+/− NA ppm)

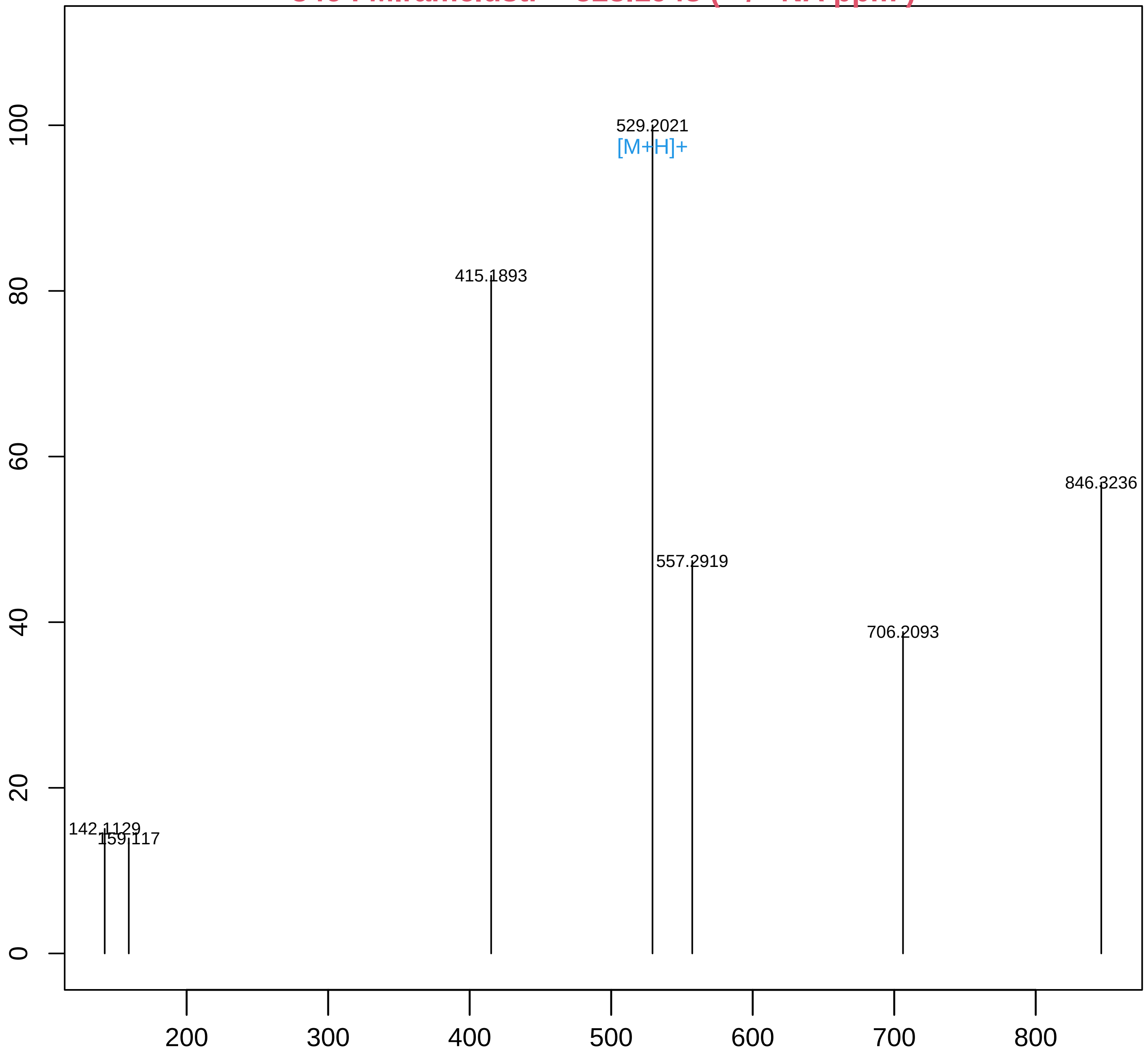

346 : M.findmain = 528.1948 (+/− 20 ppm)

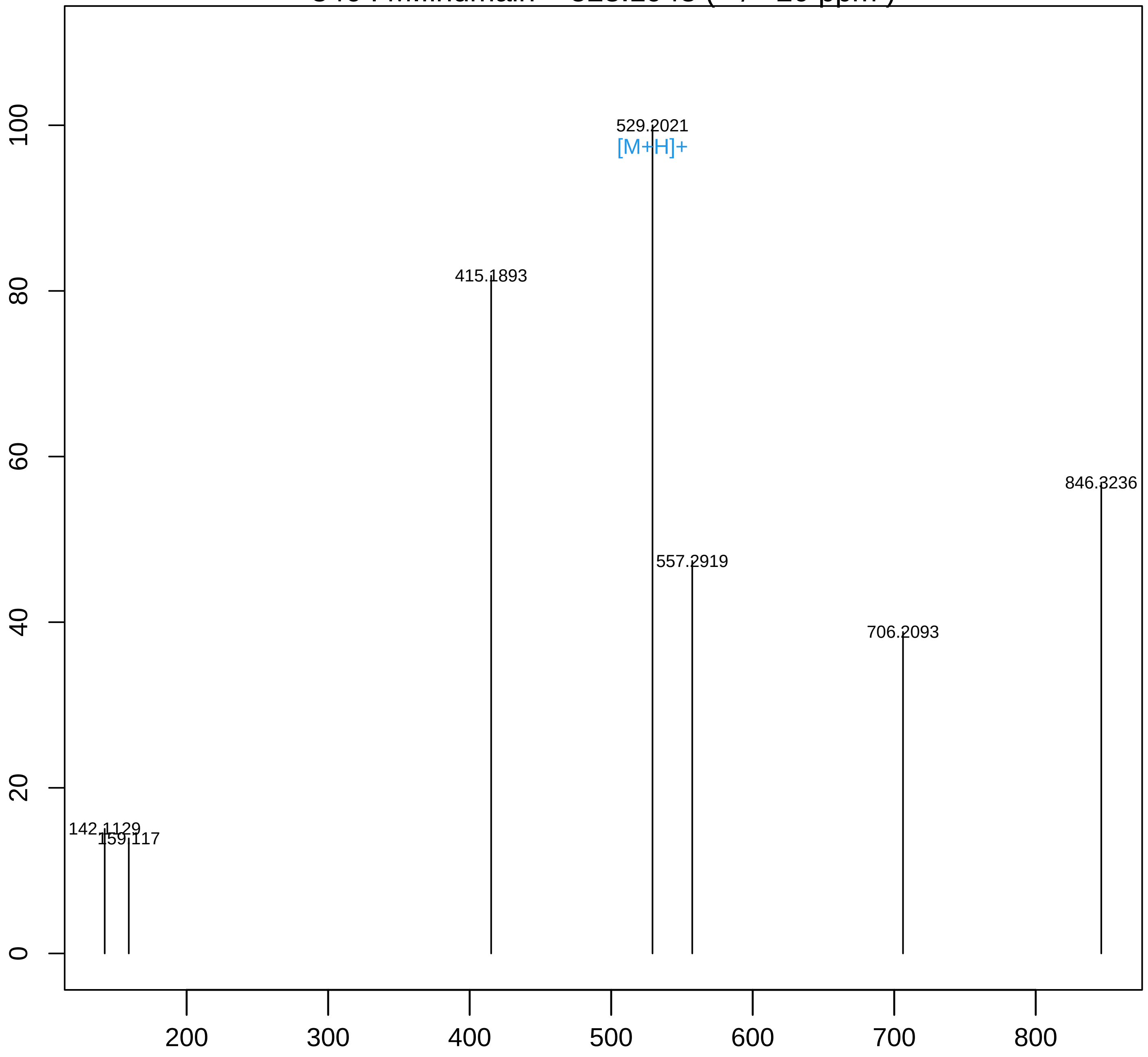

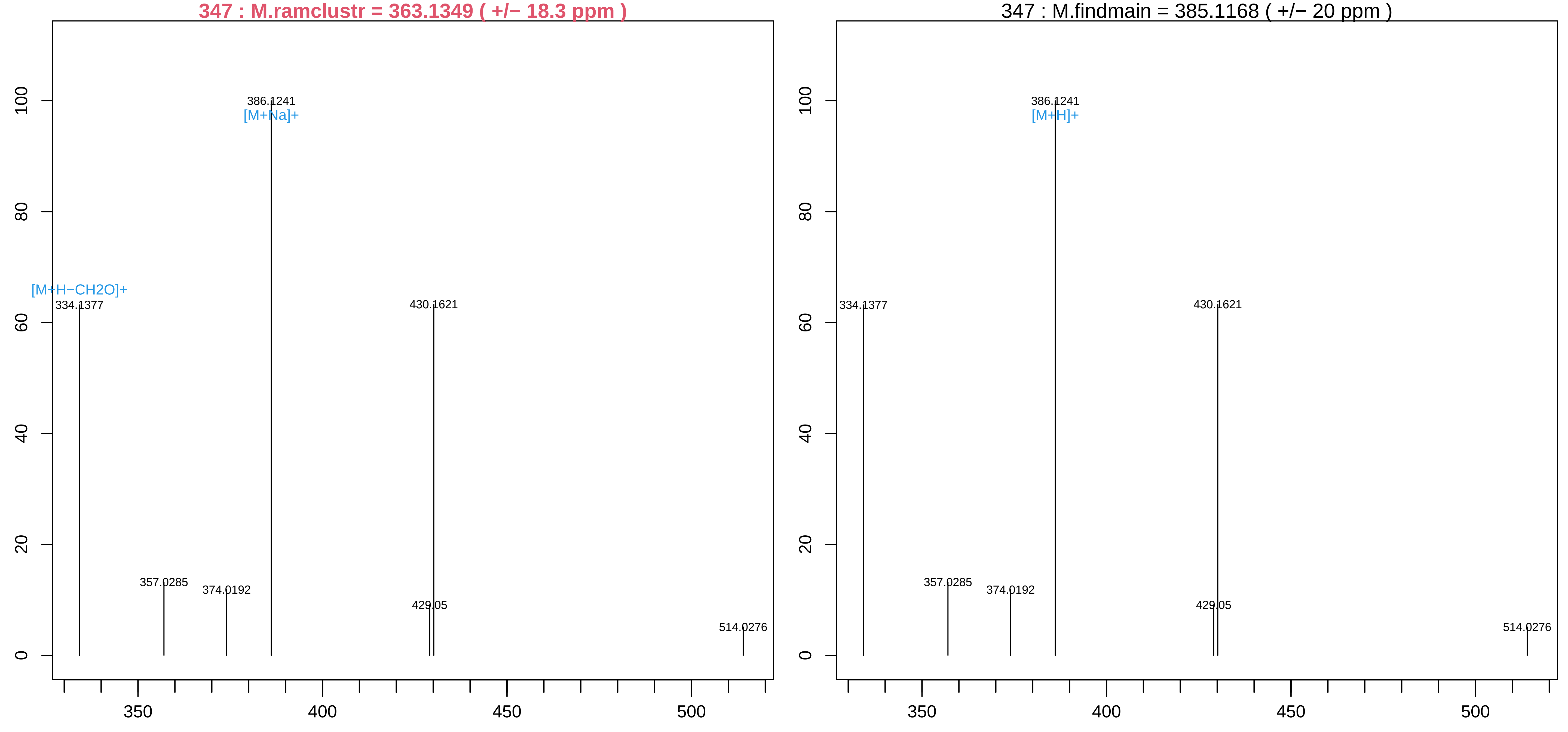
347 : M.ramclustr = 363.1349 (+/− 18.3 ppm)
386.1241
[M+Na]+
[M+H−CH2O]+
334.1377
430.1621
357.0285
374.0192
429.05
514.0276
0
20
40
60
80
100
350
400
450
500
347 : M.findmain = 385.1168 (+/− 20 ppm)
386.1241
[M+H]+
334.1377
430.1621
357.0285
374.0192
429.05
514.0276
0
20
40
60
80
100
350
400
450
500

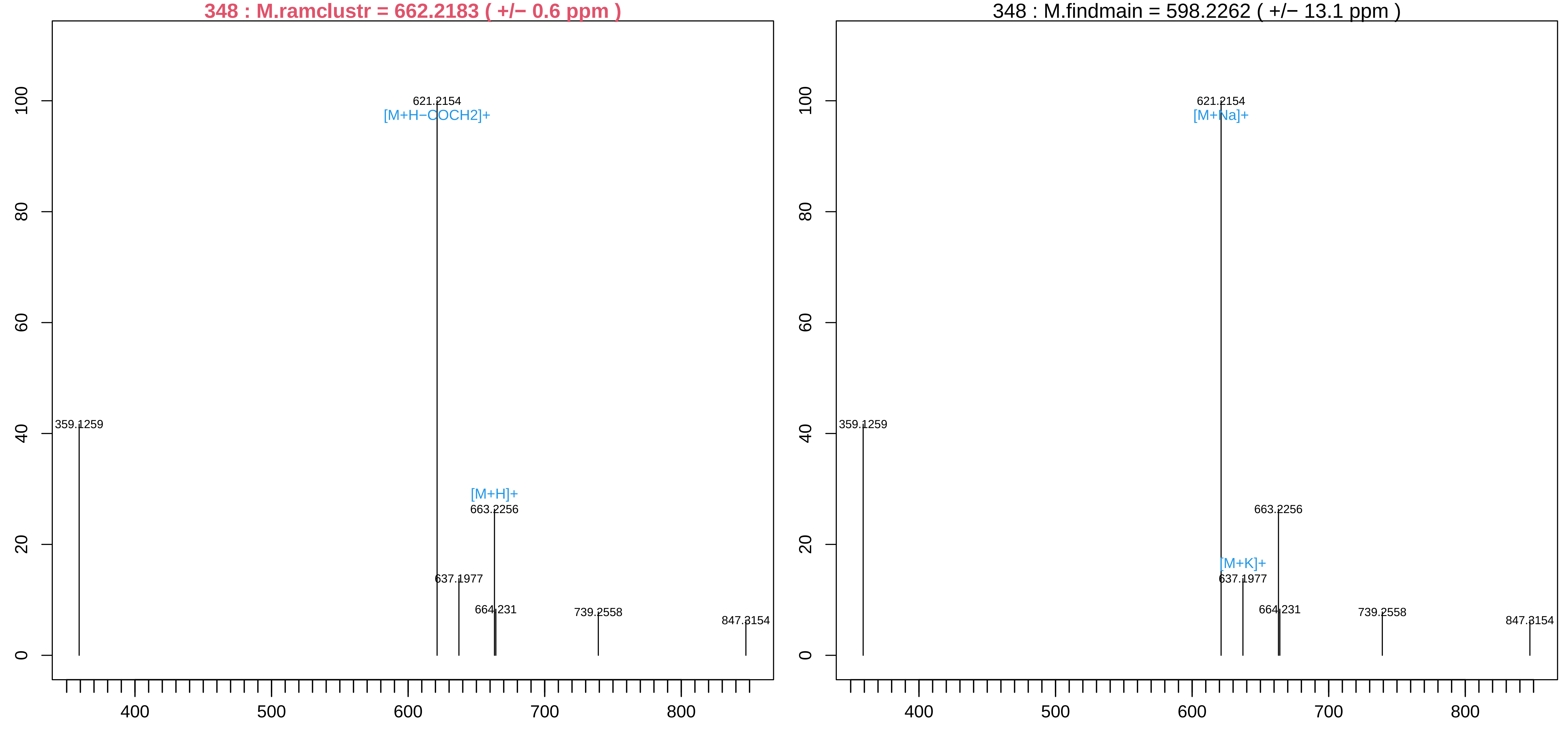
348 : M.ramclustr = 662.2183 (+/− 0.6 ppm)
621.2154
[M+H−COCH2]+
359.1259
[M+H]+
663.2256
637.1977
664.231
739.2558
847.3154
348 : M.findmain = 598.2262 (+/− 13.1 ppm)
621.2154
[M+Na]+
359.1259
663.2256
[M+K]+
637.1977
664.231
739.2558
847.3154

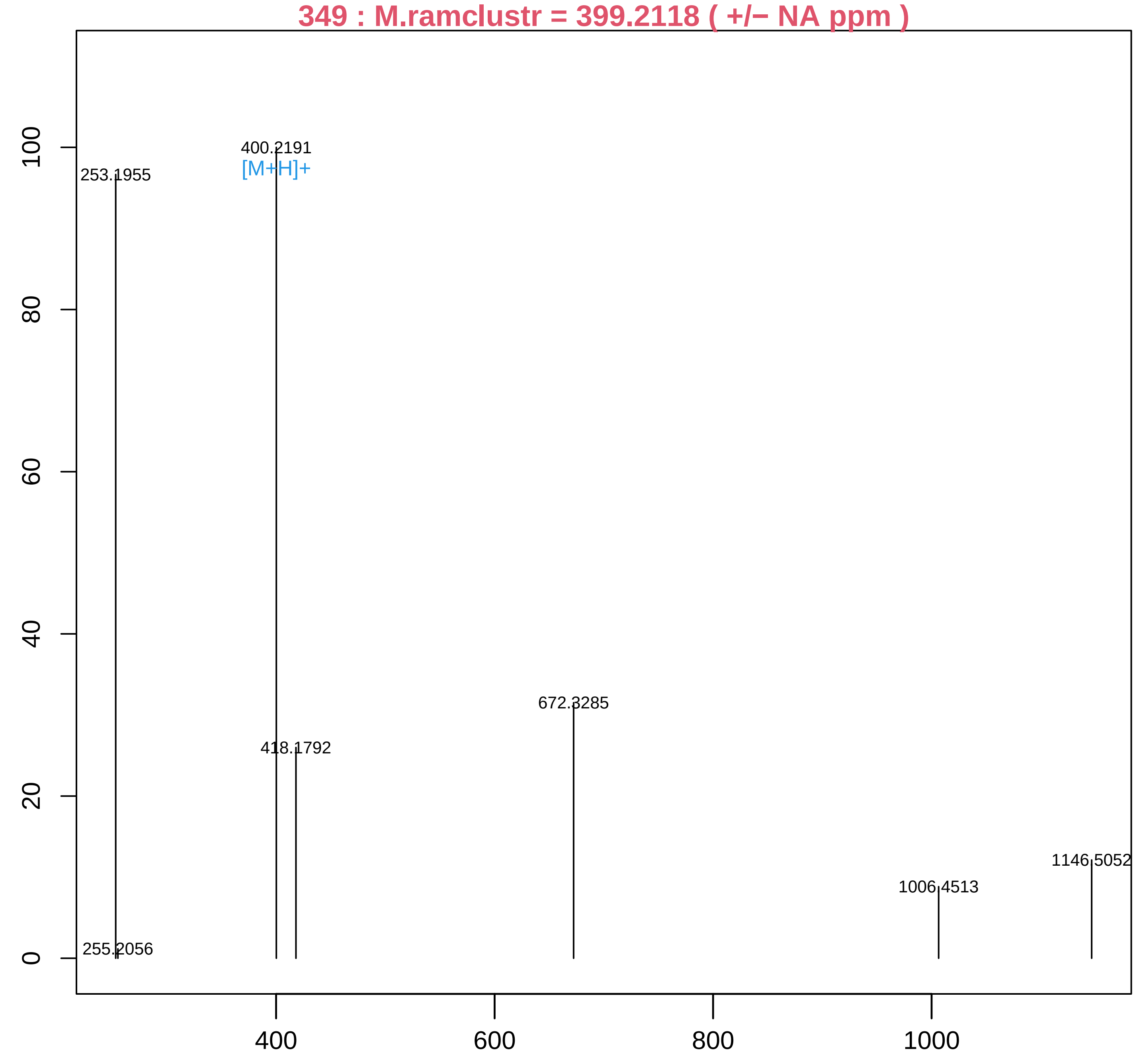
349 : M.ramclustr = 399.2118 (+/− NA ppm)
400.2191
[M+H]+
253.1955
418.1792
672.3285
1146.5052
1006.4513
255.2056
0
20
40
60
80
100
400
600
800
1000

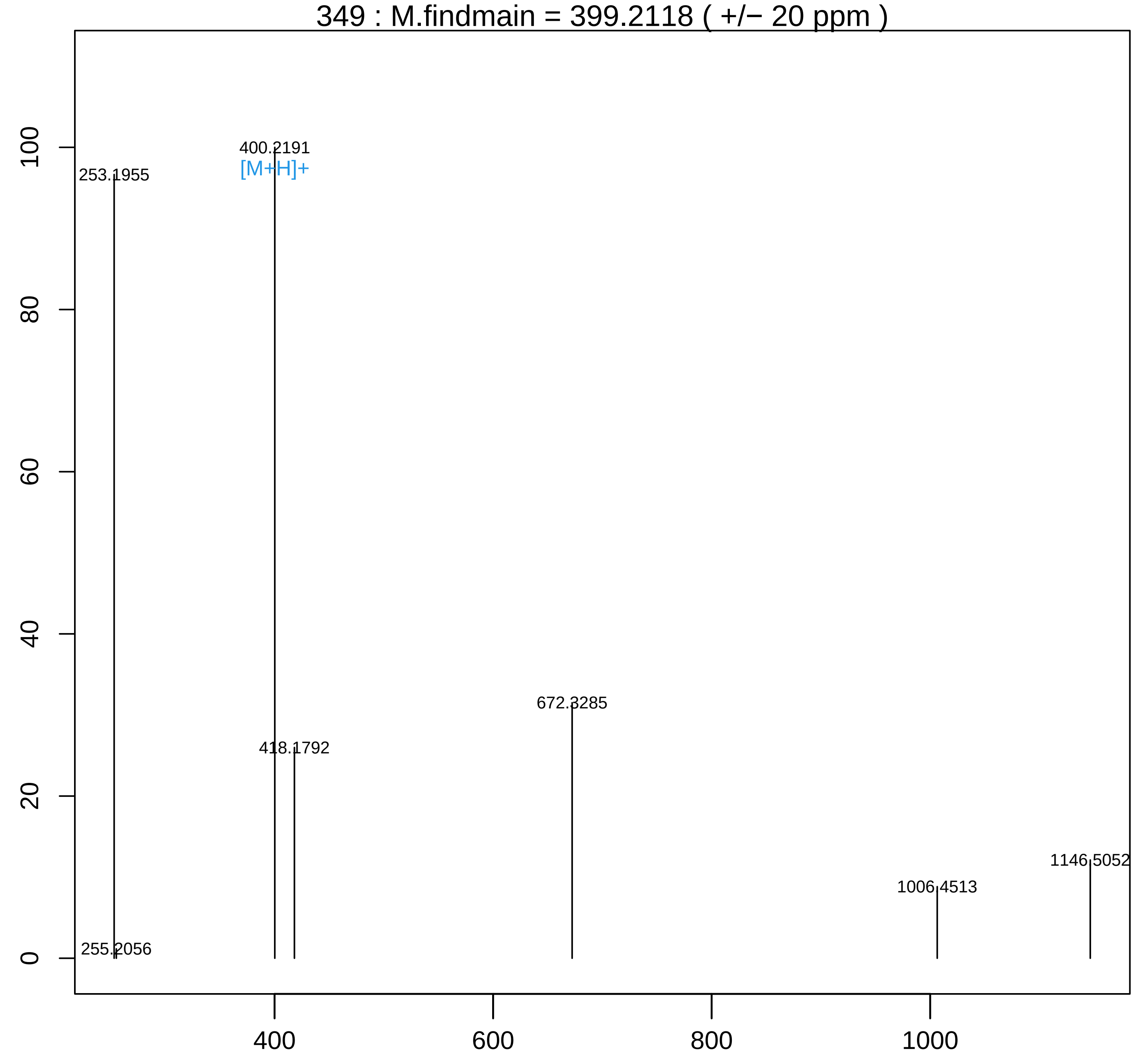
349 : M.findmain = 399.2118 (+/− 20 ppm)
400.2191
[M+H]+
253.1955
418.1792
672.3285
1146.5052
1006.4513
255.2056
0
20
40
60
80
100
400
600
800
1000

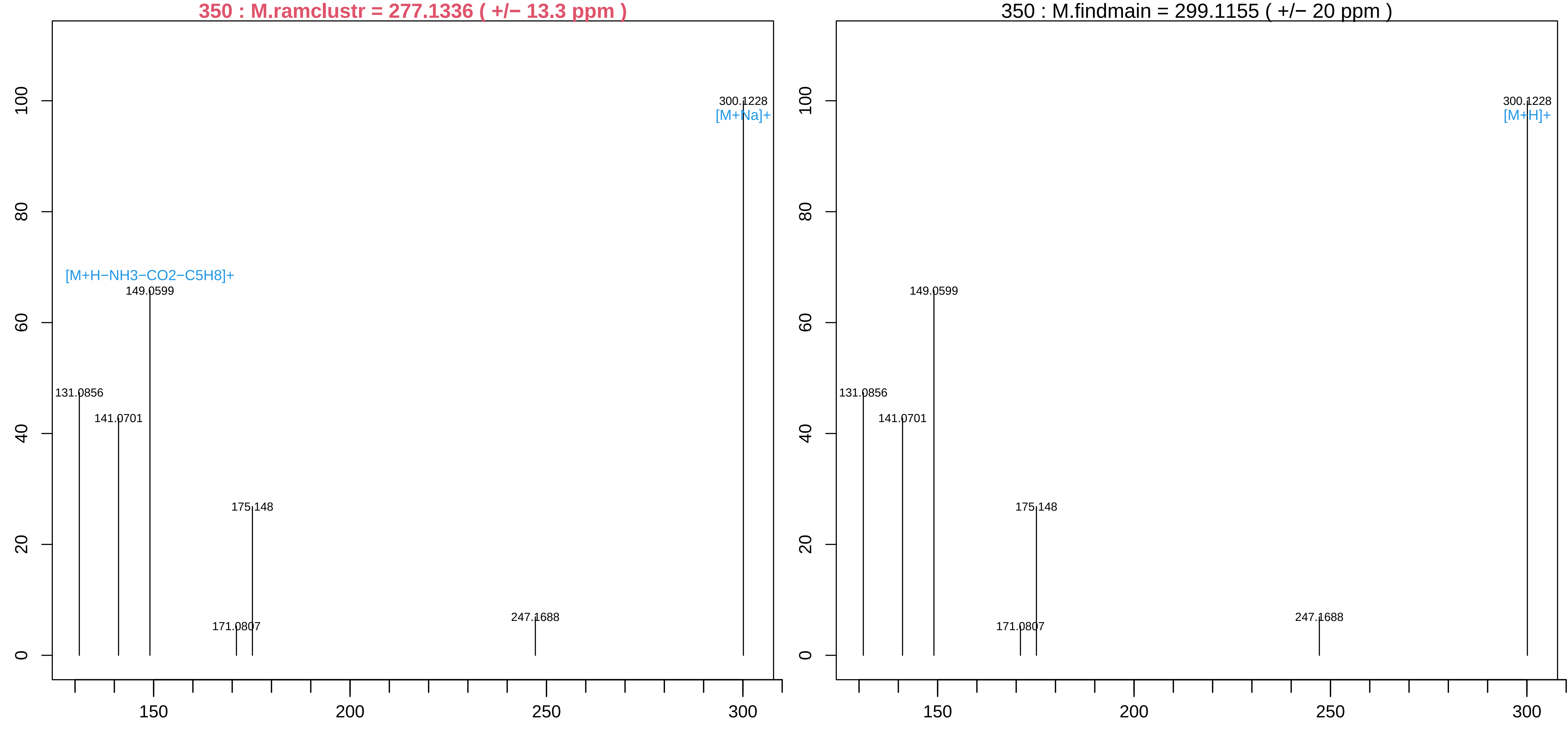

350 : M.ramclustr = 277.1336 (+/− 13.3 ppm)
300.1228
[M+Na]+
[M+H−NH3−CO2−C5H8]+
149.0599
131.0856
141.0701
175.148
171.0807
247.1688
350 : M.findmain = 299.1155 (+/− 20 ppm)
300.1228
[M+H]+
149.0599
131.0856
141.0701
175.148
171.0807
247.1688

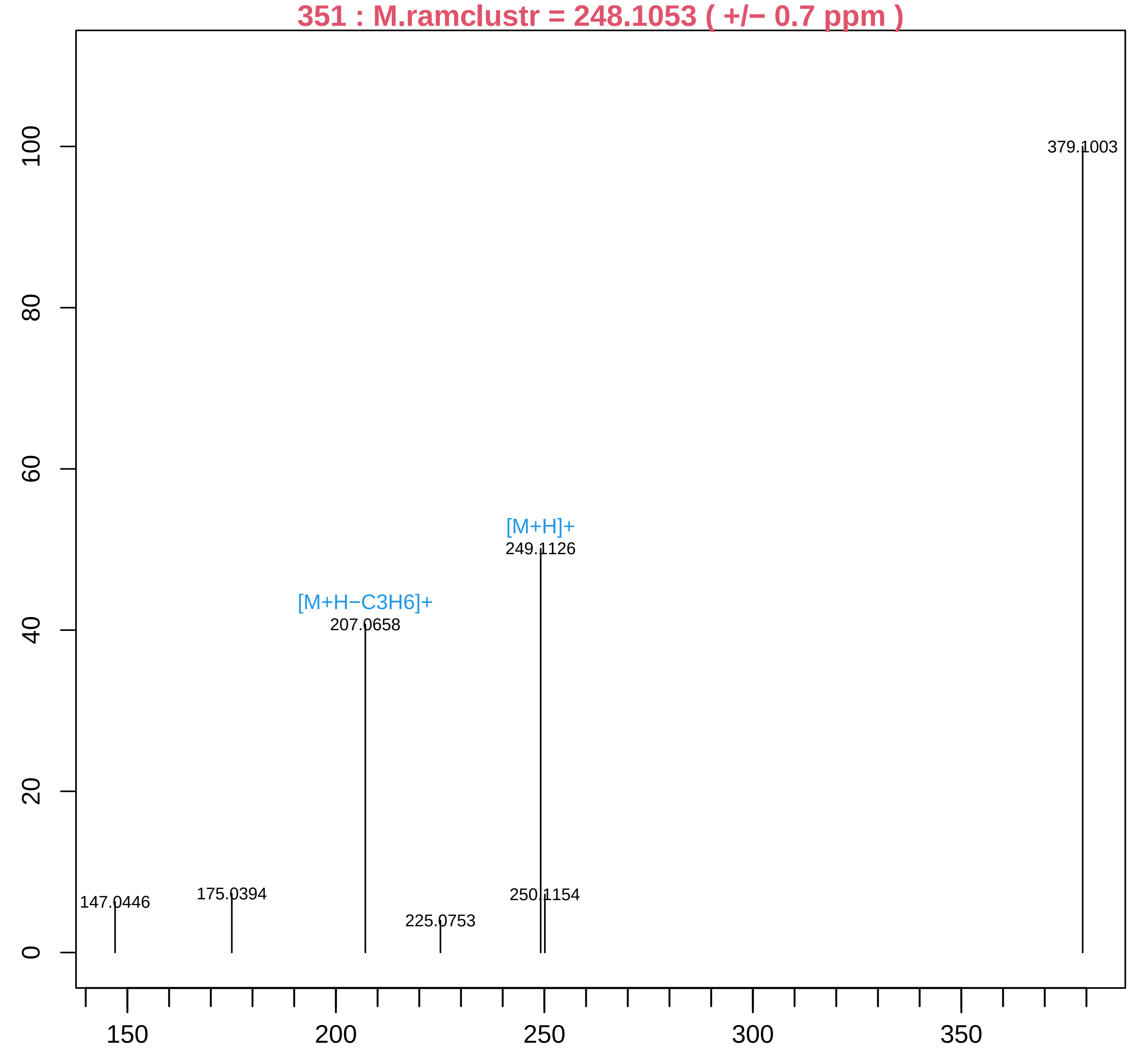
351 : M.ramclustr = 248.1053 (+/− 0.7 ppm)
379.1003
[M+H]+
249.1126
[M+H−C3H6]+
207.0658
147.0446
175.0394
225.0753
250.1154
0
20
40
60
80
100
150
200
250
300
350

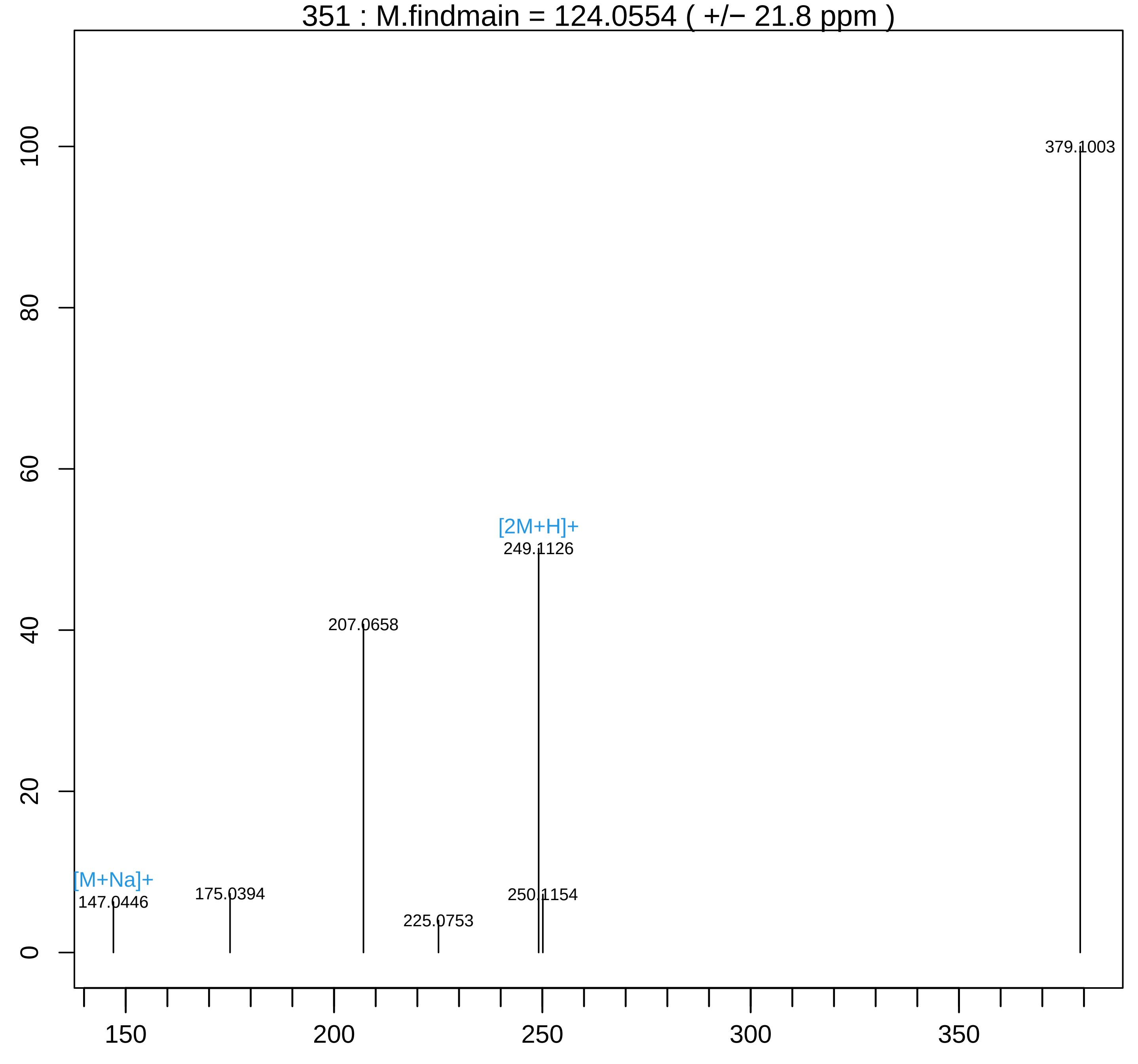
351 : M.findmain = 124.0554 (+/− 21.8 ppm)
379.1003
[2M+H]+
249.1126
207.0658
[M+Na]+
147.0446
175.0394
225.0753
250.1154
0
20
40
60
80
100
150
200
250
300
350

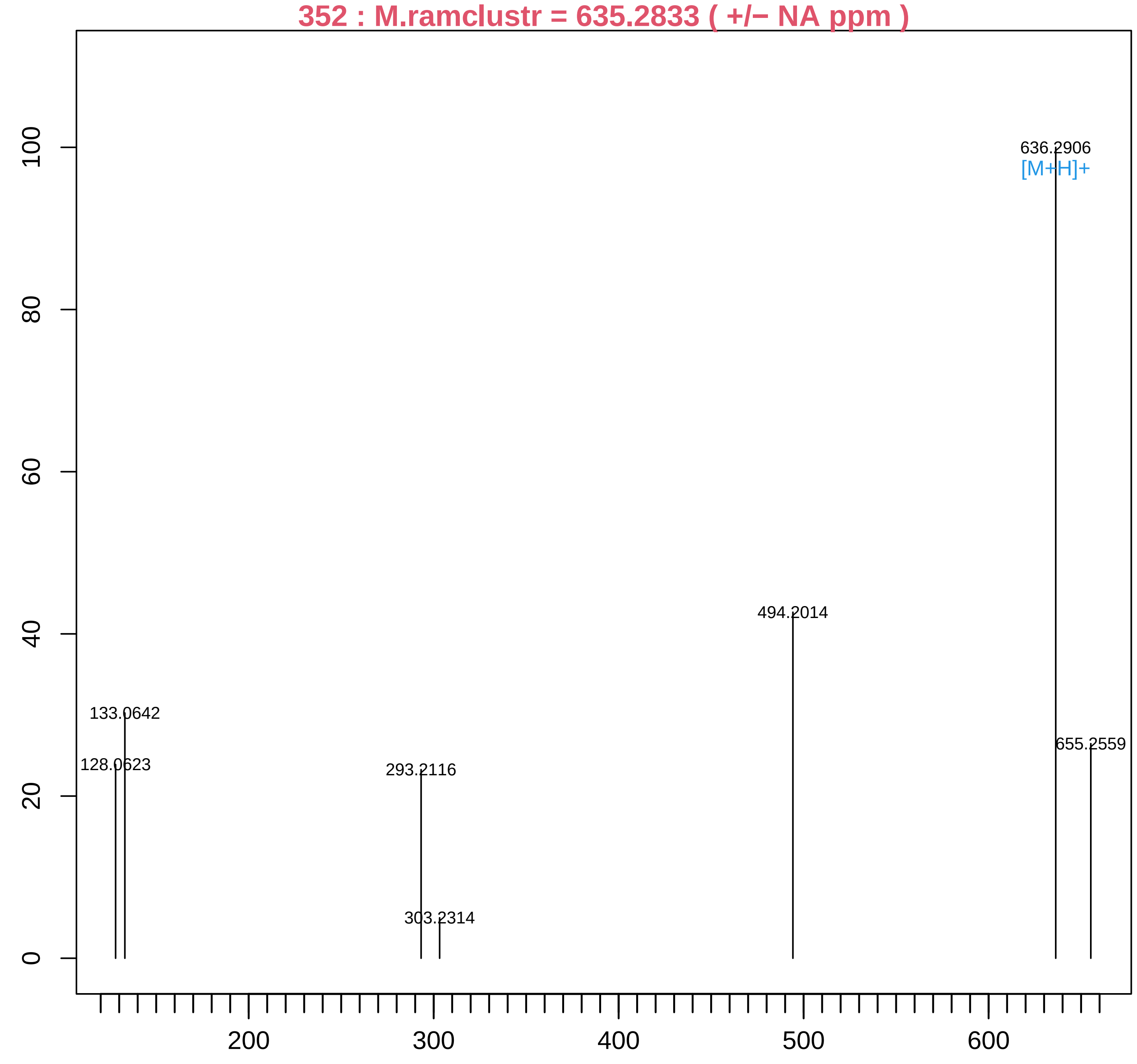

352 : M.ramclustr = 635.2833 (+/− NA ppm)
636.2906
[M+H]+
494.2014
133.0642
128.0623
293.2116
303.2314
655.2559
0
20
40
60
80
100
200
300
400
500
600

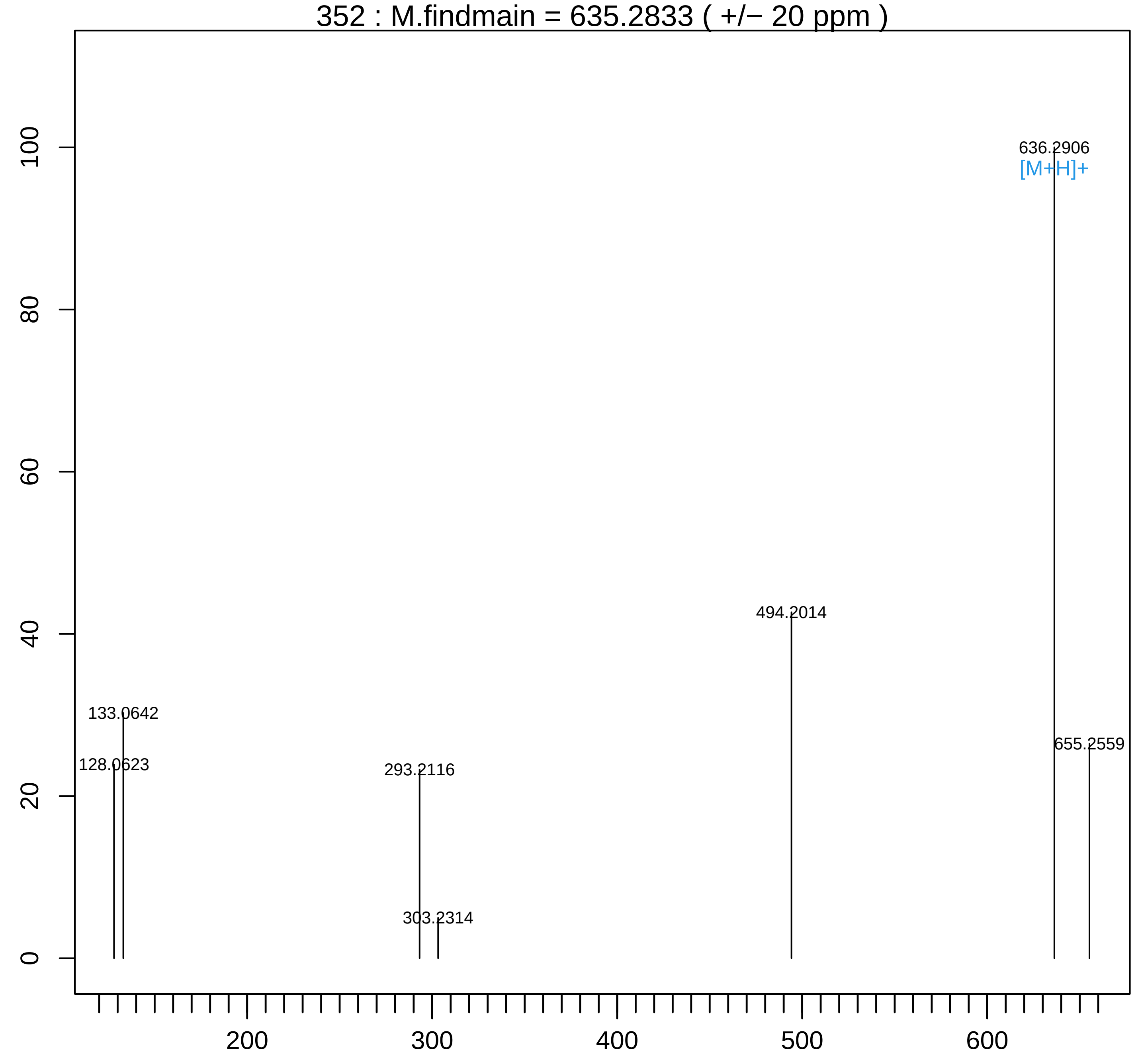

352 : M.findmain = 635.2833 (+/− 20 ppm)
636.2906
[M+H]+
494.2014
133.0642
128.0623
293.2116
303.2314
655.2559
0
20
40
60
80
100
200
300
400
500
600

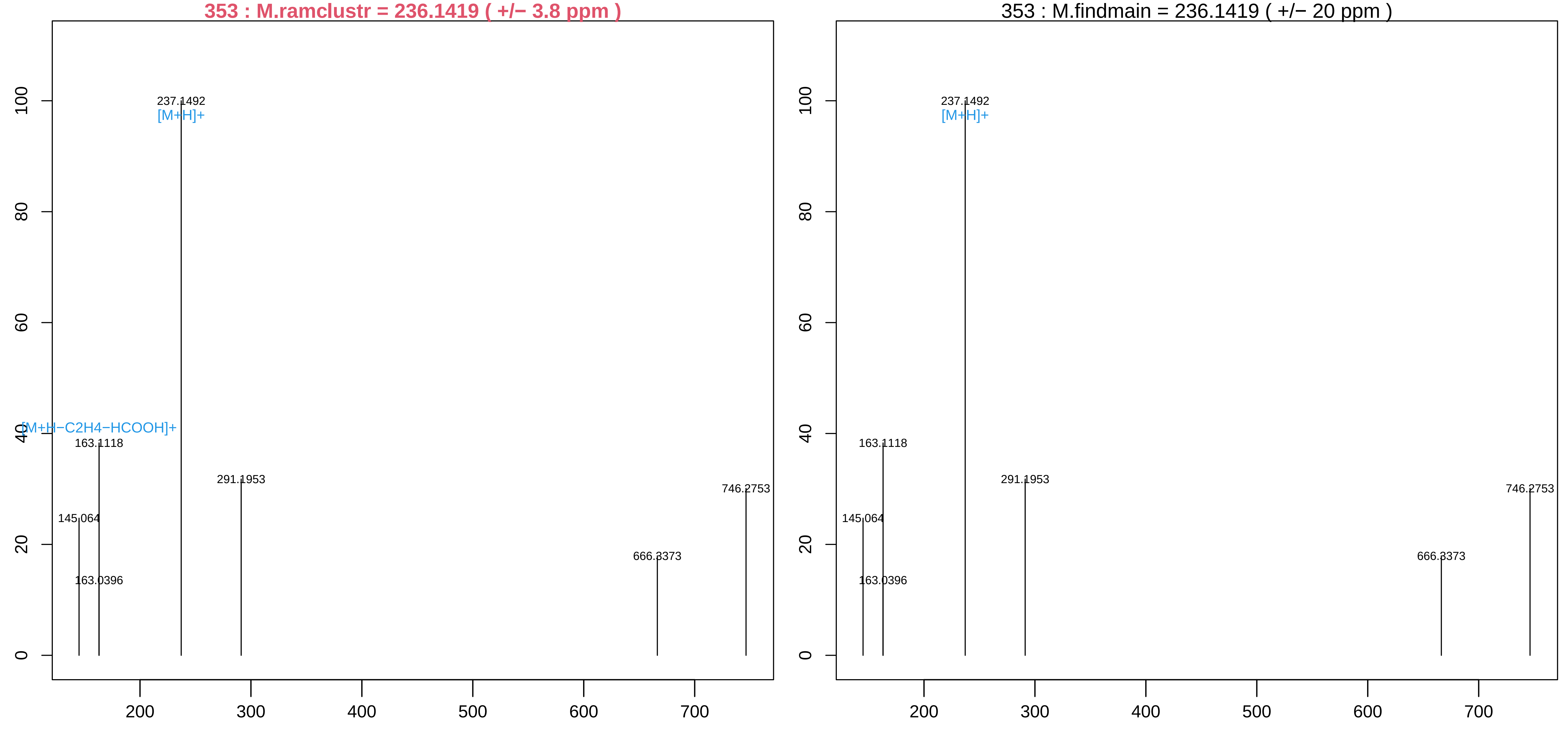
353 : M.ramclustr = 236.1419 (+/− 3.8 ppm)
237.1492
[M+H]+
[M+H−C2H4−HCOOH]+
163.1118
291.1953
746.2753
145.064
666.3373
163.0396
353 : M.findmain = 236.1419 (+/− 20 ppm)
237.1492
[M+H]+
163.1118
291.1953
746.2753
145.064
666.3373
163.0396
0
20
40
60
80
100
200
300
400
500
600
700

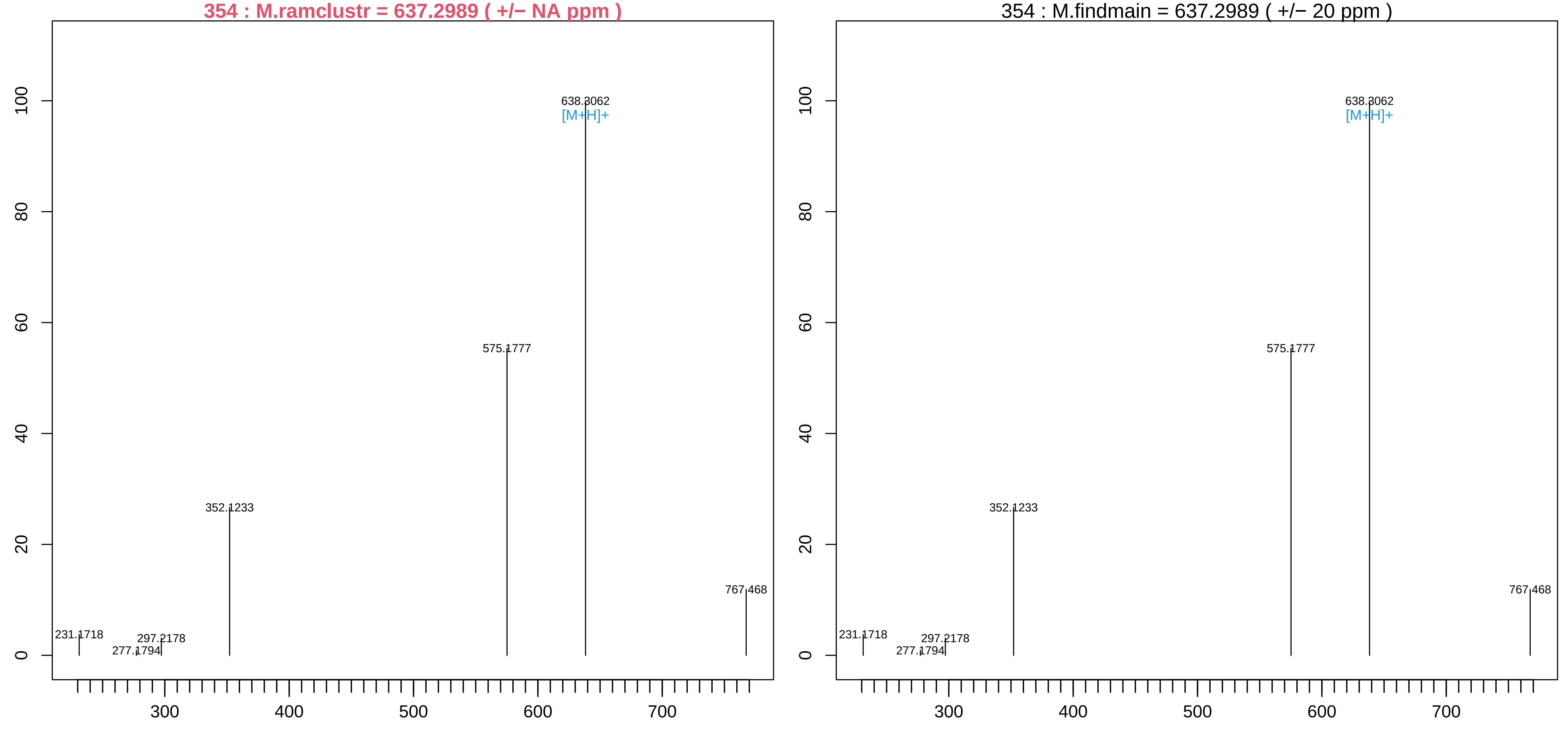
354 : M.ramclustr = 637.2989 (+/− NA ppm)
354 : M.findmain = 637.2989 (+/− 20 ppm)
638.3062
[M+H]+
575.1777
352.1233
767.468
231.1718
297.2178
277.1794
0
20
40
60
80
100
300
400
500
600
700

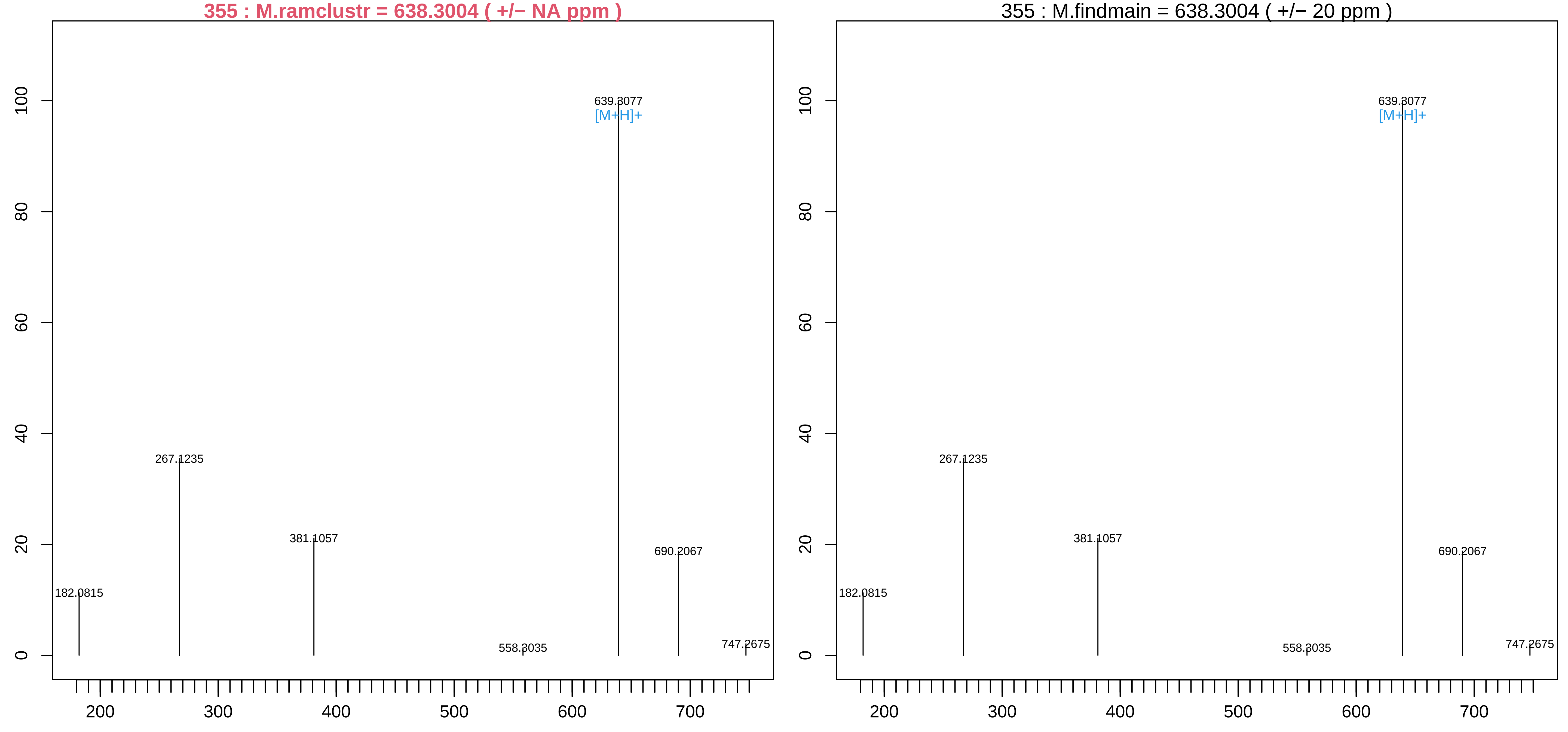
355 : M.ramclustr = 638.3004 (+/− NA ppm)
355 : M.findmain = 638.3004 (+/− 20 ppm)
639.3077
[M+H]+
267.1235
381.1057
690.2067
182.0815
558.3035
747.2675
0
20
40
60
80
100
200
300
400
500
600
700

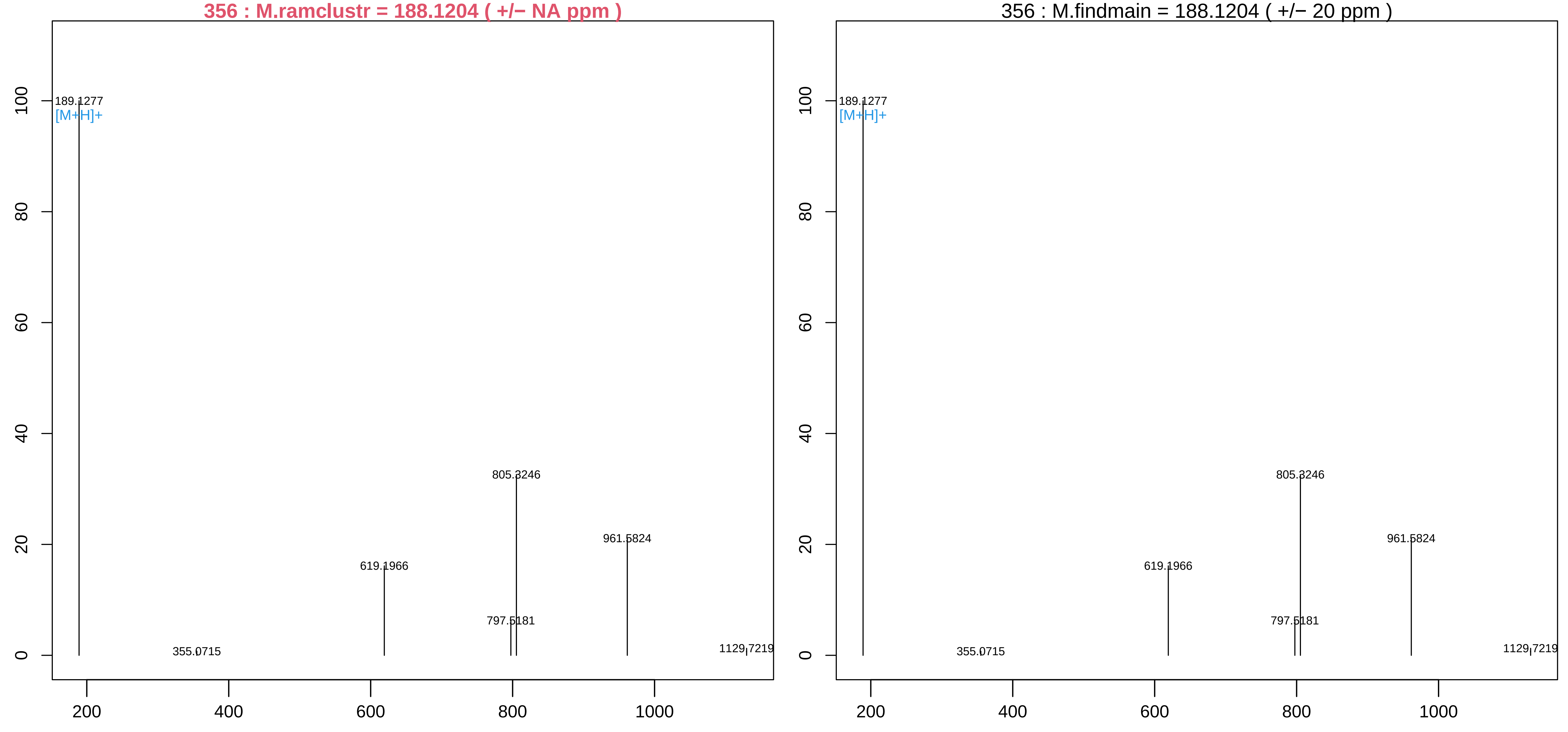
356 : M.ramclustr = 188.1204 (+/− NA ppm)
189.1277
[M+H]+
805.3246
961.5824
619.1966
797.5181
355.0715
1129.7219
356 : M.findmain = 188.1204 (+/− 20 ppm)
189.1277
[M+H]+
805.3246
961.5824
619.1966
797.5181
355.0715
1129.7219
0
20
40
60
80
100
200
400
600
800
1000

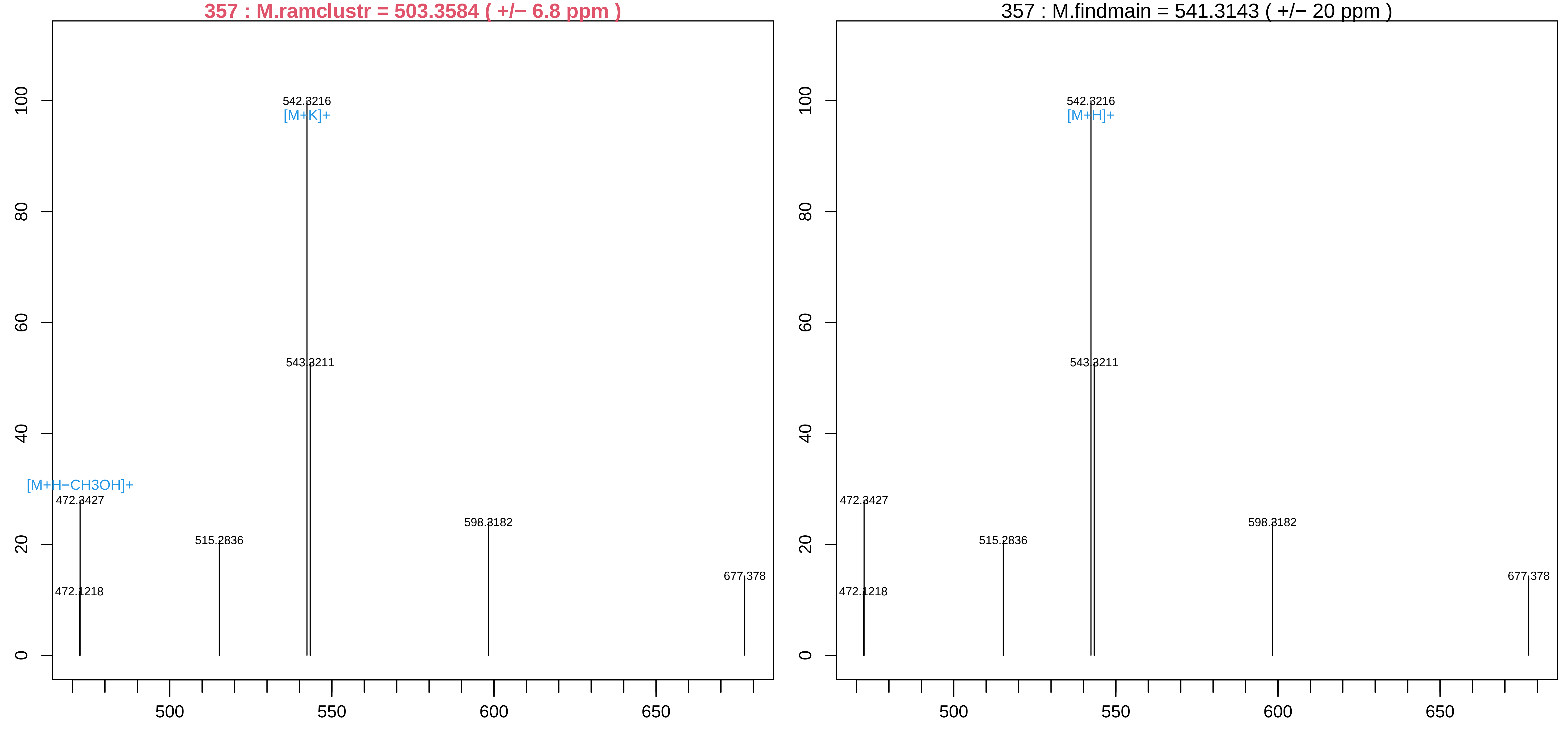
357 : M.ramclustr = 503.3584 (+/− 6.8 ppm)
542.3216
[M+K]+
543.3211
[M+H−CH3OH]+
472.3427
472.1218
515.2836
598.3182
677.378
357 : M.findmain = 541.3143 (+/− 20 ppm)
542.3216
[M+H]+
543.3211
472.3427
472.1218
515.2836
598.3182
677.378

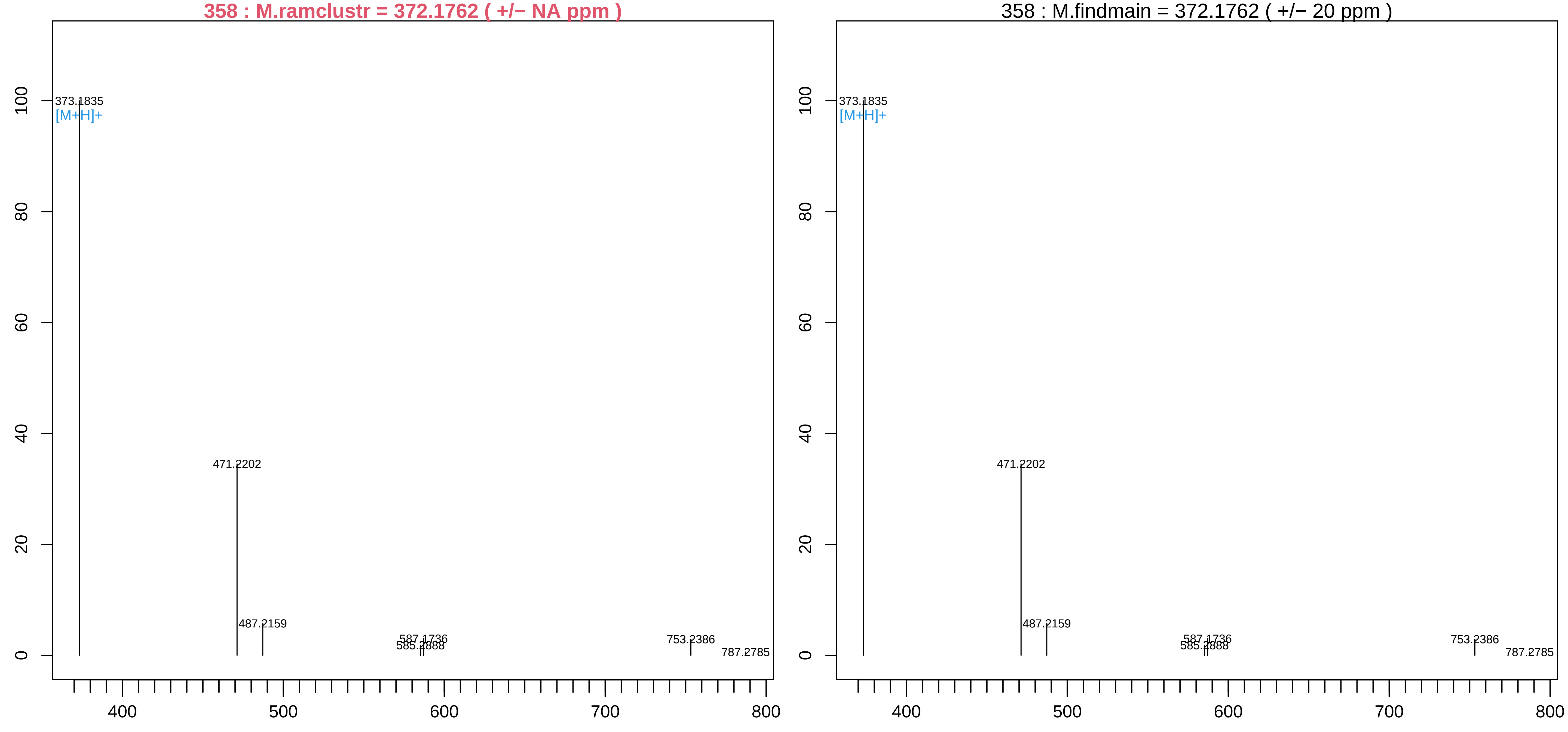
358 : M.ramclustr = 372.1762 (+/− NA ppm)
358 : M.findmain = 372.1762 (+/− 20 ppm)
373.1835
[M+H]+
471.2202
487.2159
587.1736
585.2888
753.2386
787.2785
0
20
40
60
80
100
400
500
600
700
800

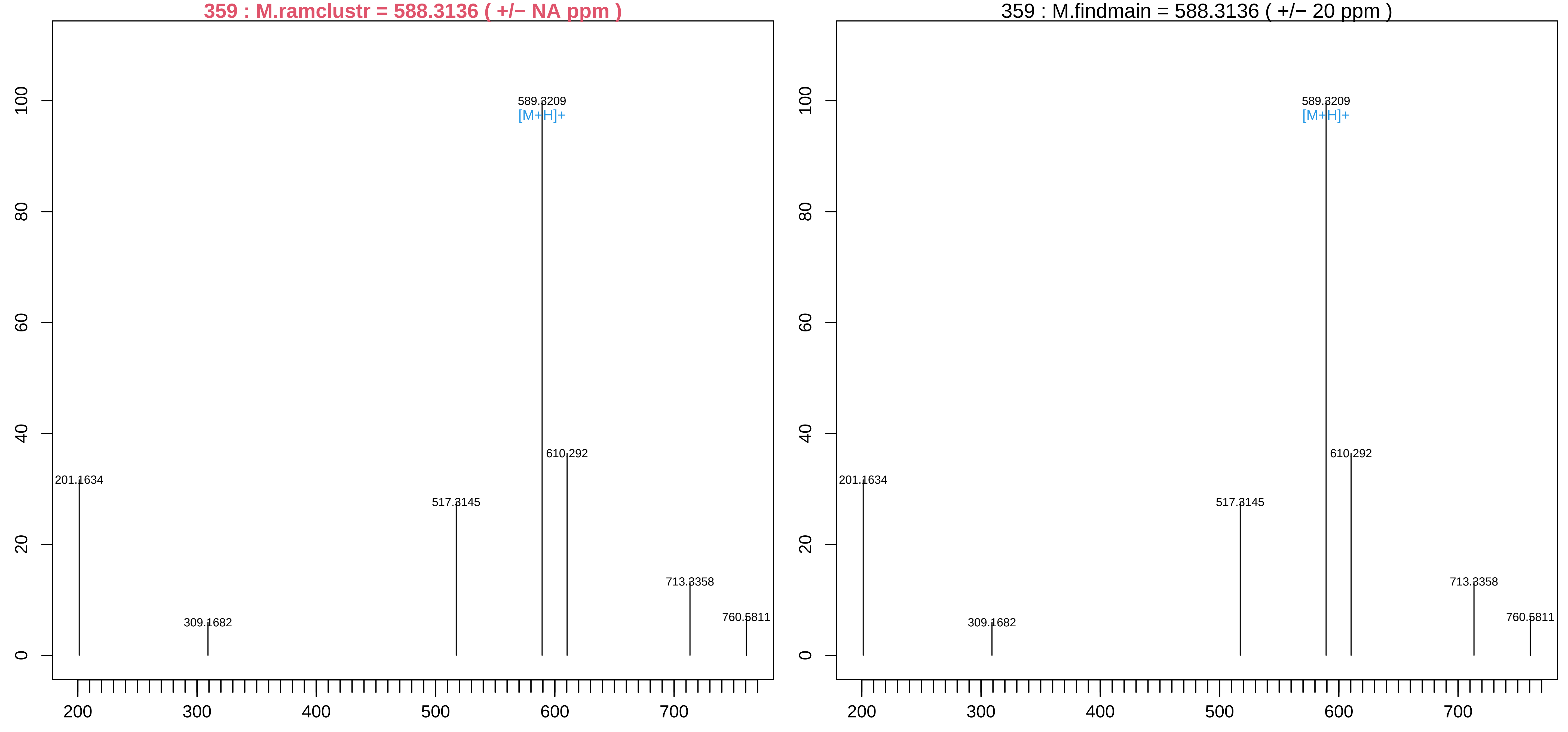

359 : M.ramclustr = 588.3136 (+/− NA ppm)
589.3209
[M+H]+
610.292
201.1634
517.3145
713.3358
760.5811
309.1682
0
20
40
60
80
100
200
300
400
500
600
700
359 : M.findmain = 588.3136 (+/− 20 ppm)
589.3209
[M+H]+
610.292
201.1634
517.3145
713.3358
760.5811
309.1682
0
20
40
60
80
100
200
300
400
500
600
700

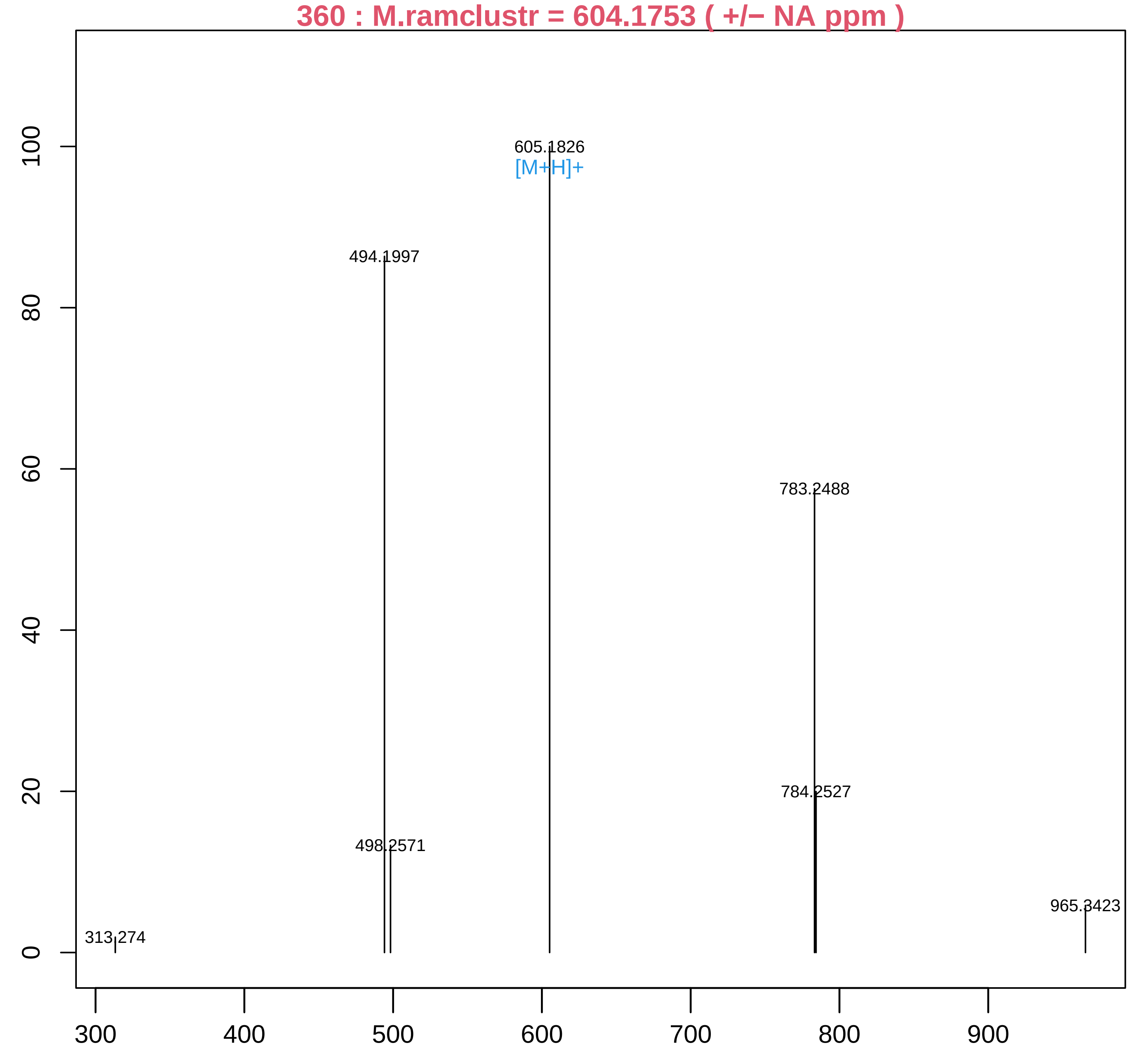
360 : M.ramclustr = 604.1753 (+/− NA ppm)
605.1826
[M+H]+
494.1997
783.2488
784.2527
498.2571
965.3423
313.274
0
20
40
60
80
100
300
400
500
600
700
800
900

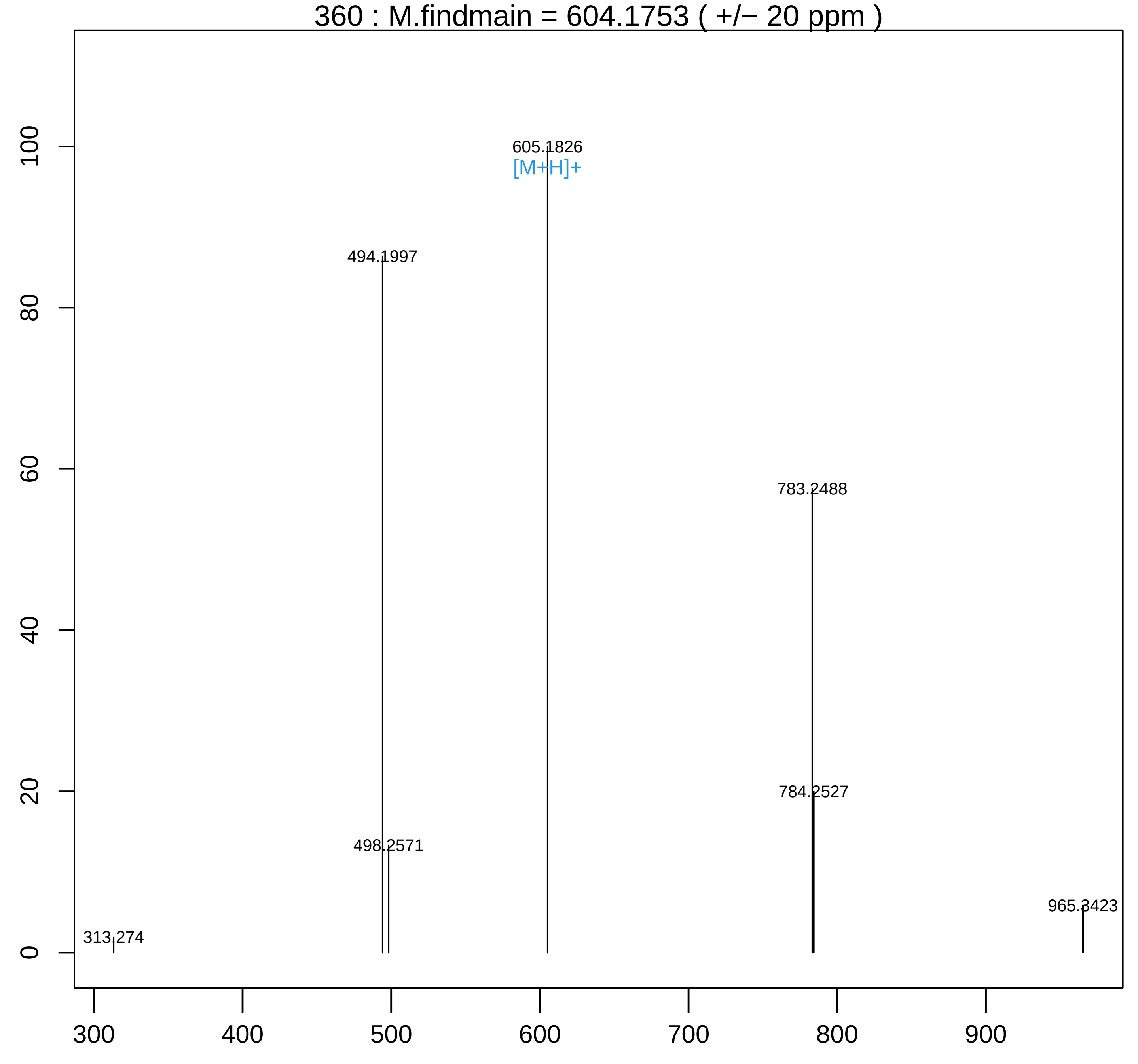
360 : M.findmain = 604.1753 (+/− 20 ppm)
605.1826
[M+H]+
494.1997
783.2488
784.2527
498.2571
965.3423
313.274
0
20
40
60
80
100
300
400
500
600
700
800
900

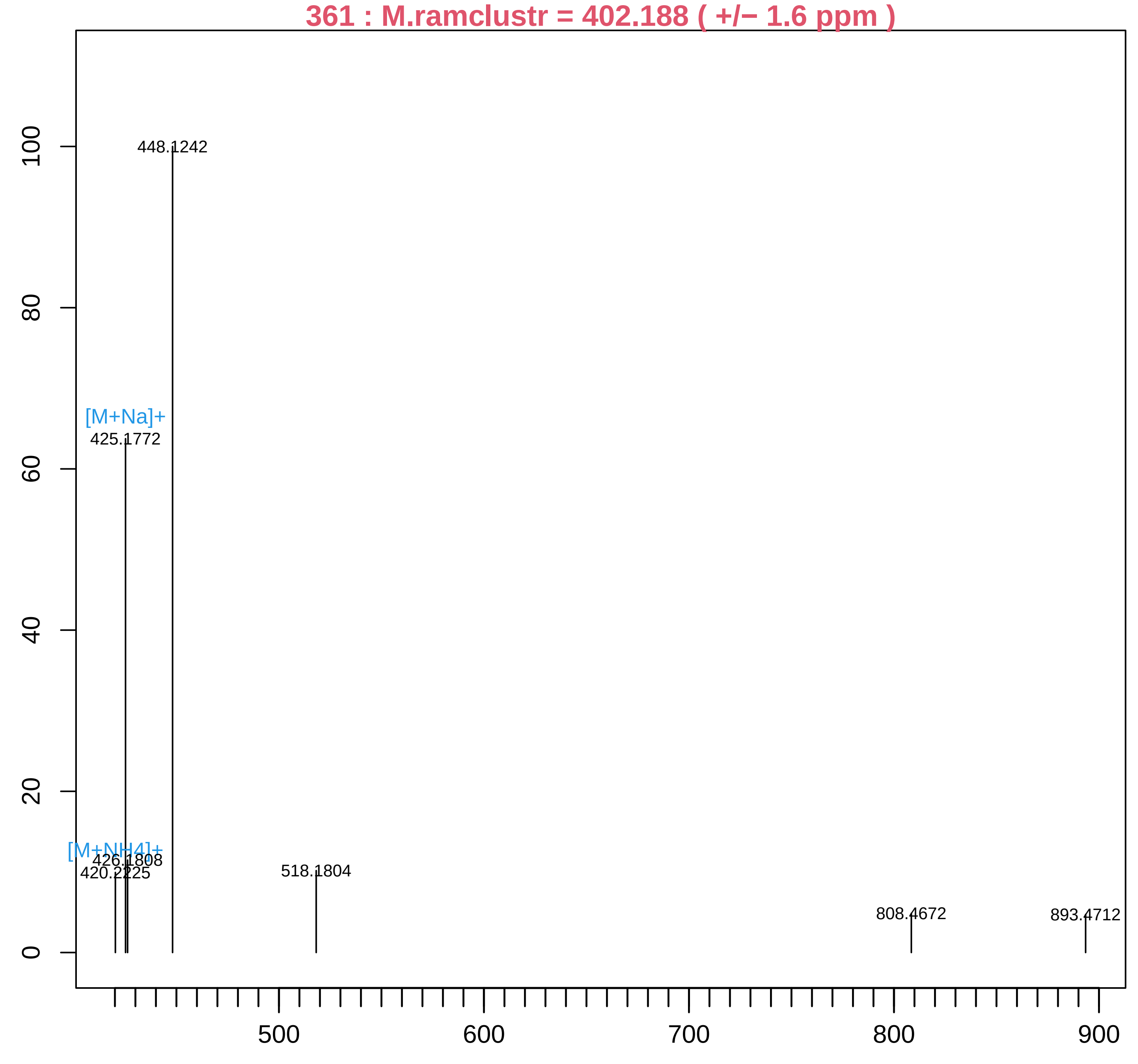

361 : M.ramclustr = 402.188 (+/− 1.6 ppm)
448.1242
[M+Na]+
425.1772
[M+NH4]+
426.1808
420.2225
518.1804
808.4672
893.4712
0
20
40
60
80
100
500
600
700
800
900

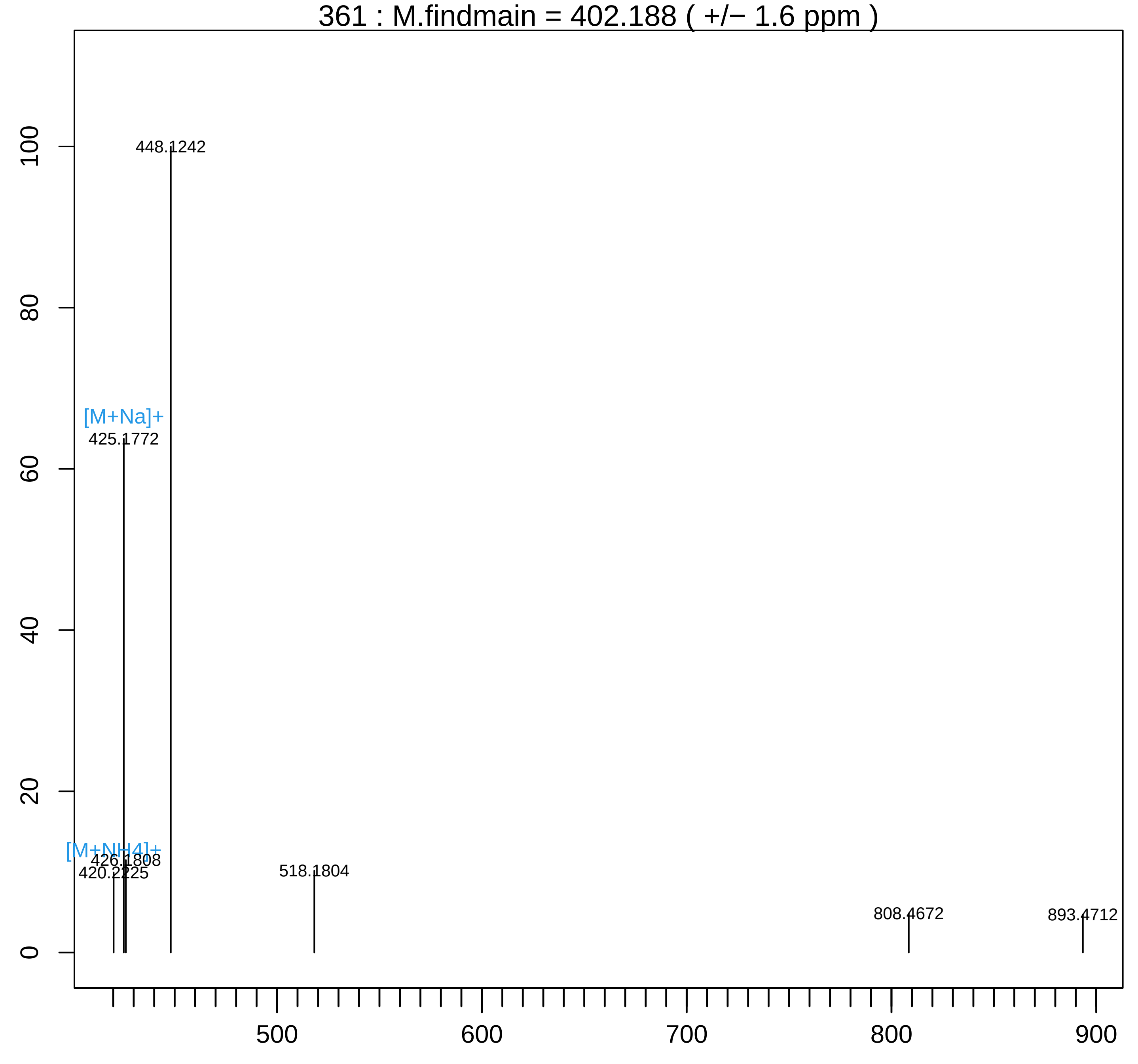

361 : M.findmain = 402.188 (+/− 1.6 ppm)
448.1242
[M+Na]+
425.1772
[M+NH4]+
426.1808
420.2225
518.1804
808.4672
893.4712
0
20
40
60
80
100
500
600
700
800
900

362 : M.ramclustr = 118.0284 (+/− 1.2 ppm)

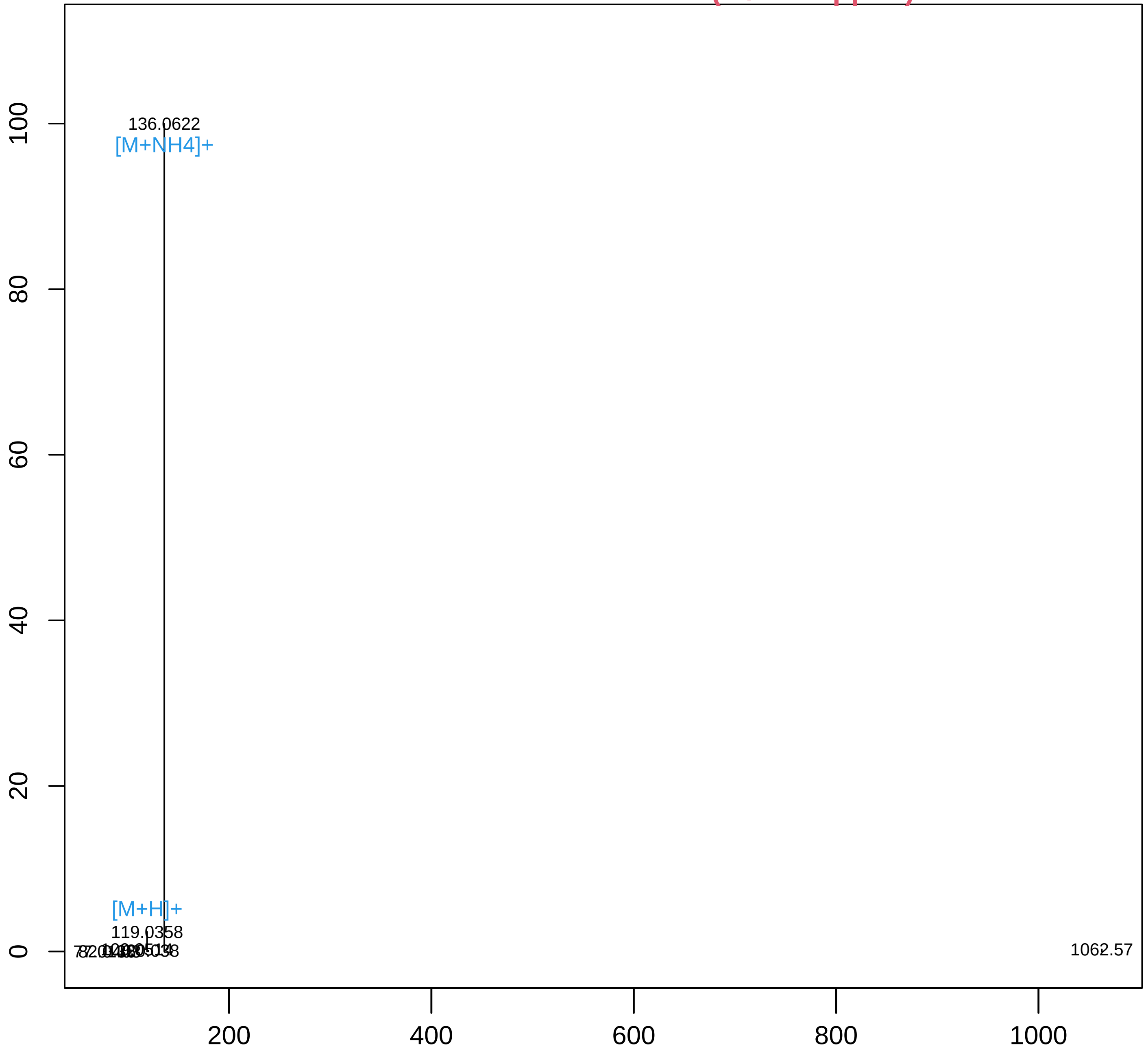

362 : M.findmain = 118.0284 (+/− 1.2 ppm)

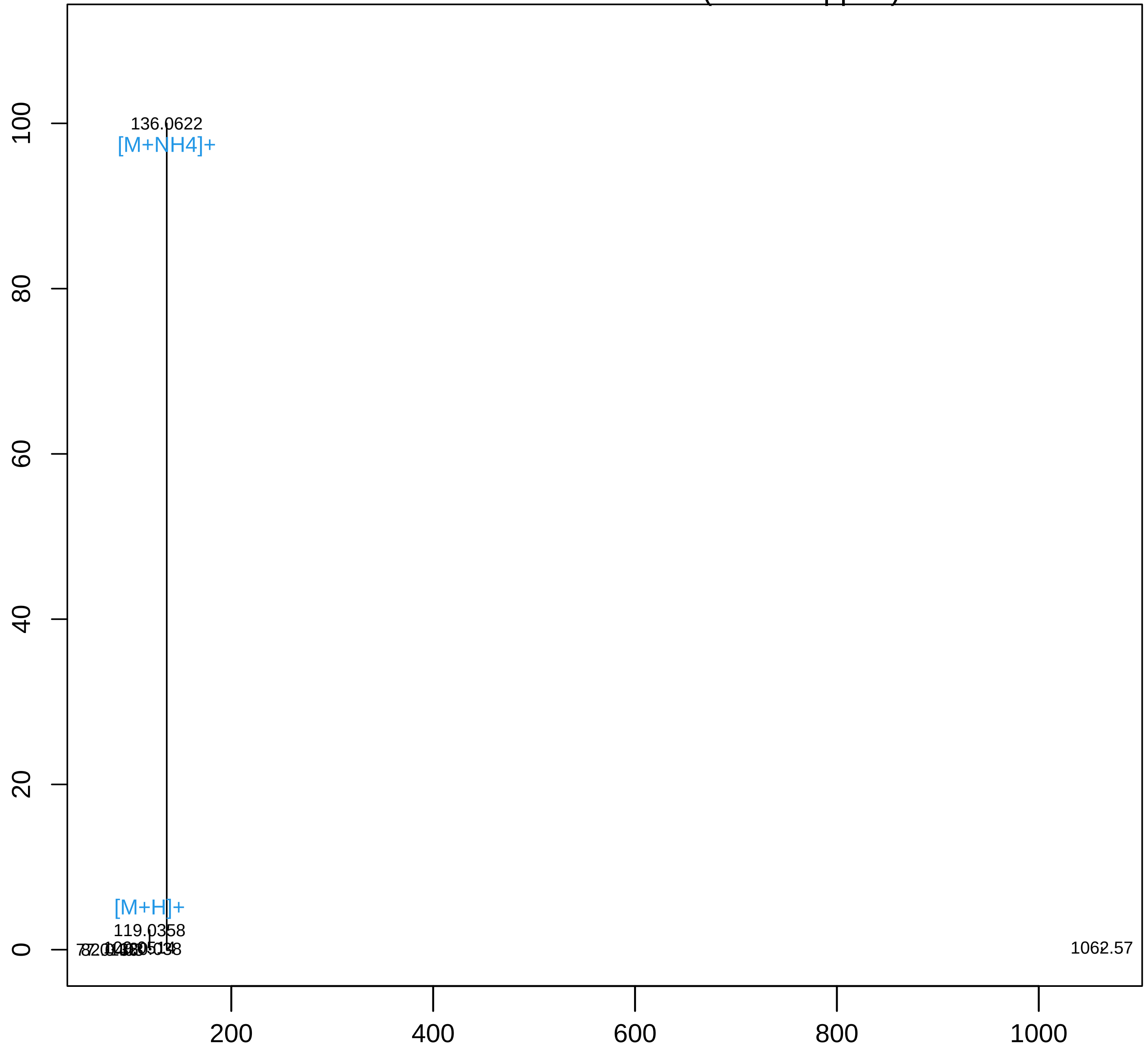

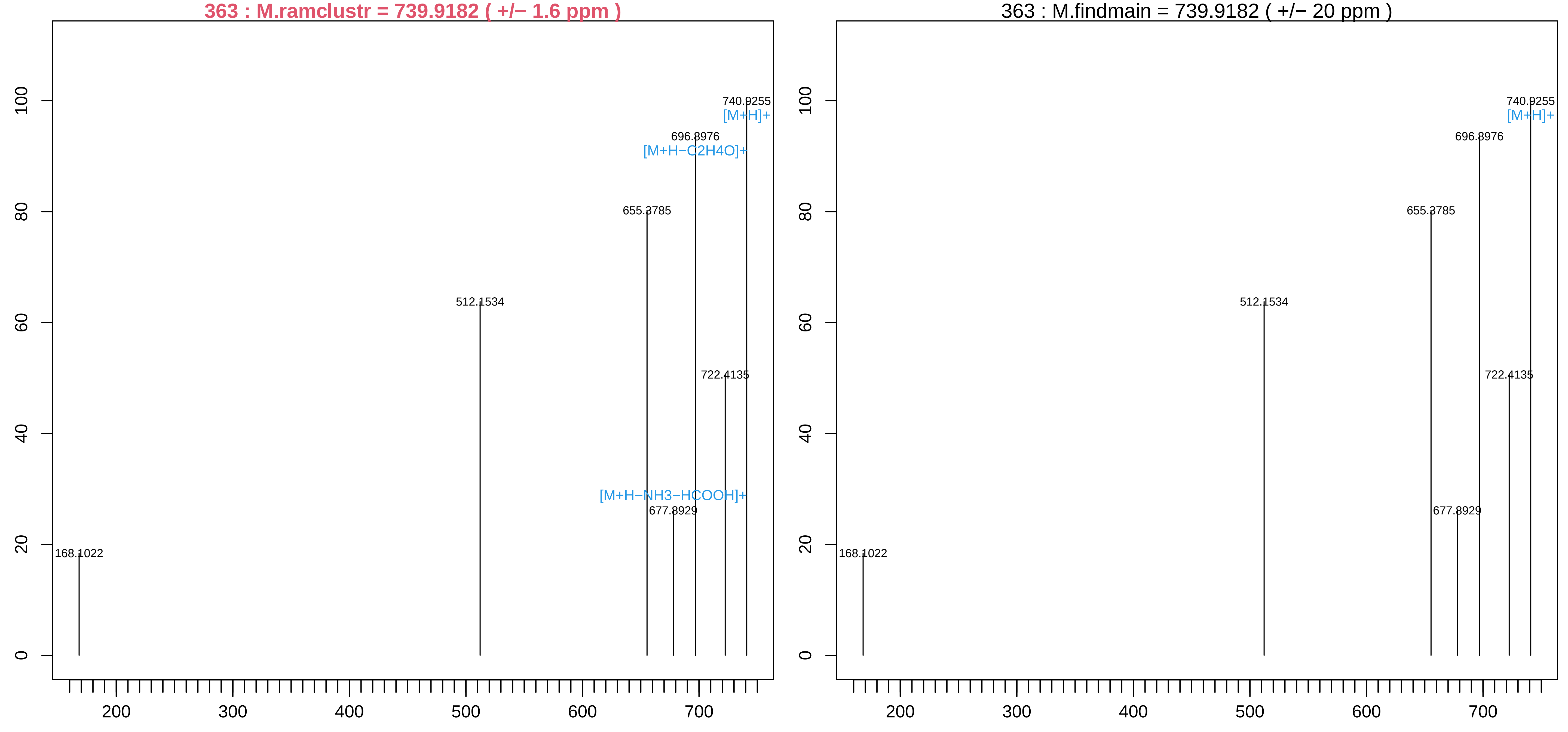
363 : M.ramclustr = 739.9182 (+/− 1.6 ppm)
740.9255
[M+H]+
696.8976
[M+H−C2H4O]+
655.3785
512.1534
722.4135
[M+H−NH3−HCOOH]+
677.8929
168.1022
0
20
40
60
80
100
200
300
400
500
600
700
363 : M.findmain = 739.9182 (+/− 20 ppm)
740.9255
[M+H]+
696.8976
655.3785
512.1534
722.4135
677.8929
168.1022

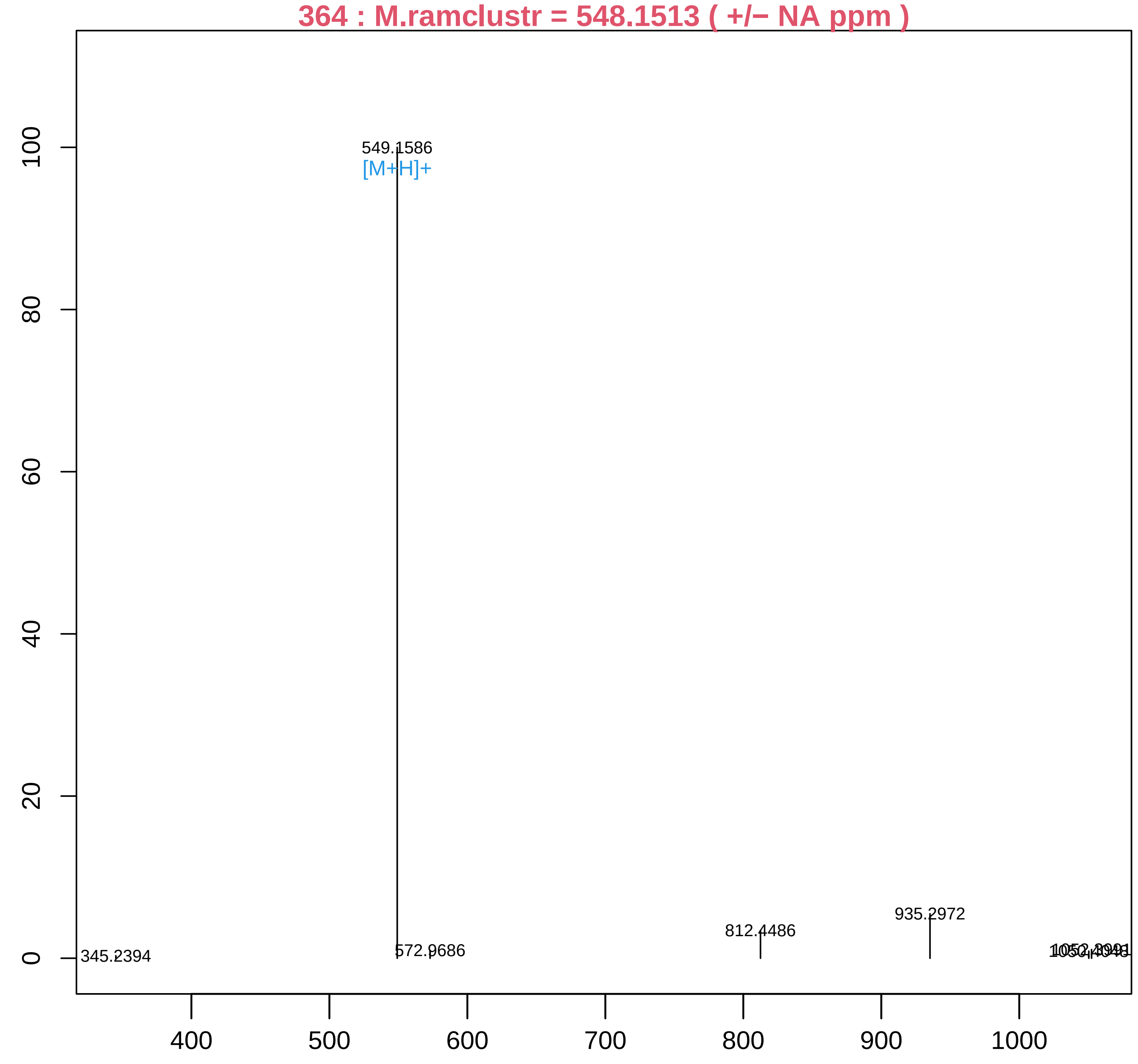
364 : M.ramclustr = 548.1513 (+/− NA ppm)
549.1586
[M+H]+
345.2394
572.9686
812.4486
935.2972
0
20
40
60
80
100
400
500
600
700
800
900
1000

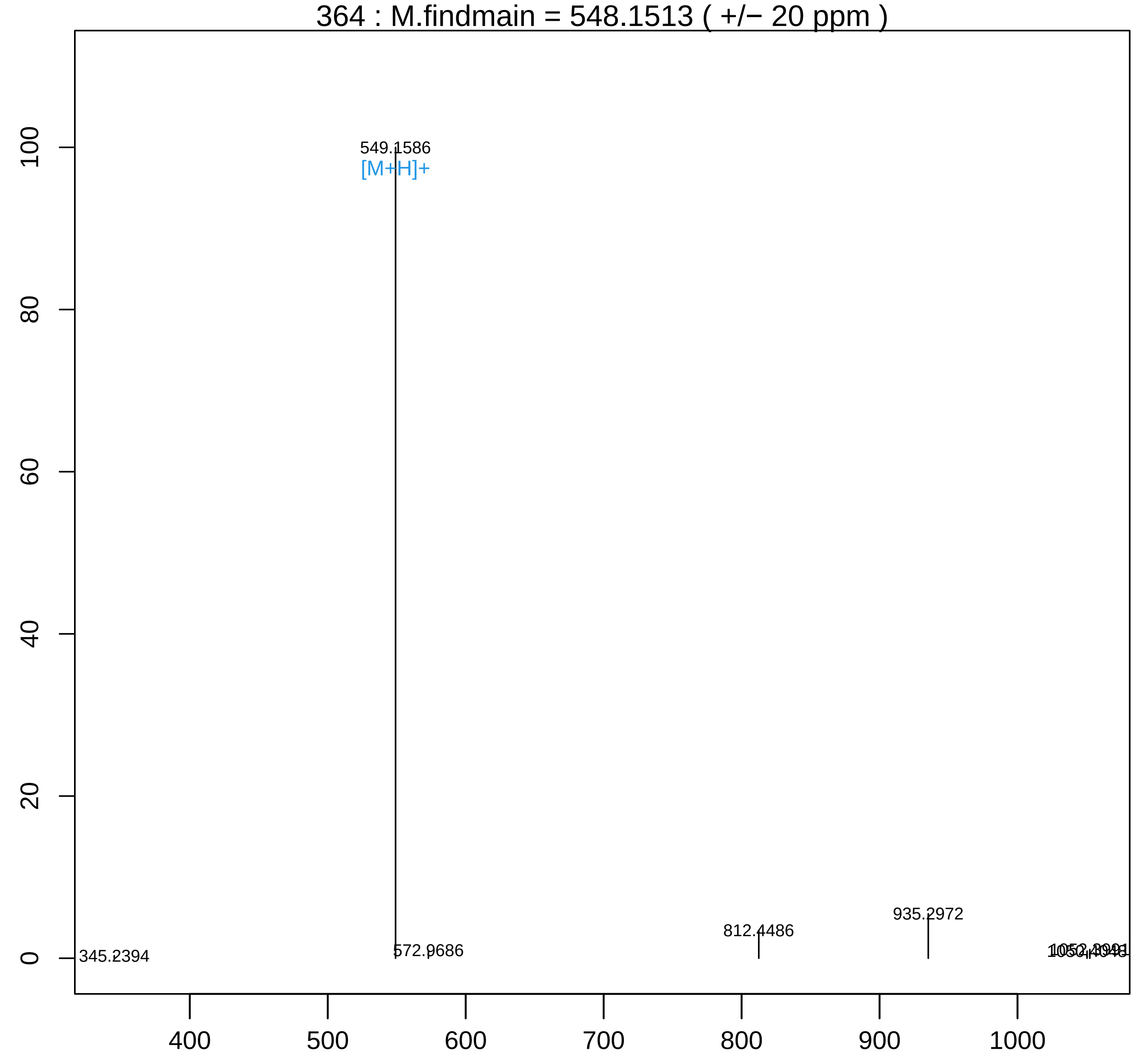
364 : M.findmain = 548.1513 (+/− 20 ppm)
549.1586
[M+H]+
345.2394
572.9686
812.4486
935.2972
0
20
40
60
80
100
400
500
600
700
800
900
1000

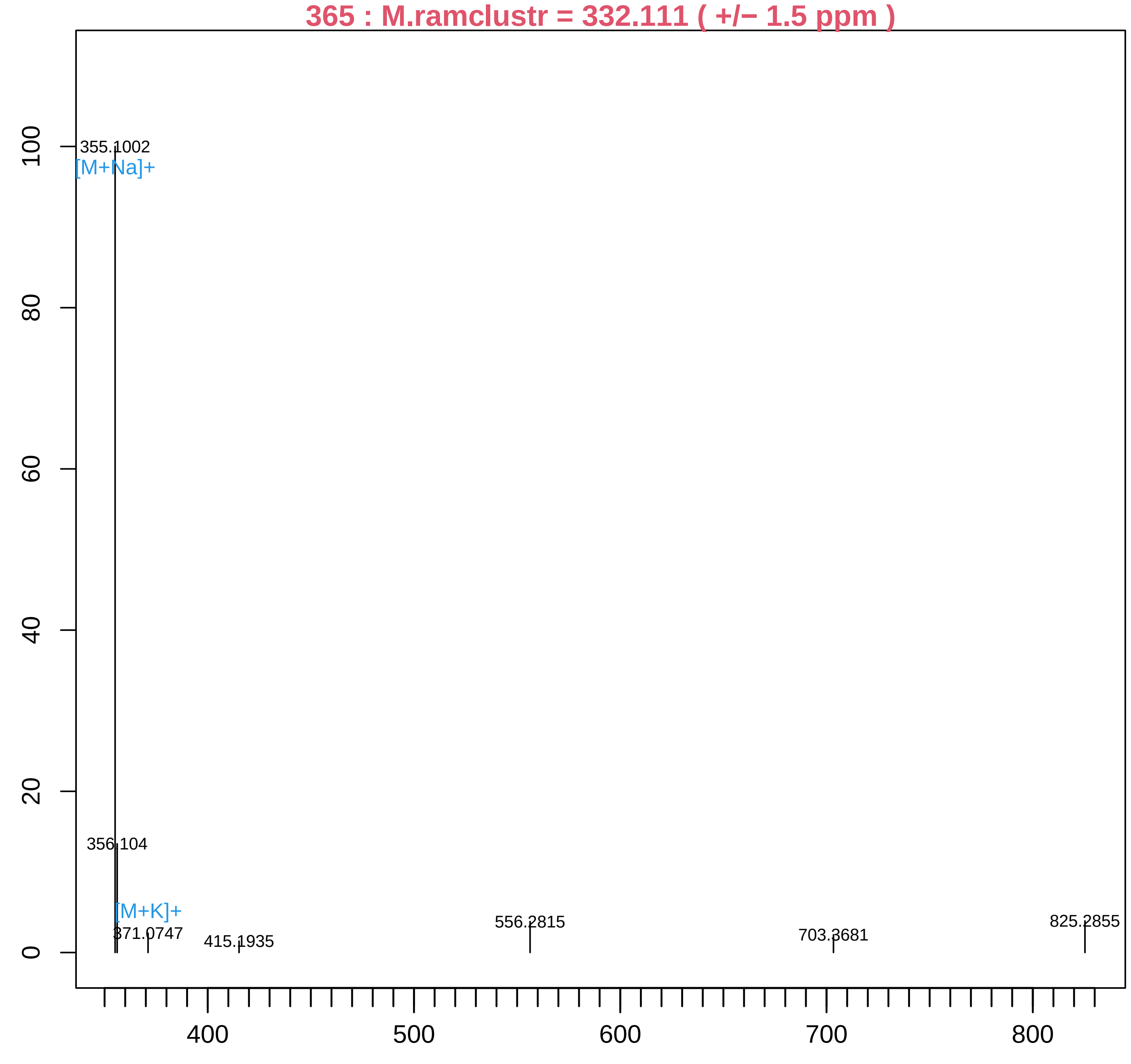
365 : M.ramclustr = 332.111 (+/− 1.5 ppm)
355.1002
[M+Na]+
356.104
[M+K]+
371.0747
415.1935
556.2815
703.3681
825.2855
0
20
40
60
80
100
400
500
600
700
800

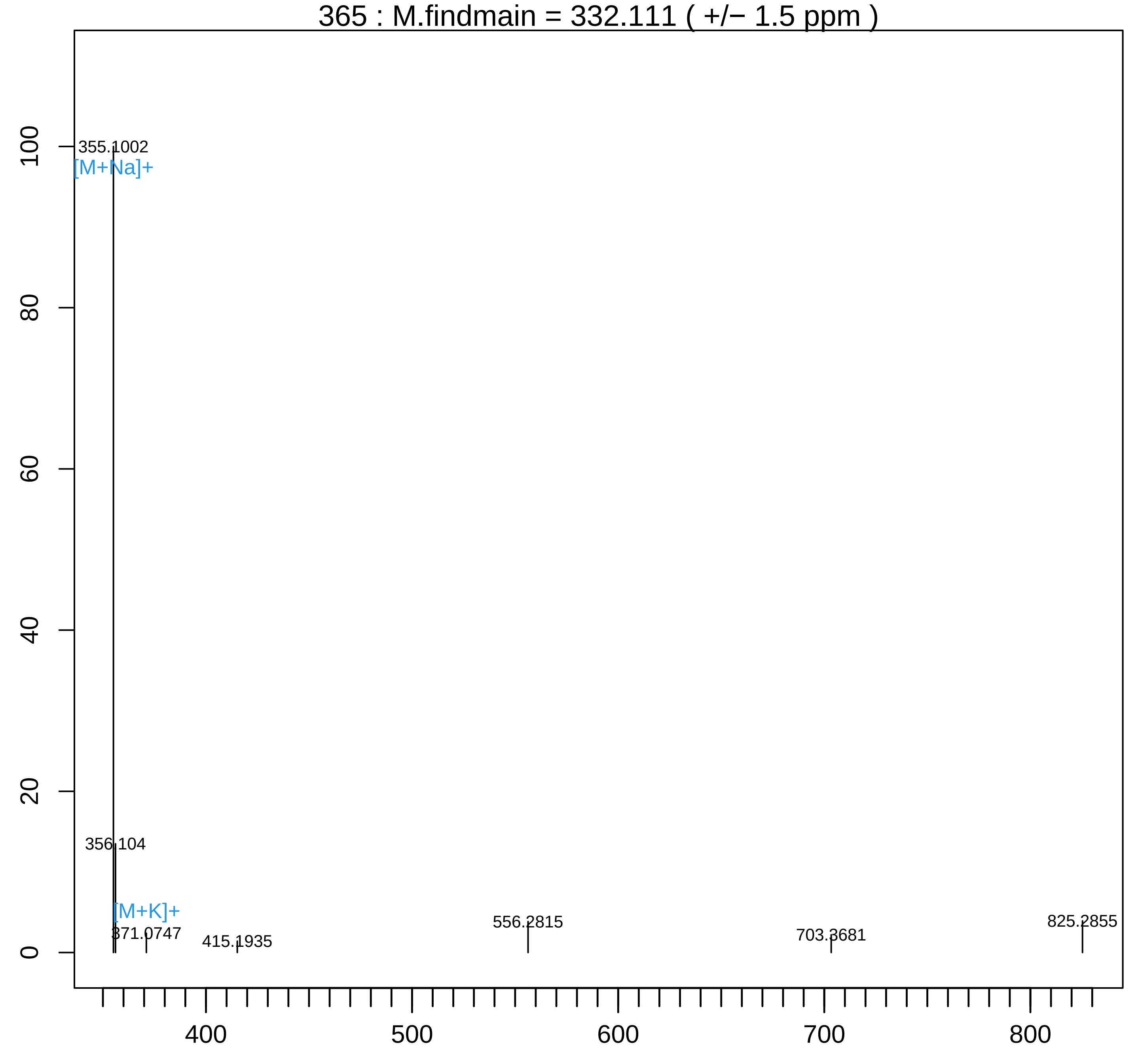
365 : M.findmain = 332.111 (+/− 1.5 ppm)
355.1002
[M+Na]+
356.104
[M+K]+
371.0747
415.1935
556.2815
703.3681
825.2855
0
20
40
60
80
100
400
500
600
700
800

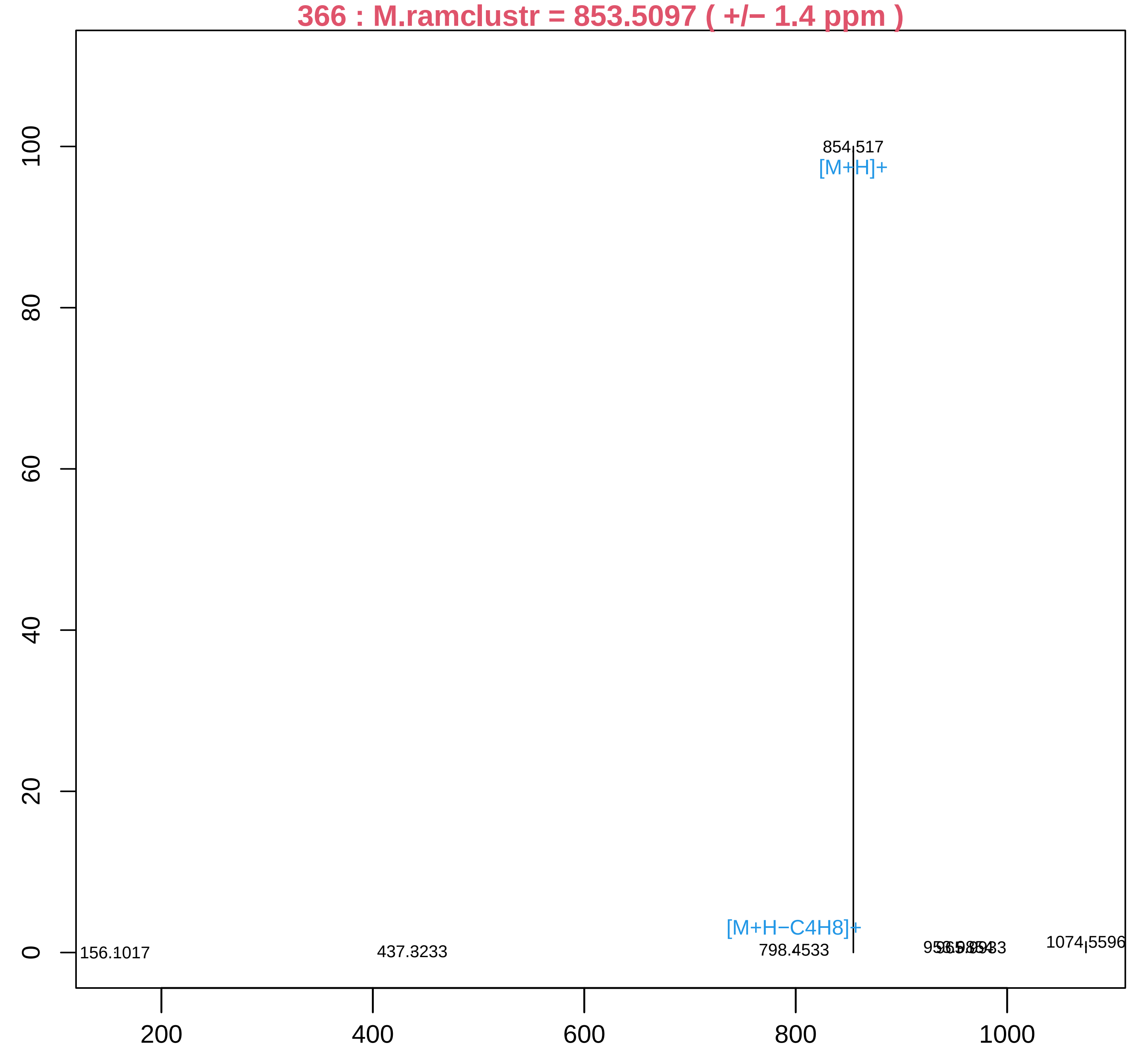
366 : M.ramclustr = 853.5097 (+/− 1.4 ppm)
854.517
[M+H]+
[M+H−C4H8]+
156.1017
437.3233
798.4533
1074.5596
0
20
40
60
80
100
200
400
600
800
1000

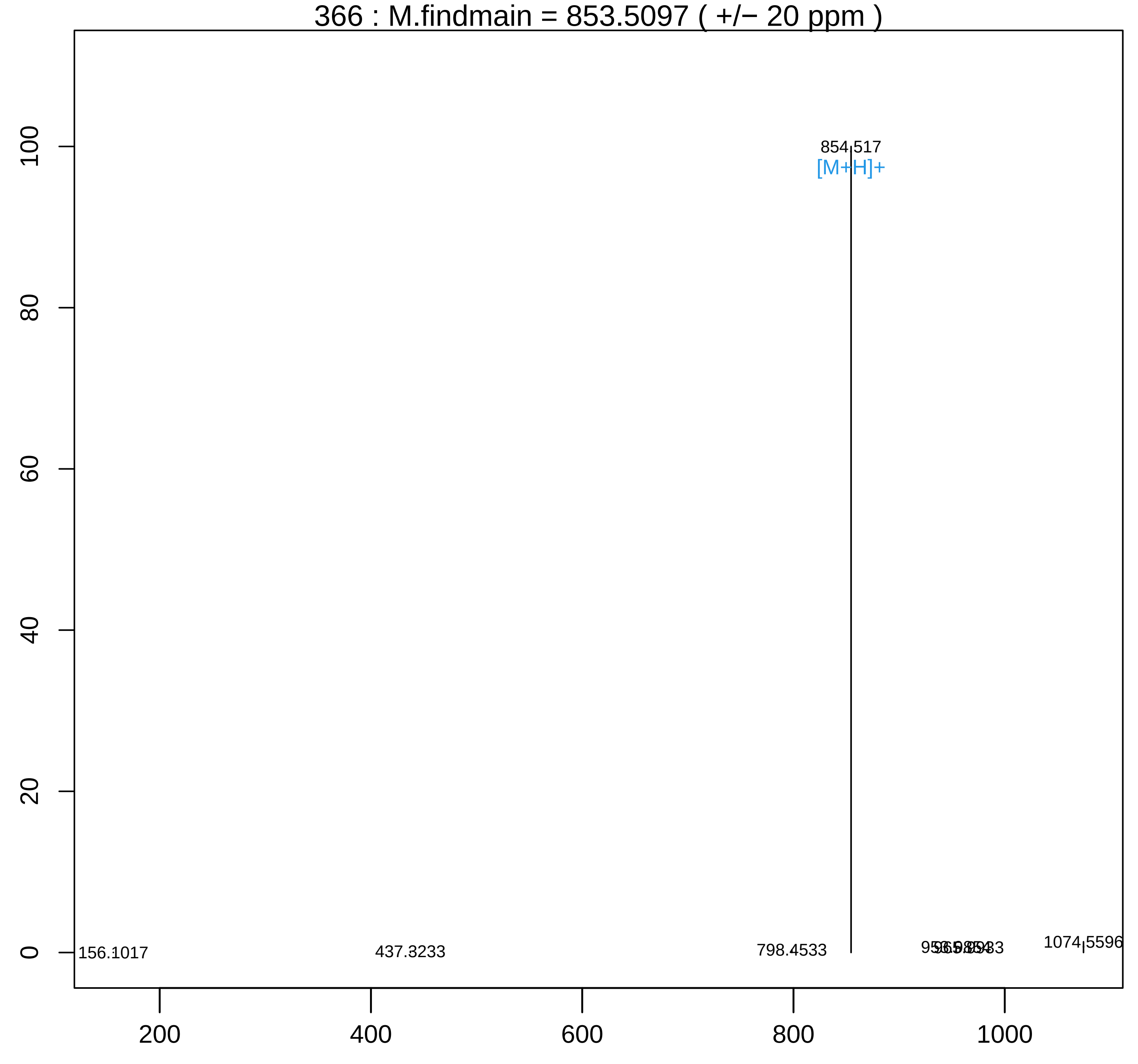
366 : M.findmain = 853.5097 (+/− 20 ppm)
854.517
[M+H]+
156.1017
437.3233
798.4533
1074.5596
0
20
40
60
80
100
200
400
600
800
1000

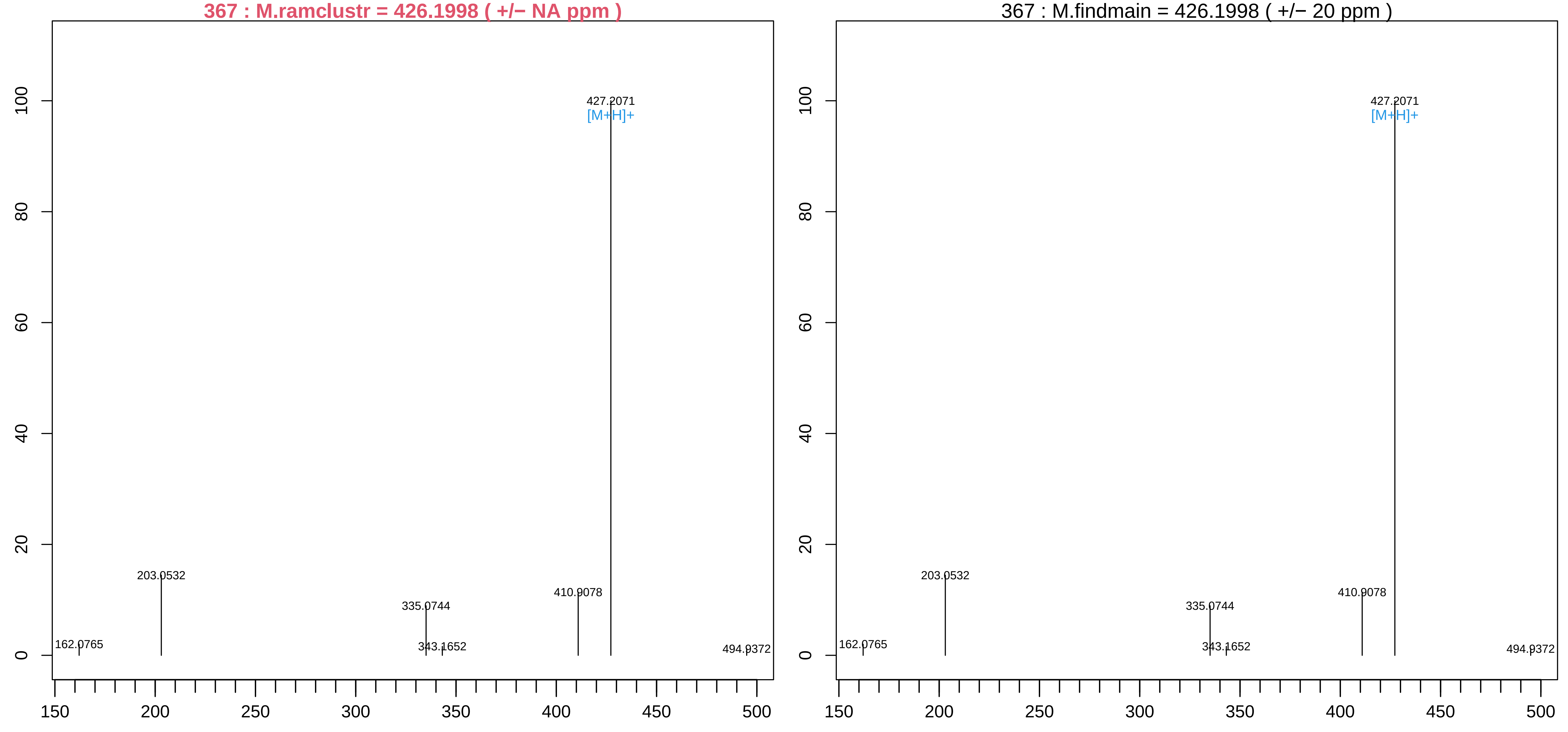
367 : M.ramclustr = 426.1998 (+/− NA ppm)
427.2071
[M+H]+
203.0532
410.9078
335.0744
343.1652
162.0765
494.9372
0
20
40
60
80
100
150
200
250
300
350
400
450
500
367 : M.findmain = 426.1998 (+/− 20 ppm)
427.2071
[M+H]+
203.0532
410.9078
335.0744
343.1652
162.0765
494.9372
0
20
40
60
80
100
150
200
250
300
350
400
450
500

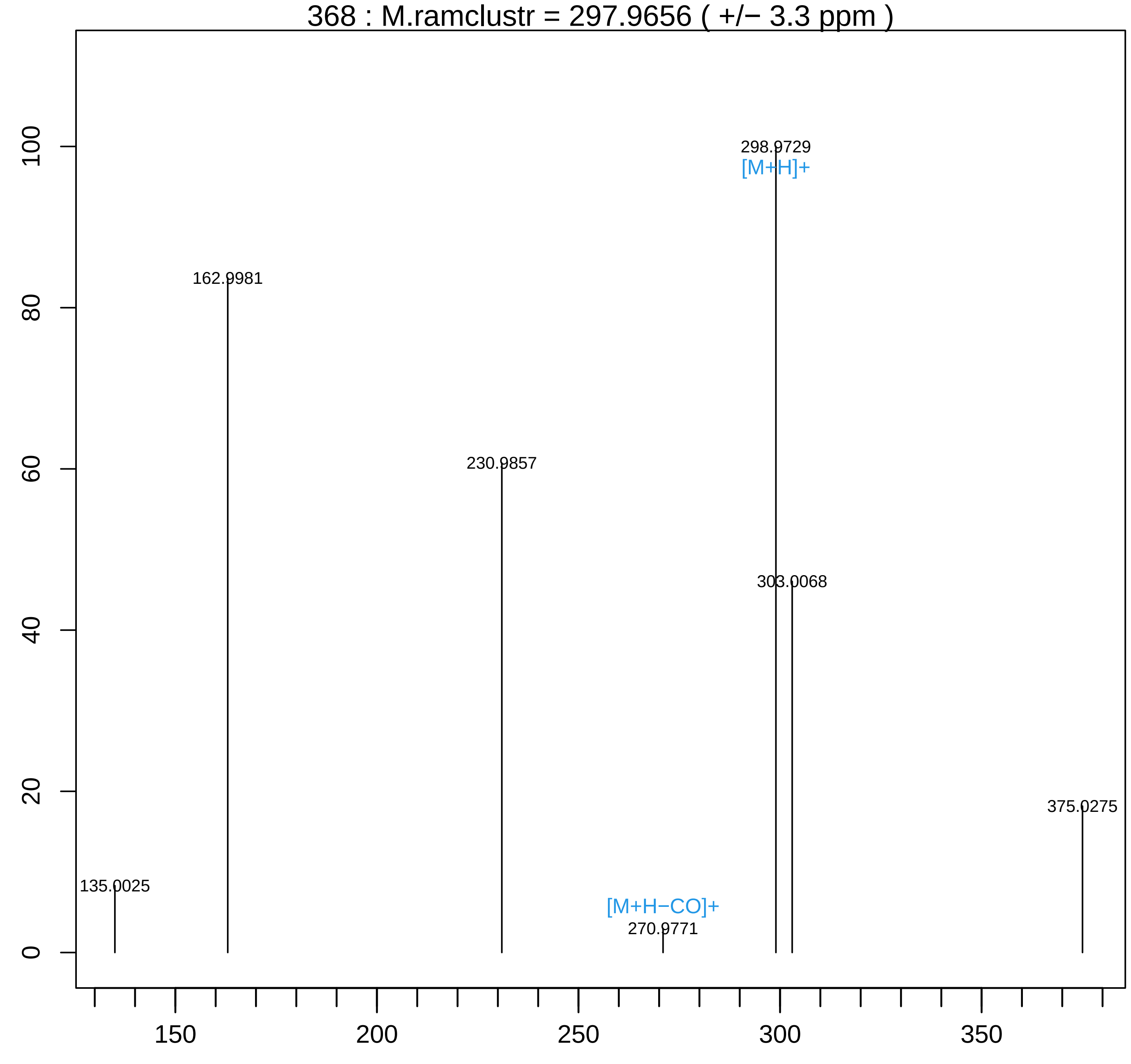

368 : M.ramclustr = 297.9656 (+/− 3.3 ppm)
298.9729
[M+H]+
162.9981
230.9857
303.0068
375.0275
135.0025
[M+H−CO]+
270.9771
0
20
40
60
80
100
150
200
250
300
350

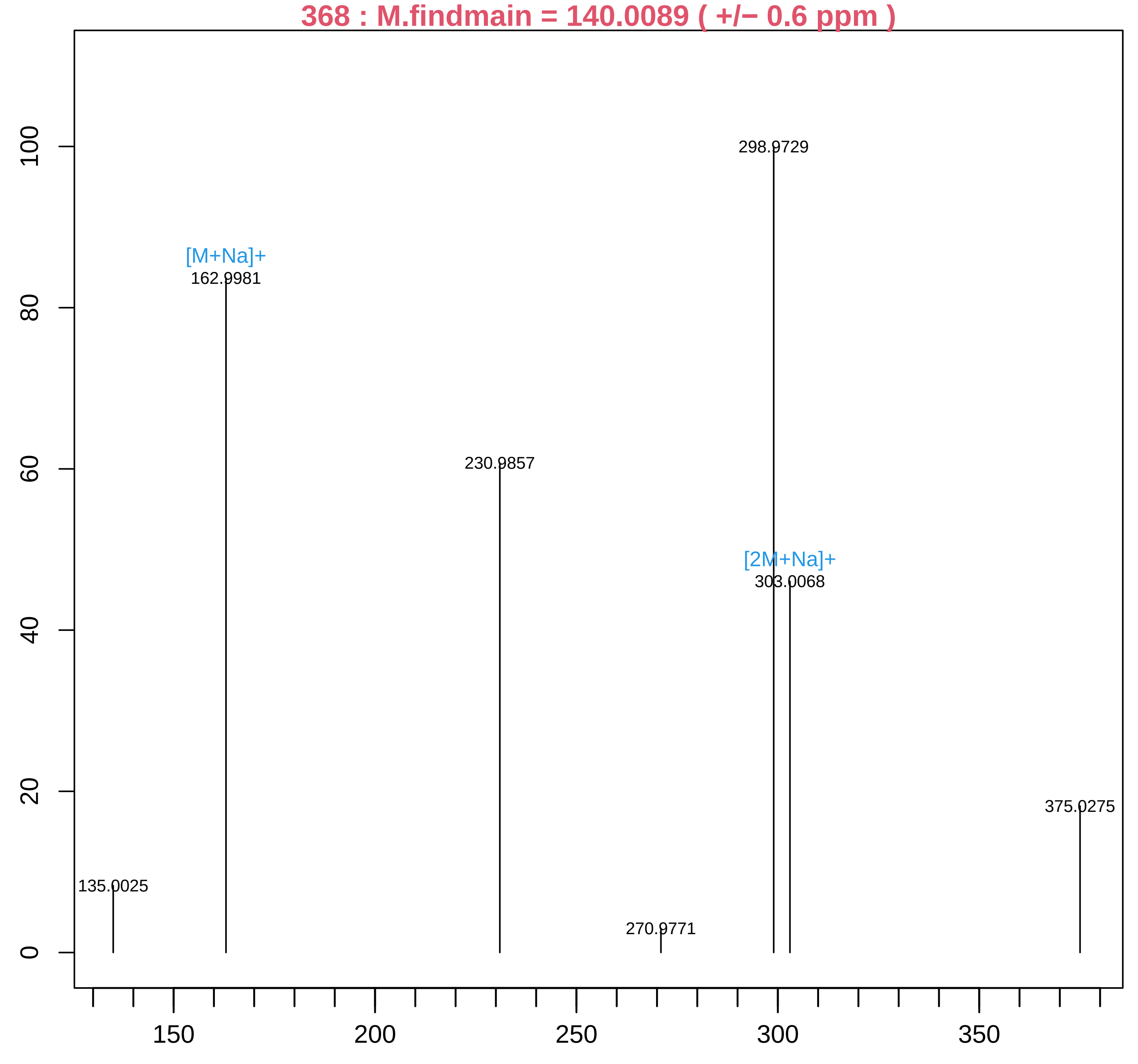

368 : M.findmain = 140.0089 (+/− 0.6 ppm)
298.9729
[M+Na]+
162.9981
230.9857
[2M+Na]+
303.0068
375.0275
135.0025
270.9771
0
20
40
60
80
100
150
200
250
300
350

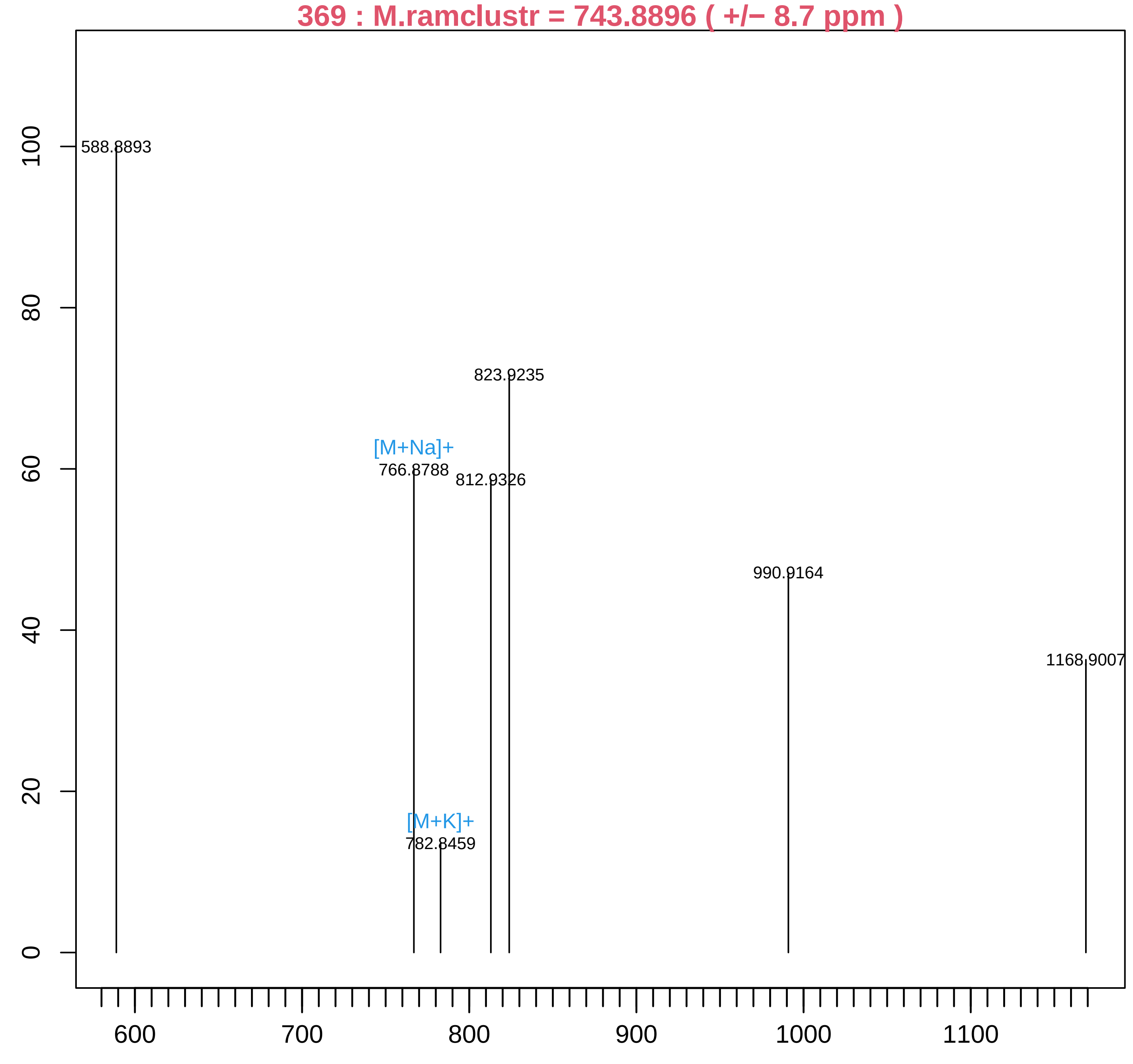
369 : M.ramclustr = 743.8896 (+/− 8.7 ppm)
588.8893
823.9235
[M+Na]+
766.8788
812.9326
990.9164
1168.9007
[M+K]+
782.8459
0
20
40
60
80
100
600
700
800
900
1000
1100

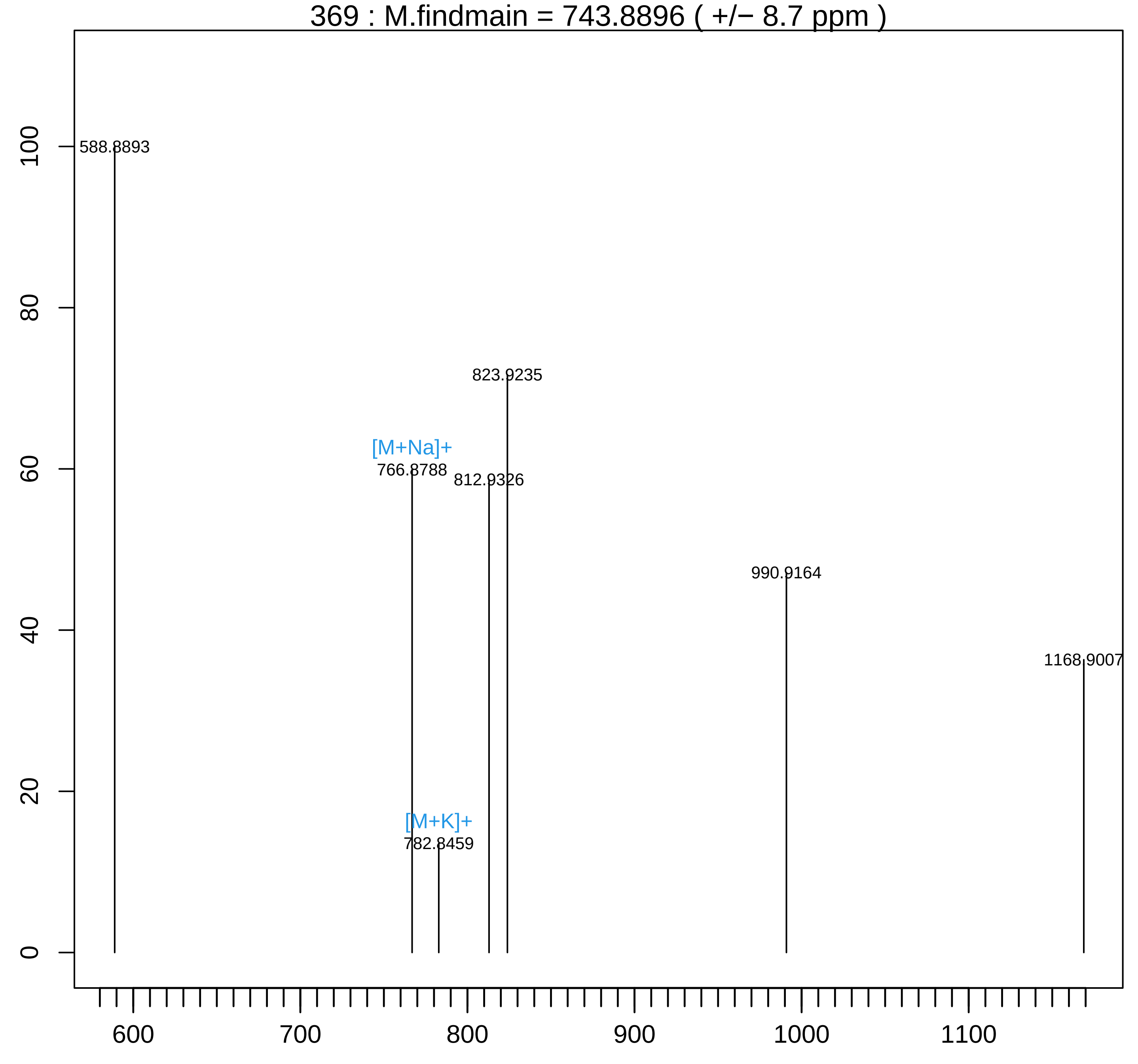
369 : M.findmain = 743.8896 (+/− 8.7 ppm)
588.8893
823.9235
[M+Na]+
766.8788
812.9326
990.9164
1168.9007
[M+K]+
782.8459
0
20
40
60
80
100
600
700
800
900
1000
1100

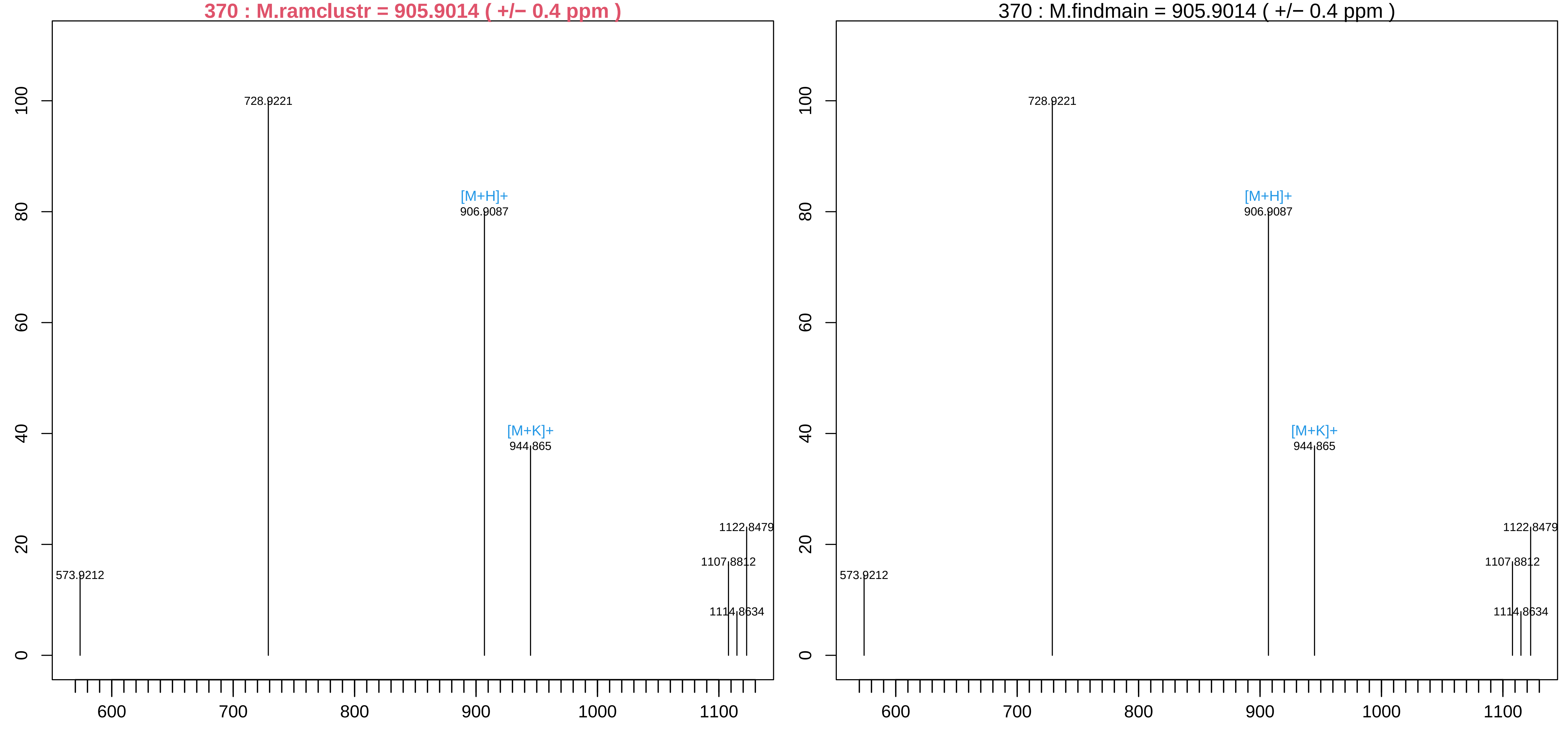
370 : M.ramclustr = 905.9014 (+/− 0.4 ppm)
728.9221
[M+H]+
906.9087
[M+K]+
944.865
573.9212
1122.8479
1107.8812
1114.8634
370 : M.findmain = 905.9014 (+/− 0.4 ppm)
728.9221
[M+H]+
906.9087
[M+K]+
944.865
573.9212
1122.8479
1107.8812
1114.8634

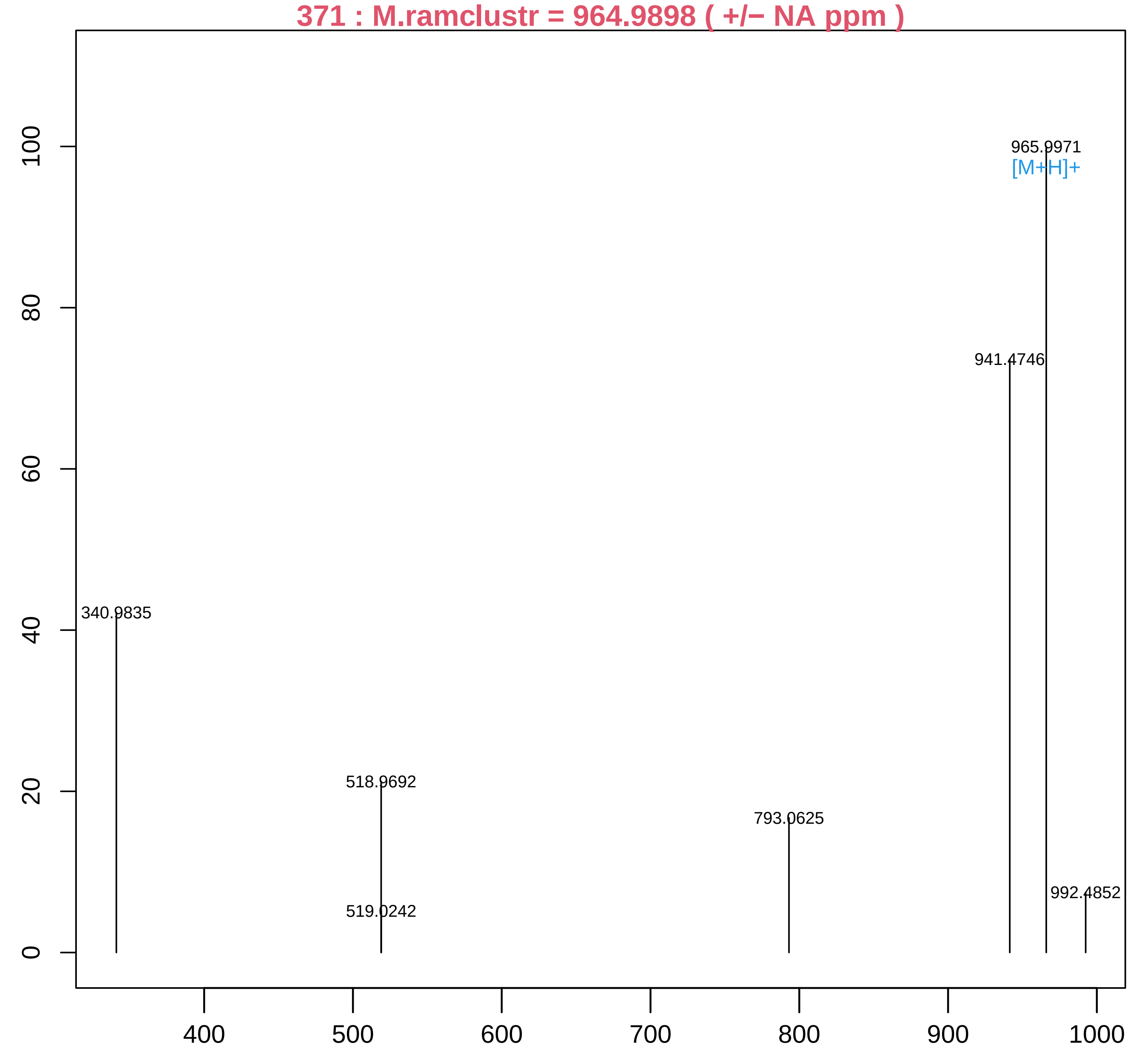
371 : M.ramclustr = 964.9898 (+/− NA ppm)
965.9971
[M+H]+
941.4746
340.9835
518.9692
519.0242
793.0625
992.4852
0
20
40
60
80
100
400
500
600
700
800
900
1000

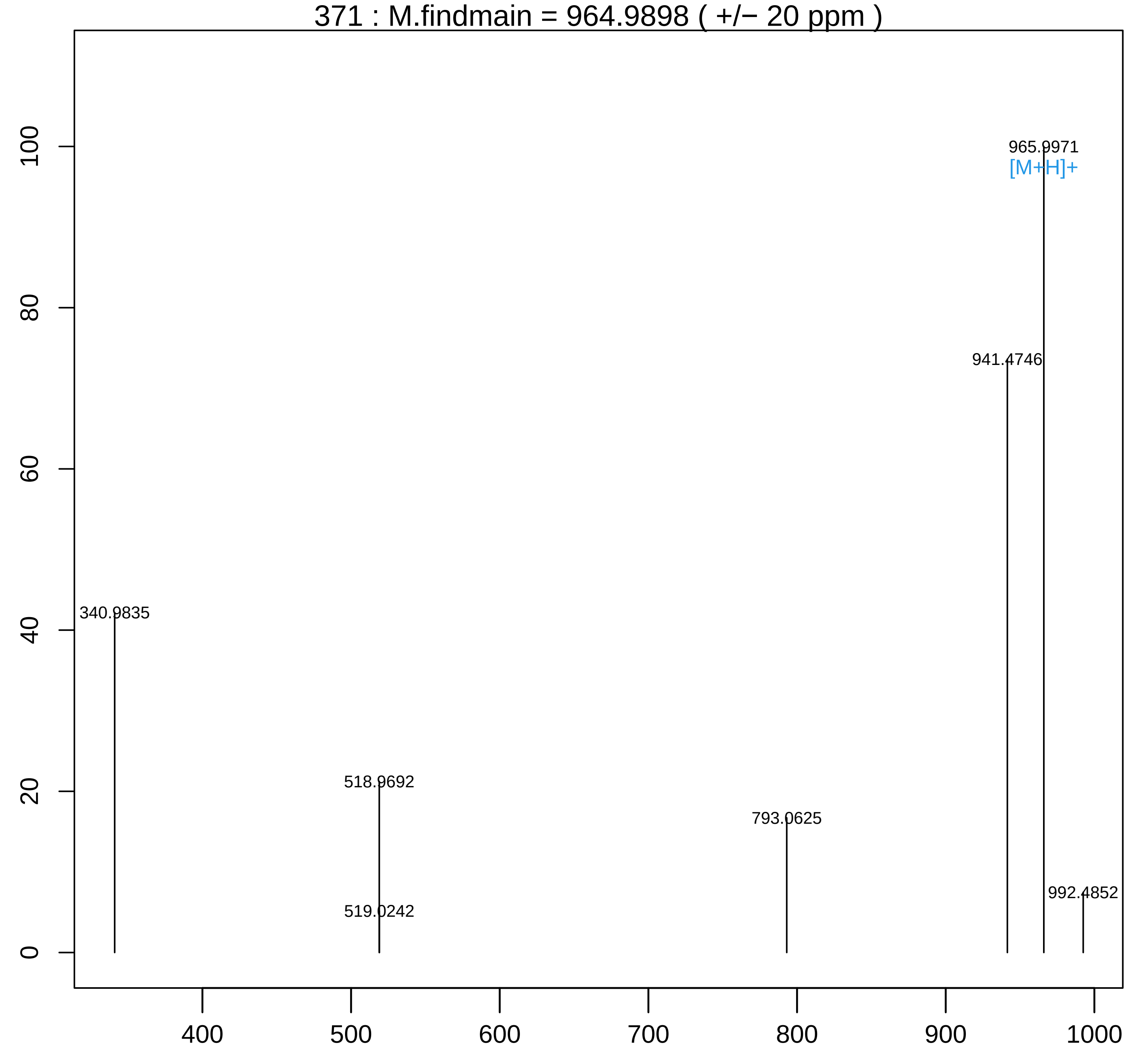
371 : M.findmain = 964.9898 (+/− 20 ppm)
965.9971
[M+H]+
941.4746
340.9835
518.9692
519.0242
793.0625
992.4852
0
20
40
60
80
100
400
500
600
700
800
900
1000

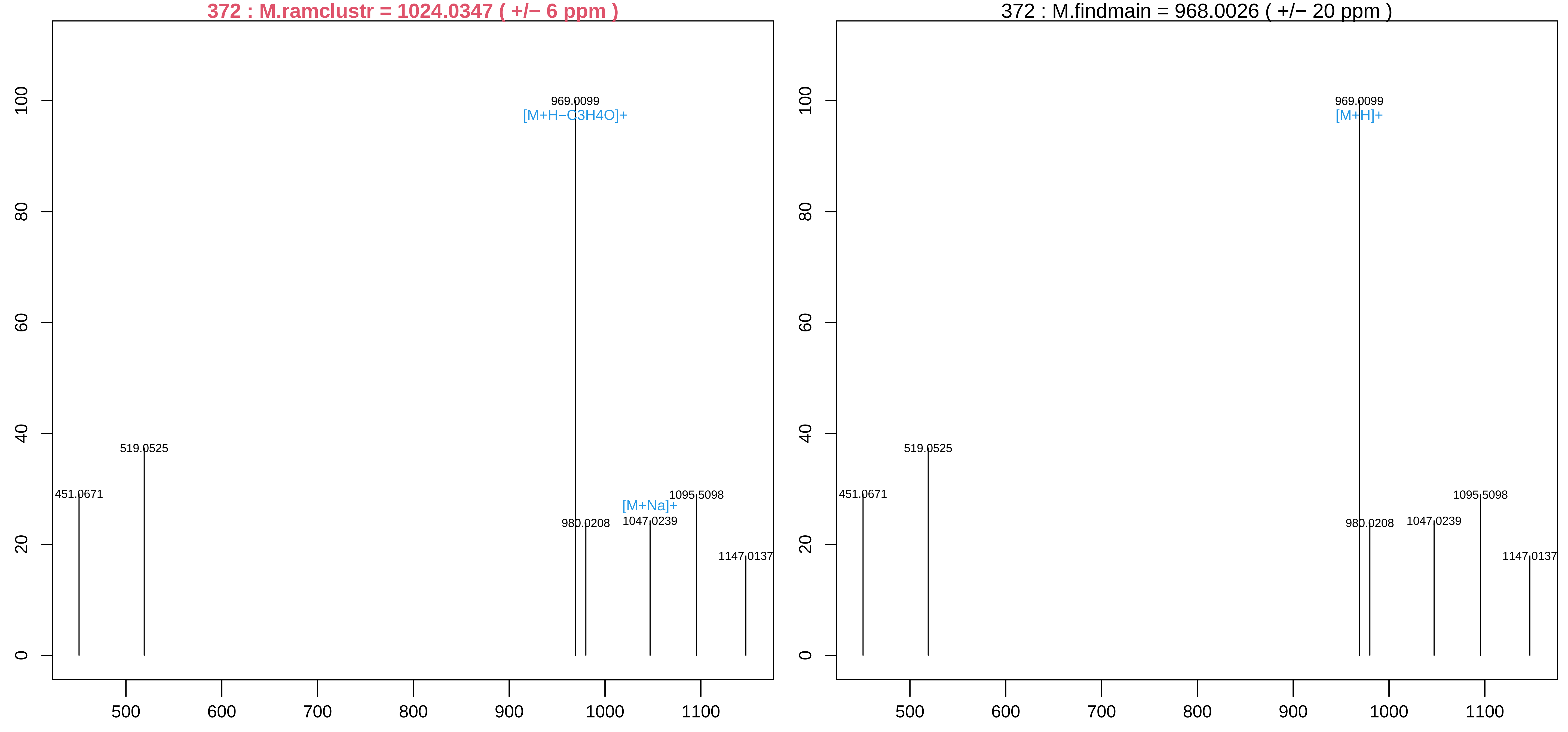
372 : M.ramclustr = 1024.0347 (+/− 6 ppm)
969.0099
[M+H−C3H4O]+
519.0525
451.0671
[M+Na]+
1095.5098
980.0208
1047.0239
1147.0137
372 : M.findmain = 968.0026 (+/− 20 ppm)
969.0099
[M+H]+
519.0525
451.0671
1095.5098
980.0208
1047.0239
1147.0137

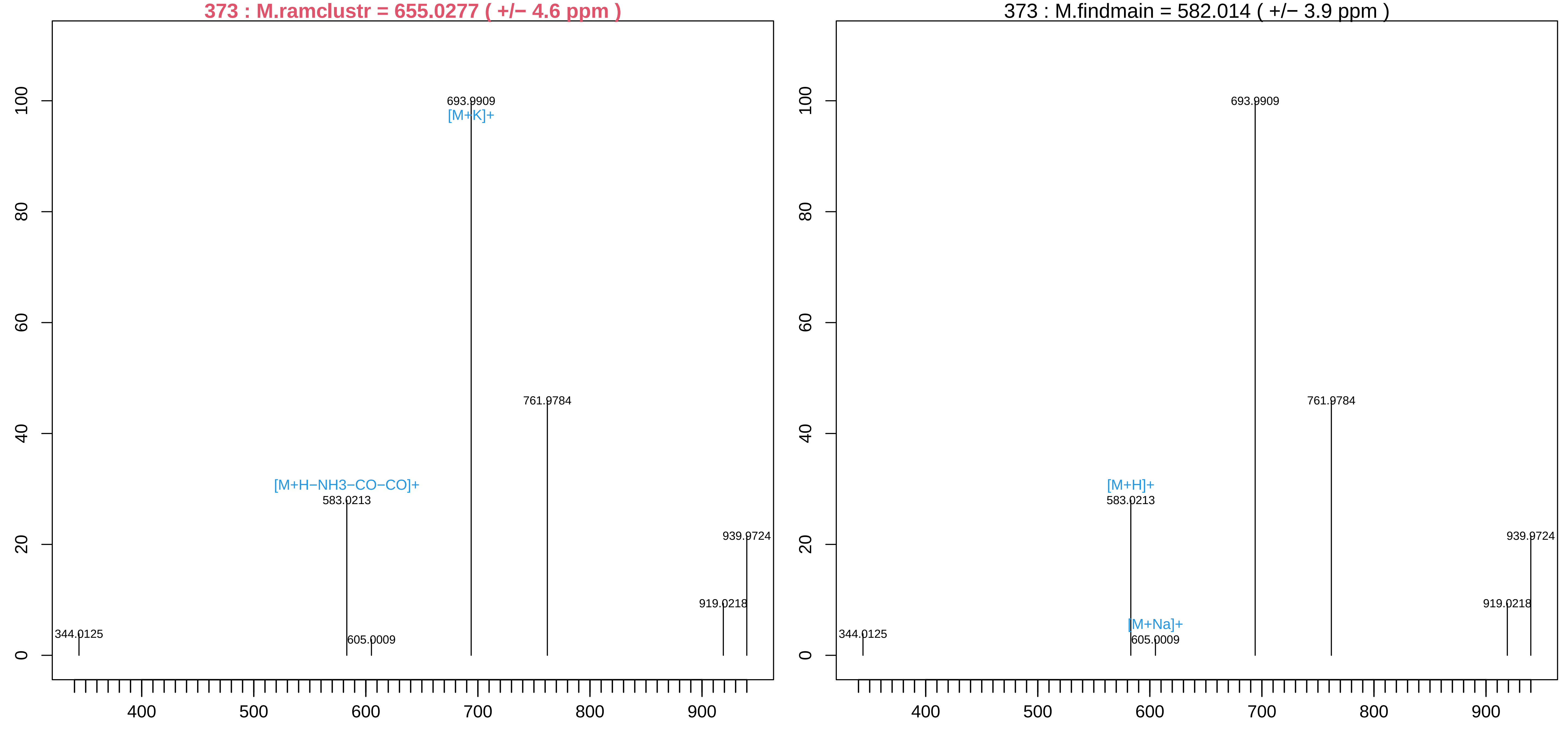

373 : M.ramclustr = 655.0277 (+/− 4.6 ppm)
693.9909
[M+K]+
761.9784
[M+H−NH3−CO−CO]+
583.0213
939.9724
919.0218
344.0125
605.0009
0
20
40
60
80
100
400
500
600
700
800
900
373 : M.findmain = 582.014 (+/− 3.9 ppm)
693.9909
761.9784
[M+H]+
583.0213
939.9724
919.0218
[M+Na]+
344.0125
605.0009
0
20
40
60
80
100
400
500
600
700
800
900

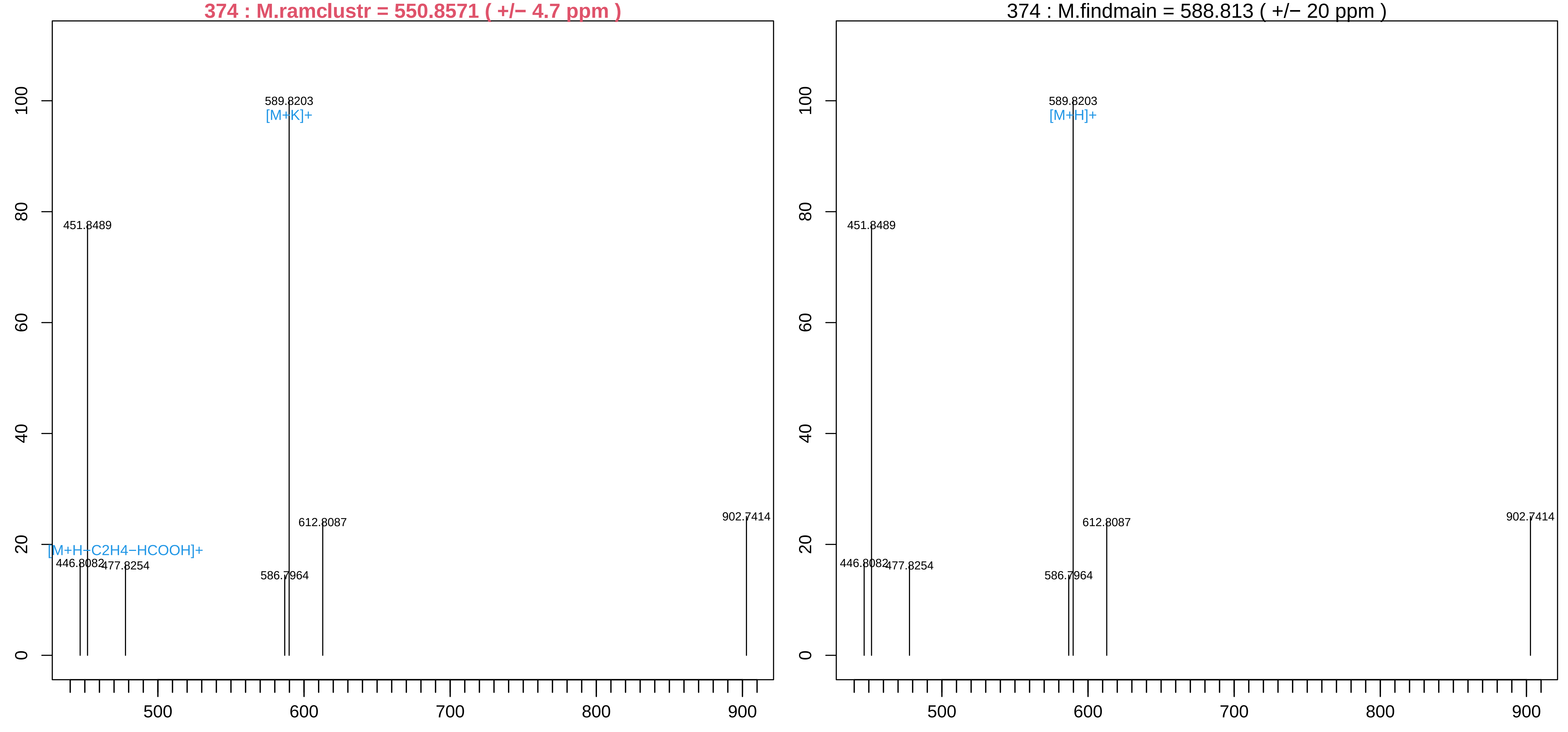
374 : M.ramclustr = 550.8571 (+/− 4.7 ppm)
589.8203
[M+K]+
451.8489
612.8087
902.7414
[M+H−C2H4−HCOOH]+
446.8082
477.8254
586.7964
374 : M.findmain = 588.813 (+/− 20 ppm)
589.8203
[M+H]+
451.8489
612.8087
902.7414
446.8082
477.8254
586.7964
0
20
40
60
80
100
500
600
700
800
900

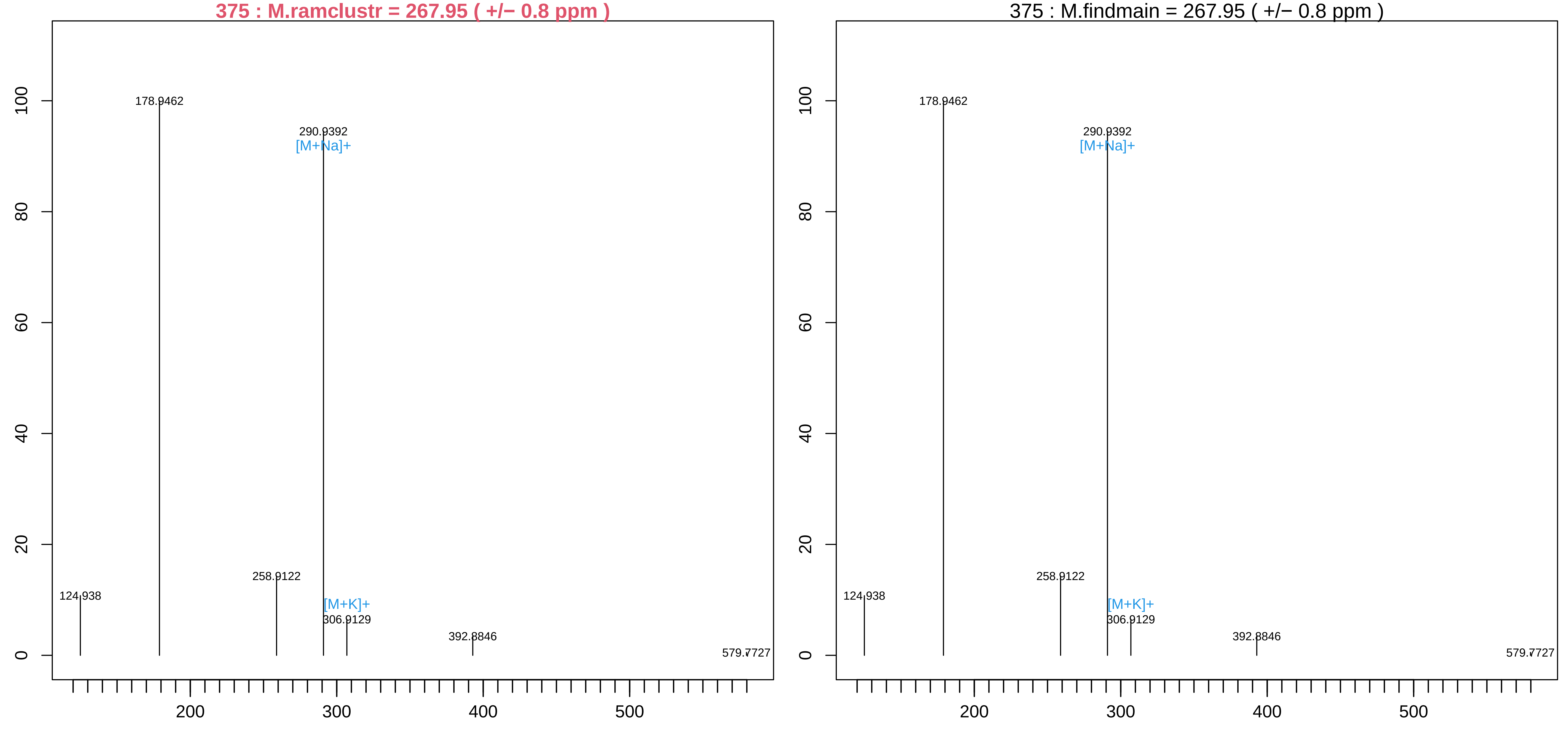
375 : M.ramclustr = 267.95 (+/− 0.8 ppm)
375 : M.findmain = 267.95 (+/− 0.8 ppm)
178.9462
290.9392
[M+Na]+
258.9122
124.938
[M+K]+
306.9129
392.8846
579.7727
0
20
40
60
80
100
200
300
400
500

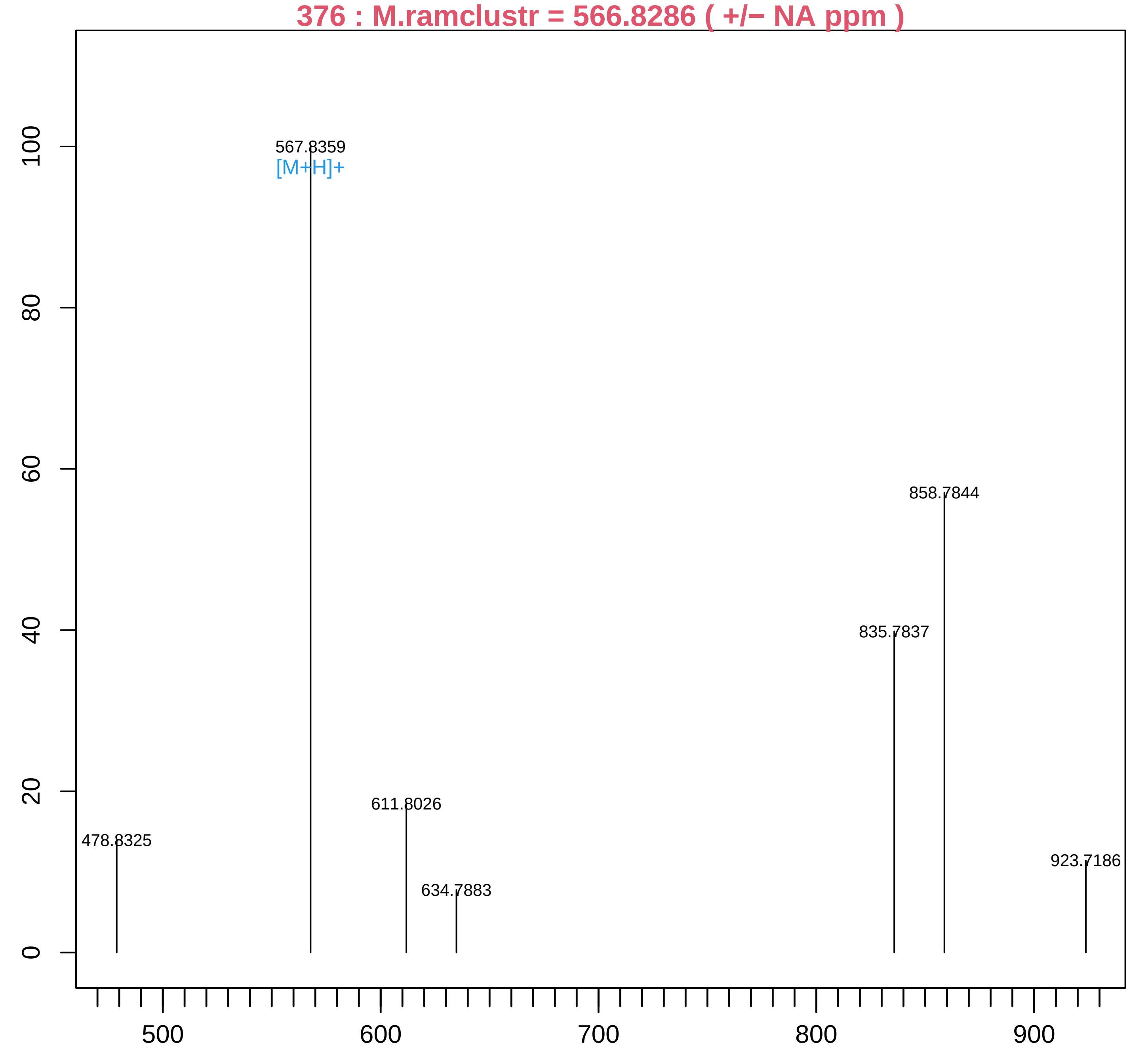
376 : M.ramclustr = 566.8286 (+/− NA ppm)
567.8359
[M+H]+
478.8325
611.8026
634.7883
835.7837
858.7844
923.7186
0
20
40
60
80
100
500
600
700
800
900
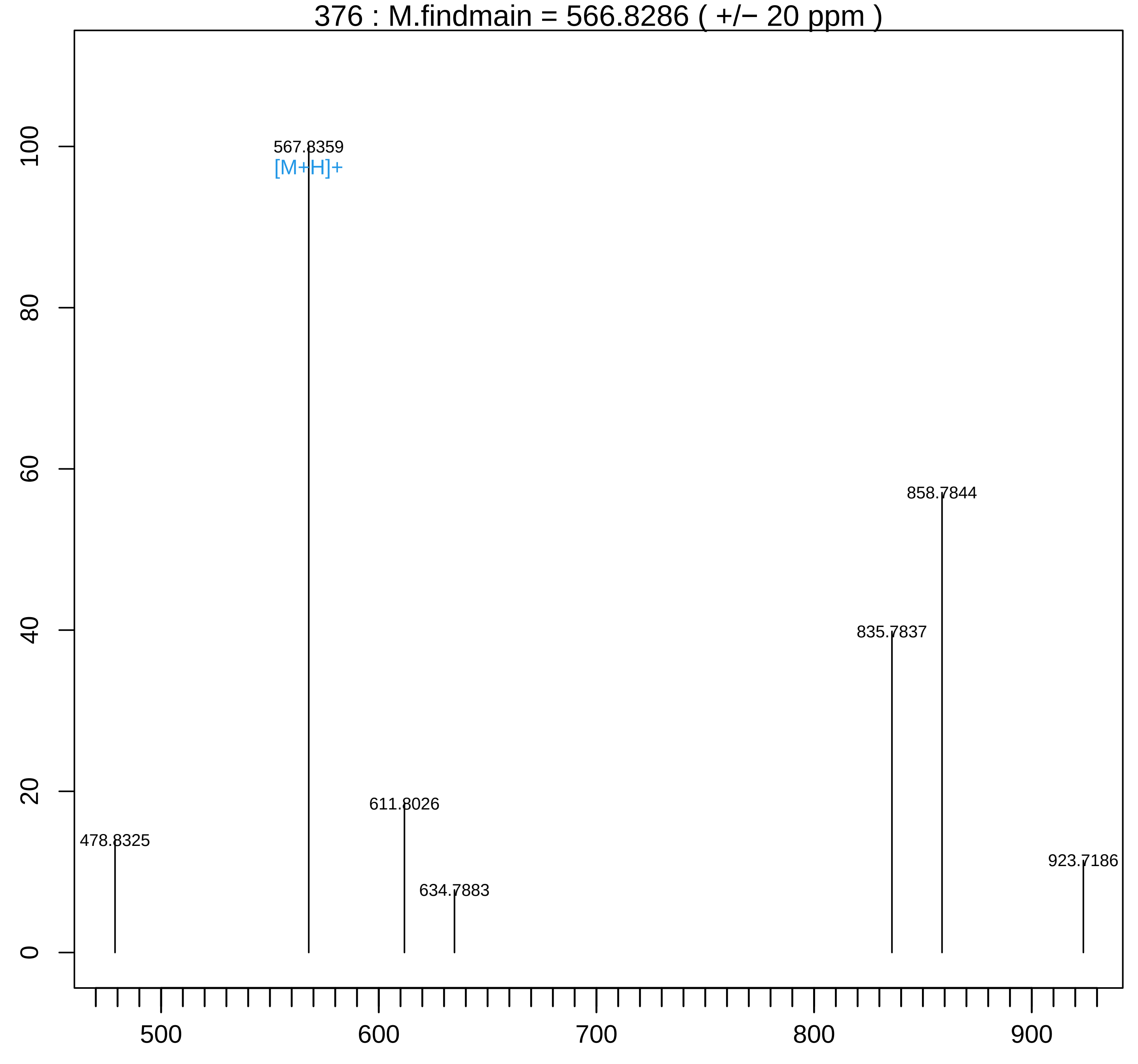
376 : M.findmain = 566.8286 (+/− 20 ppm)
567.8359
[M+H]+
478.8325
611.8026
634.7883
835.7837
858.7844
923.7186
0
20
40
60
80
100
500
600
700
800
900

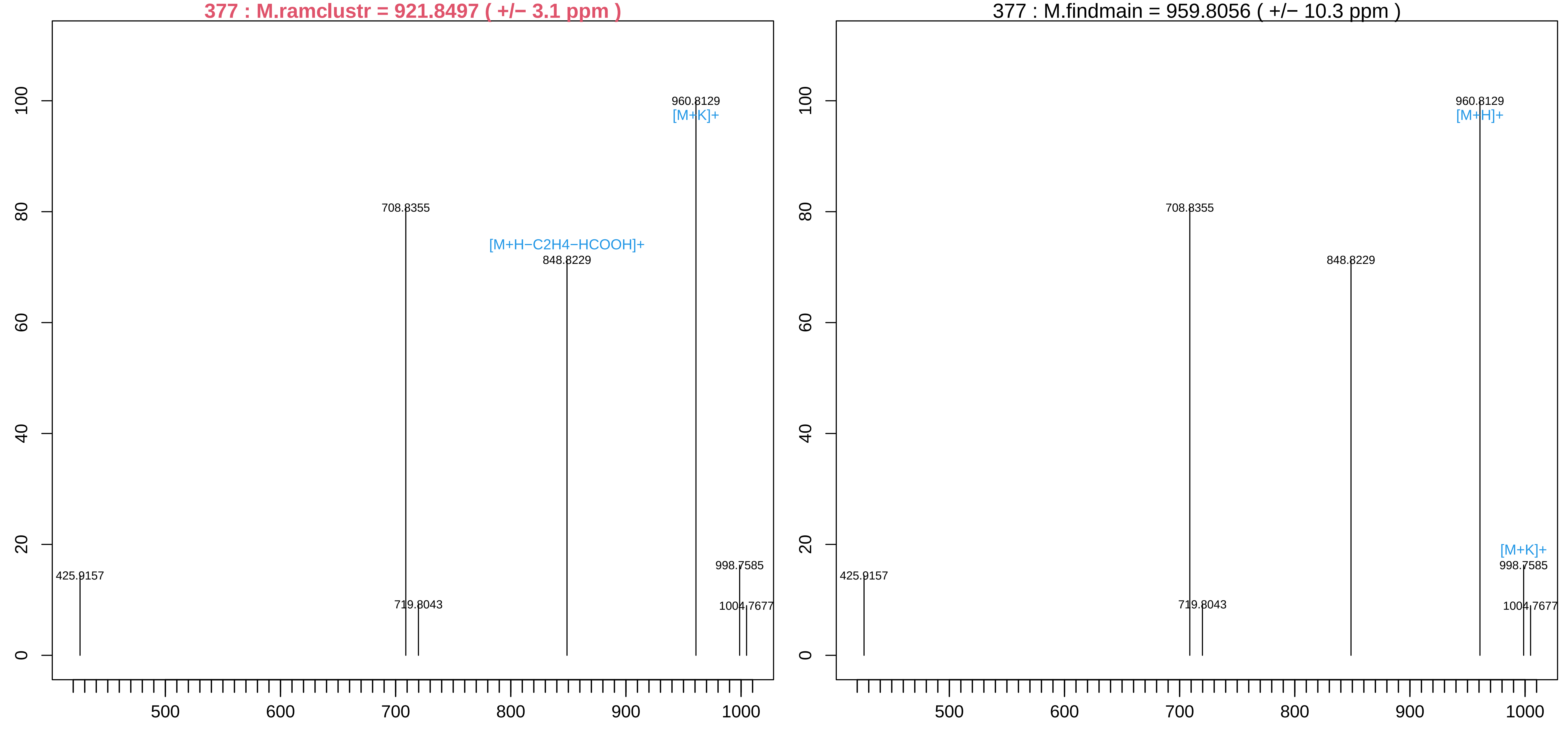

377 : M.ramclustr = 921.8497 (+/− 3.1 ppm)
960.8129
[M+K]+
708.8355
[M+H−C2H4−HCOOH]+
848.8229
998.7585
425.9157
719.8043
1004.7677
0
20
40
60
80
100
500
600
700
800
900
1000
377 : M.findmain = 959.8056 (+/− 10.3 ppm)
960.8129
[M+H]+
708.8355
848.8229
[M+K]+
998.7585
425.9157
719.8043
1004.7677

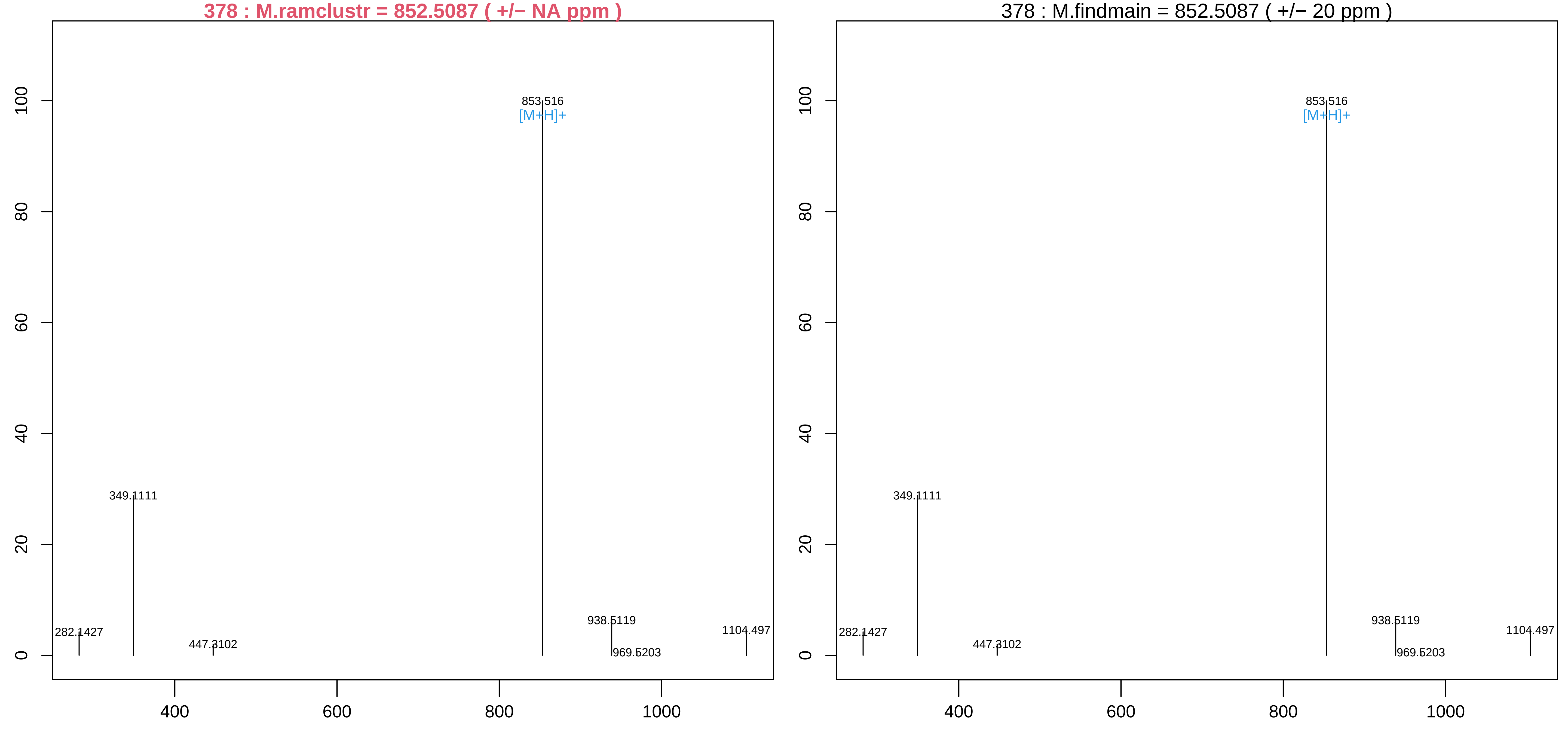
378 : M.ramclustr = 852.5087 (+/− NA ppm)
378 : M.findmain = 852.5087 (+/− 20 ppm)
853.516
[M+H]+
349.1111
282.1427
447.3102
938.5119
969.5203
1104.497
0
20
40
60
80
100
400
600
800
1000

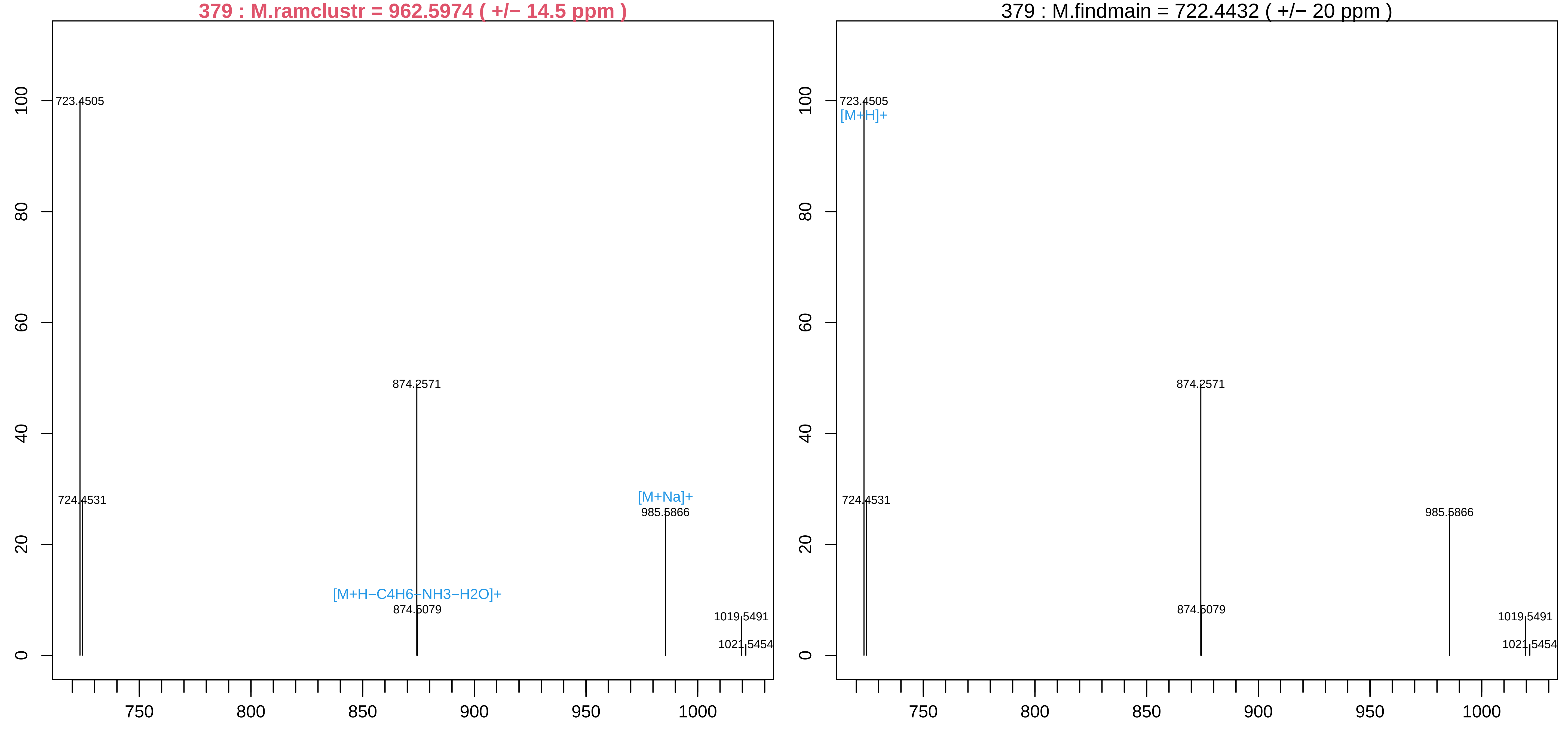

379 : M.ramclustr = 962.5974 (+/− 14.5 ppm)
723.4505
724.4531
874.2571
[M+H−C4H6−NH3−H2O]+
874.5079
[M+Na]+
985.5866
1019.5491
1021.5454
379 : M.findmain = 722.4432 (+/− 20 ppm)
723.4505
[M+H]+
724.4531
874.2571
874.5079
985.5866
1019.5491
1021.5454
0
20
40
60
80
100
750
800
850
900
950
1000

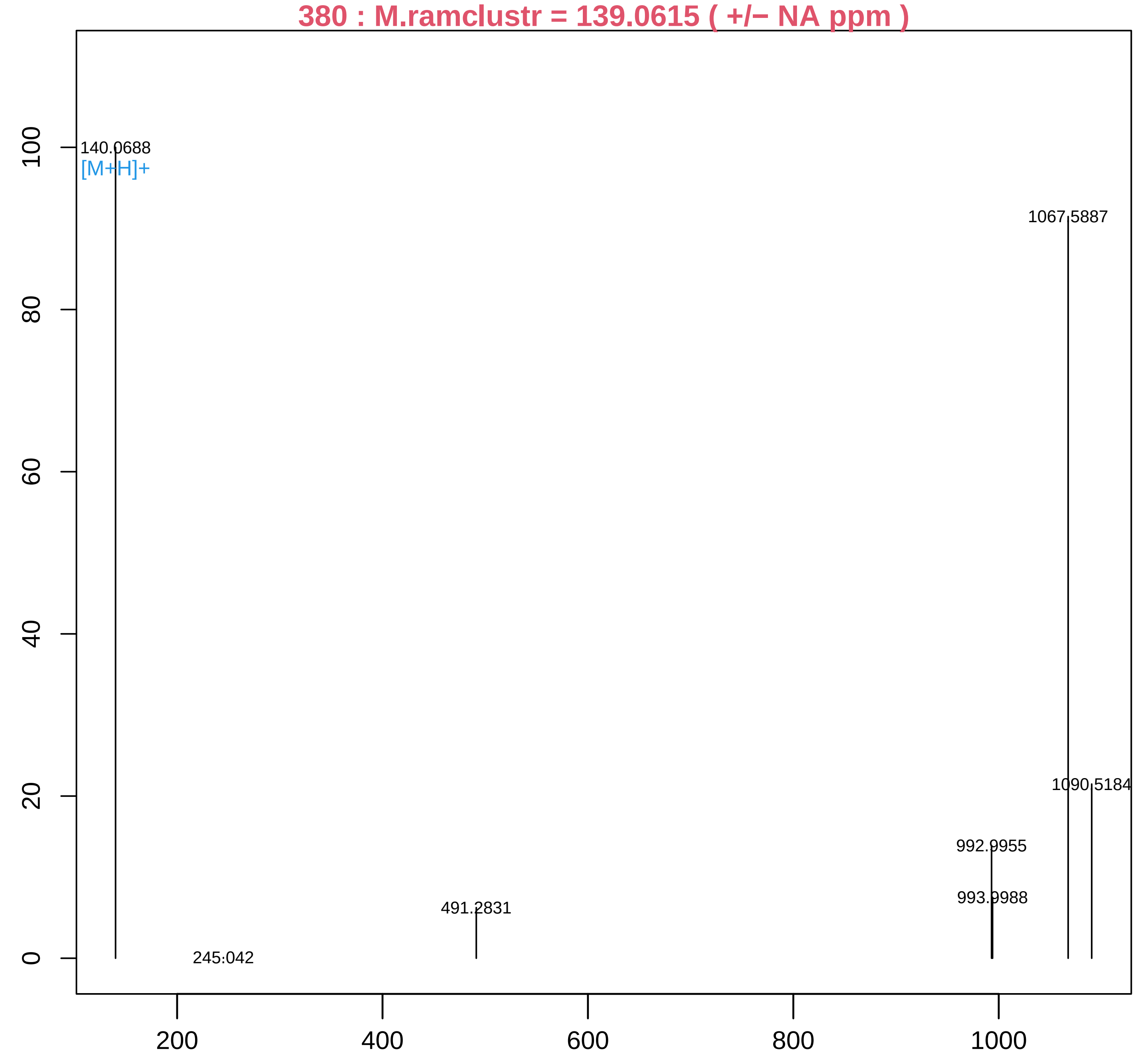
380 : M.ramclustr = 139.0615 (+/− NA ppm)
140.0688
[M+H]+
1067.5887
1090.5184
992.9955
993.9988
491.2831
245.042
0
20
40
60
80
100
200
400
600
800
1000

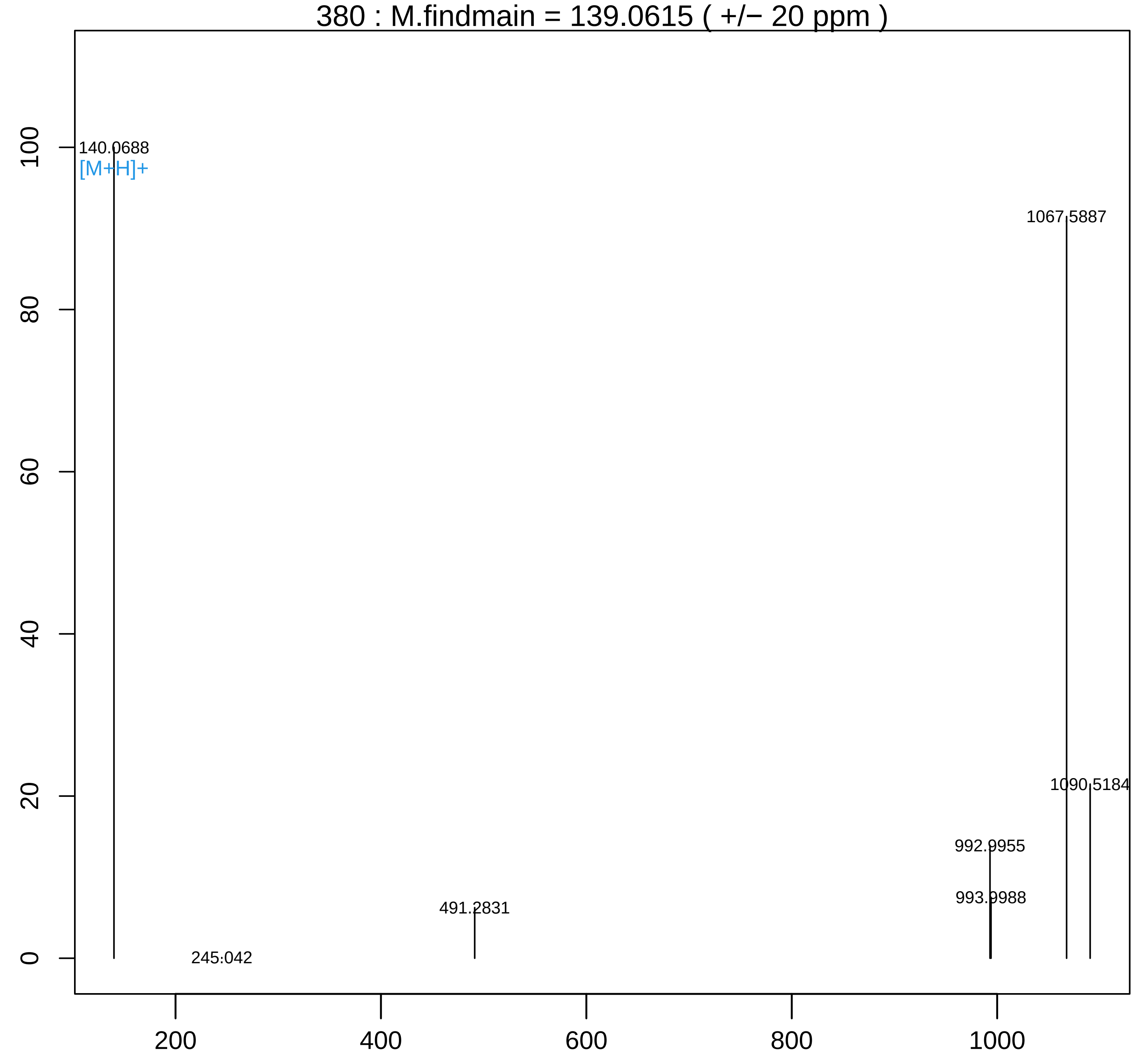
380 : M.findmain = 139.0615 (+/− 20 ppm)
140.0688
[M+H]+
1067.5887
1090.5184
992.9955
993.9988
491.2831
245.042
0
20
40
60
80
100
200
400
600
800
1000

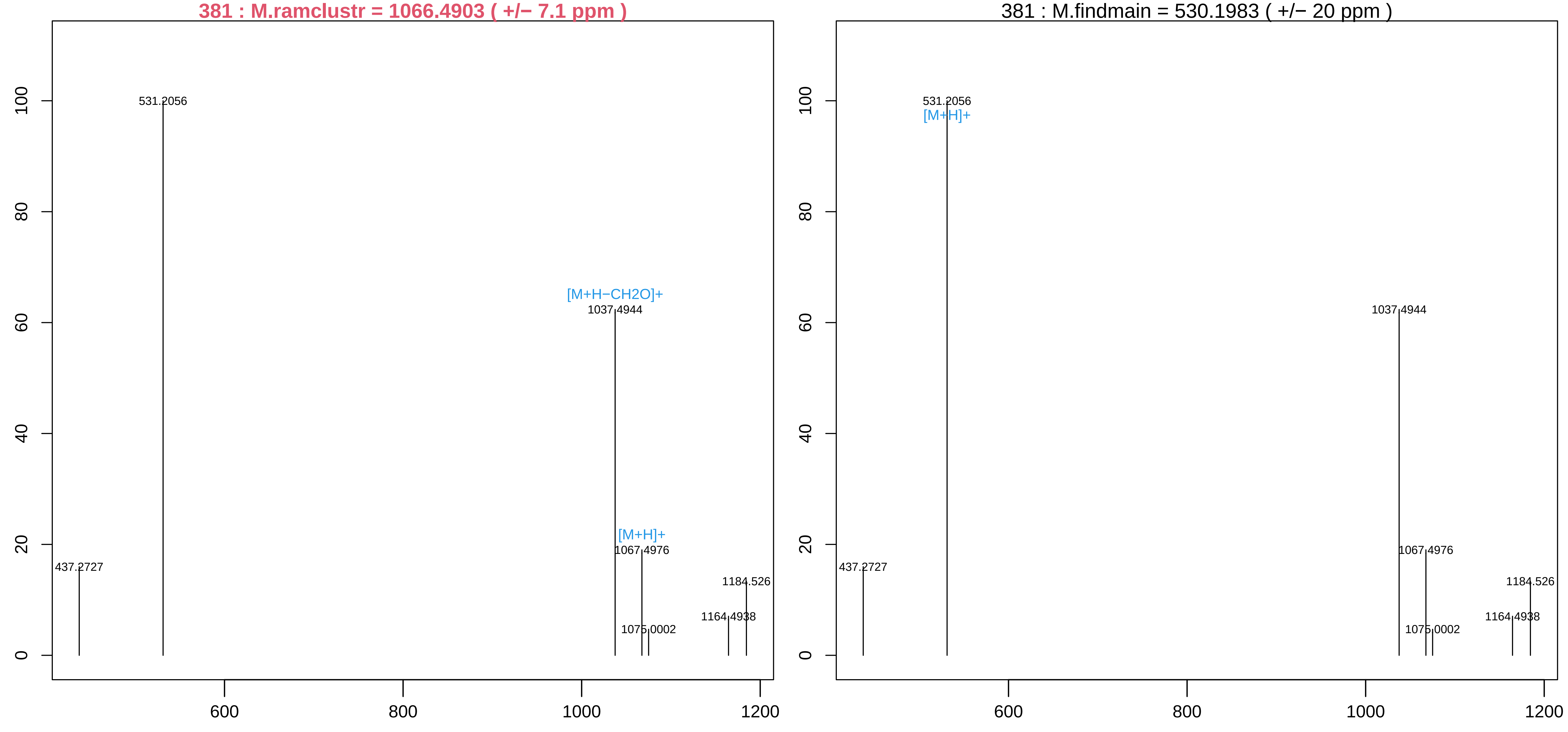
381 : M.ramclustr = 1066.4903 (+/− 7.1 ppm)
531.2056
437.2727
[M+H−CH2O]+
1037.4944
[M+H]+
1067.4976
1075.0002
1164.4938
1184.526
381 : M.findmain = 530.1983 (+/− 20 ppm)
531.2056
[M+H]+
437.2727
1037.4944
1067.4976
1075.0002
1164.4938
1184.526
0
20
40
60
80
100
600
800
1000
1200

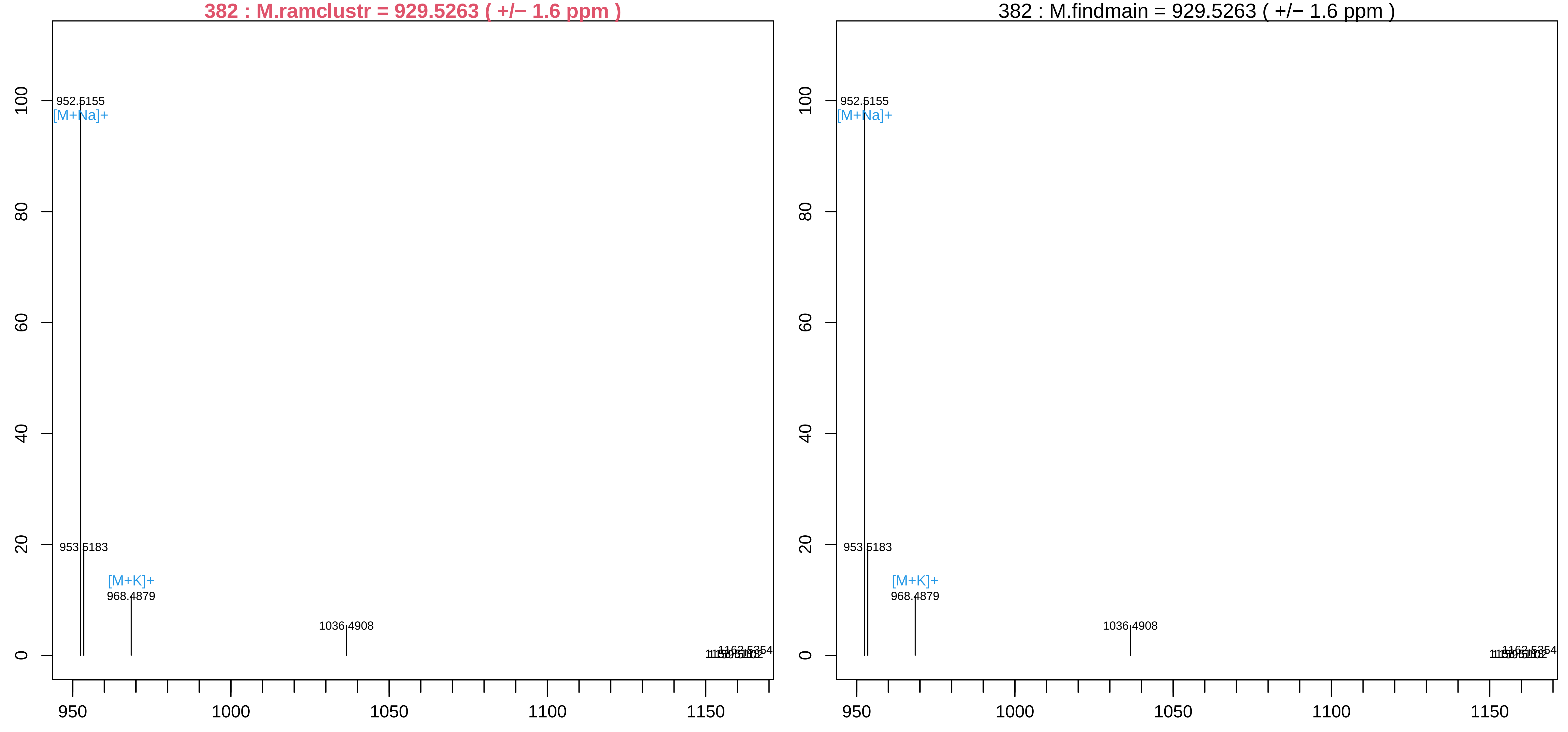
382 : M.ramclustr = 929.5263 (+/− 1.6 ppm)
952.5155
[M+Na]+
953.5183
[M+K]+
968.4879
1036.4908
1162.5354
382 : M.findmain = 929.5263 (+/− 1.6 ppm)
952.5155
[M+Na]+
953.5183
[M+K]+
968.4879
1036.4908
1162.5354

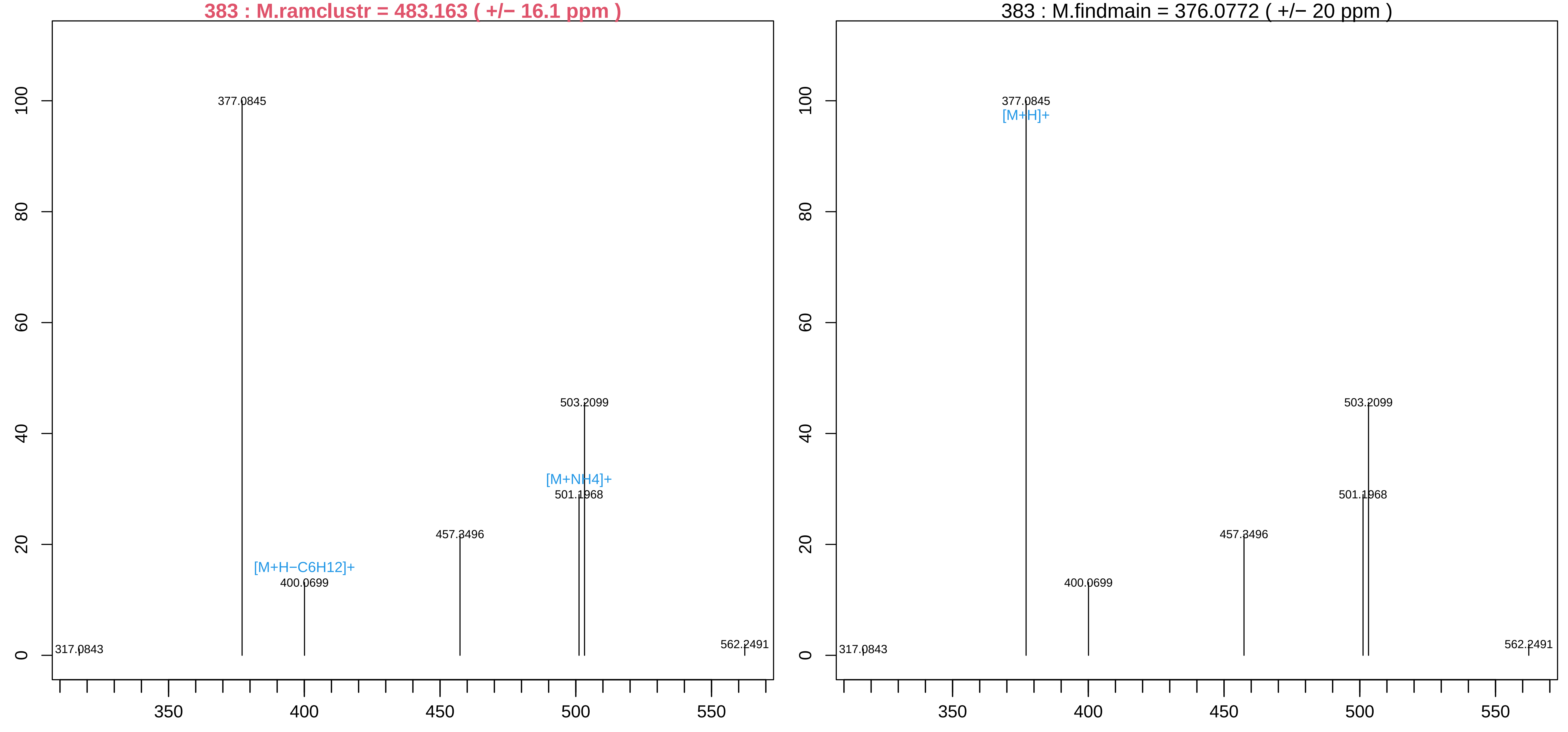
383 : M.ramclustr = 483.163 (+/− 16.1 ppm)
377.0845
503.2099
[M+NH4]+
501.1968
457.3496
[M+H−C6H12]+
400.0699
317.0843
562.2491
0
20
40
60
80
100
350
400
450
500
550
383 : M.findmain = 376.0772 (+/− 20 ppm)
377.0845
[M+H]+
503.2099
501.1968
457.3496
400.0699
317.0843
562.2491

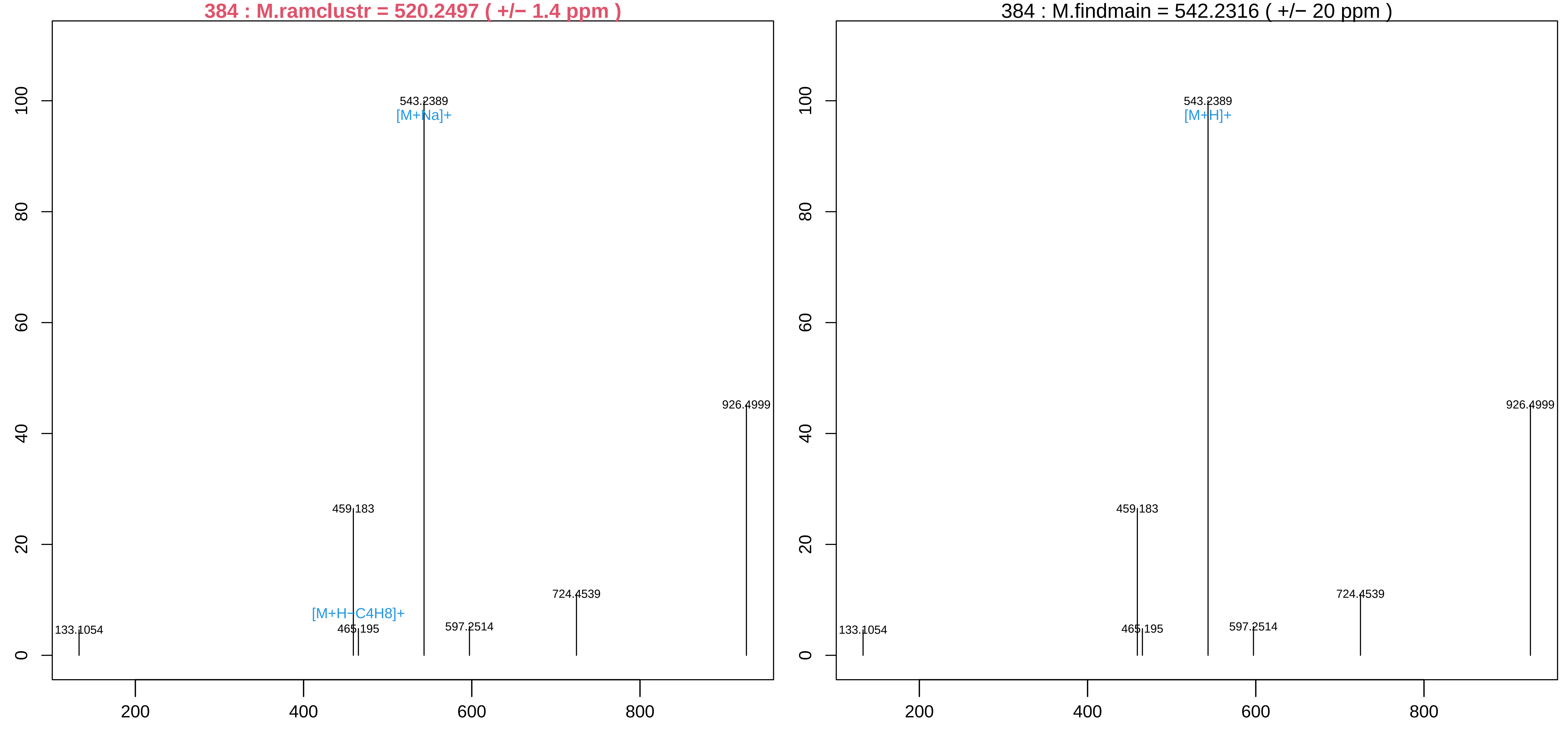
384 : M.ramclustr = 520.2497 (+/− 1.4 ppm)
543.2389
[M+Na]+
926.4999
459.183
724.4539
[M+H−C4H8]+
133.1054
465.195
597.2514
384 : M.findmain = 542.2316 (+/− 20 ppm)
543.2389
[M+H]+
926.4999
459.183
724.4539
133.1054
465.195
597.2514

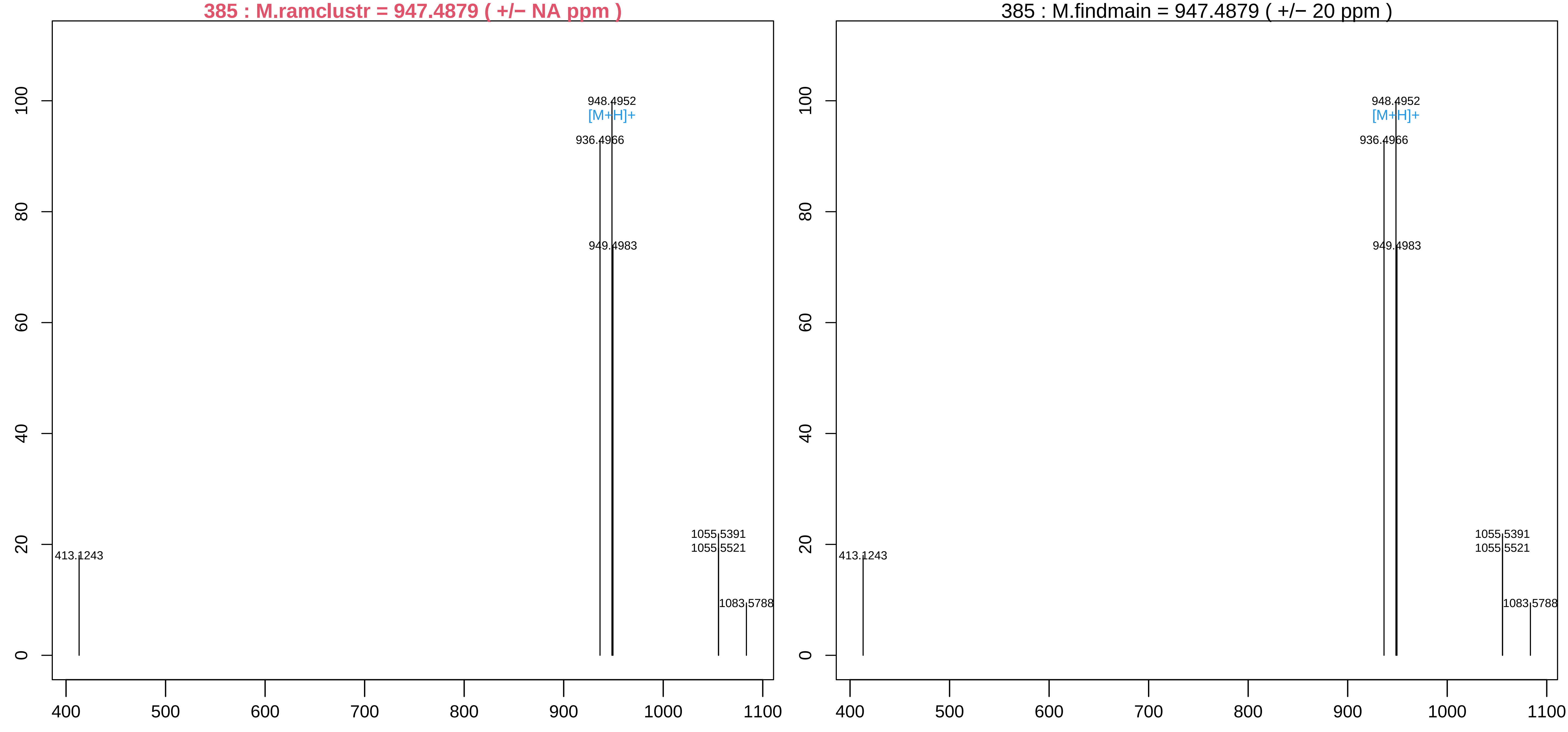
385 : M.ramclustr = 947.4879 (+/− NA ppm)
948.4952
[M+H]+
936.4966
949.4983
1055.5391
1055.5521
413.1243
1083.5788
385 : M.findmain = 947.4879 (+/− 20 ppm)
948.4952
[M+H]+
936.4966
949.4983
1055.5391
1055.5521
413.1243
1083.5788

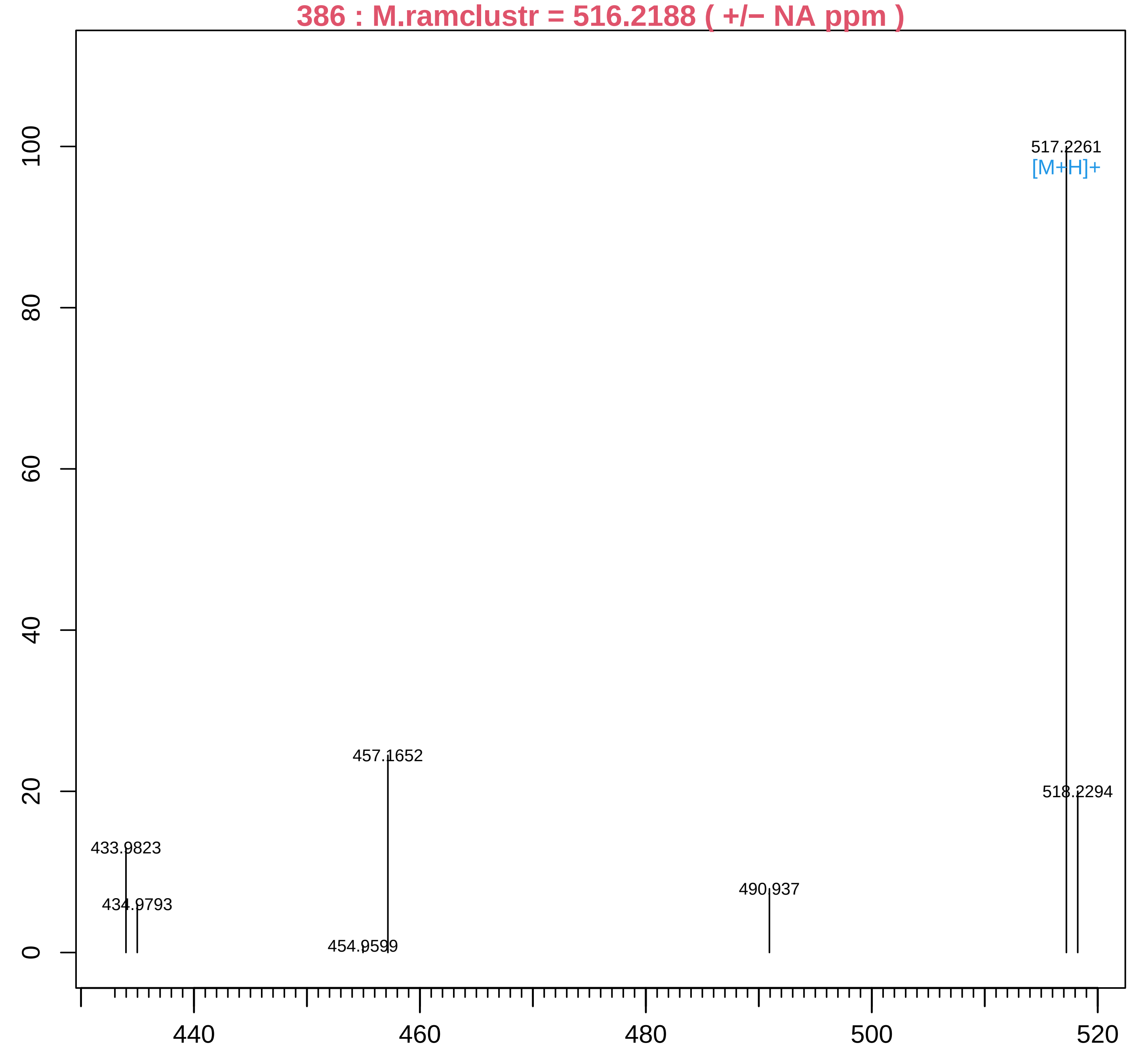
386 : M.ramclustr = 516.2188 (+/− NA ppm)
517.2261
[M+H]+
457.1652
518.2294
433.9823
434.9793
454.9599
490.937
0
20
40
60
80
100
440
460
480
500
520

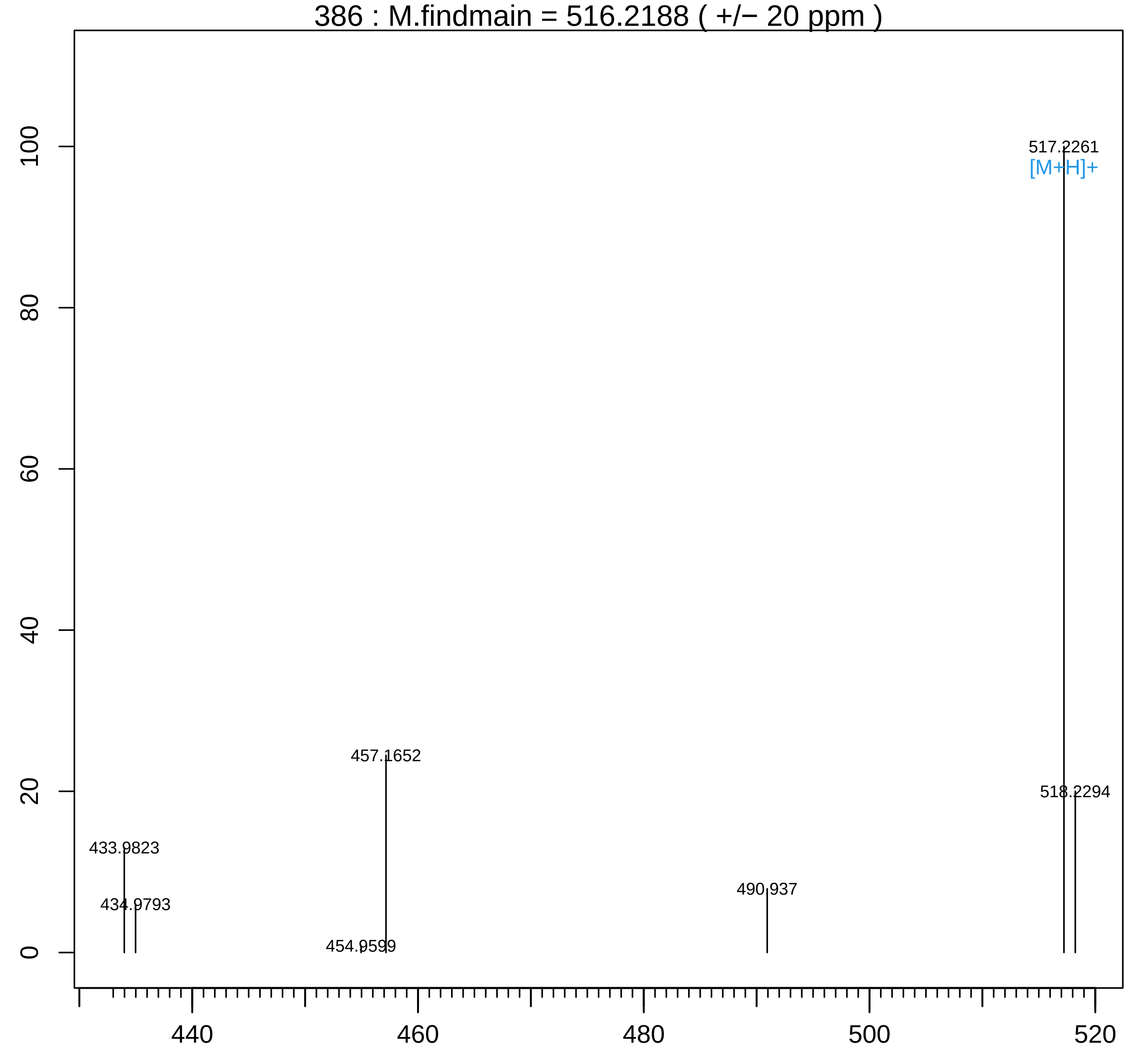
386 : M.findmain = 516.2188 (+/− 20 ppm)
517.2261
[M+H]+
457.1652
518.2294
433.9823
434.9793
454.9599
490.937
0
20
40
60
80
100
440
460
480
500
520

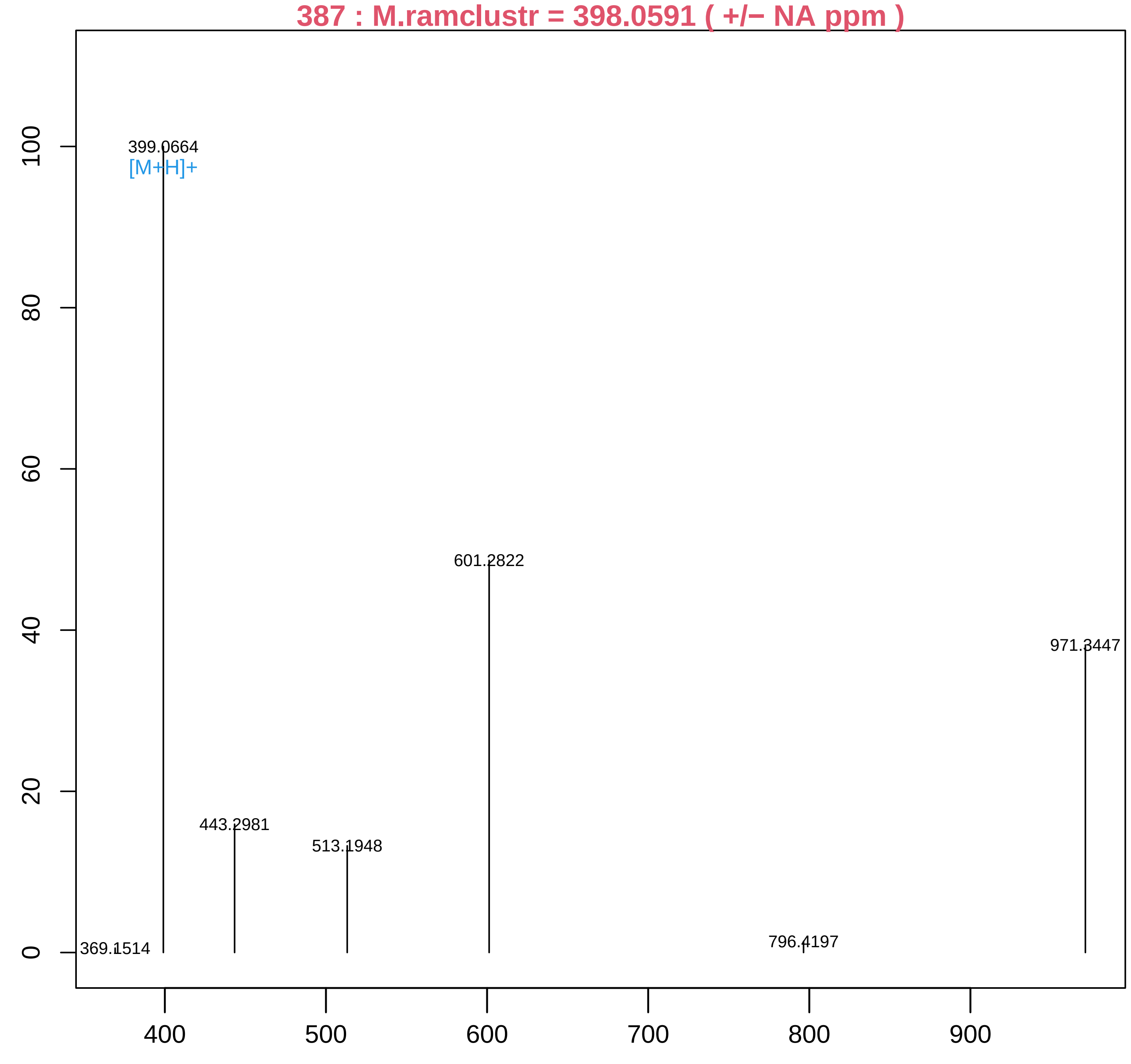
387 : M.ramclustr = 398.0591 (+/− NA ppm)
399.0664
[M+H]+
601.2822
971.3447
443.2981
513.1948
369.1514
796.4197
0
20
40
60
80
100
400
500
600
700
800
900

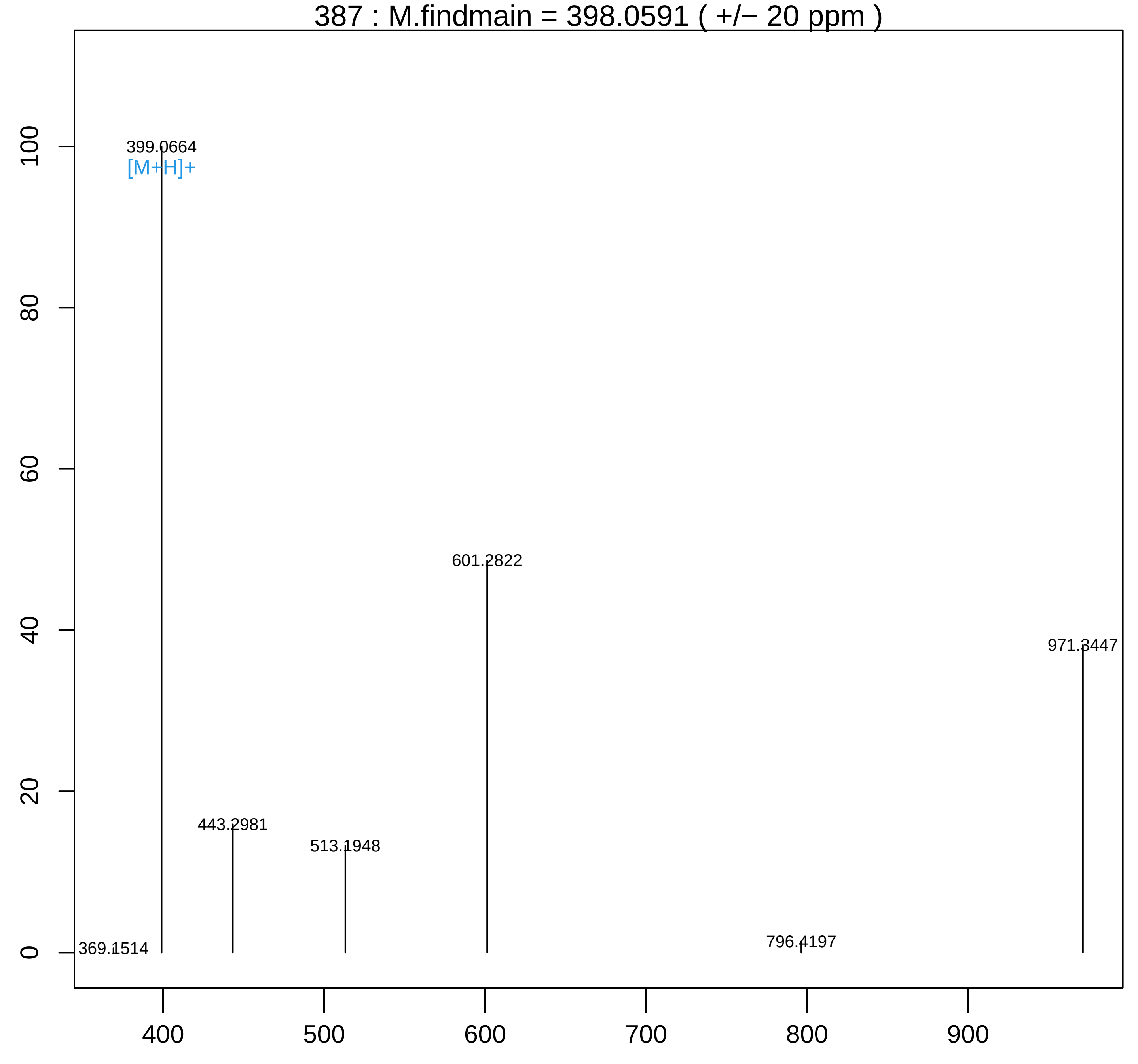
387 : M.findmain = 398.0591 (+/− 20 ppm)
399.0664
[M+H]+
601.2822
971.3447
443.2981
513.1948
369.1514
796.4197
0
20
40
60
80
100
400
500
600
700
800
900

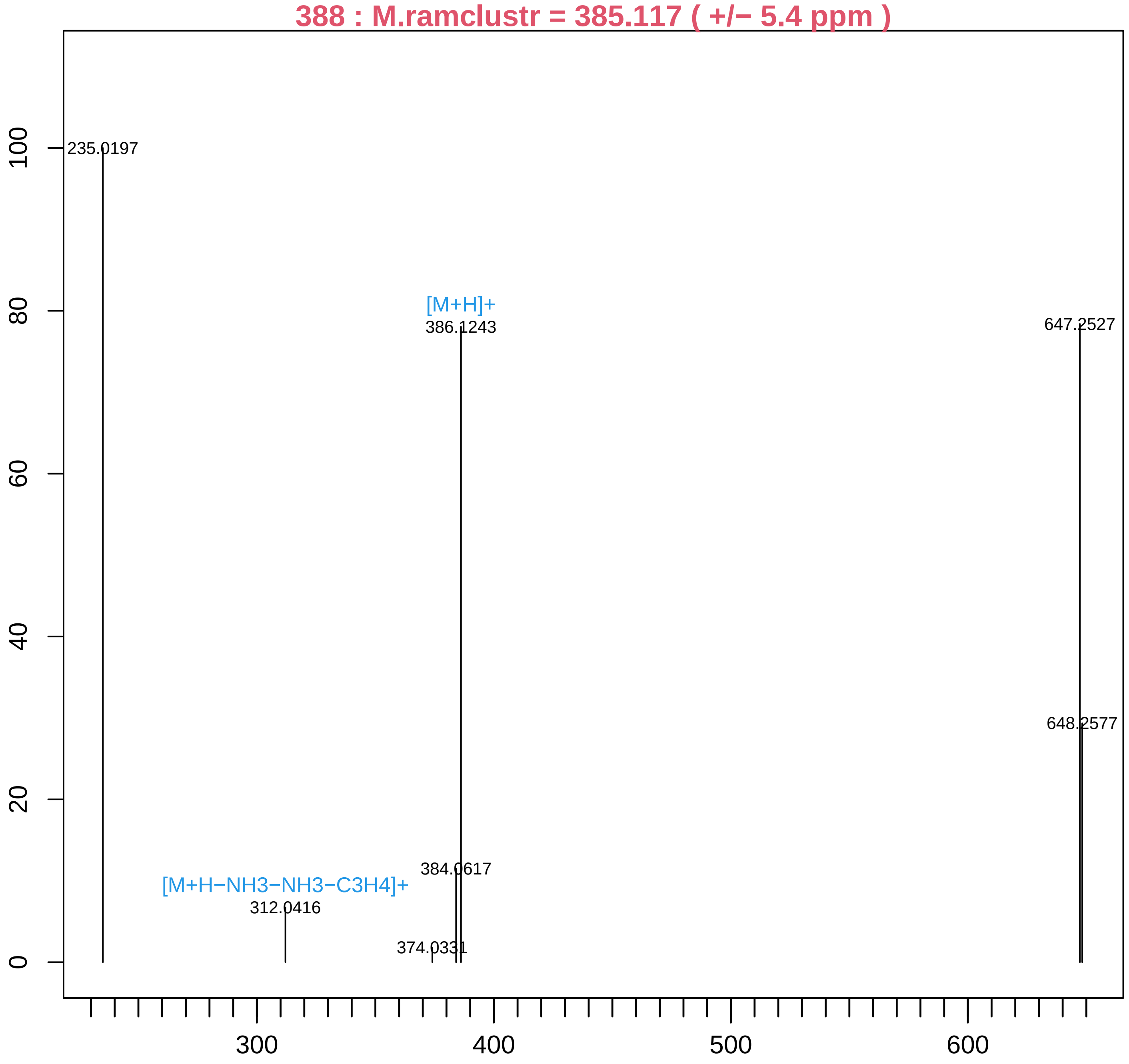
388 : M.ramclustr = 385.117 (+/− 5.4 ppm)
235.0197
[M+H]+
386.1243
647.2527
648.2577
384.0617
[M+H−NH3−NH3−C3H4]+
312.0416
374.0331
0
20
40
60
80
100
300
400
500
600
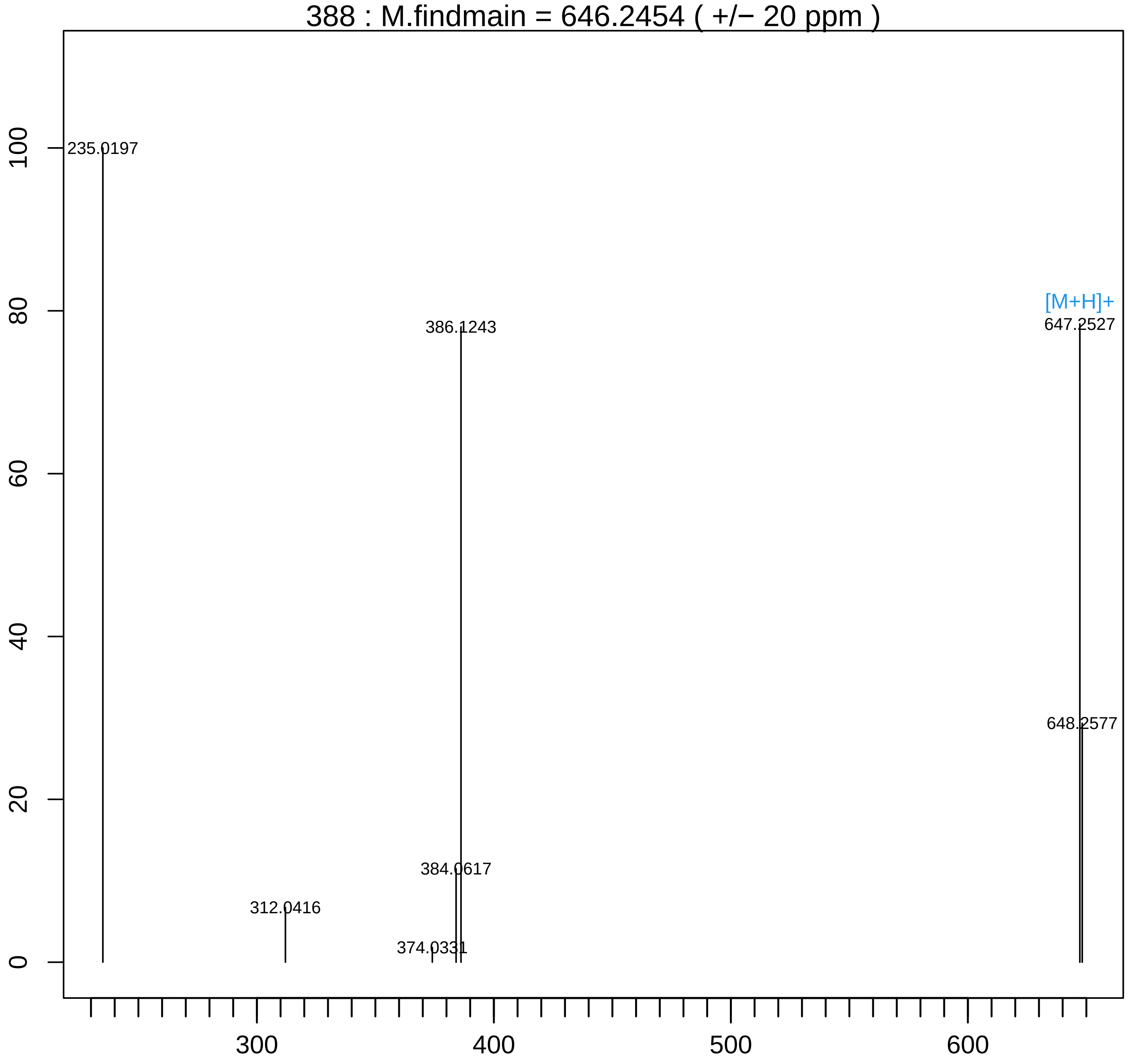
388 : M.findmain = 646.2454 (+/− 20 ppm)
235.0197
386.1243
[M+H]+
647.2527
648.2577
384.0617
312.0416
374.0331
0
20
40
60
80
100
300
400
500
600

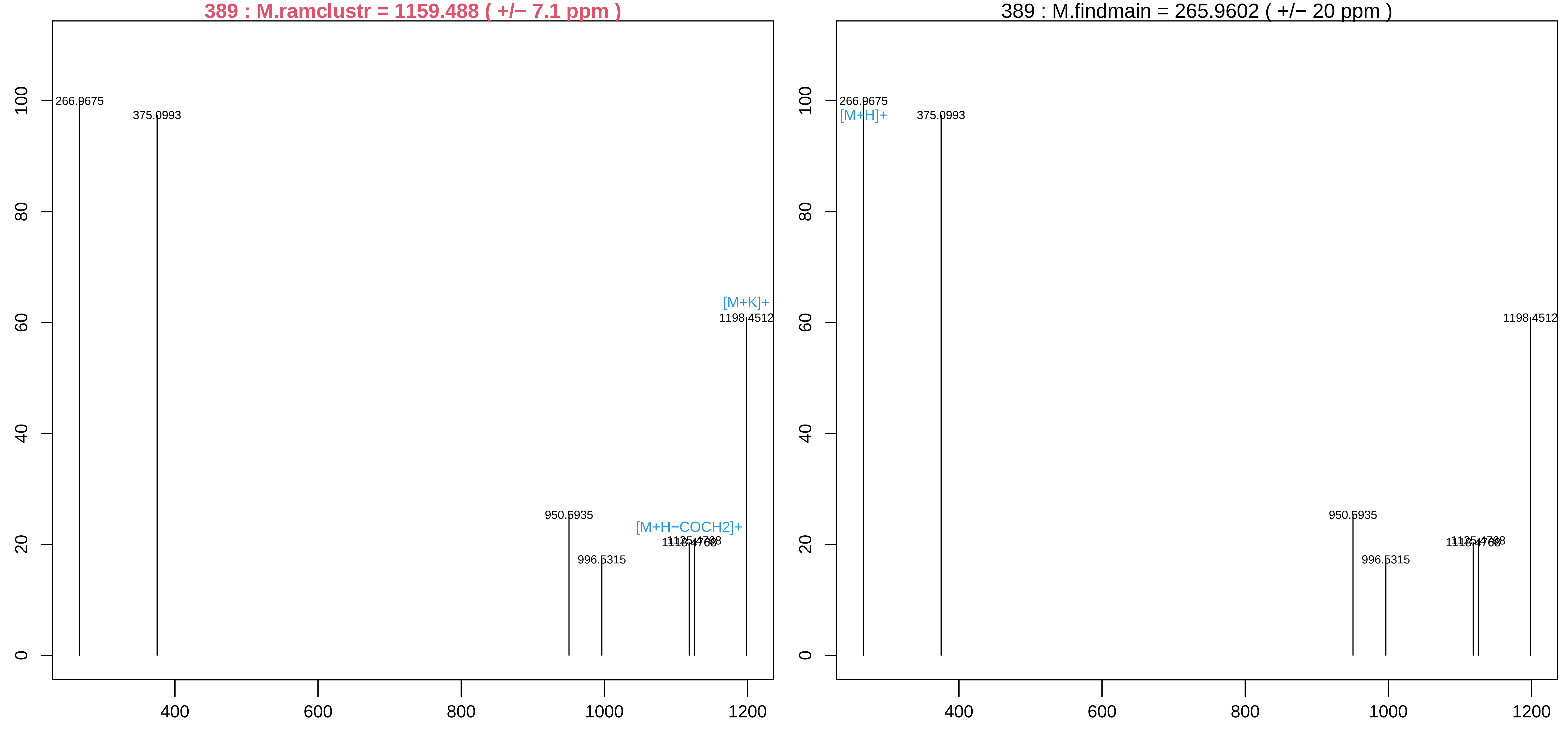
389 : M.ramclustr = 1159.488 (+/− 7.1 ppm)
266.9675
375.0993
[M+K]+
1198.4512
950.5935
996.5315
[M+H−COCH2]+
1125.4788
1118.4768
389 : M.findmain = 265.9602 (+/− 20 ppm)
266.9675
[M+H]+
375.0993
1198.4512
950.5935
996.5315
1125.4788
1118.4768
0
20
40
60
80
100
400
600
800
1000
1200

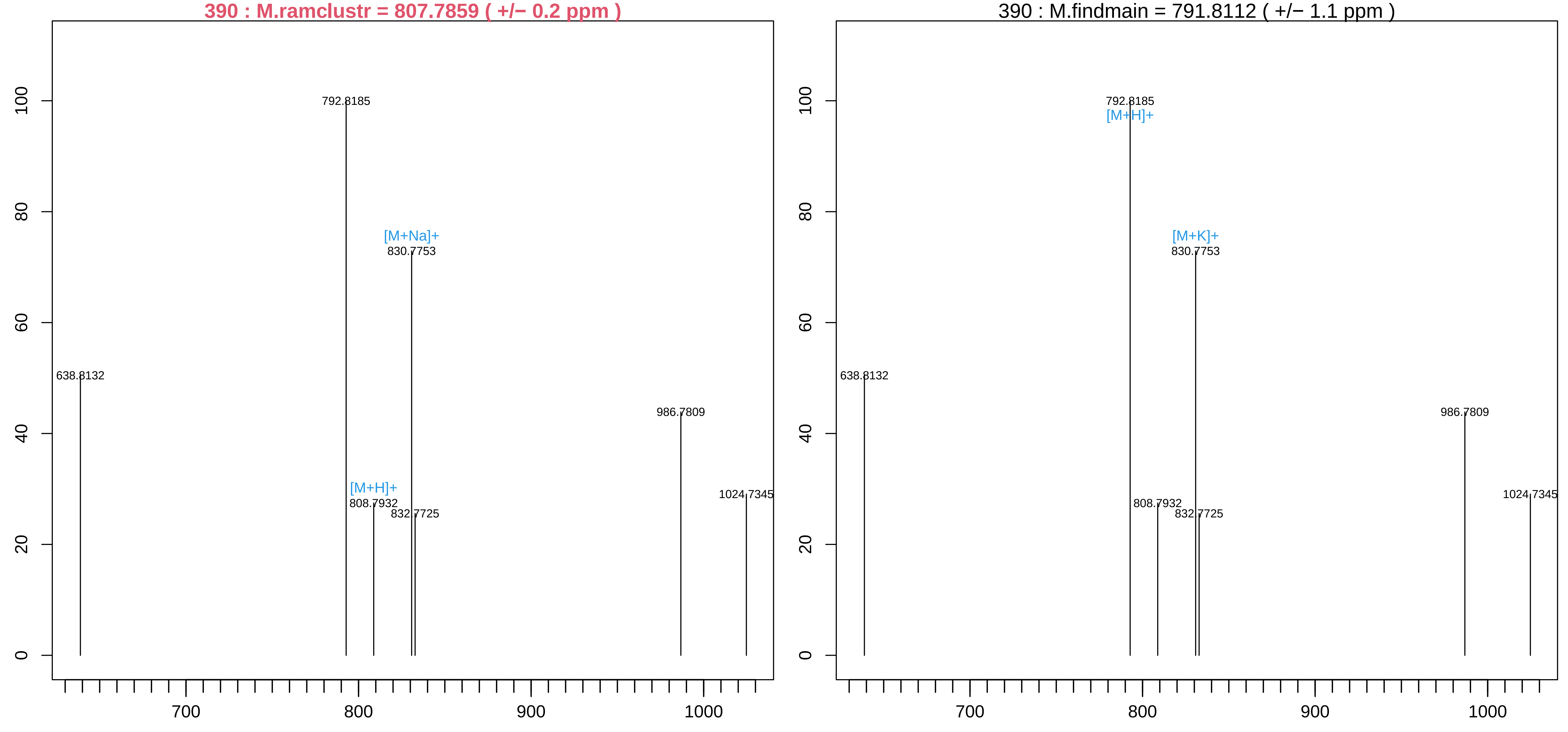
390 : M.ramclustr = 807.7859 (+/− 0.2 ppm)
792.8185
[M+Na]+
830.7753
638.8132
986.7809
[M+H]+
808.7932
832.7725
1024.7345
0
20
40
60
80
100
700
800
900
1000
390 : M.findmain = 791.8112 (+/− 1.1 ppm)
792.8185
[M+H]+
[M+K]+
830.7753
638.8132
986.7809
808.7932
832.7725
1024.7345

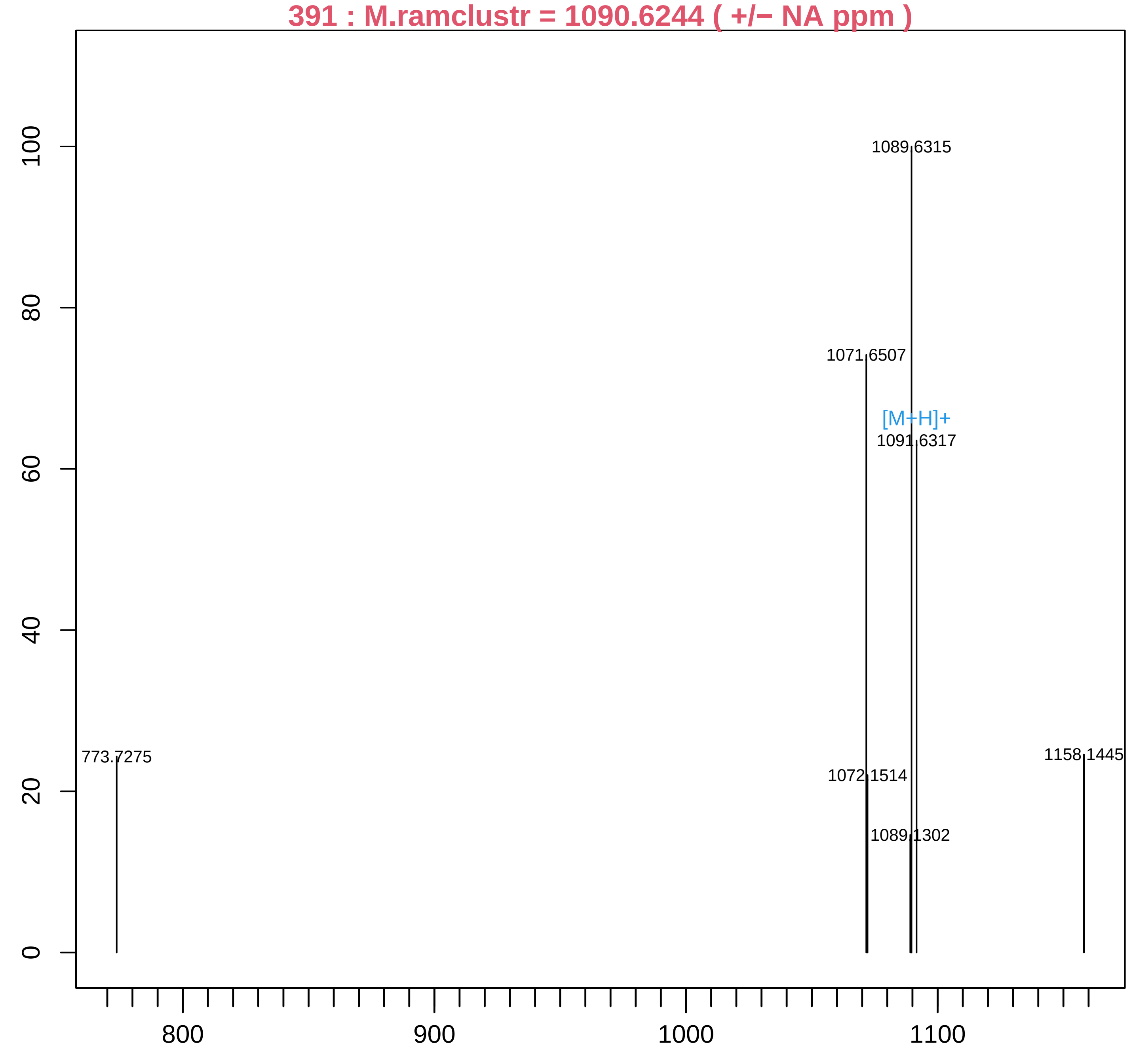
391 : M.ramclustr = 1090.6244 (+/− NA ppm)
1089.6315
1071.6507
[M+H]+
1091.6317
773.7275
1158.1445
1072.1514
1089.1302
0
20
40
60
80
100
800
900
1000
1100

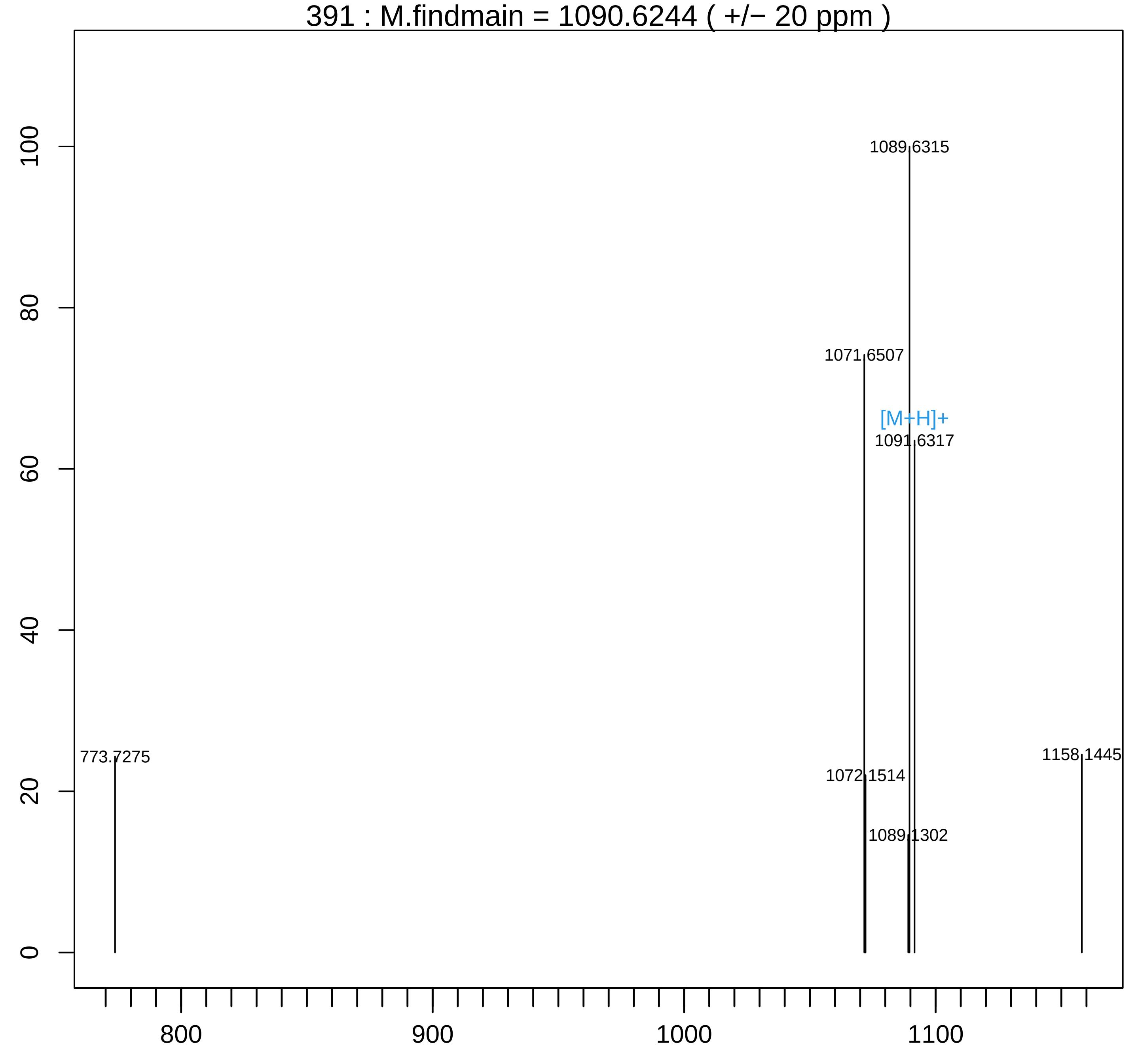
391 : M.findmain = 1090.6244 (+/− 20 ppm)
1089.6315
1071.6507
[M+H]+
1091.6317
773.7275
1158.1445
1072.1514
1089.1302
0
20
40
60
80
100
800
900
1000
1100

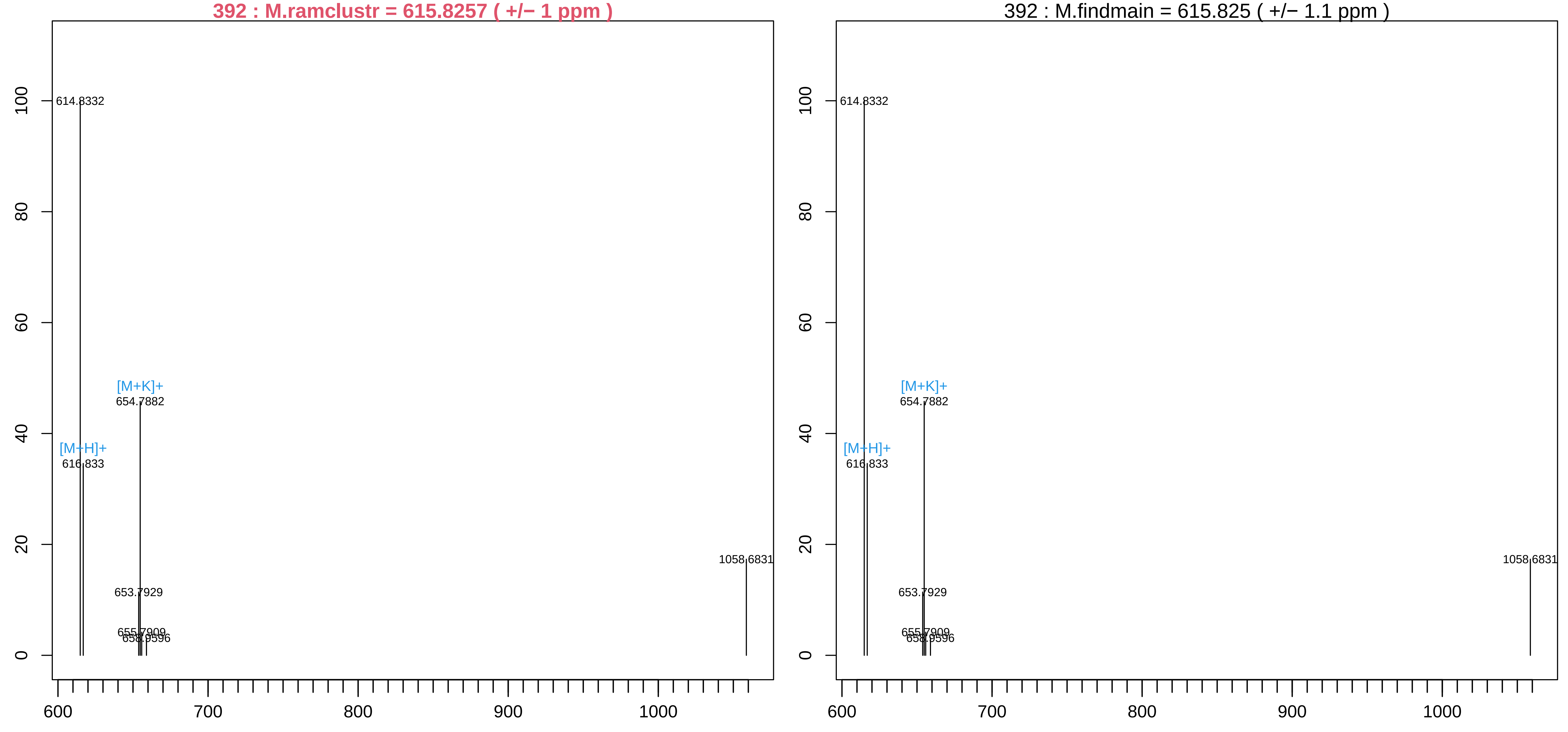
392 : M.ramclustr = 615.8257 (+/− 1 ppm)
614.8332
[M+K]+
654.7882
[M+H]+
616.833
653.7929
655.7909
658.9596
1058.6831
0
20
40
60
80
100
600
700
800
900
1000
392 : M.findmain = 615.825 (+/− 1.1 ppm)
614.8332
[M+K]+
654.7882
[M+H]+
616.833
653.7929
655.7909
658.9596
1058.6831
0
20
40
60
80
100
600
700
800
900
1000

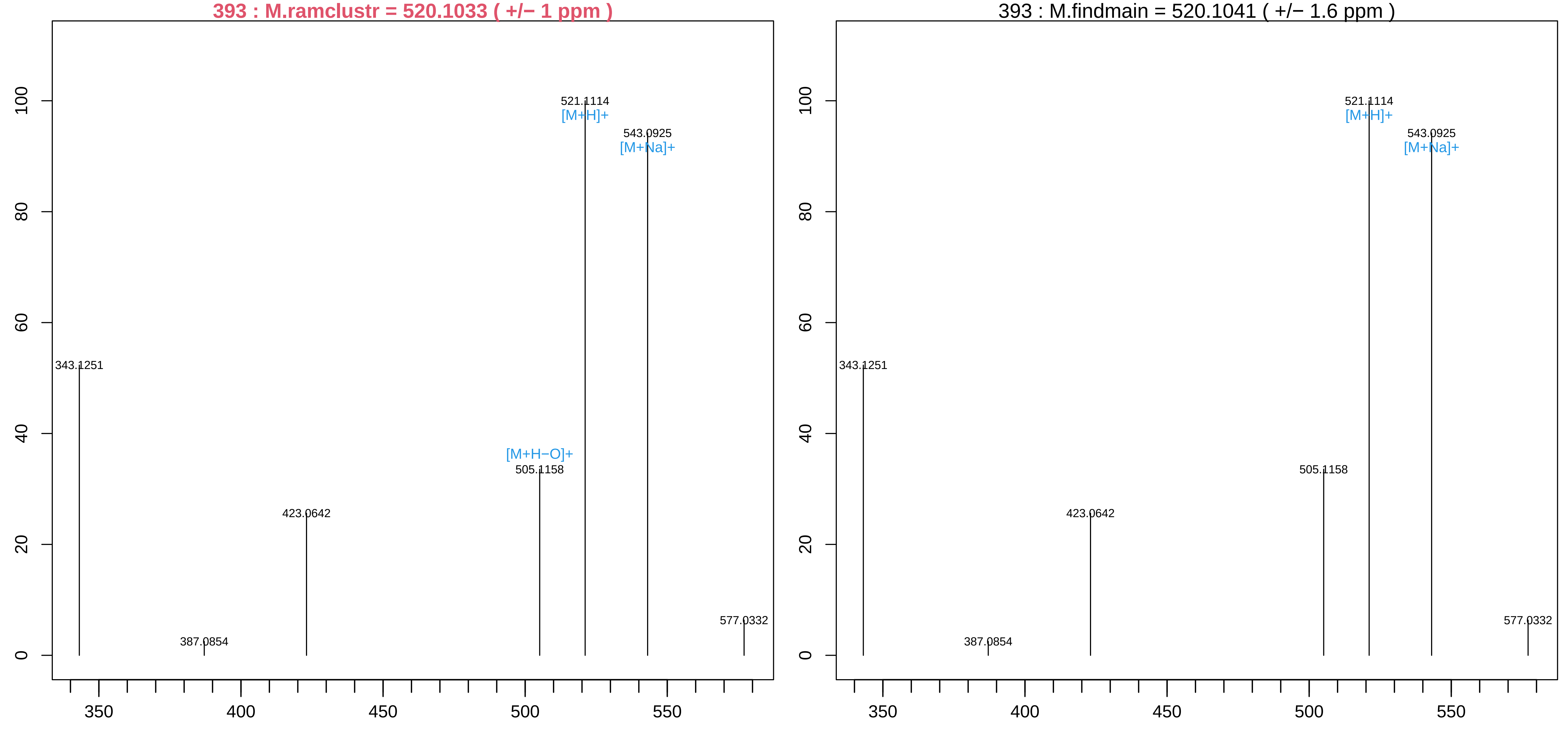
393 : M.ramclustr = 520.1033 (+/− 1 ppm)
393 : M.findmain = 520.1041 (+/− 1.6 ppm)
521.1114
[M+H]+
543.0925
[M+Na]+
343.1251
[M+H−O]+
505.1158
423.0642
387.0854
577.0332
0
20
40
60
80
100
350
400
450
500
550

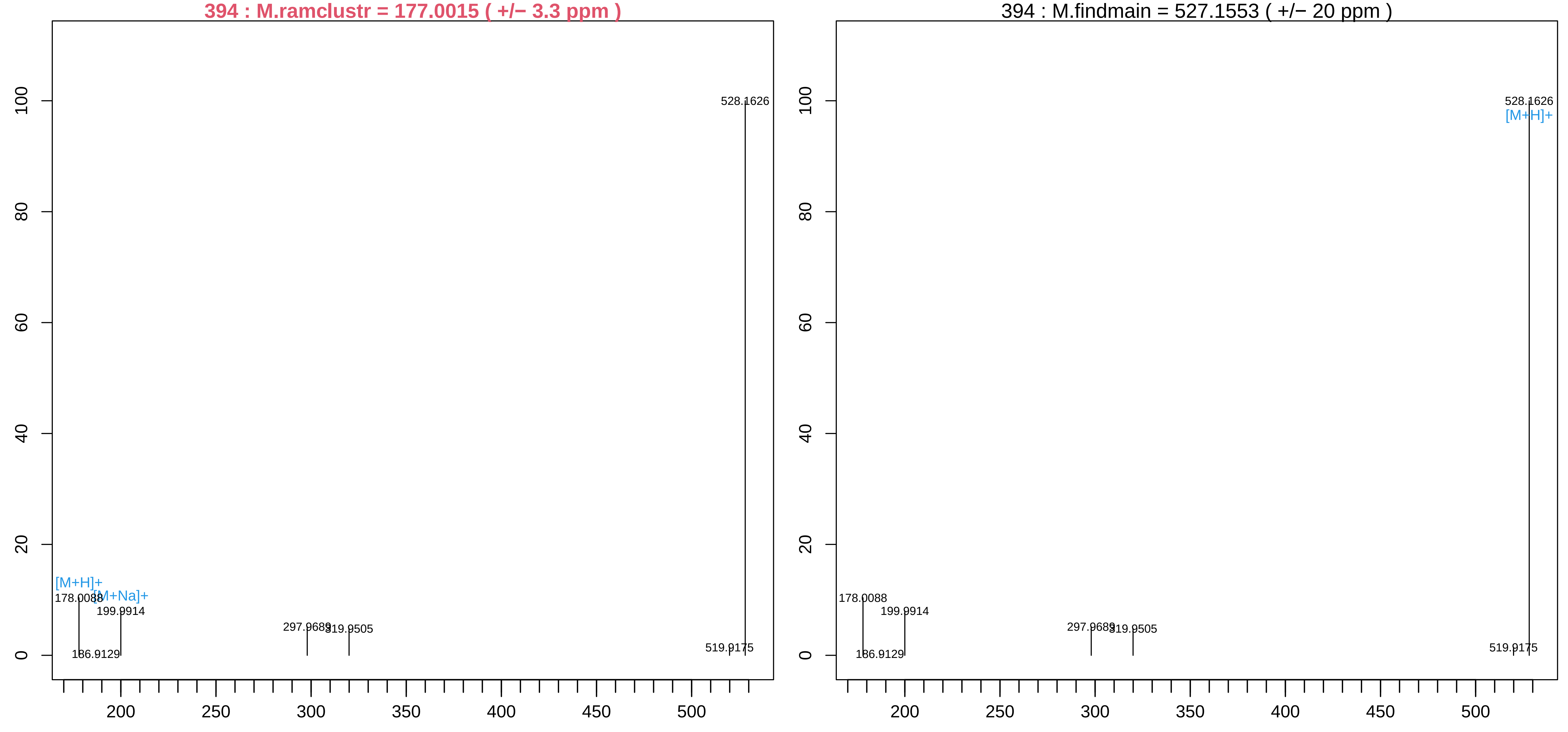
394 : M.ramclustr = 177.0015 (+/− 3.3 ppm)
528.1626
[M+H]+
178.0088
[M+Na]+
199.9914
186.9129
297.9689
319.9505
519.9175
394 : M.findmain = 527.1553 (+/− 20 ppm)
528.1626
[M+H]+
178.0088
199.9914
186.9129
297.9689
319.9505
519.9175
0
20
40
60
80
100
200
250
300
350
400
450
500

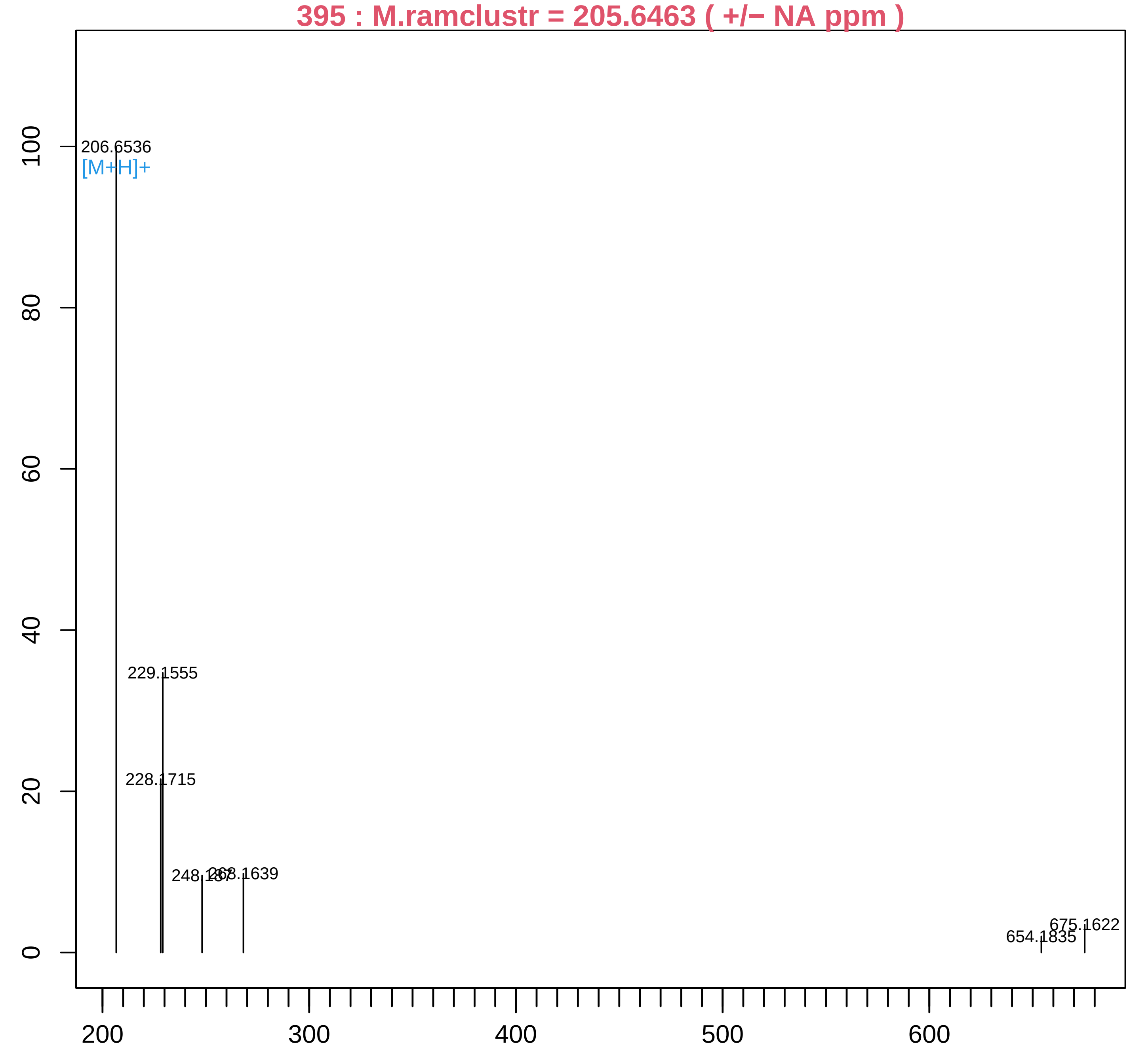
395 : M.ramclustr = 205.6463 (+/− NA ppm)
206.6536
[M+H]+
229.1555
228.1715
248.187
268.1639
654.1835
675.1622
0
20
40
60
80
100
200
300
400
500
600

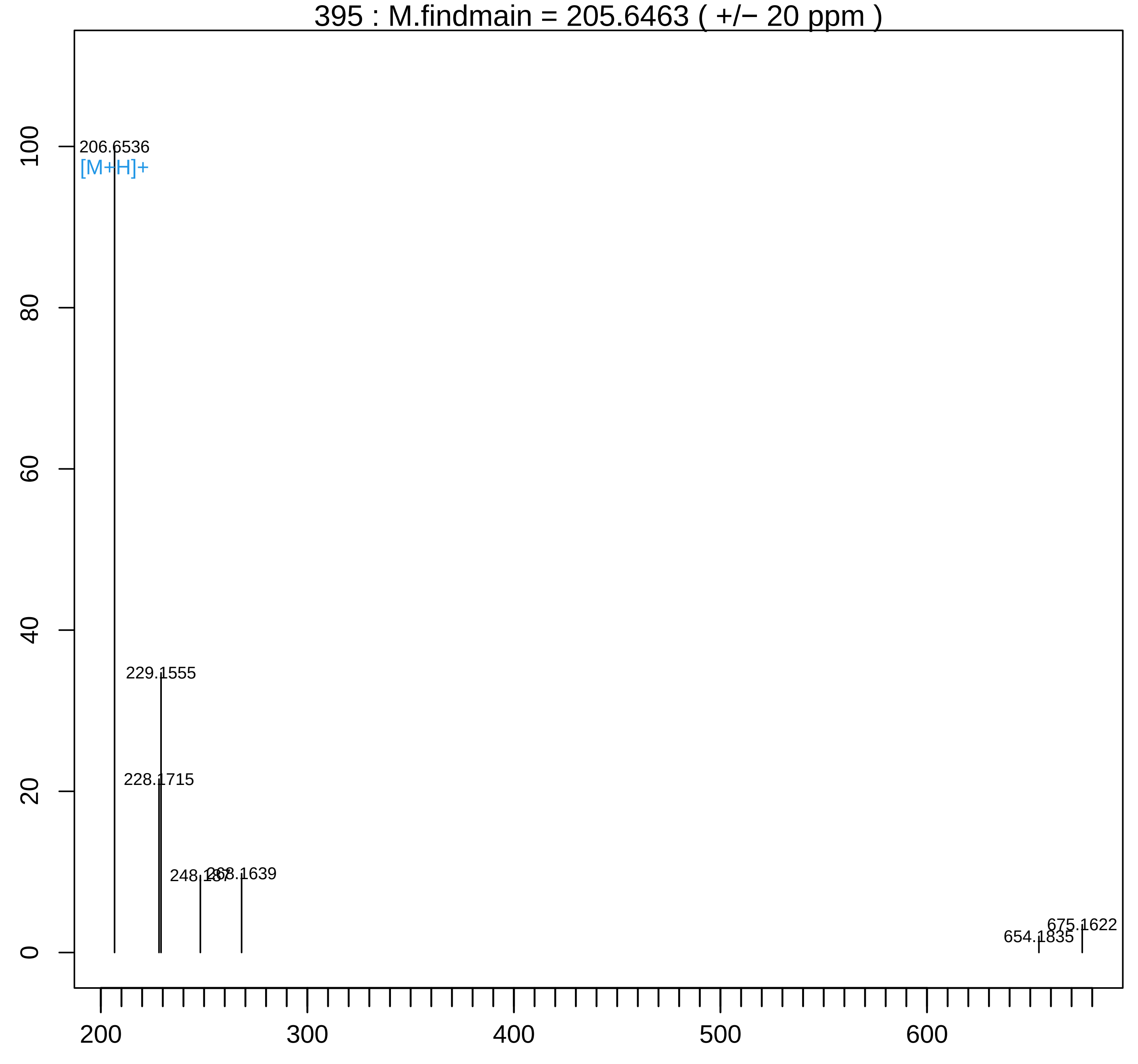
395 : M.findmain = 205.6463 (+/− 20 ppm)
206.6536
[M+H]+
229.1555
228.1715
248.187
268.1639
654.1835
675.1622
0
20
40
60
80
100
200
300
400
500
600

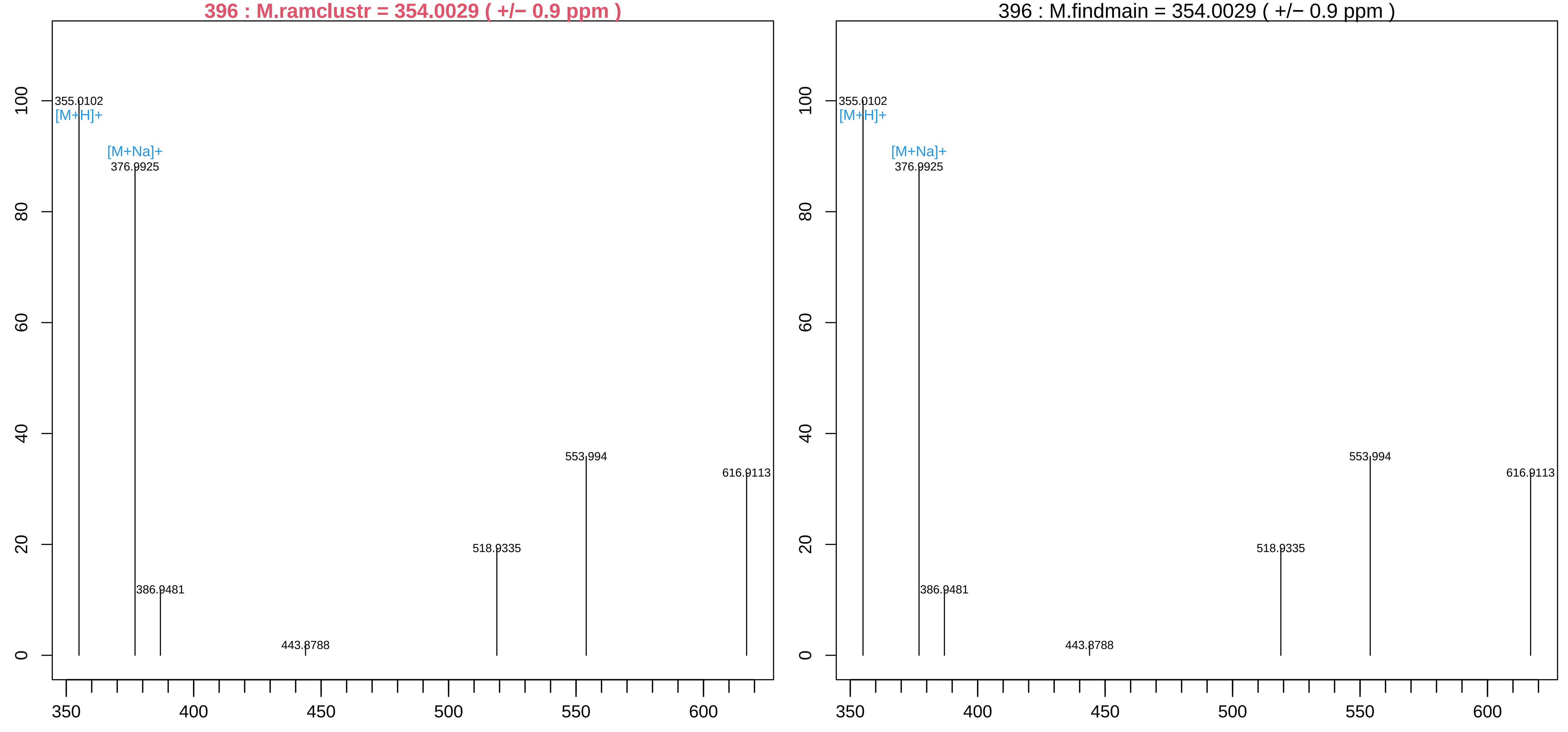
396 : M.ramclustr = 354.0029 (+/− 0.9 ppm)
396 : M.findmain = 354.0029 (+/− 0.9 ppm)
355.0102
[M+H]+
[M+Na]+
376.9925
386.9481
443.8788
518.9335
553.994
616.9113
0
20
40
60
80
100
350
400
450
500
550
600

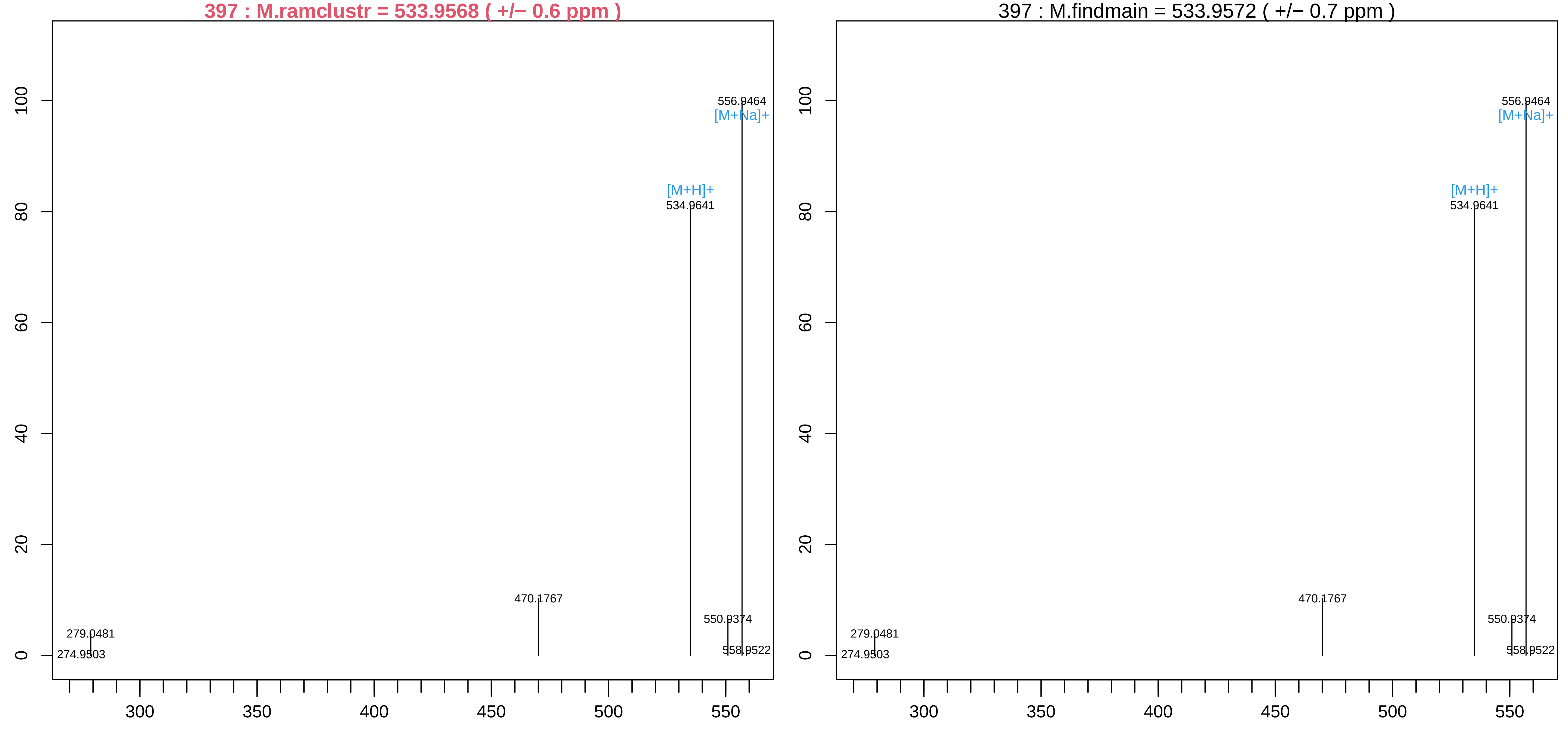

397 : M.ramclustr = 533.9568 (+/− 0.6 ppm)
556.9464
[M+Na]+
[M+H]+
534.9641
470.1767
550.9374
279.0481
274.9503
558.9522
0
20
40
60
80
100
300
350
400
450
500
550
397 : M.findmain = 533.9572 (+/− 0.7 ppm)
556.9464
[M+Na]+
[M+H]+
534.9641
470.1767
550.9374
279.0481
274.9503
558.9522
0
20
40
60
80
100
300
350
400
450
500
550

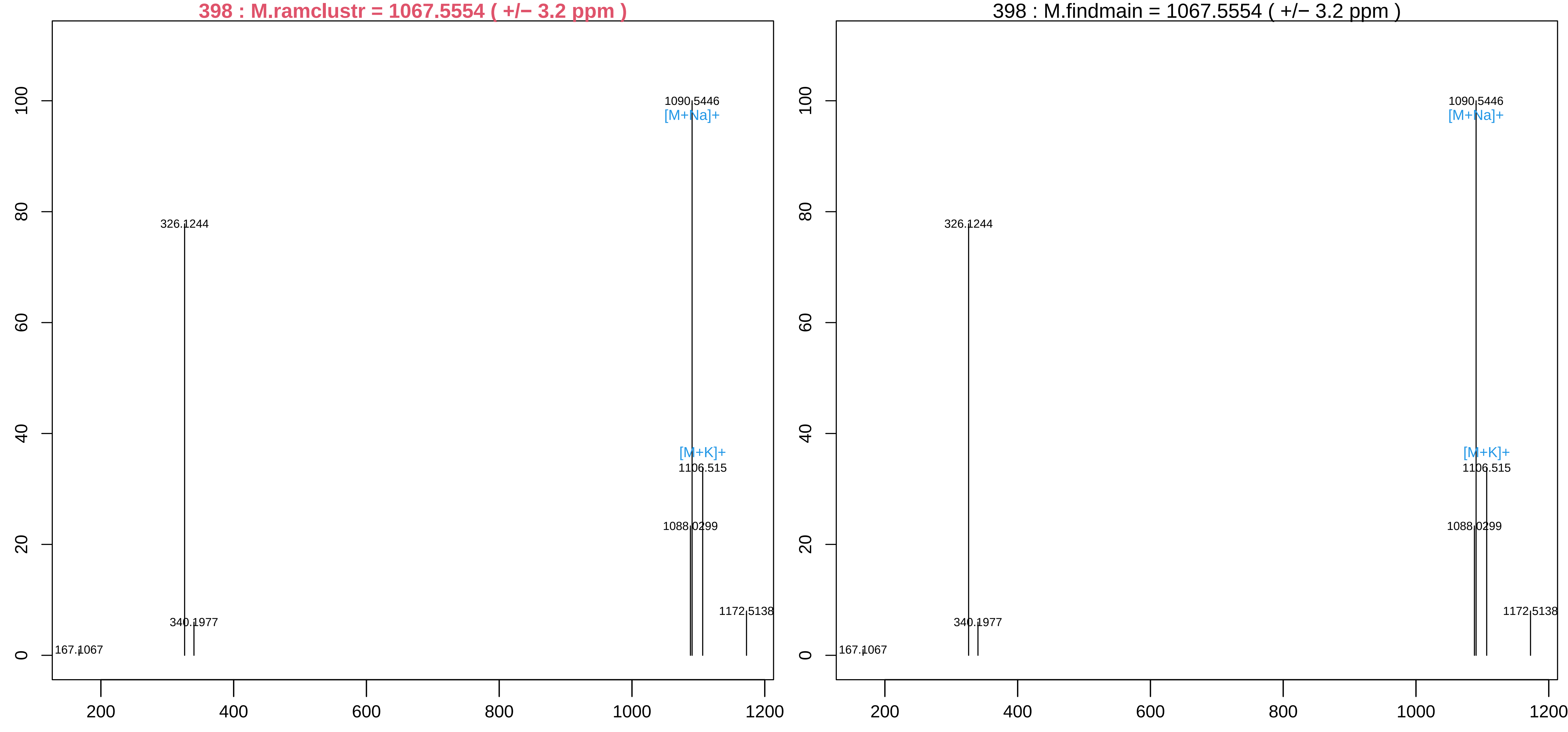

398 : M.ramclustr = 1067.5554 (+/− 3.2 ppm)
1090.5446
[M+Na]+
326.1244
[M+K]+
1106.515
1088.0299
1172.5138
340.1977
167.1067
398 : M.findmain = 1067.5554 (+/− 3.2 ppm)
1090.5446
[M+Na]+
326.1244
[M+K]+
1106.515
1088.0299
1172.5138
340.1977
167.1067

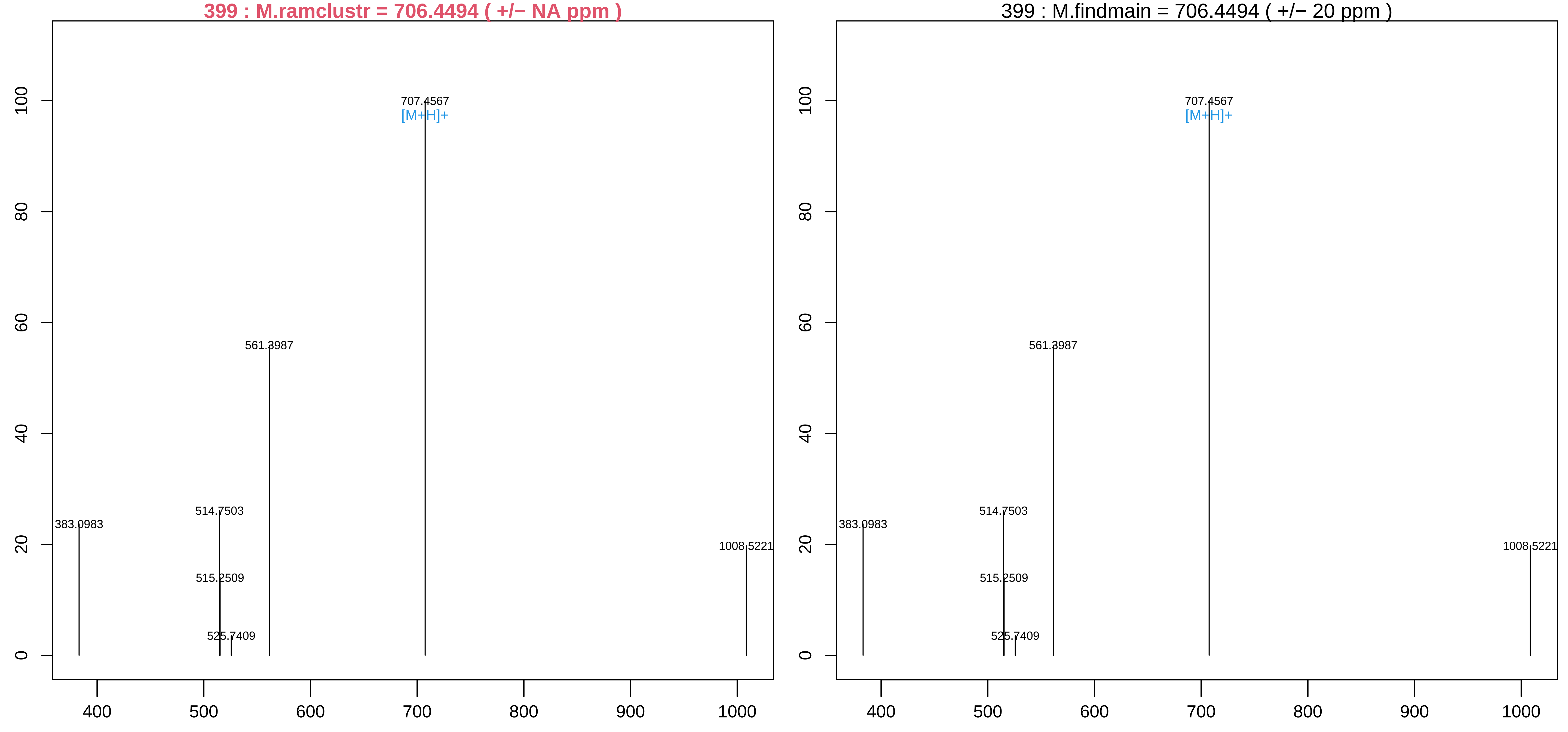
399 : M.ramclustr = 706.4494 (+/− NA ppm)
399 : M.findmain = 706.4494 (+/− 20 ppm)
707.4567
[M+H]+
561.3987
514.7503
383.0983
515.2509
525.7409
1008.5221
0
20
40
60
80
100
400
500
600
700
800
900
1000

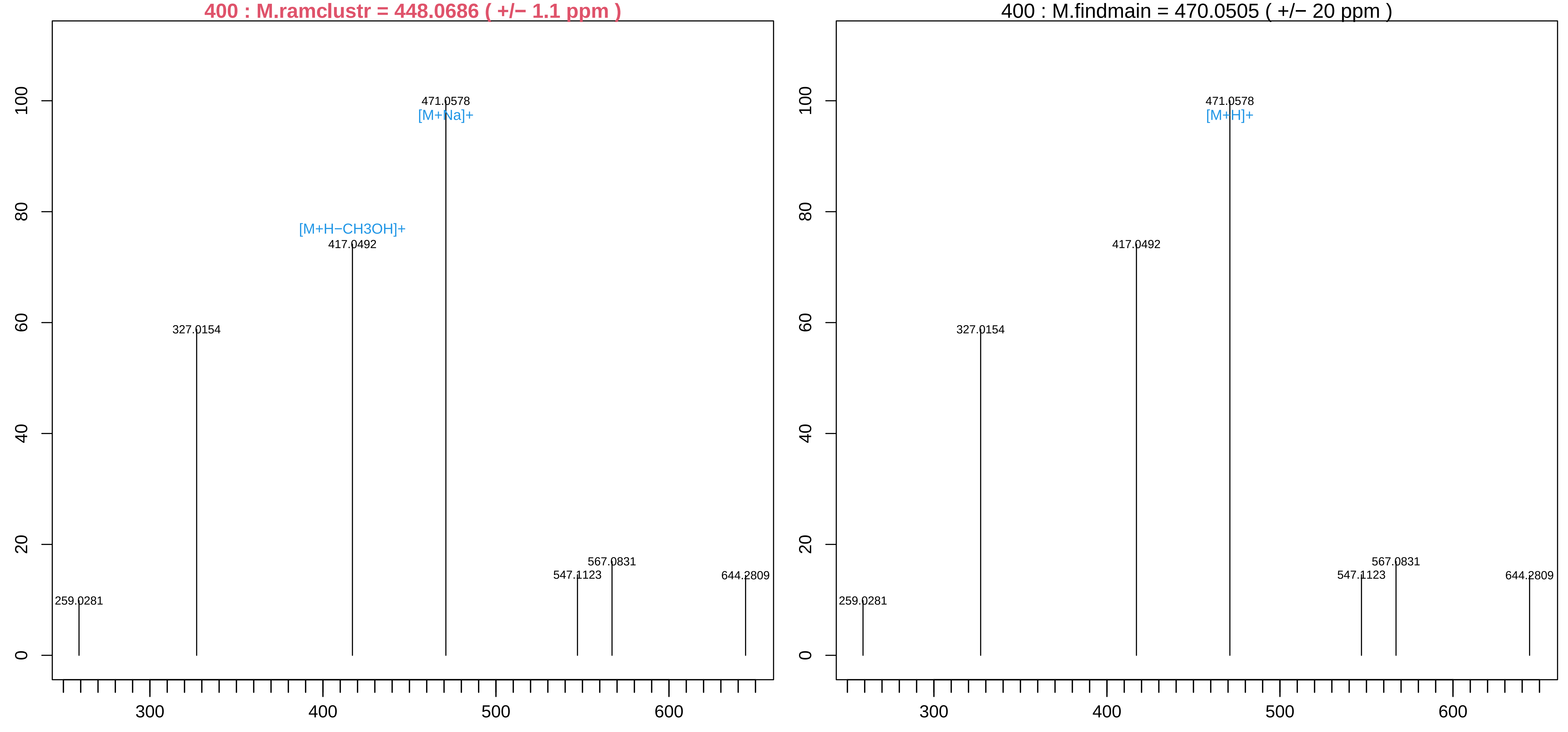

400 : M.ramclustr = 448.0686 (+/− 1.1 ppm)
471.0578
[M+Na]+
[M+H−CH3OH]+
417.0492
327.0154
259.0281
547.1123
567.0831
644.2809
400 : M.findmain = 470.0505 (+/− 20 ppm)
471.0578
[M+H]+
417.0492
327.0154
259.0281
547.1123
567.0831
644.2809

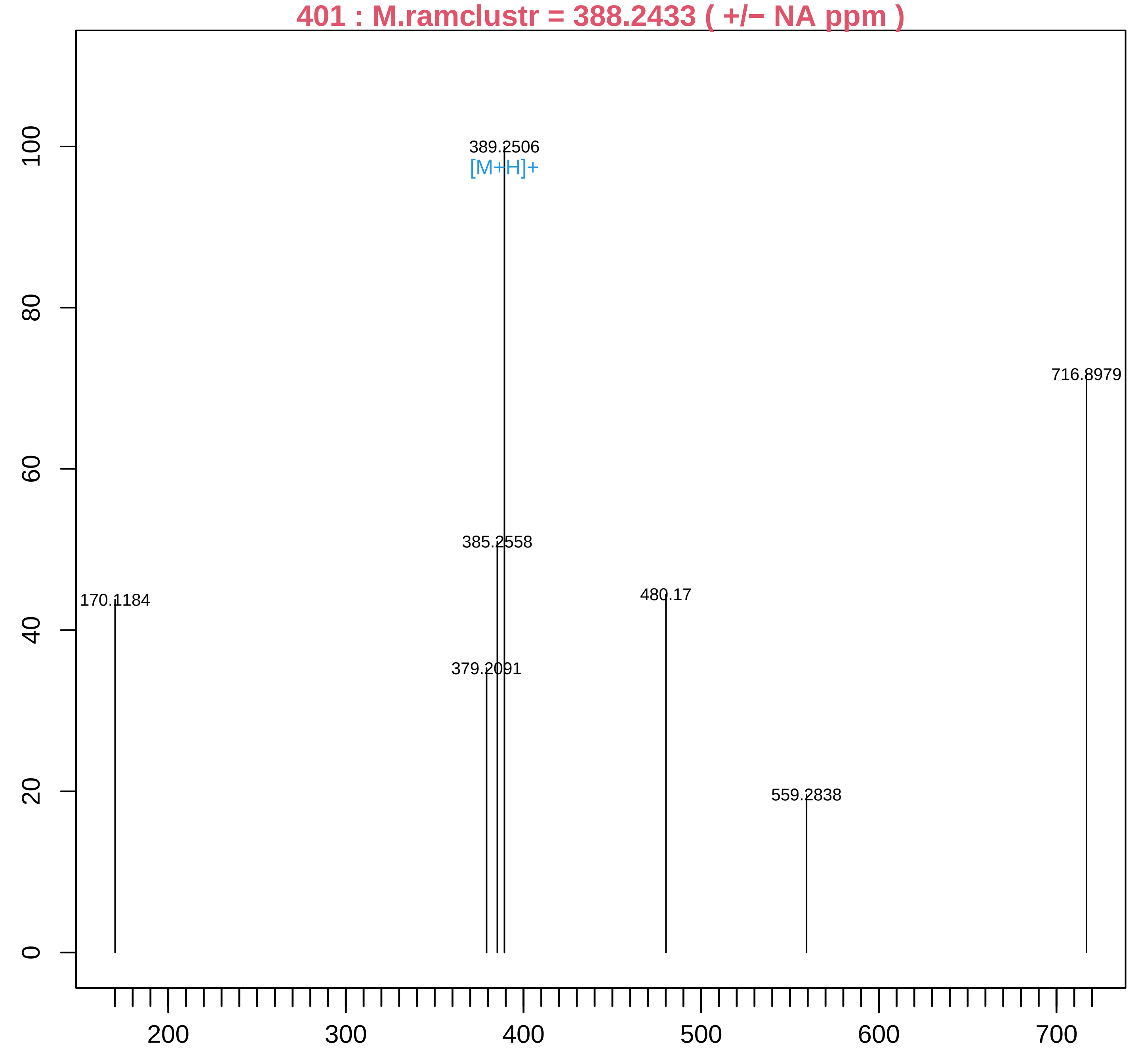
401 : M.ramclustr = 388.2433 (+/− NA ppm)
389.2506
[M+H]+
716.8979
385.2558
170.1184
480.17
379.2091
559.2838
0
20
40
60
80
100
200
300
400
500
600
700

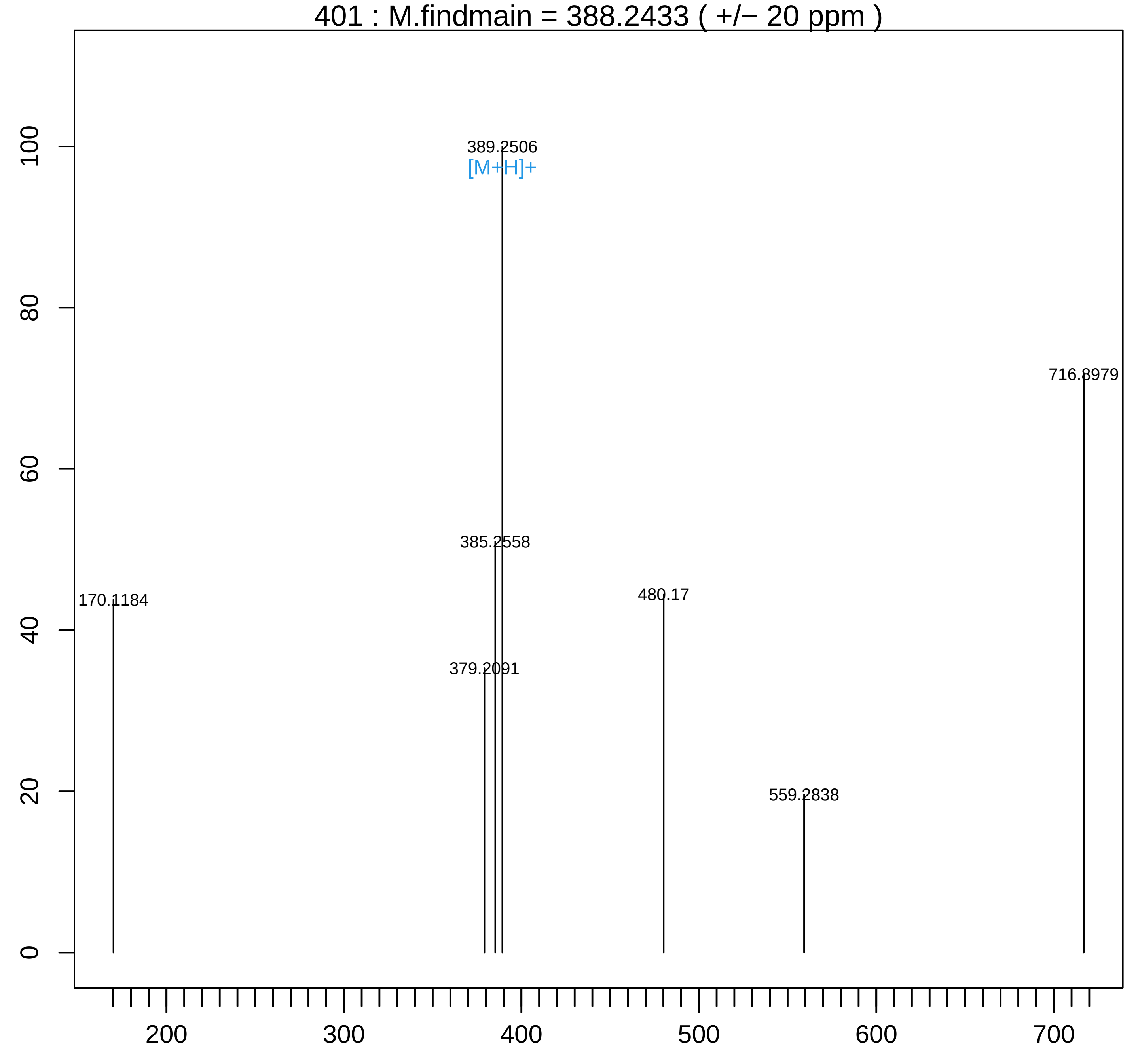
401 : M.findmain = 388.2433 (+/− 20 ppm)
389.2506
[M+H]+
716.8979
385.2558
170.1184
480.17
379.2091
559.2838
0
20
40
60
80
100
200
300
400
500
600
700

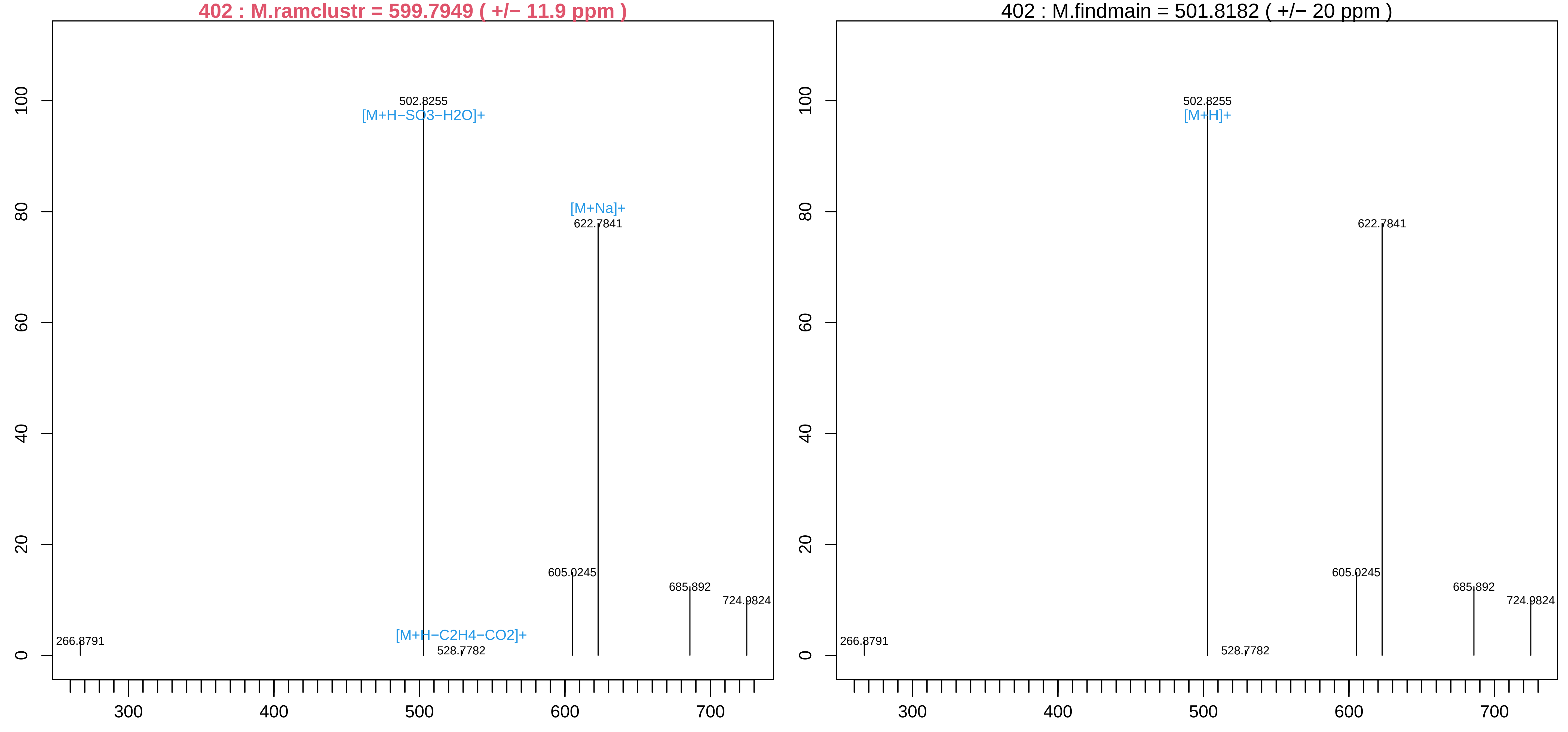
402 : M.ramclustr = 599.7949 (+/− 11.9 ppm)
502.8255
[M+H−SO3−H2O]+
[M+Na]+
622.7841
605.0245
685.892
724.9824
266.8791
[M+H−C2H4−CO2]+
528.7782
402 : M.findmain = 501.8182 (+/− 20 ppm)
502.8255
[M+H]+
622.7841
605.0245
685.892
724.9824
266.8791
528.7782
0
20
40
60
80
100
300
400
500
600
700

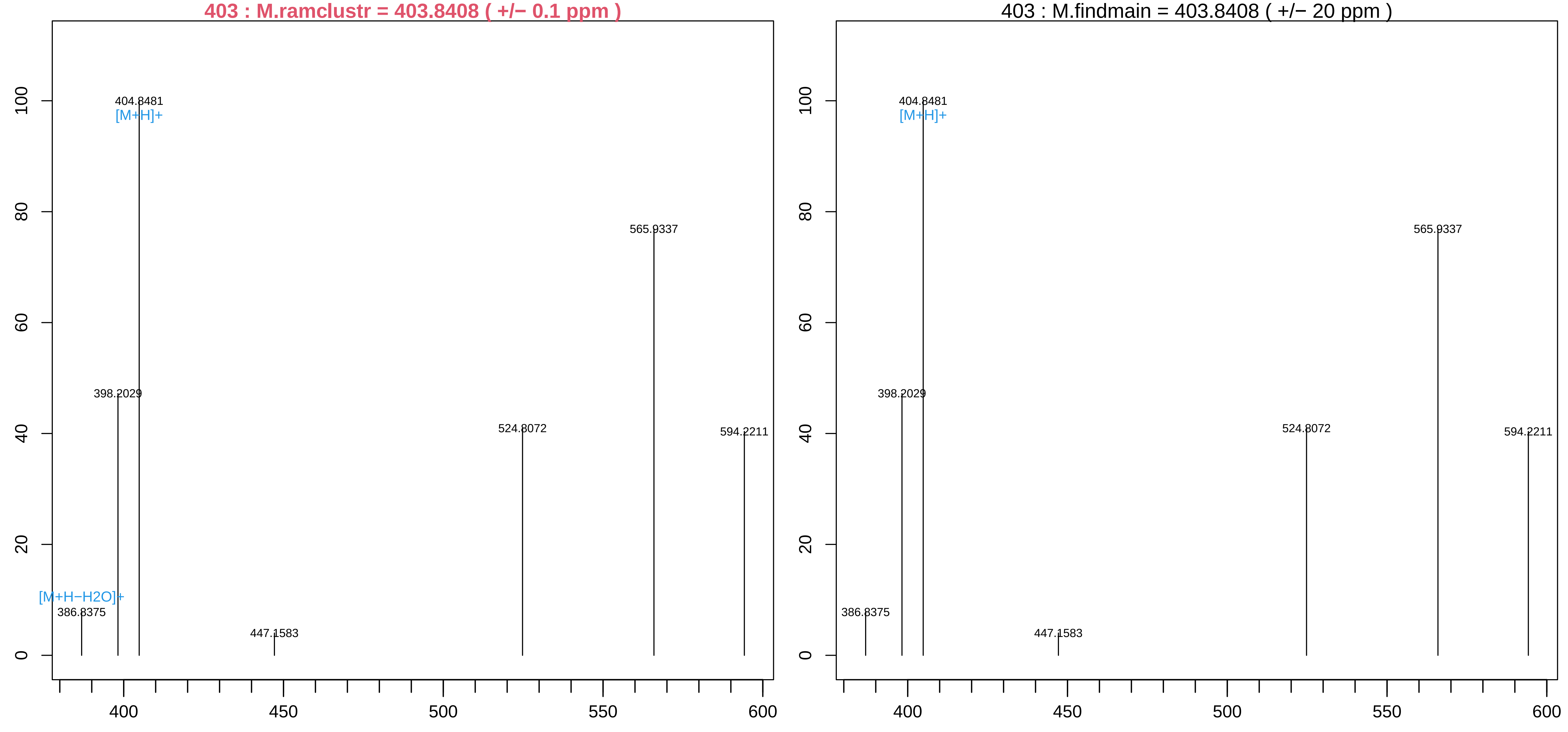
403 : M.ramclustr = 403.8408 (+/− 0.1 ppm)
404.8481
[M+H]+
565.9337
398.2029
524.8072
594.2211
[M+H−H2O]+
386.8375
447.1583
0
20
40
60
80
100
400
450
500
550
600
403 : M.findmain = 403.8408 (+/− 20 ppm)
404.8481
[M+H]+
565.9337
398.2029
524.8072
594.2211
386.8375
447.1583
0
20
40
60
80
100
400
450
500
550
600

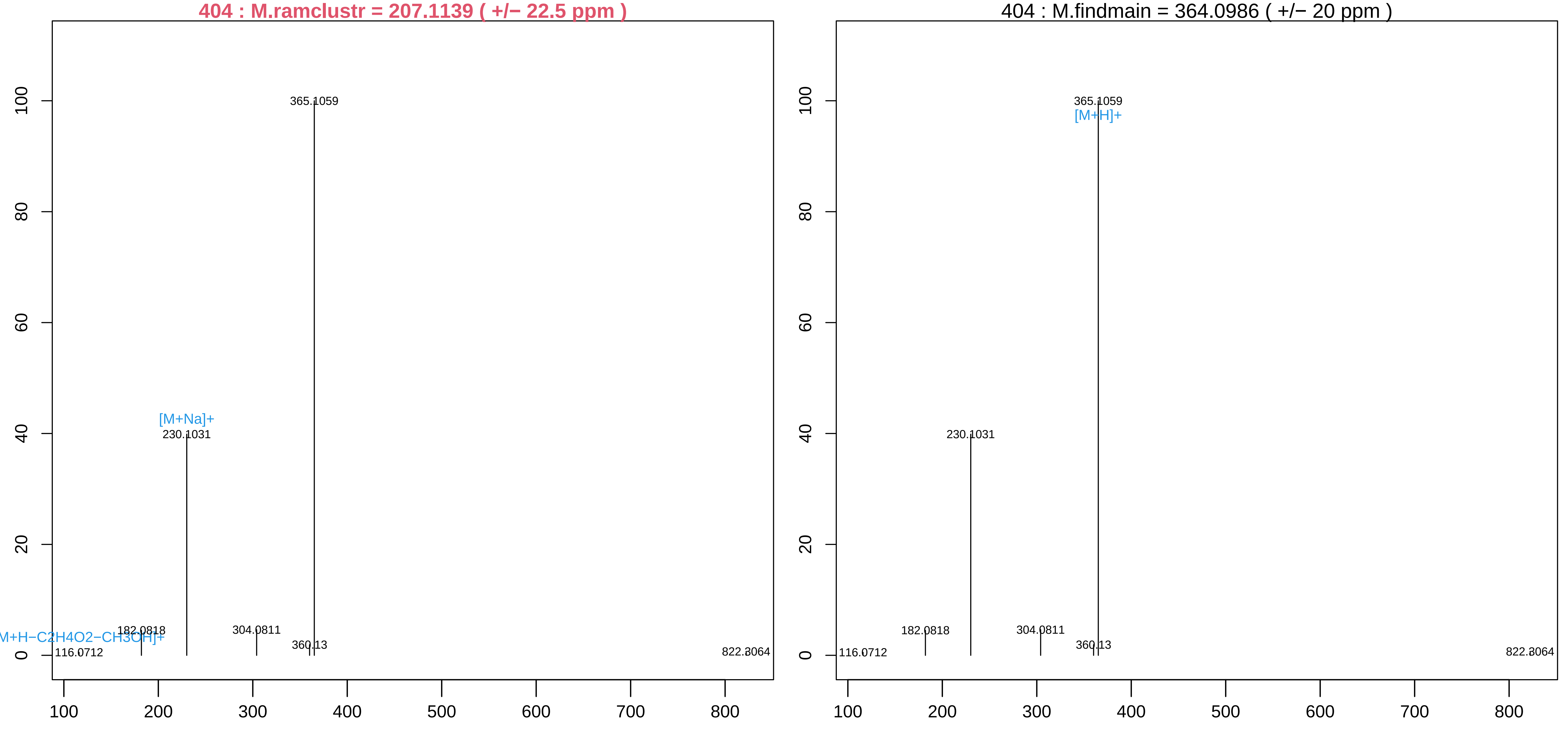
404 : M.ramclustr = 207.1139 (+/− 22.5 ppm)
365.1059
[M+Na]+
230.1031
182.0818
[M+H−C2H4O2−CH3OH]+
116.0712
304.0811
360.13
822.3064
404 : M.findmain = 364.0986 (+/− 20 ppm)
365.1059
[M+H]+
230.1031
182.0818
116.0712
304.0811
360.13
822.3064
0
20
40
60
80
100
100
200
300
400
500
600
700
800

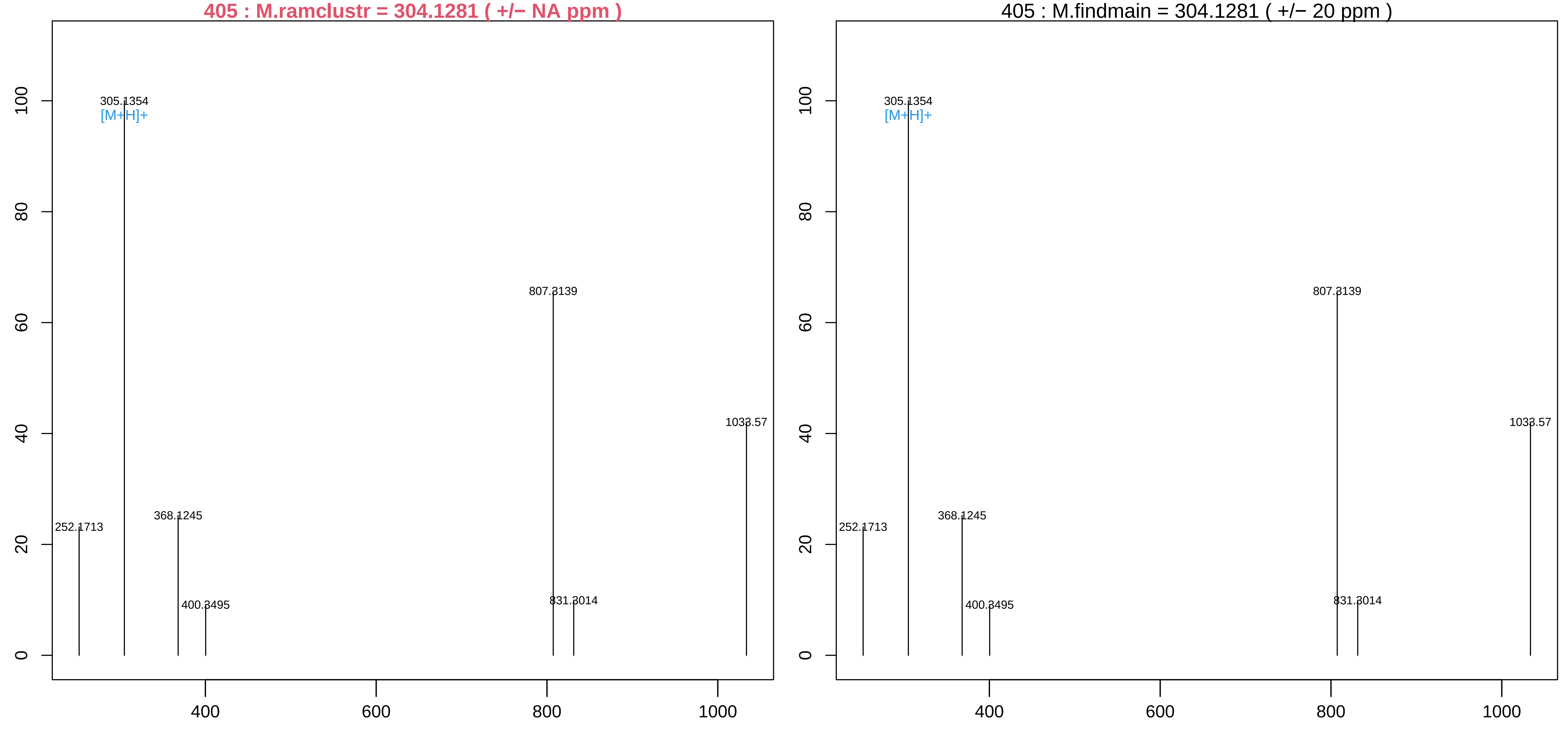
405 : M.ramclustr = 304.1281 (+/− NA ppm)
305.1354
[M+H]+
807.3139
1033.57
368.1245
252.1713
400.3495
831.3014
405 : M.findmain = 304.1281 (+/− 20 ppm)
305.1354
[M+H]+
807.3139
1033.57
368.1245
252.1713
400.3495
831.3014

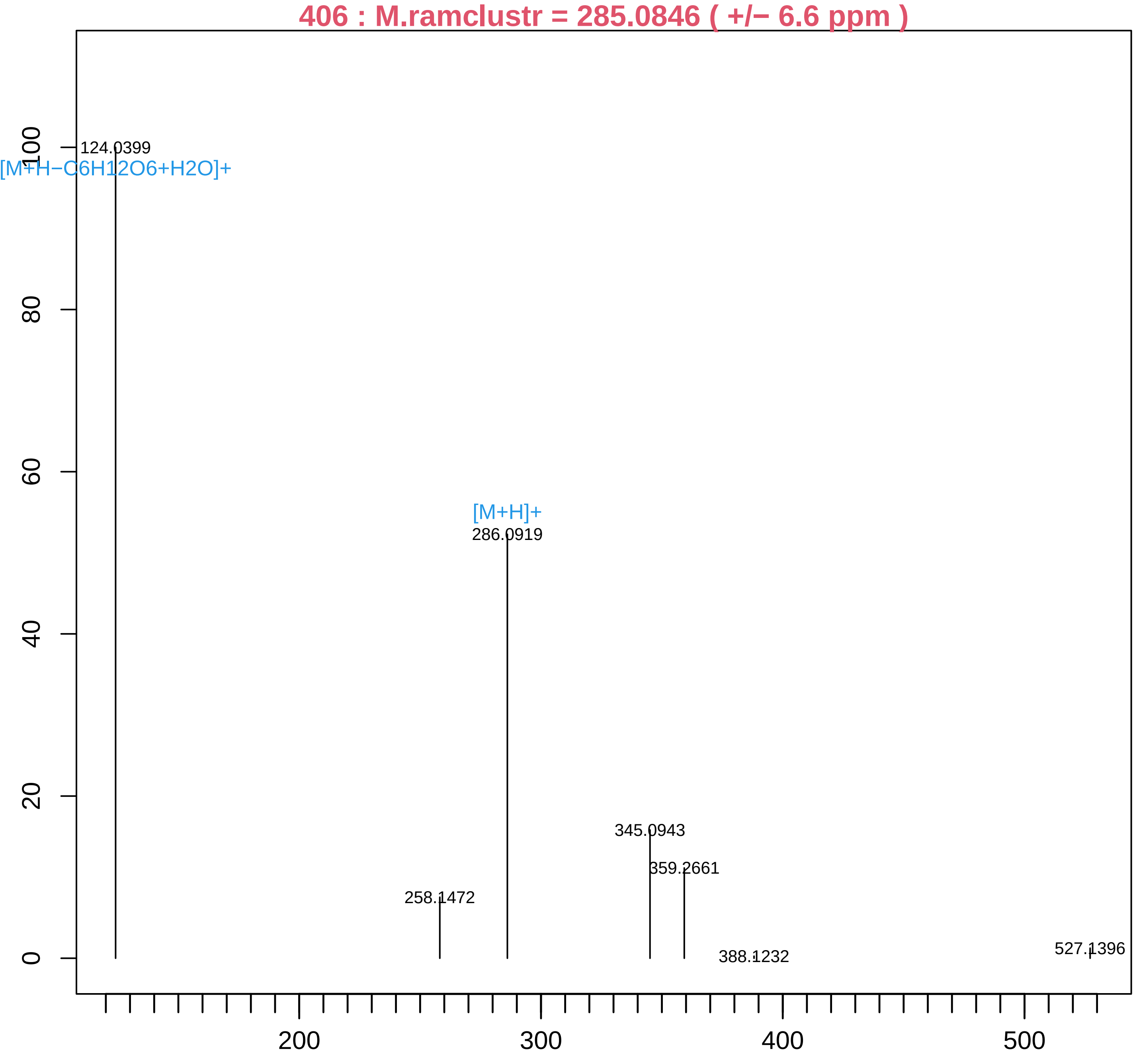
406 : M.ramclustr = 285.0846 (+/− 6.6 ppm)
124.0399
[M+H−C6H12O6+H2O]+
[M+H]+
286.0919
258.1472
345.0943
359.2661
388.1232
527.1396
0
20
40
60
80
100
200
300
400
500

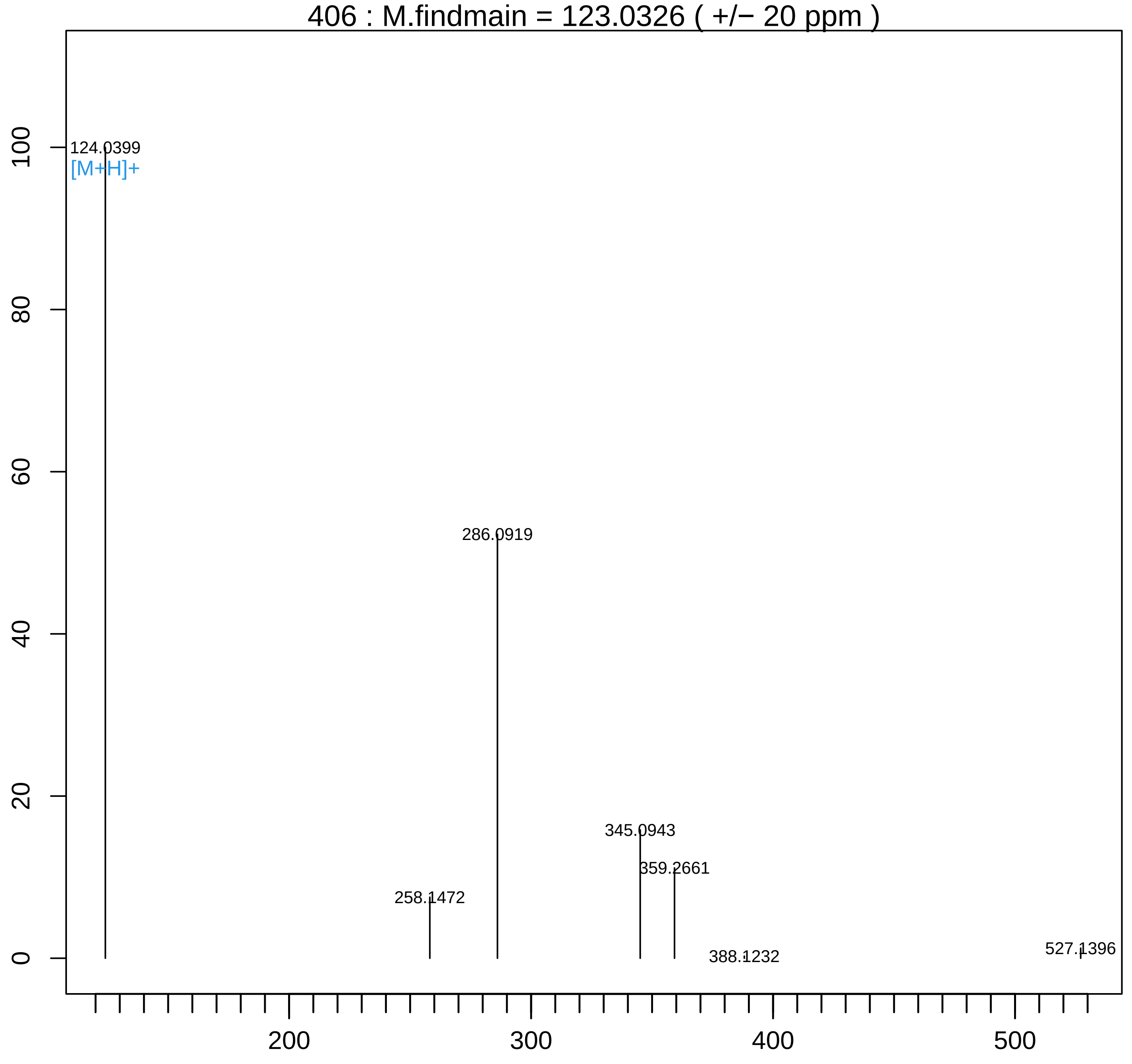
406 : M.findmain = 123.0326 (+/− 20 ppm)
124.0399
[M+H]+
286.0919
258.1472
345.0943
359.2661
388.1232
527.1396
0
20
40
60
80
100
200
300
400
500

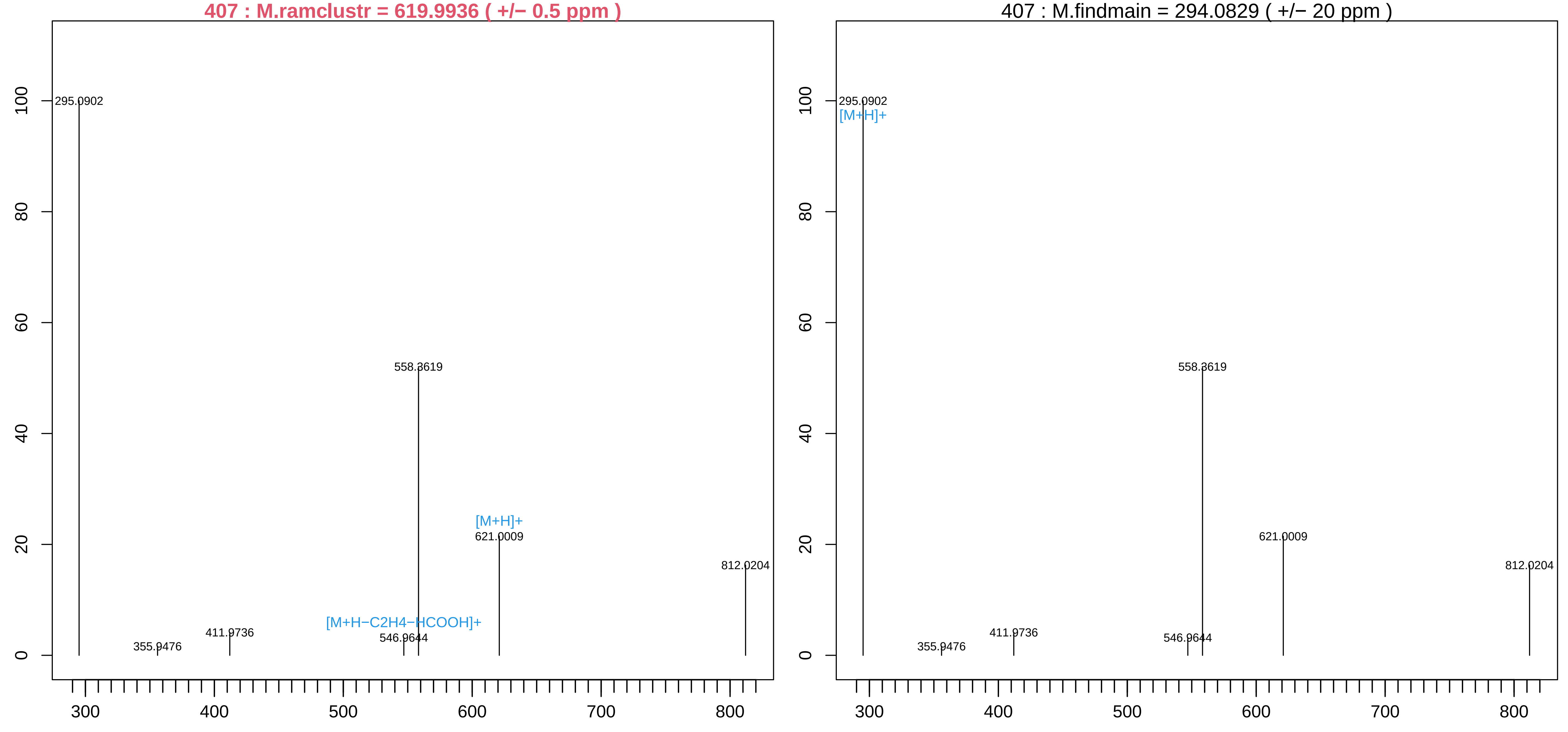

407 : M.ramclustr = 619.9936 (+/− 0.5 ppm)
295.0902
355.9476
411.9736
[M+H−C2H4−HCOOH]+
546.9644
558.3619
[M+H]+
621.0009
812.0204
0
20
40
60
80
100
300
400
500
600
700
800
407 : M.findmain = 294.0829 (+/− 20 ppm)
295.0902
[M+H]+
355.9476
411.9736
546.9644
558.3619
621.0009
812.0204

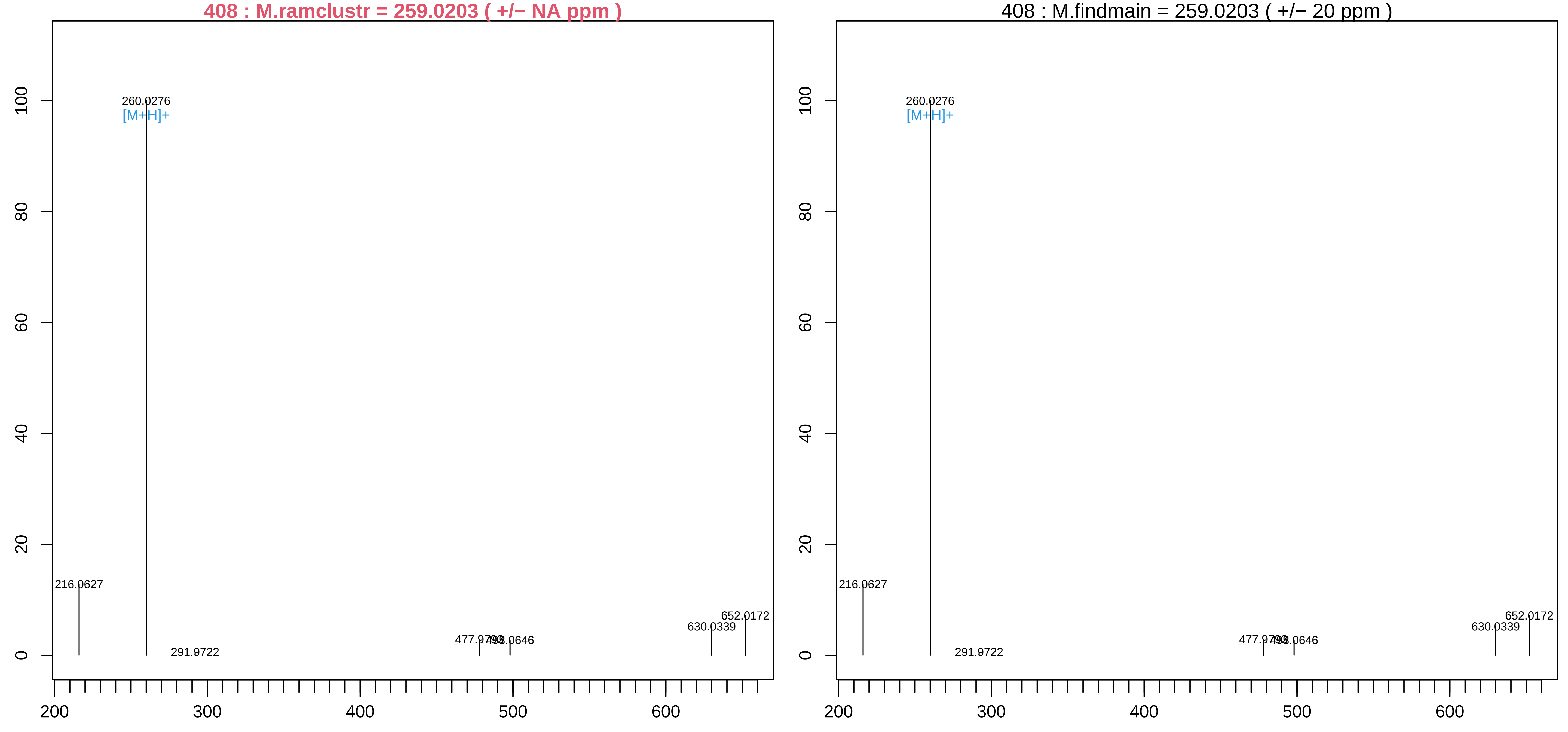

408 : M.ramclustr = 259.0203 (+/− NA ppm)
260.0276
[M+H]+
216.0627
291.9722
477.9798
498.0646
630.0339
652.0172
408 : M.findmain = 259.0203 (+/− 20 ppm)
260.0276
[M+H]+
216.0627
291.9722
477.9798
498.0646
630.0339
652.0172

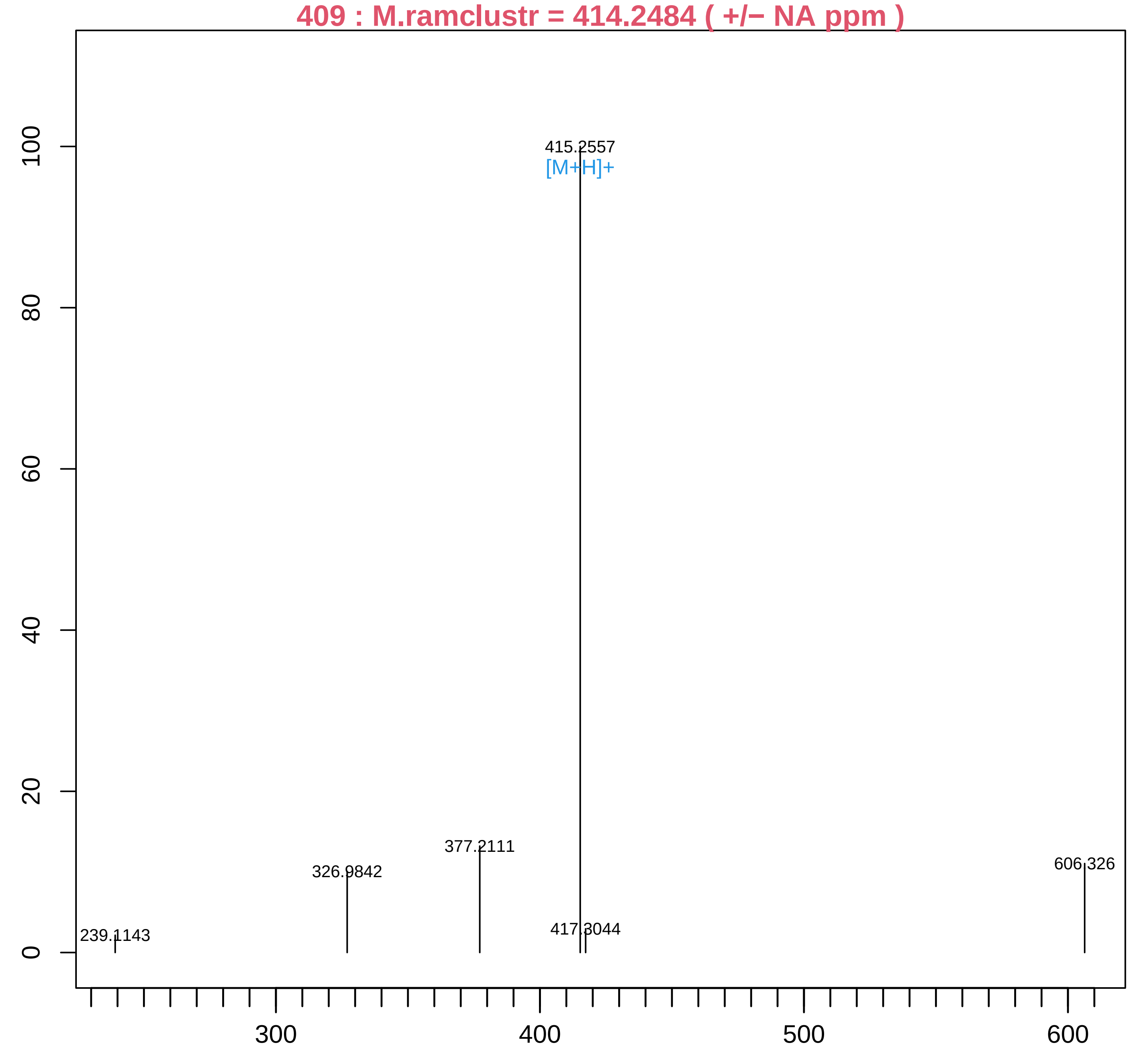
409 : M.ramclustr = 414.2484 (+/− NA ppm)
415.2557
[M+H]+
377.2111
326.9842
606.326
239.1143
417.3044
0
20
40
60
80
100
300
400
500
600

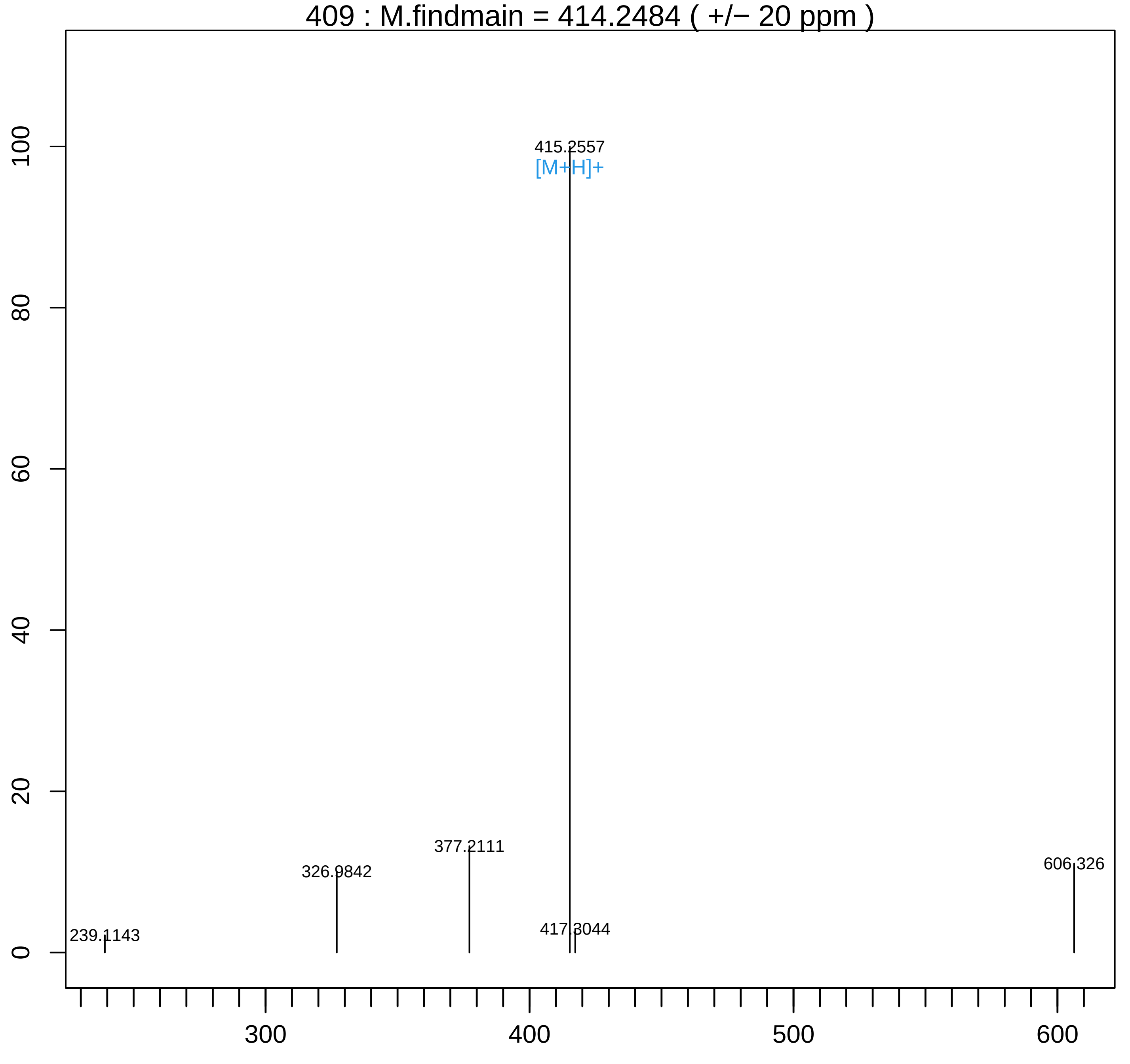
409 : M.findmain = 414.2484 (+/− 20 ppm)
415.2557
[M+H]+
377.2111
326.9842
606.326
239.1143
417.3044
0
20
40
60
80
100
300
400
500
600

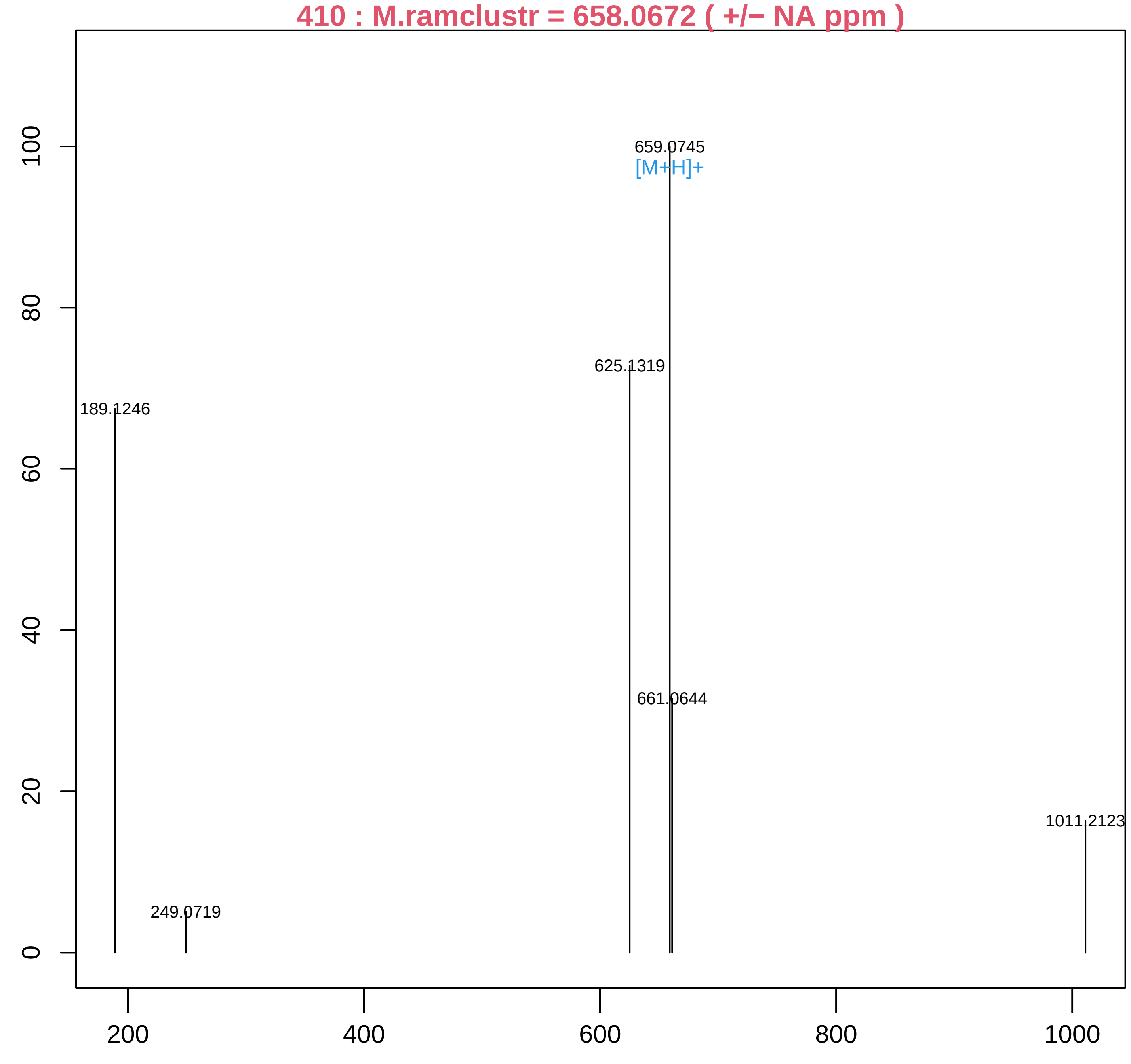
410 : M.ramclustr = 658.0672 (+/− NA ppm)
659.0745
[M+H]+
625.1319
189.1246
661.0644
1011.2123
249.0719
0
20
40
60
80
100
200
400
600
800
1000

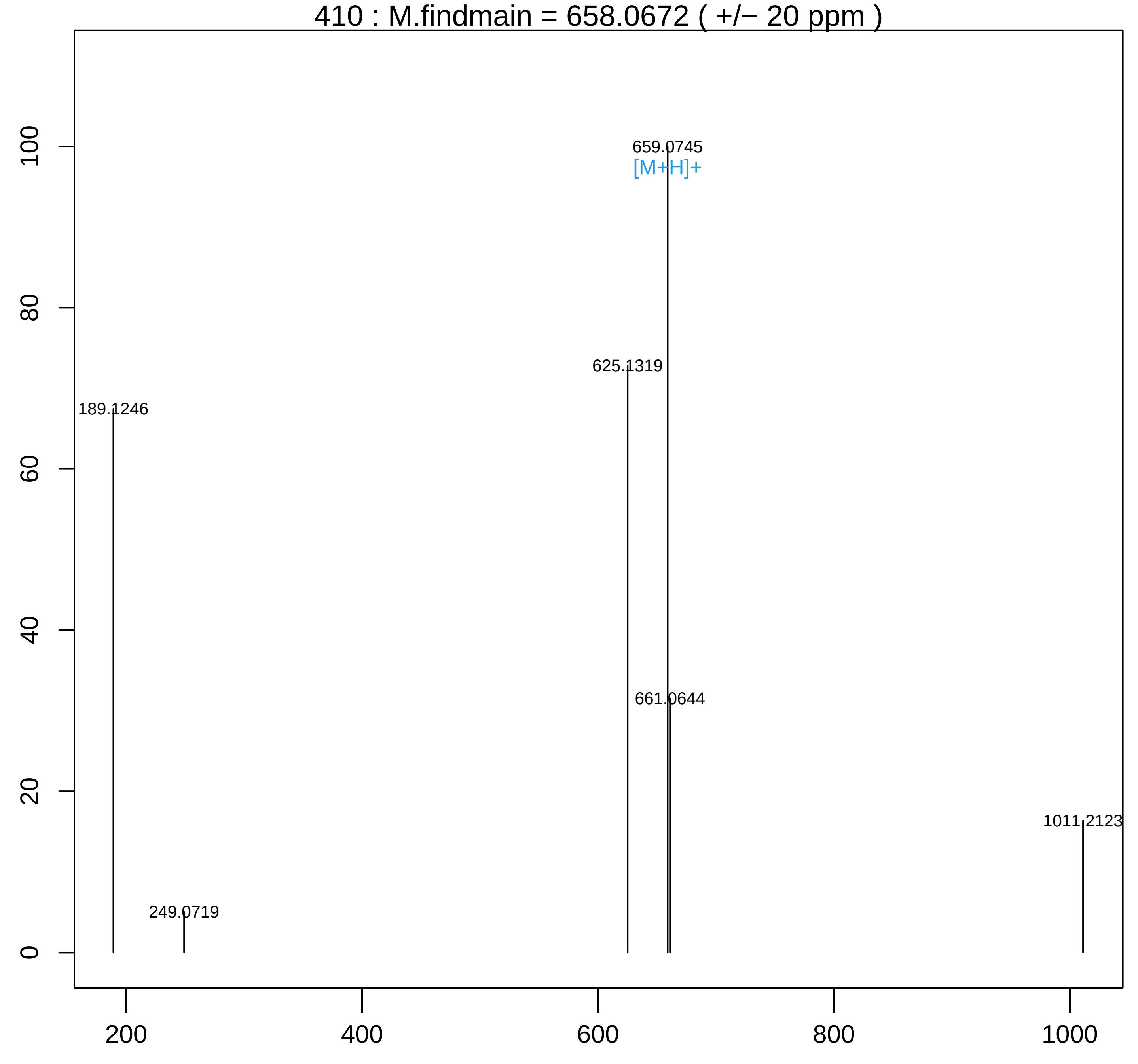
410 : M.findmain = 658.0672 (+/− 20 ppm)
659.0745
[M+H]+
625.1319
189.1246
661.0644
1011.2123
249.0719
0
20
40
60
80
100
200
400
600
800
1000

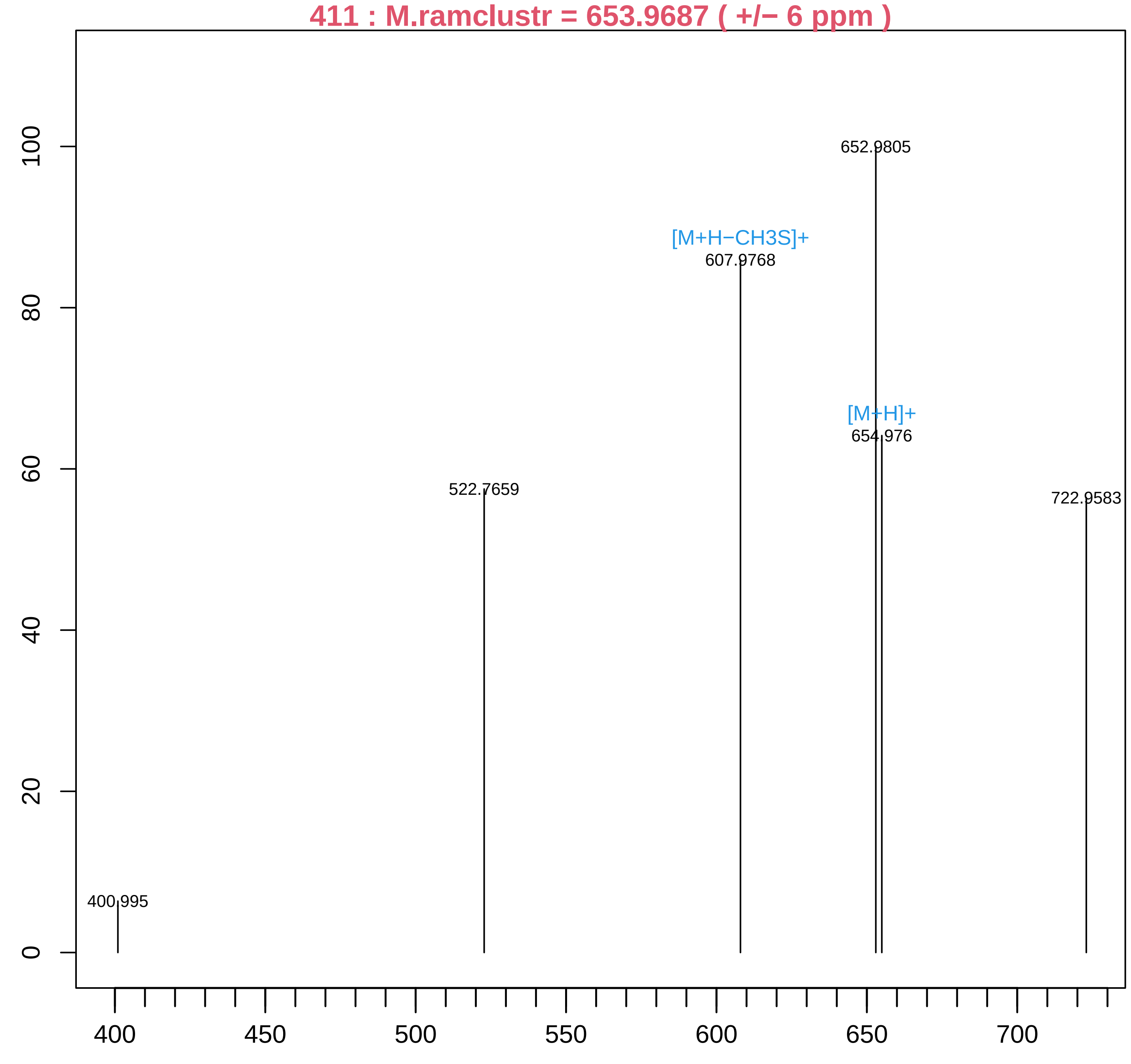

411 : M.ramclustr = 653.9687 (+/− 6 ppm)
[M+H−CH3S]+
607.9768
652.9805
[M+H]+
654.976
522.7659
722.9583
400.995
0
20
40
60
80
100
400
450
500
550
600
650
700

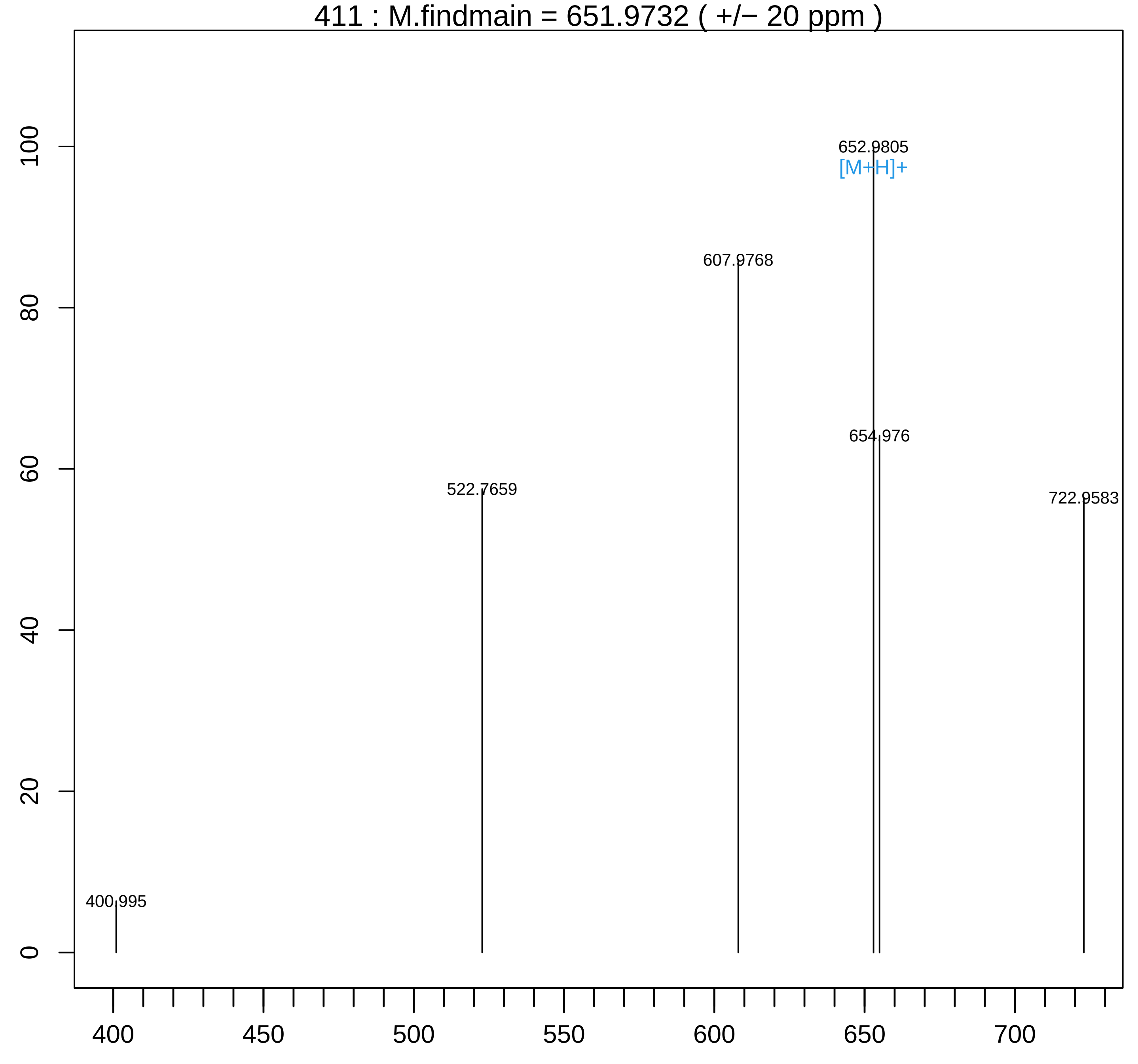

411 : M.findmain = 651.9732 (+/− 20 ppm)
652.9805
[M+H]+
607.9768
654.976
522.7659
722.9583
400.995
0
20
40
60
80
100
400
450
500
550
600
650
700

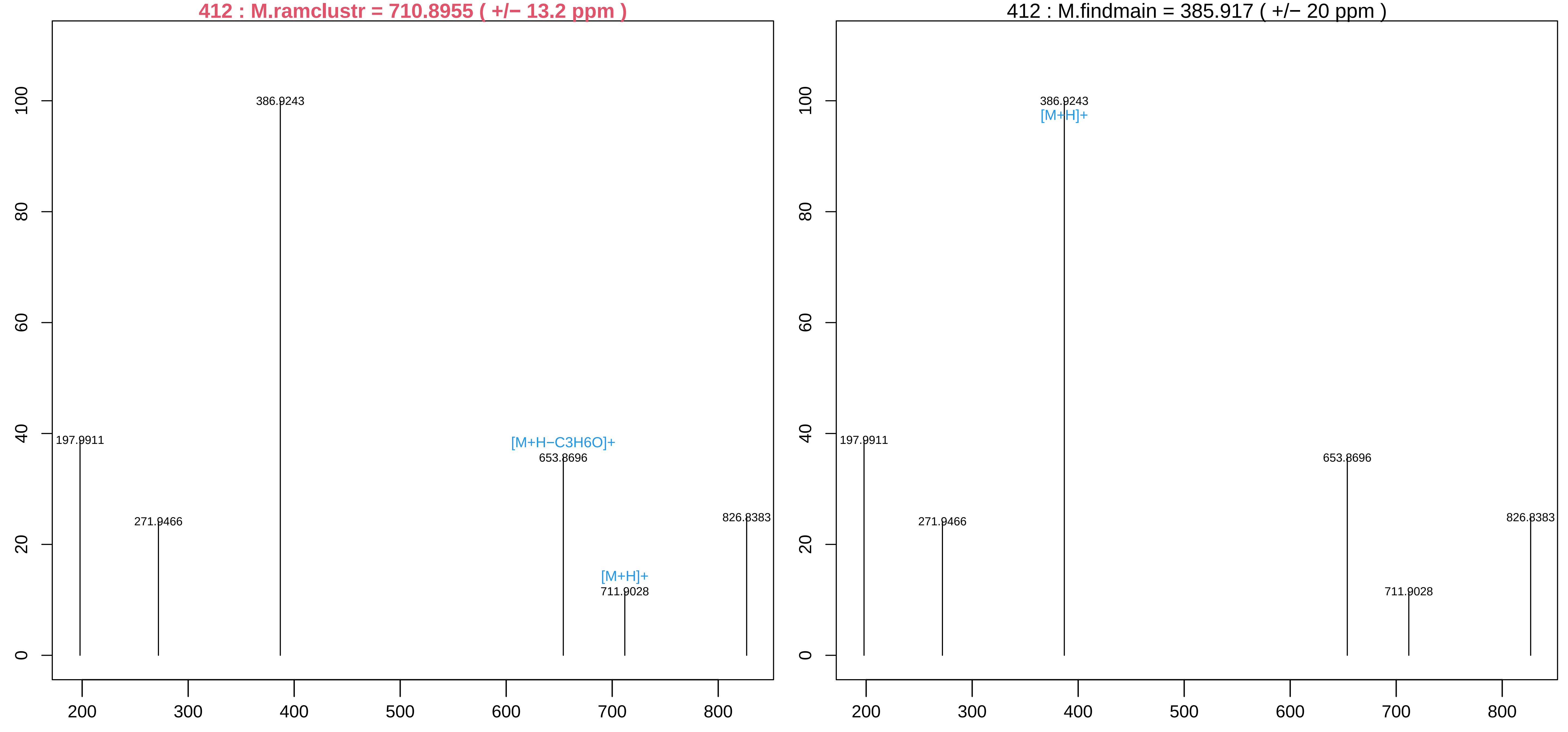
412 : M.ramclustr = 710.8955 (+/− 13.2 ppm)
386.9243
197.9911
271.9466
[M+H−C3H6O]+
653.8696
[M+H]+
711.9028
826.8383
412 : M.findmain = 385.917 (+/− 20 ppm)
386.9243
[M+H]+
197.9911
271.9466
653.8696
711.9028
826.8383

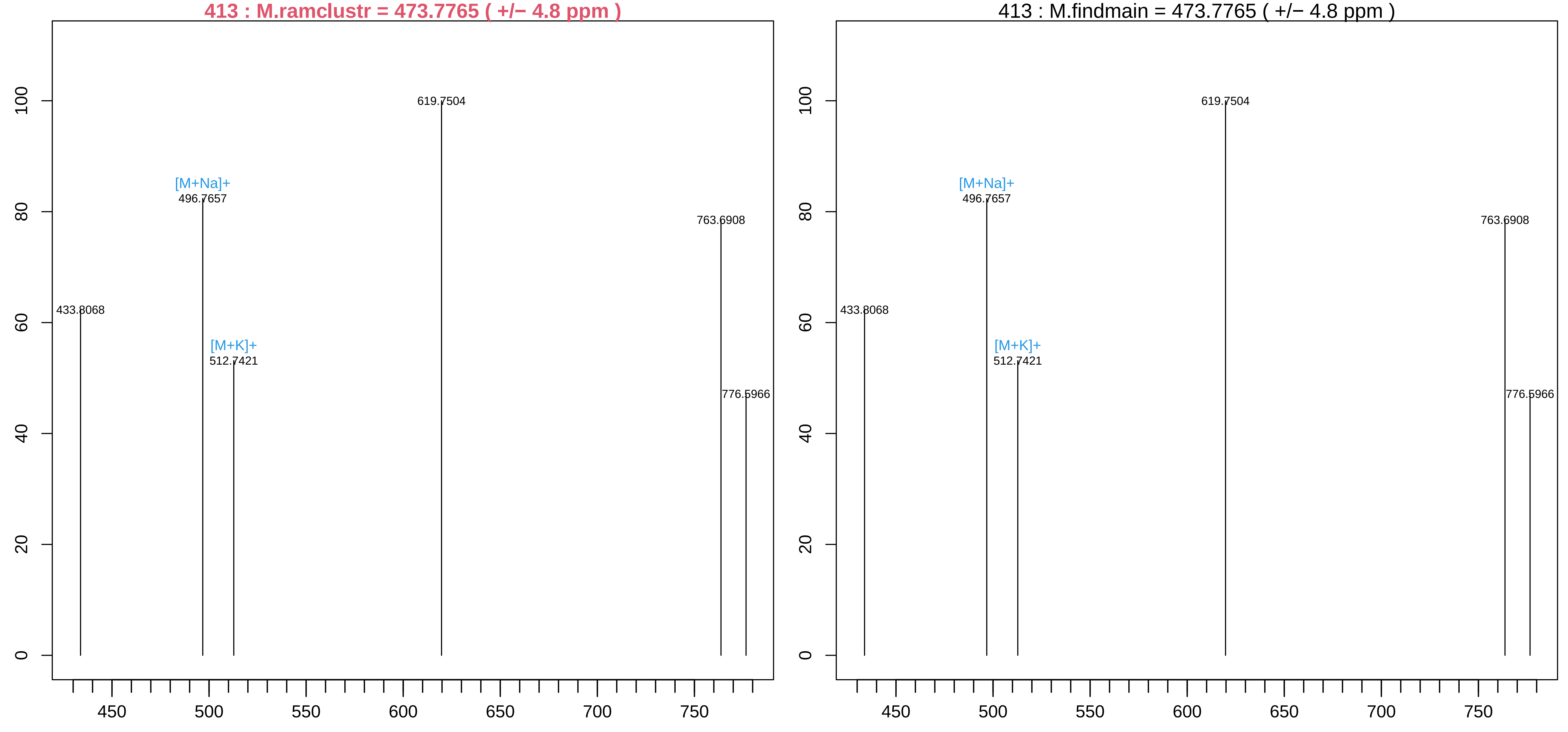

413 : M.ramclustr = 473.7765 (+/− 4.8 ppm)
413 : M.findmain = 473.7765 (+/− 4.8 ppm)
619.7504
[M+Na]+
496.7657
763.6908
433.8068
[M+K]+
512.7421
776.5966
0
20
40
60
80
100
450
500
550
600
650
700
750

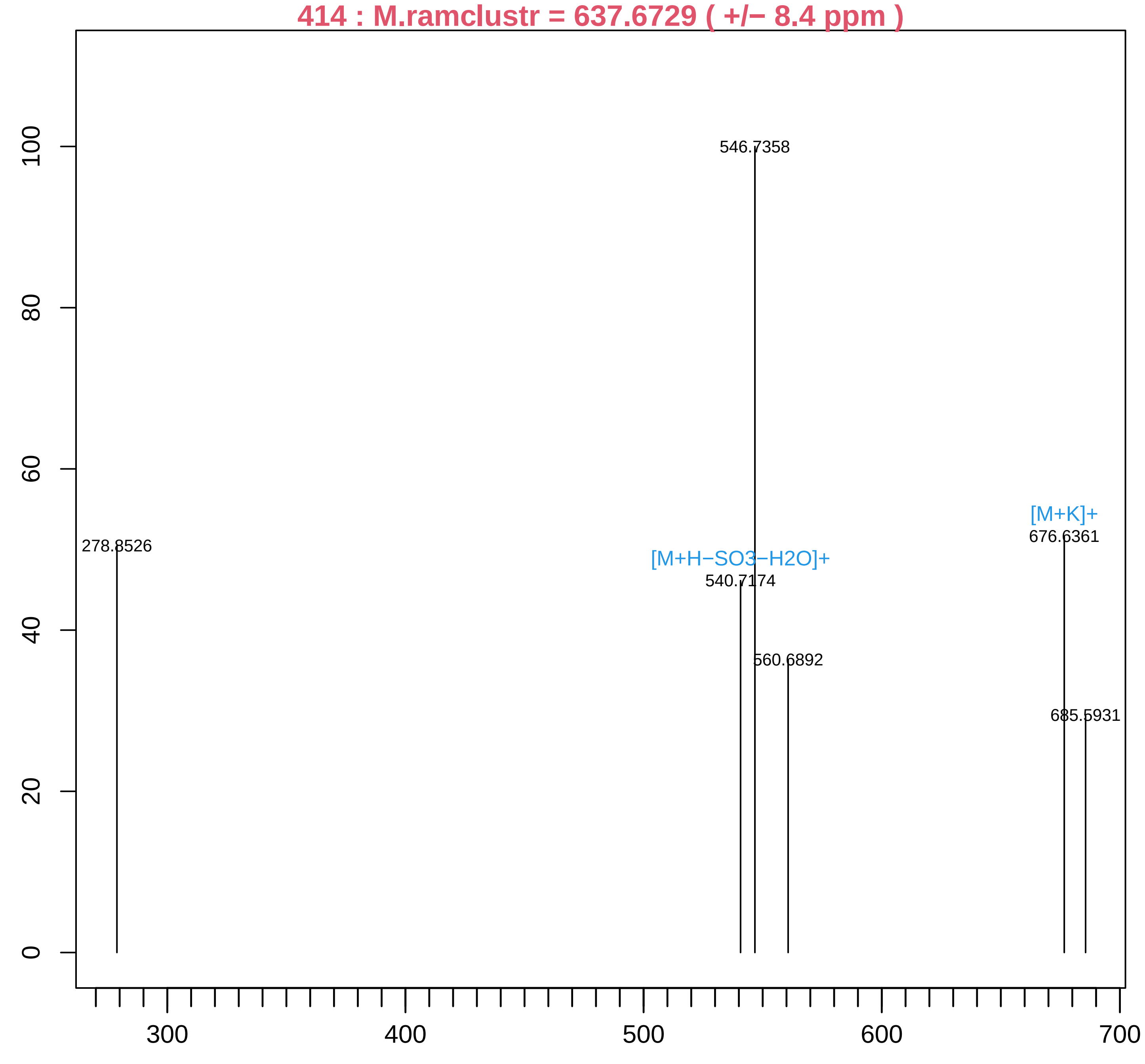
414 : M.ramclustr = 637.6729 (+/− 8.4 ppm)
546.7358
[M+H−SO3−H2O]+
540.7174
560.6892
278.8526
[M+K]+
676.6361
685.5931
0
20
40
60
80
100
300
400
500
600
700
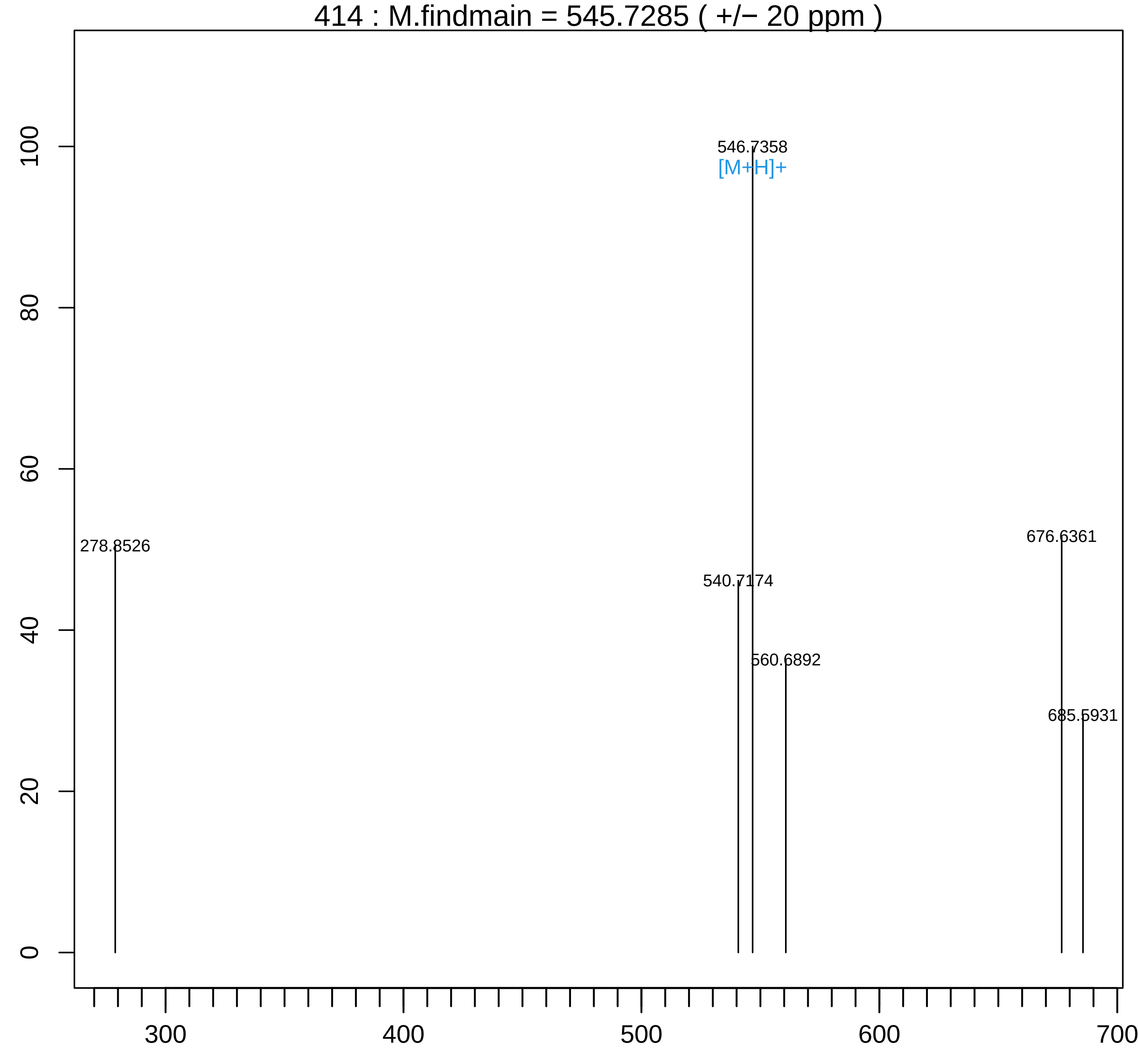
414 : M.findmain = 545.7285 (+/− 20 ppm)
546.7358
[M+H]+
540.7174
560.6892
278.8526
676.6361
685.5931
0
20
40
60
80
100
300
400
500
600
700

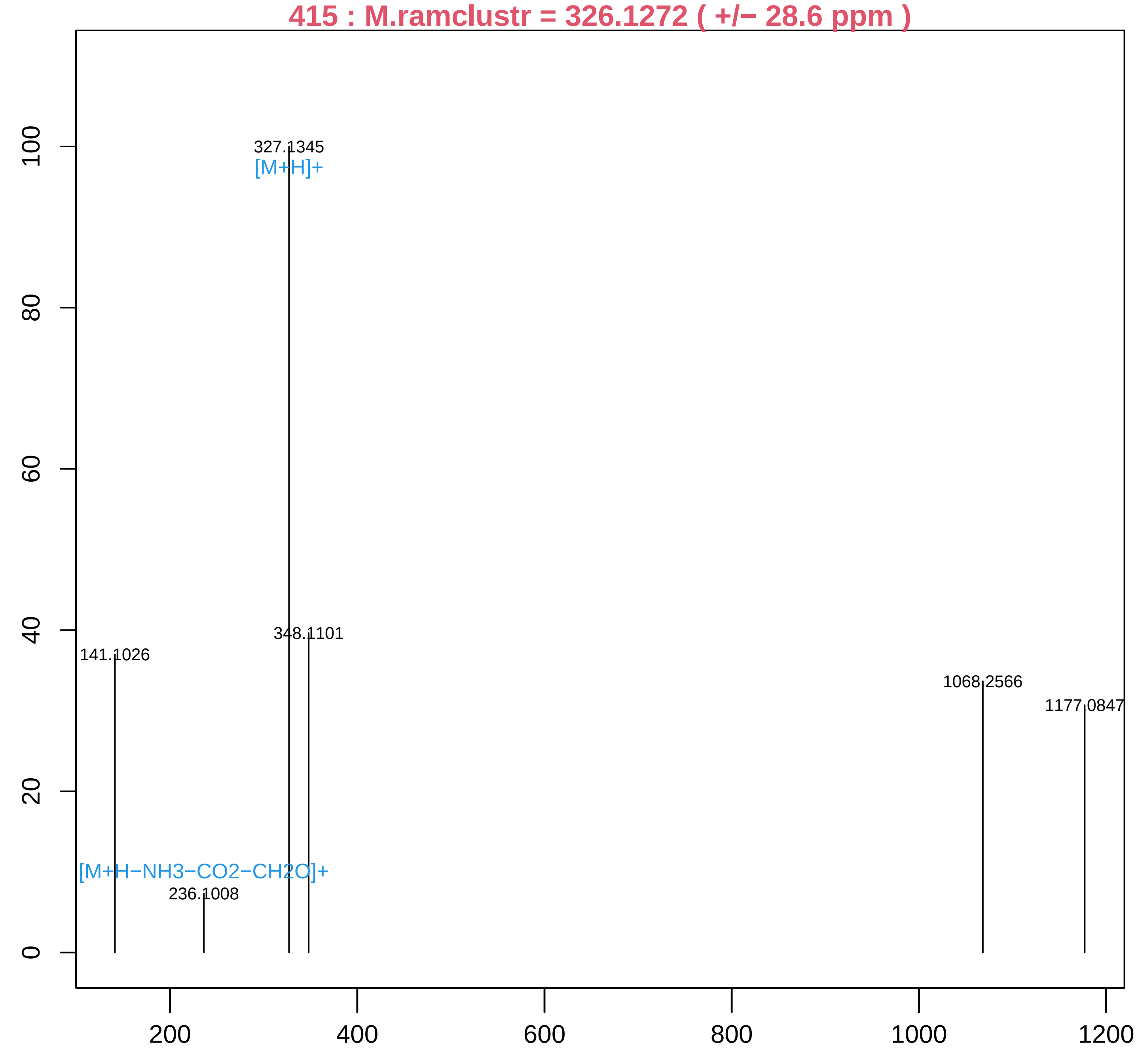
415 : M.ramclustr = 326.1272 (+/− 28.6 ppm)
327.1345
[M+H]+
348.1101
141.1026
1068.2566
1177.0847
[M+H−NH3−CO2−CH2O]+
236.1008
0
20
40
60
80
100
200
400
600
800
1000
1200

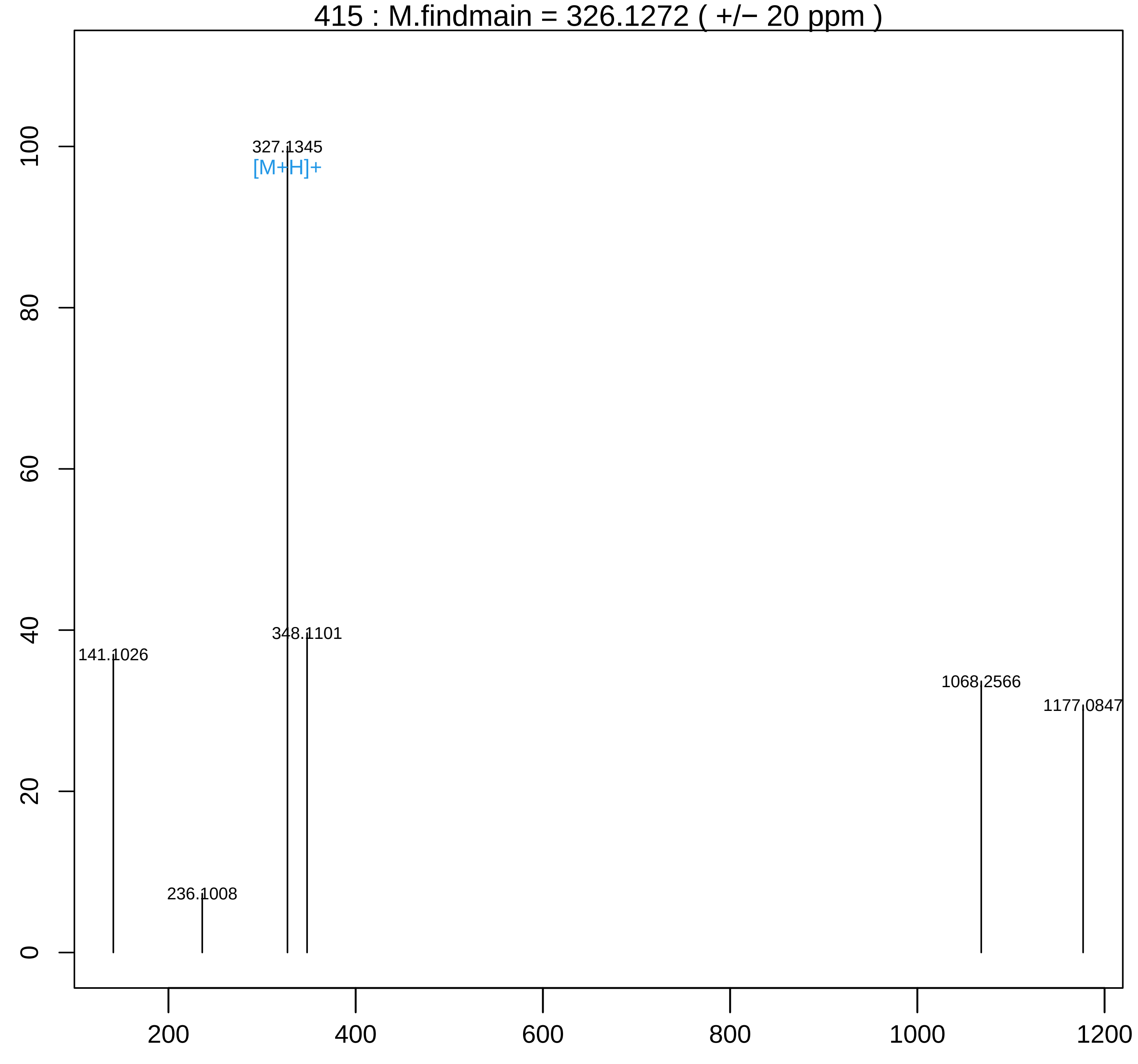
415 : M.findmain = 326.1272 (+/− 20 ppm)
327.1345
[M+H]+
348.1101
141.1026
1068.2566
1177.0847
236.1008
0
20
40
60
80
100
200
400
600
800
1000
1200

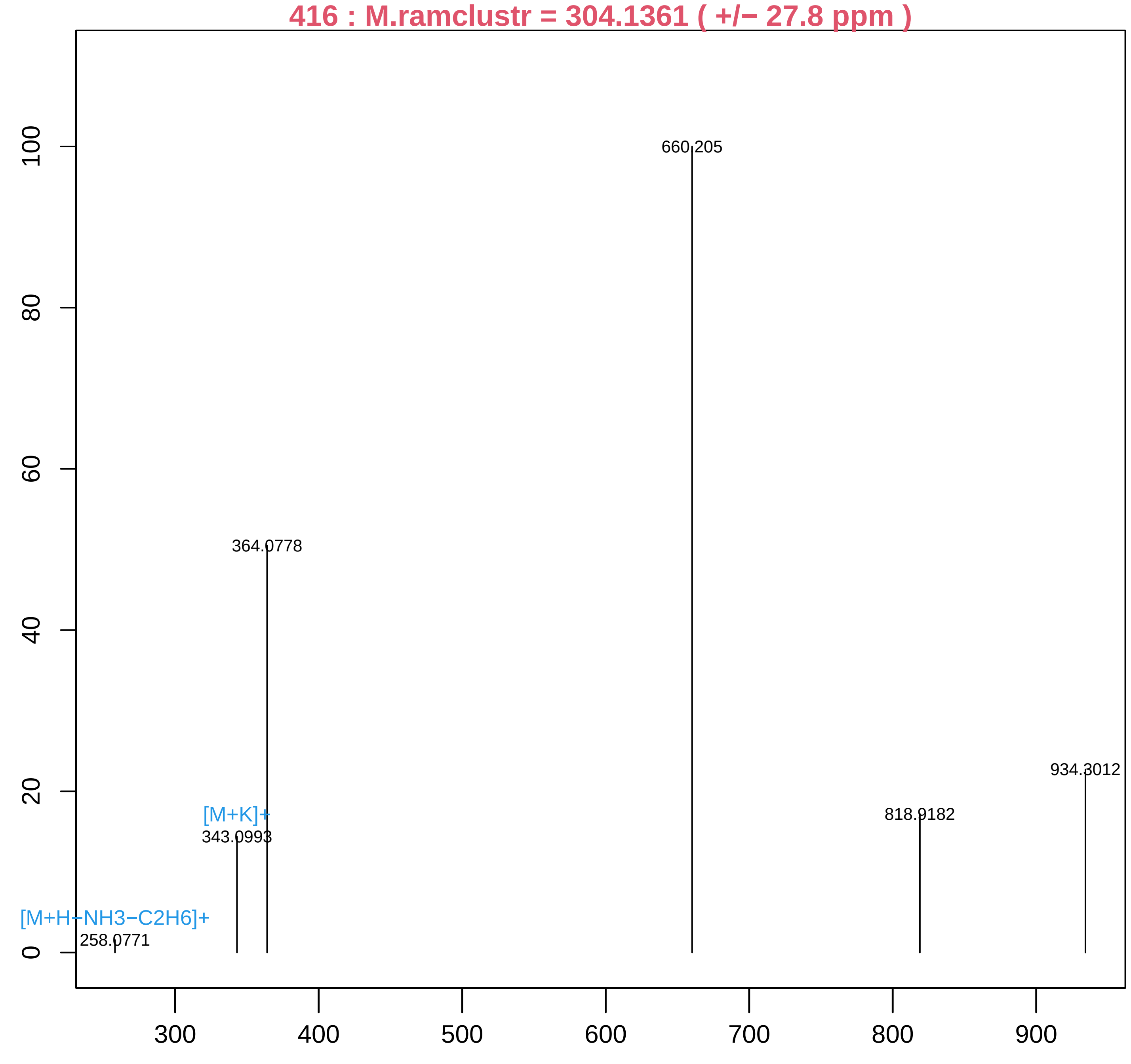
416 : M.ramclustr = 304.1361 (+/− 27.8 ppm)
660.205
364.0778
934.3012
818.9182
[M+K]+
343.0993
[M+H−NH3−C2H6]+
258.0771
0
20
40
60
80
100
300
400
500
600
700
800
900

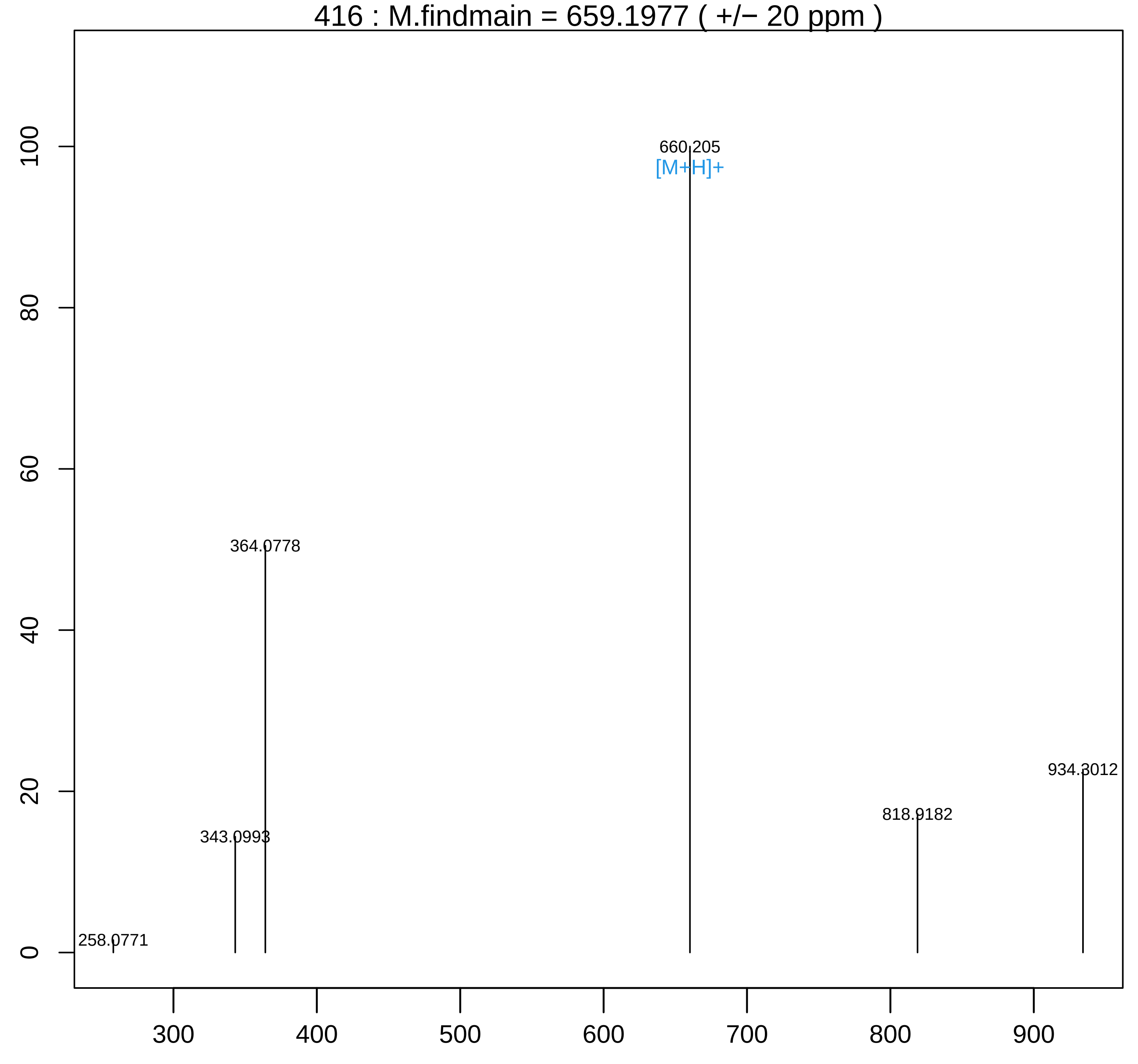
416 : M.findmain = 659.1977 (+/− 20 ppm)
660.205
[M+H]+
364.0778
934.3012
818.9182
343.0993
258.0771
0
20
40
60
80
100
300
400
500
600
700
800
900

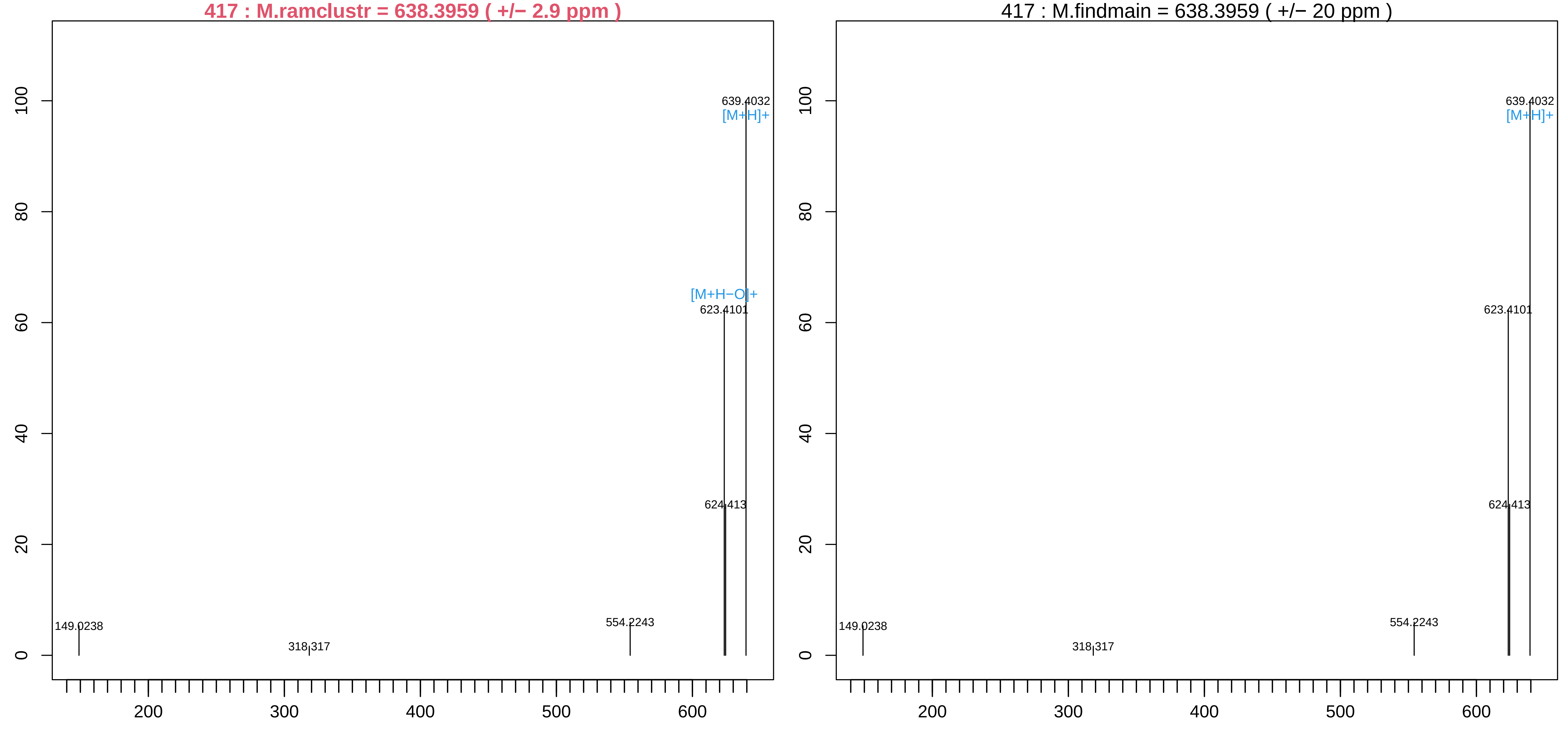

417 : M.ramclustr = 638.3959 (+/− 2.9 ppm)
639.4032
[M+H]+
[M+H−O]+
623.4101
624.413
149.0238
318.317
554.2243
0
20
40
60
80
100
200
300
400
500
600
417 : M.findmain = 638.3959 (+/− 20 ppm)
639.4032
[M+H]+
623.4101
624.413
149.0238
318.317
554.2243
0
20
40
60
80
100
200
300
400
500
600

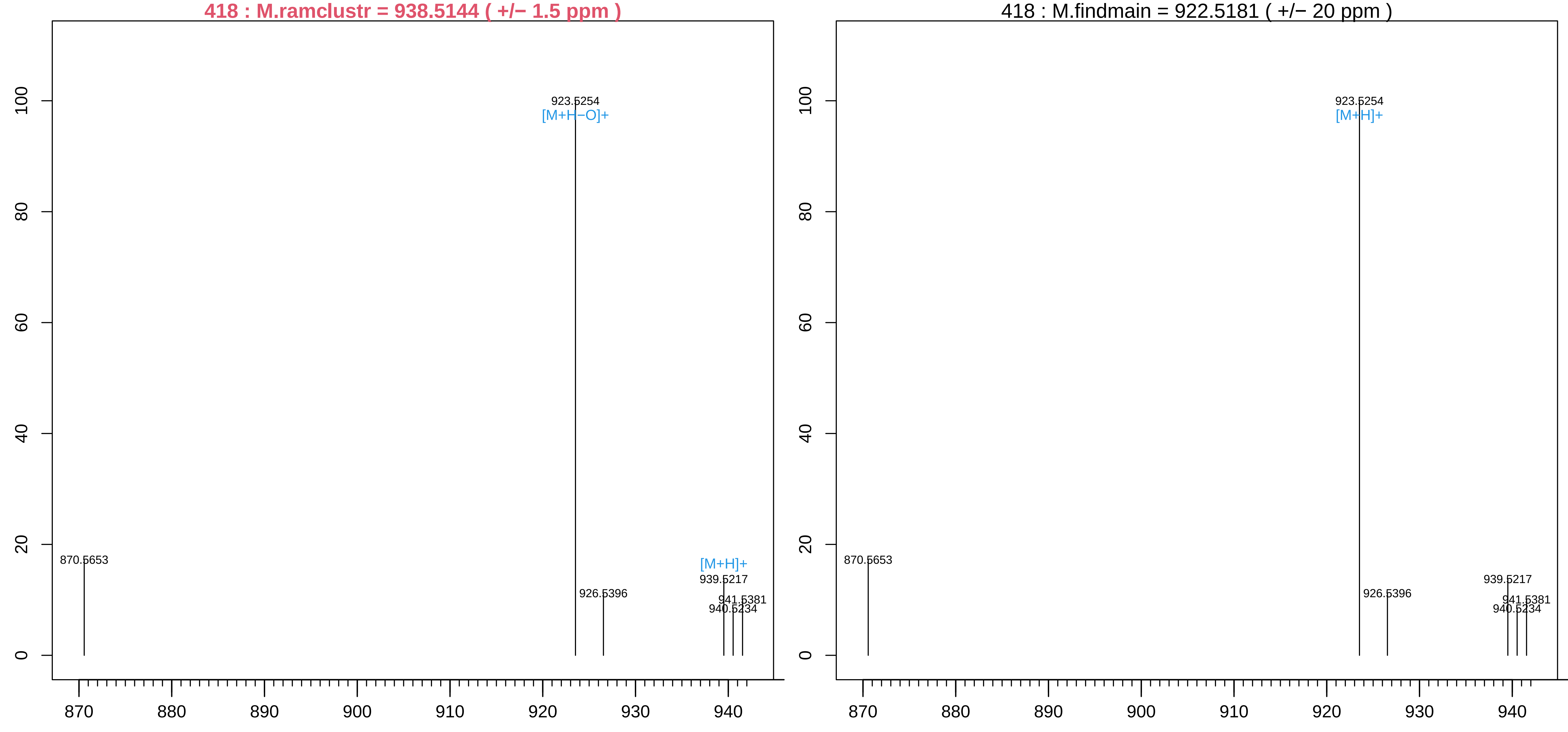

418 : M.ramclustr = 938.5144 (+/− 1.5 ppm)
923.5254
[M+H−O]+
870.5653
[M+H]+
939.5217
926.5396
941.5381
940.5234
418 : M.findmain = 922.5181 (+/− 20 ppm)
923.5254
[M+H]+
870.5653
939.5217
926.5396
941.5381
940.5234

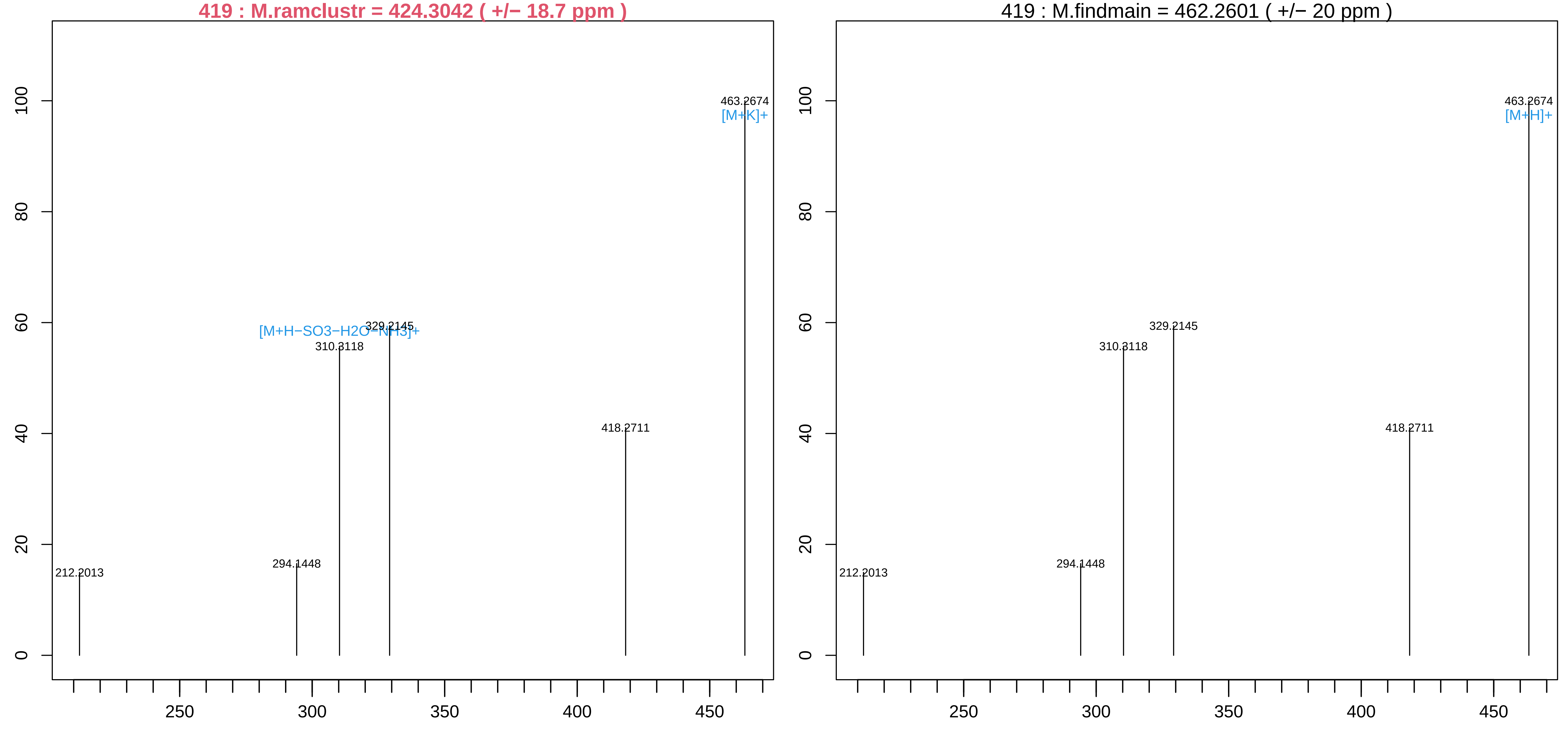

419 : M.ramclustr = 424.3042 (+/− 18.7 ppm)
463.2674
[M+K]+
[M+H−SO3−H2O−NH3]+
329.2145
310.3118
418.2711
294.1448
212.2013
0
20
40
60
80
100
250
300
350
400
450
419 : M.findmain = 462.2601 (+/− 20 ppm)
463.2674
[M+H]+
329.2145
310.3118
418.2711
294.1448
212.2013
0
20
40
60
80
100
250
300
350
400
450

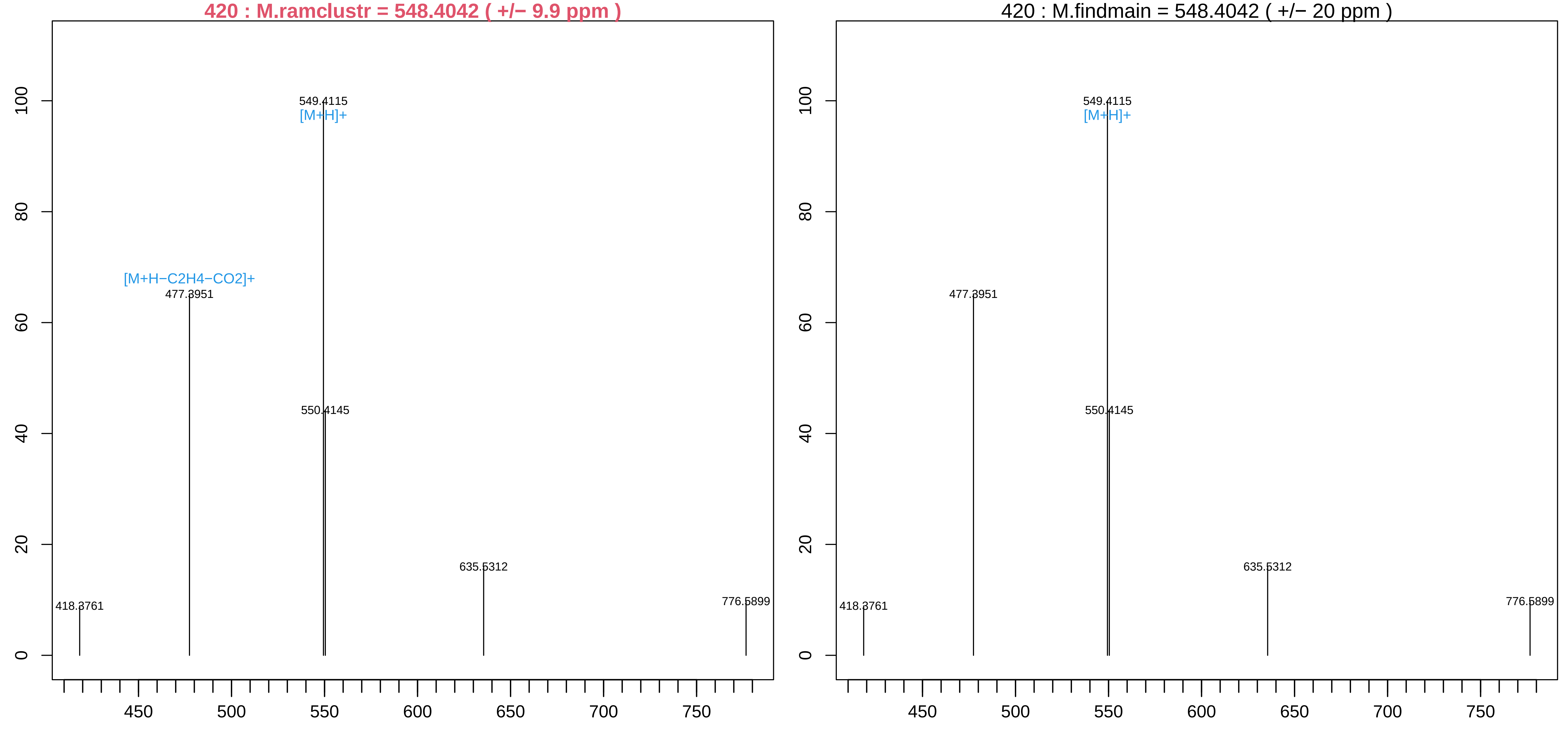

420 : M.ramclustr = 548.4042 (+/− 9.9 ppm)
549.4115
[M+H]+
[M+H−C2H4−CO2]+
477.3951
550.4145
635.5312
418.3761
776.5899
0
20
40
60
80
100
450
500
550
600
650
700
750
420 : M.findmain = 548.4042 (+/− 20 ppm)
549.4115
[M+H]+
477.3951
550.4145
635.5312
418.3761
776.5899

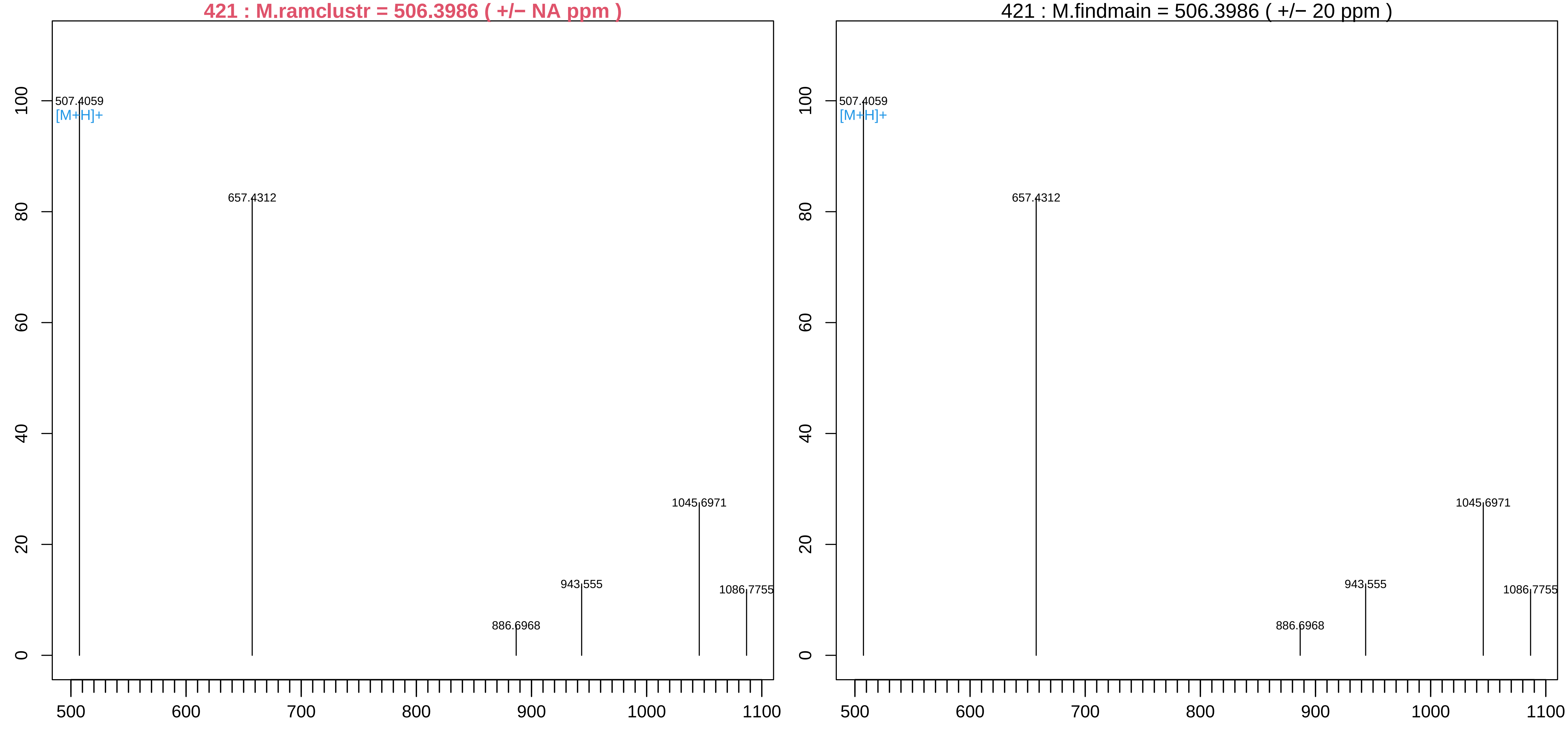

421 : M.ramclustr = 506.3986 (+/− NA ppm)
421 : M.findmain = 506.3986 (+/− 20 ppm)
507.4059
[M+H]+
657.4312
886.6968
943.555
1045.6971
1086.7755
0
20
40
60
80
100
500
600
700
800
900
1000
1100

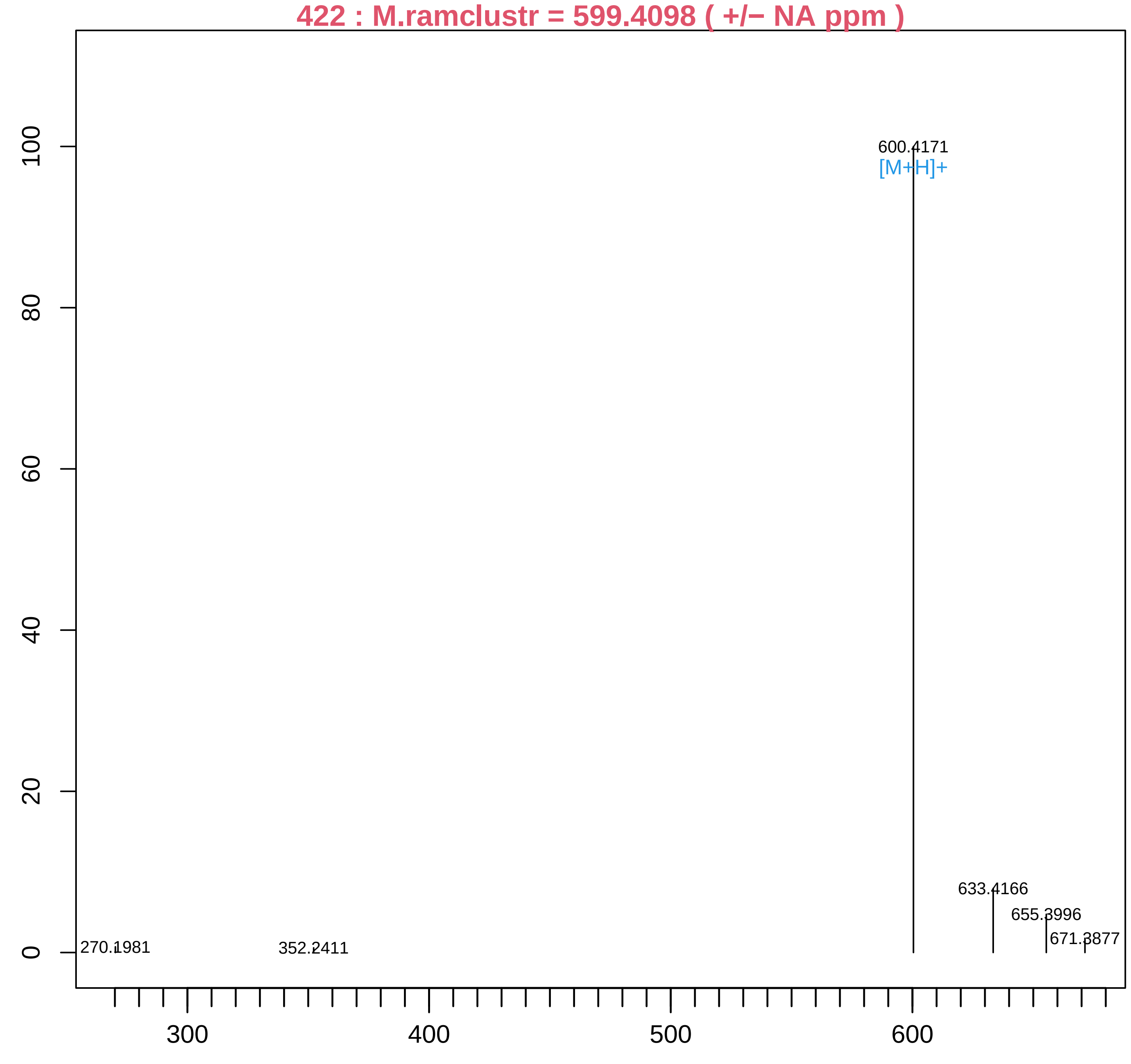

422 : M.ramclustr = 599.4098 (+/− NA ppm)
600.4171
[M+H]+
633.4166
655.3996
671.3877
270.1981
352.2411
0
20
40
60
80
100
300
400
500
600

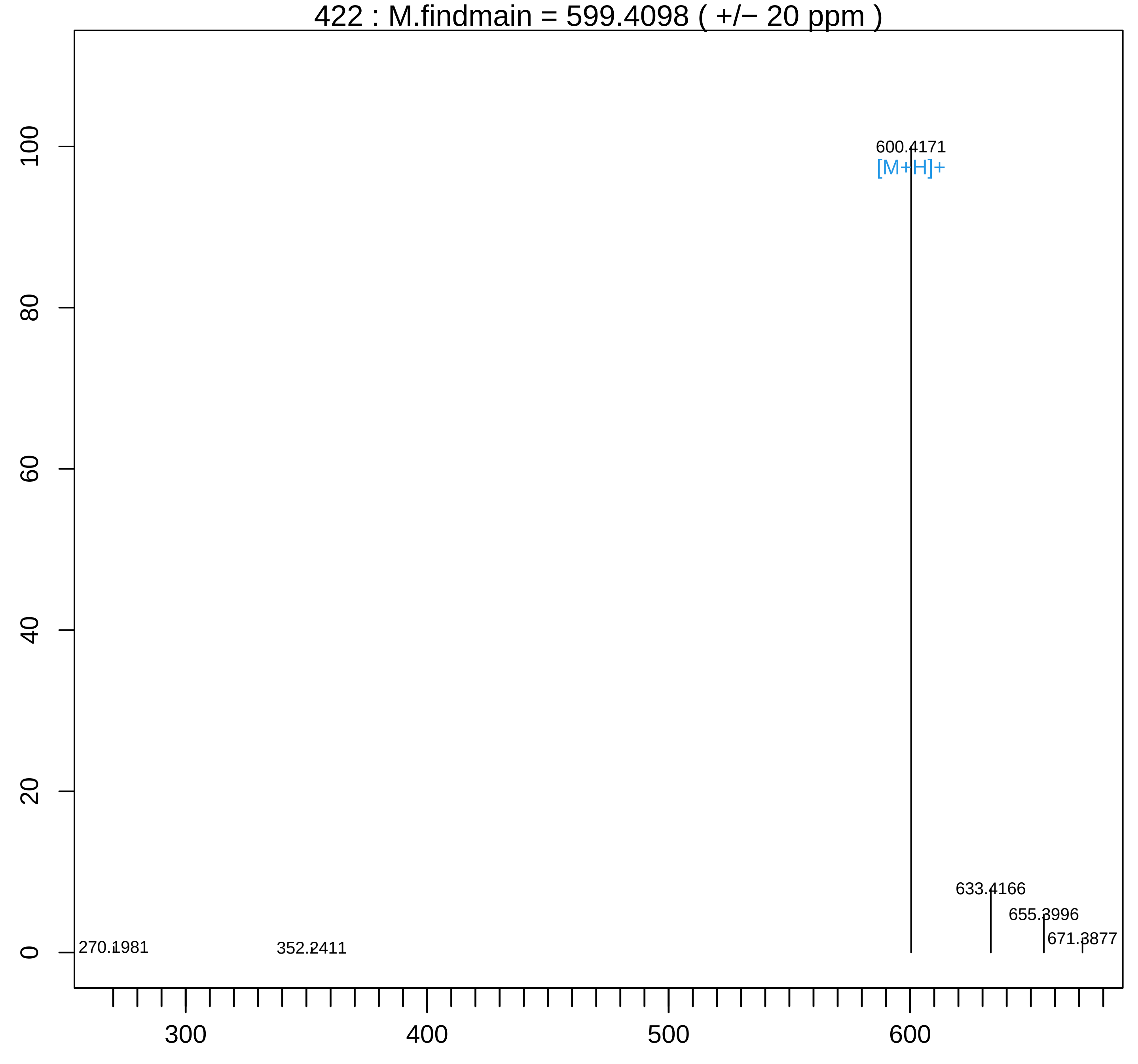

422 : M.findmain = 599.4098 (+/− 20 ppm)
600.4171
[M+H]+
633.4166
655.3996
671.3877
270.1981
352.2411
0
20
40
60
80
100
300
400
500
600

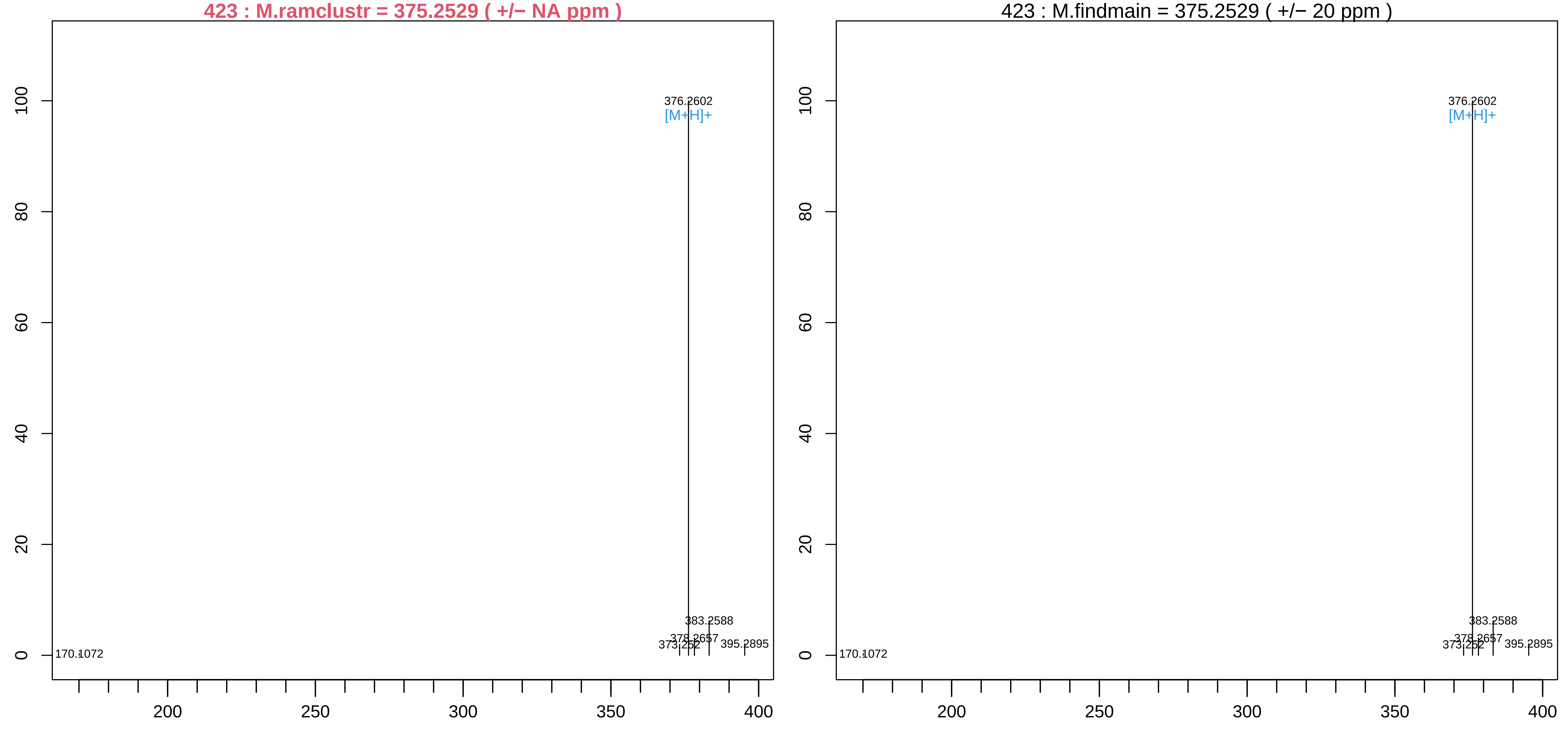
423 : M.ramclustr = 375.2529 (+/− NA ppm)
376.2602
[M+H]+
383.2588
378.2657
373.252
395.2895
170.1072
423 : M.findmain = 375.2529 (+/− 20 ppm)
376.2602
[M+H]+
383.2588
378.2657
373.252
395.2895
170.1072

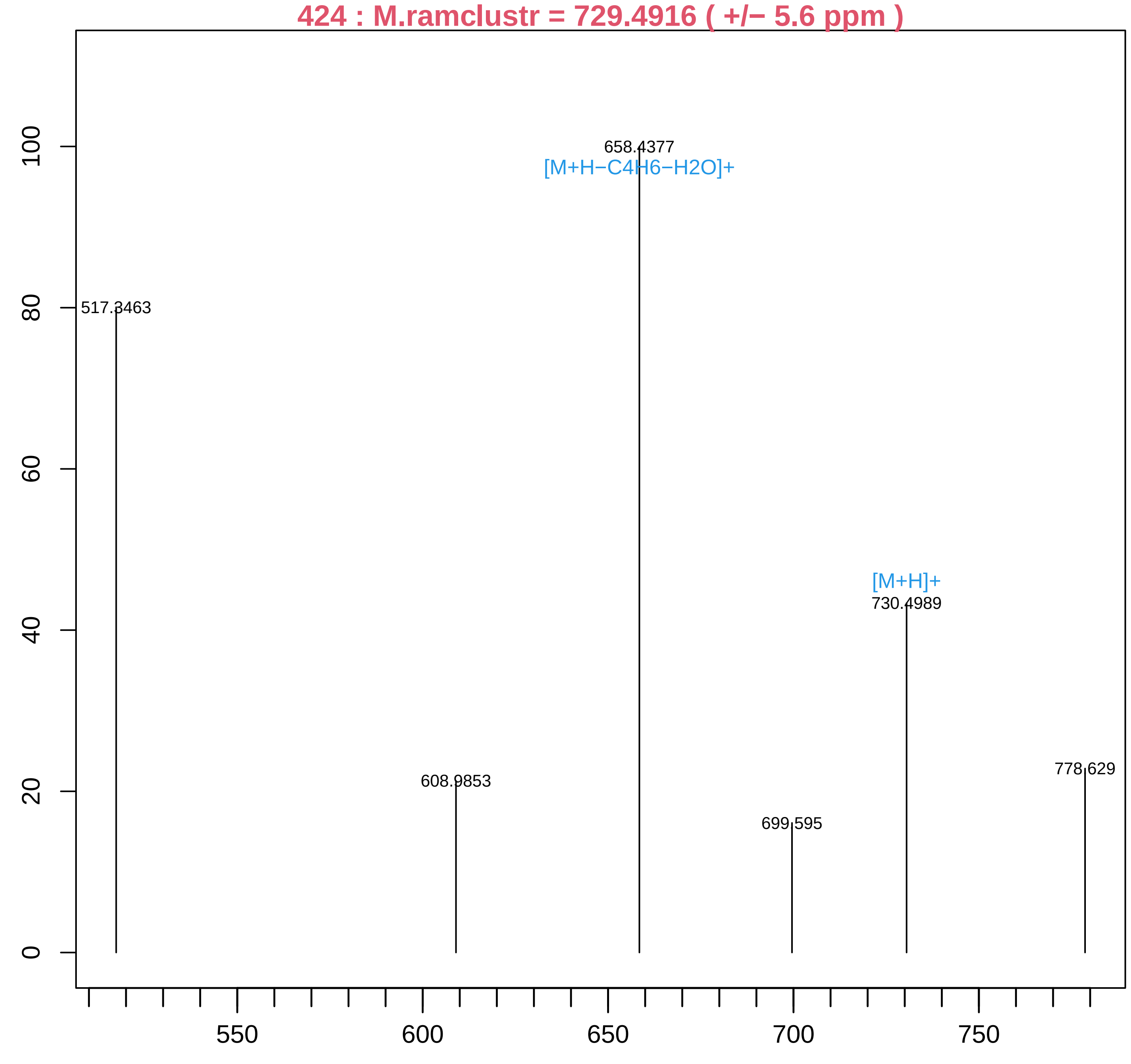
424 : M.ramclustr = 729.4916 (+/− 5.6 ppm)
517.3463
608.9853
658.4377
[M+H−C4H6−H2O]+
699.595
[M+H]+
730.4989
778.629
0
20
40
60
80
100
550
600
650
700
750

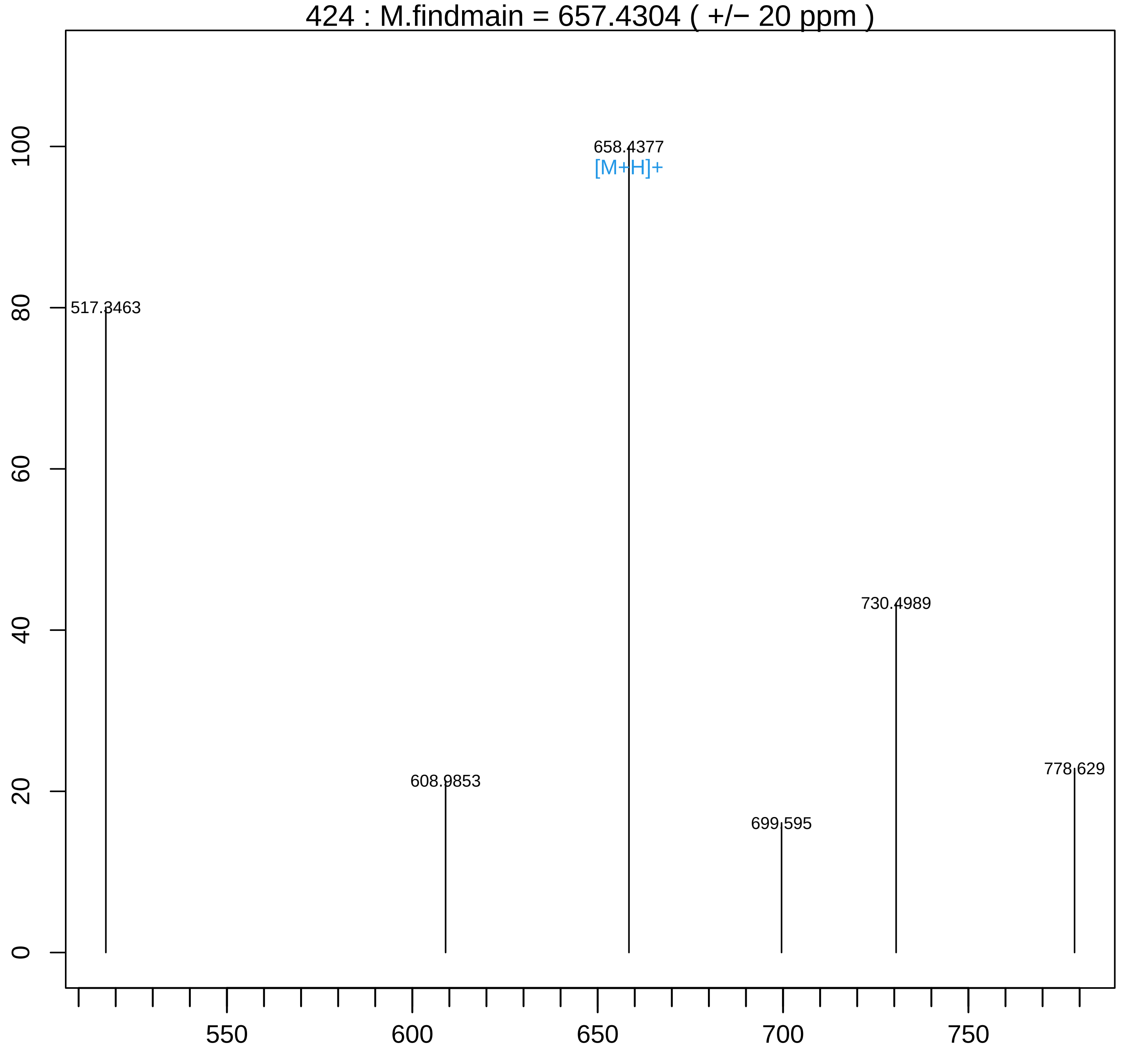
424 : M.findmain = 657.4304 (+/− 20 ppm)
517.3463
608.9853
658.4377
[M+H]+
699.595
730.4989
778.629
0
20
40
60
80
100
550
600
650
700
750

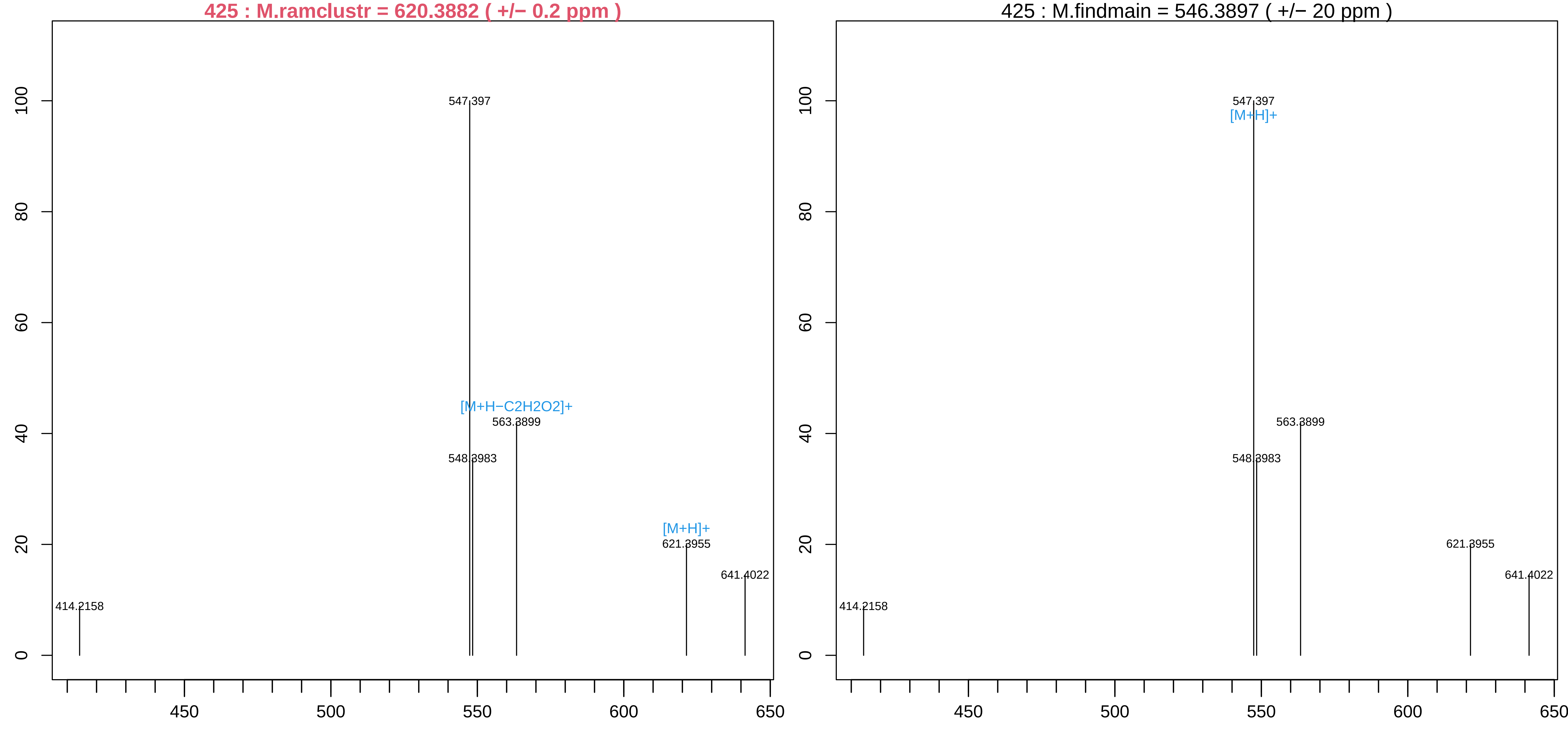

425 : M.ramclustr = 620.3882 (+/− 0.2 ppm)
547.397
[M+H−C2H2O2]+
563.3899
548.3983
[M+H]+
621.3955
641.4022
414.2158
425 : M.findmain = 546.3897 (+/− 20 ppm)
547.397
[M+H]+
563.3899
548.3983
621.3955
641.4022
414.2158
0
20
40
60
80
100
450
500
550
600
650

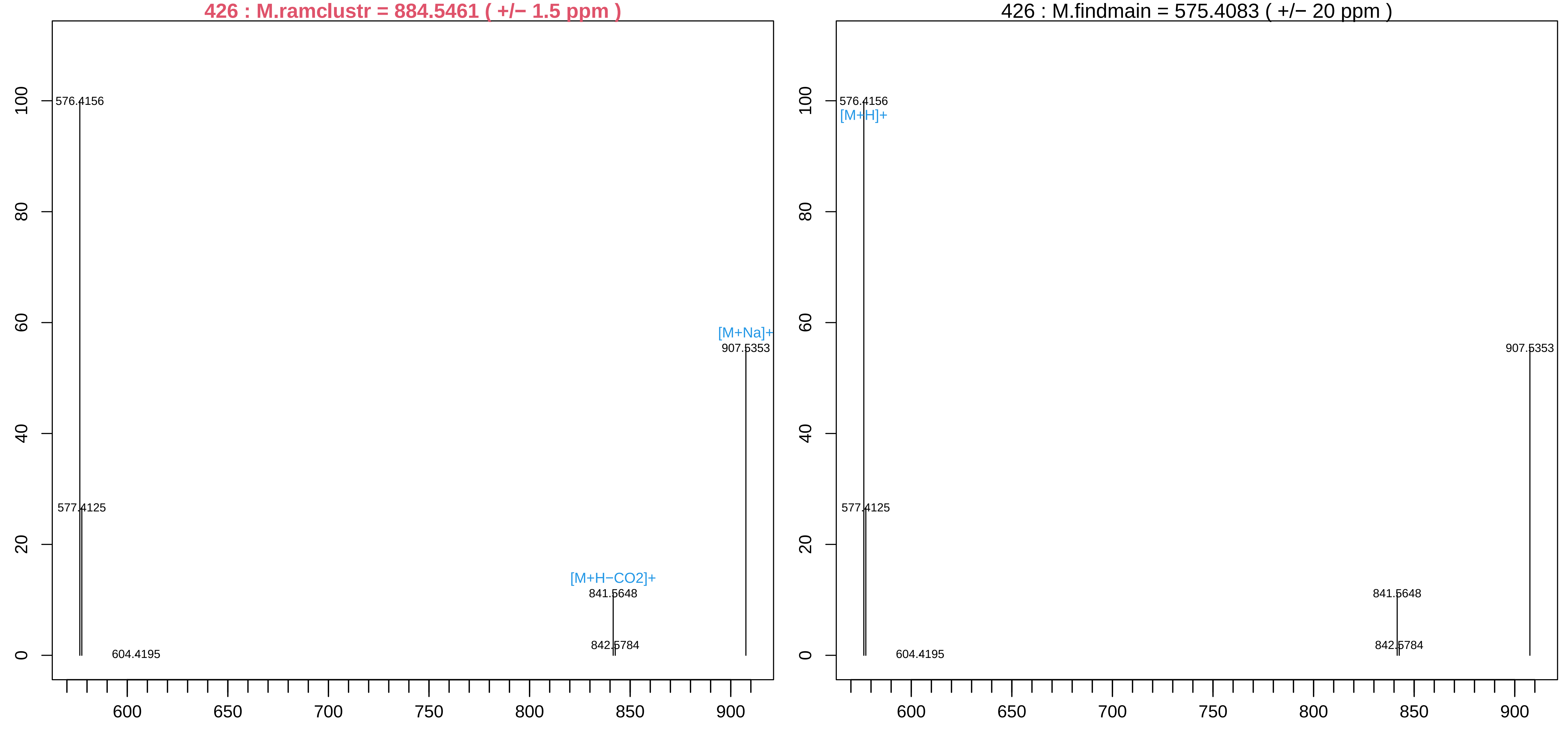
426 : M.ramclustr = 884.5461 (+/− 1.5 ppm)
576.4156
577.4125
604.4195
[M+H−CO2]+
841.5648
842.5784
[M+Na]+
907.5353
0
20
40
60
80
100
600
650
700
750
800
850
900
426 : M.findmain = 575.4083 (+/− 20 ppm)
576.4156
[M+H]+
577.4125
604.4195
841.5648
842.5784
907.5353

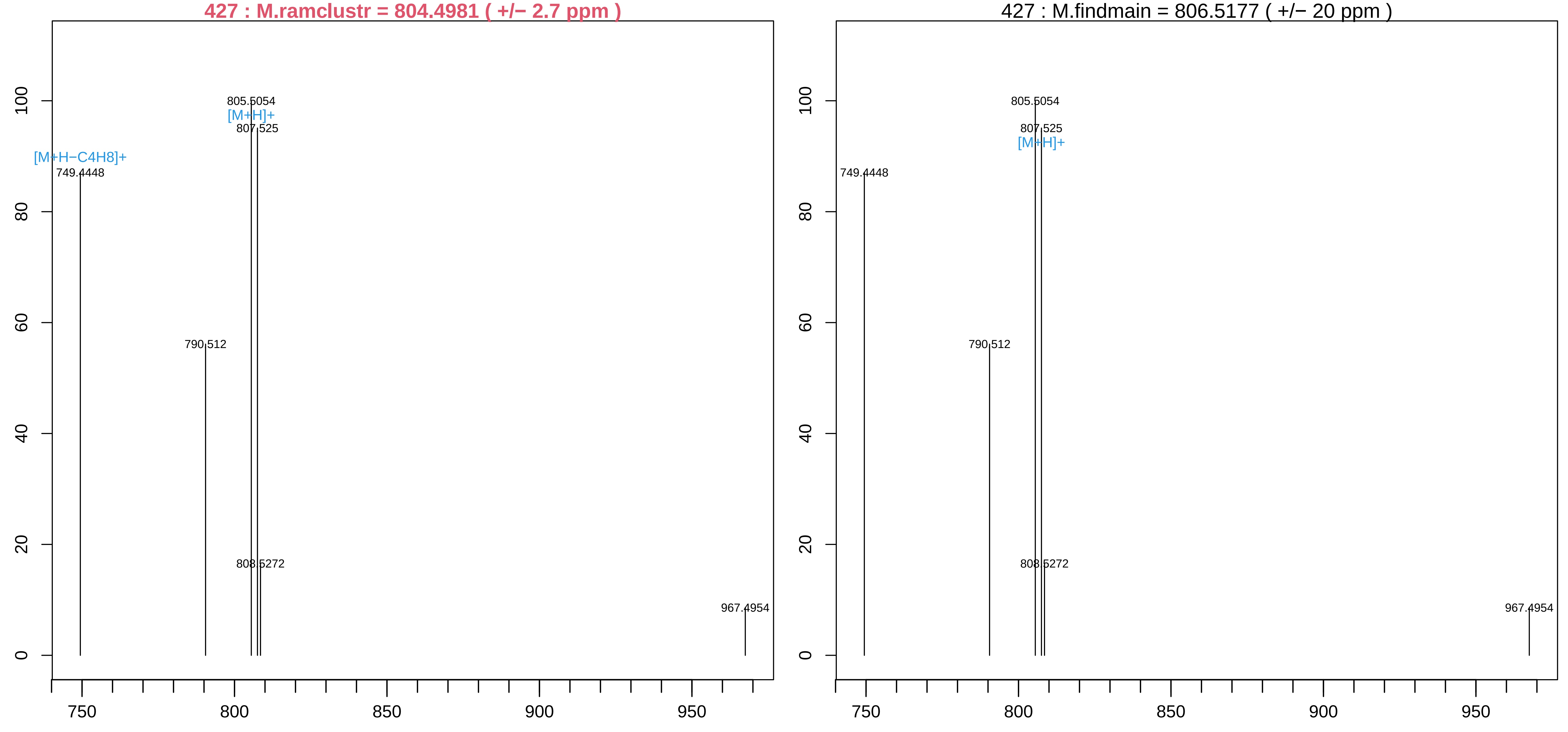
427 : M.ramclustr = 804.4981 (+/− 2.7 ppm)
805.5054
[M+H]+
807.525
[M+H−C4H8]+
749.4448
790.512
808.5272
967.4954
0
20
40
60
80
100
750
800
850
900
950
427 : M.findmain = 806.5177 (+/− 20 ppm)
805.5054
807.525
[M+H]+
749.4448
790.512
808.5272
967.4954
0
20
40
60
80
100
750
800
850
900
950

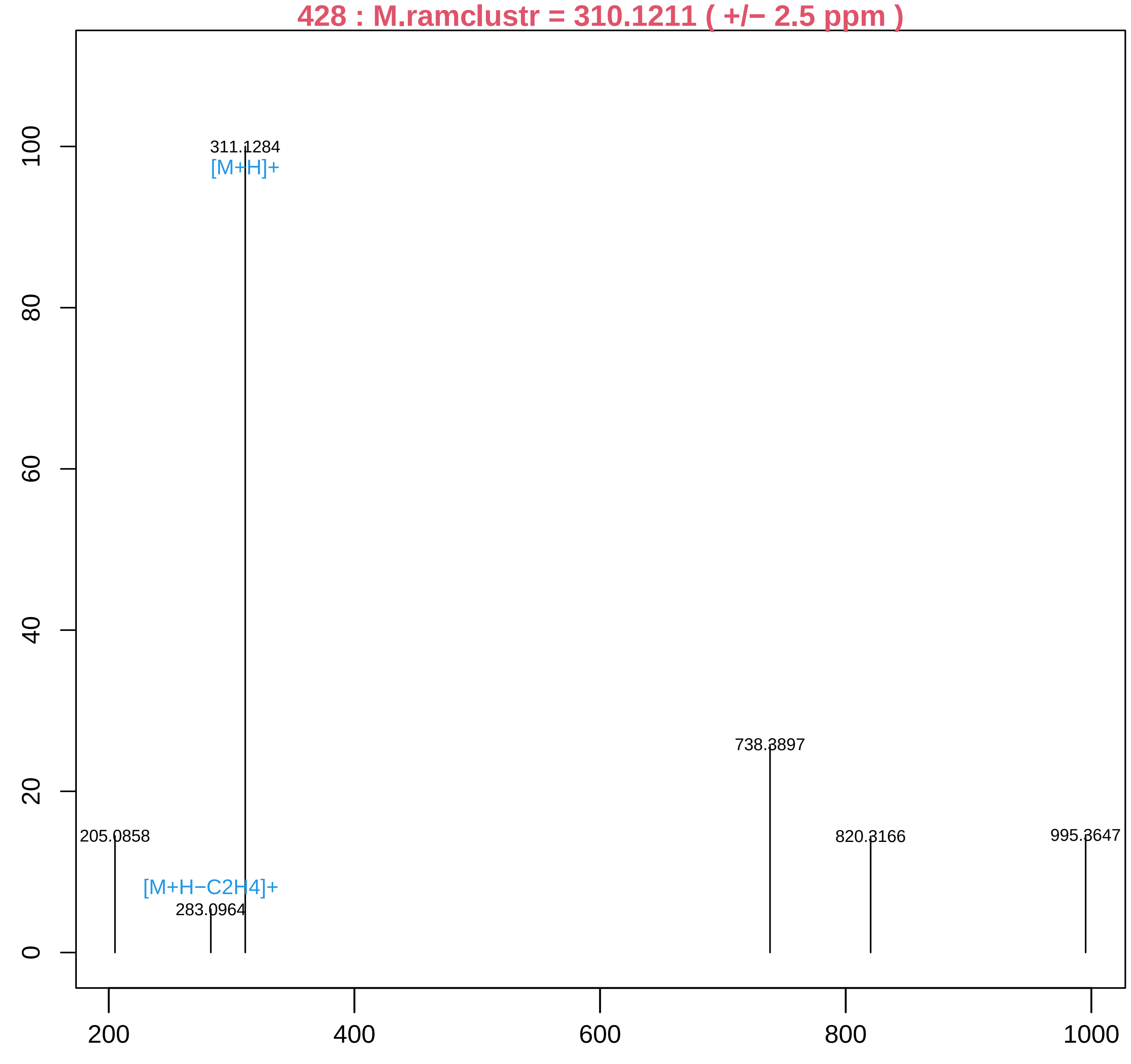
428 : M.ramclustr = 310.1211 (+/− 2.5 ppm)
311.1284
[M+H]+
205.0858
[M+H−C2H4]+
283.0964
738.3897
820.3166
995.3647
0
20
40
60
80
100
200
400
600
800
1000

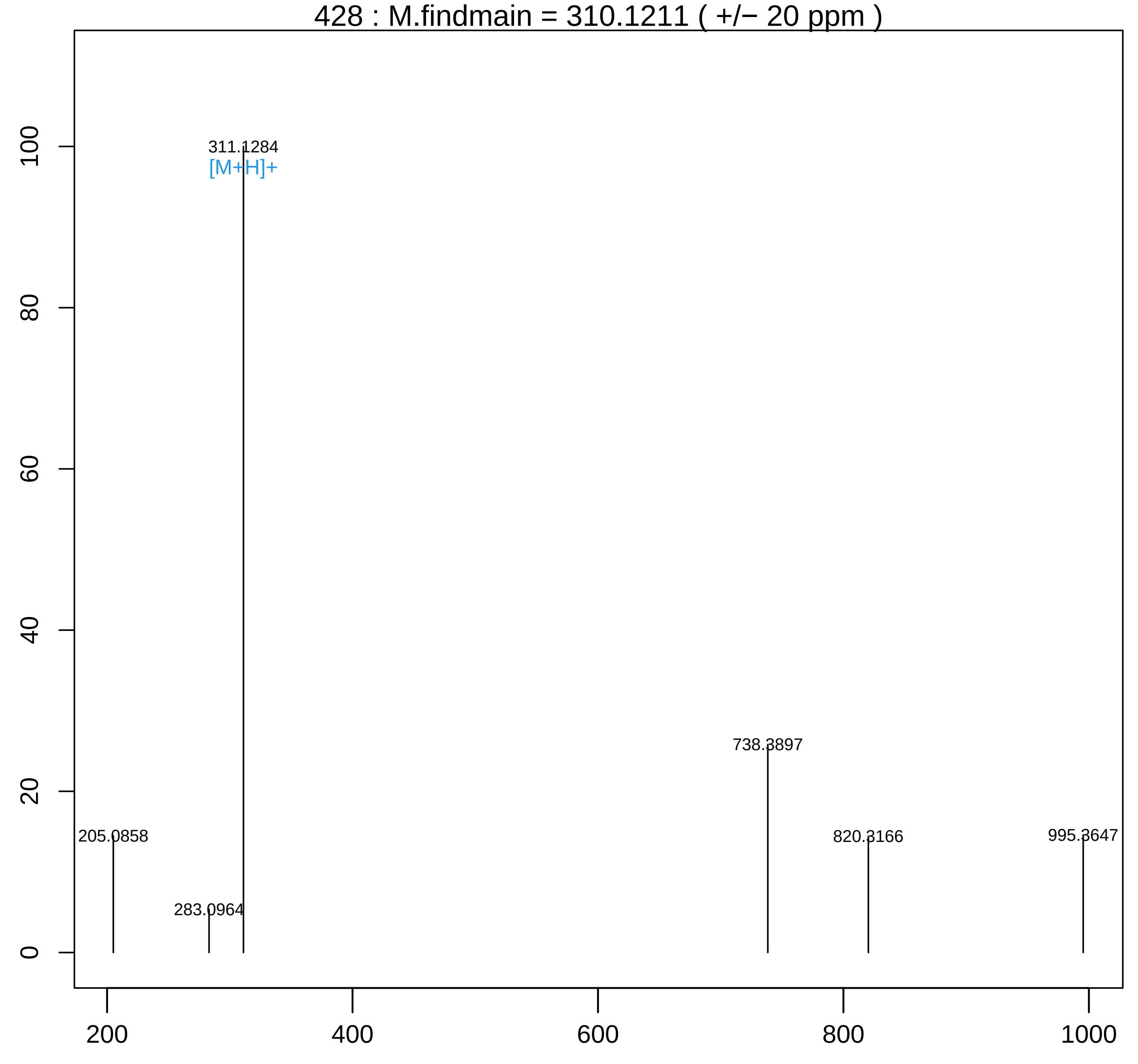
428 : M.findmain = 310.1211 (+/− 20 ppm)
311.1284
[M+H]+
205.0858
283.0964
738.3897
820.3166
995.3647
0
20
40
60
80
100
200
400
600
800
1000

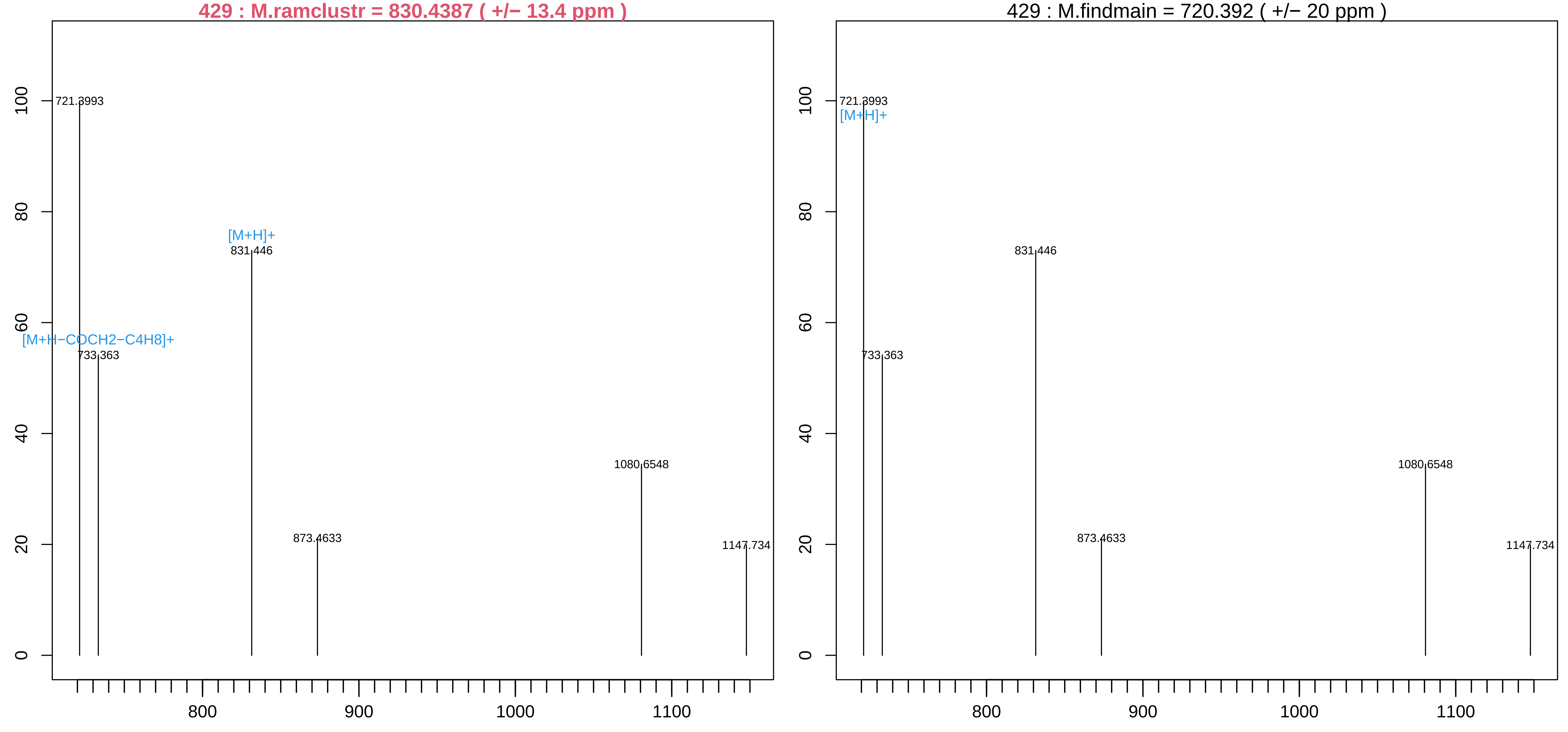

429 : M.ramclustr = 830.4387 (+/− 13.4 ppm)
721.3993
[M+H−COCH2−C4H8]+
733.363
[M+H]+
831.446
873.4633
1080.6548
1147.734
0
20
40
60
80
100
800
900
1000
1100
429 : M.findmain = 720.392 (+/− 20 ppm)
721.3993
[M+H]+
733.363
831.446
873.4633
1080.6548
1147.734

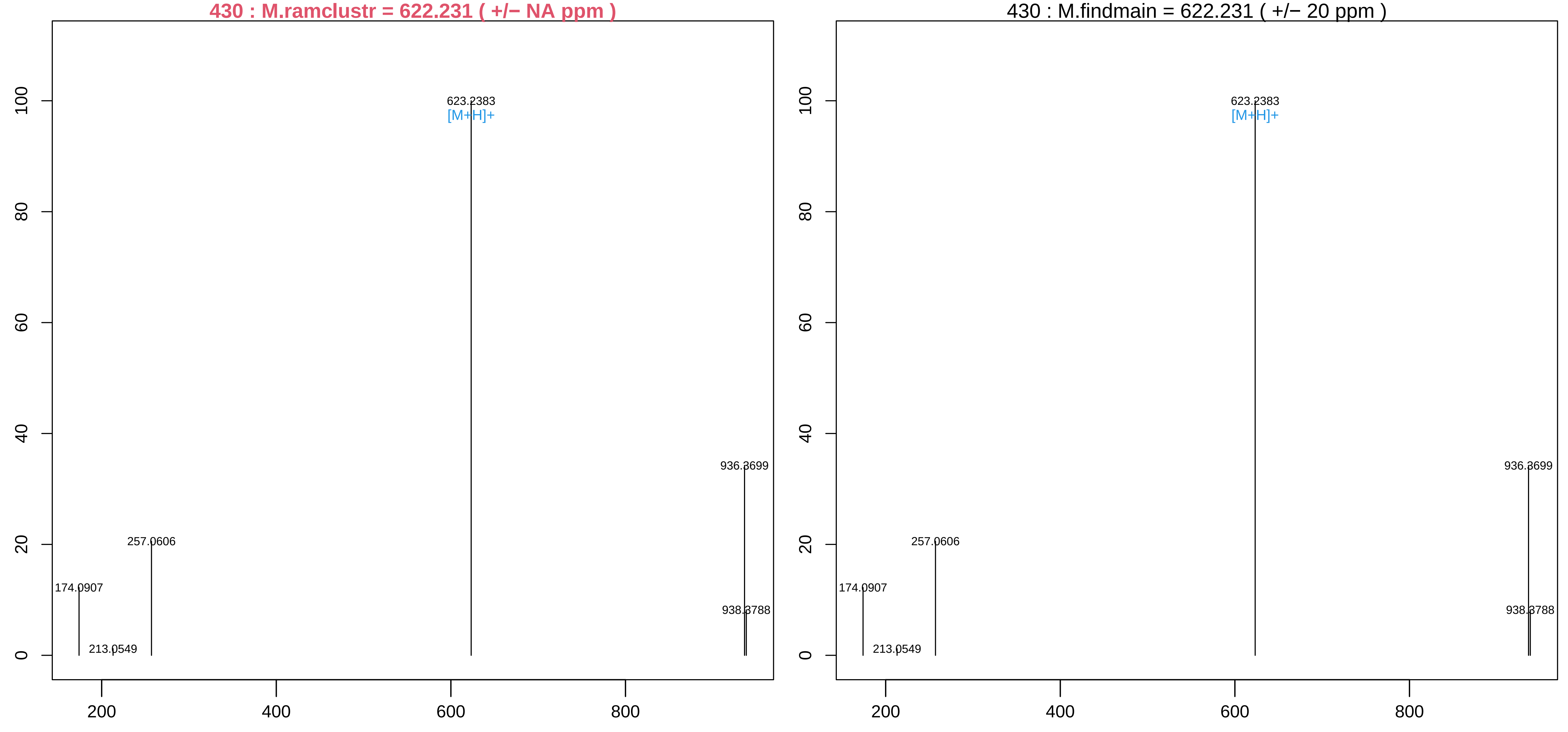
430 : M.ramclustr = 622.231 (+/− NA ppm)
430 : M.findmain = 622.231 (+/− 20 ppm)
623.2383
[M+H]+
936.3699
938.3788
257.0606
174.0907
213.0549
623.2383
[M+H]+
936.3699
938.3788
257.0606
174.0907
213.0549
0
20
40
60
80
100
200
400
600
800

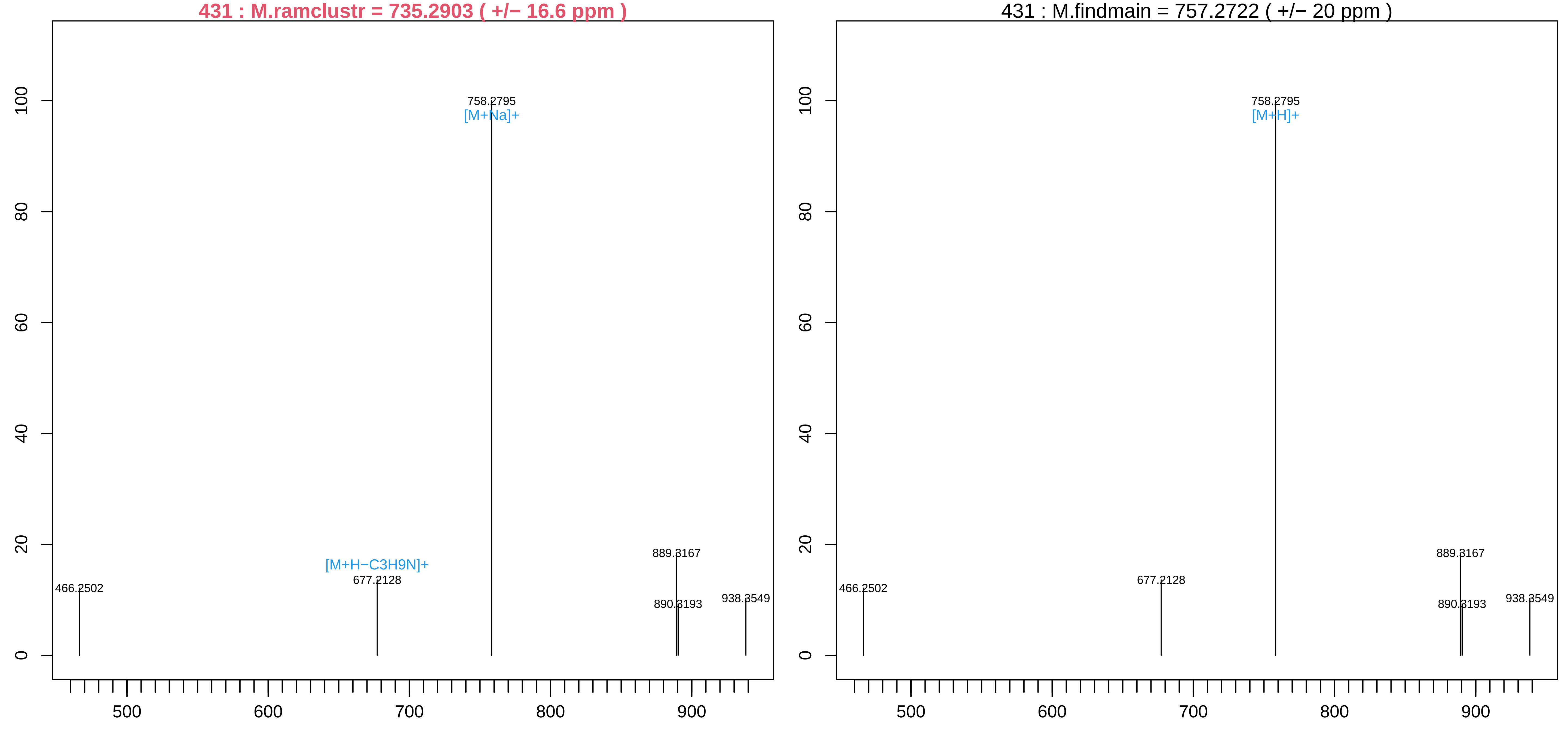
431 : M.ramclustr = 735.2903 (+/− 16.6 ppm)
758.2795
[M+Na]+
[M+H−C3H9N]+
677.2128
466.2502
889.3167
890.3193
938.3549
431 : M.findmain = 757.2722 (+/− 20 ppm)
758.2795
[M+H]+
677.2128
466.2502
889.3167
890.3193
938.3549
0
20
40
60
80
100
500
600
700
800
900

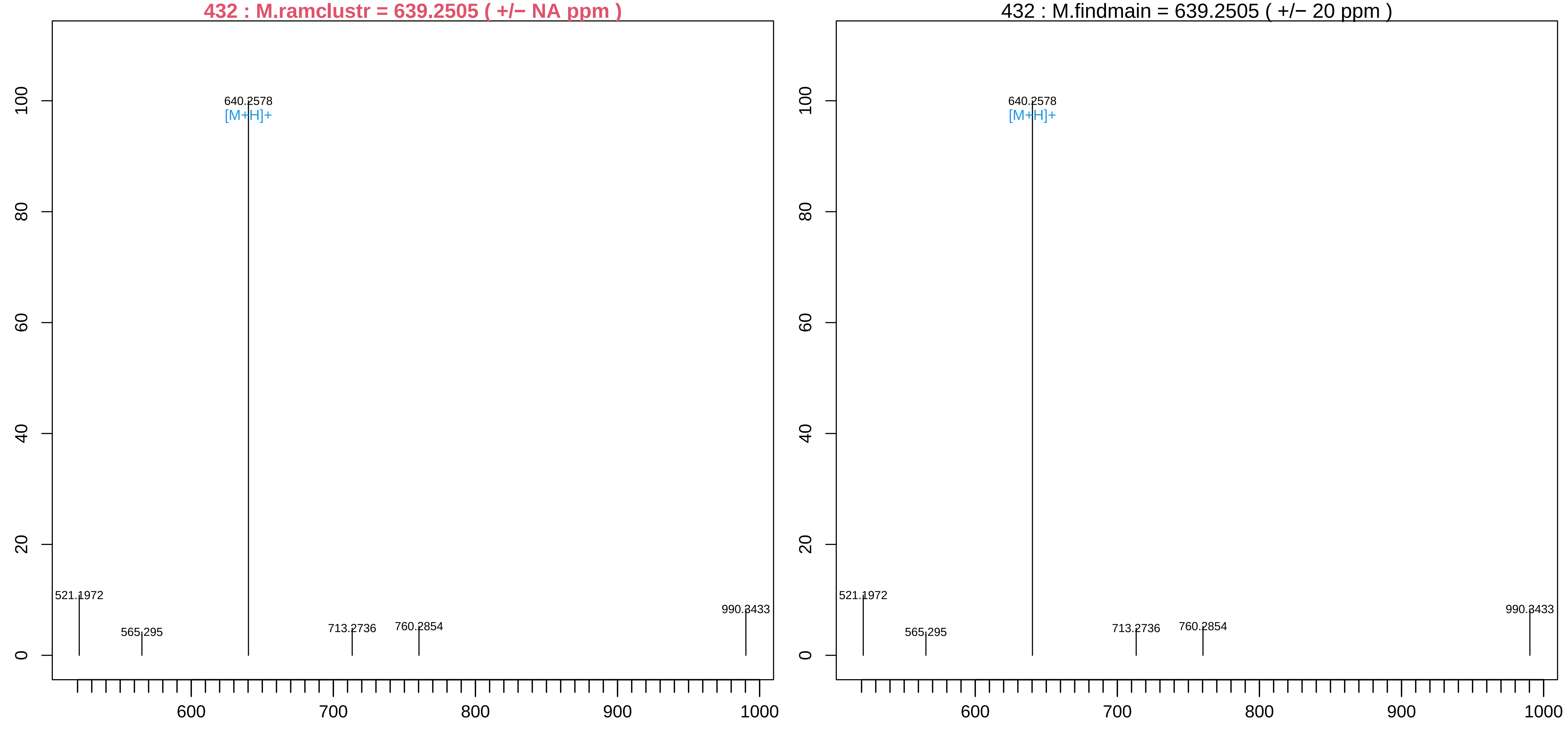
432 : M.ramclustr = 639.2505 (+/− NA ppm)
432 : M.findmain = 639.2505 (+/− 20 ppm)
640.2578
[M+H]+
521.1972
565.295
713.2736
760.2854
990.3433
0
20
40
60
80
100
600
700
800
900
1000

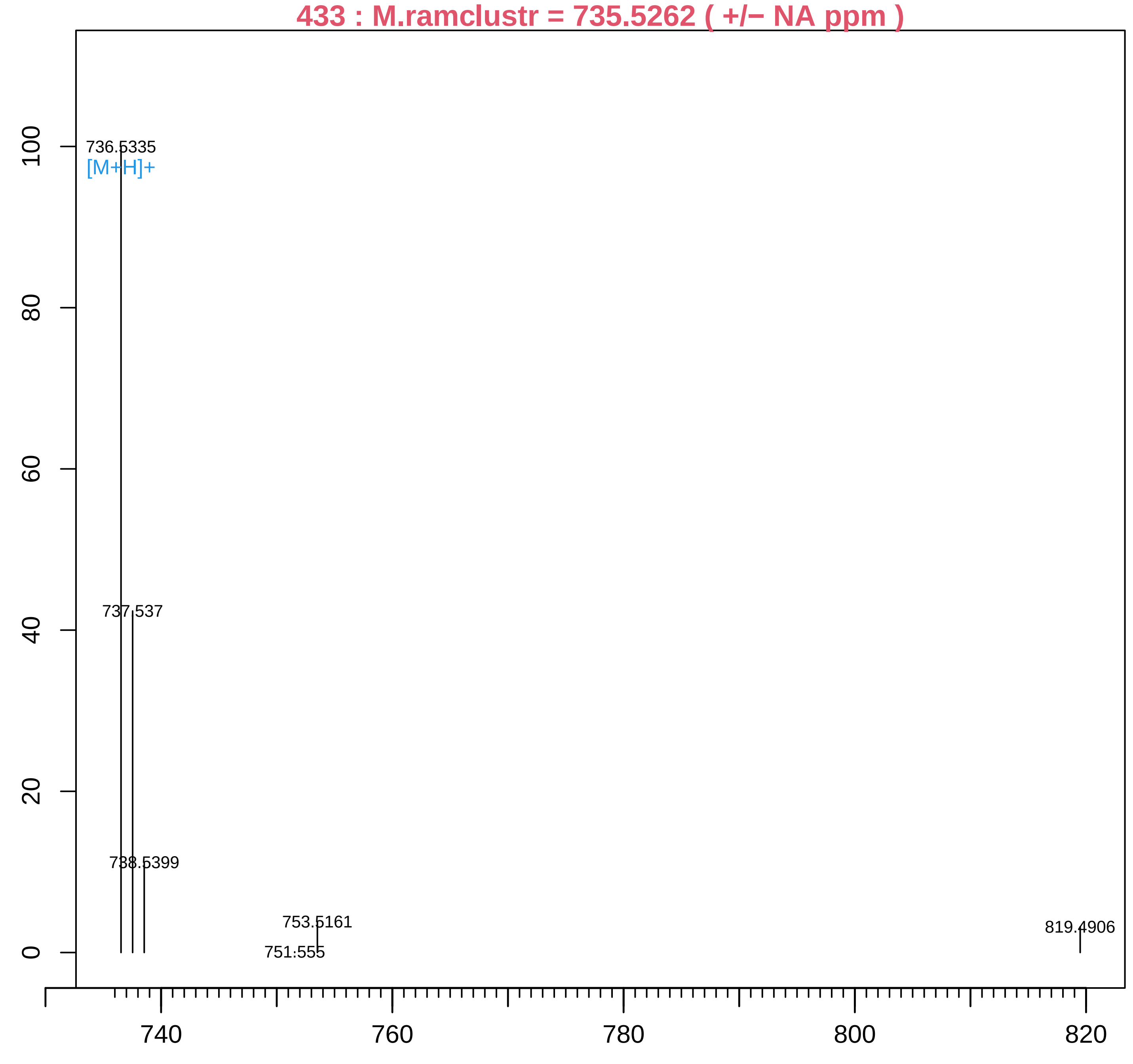
433 : M.ramclustr = 735.5262 (+/− NA ppm)
736.5335
[M+H]+
737.537
738.5399
753.5161
751.555
819.4906
0
20
40
60
80
100
740
760
780
800
820

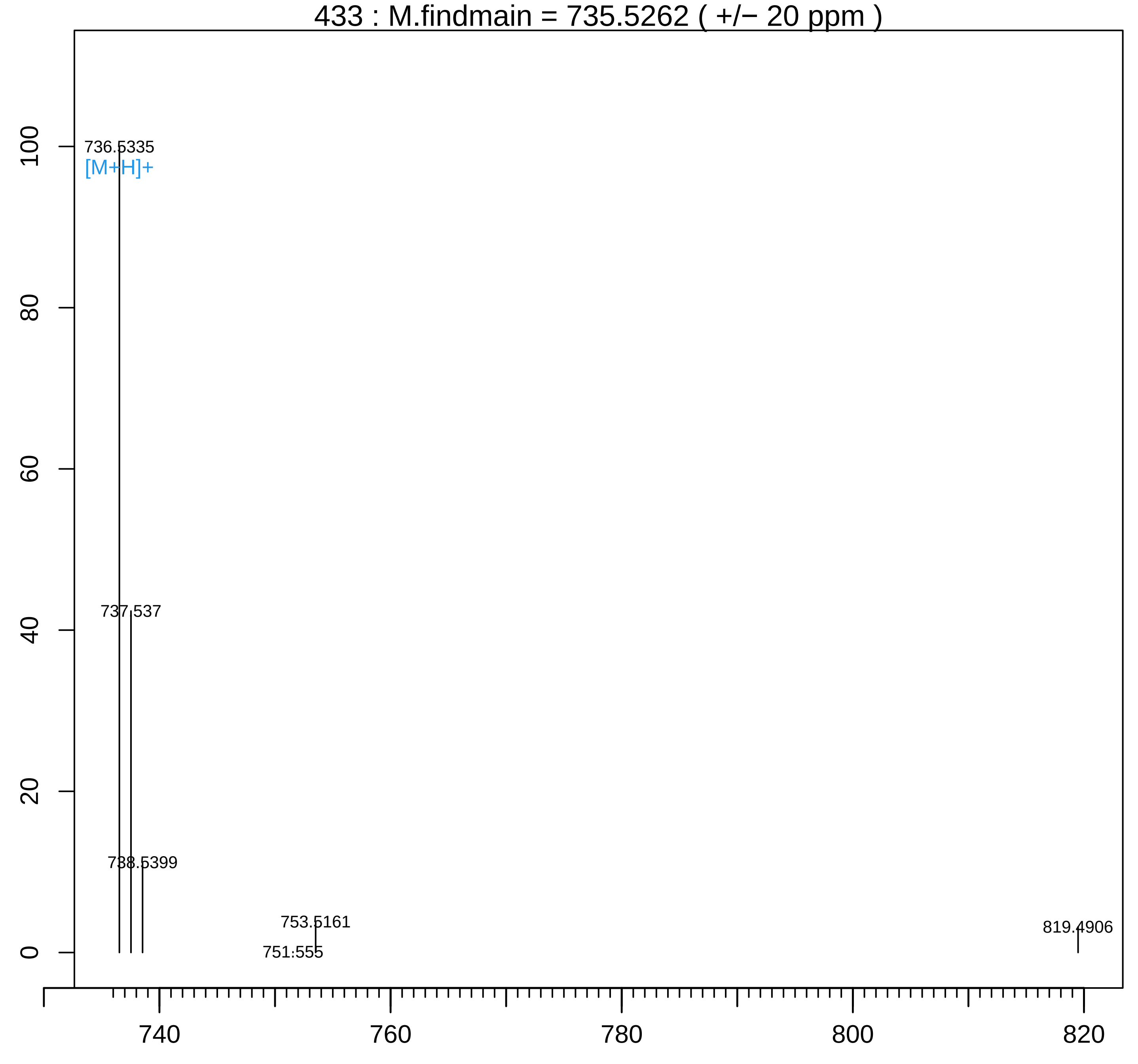
433 : M.findmain = 735.5262 (+/− 20 ppm)
736.5335
[M+H]+
737.537
738.5399
753.5161
751.555
819.4906
0
20
40
60
80
100
740
760
780
800
820

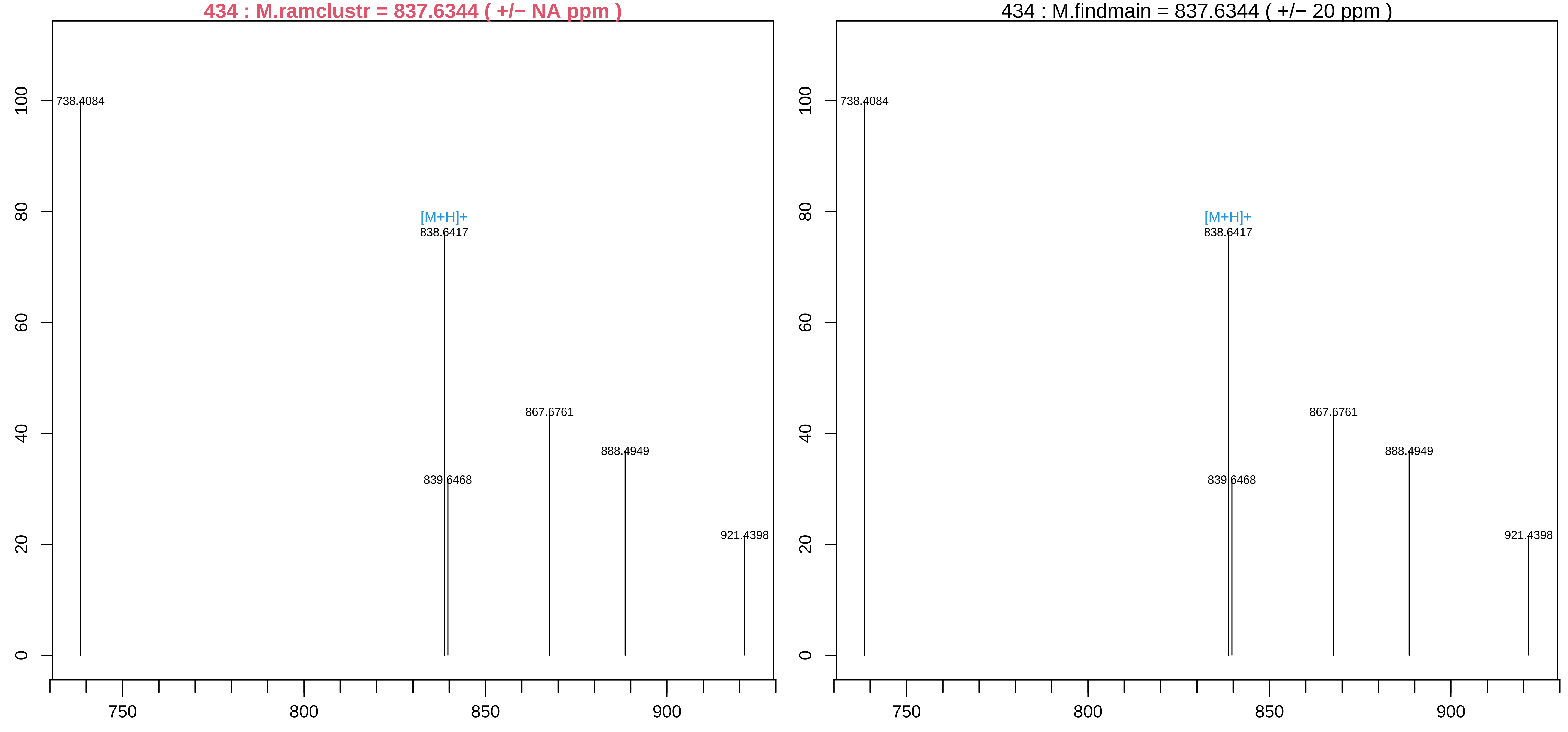

434 : M.ramclustr = 837.6344 (+/− NA ppm)
738.4084
[M+H]+
838.6417
839.6468
867.6761
888.4949
921.4398
0
20
40
60
80
100
750
800
850
900
434 : M.findmain = 837.6344 (+/− 20 ppm)
738.4084
[M+H]+
838.6417
839.6468
867.6761
888.4949
921.4398
0
20
40
60
80
100
750
800
850
900

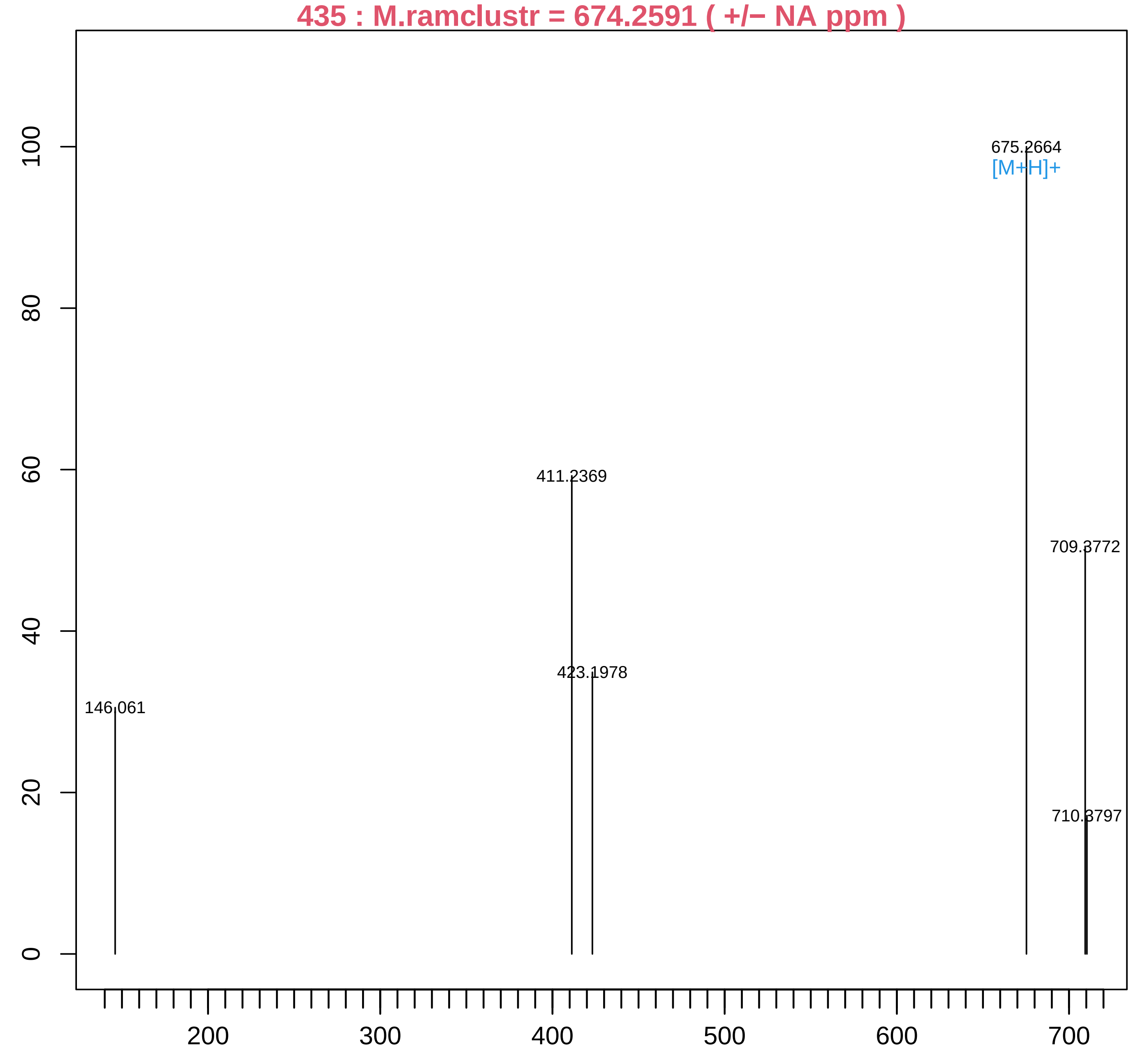
435 : M.ramclustr = 674.2591 (+/− NA ppm)
675.2664
[M+H]+
411.2369
709.3772
423.1978
146.061
710.3797
0
20
40
60
80
100
200
300
400
500
600
700

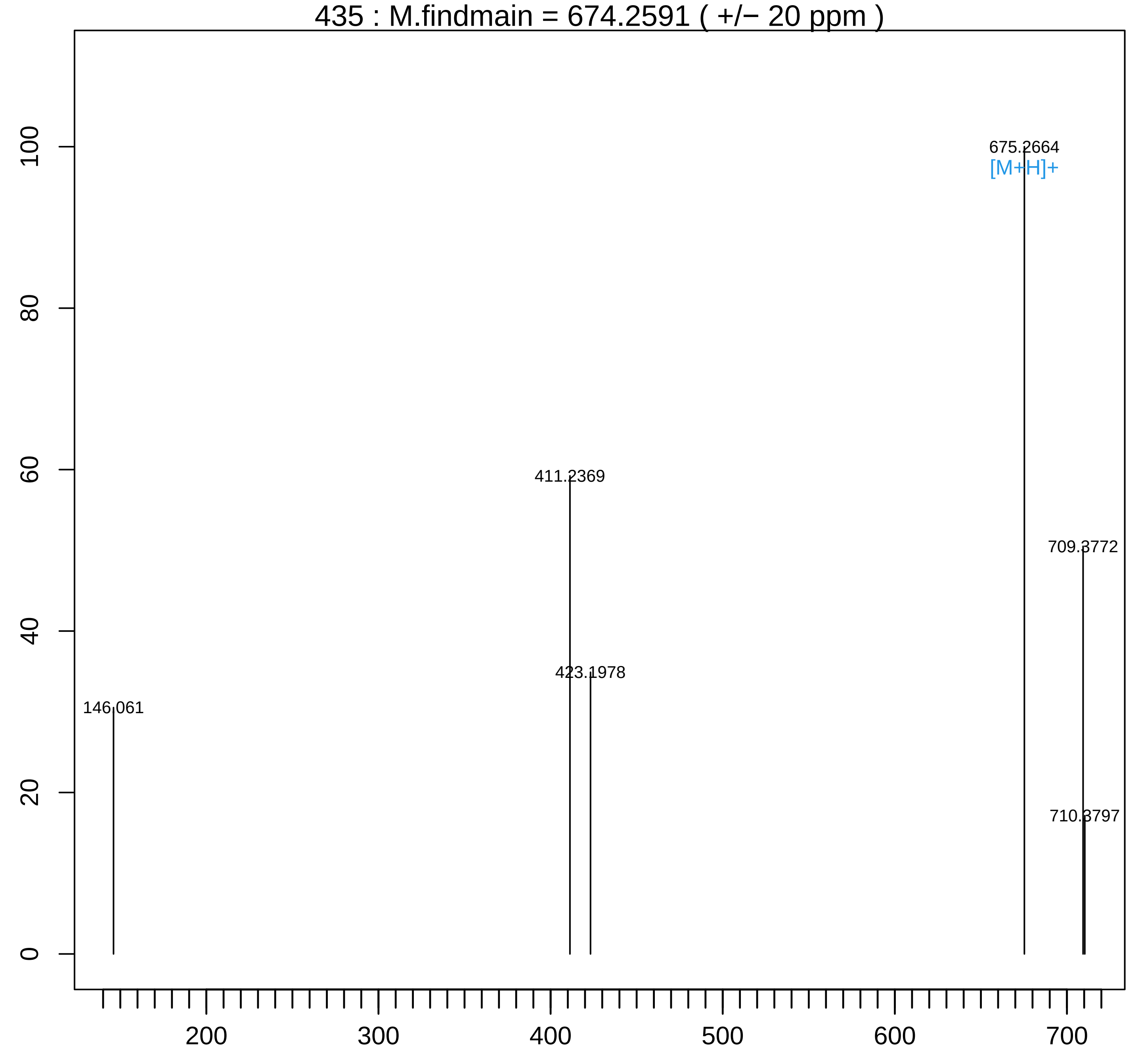
435 : M.findmain = 674.2591 (+/− 20 ppm)
675.2664
[M+H]+
411.2369
709.3772
423.1978
146.061
710.3797
0
20
40
60
80
100
200
300
400
500
600
700

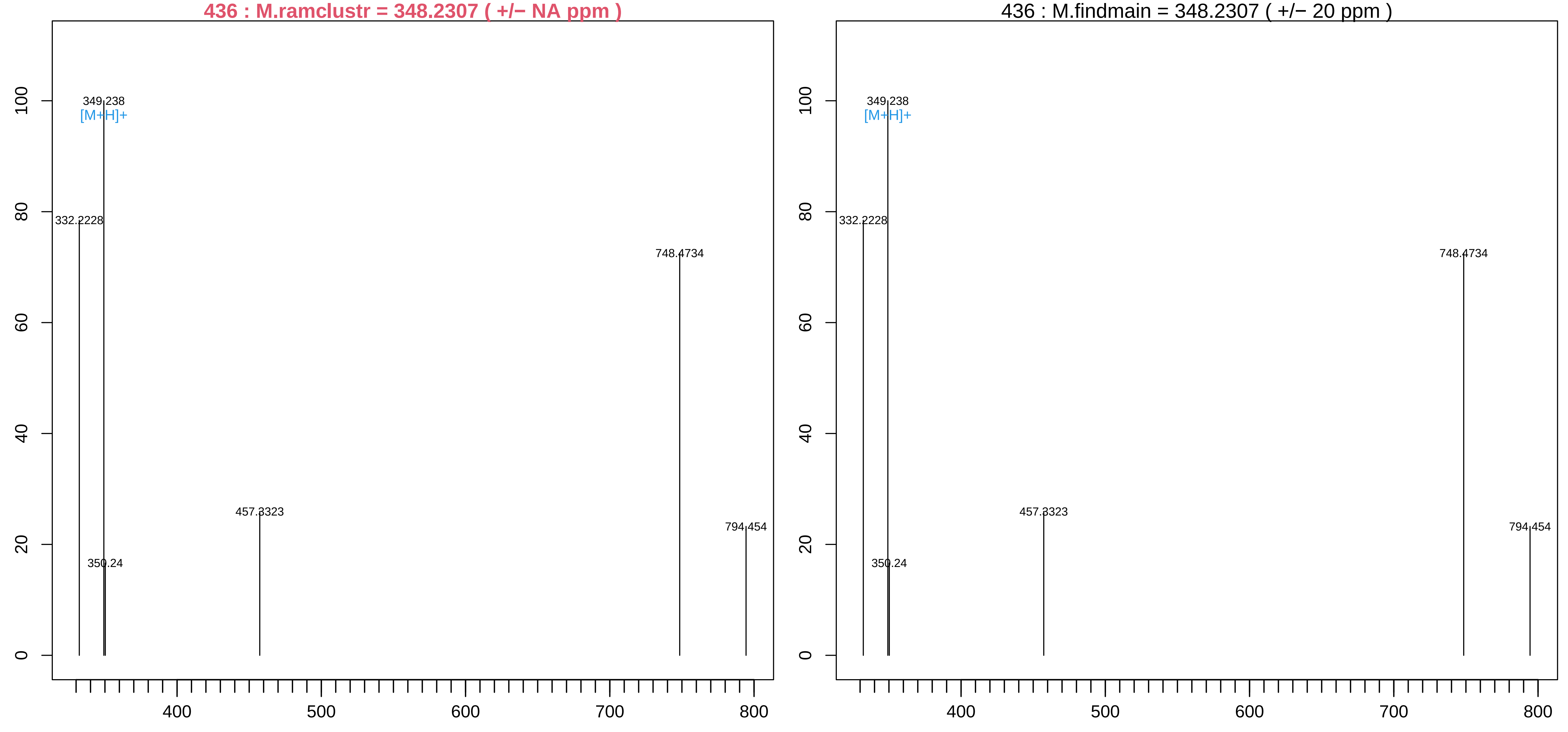

436 : M.ramclustr = 348.2307 (+/− NA ppm)
349.238
[M+H]+
332.2228
748.4734
457.3323
794.454
350.24
436 : M.findmain = 348.2307 (+/− 20 ppm)
349.238
[M+H]+
332.2228
748.4734
457.3323
794.454
350.24

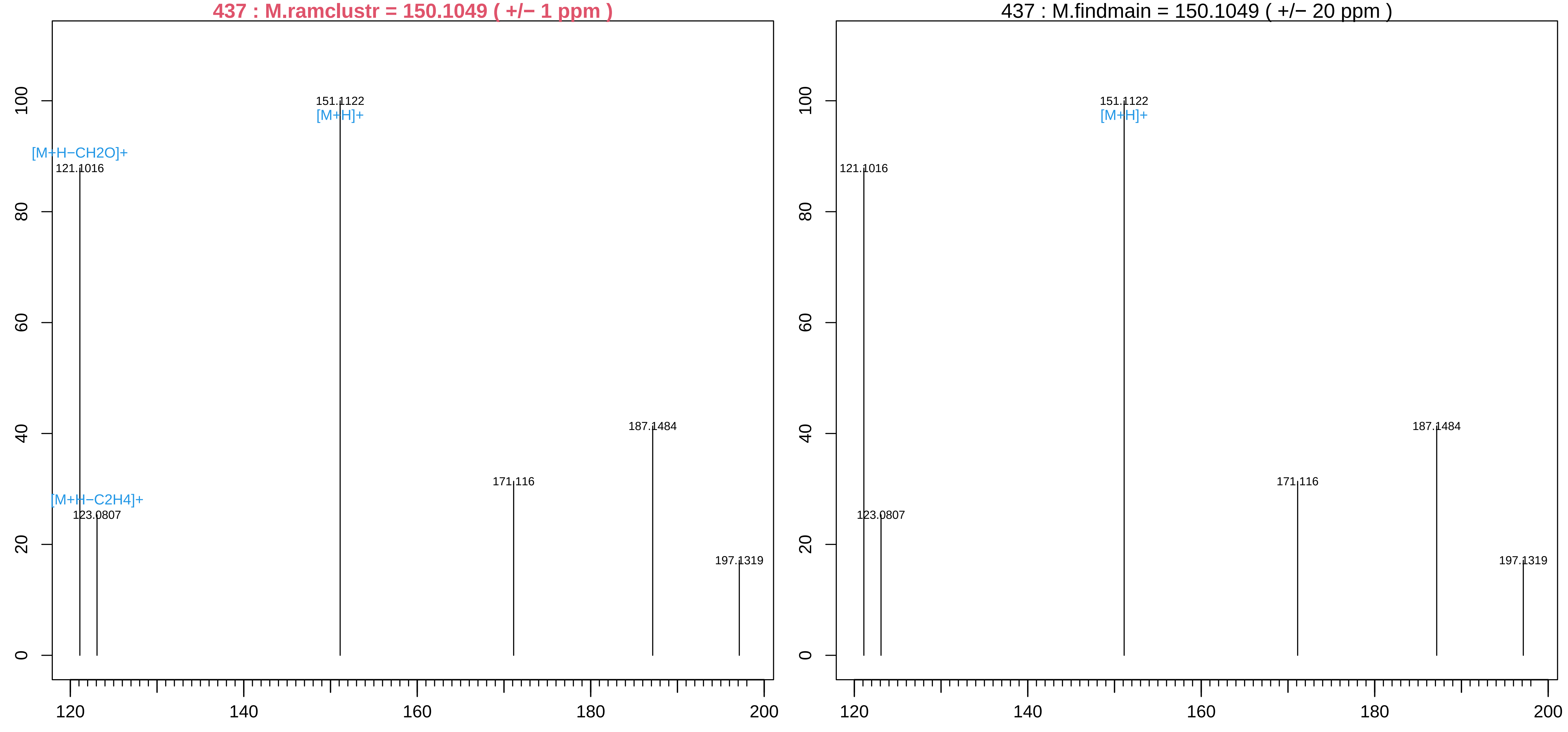

437 : M.ramclustr = 150.1049 (+/− 1 ppm)
151.1122
[M+H]+
[M+H−CH2O]+
121.1016
[M+H−C2H4]+
123.0807
171.116
187.1484
197.1319
437 : M.findmain = 150.1049 (+/− 20 ppm)
151.1122
[M+H]+
121.1016
123.0807
171.116
187.1484
197.1319

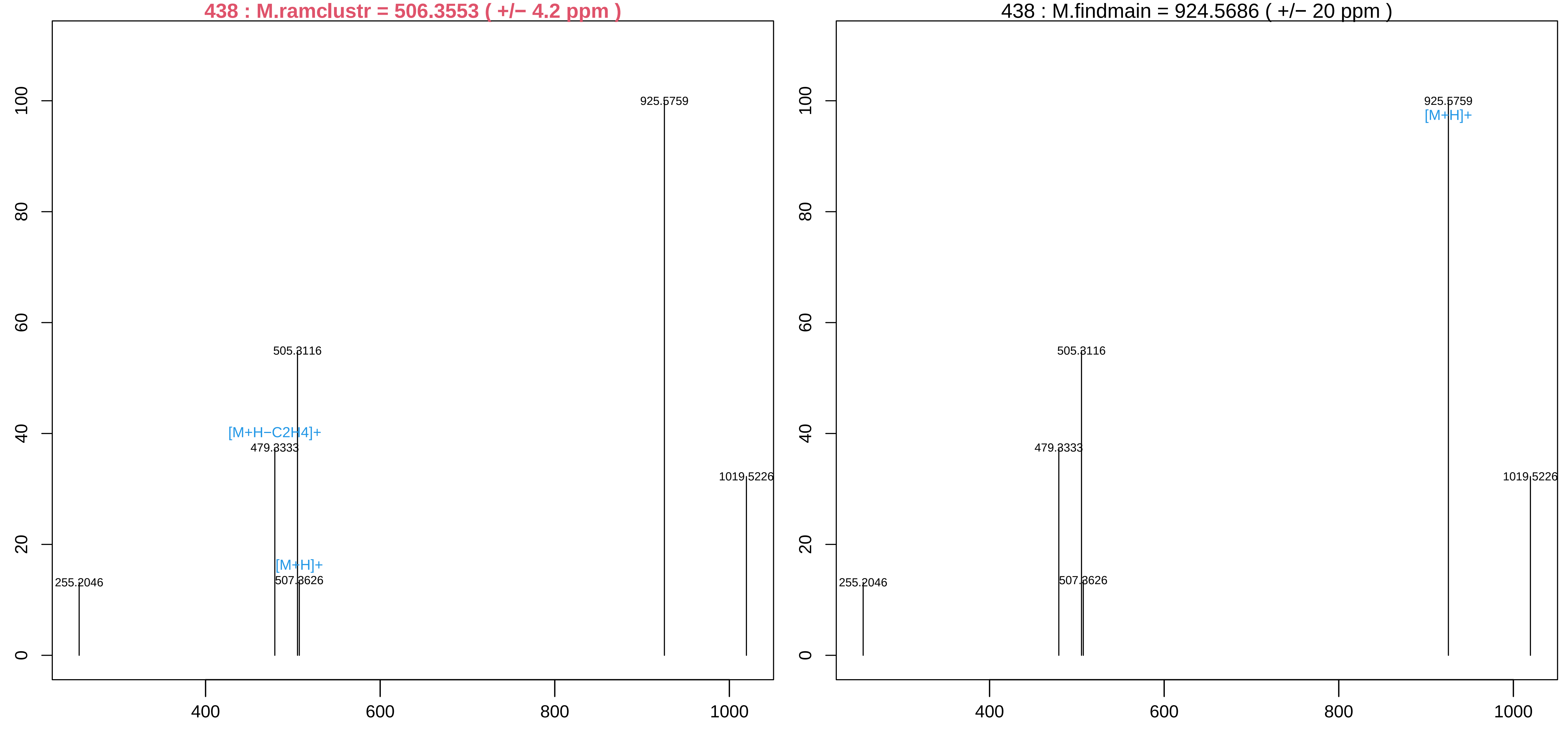

438 : M.ramclustr = 506.3553 (+/− 4.2 ppm)
925.5759
505.3116
[M+H−C2H4]+
479.3333
1019.5226
[M+H]+
507.3626
255.2046
438 : M.findmain = 924.5686 (+/− 20 ppm)
925.5759
[M+H]+
505.3116
479.3333
1019.5226
507.3626
255.2046

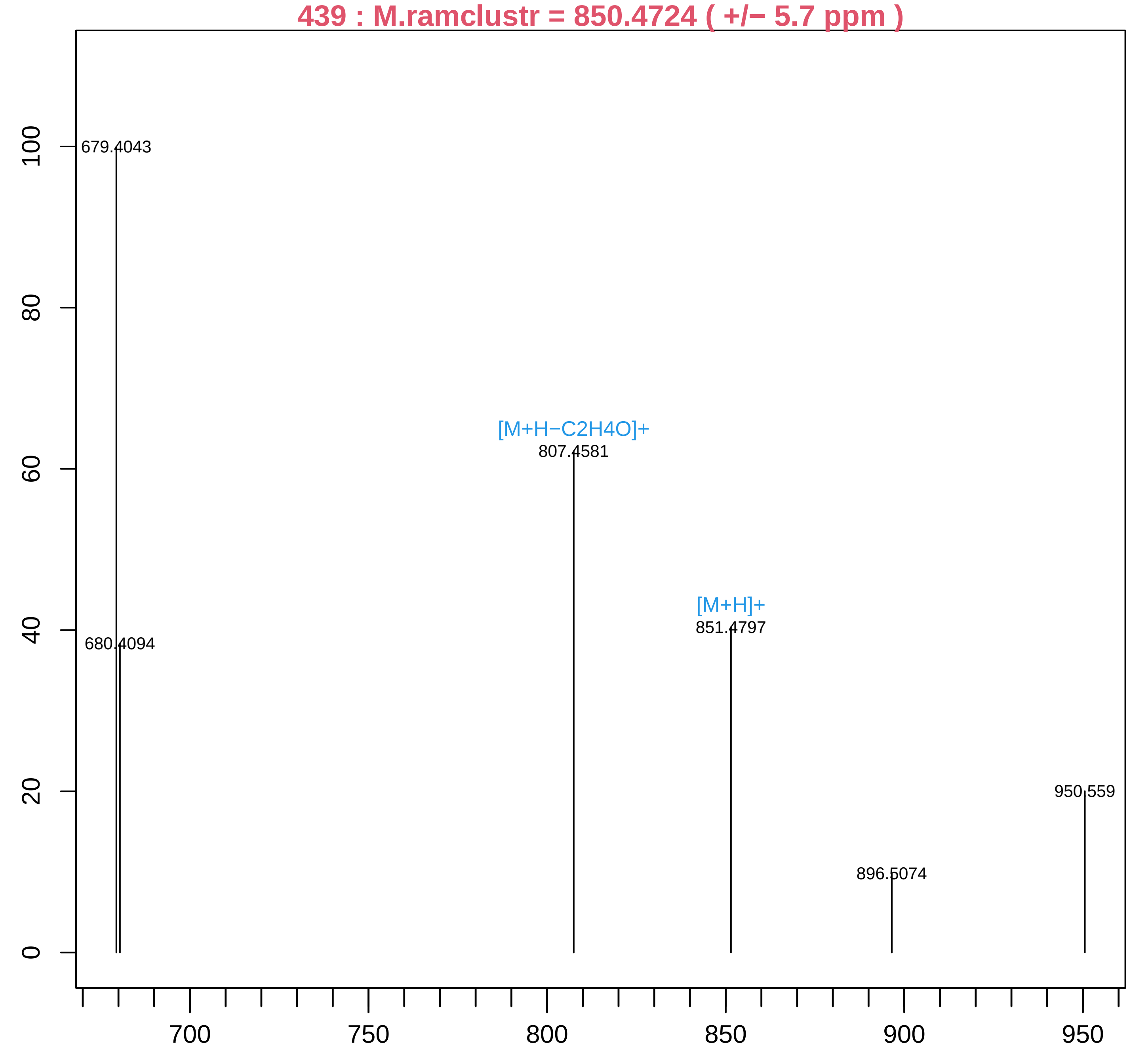
439 : M.ramclustr = 850.4724 (+/− 5.7 ppm)
679.4043
680.4094
[M+H−C2H4O]+
807.4581
[M+H]+
851.4797
896.5074
950.559
0
20
40
60
80
100
700
750
800
850
900
950

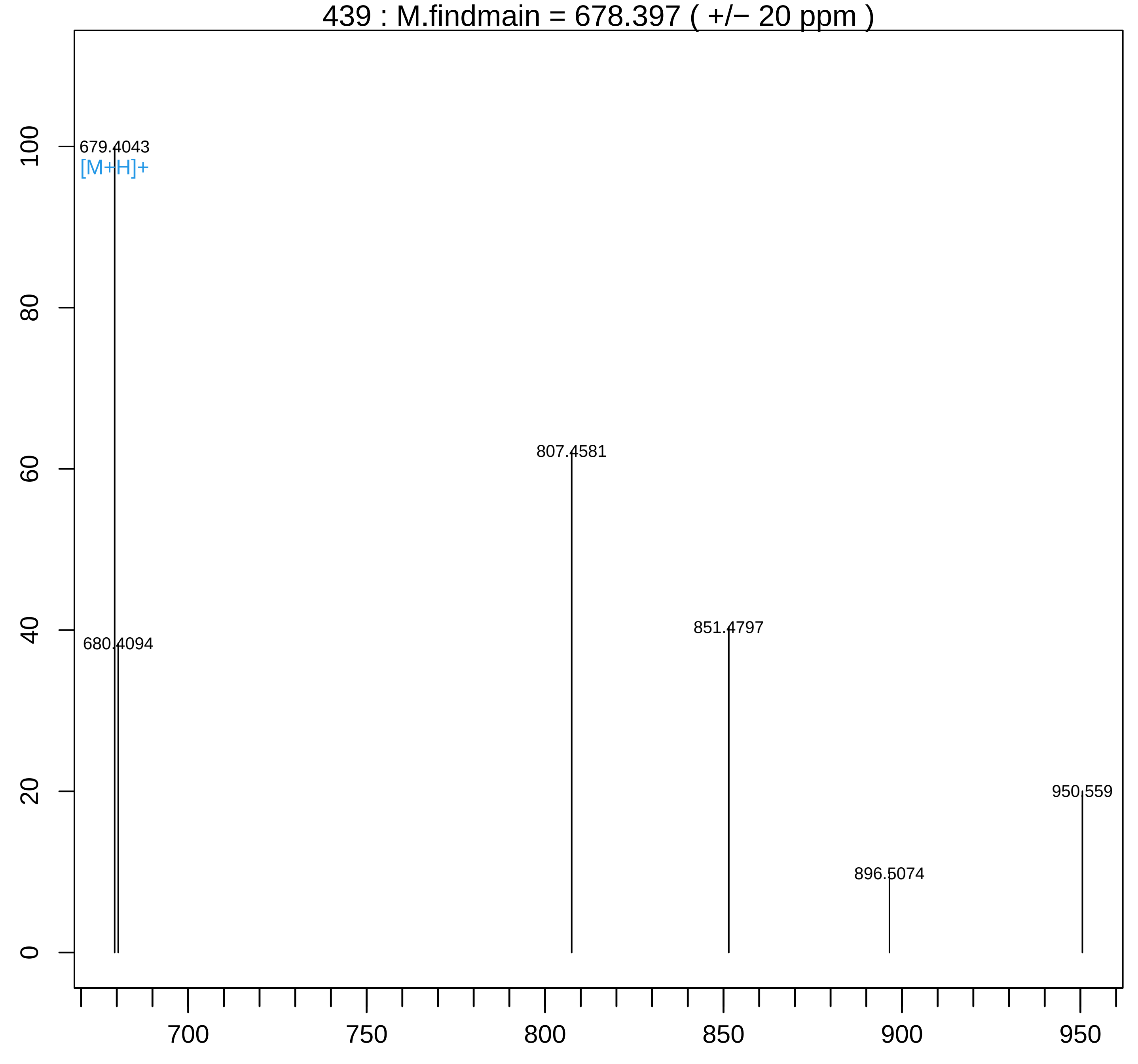
439 : M.findmain = 678.397 (+/− 20 ppm)
679.4043
[M+H]+
680.4094
807.4581
851.4797
896.5074
950.559
0
20
40
60
80
100
700
750
800
850
900
950

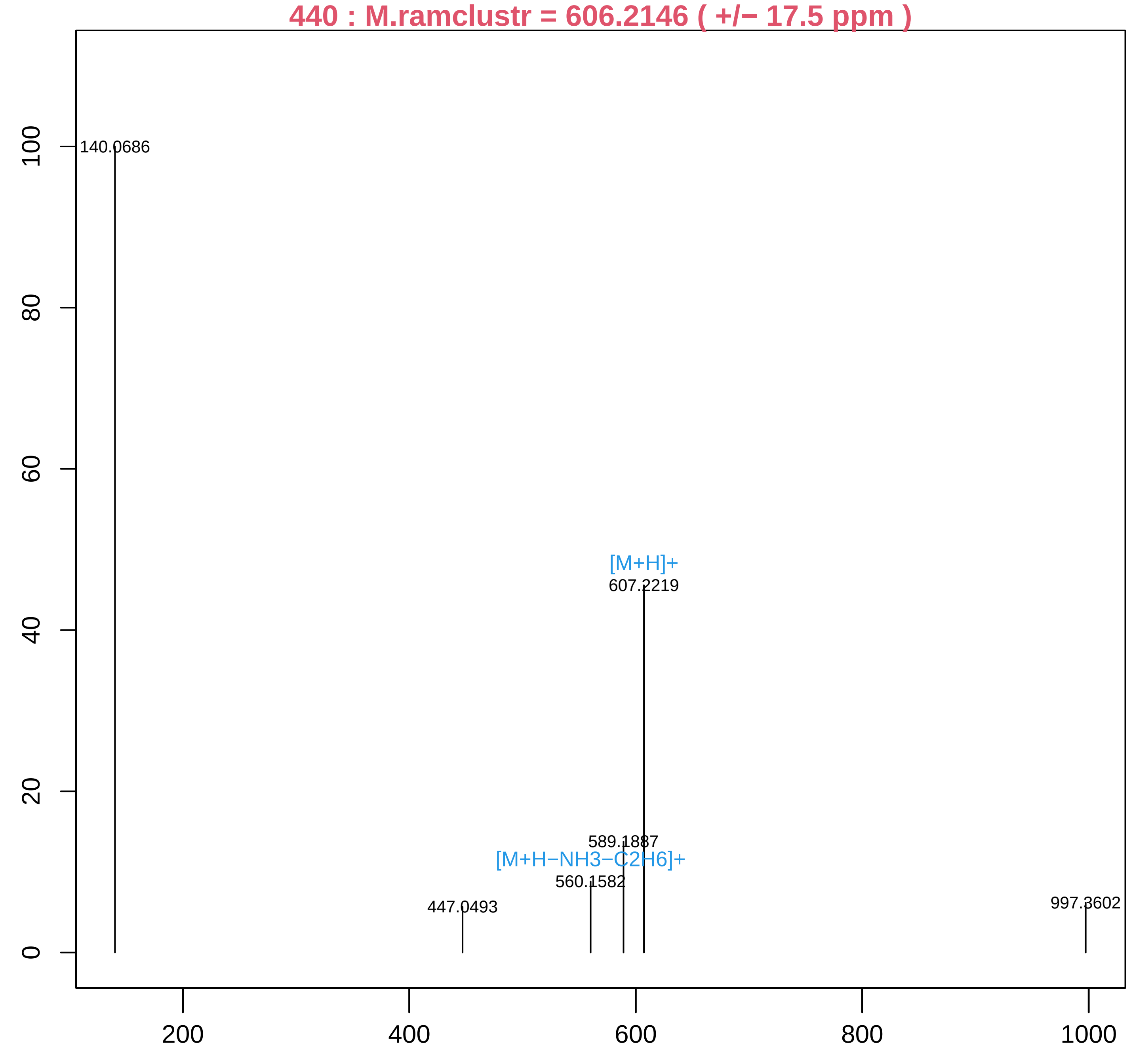
440 : M.ramclustr = 606.2146 (+/− 17.5 ppm)
140.0686
[M+H]+
607.2219
589.1887
[M+H−NH3−C2H6]+
560.1582
447.0493
997.3602
0
20
40
60
80
100
200
400
600
800
1000

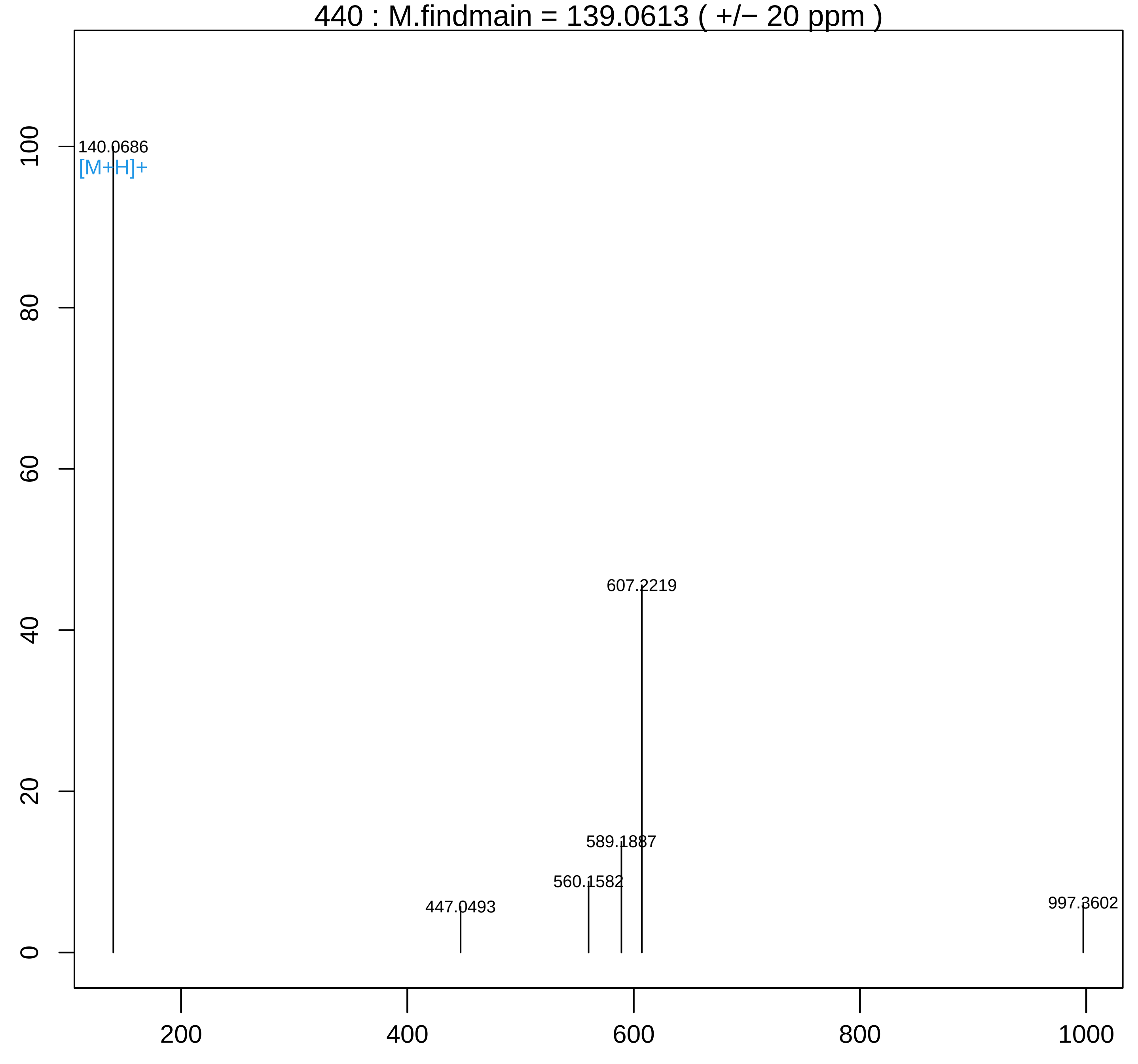
440 : M.findmain = 139.0613 (+/− 20 ppm)
140.0686
[M+H]+
607.2219
589.1887
560.1582
447.0493
997.3602
0
20
40
60
80
100
200
400
600
800
1000

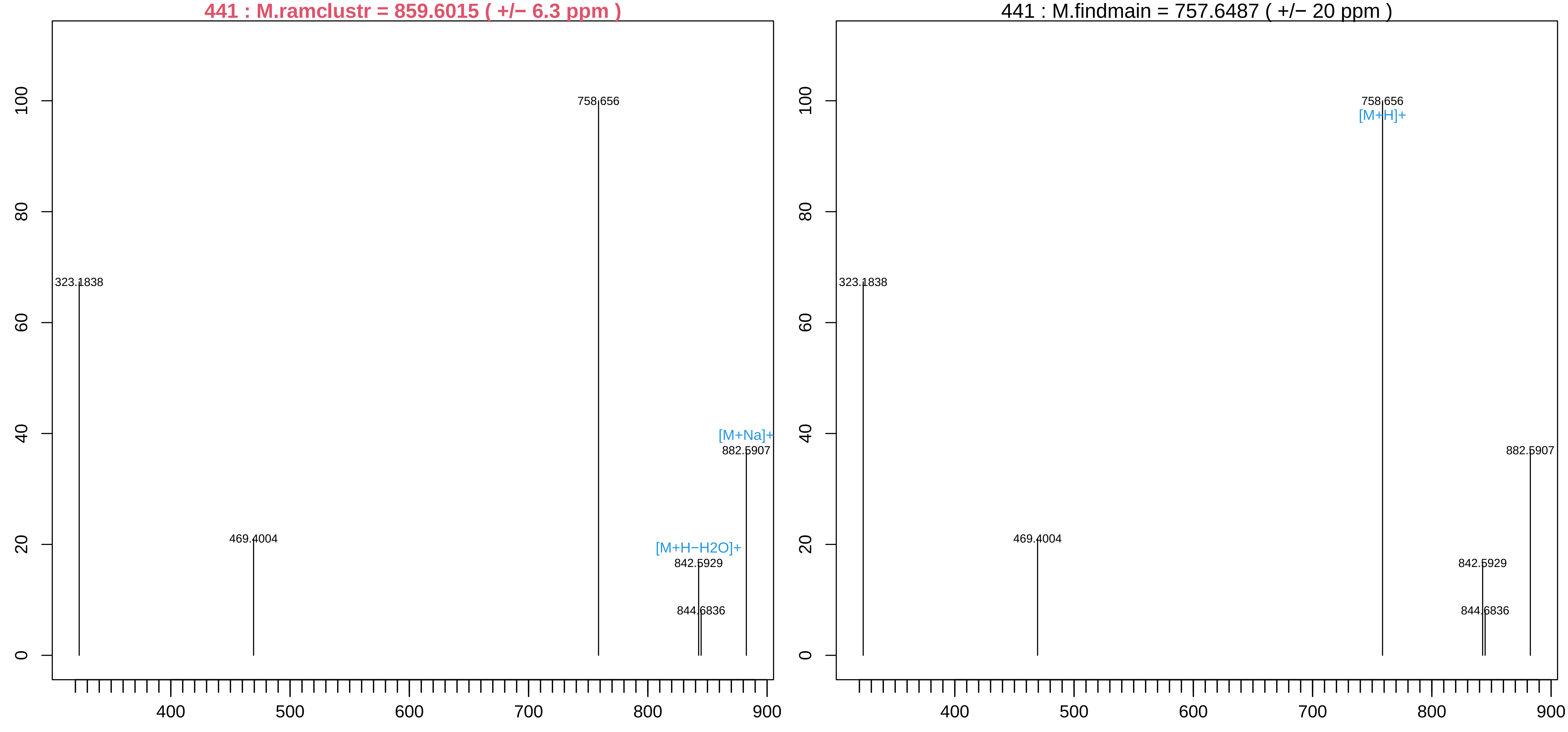

441 : M.ramclustr = 859.6015 (+/− 6.3 ppm)
758.656
323.1838
469.4004
[M+Na]+
882.5907
[M+H−H2O]+
842.5929
844.6836
441 : M.findmain = 757.6487 (+/− 20 ppm)
758.656
[M+H]+
323.1838
469.4004
882.5907
842.5929
844.6836
0
20
40
60
80
100
400
500
600
700
800
900

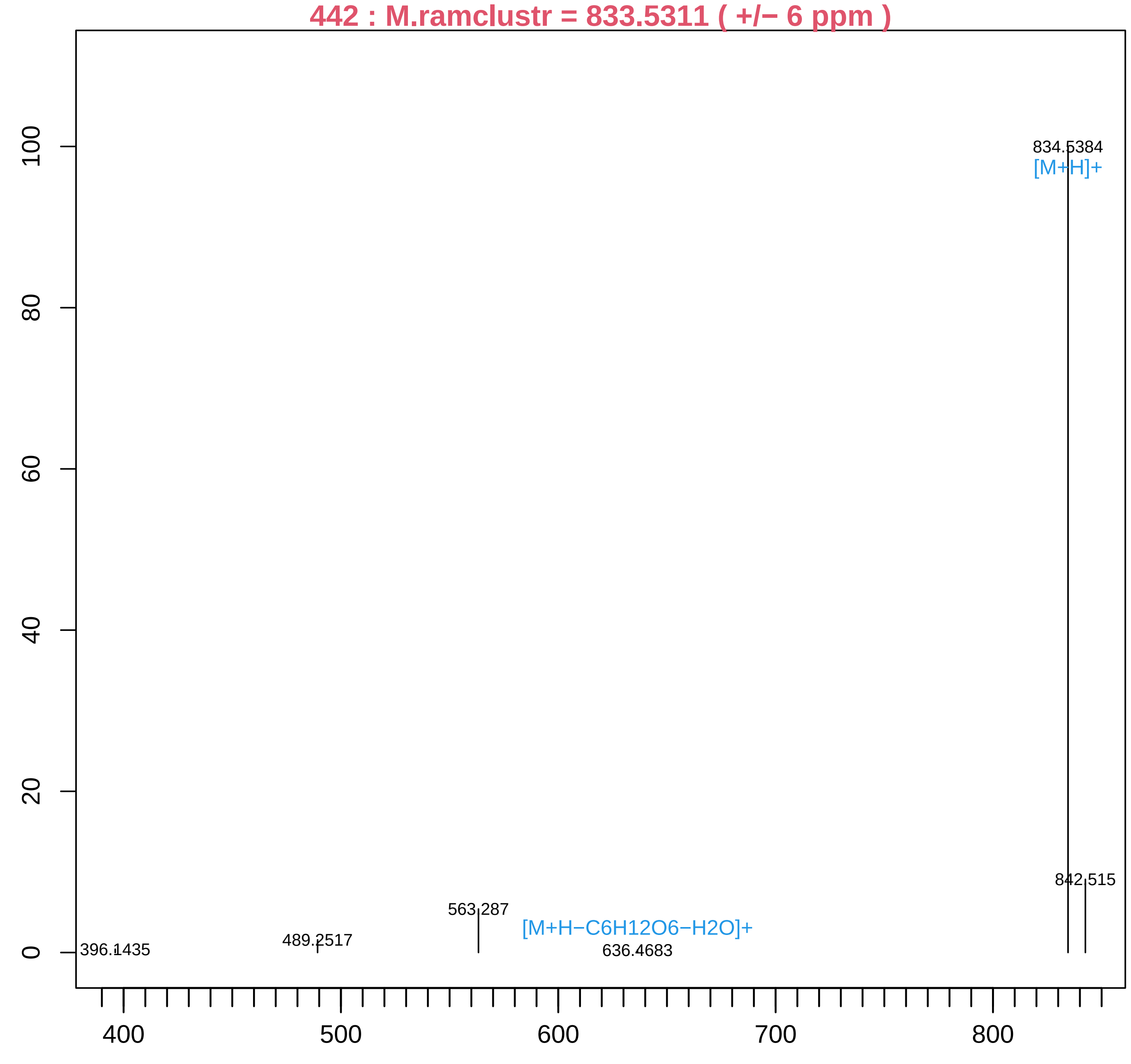

442 : M.ramclustr = 833.5311 (+/− 6 ppm)
834.5384
[M+H]+
842.515
563.287
[M+H−C6H12O6−H2O]+
489.2517
396.1435
636.4683
0
20
40
60
80
100
400
500
600
700
800

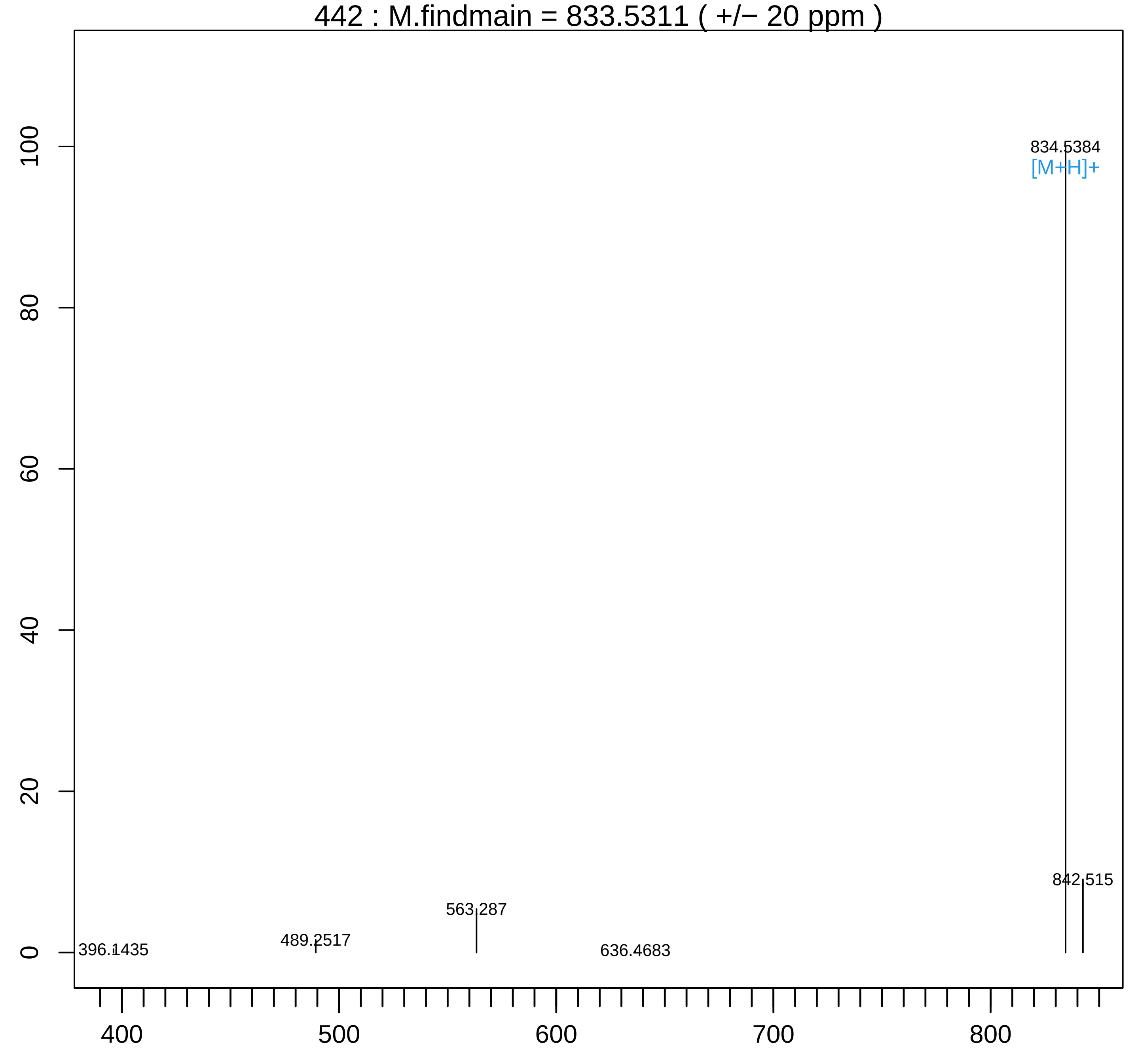

442 : M.findmain = 833.5311 (+/− 20 ppm)
834.5384
[M+H]+
842.515
563.287
489.2517
396.1435
636.4683
0
20
40
60
80
100
400
500
600
700
800

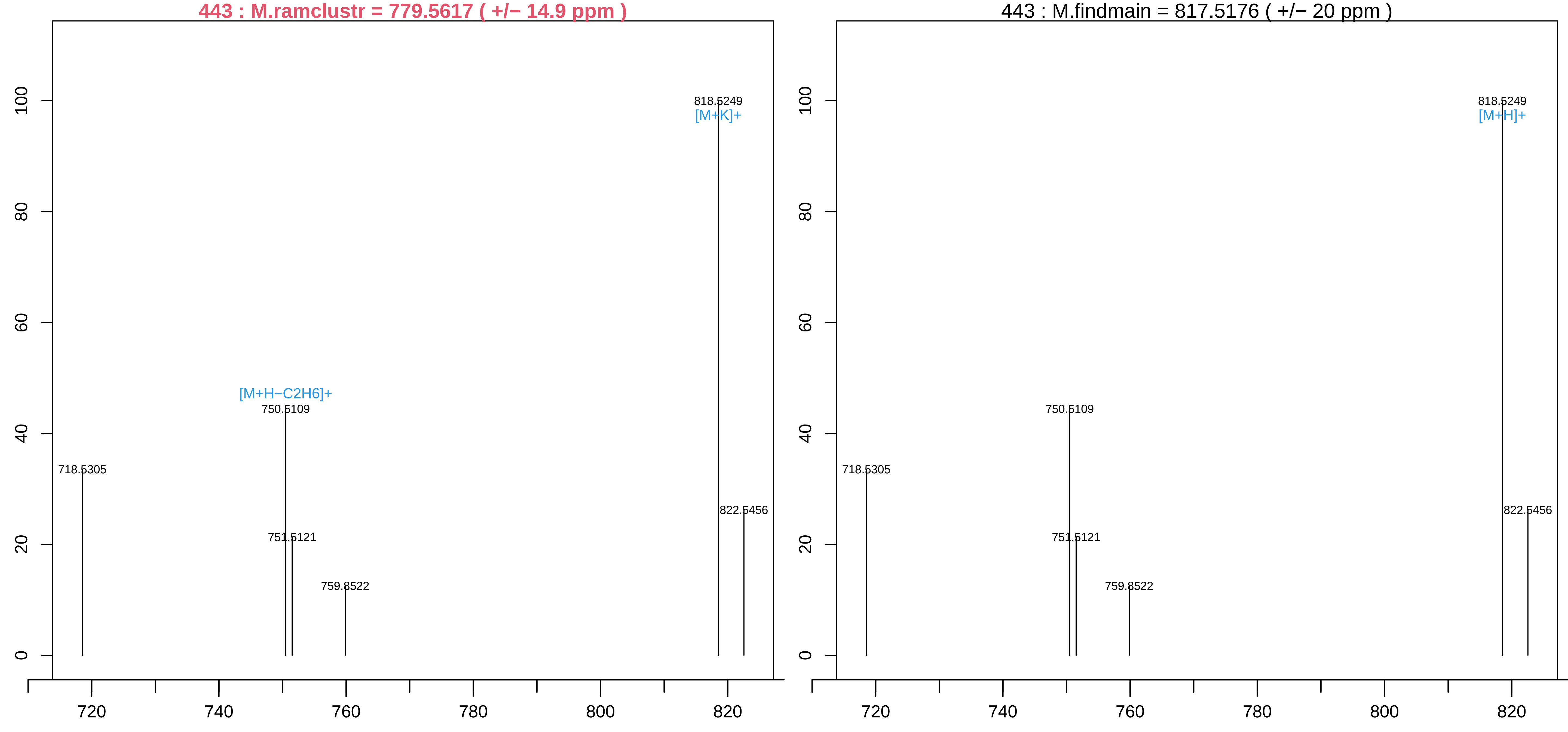

443 : M.ramclustr = 779.5617 (+/− 14.9 ppm)
818.5249
[M+K]+
[M+H−C2H6]+
750.5109
718.5305
822.5456
751.5121
759.8522
0
20
40
60
80
100
720
740
760
780
800
820
443 : M.findmain = 817.5176 (+/− 20 ppm)
818.5249
[M+H]+
750.5109
718.5305
822.5456
751.5121
759.8522

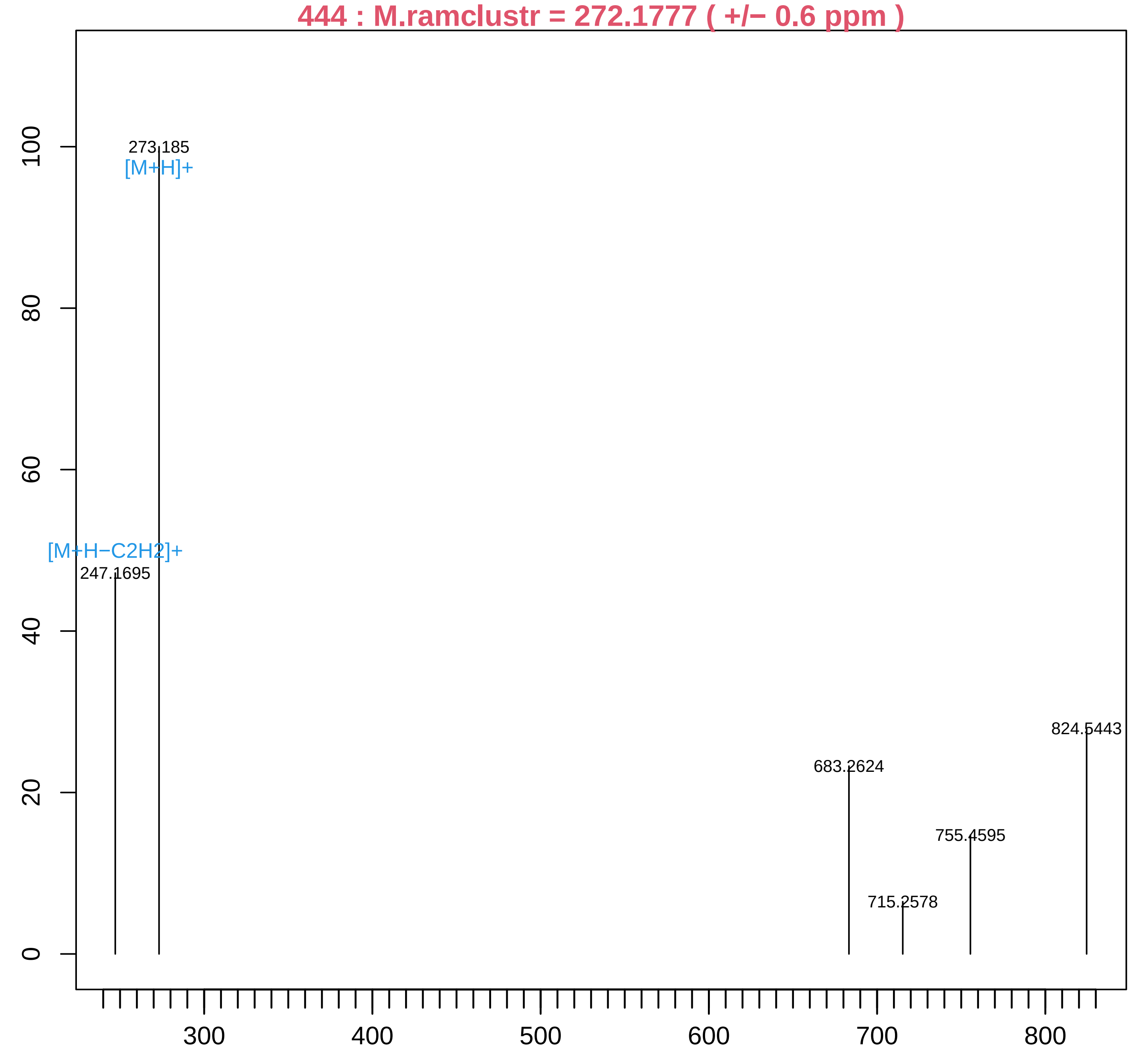

444 : M.ramclustr = 272.1777 (+/− 0.6 ppm)
273.185
[M+H]+
[M+H−C2H2]+
247.1695
683.2624
715.2578
755.4595
824.5443
0
20
40
60
80
100
300
400
500
600
700
800

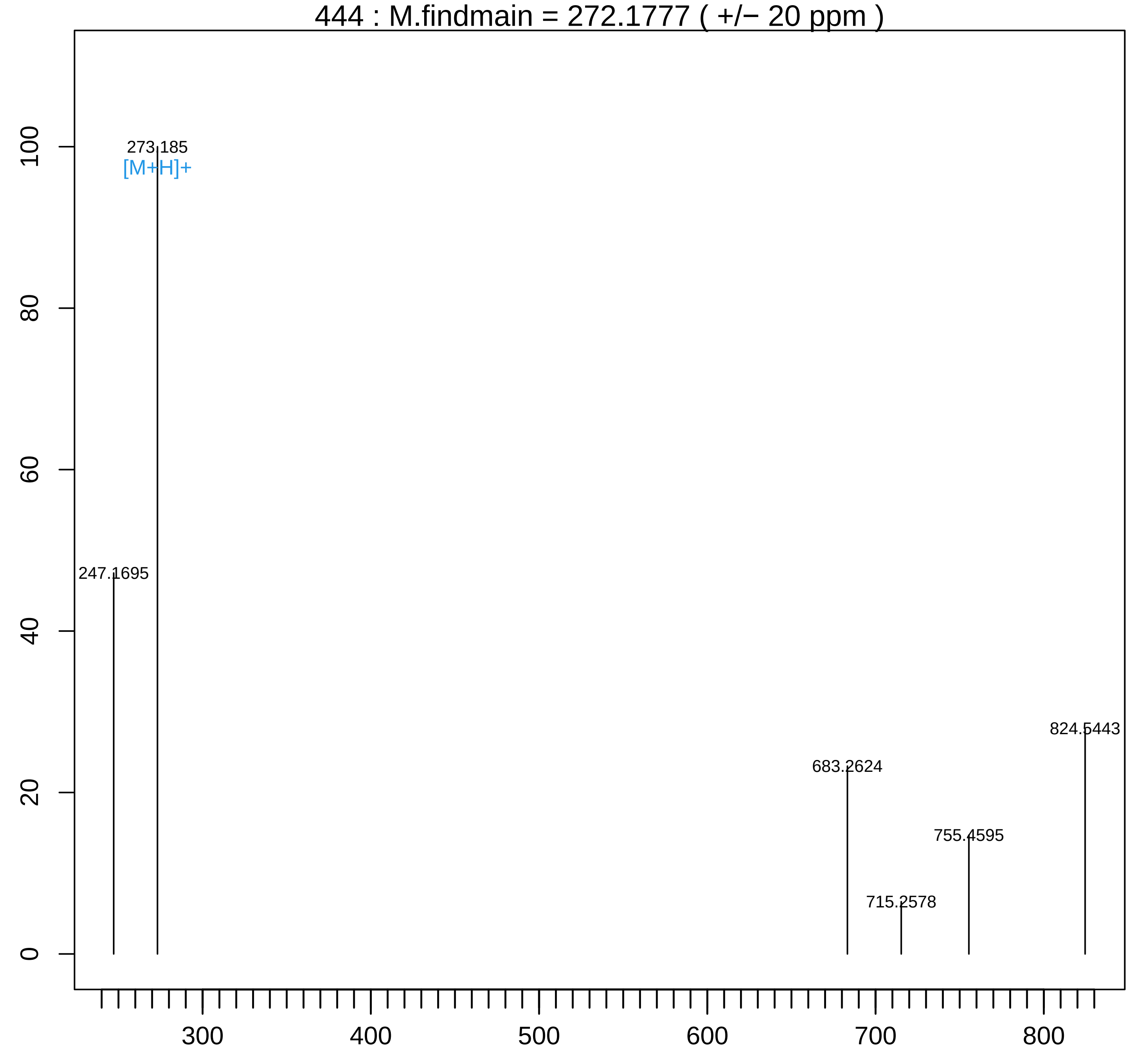

444 : M.findmain = 272.1777 (+/− 20 ppm)
273.185
[M+H]+
247.1695
683.2624
715.2578
755.4595
824.5443
0
20
40
60
80
100
300
400
500
600
700
800

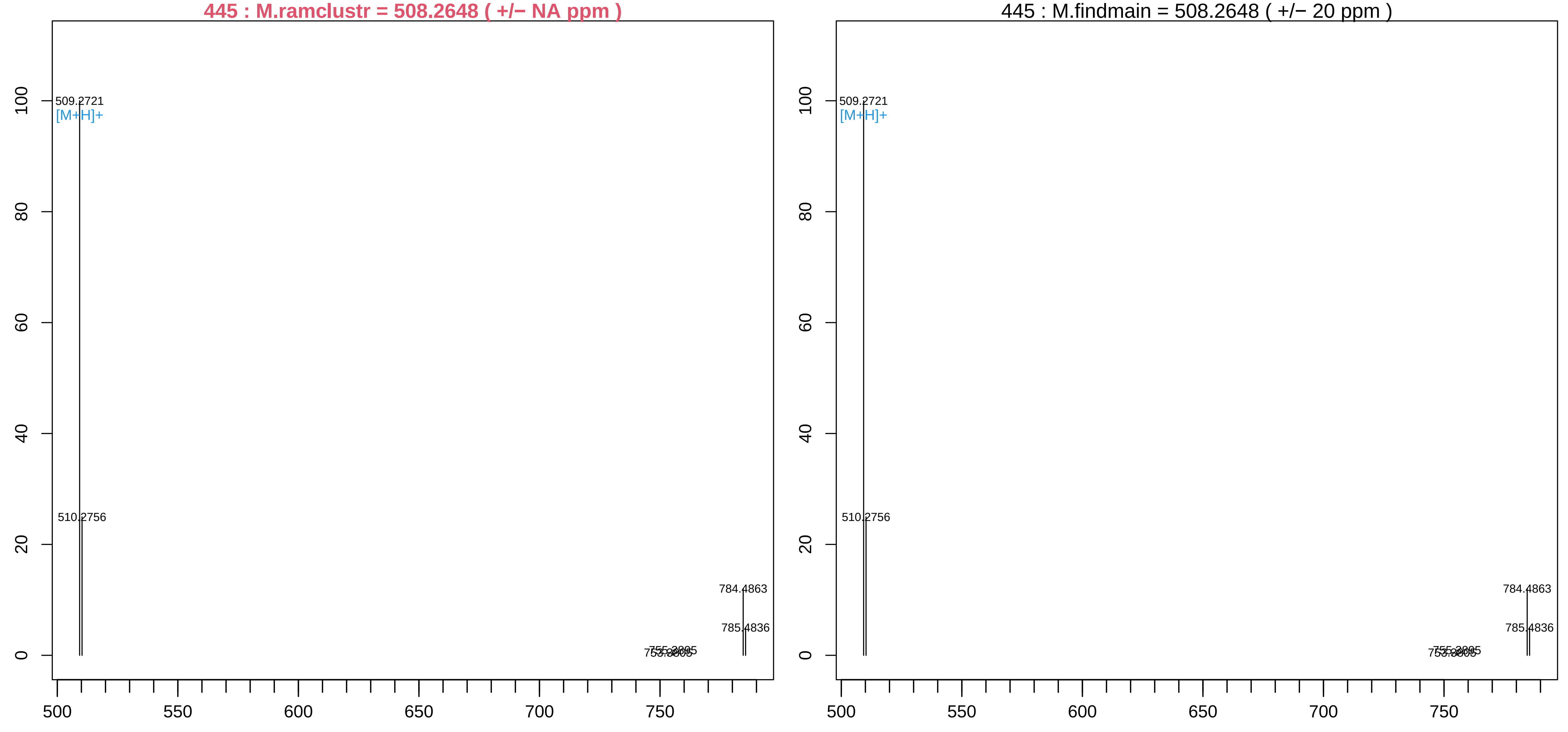

445 : M.ramclustr = 508.2648 (+/− NA ppm)
509.2721
[M+H]+
510.2756
784.4863
785.4836
755.3895
753.8805
445 : M.findmain = 508.2648 (+/− 20 ppm)
509.2721
[M+H]+
510.2756
784.4863
785.4836
755.3895
753.8805

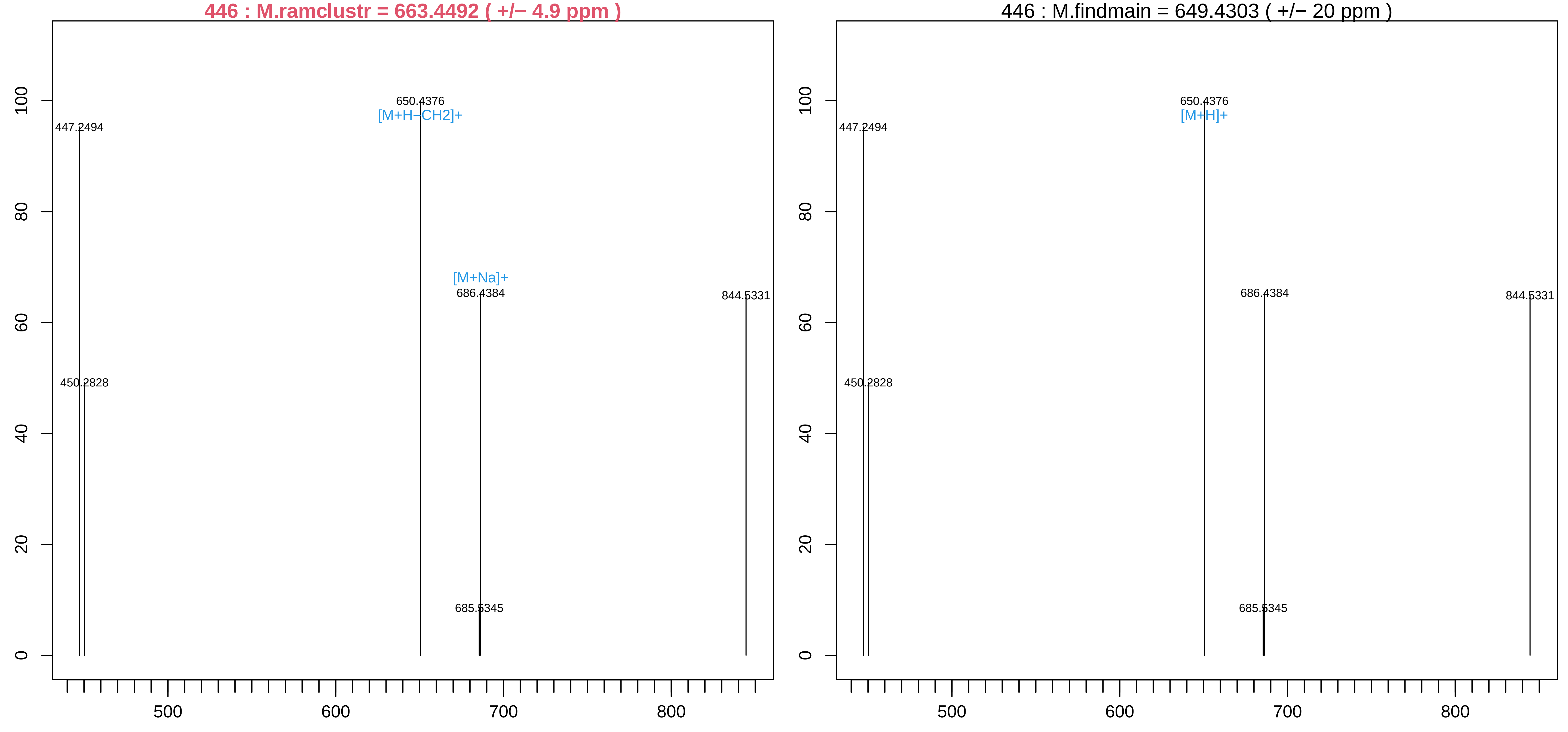

446 : M.ramclustr = 663.4492 (+/− 4.9 ppm)
650.4376
[M+H−CH2]+
447.2494
[M+Na]+
686.4384
844.5331
450.2828
685.5345
446 : M.findmain = 649.4303 (+/− 20 ppm)
650.4376
[M+H]+
447.2494
686.4384
844.5331
450.2828
685.5345

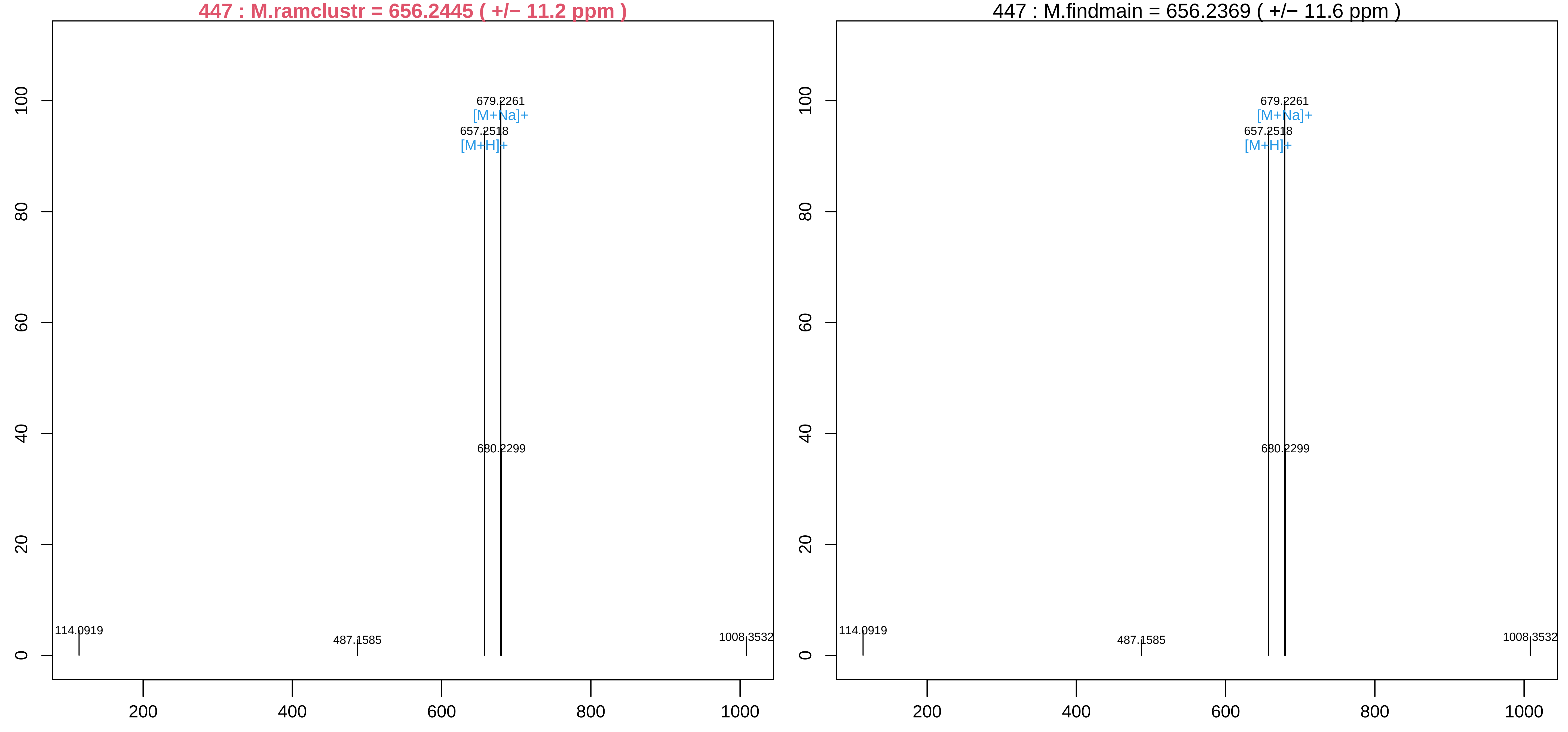
447 : M.ramclustr = 656.2445 (+/− 11.2 ppm)
679.2261
[M+Na]+
657.2518
[M+H]+
680.2299
114.0919
487.1585
1008.3532
0
20
40
60
80
100
200
400
600
800
1000
447 : M.findmain = 656.2369 (+/− 11.6 ppm)
679.2261
[M+Na]+
657.2518
[M+H]+
680.2299
114.0919
487.1585
1008.3532
0
20
40
60
80
100
200
400
600
800
1000

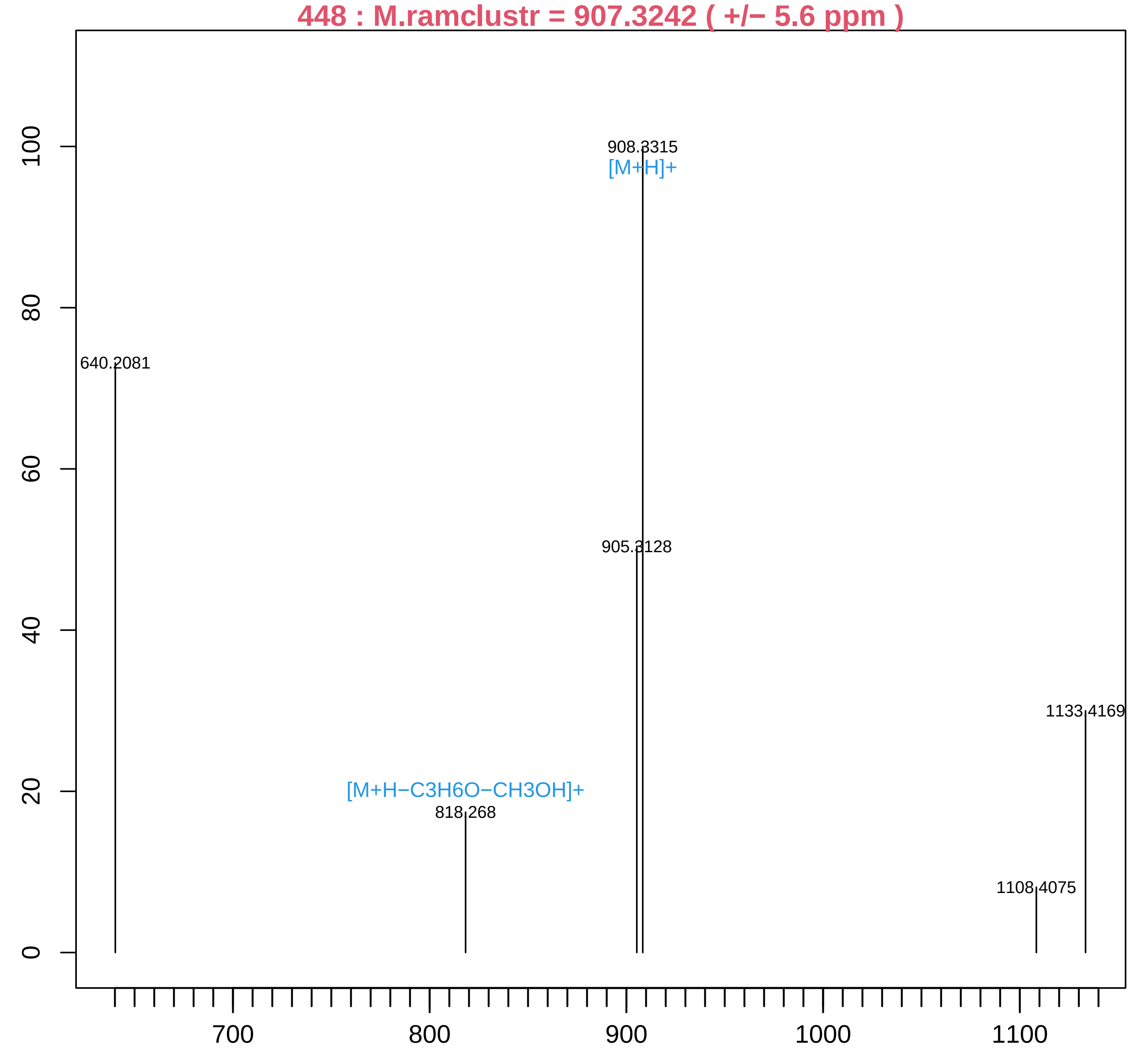
448 : M.ramclustr = 907.3242 (+/− 5.6 ppm)
908.3315
[M+H]+
640.2081
905.3128
1133.4169
[M+H−C3H6O−CH3OH]+
818.268
1108.4075
0
20
40
60
80
100
700
800
900
1000
1100

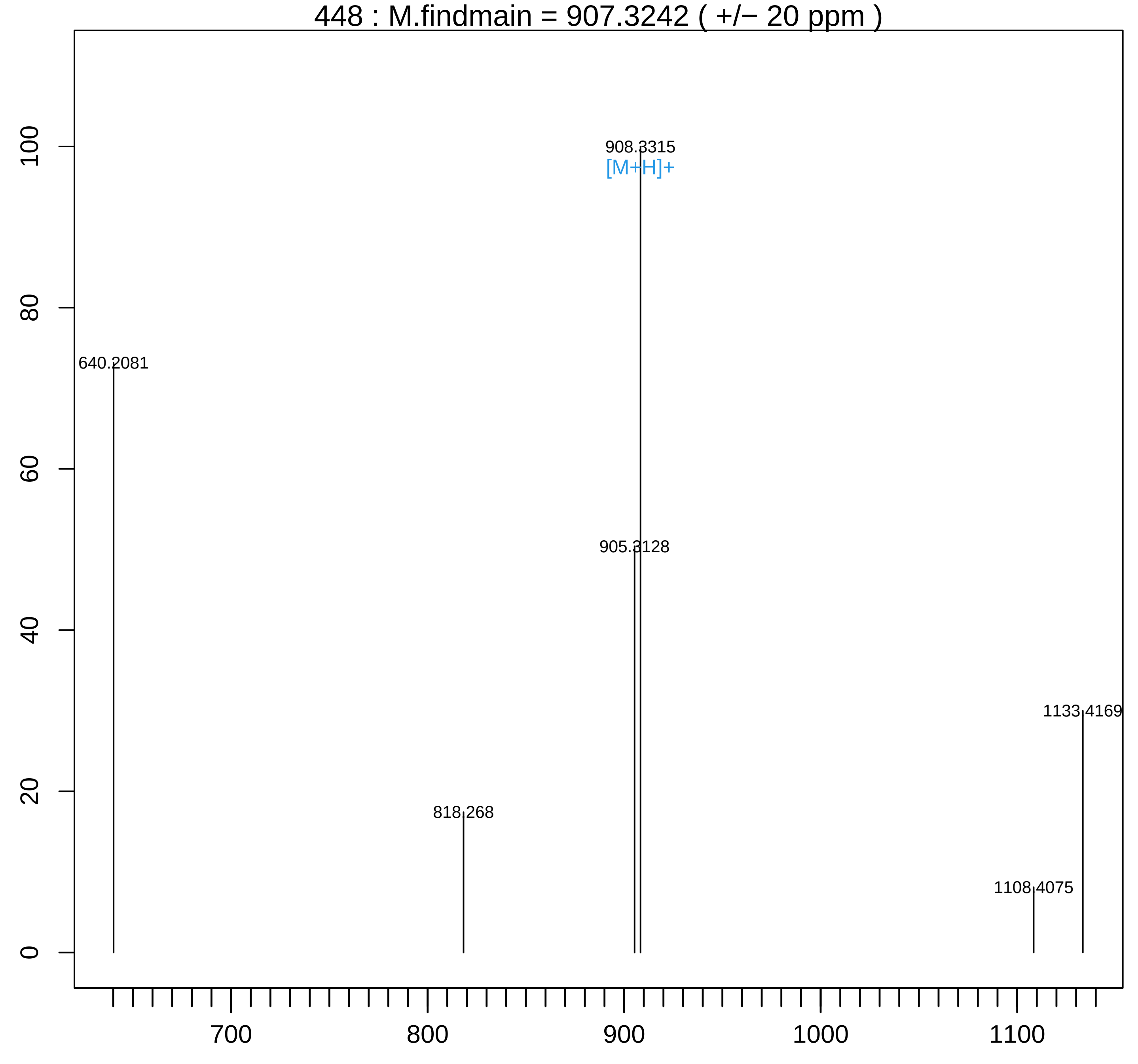
448 : M.findmain = 907.3242 (+/− 20 ppm)
908.3315
[M+H]+
640.2081
905.3128
1133.4169
818.268
1108.4075
0
20
40
60
80
100
700
800
900
1000
1100

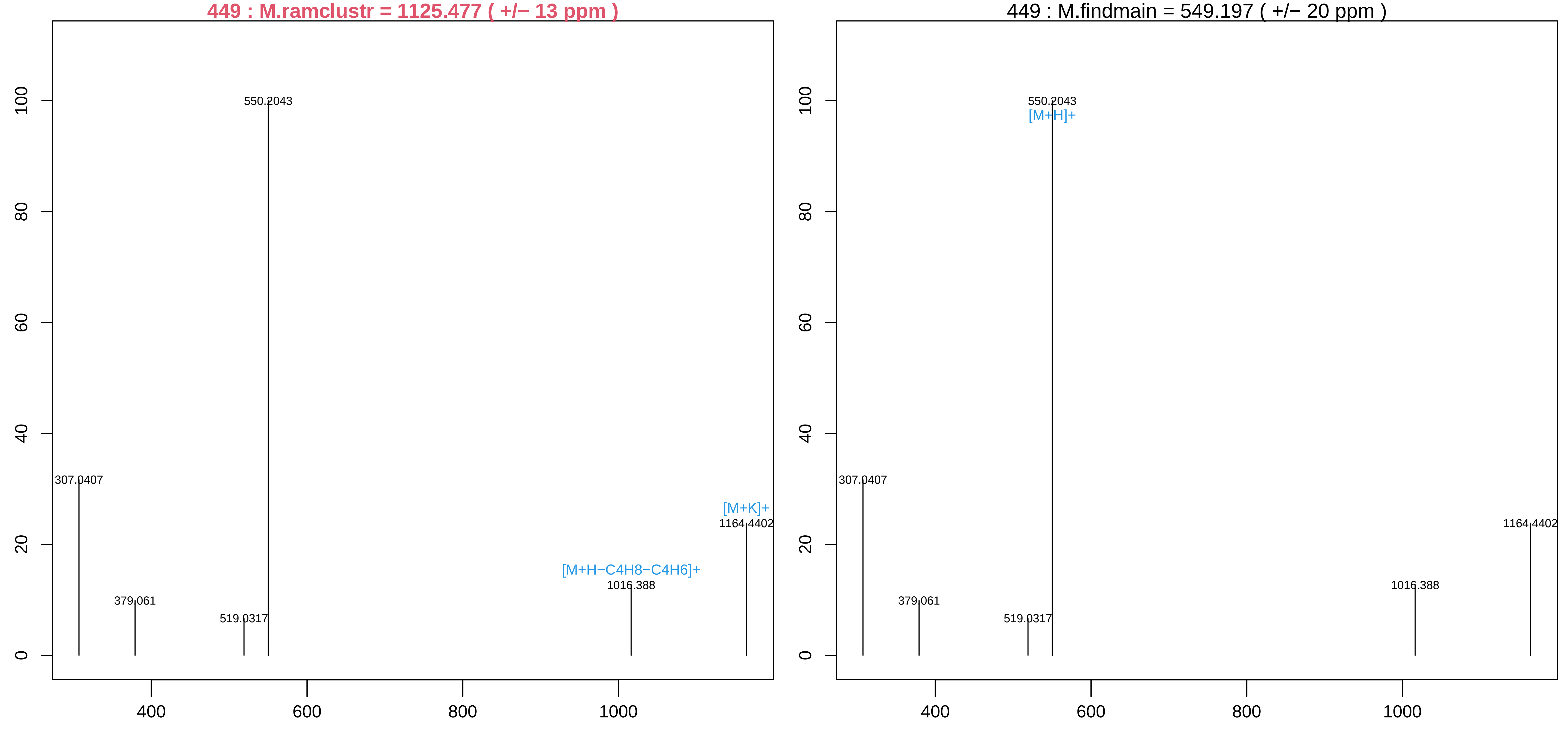
449 : M.ramclustr = 1125.477 (+/− 13 ppm)
550.2043
307.0407
379.061
519.0317
[M+H−C4H8−C4H6]+
1016.388
[M+K]+
1164.4402
0
20
40
60
80
100
400
600
800
1000
449 : M.findmain = 549.197 (+/− 20 ppm)
550.2043
[M+H]+
307.0407
379.061
519.0317
1016.388
1164.4402

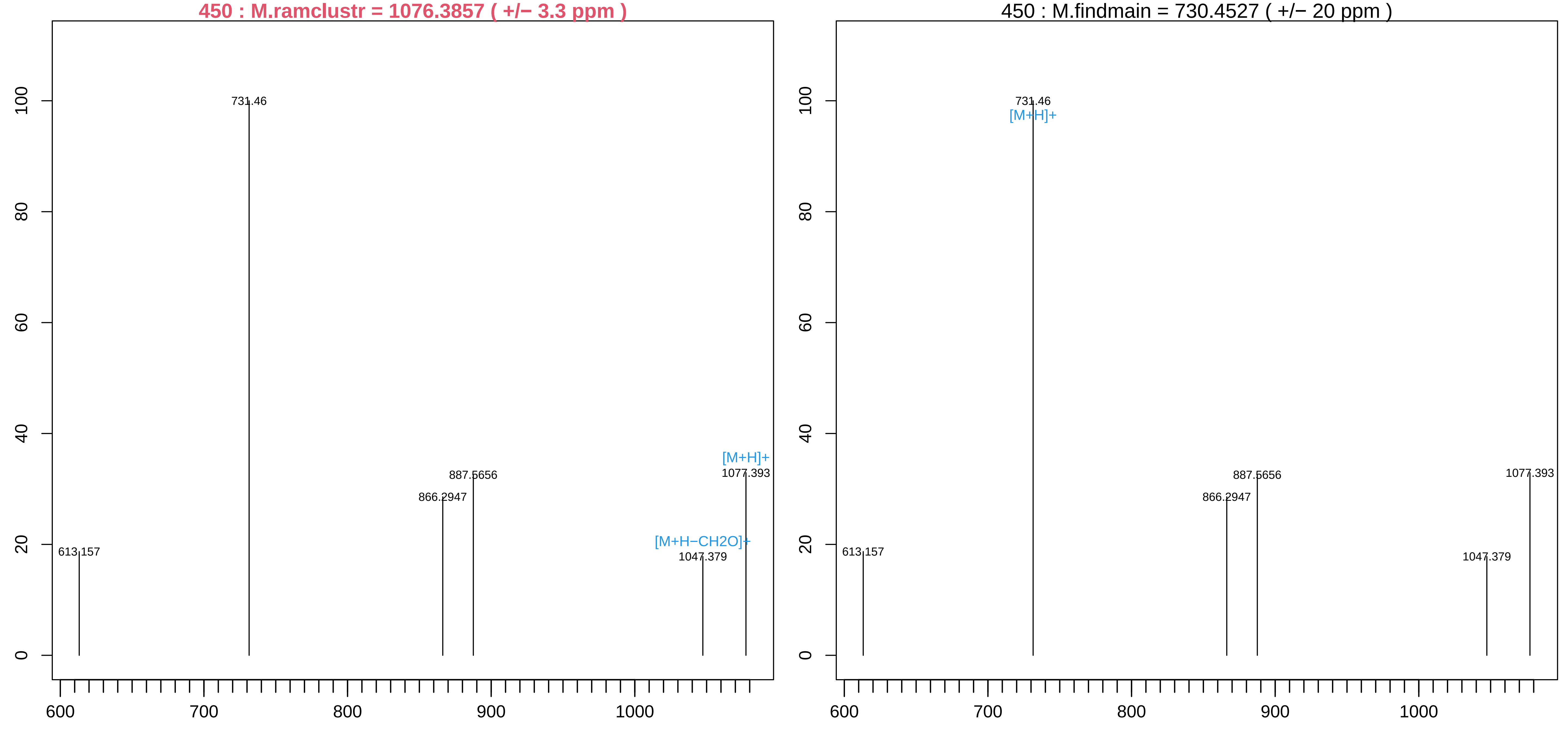
450 : M.ramclustr = 1076.3857 (+/− 3.3 ppm)
731.46
887.5656
866.2947
[M+H]+
1077.393
[M+H−CH2O]+
1047.379
613.157
450 : M.findmain = 730.4527 (+/− 20 ppm)
731.46
[M+H]+
887.5656
866.2947
1077.393
1047.379
613.157
0
20
40
60
80
100
600
700
800
900
1000

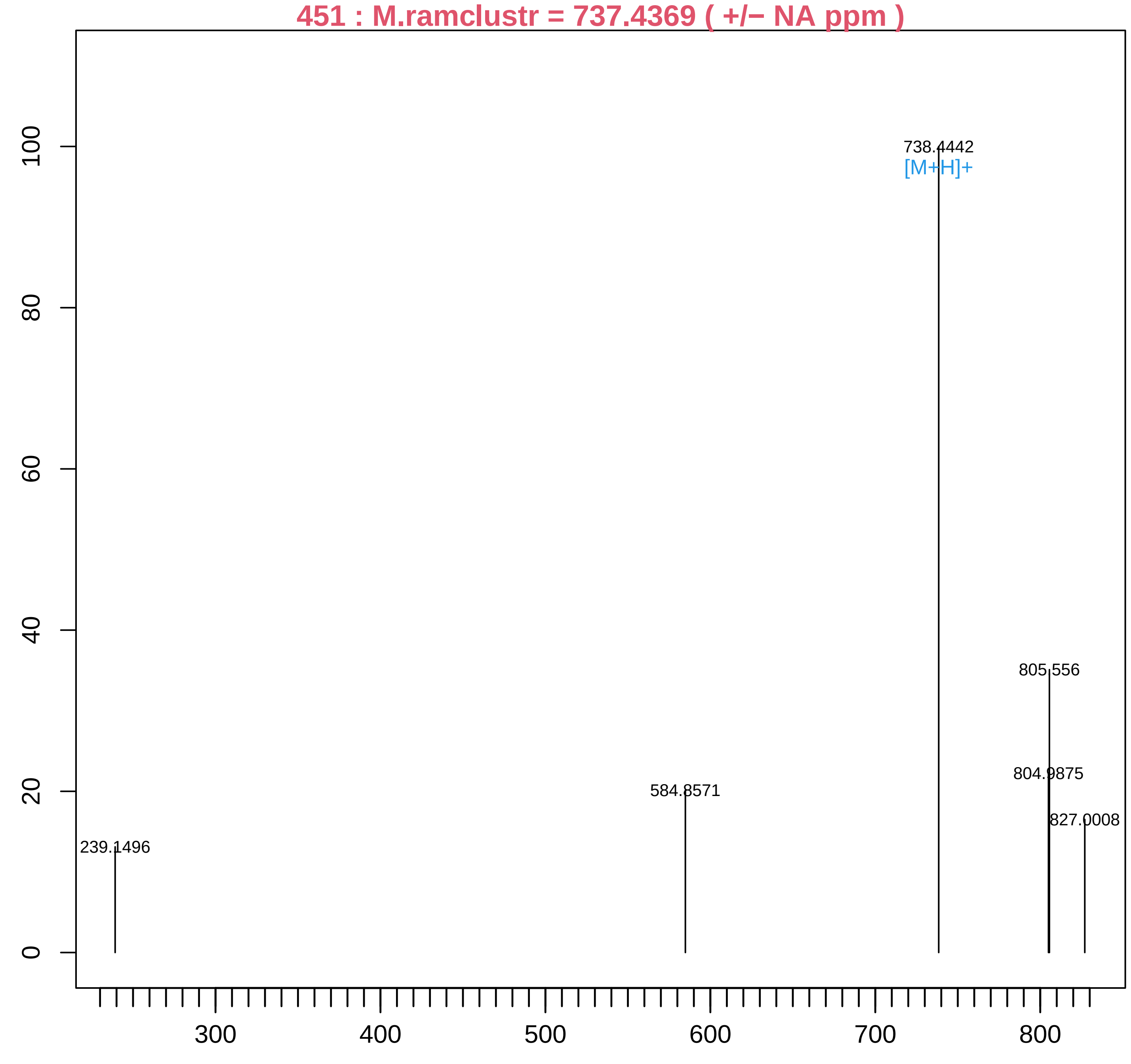
451 : M.ramclustr = 737.4369 (+/− NA ppm)
738.4442
[M+H]+
805.556
804.9875
827.0008
584.8571
239.1496
0
20
40
60
80
100
300
400
500
600
700
800

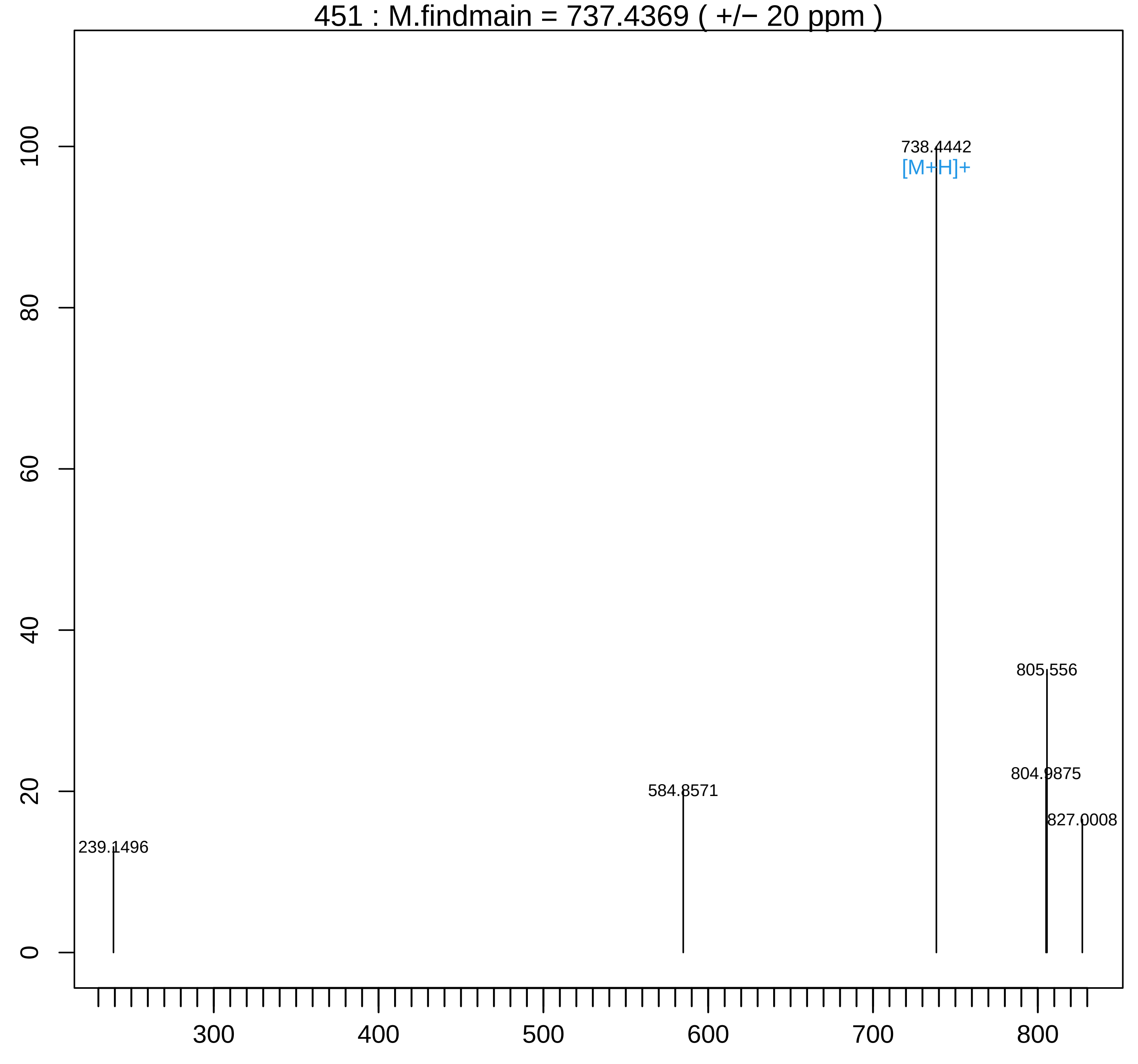
451 : M.findmain = 737.4369 (+/− 20 ppm)
738.4442
[M+H]+
805.556
804.9875
827.0008
584.8571
239.1496
0
20
40
60
80
100
300
400
500
600
700
800

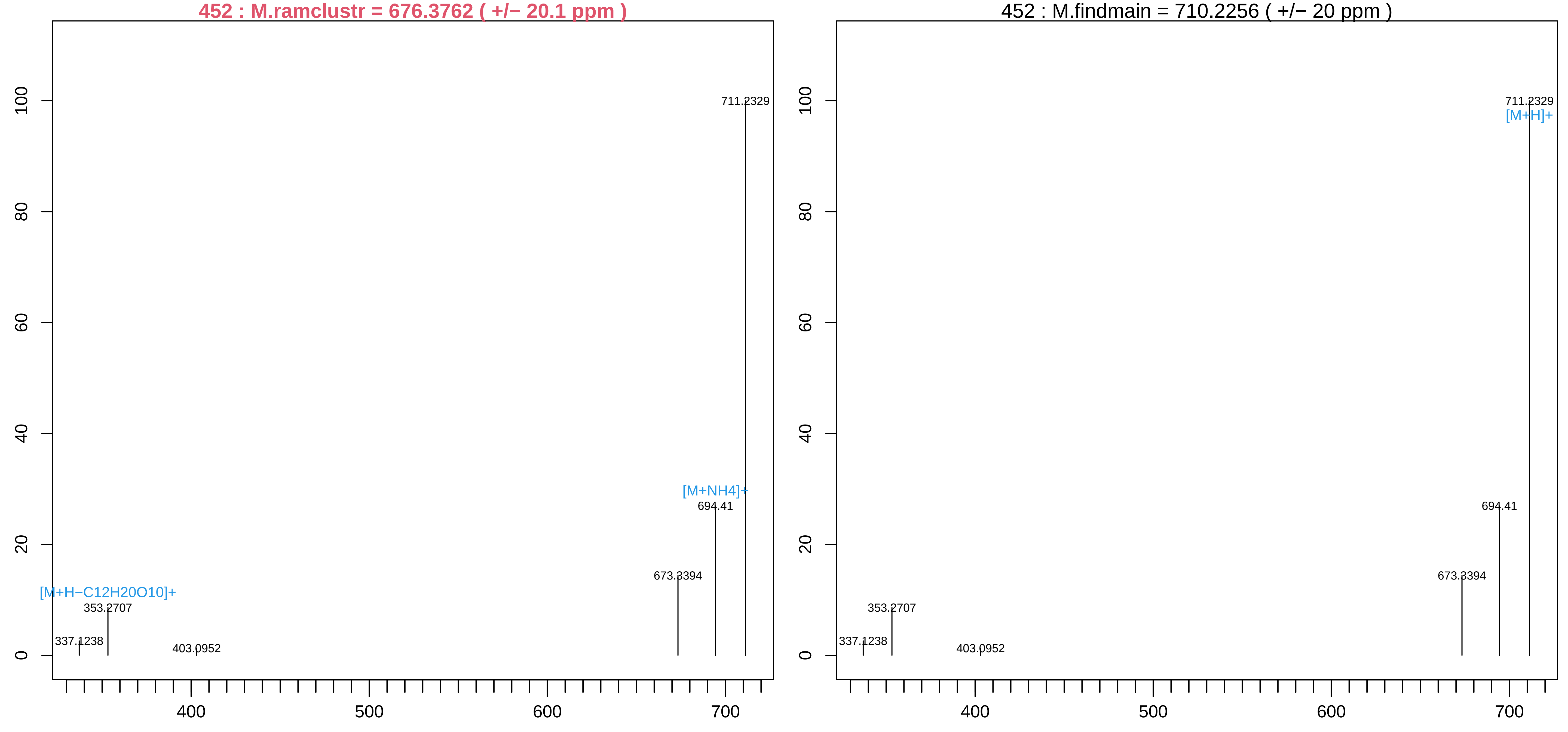

452 : M.ramclustr = 676.3762 (+/− 20.1 ppm)
711.2329
[M+NH4]+
694.41
673.3394
[M+H−C12H20O10]+
353.2707
337.1238
403.0952
452 : M.findmain = 710.2256 (+/− 20 ppm)
711.2329
[M+H]+
694.41
673.3394
353.2707
337.1238
403.0952
0
20
40
60
80
100
400
500
600
700

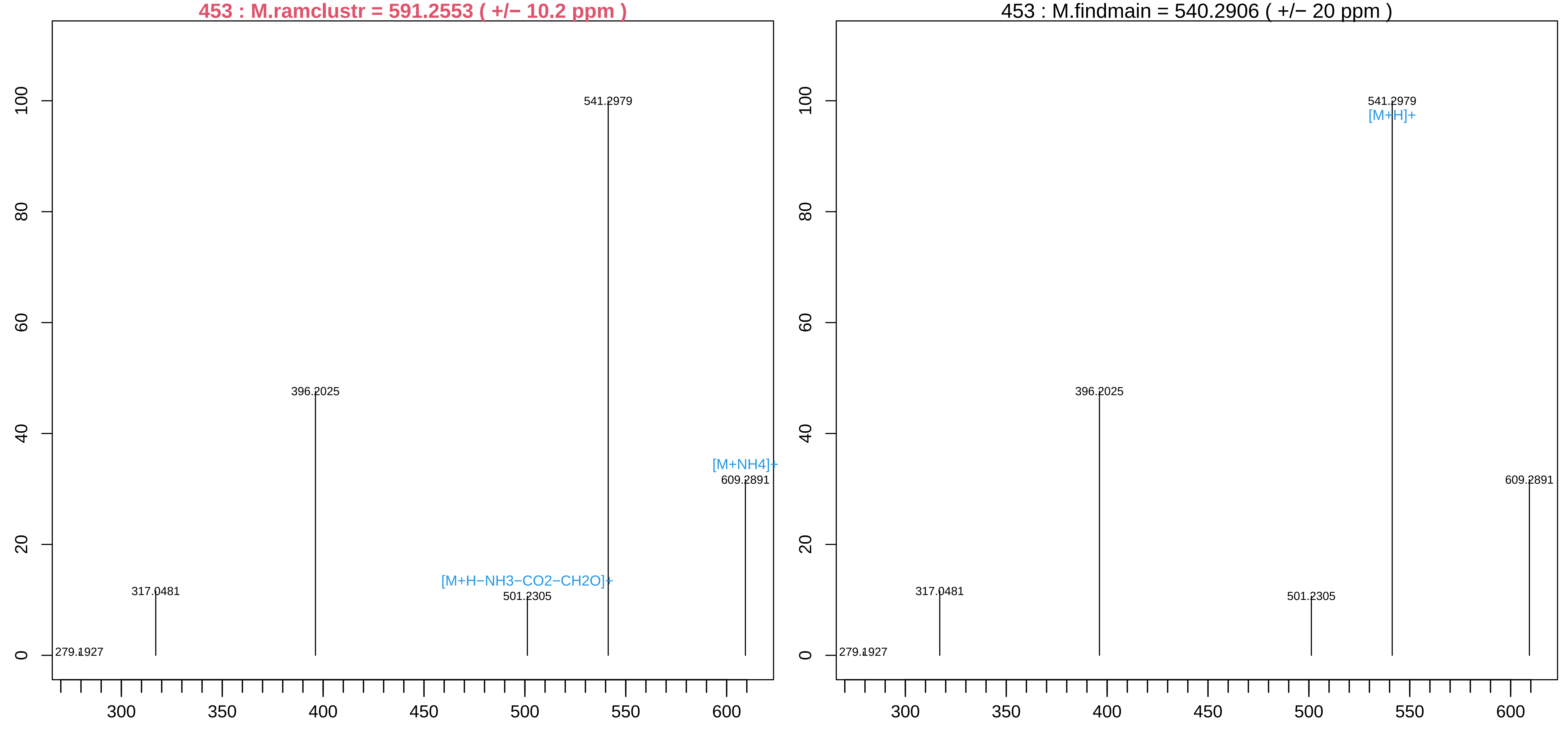

453 : M.ramclustr = 591.2553 (+/− 10.2 ppm)
541.2979
396.2025
[M+NH4]+
609.2891
[M+H−NH3−CO2−CH2O]+
501.2305
317.0481
279.1927
0
20
40
60
80
100
300
350
400
450
500
550
600
453 : M.findmain = 540.2906 (+/− 20 ppm)
541.2979
[M+H]+
396.2025
609.2891
317.0481
501.2305
279.1927

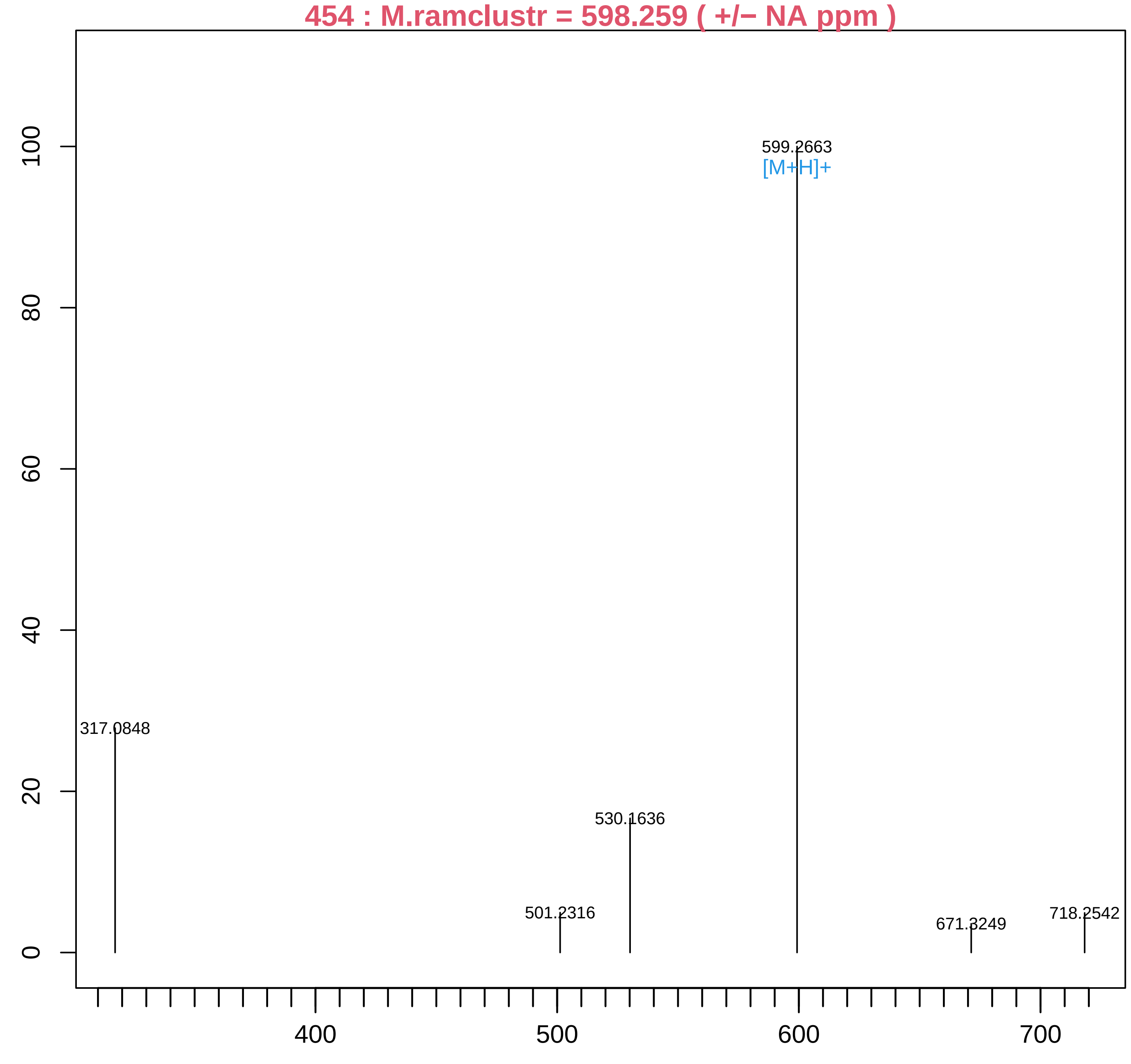
454 : M.ramclustr = 598.259 (+/− NA ppm)
599.2663
[M+H]+
317.0848
530.1636
501.2316
671.3249
718.2542
0
20
40
60
80
100
400
500
600
700

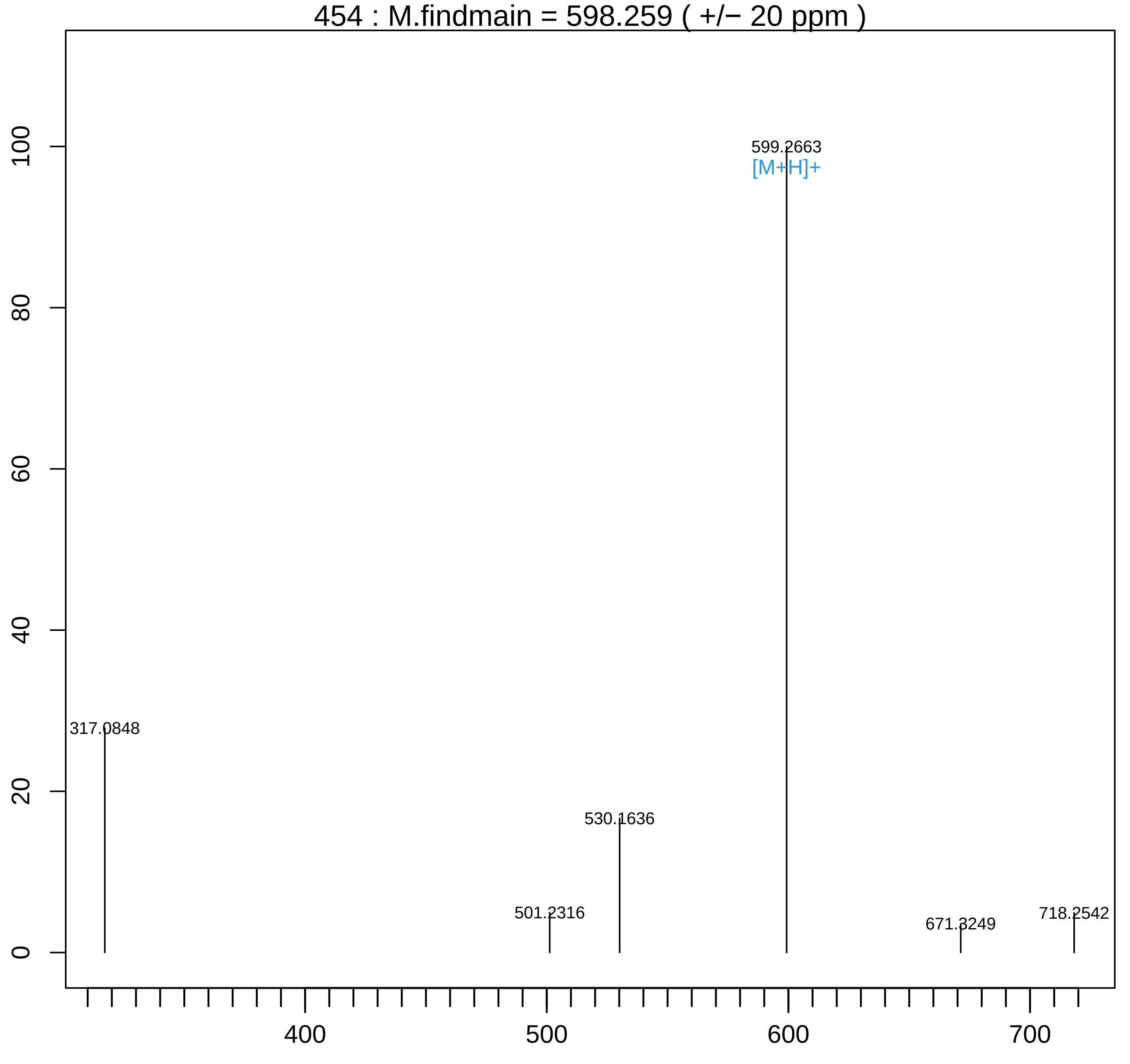
454 : M.findmain = 598.259 (+/− 20 ppm)
599.2663
[M+H]+
317.0848
530.1636
501.2316
671.3249
718.2542
0
20
40
60
80
100
400
500
600
700

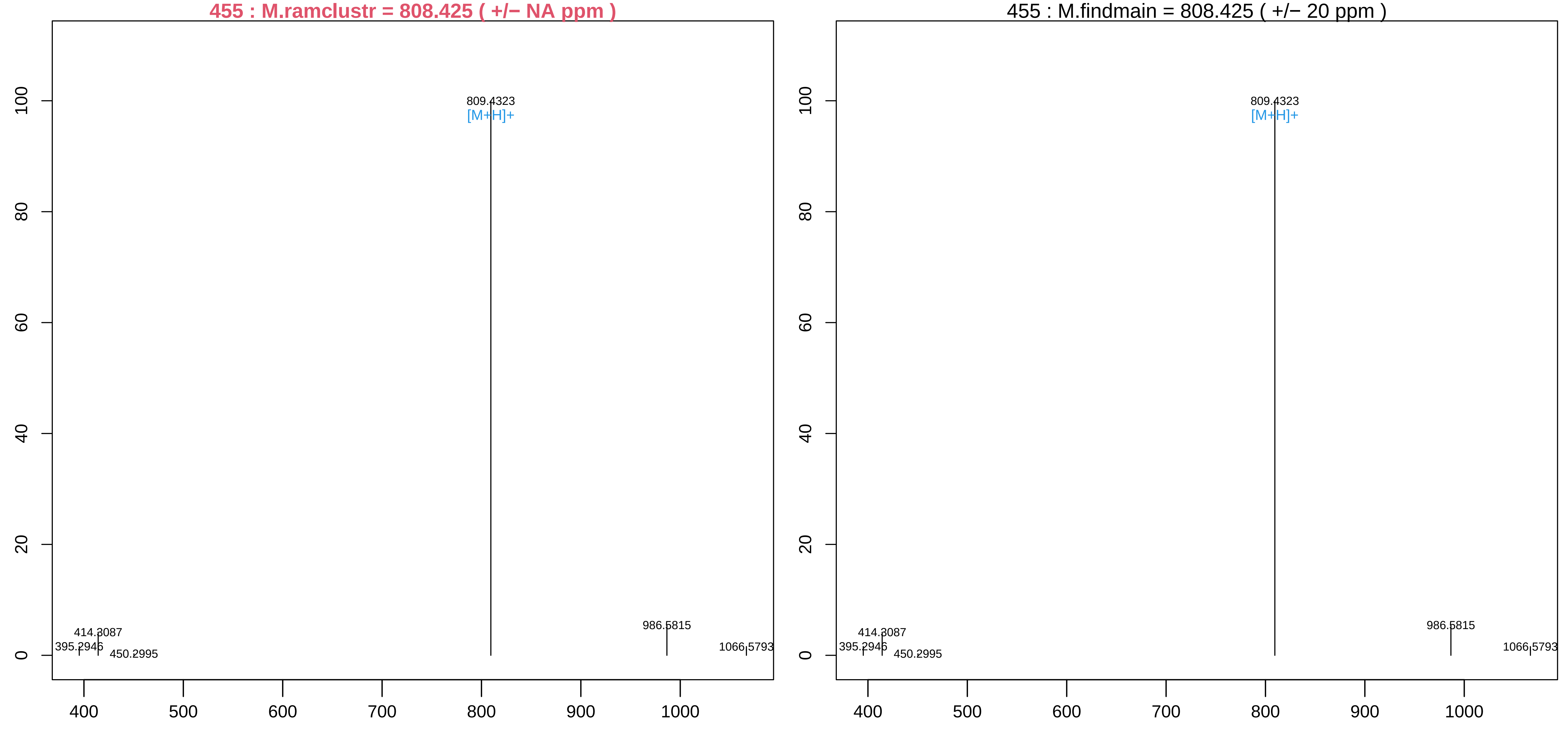
455 : M.ramclustr = 808.425 (+/− NA ppm)
809.4323
[M+H]+
414.3087
395.2946
450.2995
986.5815
1066.5793
455 : M.findmain = 808.425 (+/− 20 ppm)
809.4323
[M+H]+
414.3087
395.2946
450.2995
986.5815
1066.5793

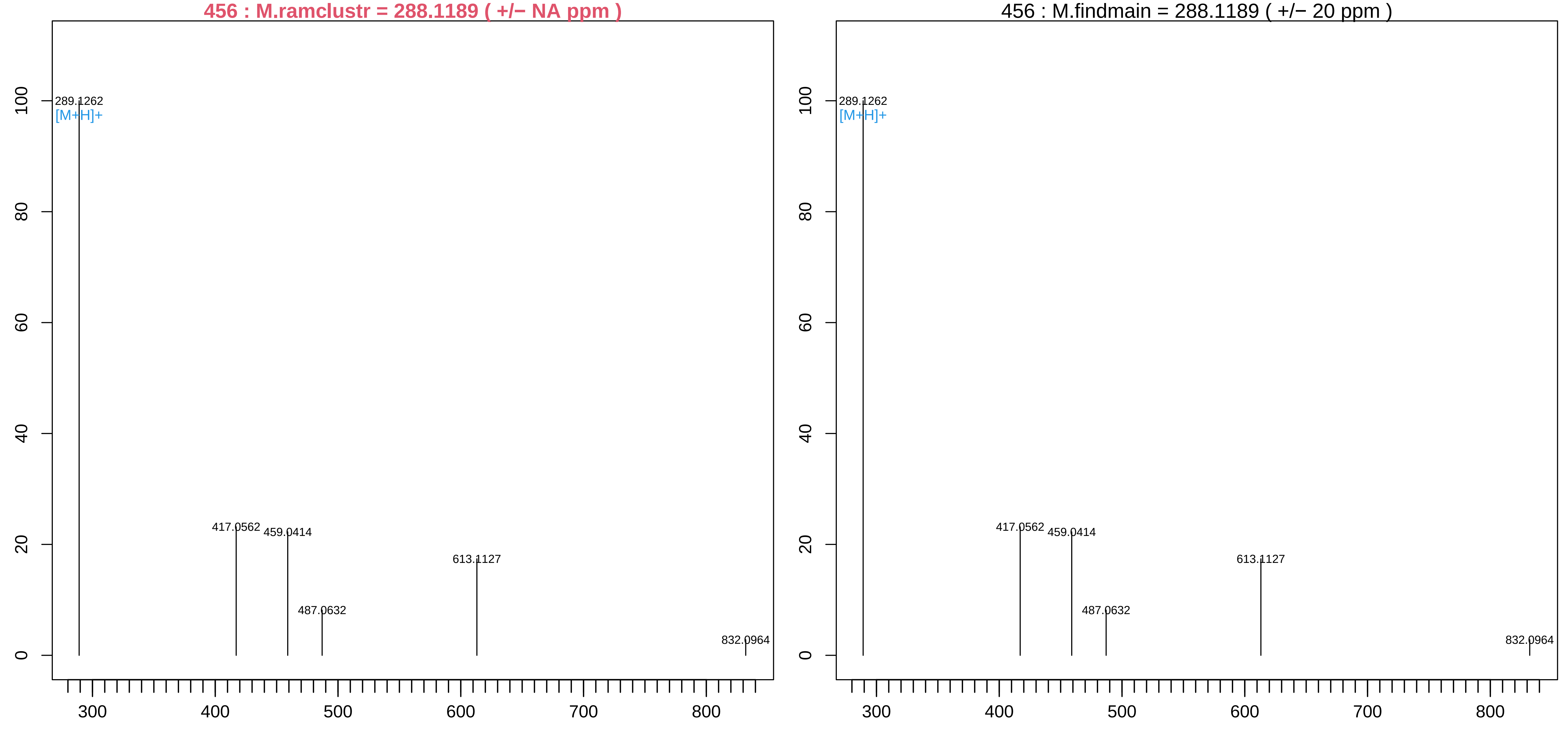

456 : M.ramclustr = 288.1189 (+/− NA ppm)
289.1262
[M+H]+
417.0562
459.0414
487.0632
613.1127
832.0964
0
20
40
60
80
100
300
400
500
600
700
800
456 : M.findmain = 288.1189 (+/− 20 ppm)
289.1262
[M+H]+
417.0562
459.0414
487.0632
613.1127
832.0964
0
20
40
60
80
100
300
400
500
600
700
800

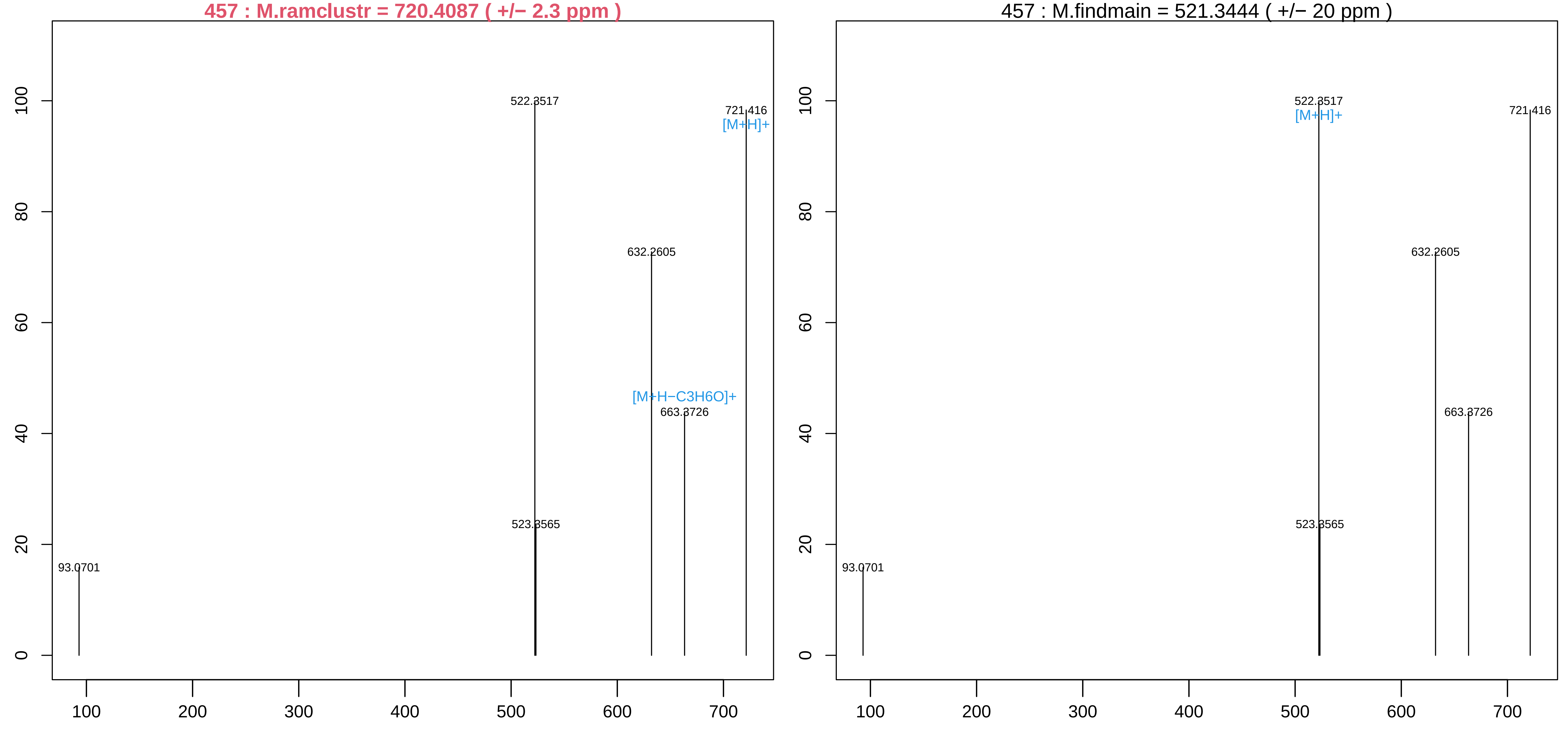

457 : M.ramclustr = 720.4087 (+/− 2.3 ppm)
522.3517
721.416
[M+H]+
632.2605
[M+H−C3H6O]+
663.3726
523.3565
93.0701
457 : M.findmain = 521.3444 (+/− 20 ppm)
522.3517
[M+H]+
721.416
632.2605
663.3726
523.3565
93.0701

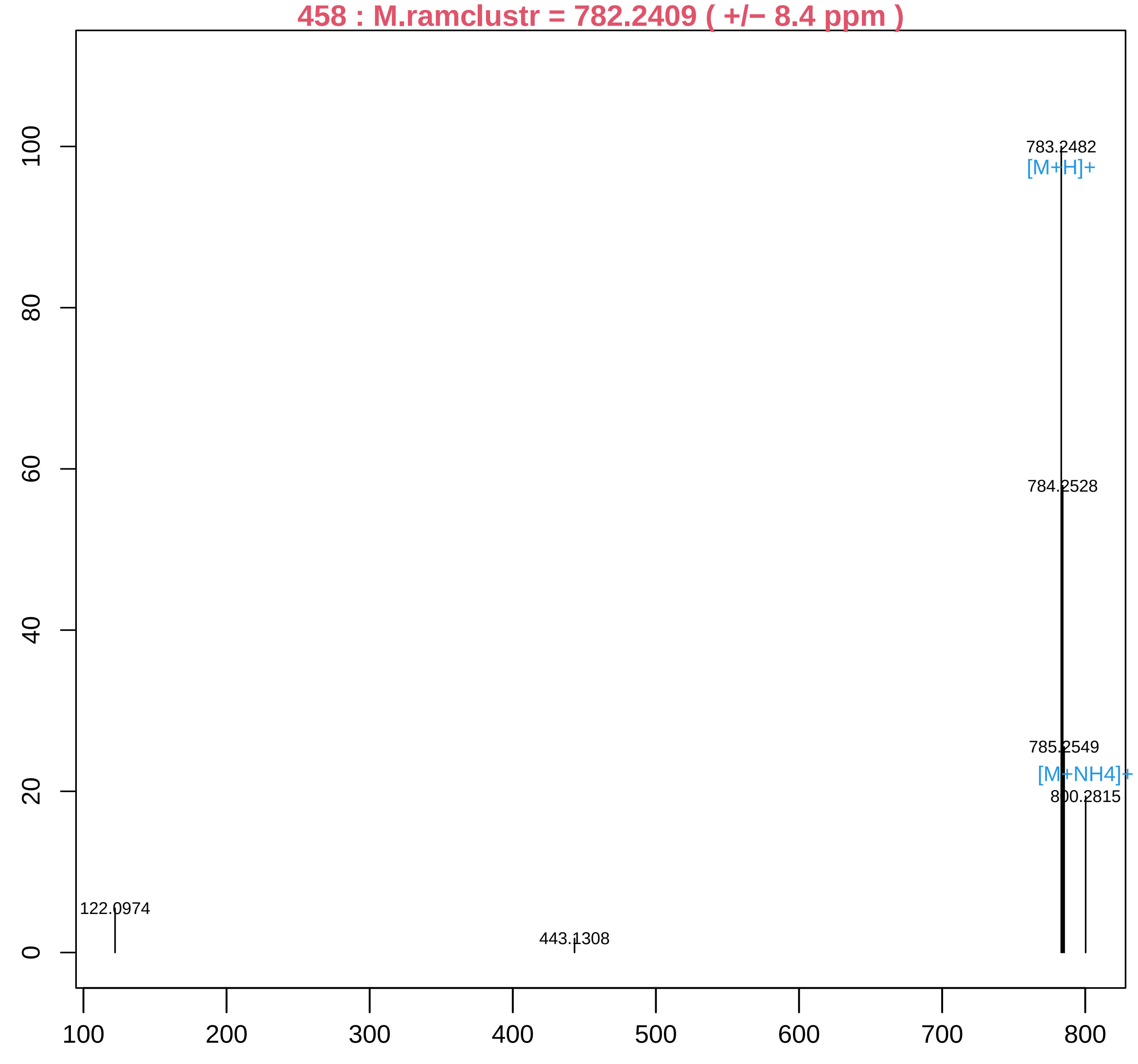
458 : M.ramclustr = 782.2409 (+/− 8.4 ppm)
783.2482
[M+H]+
784.2528
785.2549
[M+NH4]+
800.2815
122.0974
443.1308
0
20
40
60
80
100
100
200
300
400
500
600
700
800

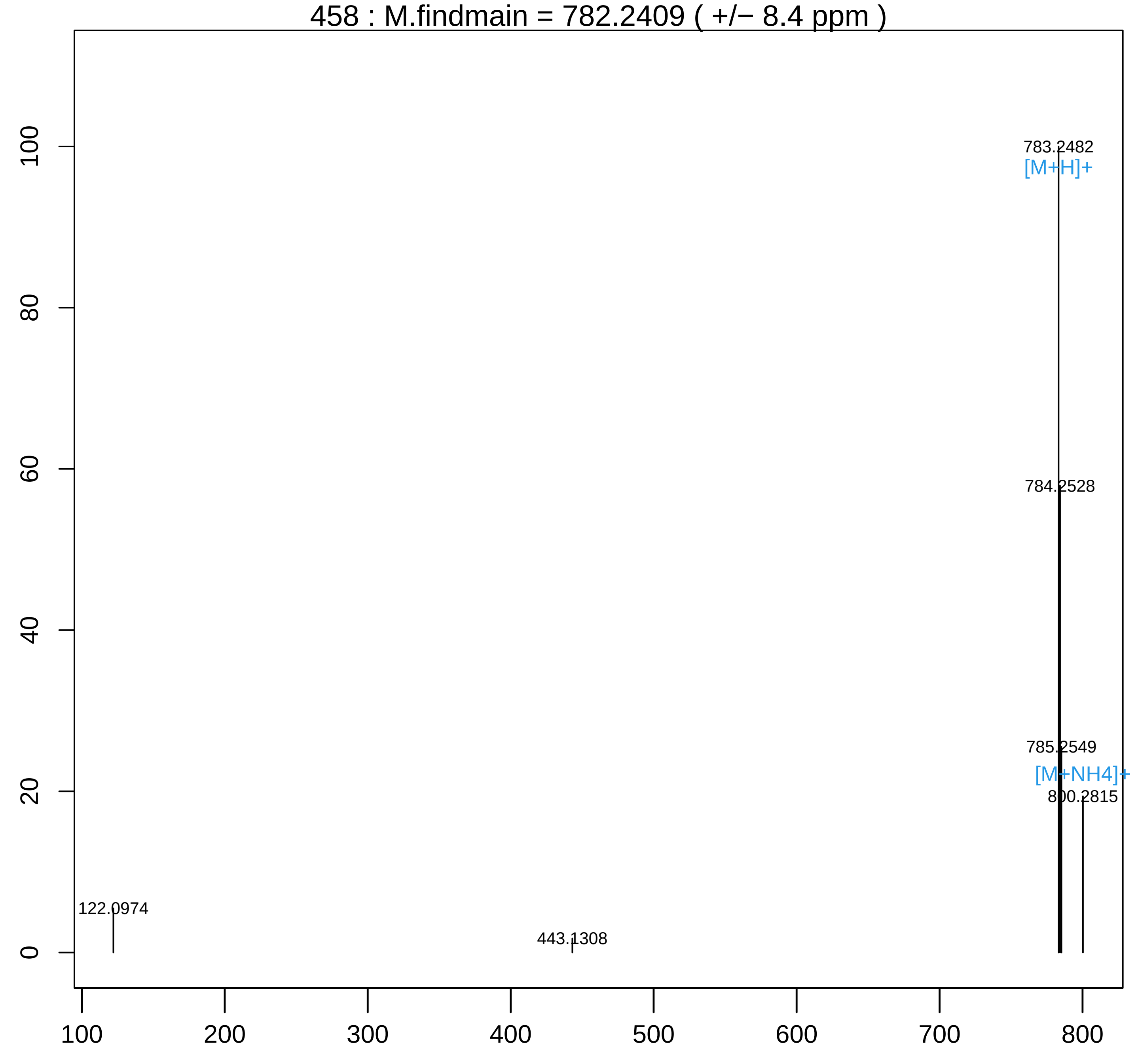
458 : M.findmain = 782.2409 (+/− 8.4 ppm)
783.2482
[M+H]+
784.2528
785.2549
[M+NH4]+
800.2815
122.0974
443.1308
0
20
40
60
80
100
100
200
300
400
500
600
700
800

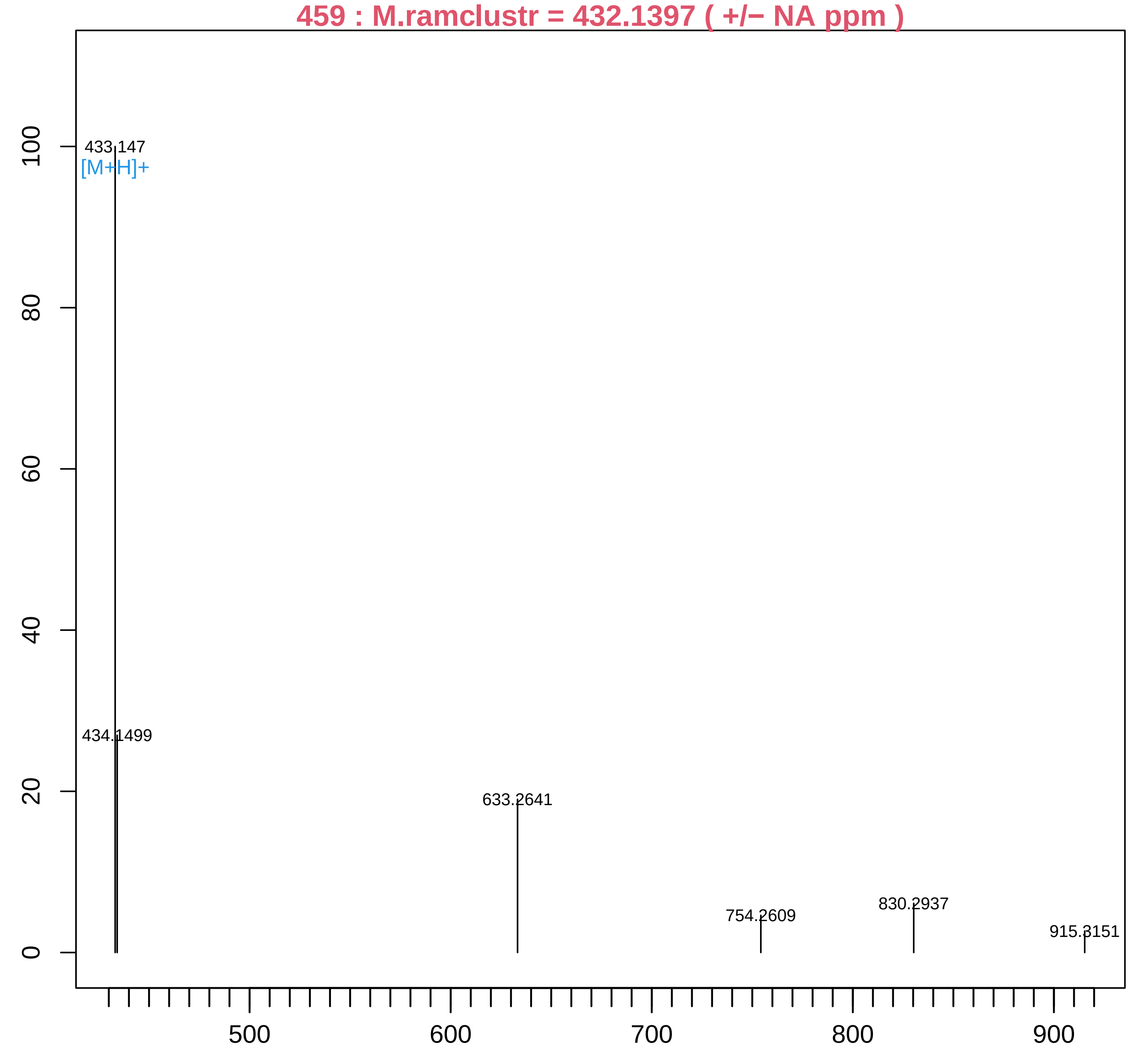
459 : M.ramclustr = 432.1397 (+/− NA ppm)
433.147
[M+H]+
434.1499
633.2641
754.2609
830.2937
915.3151
0
20
40
60
80
100
500
600
700
800
900

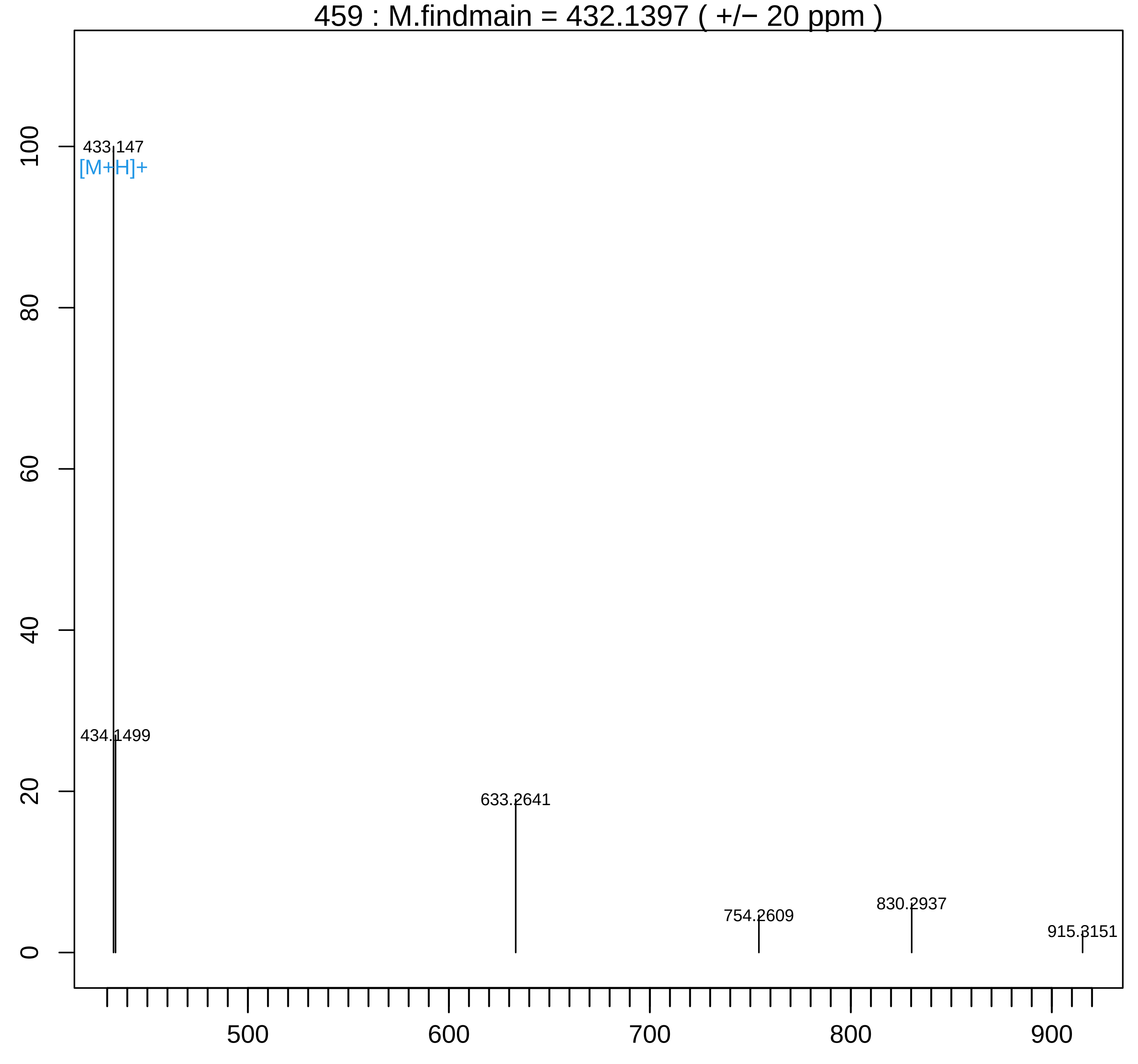
459 : M.findmain = 432.1397 (+/− 20 ppm)
433.147
[M+H]+
434.1499
633.2641
754.2609
830.2937
915.3151
0
20
40
60
80
100
500
600
700
800
900

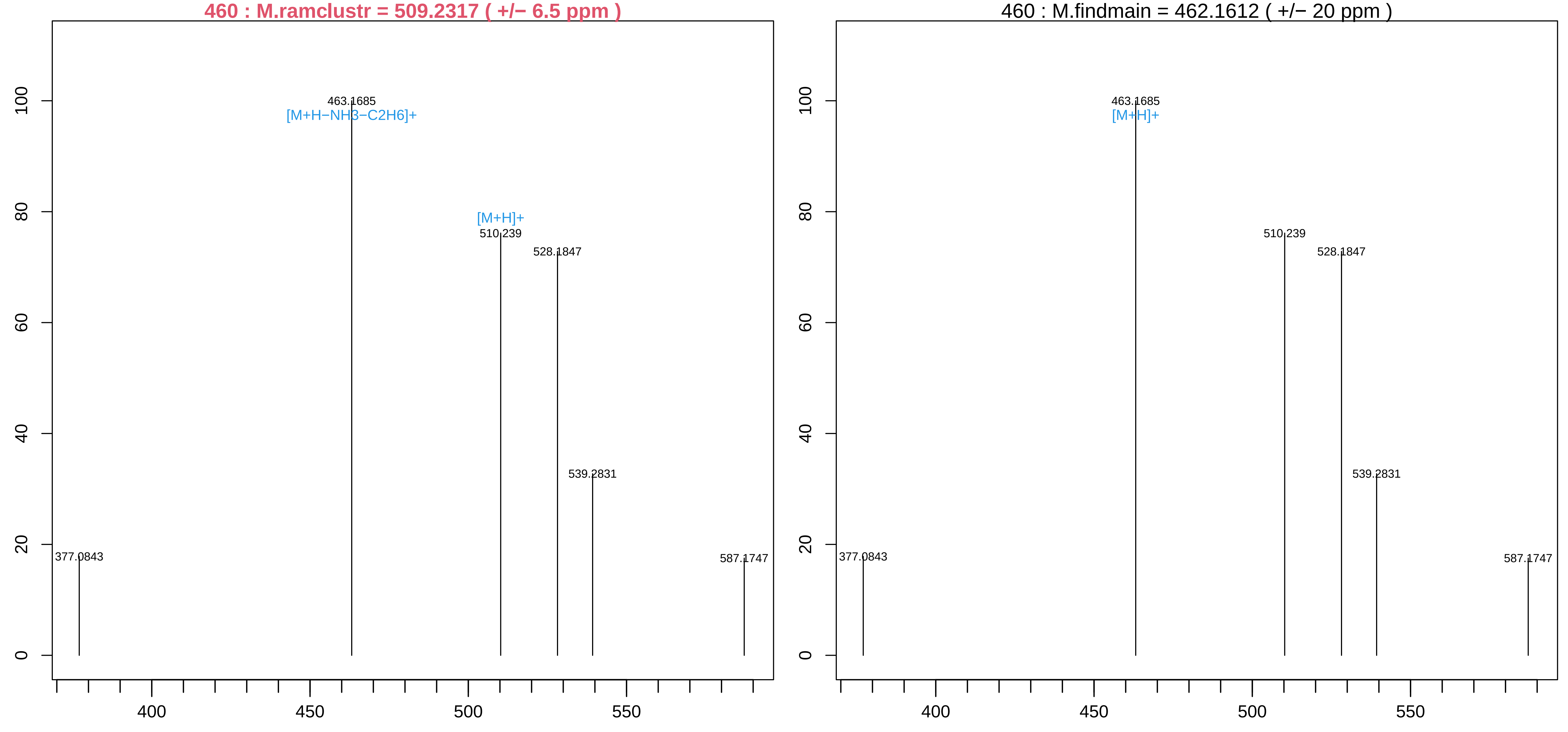
460 : M.ramclustr = 509.2317 (+/− 6.5 ppm)
463.1685
[M+H−NH3−C2H6]+
[M+H]+
510.239
528.1847
539.2831
377.0843
587.1747
460 : M.findmain = 462.1612 (+/− 20 ppm)
463.1685
[M+H]+
510.239
528.1847
539.2831
377.0843
587.1747

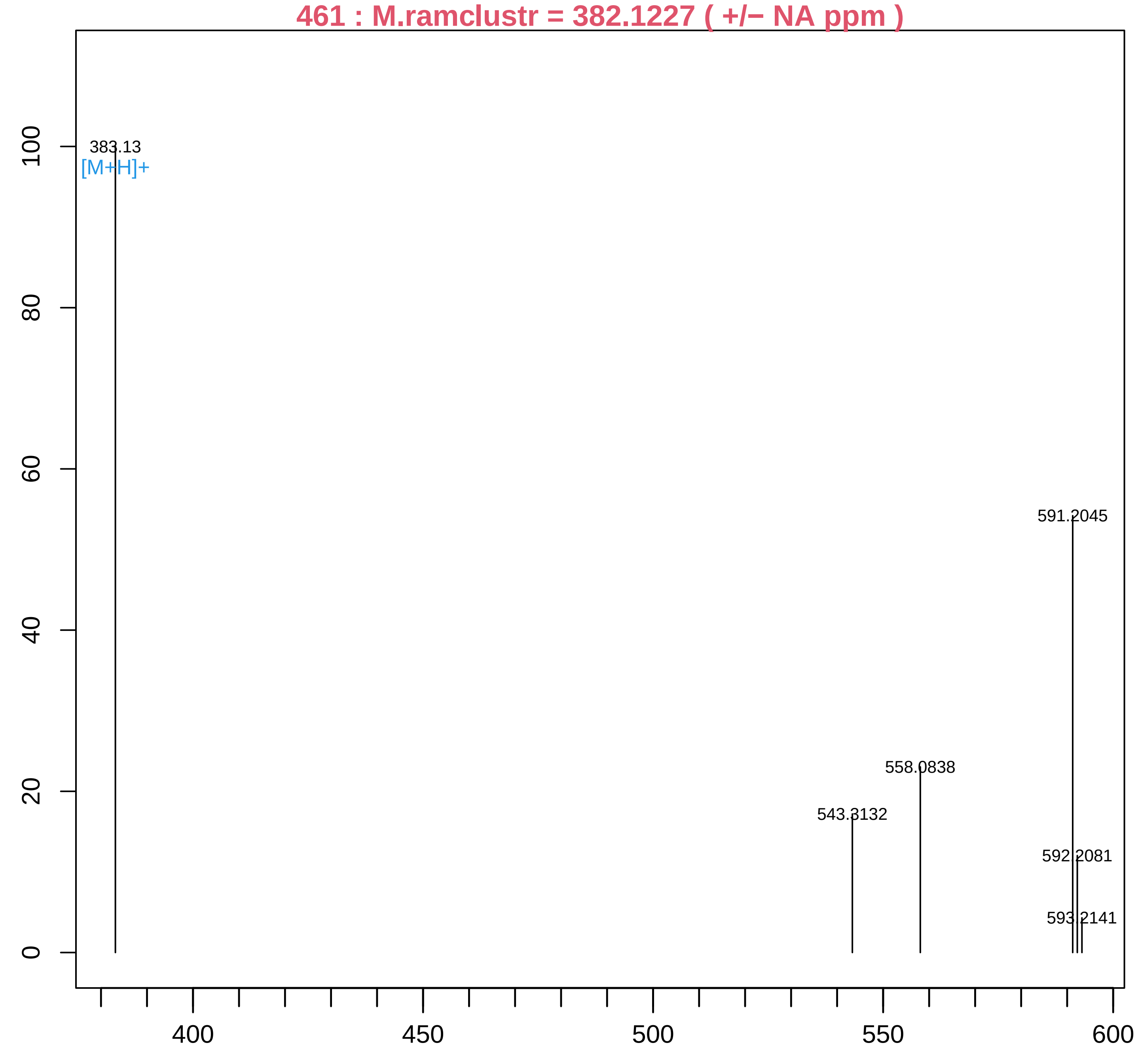
461 : M.ramclustr = 382.1227 (+/− NA ppm)
383.13
[M+H]+
591.2045
558.0838
543.3132
592.2081
593.2141
0
20
40
60
80
100
400
450
500
550
600

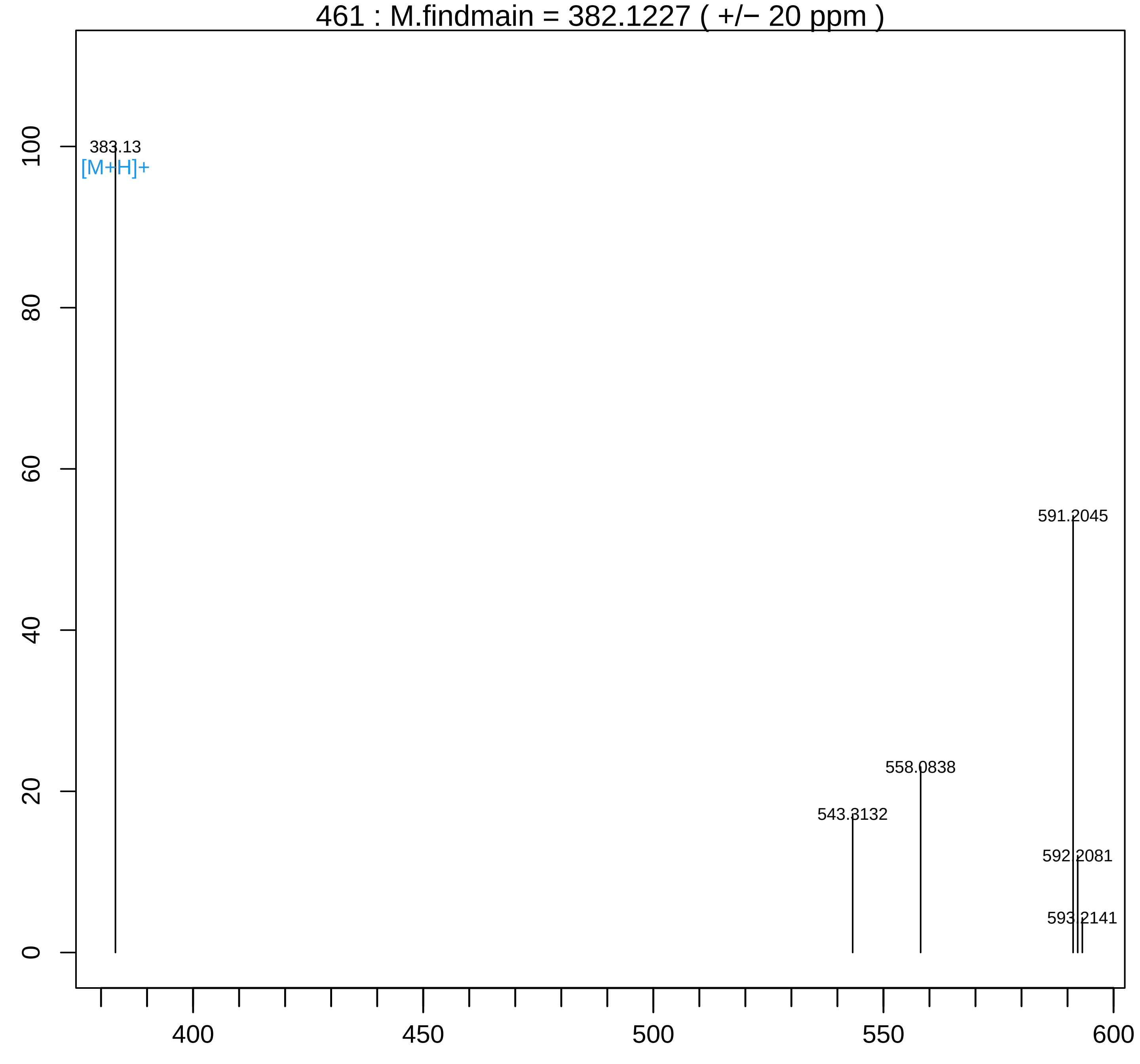
461 : M.findmain = 382.1227 (+/− 20 ppm)
383.13
[M+H]+
591.2045
558.0838
543.3132
592.2081
593.2141
0
20
40
60
80
100
400
450
500
550
600

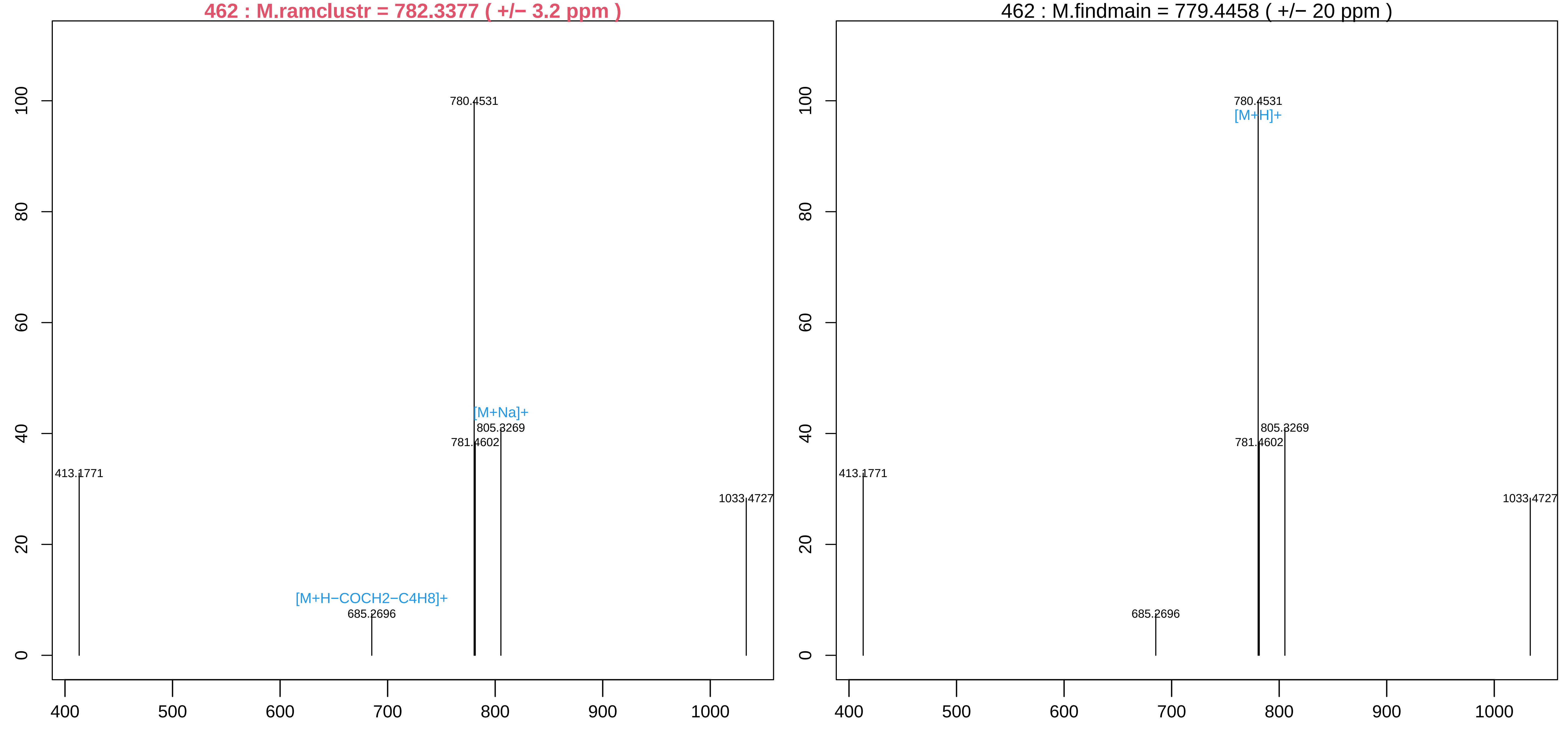
462 : M.ramclustr = 782.3377 (+/− 3.2 ppm)
780.4531
[M+Na]+
805.3269
781.4602
413.1771
1033.4727
[M+H−COCH2−C4H8]+
685.2696
462 : M.findmain = 779.4458 (+/− 20 ppm)
780.4531
[M+H]+
805.3269
781.4602
413.1771
1033.4727
685.2696

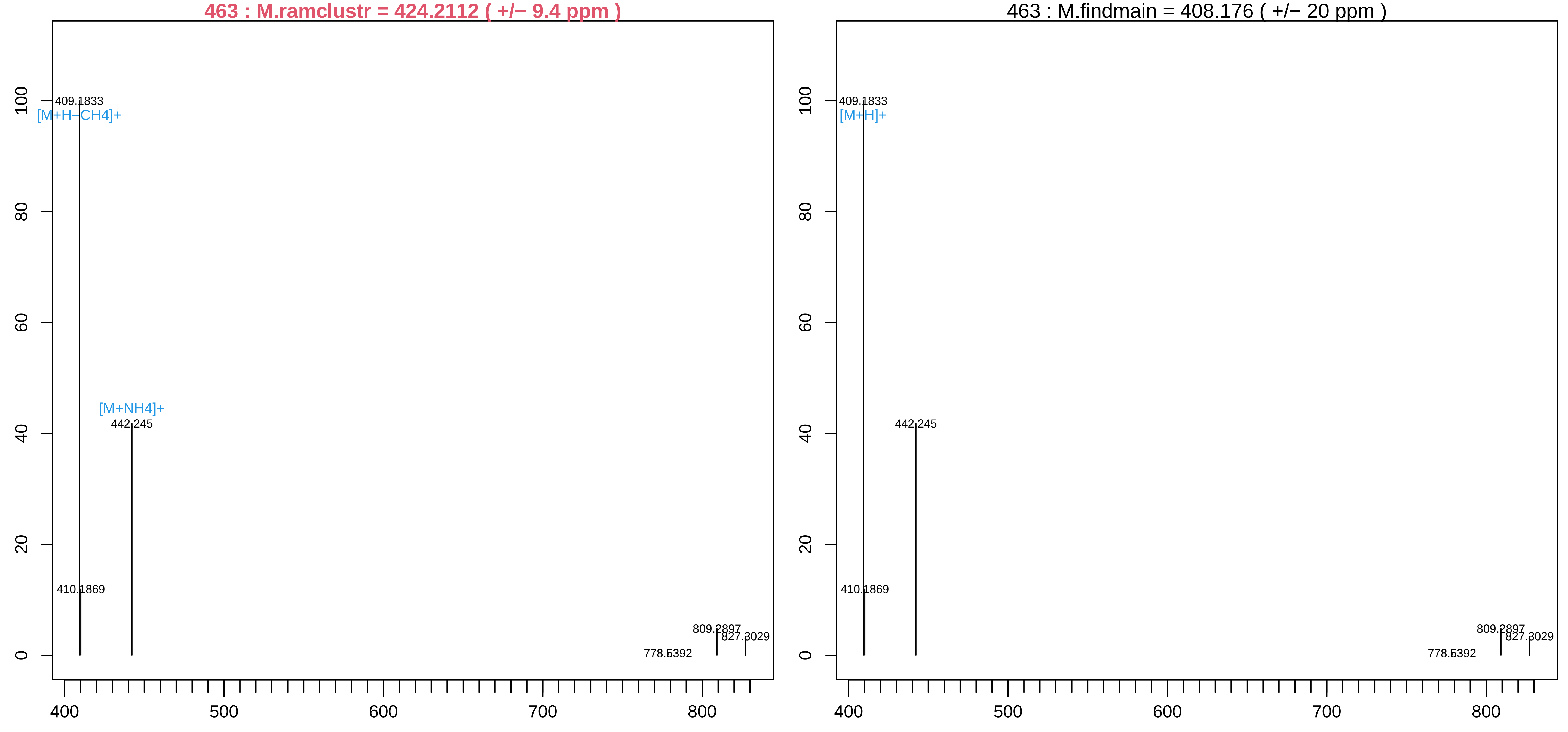
463 : M.ramclustr = 424.2112 (+/− 9.4 ppm)
409.1833
[M+H−CH4]+
[M+NH4]+
442.245
410.1869
809.2897
827.3029
778.5392
463 : M.findmain = 408.176 (+/− 20 ppm)
409.1833
[M+H]+
442.245
410.1869
809.2897
827.3029
778.5392

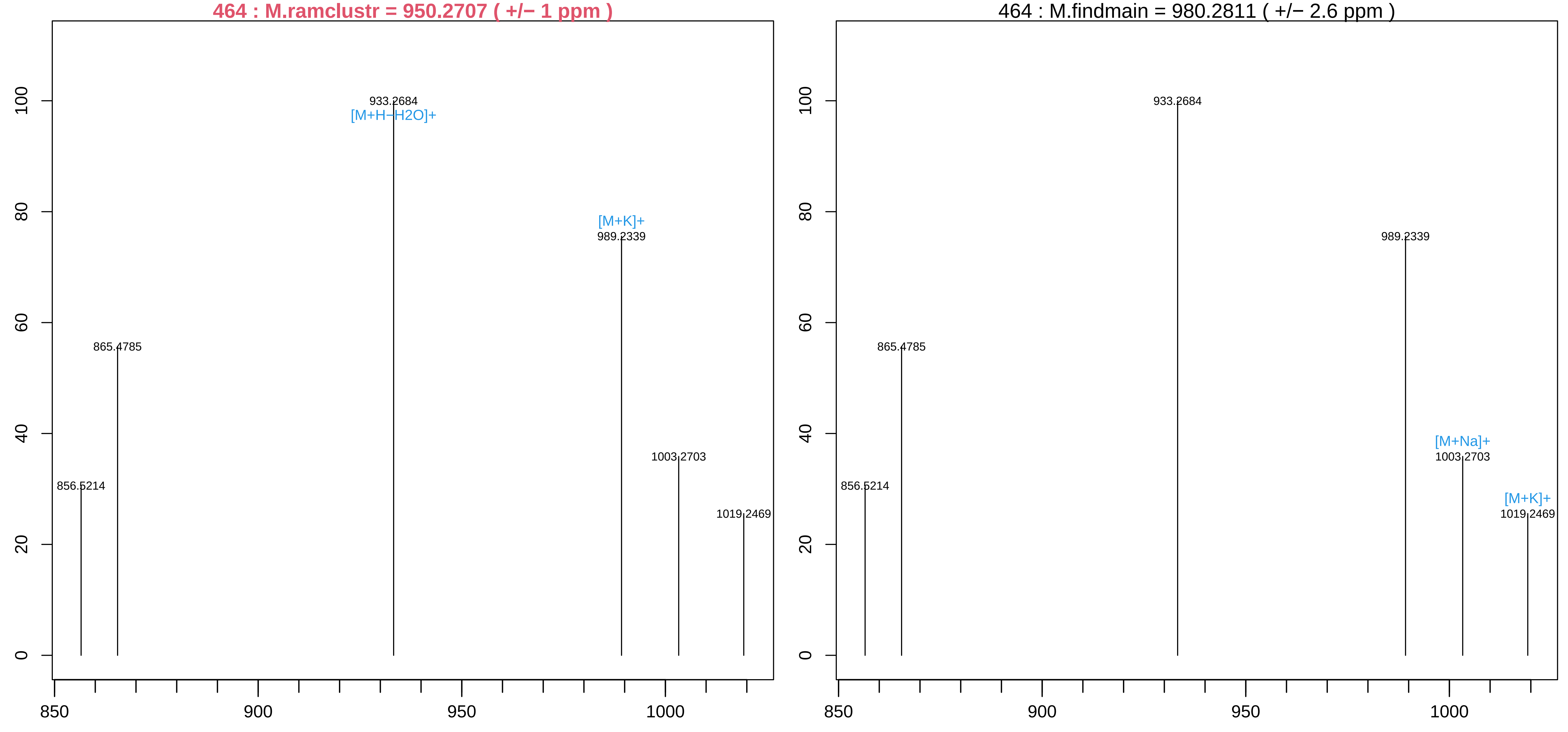

464 : M.ramclustr = 950.2707 (+/− 1 ppm)
933.2684
[M+H−H2O]+
[M+K]+
989.2339
865.4785
1003.2703
856.5214
1019.2469
0
20
40
60
80
100
850
900
950
1000
464 : M.findmain = 980.2811 (+/− 2.6 ppm)
933.2684
989.2339
865.4785
[M+Na]+
1003.2703
856.5214
[M+K]+
1019.2469
0
20
40
60
80
100
850
900
950
1000

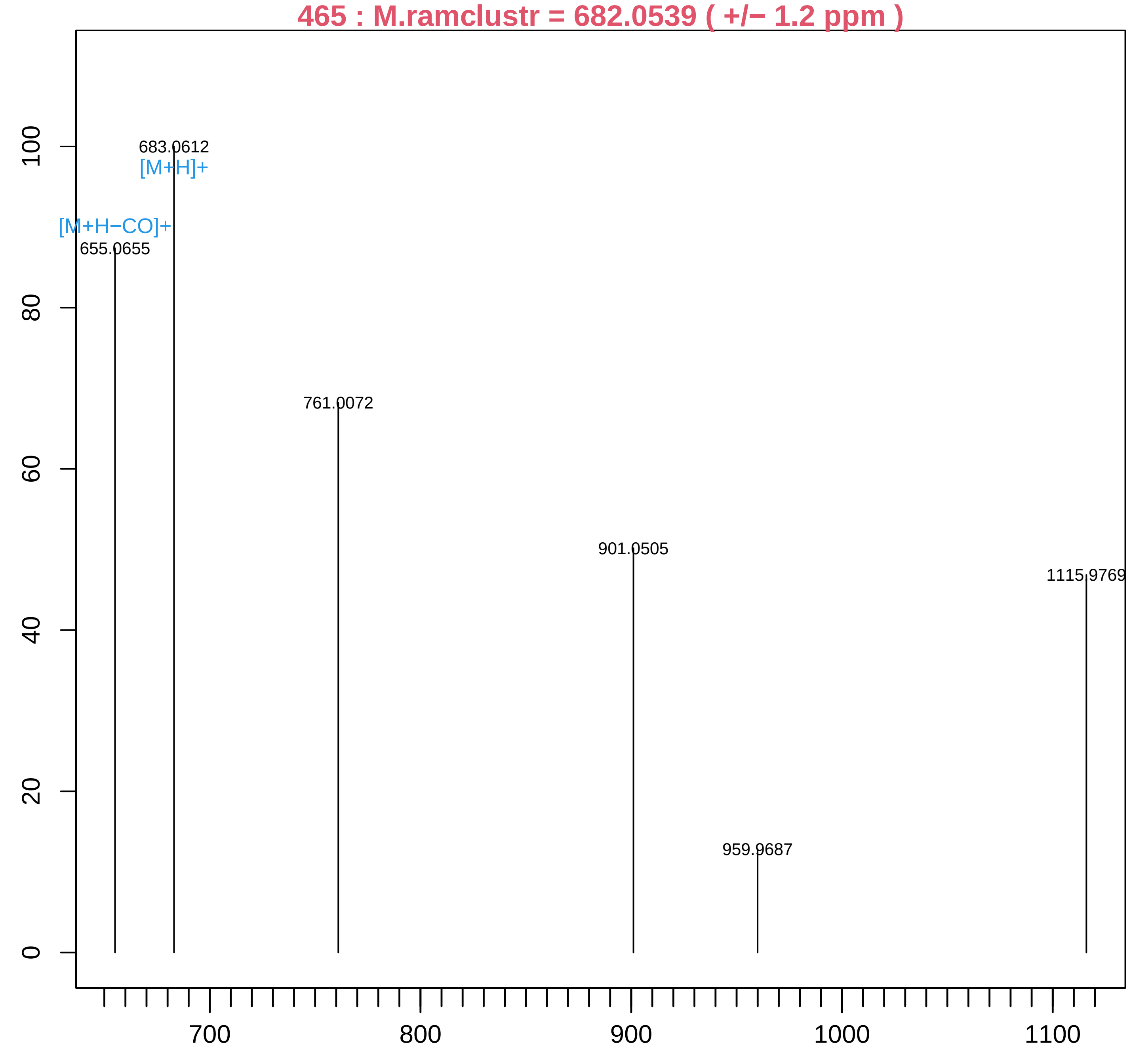
465 : M.ramclustr = 682.0539 (+/− 1.2 ppm)
683.0612
[M+H]+
[M+H−CO]+
655.0655
761.0072
901.0505
959.9687
1115.9769
0
20
40
60
80
100
700
800
900
1000
1100
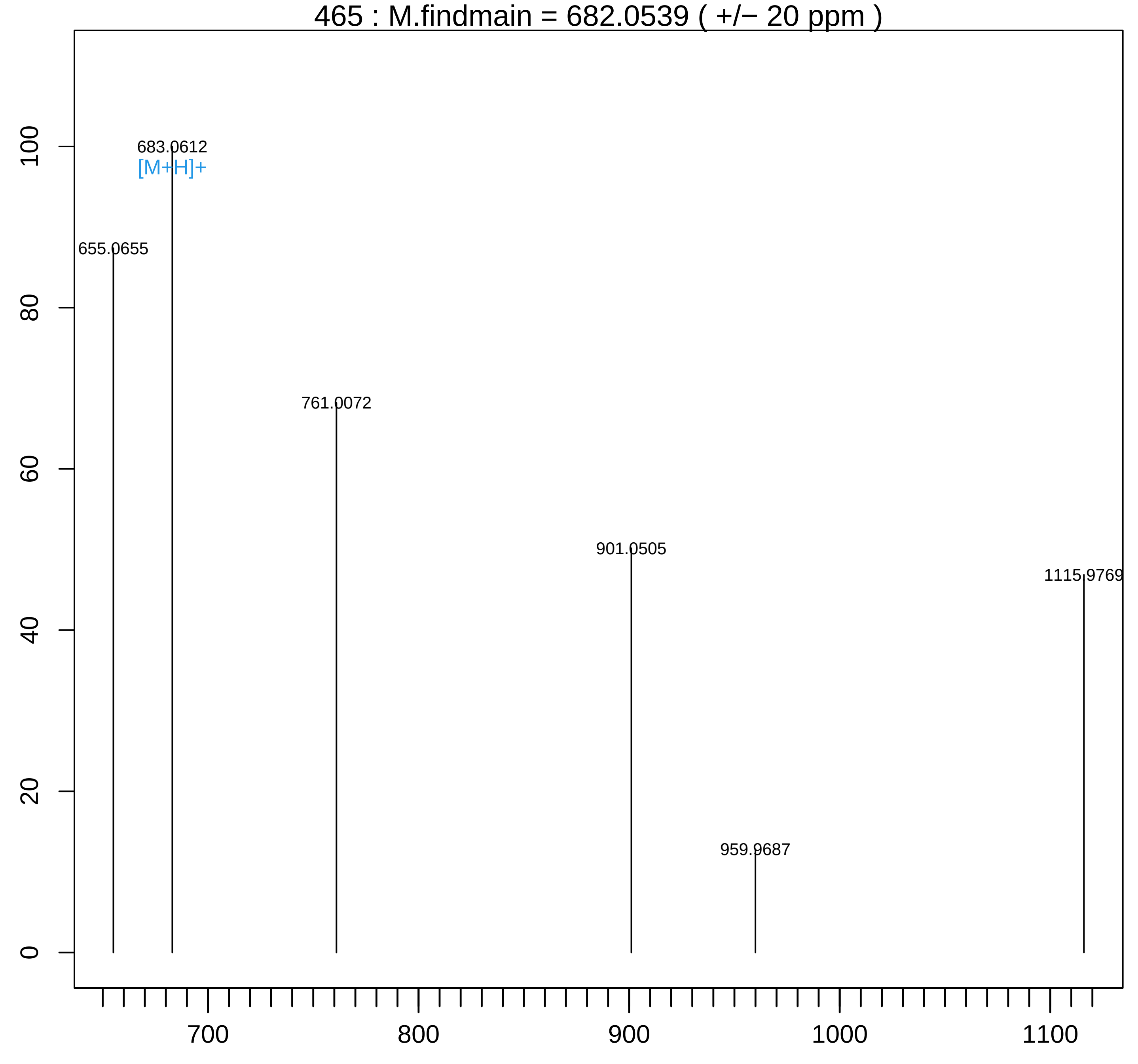
465 : M.findmain = 682.0539 (+/− 20 ppm)
683.0612
[M+H]+
655.0655
761.0072
901.0505
959.9687
1115.9769
0
20
40
60
80
100
700
800
900
1000
1100

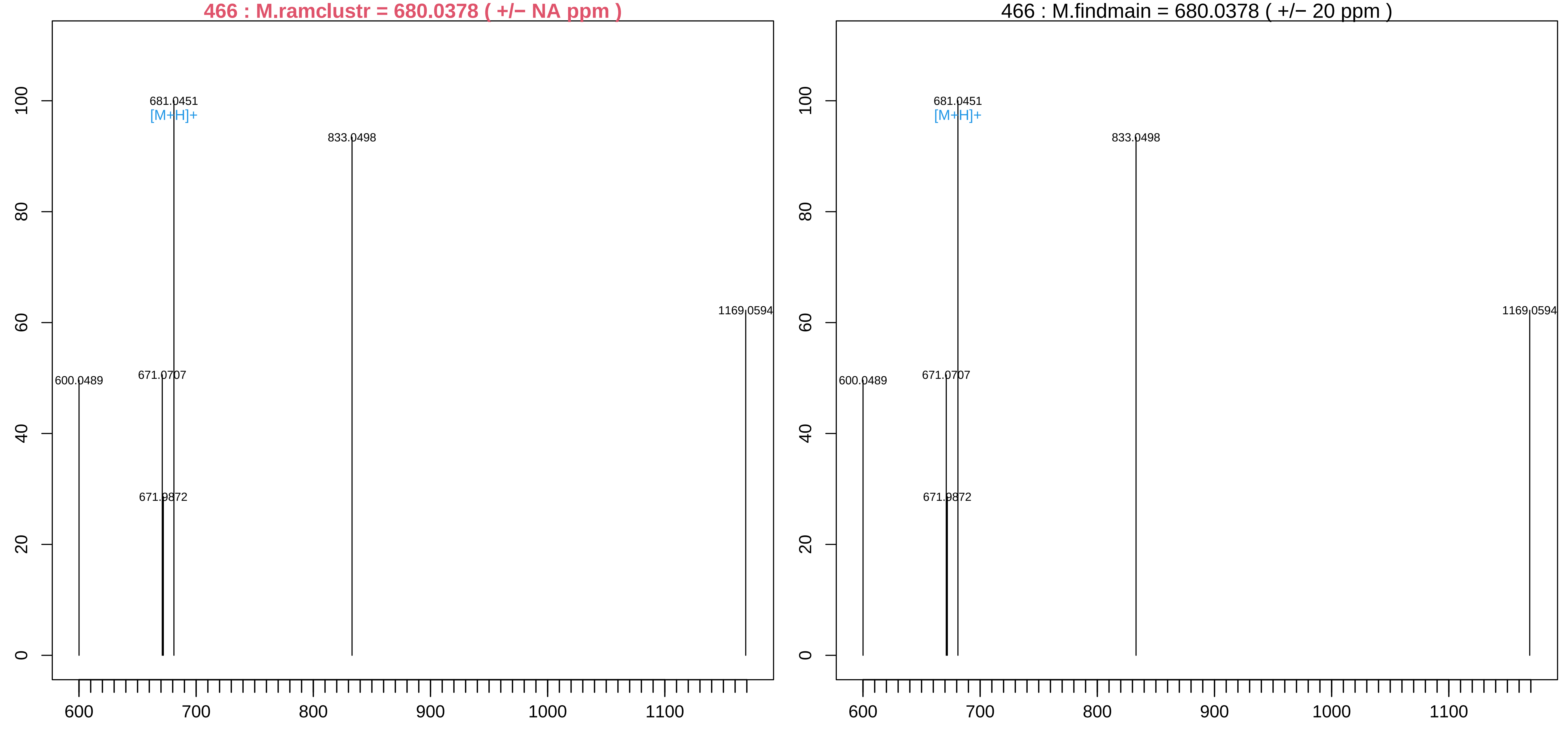
466 : M.ramclustr = 680.0378 (+/− NA ppm)
681.0451
[M+H]+
833.0498
1169.0594
600.0489
671.0707
671.9872
466 : M.findmain = 680.0378 (+/− 20 ppm)
681.0451
[M+H]+
833.0498
1169.0594
600.0489
671.0707
671.9872

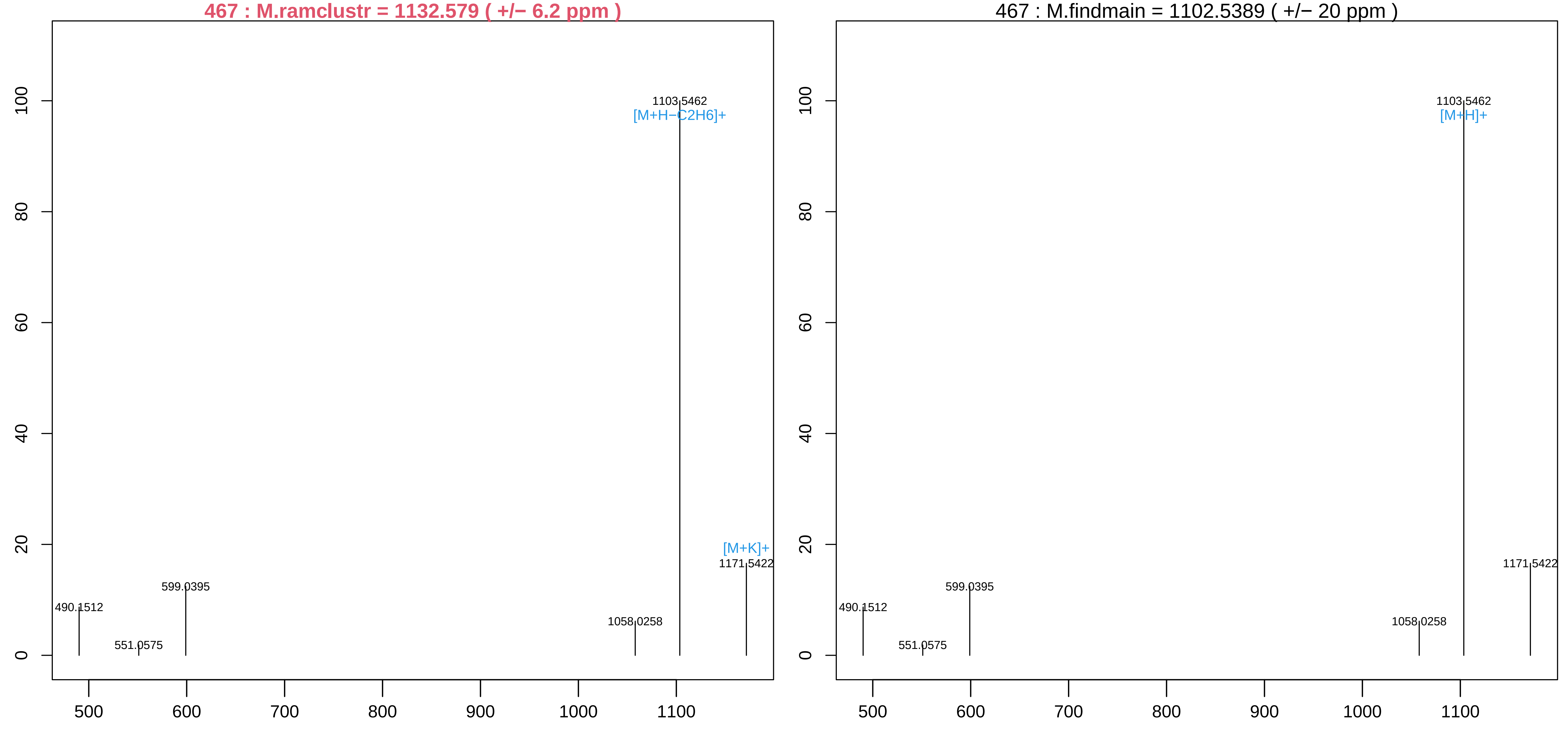
467 : M.ramclustr = 1132.579 (+/− 6.2 ppm)
1103.5462
[M+H−C2H6]+
[M+K]+
1171.5422
599.0395
490.1512
551.0575
1058.0258
467 : M.findmain = 1102.5389 (+/− 20 ppm)
1103.5462
[M+H]+
1171.5422
599.0395
490.1512
551.0575
1058.0258
0
20
40
60
80
100
500
600
700
800
900
1000
1100

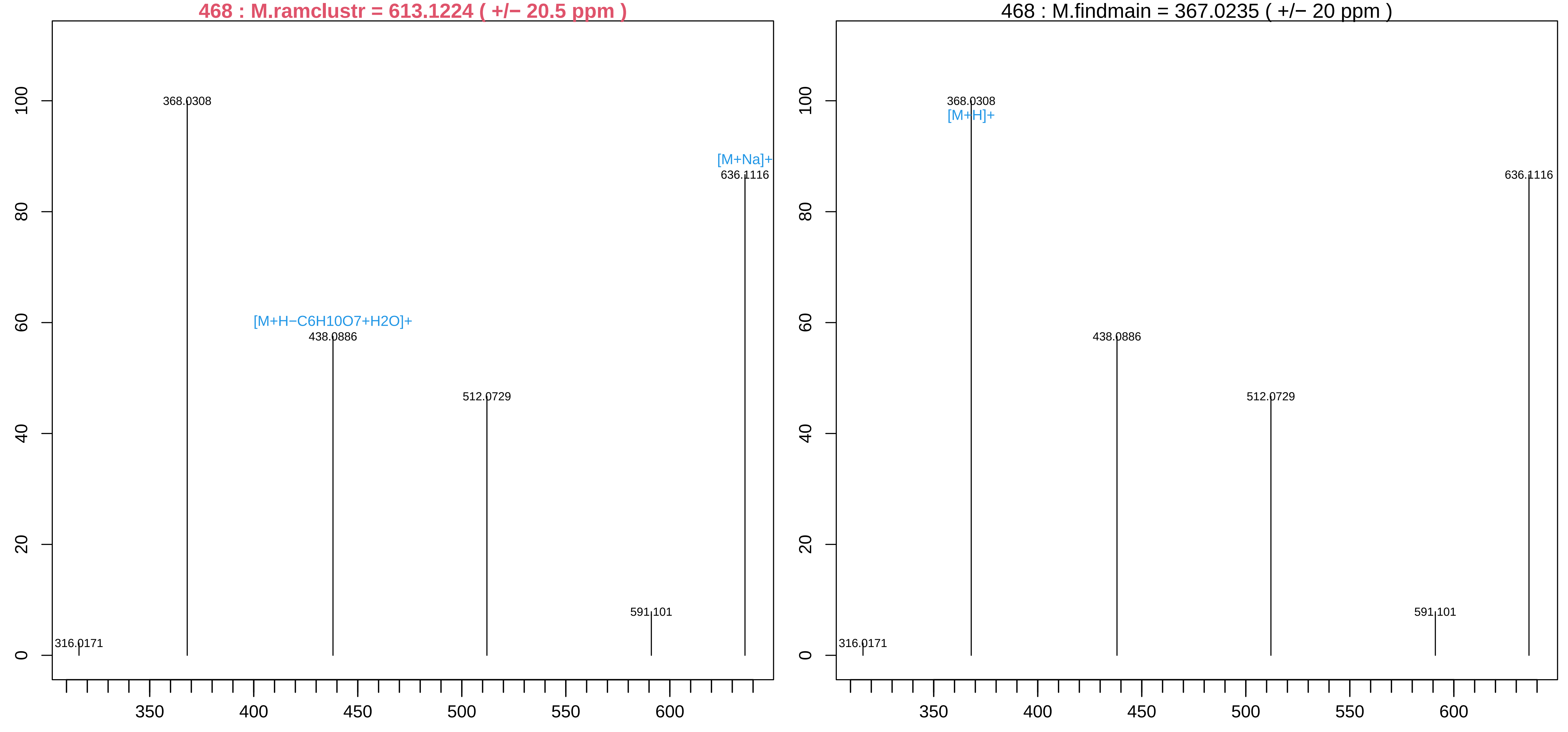
468 : M.ramclustr = 613.1224 (+/− 20.5 ppm)
368.0308
[M+Na]+
636.1116
[M+H−C6H10O7+H2O]+
438.0886
512.0729
591.101
316.0171
0
20
40
60
80
100
350
400
450
500
550
600
468 : M.findmain = 367.0235 (+/− 20 ppm)
368.0308
[M+H]+
636.1116
438.0886
512.0729
591.101
316.0171

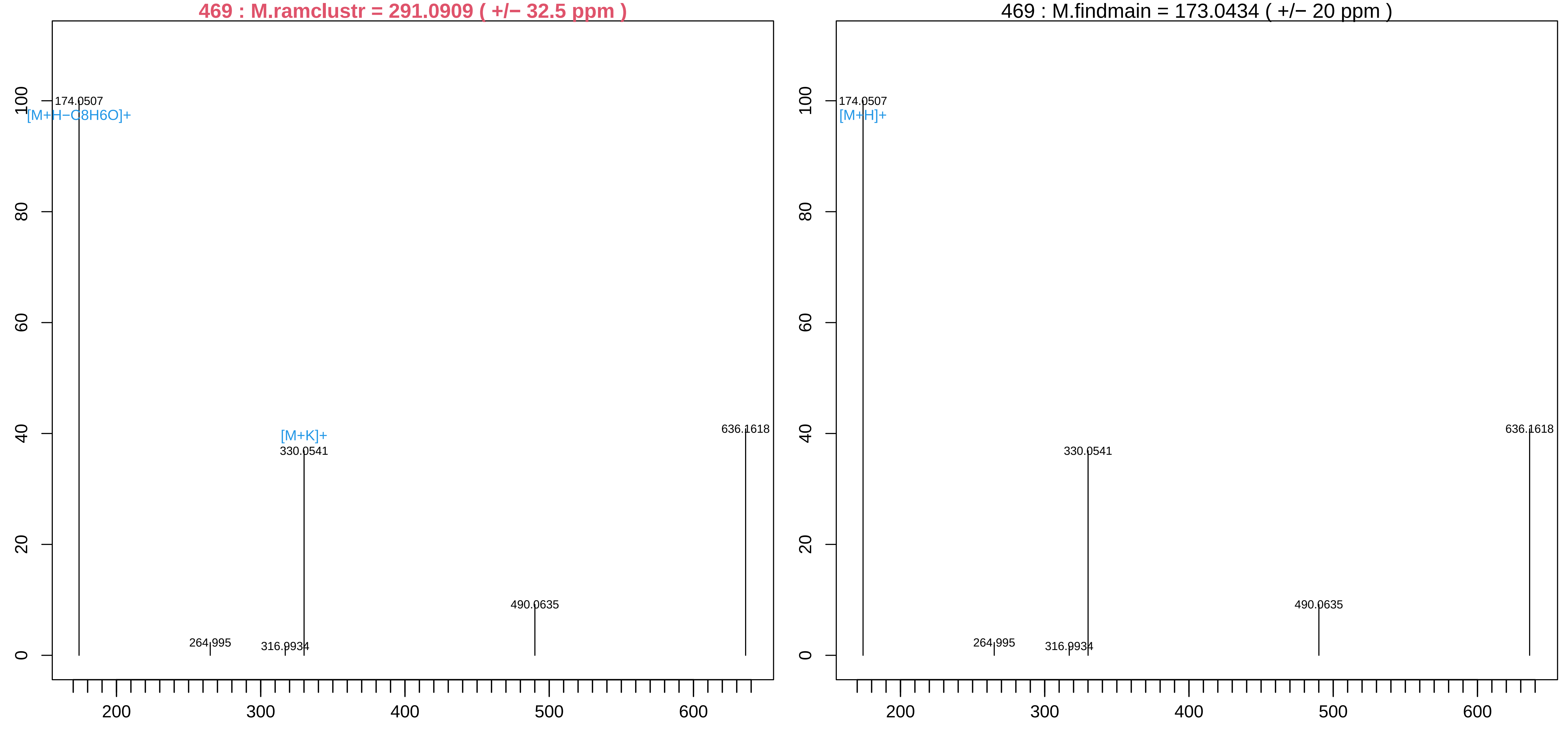
469 : M.ramclustr = 291.0909 (+/− 32.5 ppm)
174.0507
[M+H−C8H6O]+
[M+K]+
330.0541
264.995
316.9934
490.0635
636.1618
469 : M.findmain = 173.0434 (+/− 20 ppm)
174.0507
[M+H]+
330.0541
264.995
316.9934
490.0635
636.1618

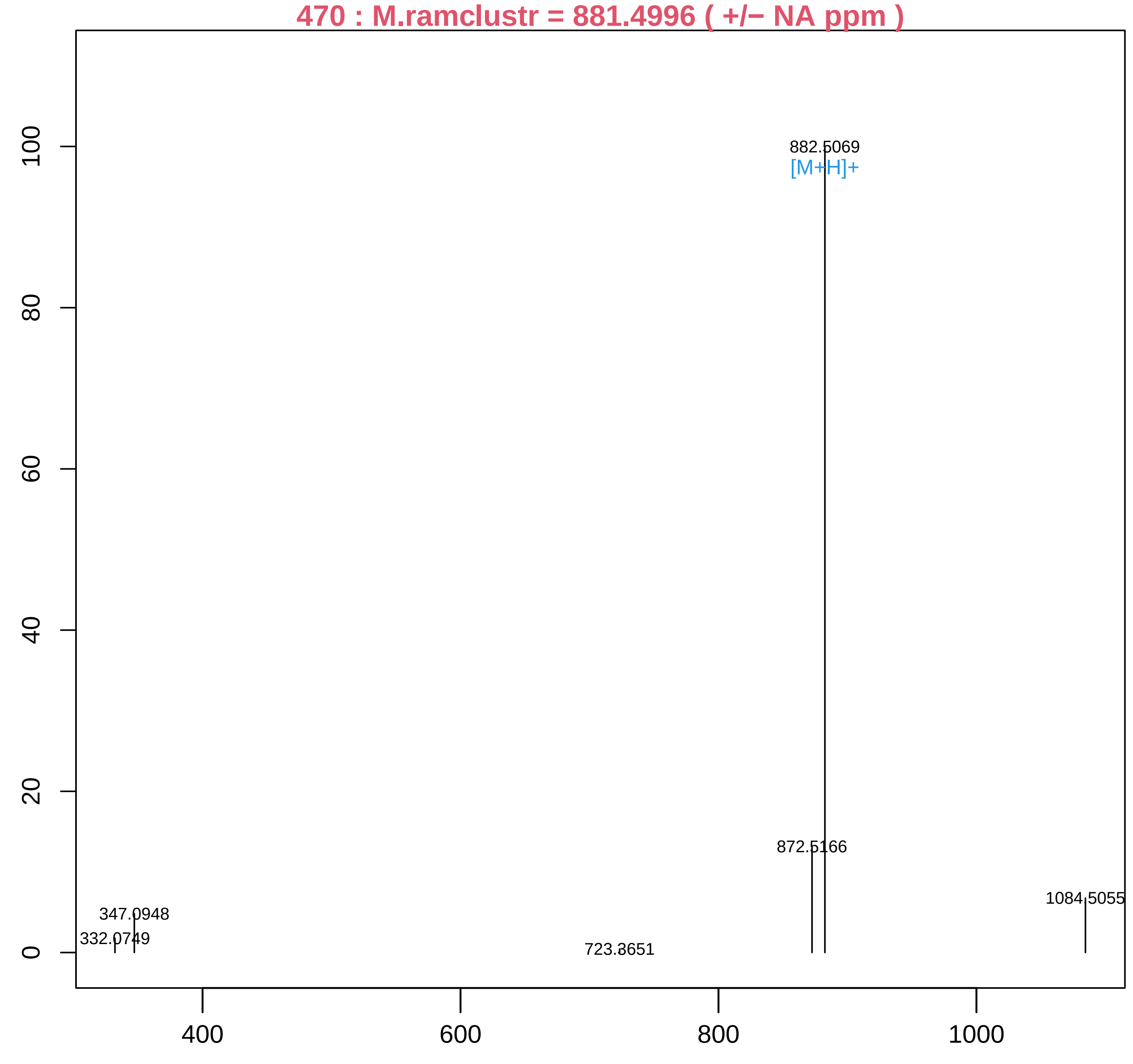
470 : M.ramclustr = 881.4996 (+/− NA ppm)
882.5069
[M+H]+
872.5166
1084.5055
347.0948
332.0749
723.3651
0
20
40
60
80
100
400
600
800
1000

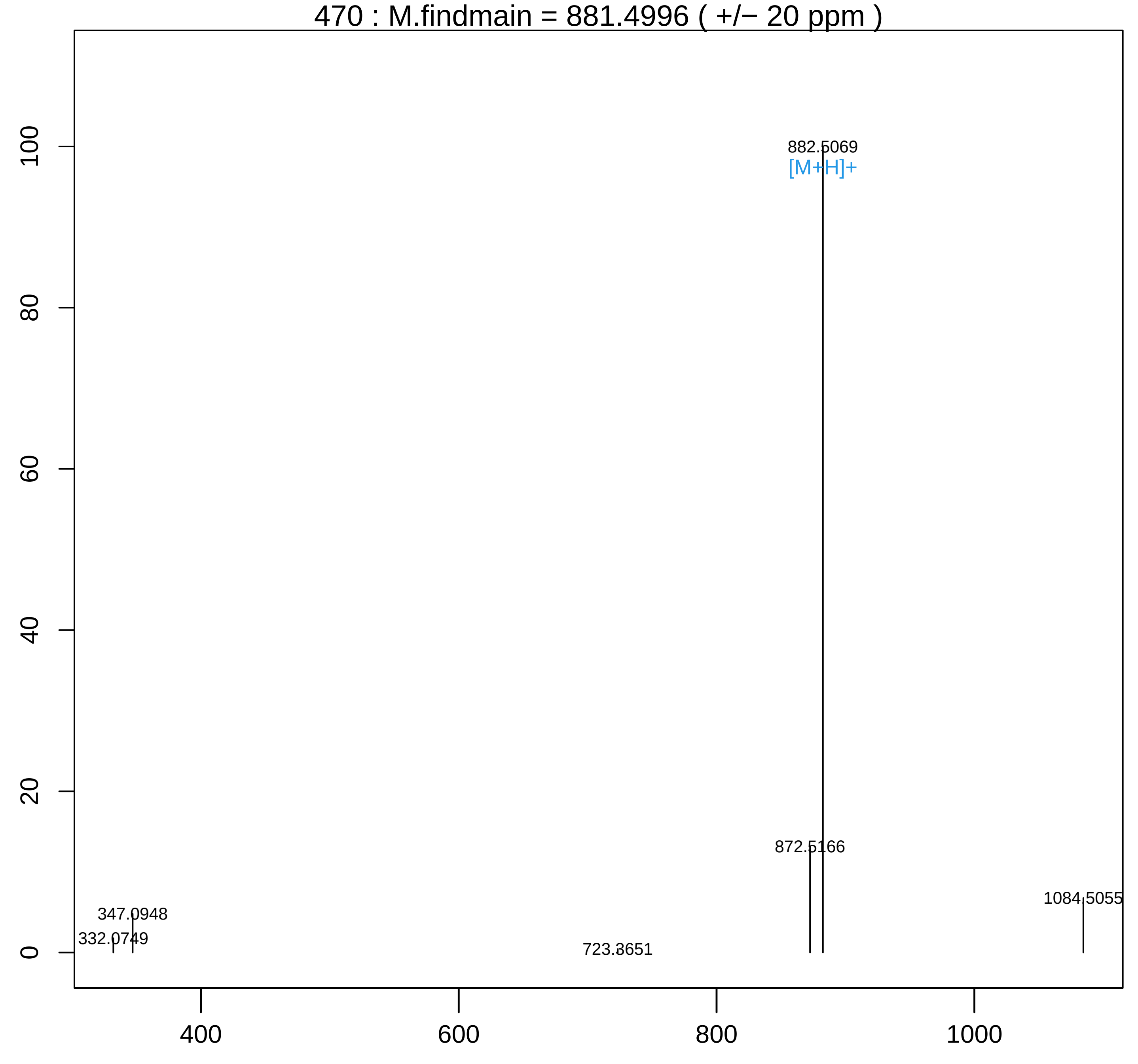
470 : M.findmain = 881.4996 (+/− 20 ppm)
882.5069
[M+H]+
872.5166
1084.5055
347.0948
332.0749
723.3651
0
20
40
60
80
100
400
600
800
1000

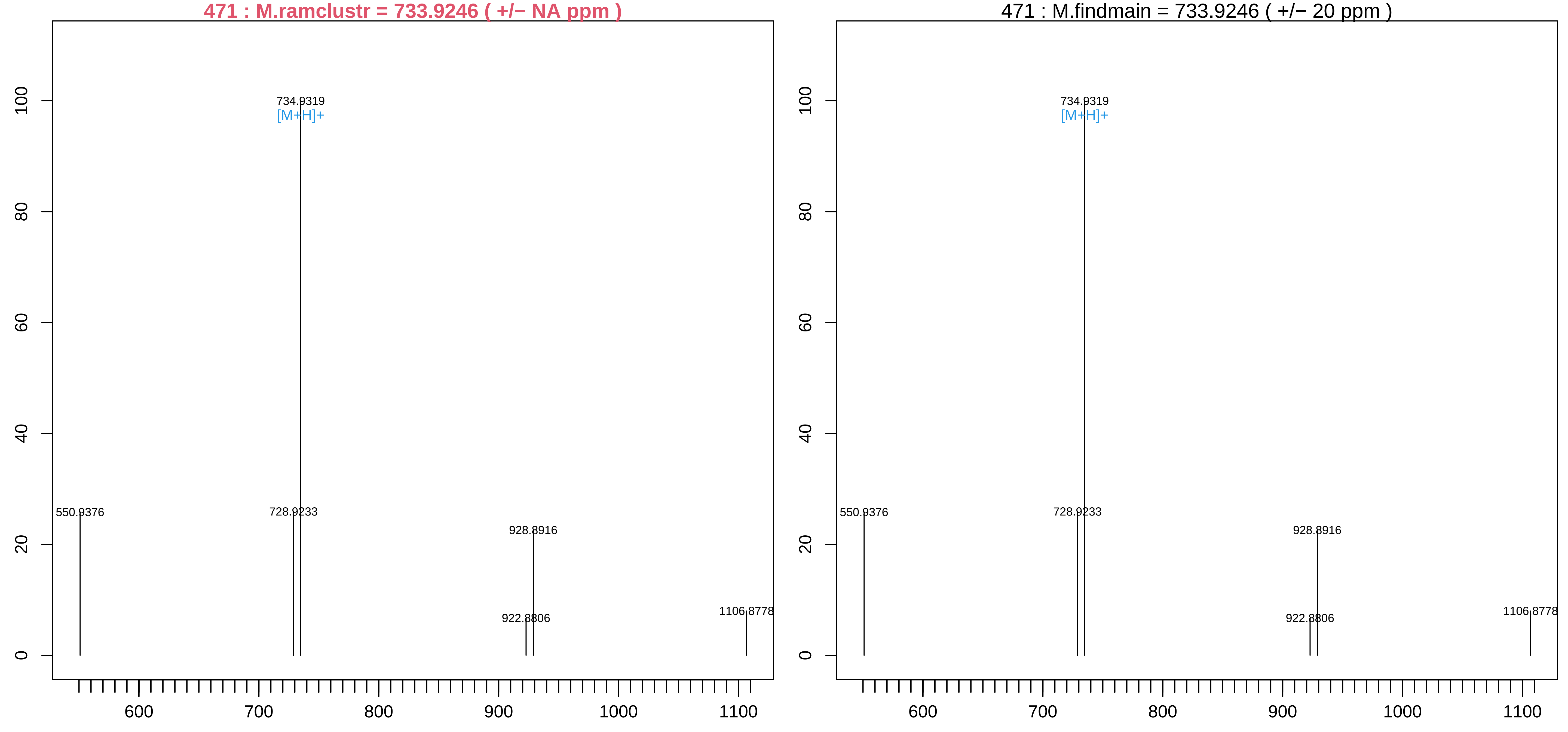
471 : M.ramclustr = 733.9246 (+/− NA ppm)
471 : M.findmain = 733.9246 (+/− 20 ppm)
734.9319
[M+H]+
550.9376
728.9233
928.8916
922.8806
1106.8778
0
20
40
60
80
100
600
700
800
900
1000
1100

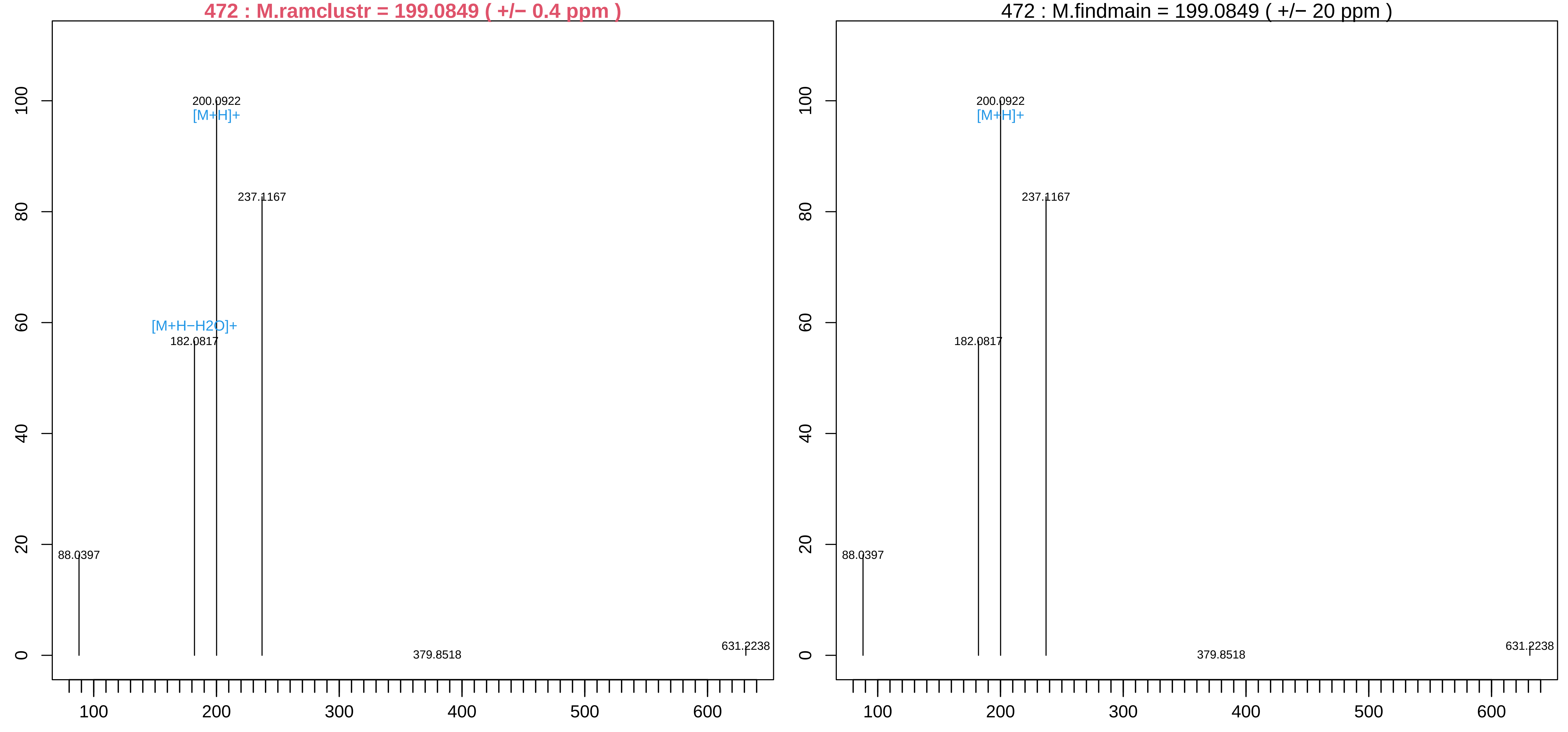
472 : M.ramclustr = 199.0849 (+/− 0.4 ppm)
200.0922
[M+H]+
237.1167
[M+H−H2O]+
182.0817
88.0397
379.8518
631.2238
472 : M.findmain = 199.0849 (+/− 20 ppm)
200.0922
[M+H]+
237.1167
182.0817
88.0397
379.8518
631.2238

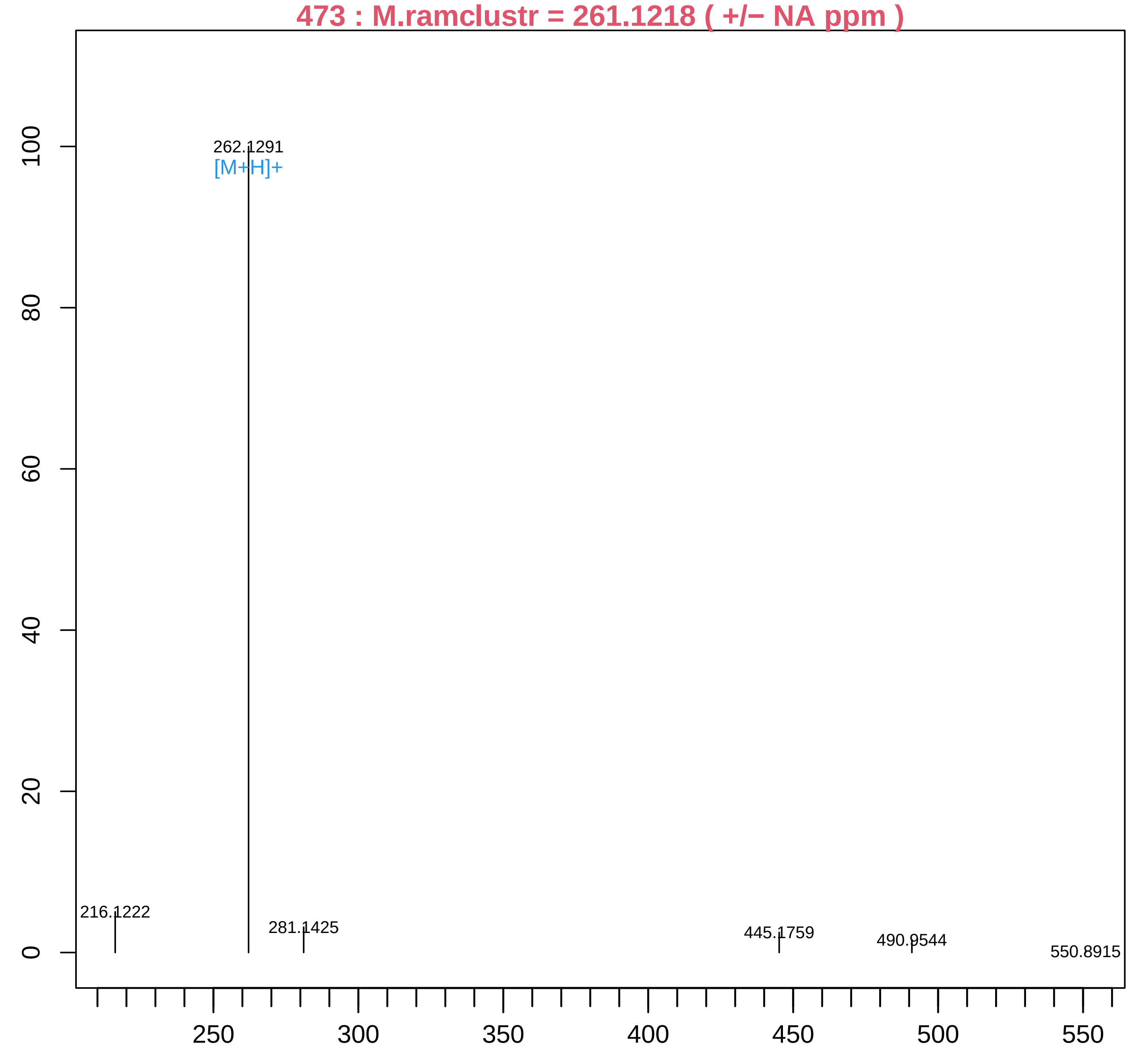
473 : M.ramclustr = 261.1218 (+/− NA ppm)
262.1291
[M+H]+
216.1222
281.1425
445.1759
490.9544
550.8915
0
20
40
60
80
100
250
300
350
400
450
500
550

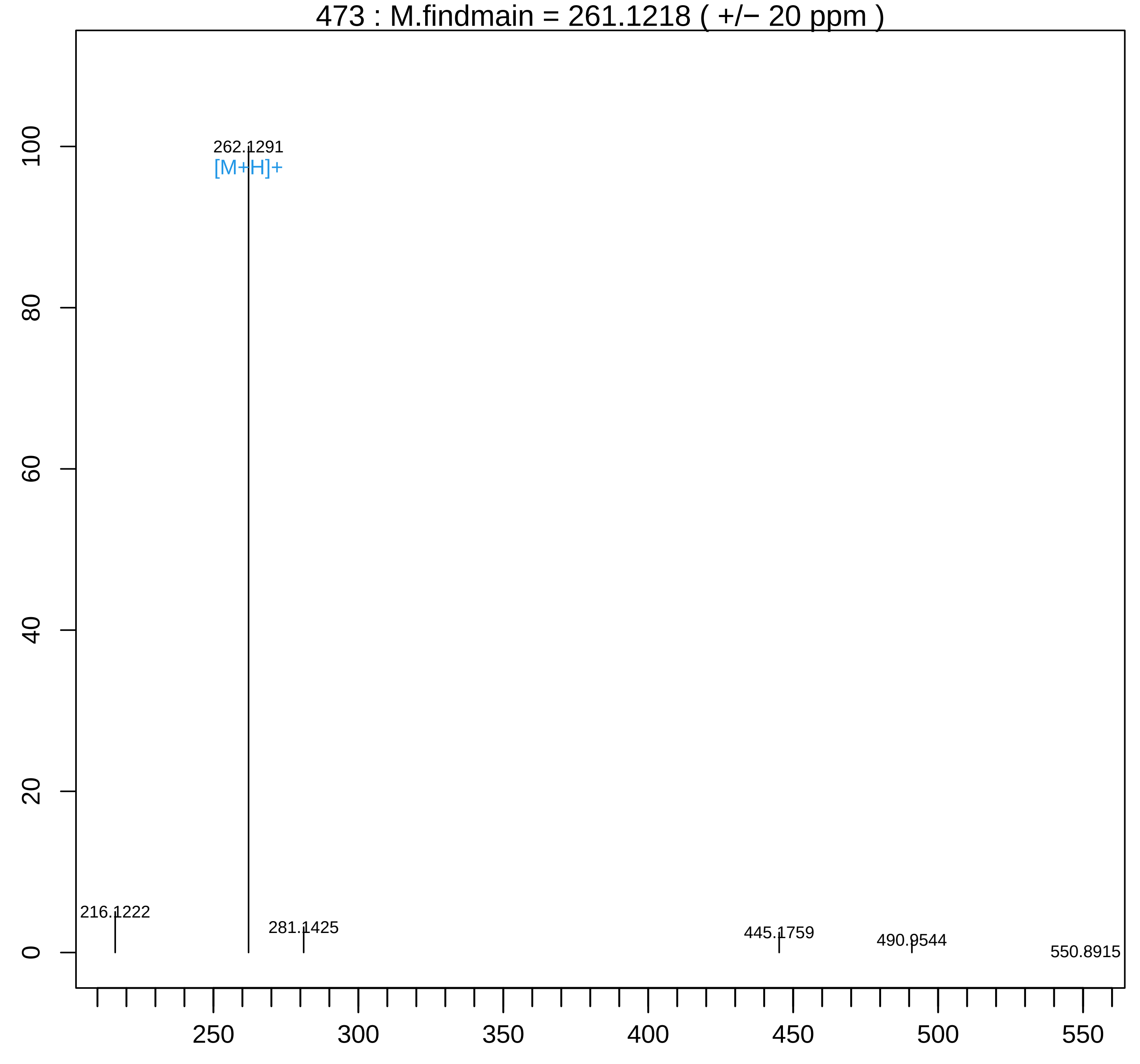
473 : M.findmain = 261.1218 (+/− 20 ppm)
262.1291
[M+H]+
216.1222
281.1425
445.1759
490.9544
550.8915
0
20
40
60
80
100
250
300
350
400
450
500
550

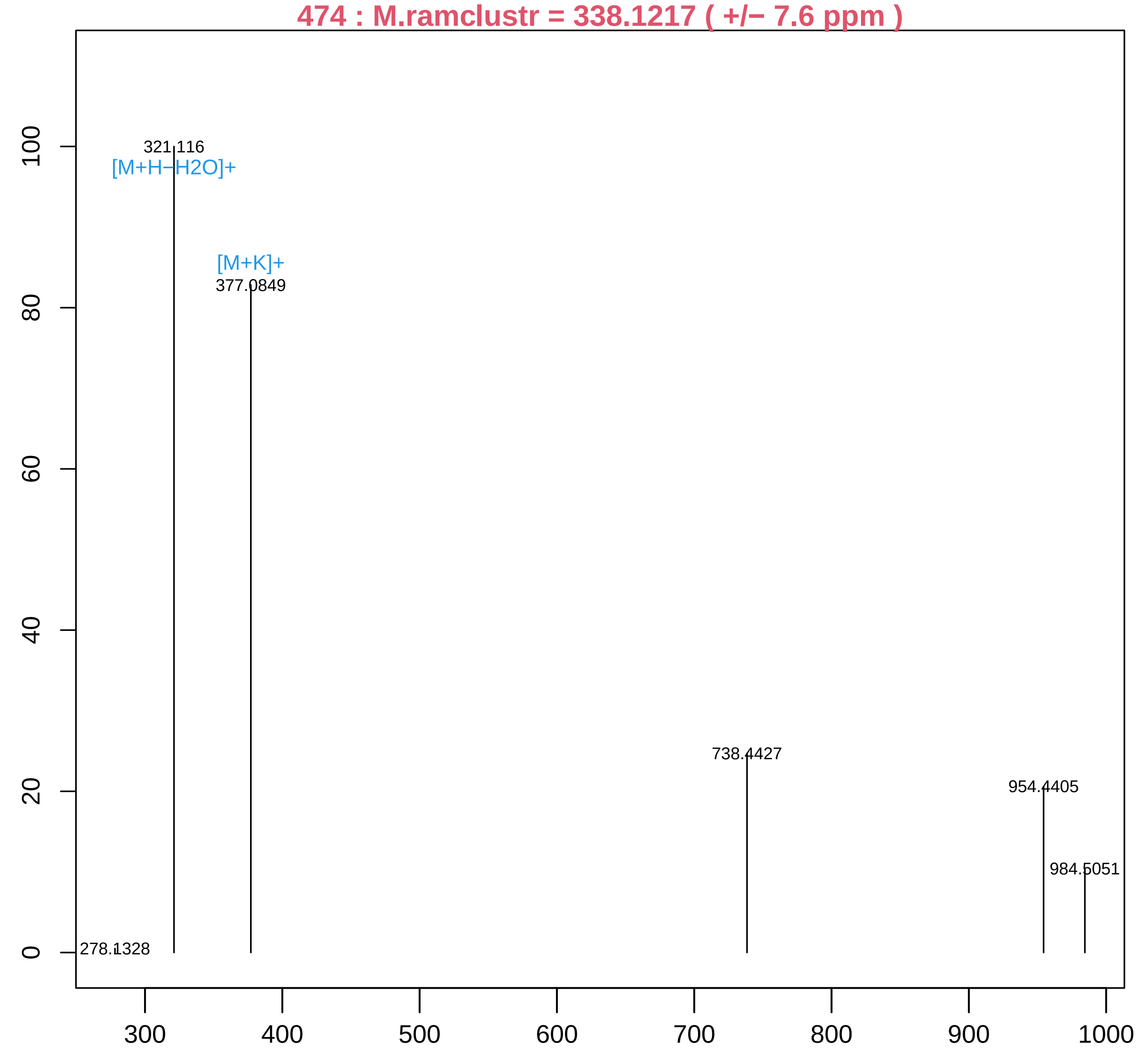
474 : M.ramclustr = 338.1217 (+/− 7.6 ppm)
321.116
[M+H−H2O]+
[M+K]+
377.0849
738.4427
954.4405
984.5051
278.1328
0
20
40
60
80
100
300
400
500
600
700
800
900
1000

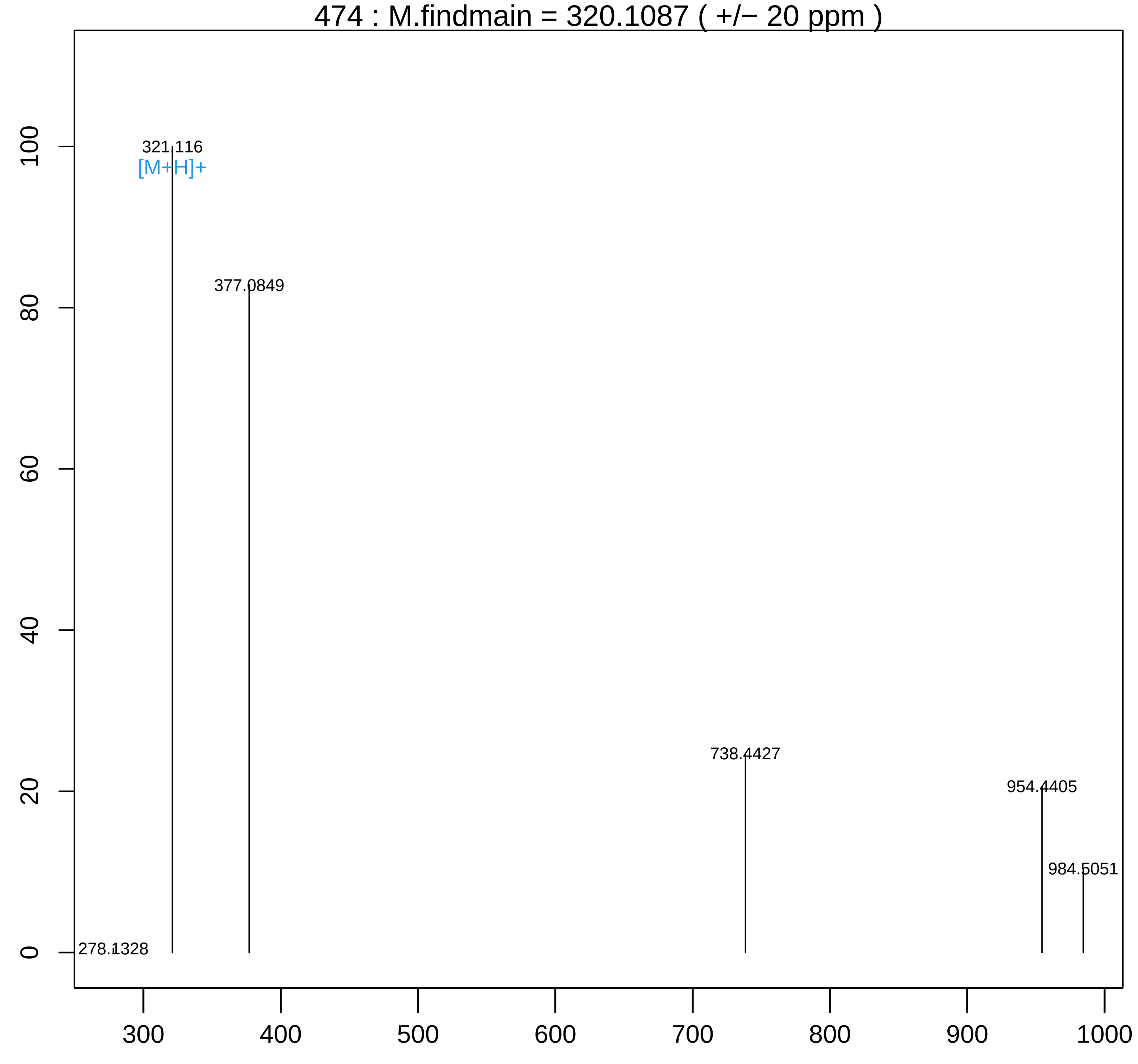
474 : M.findmain = 320.1087 (+/− 20 ppm)
321.116
[M+H]+
377.0849
738.4427
954.4405
984.5051
278.1328
0
20
40
60
80
100
300
400
500
600
700
800
900
1000

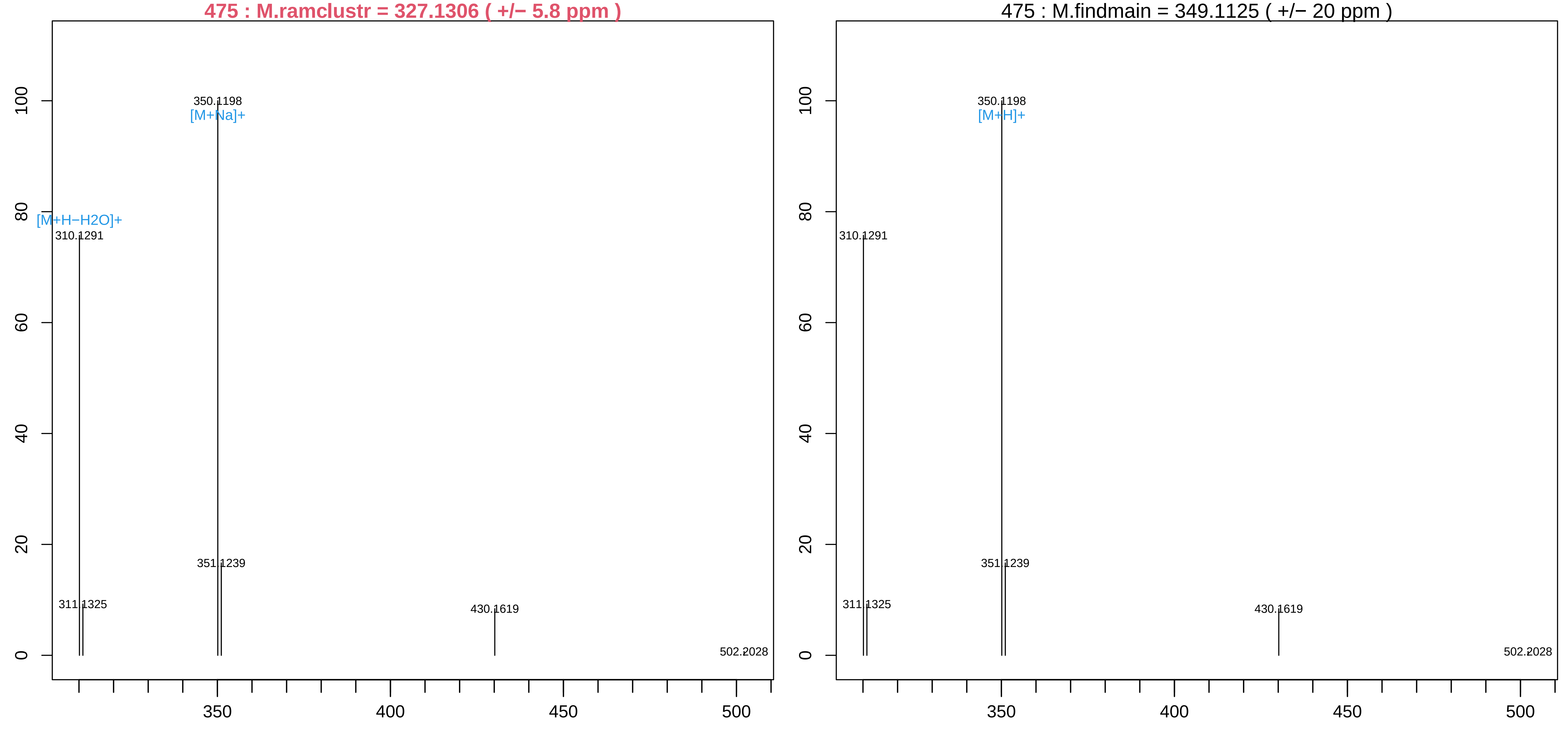
475 : M.ramclustr = 327.1306 (+/− 5.8 ppm)
350.1198
[M+Na]+
[M+H−H2O]+
310.1291
351.1239
311.1325
430.1619
502.2028
0
20
40
60
80
100
350
400
450
500
475 : M.findmain = 349.1125 (+/− 20 ppm)
350.1198
[M+H]+
310.1291
351.1239
311.1325
430.1619
502.2028

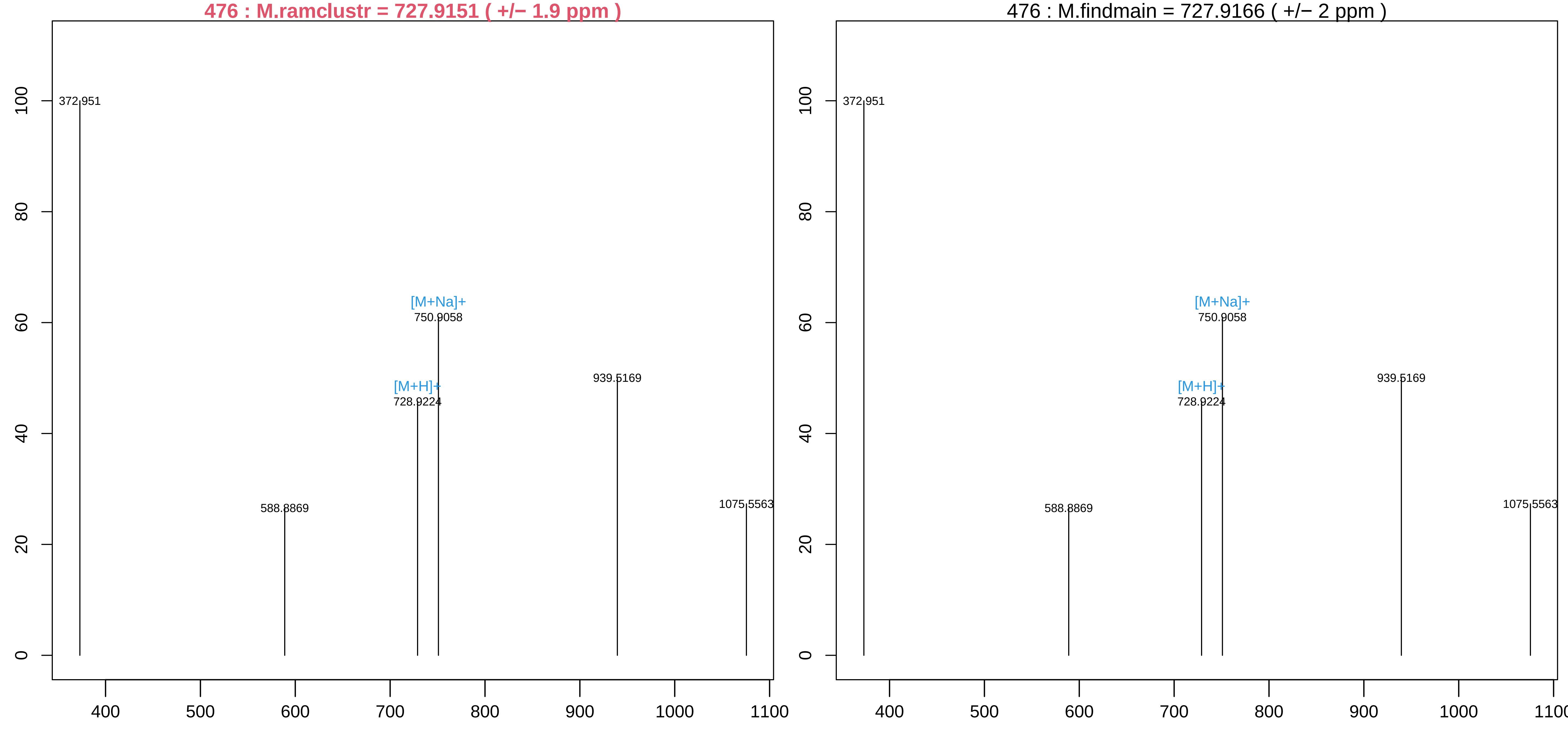
476 : M.ramclustr = 727.9151 (+/− 1.9 ppm)
476 : M.findmain = 727.9166 (+/− 2 ppm)
372.951
588.8869
[M+H]+
728.9224
[M+Na]+
750.9058
939.5169
1075.5563
0
20
40
60
80
100
400
500
600
700
800
900
1000
1100

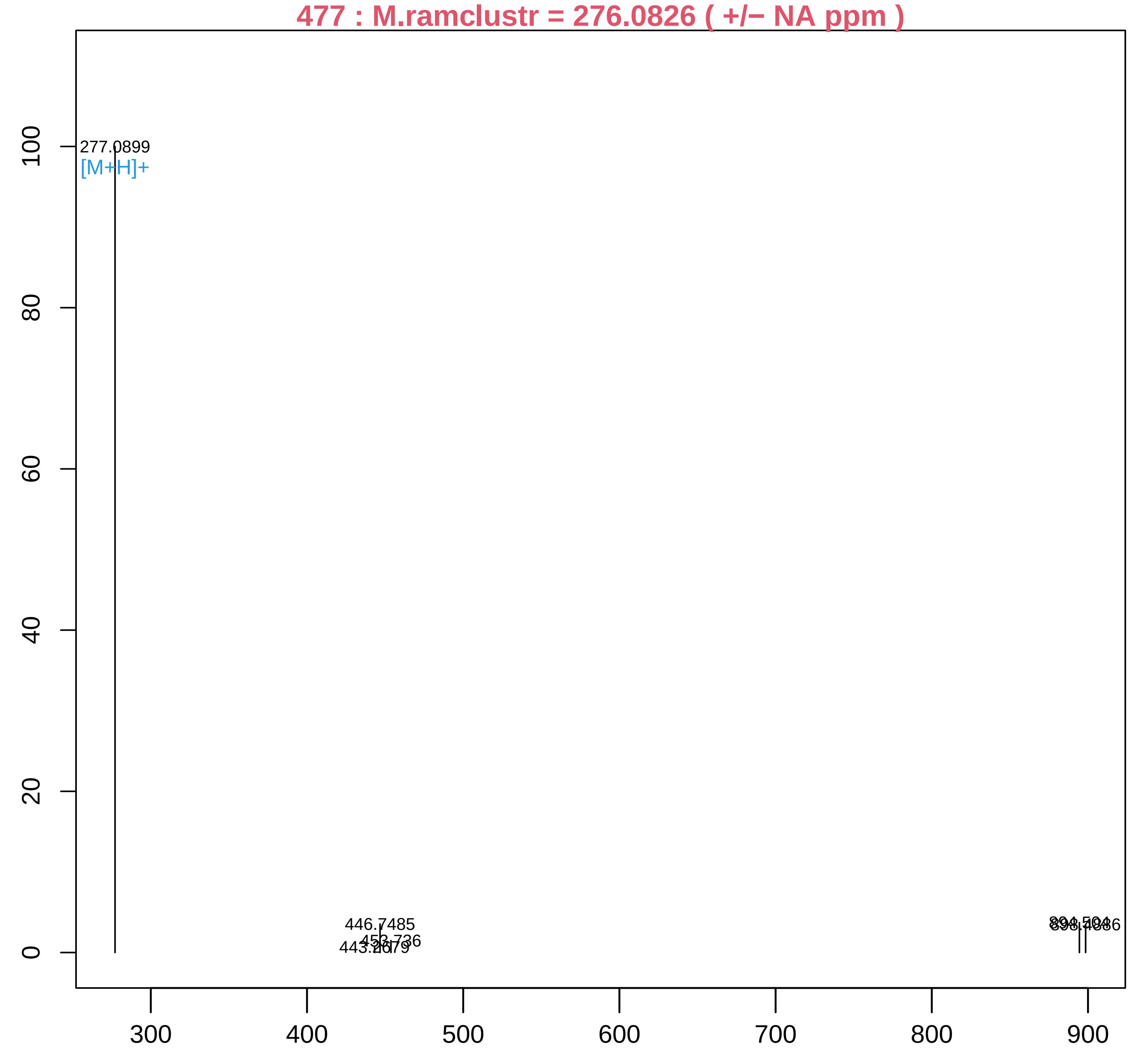
477 : M.ramclustr = 276.0826 (+/− NA ppm)
277.0899
[M+H]+
446.7485
0
20
40
60
80
100
300
400
500
600
700
800
900

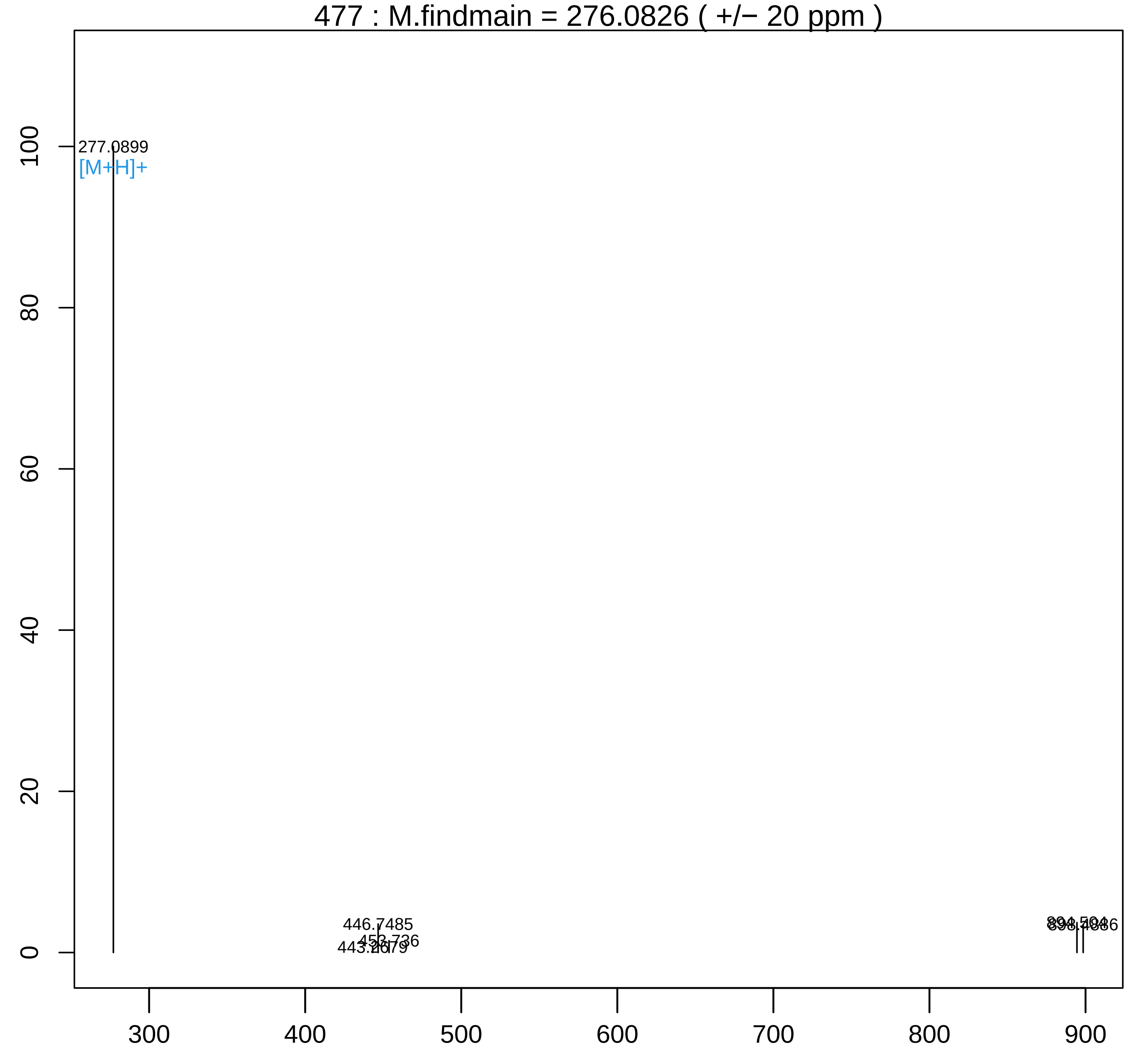
477 : M.findmain = 276.0826 (+/− 20 ppm)
277.0899
[M+H]+
446.7485
0
20
40
60
80
100
300
400
500
600
700
800
900

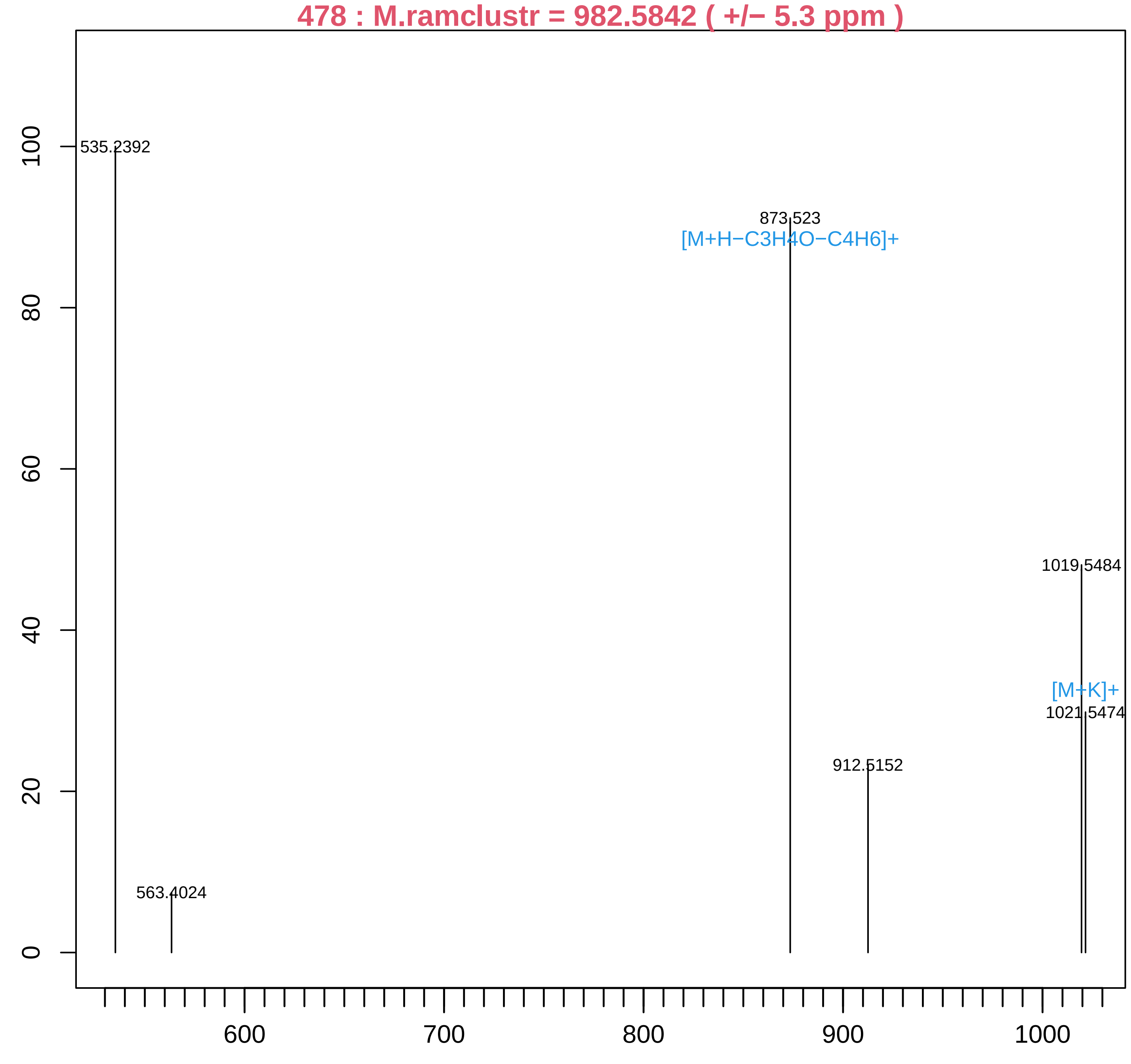
478 : M.ramclustr = 982.5842 (+/− 5.3 ppm)
535.2392
563.4024
873.523
[M+H−C3H4O−C4H6]+
912.5152
1019.5484
[M+K]+
1021.5474
0
20
40
60
80
100
600
700
800
900
1000

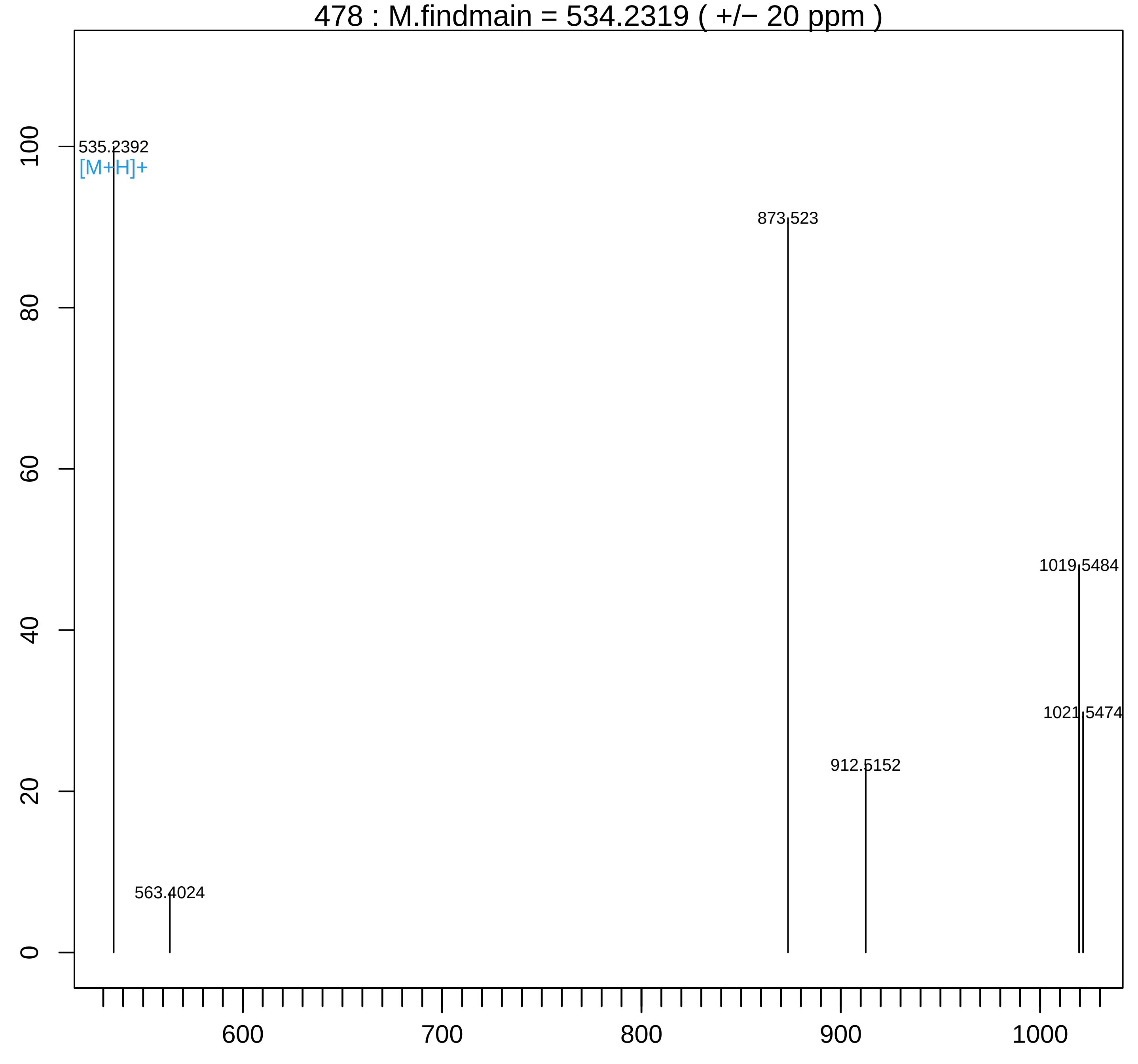
478 : M.findmain = 534.2319 (+/− 20 ppm)
535.2392
[M+H]+
563.4024
873.523
912.5152
1019.5484
1021.5474
0
20
40
60
80
100
600
700
800
900
1000

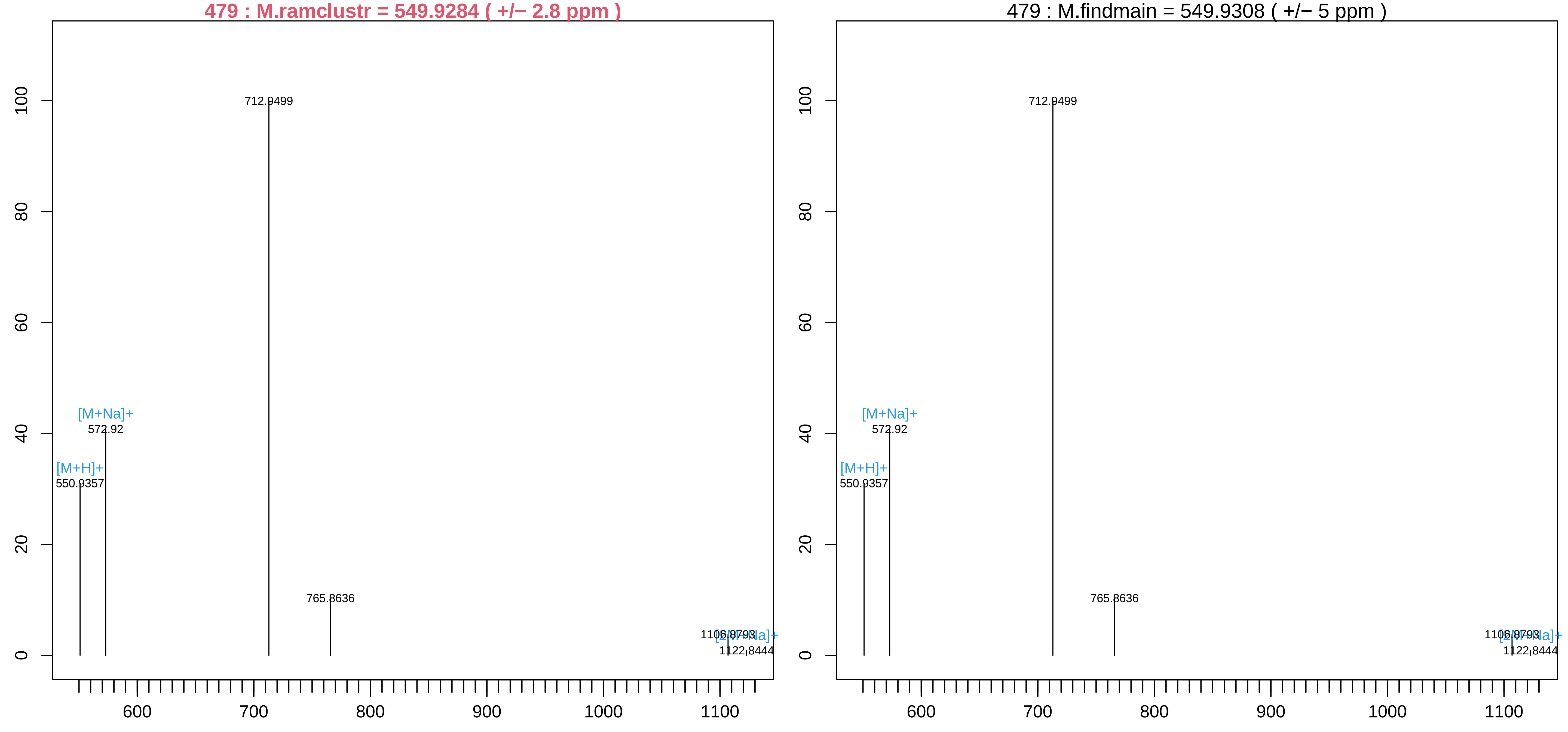
479 : M.ramclustr = 549.9284 (+/− 2.8 ppm)
479 : M.findmain = 549.9308 (+/− 5 ppm)
712.9499
765.8636
[M+Na]+
572.92
[M+H]+
550.9357
1106.8793
[2M+Na]+
1122.8444
0
20
40
60
80
100
600
700
800
900
1000
1100

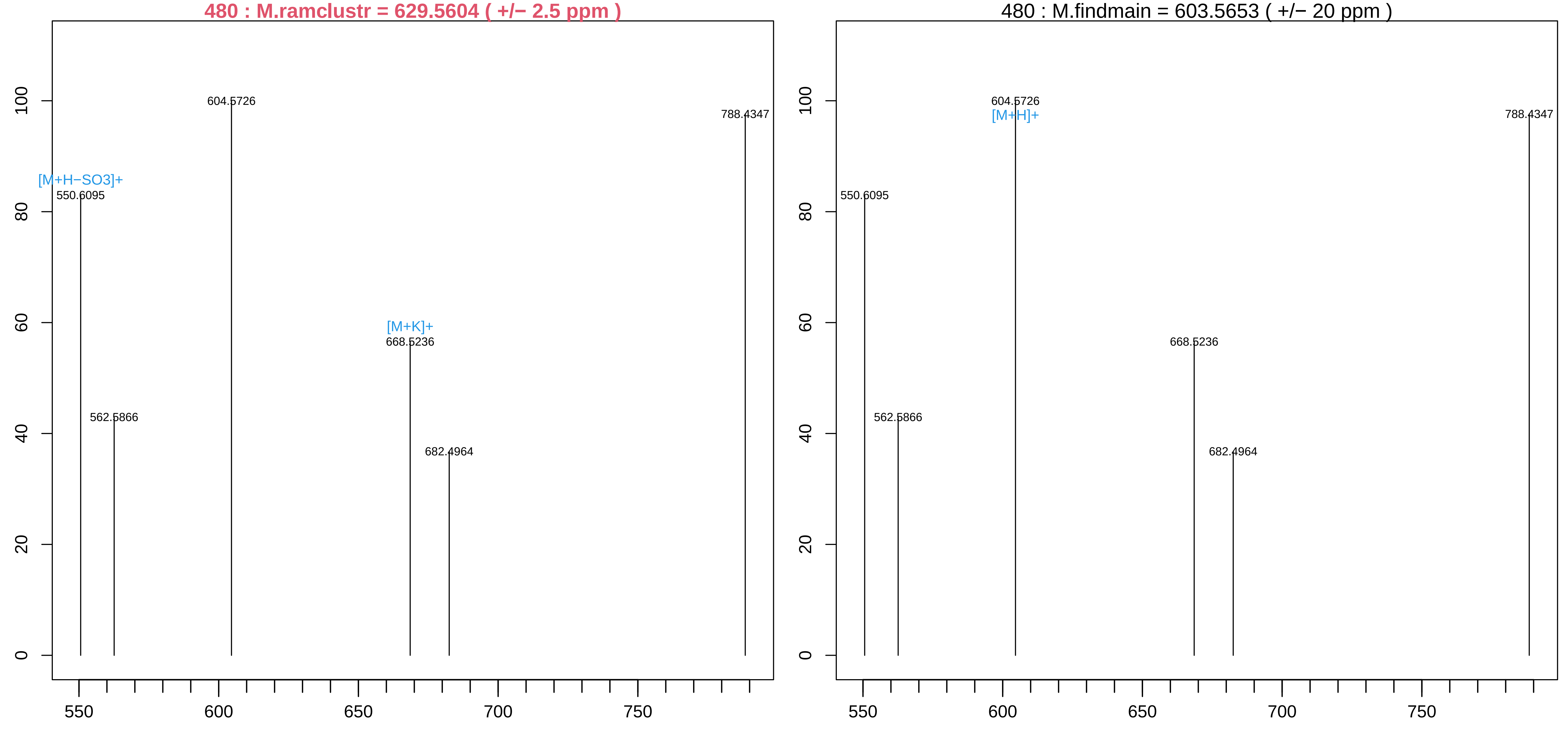

480 : M.ramclustr = 629.5604 (+/− 2.5 ppm)
[M+H−SO3]+
550.6095
562.5866
604.5726
[M+K]+
668.5236
682.4964
788.4347
0
20
40
60
80
100
550
600
650
700
750
480 : M.findmain = 603.5653 (+/− 20 ppm)
604.5726
[M+H]+
550.6095
562.5866
668.5236
682.4964
788.4347
0
20
40
60
80
100
550
600
650
700
750

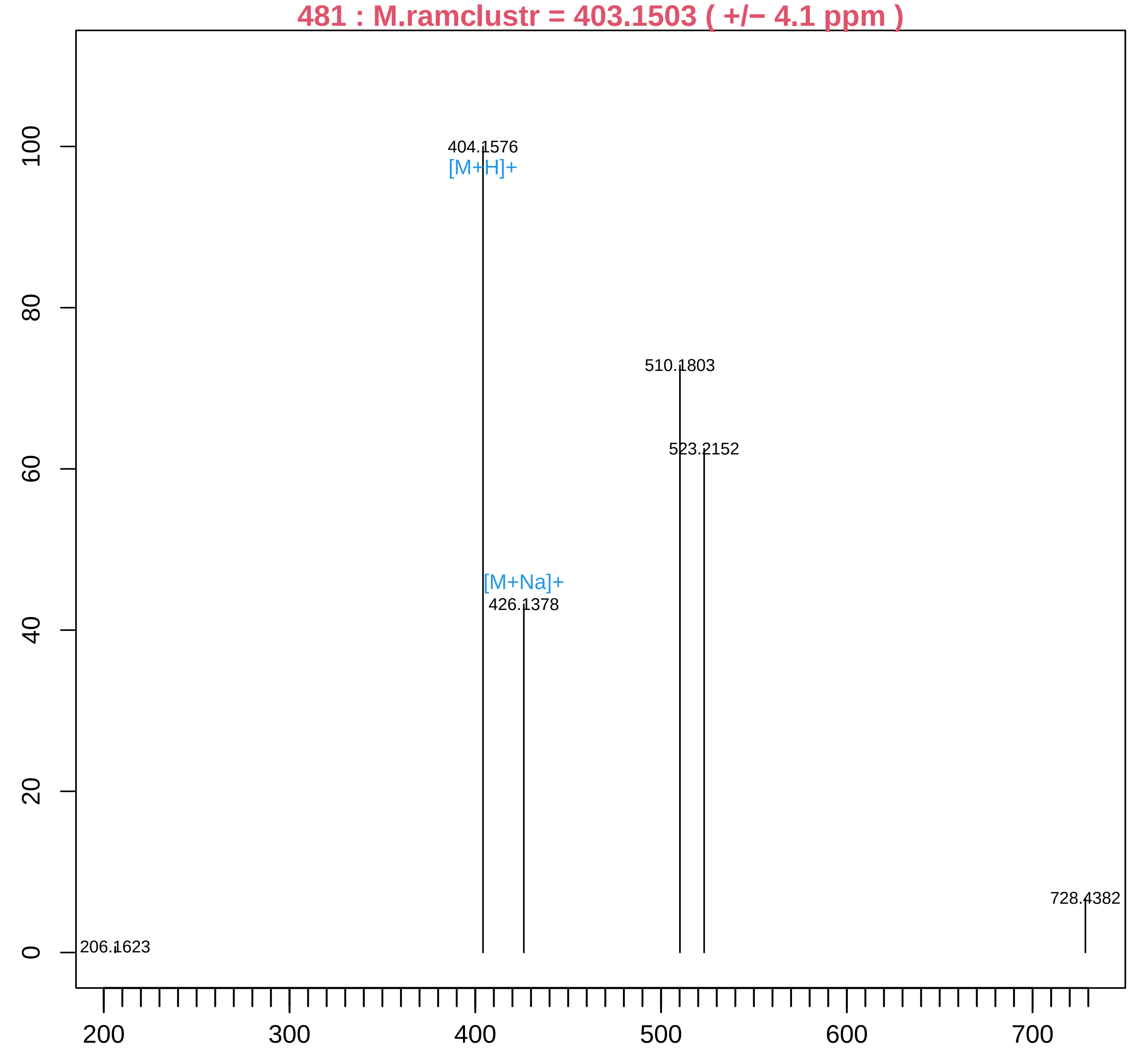
481 : M.ramclustr = 403.1503 (+/− 4.1 ppm)
404.1576
[M+H]+
[M+Na]+
426.1378
510.1803
523.2152
728.4382
206.1623
0
20
40
60
80
100
200
300
400
500
600
700

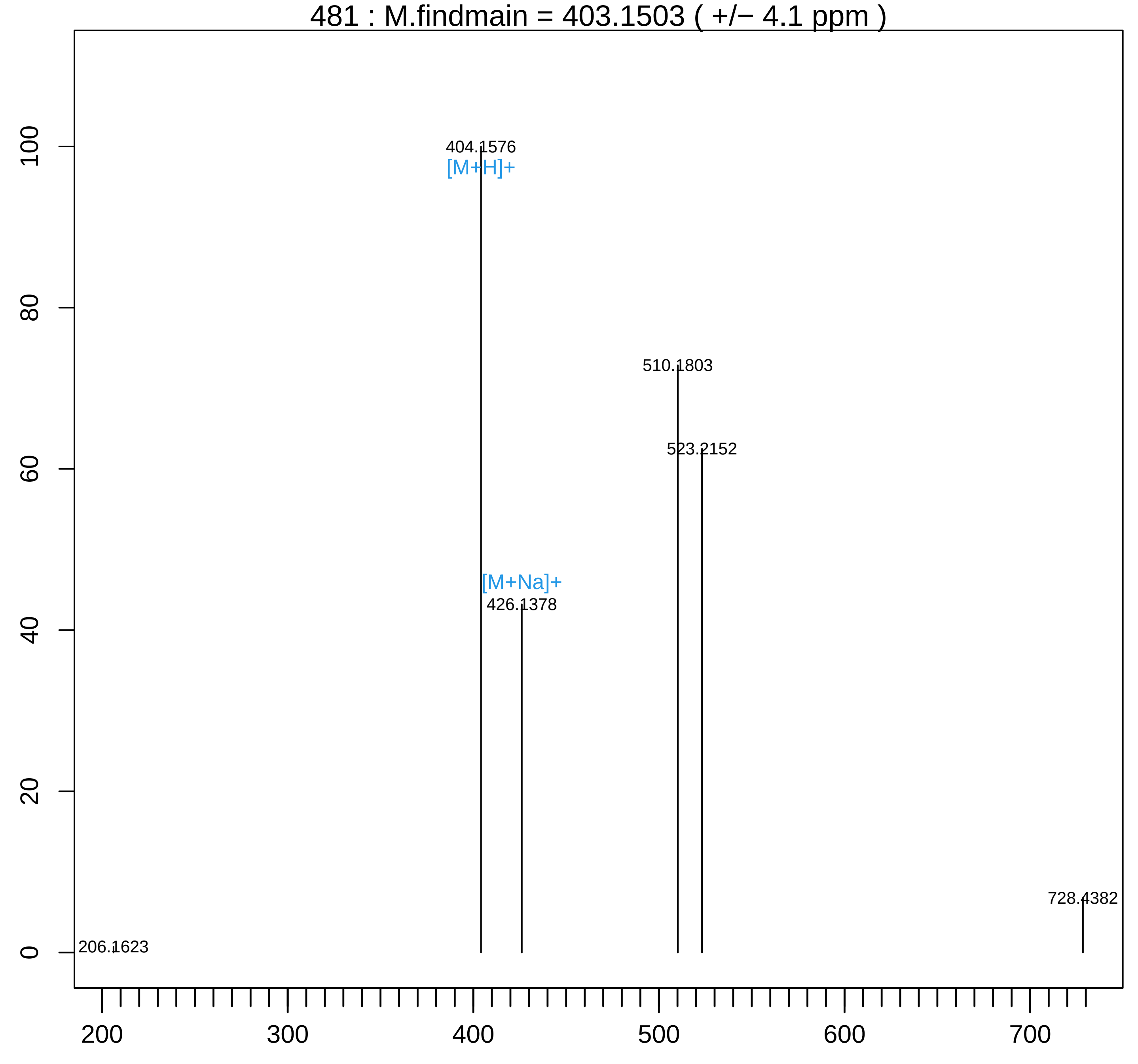
481 : M.findmain = 403.1503 (+/− 4.1 ppm)
404.1576
[M+H]+
[M+Na]+
426.1378
510.1803
523.2152
728.4382
206.1623
0
20
40
60
80
100
200
300
400
500
600
700

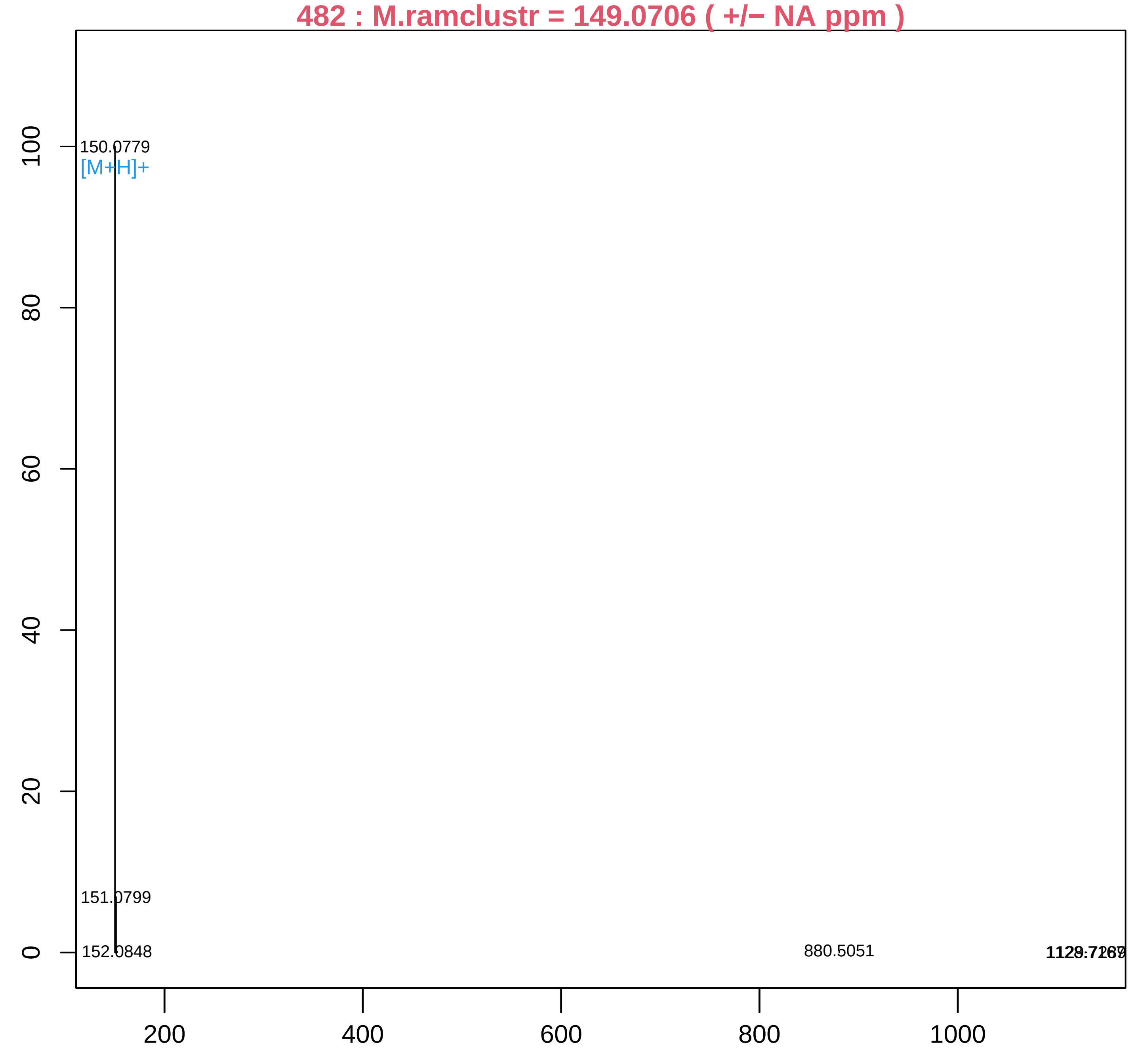
482 : M.ramclustr = 149.0706 (+/− NA ppm)
150.0779
[M+H]+
151.0799
152.0848
880.5051
0
20
40
60
80
100
200
400
600
800
1000

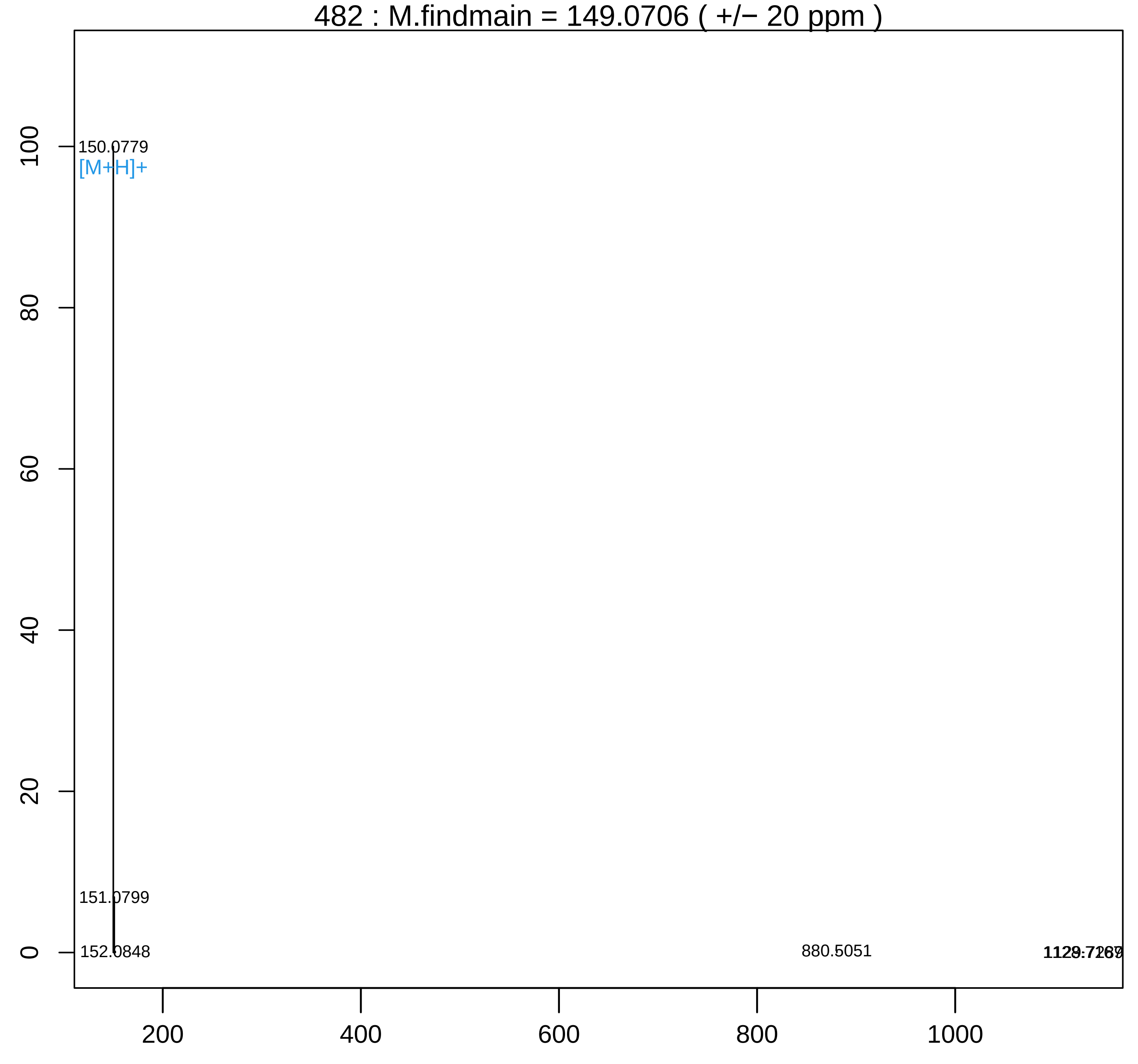
482 : M.findmain = 149.0706 (+/− 20 ppm)
150.0779
[M+H]+
151.0799
152.0848
880.5051
0
20
40
60
80
100
200
400
600
800
1000

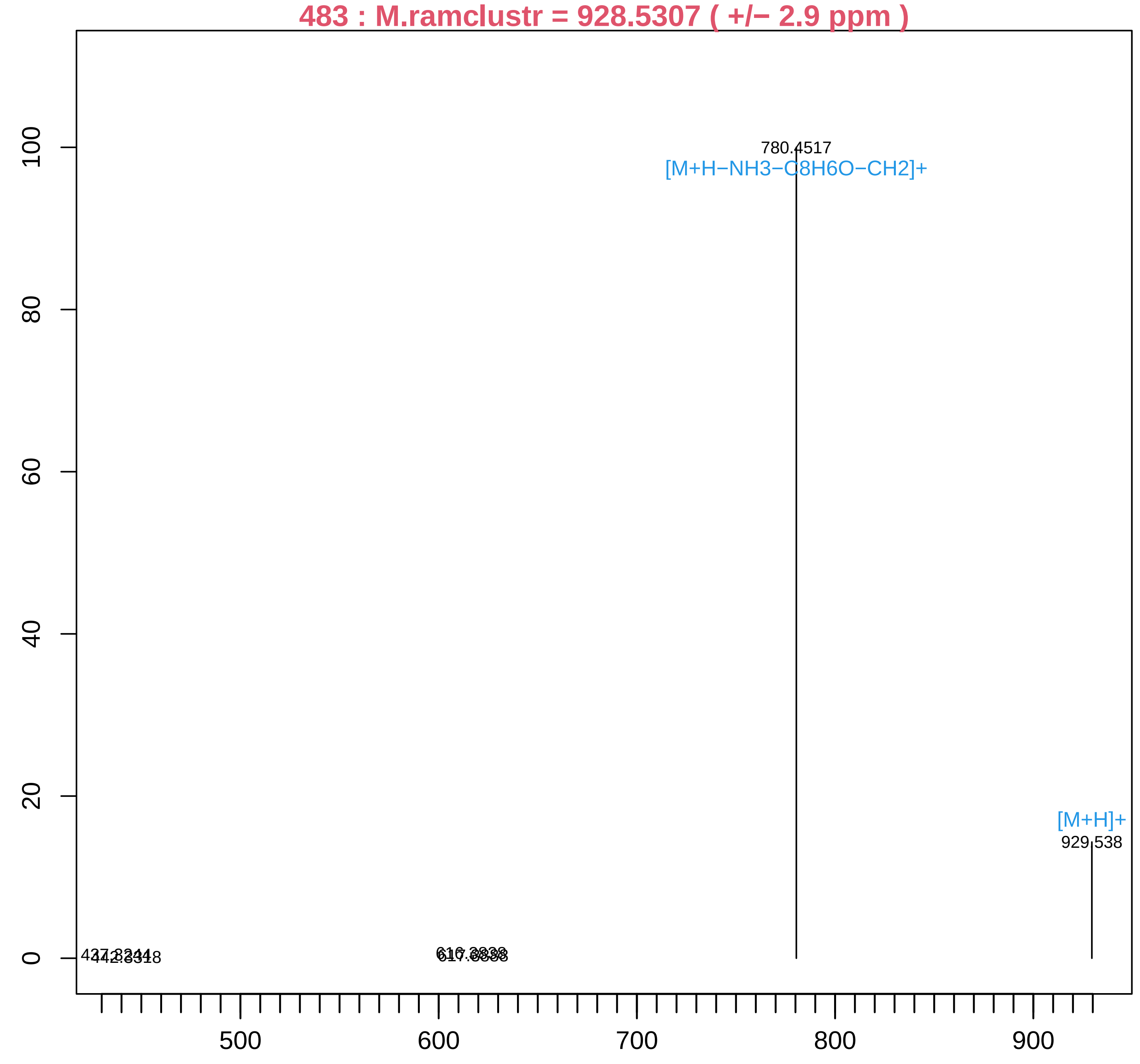
483 : M.ramclustr = 928.5307 (+/− 2.9 ppm)
780.4517
[M+H−NH3−C8H6O−CH2]+
[M+H]+
929.538
0
20
40
60
80
100
500
600
700
800
900

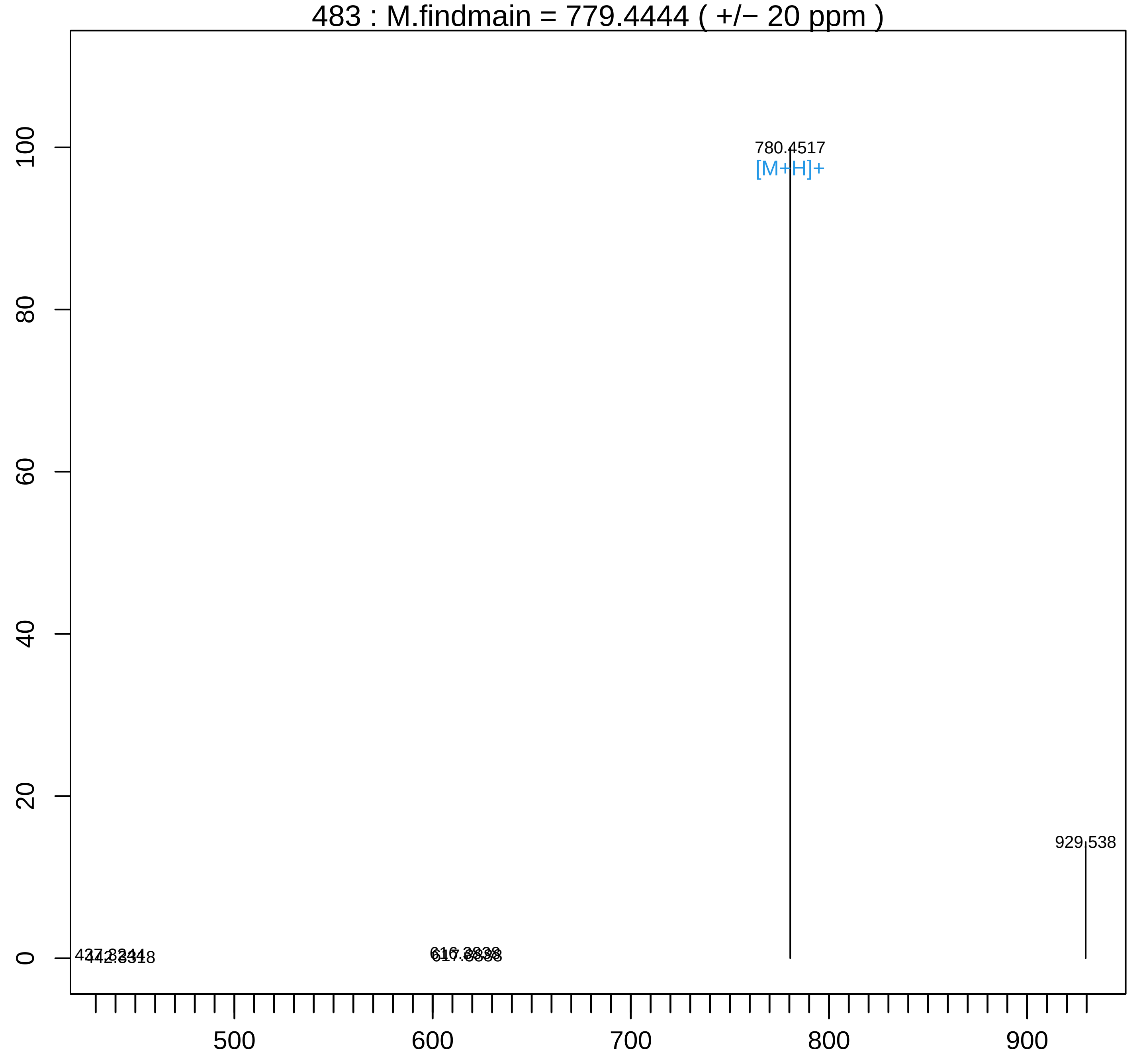
483 : M.findmain = 779.4444 (+/− 20 ppm)
780.4517
[M+H]+
929.538
0
20
40
60
80
100
500
600
700
800
900

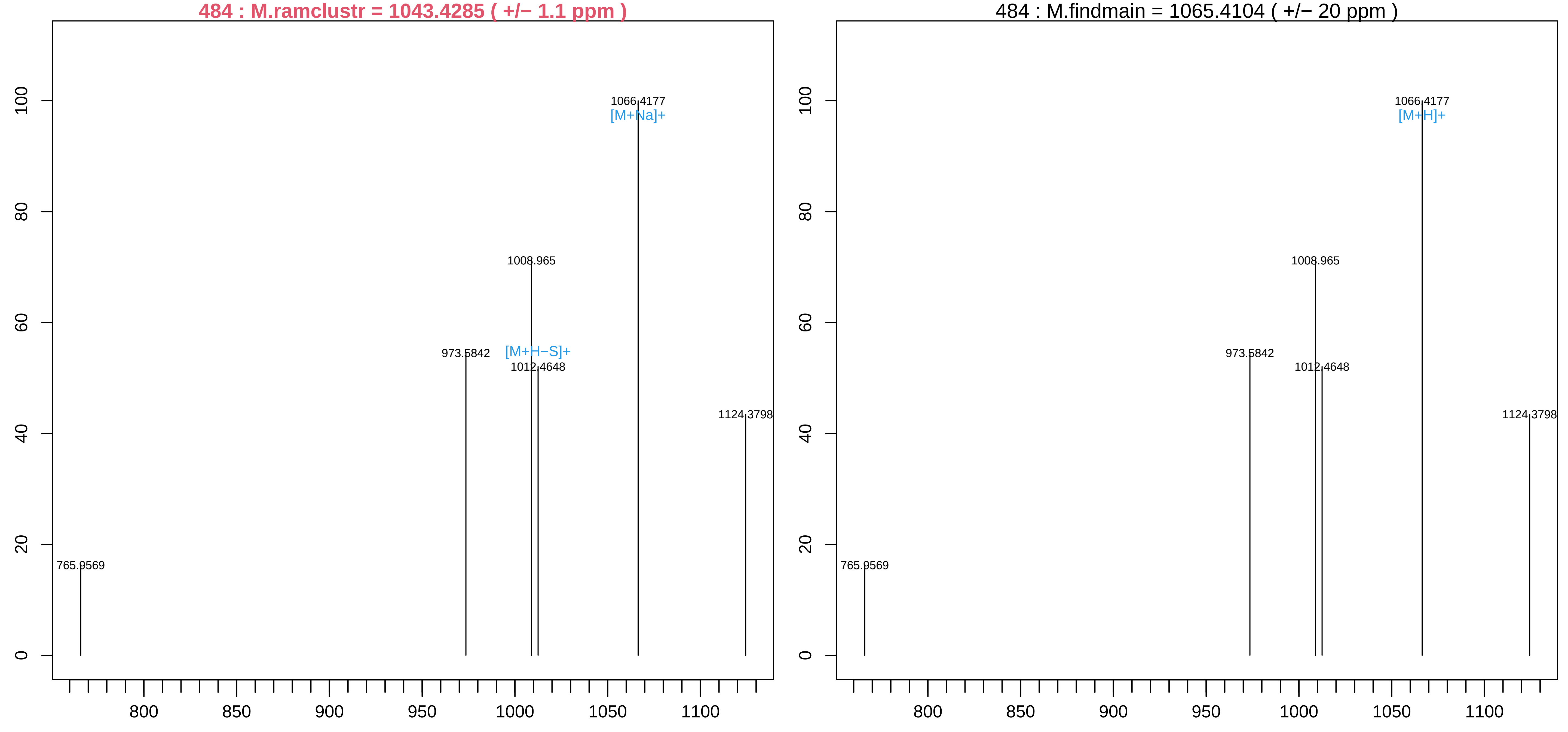
484 : M.ramclustr = 1043.4285 (+/− 1.1 ppm)
765.9569
973.5842
1008.965
[M+H−S]+
1012.4648
1066.4177
[M+Na]+
1124.3798
484 : M.findmain = 1065.4104 (+/− 20 ppm)
765.9569
973.5842
1008.965
1012.4648
1066.4177
[M+H]+
1124.3798
0
20
40
60
80
100
800
850
900
950
1000
1050
1100

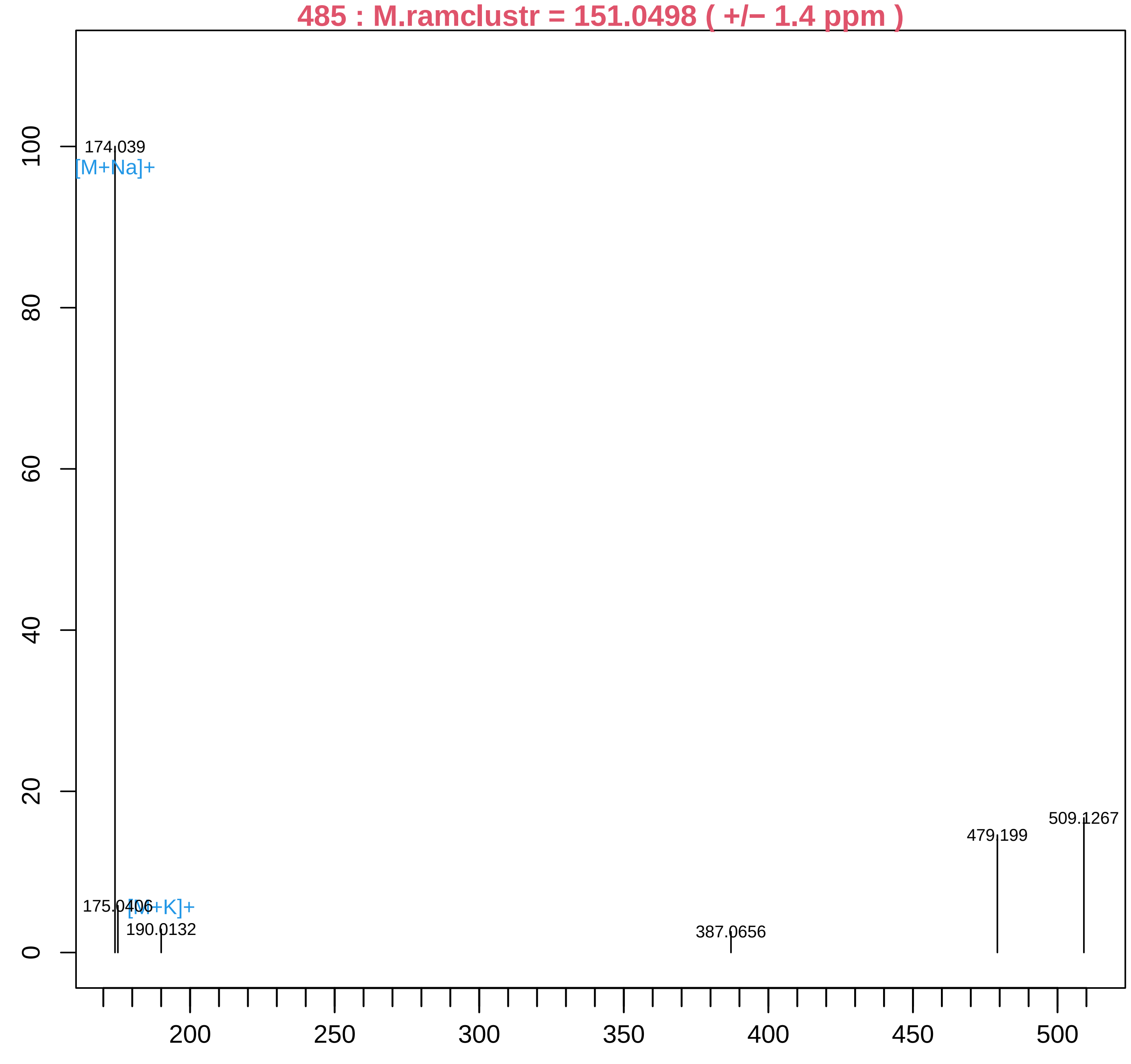
485 : M.ramclustr = 151.0498 (+/− 1.4 ppm)
174.039
[M+Na]+
175.0406
[M+K]+
190.0132
387.0656
479.199
509.1267
0
20
40
60
80
100
200
250
300
350
400
450
500

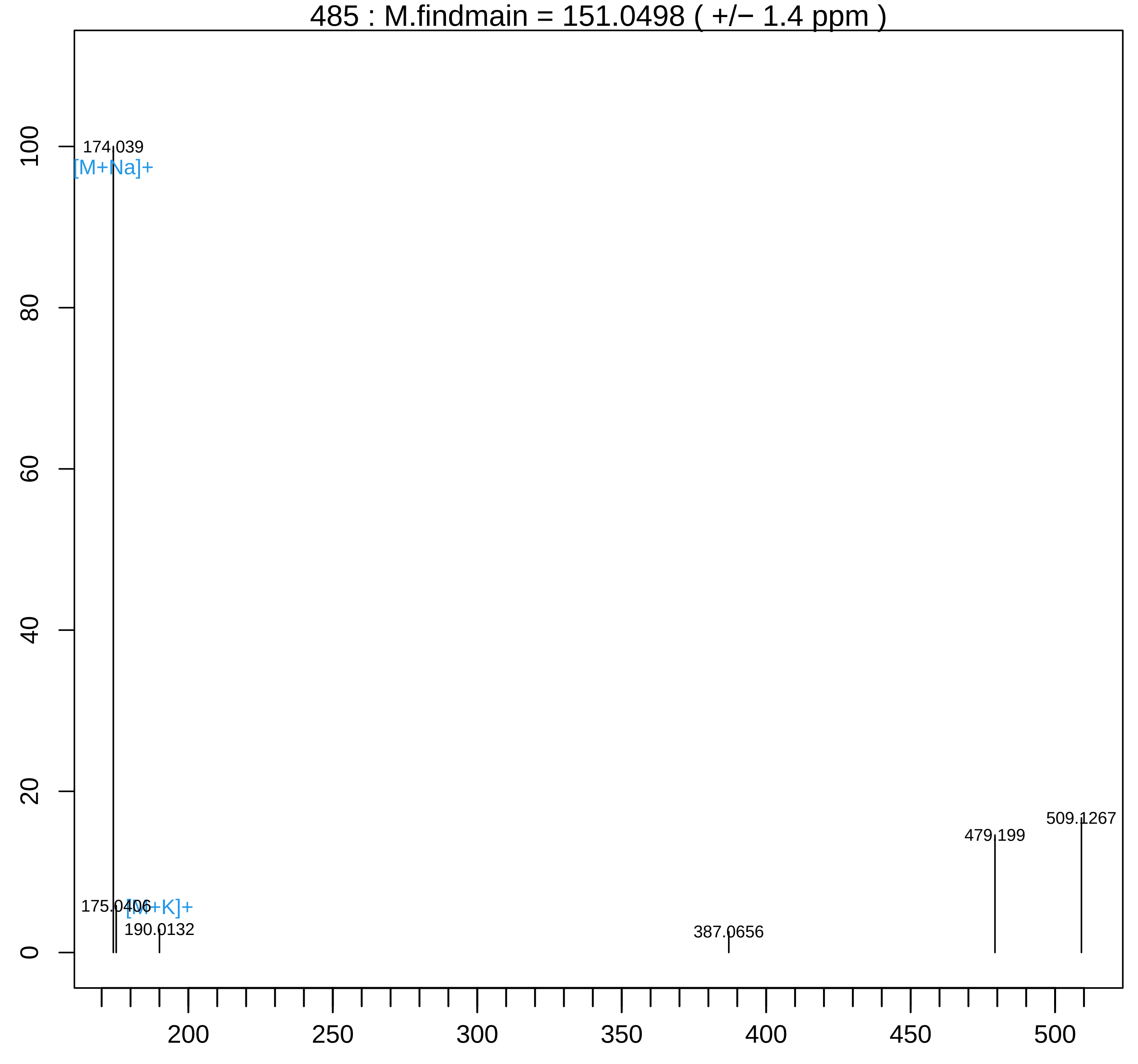
485 : M.findmain = 151.0498 (+/− 1.4 ppm)
174.039
[M+Na]+
175.0406
[M+K]+
190.0132
387.0656
479.199
509.1267
0
20
40
60
80
100
200
250
300
350
400
450
500

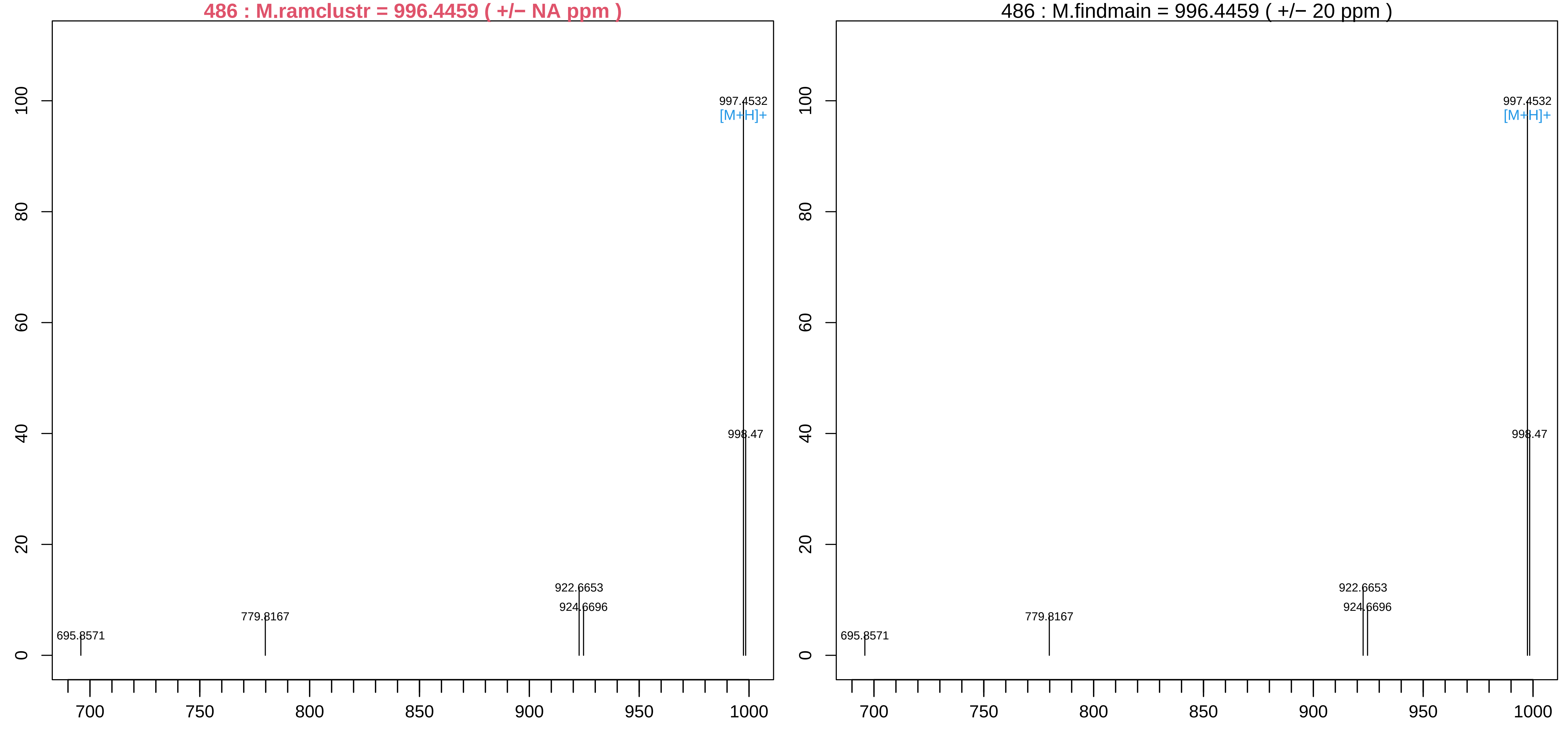
486 : M.ramclustr = 996.4459 (+/− NA ppm)
997.4532
[M+H]+
998.47
922.6653
924.6696
779.8167
695.8571
486 : M.findmain = 996.4459 (+/− 20 ppm)
997.4532
[M+H]+
998.47
922.6653
924.6696
779.8167
695.8571

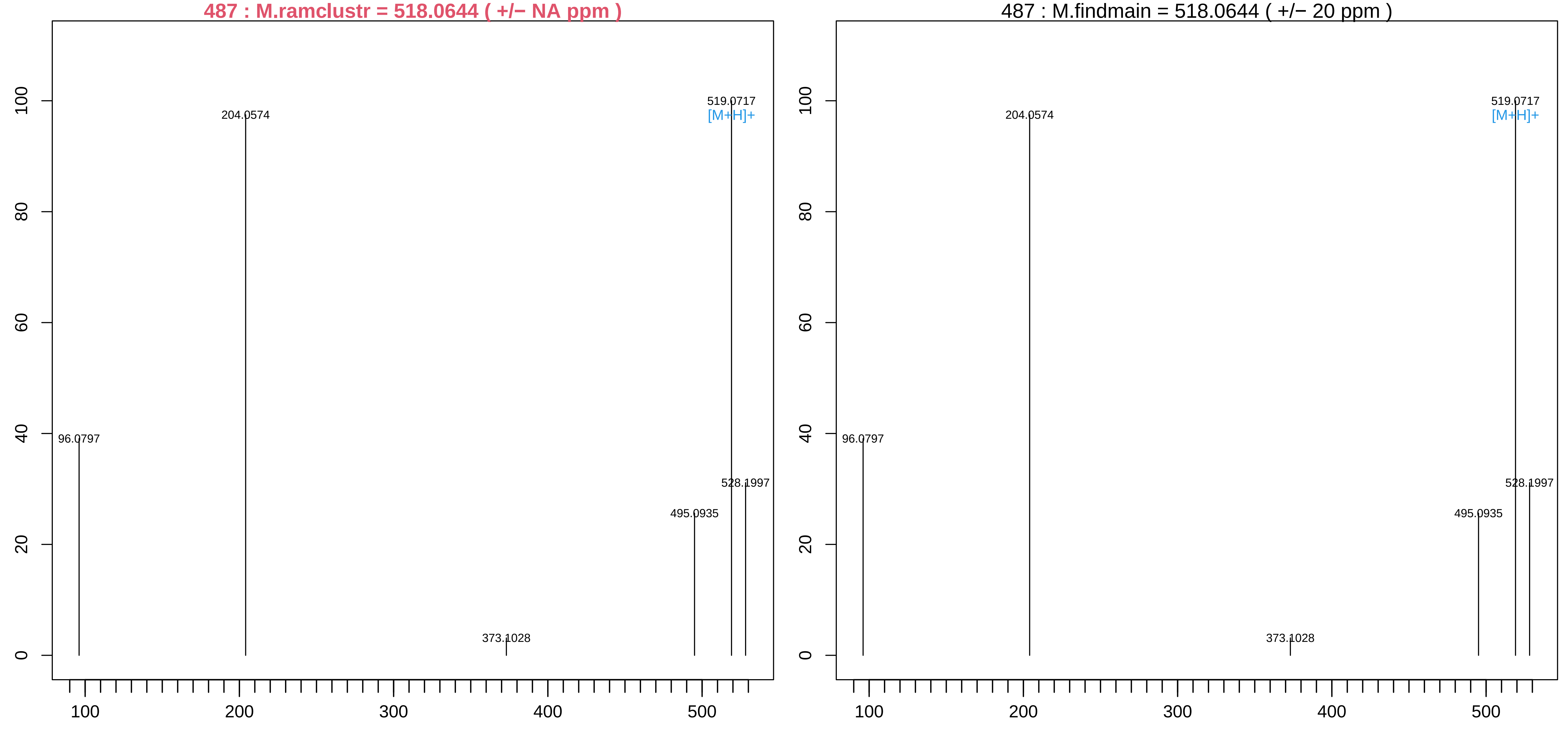
487 : M.ramclustr = 518.0644 (+/− NA ppm)
519.0717
[M+H]+
204.0574
96.0797
528.1997
495.0935
373.1028
0
20
40
60
80
100
100
200
300
400
500
487 : M.findmain = 518.0644 (+/− 20 ppm)
519.0717
[M+H]+
204.0574
96.0797
528.1997
495.0935
373.1028
0
20
40
60
80
100
100
200
300
400
500

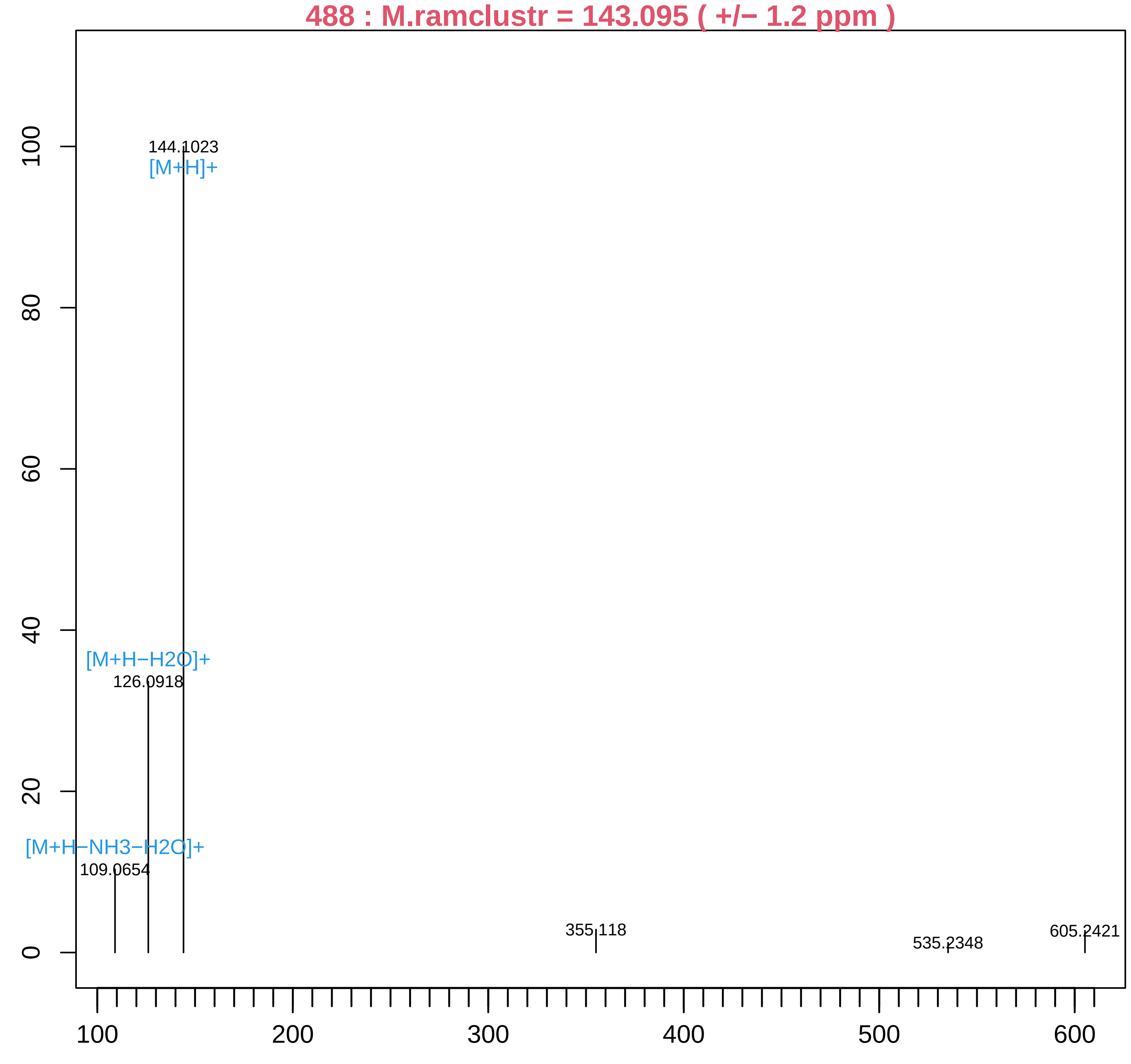

488 : M.ramclustr = 143.095 (+/− 1.2 ppm)
144.1023
[M+H]+
[M+H−H2O]+
126.0918
[M+H−NH3−H2O]+
109.0654
355.118
535.2348
605.2421
0
20
40
60
80
100
100
200
300
400
500
600

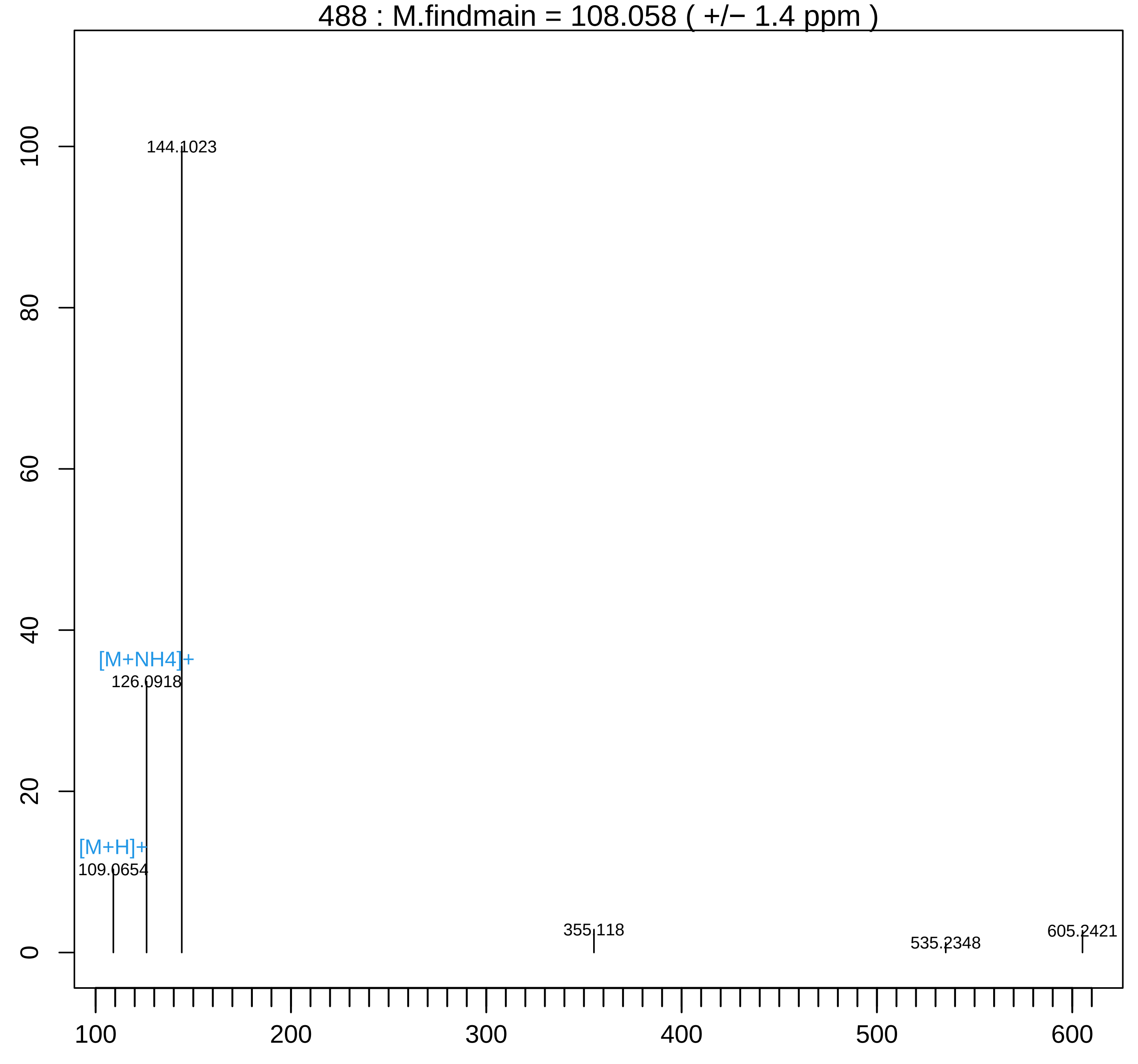

488 : M.findmain = 108.058 (+/− 1.4 ppm)
144.1023
[M+NH4]+
126.0918
[M+H]+
109.0654
355.118
535.2348
605.2421
0
20
40
60
80
100
100
200
300
400
500
600

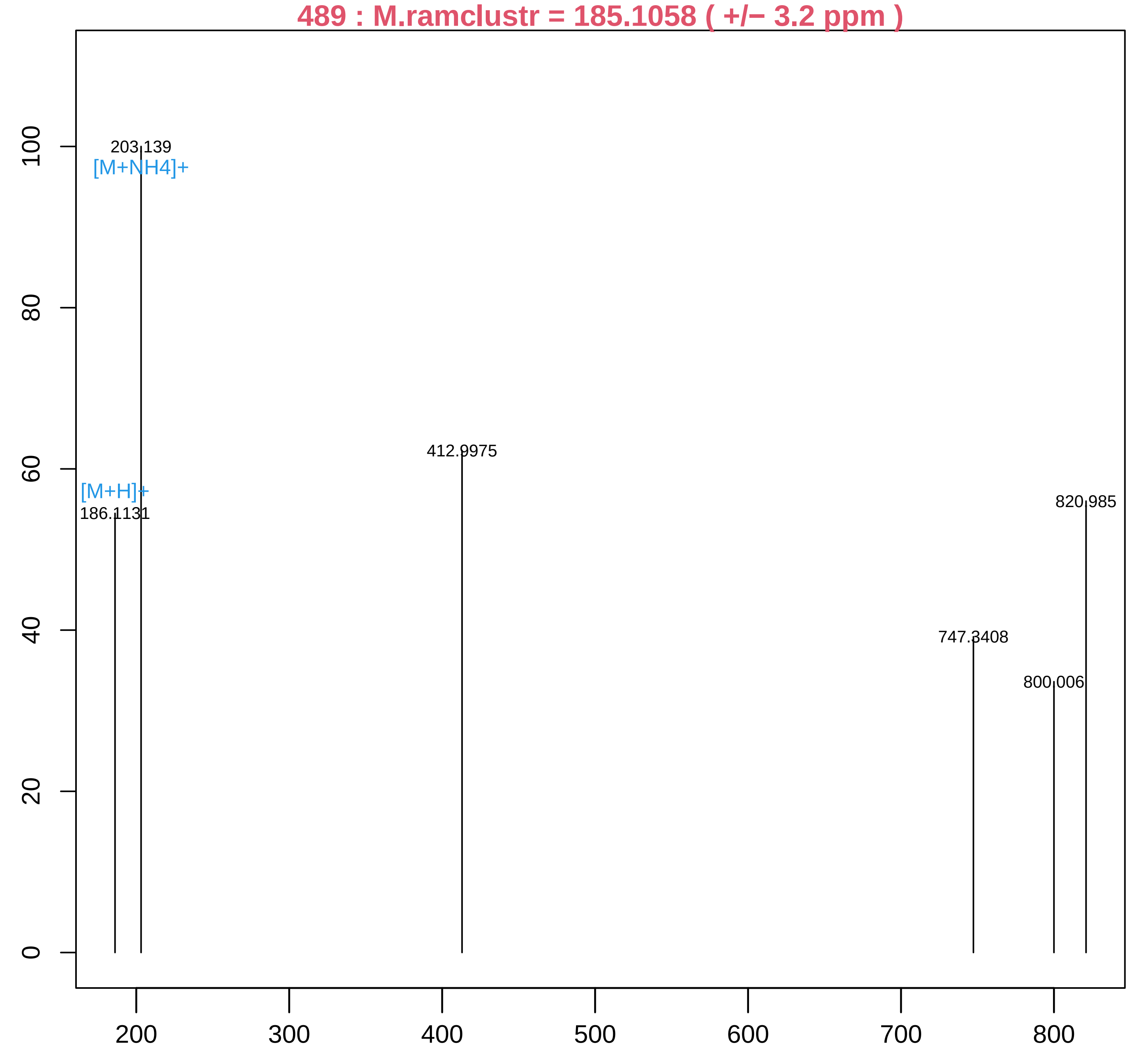

489 : M.ramclustr = 185.1058 (+/− 3.2 ppm)
203.139
[M+NH4]+
412.9975
[M+H]+
186.1131
820.985
747.3408
800.006
0
20
40
60
80
100
200
300
400
500
600
700
800

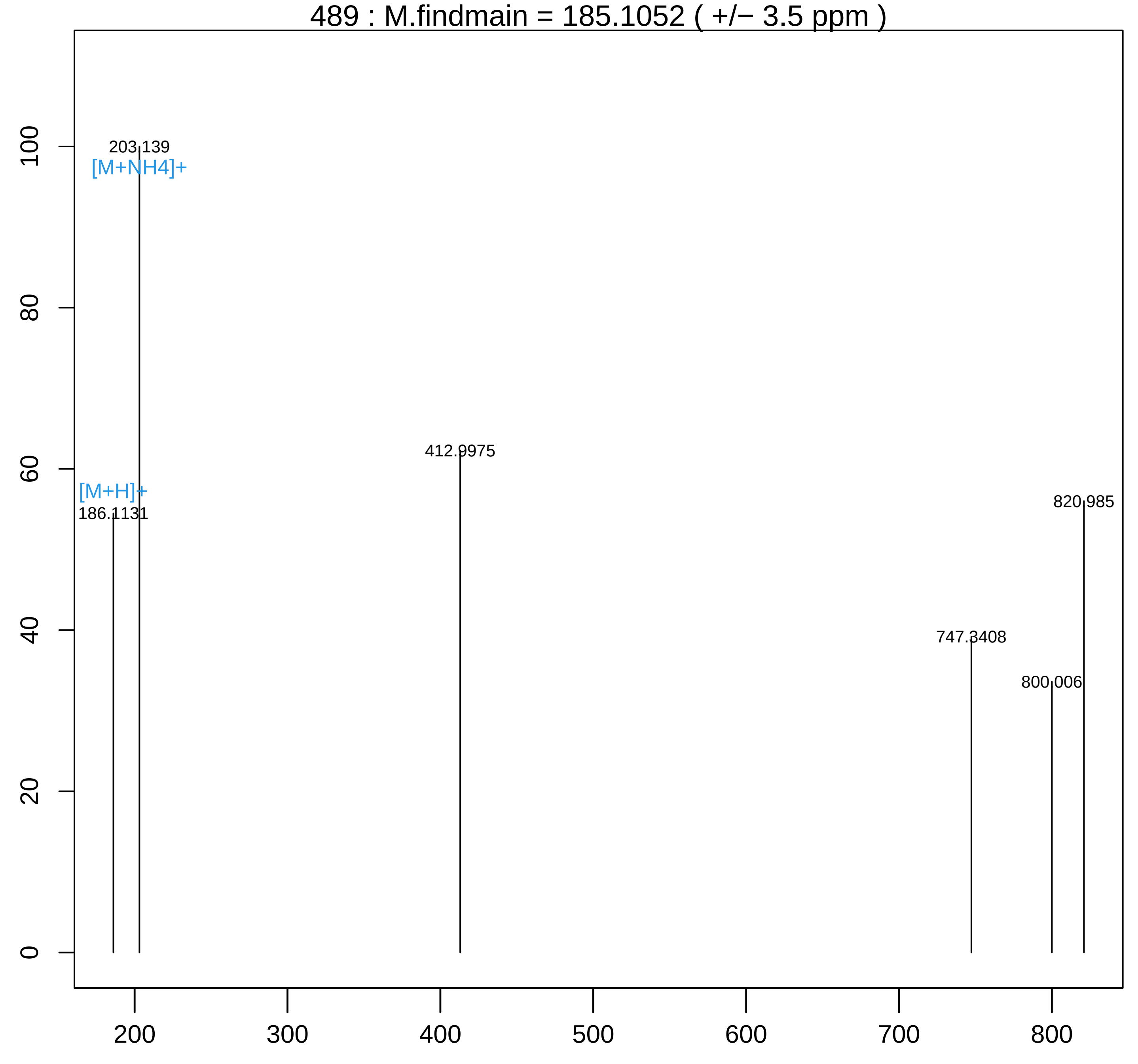

489 : M.findmain = 185.1052 (+/− 3.5 ppm)
203.139
[M+NH4]+
412.9975
[M+H]+
186.1131
820.985
747.3408
800.006
0
20
40
60
80
100
200
300
400
500
600
700
800

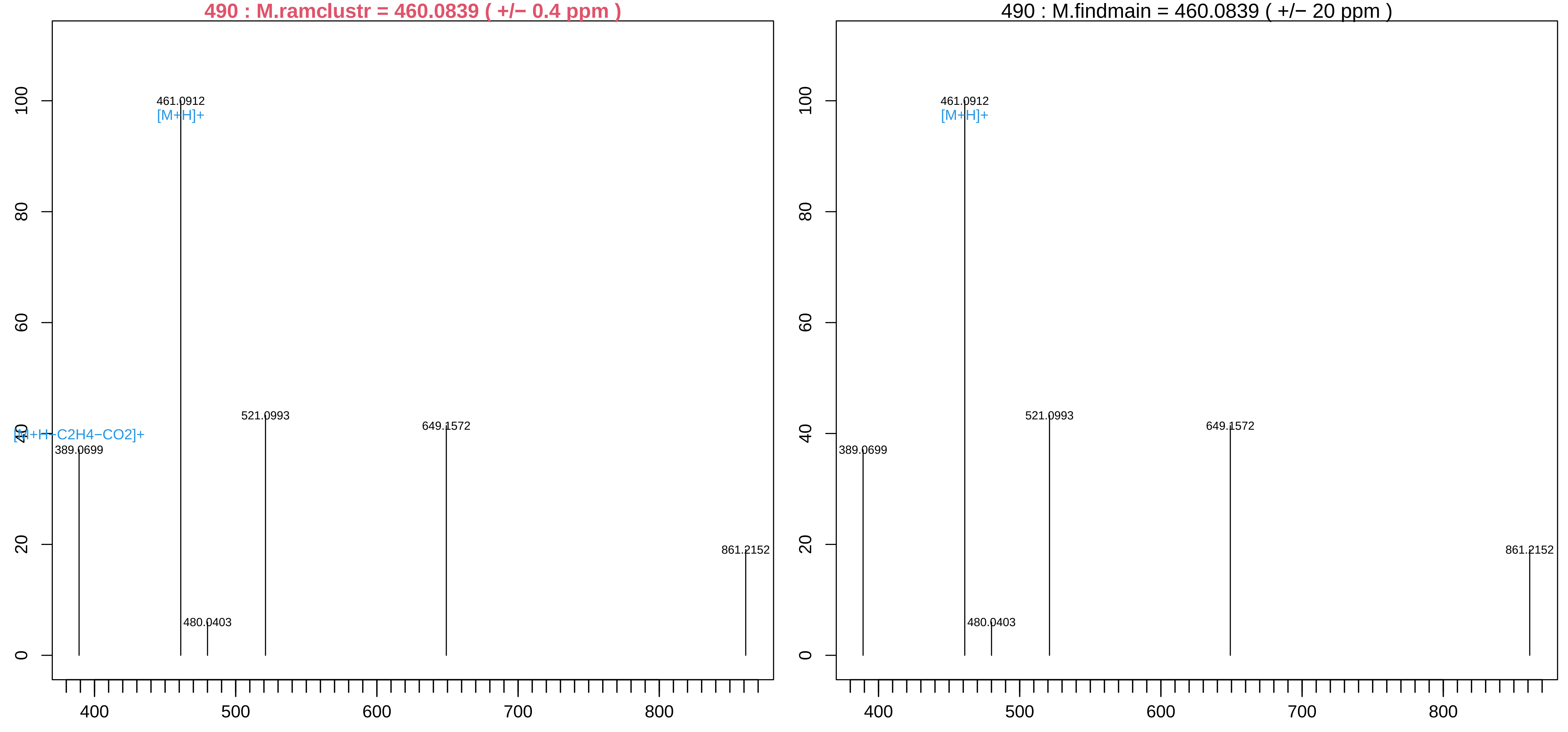
490 : M.ramclustr = 460.0839 (+/− 0.4 ppm)
461.0912
[M+H]+
[M+H−C2H4−CO2]+
389.0699
480.0403
521.0993
649.1572
861.2152
490 : M.findmain = 460.0839 (+/− 20 ppm)
461.0912
[M+H]+
389.0699
480.0403
521.0993
649.1572
861.2152
0
20
40
60
80
100
400
500
600
700
800

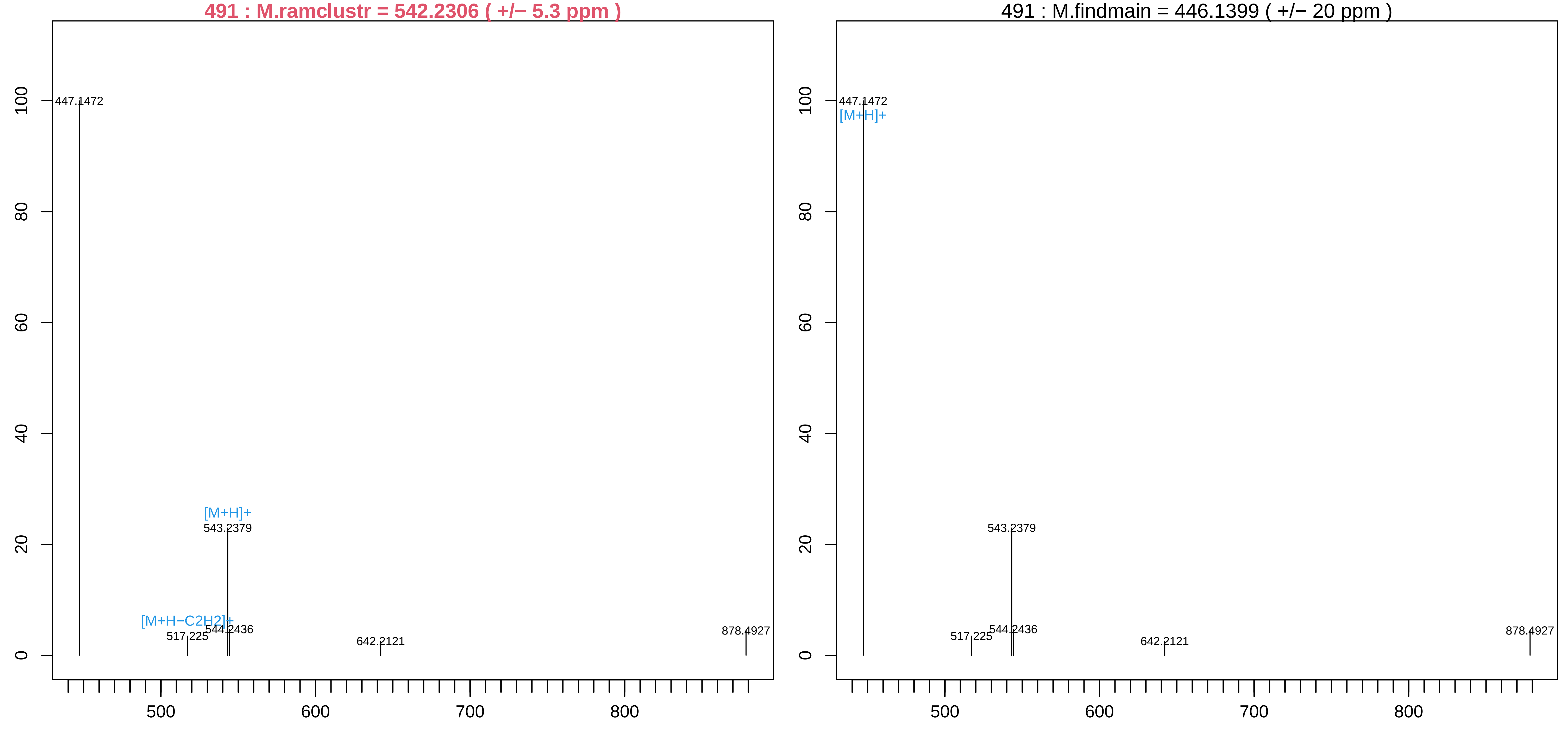
491 : M.ramclustr = 542.2306 (+/− 5.3 ppm)
447.1472
[M+H]+
543.2379
[M+H−C2H2]+
517.225
544.2436
642.2121
878.4927
0
20
40
60
80
100
500
600
700
800
491 : M.findmain = 446.1399 (+/− 20 ppm)
447.1472
[M+H]+
543.2379
517.225
544.2436
642.2121
878.4927
0
20
40
60
80
100
500
600
700
800

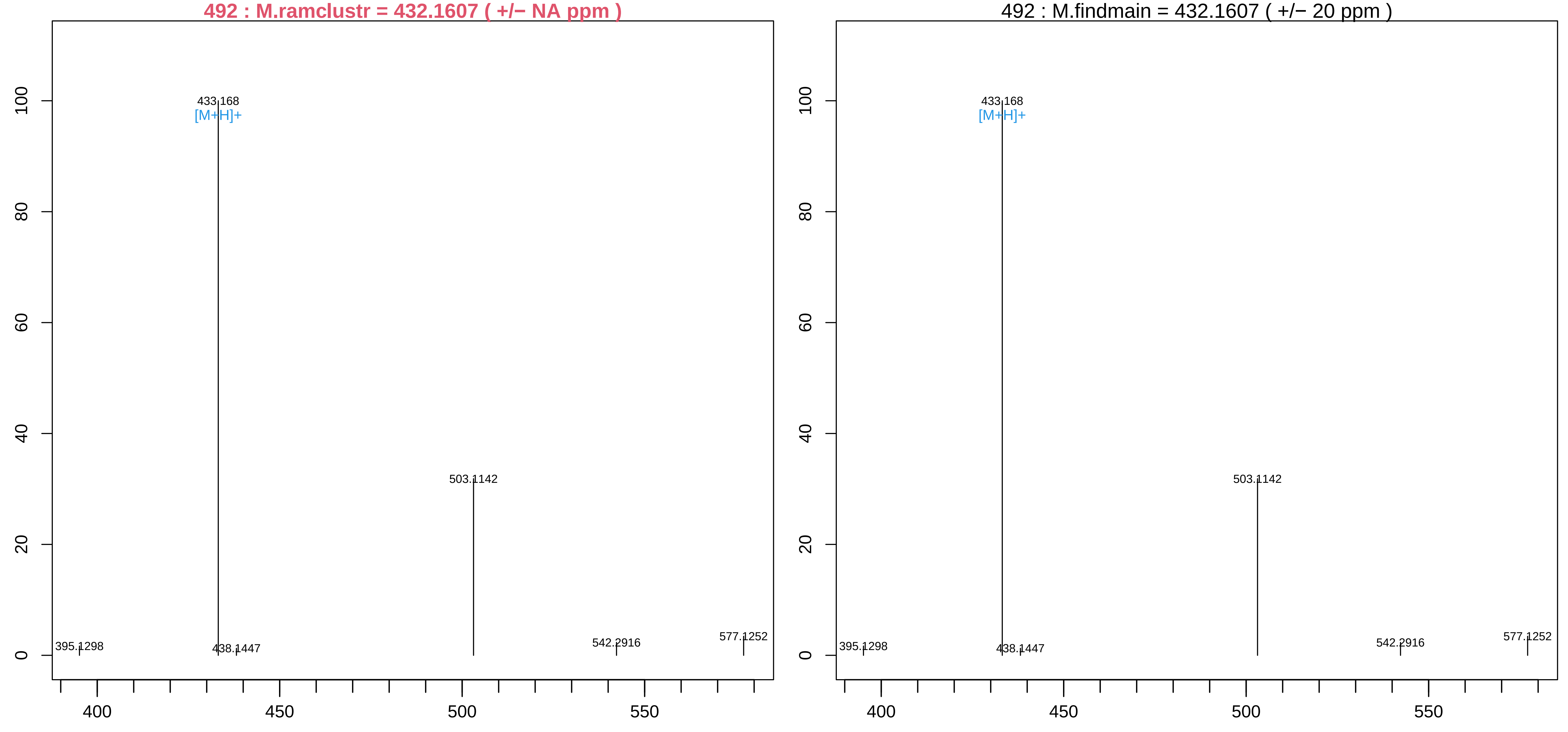

492 : M.ramclustr = 432.1607 (+/− NA ppm)
433.168
[M+H]+
503.1142
395.1298
438.1447
542.2916
577.1252
492 : M.findmain = 432.1607 (+/− 20 ppm)
433.168
[M+H]+
503.1142
395.1298
438.1447
542.2916
577.1252
0
20
40
60
80
100
400
450
500
550

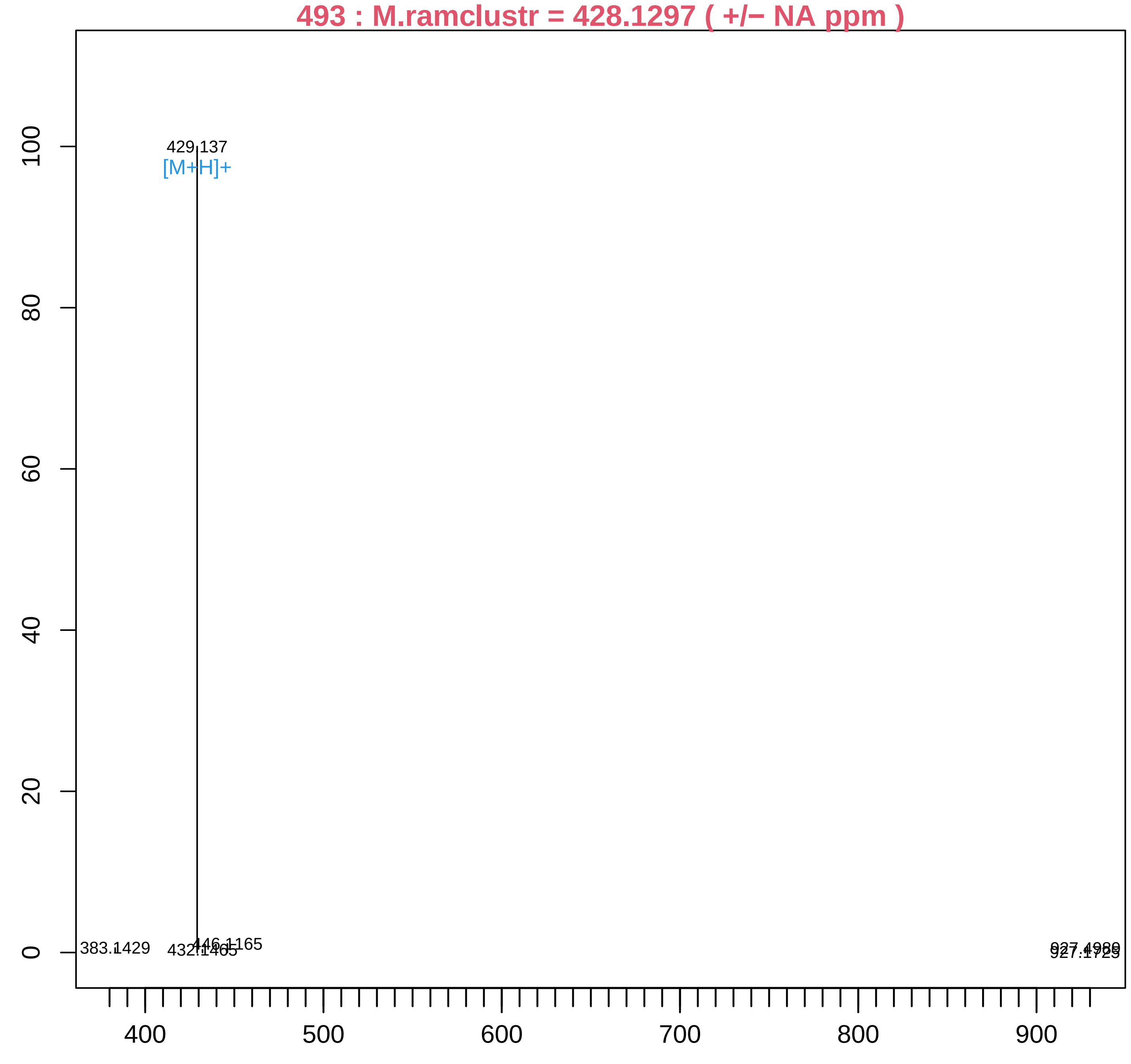
493 : M.ramclustr = 428.1297 (+/− NA ppm)
429.137
[M+H]+
383.1429
432.1405
446.1165
927.4989
927.1723

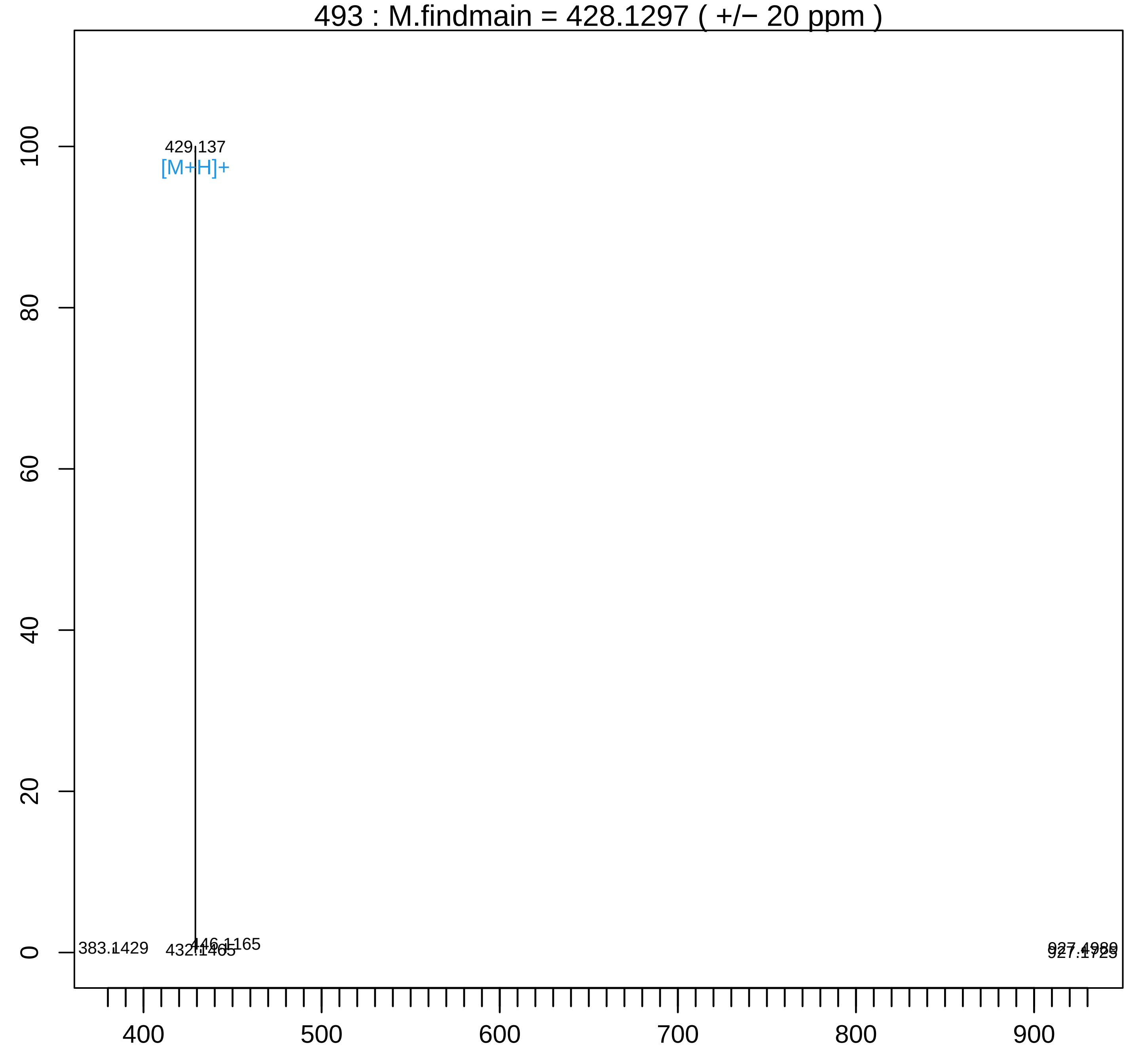
493 : M.findmain = 428.1297 (+/− 20 ppm)
429.137
[M+H]+
383.1429
432.1405
446.1165
927.4989
927.1723

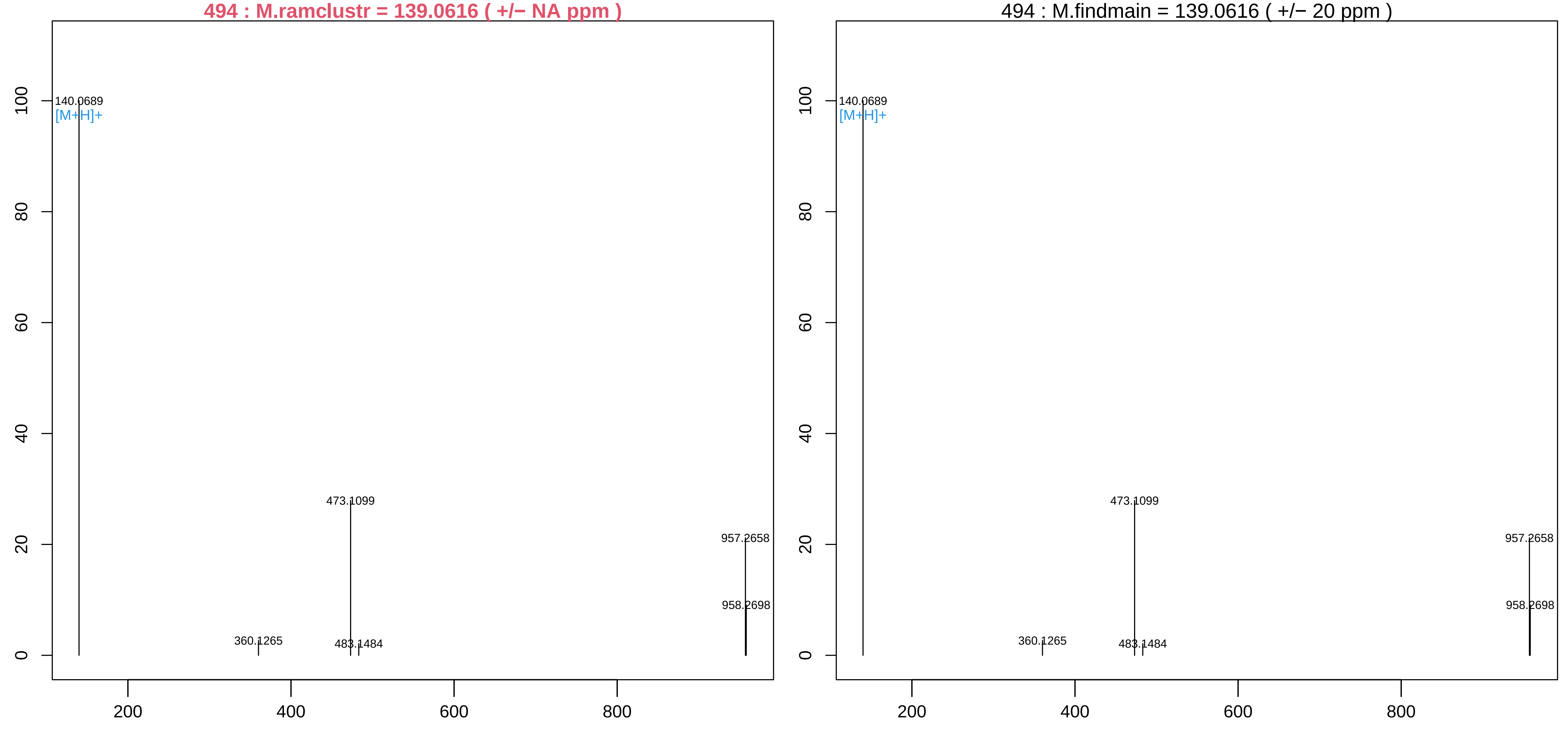

494 : M.ramclustr = 139.0616 (+/− NA ppm)
140.0689
[M+H]+
473.1099
957.2658
958.2698
360.1265
483.1484
0
20
40
60
80
100
200
400
600
800
494 : M.findmain = 139.0616 (+/− 20 ppm)
140.0689
[M+H]+
473.1099
957.2658
958.2698
360.1265
483.1484
0
20
40
60
80
100
200
400
600
800

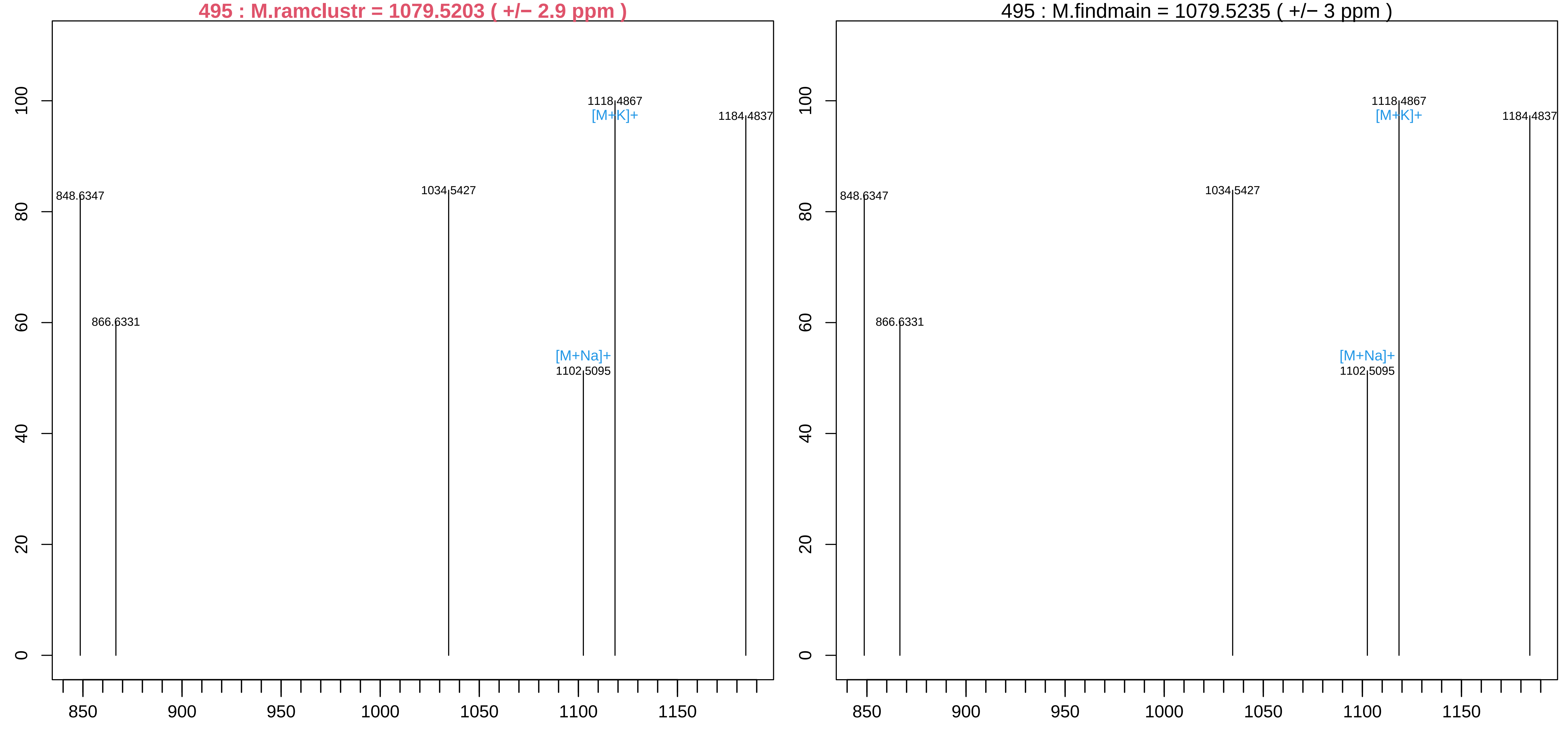
495 : M.ramclustr = 1079.5203 (+/− 2.9 ppm)
848.6347
866.6331
1034.5427
[M+Na]+
1102.5095
1118.4867
[M+K]+
1184.4837
495 : M.findmain = 1079.5235 (+/− 3 ppm)
848.6347
866.6331
1034.5427
[M+Na]+
1102.5095
1118.4867
[M+K]+
1184.4837

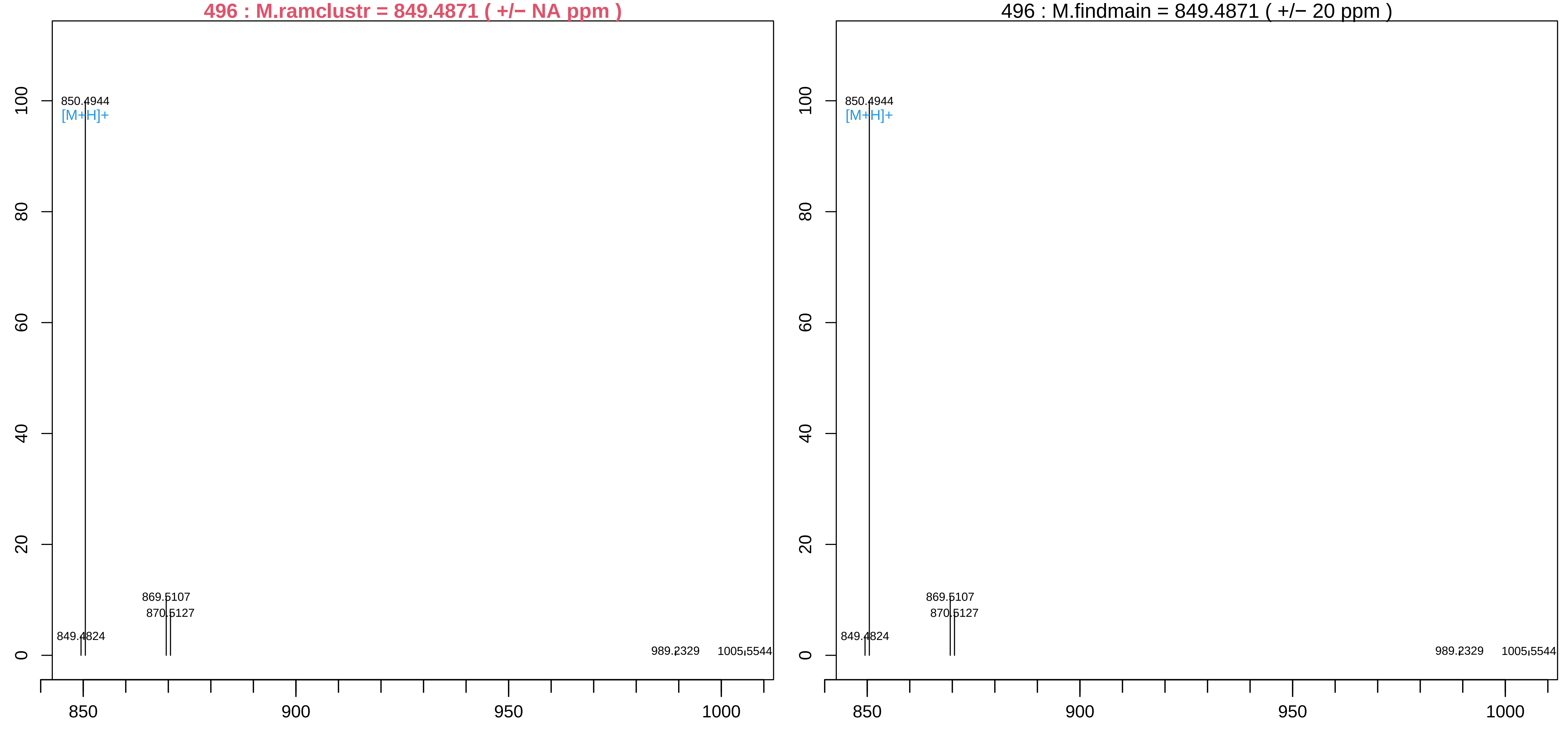

496 : M.ramclustr = 849.4871 (+/− NA ppm)
850.4944
[M+H]+
869.5107
870.5127
849.4824
989.2329
1005.5544
496 : M.findmain = 849.4871 (+/− 20 ppm)
850.4944
[M+H]+
869.5107
870.5127
849.4824
989.2329
1005.5544

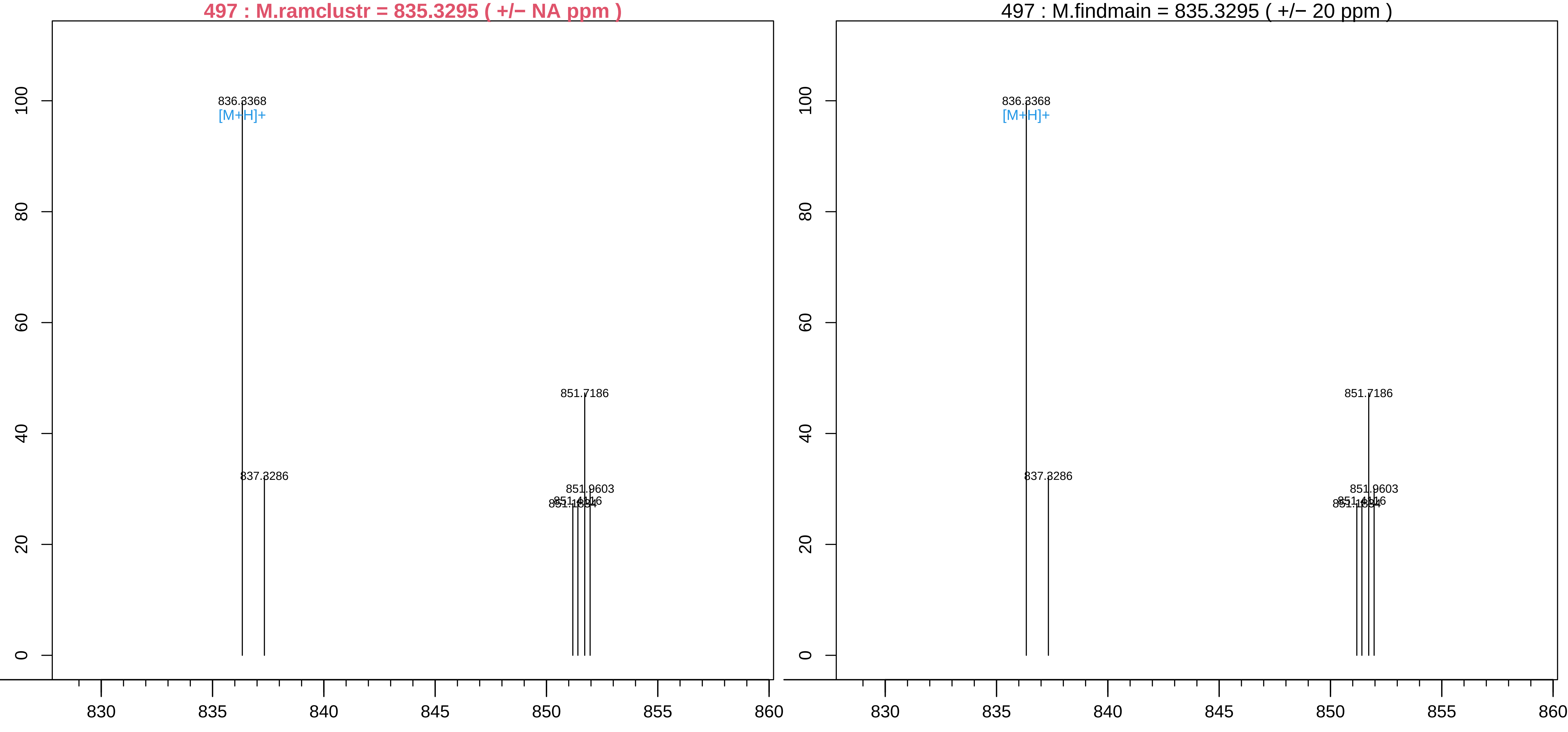
497 : M.ramclustr = 835.3295 (+/− NA ppm)
836.3368
[M+H]+
837.3286
851.7186
851.9603
851.4116
851.1854
497 : M.findmain = 835.3295 (+/− 20 ppm)
836.3368
[M+H]+
837.3286
851.7186
851.9603
851.4116
851.1854

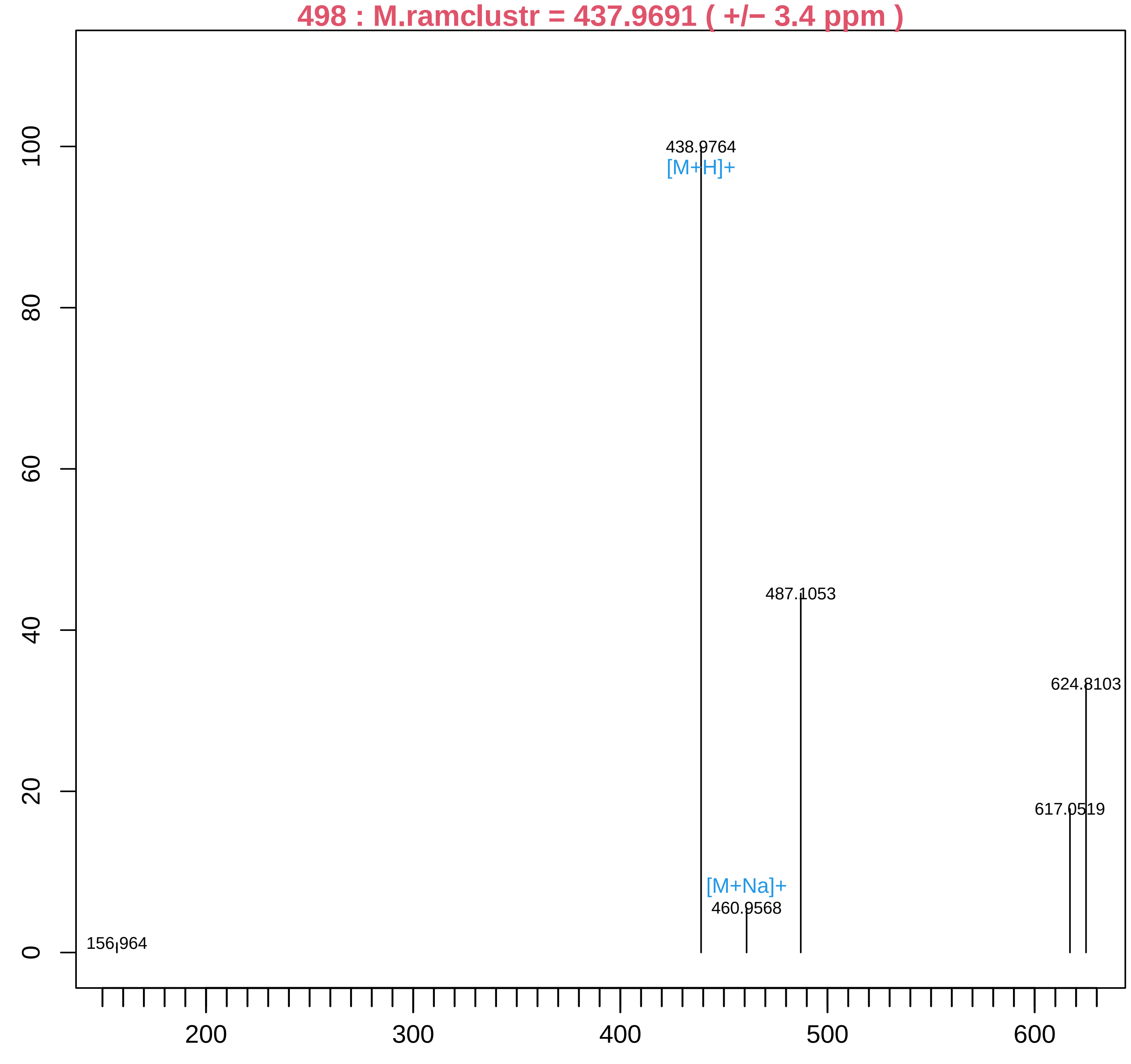

498 : M.ramclustr = 437.9691 (+/− 3.4 ppm)
438.9764
[M+H]+
487.1053
624.8103
617.0519
[M+Na]+
460.9568
156.964
0
20
40
60
80
100
200
300
400
500
600

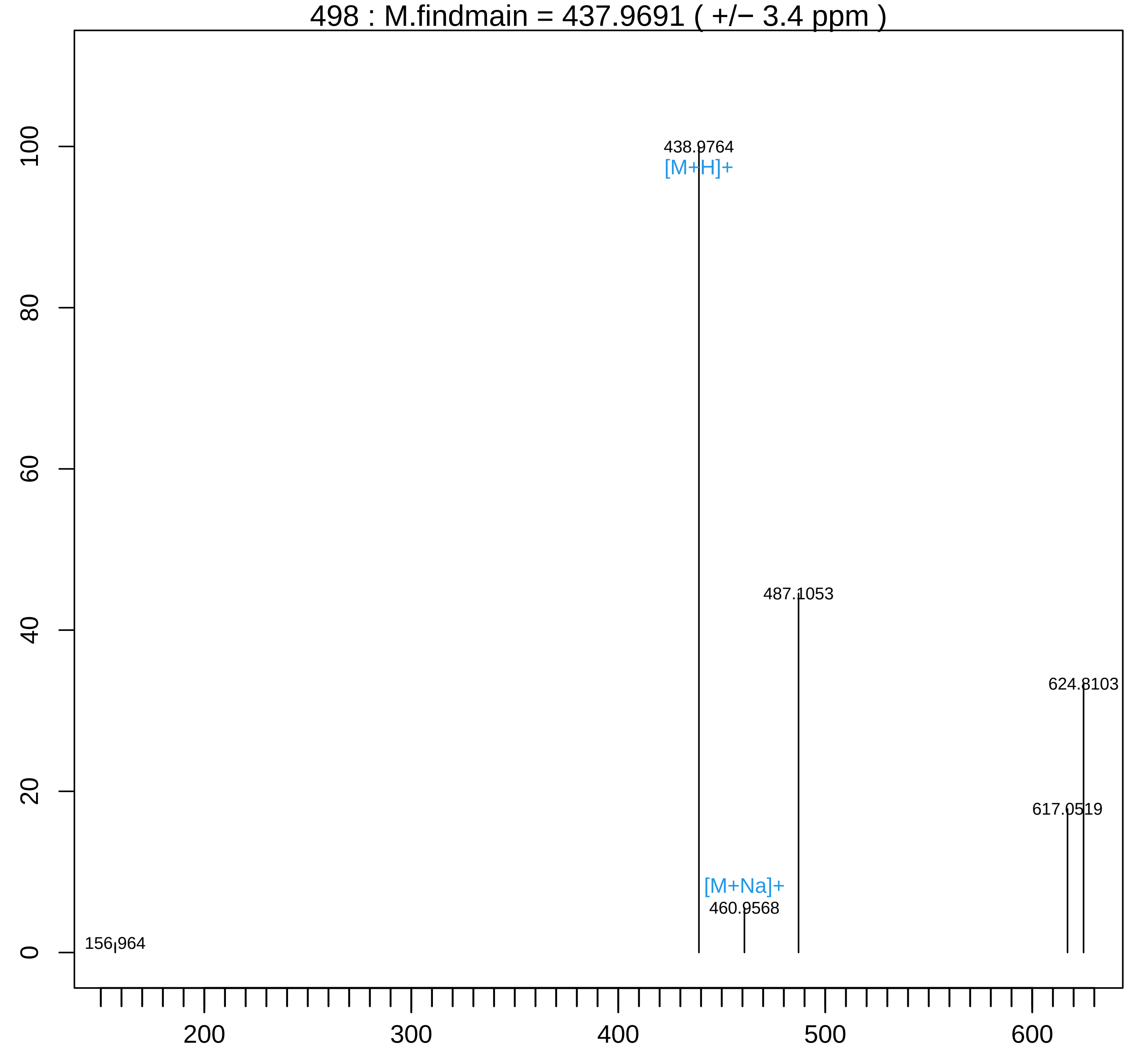

498 : M.findmain = 437.9691 (+/− 3.4 ppm)
438.9764
[M+H]+
487.1053
624.8103
617.0519
[M+Na]+
460.9568
156.964
0
20
40
60
80
100
200
300
400
500
600

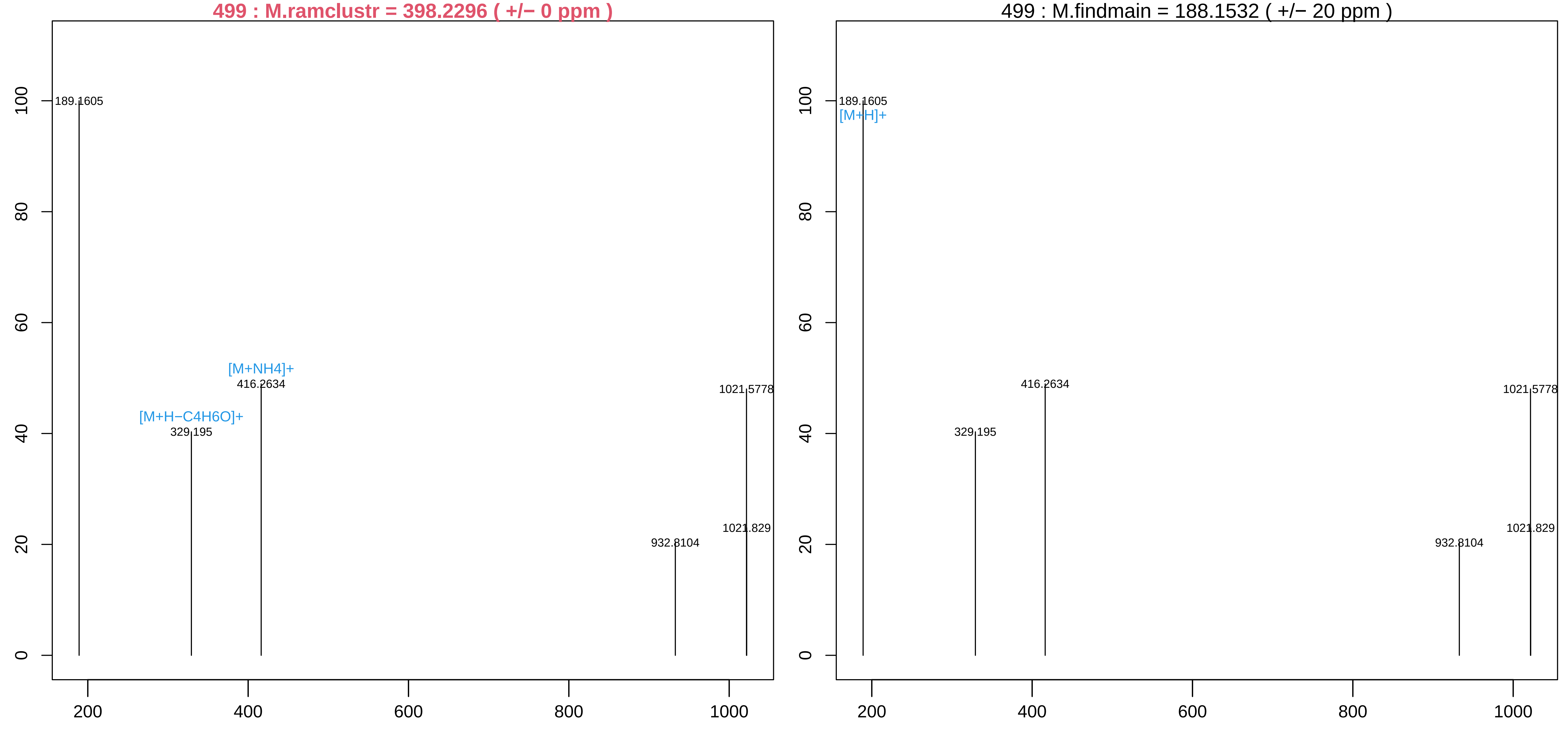

499 : M.ramclustr = 398.2296 (+/− 0 ppm)
189.1605
[M+NH4]+
416.2634
[M+H−C4H6O]+
329.195
1021.5778
1021.829
932.8104
0
20
40
60
80
100
200
400
600
800
1000
499 : M.findmain = 188.1532 (+/− 20 ppm)
189.1605
[M+H]+
416.2634
329.195
1021.5778
1021.829
932.8104

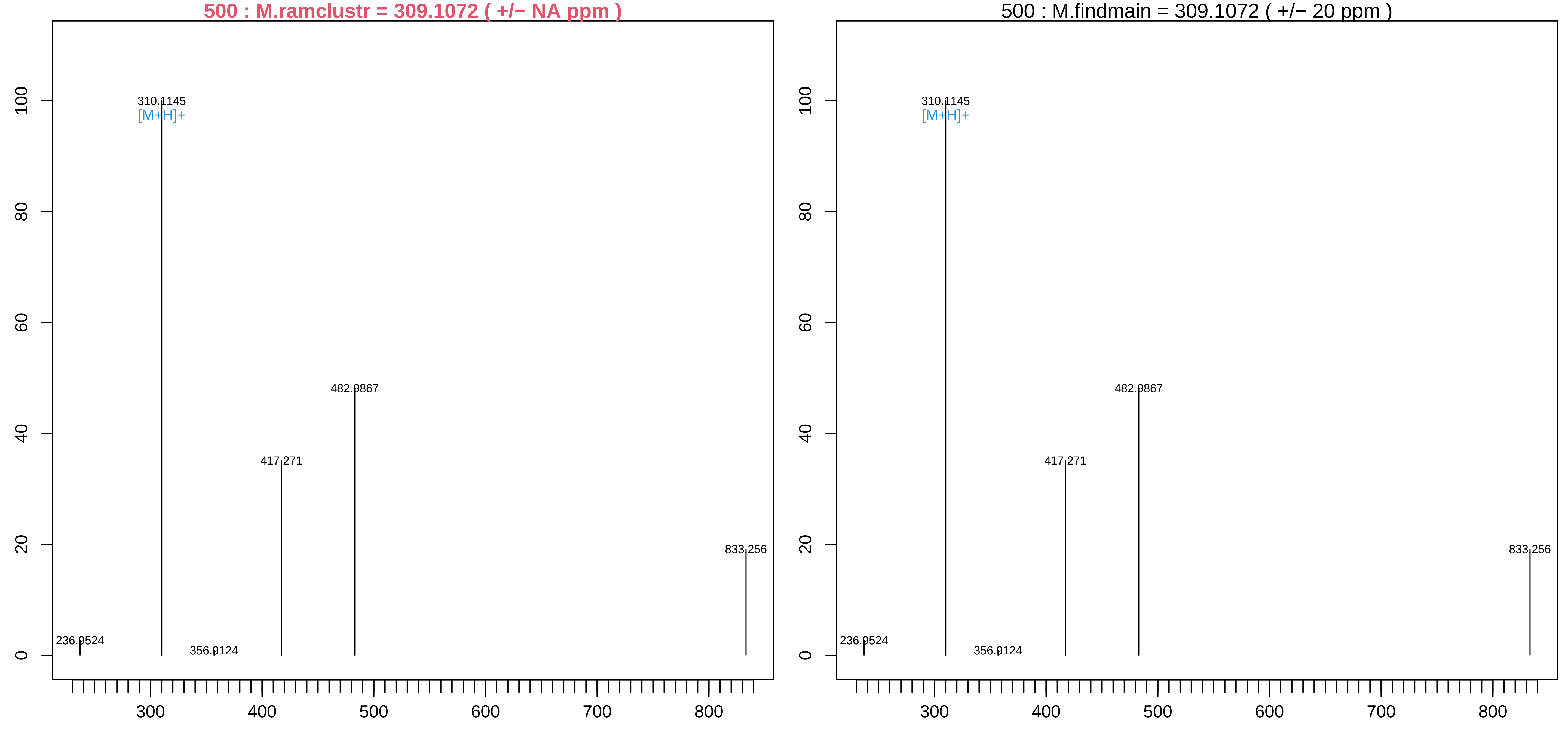
500 : M.ramclustr = 309.1072 (+/− NA ppm)
310.1145
[M+H]+
482.9867
417.271
833.256
236.9524
356.9124
500 : M.findmain = 309.1072 (+/− 20 ppm)
310.1145
[M+H]+
482.9867
417.271
833.256
236.9524
356.9124
0
20
40
60
80
100
300
400
500
600
700
800

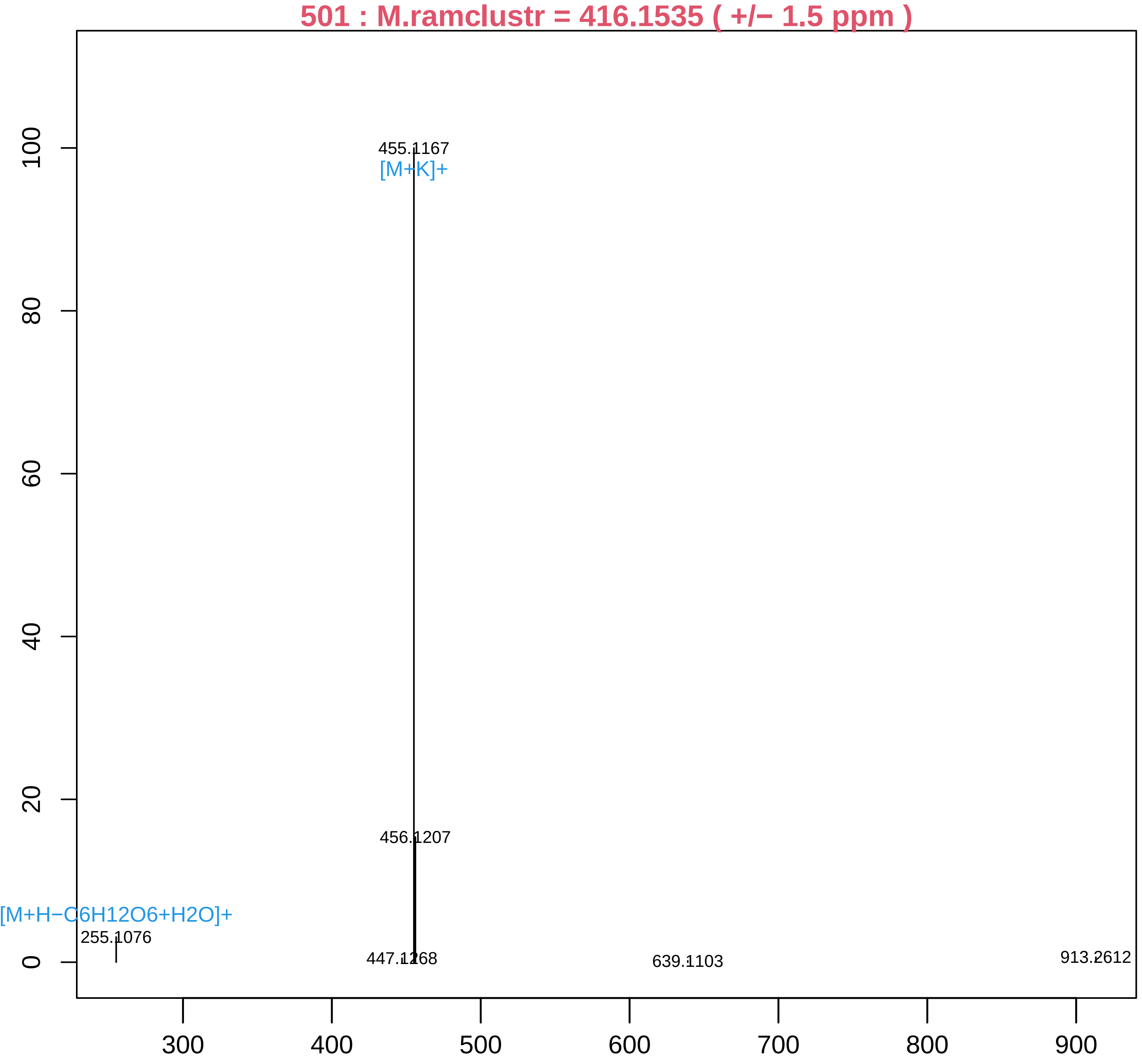
501 : M.ramclustr = 416.1535 (+/− 1.5 ppm)
455.1167
[M+K]+
456.1207
[M+H−C6H12O6+H2O]+
255.1076
447.1268
639.1103
913.2612
0
20
40
60
80
100
300
400
500
600
700
800
900

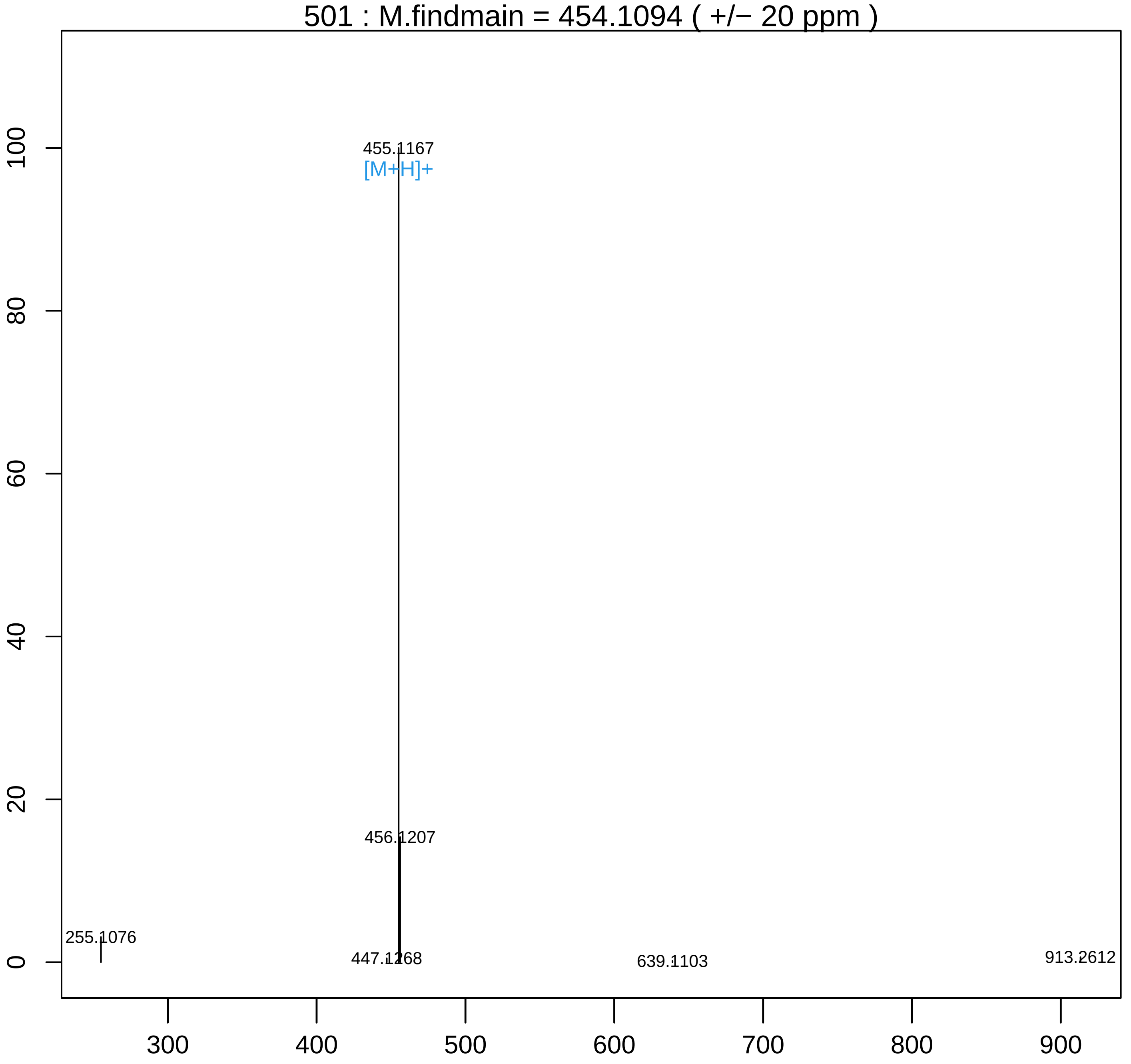
501 : M.findmain = 454.1094 (+/− 20 ppm)
455.1167
[M+H]+
456.1207
255.1076
447.1268
639.1103
913.2612
0
20
40
60
80
100
300
400
500
600
700
800
900

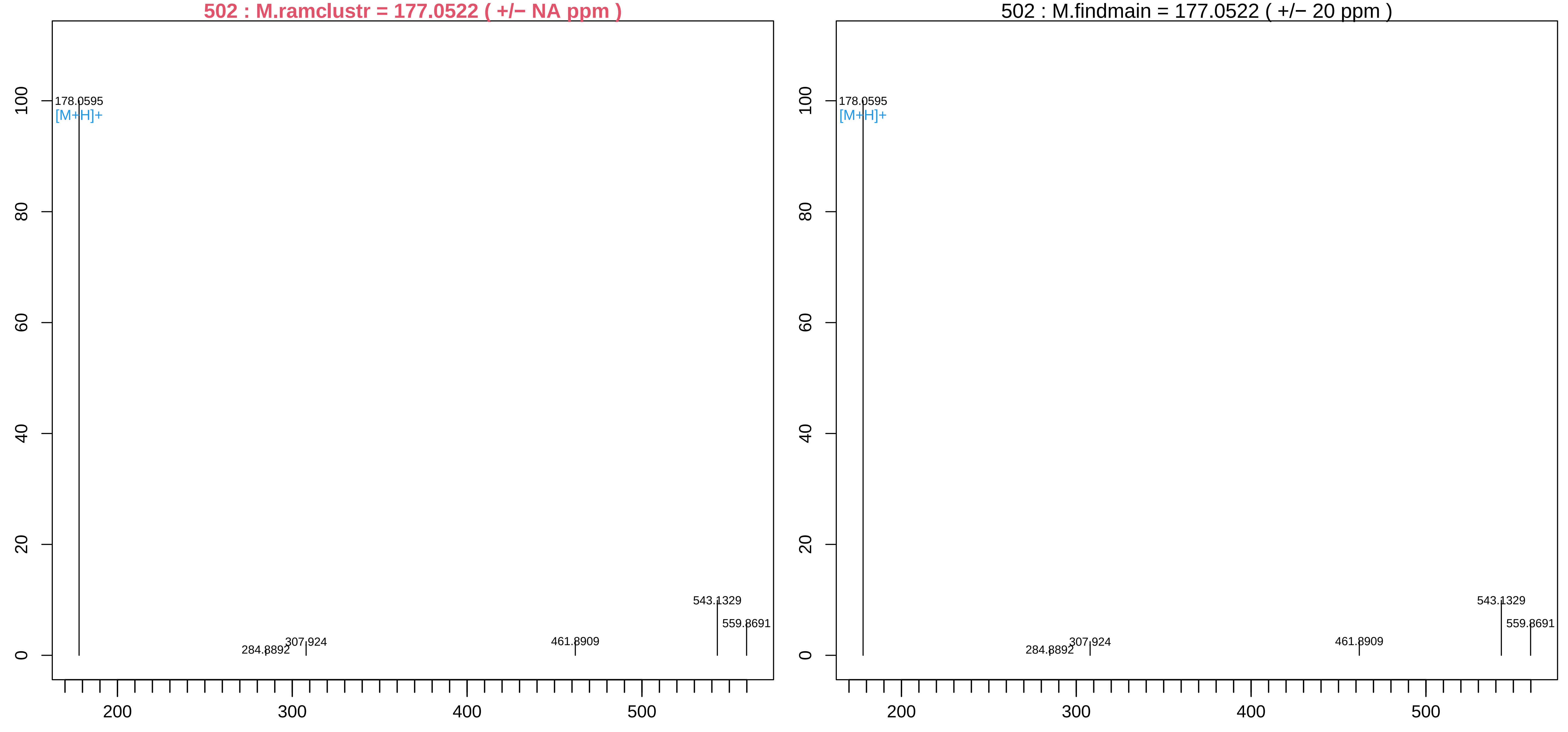
502 : M.ramclustr = 177.0522 (+/− NA ppm)
178.0595
[M+H]+
284.8892
307.924
461.8909
543.1329
559.8691
0
20
40
60
80
100
200
300
400
500
502 : M.findmain = 177.0522 (+/− 20 ppm)
178.0595
[M+H]+
284.8892
307.924
461.8909
543.1329
559.8691
0
20
40
60
80
100
200
300
400
500

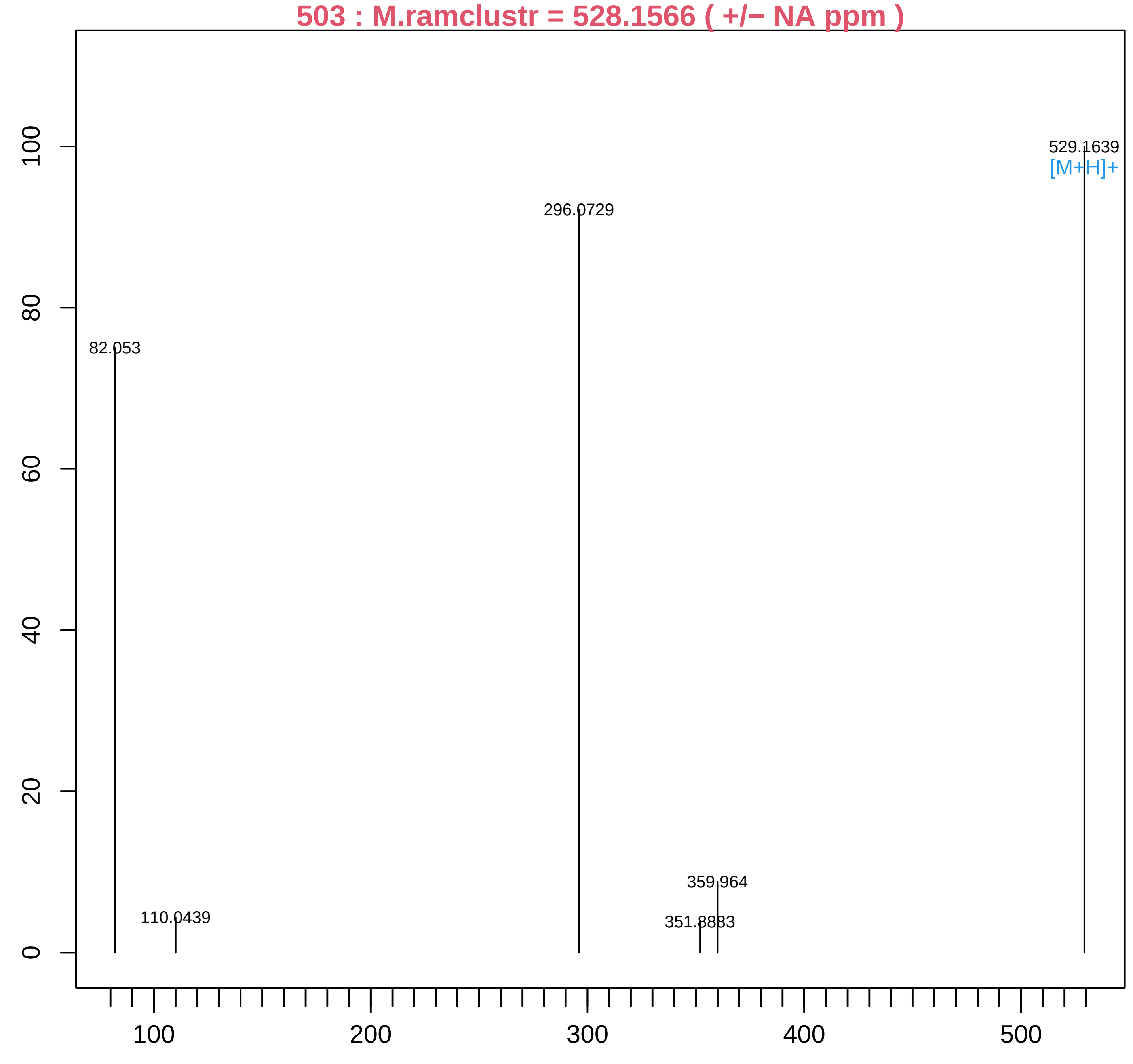
503 : M.ramclustr = 528.1566 (+/− NA ppm)
529.1639
[M+H]+
296.0729
82.053
110.0439
359.964
351.8883
0
20
40
60
80
100
100
200
300
400
500

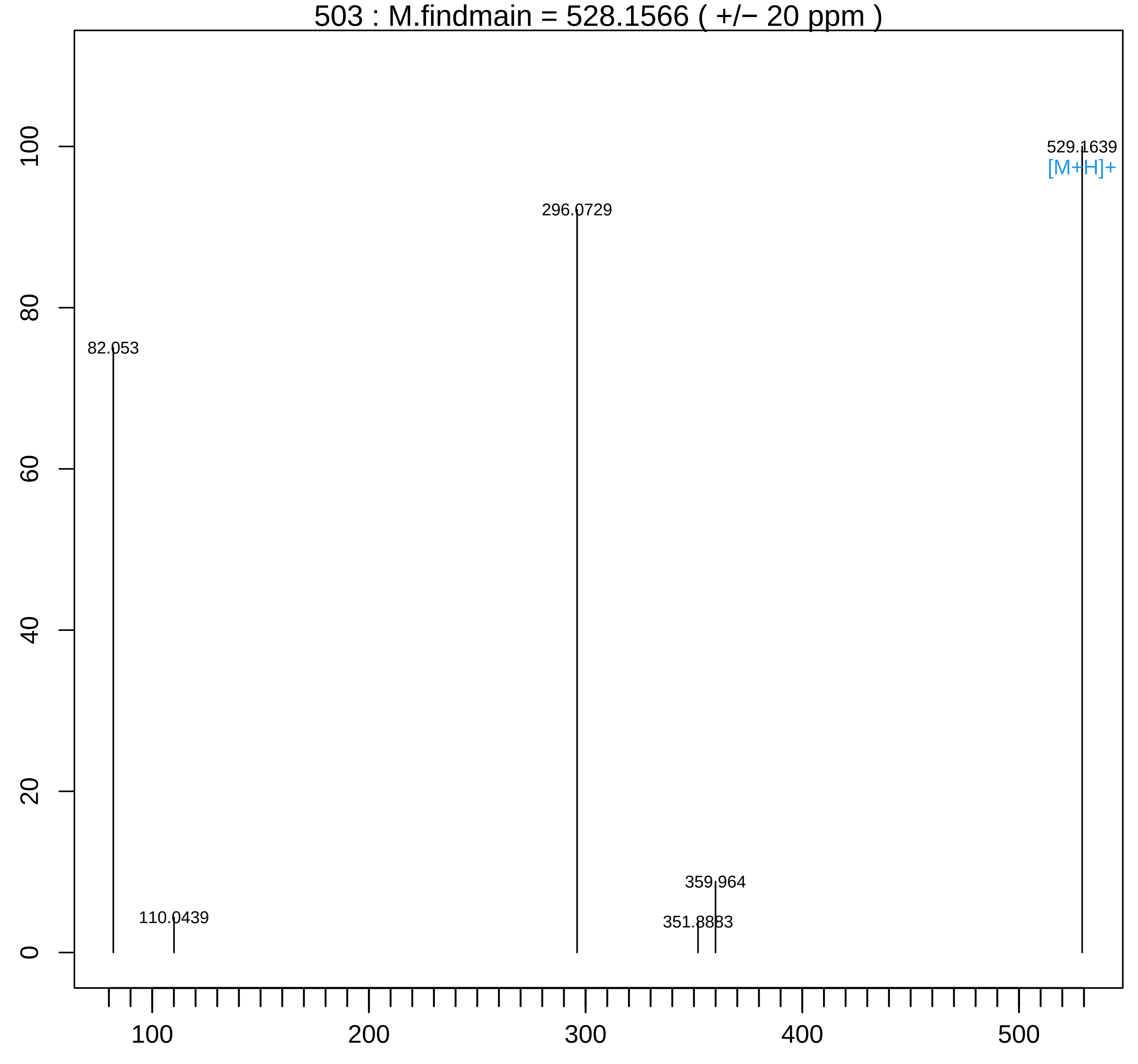
503 : M.findmain = 528.1566 (+/− 20 ppm)
529.1639
[M+H]+
296.0729
82.053
110.0439
359.964
351.8883
0
20
40
60
80
100
100
200
300
400
500

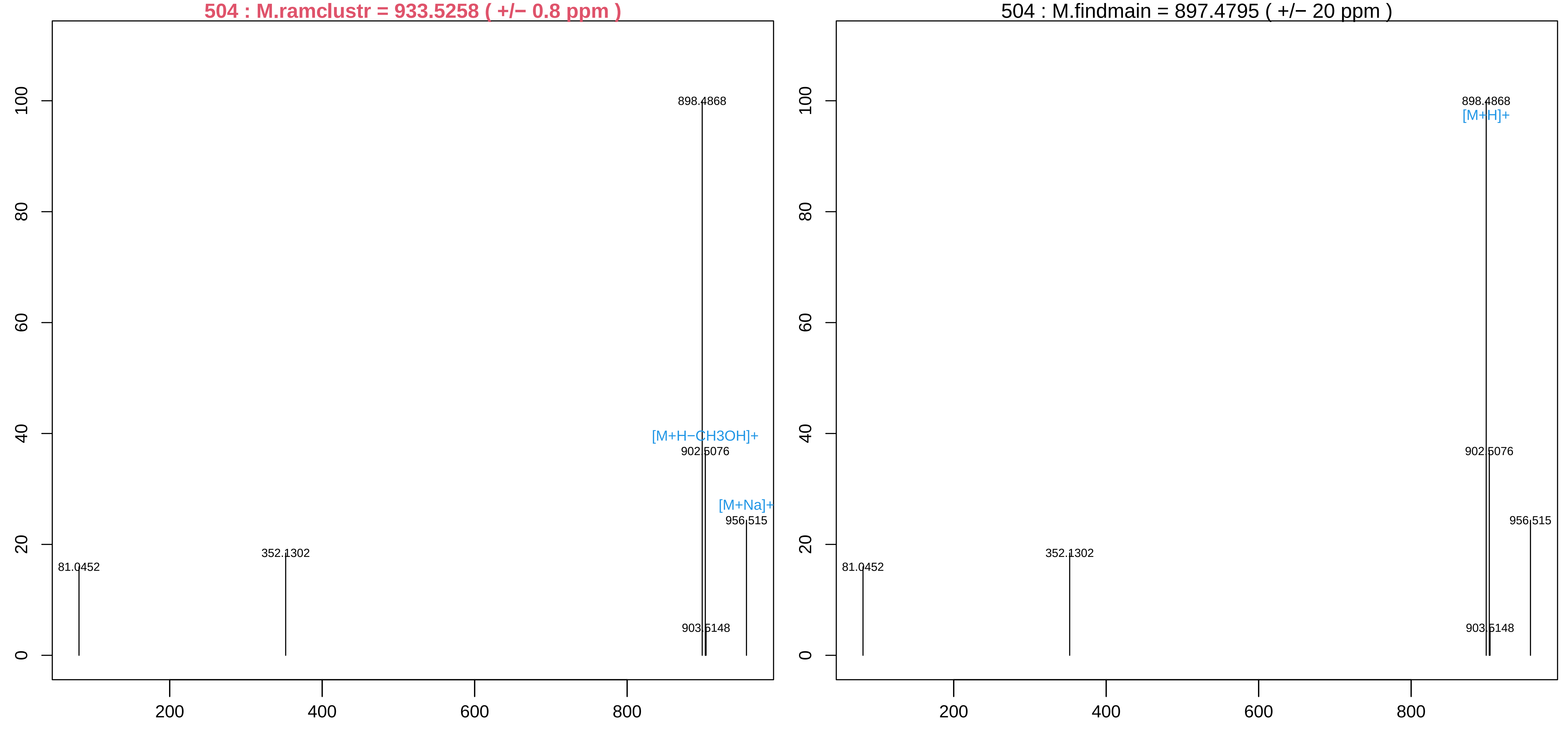
504 : M.ramclustr = 933.5258 (+/− 0.8 ppm)
898.4868
[M+H−CH3OH]+
902.5076
[M+Na]+
956.515
352.1302
81.0452
903.5148
504 : M.findmain = 897.4795 (+/− 20 ppm)
898.4868
[M+H]+
902.5076
956.515
352.1302
81.0452
903.5148
0
20
40
60
80
100
200
400
600
800

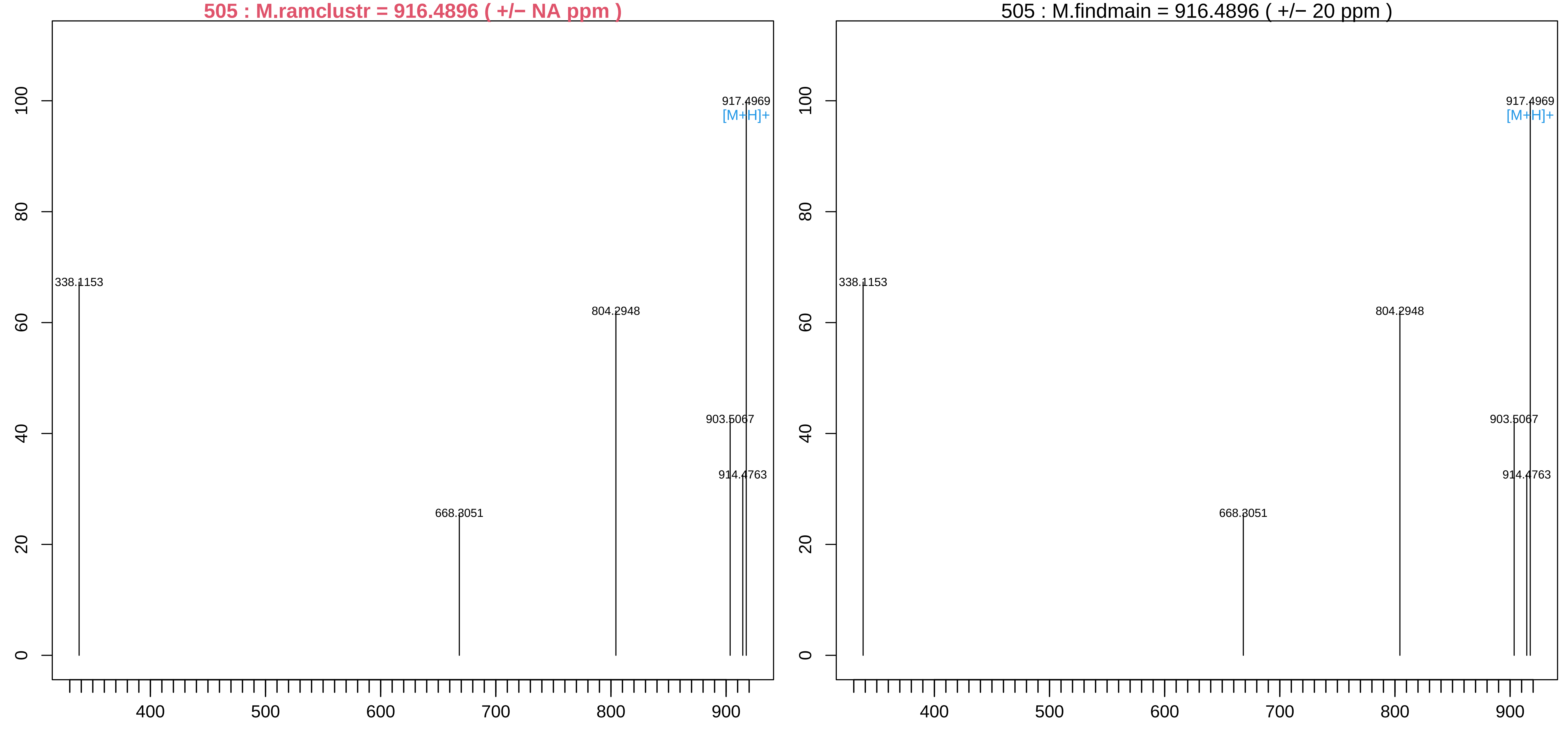
505 : M.ramclustr = 916.4896 (+/− NA ppm)
338.1153
668.3051
804.2948
903.5067
914.4763
917.4969
[M+H]+
0
20
40
60
80
100
400
500
600
700
800
900
505 : M.findmain = 916.4896 (+/− 20 ppm)
338.1153
668.3051
804.2948
903.5067
914.4763
917.4969
[M+H]+
0
20
40
60
80
100
400
500
600
700
800
900

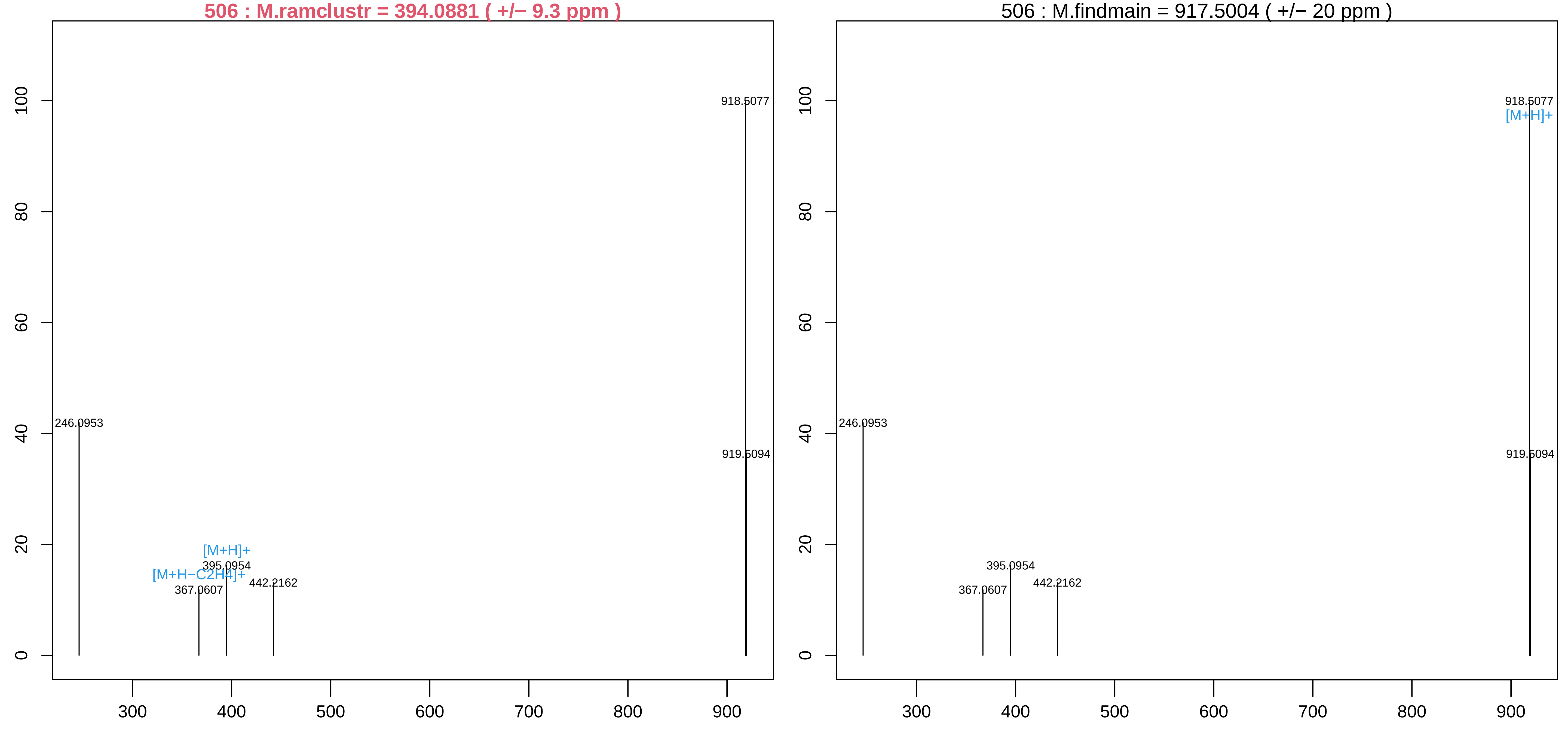
506 : M.ramclustr = 394.0881 (+/− 9.3 ppm)
918.5077
919.5094
246.0953
[M+H]+
395.0954
[M+H−C2H4]+
367.0607
442.2162
506 : M.findmain = 917.5004 (+/− 20 ppm)
918.5077
[M+H]+
919.5094
246.0953
395.0954
367.0607
442.2162

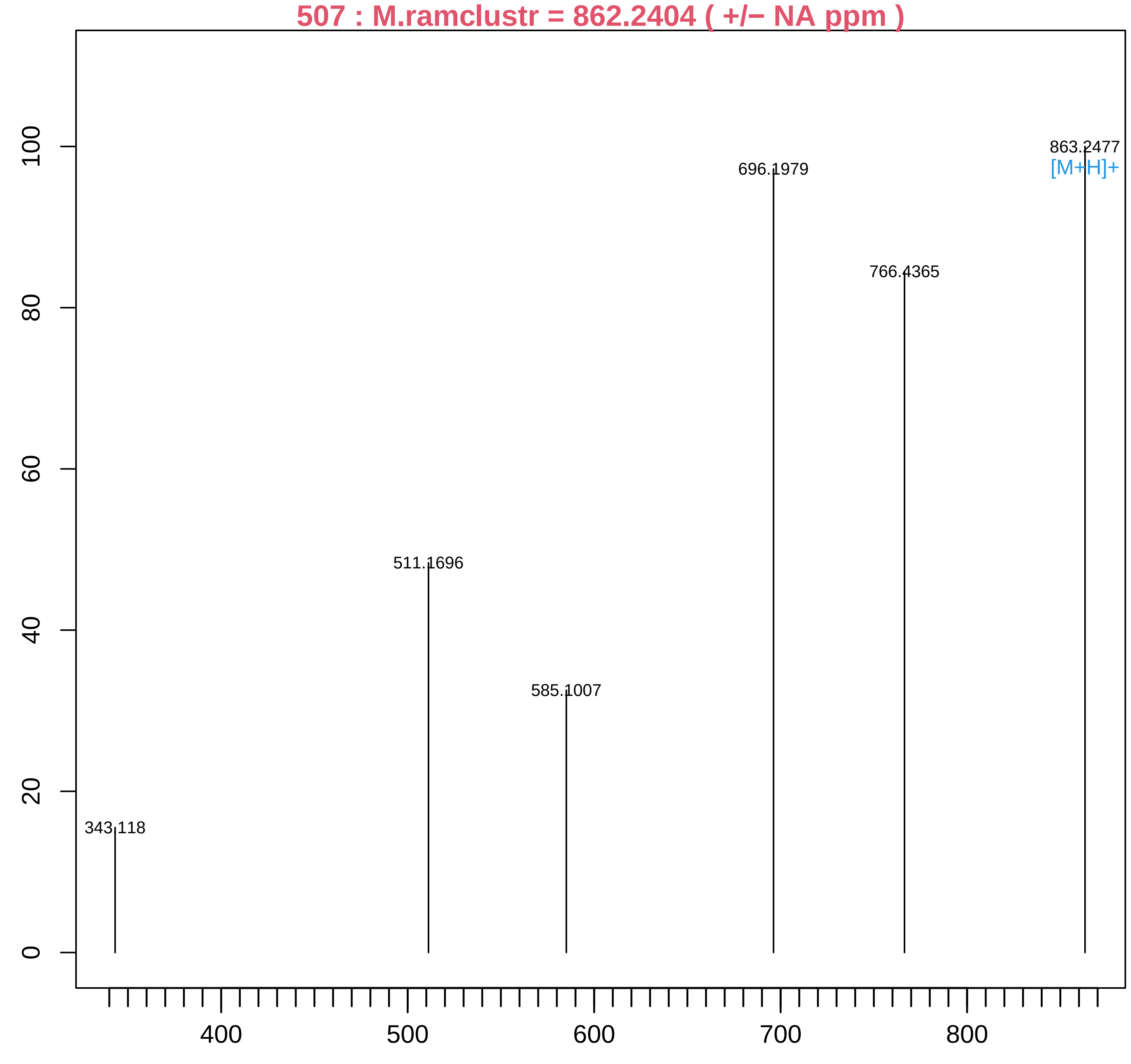
507 : M.ramclustr = 862.2404 (+/− NA ppm)
863.2477
[M+H]+
696.1979
766.4365
511.1696
585.1007
343.118
0
20
40
60
80
100
400
500
600
700
800

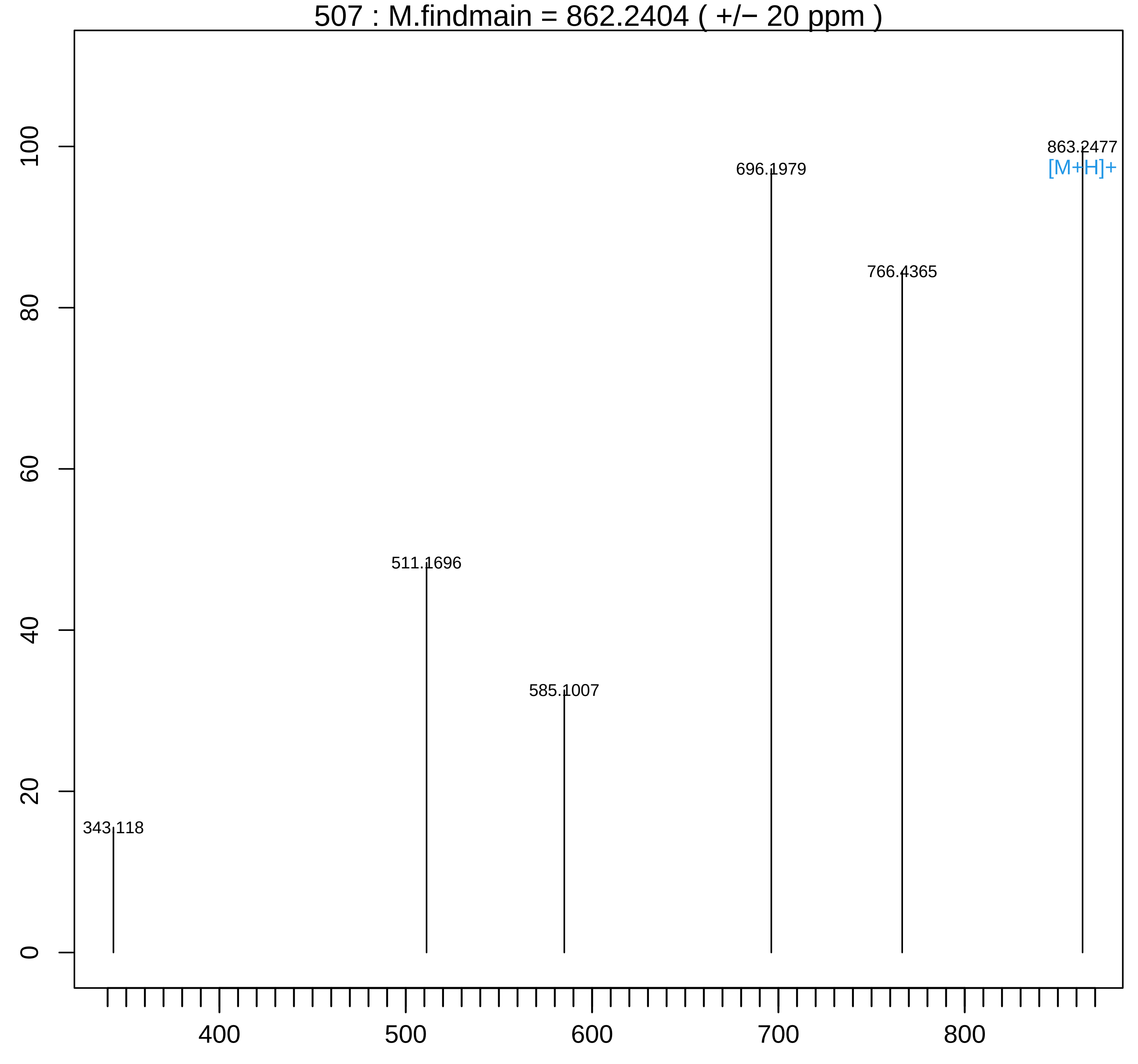
507 : M.findmain = 862.2404 (+/− 20 ppm)
863.2477
[M+H]+
696.1979
766.4365
511.1696
585.1007
343.118
0
20
40
60
80
100
400
500
600
700
800

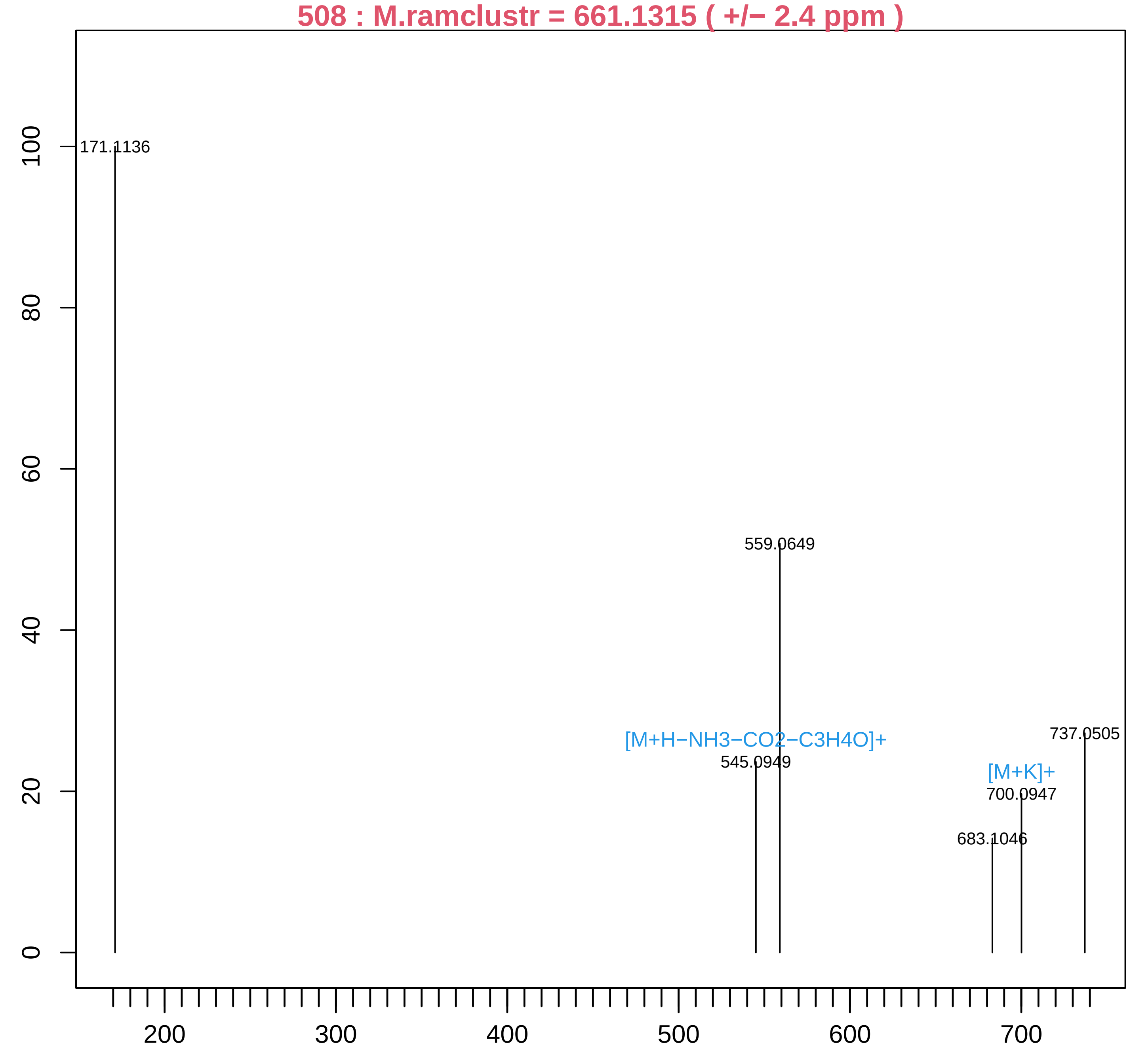
508 : M.ramclustr = 661.1315 (+/− 2.4 ppm)
171.1136
559.0649
[M+H−NH3−CO2−C3H4O]+
545.0949
737.0505
[M+K]+
700.0947
683.1046

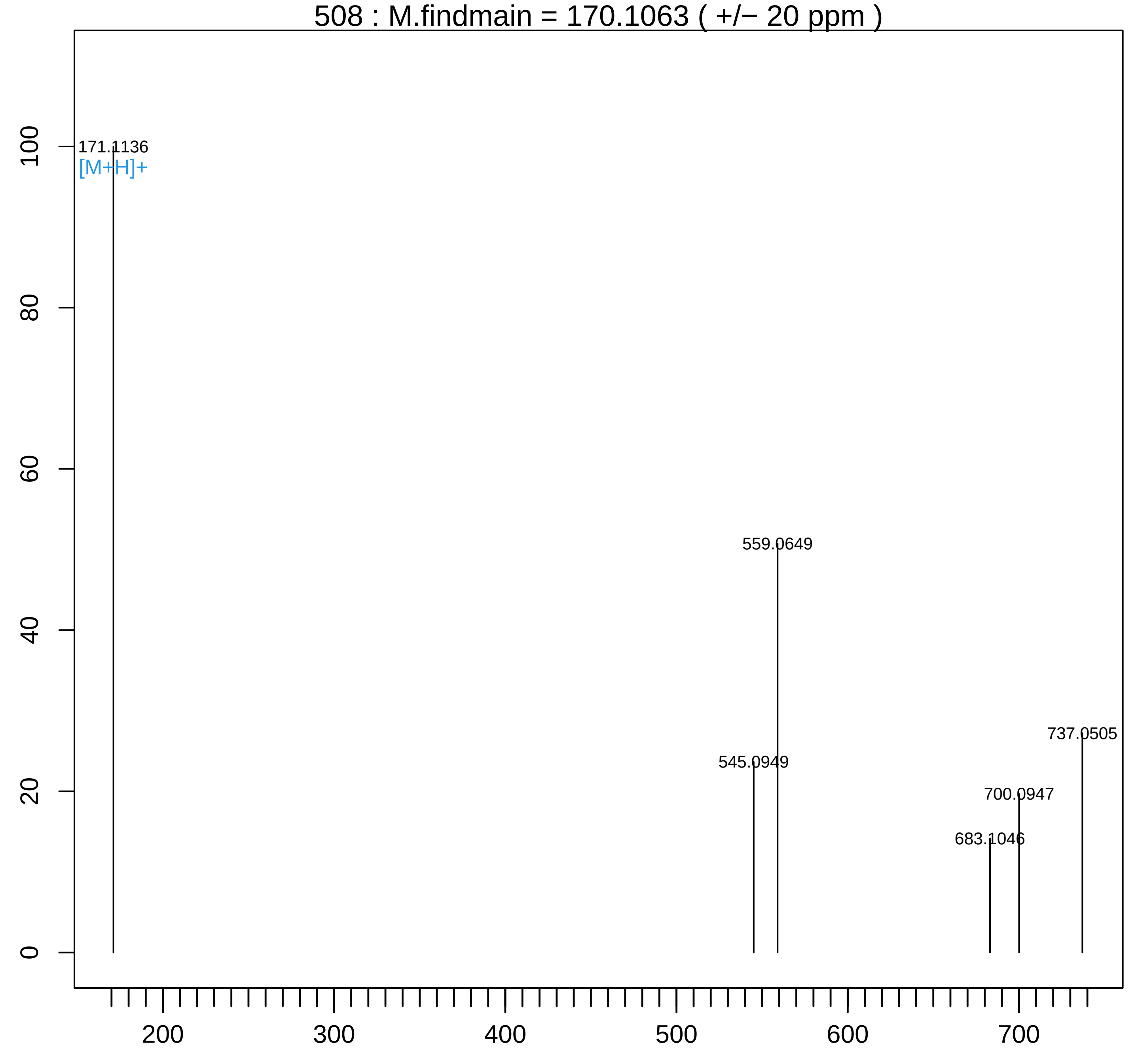
508 : M.findmain = 170.1063 (+/− 20 ppm)
171.1136
[M+H]+
559.0649
545.0949
737.0505
700.0947
683.1046

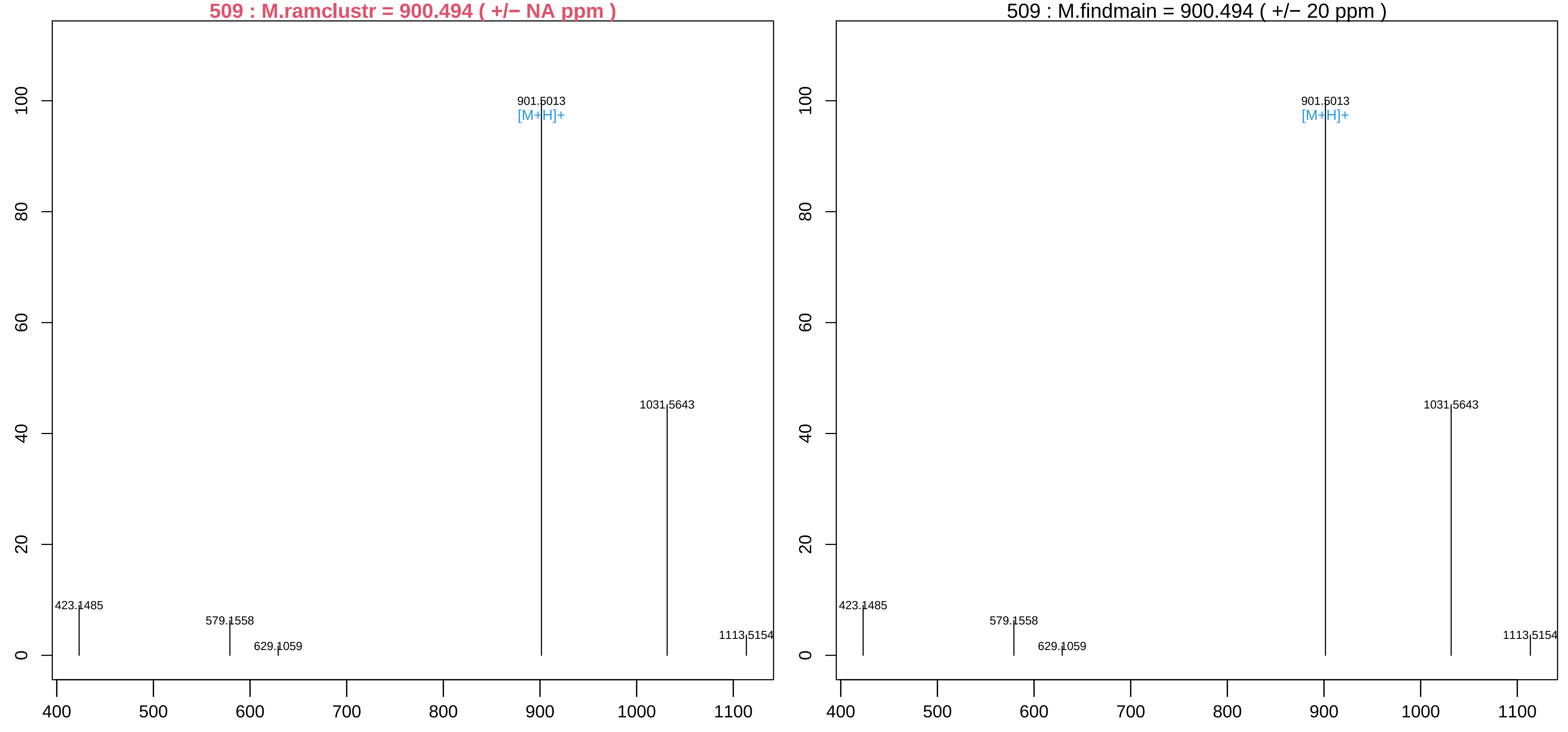

509 : M.ramclustr = 900.494 (+/− NA ppm)
901.5013
[M+H]+
1031.5643
423.1485
579.1558
629.1059
1113.5154
0
20
40
60
80
100
400
500
600
700
800
900
1000
1100
509 : M.findmain = 900.494 (+/− 20 ppm)
901.5013
[M+H]+
1031.5643
423.1485
579.1558
629.1059
1113.5154
0
20
40
60
80
100
400
500
600
700
800
900
1000
1100

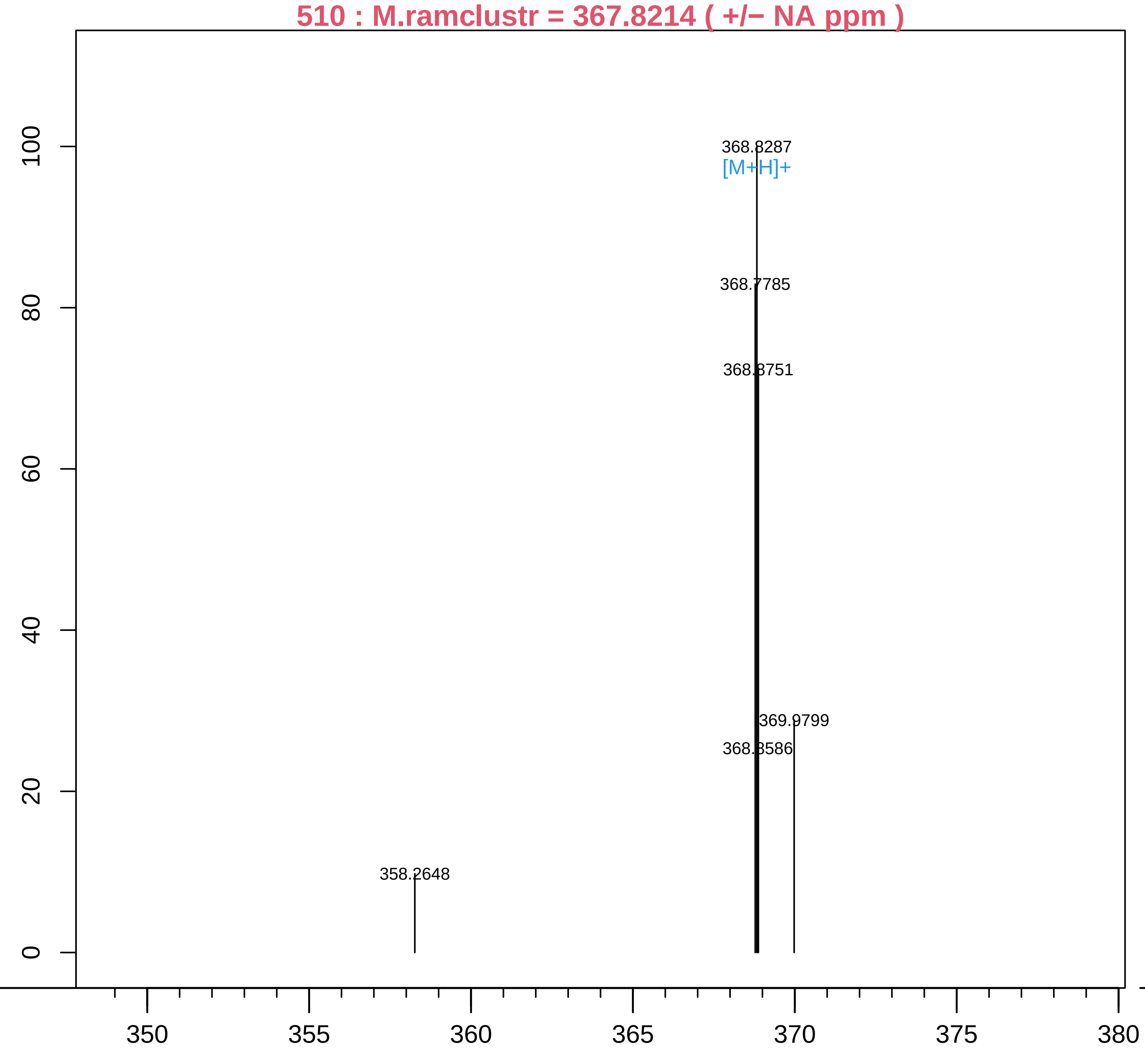
510 : M.ramclustr = 367.8214 (+/− NA ppm)
368.8287
[M+H]+
368.7785
368.8751
369.9799
368.8586
358.2648

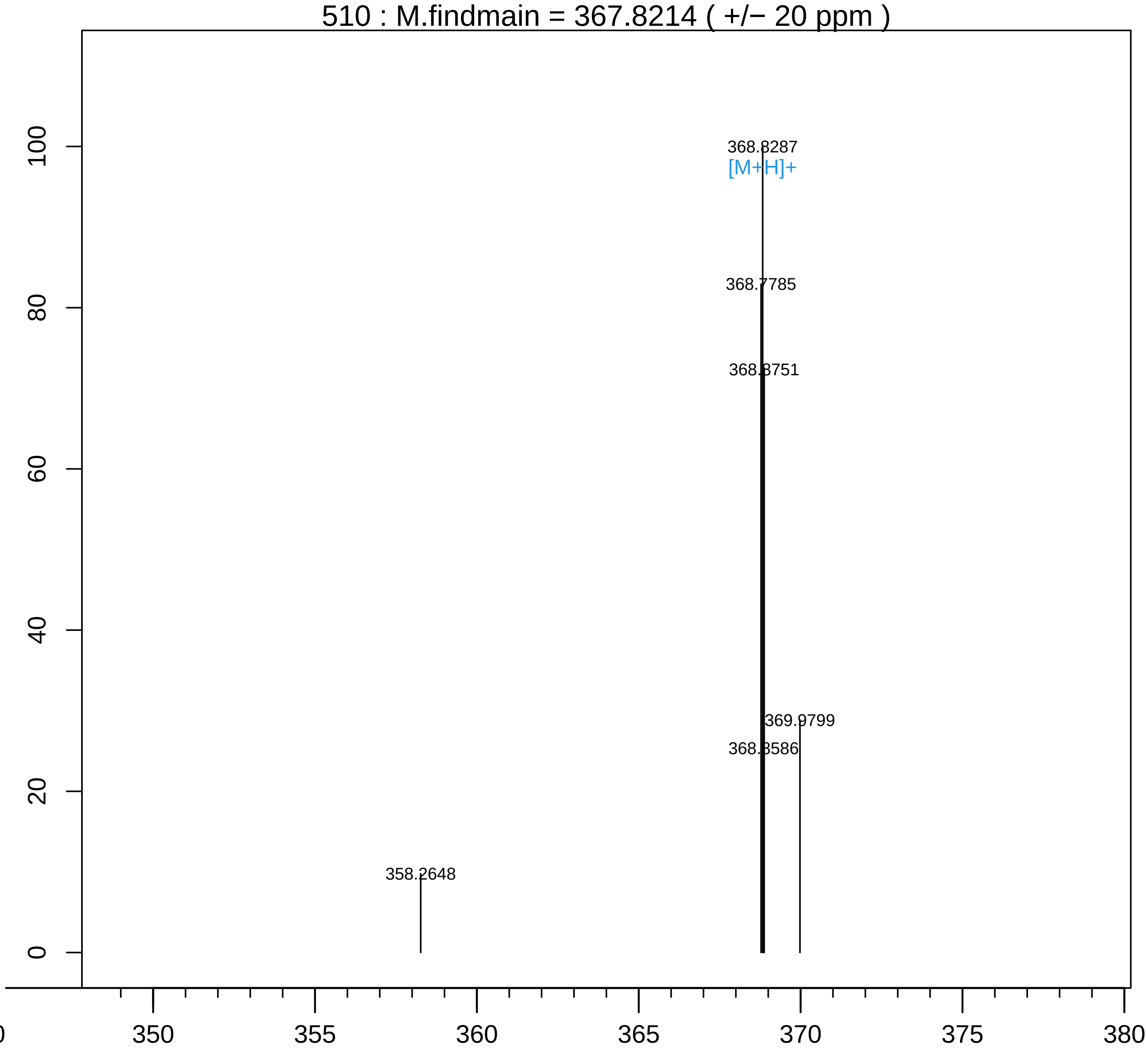
510 : M.findmain = 367.8214 (+/− 20 ppm)
368.8287
[M+H]+
368.7785
368.8751
369.9799
368.8586
358.2648

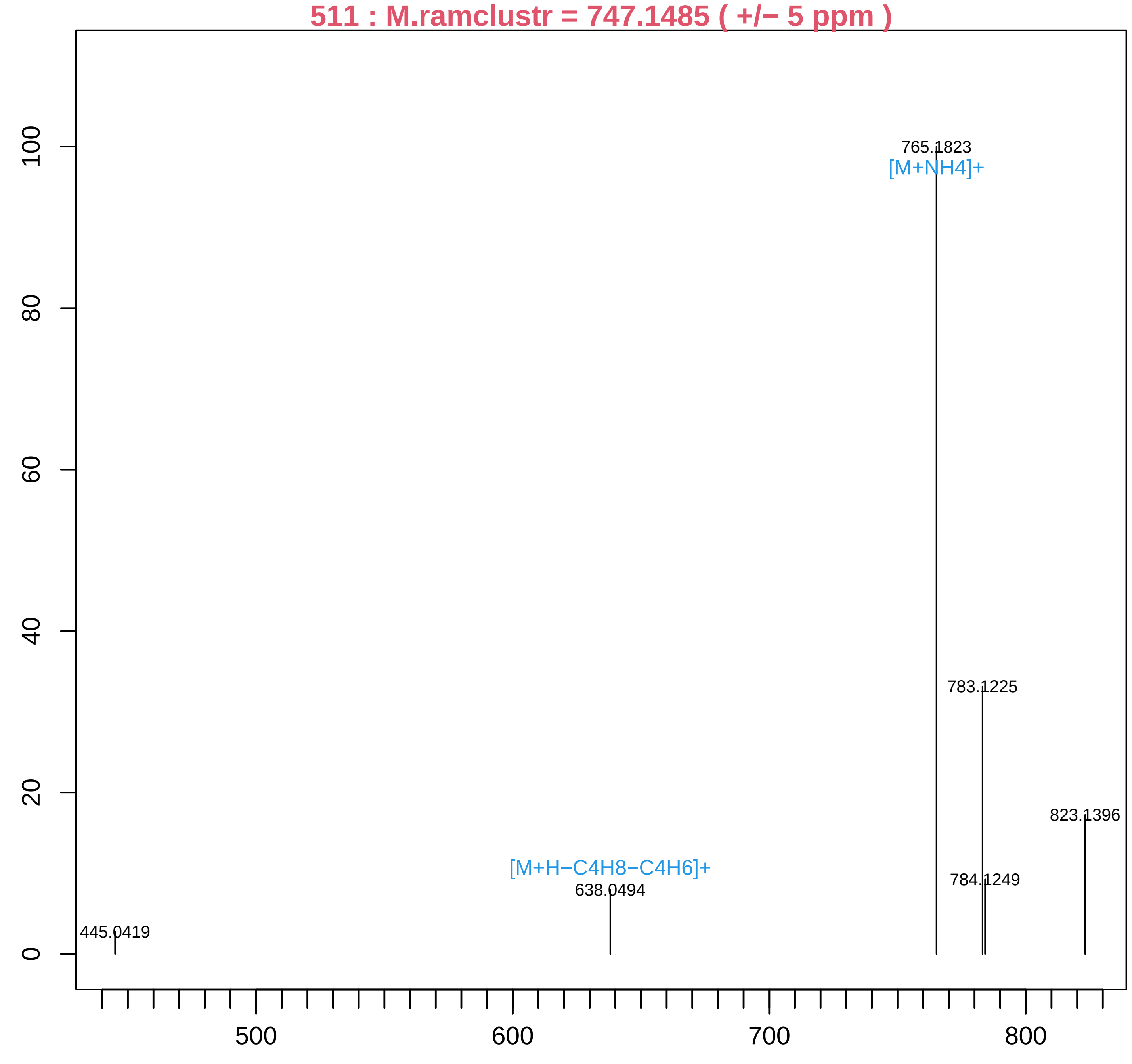
511 : M.ramclustr = 747.1485 (+/− 5 ppm)
765.1823
[M+NH4]+
783.1225
823.1396
[M+H−C4H8−C4H6]+
638.0494
784.1249
445.0419
0
20
40
60
80
100
500
600
700
800

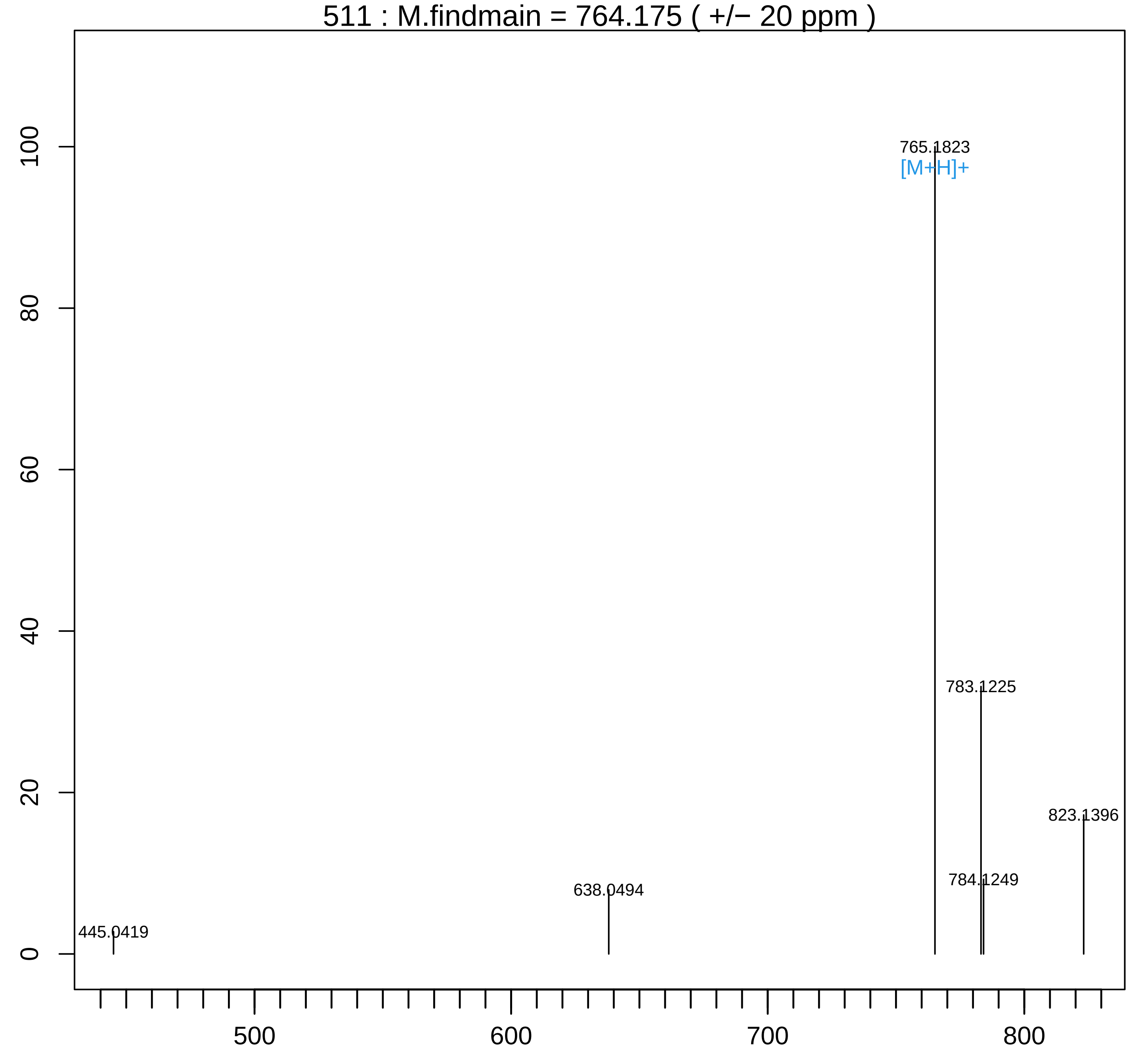
511 : M.findmain = 764.175 (+/− 20 ppm)
765.1823
[M+H]+
783.1225
823.1396
638.0494
784.1249
445.0419
0
20
40
60
80
100
500
600
700
800

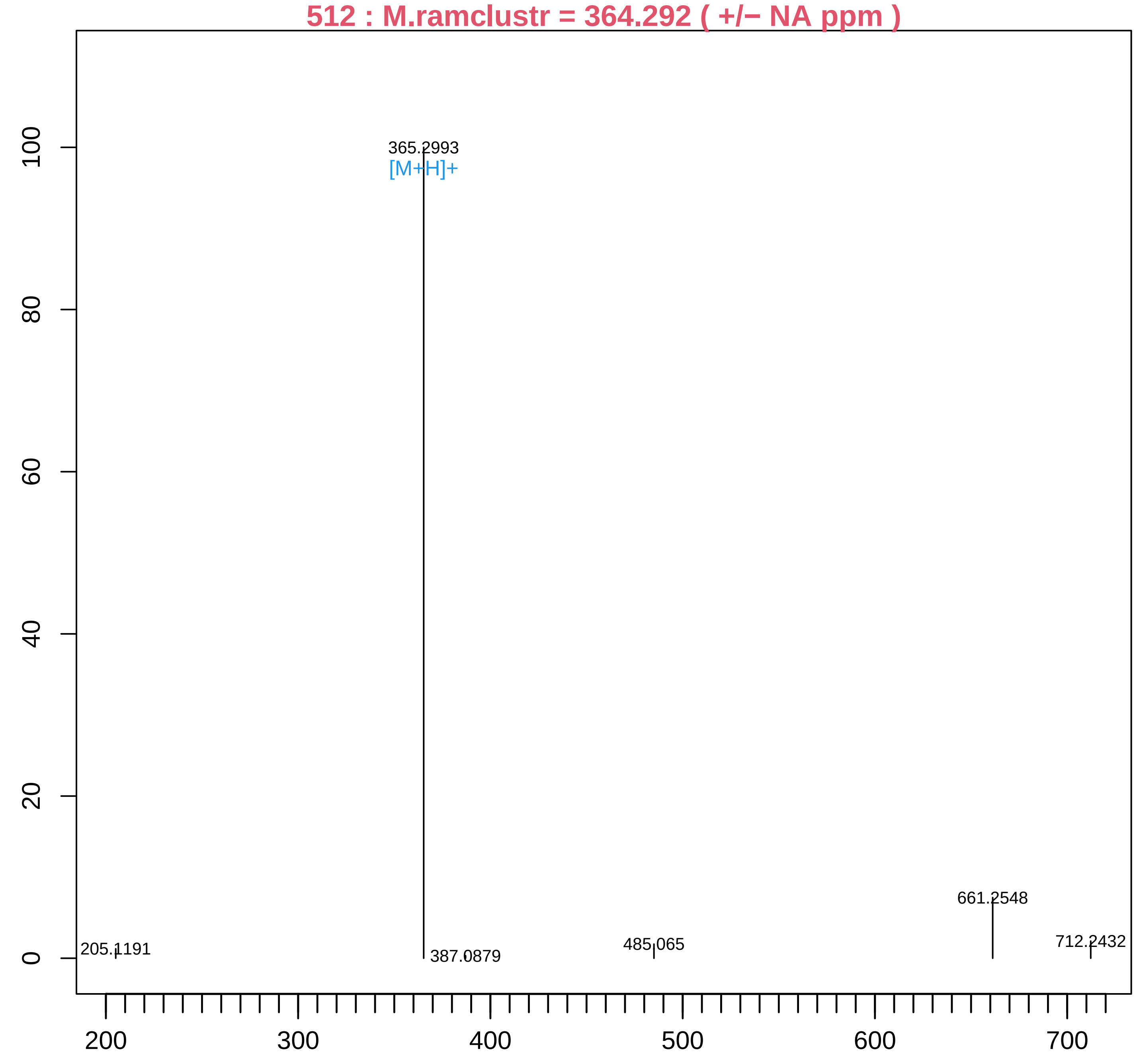
512 : M.ramclustr = 364.292 (+/− NA ppm)
365.2993
[M+H]+
205.1191
387.0879
485.065
661.2548
712.2432
0
20
40
60
80
100
200
300
400
500
600
700
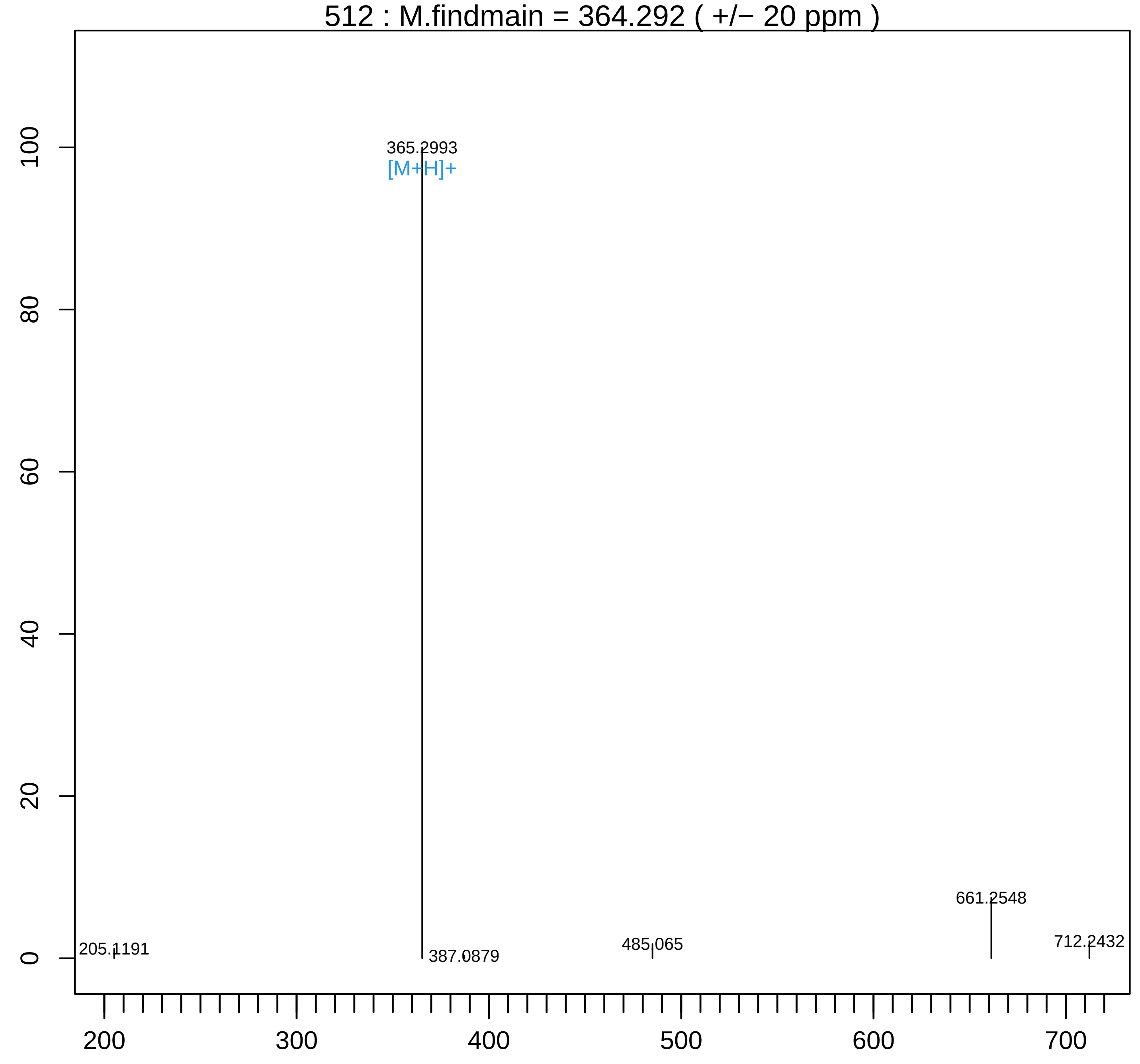
512 : M.findmain = 364.292 (+/− 20 ppm)
365.2993
[M+H]+
205.1191
387.0879
485.065
661.2548
712.2432
0
20
40
60
80
100
200
300
400
500
600
700

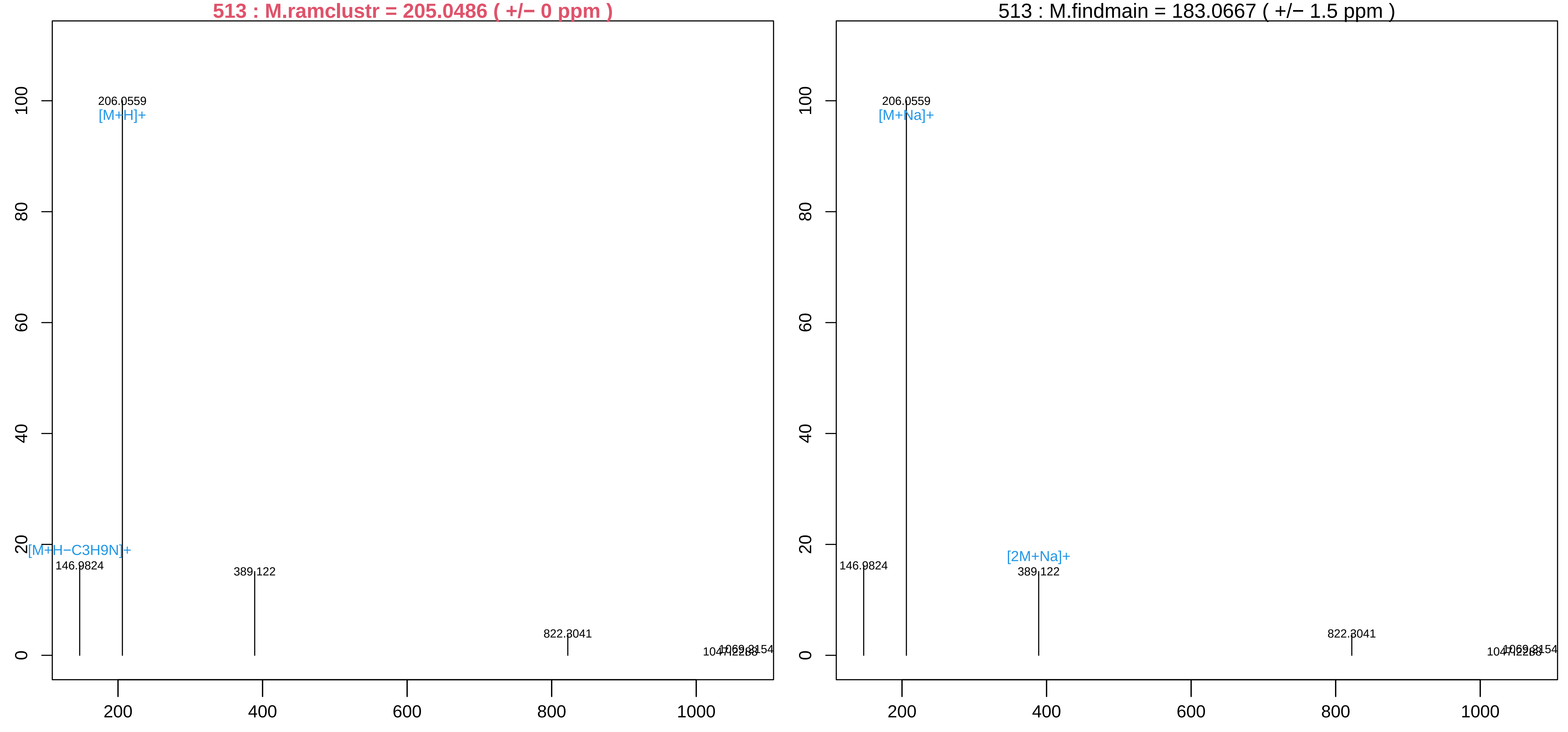
513 : M.ramclustr = 205.0486 (+/− 0 ppm)
206.0559
[M+H]+
[M+H−C3H9N]+
146.9824
389.122
822.3041
1047.2288
1069.2154
0
20
40
60
80
100
200
400
600
800
1000
513 : M.findmain = 183.0667 (+/− 1.5 ppm)
206.0559
[M+Na]+
146.9824
[2M+Na]+
389.122
822.3041
1047.2288
1069.2154
0
20
40
60
80
100
200
400
600
800
1000

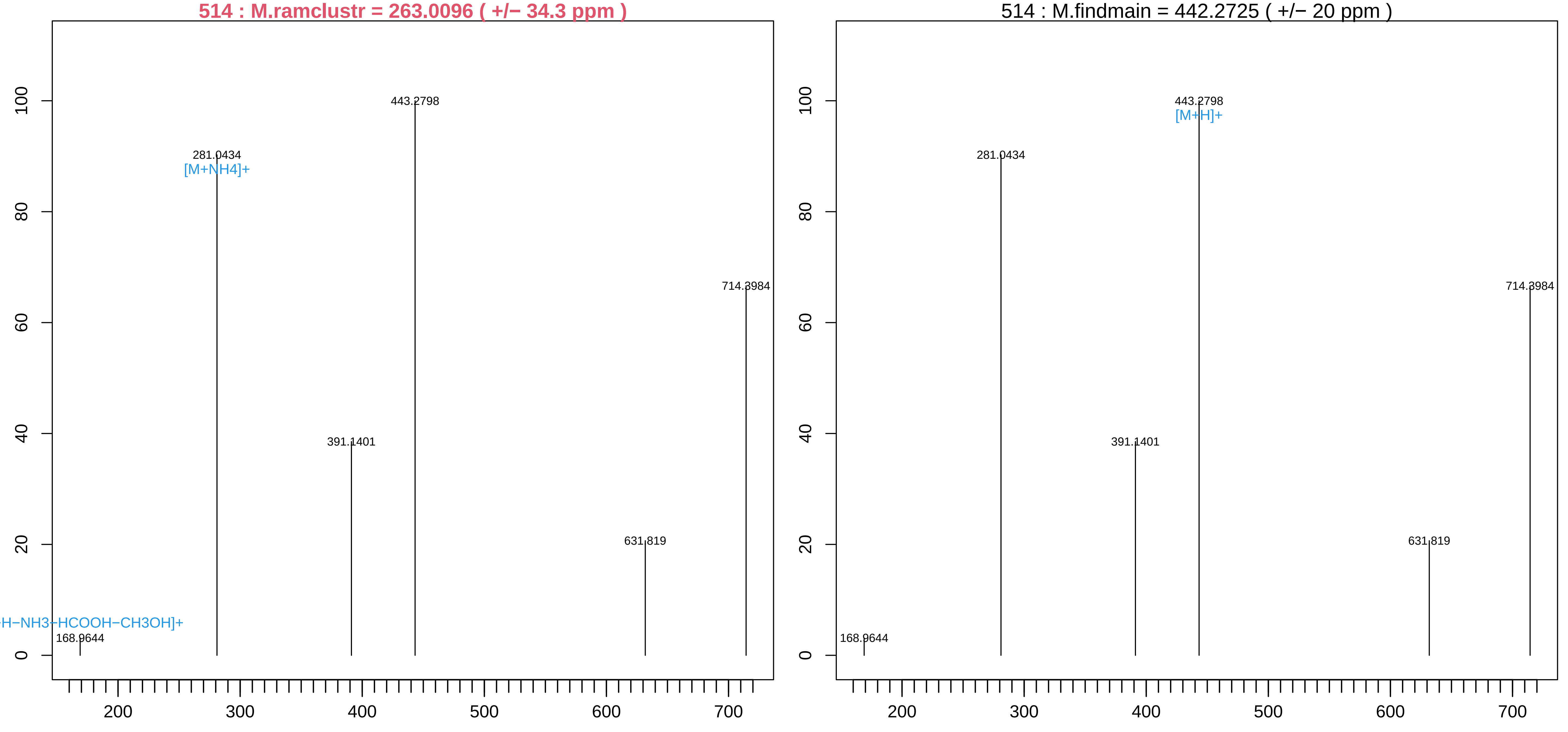

514 : M.ramclustr = 263.0096 (+/− 34.3 ppm)
281.0434
[M+NH4]+
443.2798
391.1401
631.819
714.3984
168.9644
H−NH3−HCOOH−CH3OH]+
514 : M.findmain = 442.2725 (+/− 20 ppm)
443.2798
[M+H]+
281.0434
391.1401
631.819
714.3984
168.9644

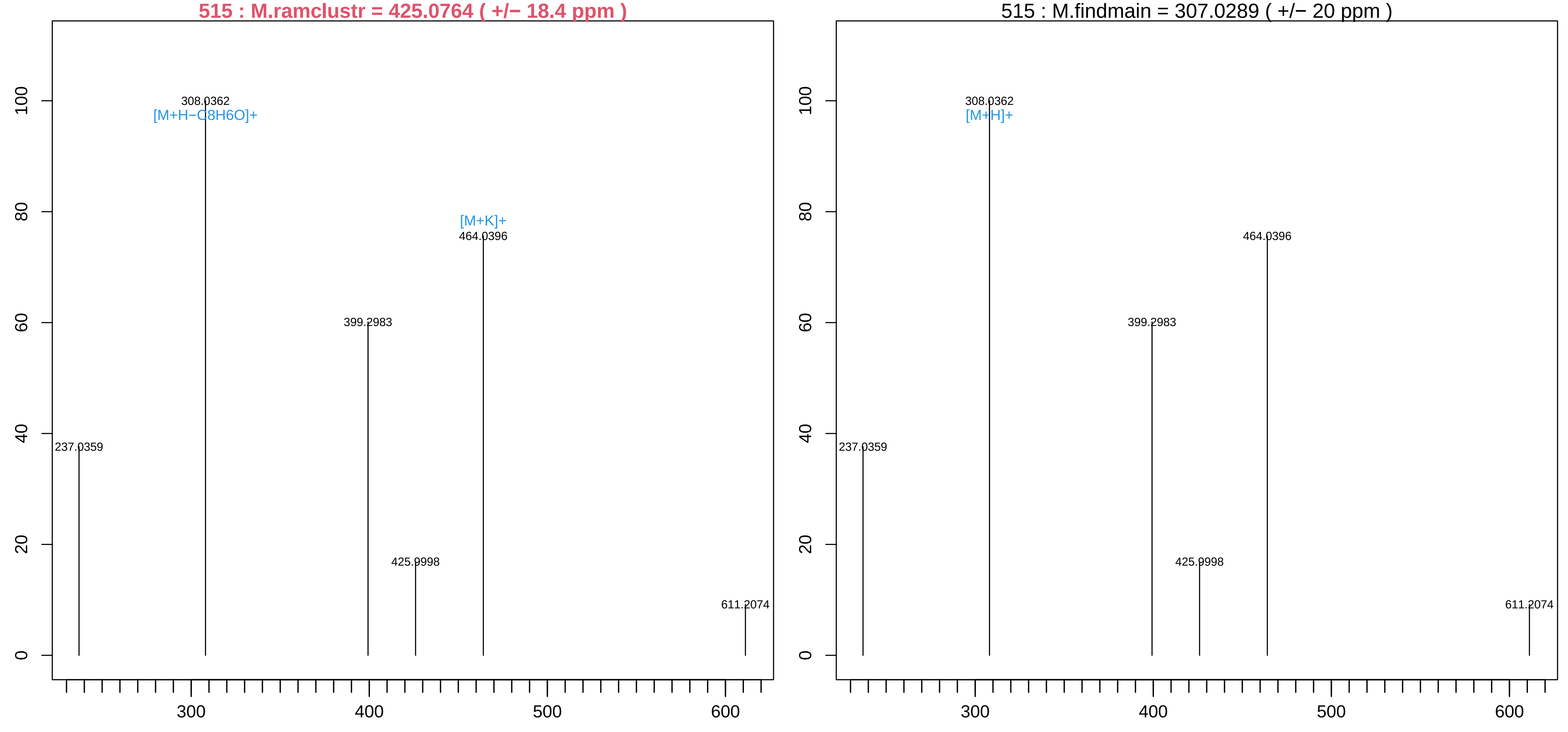

515 : M.ramclustr = 425.0764 (+/− 18.4 ppm)
308.0362
[M+H−C8H6O]+
[M+K]+
464.0396
399.2983
237.0359
425.9998
611.2074
0
20
40
60
80
100
300
400
500
600
515 : M.findmain = 307.0289 (+/− 20 ppm)
308.0362
[M+H]+
464.0396
399.2983
237.0359
425.9998
611.2074
0
20
40
60
80
100
300
400
500
600

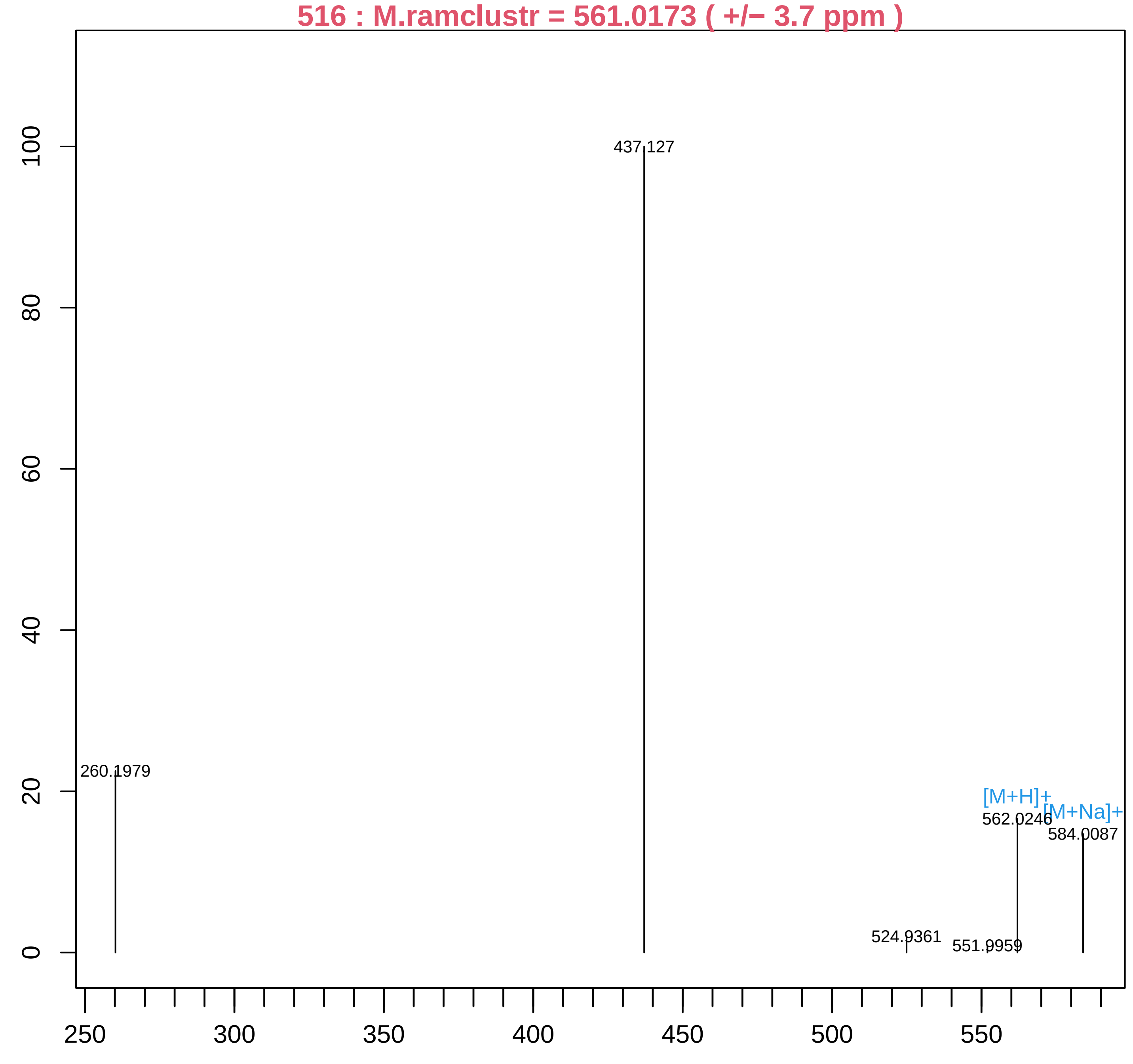
516 : M.ramclustr = 561.0173 (+/− 3.7 ppm)
437.127
260.1979
[M+H]+
562.0246
[M+Na]+
584.0087
524.9361
551.9959
0
20
40
60
80
100
250
300
350
400
450
500
550

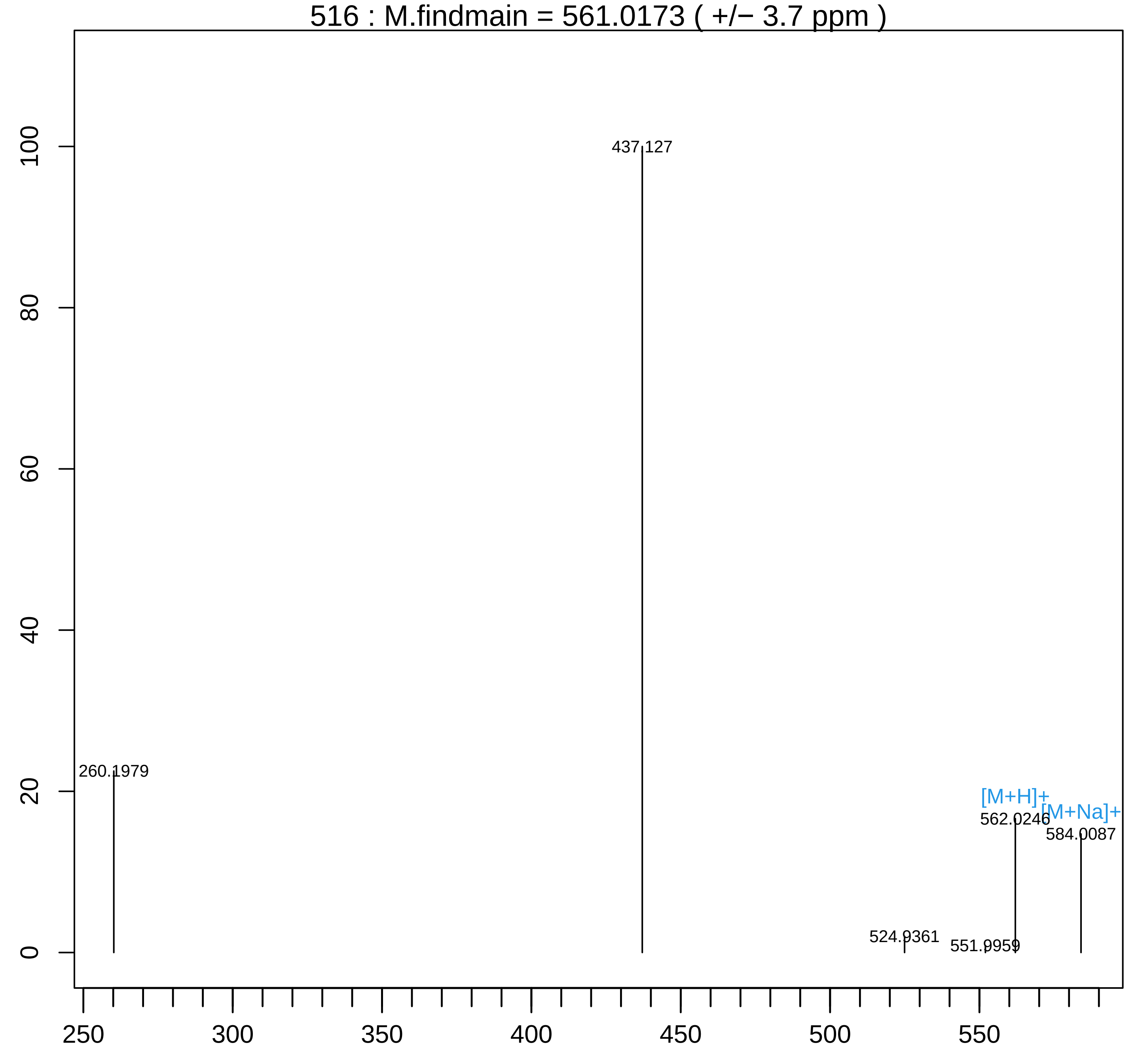
516 : M.findmain = 561.0173 (+/− 3.7 ppm)
437.127
260.1979
[M+H]+
562.0246
[M+Na]+
584.0087
524.9361
551.9959
0
20
40
60
80
100
250
300
350
400
450
500
550

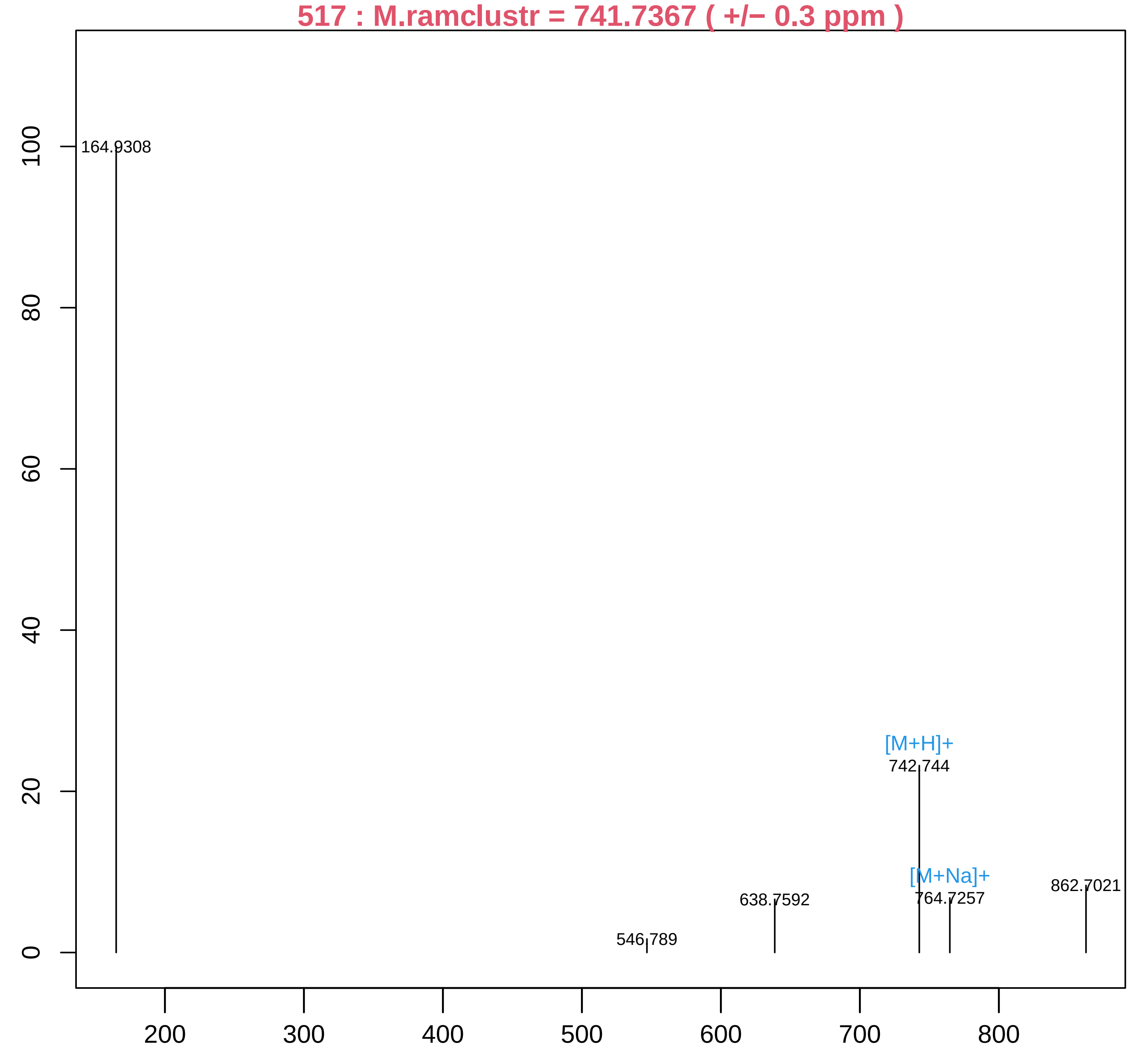
517 : M.ramclustr = 741.7367 (+/− 0.3 ppm)
164.9308
546.789
638.7592
[M+H]+
742.744
[M+Na]+
764.7257
862.7021
0
20
40
60
80
100
200
300
400
500
600
700
800

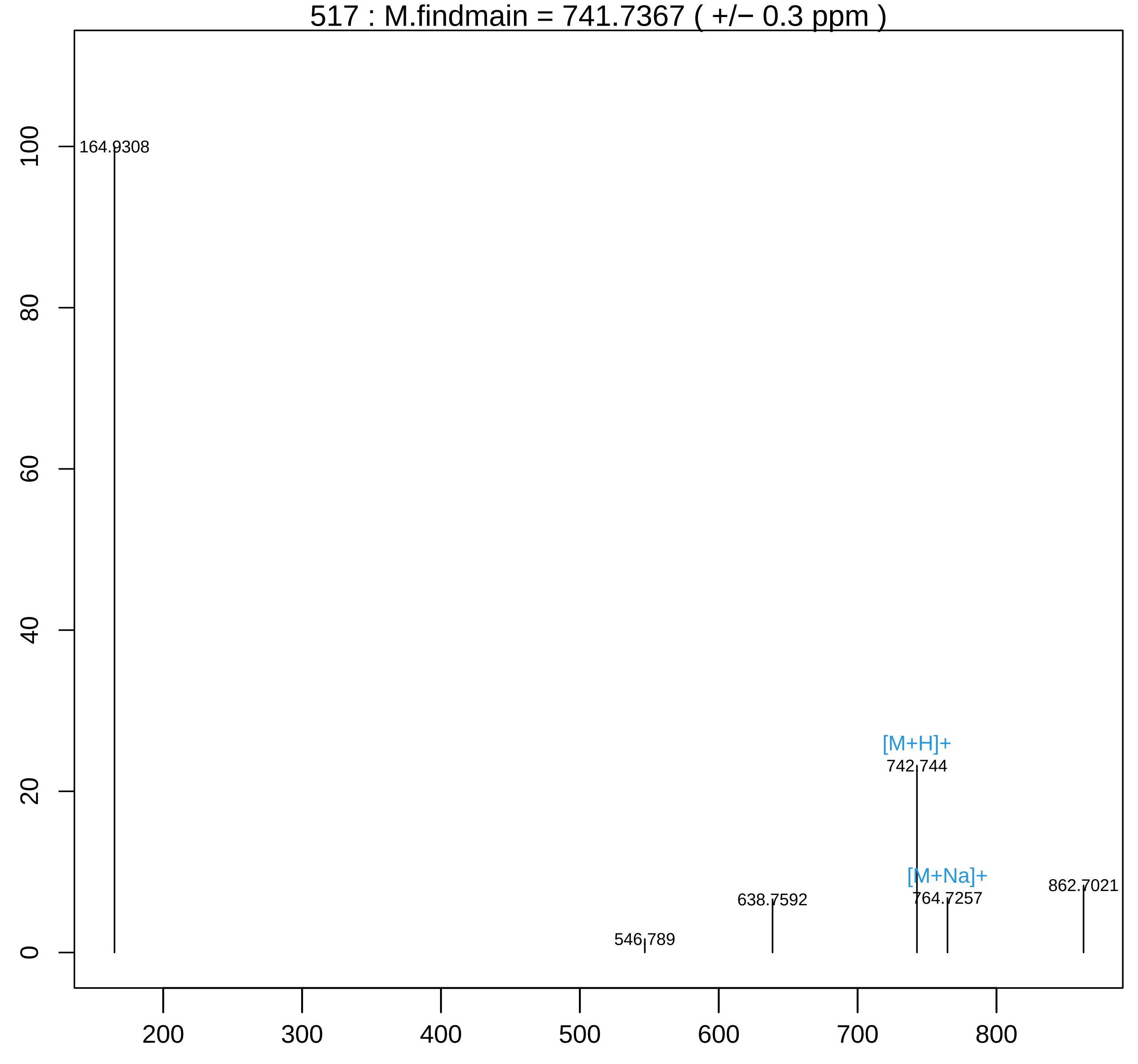
517 : M.findmain = 741.7367 (+/− 0.3 ppm)
164.9308
546.789
638.7592
[M+H]+
742.744
[M+Na]+
764.7257
862.7021
0
20
40
60
80
100
200
300
400
500
600
700
800

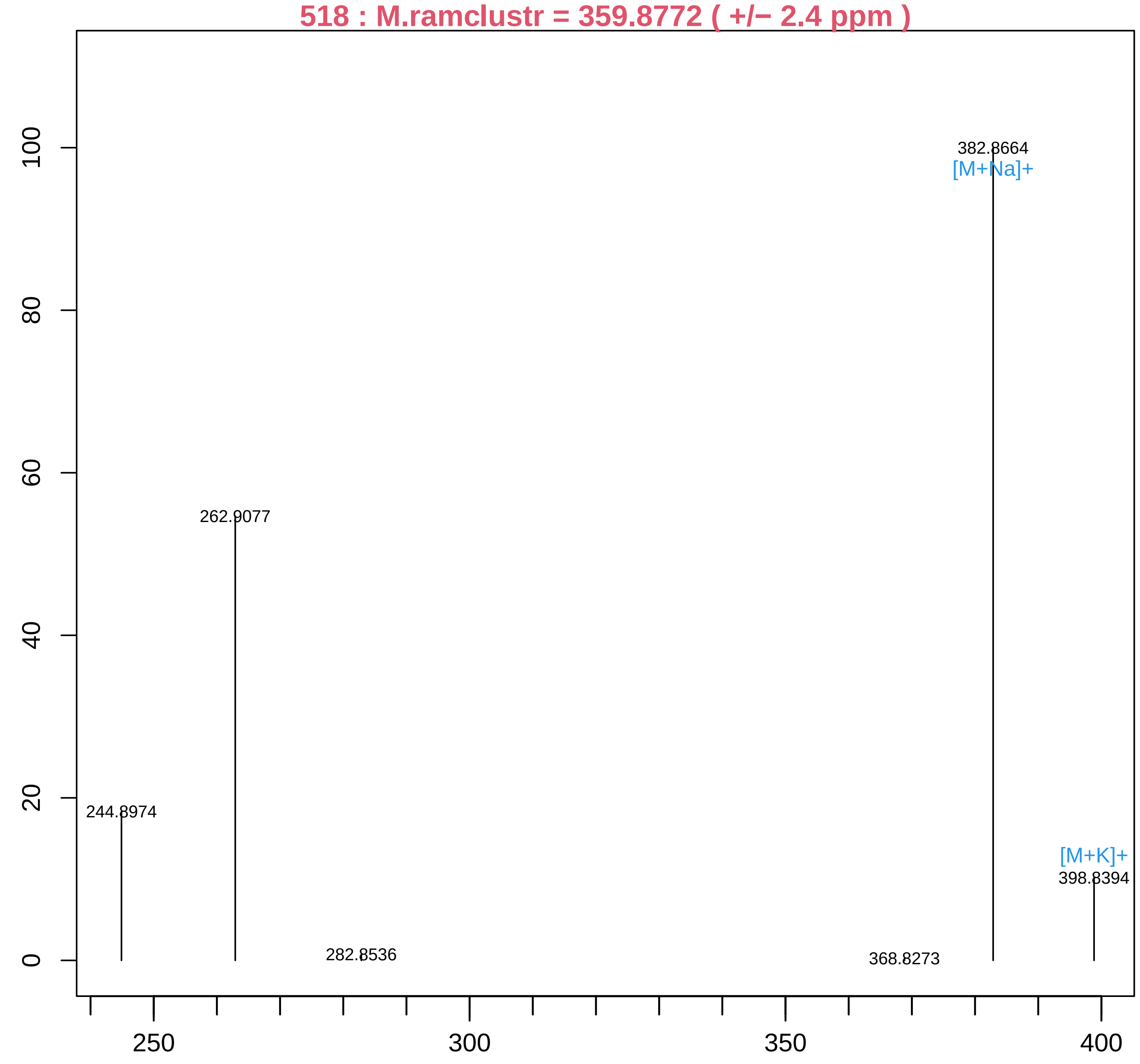
518 : M.ramclustr = 359.8772 (+/− 2.4 ppm)
382.8664
[M+Na]+
262.9077
244.8974
[M+K]+
398.8394
282.8536
368.8273
0
20
40
60
80
100
250
300
350
400

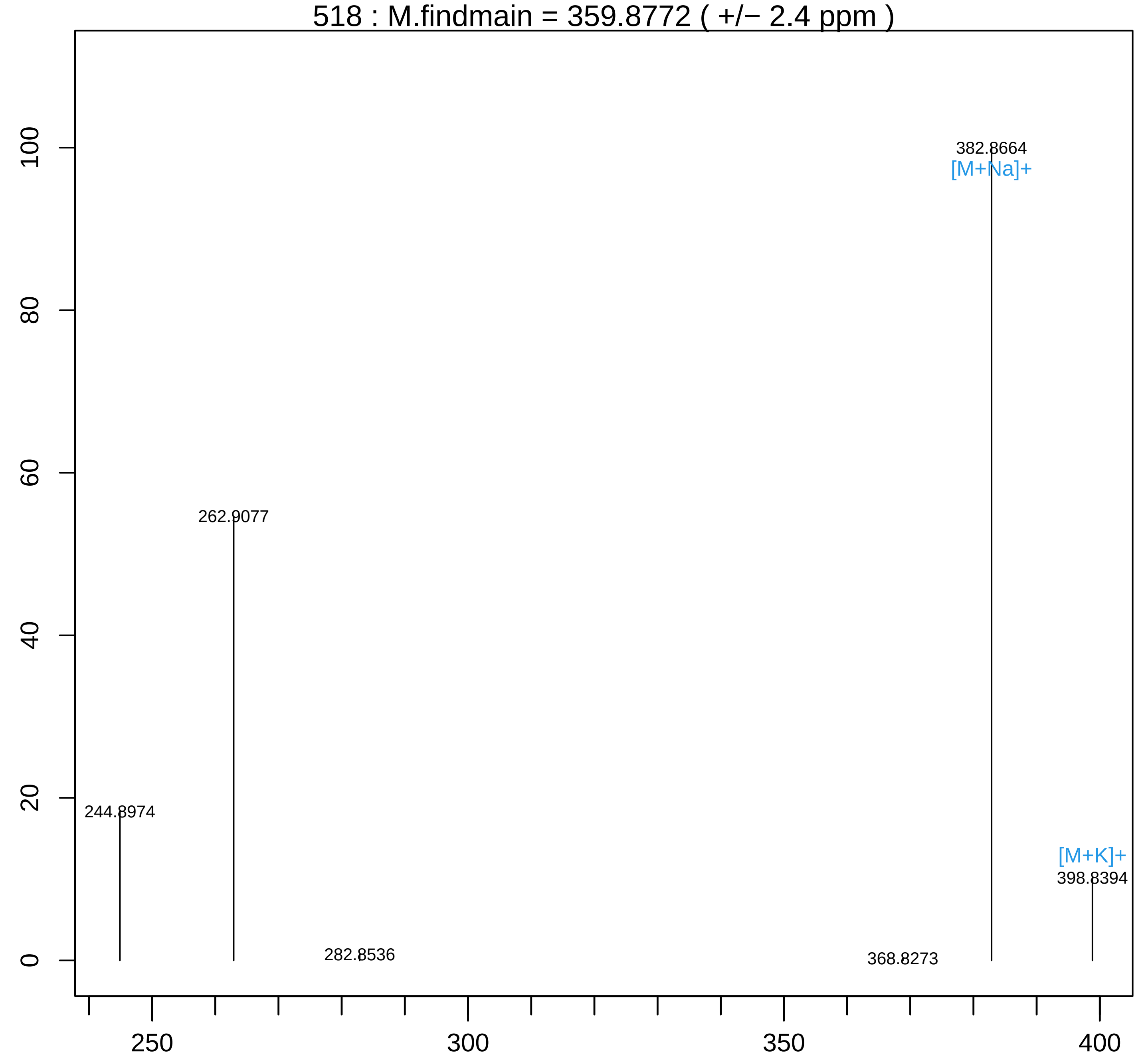
518 : M.findmain = 359.8772 (+/− 2.4 ppm)
382.8664
[M+Na]+
262.9077
244.8974
[M+K]+
398.8394
282.8536
368.8273
0
20
40
60
80
100
250
300
350
400

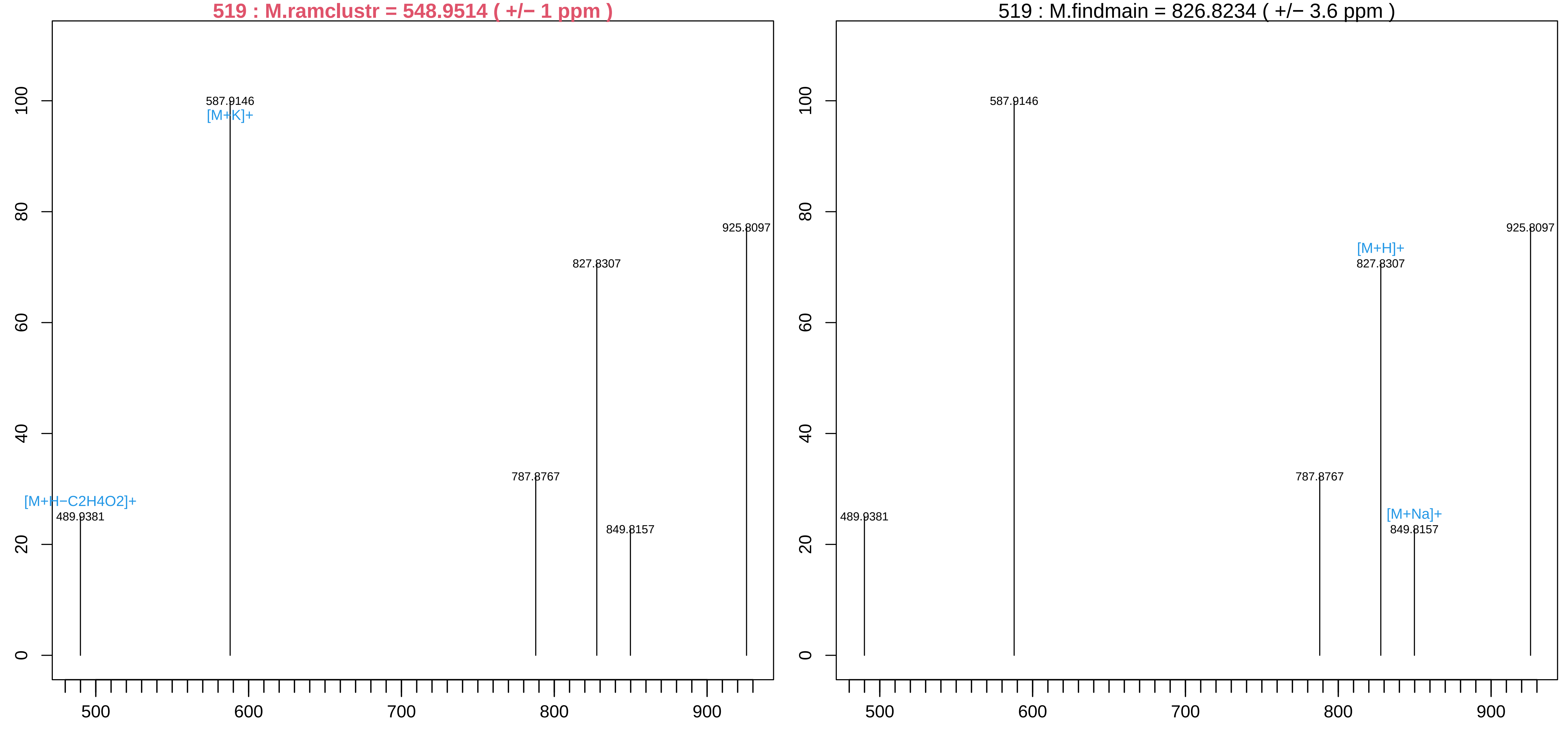

519 : M.ramclustr = 548.9514 (+/− 1 ppm)
587.9146
[M+K]+
925.8097
827.8307
787.8767
[M+H−C2H4O2]+
489.9381
849.8157
519 : M.findmain = 826.8234 (+/− 3.6 ppm)
587.9146
925.8097
[M+H]+
827.8307
787.8767
489.9381
[M+Na]+
849.8157

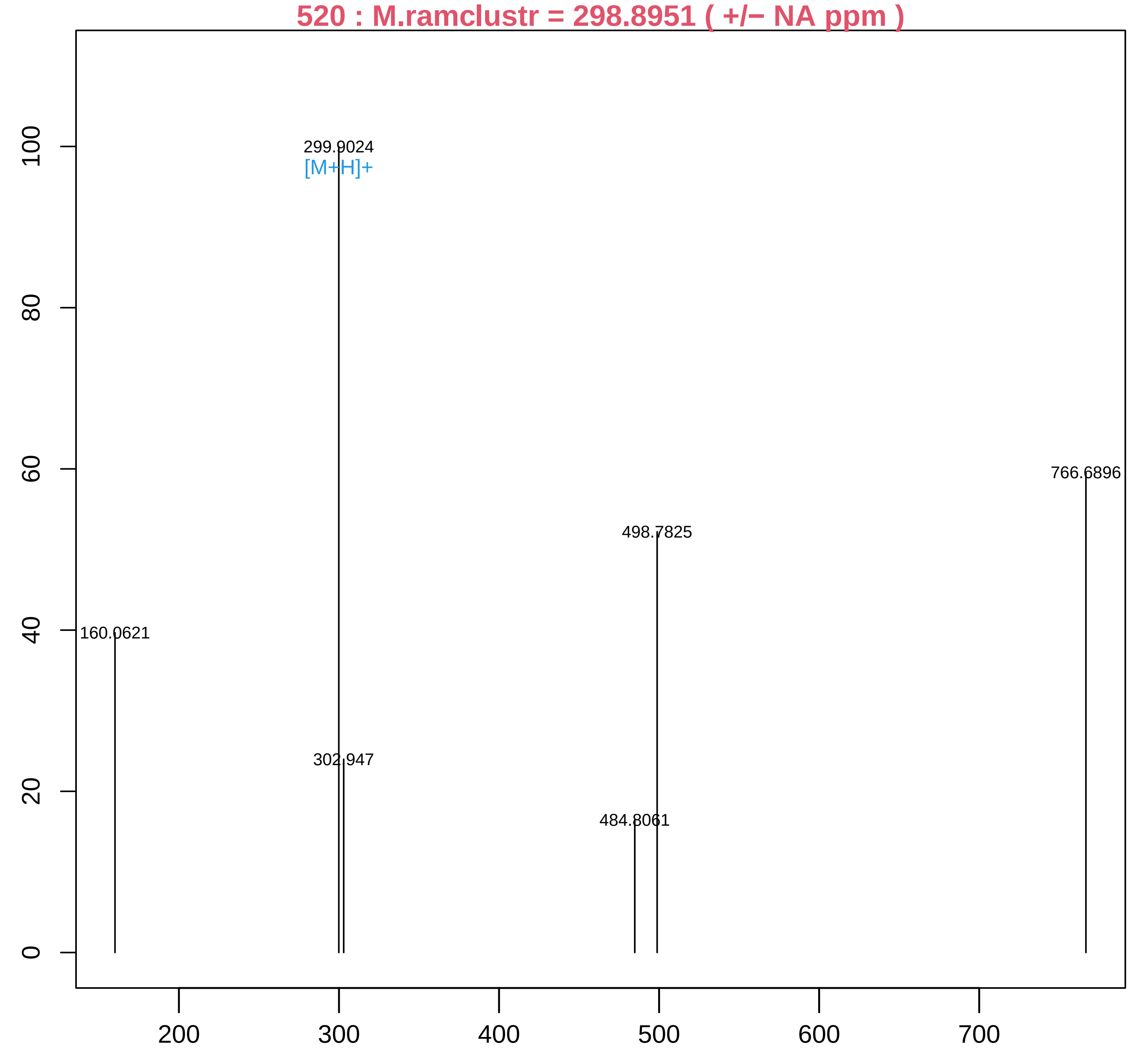
520 : M.ramclustr = 298.8951 (+/− NA ppm)
299.9024
[M+H]+
766.6896
498.7825
160.0621
302.947
484.8061
0
20
40
60
80
100
200
300
400
500
600
700

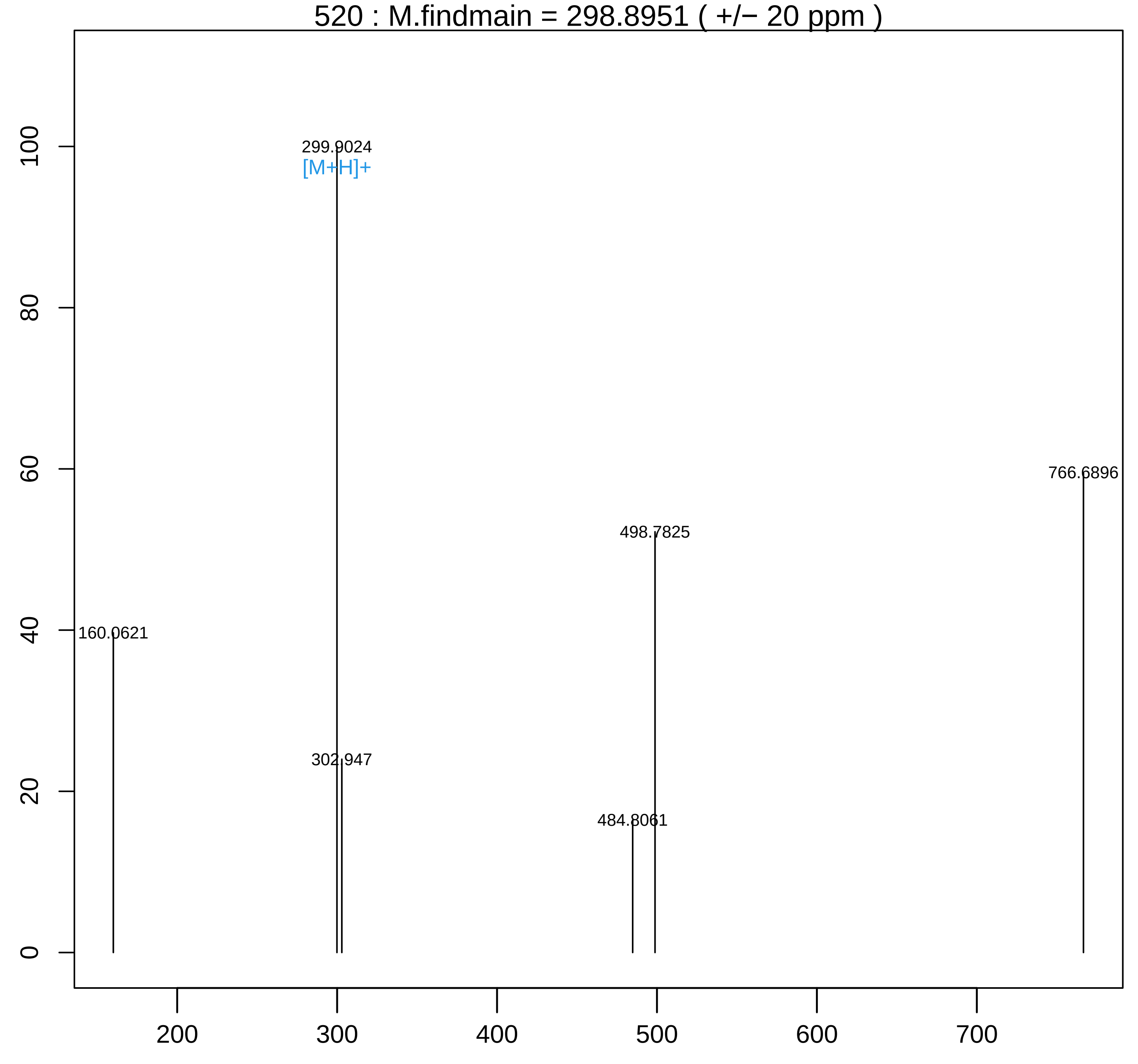
520 : M.findmain = 298.8951 (+/− 20 ppm)
299.9024
[M+H]+
766.6896
498.7825
160.0621
302.947
484.8061
0
20
40
60
80
100
200
300
400
500
600
700

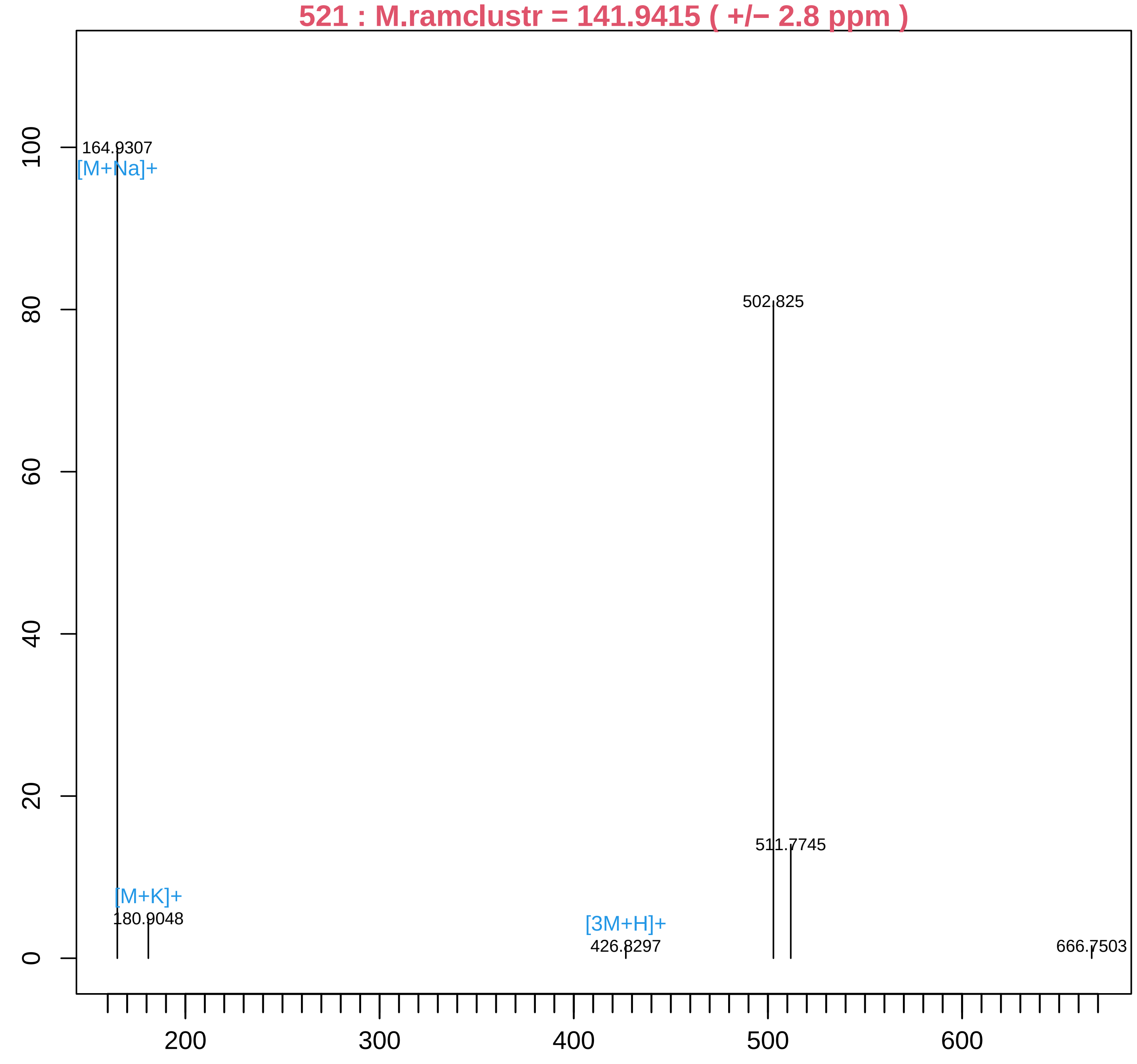
521 : M.ramclustr = 141.9415 (+/− 2.8 ppm)
164.9307
[M+Na]+
502.825
511.7745
[M+K]+
180.9048
[3M+H]+
426.8297
666.7503
0
20
40
60
80
100
200
300
400
500
600

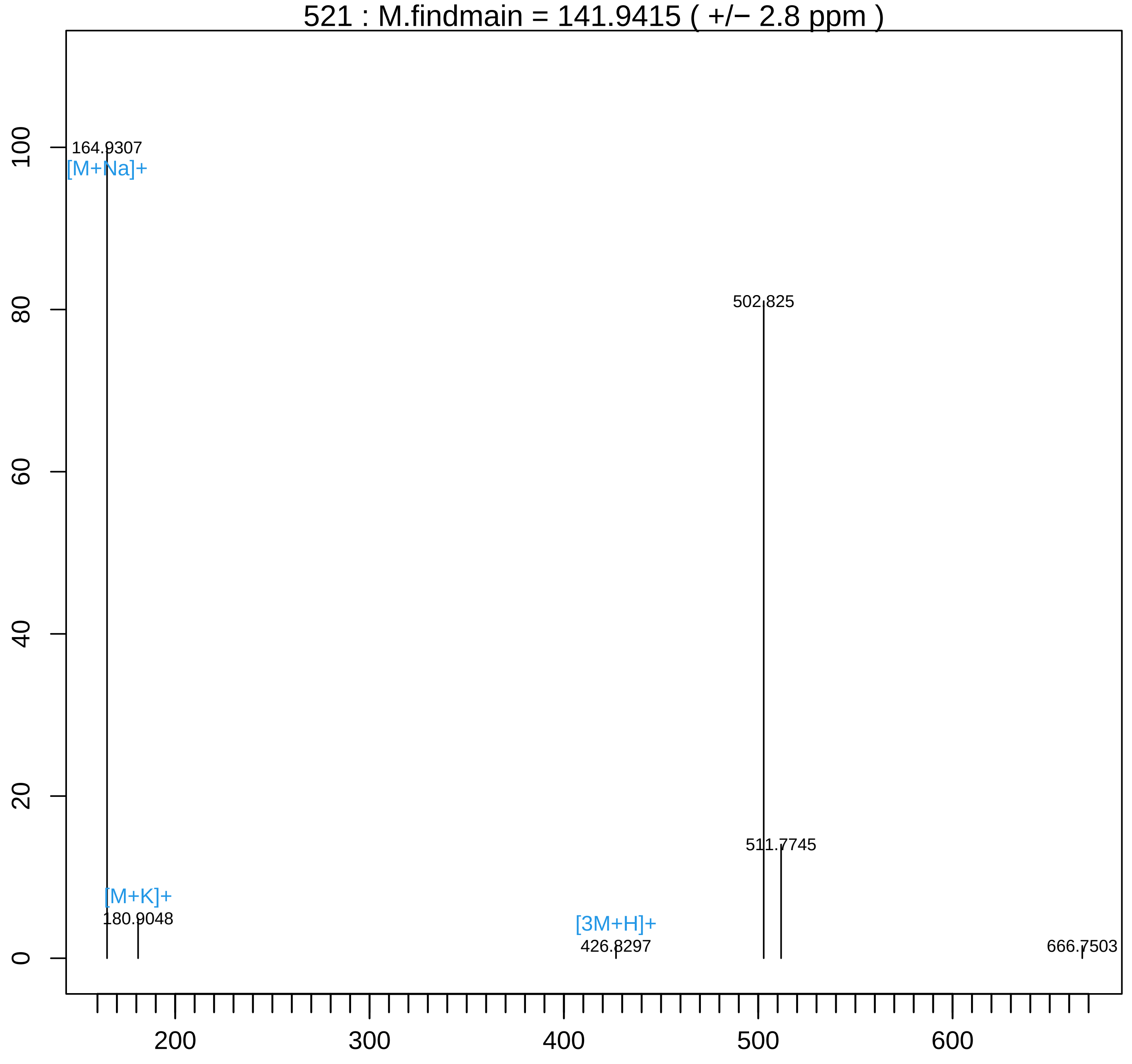
521 : M.findmain = 141.9415 (+/− 2.8 ppm)
164.9307
[M+Na]+
502.825
511.7745
[M+K]+
180.9048
[3M+H]+
426.8297
666.7503
0
20
40
60
80
100
200
300
400
500
600

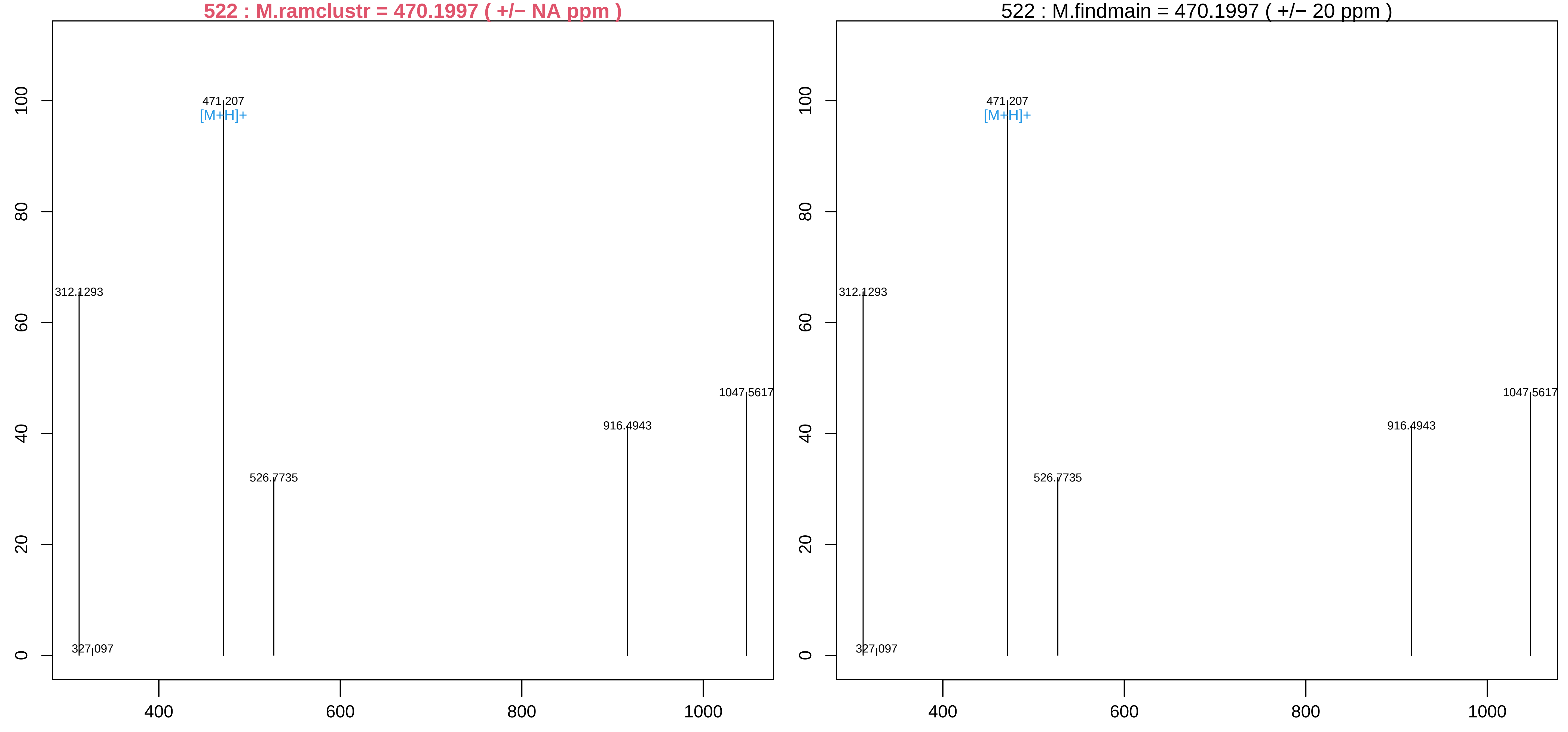
522 : M.ramclustr = 470.1997 (+/− NA ppm)
522 : M.findmain = 470.1997 (+/− 20 ppm)
471.207
[M+H]+
312.1293
1047.5617
916.4943
526.7735
327.097
0
20
40
60
80
100
400
600
800
1000

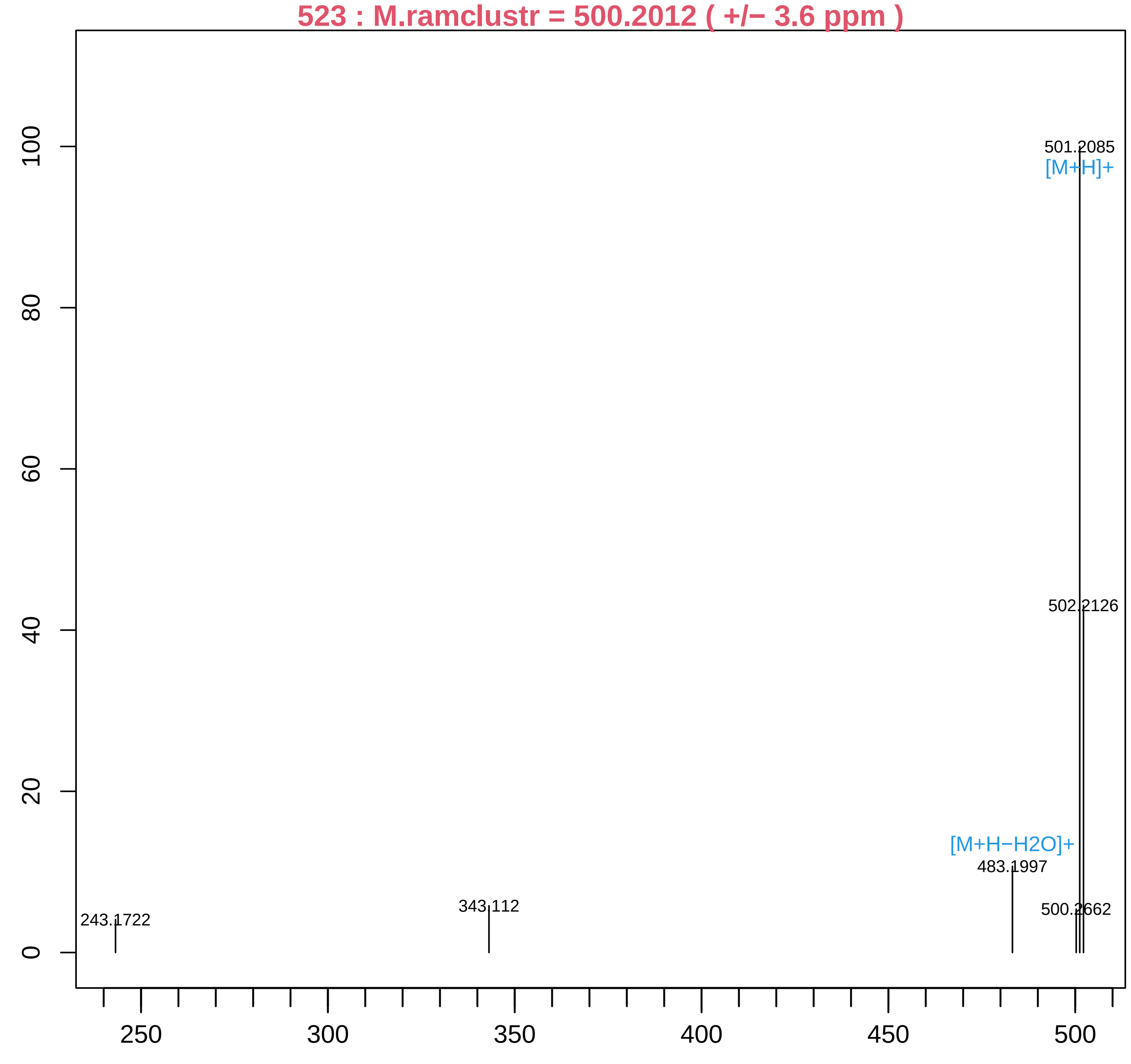

523 : M.ramclustr = 500.2012 (+/− 3.6 ppm)
501.2085
[M+H]+
502.2126
[M+H−H2O]+
483.1997
500.2662
343.112
243.1722
0
20
40
60
80
100
250
300
350
400
450
500

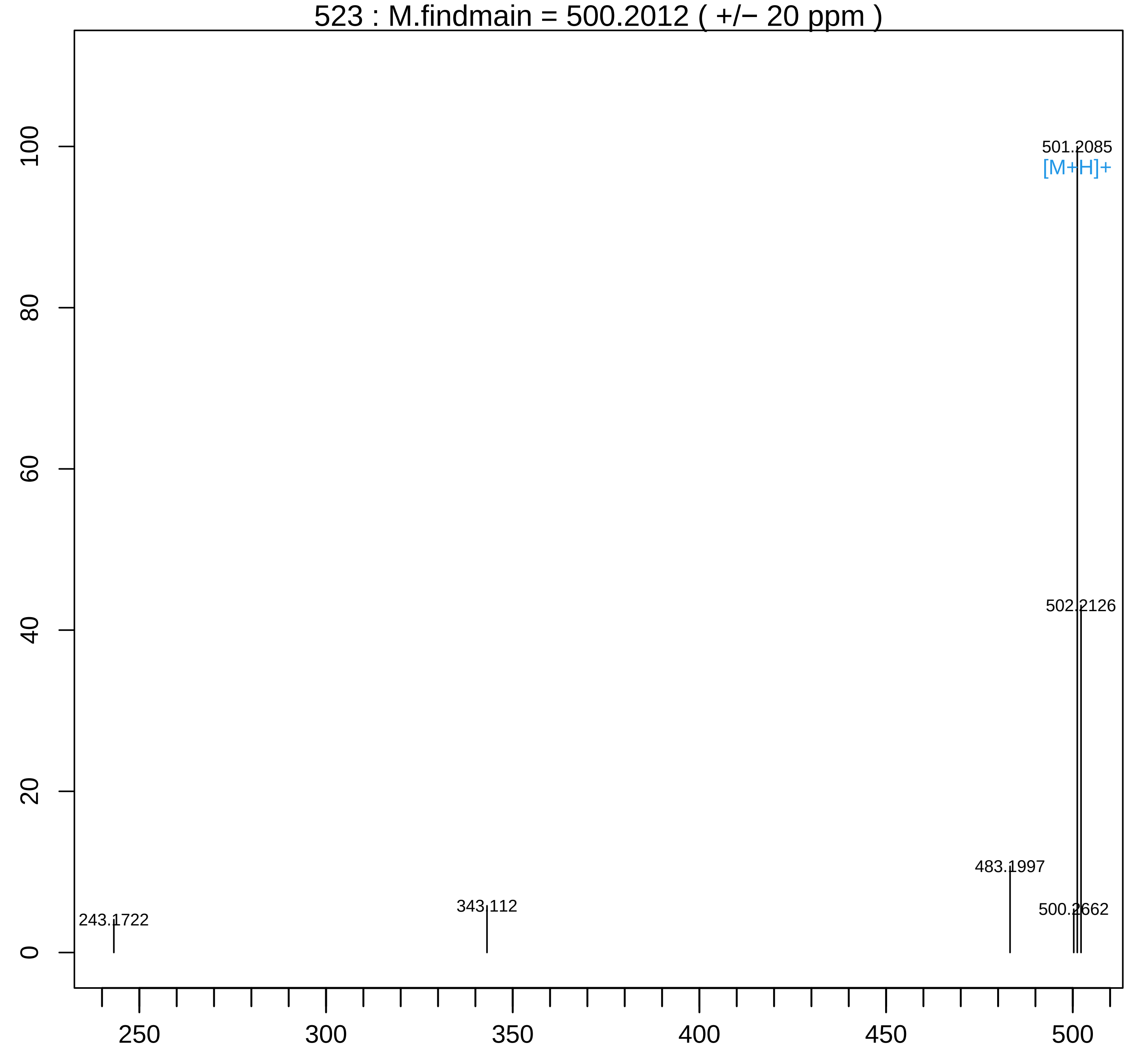

523 : M.findmain = 500.2012 (+/− 20 ppm)
501.2085
[M+H]+
502.2126
483.1997
500.2662
343.112
243.1722
0
20
40
60
80
100
250
300
350
400
450
500

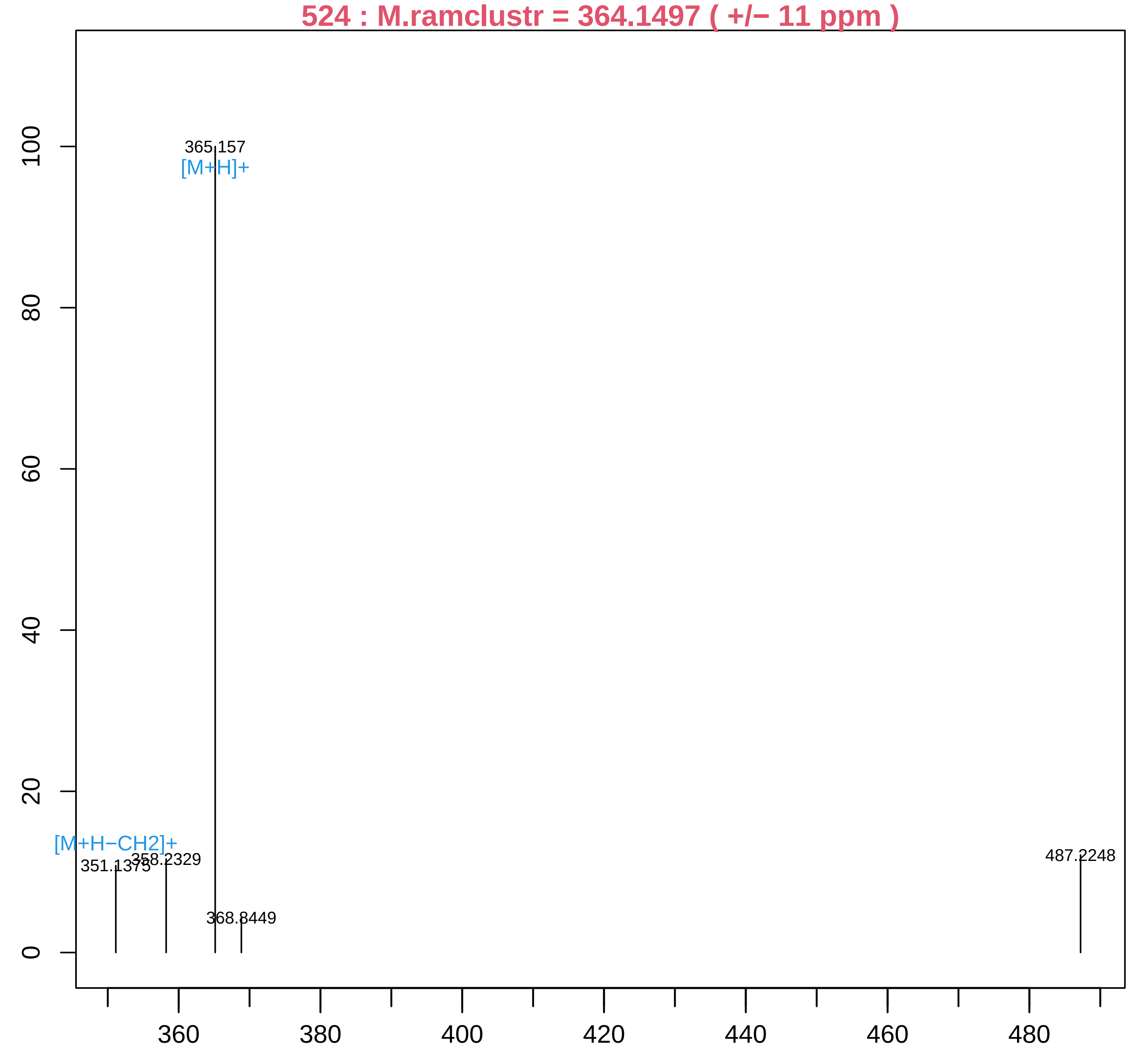

524 : M.ramclustr = 364.1497 (+/− 11 ppm)
365.157
[M+H]+
[M+H−CH2]+
351.1375
358.2329
368.8449
487.2248
0
20
40
60
80
100
360
380
400
420
440
460
480

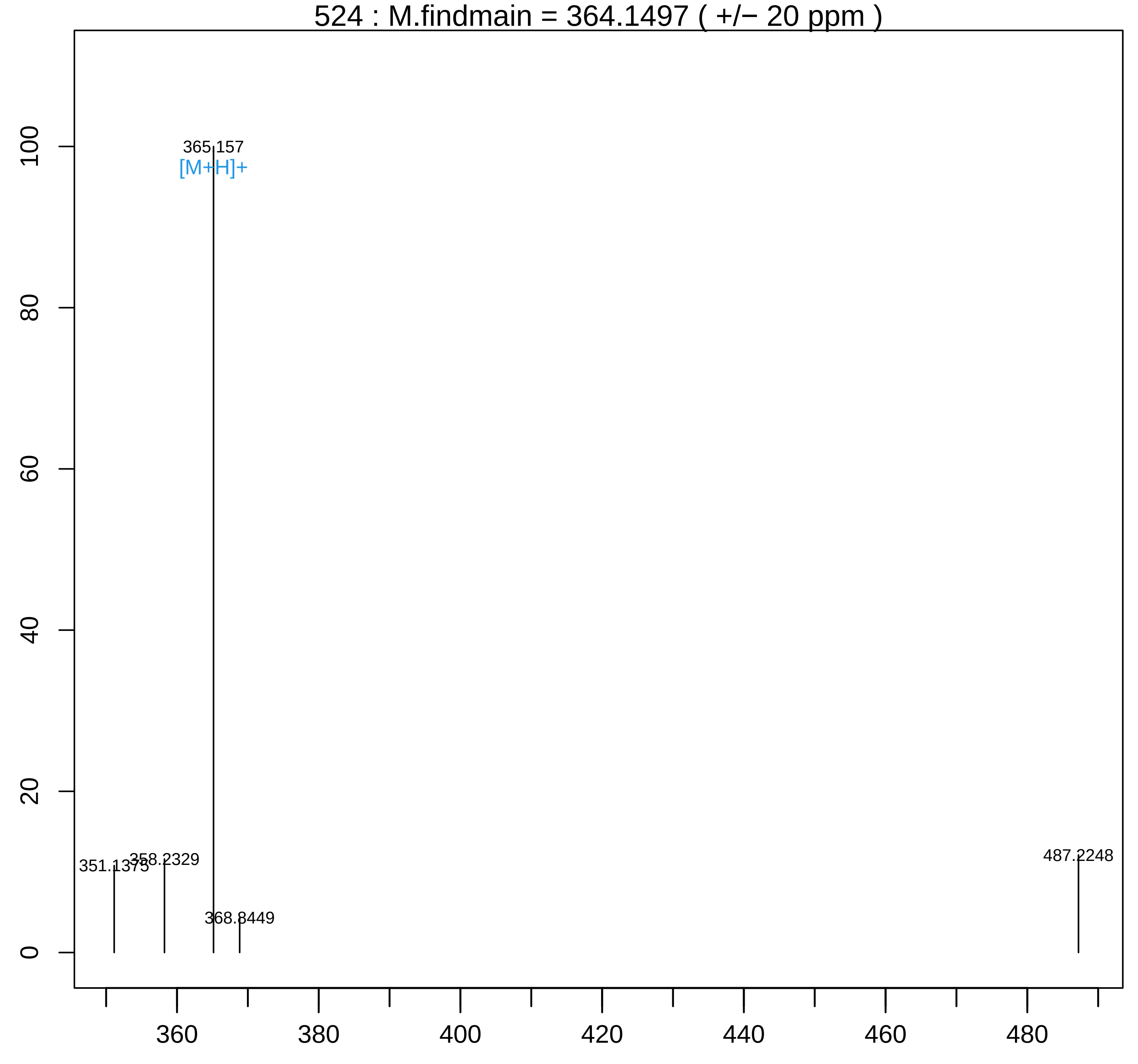

524 : M.findmain = 364.1497 (+/− 20 ppm)
365.157
[M+H]+
351.1375
358.2329
368.8449
487.2248
0
20
40
60
80
100
360
380
400
420
440
460
480

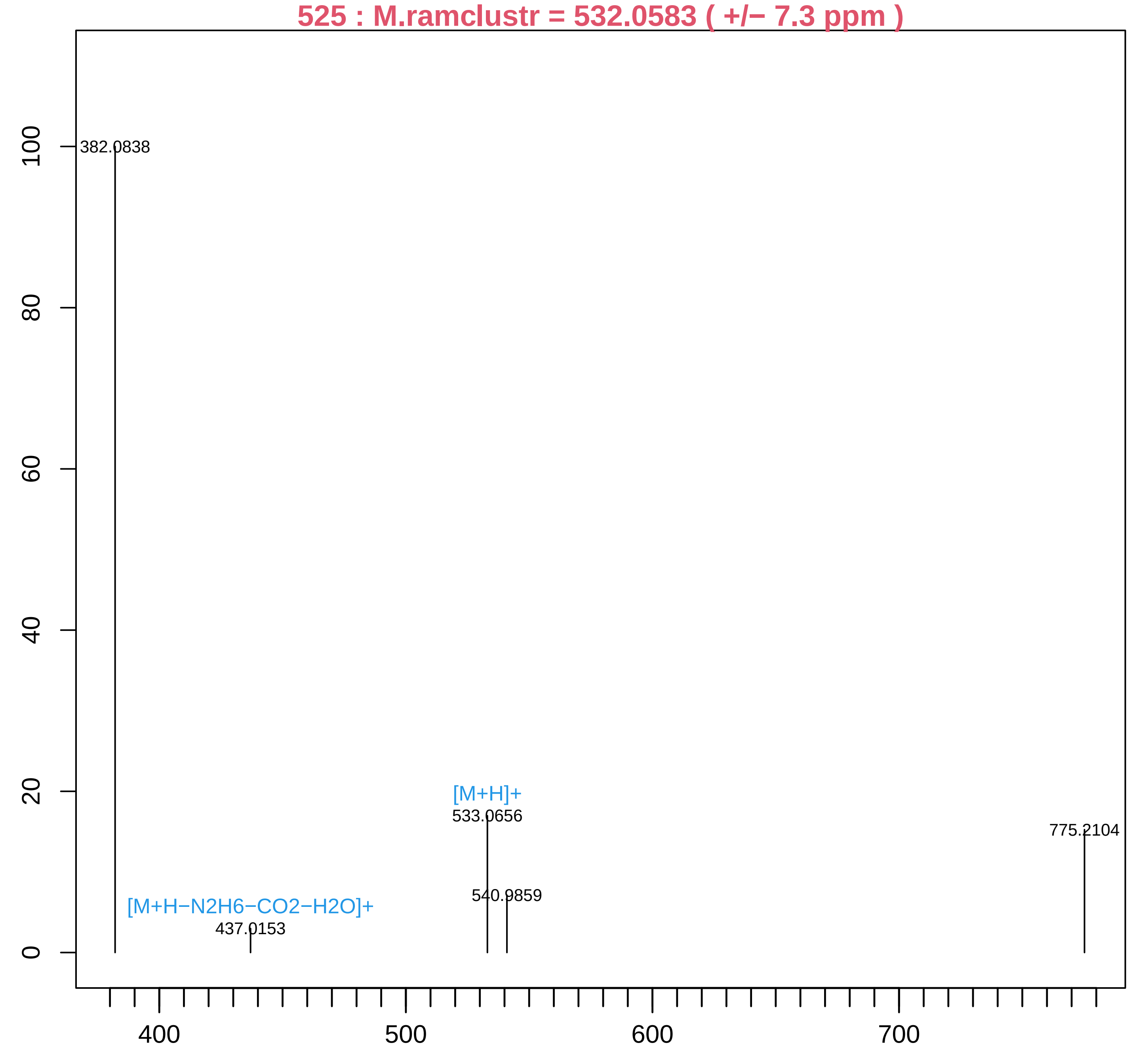
525 : M.ramclustr = 532.0583 (+/− 7.3 ppm)
382.0838
[M+H−N2H6−CO2−H2O]+
437.0153
[M+H]+
533.0656
540.9859
775.2104
0
20
40
60
80
100
400
500
600
700

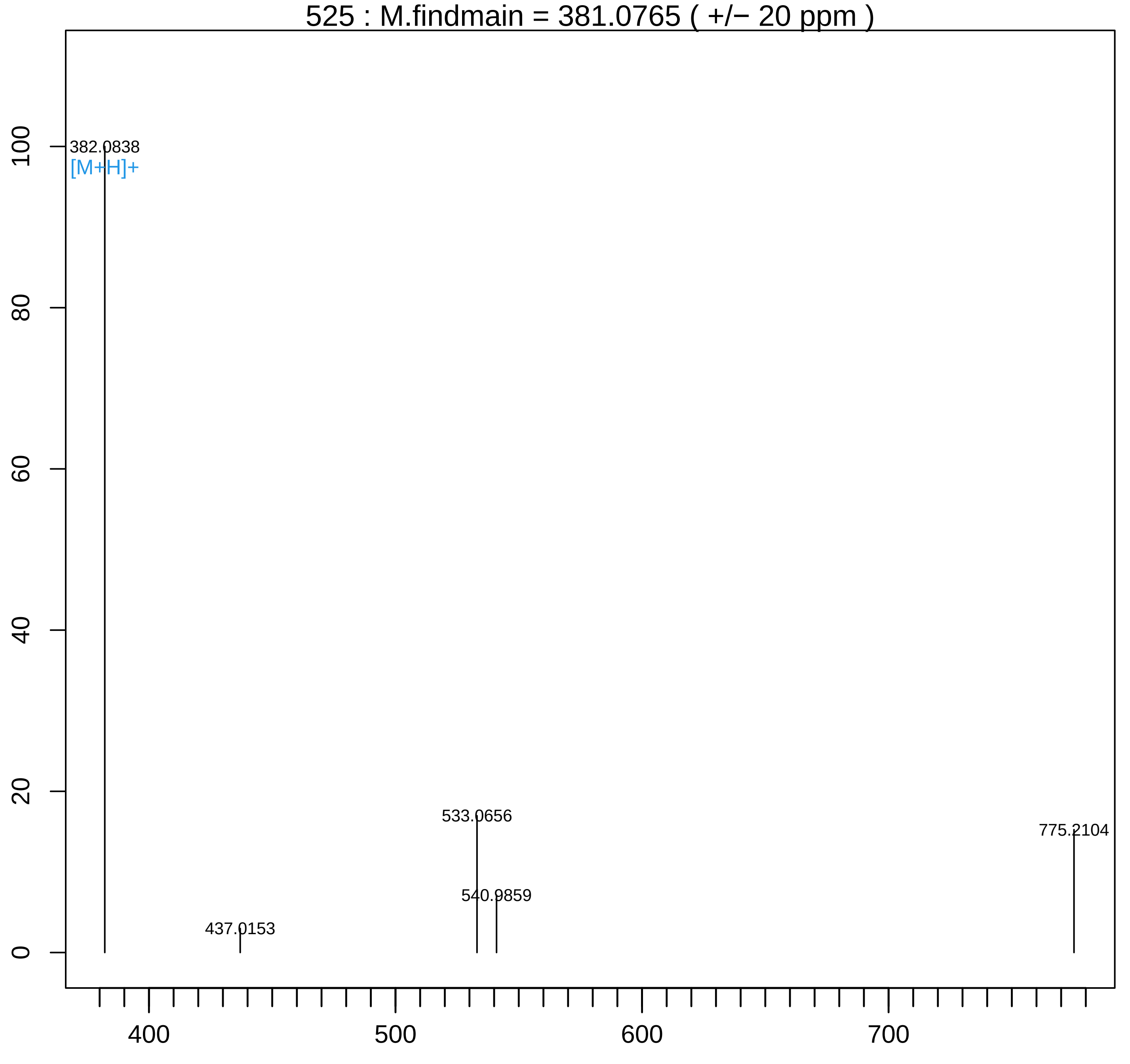
525 : M.findmain = 381.0765 (+/− 20 ppm)
382.0838
[M+H]+
437.0153
533.0656
540.9859
775.2104
0
20
40
60
80
100
400
500
600
700

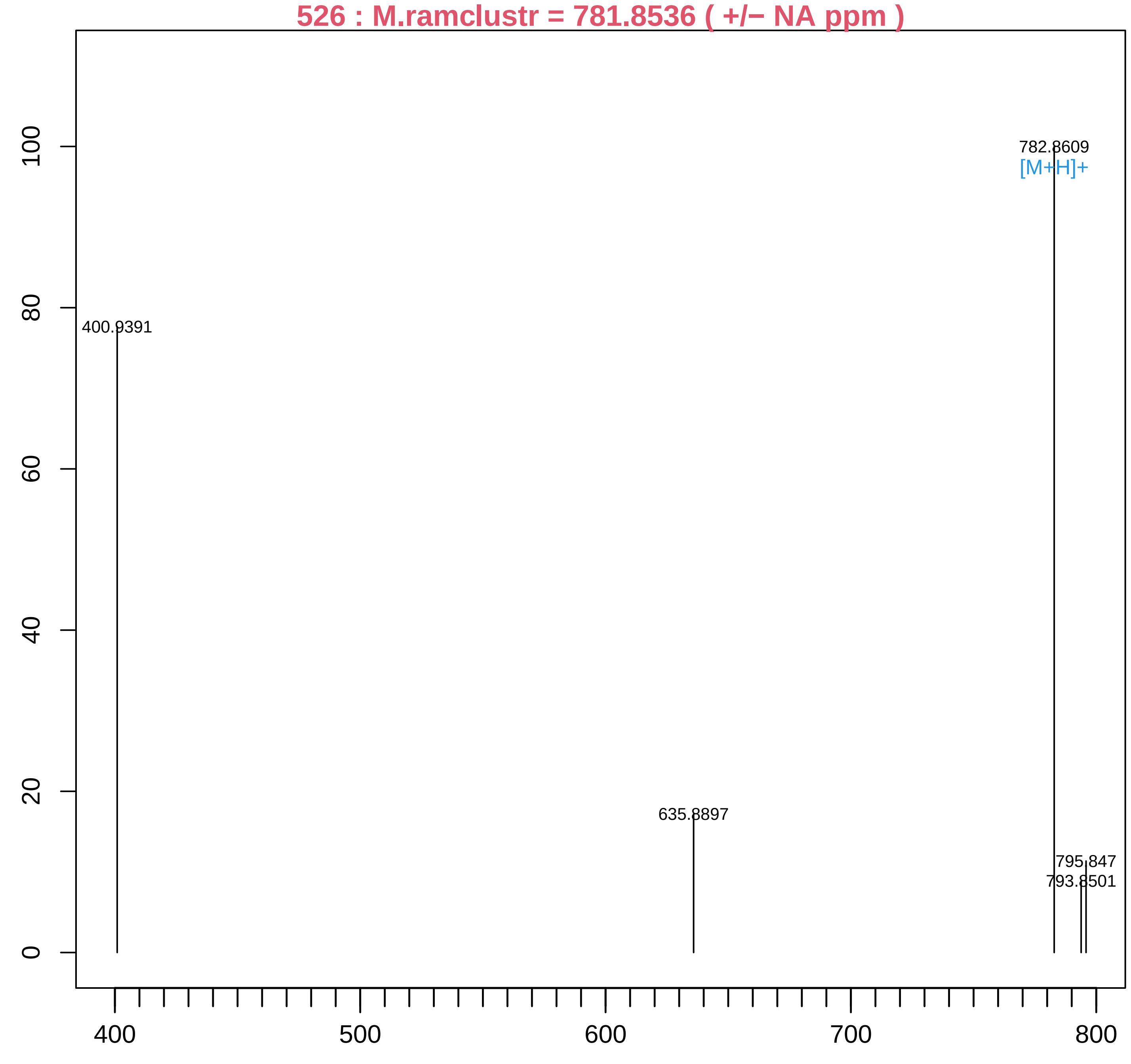
526 : M.ramclustr = 781.8536 (+/− NA ppm)
782.8609
[M+H]+
400.9391
635.8897
795.847
793.8501
0
20
40
60
80
100
400
500
600
700
800

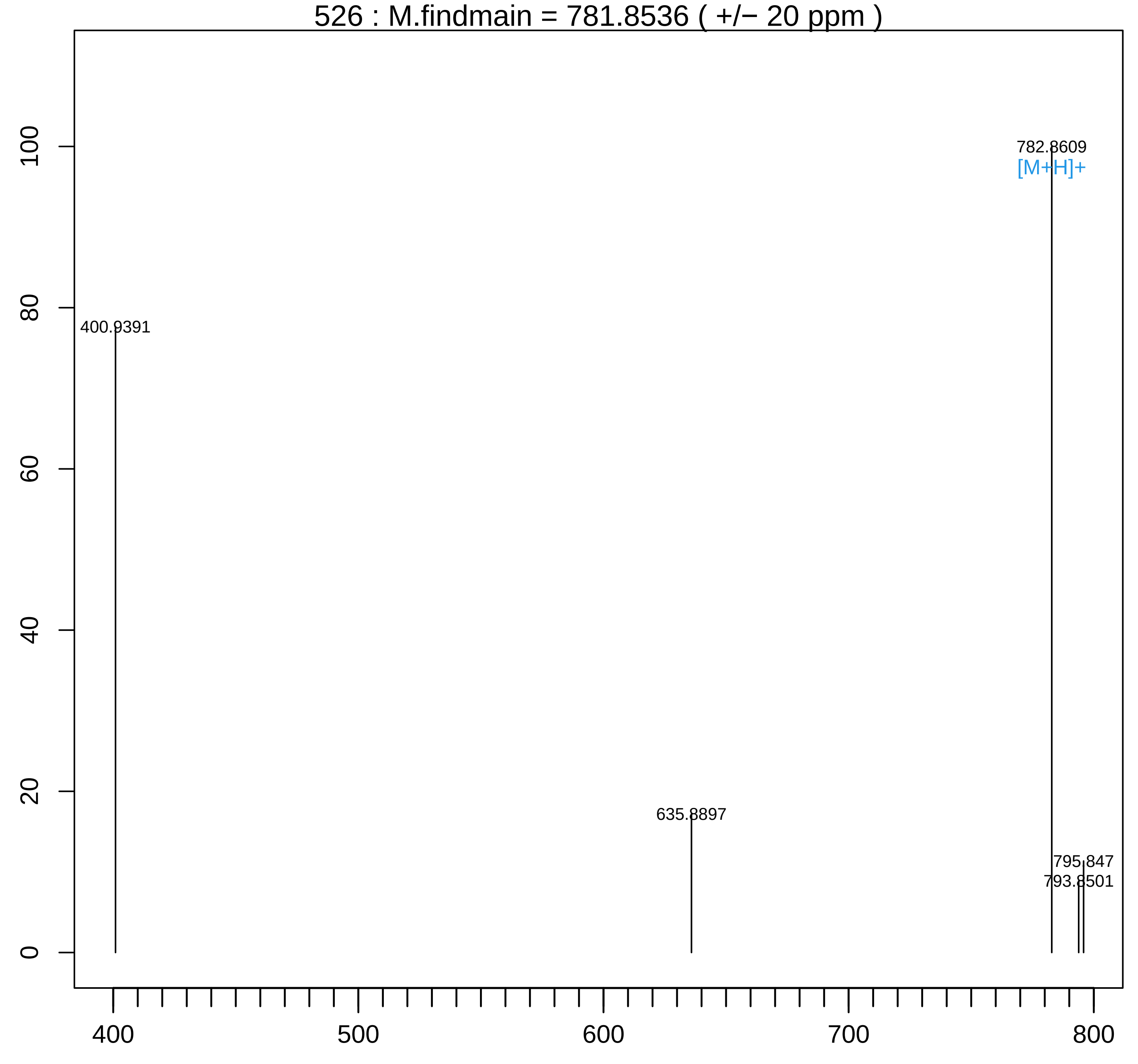
526 : M.findmain = 781.8536 (+/− 20 ppm)
782.8609
[M+H]+
400.9391
635.8897
795.847
793.8501
0
20
40
60
80
100
400
500
600
700
800

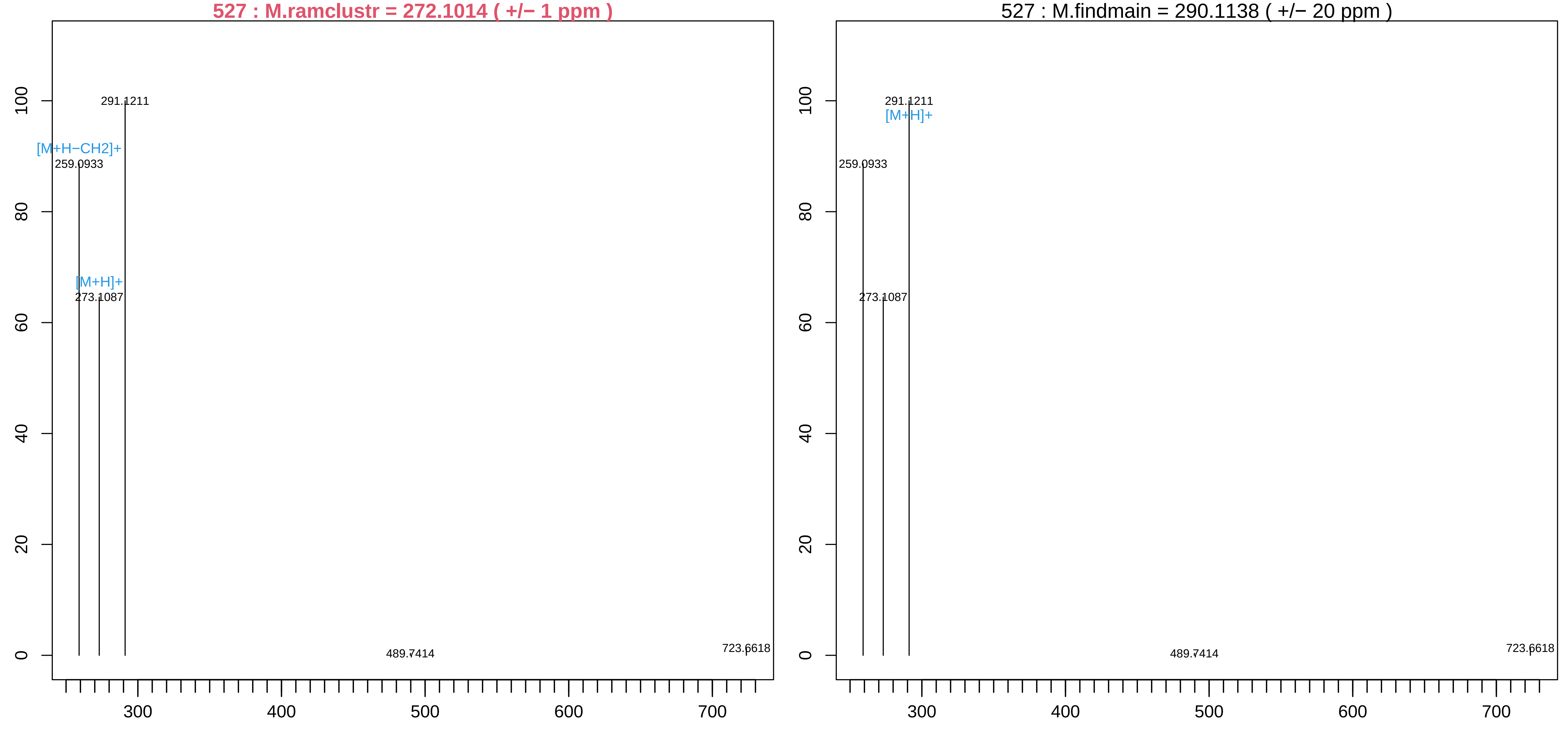
527 : M.ramclustr = 272.1014 (+/− 1 ppm)
291.1211
[M+H−CH2]+
259.0933
[M+H]+
273.1087
489.7414
723.6618
0
20
40
60
80
100
300
400
500
600
700
527 : M.findmain = 290.1138 (+/− 20 ppm)
291.1211
[M+H]+
259.0933
273.1087
489.7414
723.6618
0
20
40
60
80
100
300
400
500
600
700

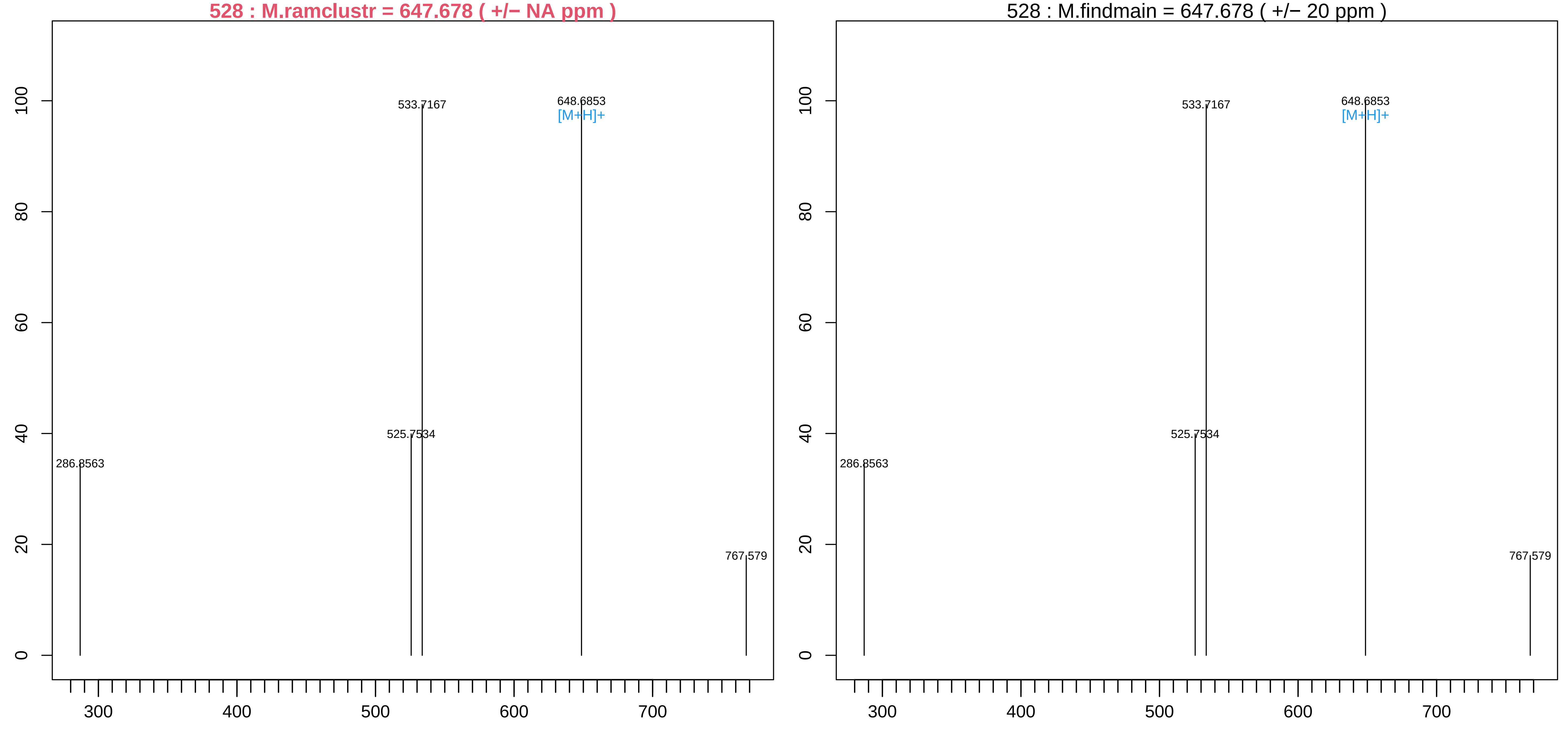
528 : M.ramclustr = 647.678 (+/− NA ppm)
528 : M.findmain = 647.678 (+/− 20 ppm)
533.7167
648.6853
[M+H]+
525.7534
286.8563
767.579
0
20
40
60
80
100
300
400
500
600
700

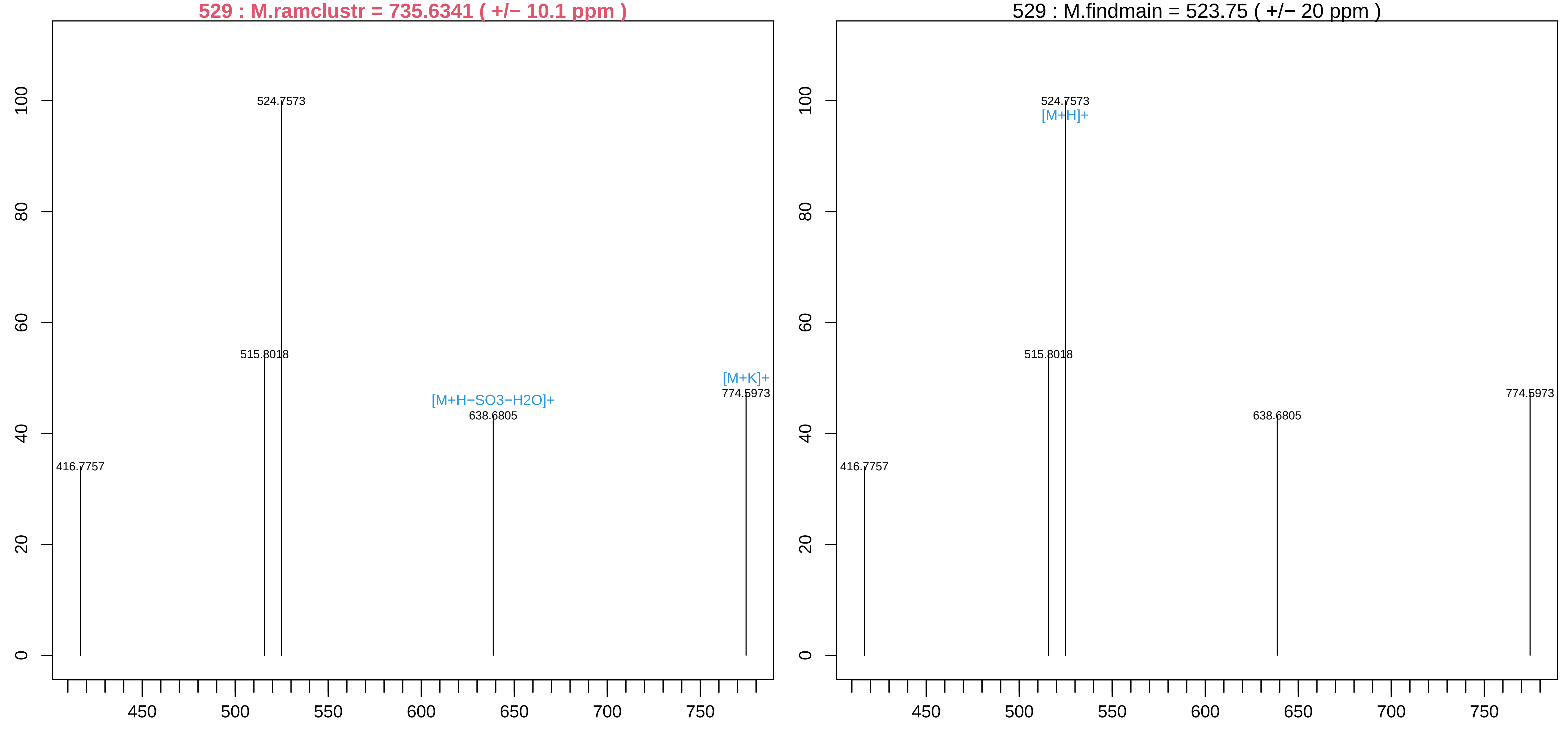
529 : M.ramclustr = 735.6341 (+/− 10.1 ppm)
524.7573
515.8018
[M+K]+
774.5973
[M+H−SO3−H2O]+
638.6805
416.7757
0
20
40
60
80
100
450
500
550
600
650
700
750
529 : M.findmain = 523.75 (+/− 20 ppm)
524.7573
[M+H]+
515.8018
774.5973
638.6805
416.7757
0
20
40
60
80
100
450
500
550
600
650
700
750

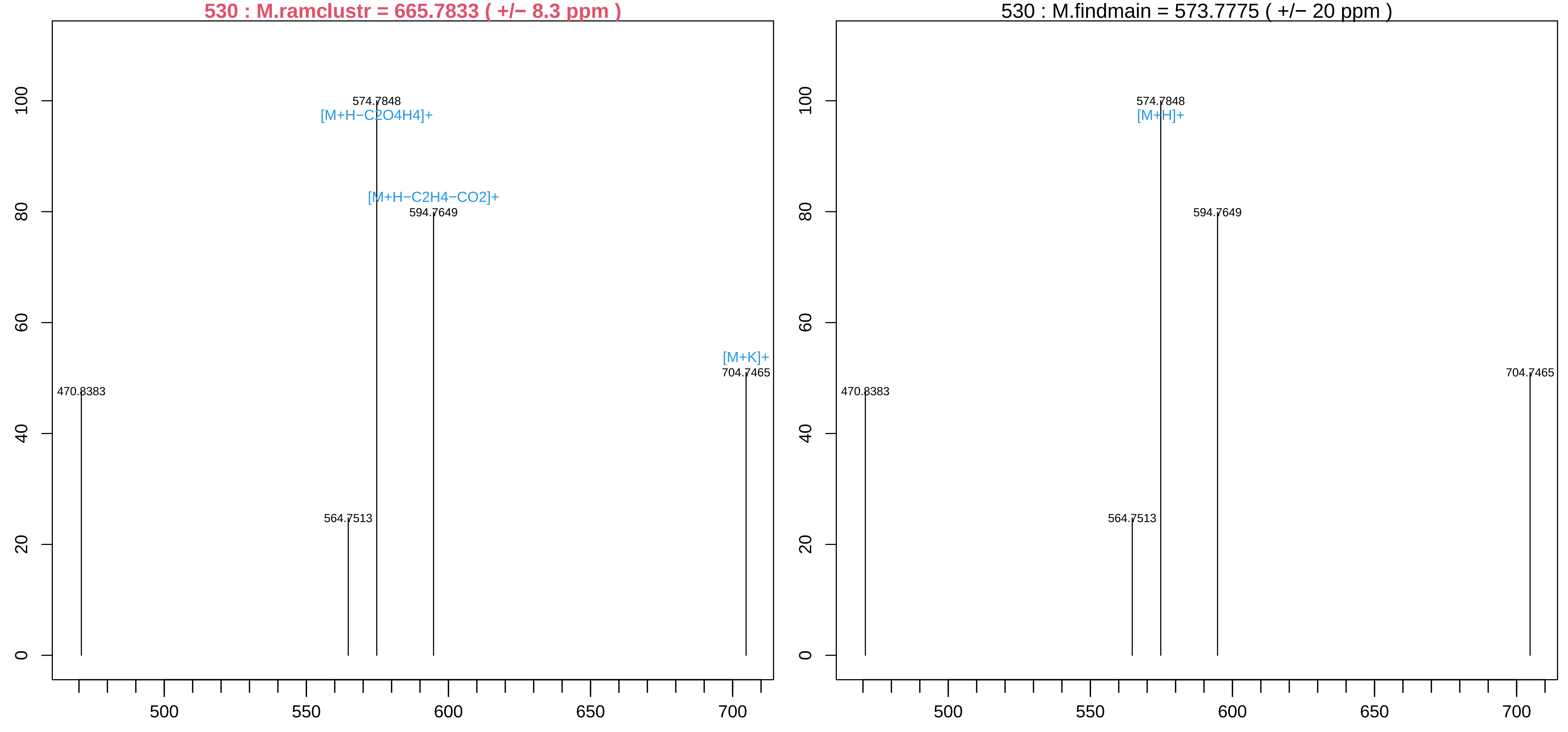
530 : M.ramclustr = 665.7833 (+/− 8.3 ppm)
574.7848
[M+H−C2O4H4]+
[M+H−C2H4−CO2]+
594.7649
[M+K]+
704.7465
470.8383
564.7513
530 : M.findmain = 573.7775 (+/− 20 ppm)
574.7848
[M+H]+
594.7649
470.8383
704.7465
564.7513

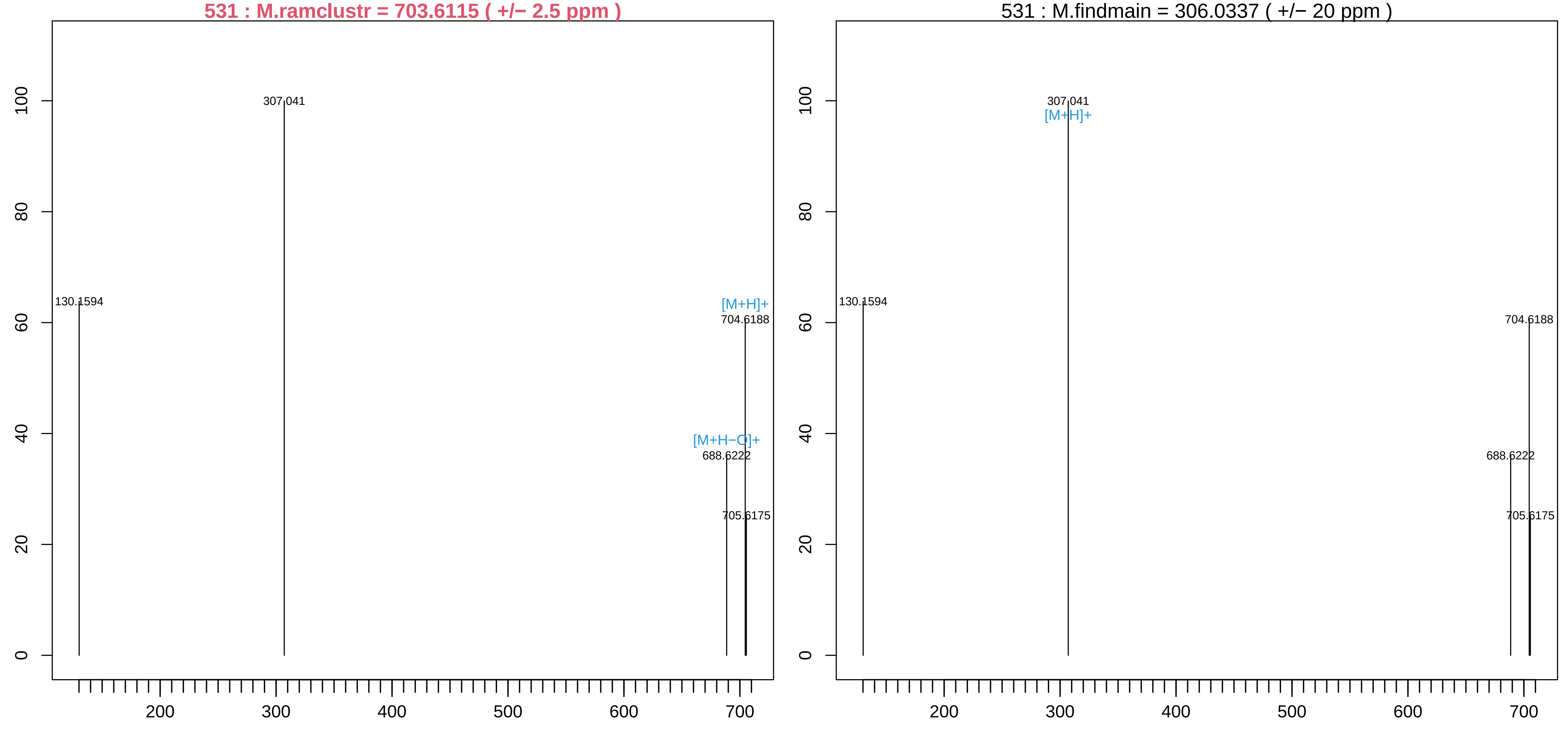

531 : M.ramclustr = 703.6115 (+/− 2.5 ppm)
130.1594
307.041
[M+H]+
704.6188
[M+H−O]+
688.6222
705.6175
531 : M.findmain = 306.0337 (+/− 20 ppm)
130.1594
307.041
[M+H]+
704.6188
688.6222
705.6175
0
20
40
60
80
100
200
300
400
500
600
700

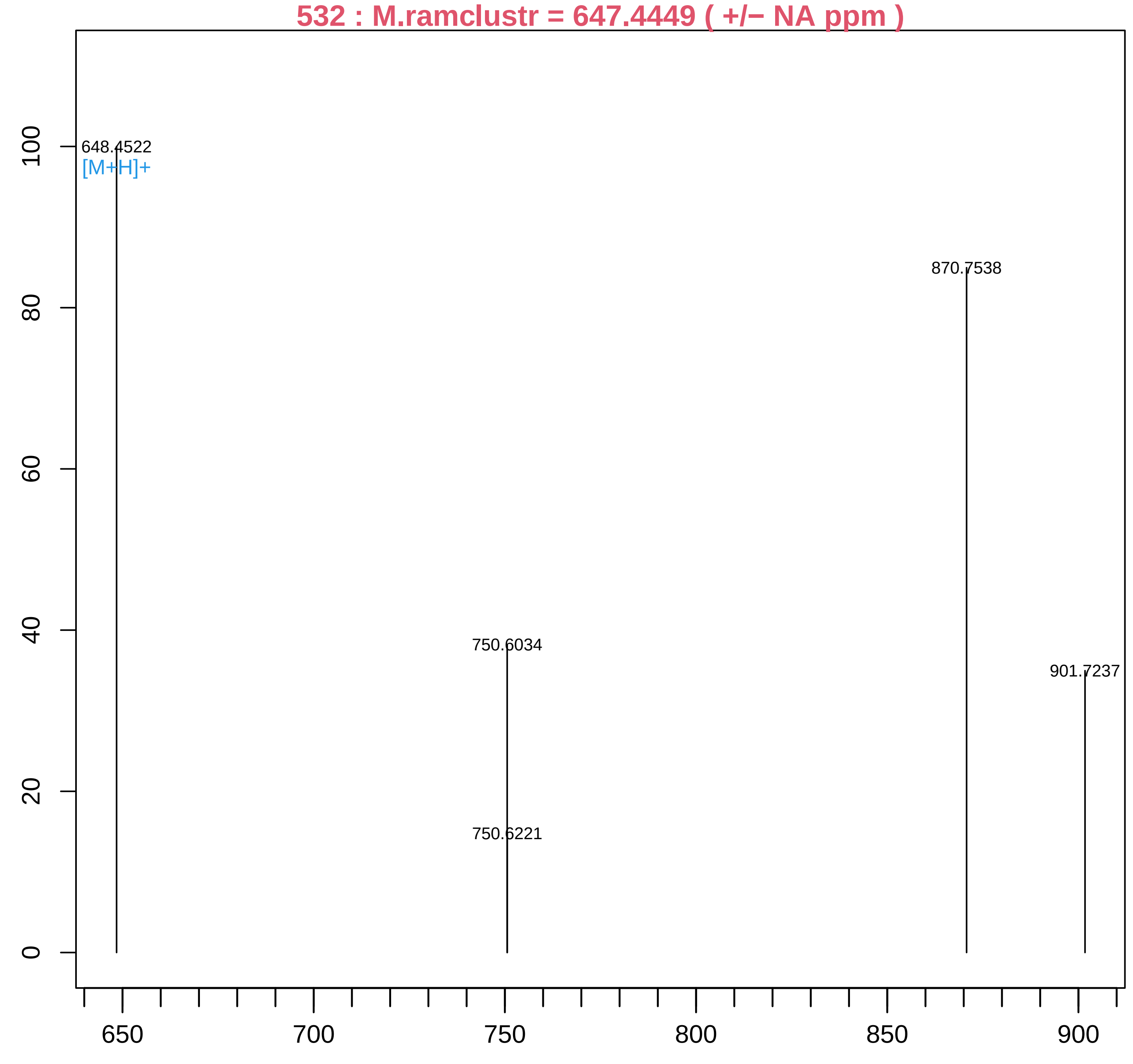

532 : M.ramclustr = 647.4449 (+/− NA ppm)
648.4522
[M+H]+
870.7538
750.6034
901.7237
750.6221
0
20
40
60
80
100
650
700
750
800
850
900

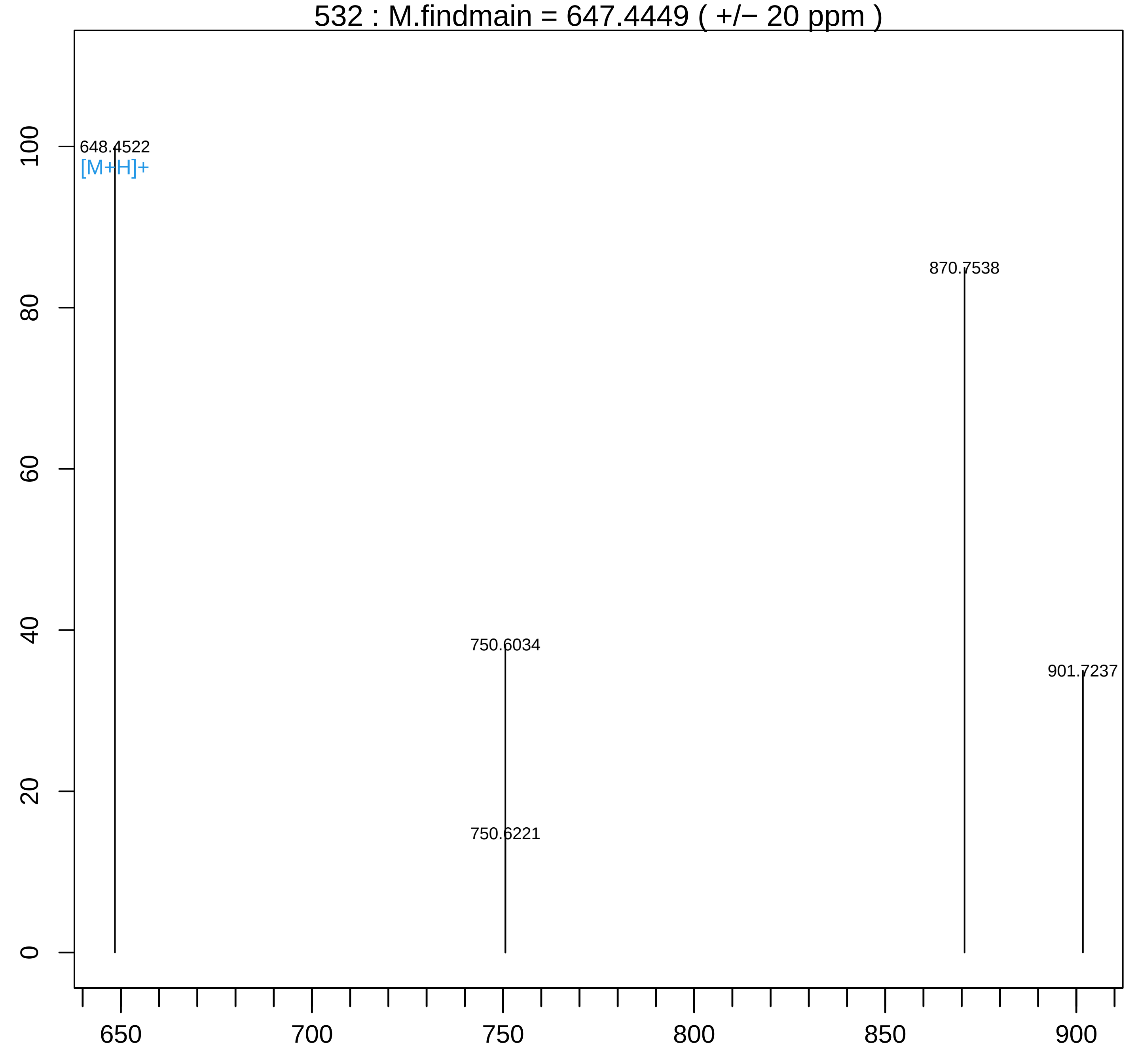

532 : M.findmain = 647.4449 (+/− 20 ppm)
648.4522
[M+H]+
870.7538
750.6034
901.7237
750.6221
0
20
40
60
80
100
650
700
750
800
850
900

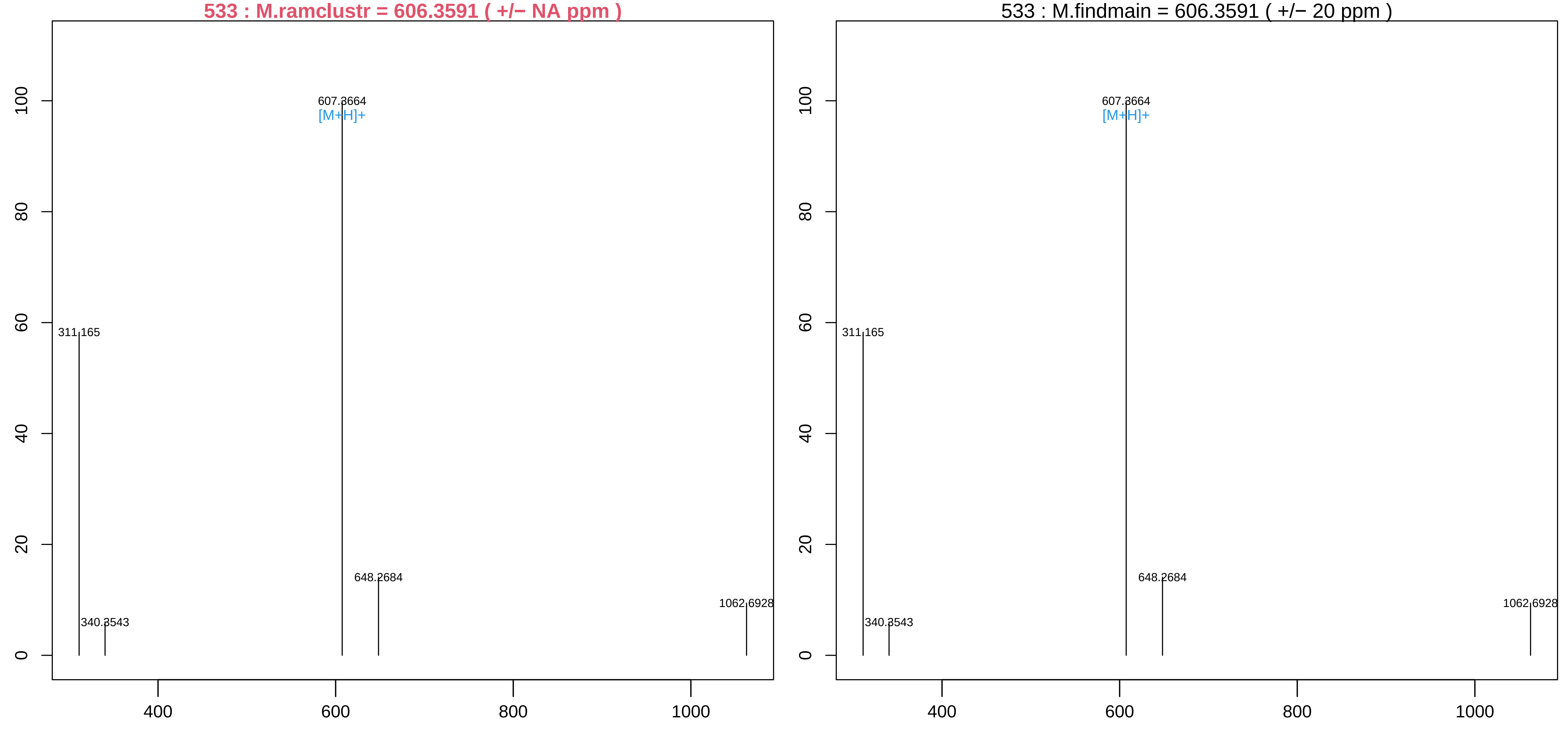
533 : M.ramclustr = 606.3591 (+/− NA ppm)
607.3664
[M+H]+
311.165
648.2684
1062.6928
340.3543
0
20
40
60
80
100
400
600
800
1000
533 : M.findmain = 606.3591 (+/− 20 ppm)
607.3664
[M+H]+
311.165
648.2684
1062.6928
340.3543
0
20
40
60
80
100
400
600
800
1000

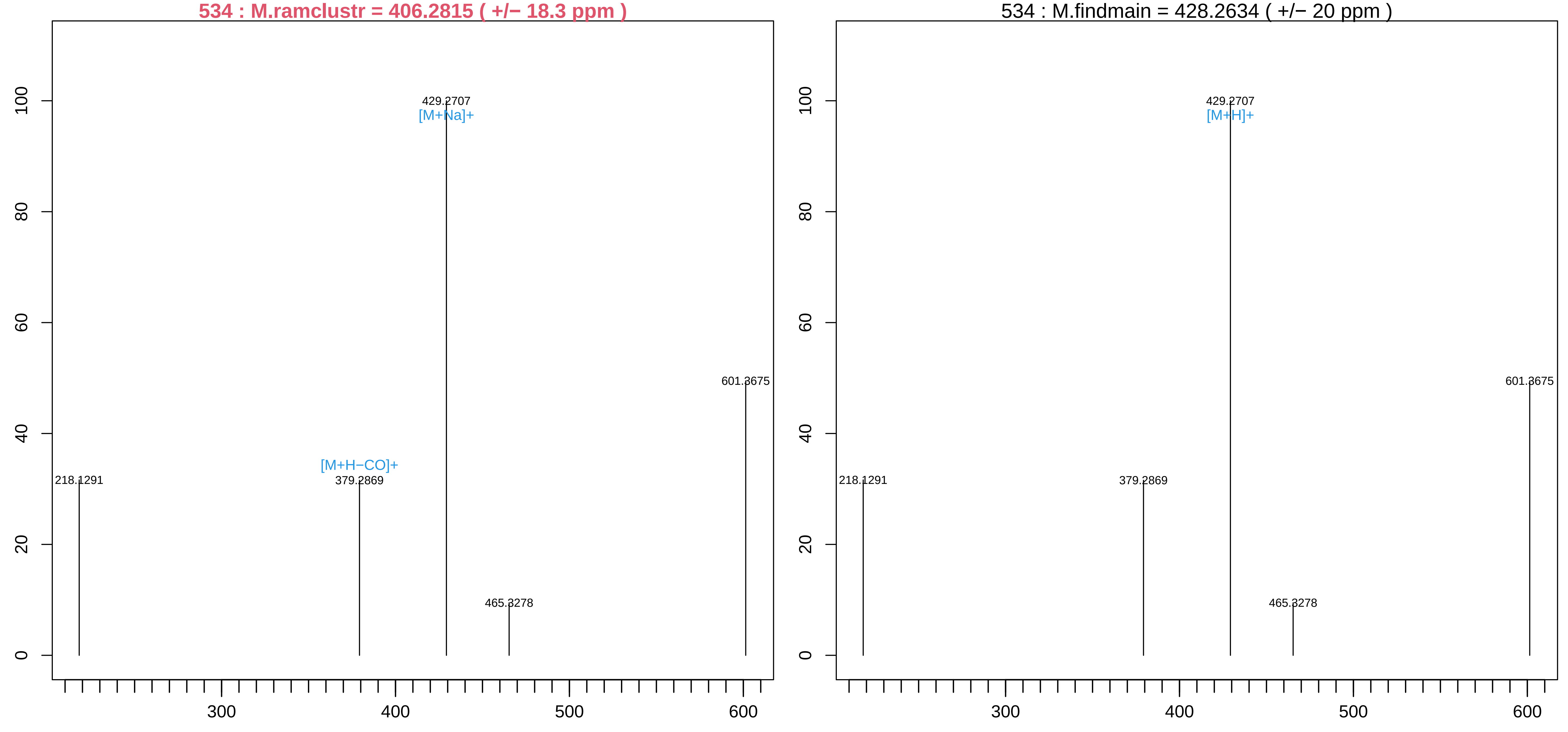
534 : M.ramclustr = 406.2815 (+/− 18.3 ppm)
429.2707
[M+Na]+
601.3675
[M+H−CO]+
218.1291
379.2869
465.3278
534 : M.findmain = 428.2634 (+/− 20 ppm)
429.2707
[M+H]+
601.3675
218.1291
379.2869
465.3278
0
20
40
60
80
100
300
400
500
600

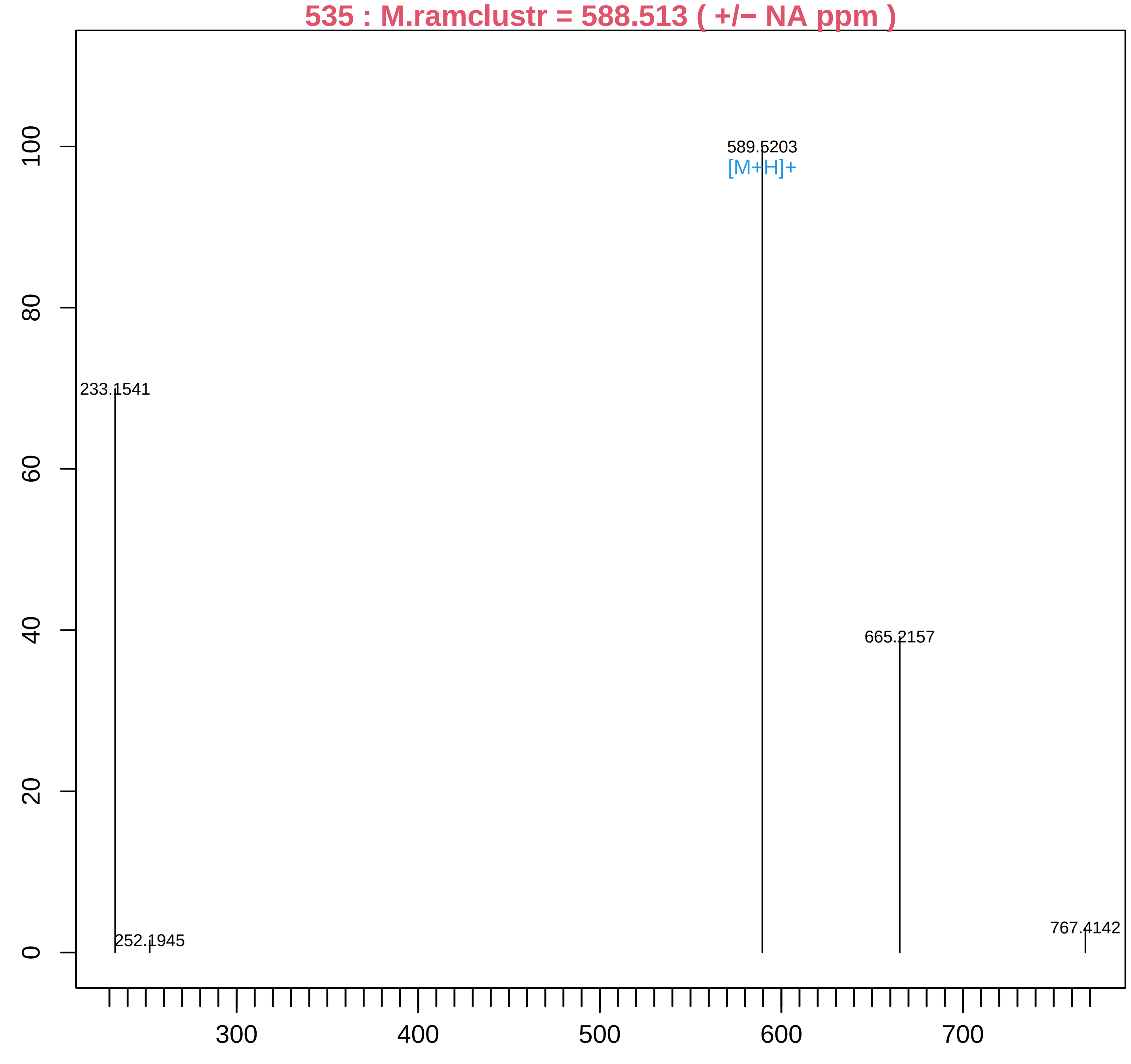
535 : M.ramclustr = 588.513 (+/− NA ppm)
589.5203
[M+H]+
233.1541
665.2157
252.1945
767.4142
0
20
40
60
80
100
300
400
500
600
700

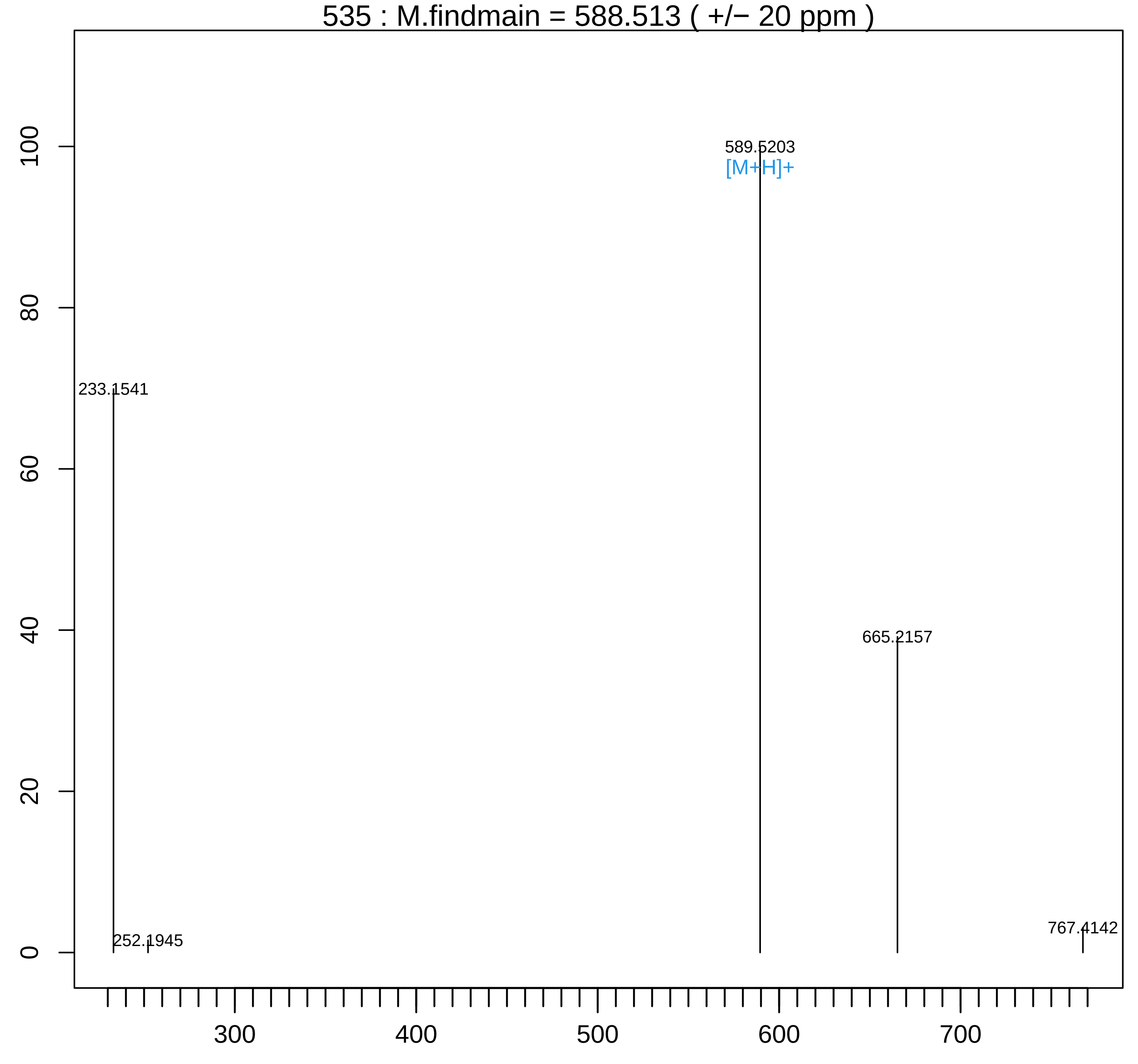
535 : M.findmain = 588.513 (+/− 20 ppm)
589.5203
[M+H]+
233.1541
665.2157
252.1945
767.4142
0
20
40
60
80
100
300
400
500
600
700

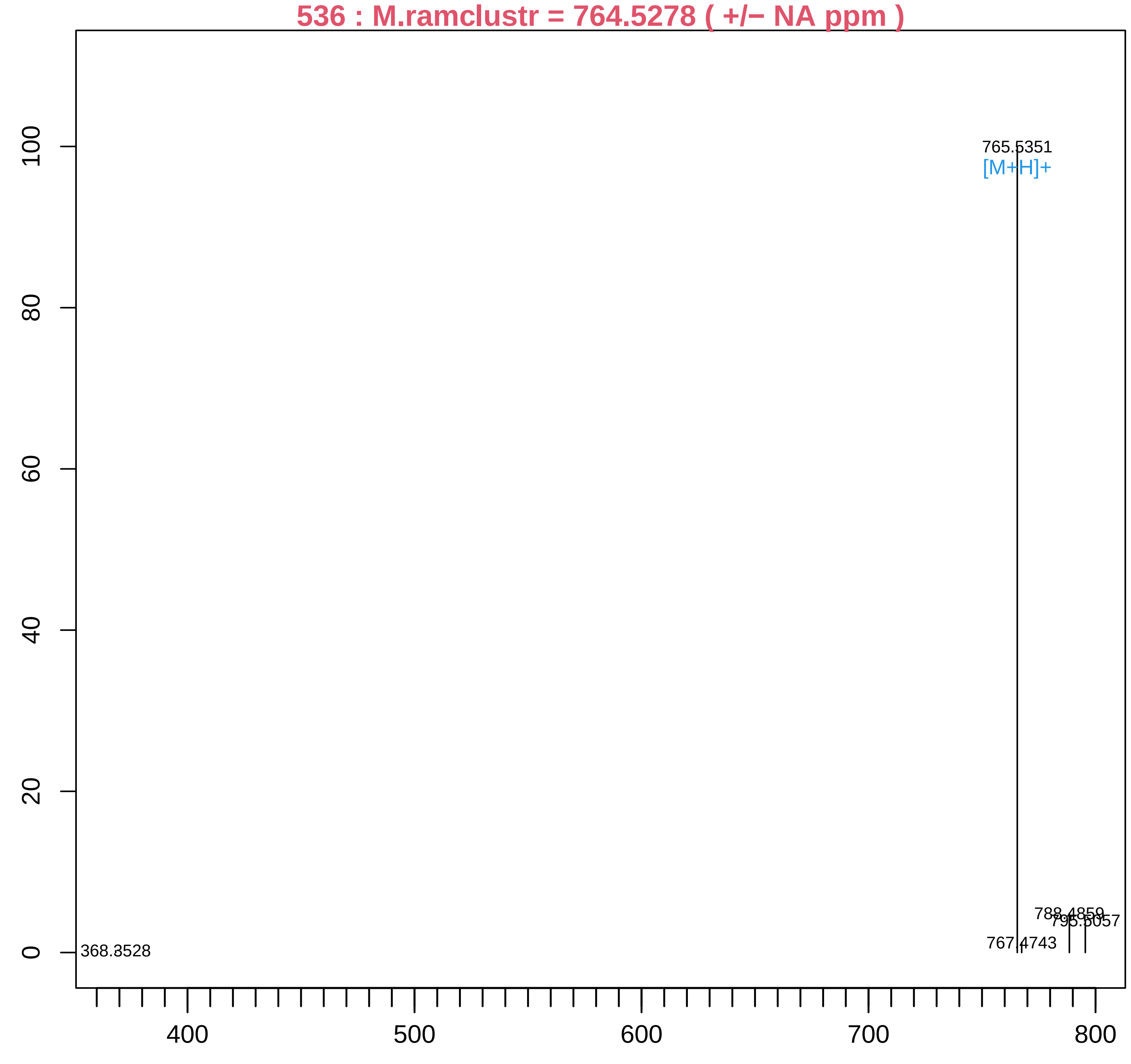
536 : M.ramclustr = 764.5278 (+/− NA ppm)
765.5351
[M+H]+
788.4859
795.5057
767.4743
368.3528
0
20
40
60
80
100
400
500
600
700
800

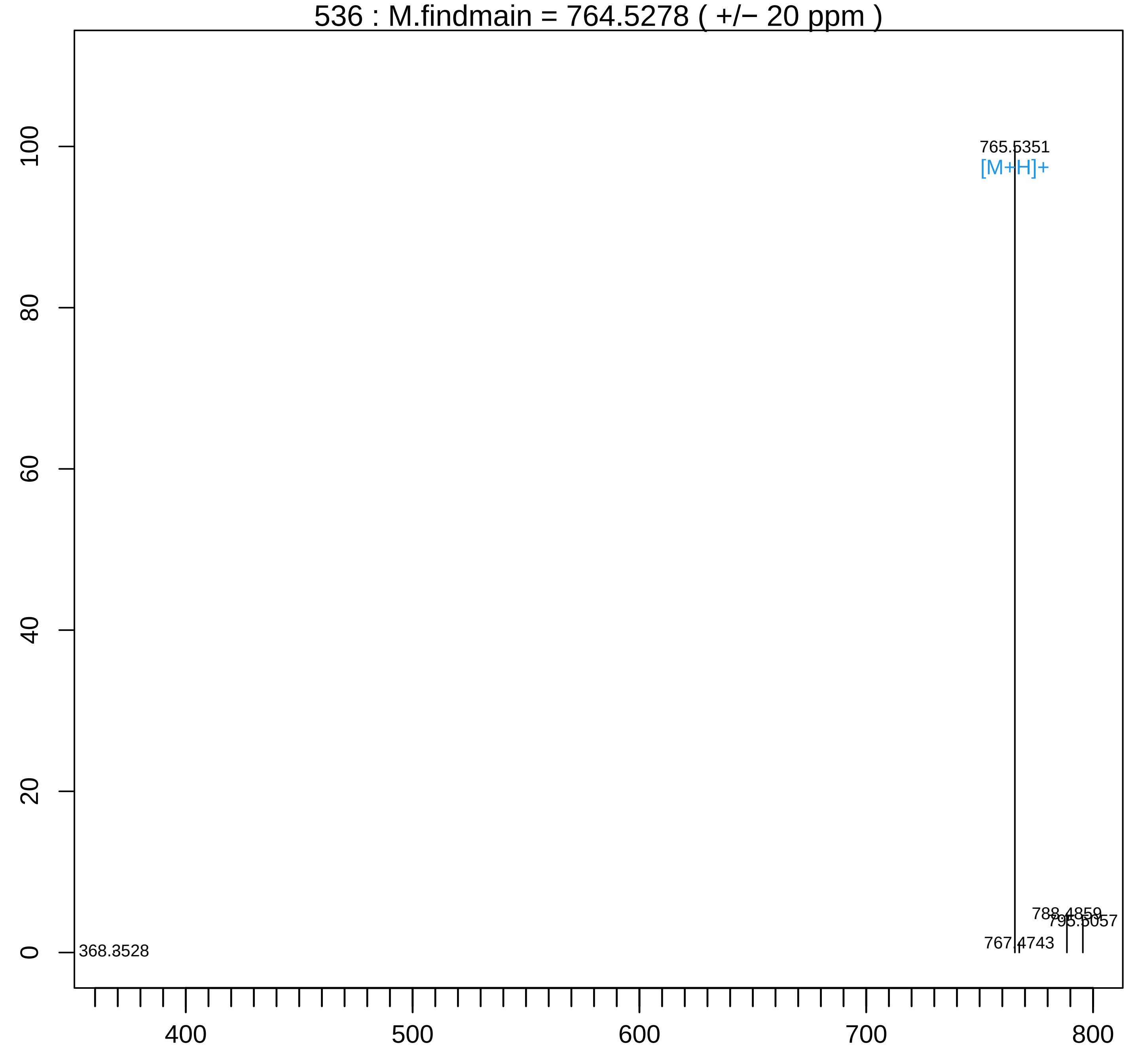
536 : M.findmain = 764.5278 (+/− 20 ppm)
765.5351
[M+H]+
788.4859
795.5057
767.4743
368.3528
0
20
40
60
80
100
400
500
600
700
800

537 : M.ramclustr = 598.4042 (+/− NA ppm)

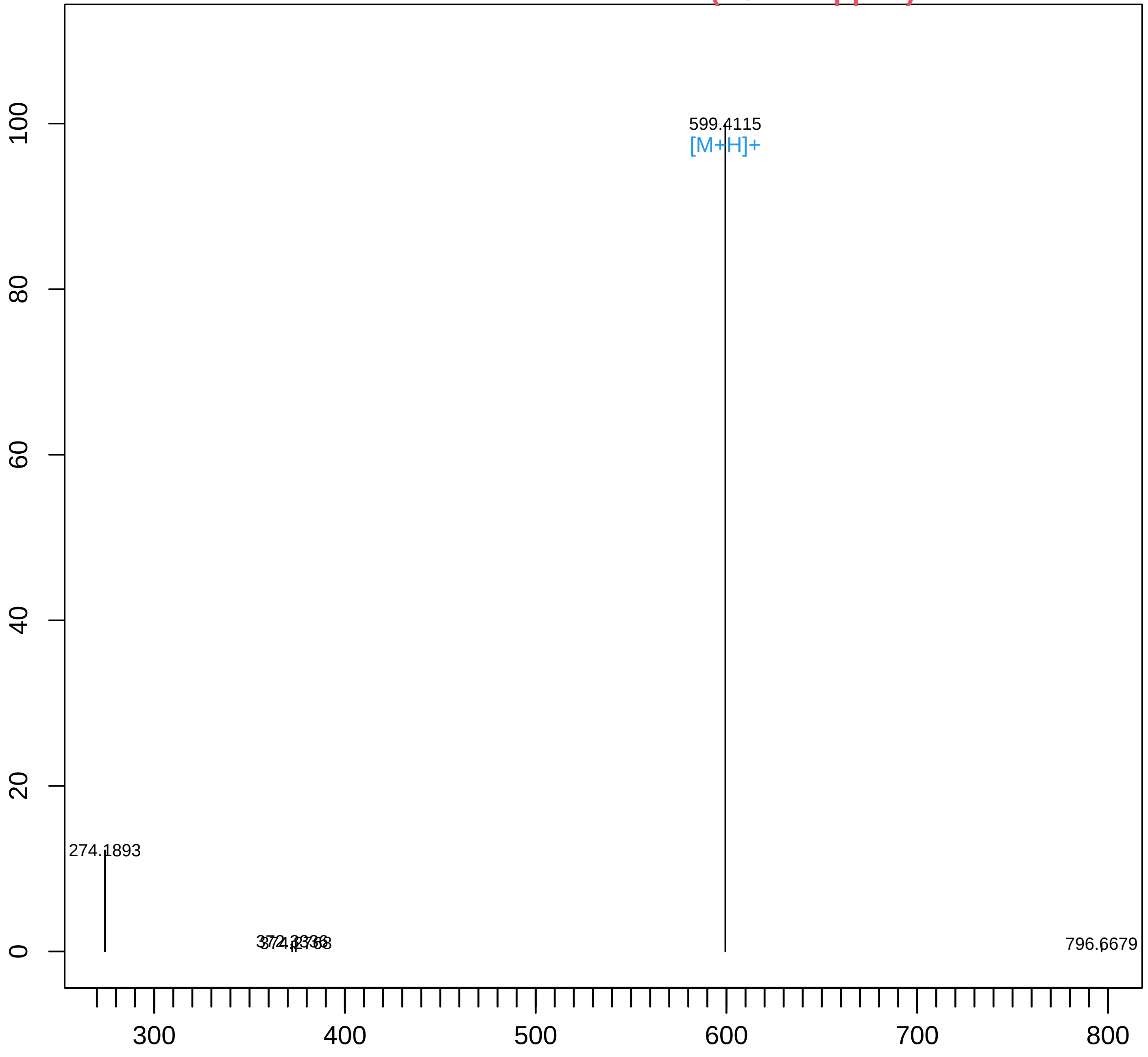

537 : M.findmain = 598.4042 (+/− 20 ppm)

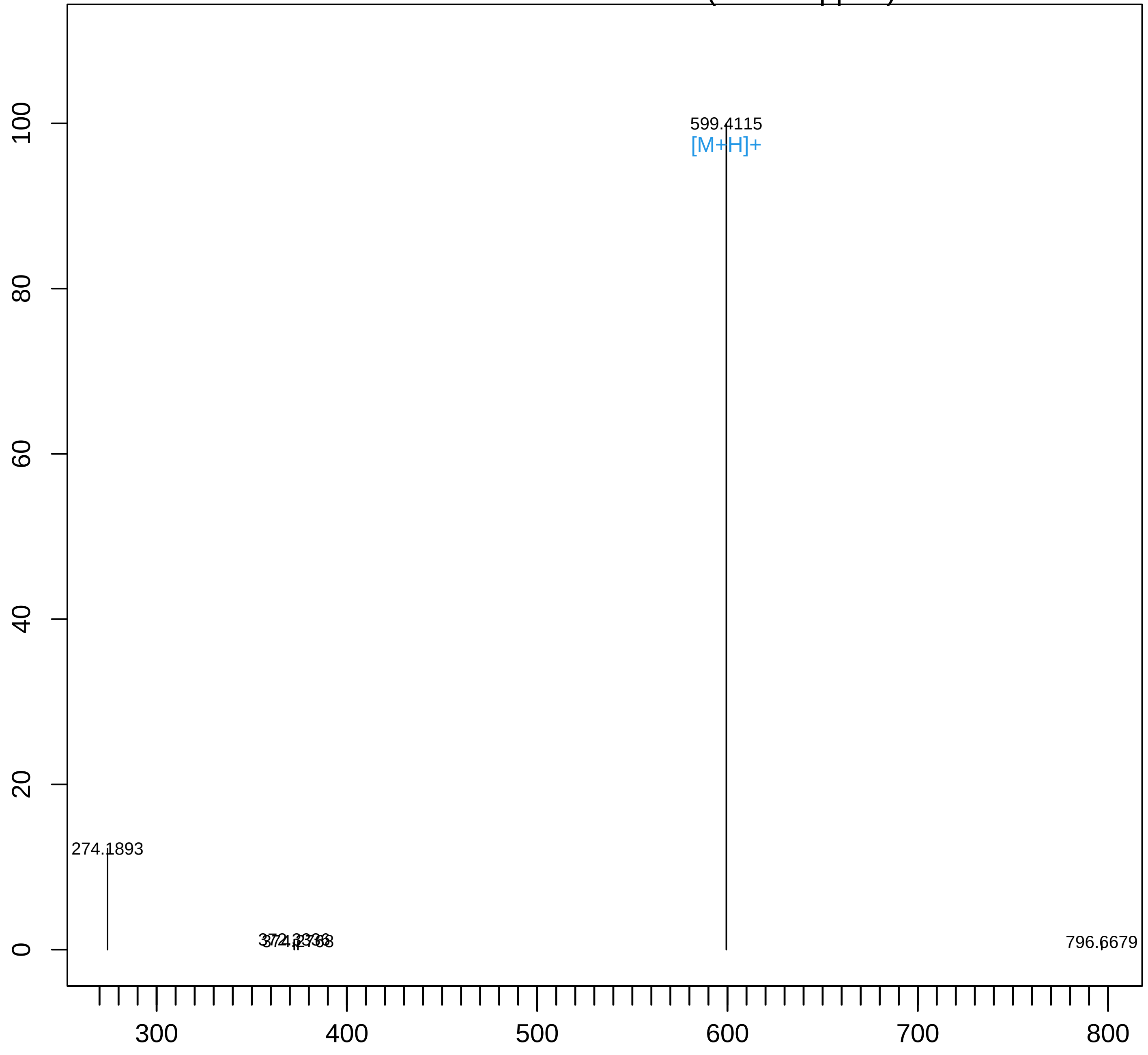

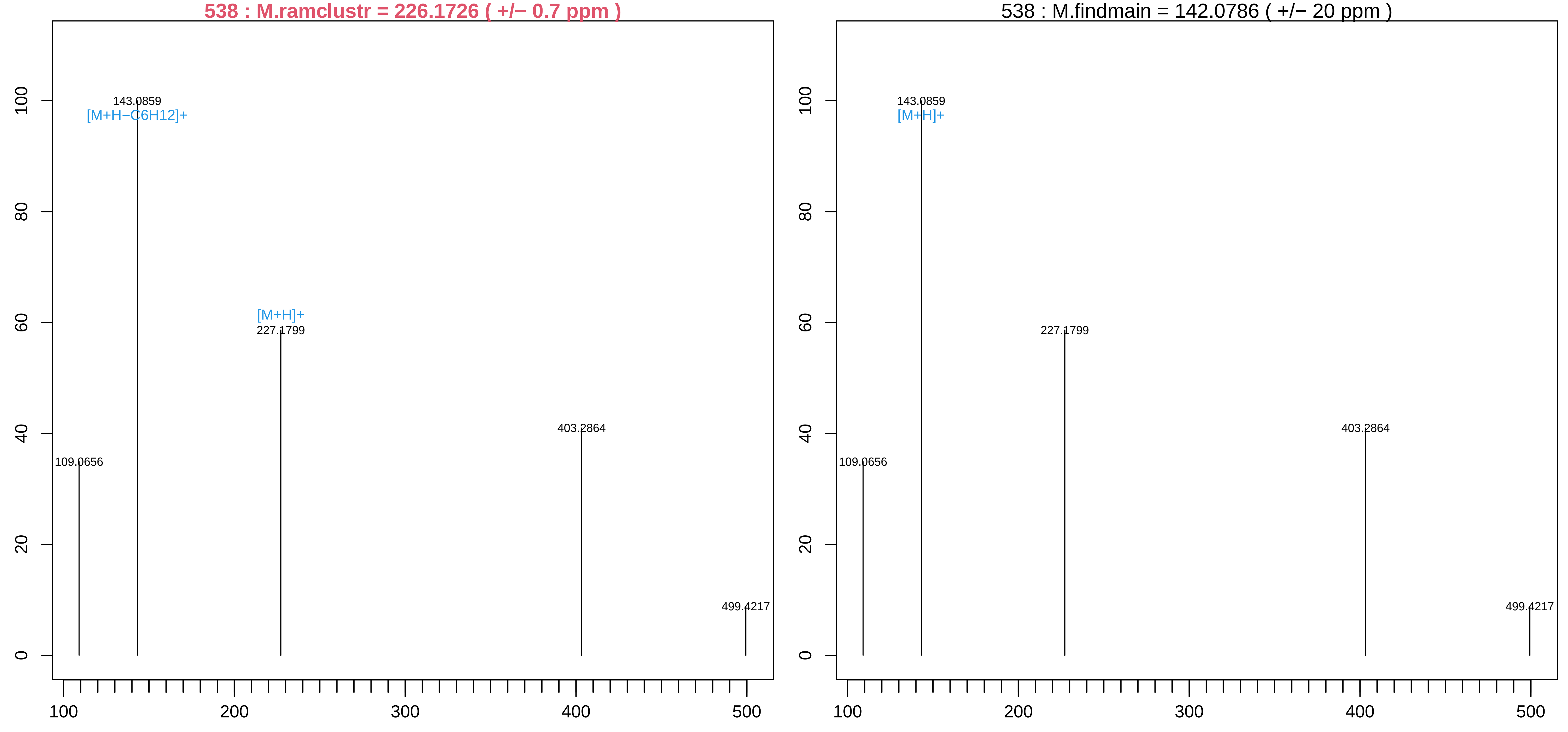
538 : M.ramclustr = 226.1726 (+/− 0.7 ppm)
143.0859
[M+H−C6H12]+
[M+H]+
227.1799
403.2864
109.0656
499.4217
538 : M.findmain = 142.0786 (+/− 20 ppm)
143.0859
[M+H]+
227.1799
403.2864
109.0656
499.4217

539 : M.ramclustr = 855.577 (+/− NA ppm)

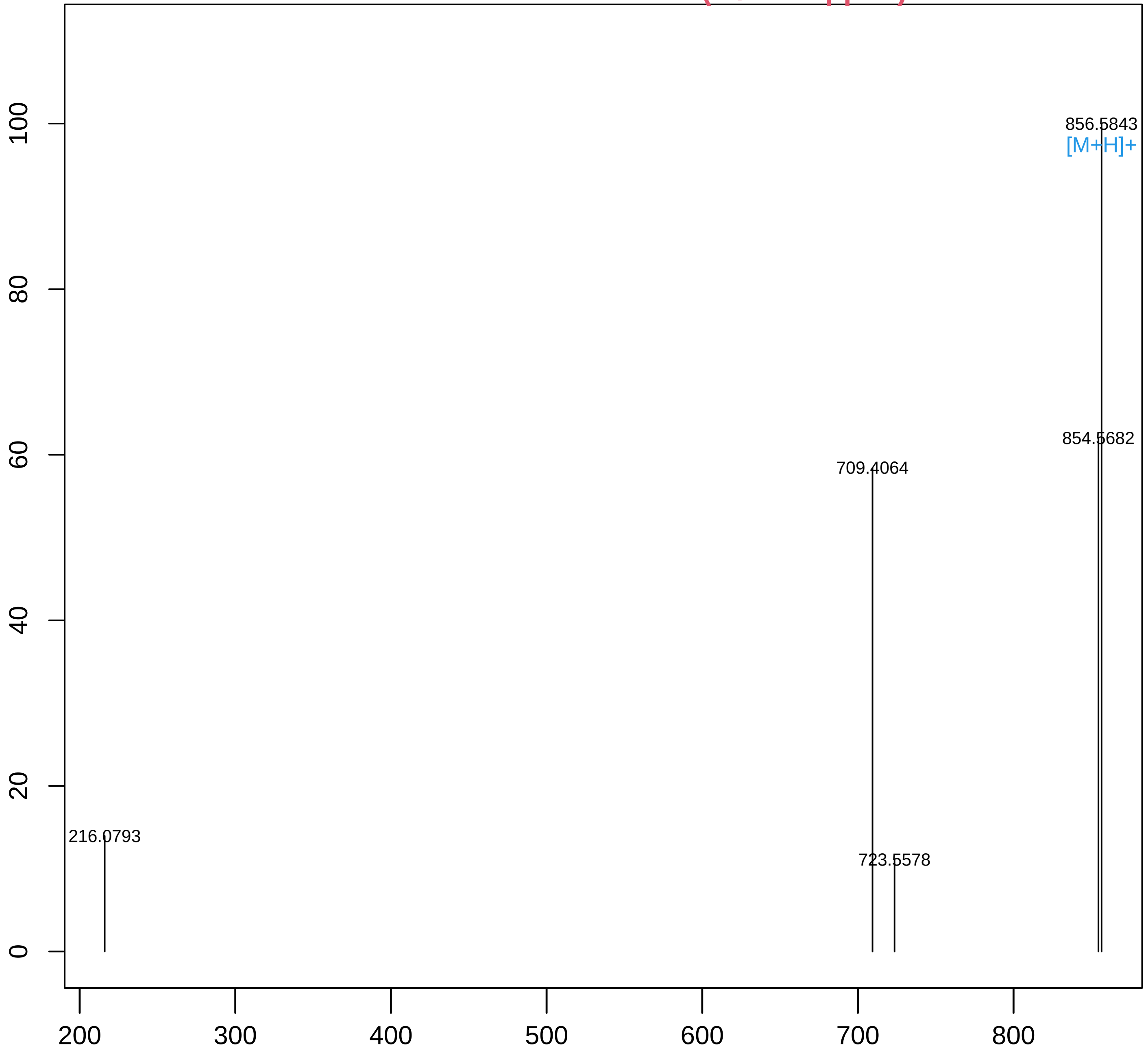

539 : M.findmain = 855.577 (+/− 20 ppm)

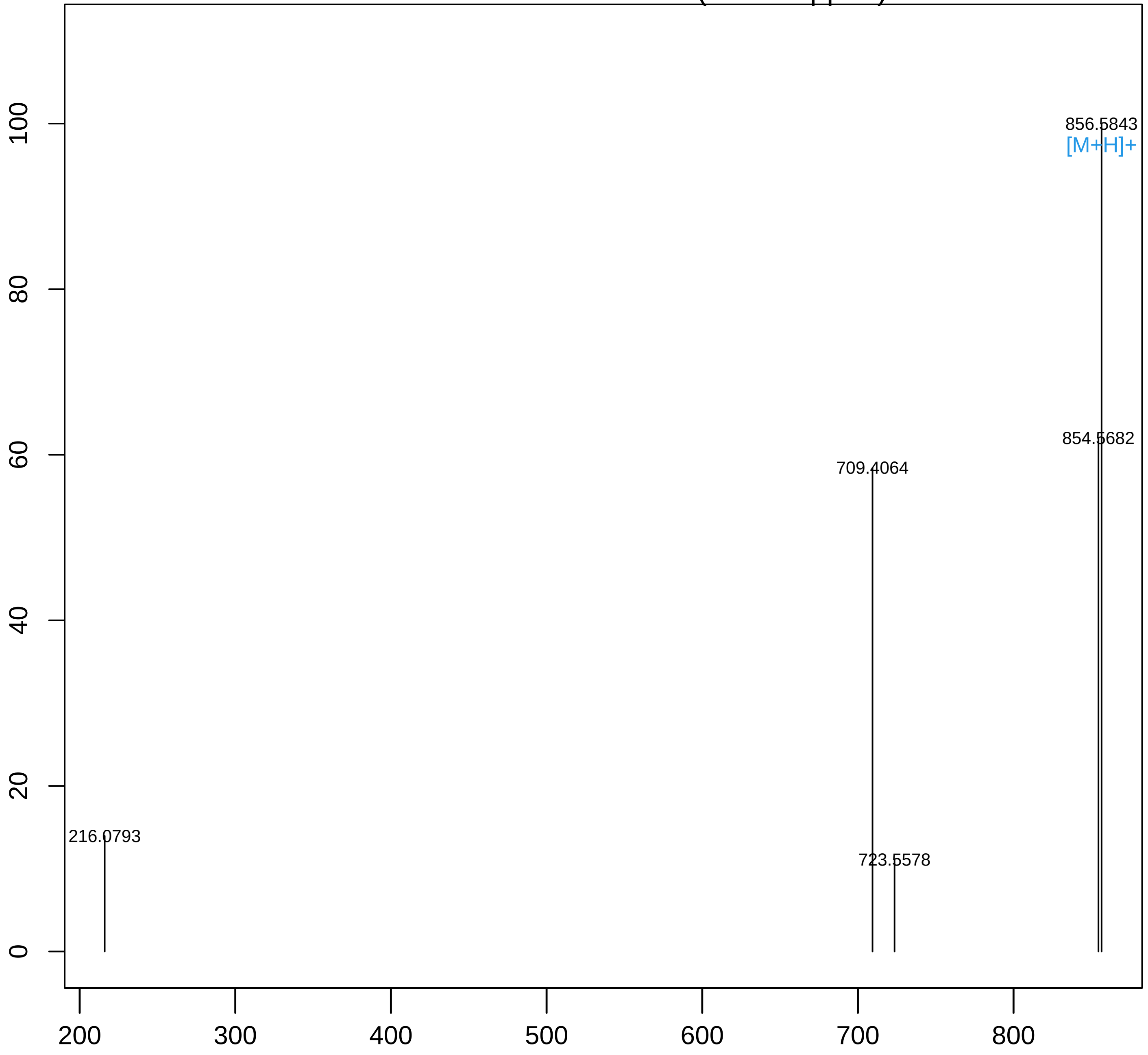

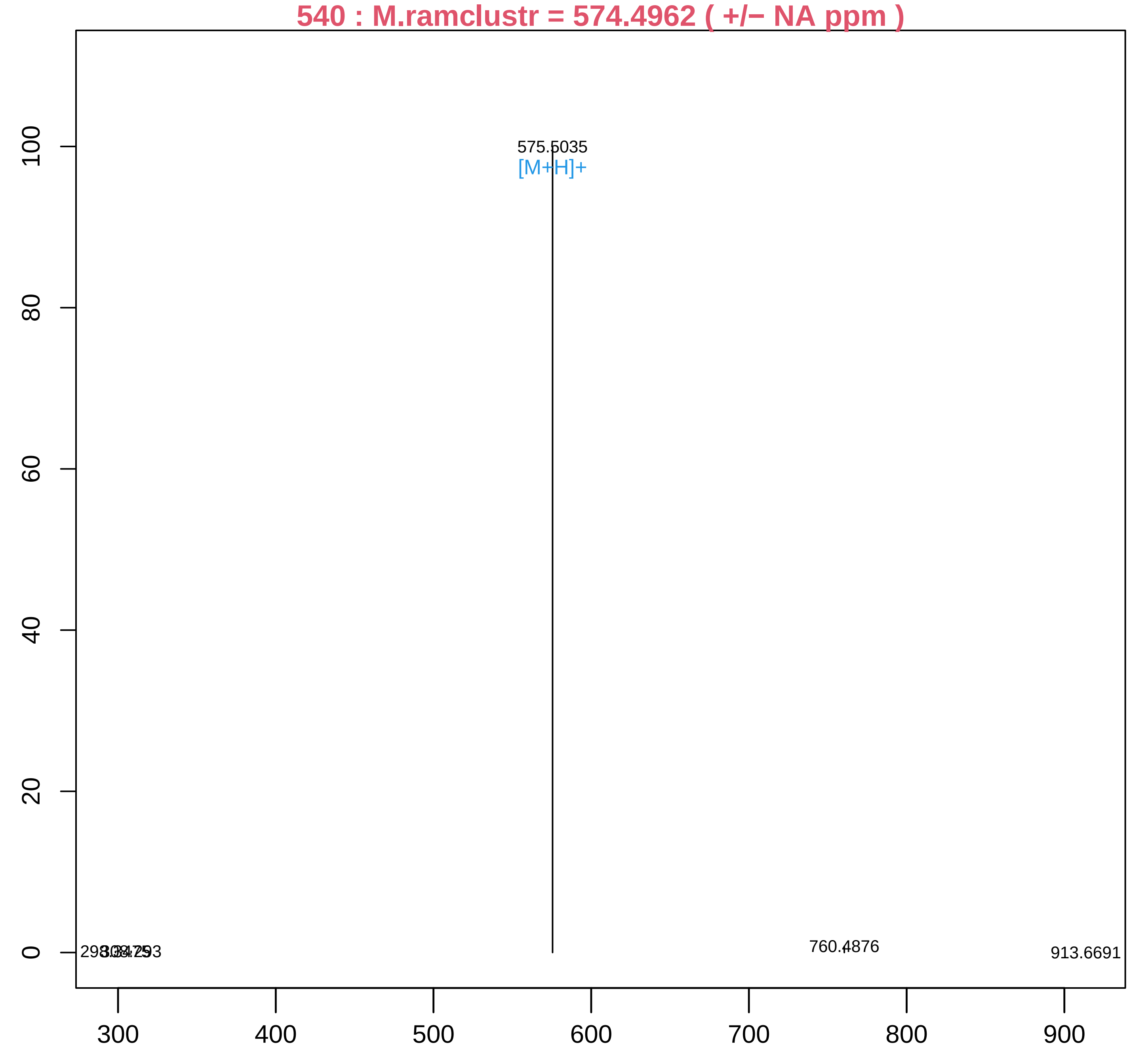

540 : M.ramclustr = 574.4962 (+/− NA ppm)
575.5035
[M+H]+
760.4876
913.6691

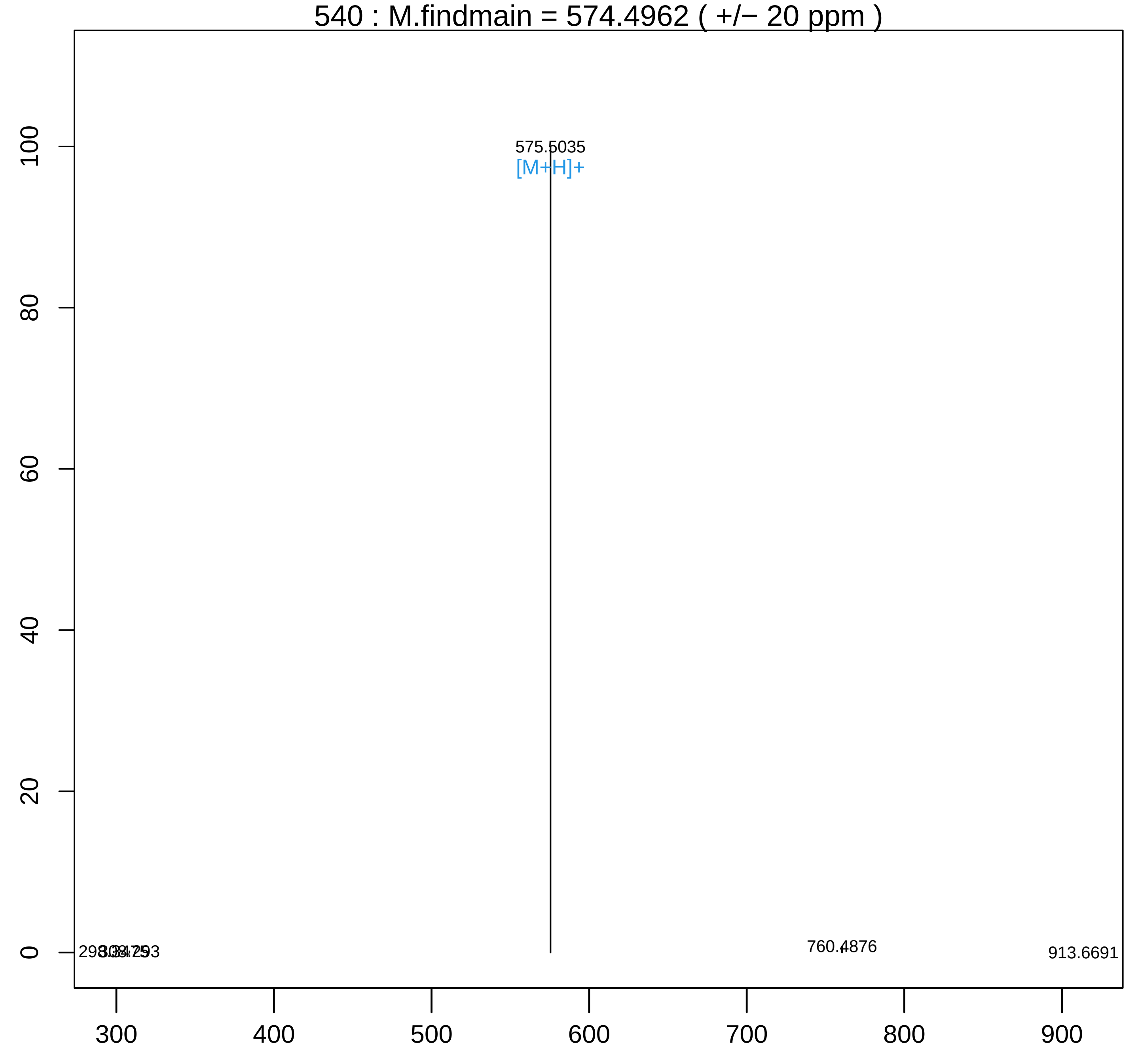

540 : M.findmain = 574.4962 (+/− 20 ppm)
575.5035
[M+H]+
760.4876
913.6691

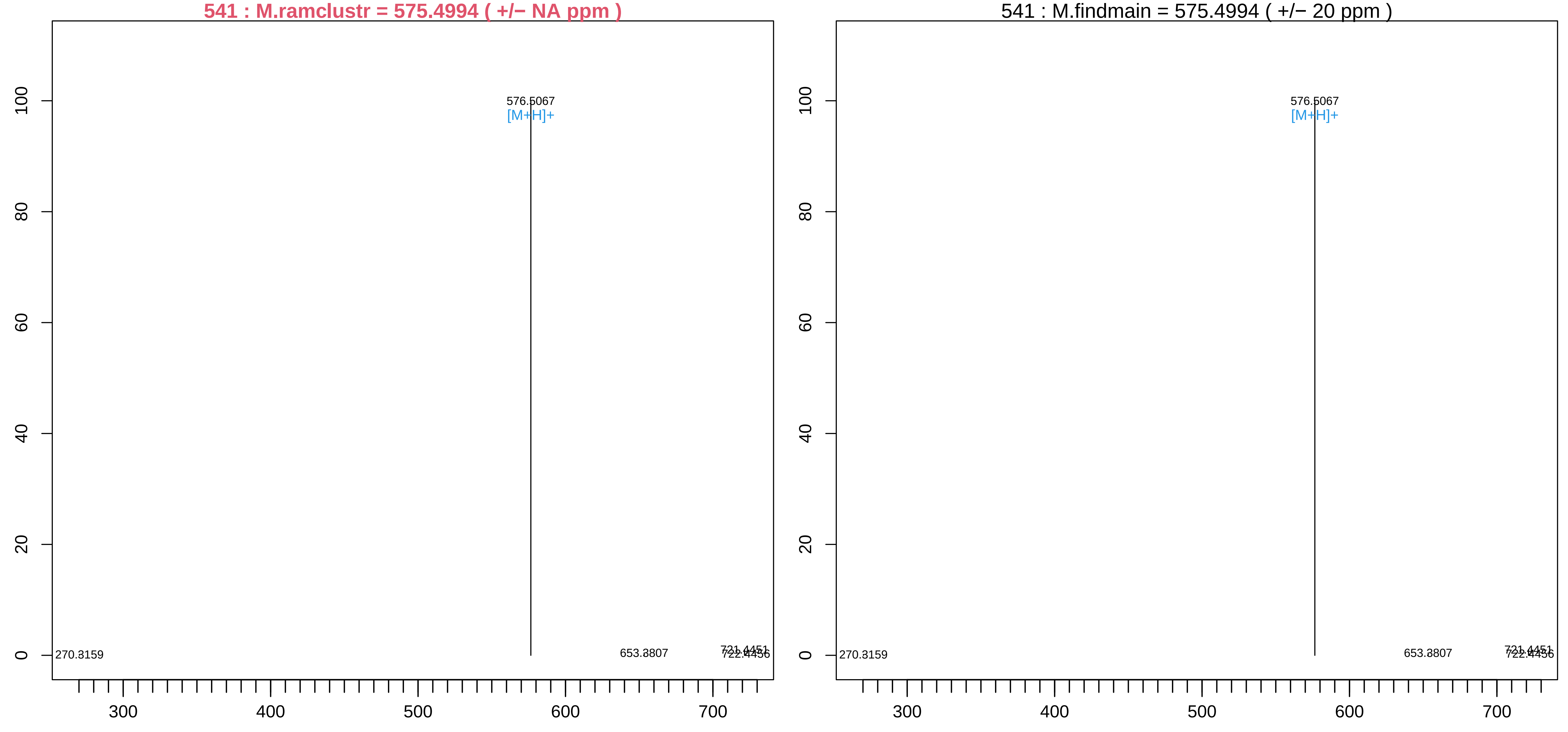

541 : M.ramclustr = 575.4994 (+/− NA ppm)
576.5067
[M+H]+
270.3159
653.3807
721.4451
722.4436
541 : M.findmain = 575.4994 (+/− 20 ppm)
576.5067
[M+H]+
270.3159
653.3807
721.4451
722.4436

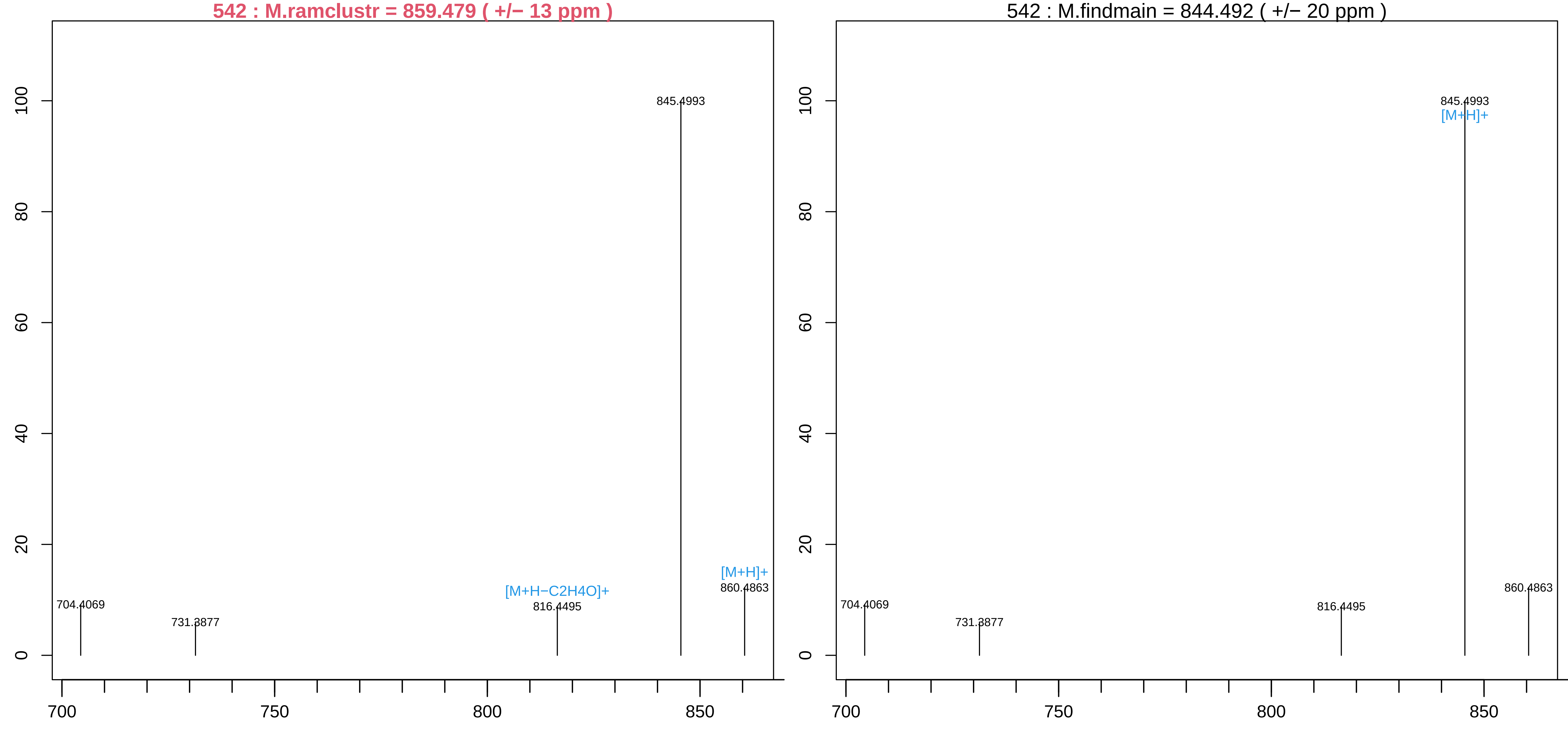

542 : M.ramclustr = 859.479 (+/− 13 ppm)
845.4993
[M+H]+
860.4863
[M+H−C2H4O]+
816.4495
704.4069
731.3877
542 : M.findmain = 844.492 (+/− 20 ppm)
845.4993
[M+H]+
704.4069
731.3877
816.4495
860.4863
0
20
40
60
80
100
700
750
800
850

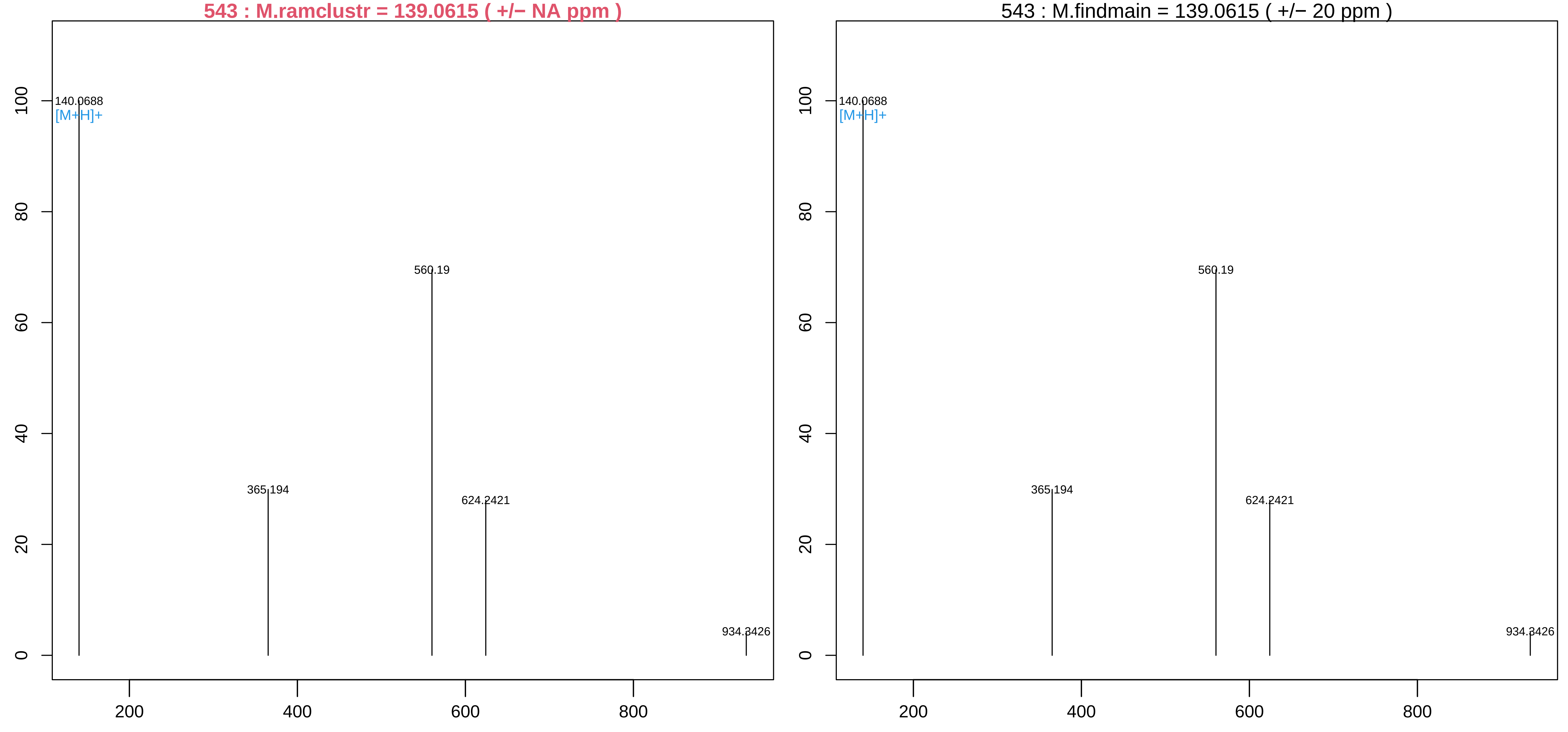

543 : M.ramclustr = 139.0615 (+/− NA ppm)
140.0688
[M+H]+
365.194
560.19
624.2421
934.3426
543 : M.findmain = 139.0615 (+/− 20 ppm)
140.0688
[M+H]+
365.194
560.19
624.2421
934.3426

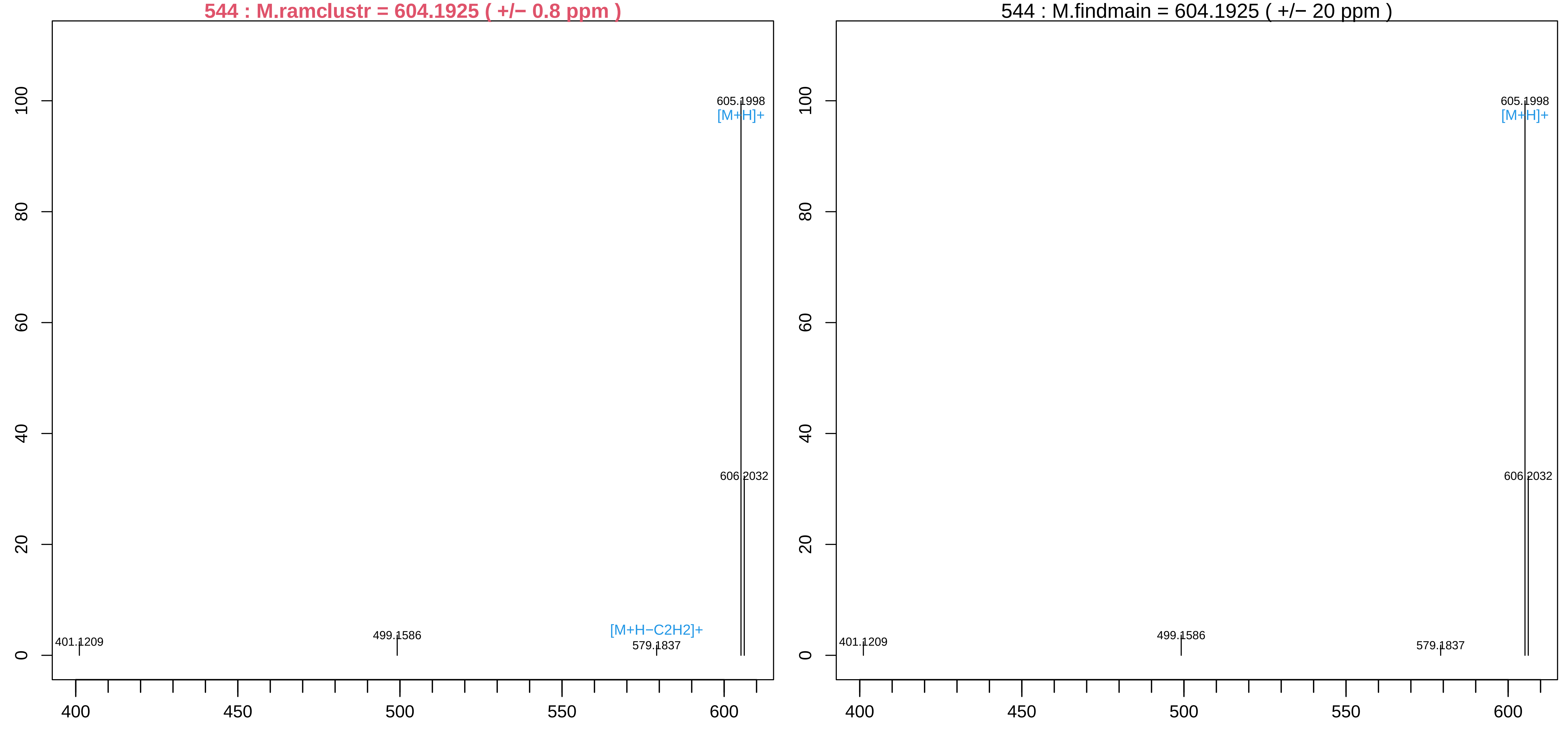

544 : M.ramclustr = 604.1925 (+/− 0.8 ppm)
605.1998
[M+H]+
606.2032
[M+H−C2H2]+
401.1209
499.1586
579.1837
544 : M.findmain = 604.1925 (+/− 20 ppm)
605.1998
[M+H]+
606.2032
401.1209
499.1586
579.1837

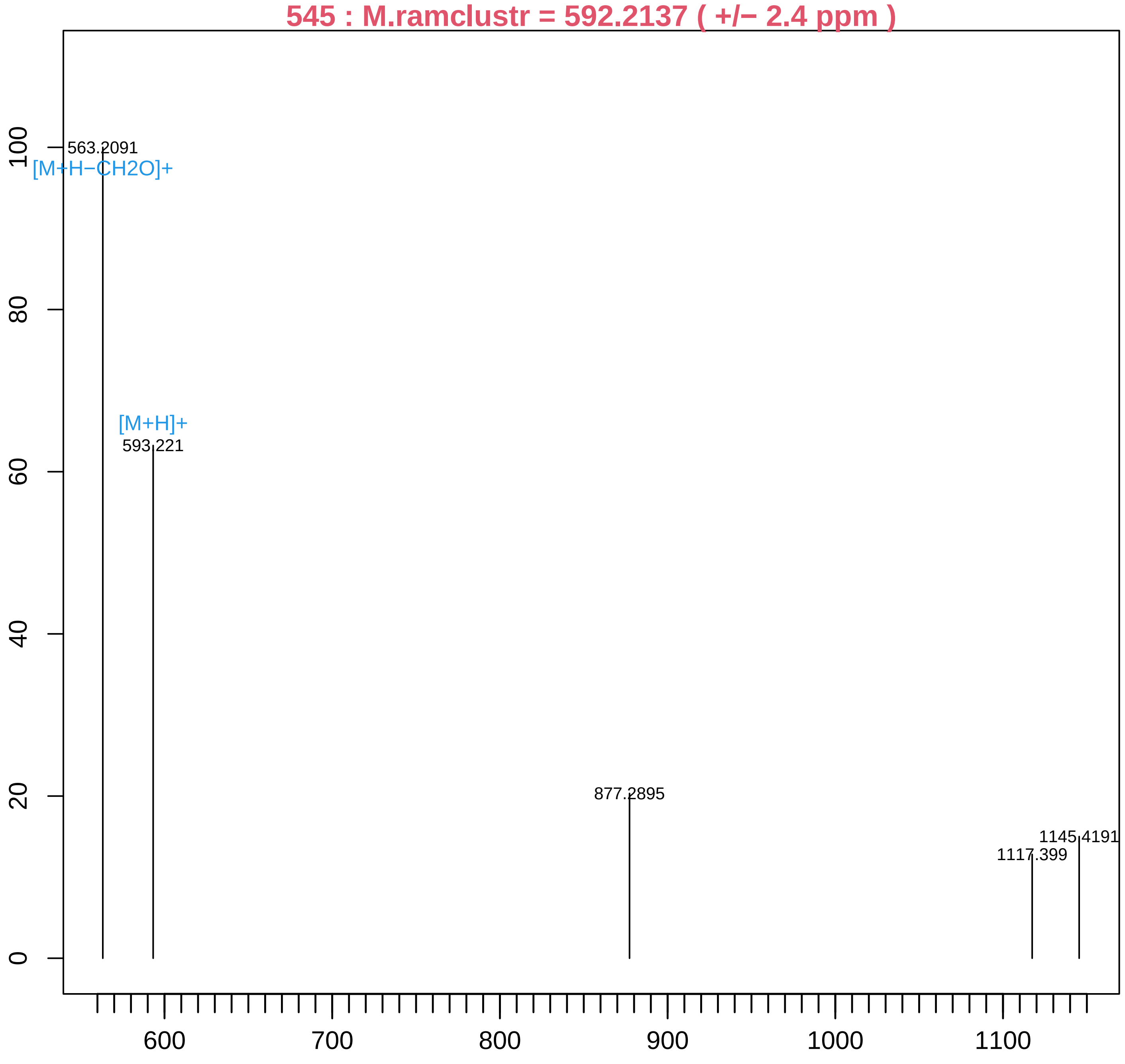
545 : M.ramclustr = 592.2137 (+/− 2.4 ppm)
563.2091
[M+H−CH2O]+
[M+H]+
593.221
877.2895
1117.399
1145.4191
0
20
40
60
80
100
600
700
800
900
1000
1100

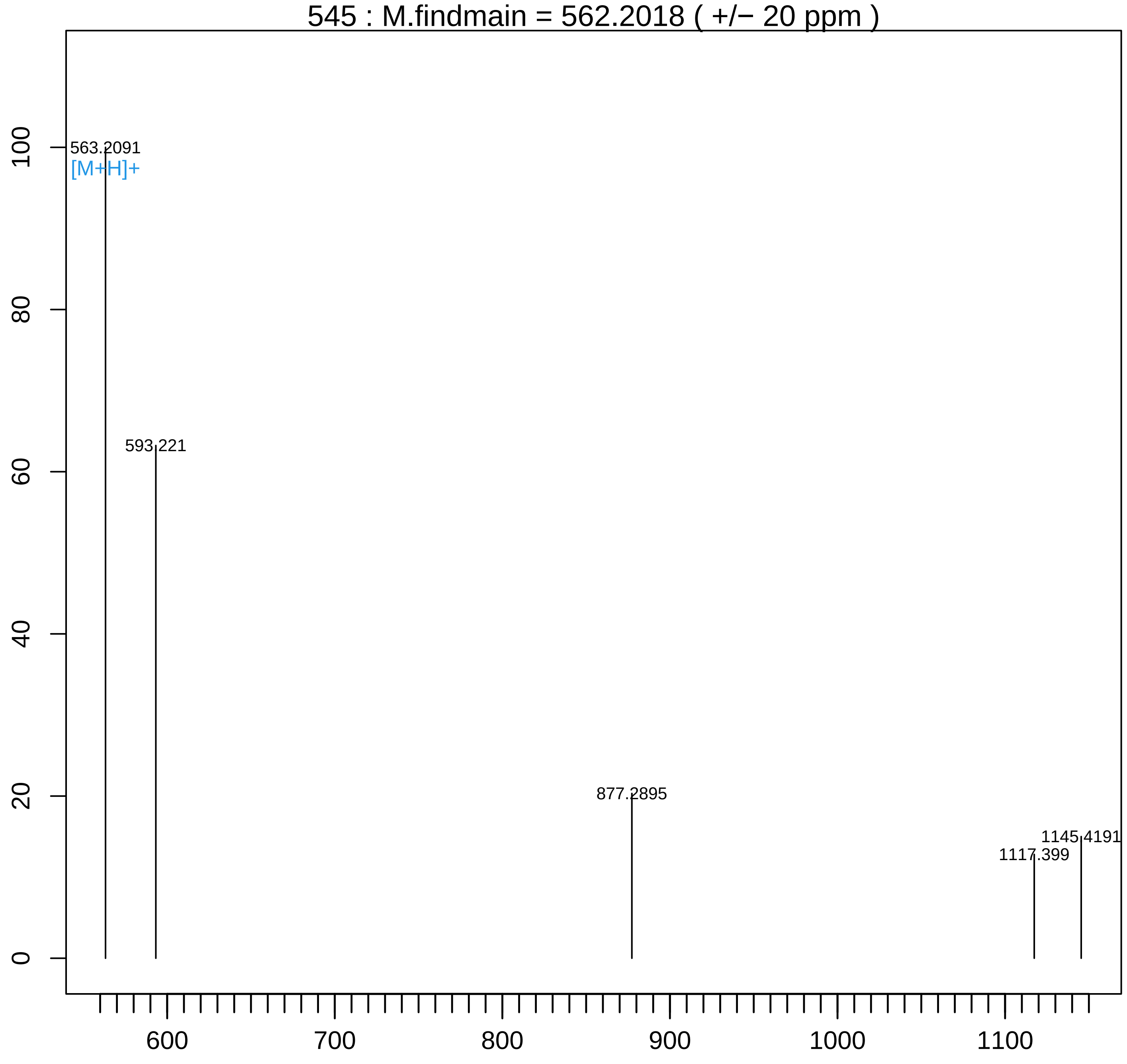
545 : M.findmain = 562.2018 (+/− 20 ppm)
563.2091
[M+H]+
593.221
877.2895
1117.399
1145.4191
0
20
40
60
80
100
600
700
800
900
1000
1100

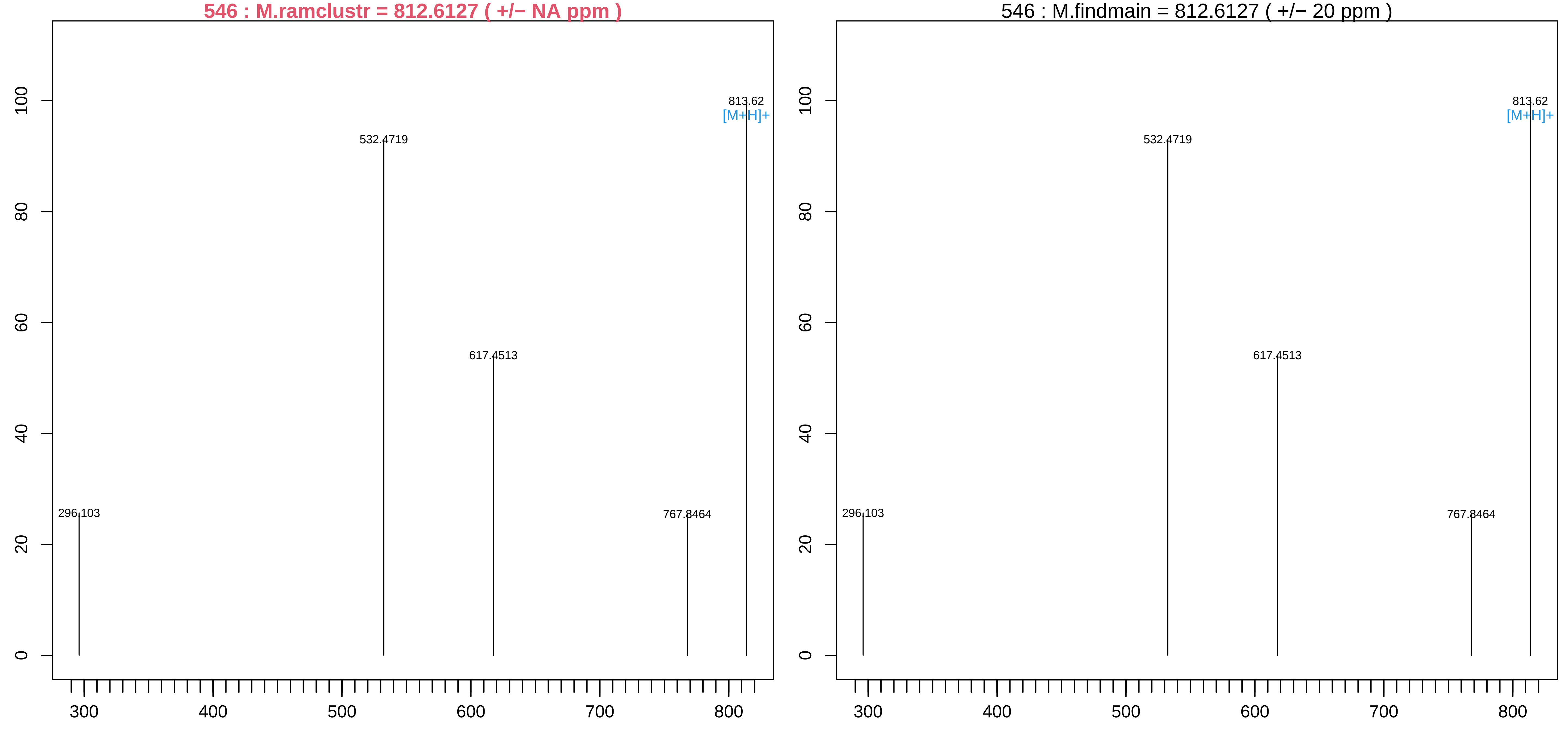
546 : M.ramclustr = 812.6127 (+/− NA ppm)
546 : M.findmain = 812.6127 (+/− 20 ppm)
813.62
[M+H]+
532.4719
617.4513
296.103
767.8464
0
20
40
60
80
100
300
400
500
600
700
800

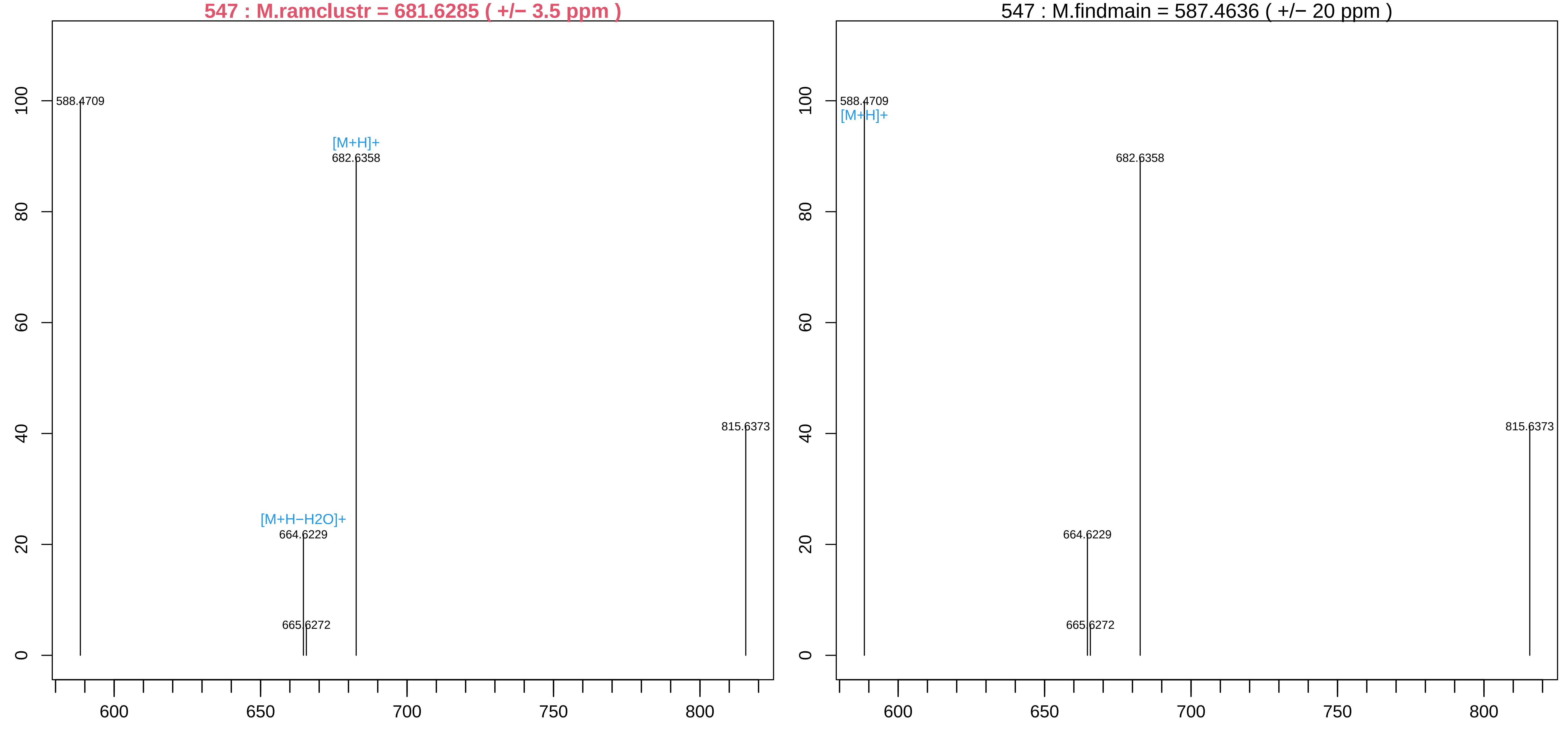

547 : M.ramclustr = 681.6285 (+/− 3.5 ppm)
588.4709
[M+H]+
682.6358
[M+H−H2O]+
664.6229
665.6272
815.6373
547 : M.findmain = 587.4636 (+/− 20 ppm)
588.4709
[M+H]+
682.6358
664.6229
665.6272
815.6373

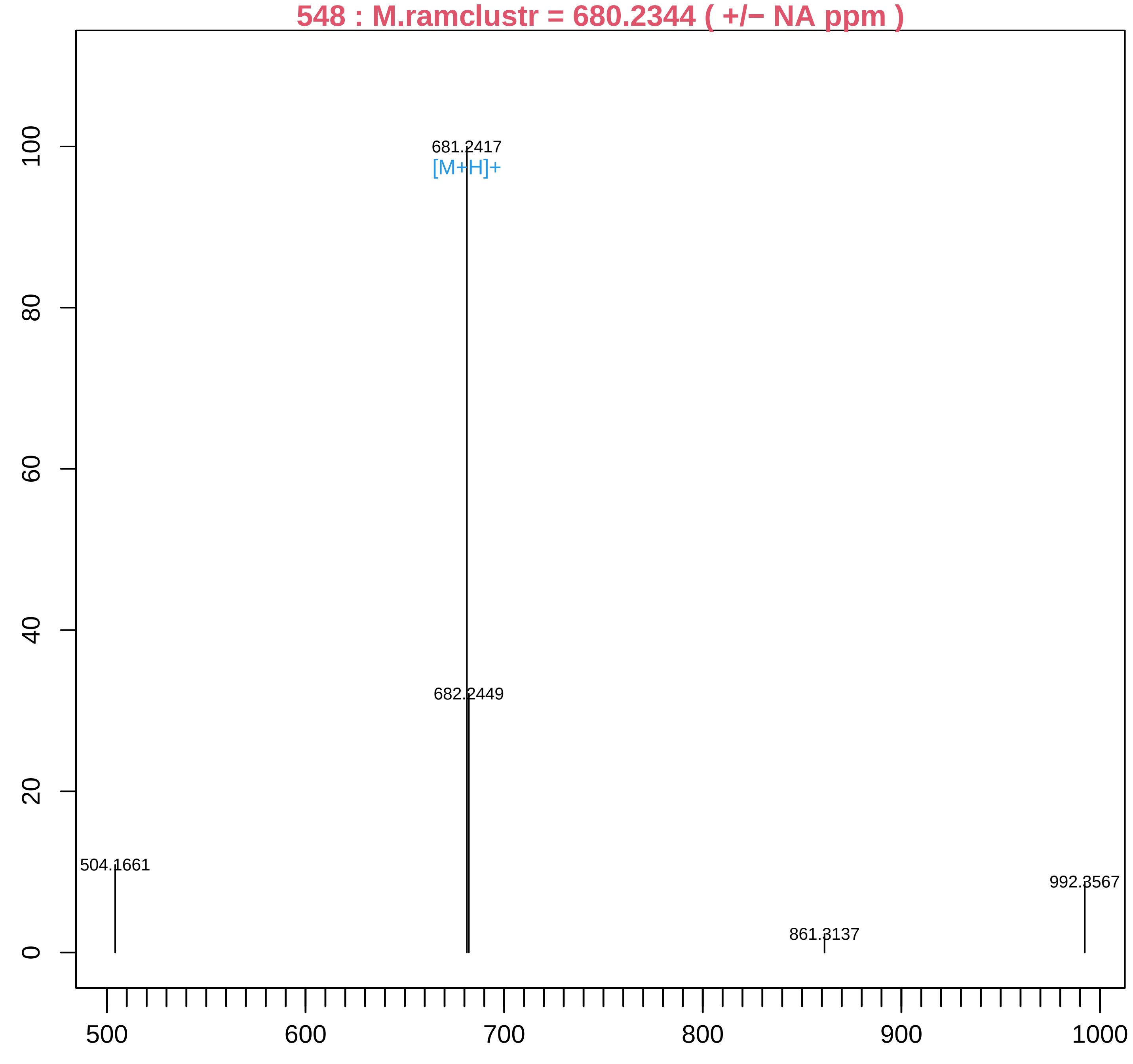
548 : M.ramclustr = 680.2344 (+/− NA ppm)
681.2417
[M+H]+
682.2449
504.1661
992.3567
861.3137

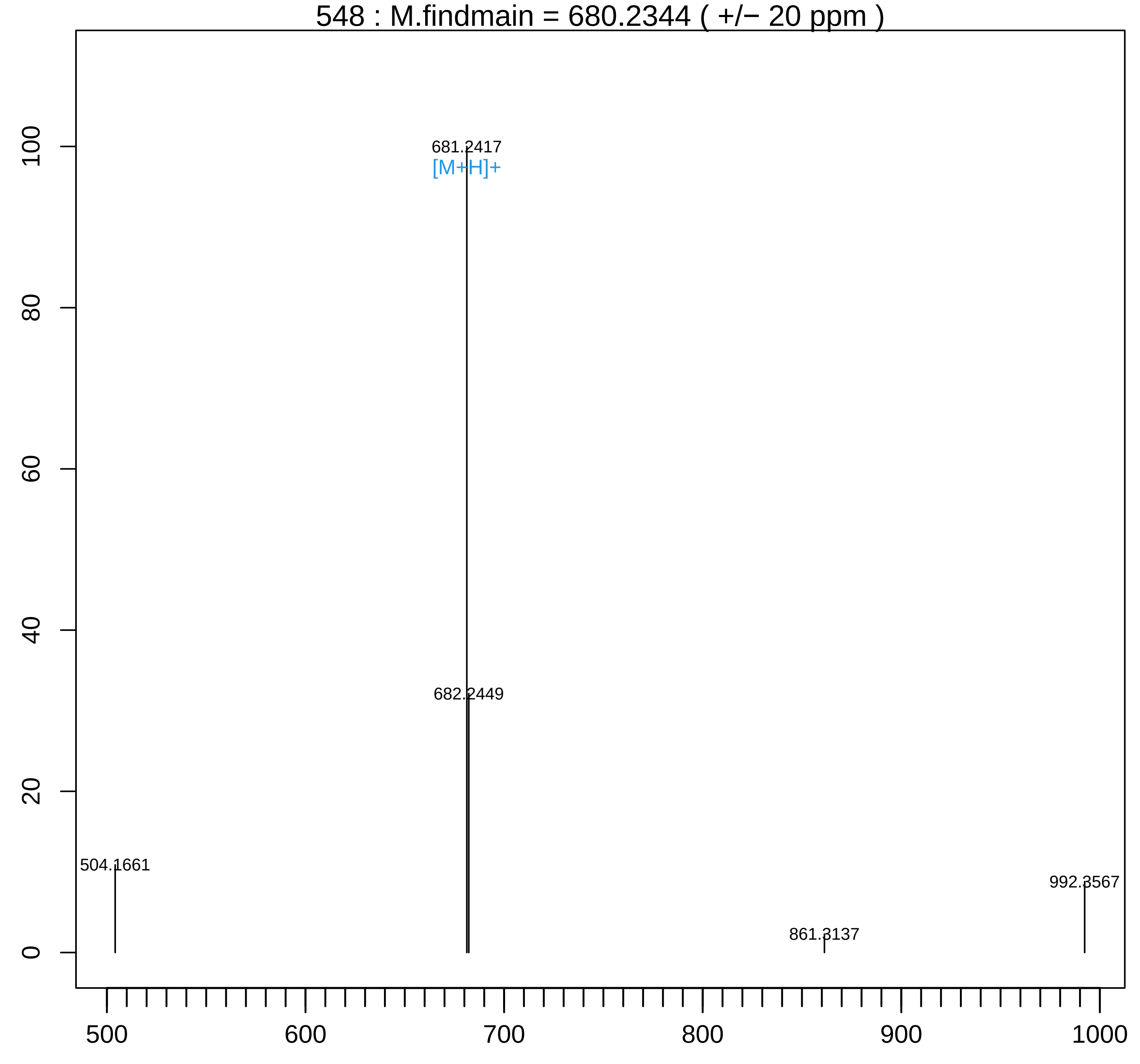
548 : M.findmain = 680.2344 (+/− 20 ppm)
681.2417
[M+H]+
682.2449
504.1661
992.3567
861.3137

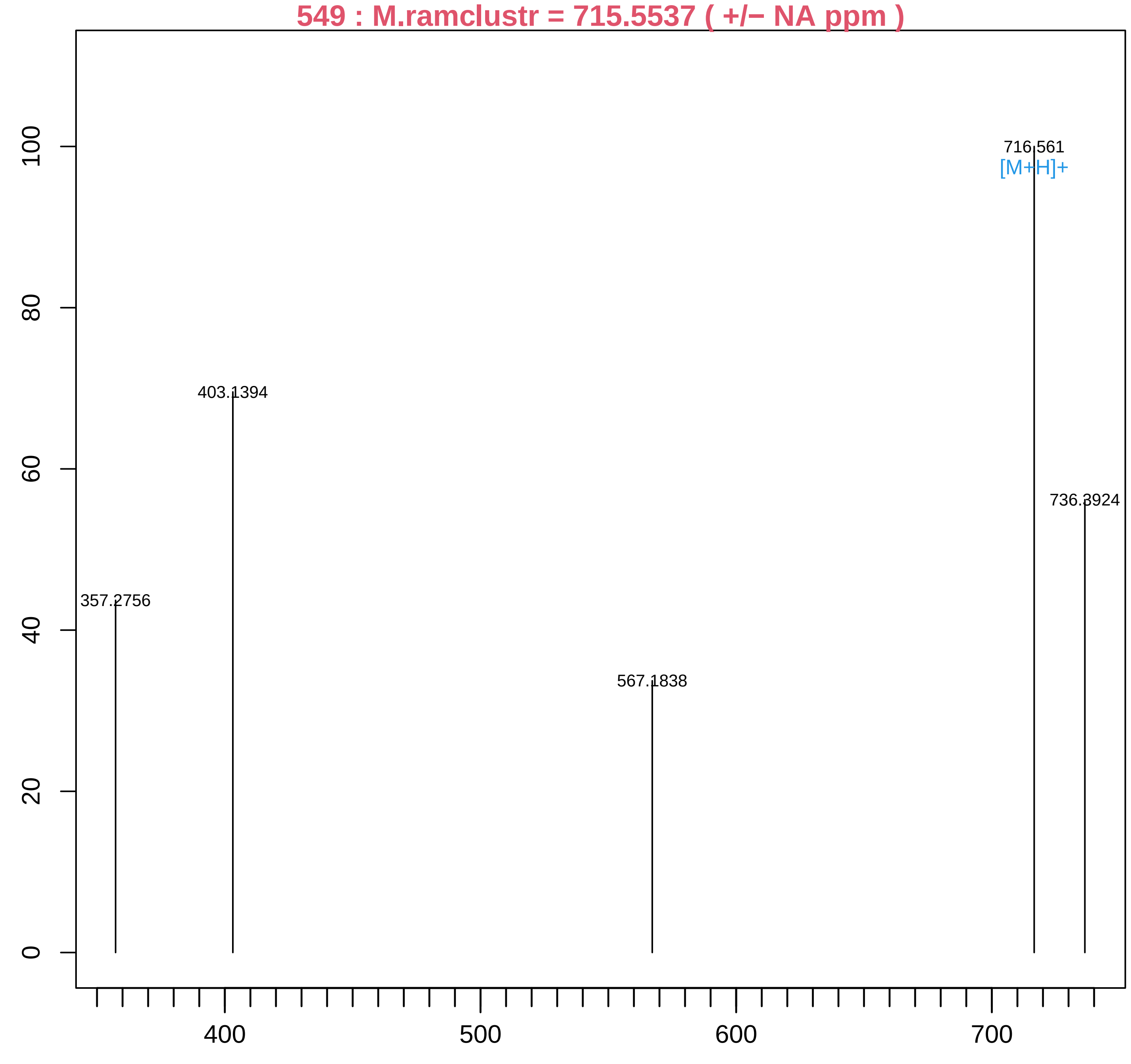
549 : M.ramclustr = 715.5537 (+/− NA ppm)
716.561
[M+H]+
403.1394
736.3924
357.2756
567.1838
0
20
40
60
80
100
400
500
600
700

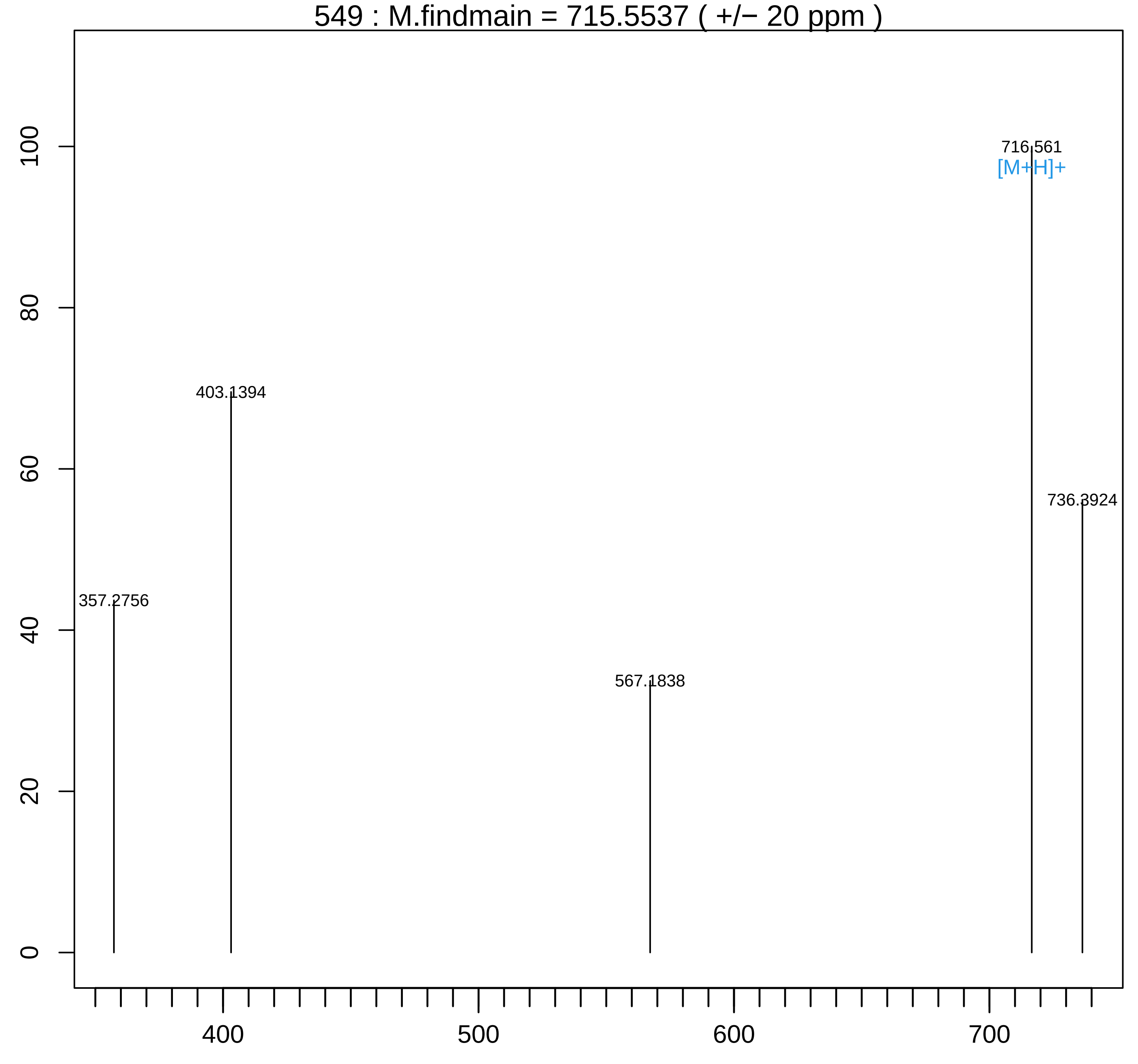
549 : M.findmain = 715.5537 (+/− 20 ppm)
716.561
[M+H]+
403.1394
736.3924
357.2756
567.1838
0
20
40
60
80
100
400
500
600
700

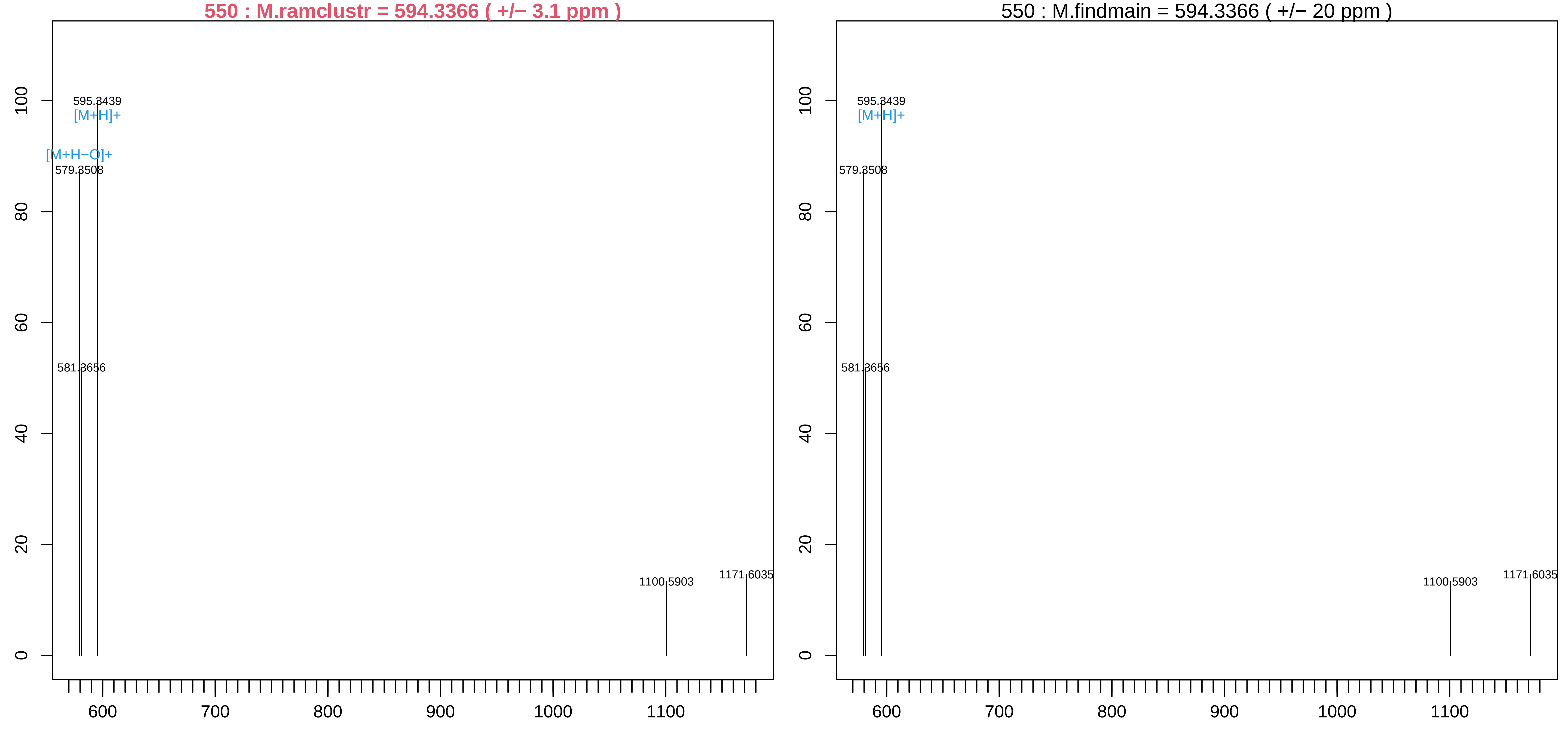
550 : M.ramclustr = 594.3366 (+/− 3.1 ppm)
595.3439
[M+H]+
[M+H−O]+
579.3508
581.3656
1100.5903
1171.6035
0
20
40
60
80
100
600
700
800
900
1000
1100
550 : M.findmain = 594.3366 (+/− 20 ppm)
595.3439
[M+H]+
579.3508
581.3656
1100.5903
1171.6035
0
20
40
60
80
100
600
700
800
900
1000
1100

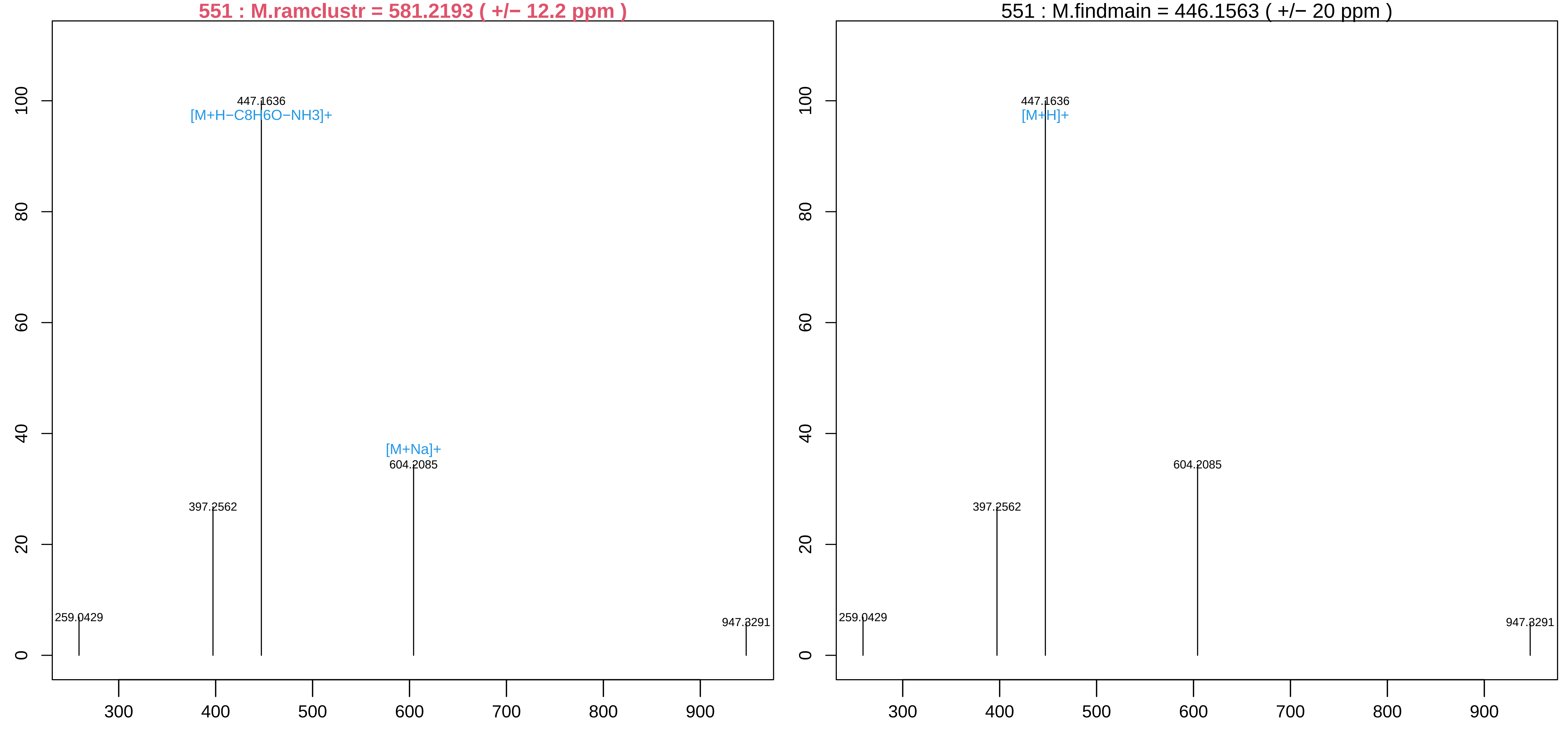
551 : M.ramclustr = 581.2193 (+/− 12.2 ppm)
447.1636
[M+H−C8H6O−NH3]+
[M+Na]+
604.2085
397.2562
259.0429
947.3291
551 : M.findmain = 446.1563 (+/− 20 ppm)
447.1636
[M+H]+
604.2085
397.2562
259.0429
947.3291

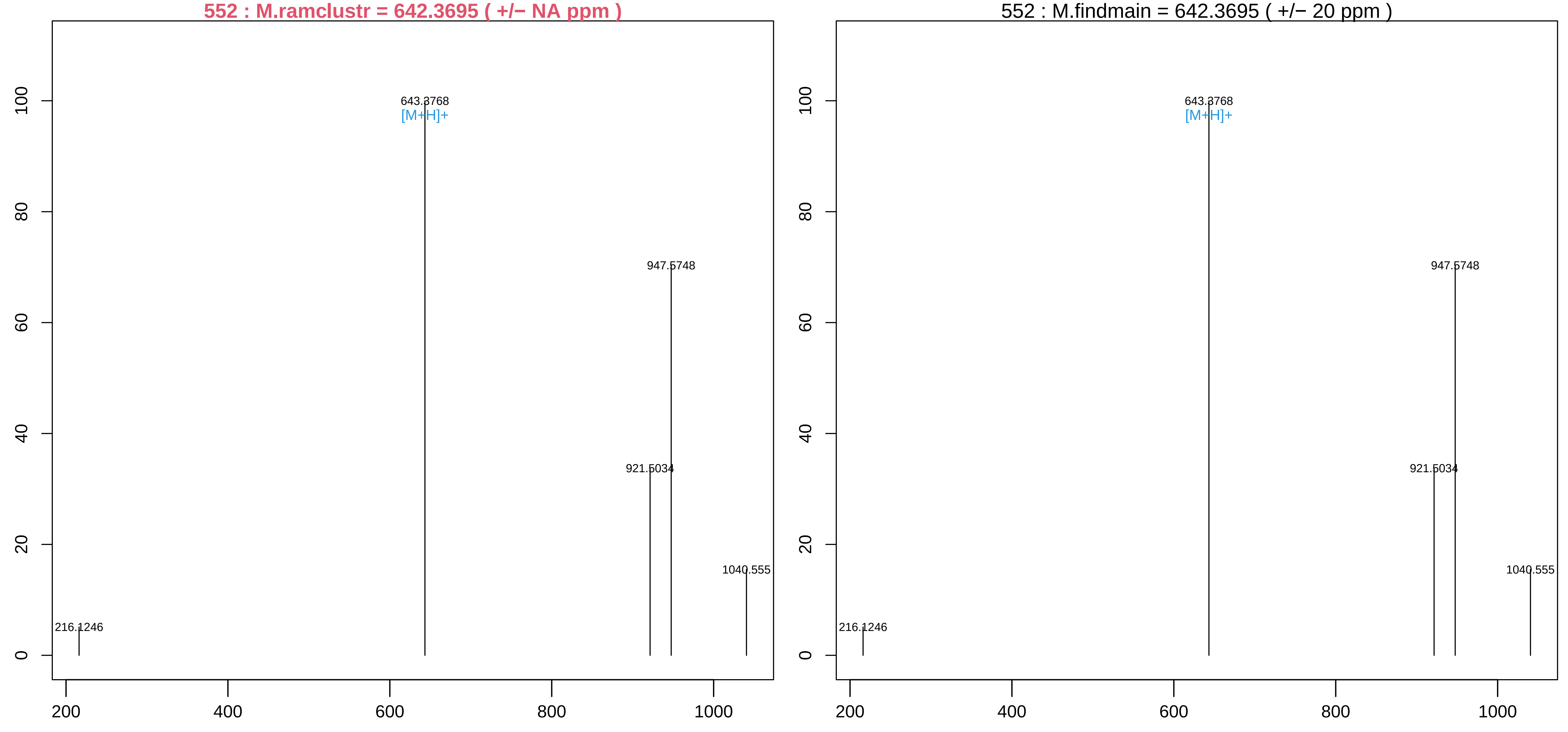
552 : M.ramclustr = 642.3695 (+/− NA ppm)
643.3768
[M+H]+
947.5748
921.5034
1040.555
216.1246
552 : M.findmain = 642.3695 (+/− 20 ppm)
643.3768
[M+H]+
947.5748
921.5034
1040.555
216.1246

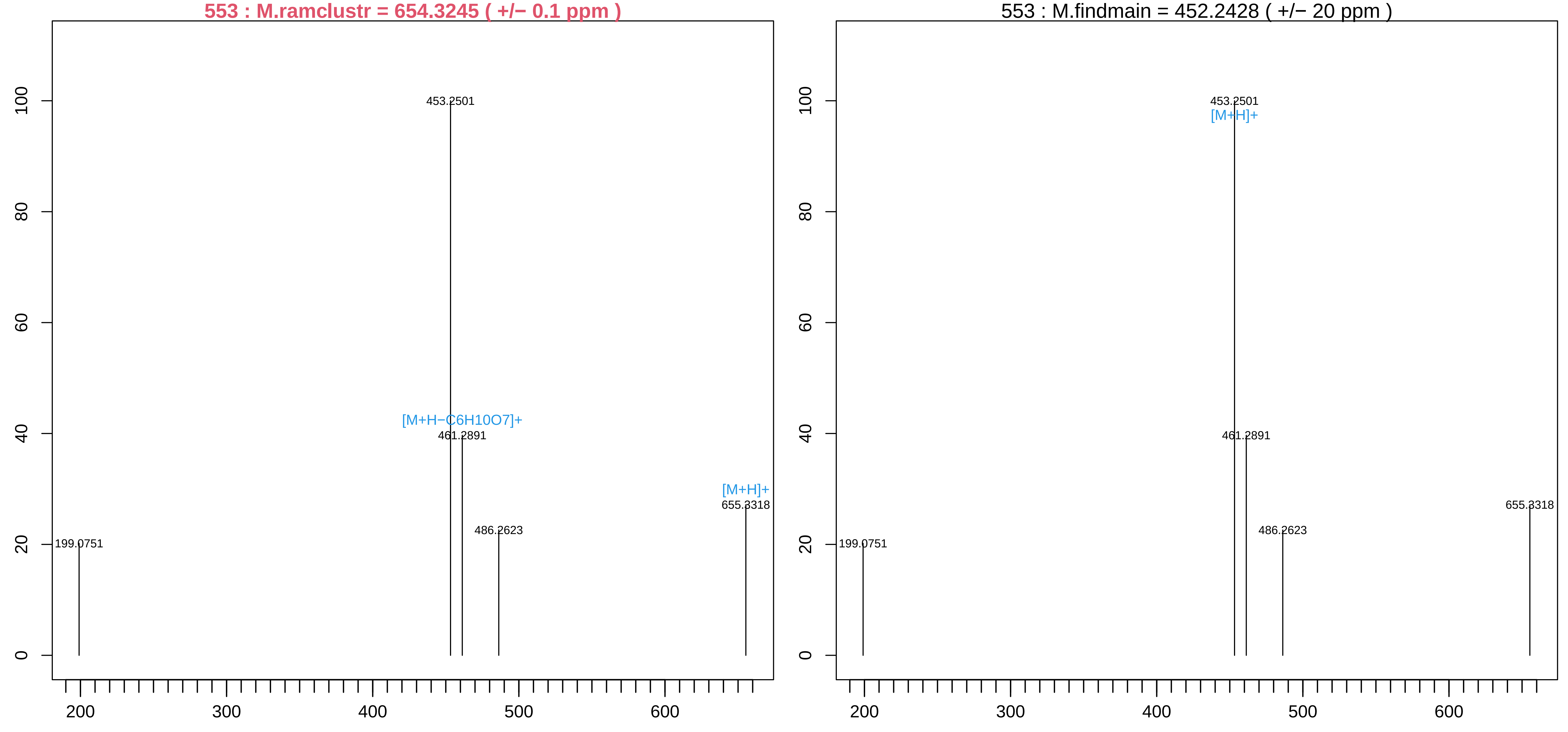
553 : M.ramclustr = 654.3245 (+/− 0.1 ppm)
453.2501
[M+H−C6H10O7]+
461.2891
486.2623
[M+H]+
655.3318
199.0751
553 : M.findmain = 452.2428 (+/− 20 ppm)
453.2501
[M+H]+
461.2891
486.2623
655.3318
199.0751
0
20
40
60
80
100
200
300
400
500
600

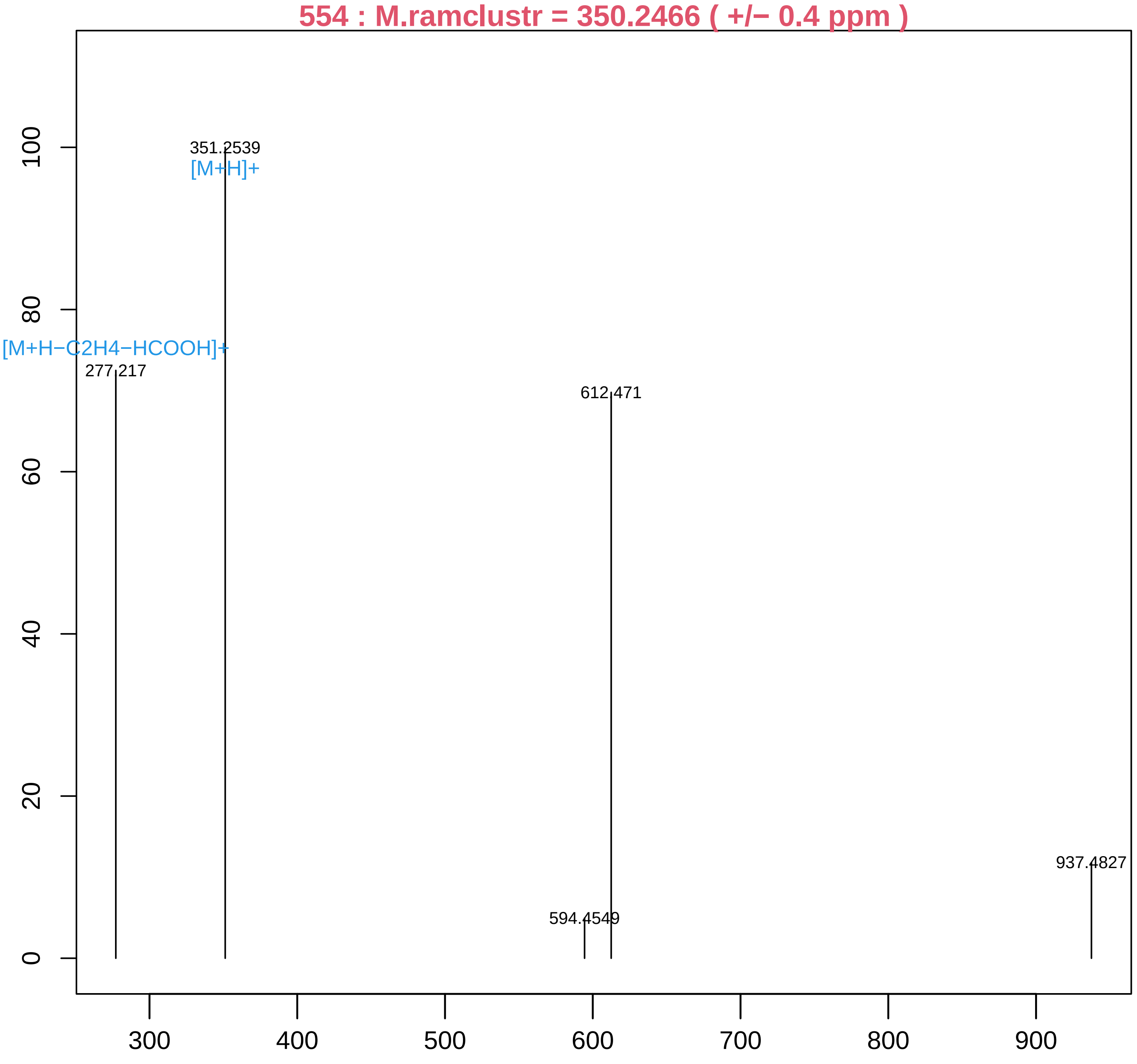
554 : M.ramclustr = 350.2466 (+/− 0.4 ppm)
351.2539
[M+H]+
[M+H−C2H4−HCOOH]+
277.217
612.471
937.4827
594.4549
0
20
40
60
80
100
300
400
500
600
700
800
900

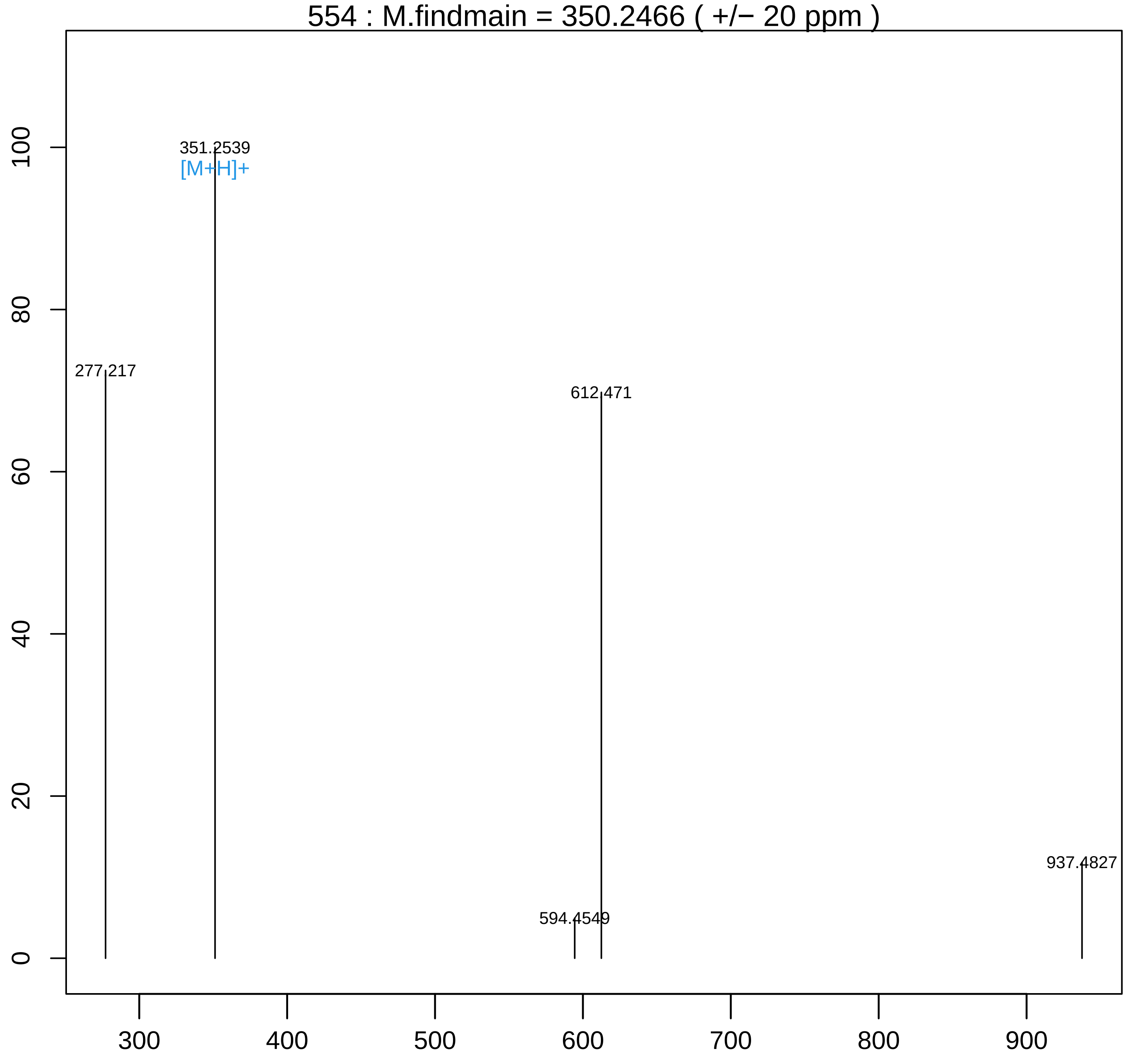
554 : M.findmain = 350.2466 (+/− 20 ppm)
351.2539
[M+H]+
277.217
612.471
937.4827
594.4549
0
20
40
60
80
100
300
400
500
600
700
800
900

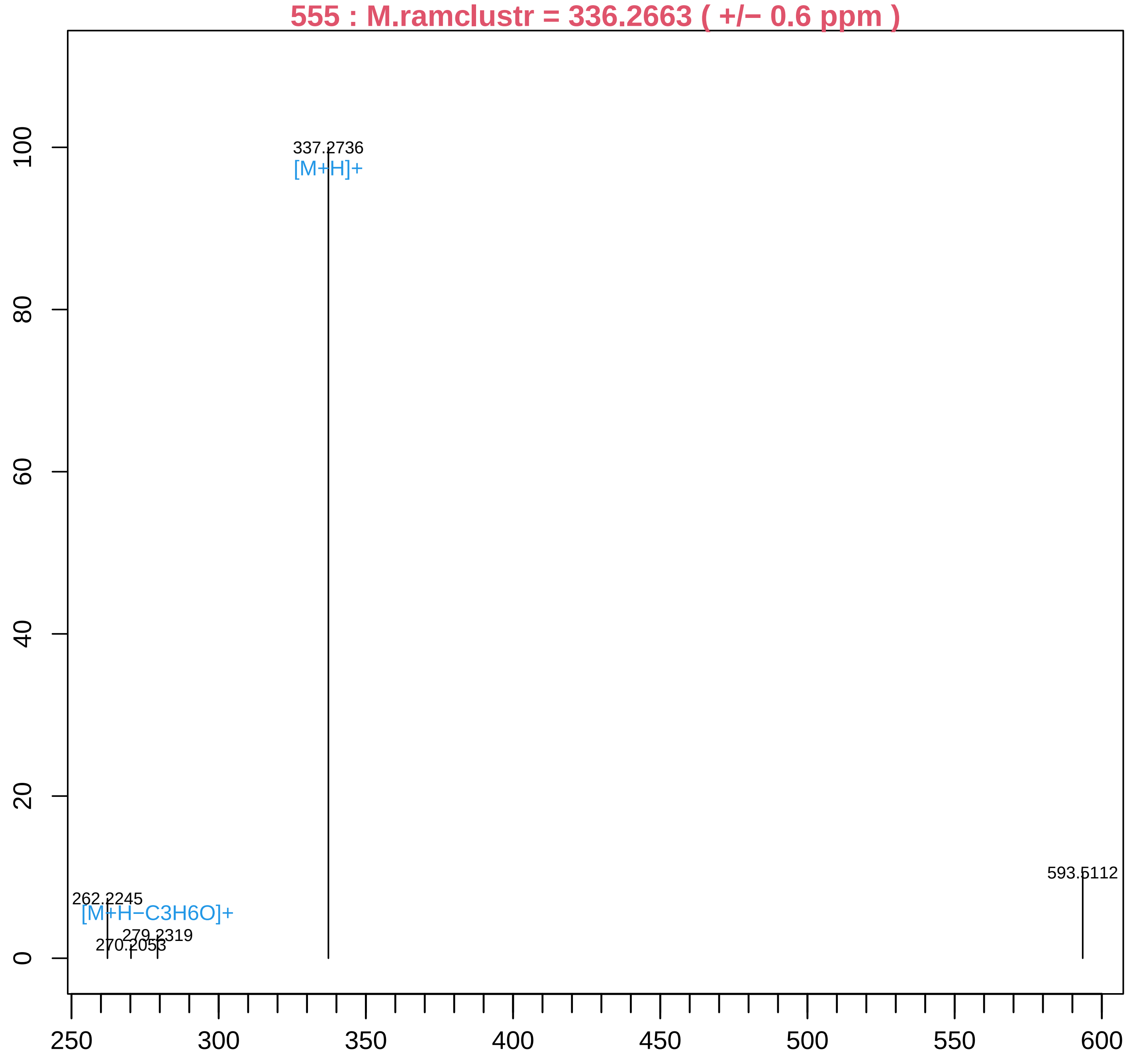
555 : M.ramclustr = 336.2663 (+/− 0.6 ppm)
337.2736
[M+H]+
262.2245
[M+H−C3H6O]+
279.2319
270.2053
593.5112
0
20
40
60
80
100
250
300
350
400
450
500
550
600

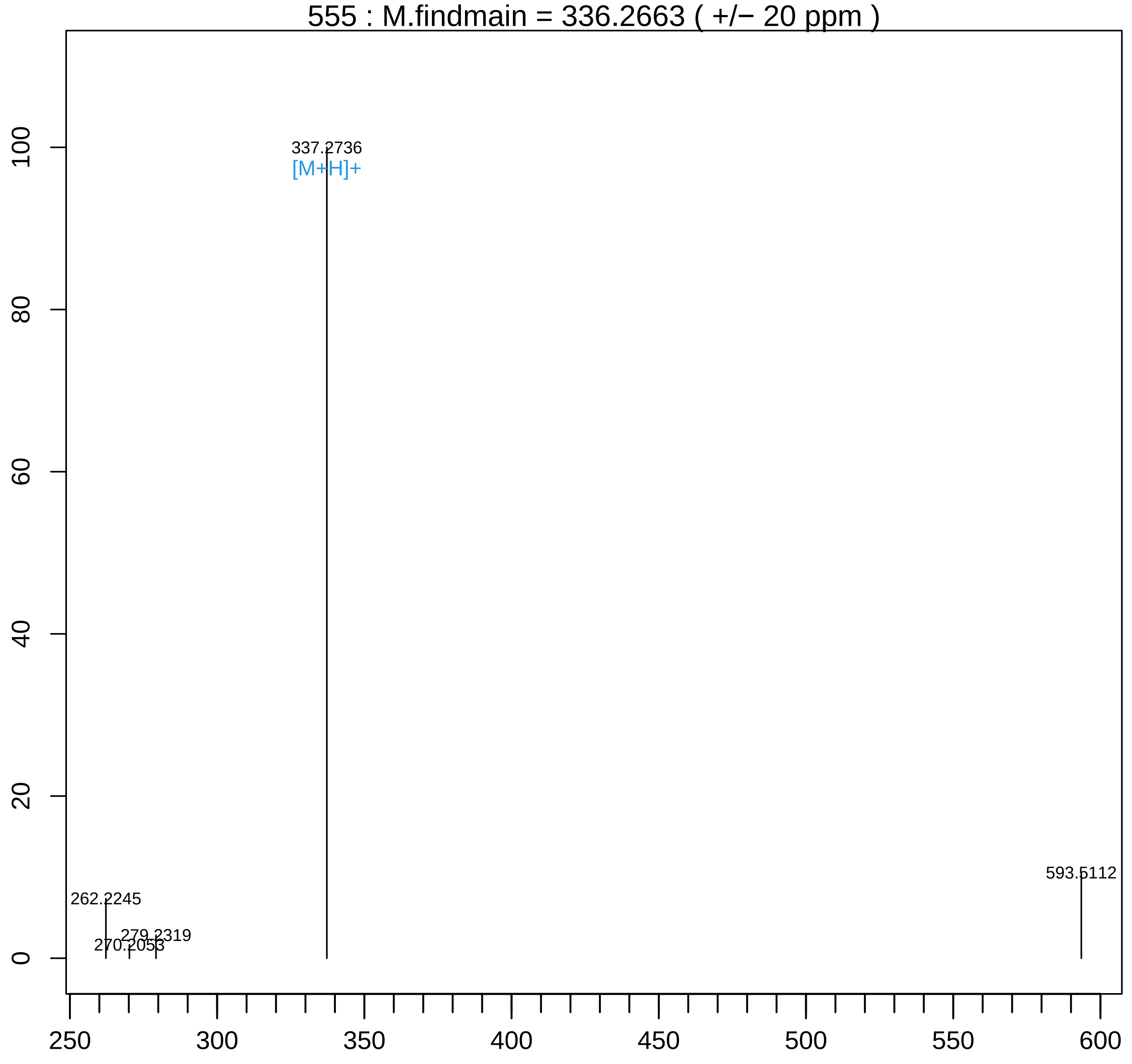
555 : M.findmain = 336.2663 (+/− 20 ppm)
337.2736
[M+H]+
262.2245
279.2319
270.2053
593.5112
0
20
40
60
80
100
250
300
350
400
450
500
550
600

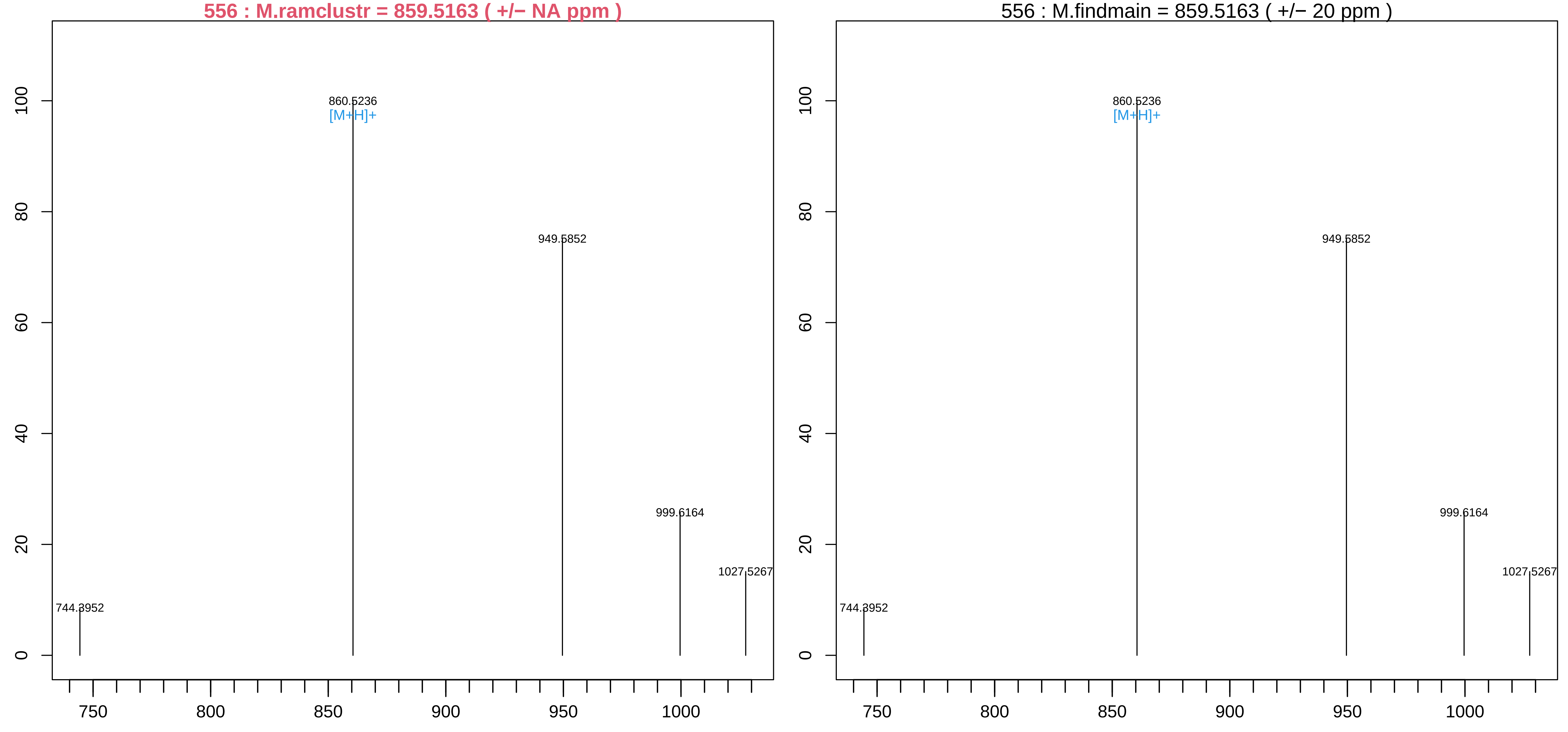

556 : M.ramclustr = 859.5163 (+/− NA ppm)
860.5236
[M+H]+
949.5852
999.6164
1027.5267
744.3952
556 : M.findmain = 859.5163 (+/− 20 ppm)
860.5236
[M+H]+
949.5852
999.6164
1027.5267
744.3952

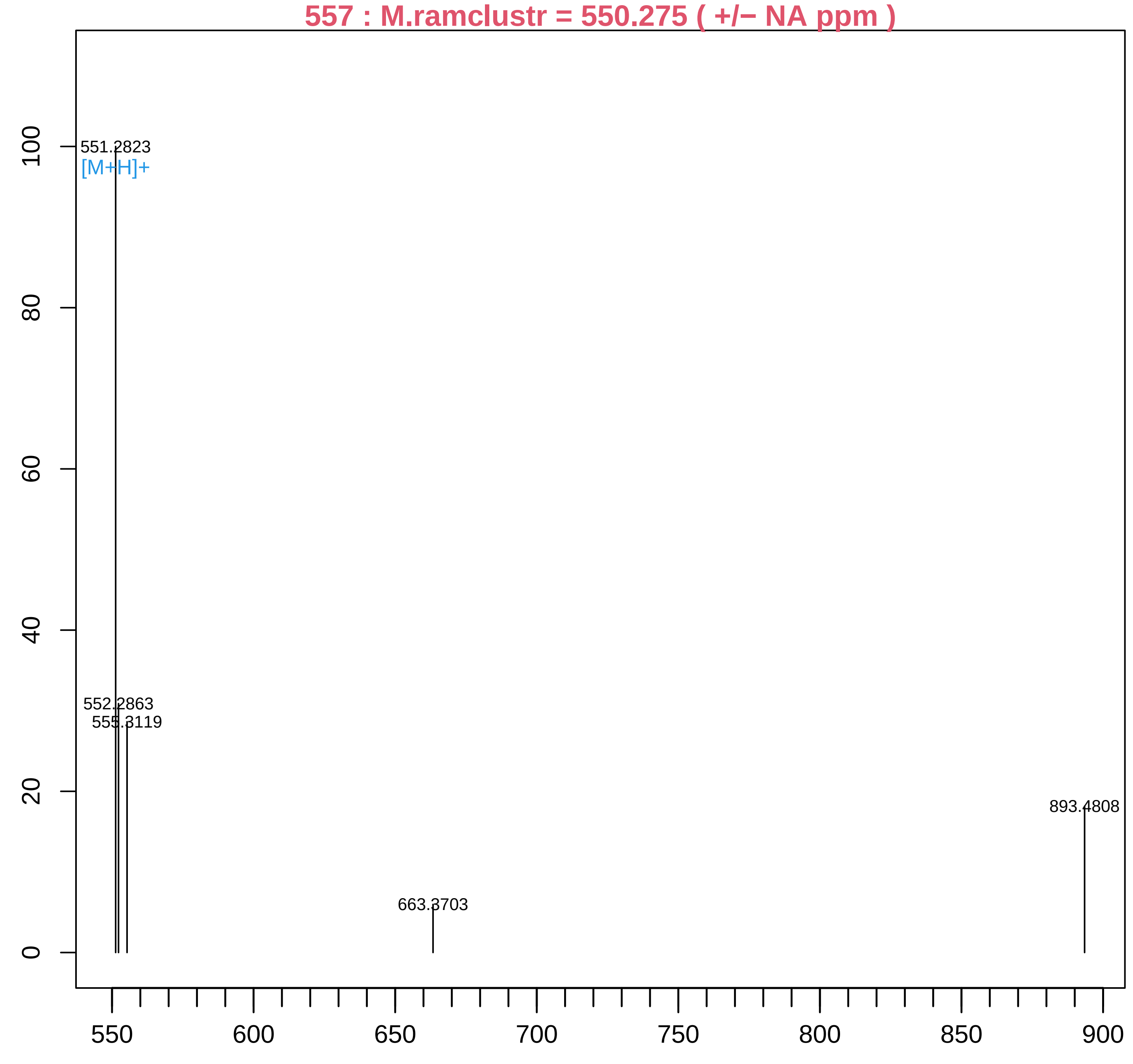
557 : M.ramclustr = 550.275 (+/− NA ppm)
551.2823
[M+H]+
552.2863
555.3119
663.3703
893.4808
0
20
40
60
80
100
550
600
650
700
750
800
850
900
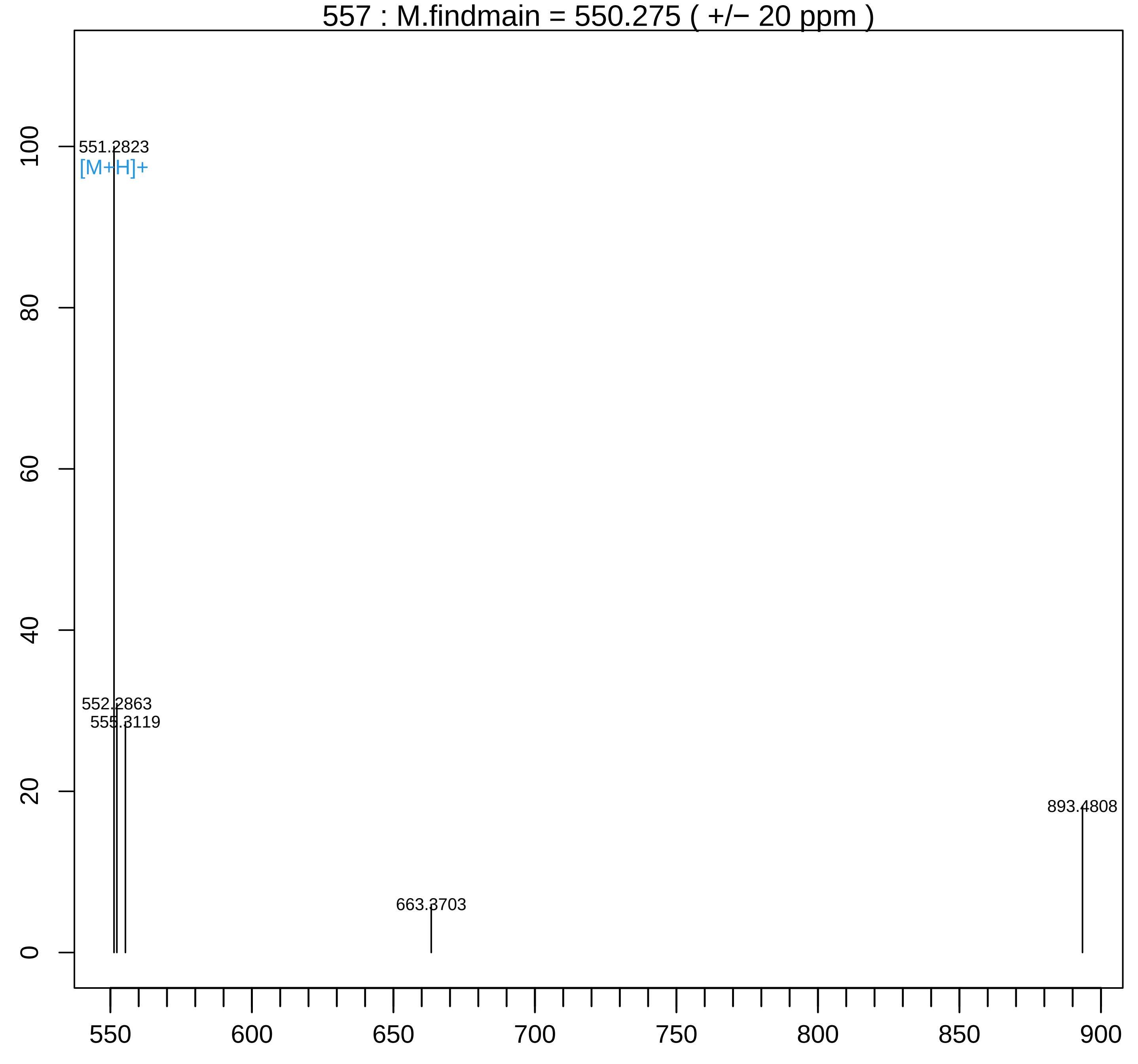
557 : M.findmain = 550.275 (+/− 20 ppm)
551.2823
[M+H]+
552.2863
555.3119
663.3703
893.4808
0
20
40
60
80
100
550
600
650
700
750
800
850
900

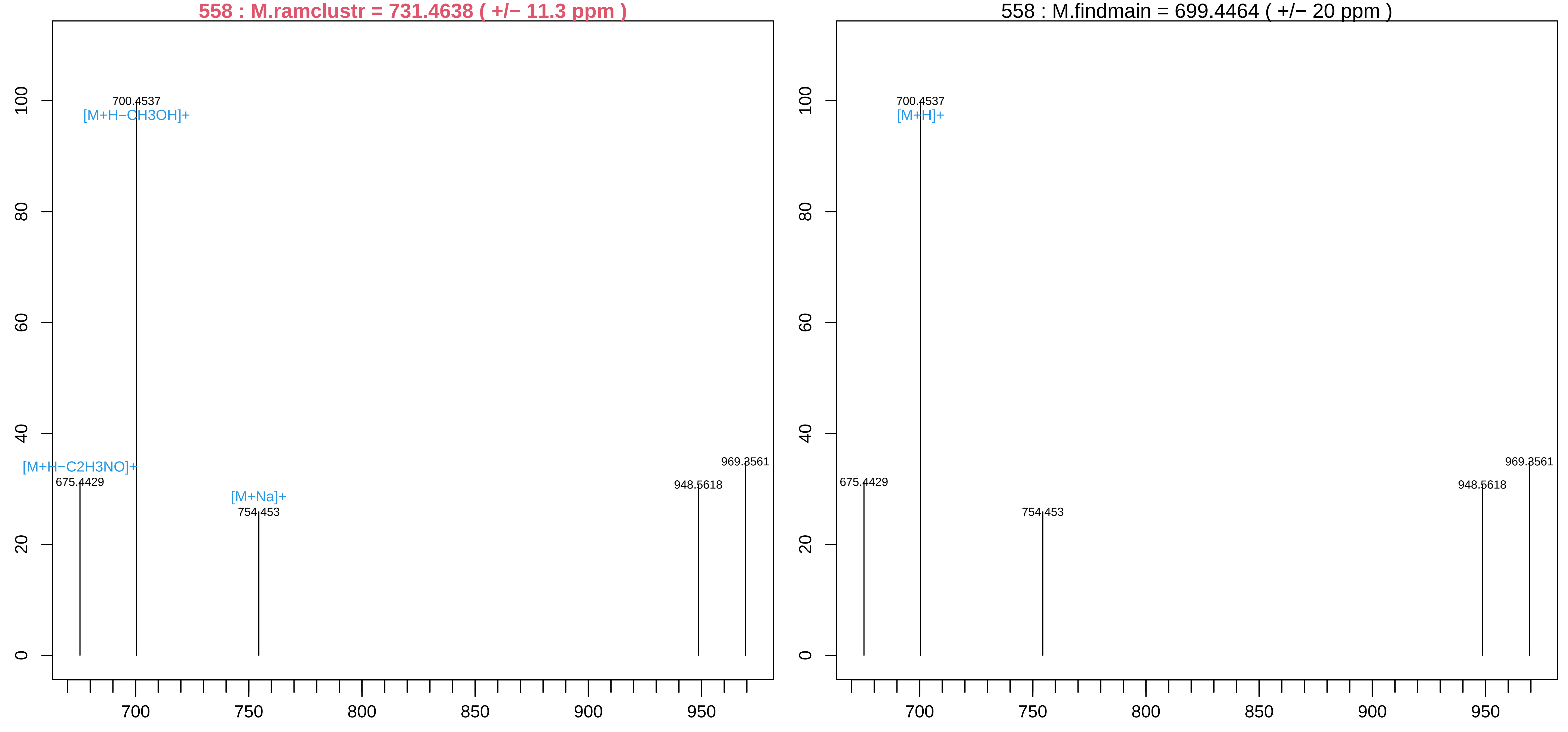
558 : M.ramclustr = 731.4638 (+/− 11.3 ppm)
700.4537
[M+H−CH3OH]+
[M+H−C2H3NO]+
675.4429
[M+Na]+
754.453
948.5618
969.3561
0
20
40
60
80
100
700
750
800
850
900
950
558 : M.findmain = 699.4464 (+/− 20 ppm)
700.4537
[M+H]+
675.4429
754.453
948.5618
969.3561
0
20
40
60
80
100
700
750
800
850
900
950

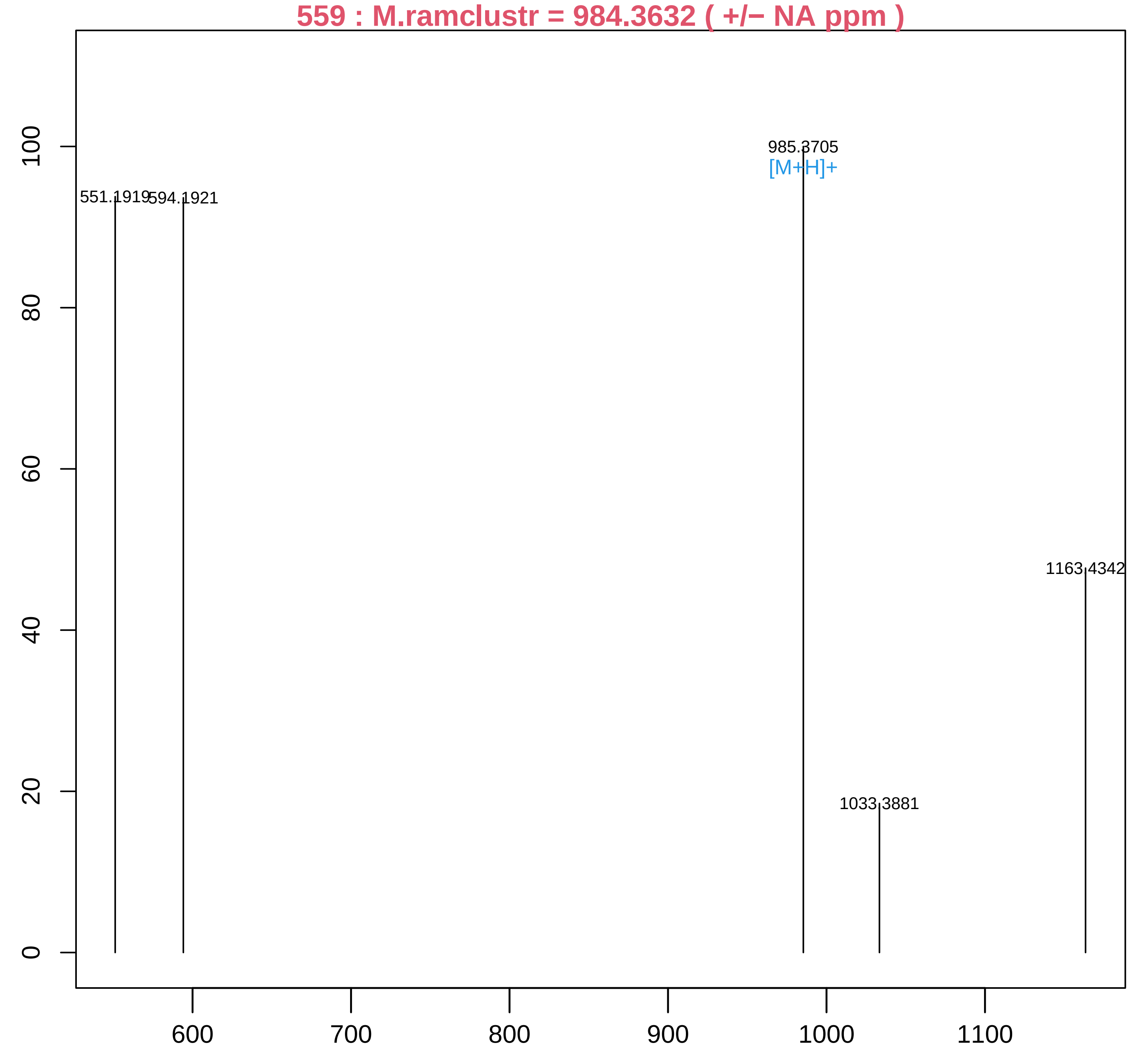
559 : M.ramclustr = 984.3632 (+/− NA ppm)
985.3705
[M+H]+
551.1919
594.1921
1163.4342
1033.3881
0
20
40
60
80
100
600
700
800
900
1000
1100

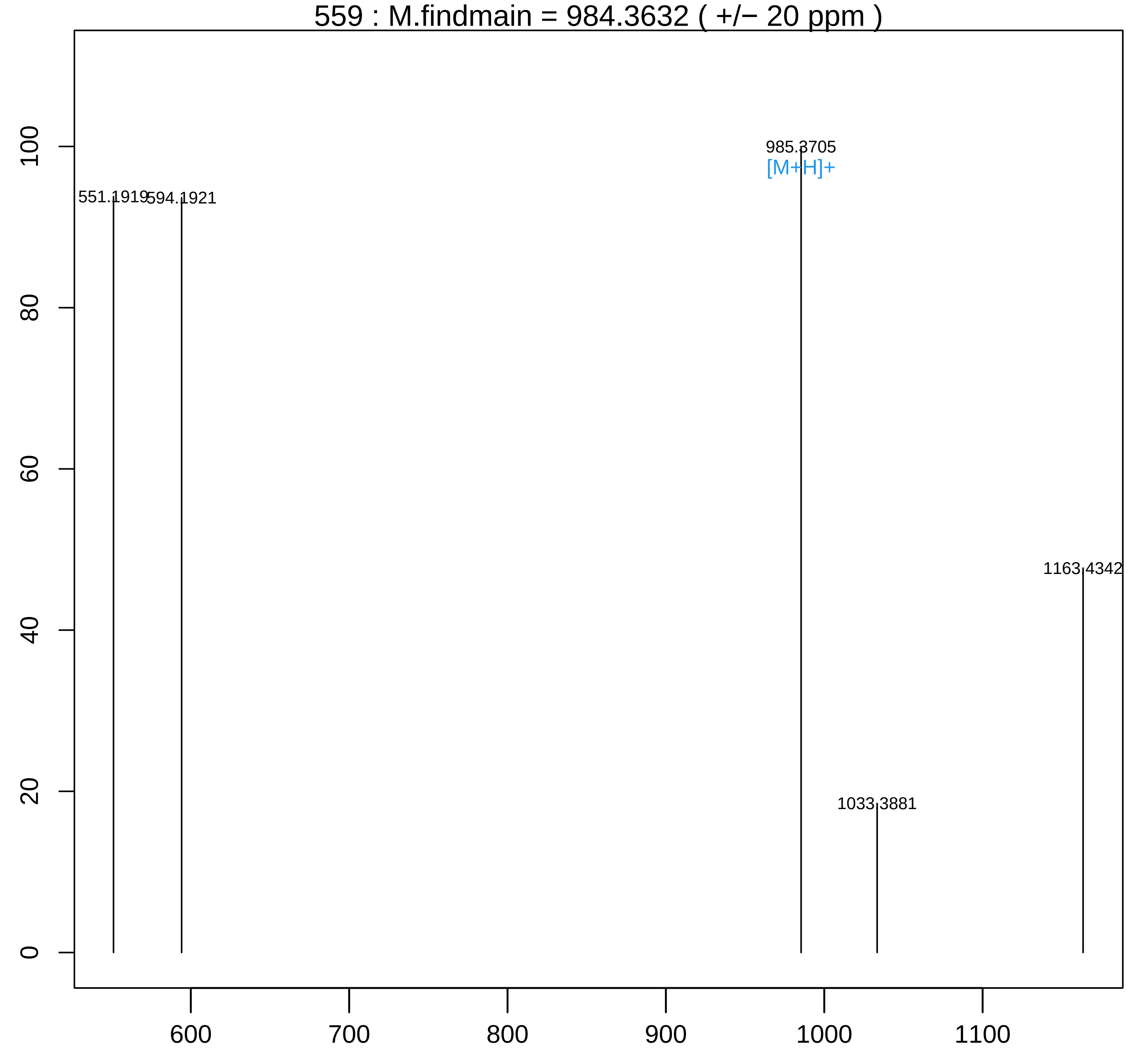
559 : M.findmain = 984.3632 (+/− 20 ppm)
985.3705
[M+H]+
551.1919
594.1921
1163.4342
1033.3881
0
20
40
60
80
100
600
700
800
900
1000
1100

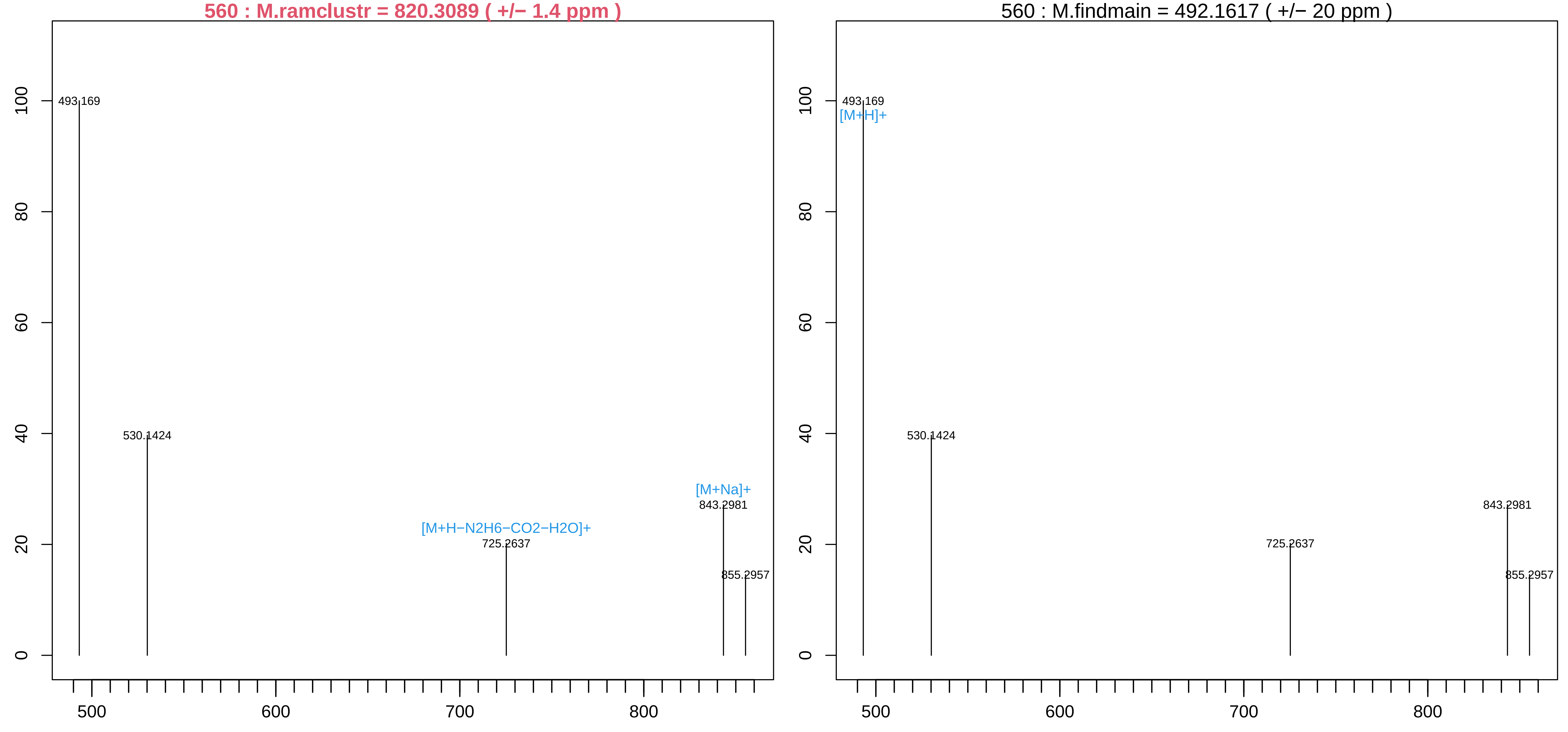
560 : M.ramclustr = 820.3089 (+/− 1.4 ppm)
493.169
530.1424
[M+H−N2H6−CO2−H2O]+
725.2637
[M+Na]+
843.2981
855.2957
0
20
40
60
80
100
500
600
700
800
560 : M.findmain = 492.1617 (+/− 20 ppm)
493.169
[M+H]+
530.1424
725.2637
843.2981
855.2957
0
20
40
60
80
100
500
600
700
800

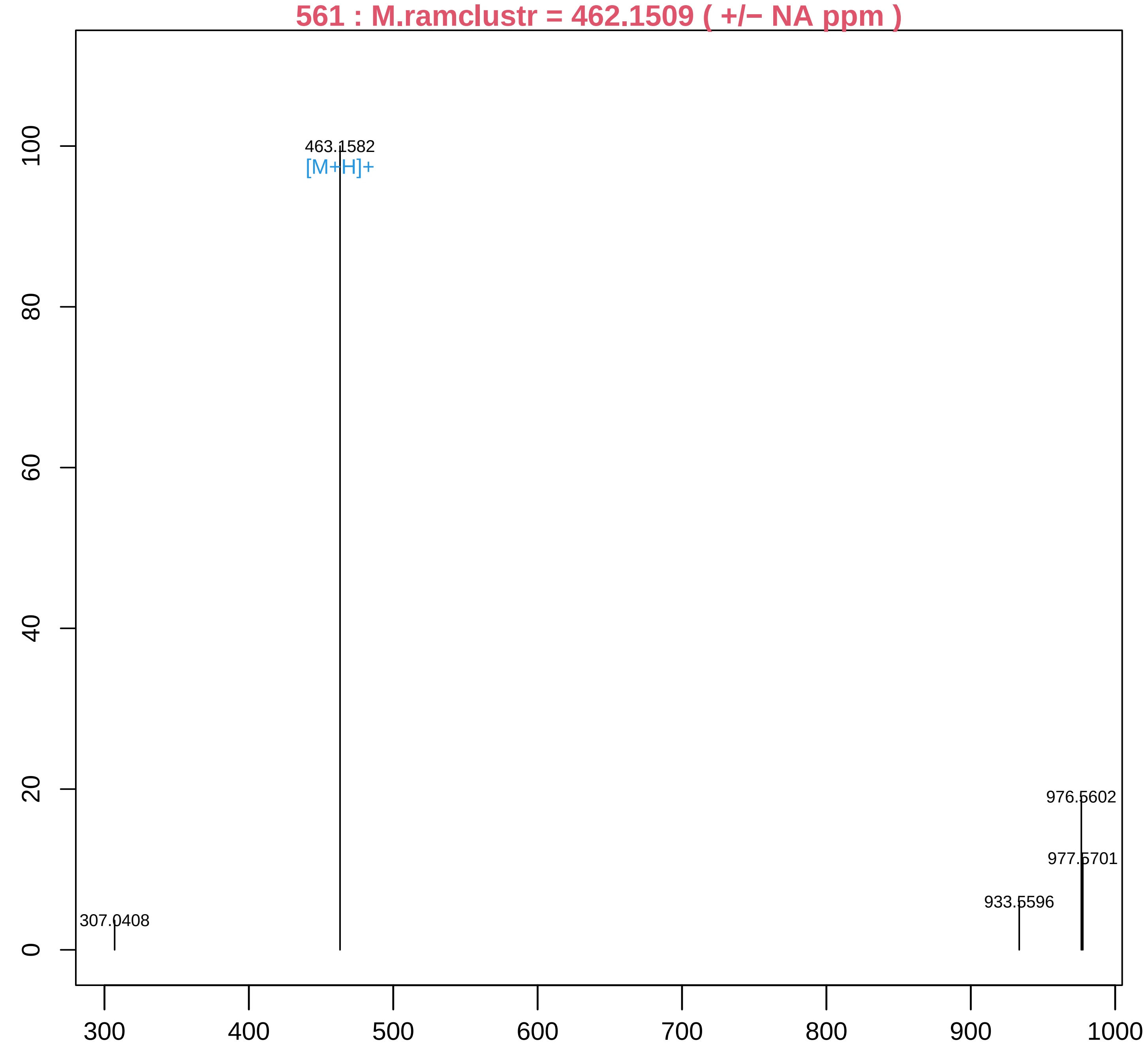
561 : M.ramclustr = 462.1509 (+/− NA ppm)
463.1582
[M+H]+
307.0408
933.5596
976.5602
977.5701
0
20
40
60
80
100
300
400
500
600
700
800
900
1000
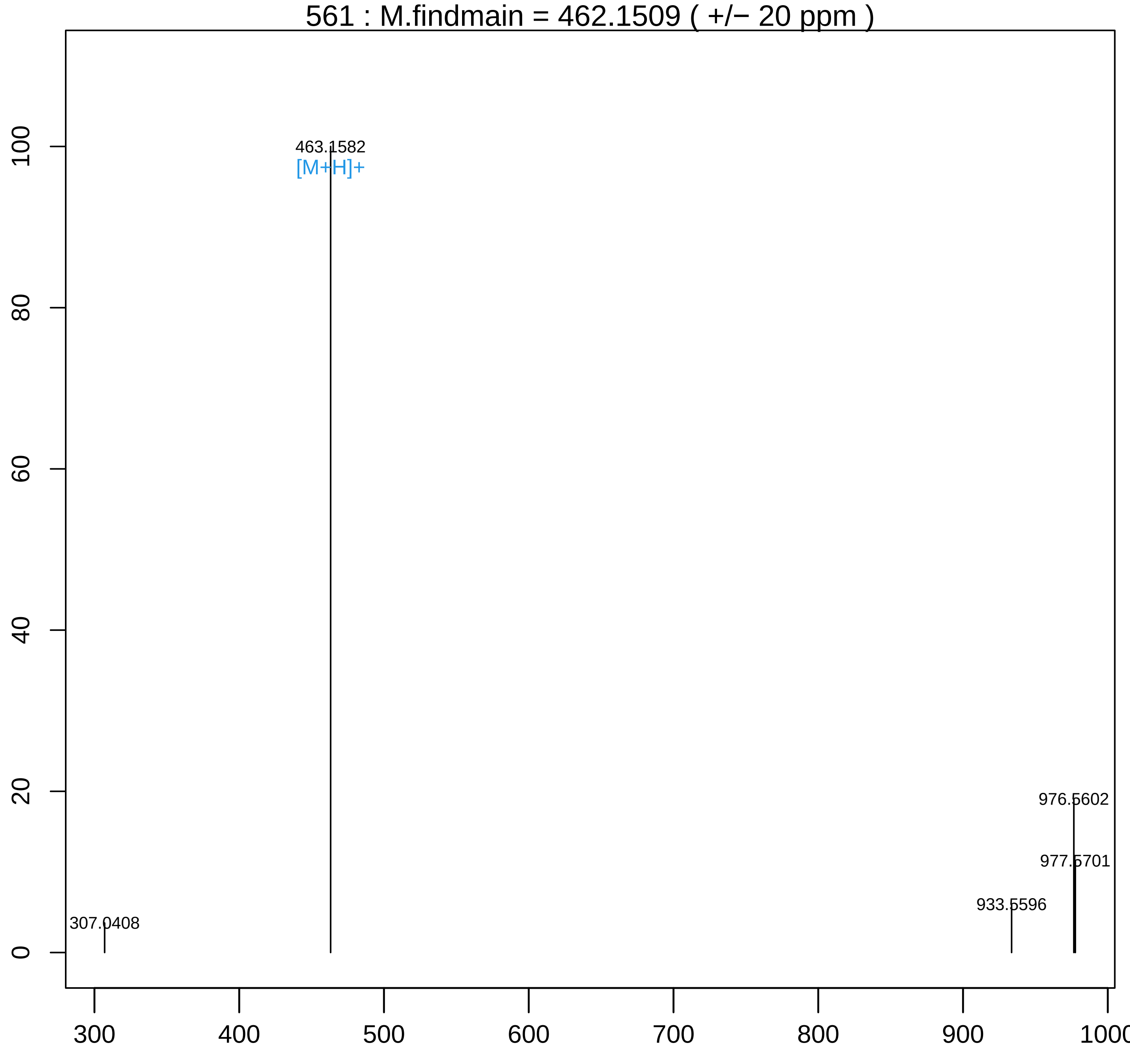
561 : M.findmain = 462.1509 (+/− 20 ppm)
463.1582
[M+H]+
307.0408
933.5596
976.5602
977.5701
0
20
40
60
80
100
300
400
500
600
700
800
900

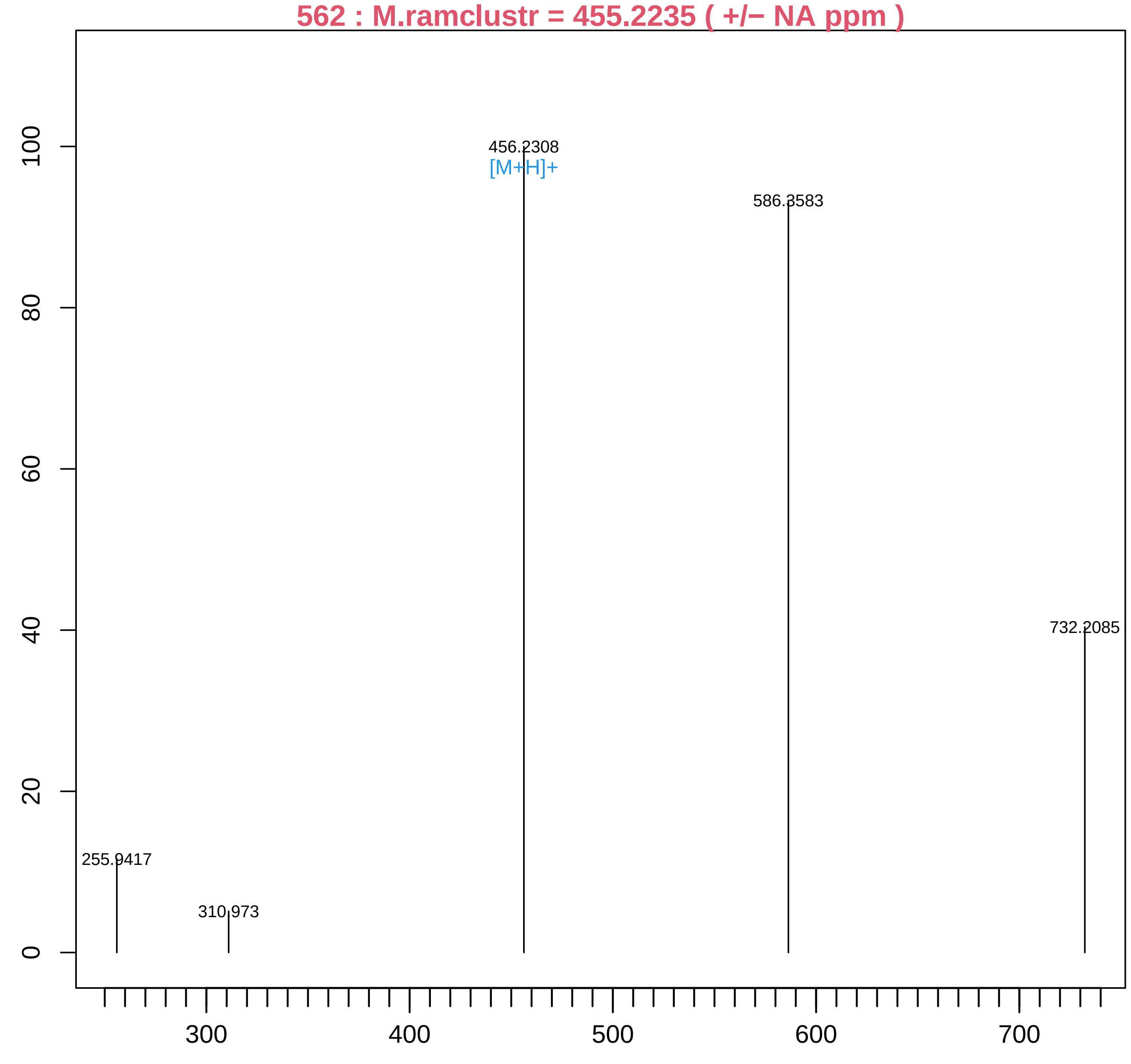
562 : M.ramclustr = 455.2235 (+/− NA ppm)
456.2308
[M+H]+
586.3583
732.2085
255.9417
310.973
0
20
40
60
80
100
300
400
500
600
700

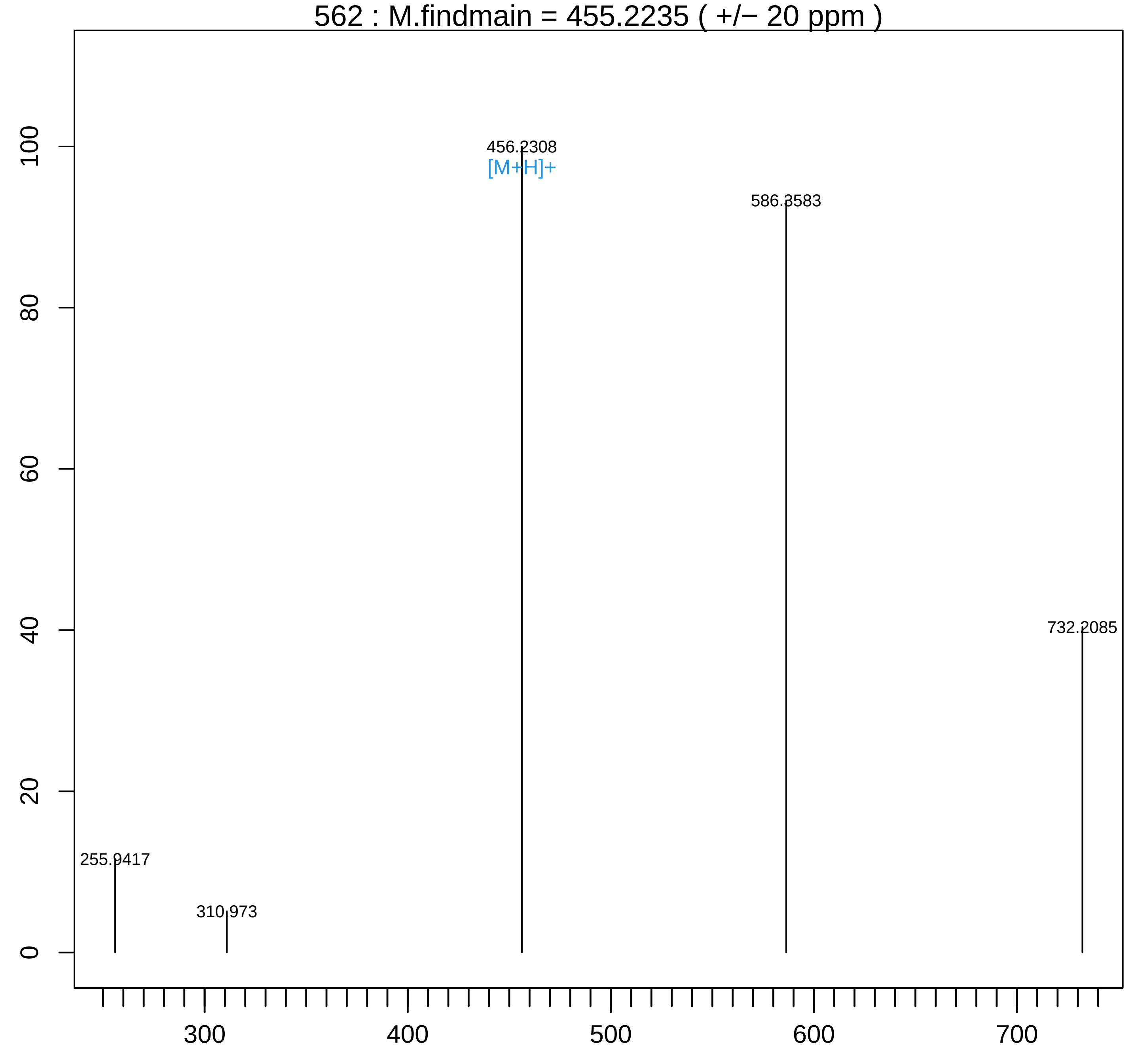
562 : M.findmain = 455.2235 (+/− 20 ppm)
456.2308
[M+H]+
586.3583
732.2085
255.9417
310.973
0
20
40
60
80
100
300
400
500
600
700

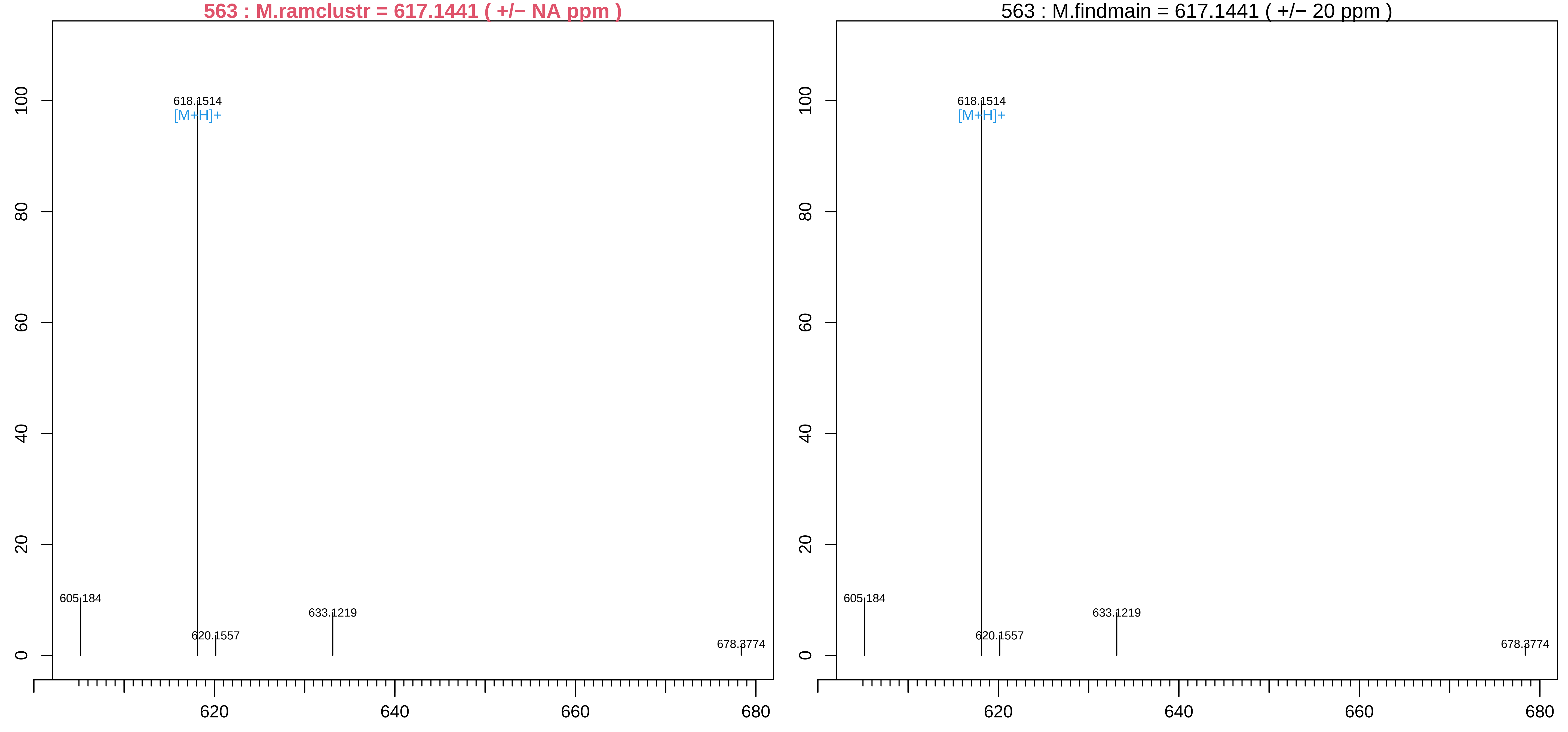
563 : M.ramclustr = 617.1441 (+/− NA ppm)
618.1514
[M+H]+
605.184
620.1557
633.1219
678.3774
0
20
40
60
80
100
620
640
660
680
563 : M.findmain = 617.1441 (+/− 20 ppm)
618.1514
[M+H]+
605.184
620.1557
633.1219
678.3774
0
20
40
60
80
100
620
640
660
680

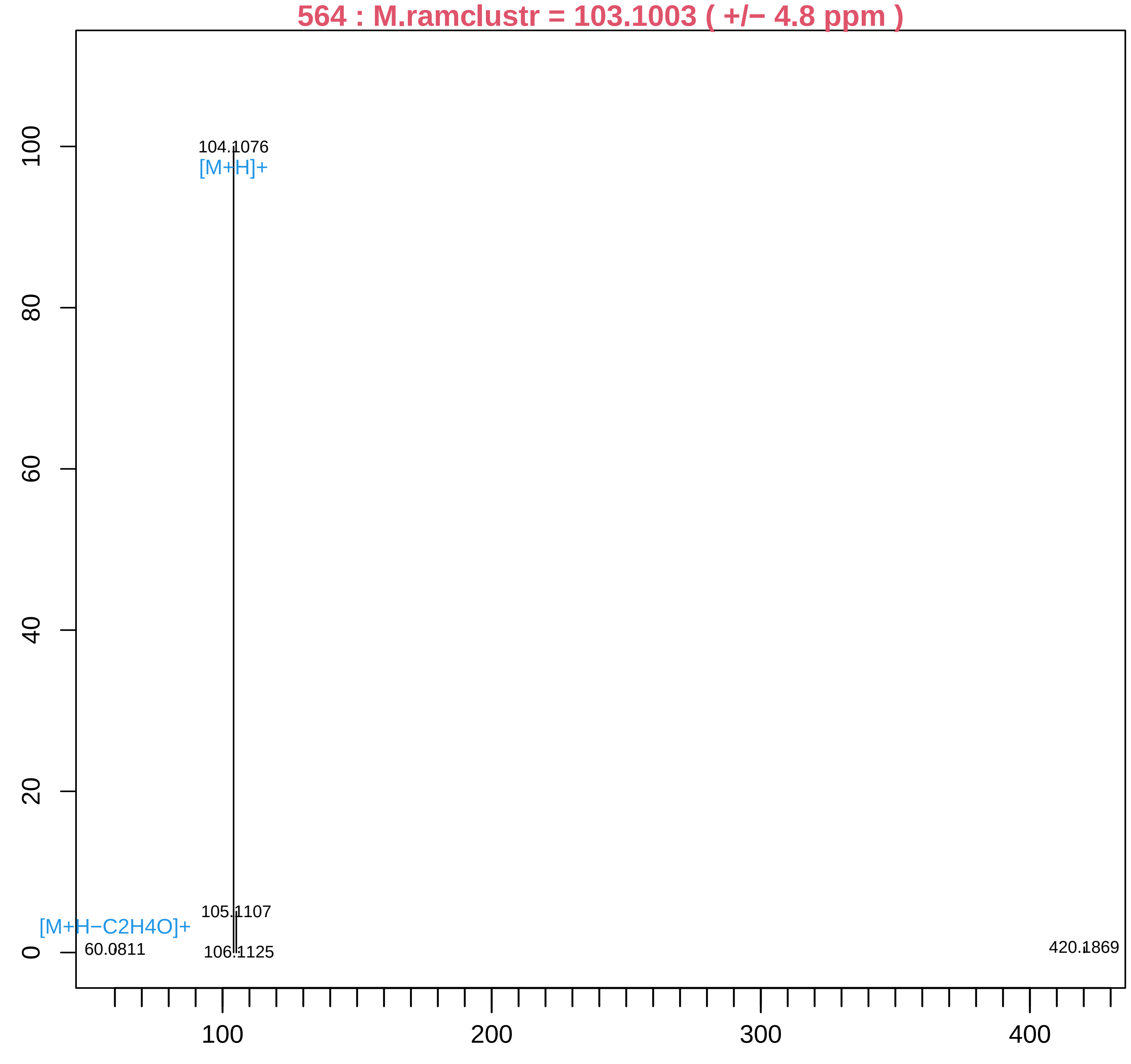
564 : M.ramclustr = 103.1003 (+/− 4.8 ppm)
104.1076
[M+H]+
105.1107
[M+H−C2H4O]+
60.0811
106.1125
420.1869
0
20
40
60
80
100
100
200
300
400

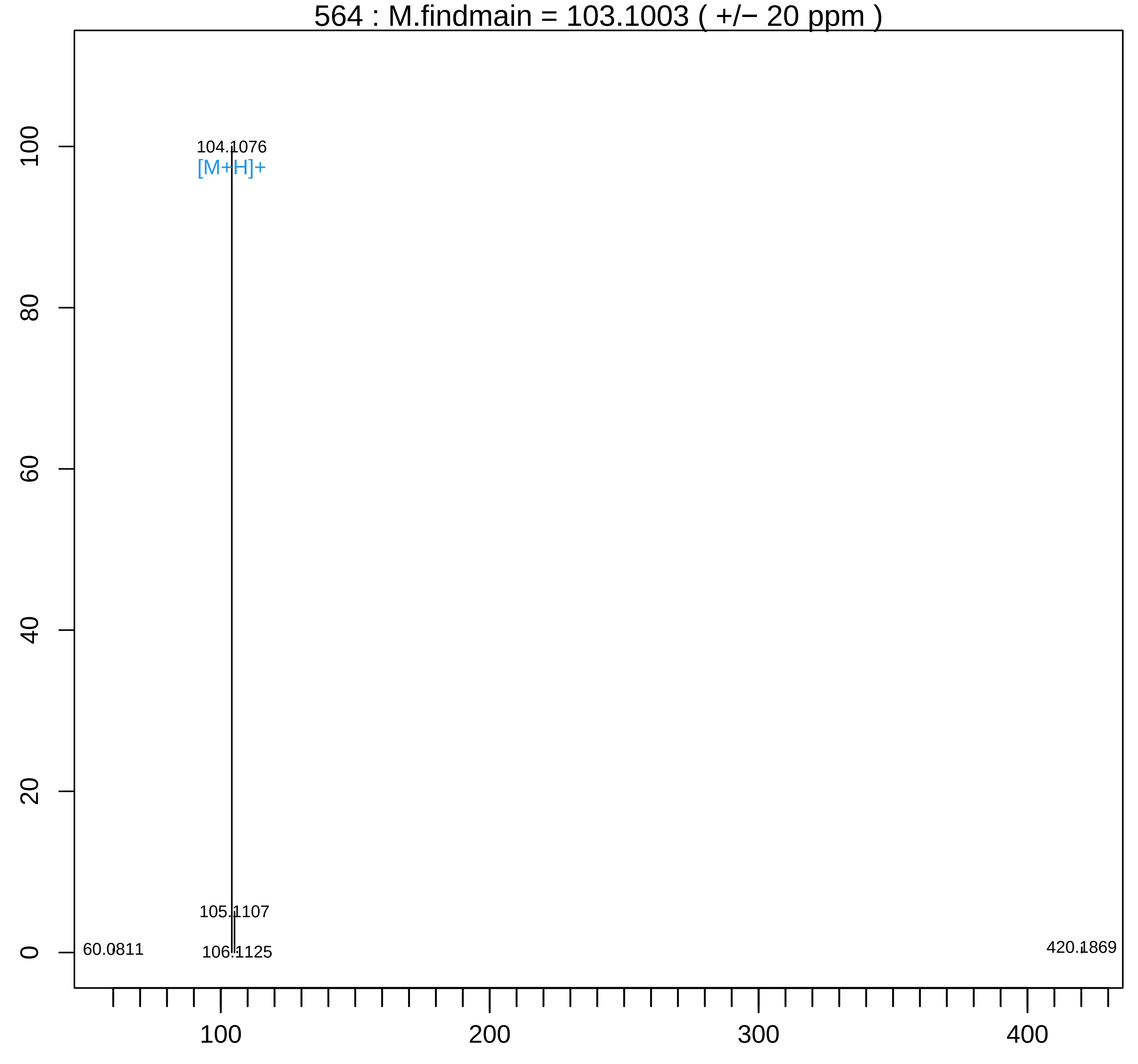
564 : M.findmain = 103.1003 (+/− 20 ppm)
104.1076
[M+H]+
105.1107
60.0811
106.1125
420.1869
0
20
40
60
80
100
100
200
300
400

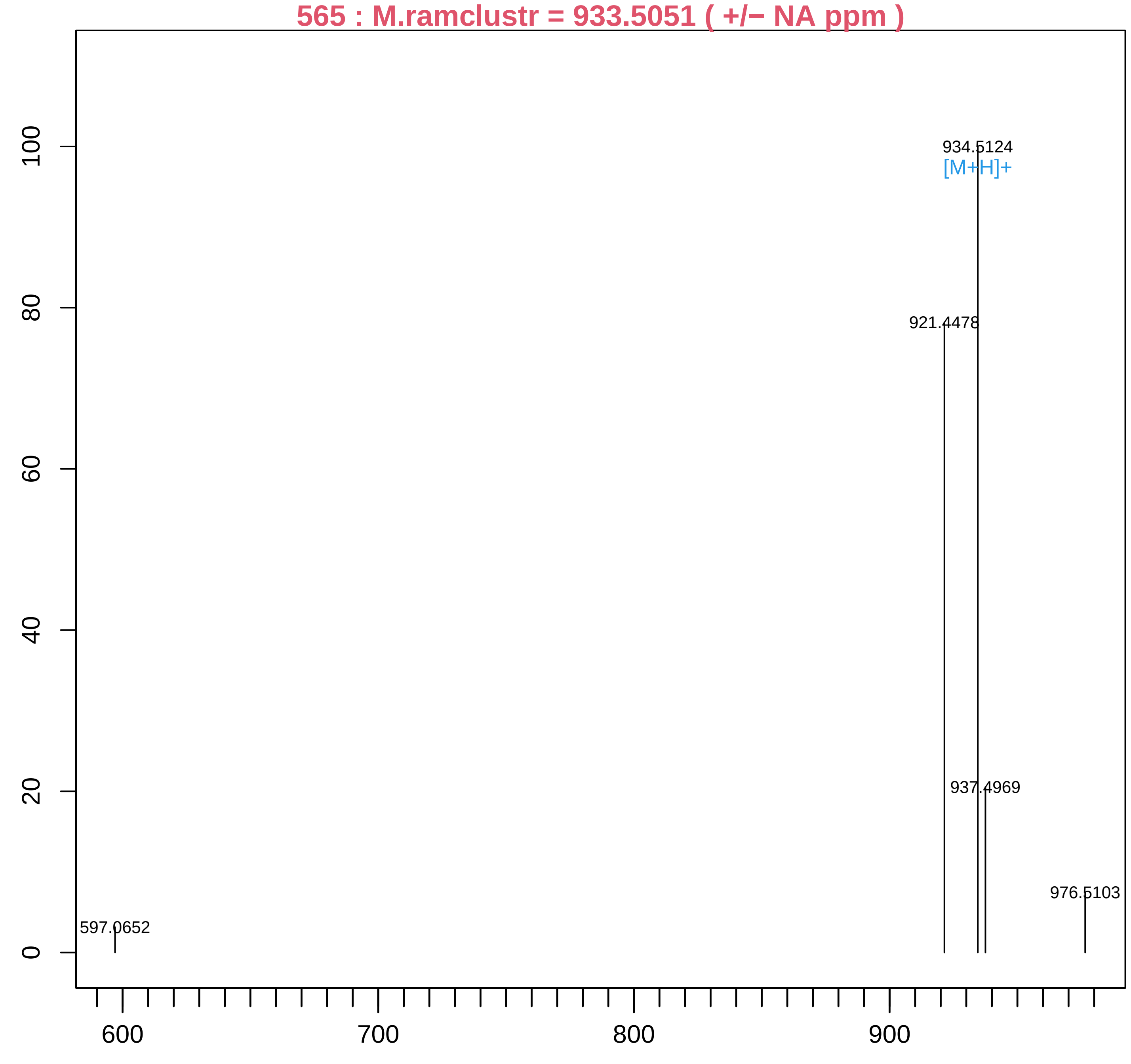
565 : M.ramclustr = 933.5051 (+/− NA ppm)
934.5124
[M+H]+
921.4478
937.4969
976.5103
597.0652
0
20
40
60
80
100
600
700
800
900

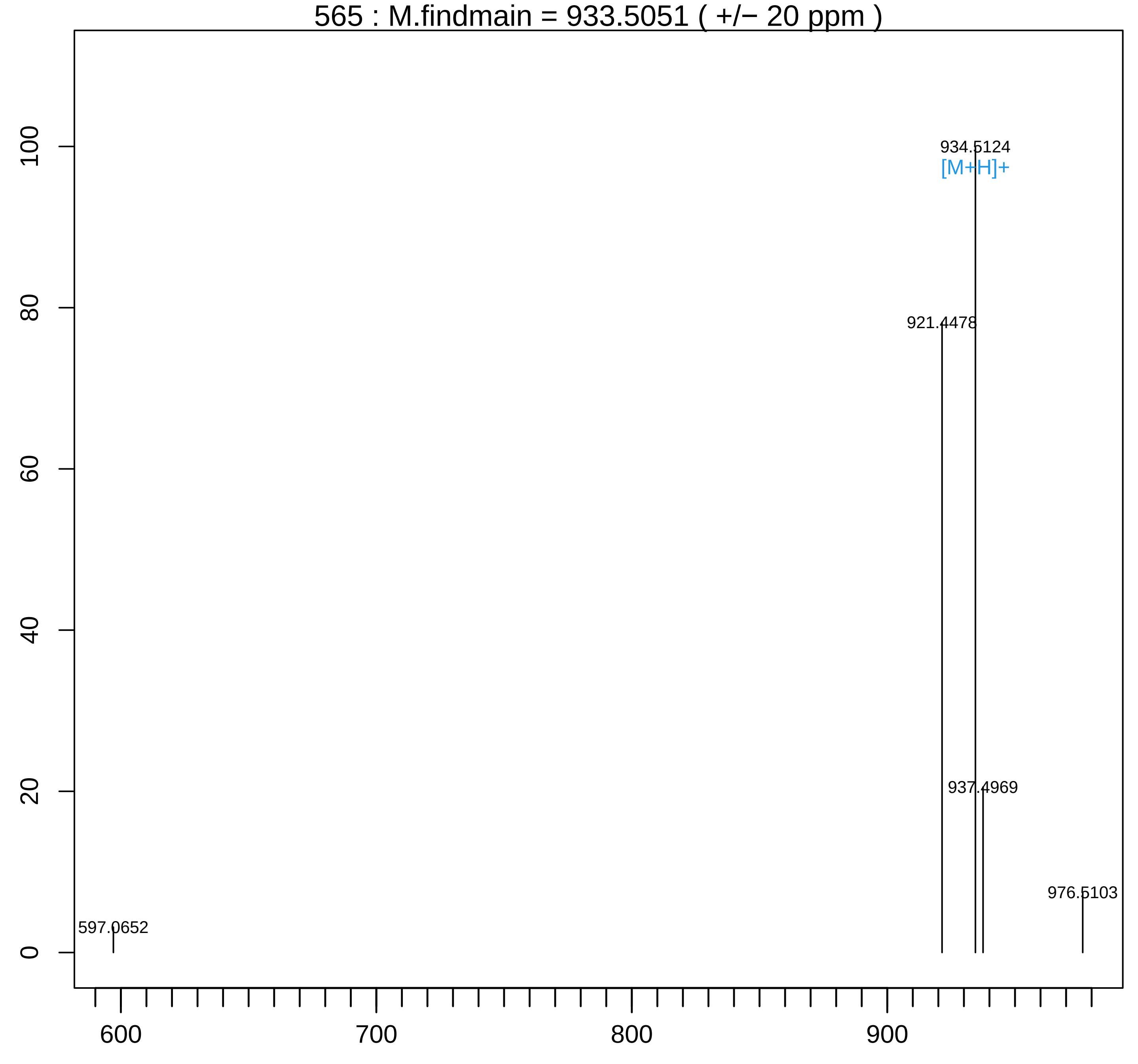
565 : M.findmain = 933.5051 (+/− 20 ppm)
934.5124
[M+H]+
921.4478
937.4969
976.5103
597.0652
0
20
40
60
80
100
600
700
800
900

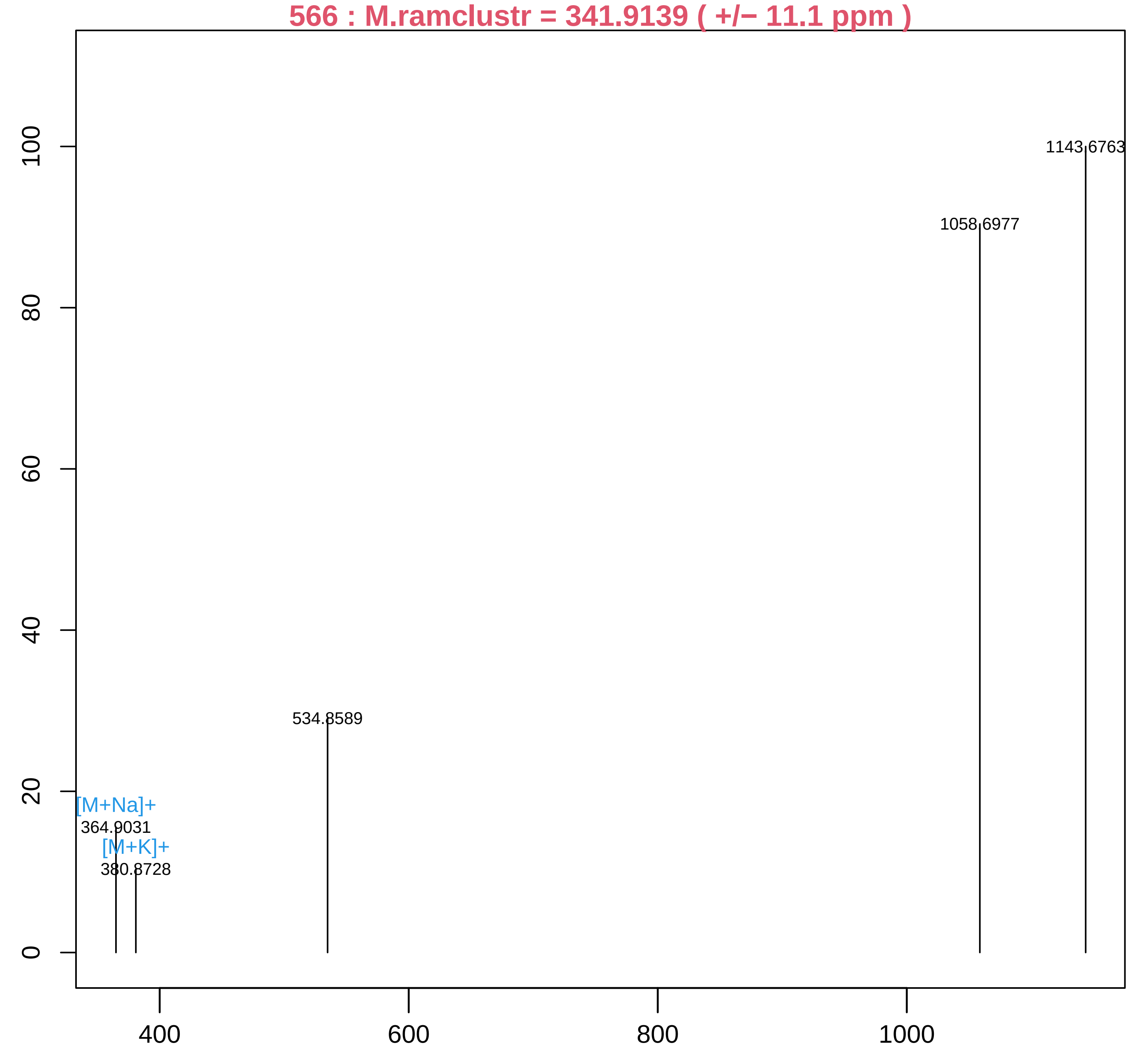
566 : M.ramclustr = 341.9139 (+/− 11.1 ppm)
0
20
40
60
80
100
400
600
800
1000
[M+Na]+
364.9031
[M+K]+
380.8728
534.8589
1058.6977
1143.6763

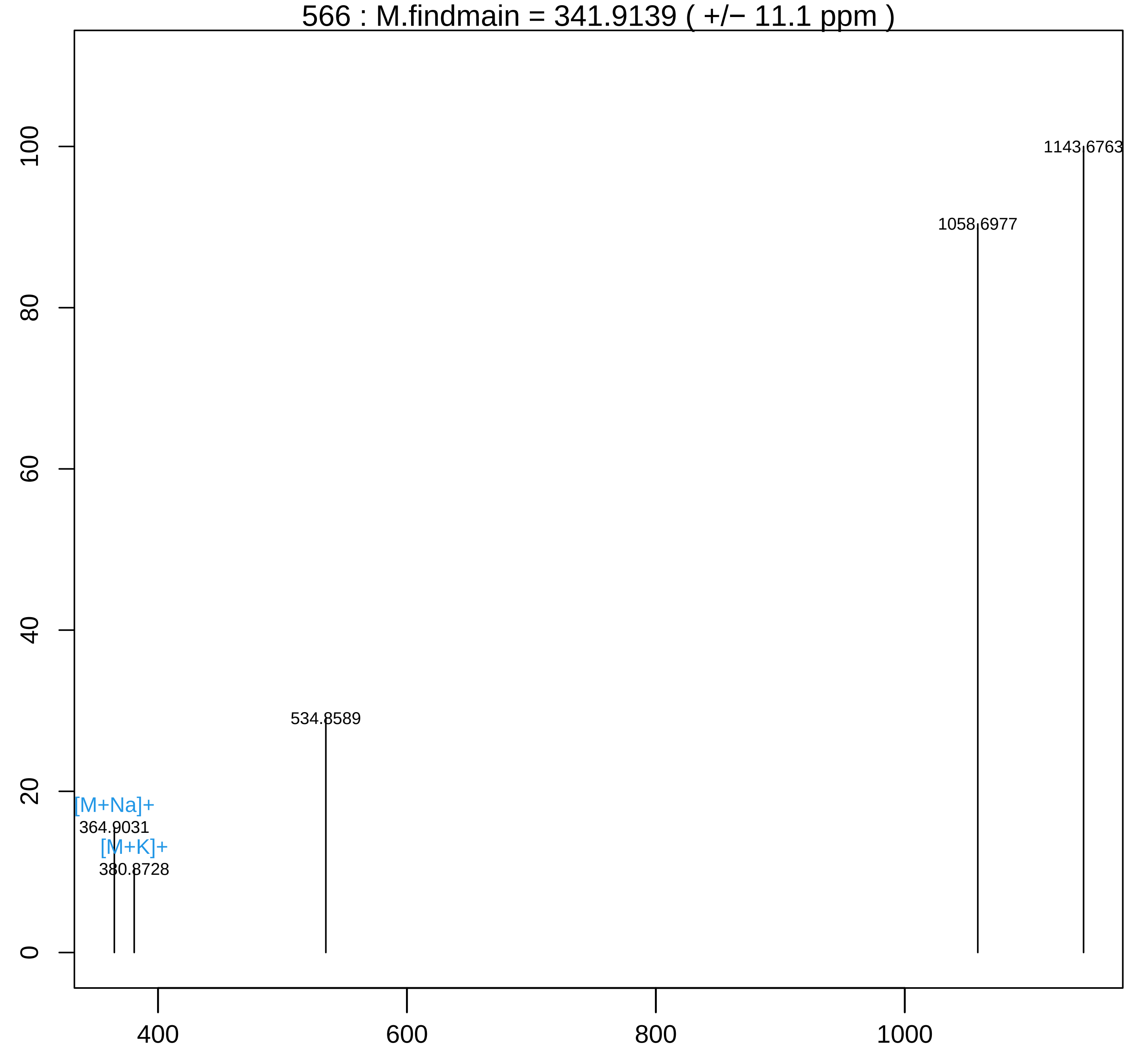
566 : M.findmain = 341.9139 (+/− 11.1 ppm)
0
20
40
60
80
100
400
600
800
1000
[M+Na]+
364.9031
[M+K]+
380.8728
534.8589
1058.6977
1143.6763

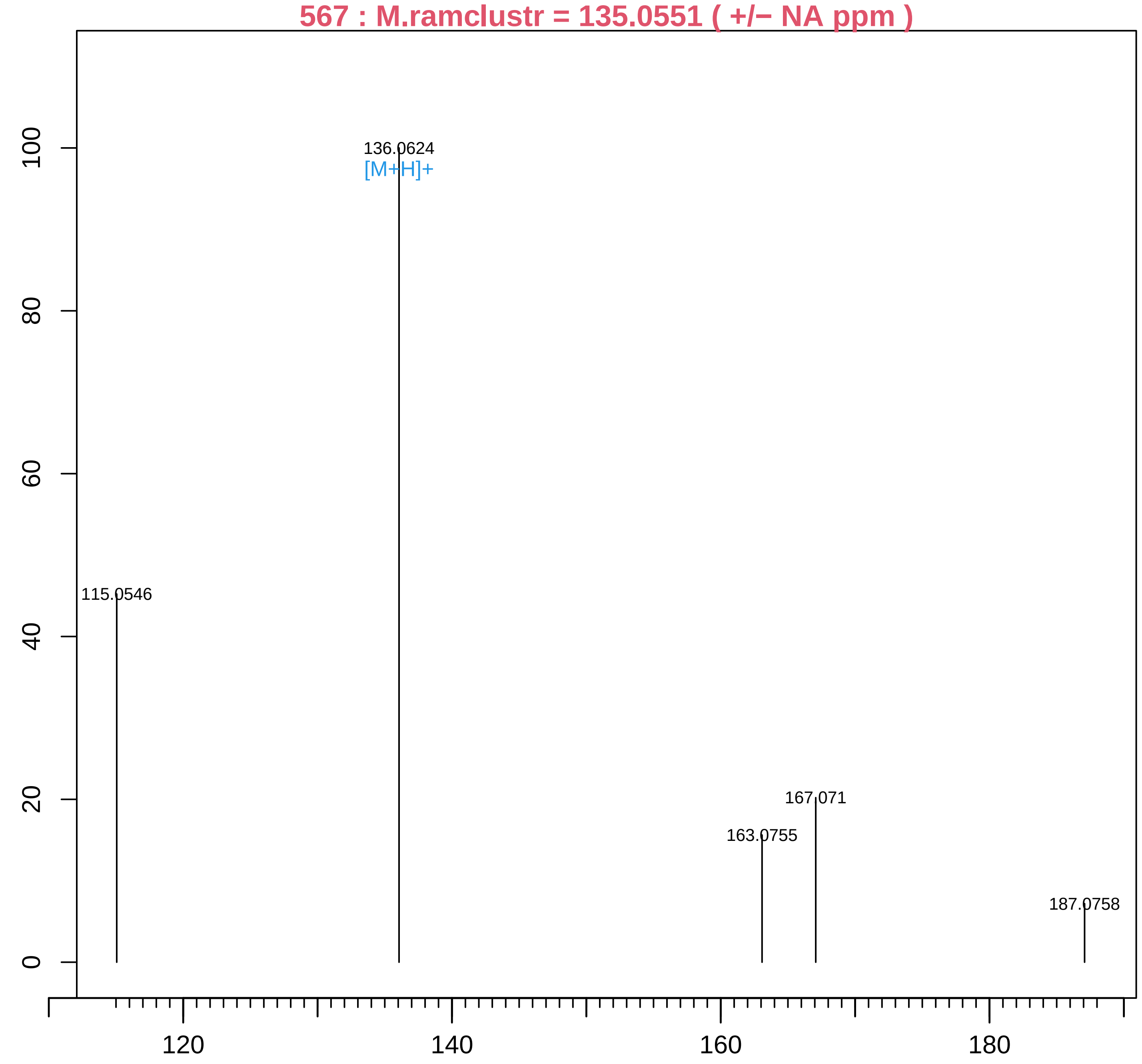
567 : M.ramclustr = 135.0551 (+/− NA ppm)
136.0624
[M+H]+
115.0546
163.0755
167.071
187.0758
0
20
40
60
80
100
120
140
160
180

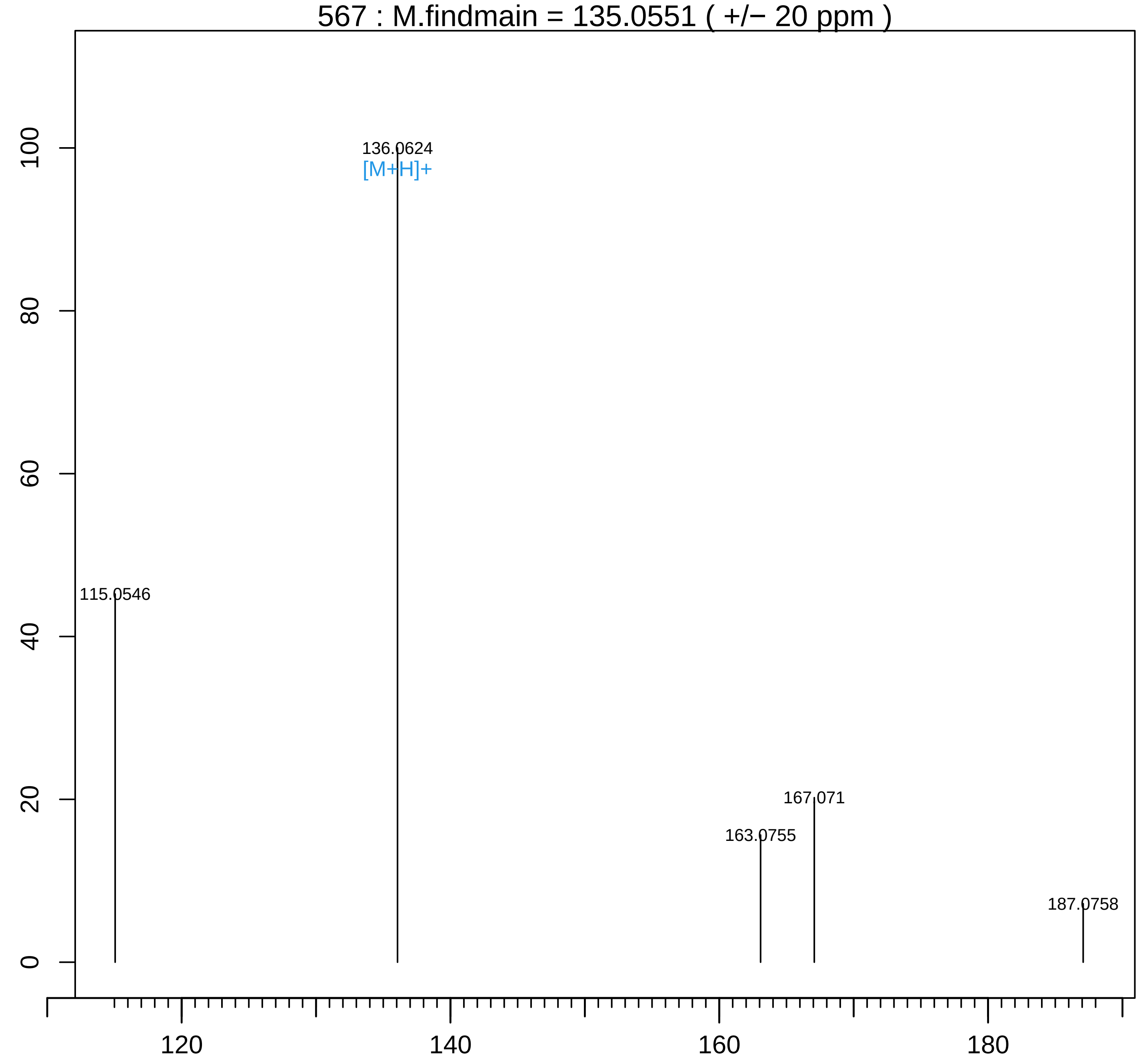
567 : M.findmain = 135.0551 (+/− 20 ppm)
136.0624
[M+H]+
115.0546
163.0755
167.071
187.0758
0
20
40
60
80
100
120
140
160
180

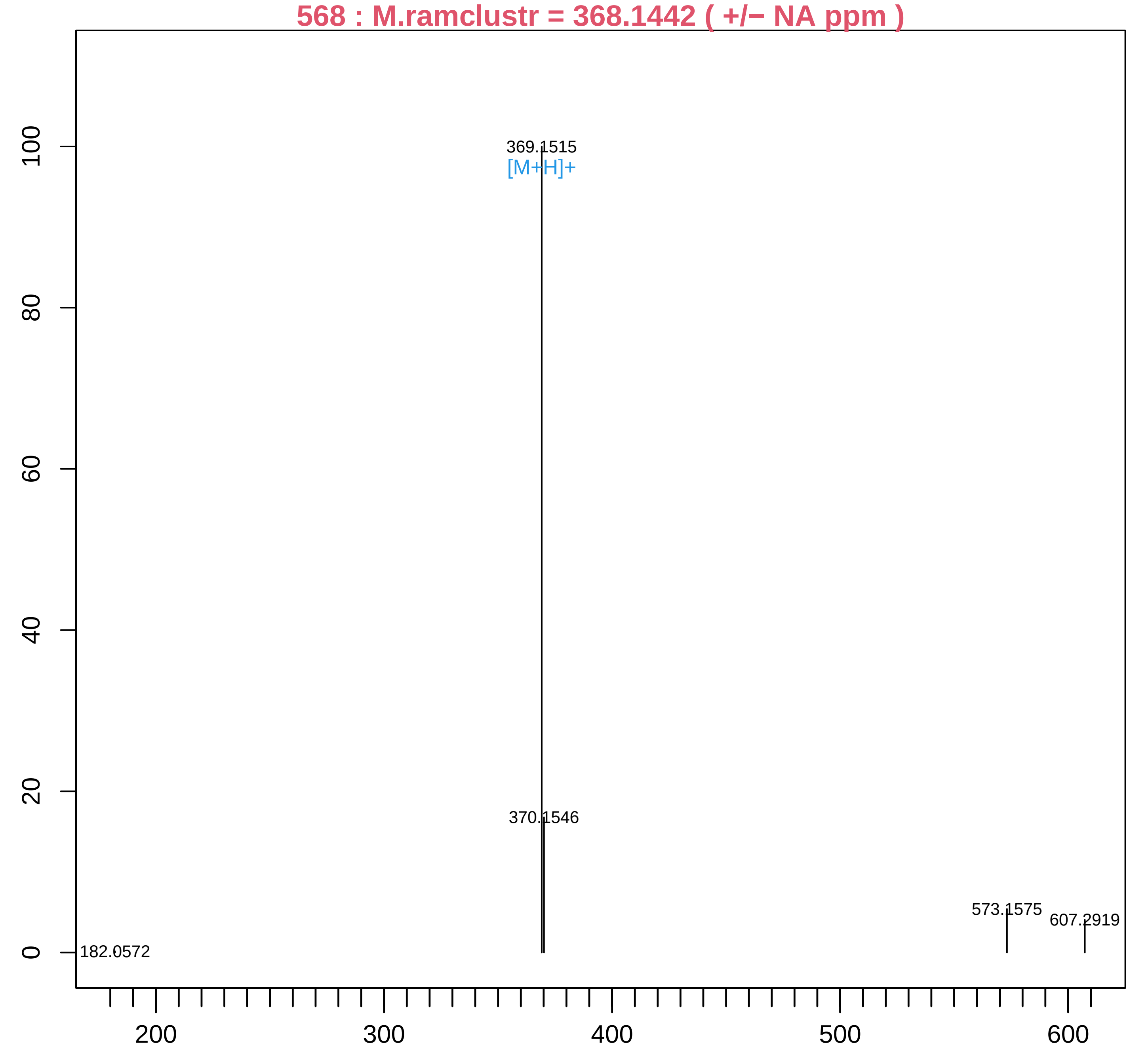
568 : M.ramclustr = 368.1442 (+/− NA ppm)
369.1515
[M+H]+
370.1546
573.1575
607.2919
182.0572
0
20
40
60
80
100
200
300
400
500
600

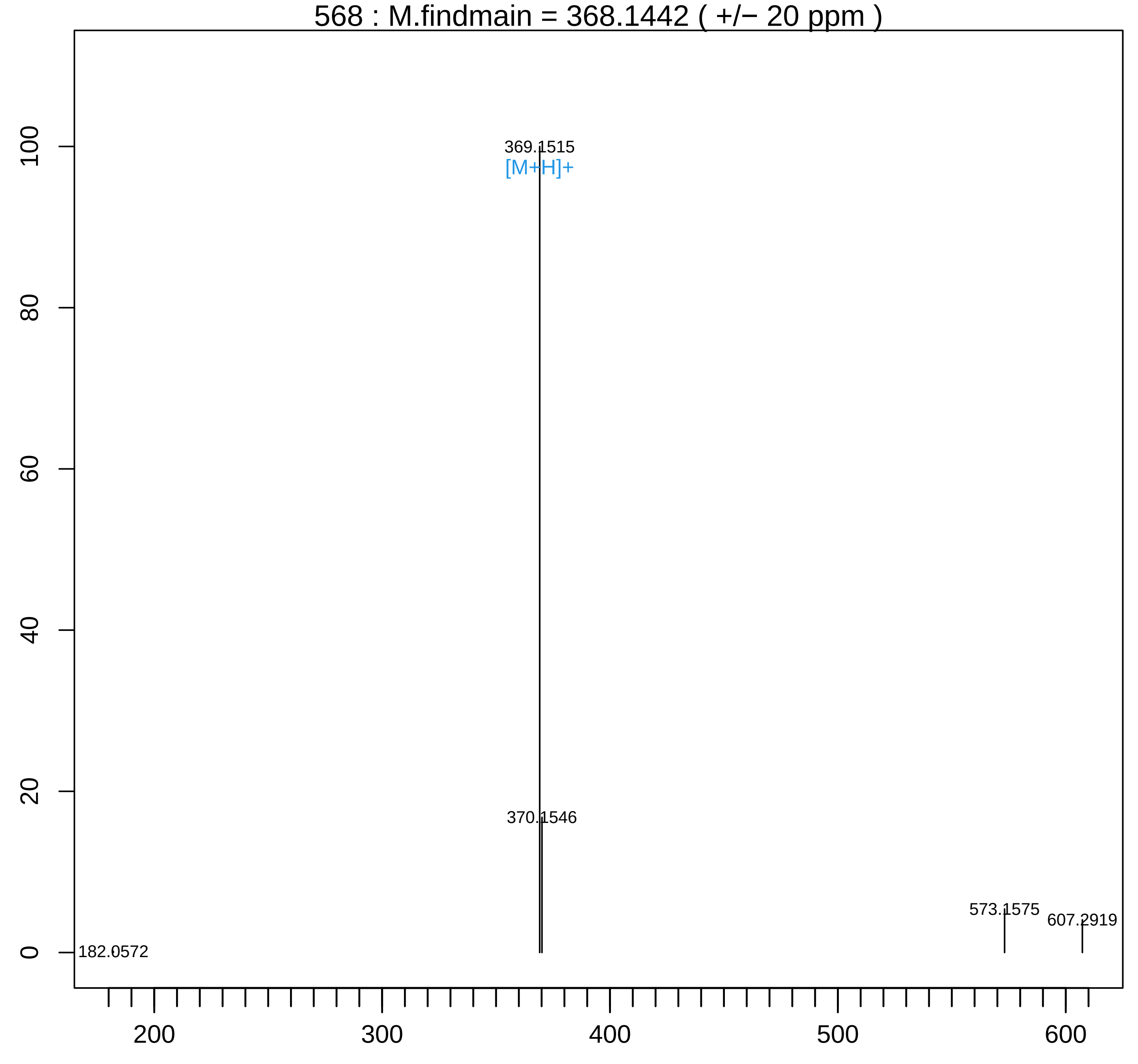
568 : M.findmain = 368.1442 (+/− 20 ppm)
369.1515
[M+H]+
370.1546
573.1575
607.2919
182.0572
0
20
40
60
80
100
200
300
400
500
600

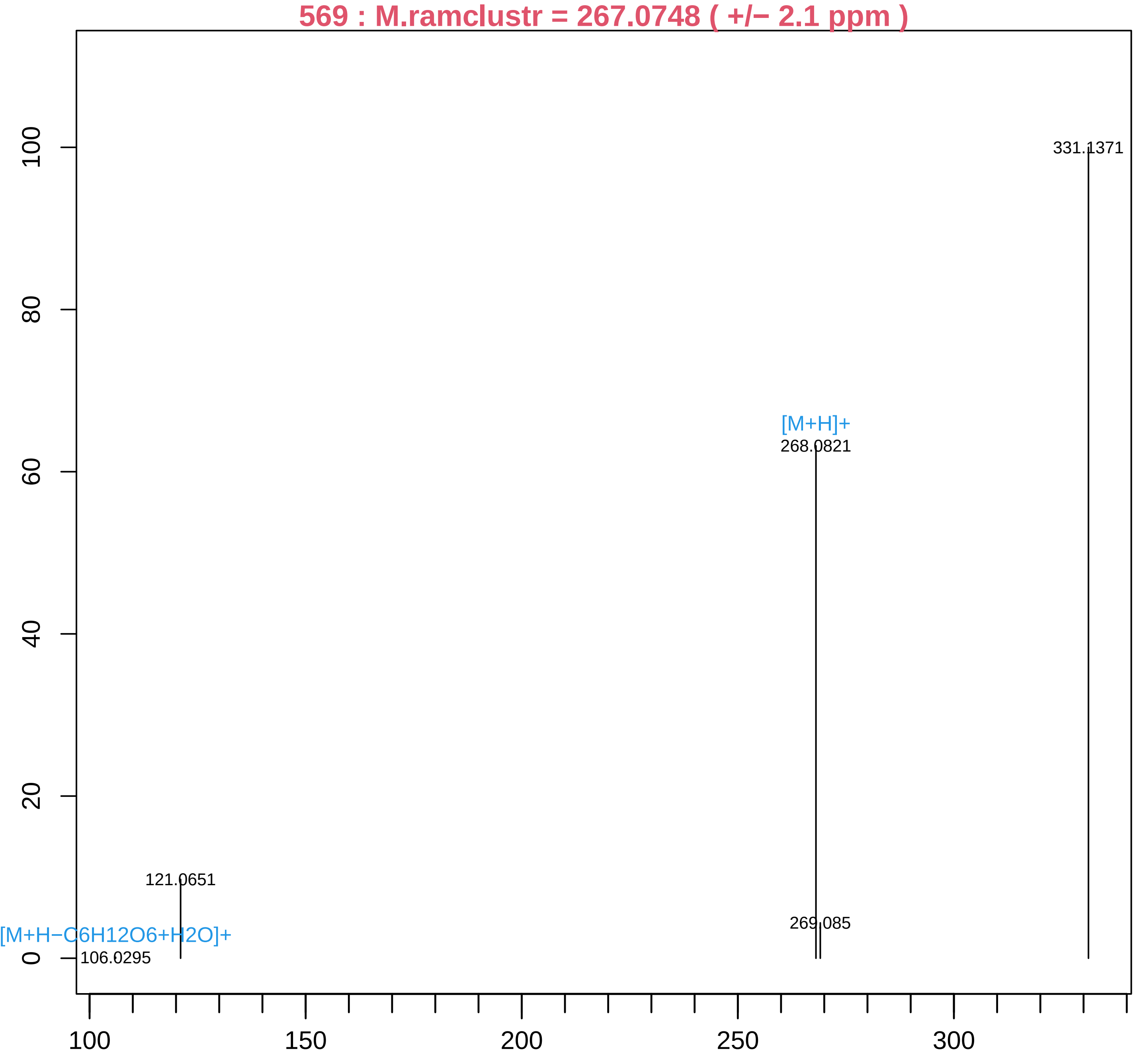
569 : M.ramclustr = 267.0748 (+/− 2.1 ppm)
331.1371
[M+H]+
268.0821
121.0651
269.085
[M+H−C6H12O6+H2O]+
106.0295
0
20
40
60
80
100
150
200
250
300

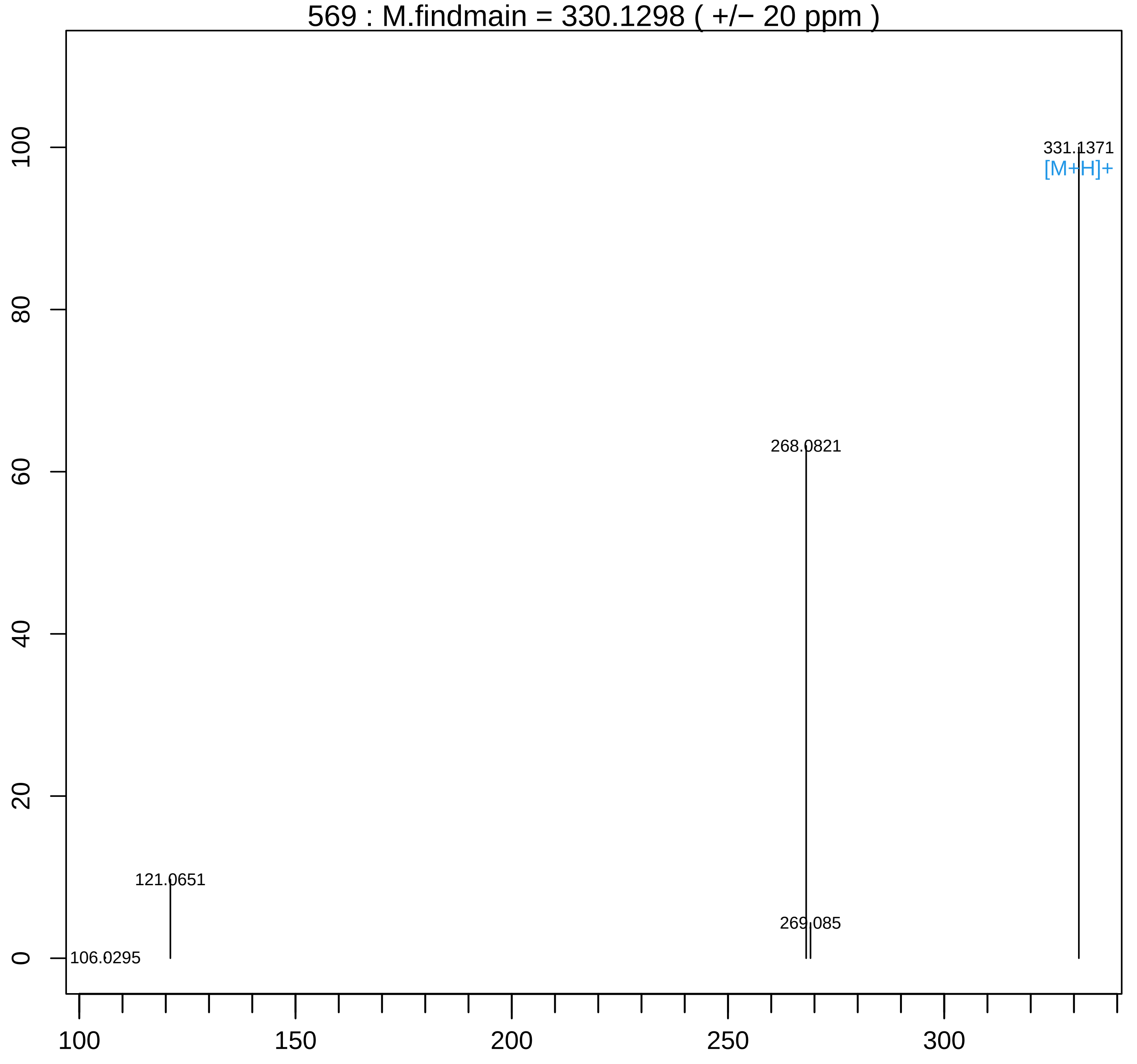
569 : M.findmain = 330.1298 (+/− 20 ppm)
331.1371
[M+H]+
268.0821
121.0651
269.085
106.0295
0
20
40
60
80
100
150
200
250
300

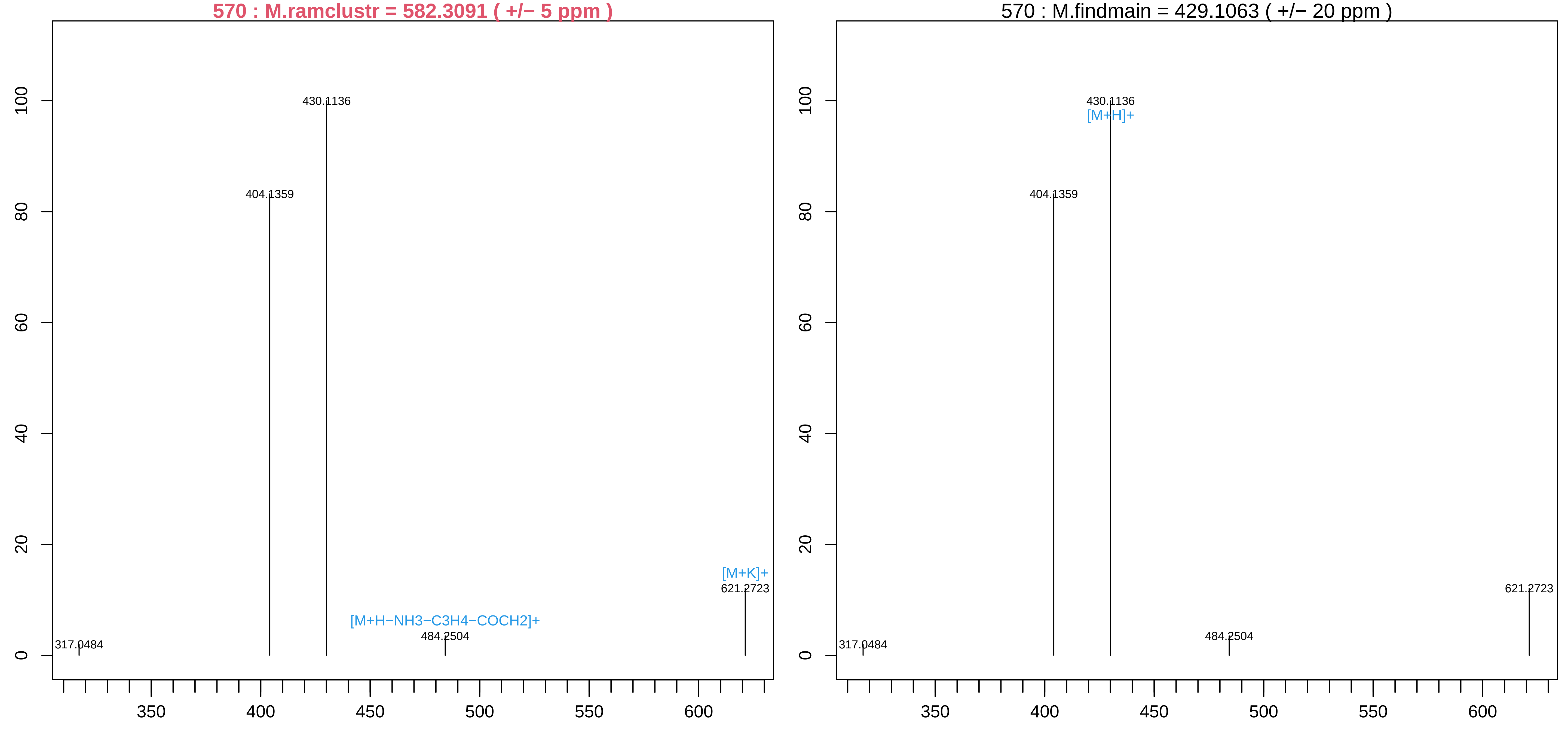

570 : M.ramclustr = 582.3091 (+/− 5 ppm)
430.1136
404.1359
[M+K]+
621.2723
[M+H−NH3−C3H4−COCH2]+
484.2504
317.0484
570 : M.findmain = 429.1063 (+/− 20 ppm)
430.1136
[M+H]+
404.1359
621.2723
484.2504
317.0484

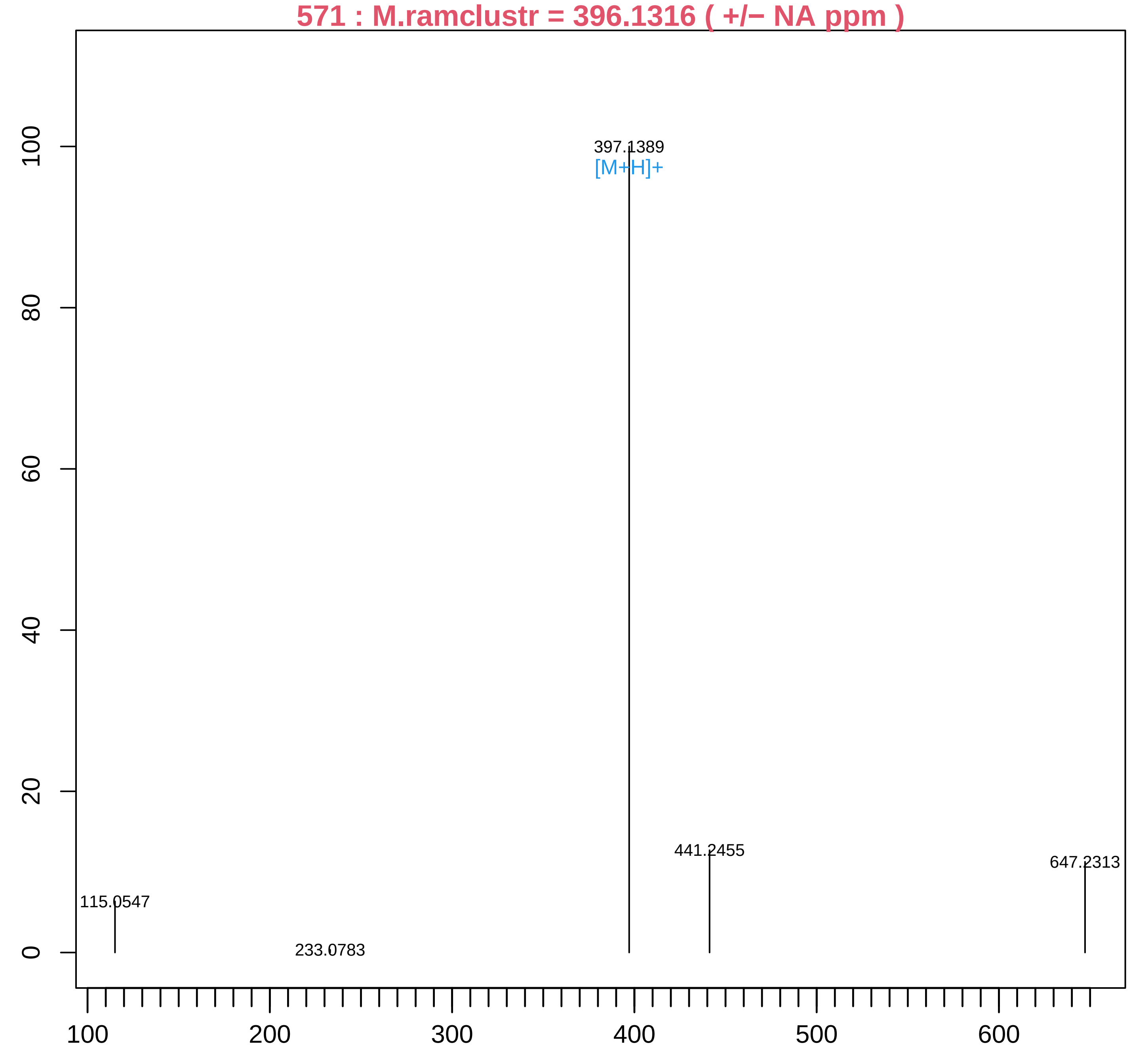
571 : M.ramclustr = 396.1316 (+/− NA ppm)
397.1389
[M+H]+
441.2455
647.2313
115.0547
233.0783
0
20
40
60
80
100
200
300
400
500
600

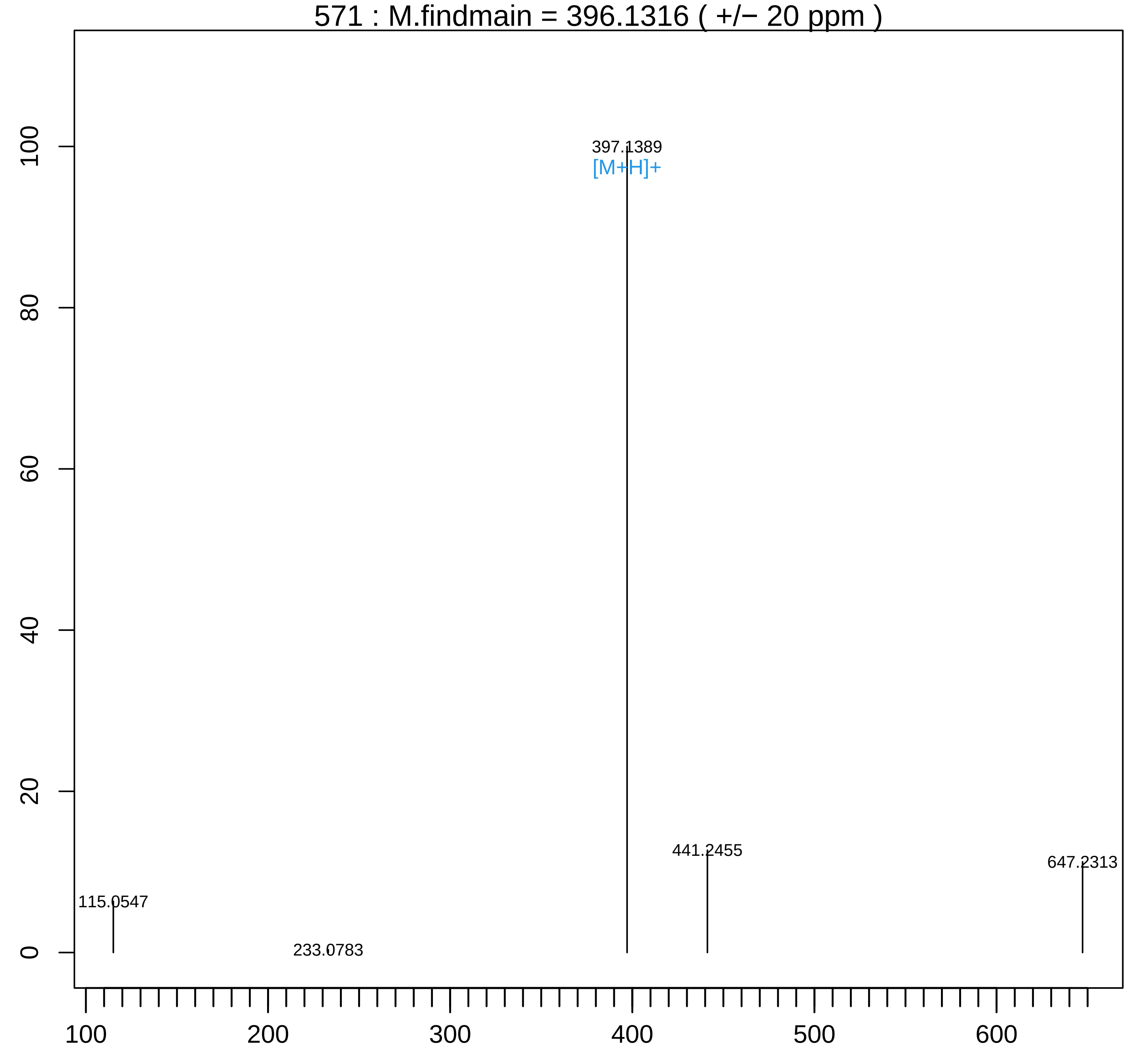
571 : M.findmain = 396.1316 (+/− 20 ppm)
397.1389
[M+H]+
441.2455
647.2313
115.0547
233.0783
0
20
40
60
80
100
200
300
400
500
600

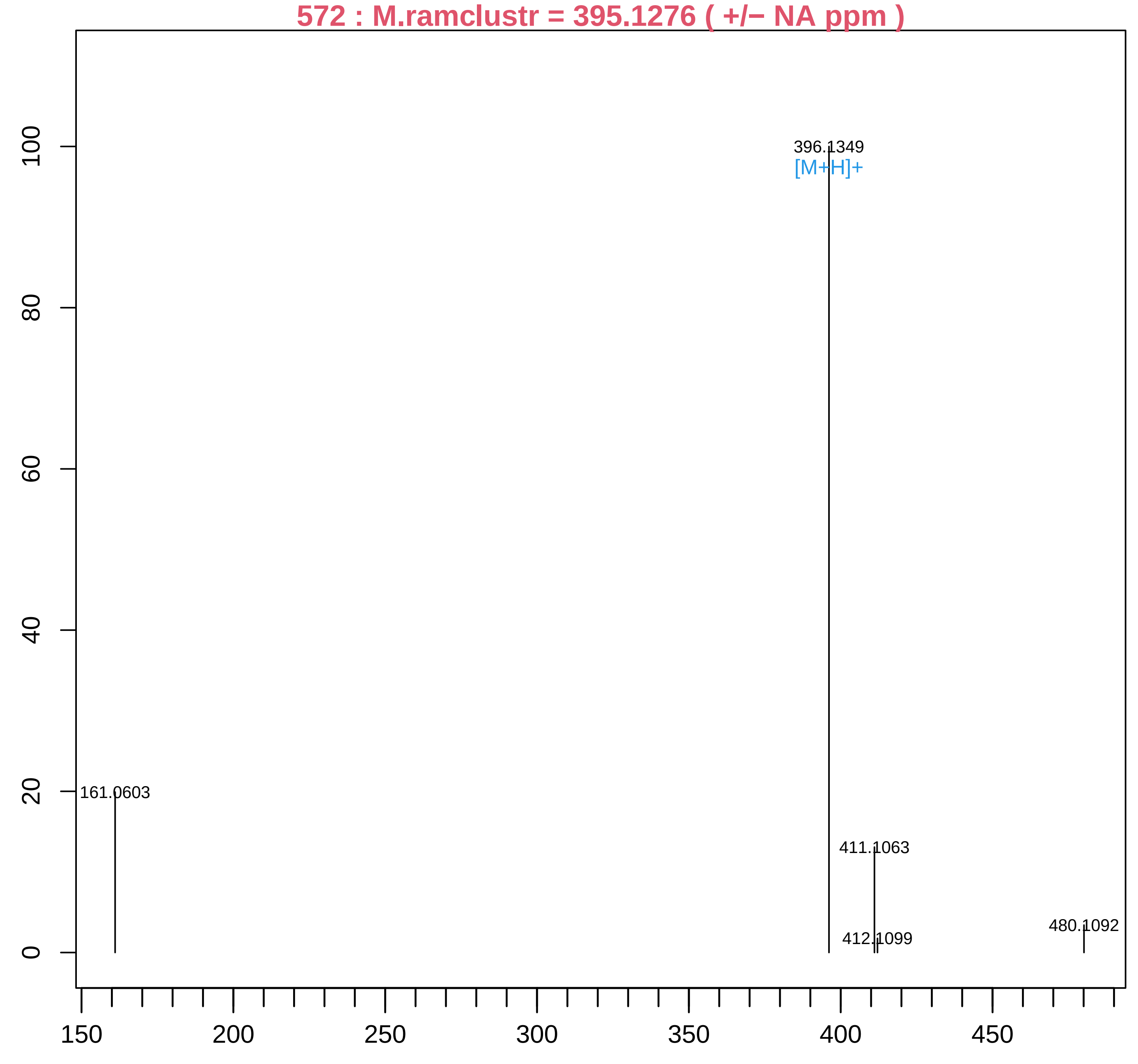
572 : M.ramclustr = 395.1276 (+/− NA ppm)
396.1349
[M+H]+
161.0603
411.1063
412.1099
480.1092
0
20
40
60
80
100
150
200
250
300
350
400
450

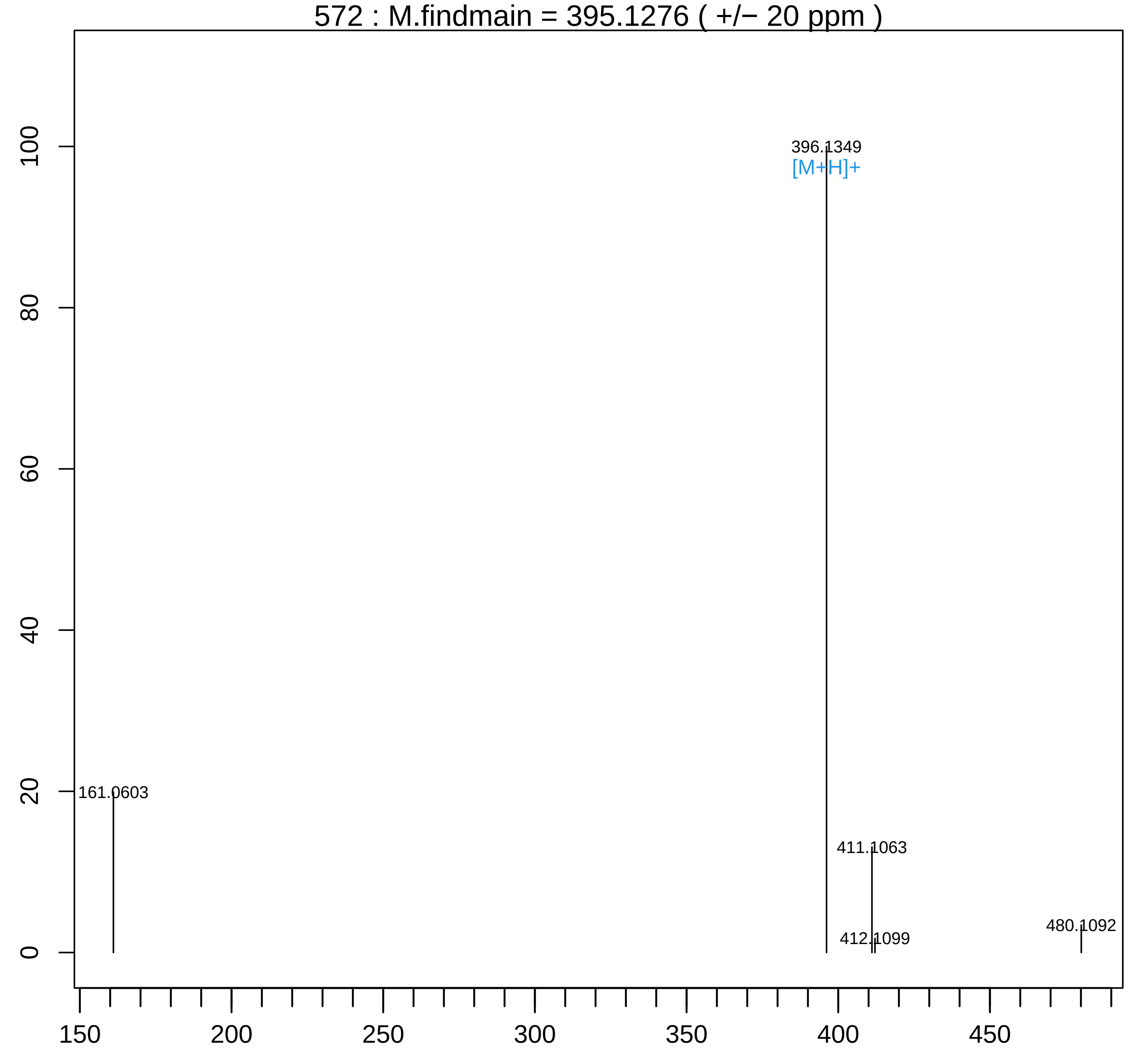
572 : M.findmain = 395.1276 (+/− 20 ppm)
396.1349
[M+H]+
161.0603
411.1063
412.1099
480.1092
0
20
40
60
80
100
150
200
250
300
350
400
450

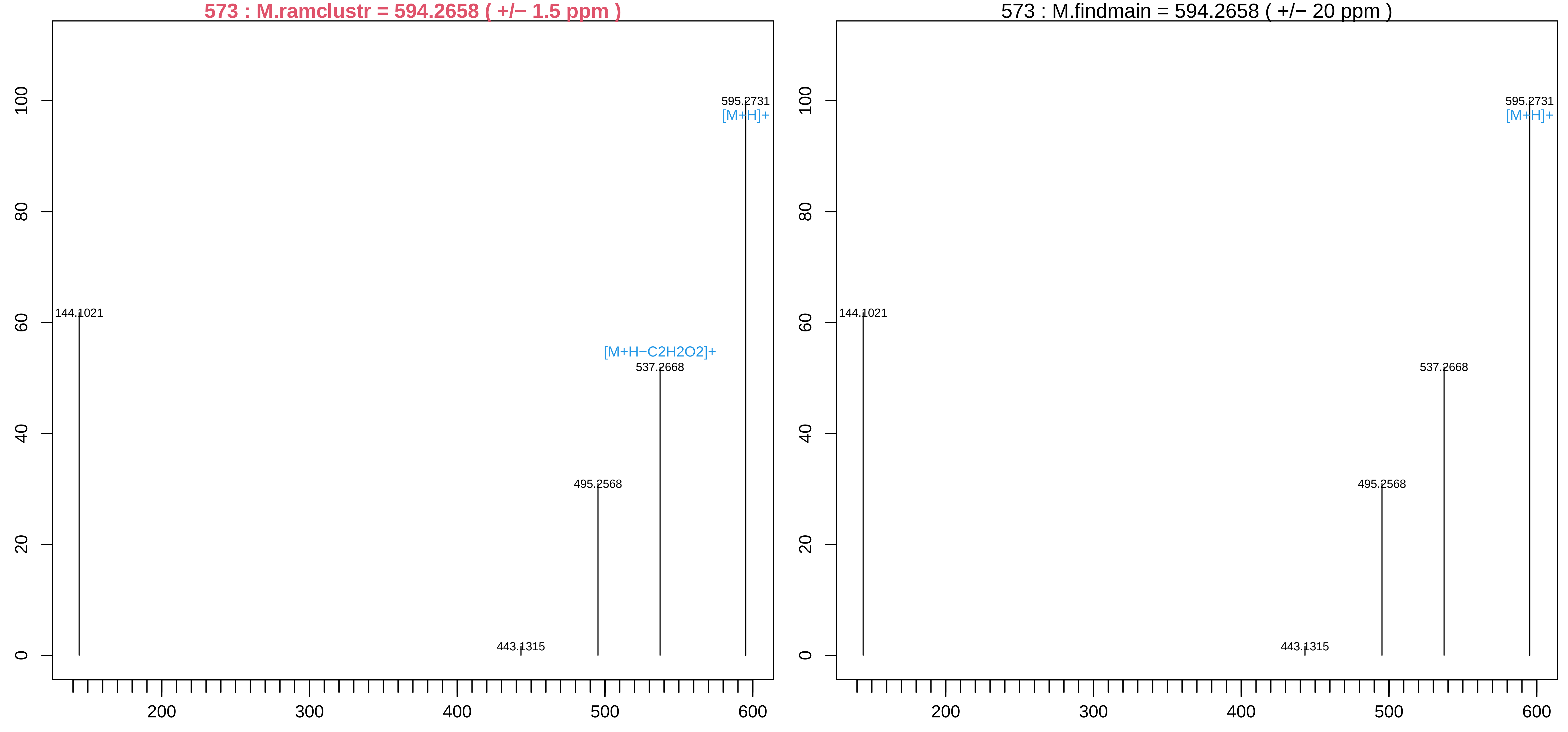
573 : M.ramclustr = 594.2658 (+/− 1.5 ppm)
595.2731
[M+H]+
144.1021
[M+H−C2H2O2]+
537.2668
495.2568
443.1315
0
20
40
60
80
100
200
300
400
500
600
573 : M.findmain = 594.2658 (+/− 20 ppm)
595.2731
[M+H]+
144.1021
537.2668
495.2568
443.1315
0
20
40
60
80
100
200
300
400
500
600

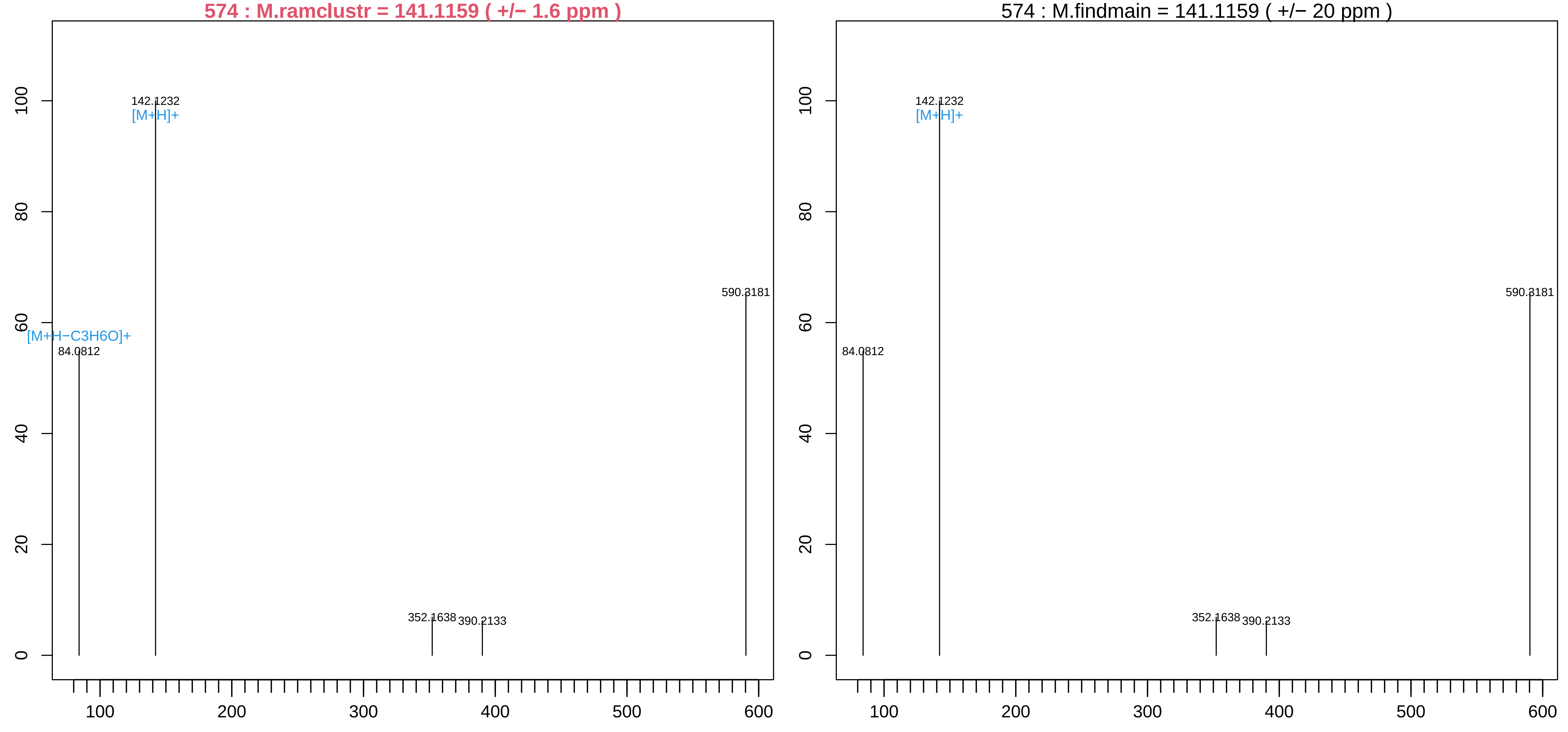
574 : M.ramclustr = 141.1159 (+/− 1.6 ppm)
142.1232
[M+H]+
[M+H−C3H6O]+
84.0812
590.3181
352.1638
390.2133
0
20
40
60
80
100
100
200
300
400
500
600
574 : M.findmain = 141.1159 (+/− 20 ppm)
142.1232
[M+H]+
84.0812
590.3181
352.1638
390.2133
0
20
40
60
80
100
100
200
300
400
500
600

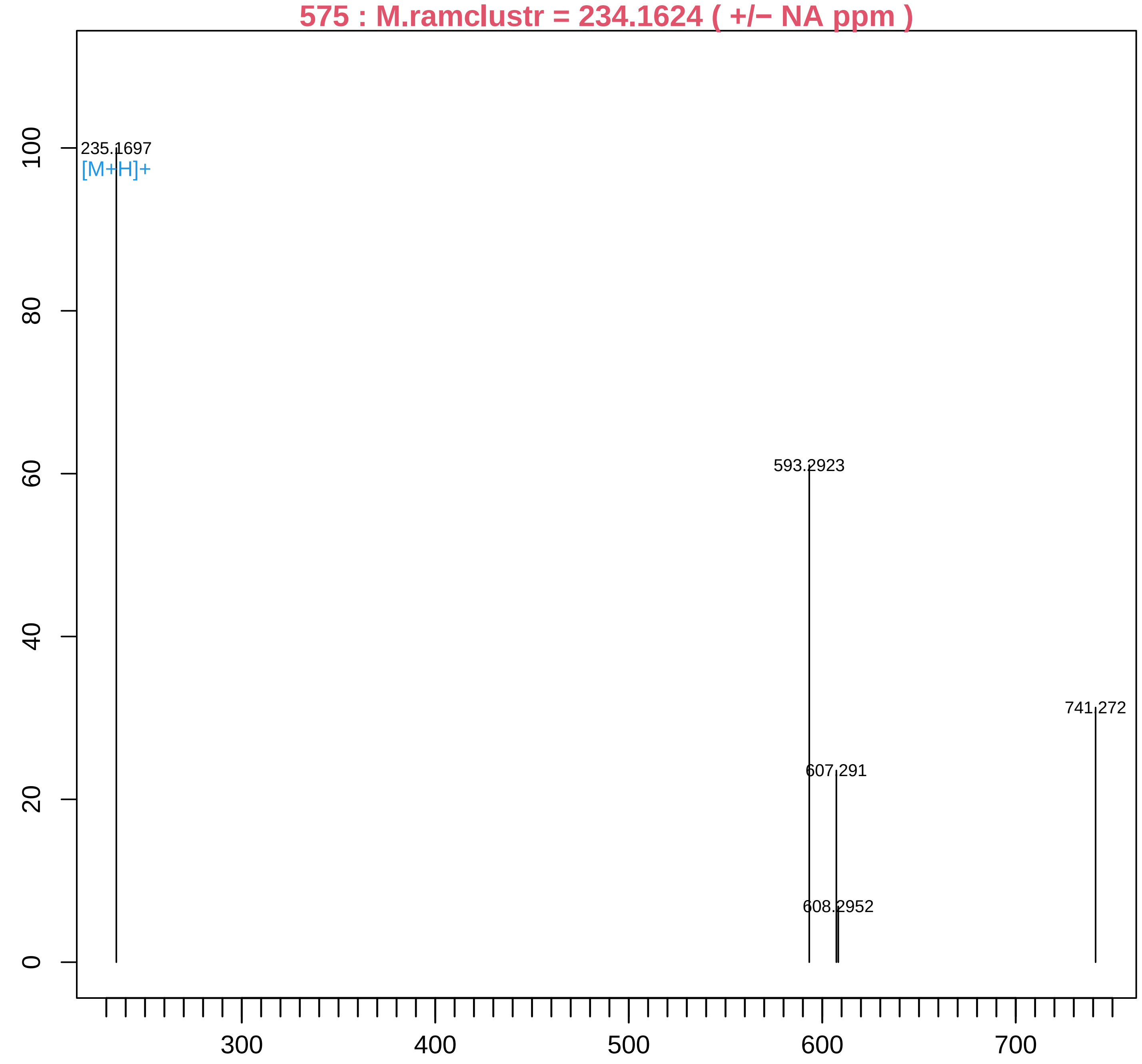
575 : M.ramclustr = 234.1624 (+/− NA ppm)
235.1697
[M+H]+
593.2923
607.291
608.2952
741.272
0
20
40
60
80
100
300
400
500
600
700
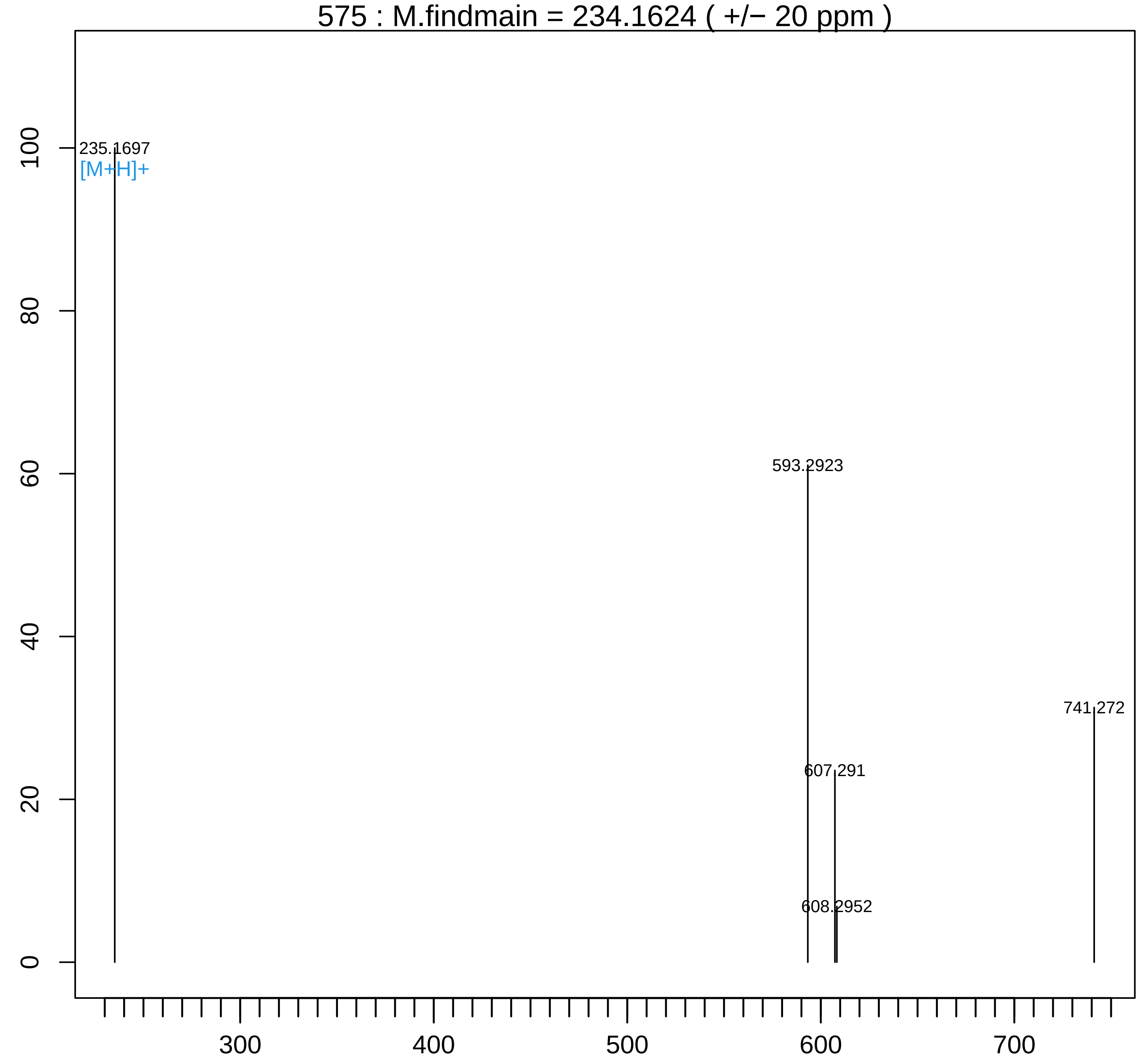
575 : M.findmain = 234.1624 (+/− 20 ppm)
235.1697
[M+H]+
593.2923
607.291
608.2952
741.272
0
20
40
60
80
100
300
400
500
600
700

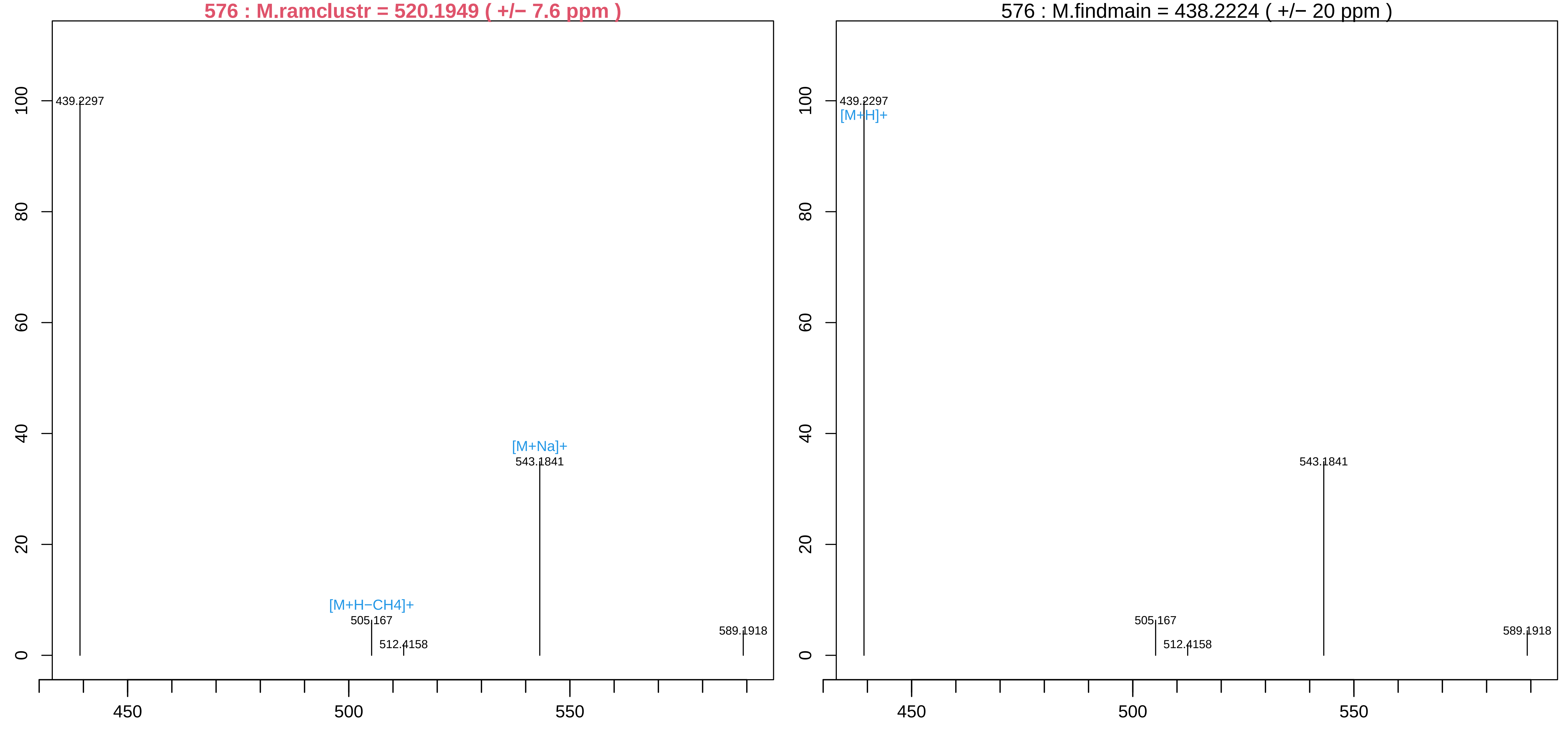
576 : M.ramclustr = 520.1949 (+/− 7.6 ppm)
439.2297
[M+H−CH4]+
505.167
512.4158
[M+Na]+
543.1841
589.1918
0
20
40
60
80
100
450
500
550
576 : M.findmain = 438.2224 (+/− 20 ppm)
439.2297
[M+H]+
505.167
512.4158
543.1841
589.1918
0
20
40
60
80
100
450
500
550

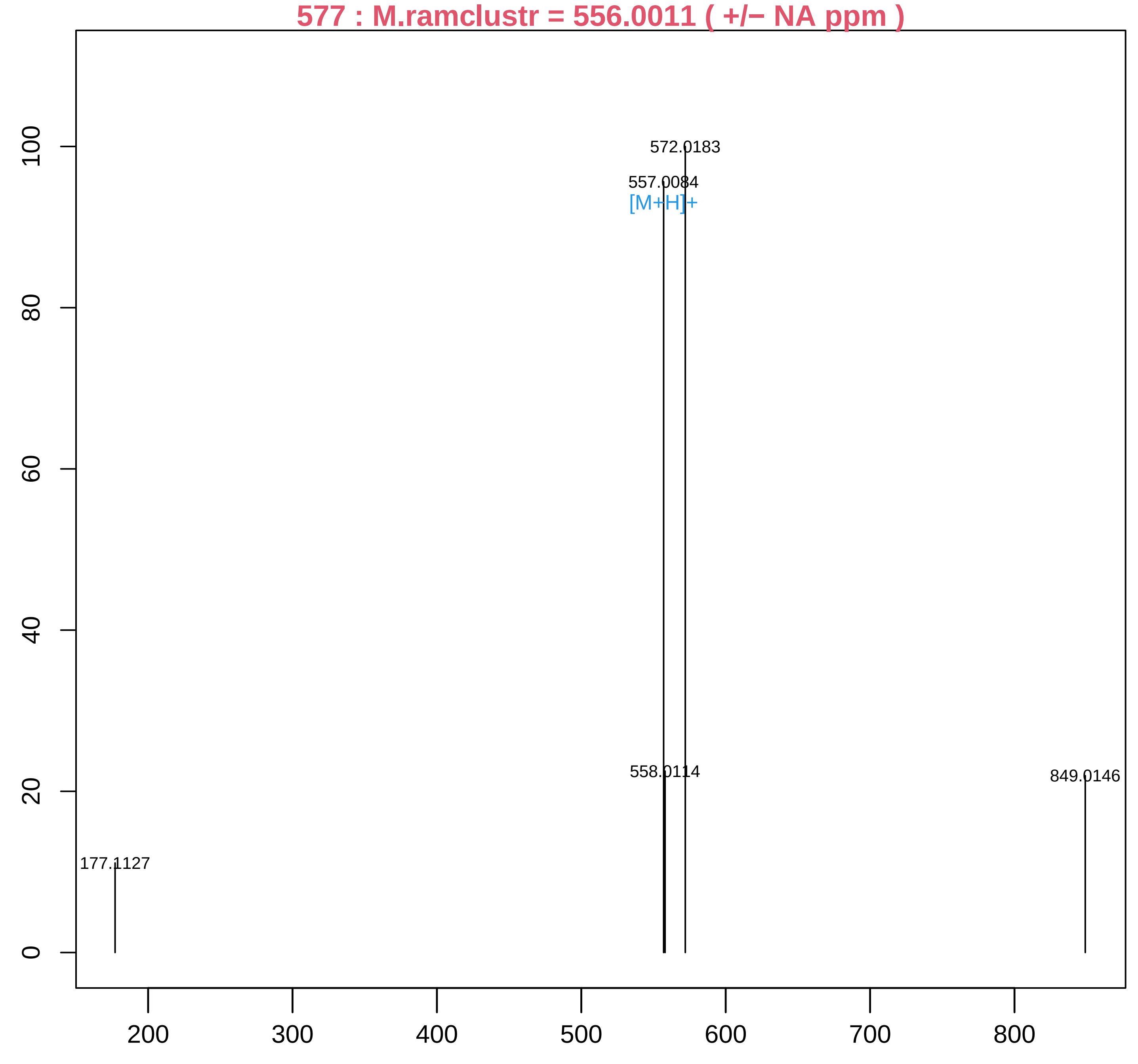
577 : M.ramclustr = 556.0011 (+/− NA ppm)
572.0183
557.0084
[M+H]+
558.0114
849.0146
177.1127
0
20
40
60
80
100
200
300
400
500
600
700
800
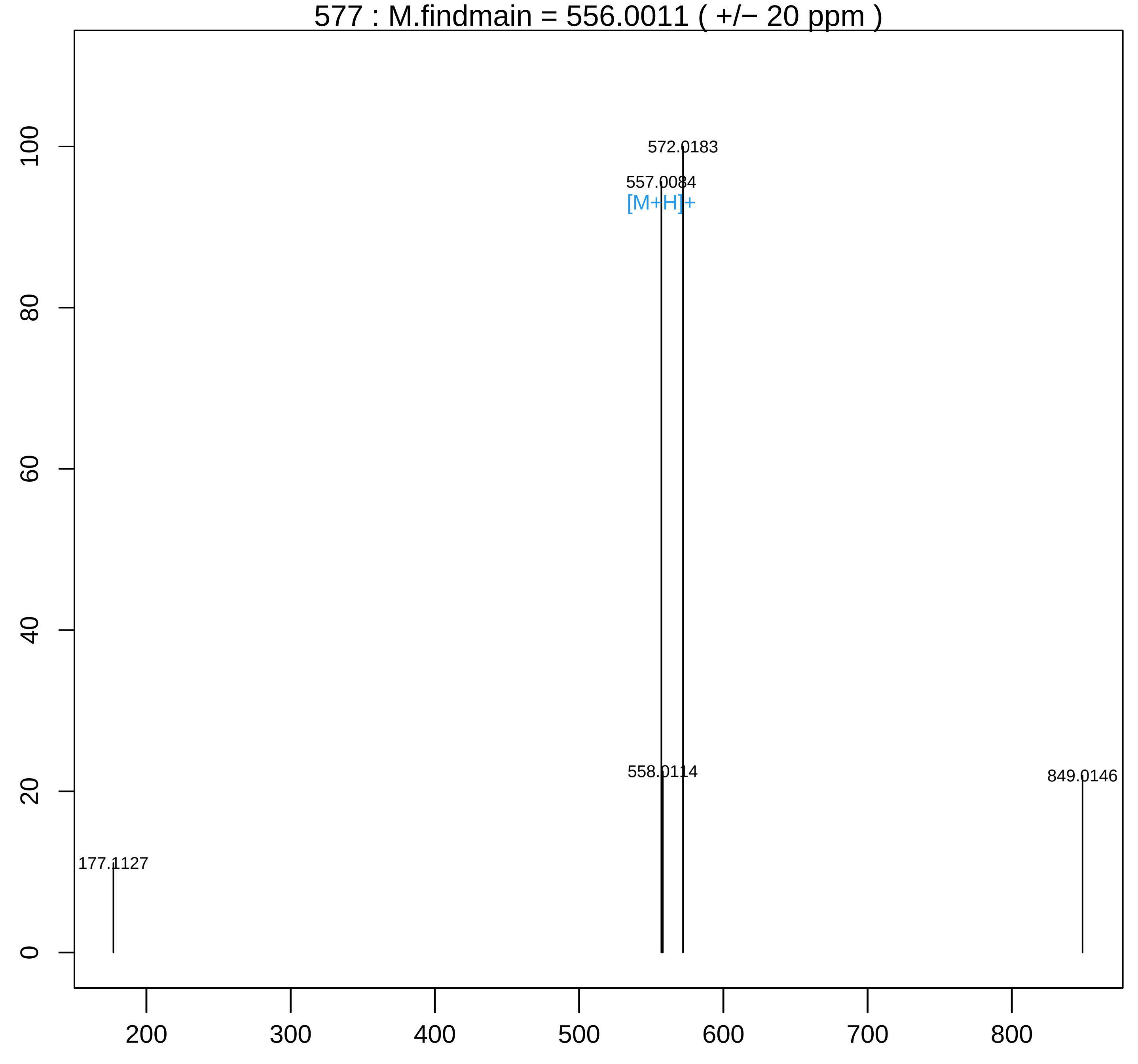
577 : M.findmain = 556.0011 (+/− 20 ppm)
572.0183
557.0084
[M+H]+
558.0114
849.0146
177.1127
0
20
40
60
80
100
200
300
400
500
600
700
800

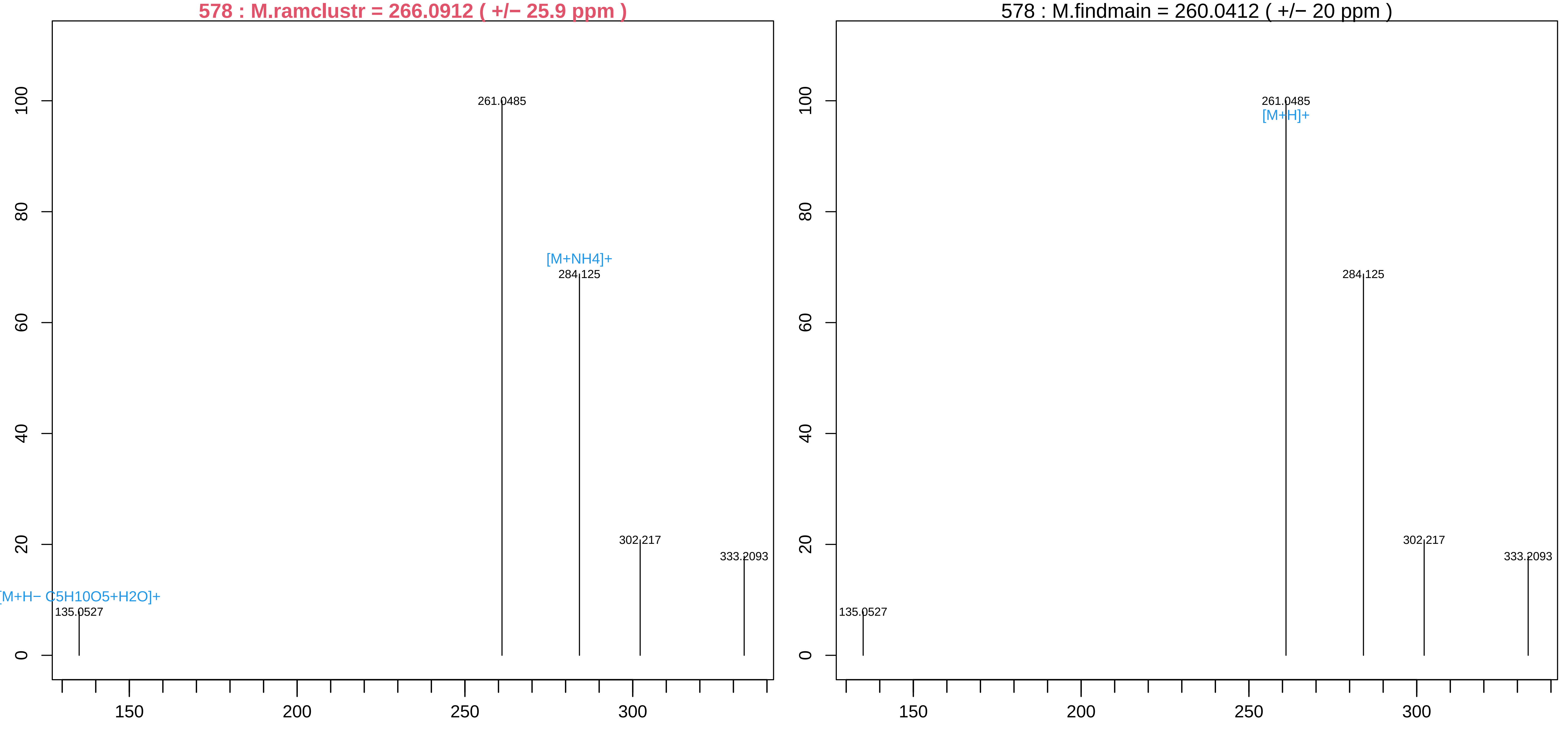
578 : M.ramclustr = 266.0912 (+/− 25.9 ppm)
261.0485
[M+NH4]+
284.125
302.217
333.2093
[M+H− C5H10O5+H2O]+
135.0527
578 : M.findmain = 260.0412 (+/− 20 ppm)
261.0485
[M+H]+
284.125
302.217
333.2093
135.0527
0
20
40
60
80
100
150
200
250
300

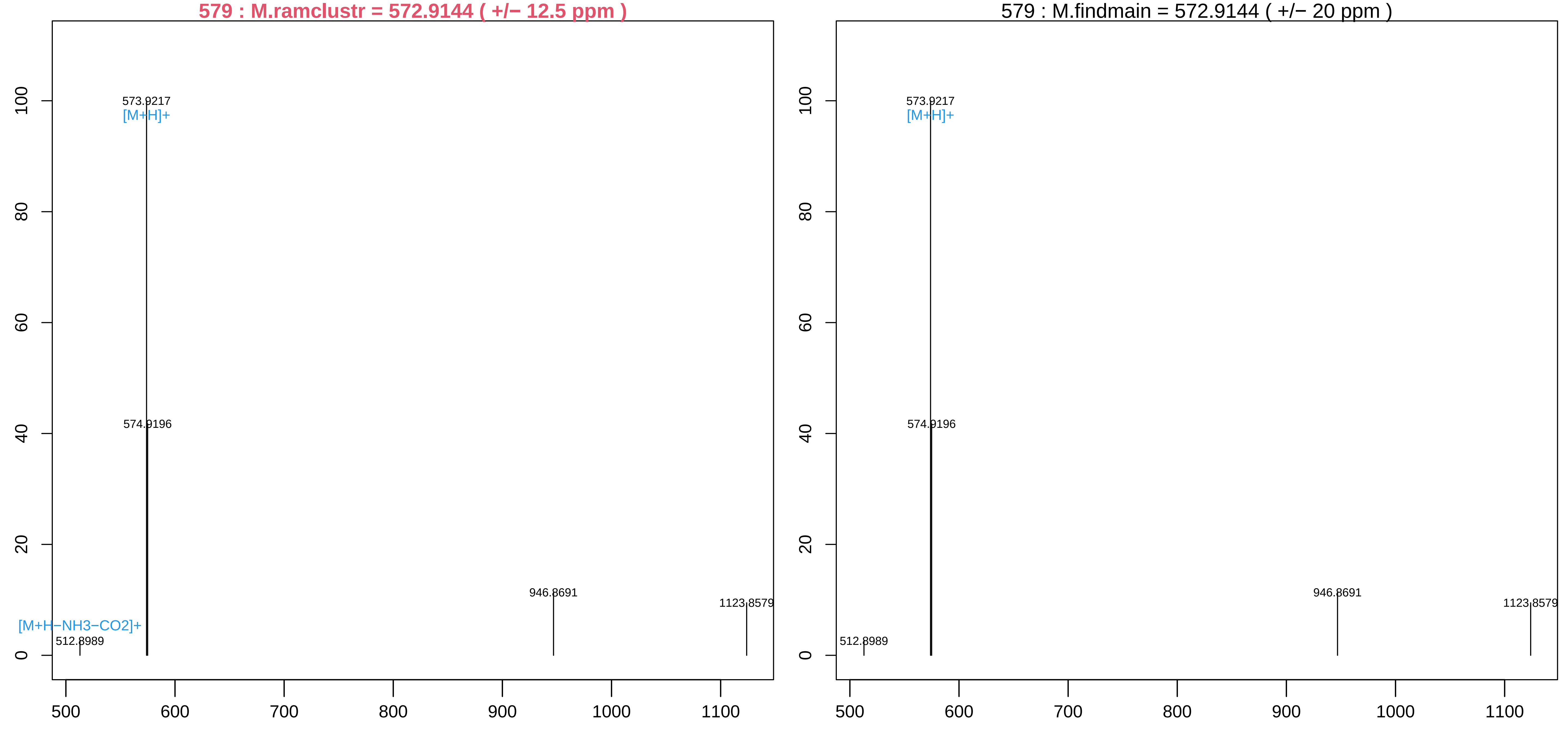
579 : M.ramclustr = 572.9144 (+/− 12.5 ppm)
573.9217
[M+H]+
574.9196
946.8691
1123.8579
[M+H−NH3−CO2]+
512.8989
0
20
40
60
80
100
500
600
700
800
900
1000
1100
579 : M.findmain = 572.9144 (+/− 20 ppm)
573.9217
[M+H]+
574.9196
946.8691
1123.8579
512.8989
0
20
40
60
80
100
500
600
700
800
900
1000
1100

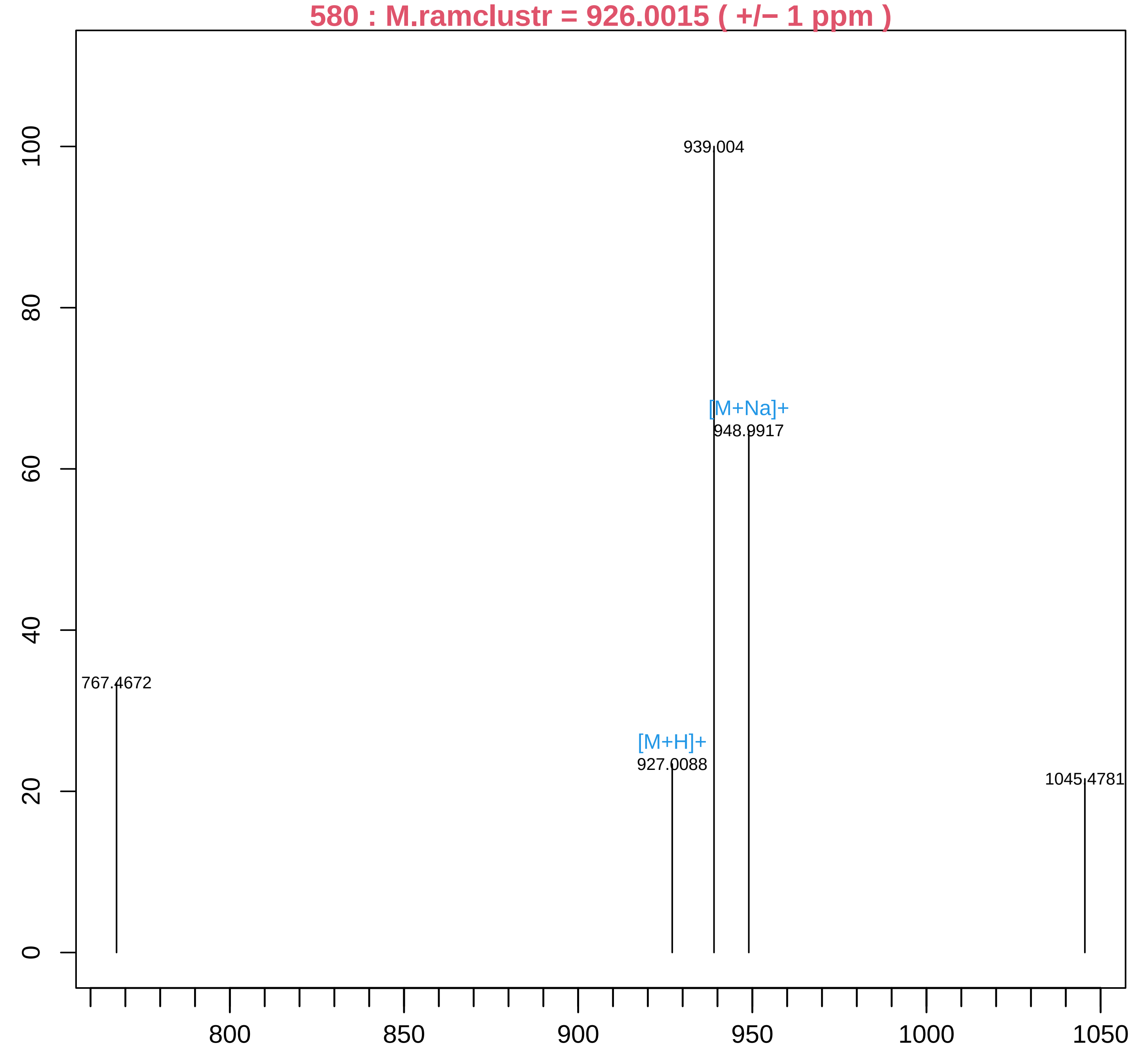
580 : M.ramclustr = 926.0015 (+/− 1 ppm)
939.004
[M+Na]+
948.9917
767.4672
[M+H]+
927.0088
1045.4781
0
20
40
60
80
100
800
850
900
950
1000
1050
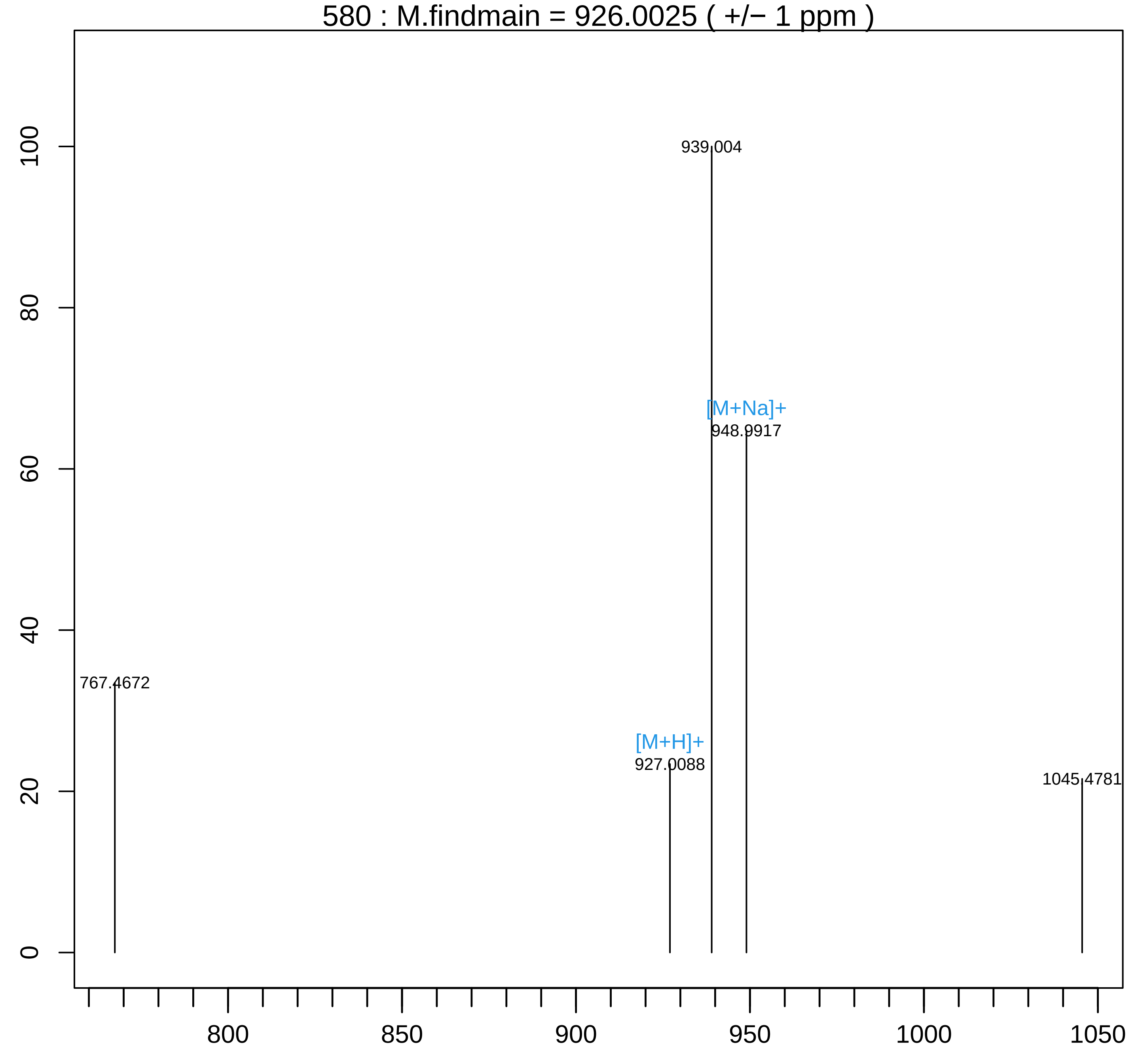
580 : M.findmain = 926.0025 (+/− 1 ppm)
939.004
[M+Na]+
948.9917
767.4672
[M+H]+
927.0088
1045.4781
0
20
40
60
80
100
800
850
900
950
1000
1050

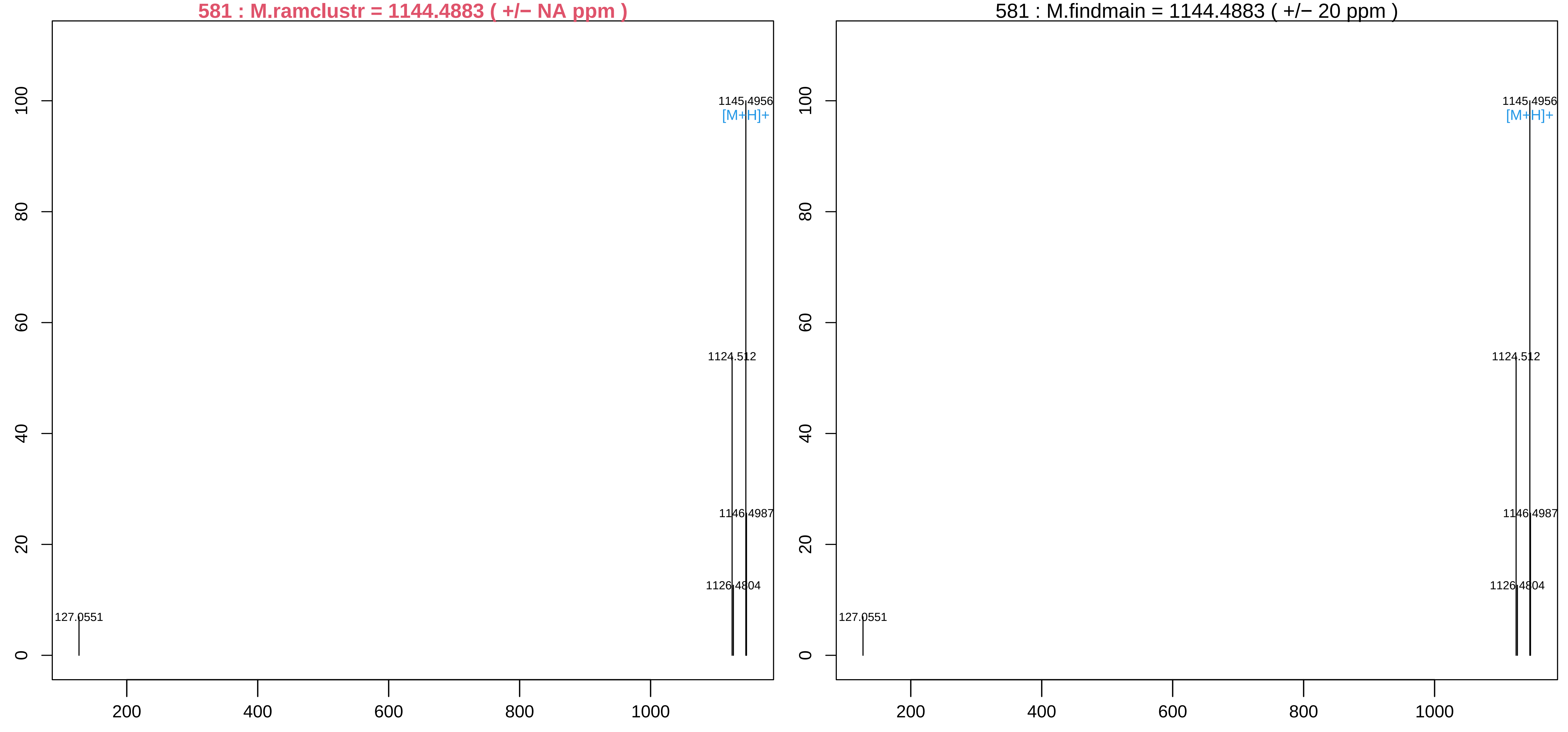
581 : M.ramclustr = 1144.4883 (+/− NA ppm)
1145.4956
[M+H]+
1124.512
1146.4987
1126.4804
127.0551
581 : M.findmain = 1144.4883 (+/− 20 ppm)
1145.4956
[M+H]+
1124.512
1146.4987
1126.4804
127.0551

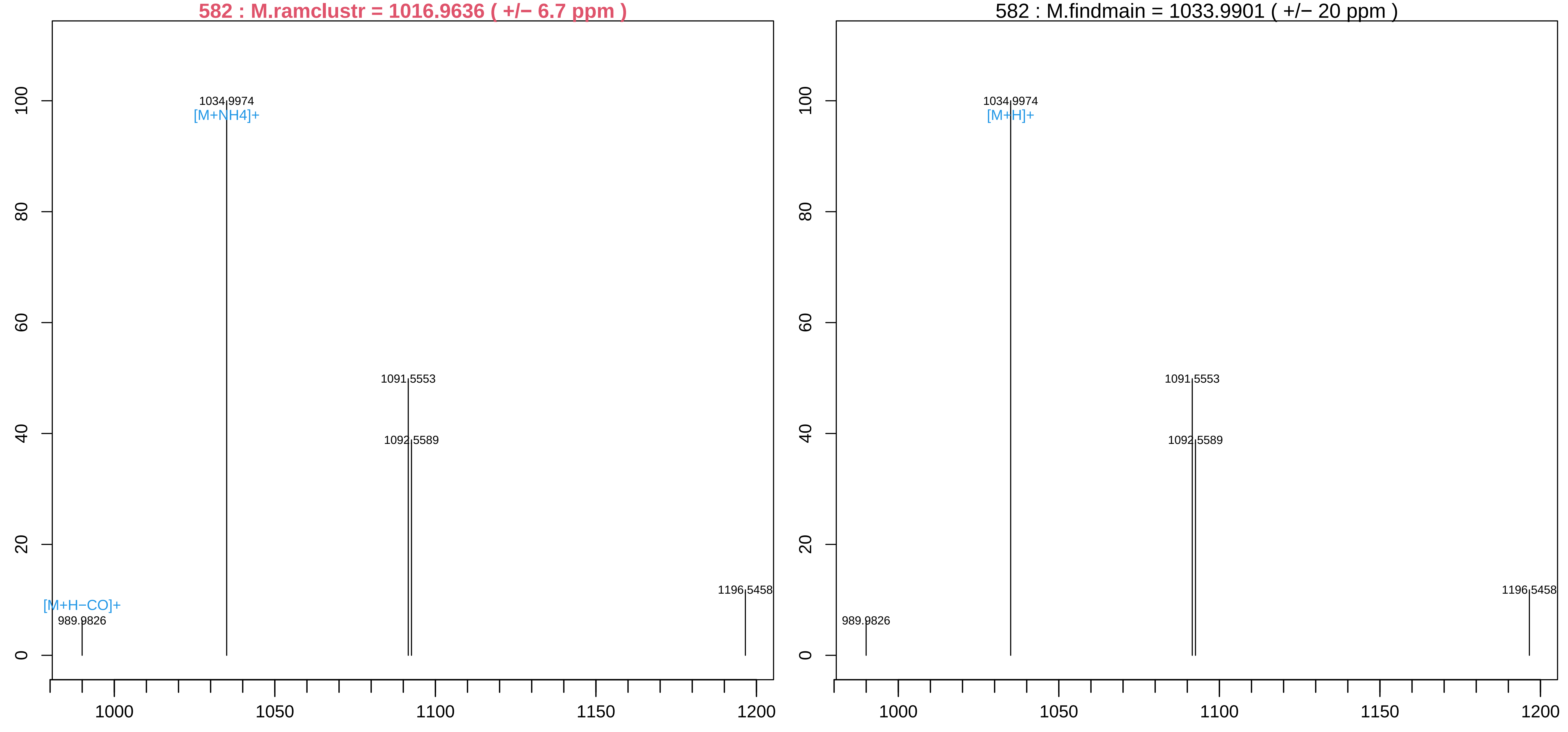

582 : M.ramclustr = 1016.9636 (+/− 6.7 ppm)
1034.9974
[M+NH4]+
1091.5553
1092.5589
1196.5458
[M+H−CO]+
989.9826
582 : M.findmain = 1033.9901 (+/− 20 ppm)
1034.9974
[M+H]+
1091.5553
1092.5589
1196.5458
989.9826
0
20
40
60
80
100
1000
1050
1100
1150
1200

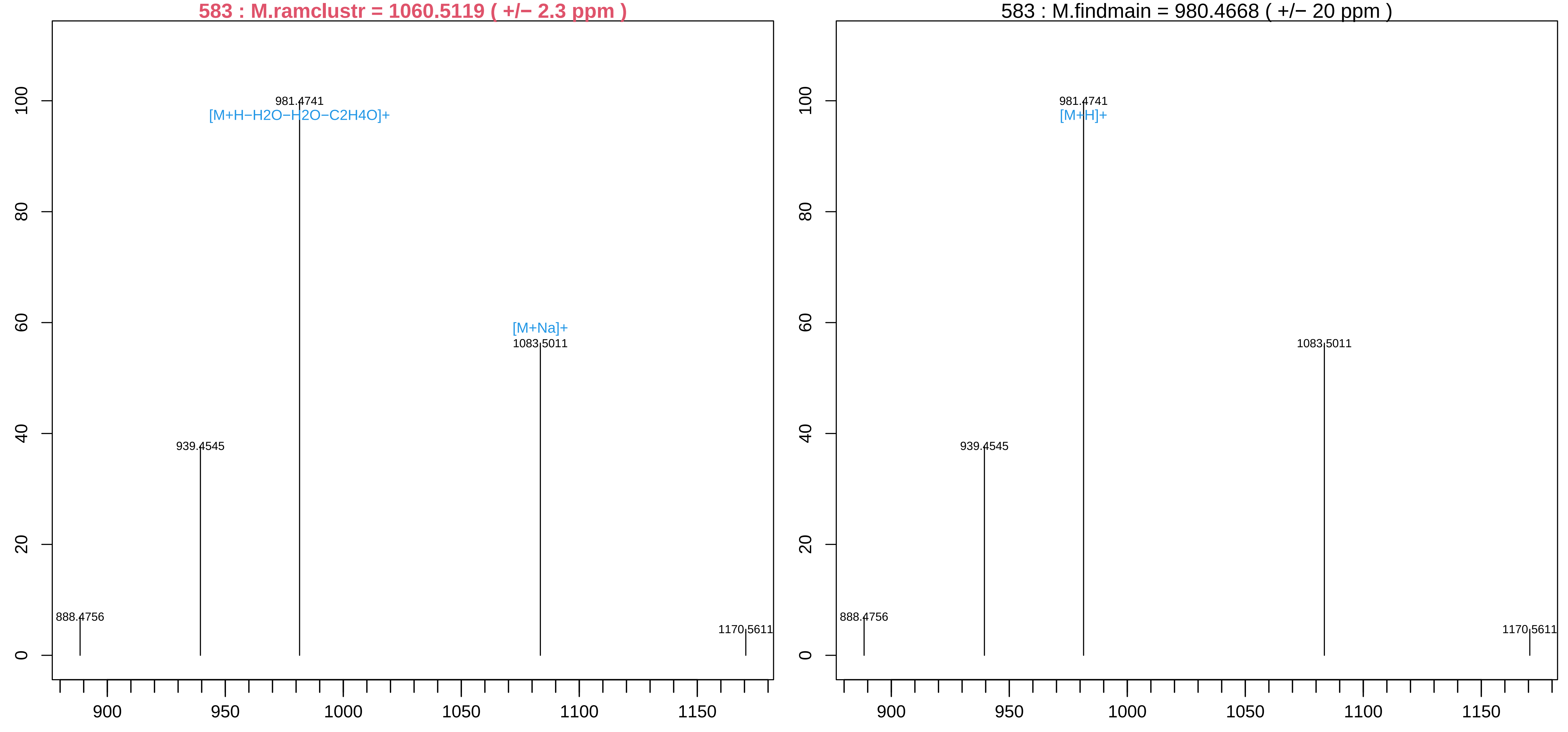
583 : M.ramclustr = 1060.5119 (+/− 2.3 ppm)
981.4741
[M+H−H2O−H2O−C2H4O]+
[M+Na]+
1083.5011
939.4545
888.4756
1170.5611
583 : M.findmain = 980.4668 (+/− 20 ppm)
981.4741
[M+H]+
1083.5011
939.4545
888.4756
1170.5611
0
20
40
60
80
100
900
950
1000
1050
1100
1150

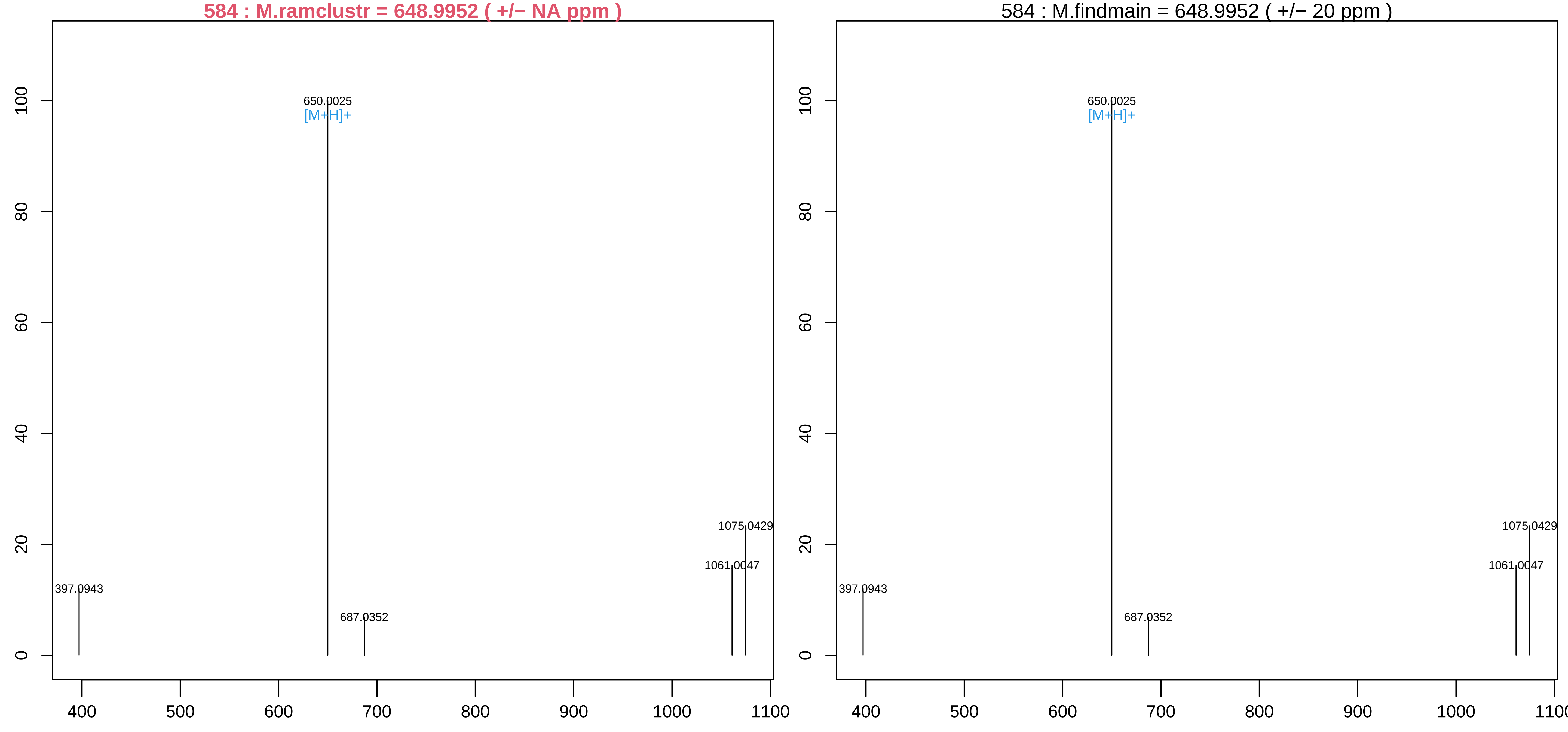
584 : M.ramclustr = 648.9952 (+/− NA ppm)
650.0025
[M+H]+
397.0943
687.0352
1061.0047
1075.0429
0
20
40
60
80
100
400
500
600
700
800
900
1000
1100
584 : M.findmain = 648.9952 (+/− 20 ppm)
650.0025
[M+H]+
397.0943
687.0352
1061.0047
1075.0429

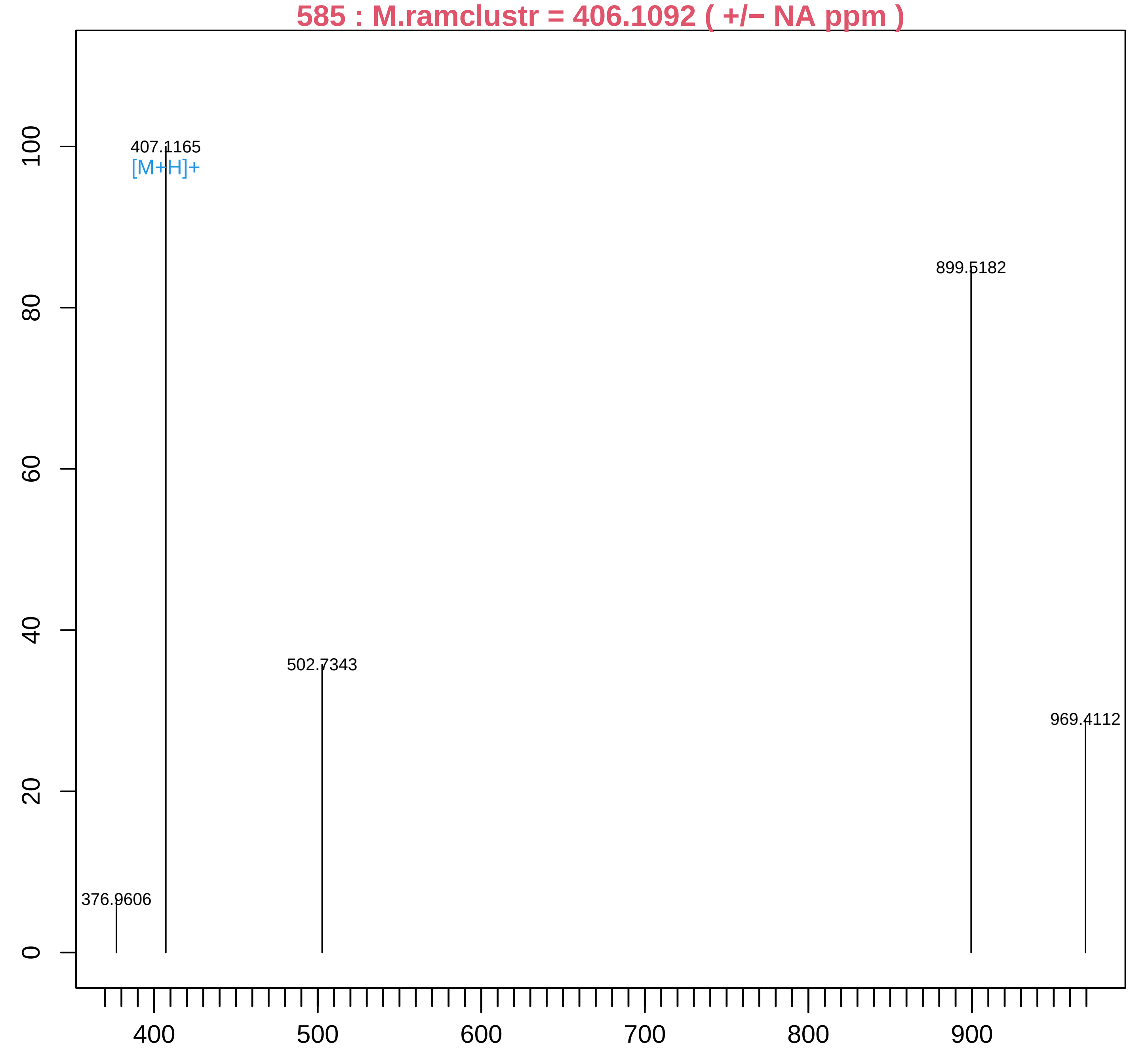
585 : M.ramclustr = 406.1092 (+/− NA ppm)
407.1165
[M+H]+
899.5182
502.7343
969.4112
376.9606
0
20
40
60
80
100
400
500
600
700
800
900

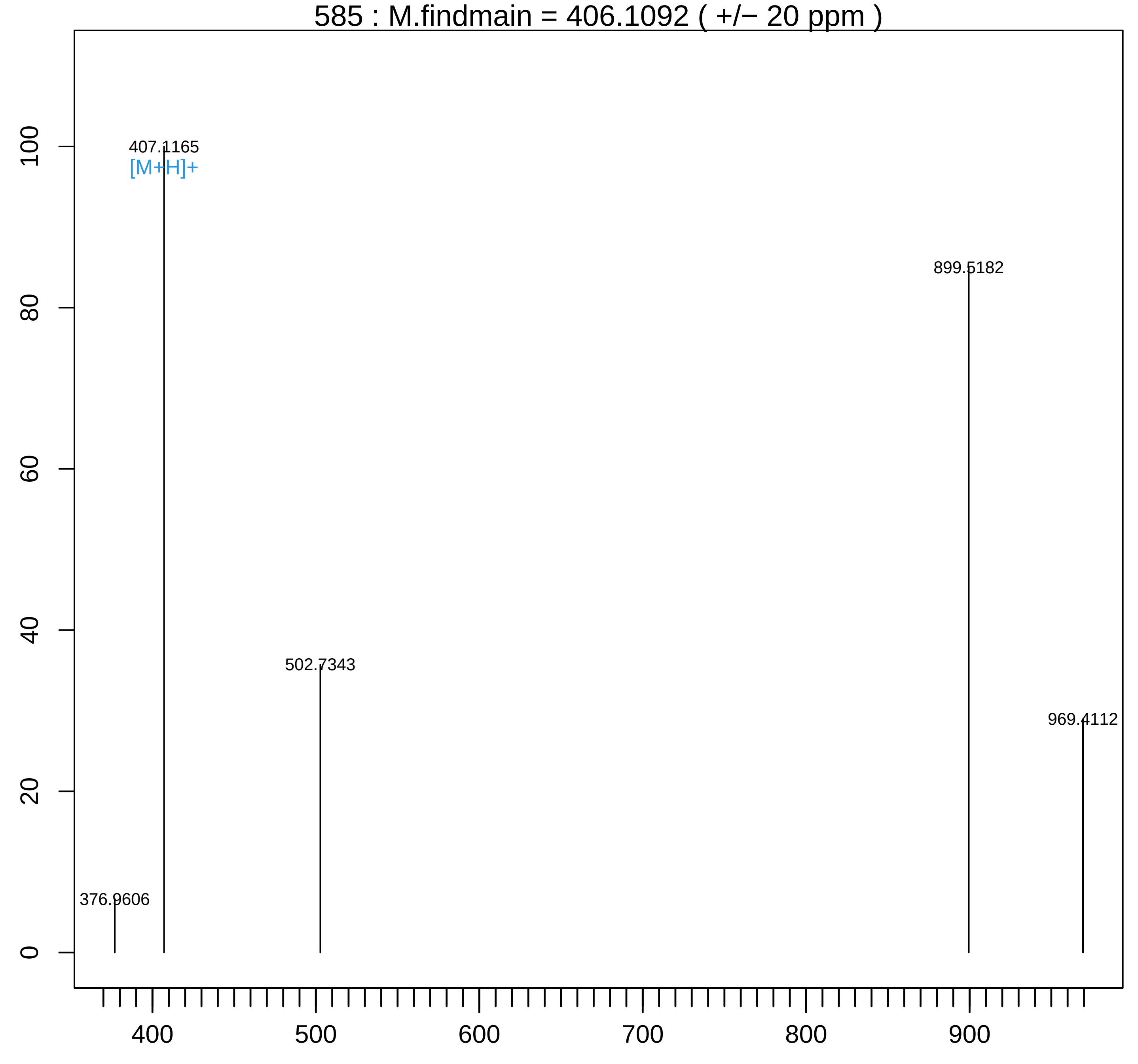
585 : M.findmain = 406.1092 (+/− 20 ppm)
407.1165
[M+H]+
899.5182
502.7343
969.4112
376.9606
0
20
40
60
80
100
400
500
600
700
800
900

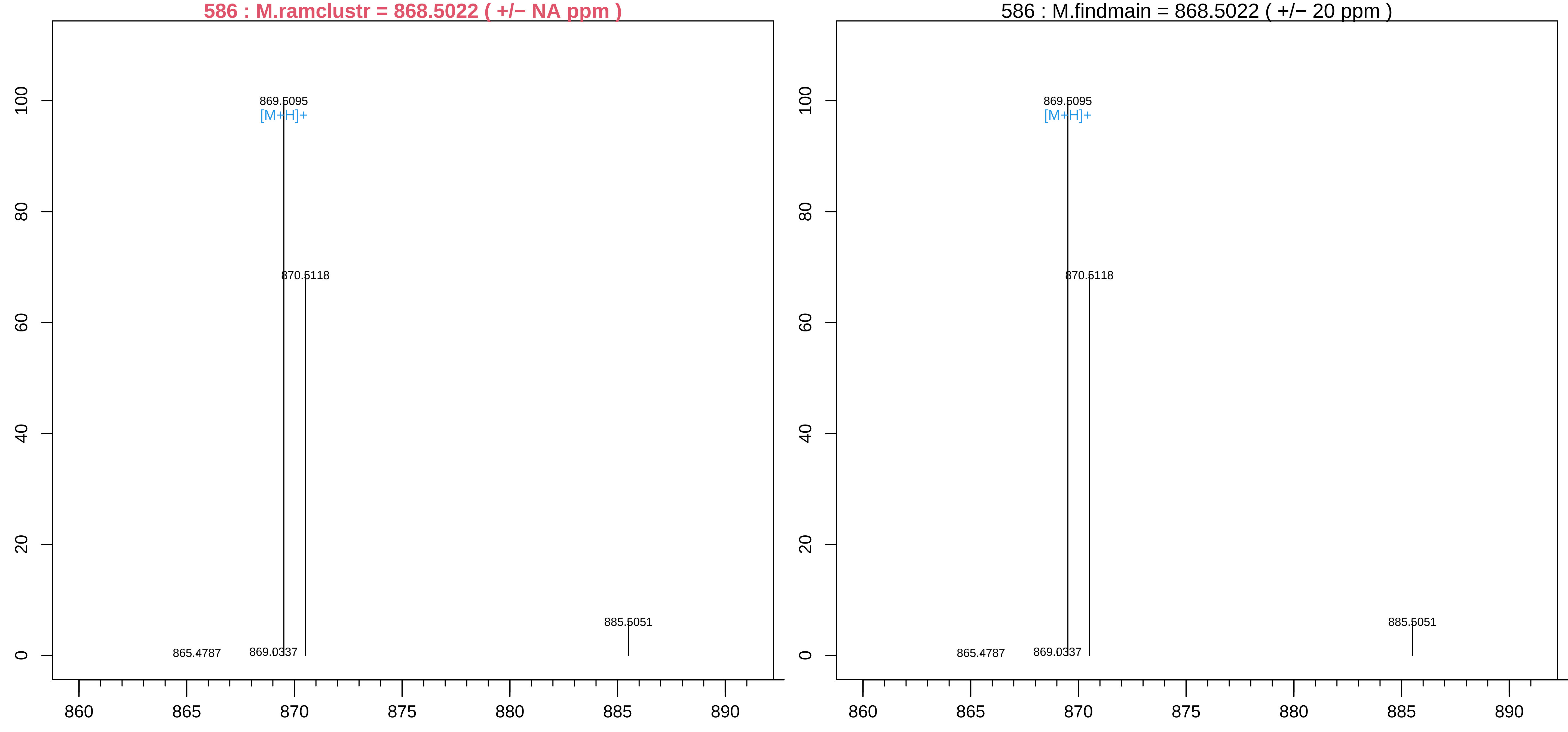
586 : M.ramclustr = 868.5022 (+/− NA ppm)
869.5095
[M+H]+
870.5118
885.5051
865.4787
869.0337
586 : M.findmain = 868.5022 (+/− 20 ppm)
869.5095
[M+H]+
870.5118
885.5051
865.4787
869.0337

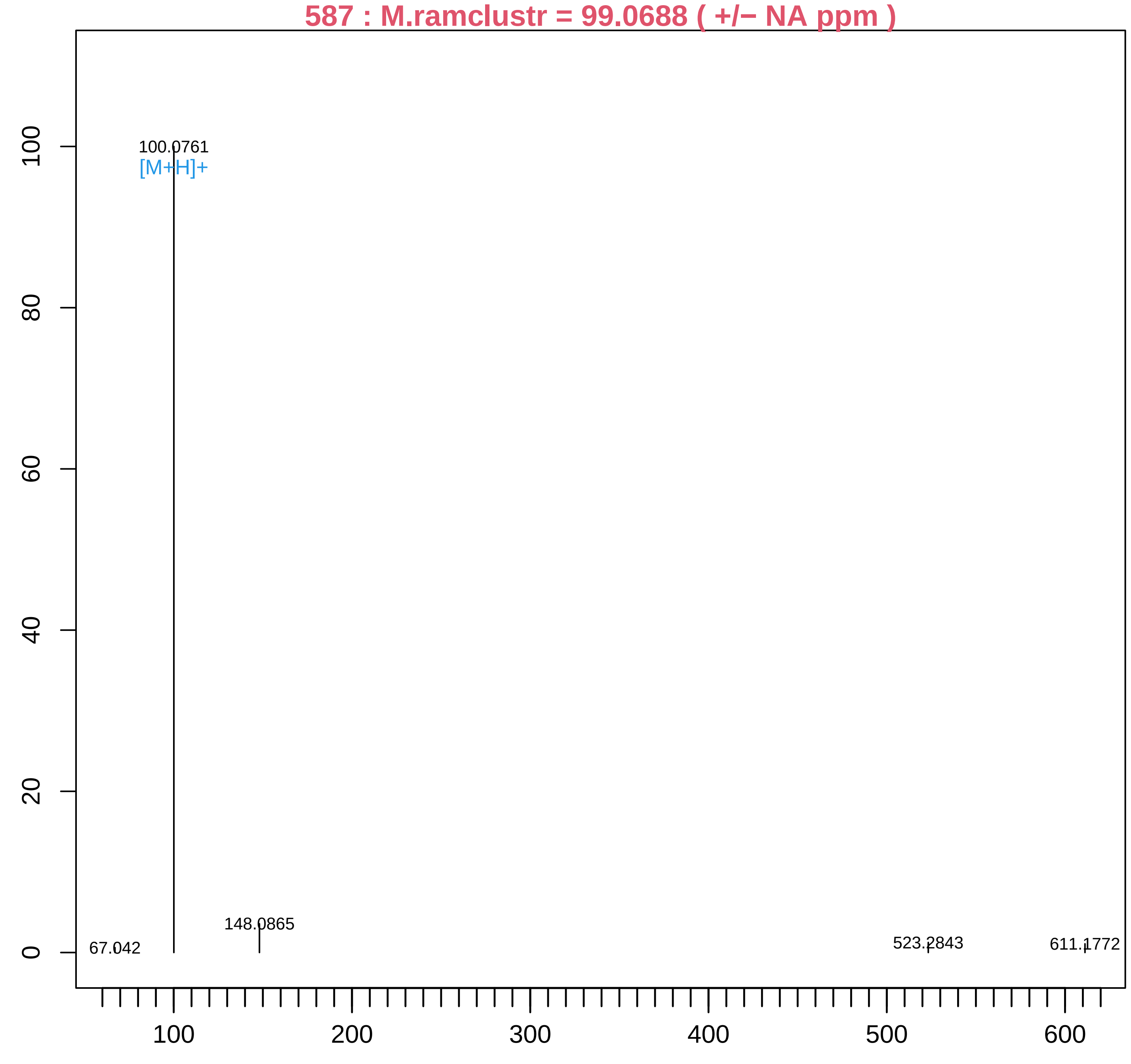
587 : M.ramclustr = 99.0688 (+/− NA ppm)
100.0761
[M+H]+
148.0865
67.042
523.2843
611.1772
0
20
40
60
80
100
100
200
300
400
500
600

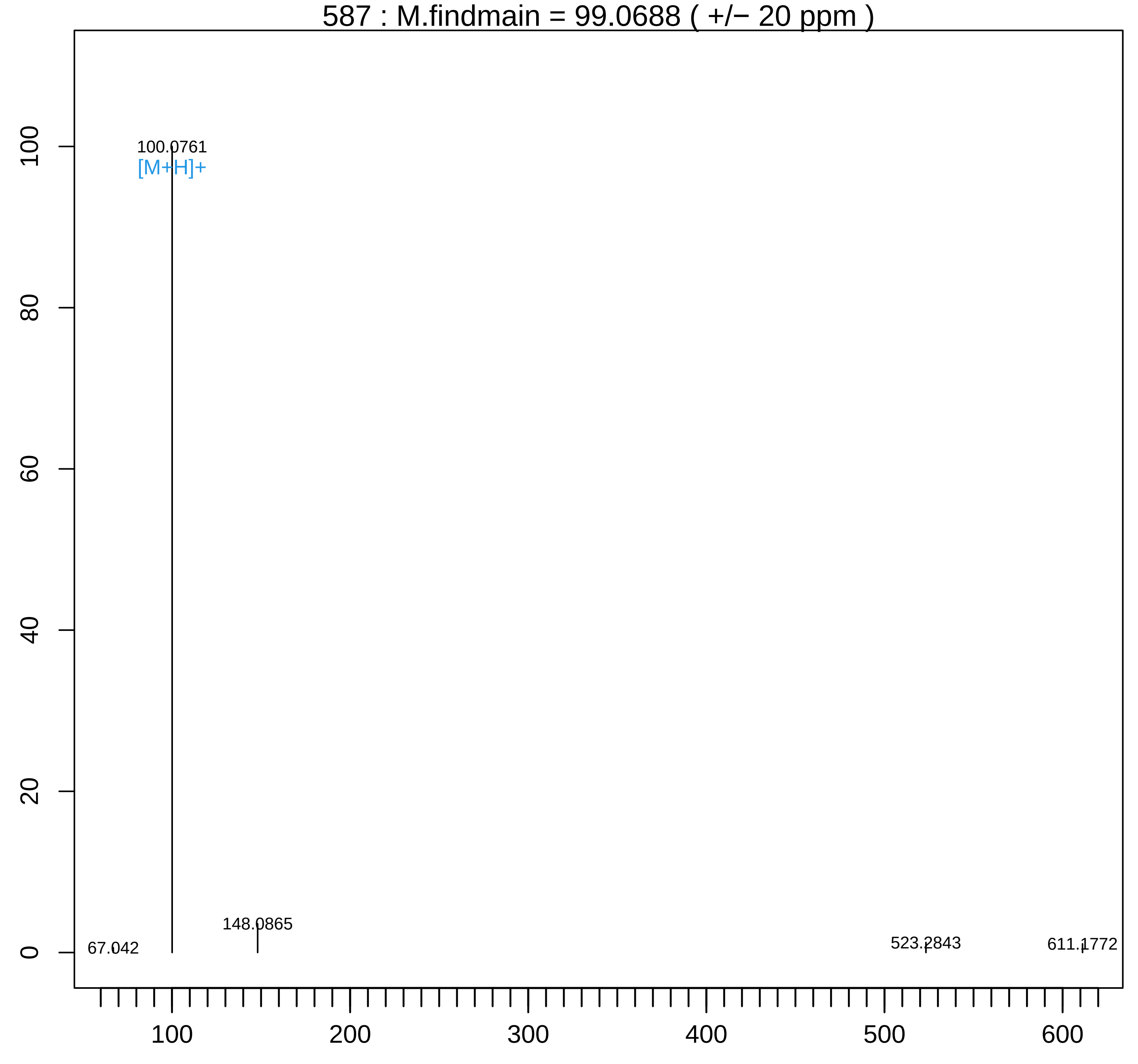
587 : M.findmain = 99.0688 (+/− 20 ppm)
100.0761
[M+H]+
148.0865
67.042
523.2843
611.1772
0
20
40
60
80
100
100
200
300
400
500
600

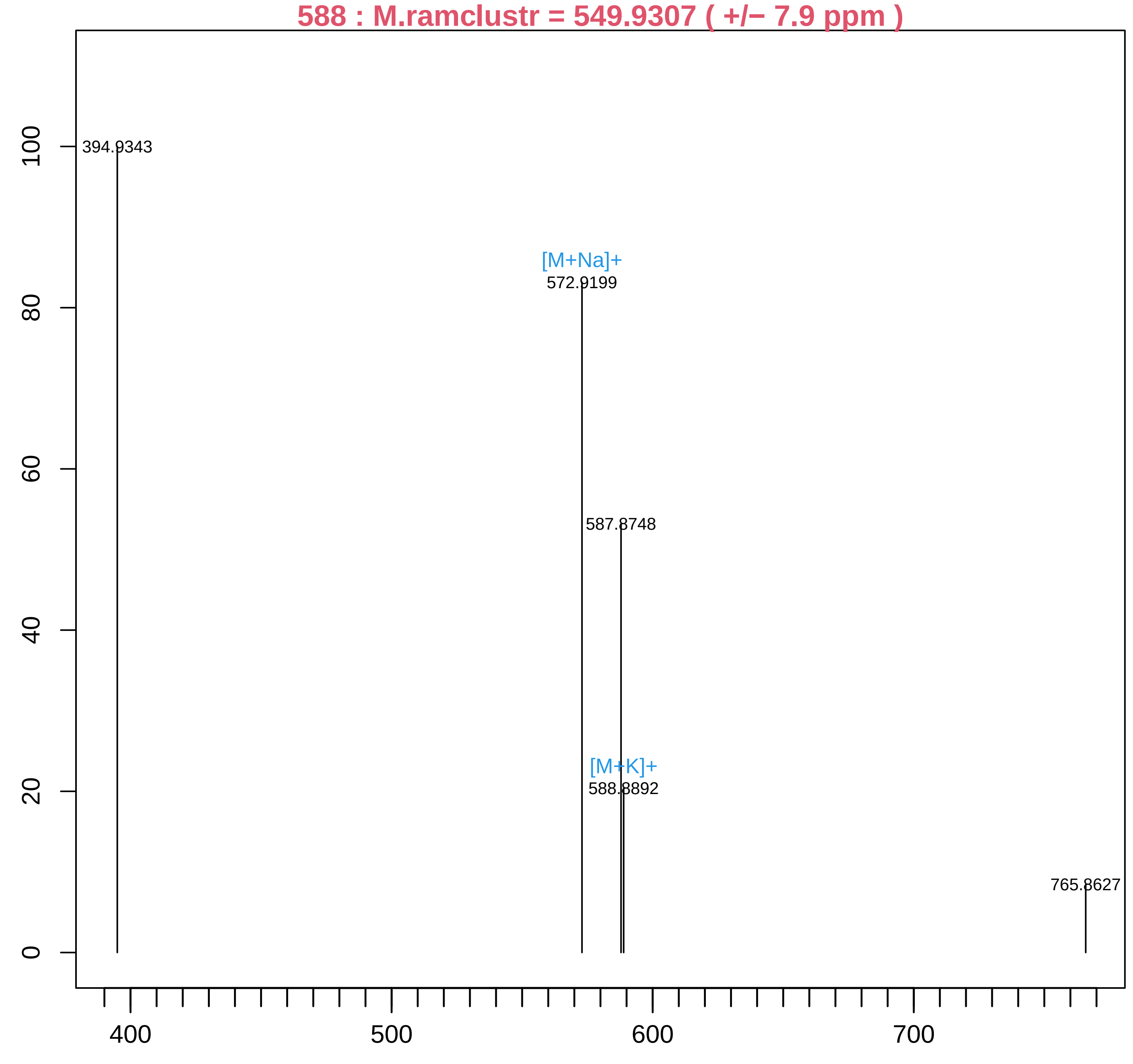

588 : M.ramclustr = 549.9307 (+/− 7.9 ppm)
394.9343
[M+Na]+
572.9199
587.8748
[M+K]+
588.8892
765.8627
0
20
40
60
80
100
400
500
600
700

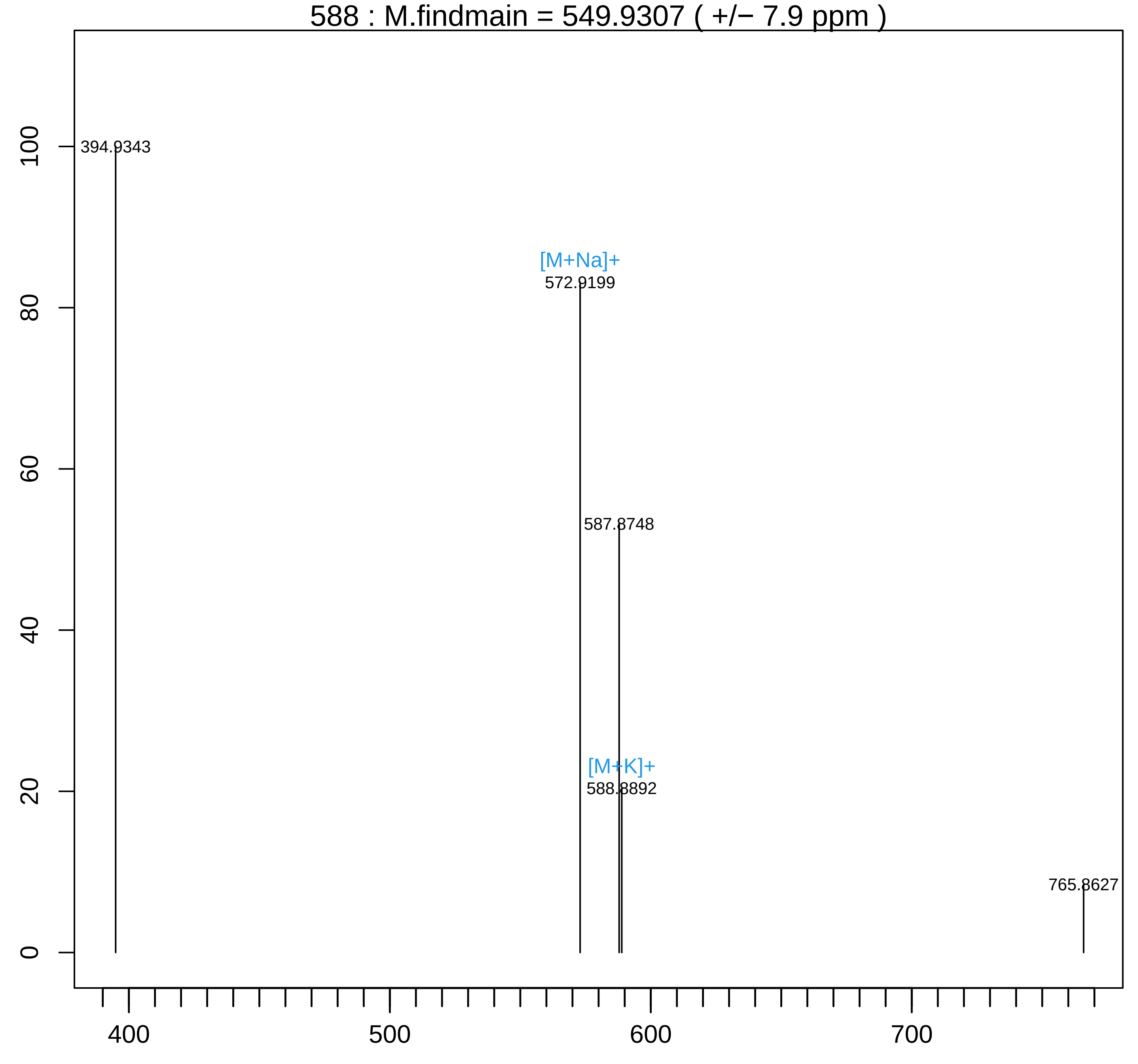

588 : M.findmain = 549.9307 (+/− 7.9 ppm)
394.9343
[M+Na]+
572.9199
587.8748
[M+K]+
588.8892
765.8627
0
20
40
60
80
100
400
500
600
700

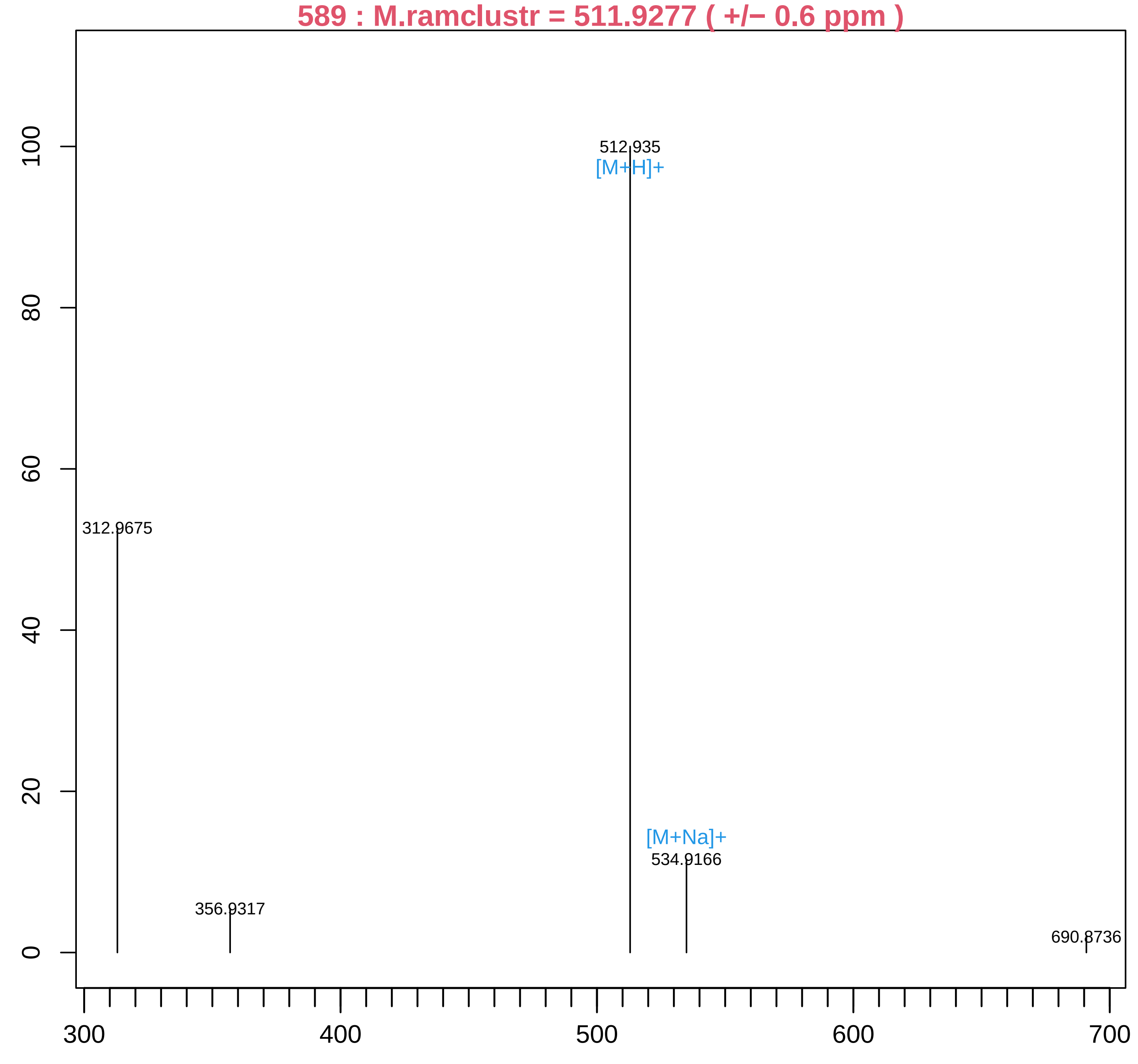
589 : M.ramclustr = 511.9277 (+/− 0.6 ppm)
512.935
[M+H]+
312.9675
356.9317
[M+Na]+
534.9166
690.8736
0
20
40
60
80
100
300
400
500
600
700

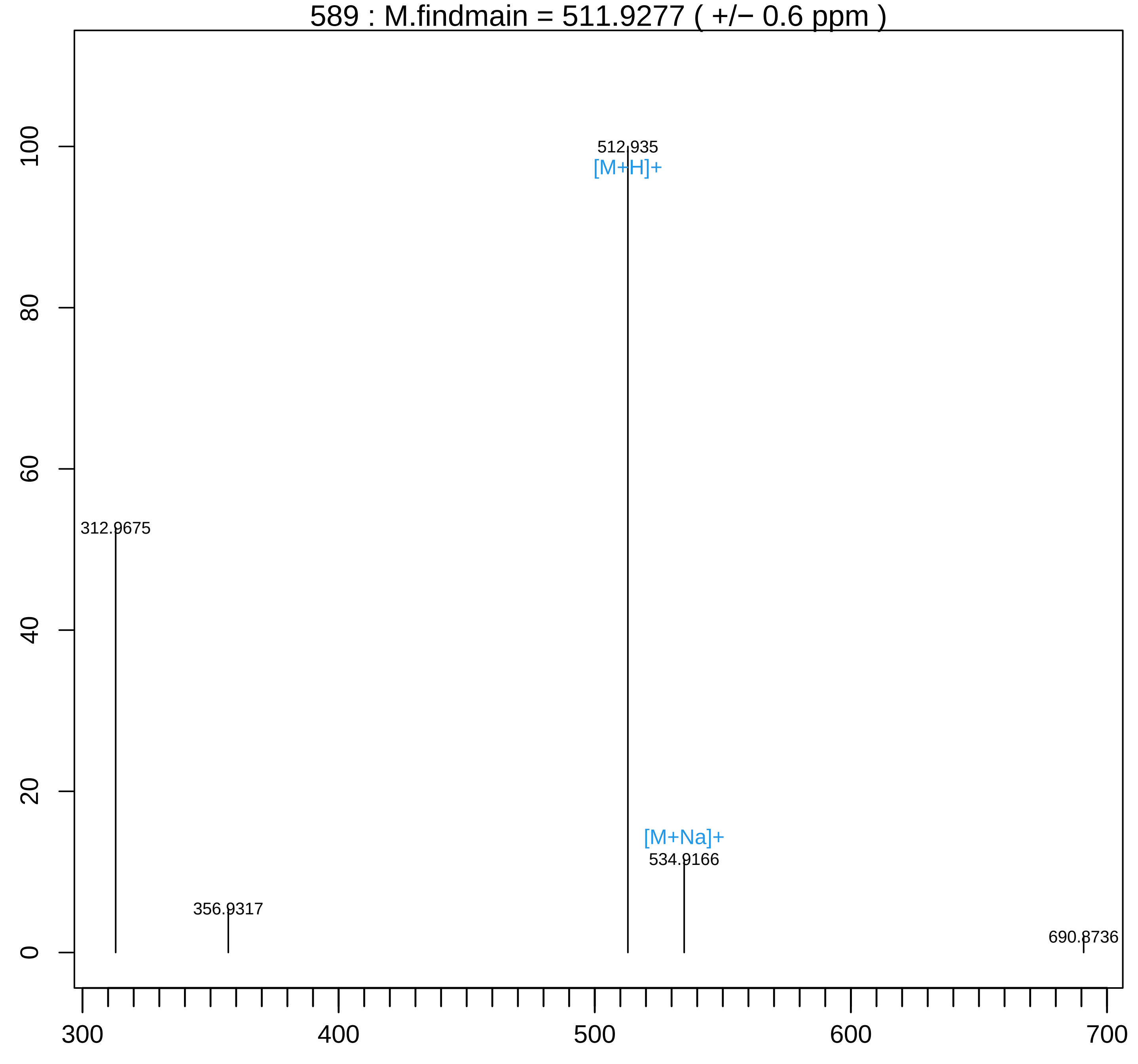
589 : M.findmain = 511.9277 (+/− 0.6 ppm)
512.935
[M+H]+
312.9675
356.9317
[M+Na]+
534.9166
690.8736
0
20
40
60
80
100
300
400
500
600
700

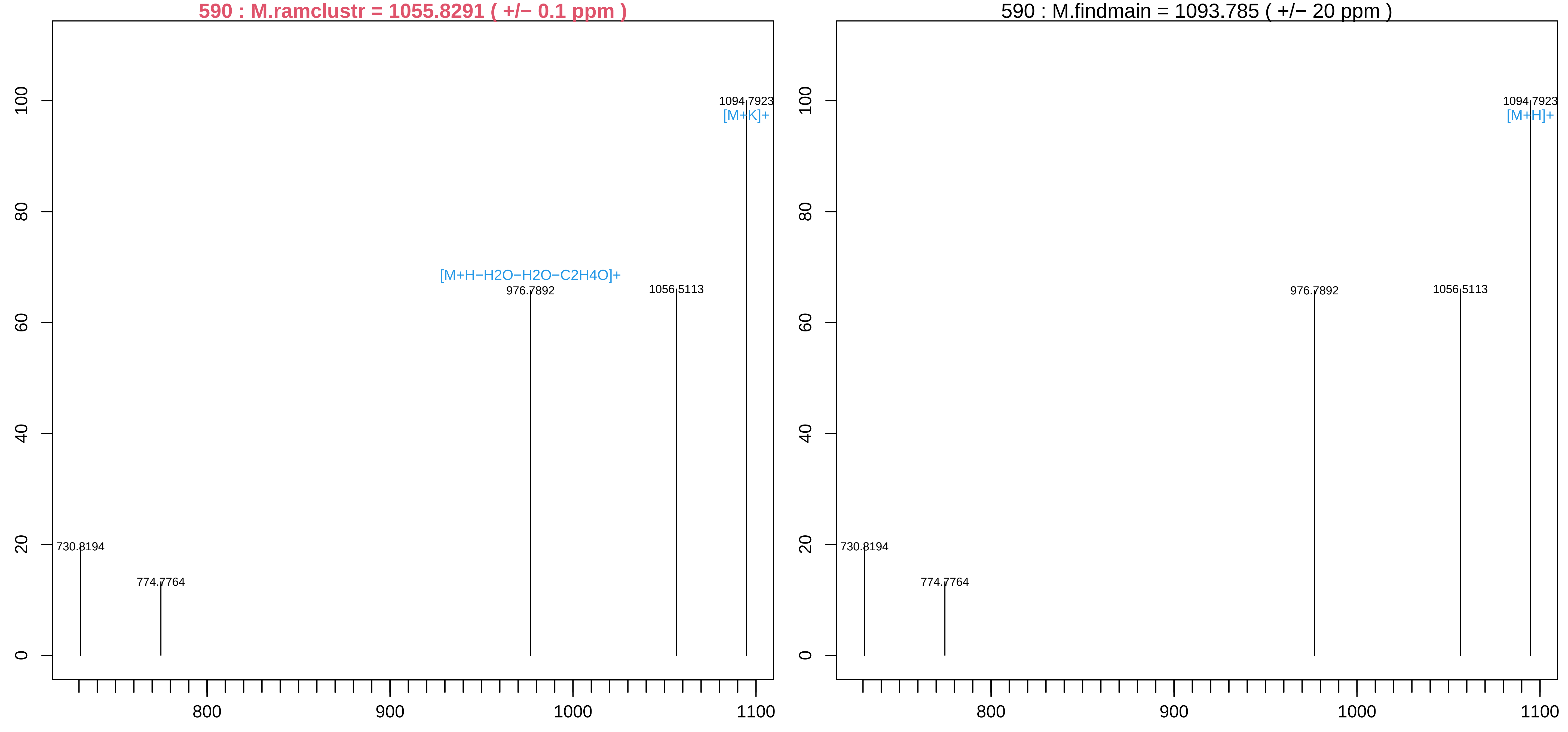
590 : M.ramclustr = 1055.8291 (+/− 0.1 ppm)
1094.7923
[M+K]+
[M+H−H2O−H2O−C2H4O]+
976.7892
1056.5113
730.8194
774.7764
590 : M.findmain = 1093.785 (+/− 20 ppm)
1094.7923
[M+H]+
976.7892
1056.5113
730.8194
774.7764
0
20
40
60
80
100
800
900
1000
1100

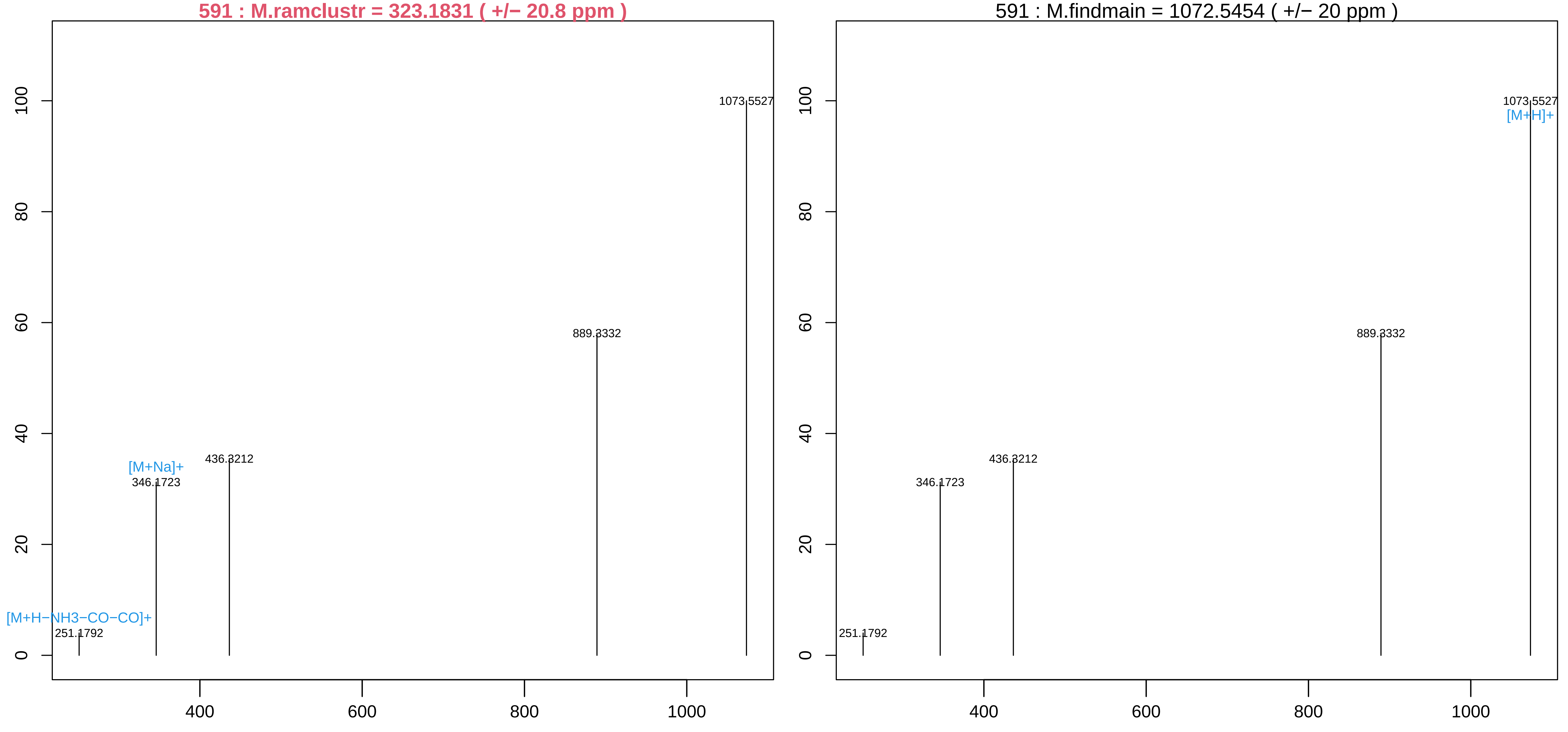
591 : M.ramclustr = 323.1831 (+/− 20.8 ppm)
1073.5527
889.3332
436.3212
[M+Na]+
346.1723
[M+H−NH3−CO−CO]+
251.1792
591 : M.findmain = 1072.5454 (+/− 20 ppm)
1073.5527
[M+H]+
889.3332
436.3212
346.1723
251.1792

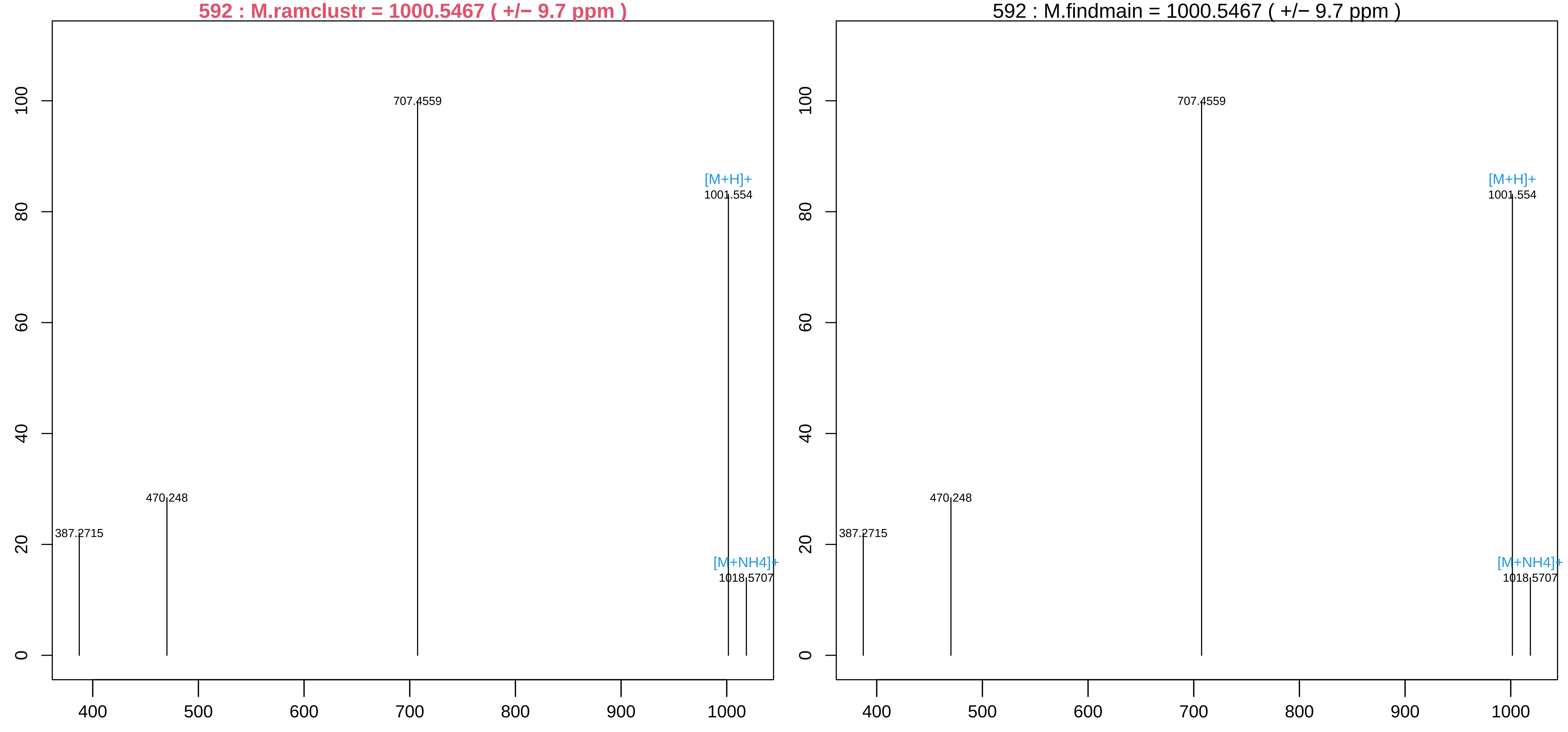

592 : M.ramclustr = 1000.5467 (+/− 9.7 ppm)
592 : M.findmain = 1000.5467 (+/− 9.7 ppm)
707.4559
[M+H]+
1001.554
470.248
387.2715
[M+NH4]+
1018.5707
0
20
40
60
80
100
400
500
600
700
800
900
1000

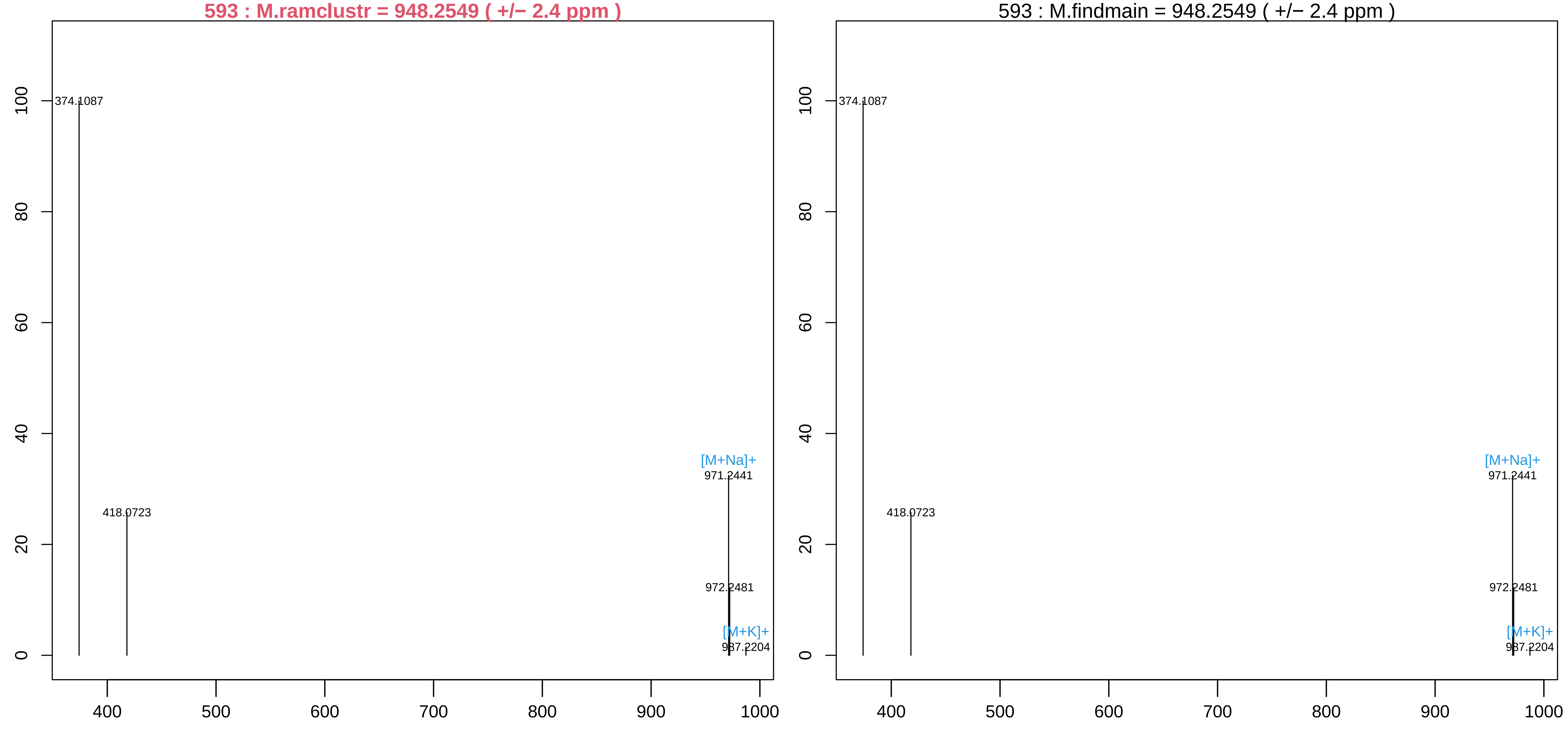
593 : M.ramclustr = 948.2549 (+/− 2.4 ppm)
374.1087
418.0723
[M+Na]+
971.2441
972.2481
[M+K]+
987.2204
0
20
40
60
80
100
400
500
600
700
800
900
1000
593 : M.findmain = 948.2549 (+/− 2.4 ppm)
374.1087
418.0723
[M+Na]+
971.2441
972.2481
[M+K]+
987.2204
0
20
40
60
80
100
400
500
600
700
800
900
1000

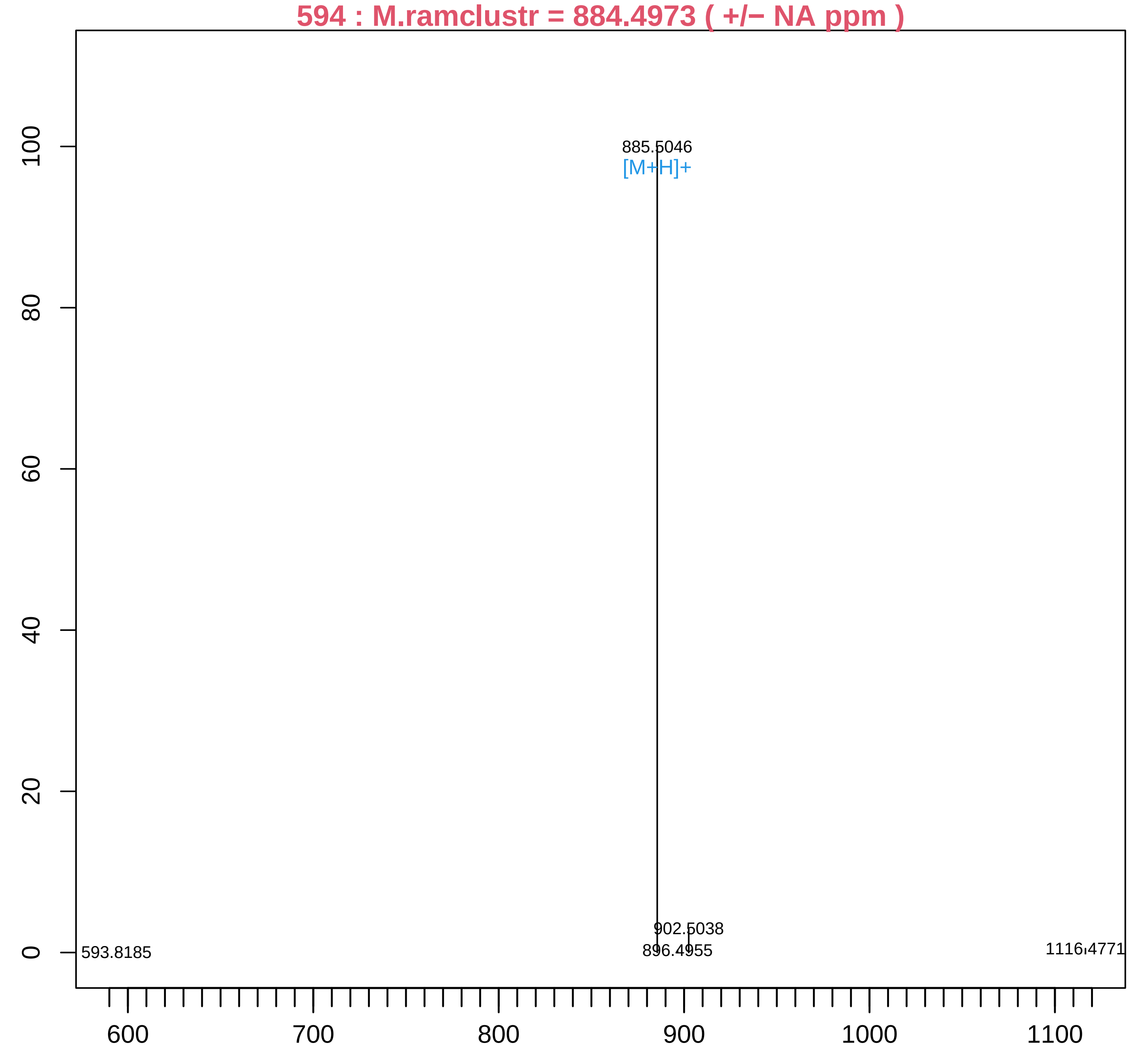
594 : M.ramclustr = 884.4973 (+/− NA ppm)
885.5046
[M+H]+
902.5038
896.4955
593.8185
1116.4771
0
20
40
60
80
100
600
700
800
900
1000
1100

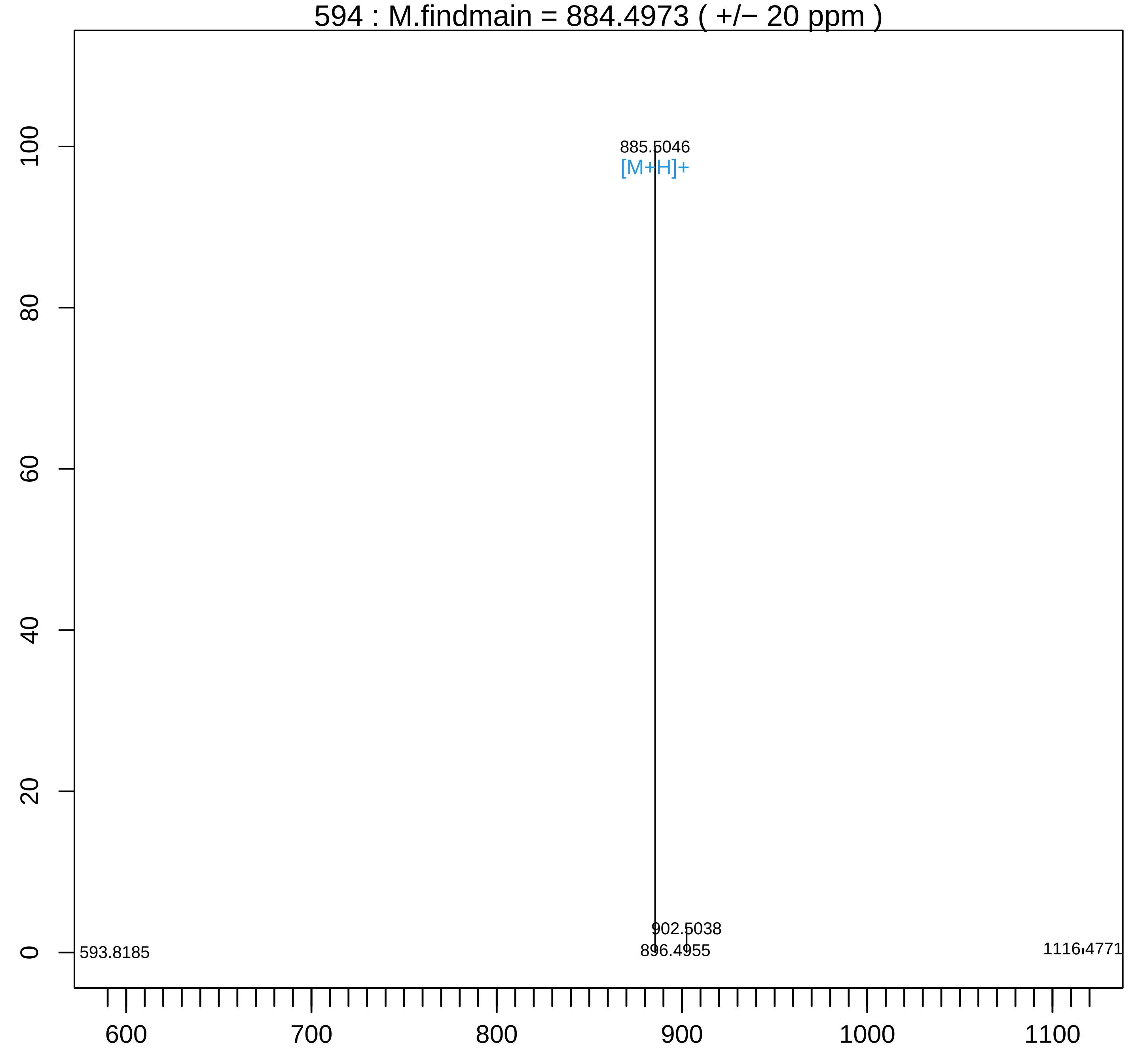
594 : M.findmain = 884.4973 (+/− 20 ppm)
885.5046
[M+H]+
902.5038
896.4955
593.8185
1116.4771
0
20
40
60
80
100
600
700
800
900
1000
1100

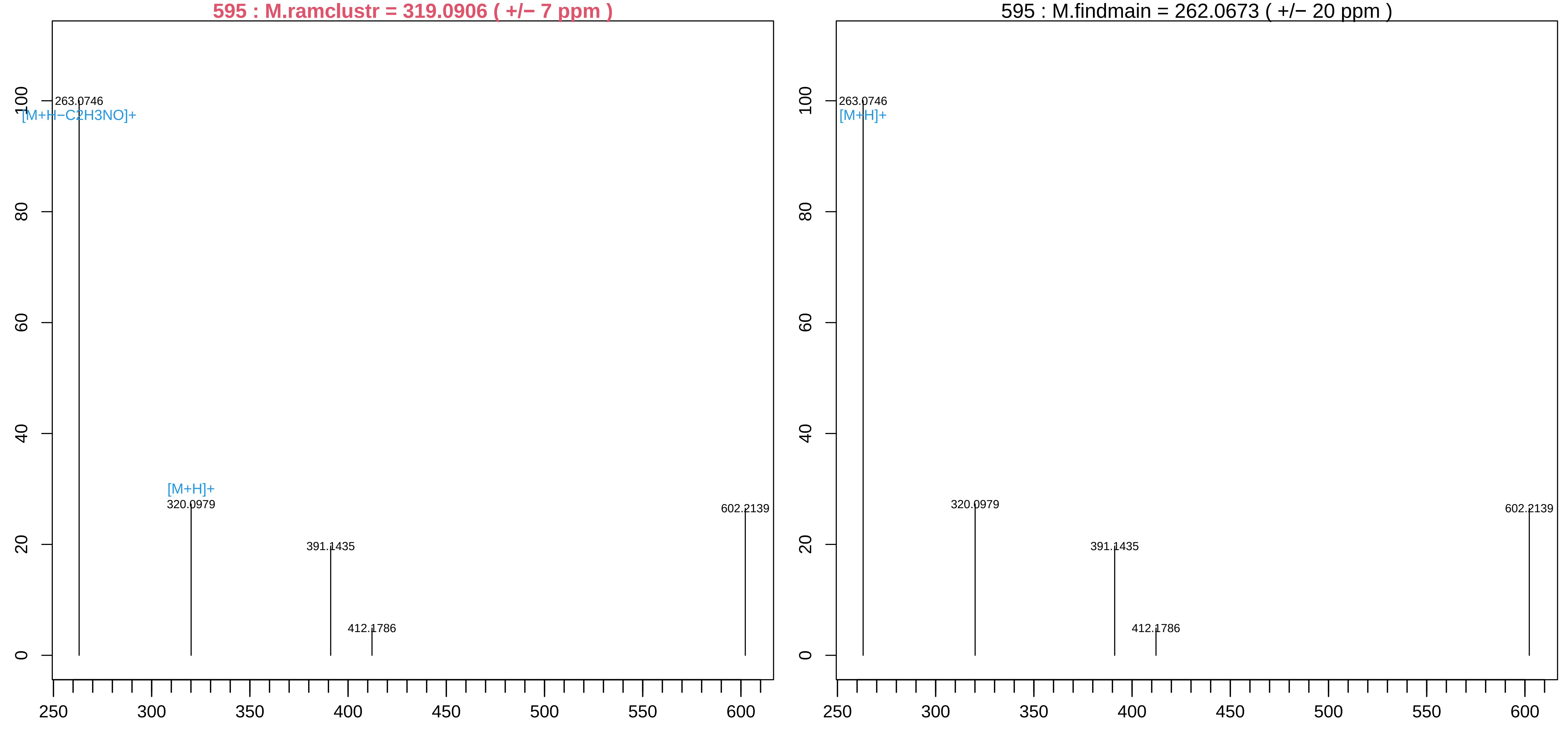

595 : M.ramclustr = 319.0906 (+/− 7 ppm)
263.0746
[M+H−C2H3NO]+
[M+H]+
320.0979
391.1435
412.1786
602.2139
0
20
40
60
80
100
250
300
350
400
450
500
550
600
595 : M.findmain = 262.0673 (+/− 20 ppm)
263.0746
[M+H]+
320.0979
391.1435
412.1786
602.2139
0
20
40
60
80
100
250
300
350
400
450
500
550
600

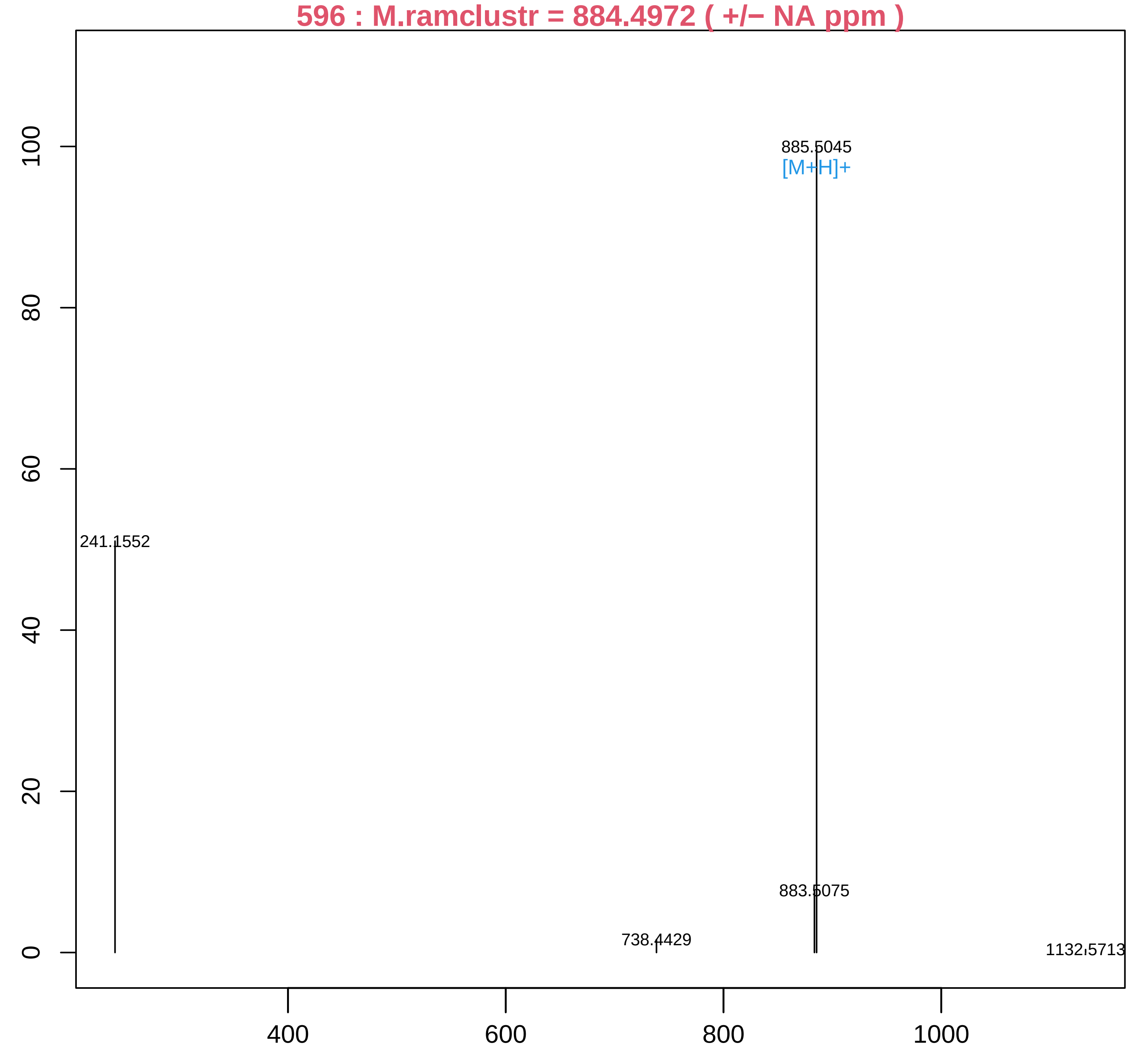
596 : M.ramclustr = 884.4972 (+/− NA ppm)
885.5045
[M+H]+
241.1552
883.5075
738.4429
1132.5713
0
20
40
60
80
100
400
600
800
1000

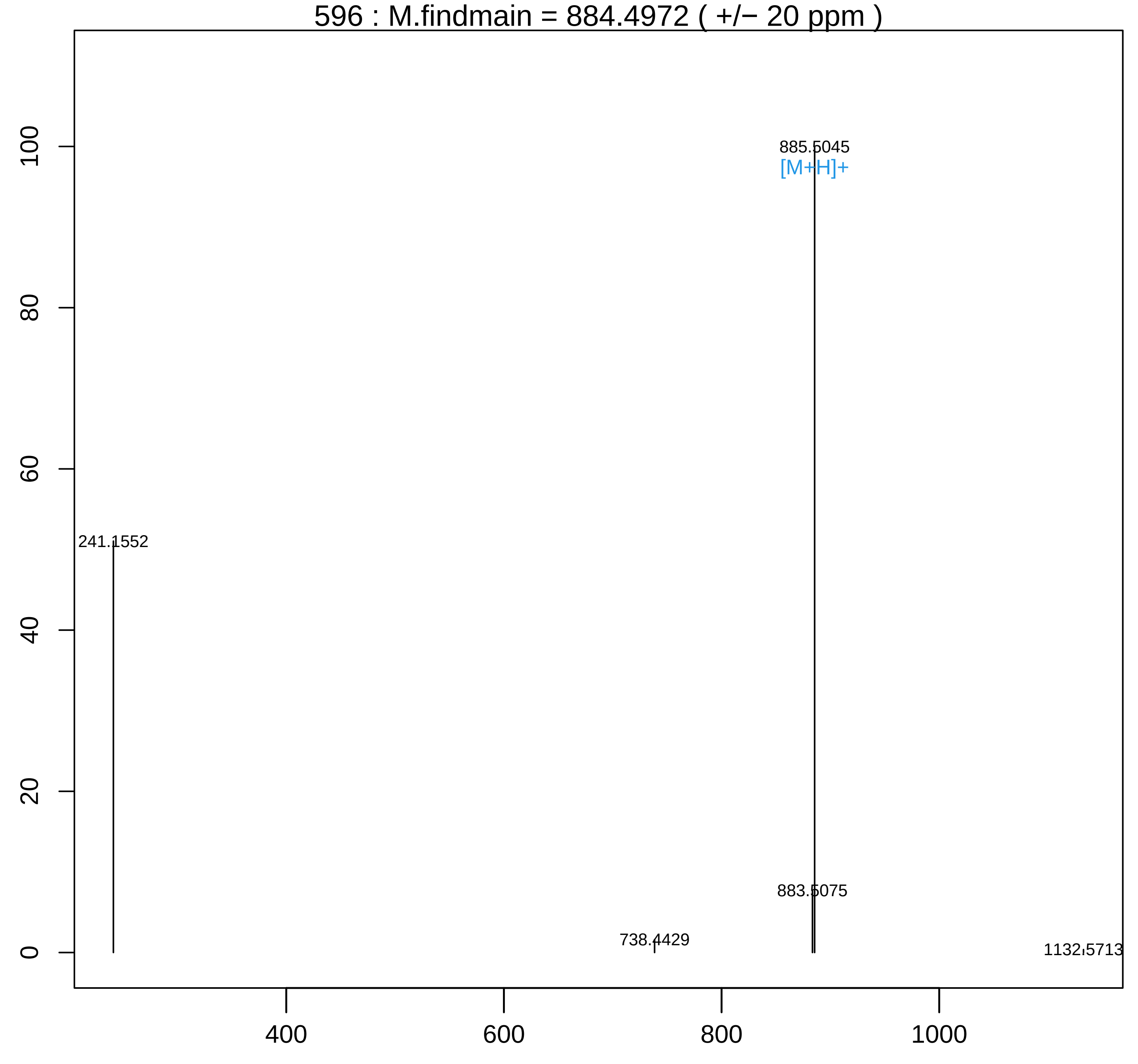
596 : M.findmain = 884.4972 (+/− 20 ppm)
885.5045
[M+H]+
241.1552
883.5075
738.4429
1132.5713
0
20
40
60
80
100
400
600
800
1000

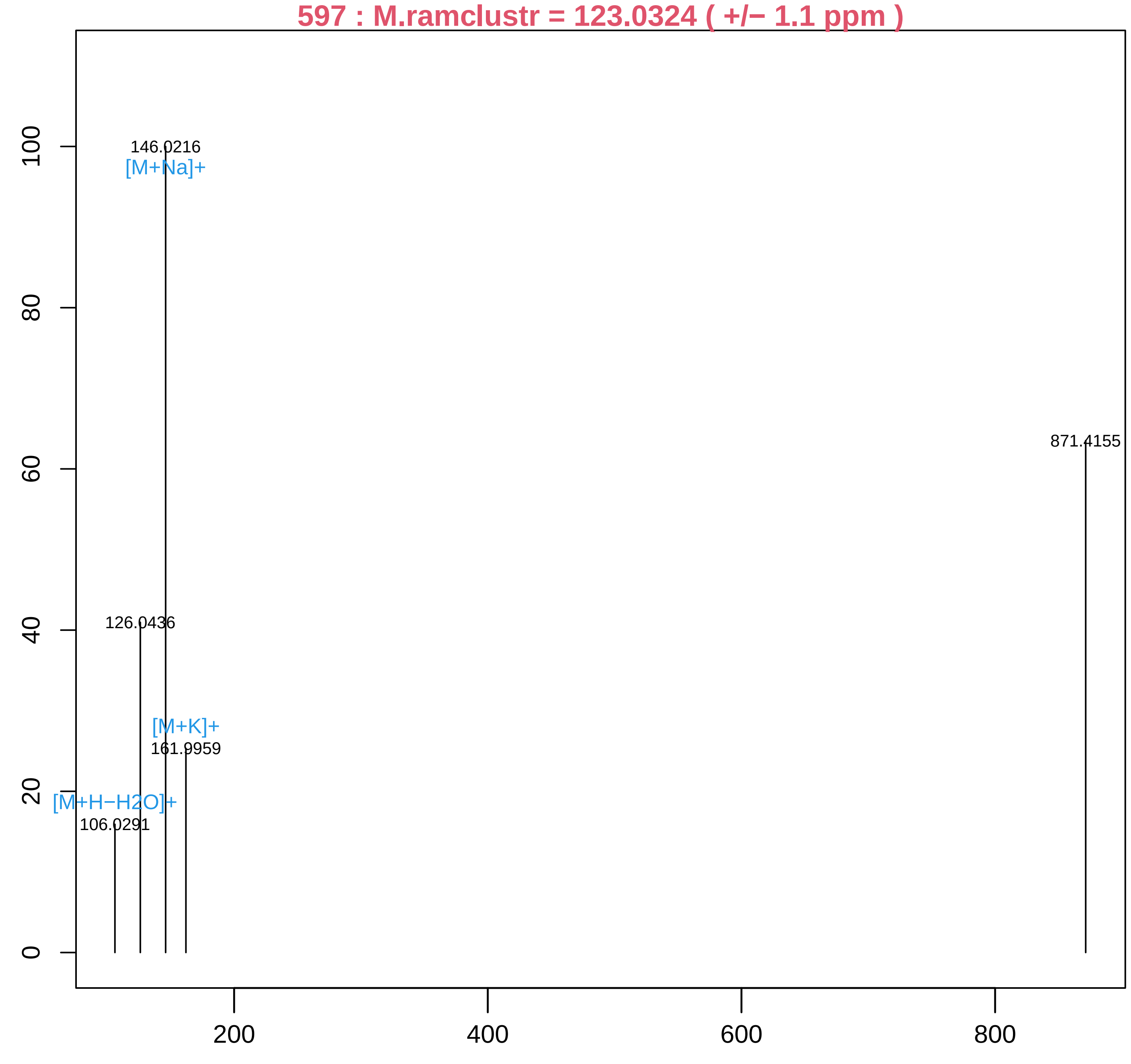

597 : M.ramclustr = 123.0324 (+/− 1.1 ppm)
146.0216
[M+Na]+
126.0436
[M+K]+
161.9959
[M+H−H2O]+
106.0291
871.4155
0
20
40
60
80
100
200
400
600
800

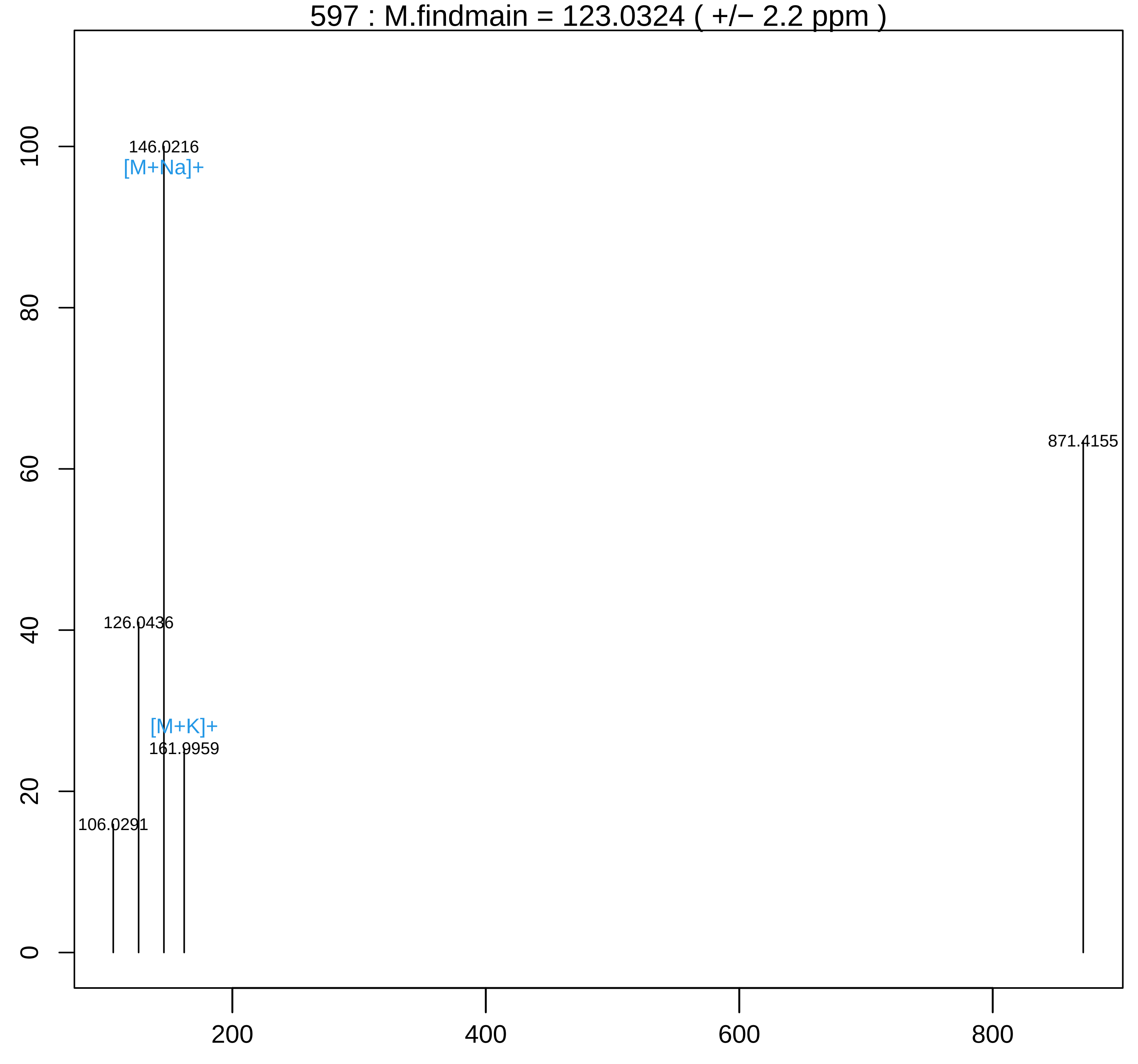

597 : M.findmain = 123.0324 (+/− 2.2 ppm)
146.0216
[M+Na]+
126.0436
[M+K]+
161.9959
106.0291
871.4155
0
20
40
60
80
100
200
400
600
800

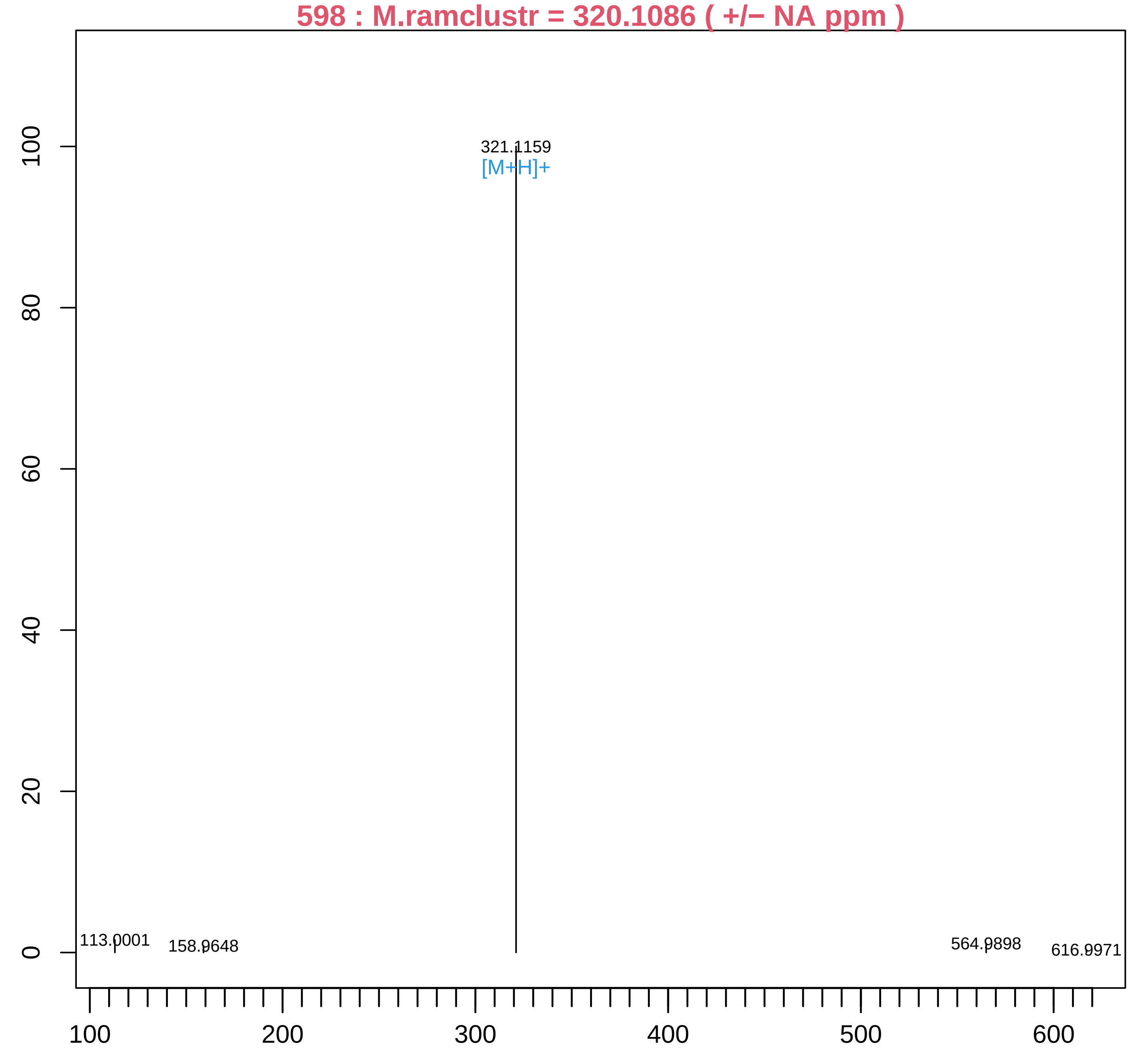
598 : M.ramclustr = 320.1086 (+/− NA ppm)
321.1159
[M+H]+
113.0001
158.9648
564.9898
616.9971
0
20
40
60
80
100
100
200
300
400
500
600

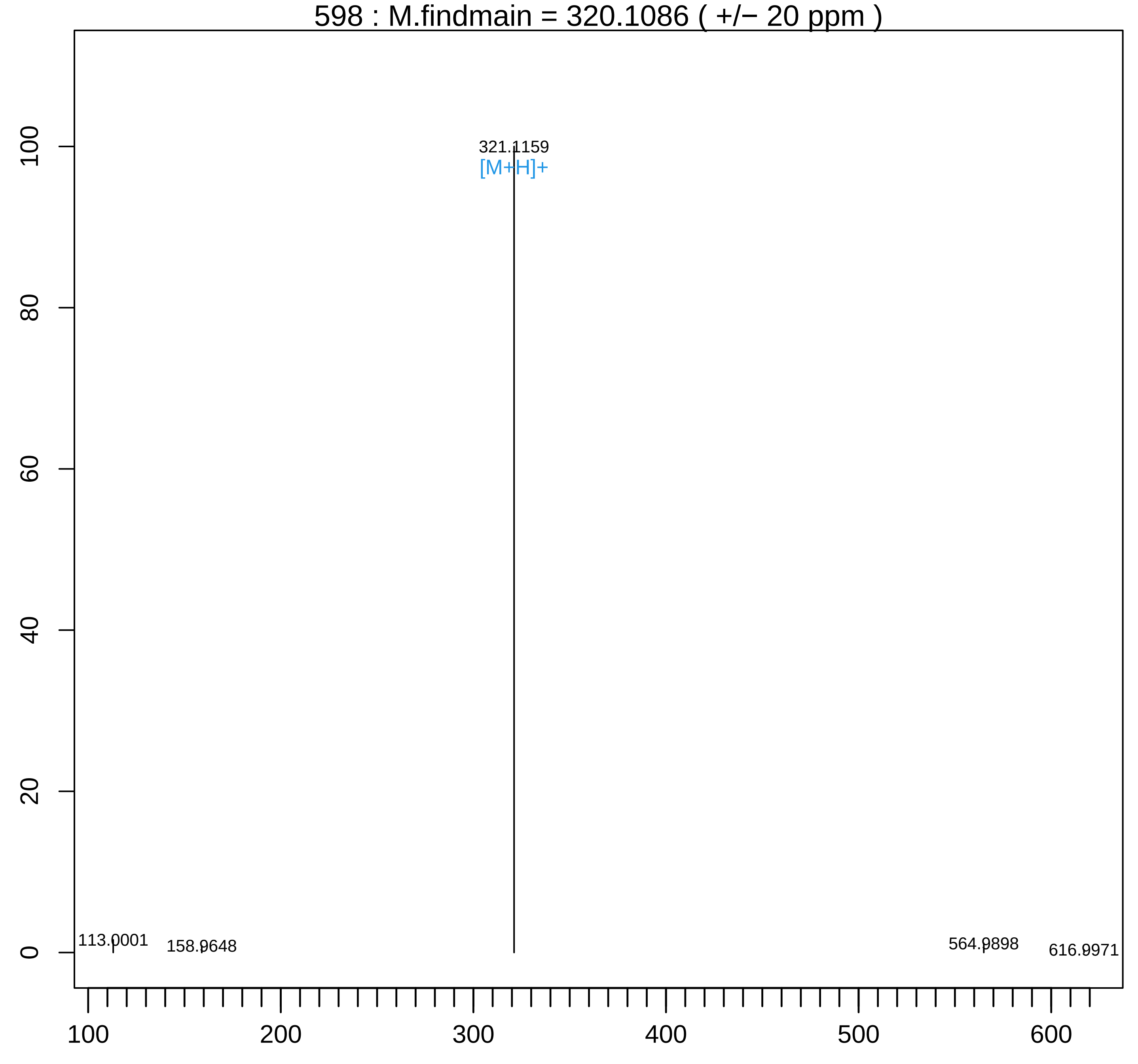
598 : M.findmain = 320.1086 (+/− 20 ppm)
321.1159
[M+H]+
113.0001
158.9648
564.9898
616.9971
0
20
40
60
80
100
100
200
300
400
500
600

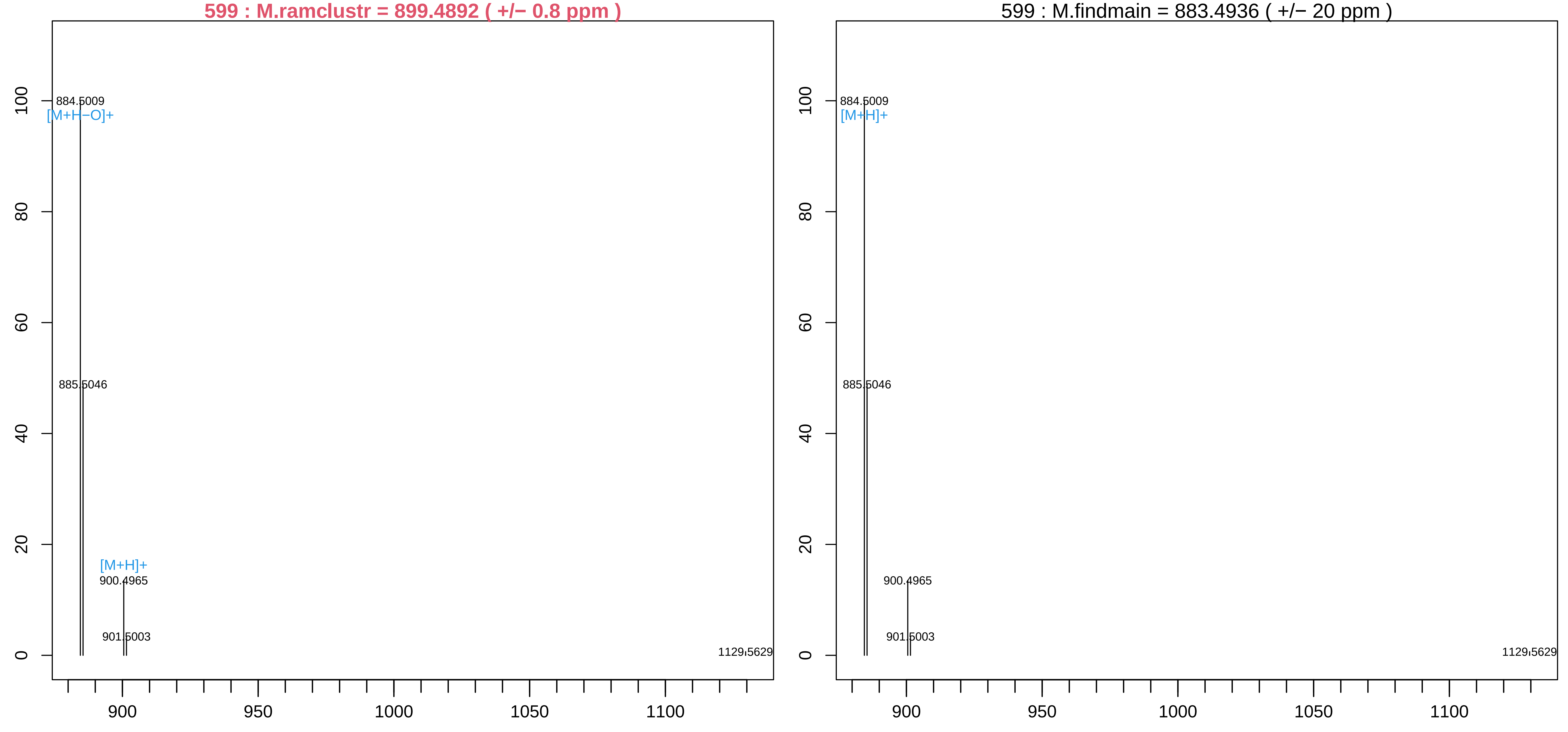

599 : M.ramclustr = 899.4892 (+/− 0.8 ppm)
884.5009
[M+H−O]+
885.5046
[M+H]+
900.4965
901.5003
1129.5629
599 : M.findmain = 883.4936 (+/− 20 ppm)
884.5009
[M+H]+
885.5046
900.4965
901.5003
1129.5629
0
20
40
60
80
100
900
950
1000
1050
1100

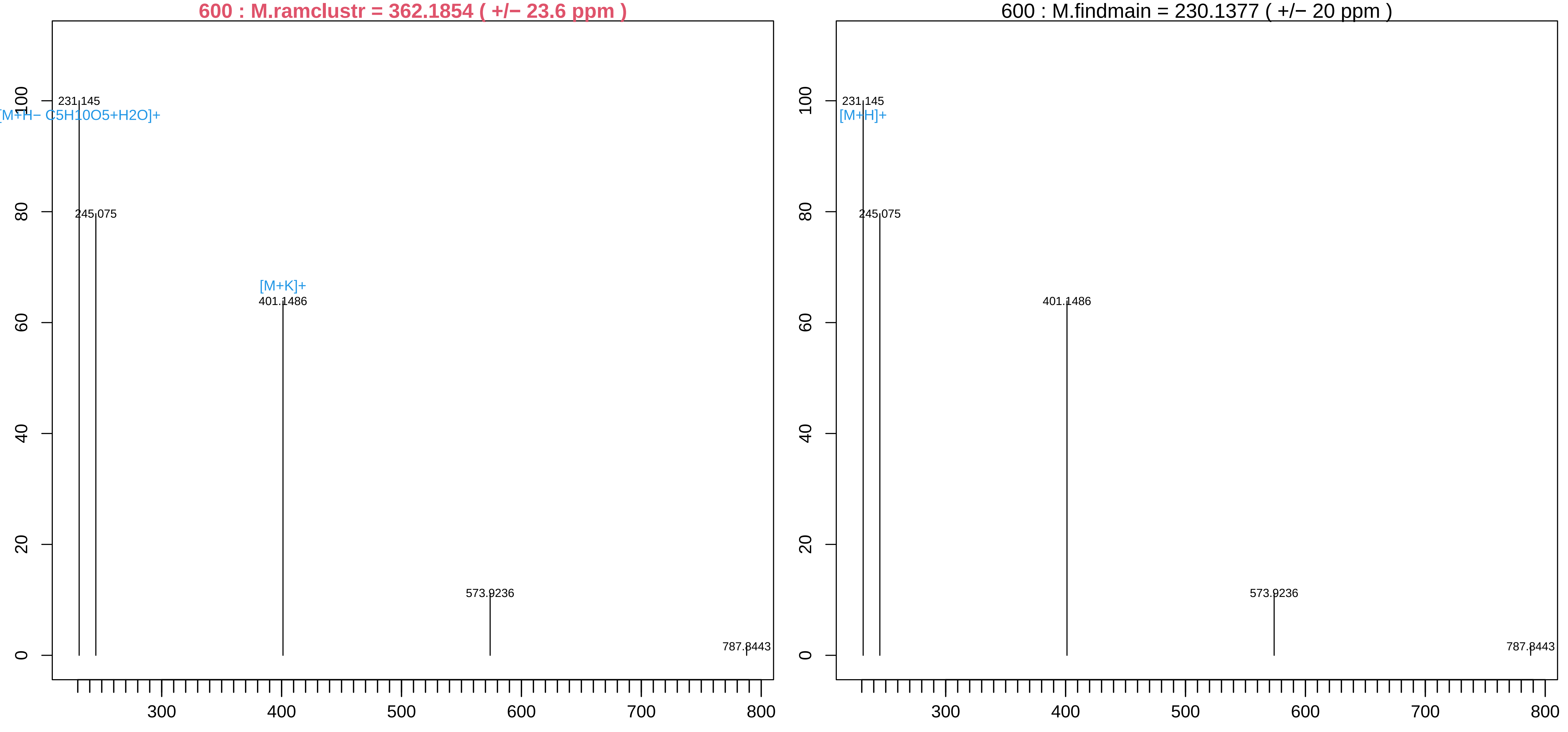
600 : M.ramclustr = 362.1854 (+/− 23.6 ppm)
231.145
[M+H− C5H10O5+H2O]+
245.075
[M+K]+
401.1486
573.9236
787.8443
0
20
40
60
80
100
300
400
500
600
700
800
600 : M.findmain = 230.1377 (+/− 20 ppm)
231.145
[M+H]+
245.075
401.1486
573.9236
787.8443
0
20
40
60
80
100
300
400
500
600
700
800

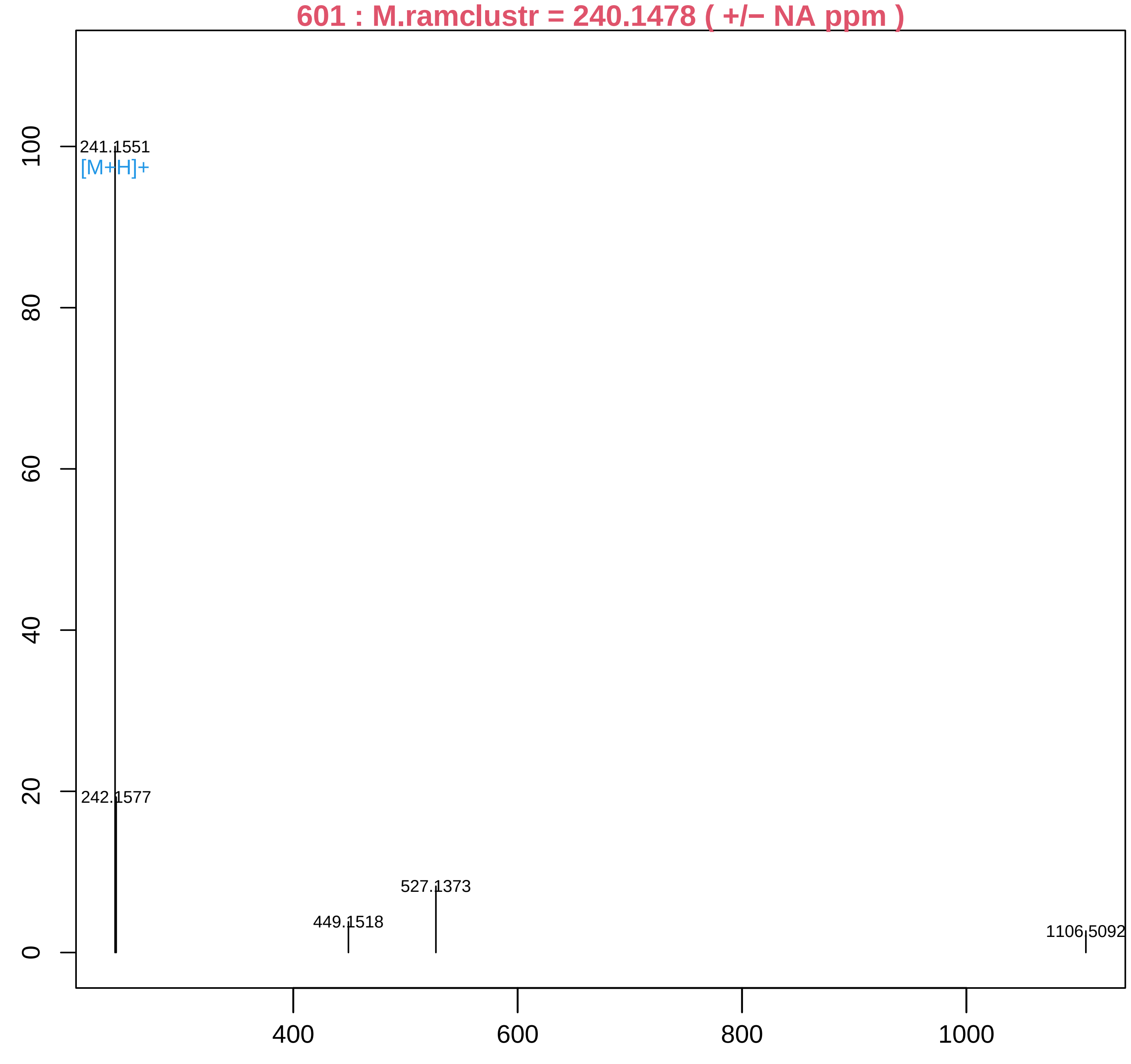

601 : M.ramclustr = 240.1478 (+/− NA ppm)
241.1551
[M+H]+
242.1577
449.1518
527.1373
1106.5092
0
20
40
60
80
100
400
600
800
1000

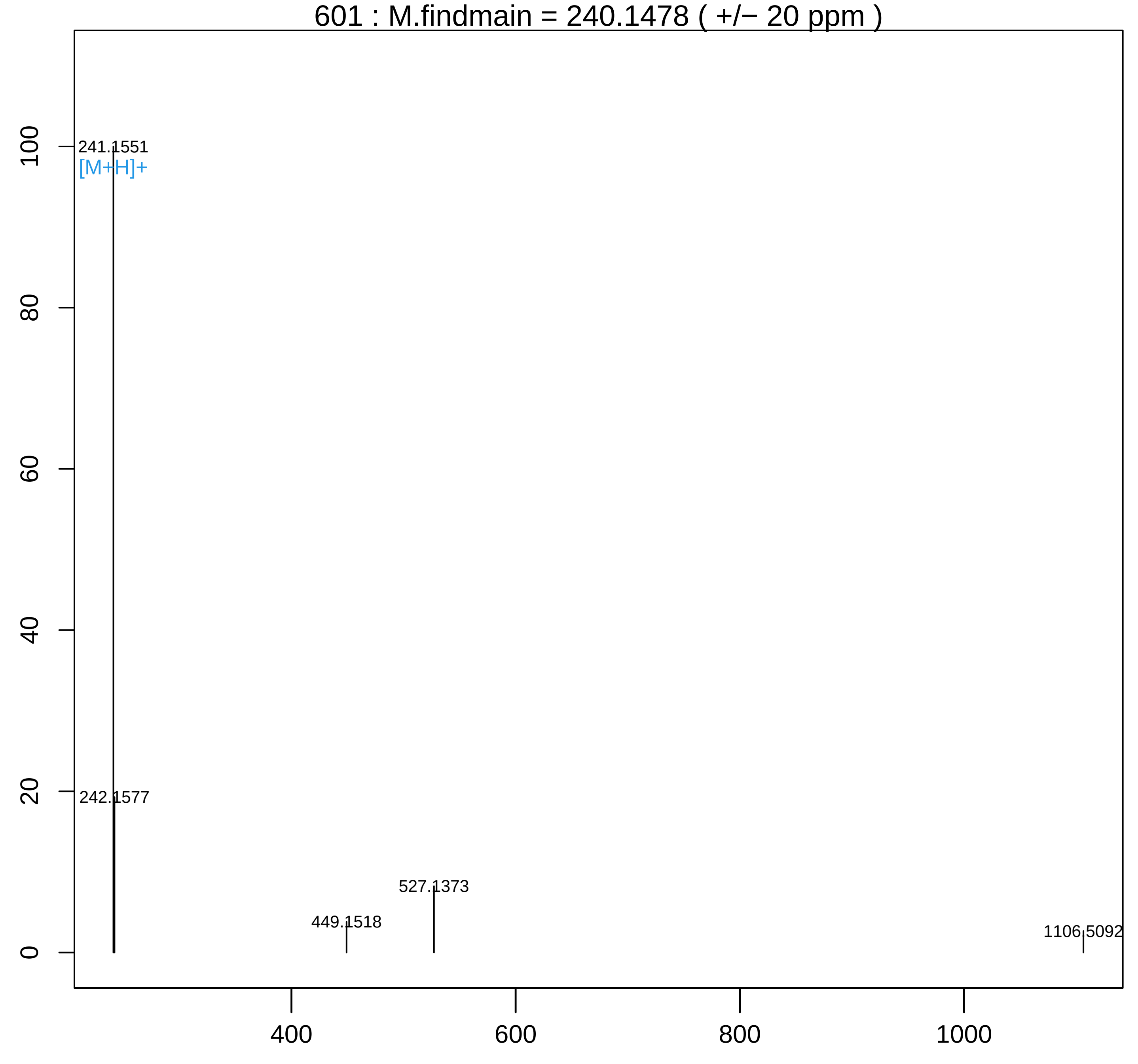

601 : M.findmain = 240.1478 (+/− 20 ppm)
241.1551
[M+H]+
242.1577
449.1518
527.1373
1106.5092
0
20
40
60
80
100
400
600
800
1000

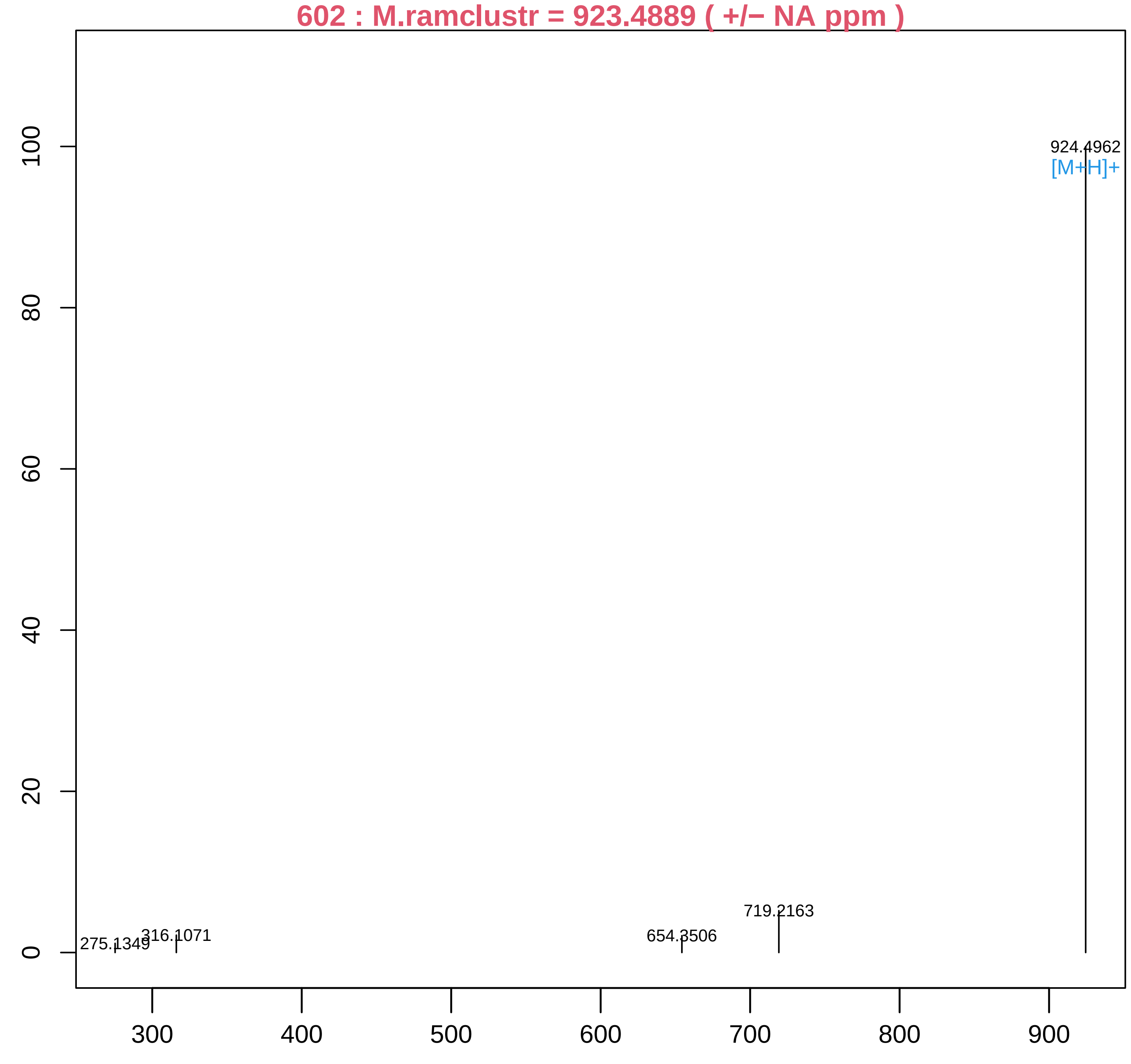
602 : M.ramclustr = 923.4889 (+/− NA ppm)
924.4962
[M+H]+
719.2163
654.3506
316.1071
275.1349
0
20
40
60
80
100
300
400
500
600
700
800
900

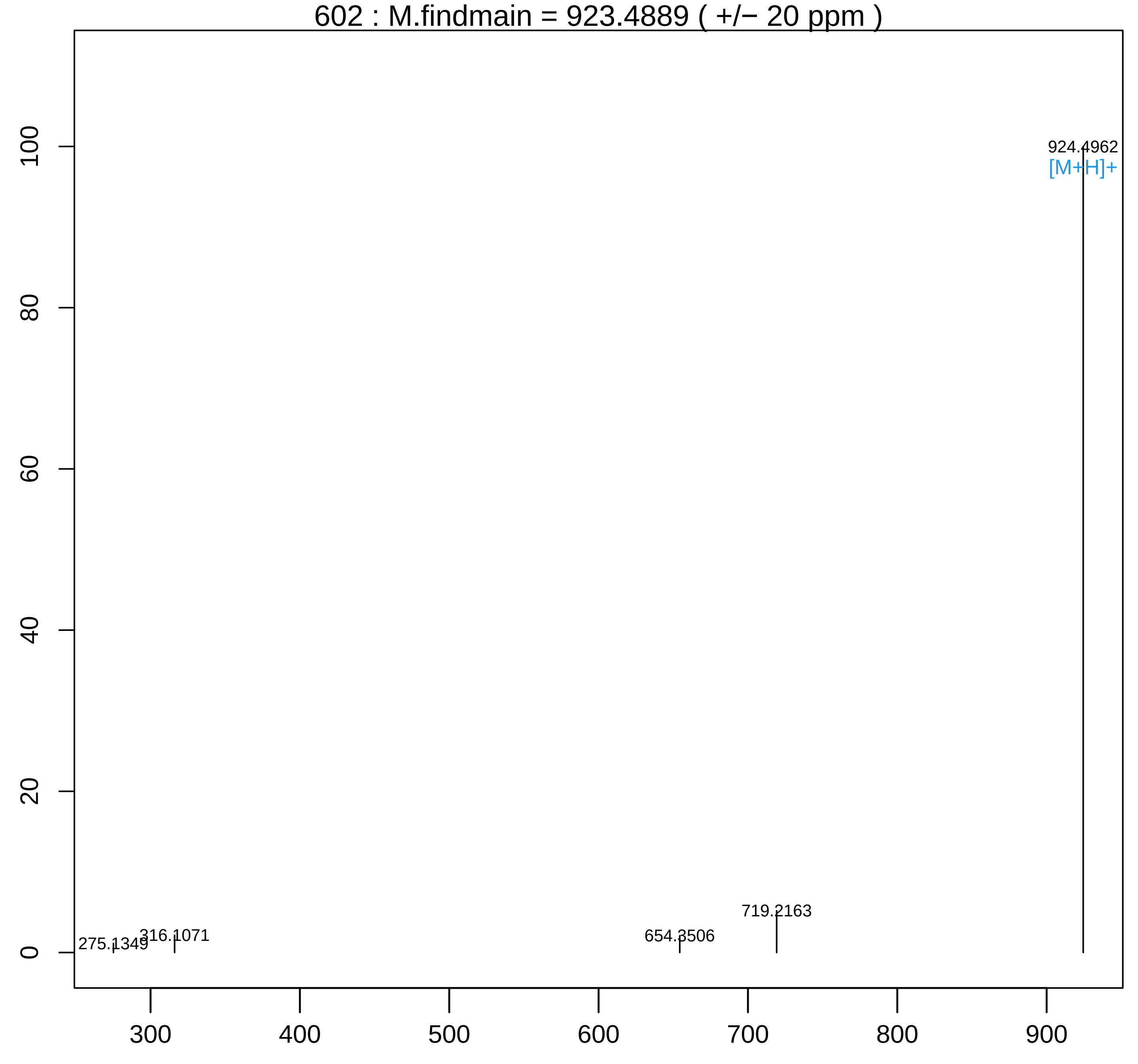
602 : M.findmain = 923.4889 (+/− 20 ppm)
924.4962
[M+H]+
719.2163
654.3506
316.1071
275.1349
0
20
40
60
80
100
300
400
500
600
700
800
900

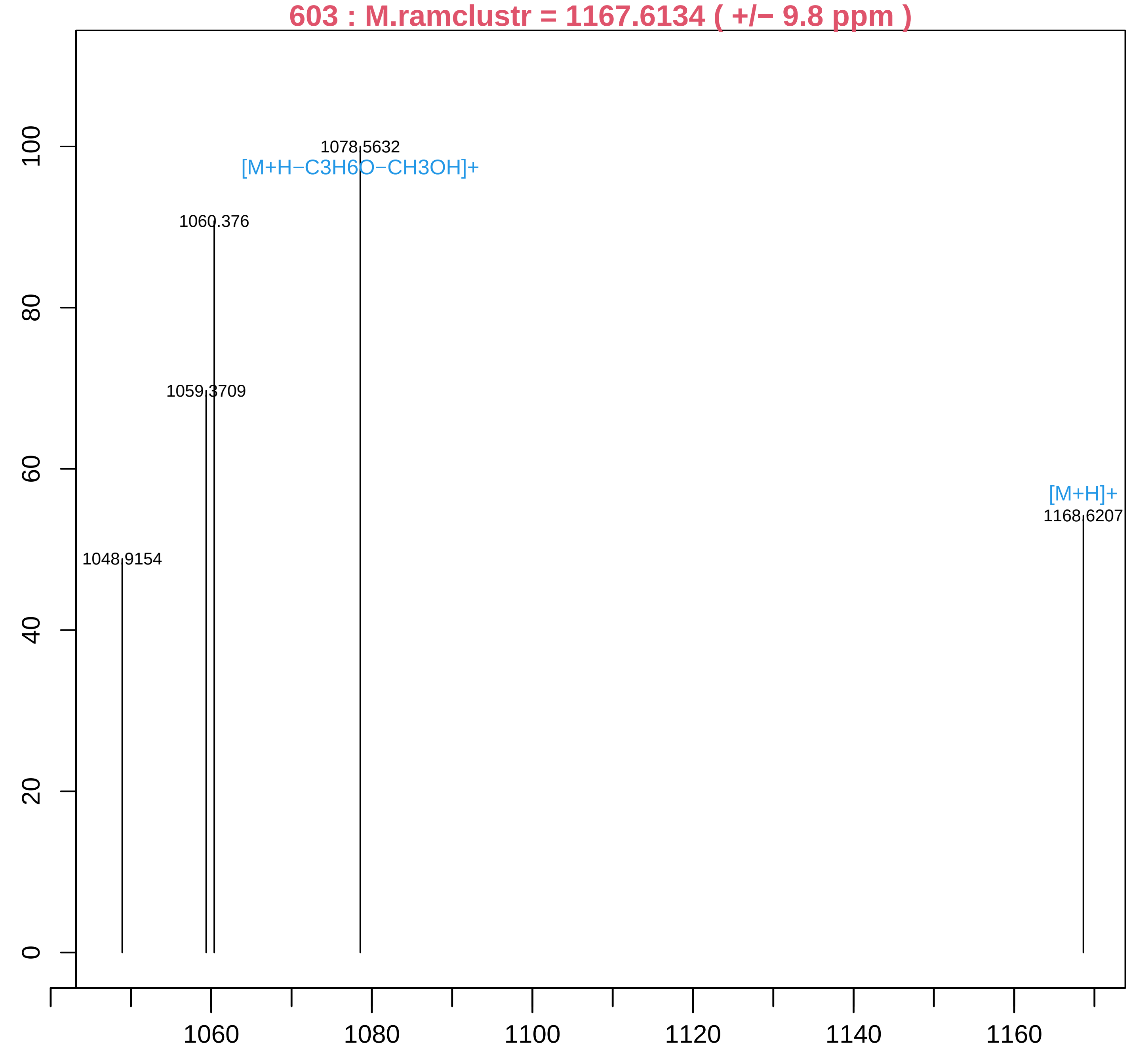
603 : M.ramclustr = 1167.6134 (+/− 9.8 ppm)
1078.5632
[M+H−C3H6O−CH3OH]+
1060.376
1059.3709
1048.9154
[M+H]+
1168.6207
0
20
40
60
80
100
1060
1080
1100
1120
1140
1160

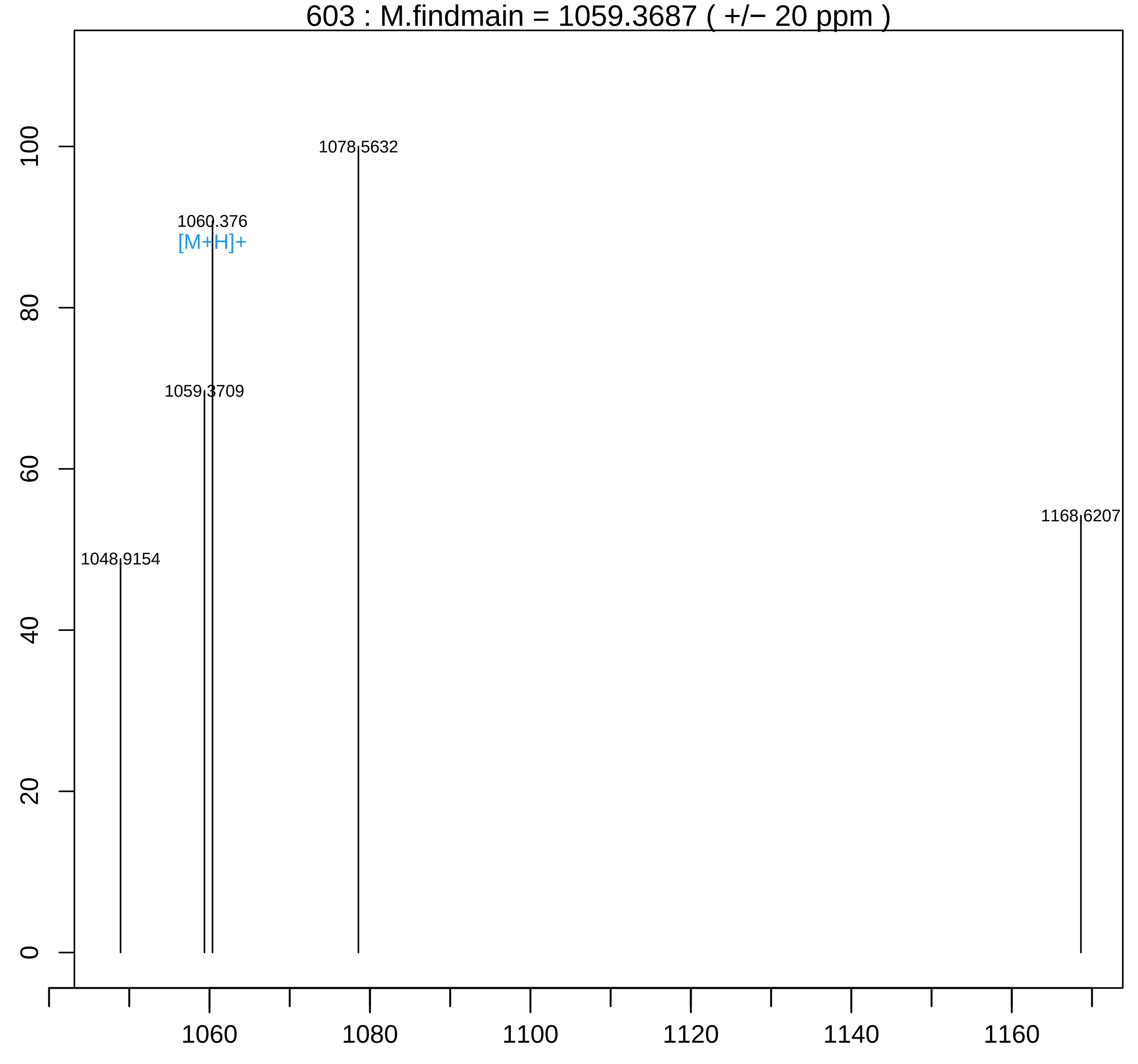
603 : M.findmain = 1059.3687 (+/− 20 ppm)
1078.5632
1060.376
[M+H]+
1059.3709
1048.9154
1168.6207
0
20
40
60
80
100
1060
1080
1100
1120
1140
1160

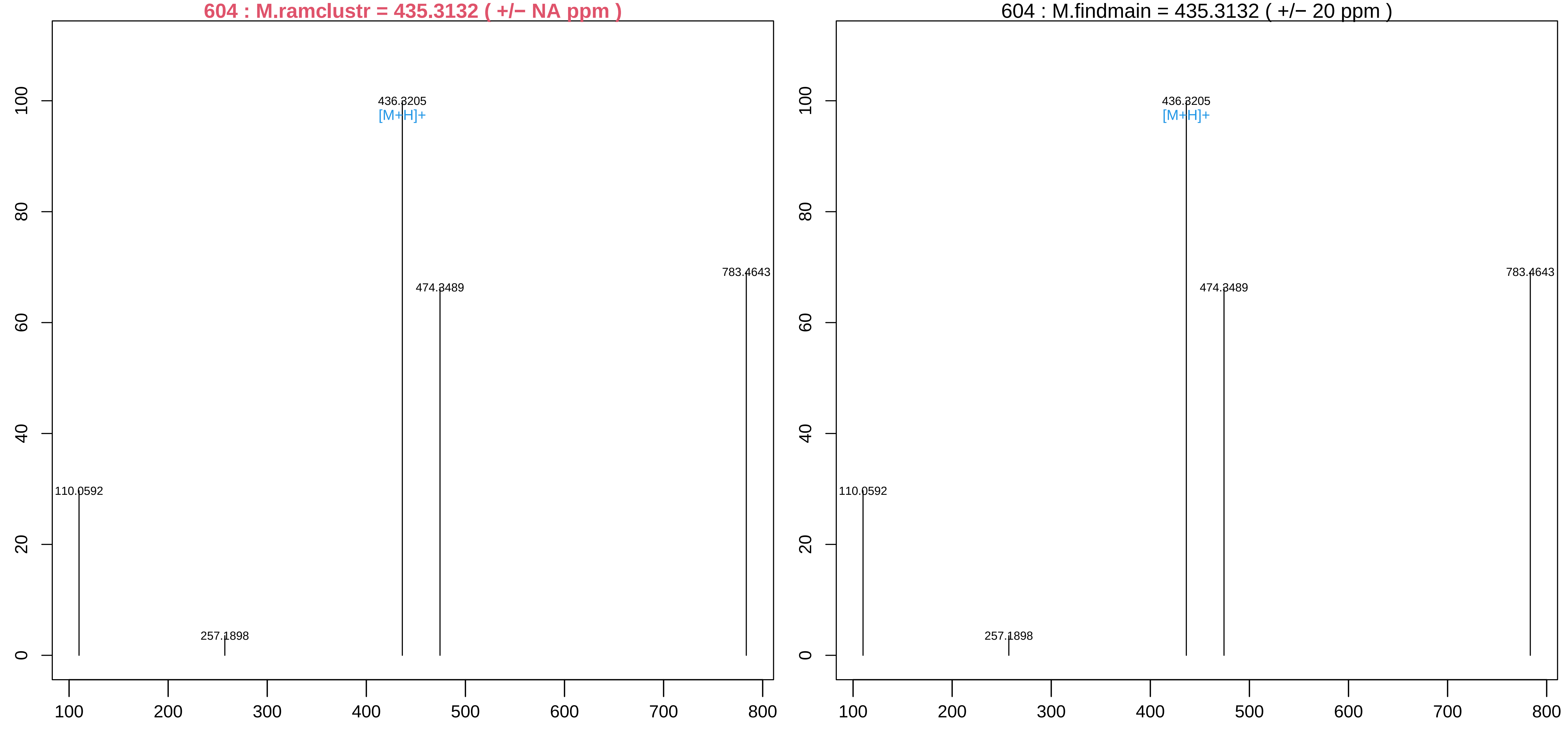
604 : M.ramclustr = 435.3132 (+/− NA ppm)
436.3205
[M+H]+
474.3489
783.4643
110.0592
257.1898
0
20
40
60
80
100
100
200
300
400
500
600
700
800
604 : M.findmain = 435.3132 (+/− 20 ppm)
436.3205
[M+H]+
474.3489
783.4643
110.0592
257.1898
0
20
40
60
80
100
100
200
300
400
500
600
700
800

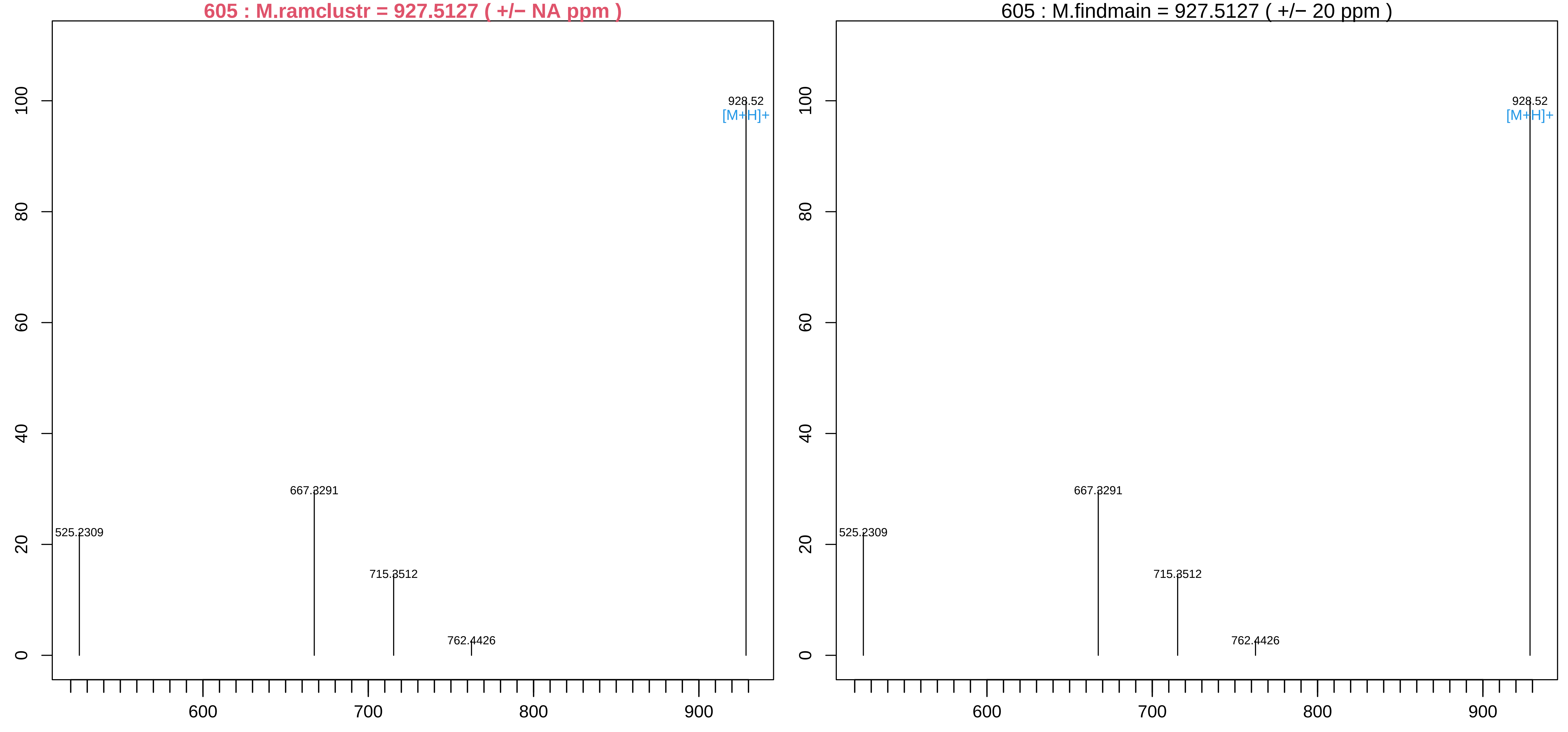
605 : M.ramclustr = 927.5127 (+/− NA ppm)
605 : M.findmain = 927.5127 (+/− 20 ppm)
928.52
[M+H]+
667.3291
525.2309
715.3512
762.4426
0
20
40
60
80
100
600
700
800
900

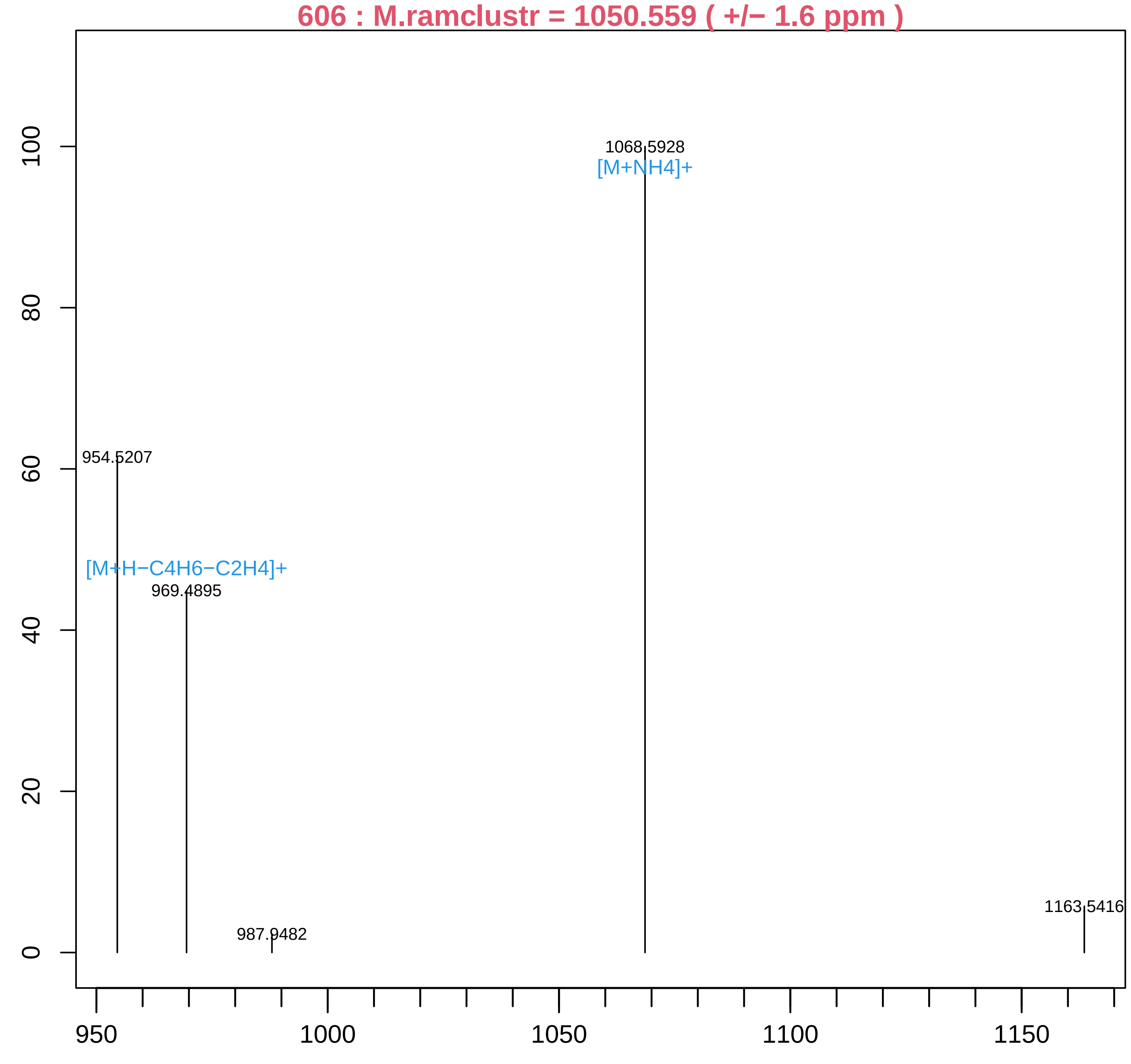
606 : M.ramclustr = 1050.559 (+/− 1.6 ppm)
1068.5928
[M+NH4]+
954.5207
[M+H−C4H6−C2H4]+
969.4895
987.9482
1163.5416
0
20
40
60
80
100
950
1000
1050
1100
1150

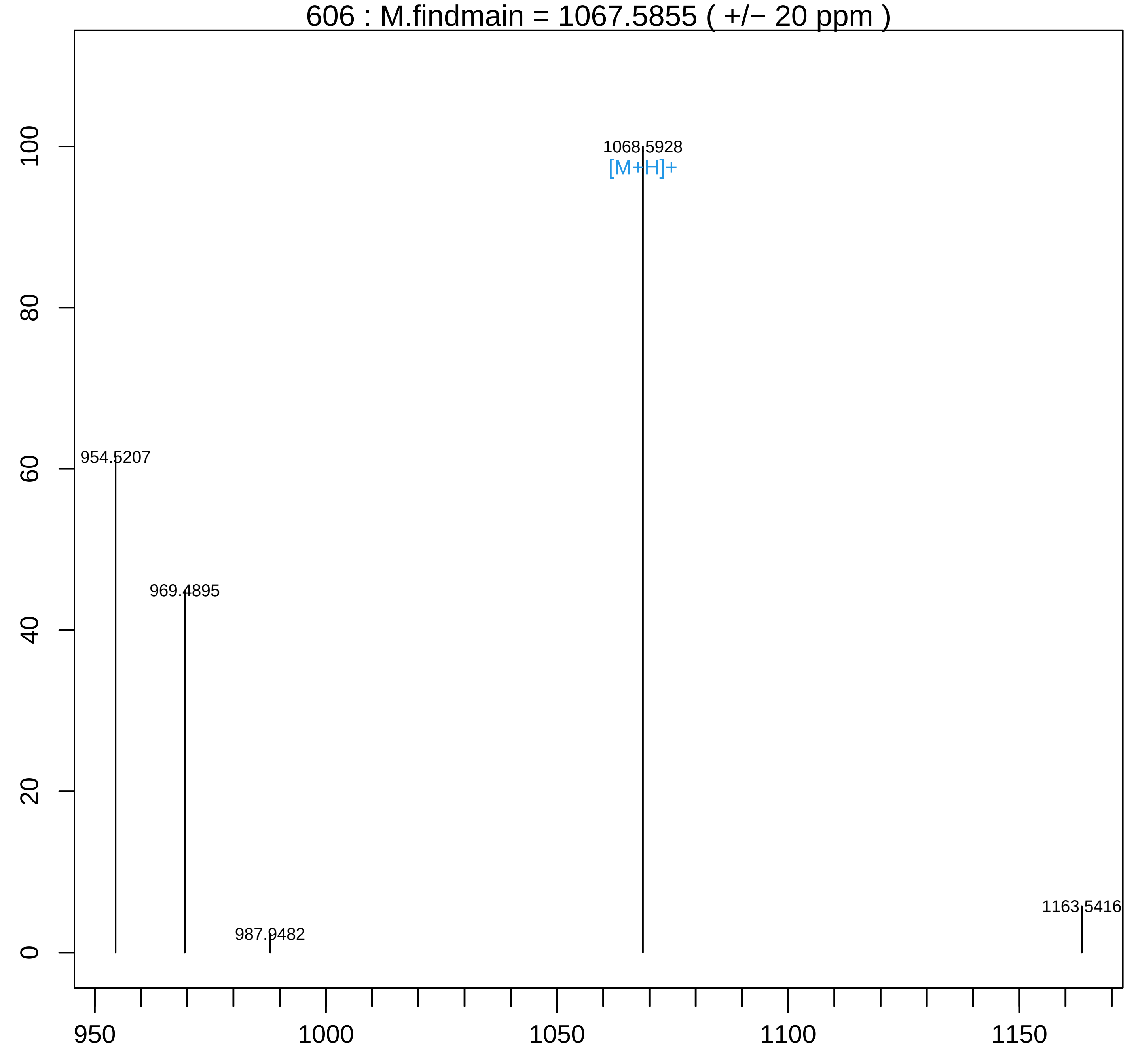
606 : M.findmain = 1067.5855 (+/− 20 ppm)
1068.5928
[M+H]+
954.5207
969.4895
987.9482
1163.5416
0
20
40
60
80
100
950
1000
1050
1100
1150

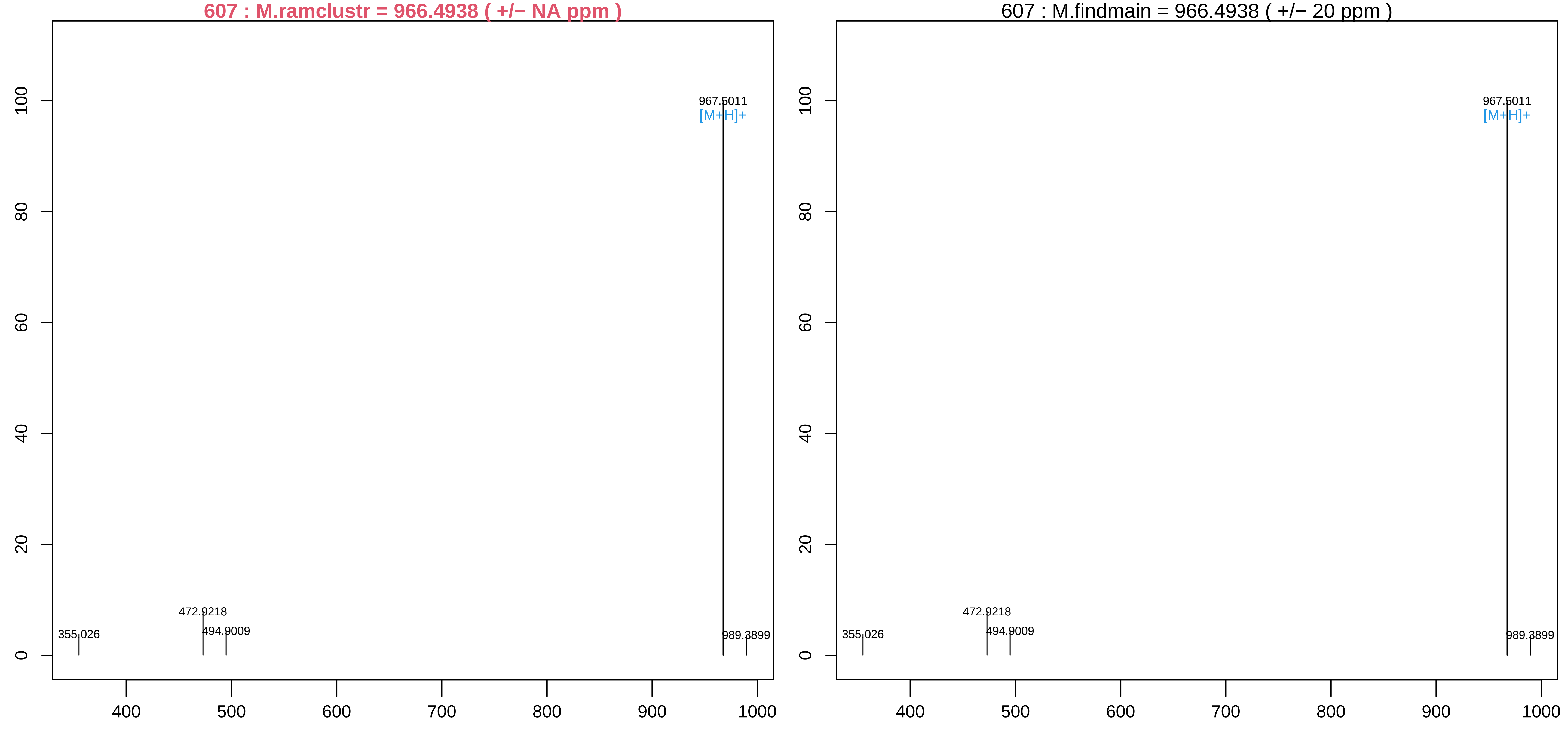
607 : M.ramclustr = 966.4938 (+/− NA ppm)
967.5011
[M+H]+
472.9218
494.9009
355.026
989.3899
607 : M.findmain = 966.4938 (+/− 20 ppm)
967.5011
[M+H]+
472.9218
494.9009
355.026
989.3899

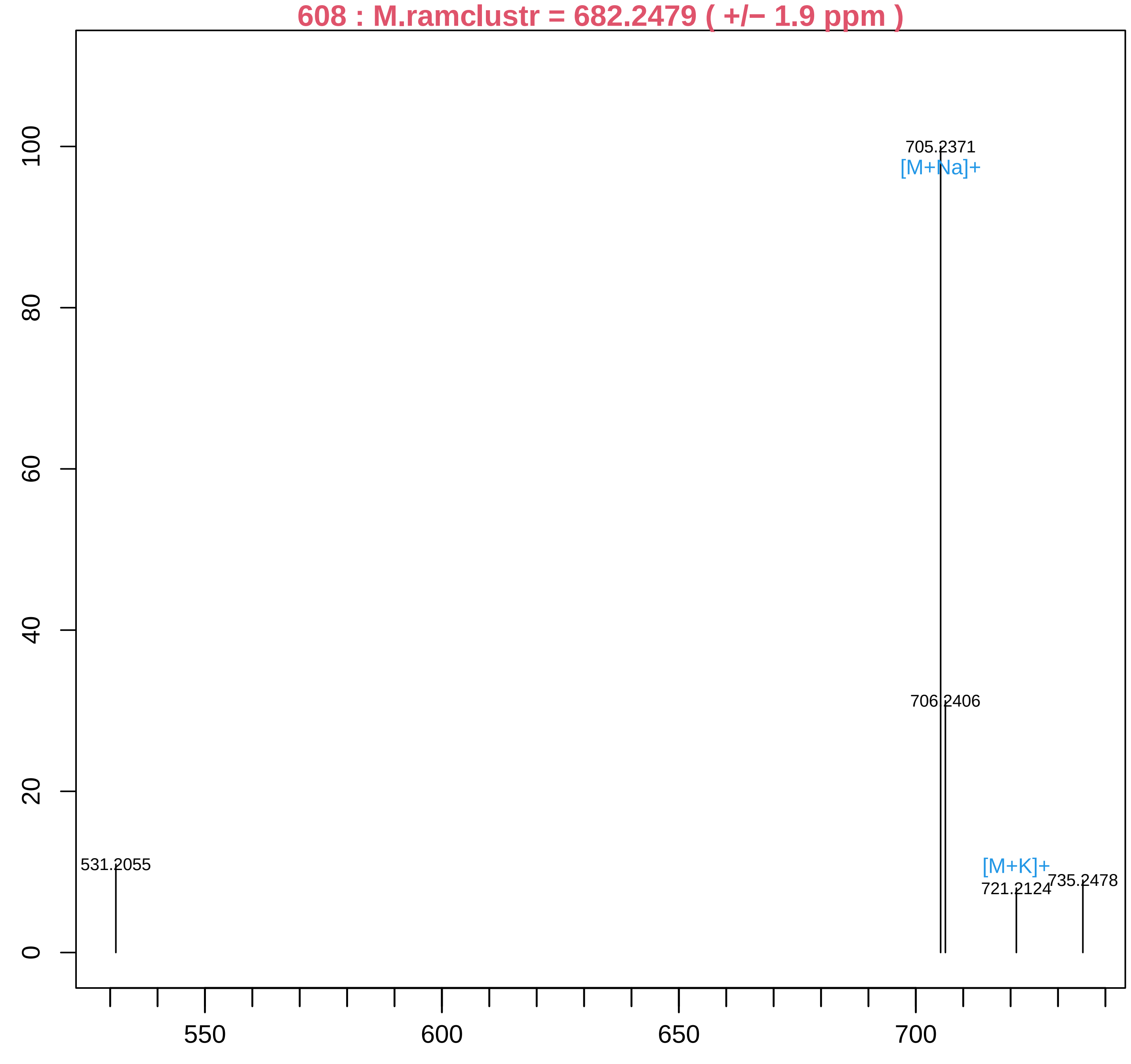
608 : M.ramclustr = 682.2479 (+/− 1.9 ppm)
705.2371
[M+Na]+
706.2406
531.2055
[M+K]+
721.2124
735.2478
0
20
40
60
80
100
550
600
650
700

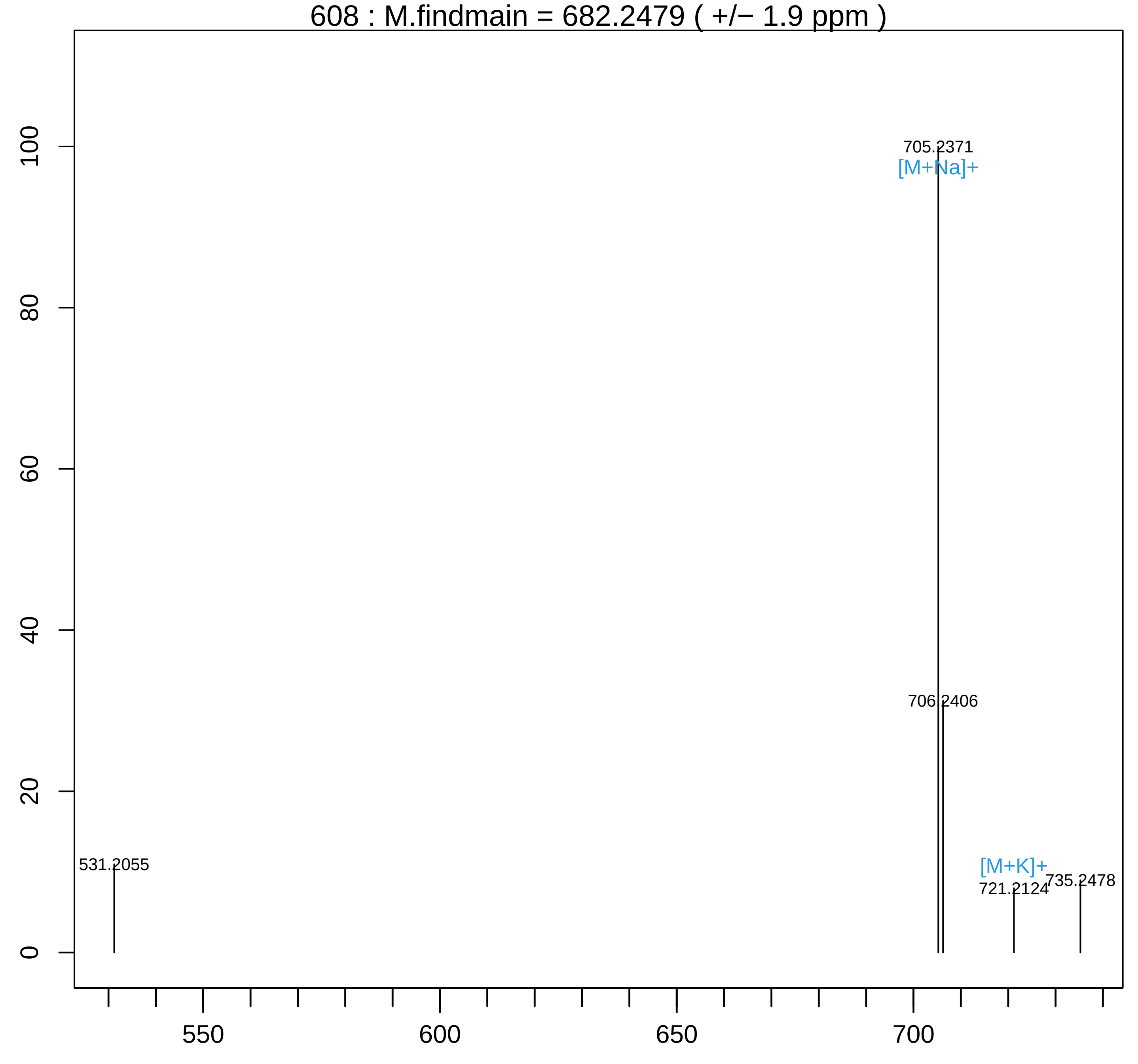
608 : M.findmain = 682.2479 (+/− 1.9 ppm)
705.2371
[M+Na]+
706.2406
531.2055
[M+K]+
721.2124
735.2478
0
20
40
60
80
100
550
600
650
700

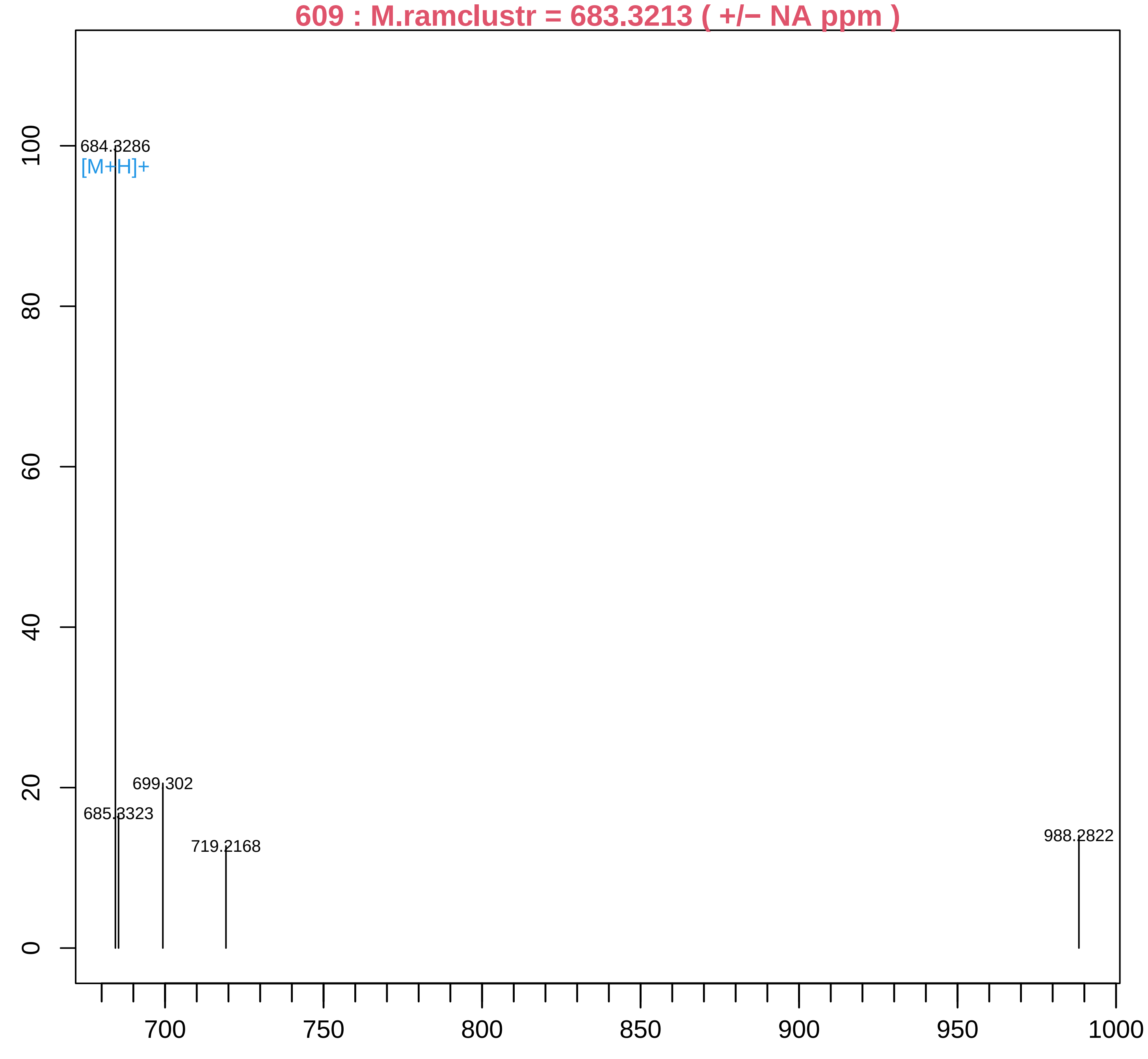

609 : M.ramclustr = 683.3213 (+/− NA ppm)
684.3286
[M+H]+
699.302
685.3323
719.2168
988.2822
0
20
40
60
80
100
700
750
800
850
900
950
1000

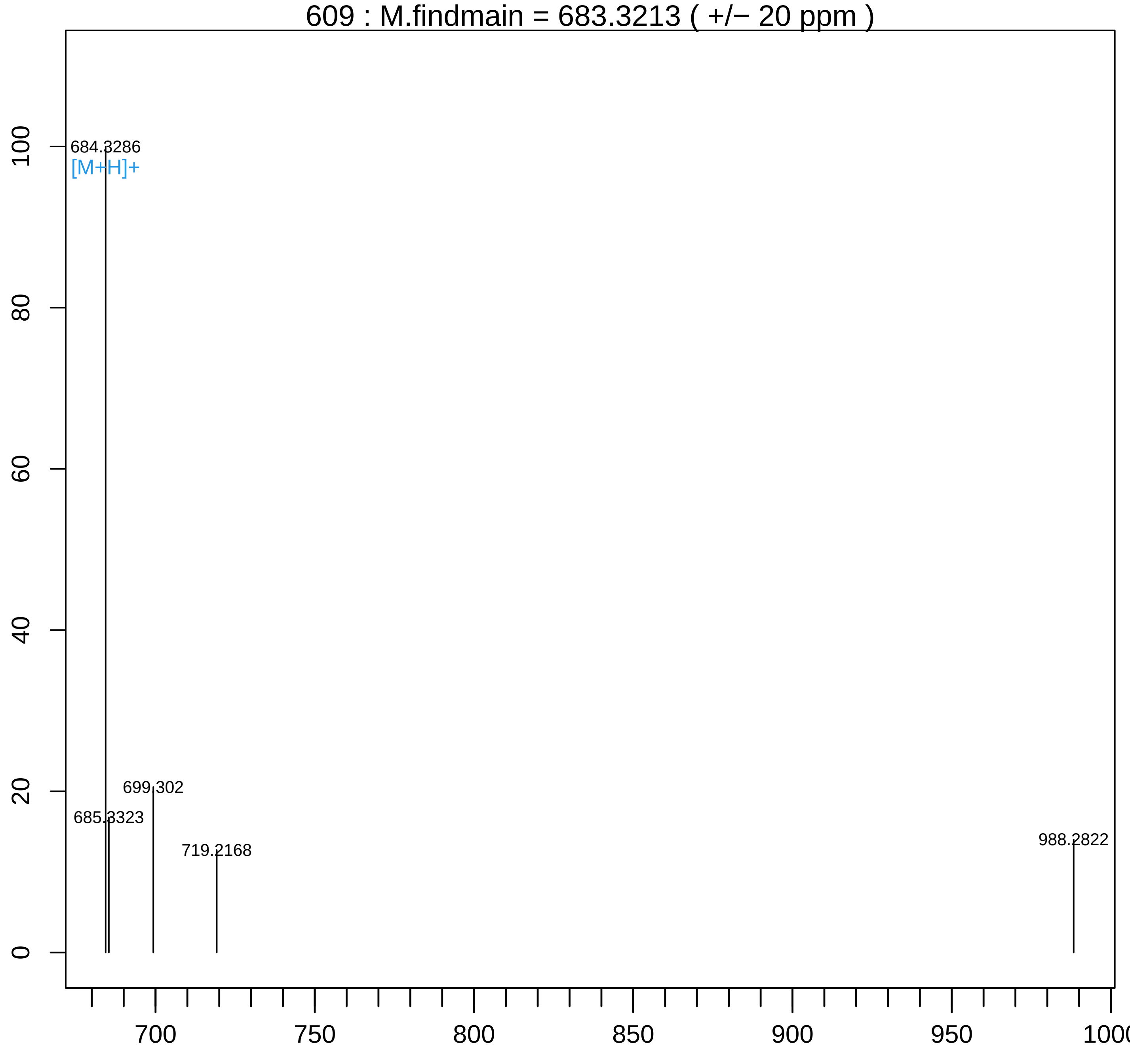

609 : M.findmain = 683.3213 (+/− 20 ppm)
684.3286
[M+H]+
699.302
685.3323
719.2168
988.2822
0
20
40
60
80
100
700
750
800
850
900
950
1000

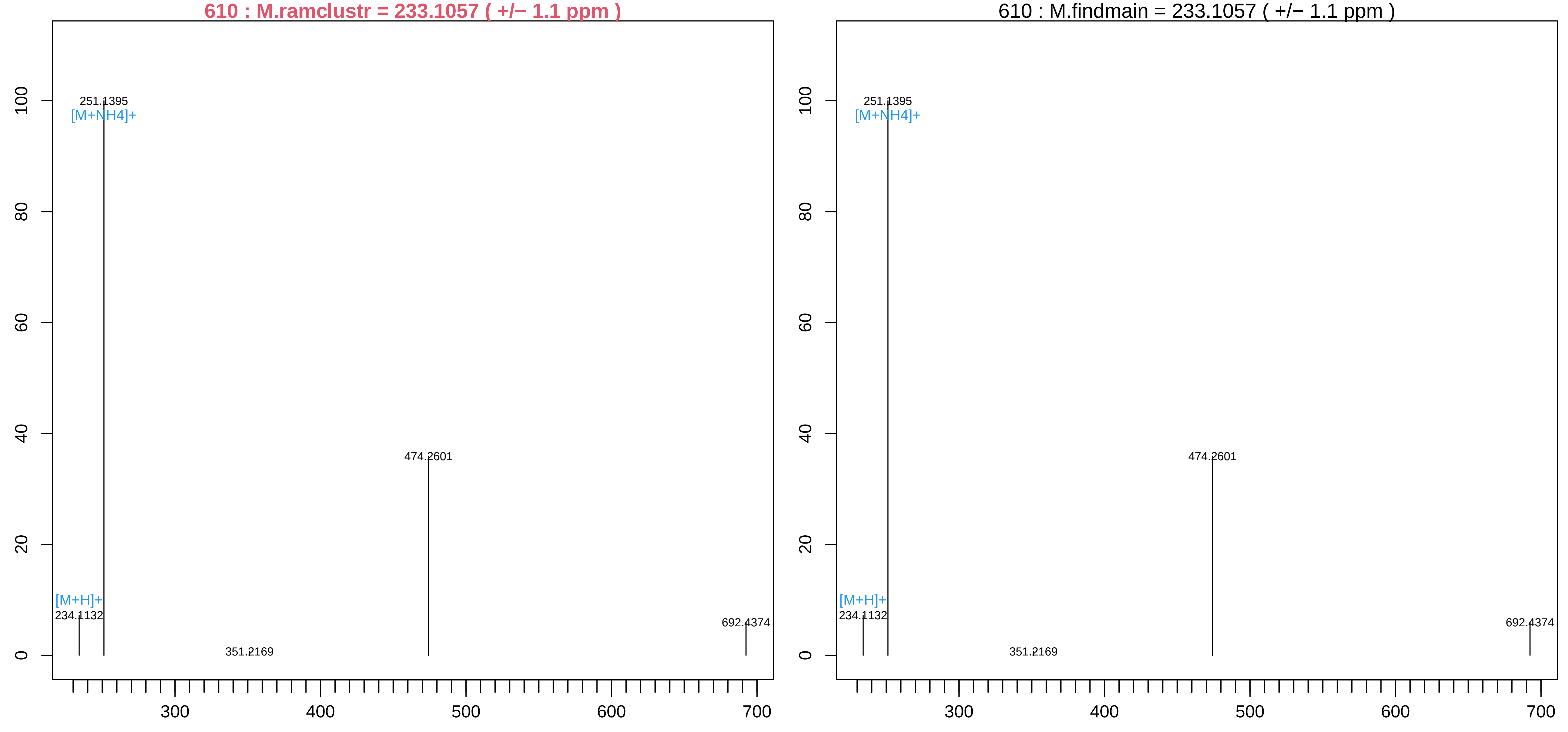
610 : M.ramclustr = 233.1057 (+/− 1.1 ppm)
251.1395
[M+NH4]+
474.2601
[M+H]+
234.1132
692.4374
351.2169
0
20
40
60
80
100
300
400
500
600
700
610 : M.findmain = 233.1057 (+/− 1.1 ppm)
251.1395
[M+NH4]+
474.2601
[M+H]+
234.1132
692.4374
351.2169
0
20
40
60
80
100
300
400
500
600
700

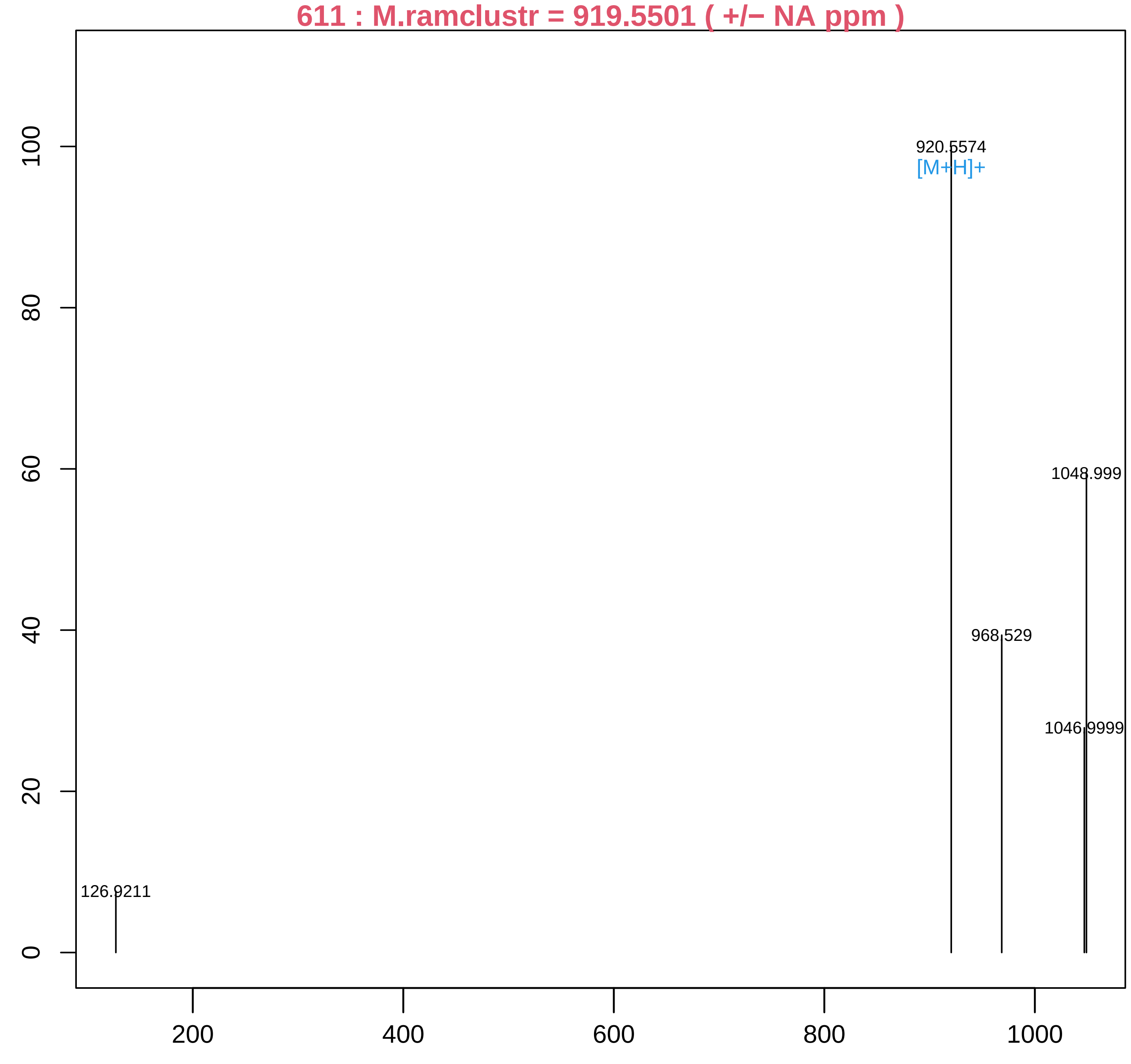

611 : M.ramclustr = 919.5501 (+/− NA ppm)
920.5574
[M+H]+
1048.999
968.529
1046.9999
126.9211
0
20
40
60
80
100
200
400
600
800
1000

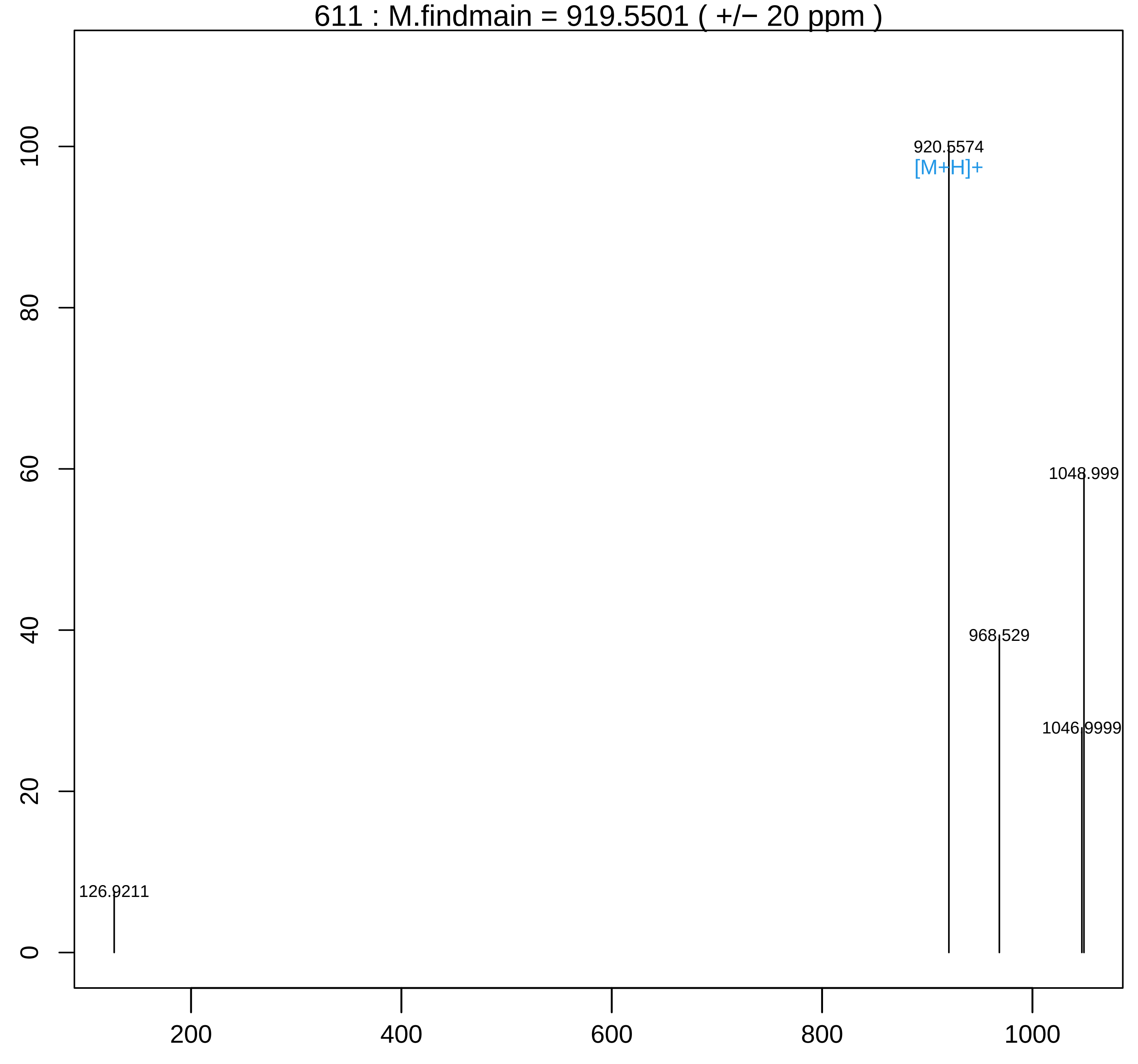

611 : M.findmain = 919.5501 (+/− 20 ppm)
920.5574
[M+H]+
1048.999
968.529
1046.9999
126.9211
0
20
40
60
80
100
200
400
600
800
1000

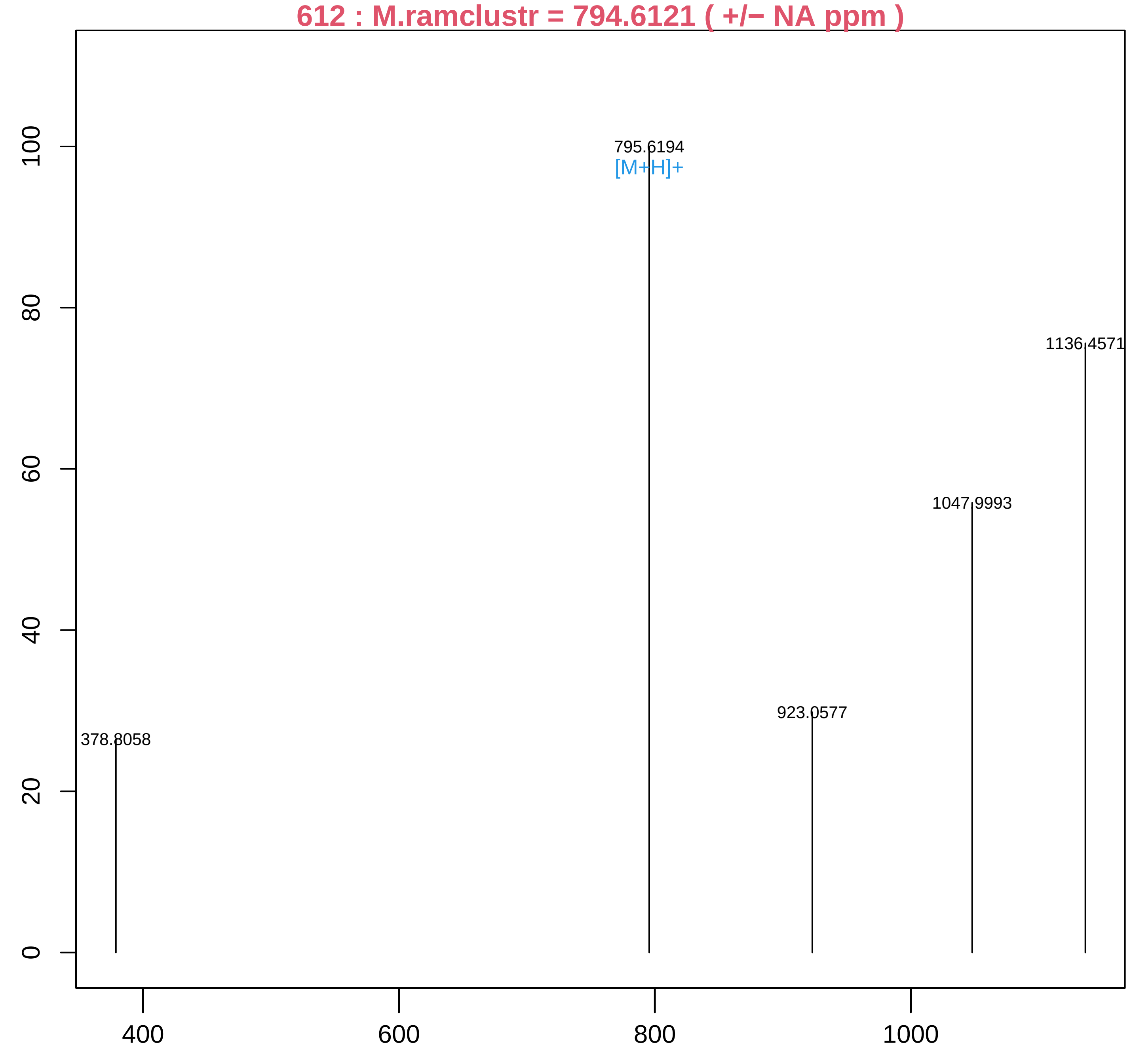
612 : M.ramclustr = 794.6121 (+/− NA ppm)
795.6194
[M+H]+
1136.4571
1047.9993
923.0577
378.8058
0
20
40
60
80
100
400
600
800
1000

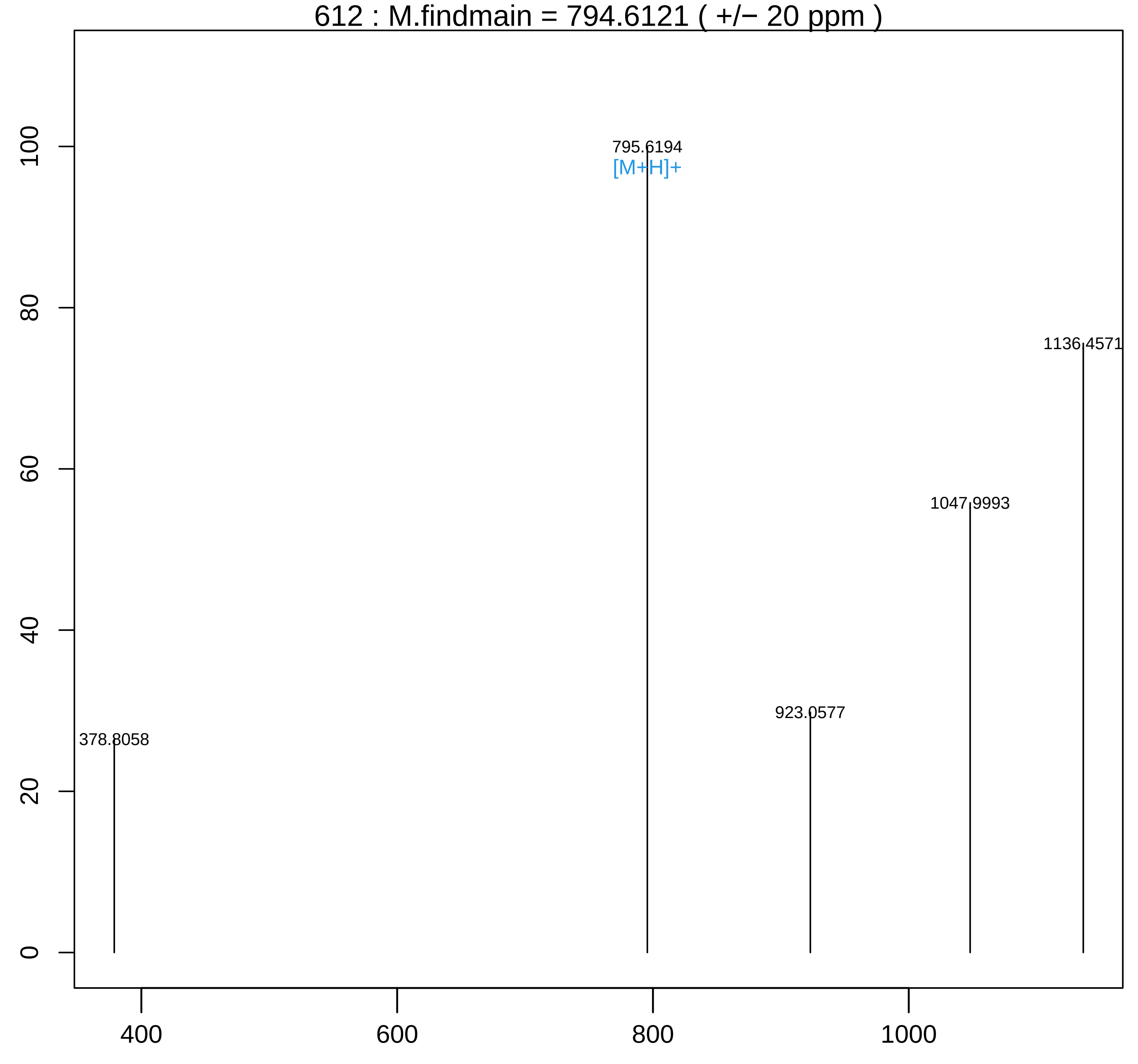
612 : M.findmain = 794.6121 (+/− 20 ppm)
795.6194
[M+H]+
1136.4571
1047.9993
923.0577
378.8058
0
20
40
60
80
100
400
600
800
1000

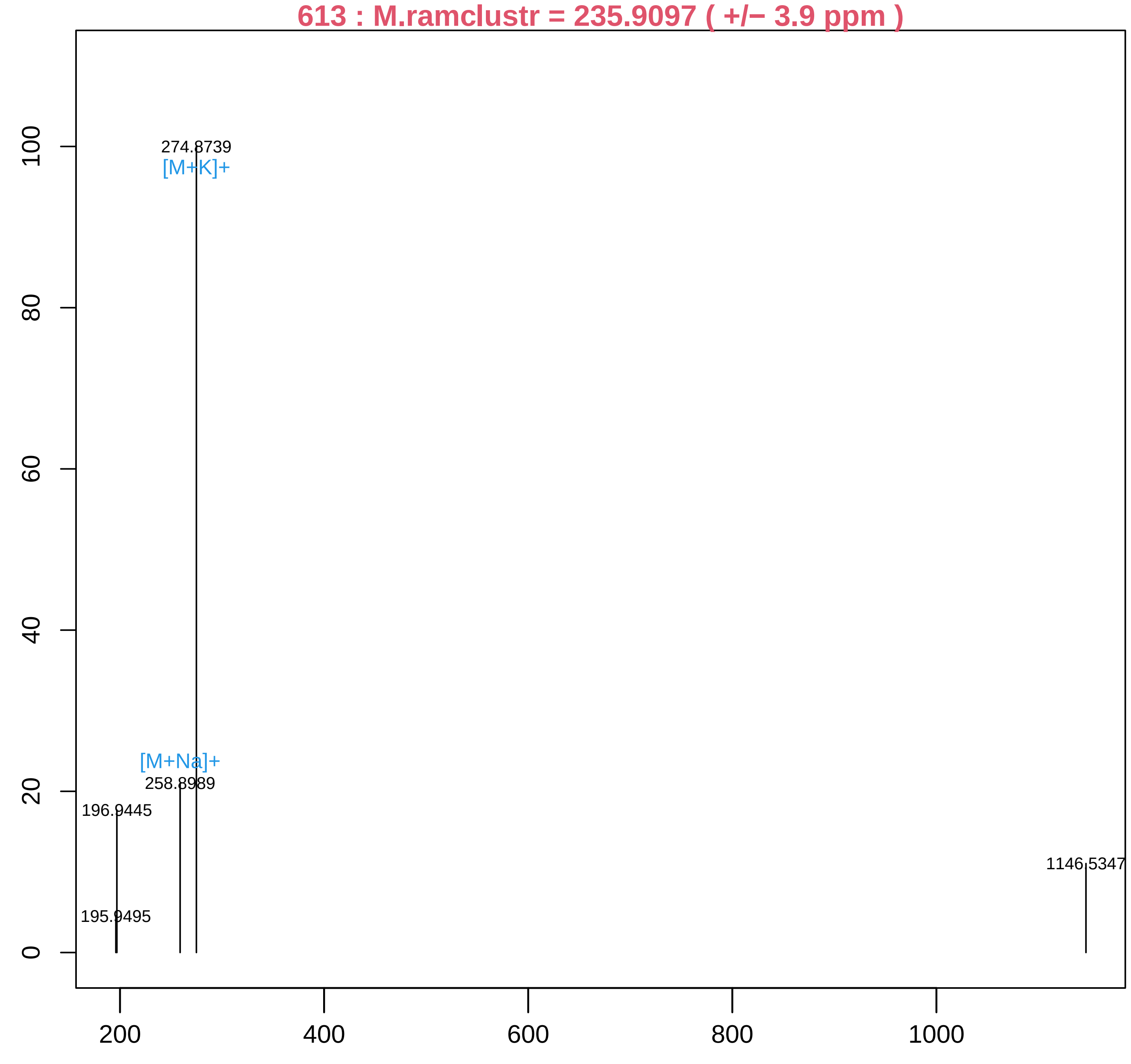
613 : M.ramclustr = 235.9097 (+/− 3.9 ppm)
274.8739
[M+K]+
[M+Na]+
258.8989
196.9445
195.9495
1146.5347
0
20
40
60
80
100
200
400
600
800
1000

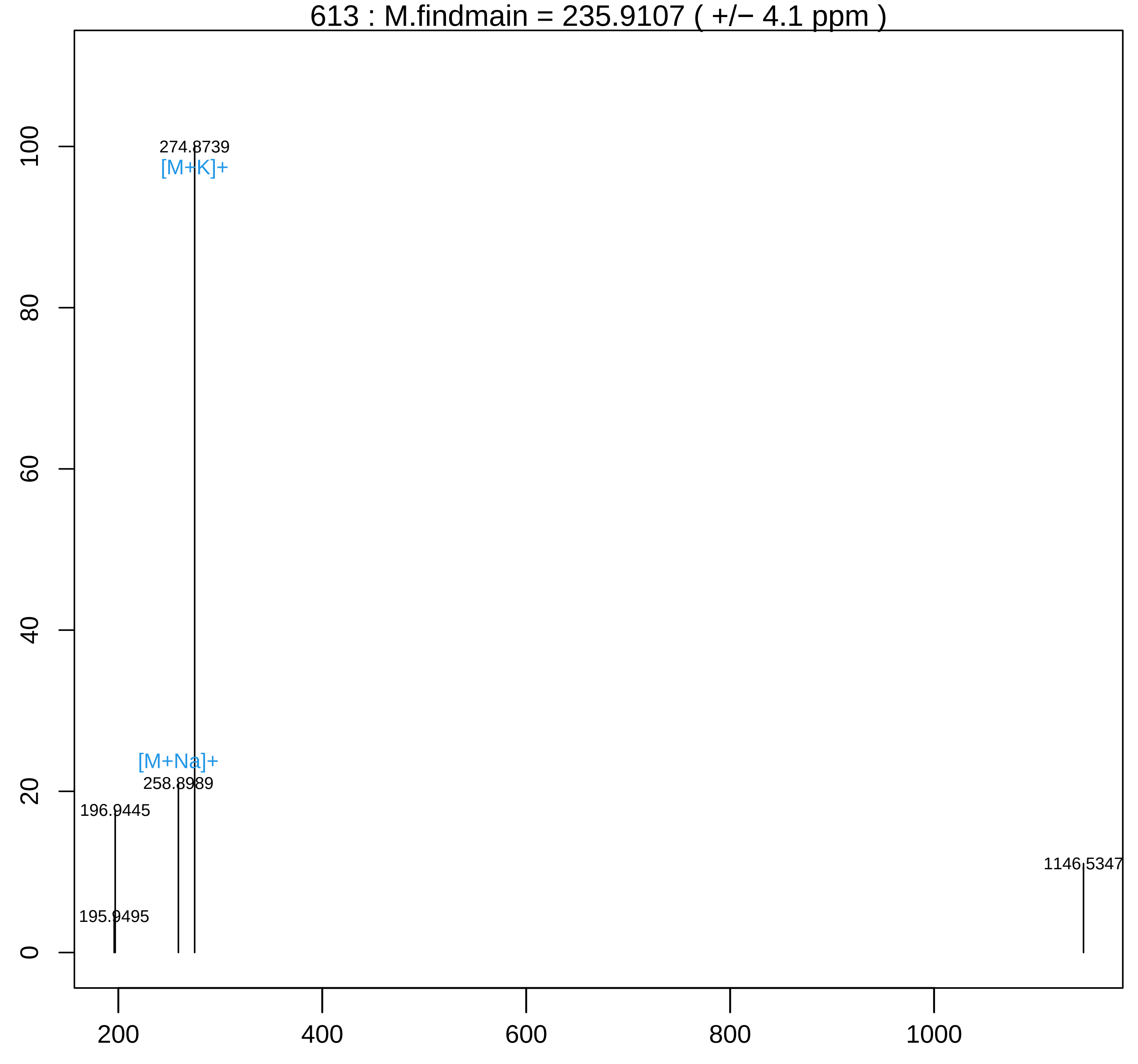
613 : M.findmain = 235.9107 (+/− 4.1 ppm)
274.8739
[M+K]+
[M+Na]+
258.8989
196.9445
195.9495
1146.5347
0
20
40
60
80
100
200
400
600
800
1000

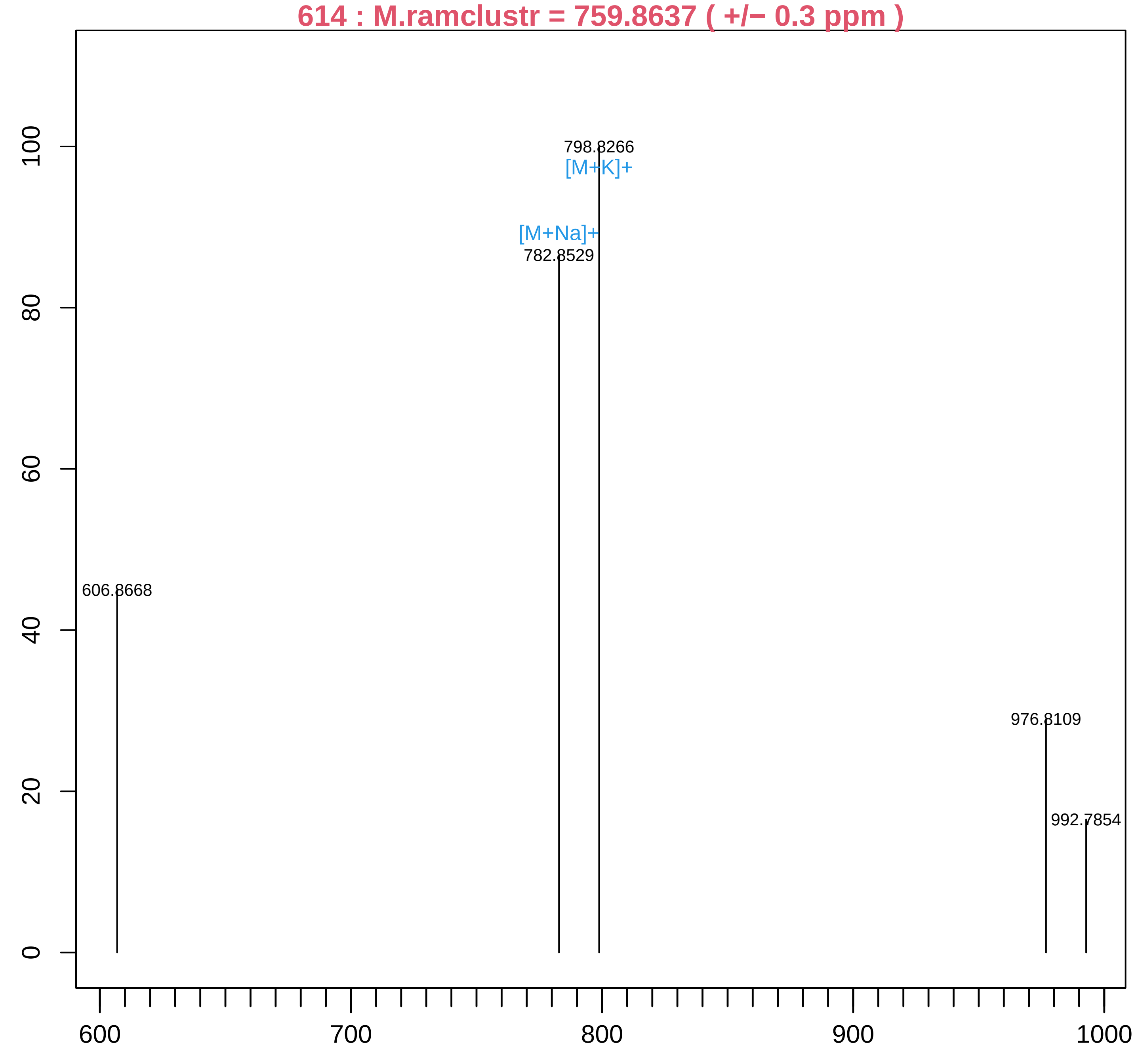
614 : M.ramclustr = 759.8637 (+/− 0.3 ppm)
798.8266
[M+K]+
[M+Na]+
782.8529
606.8668
976.8109
992.7854
0
20
40
60
80
100
600
700
800
900
1000

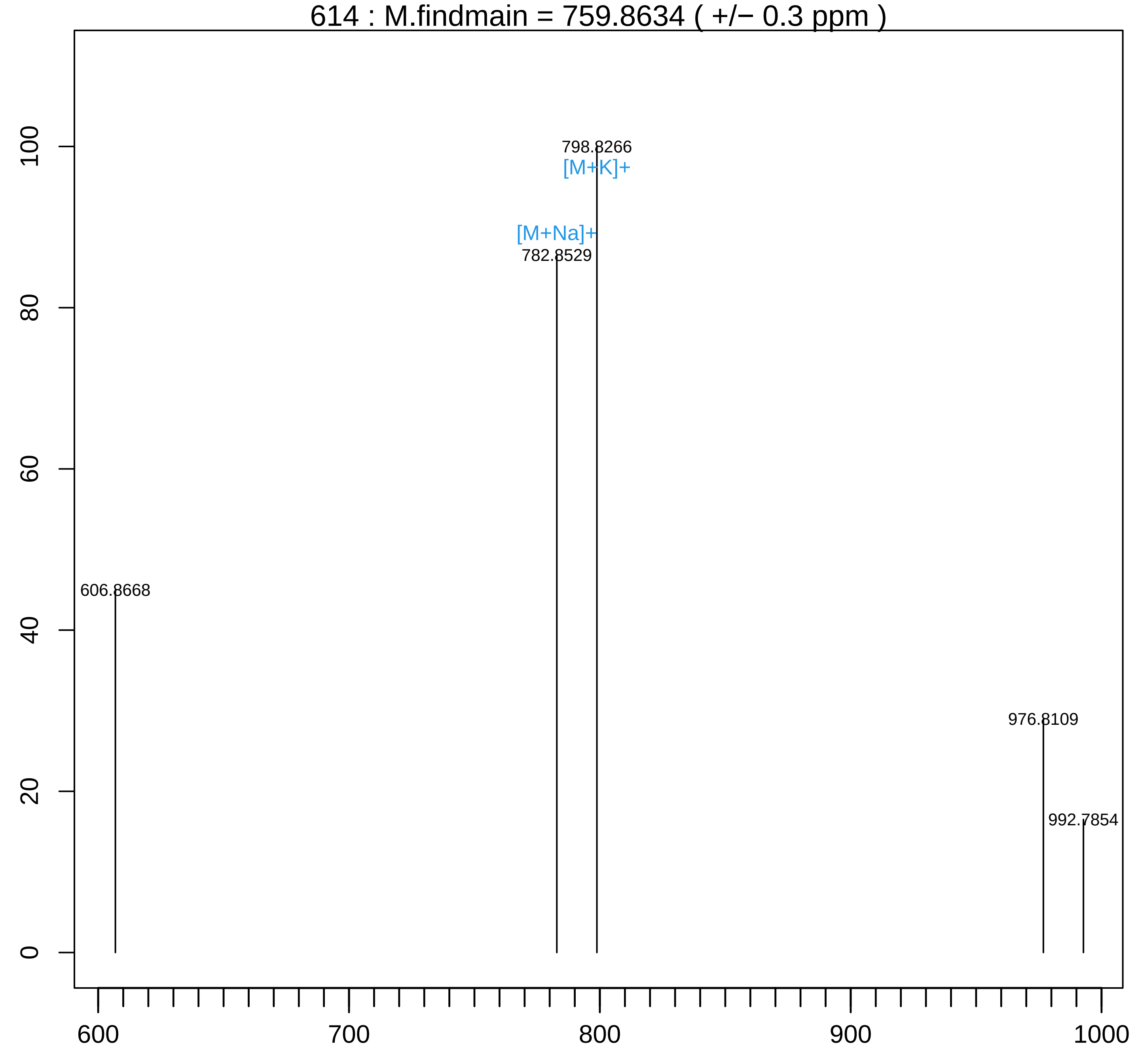
614 : M.findmain = 759.8634 (+/− 0.3 ppm)
798.8266
[M+K]+
[M+Na]+
782.8529
606.8668
976.8109
992.7854
0
20
40
60
80
100
600
700
800
900
1000

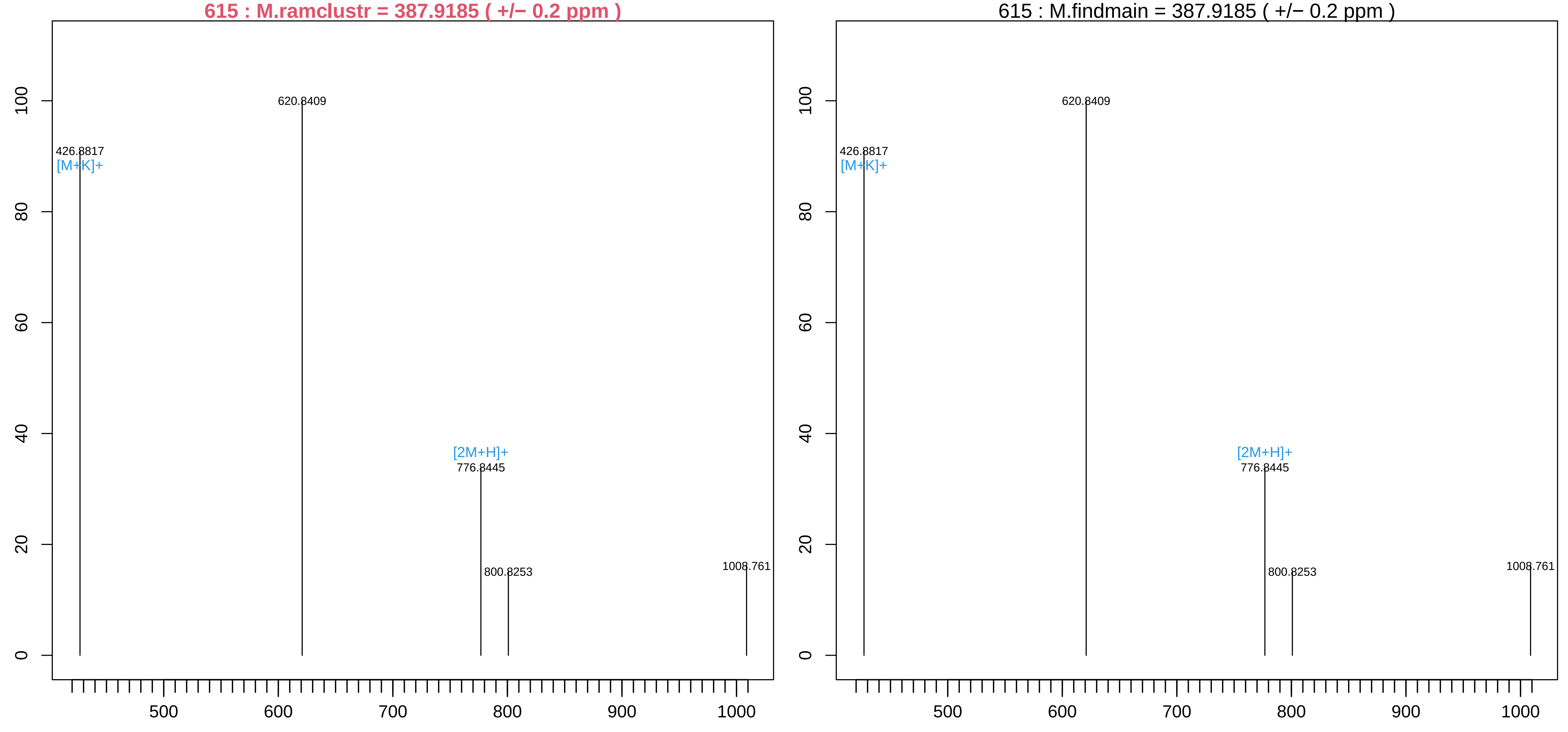
615 : M.ramclustr = 387.9185 (+/− 0.2 ppm)
426.8817
[M+K]+
620.8409
[2M+H]+
776.8445
800.8253
1008.761
0
20
40
60
80
100
500
600
700
800
900
1000
615 : M.findmain = 387.9185 (+/− 0.2 ppm)
426.8817
[M+K]+
620.8409
[2M+H]+
776.8445
800.8253
1008.761
0
20
40
60
80
100
500
600
700
800
900
1000

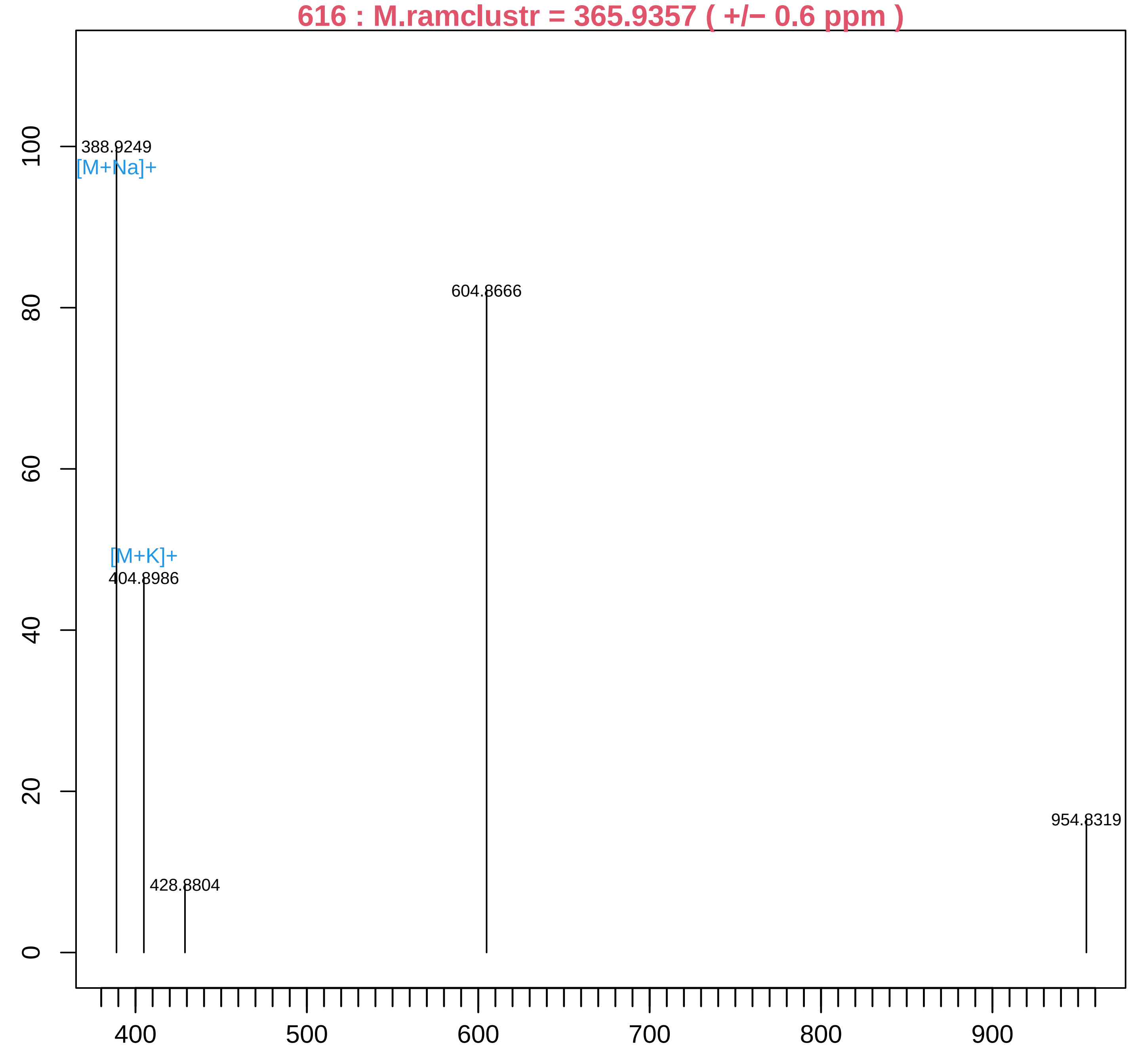
616 : M.ramclustr = 365.9357 (+/− 0.6 ppm)
388.9249
[M+Na]+
604.8666
[M+K]+
404.8986
428.8804
954.8319
0
20
40
60
80
100
400
500
600
700
800
900

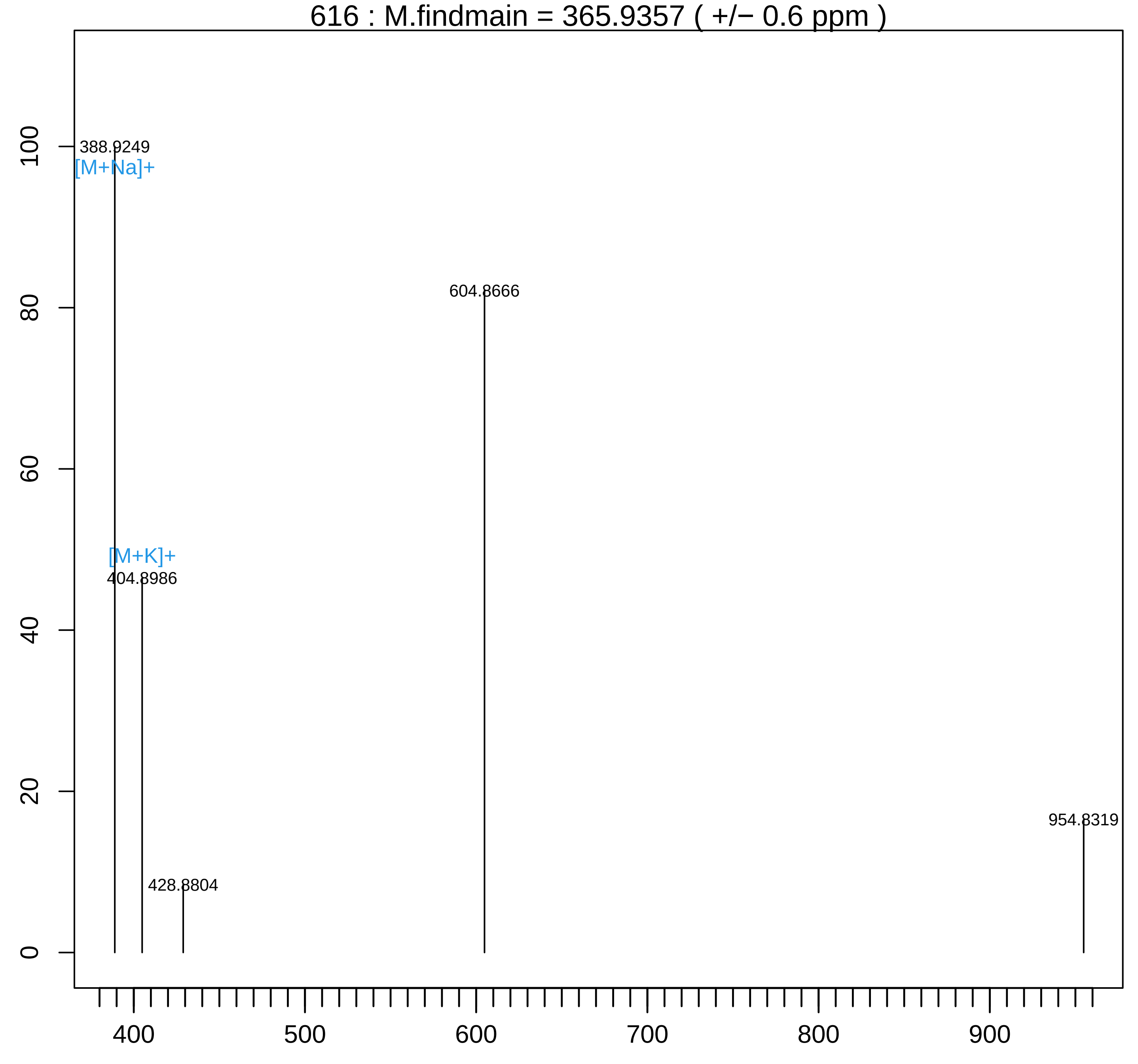
616 : M.findmain = 365.9357 (+/− 0.6 ppm)
388.9249
[M+Na]+
604.8666
[M+K]+
404.8986
428.8804
954.8319
0
20
40
60
80
100
400
500
600
700
800
900

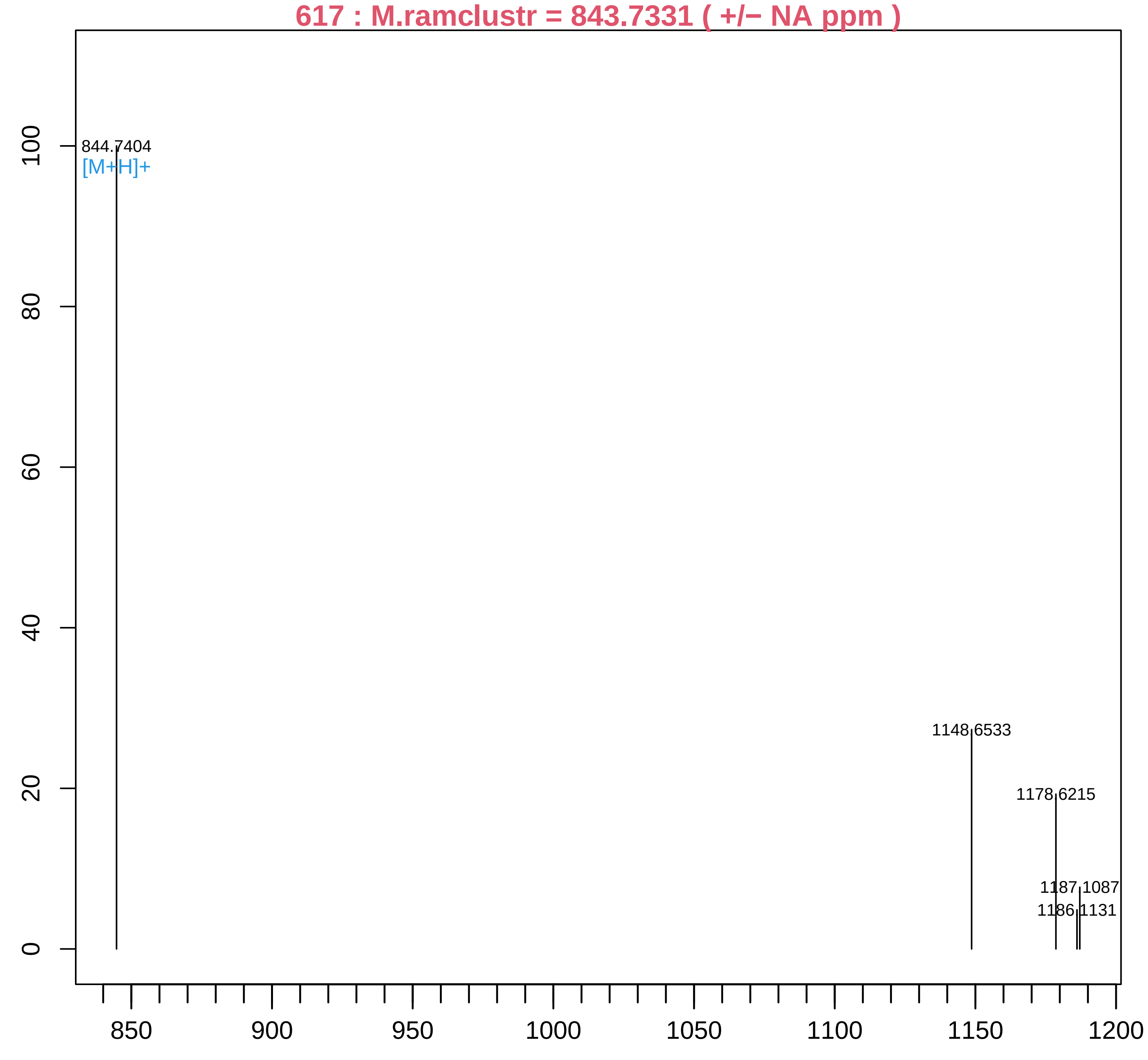

617 : M.ramclustr = 843.7331 (+/− NA ppm)
844.7404
[M+H]+
1148.6533
1178.6215
1187.1087
1186.1131
0
20
40
60
80
100
850
900
950
1000
1050
1100
1150
1200

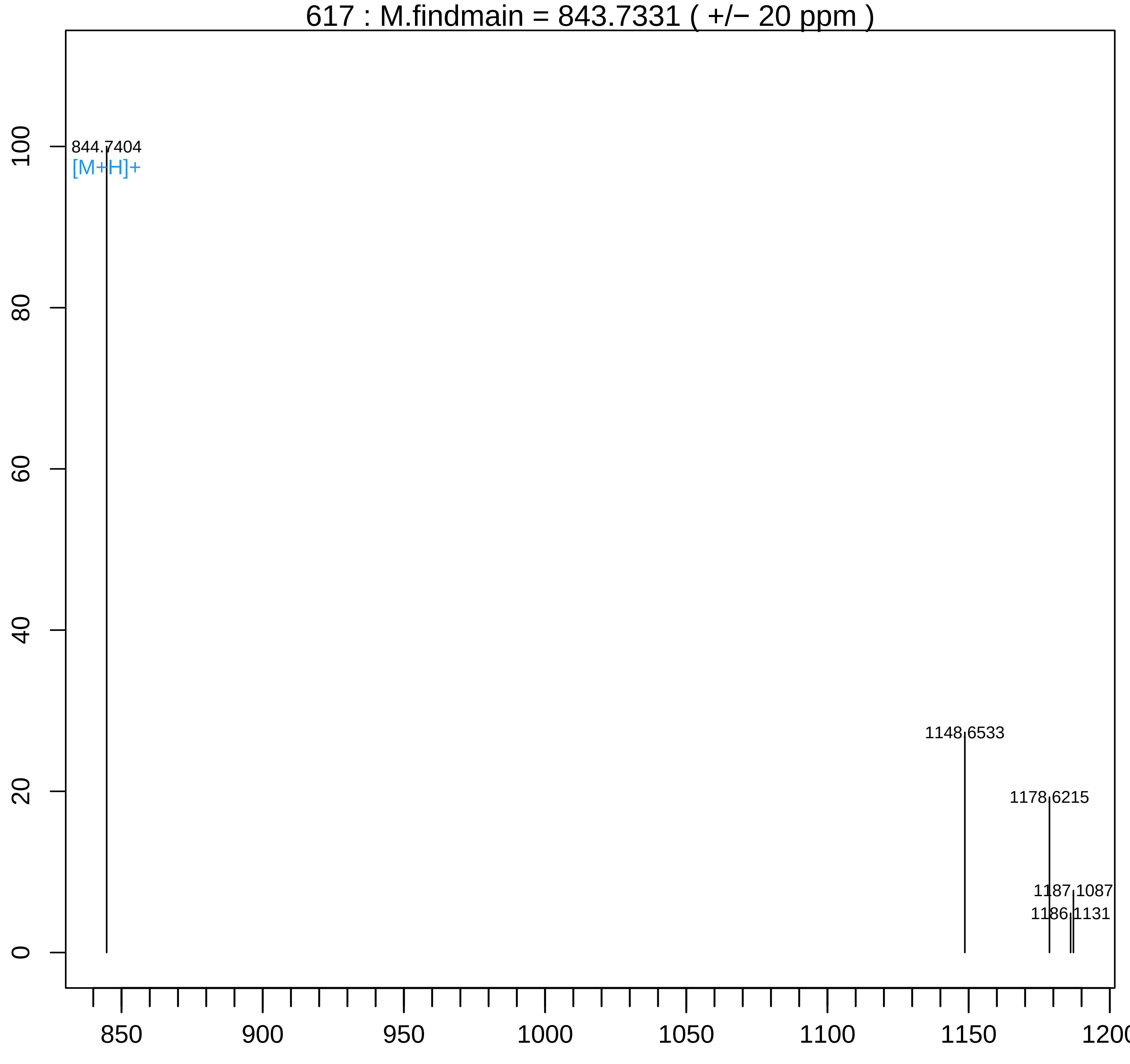

617 : M.findmain = 843.7331 (+/− 20 ppm)
844.7404
[M+H]+
1148.6533
1178.6215
1187.1087
1186.1131
0
20
40
60
80
100
850
900
950
1000
1050
1100
1150
1200

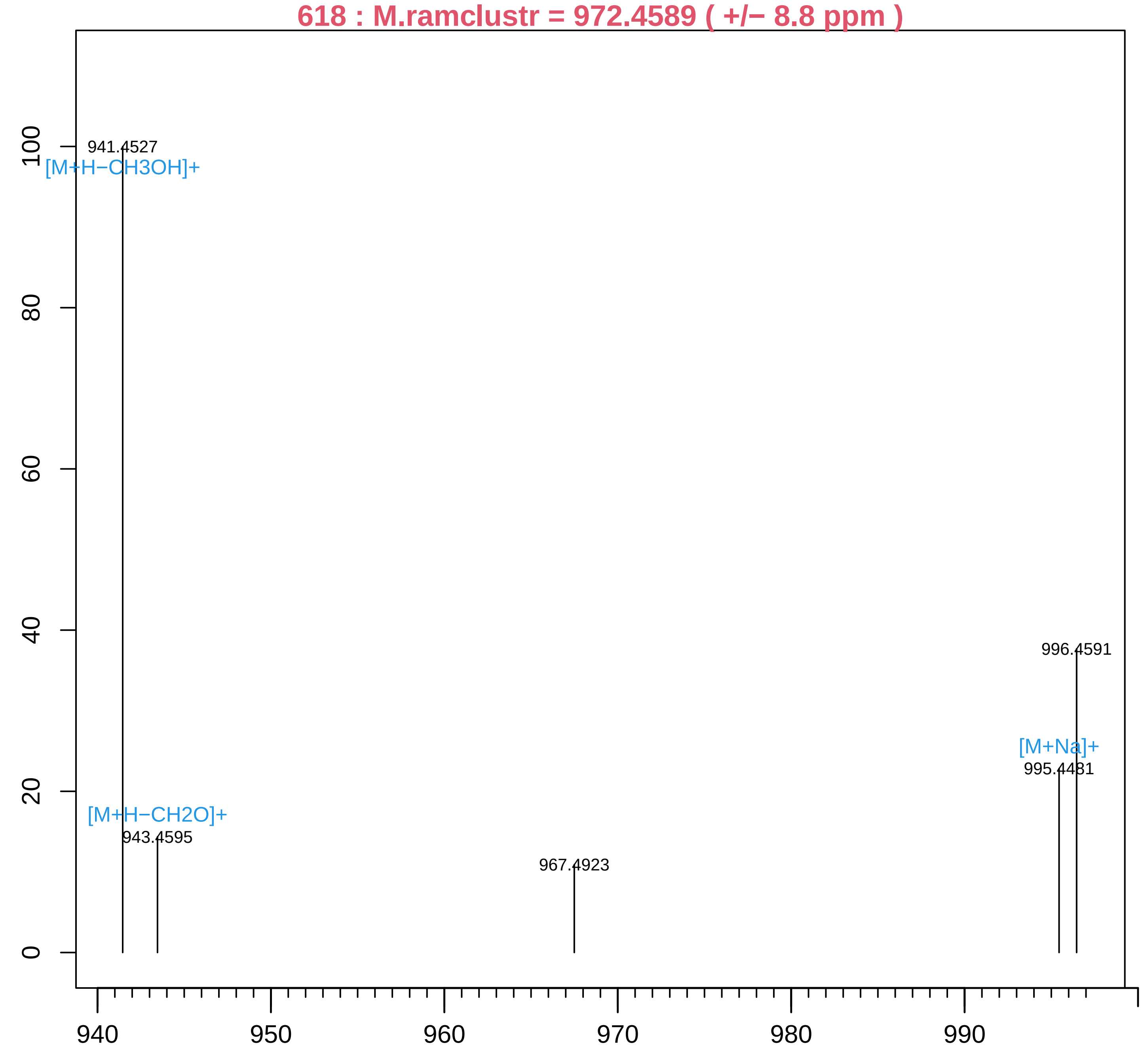
618 : M.ramclustr = 972.4589 (+/− 8.8 ppm)
941.4527
[M+H−CH3OH]+
[M+H−CH2O]+
943.4595
967.4923
996.4591
[M+Na]+
995.4481
0
20
40
60
80
100
940
950
960
970
980
990

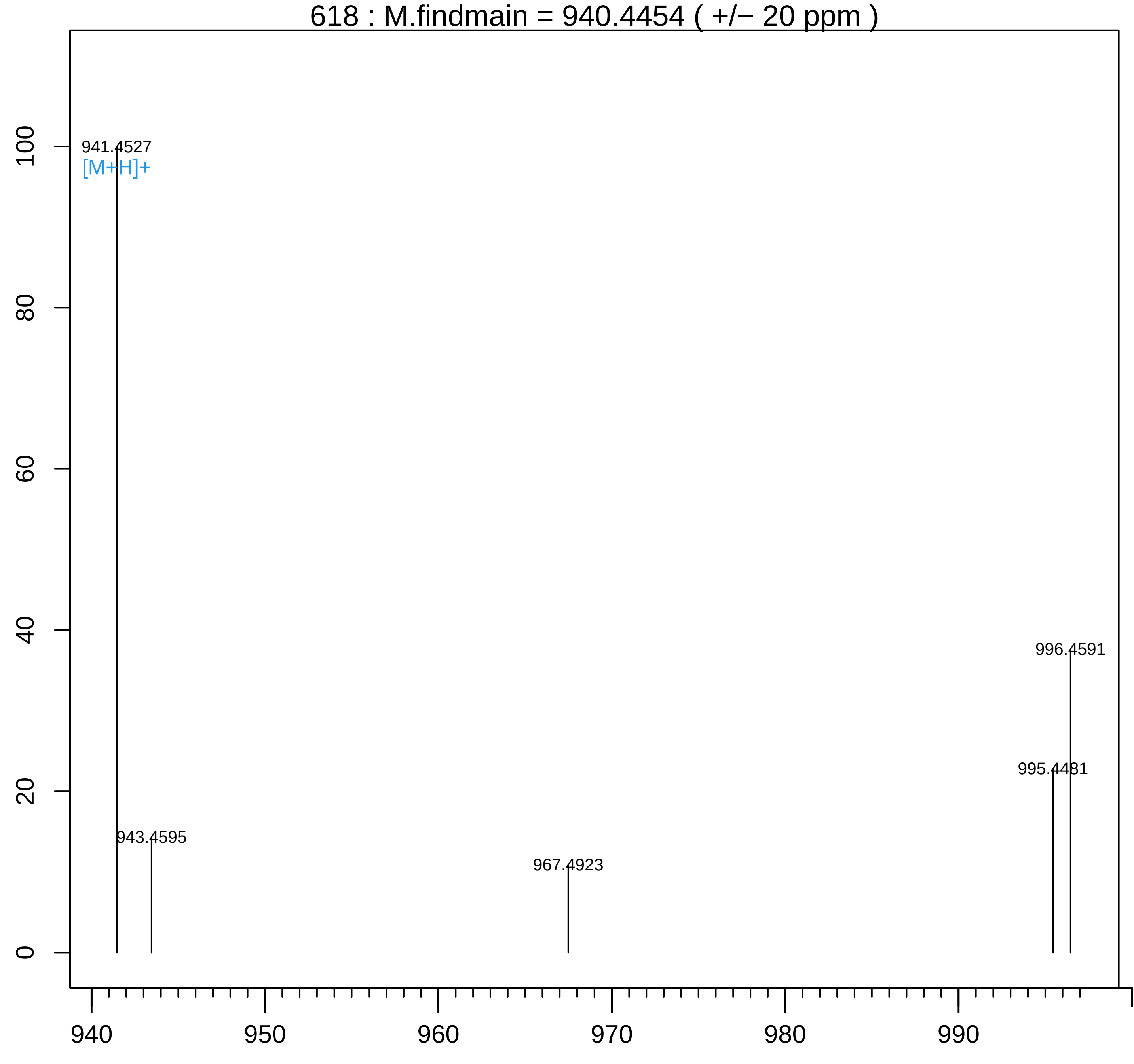
618 : M.findmain = 940.4454 (+/− 20 ppm)
941.4527
[M+H]+
943.4595
967.4923
996.4591
995.4481
0
20
40
60
80
100
940
950
960
970
980
990

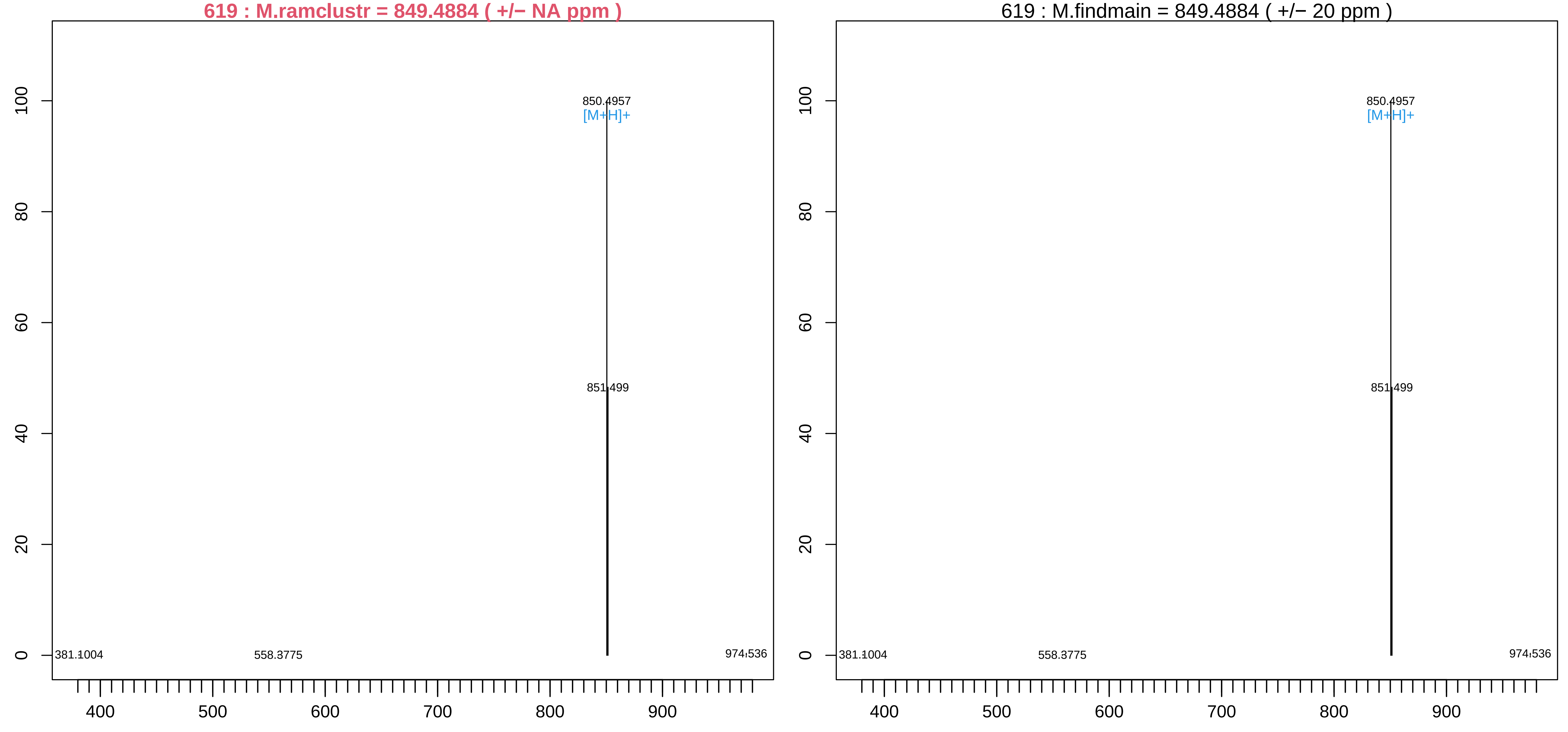

619 : M.ramclustr = 849.4884 (+/− NA ppm)
850.4957
[M+H]+
851.499
381.1004
558.3775
974.536
0
20
40
60
80
100
400
500
600
700
800
900
619 : M.findmain = 849.4884 (+/− 20 ppm)
850.4957
[M+H]+
851.499
381.1004
558.3775
974.536
0
20
40
60
80
100
400
500
600
700
800
900

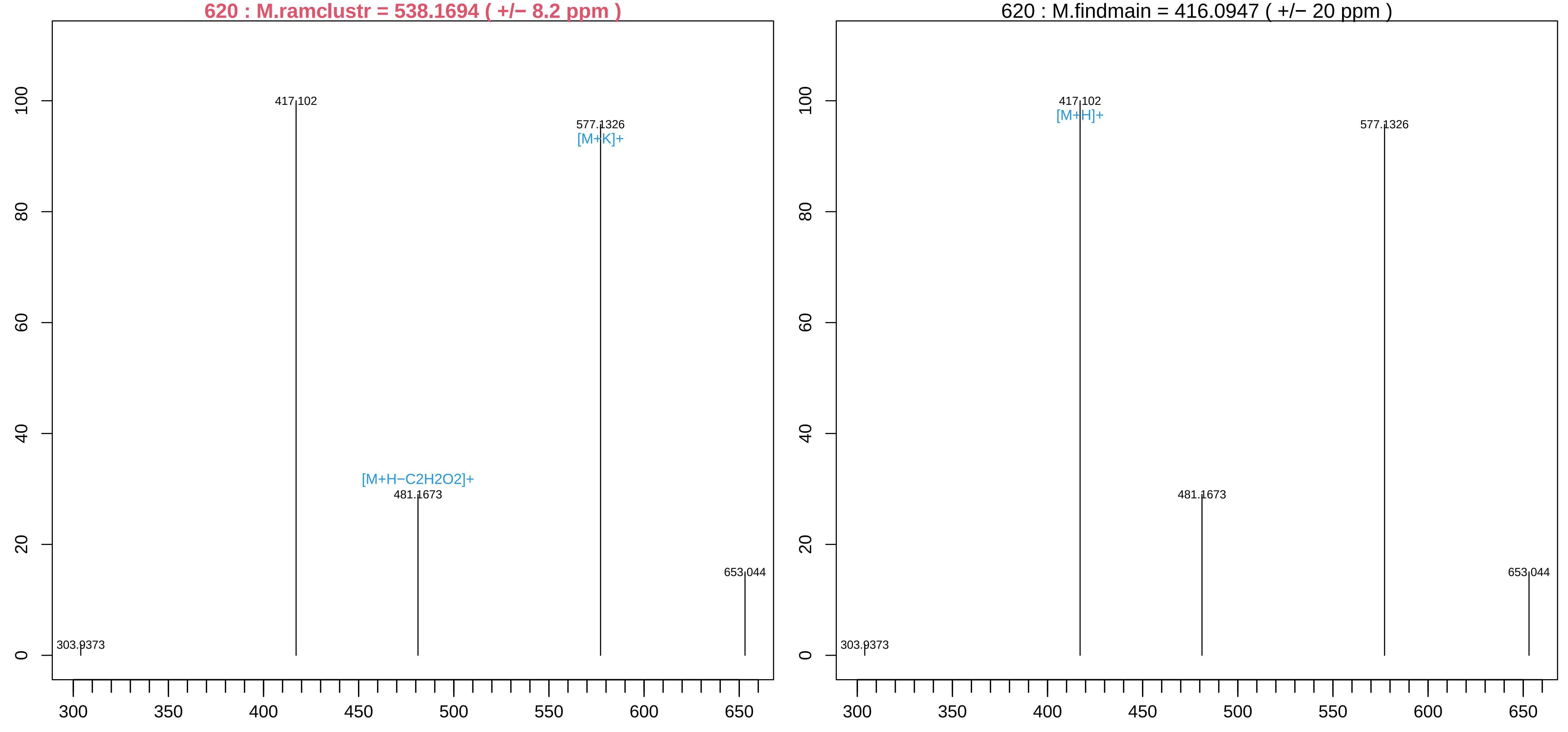
620 : M.ramclustr = 538.1694 (+/− 8.2 ppm)
417.102
577.1326
[M+K]+
[M+H−C2H2O2]+
481.1673
653.044
303.9373
620 : M.findmain = 416.0947 (+/− 20 ppm)
417.102
[M+H]+
577.1326
481.1673
653.044
303.9373
0
20
40
60
80
100
300
350
400
450
500
550
600
650

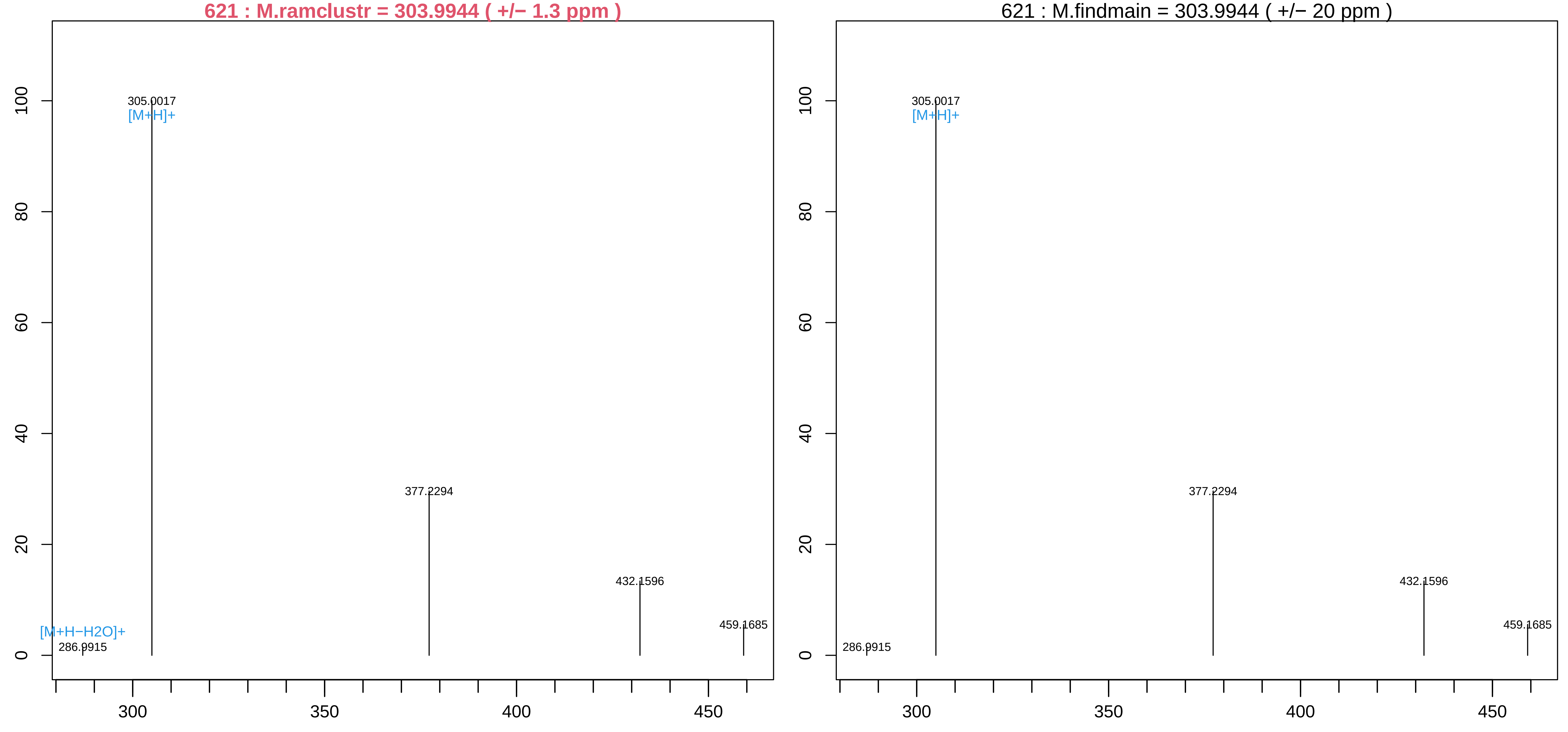
621 : M.ramclustr = 303.9944 (+/− 1.3 ppm)
305.0017
[M+H]+
377.2294
432.1596
459.1685
[M+H−H2O]+
286.9915
621 : M.findmain = 303.9944 (+/− 20 ppm)
305.0017
[M+H]+
377.2294
432.1596
459.1685
286.9915

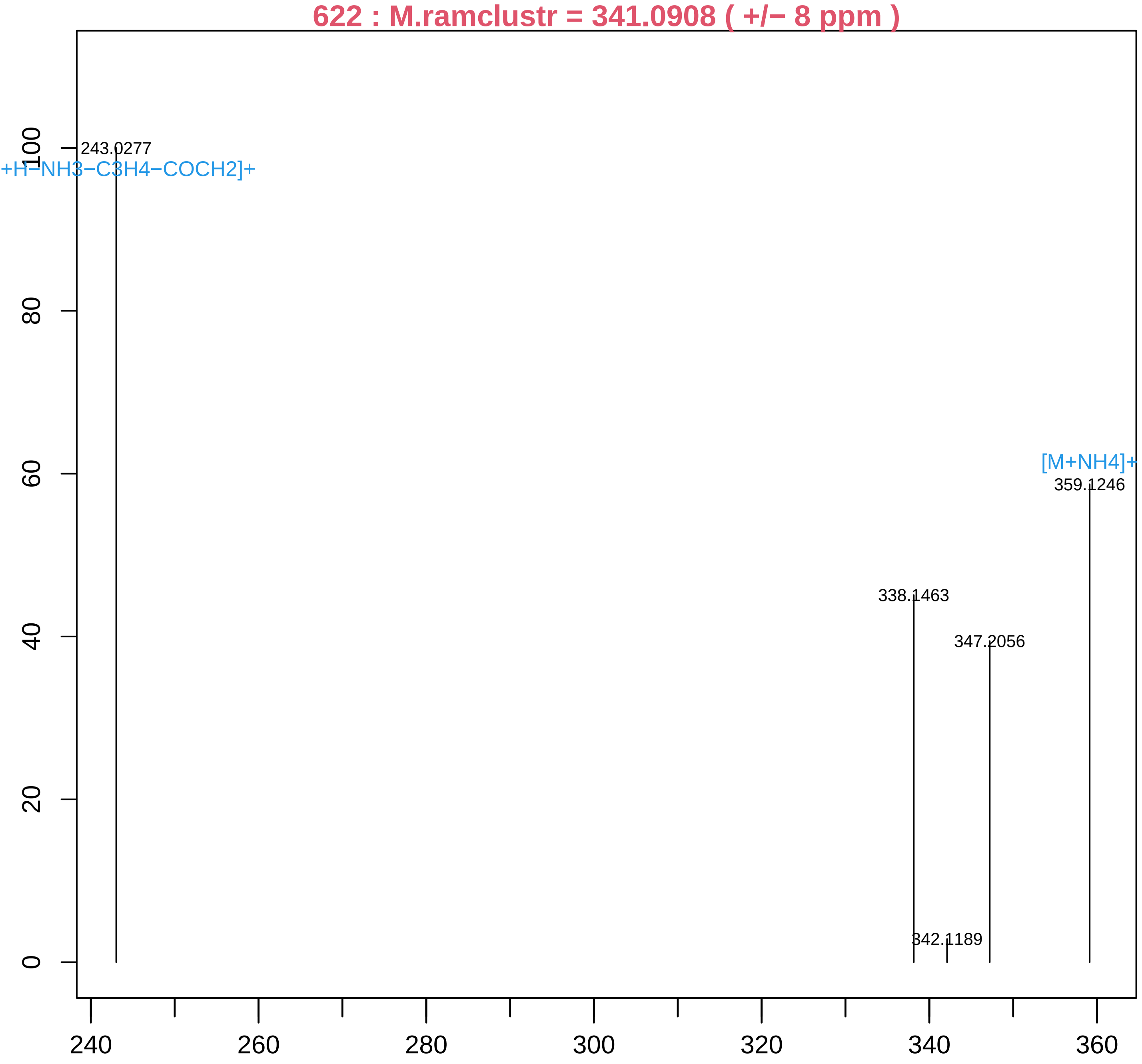
622 : M.ramclustr = 341.0908 (+/− 8 ppm)
243.0277
+H−NH3−C3H4−COCH2]+
[M+NH4]+
359.1246
338.1463
347.2056
342.1189
0
20
40
60
80
100
240
260
280
300
320
340
360

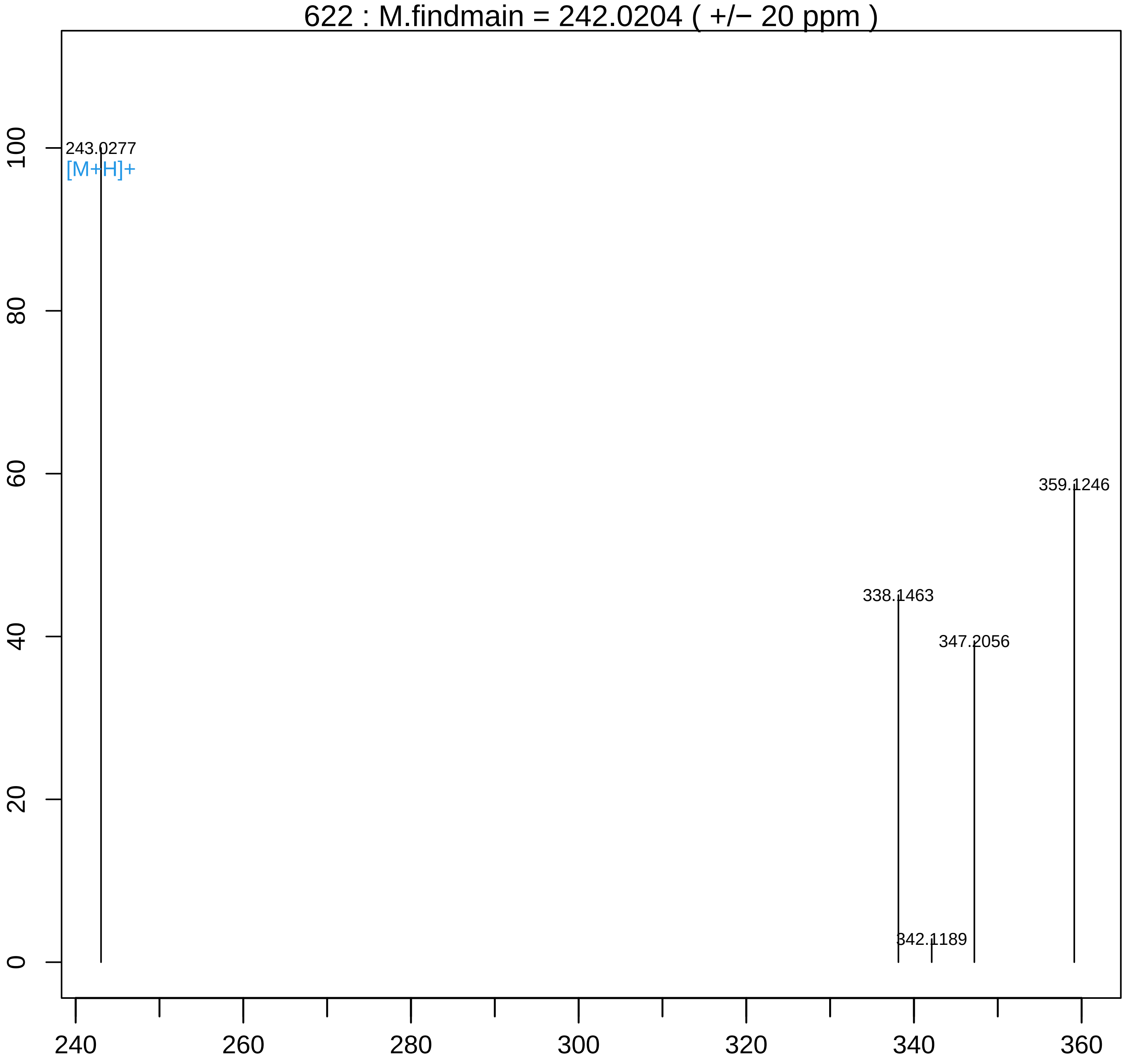
622 : M.findmain = 242.0204 (+/− 20 ppm)
243.0277
[M+H]+
359.1246
338.1463
347.2056
342.1189
0
20
40
60
80
100
240
260
280
300
320
340
360

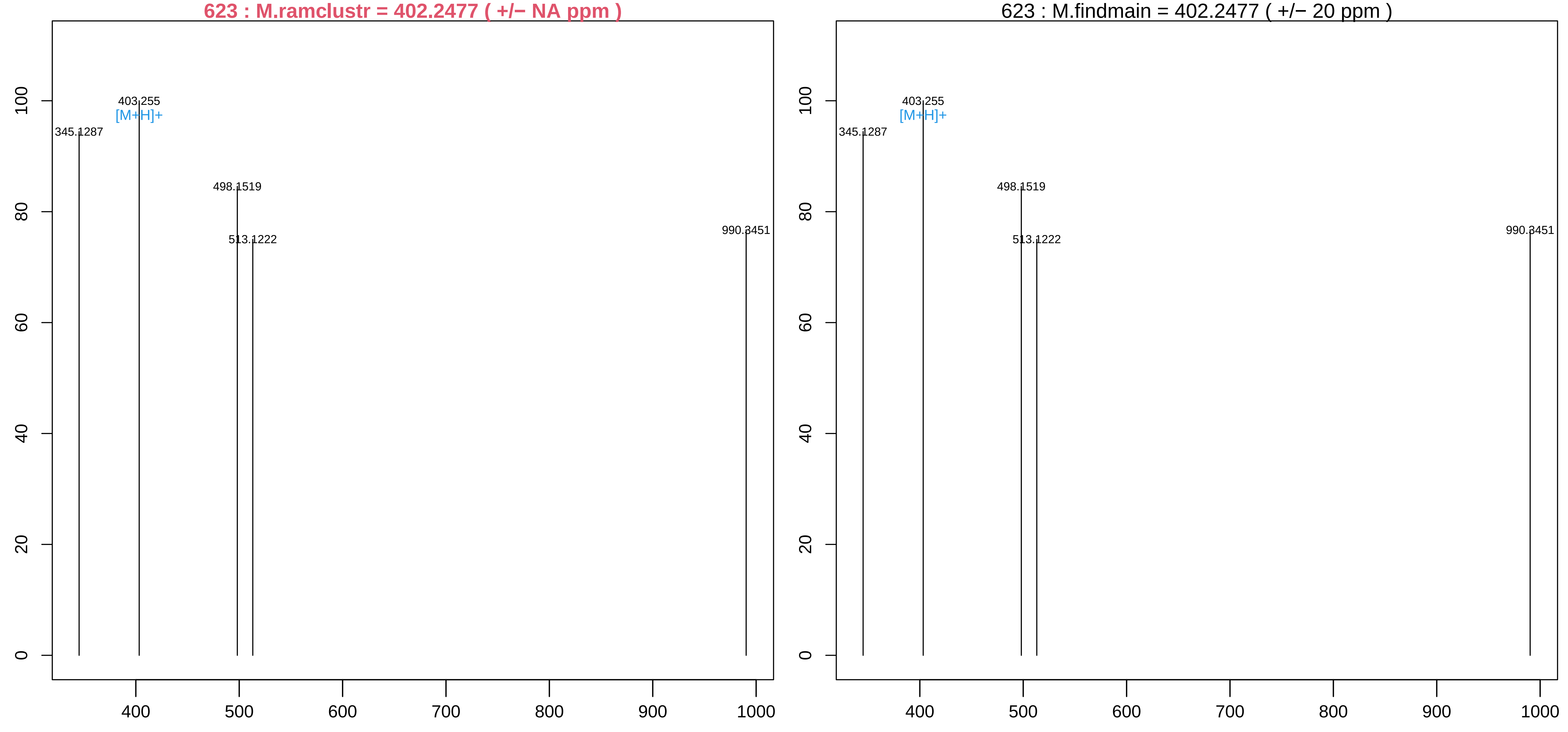
623 : M.ramclustr = 402.2477 (+/− NA ppm)
403.255
[M+H]+
345.1287
498.1519
513.1222
990.3451
623 : M.findmain = 402.2477 (+/− 20 ppm)
403.255
[M+H]+
345.1287
498.1519
513.1222
990.3451

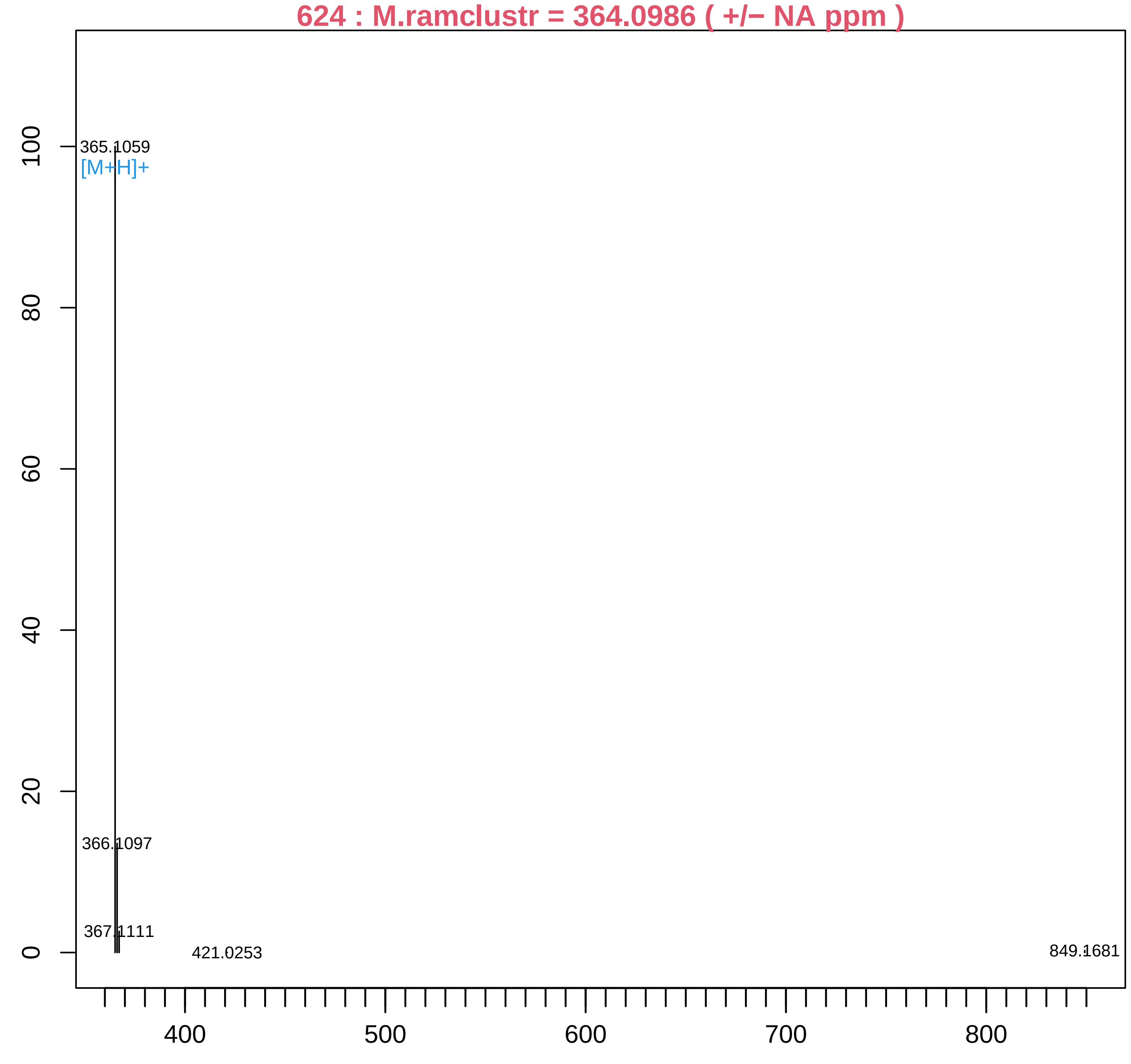
624 : M.ramclustr = 364.0986 (+/− NA ppm)
365.1059
[M+H]+
366.1097
367.1111
421.0253
849.1681
0
20
40
60
80
100
400
500
600
700
800

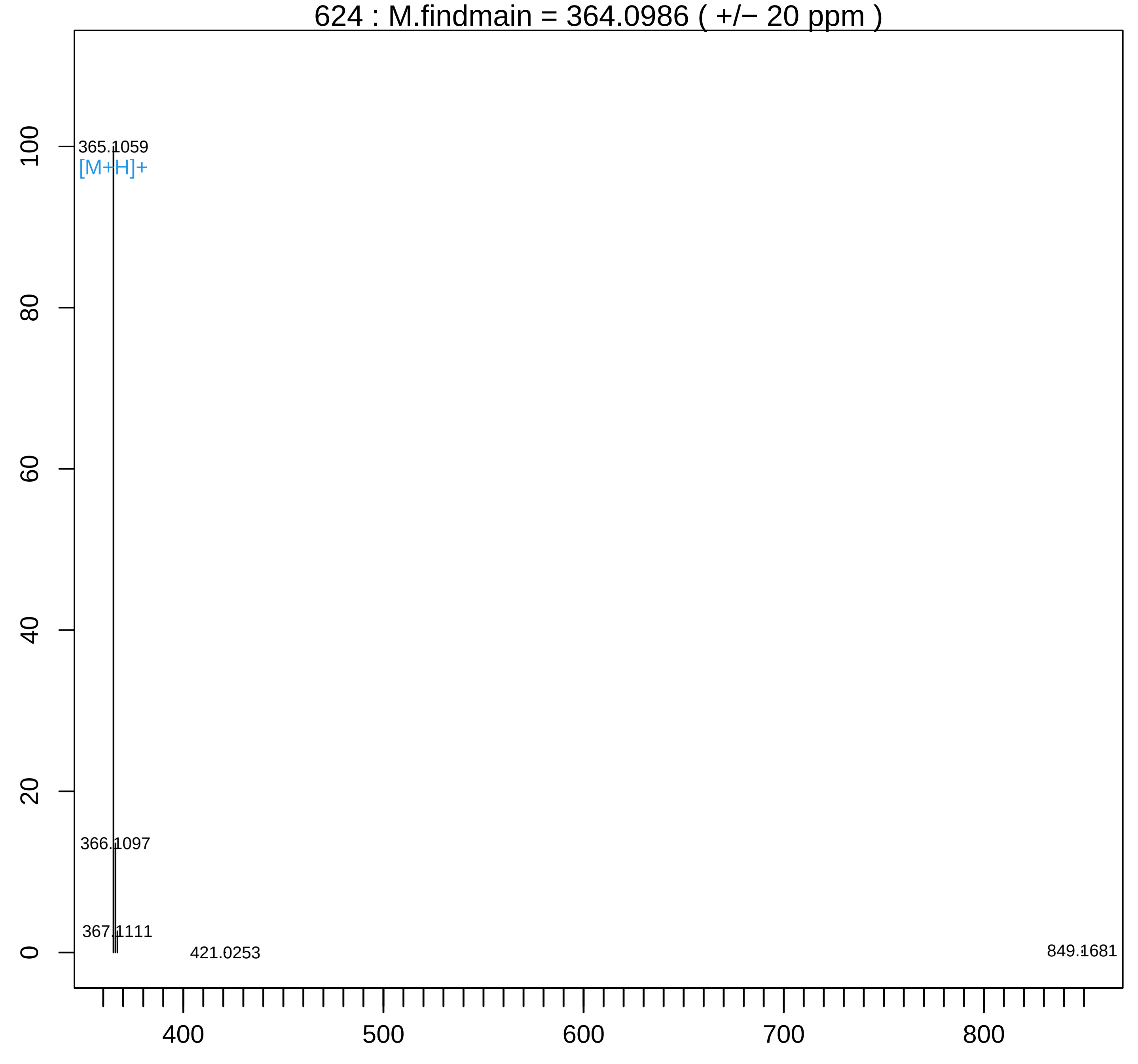
624 : M.findmain = 364.0986 (+/− 20 ppm)
365.1059
[M+H]+
366.1097
367.1111
421.0253
849.1681
0
20
40
60
80
100
400
500
600
700
800

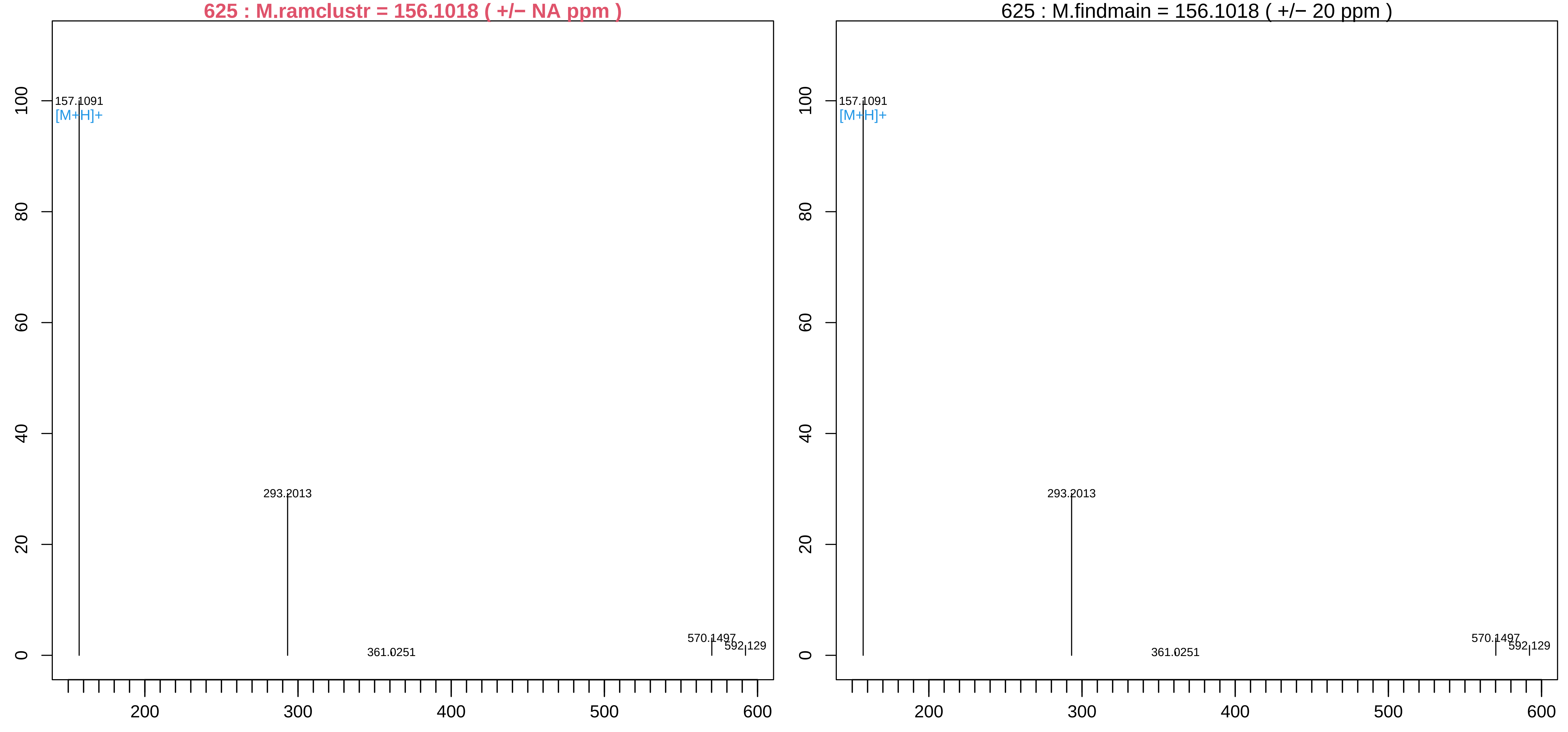
625 : M.ramclustr = 156.1018 (+/− NA ppm)
157.1091
[M+H]+
293.2013
361.0251
570.1497
592.129
625 : M.findmain = 156.1018 (+/− 20 ppm)
157.1091
[M+H]+
293.2013
361.0251
570.1497
592.129

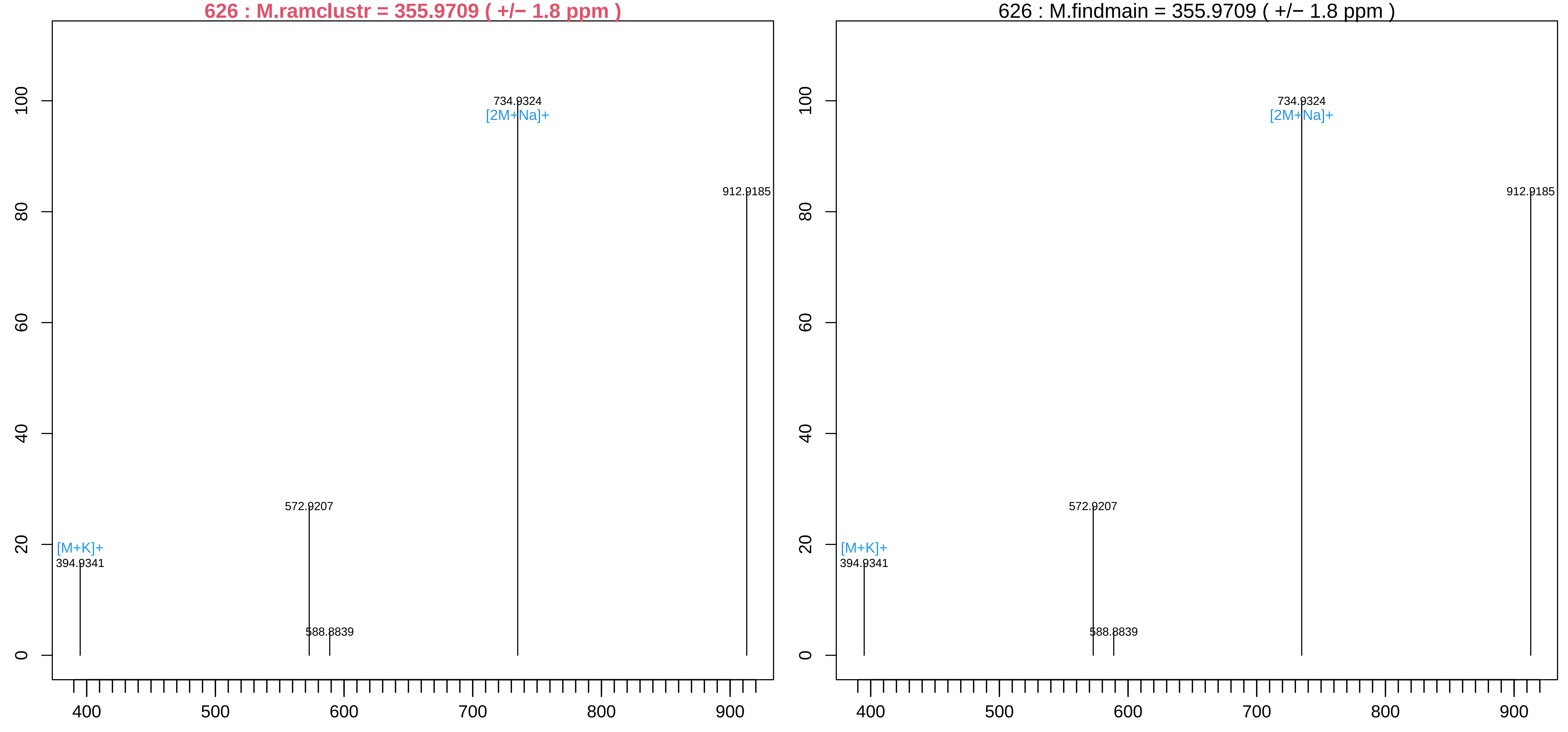

626 : M.ramclustr = 355.9709 (+/− 1.8 ppm)
734.9324
[2M+Na]+
912.9185
572.9207
[M+K]+
394.9341
588.8839
626 : M.findmain = 355.9709 (+/− 1.8 ppm)
734.9324
[2M+Na]+
912.9185
572.9207
[M+K]+
394.9341
588.8839
0
20
40
60
80
100
400
500
600
700
800
900

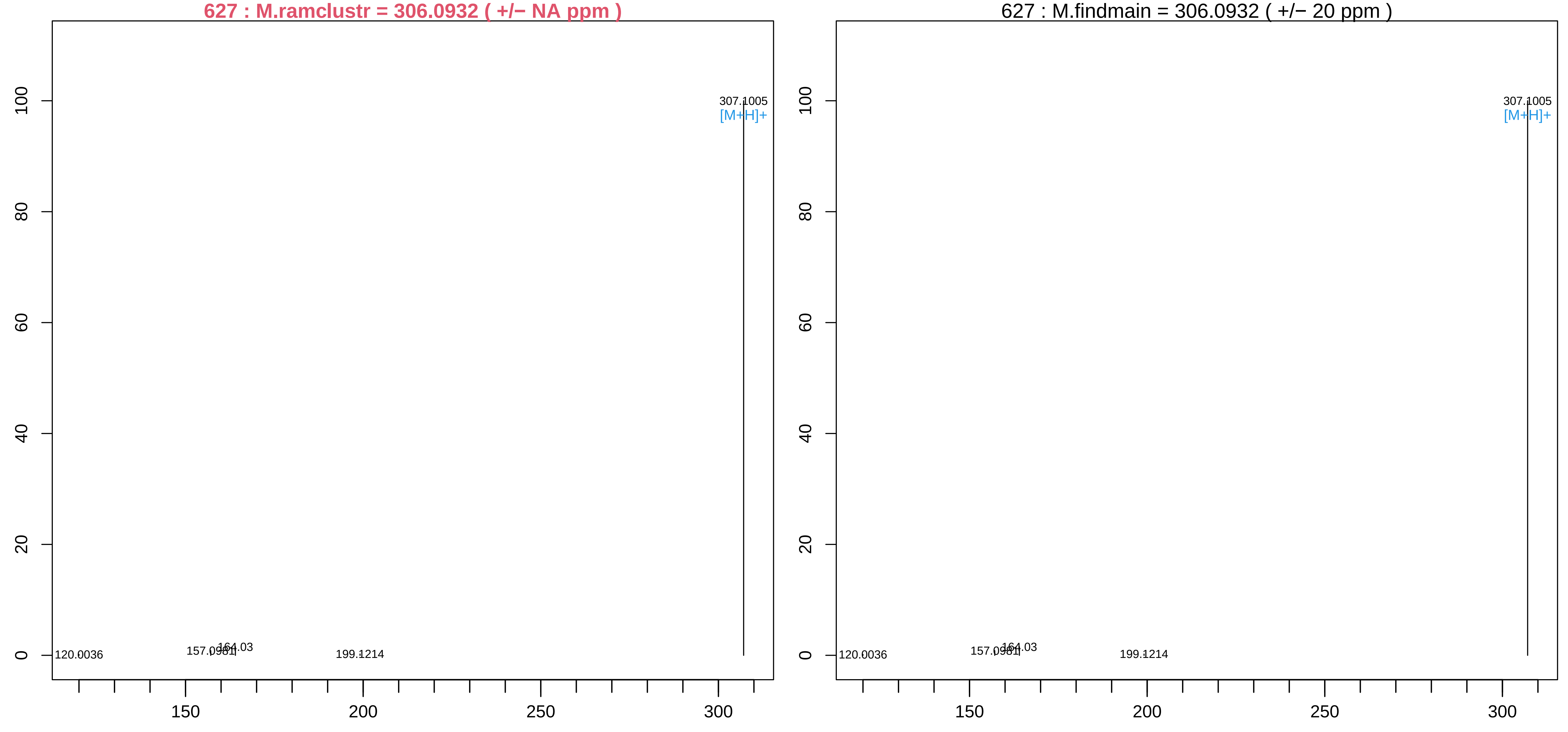

627 : M.ramclustr = 306.0932 (+/− NA ppm)
307.1005
[M+H]+
120.0036
157.0981
164.03
199.1214
0
20
40
60
80
100
150
200
250
300
627 : M.findmain = 306.0932 (+/− 20 ppm)
307.1005
[M+H]+
120.0036
157.0981
164.03
199.1214
0
20
40
60
80
100
150
200
250
300

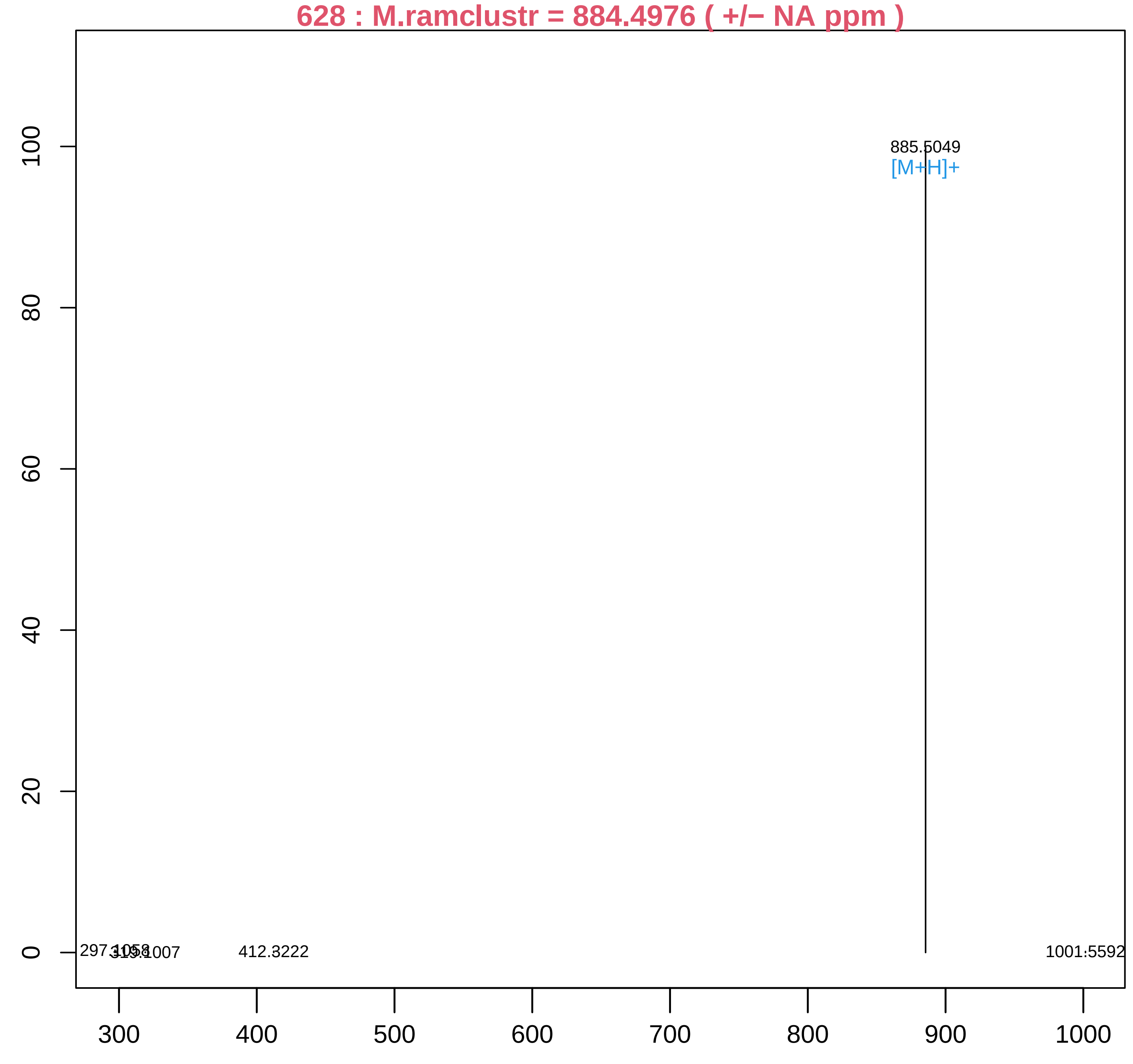
628 : M.ramclustr = 884.4976 (+/− NA ppm)
885.5049
[M+H]+
297.1058
313.1007
412.3222
1001.5592
0
20
40
60
80
100
300
400
500
600
700
800
900
1000

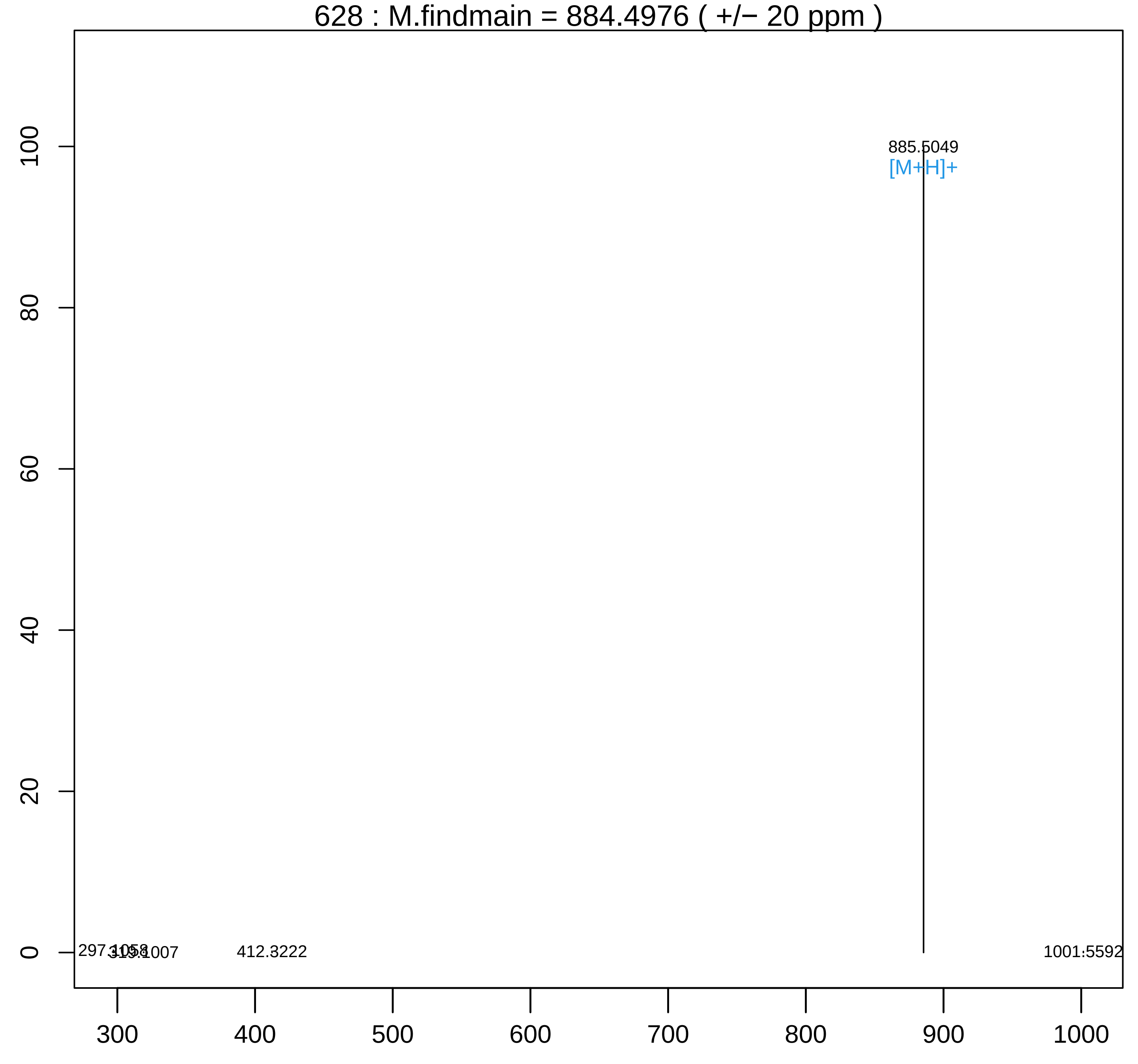
628 : M.findmain = 884.4976 (+/− 20 ppm)
885.5049
[M+H]+
297.1058
313.1007
412.3222
1001.5592
0
20
40
60
80
100
300
400
500
600
700
800
900
1000

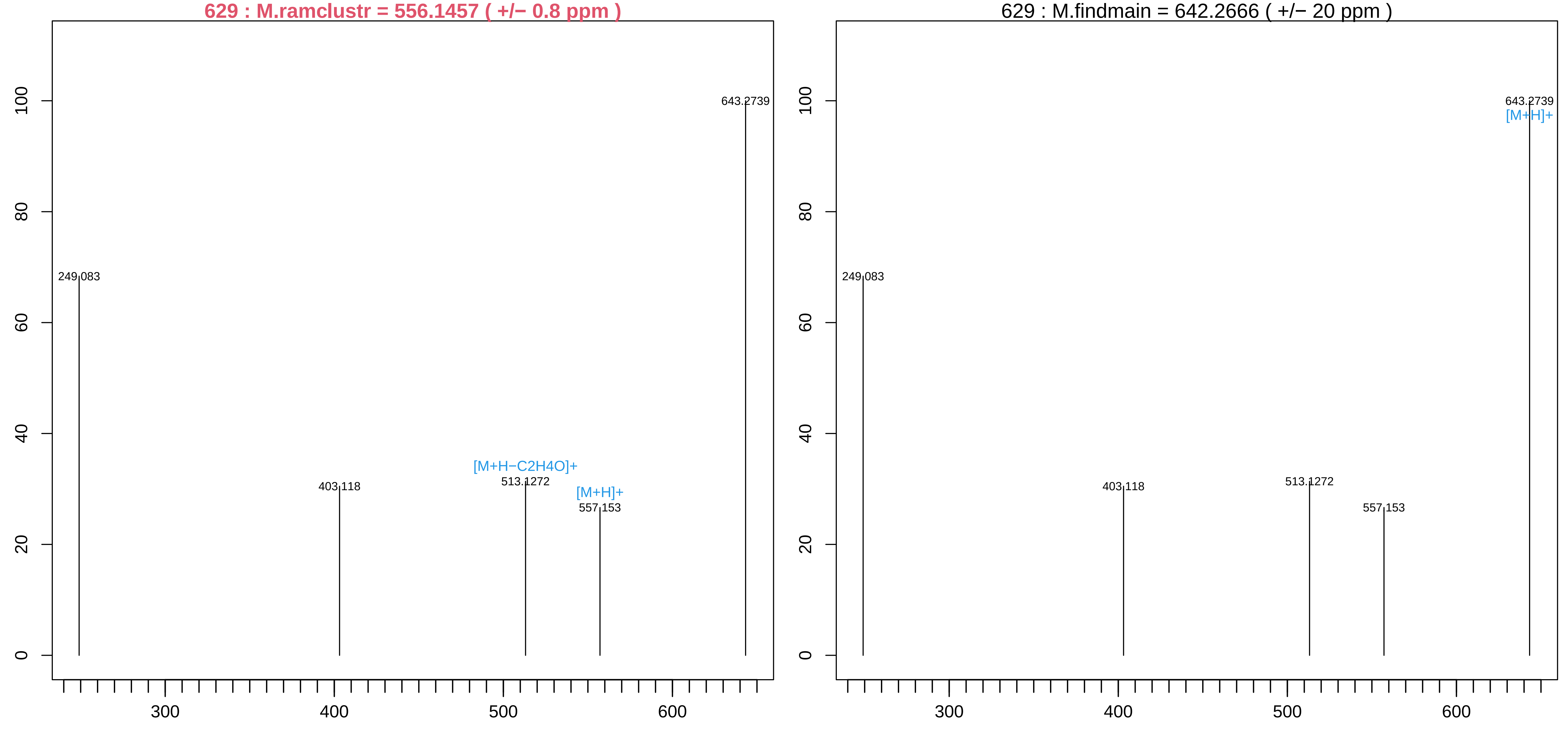
629 : M.ramclustr = 556.1457 (+/− 0.8 ppm)
643.2739
249.083
403.118
[M+H−C2H4O]+
513.1272
[M+H]+
557.153
629 : M.findmain = 642.2666 (+/− 20 ppm)
643.2739
[M+H]+
249.083
403.118
513.1272
557.153
0
20
40
60
80
100
300
400
500
600

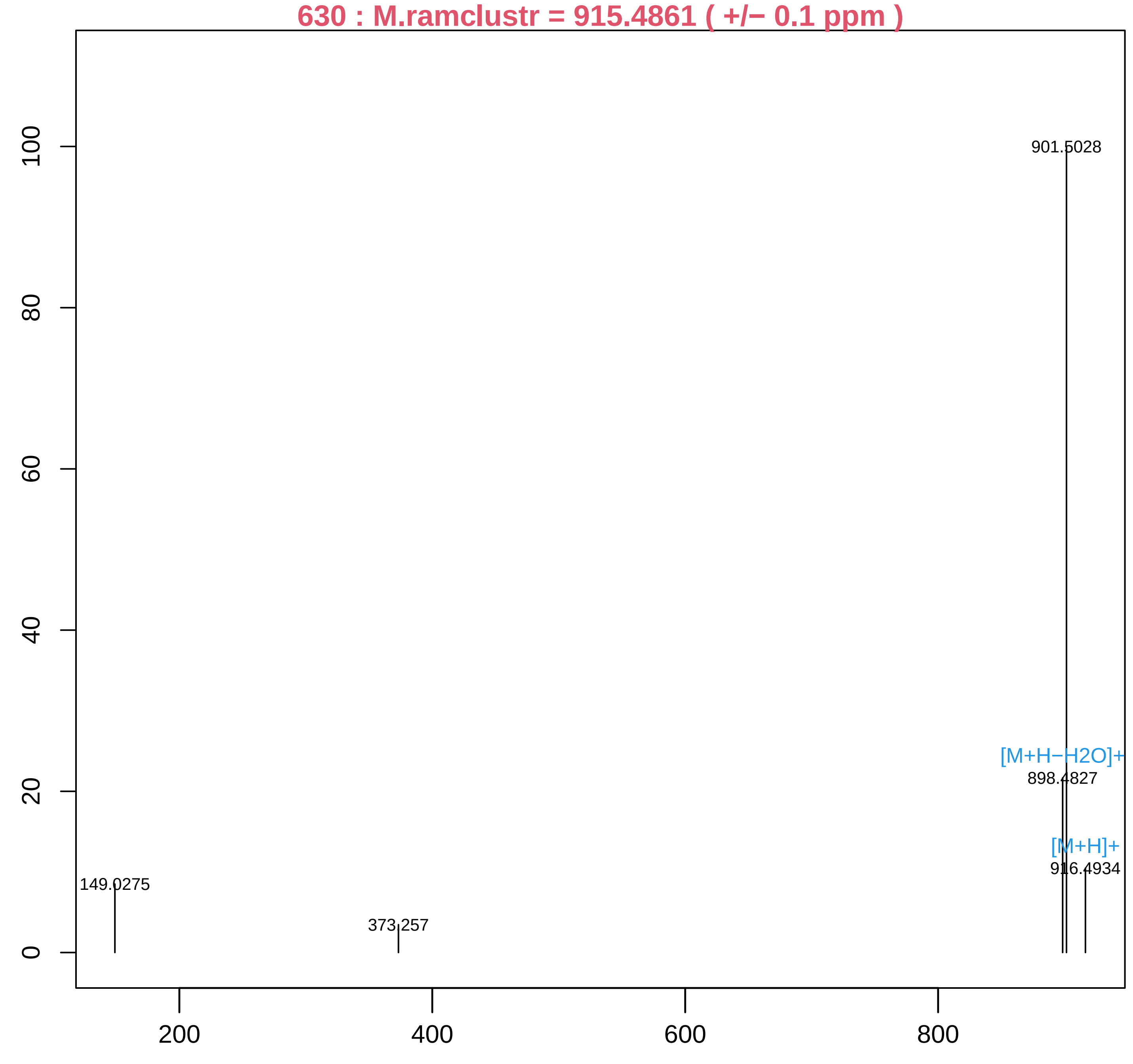

630 : M.ramclustr = 915.4861 (+/− 0.1 ppm)
0
20
40
60
80
100
200
400
600
800
901.5028
[M+H−H2O]+
898.4827
[M+H]+
916.4934
149.0275
373.257

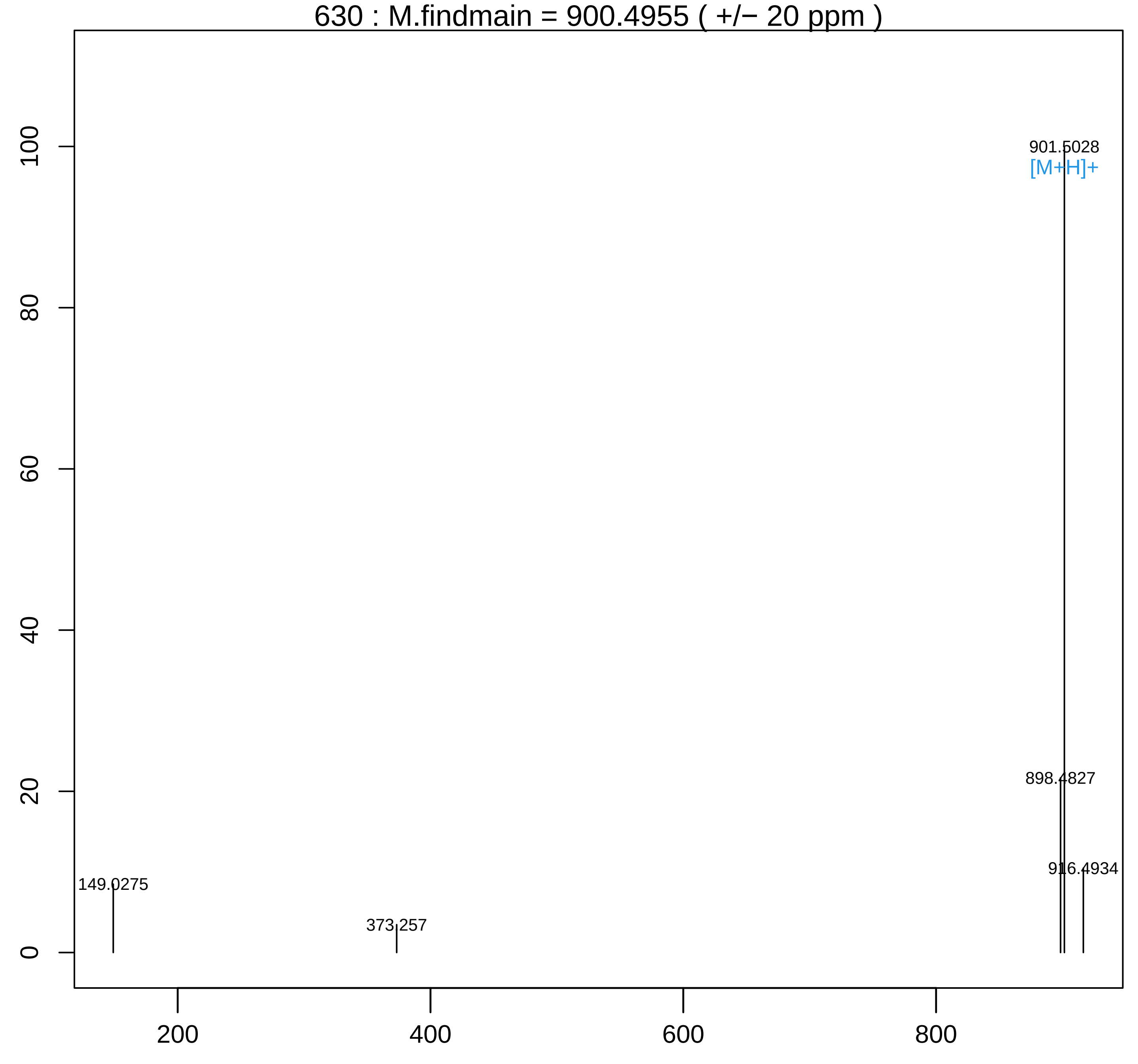

630 : M.findmain = 900.4955 (+/− 20 ppm)
0
20
40
60
80
100
200
400
600
800
901.5028
[M+H]+
898.4827
916.4934
149.0275
373.257

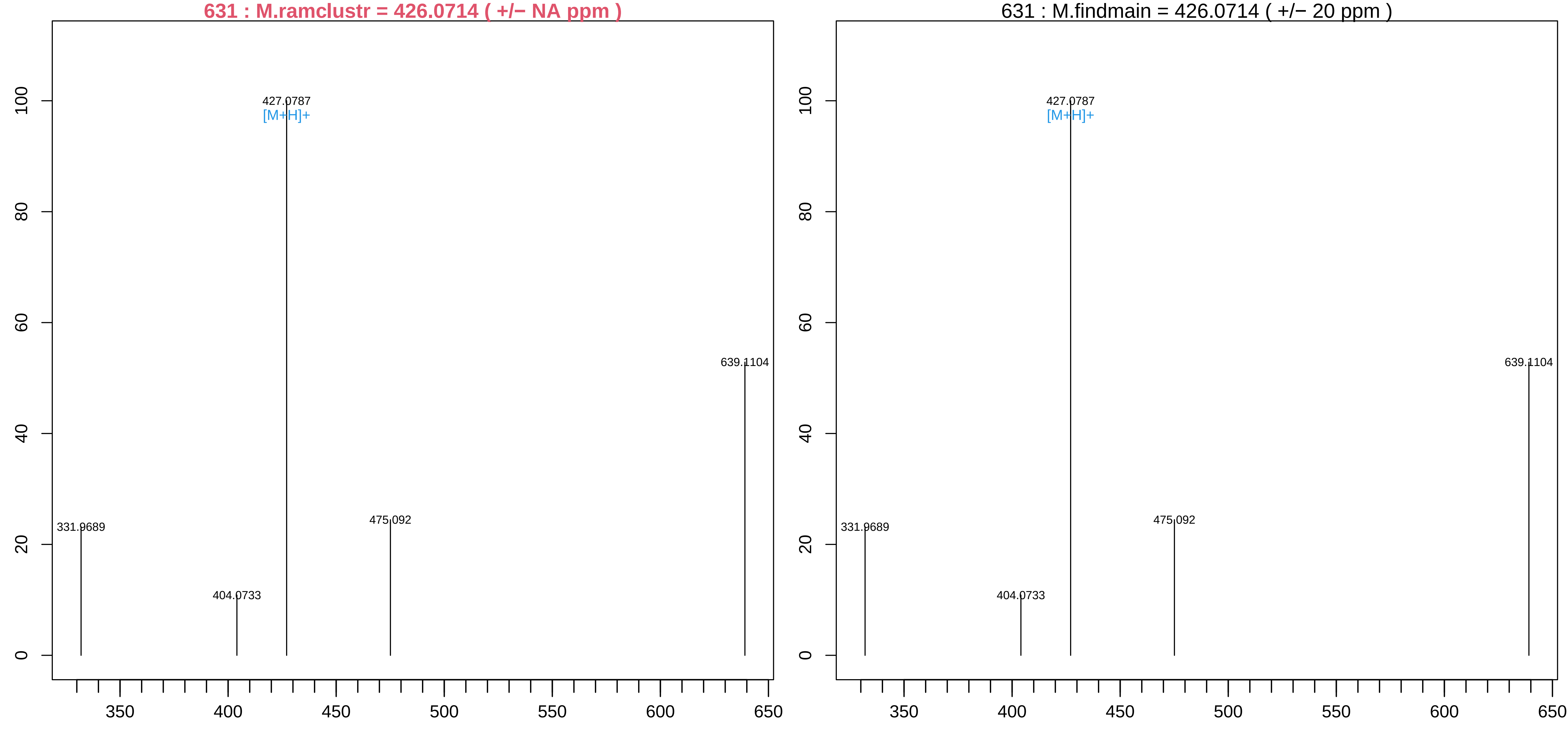
631 : M.ramclustr = 426.0714 (+/− NA ppm)
427.0787
[M+H]+
639.1104
331.9689
475.092
404.0733
631 : M.findmain = 426.0714 (+/− 20 ppm)
427.0787
[M+H]+
639.1104
331.9689
475.092
404.0733
0
20
40
60
80
100
350
400
450
500
550
600
650

632 : M.ramclustr = 999.5425 (+/− NA ppm)

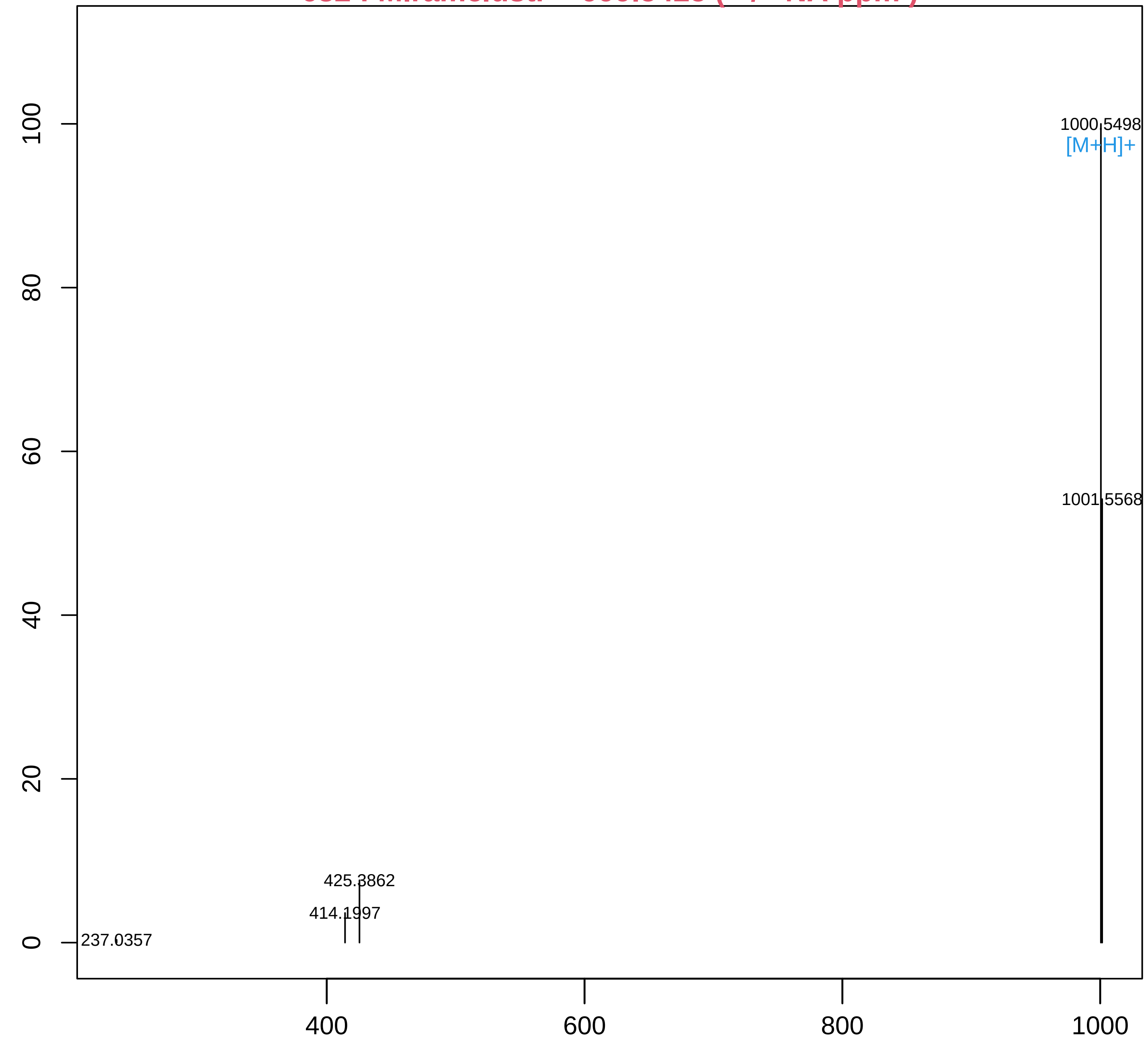

632 : M.findmain = 999.5425 (+/− 20 ppm)

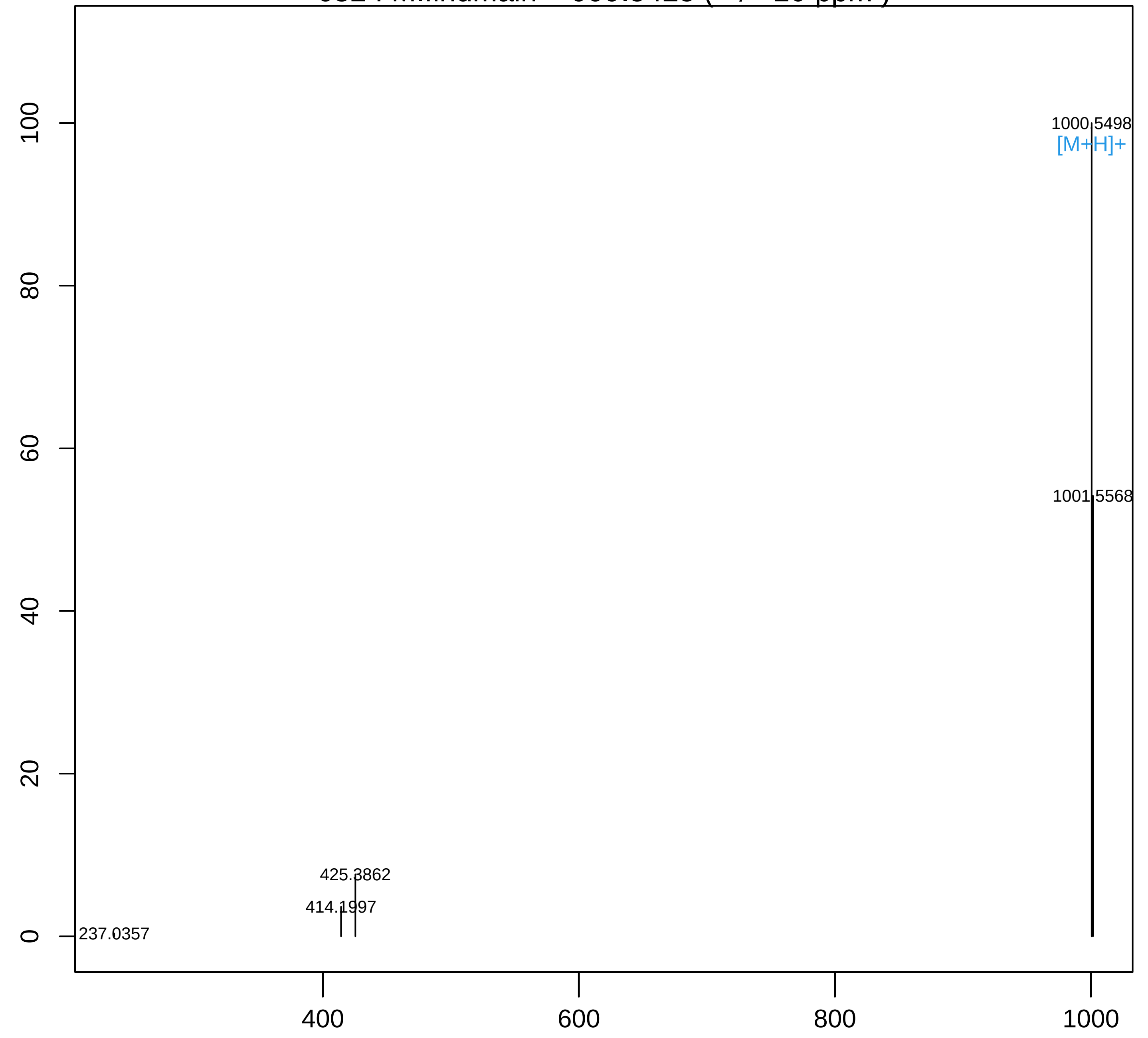

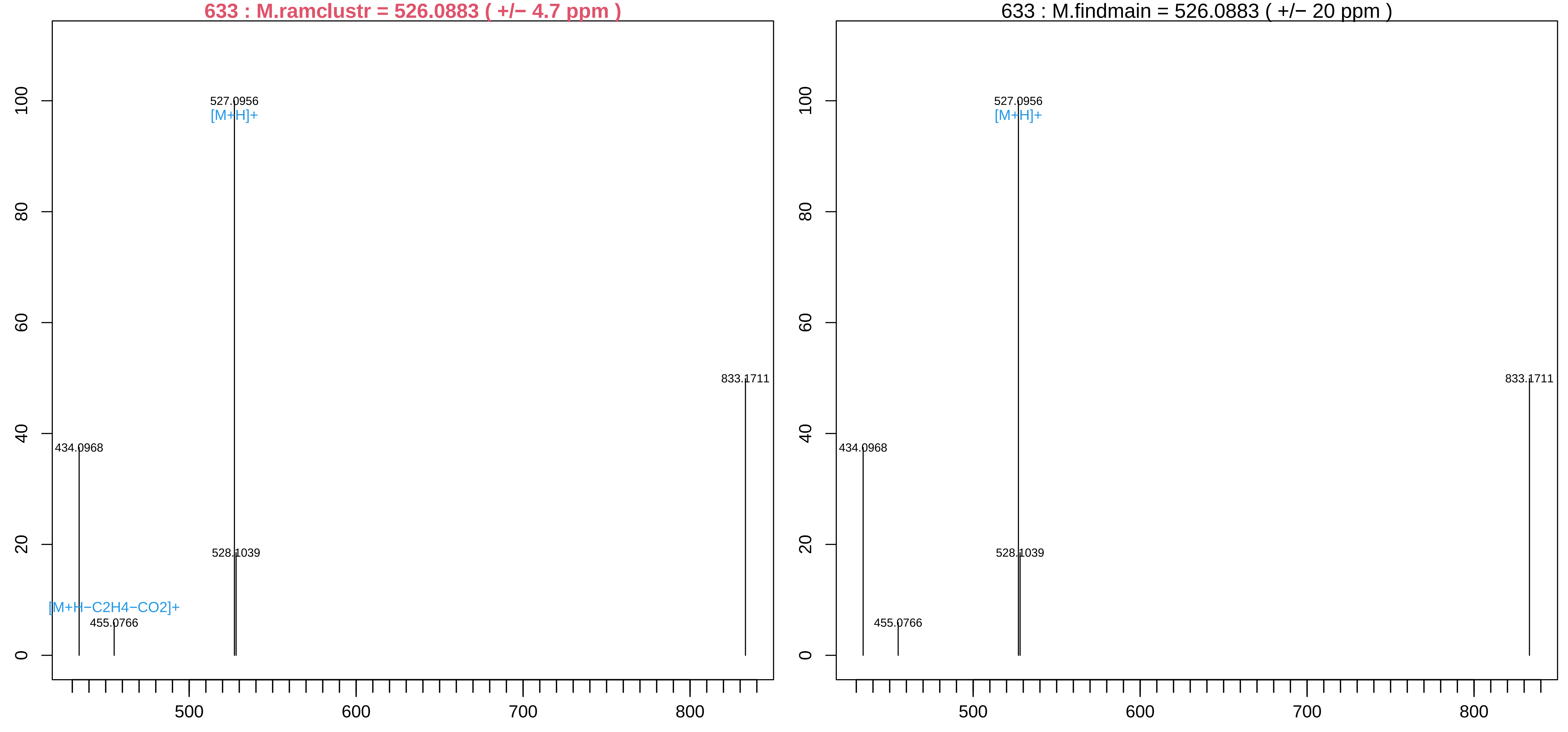
633 : M.ramclustr = 526.0883 (+/− 4.7 ppm)
527.0956
[M+H]+
833.1711
434.0968
528.1039
[M+H−C2H4−CO2]+
455.0766
633 : M.findmain = 526.0883 (+/− 20 ppm)
527.0956
[M+H]+
833.1711
434.0968
528.1039
455.0766

634 : M.ramclustr = 352.3207 (+/− NA ppm)

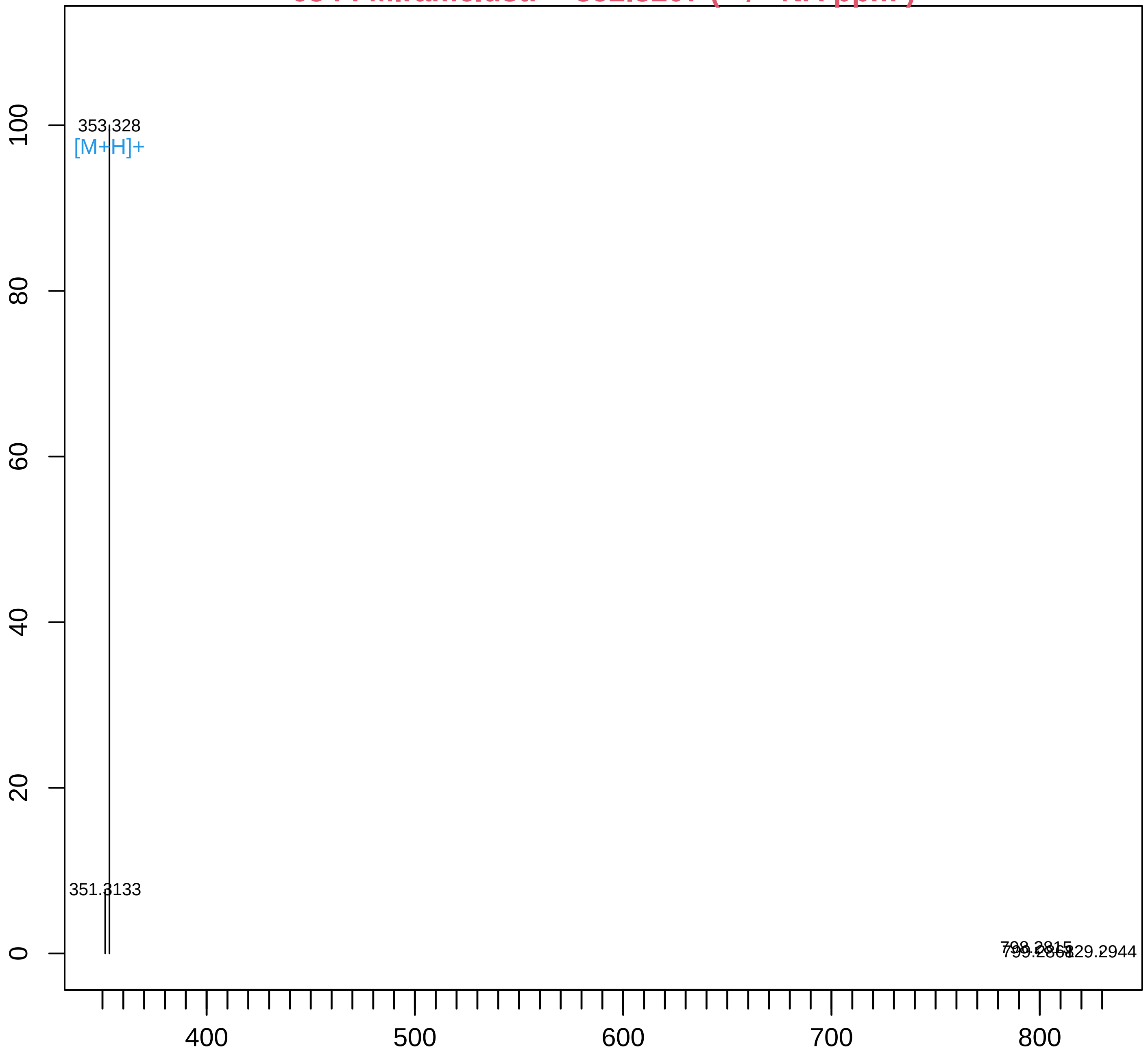

634 : M.findmain = 352.3207 (+/− 20 ppm)

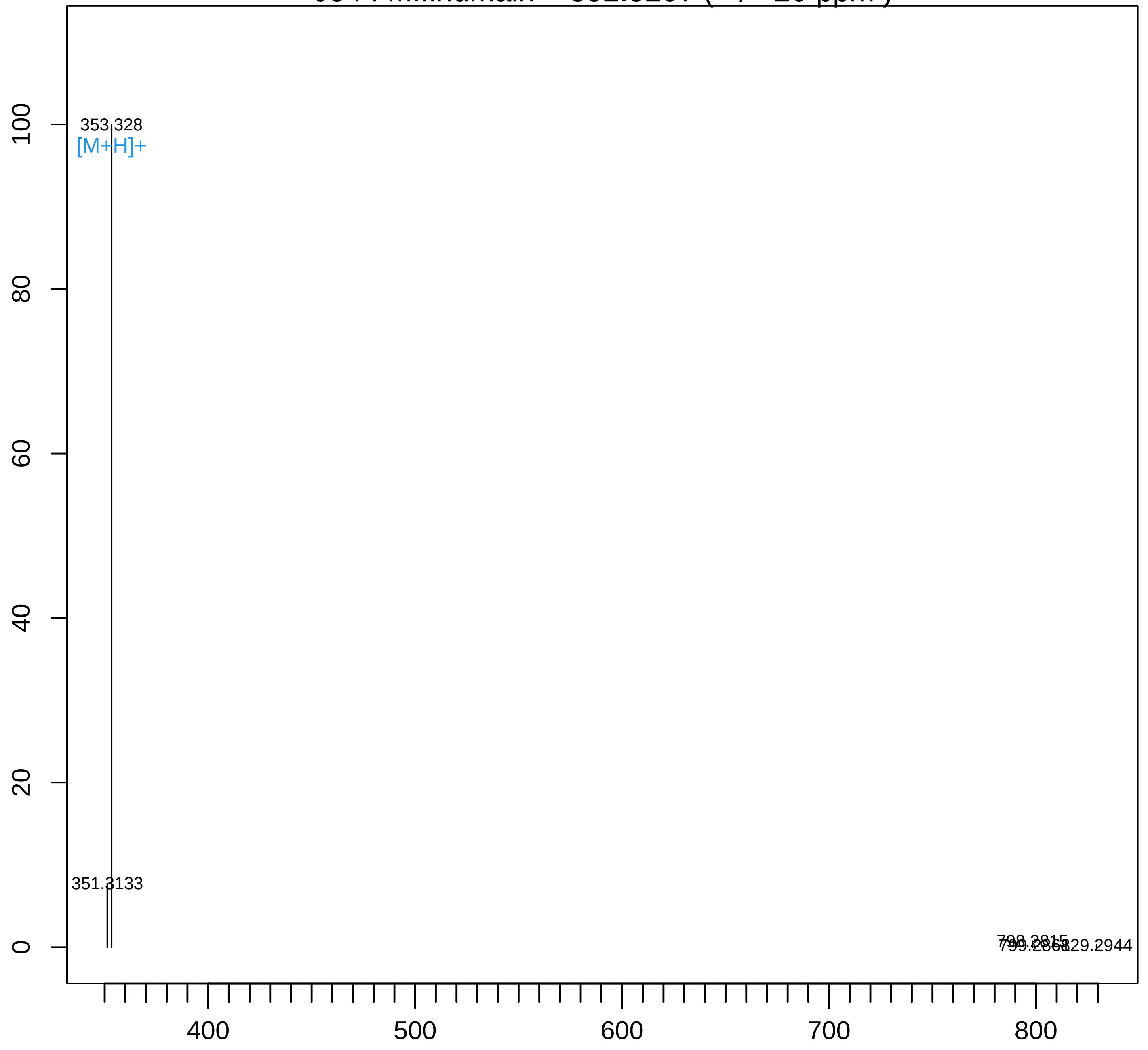

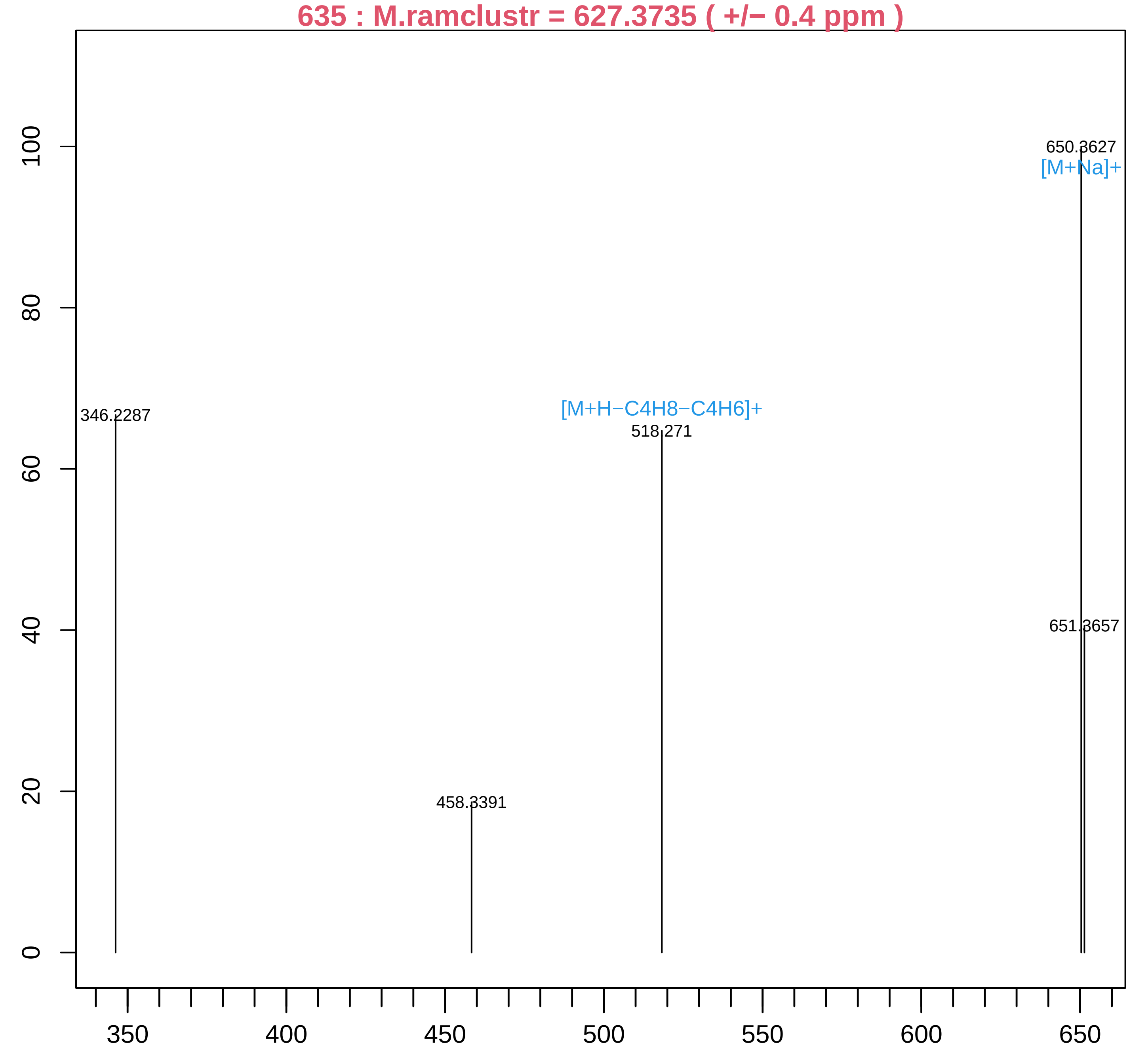

635 : M.ramclustr = 627.3735 (+/− 0.4 ppm)
346.2287
458.3391
[M+H−C4H8−C4H6]+
518.271
650.3627
[M+Na]+
651.3657
0
20
40
60
80
100
350
400
450
500
550
600
650

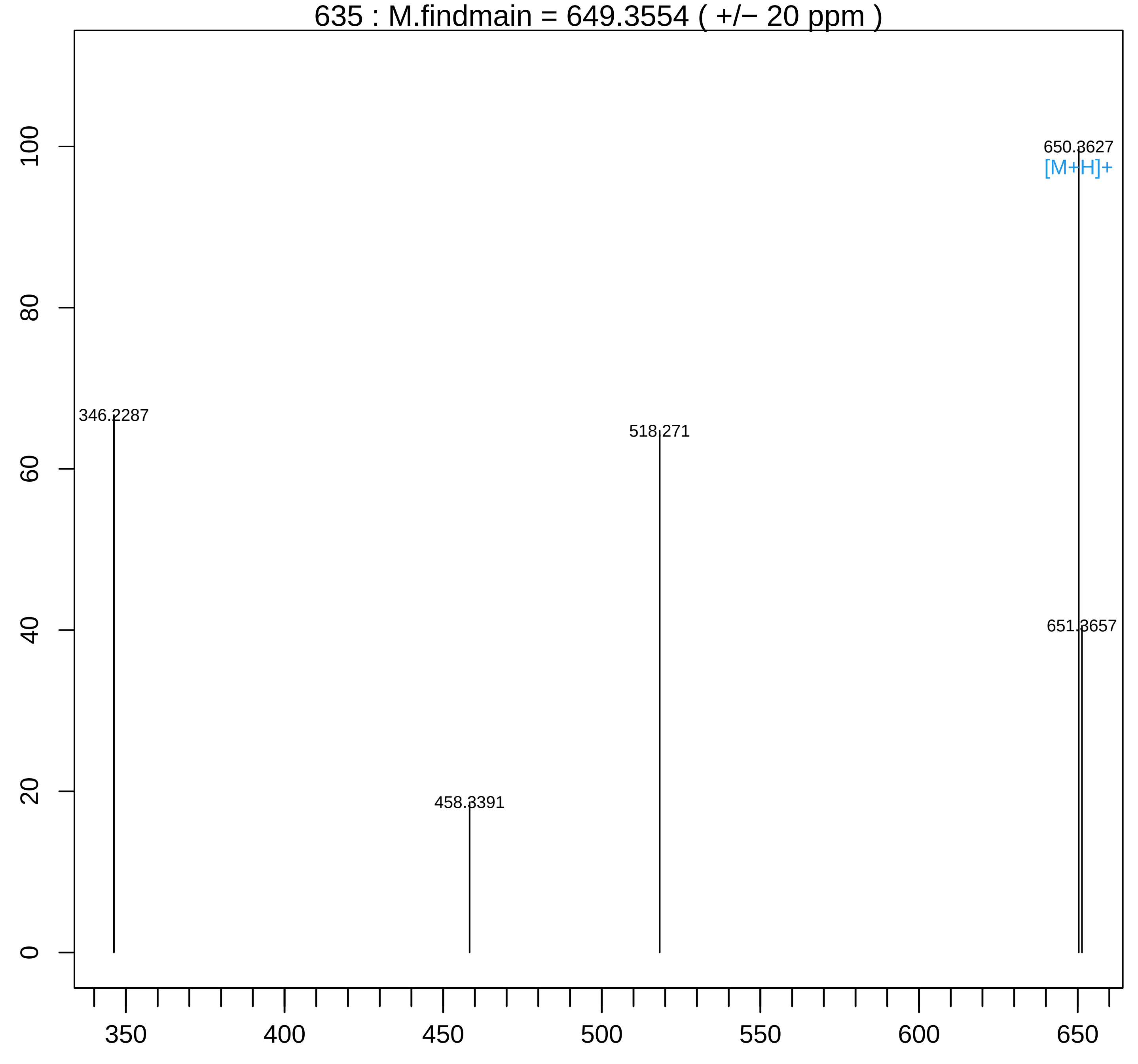

635 : M.findmain = 649.3554 (+/− 20 ppm)
346.2287
458.3391
518.271
650.3627
[M+H]+
651.3657
0
20
40
60
80
100
350
400
450
500
550
600
650

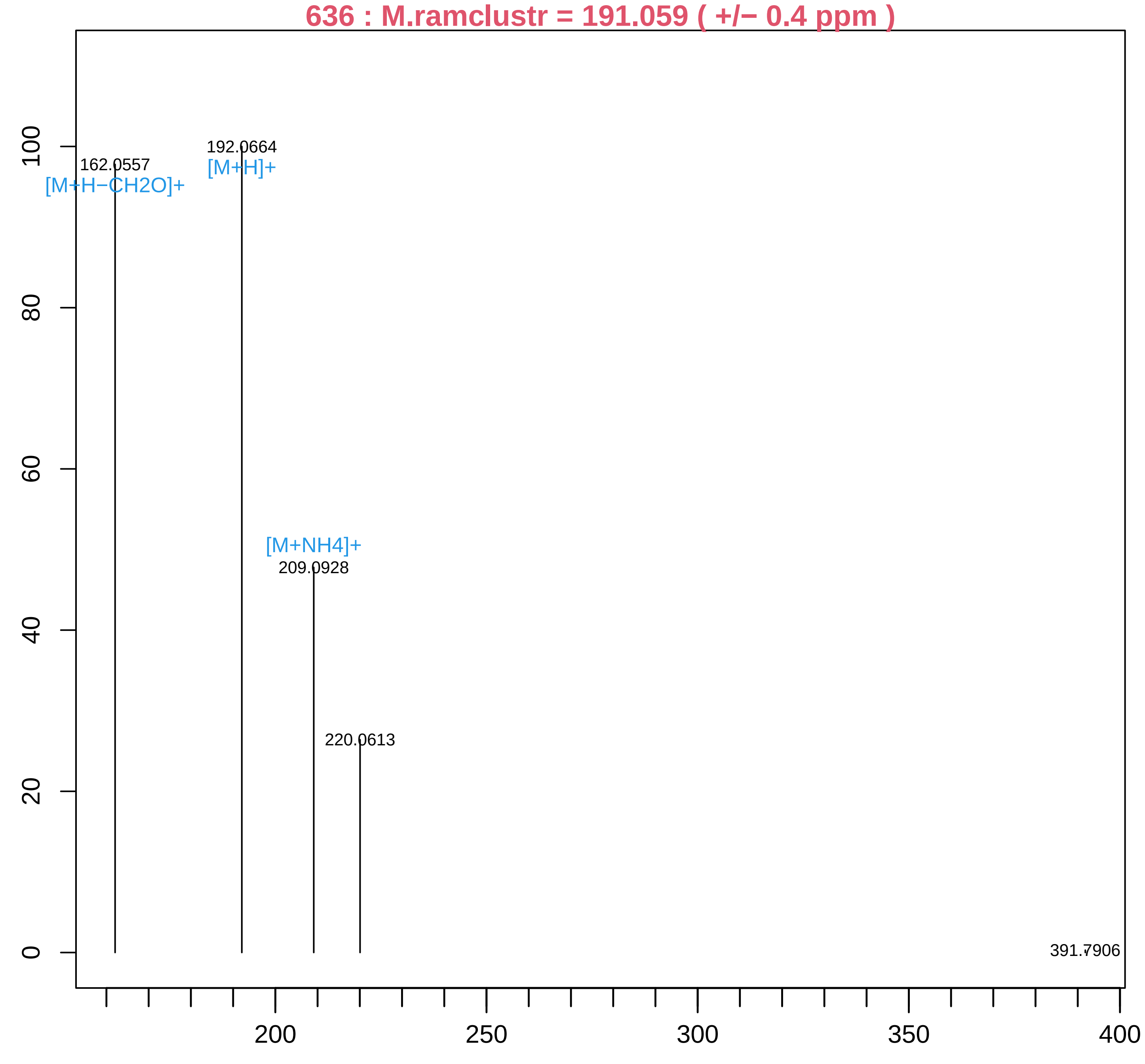
636 : M.ramclustr = 191.059 (+/− 0.4 ppm)
162.0557
[M+H−CH2O]+
192.0664
[M+H]+
[M+NH4]+
209.0928
220.0613
391.7906
0
20
40
60
80
100
200
250
300
350
400

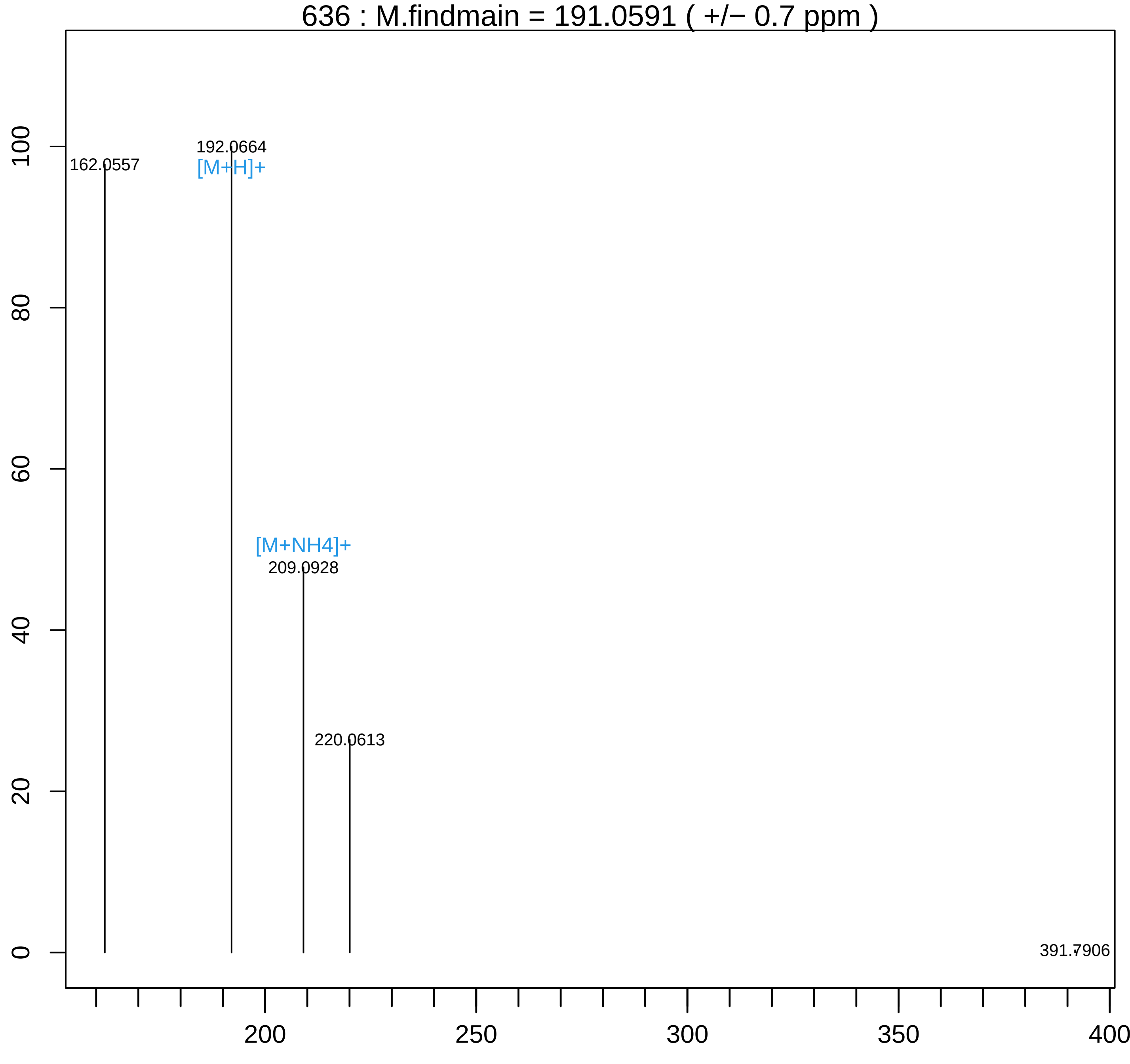
636 : M.findmain = 191.0591 (+/− 0.7 ppm)
162.0557
192.0664
[M+H]+
[M+NH4]+
209.0928
220.0613
391.7906
0
20
40
60
80
100
200
250
300
350
400

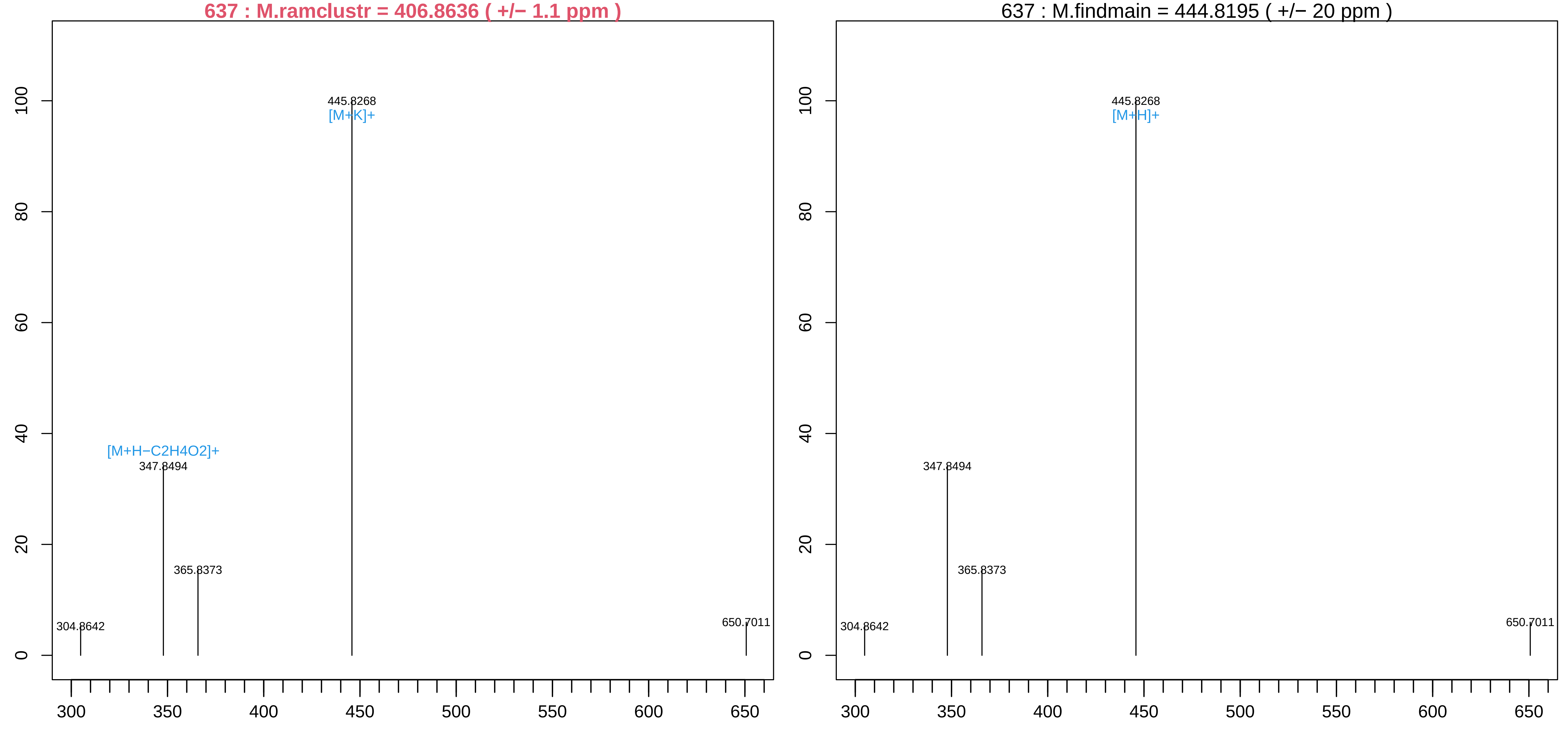
637 : M.ramclustr = 406.8636 (+/− 1.1 ppm)
445.8268
[M+K]+
[M+H−C2H4O2]+
347.8494
365.8373
304.8642
650.7011
637 : M.findmain = 444.8195 (+/− 20 ppm)
445.8268
[M+H]+
347.8494
365.8373
304.8642
650.7011

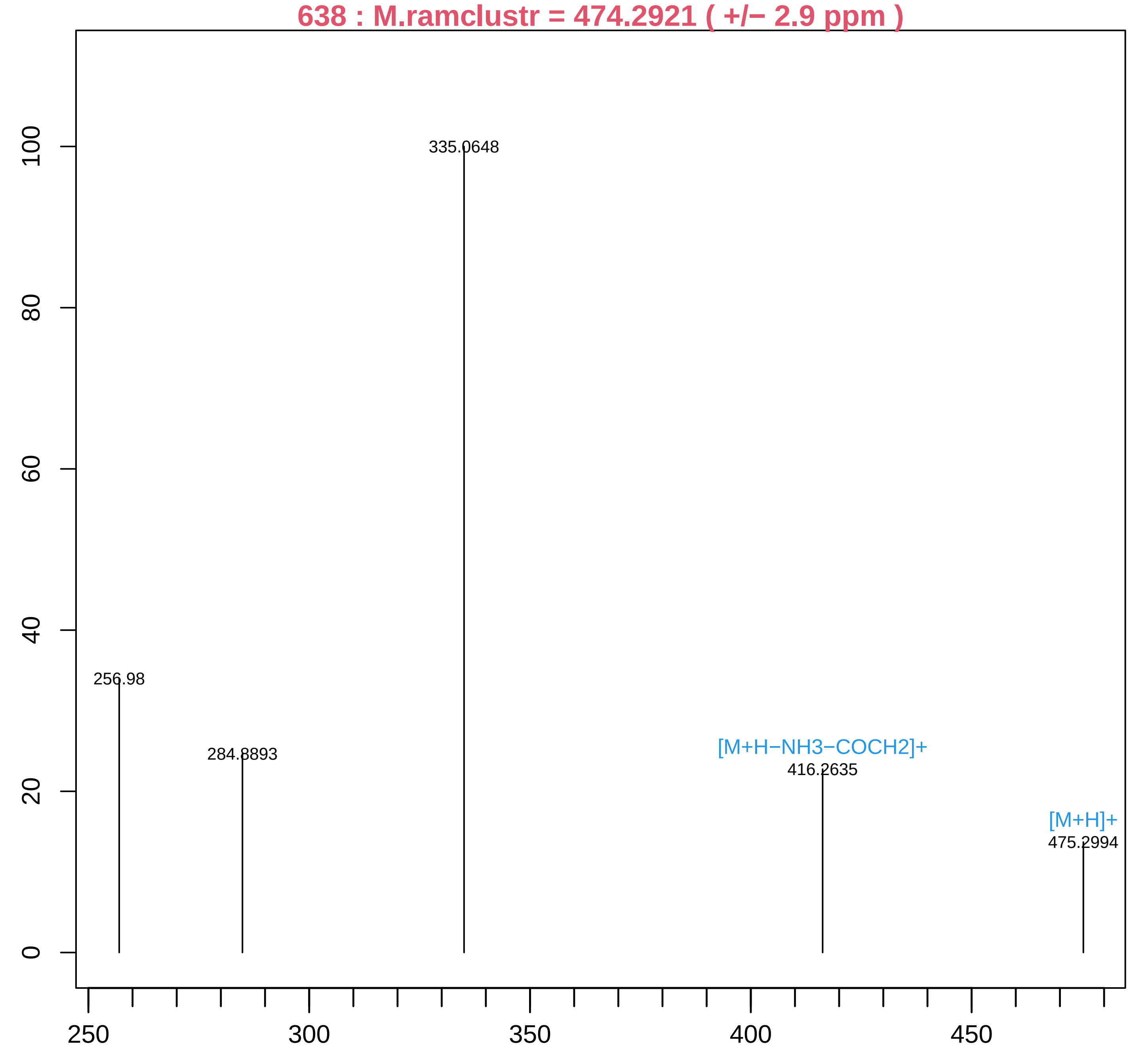

638 : M.ramclustr = 474.2921 (+/− 2.9 ppm)
335.0648
256.98
284.8893
[M+H−NH3−COCH2]+
416.2635
[M+H]+
475.2994
0
20
40
60
80
100
250
300
350
400
450

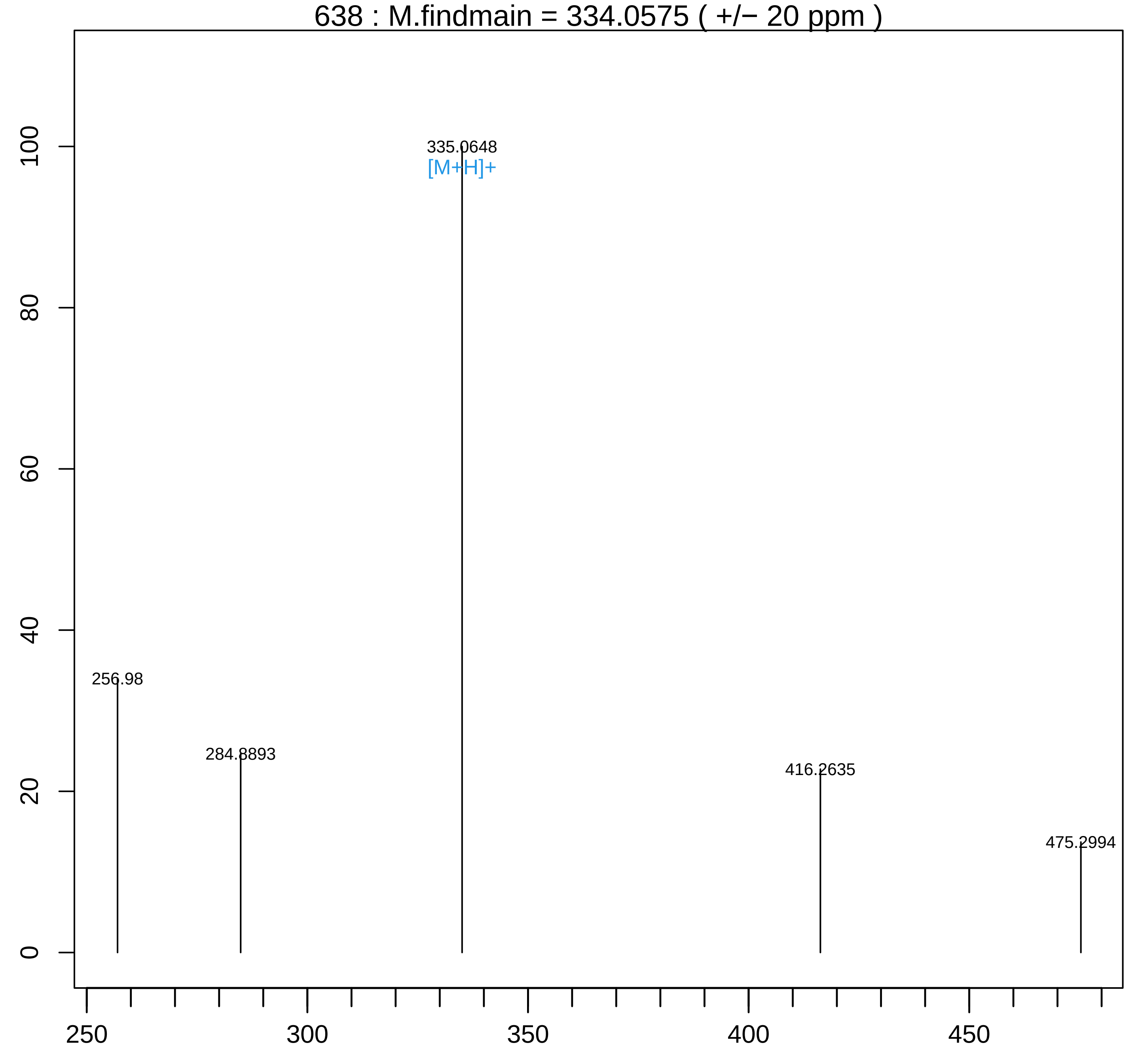

638 : M.findmain = 334.0575 (+/− 20 ppm)
335.0648
[M+H]+
256.98
284.8893
416.2635
475.2994
0
20
40
60
80
100
250
300
350
400
450

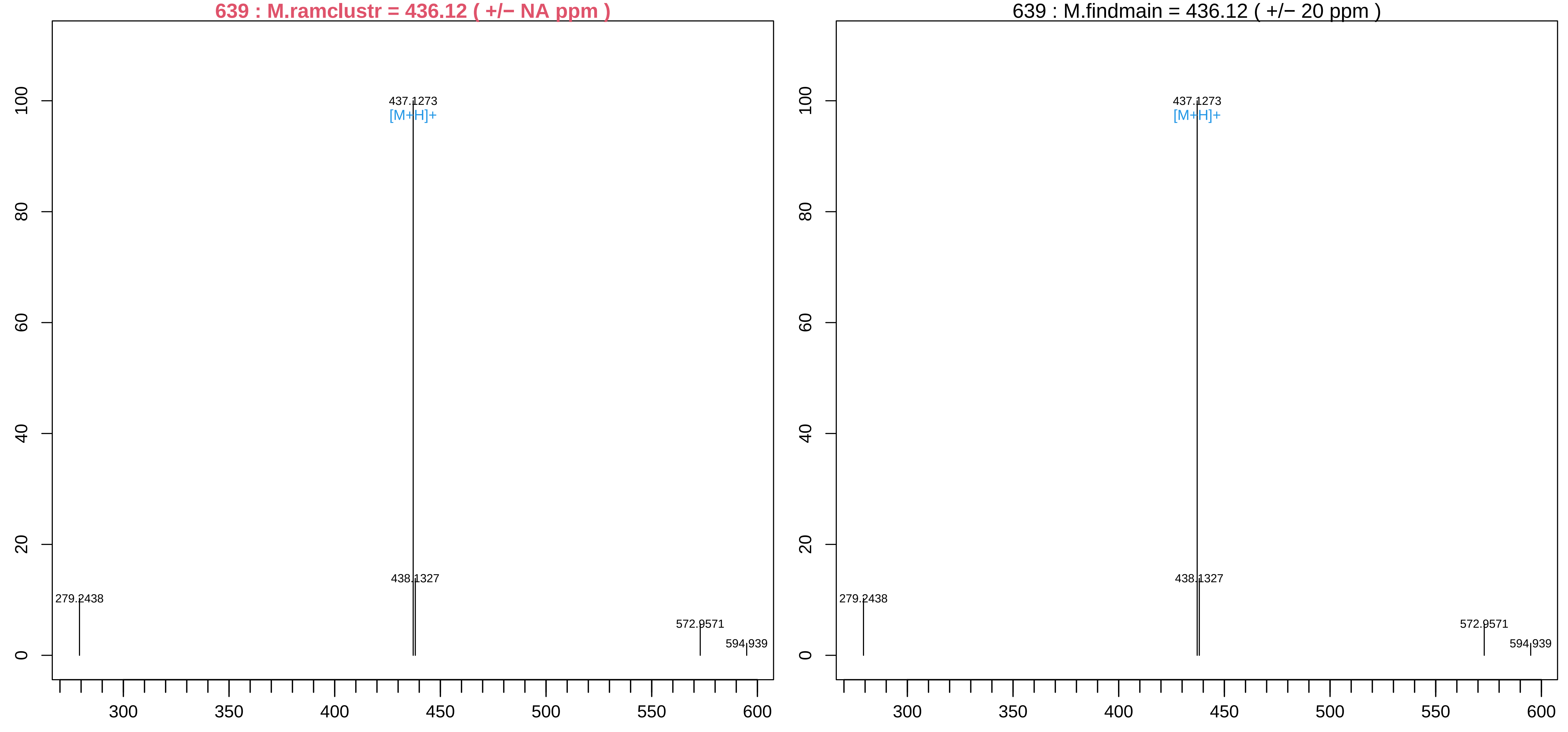

639 : M.ramclustr = 436.12 (+/− NA ppm)
437.1273
[M+H]+
438.1327
279.2438
572.9571
594.939
0
20
40
60
80
100
300
350
400
450
500
550
600
639 : M.findmain = 436.12 (+/− 20 ppm)
437.1273
[M+H]+
438.1327
279.2438
572.9571
594.939
0
20
40
60
80
100
300
350
400
450
500
550
600

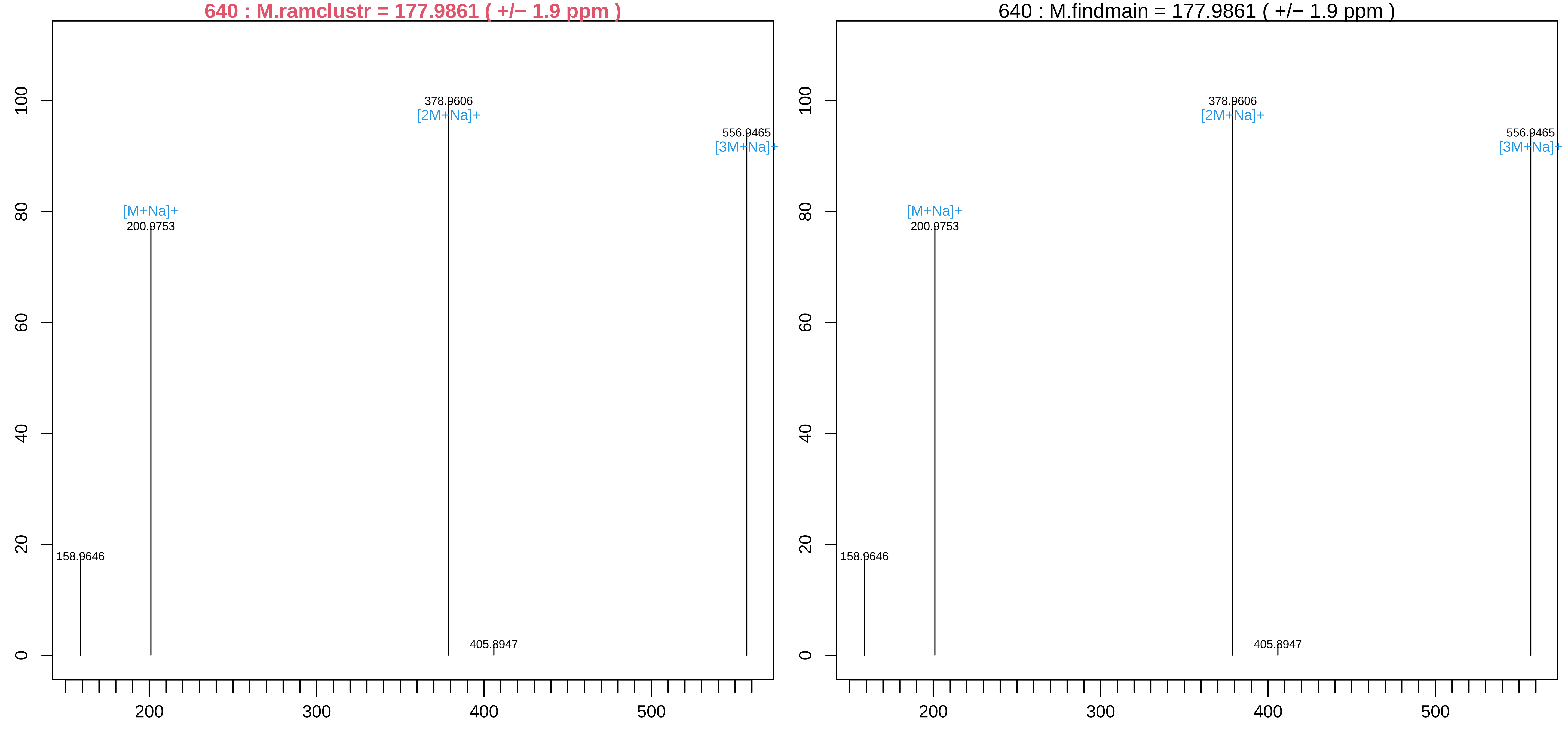

640 : M.ramclustr = 177.9861 (+/− 1.9 ppm)
640 : M.findmain = 177.9861 (+/− 1.9 ppm)
158.9646
[M+Na]+
200.9753
378.9606
[2M+Na]+
405.8947
556.9465
[3M+Na]+
0
20
40
60
80
100
200
300
400
500

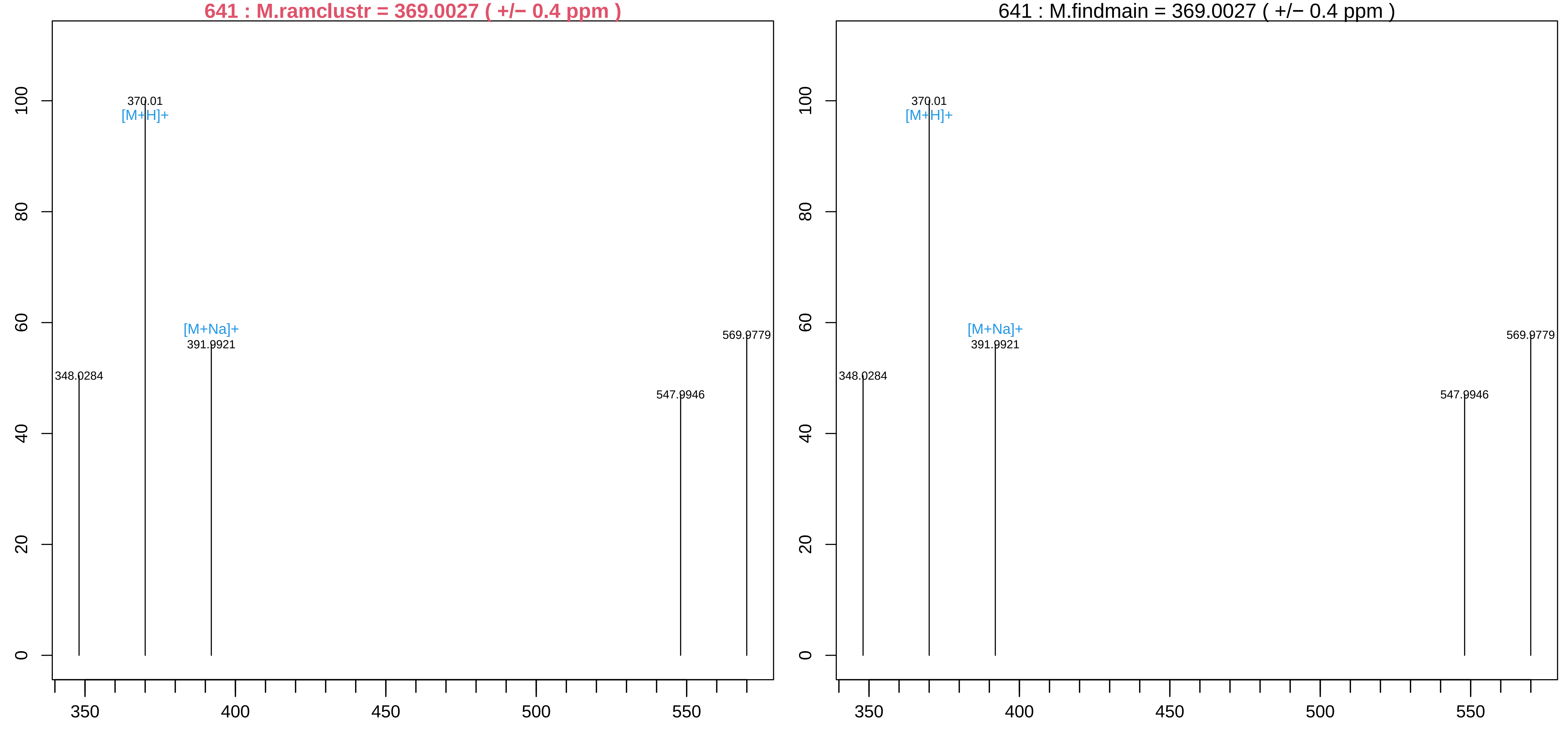

641 : M.ramclustr = 369.0027 (+/− 0.4 ppm)
370.01
[M+H]+
[M+Na]+
391.9921
348.0284
547.9946
569.9779
0
20
40
60
80
100
350
400
450
500
550
641 : M.findmain = 369.0027 (+/− 0.4 ppm)
370.01
[M+H]+
[M+Na]+
391.9921
348.0284
547.9946
569.9779
0
20
40
60
80
100
350
400
450
500
550

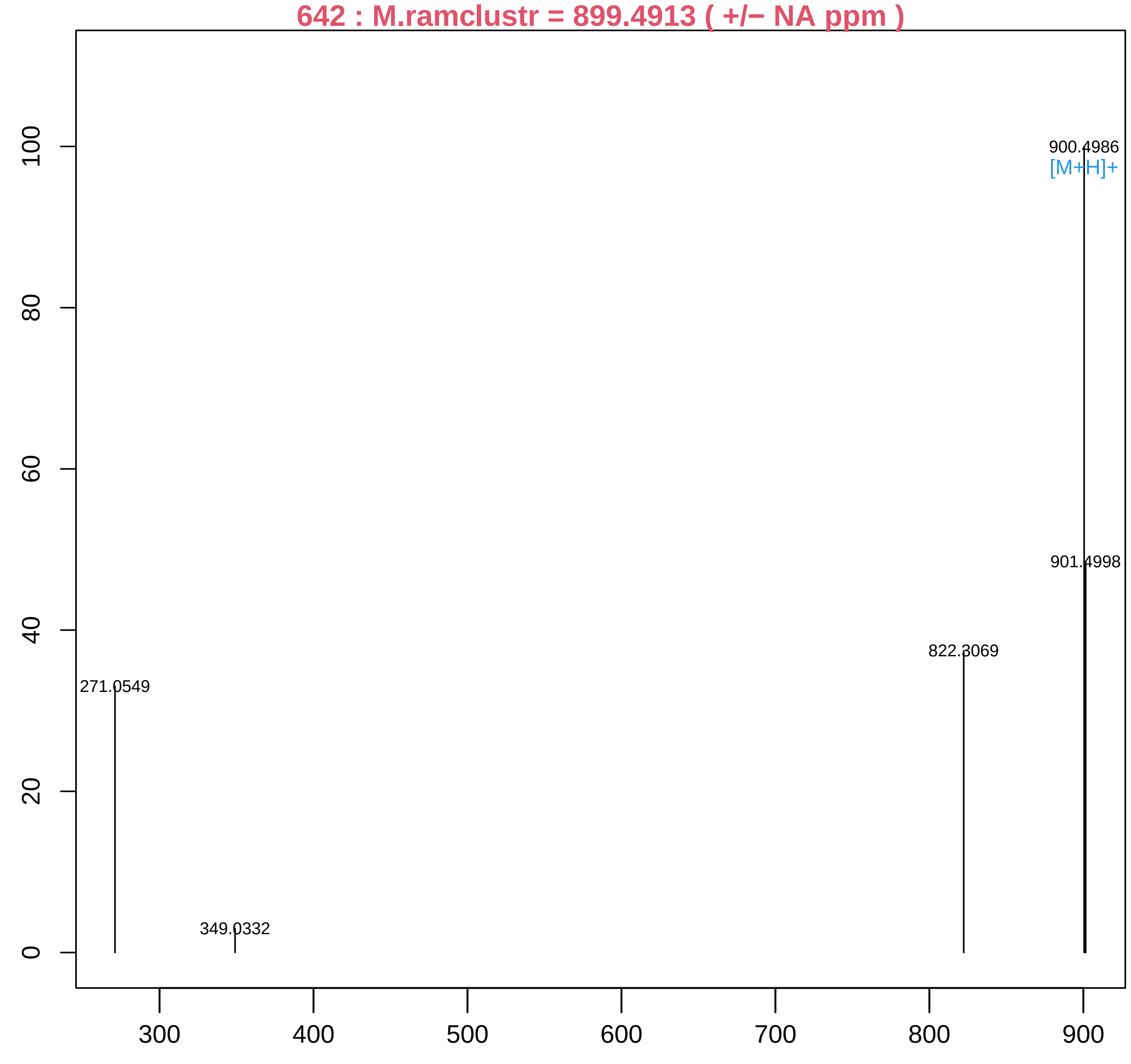

642 : M.ramclustr = 899.4913 (+/− NA ppm)
0
20
40
60
80
100
300
400
500
600
700
800
900
900.4986
[M+H]+
901.4998
822.3069
271.0549
349.0332

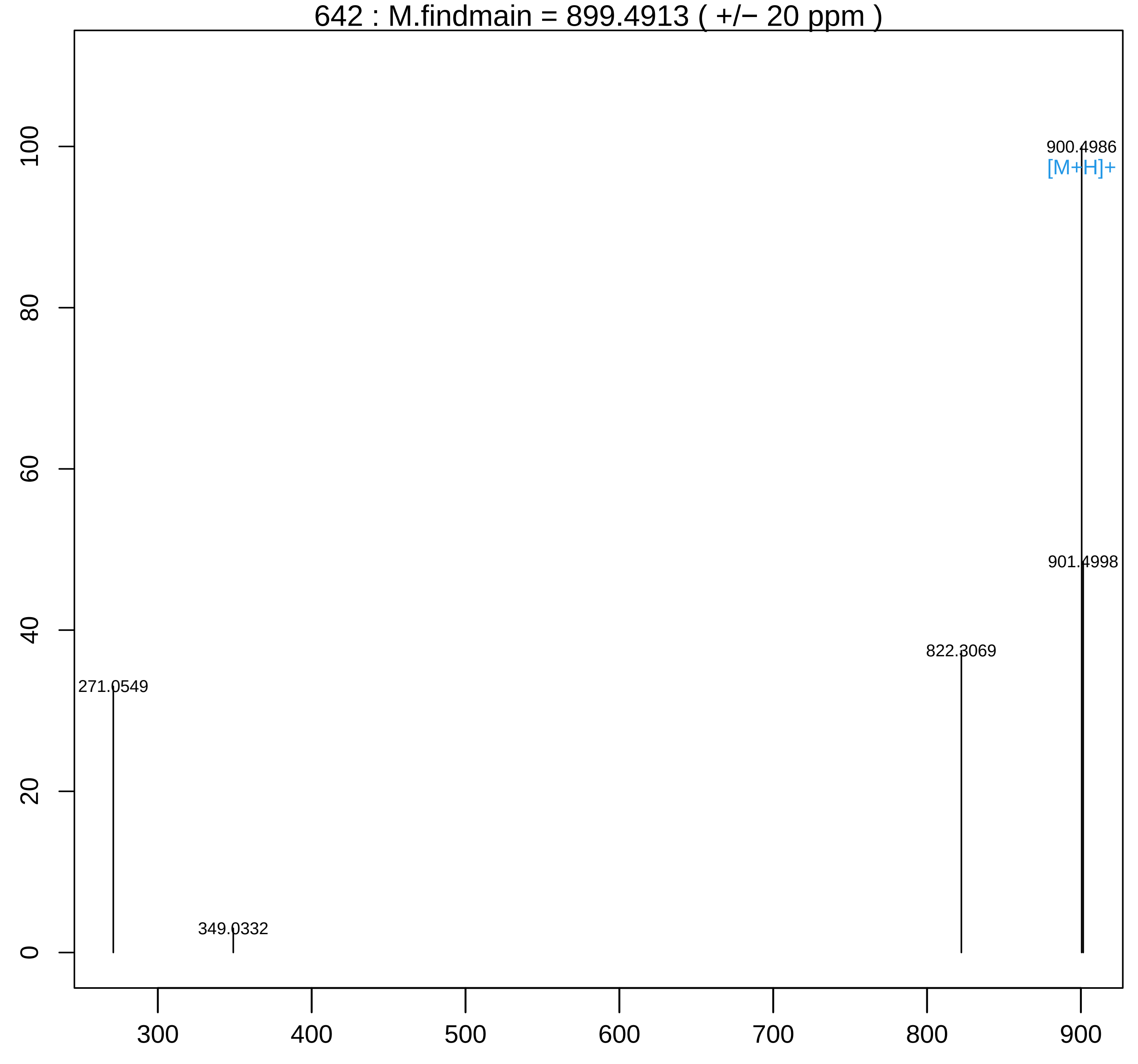

642 : M.findmain = 899.4913 (+/− 20 ppm)
0
20
40
60
80
100
300
400
500
600
700
800
900
900.4986
[M+H]+
901.4998
822.3069
271.0549
349.0332

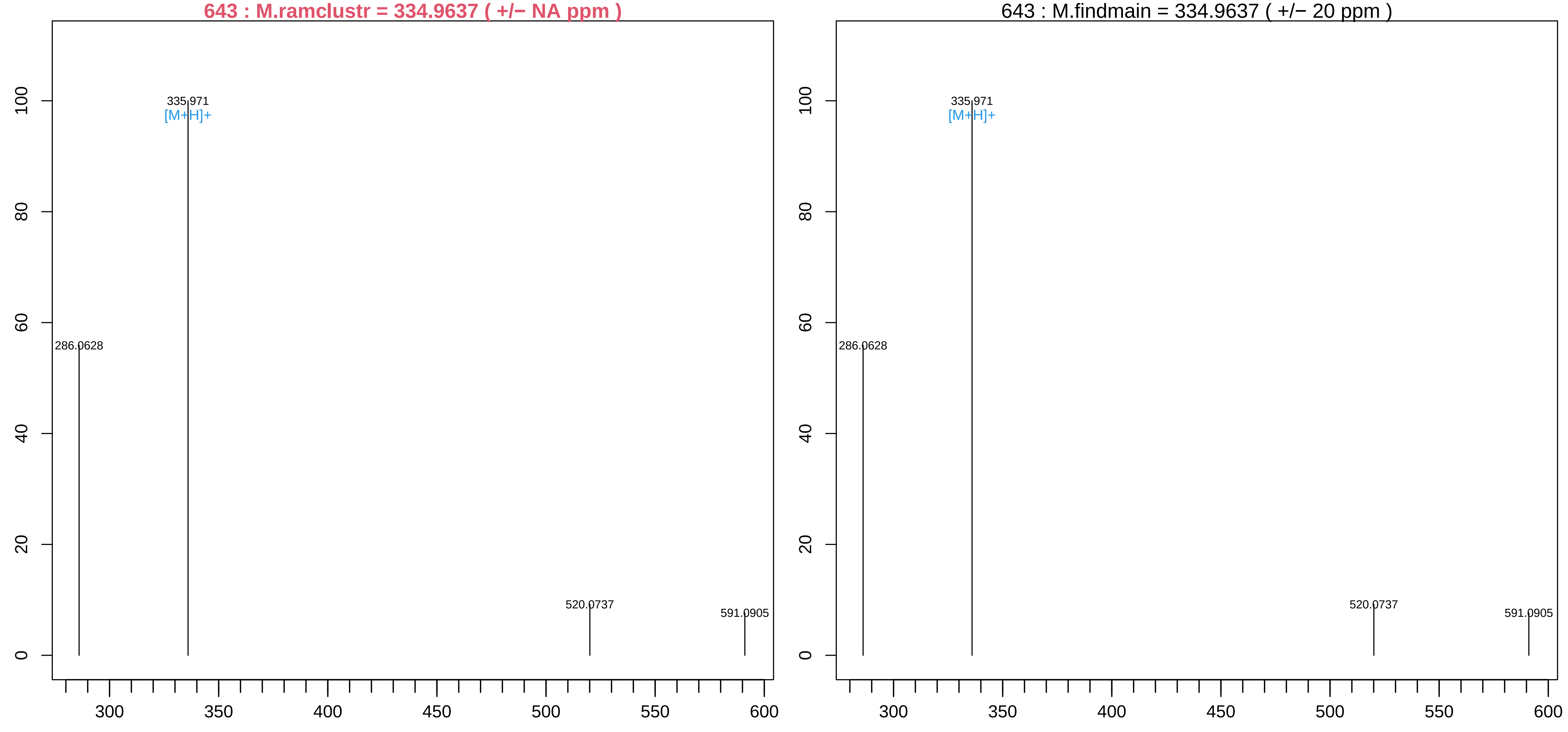

643 : M.ramclustr = 334.9637 (+/− NA ppm)
335.971
[M+H]+
286.0628
520.0737
591.0905
643 : M.findmain = 334.9637 (+/− 20 ppm)
335.971
[M+H]+
286.0628
520.0737
591.0905

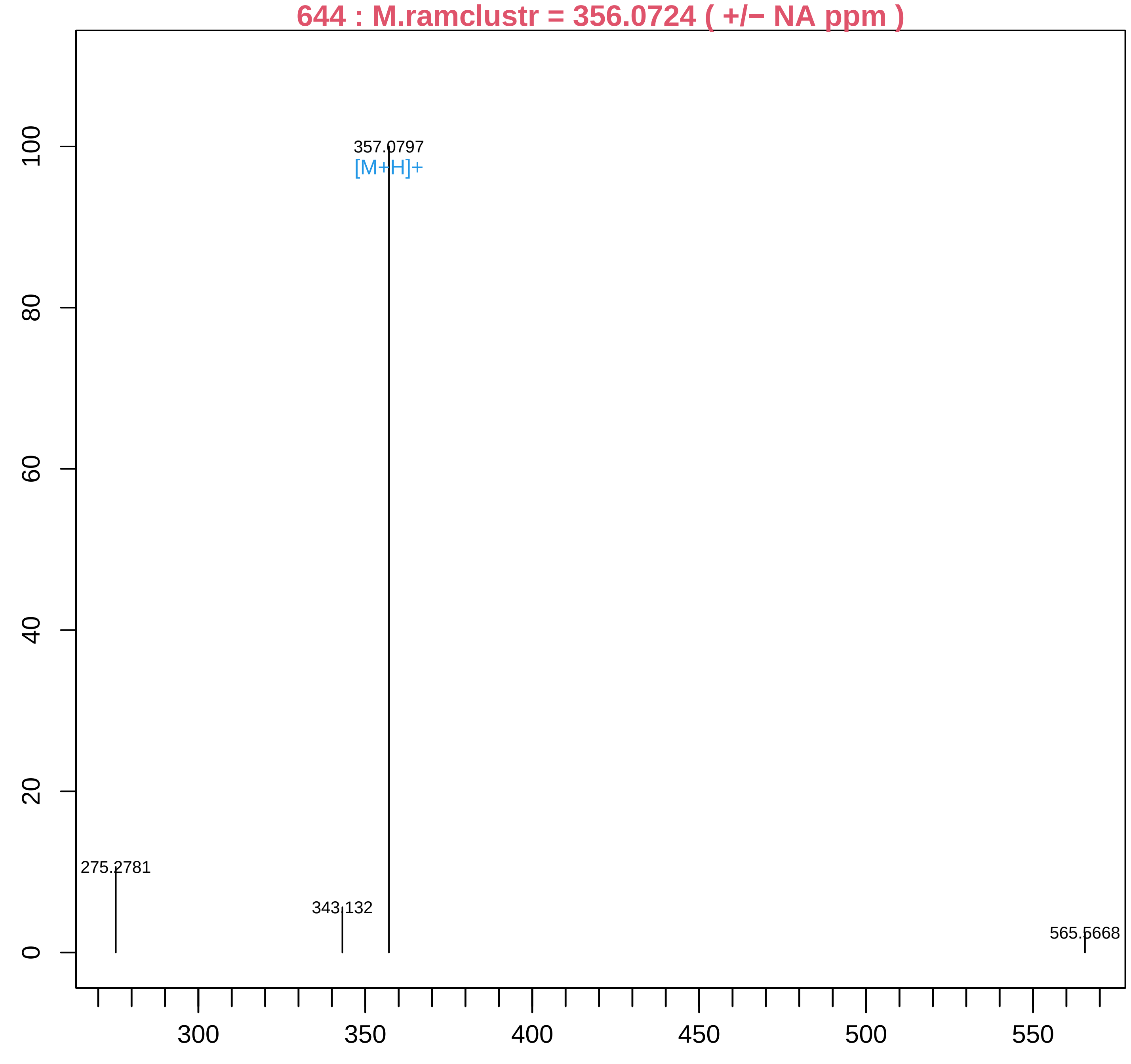
644 : M.ramclustr = 356.0724 (+/− NA ppm)
357.0797
[M+H]+
275.2781
343.132
565.5668
0
20
40
60
80
100
300
350
400
450
500
550

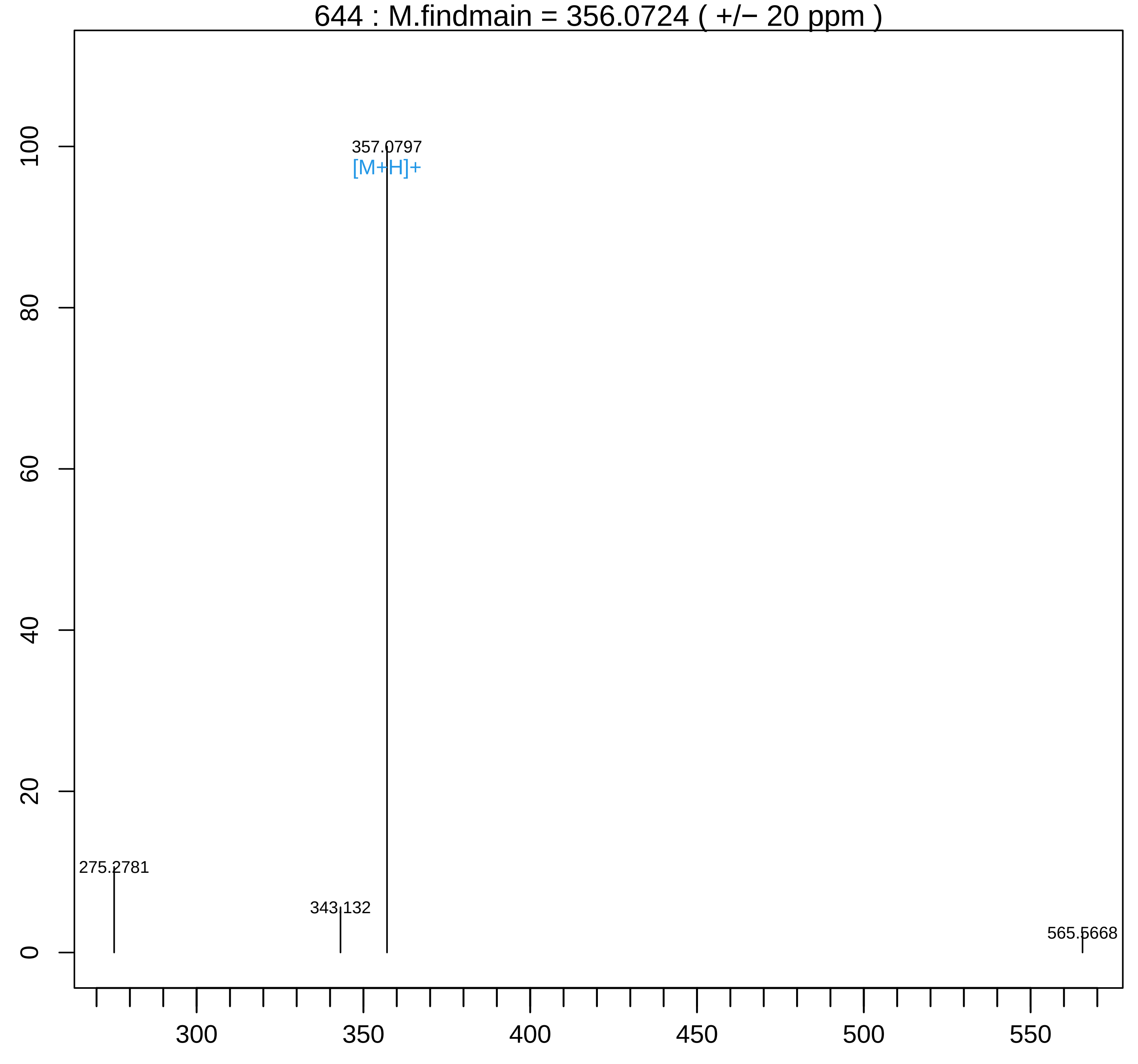
644 : M.findmain = 356.0724 (+/− 20 ppm)
357.0797
[M+H]+
275.2781
343.132
565.5668
0
20
40
60
80
100
300
350
400
450
500
550

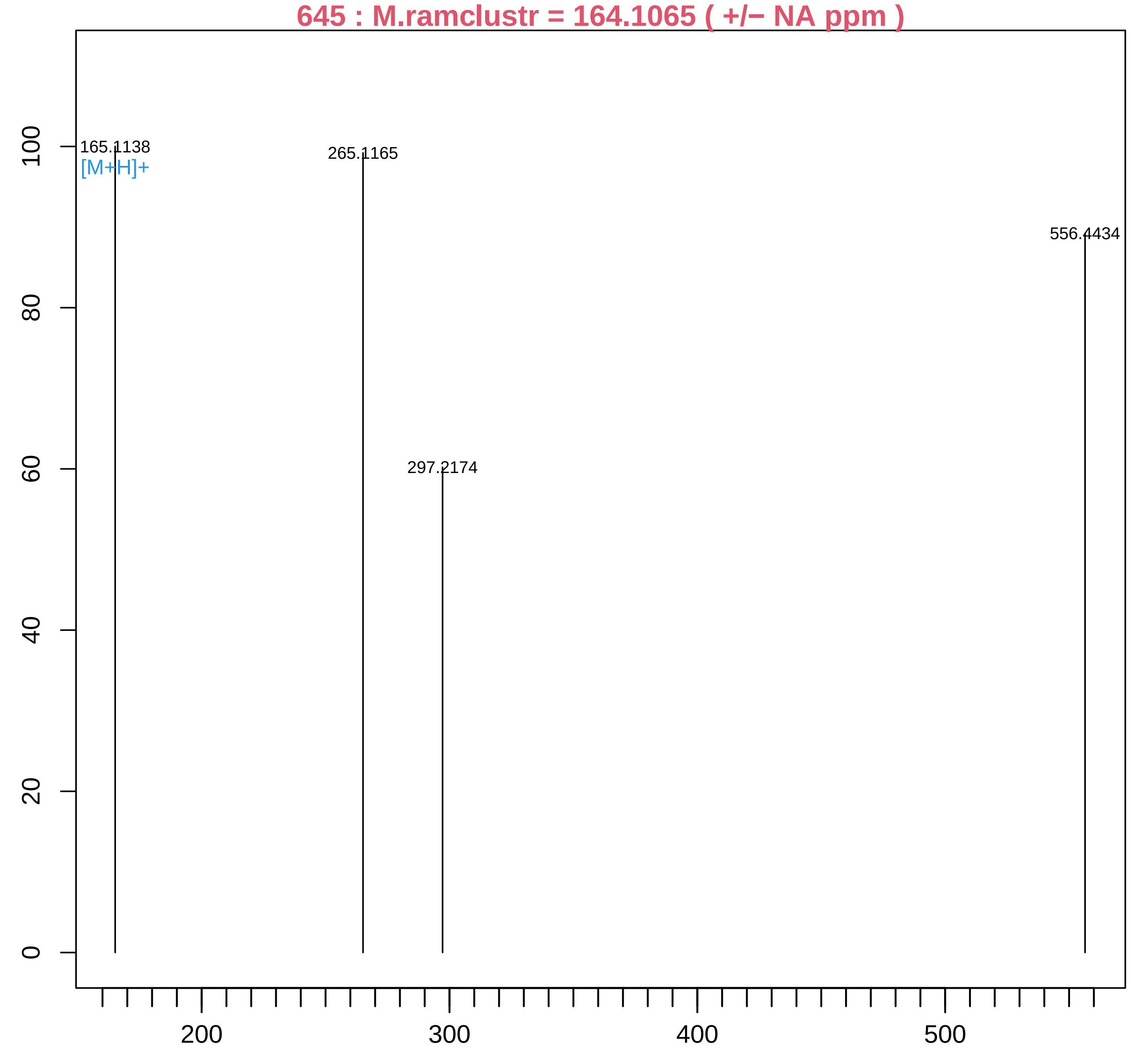
645 : M.ramclustr = 164.1065 (+/− NA ppm)
165.1138
[M+H]+
265.1165
297.2174
556.4434
0
20
40
60
80
100
200
300
400
500

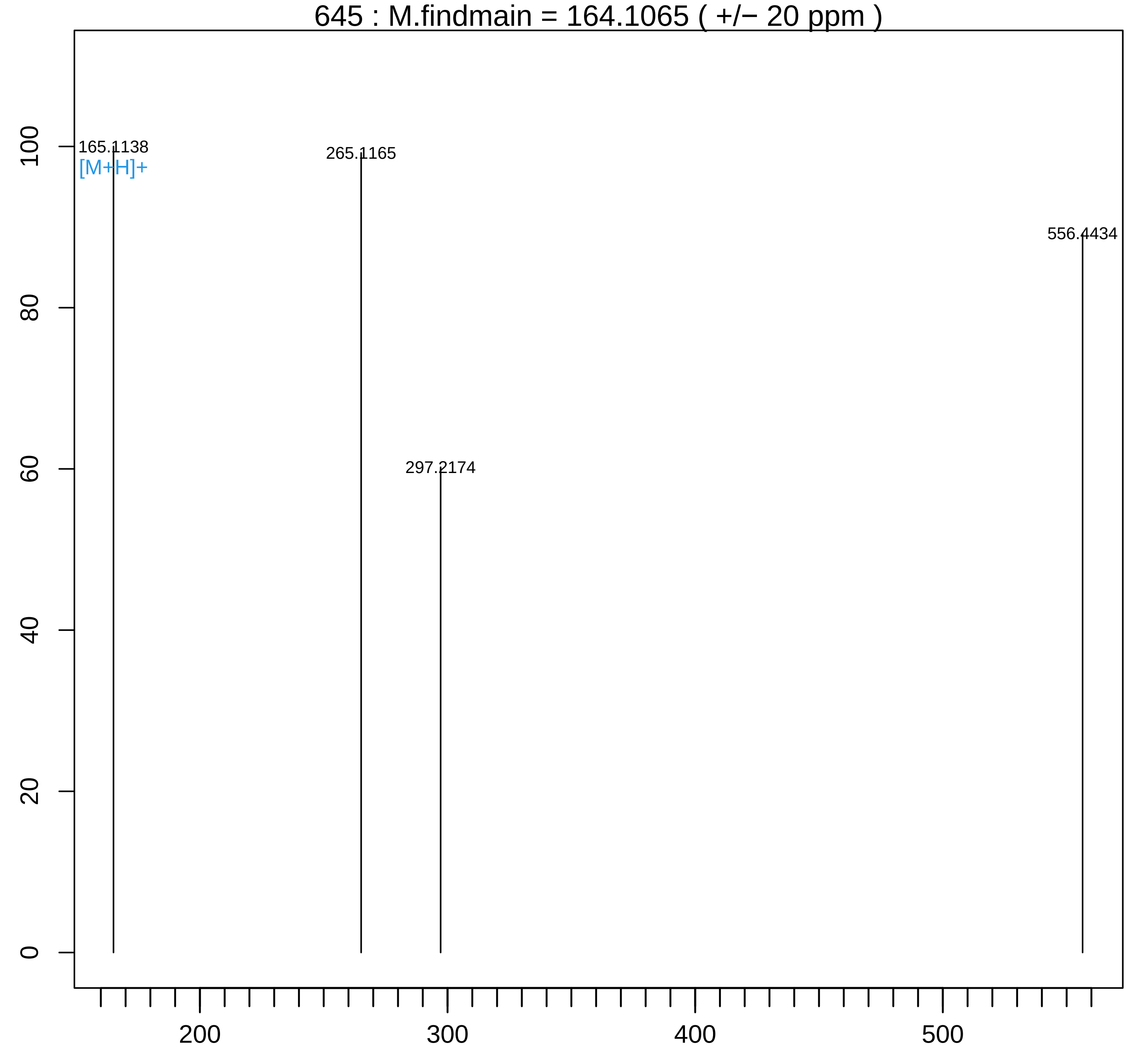
645 : M.findmain = 164.1065 (+/− 20 ppm)
165.1138
[M+H]+
265.1165
297.2174
556.4434
0
20
40
60
80
100
200
300
400
500

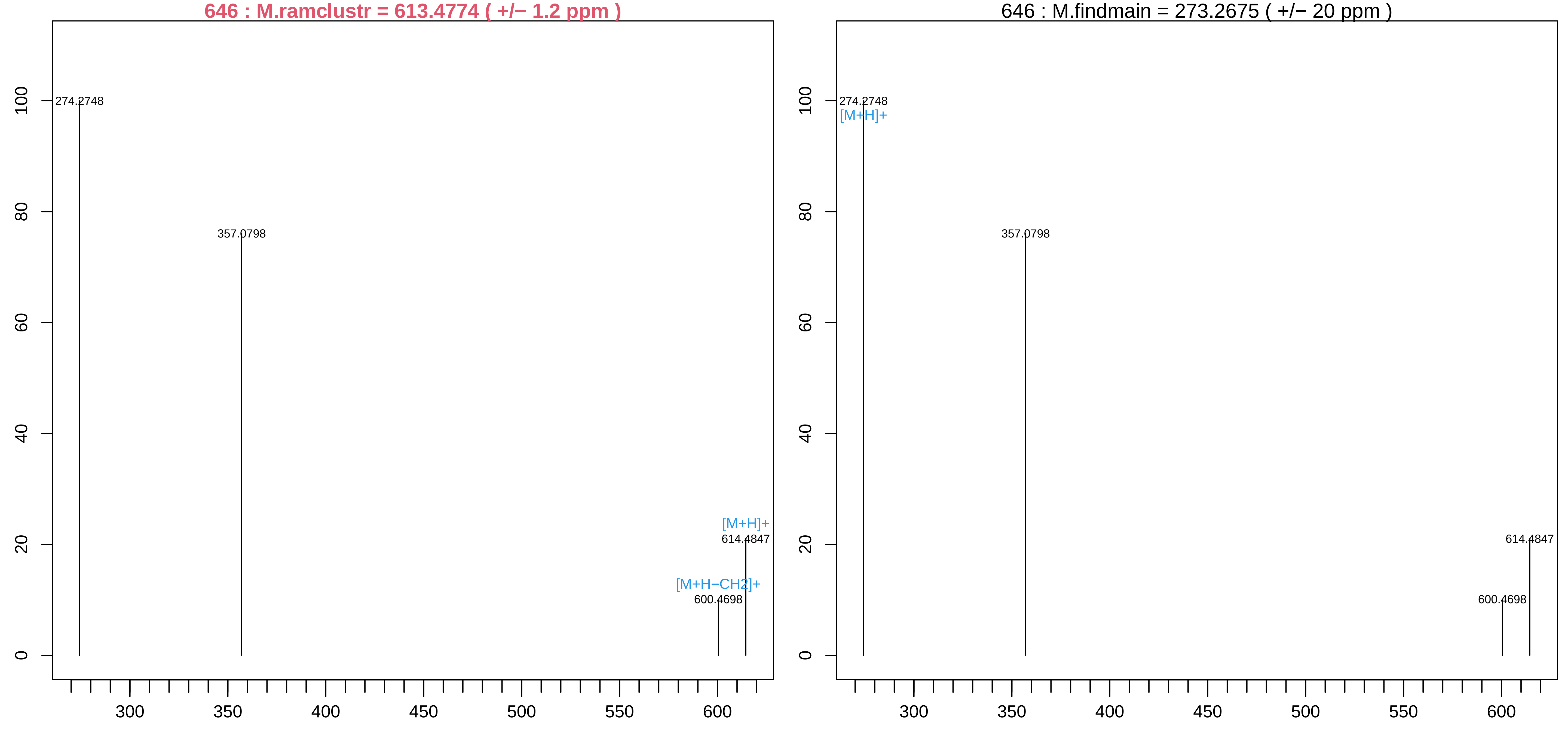

646 : M.ramclustr = 613.4774 (+/− 1.2 ppm)
274.2748
357.0798
[M+H]+
614.4847
[M+H−CH2]+
600.4698
646 : M.findmain = 273.2675 (+/− 20 ppm)
274.2748
[M+H]+
357.0798
614.4847
600.4698
0
20
40
60
80
100
300
350
400
450
500
550
600

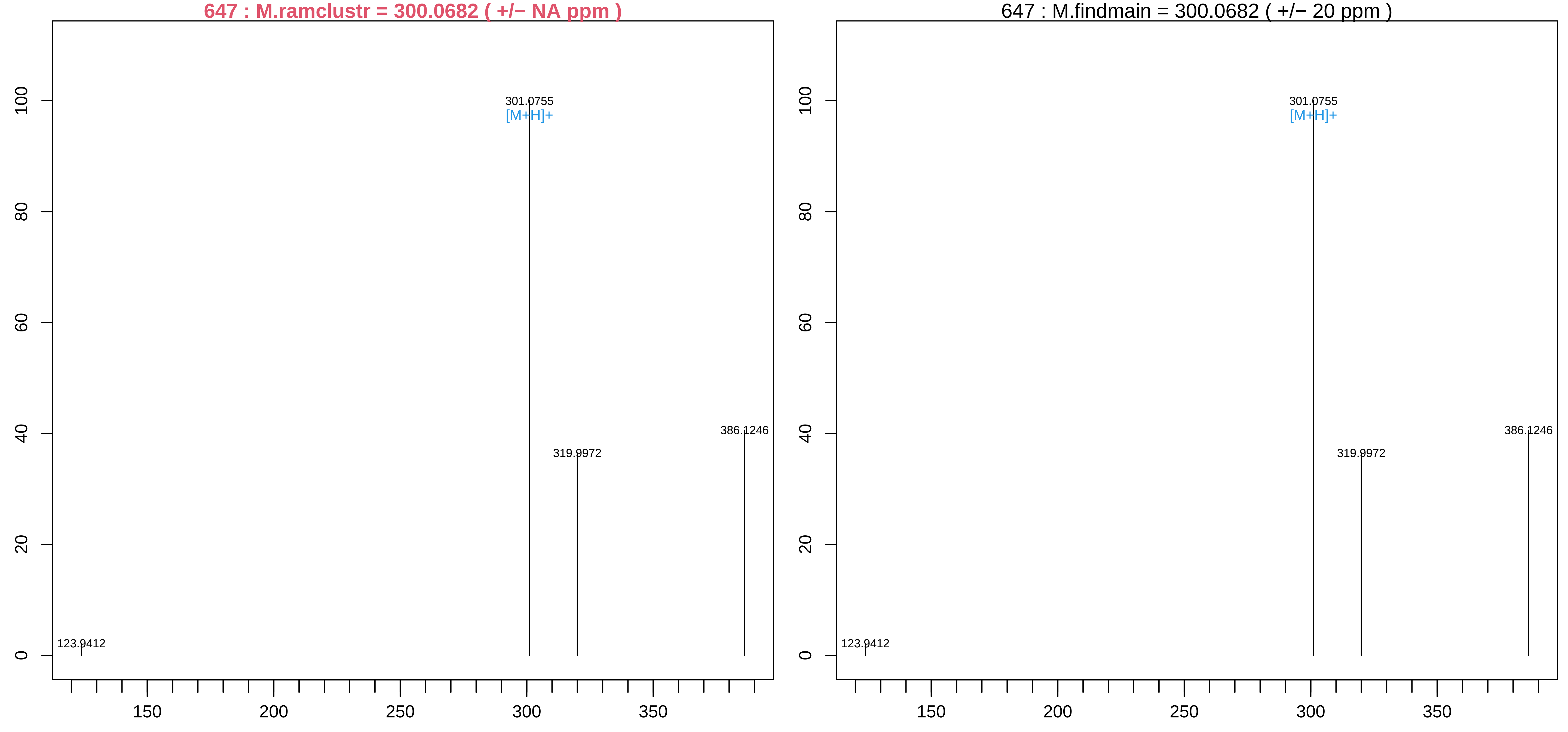

647 : M.ramclustr = 300.0682 (+/− NA ppm)
301.0755
[M+H]+
319.9972
386.1246
123.9412
0
20
40
60
80
100
150
200
250
300
350
647 : M.findmain = 300.0682 (+/− 20 ppm)
301.0755
[M+H]+
319.9972
386.1246
123.9412
0
20
40
60
80
100
150
200
250
300
350

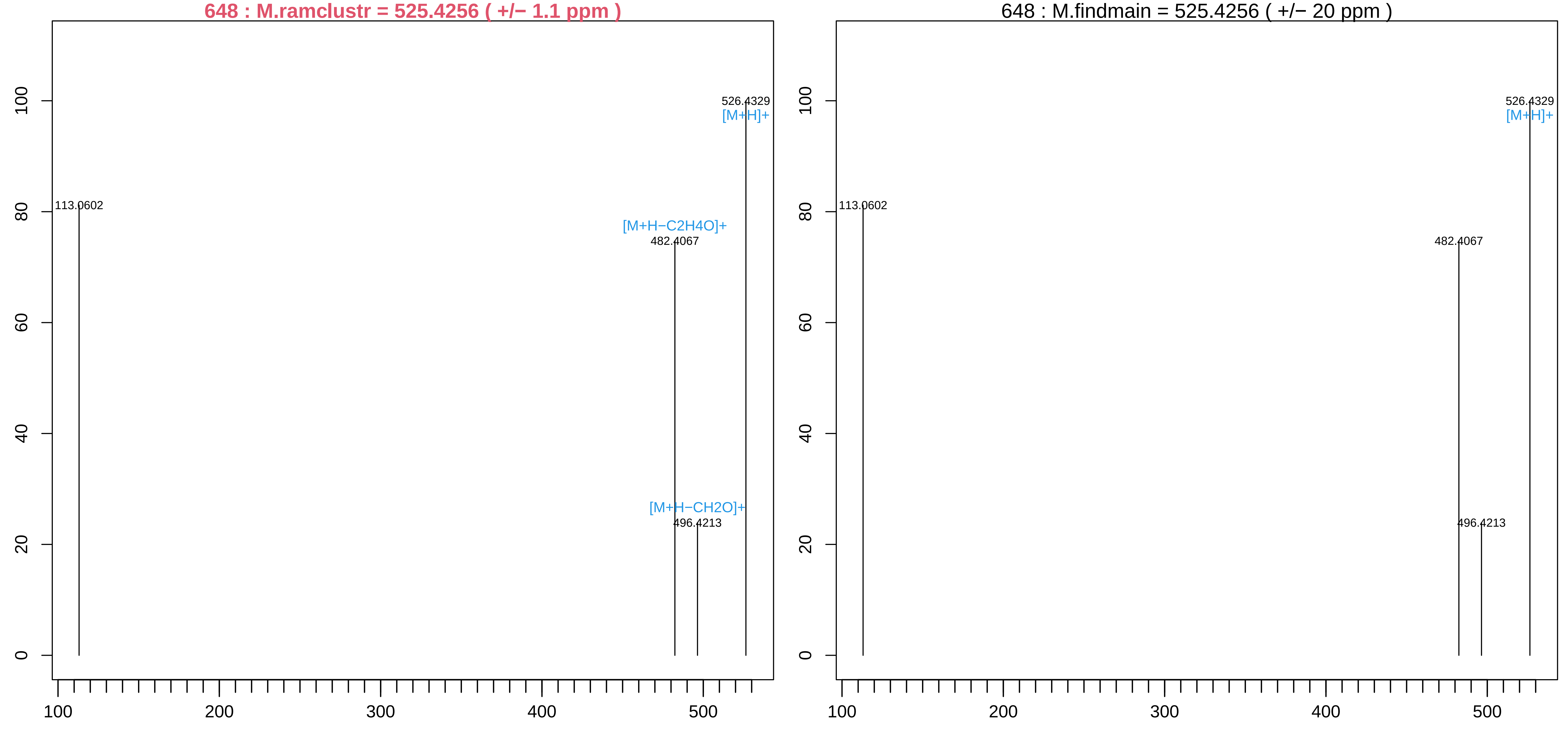

648 : M.ramclustr = 525.4256 (+/− 1.1 ppm)
526.4329
[M+H]+
113.0602
[M+H−C2H4O]+
482.4067
[M+H−CH2O]+
496.4213
0
20
40
60
80
100
100
200
300
400
500
648 : M.findmain = 525.4256 (+/− 20 ppm)
526.4329
[M+H]+
113.0602
482.4067
496.4213
0
20
40
60
80
100
100
200
300
400
500

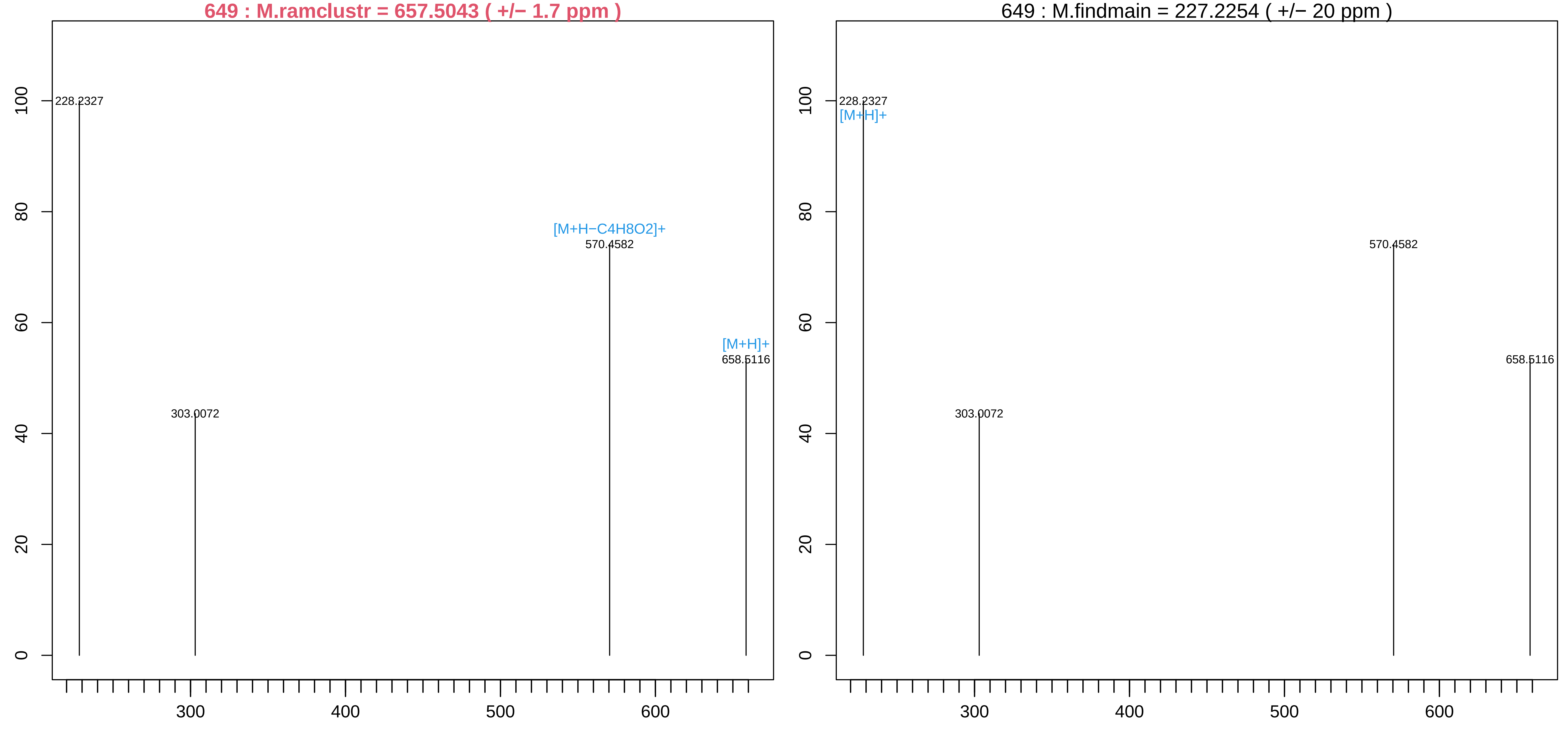
649 : M.ramclustr = 657.5043 (+/− 1.7 ppm)
228.2327
303.0072
[M+H−C4H8O2]+
570.4582
[M+H]+
658.5116
649 : M.findmain = 227.2254 (+/− 20 ppm)
228.2327
[M+H]+
303.0072
570.4582
658.5116
0
20
40
60
80
100
300
400
500
600

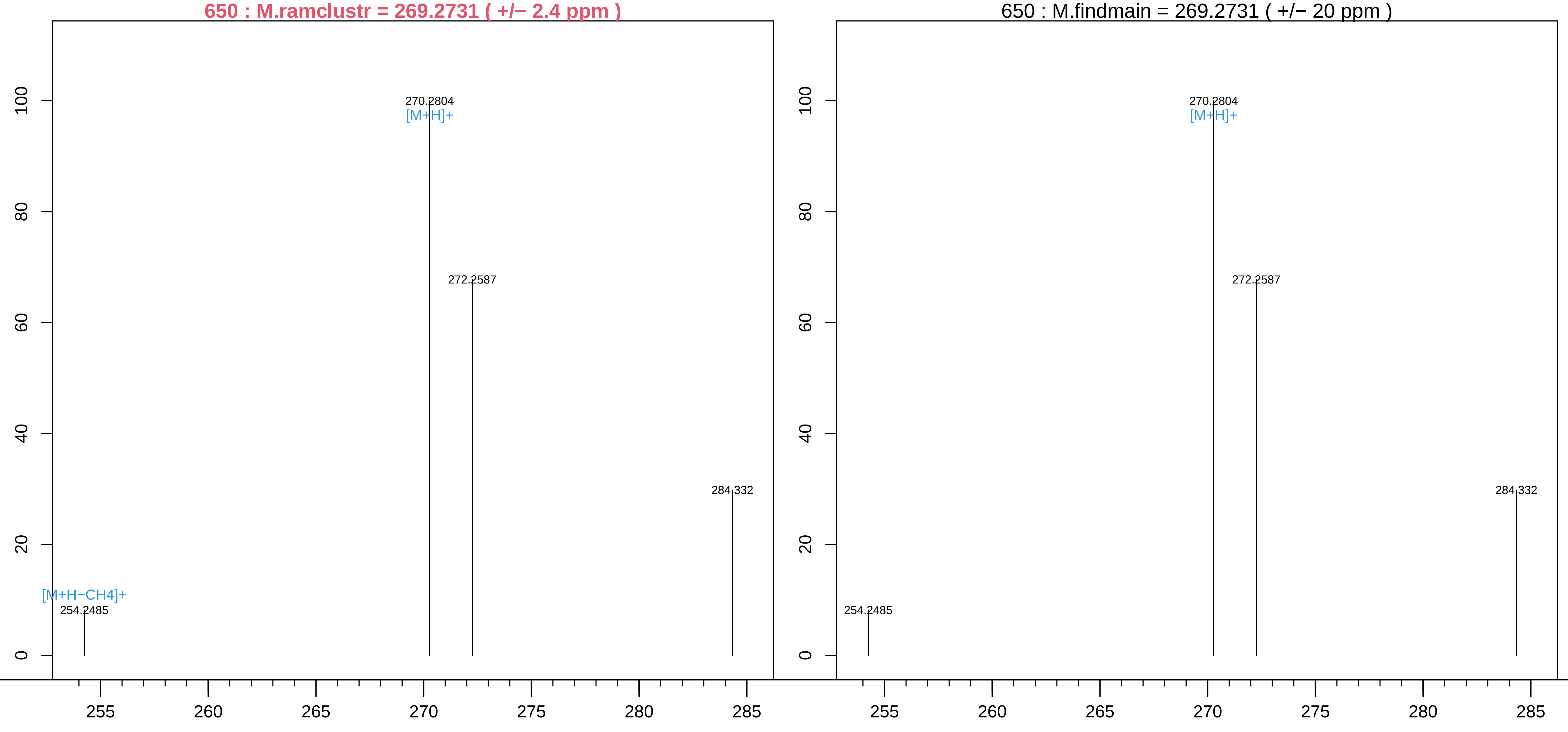

650 : M.ramclustr = 269.2731 (+/− 2.4 ppm)
270.2804
[M+H]+
272.2587
284.332
[M+H−CH4]+
254.2485
650 : M.findmain = 269.2731 (+/− 20 ppm)
270.2804
[M+H]+
272.2587
284.332
254.2485

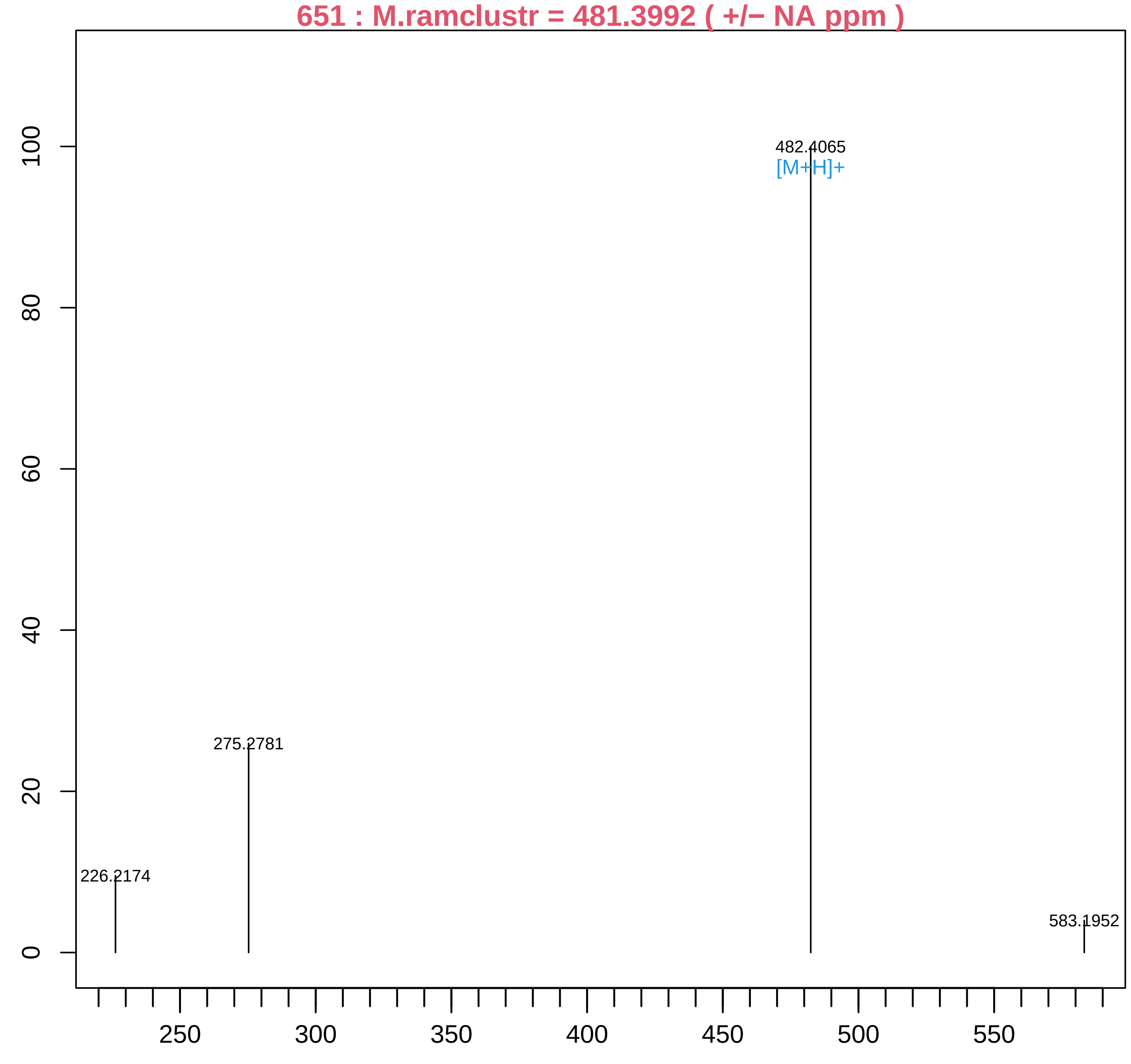

651 : M.ramclustr = 481.3992 (+/− NA ppm)
482.4065
[M+H]+
275.2781
226.2174
583.1952
0
20
40
60
80
100
250
300
350
400
450
500
550

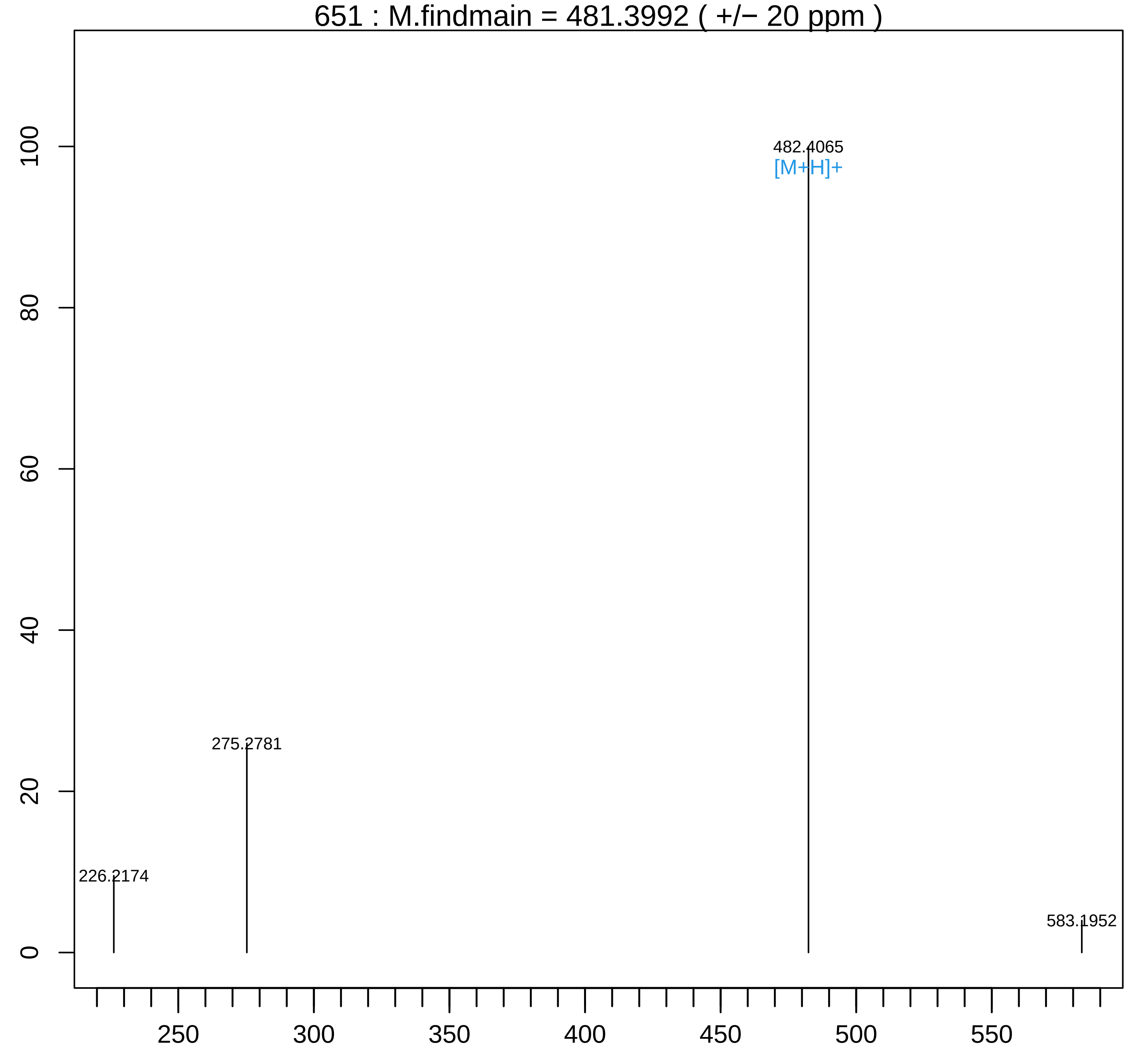

651 : M.findmain = 481.3992 (+/− 20 ppm)
482.4065
[M+H]+
275.2781
226.2174
583.1952
0
20
40
60
80
100
250
300
350
400
450
500
550

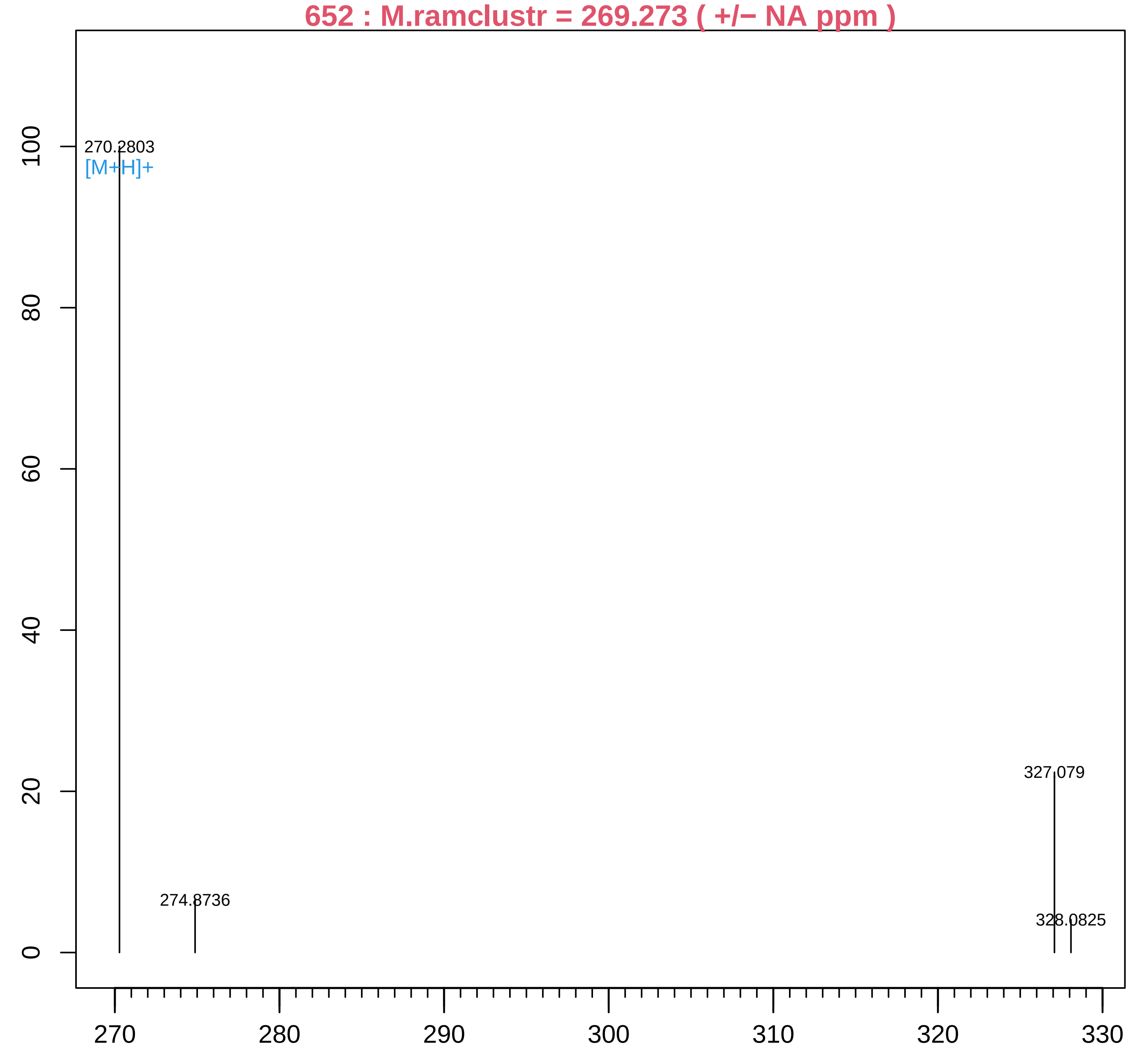
652 : M.ramclustr = 269.273 (+/− NA ppm)
270.2803
[M+H]+
274.8736
327.079
328.0825

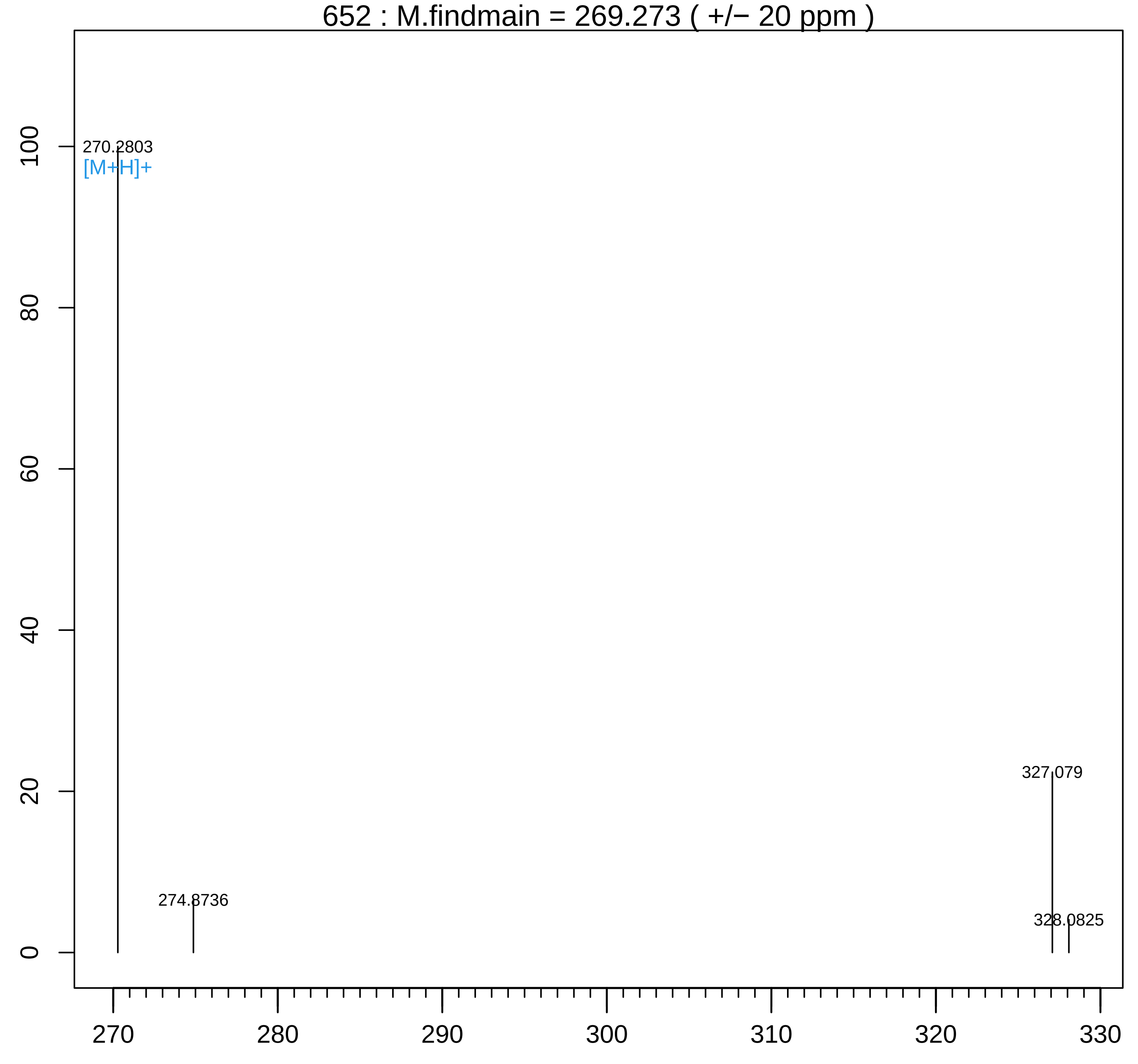
652 : M.findmain = 269.273 (+/− 20 ppm)
270.2803
[M+H]+
274.8736
327.079
328.0825

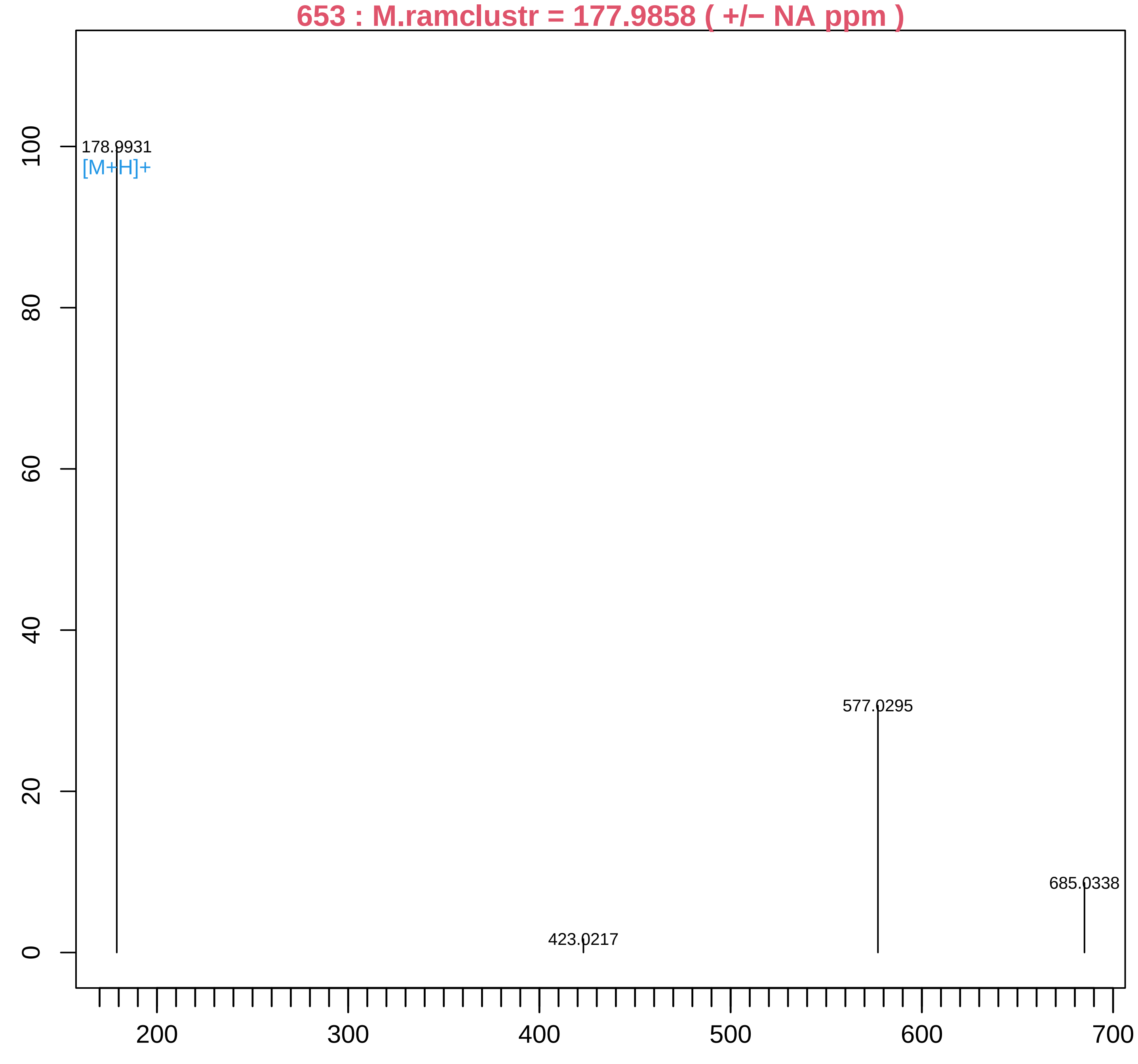
653 : M.ramclustr = 177.9858 (+/− NA ppm)
178.9931
[M+H]+
577.0295
685.0338
423.0217
0
20
40
60
80
100
200
300
400
500
600
700

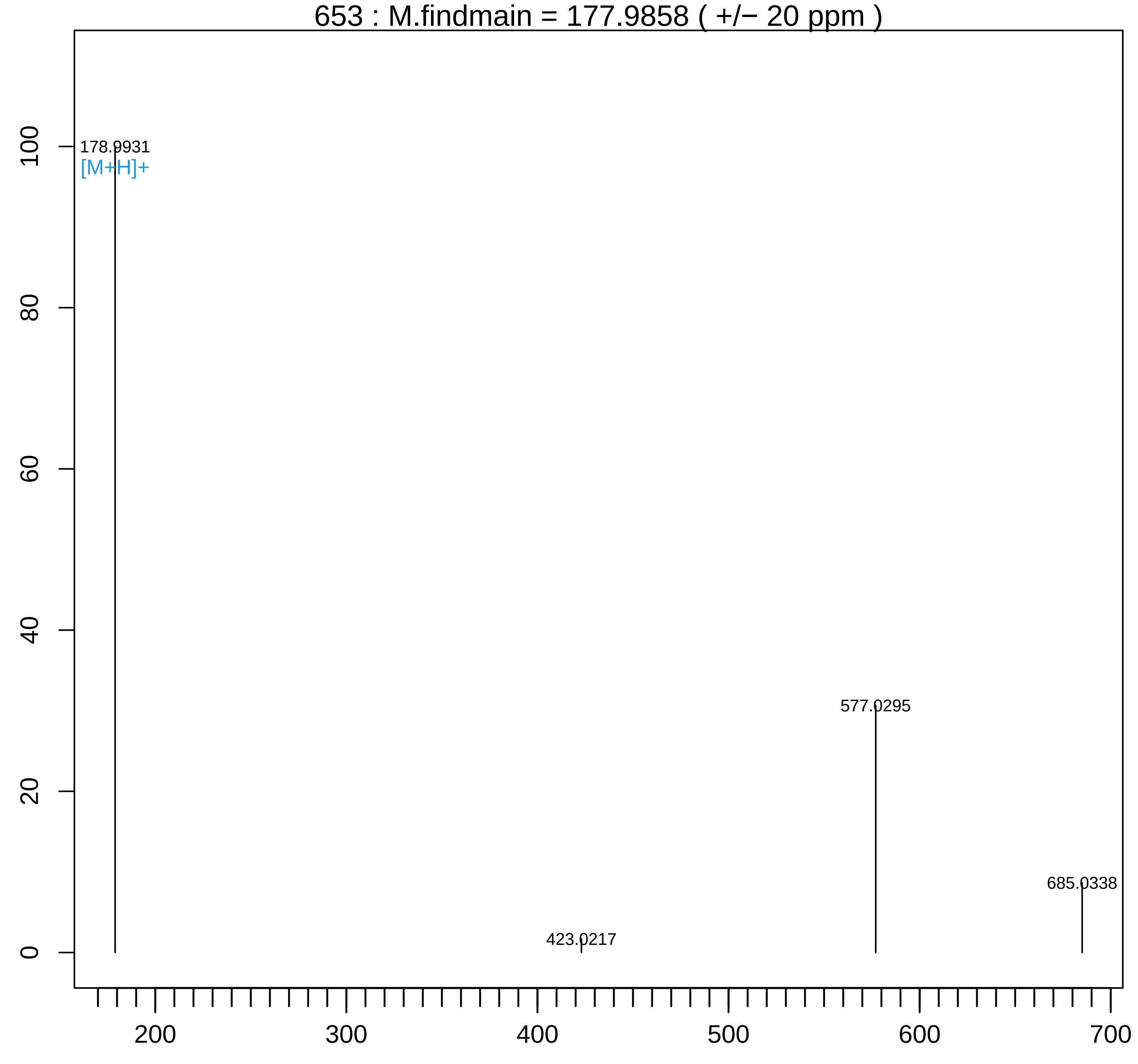
653 : M.findmain = 177.9858 (+/− 20 ppm)
178.9931
[M+H]+
577.0295
685.0338
423.0217
0
20
40
60
80
100
200
300
400
500
600
700

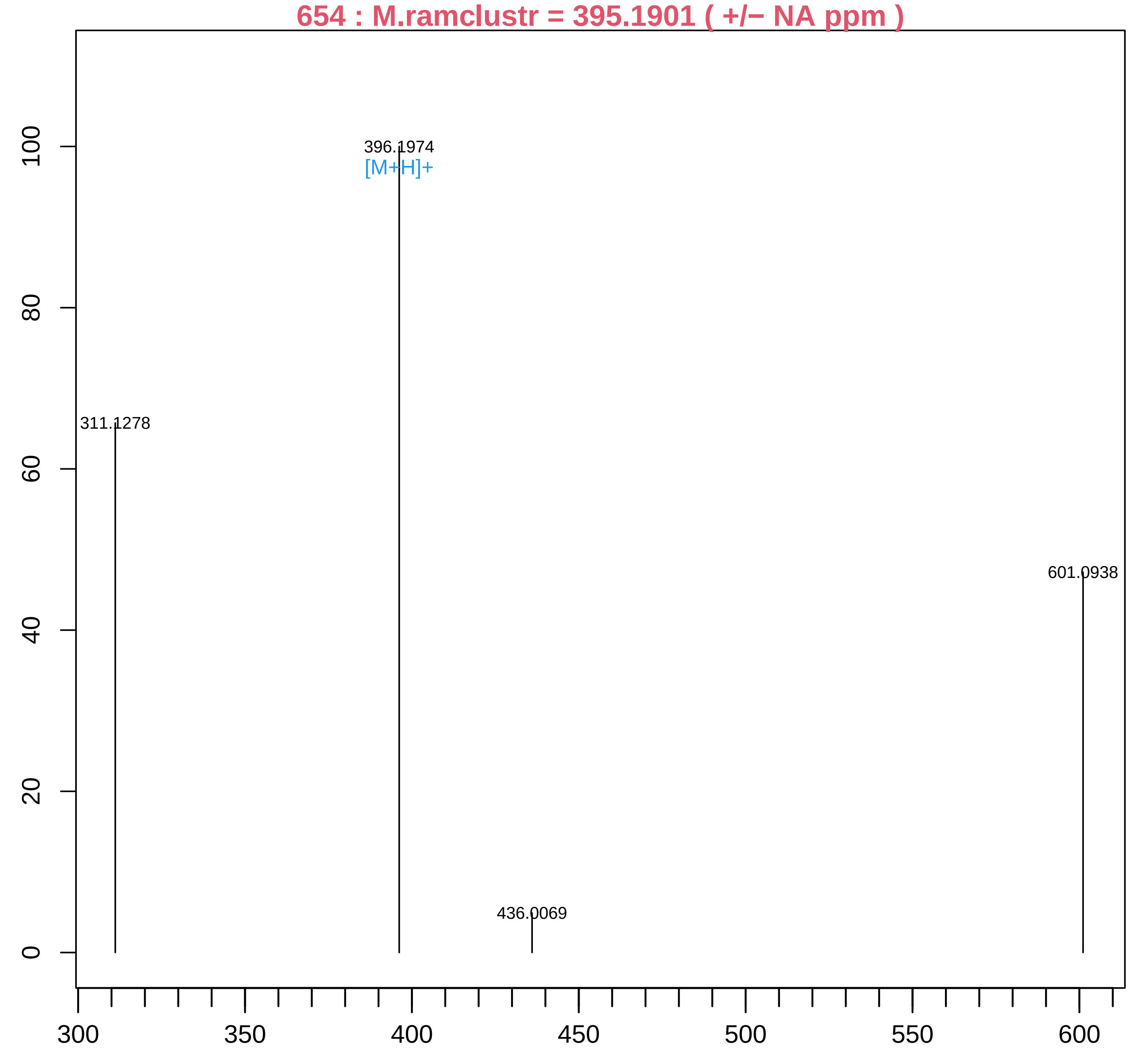

654 : M.ramclustr = 395.1901 (+/− NA ppm)
396.1974
[M+H]+
311.1278
601.0938
436.0069
0
20
40
60
80
100
300
350
400
450
500
550
600

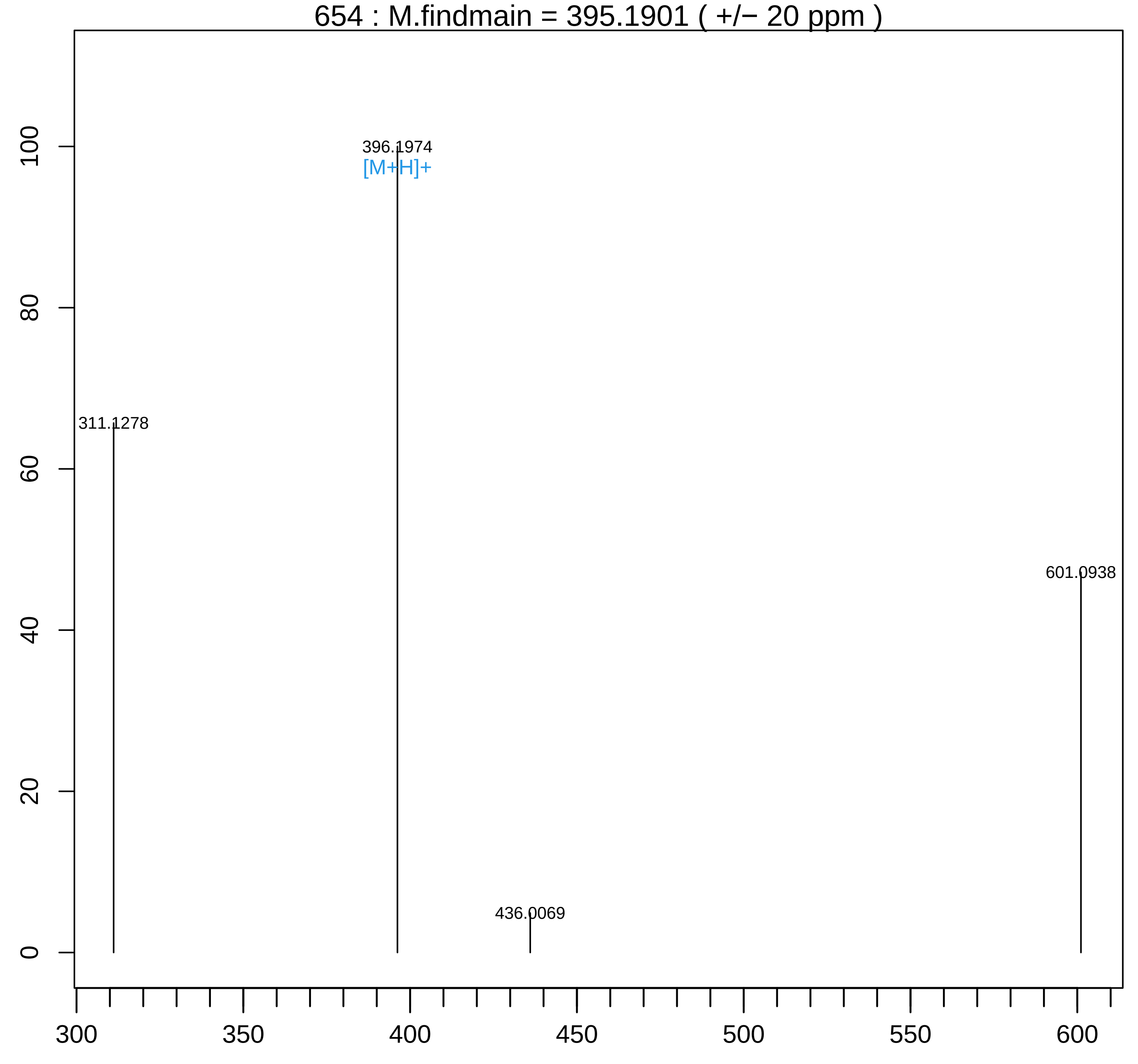

654 : M.findmain = 395.1901 (+/− 20 ppm)
396.1974
[M+H]+
311.1278
601.0938
436.0069
0
20
40
60
80
100
300
350
400
450
500
550
600

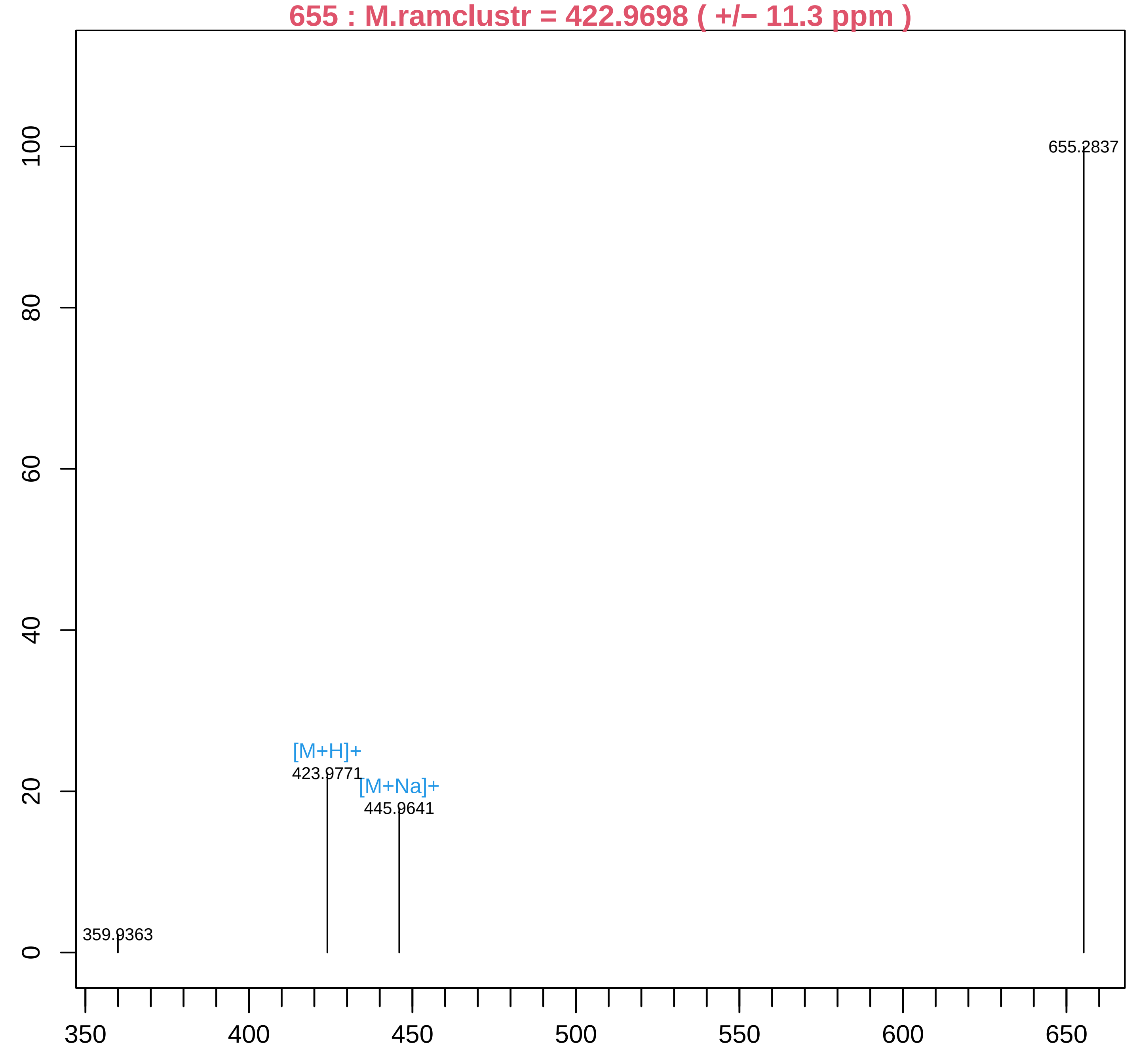
655 : M.ramclustr = 422.9698 (+/− 11.3 ppm)
655.2837
[M+H]+
423.9771
[M+Na]+
445.9641
359.9363
0
20
40
60
80
100
350
400
450
500
550
600
650

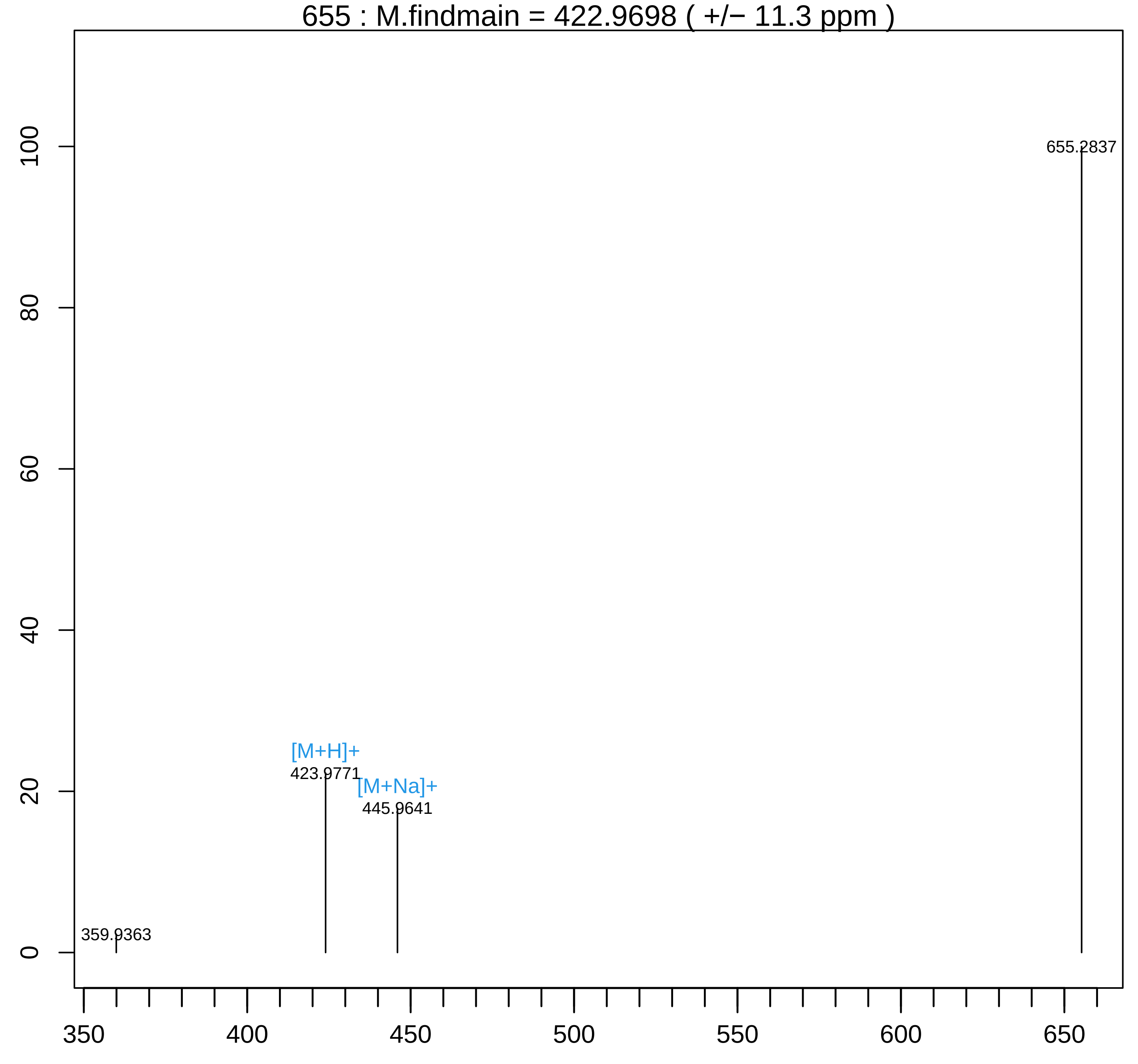
655 : M.findmain = 422.9698 (+/− 11.3 ppm)
655.2837
[M+H]+
423.9771
[M+Na]+
445.9641
359.9363
0
20
40
60
80
100
350
400
450
500
550
600
650

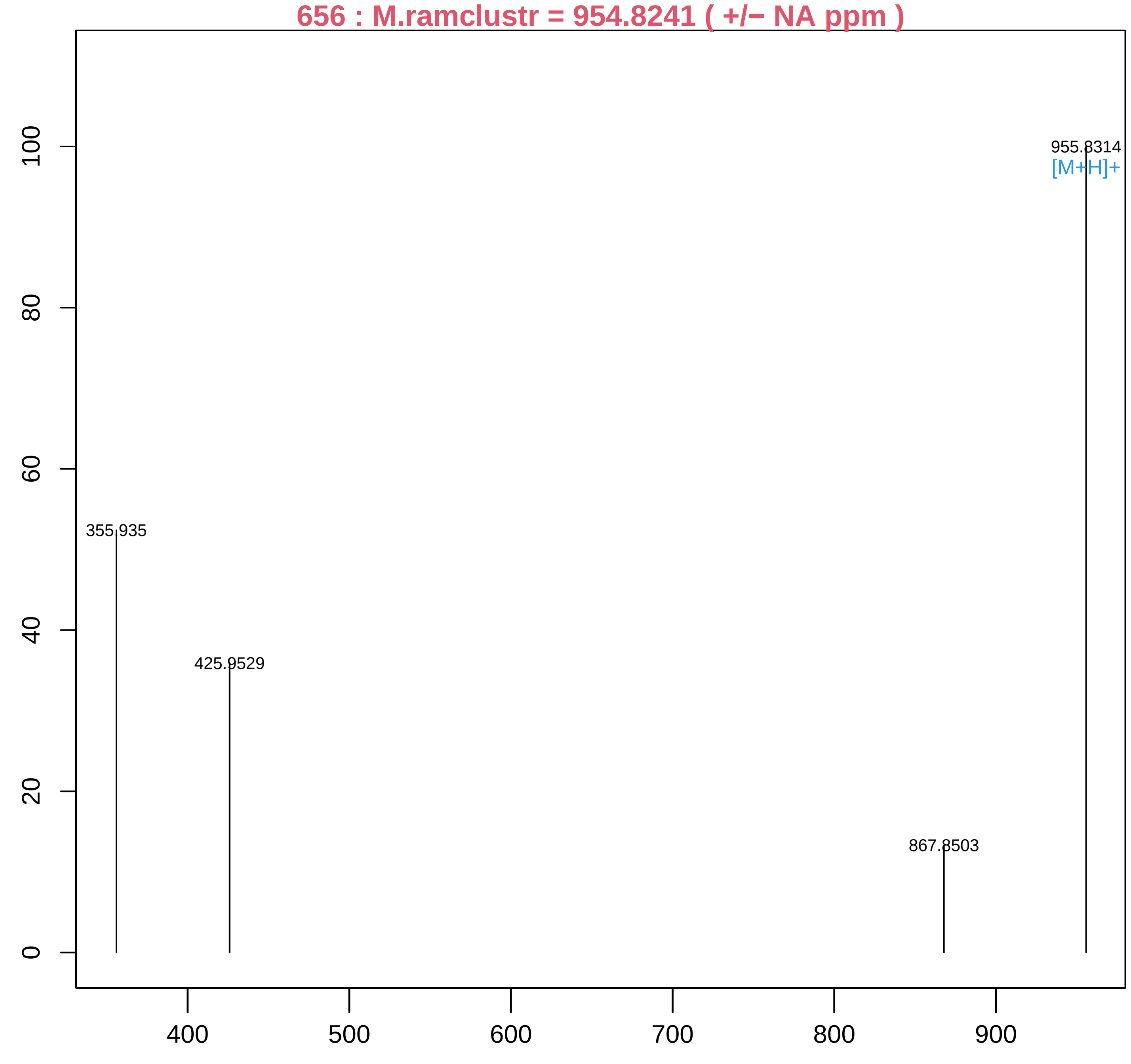
656 : M.ramclustr = 954.8241 (+/− NA ppm)
955.8314
[M+H]+
355.935
425.9529
867.8503
0
20
40
60
80
100
400
500
600
700
800
900

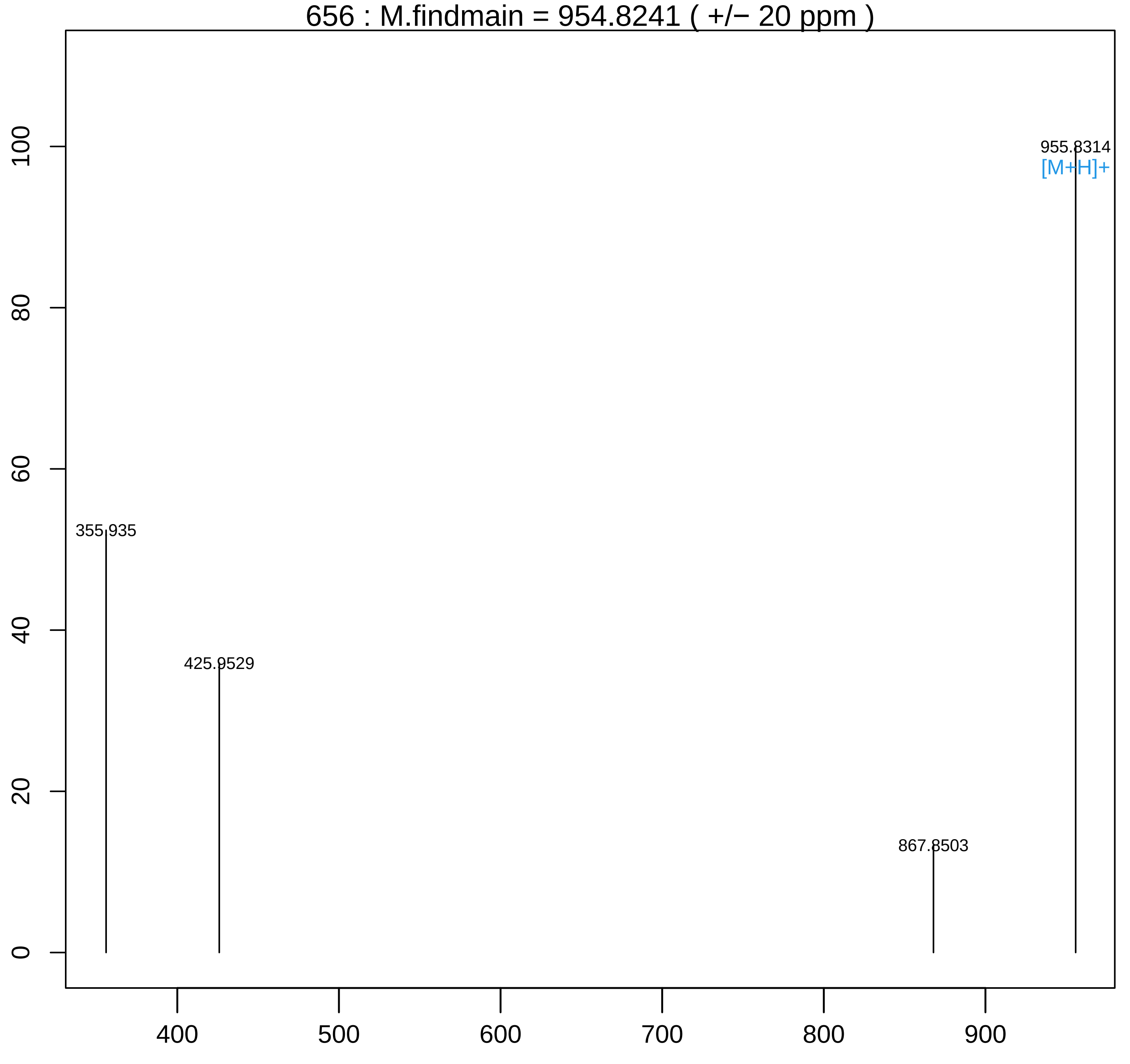
656 : M.findmain = 954.8241 (+/− 20 ppm)
955.8314
[M+H]+
355.935
425.9529
867.8503
0
20
40
60
80
100
400
500
600
700
800
900

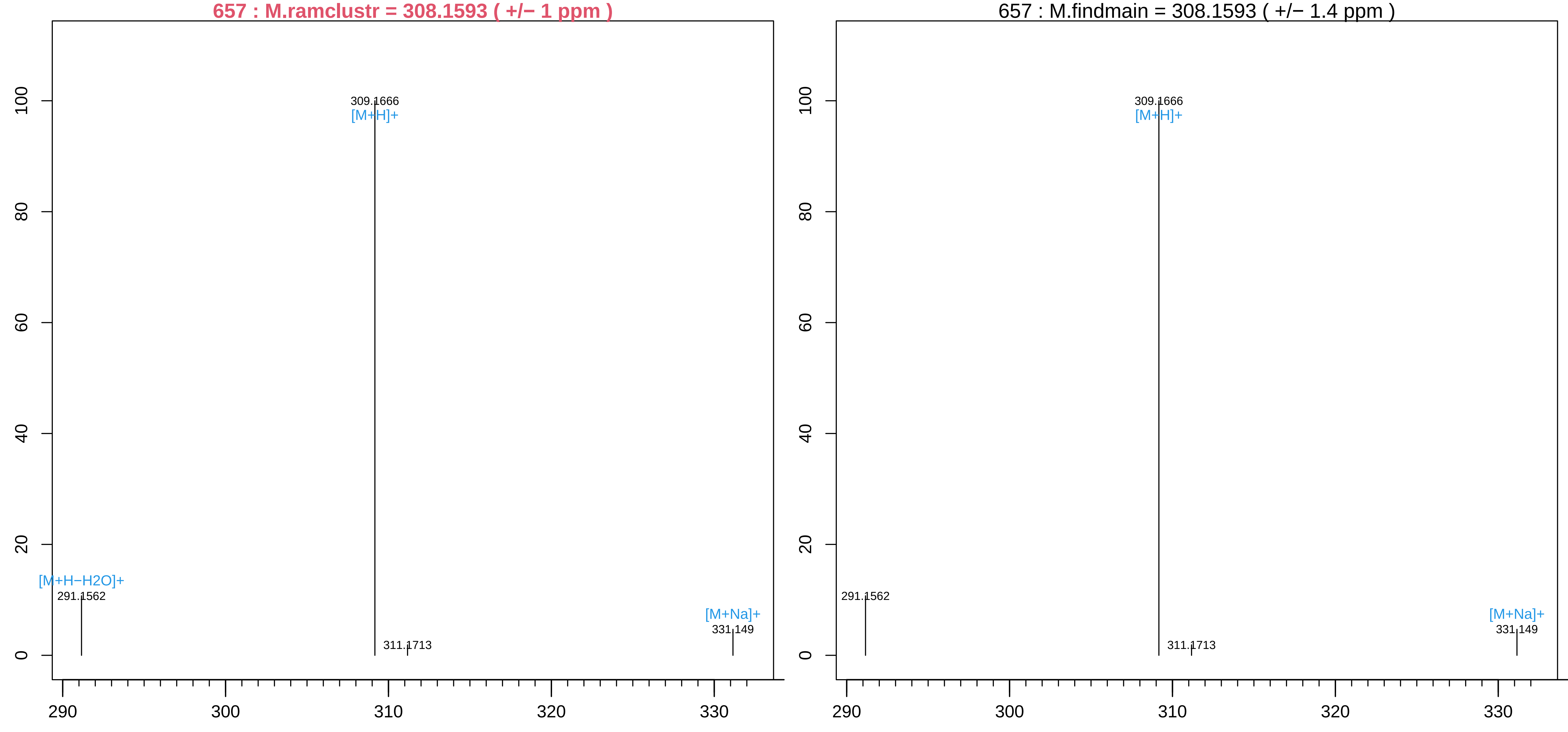

657 : M.ramclustr = 308.1593 (+/− 1 ppm)
309.1666
[M+H]+
[M+H−H2O]+
291.1562
[M+Na]+
331.149
311.1713
657 : M.findmain = 308.1593 (+/− 1.4 ppm)
309.1666
[M+H]+
291.1562
[M+Na]+
331.149
311.1713

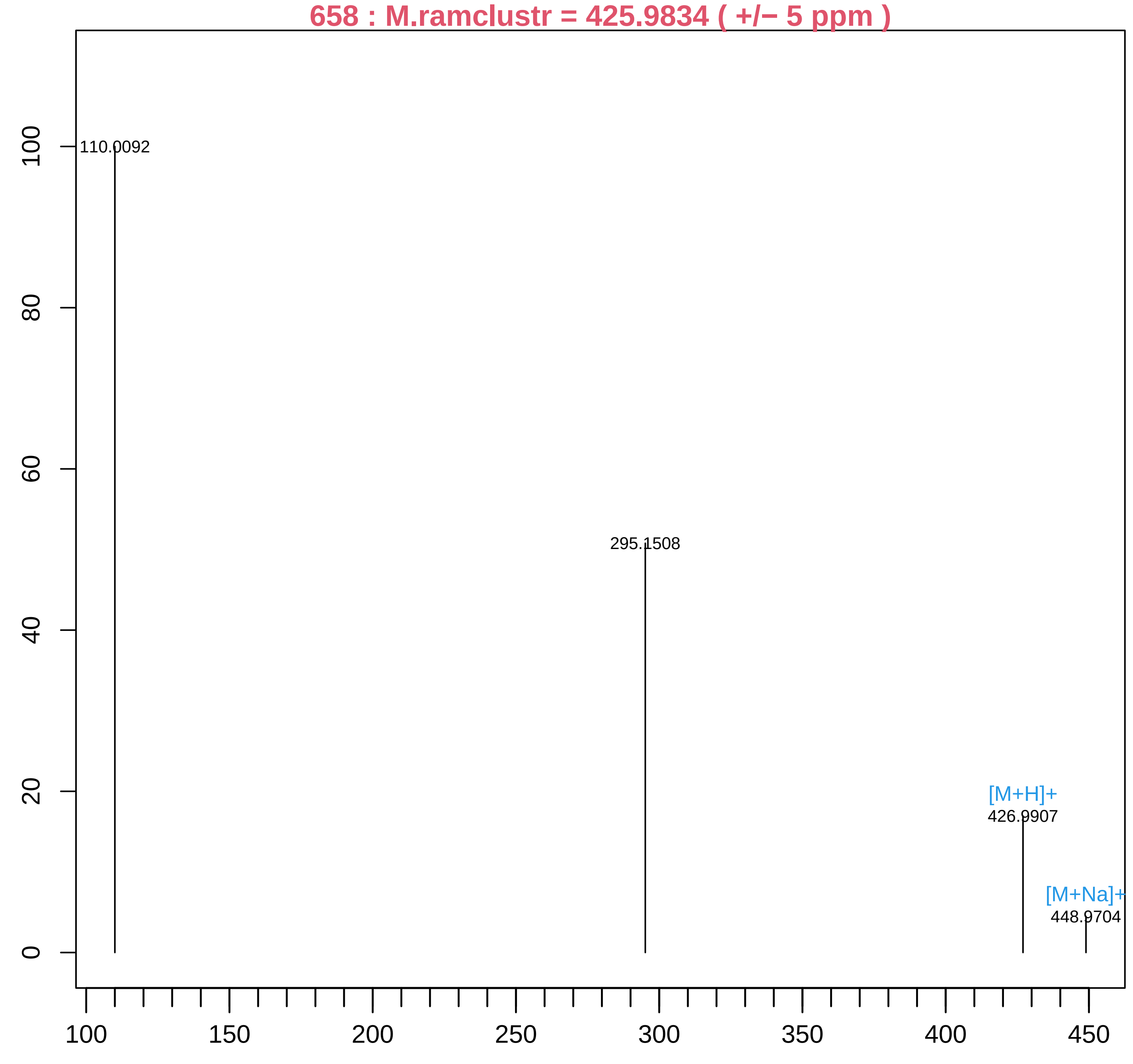
658 : M.ramclustr = 425.9834 (+/− 5 ppm)
110.0092
295.1508
[M+H]+
426.9907
[M+Na]+
448.9704
0
20
40
60
80
100
100
150
200
250
300
350
400
450
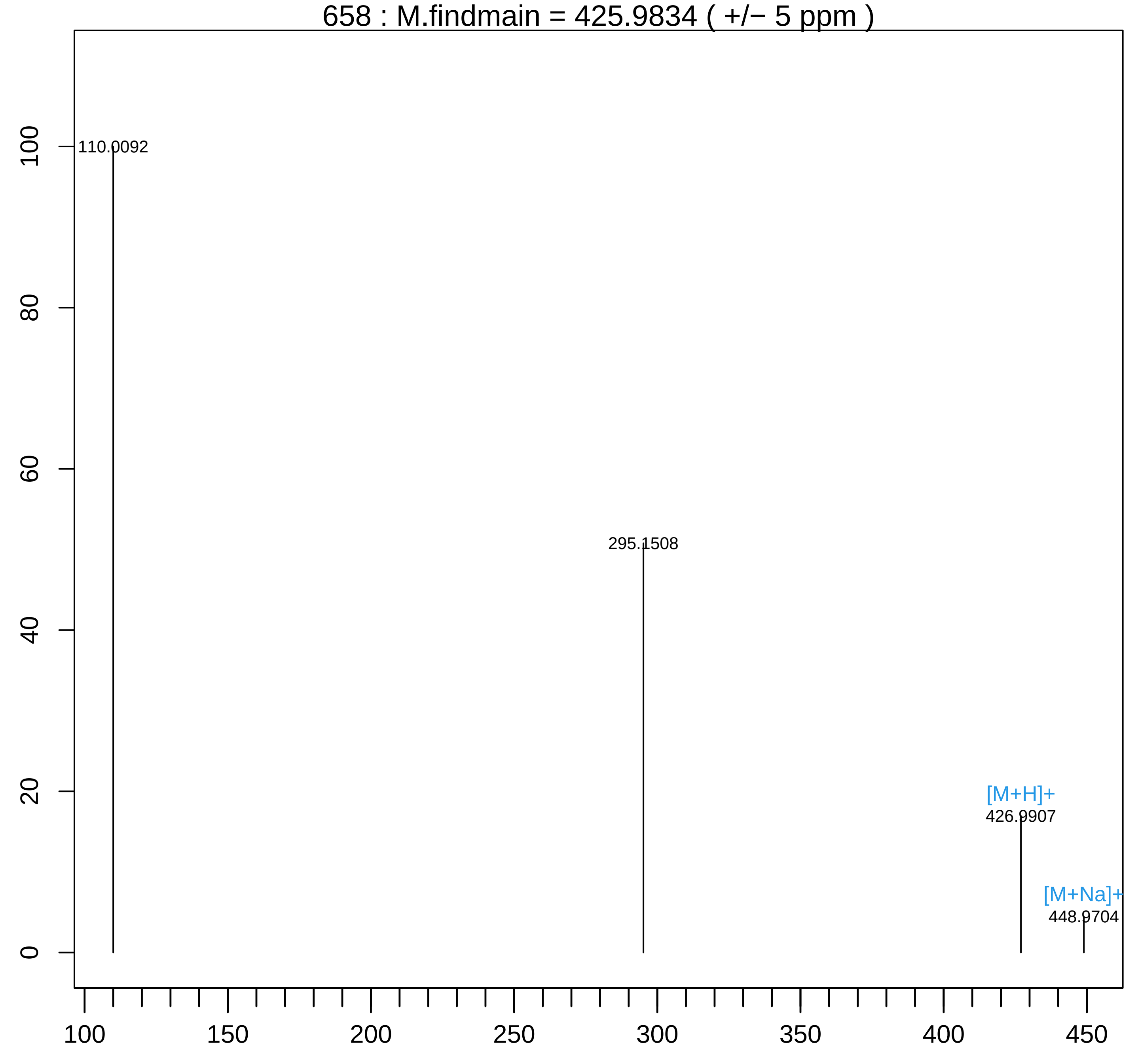
658 : M.findmain = 425.9834 (+/− 5 ppm)
110.0092
295.1508
[M+H]+
426.9907
[M+Na]+
448.9704
0
20
40
60
80
100
100
150
200
250
300
350
400
450

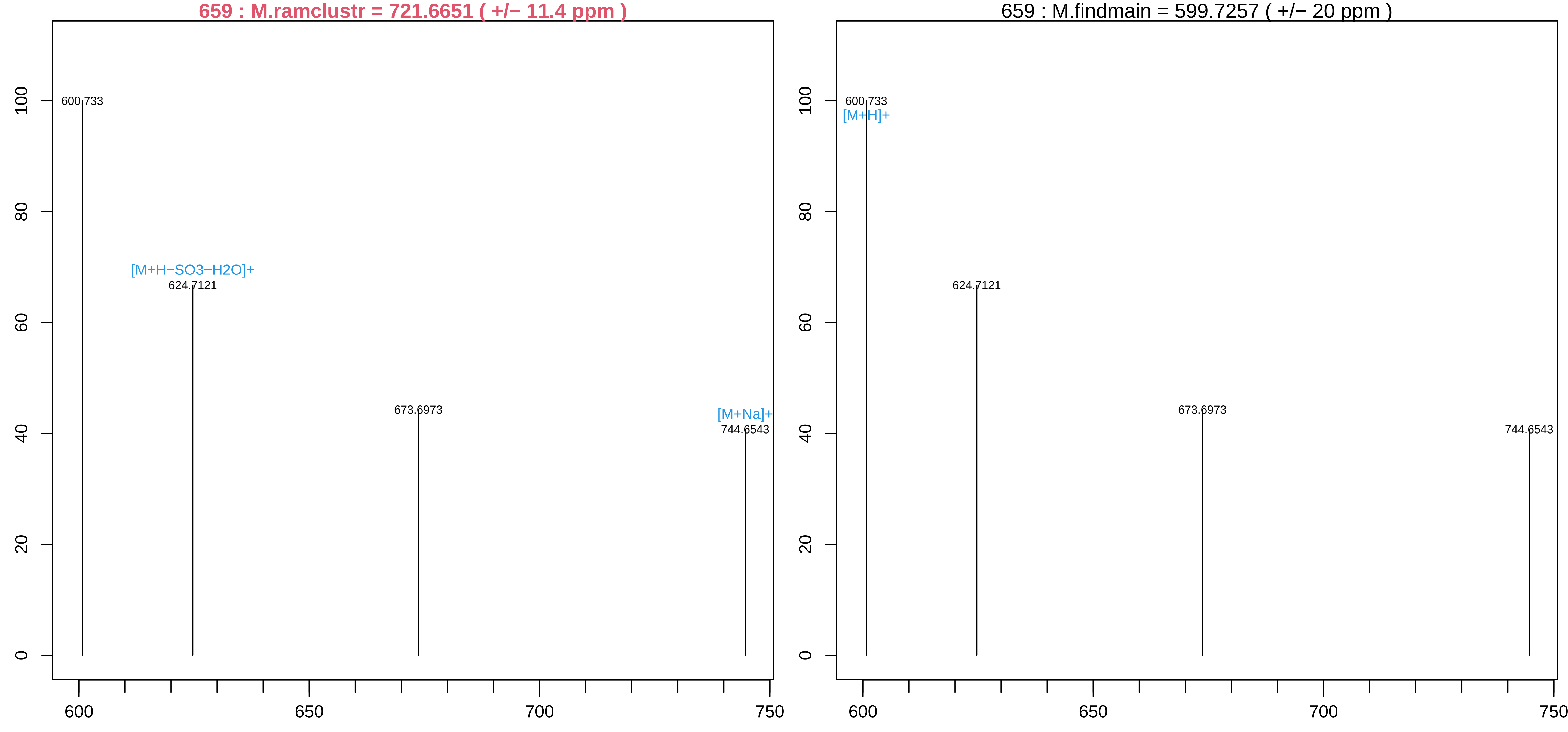

659 : M.ramclustr = 721.6651 (+/− 11.4 ppm)
600.733
[M+H−SO3−H2O]+
624.7121
673.6973
[M+Na]+
744.6543
659 : M.findmain = 599.7257 (+/− 20 ppm)
600.733
[M+H]+
624.7121
673.6973
744.6543

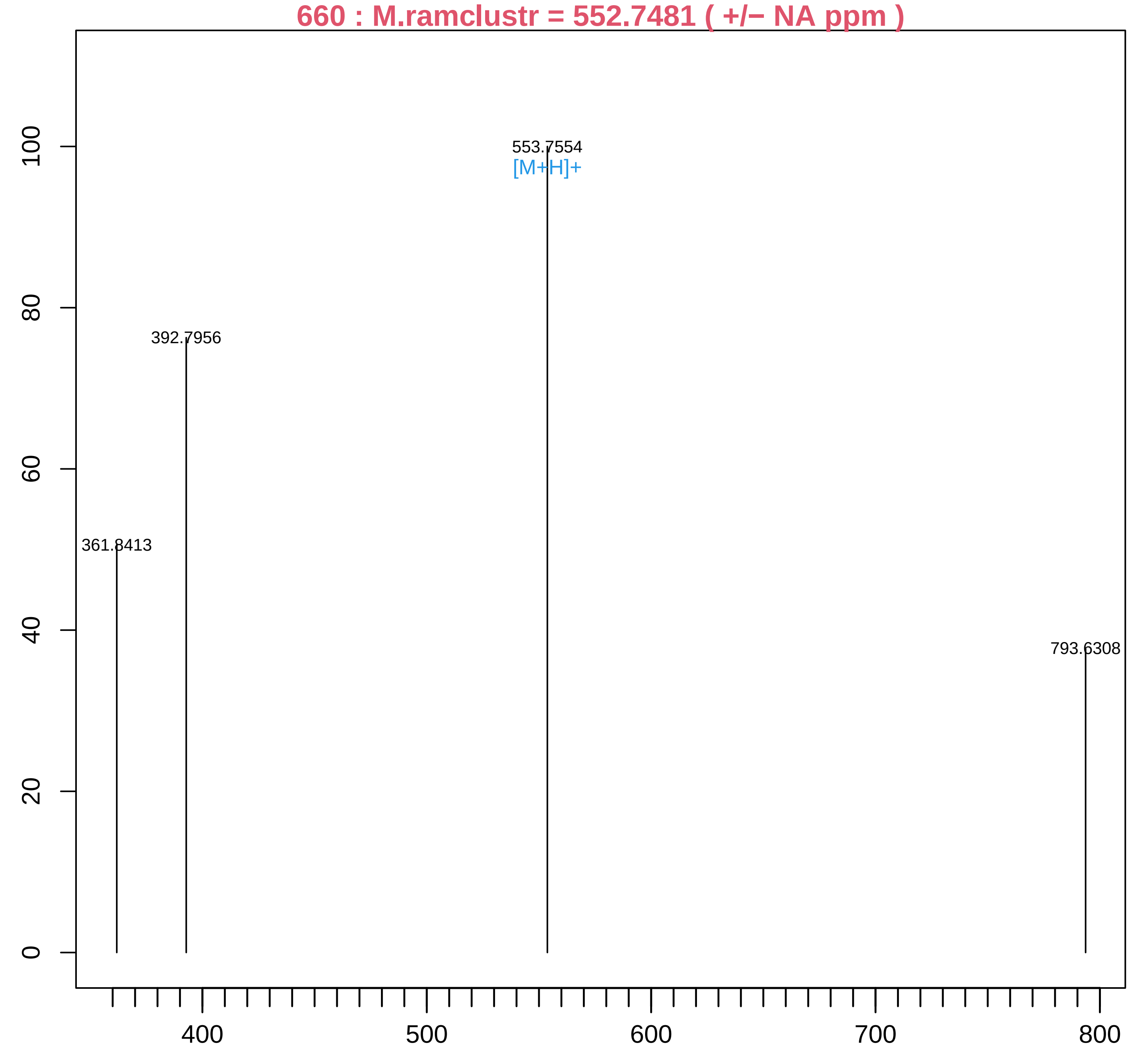

660 : M.ramclustr = 552.7481 (+/− NA ppm)
553.7554
[M+H]+
392.7956
361.8413
793.6308
0
20
40
60
80
100
400
500
600
700
800

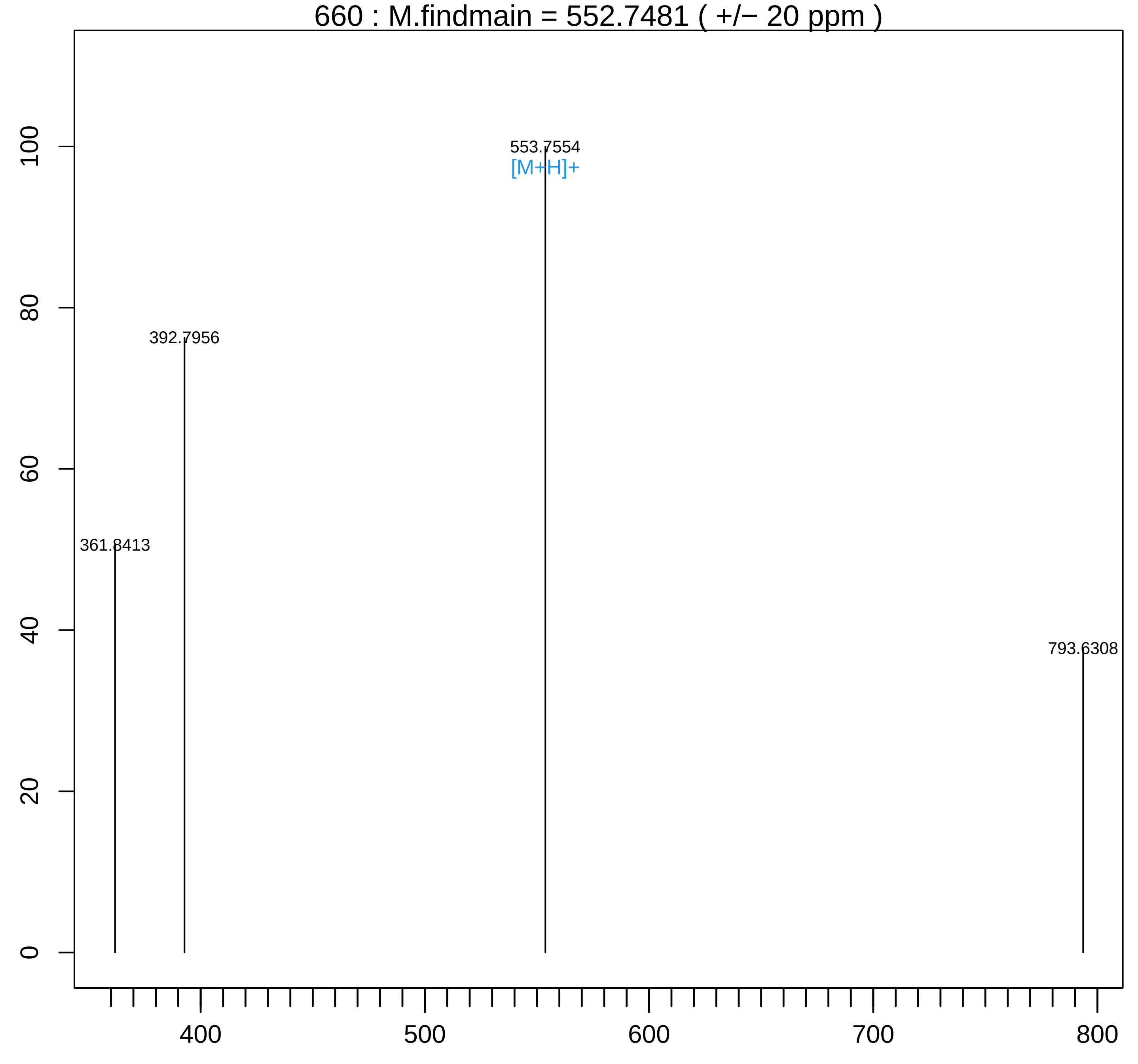

660 : M.findmain = 552.7481 (+/− 20 ppm)
553.7554
[M+H]+
392.7956
361.8413
793.6308
0
20
40
60
80
100
400
500
600
700
800

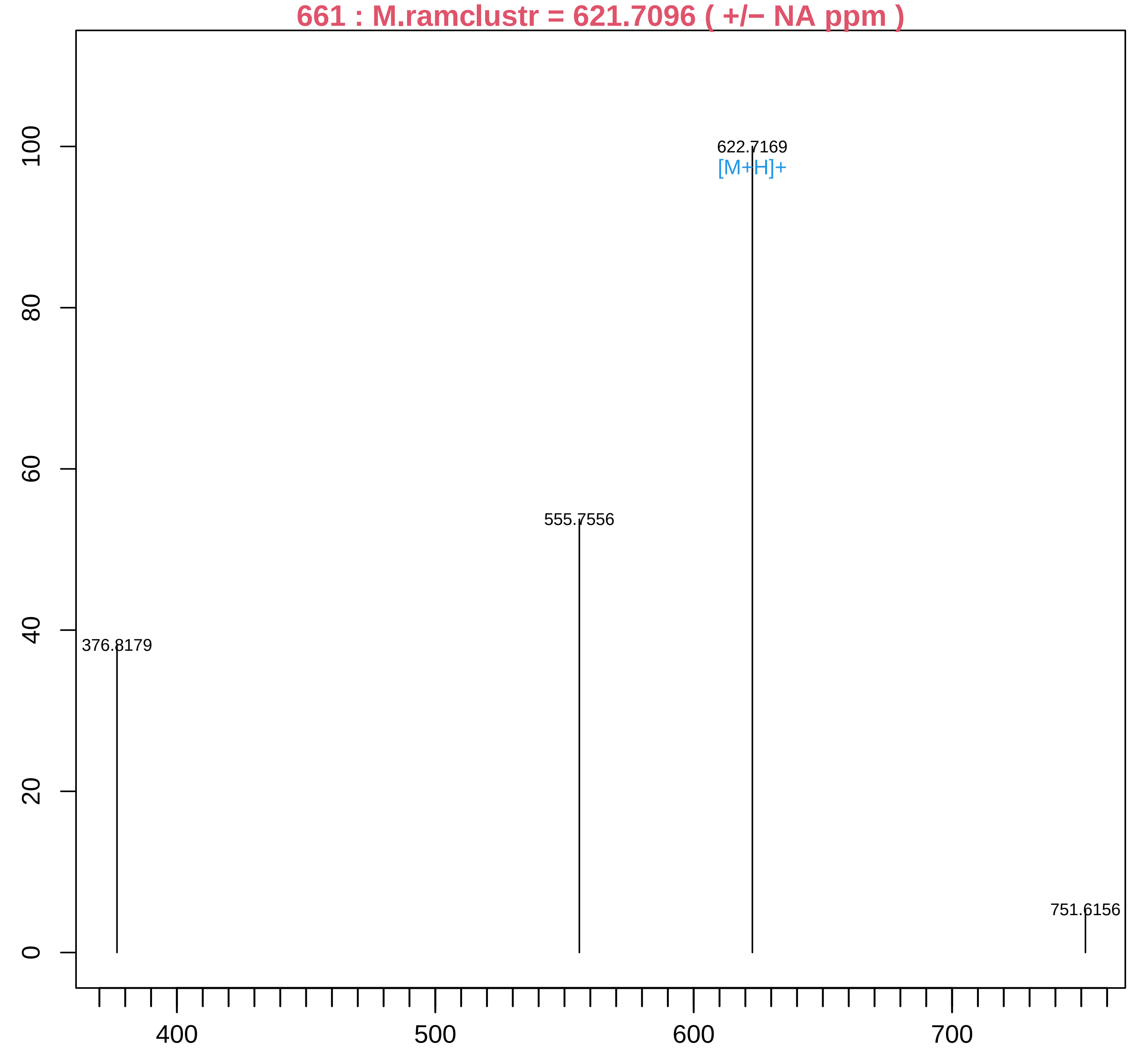
661 : M.ramclustr = 621.7096 (+/− NA ppm)
376.8179
555.7556
622.7169
[M+H]+
751.6156
0
20
40
60
80
100
400
500
600
700

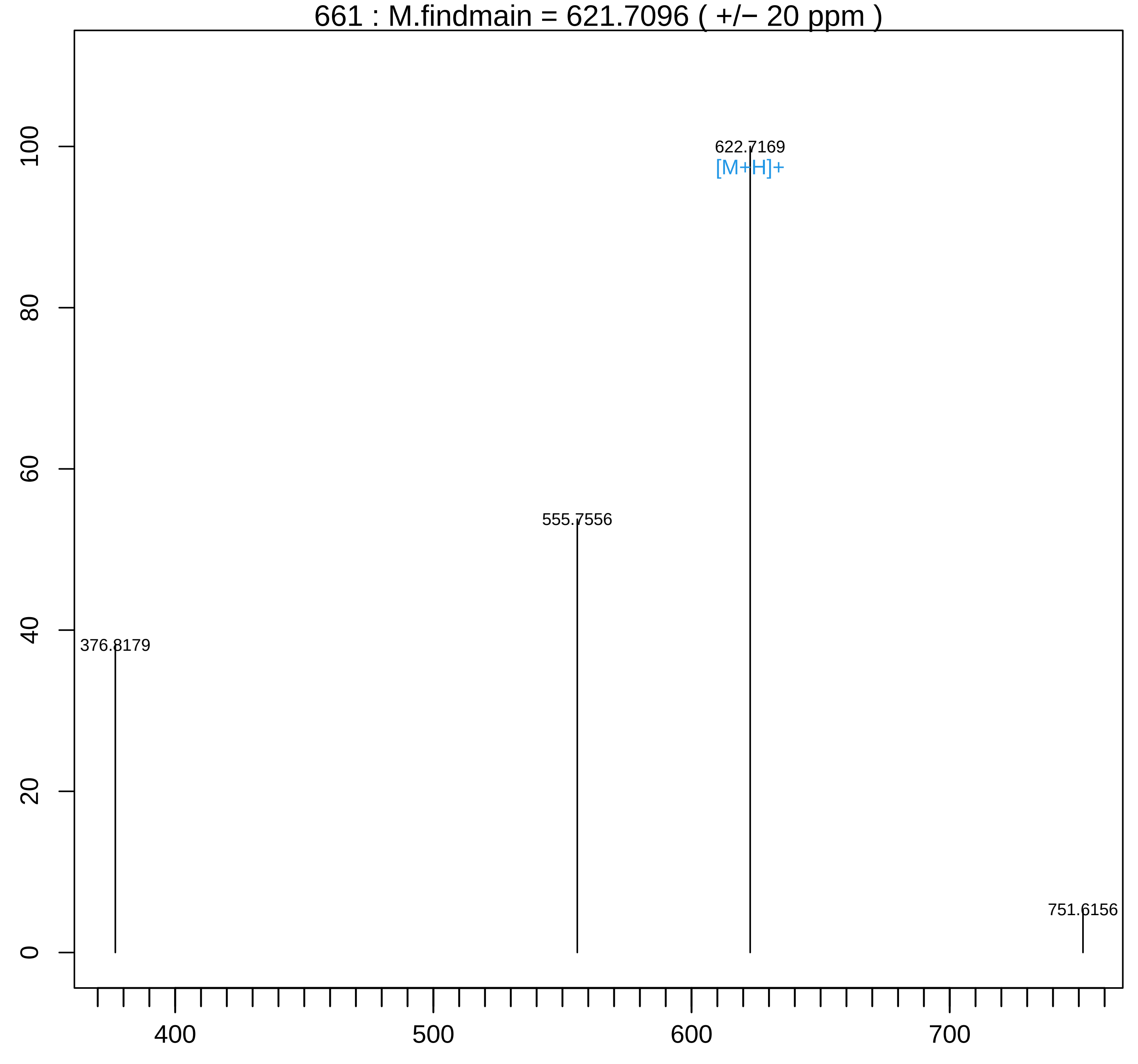
661 : M.findmain = 621.7096 (+/− 20 ppm)
376.8179
555.7556
622.7169
[M+H]+
751.6156
0
20
40
60
80
100
400
500
600
700

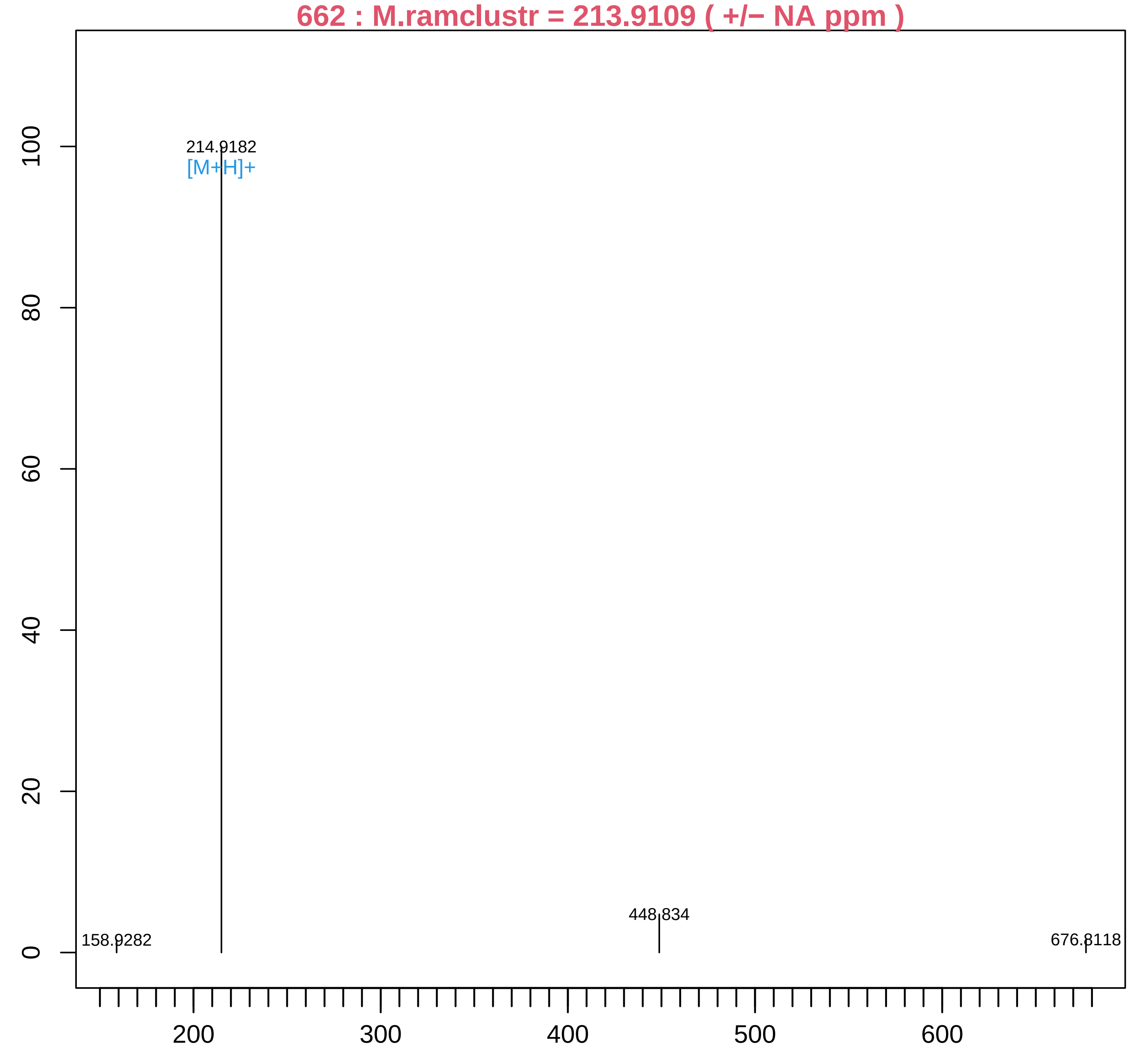
662 : M.ramclustr = 213.9109 (+/− NA ppm)
214.9182
[M+H]+
158.9282
448.834
676.8118
0
20
40
60
80
100
200
300
400
500
600

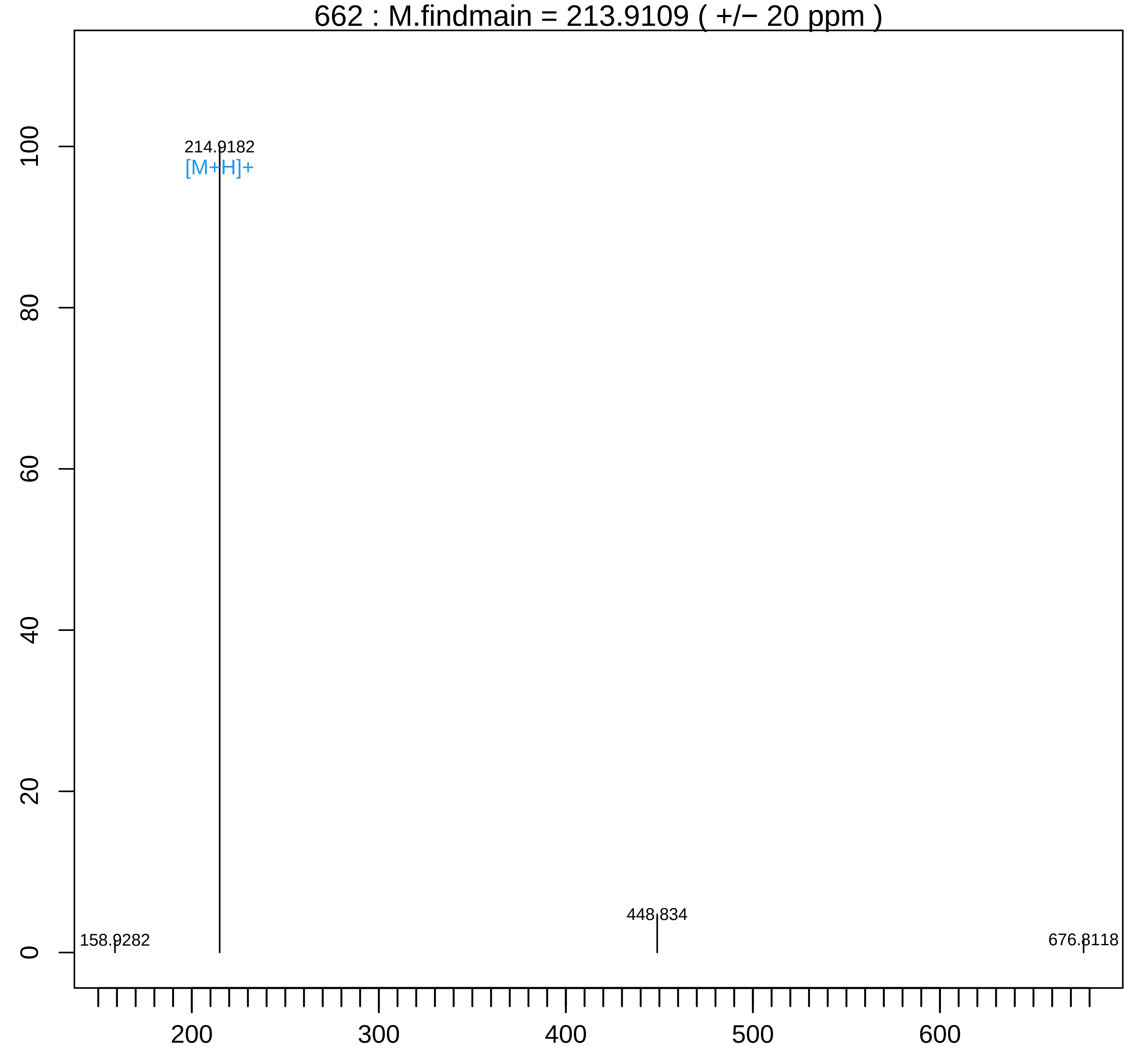
662 : M.findmain = 213.9109 (+/− 20 ppm)
214.9182
[M+H]+
158.9282
448.834
676.8118
0
20
40
60
80
100
200
300
400
500
600

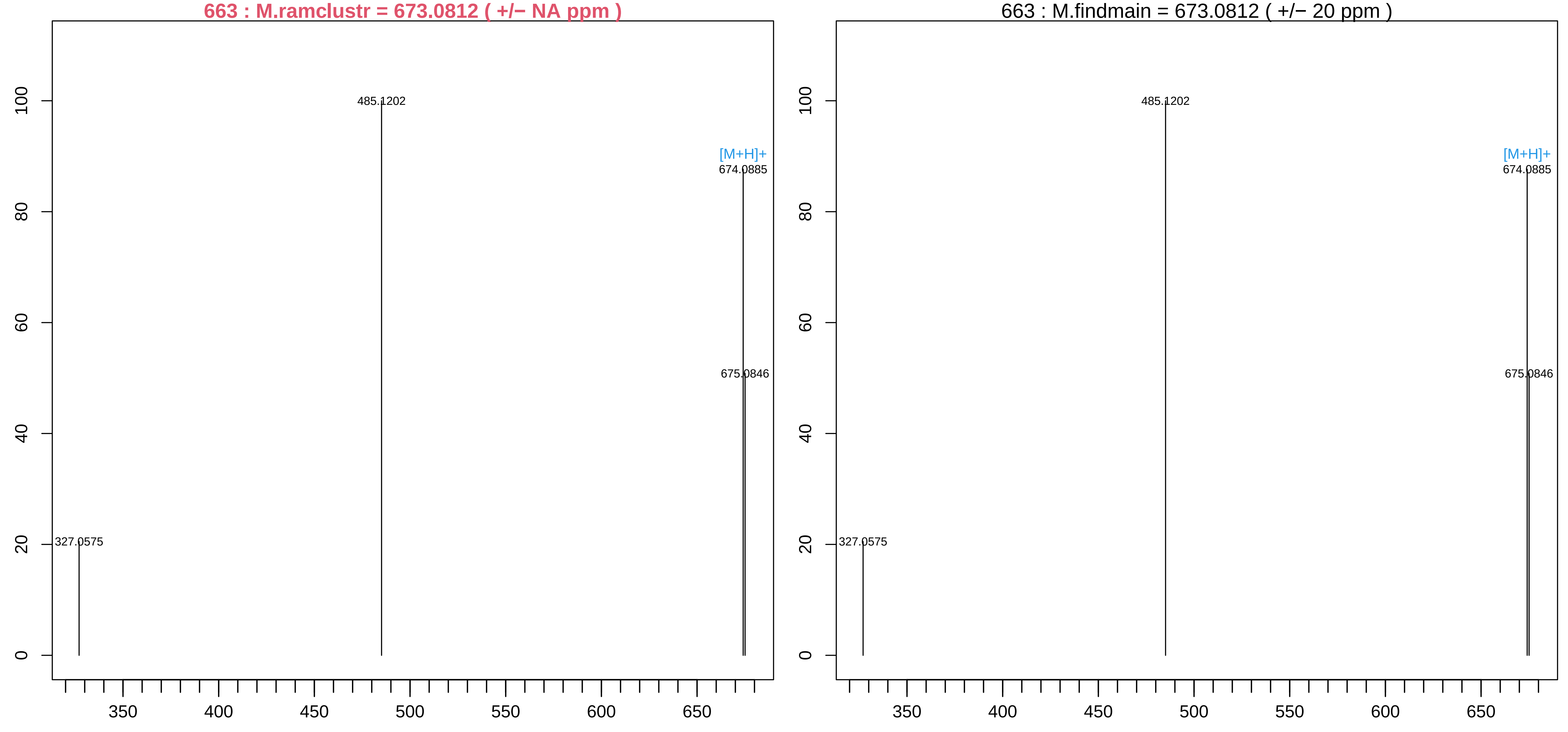

663 : M.ramclustr = 673.0812 (+/− NA ppm)
485.1202
[M+H]+
674.0885
675.0846
327.0575
0
20
40
60
80
100
350
400
450
500
550
600
650
663 : M.findmain = 673.0812 (+/− 20 ppm)
485.1202
[M+H]+
674.0885
675.0846
327.0575
0
20
40
60
80
100
350
400
450
500
550
600
650

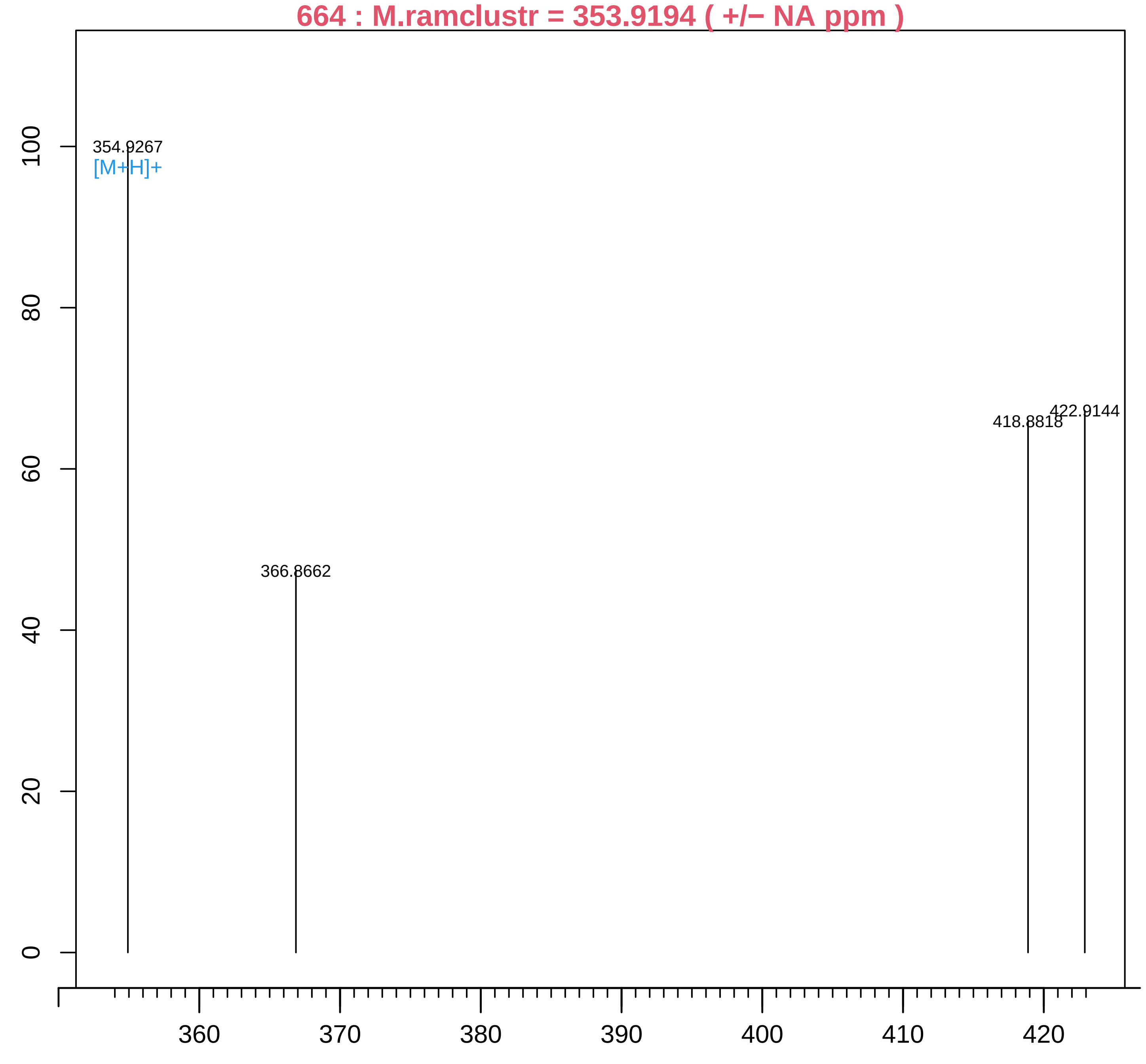
664 : M.ramclustr = 353.9194 (+/− NA ppm)
354.9267
[M+H]+
366.8662
418.8818
422.9144
0
20
40
60
80
100
360
370
380
390
400
410
420

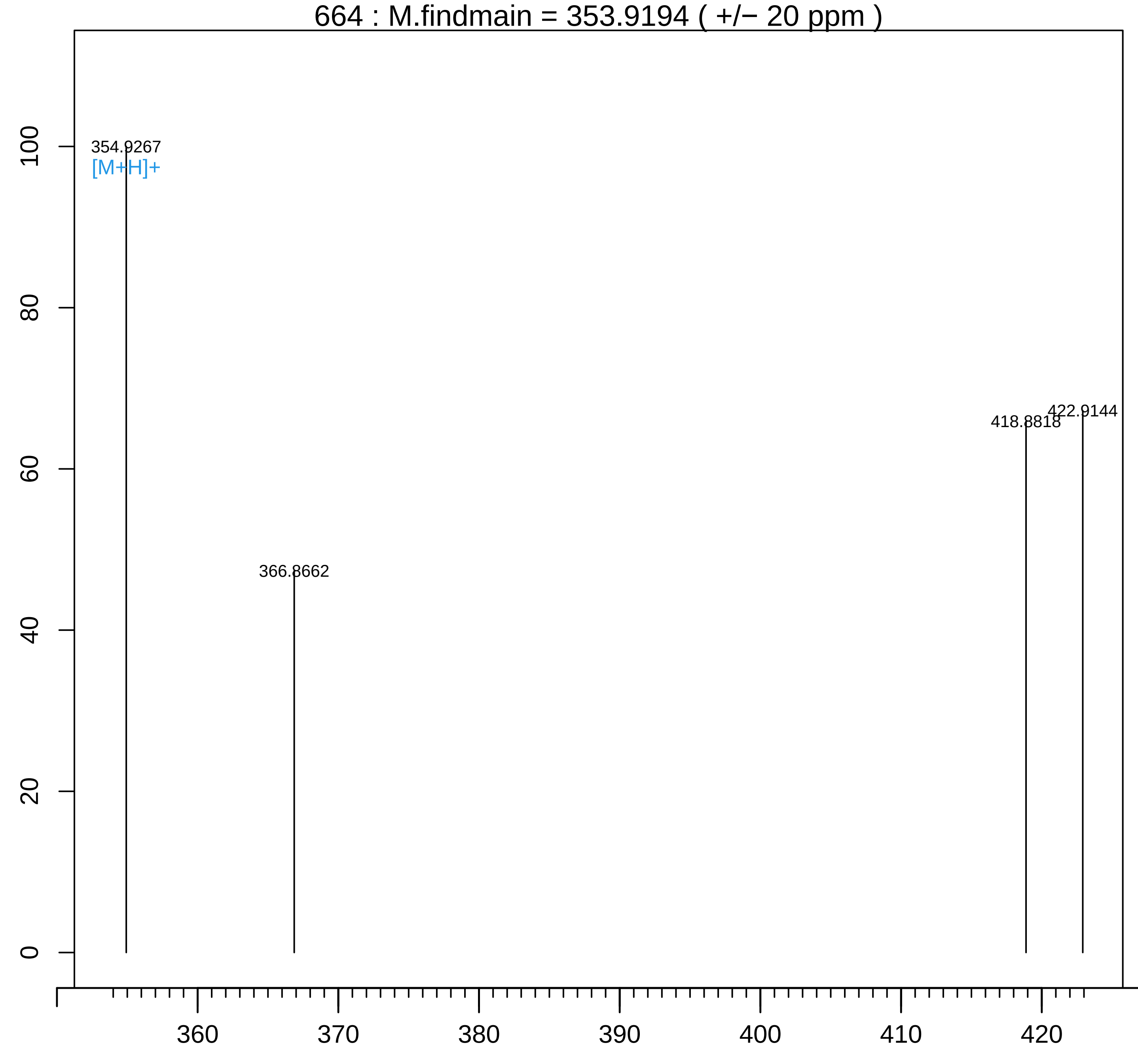
664 : M.findmain = 353.9194 (+/− 20 ppm)
354.9267
[M+H]+
366.8662
418.8818
422.9144
0
20
40
60
80
100
360
370
380
390
400
410
420

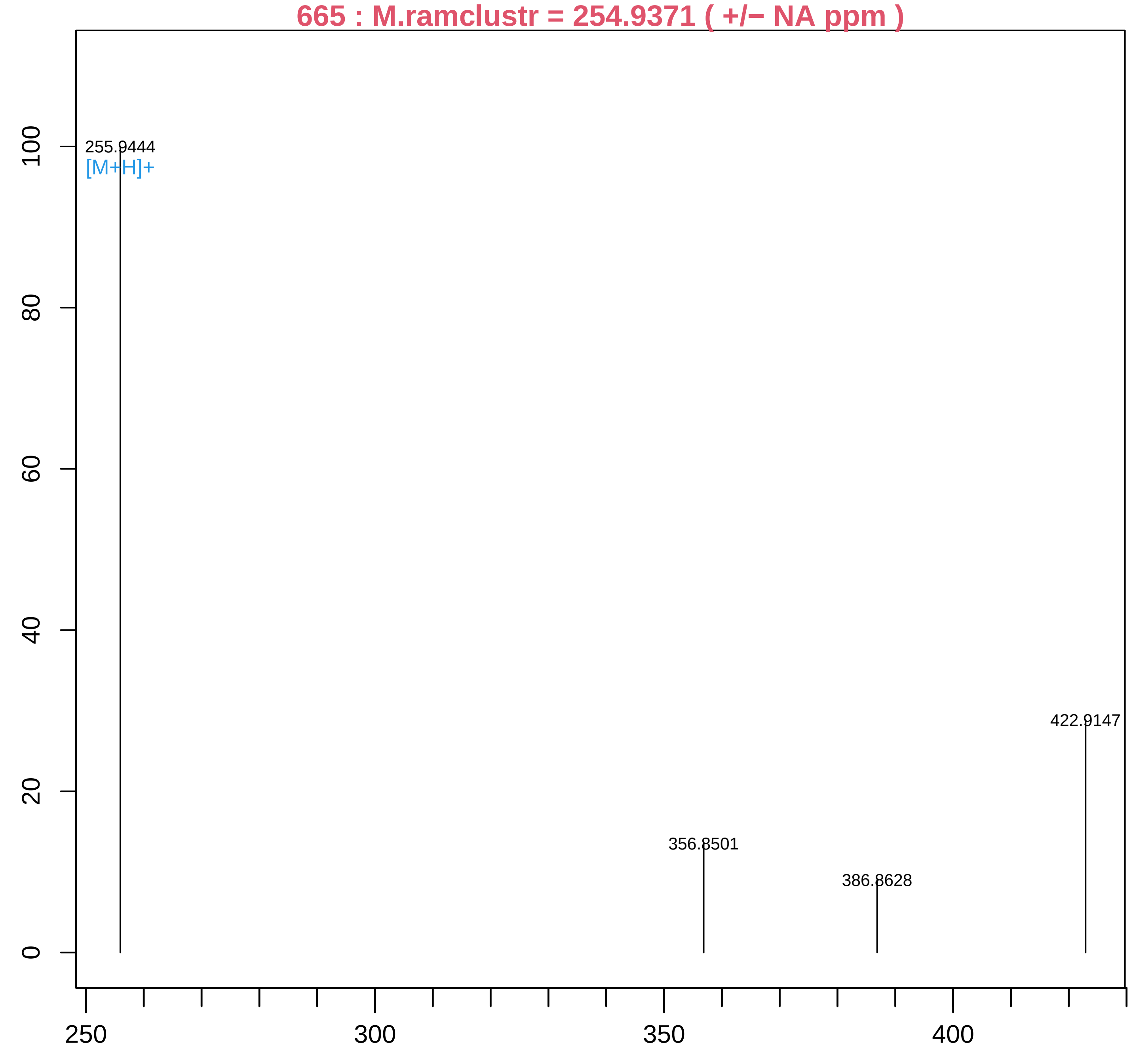
665 : M.ramclustr = 254.9371 (+/− NA ppm)
255.9444
[M+H]+
356.8501
386.8628
422.9147
0
20
40
60
80
100
250
300
350
400
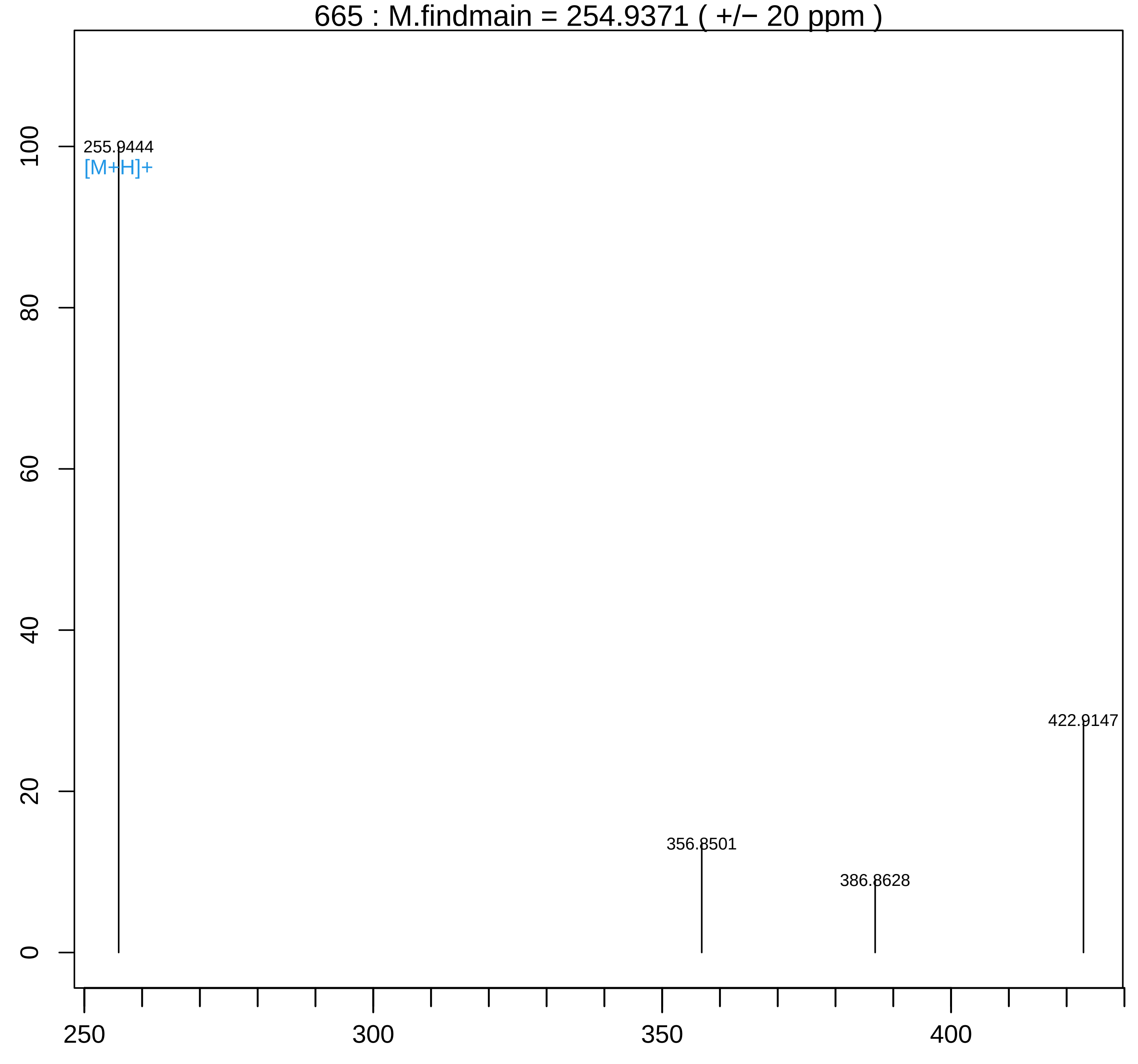
665 : M.findmain = 254.9371 (+/− 20 ppm)
255.9444
[M+H]+
356.8501
386.8628
422.9147
0
20
40
60
80
100
250
300
350
400

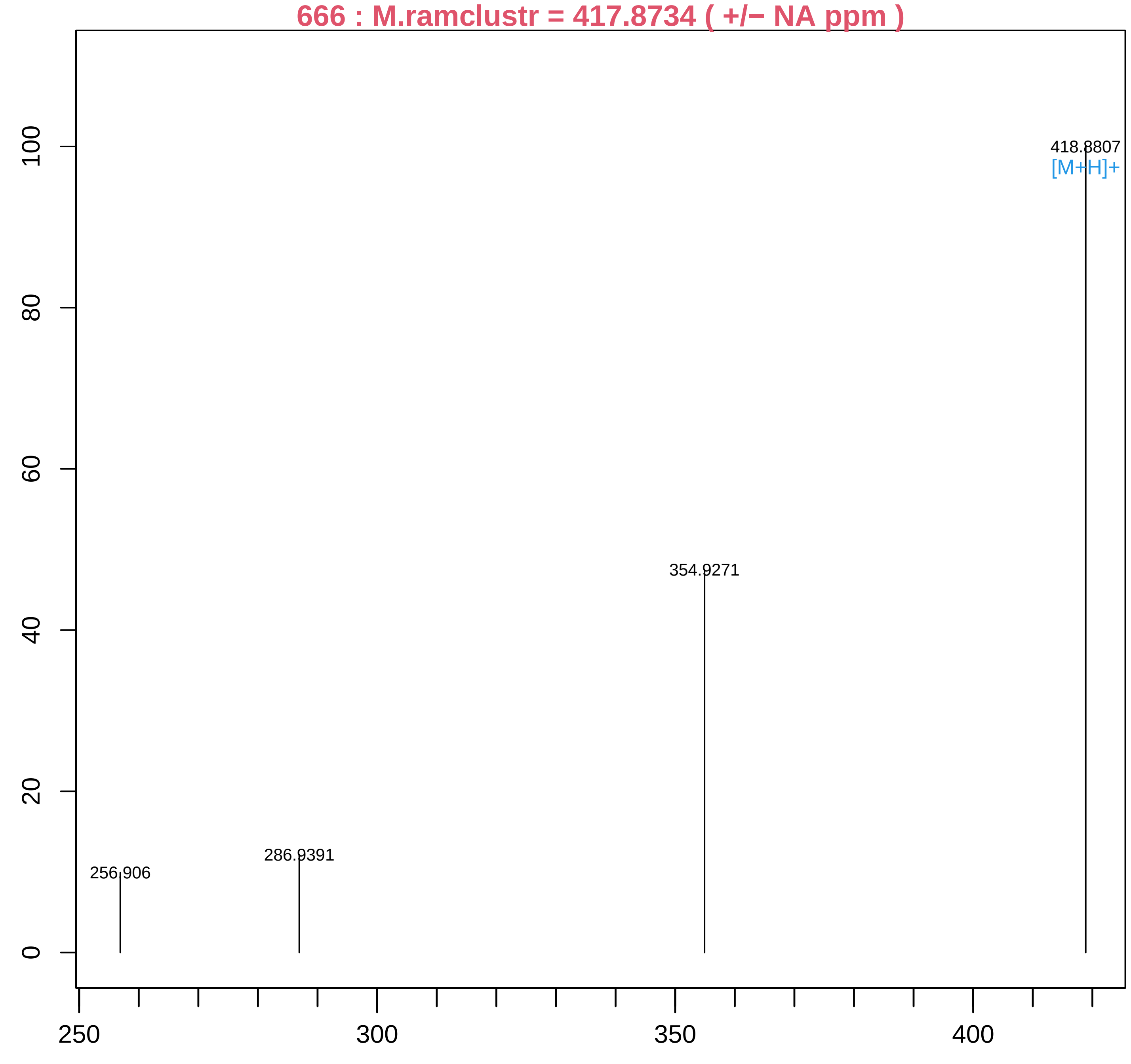
666 : M.ramclustr = 417.8734 (+/− NA ppm)
418.8807
[M+H]+
354.9271
286.9391
256.906
0
20
40
60
80
100
250
300
350
400

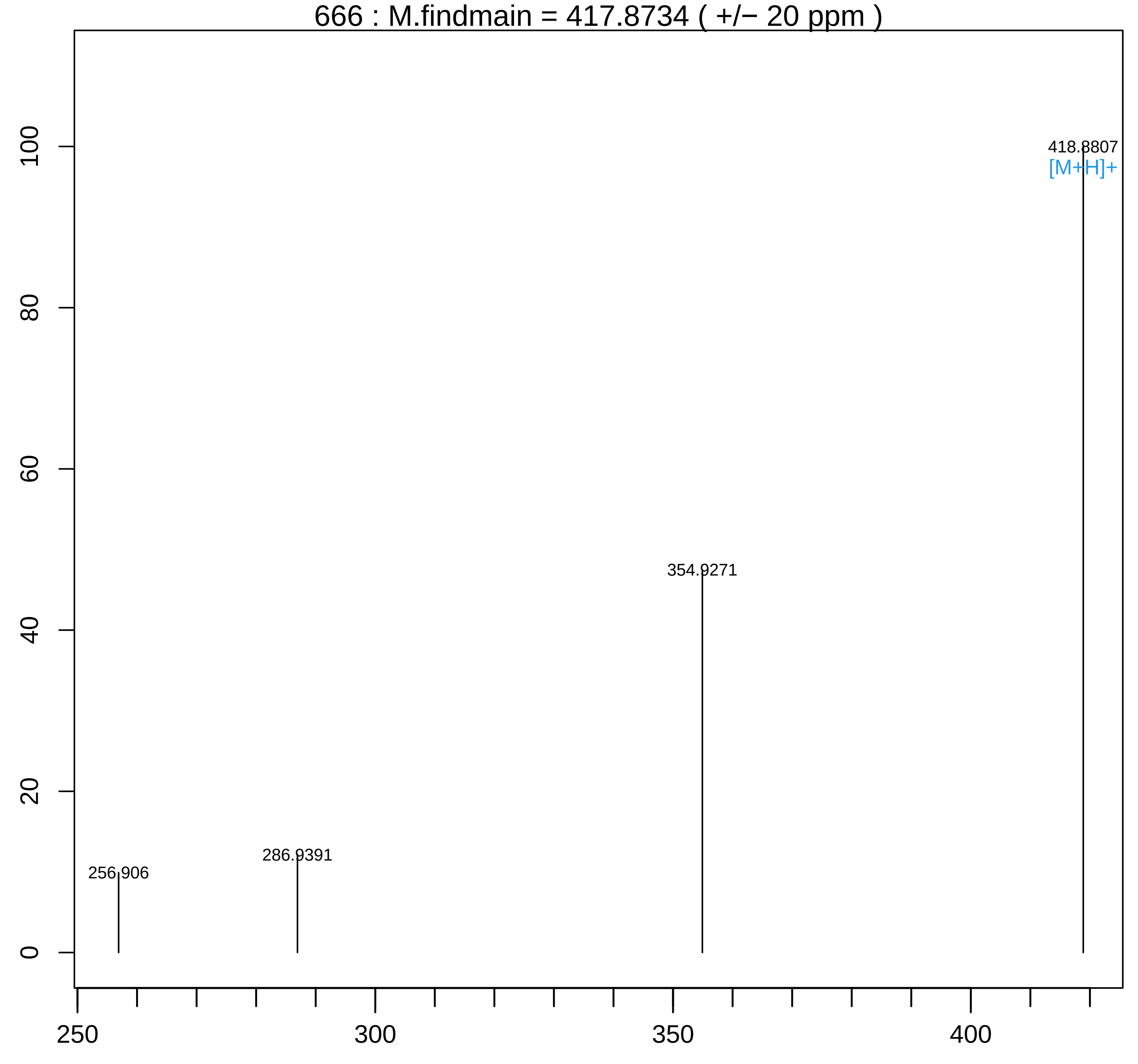
666 : M.findmain = 417.8734 (+/− 20 ppm)
418.8807
[M+H]+
354.9271
286.9391
256.906
0
20
40
60
80
100
250
300
350
400

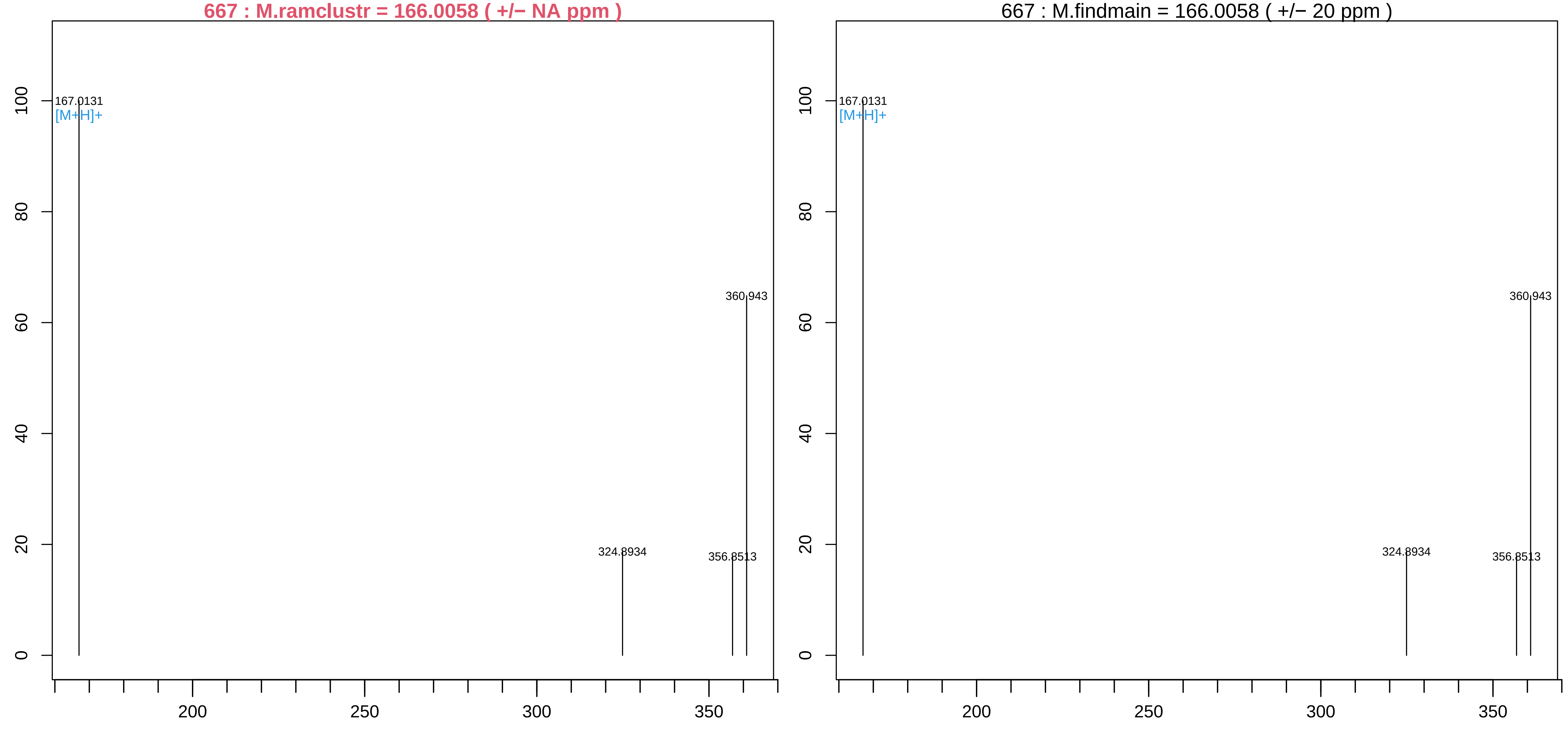

667 : M.ramclustr = 166.0058 (+/− NA ppm)
167.0131
[M+H]+
360.943
324.8934
356.8513
0
20
40
60
80
100
200
250
300
350
667 : M.findmain = 166.0058 (+/− 20 ppm)
167.0131
[M+H]+
360.943
324.8934
356.8513
0
20
40
60
80
100
200
250
300
350

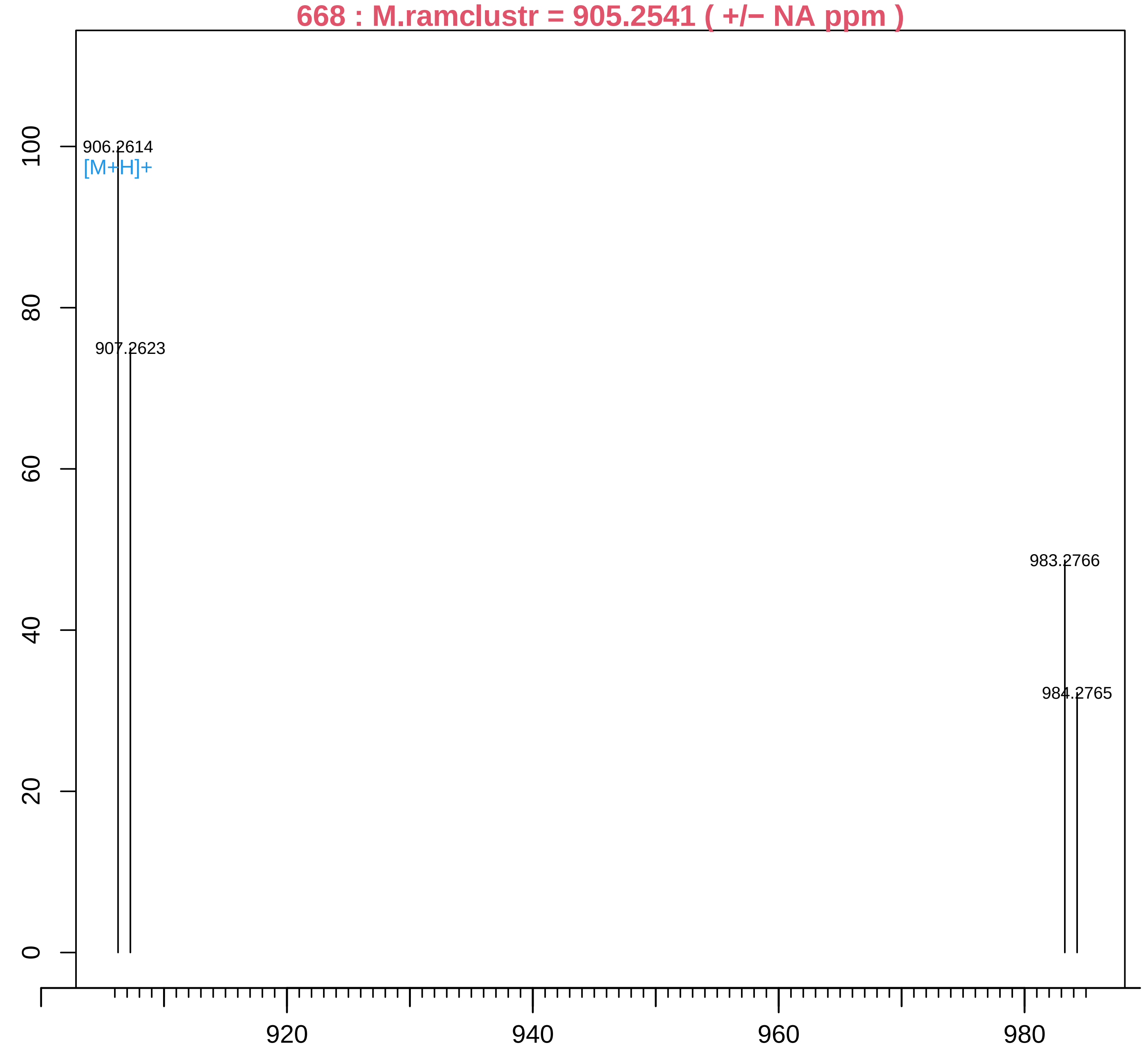

668 : M.ramclustr = 905.2541 (+/− NA ppm)
906.2614
[M+H]+
907.2623
983.2766
984.2765
0
20
40
60
80
100
920
940
960
980

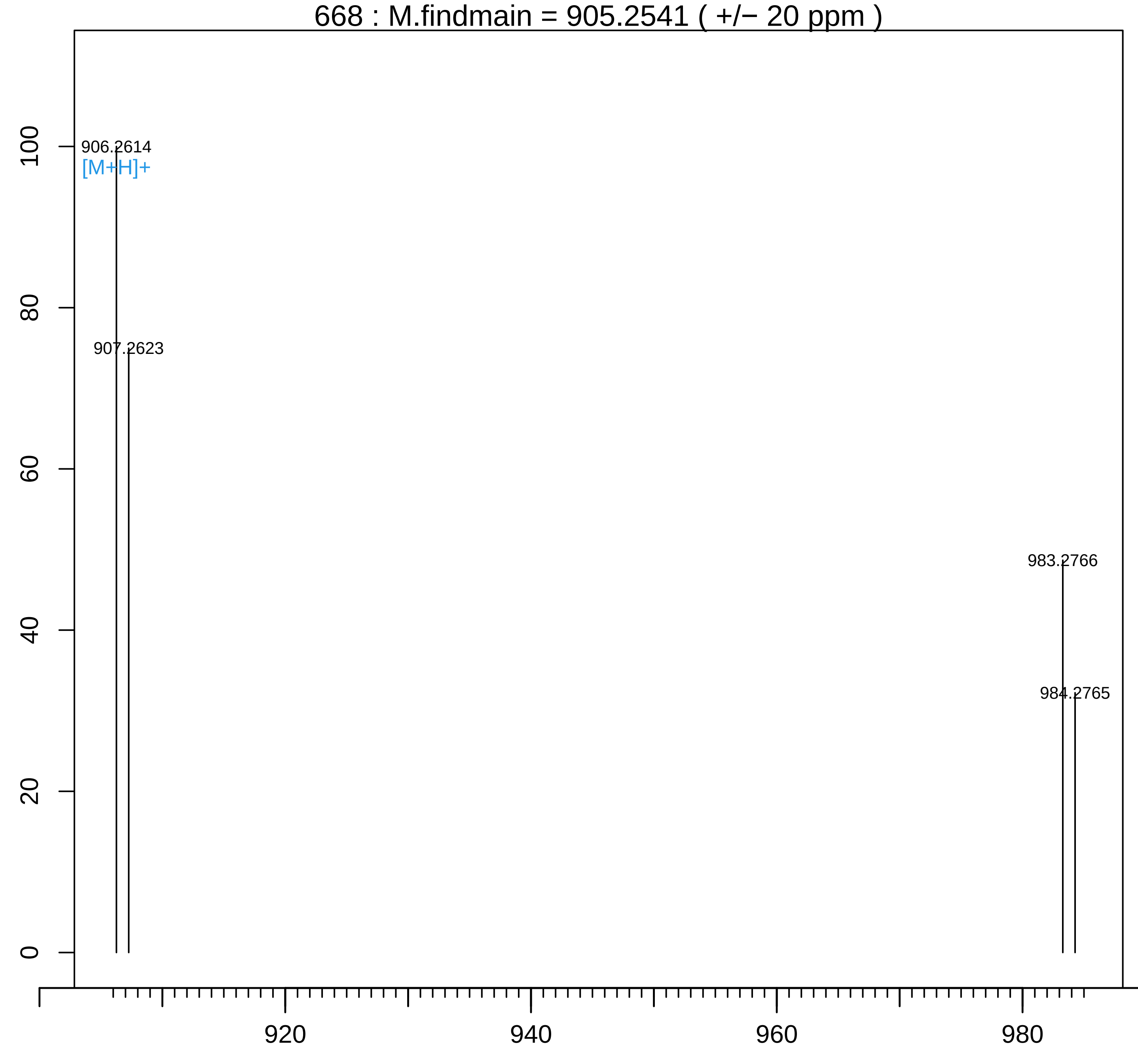

668 : M.findmain = 905.2541 (+/− 20 ppm)
906.2614
[M+H]+
907.2623
983.2766
984.2765
0
20
40
60
80
100
920
940
960
980

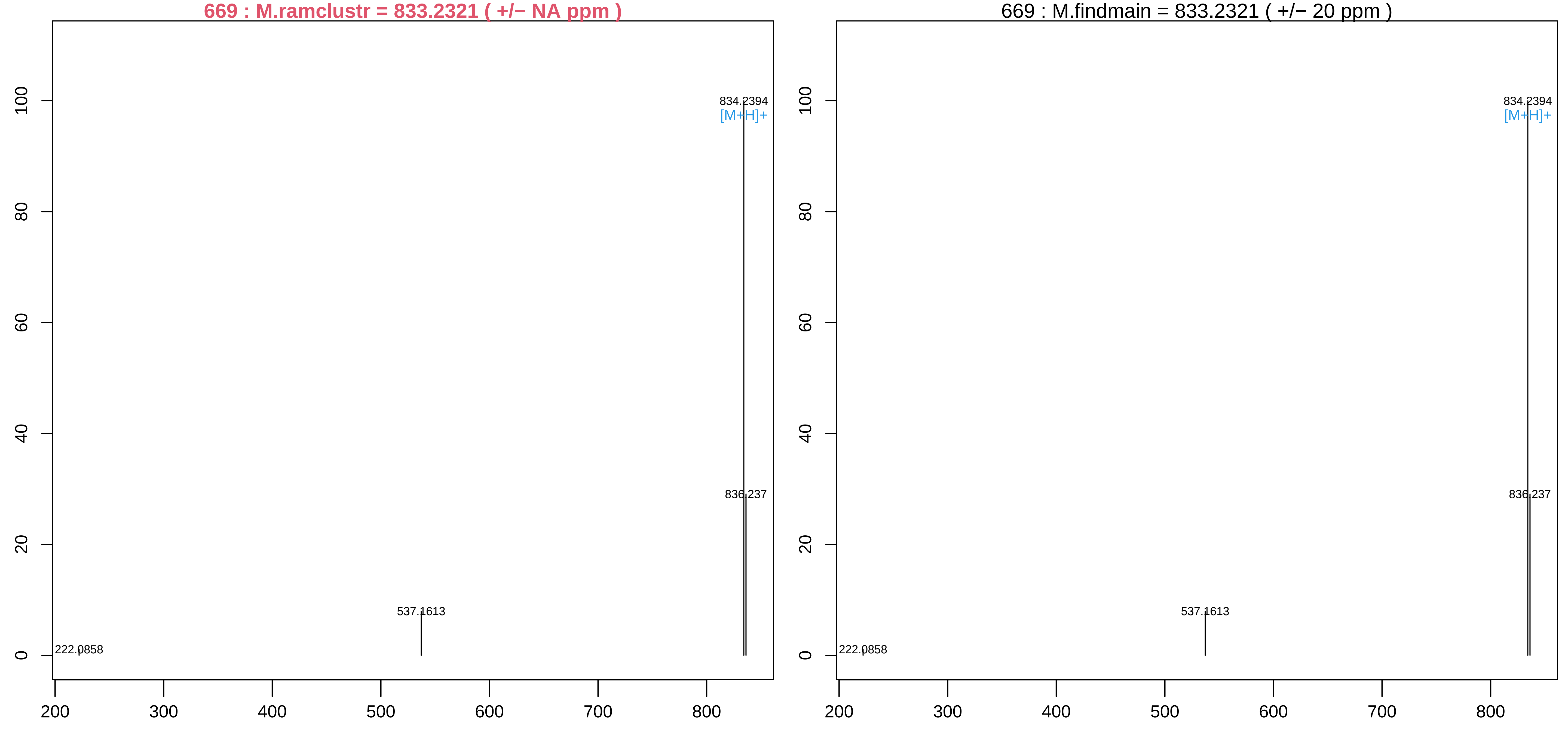
669 : M.ramclustr = 833.2321 (+/− NA ppm)
669 : M.findmain = 833.2321 (+/− 20 ppm)
834.2394
[M+H]+
836.237
537.1613
222.0858
0
20
40
60
80
100
200
300
400
500
600
700
800

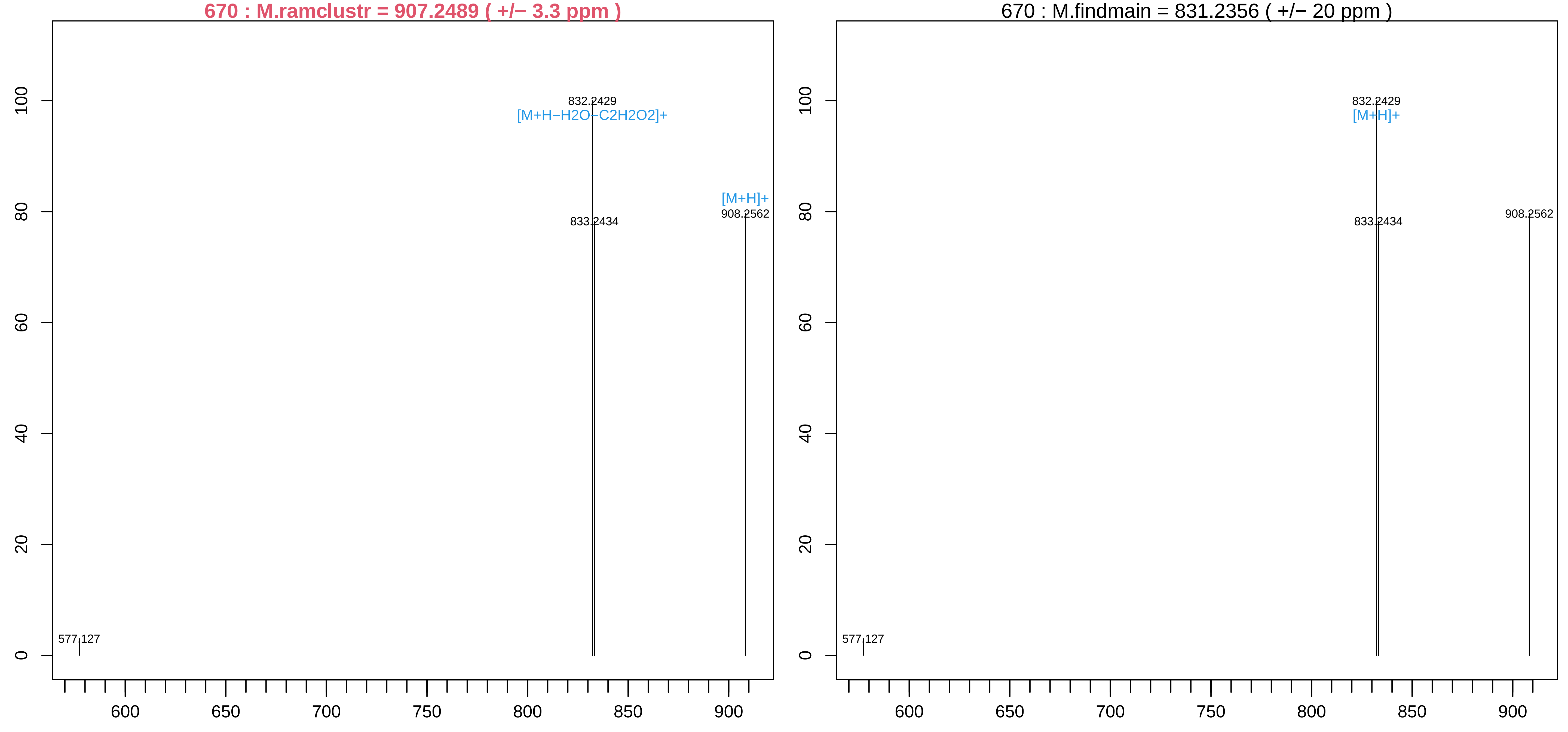
670 : M.ramclustr = 907.2489 (+/− 3.3 ppm)
832.2429
[M+H−H2O−C2H2O2]+
[M+H]+
908.2562
833.2434
577.127
670 : M.findmain = 831.2356 (+/− 20 ppm)
832.2429
[M+H]+
833.2434
908.2562
577.127

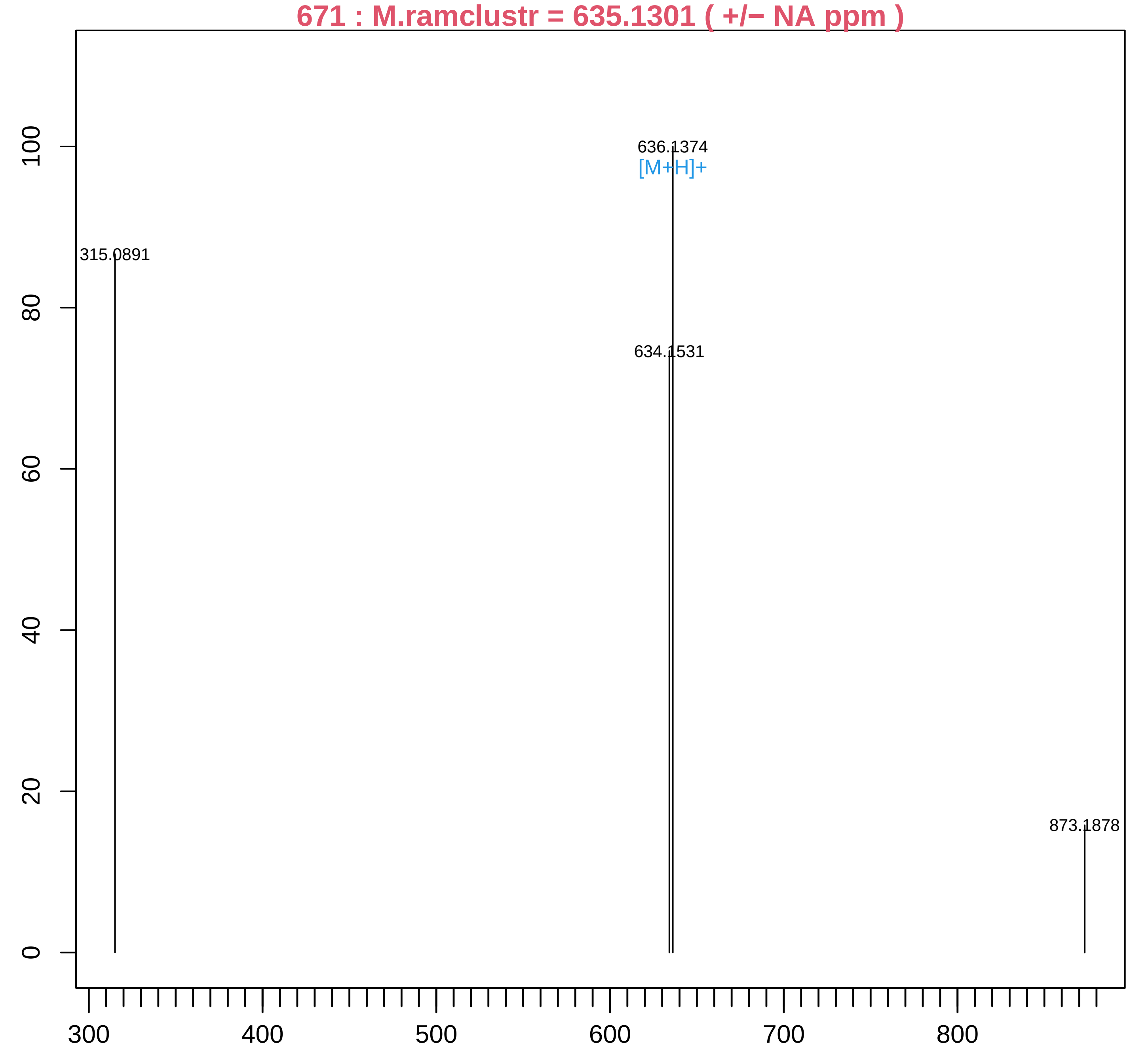
671 : M.ramclustr = 635.1301 (+/− NA ppm)
636.1374
[M+H]+
315.0891
634.1531
873.1878
0
20
40
60
80
100
300
400
500
600
700
800

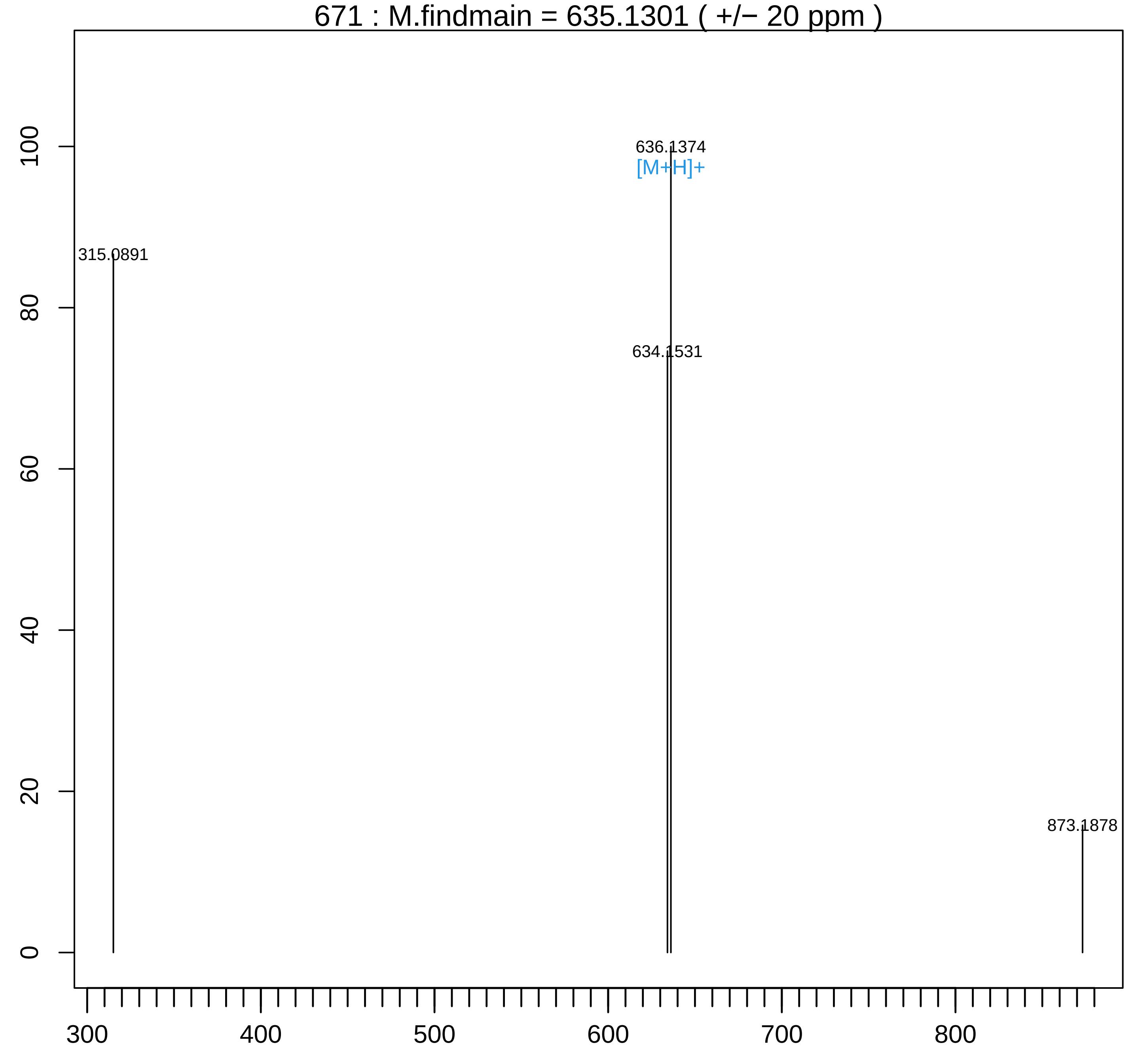
671 : M.findmain = 635.1301 (+/− 20 ppm)
636.1374
[M+H]+
315.0891
634.1531
873.1878
0
20
40
60
80
100
300
400
500
600
700
800

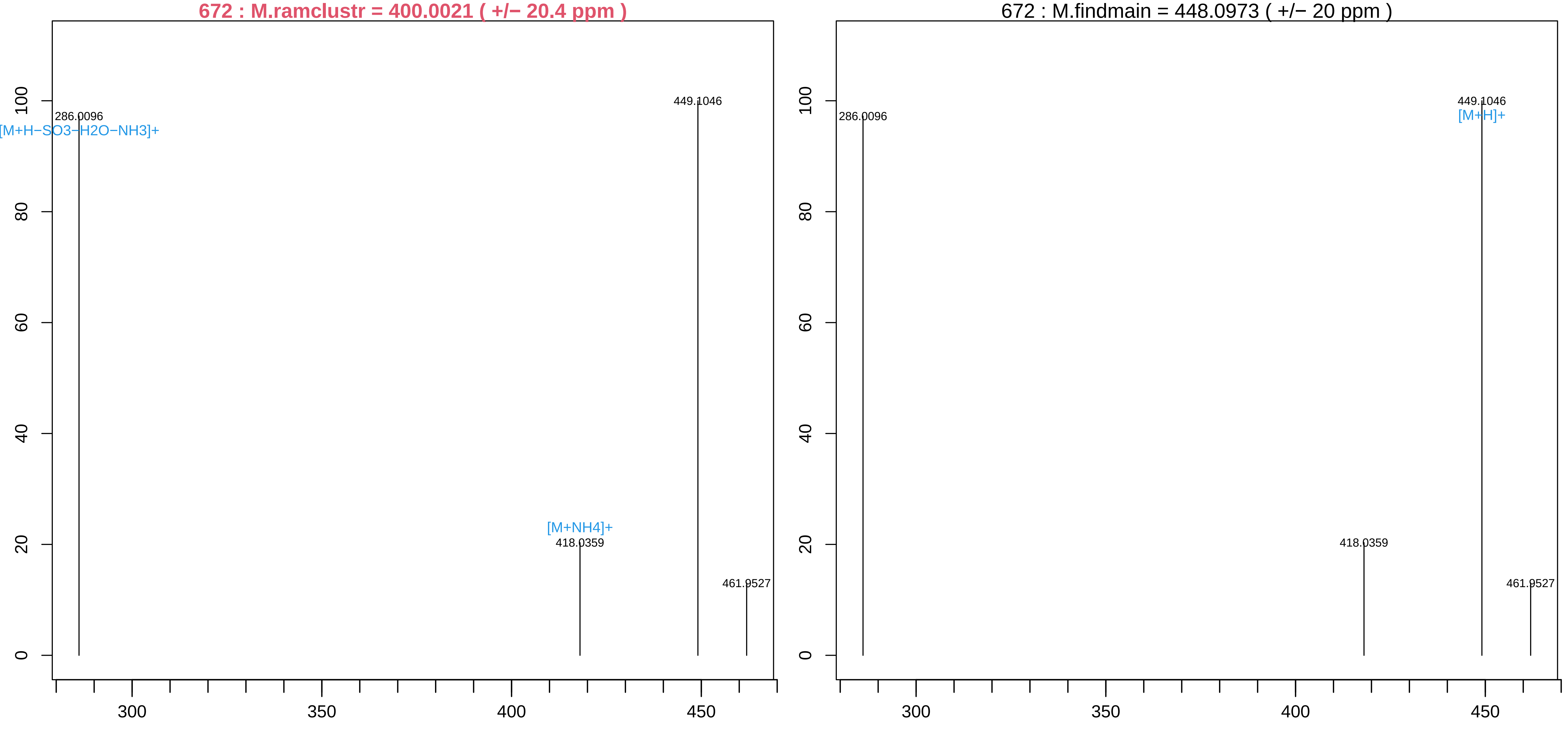
672 : M.ramclustr = 400.0021 (+/− 20.4 ppm)
286.0096
[M+H−SO3−H2O−NH3]+
449.1046
[M+NH4]+
418.0359
461.9527
672 : M.findmain = 448.0973 (+/− 20 ppm)
286.0096
449.1046
[M+H]+
418.0359
461.9527
0
20
40
60
80
100
300
350
400
450

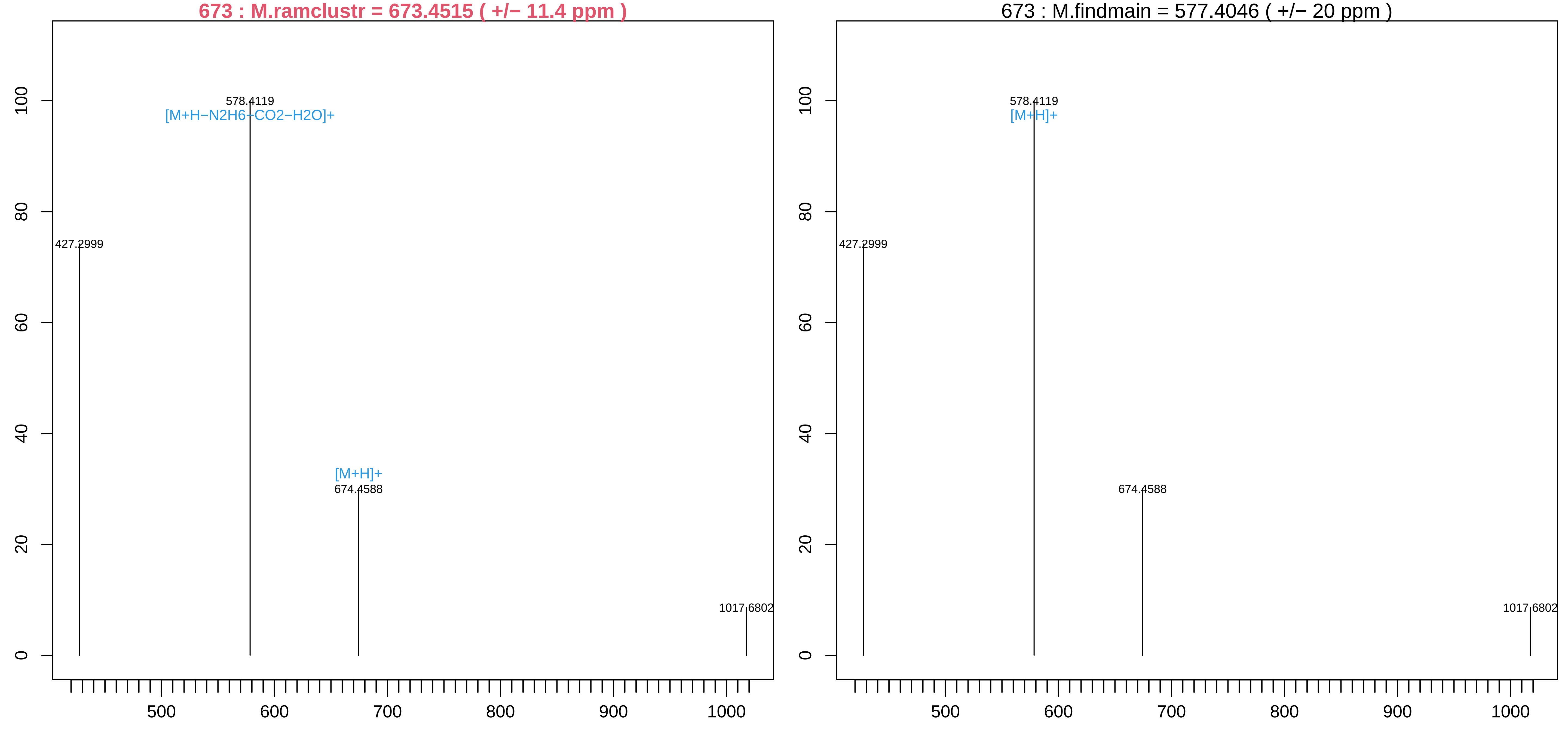
673 : M.ramclustr = 673.4515 (+/− 11.4 ppm)
578.4119
[M+H−N2H6−CO2−H2O]+
427.2999
[M+H]+
674.4588
1017.6802
673 : M.findmain = 577.4046 (+/− 20 ppm)
578.4119
[M+H]+
427.2999
674.4588
1017.6802

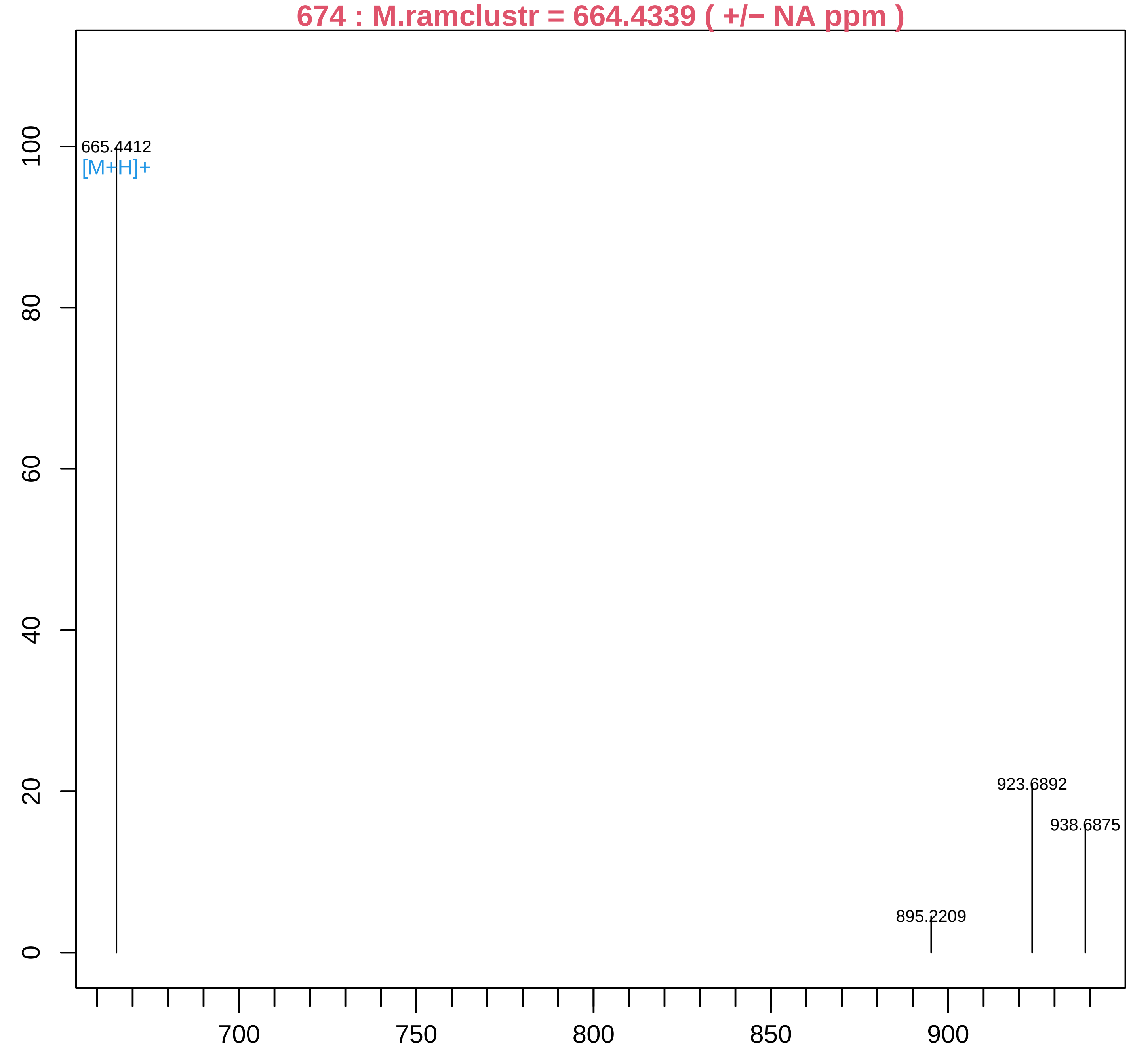

674 : M.ramclustr = 664.4339 (+/− NA ppm)
665.4412
[M+H]+
923.6892
938.6875
895.2209
0
20
40
60
80
100
700
750
800
850
900

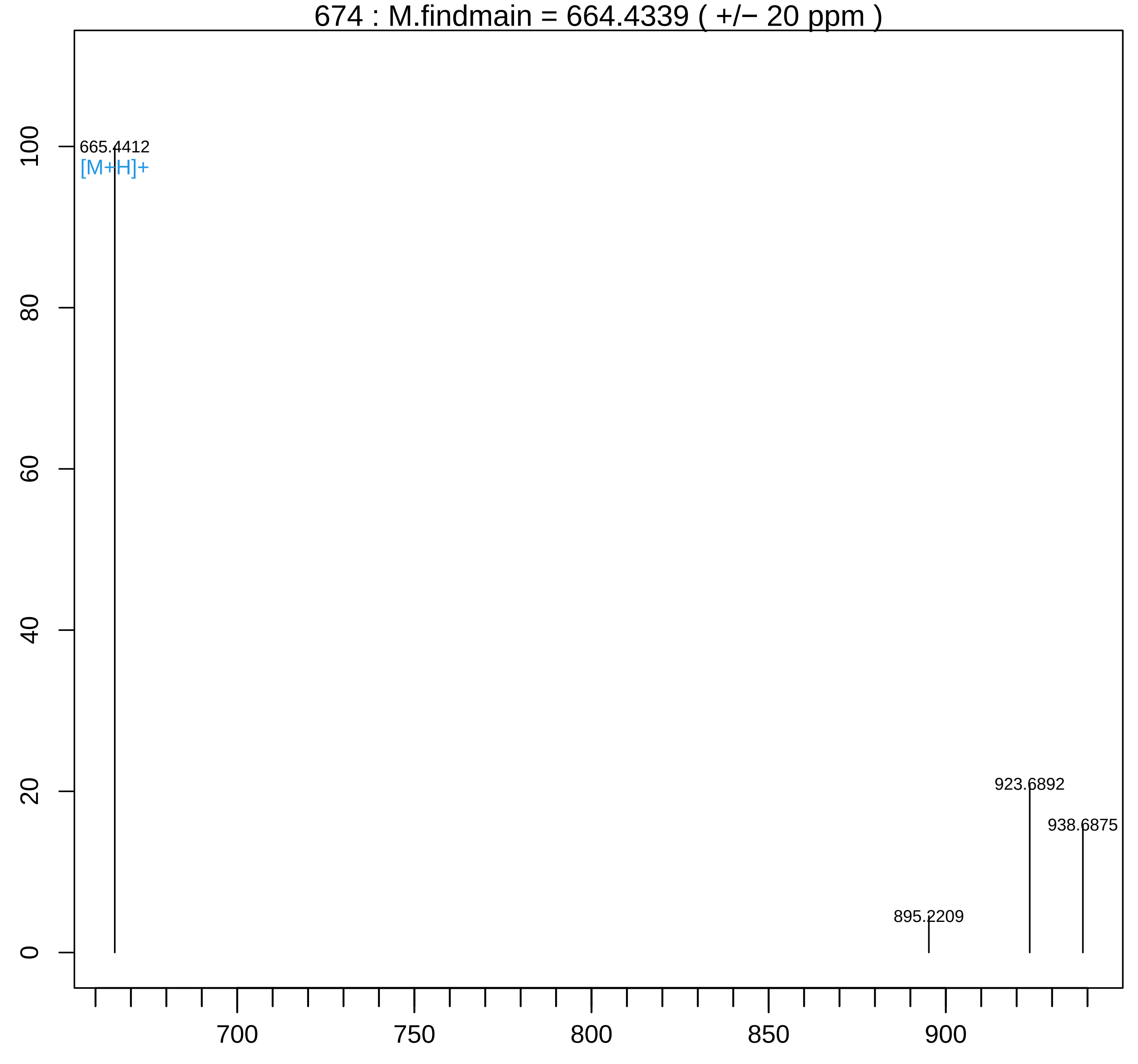

674 : M.findmain = 664.4339 (+/− 20 ppm)
665.4412
[M+H]+
923.6892
938.6875
895.2209
0
20
40
60
80
100
700
750
800
850
900

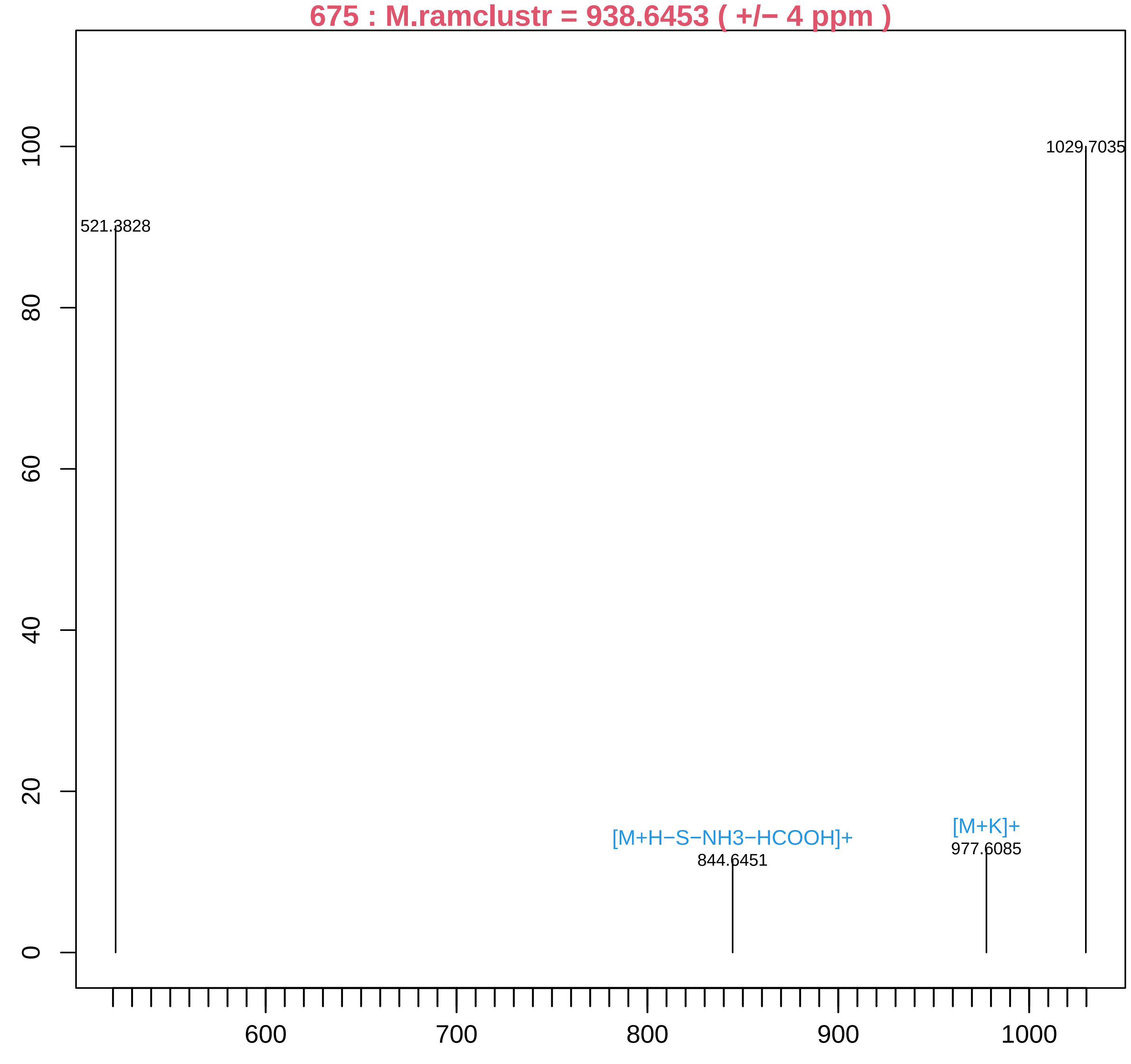

675 : M.ramclustr = 938.6453 (+/− 4 ppm)
521.3828
[M+H−S−NH3−HCOOH]+
844.6451
[M+K]+
977.6085
1029.7035
0
20
40
60
80
100
600
700
800
900
1000

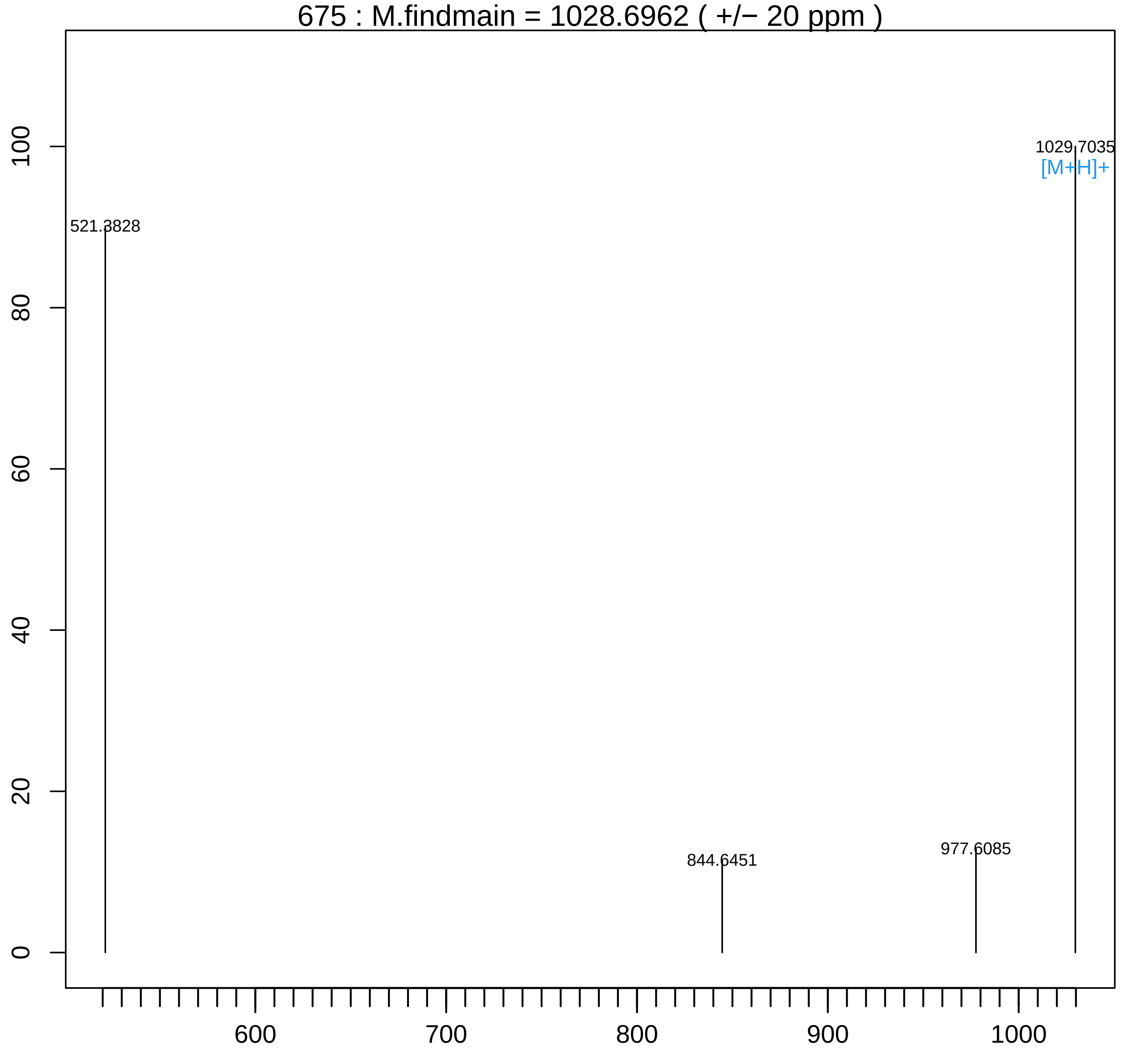

675 : M.findmain = 1028.6962 (+/− 20 ppm)
521.3828
844.6451
977.6085
1029.7035
[M+H]+
0
20
40
60
80
100
600
700
800
900
1000

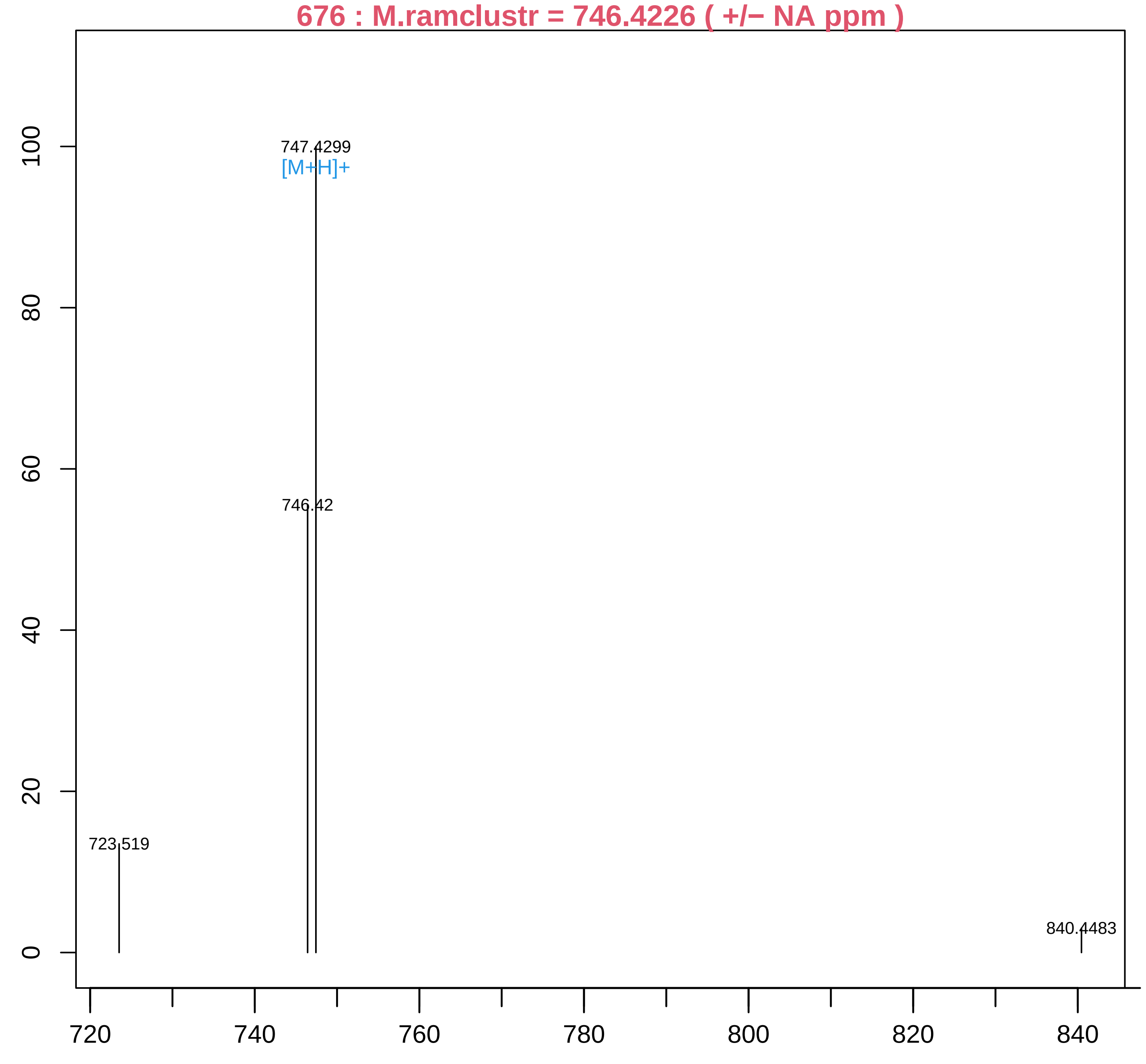

676 : M.ramclustr = 746.4226 (+/− NA ppm)
747.4299
[M+H]+
746.42
723.519
840.4483
0
20
40
60
80
100
720
740
760
780
800
820
840

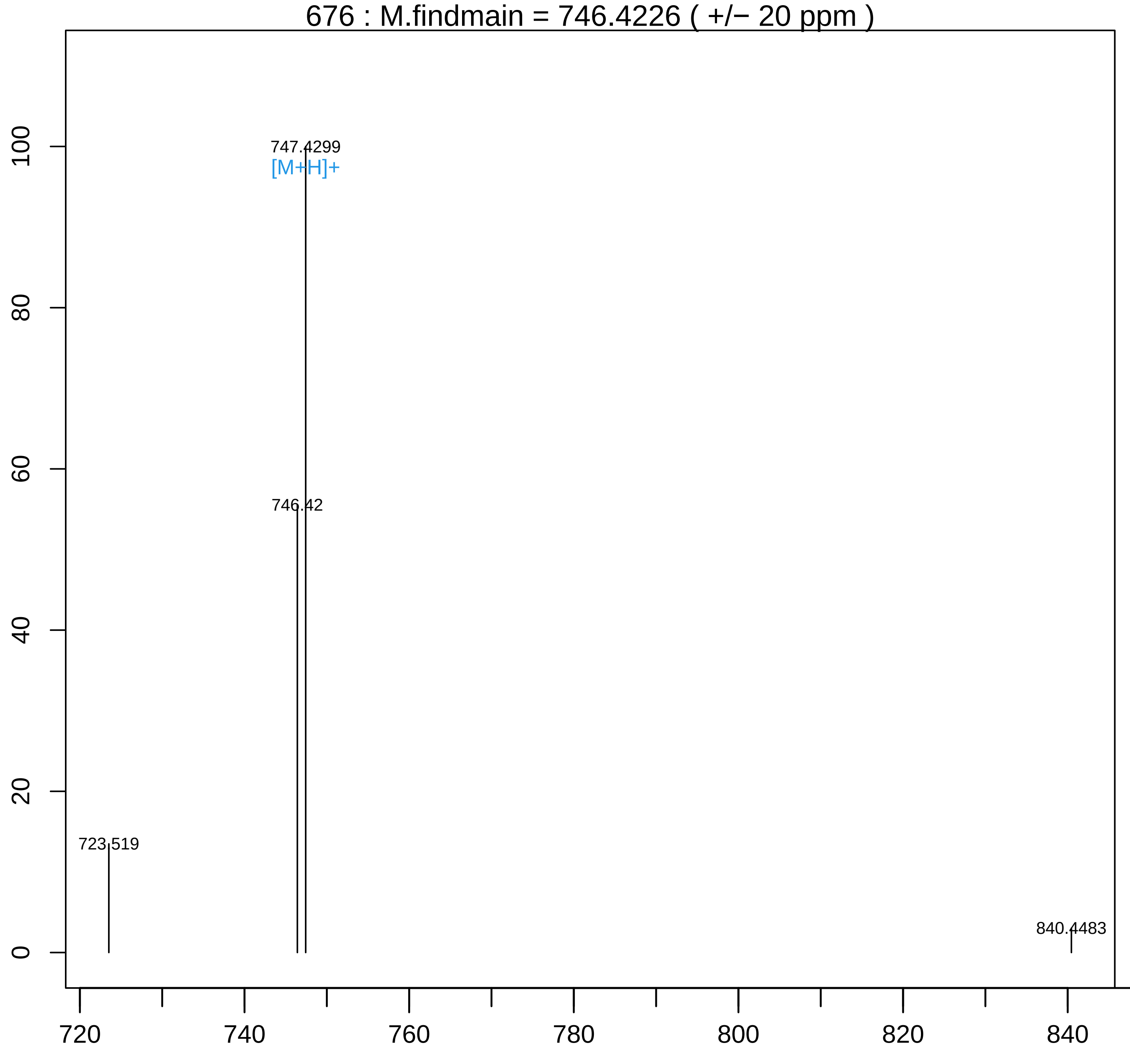

676 : M.findmain = 746.4226 (+/− 20 ppm)
747.4299
[M+H]+
746.42
723.519
840.4483
0
20
40
60
80
100
720
740
760
780
800
820
840

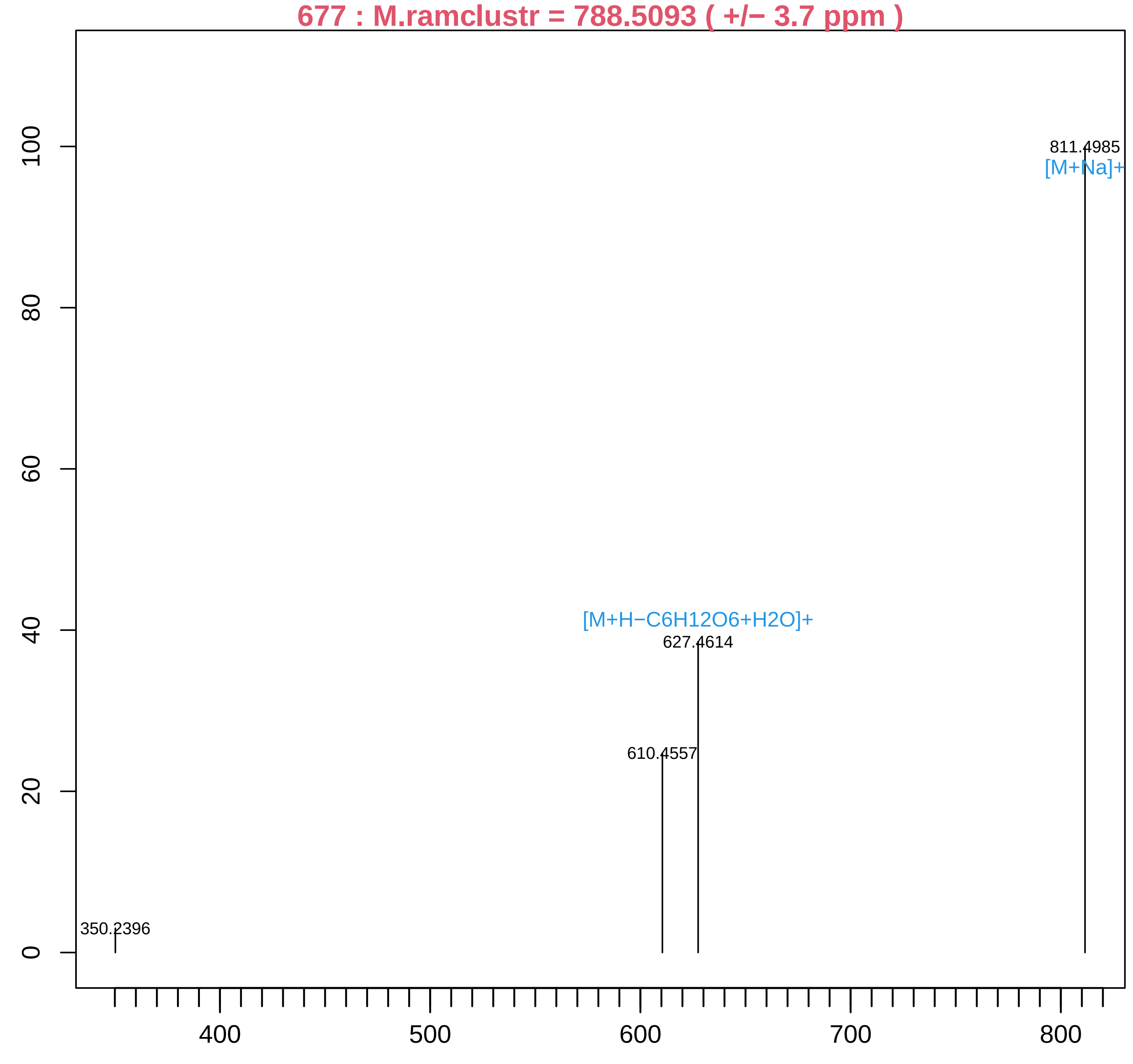
677 : M.ramclustr = 788.5093 (+/− 3.7 ppm)
811.4985
[M+Na]+
[M+H−C6H12O6+H2O]+
627.4614
610.4557
350.2396
0
20
40
60
80
100
400
500
600
700
800

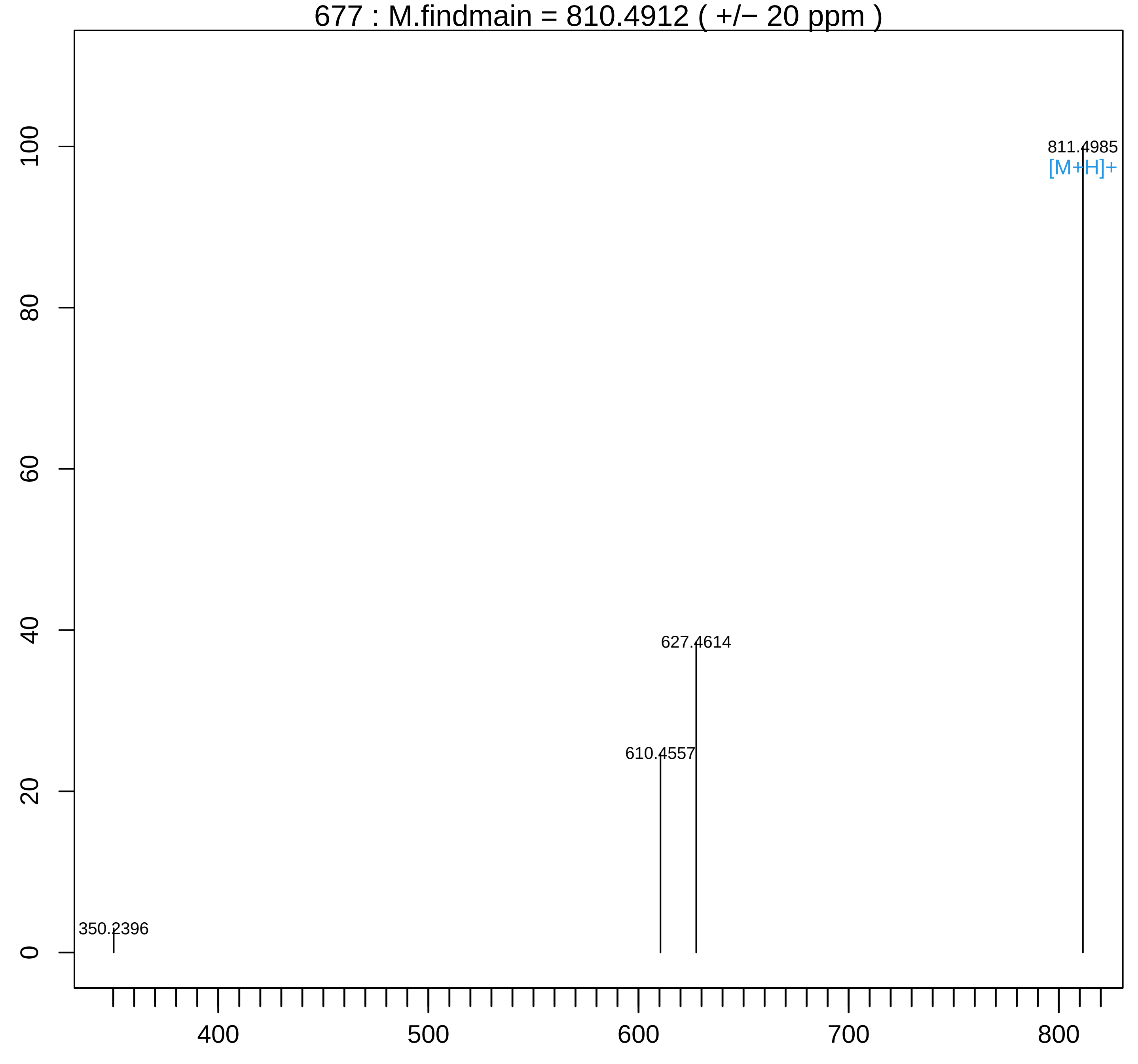
677 : M.findmain = 810.4912 (+/− 20 ppm)
811.4985
[M+H]+
627.4614
610.4557
350.2396
0
20
40
60
80
100
400
500
600
700
800

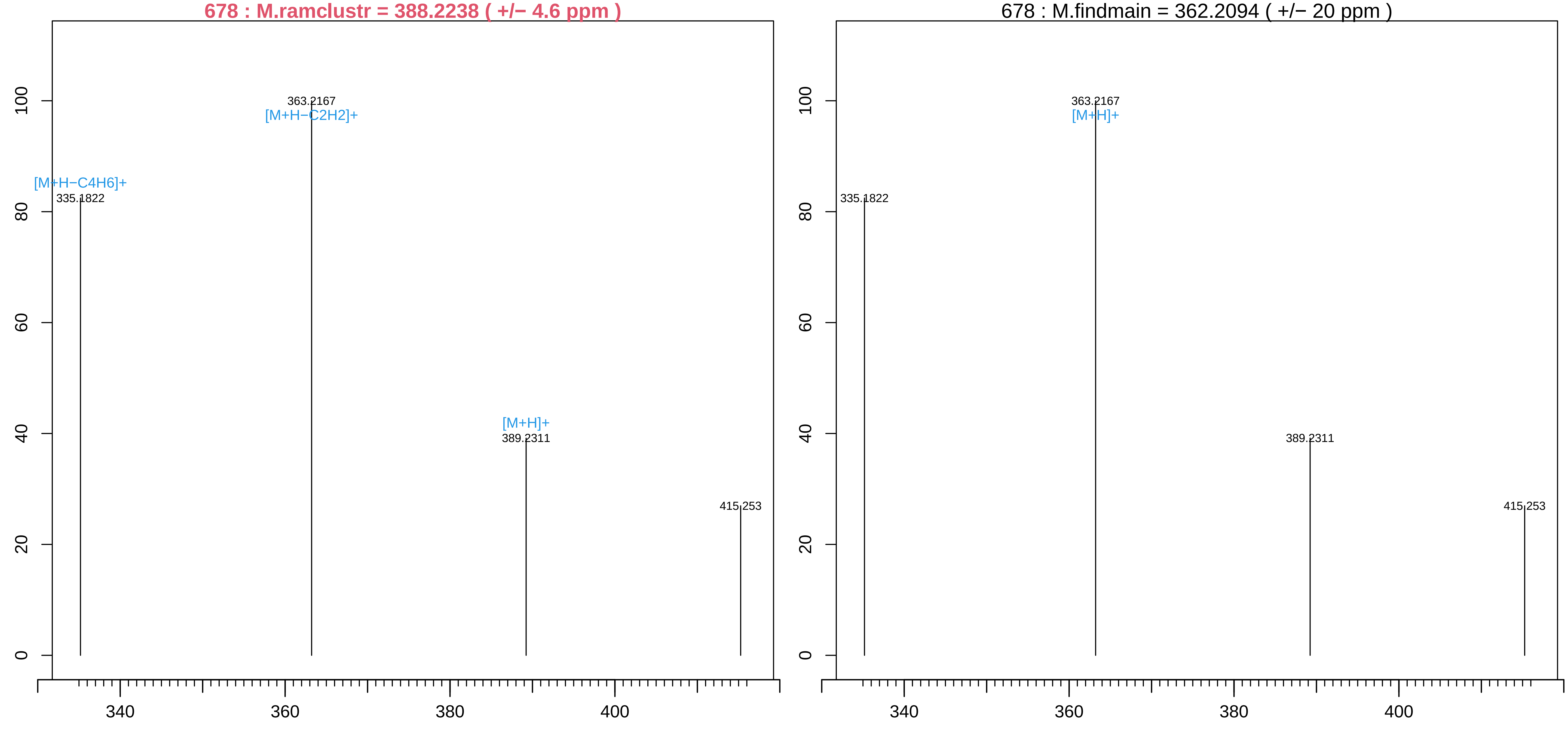
678 : M.ramclustr = 388.2238 (+/− 4.6 ppm)
363.2167
[M+H−C2H2]+
[M+H−C4H6]+
335.1822
[M+H]+
389.2311
415.253
0
20
40
60
80
100
340
360
380
400
678 : M.findmain = 362.2094 (+/− 20 ppm)
363.2167
[M+H]+
335.1822
389.2311
415.253

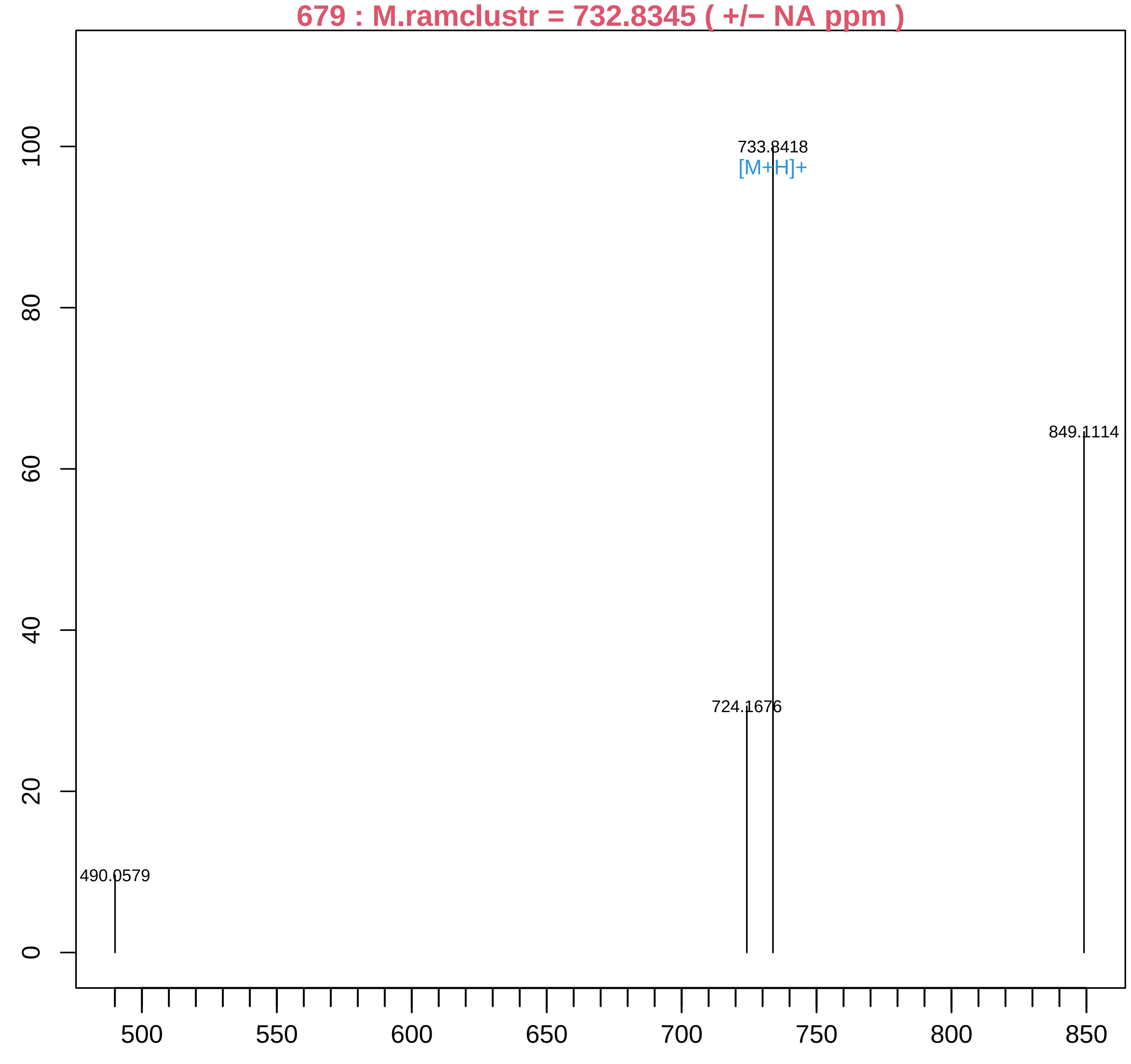
679 : M.ramclustr = 732.8345 (+/− NA ppm)
733.8418
[M+H]+
849.1114
724.1676
490.0579
0
20
40
60
80
100
500
550
600
650
700
750
800
850

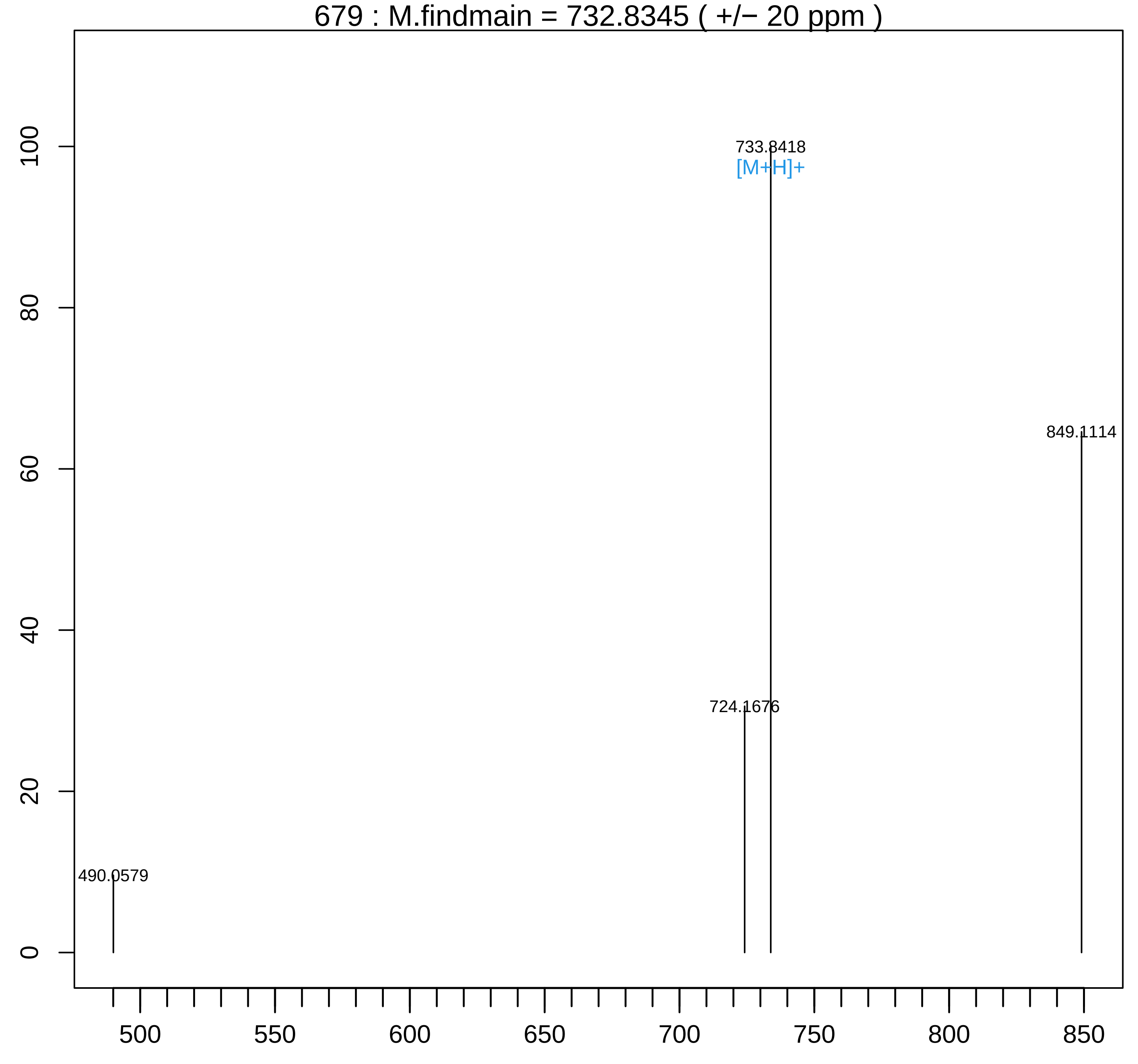
679 : M.findmain = 732.8345 (+/− 20 ppm)
733.8418
[M+H]+
849.1114
724.1676
490.0579
0
20
40
60
80
100
500
550
600
650
700
750
800
850

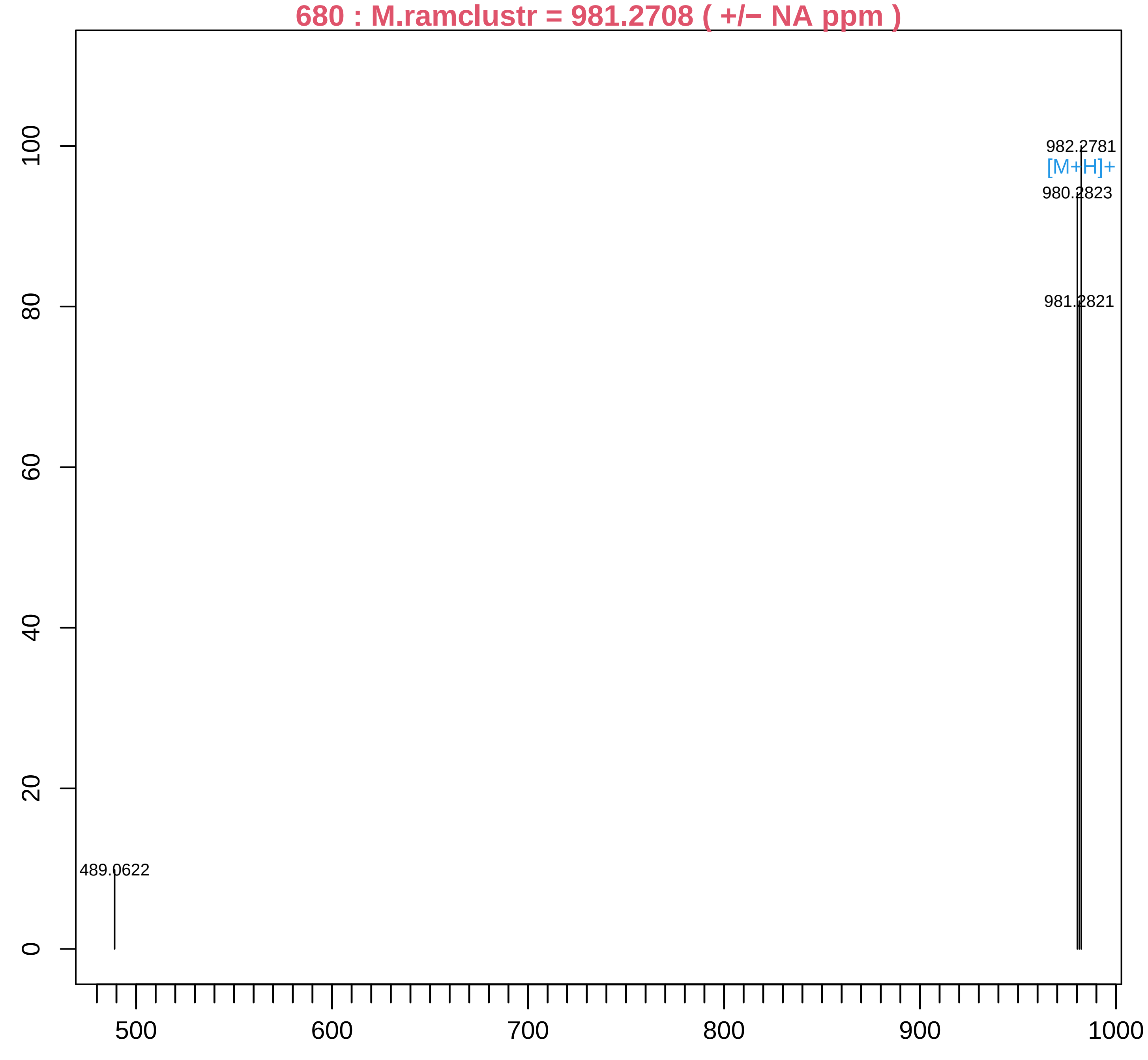
680 : M.ramclustr = 981.2708 (+/− NA ppm)
982.2781
[M+H]+
980.2823
981.2821
489.0622
0
20
40
60
80
100
500
600
700
800
900
1000

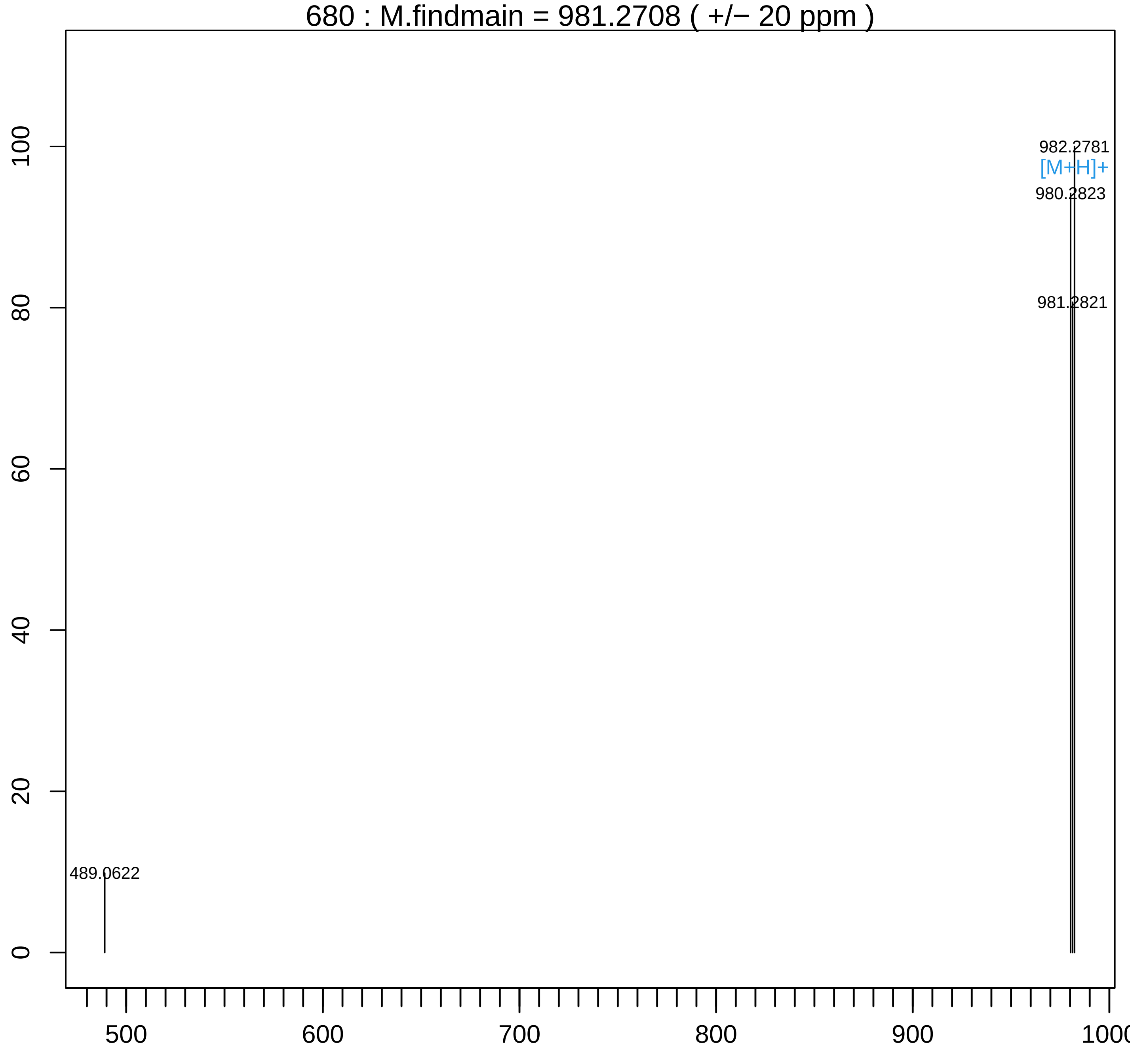
680 : M.findmain = 981.2708 (+/− 20 ppm)
982.2781
[M+H]+
980.2823
981.2821
489.0622
0
20
40
60
80
100
500
600
700
800
900
1000

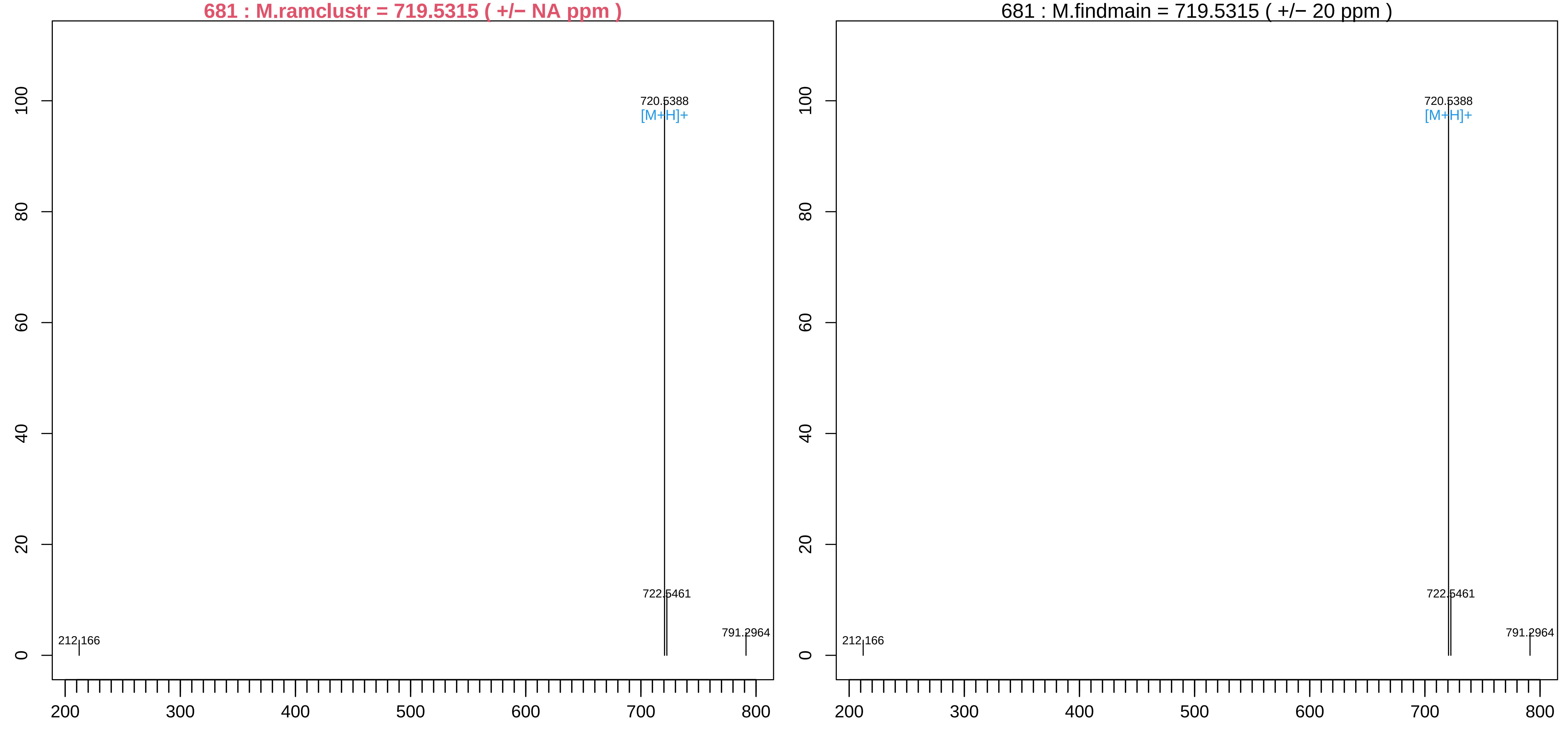
681 : M.ramclustr = 719.5315 (+/− NA ppm)
720.5388
[M+H]+
722.5461
212.166
791.2964
0
20
40
60
80
100
200
300
400
500
600
700
800
681 : M.findmain = 719.5315 (+/− 20 ppm)
720.5388
[M+H]+
722.5461
212.166
791.2964

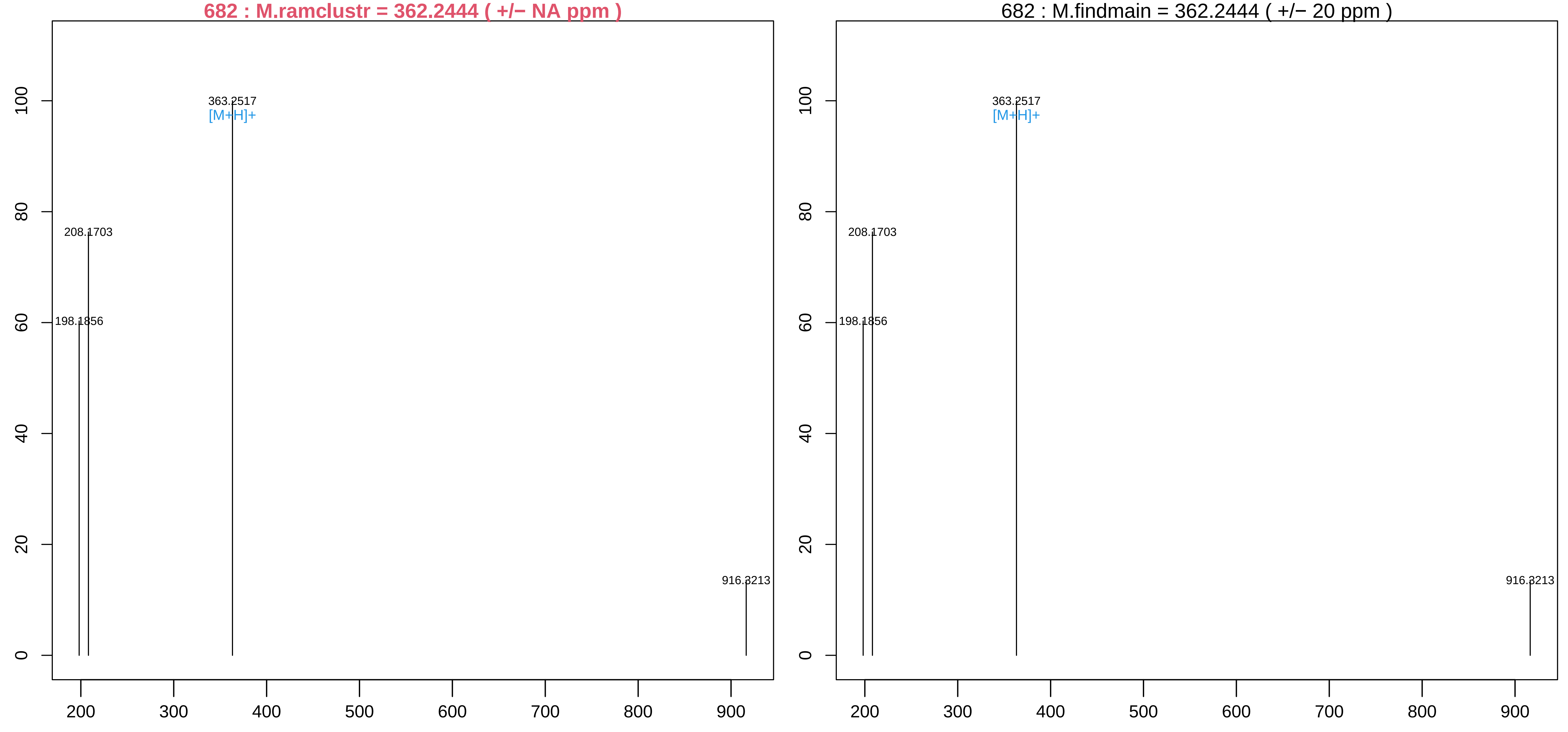

682 : M.ramclustr = 362.2444 (+/− NA ppm)
363.2517
[M+H]+
208.1703
198.1856
916.3213
0
20
40
60
80
100
200
300
400
500
600
700
800
900
682 : M.findmain = 362.2444 (+/− 20 ppm)
363.2517
[M+H]+
208.1703
198.1856
916.3213

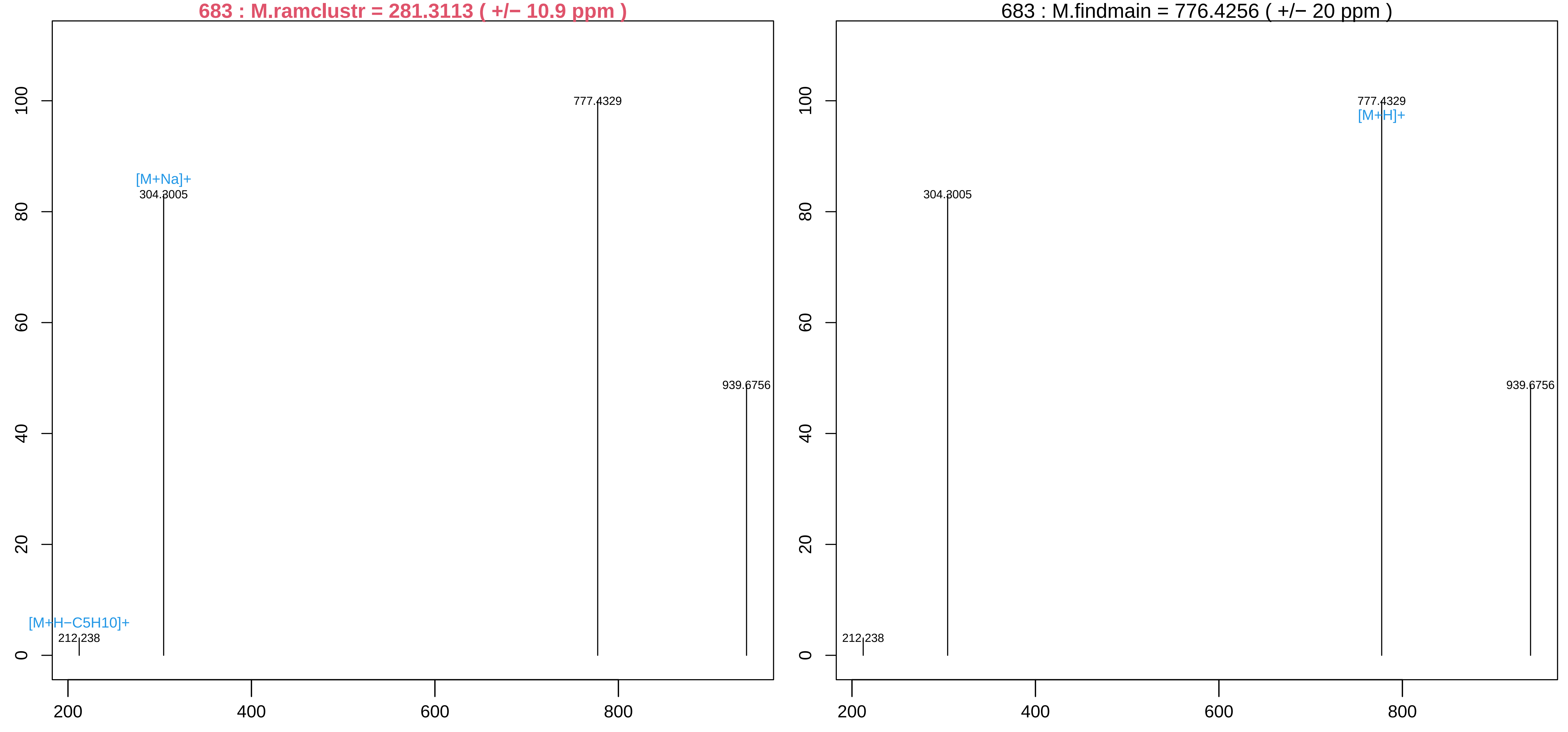

683 : M.ramclustr = 281.3113 (+/− 10.9 ppm)
[M+Na]+
304.3005
777.4329
939.6756
[M+H−C5H10]+
212.238
683 : M.findmain = 776.4256 (+/− 20 ppm)
777.4329
[M+H]+
304.3005
939.6756
212.238

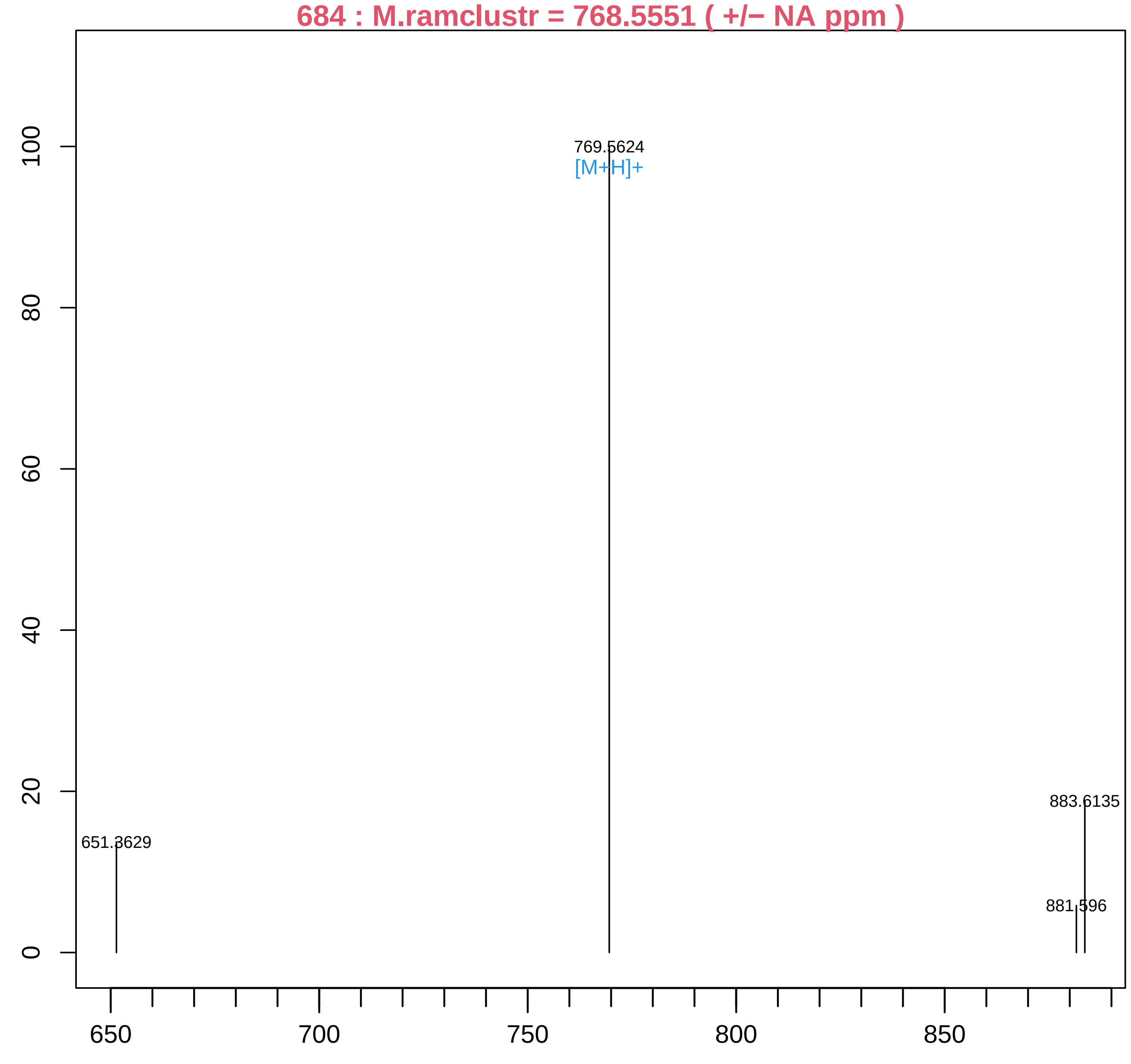
684 : M.ramclustr = 768.5551 (+/− NA ppm)
769.5624
[M+H]+
883.6135
651.3629
881.596
0
20
40
60
80
100
650
700
750
800
850

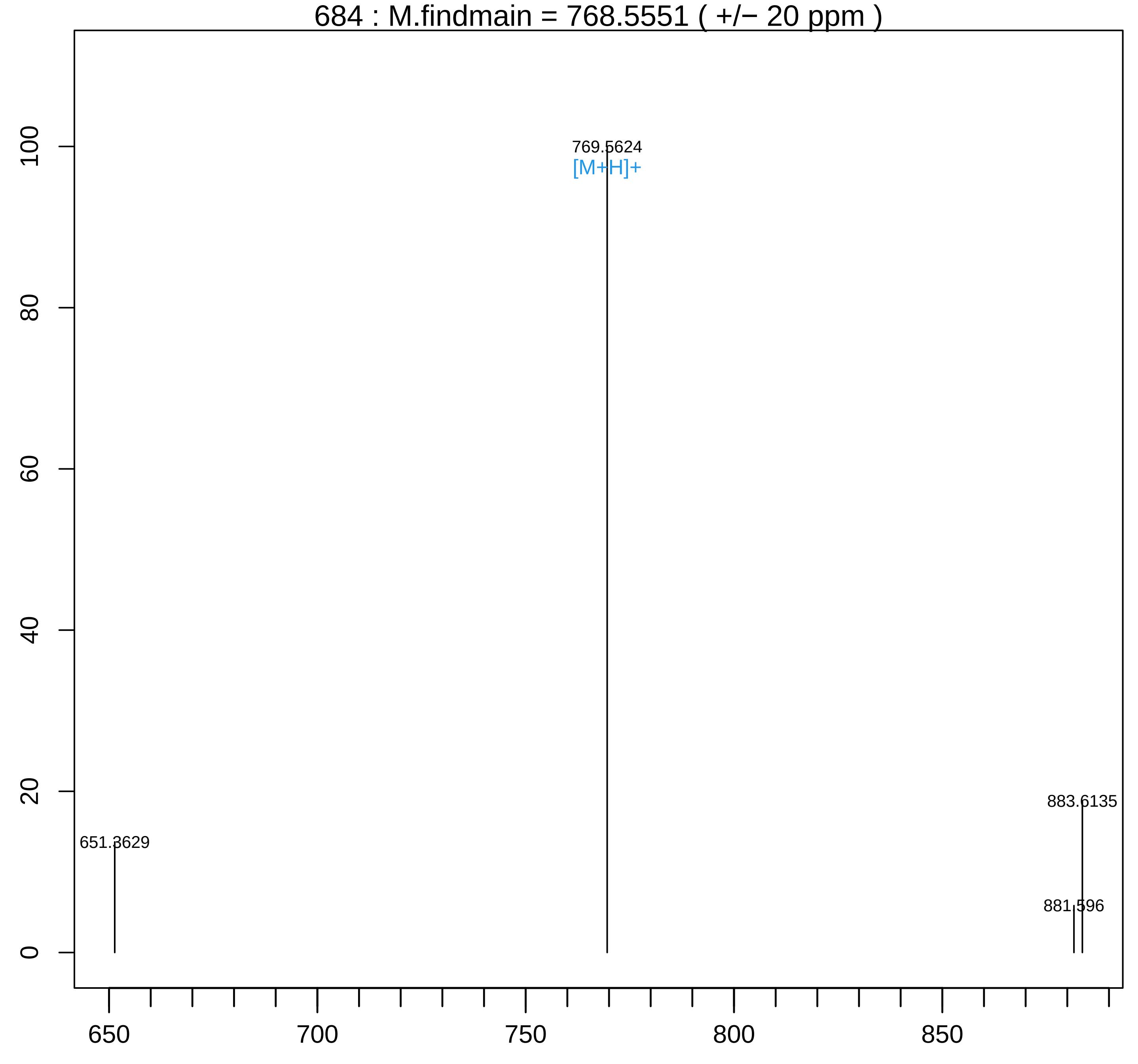
684 : M.findmain = 768.5551 (+/− 20 ppm)
769.5624
[M+H]+
883.6135
651.3629
881.596
0
20
40
60
80
100
650
700
750
800
850

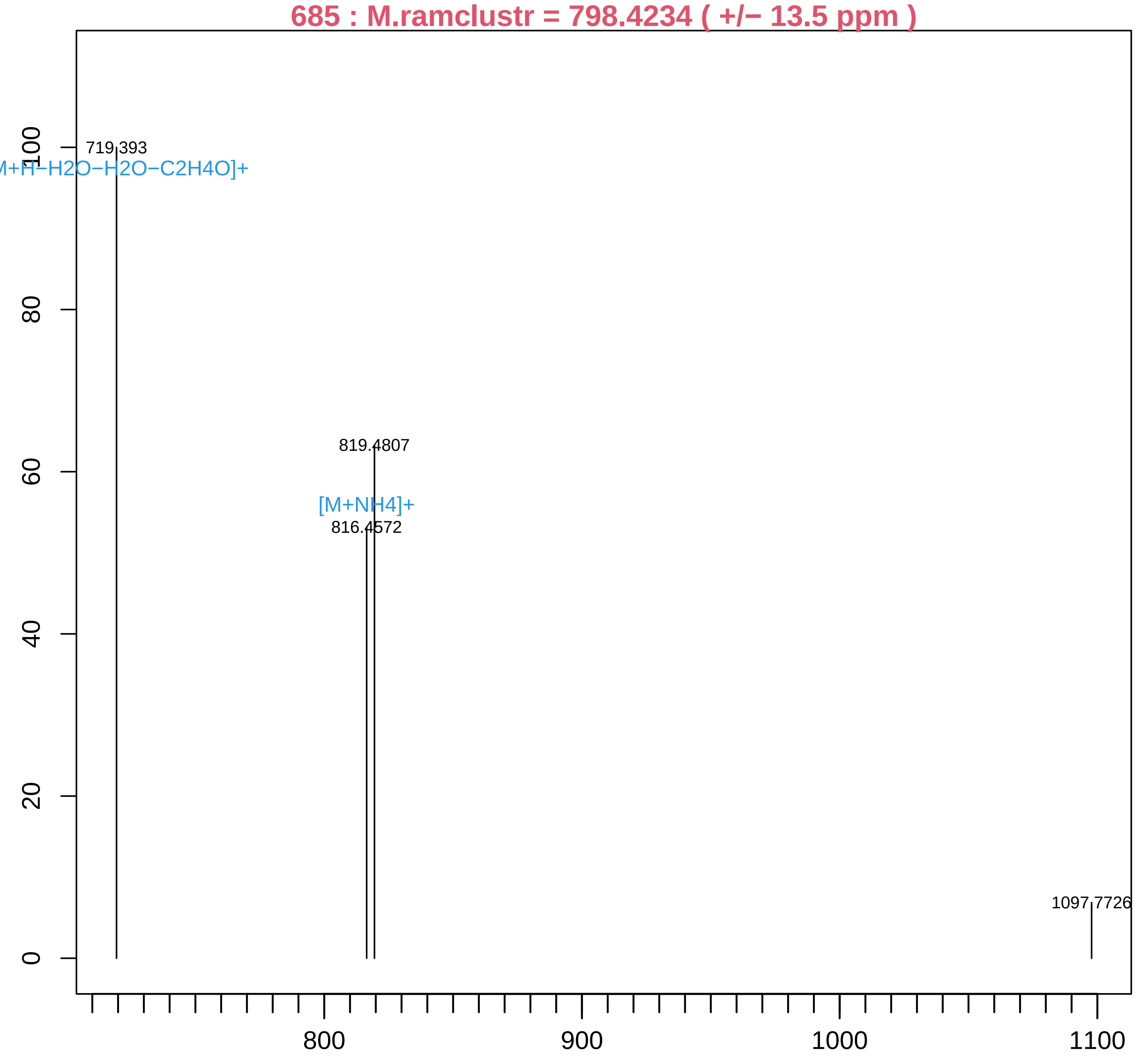
685 : M.ramclustr = 798.4234 (+/− 13.5 ppm)
719.393
[M+H−H2O−H2O−C2H4O]+
819.4807
[M+NH4]+
816.4572
1097.7726
0
20
40
60
80
100
800
900
1000
1100

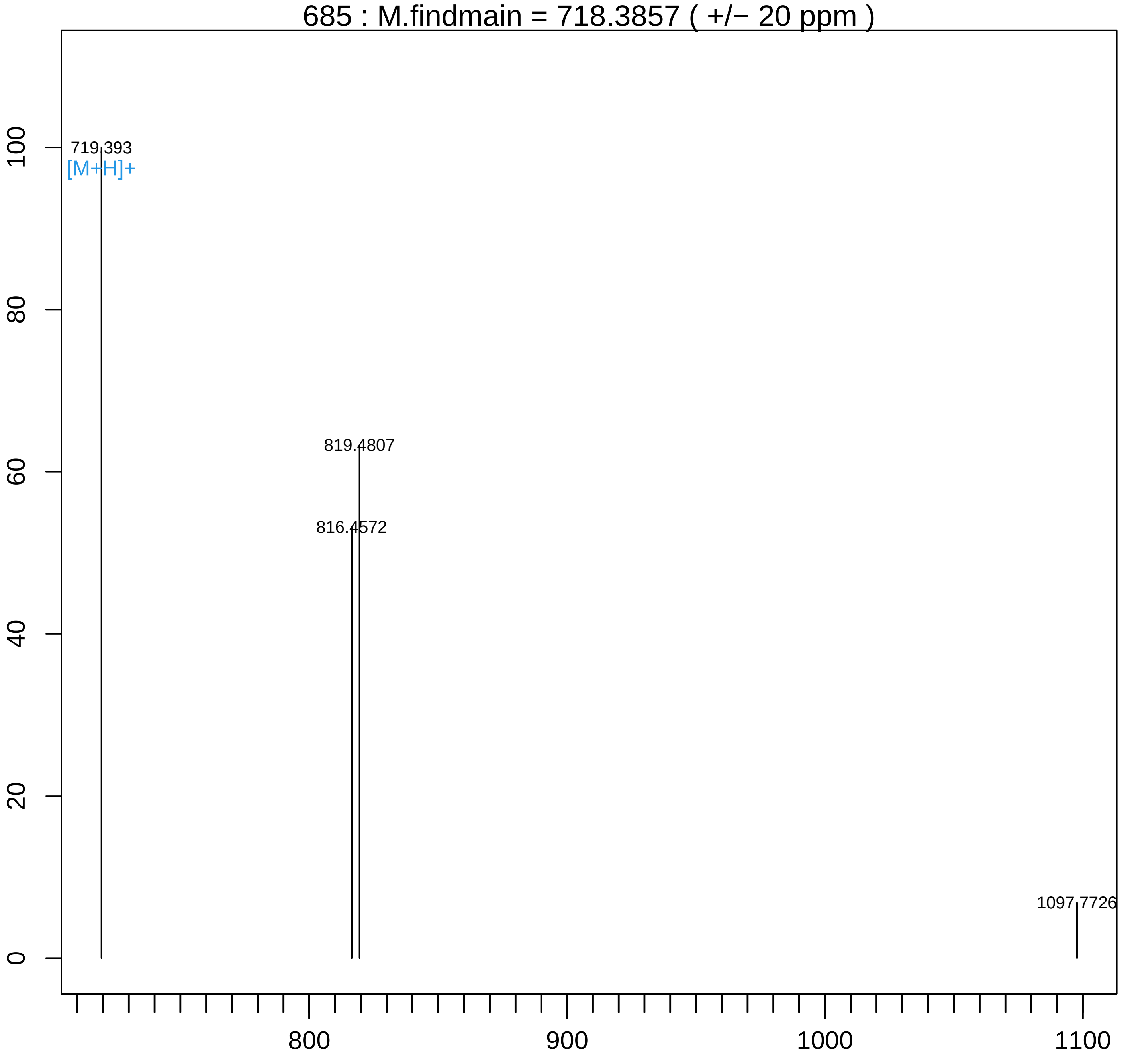
685 : M.findmain = 718.3857 (+/− 20 ppm)
719.393
[M+H]+
819.4807
816.4572
1097.7726
0
20
40
60
80
100
800
900
1000
1100

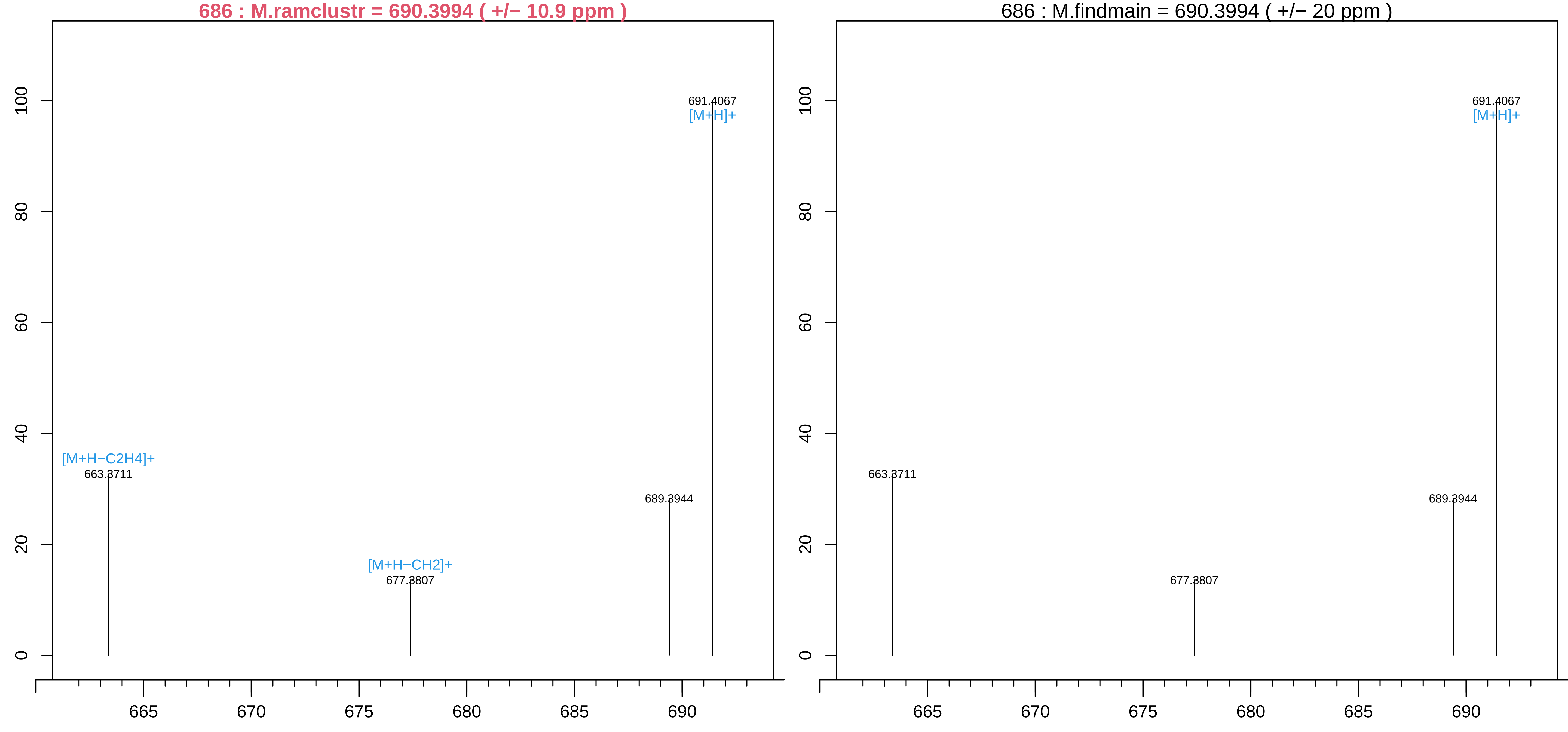
686 : M.ramclustr = 690.3994 (+/− 10.9 ppm)
691.4067
[M+H]+
[M+H−C2H4]+
663.3711
689.3944
[M+H−CH2]+
677.3807
686 : M.findmain = 690.3994 (+/− 20 ppm)
691.4067
[M+H]+
663.3711
689.3944
677.3807

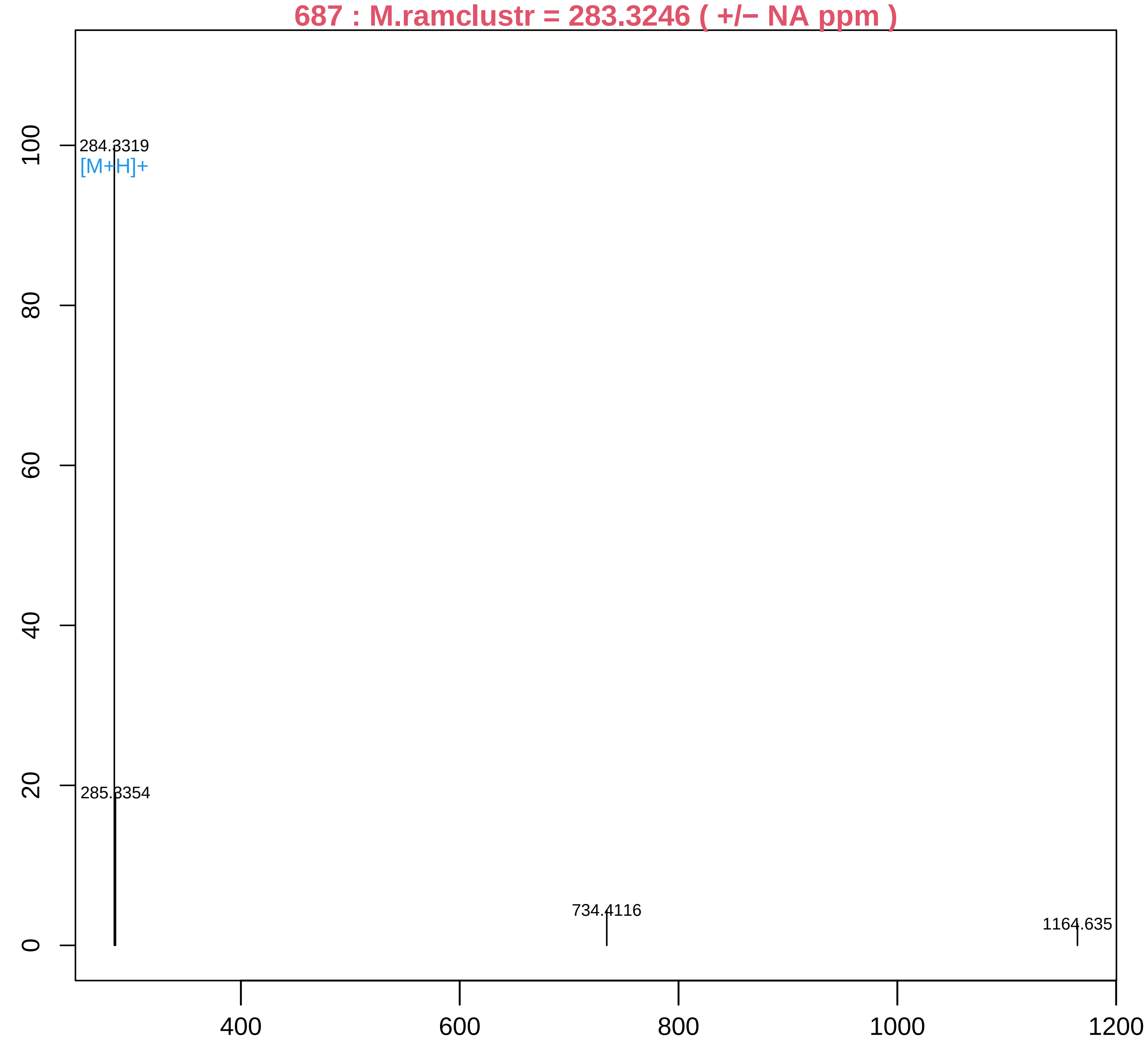
687 : M.ramclustr = 283.3246 (+/− NA ppm)
284.3319
[M+H]+
285.3354
734.4116
1164.635
0
20
40
60
80
100
400
600
800
1000
1200

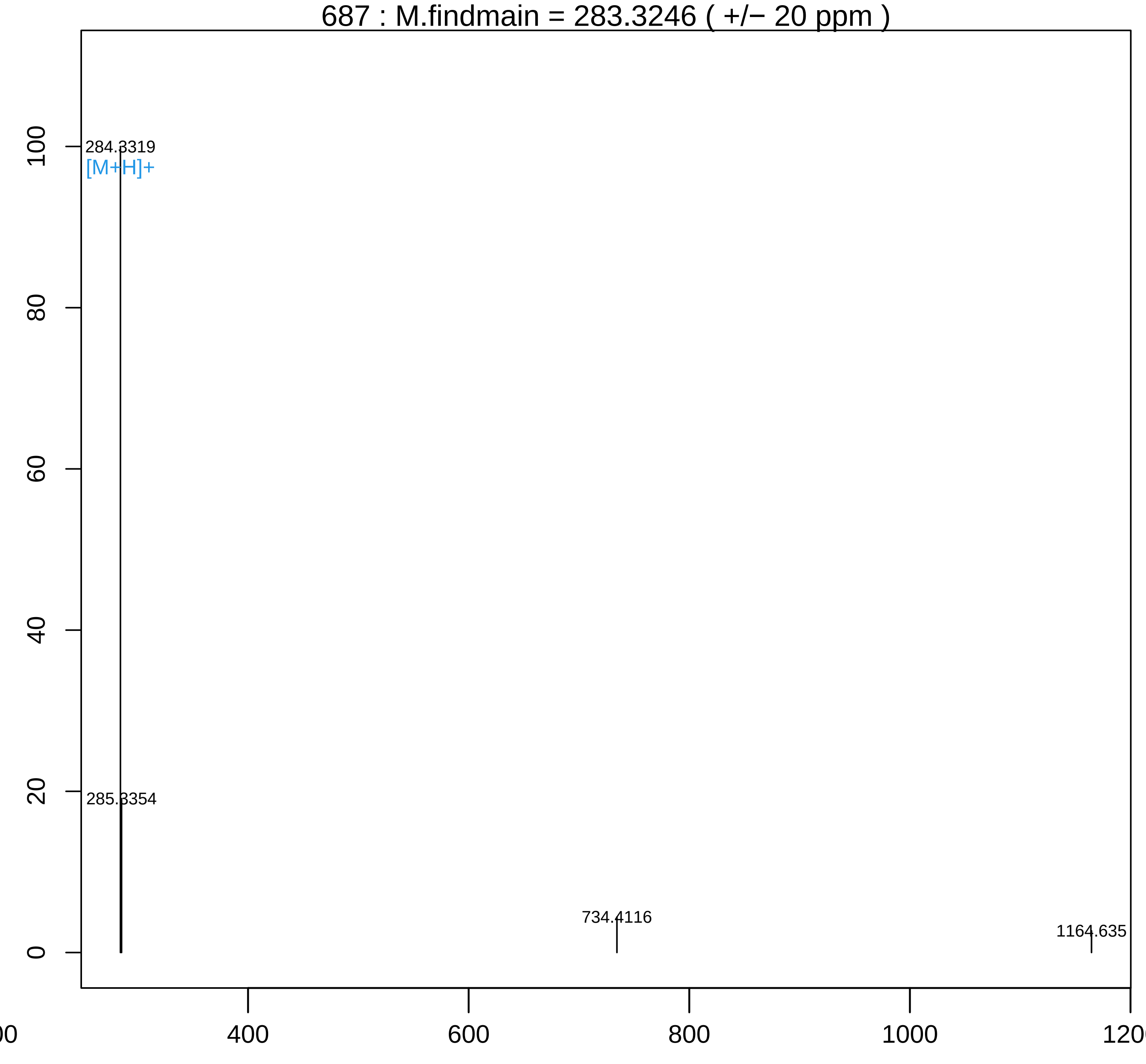
687 : M.findmain = 283.3246 (+/− 20 ppm)
284.3319
[M+H]+
285.3354
734.4116
1164.635
0
20
40
60
80
100
400
600
800
1000

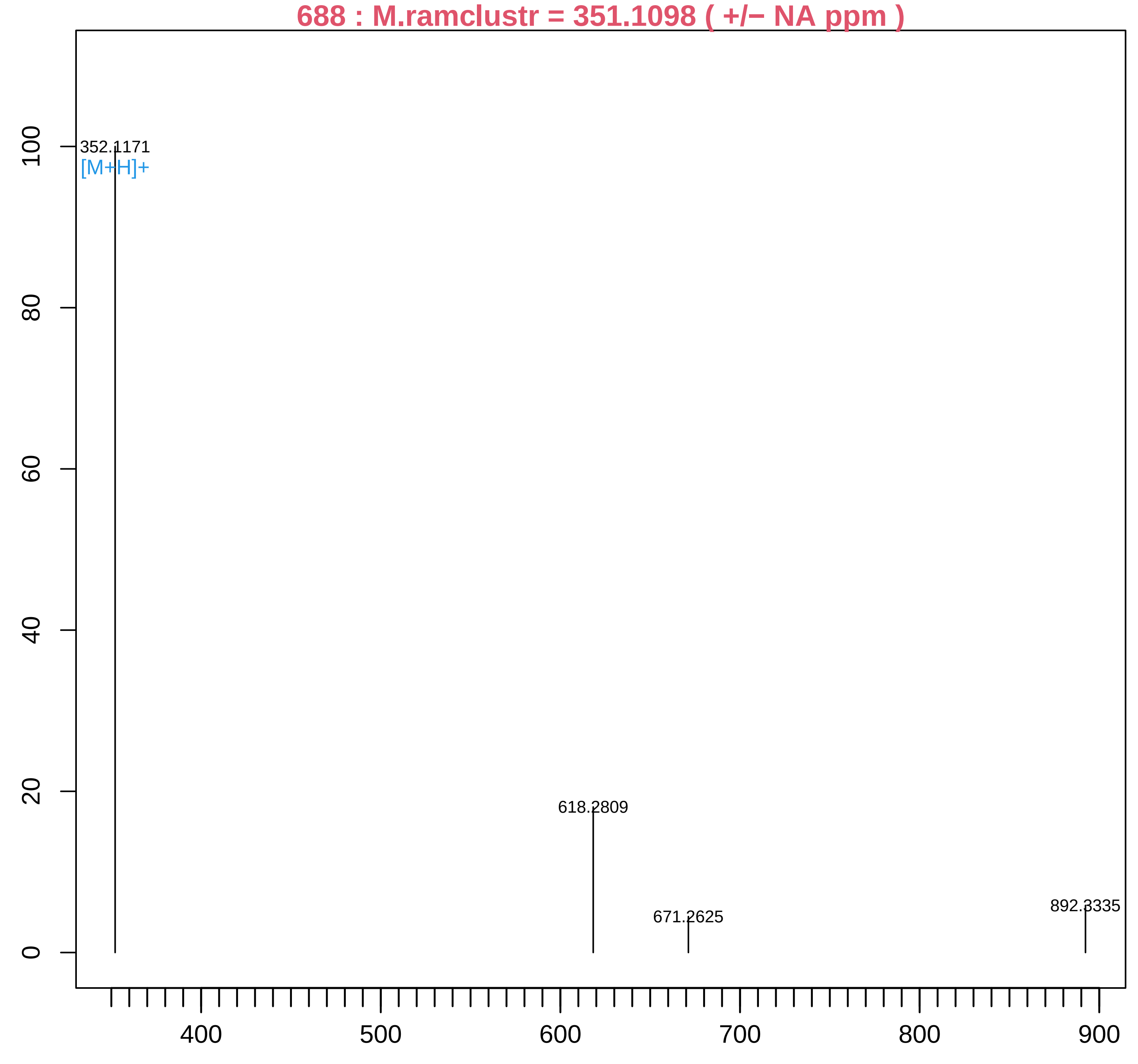

688 : M.ramclustr = 351.1098 (+/− NA ppm)
352.1171
[M+H]+
618.2809
671.2625
892.3335
0
20
40
60
80
100
400
500
600
700
800
900

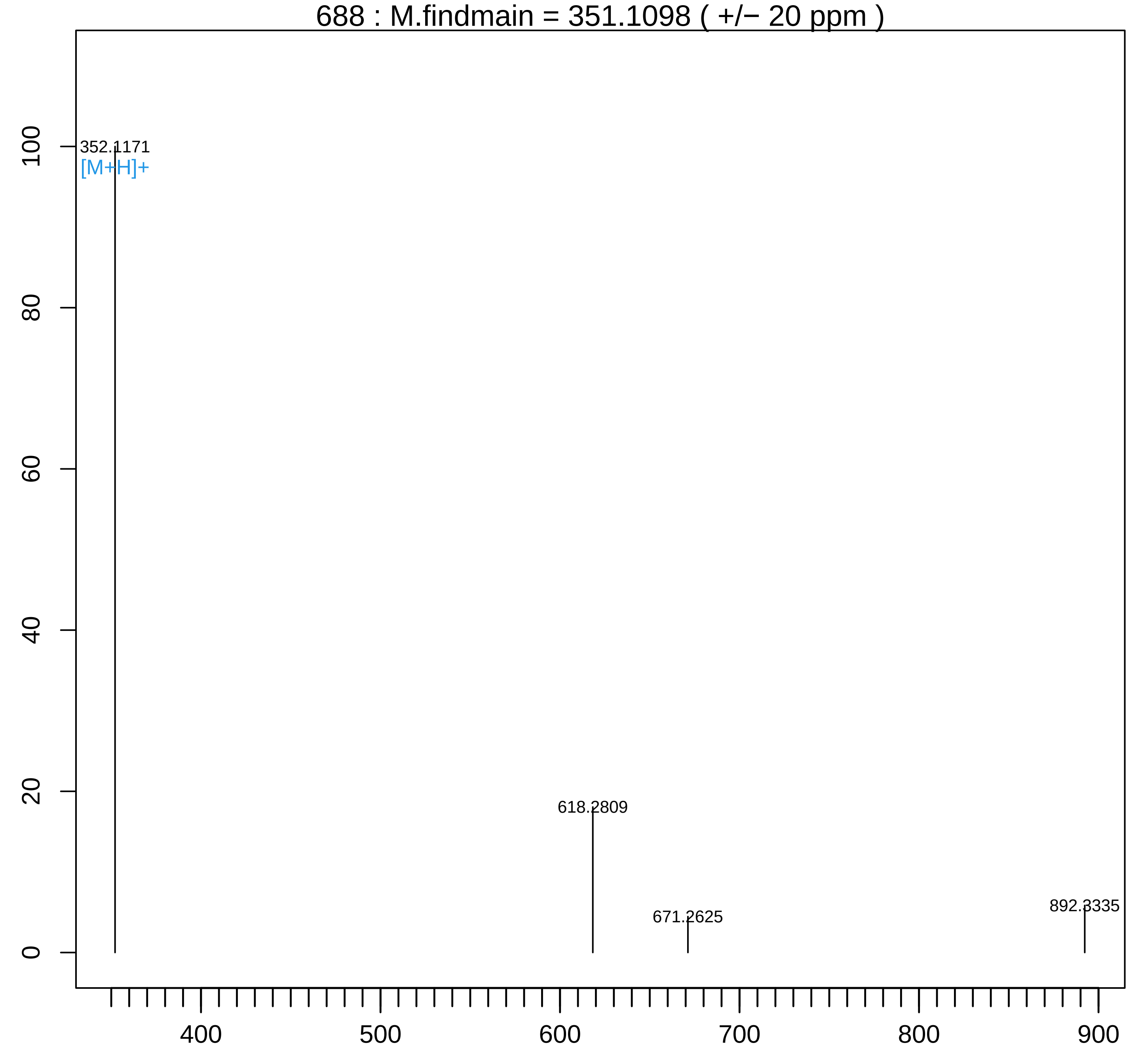

688 : M.findmain = 351.1098 (+/− 20 ppm)
352.1171
[M+H]+
618.2809
671.2625
892.3335
0
20
40
60
80
100
400
500
600
700
800
900

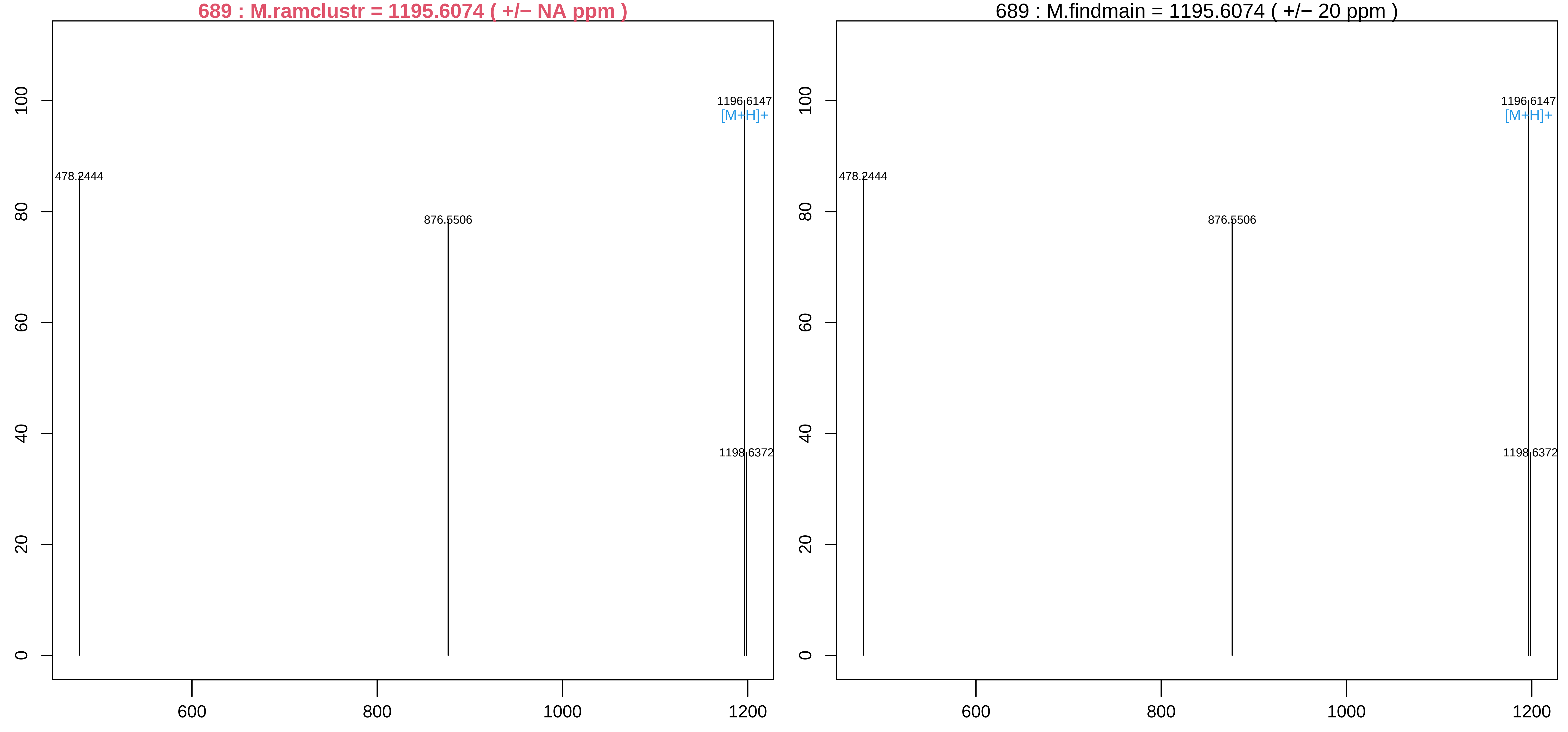

689 : M.ramclustr = 1195.6074 (+/− NA ppm)
689 : M.findmain = 1195.6074 (+/− 20 ppm)
1196.6147
[M+H]+
478.2444
876.5506
1198.6372
0
20
40
60
80
100
600
800
1000
1200

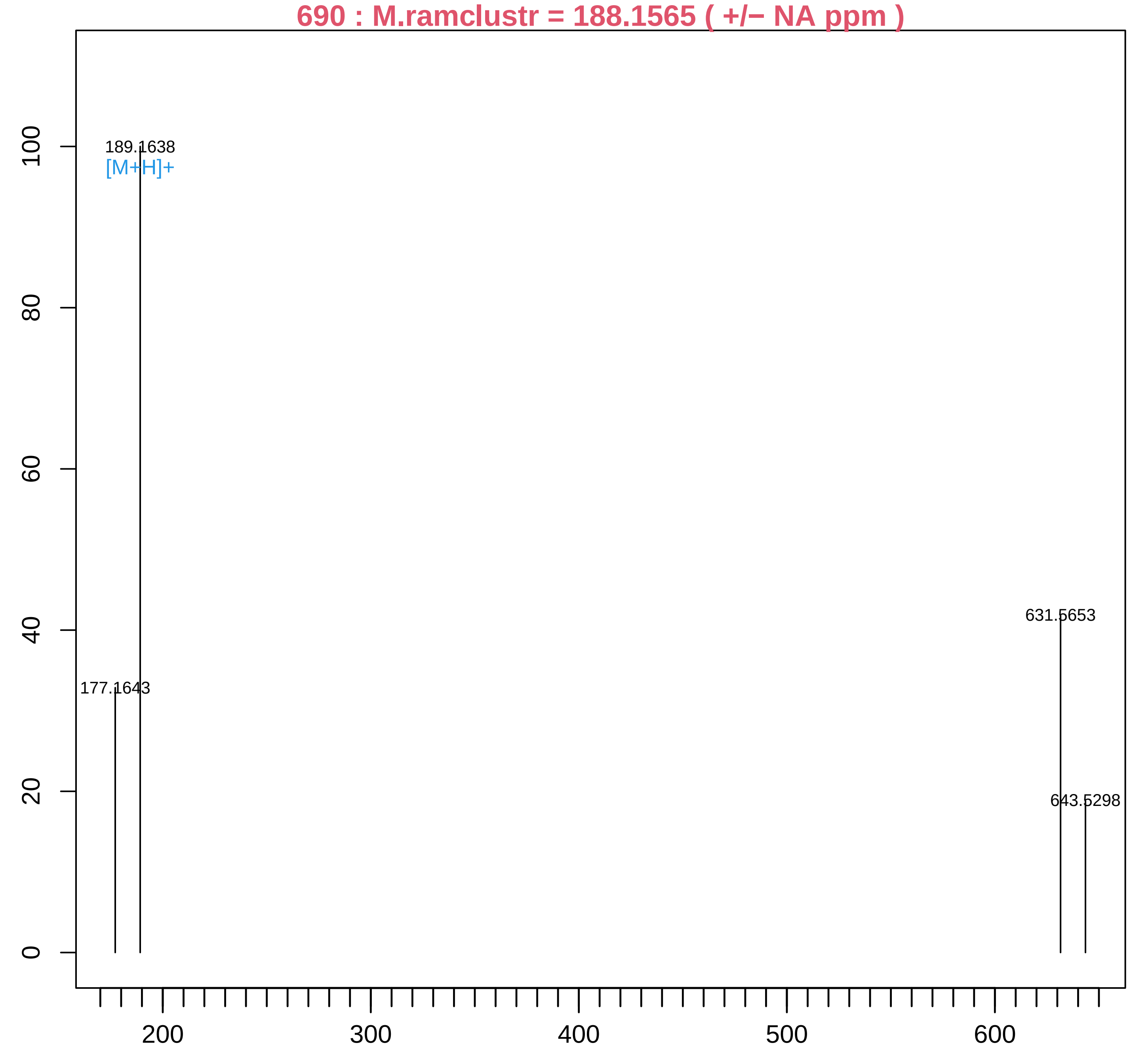
690 : M.ramclustr = 188.1565 (+/− NA ppm)
189.1638
[M+H]+
177.1643
631.5653
643.5298
0
20
40
60
80
100
200
300
400
500
600

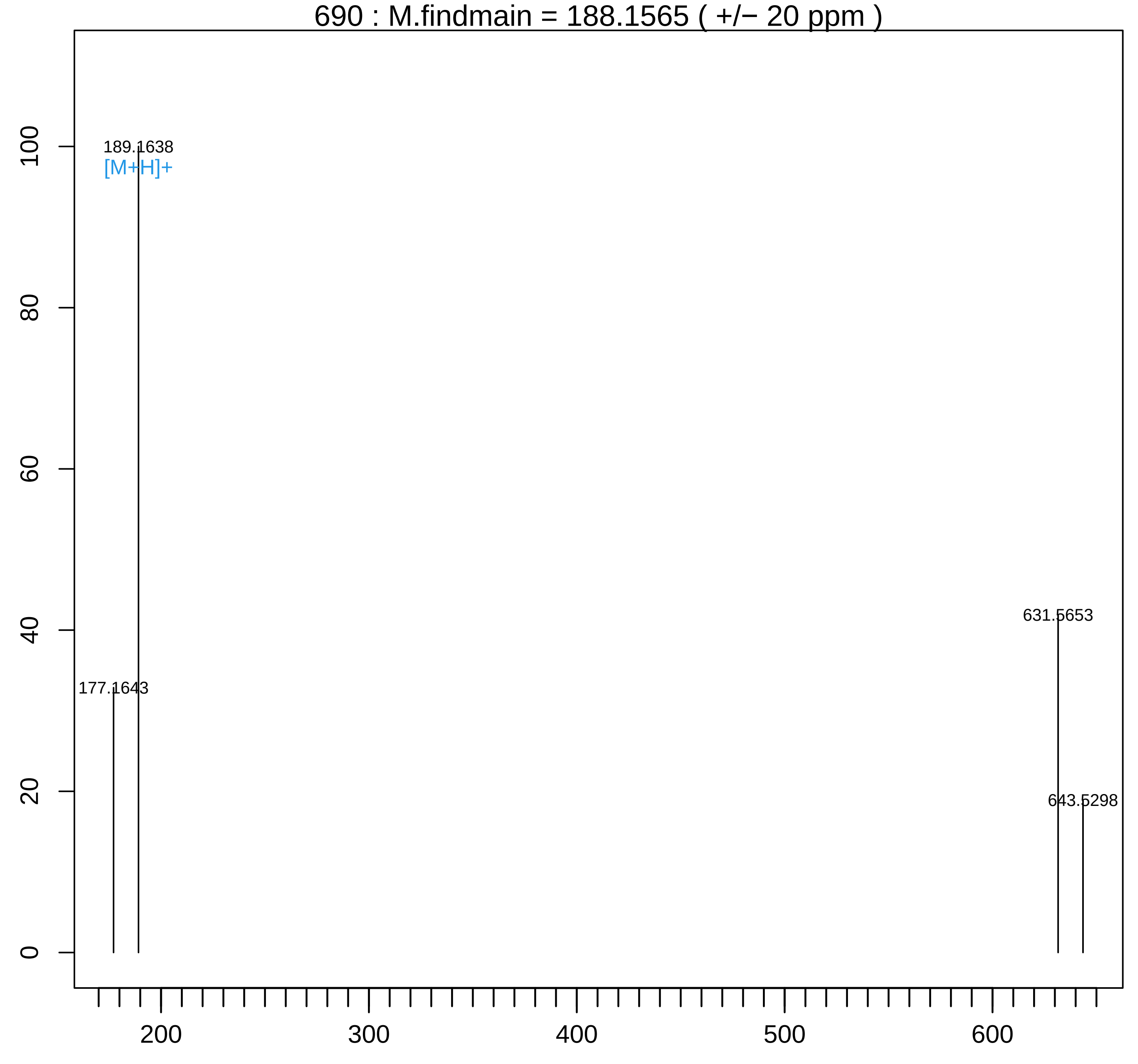
690 : M.findmain = 188.1565 (+/− 20 ppm)
189.1638
[M+H]+
177.1643
631.5653
643.5298
0
20
40
60
80
100
200
300
400
500
600

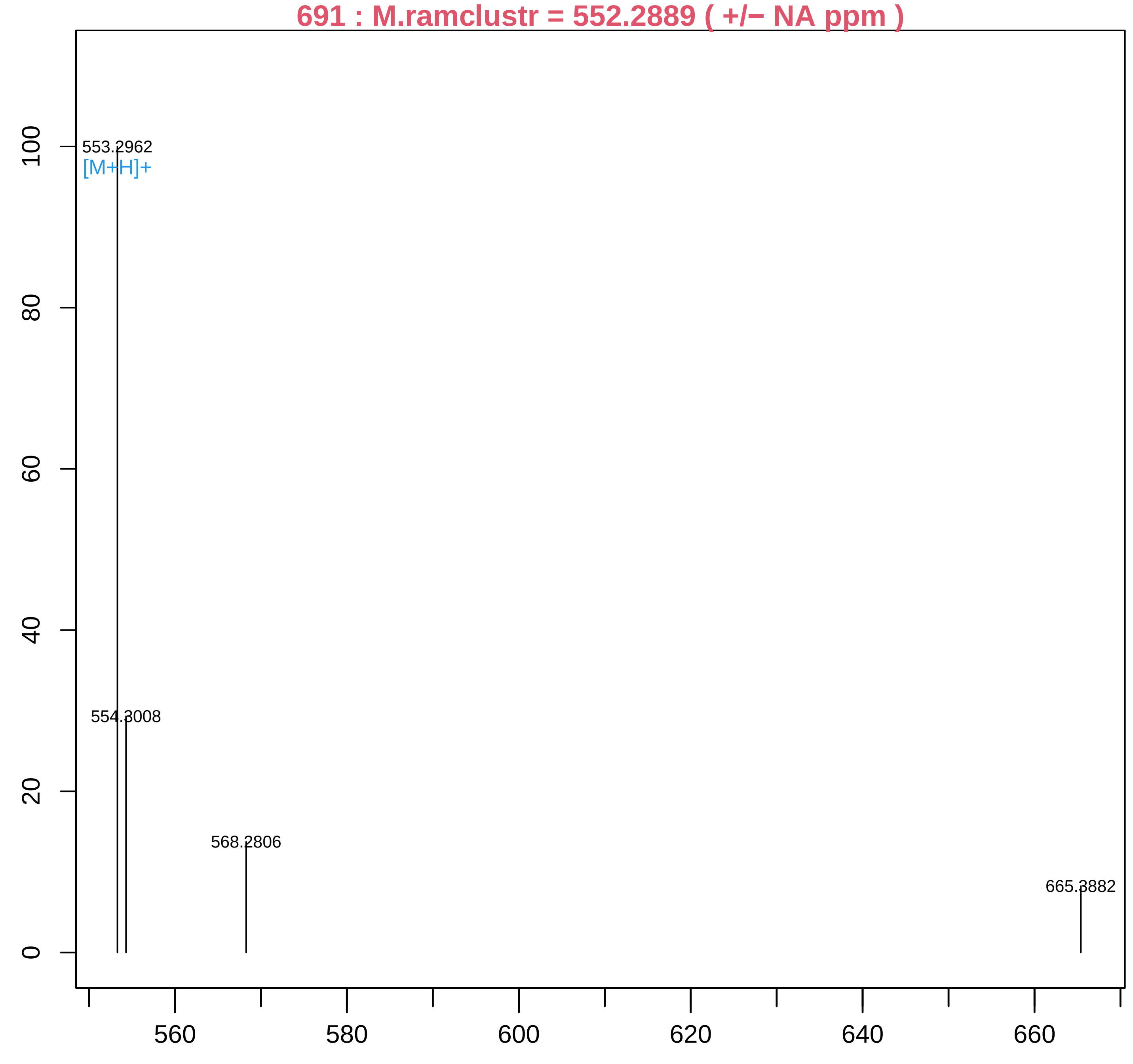
691 : M.ramclustr = 552.2889 (+/− NA ppm)
553.2962
[M+H]+
554.3008
568.2806
665.3882
0
20
40
60
80
100
560
580
600
620
640
660
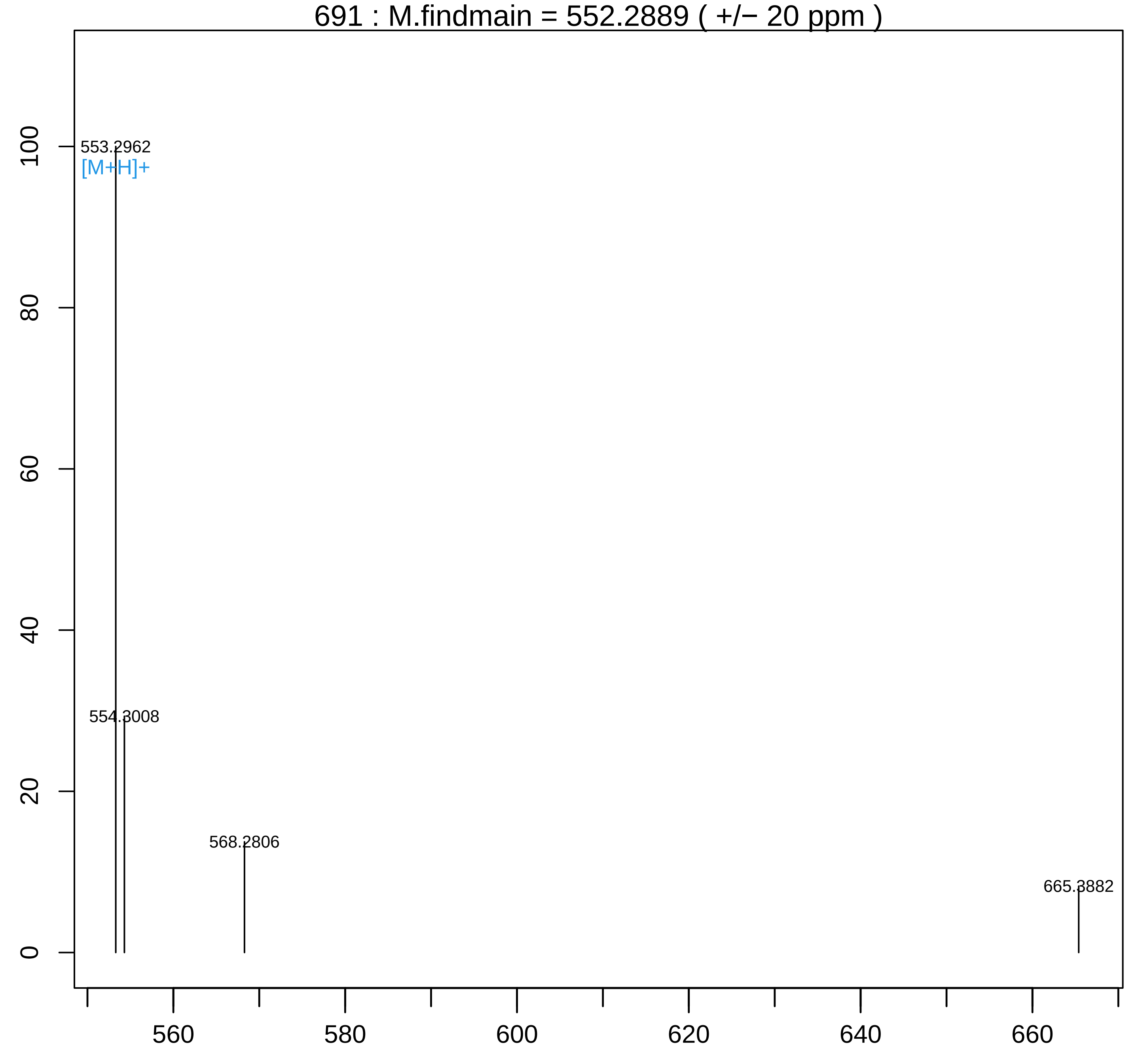
691 : M.findmain = 552.2889 (+/− 20 ppm)
553.2962
[M+H]+
554.3008
568.2806
665.3882
0
20
40
60
80
100
560
580
600
620
640
660

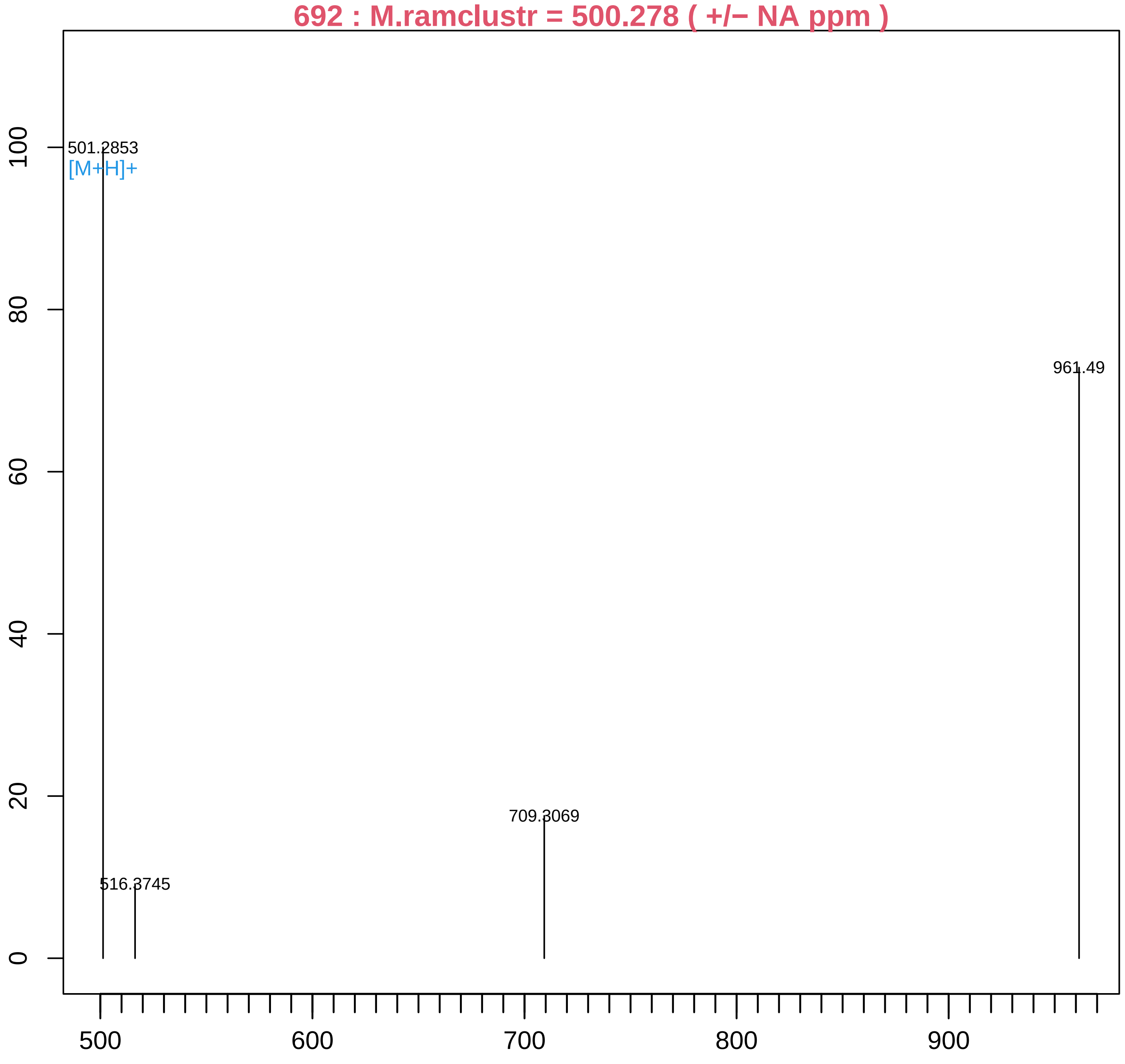

692 : M.ramclustr = 500.278 (+/− NA ppm)
501.2853
[M+H]+
961.49
709.3069
516.3745
0
20
40
60
80
100
500
600
700
800
900

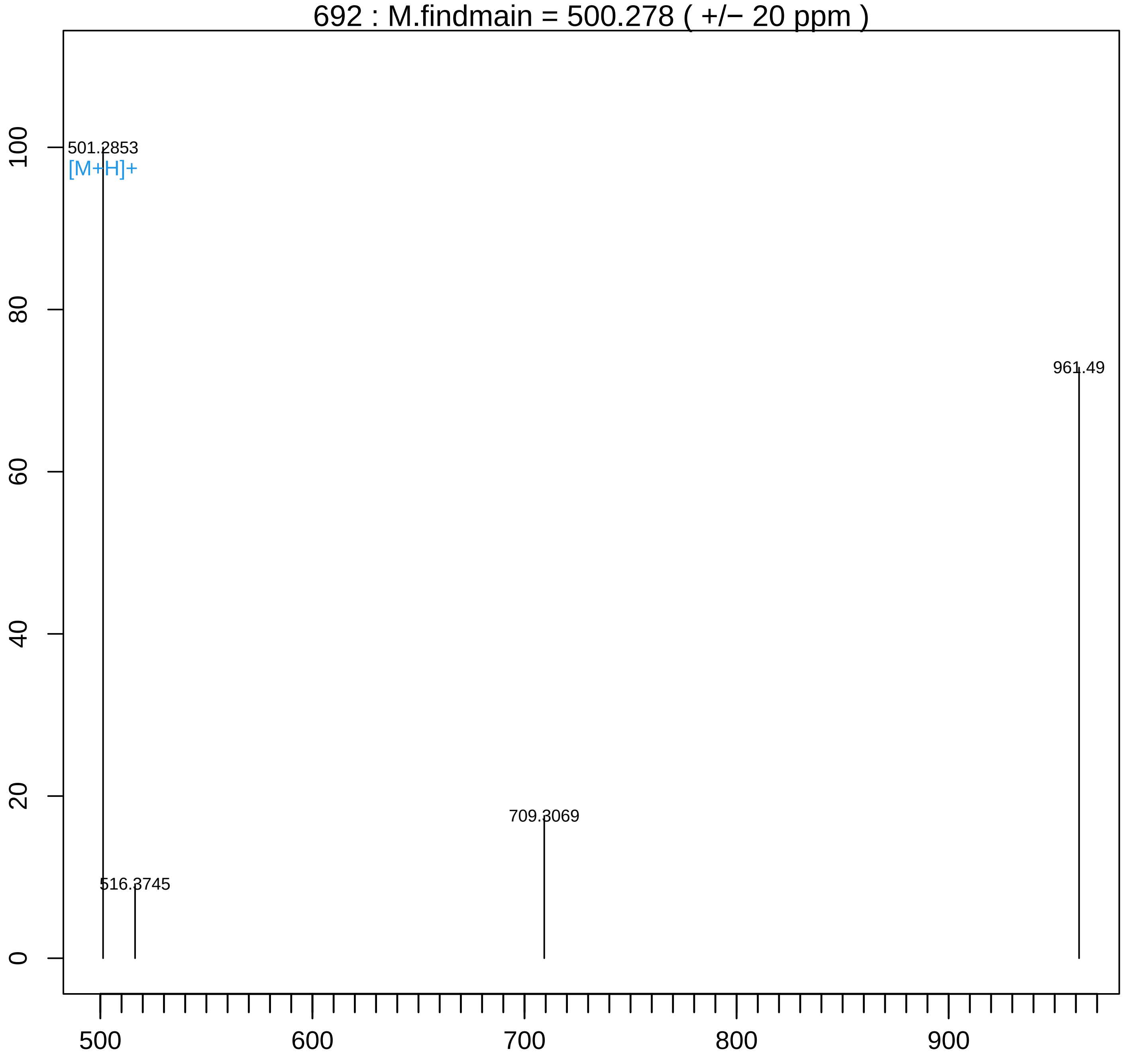

692 : M.findmain = 500.278 (+/− 20 ppm)
501.2853
[M+H]+
961.49
709.3069
516.3745
0
20
40
60
80
100
500
600
700
800
900

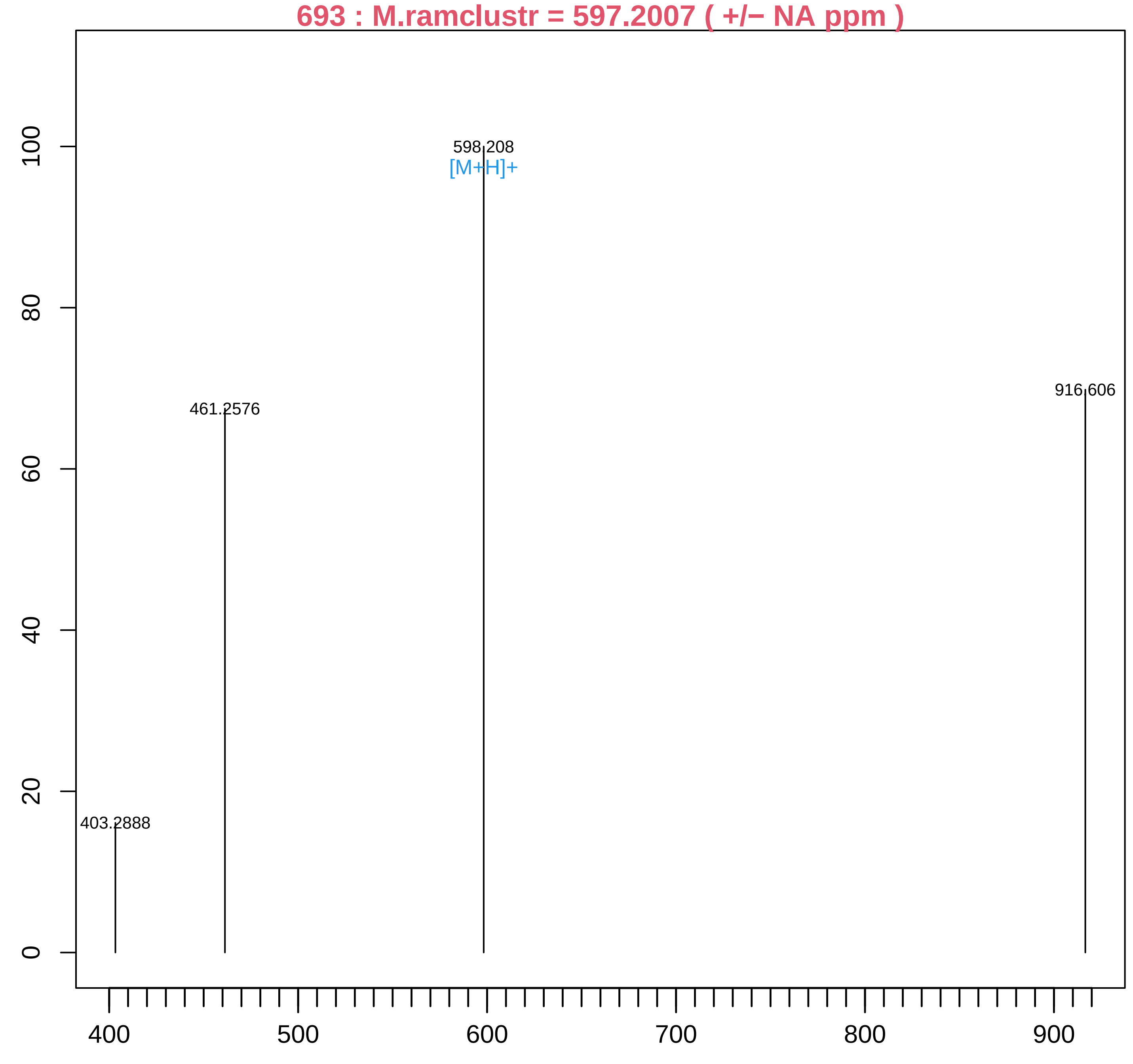
693 : M.ramclustr = 597.2007 (+/− NA ppm)
598.208
[M+H]+
461.2576
916.606
403.2888
0
20
40
60
80
100
400
500
600
700
800
900
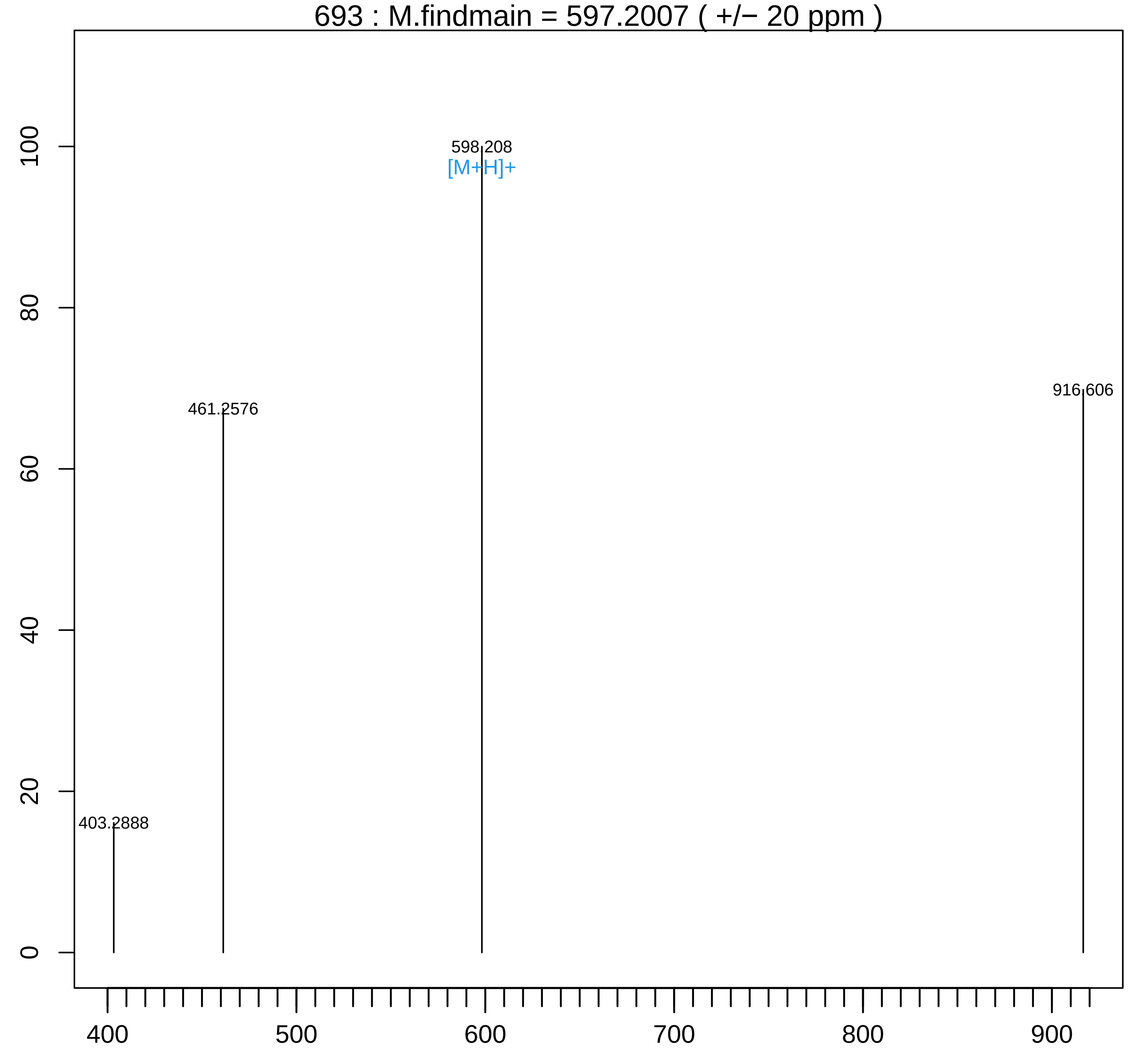
693 : M.findmain = 597.2007 (+/− 20 ppm)
598.208
[M+H]+
461.2576
916.606
403.2888
0
20
40
60
80
100
400
500
600
700
800
900

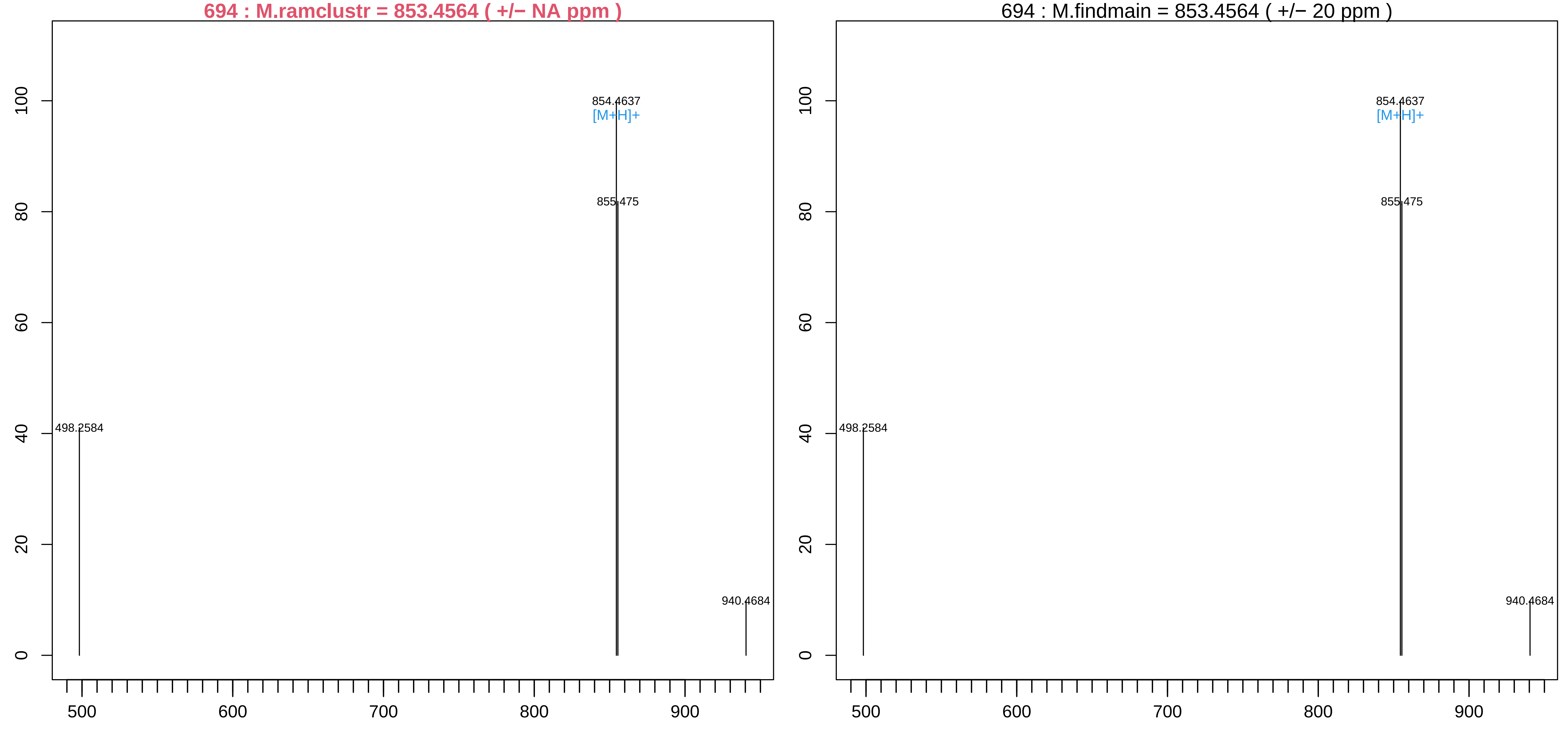

694 : M.ramclustr = 853.4564 (+/− NA ppm)
694 : M.findmain = 853.4564 (+/− 20 ppm)
854.4637
[M+H]+
855.475
498.2584
940.4684
0
20
40
60
80
100
500
600
700
800
900

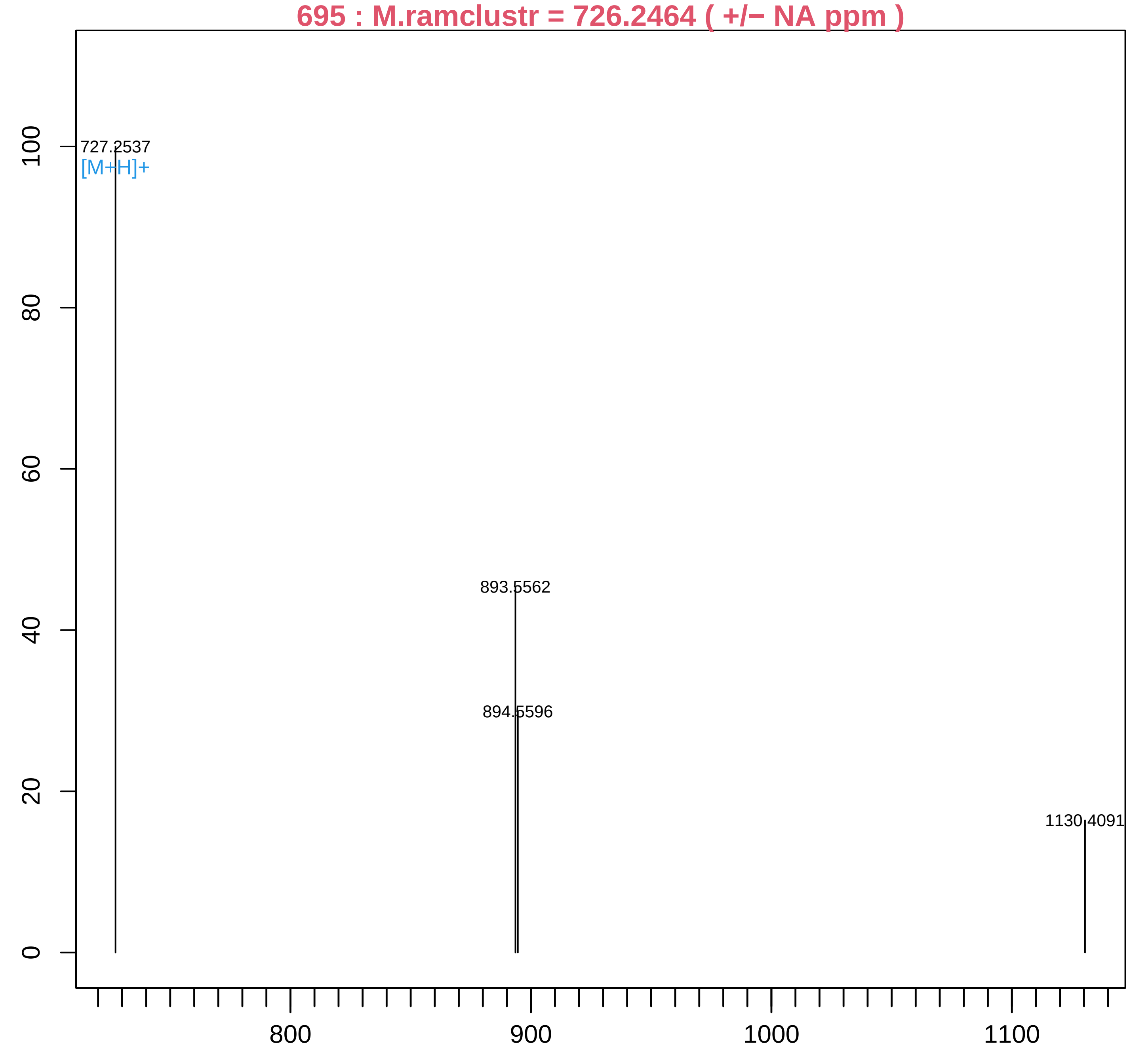
695 : M.ramclustr = 726.2464 (+/− NA ppm)
727.2537
[M+H]+
893.5562
894.5596
1130.4091
0
20
40
60
80
100
800
900
1000
1100

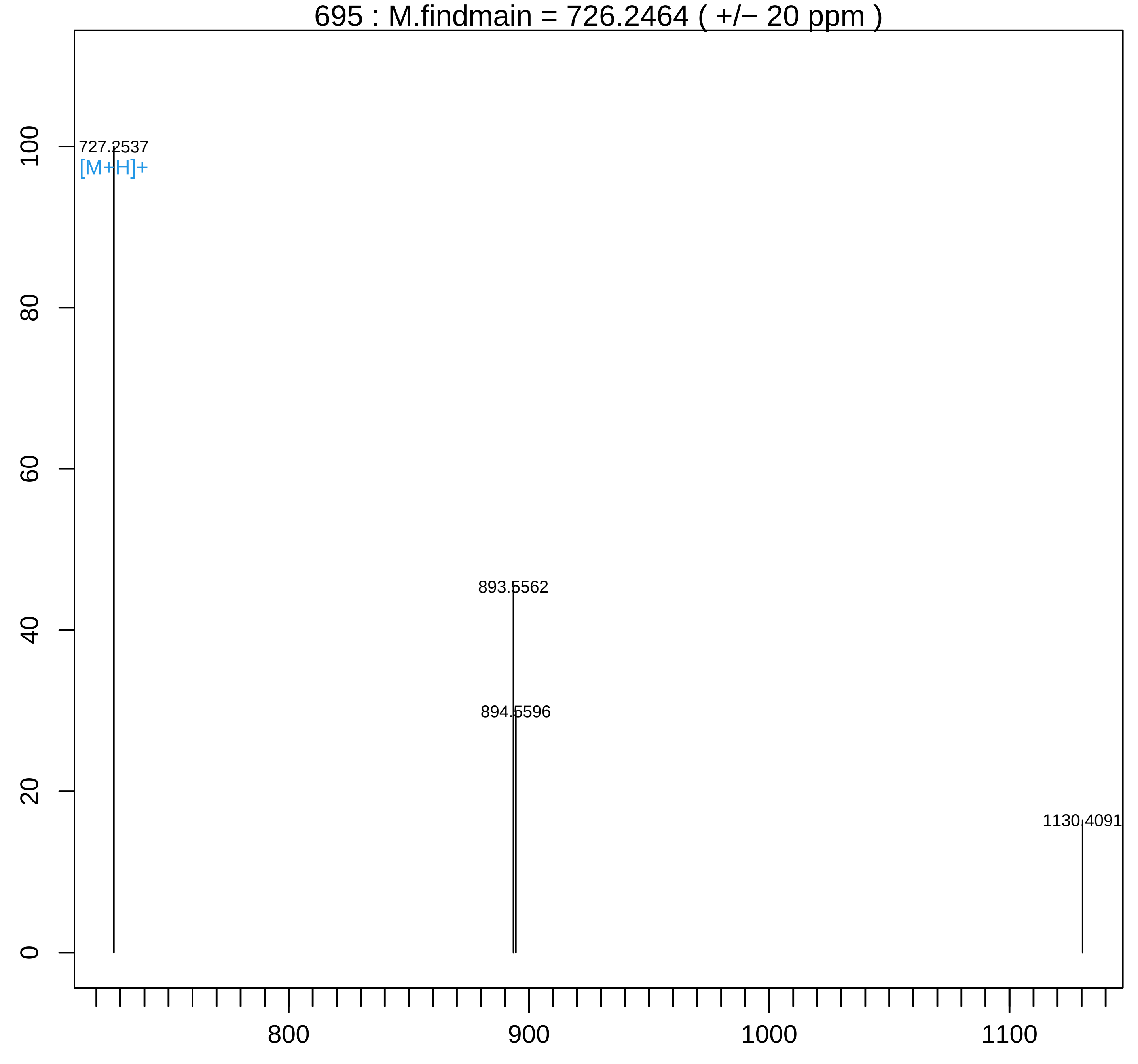
695 : M.findmain = 726.2464 (+/− 20 ppm)
727.2537
[M+H]+
893.5562
894.5596
1130.4091
0
20
40
60
80
100
800
900
1000
1100

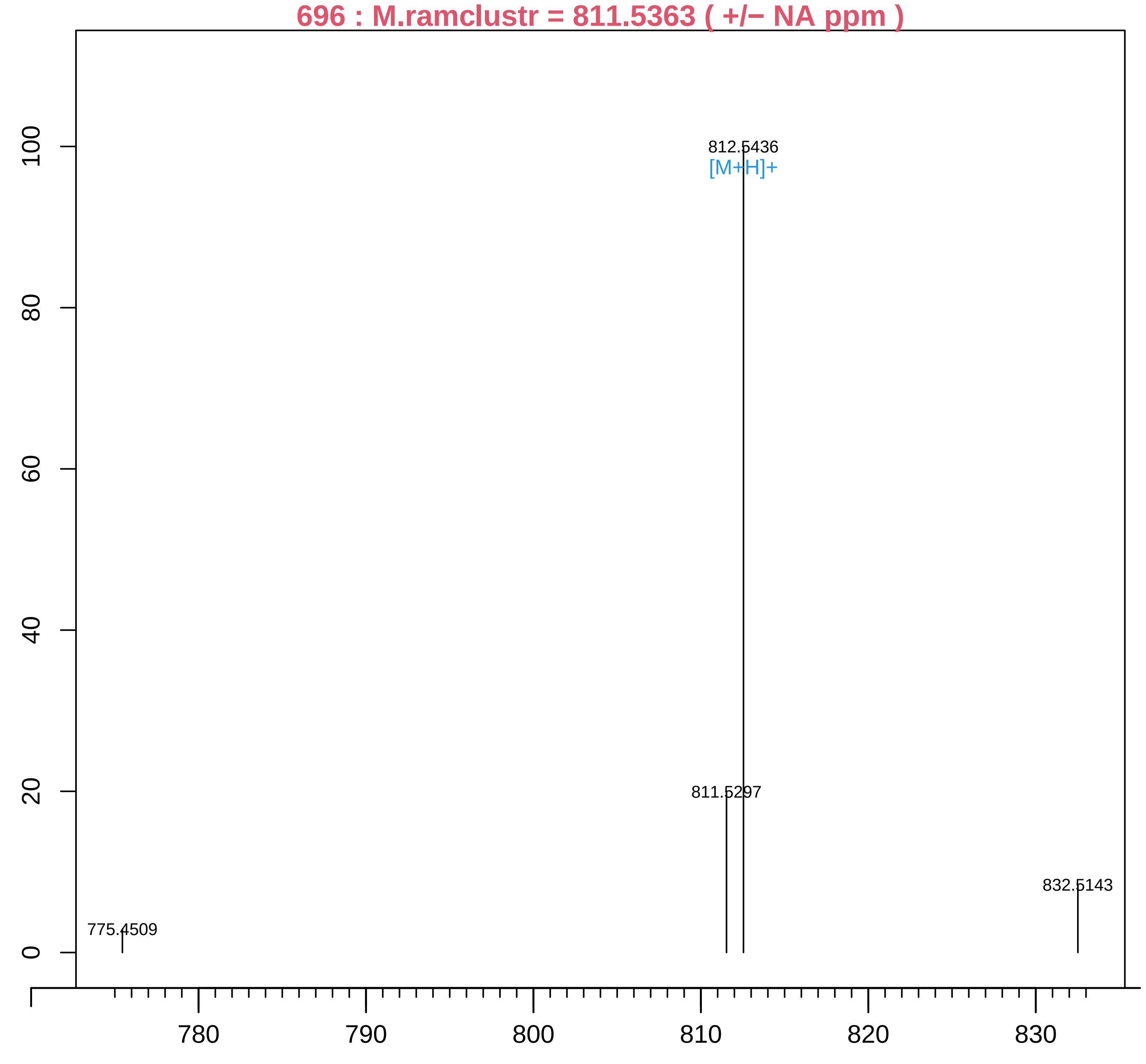
696 : M.ramclustr = 811.5363 (+/− NA ppm)
812.5436
[M+H]+
811.5297
832.5143
775.4509
0
20
40
60
80
100
780
790
800
810
820
830

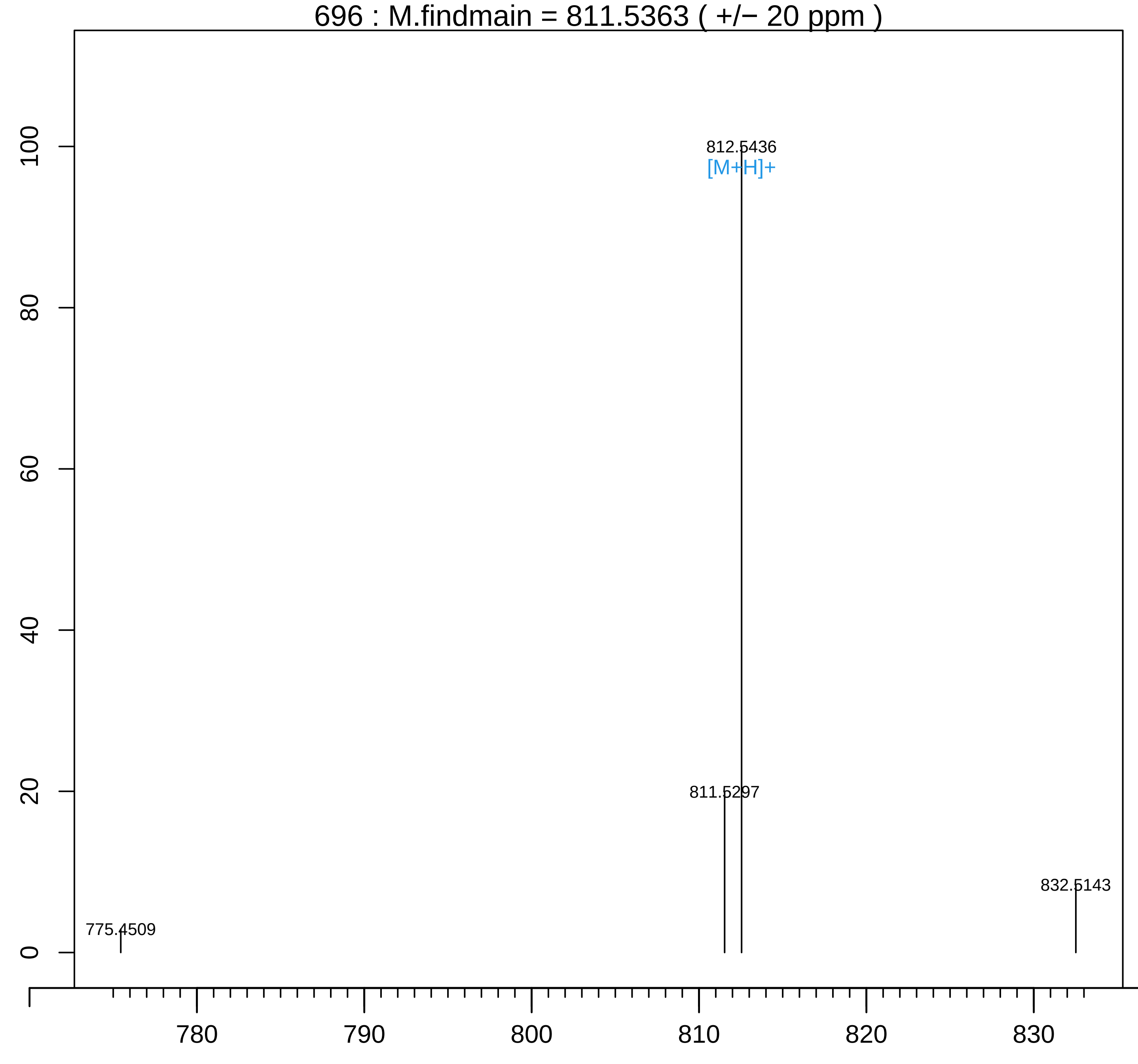
696 : M.findmain = 811.5363 (+/− 20 ppm)
812.5436
[M+H]+
811.5297
832.5143
775.4509
0
20
40
60
80
100
780
790
800
810
820
830

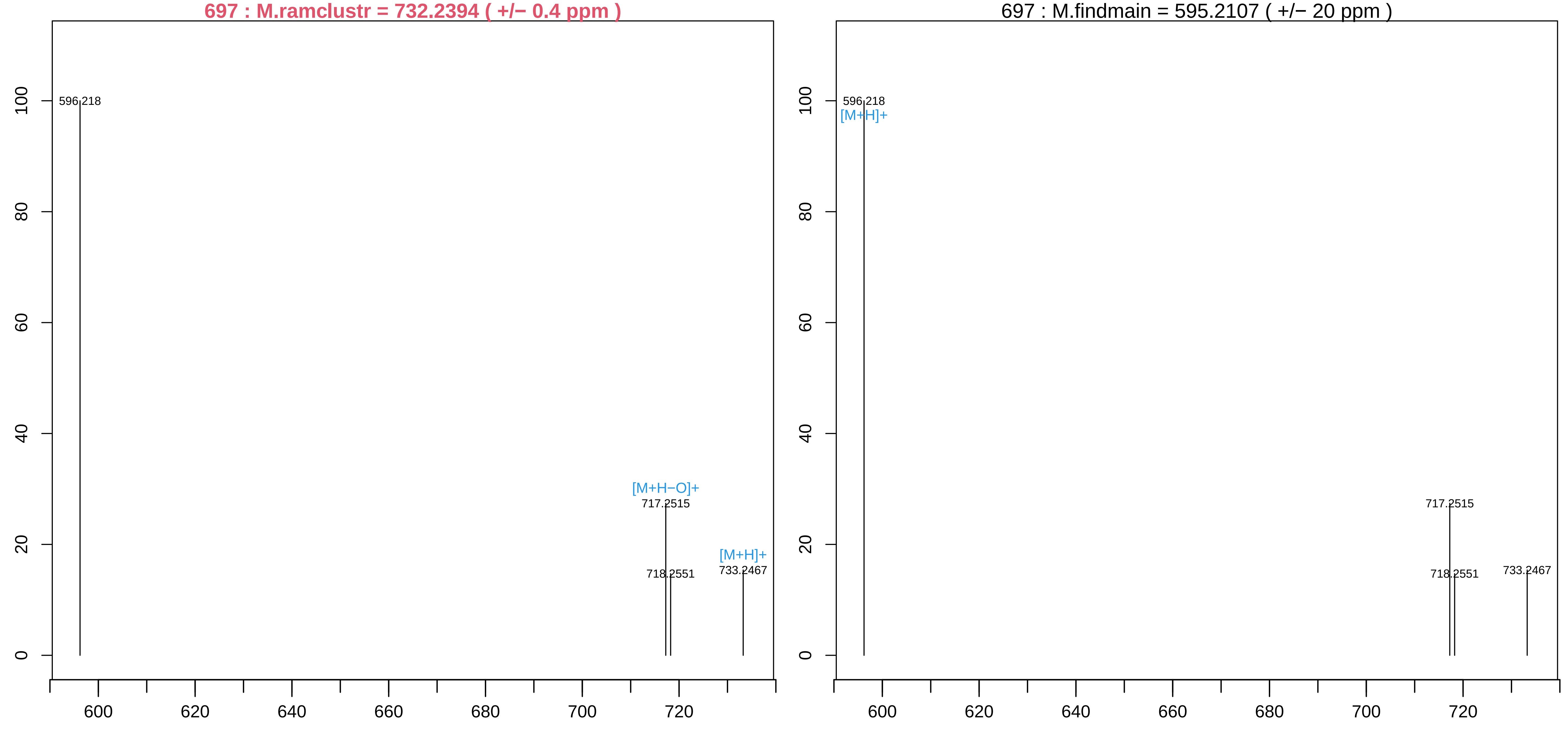

697 : M.ramclustr = 732.2394 (+/− 0.4 ppm)
596.218
[M+H−O]+
717.2515
718.2551
[M+H]+
733.2467
697 : M.findmain = 595.2107 (+/− 20 ppm)
596.218
[M+H]+
717.2515
718.2551
733.2467

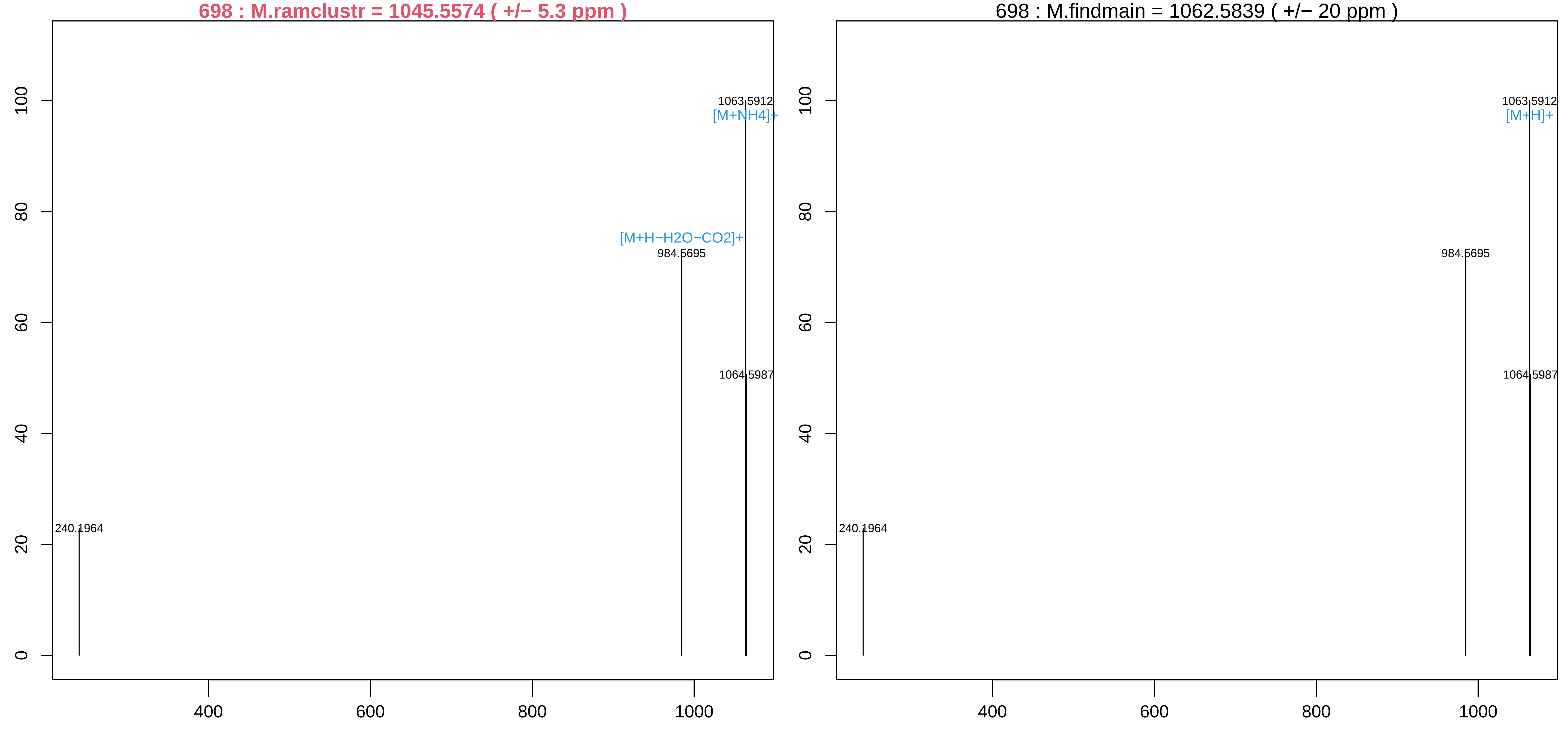

698 : M.ramclustr = 1045.5574 (+/− 5.3 ppm)
1063.5912
[M+NH4]+
[M+H−H2O−CO2]+
984.5695
1064.5987
240.1964
698 : M.findmain = 1062.5839 (+/− 20 ppm)
1063.5912
[M+H]+
984.5695
1064.5987
240.1964

699 : M.ramclustr = 540.1825 (+/− NA ppm)

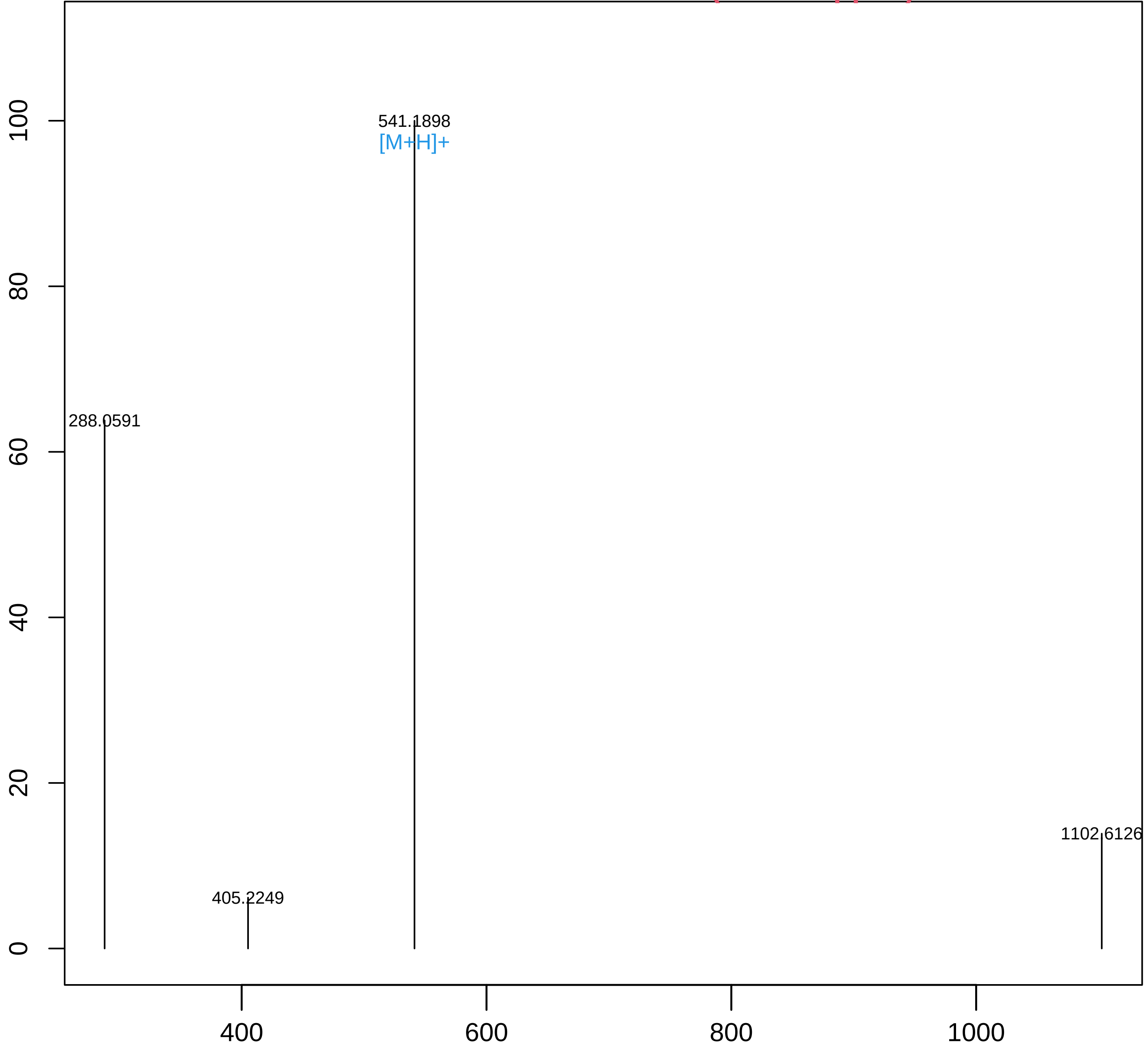

699 : M.findmain = 540.1825 (+/− 20 ppm)

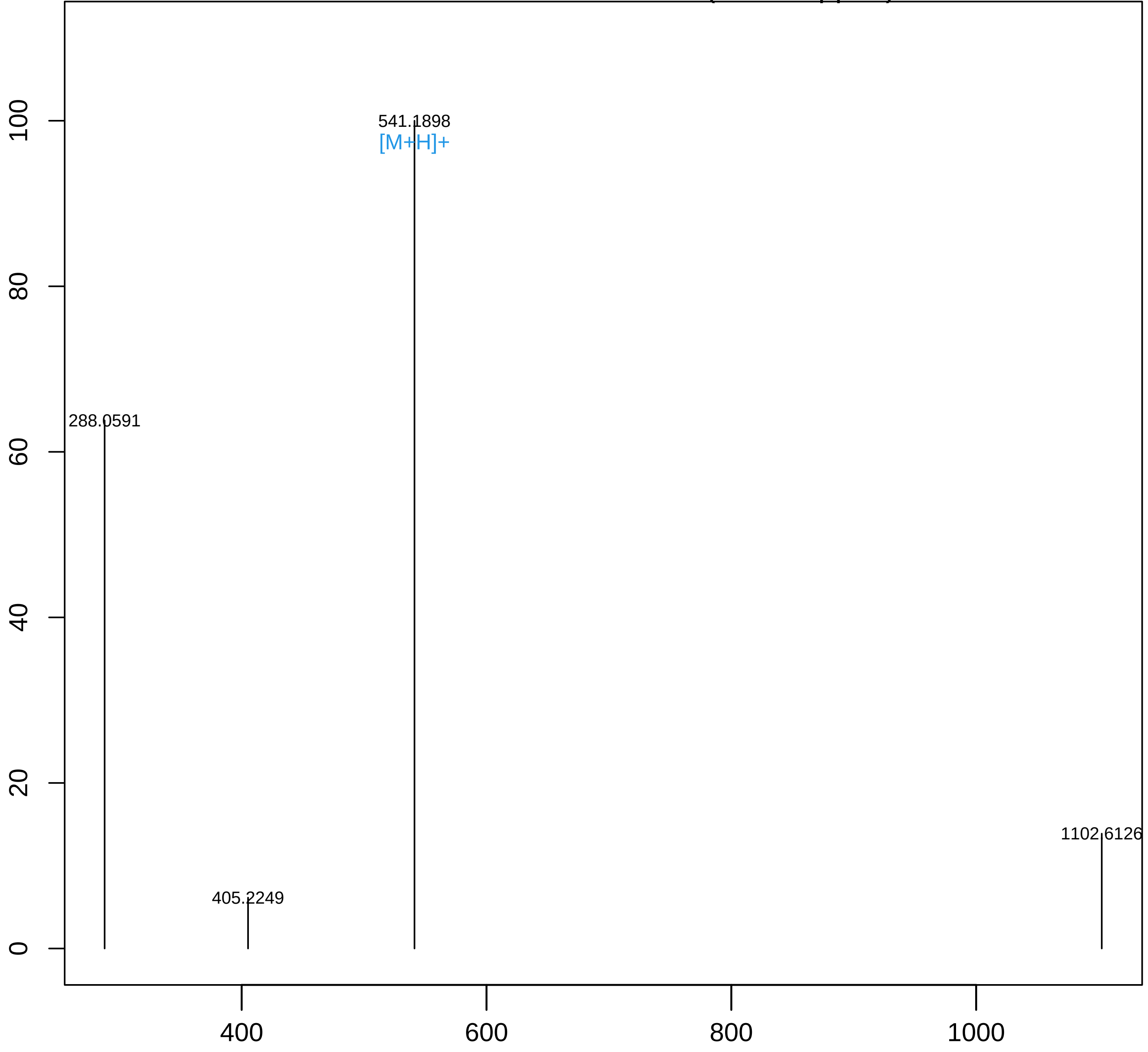

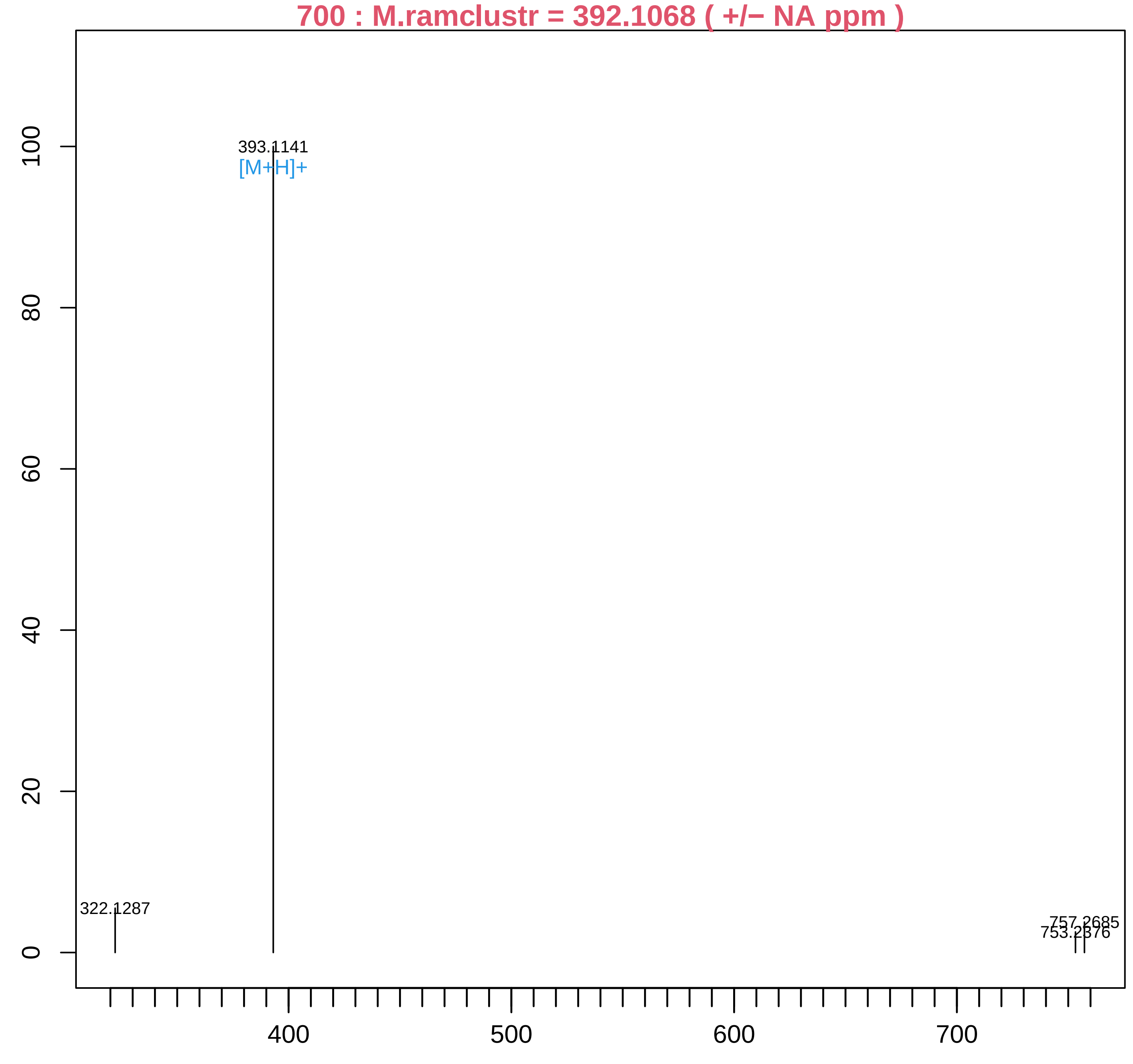
700 : M.ramclustr = 392.1068 (+/− NA ppm)
393.1141
[M+H]+
322.1287
757.2685
753.2376
0
20
40
60
80
100
400
500
600
700

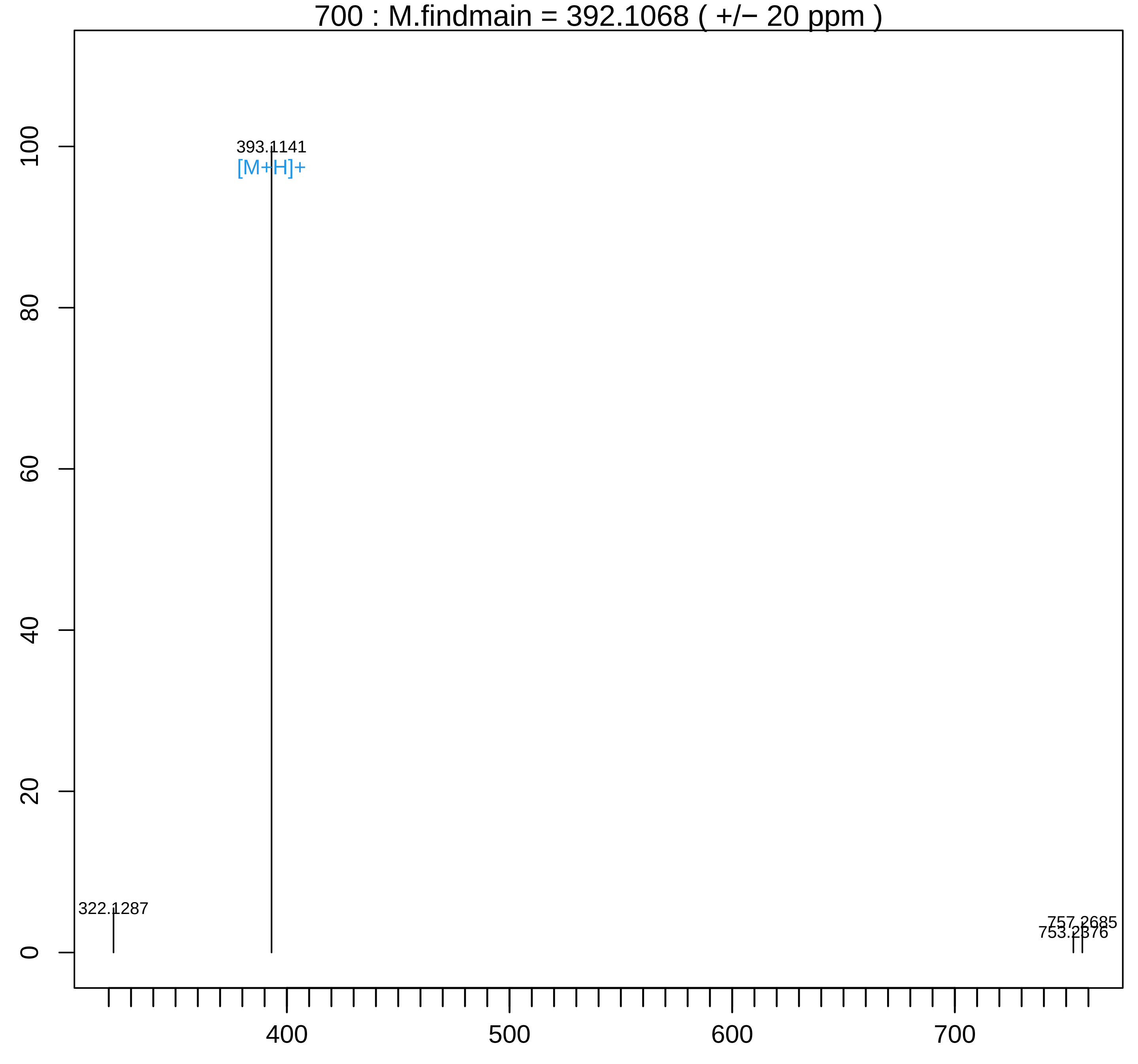
700 : M.findmain = 392.1068 (+/− 20 ppm)
393.1141
[M+H]+
322.1287
757.2685
753.2376
0
20
40
60
80
100
400
500
600
700

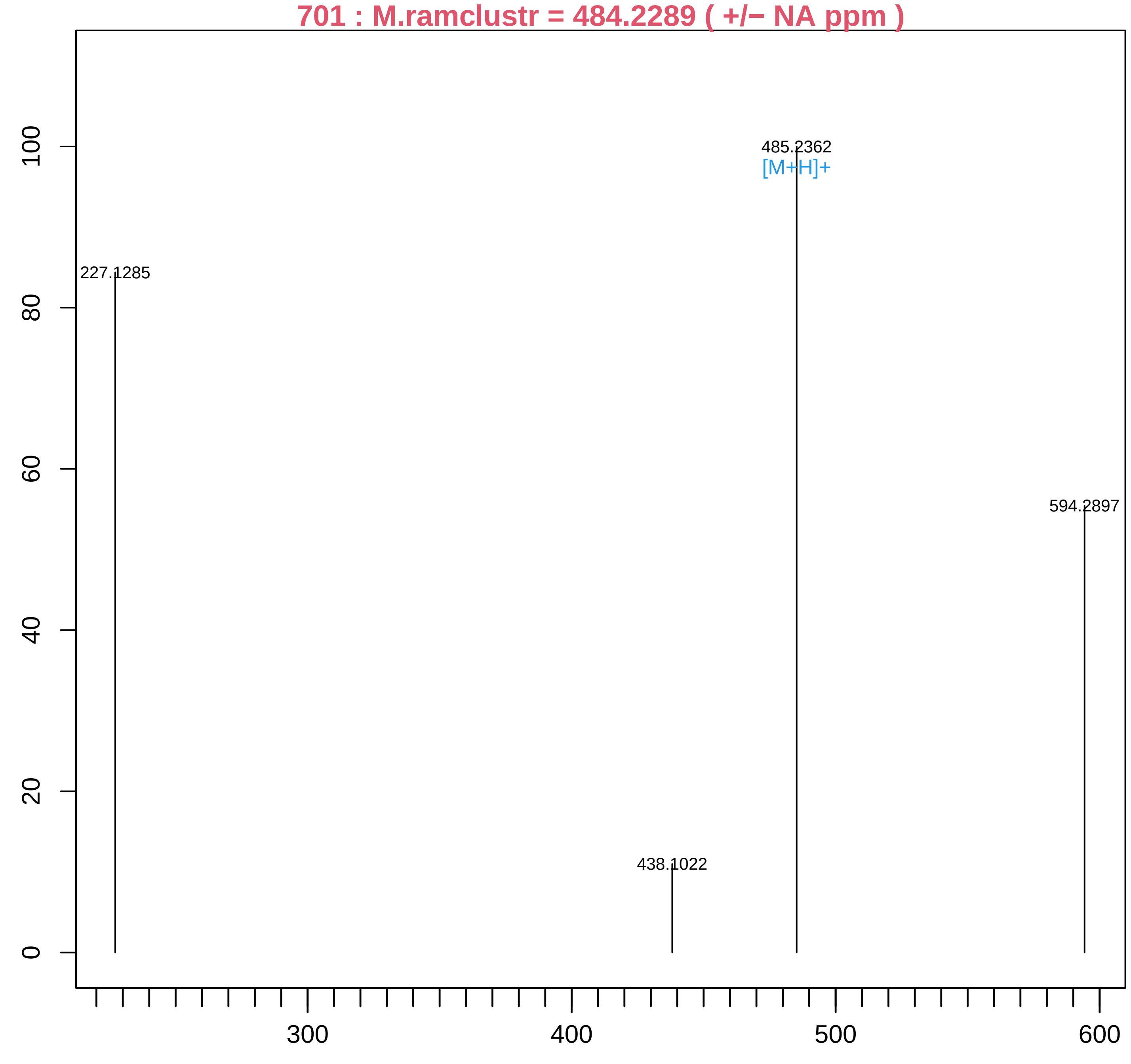

701 : M.ramclustr = 484.2289 (+/− NA ppm)
227.1285
438.1022
485.2362
[M+H]+
594.2897
0
20
40
60
80
100
300
400
500
600

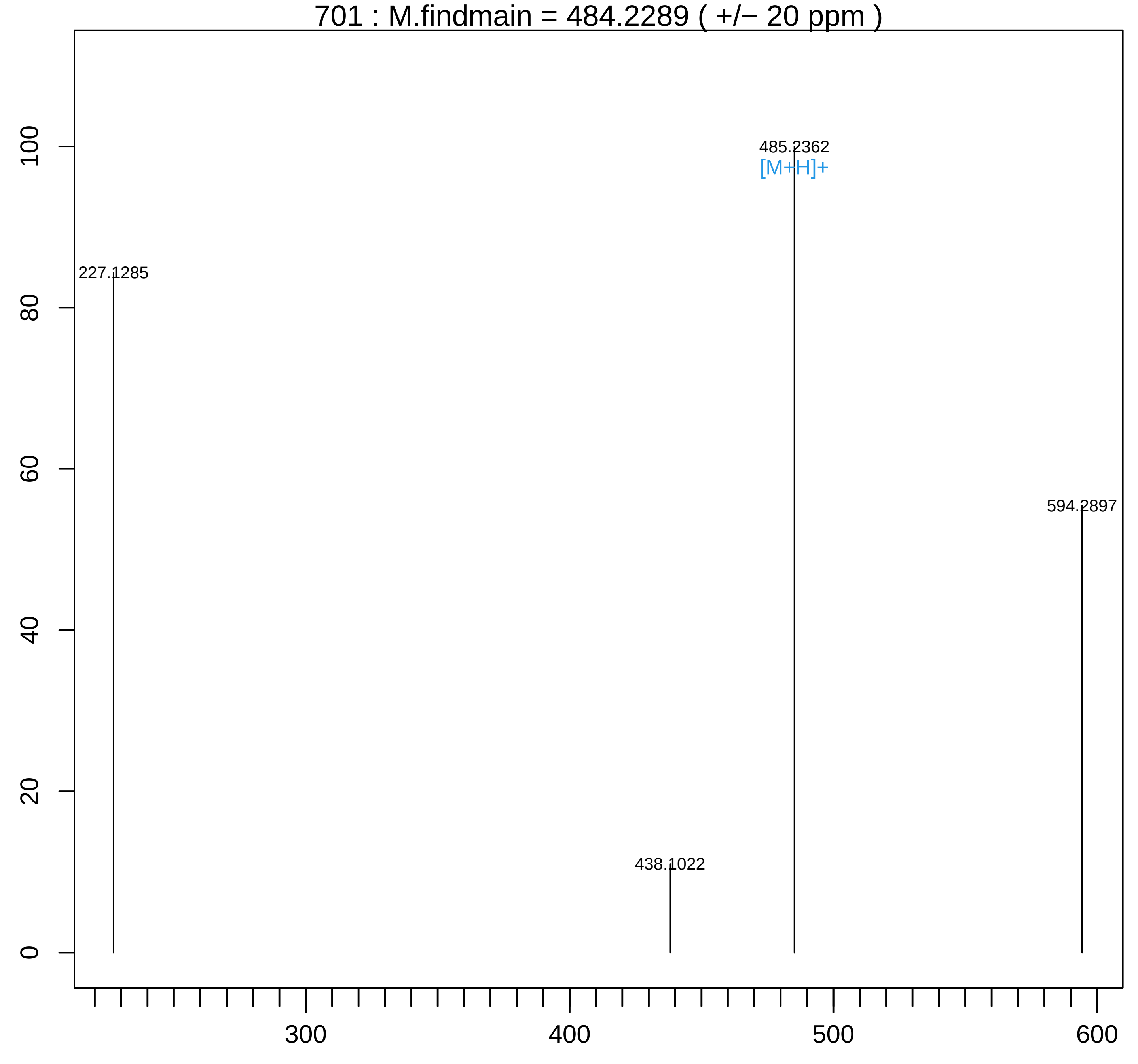

701 : M.findmain = 484.2289 (+/− 20 ppm)
227.1285
438.1022
485.2362
[M+H]+
594.2897
0
20
40
60
80
100
300
400
500
600

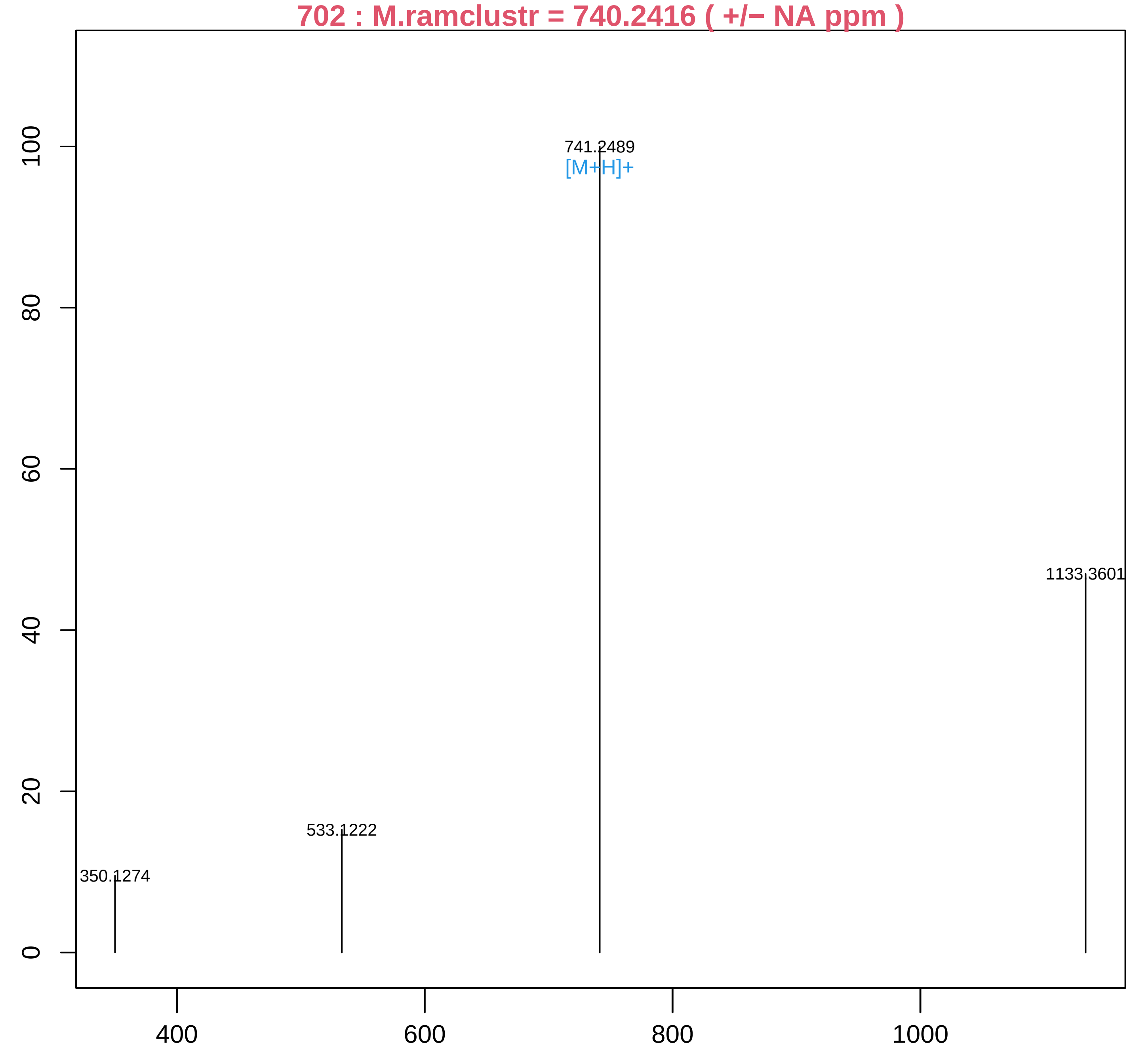
702 : M.ramclustr = 740.2416 (+/− NA ppm)
741.2489
[M+H]+
1133.3601
533.1222
350.1274
0
20
40
60
80
100
400
600
800
1000

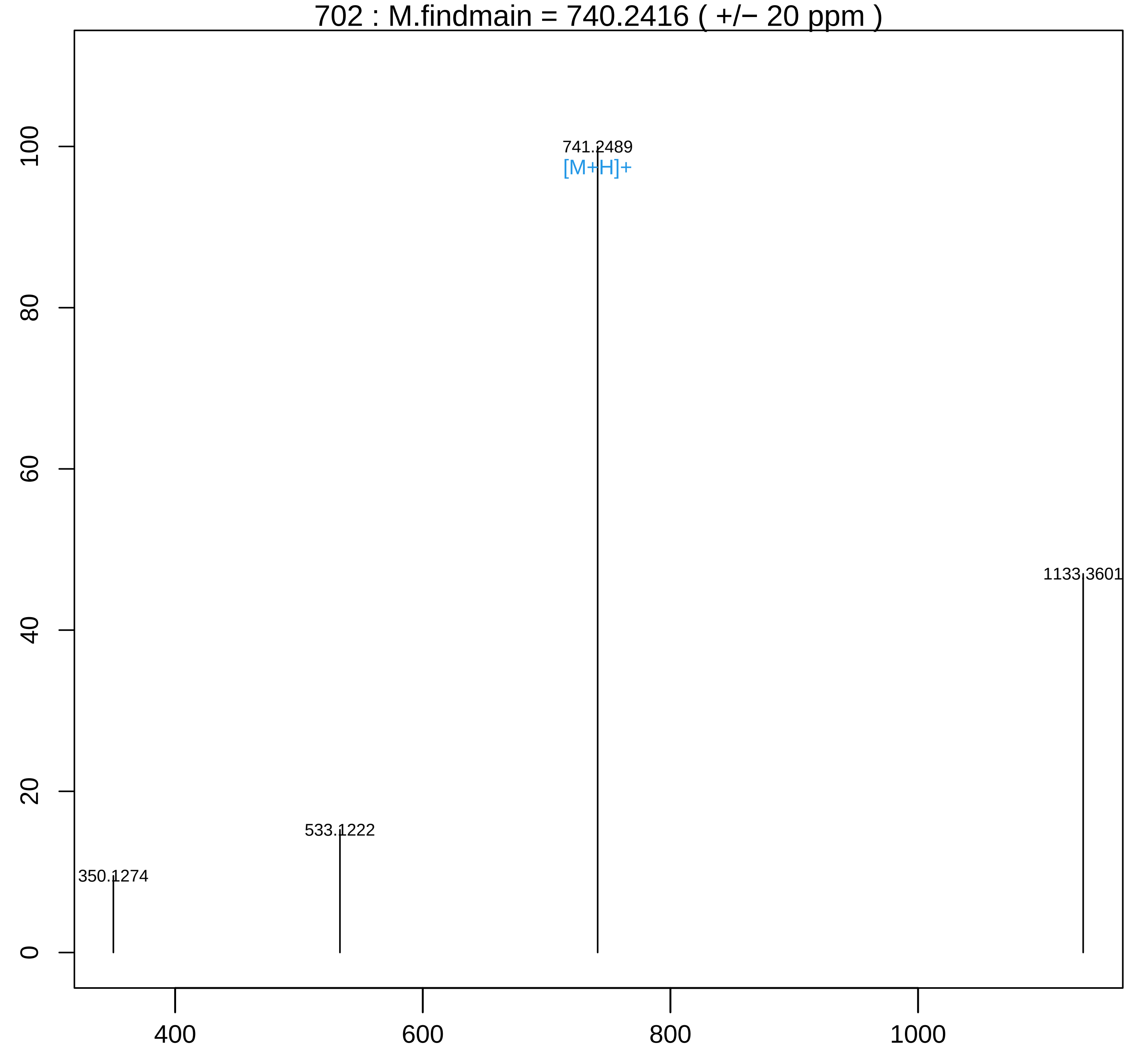
702 : M.findmain = 740.2416 (+/− 20 ppm)
741.2489
[M+H]+
1133.3601
533.1222
350.1274
0
20
40
60
80
100
400
600
800
1000

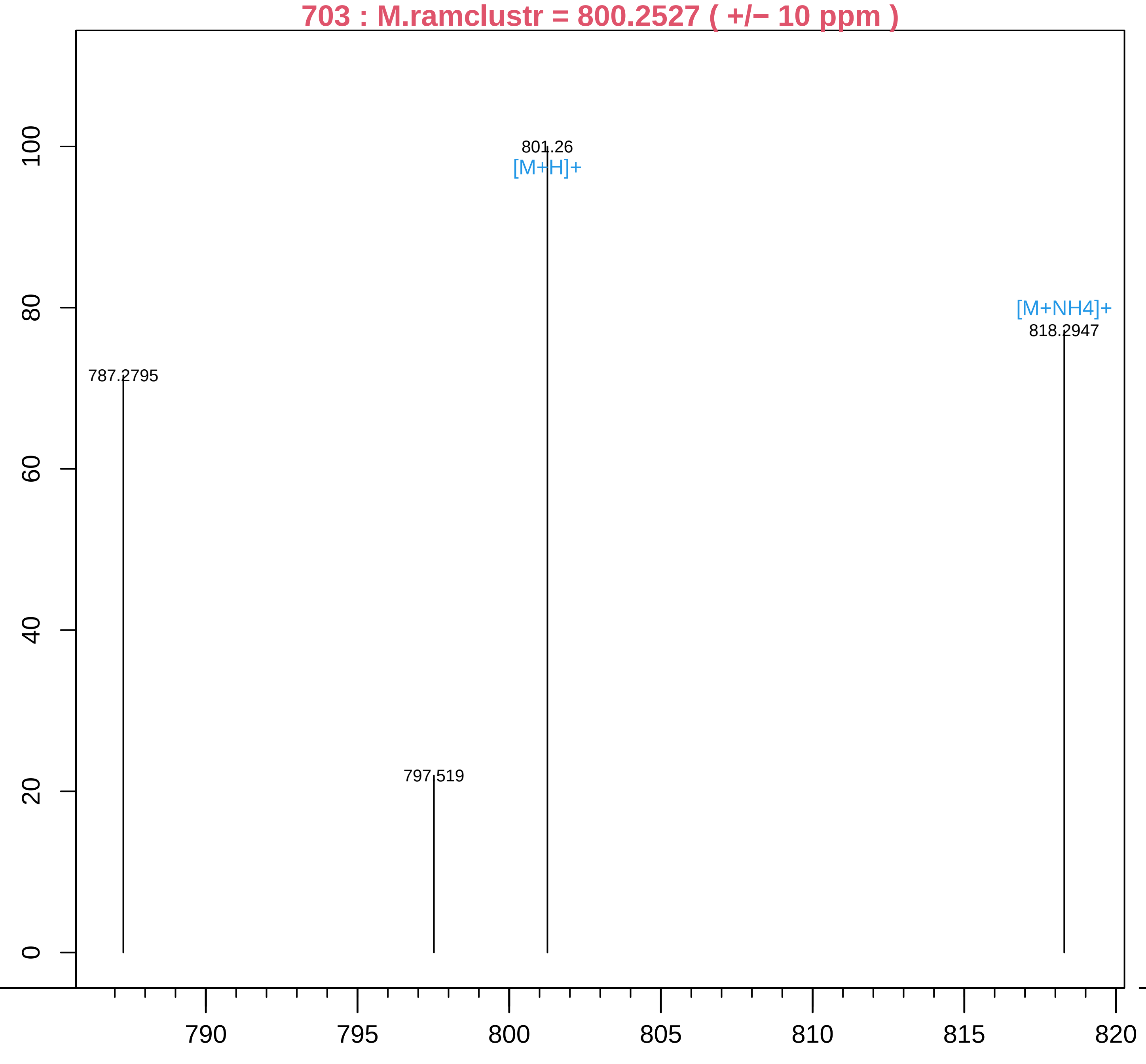
703 : M.ramclustr = 800.2527 (+/− 10 ppm)
801.26
[M+H]+
[M+NH4]+
818.2947
787.2795
797.519
0
20
40
60
80
100
790
795
800
805
810
815
820

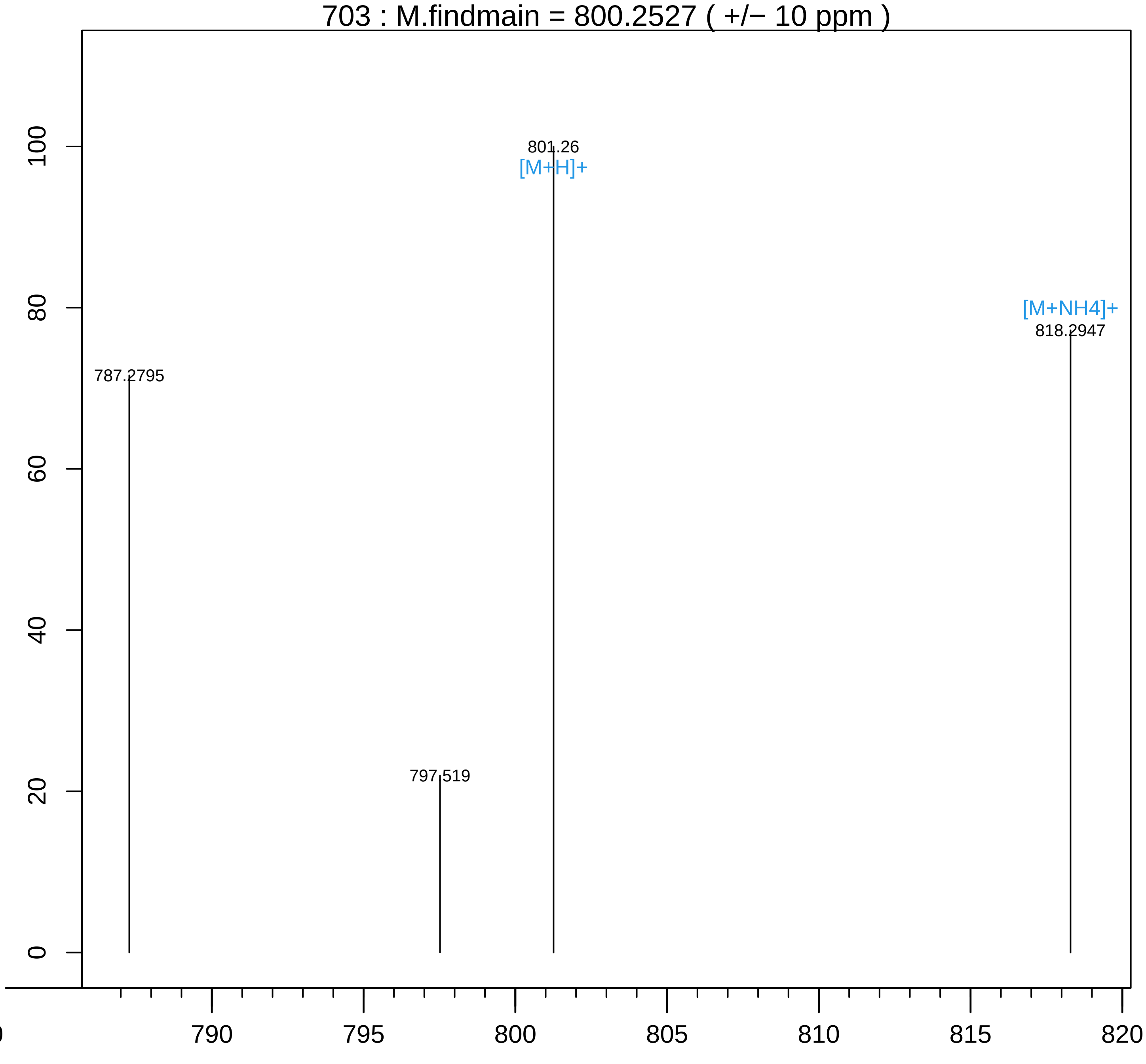
703 : M.findmain = 800.2527 (+/− 10 ppm)
801.26
[M+H]+
[M+NH4]+
818.2947
787.2795
797.519
0
20
40
60
80
100
790
795
800
805
810
815
820

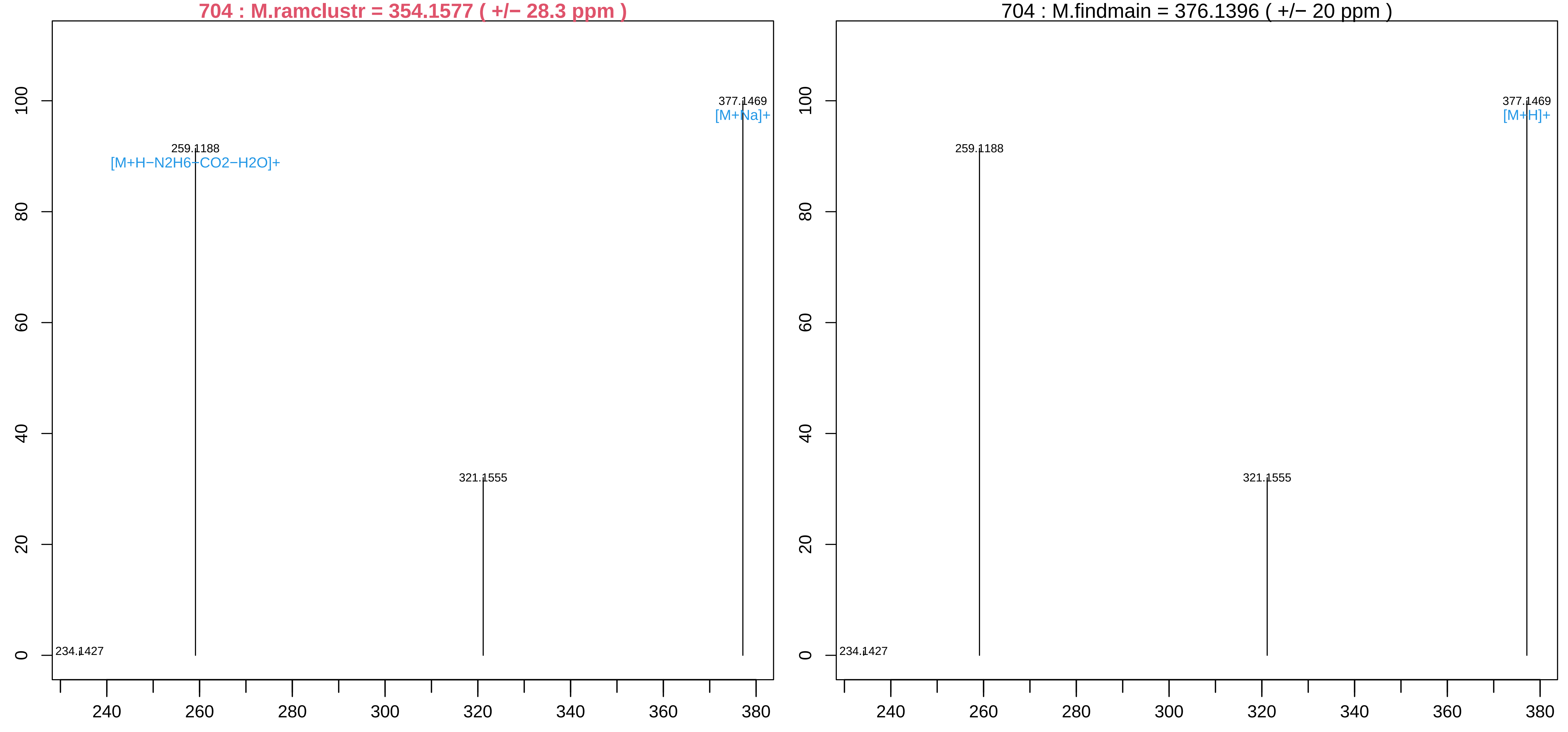

704 : M.ramclustr = 354.1577 (+/− 28.3 ppm)
377.1469
[M+Na]+
259.1188
[M+H−N2H6−CO2−H2O]+
321.1555
234.1427
0
20
40
60
80
100
240
260
280
300
320
340
360
380
704 : M.findmain = 376.1396 (+/− 20 ppm)
377.1469
[M+H]+
259.1188
321.1555
234.1427
0
20
40
60
80
100
240
260
280
300
320
340
360
380

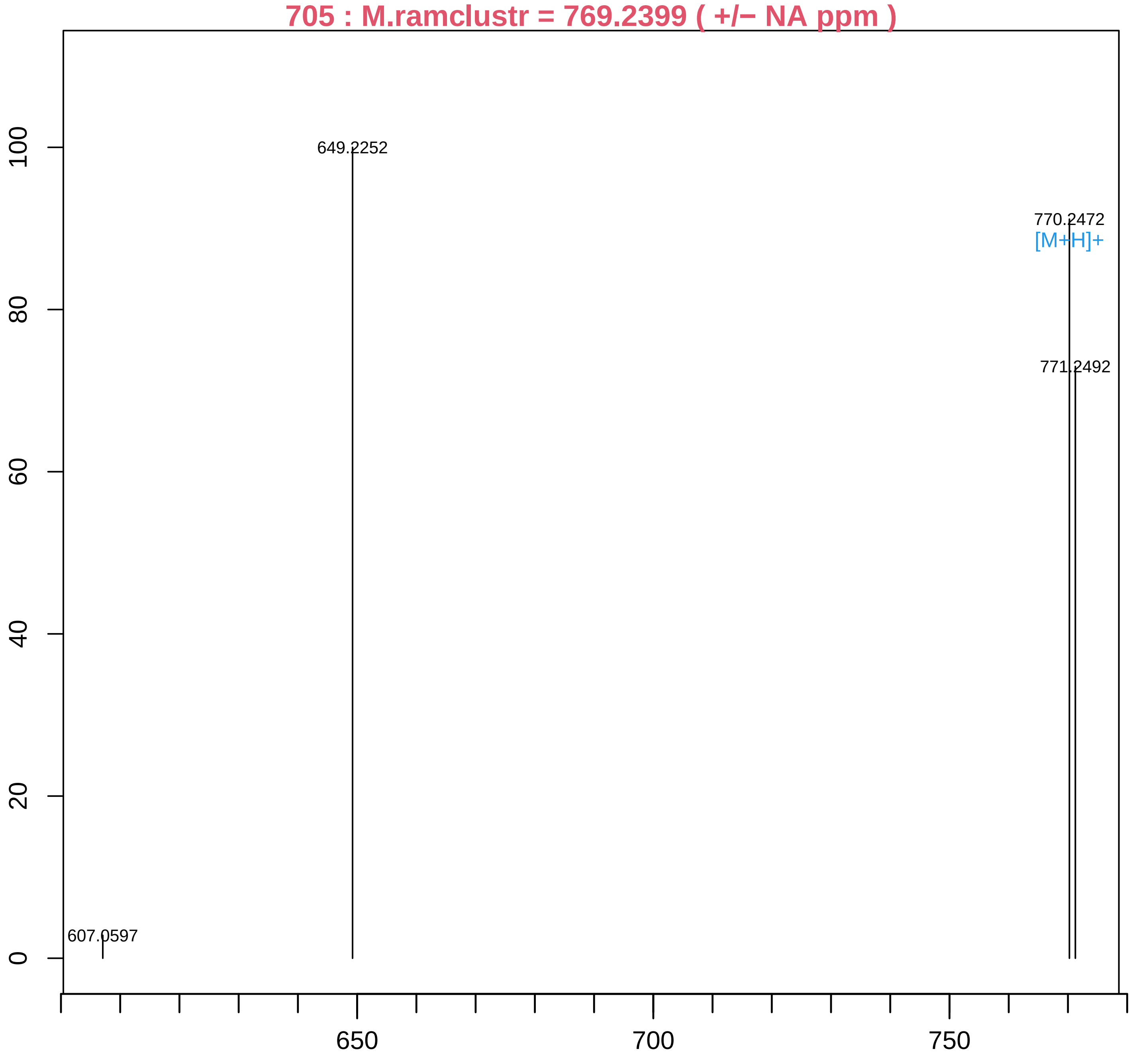
705 : M.ramclustr = 769.2399 (+/− NA ppm)
649.2252
770.2472
[M+H]+
771.2492
607.0597
0
20
40
60
80
100
650
700
750

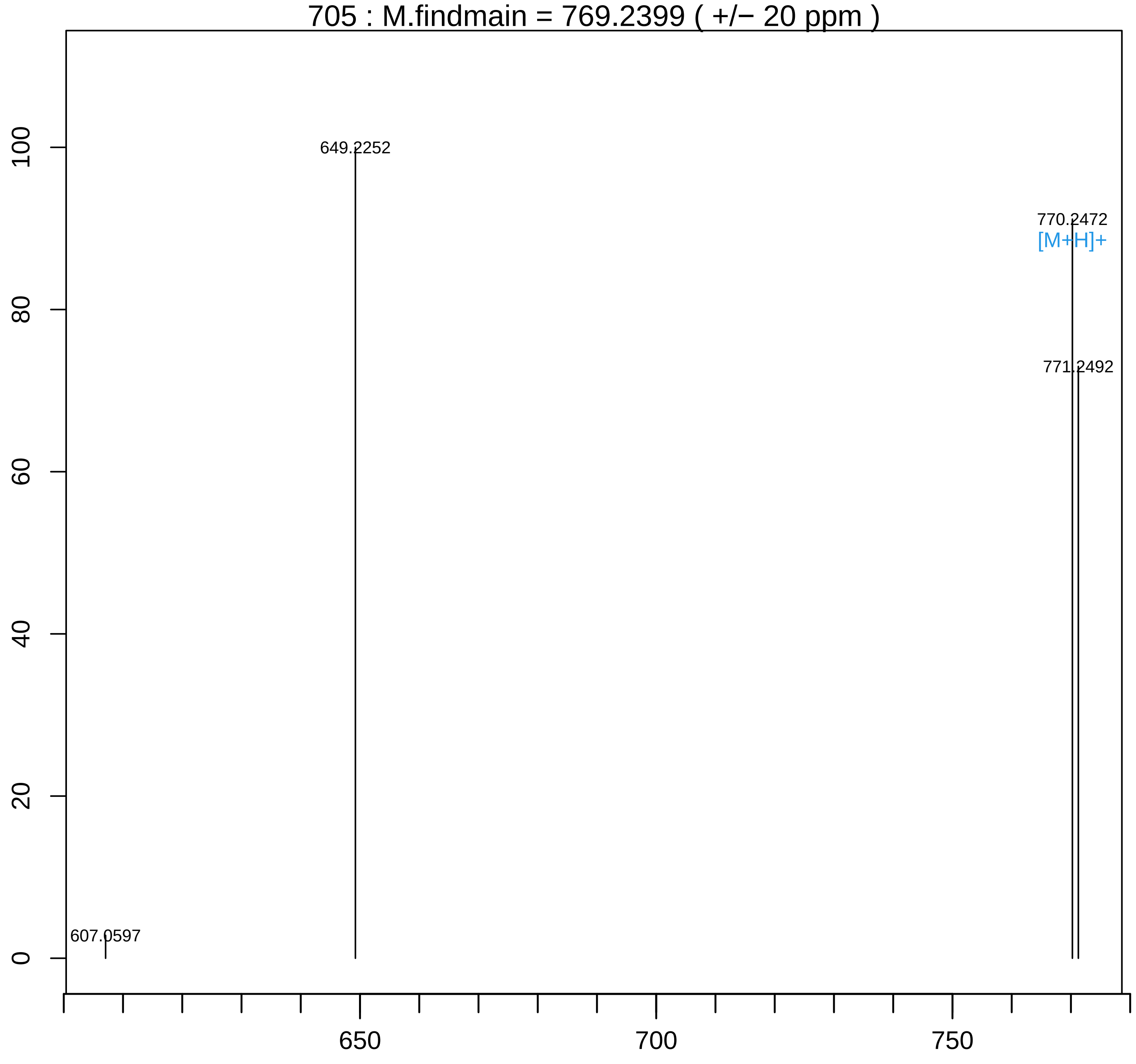
705 : M.findmain = 769.2399 (+/− 20 ppm)
649.2252
770.2472
[M+H]+
771.2492
607.0597
0
20
40
60
80
100
650
700
750

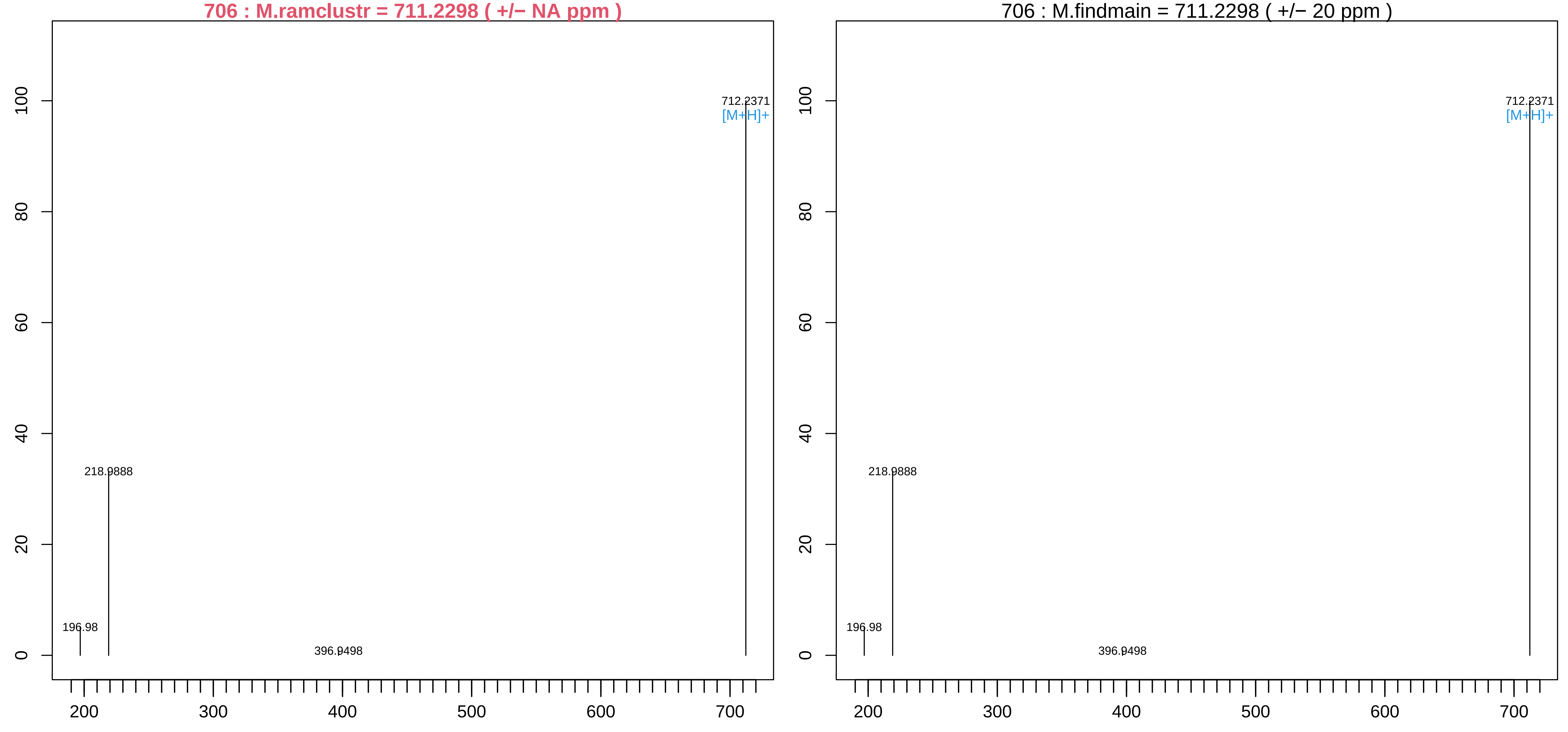

706 : M.ramclustr = 711.2298 (+/− NA ppm)
712.2371
[M+H]+
218.9888
196.98
396.9498
706 : M.findmain = 711.2298 (+/− 20 ppm)
712.2371
[M+H]+
218.9888
196.98
396.9498

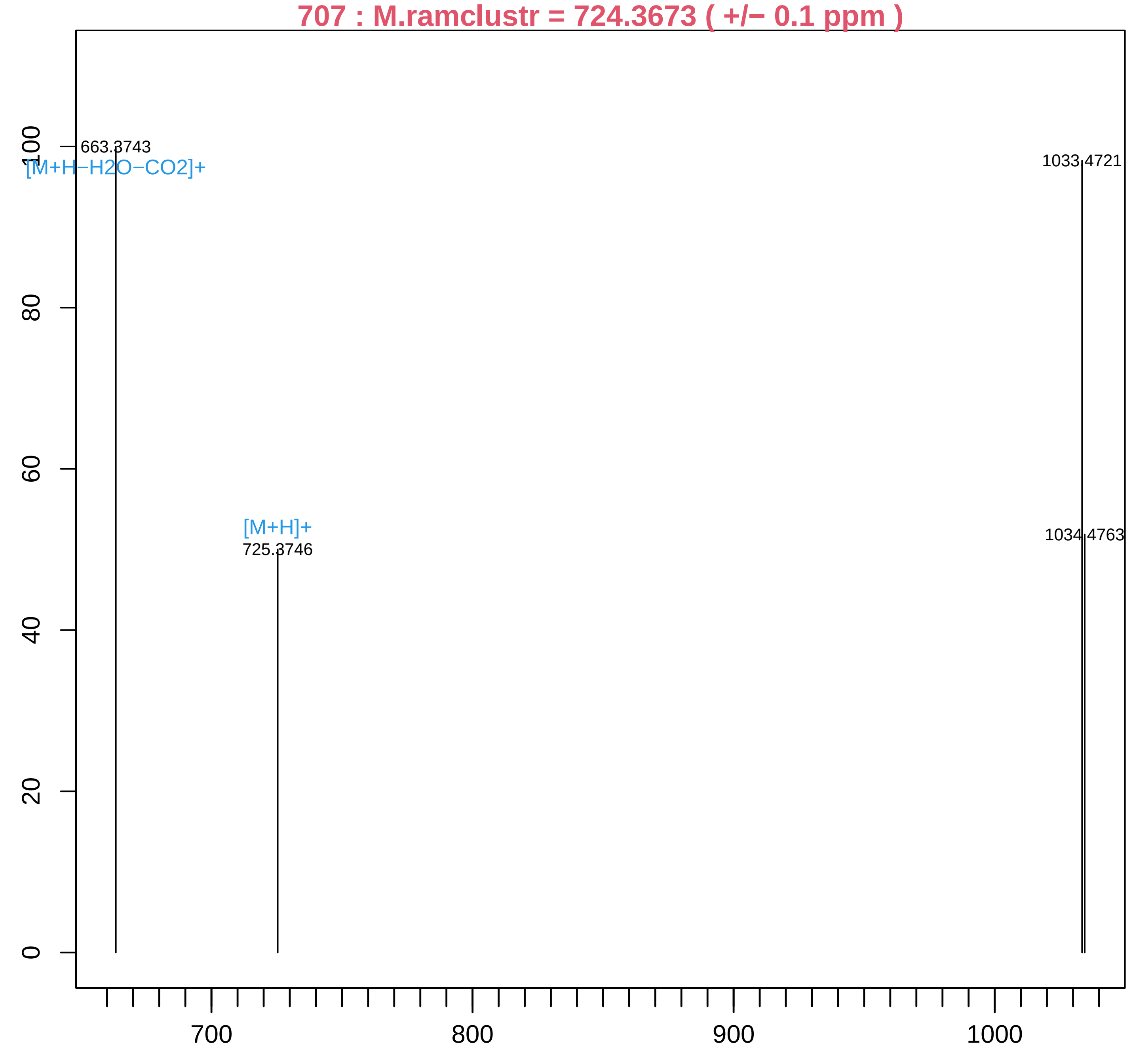
707 : M.ramclustr = 724.3673 (+/− 0.1 ppm)
663.3743
[M+H−H2O−CO2]+
[M+H]+
725.3746
1033.4721
1034.4763
0
20
40
60
80
100
700
800
900
1000

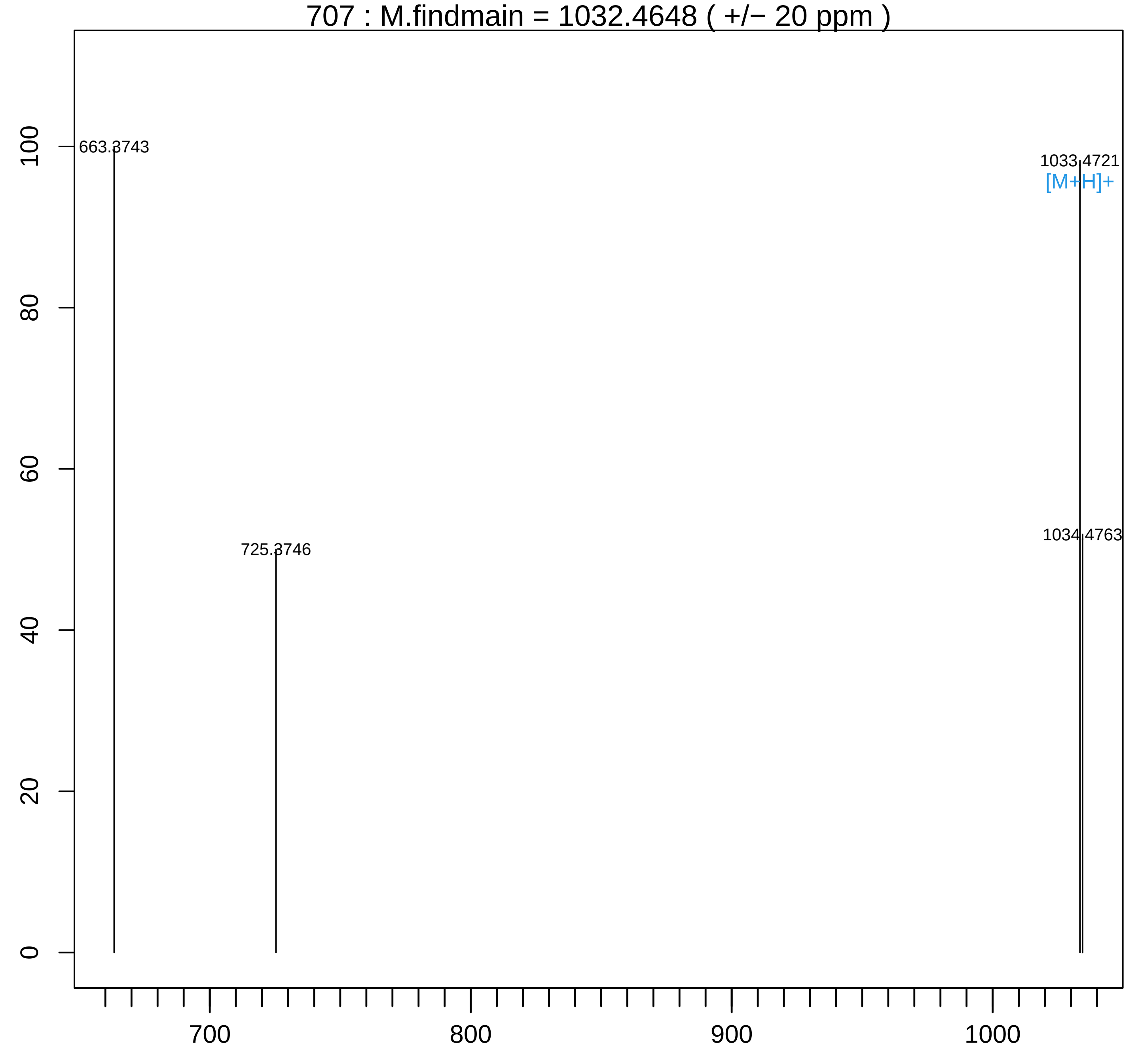
707 : M.findmain = 1032.4648 (+/− 20 ppm)
663.3743
725.3746
1033.4721
[M+H]+
1034.4763
0
20
40
60
80
100
700
800
900
1000

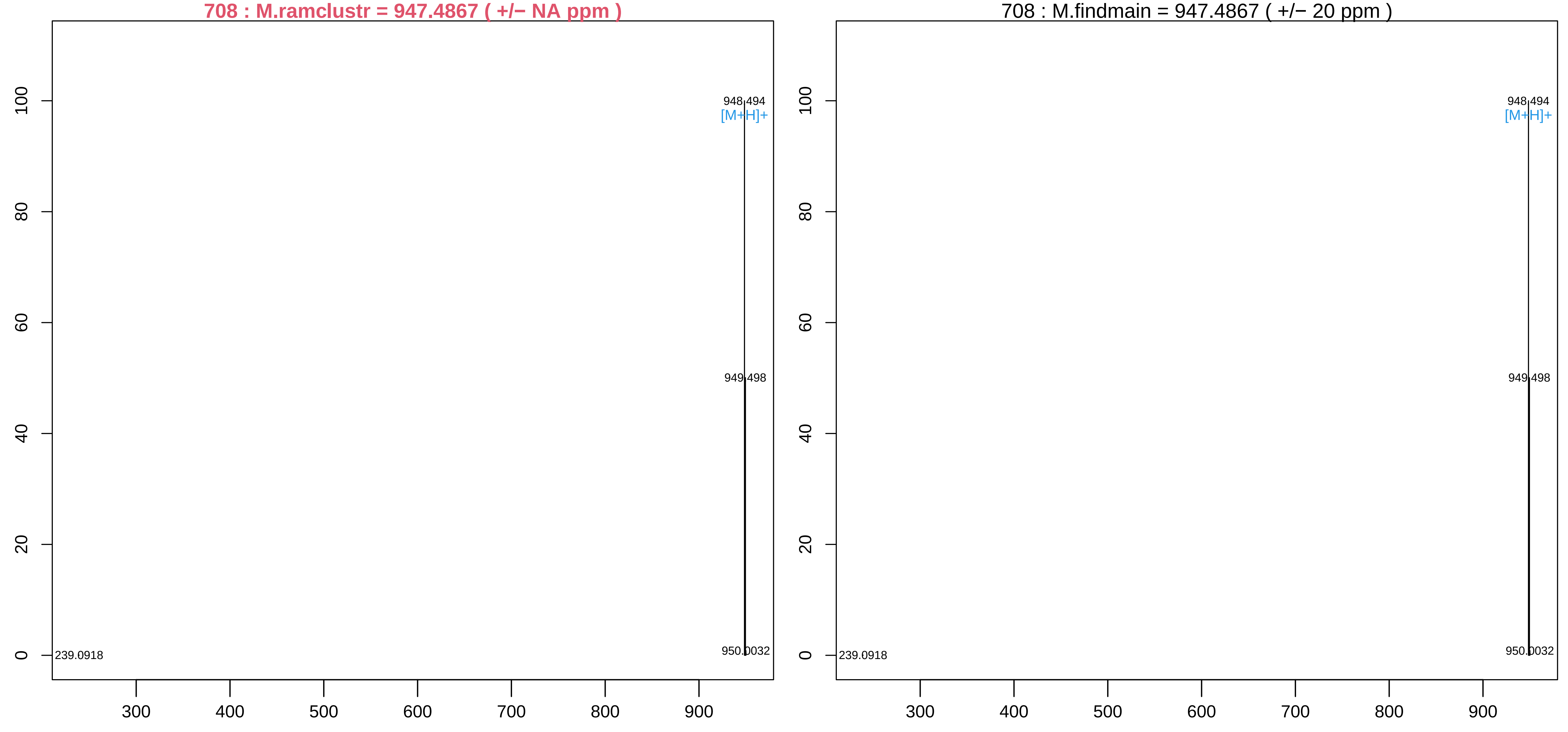

708 : M.ramclustr = 947.4867 (+/− NA ppm)
948.494
[M+H]+
949.498
239.0918
950.0032
0
20
40
60
80
100
300
400
500
600
700
800
900
708 : M.findmain = 947.4867 (+/− 20 ppm)
948.494
[M+H]+
949.498
239.0918
950.0032
0
20
40
60
80
100
300
400
500
600
700
800
900

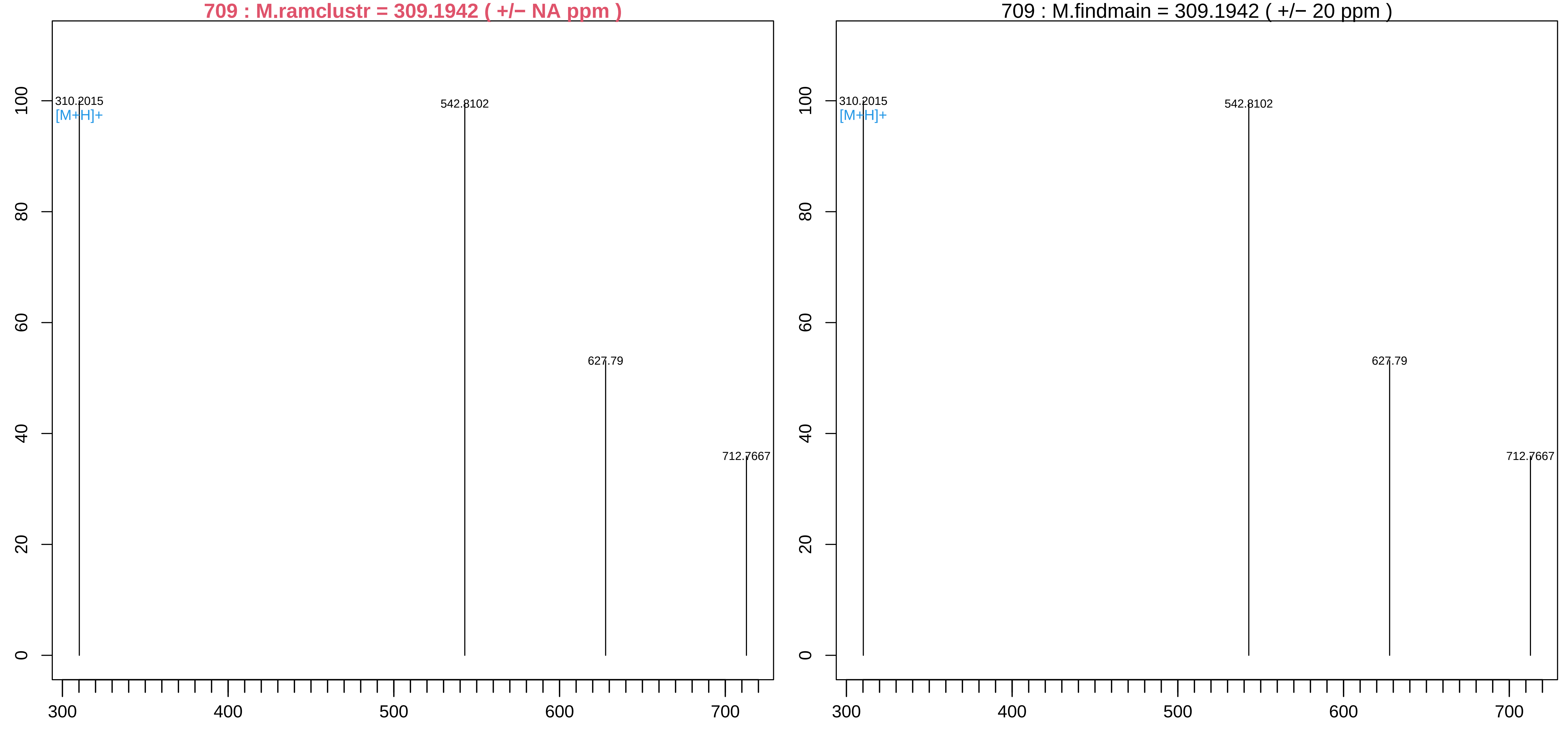

709 : M.ramclustr = 309.1942 (+/− NA ppm)
310.2015
[M+H]+
542.8102
627.79
712.7667
709 : M.findmain = 309.1942 (+/− 20 ppm)
310.2015
[M+H]+
542.8102
627.79
712.7667
0
20
40
60
80
100
300
400
500
600
700

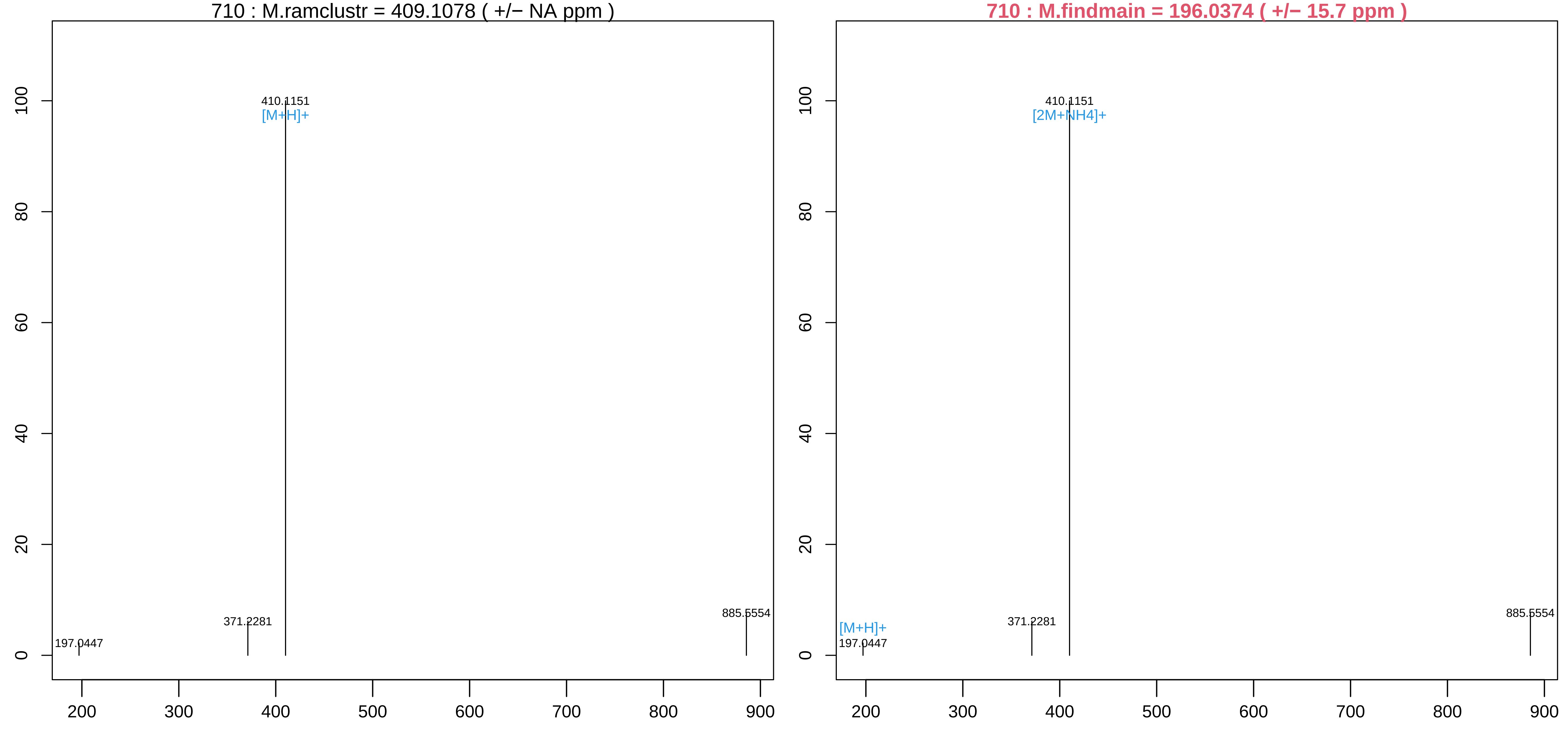
710 : M.ramclustr = 409.1078 (+/− NA ppm)
410.1151
[M+H]+
371.2281
197.0447
885.5554
710 : M.findmain = 196.0374 (+/− 15.7 ppm)
410.1151
[2M+NH4]+
[M+H]+
197.0447
371.2281
885.5554

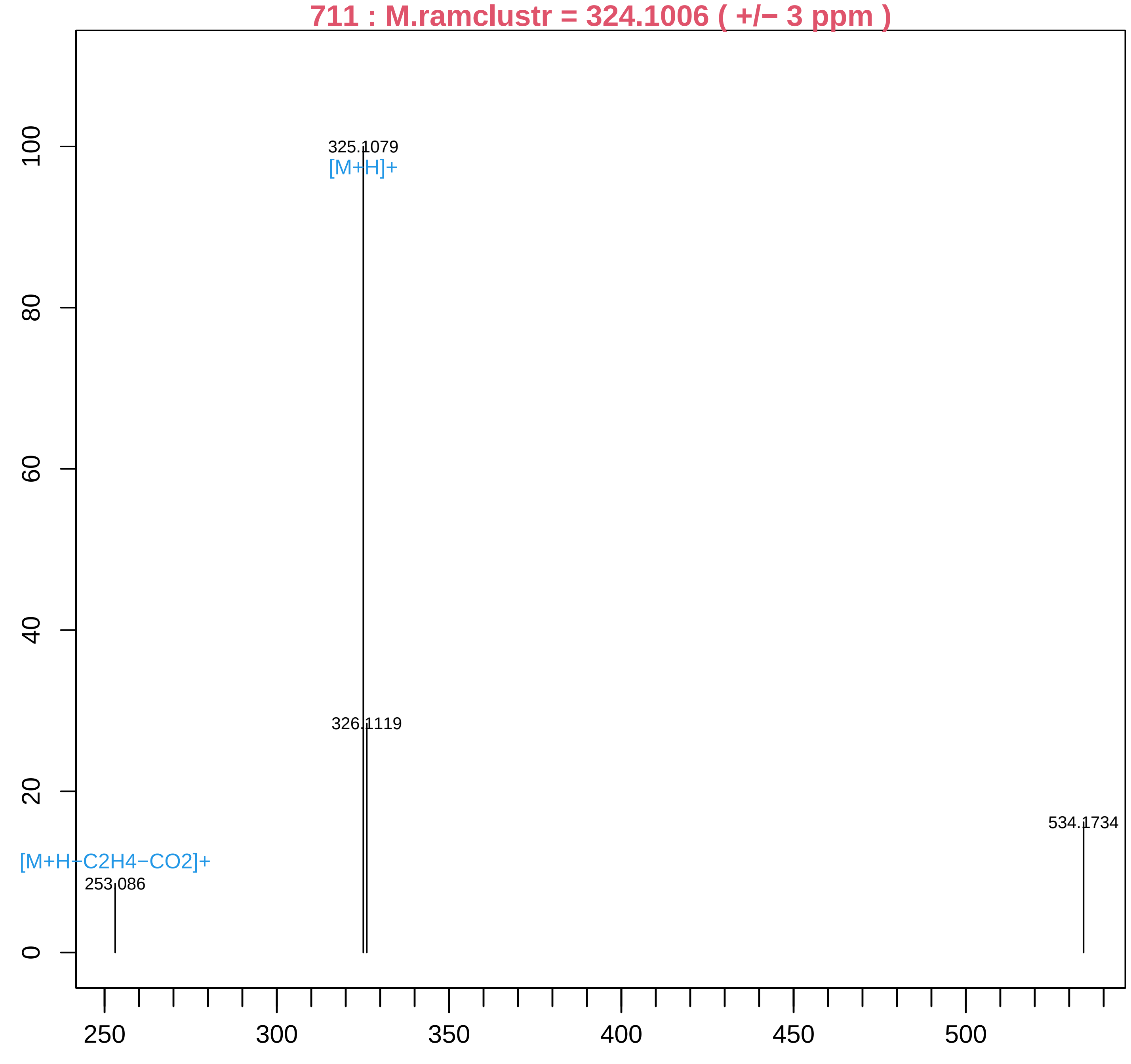

711 : M.ramclustr = 324.1006 (+/− 3 ppm)
325.1079
[M+H]+
326.1119
534.1734
[M+H−C2H4−CO2]+
253.086
0
20
40
60
80
100
250
300
350
400
450
500

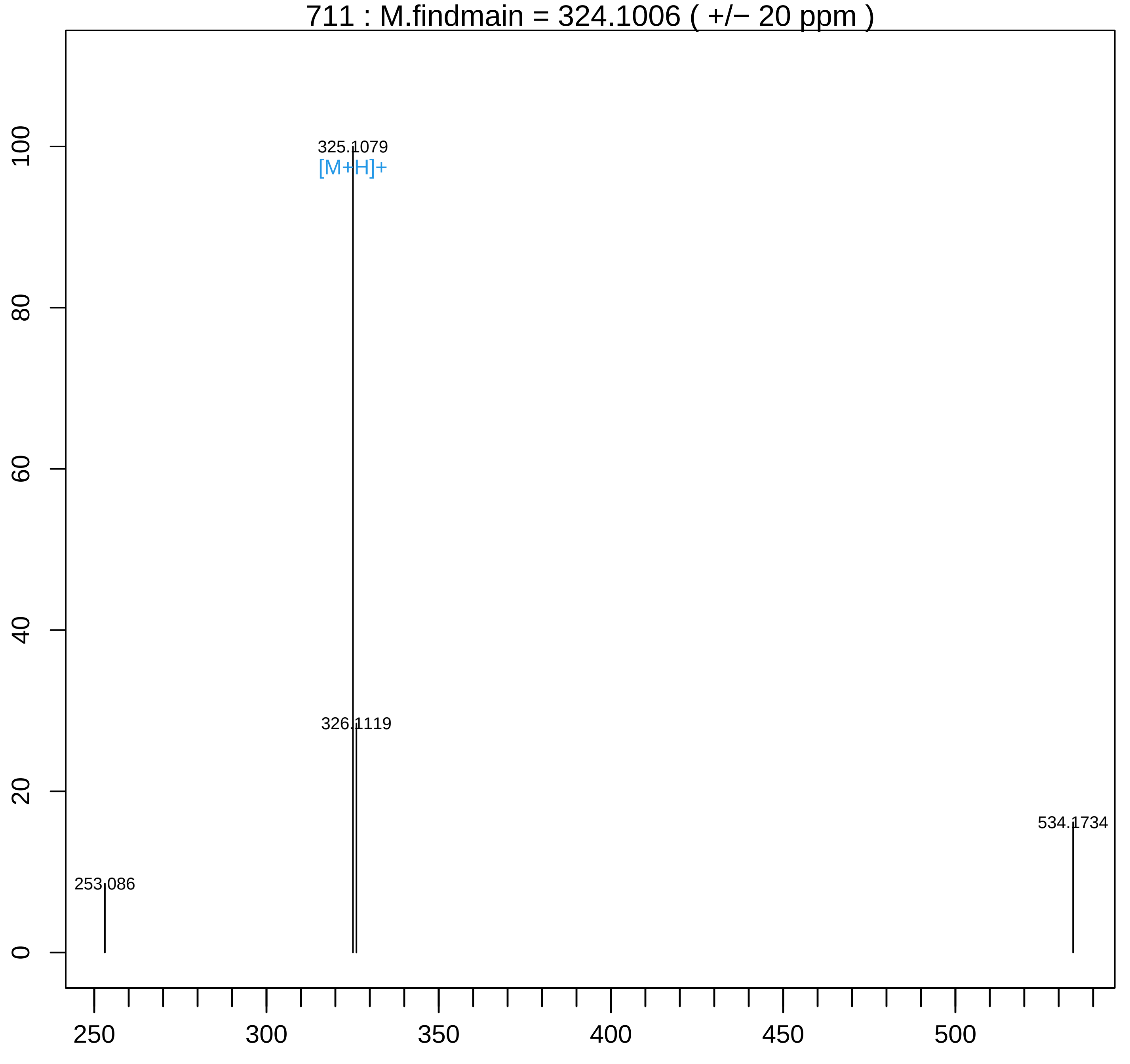

711 : M.findmain = 324.1006 (+/− 20 ppm)
325.1079
[M+H]+
326.1119
534.1734
253.086
0
20
40
60
80
100
250
300
350
400
450
500

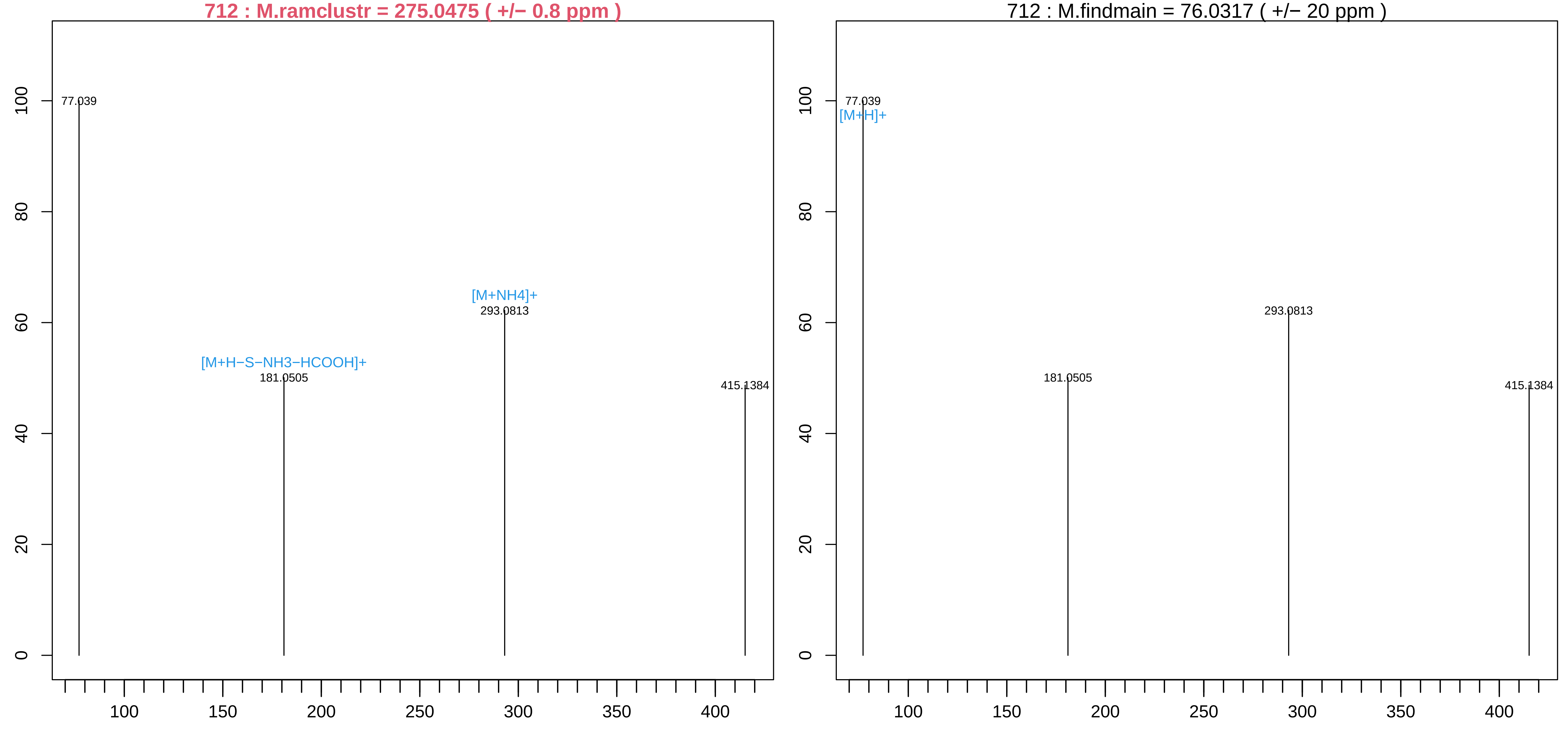
712 : M.ramclustr = 275.0475 (+/− 0.8 ppm)
77.039
[M+H−S−NH3−HCOOH]+
181.0505
[M+NH4]+
293.0813
415.1384
712 : M.findmain = 76.0317 (+/− 20 ppm)
77.039
[M+H]+
181.0505
293.0813
415.1384

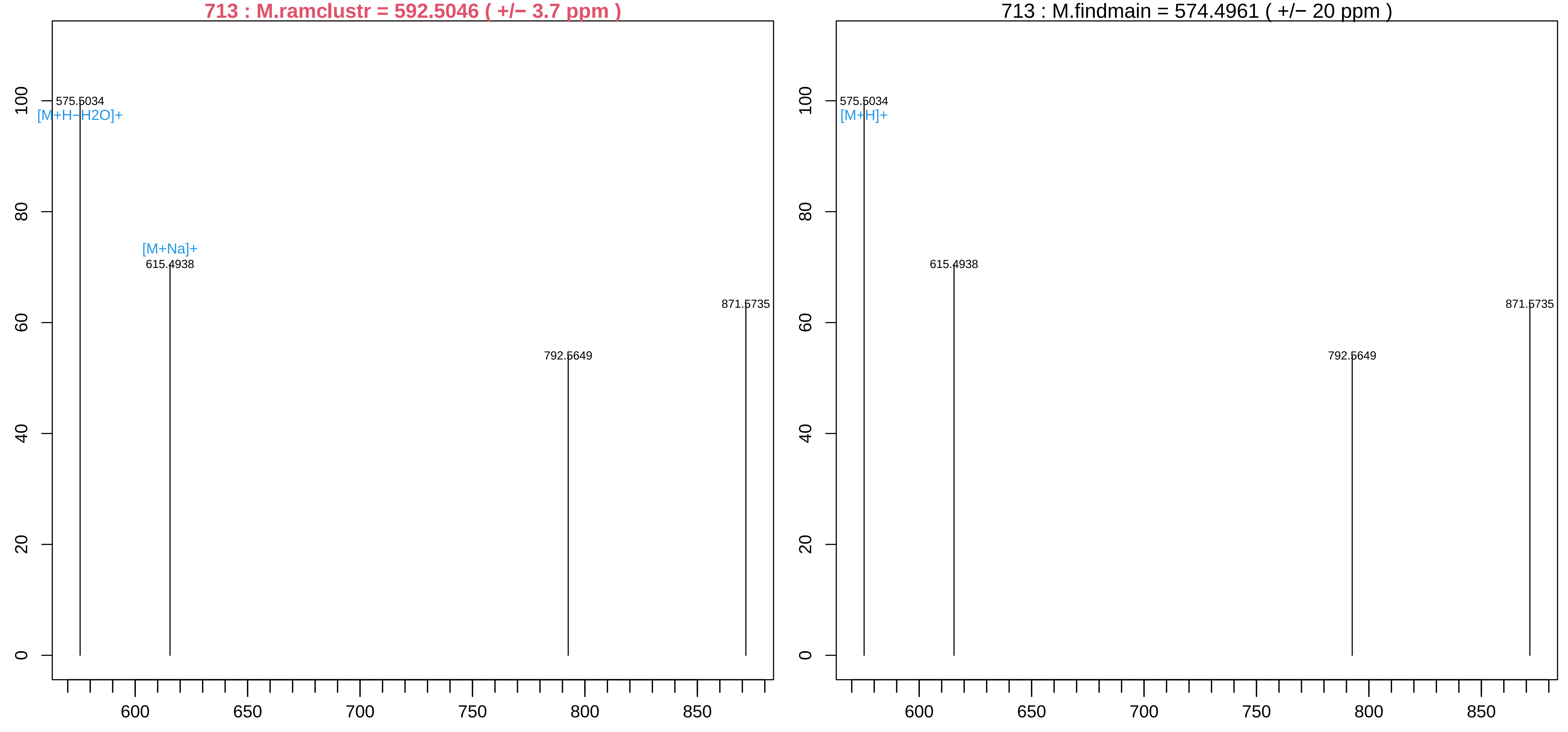

713 : M.ramclustr = 592.5046 (+/− 3.7 ppm)
575.5034
[M+H−H2O]+
[M+Na]+
615.4938
792.5649
871.5735
713 : M.findmain = 574.4961 (+/− 20 ppm)
575.5034
[M+H]+
615.4938
792.5649
871.5735
0
20
40
60
80
100
600
650
700
750
800
850

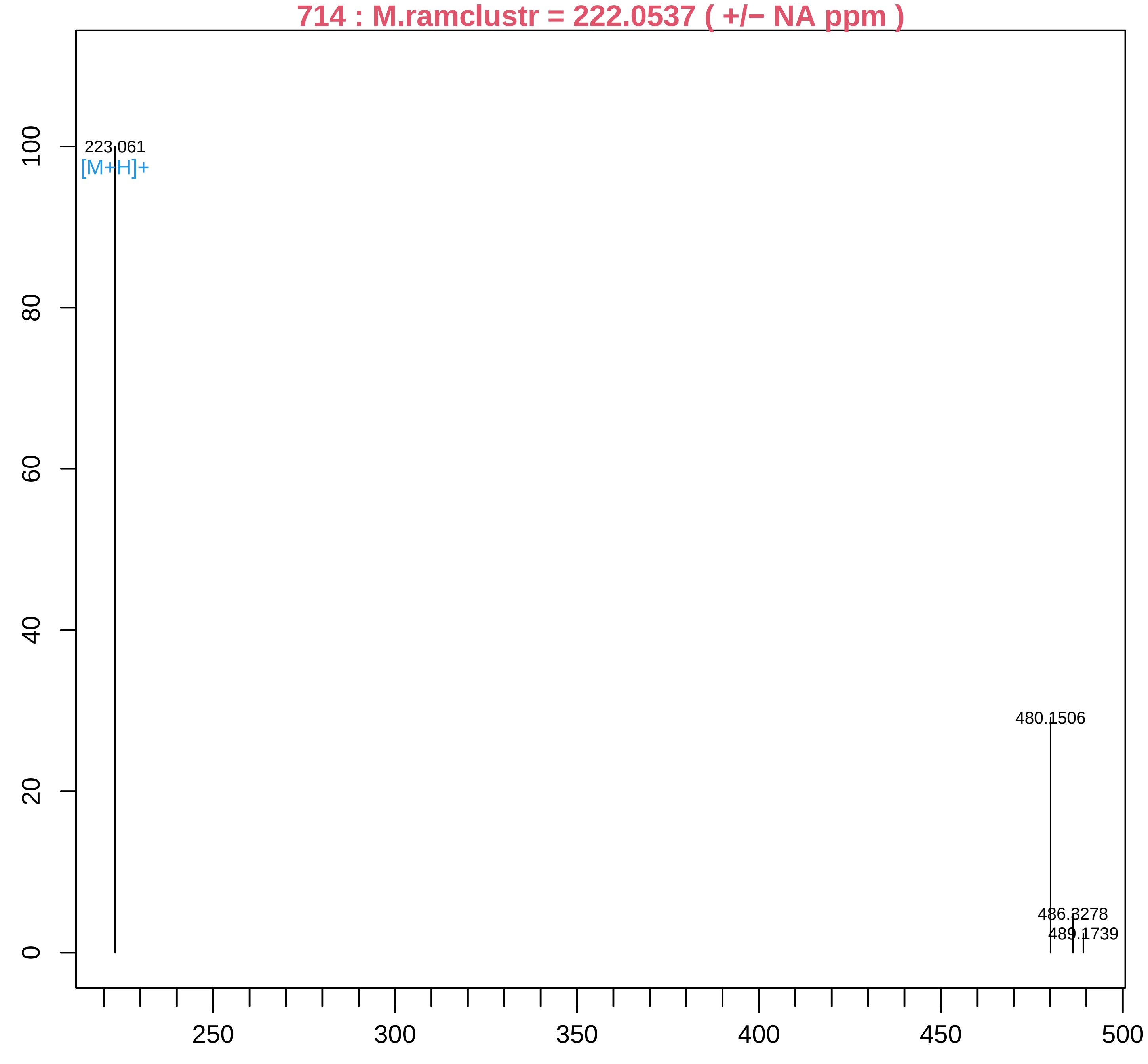
714 : M.ramclustr = 222.0537 (+/− NA ppm)
223.061
[M+H]+
480.1506
486.3278
489.1739
0
20
40
60
80
100
250
300
350
400
450
500

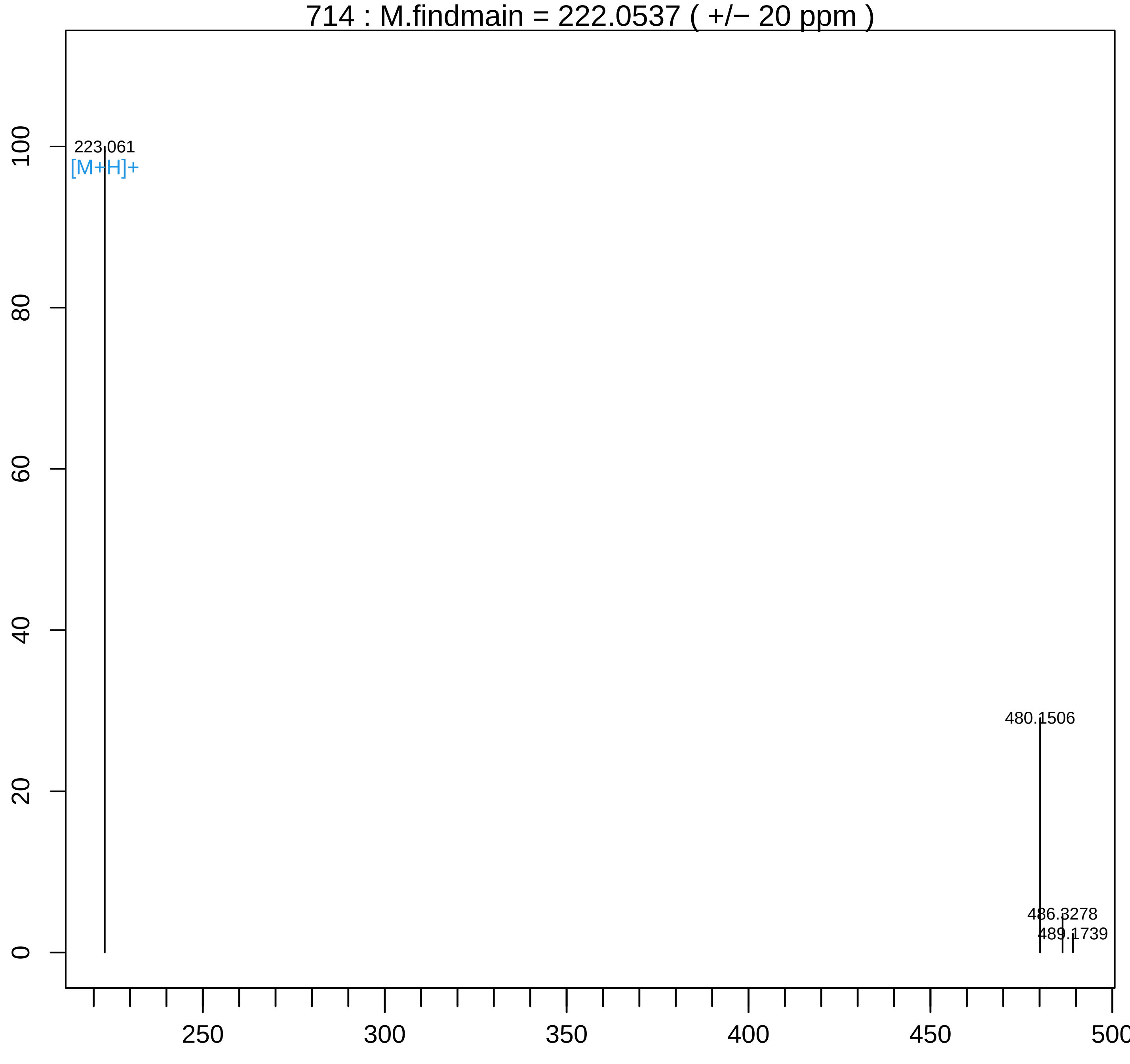
714 : M.findmain = 222.0537 (+/− 20 ppm)
223.061
[M+H]+
480.1506
486.3278
489.1739
0
20
40
60
80
100
250
300
350
400
450
500

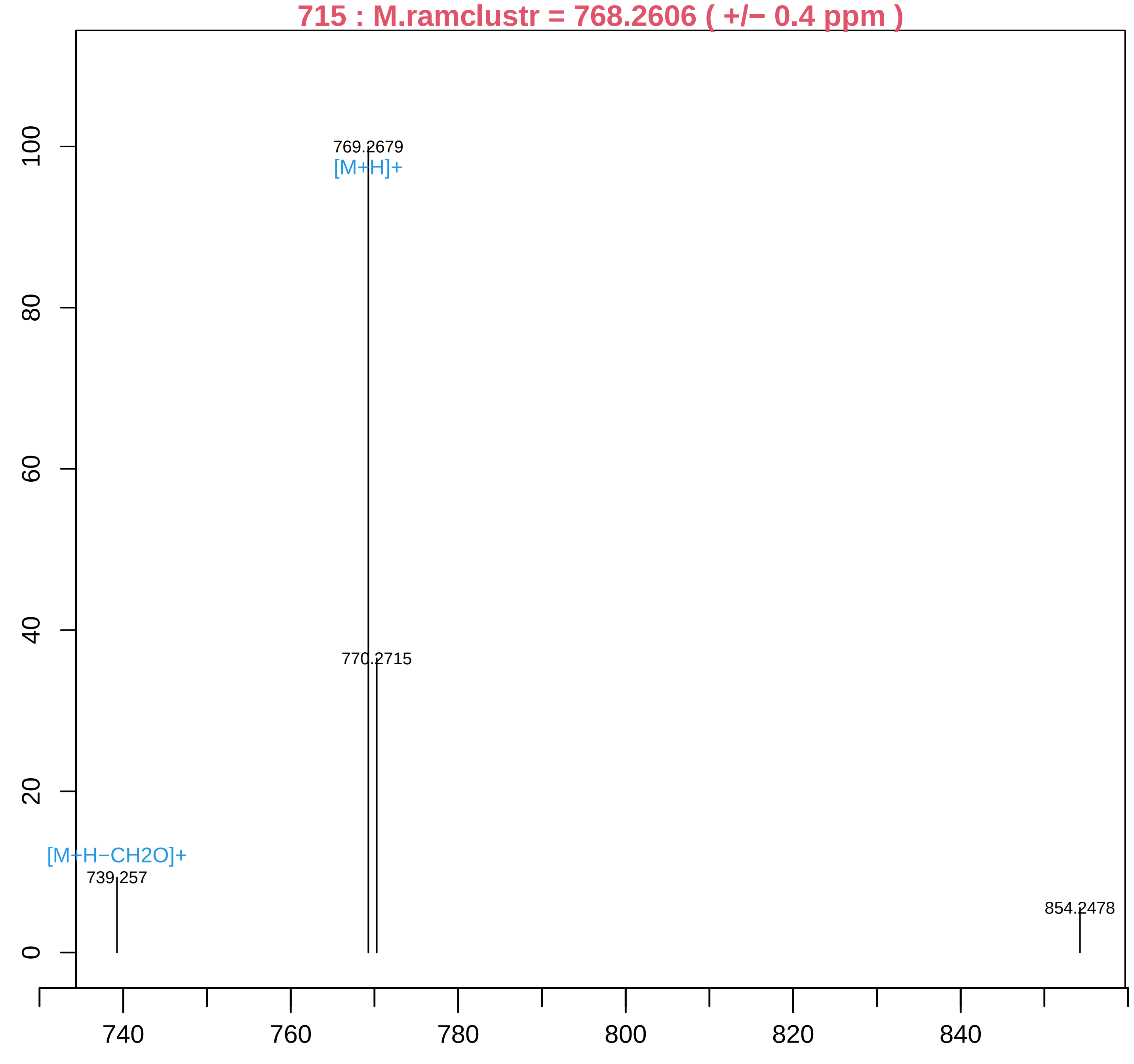
715 : M.ramclustr = 768.2606 (+/− 0.4 ppm)
769.2679
[M+H]+
770.2715
[M+H−CH2O]+
739.257
854.2478
0
20
40
60
80
100
740
760
780
800
820
840

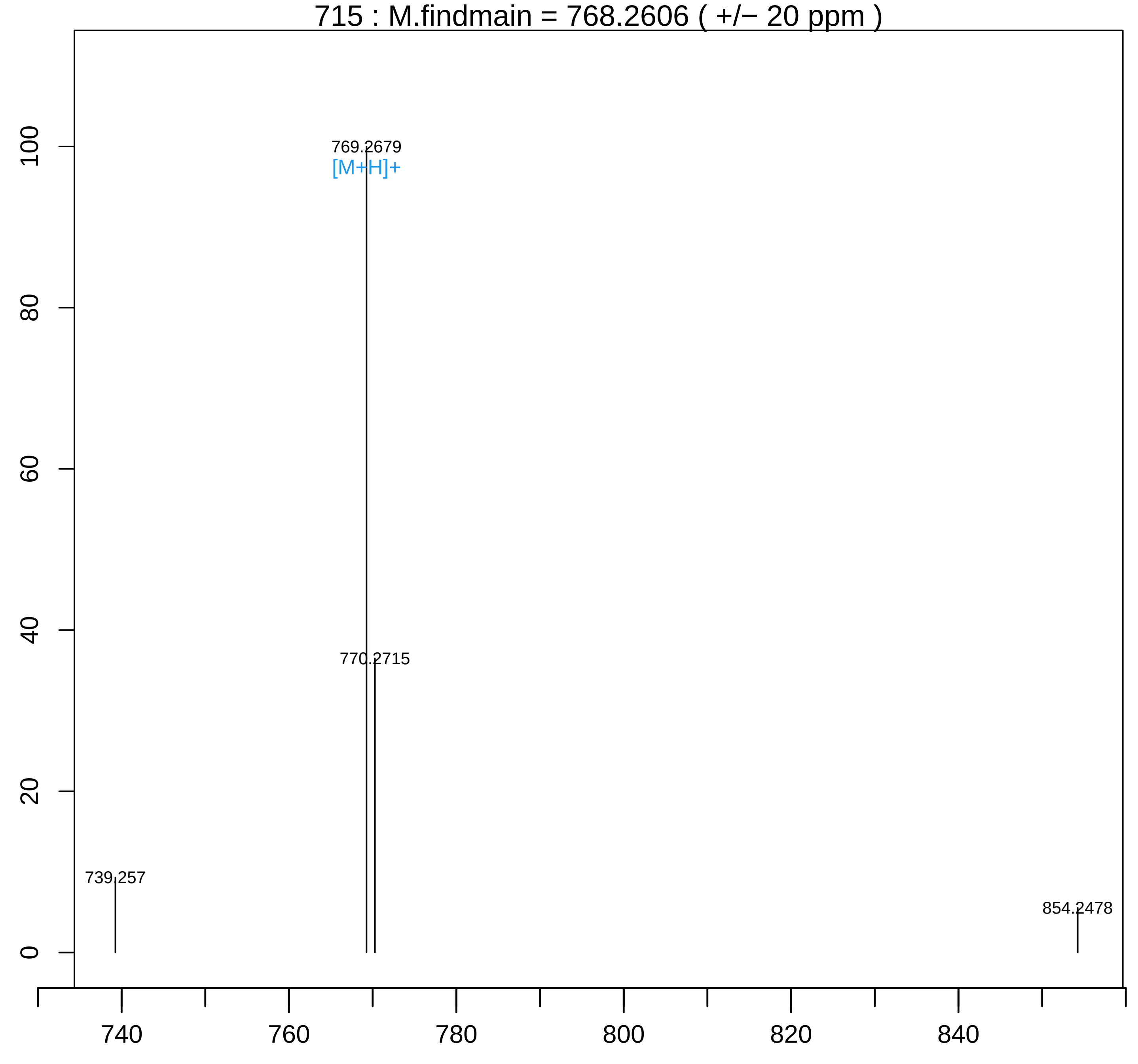
715 : M.findmain = 768.2606 (+/− 20 ppm)
769.2679
[M+H]+
770.2715
739.257
854.2478
0
20
40
60
80
100
740
760
780
800
820
840

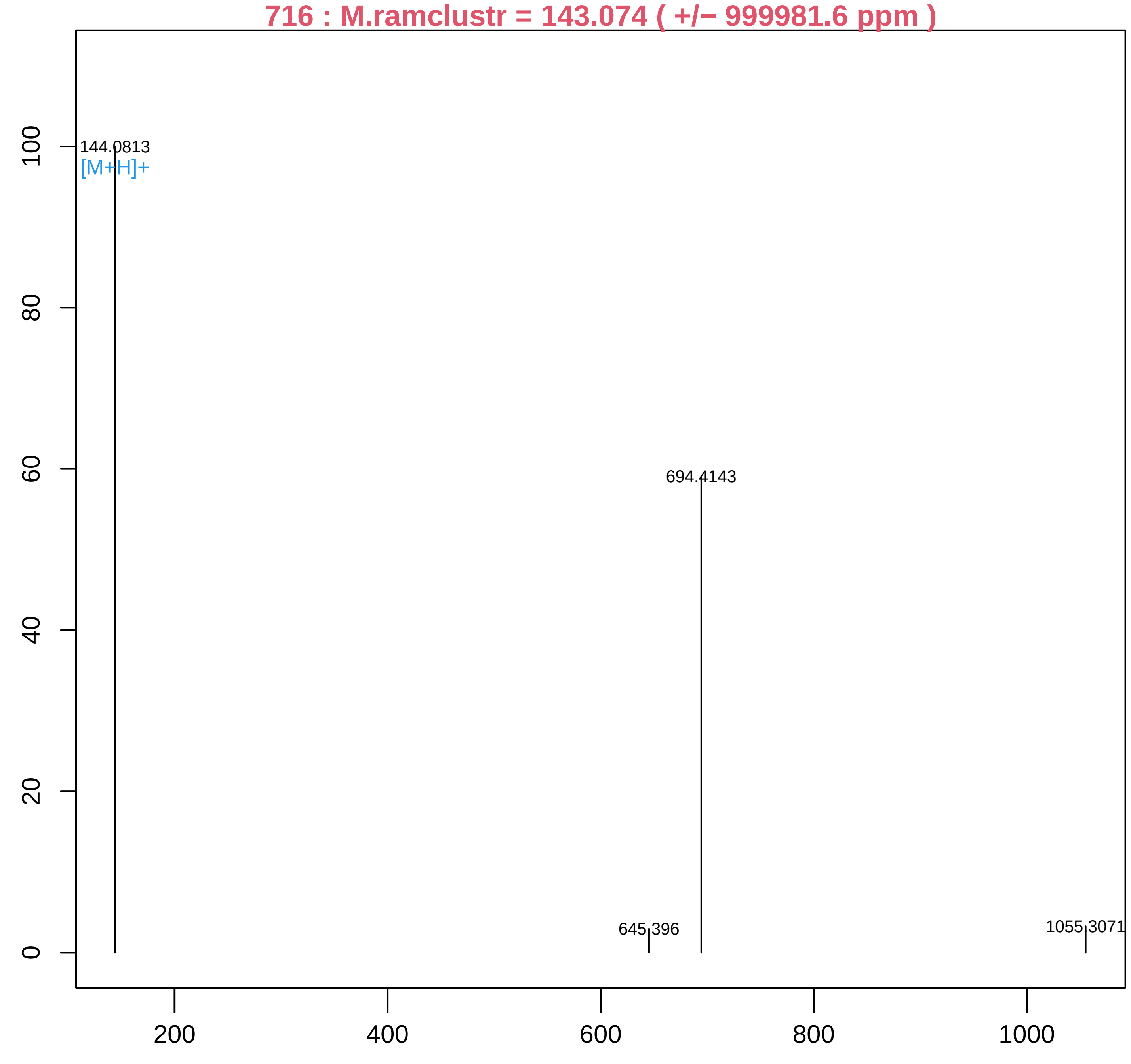
716 : M.ramclustr = 143.074 (+/− 999981.6 ppm)
144.0813
[M+H]+
694.4143
645.396
1055.3071
0
20
40
60
80
100
200
400
600
800
1000

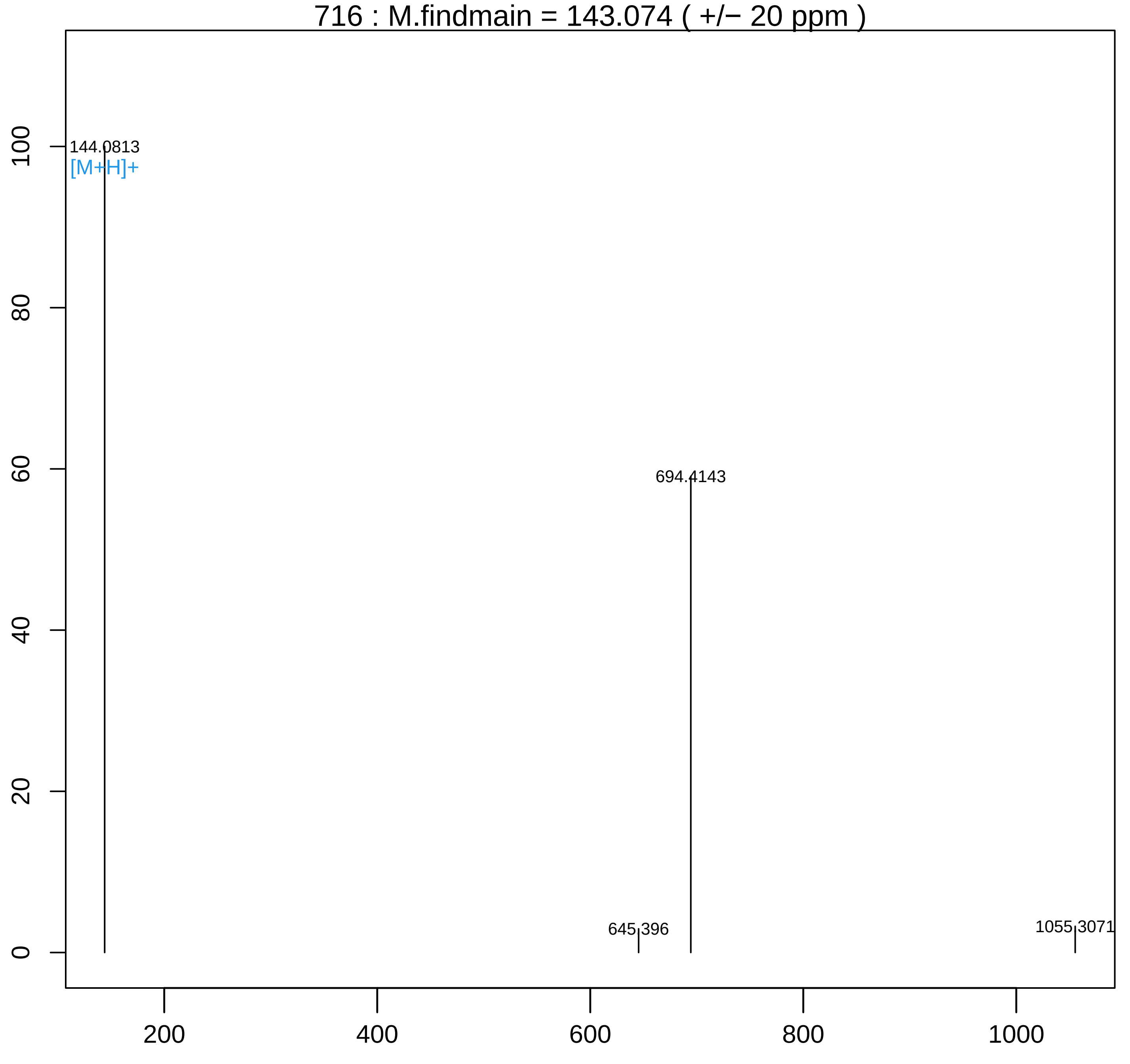
716 : M.findmain = 143.074 (+/− 20 ppm)
144.0813
[M+H]+
694.4143
645.396
1055.3071
0
20
40
60
80
100
200
400
600
800
1000

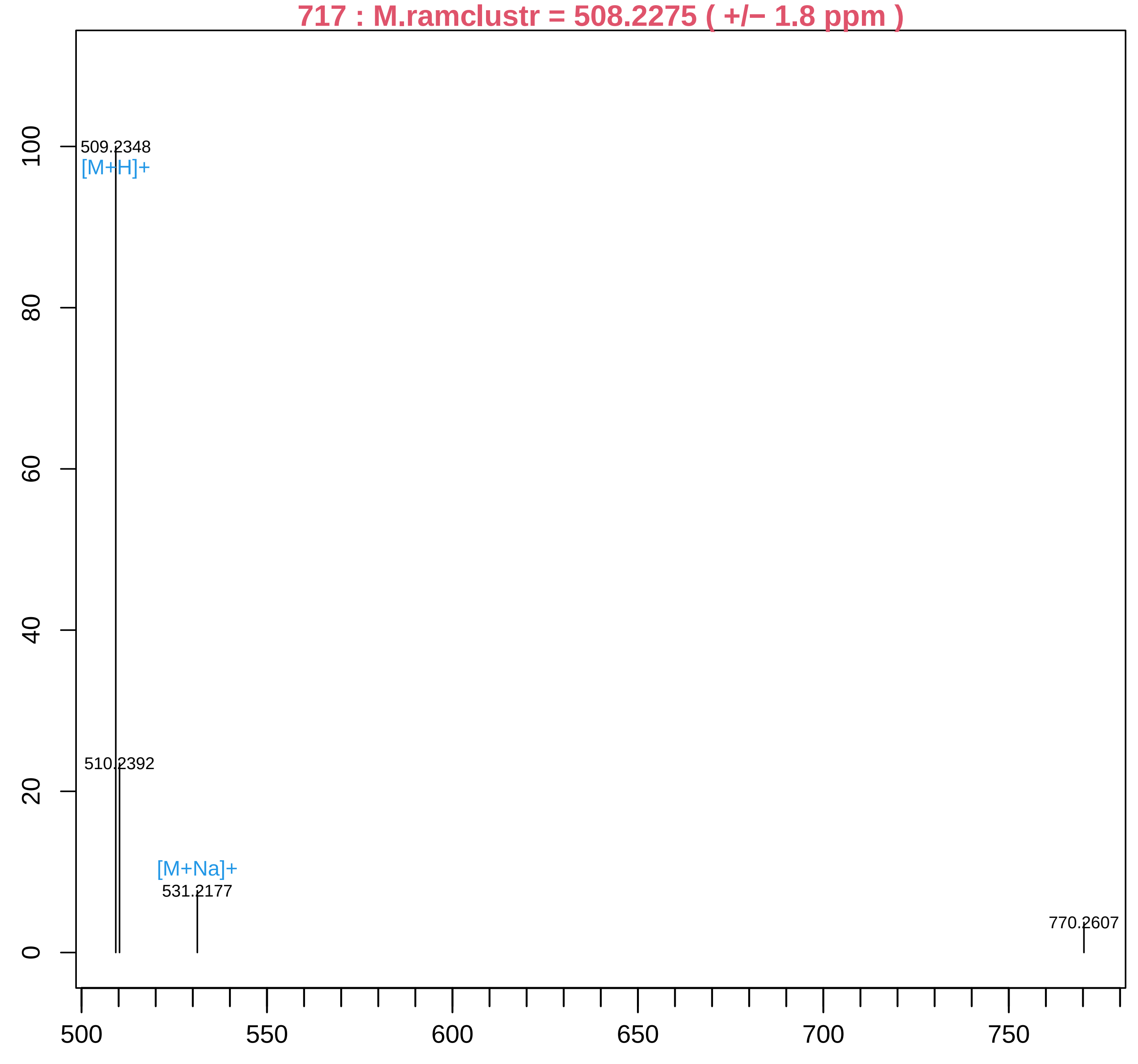
717 : M.ramclustr = 508.2275 (+/− 1.8 ppm)
509.2348
[M+H]+
510.2392
[M+Na]+
531.2177
770.2607
0
20
40
60
80
100
500
550
600
650
700
750

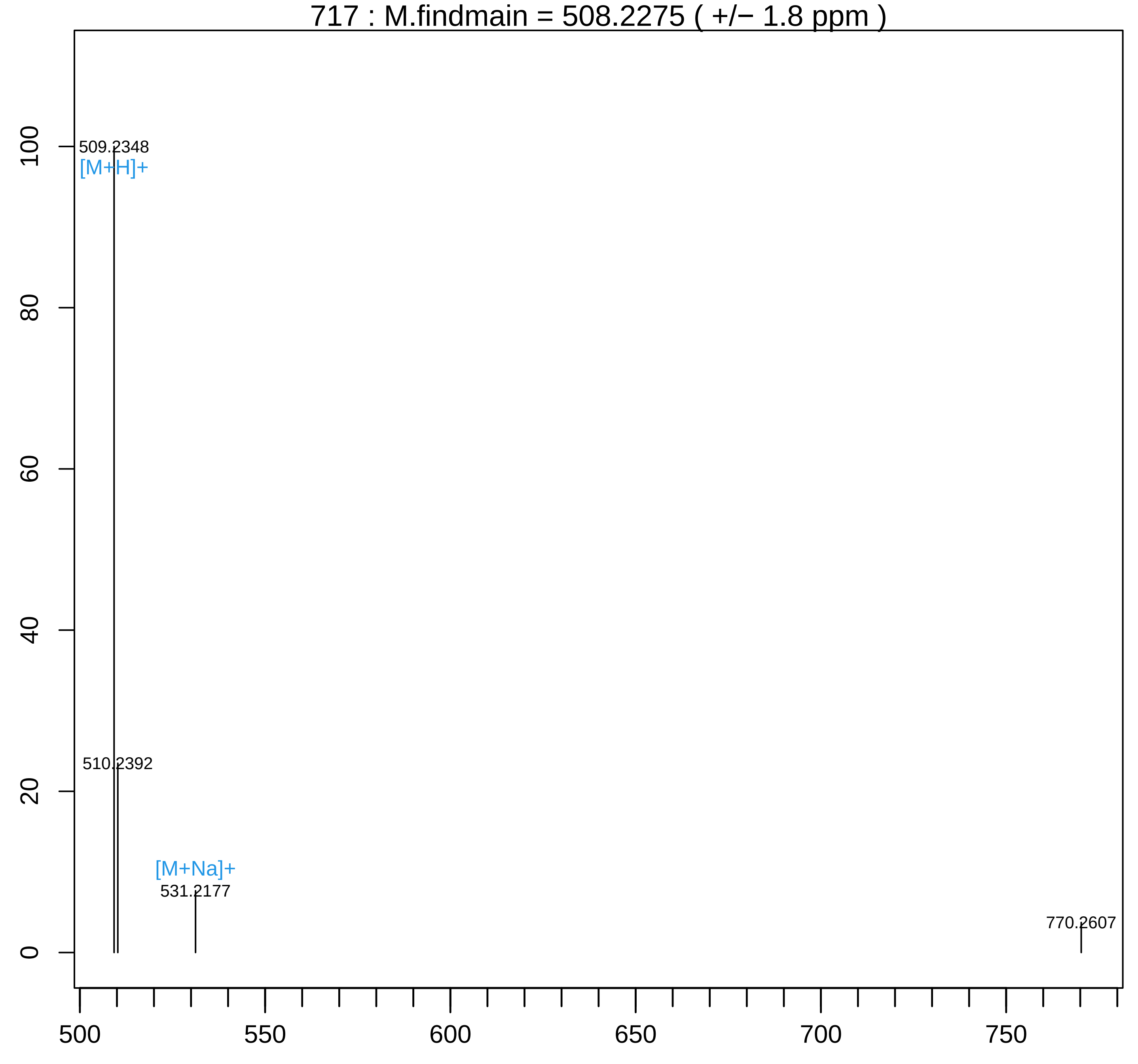
717 : M.findmain = 508.2275 (+/− 1.8 ppm)
509.2348
[M+H]+
510.2392
[M+Na]+
531.2177
770.2607
0
20
40
60
80
100
500
550
600
650
700
750

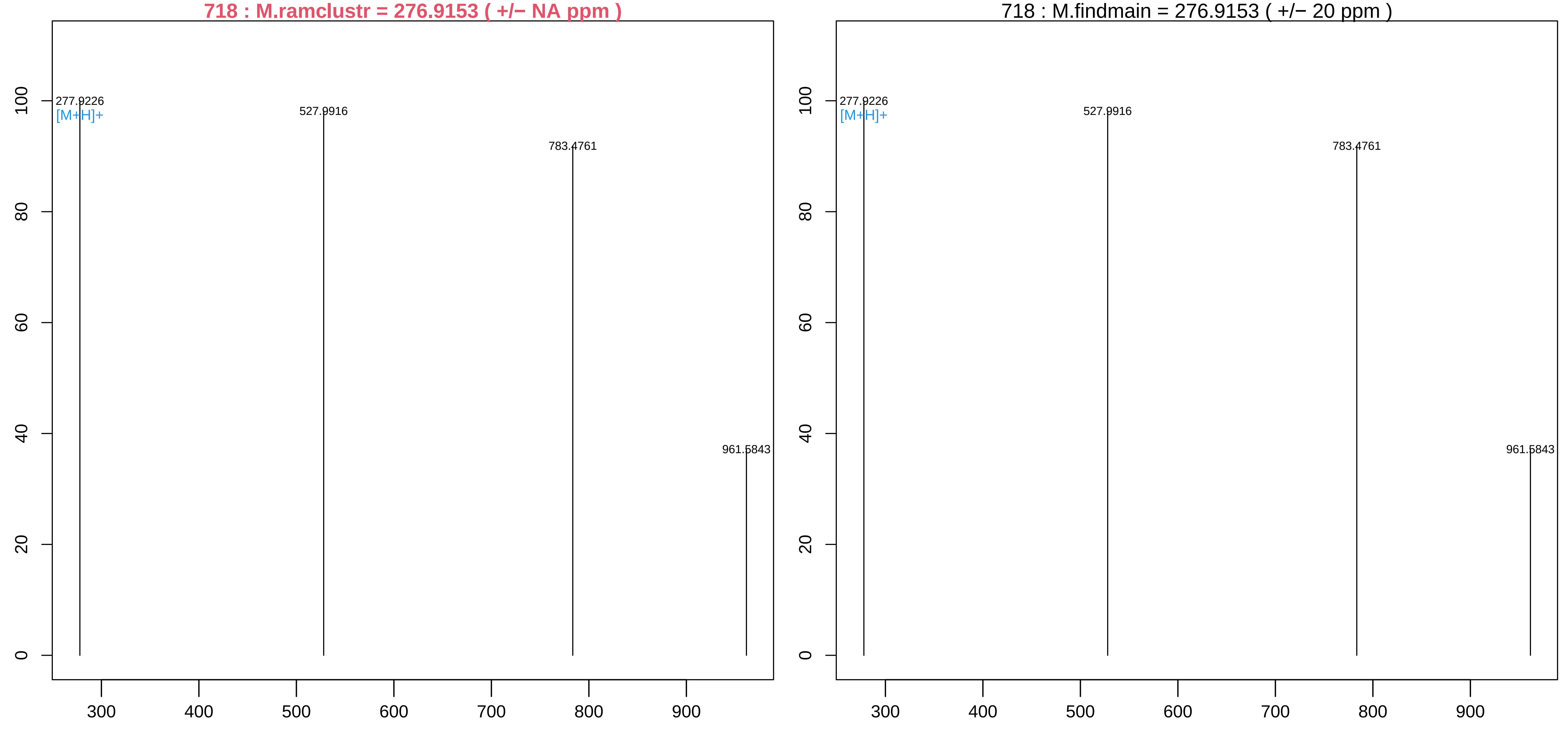
718 : M.ramclustr = 276.9153 (+/− NA ppm)
277.9226
[M+H]+
527.9916
783.4761
961.5843
718 : M.findmain = 276.9153 (+/− 20 ppm)
277.9226
[M+H]+
527.9916
783.4761
961.5843
0
20
40
60
80
100
300
400
500
600
700
800
900

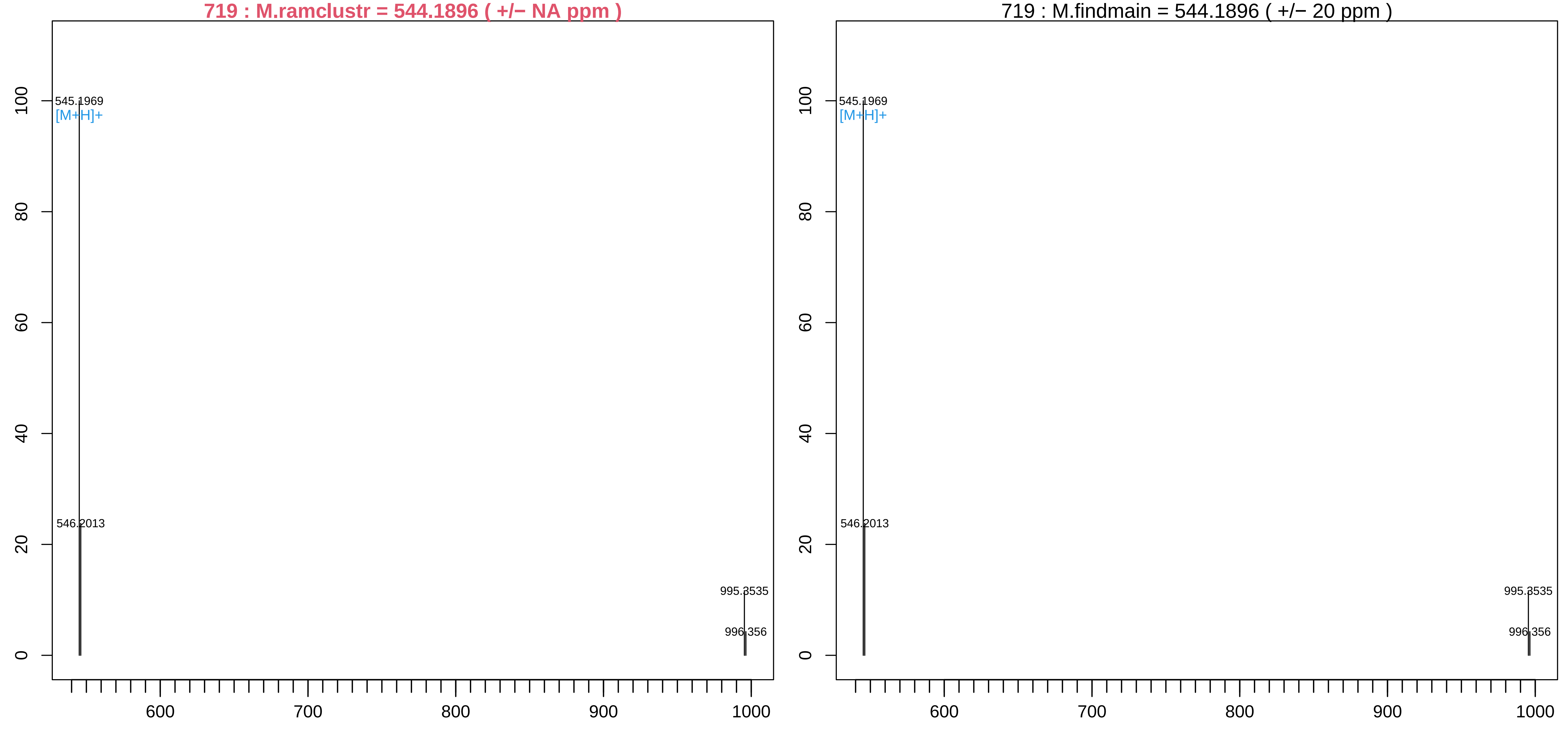
719 : M.ramclustr = 544.1896 (+/− NA ppm)
545.1969
[M+H]+
546.2013
995.3535
996.356
719 : M.findmain = 544.1896 (+/− 20 ppm)
545.1969
[M+H]+
546.2013
995.3535
996.356
0
20
40
60
80
100
600
700
800
900
1000

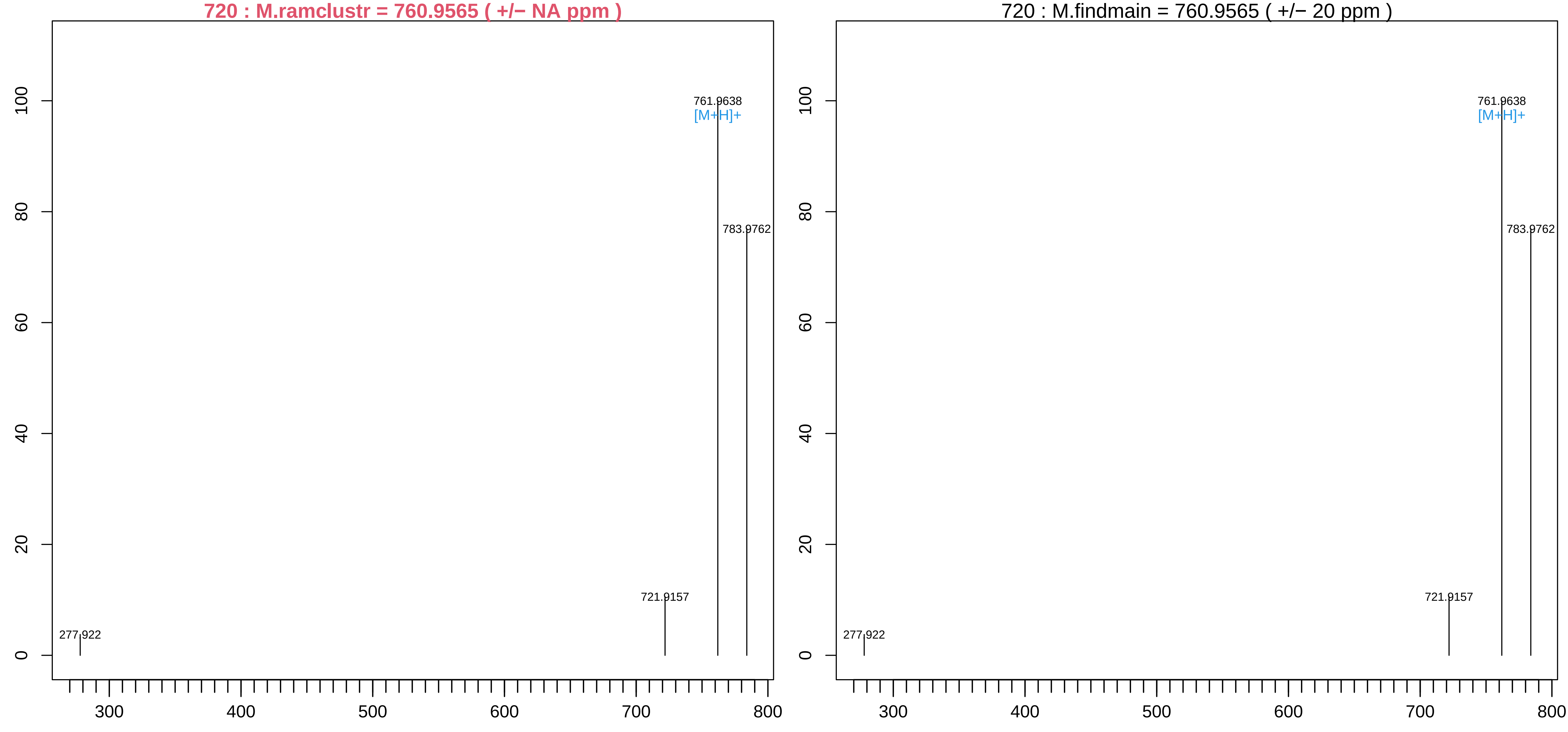
720 : M.ramclustr = 760.9565 (+/− NA ppm)
761.9638
[M+H]+
783.9762
721.9157
277.922
720 : M.findmain = 760.9565 (+/− 20 ppm)
761.9638
[M+H]+
783.9762
721.9157
277.922

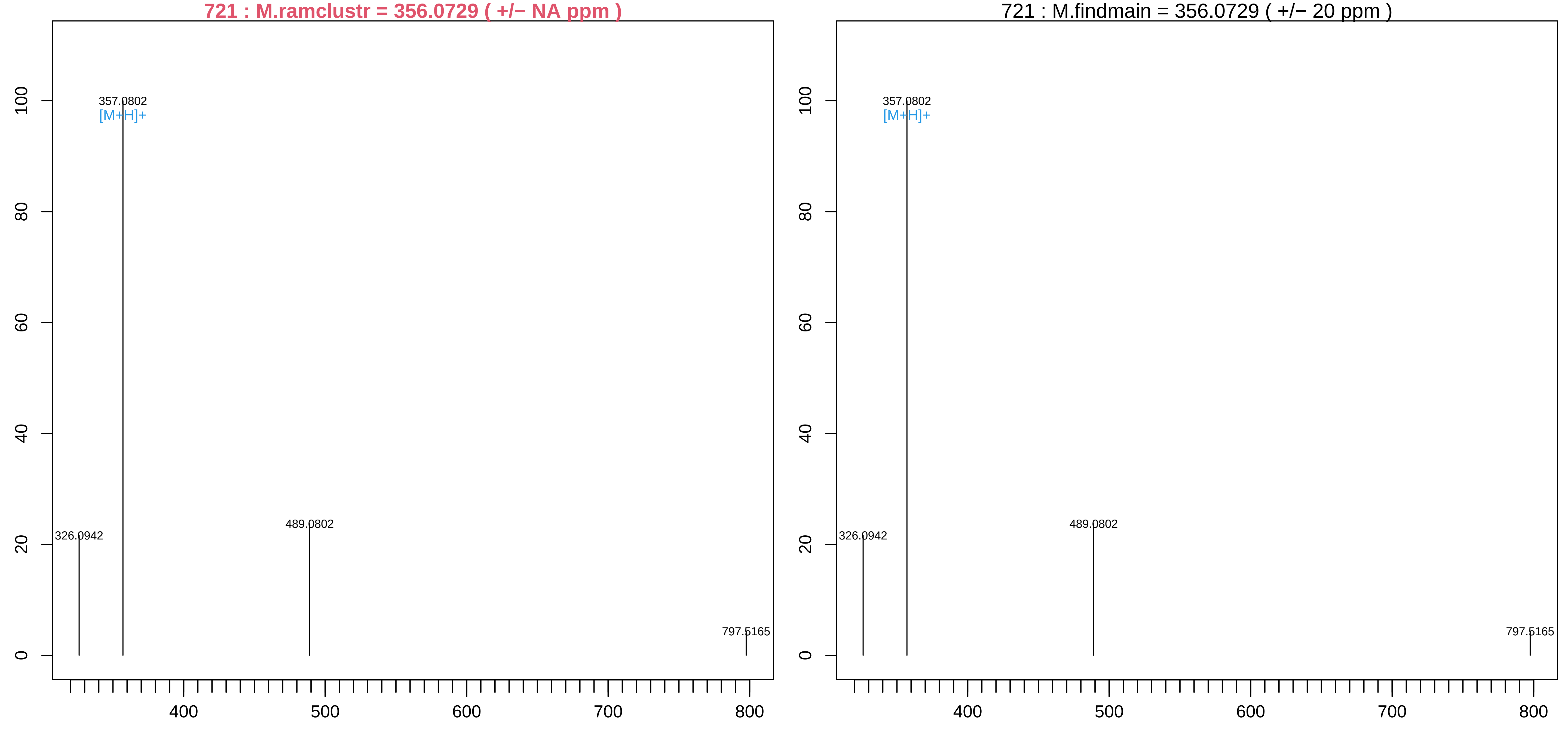

721 : M.ramclustr = 356.0729 (+/− NA ppm)
357.0802
[M+H]+
326.0942
489.0802
797.5165
0
20
40
60
80
100
400
500
600
700
800
721 : M.findmain = 356.0729 (+/− 20 ppm)
357.0802
[M+H]+
326.0942
489.0802
797.5165
0
20
40
60
80
100
400
500
600
700
800

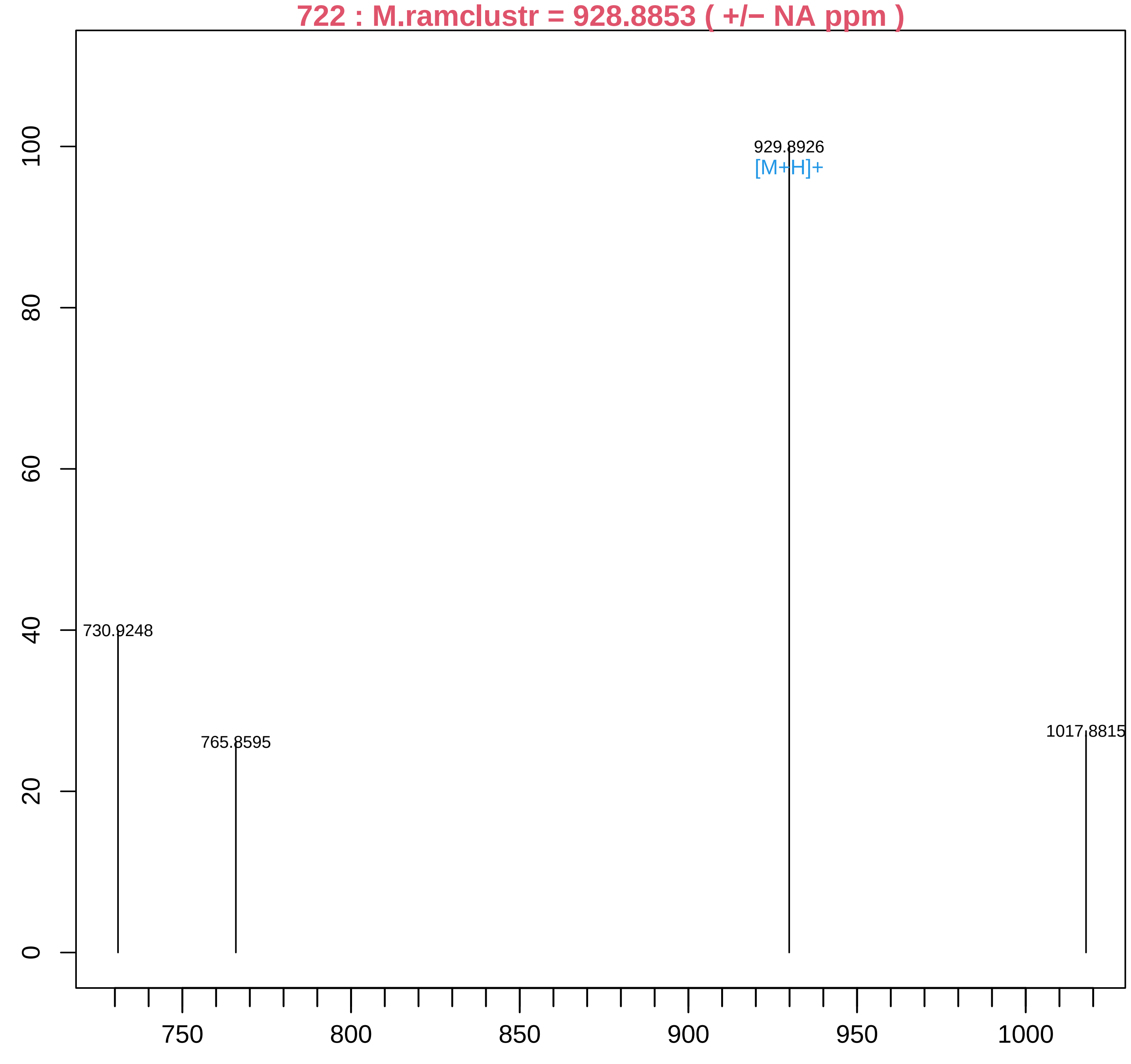

722 : M.ramclustr = 928.8853 (+/− NA ppm)
0
20
40
60
80
100
750
800
850
900
950
1000
730.9248
765.8595
929.8926
[M+H]+
1017.8815

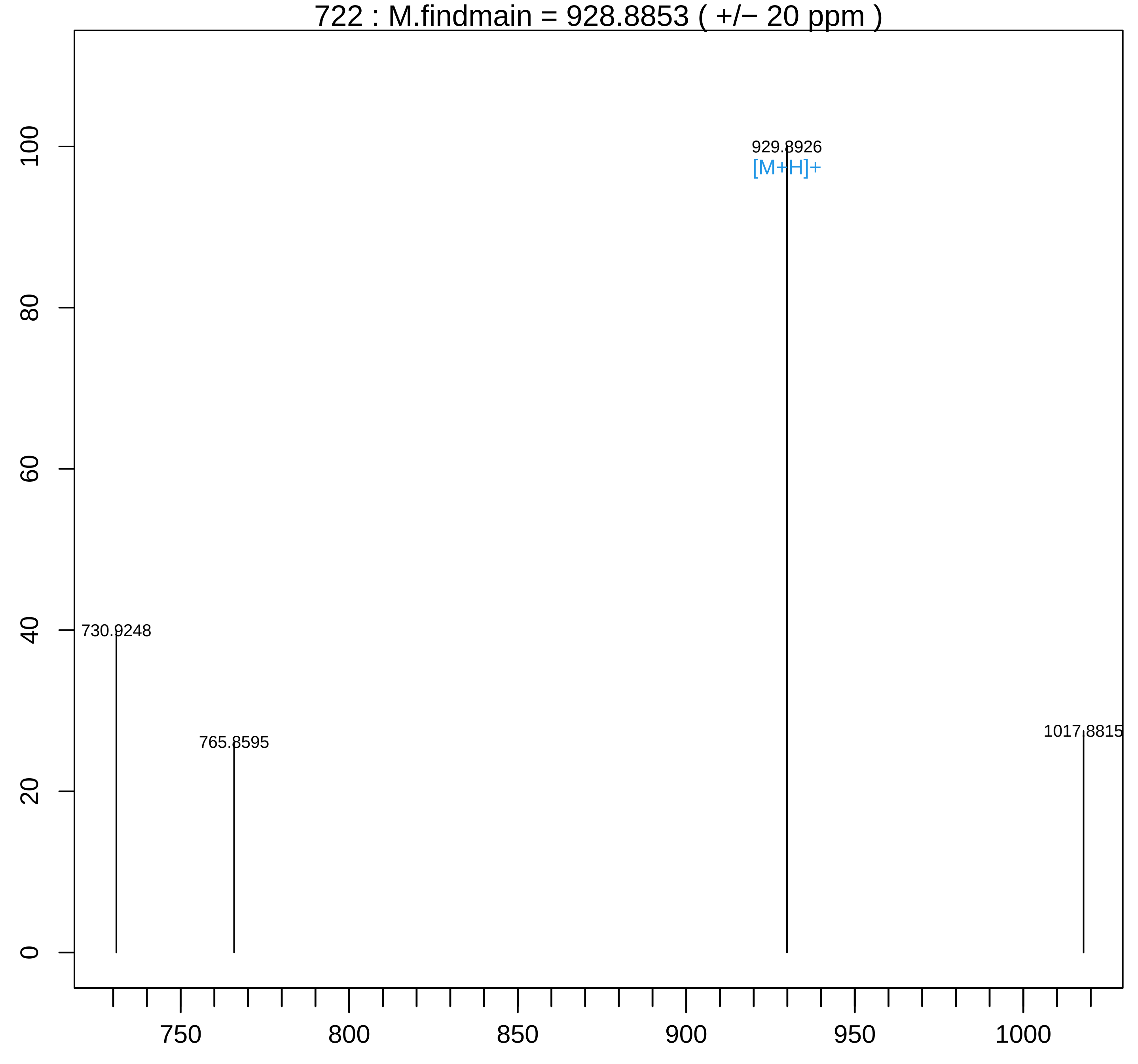

722 : M.findmain = 928.8853 (+/− 20 ppm)
0
20
40
60
80
100
750
800
850
900
950
1000
730.9248
765.8595
929.8926
[M+H]+
1017.8815

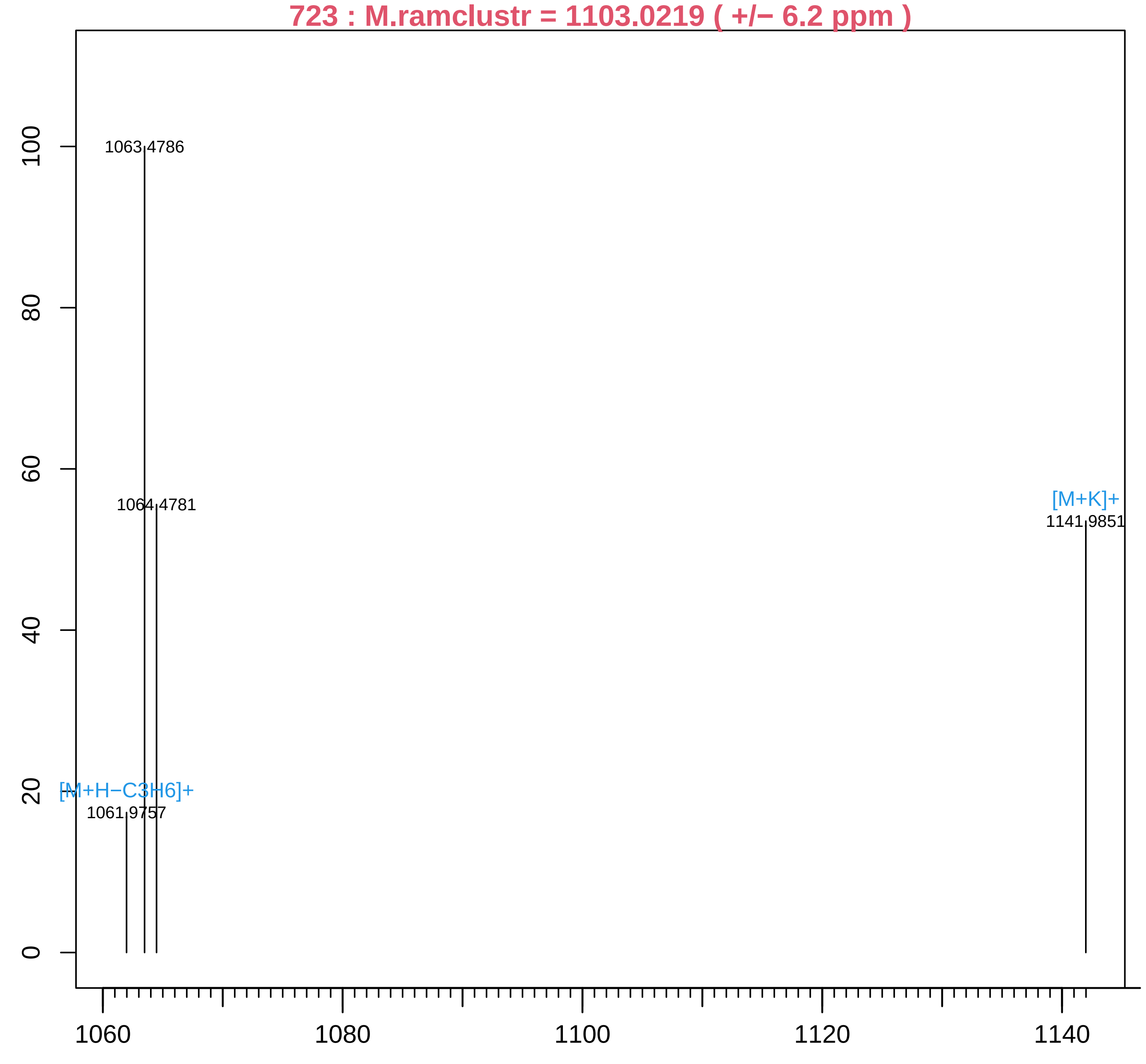
723 : M.ramclustr = 1103.0219 (+/− 6.2 ppm)
1063.4786
1064.4781
[M+K]+
1141.9851
[M+H−C3H6]+
1061.9757
0
20
40
60
80
100
1060
1080
1100
1120
1140

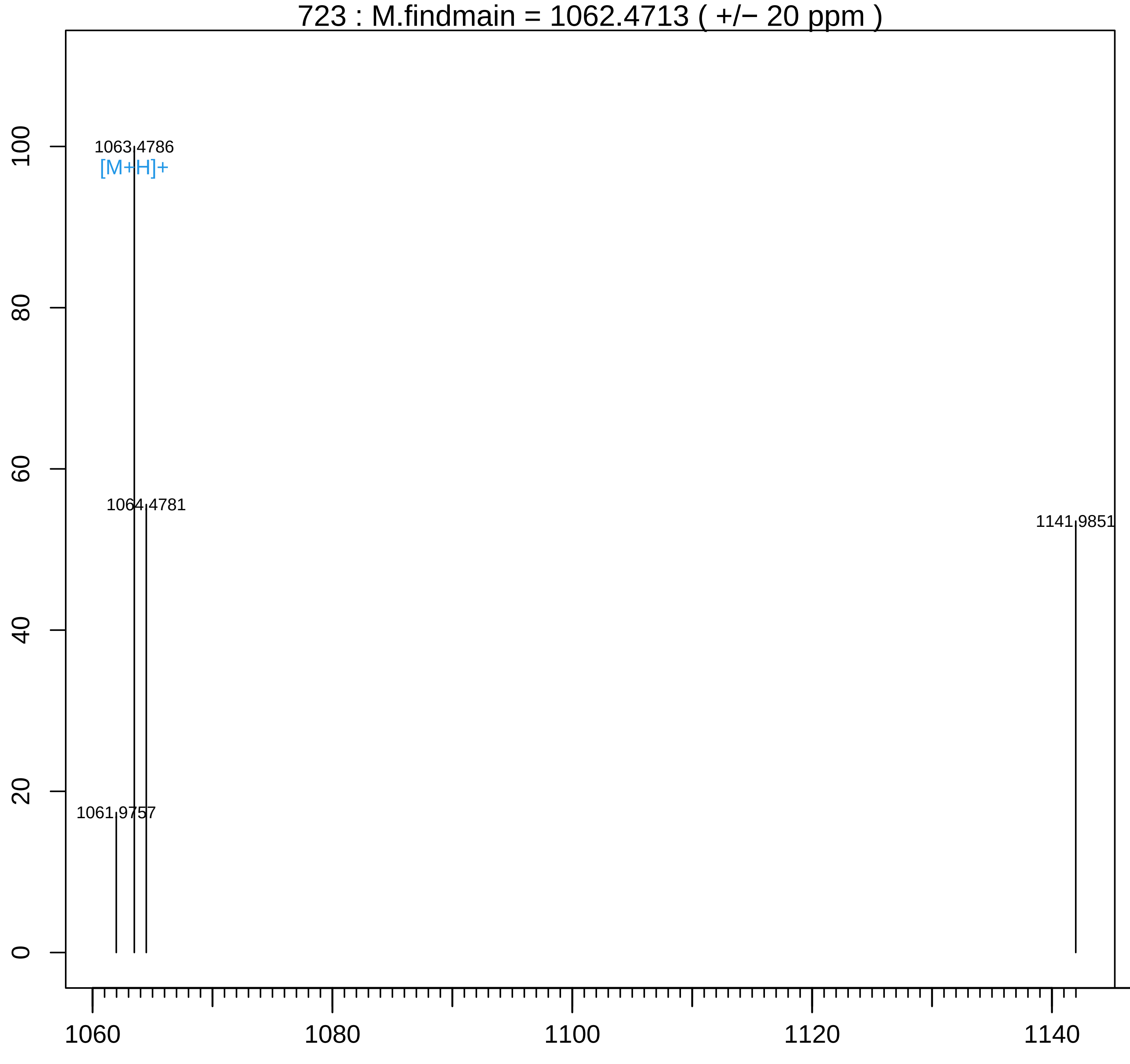
723 : M.findmain = 1062.4713 (+/− 20 ppm)
1063.4786
[M+H]+
1064.4781
1141.9851
1061.9757
0
20
40
60
80
100
1060
1080
1100
1120
1140

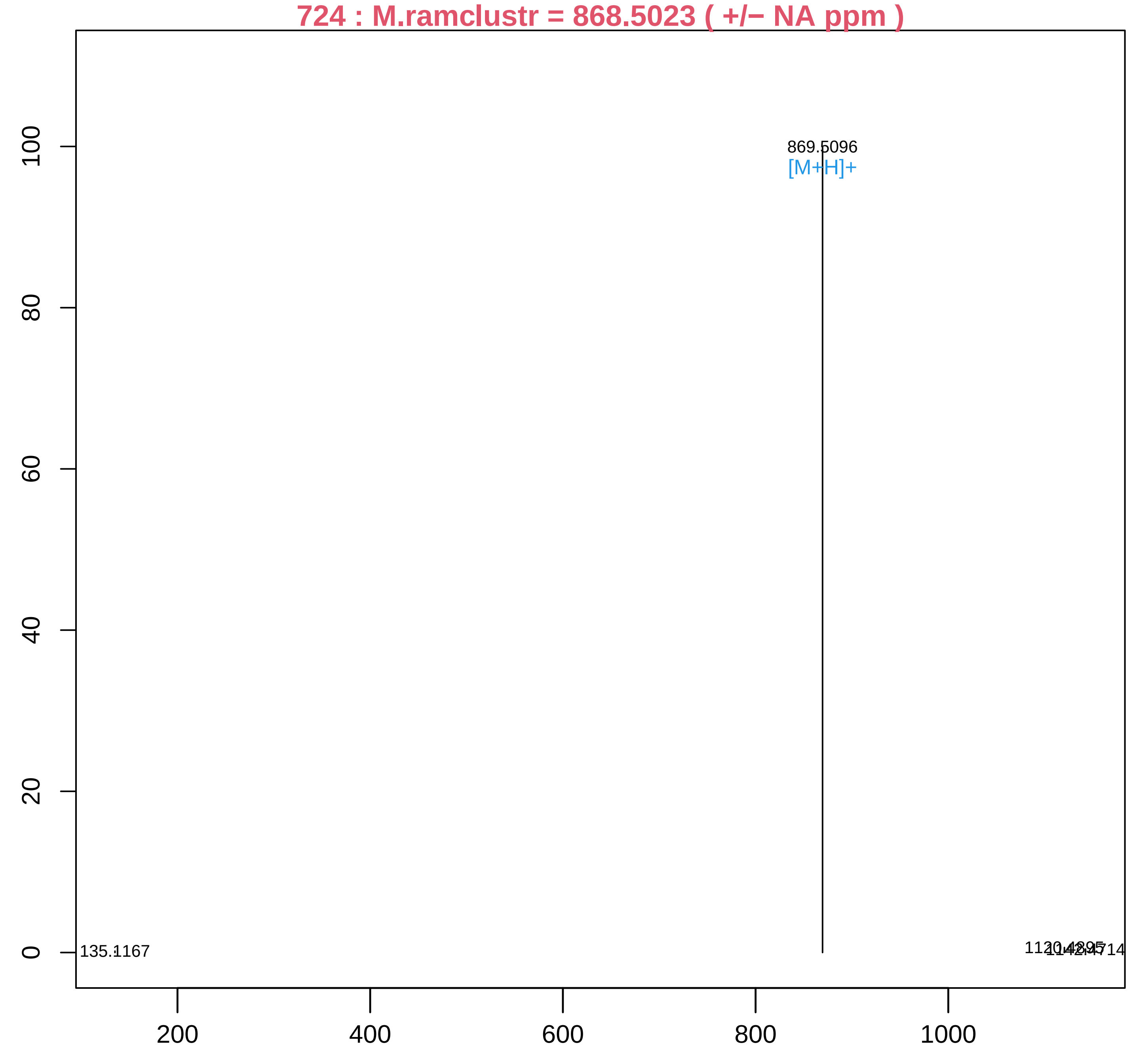
724 : M.ramclustr = 868.5023 (+/− NA ppm)
869.5096
[M+H]+
135.1167
1120.4895
1142.4714
0
20
40
60
80
100
200
400
600
800
1000
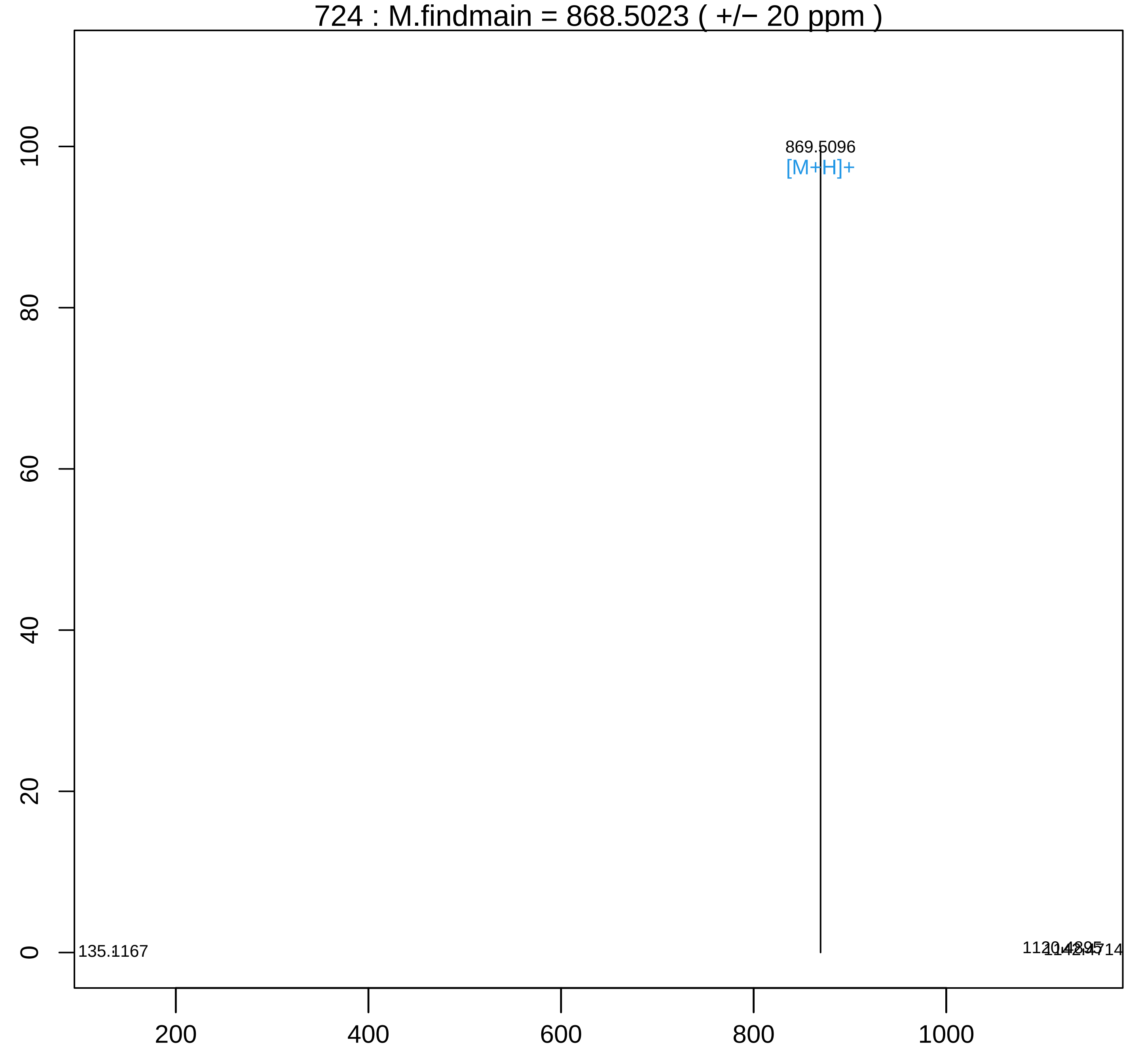
724 : M.findmain = 868.5023 (+/− 20 ppm)
869.5096
[M+H]+
135.1167
1120.4895
1142.4714
0
20
40
60
80
100
200
400
600
800
1000

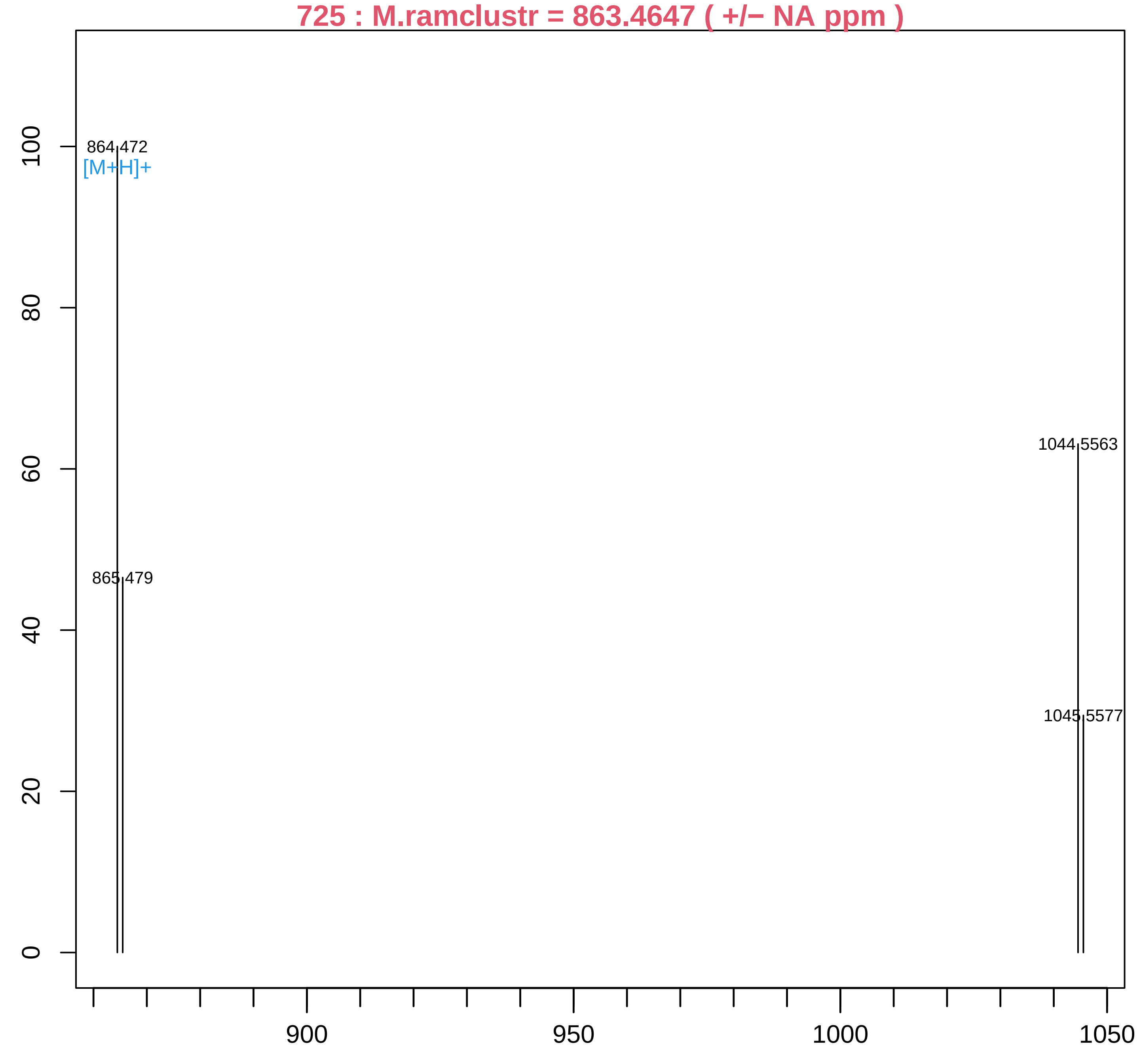
725 : M.ramclustr = 863.4647 (+/− NA ppm)
864.472
[M+H]+
865.479
1044.5563
1045.5577
0
20
40
60
80
100
900
950
1000
1050

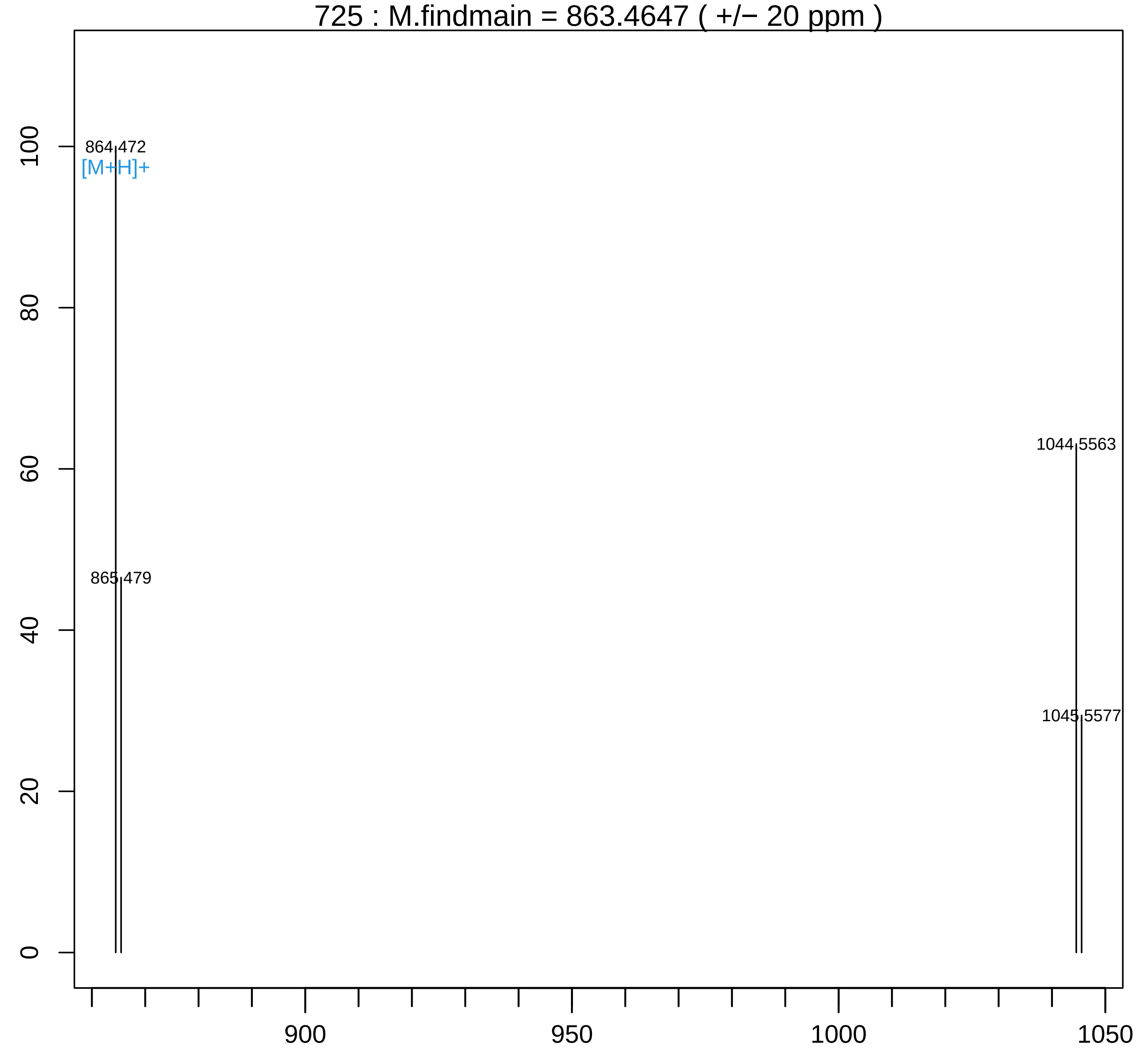
725 : M.findmain = 863.4647 (+/− 20 ppm)
864.472
[M+H]+
865.479
1044.5563
1045.5577
0
20
40
60
80
100
900
950
1000
1050

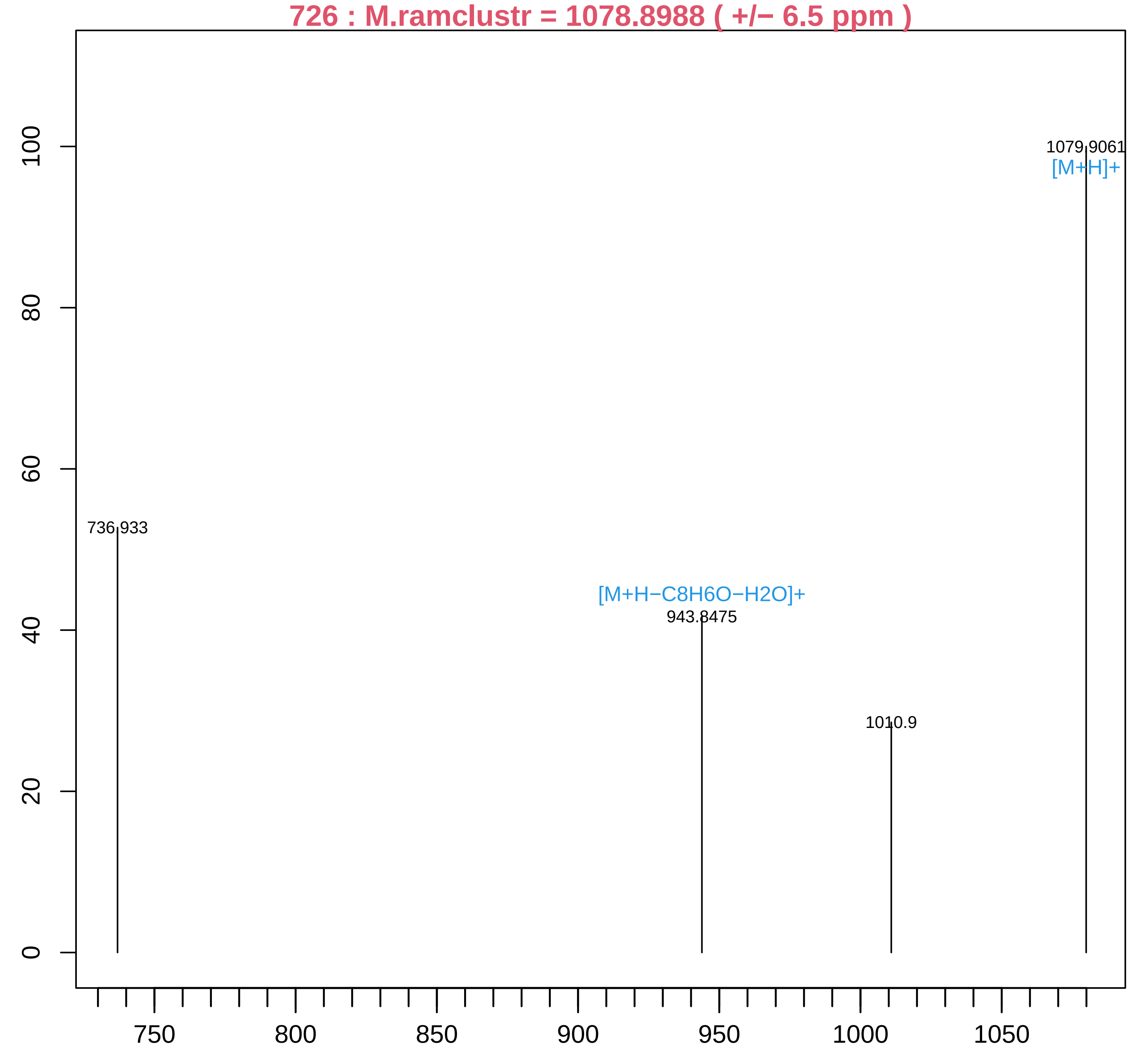

726 : M.ramclustr = 1078.8988 (+/− 6.5 ppm)
1079.9061
[M+H]+
736.933
[M+H−C8H6O−H2O]+
943.8475
1010.9
0
20
40
60
80
100
750
800
850
900
950
1000
1050

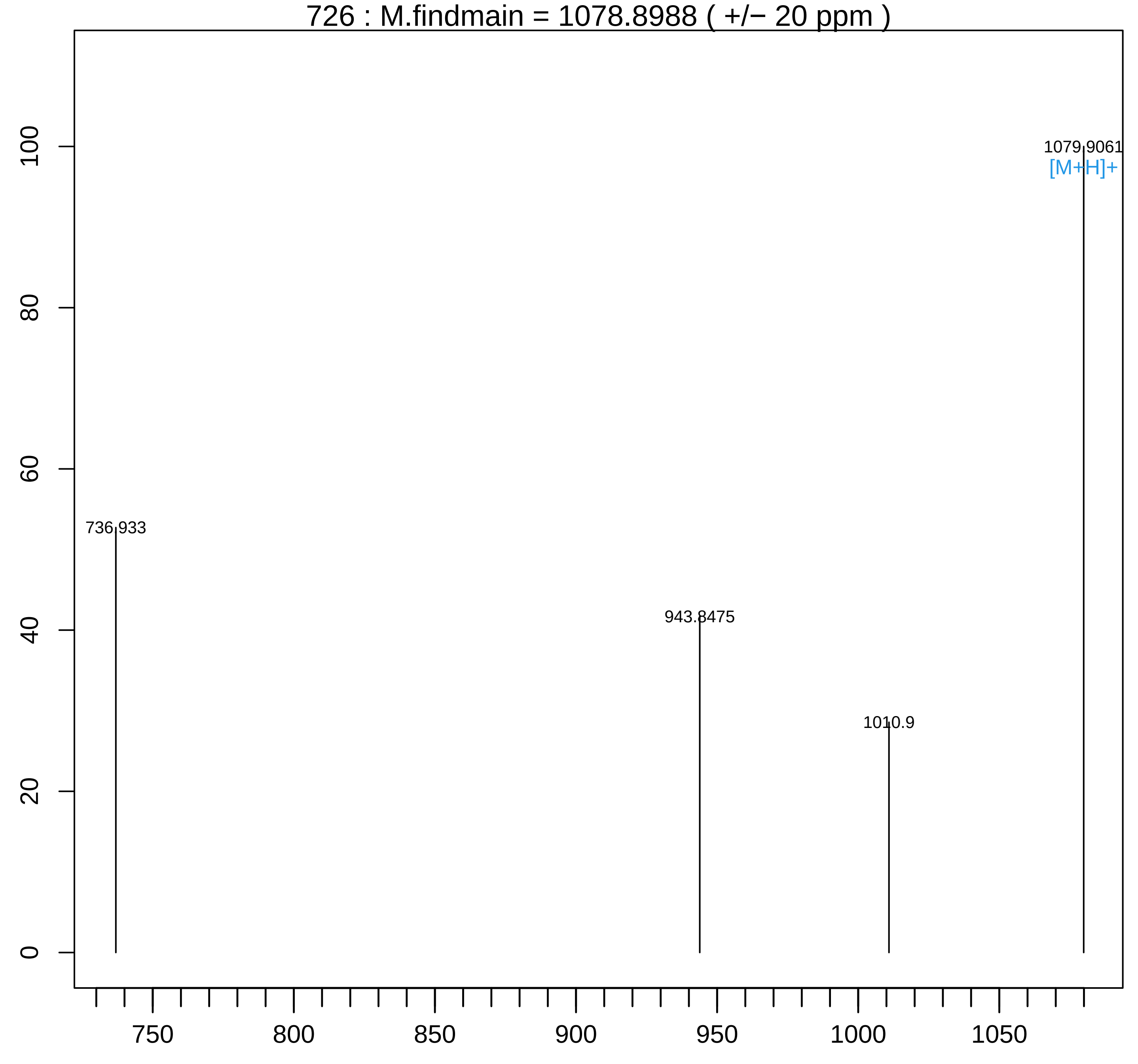

726 : M.findmain = 1078.8988 (+/− 20 ppm)
1079.9061
[M+H]+
736.933
943.8475
1010.9
0
20
40
60
80
100
750
800
850
900
950
1000
1050

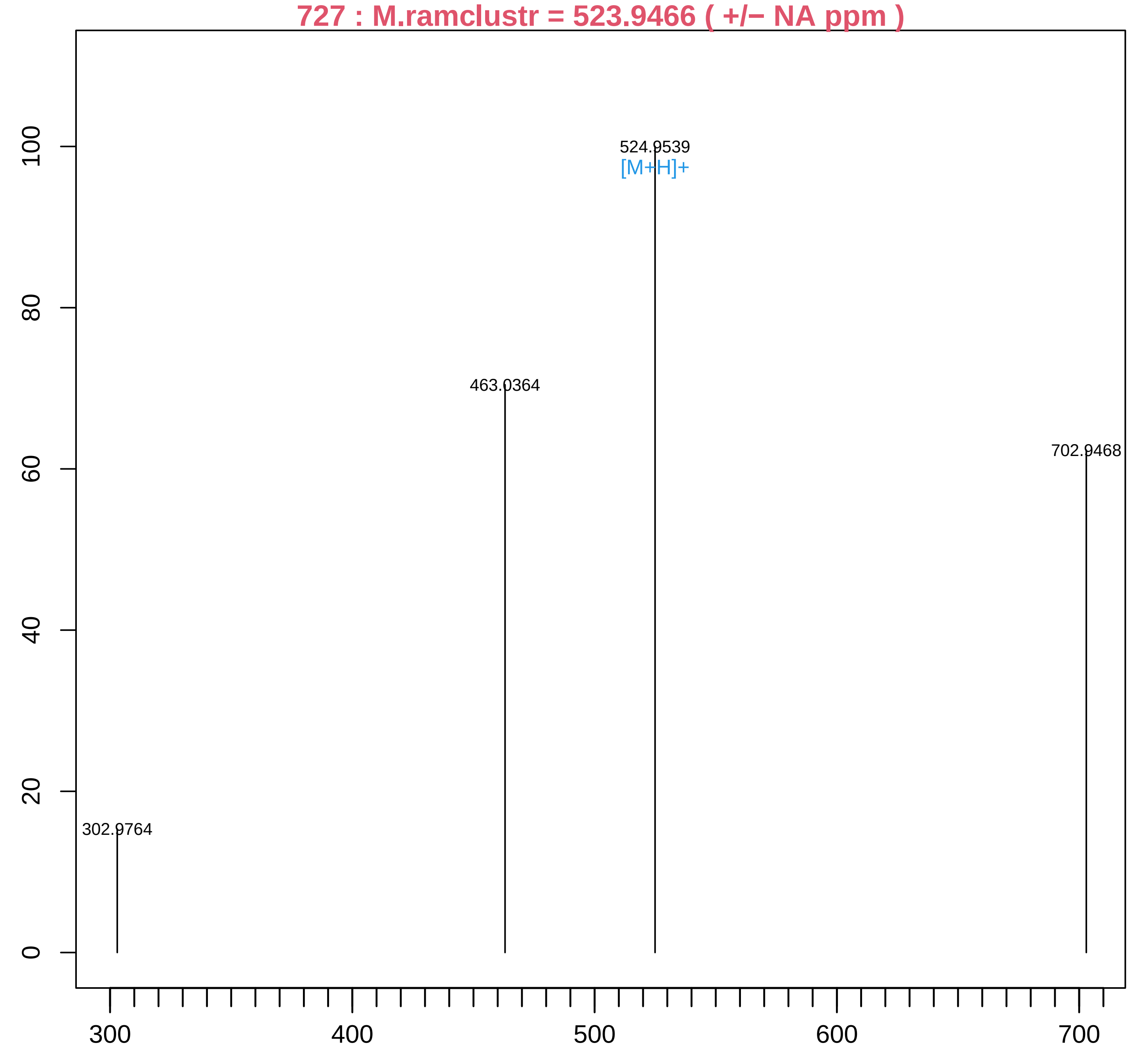
727 : M.ramclustr = 523.9466 (+/− NA ppm)
524.9539
[M+H]+
463.0364
702.9468
302.9764
0
20
40
60
80
100
300
400
500
600
700

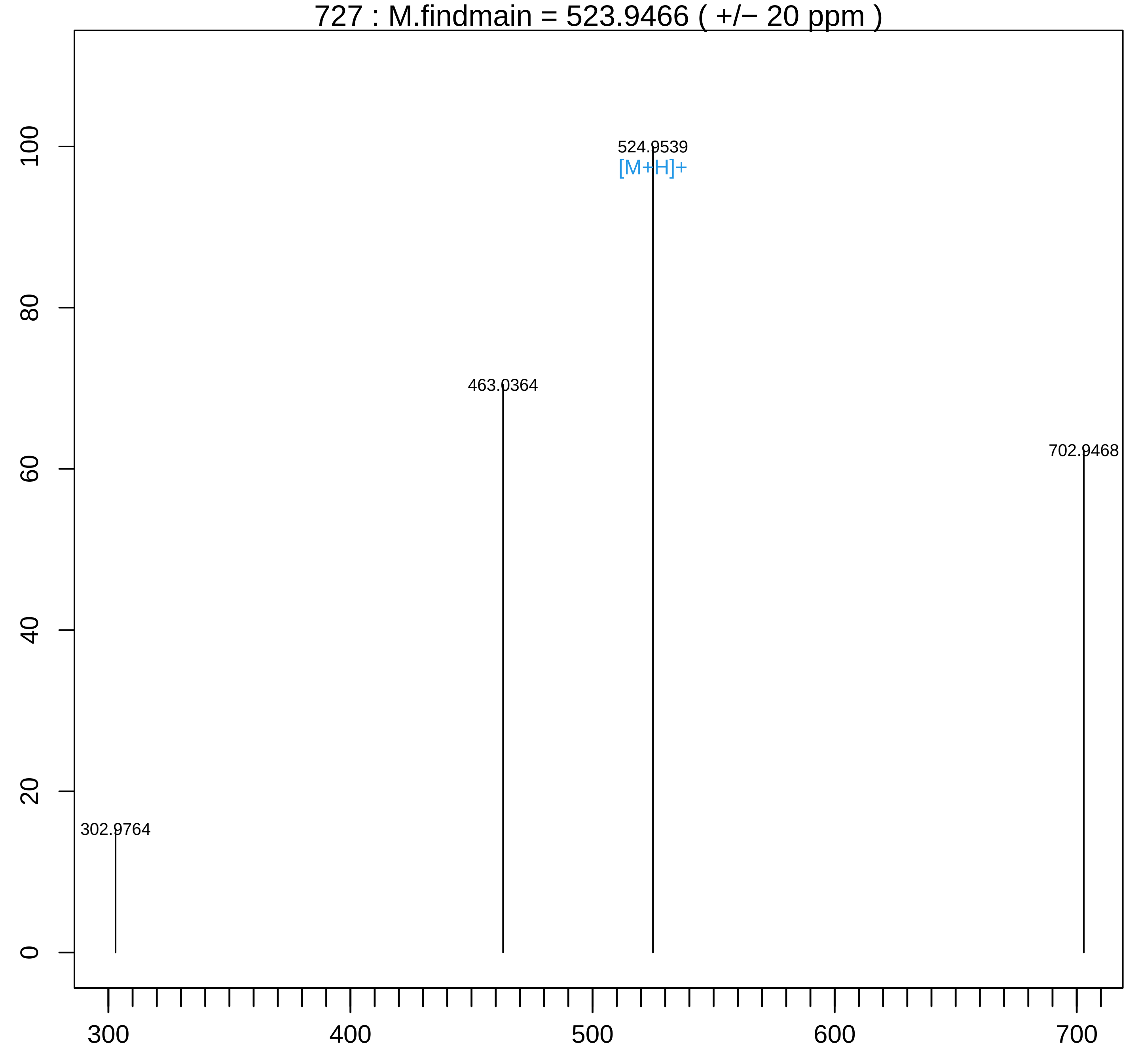
727 : M.findmain = 523.9466 (+/− 20 ppm)
524.9539
[M+H]+
463.0364
702.9468
302.9764
0
20
40
60
80
100
300
400
500
600
700

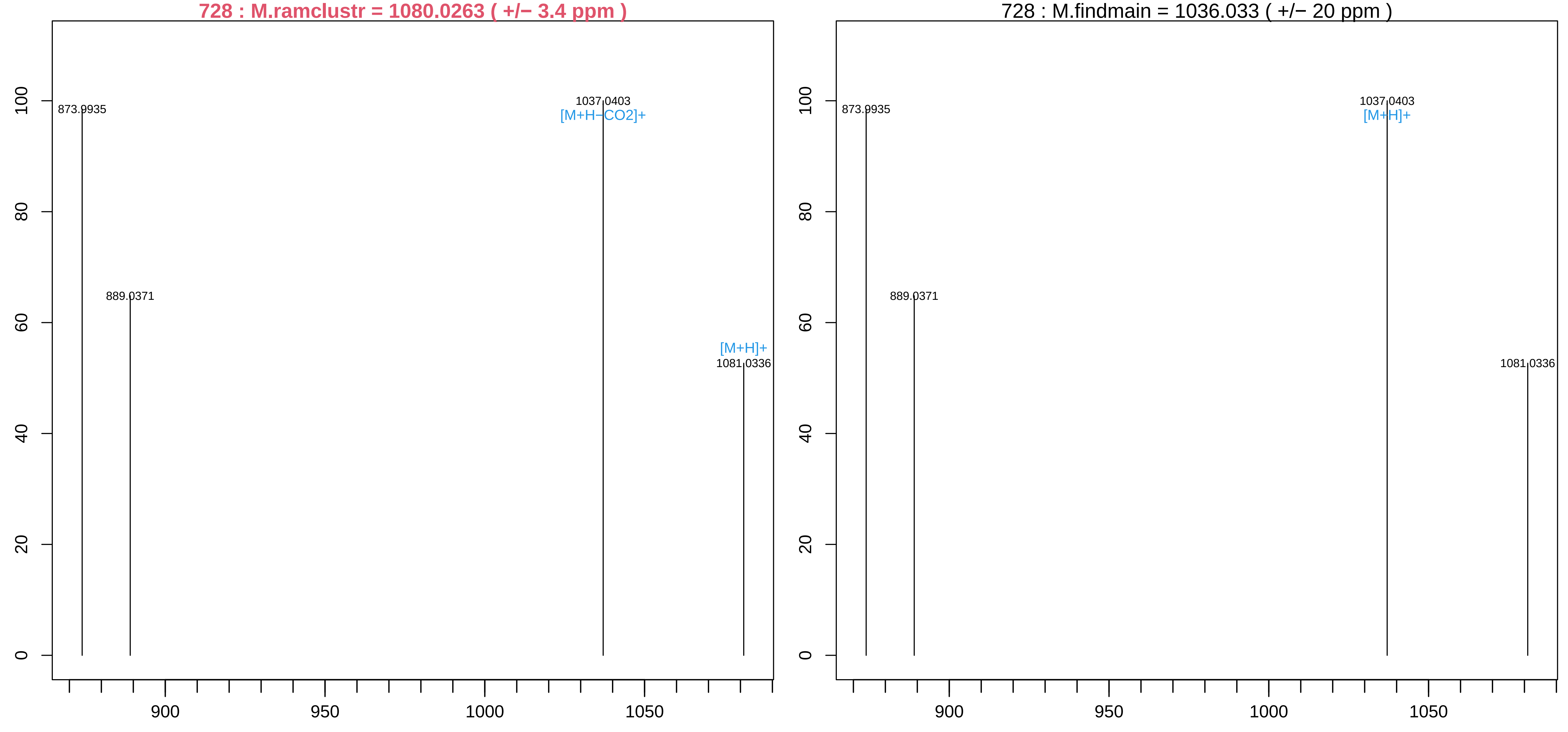

728 : M.ramclustr = 1080.0263 (+/− 3.4 ppm)
873.9935
889.0371
1037.0403
[M+H−CO2]+
[M+H]+
1081.0336
0
20
40
60
80
100
900
950
1000
1050
728 : M.findmain = 1036.033 (+/− 20 ppm)
873.9935
889.0371
1037.0403
[M+H]+
1081.0336
0
20
40
60
80
100
900
950
1000
1050

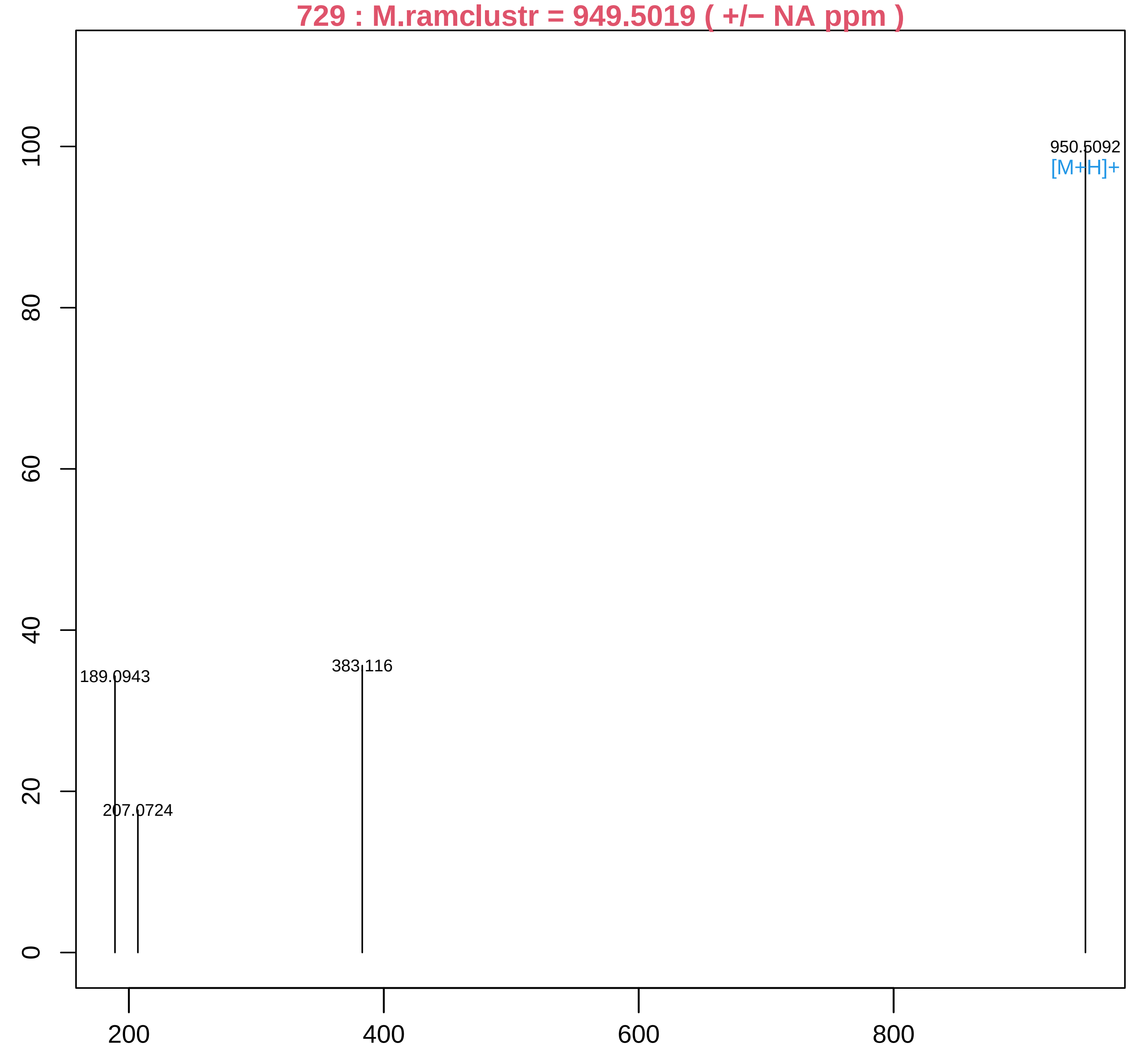
729 : M.ramclustr = 949.5019 (+/− NA ppm)
950.5092
[M+H]+
189.0943
207.0724
383.116
0
20
40
60
80
100
200
400
600
800

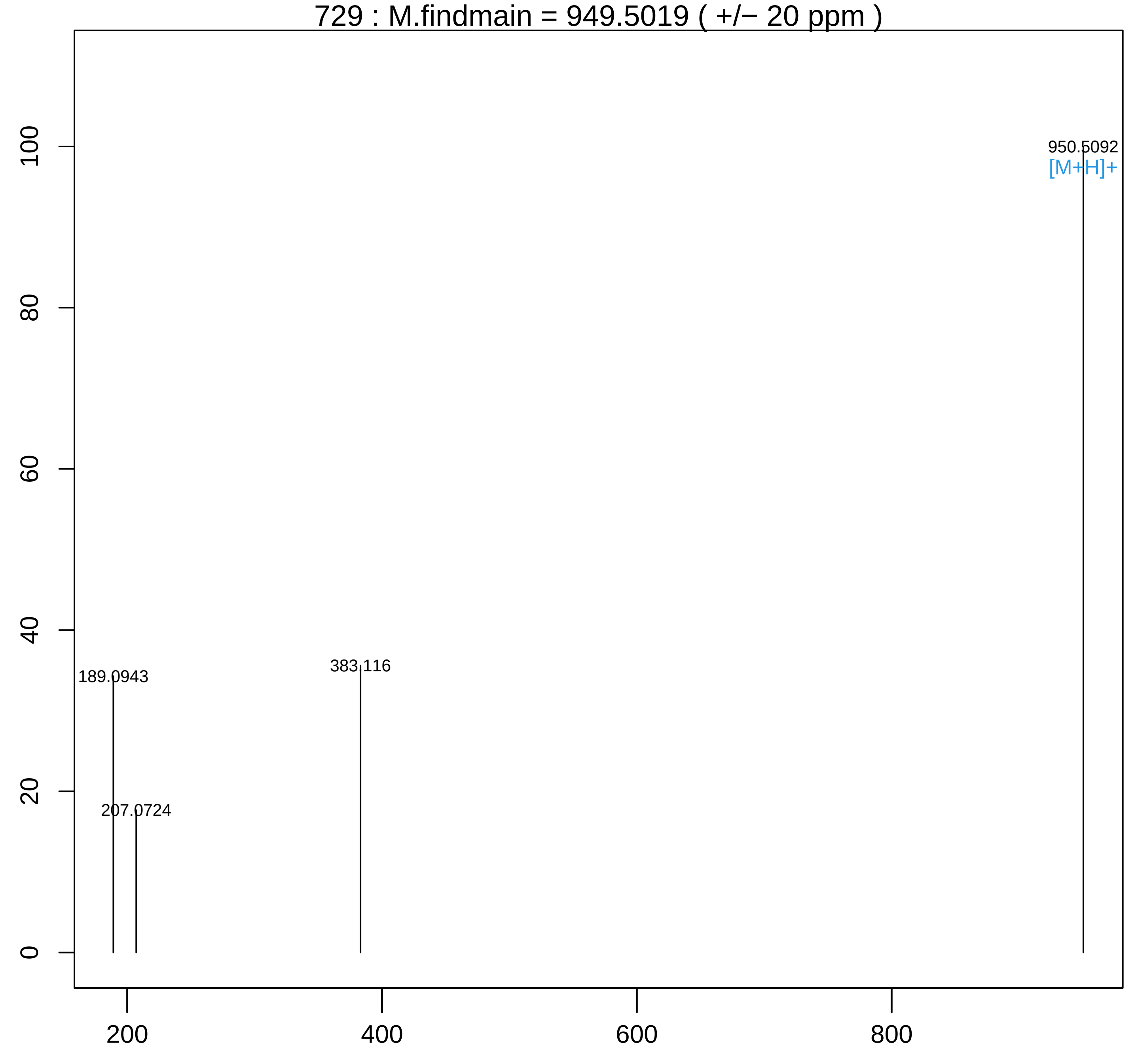
729 : M.findmain = 949.5019 (+/− 20 ppm)
950.5092
[M+H]+
189.0943
207.0724
383.116
0
20
40
60
80
100
200
400
600
800

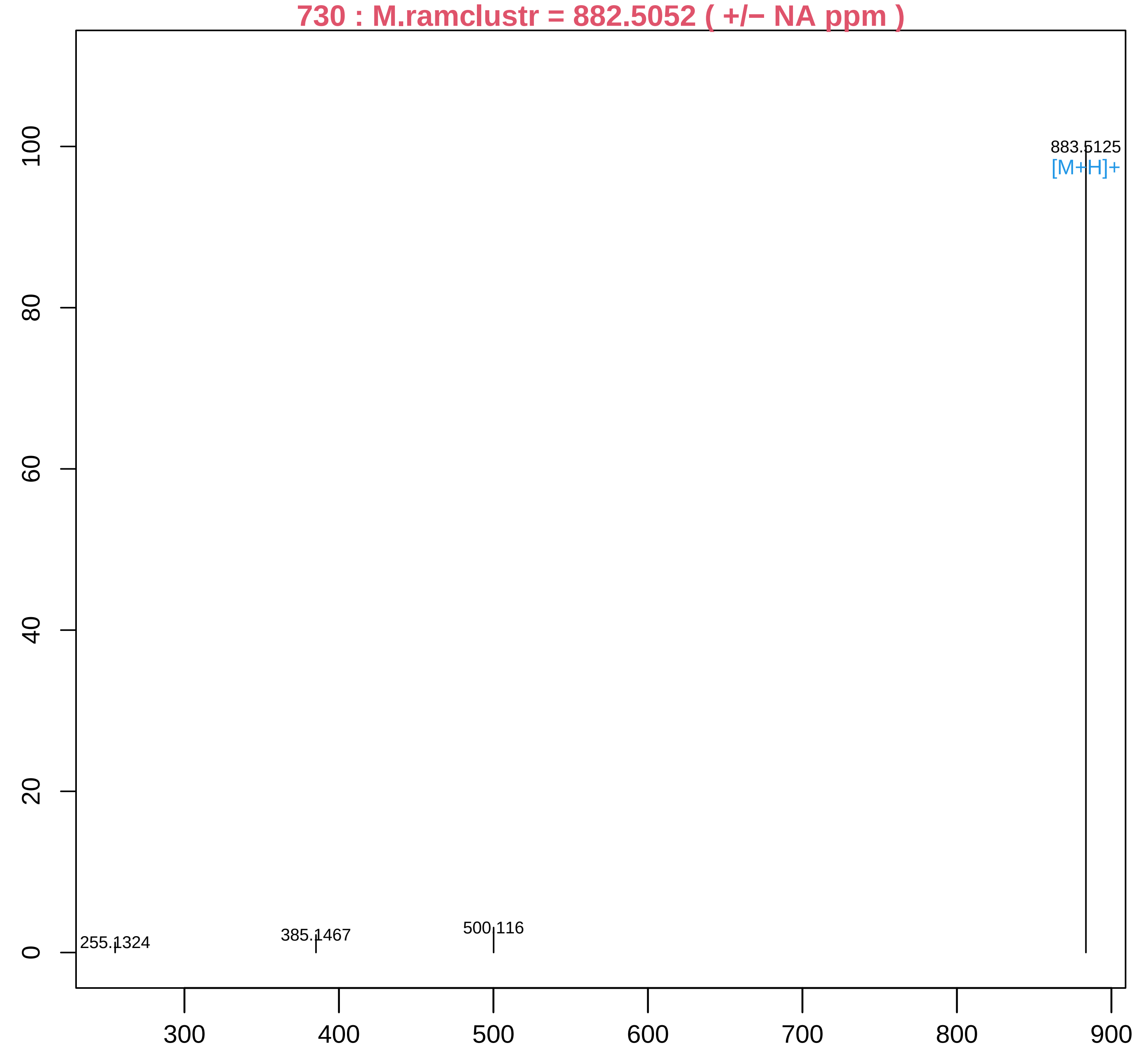
730 : M.ramclustr = 882.5052 (+/− NA ppm)
883.5125
[M+H]+
255.1324
385.1467
500.116
0
20
40
60
80
100
300
400
500
600
700
800
900

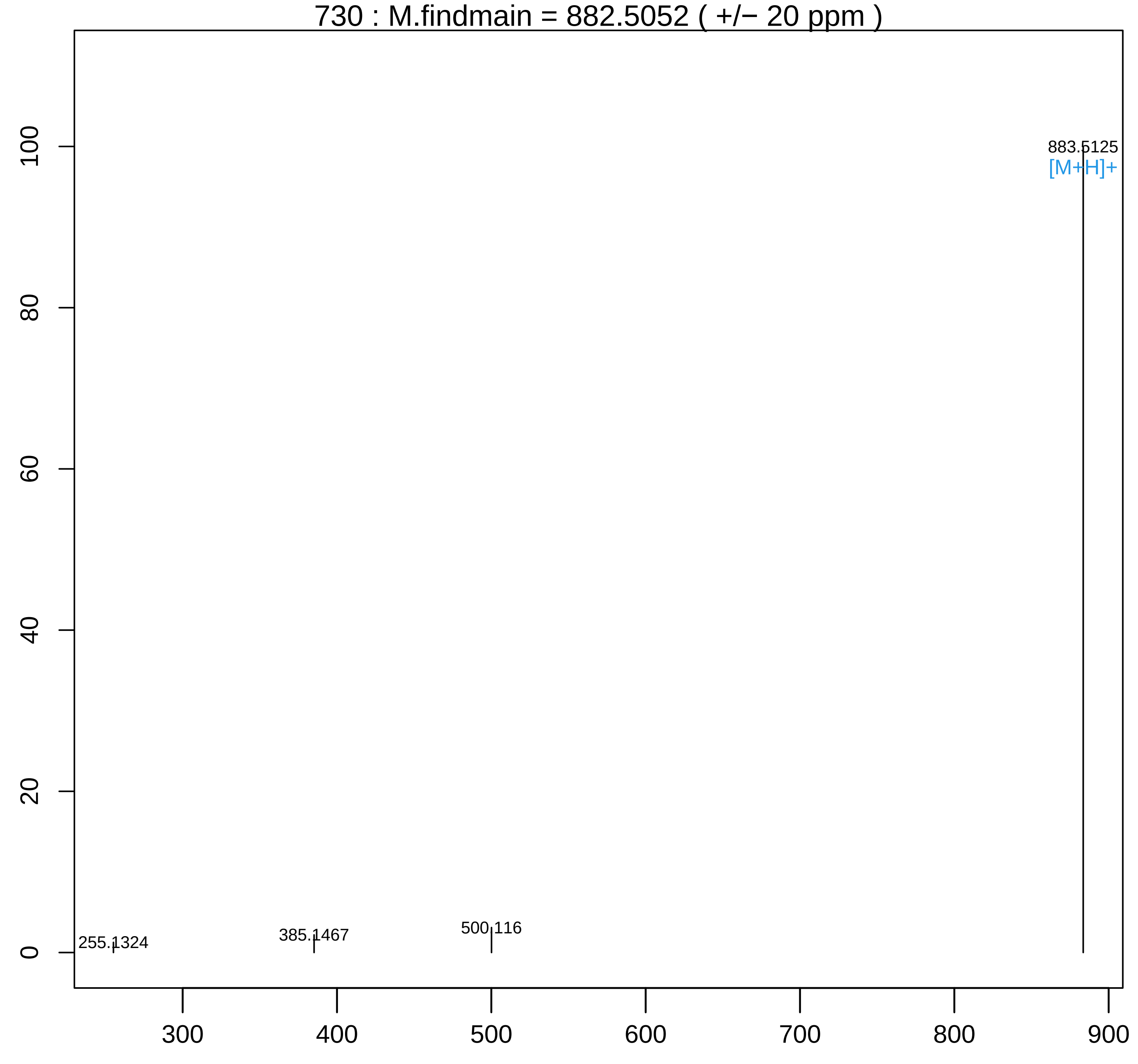
730 : M.findmain = 882.5052 (+/− 20 ppm)
883.5125
[M+H]+
255.1324
385.1467
500.116
0
20
40
60
80
100
300
400
500
600
700
800
900

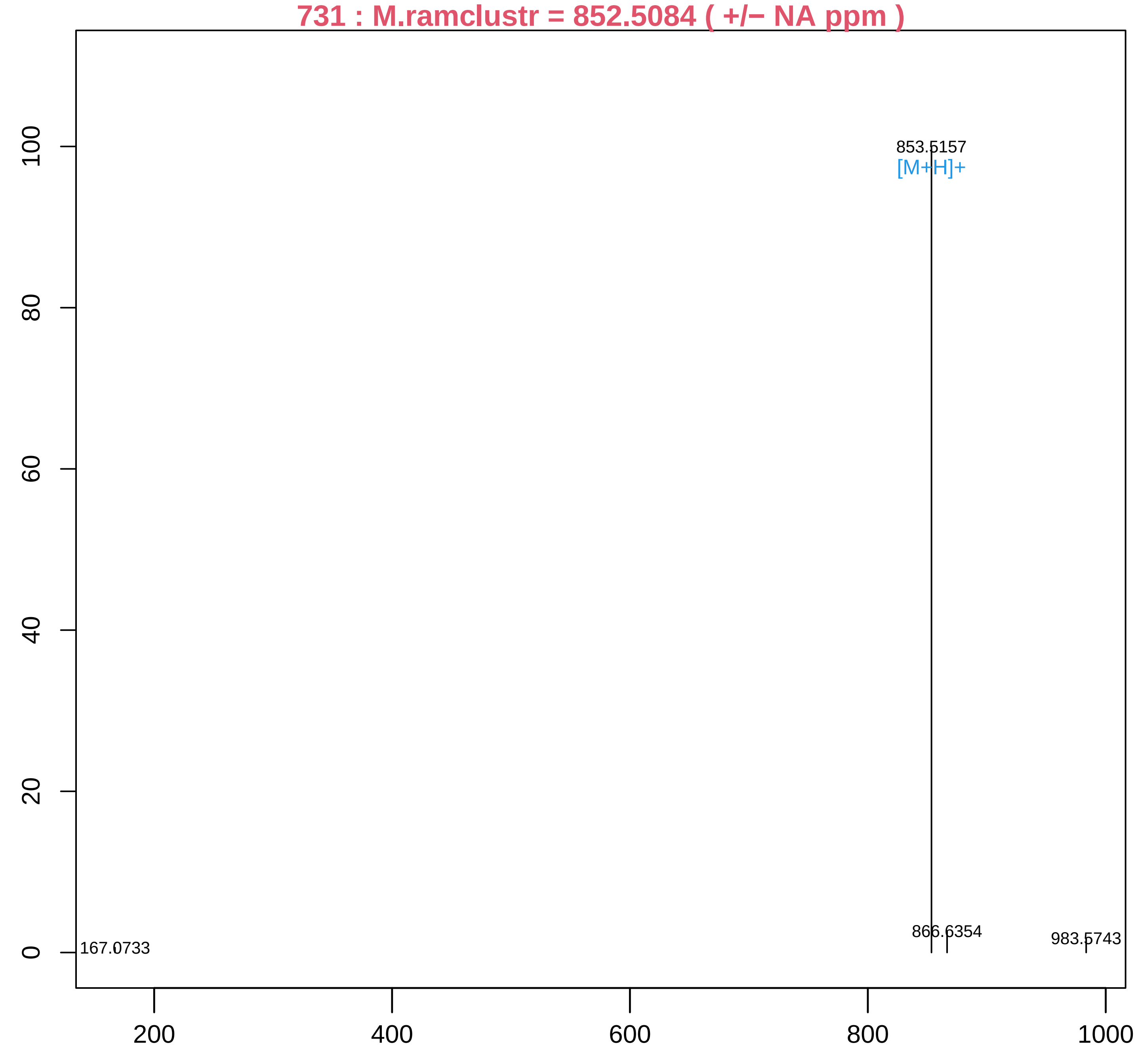
731 : M.ramclustr = 852.5084 (+/− NA ppm)
853.5157
[M+H]+
866.6354
983.5743
167.0733
0
20
40
60
80
100
200
400
600
800
1000

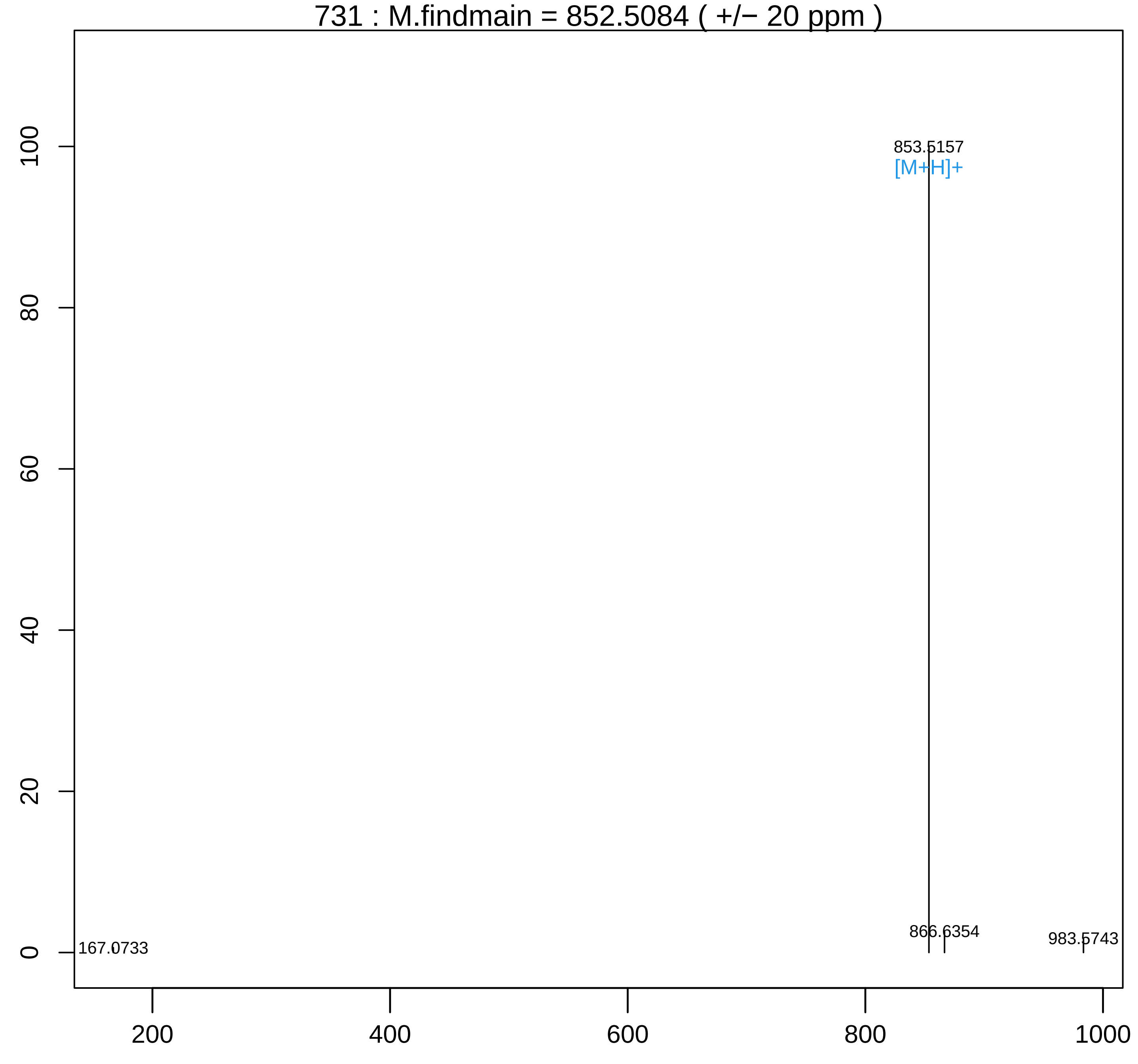
731 : M.findmain = 852.5084 (+/− 20 ppm)
853.5157
[M+H]+
866.6354
983.5743
167.0733
0
20
40
60
80
100
200
400
600
800
1000

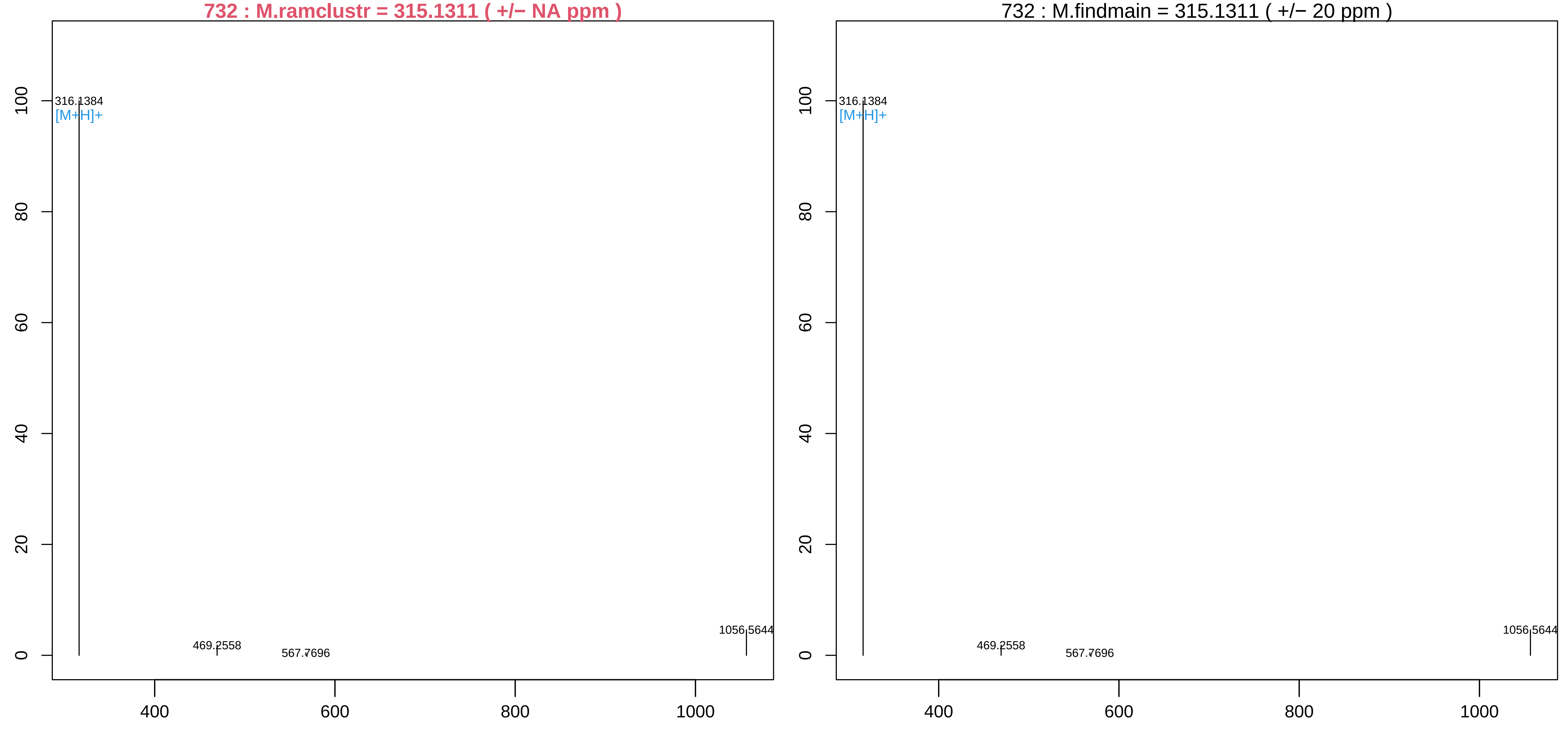
732 : M.ramclustr = 315.1311 (+/− NA ppm)
316.1384
[M+H]+
469.2558
567.7696
1056.5644
0
20
40
60
80
100
400
600
800
1000
732 : M.findmain = 315.1311 (+/− 20 ppm)
316.1384
[M+H]+
469.2558
567.7696
1056.5644
0
20
40
60
80
100
400
600
800
1000

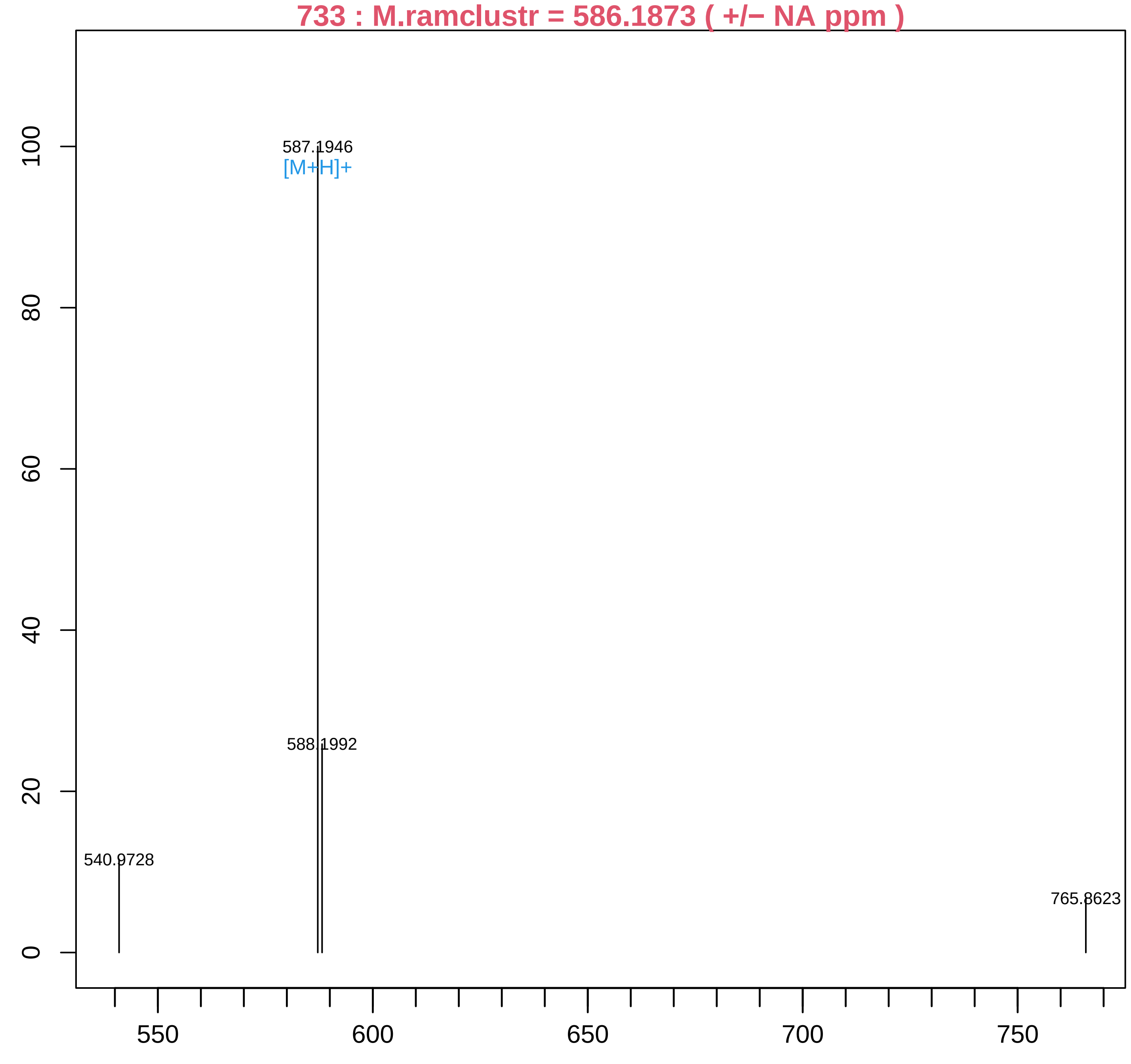
733 : M.ramclustr = 586.1873 (+/− NA ppm)
587.1946
[M+H]+
588.1992
540.9728
765.8623
0
20
40
60
80
100
550
600
650
700
750

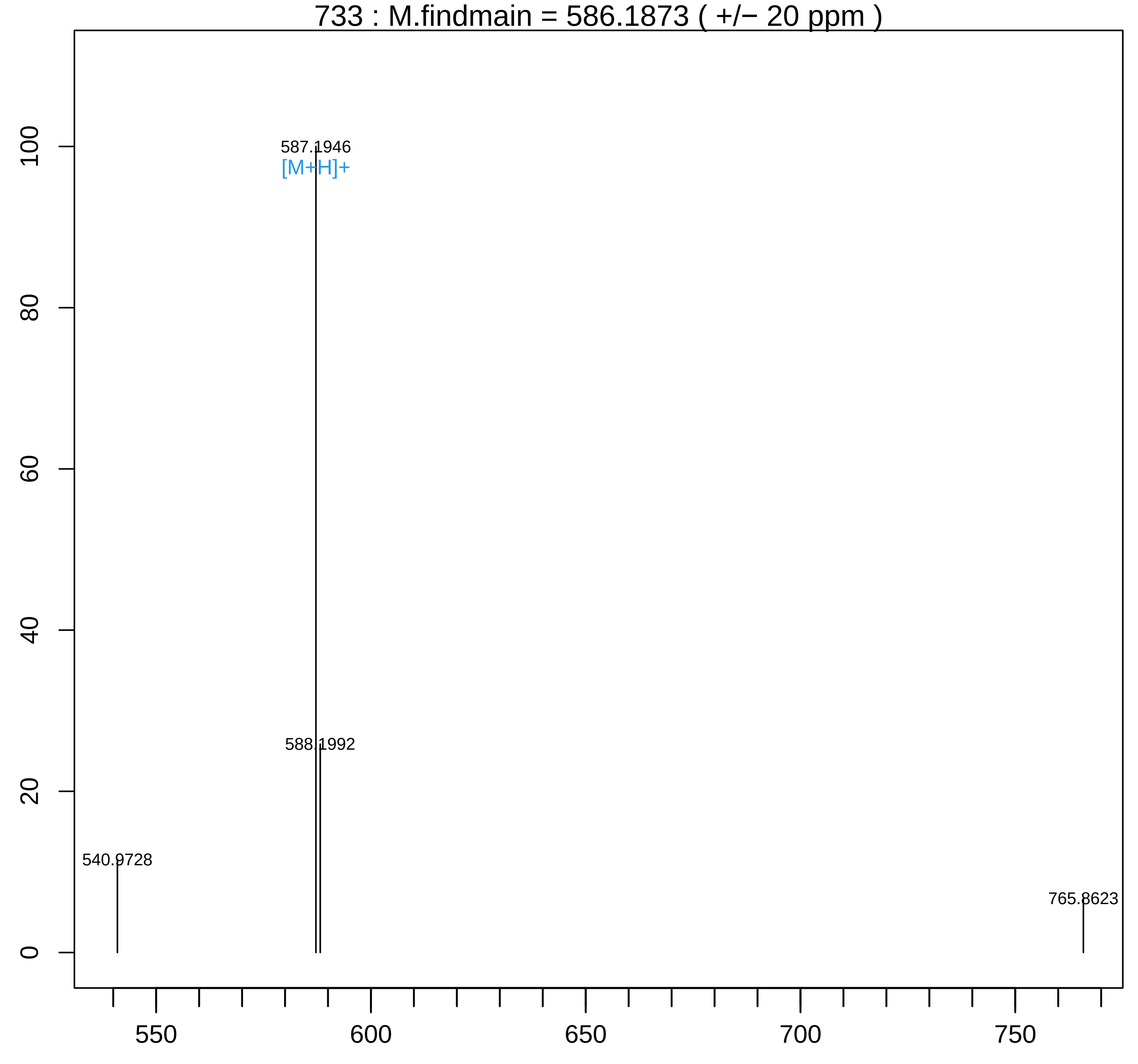
733 : M.findmain = 586.1873 (+/− 20 ppm)
587.1946
[M+H]+
588.1992
540.9728
765.8623
0
20
40
60
80
100
550
600
650
700
750

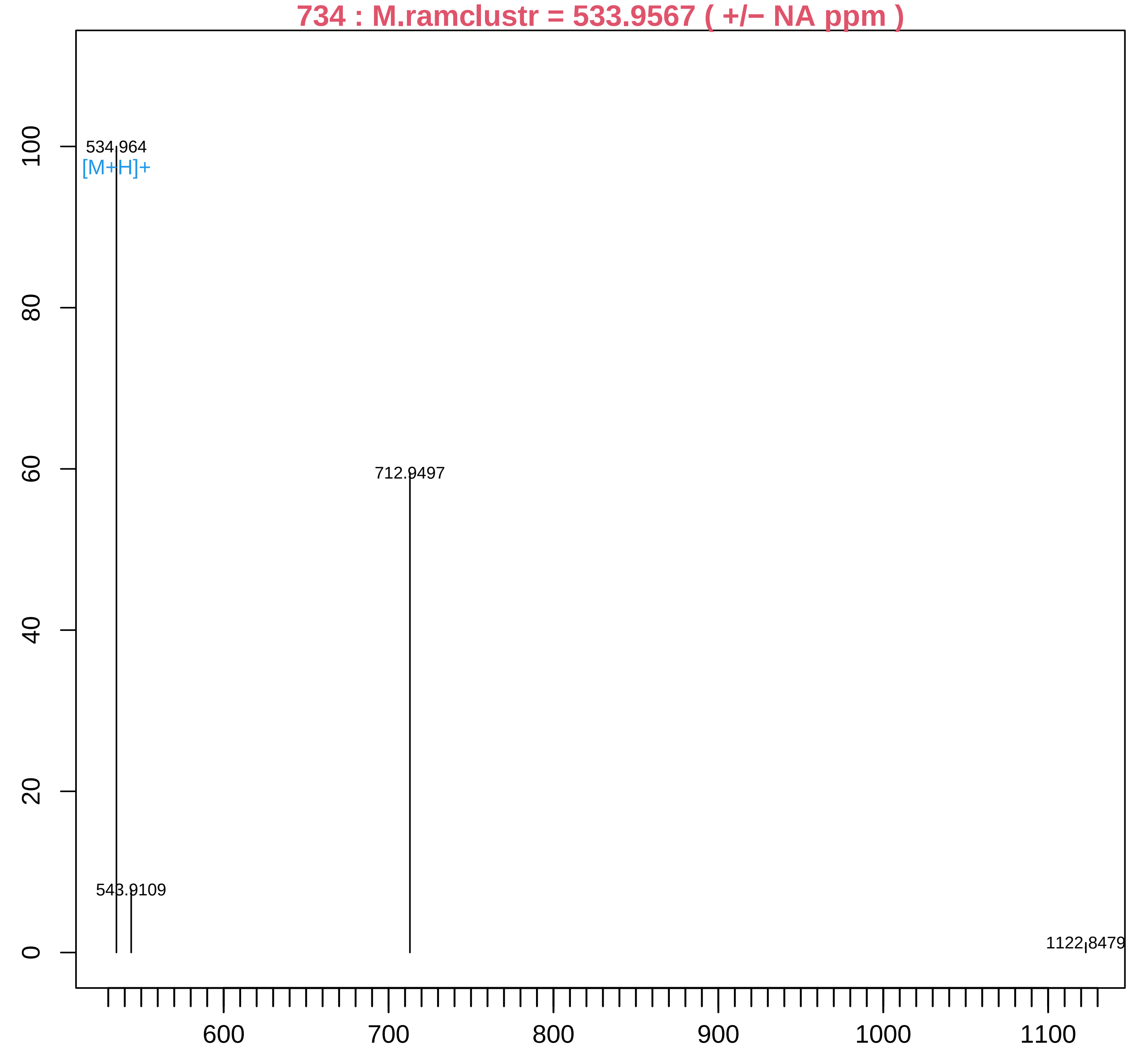
734 : M.ramclustr = 533.9567 (+/− NA ppm)
534.964
[M+H]+
543.9109
712.9497
1122.8479
0
20
40
60
80
100
600
700
800
900
1000
1100

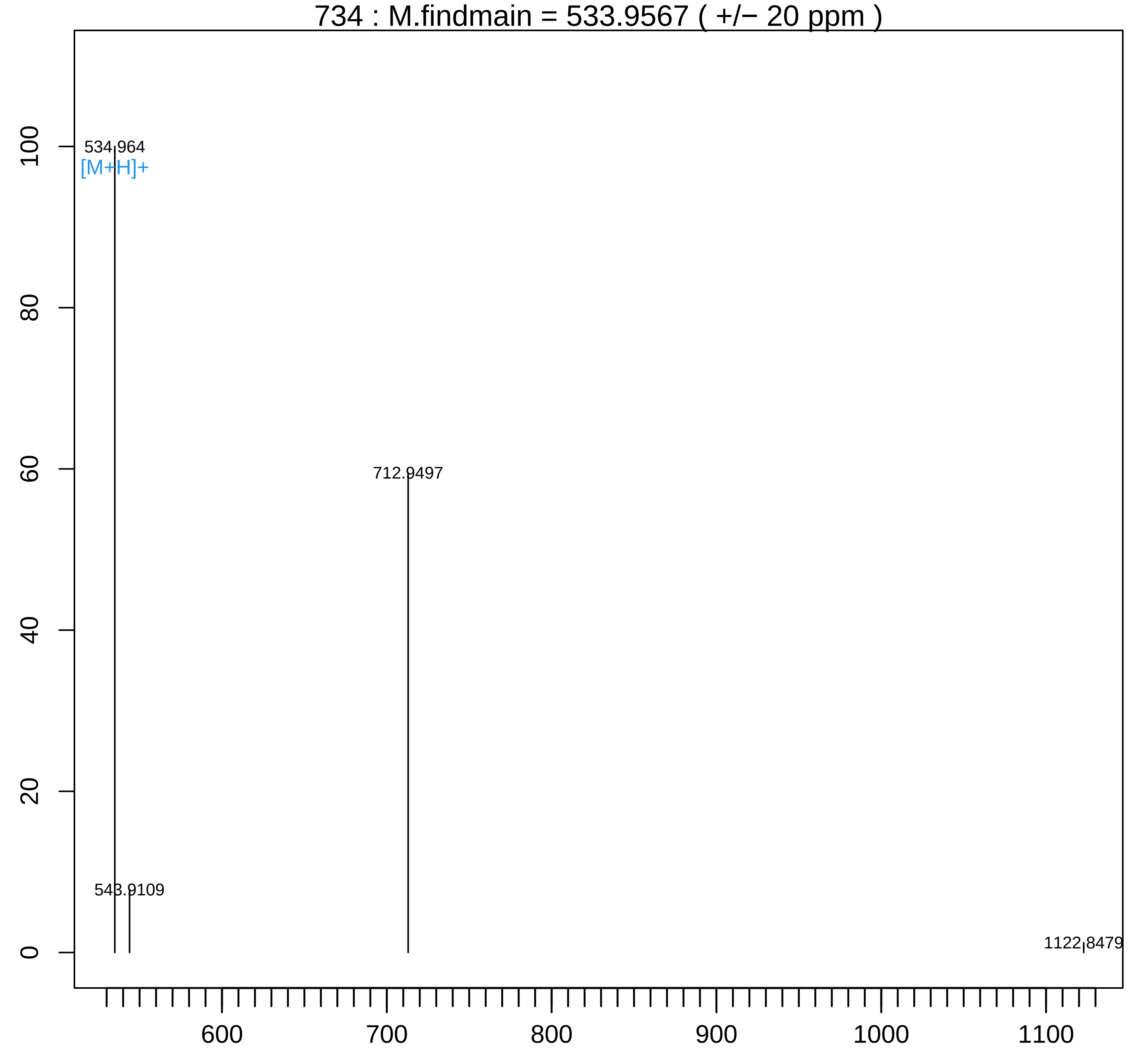
734 : M.findmain = 533.9567 (+/− 20 ppm)
534.964
[M+H]+
543.9109
712.9497
1122.8479
0
20
40
60
80
100
600
700
800
900
1000
1100

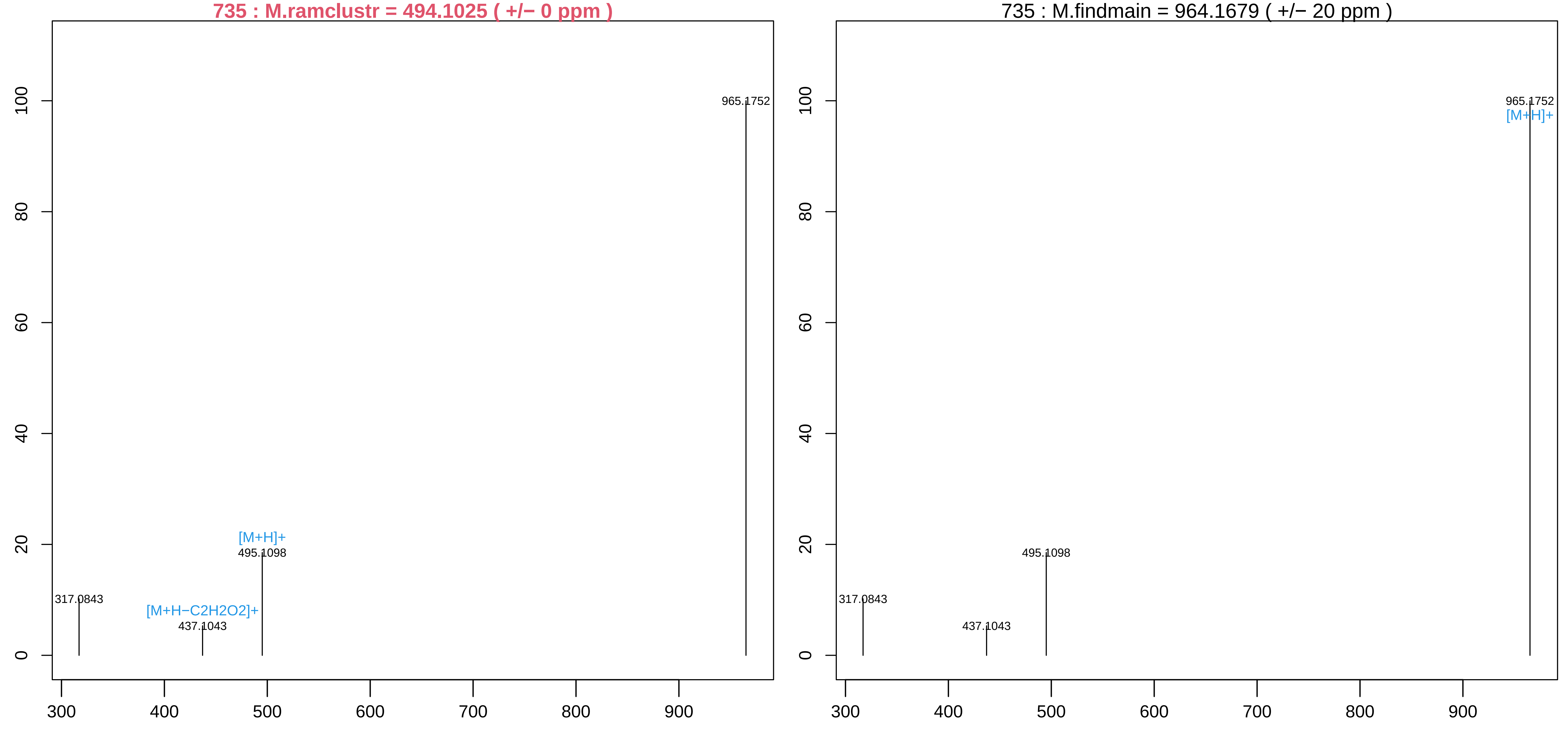
735 : M.ramclustr = 494.1025 (+/− 0 ppm)
965.1752
[M+H]+
495.1098
317.0843
[M+H−C2H2O2]+
437.1043
0
20
40
60
80
100
300
400
500
600
700
800
900
735 : M.findmain = 964.1679 (+/− 20 ppm)
965.1752
[M+H]+
495.1098
317.0843
437.1043
0
20
40
60
80
100
300
400
500
600
700
800
900

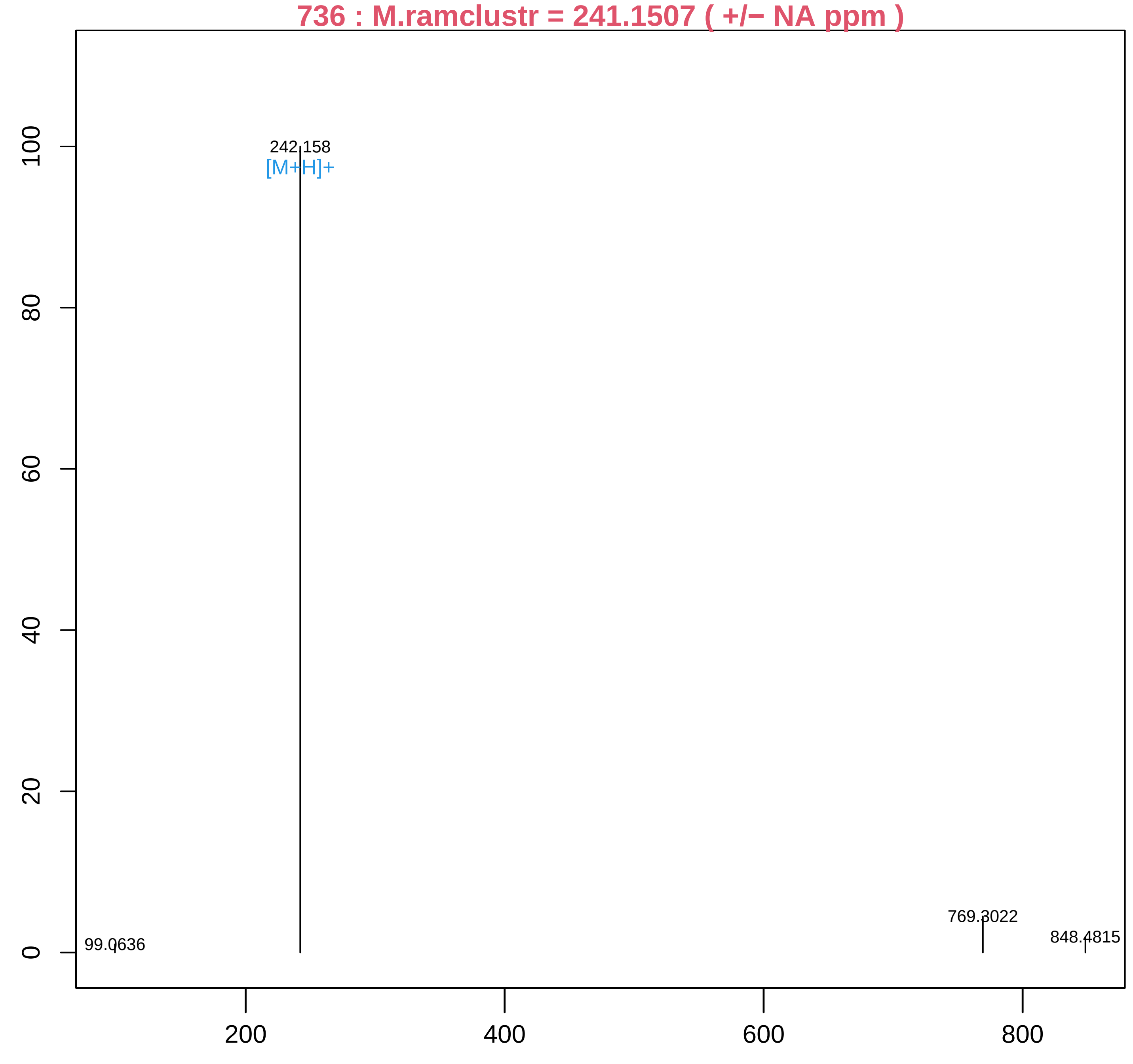
736 : M.ramclustr = 241.1507 (+/− NA ppm)
242.158
[M+H]+
99.0636
769.3022
848.4815
0
20
40
60
80
100
200
400
600
800

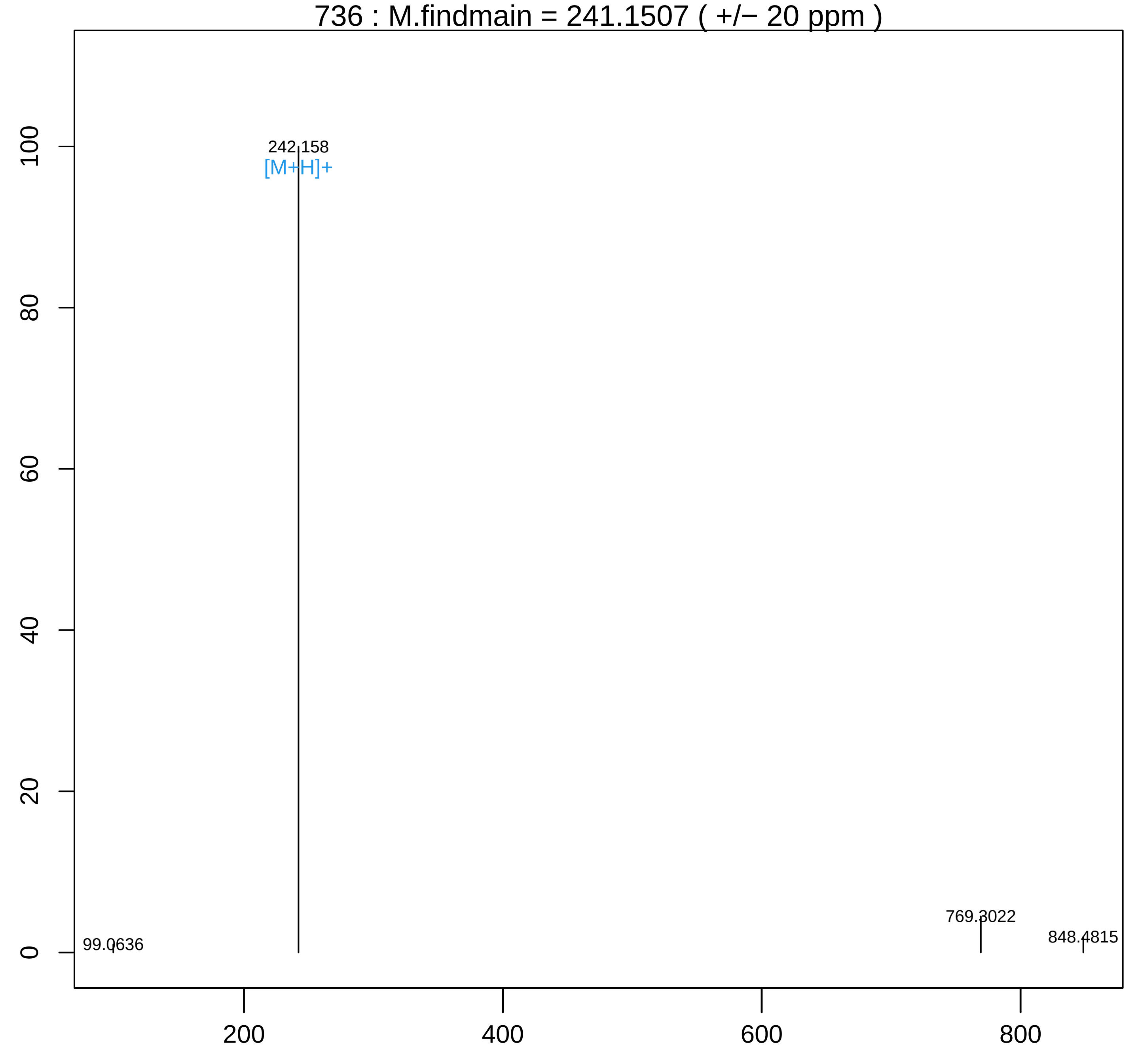
736 : M.findmain = 241.1507 (+/− 20 ppm)
242.158
[M+H]+
99.0636
769.3022
848.4815
0
20
40
60
80
100
200
400
600
800

737 : M.ramclustr = 865.4835 (+/− NA ppm)

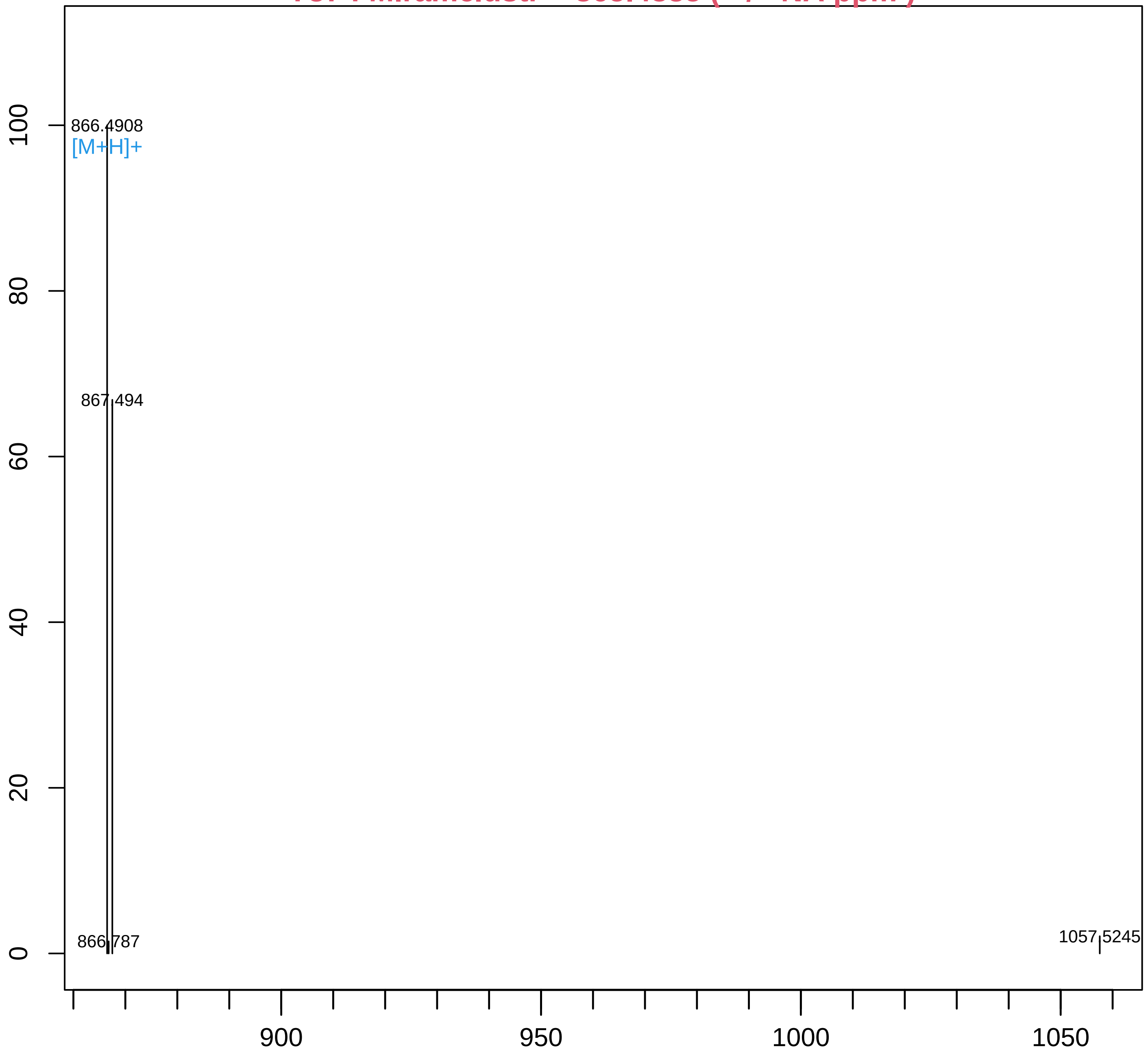

737 : M.findmain = 865.4835 (+/− 20 ppm)

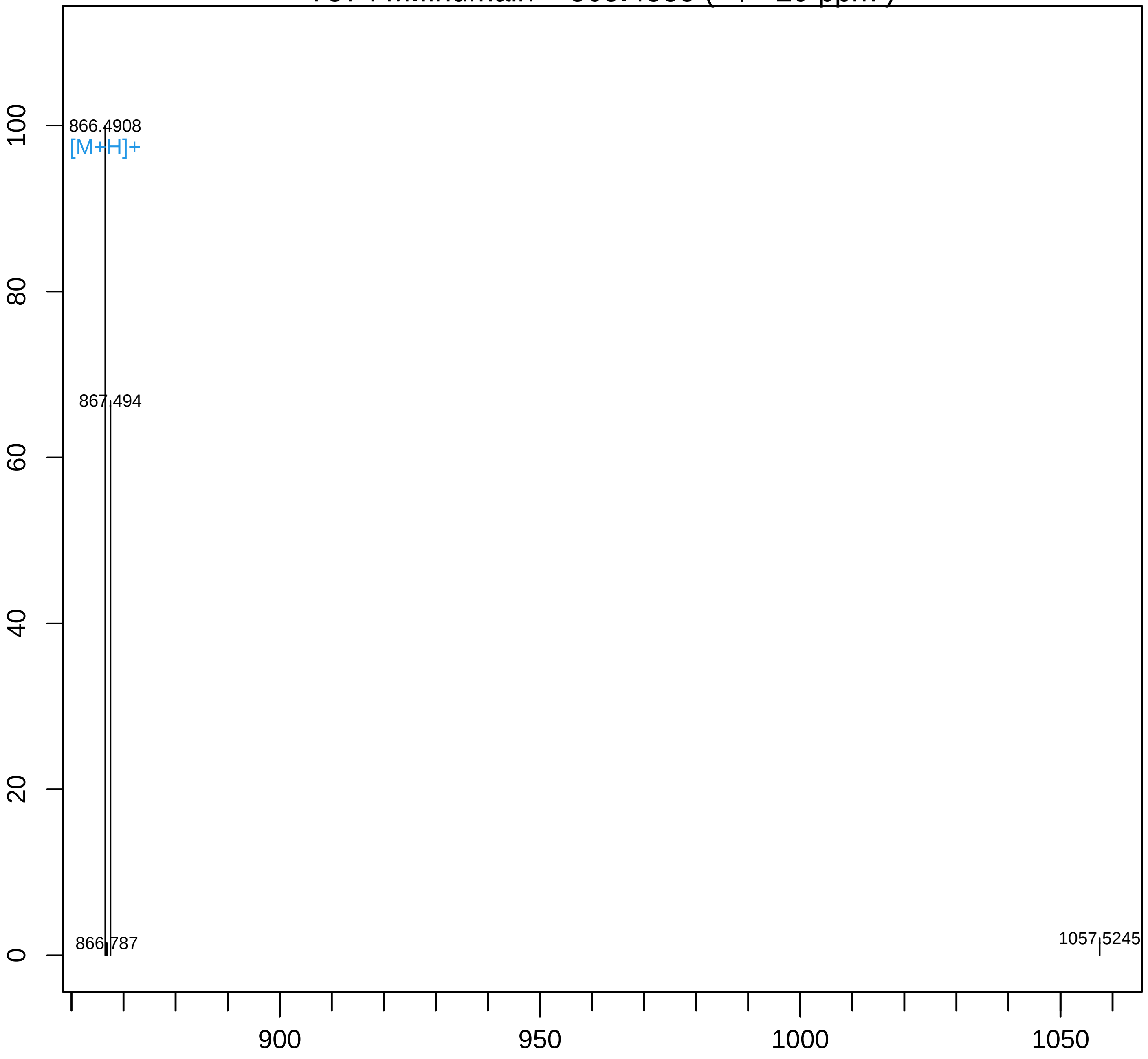

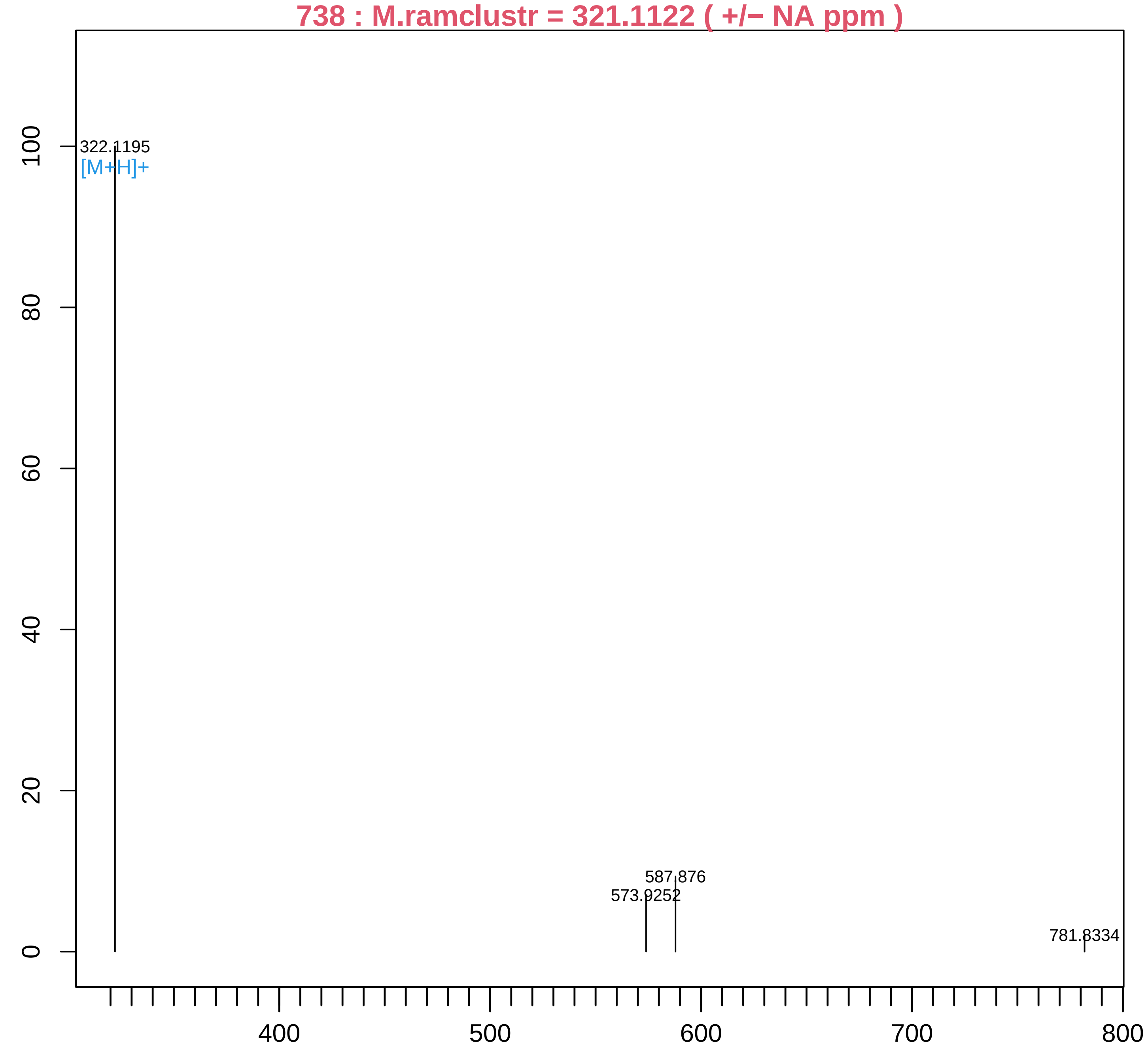
738 : M.ramclustr = 321.1122 (+/− NA ppm)
322.1195
[M+H]+
587.876
573.9252
781.8334
0
20
40
60
80
100
400
500
600
700
800
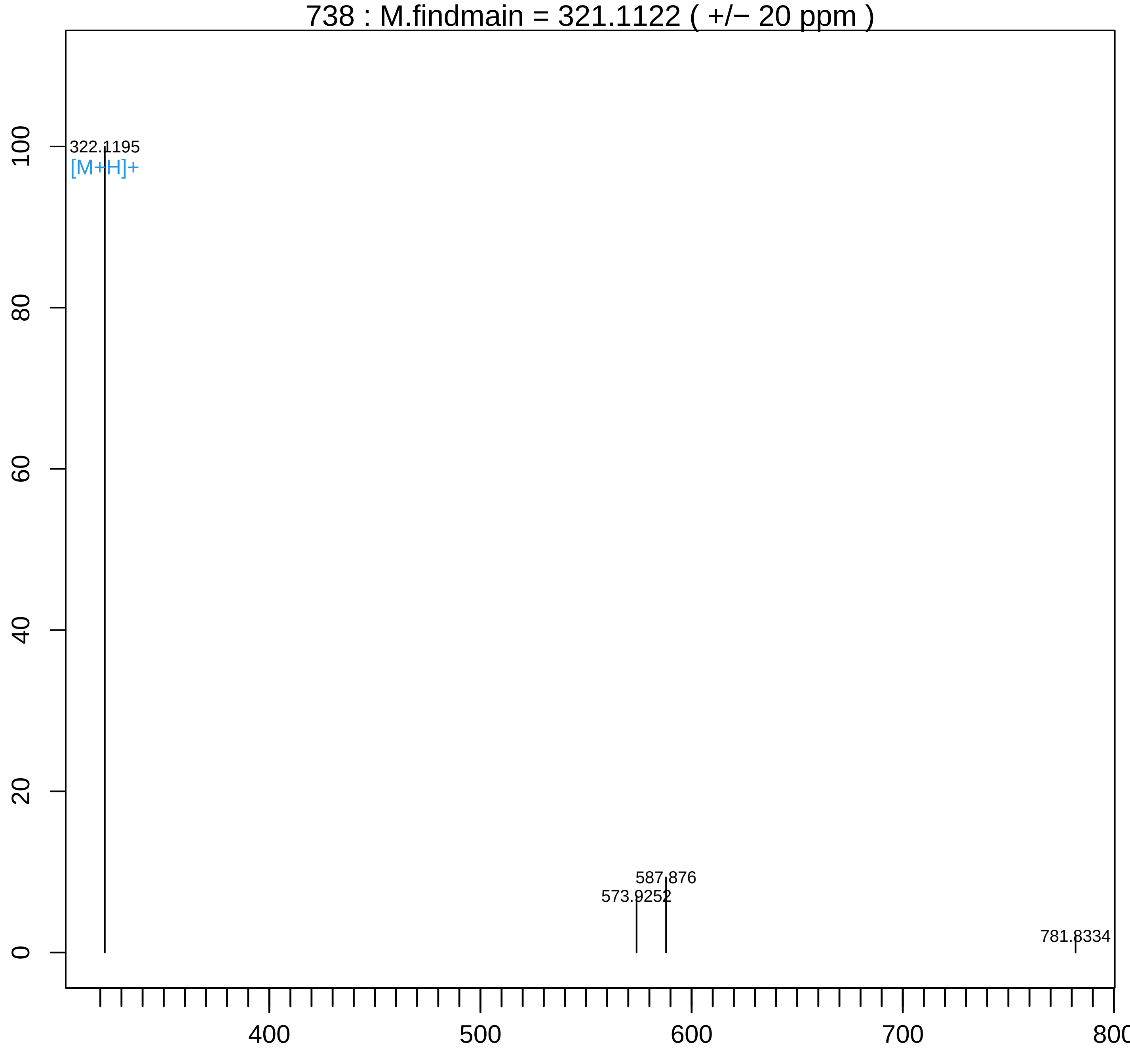
738 : M.findmain = 321.1122 (+/− 20 ppm)
322.1195
[M+H]+
587.876
573.9252
781.8334
0
20
40
60
80
100
400
500
600
700
800

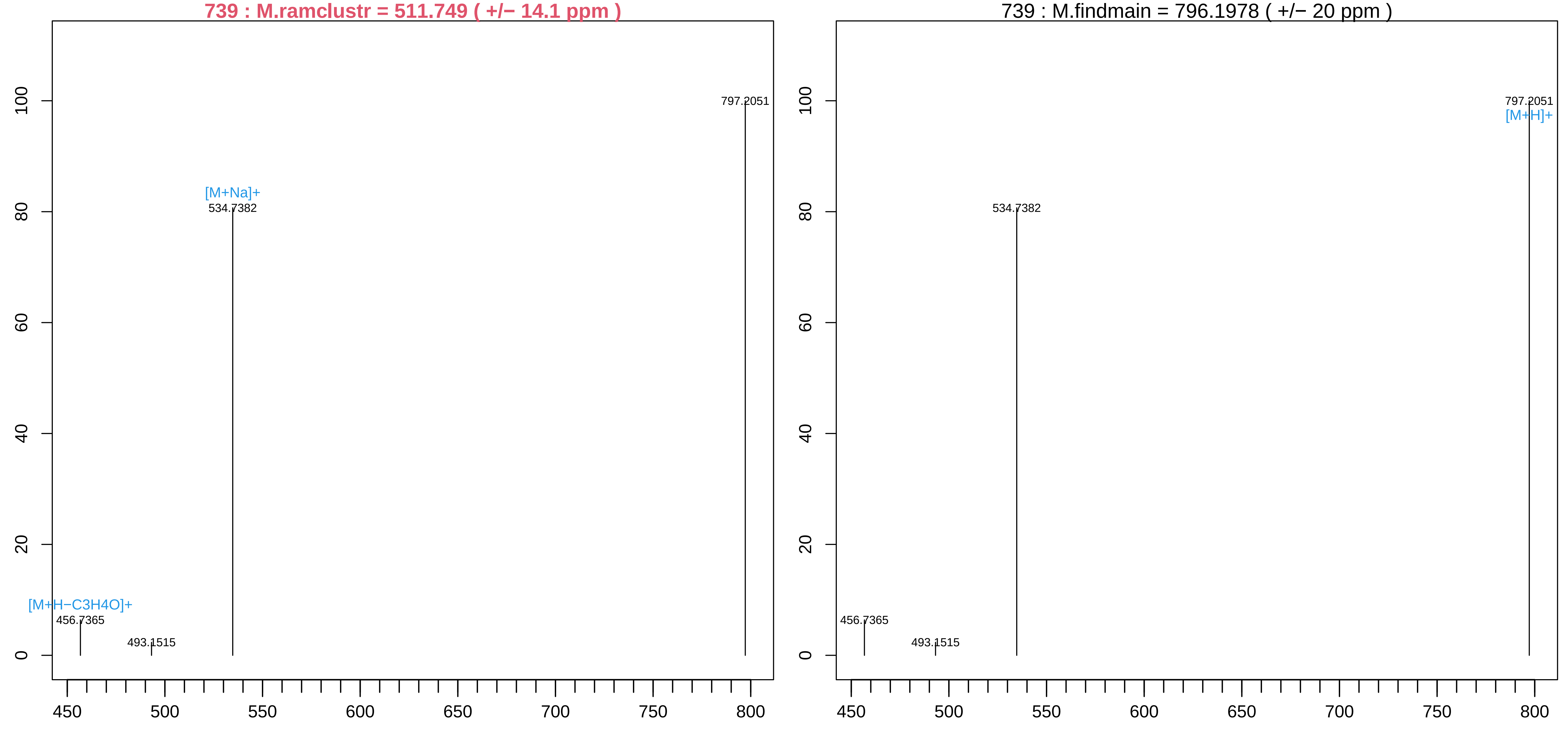

739 : M.ramclustr = 511.749 (+/− 14.1 ppm)
[M+Na]+
534.7382
797.2051
[M+H−C3H4O]+
456.7365
493.1515
739 : M.findmain = 796.1978 (+/− 20 ppm)
797.2051
[M+H]+
534.7382
456.7365
493.1515

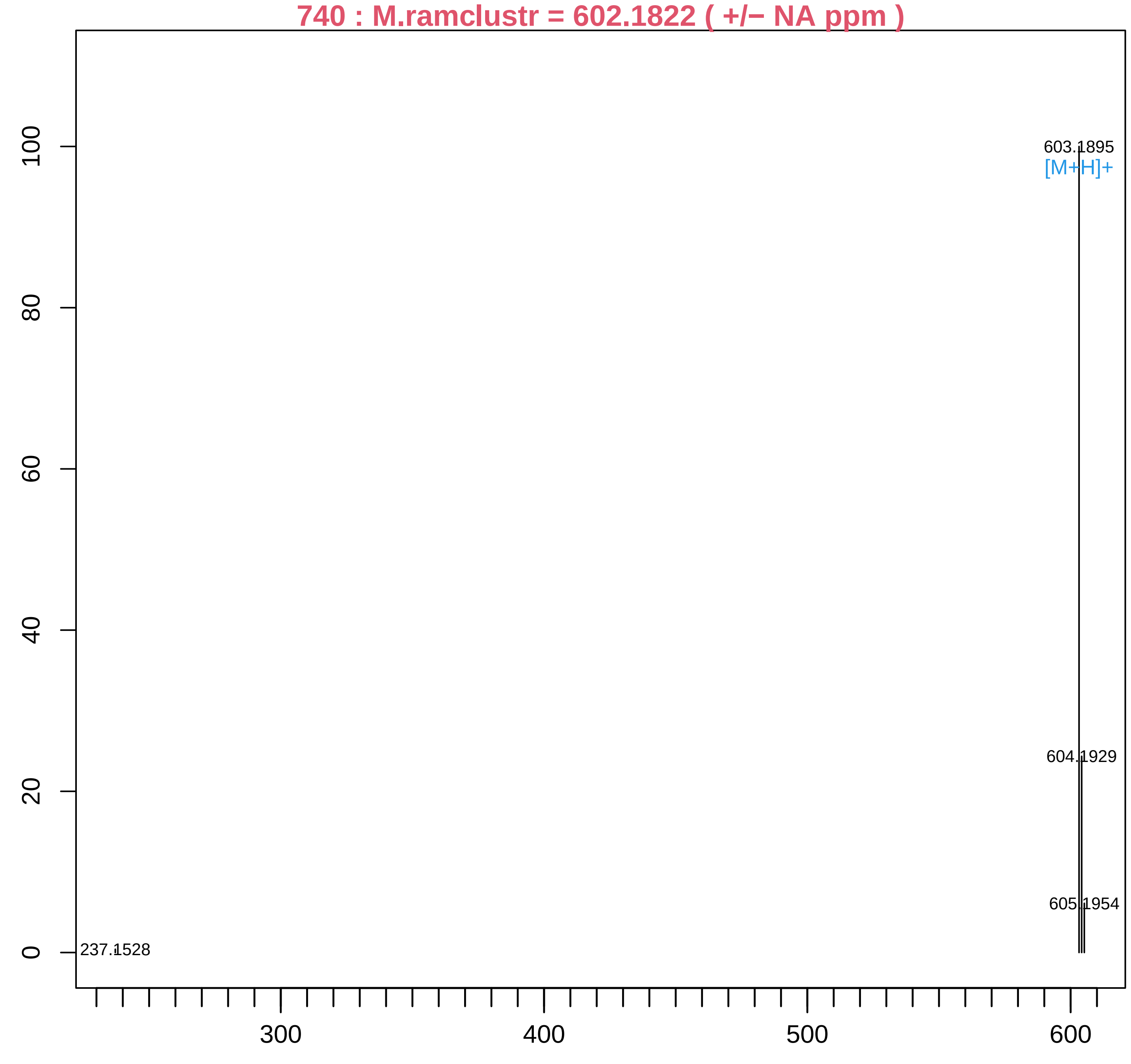
740 : M.ramclustr = 602.1822 (+/− NA ppm)
603.1895
[M+H]+
604.1929
605.1954
237.1528
0
20
40
60
80
100
300
400
500
600

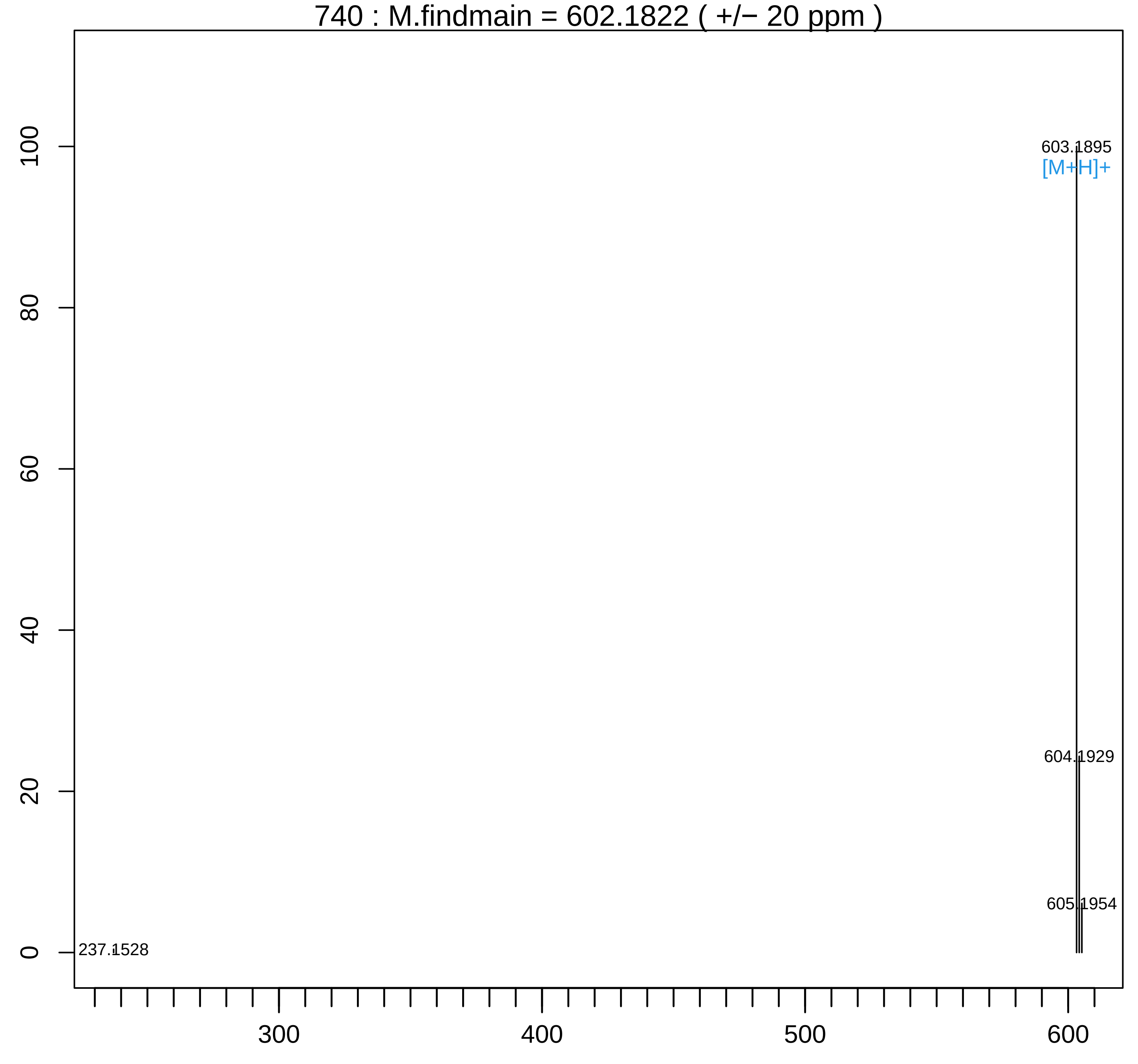
740 : M.findmain = 602.1822 (+/− 20 ppm)
603.1895
[M+H]+
604.1929
605.1954
237.1528
0
20
40
60
80
100
300
400
500
600

741 : M.ramclustr = 377.9527 (+/− NA ppm)

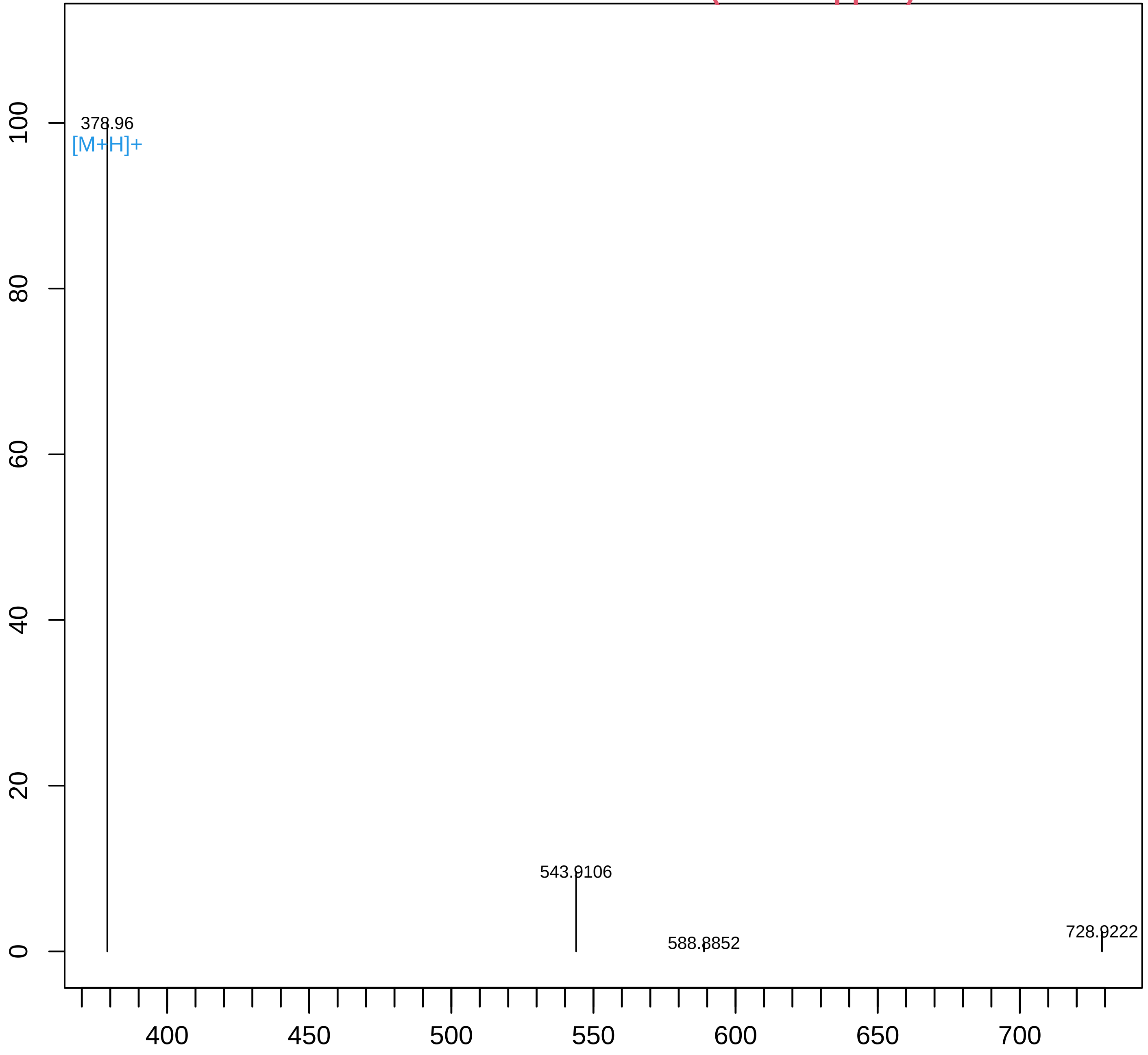

741 : M.findmain = 377.9527 (+/− 20 ppm)

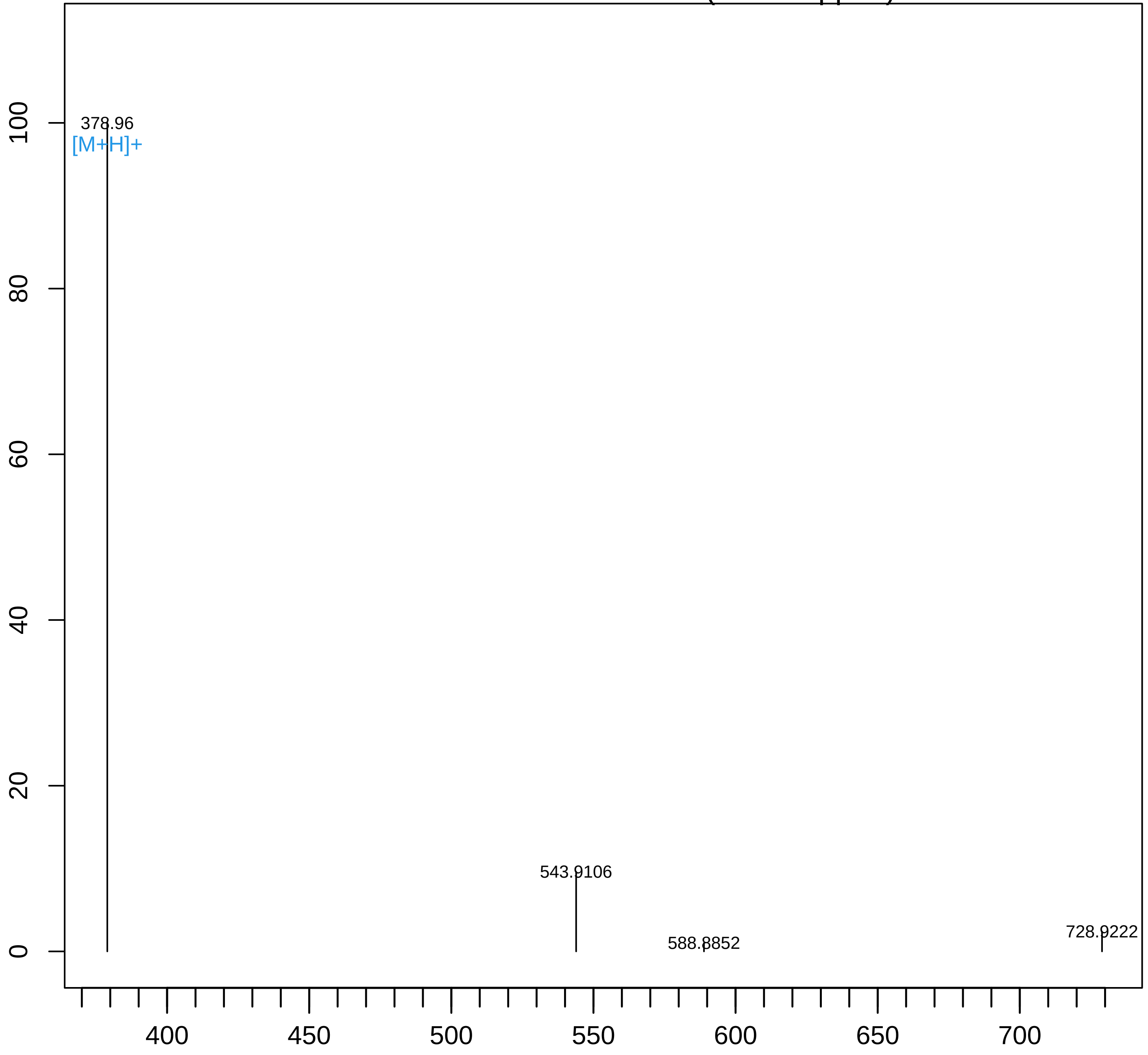

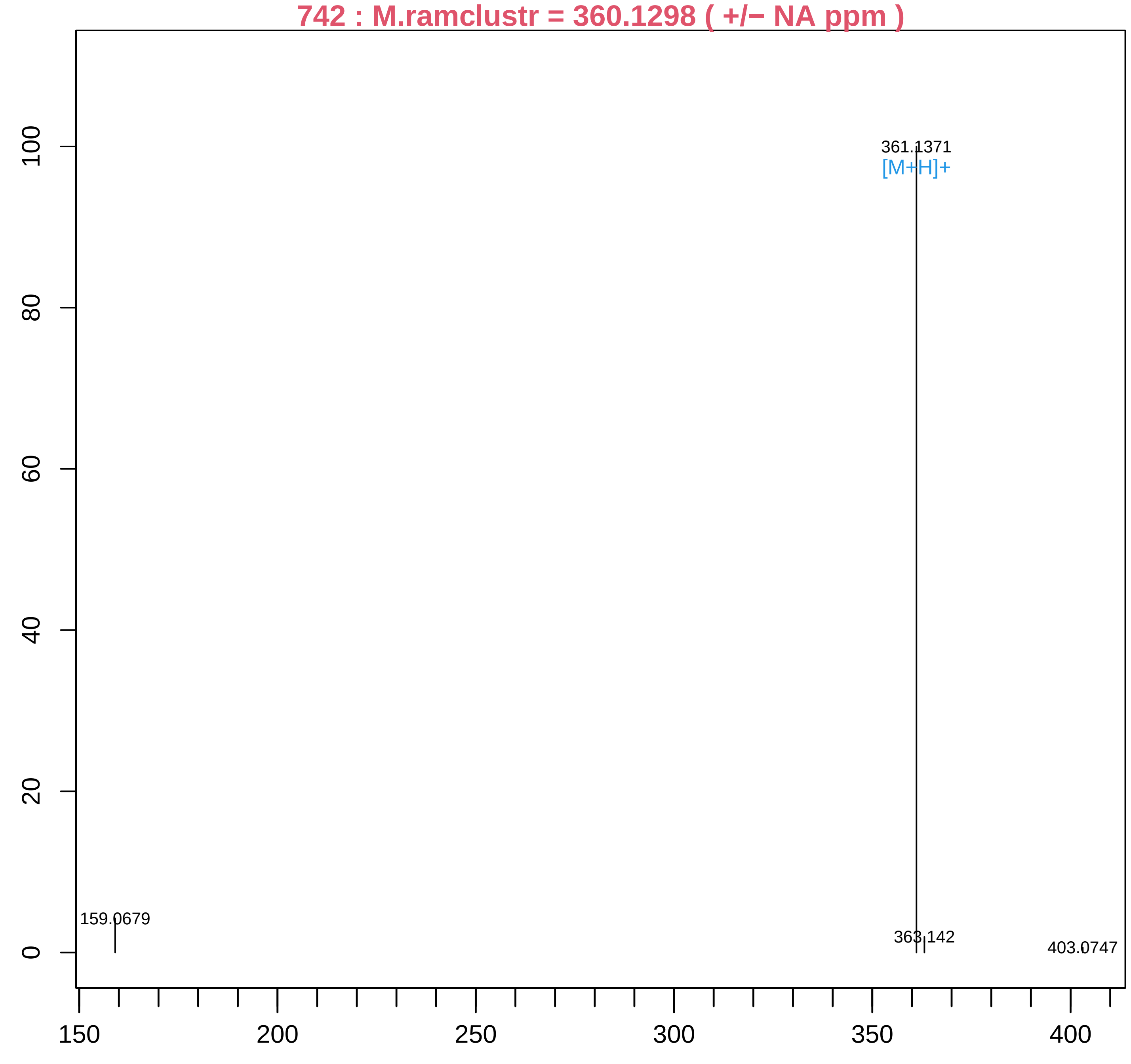
742 : M.ramclustr = 360.1298 (+/− NA ppm)
361.1371
[M+H]+
159.0679
363.142
403.0747
0
20
40
60
80
100
150
200
250
300
350
400

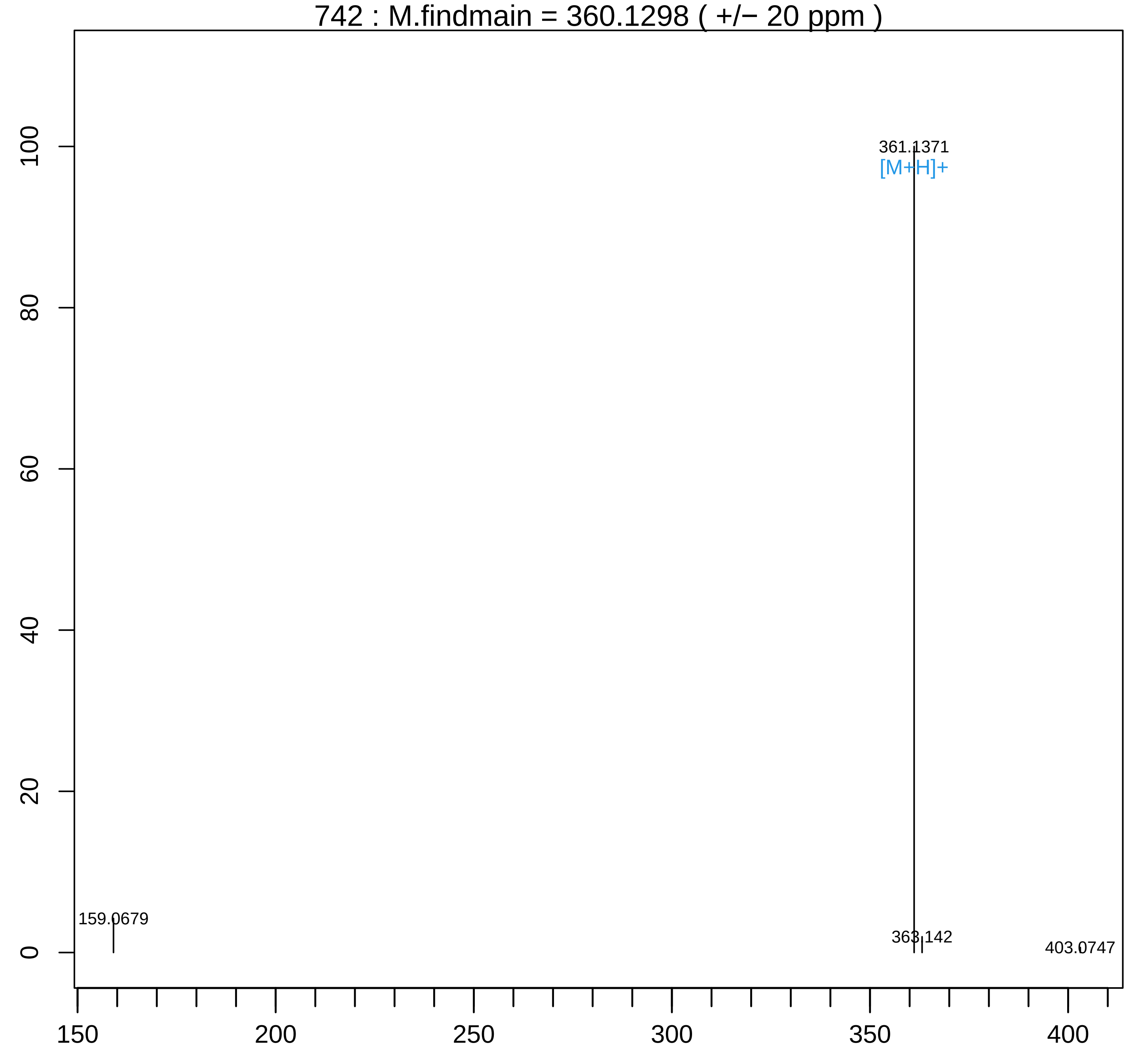
742 : M.findmain = 360.1298 (+/− 20 ppm)
361.1371
[M+H]+
159.0679
363.142
403.0747
0
20
40
60
80
100
150
200
250
300
350
400

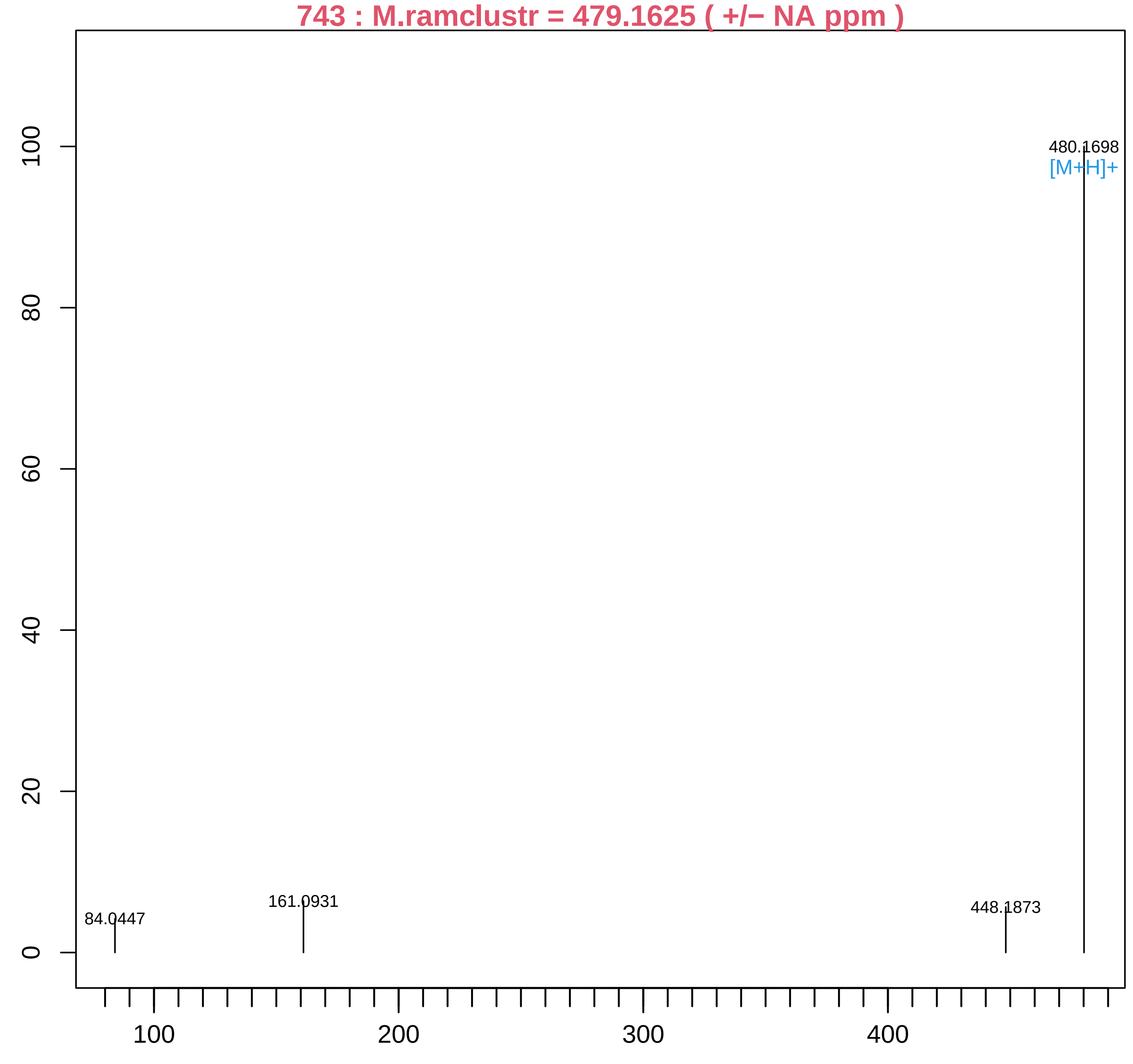
743 : M.ramclustr = 479.1625 (+/− NA ppm)
480.1698
[M+H]+
84.0447
161.0931
448.1873
0
20
40
60
80
100
100
200
300
400

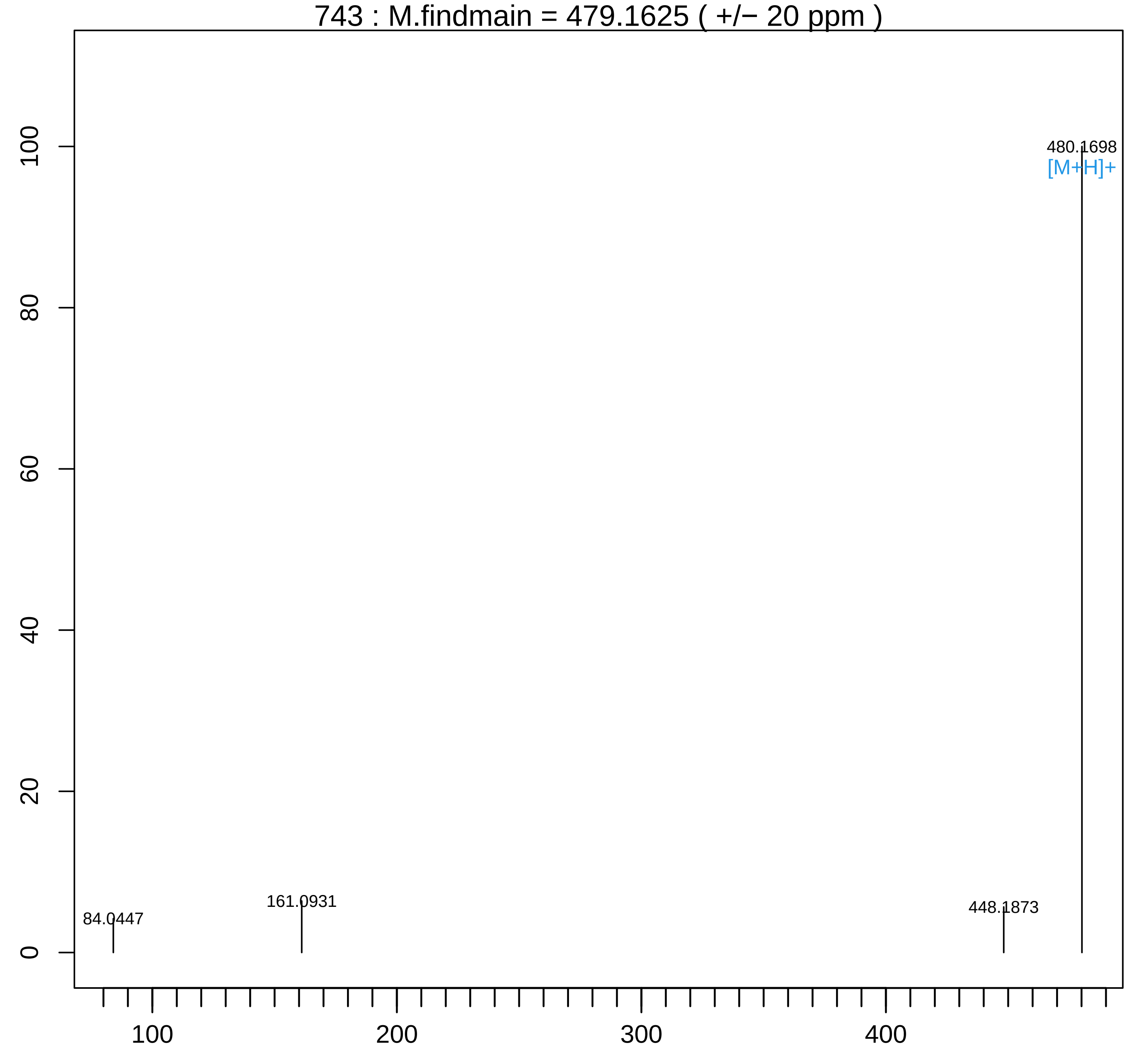
743 : M.findmain = 479.1625 (+/− 20 ppm)
480.1698
[M+H]+
84.0447
161.0931
448.1873
0
20
40
60
80
100
100
200
300
400

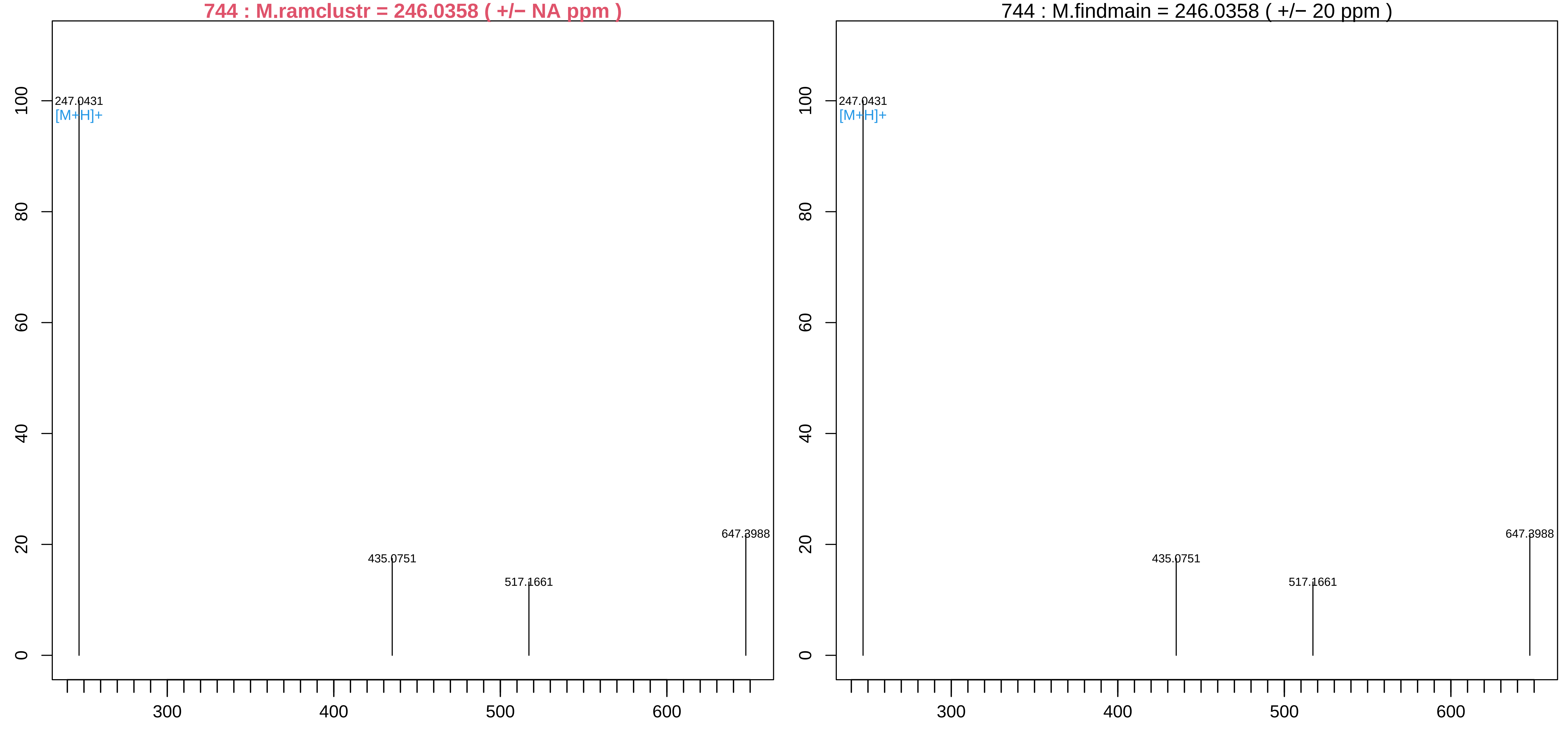

744 : M.ramclustr = 246.0358 (+/− NA ppm)
247.0431
[M+H]+
435.0751
517.1661
647.3988
744 : M.findmain = 246.0358 (+/− 20 ppm)
247.0431
[M+H]+
435.0751
517.1661
647.3988

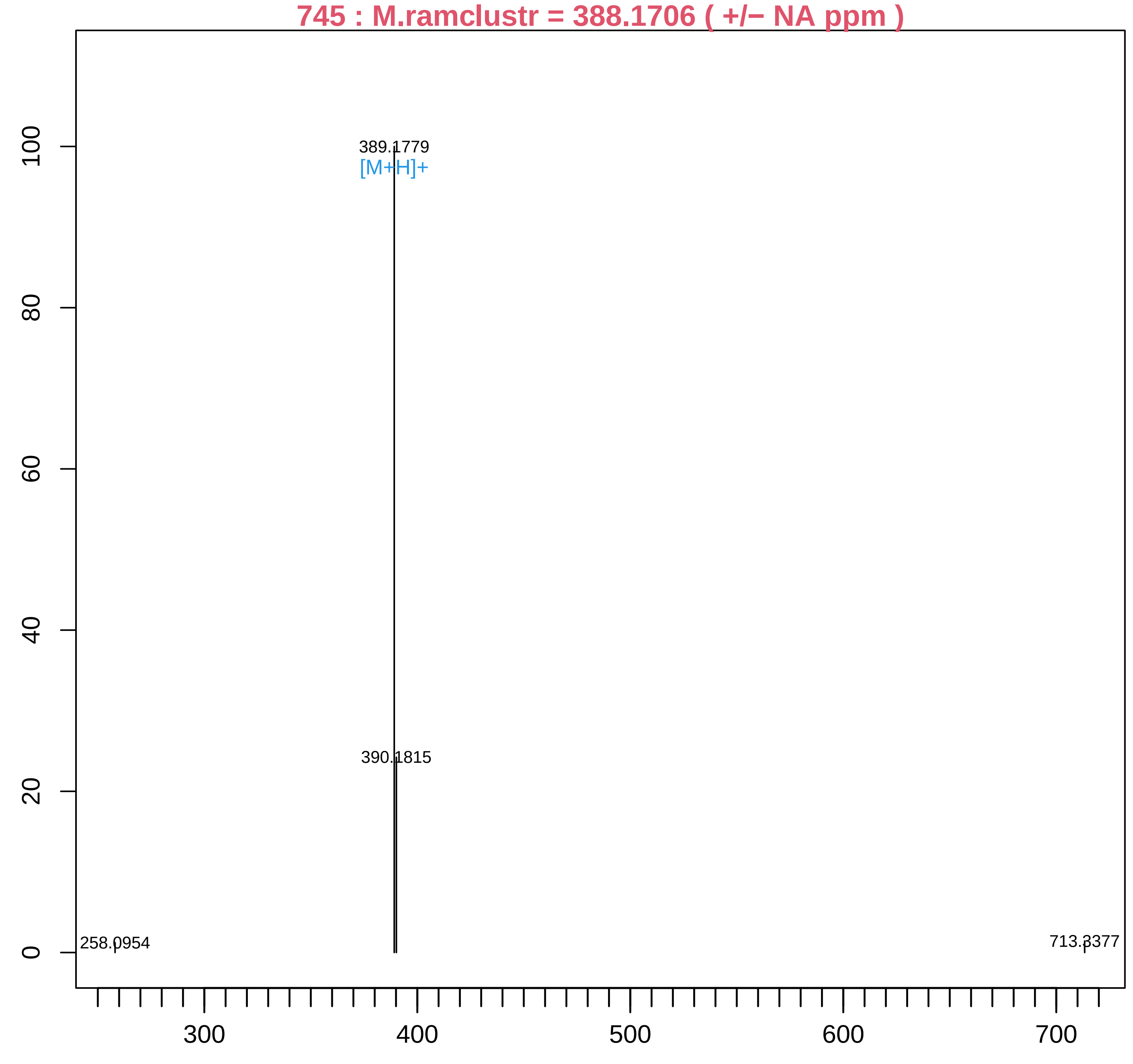
745 : M.ramclustr = 388.1706 (+/− NA ppm)
389.1779
[M+H]+
390.1815
258.0954
713.3377
0
20
40
60
80
100
300
400
500
600
700

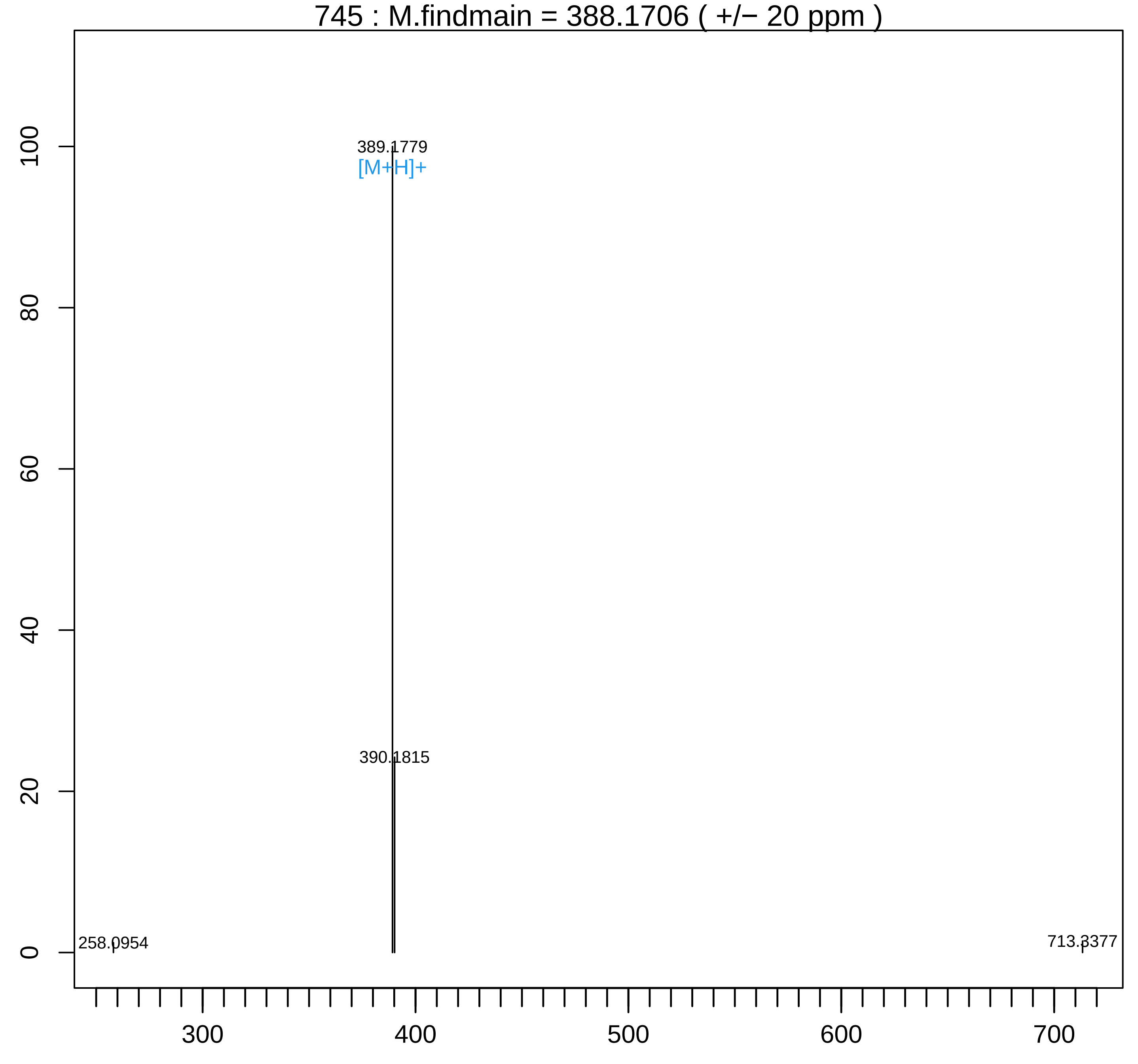
745 : M.findmain = 388.1706 (+/− 20 ppm)
389.1779
[M+H]+
390.1815
258.0954
713.3377
0
20
40
60
80
100
300
400
500
600
700

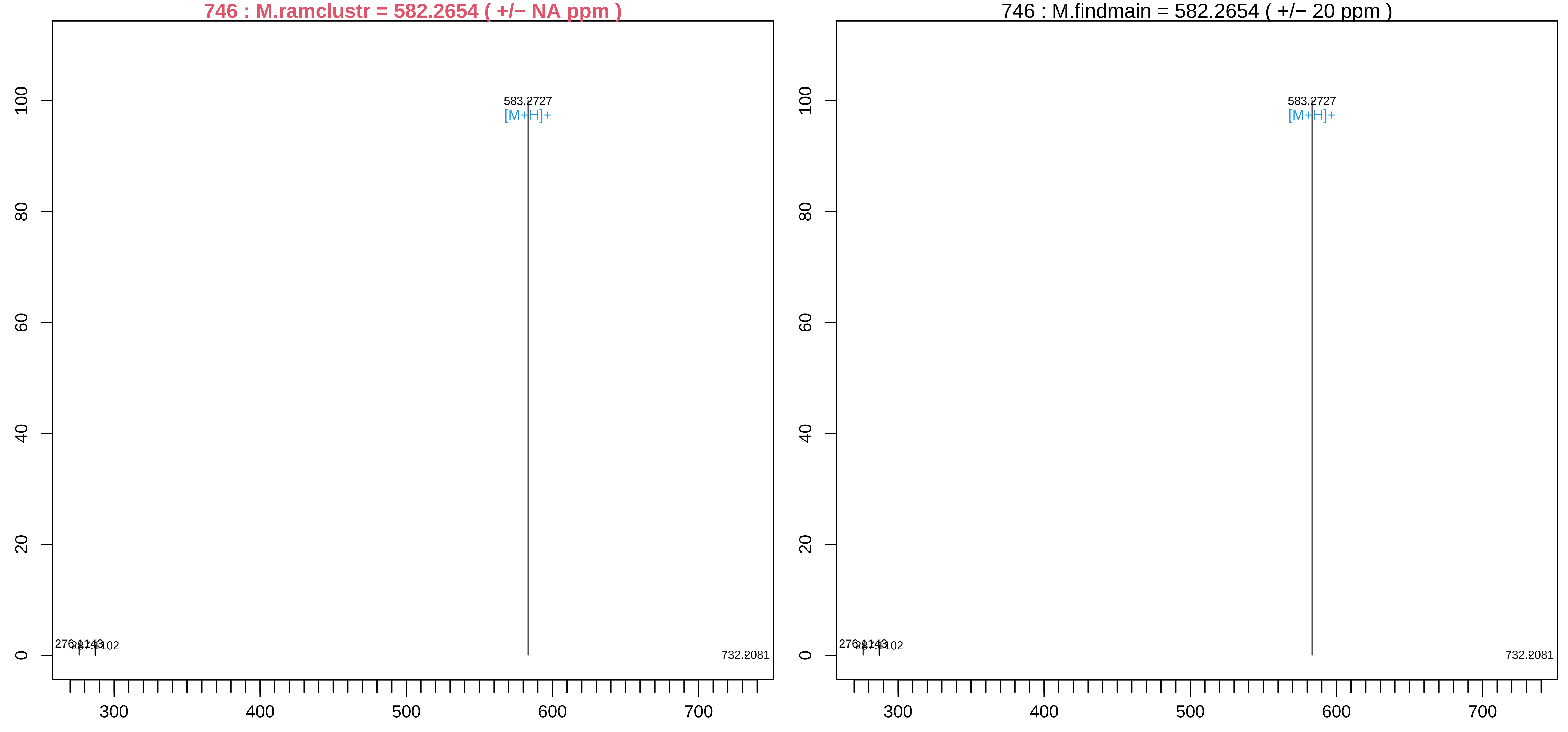

746 : M.ramclustr = 582.2654 (+/− NA ppm)
746 : M.findmain = 582.2654 (+/− 20 ppm)
583.2727
[M+H]+
276.1143
287.1102
732.2081
0
20
40
60
80
100
300
400
500
600
700

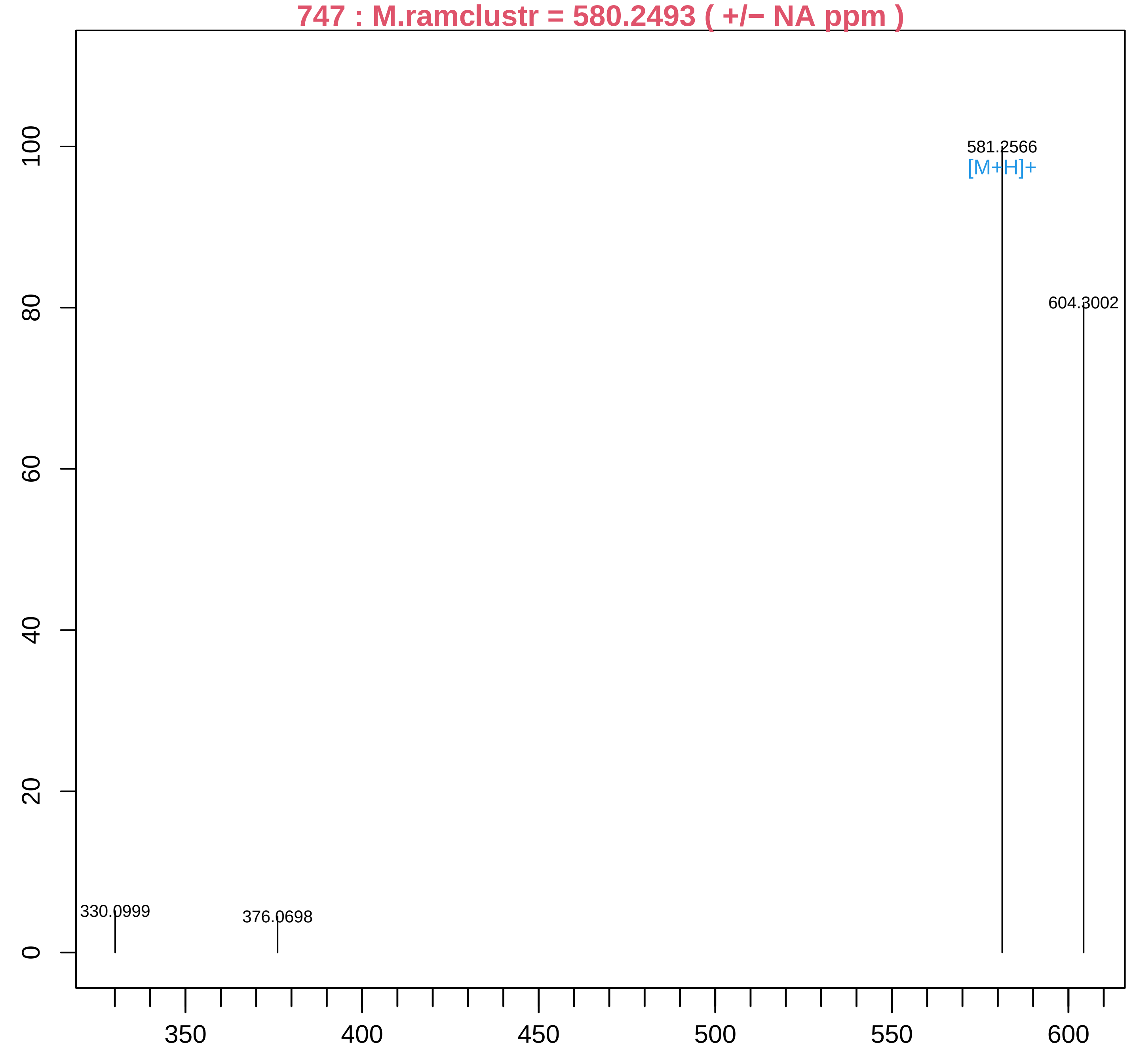

747 : M.ramclustr = 580.2493 (+/− NA ppm)
581.2566
[M+H]+
604.3002
330.0999
376.0698
0
20
40
60
80
100
350
400
450
500
550
600

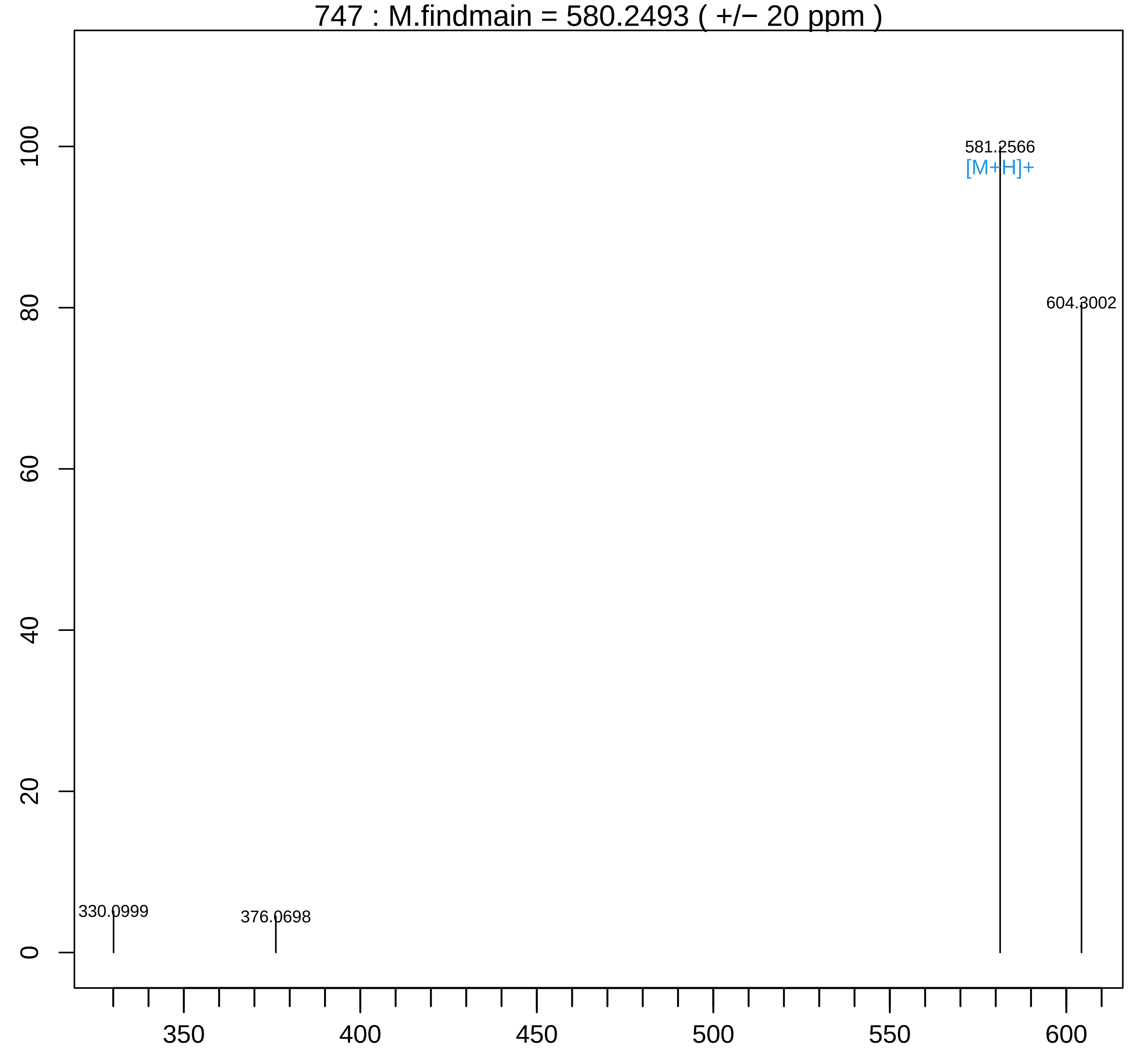

747 : M.findmain = 580.2493 (+/− 20 ppm)
581.2566
[M+H]+
604.3002
330.0999
376.0698
0
20
40
60
80
100
350
400
450
500
550
600

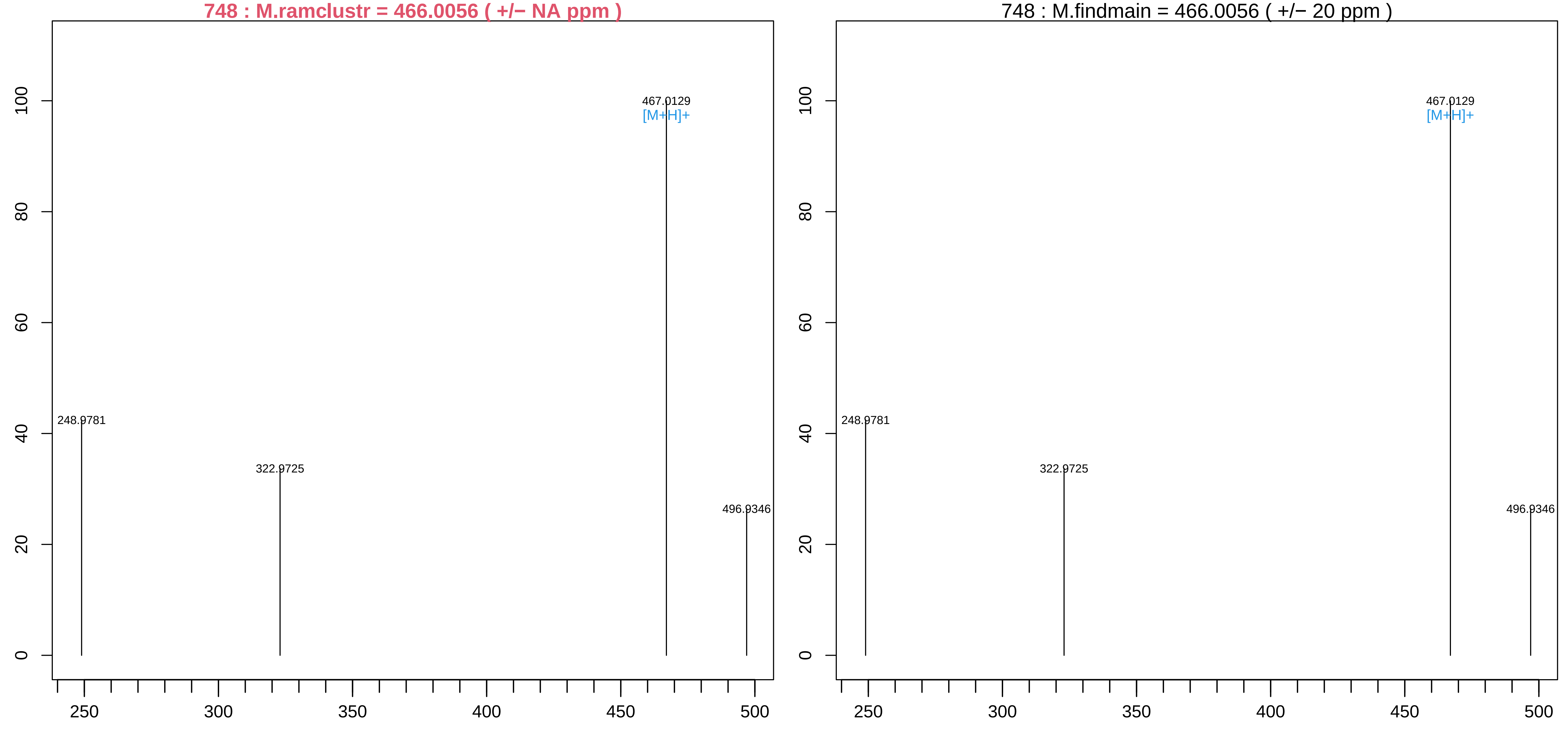

748 : M.ramclustr = 466.0056 (+/− NA ppm)
467.0129
[M+H]+
248.9781
322.9725
496.9346
748 : M.findmain = 466.0056 (+/− 20 ppm)
467.0129
[M+H]+
248.9781
322.9725
496.9346

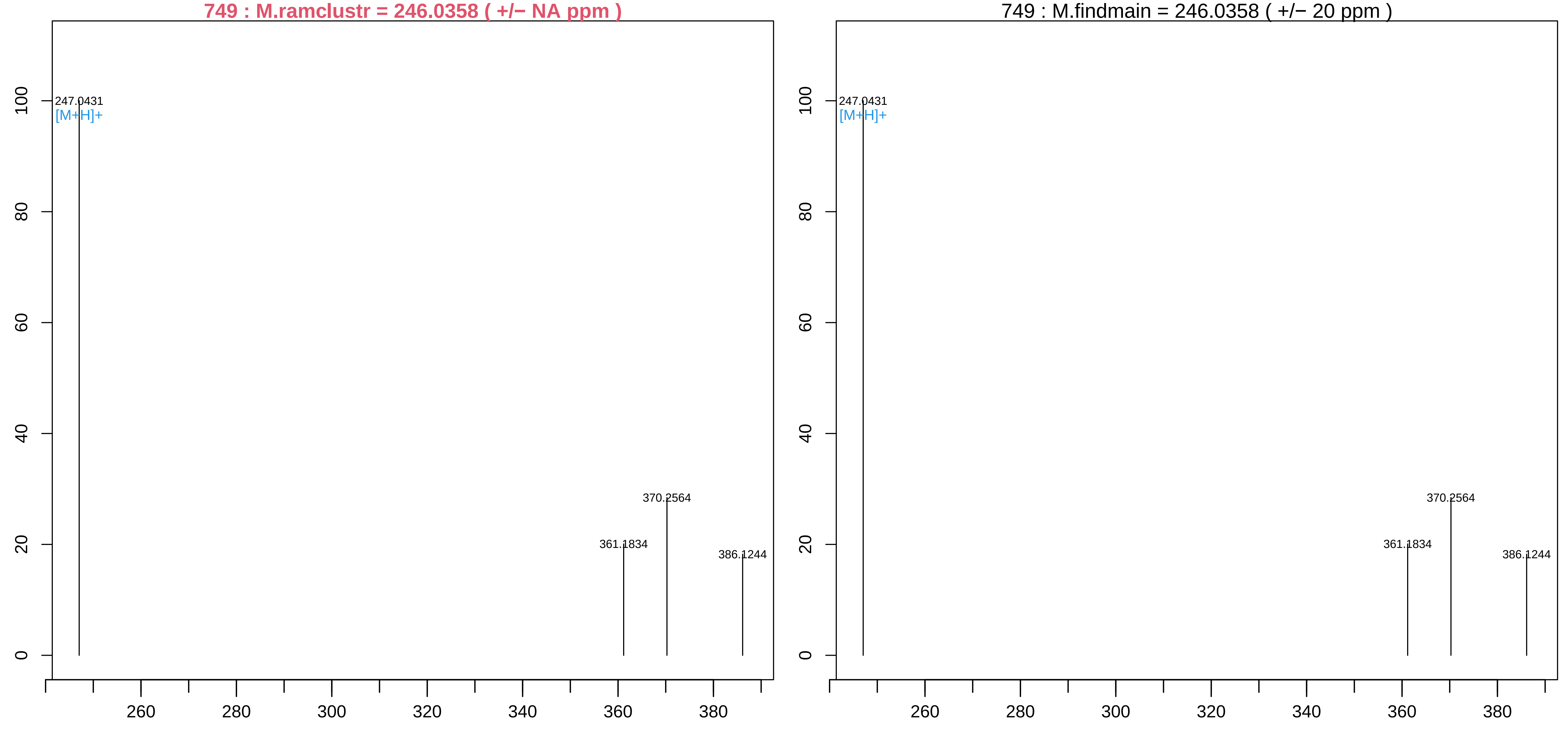

749 : M.ramclustr = 246.0358 (+/− NA ppm)
247.0431
[M+H]+
361.1834
370.2564
386.1244
0
20
40
60
80
100
260
280
300
320
340
360
380
749 : M.findmain = 246.0358 (+/− 20 ppm)
247.0431
[M+H]+
361.1834
370.2564
386.1244
0
20
40
60
80
100
260
280
300
320
340
360
380

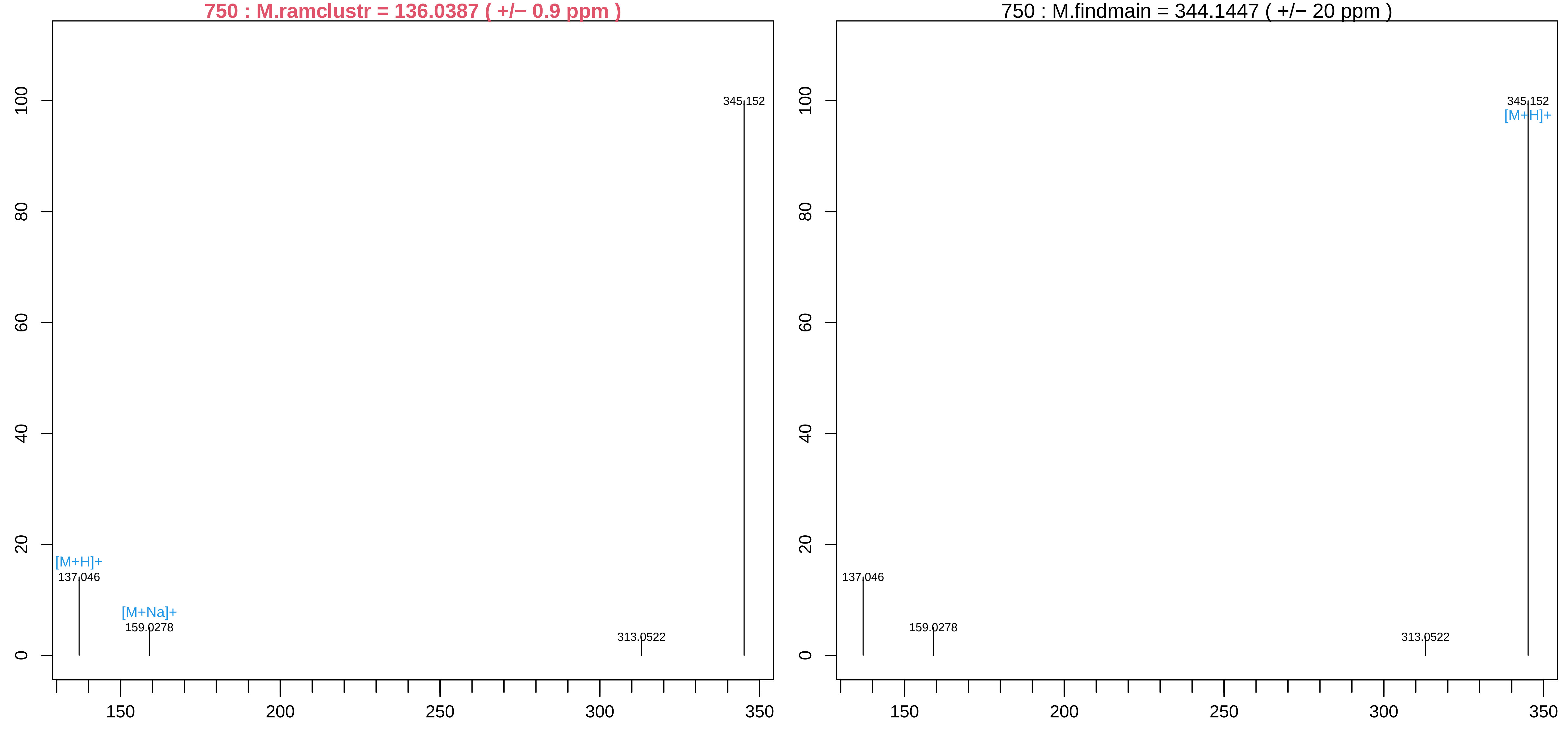

750 : M.ramclustr = 136.0387 (+/− 0.9 ppm)
[M+H]+
137.046
[M+Na]+
159.0278
313.0522
345.152
750 : M.findmain = 344.1447 (+/− 20 ppm)
137.046
159.0278
313.0522
345.152
[M+H]+
0
20
40
60
80
100
150
200
250
300
350

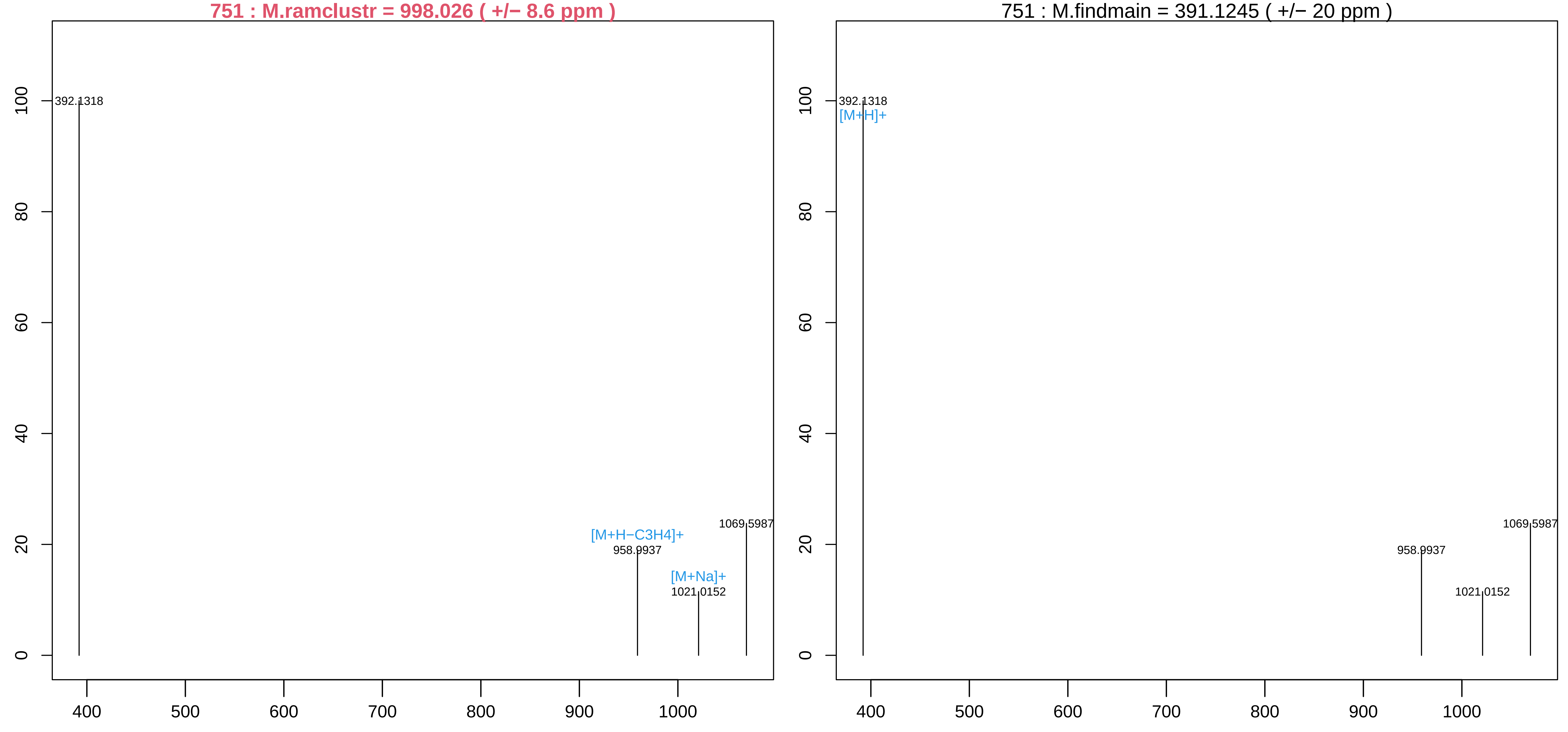
751 : M.ramclustr = 998.026 (+/− 8.6 ppm)
392.1318
[M+H−C3H4]+
958.9937
[M+Na]+
1021.0152
1069.5987
751 : M.findmain = 391.1245 (+/− 20 ppm)
392.1318
[M+H]+
958.9937
1021.0152
1069.5987
0
20
40
60
80
100
400
500
600
700
800
900
1000

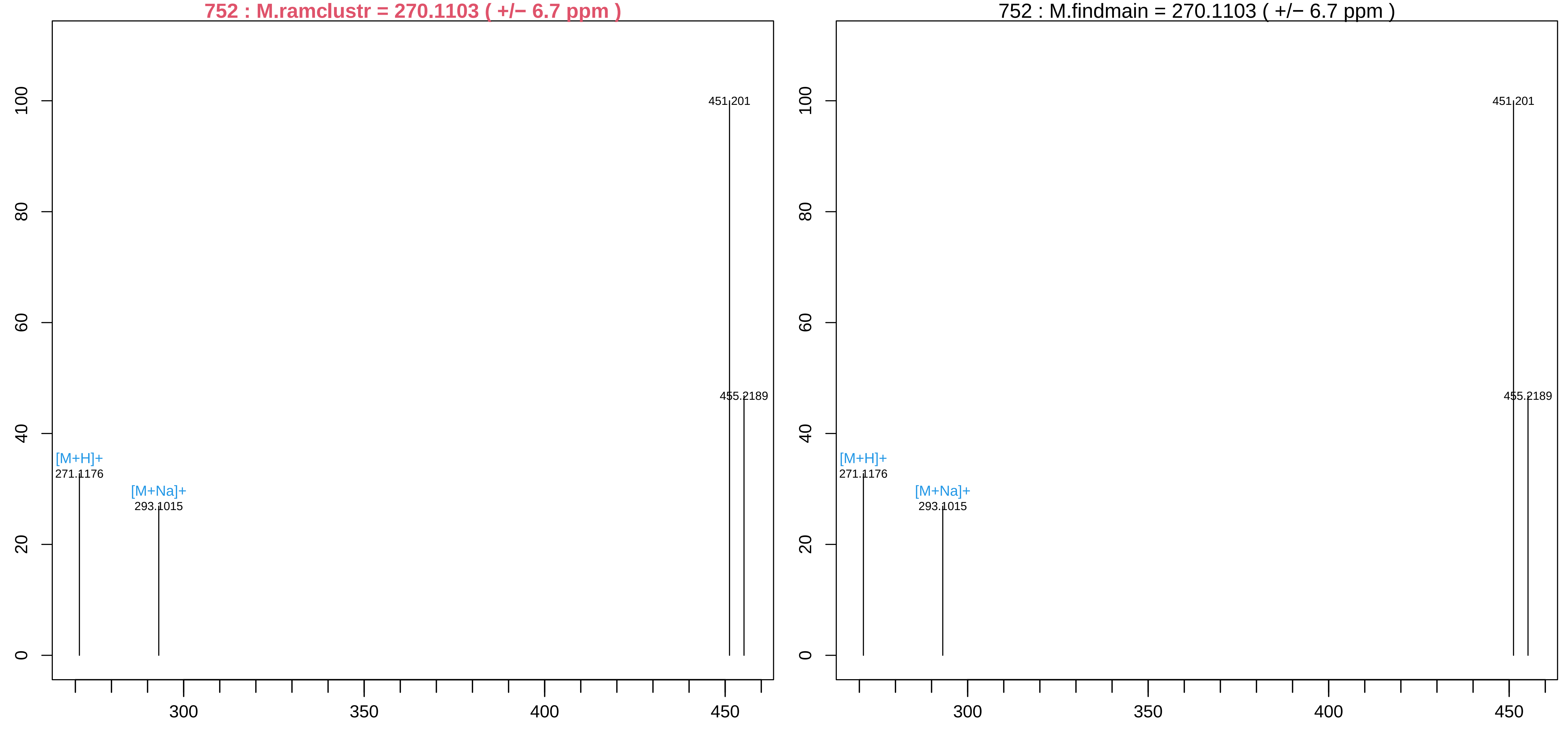
752 : M.ramclustr = 270.1103 (+/− 6.7 ppm)
752 : M.findmain = 270.1103 (+/− 6.7 ppm)
451.201
455.2189
[M+H]+
271.1176
[M+Na]+
293.1015
0
20
40
60
80
100
300
350
400
450

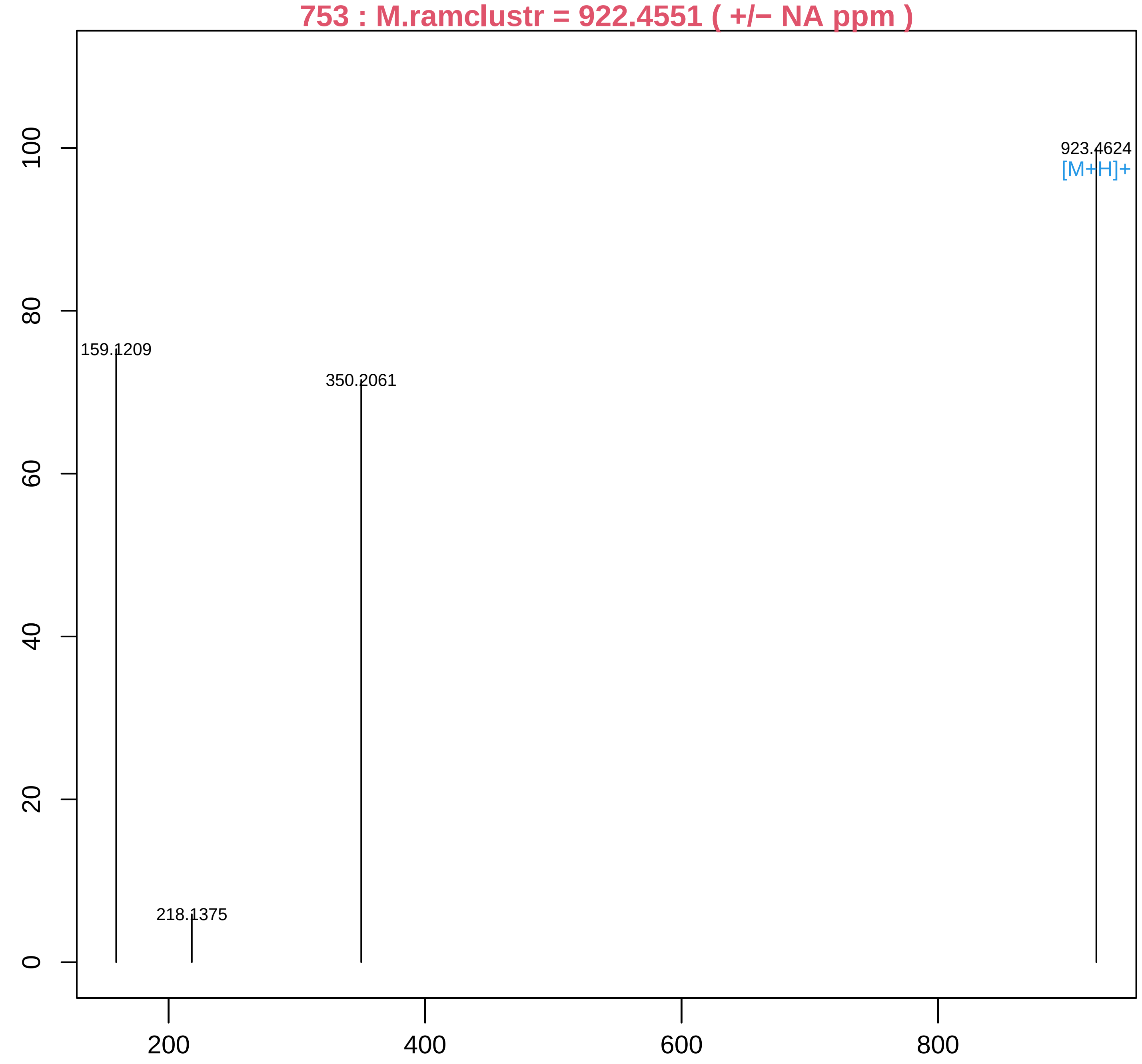
753 : M.ramclustr = 922.4551 (+/− NA ppm)
923.4624
[M+H]+
159.1209
350.2061
218.1375
0
20
40
60
80
100
200
400
600
800

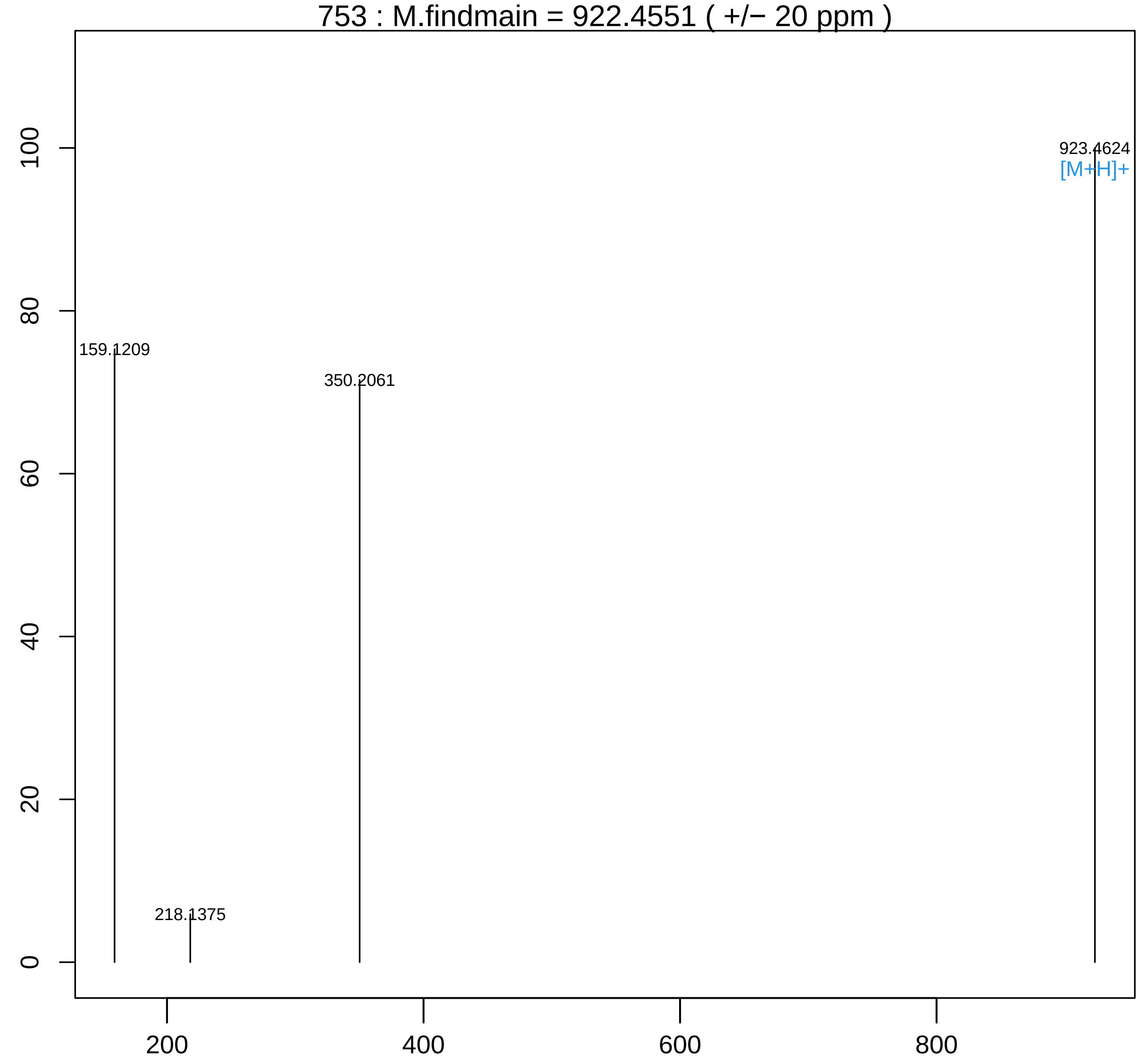
753 : M.findmain = 922.4551 (+/− 20 ppm)
923.4624
[M+H]+
159.1209
350.2061
218.1375
0
20
40
60
80
100
200
400
600
800

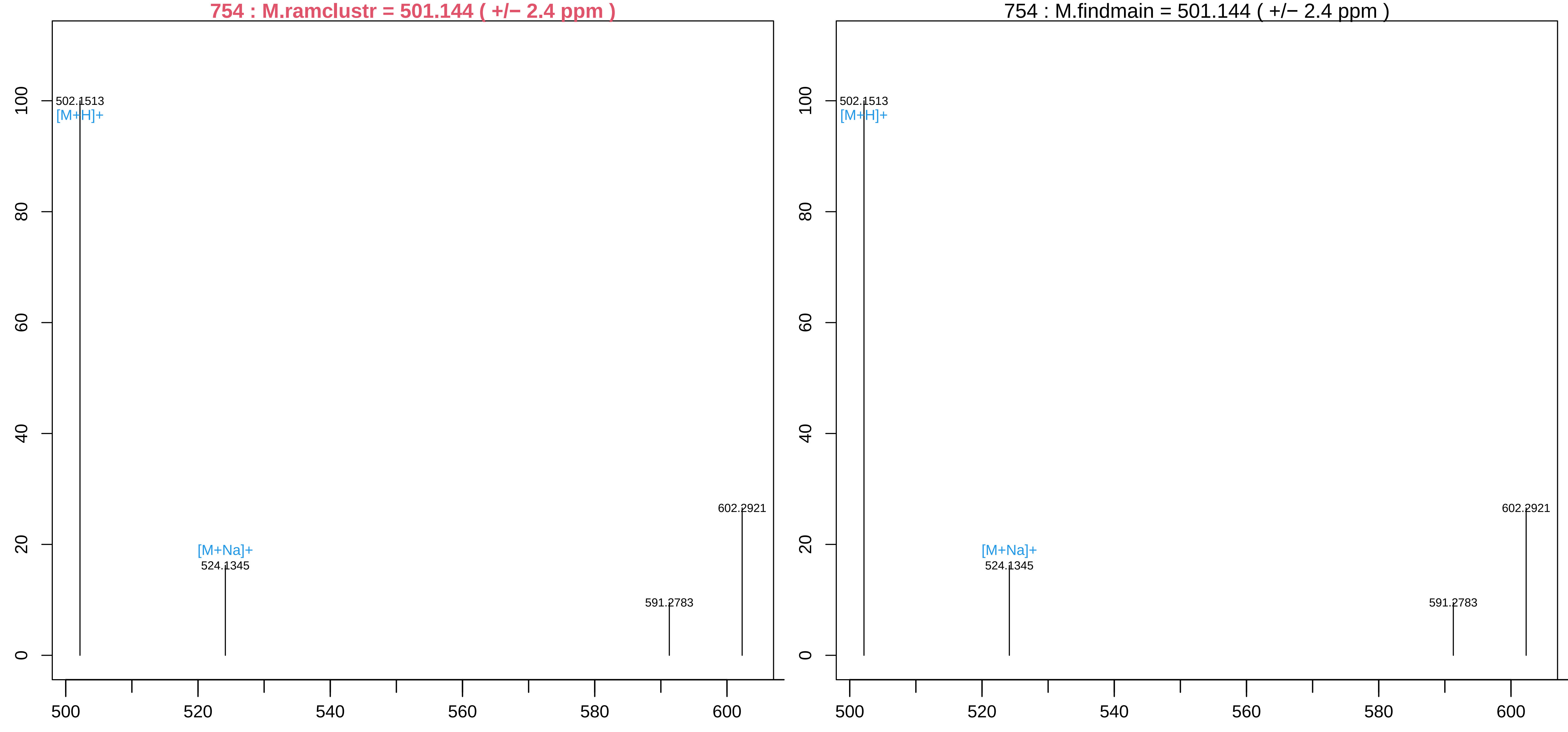
754 : M.ramclustr = 501.144 (+/− 2.4 ppm)
502.1513
[M+H]+
[M+Na]+
524.1345
591.2783
602.2921
0
20
40
60
80
100
500
520
540
560
580
600
754 : M.findmain = 501.144 (+/− 2.4 ppm)
502.1513
[M+H]+
[M+Na]+
524.1345
591.2783
602.2921
0
20
40
60
80
100
500
520
540
560
580
600

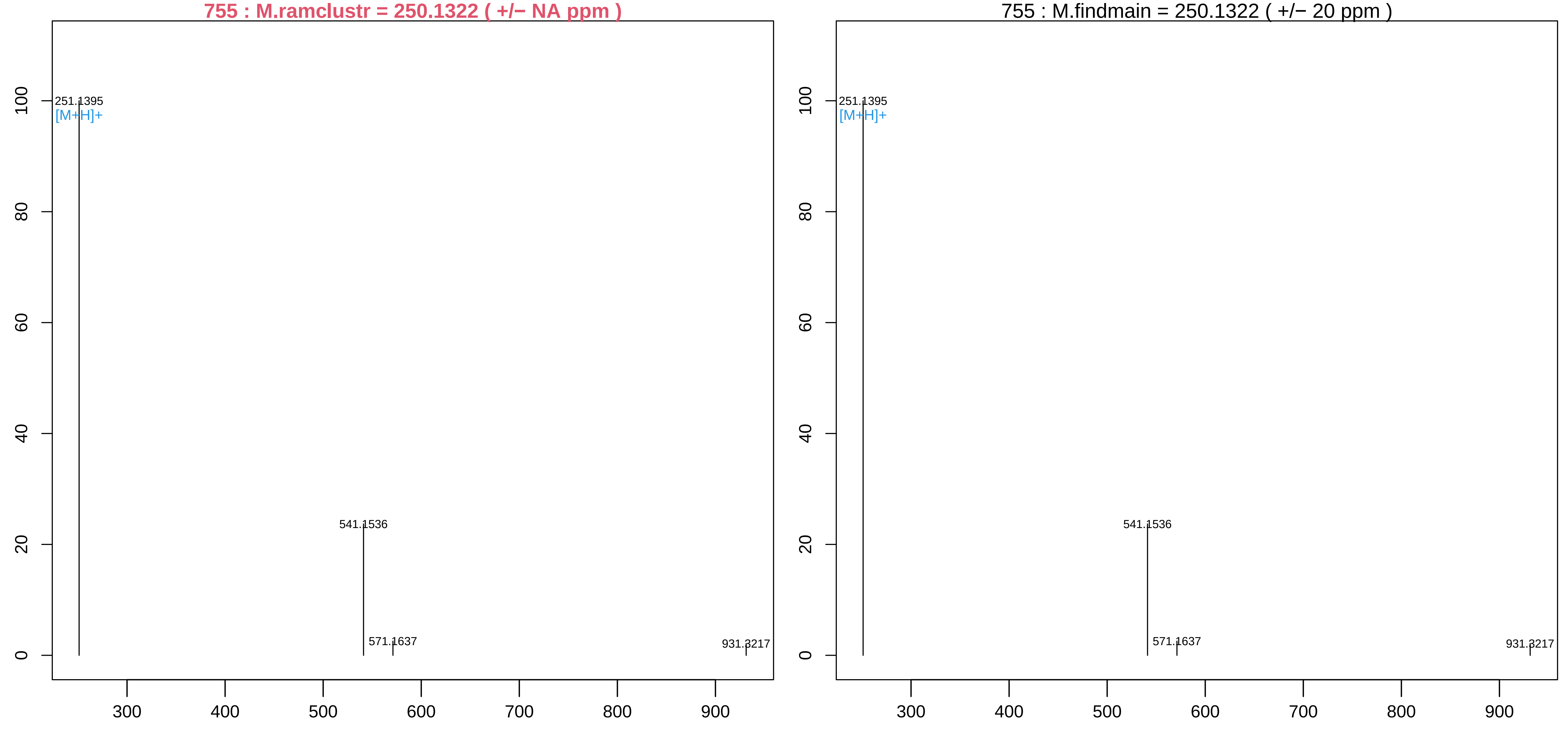
755 : M.ramclustr = 250.1322 (+/− NA ppm)
251.1395
[M+H]+
541.1536
571.1637
931.3217
755 : M.findmain = 250.1322 (+/− 20 ppm)
251.1395
[M+H]+
541.1536
571.1637
931.3217

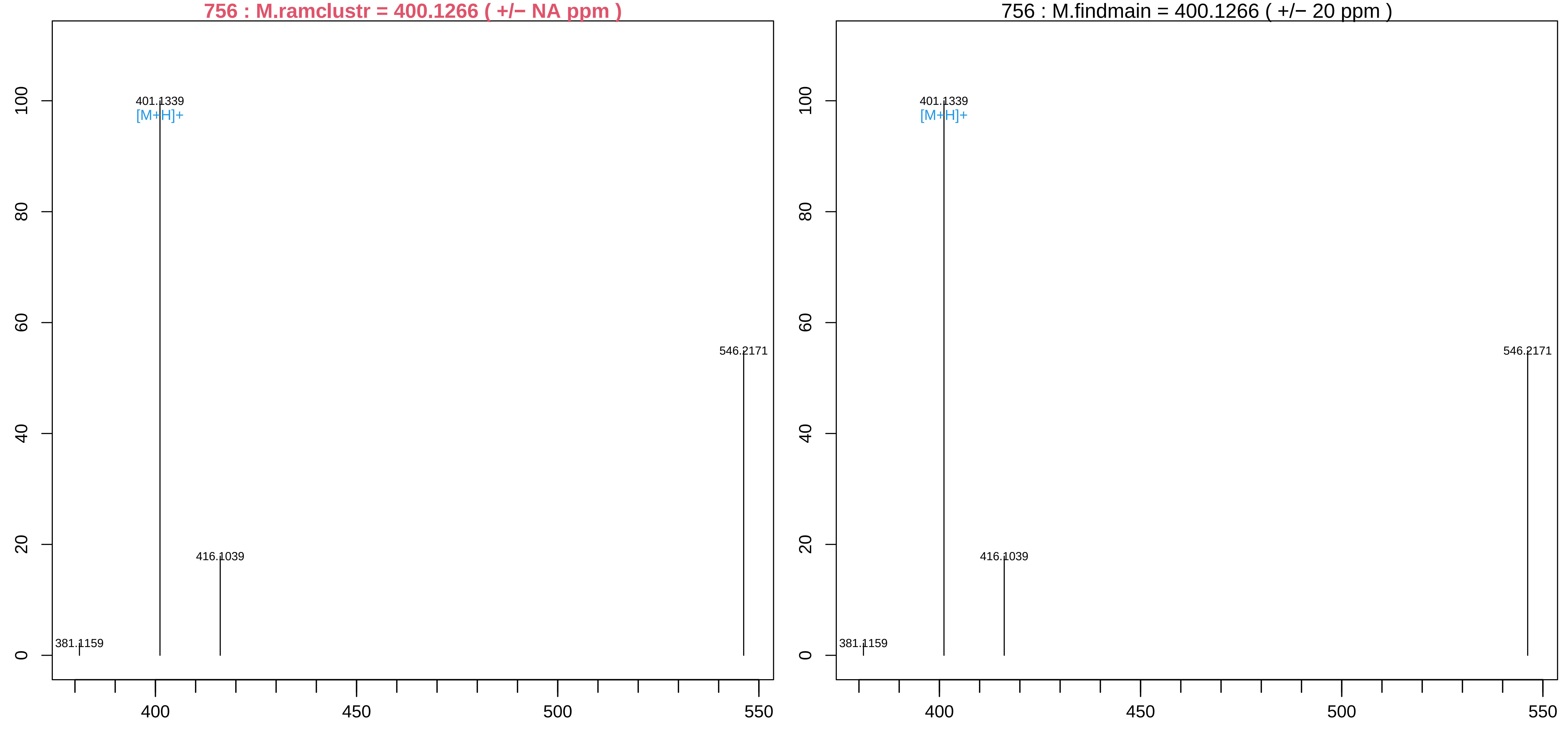
756 : M.ramclustr = 400.1266 (+/− NA ppm)
401.1339
[M+H]+
546.2171
416.1039
381.1159
756 : M.findmain = 400.1266 (+/− 20 ppm)
401.1339
[M+H]+
546.2171
416.1039
381.1159

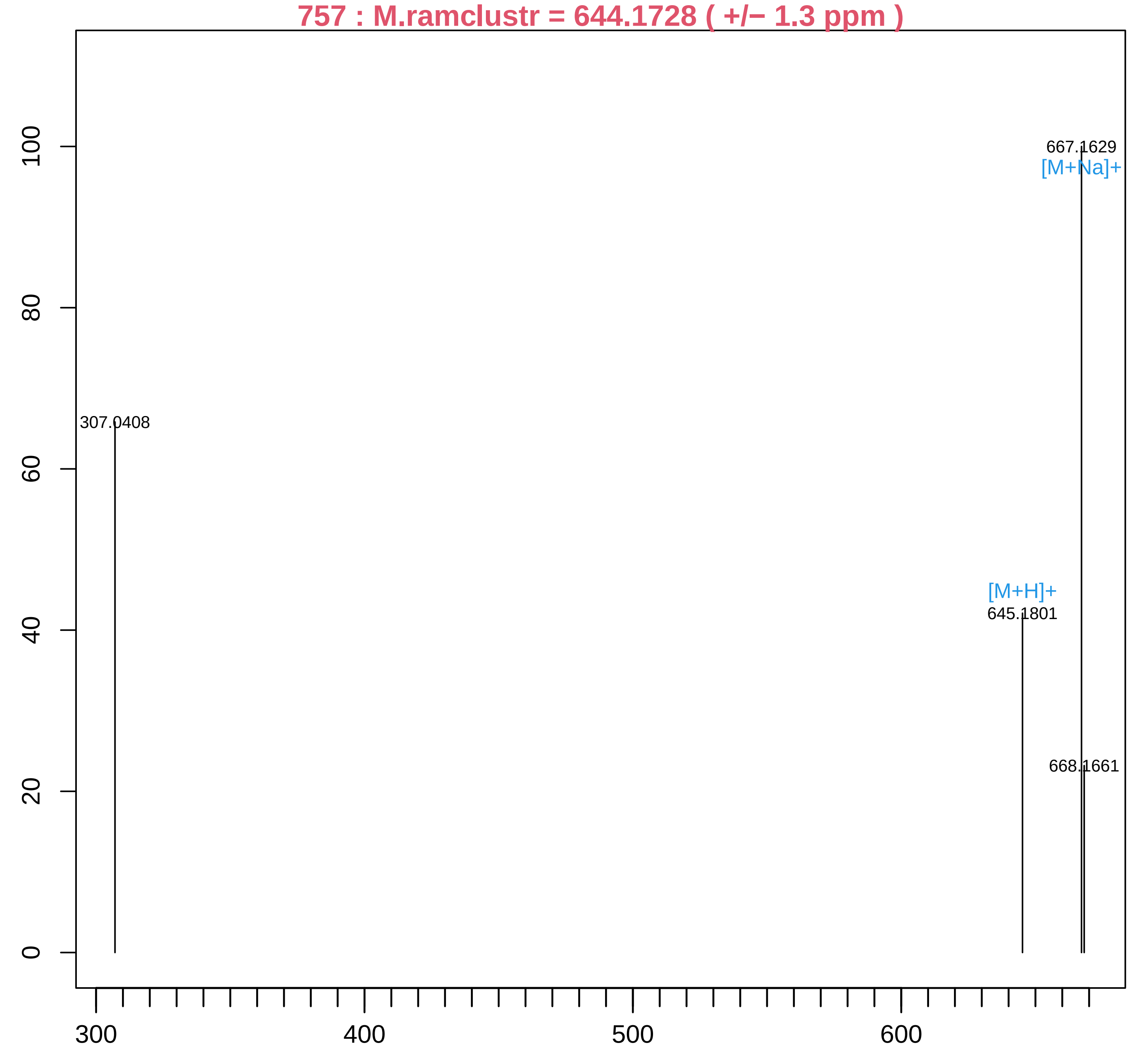
757 : M.ramclustr = 644.1728 (+/− 1.3 ppm)
667.1629
[M+Na]+
307.0408
[M+H]+
645.1801
668.1661
0
20
40
60
80
100
300
400
500
600

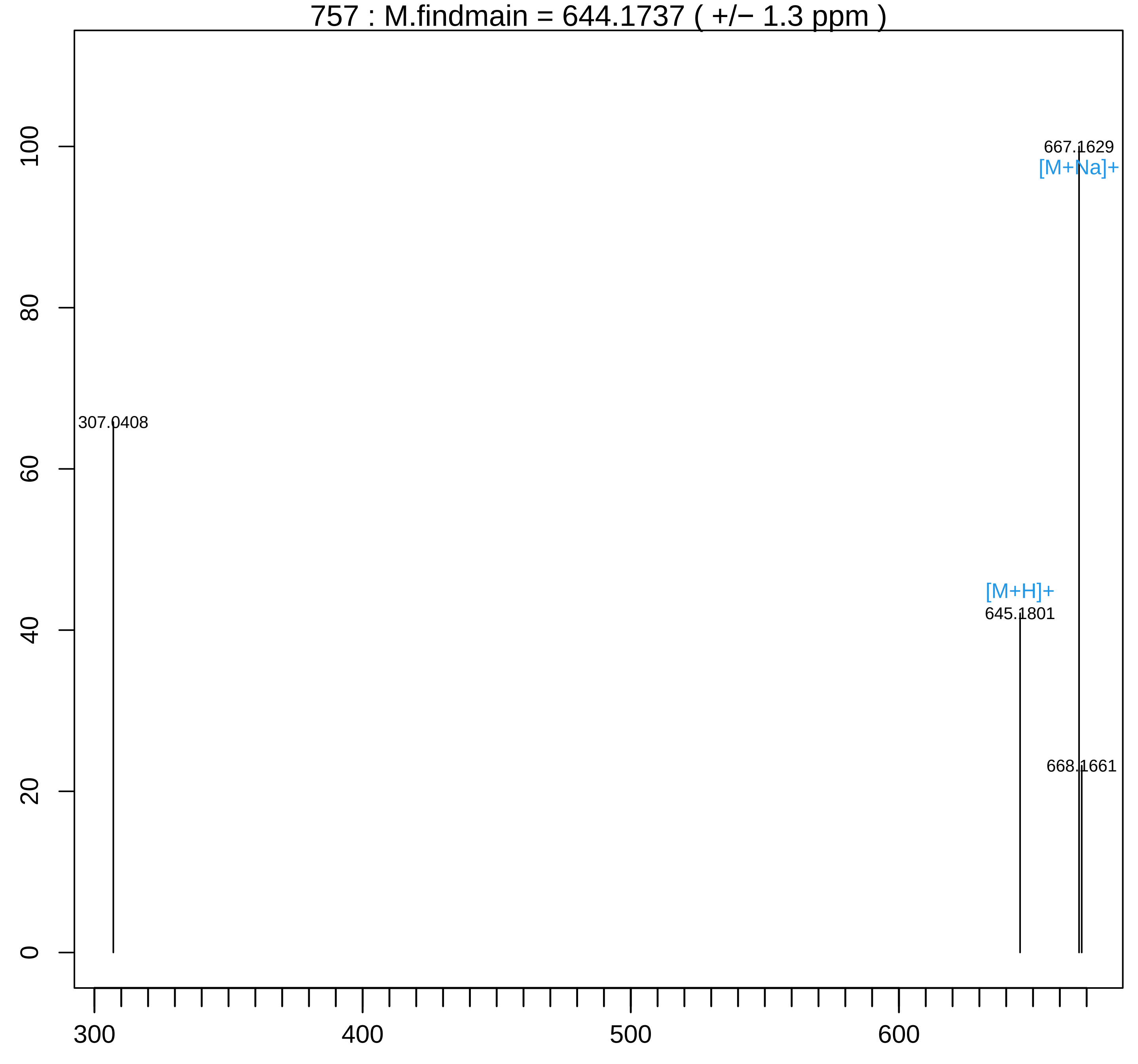
757 : M.findmain = 644.1737 (+/− 1.3 ppm)
667.1629
[M+Na]+
307.0408
[M+H]+
645.1801
668.1661
0
20
40
60
80
100
300
400
500
600

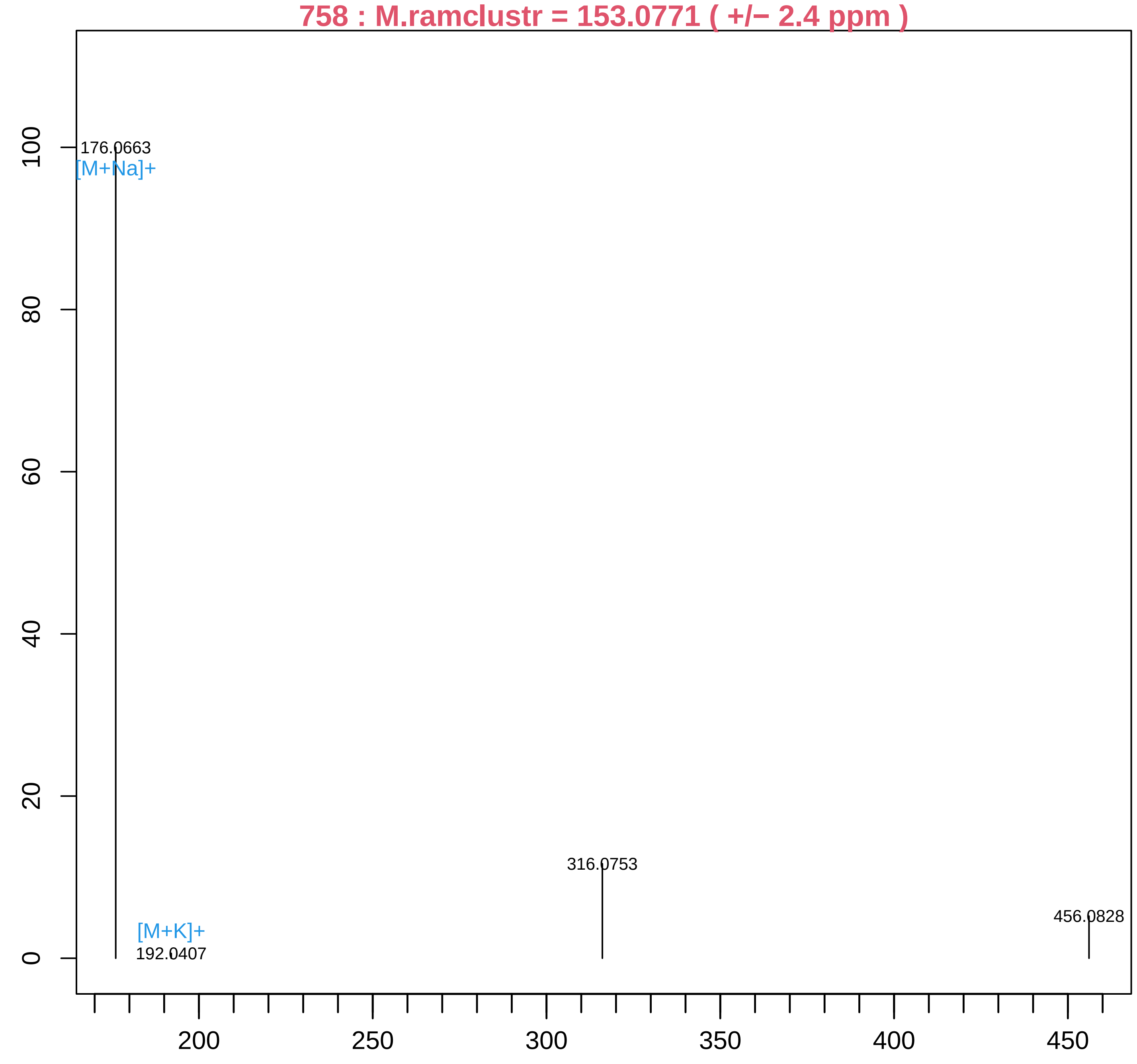
758 : M.ramclustr = 153.0771 (+/− 2.4 ppm)
176.0663
[M+Na]+
[M+K]+
192.0407
316.0753
456.0828
0
20
40
60
80
100
200
250
300
350
400
450

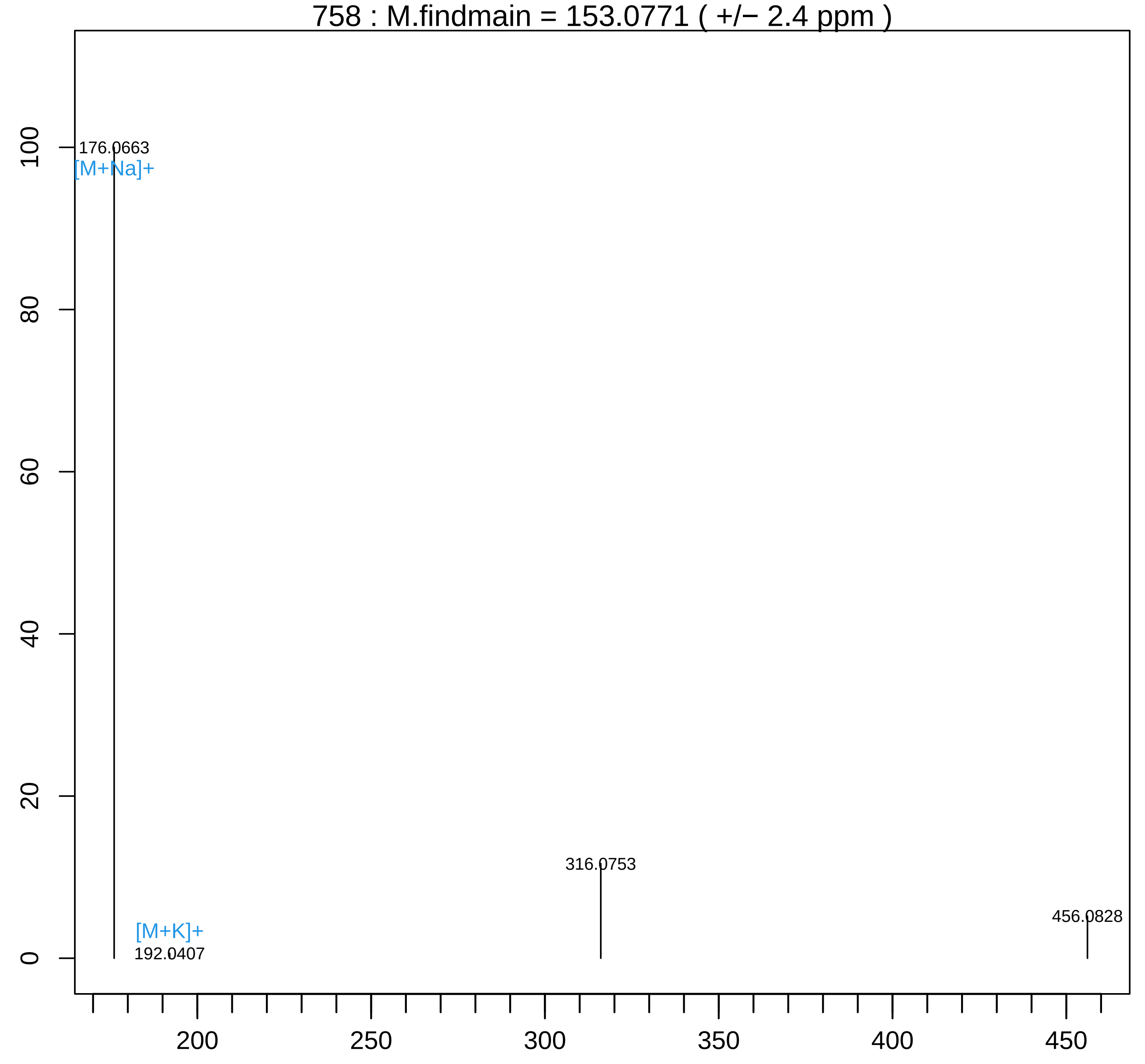
758 : M.findmain = 153.0771 (+/− 2.4 ppm)
176.0663
[M+Na]+
[M+K]+
192.0407
316.0753
456.0828
0
20
40
60
80
100
200
250
300
350
400
450

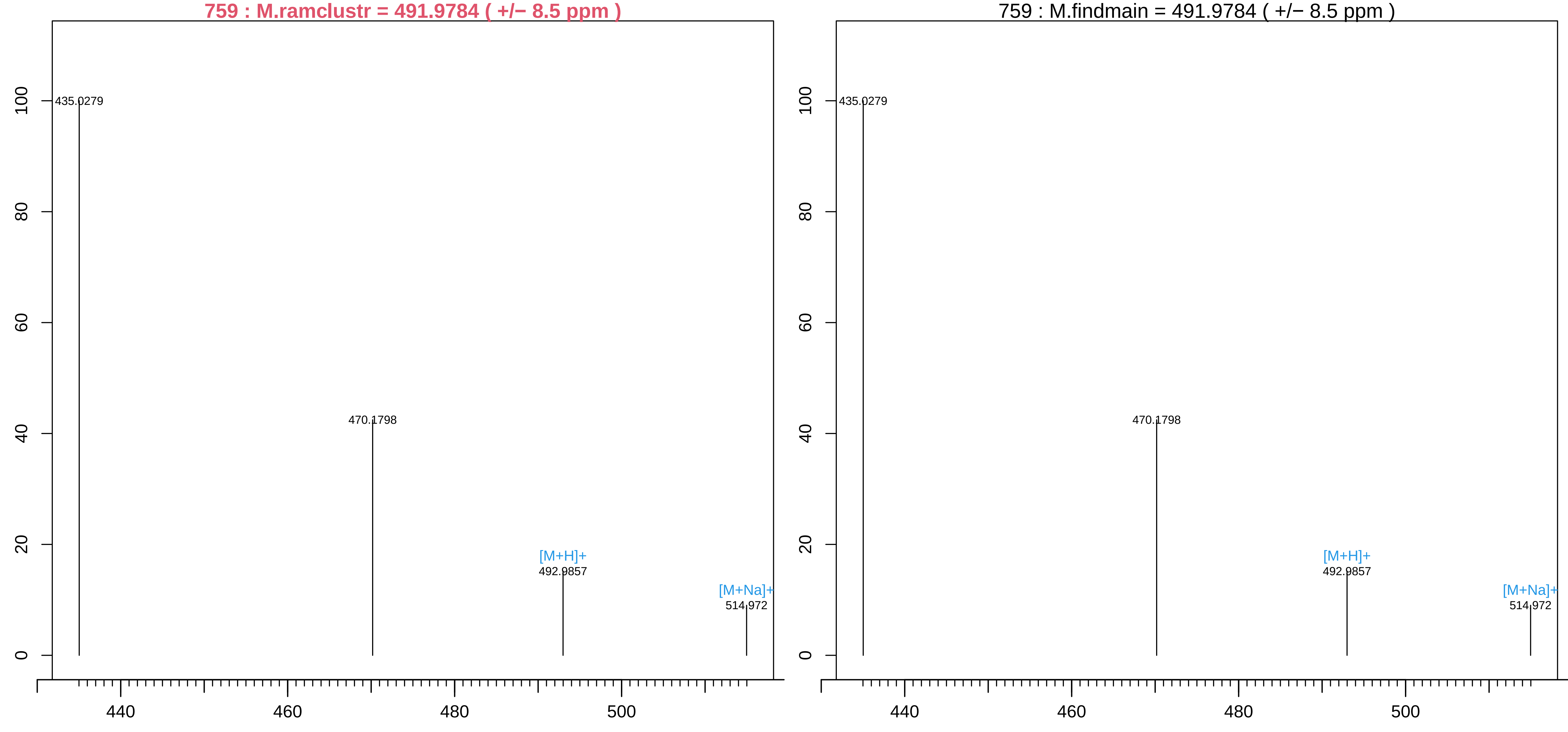
759 : M.ramclustr = 491.9784 (+/− 8.5 ppm)
435.0279
470.1798
[M+H]+
492.9857
[M+Na]+
514.972
0
20
40
60
80
100
440
460
480
500
759 : M.findmain = 491.9784 (+/− 8.5 ppm)
435.0279
470.1798
[M+H]+
492.9857
[M+Na]+
514.972
0
20
40
60
80
100
440
460
480
500

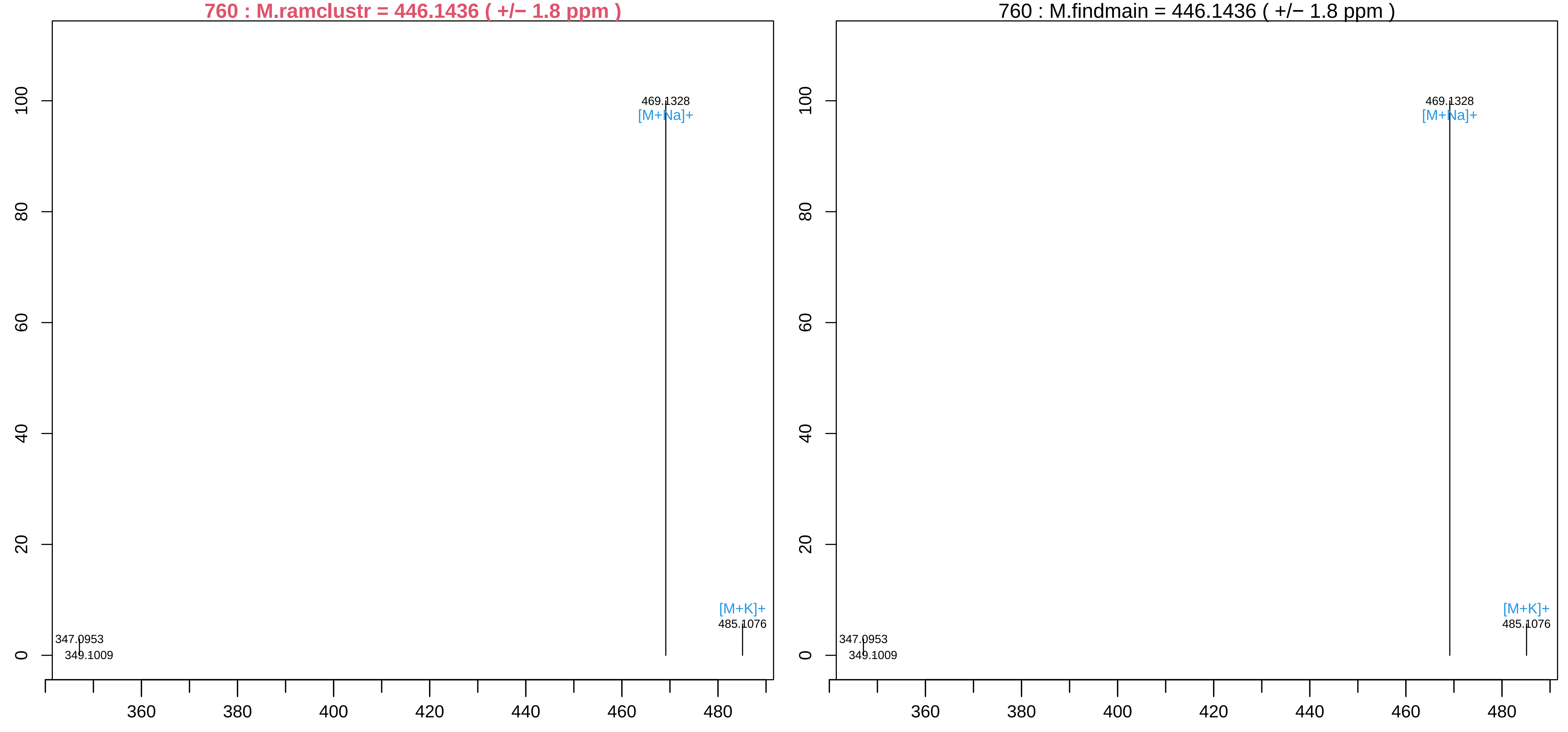

760 : M.ramclustr = 446.1436 (+/− 1.8 ppm)
469.1328
[M+Na]+
[M+K]+
485.1076
347.0953
349.1009
0
20
40
60
80
100
360
380
400
420
440
460
480
760 : M.findmain = 446.1436 (+/− 1.8 ppm)
469.1328
[M+Na]+
[M+K]+
485.1076
347.0953
349.1009
0
20
40
60
80
100
360
380
400
420
440
460
480

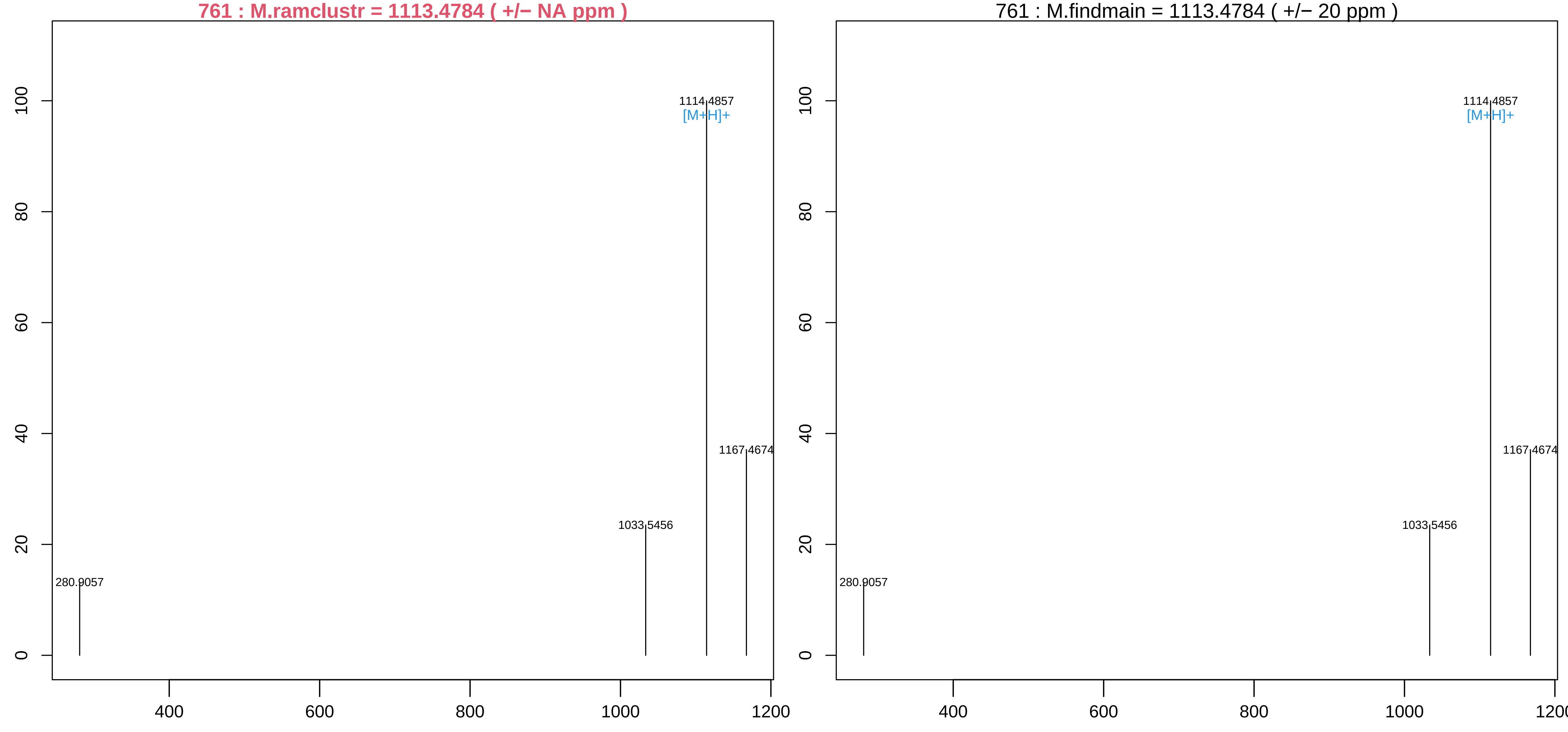

761 : M.ramclustr = 1113.4784 (+/− NA ppm)
761 : M.findmain = 1113.4784 (+/− 20 ppm)
1114.4857
[M+H]+
1167.4674
1033.5456
280.9057
0
20
40
60
80
100
400
600
800
1000
1200

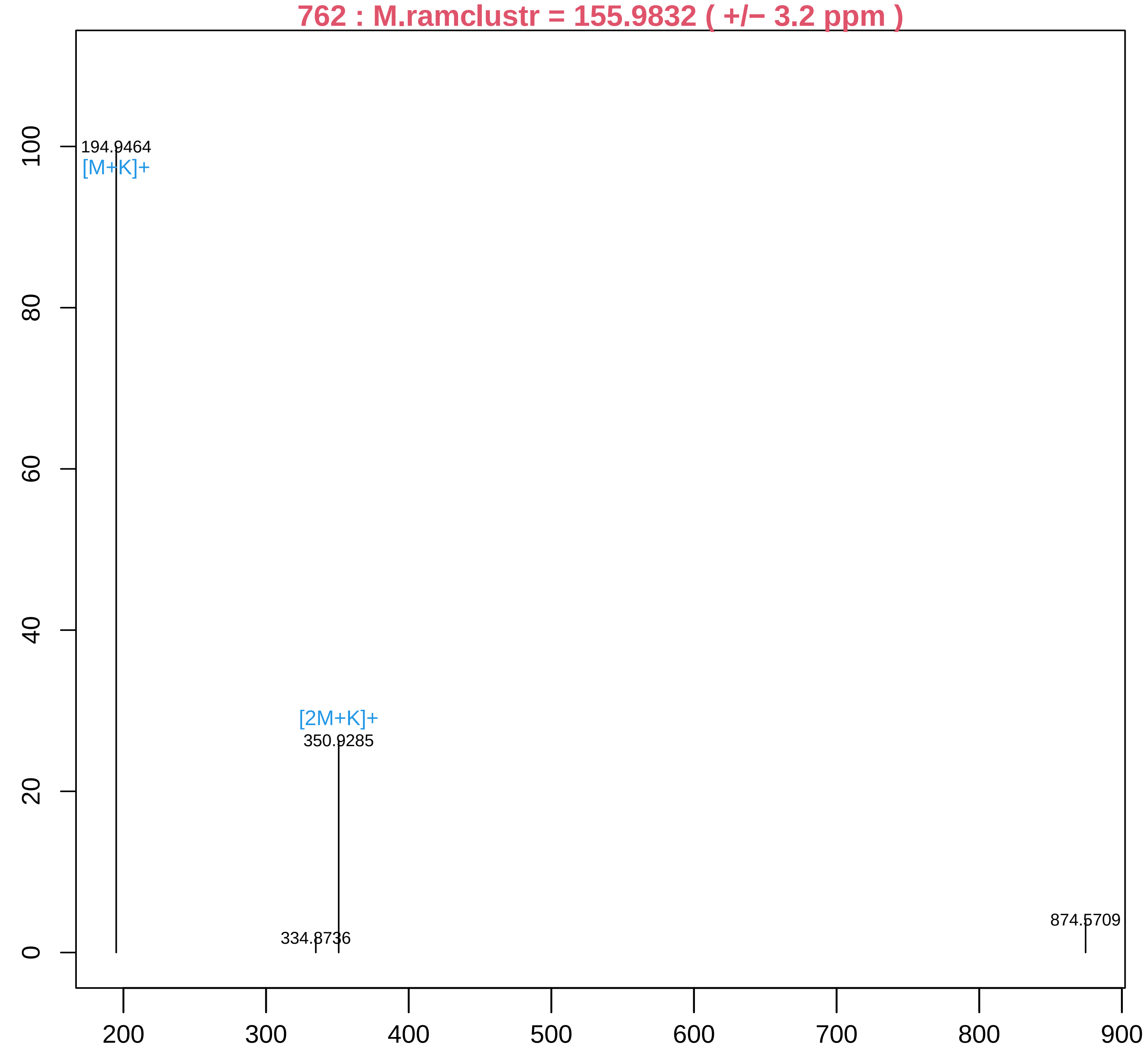

762 : M.ramclustr = 155.9832 (+/− 3.2 ppm)
194.9464
[M+K]+
[2M+K]+
350.9285
334.8736
874.5709
0
20
40
60
80
100
200
300
400
500
600
700
800
900

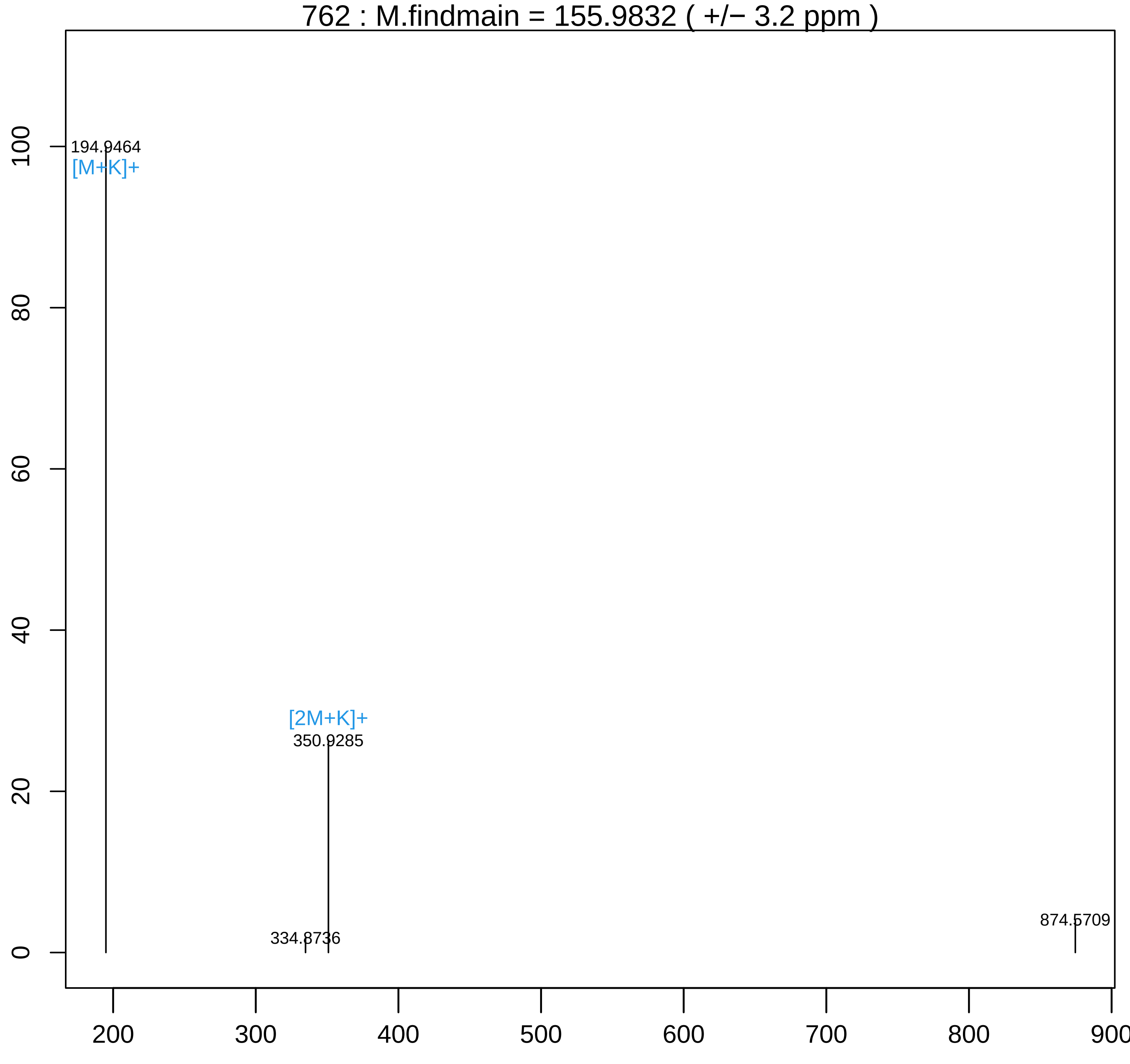

762 : M.findmain = 155.9832 (+/− 3.2 ppm)
194.9464
[M+K]+
[2M+K]+
350.9285
334.8736
874.5709
0
20
40
60
80
100
200
300
400
500
600
700
800
900

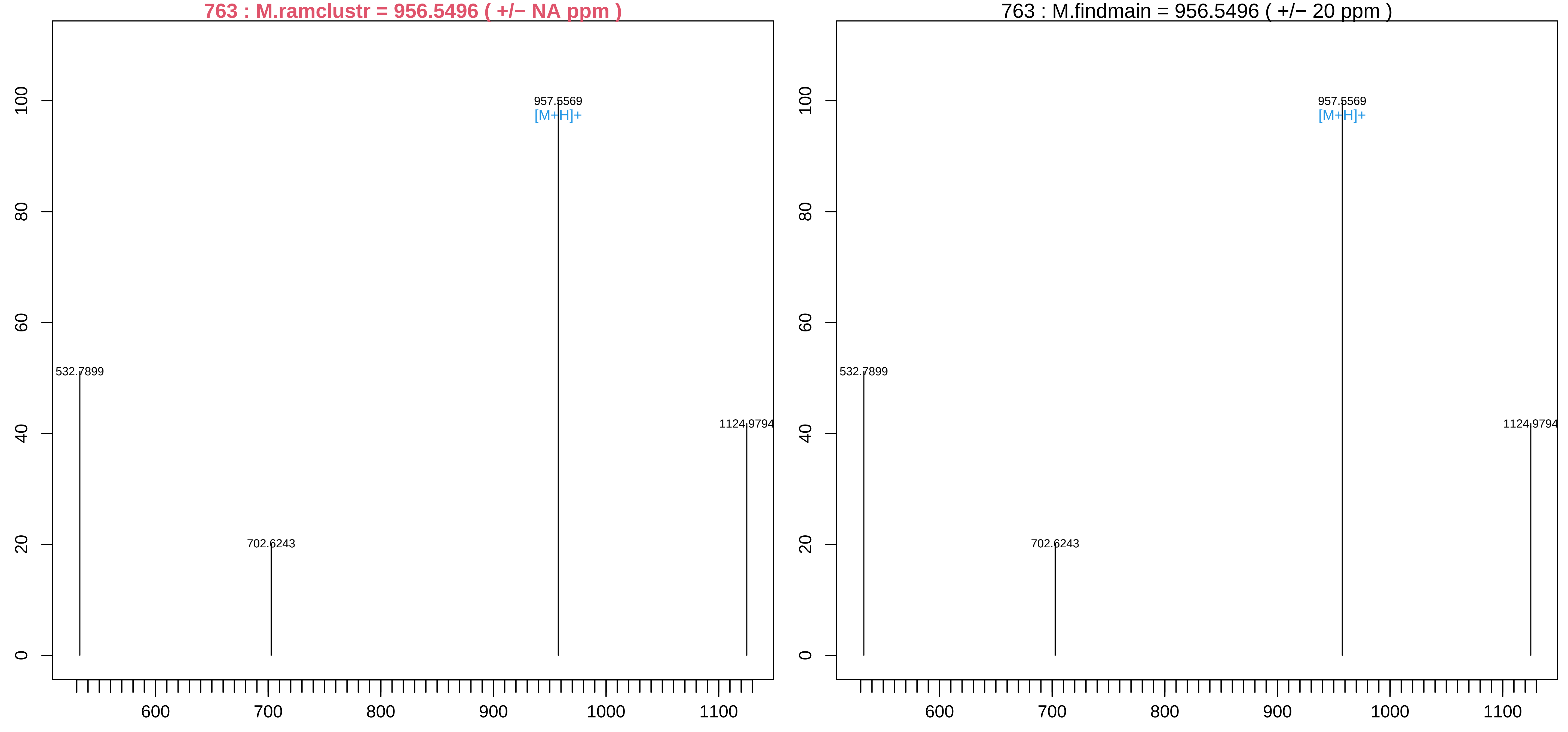
763 : M.ramclustr = 956.5496 (+/− NA ppm)
957.5569
[M+H]+
532.7899
1124.9794
702.6243
763 : M.findmain = 956.5496 (+/− 20 ppm)
957.5569
[M+H]+
532.7899
1124.9794
702.6243
0
20
40
60
80
100
600
700
800
900
1000
1100

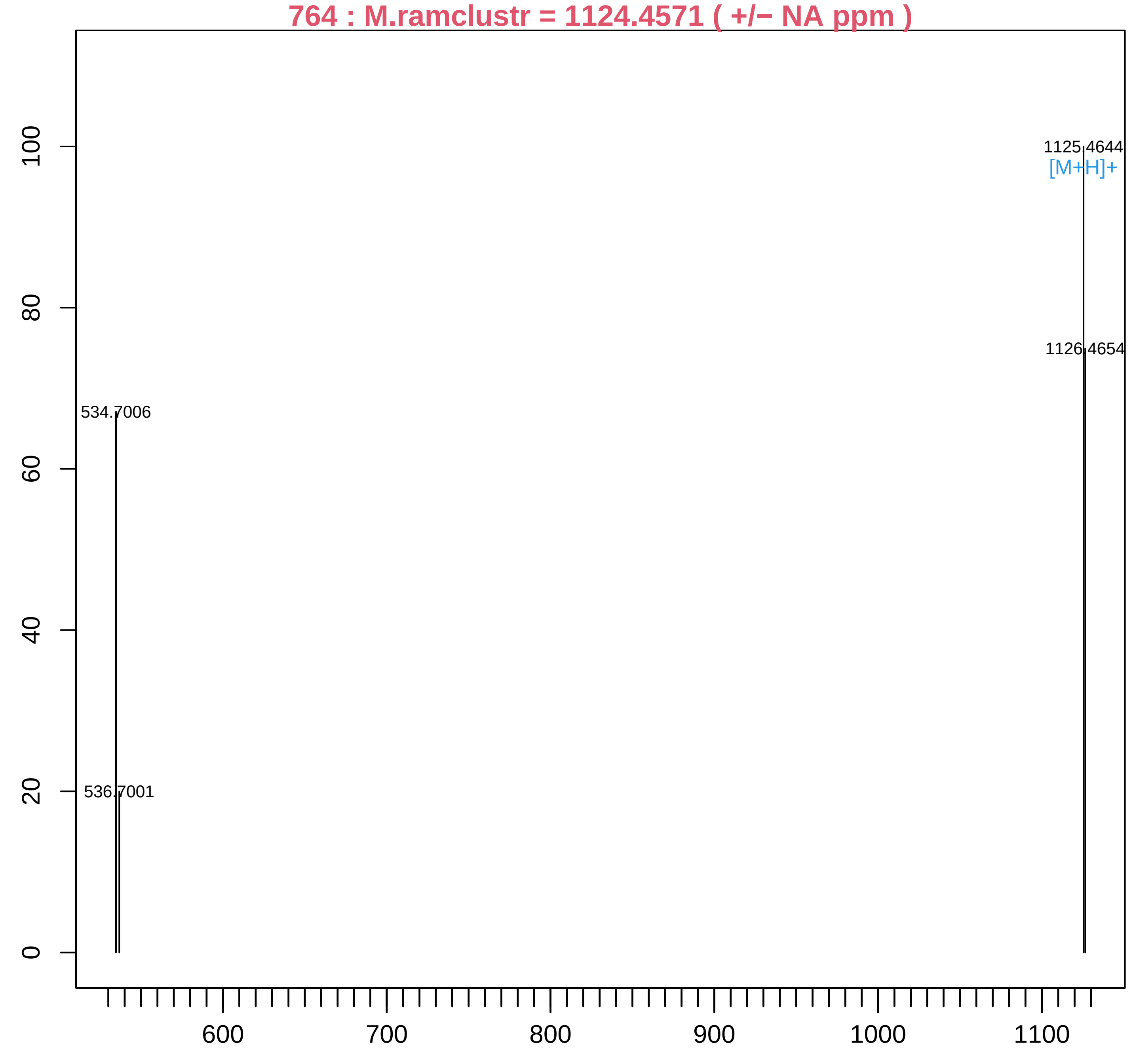
764 : M.ramclustr = 1124.4571 (+/− NA ppm)
1125.4644
[M+H]+
1126.4654
534.7006
536.7001
0
20
40
60
80
100
600
700
800
900
1000
1100

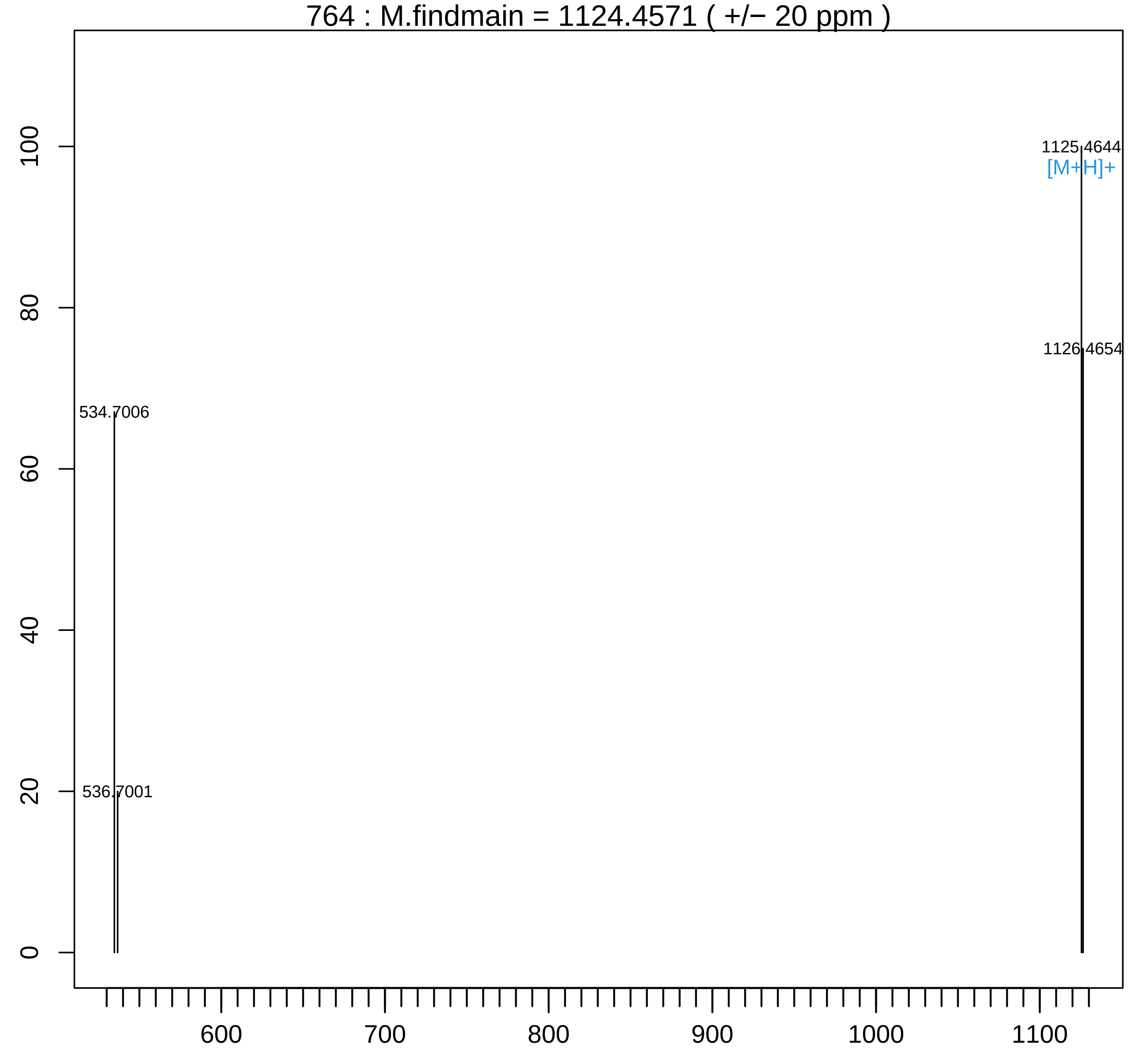
764 : M.findmain = 1124.4571 (+/− 20 ppm)
1125.4644
[M+H]+
1126.4654
534.7006
536.7001
0
20
40
60
80
100
600
700
800
900
1000
1100

765 : M.ramclustr = 1031.5201 (+/− NA ppm)

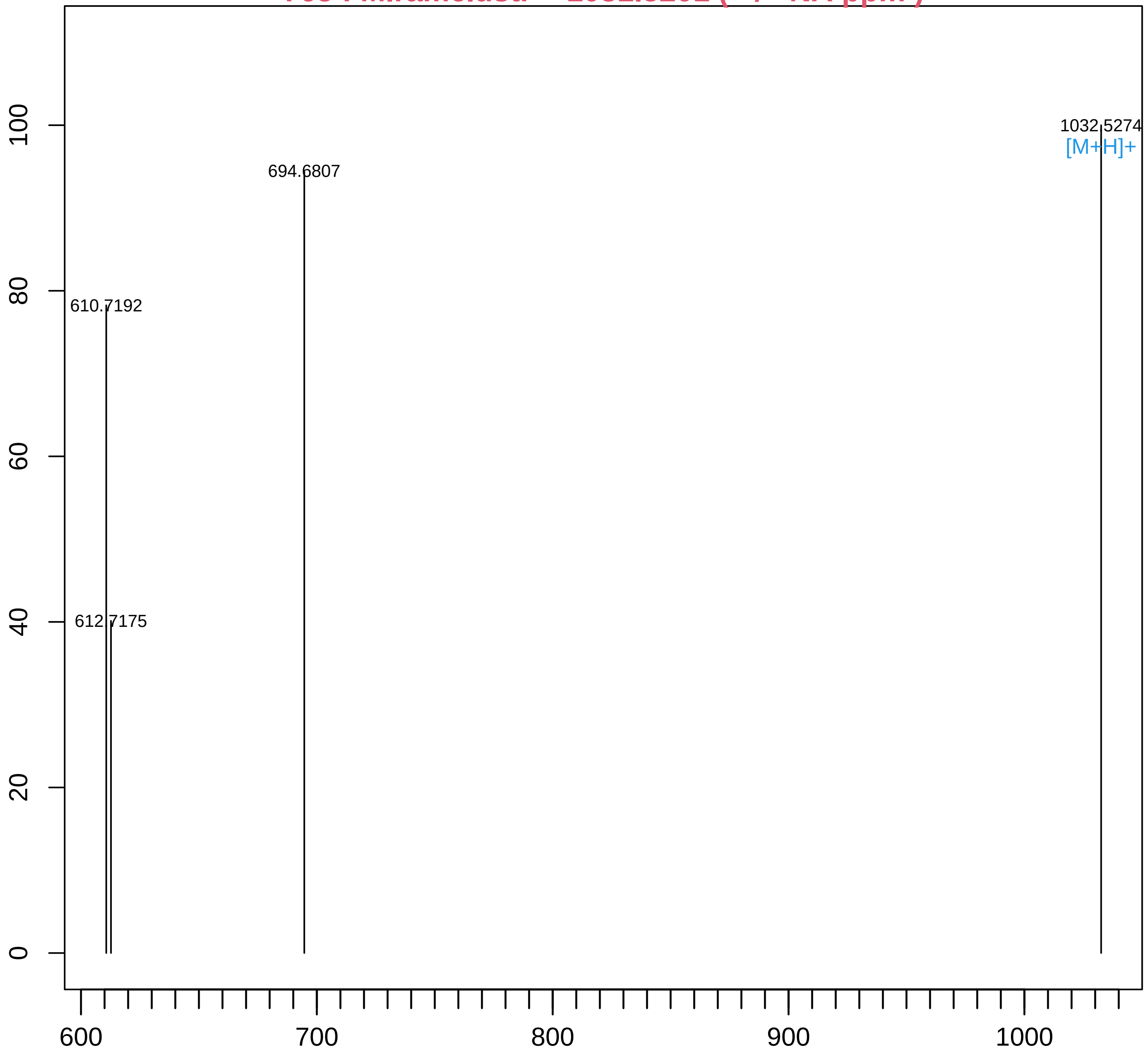

765 : M.findmain = 1031.5201 (+/− 20 ppm)

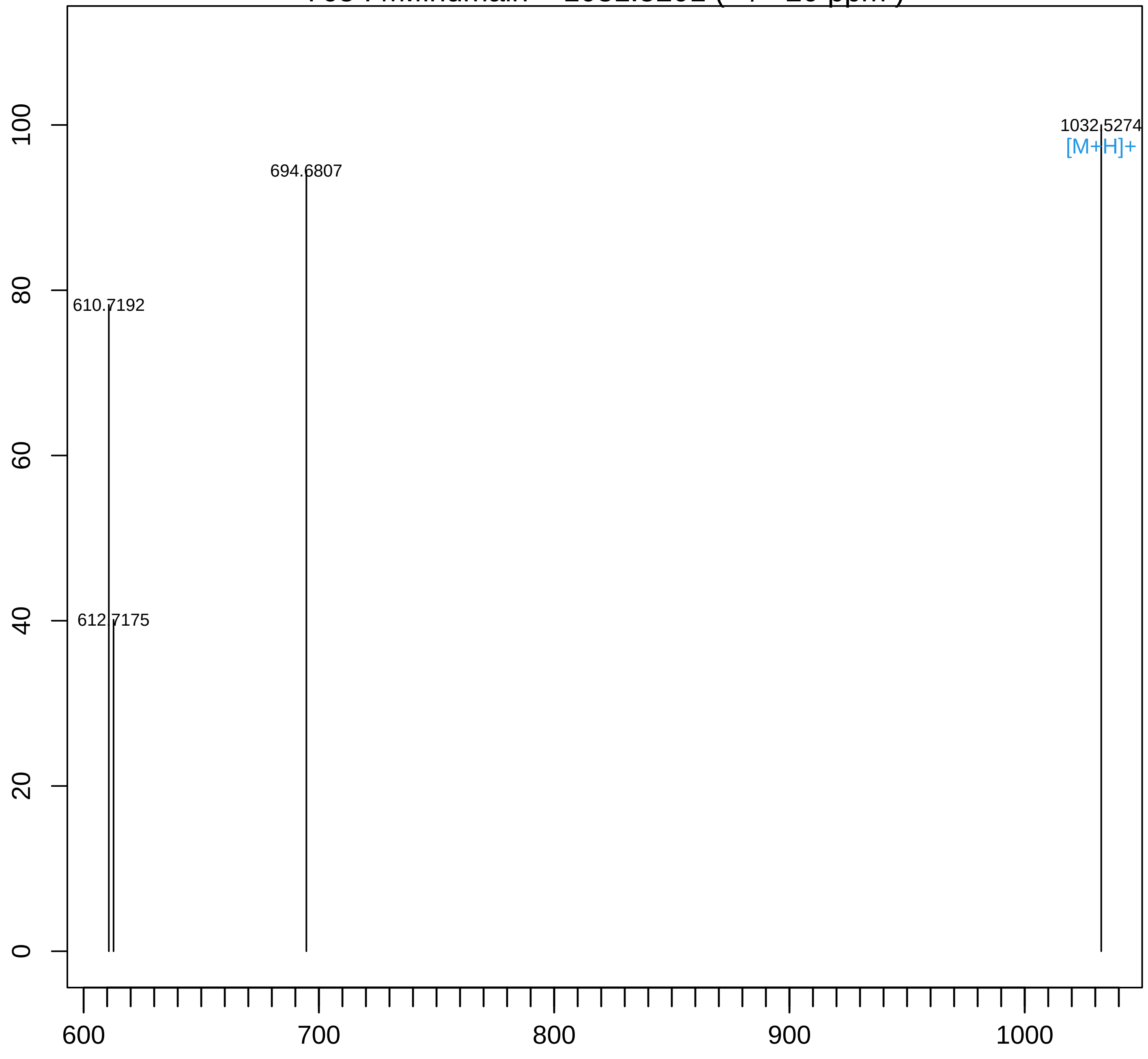

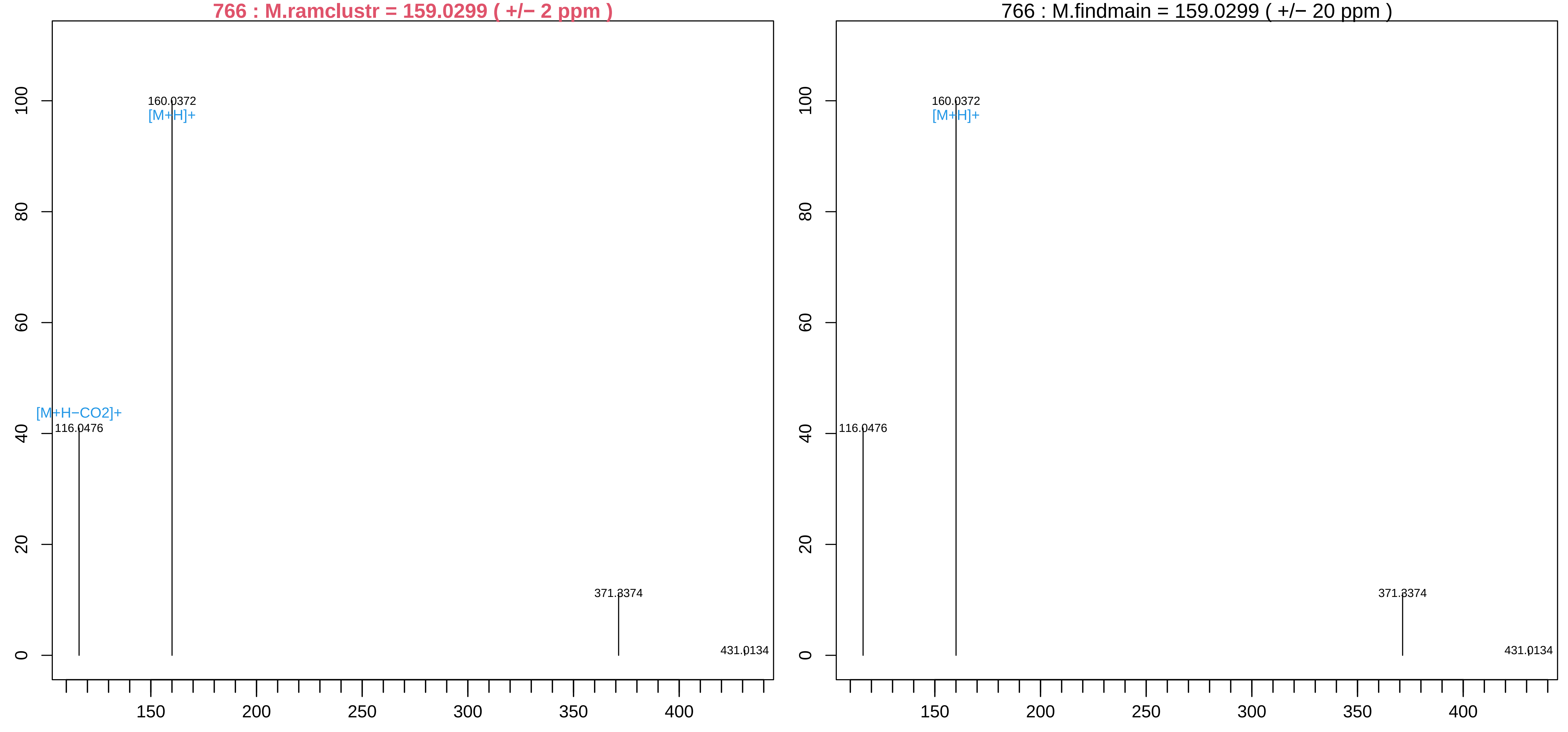
766 : M.ramclustr = 159.0299 (+/− 2 ppm)
160.0372
[M+H]+
[M+H−CO2]+
116.0476
371.3374
431.0134
0
20
40
60
80
100
150
200
250
300
350
400
766 : M.findmain = 159.0299 (+/− 20 ppm)
160.0372
[M+H]+
116.0476
371.3374
431.0134
0
20
40
60
80
100
150
200
250
300
350
400

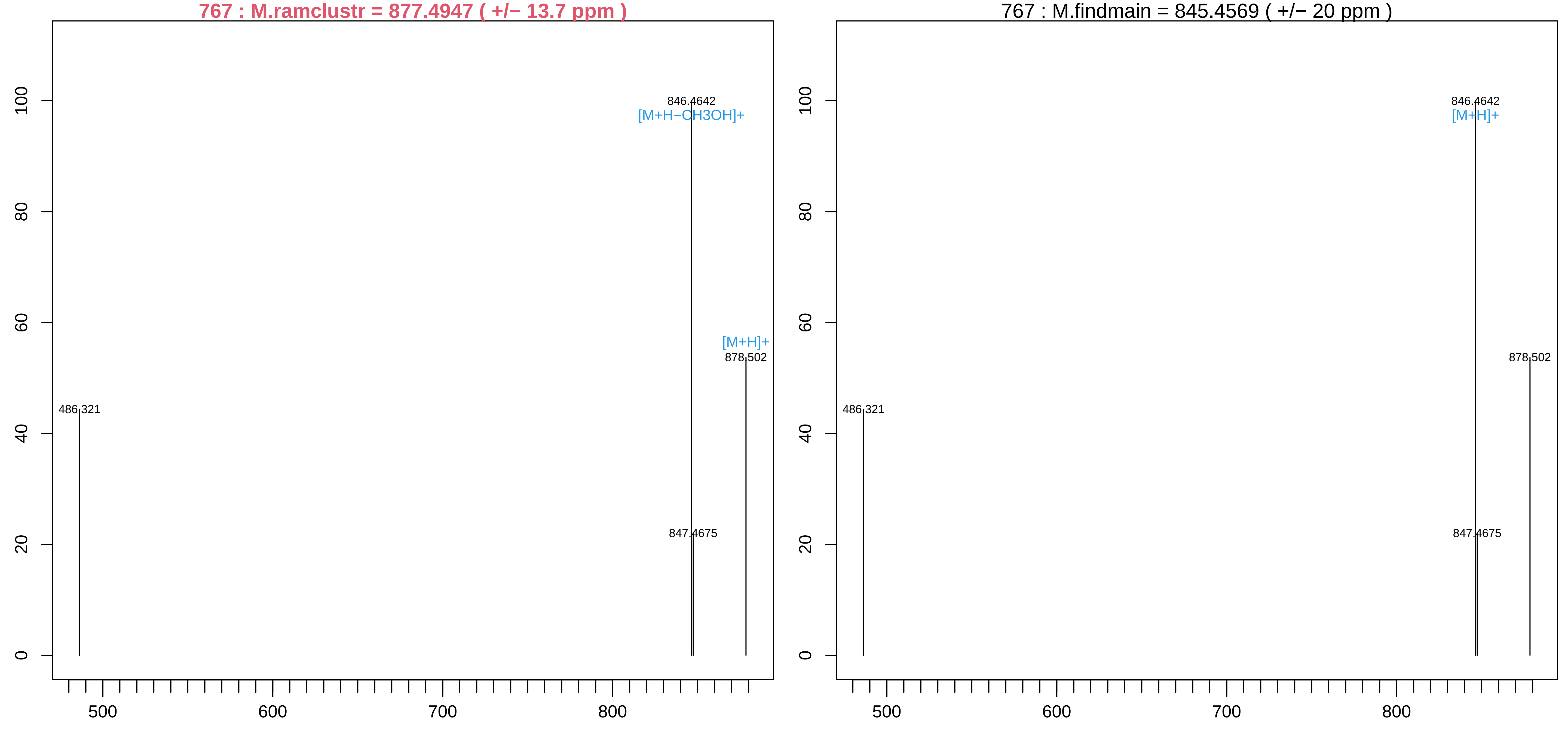

767 : M.ramclustr = 877.4947 (+/− 13.7 ppm)
846.4642
[M+H−CH3OH]+
[M+H]+
878.502
486.321
847.4675
767 : M.findmain = 845.4569 (+/− 20 ppm)
846.4642
[M+H]+
878.502
486.321
847.4675
0
20
40
60
80
100
500
600
700
800

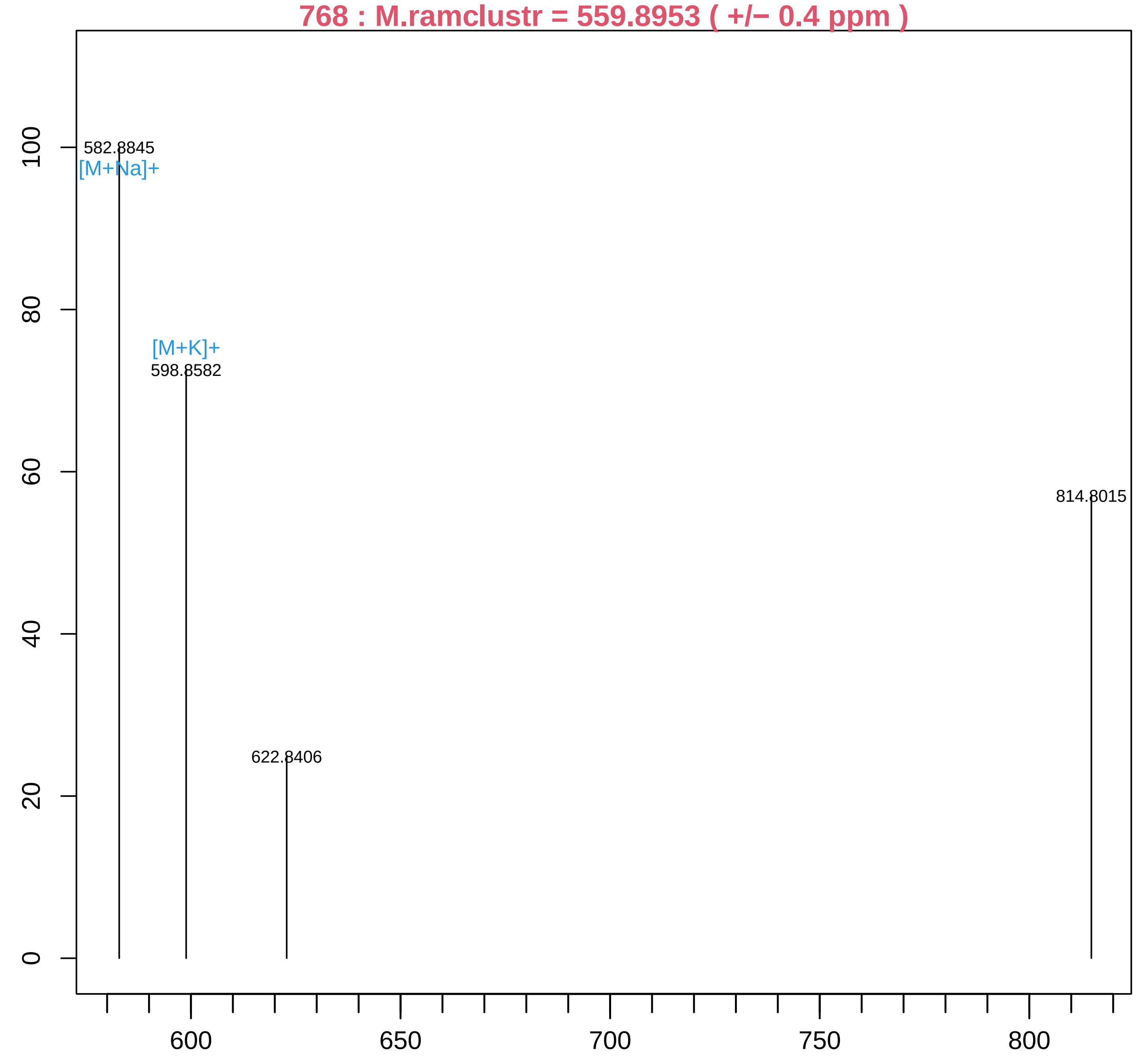
768 : M.ramclustr = 559.8953 (+/− 0.4 ppm)
582.8845
[M+Na]+
[M+K]+
598.8582
622.8406
814.8015
0
20
40
60
80
100
600
650
700
750
800
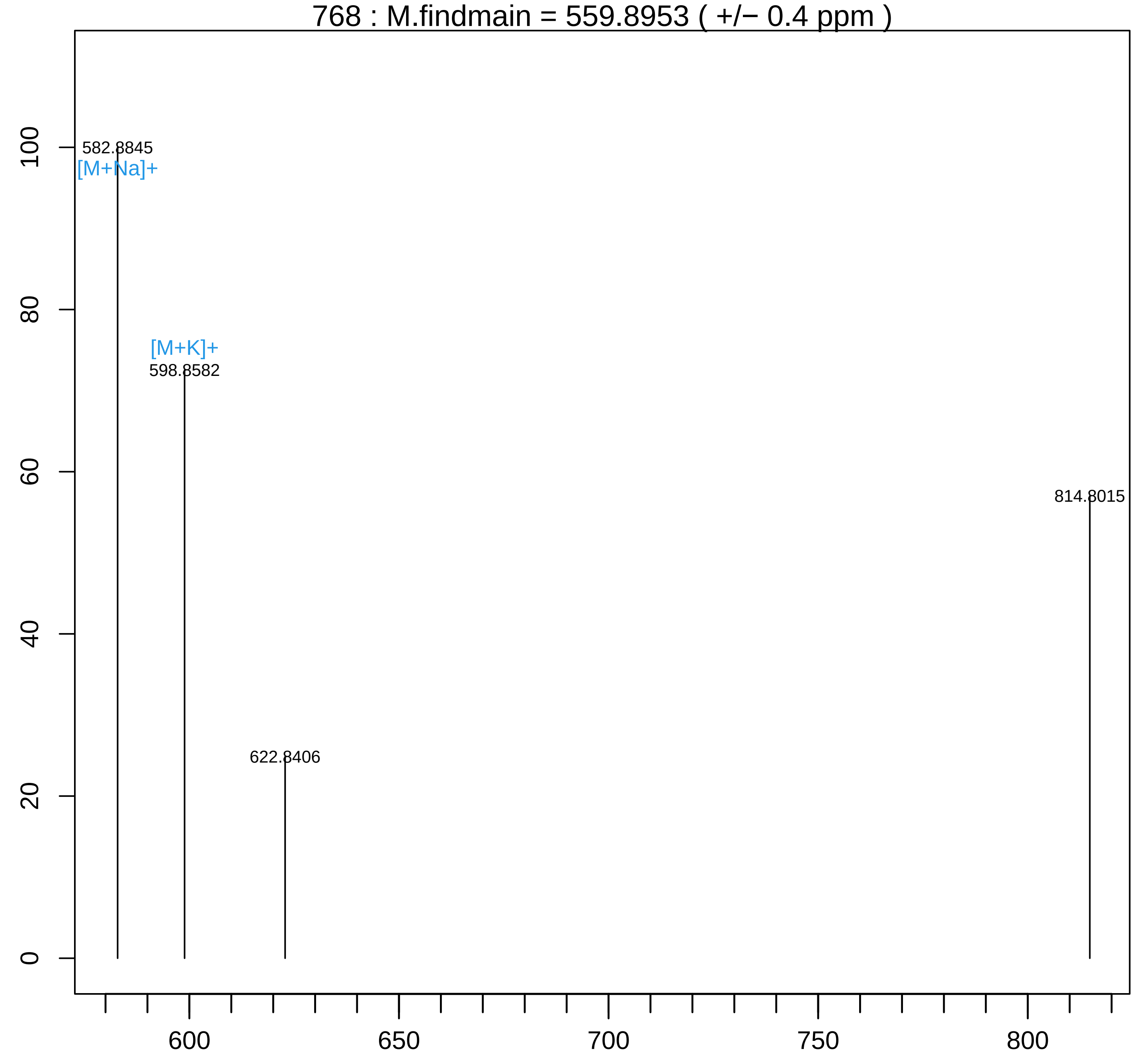
768 : M.findmain = 559.8953 (+/− 0.4 ppm)
582.8845
[M+Na]+
[M+K]+
598.8582
622.8406
814.8015
0
20
40
60
80
100
600
650
700
750
800

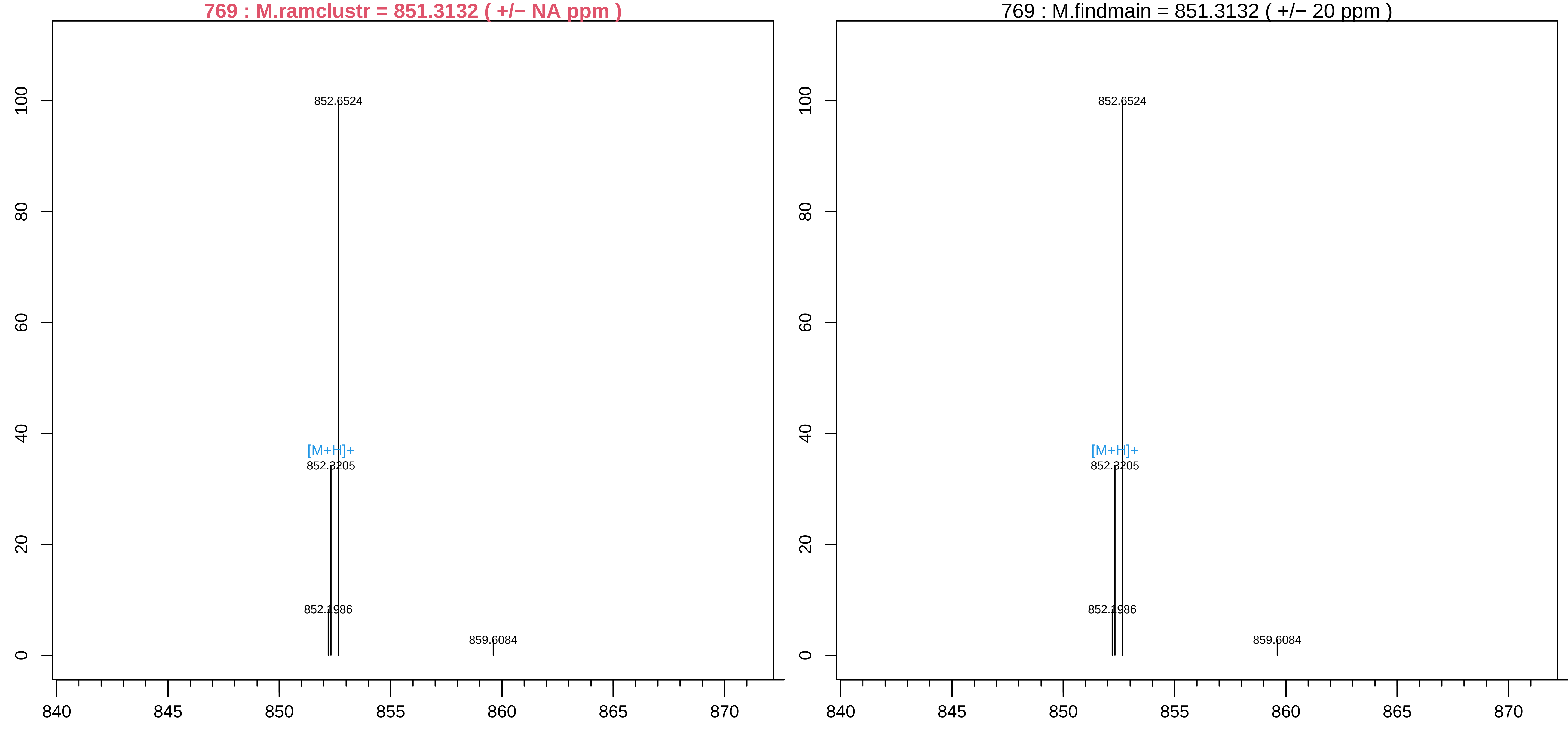
769 : M.ramclustr = 851.3132 (+/− NA ppm)
769 : M.findmain = 851.3132 (+/− 20 ppm)
852.6524
[M+H]+
852.3205
852.1986
859.6084
0
20
40
60
80
100
840
845
850
855
860
865
870

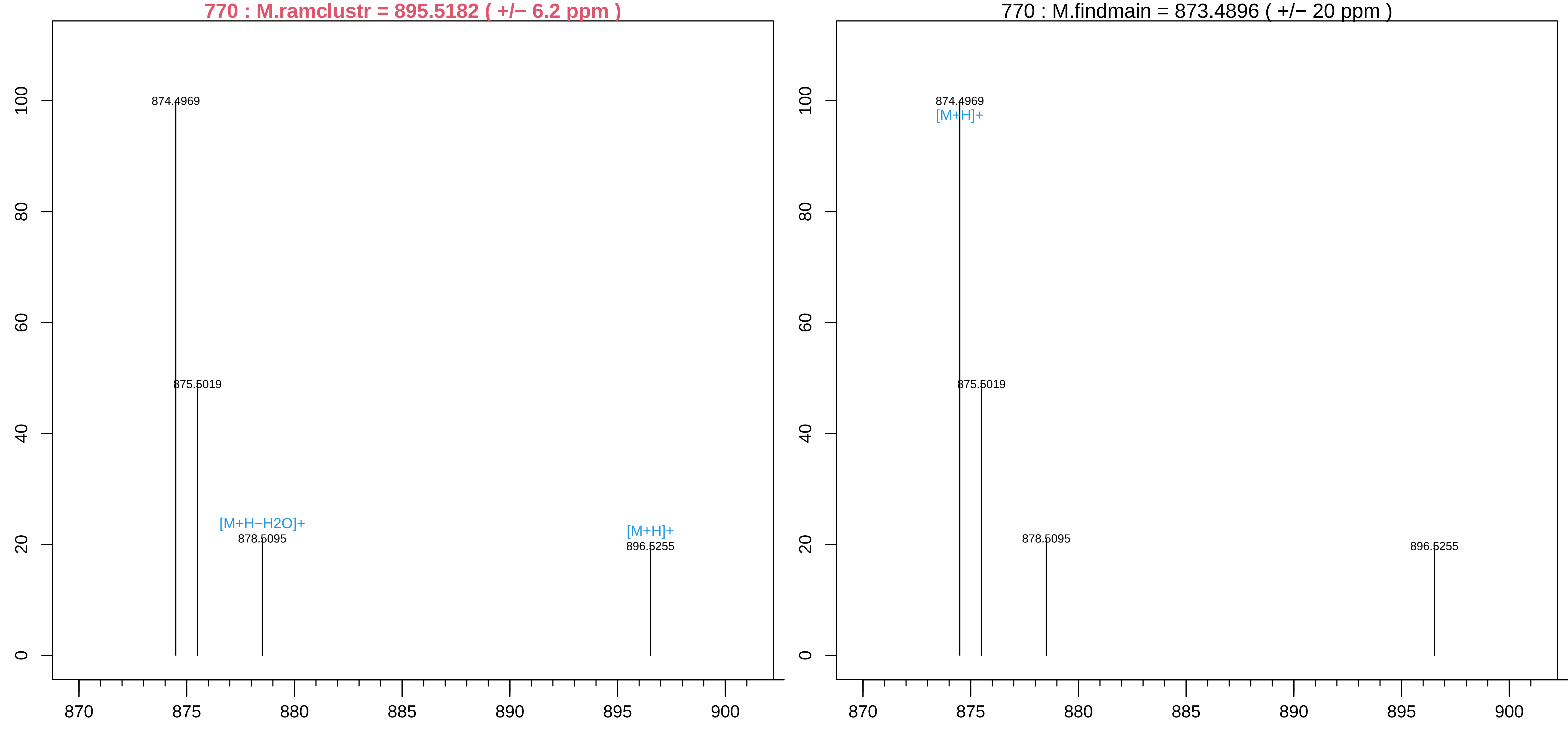

770 : M.ramclustr = 895.5182 (+/− 6.2 ppm)
874.4969
875.5019
[M+H−H2O]+
878.5095
[M+H]+
896.5255
0
20
40
60
80
100
870
875
880
885
890
895
900
770 : M.findmain = 873.4896 (+/− 20 ppm)
874.4969
[M+H]+
875.5019
878.5095
896.5255

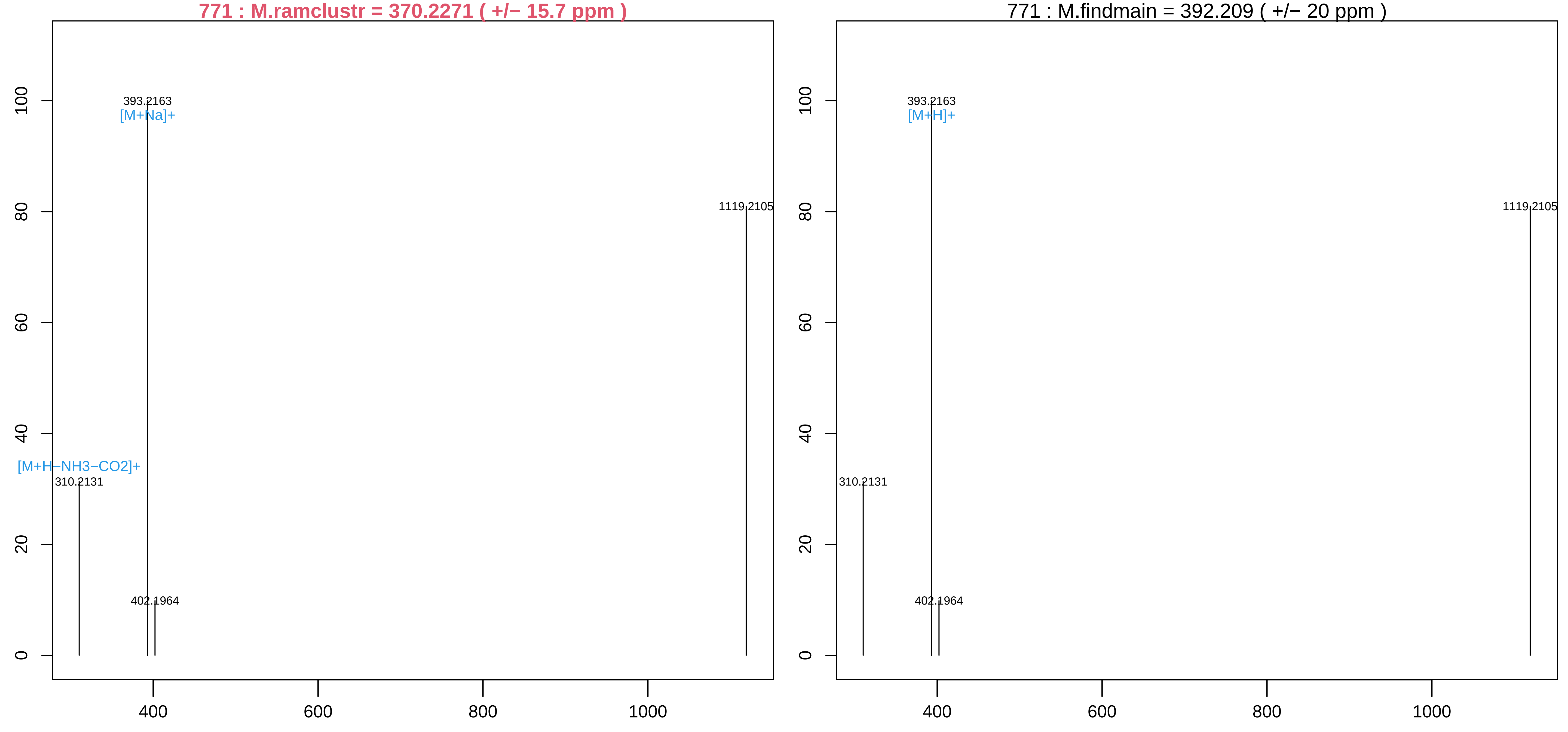
771 : M.ramclustr = 370.2271 (+/− 15.7 ppm)
393.2163
[M+Na]+
1119.2105
[M+H−NH3−CO2]+
310.2131
402.1964
771 : M.findmain = 392.209 (+/− 20 ppm)
393.2163
[M+H]+
1119.2105
310.2131
402.1964
0
20
40
60
80
100
400
600
800
1000

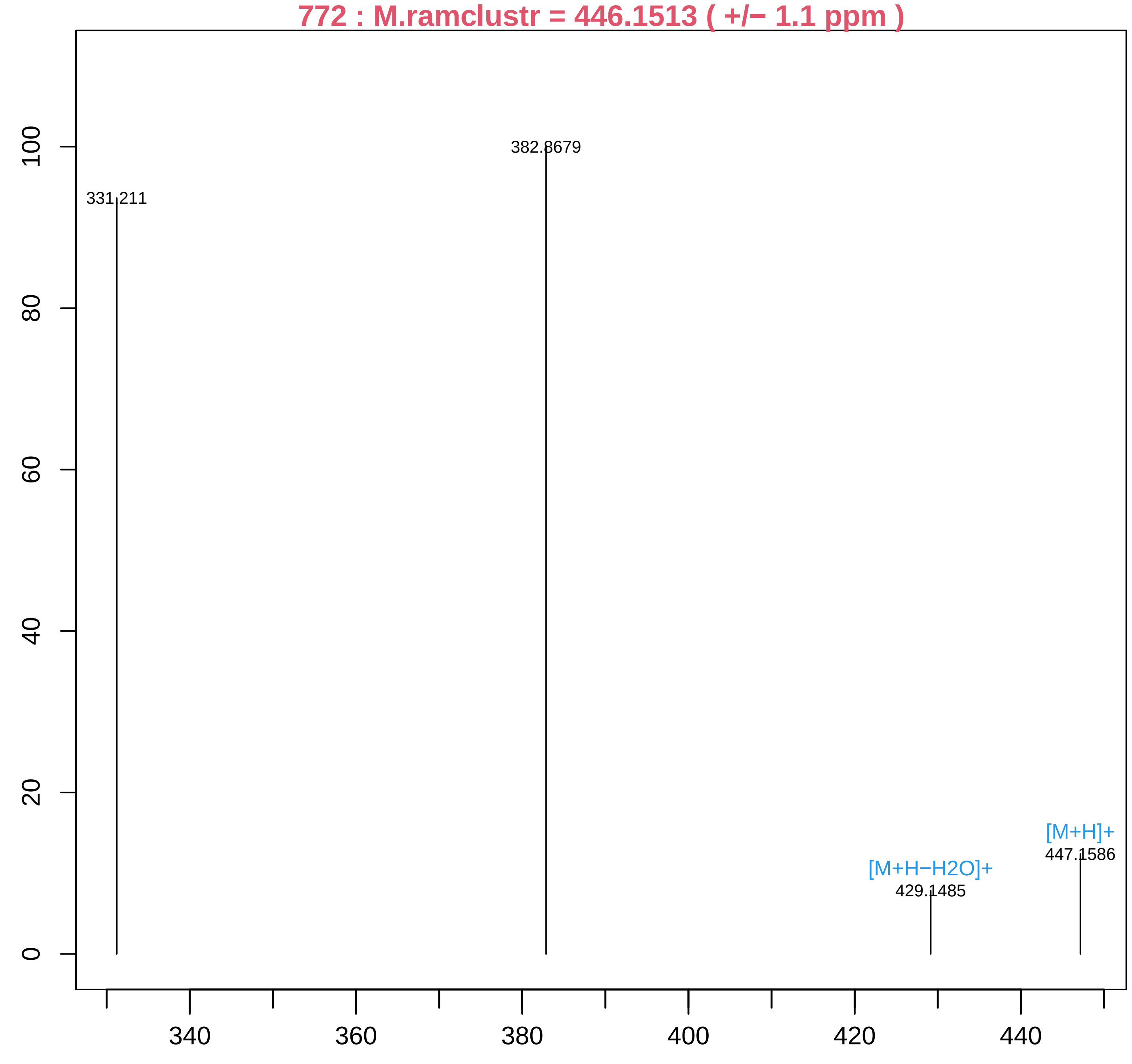
772 : M.ramclustr = 446.1513 (+/− 1.1 ppm)
331.211
382.8679
[M+H−H2O]+
429.1485
[M+H]+
447.1586
0
20
40
60
80
100
340
360
380
400
420
440

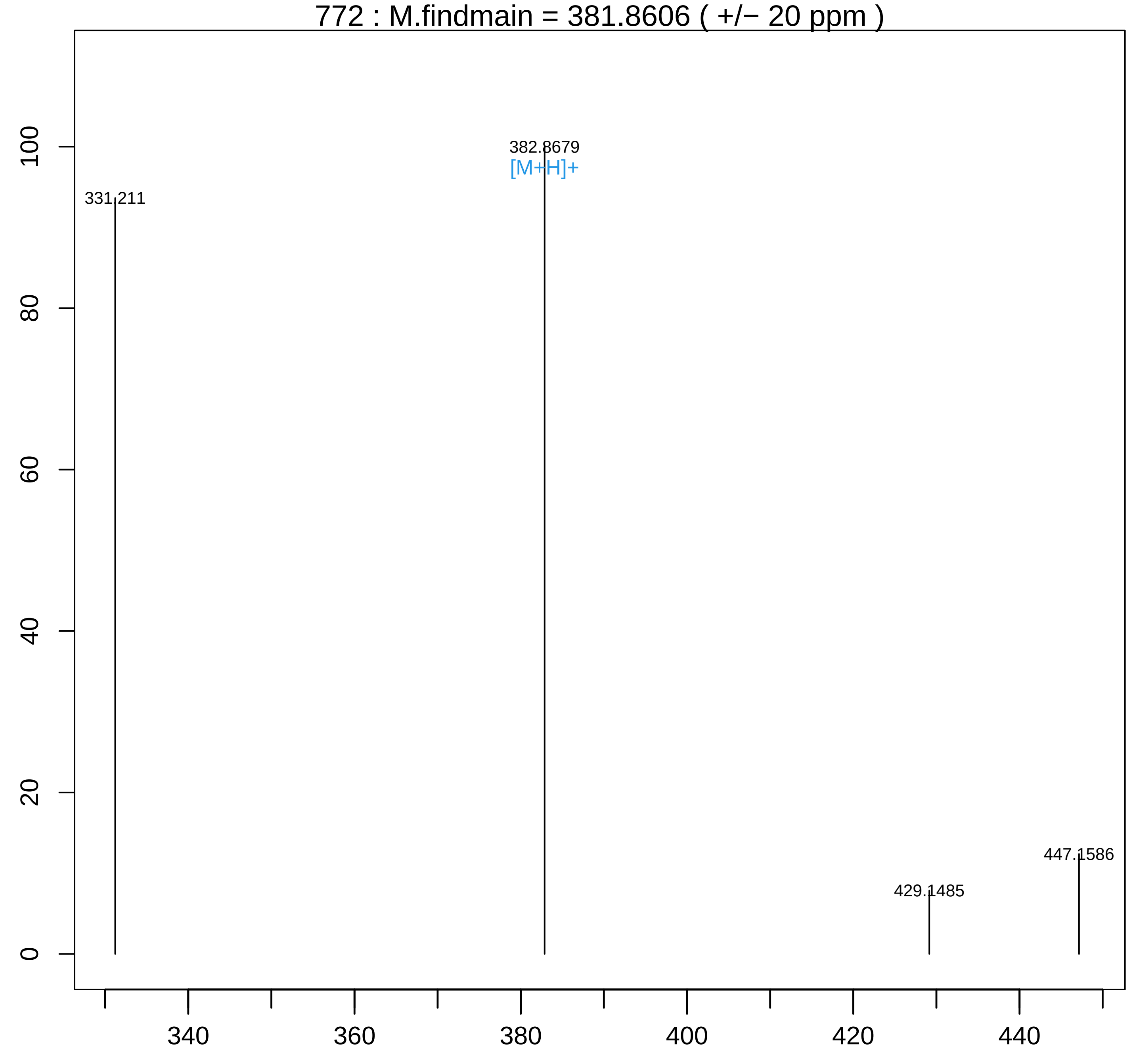
772 : M.findmain = 381.8606 (+/− 20 ppm)
331.211
382.8679
[M+H]+
429.1485
447.1586
0
20
40
60
80
100
340
360
380
400
420
440

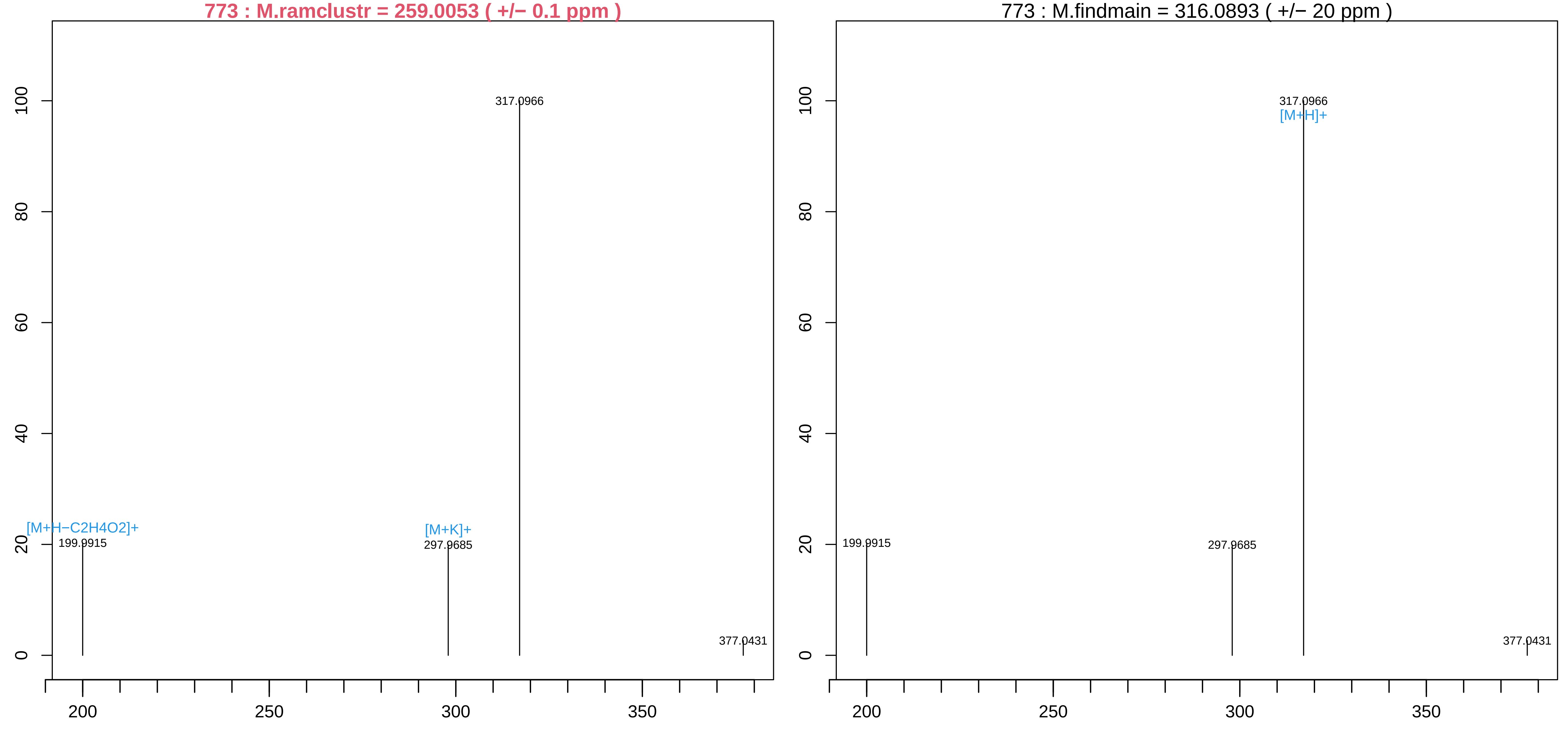

773 : M.ramclustr = 259.0053 (+/− 0.1 ppm)
317.0966
[M+H−C2H4O2]+
199.9915
[M+K]+
297.9685
377.0431
0
20
40
60
80
100
200
250
300
350
773 : M.findmain = 316.0893 (+/− 20 ppm)
317.0966
[M+H]+
199.9915
297.9685
377.0431
0
20
40
60
80
100
200
250
300
350

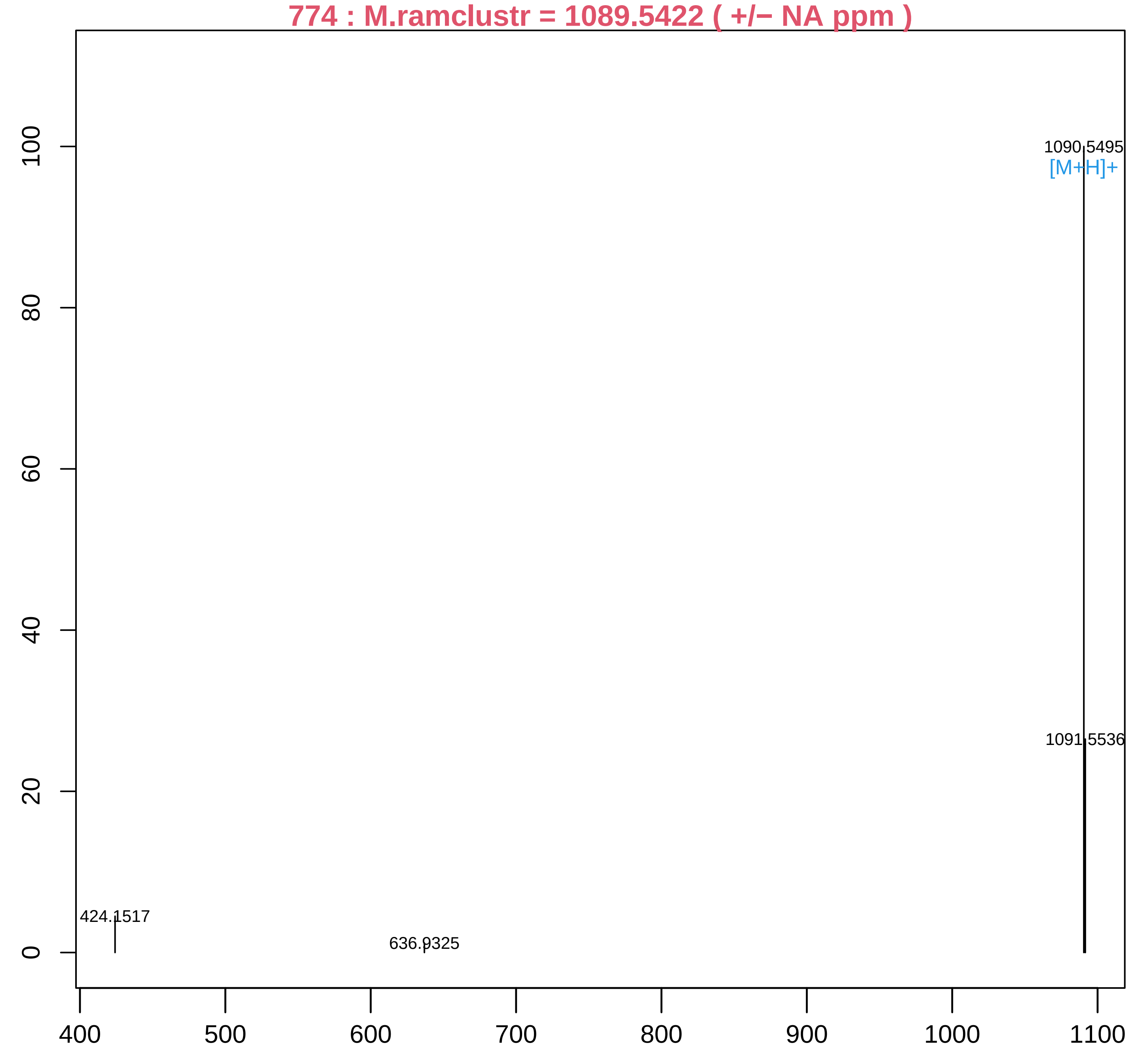

774 : M.ramclustr = 1089.5422 (+/− NA ppm)
1090.5495
[M+H]+
1091.5536
424.1517
636.9325

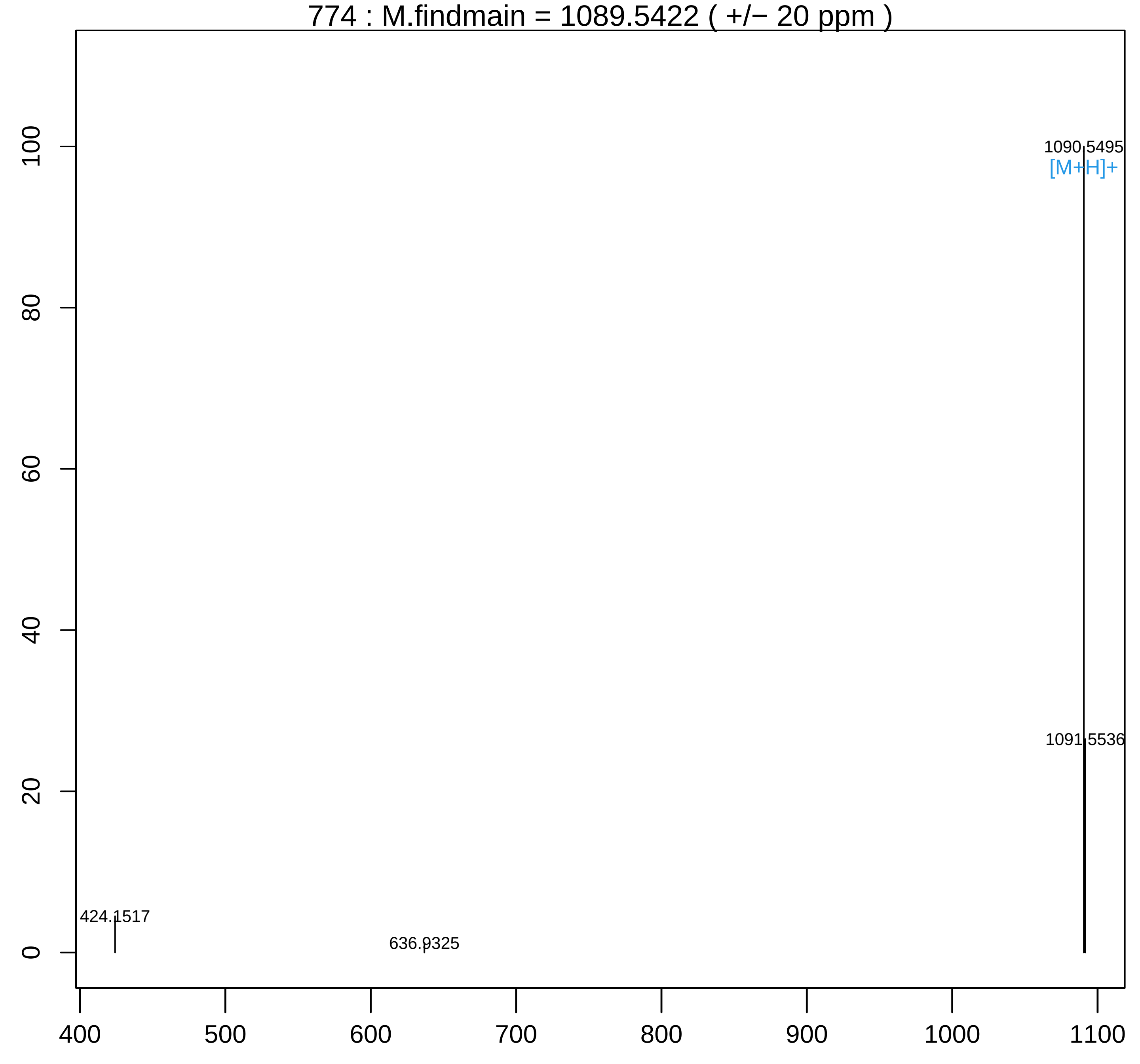

774 : M.findmain = 1089.5422 (+/− 20 ppm)
1090.5495
[M+H]+
1091.5536
424.1517
636.9325

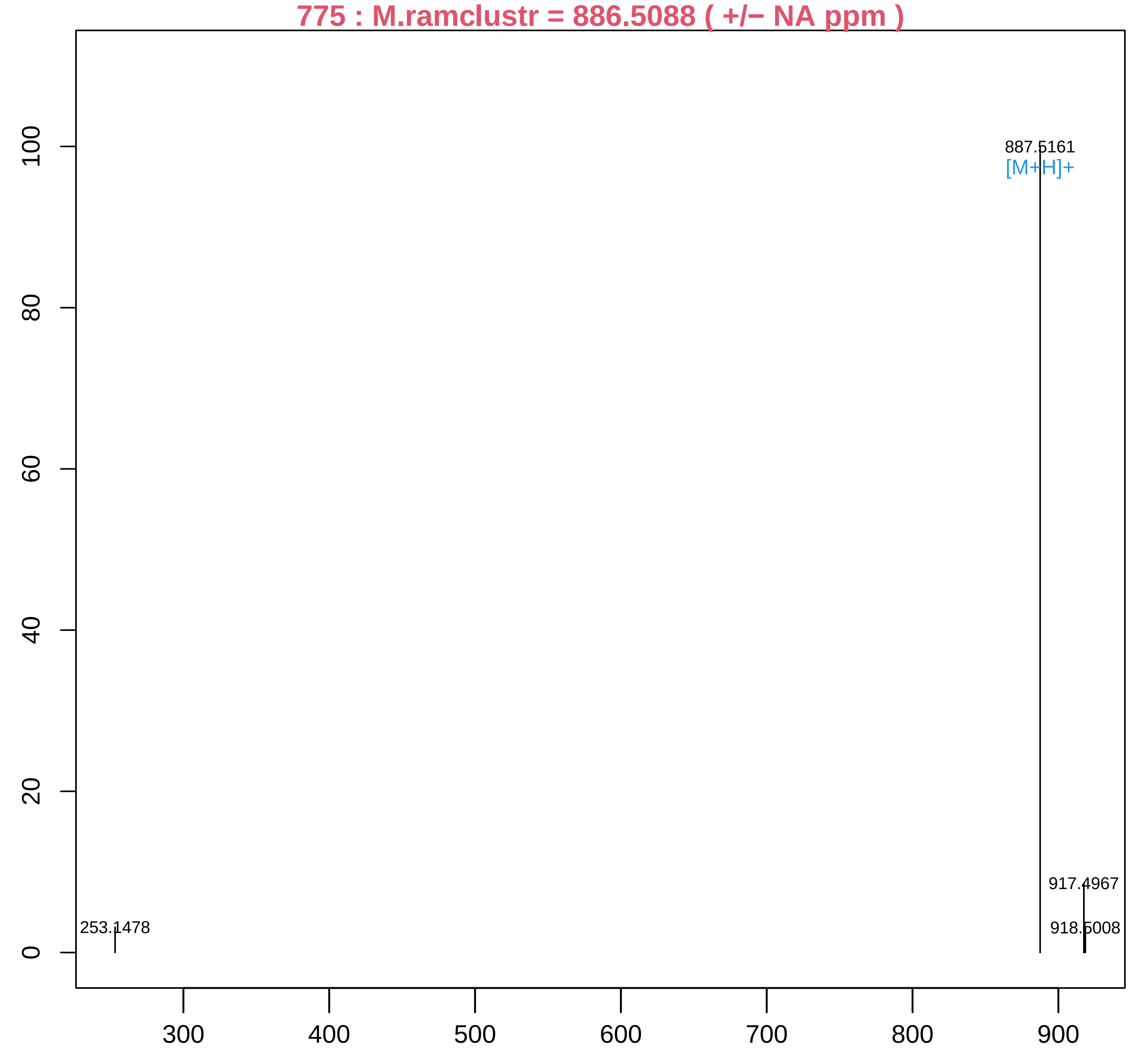
775 : M.ramclustr = 886.5088 (+/− NA ppm)
887.5161
[M+H]+
917.4967
918.5008
253.1478
0
20
40
60
80
100
300
400
500
600
700
800
900

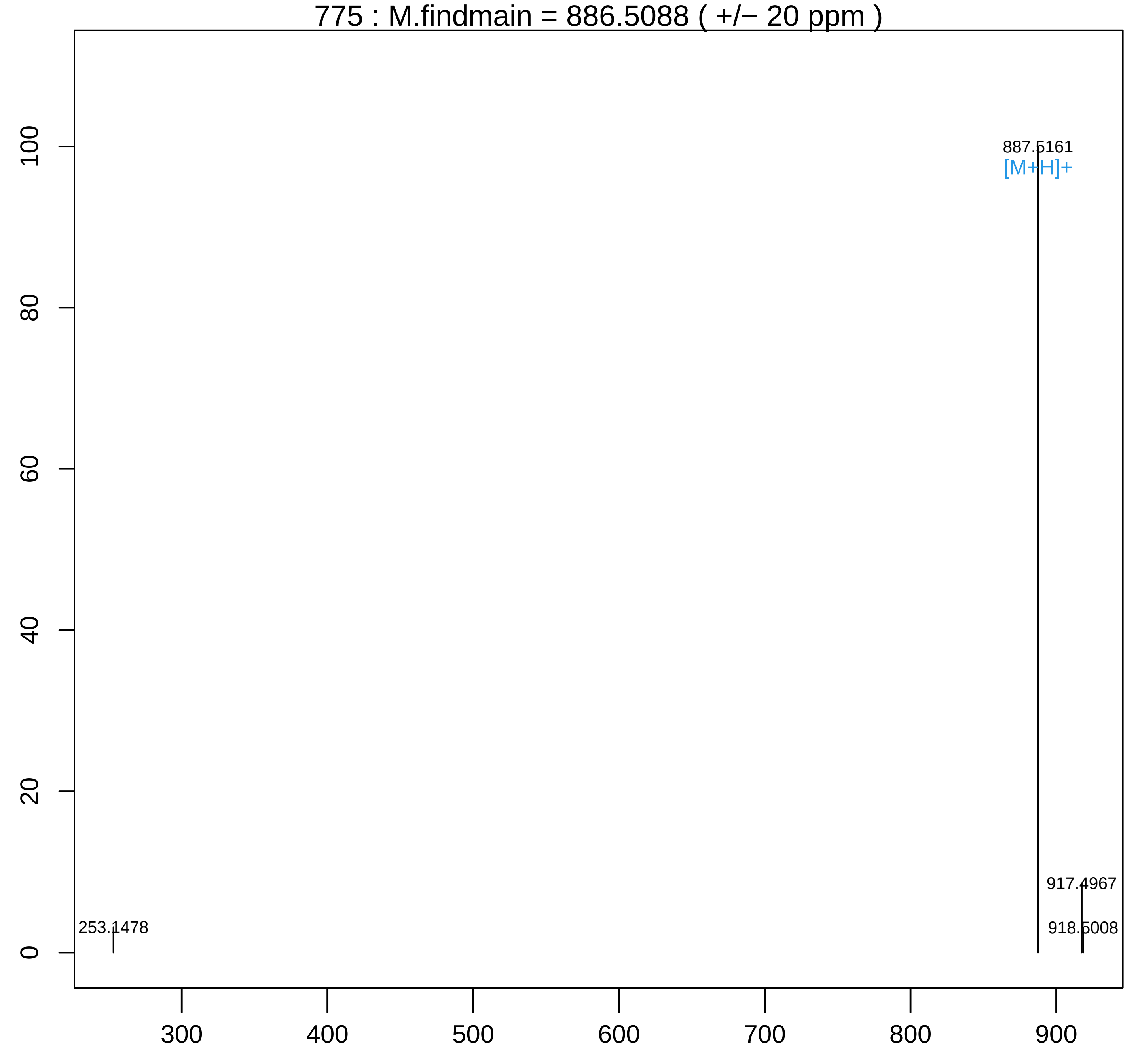
775 : M.findmain = 886.5088 (+/− 20 ppm)
887.5161
[M+H]+
917.4967
918.5008
253.1478
0
20
40
60
80
100
300
400
500
600
700
800
900

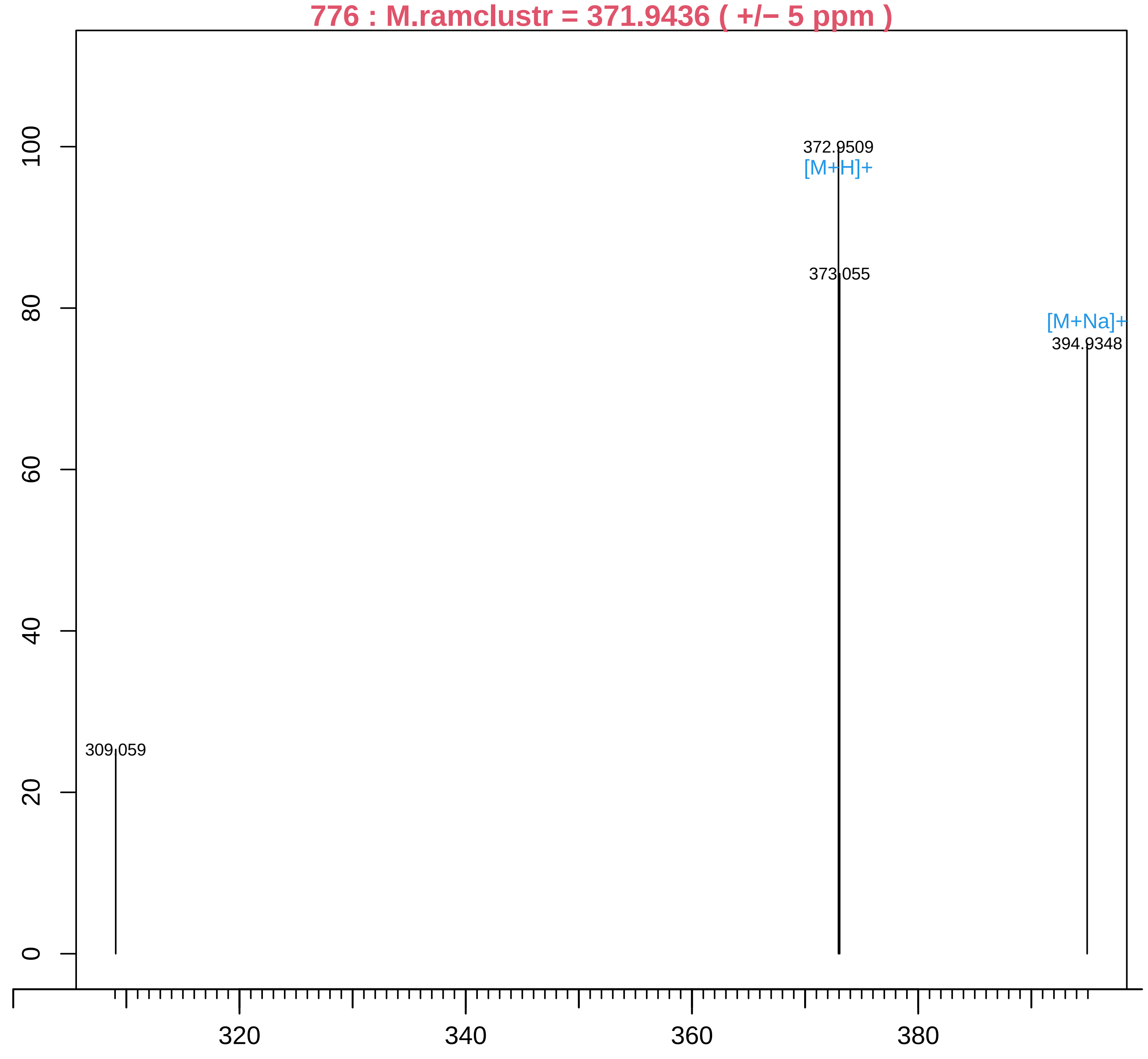
776 : M.ramclustr = 371.9436 (+/− 5 ppm)
372.9509
[M+H]+
373.055
[M+Na]+
394.9348
309.059
0
20
40
60
80
100
320
340
360
380

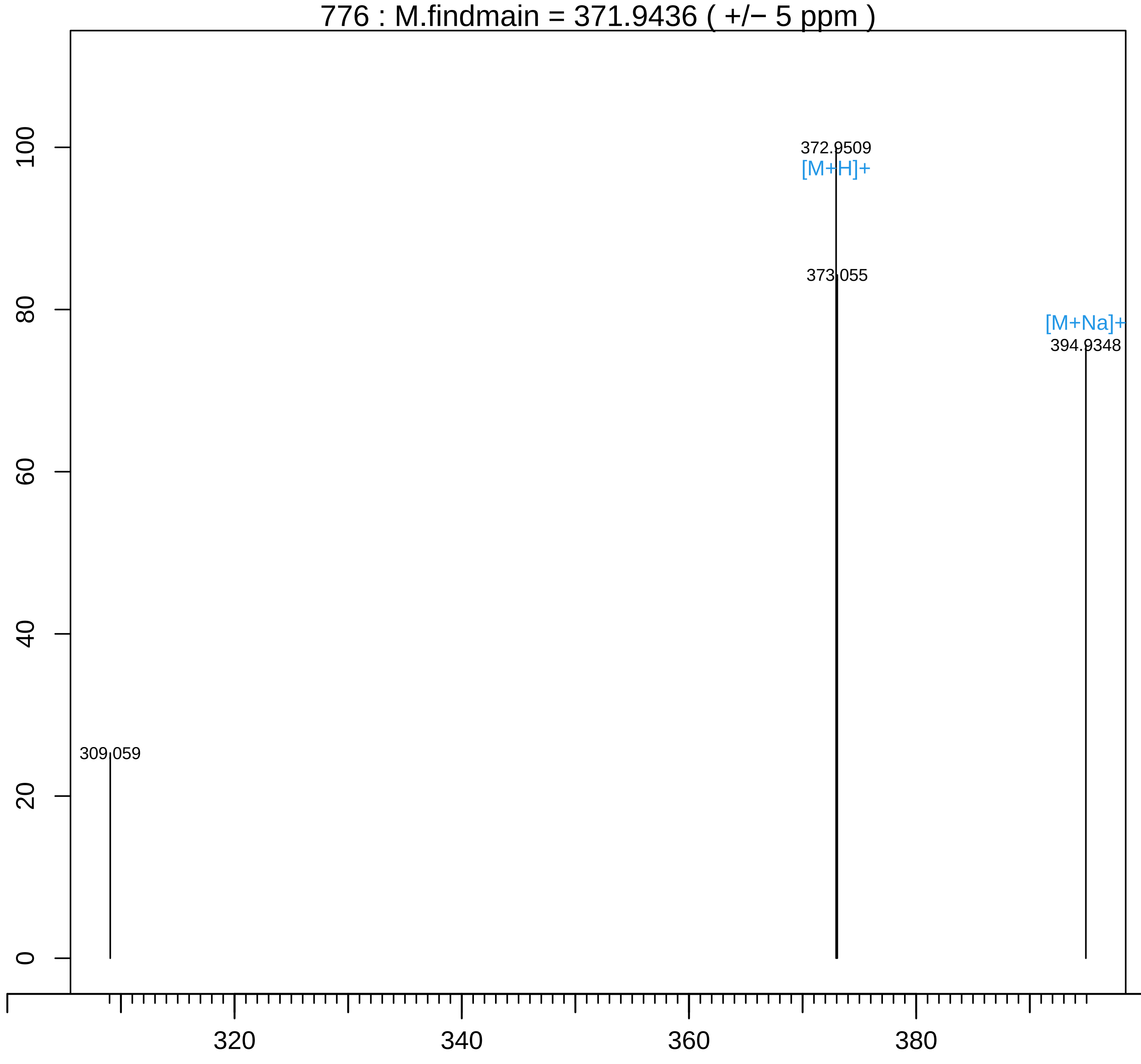
776 : M.findmain = 371.9436 (+/− 5 ppm)
372.9509
[M+H]+
373.055
[M+Na]+
394.9348
309.059
0
20
40
60
80
100
320
340
360
380

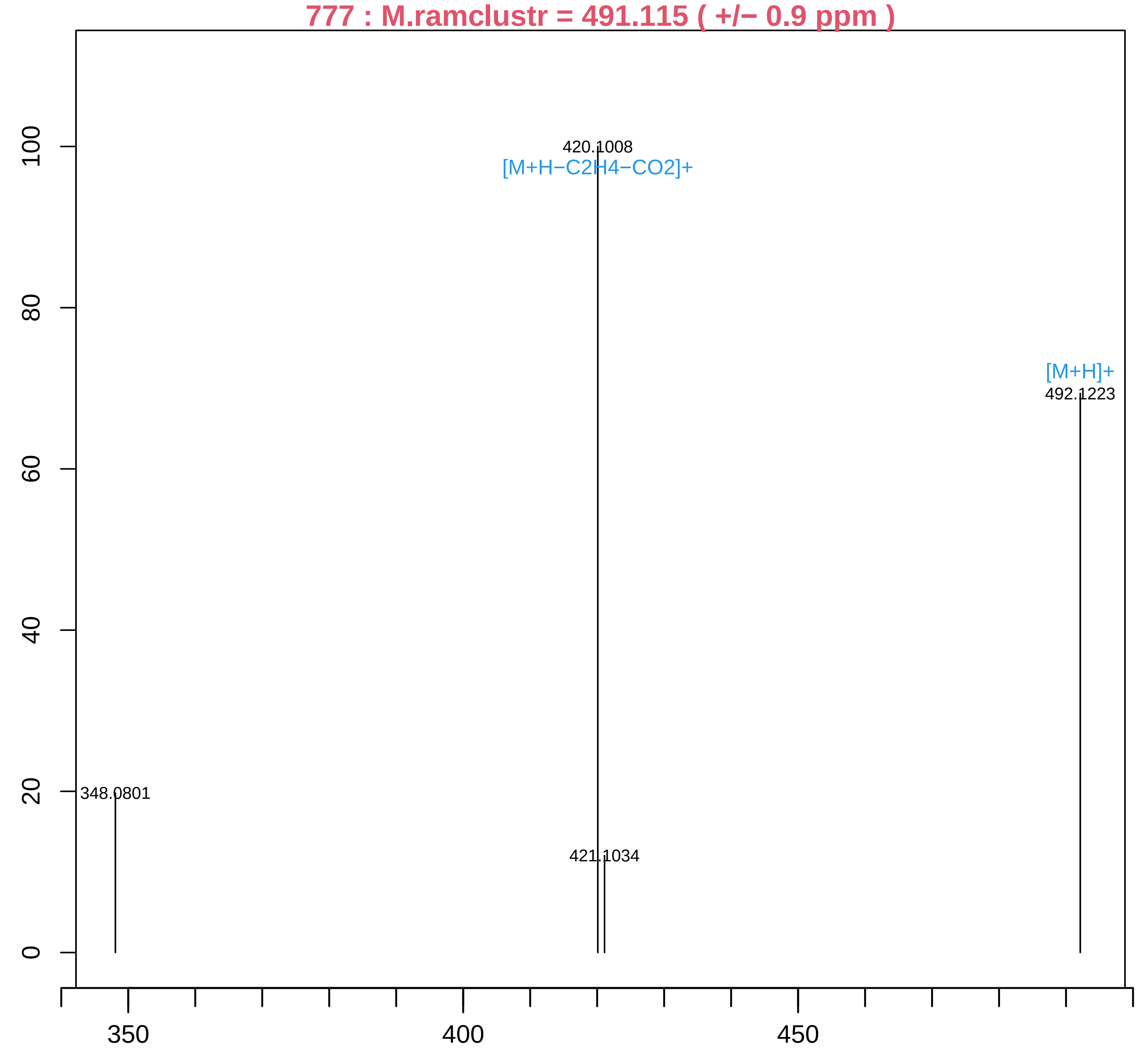
777 : M.ramclustr = 491.115 (+/− 0.9 ppm)
420.1008
[M+H−C2H4−CO2]+
[M+H]+
492.1223
348.0801
421.1034
0
20
40
60
80
100
350
400
450
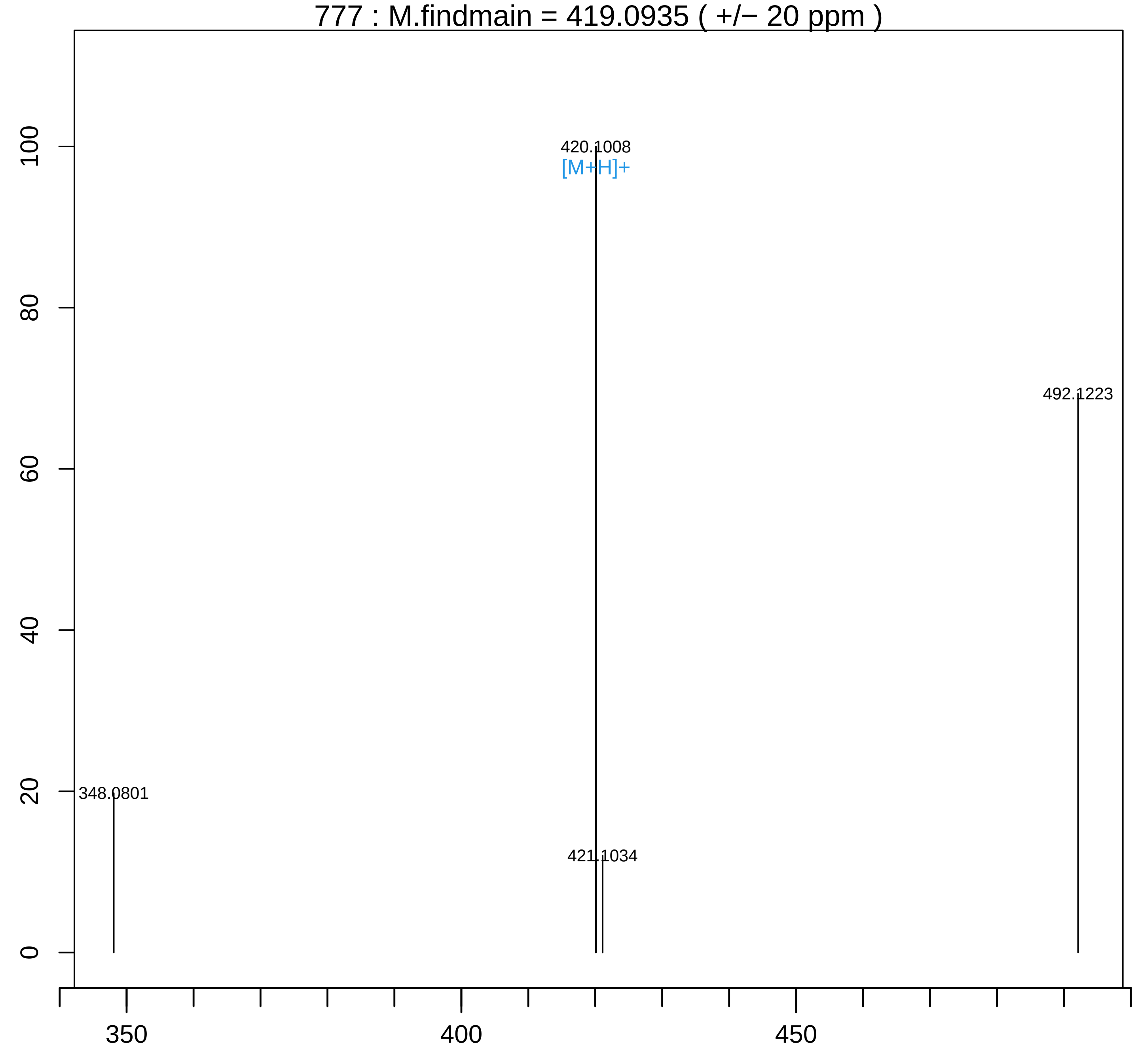
777 : M.findmain = 419.0935 (+/− 20 ppm)
420.1008
[M+H]+
492.1223
348.0801
421.1034
0
20
40
60
80
100
350
400
450

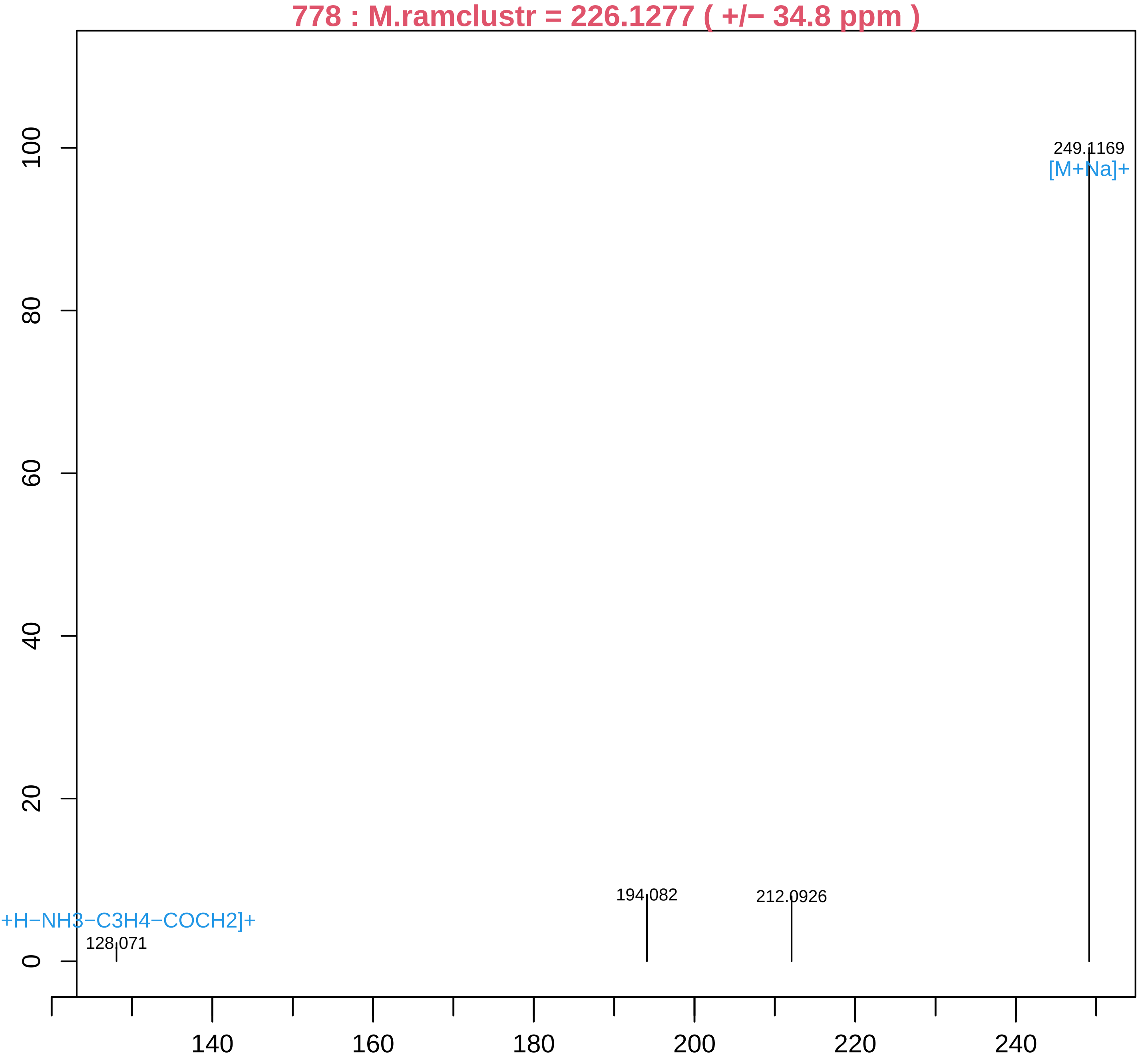
778 : M.ramclustr = 226.1277 (+/− 34.8 ppm)
249.1169
[M+Na]+
194.082
212.0926
+H−NH3−C3H4−COCH2]+
128.071
0
20
40
60
80
100
140
160
180
200
220
240

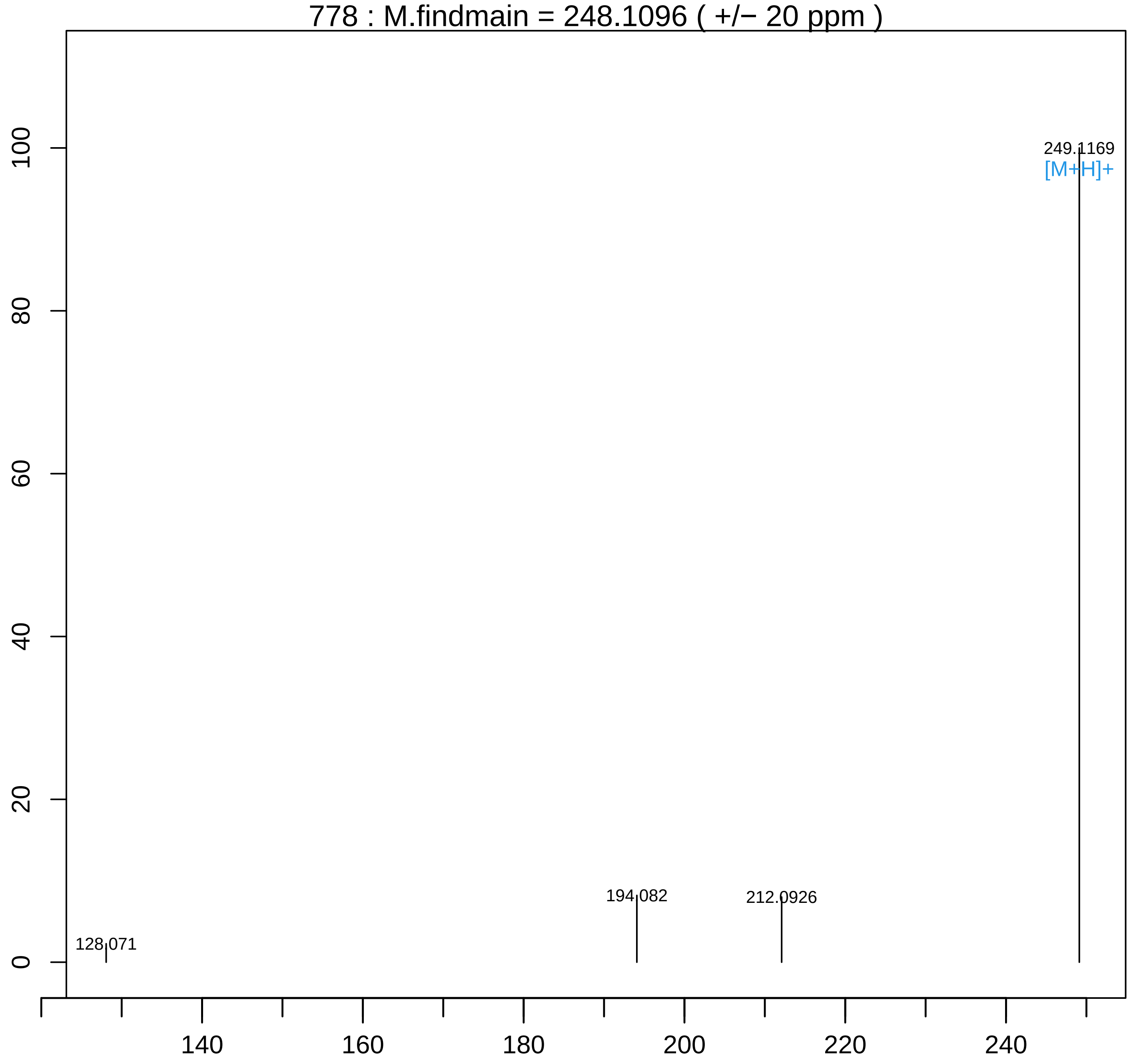
778 : M.findmain = 248.1096 (+/− 20 ppm)
249.1169
[M+H]+
194.082
212.0926
128.071
0
20
40
60
80
100
140
160
180
200
220
240

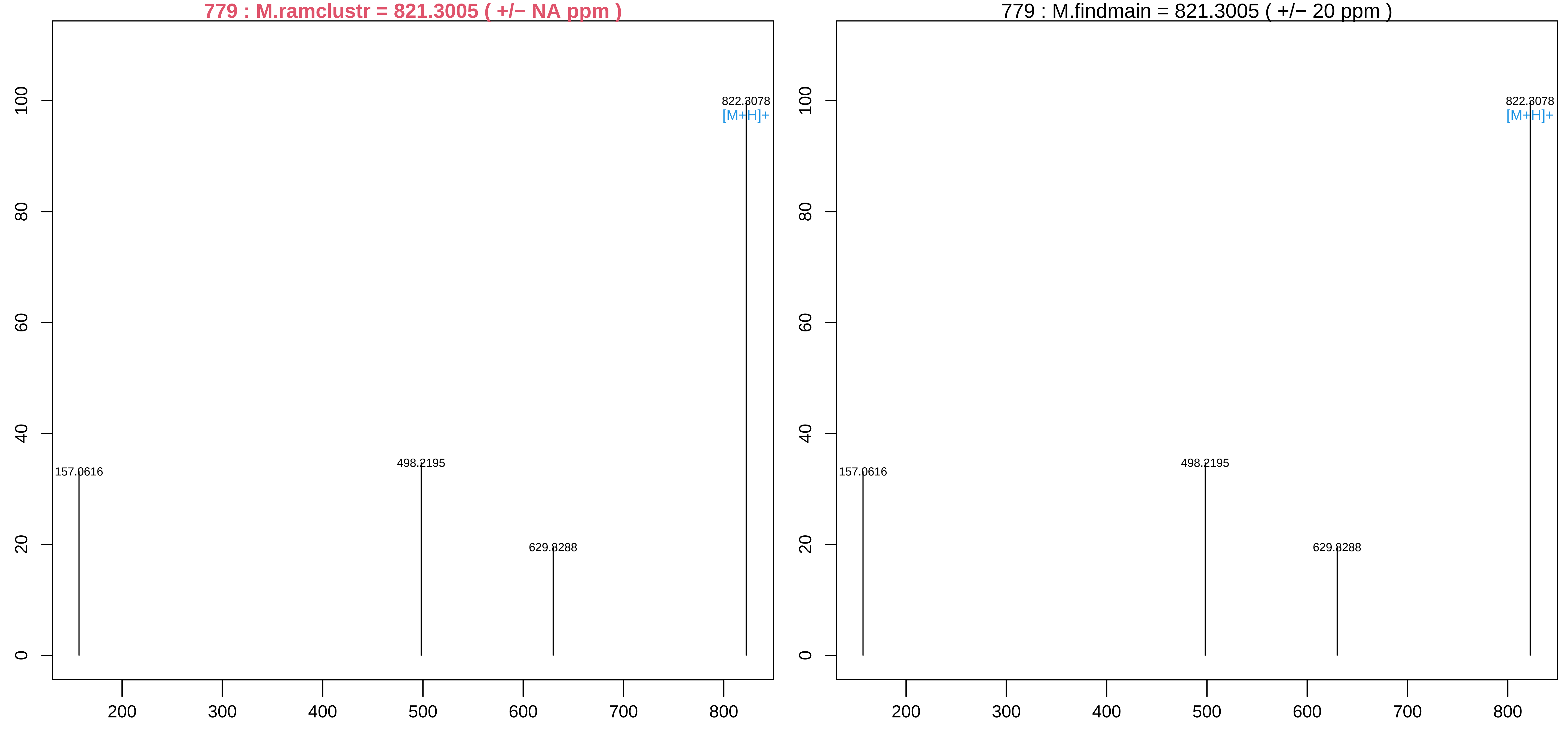
779 : M.ramclustr = 821.3005 (+/− NA ppm)
822.3078
[M+H]+
157.0616
498.2195
629.8288
0
20
40
60
80
100
200
300
400
500
600
700
800
779 : M.findmain = 821.3005 (+/− 20 ppm)
822.3078
[M+H]+
157.0616
498.2195
629.8288
0
20
40
60
80
100
200
300
400
500
600
700
800

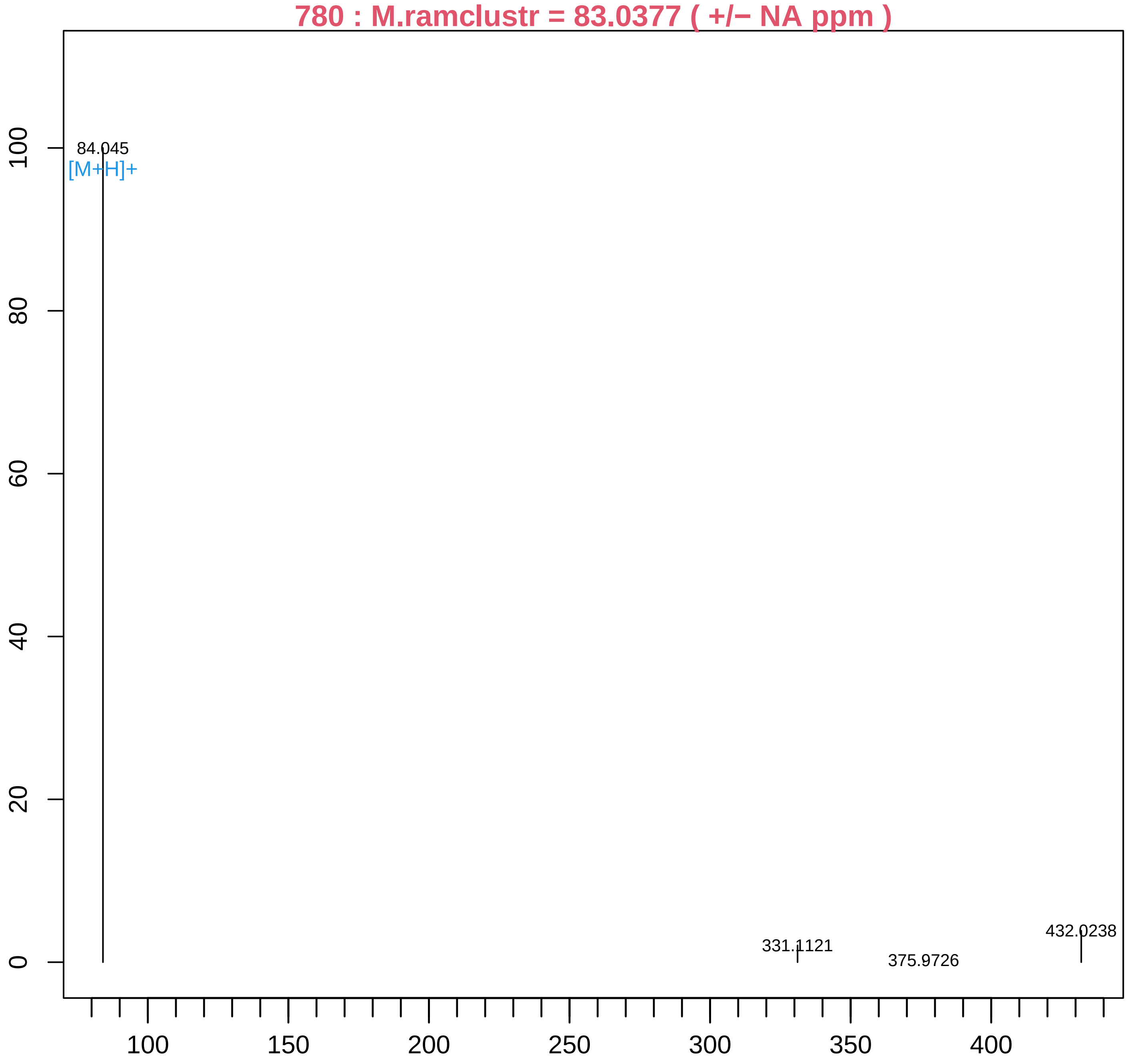
780 : M.ramclustr = 83.0377 (+/− NA ppm)
84.045
[M+H]+
331.1121
375.9726
432.0238
0
20
40
60
80
100
100
150
200
250
300
350
400

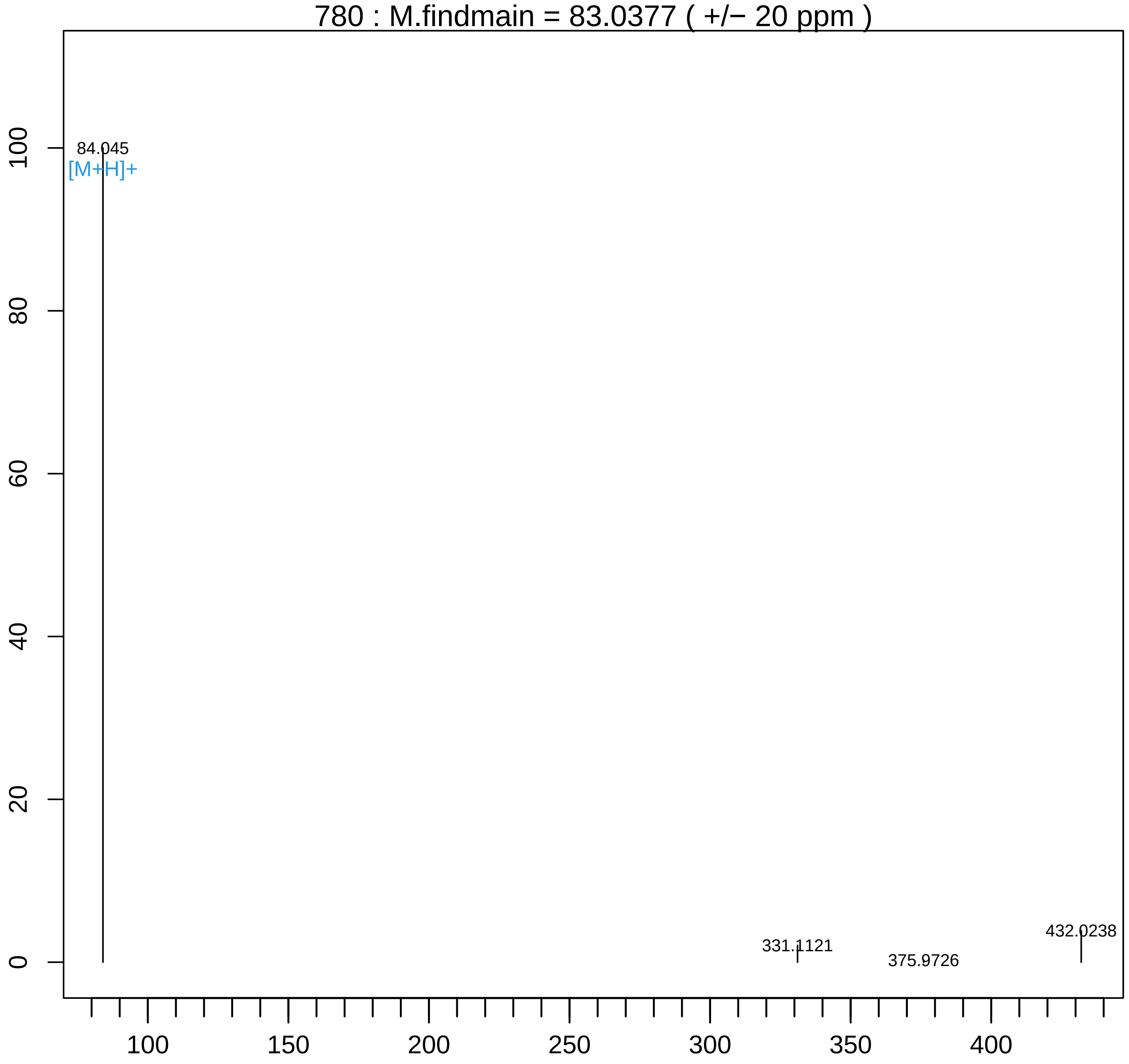
780 : M.findmain = 83.0377 (+/− 20 ppm)
84.045
[M+H]+
331.1121
375.9726
432.0238
0
20
40
60
80
100
100
150
200
250
300
350
400

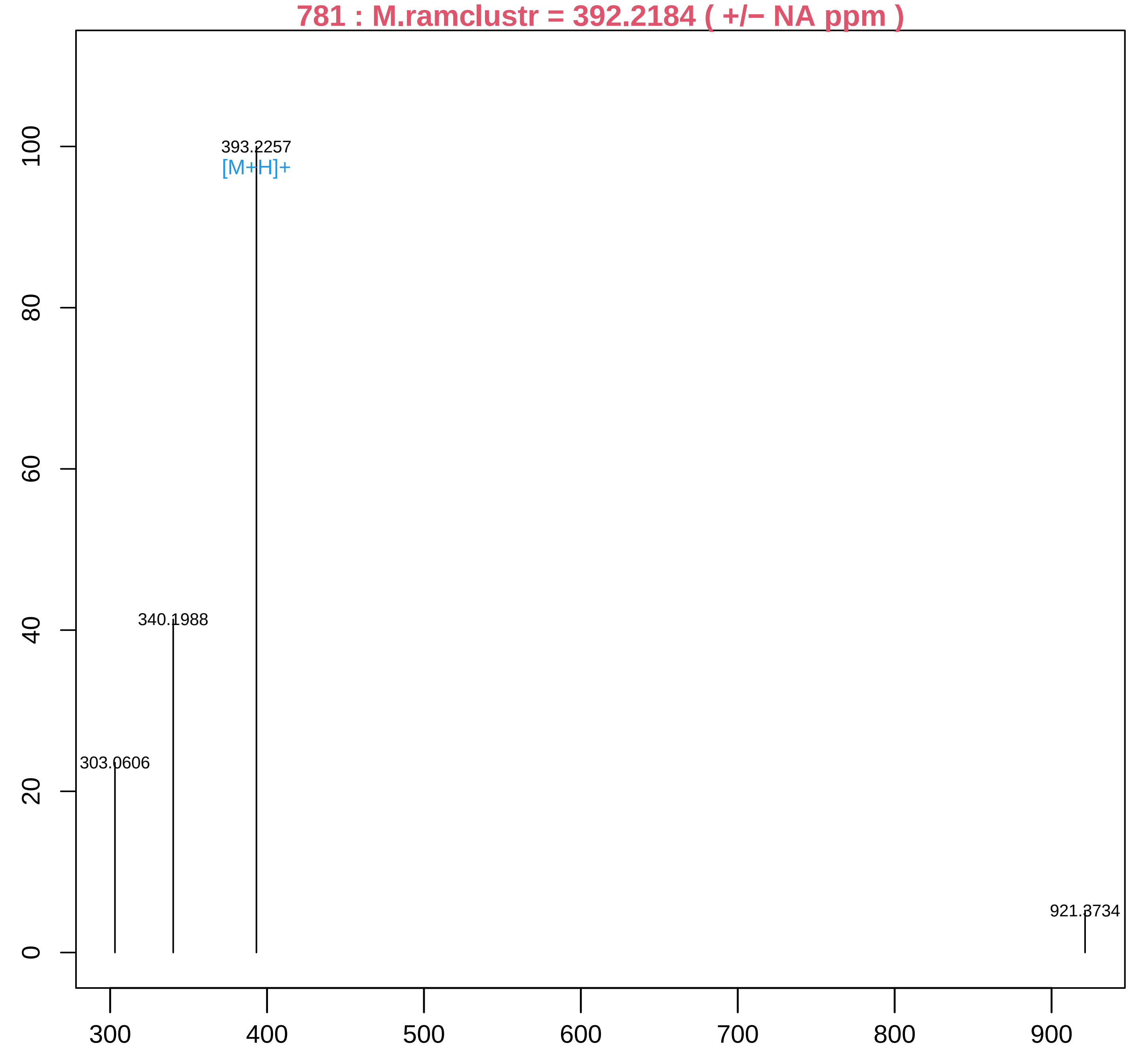
781 : M.ramclustr = 392.2184 (+/− NA ppm)
393.2257
[M+H]+
340.1988
303.0606
921.3734
0
20
40
60
80
100
300
400
500
600
700
800
900
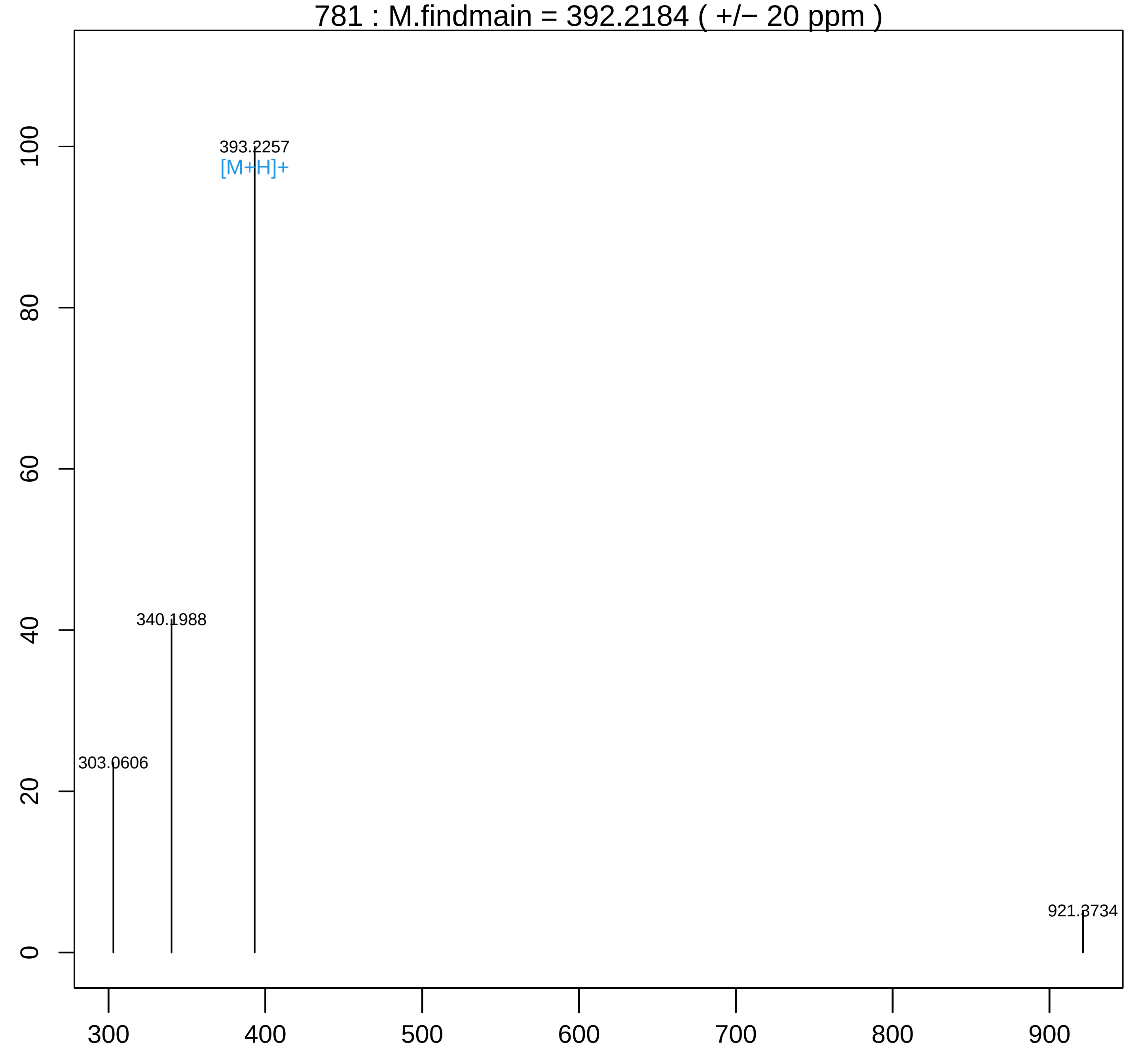
781 : M.findmain = 392.2184 (+/− 20 ppm)
393.2257
[M+H]+
340.1988
303.0606
921.3734
0
20
40
60
80
100
300
400
500
600
700
800
900

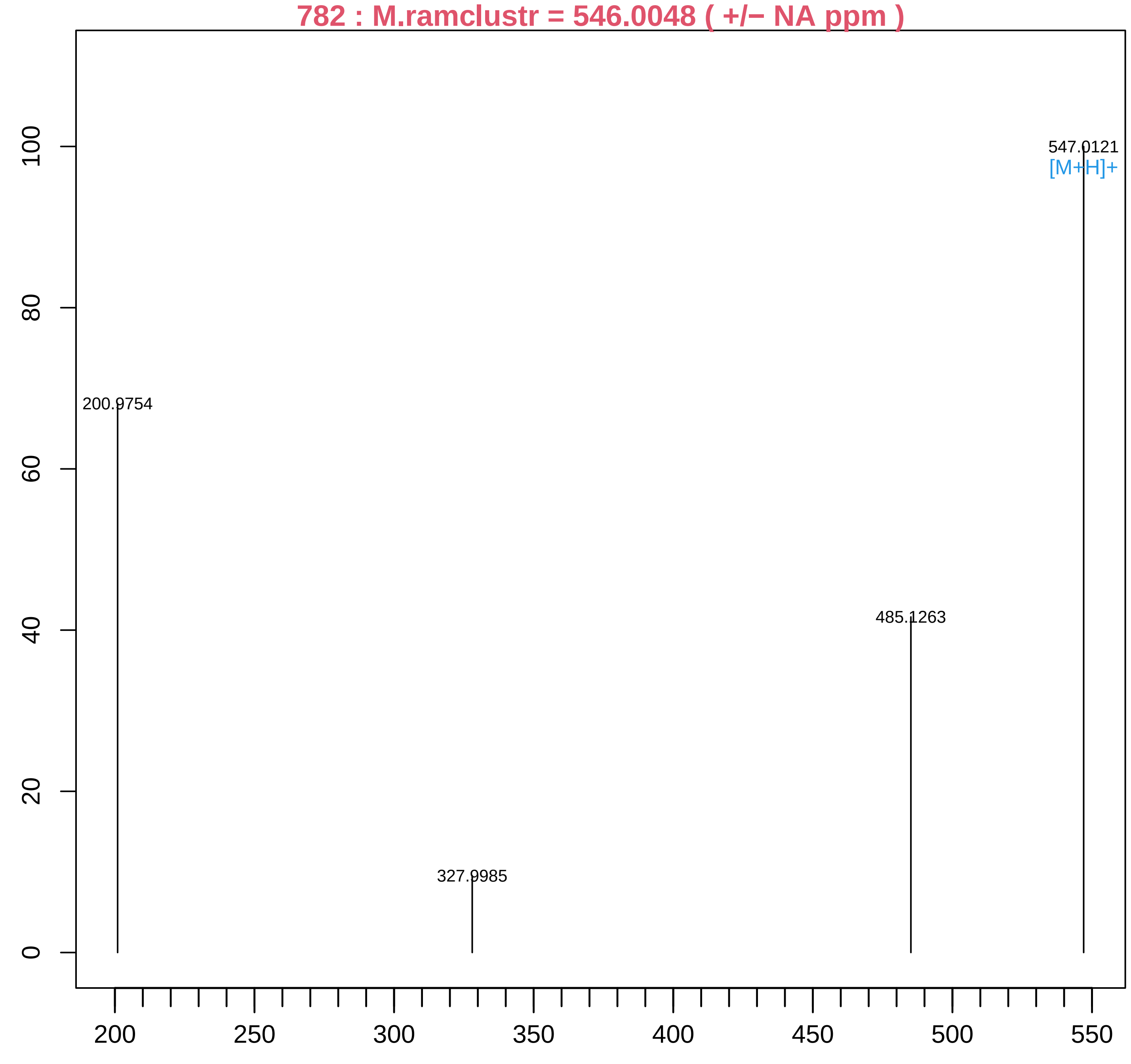
782 : M.ramclustr = 546.0048 (+/− NA ppm)
547.0121
[M+H]+
200.9754
485.1263
327.9985
0
20
40
60
80
100
200
250
300
350
400
450
500
550

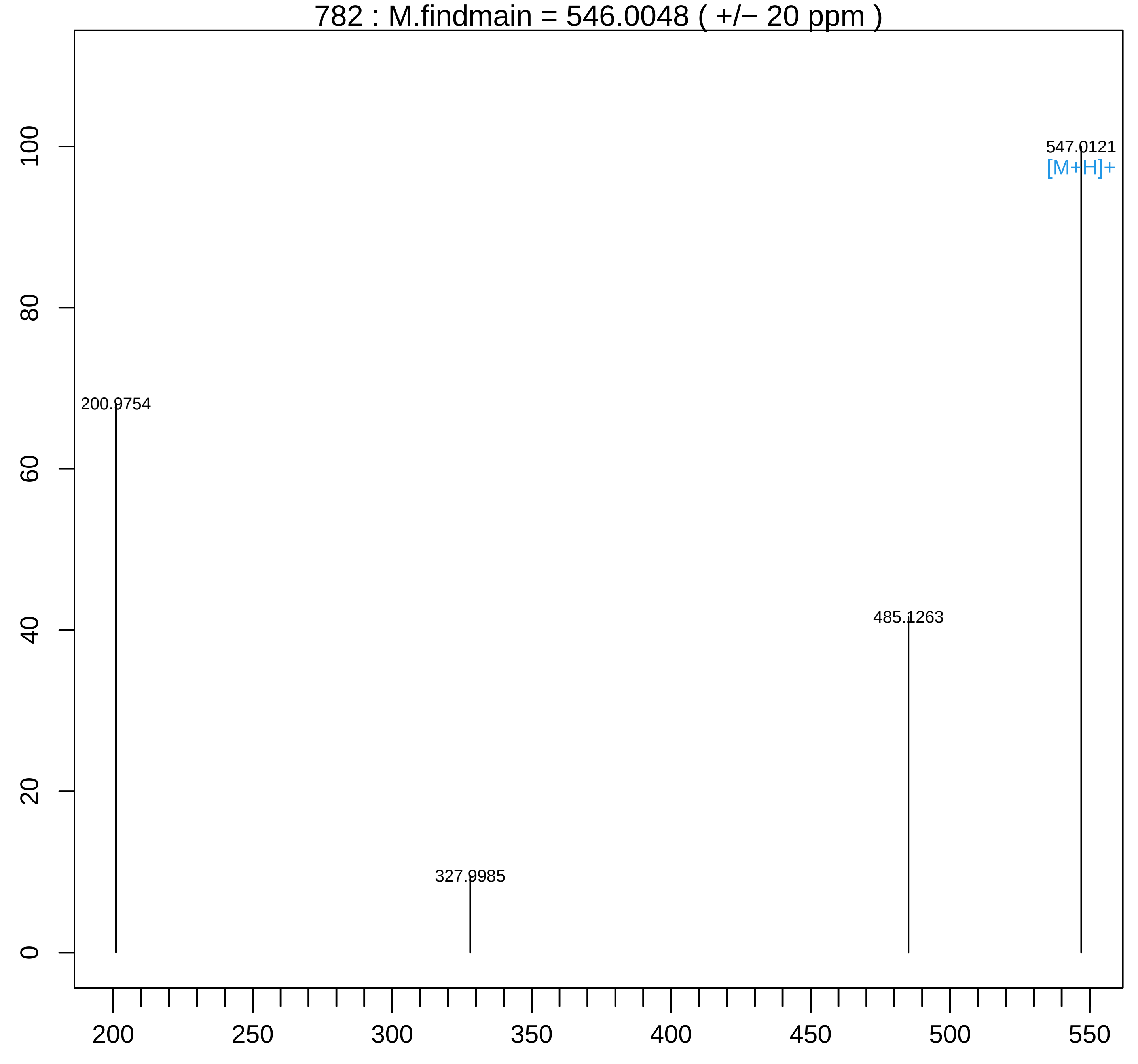
782 : M.findmain = 546.0048 (+/− 20 ppm)
547.0121
[M+H]+
200.9754
485.1263
327.9985
0
20
40
60
80
100
200
250
300
350
400
450
500
550

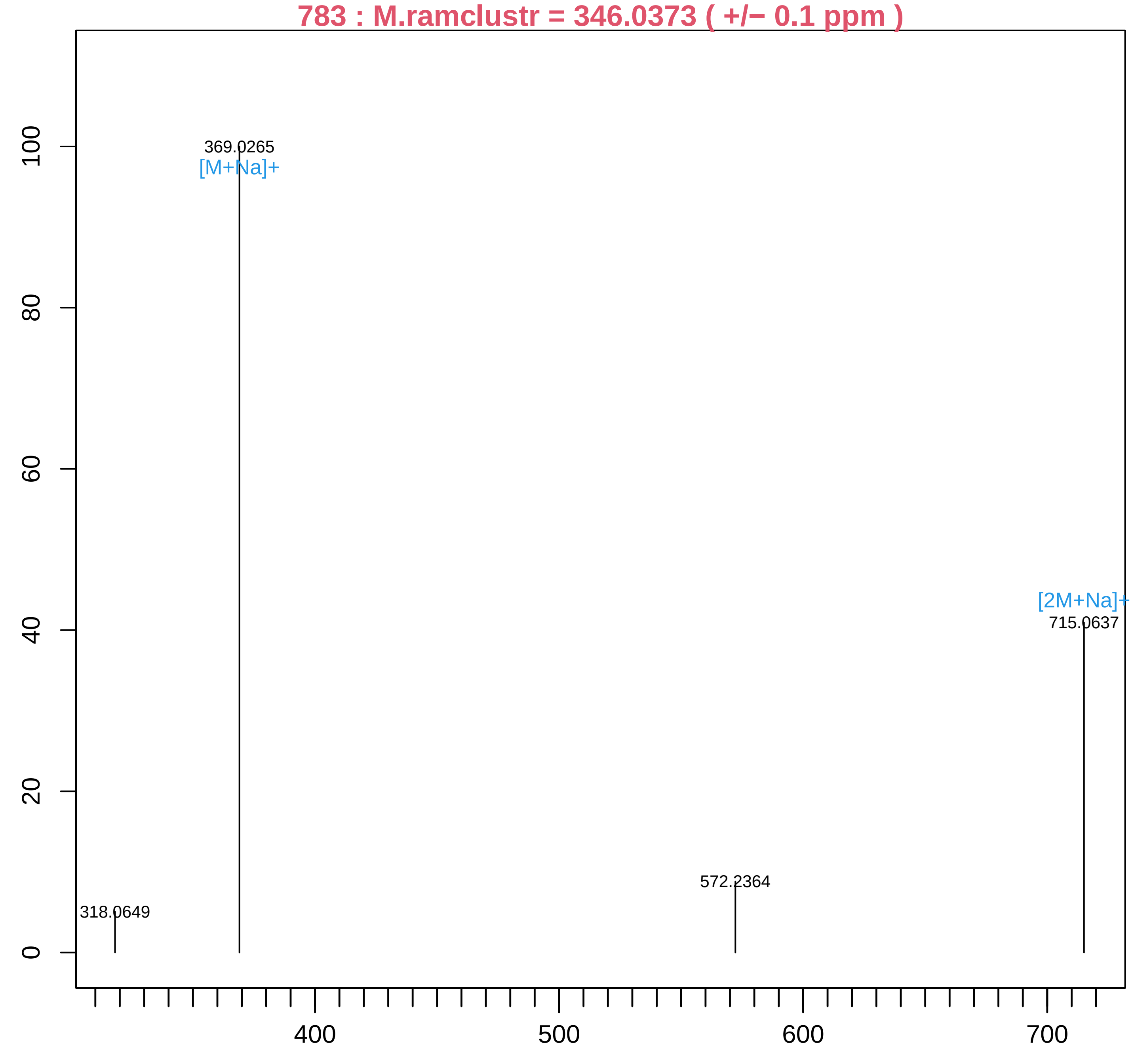

783 : M.ramclustr = 346.0373 (+/− 0.1 ppm)
369.0265
[M+Na]+
[2M+Na]+
715.0637
572.2364
318.0649
0
20
40
60
80
100
400
500
600
700

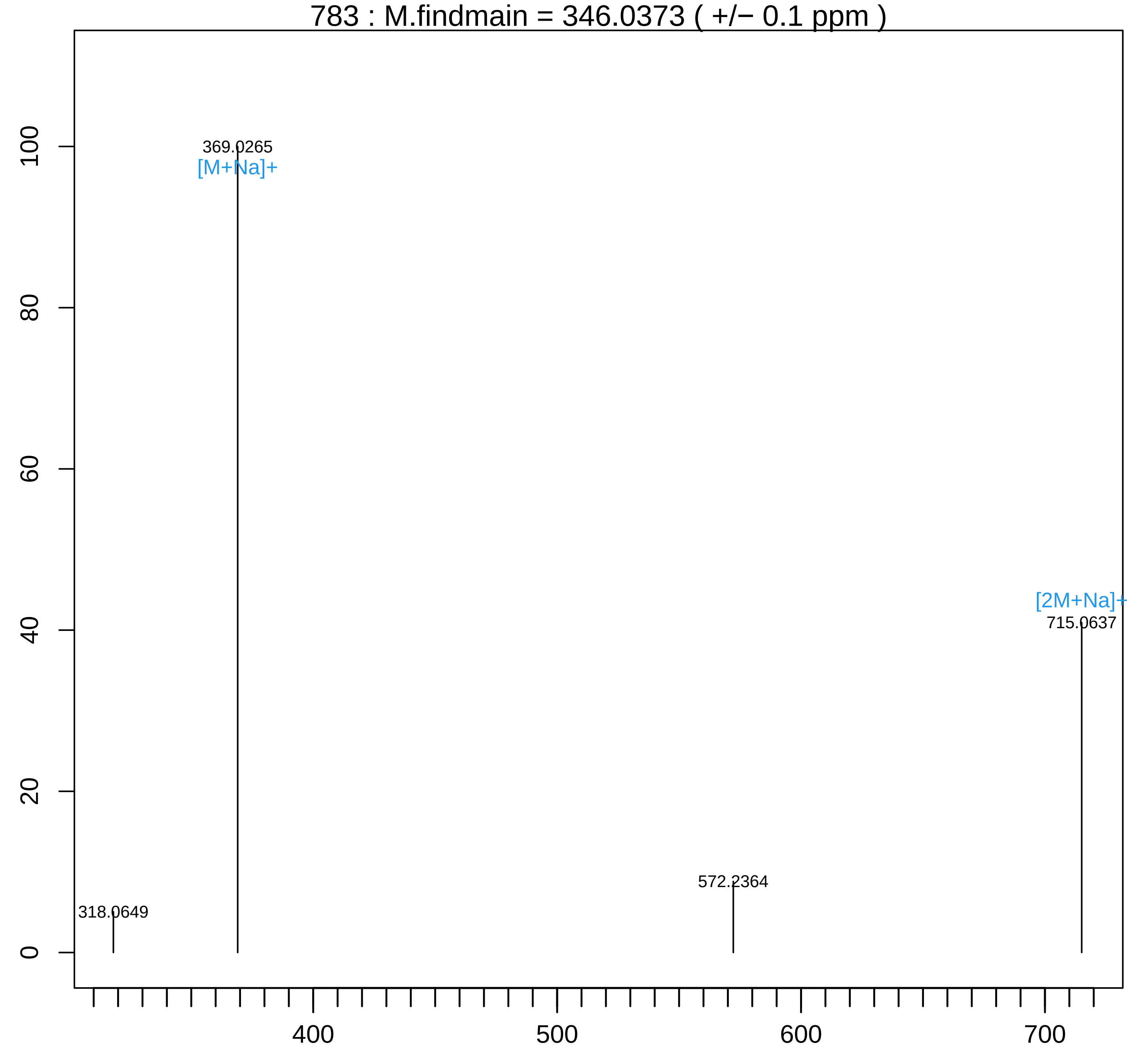

783 : M.findmain = 346.0373 (+/− 0.1 ppm)
369.0265
[M+Na]+
[2M+Na]+
715.0637
572.2364
318.0649
0
20
40
60
80
100
400
500
600
700

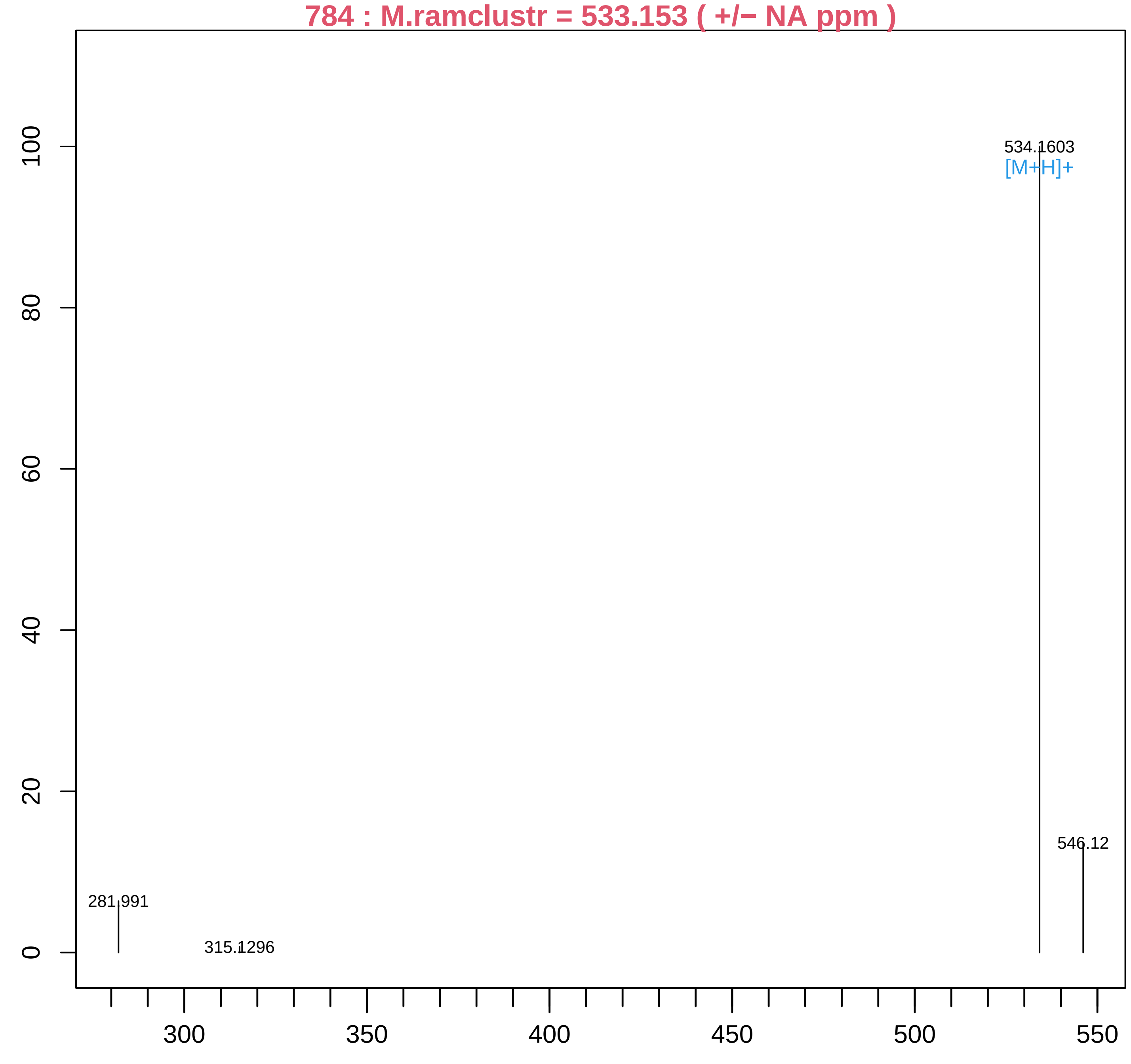
784 : M.ramclustr = 533.153 (+/− NA ppm)
534.1603
[M+H]+
546.12
281.991
315.1296
0
20
40
60
80
100
300
350
400
450
500
550

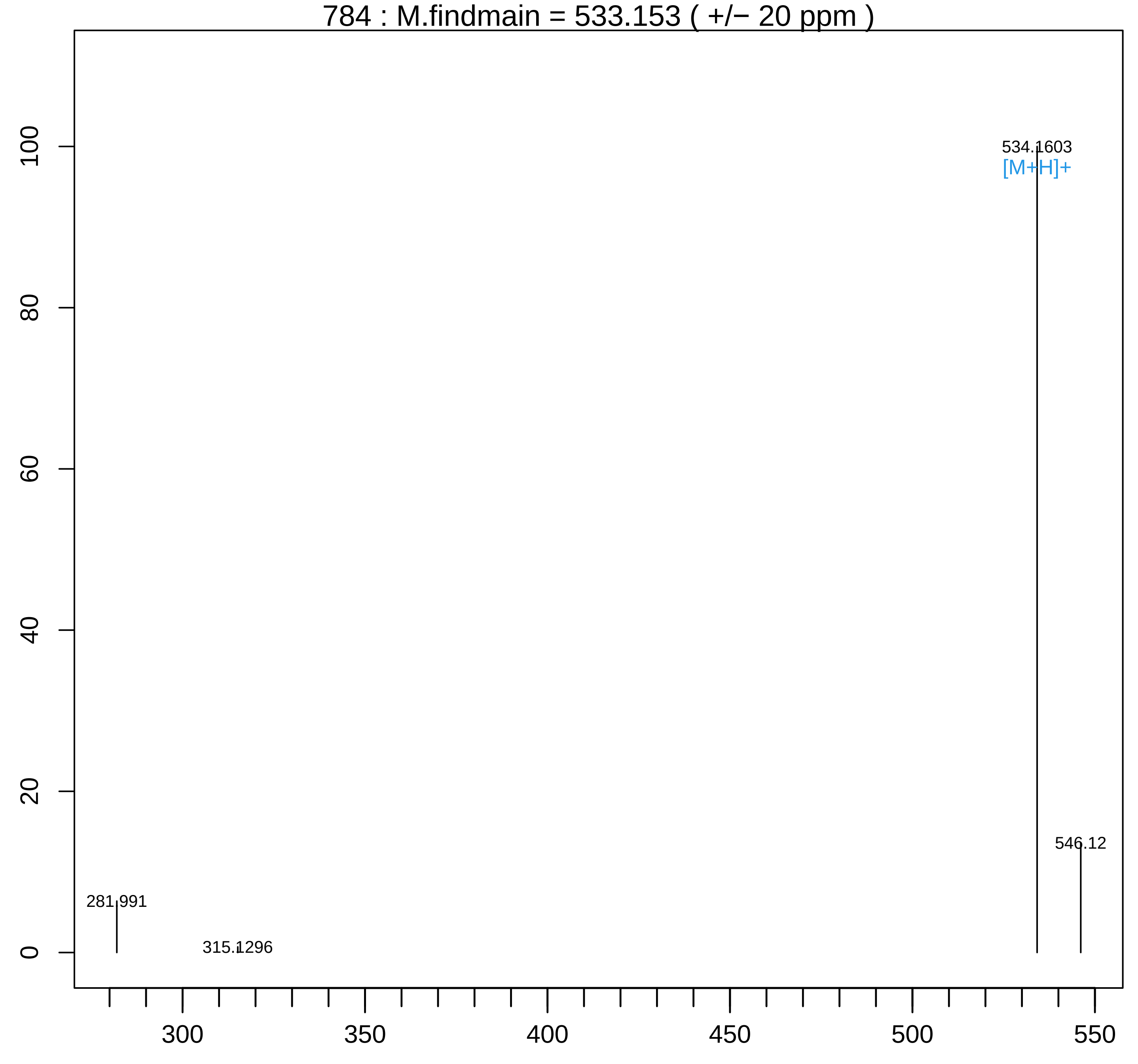
784 : M.findmain = 533.153 (+/− 20 ppm)
534.1603
[M+H]+
546.12
281.991
315.1296
0
20
40
60
80
100
300
350
400
450
500
550

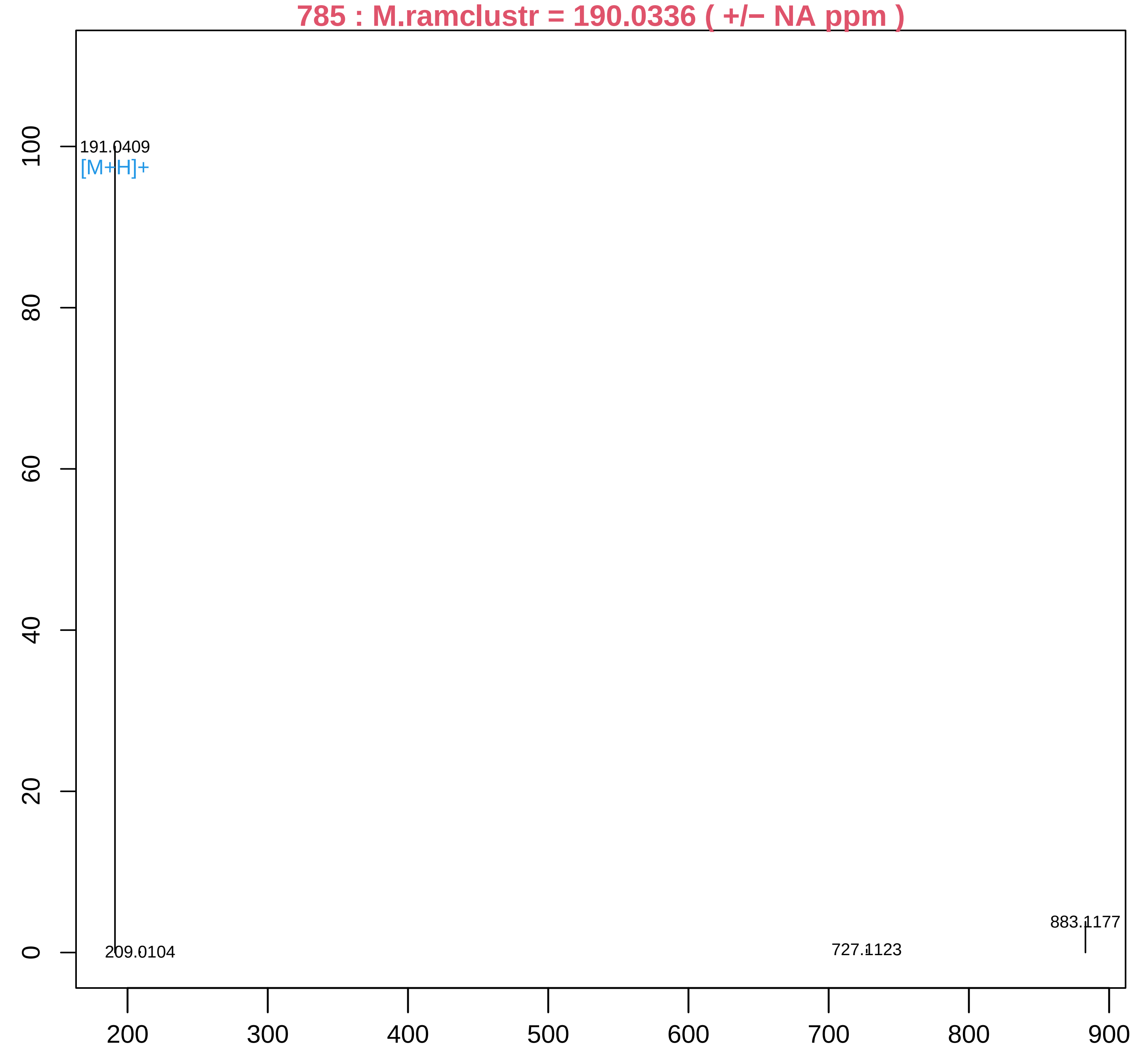

785 : M.ramclustr = 190.0336 (+/− NA ppm)
191.0409
[M+H]+
209.0104
727.1123
883.1177
0
20
40
60
80
100
200
300
400
500
600
700
800
900

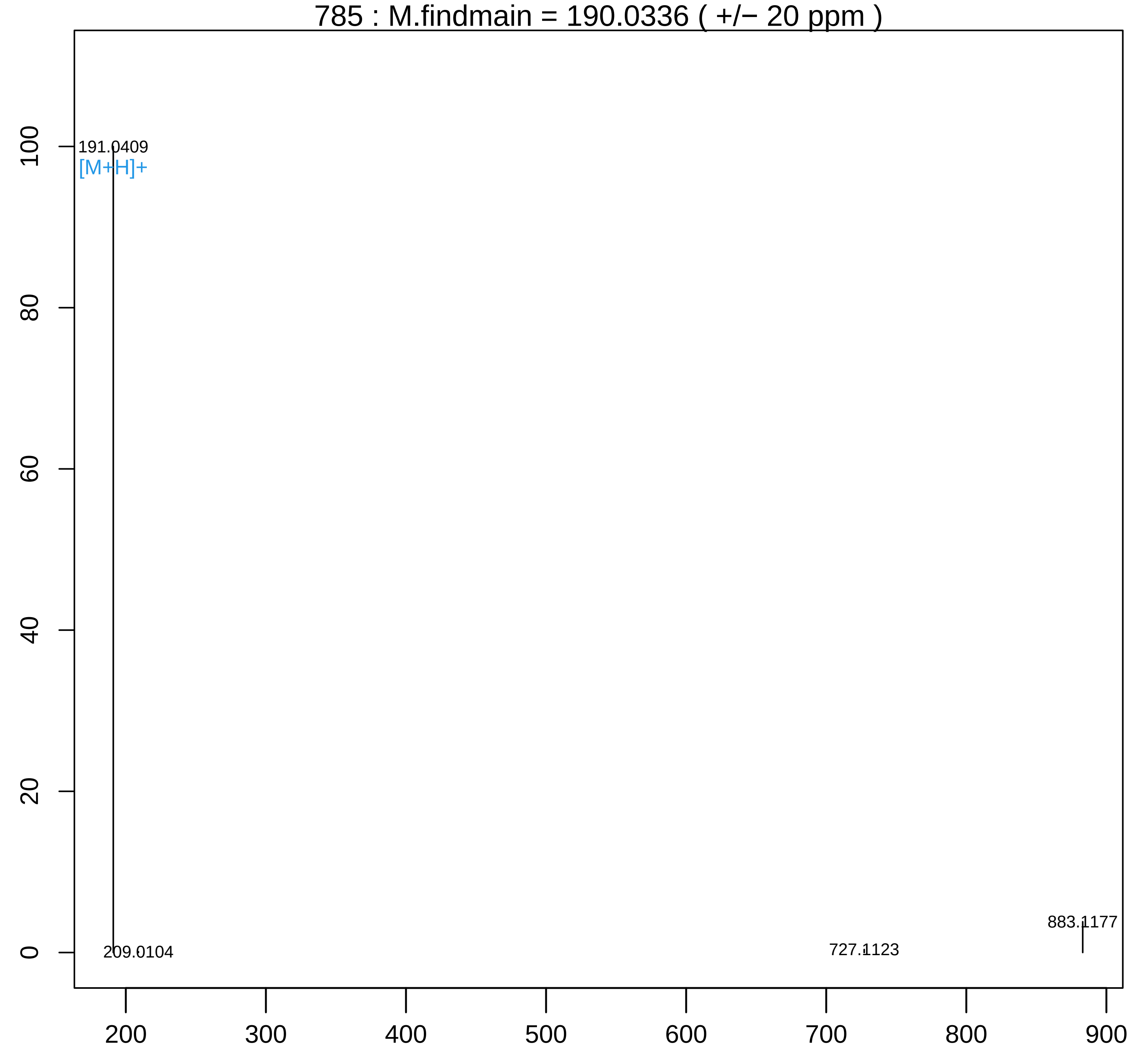

785 : M.findmain = 190.0336 (+/− 20 ppm)
191.0409
[M+H]+
209.0104
727.1123
883.1177
0
20
40
60
80
100
200
300
400
500
600
700
800
900

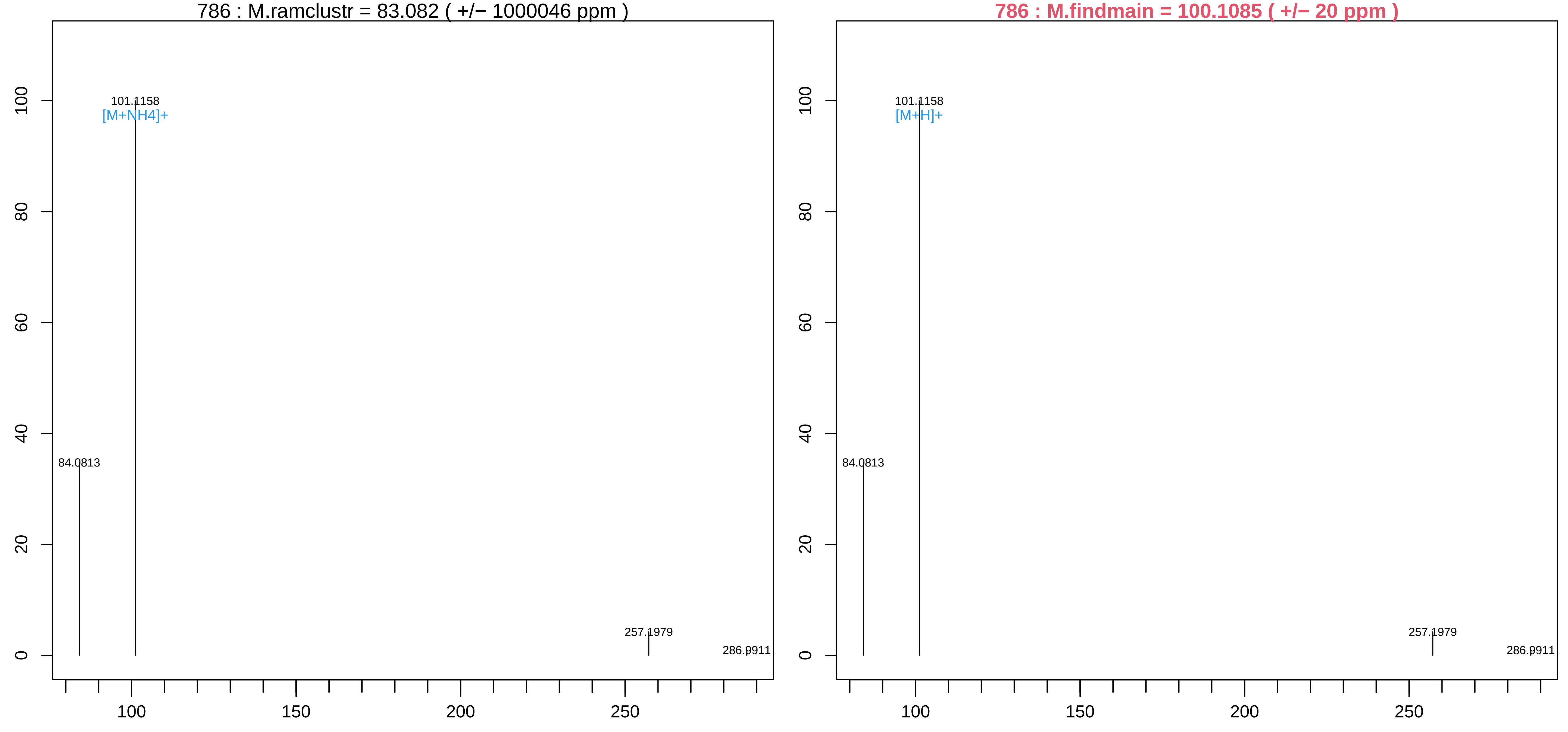
786 : M.ramclustr = 83.082 (+/− 1000046 ppm)
101.1158
[M+NH4]+
84.0813
257.1979
286.9911
786 : M.findmain = 100.1085 (+/− 20 ppm)
101.1158
[M+H]+
84.0813
257.1979
286.9911

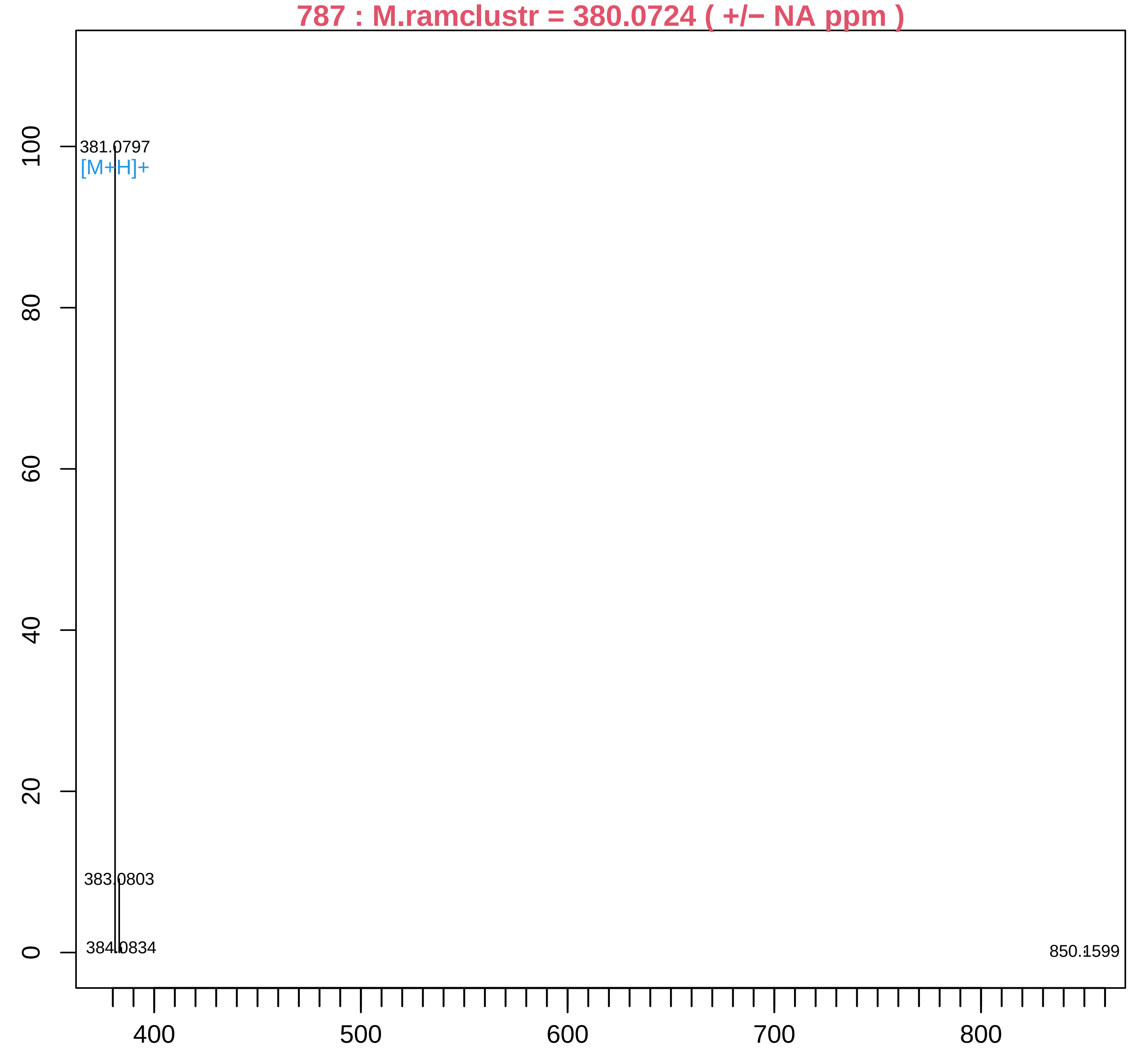
787 : M.ramclustr = 380.0724 (+/− NA ppm)
381.0797
[M+H]+
383.0803
384.0834
850.1599
0
20
40
60
80
100
400
500
600
700
800

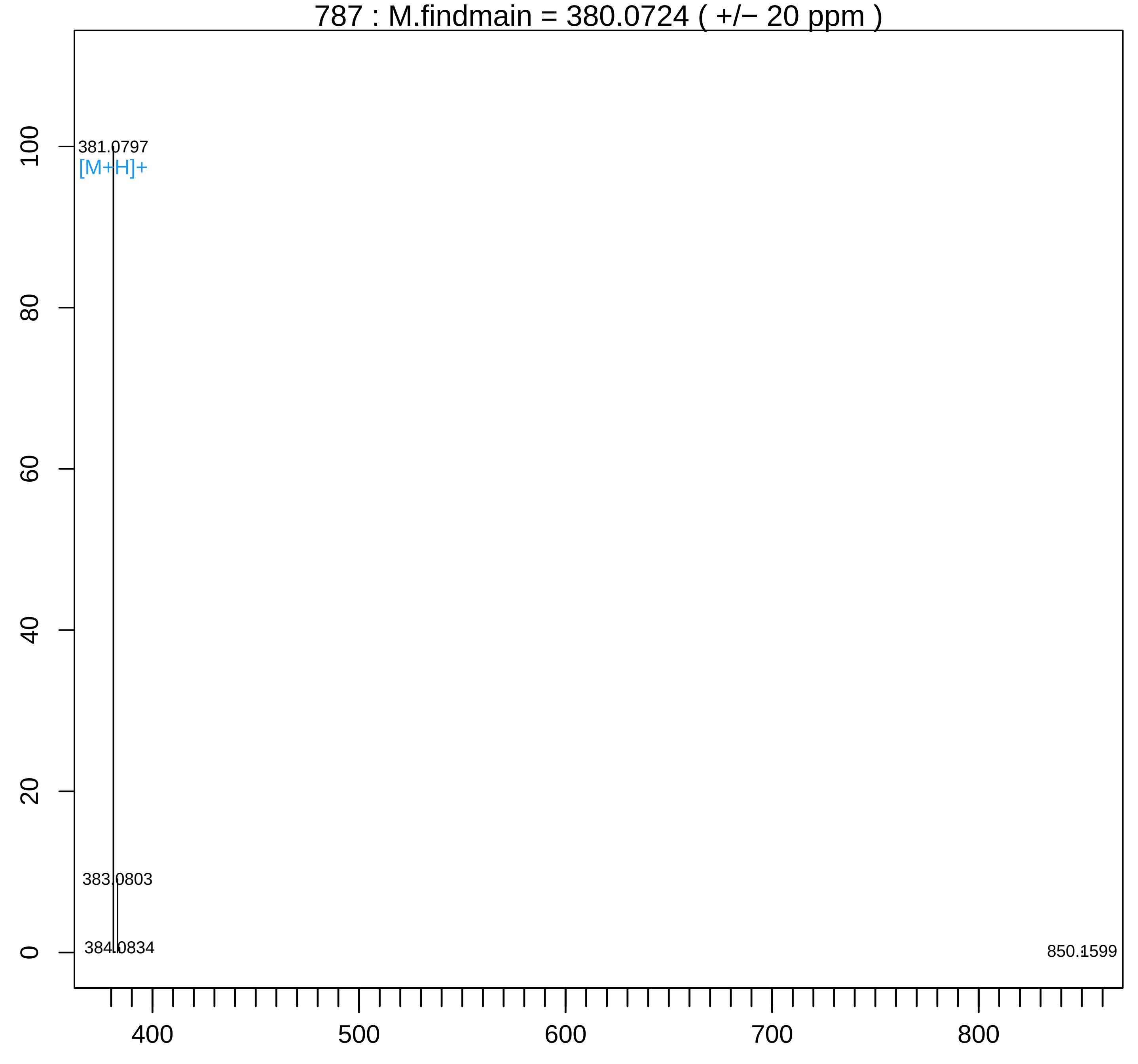
787 : M.findmain = 380.0724 (+/− 20 ppm)
381.0797
[M+H]+
383.0803
384.0834
850.1599
0
20
40
60
80
100
400
500
600
700
800

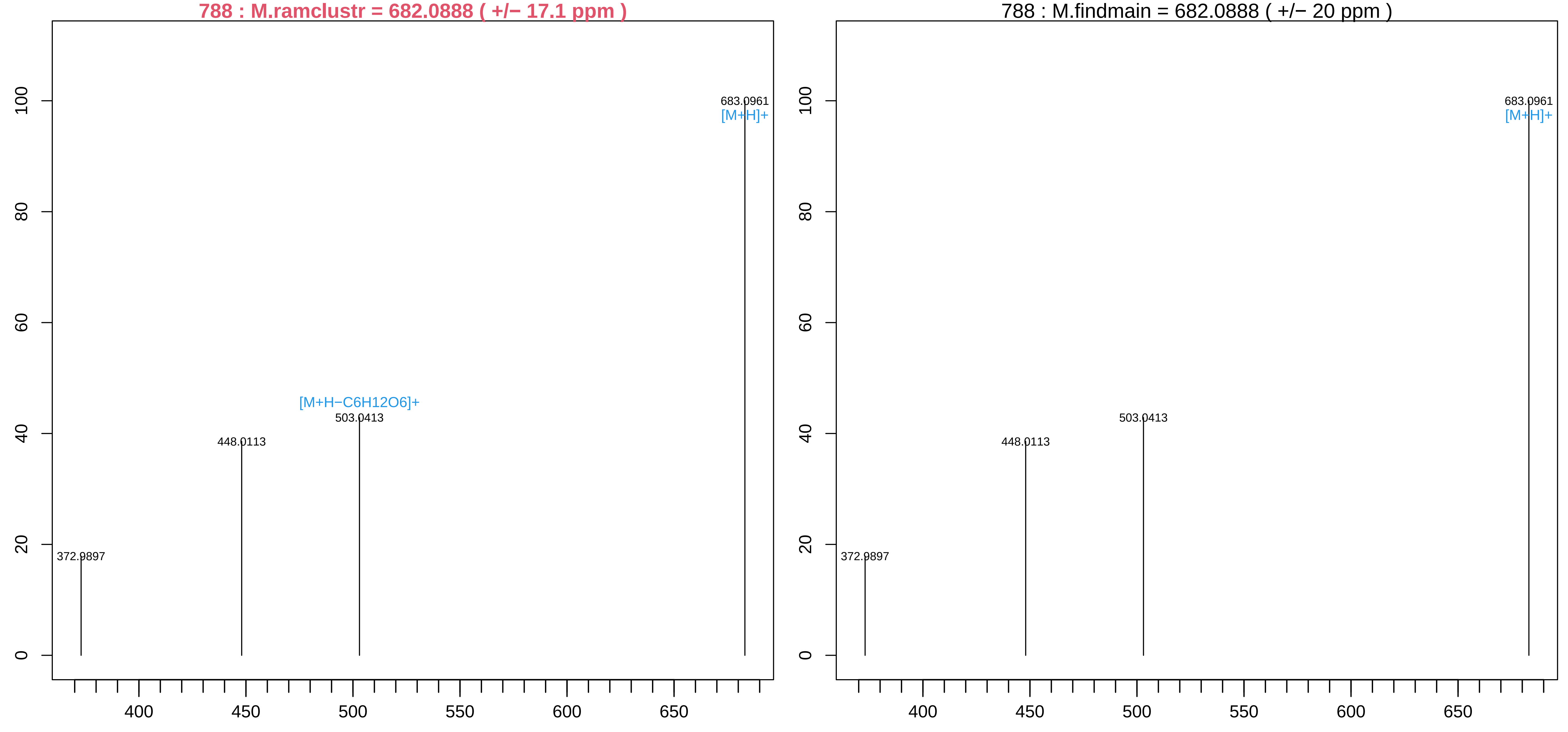

788 : M.ramclustr = 682.0888 (+/− 17.1 ppm)
683.0961
[M+H]+
[M+H−C6H12O6]+
503.0413
448.0113
372.9897
788 : M.findmain = 682.0888 (+/− 20 ppm)
683.0961
[M+H]+
503.0413
448.0113
372.9897

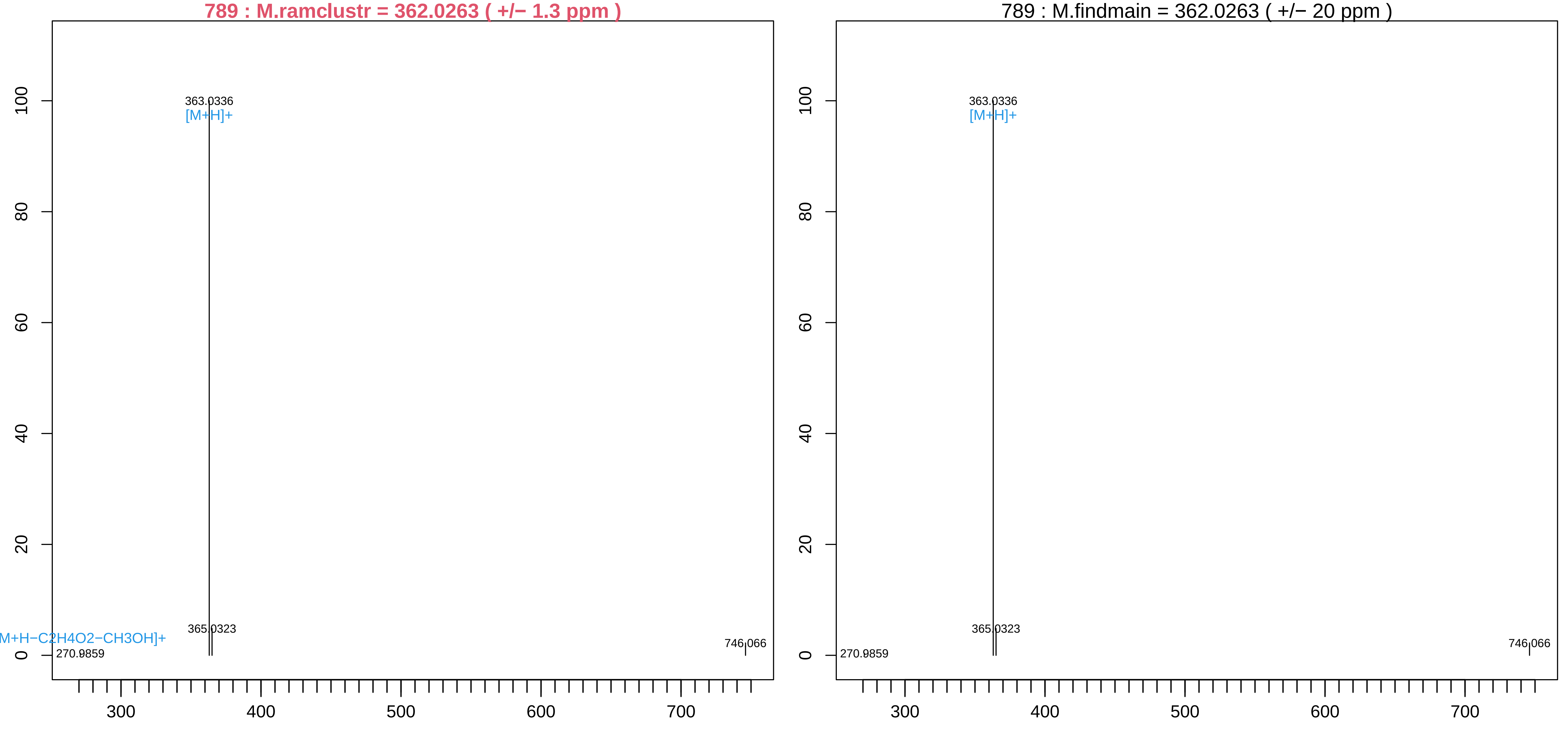

789 : M.ramclustr = 362.0263 (+/− 1.3 ppm)
363.0336
[M+H]+
365.0323
M+H−C2H4O2−CH3OH]+
270.9859
746.066
789 : M.findmain = 362.0263 (+/− 20 ppm)
363.0336
[M+H]+
365.0323
270.9859
746.066

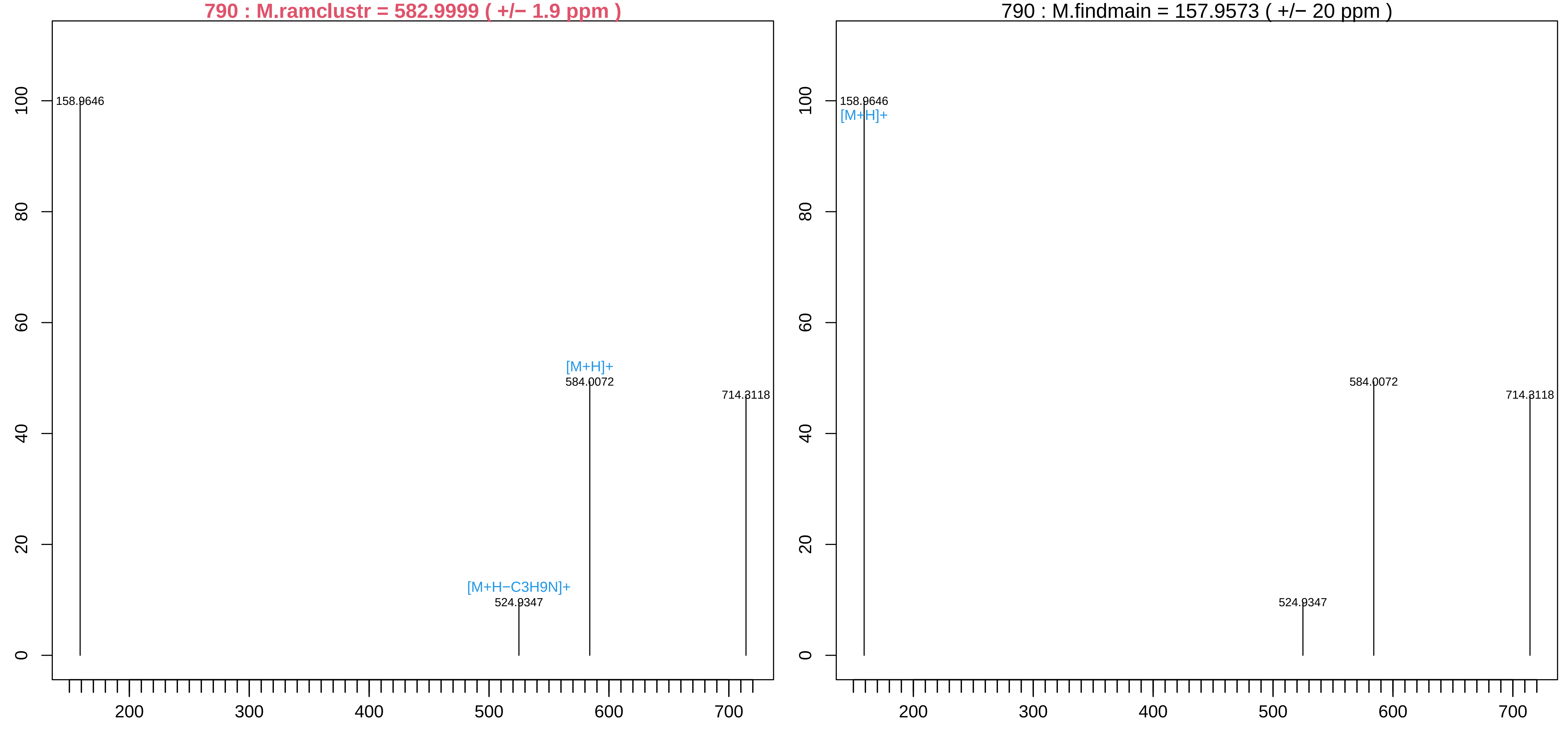
790 : M.ramclustr = 582.9999 (+/− 1.9 ppm)
158.9646
[M+H]+
584.0072
714.3118
[M+H−C3H9N]+
524.9347
790 : M.findmain = 157.9573 (+/− 20 ppm)
158.9646
[M+H]+
584.0072
714.3118
524.9347

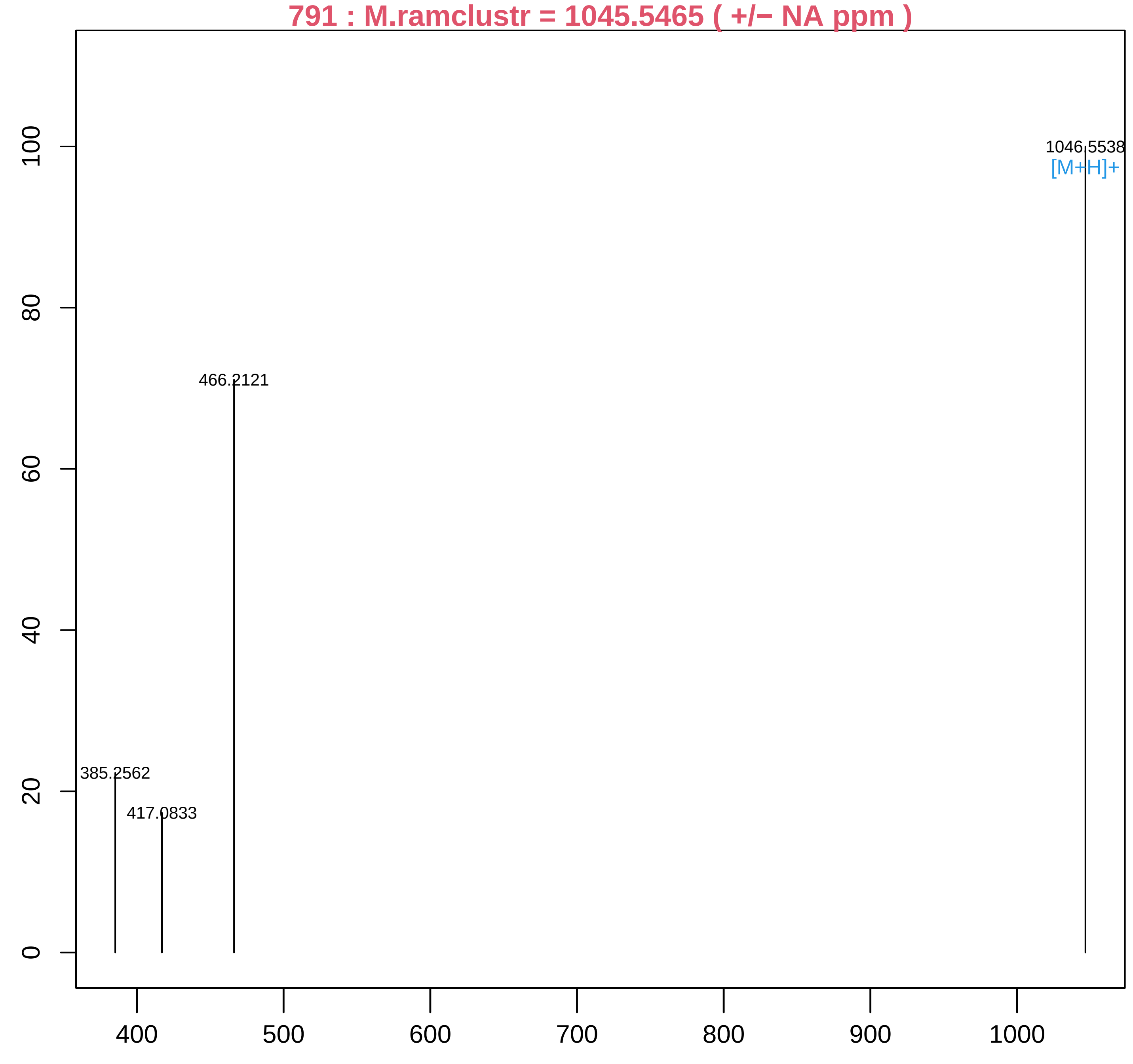
791 : M.ramclustr = 1045.5465 (+/− NA ppm)
1046.5538
[M+H]+
466.2121
385.2562
417.0833
0
20
40
60
80
100
400
500
600
700
800
900
1000

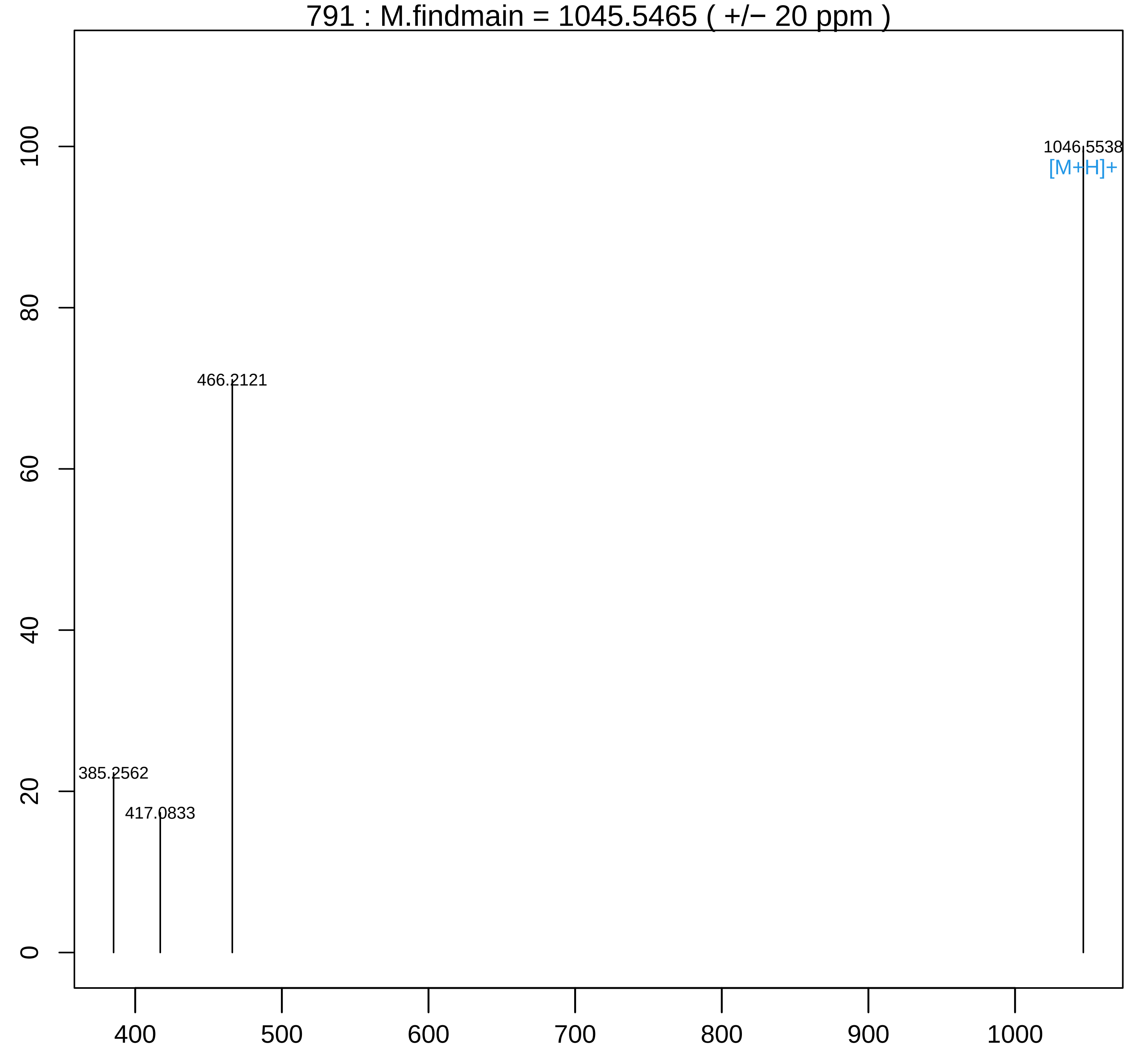
791 : M.findmain = 1045.5465 (+/− 20 ppm)
1046.5538
[M+H]+
466.2121
385.2562
417.0833
0
20
40
60
80
100
400
500
600
700
800
900
1000

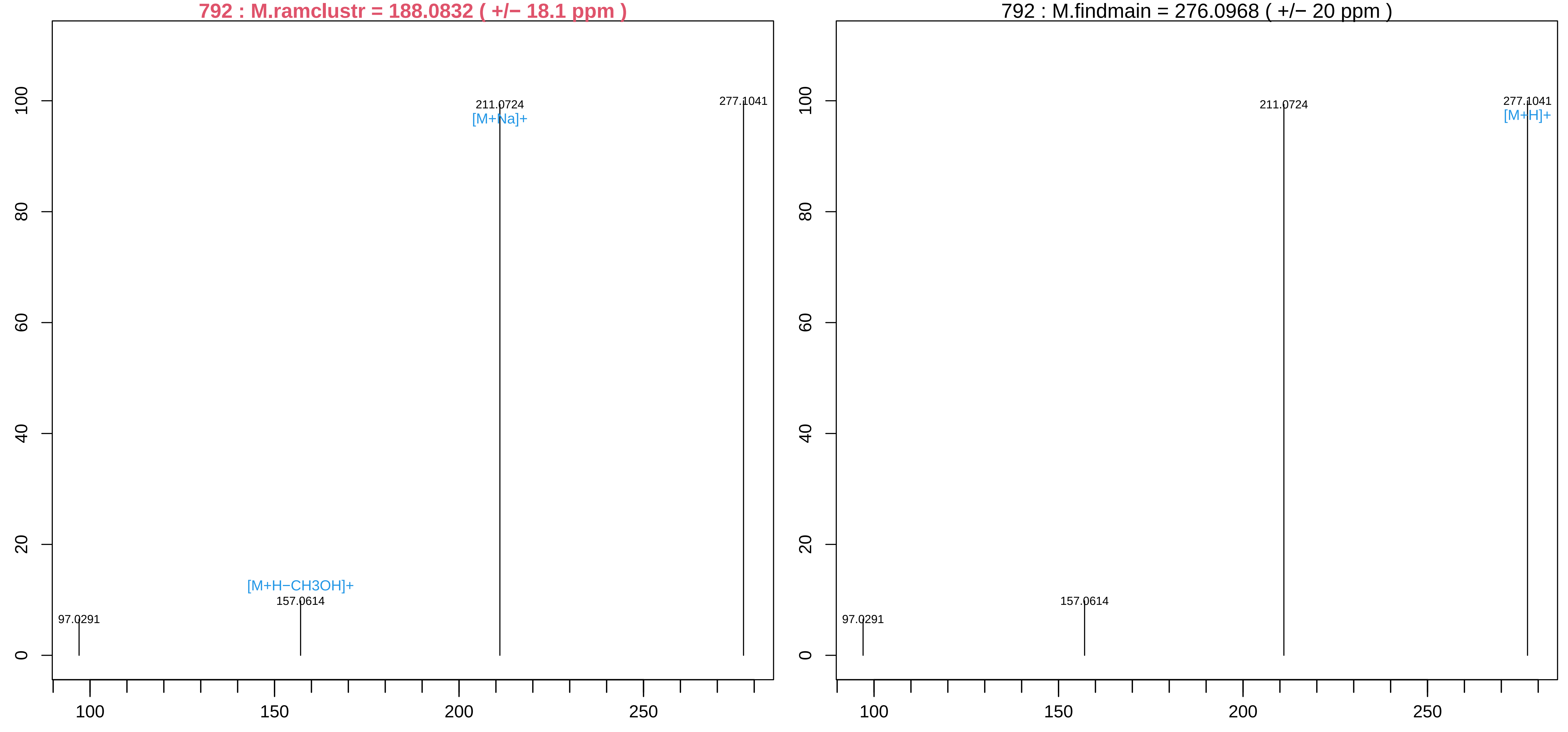

792 : M.ramclustr = 188.0832 (+/− 18.1 ppm)
97.0291
157.0614
[M+H−CH3OH]+
211.0724
[M+Na]+
277.1041
792 : M.findmain = 276.0968 (+/− 20 ppm)
97.0291
157.0614
211.0724
277.1041
[M+H]+

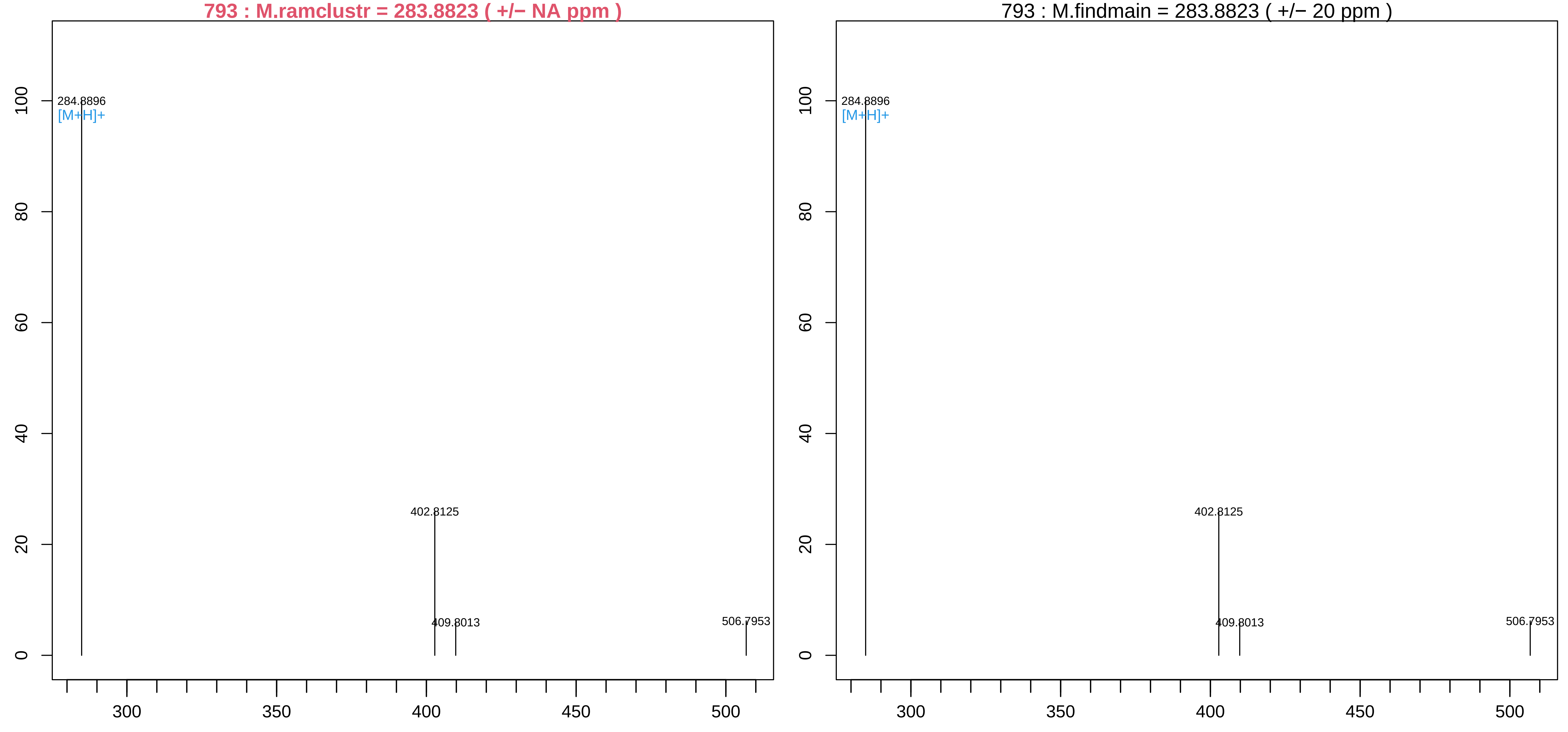

793 : M.ramclustr = 283.8823 (+/− NA ppm)
284.8896
[M+H]+
402.8125
409.8013
506.7953
793 : M.findmain = 283.8823 (+/− 20 ppm)
284.8896
[M+H]+
402.8125
409.8013
506.7953

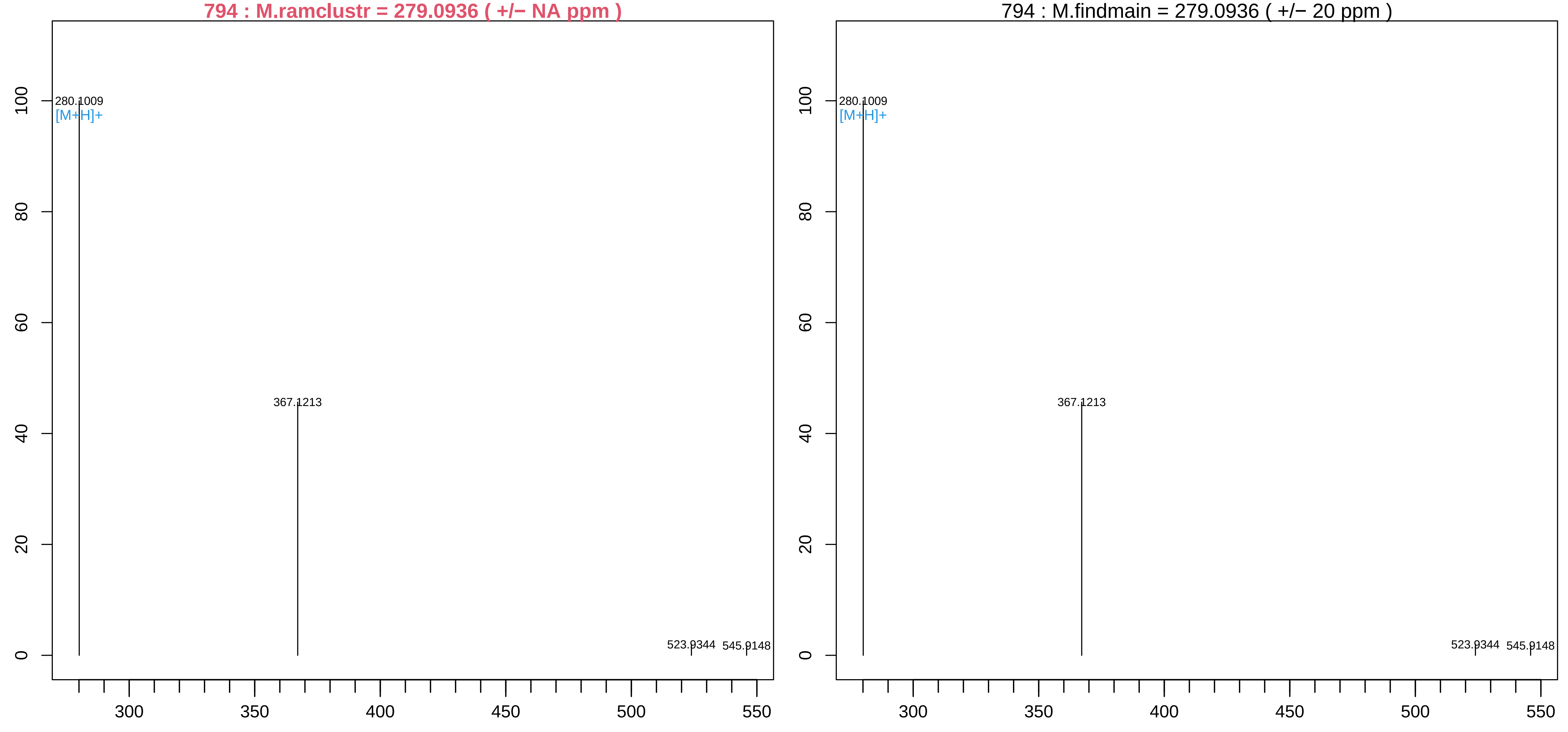

794 : M.ramclustr = 279.0936 (+/− NA ppm)
280.1009
[M+H]+
367.1213
523.9344
545.9148
794 : M.findmain = 279.0936 (+/− 20 ppm)
280.1009
[M+H]+
367.1213
523.9344
545.9148
0
20
40
60
80
100
300
350
400
450
500
550

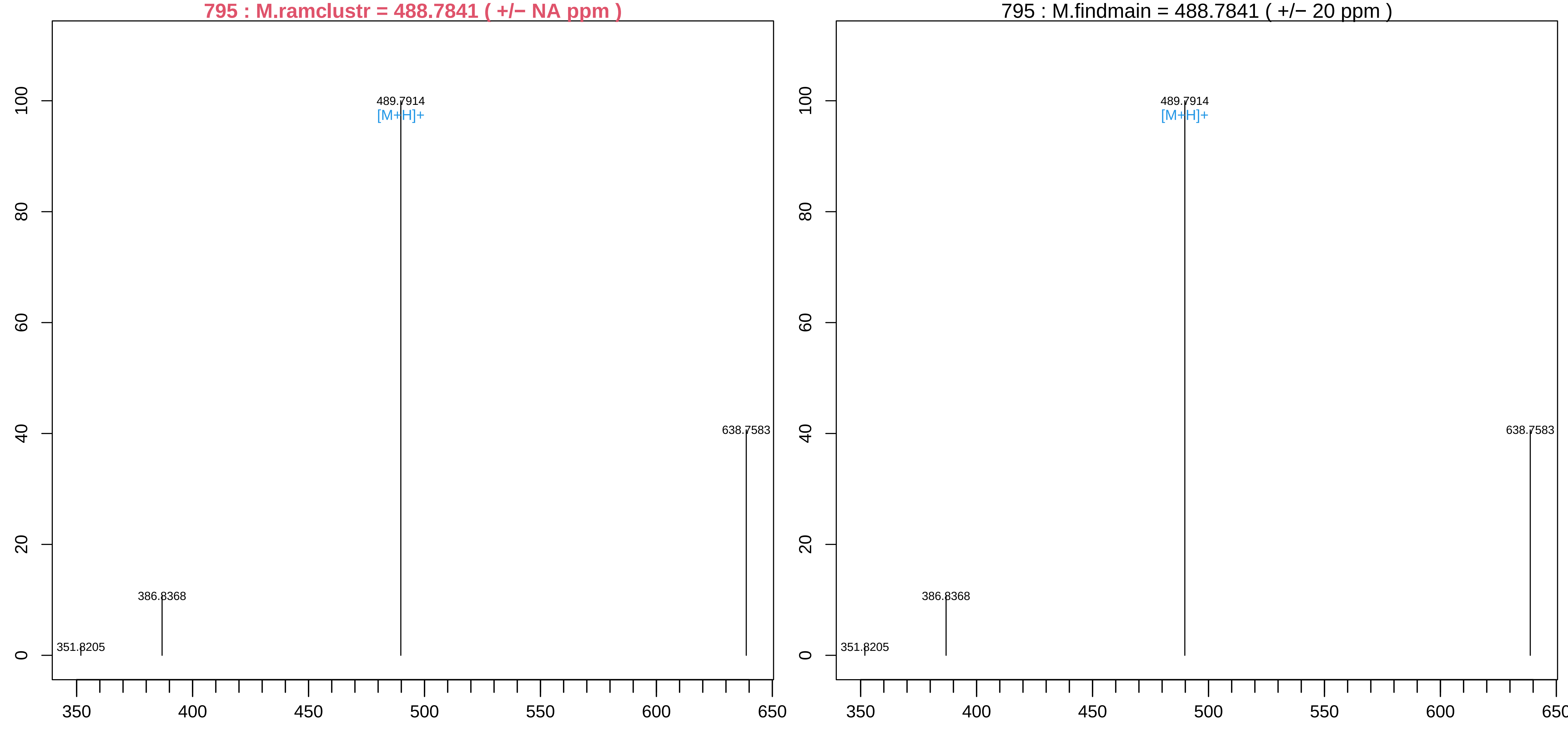
795 : M.ramclustr = 488.7841 (+/− NA ppm)
489.7914
[M+H]+
638.7583
386.8368
351.8205
795 : M.findmain = 488.7841 (+/− 20 ppm)
489.7914
[M+H]+
638.7583
386.8368
351.8205

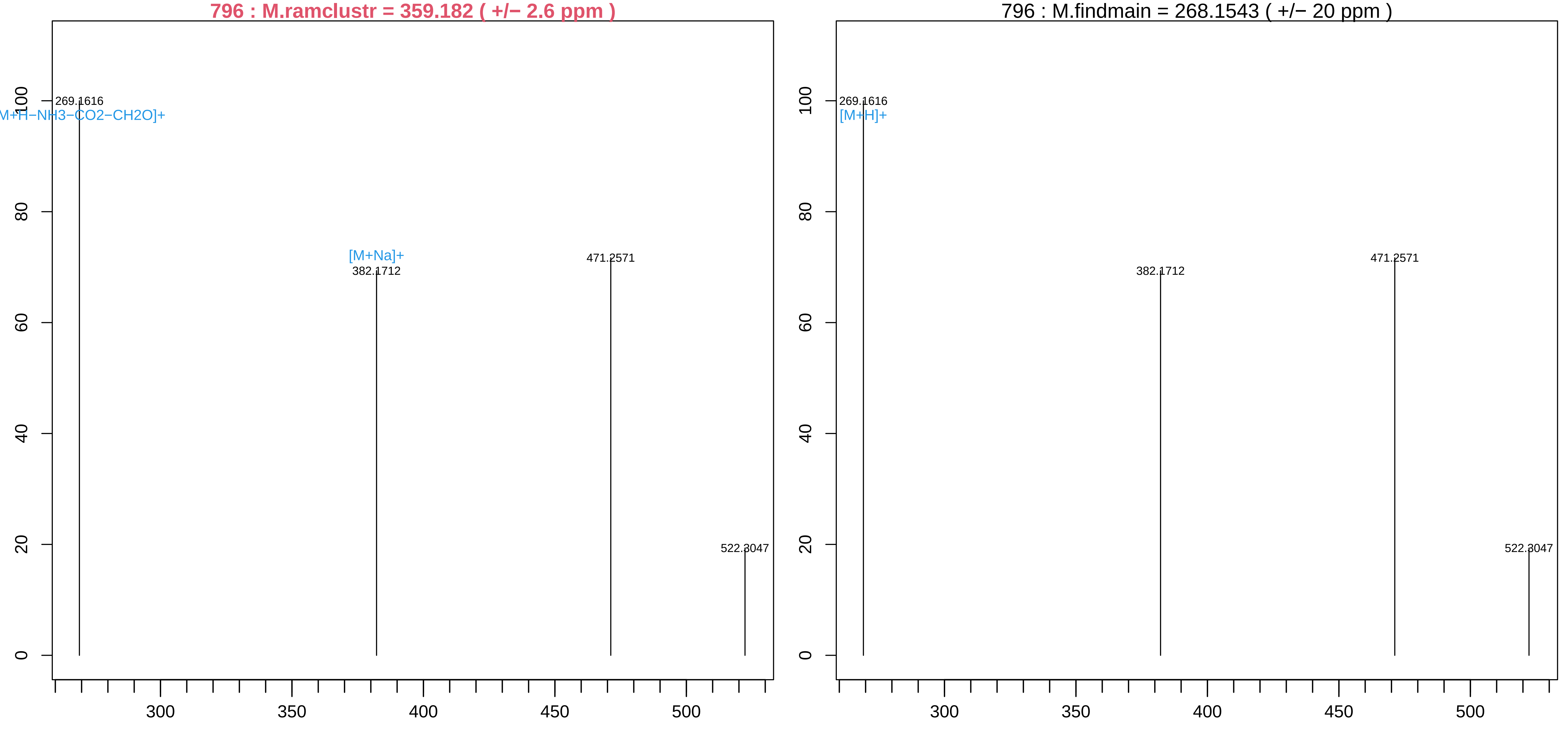
796 : M.ramclustr = 359.182 (+/− 2.6 ppm)
269.1616
M+H−NH3−CO2−CH2O]+
[M+Na]+
382.1712
471.2571
522.3047
0
20
40
60
80
100
300
350
400
450
500
796 : M.findmain = 268.1543 (+/− 20 ppm)
269.1616
[M+H]+
382.1712
471.2571
522.3047

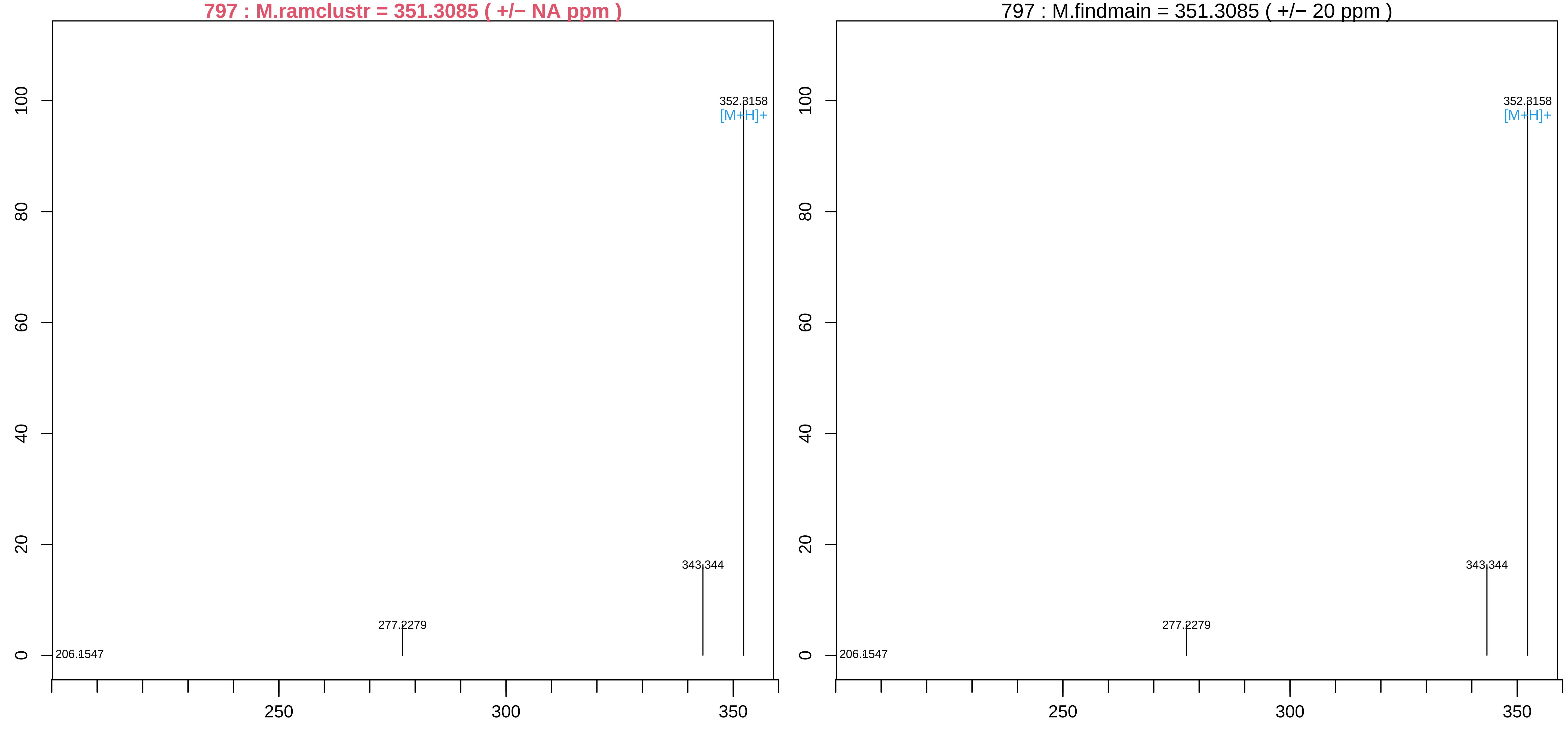
797 : M.ramclustr = 351.3085 (+/− NA ppm)
352.3158
[M+H]+
343.344
277.2279
206.1547
0
20
40
60
80
100
250
300
350
797 : M.findmain = 351.3085 (+/− 20 ppm)
352.3158
[M+H]+
343.344
277.2279
206.1547
0
20
40
60
80
100
250
300
350

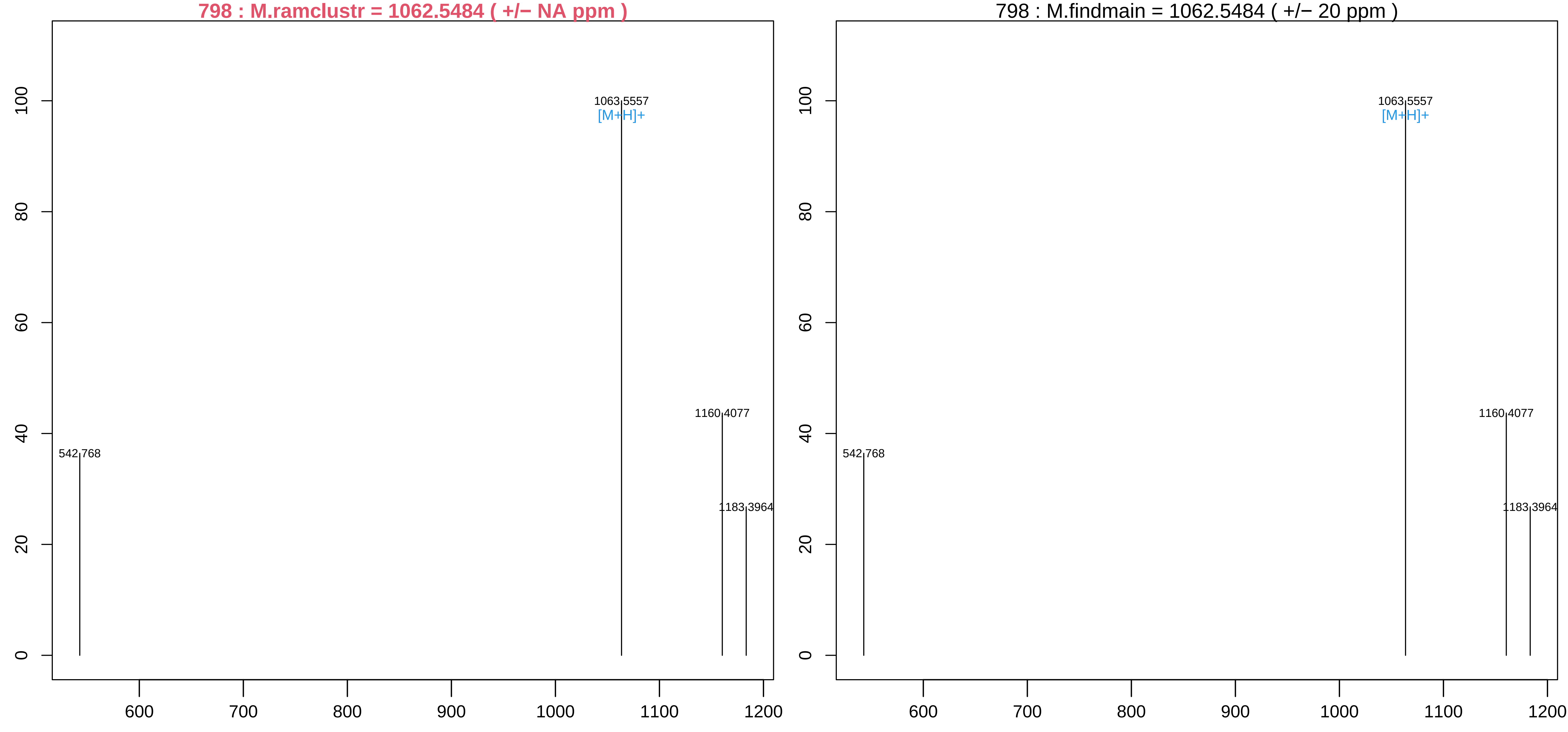

798 : M.ramclustr = 1062.5484 (+/− NA ppm)
798 : M.findmain = 1062.5484 (+/− 20 ppm)
1063.5557
[M+H]+
1160.4077
542.768
1183.3964
0
20
40
60
80
100
600
700
800
900
1000
1100
1200

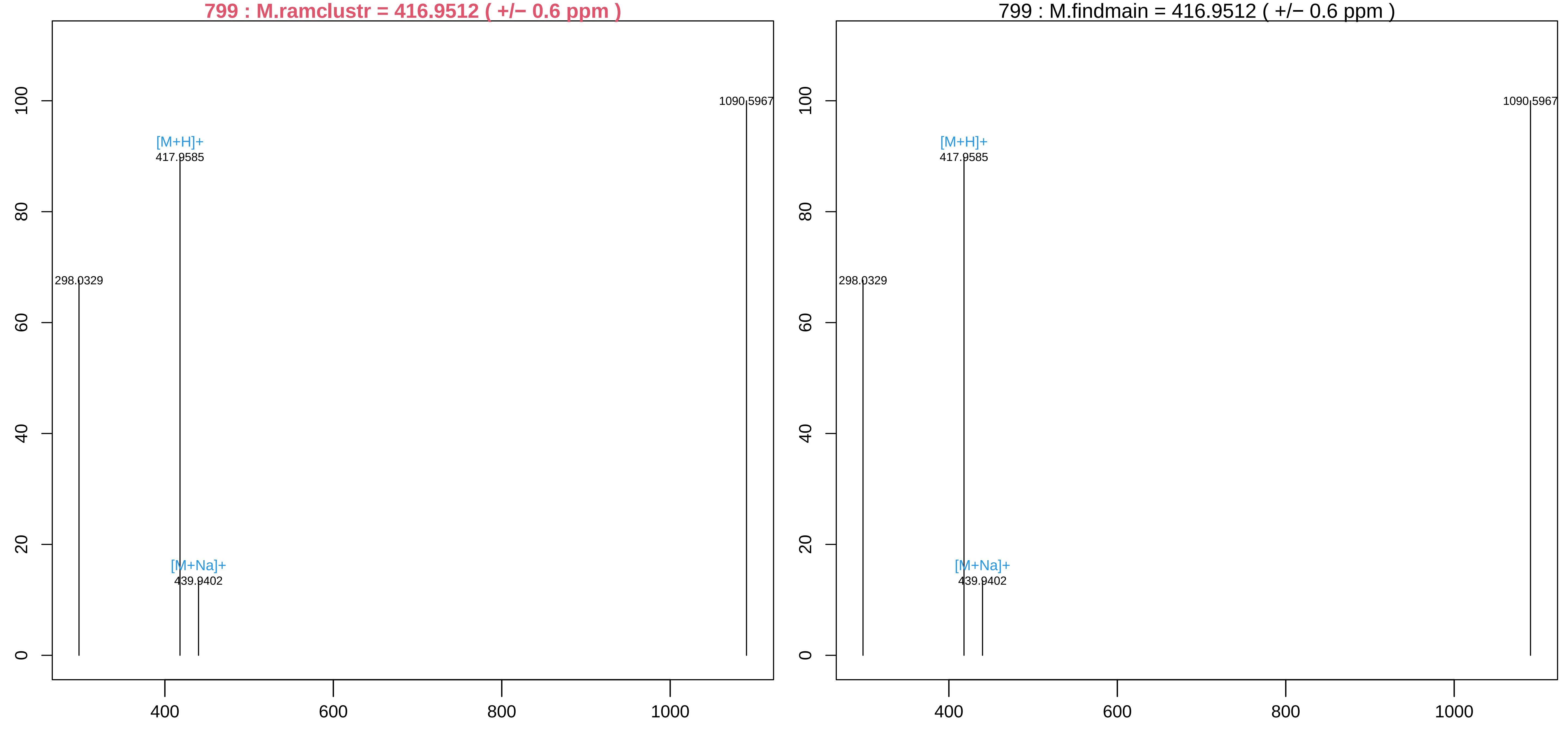
799 : M.ramclustr = 416.9512 (+/− 0.6 ppm)
[M+H]+
417.9585
298.0329
[M+Na]+
439.9402
1090.5967
799 : M.findmain = 416.9512 (+/− 0.6 ppm)
[M+H]+
417.9585
298.0329
[M+Na]+
439.9402
1090.5967
0
20
40
60
80
100
400
600
800
1000

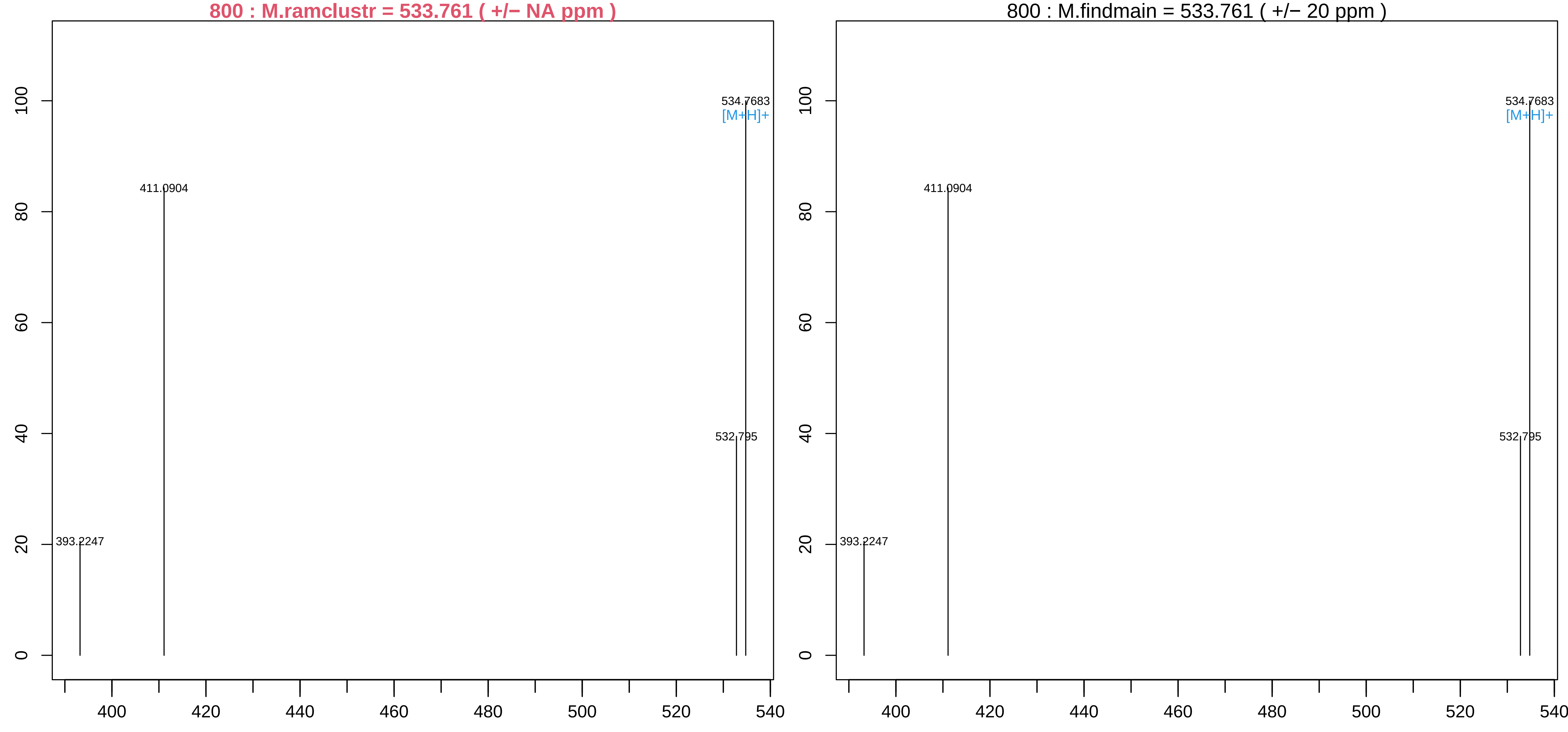

800 : M.ramclustr = 533.761 (+/− NA ppm)
800 : M.findmain = 533.761 (+/− 20 ppm)
534.7683
[M+H]+
411.0904
532.795
393.2247
0
20
40
60
80
100
400
420
440
460
480
500
520
540

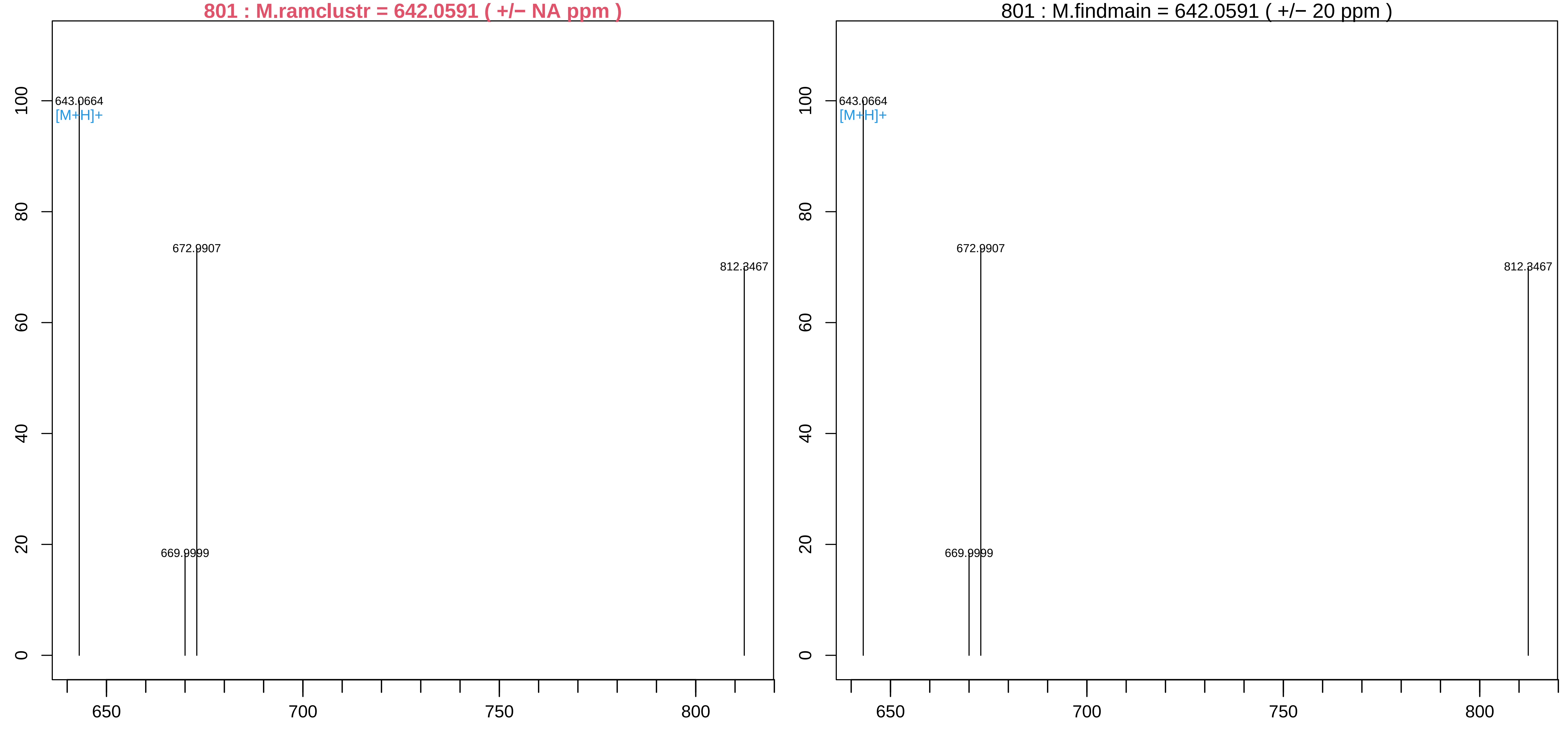
801 : M.ramclustr = 642.0591 (+/− NA ppm)
801 : M.findmain = 642.0591 (+/− 20 ppm)
643.0664
[M+H]+
672.9907
669.9999
812.3467
0
20
40
60
80
100
650
700
750
800

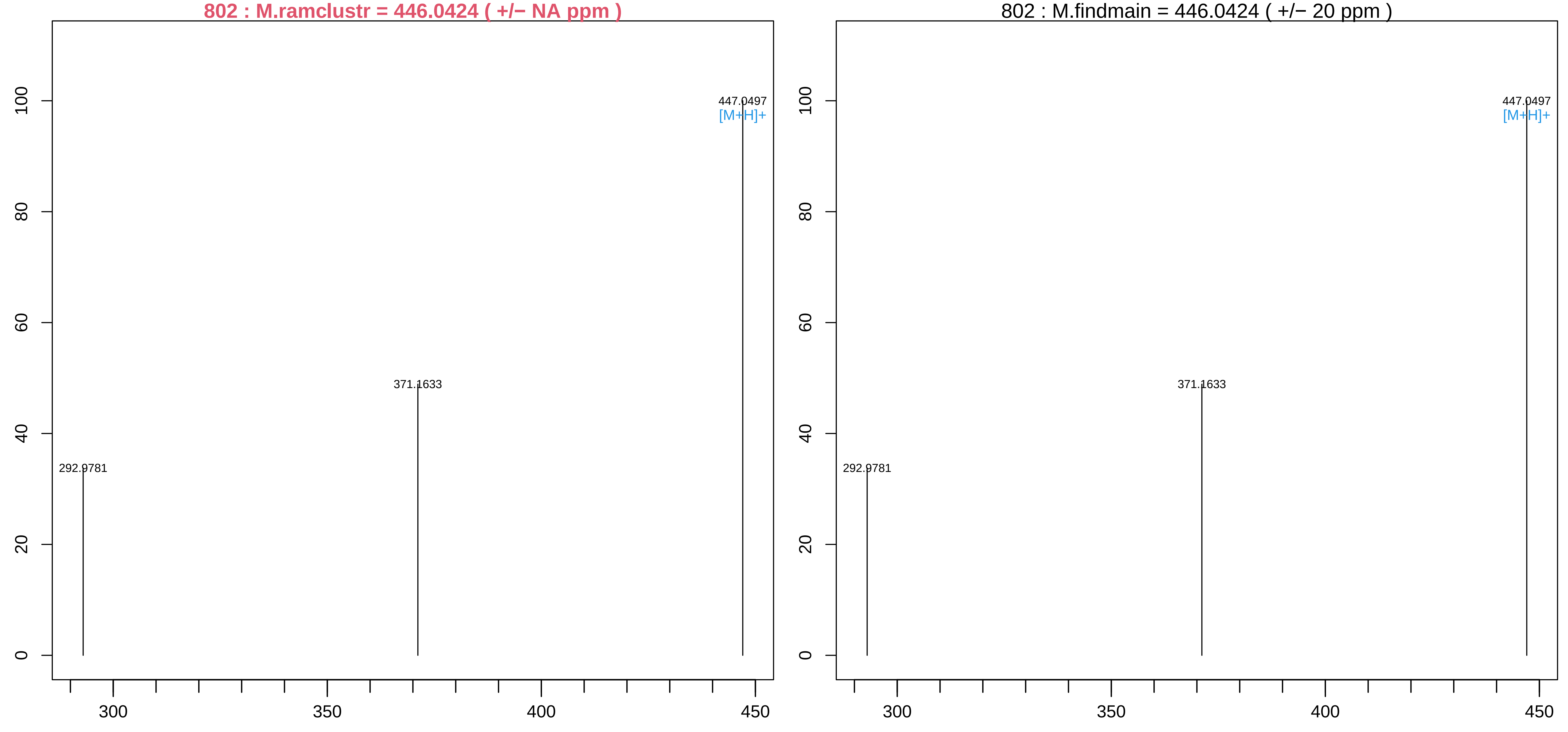

802 : M.ramclustr = 446.0424 (+/− NA ppm)
802 : M.findmain = 446.0424 (+/− 20 ppm)
447.0497
[M+H]+
371.1633
292.9781
0
20
40
60
80
100
300
350
400
450

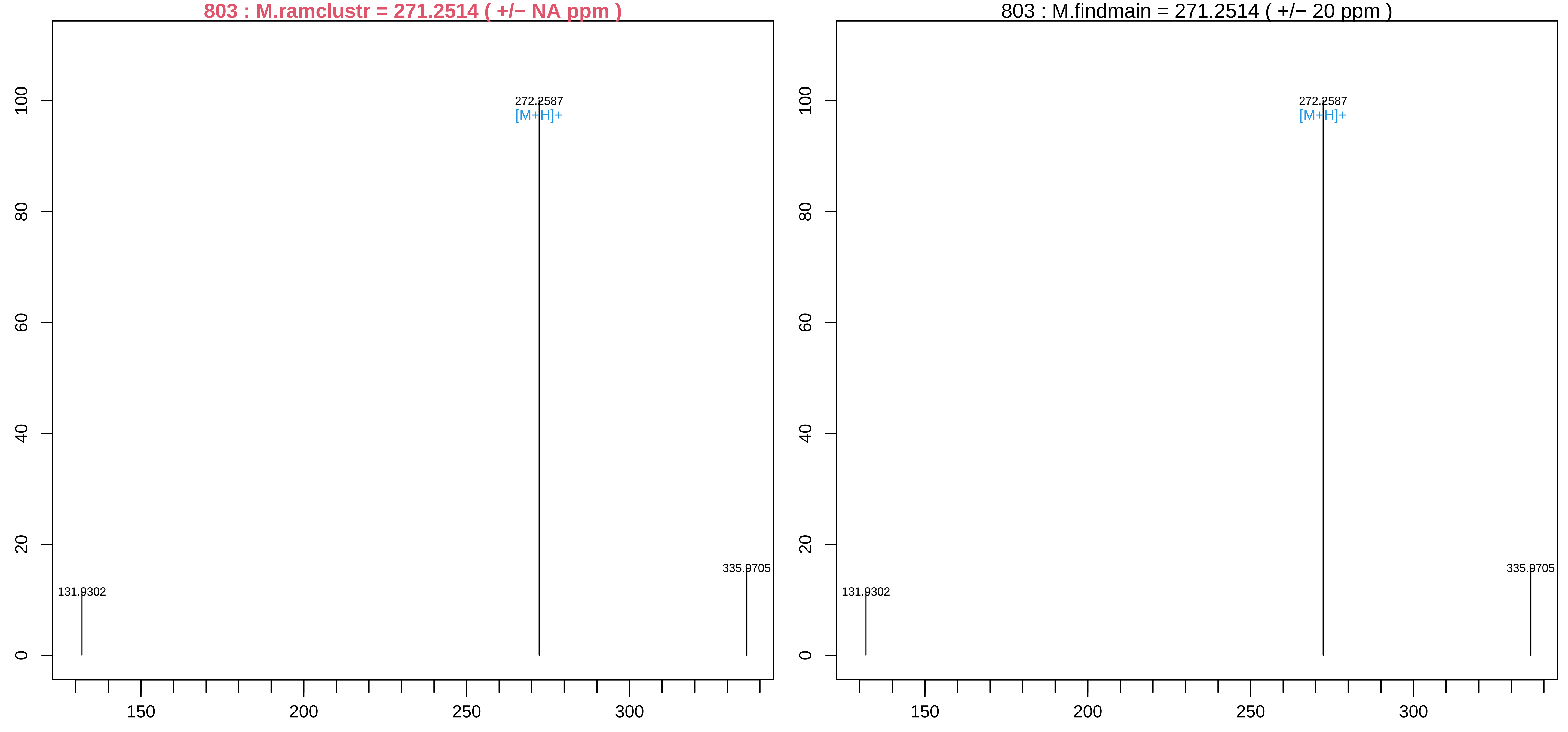

803 : M.ramclustr = 271.2514 (+/− NA ppm)
272.2587
[M+H]+
131.9302
335.9705
803 : M.findmain = 271.2514 (+/− 20 ppm)
272.2587
[M+H]+
131.9302
335.9705

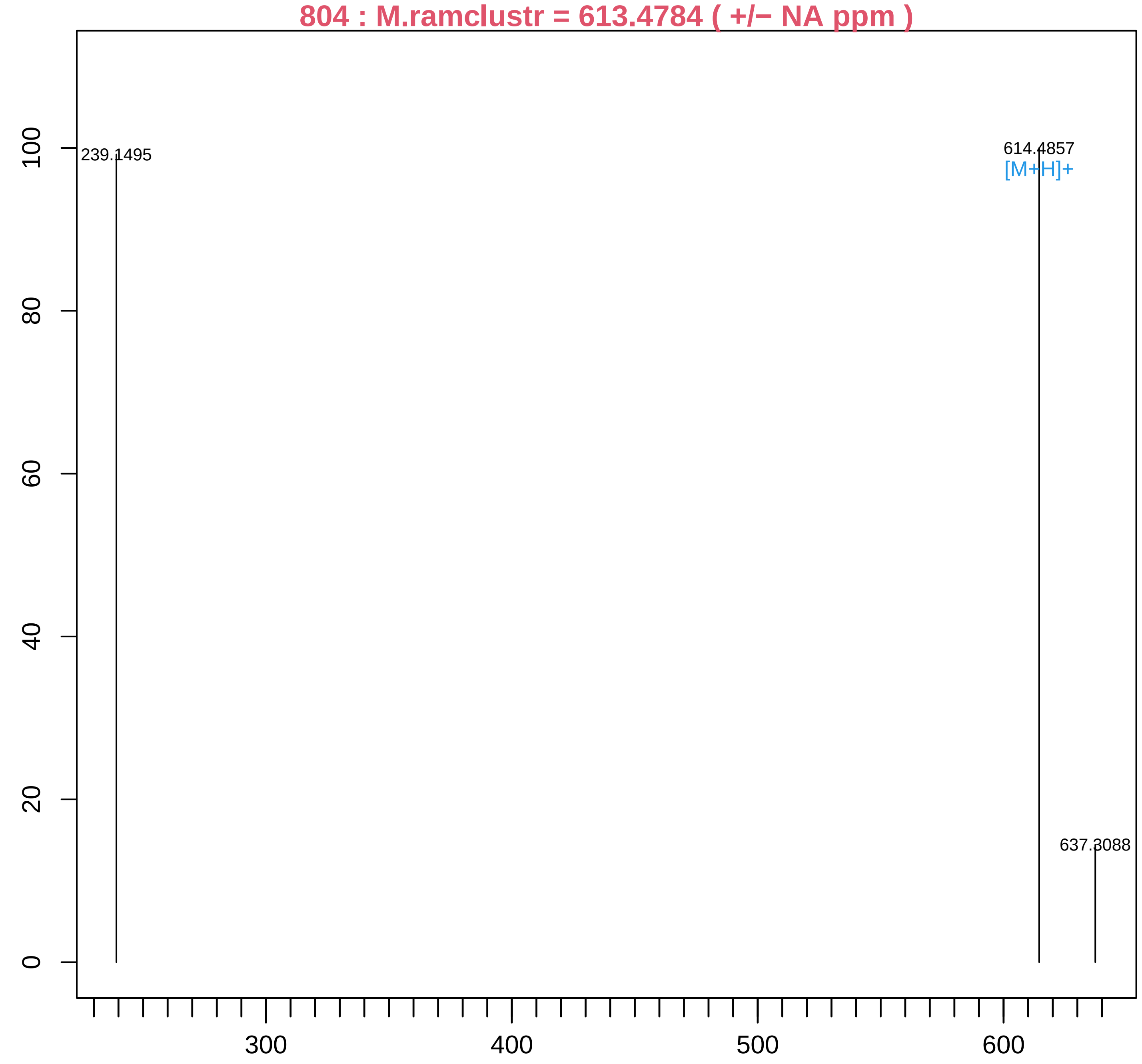
804 : M.ramclustr = 613.4784 (+/− NA ppm)
239.1495
614.4857
[M+H]+
637.3088
0
20
40
60
80
100
300
400
500
600

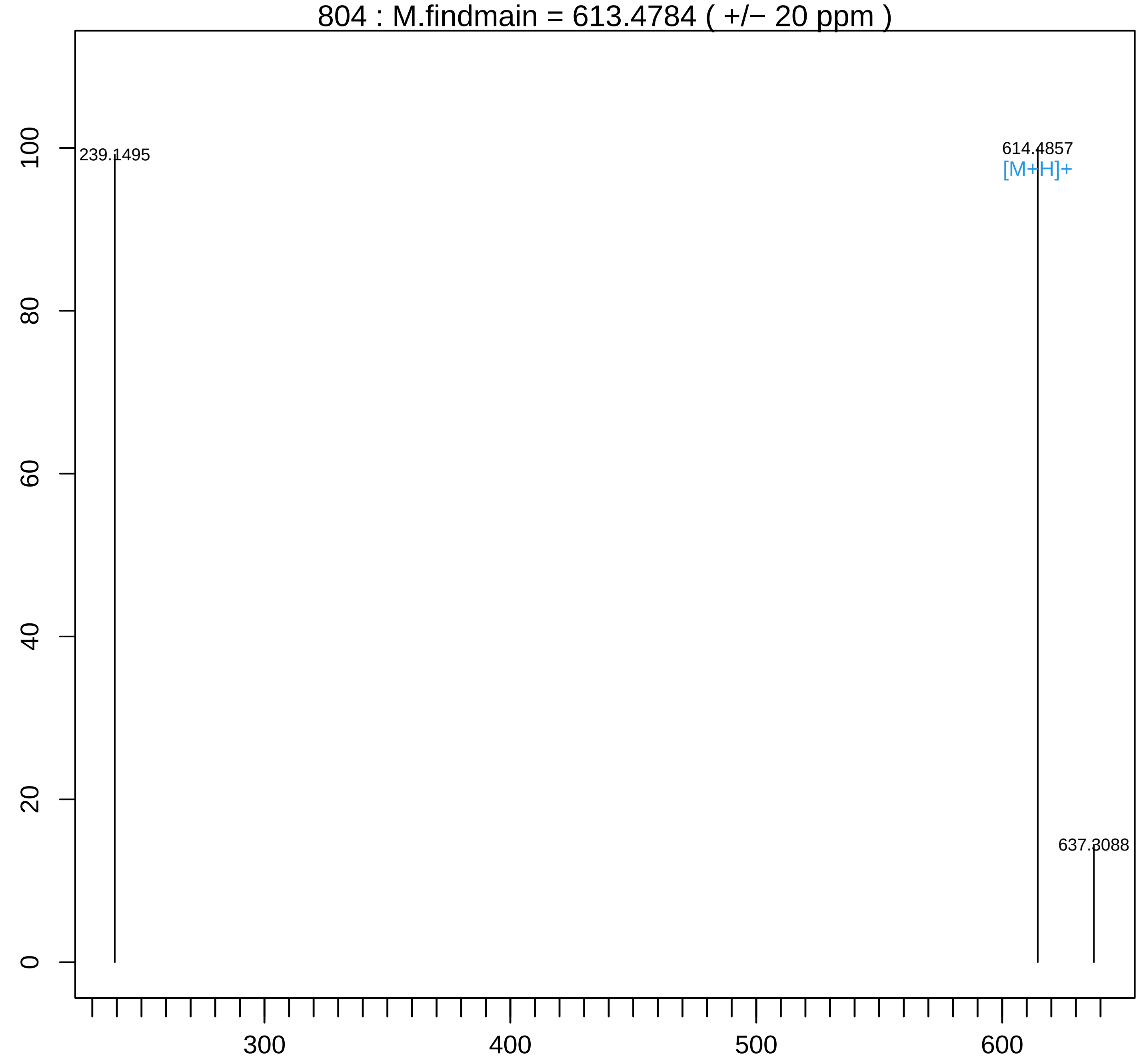
804 : M.findmain = 613.4784 (+/− 20 ppm)
239.1495
614.4857
[M+H]+
637.3088
0
20
40
60
80
100
300
400
500
600

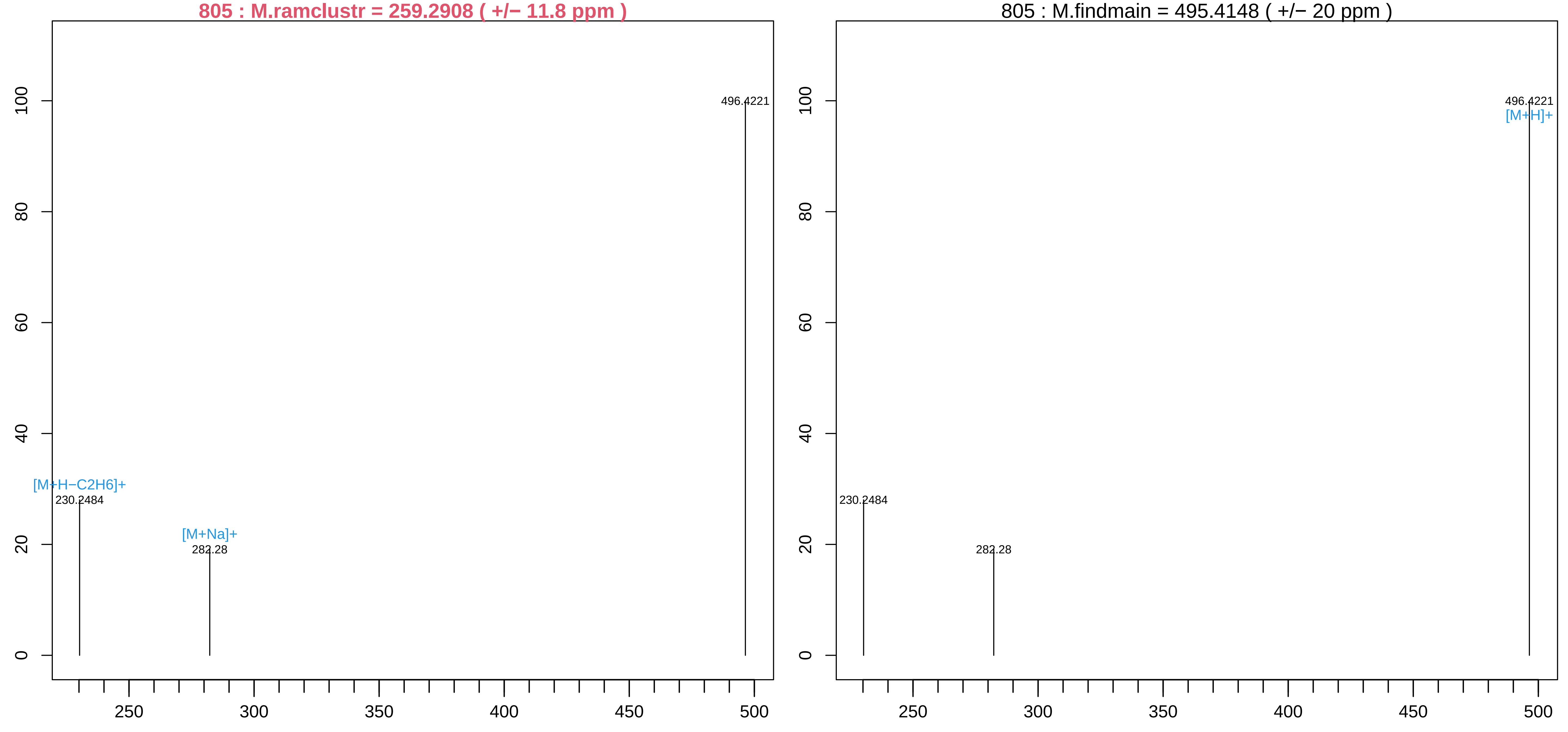
805 : M.ramclustr = 259.2908 (+/− 11.8 ppm)
496.4221
[M+H−C2H6]+
230.2484
[M+Na]+
282.28
805 : M.findmain = 495.4148 (+/− 20 ppm)
496.4221
[M+H]+
230.2484
282.28
0
20
40
60
80
100
250
300
350
400
450
500

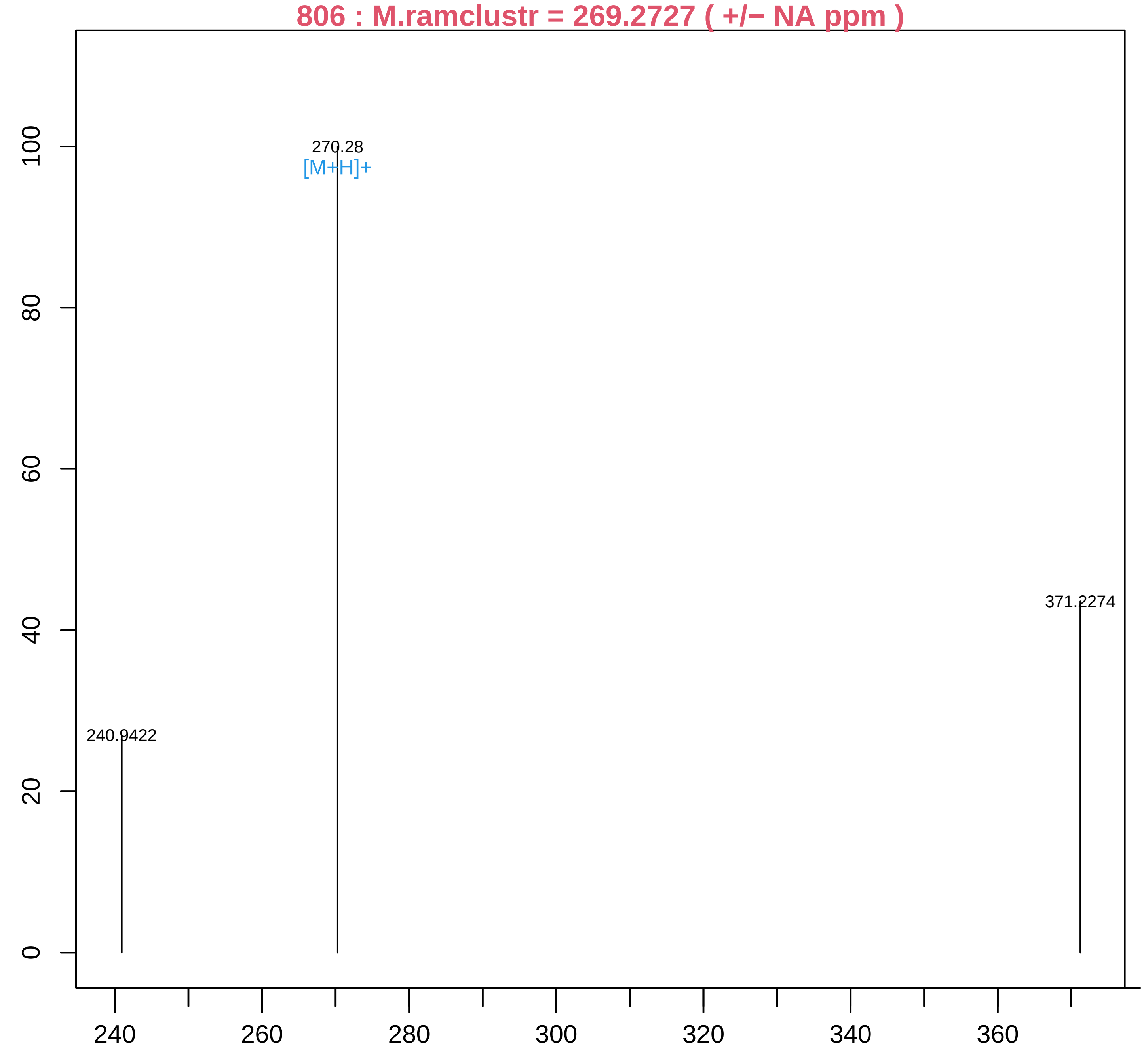

806 : M.ramclustr = 269.2727 (+/− NA ppm)
270.28
[M+H]+
371.2274
240.9422
0
20
40
60
80
100
240
260
280
300
320
340
360

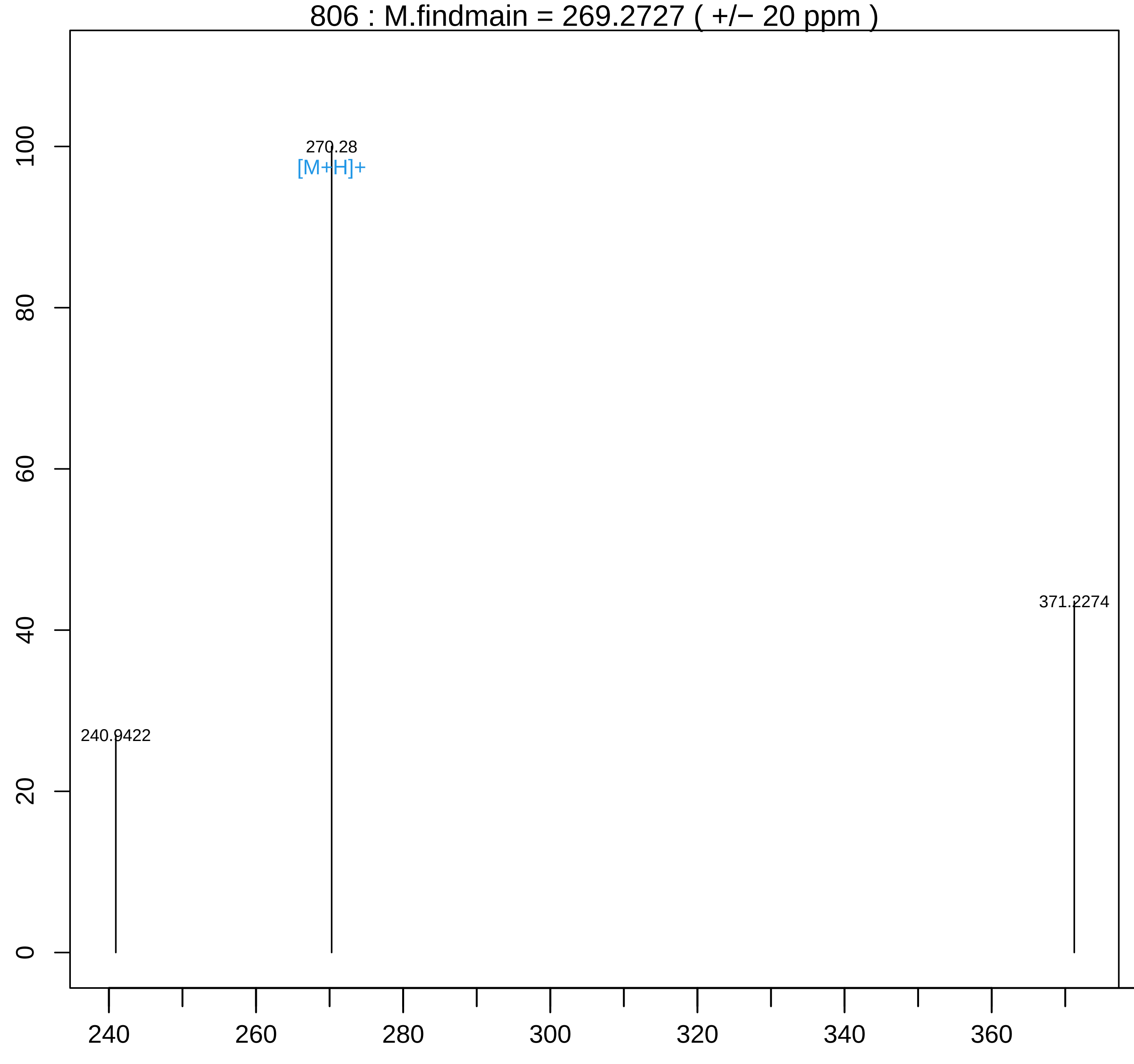

806 : M.findmain = 269.2727 (+/− 20 ppm)
270.28
[M+H]+
371.2274
240.9422
0
20
40
60
80
100
240
260
280
300
320
340
360

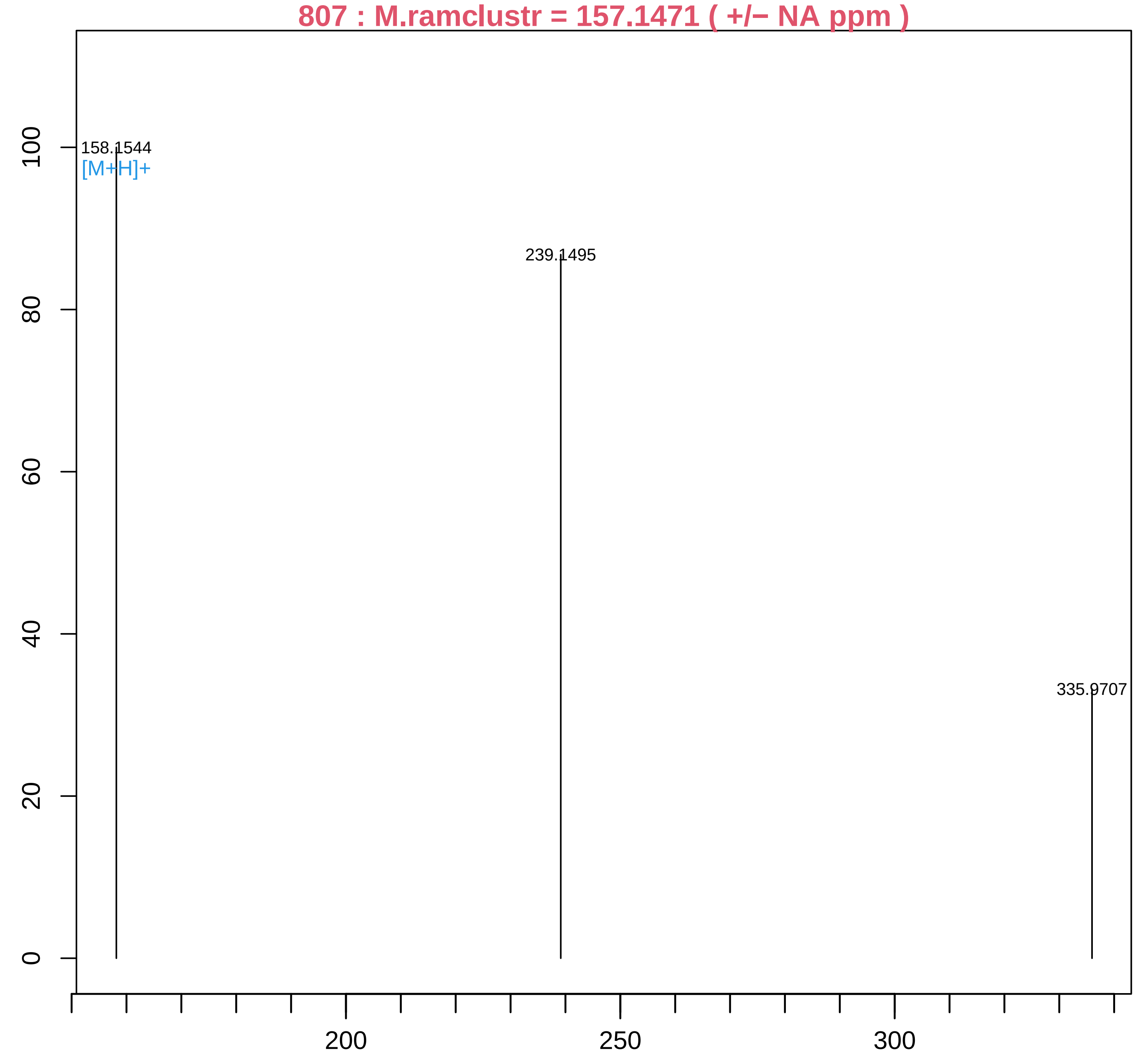
807 : M.ramclustr = 157.1471 (+/− NA ppm)
158.1544
[M+H]+
239.1495
335.9707
0
20
40
60
80
100
200
250
300

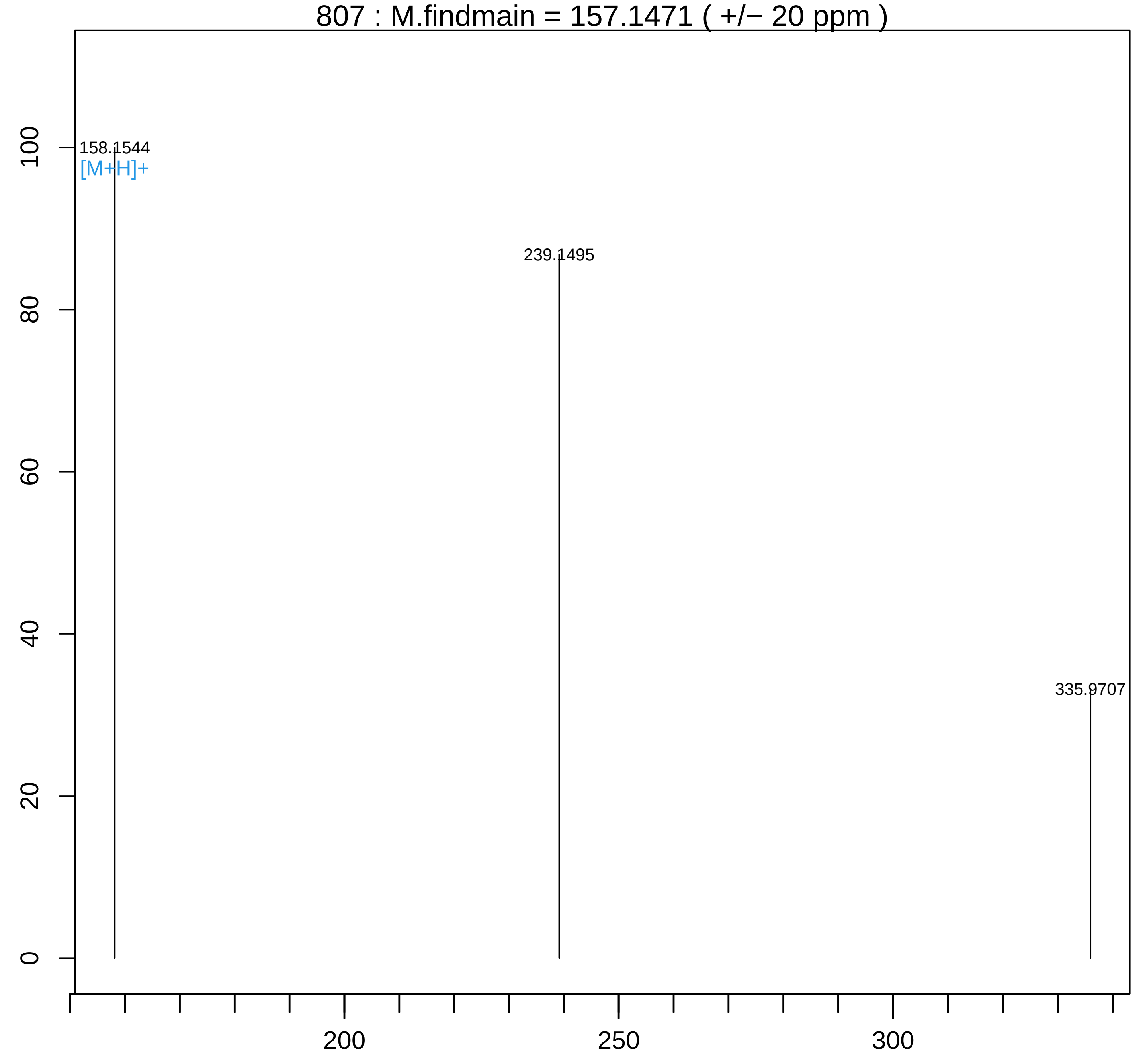
807 : M.findmain = 157.1471 (+/− 20 ppm)
158.1544
[M+H]+
239.1495
335.9707
0
20
40
60
80
100
200
250
300

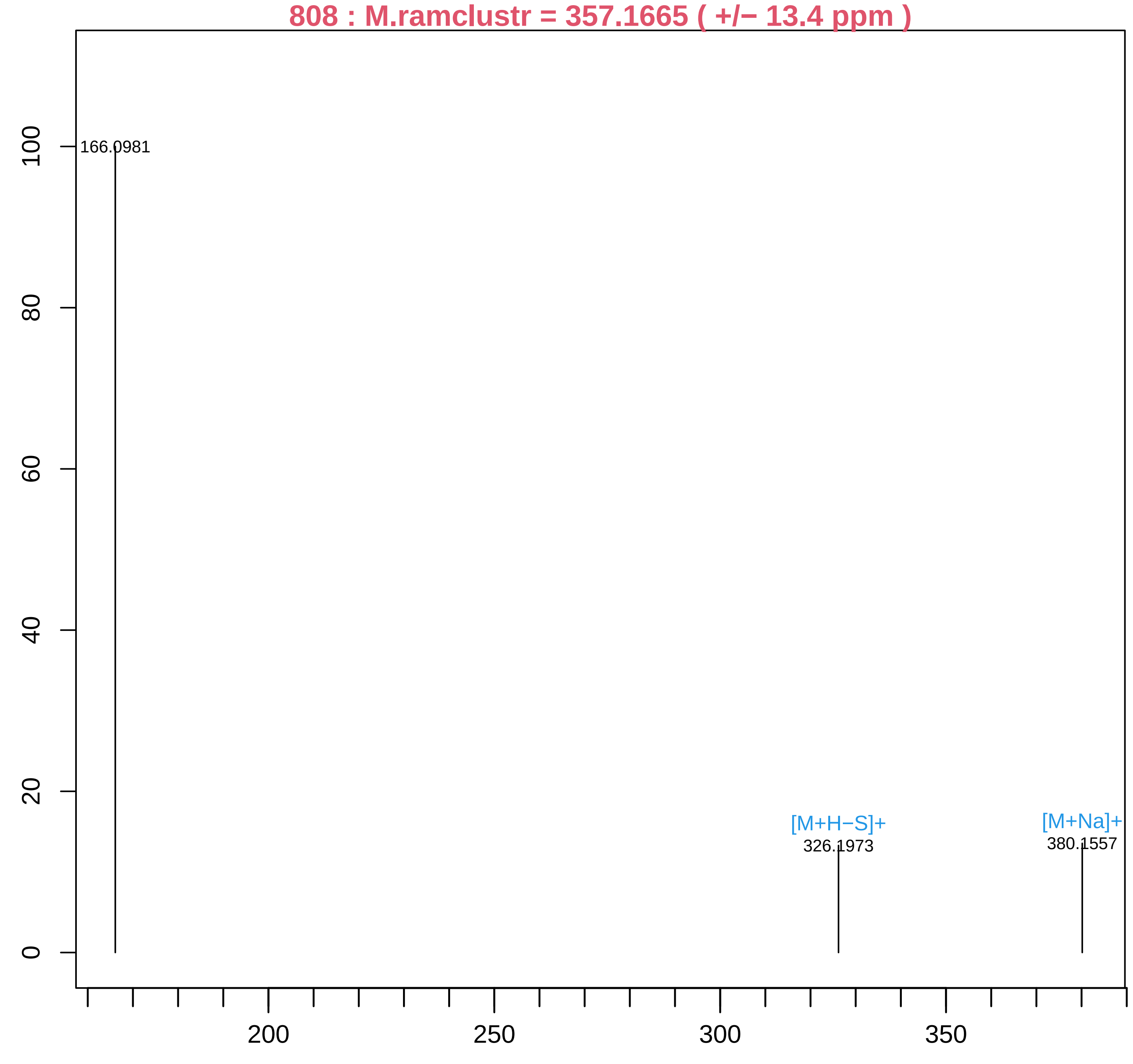
808 : M.ramclustr = 357.1665 (+/− 13.4 ppm)
0
20
40
60
80
100
166.0981
[M+H−S]+
326.1973
[M+Na]+
380.1557
200
250
300
350

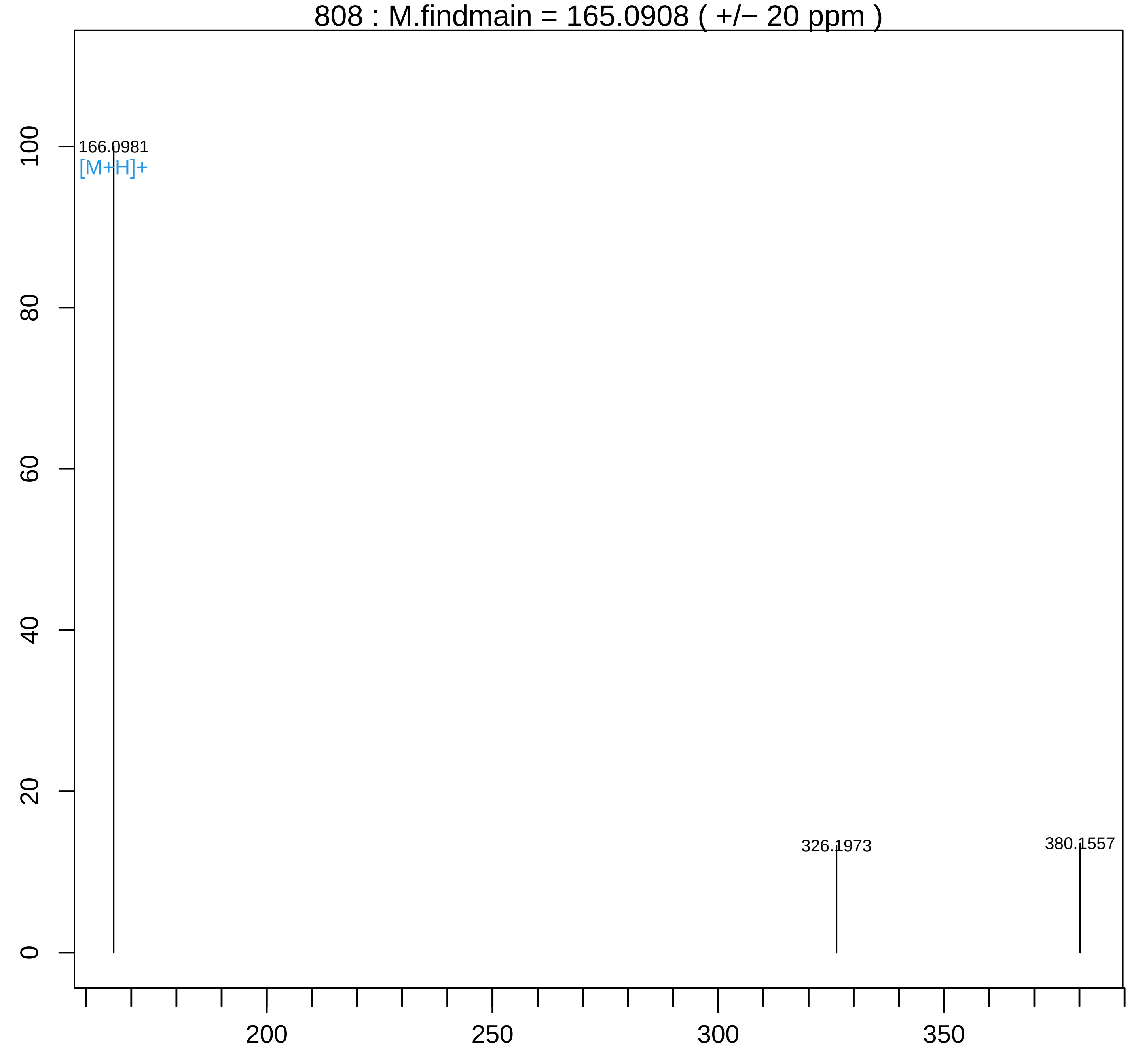
808 : M.findmain = 165.0908 (+/− 20 ppm)
0
20
40
60
80
100
166.0981
[M+H]+
326.1973
380.1557
200
250
300
350

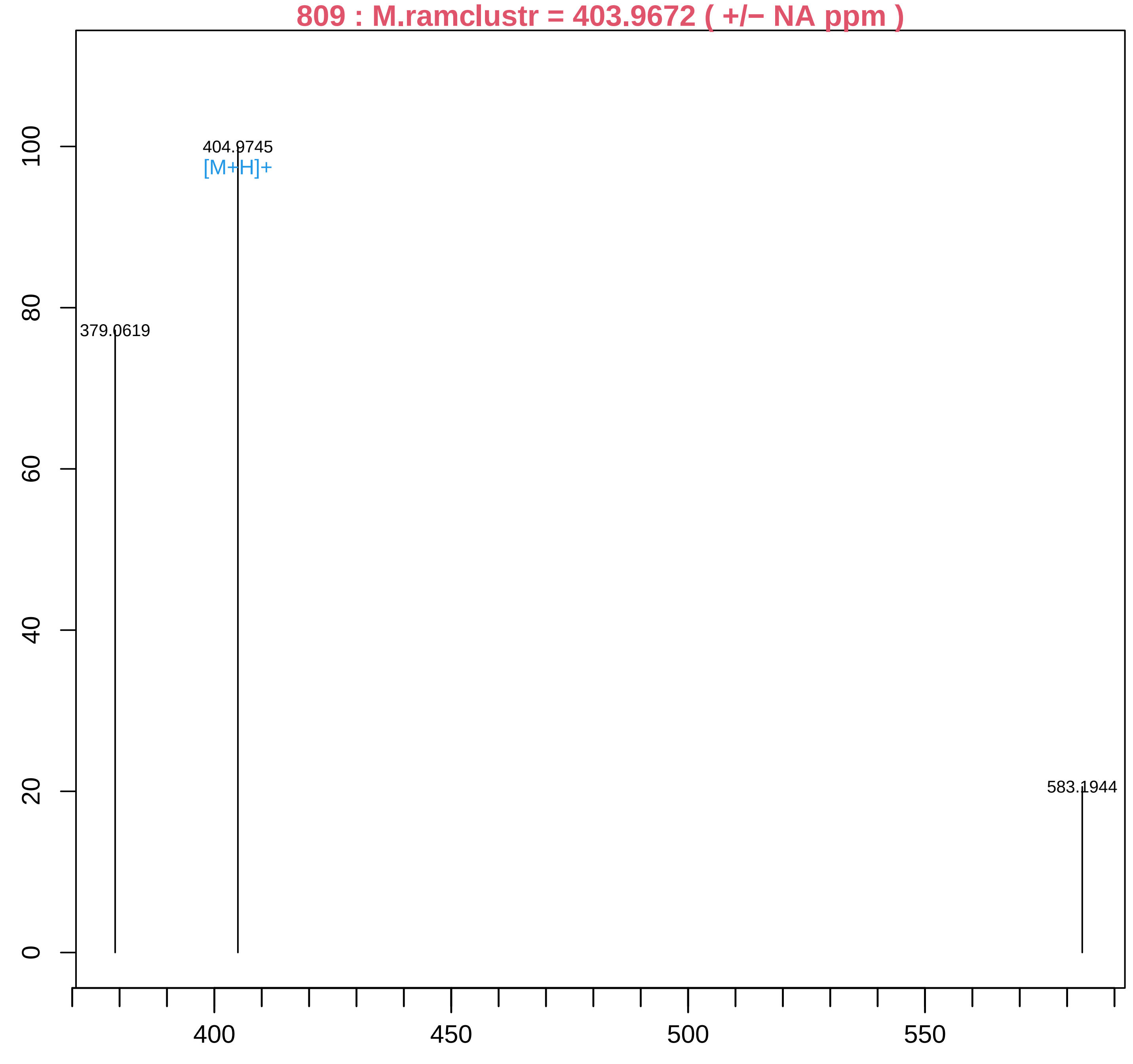

809 : M.ramclustr = 403.9672 (+/− NA ppm)
404.9745
[M+H]+
379.0619
583.1944
0
20
40
60
80
100
400
450
500
550

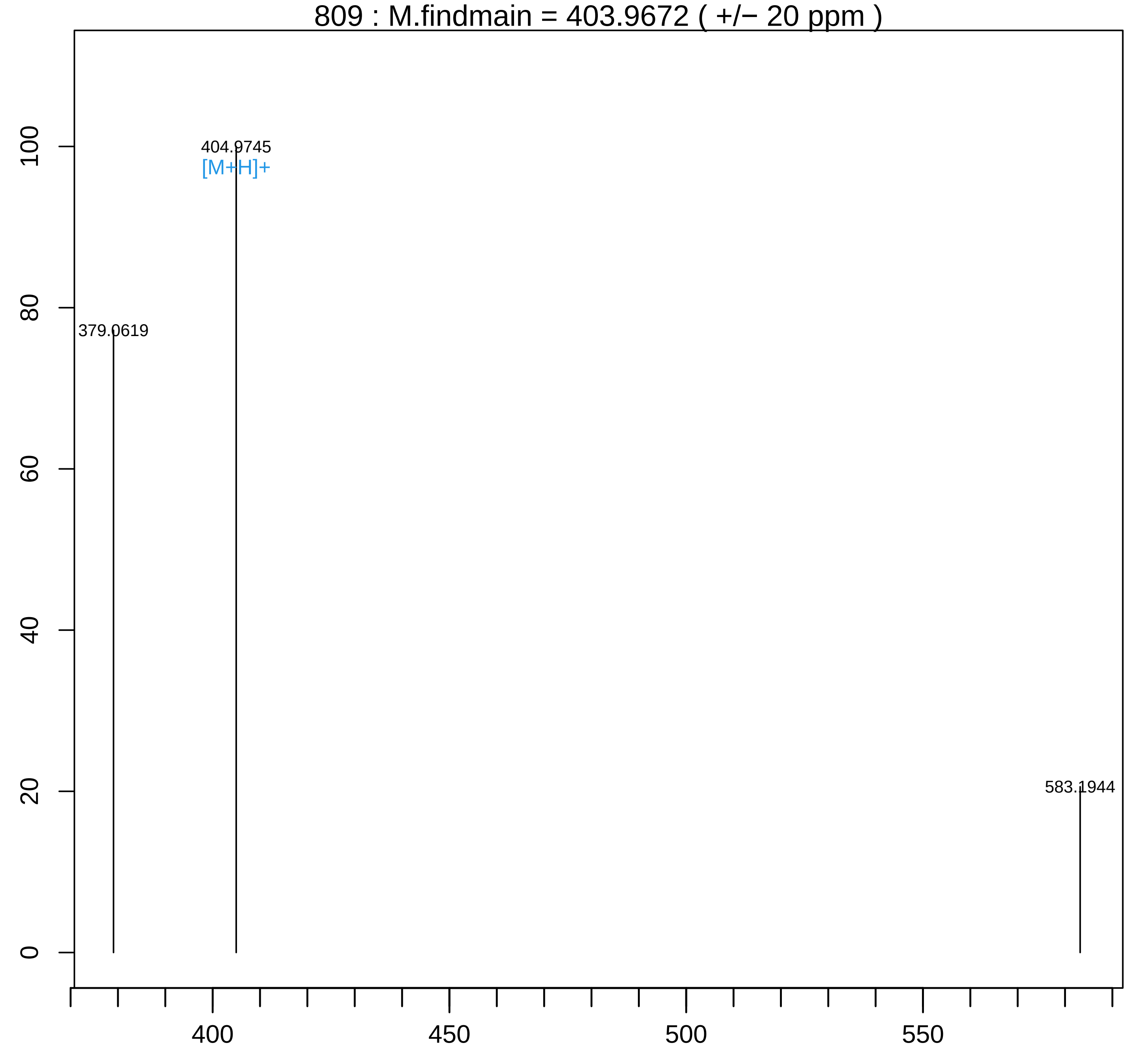

809 : M.findmain = 403.9672 (+/− 20 ppm)
404.9745
[M+H]+
379.0619
583.1944
0
20
40
60
80
100
400
450
500
550

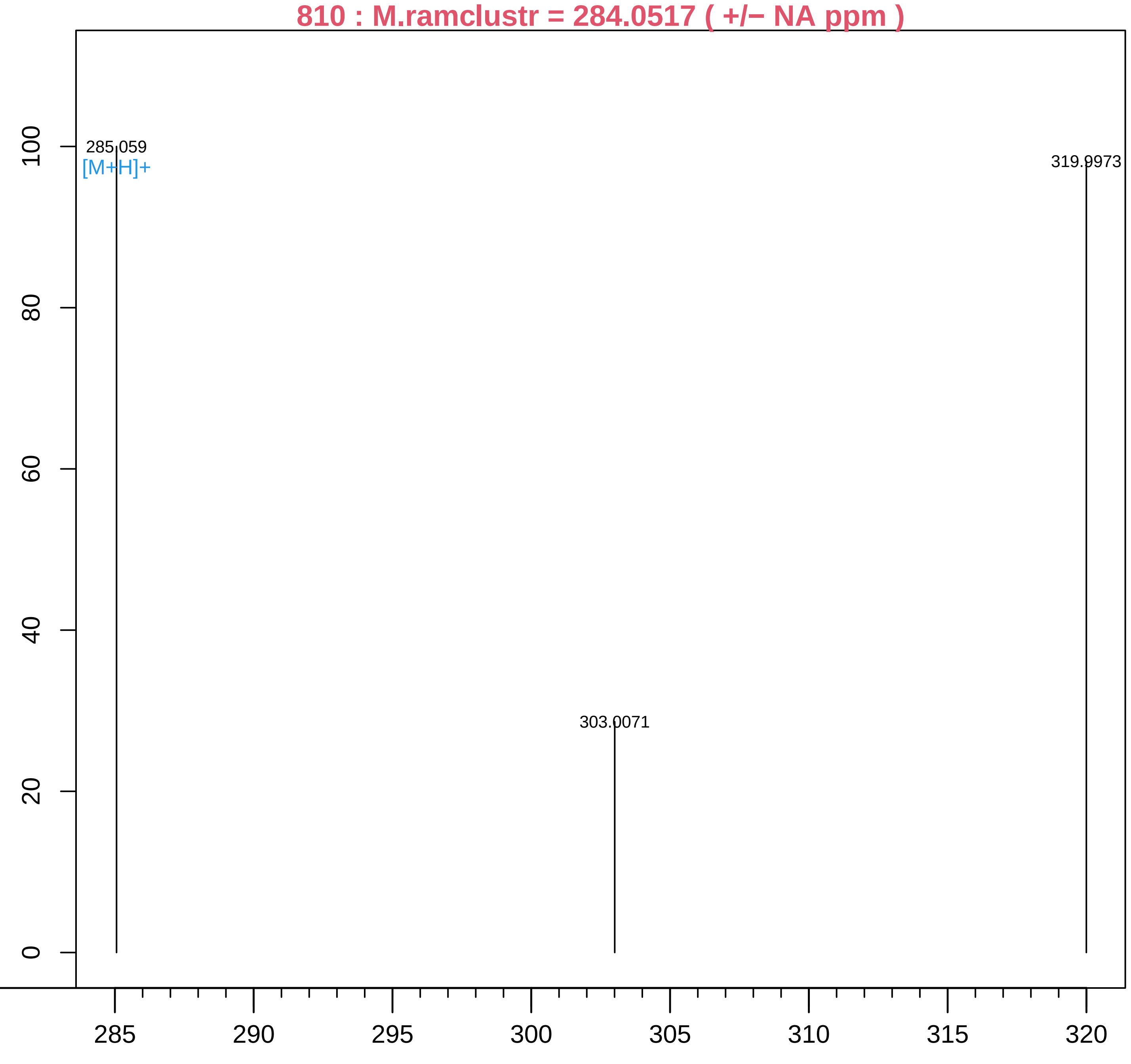
810 : M.ramclustr = 284.0517 (+/− NA ppm)
285.059
[M+H]+
303.0071
319.9973
0
20
40
60
80
100
285
290
295
300
305
310
315
320
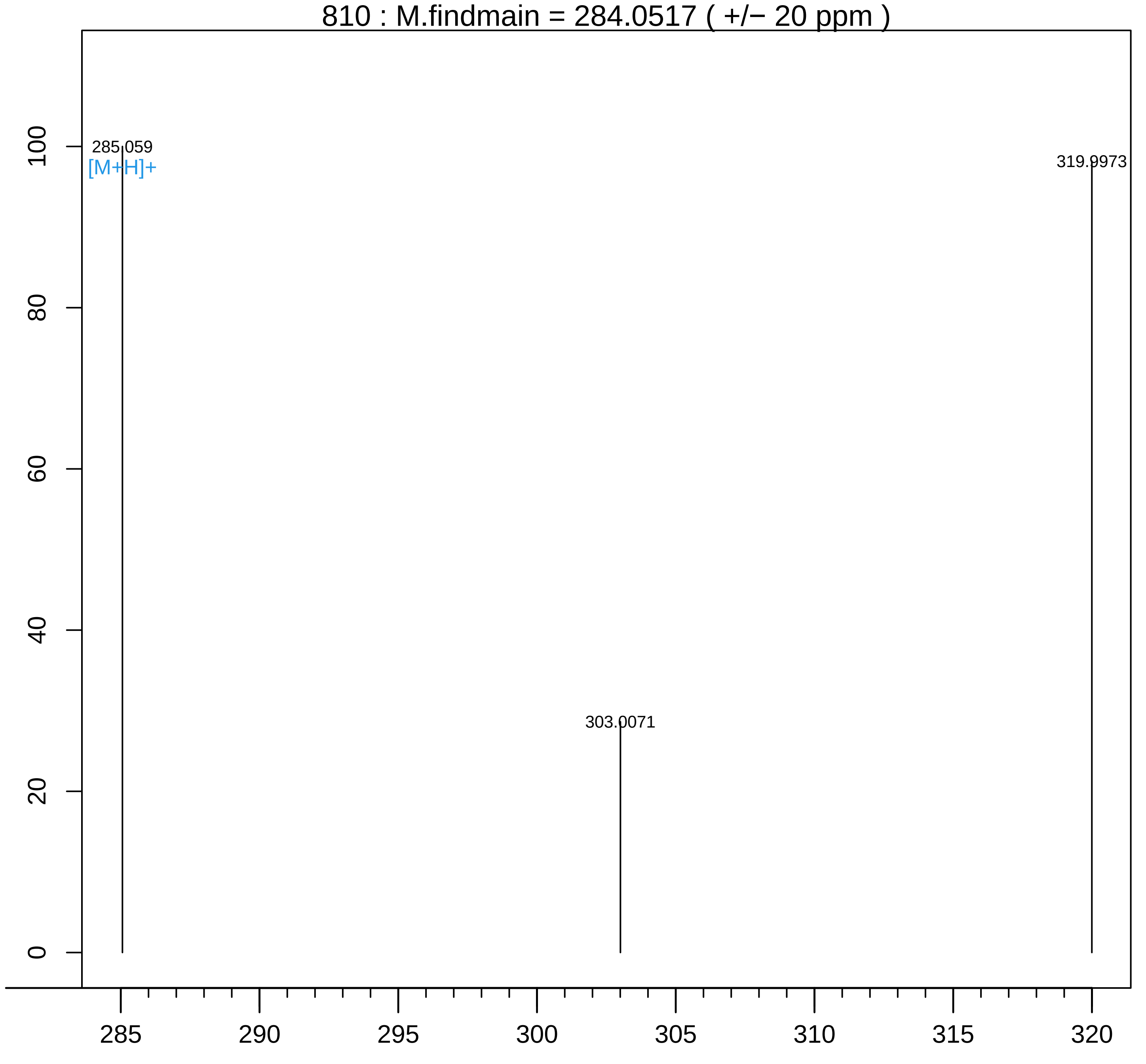
810 : M.findmain = 284.0517 (+/− 20 ppm)
285.059
[M+H]+
303.0071
319.9973
0
20
40
60
80
100
285
290
295
300
305
310
315
320

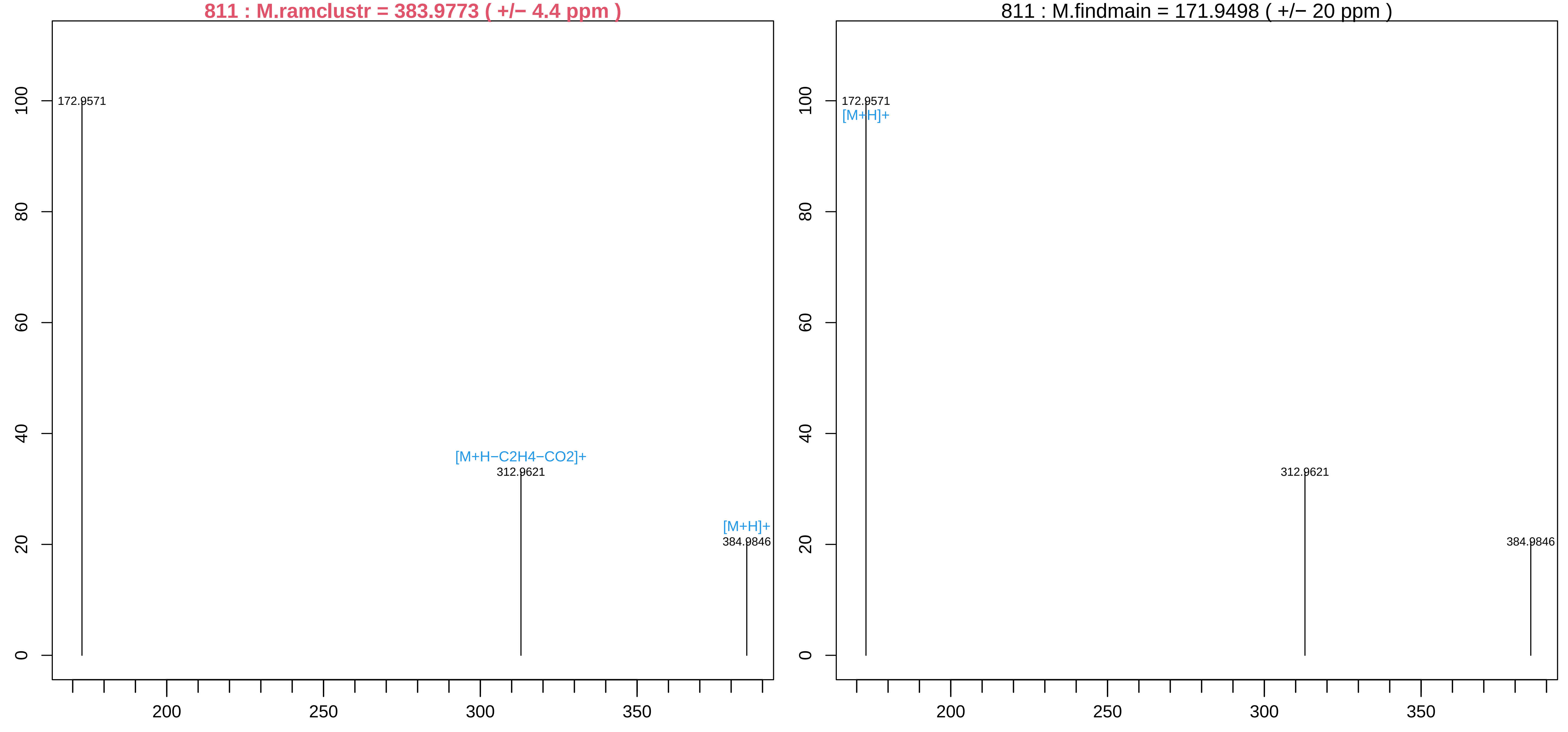

811 : M.ramclustr = 383.9773 (+/− 4.4 ppm)
172.9571
[M+H−C2H4−CO2]+
312.9621
[M+H]+
384.9846
811 : M.findmain = 171.9498 (+/− 20 ppm)
172.9571
[M+H]+
312.9621
384.9846
0
20
40
60
80
100
200
250
300
350

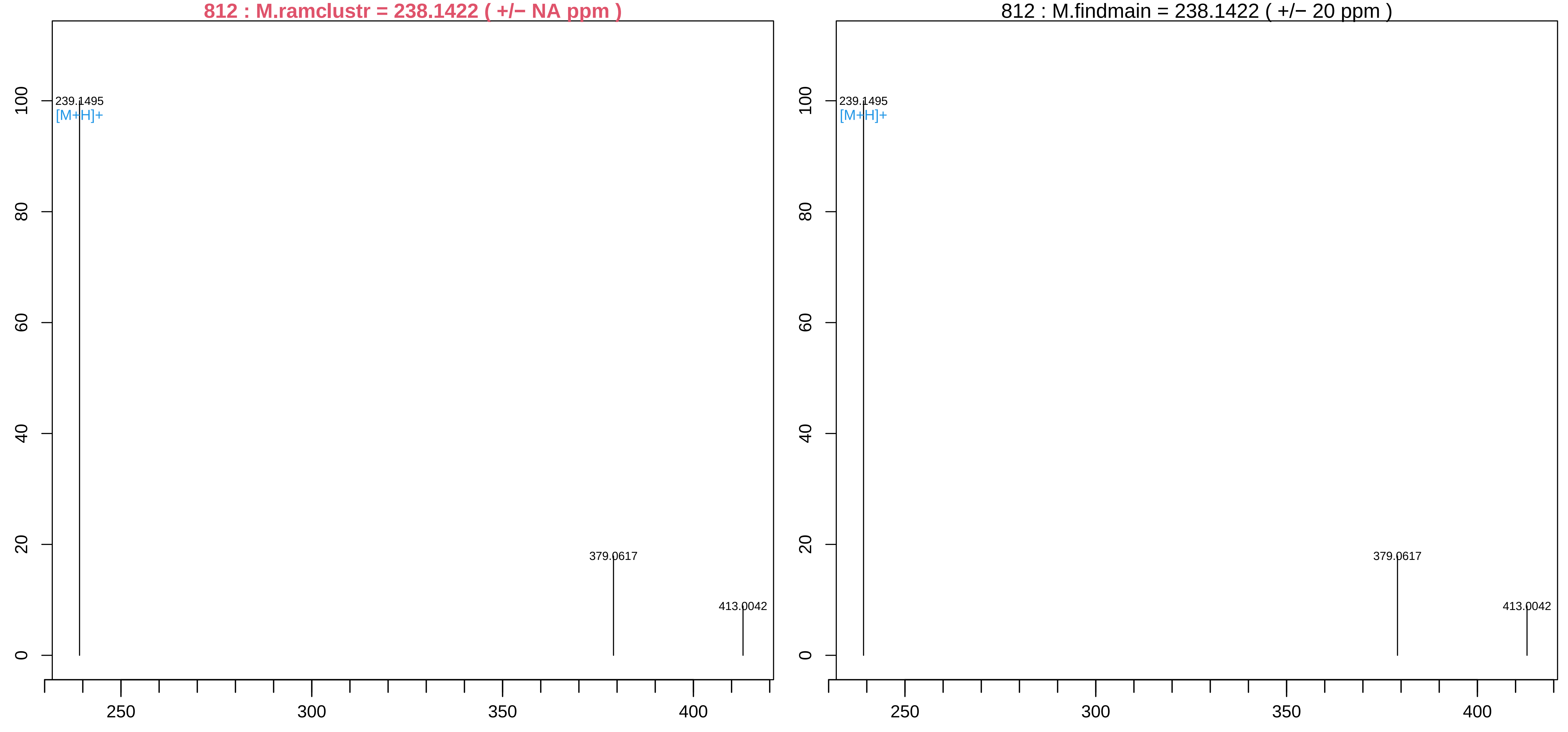

812 : M.ramclustr = 238.1422 (+/− NA ppm)
239.1495
[M+H]+
379.0617
413.0042
0
20
40
60
80
100
250
300
350
400
812 : M.findmain = 238.1422 (+/− 20 ppm)
239.1495
[M+H]+
379.0617
413.0042
0
20
40
60
80
100
250
300
350
400

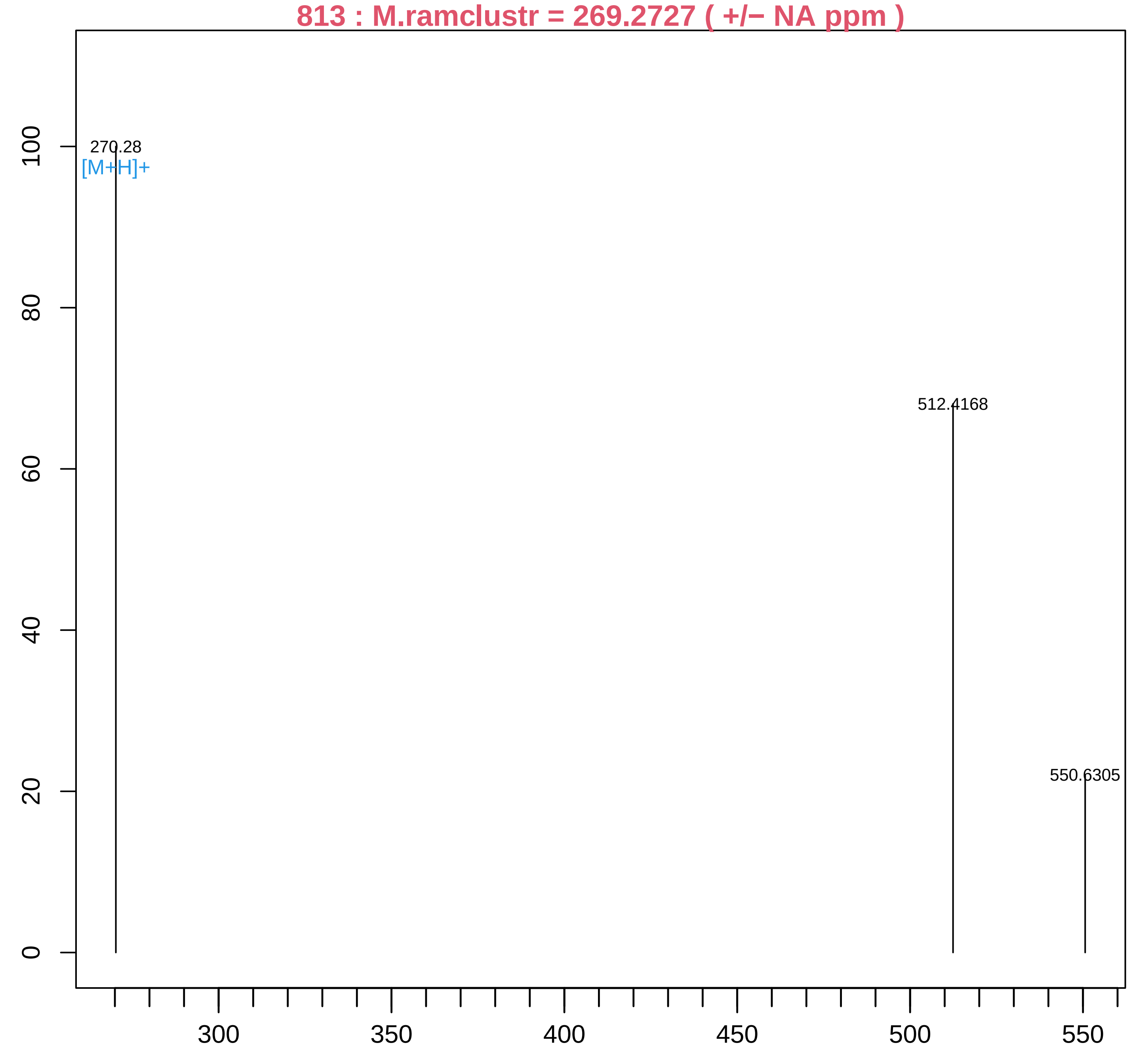
813 : M.ramclustr = 269.2727 (+/− NA ppm)
270.28
[M+H]+
512.4168
550.6305
0
20
40
60
80
100
300
350
400
450
500
550

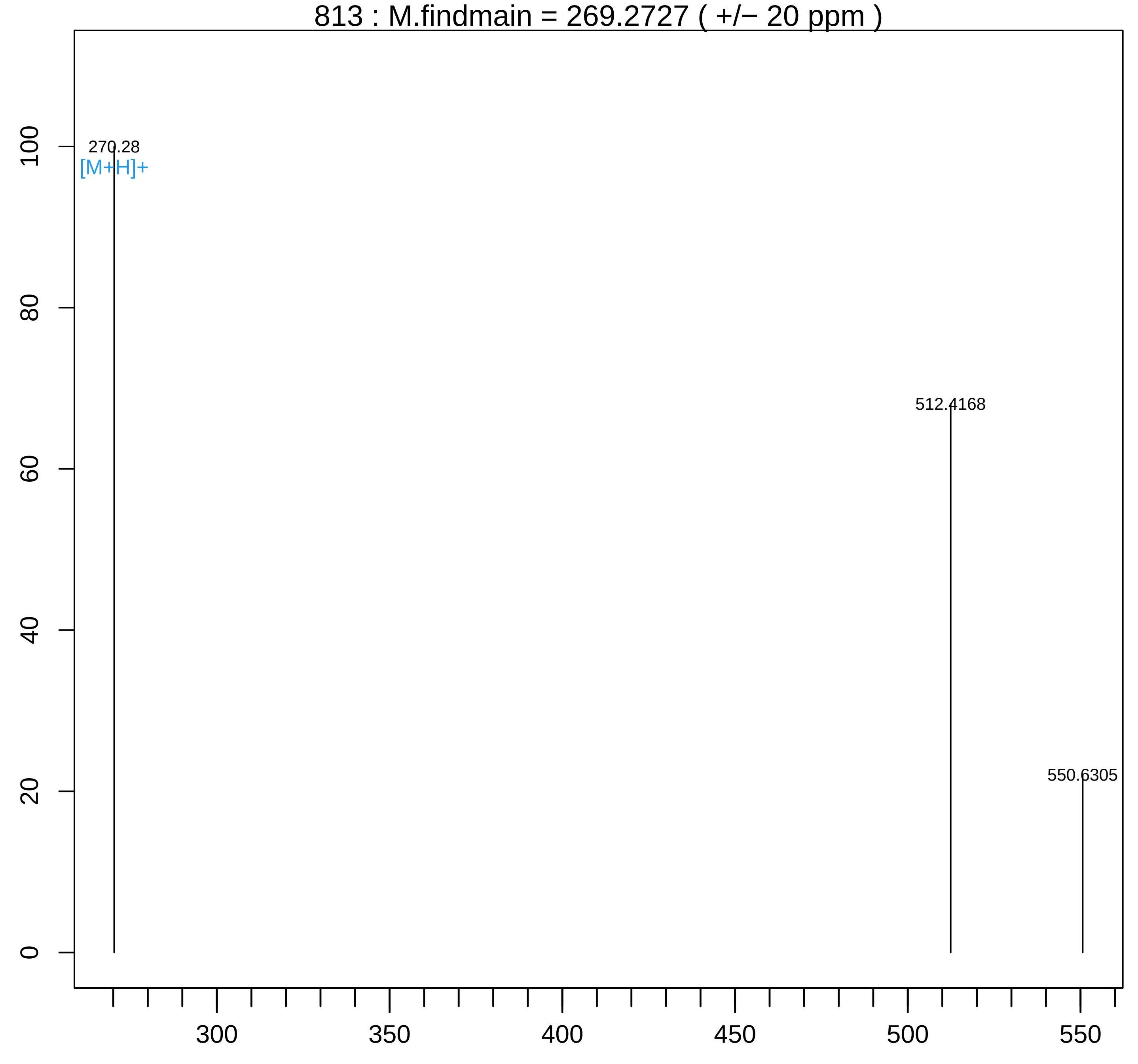
813 : M.findmain = 269.2727 (+/− 20 ppm)
270.28
[M+H]+
512.4168
550.6305
0
20
40
60
80
100
300
350
400
450
500
550

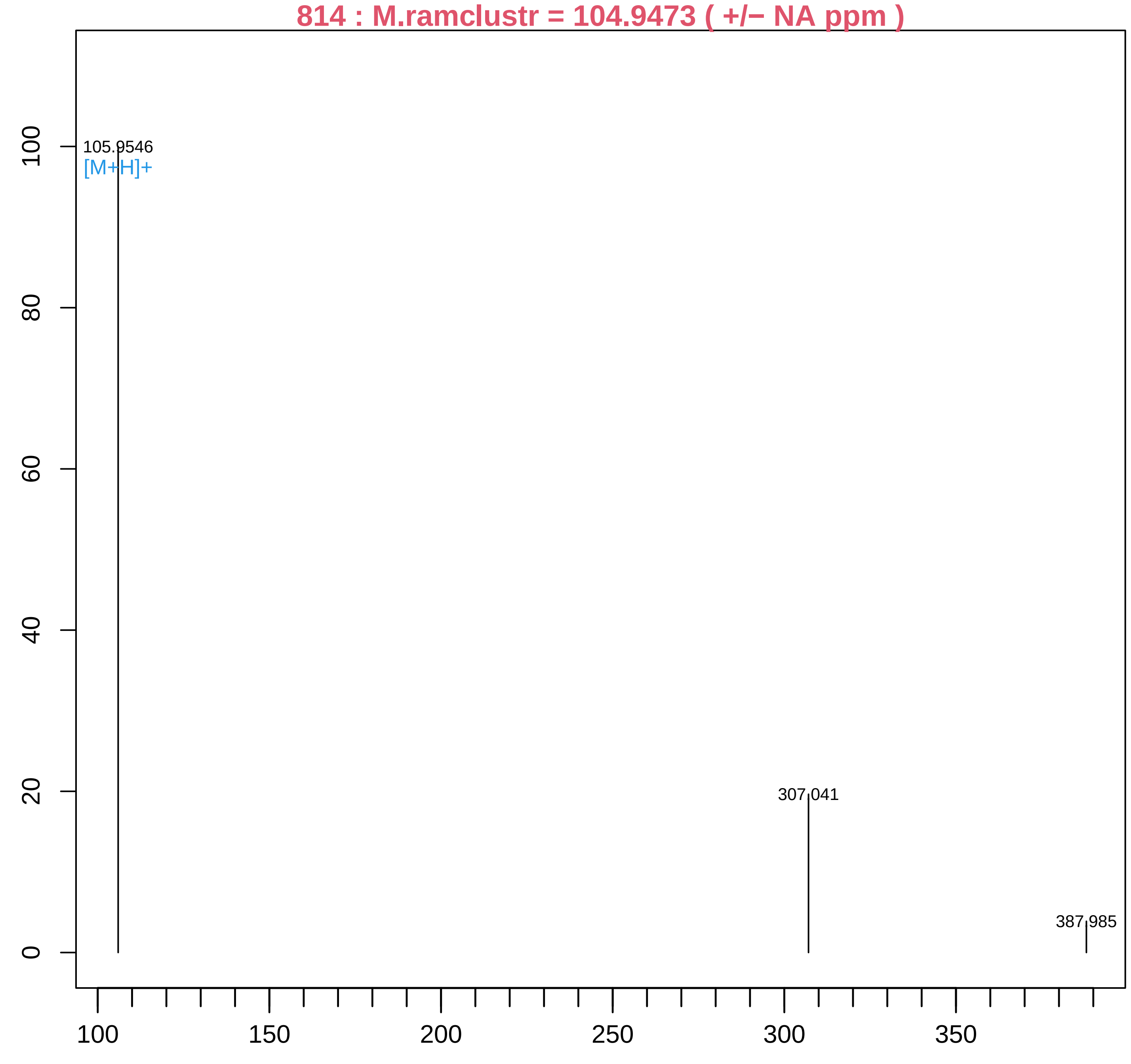
814 : M.ramclustr = 104.9473 (+/− NA ppm)
105.9546
[M+H]+
307.041
387.985

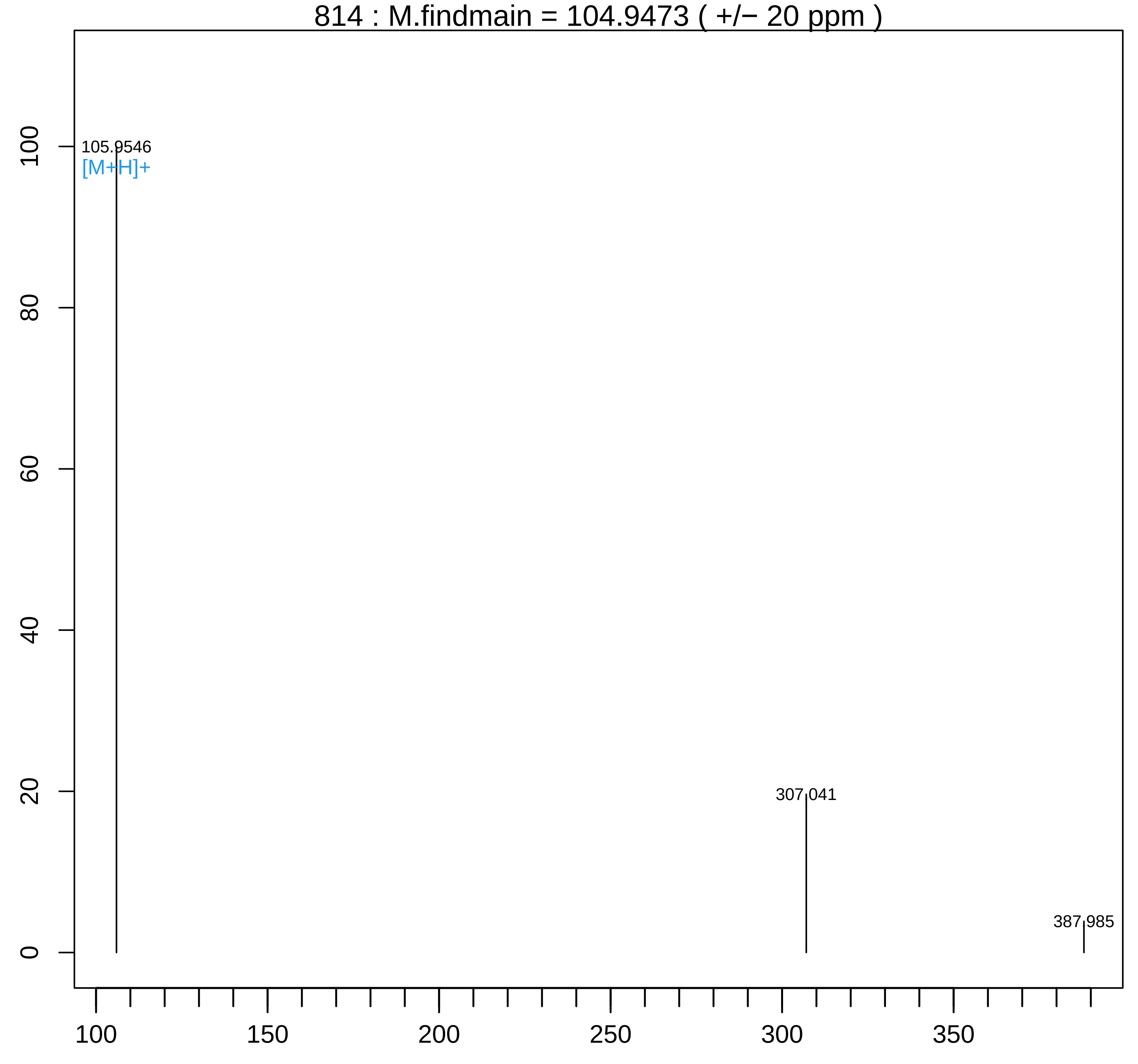
814 : M.findmain = 104.9473 (+/− 20 ppm)
105.9546
[M+H]+
307.041
387.985

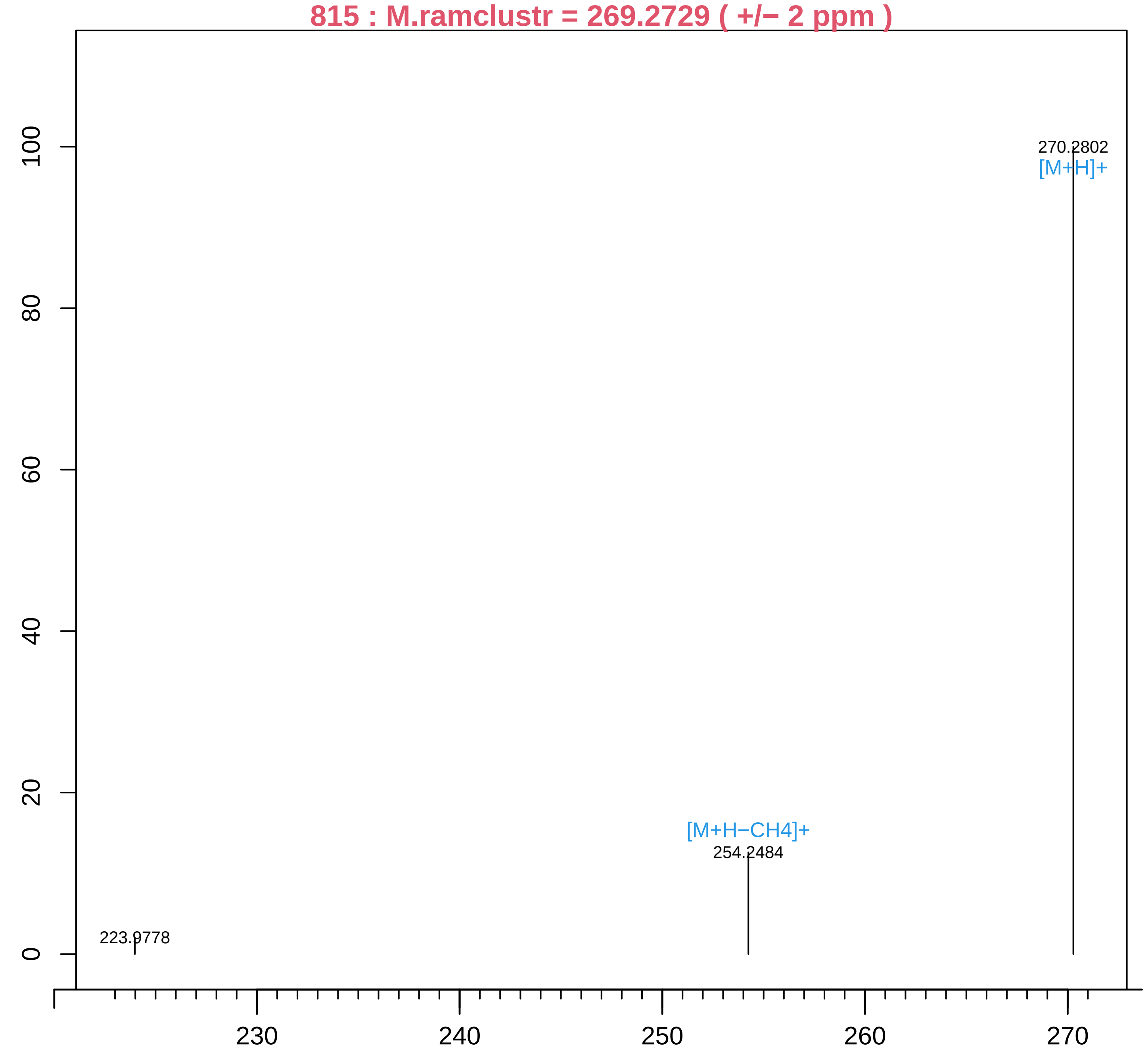

815 : M.ramclustr = 269.2729 (+/− 2 ppm)
270.2802
[M+H]+
[M+H−CH4]+
254.2484
223.9778
0
20
40
60
80
100
230
240
250
260
270

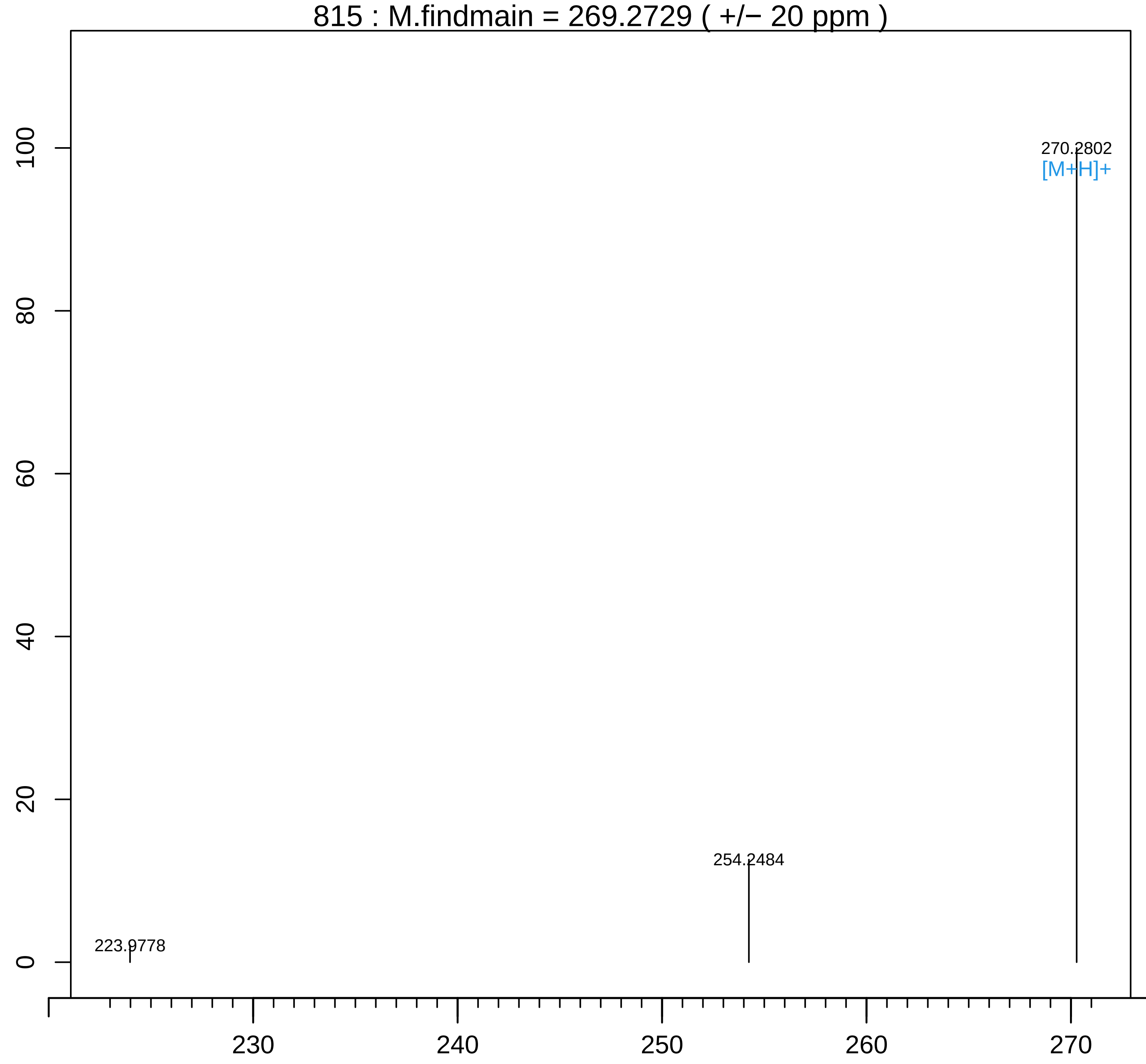

815 : M.findmain = 269.2729 (+/− 20 ppm)
270.2802
[M+H]+
254.2484
223.9778
0
20
40
60
80
100
230
240
250
260
270

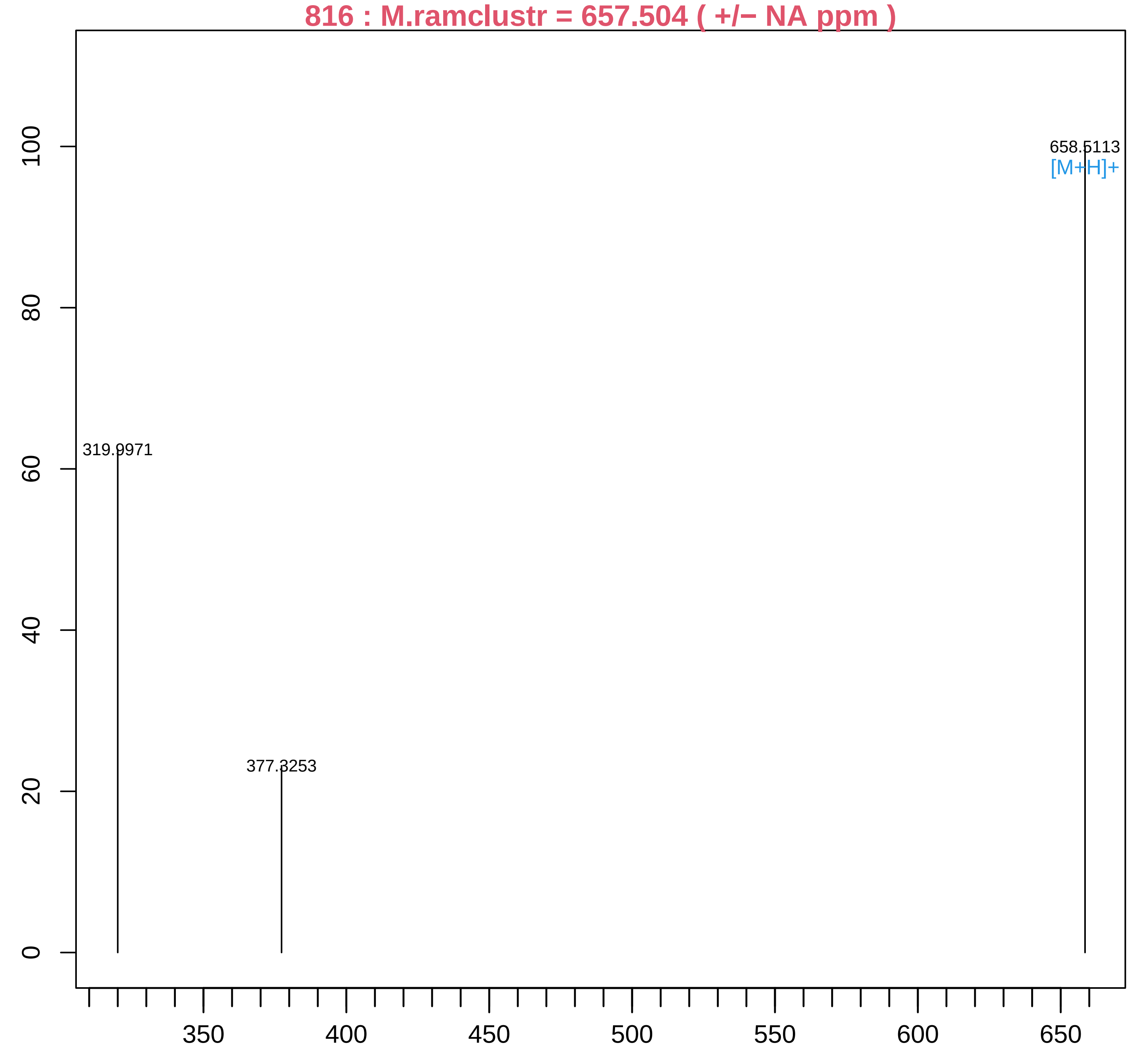
816 : M.ramclustr = 657.504 (+/− NA ppm)
658.5113
[M+H]+
319.9971
377.3253
0
20
40
60
80
100
350
400
450
500
550
600
650

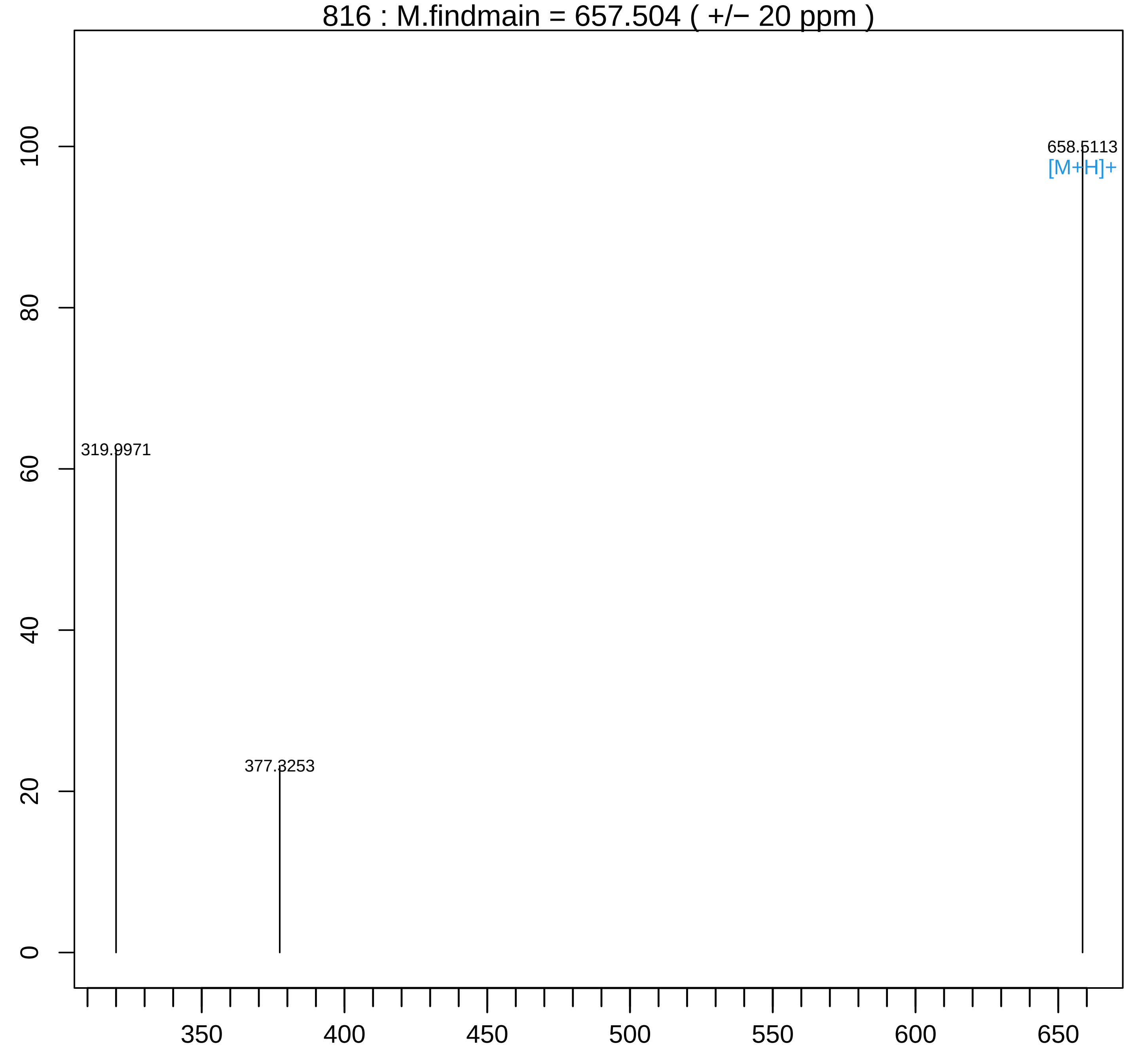
816 : M.findmain = 657.504 (+/− 20 ppm)
658.5113
[M+H]+
319.9971
377.3253
0
20
40
60
80
100
350
400
450
500
550
600
650

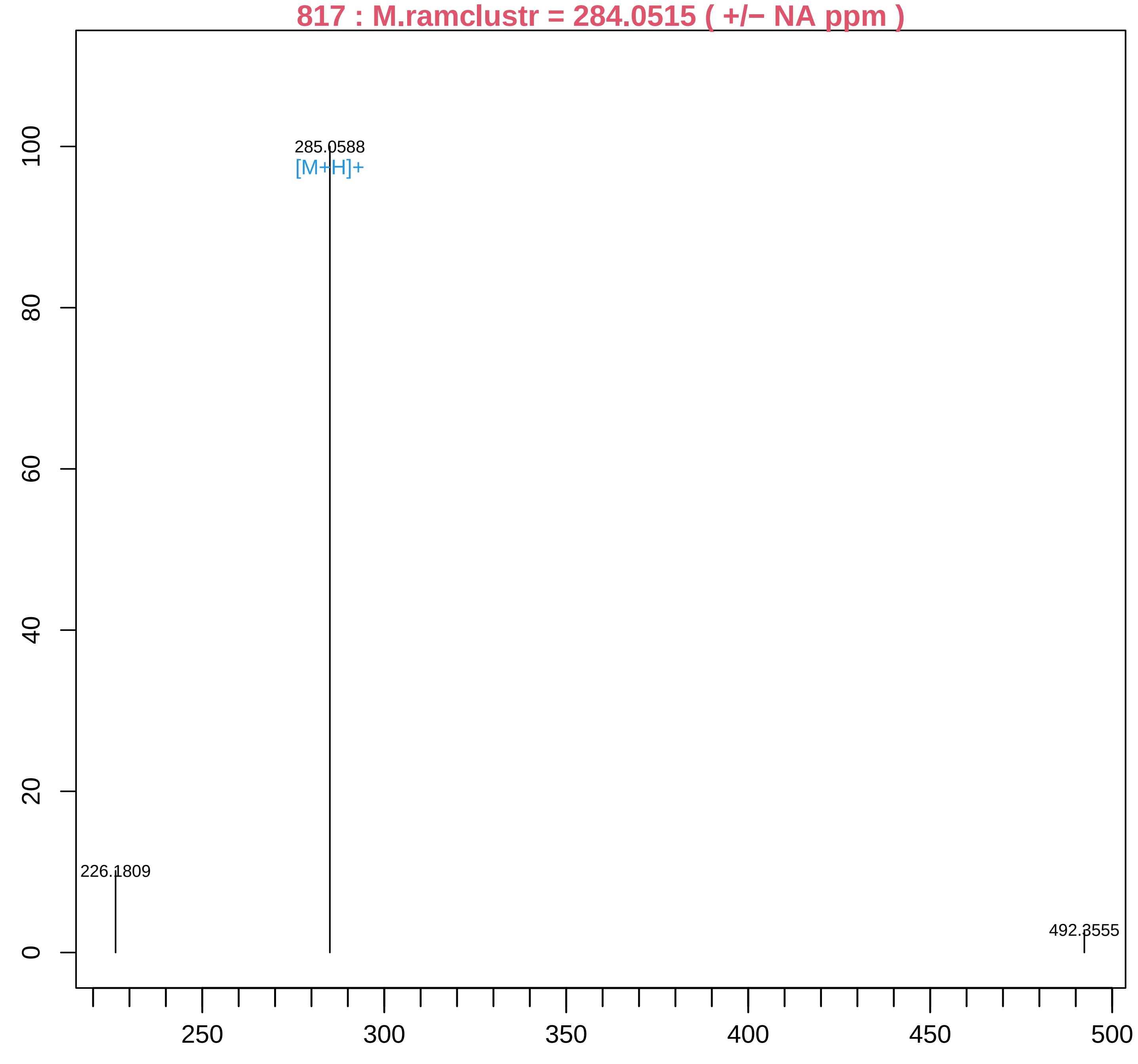
817 : M.ramclustr = 284.0515 (+/− NA ppm)
285.0588
[M+H]+
226.1809
492.3555

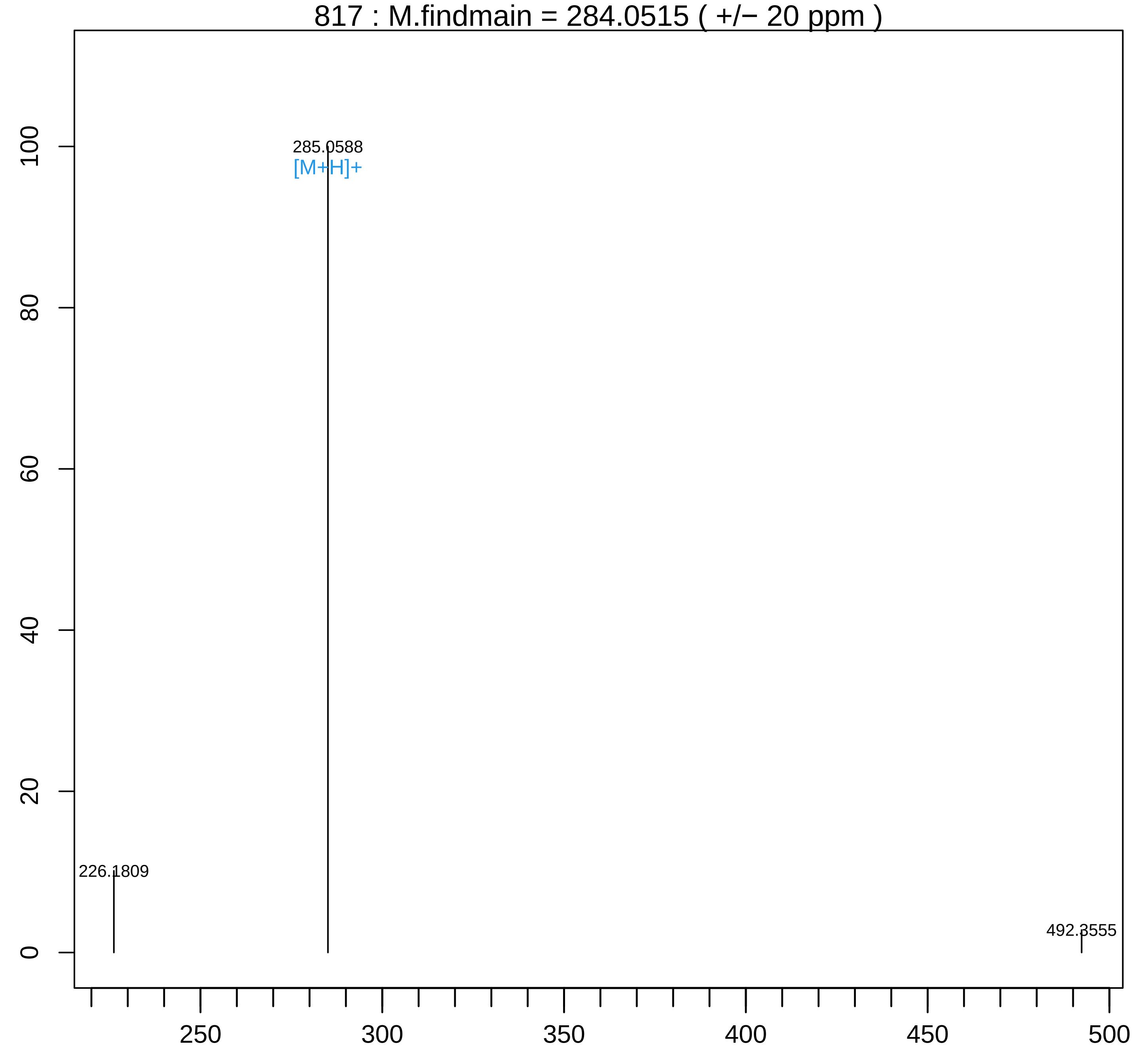
817 : M.findmain = 284.0515 (+/− 20 ppm)
285.0588
[M+H]+
226.1809
492.3555

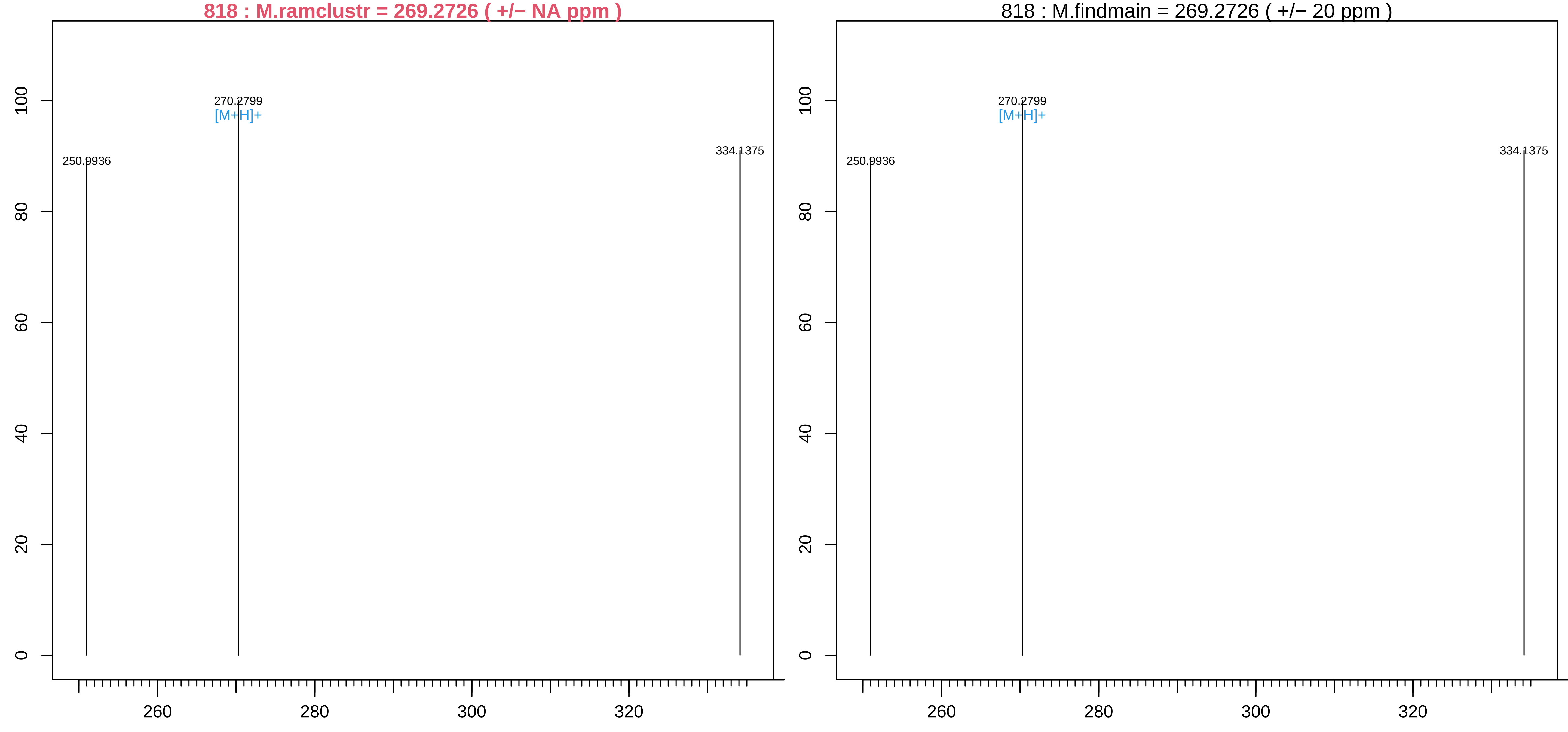

818 : M.ramclustr = 269.2726 (+/− NA ppm)
270.2799
[M+H]+
250.9936
334.1375
818 : M.findmain = 269.2726 (+/− 20 ppm)
270.2799
[M+H]+
250.9936
334.1375

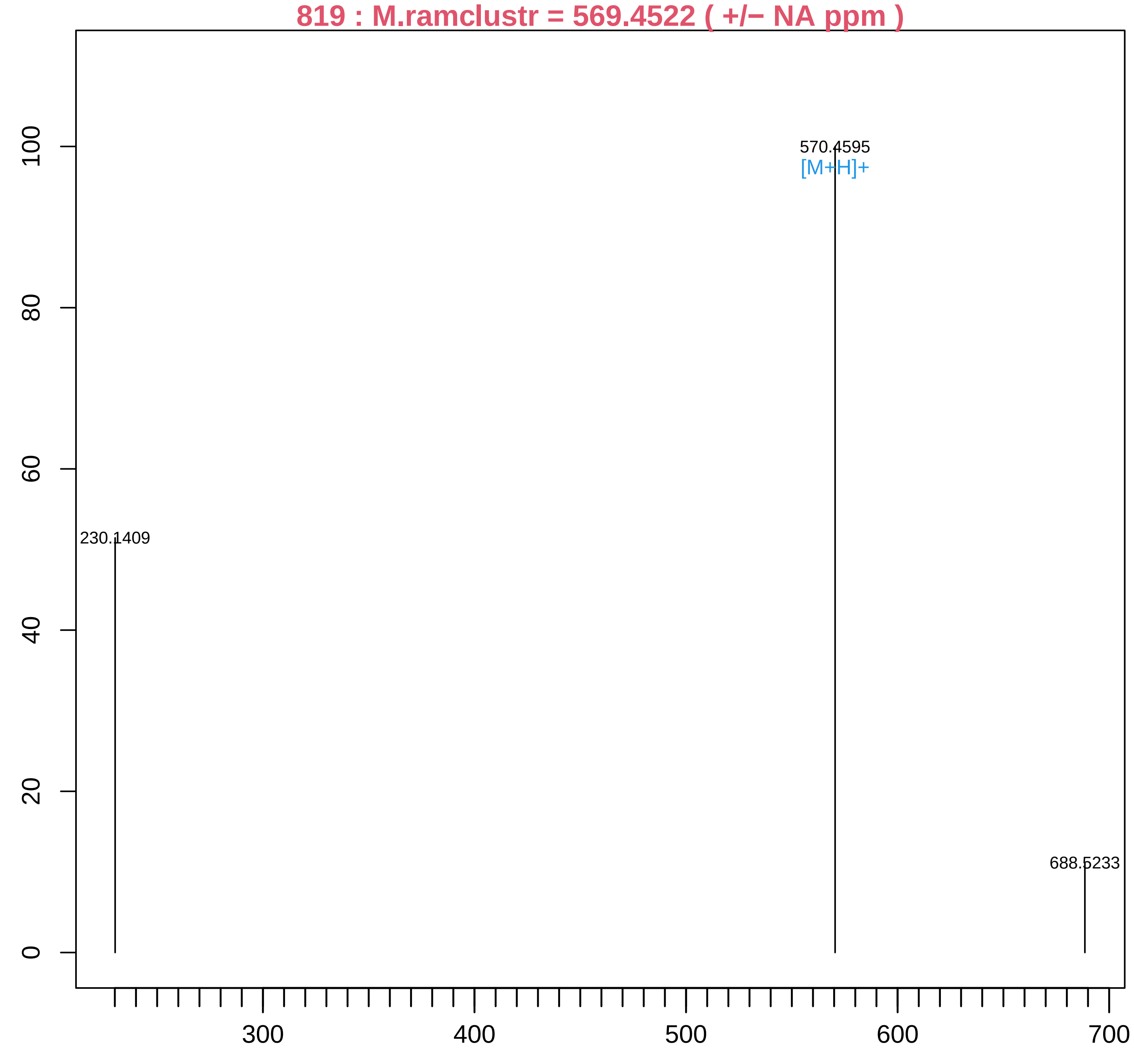

819 : M.ramclustr = 569.4522 (+/− NA ppm)
570.4595
[M+H]+
230.1409
688.5233
0
20
40
60
80
100
300
400
500
600
700

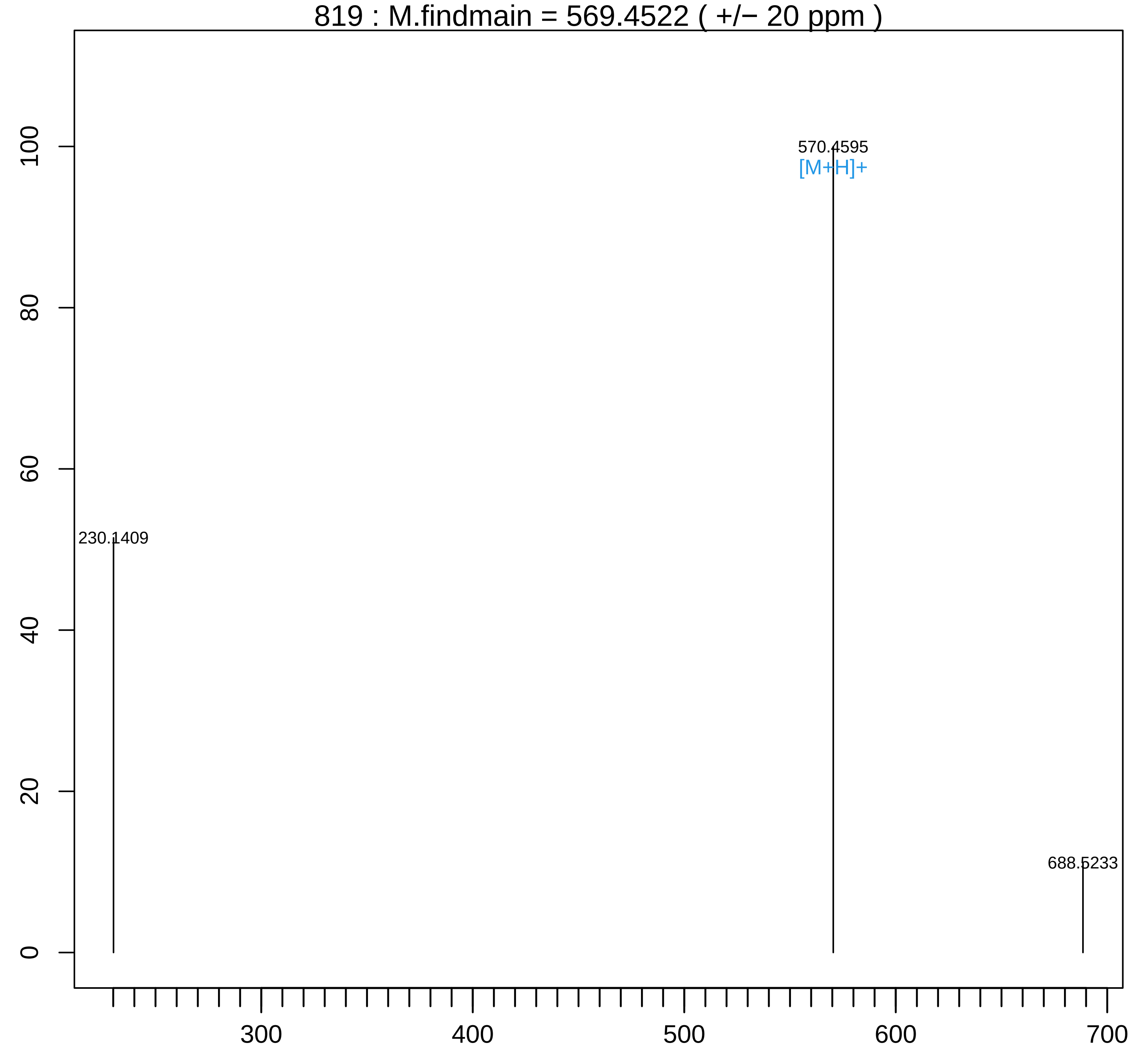

819 : M.findmain = 569.4522 (+/− 20 ppm)
570.4595
[M+H]+
230.1409
688.5233
0
20
40
60
80
100
300
400
500
600
700

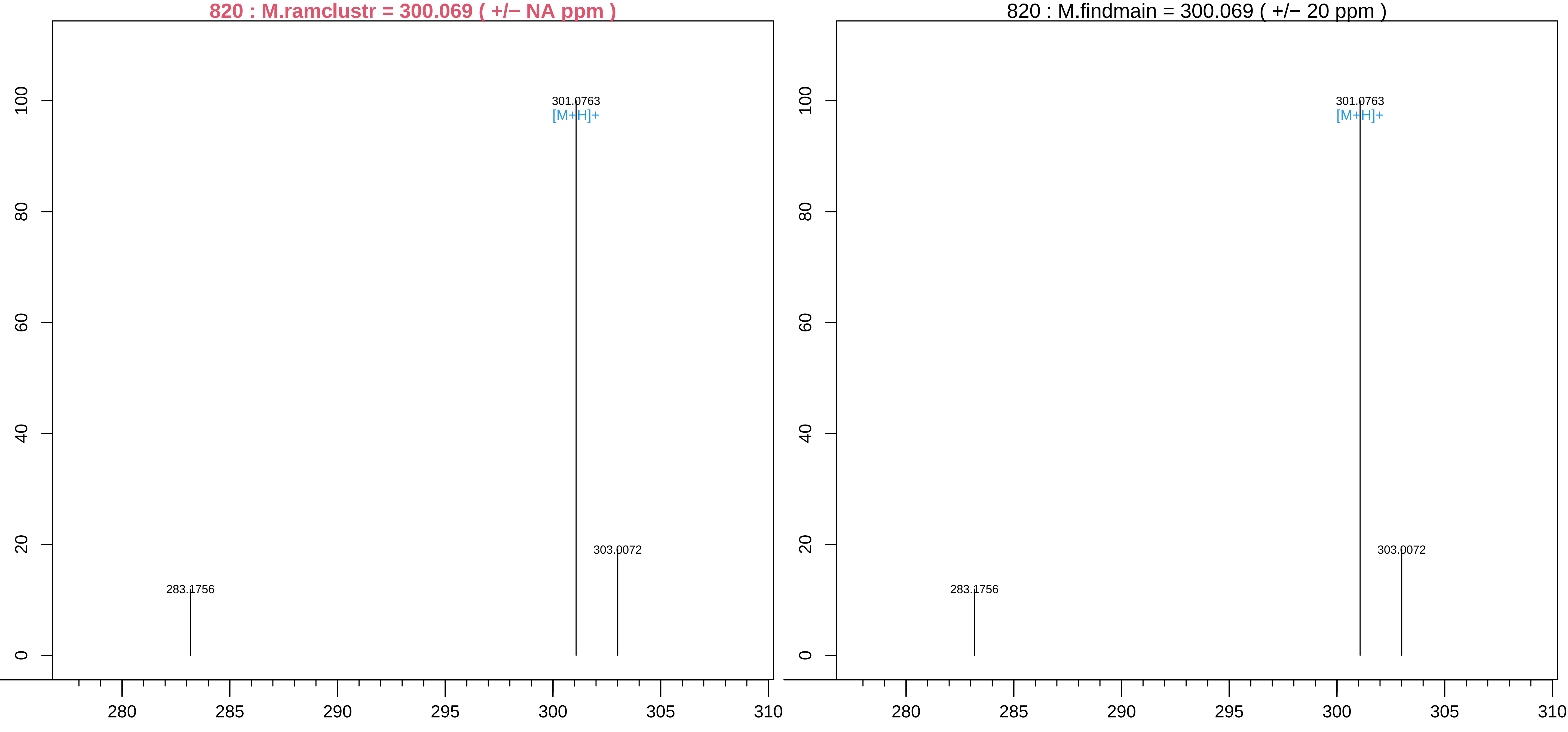

820 : M.ramclustr = 300.069 (+/− NA ppm)
301.0763
[M+H]+
303.0072
283.1756
820 : M.findmain = 300.069 (+/− 20 ppm)
301.0763
[M+H]+
303.0072
283.1756

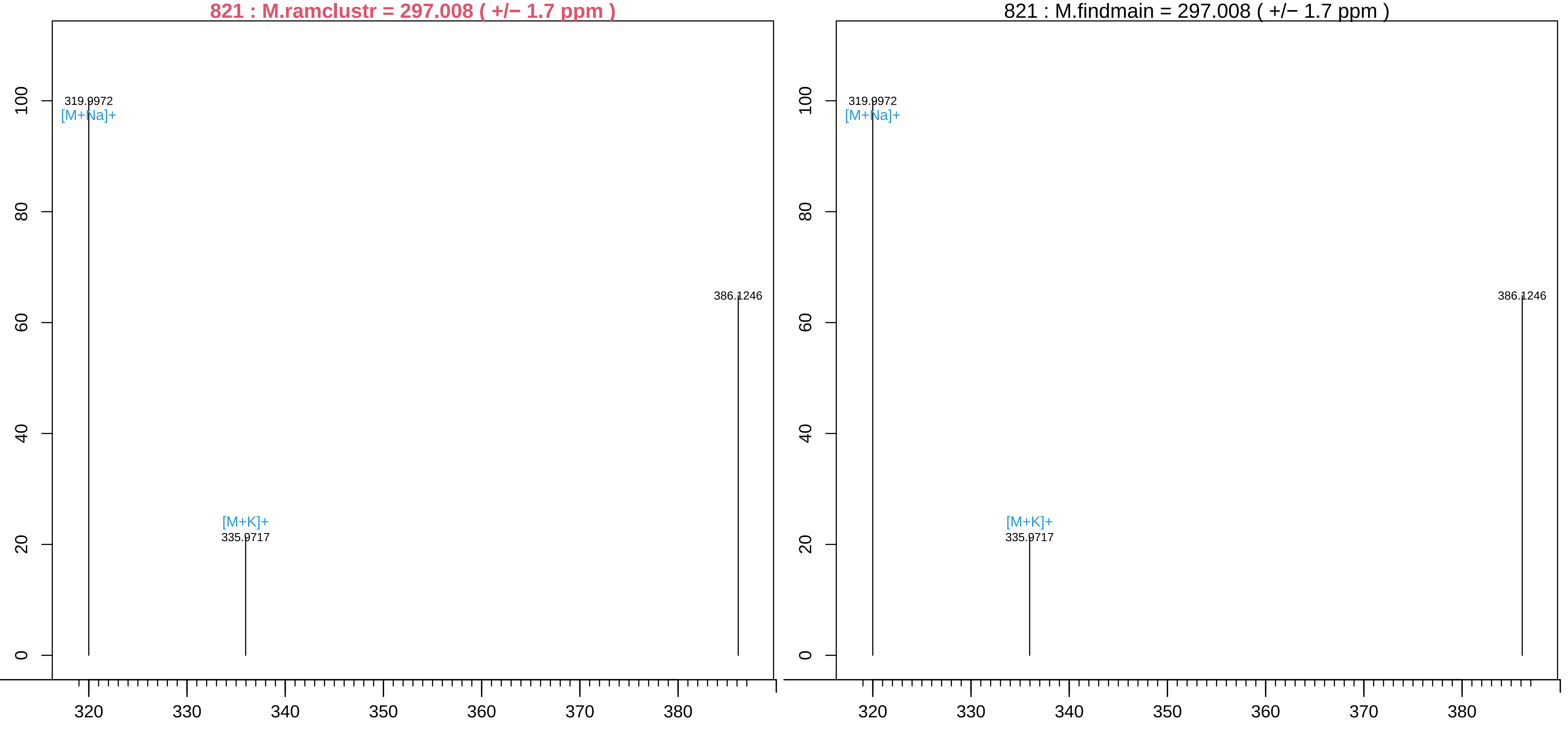
821 : M.ramclustr = 297.008 (+/− 1.7 ppm)
319.9972
[M+Na]+
386.1246
[M+K]+
335.9717
821 : M.findmain = 297.008 (+/− 1.7 ppm)
319.9972
[M+Na]+
386.1246
[M+K]+
335.9717
0
20
40
60
80
100
320
330
340
350
360
370
380

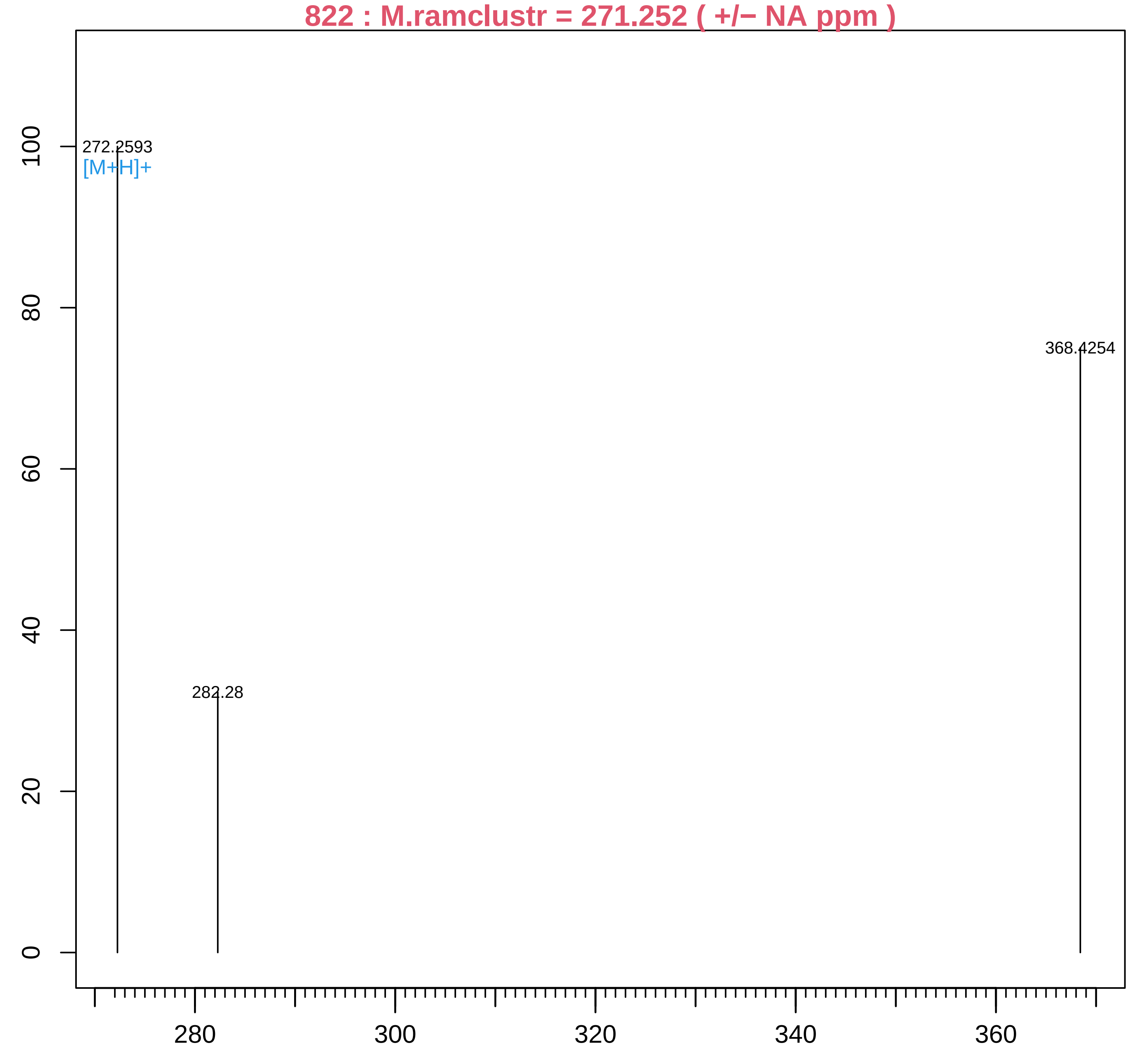
822 : M.ramclustr = 271.252 (+/− NA ppm)
272.2593
[M+H]+
282.28
368.4254
0
20
40
60
80
100
280
300
320
340
360

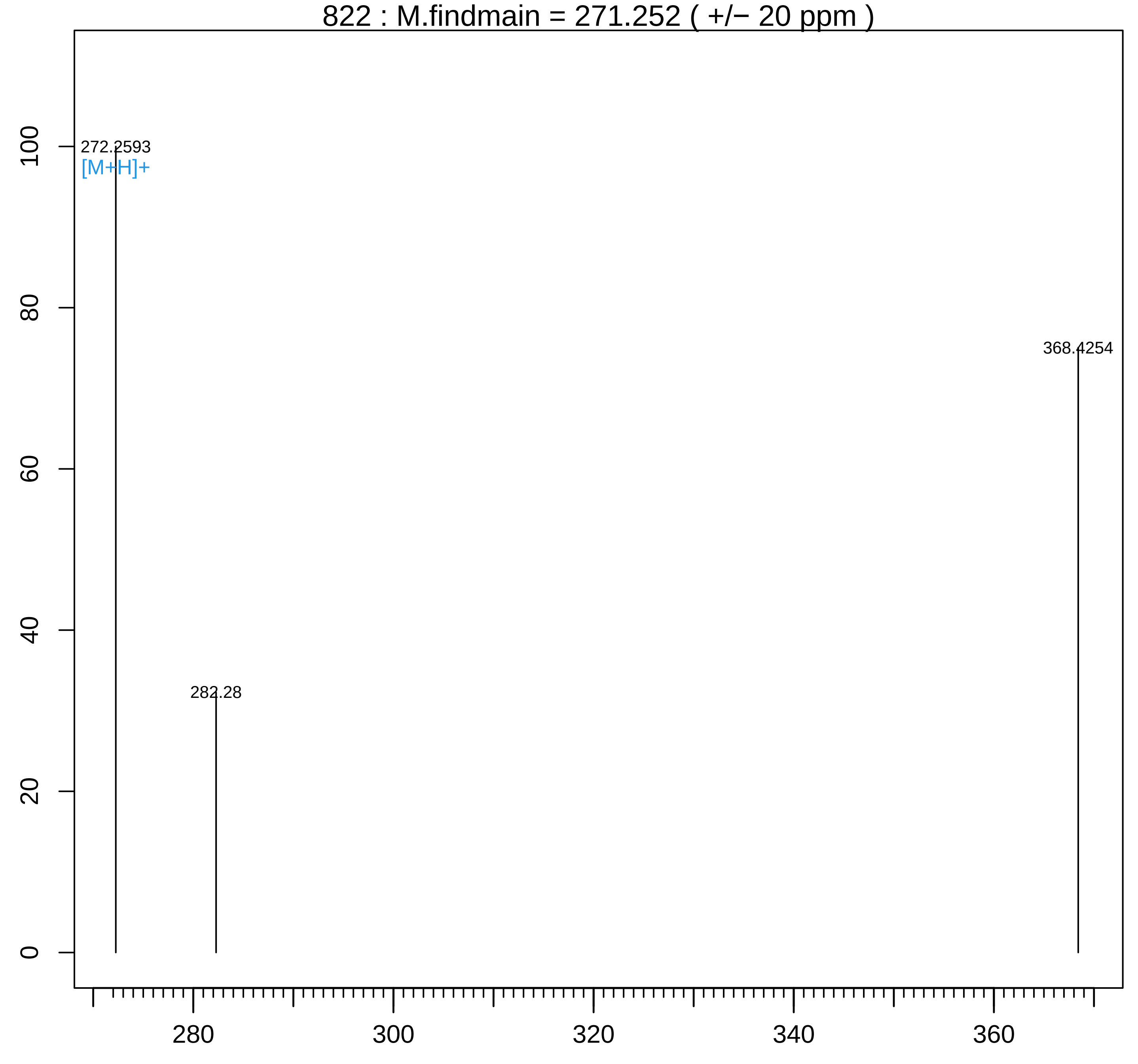
822 : M.findmain = 271.252 (+/− 20 ppm)
272.2593
[M+H]+
282.28
368.4254
0
20
40
60
80
100
280
300
320
340
360

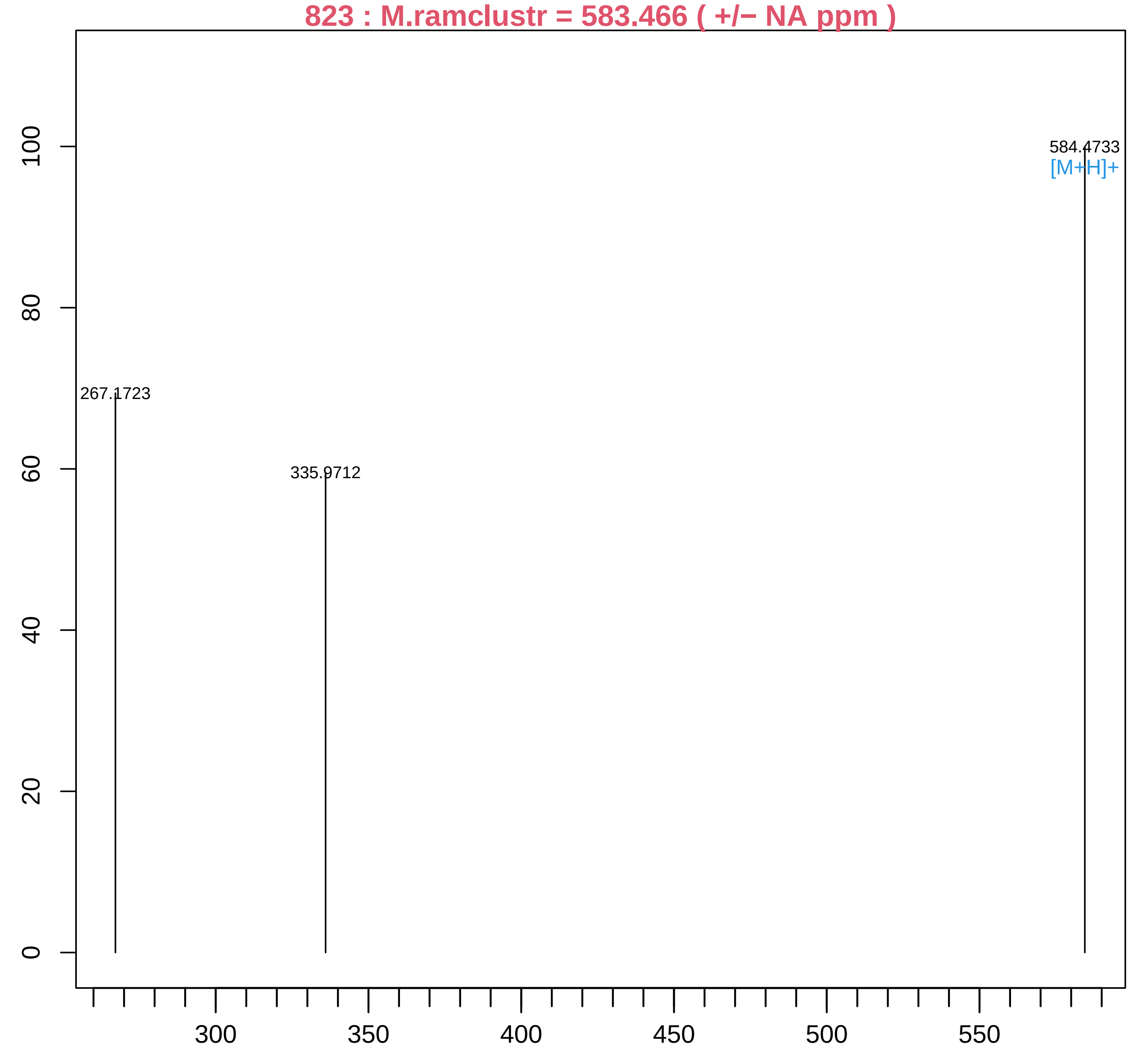
823 : M.ramclustr = 583.466 (+/− NA ppm)
584.4733
[M+H]+
267.1723
335.9712
0
20
40
60
80
100
300
350
400
450
500
550
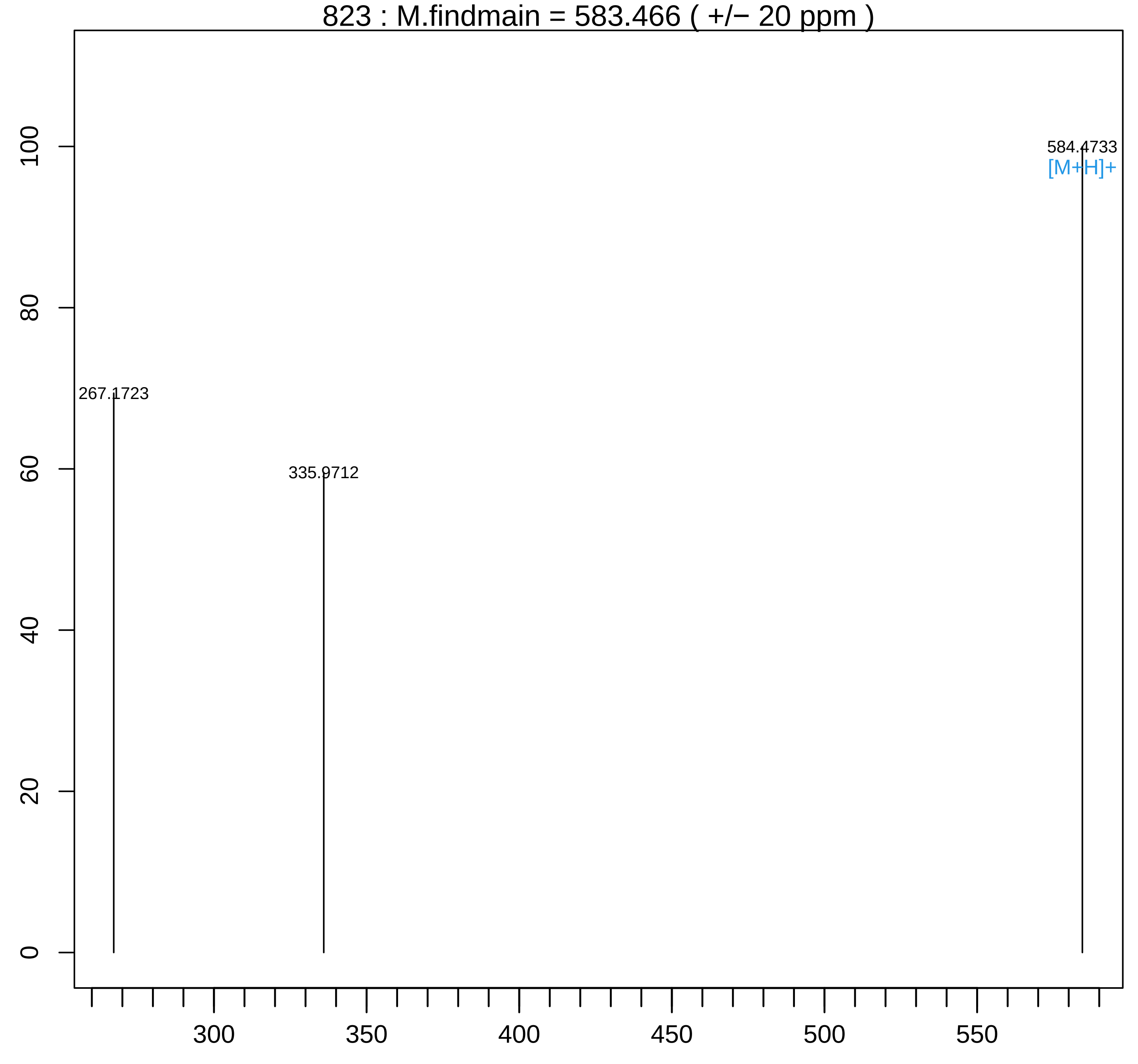
823 : M.findmain = 583.466 (+/− 20 ppm)
584.4733
[M+H]+
267.1723
335.9712
0
20
40
60
80
100
300
350
400
450
500
550

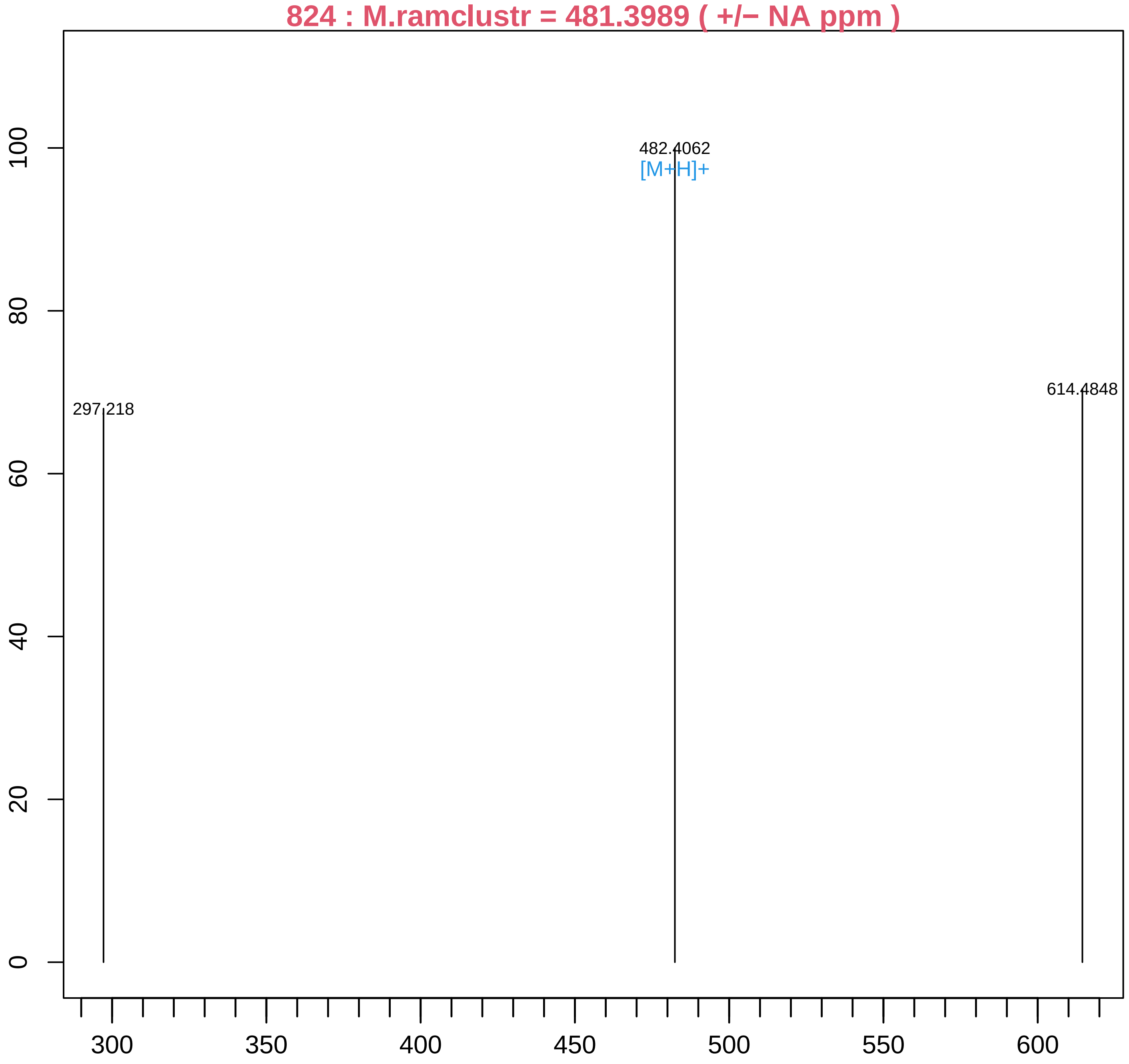
824 : M.ramclustr = 481.3989 (+/− NA ppm)
482.4062
[M+H]+
614.4848
297.218
0
20
40
60
80
100
300
350
400
450
500
550
600

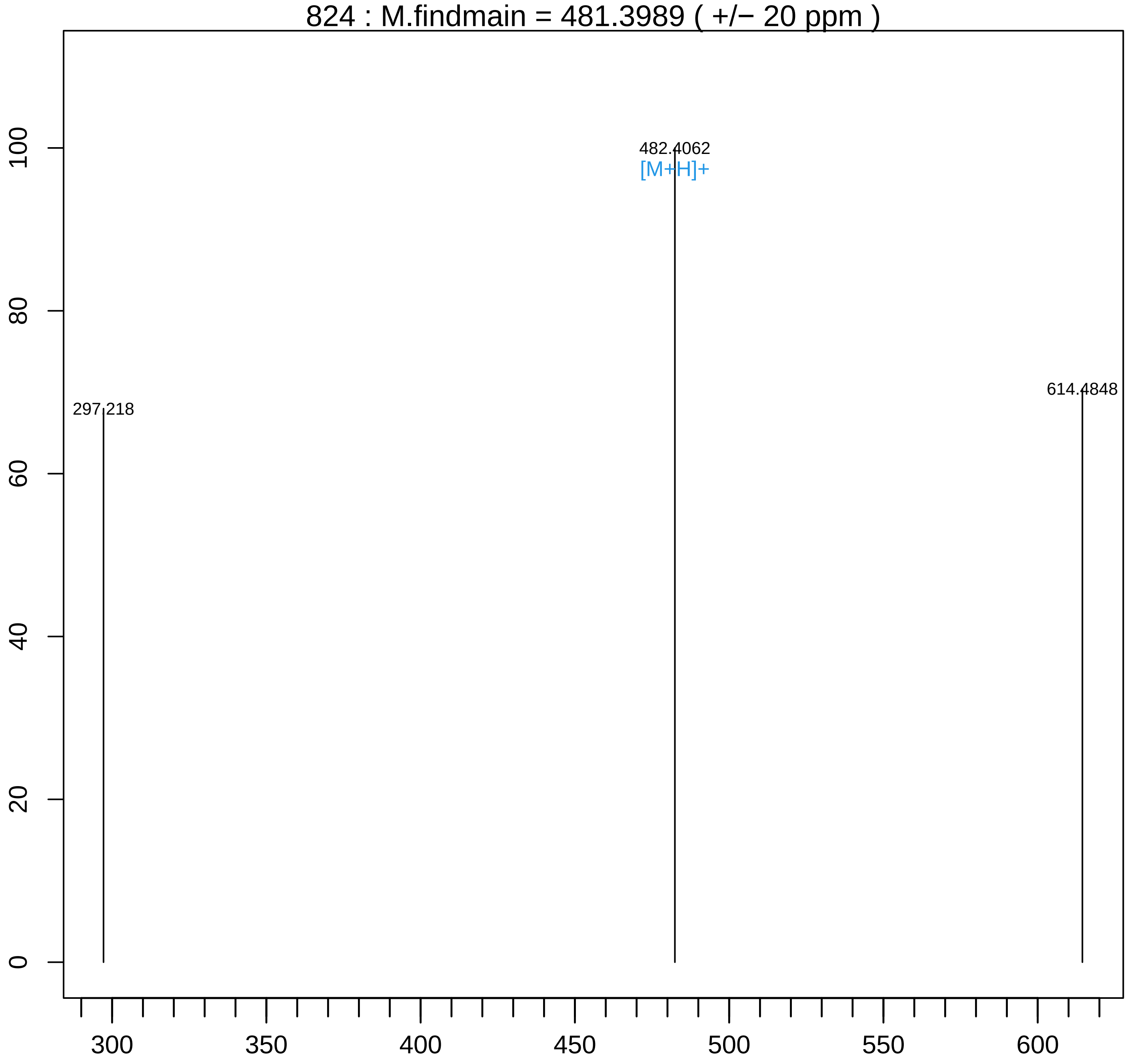
824 : M.findmain = 481.3989 (+/− 20 ppm)
482.4062
[M+H]+
614.4848
297.218
0
20
40
60
80
100
300
350
400
450
500
550
600

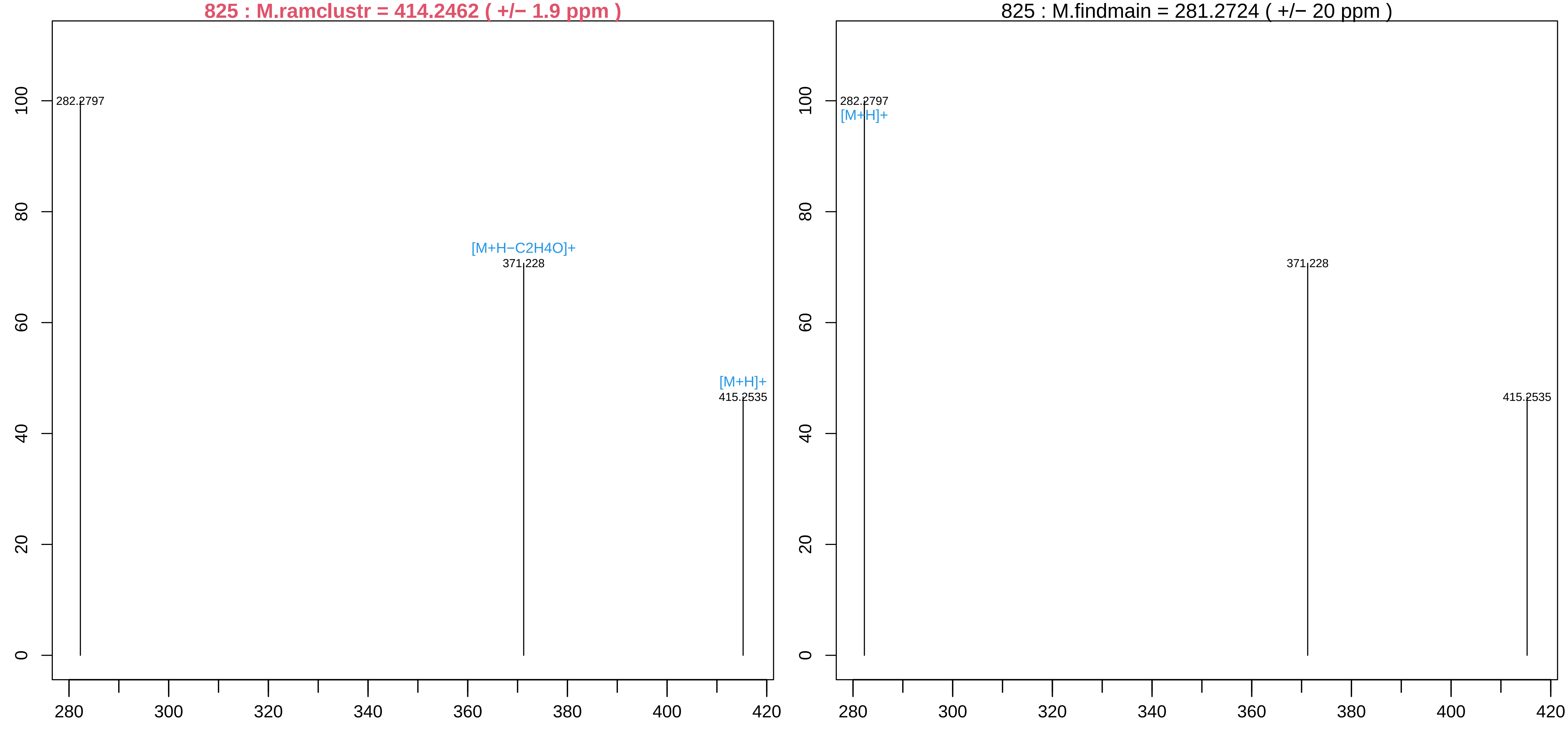
825 : M.ramclustr = 414.2462 (+/− 1.9 ppm)
282.2797
[M+H−C2H4O]+
371.228
[M+H]+
415.2535
825 : M.findmain = 281.2724 (+/− 20 ppm)
282.2797
[M+H]+
371.228
415.2535
0
20
40
60
80
100
280
300
320
340
360
380
400
420

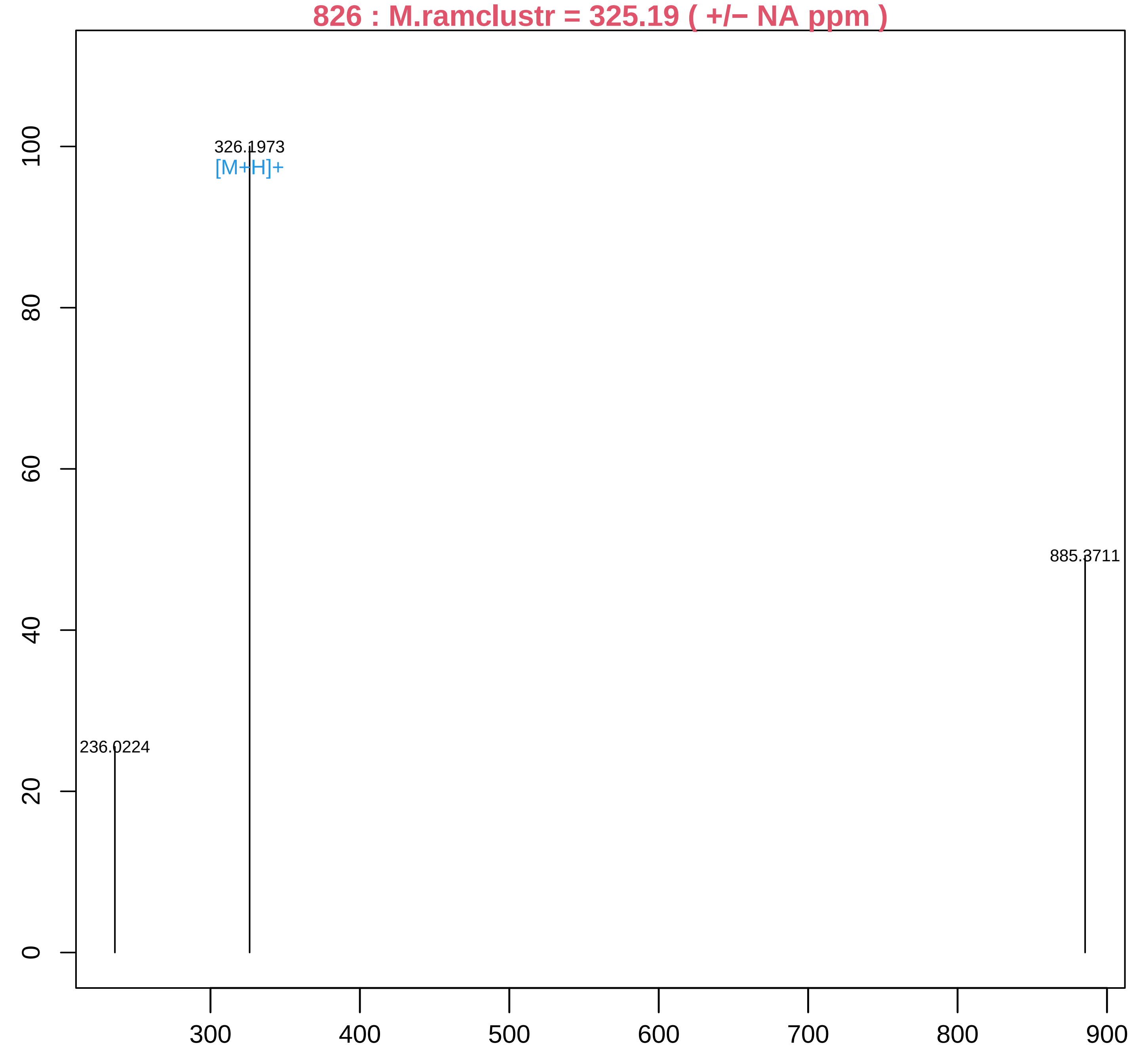

826 : M.ramclustr = 325.19 (+/− NA ppm)
326.1973
[M+H]+
885.3711
236.0224
0
20
40
60
80
100
300
400
500
600
700
800
900

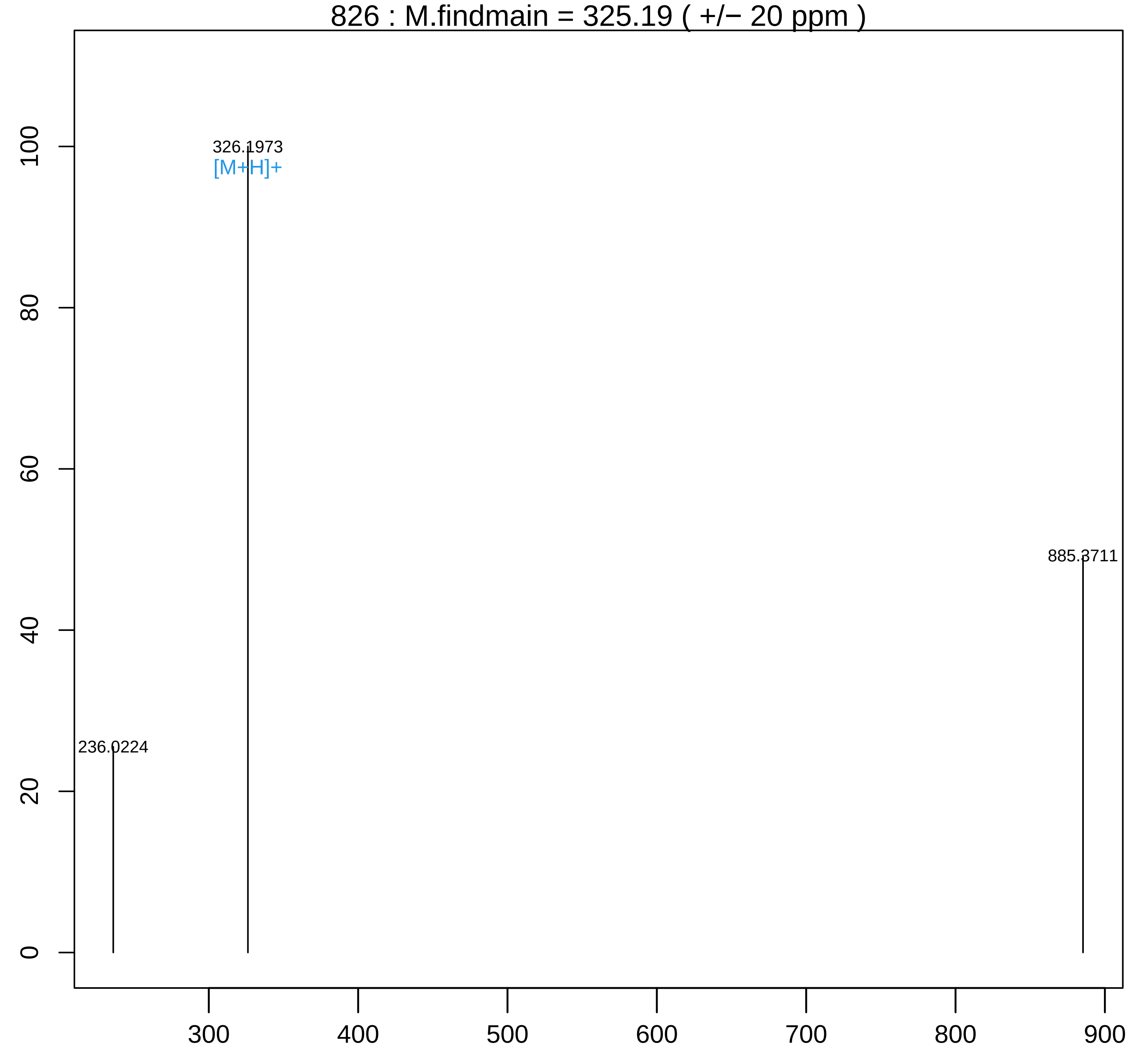

826 : M.findmain = 325.19 (+/− 20 ppm)
326.1973
[M+H]+
885.3711
236.0224
0
20
40
60
80
100
300
400
500
600
700
800
900

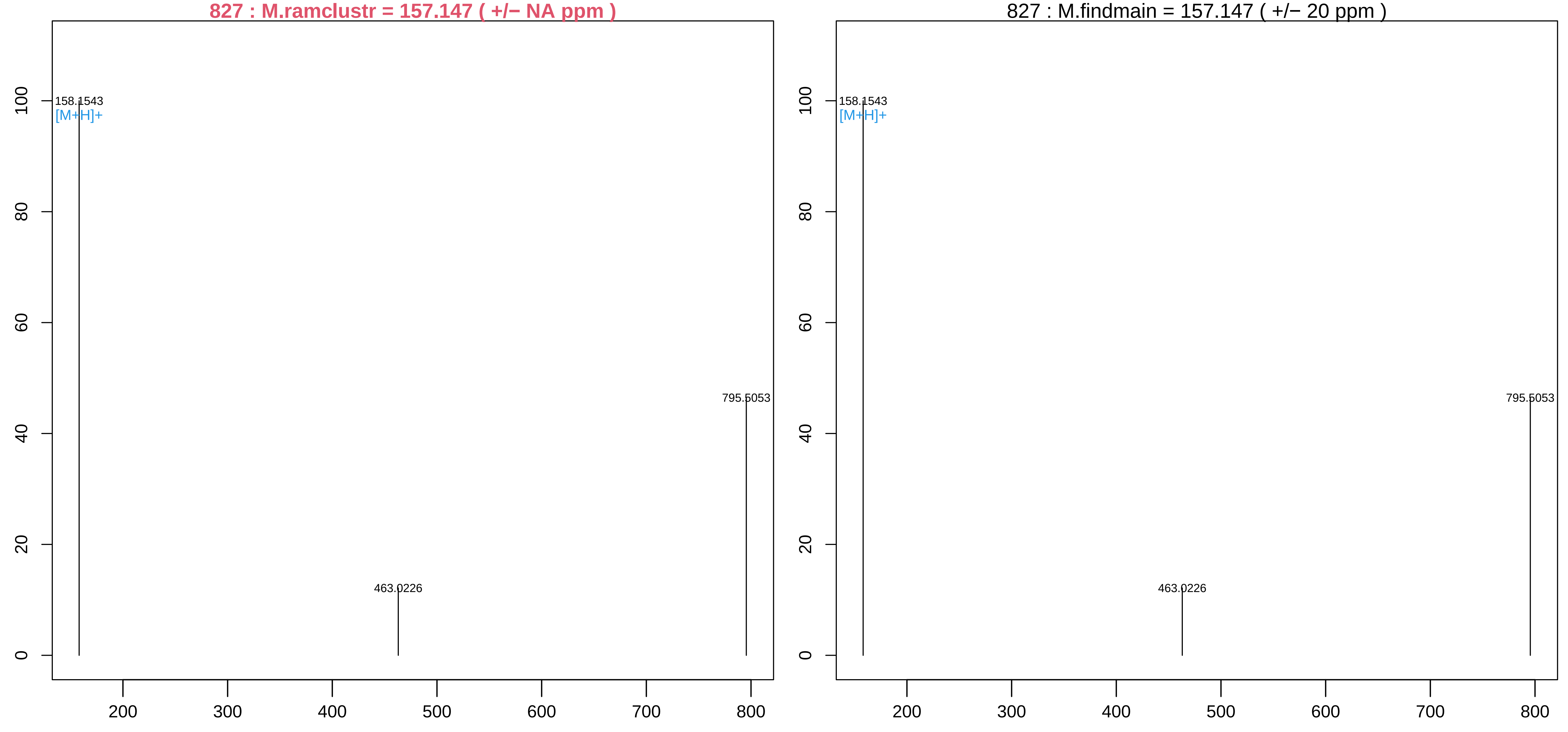
827 : M.ramclustr = 157.147 (+/− NA ppm)
158.1543
[M+H]+
463.0226
795.5053
0
20
40
60
80
100
200
300
400
500
600
700
800
827 : M.findmain = 157.147 (+/− 20 ppm)
158.1543
[M+H]+
463.0226
795.5053

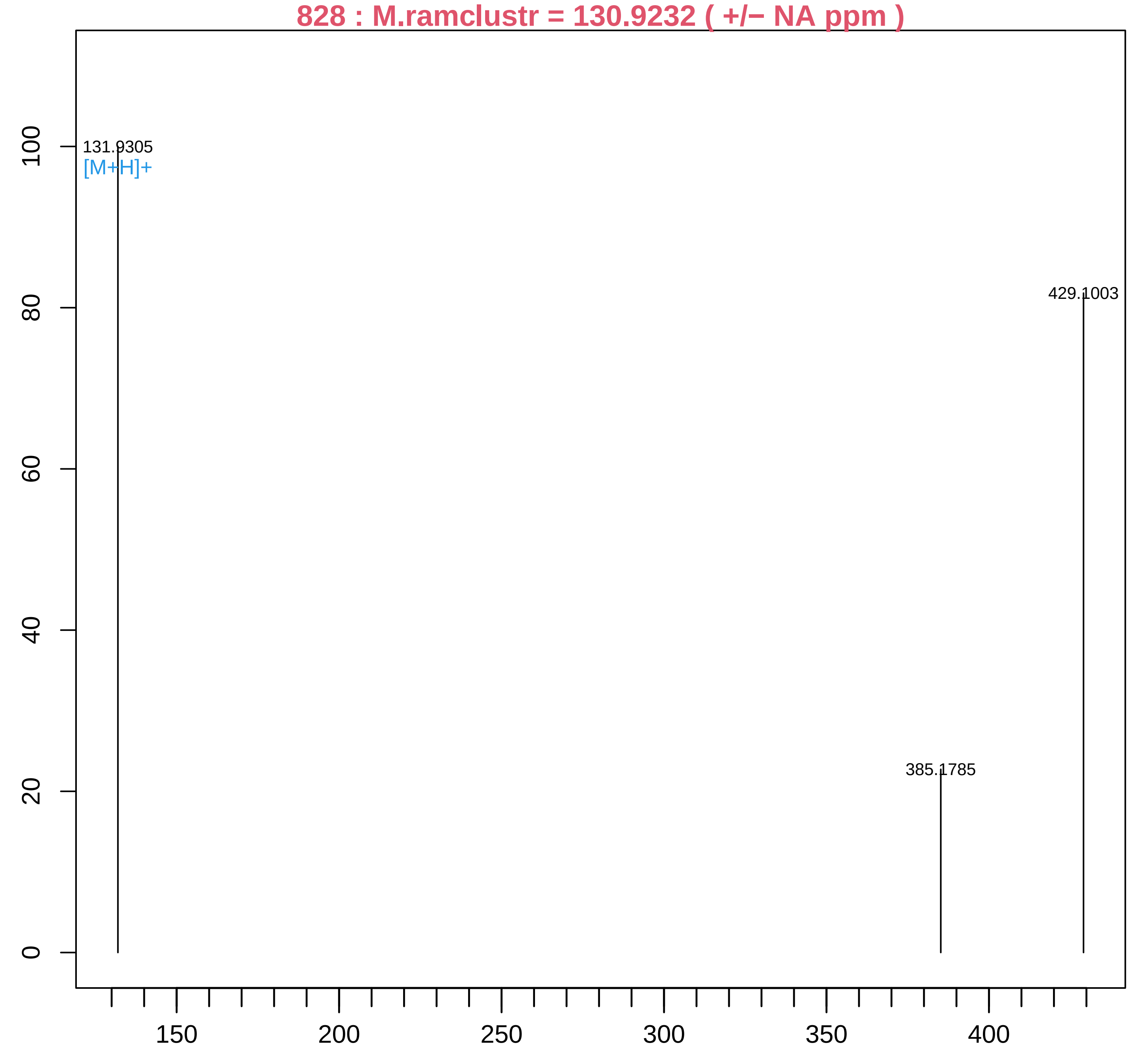
828 : M.ramclustr = 130.9232 (+/− NA ppm)
131.9305
[M+H]+
429.1003
385.1785
0
20
40
60
80
100
150
200
250
300
350
400

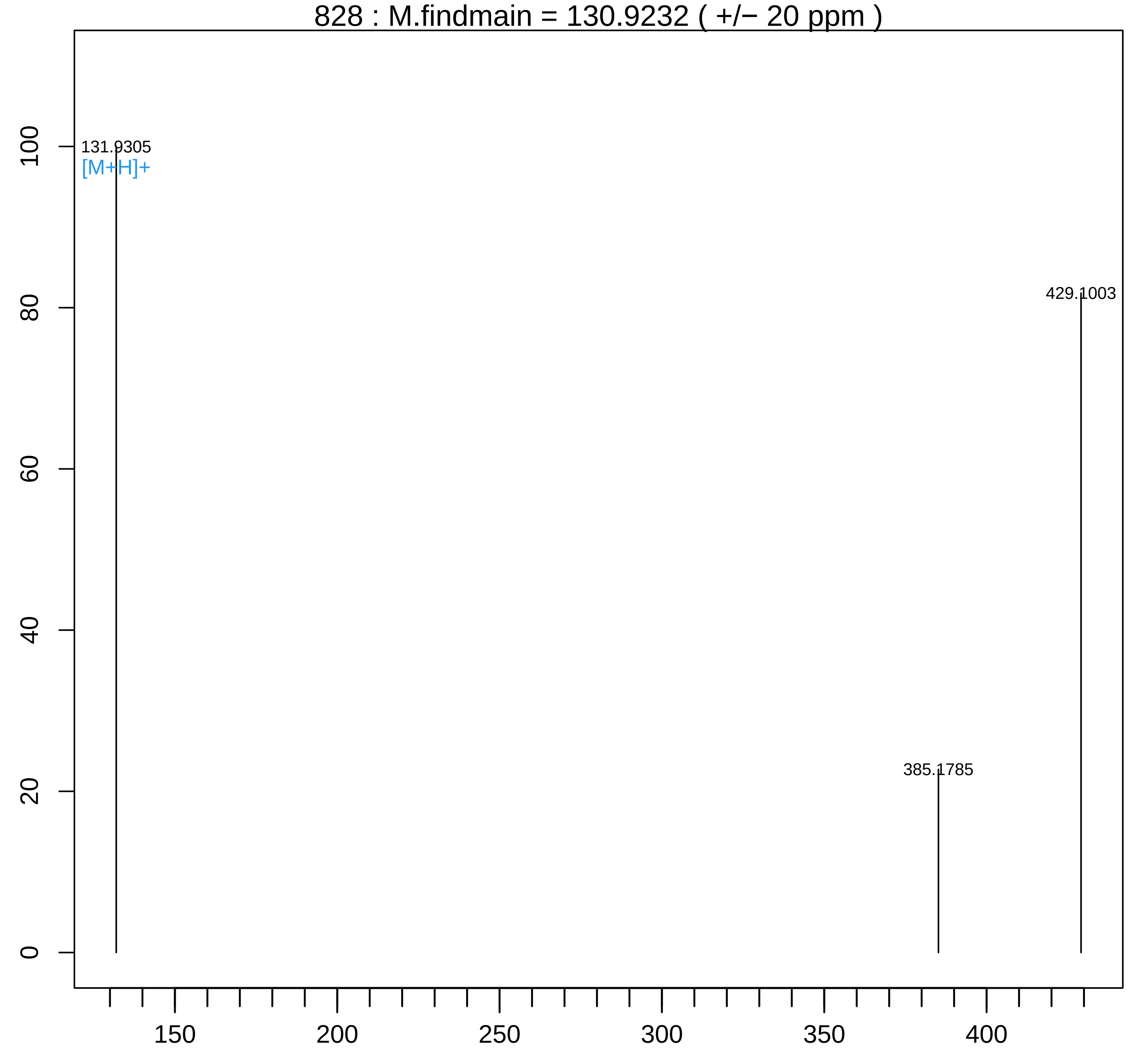
828 : M.findmain = 130.9232 (+/− 20 ppm)
131.9305
[M+H]+
429.1003
385.1785
0
20
40
60
80
100
150
200
250
300
350
400

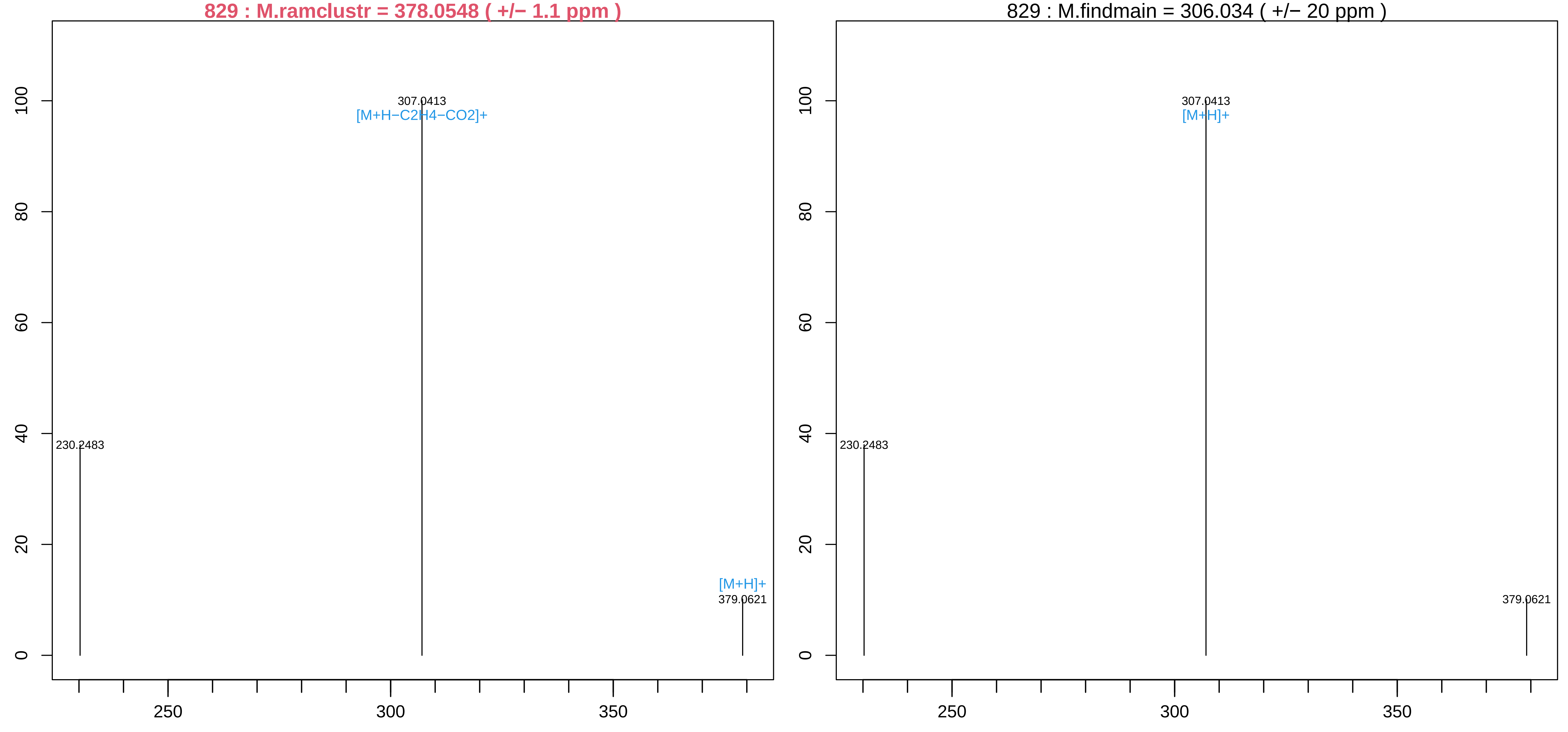
829 : M.ramclustr = 378.0548 (+/− 1.1 ppm)
307.0413
[M+H−C2H4−CO2]+
230.2483
[M+H]+
379.0621
0
20
40
60
80
100
250
300
350
829 : M.findmain = 306.034 (+/− 20 ppm)
307.0413
[M+H]+
230.2483
379.0621
0
20
40
60
80
100
250
300
350

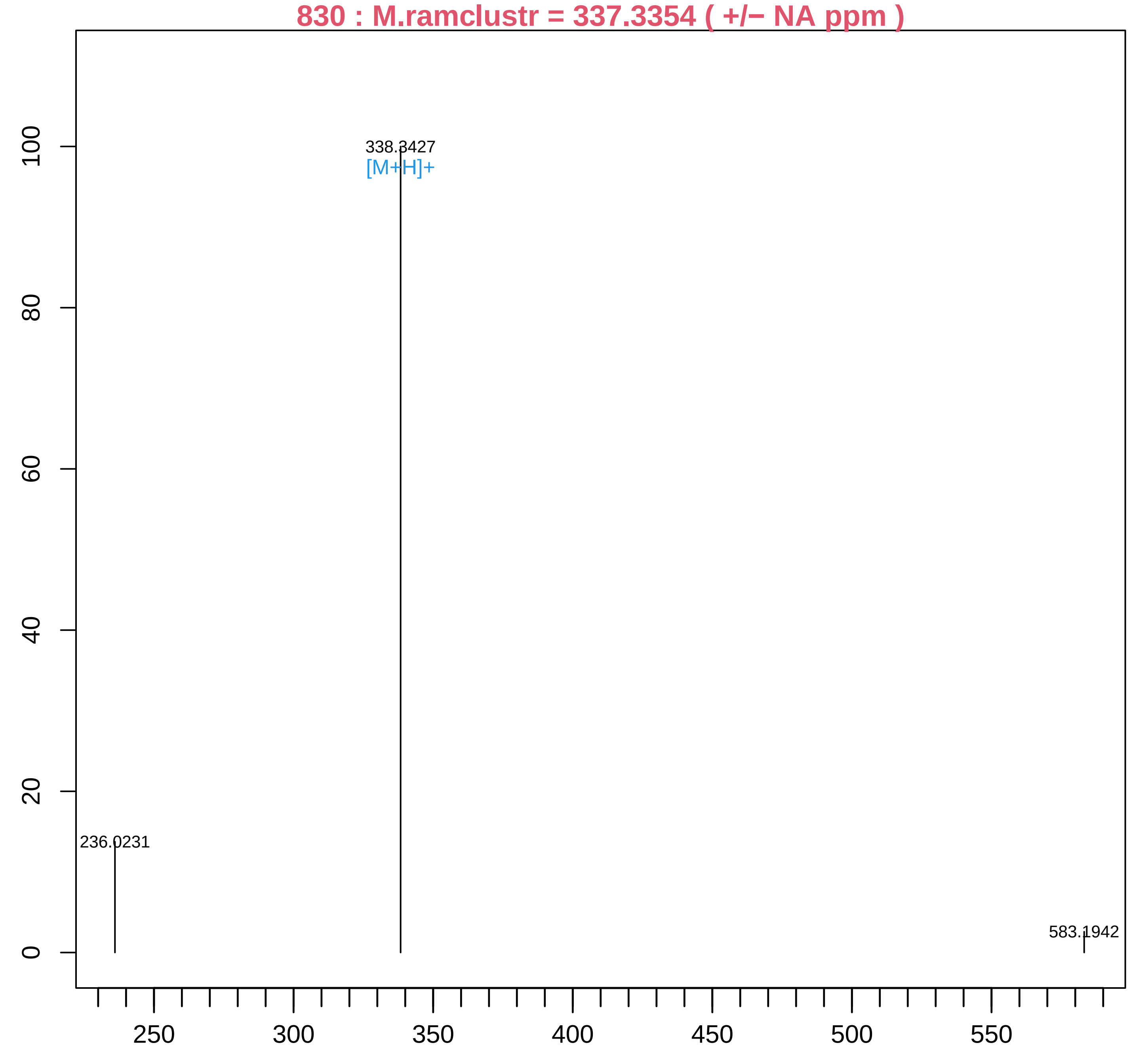

830 : M.ramclustr = 337.3354 (+/− NA ppm)
338.3427
[M+H]+
236.0231
583.1942
0
20
40
60
80
100
250
300
350
400
450
500
550

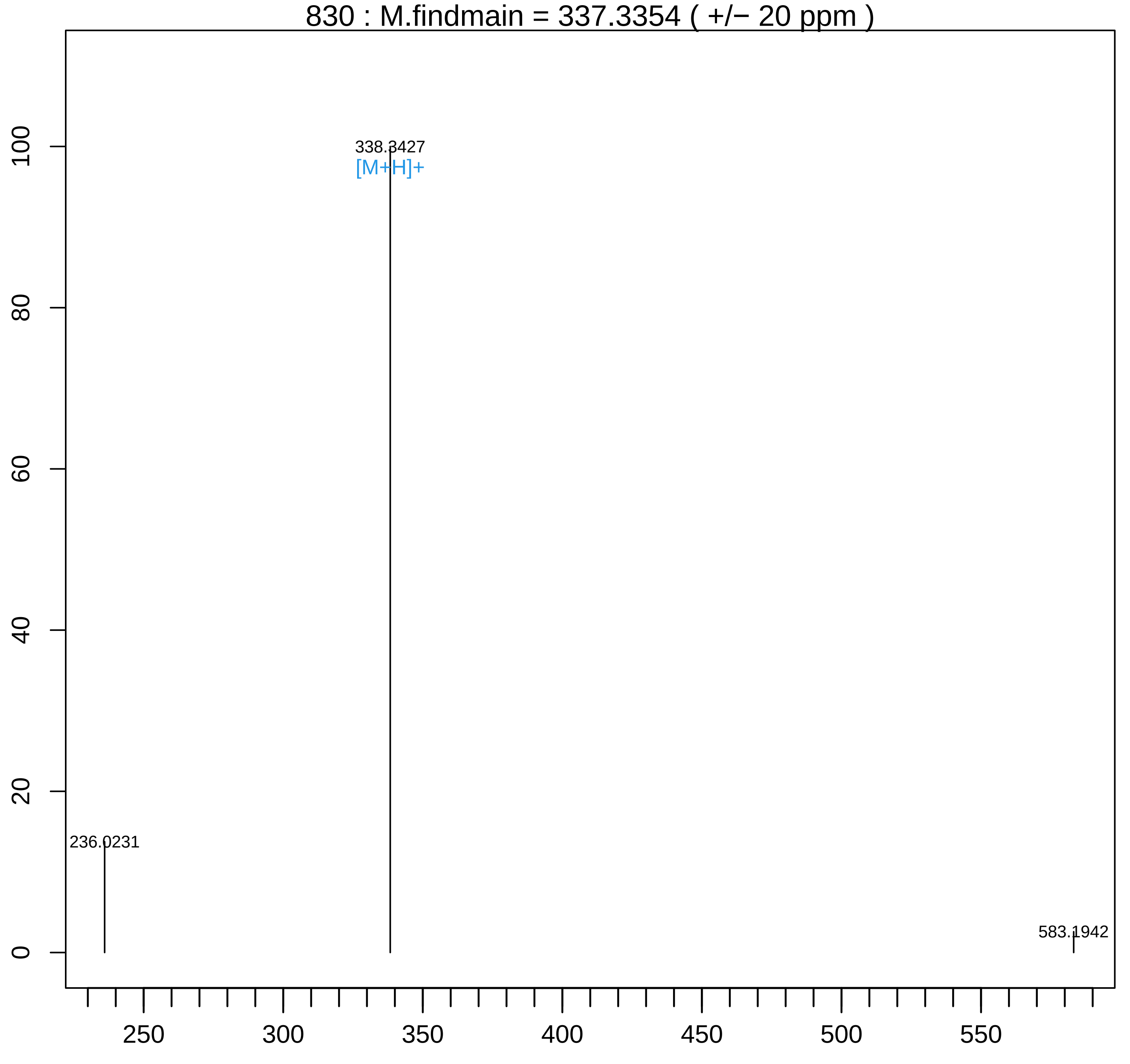

830 : M.findmain = 337.3354 (+/− 20 ppm)
338.3427
[M+H]+
236.0231
583.1942
0
20
40
60
80
100
250
300
350
400
450
500
550

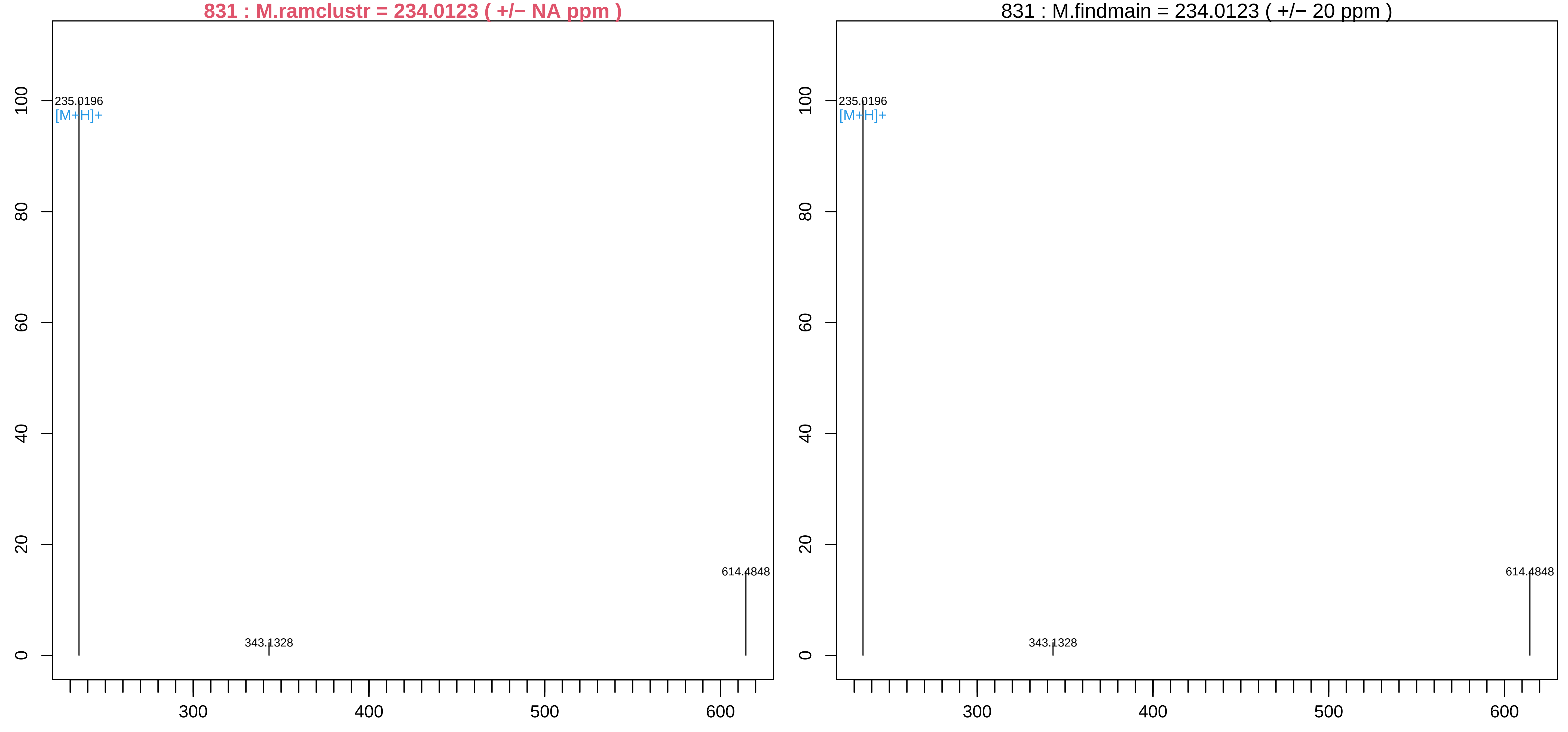

831 : M.ramclustr = 234.0123 (+/− NA ppm)
235.0196
[M+H]+
343.1328
614.4848
0
20
40
60
80
100
300
400
500
600
831 : M.findmain = 234.0123 (+/− 20 ppm)
235.0196
[M+H]+
343.1328
614.4848
0
20
40
60
80
100
300
400
500
600

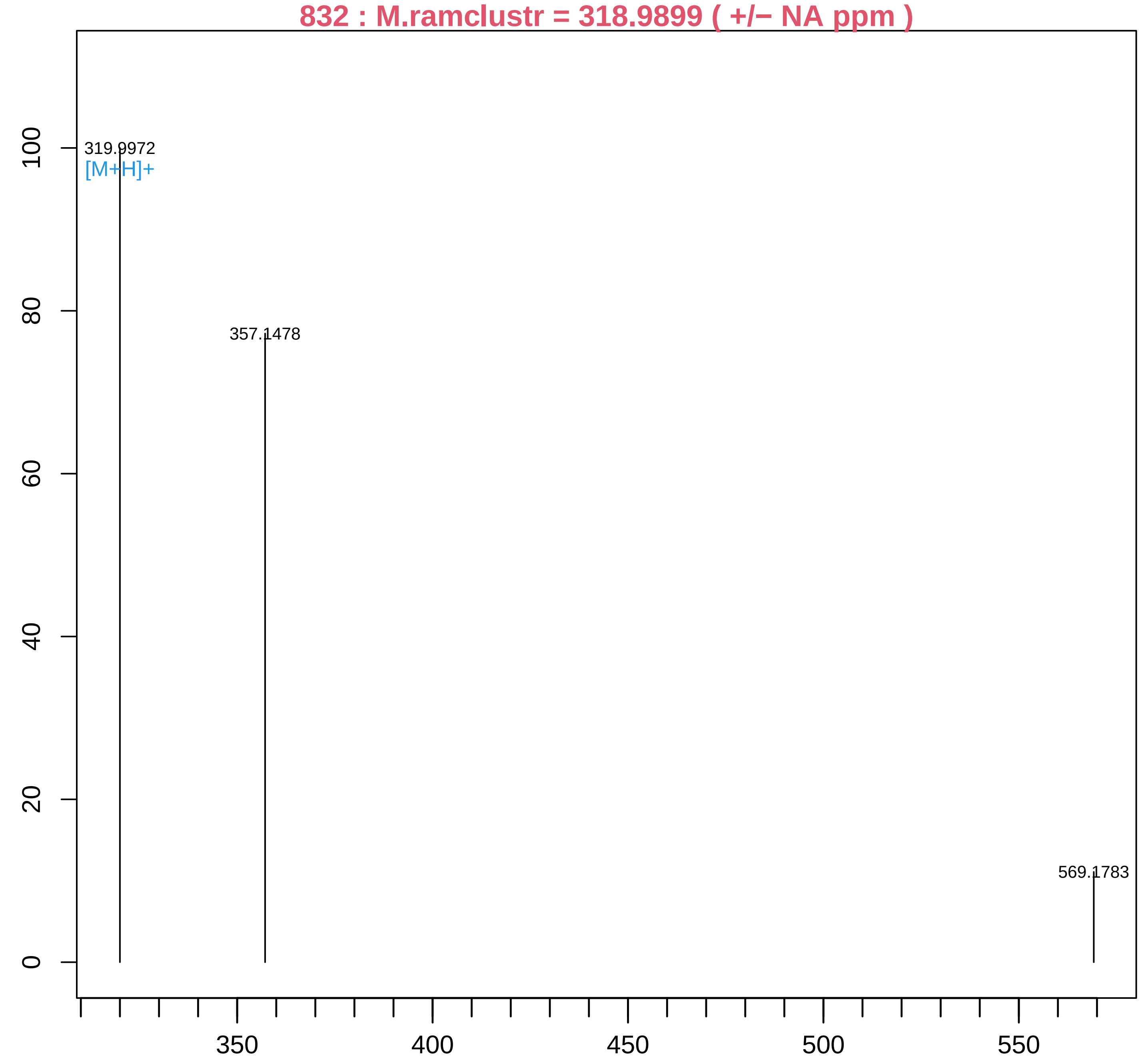

832 : M.ramclustr = 318.9899 (+/− NA ppm)
319.9972
[M+H]+
357.1478
569.1783
0
20
40
60
80
100
350
400
450
500
550

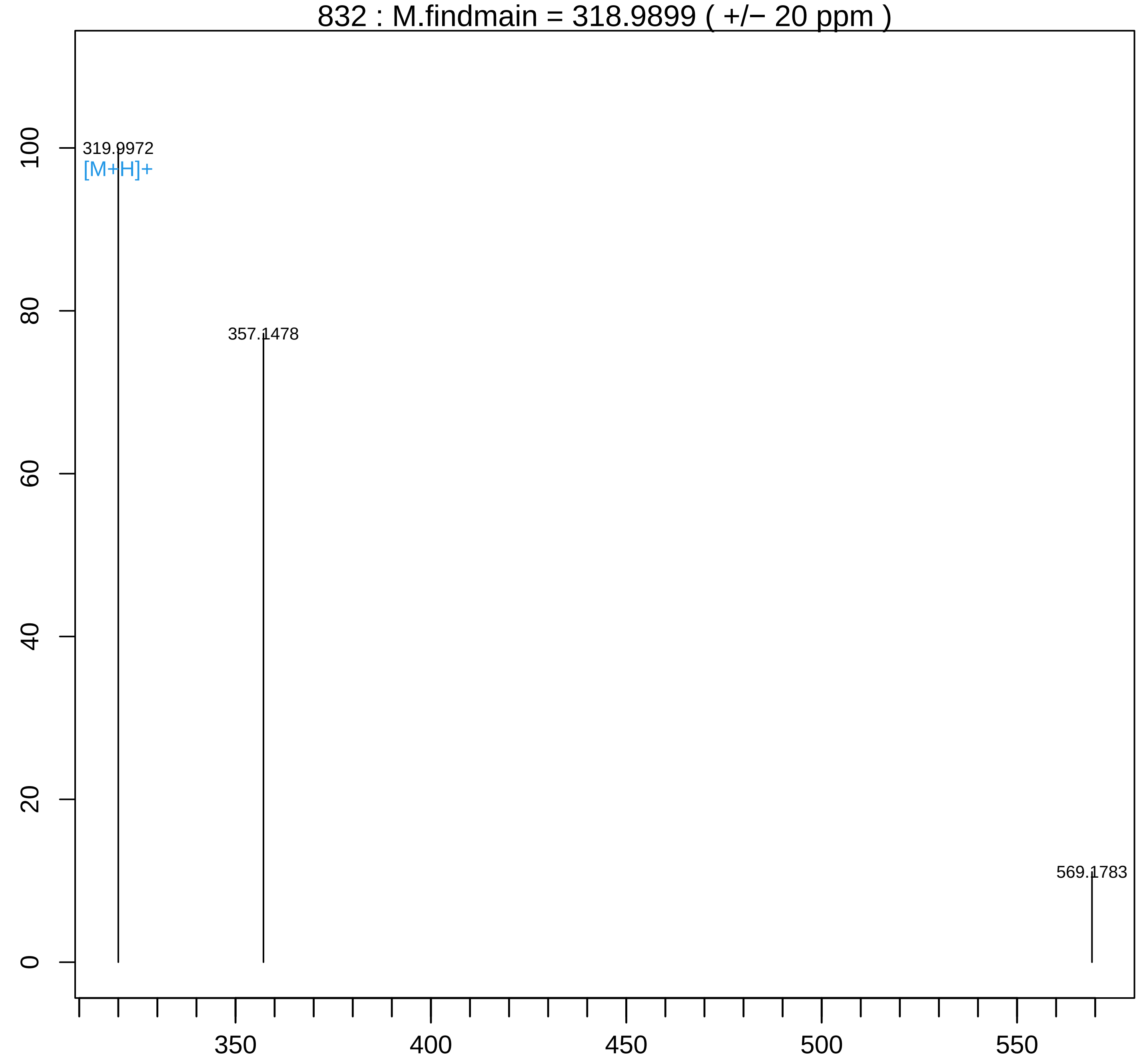

832 : M.findmain = 318.9899 (+/− 20 ppm)
319.9972
[M+H]+
357.1478
569.1783
0
20
40
60
80
100
350
400
450
500
550

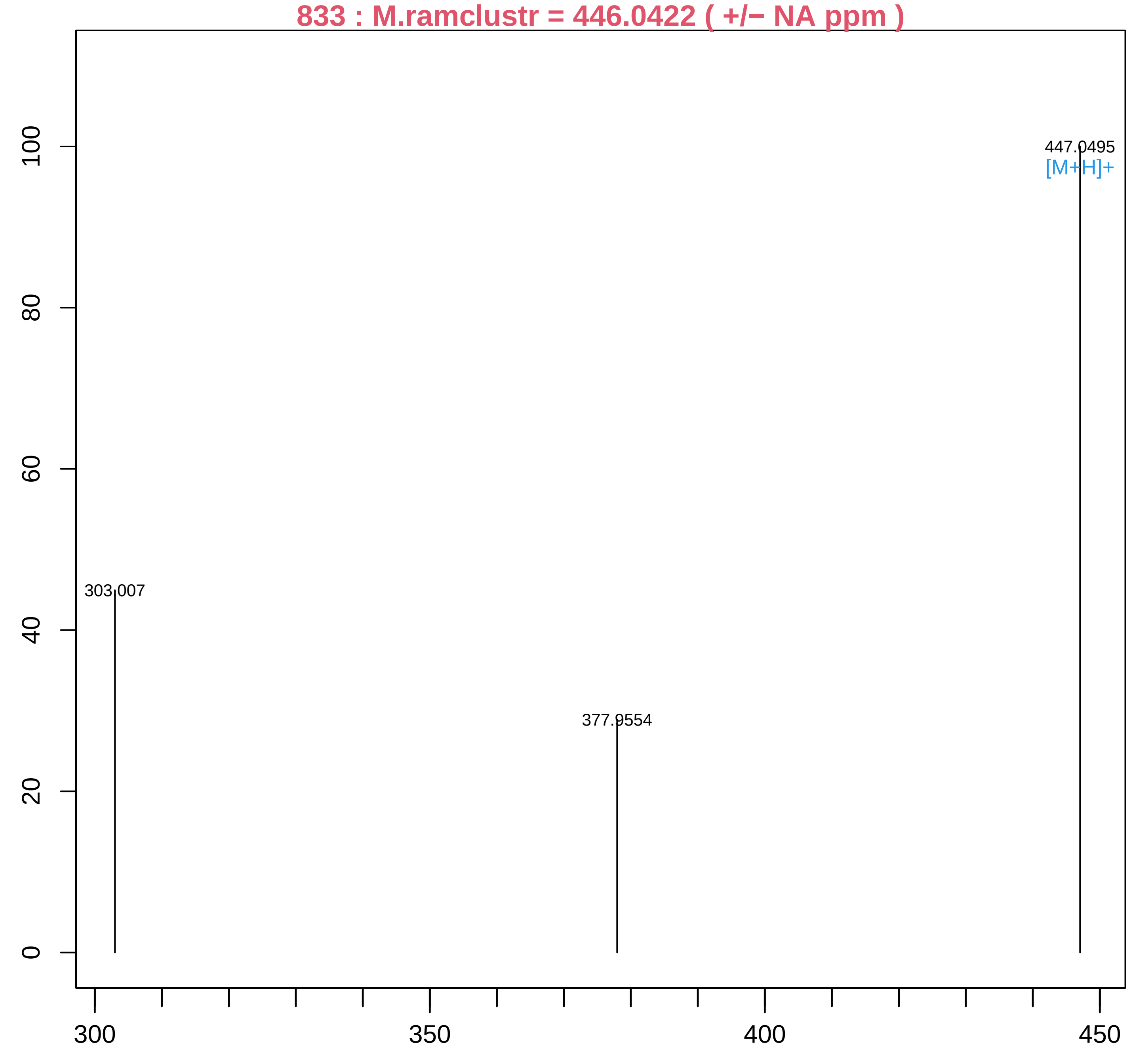

833 : M.ramclustr = 446.0422 (+/− NA ppm)
447.0495
[M+H]+
303.007
377.9554
0
20
40
60
80
100
300
350
400
450

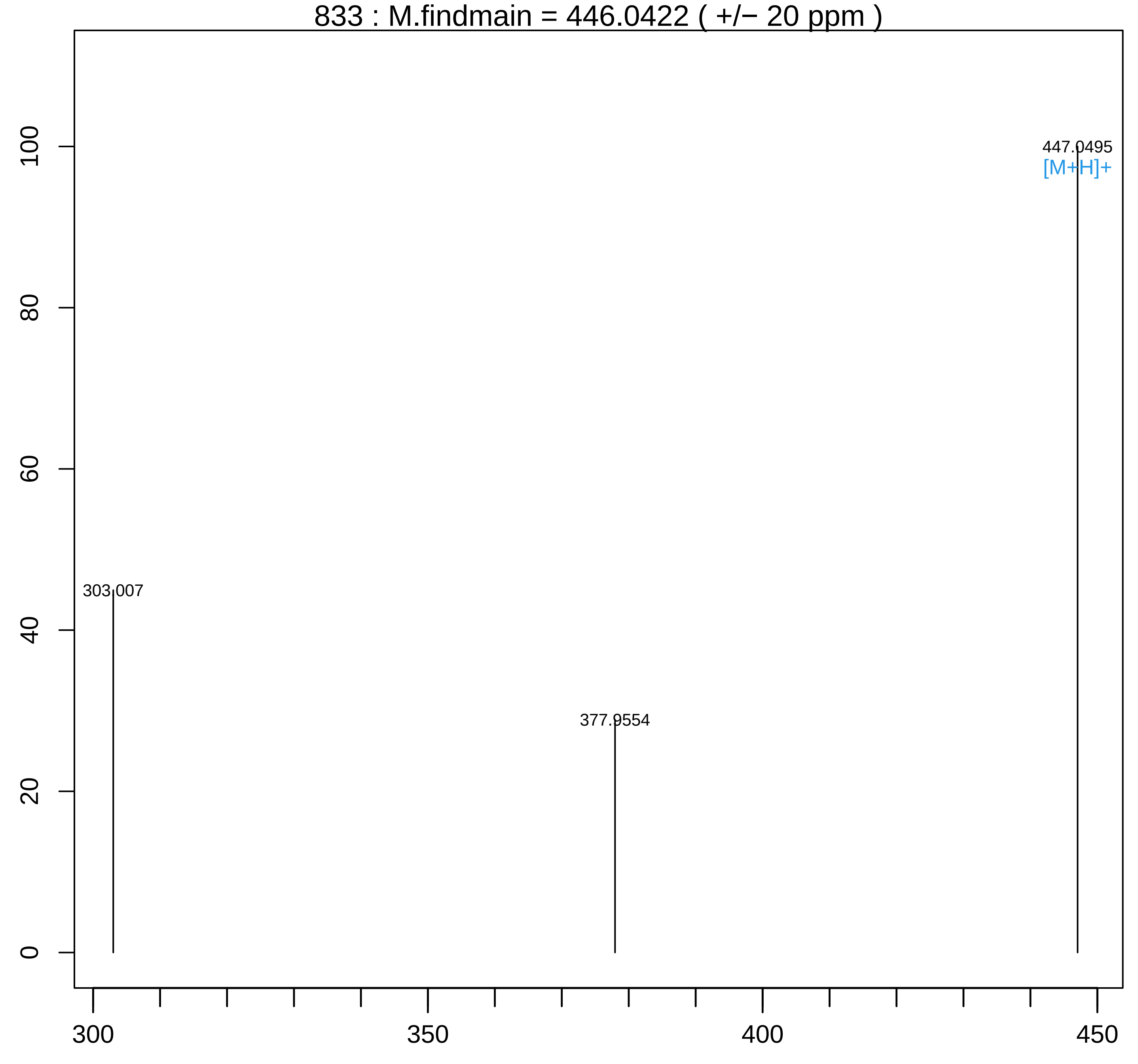

833 : M.findmain = 446.0422 (+/− 20 ppm)
447.0495
[M+H]+
303.007
377.9554
0
20
40
60
80
100
300
350
400
450

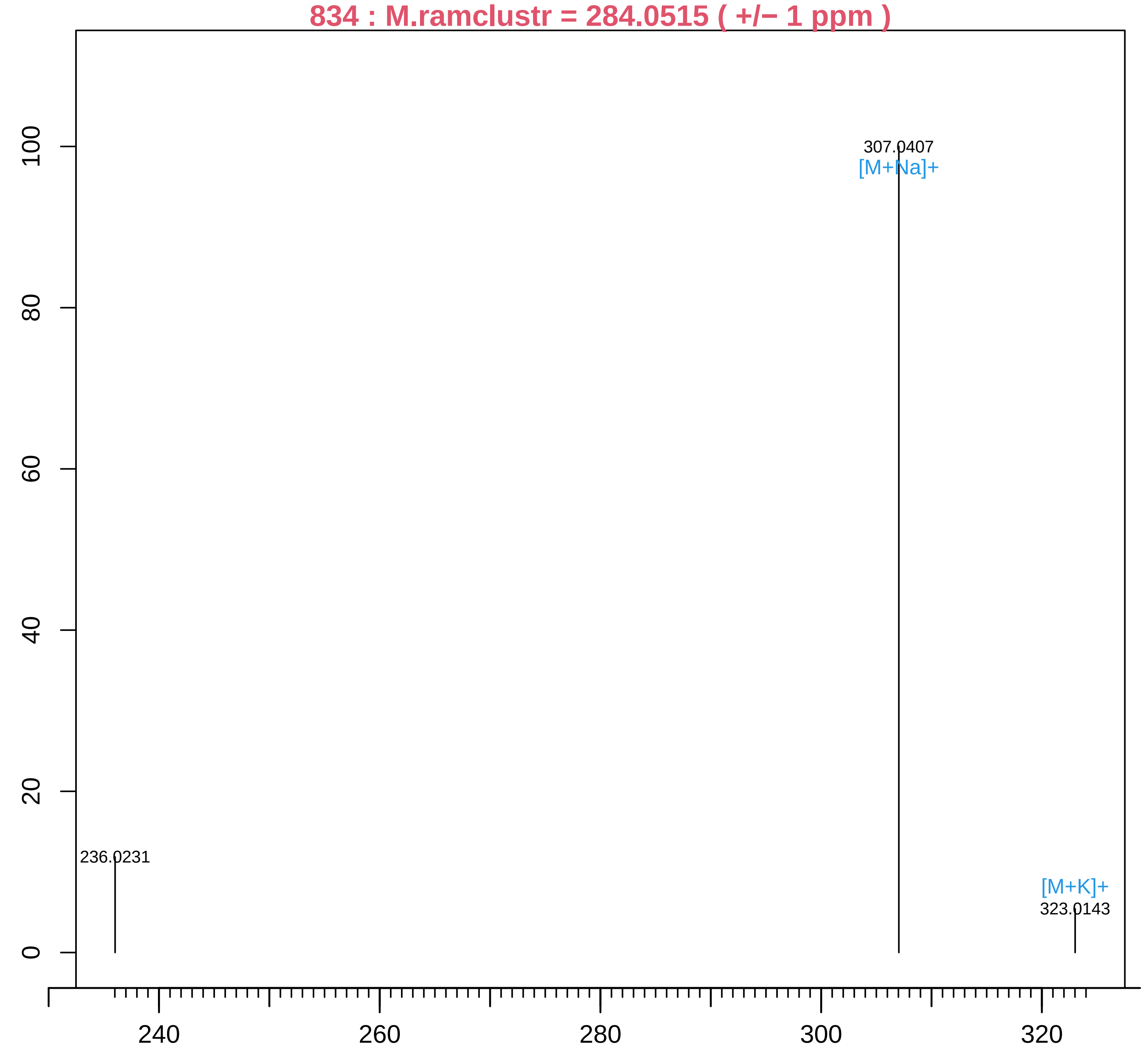

834 : M.ramclustr = 284.0515 (+/− 1 ppm)
307.0407
[M+Na]+
236.0231
[M+K]+
323.0143
0
20
40
60
80
100
240
260
280
300
320

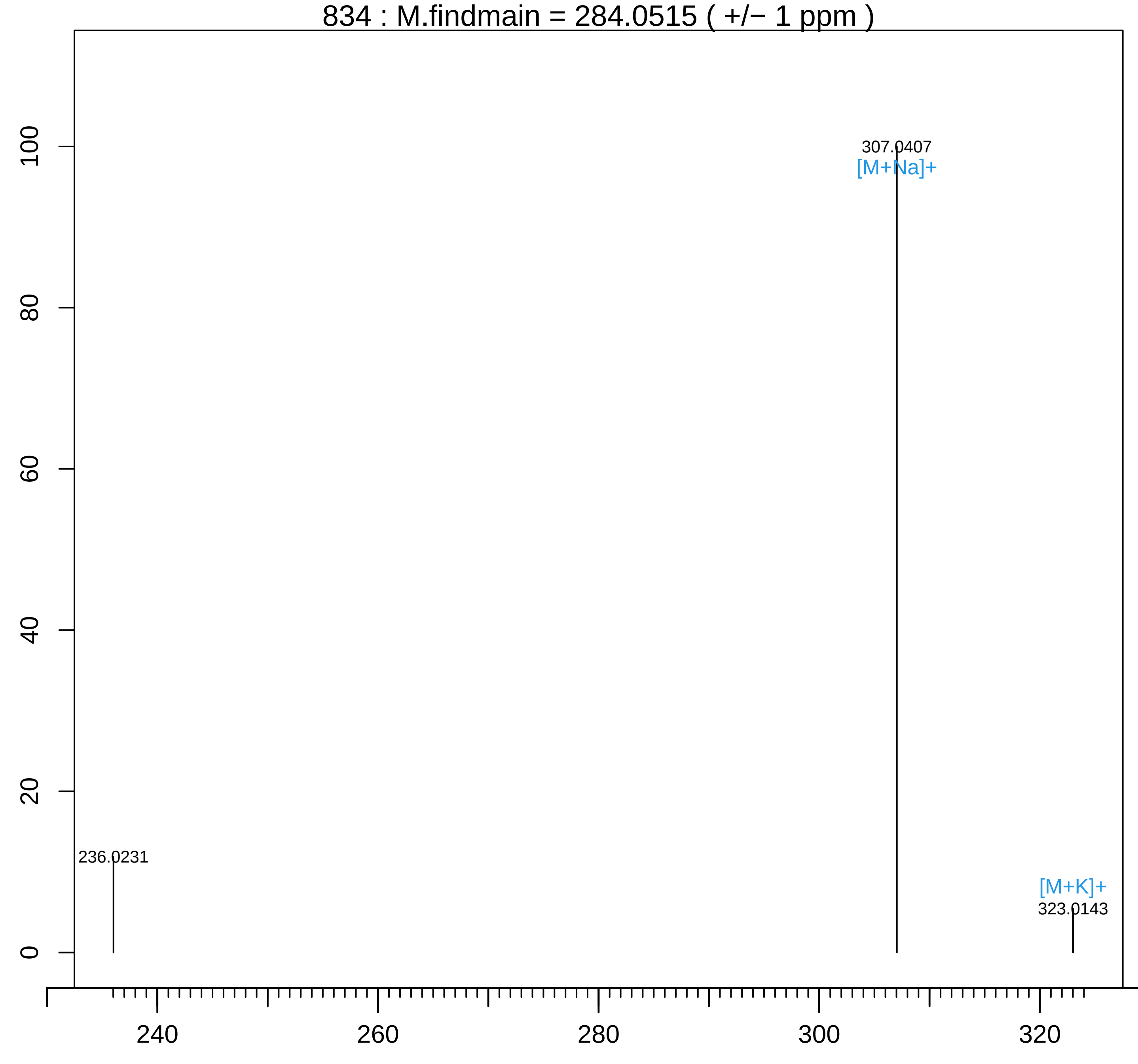

834 : M.findmain = 284.0515 (+/− 1 ppm)
307.0407
[M+Na]+
236.0231
[M+K]+
323.0143
0
20
40
60
80
100
240
260
280
300
320

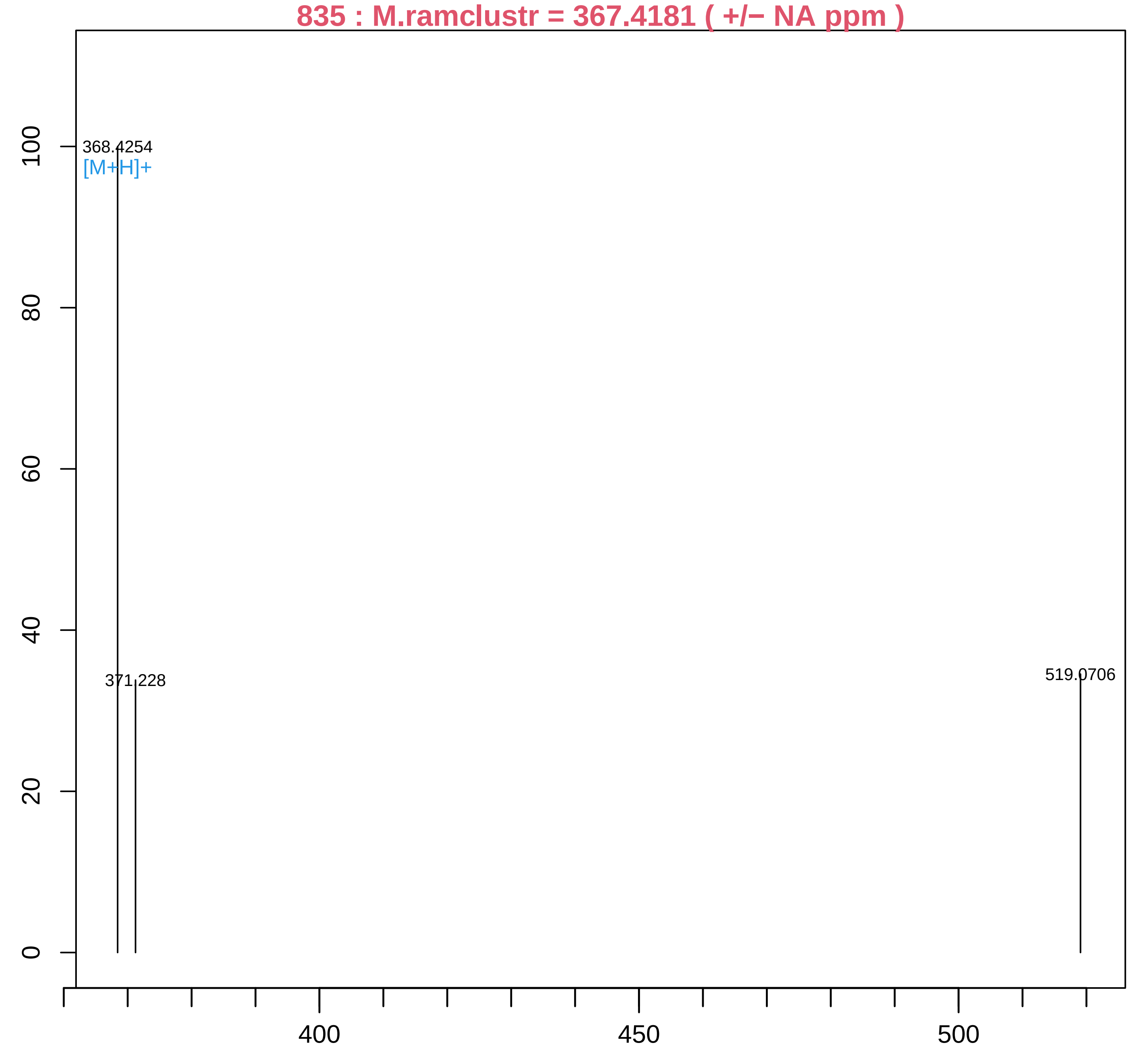
835 : M.ramclustr = 367.4181 (+/− NA ppm)
368.4254
[M+H]+
371.228
519.0706
0
20
40
60
80
100
400
450
500

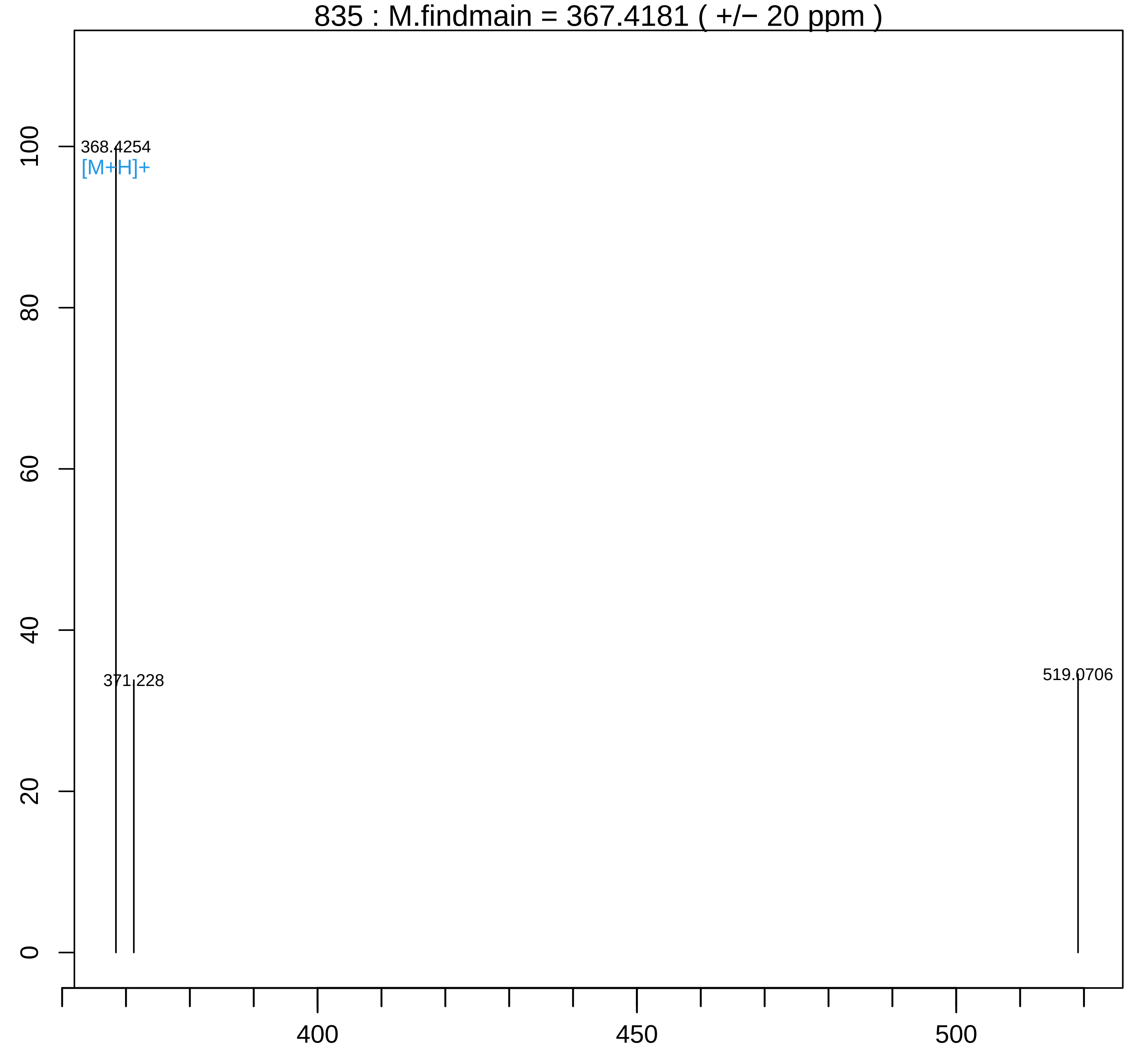
835 : M.findmain = 367.4181 (+/− 20 ppm)
368.4254
[M+H]+
371.228
519.0706
0
20
40
60
80
100
400
450
500

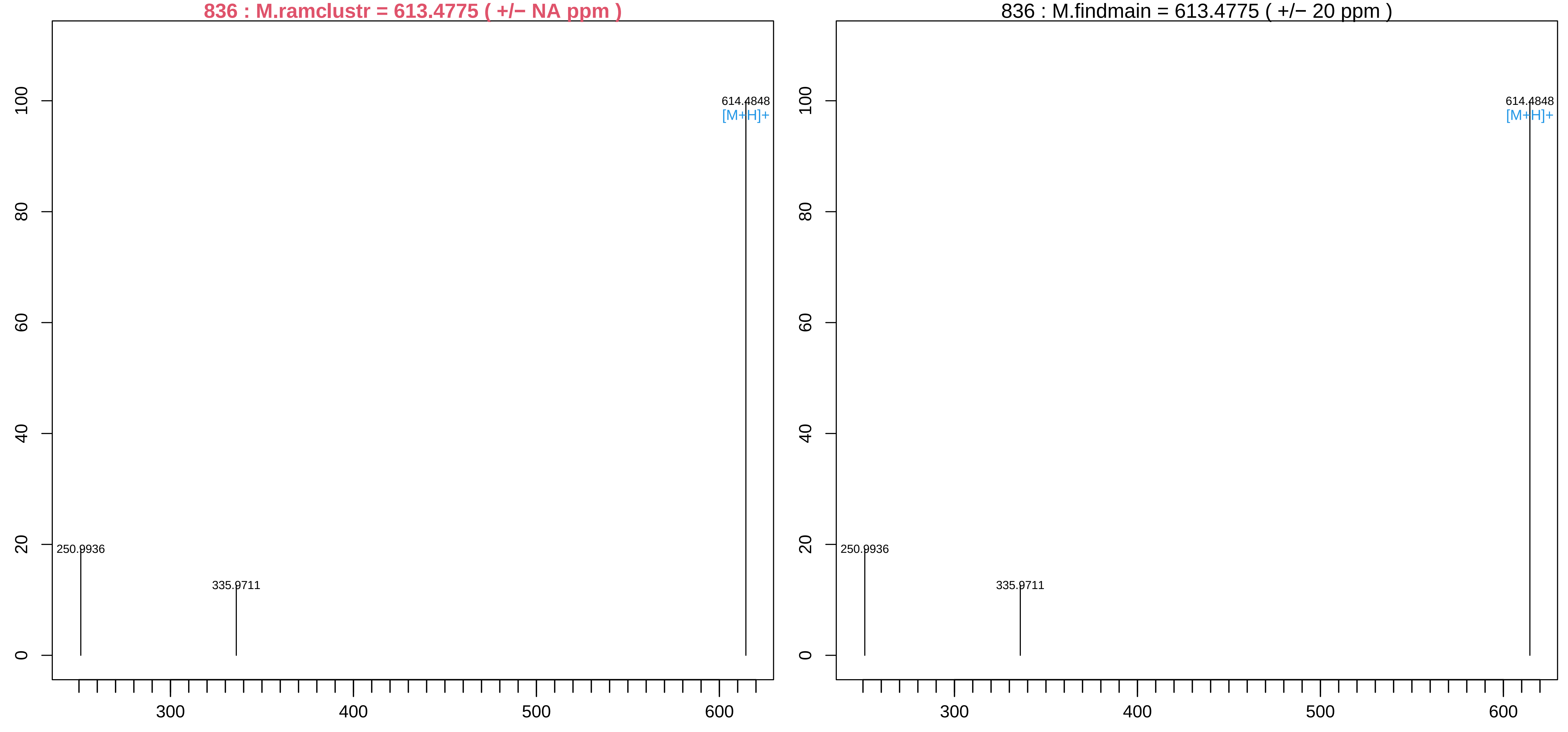

836 : M.ramclustr = 613.4775 (+/− NA ppm)
614.4848
[M+H]+
250.9936
335.9711
0
20
40
60
80
100
300
400
500
600
836 : M.findmain = 613.4775 (+/− 20 ppm)
614.4848
[M+H]+
250.9936
335.9711
0
20
40
60
80
100
300
400
500
600

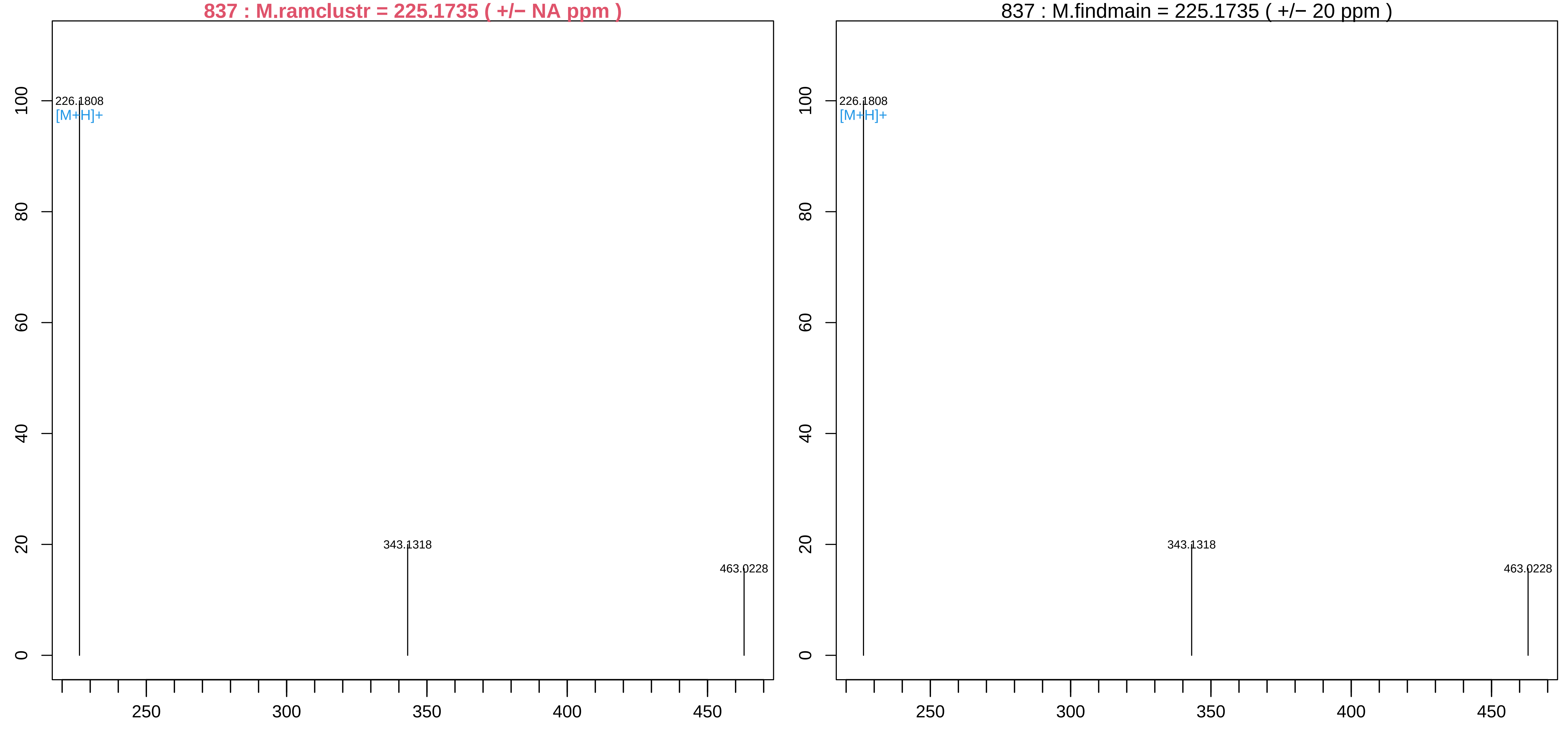
837 : M.ramclustr = 225.1735 (+/− NA ppm)
837 : M.findmain = 225.1735 (+/− 20 ppm)
226.1808
[M+H]+
343.1318
463.0228
226.1808
[M+H]+
343.1318
463.0228
0
20
40
60
80
100
250
300
350
400
450

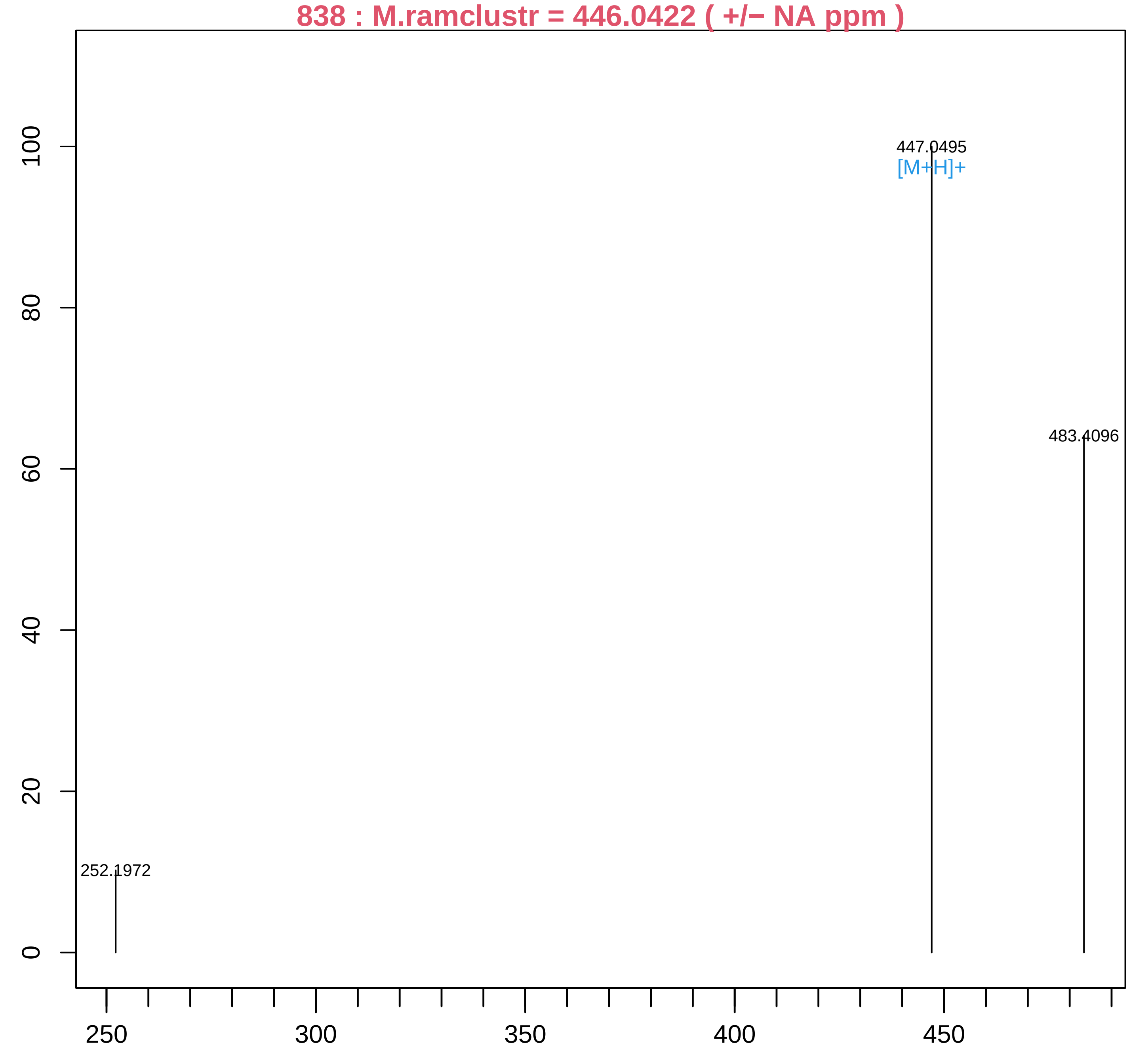
838 : M.ramclustr = 446.0422 (+/− NA ppm)
447.0495
[M+H]+
483.4096
252.1972
0
20
40
60
80
100
250
300
350
400
450

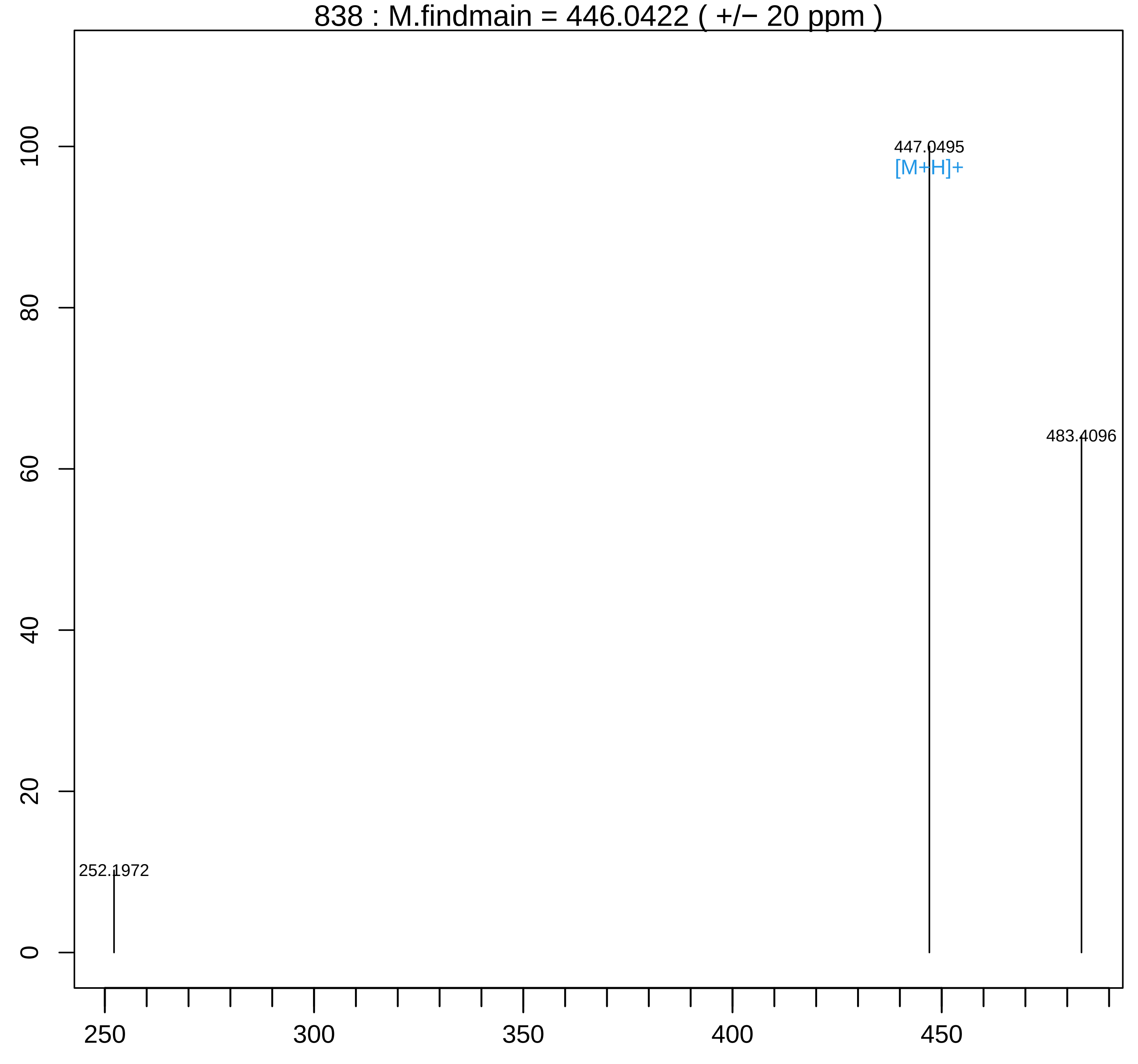
838 : M.findmain = 446.0422 (+/− 20 ppm)
447.0495
[M+H]+
483.4096
252.1972
0
20
40
60
80
100
250
300
350
400
450

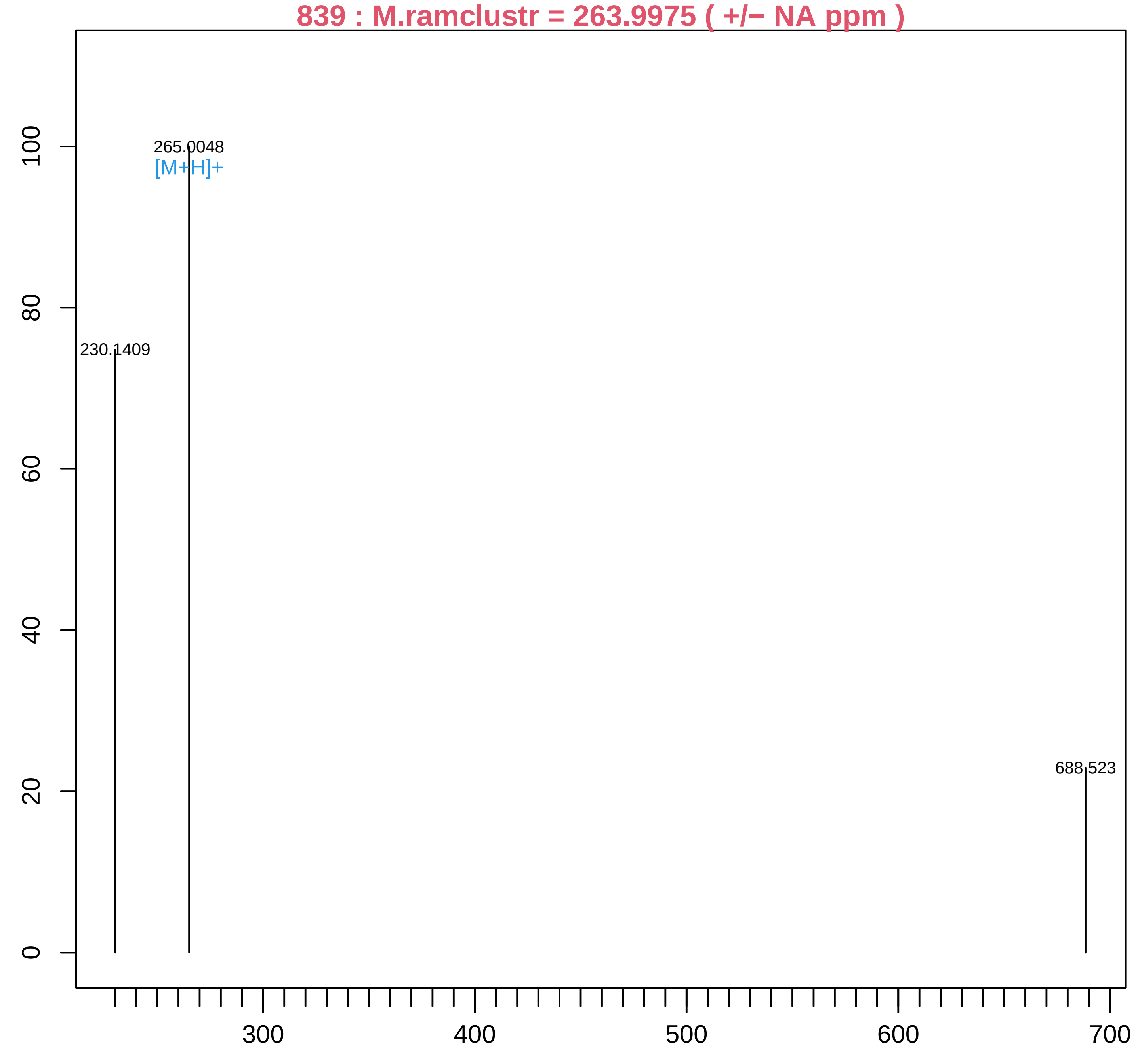
839 : M.ramclustr = 263.9975 (+/− NA ppm)
265.0048
[M+H]+
230.1409
688.523
0
20
40
60
80
100
300
400
500
600
700
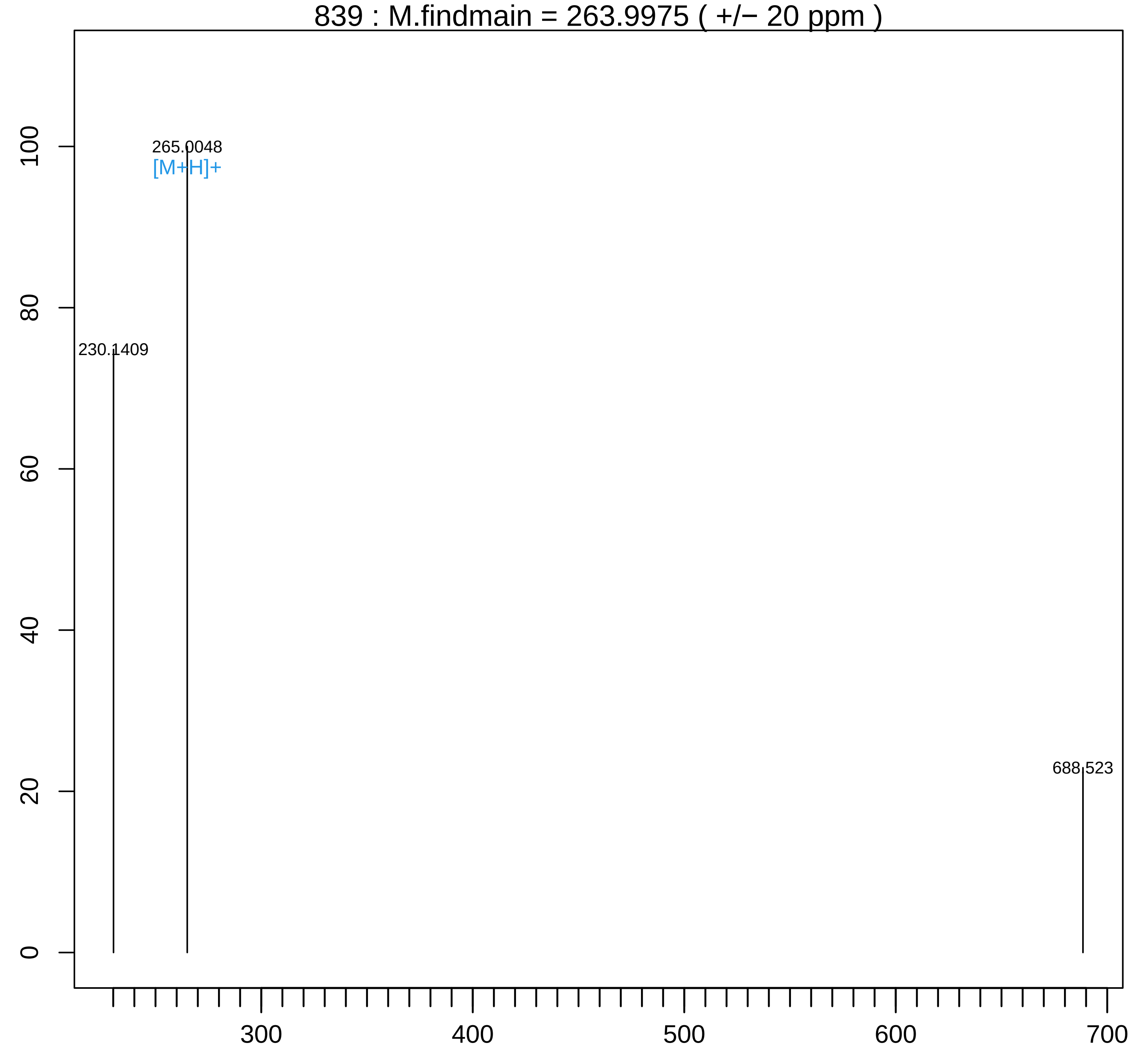
839 : M.findmain = 263.9975 (+/− 20 ppm)
265.0048
[M+H]+
230.1409
688.523
0
20
40
60
80
100
300
400
500
600
700

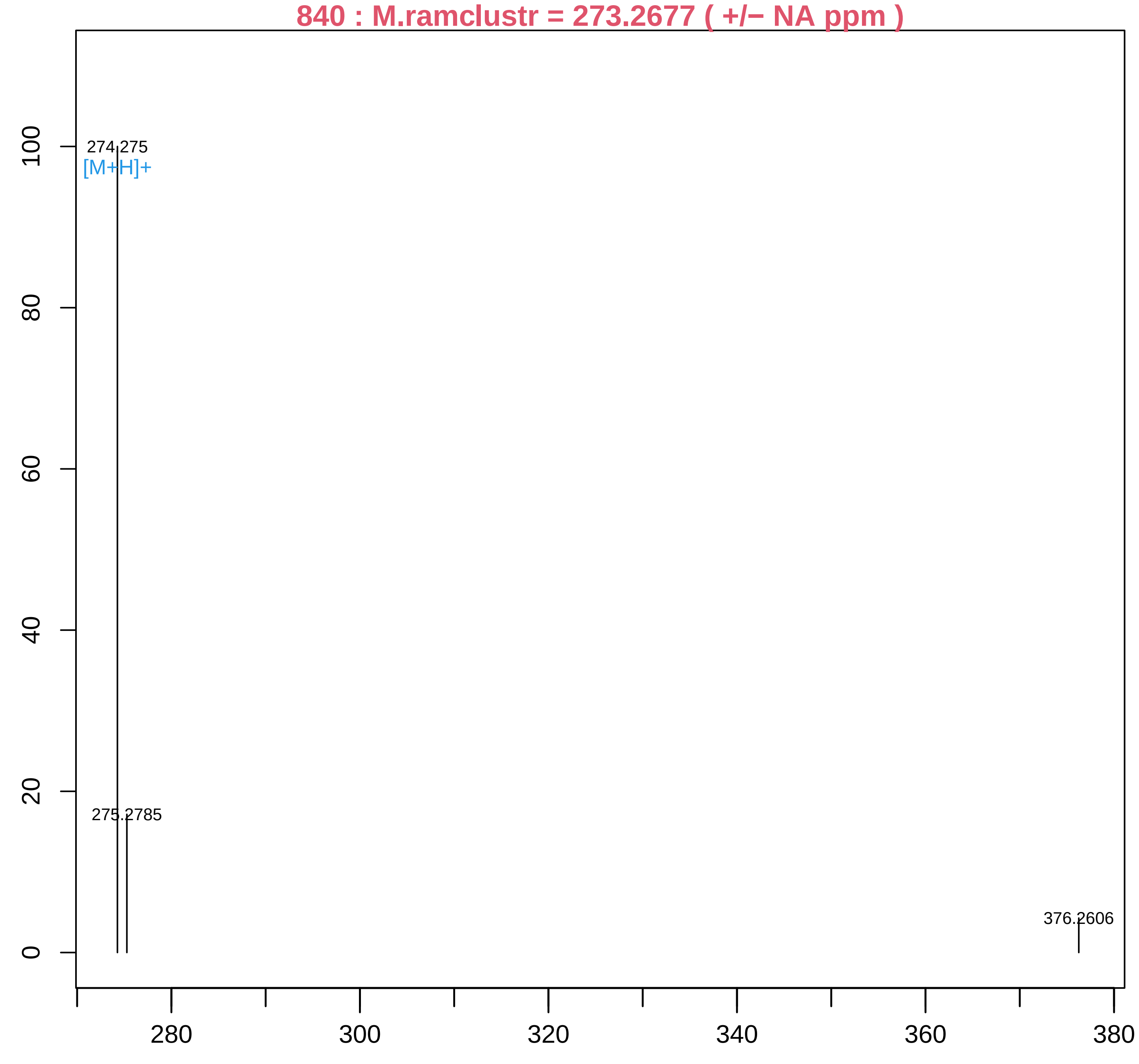
840 : M.ramclustr = 273.2677 (+/− NA ppm)
274.275
[M+H]+
275.2785
376.2606
0
20
40
60
80
100
280
300
320
340
360
380

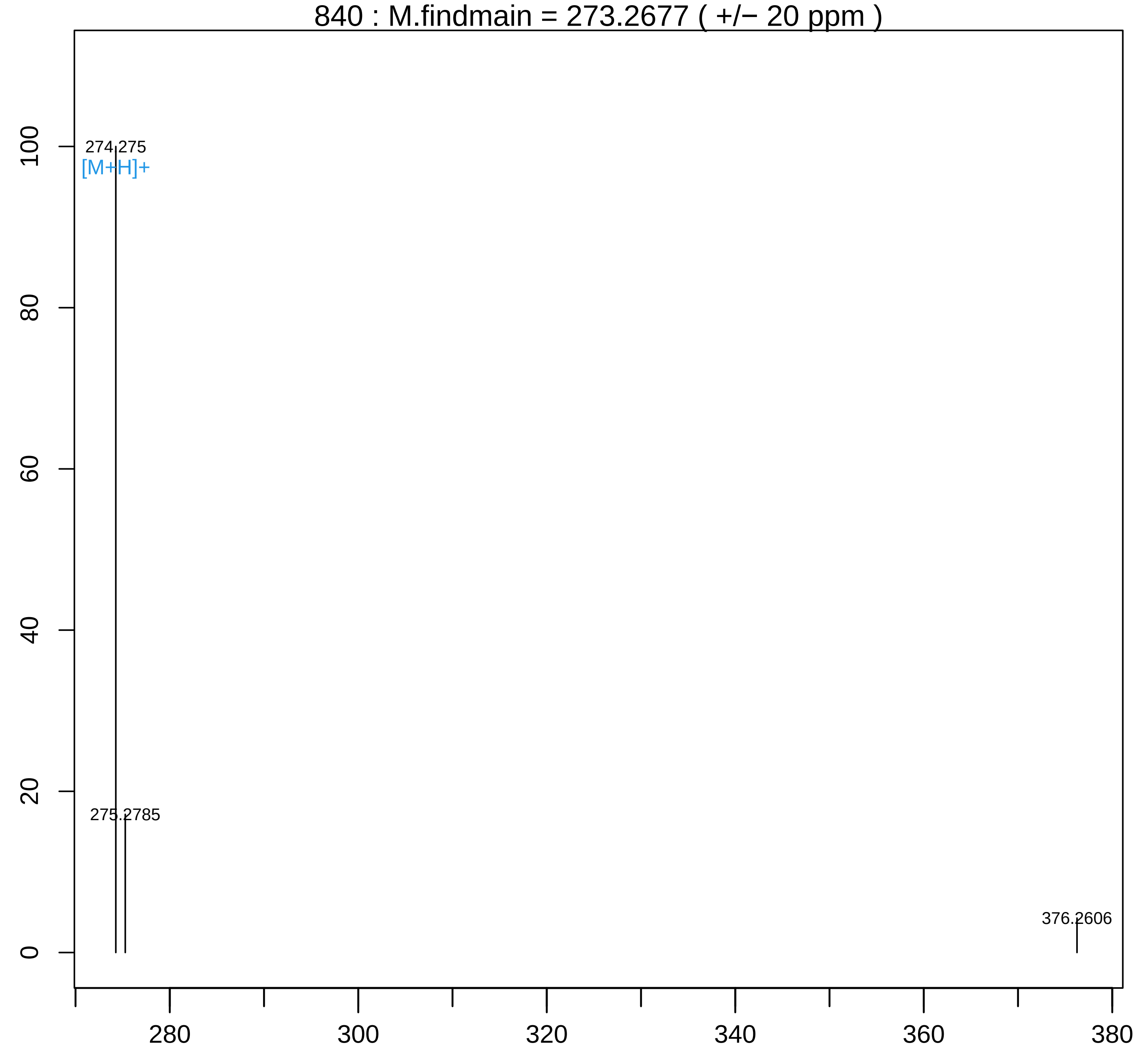
840 : M.findmain = 273.2677 (+/− 20 ppm)
274.275
[M+H]+
275.2785
376.2606
0
20
40
60
80
100
280
300
320
340
360
380

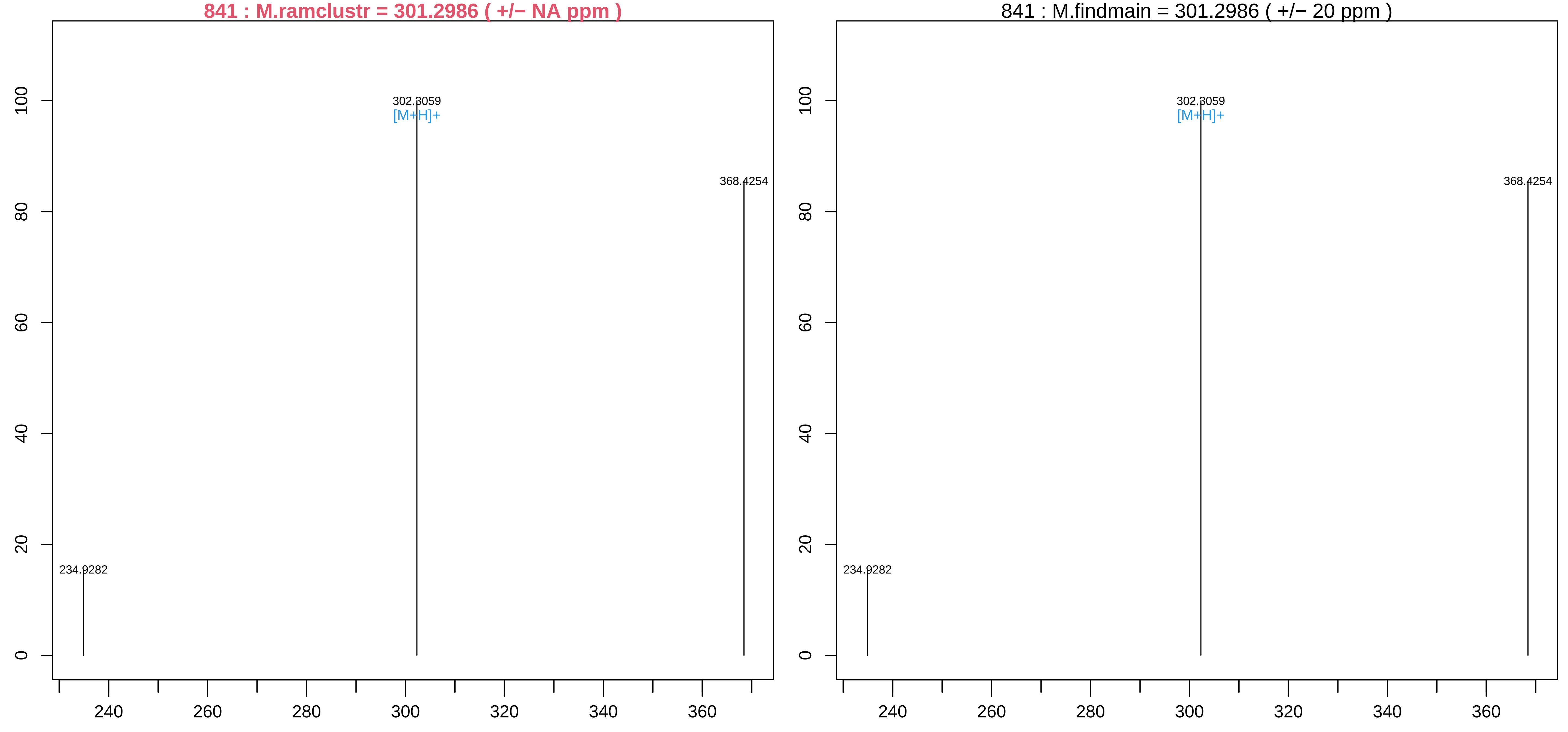
841 : M.ramclustr = 301.2986 (+/− NA ppm)
302.3059
[M+H]+
368.4254
234.9282
841 : M.findmain = 301.2986 (+/− 20 ppm)
302.3059
[M+H]+
368.4254
234.9282
0
20
40
60
80
100
240
260
280
300
320
340
360

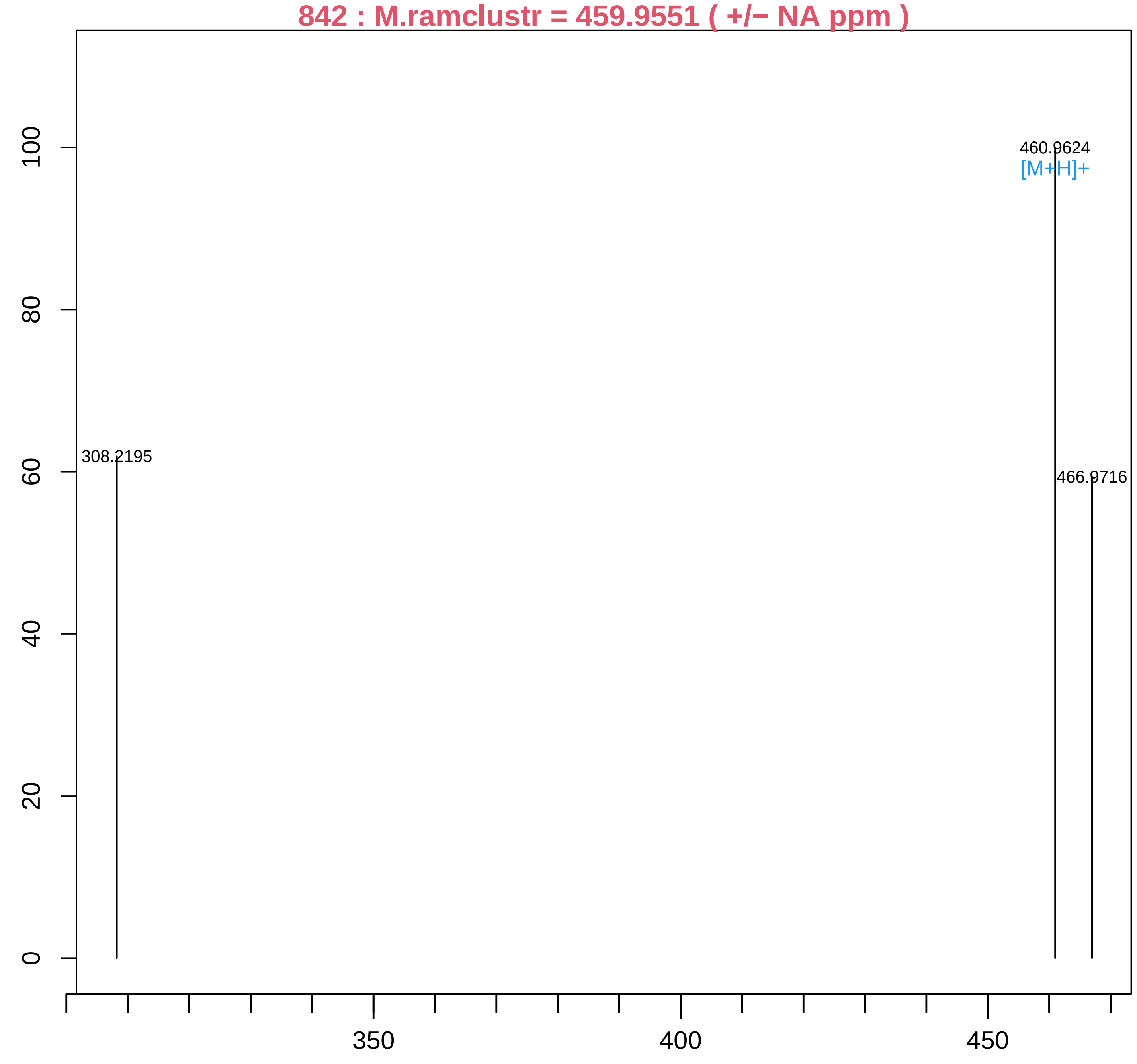
842 : M.ramclustr = 459.9551 (+/− NA ppm)
100
80
60
40
20
0
460.9624
[M+H]+
308.2195
466.9716
350
400
450

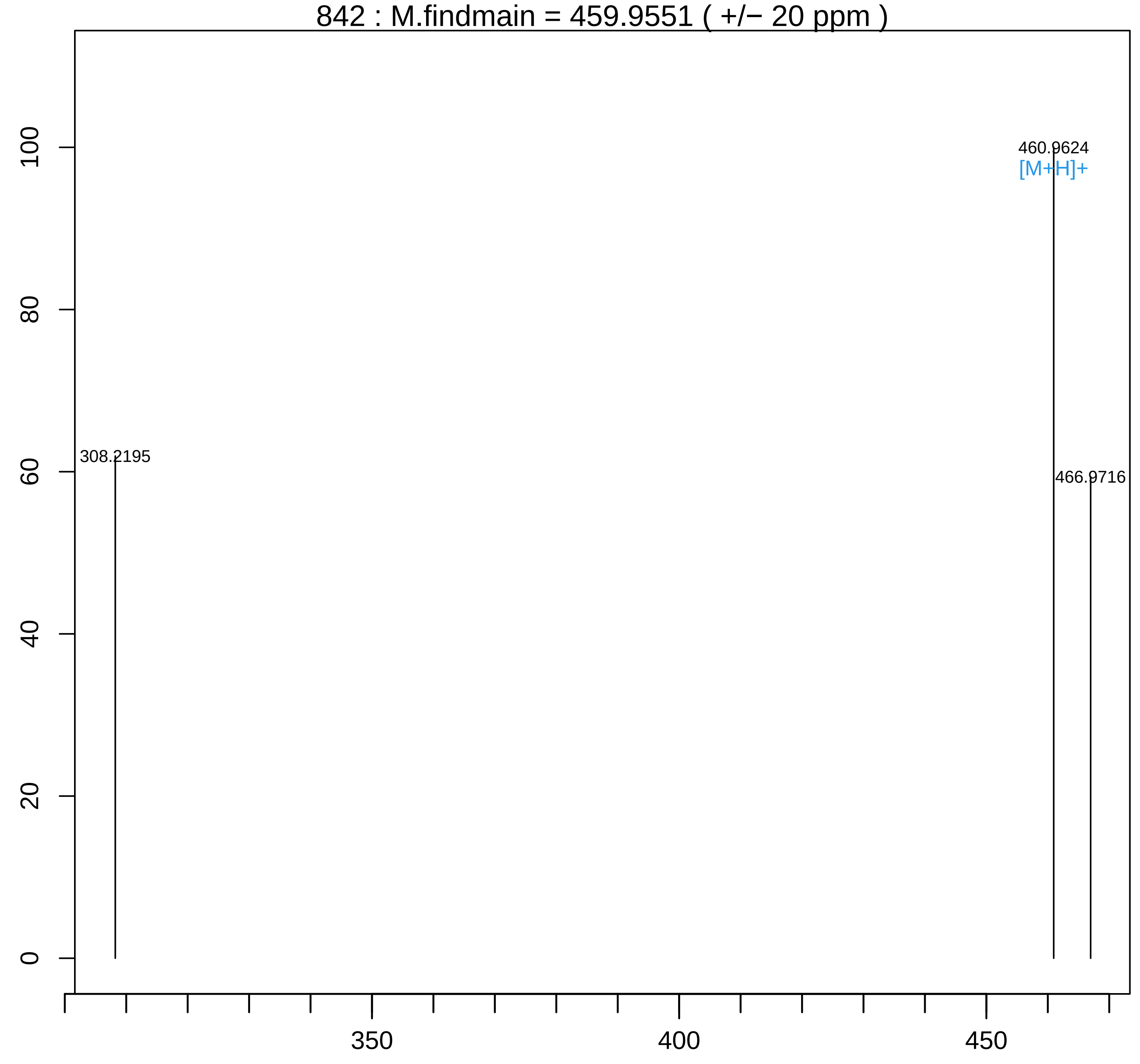
842 : M.findmain = 459.9551 (+/− 20 ppm)
100
80
60
40
20
0
460.9624
[M+H]+
308.2195
466.9716
350
400
450

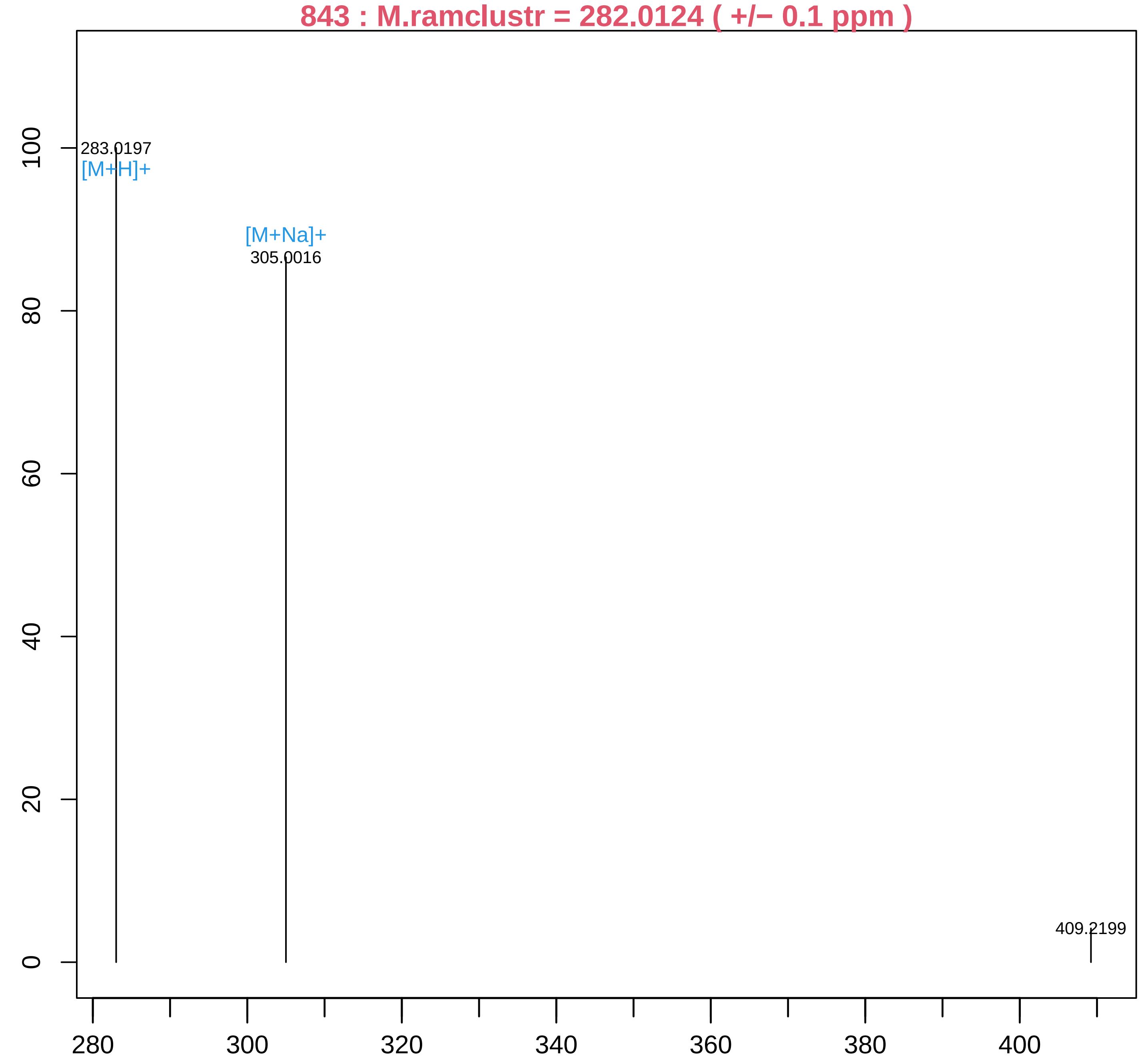
843 : M.ramclustr = 282.0124 (+/− 0.1 ppm)
283.0197
[M+H]+
[M+Na]+
305.0016
409.2199
0
20
40
60
80
100
280
300
320
340
360
380
400

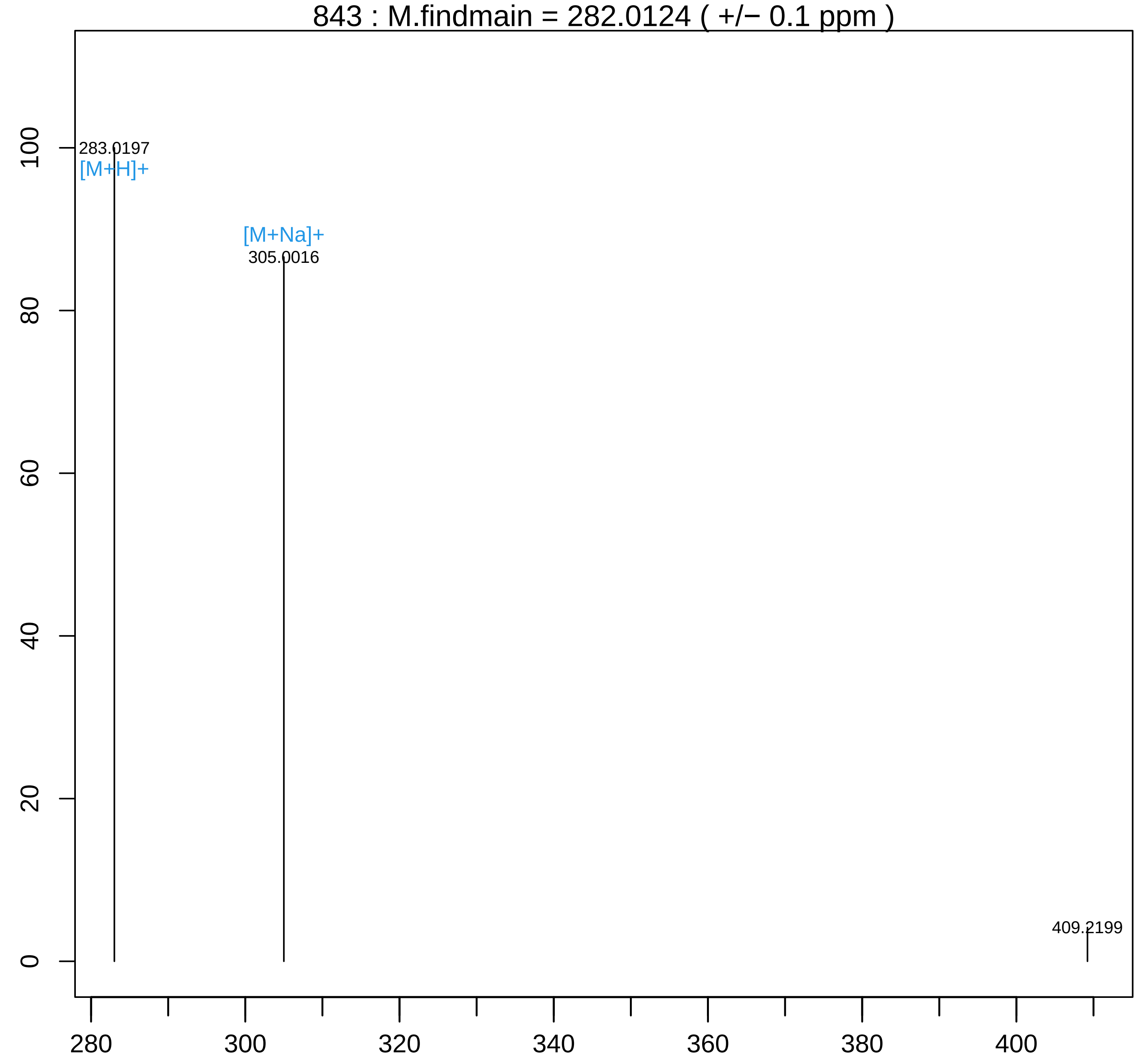
843 : M.findmain = 282.0124 (+/− 0.1 ppm)
283.0197
[M+H]+
[M+Na]+
305.0016
409.2199
0
20
40
60
80
100
280
300
320
340
360
380
400

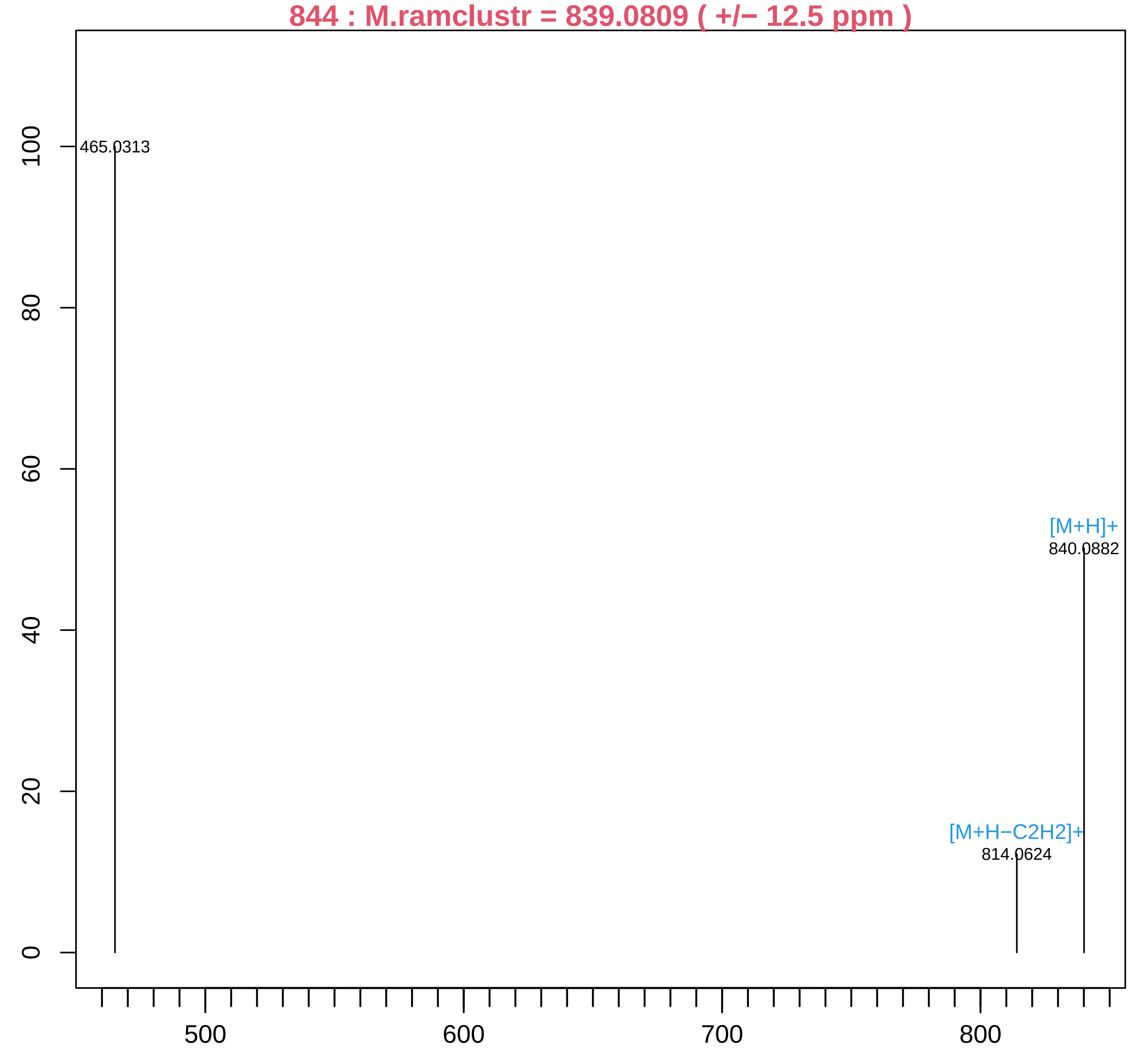
844 : M.ramclustr = 839.0809 (+/− 12.5 ppm)
465.0313
[M+H]+
840.0882
[M+H−C2H2]+
814.0624
0
20
40
60
80
100
500
600
700
800

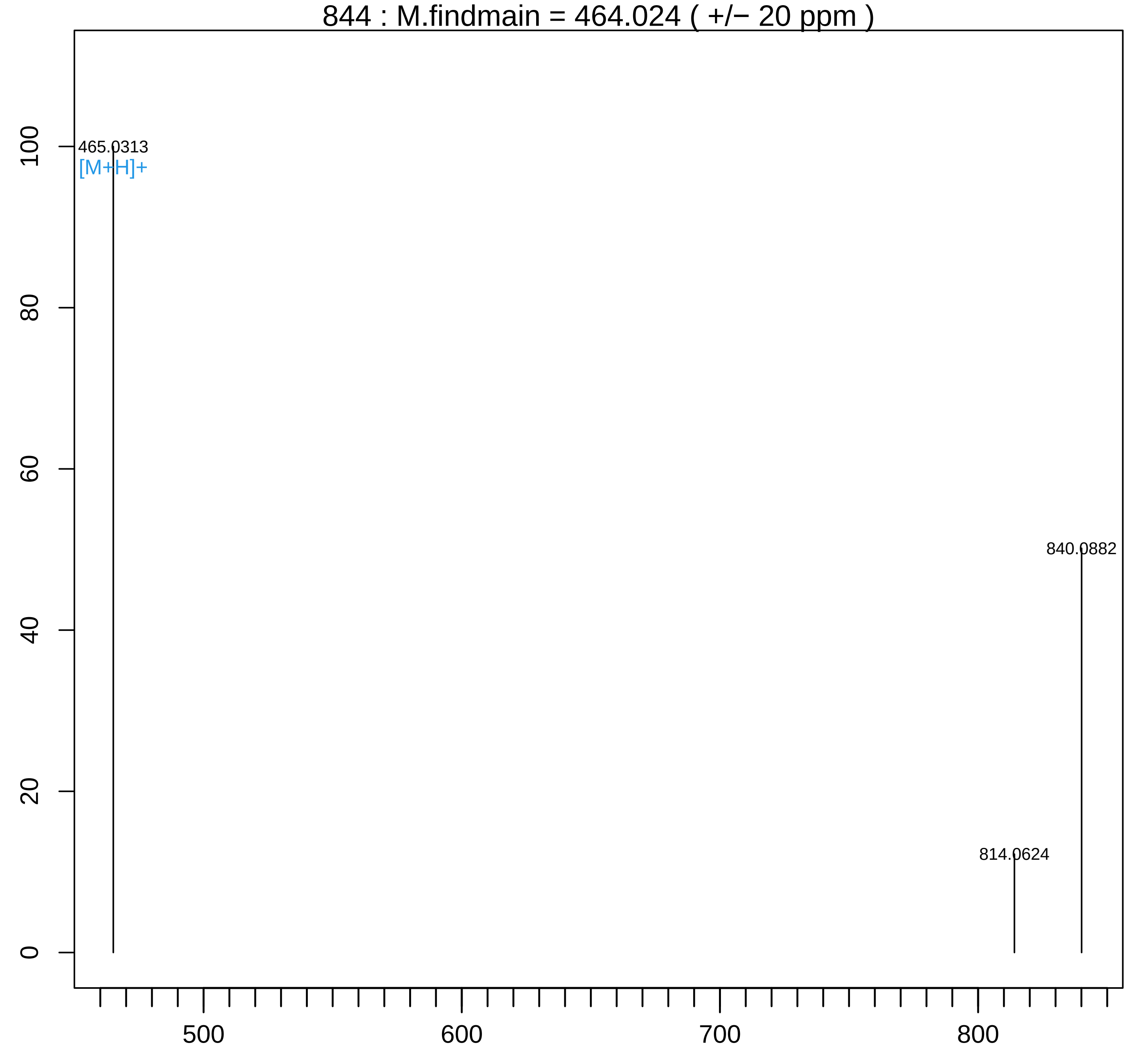
844 : M.findmain = 464.024 (+/− 20 ppm)
465.0313
[M+H]+
840.0882
814.0624
0
20
40
60
80
100
500
600
700
800

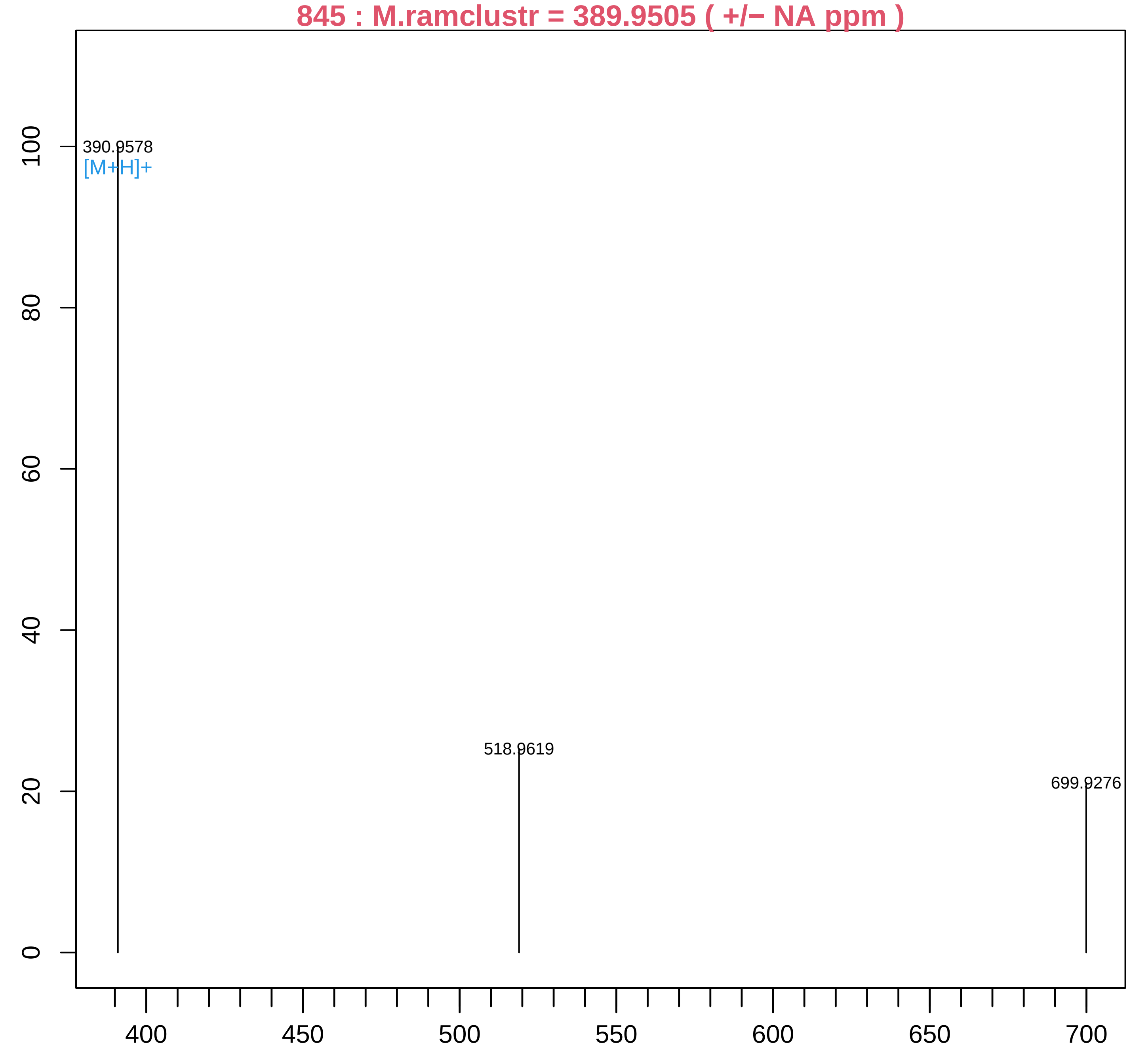
845 : M.ramclustr = 389.9505 (+/− NA ppm)
390.9578
[M+H]+
518.9619
699.9276
0
20
40
60
80
100
400
450
500
550
600
650
700

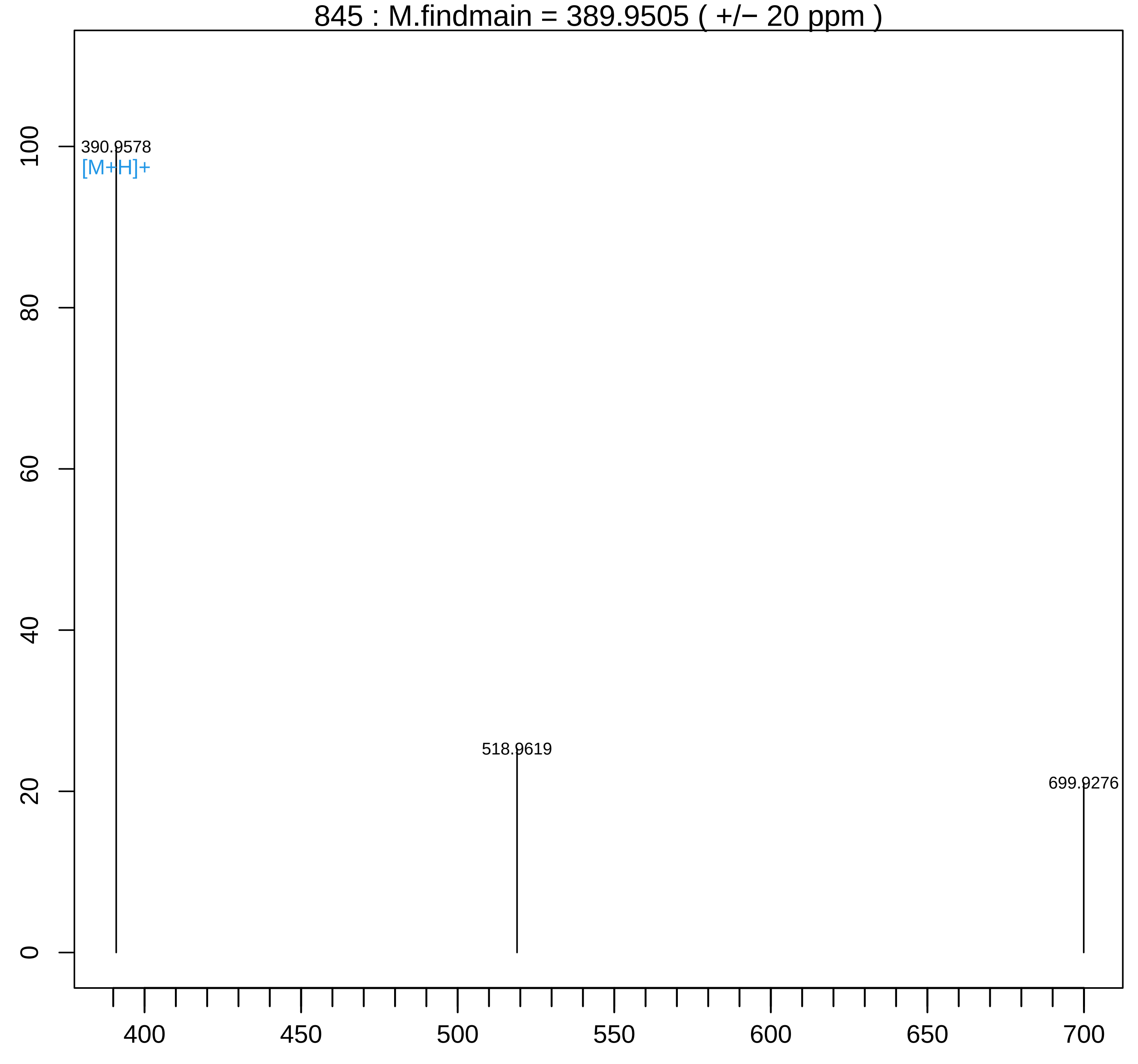
845 : M.findmain = 389.9505 (+/− 20 ppm)
390.9578
[M+H]+
518.9619
699.9276
0
20
40
60
80
100
400
450
500
550
600
650
700

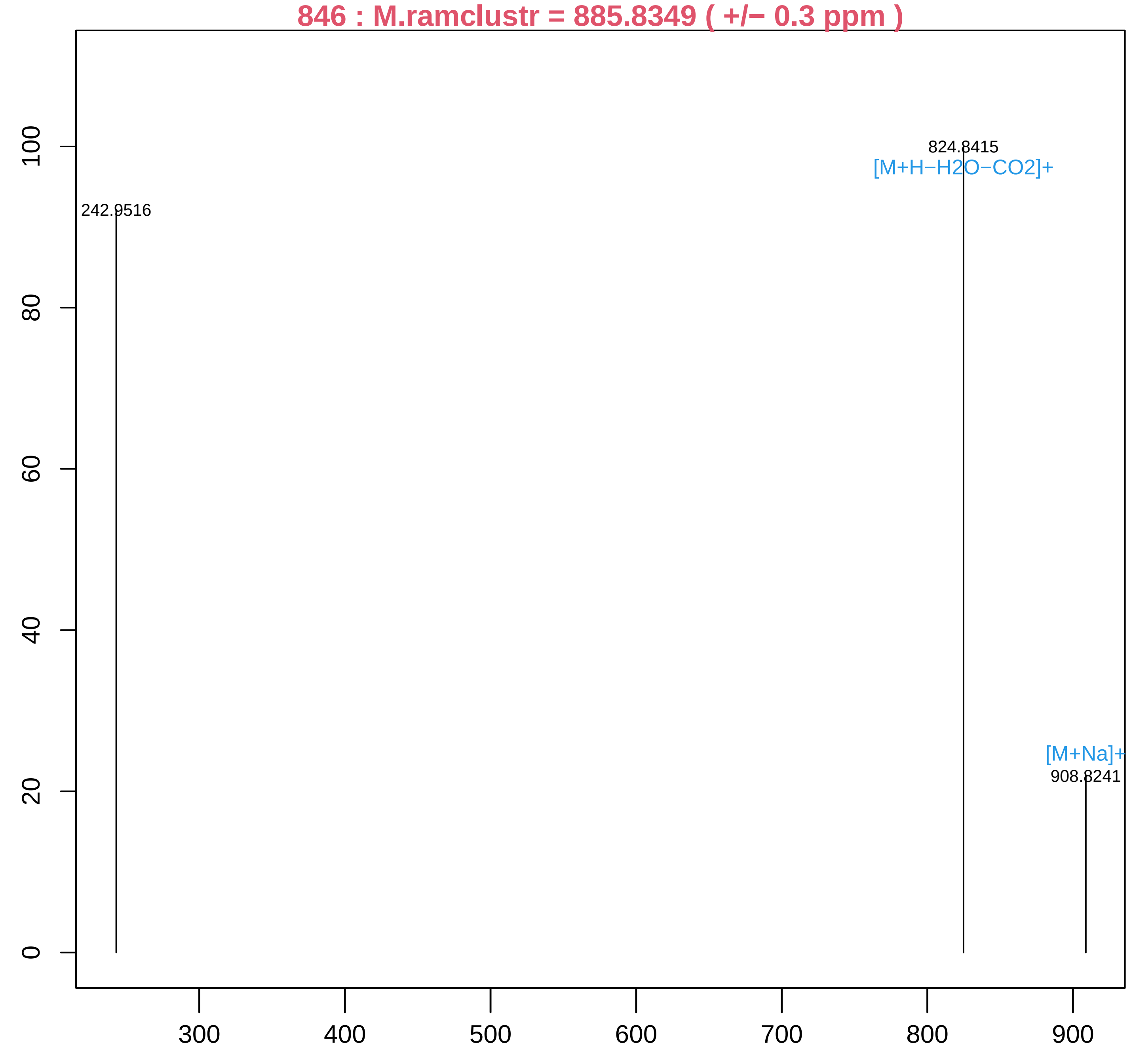
846 : M.ramclustr = 885.8349 (+/− 0.3 ppm)
824.8415
[M+H−H2O−CO2]+
242.9516
[M+Na]+
908.8241
0
20
40
60
80
100
300
400
500
600
700
800
900

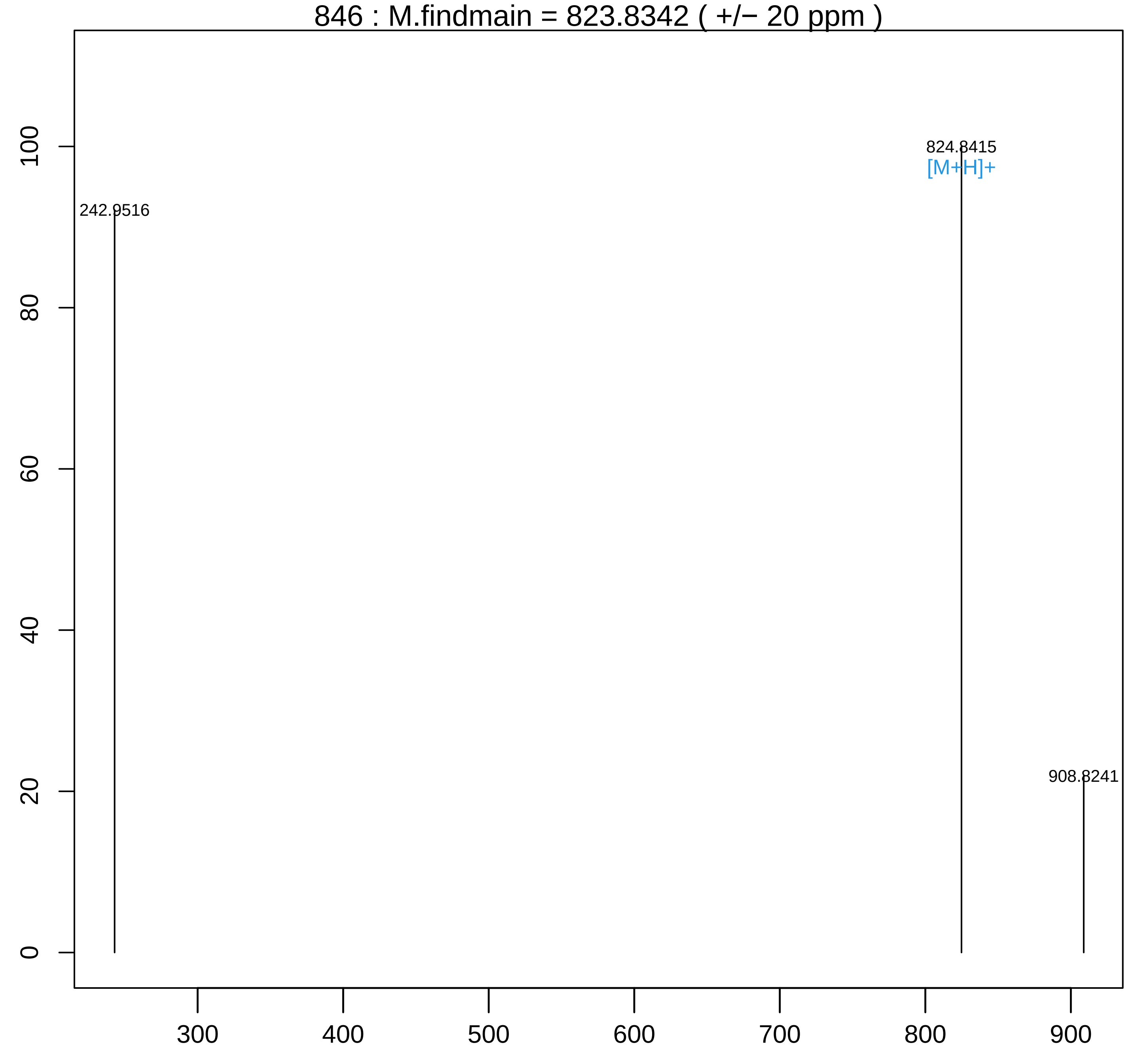
846 : M.findmain = 823.8342 (+/− 20 ppm)
824.8415
[M+H]+
242.9516
908.8241
0
20
40
60
80
100
300
400
500
600
700
800
900

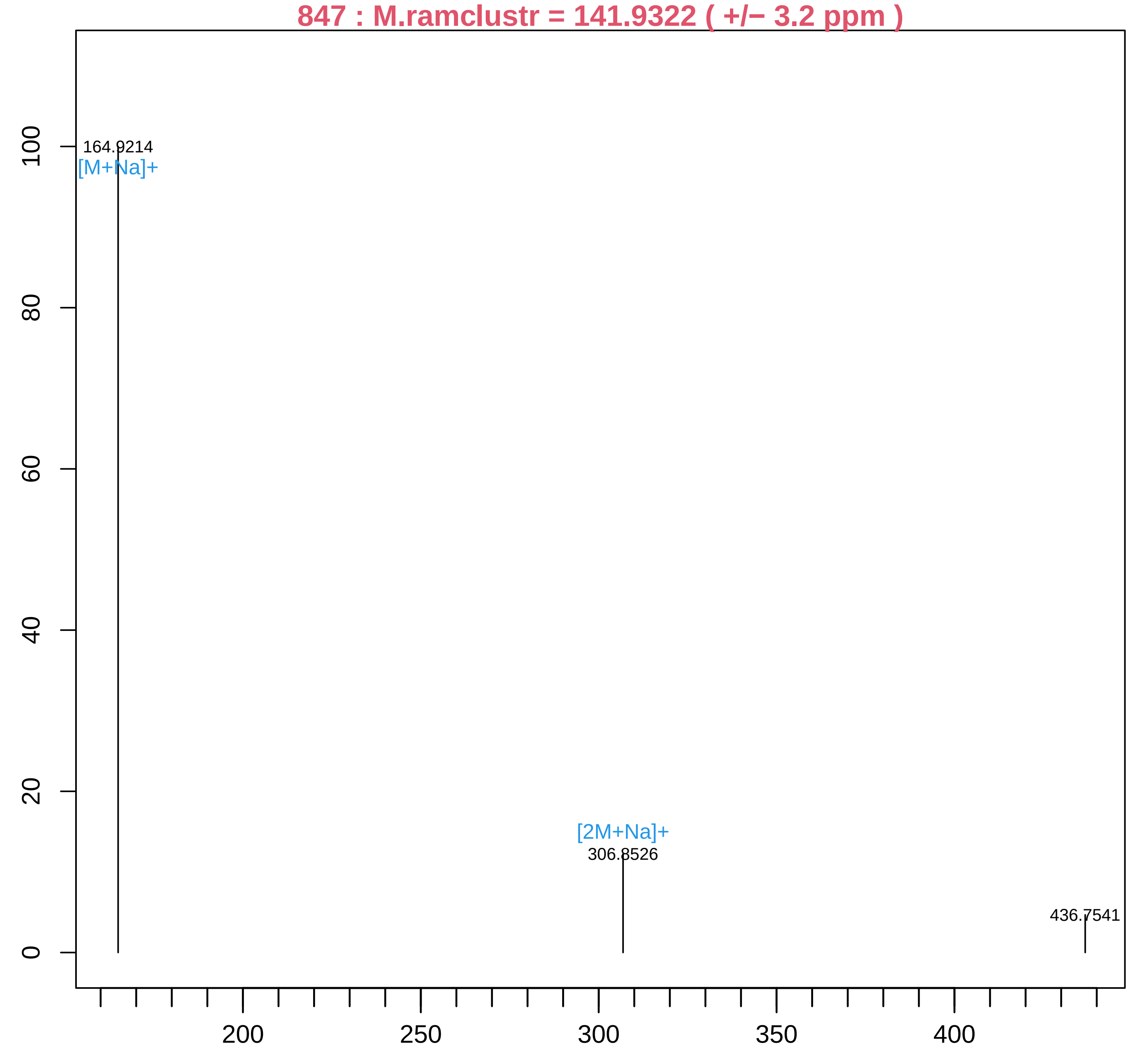
847 : M.ramclustr = 141.9322 (+/− 3.2 ppm)
164.9214
[M+Na]+
[2M+Na]+
306.8526
436.7541
0
20
40
60
80
100
200
250
300
350
400

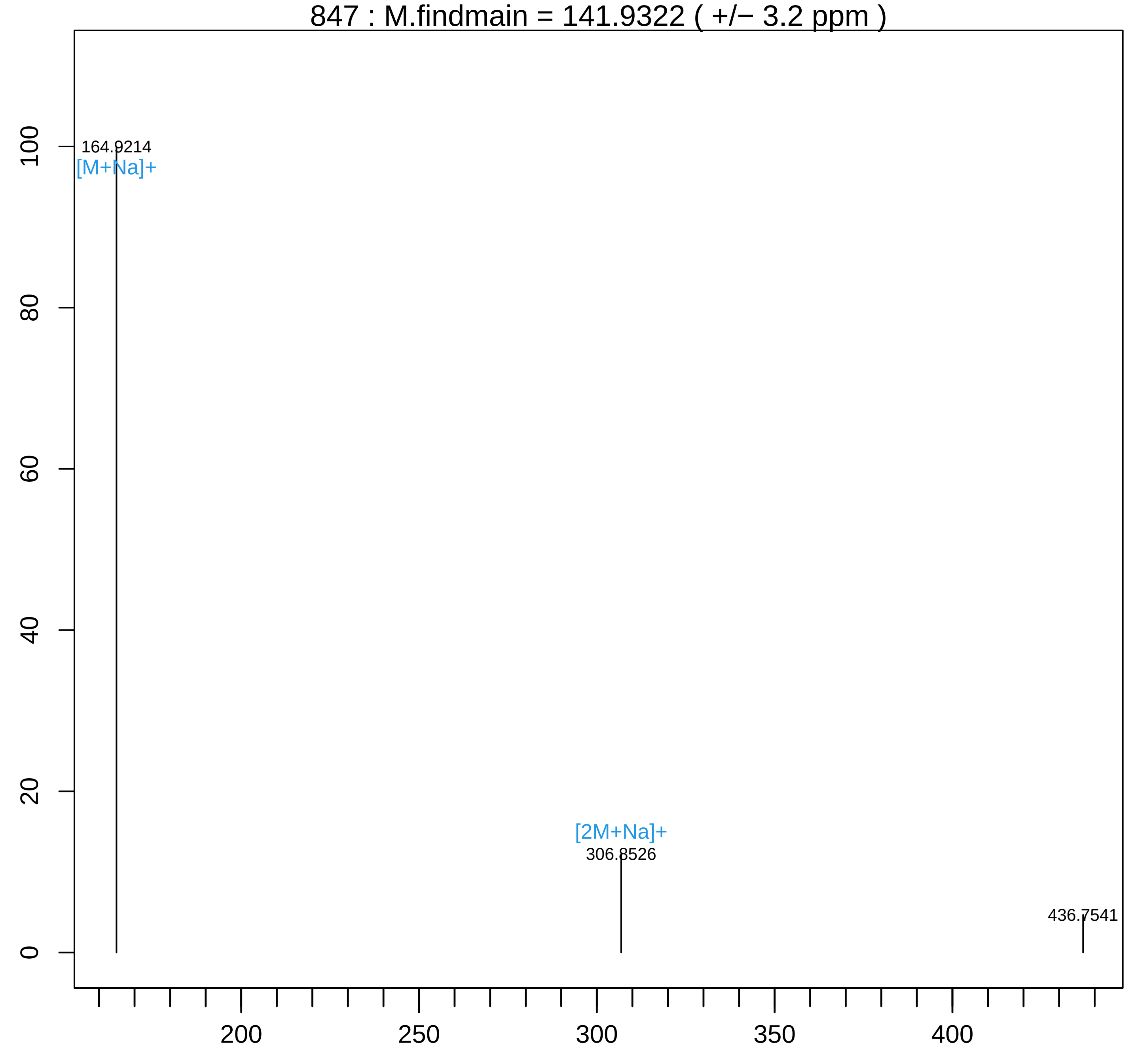
847 : M.findmain = 141.9322 (+/− 3.2 ppm)
164.9214
[M+Na]+
[2M+Na]+
306.8526
436.7541
0
20
40
60
80
100
200
250
300
350
400

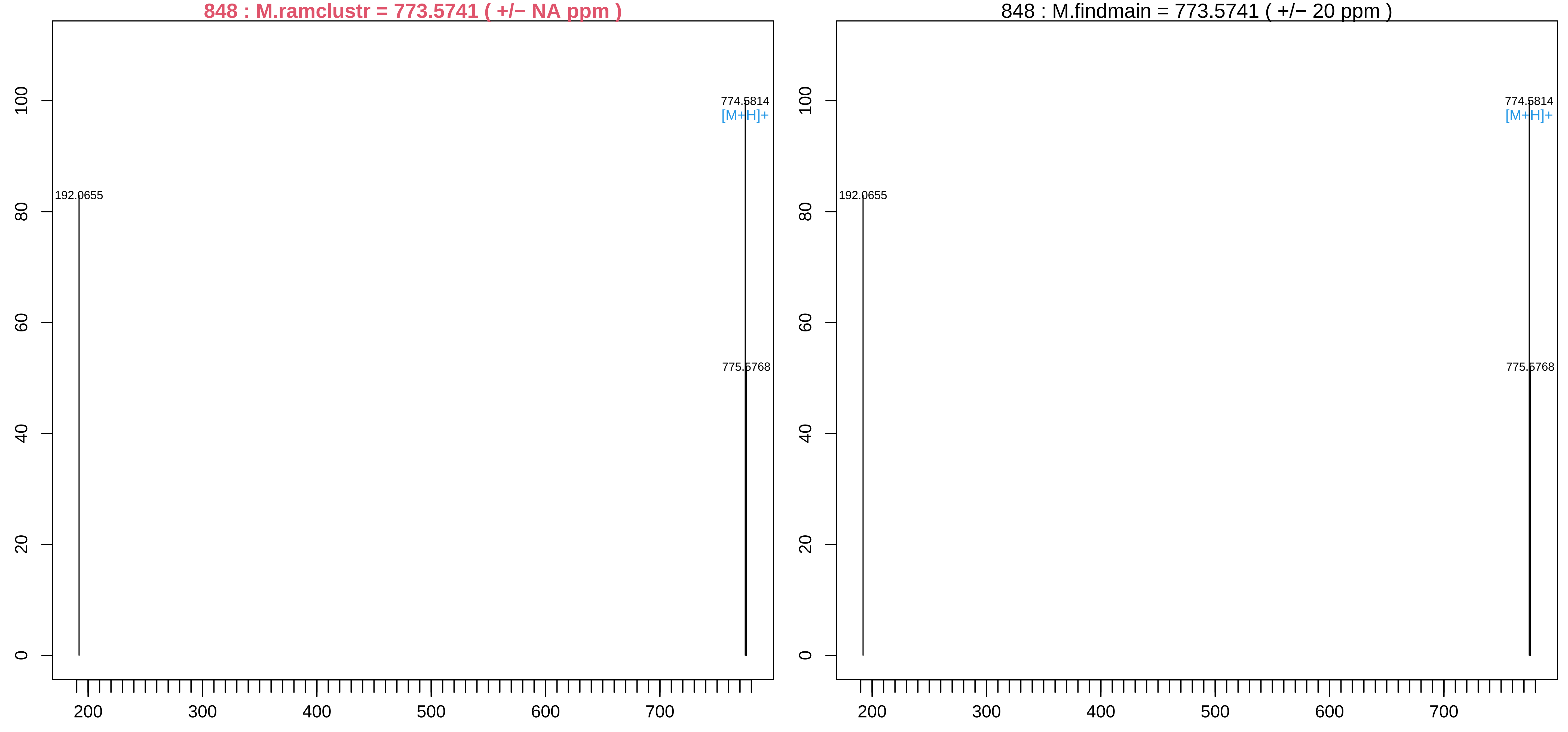

848 : M.ramclustr = 773.5741 (+/− NA ppm)
848 : M.findmain = 773.5741 (+/− 20 ppm)
774.5814
[M+H]+
192.0655
775.5768
0
20
40
60
80
100
200
300
400
500
600
700

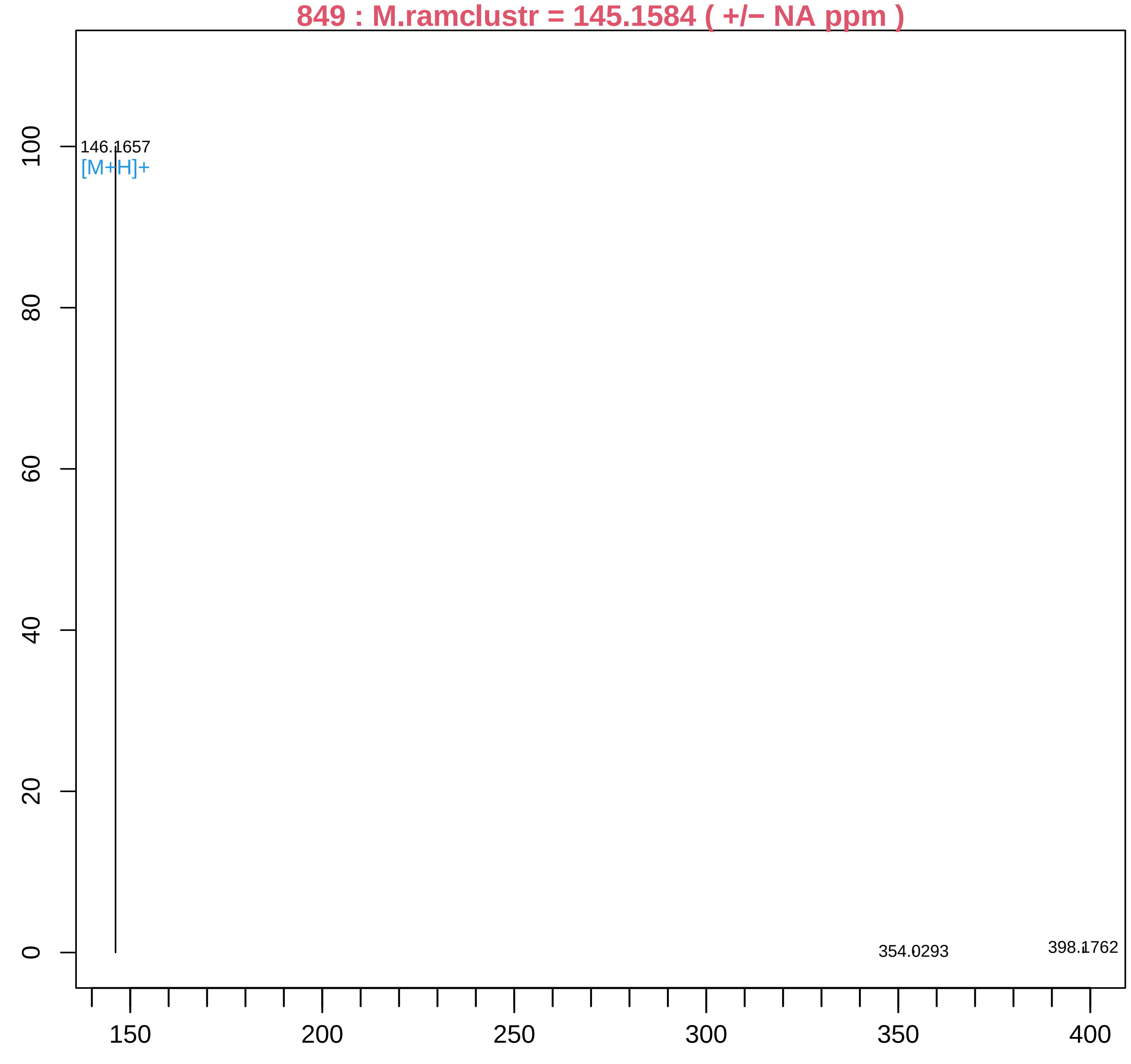
849 : M.ramclustr = 145.1584 (+/− NA ppm)
146.1657
[M+H]+
354.0293
398.1762
0
20
40
60
80
100
150
200
250
300
350
400
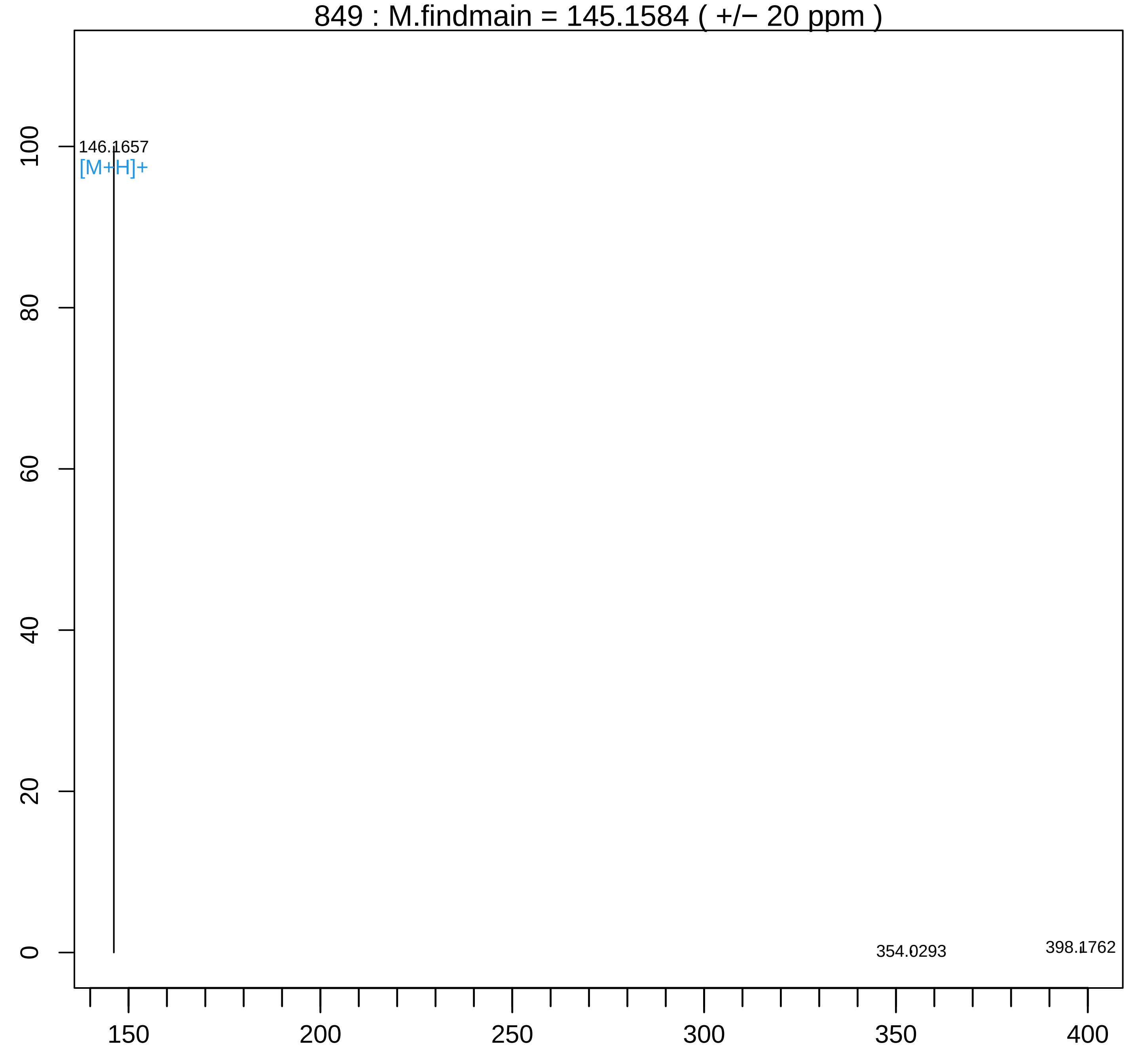
849 : M.findmain = 145.1584 (+/− 20 ppm)
146.1657
[M+H]+
354.0293
398.1762
0
20
40
60
80
100
150
200
250
300
350
400

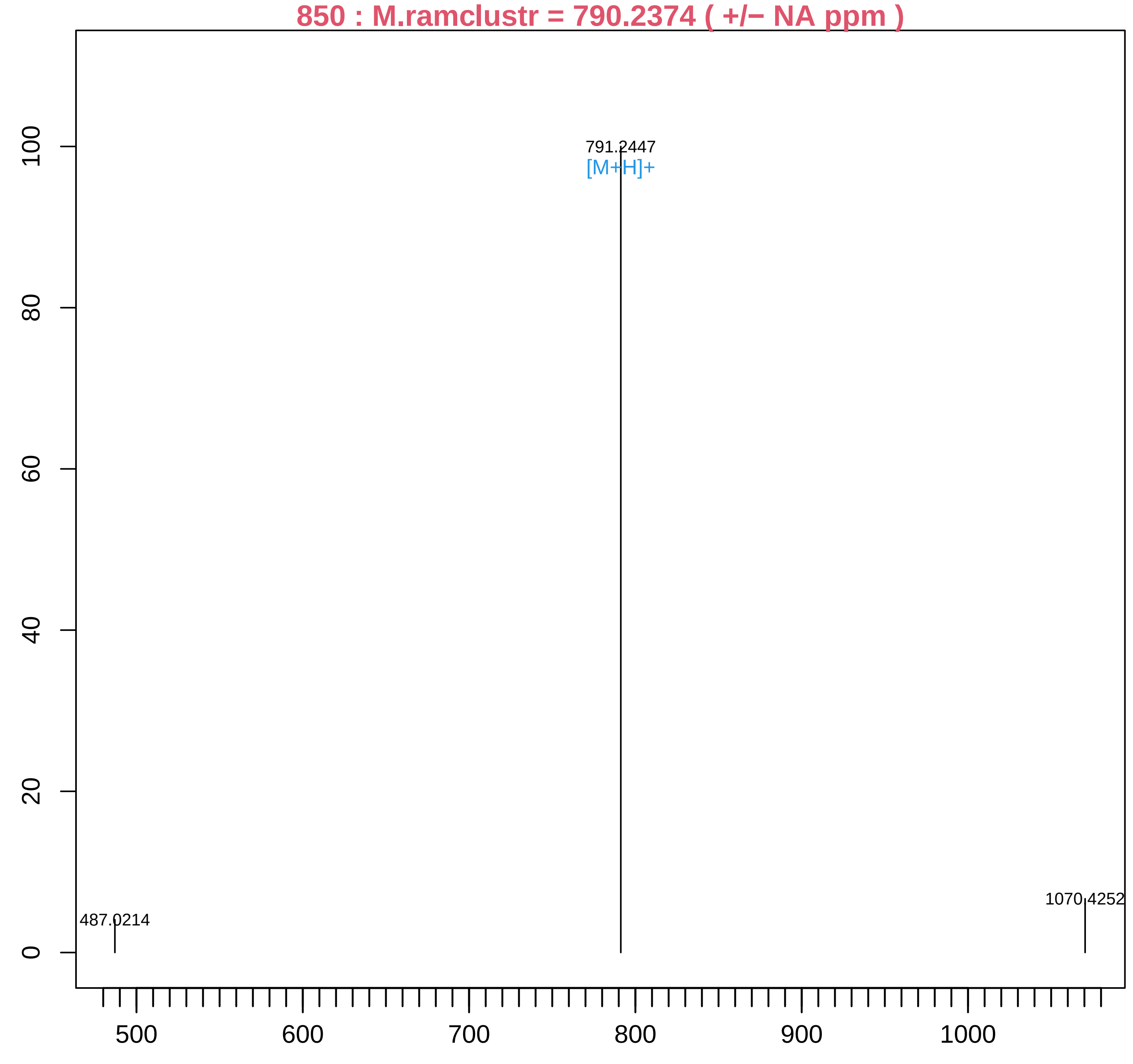
850 : M.ramclustr = 790.2374 (+/− NA ppm)
791.2447
[M+H]+
487.0214
1070.4252
0
20
40
60
80
100
500
600
700
800
900
1000

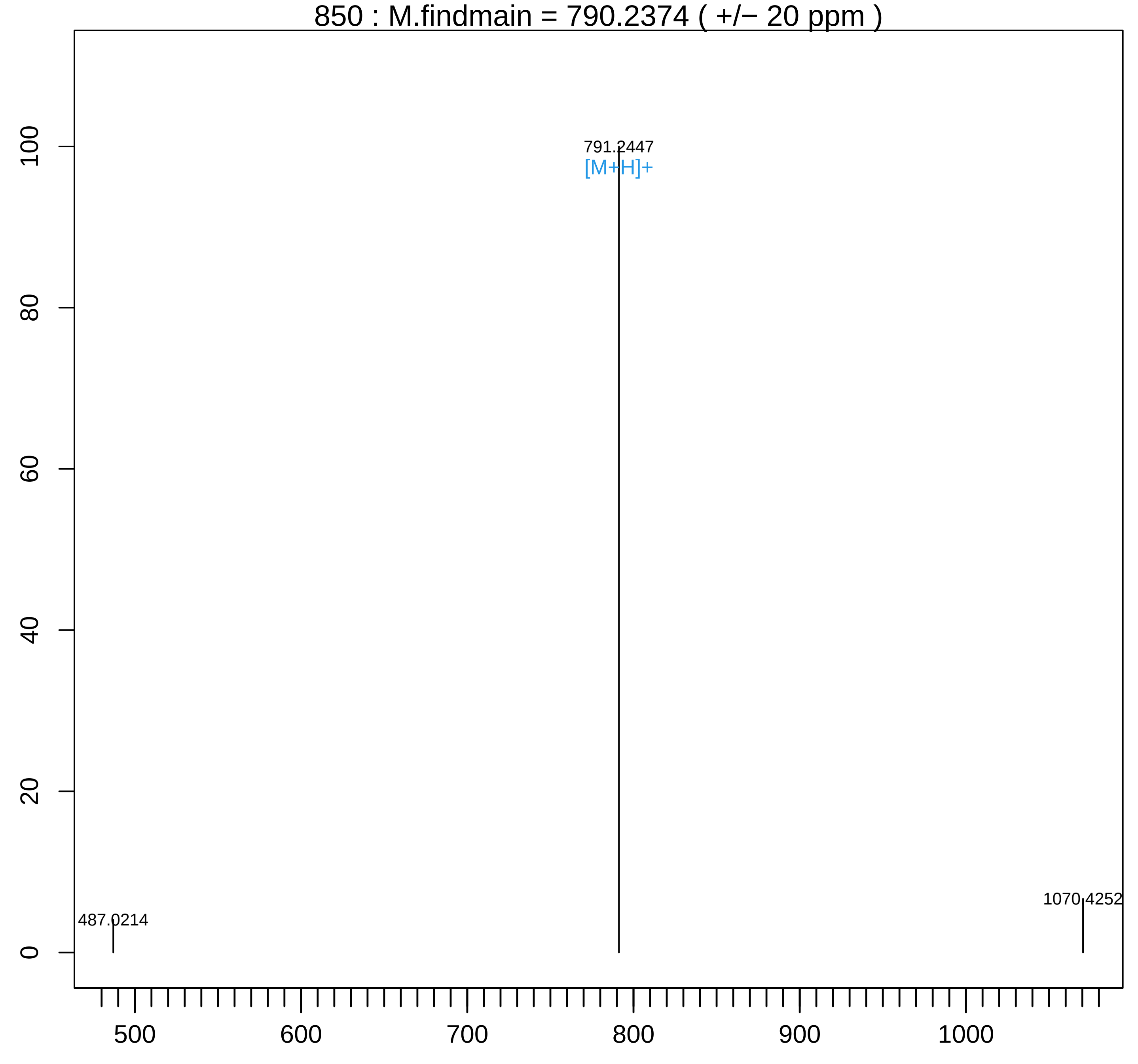
850 : M.findmain = 790.2374 (+/− 20 ppm)
791.2447
[M+H]+
487.0214
1070.4252
0
20
40
60
80
100
500
600
700
800
900
1000

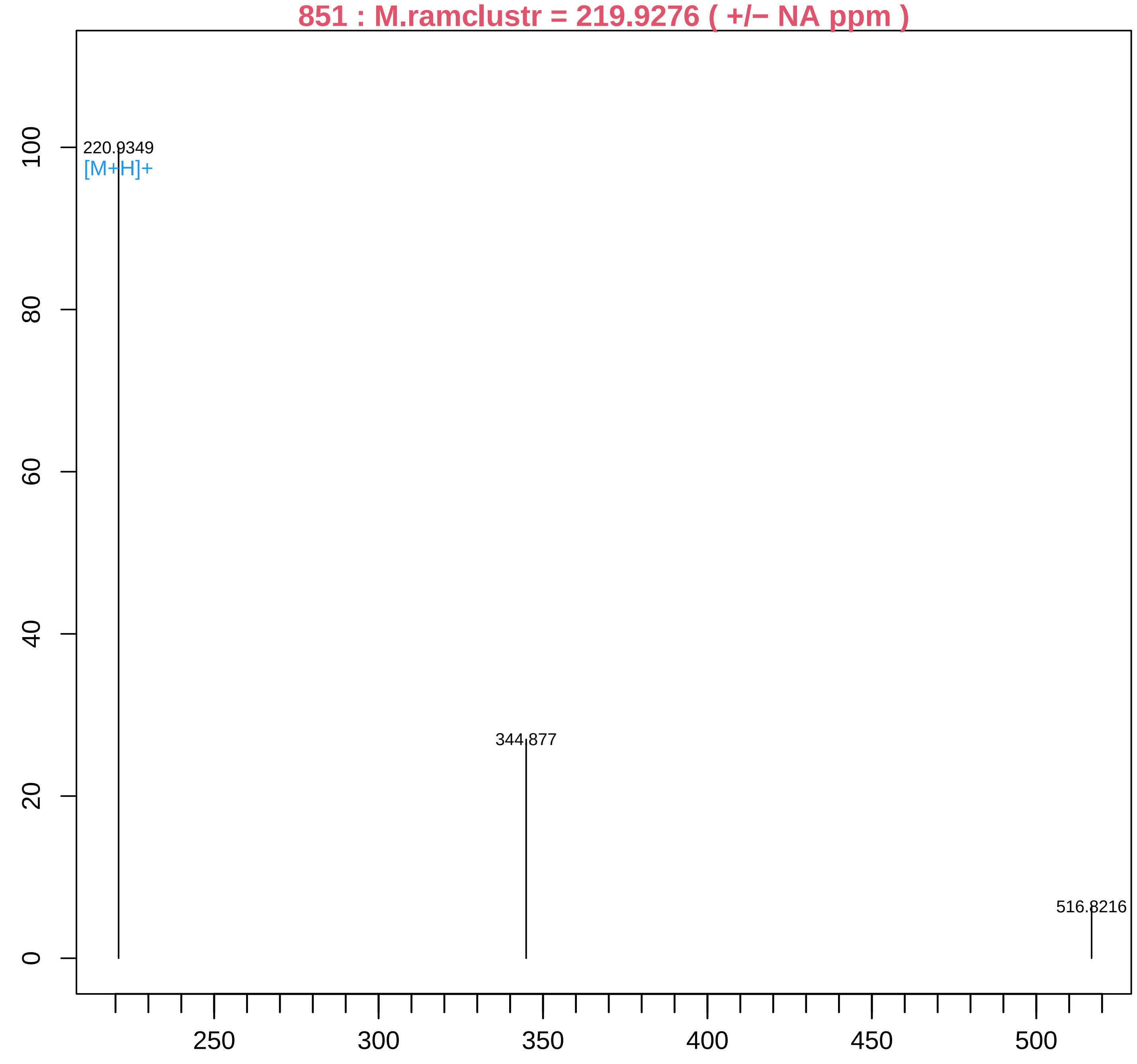
851 : M.ramclustr = 219.9276 (+/− NA ppm)
220.9349
[M+H]+
344.877
516.8216
0
20
40
60
80
100
250
300
350
400
450
500

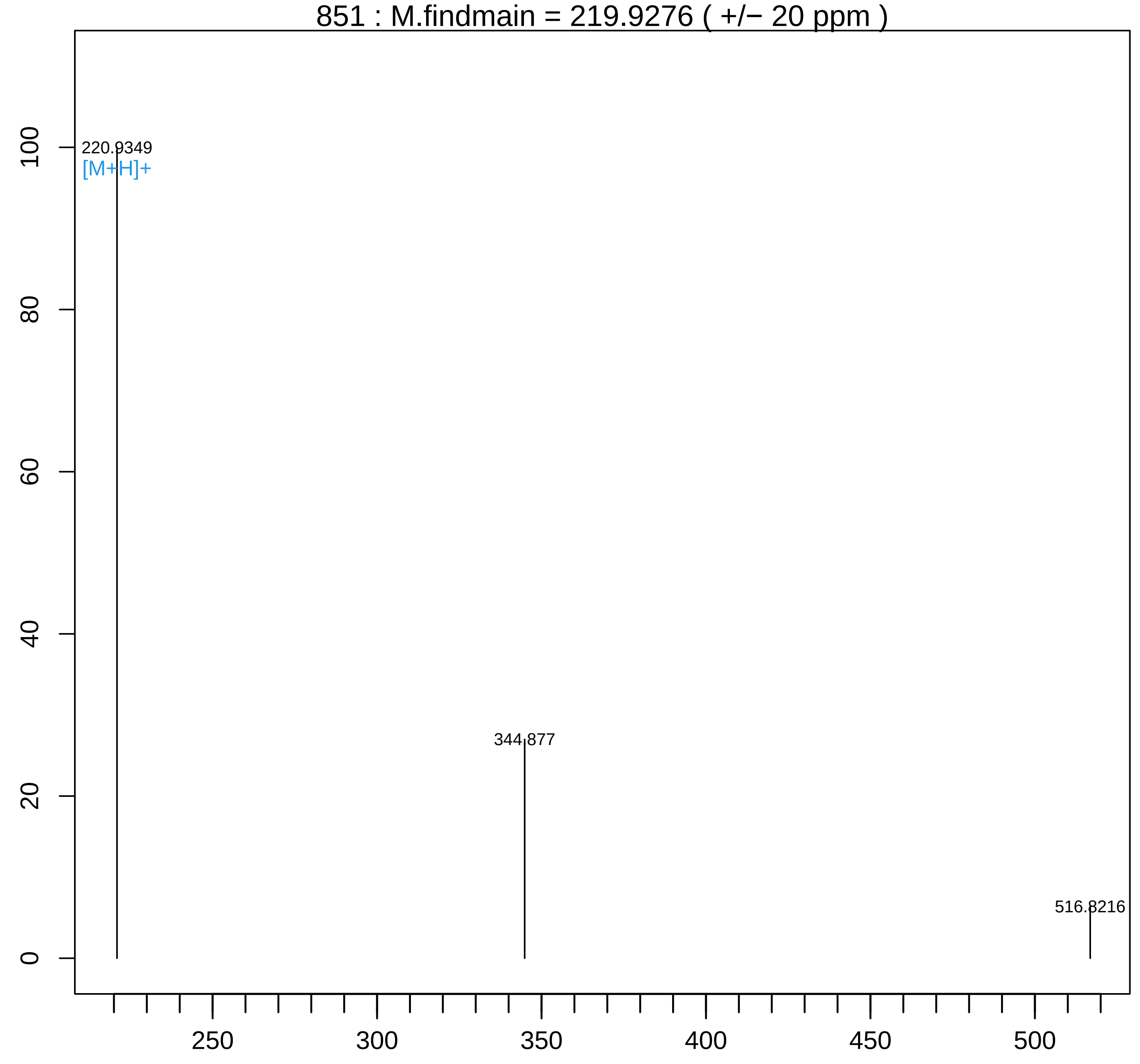
851 : M.findmain = 219.9276 (+/− 20 ppm)
220.9349
[M+H]+
344.877
516.8216
0
20
40
60
80
100
250
300
350
400
450
500

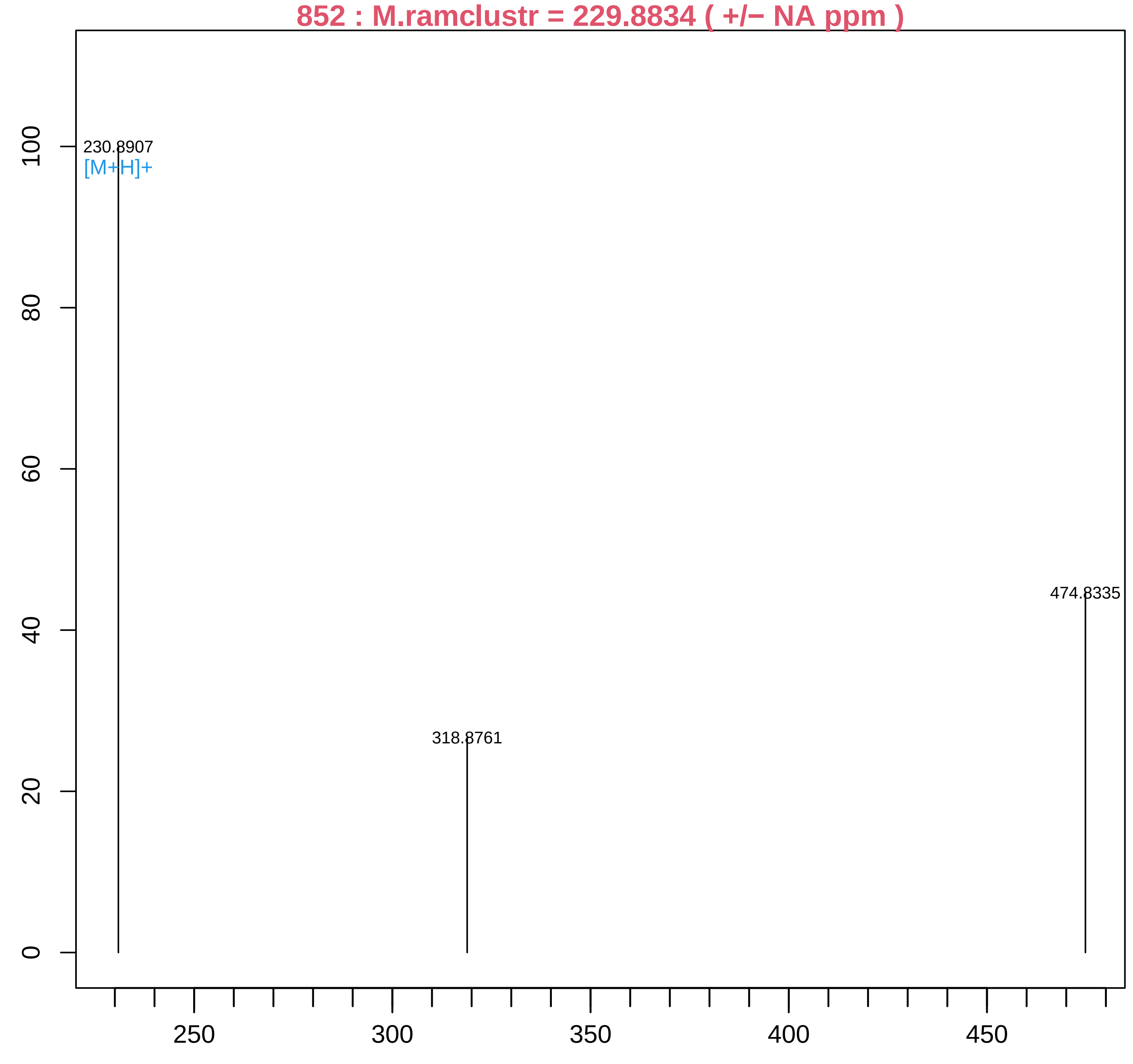

852 : M.ramclustr = 229.8834 (+/− NA ppm)
230.8907
[M+H]+
318.8761
474.8335
0
20
40
60
80
100
250
300
350
400
450

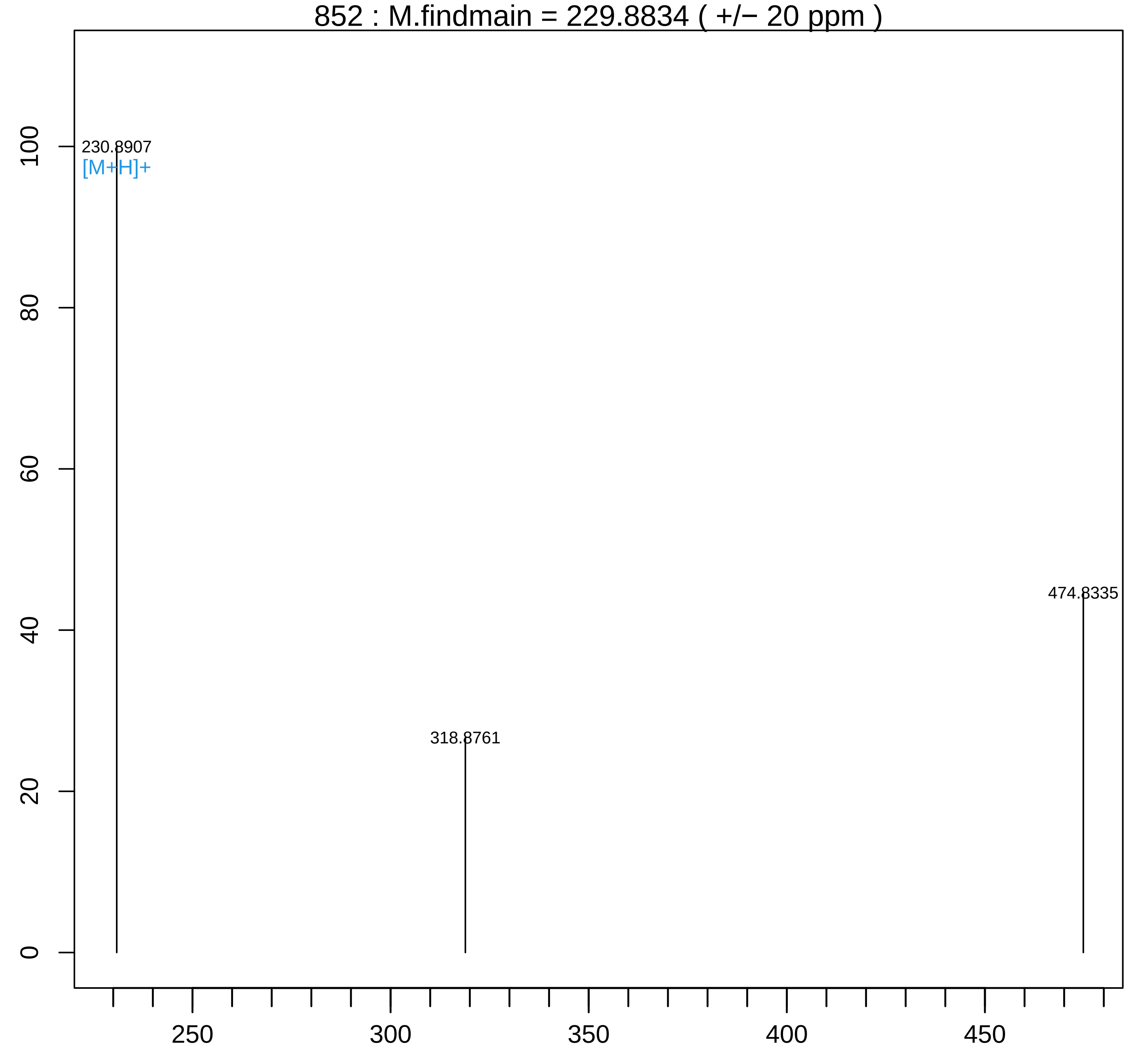

852 : M.findmain = 229.8834 (+/− 20 ppm)
230.8907
[M+H]+
318.8761
474.8335
0
20
40
60
80
100
250
300
350
400
450

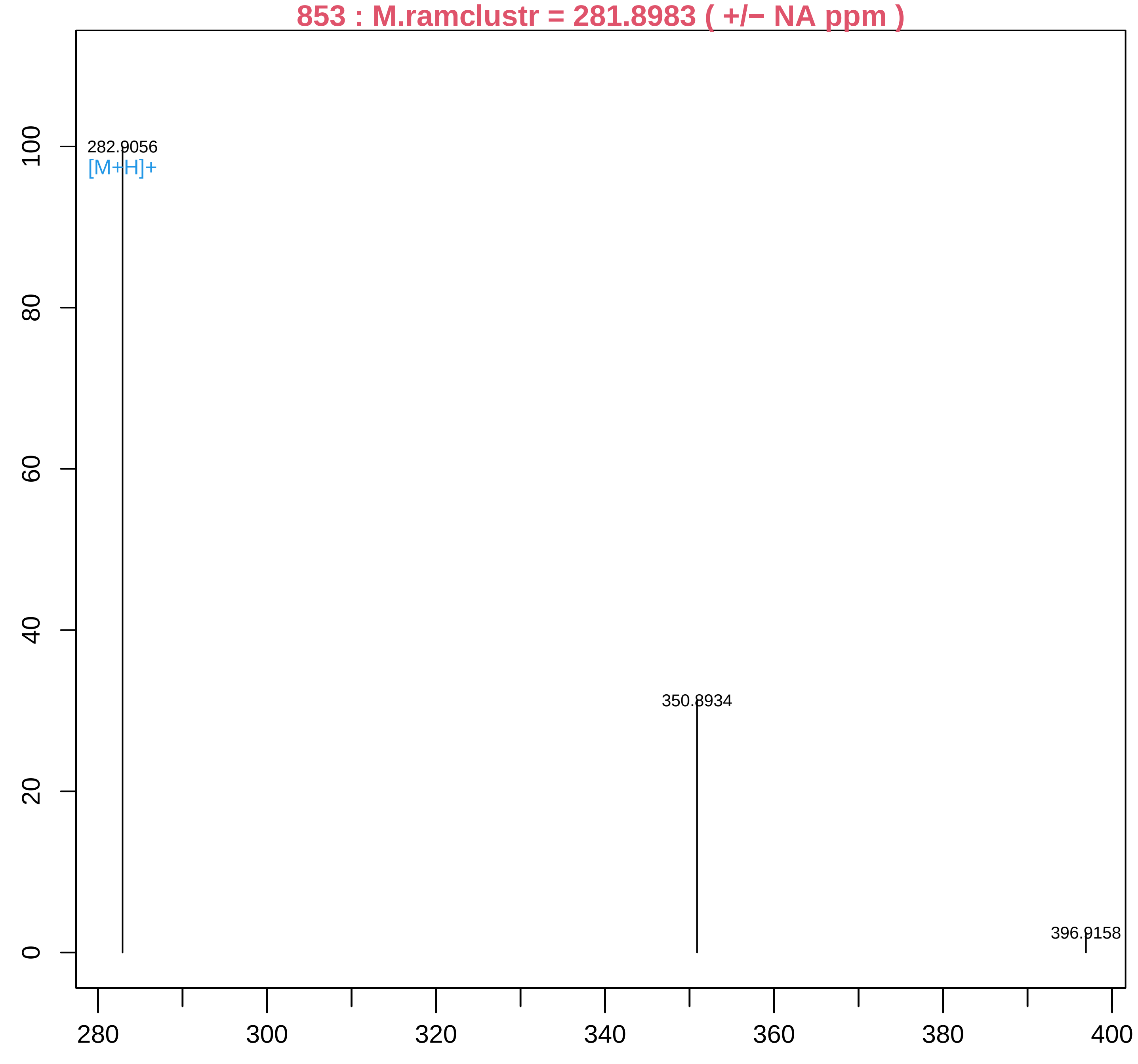
853 : M.ramclustr = 281.8983 (+/− NA ppm)
282.9056
[M+H]+
350.8934
396.9158
0
20
40
60
80
100
280
300
320
340
360
380
400
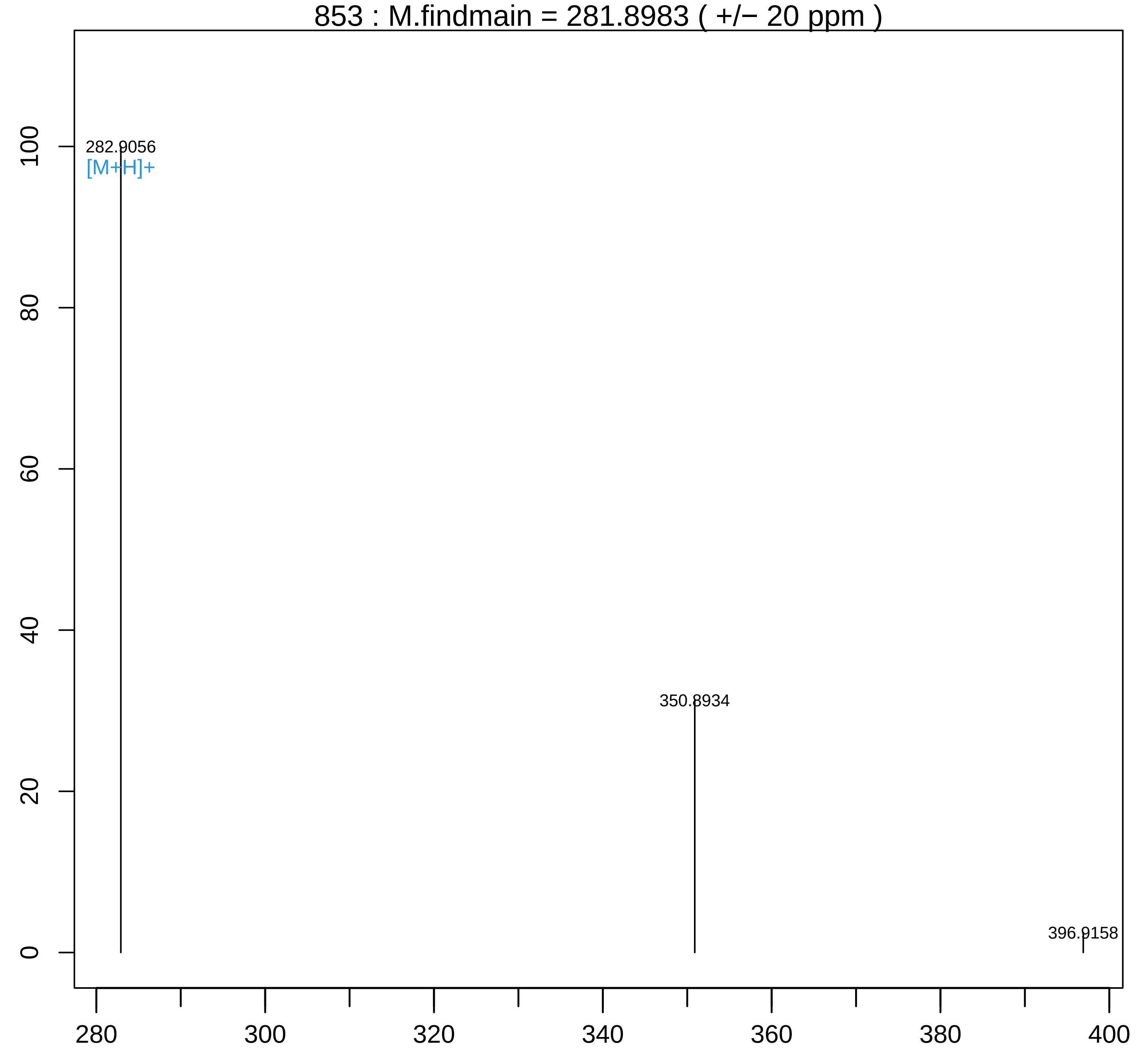
853 : M.findmain = 281.8983 (+/− 20 ppm)
282.9056
[M+H]+
350.8934
396.9158
0
20
40
60
80
100
280
300
320
340
360
380
400

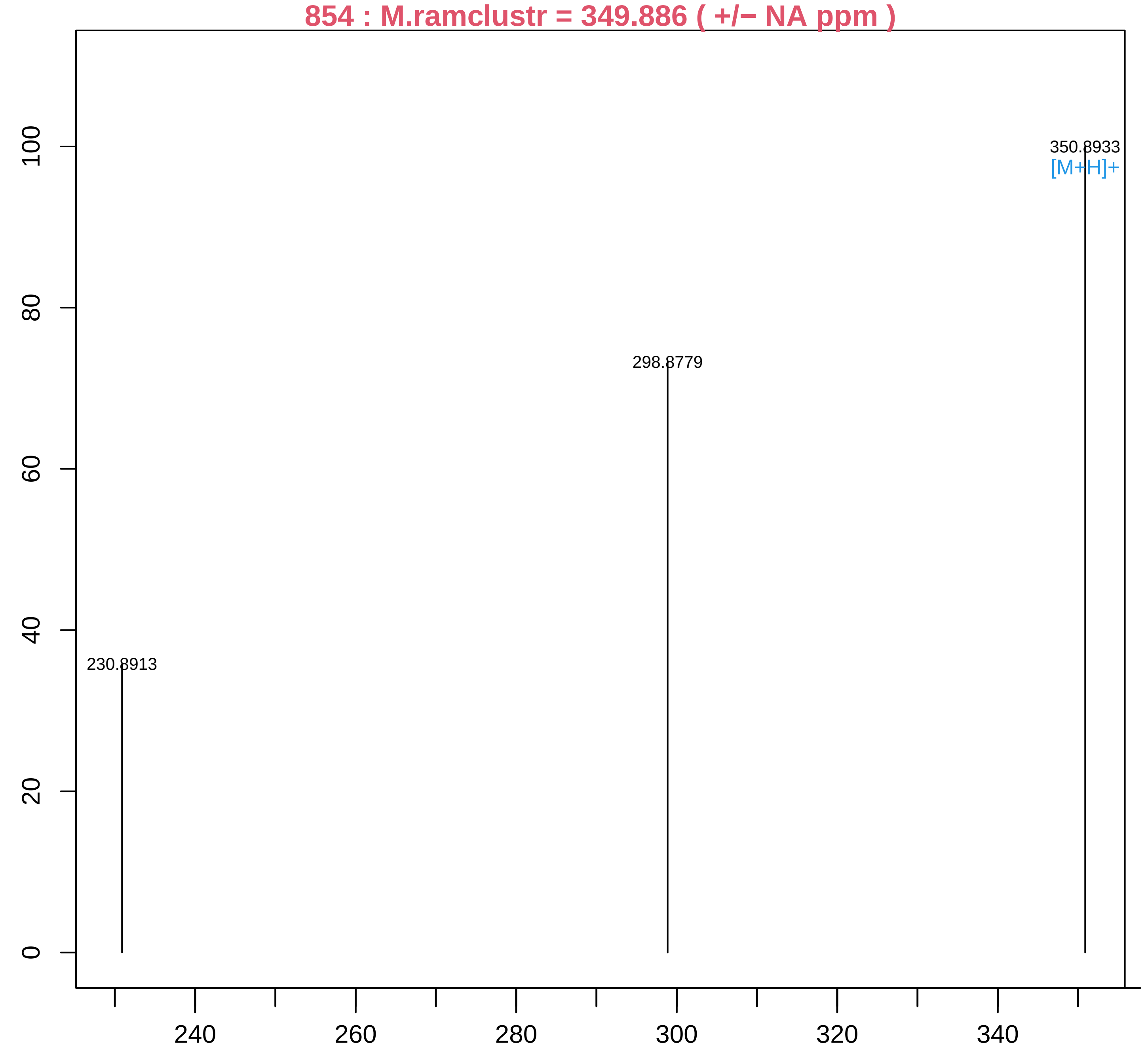
854 : M.ramclustr = 349.886 (+/− NA ppm)
350.8933
[M+H]+
298.8779
230.8913
0
20
40
60
80
100
240
260
280
300
320
340

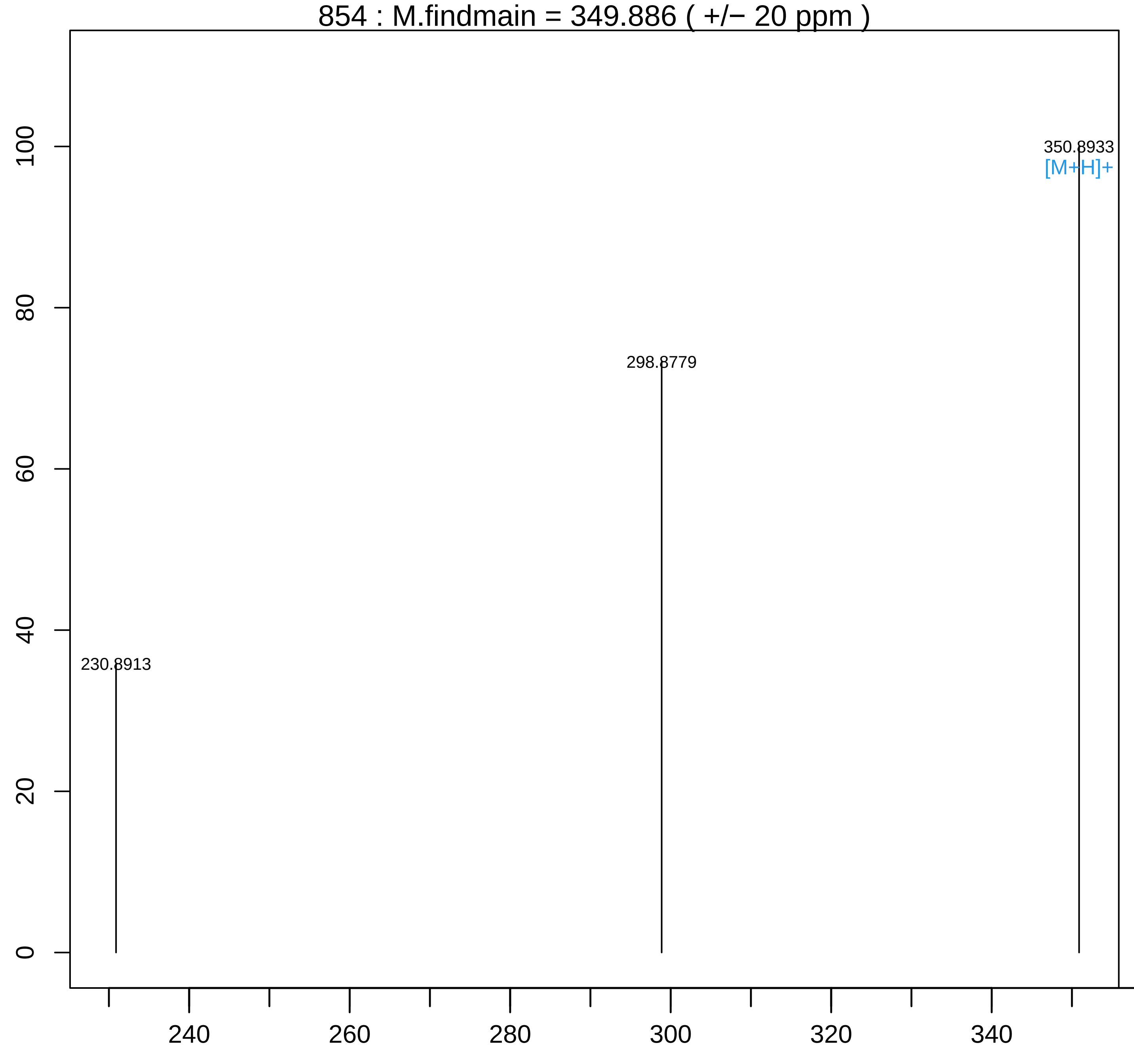
854 : M.findmain = 349.886 (+/− 20 ppm)
350.8933
[M+H]+
298.8779
230.8913
0
20
40
60
80
100
240
260
280
300
320
340

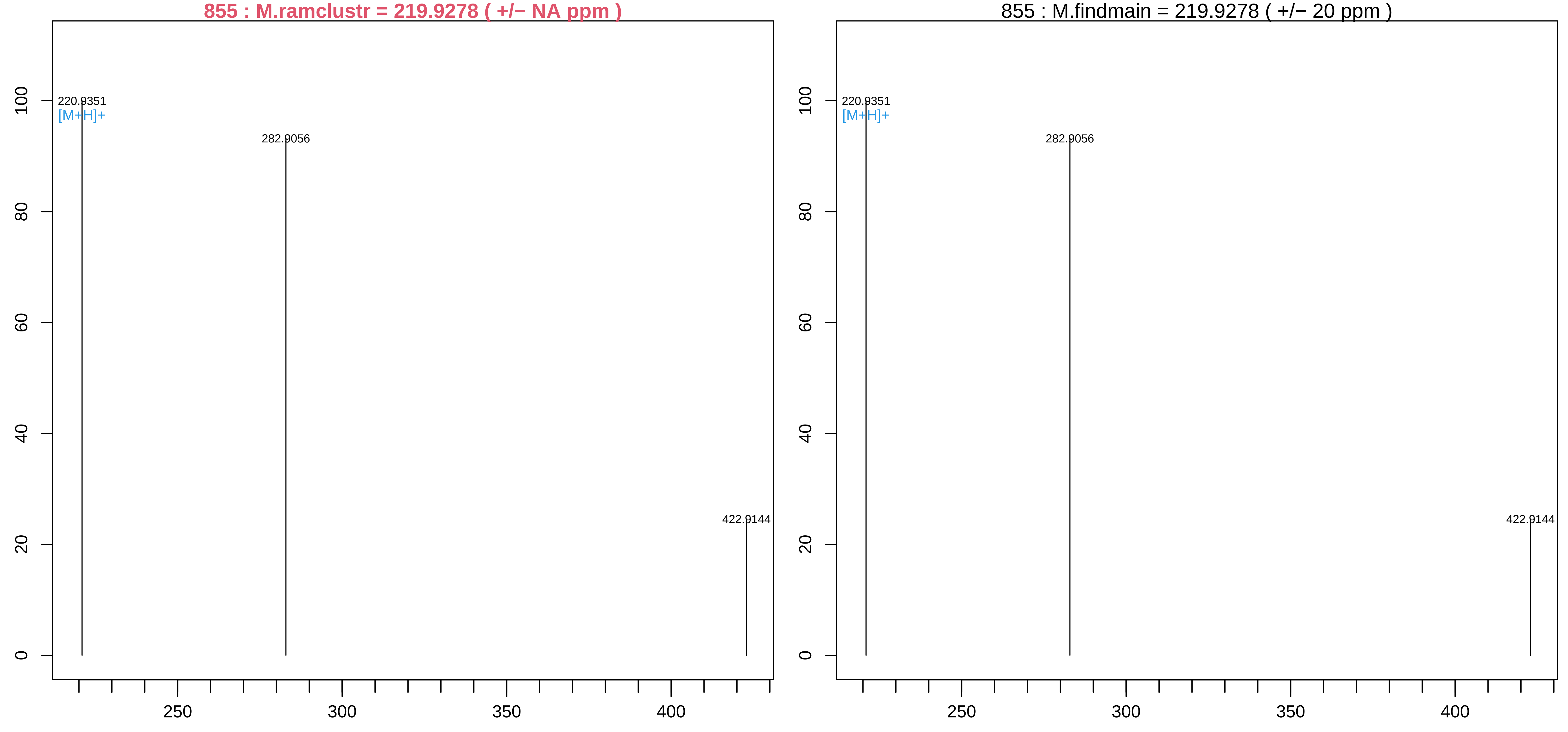
855 : M.ramclustr = 219.9278 (+/− NA ppm)
220.9351
[M+H]+
282.9056
422.9144
0
20
40
60
80
100
250
300
350
400
855 : M.findmain = 219.9278 (+/− 20 ppm)
220.9351
[M+H]+
282.9056
422.9144
0
20
40
60
80
100
250
300
350
400

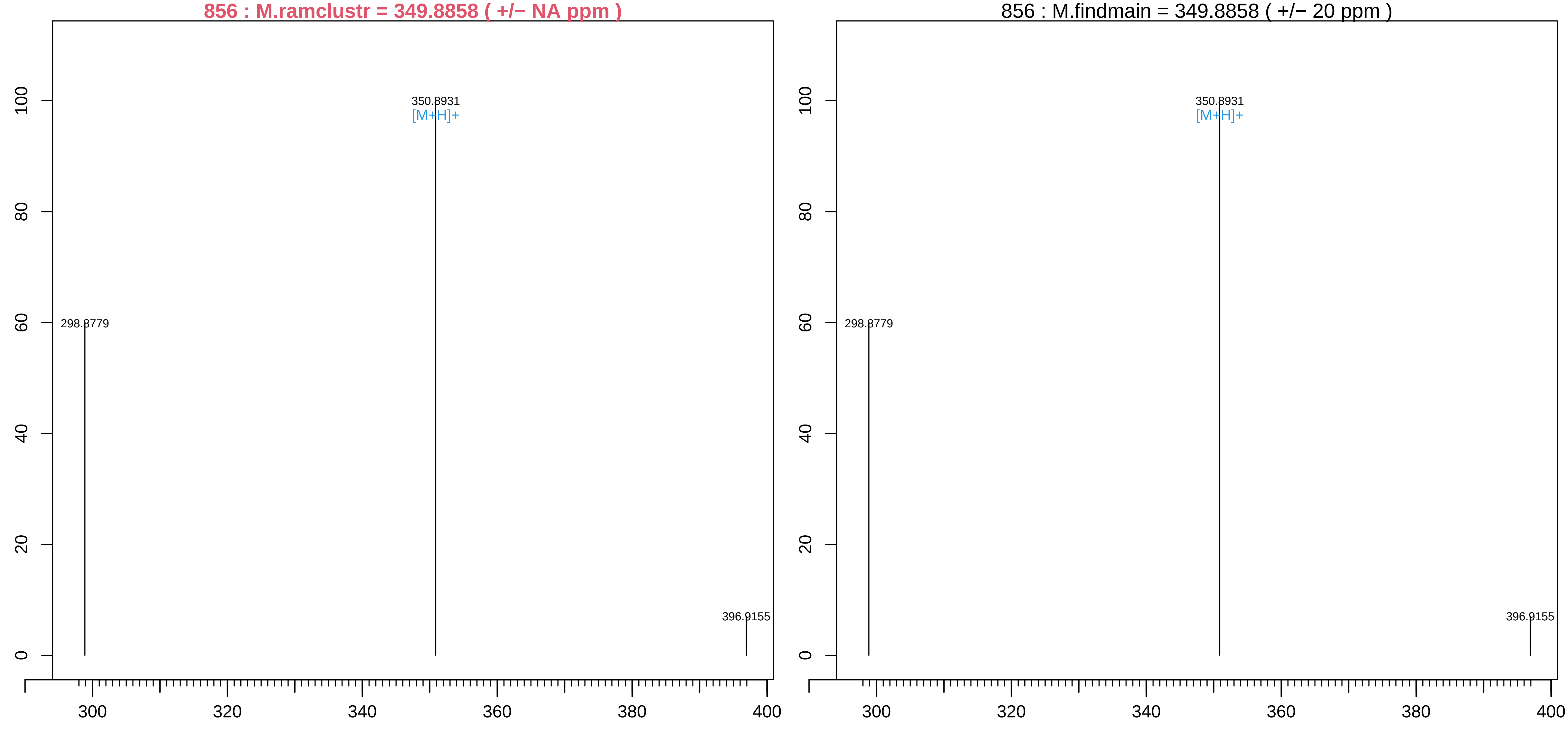
856 : M.ramclustr = 349.8858 (+/− NA ppm)
298.8779
350.8931
[M+H]+
396.9155
0
20
40
60
80
100
300
320
340
360
380
400
856 : M.findmain = 349.8858 (+/− 20 ppm)
298.8779
350.8931
[M+H]+
396.9155
0
20
40
60
80
100
300
320
340
360
380
400

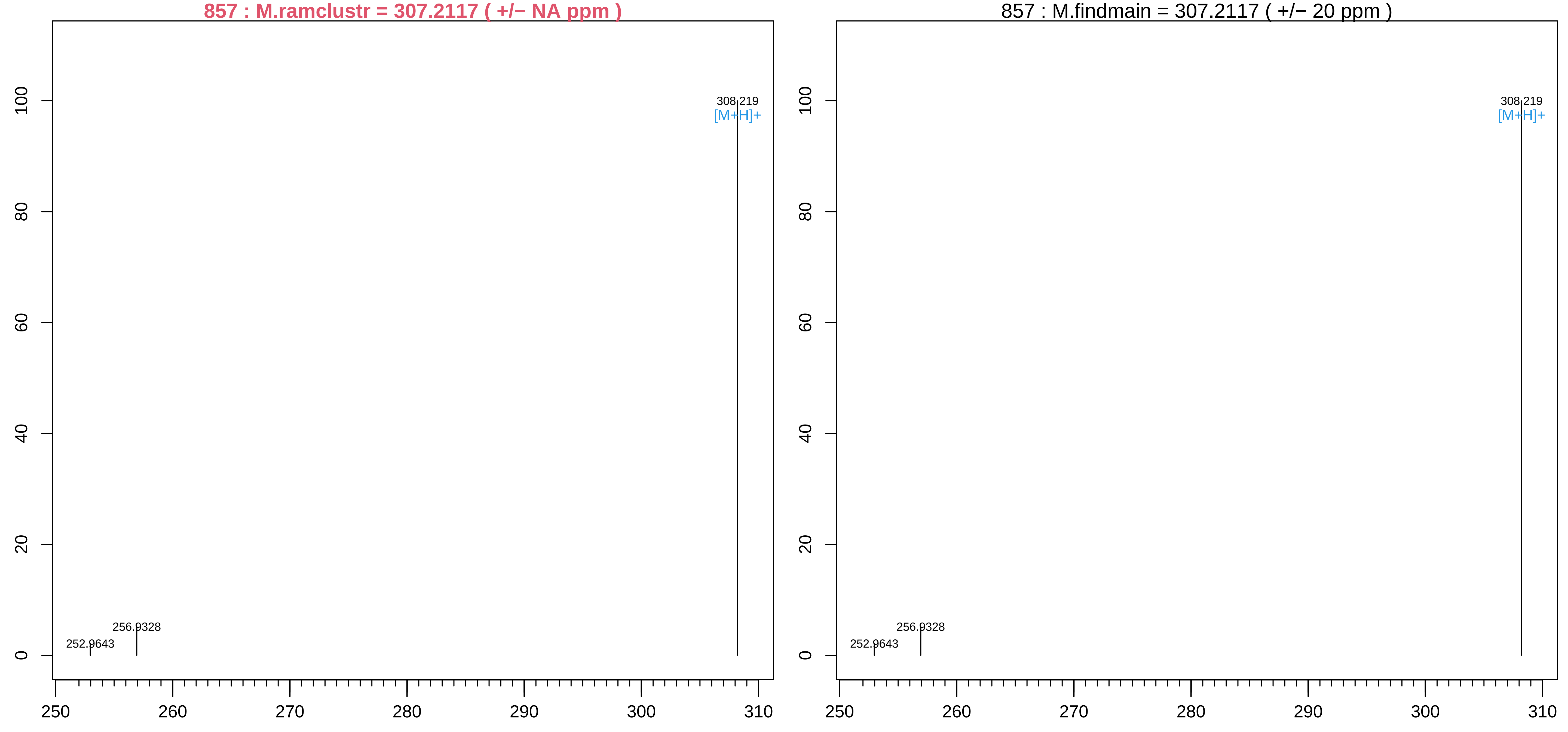
857 : M.ramclustr = 307.2117 (+/− NA ppm)
308.219
[M+H]+
256.9328
252.9643
857 : M.findmain = 307.2117 (+/− 20 ppm)
308.219
[M+H]+
256.9328
252.9643

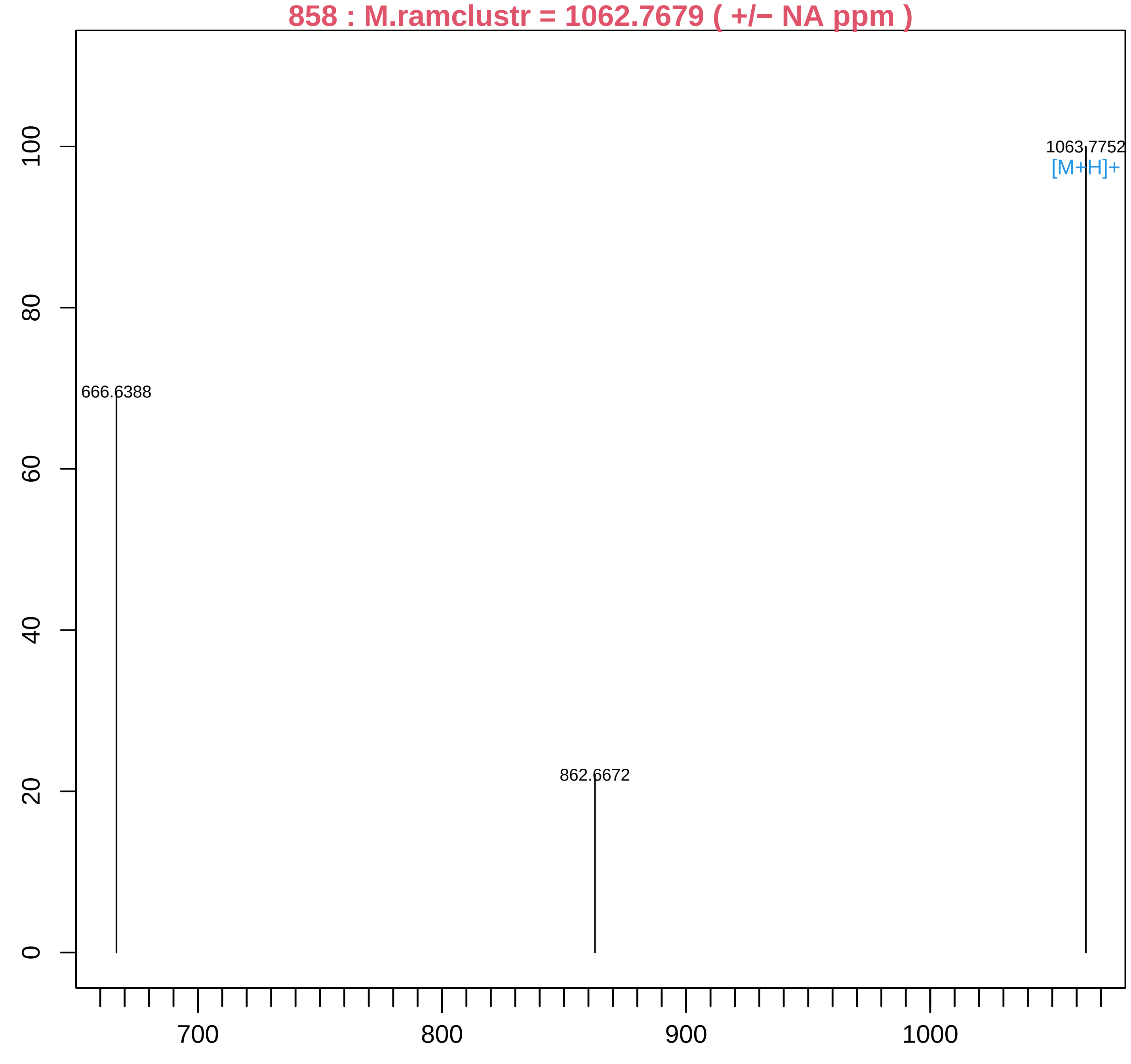
858 : M.ramclustr = 1062.7679 (+/− NA ppm)
1063.7752
[M+H]+
666.6388
862.6672
0
20
40
60
80
100
700
800
900
1000

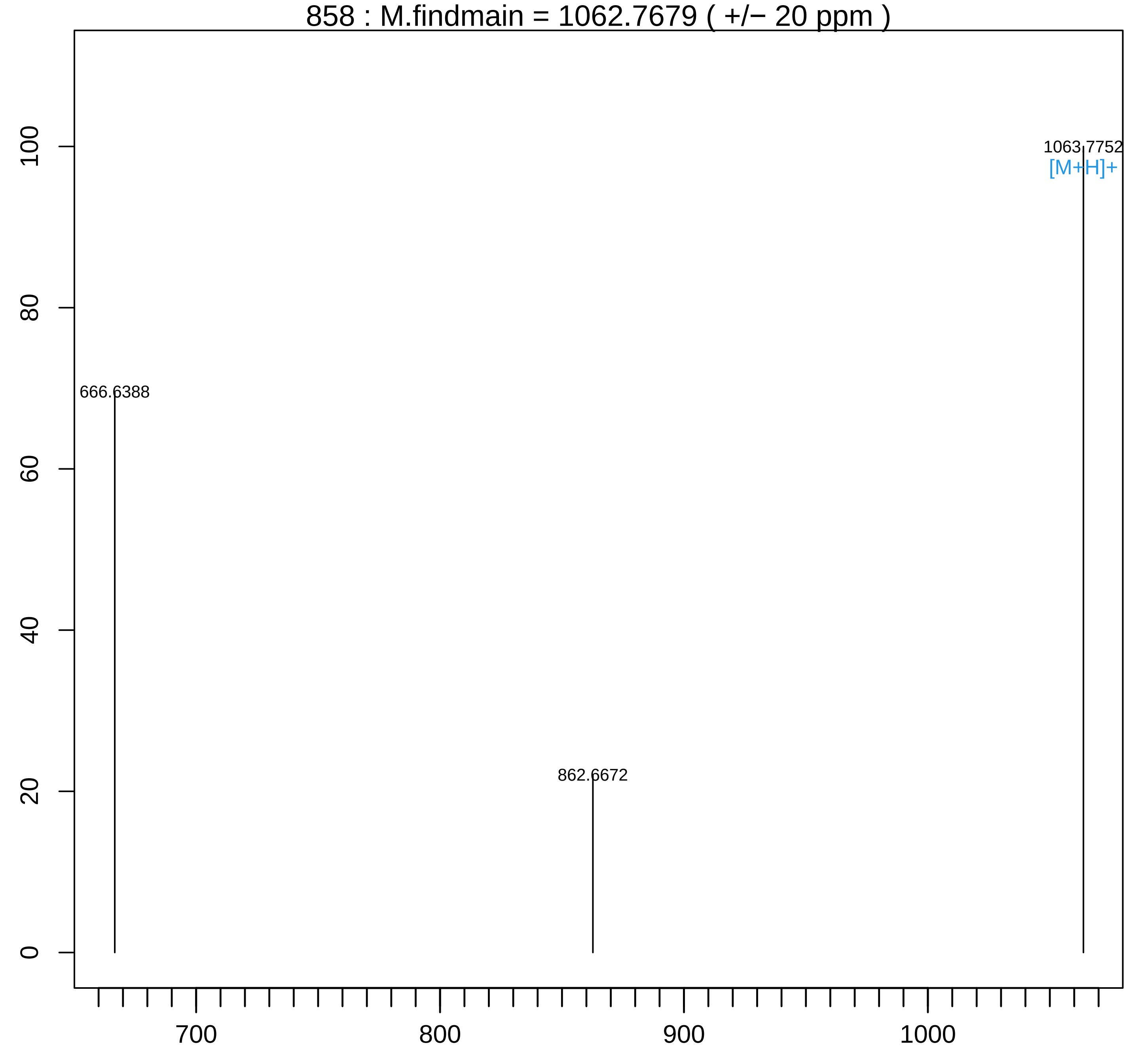
858 : M.findmain = 1062.7679 (+/− 20 ppm)
1063.7752
[M+H]+
666.6388
862.6672
0
20
40
60
80
100
700
800
900
1000

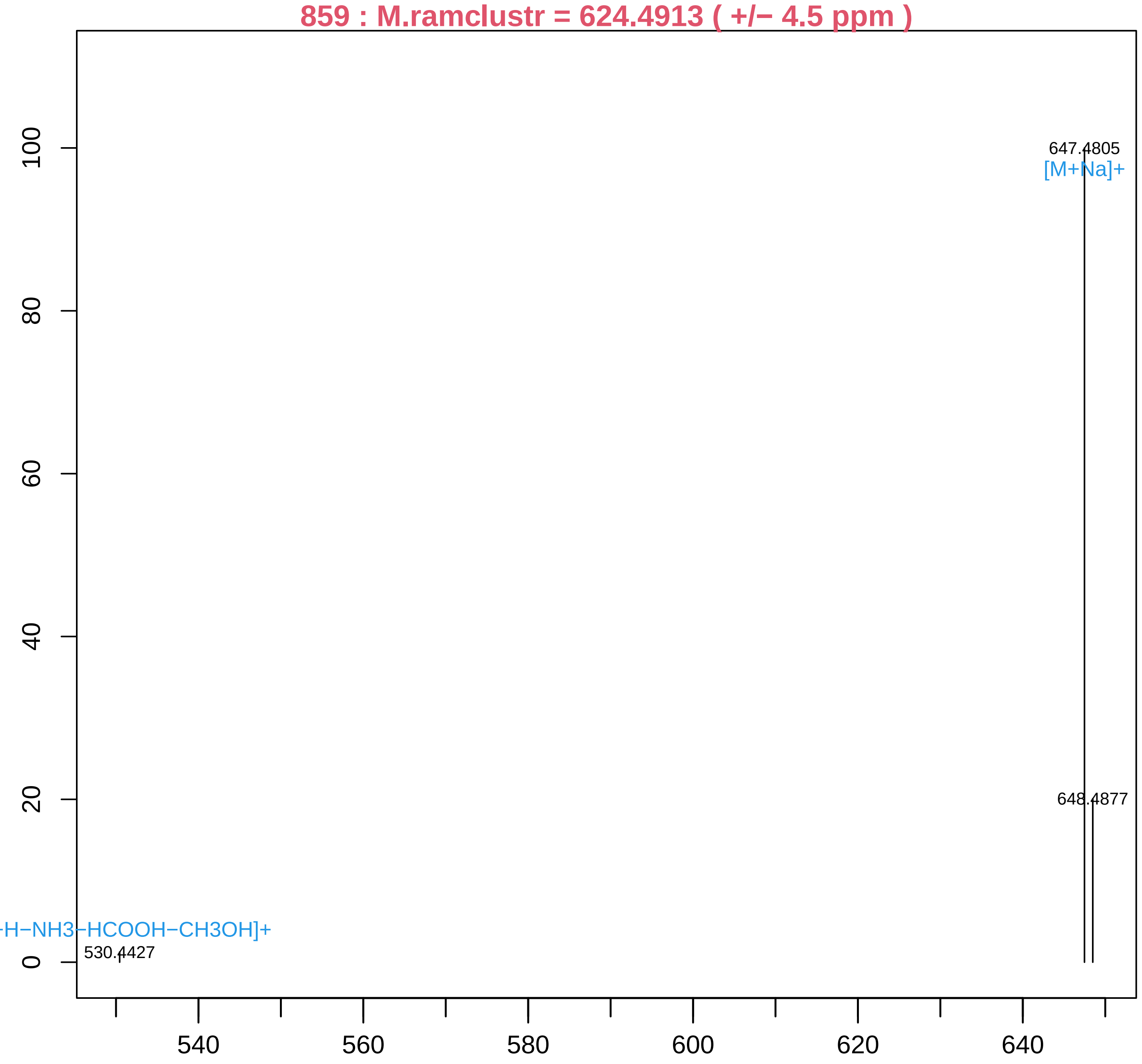

859 : M.ramclustr = 624.4913 (+/− 4.5 ppm)
647.4805
[M+Na]+
648.4877
-H−NH3−HCOOH−CH3OH]+
530.4427
100
80
60
40
20
0
540
560
580
600
620
640

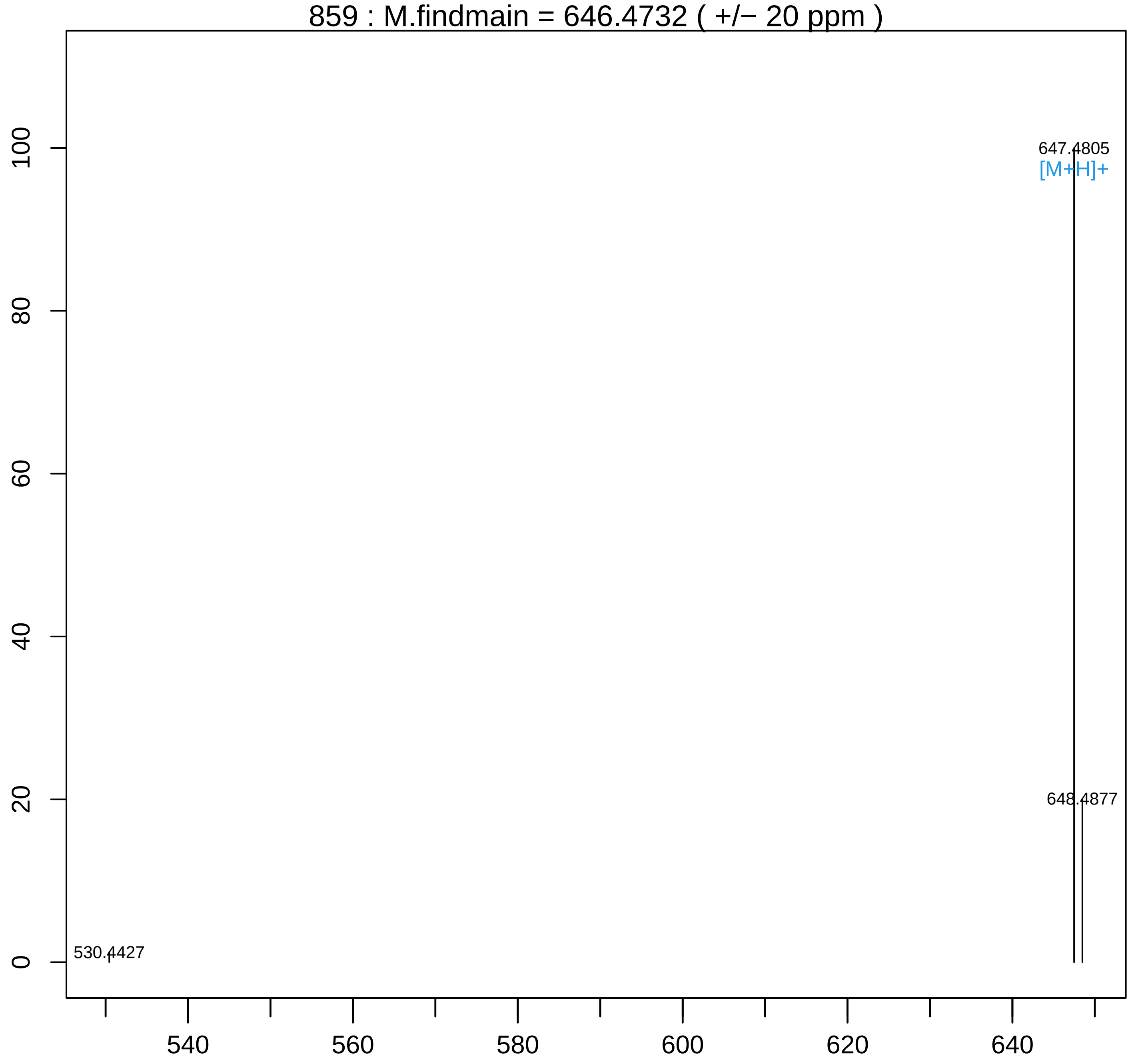

859 : M.findmain = 646.4732 (+/− 20 ppm)
647.4805
[M+H]+
648.4877
530.4427
100
80
60
40
20
0
540
560
580
600
620
640

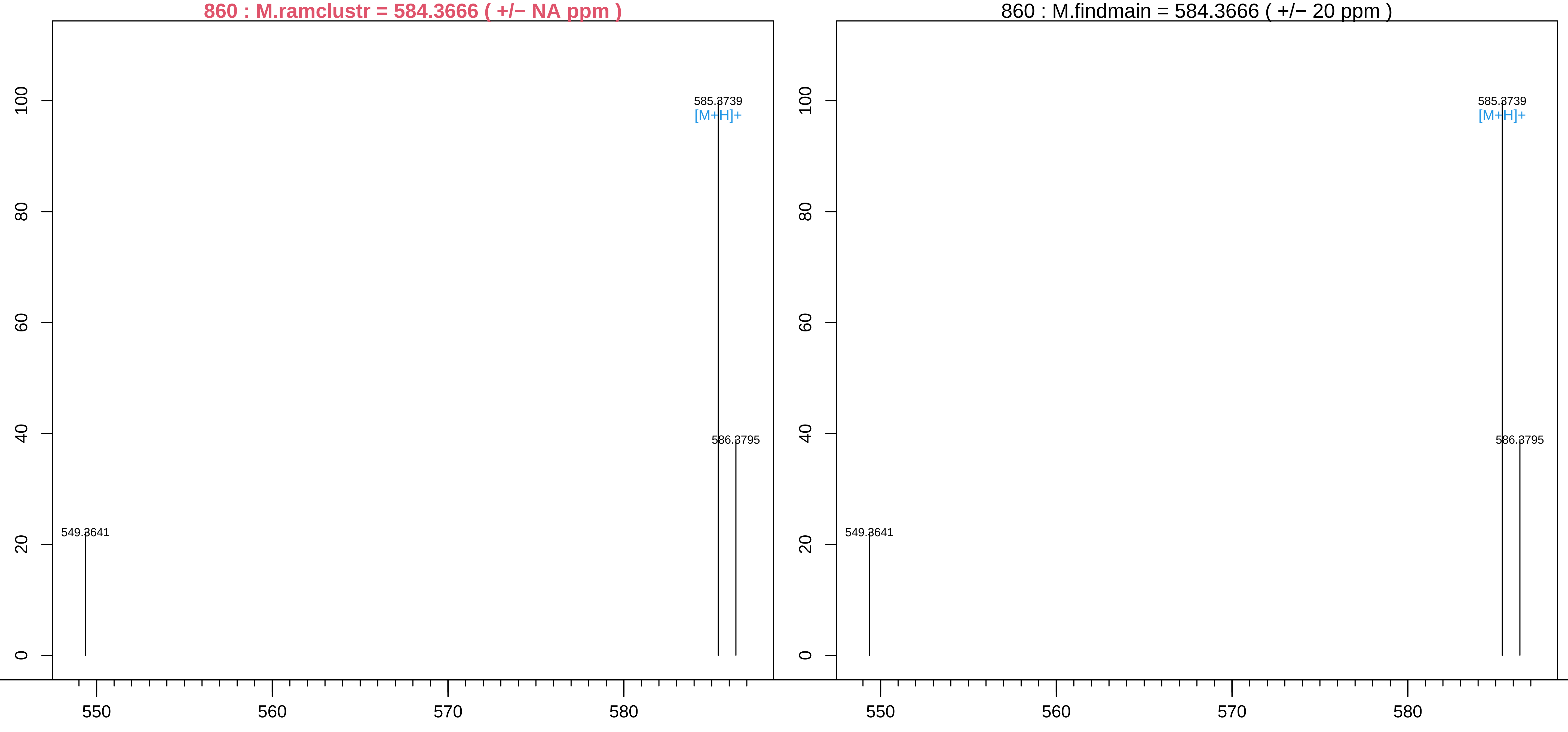

860 : M.ramclustr = 584.3666 (+/− NA ppm)
860 : M.findmain = 584.3666 (+/− 20 ppm)
585.3739
[M+H]+
586.3795
549.3641
0
20
40
60
80
100
550
560
570
580

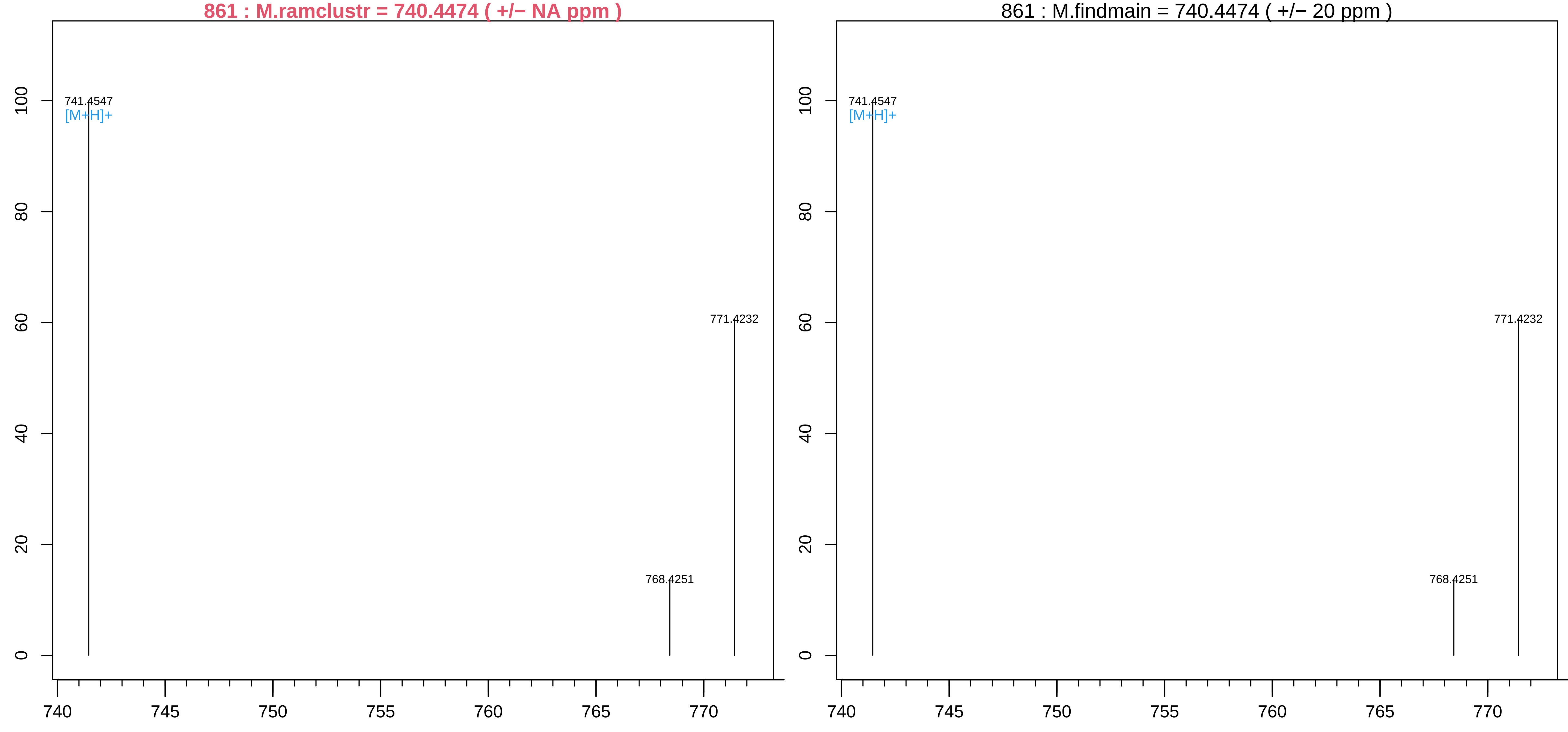
861 : M.ramclustr = 740.4474 (+/− NA ppm)
741.4547
[M+H]+
768.4251
771.4232
861 : M.findmain = 740.4474 (+/− 20 ppm)
741.4547
[M+H]+
768.4251
771.4232

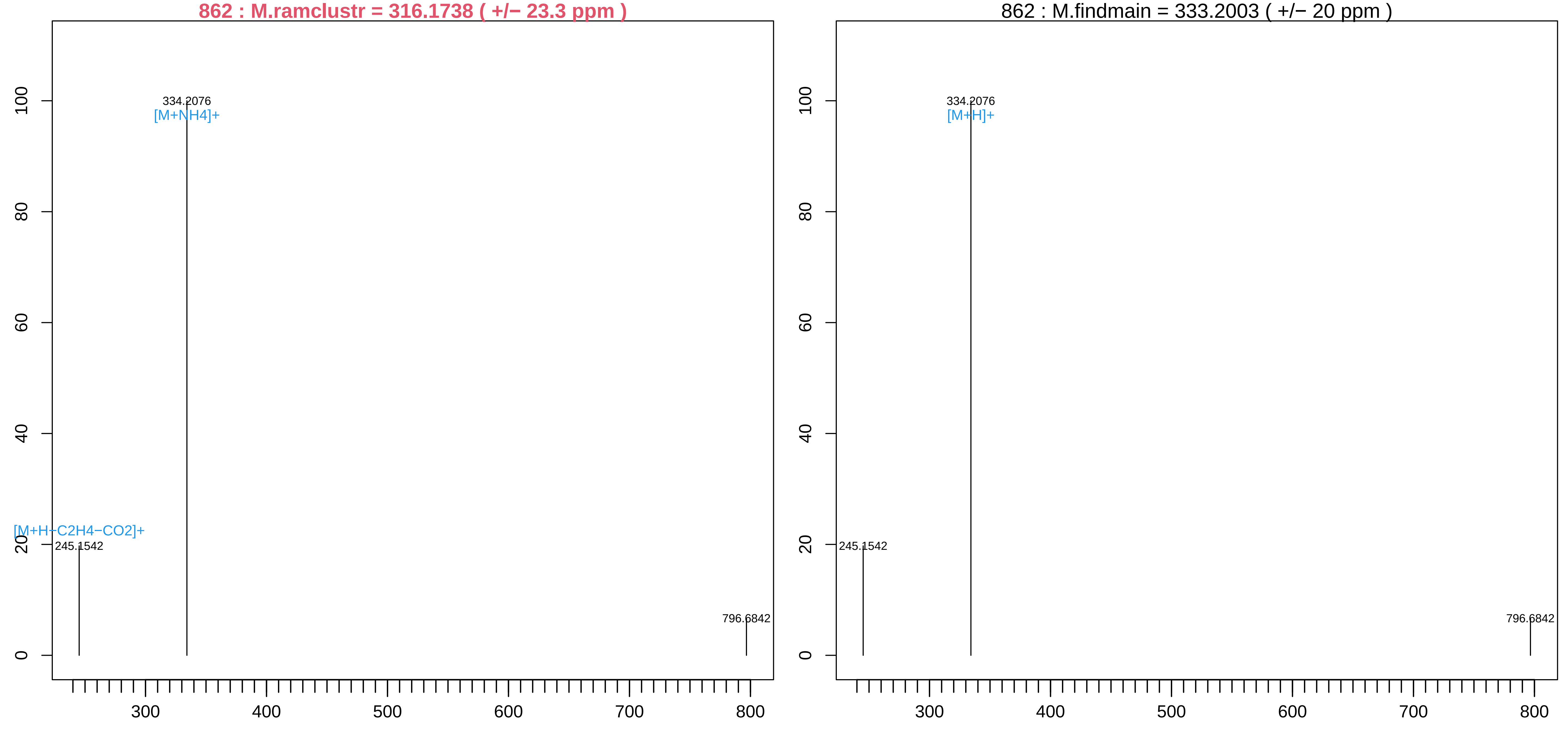
862 : M.ramclustr = 316.1738 (+/− 23.3 ppm)
334.2076
[M+NH4]+
[M+H−C2H4−CO2]+
245.1542
796.6842
862 : M.findmain = 333.2003 (+/− 20 ppm)
334.2076
[M+H]+
245.1542
796.6842

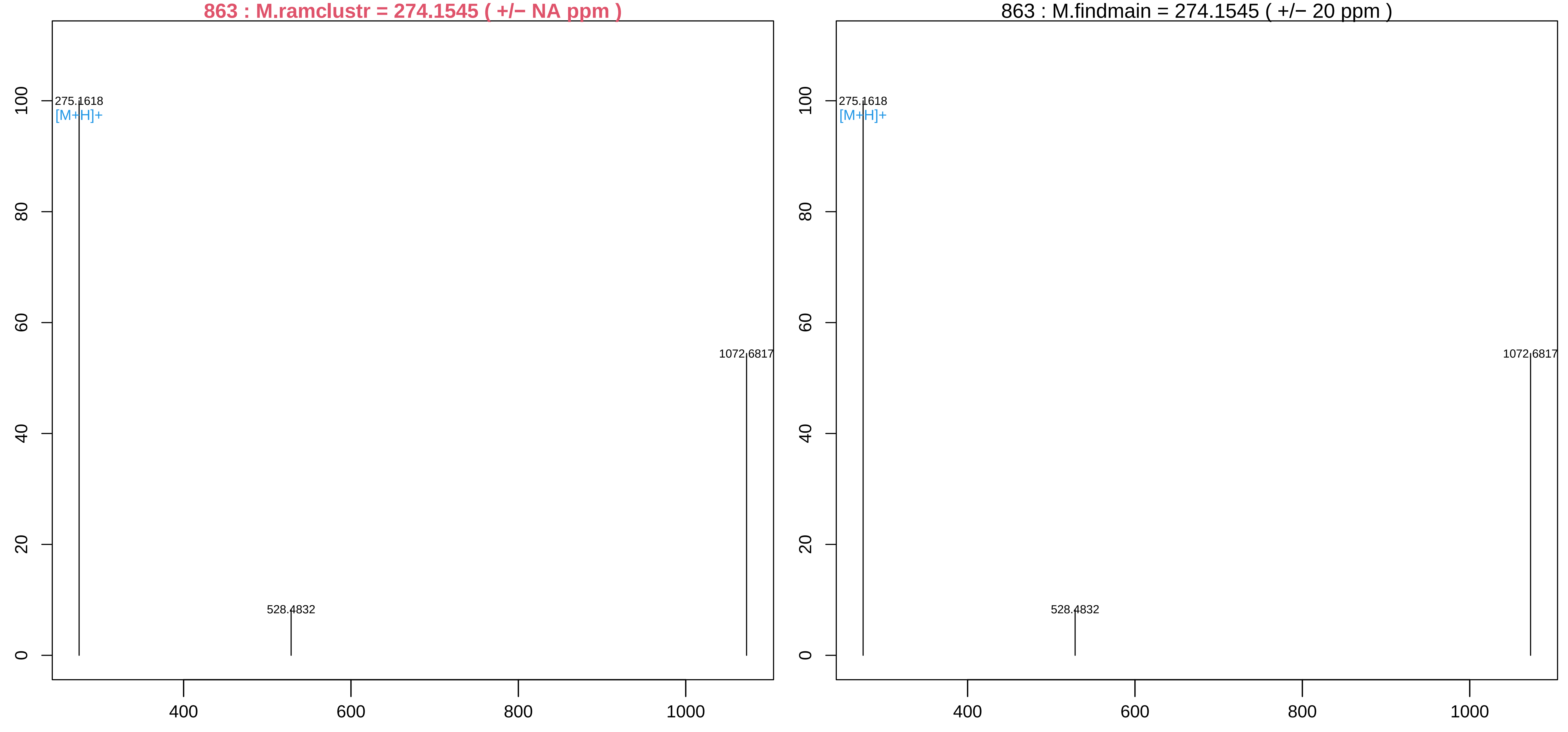
863 : M.ramclustr = 274.1545 (+/− NA ppm)
275.1618
[M+H]+
528.4832
1072.6817
863 : M.findmain = 274.1545 (+/− 20 ppm)
275.1618
[M+H]+
528.4832
1072.6817
0
20
40
60
80
100
400
600
800
1000

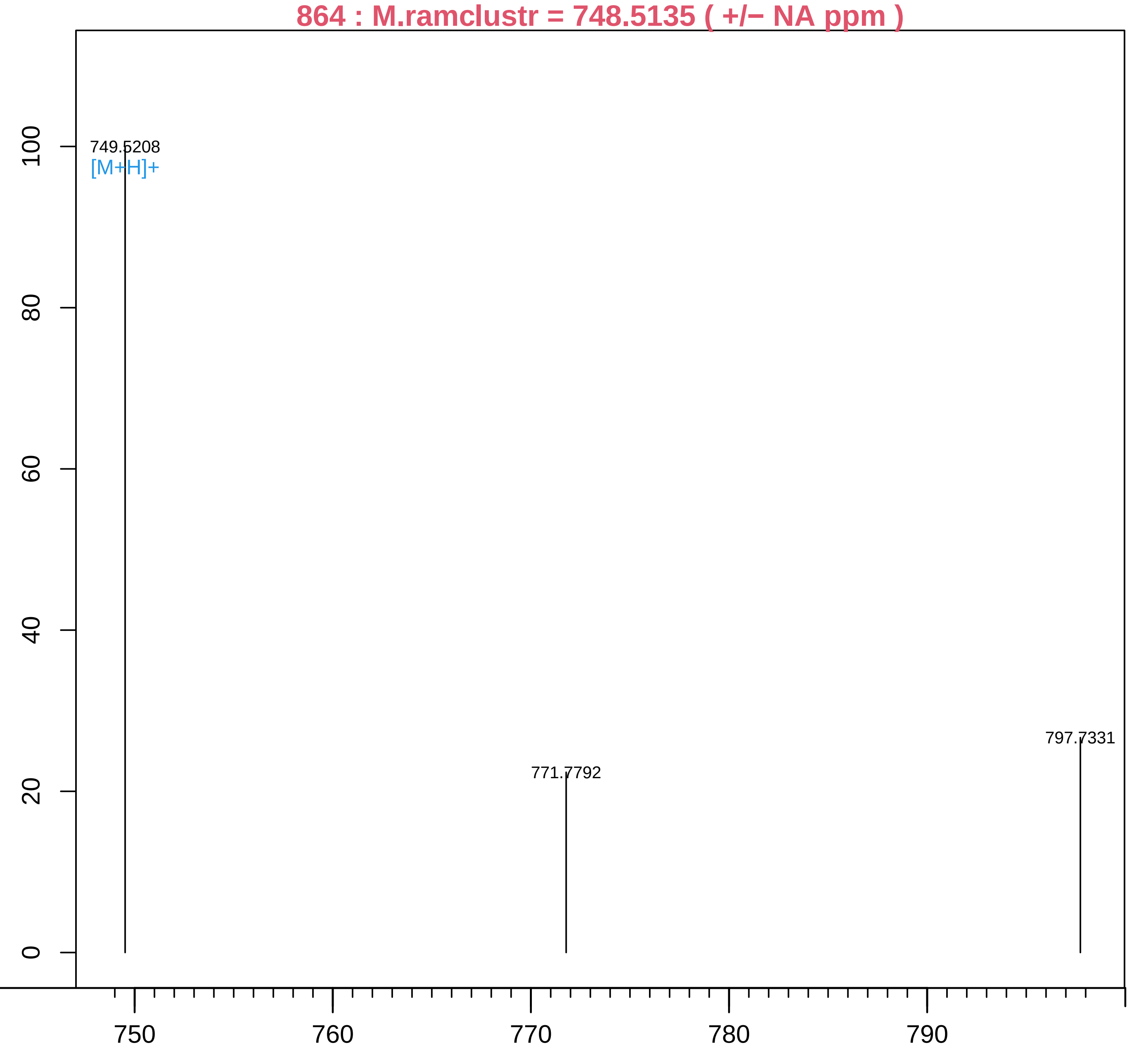
864 : M.ramclustr = 748.5135 (+/− NA ppm)
749.5208
[M+H]+
771.7792
797.7331
0
20
40
60
80
100
750
760
770
780
790

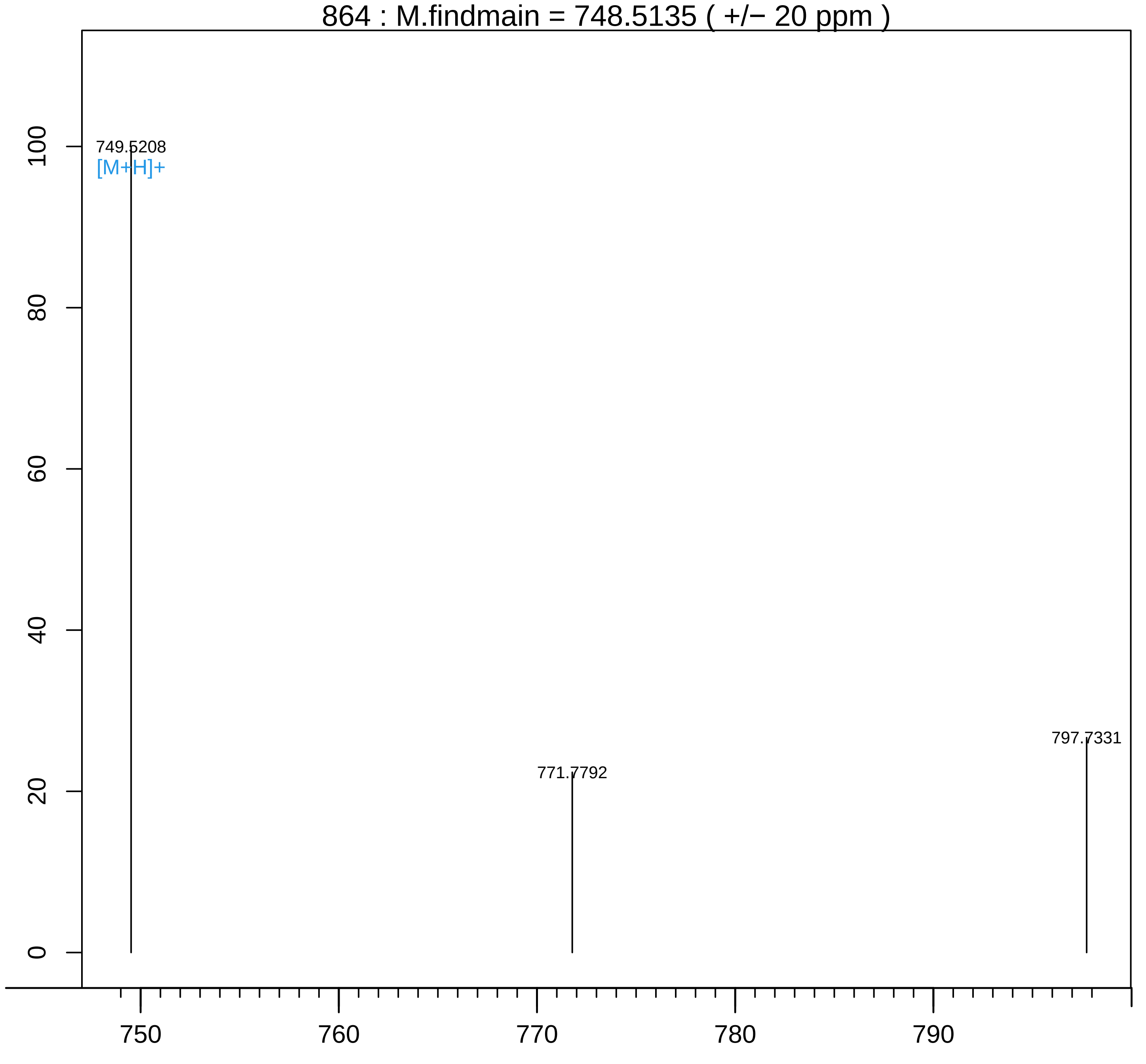
864 : M.findmain = 748.5135 (+/− 20 ppm)
749.5208
[M+H]+
771.7792
797.7331
0
20
40
60
80
100
750
760
770
780
790

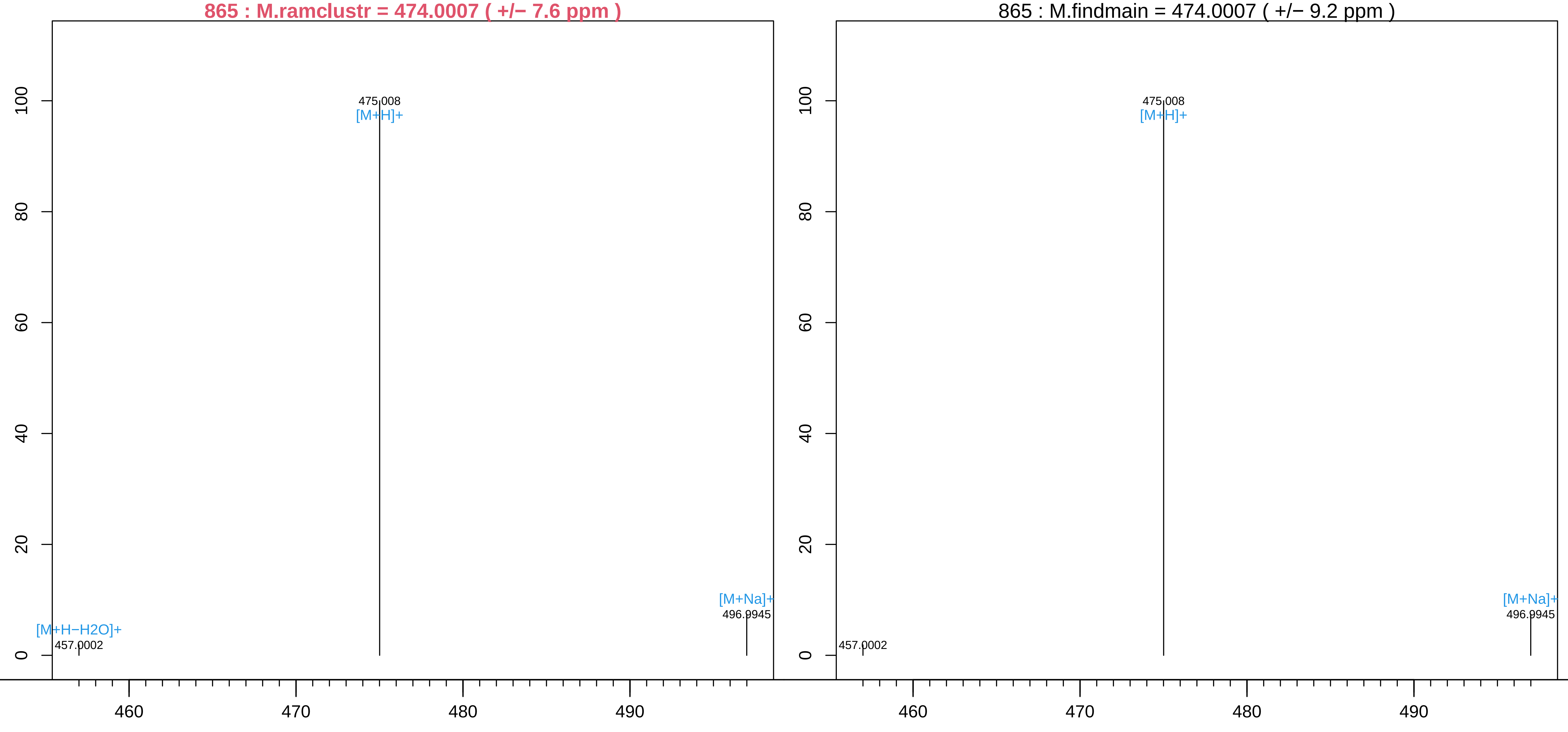

865 : M.ramclustr = 474.0007 (+/− 7.6 ppm)
475.008
[M+H]+
[M+Na]+
496.9945
[M+H−H2O]+
457.0002
865 : M.findmain = 474.0007 (+/− 9.2 ppm)
475.008
[M+H]+
[M+Na]+
496.9945
457.0002
0
20
40
60
80
100
460
470
480
490

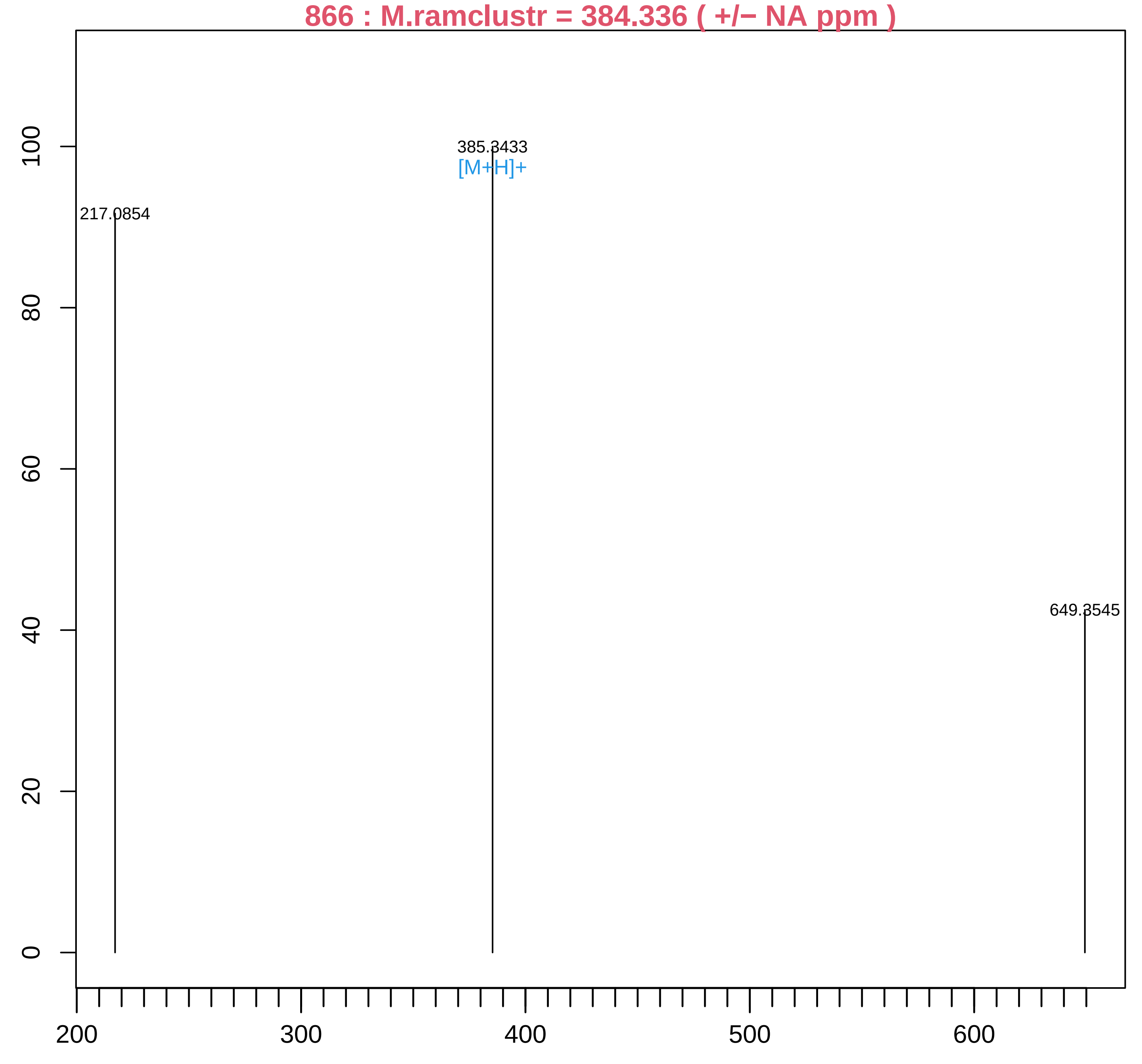
866 : M.ramclustr = 384.336 (+/− NA ppm)
217.0854
385.3433
[M+H]+
649.3545
0
20
40
60
80
100
200
300
400
500
600

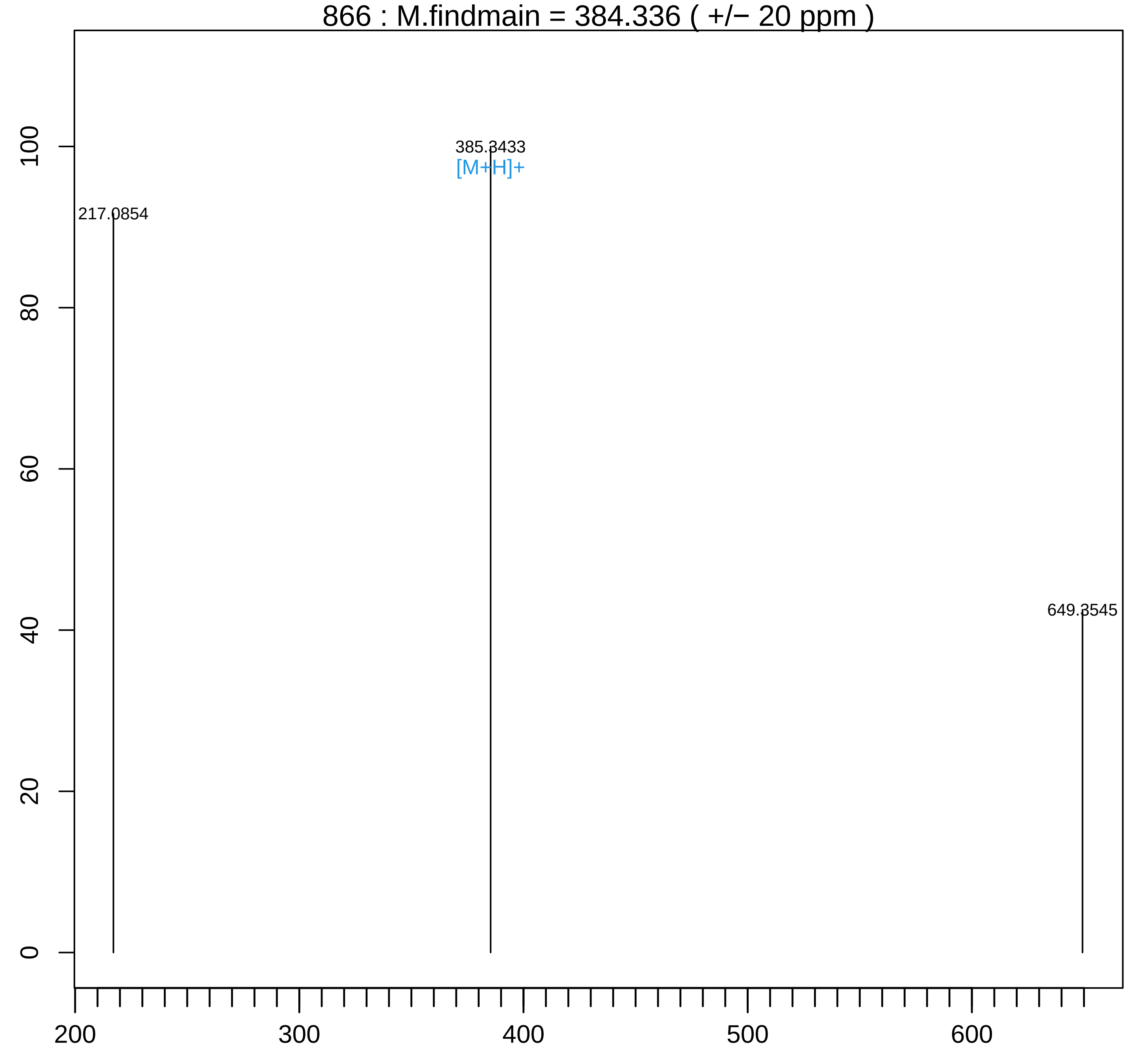
866 : M.findmain = 384.336 (+/− 20 ppm)
217.0854
385.3433
[M+H]+
649.3545
0
20
40
60
80
100
200
300
400
500
600

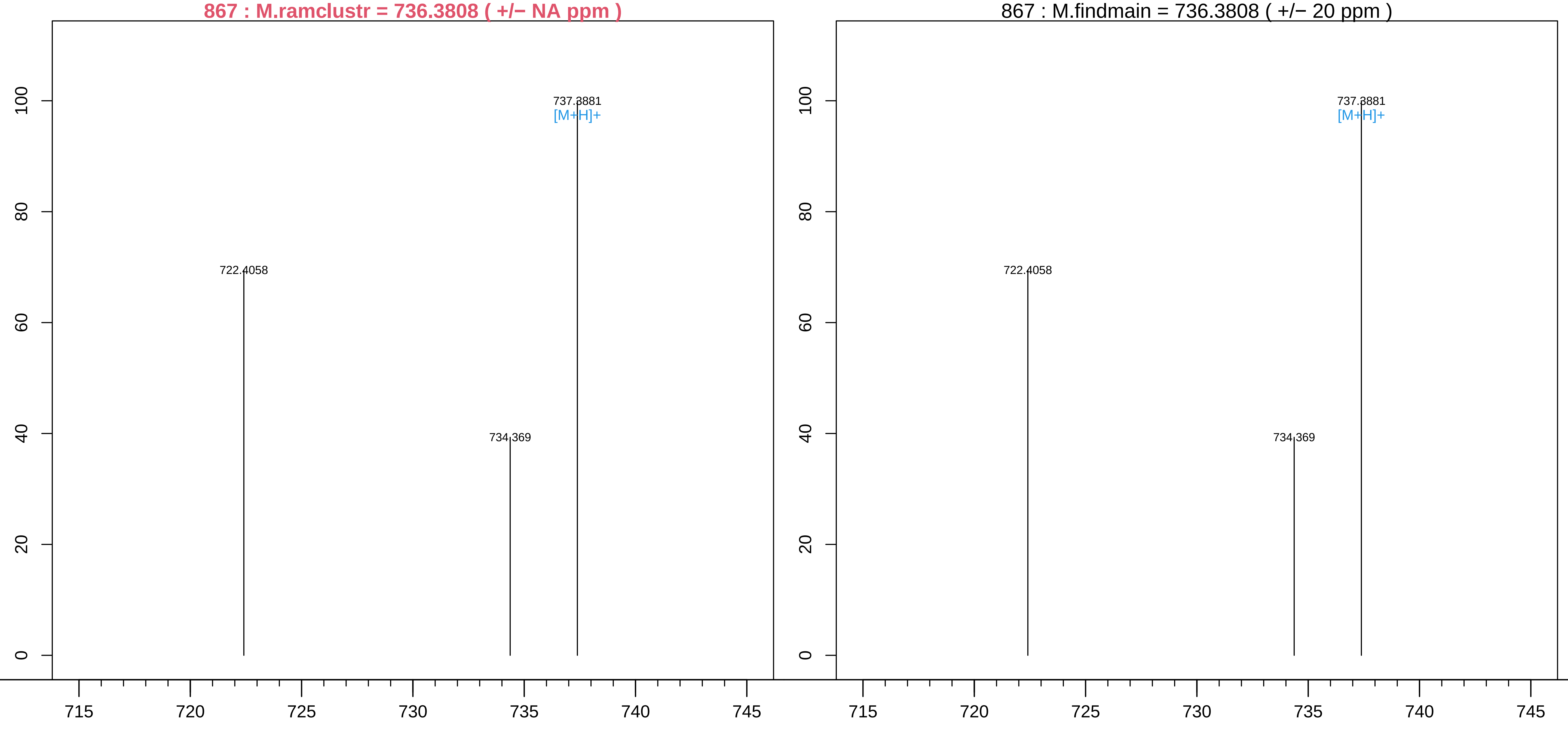

867 : M.ramclustr = 736.3808 (+/− NA ppm)
737.3881
[M+H]+
722.4058
734.369
867 : M.findmain = 736.3808 (+/− 20 ppm)
737.3881
[M+H]+
722.4058
734.369

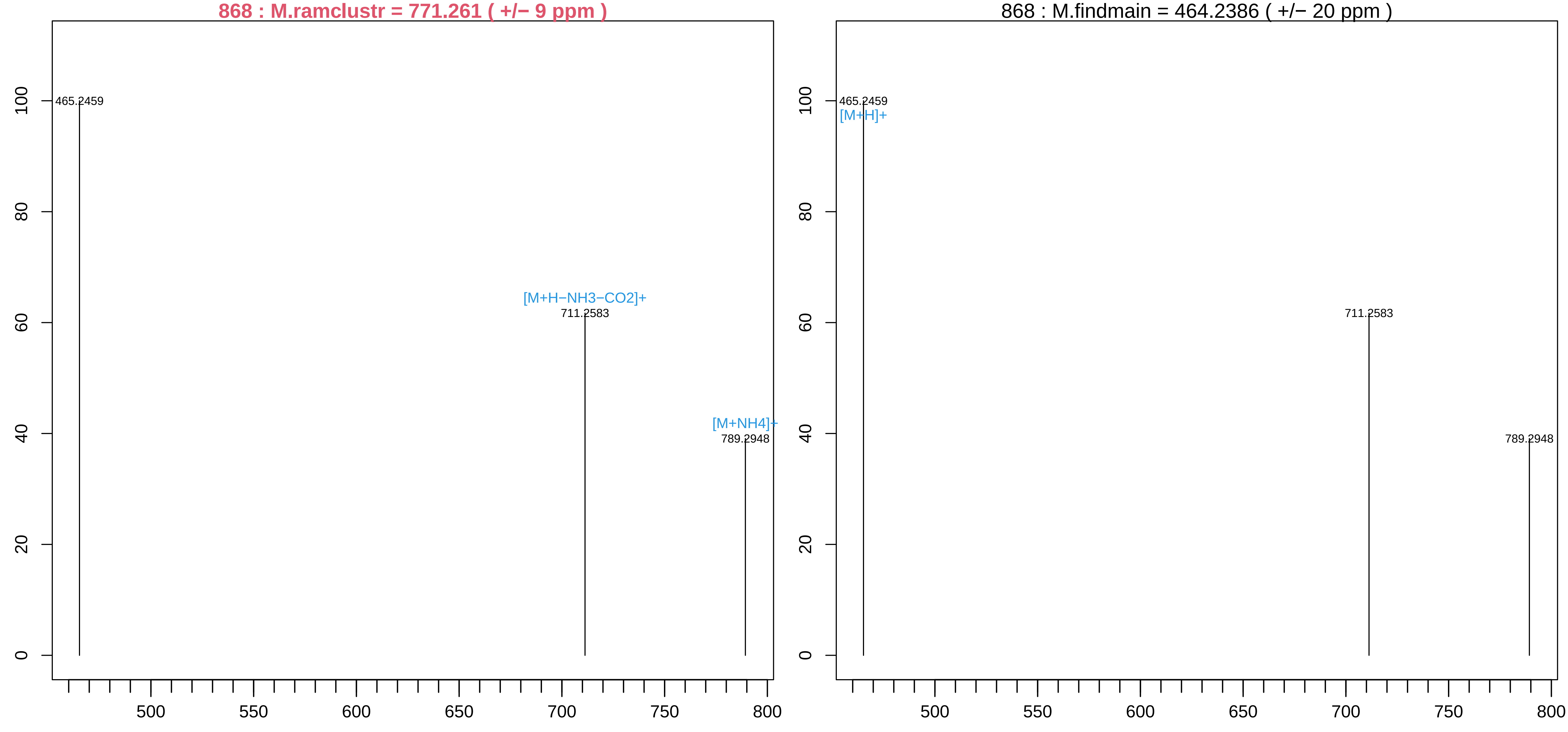

868 : M.ramclustr = 771.261 (+/− 9 ppm)
465.2459
[M+H−NH3−CO2]+
711.2583
[M+NH4]+
789.2948
868 : M.findmain = 464.2386 (+/− 20 ppm)
465.2459
[M+H]+
711.2583
789.2948
0
20
40
60
80
100
500
550
600
650
700
750
800

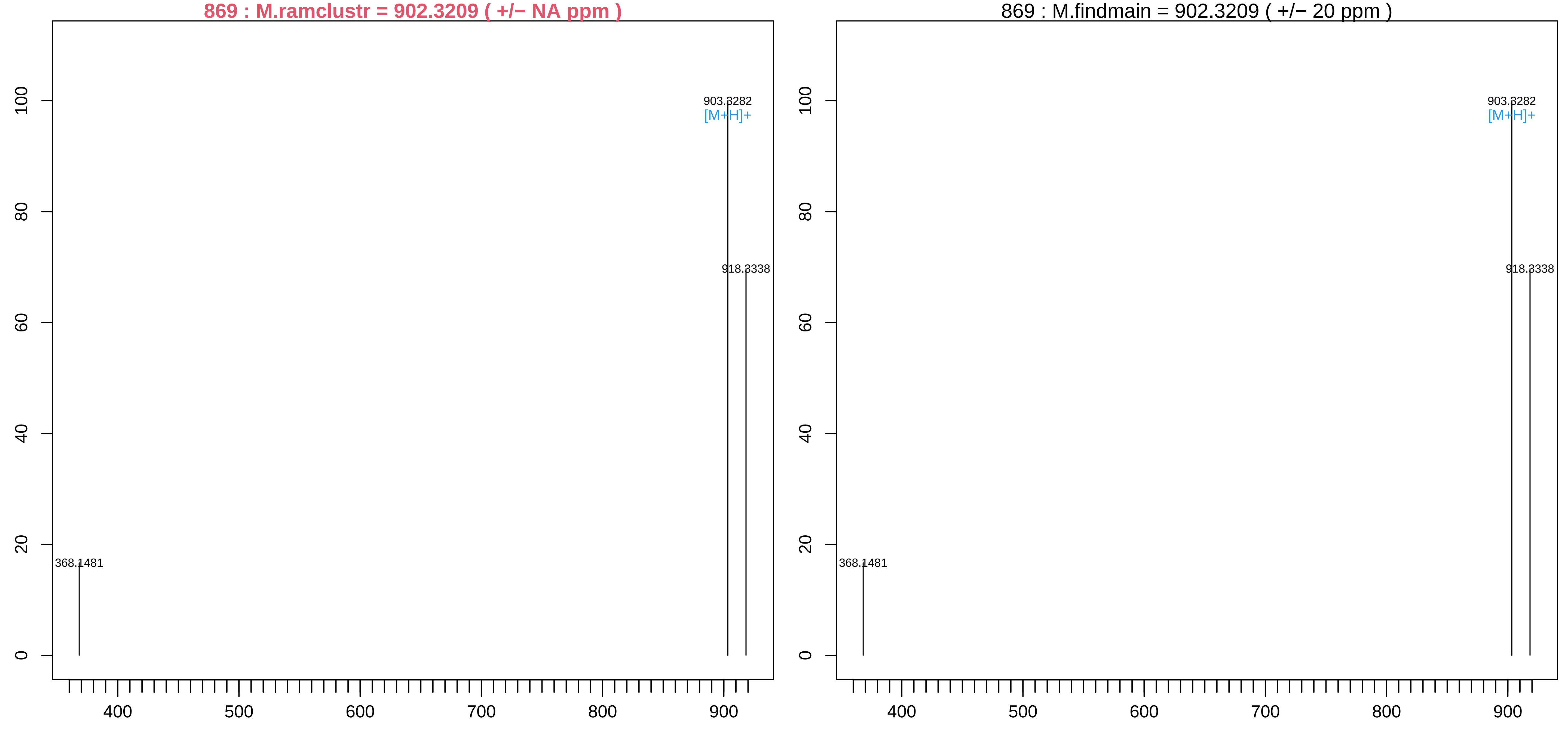

869 : M.ramclustr = 902.3209 (+/− NA ppm)
903.3282
[M+H]+
918.3338
368.1481
869 : M.findmain = 902.3209 (+/− 20 ppm)
903.3282
[M+H]+
918.3338
368.1481

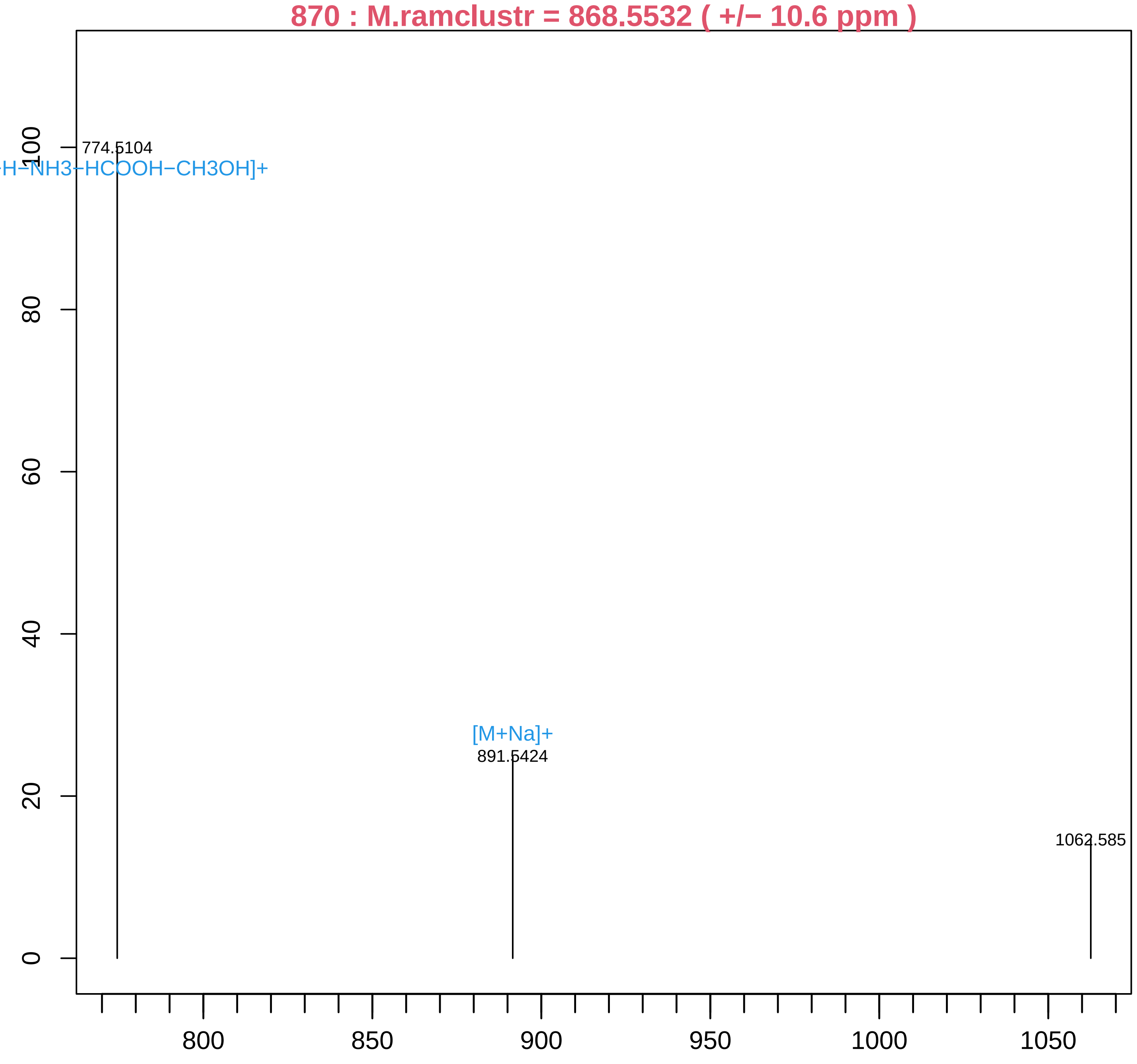
870 : M.ramclustr = 868.5532 (+/− 10.6 ppm)
774.5104
H−NH3−HCOOH−CH3OH]+
[M+Na]+
891.5424
1062.585
0
20
40
60
80
100
800
850
900
950
1000
1050
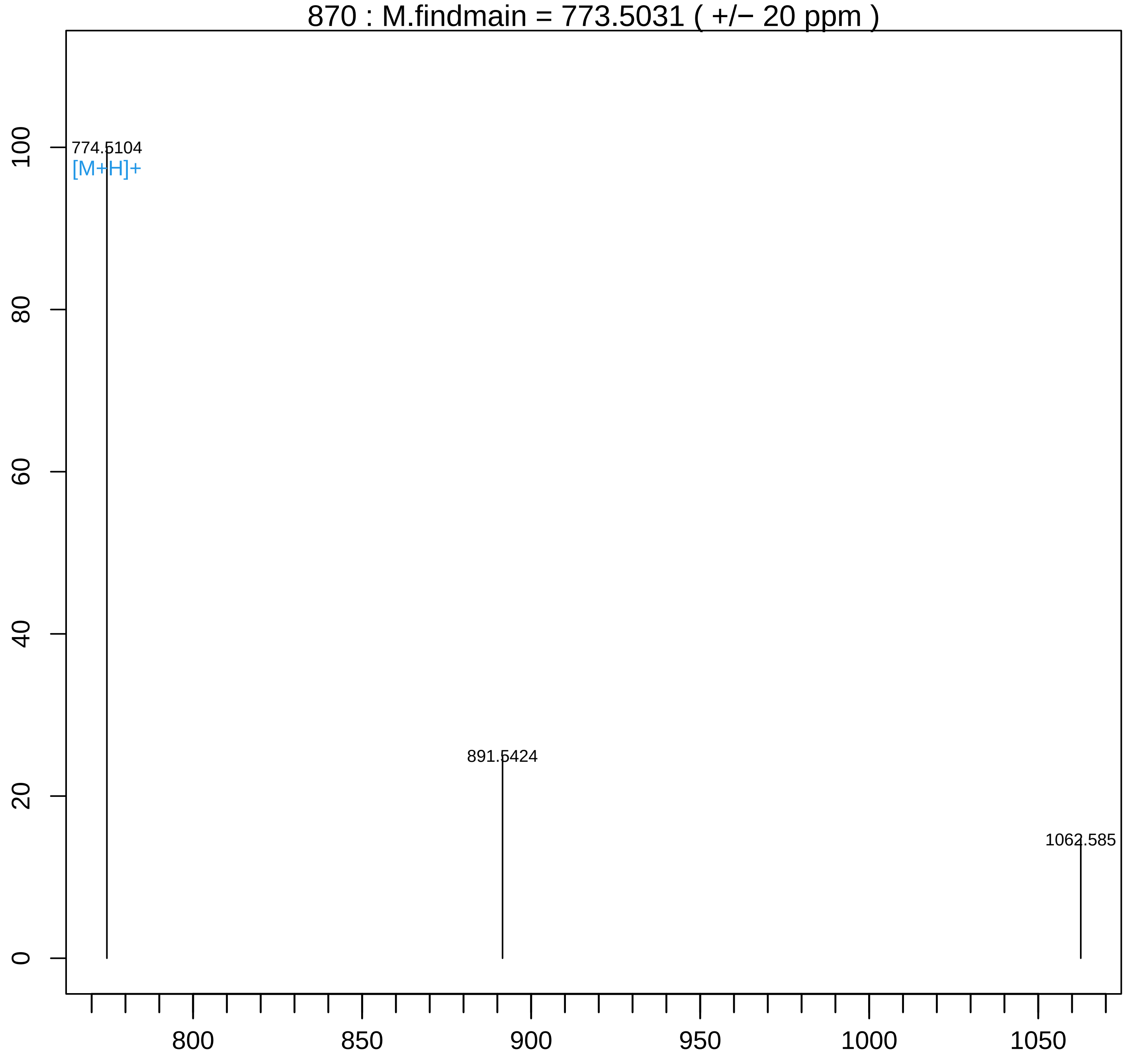
870 : M.findmain = 773.5031 (+/− 20 ppm)
774.5104
[M+H]+
891.5424
1062.585
0
20
40
60
80
100
800
850
900
950
1000
1050

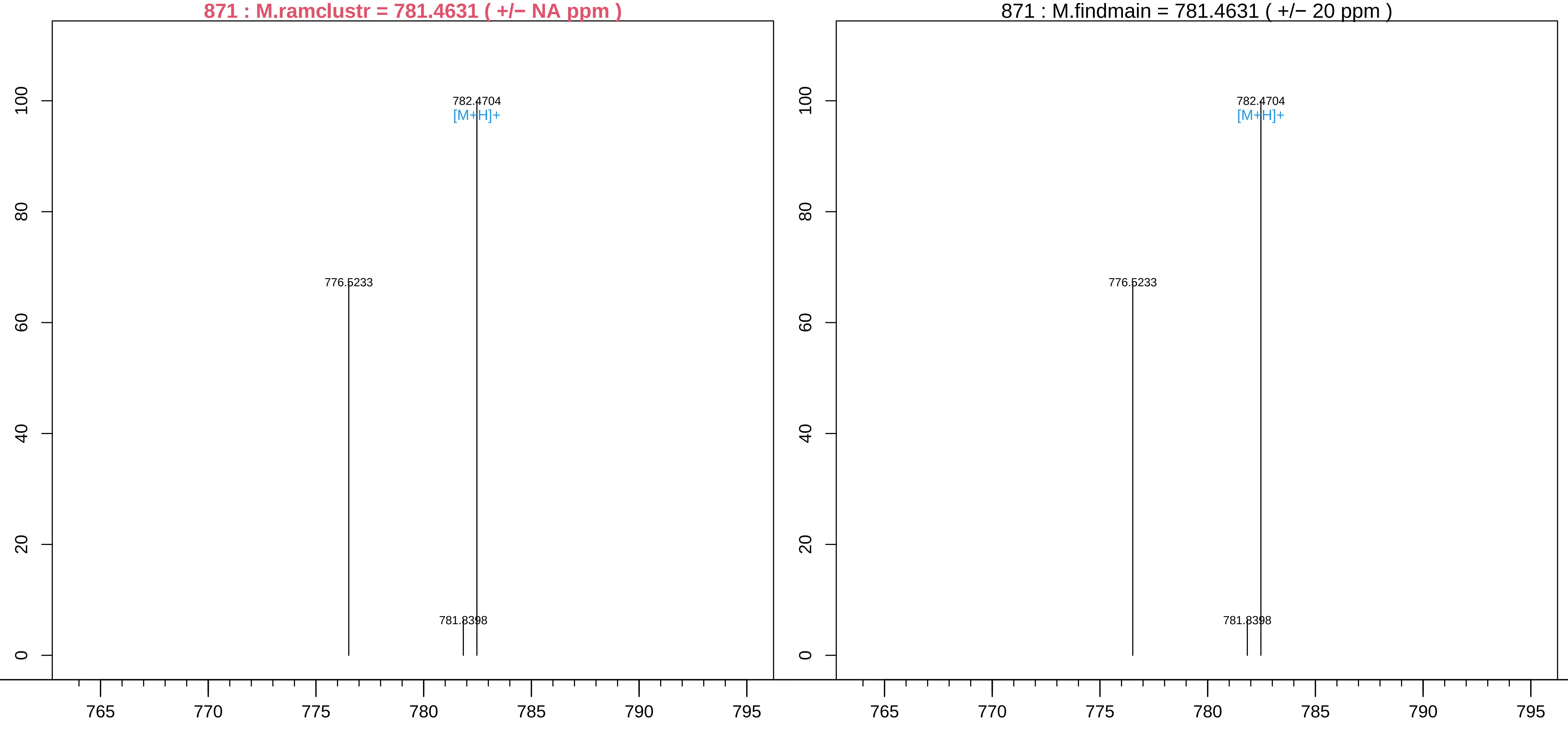
871 : M.ramclustr = 781.4631 (+/− NA ppm)
782.4704
[M+H]+
776.5233
781.8398
0
20
40
60
80
100
765
770
775
780
785
790
795
871 : M.findmain = 781.4631 (+/− 20 ppm)
782.4704
[M+H]+
776.5233
781.8398
0
20
40
60
80
100
765
770
775
780
785
790
795

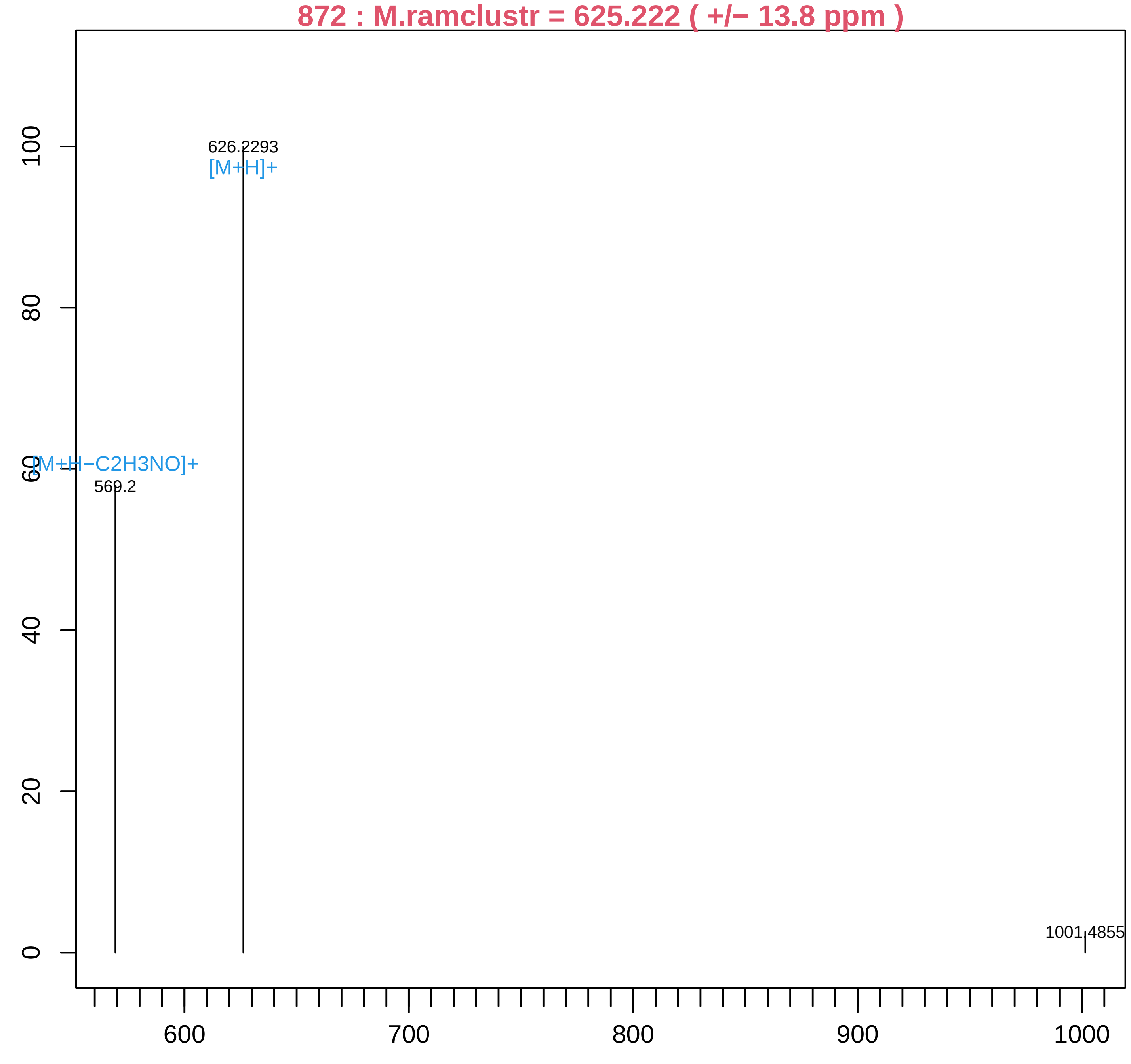

872 : M.ramclustr = 625.222 (+/− 13.8 ppm)
626.2293
[M+H]+
[M+H−C2H3NO]+
569.2
1001.4855

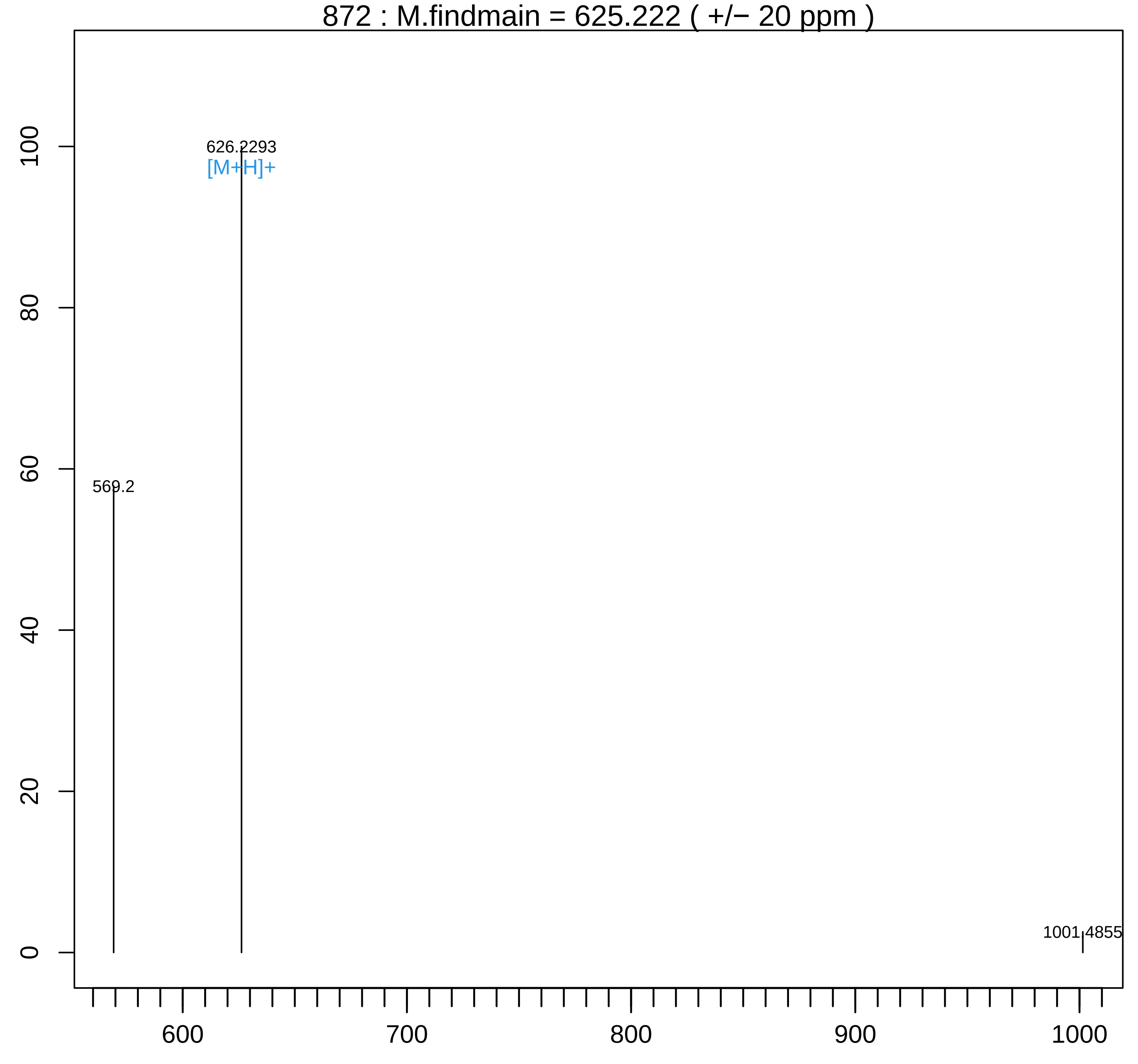

872 : M.findmain = 625.222 (+/− 20 ppm)
626.2293
[M+H]+
569.2
1001.4855

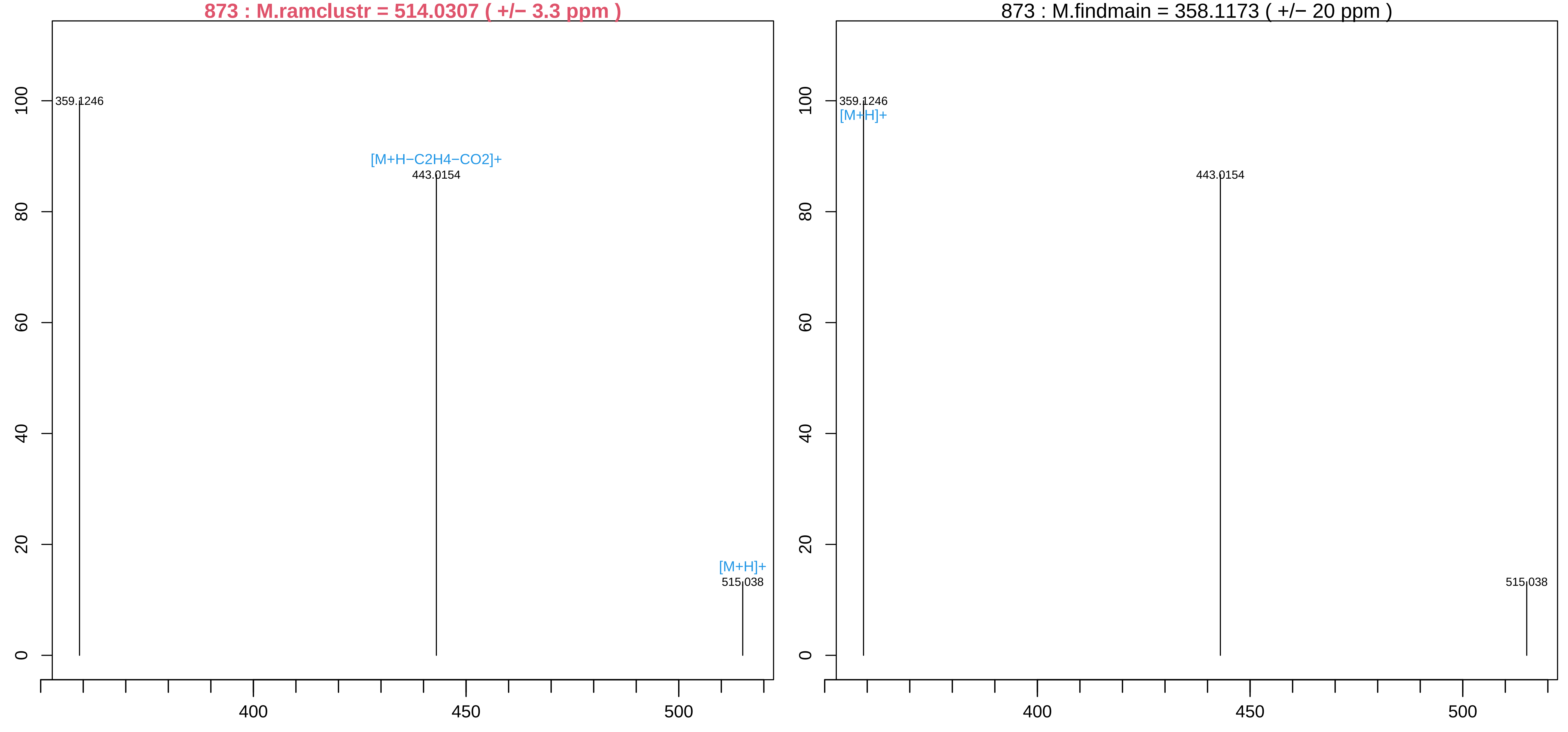

873 : M.ramclustr = 514.0307 (+/− 3.3 ppm)
359.1246
[M+H−C2H4−CO2]+
443.0154
[M+H]+
515.038
0
20
40
60
80
100
400
450
500
873 : M.findmain = 358.1173 (+/− 20 ppm)
359.1246
[M+H]+
443.0154
515.038
0
20
40
60
80
100
400
450
500

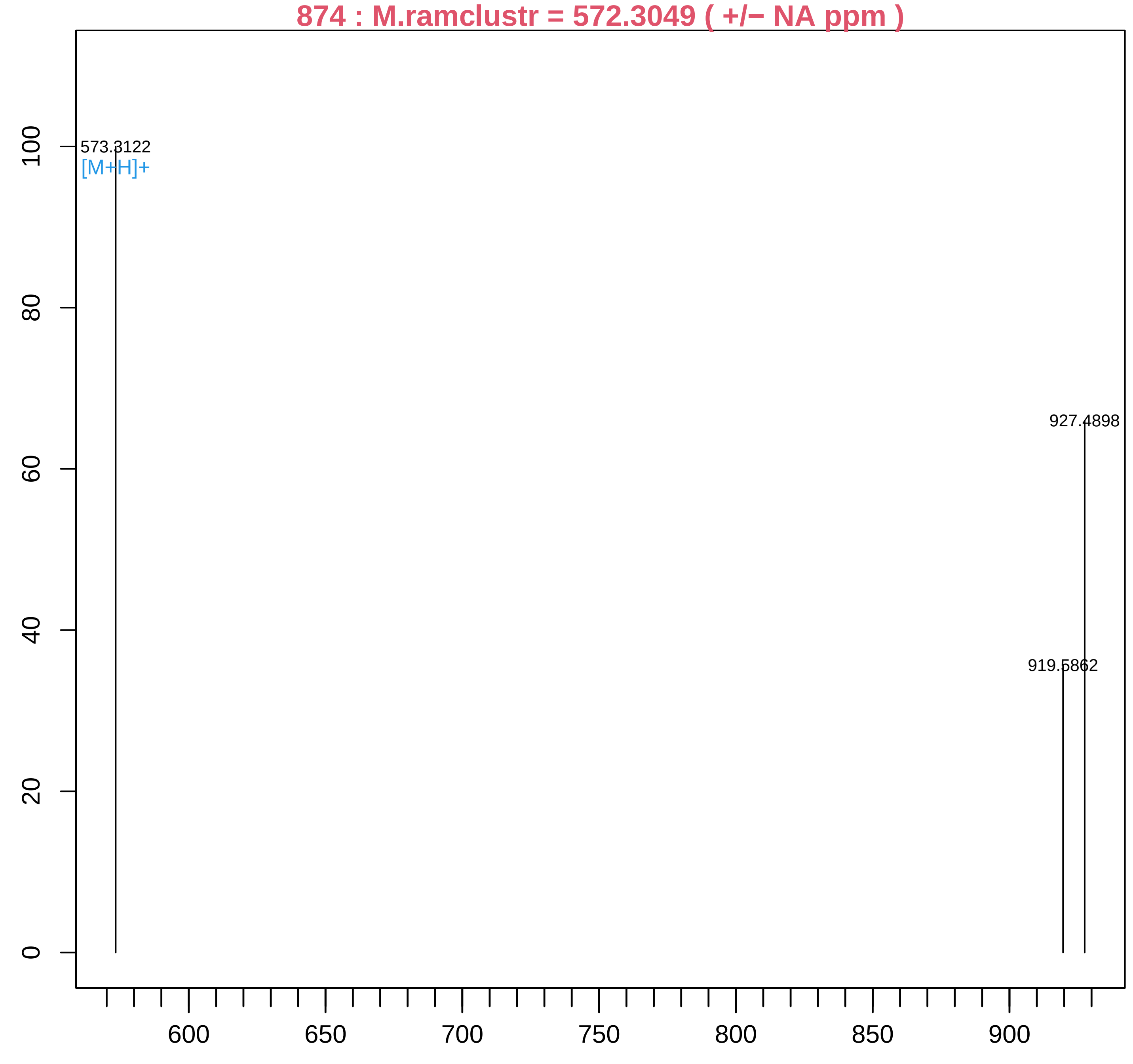
874 : M.ramclustr = 572.3049 (+/− NA ppm)
573.3122
[M+H]+
927.4898
919.5862

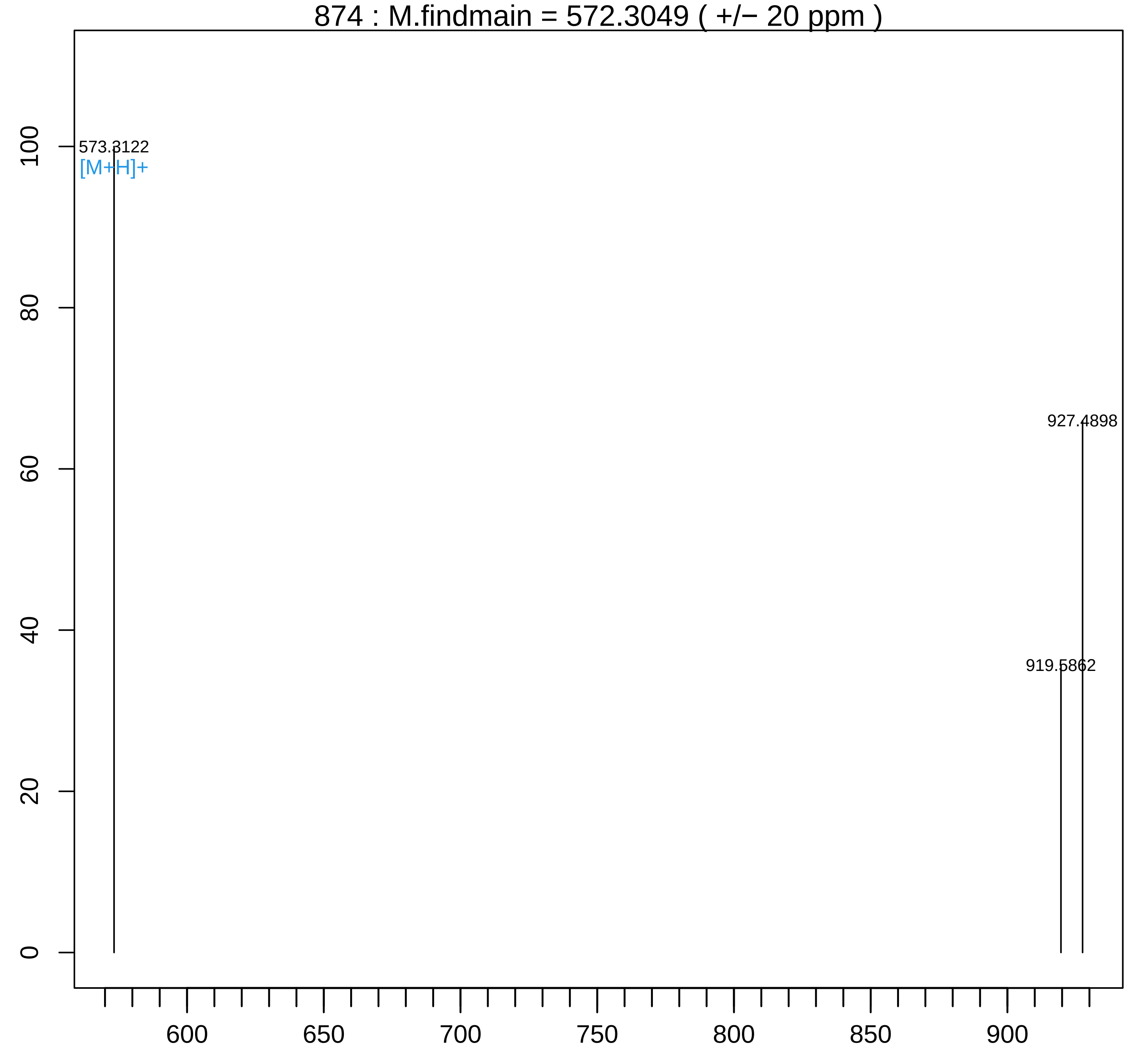
874 : M.findmain = 572.3049 (+/− 20 ppm)
573.3122
[M+H]+
927.4898
919.5862

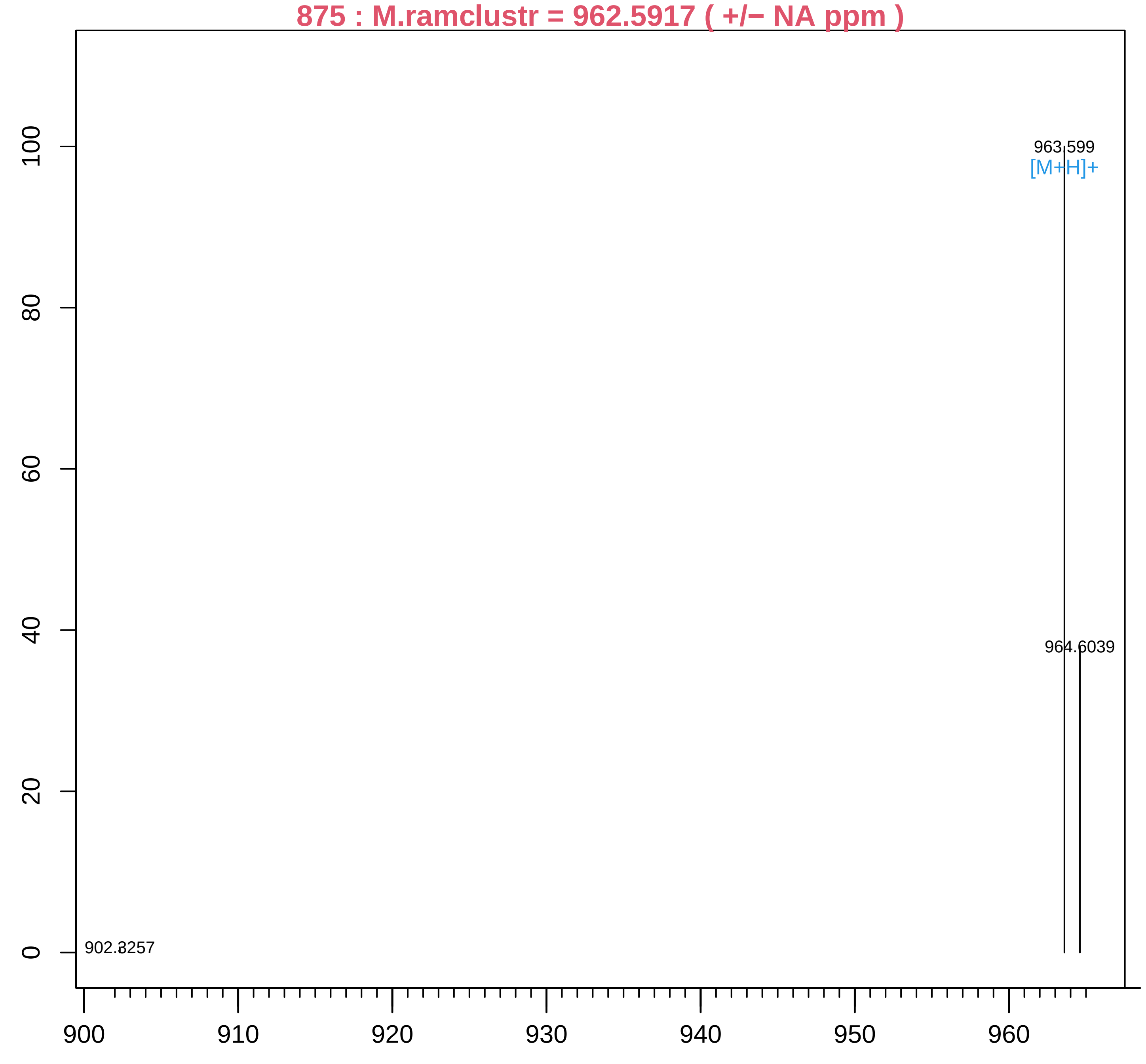
875 : M.ramclustr = 962.5917 (+/− NA ppm)
963.599
[M+H]+
964.6039
902.8257
0
20
40
60
80
100
900
910
920
930
940
950
960
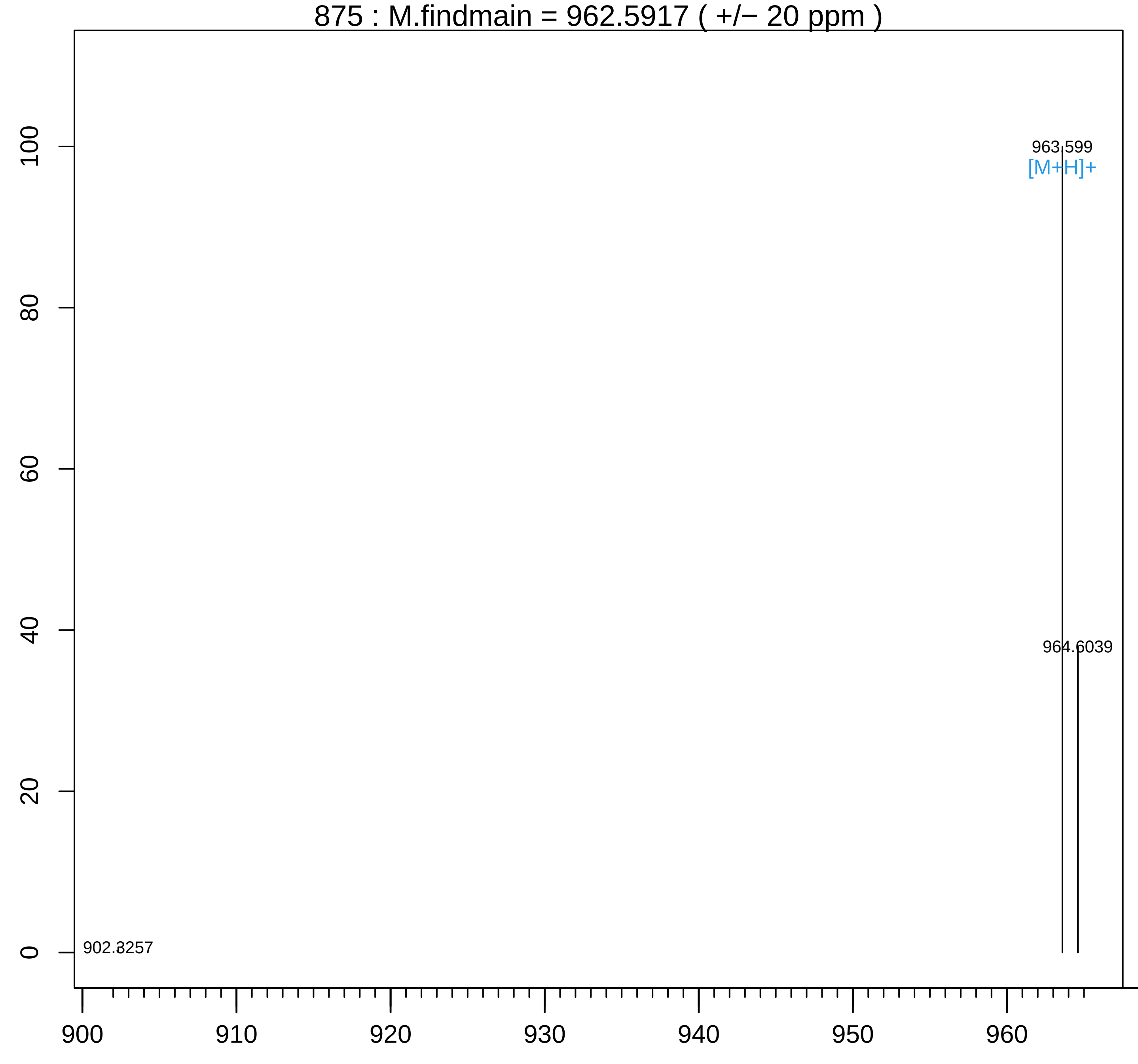
875 : M.findmain = 962.5917 (+/− 20 ppm)
963.599
[M+H]+
964.6039
902.8257
0
20
40
60
80
100
900
910
920
930
940
950
960

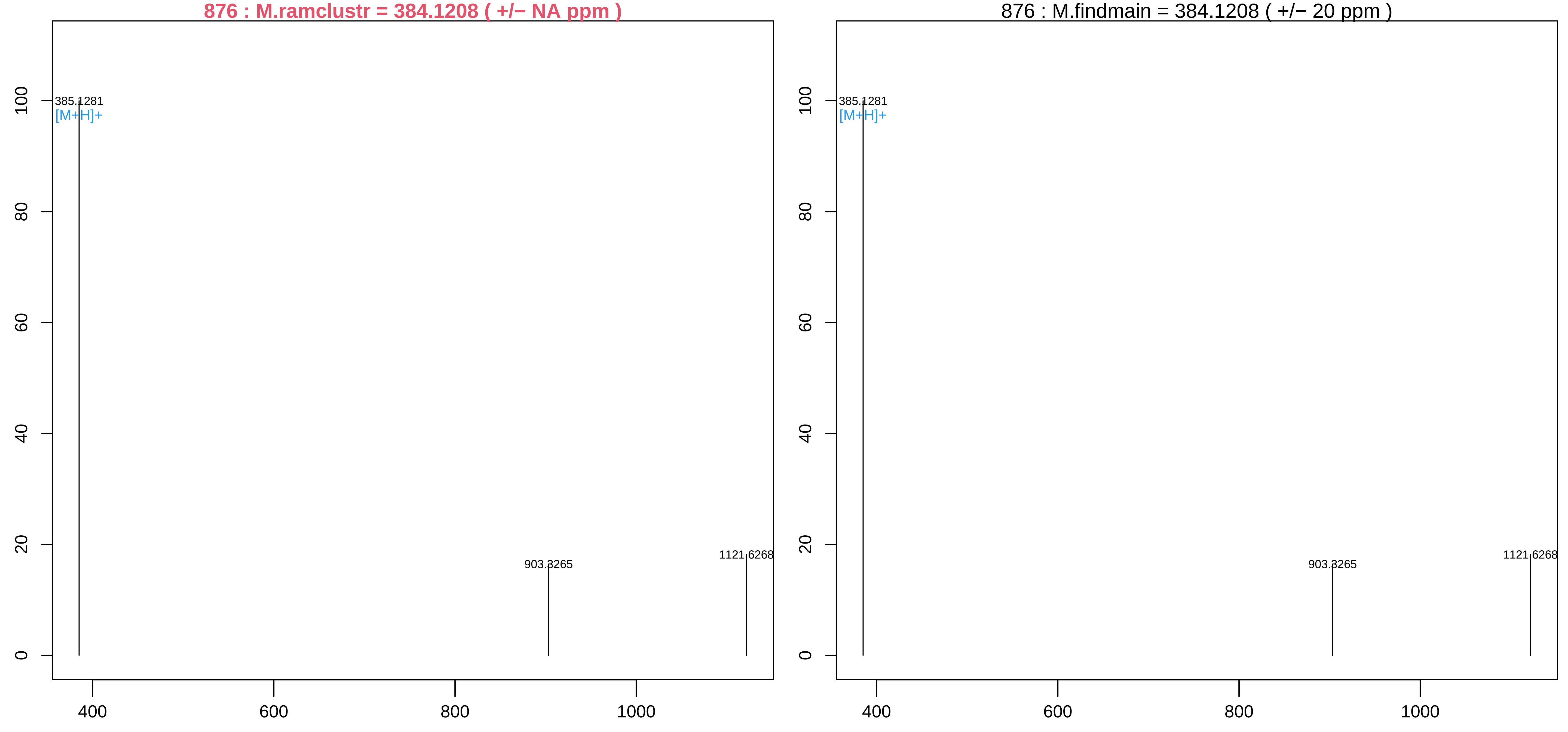

876 : M.ramclustr = 384.1208 (+/− NA ppm)
876 : M.findmain = 384.1208 (+/− 20 ppm)
385.1281
[M+H]+
903.3265
1121.6268
0
20
40
60
80
100
400
600
800
1000

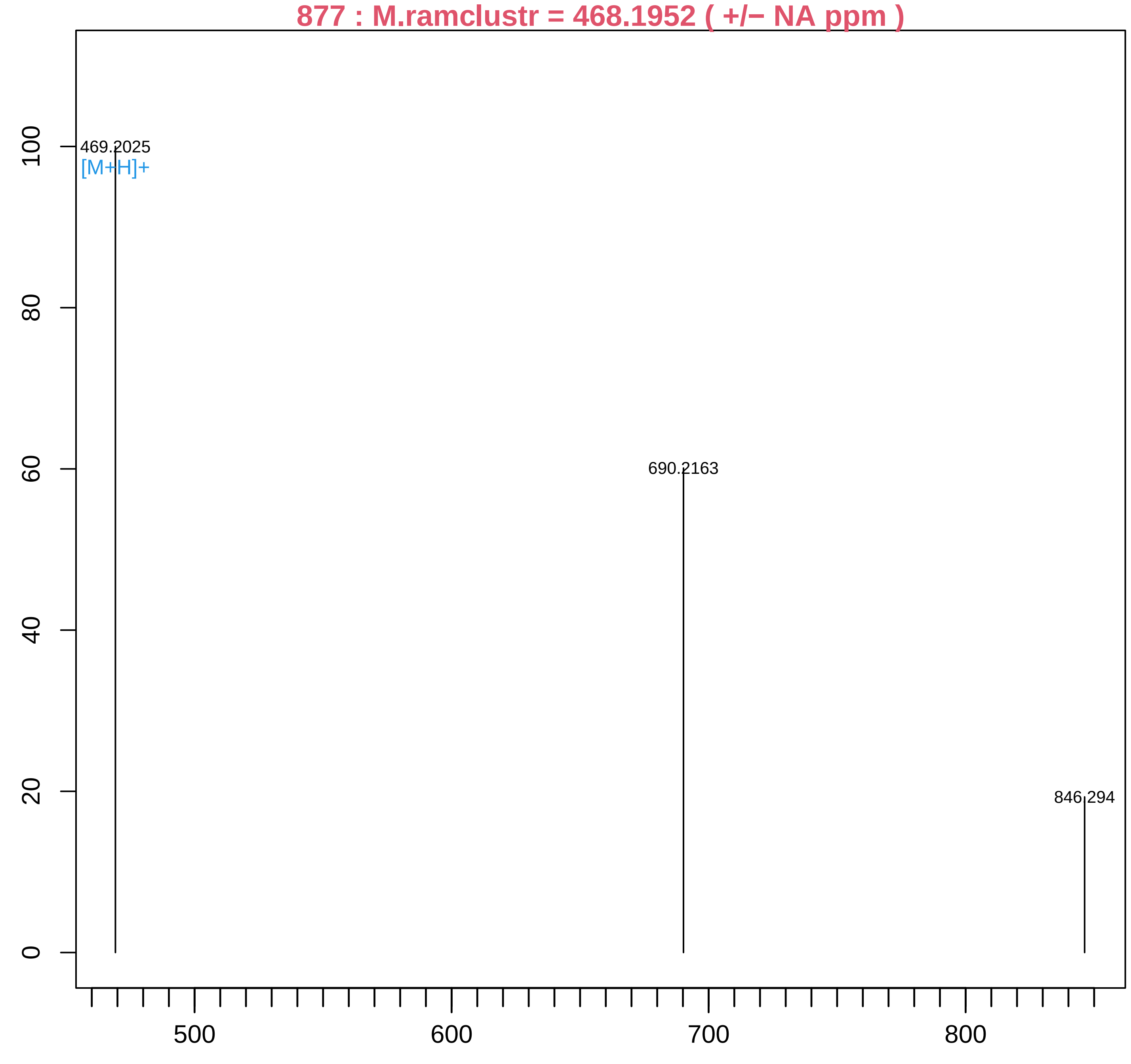
877 : M.ramclustr = 468.1952 (+/− NA ppm)
469.2025
[M+H]+
690.2163
846.294
0
20
40
60
80
100
500
600
700
800
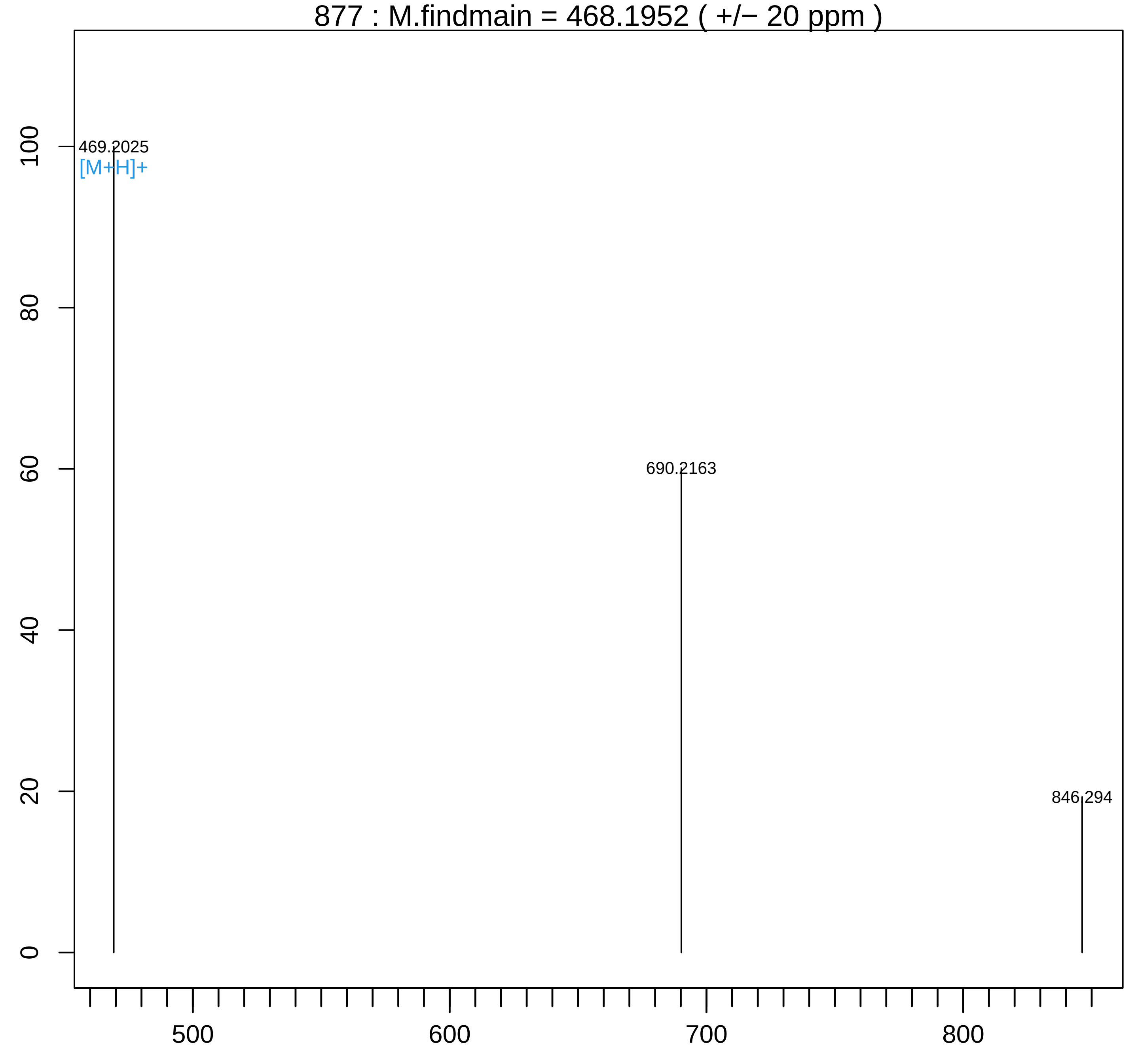
877 : M.findmain = 468.1952 (+/− 20 ppm)
469.2025
[M+H]+
690.2163
846.294
0
20
40
60
80
100
500
600
700
800

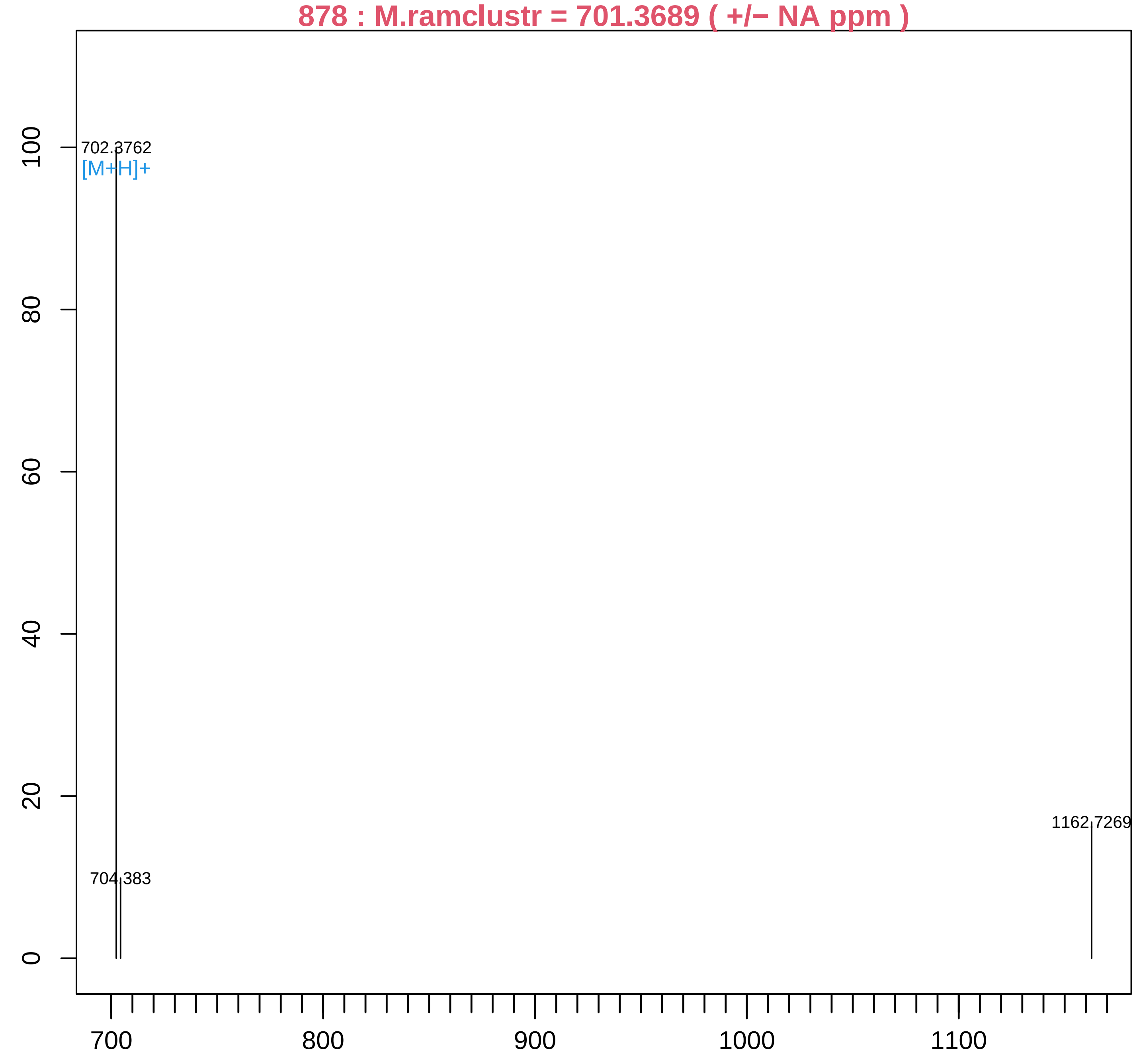
878 : M.ramclustr = 701.3689 (+/− NA ppm)
702.3762
[M+H]+
704.383
1162.7269
0
20
40
60
80
100
700
800
900
1000
1100

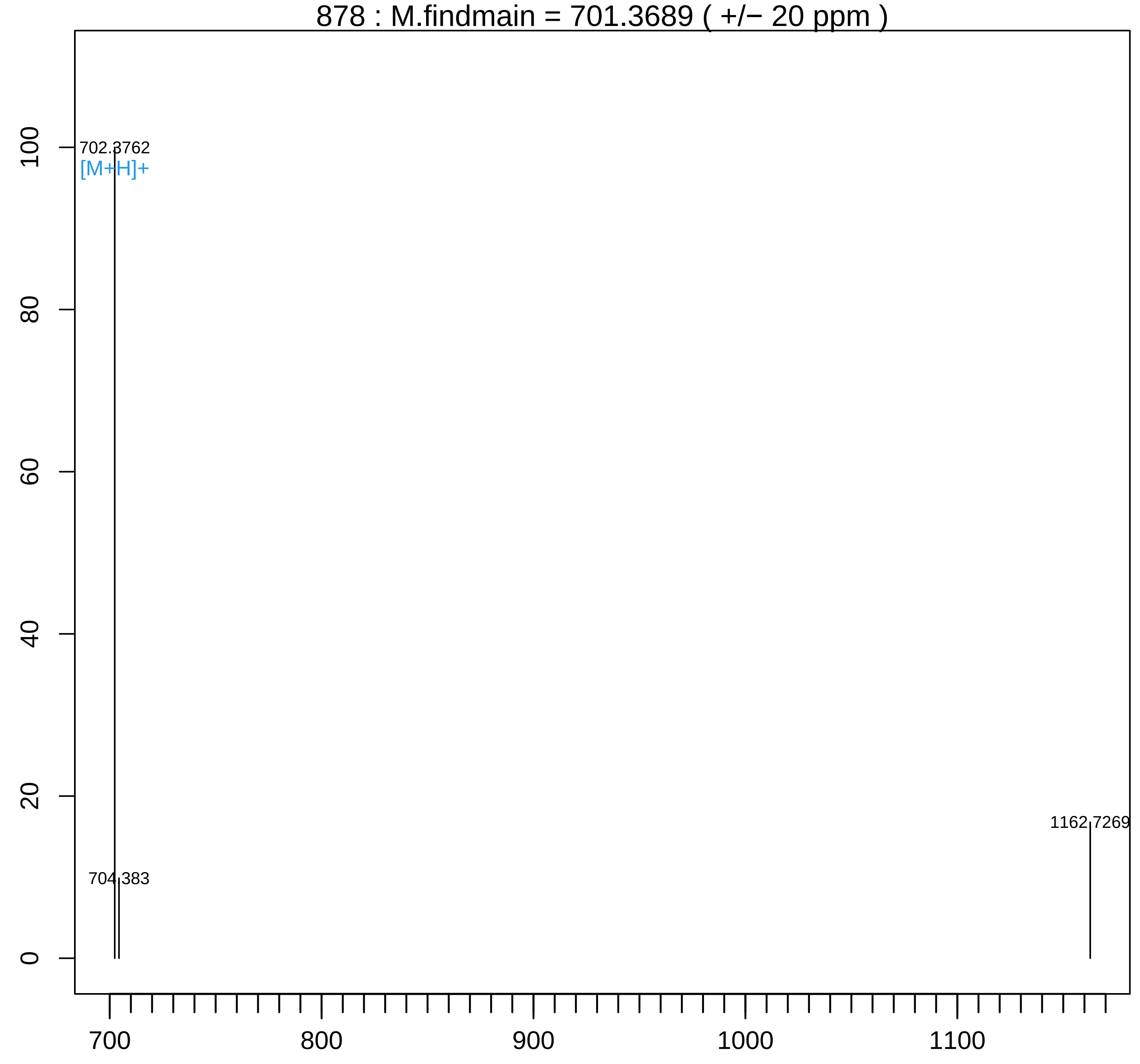
878 : M.findmain = 701.3689 (+/− 20 ppm)
702.3762
[M+H]+
704.383
1162.7269
0
20
40
60
80
100
700
800
900
1000
1100

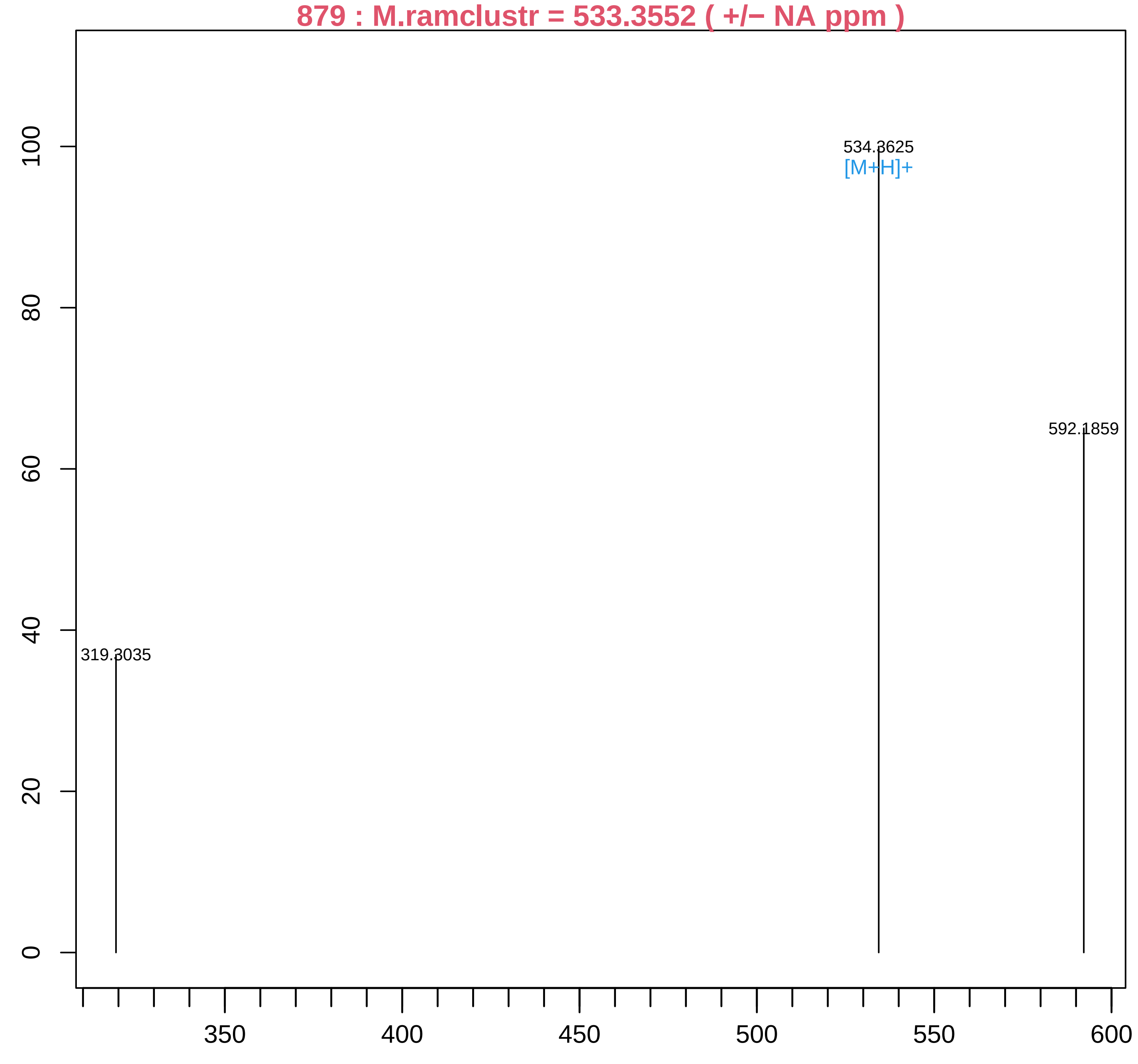
879 : M.ramclustr = 533.3552 (+/− NA ppm)
534.3625
[M+H]+
592.1859
319.3035
0
20
40
60
80
100
350
400
450
500
550
600

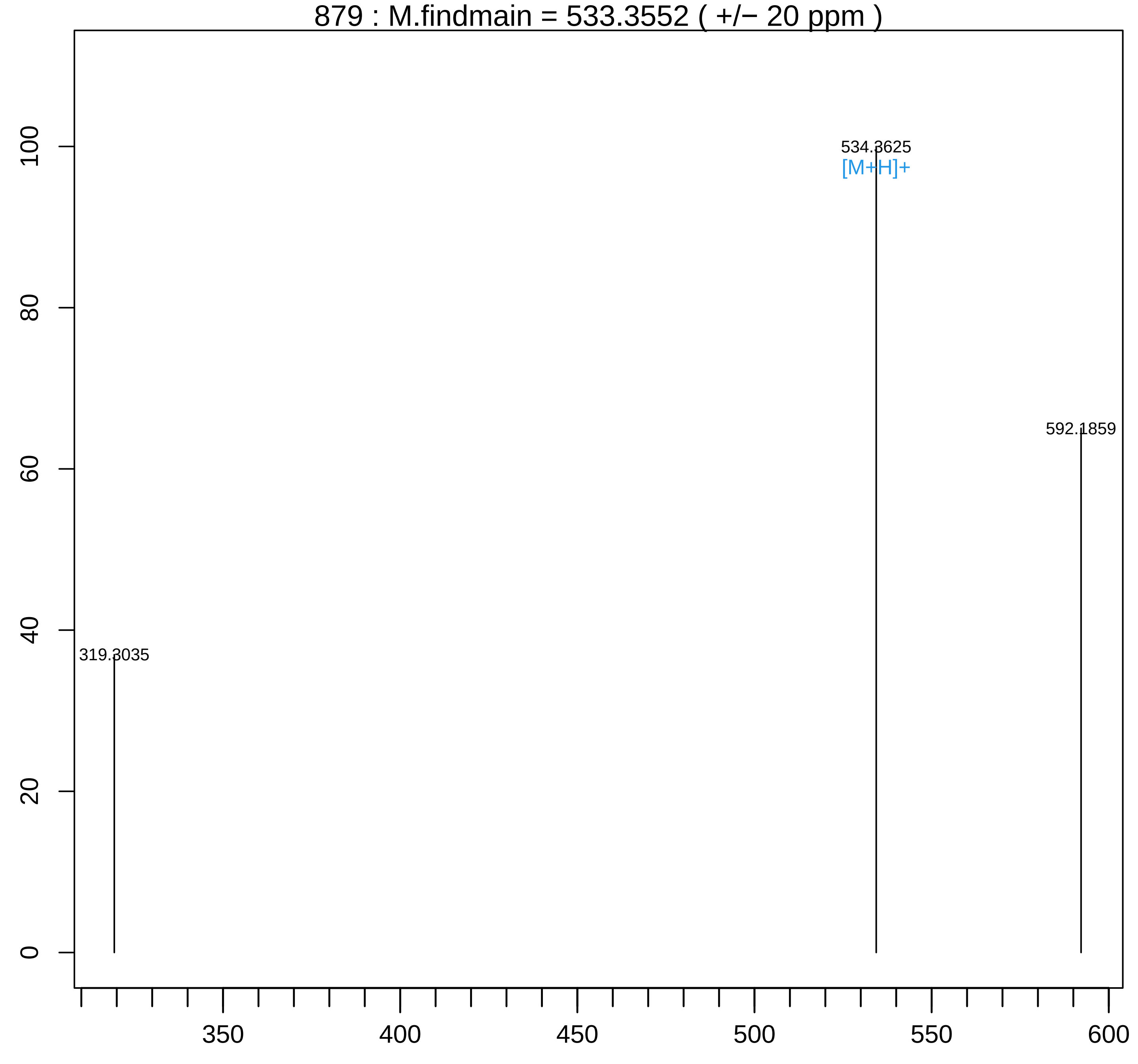
879 : M.findmain = 533.3552 (+/− 20 ppm)
534.3625
[M+H]+
592.1859
319.3035
0
20
40
60
80
100
350
400
450
500
550
600

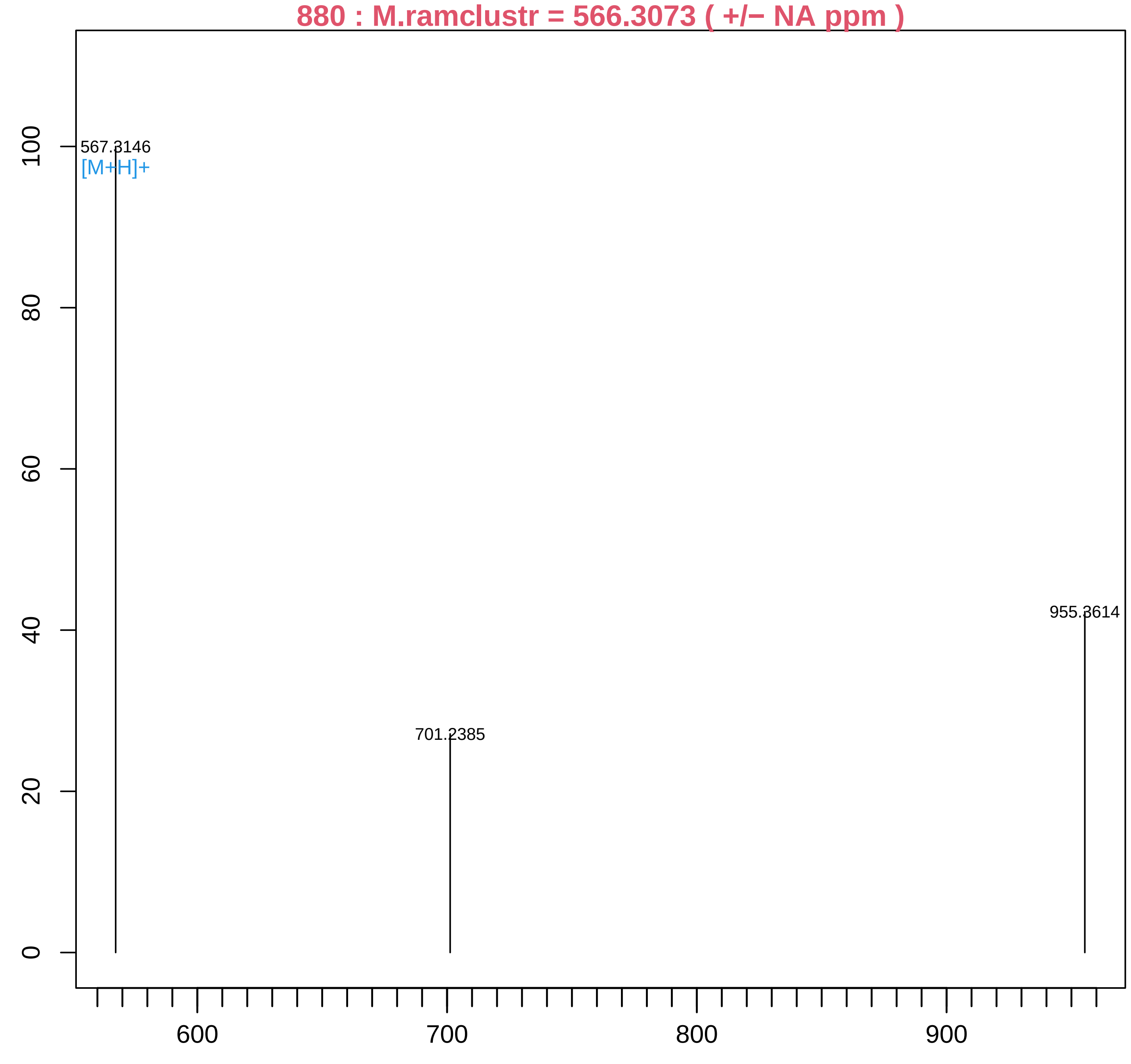
880 : M.ramclustr = 566.3073 (+/− NA ppm)
567.3146
[M+H]+
701.2385
955.3614
0
20
40
60
80
100
600
700
800
900

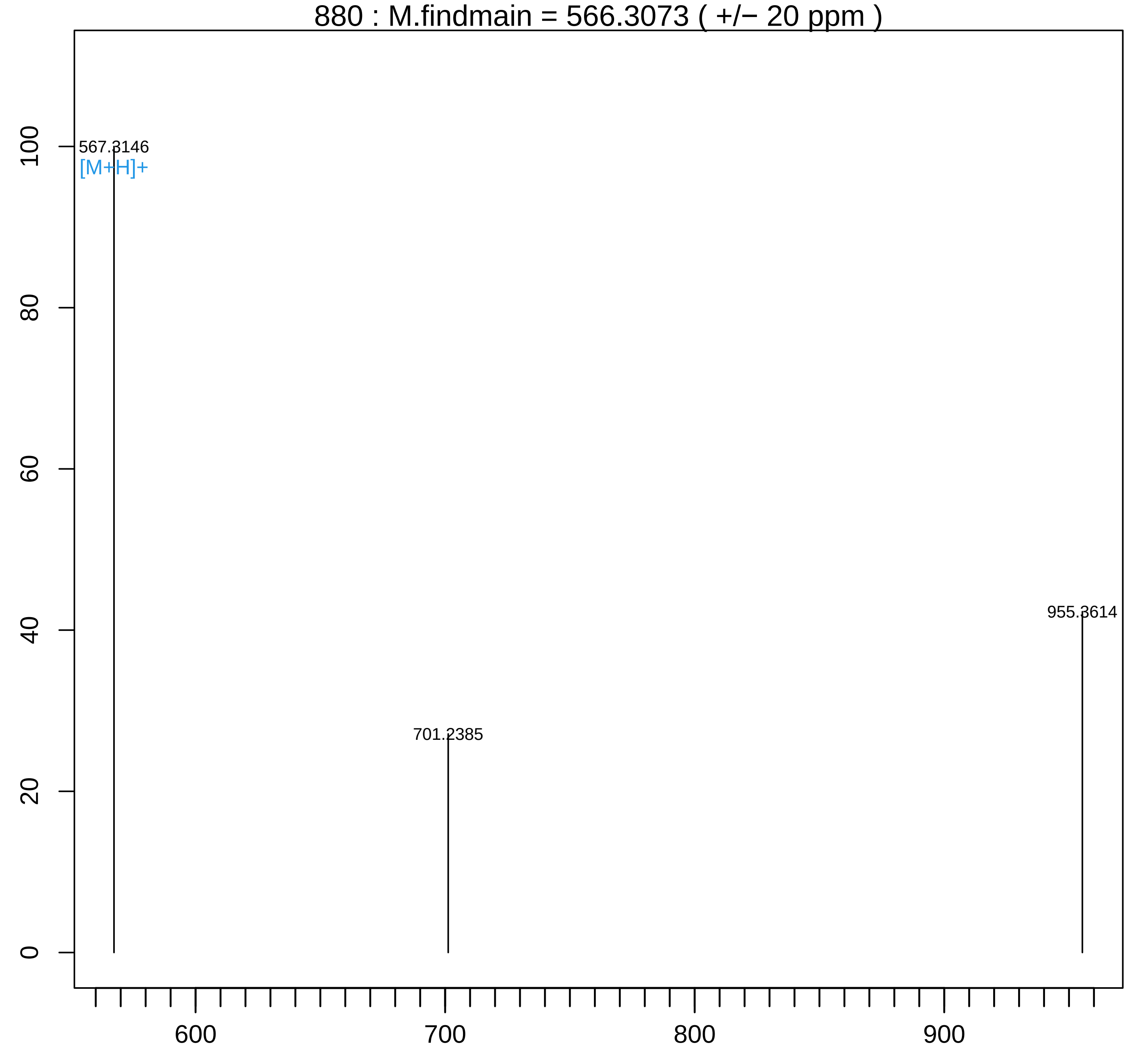
880 : M.findmain = 566.3073 (+/− 20 ppm)
567.3146
[M+H]+
701.2385
955.3614
0
20
40
60
80
100
600
700
800
900

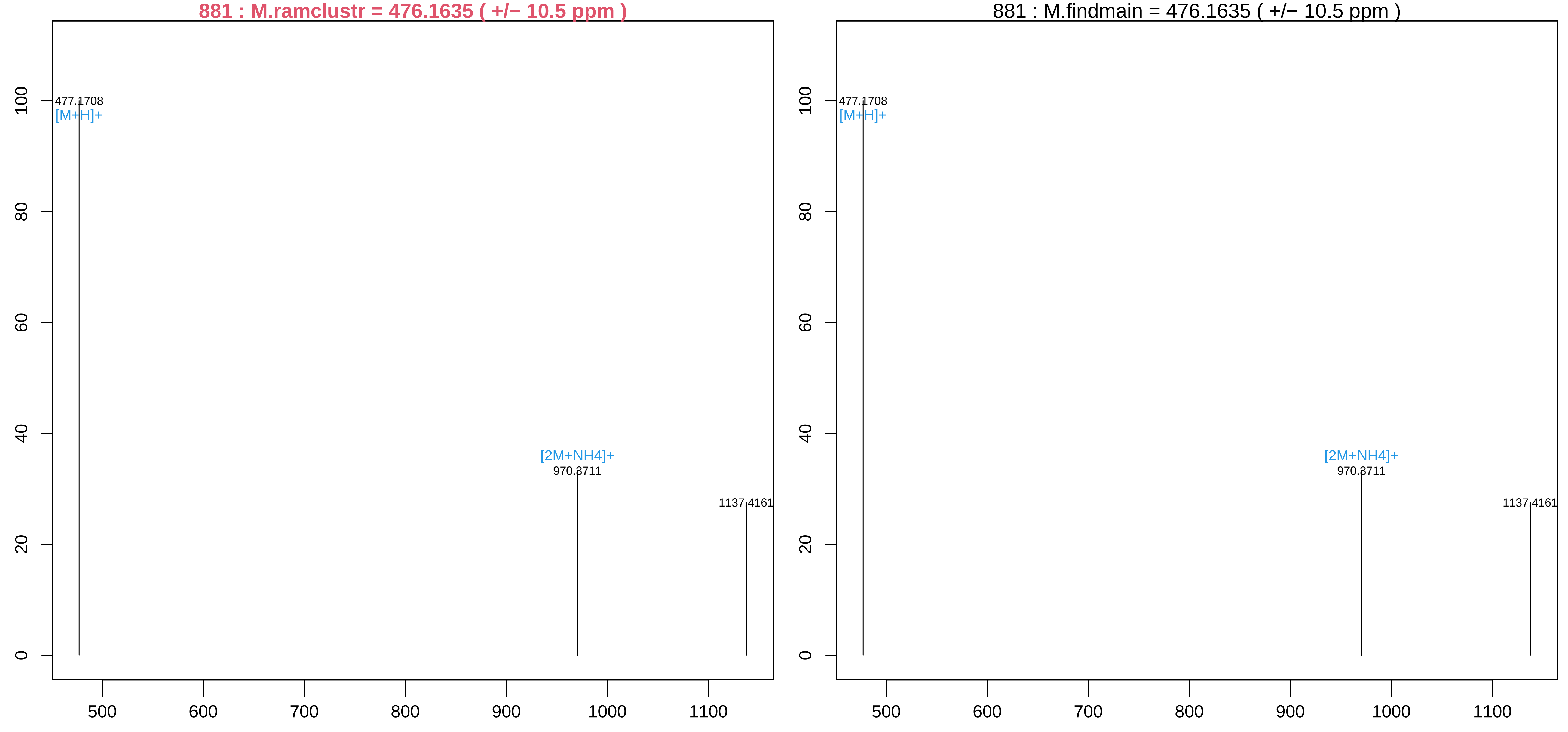
881 : M.ramclustr = 476.1635 (+/− 10.5 ppm)
477.1708
[M+H]+
[2M+NH4]+
970.3711
1137.4161
0
20
40
60
80
100
500
600
700
800
900
1000
1100
881 : M.findmain = 476.1635 (+/− 10.5 ppm)
477.1708
[M+H]+
[2M+NH4]+
970.3711
1137.4161
0
20
40
60
80
100
500
600
700
800
900
1000
1100

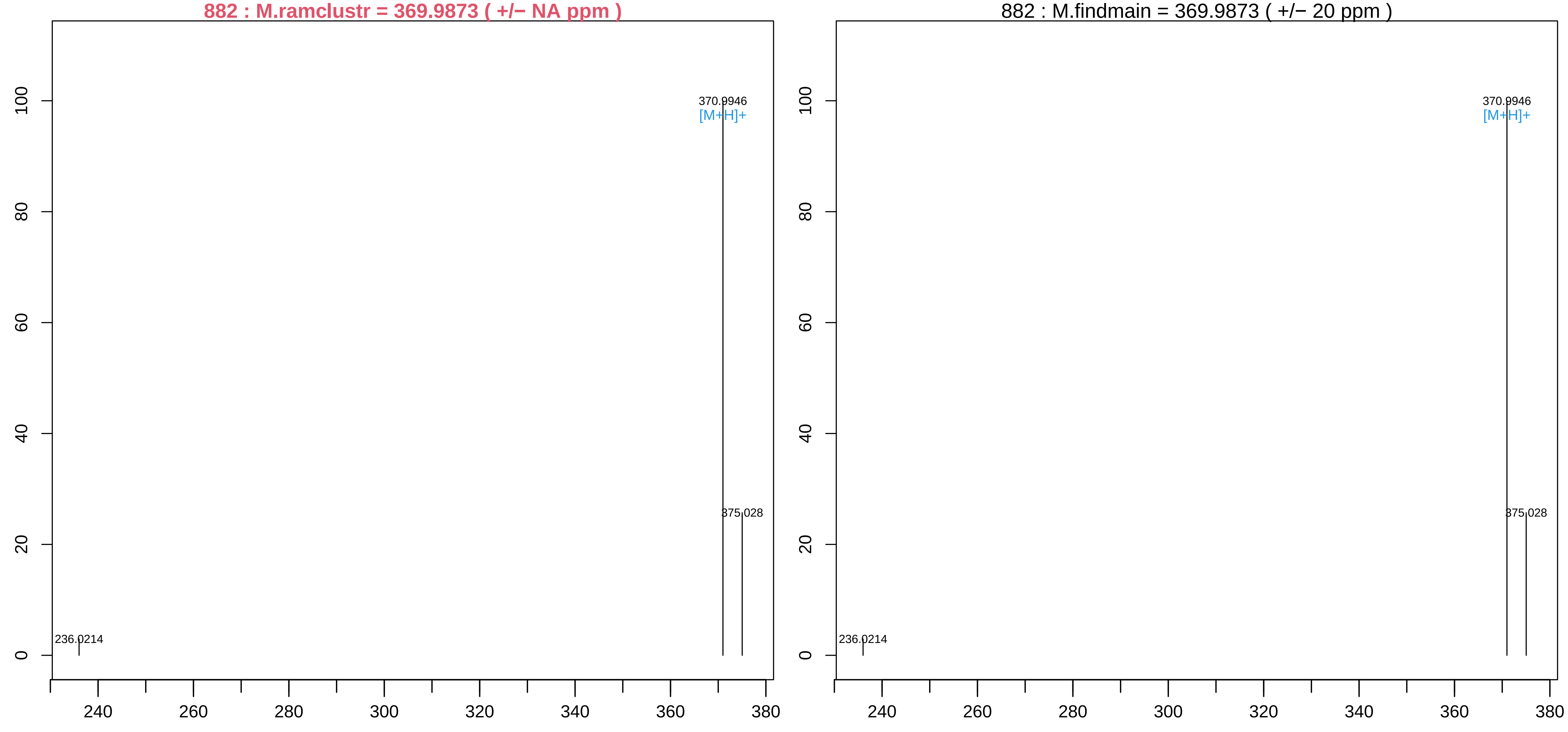
882 : M.ramclustr = 369.9873 (+/− NA ppm)
370.9946
[M+H]+
375.028
236.0214
882 : M.findmain = 369.9873 (+/− 20 ppm)
370.9946
[M+H]+
375.028
236.0214
0
20
40
60
80
100
240
260
280
300
320
340
360
380

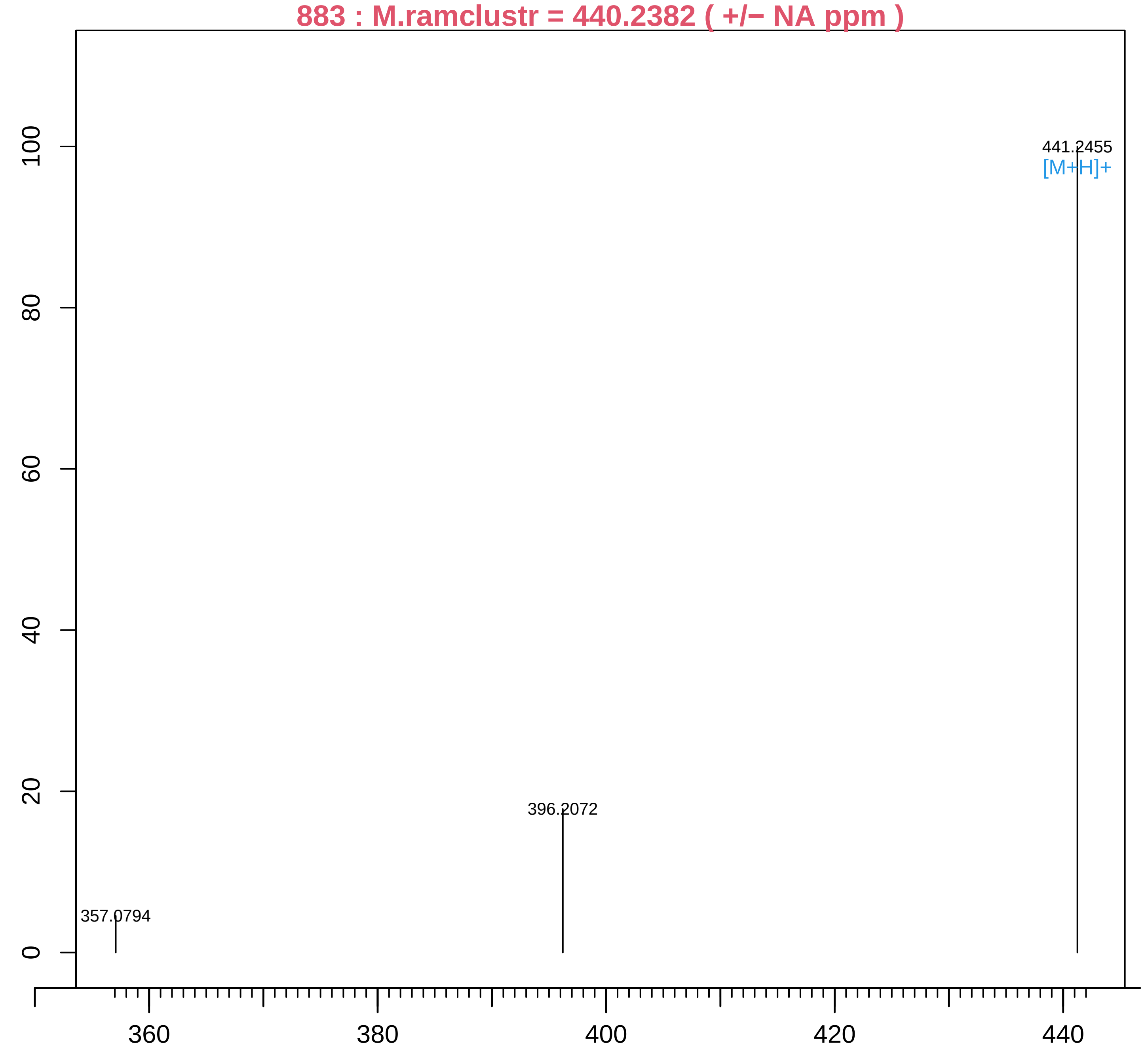
883 : M.ramclustr = 440.2382 (+/− NA ppm)
441.2455
[M+H]+
396.2072
357.0794
0
20
40
60
80
100
360
380
400
420
440

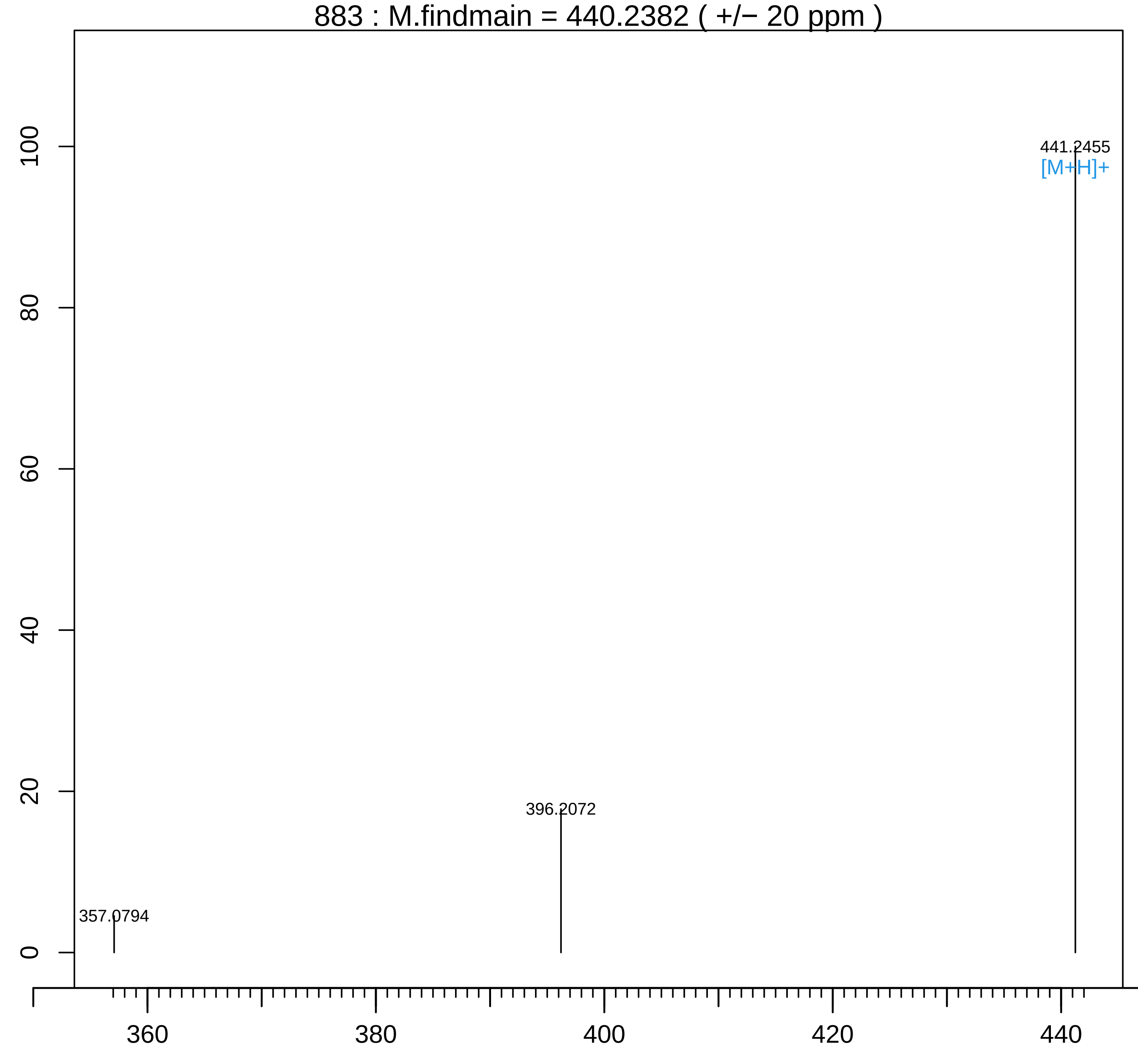
883 : M.findmain = 440.2382 (+/− 20 ppm)
441.2455
[M+H]+
396.2072
357.0794
0
20
40
60
80
100
360
380
400
420
440

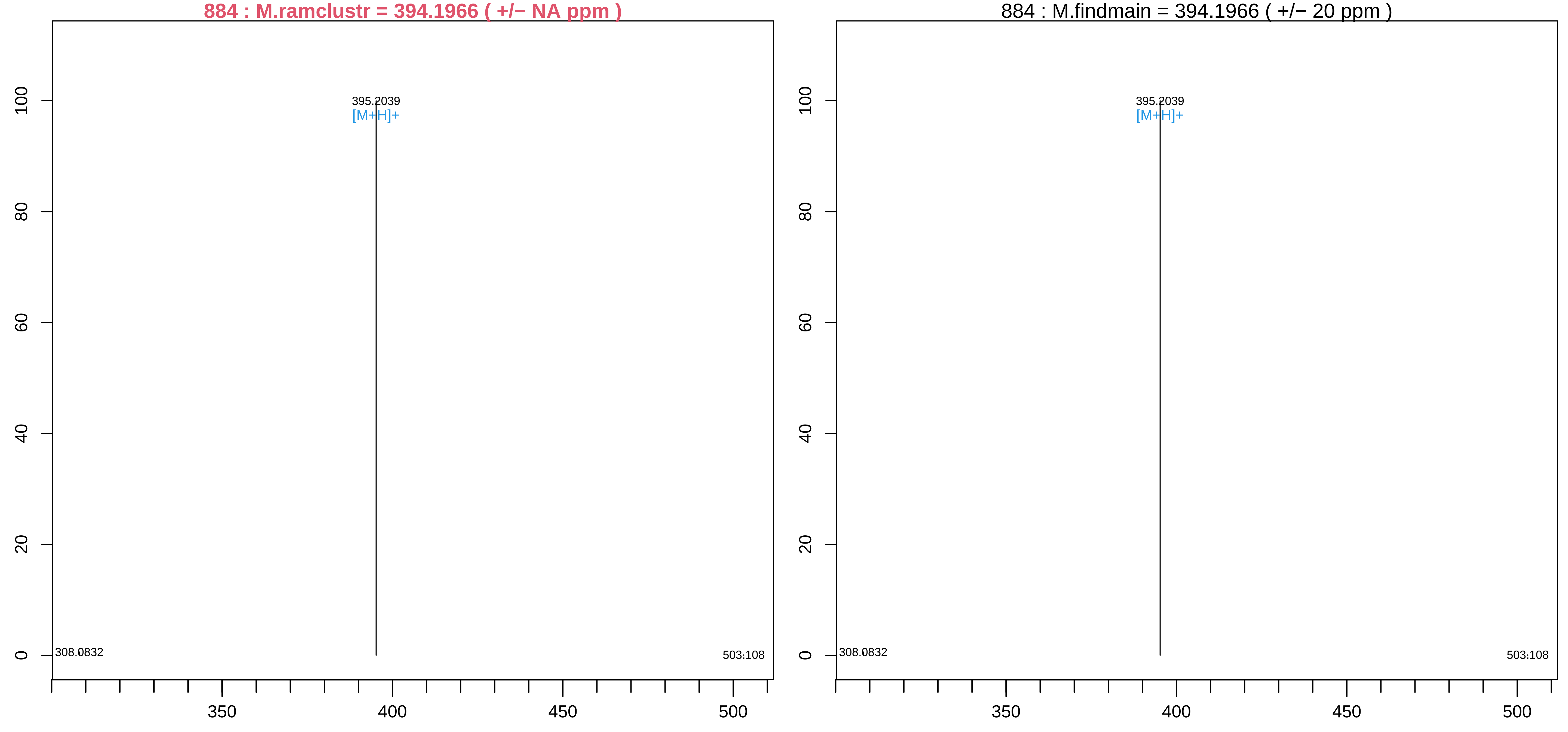

884 : M.ramclustr = 394.1966 (+/− NA ppm)
395.2039
[M+H]+
308.0832
503:108
0
20
40
60
80
100
350
400
450
500
884 : M.findmain = 394.1966 (+/− 20 ppm)
395.2039
[M+H]+
308.0832
503:108
0
20
40
60
80
100
350
400
450
500

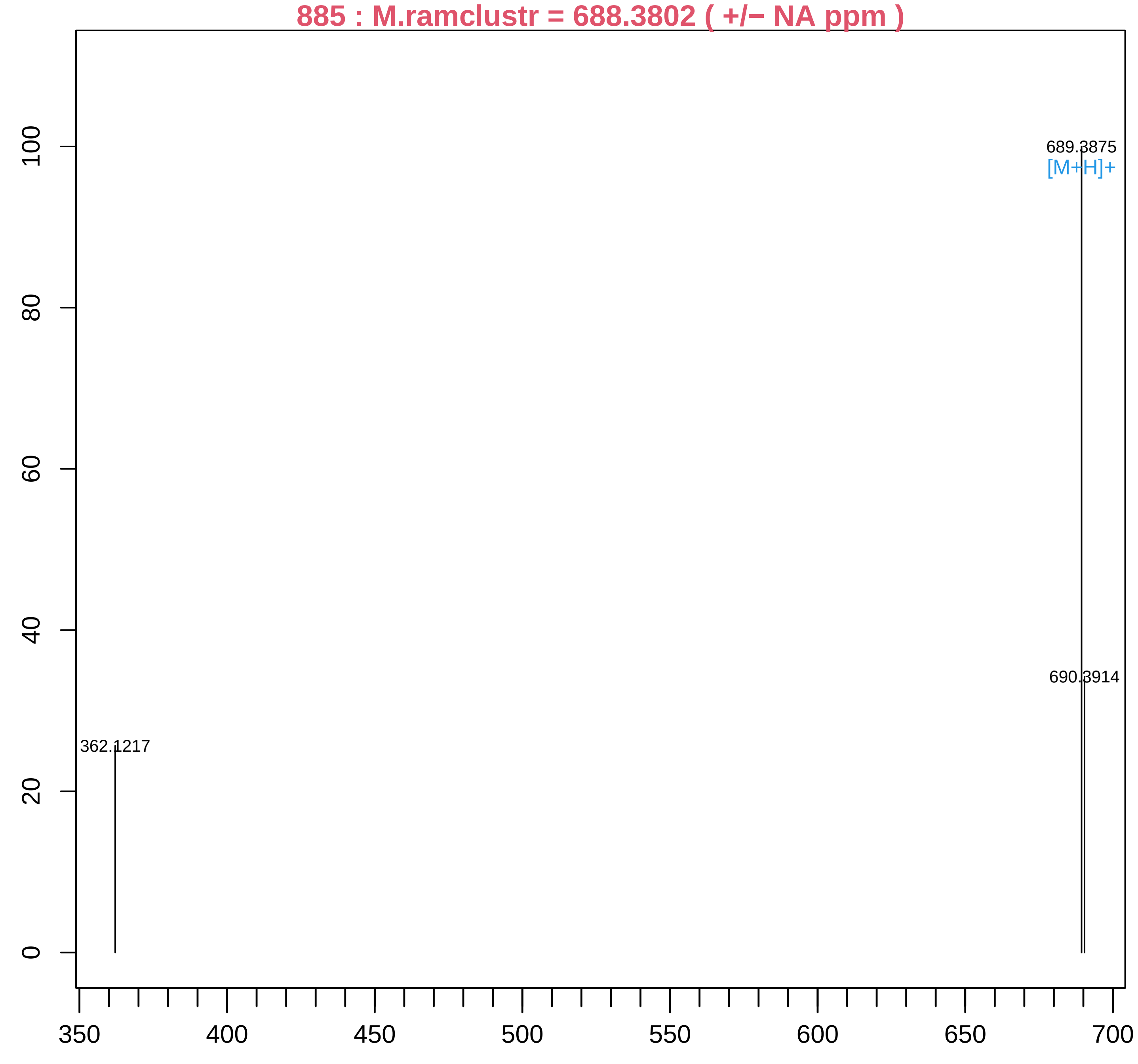
885 : M.ramclustr = 688.3802 (+/− NA ppm)
689.3875
[M+H]+
690.3914
362.1217
0
20
40
60
80
100
350
400
450
500
550
600
650
700

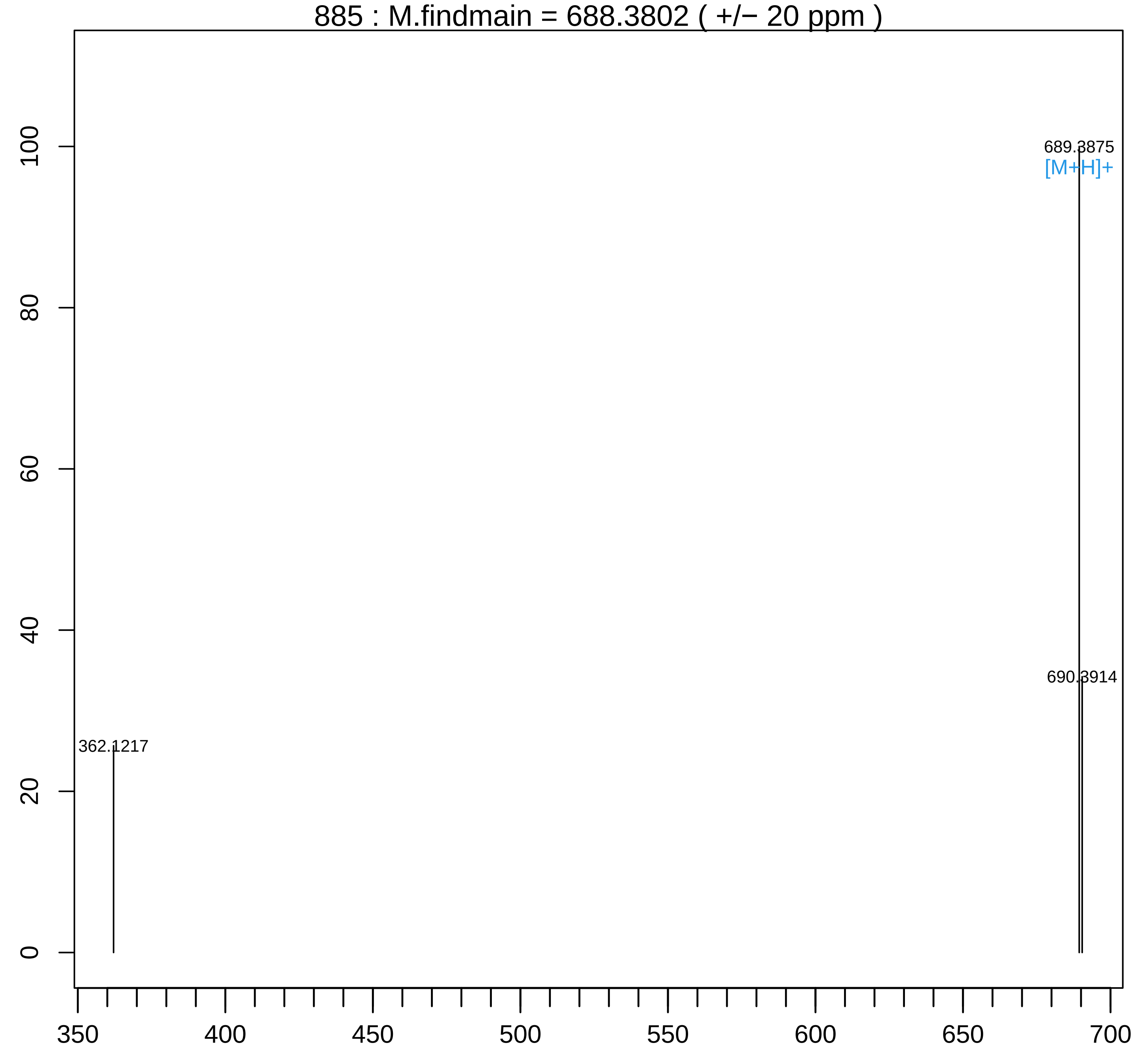
885 : M.findmain = 688.3802 (+/− 20 ppm)
689.3875
[M+H]+
690.3914
362.1217
0
20
40
60
80
100
350
400
450
500
550
600
650
700

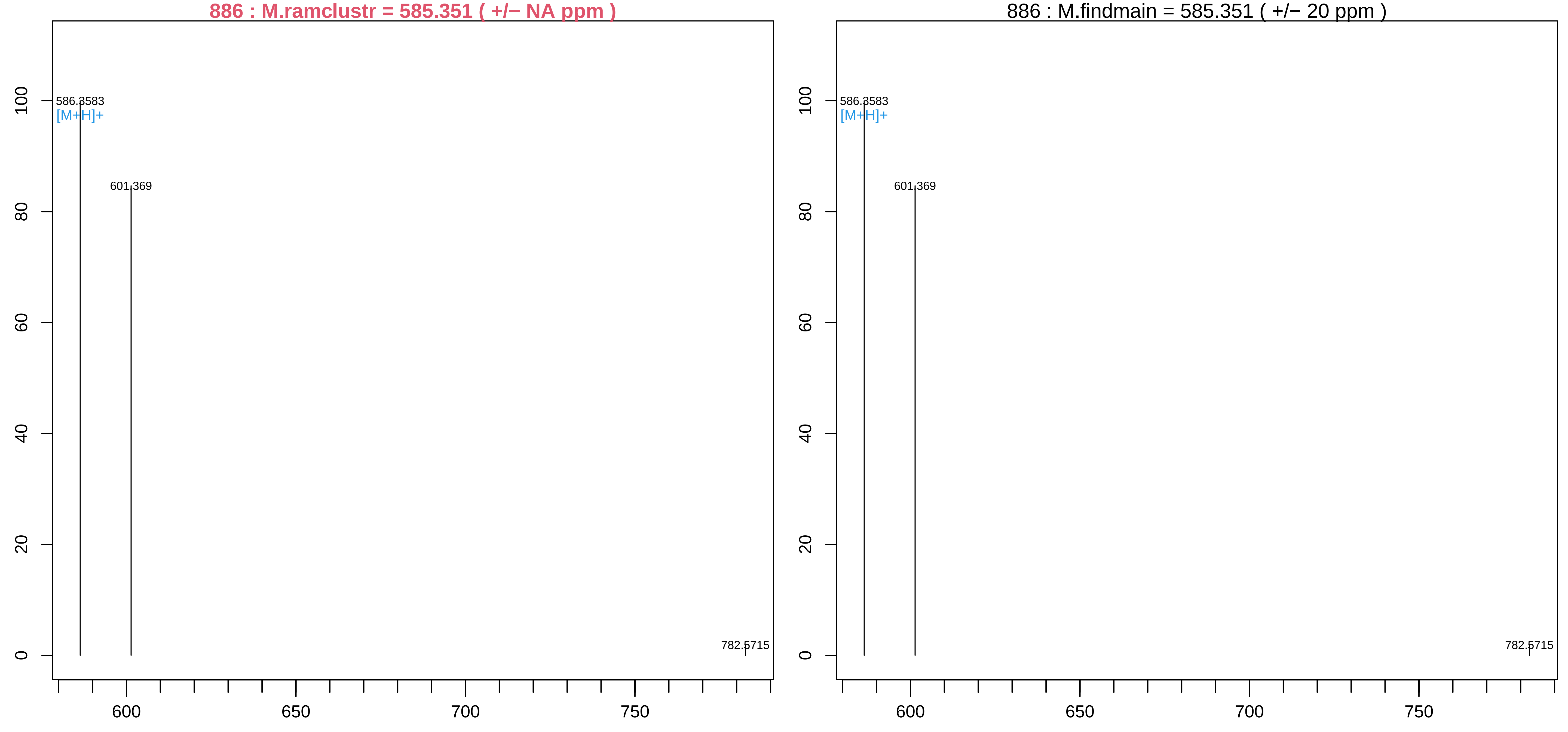
886 : M.ramclustr = 585.351 (+/− NA ppm)
886 : M.findmain = 585.351 (+/− 20 ppm)
586.3583
[M+H]+
601.369
782.5715
0
20
40
60
80
100
600
650
700
750

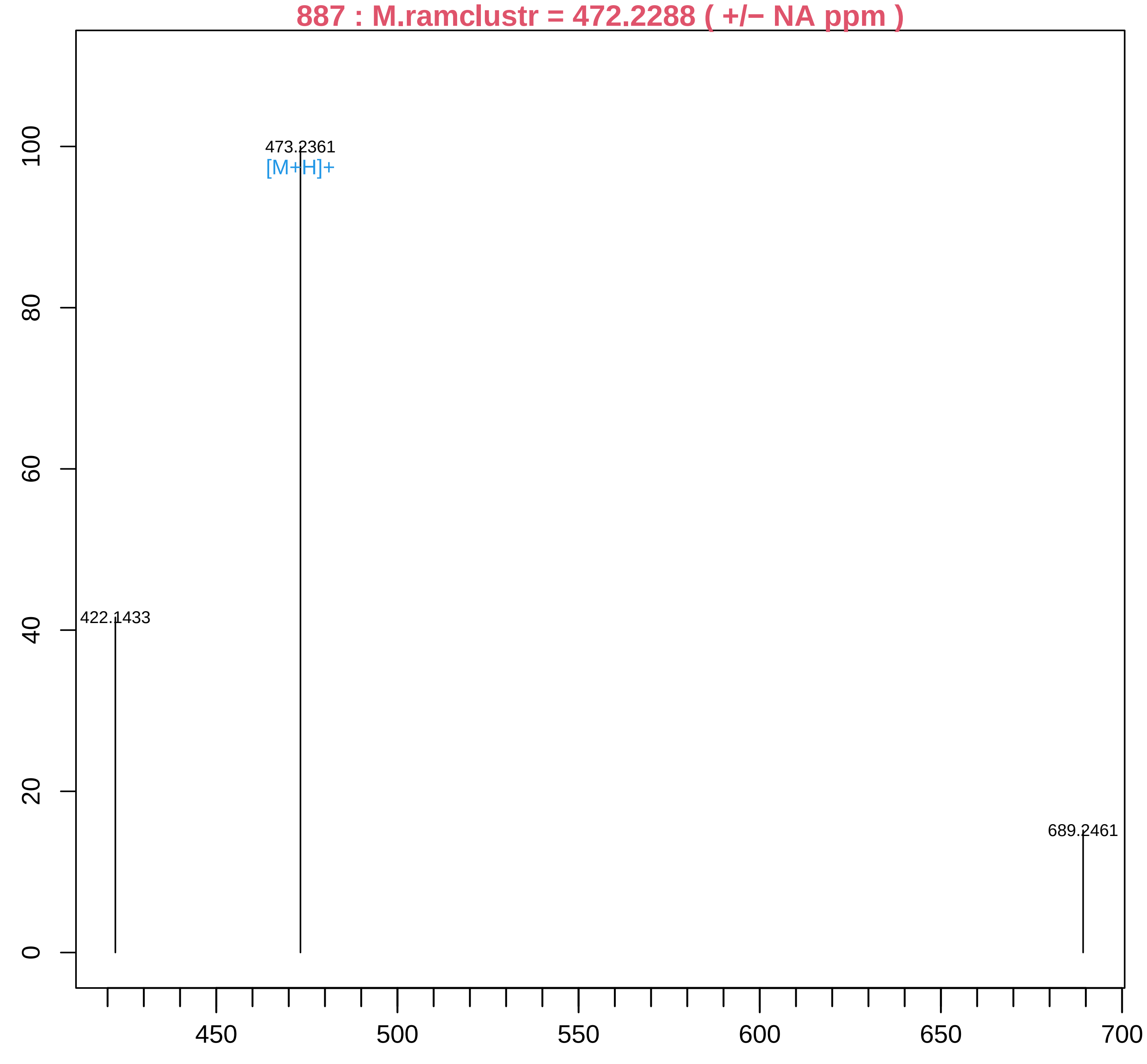
887 : M.ramclustr = 472.2288 (+/− NA ppm)
473.2361
[M+H]+
422.1433
689.2461
0
20
40
60
80
100
450
500
550
600
650
700

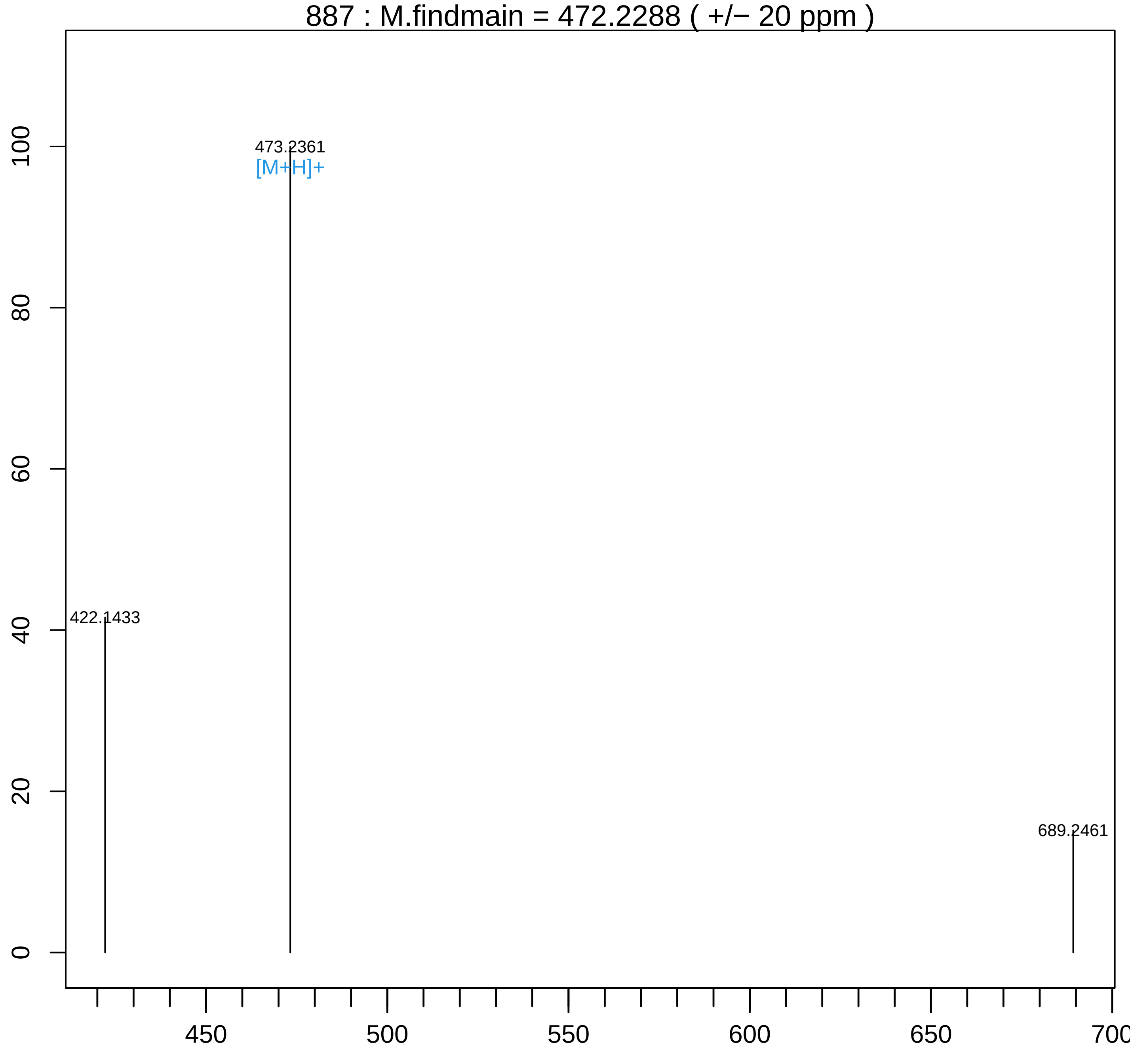
887 : M.findmain = 472.2288 (+/− 20 ppm)
473.2361
[M+H]+
422.1433
689.2461
0
20
40
60
80
100
450
500
550
600
650
700

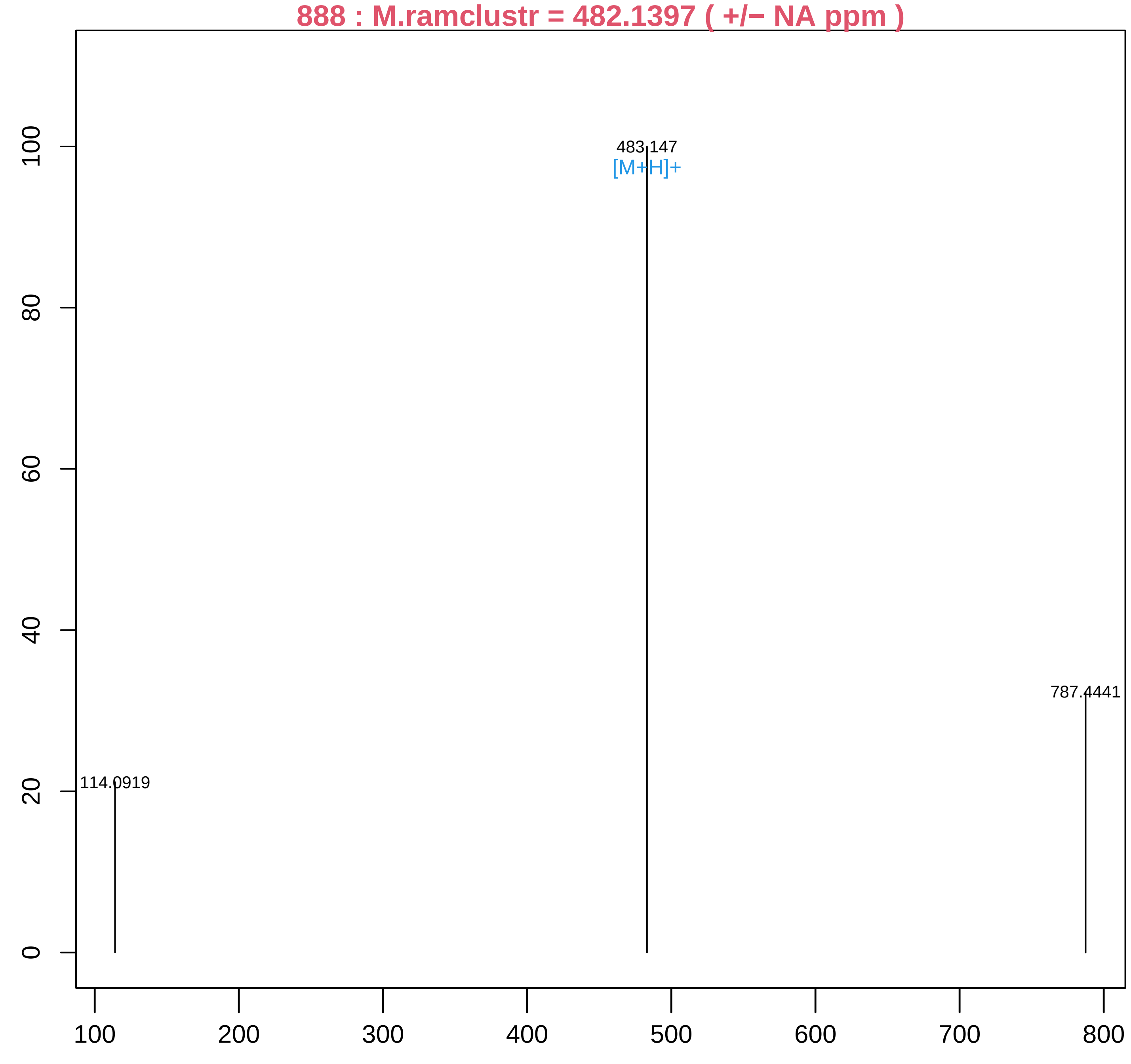
888 : M.ramclustr = 482.1397 (+/− NA ppm)
483.147
[M+H]+
787.4441
114.0919
0
20
40
60
80
100
100
200
300
400
500
600
700
800

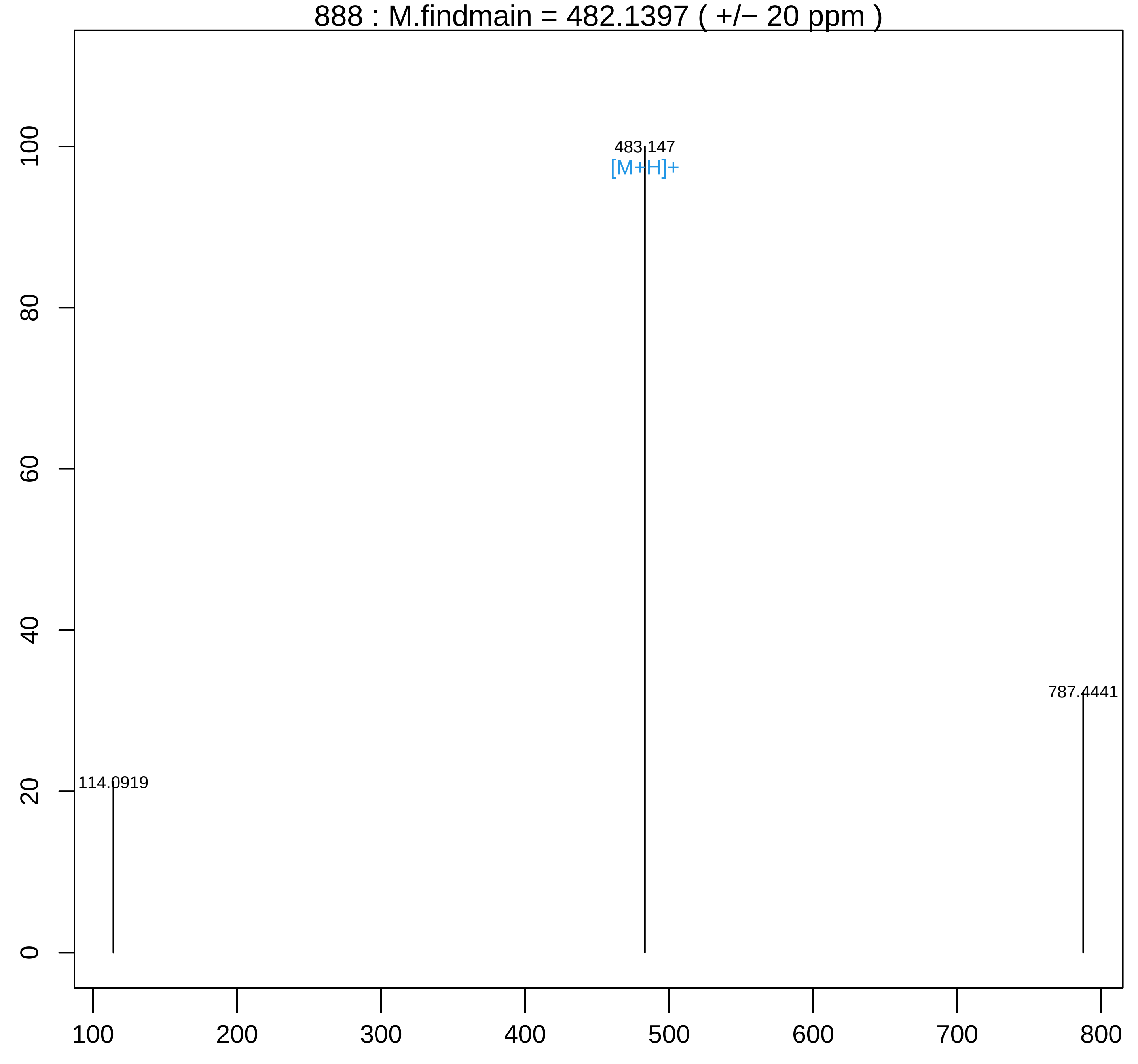
888 : M.findmain = 482.1397 (+/− 20 ppm)
483.147
[M+H]+
787.4441
114.0919
0
20
40
60
80
100
100
200
300
400
500
600
700
800

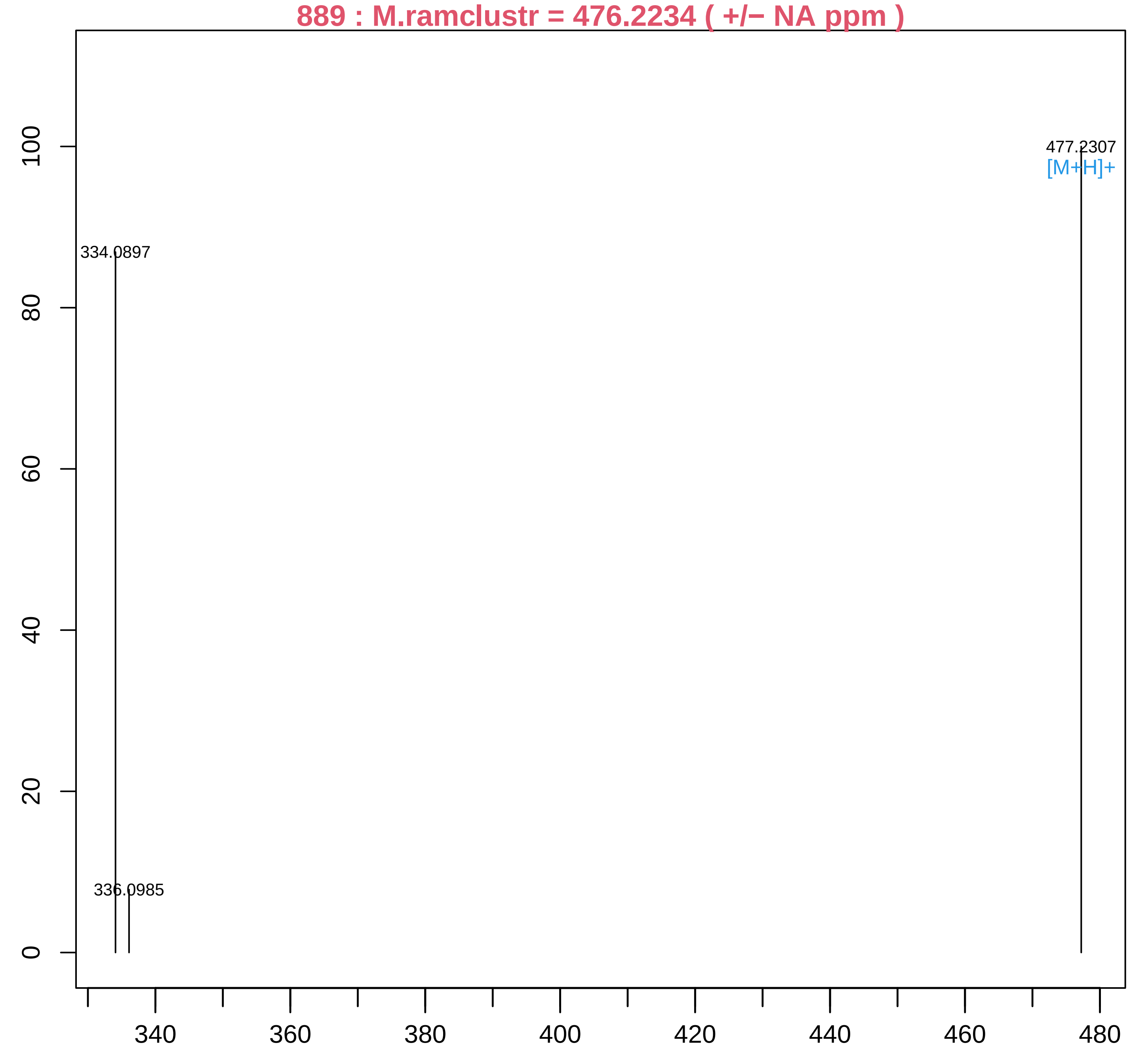

889 : M.ramclustr = 476.2234 (+/− NA ppm)
334.0897
336.0985
477.2307
[M+H]+
0
20
40
60
80
100
340
360
380
400
420
440
460
480

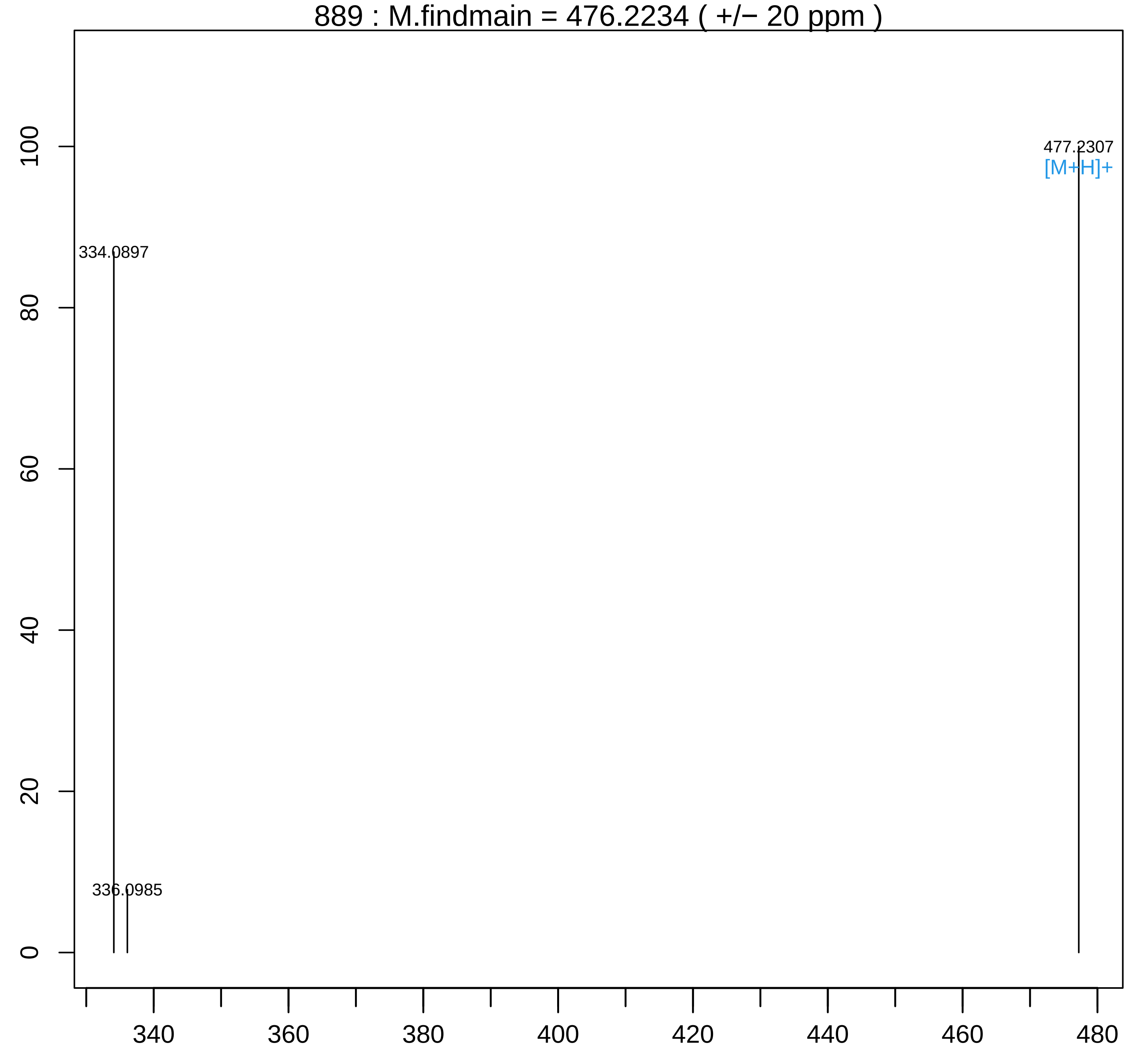

889 : M.findmain = 476.2234 (+/− 20 ppm)
334.0897
336.0985
477.2307
[M+H]+
0
20
40
60
80
100
340
360
380
400
420
440
460
480

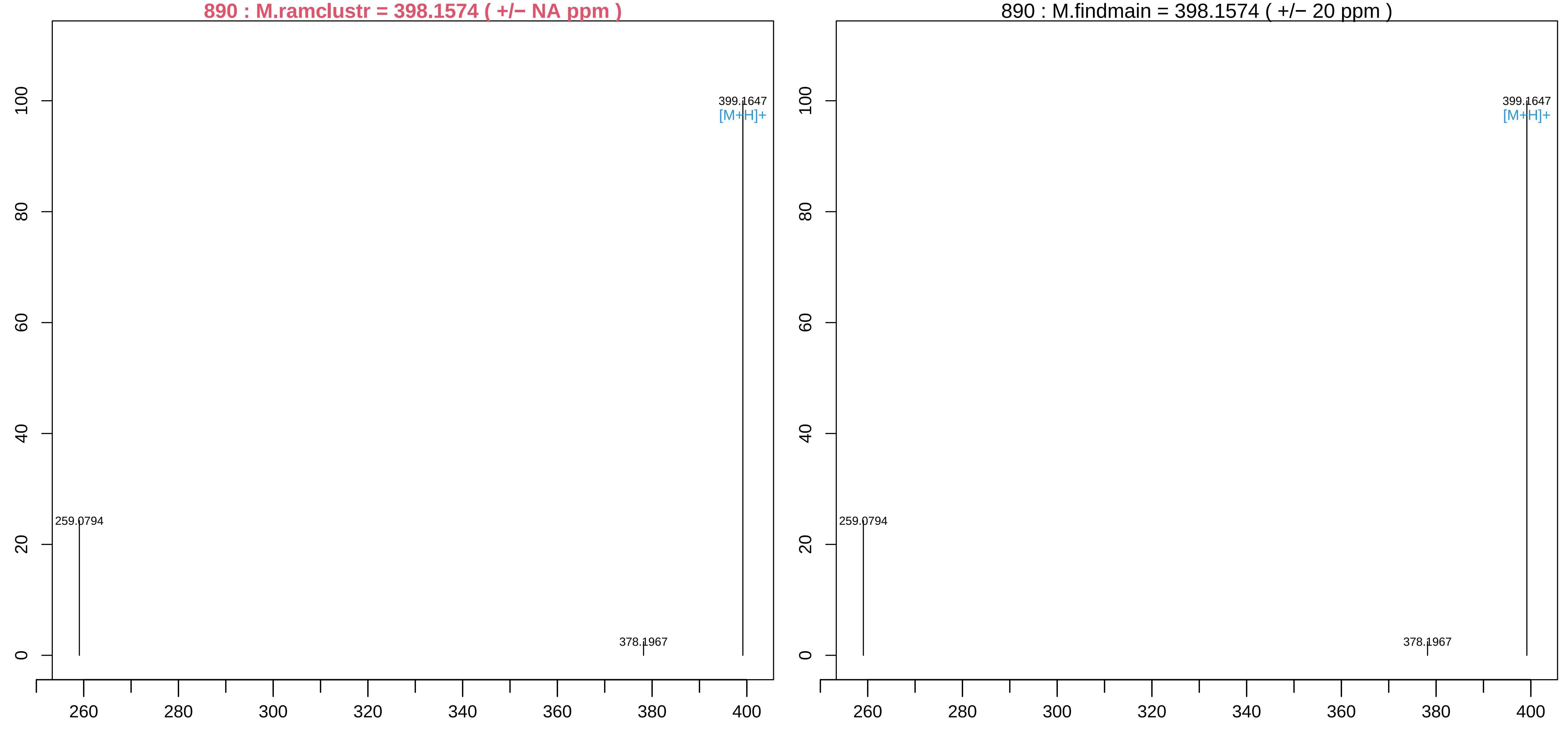
890 : M.ramclustr = 398.1574 (+/− NA ppm)
399.1647
[M+H]+
259.0794
378.1967
890 : M.findmain = 398.1574 (+/− 20 ppm)
399.1647
[M+H]+
259.0794
378.1967
0
20
40
60
80
100
260
280
300
320
340
360
380
400

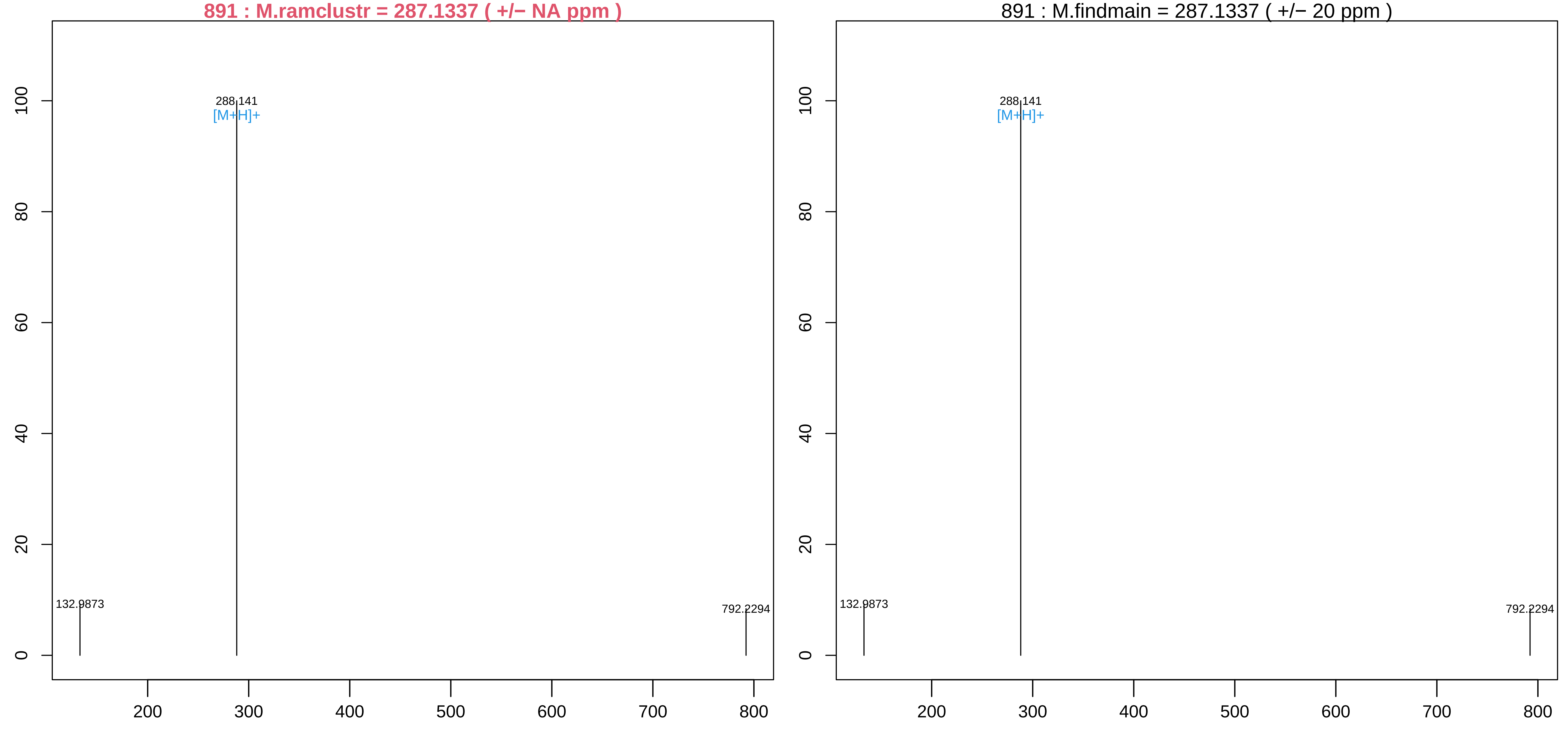

891 : M.ramclustr = 287.1337 (+/− NA ppm)
288.141
[M+H]+
132.9873
792.2294
0
20
40
60
80
100
200
300
400
500
600
700
800
891 : M.findmain = 287.1337 (+/− 20 ppm)
288.141
[M+H]+
132.9873
792.2294
0
20
40
60
80
100
200
300
400
500
600
700
800

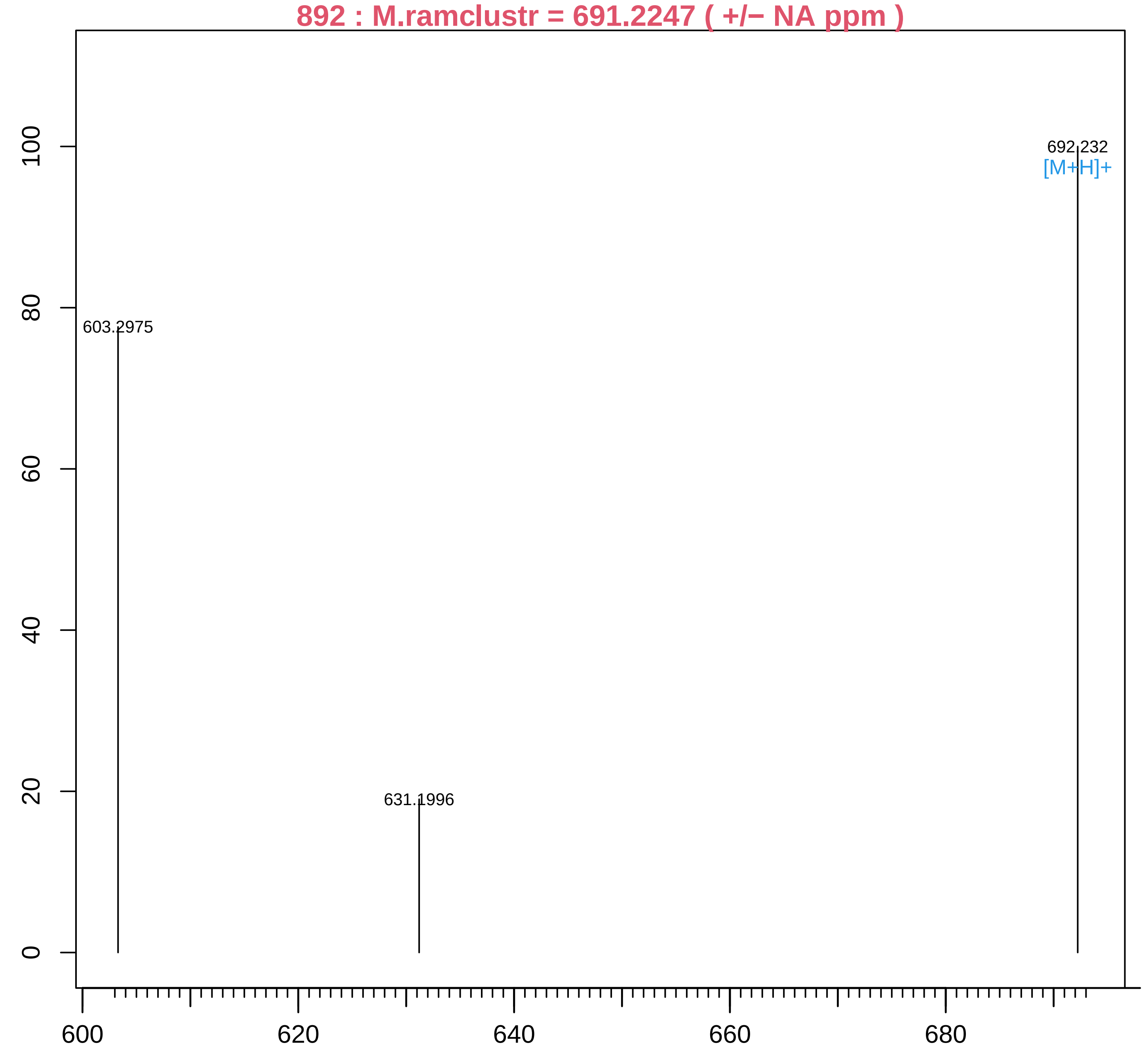
892 : M.ramclustr = 691.2247 (+/− NA ppm)
692.232
[M+H]+
603.2975
631.1996
0
20
40
60
80
100
600
620
640
660
680

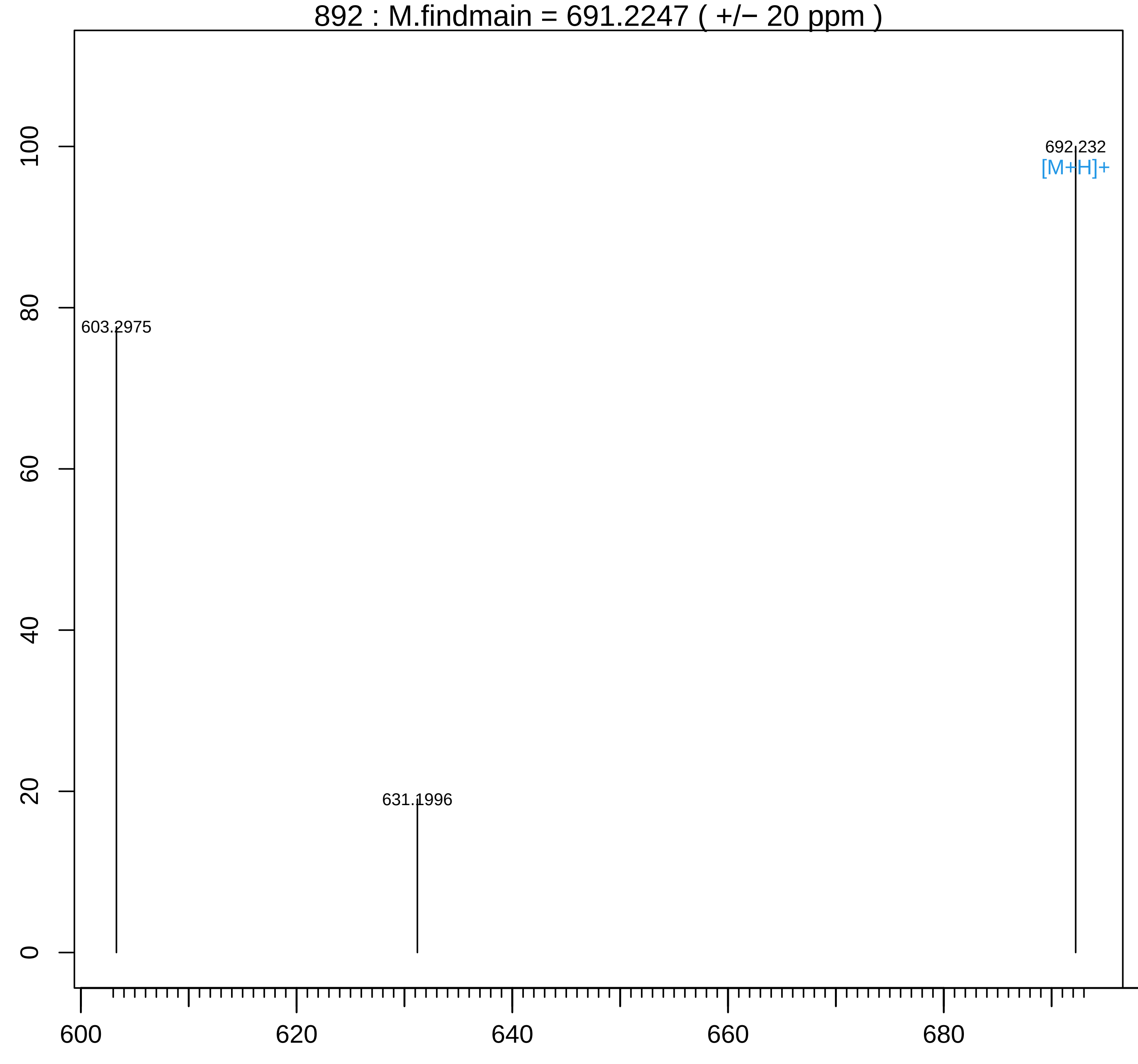
892 : M.findmain = 691.2247 (+/− 20 ppm)
692.232
[M+H]+
603.2975
631.1996
0
20
40
60
80
100
600
620
640
660
680

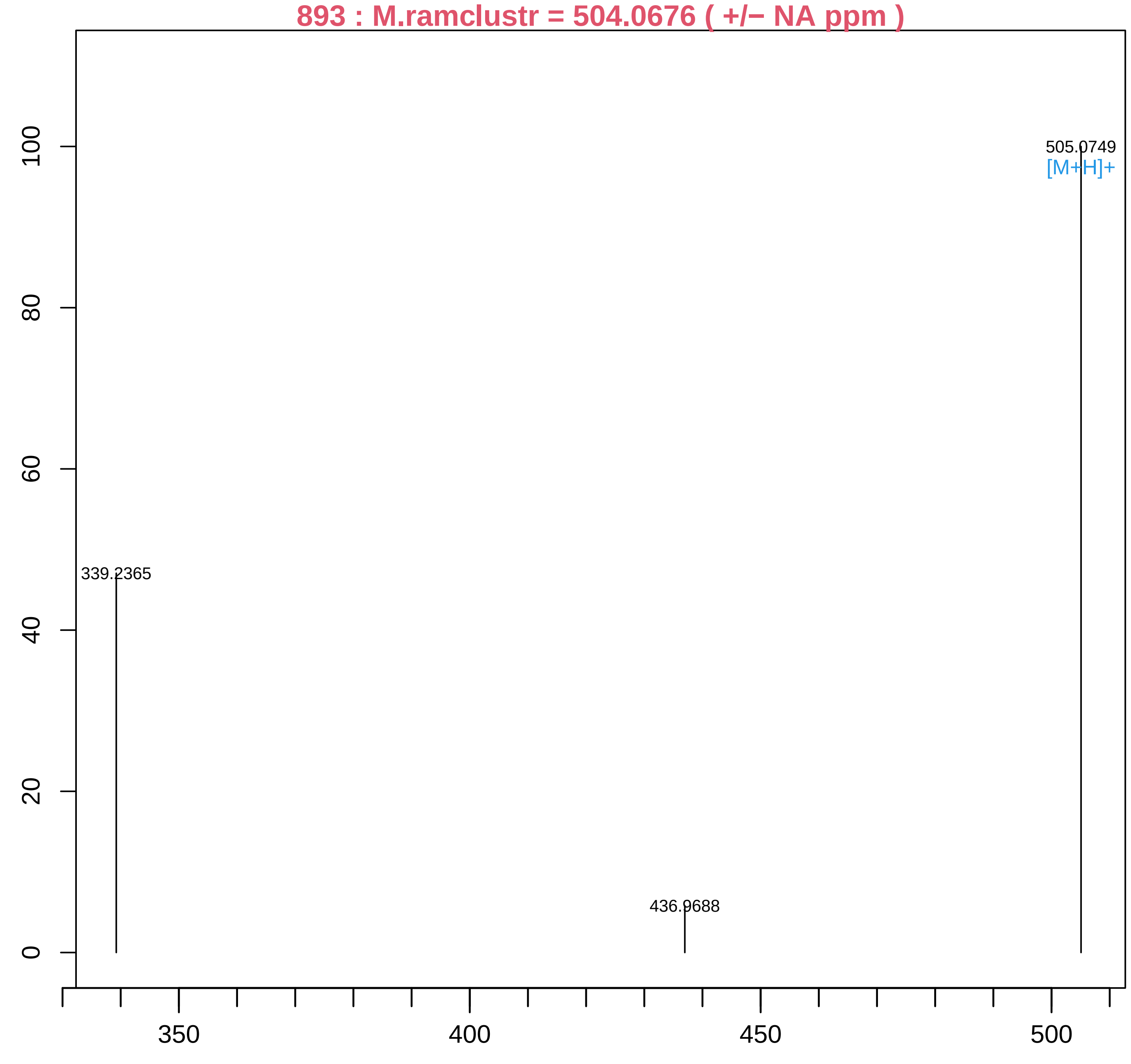

893 : M.ramclustr = 504.0676 (+/− NA ppm)
505.0749
[M+H]+
339.2365
436.9688
0
20
40
60
80
100
350
400
450
500

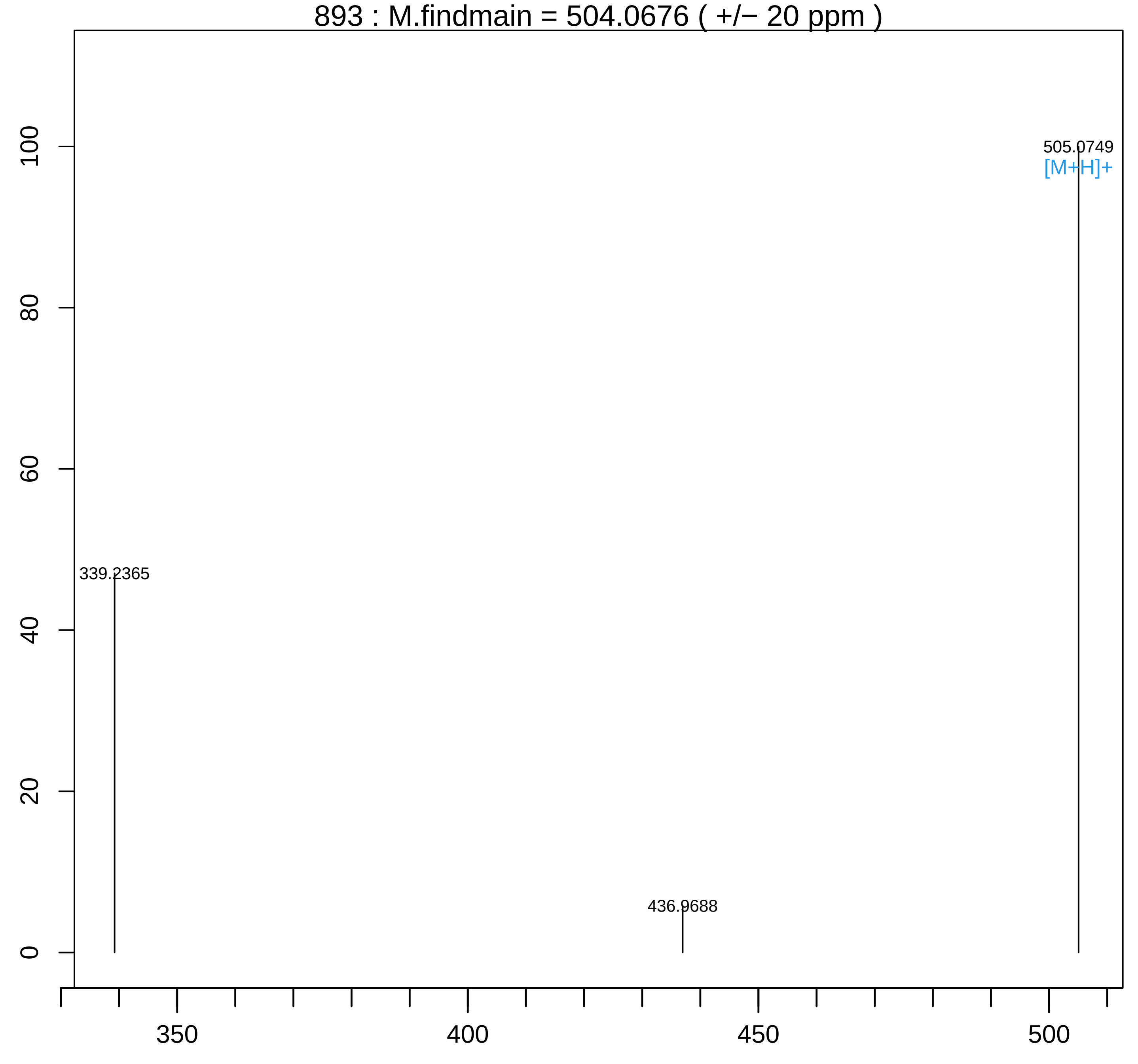

893 : M.findmain = 504.0676 (+/− 20 ppm)
505.0749
[M+H]+
339.2365
436.9688
0
20
40
60
80
100
350
400
450
500

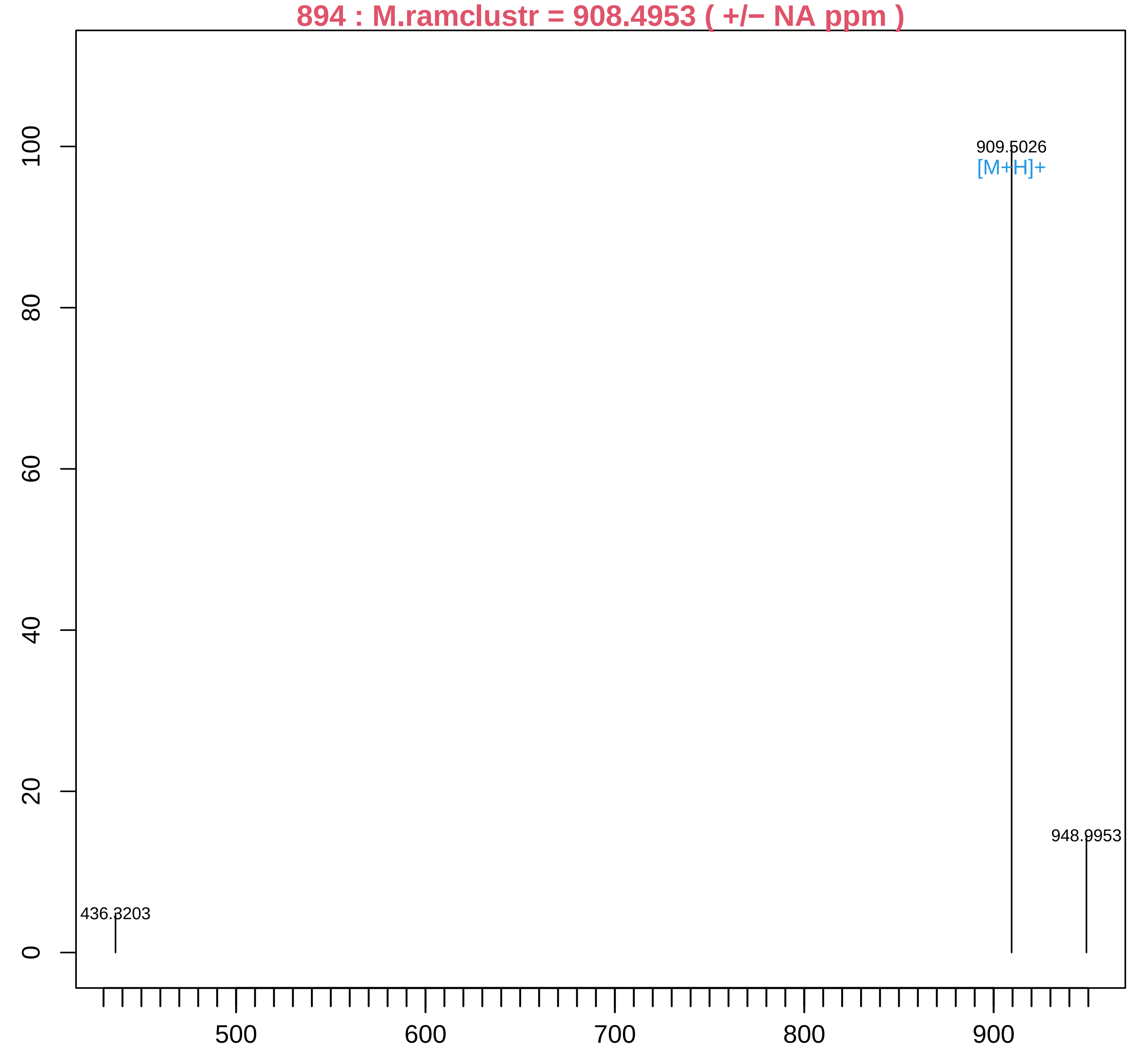
894 : M.ramclustr = 908.4953 (+/− NA ppm)
909.5026
[M+H]+
948.9953
436.3203
0
20
40
60
80
100
500
600
700
800
900

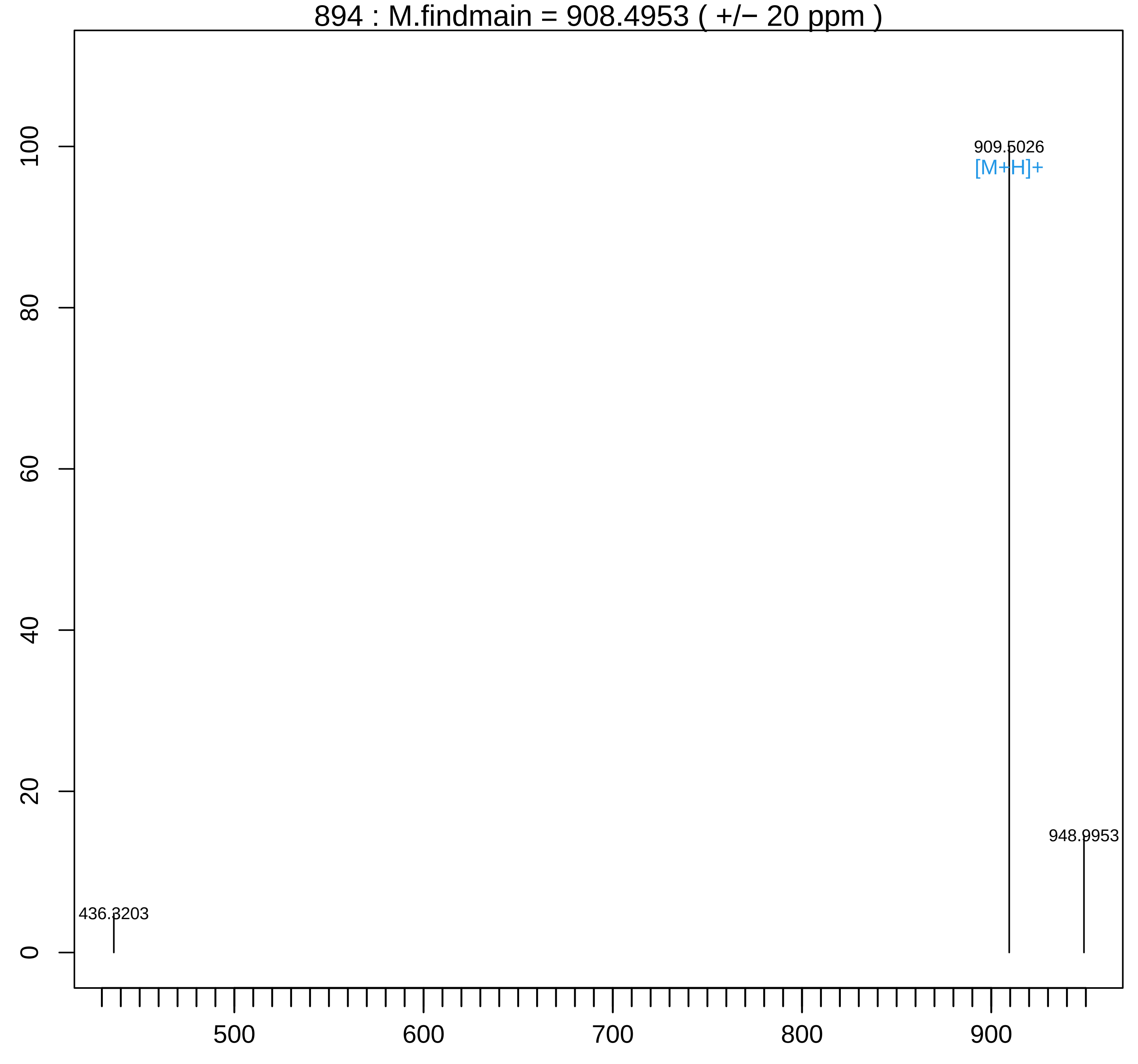
894 : M.findmain = 908.4953 (+/− 20 ppm)
909.5026
[M+H]+
948.9953
436.3203
0
20
40
60
80
100
500
600
700
800
900

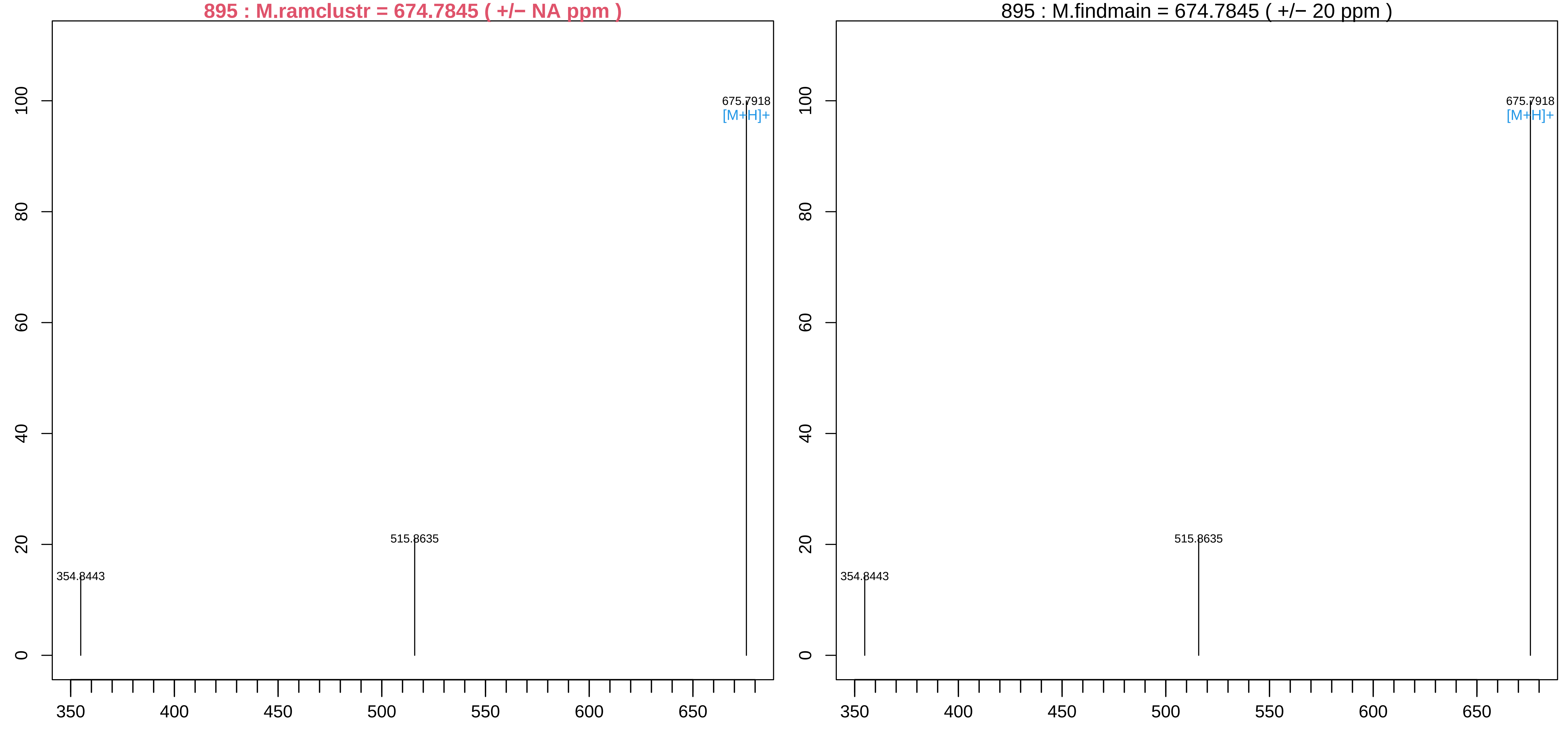

895 : M.ramclustr = 674.7845 (+/− NA ppm)
675.7918
[M+H]+
515.8635
354.8443
0
20
40
60
80
100
350
400
450
500
550
600
650
895 : M.findmain = 674.7845 (+/− 20 ppm)
675.7918
[M+H]+
515.8635
354.8443
0
20
40
60
80
100
350
400
450
500
550
600
650

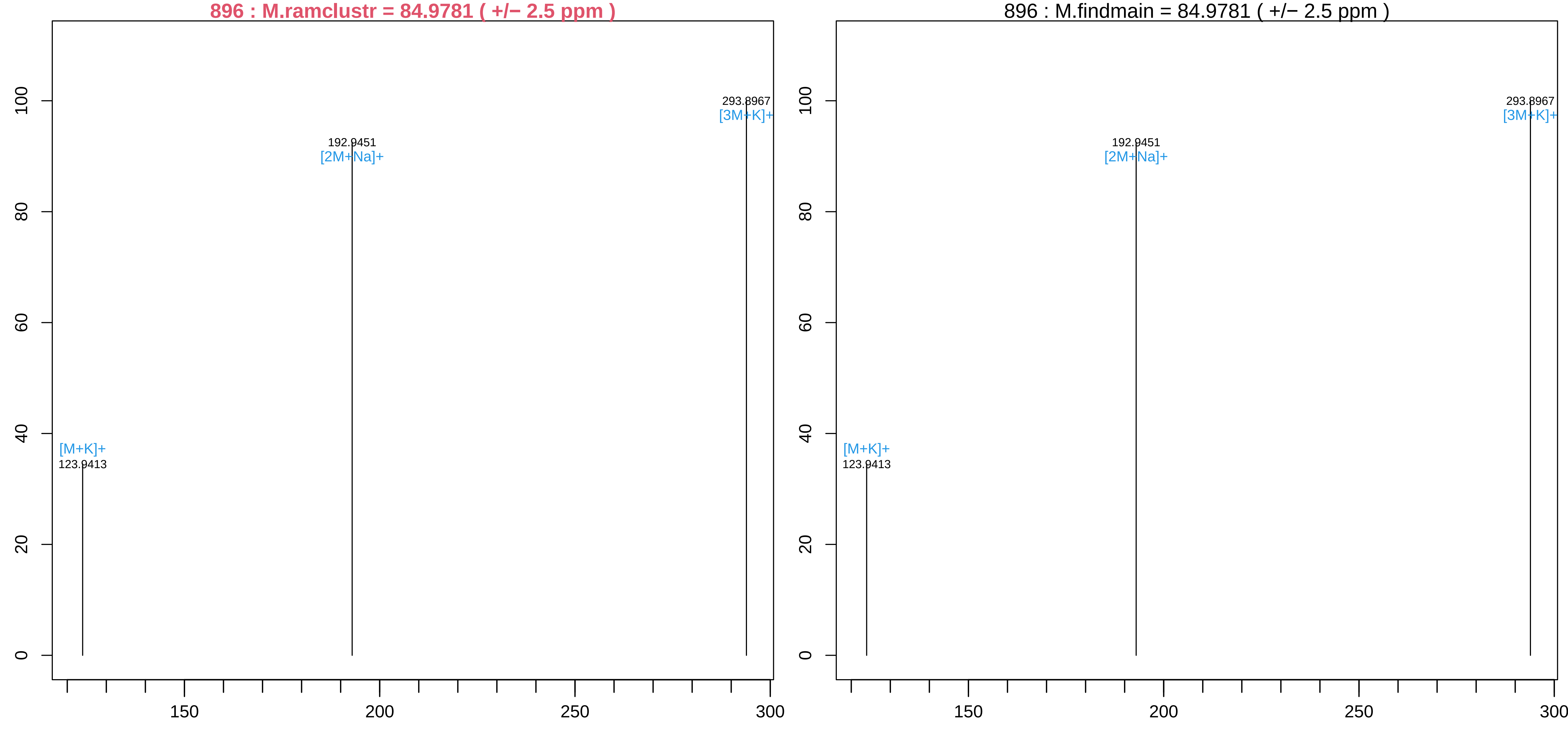
896 : M.ramclustr = 84.9781 (+/− 2.5 ppm)
293.8967
[3M+K]+
192.9451
[2M+Na]+
[M+K]+
123.9413
0
20
40
60
80
100
150
200
250
300
896 : M.findmain = 84.9781 (+/− 2.5 ppm)
293.8967
[3M+K]+
192.9451
[2M+Na]+
[M+K]+
123.9413
0
20
40
60
80
100
150
200
250
300

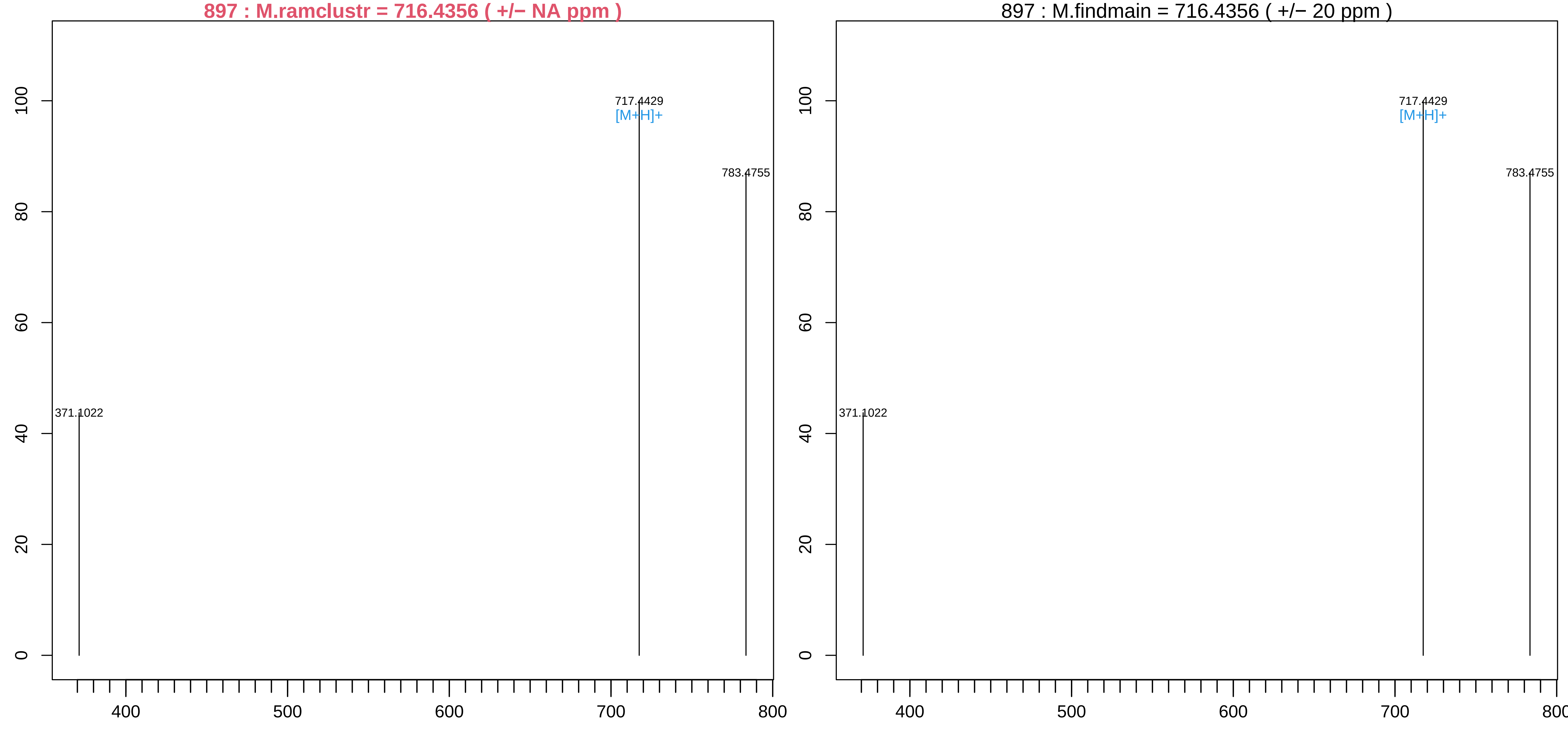
897 : M.ramclustr = 716.4356 (+/− NA ppm)
371.1022
717.4429
[M+H]+
783.4755
897 : M.findmain = 716.4356 (+/− 20 ppm)
371.1022
717.4429
[M+H]+
783.4755

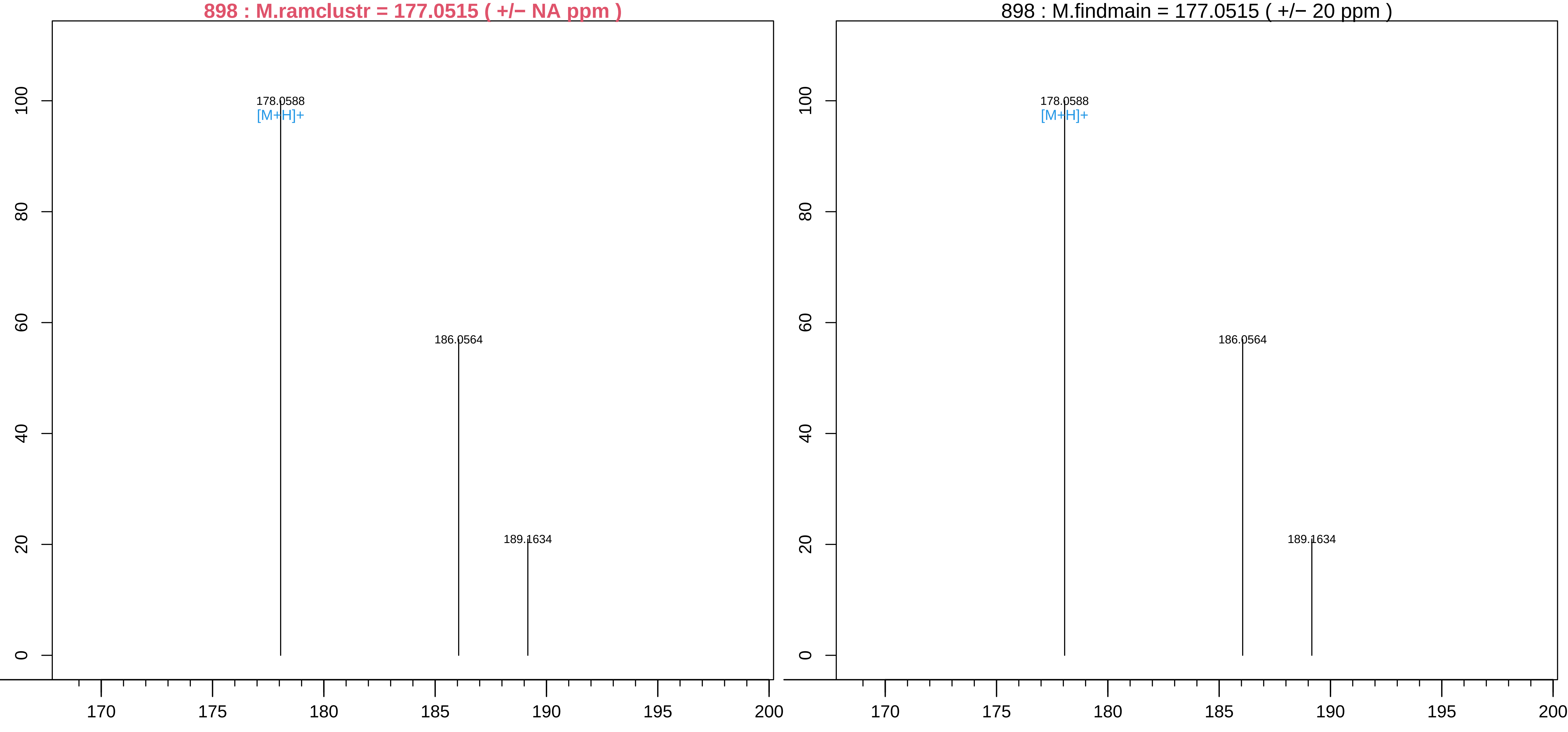
898 : M.ramclustr = 177.0515 (+/− NA ppm)
178.0588
[M+H]+
186.0564
189.1634
898 : M.findmain = 177.0515 (+/− 20 ppm)
178.0588
[M+H]+
186.0564
189.1634

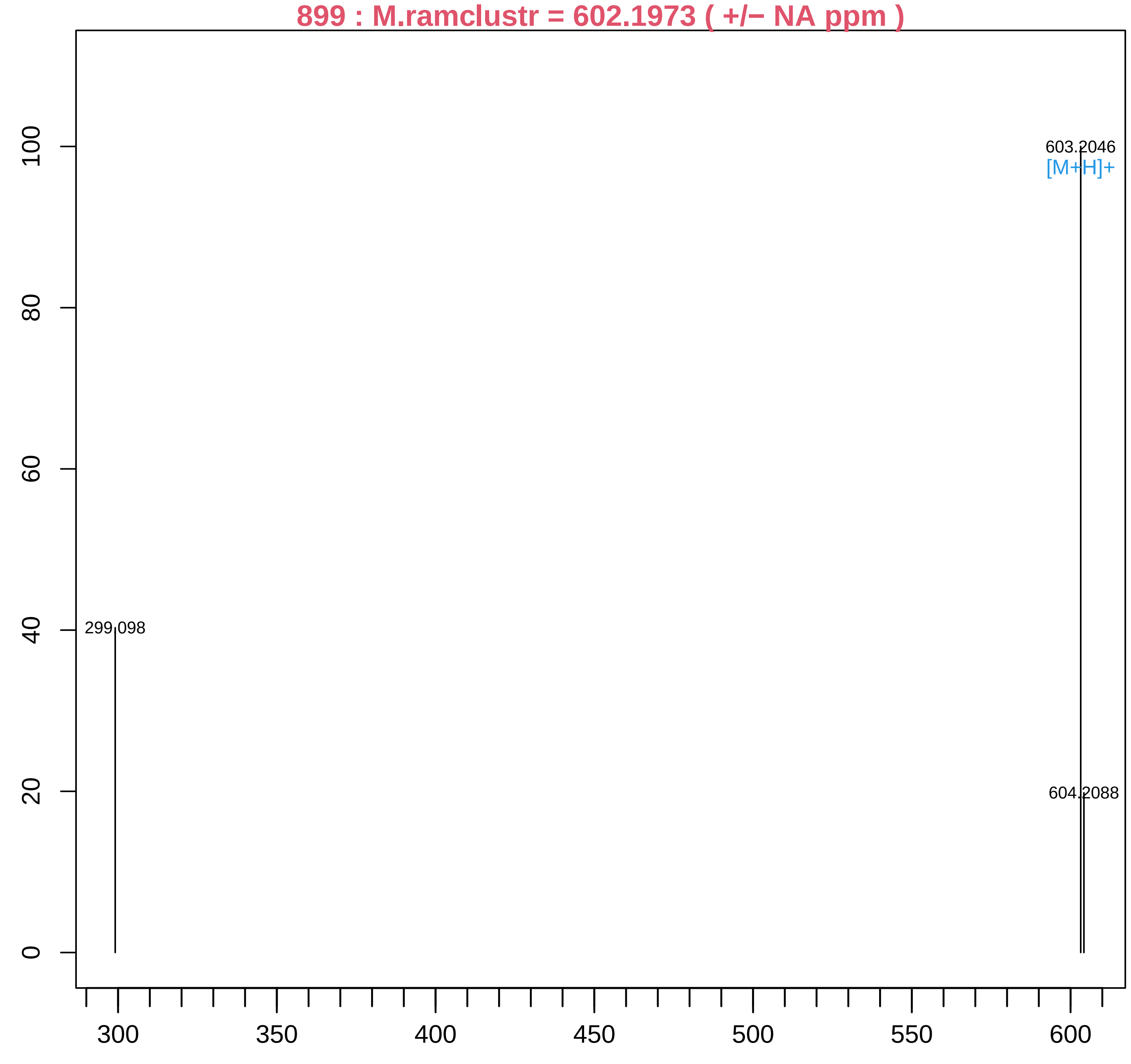
899 : M.ramclustr = 602.1973 (+/− NA ppm)
603.2046
[M+H]+
299.098
604.2088
0
20
40
60
80
100
300
350
400
450
500
550
600
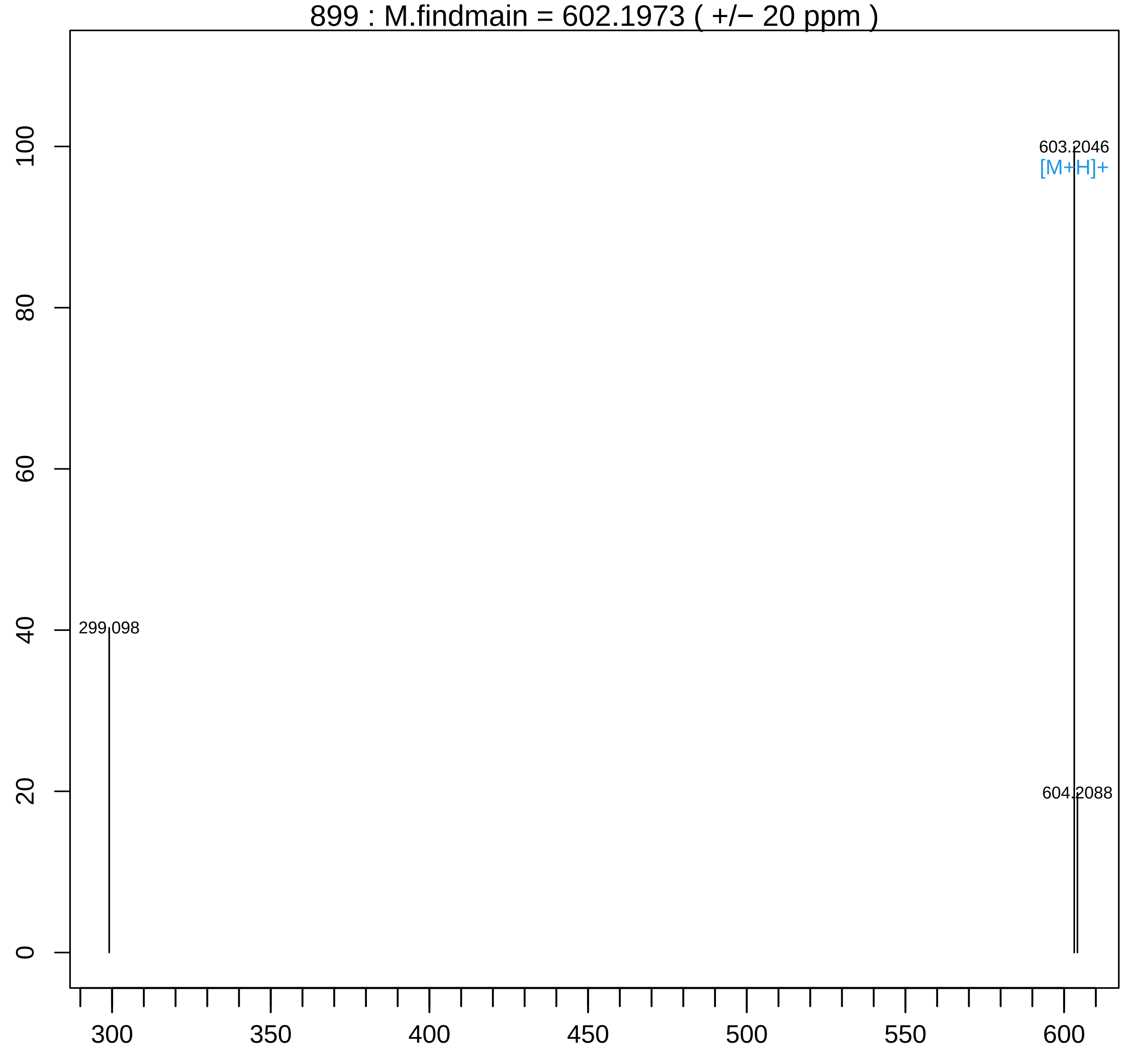
899 : M.findmain = 602.1973 (+/− 20 ppm)
603.2046
[M+H]+
299.098
604.2088
0
20
40
60
80
100
300
350
400
450
500
550
600

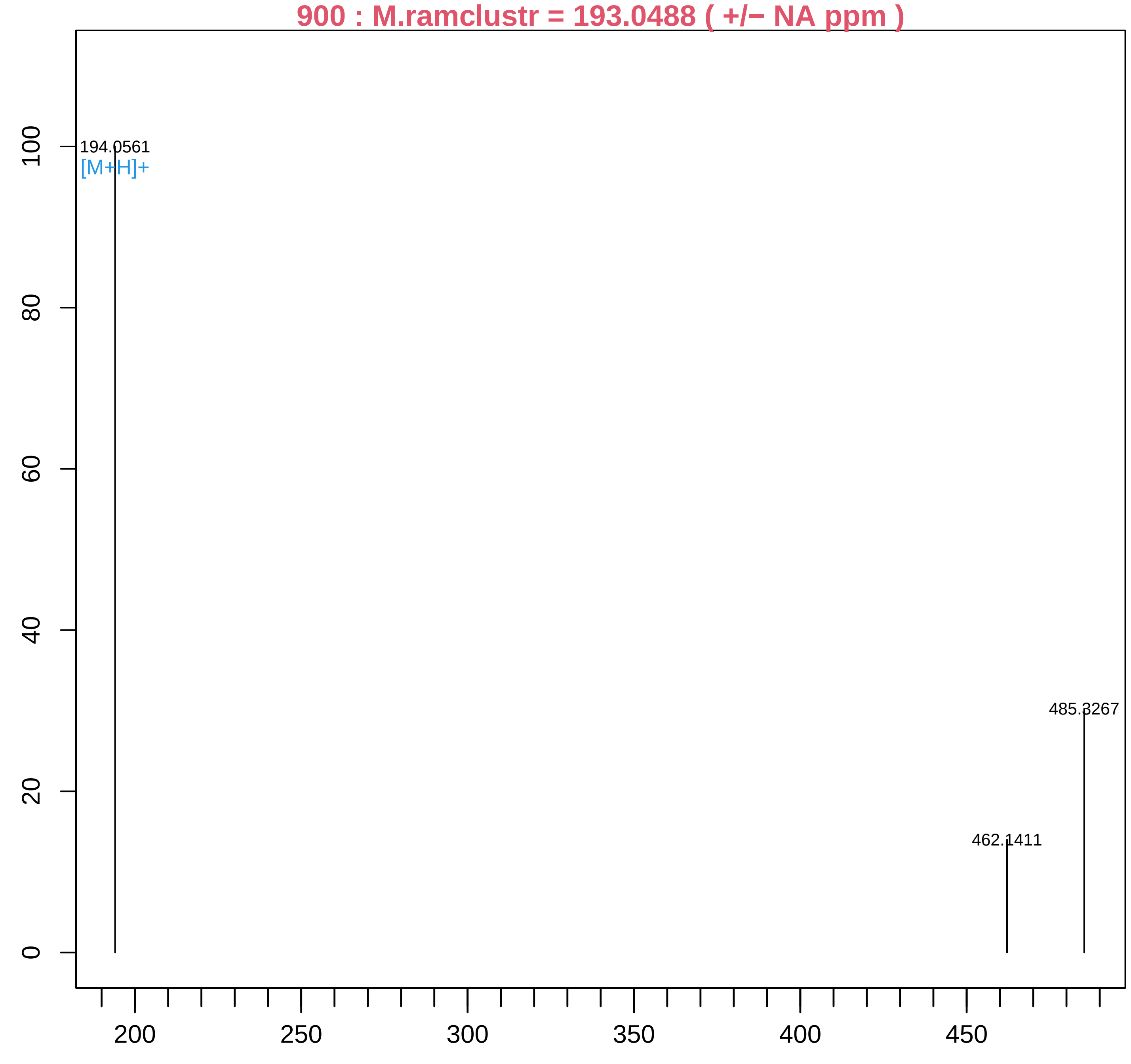
900 : M.ramclustr = 193.0488 (+/− NA ppm)
194.0561
[M+H]+
462.1411
485.3267
0
20
40
60
80
100
200
250
300
350
400
450

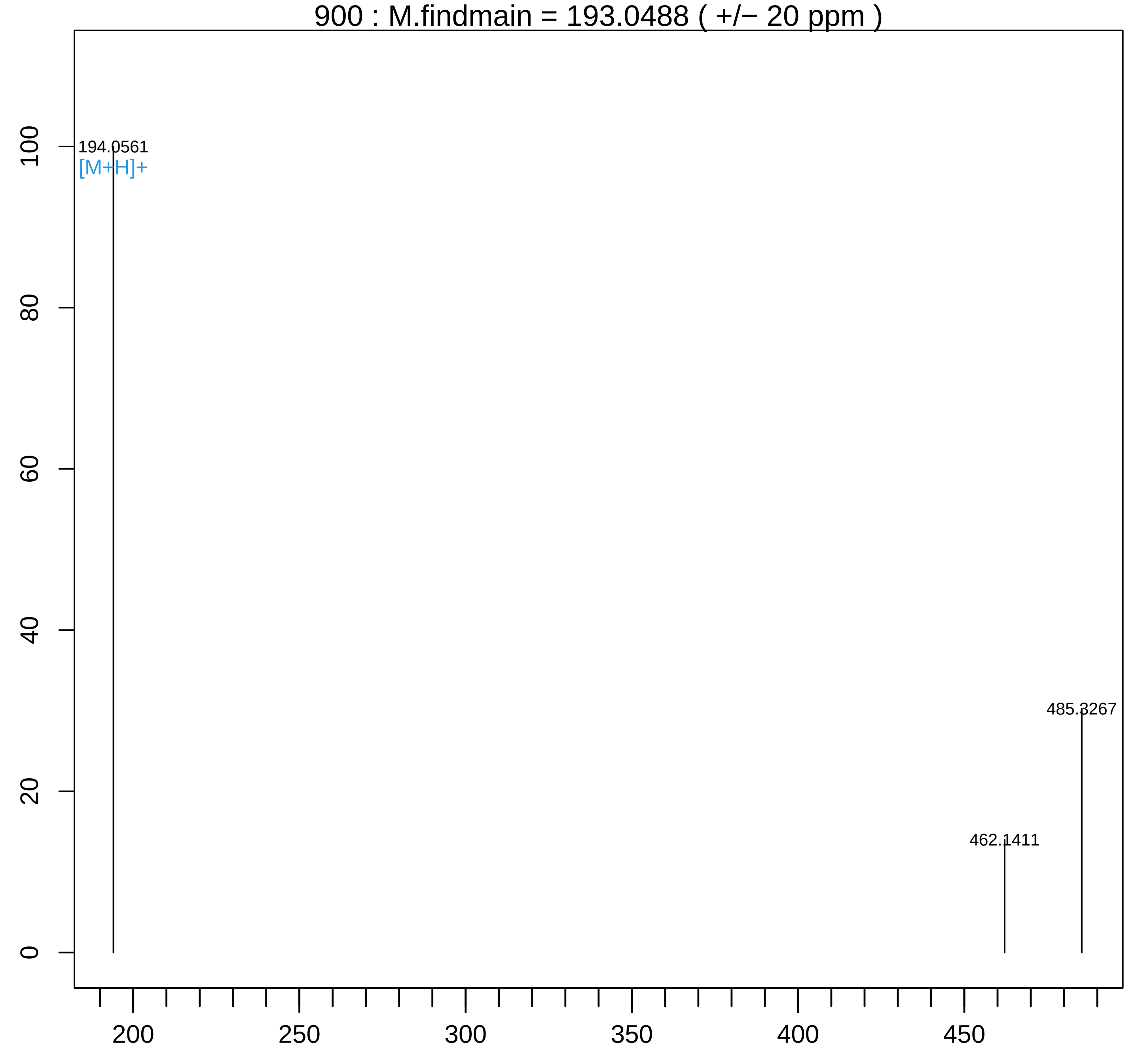
900 : M.findmain = 193.0488 (+/− 20 ppm)
194.0561
[M+H]+
462.1411
485.3267
0
20
40
60
80
100
200
250
300
350
400
450

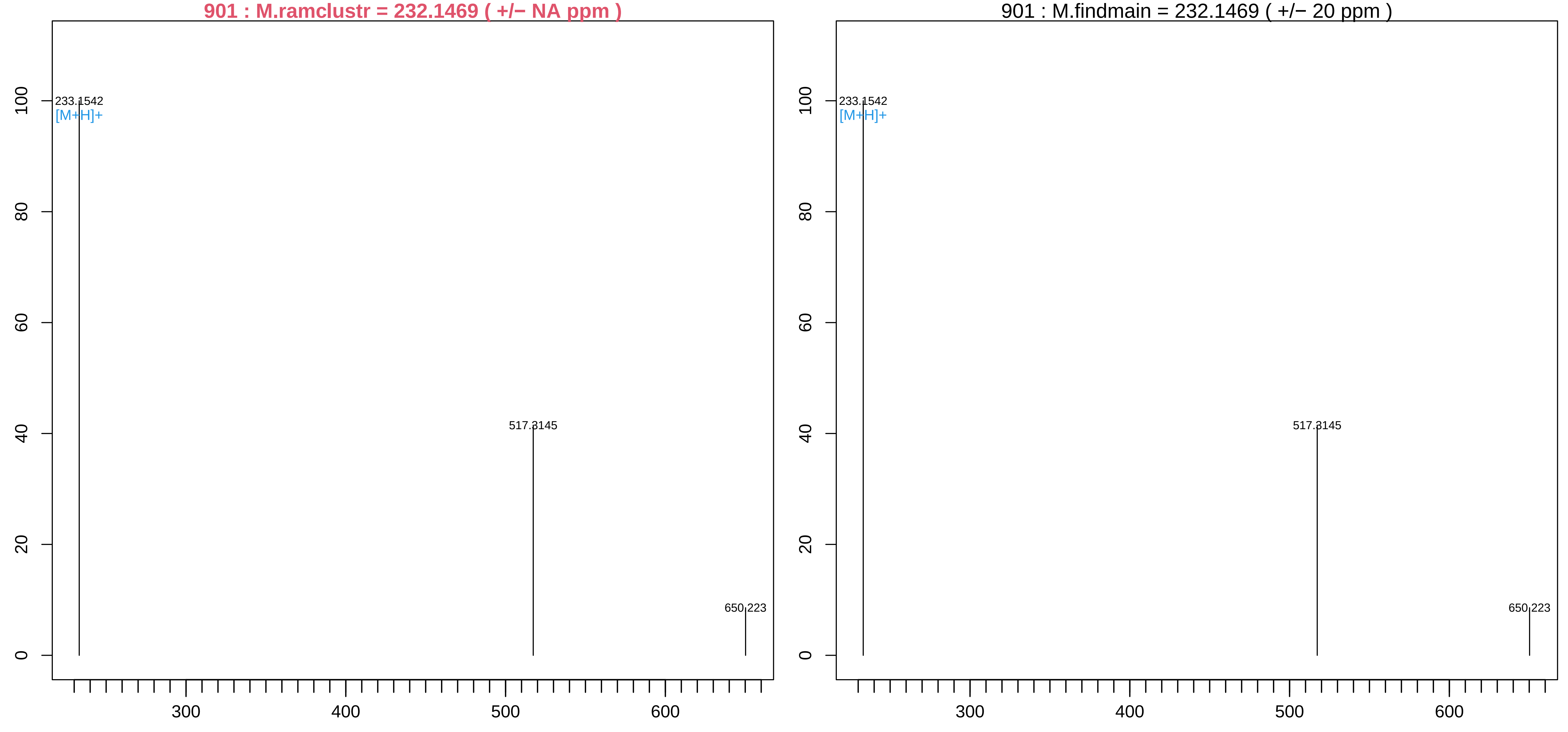

901 : M.ramclustr = 232.1469 (+/− NA ppm)
233.1542
[M+H]+
517.3145
650.223
0
20
40
60
80
100
300
400
500
600
901 : M.findmain = 232.1469 (+/− 20 ppm)
233.1542
[M+H]+
517.3145
650.223
0
20
40
60
80
100
300
400
500
600

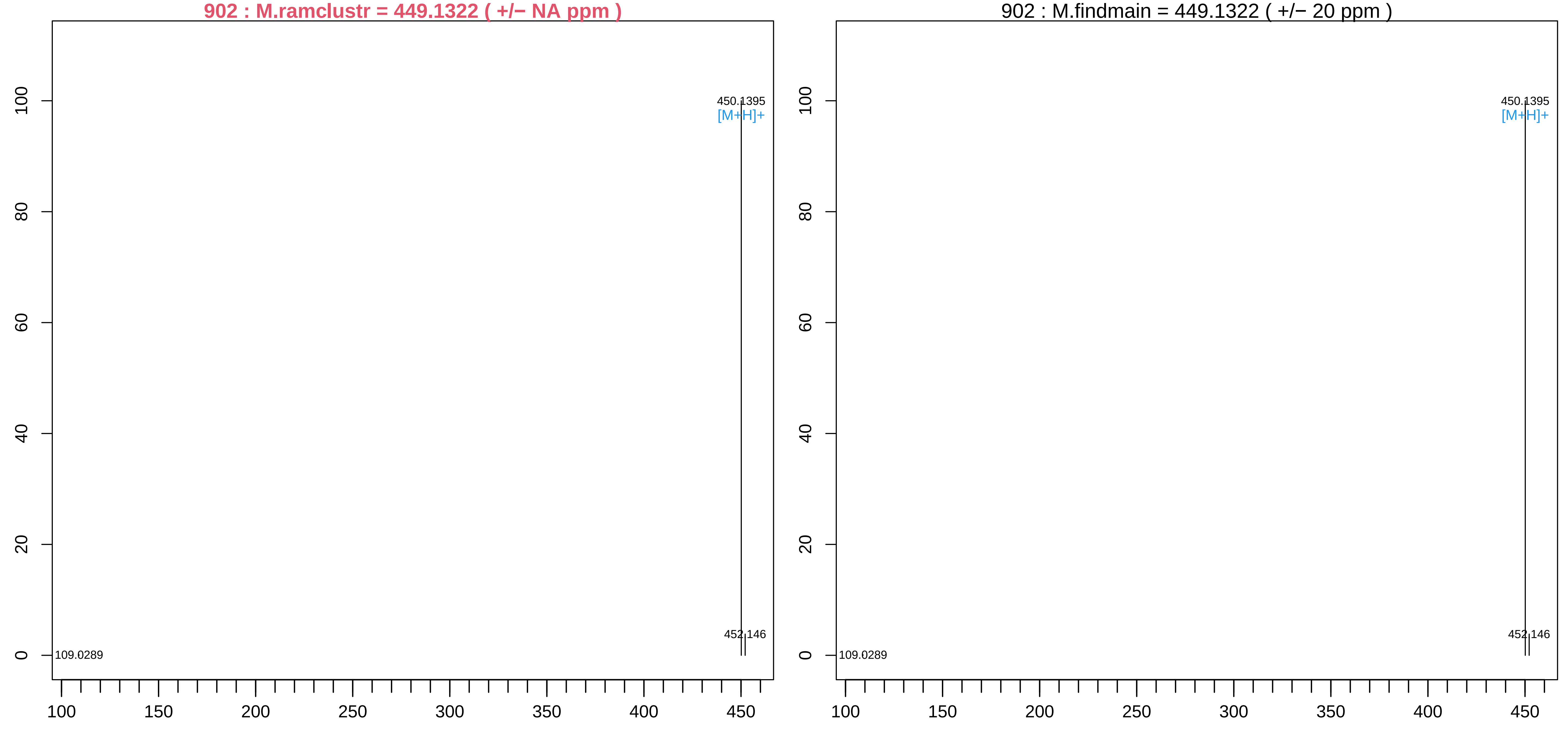

902 : M.ramclustr = 449.1322 (+/− NA ppm)
450.1395
[M+H]+
452.146
109.0289
0
20
40
60
80
100
100
150
200
250
300
350
400
450
902 : M.findmain = 449.1322 (+/− 20 ppm)
450.1395
[M+H]+
452.146
109.0289

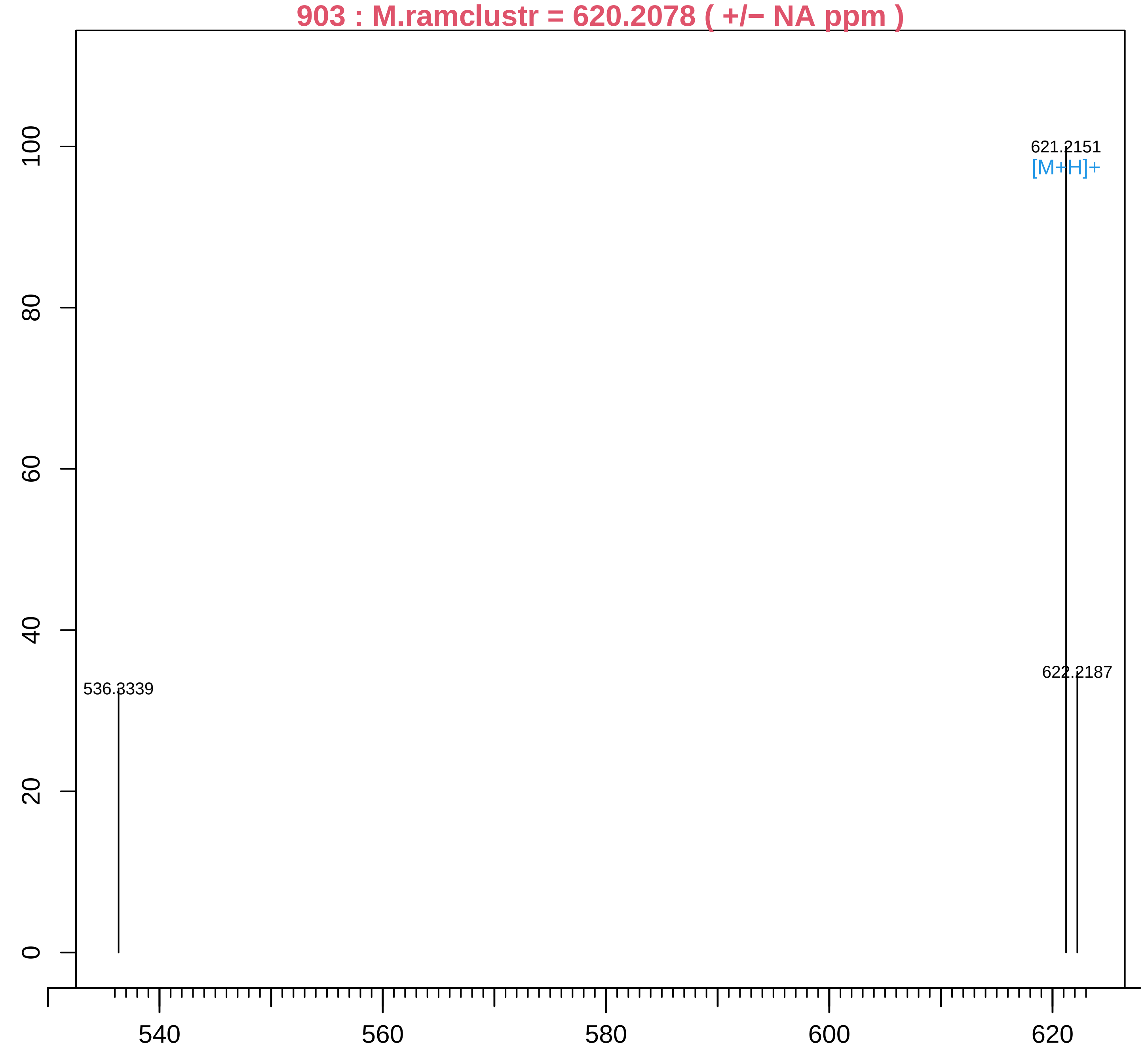

903 : M.ramclustr = 620.2078 (+/− NA ppm)
621.2151
[M+H]+
622.2187
536.3339
0
20
40
60
80
100
540
560
580
600
620

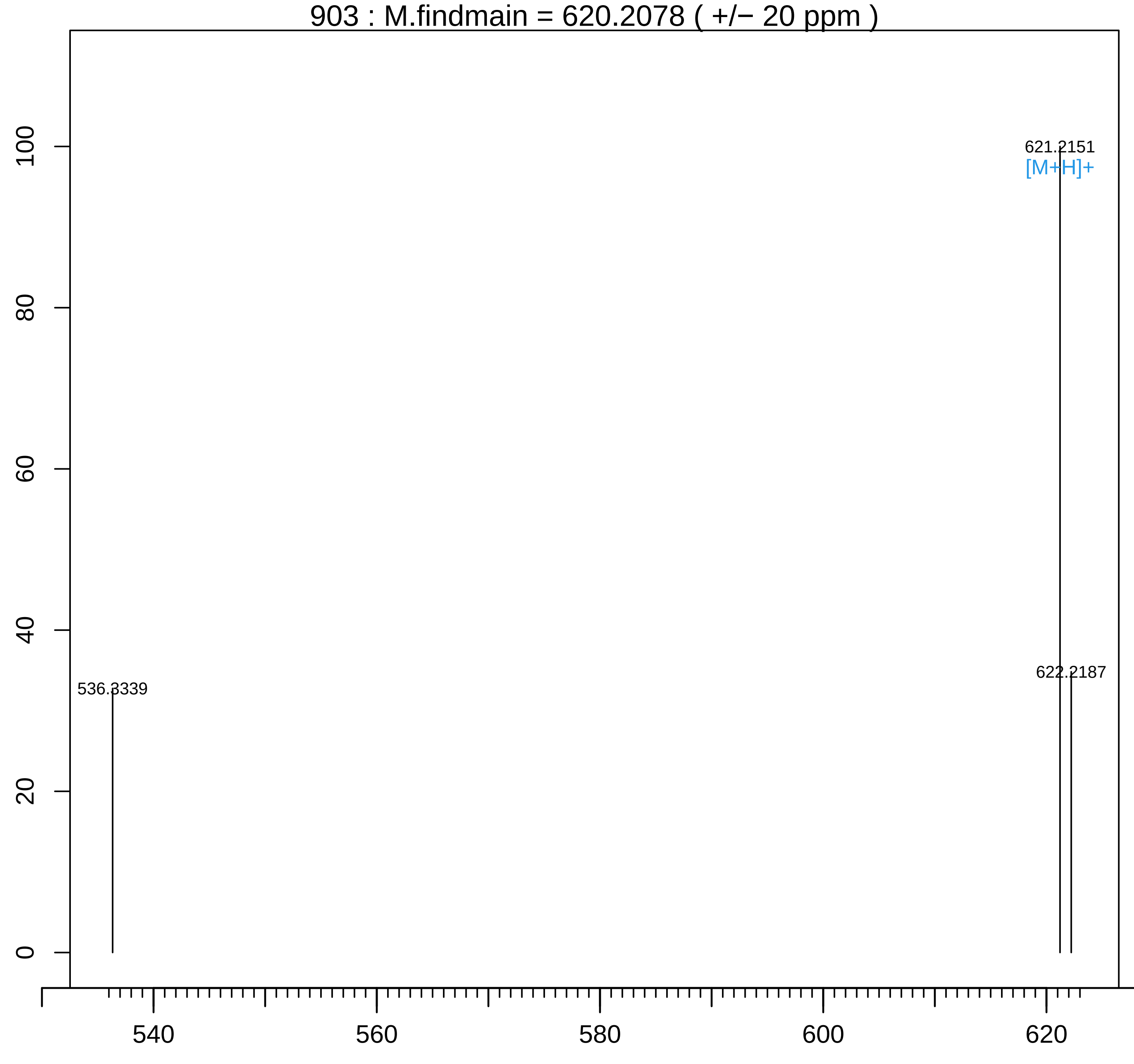

903 : M.findmain = 620.2078 (+/− 20 ppm)
621.2151
[M+H]+
622.2187
536.3339
0
20
40
60
80
100
540
560
580
600
620

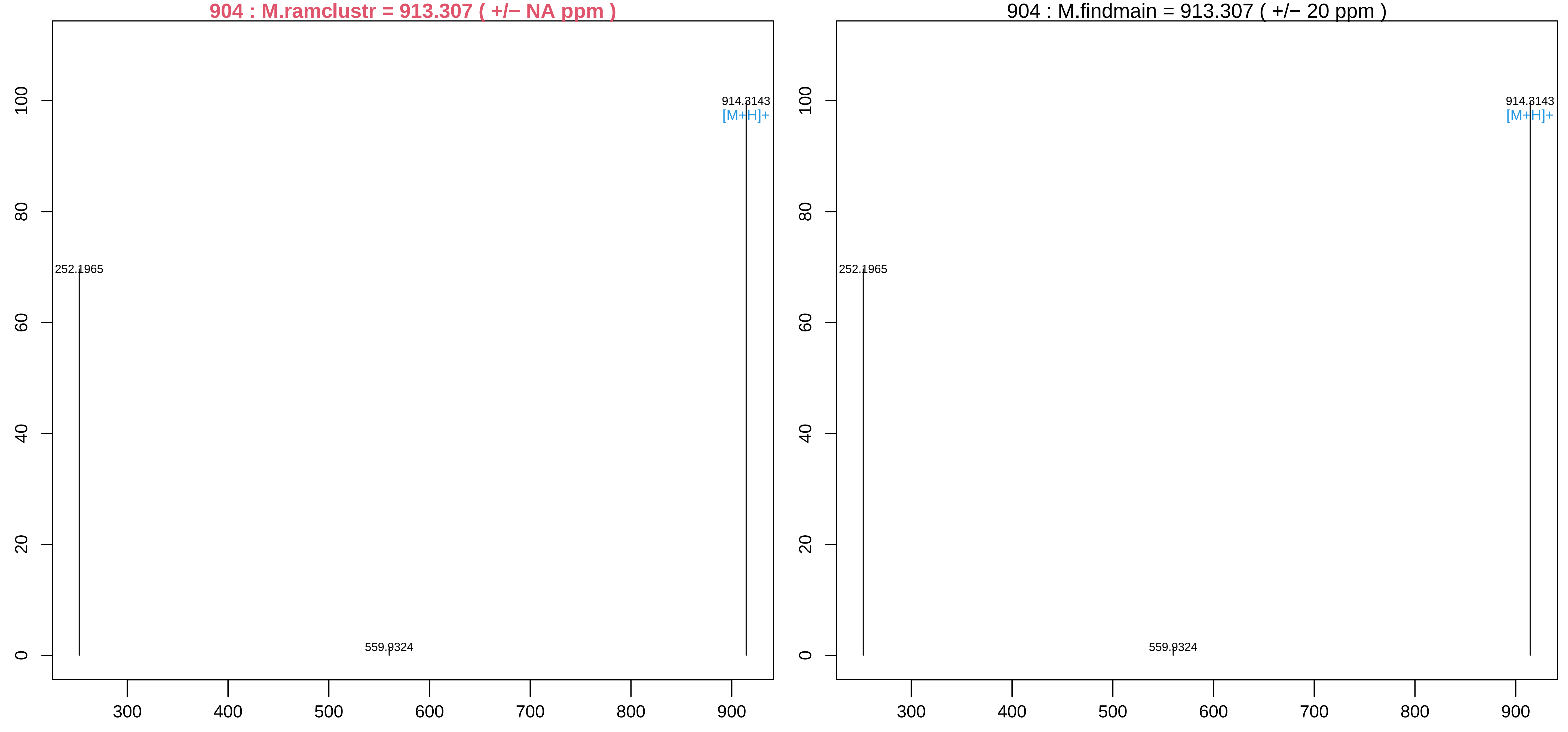

904 : M.ramclustr = 913.307 (+/− NA ppm)
914.3143
[M+H]+
252.1965
559.9324
0
20
40
60
80
100
300
400
500
600
700
800
900
904 : M.findmain = 913.307 (+/− 20 ppm)
914.3143
[M+H]+
252.1965
559.9324
0
20
40
60
80
100
300
400
500
600
700
800
900

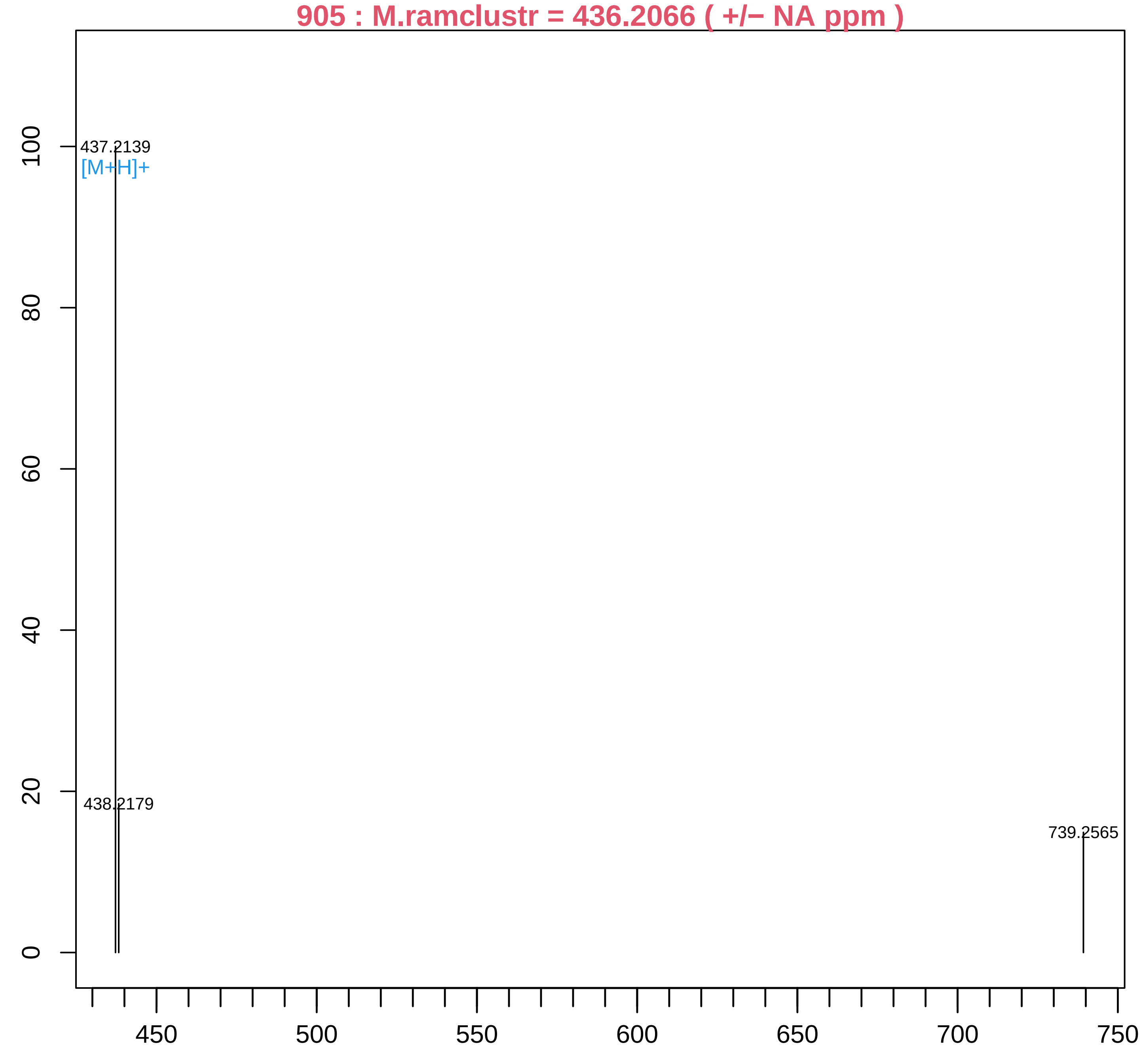

905 : M.ramclustr = 436.2066 (+/− NA ppm)
437.2139
[M+H]+
438.2179
739.2565
0
20
40
60
80
100
450
500
550
600
650
700
750

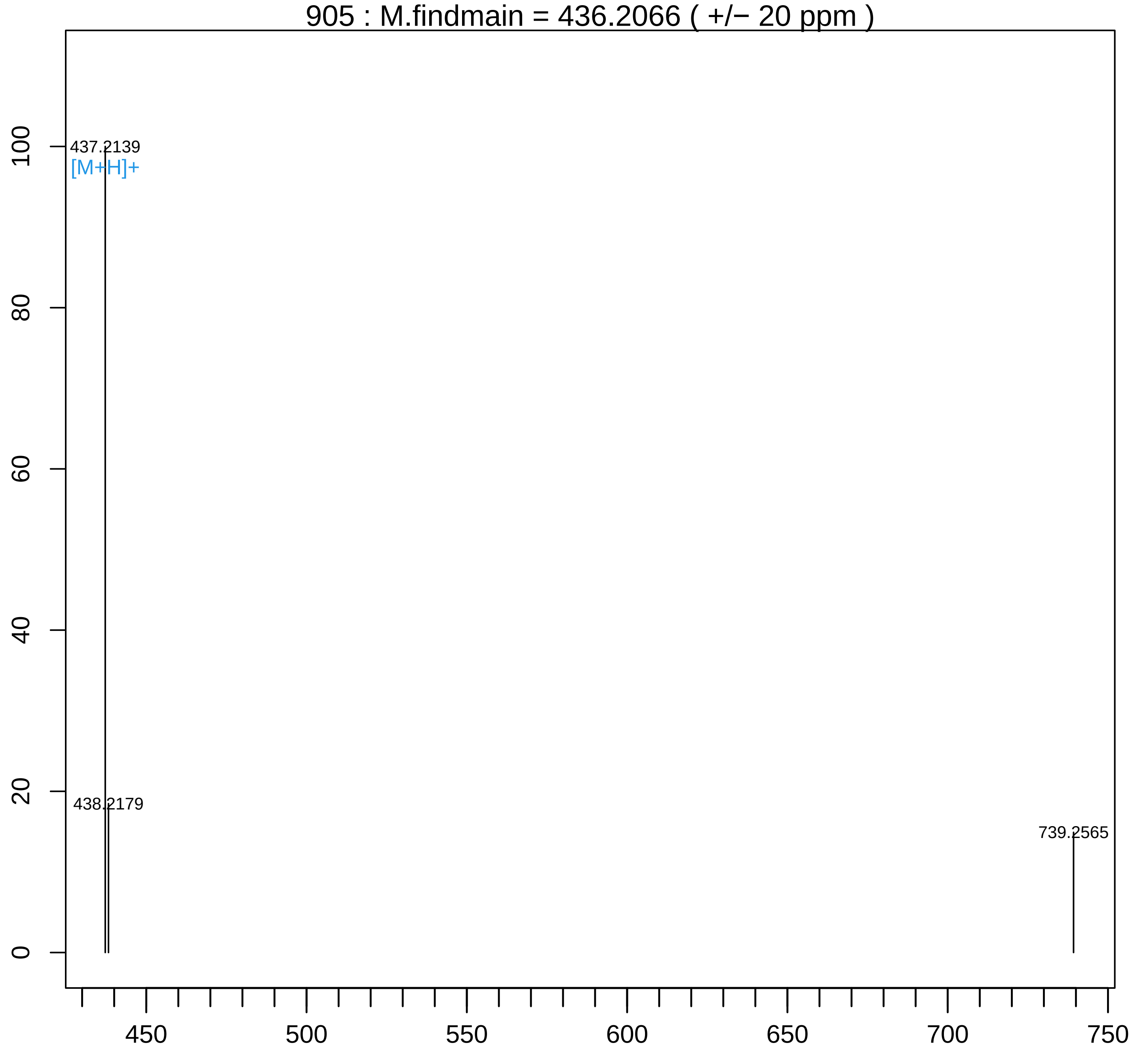

905 : M.findmain = 436.2066 (+/− 20 ppm)
437.2139
[M+H]+
438.2179
739.2565
0
20
40
60
80
100
450
500
550
600
650
700
750

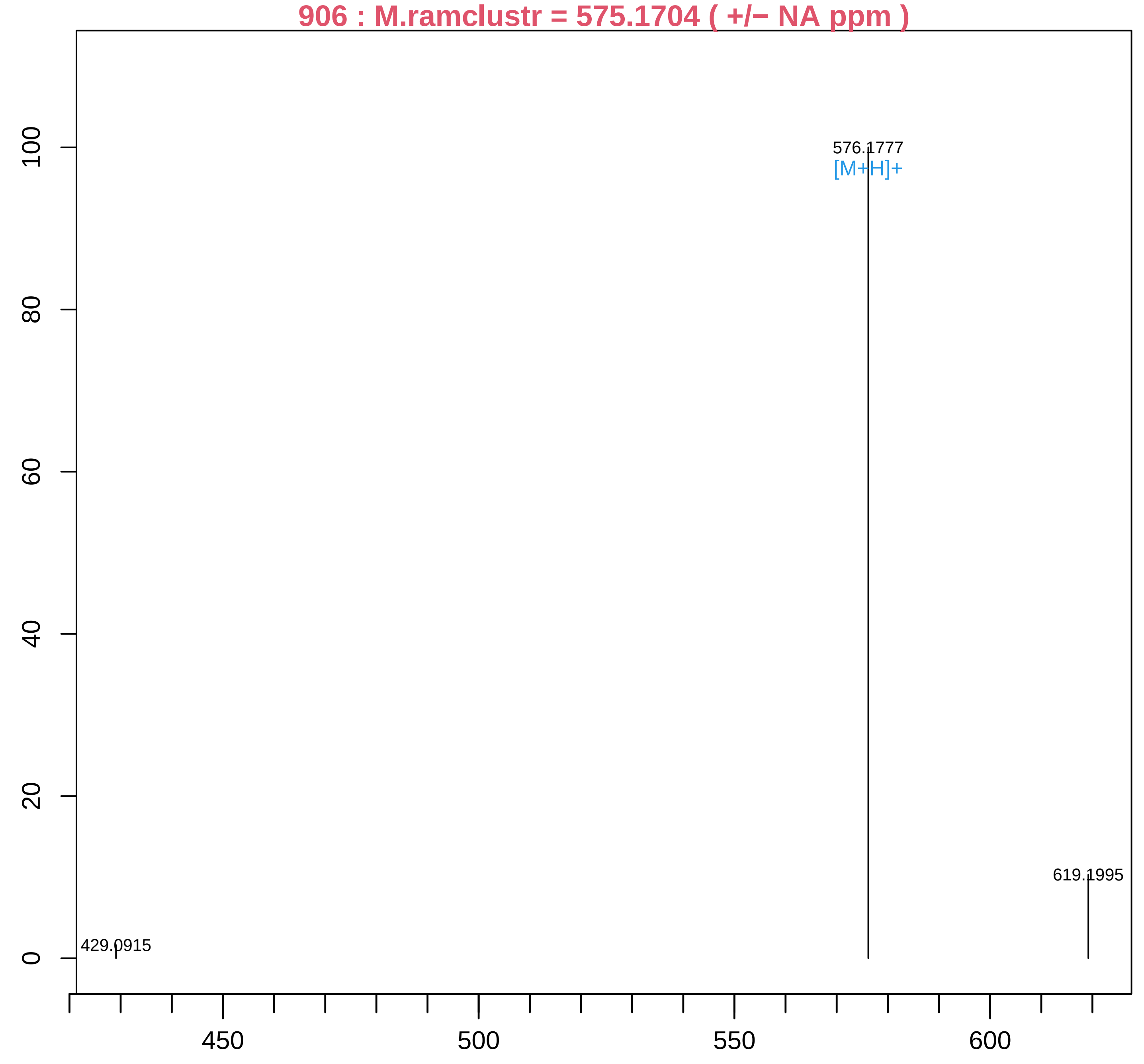
906 : M.ramclustr = 575.1704 (+/− NA ppm)
576.1777
[M+H]+
619.1995
429.0915
0
20
40
60
80
100
450
500
550
600

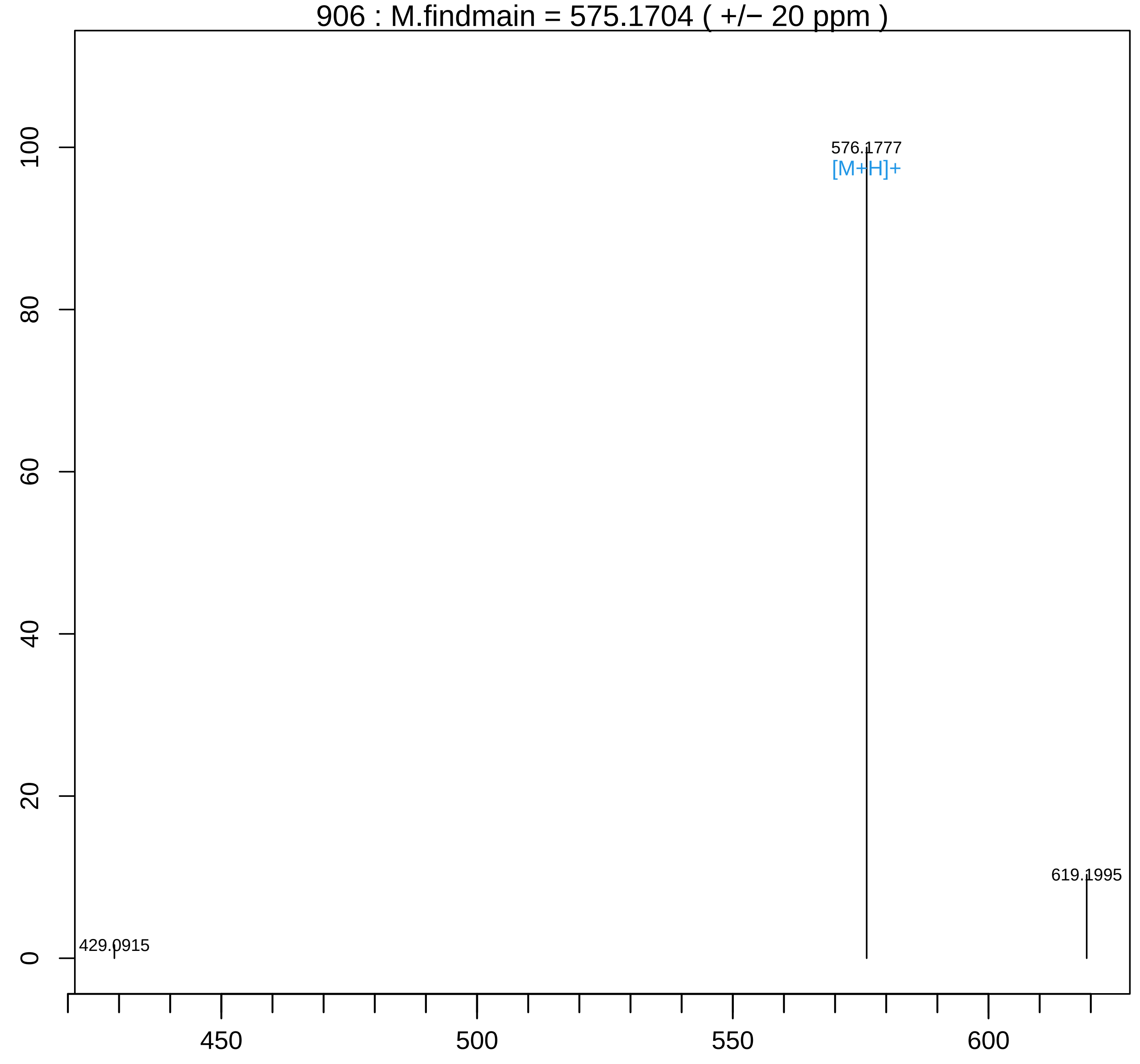
906 : M.findmain = 575.1704 (+/− 20 ppm)
576.1777
[M+H]+
619.1995
429.0915
0
20
40
60
80
100
450
500
550
600

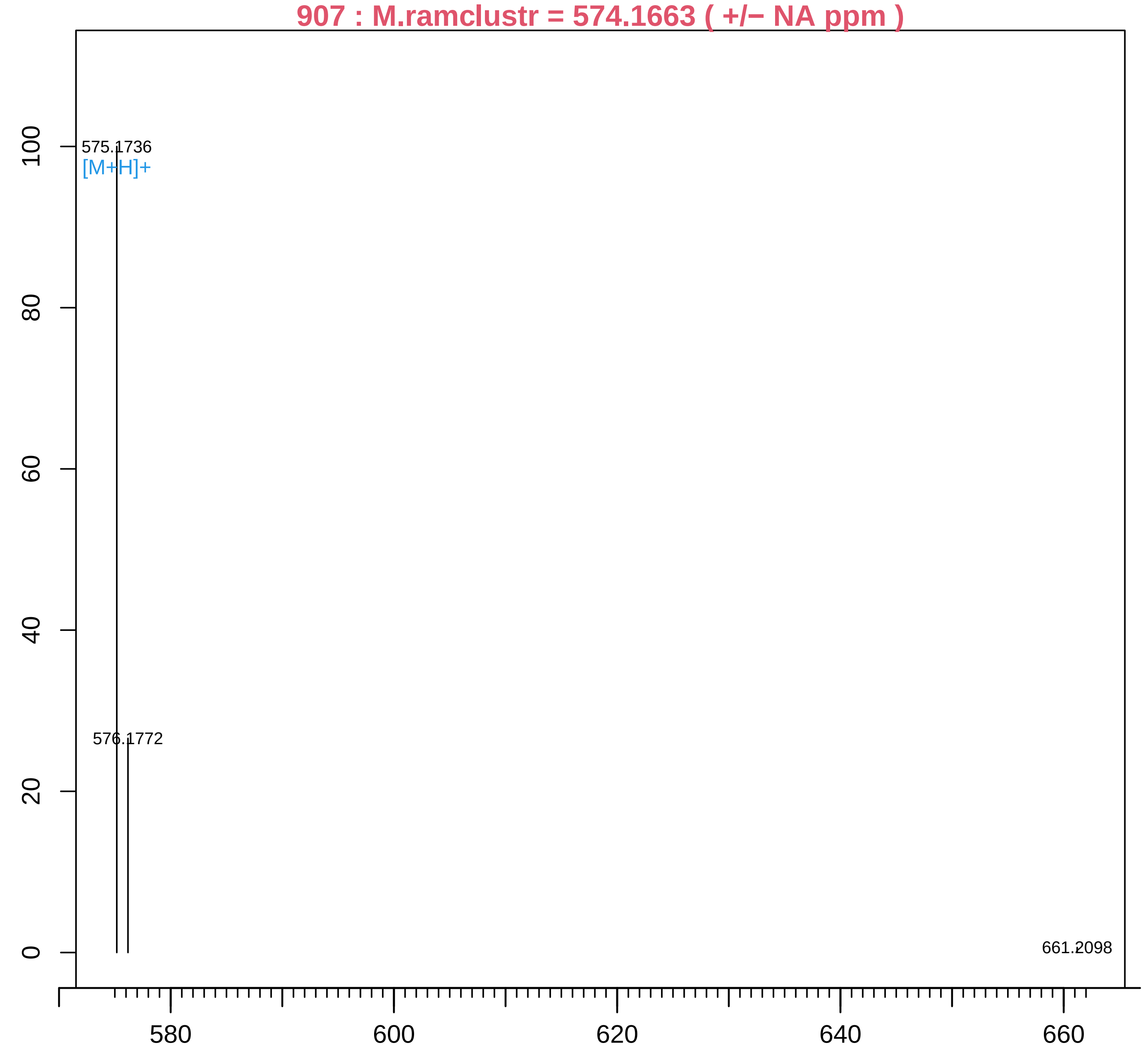
907 : M.ramclustr = 574.1663 (+/− NA ppm)
575.1736
[M+H]+
576.1772
661.2098
0
20
40
60
80
100
580
600
620
640
660

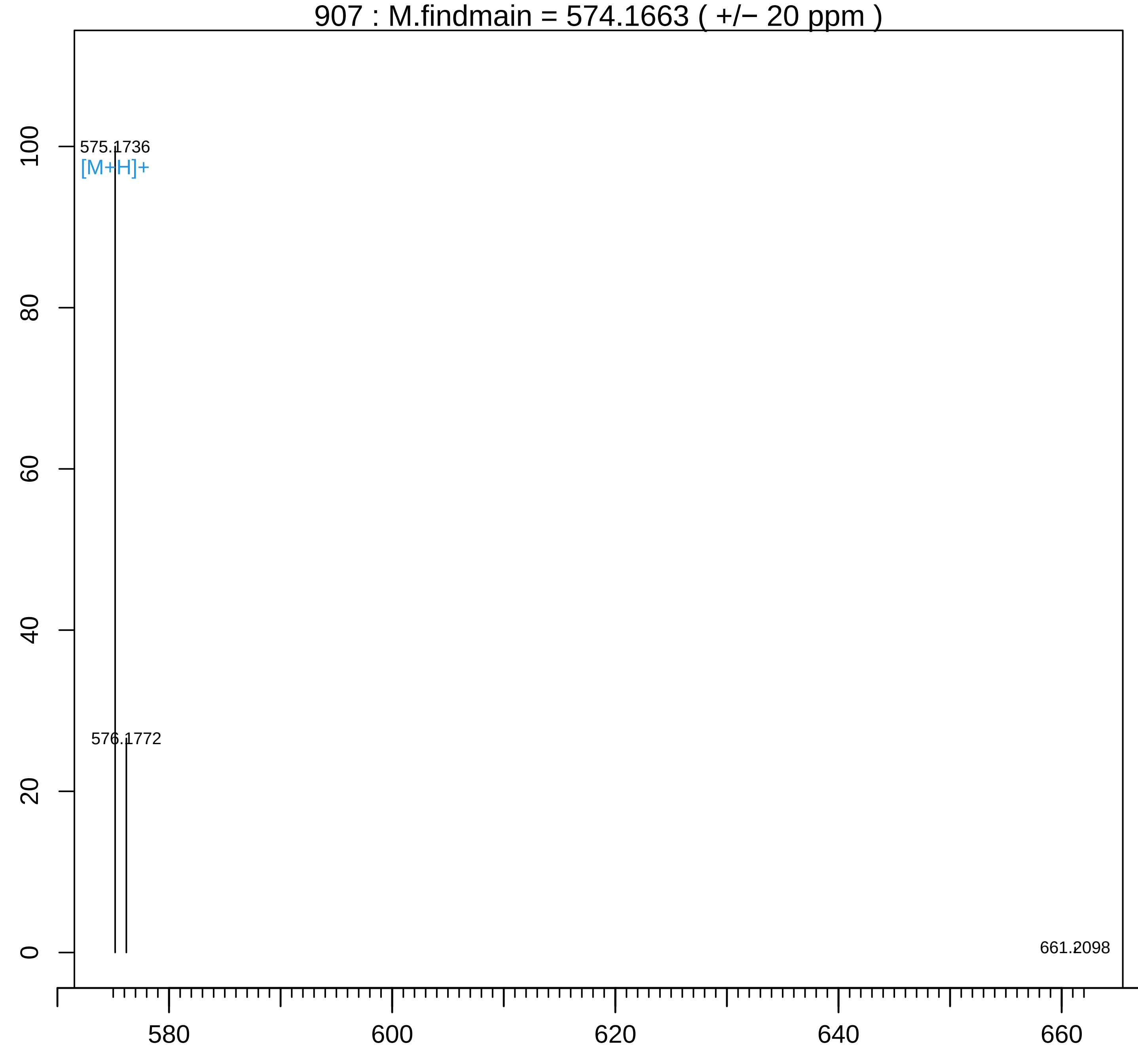
907 : M.findmain = 574.1663 (+/− 20 ppm)
575.1736
[M+H]+
576.1772
661.2098
0
20
40
60
80
100
580
600
620
640
660

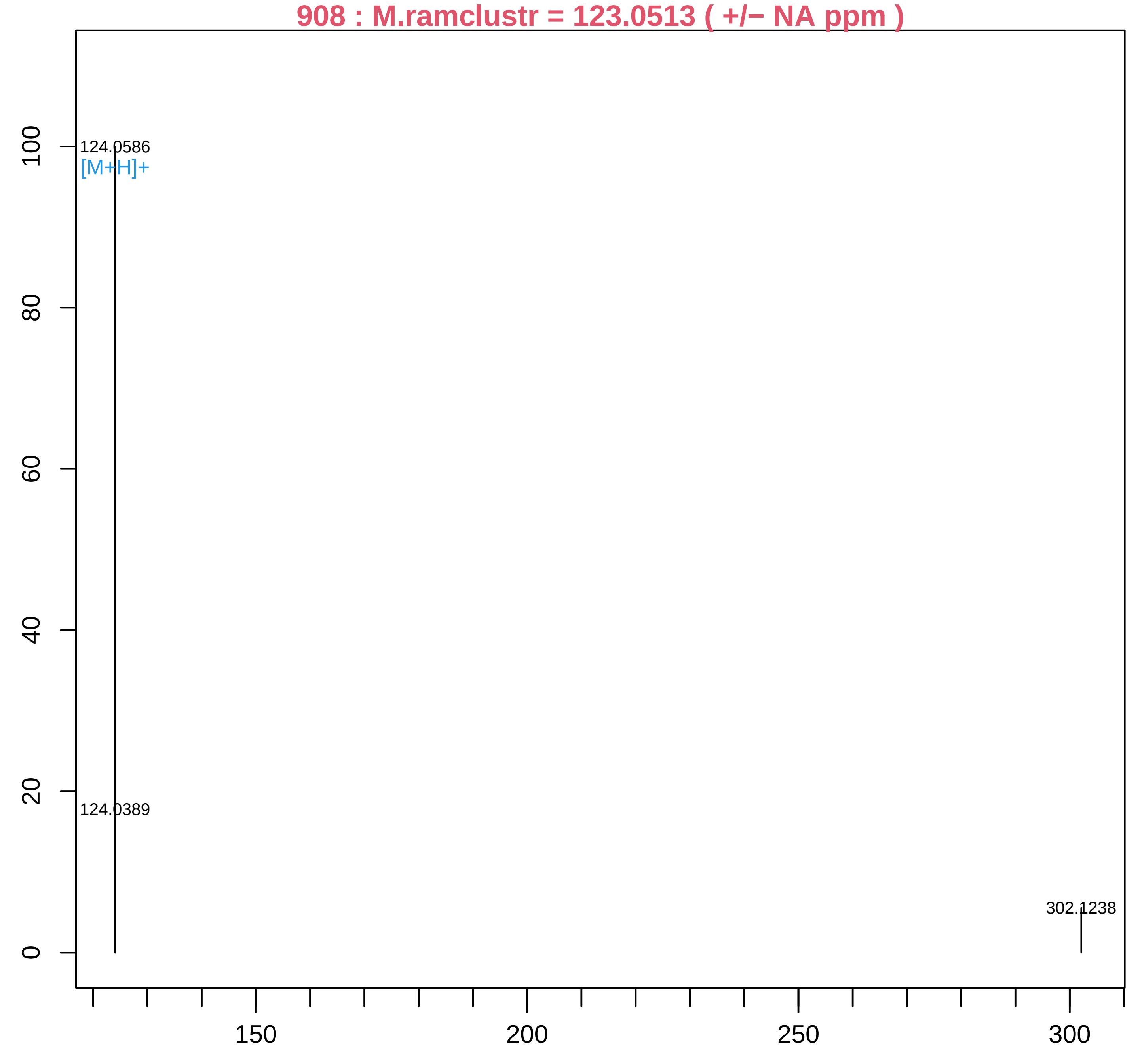

908 : M.ramclustr = 123.0513 (+/− NA ppm)
124.0586
[M+H]+
124.0389
302.1238
0
20
40
60
80
100
150
200
250
300

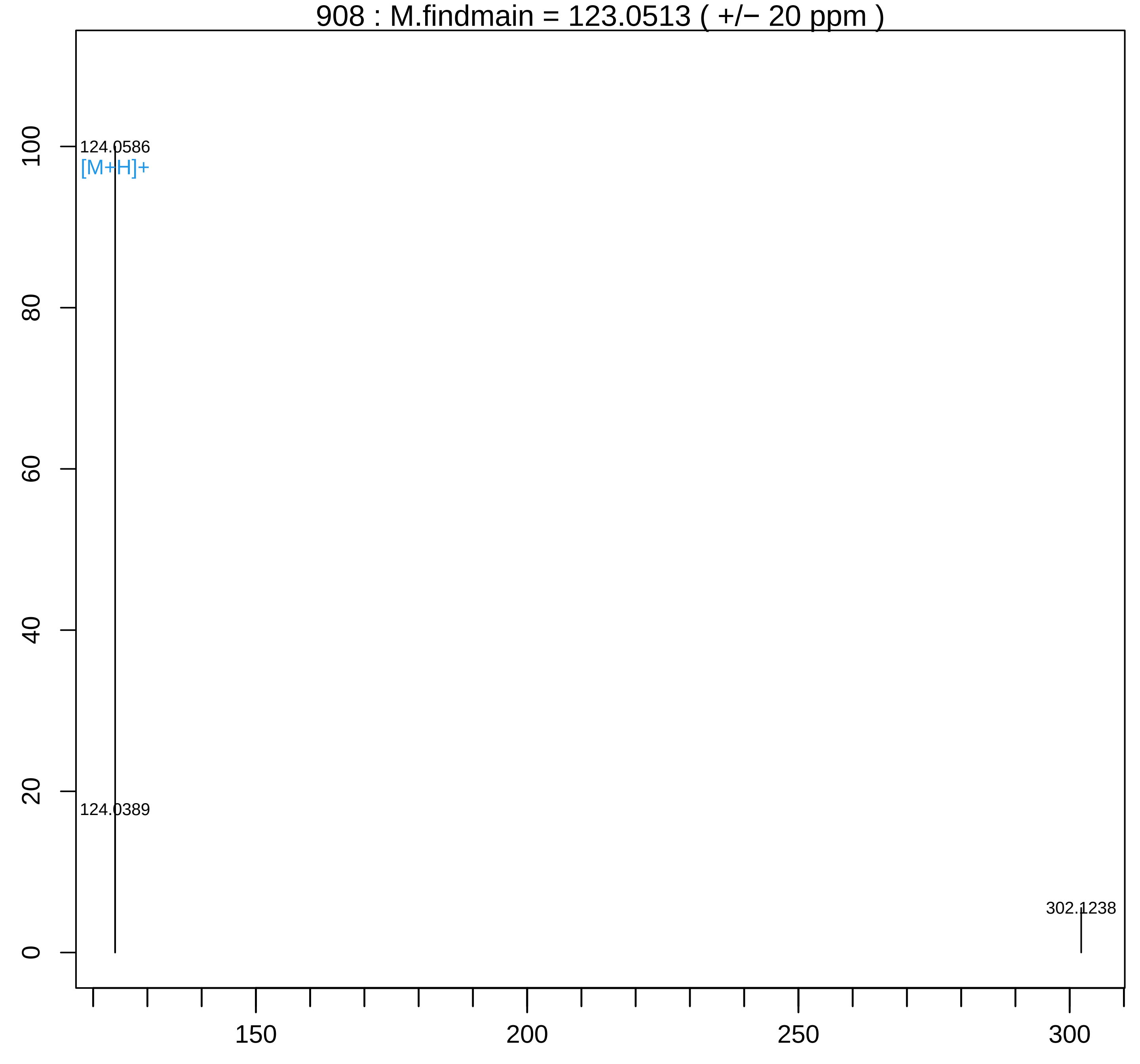

908 : M.findmain = 123.0513 (+/− 20 ppm)
124.0586
[M+H]+
124.0389
302.1238
0
20
40
60
80
100
150
200
250
300

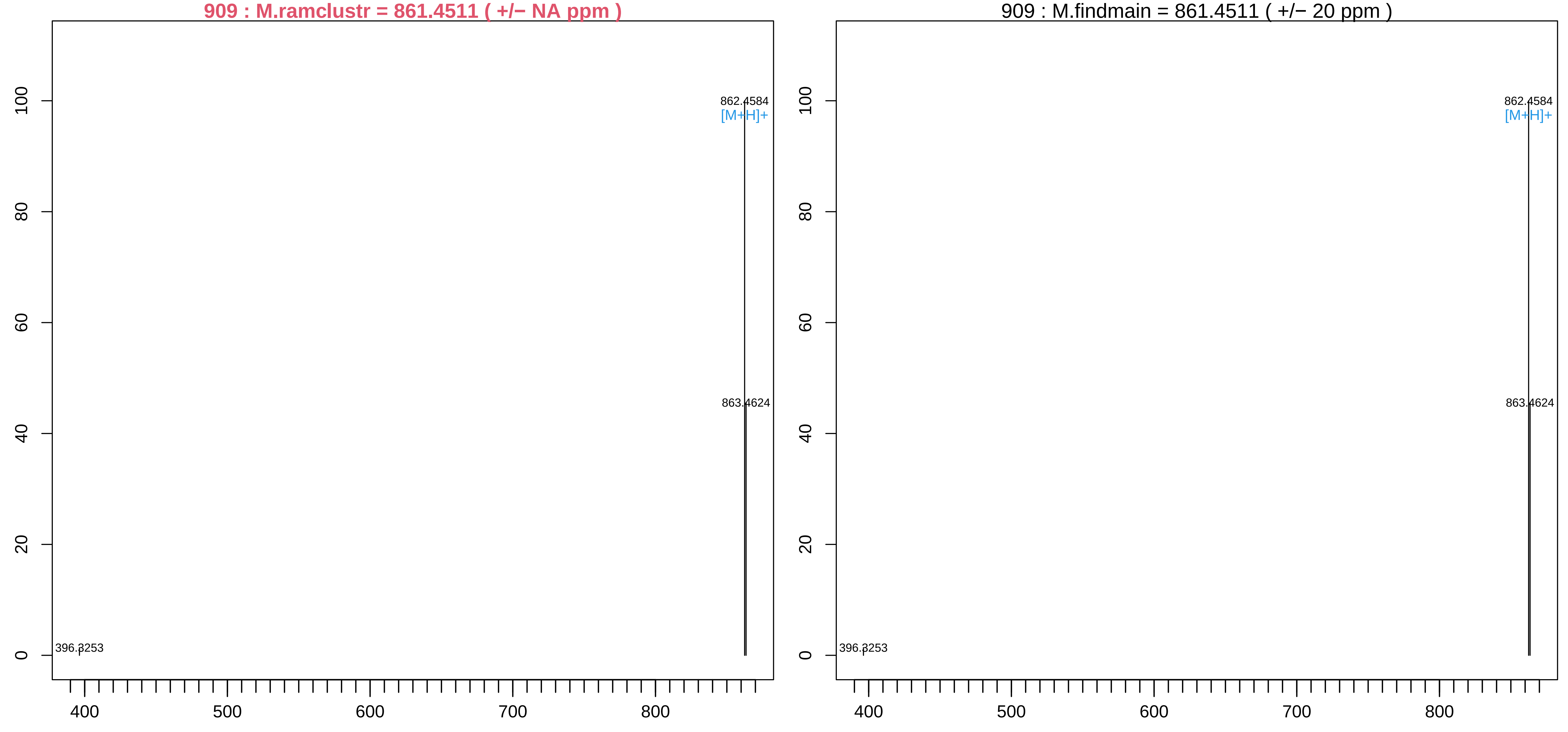

909 : M.ramclustr = 861.4511 (+/− NA ppm)
862.4584
[M+H]+
863.4624
396.3253
909 : M.findmain = 861.4511 (+/− 20 ppm)
862.4584
[M+H]+
863.4624
396.3253

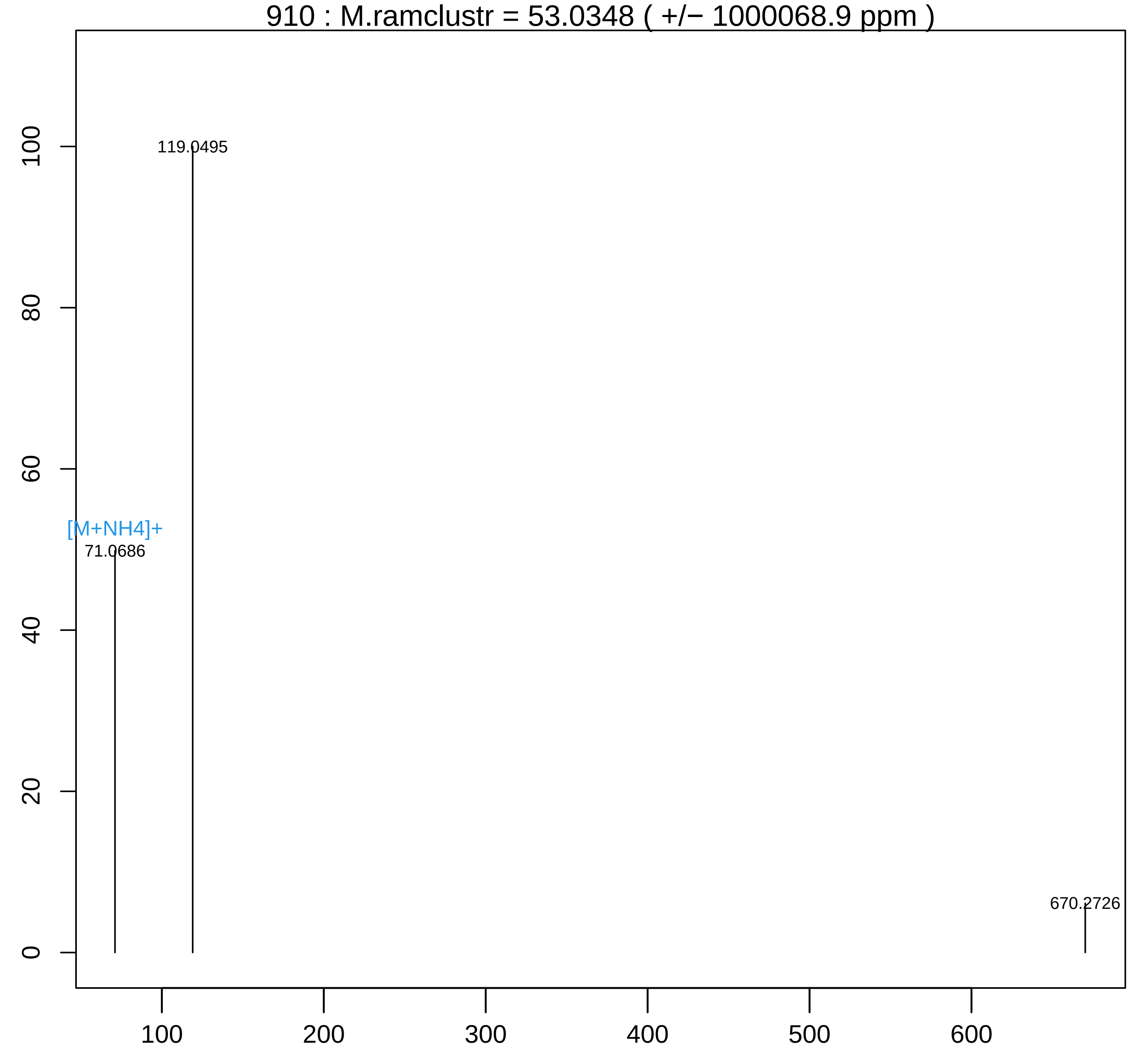

910 : M.ramclustr = 53.0348 (+/− 1000068.9 ppm)
119.0495
[M+NH4]+
71.0686
670.2726
0
20
40
60
80
100
100
200
300
400
500
600

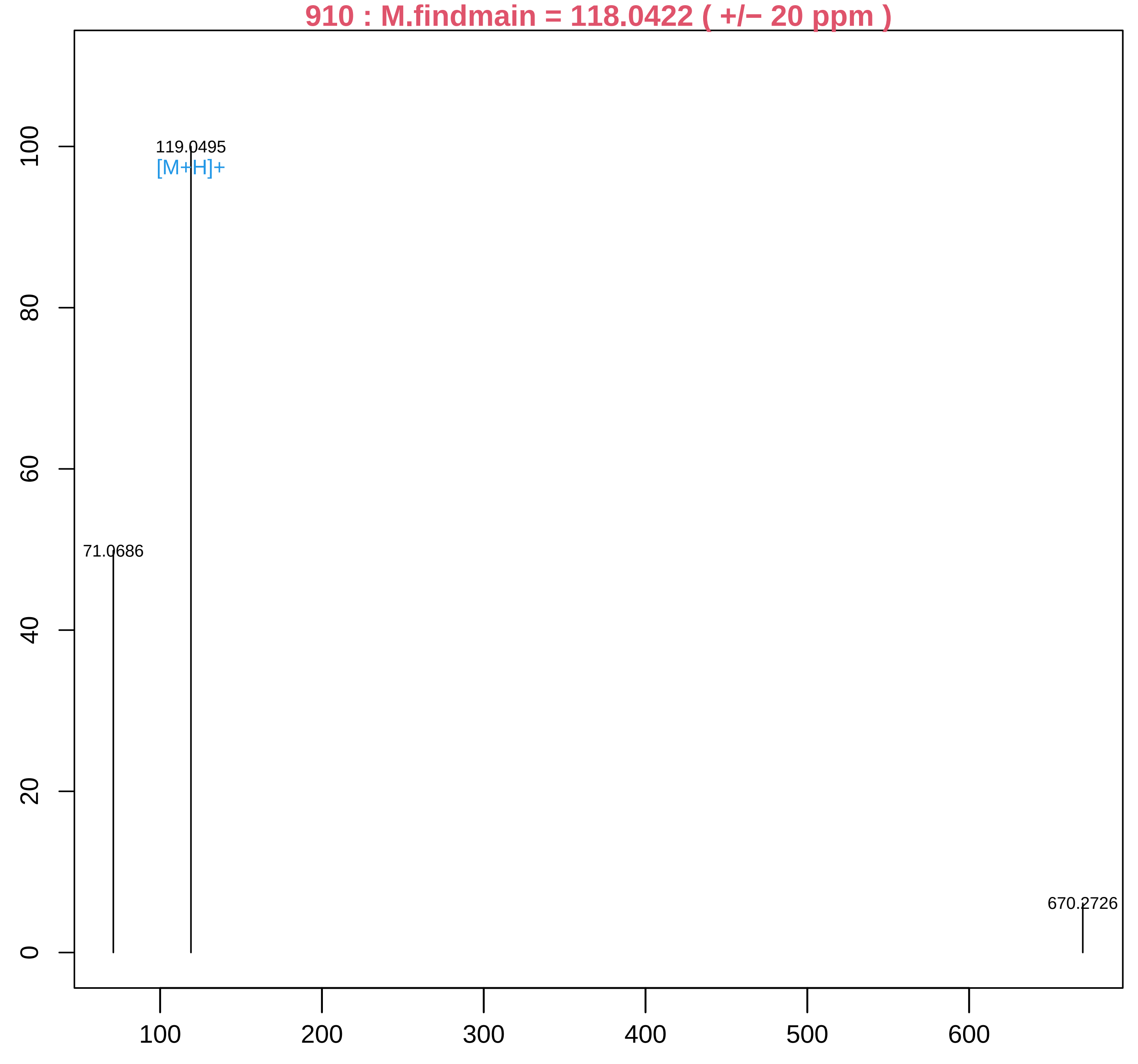

910 : M.findmain = 118.0422 (+/− 20 ppm)
119.0495
[M+H]+
71.0686
670.2726
0
20
40
60
80
100
100
200
300
400
500
600

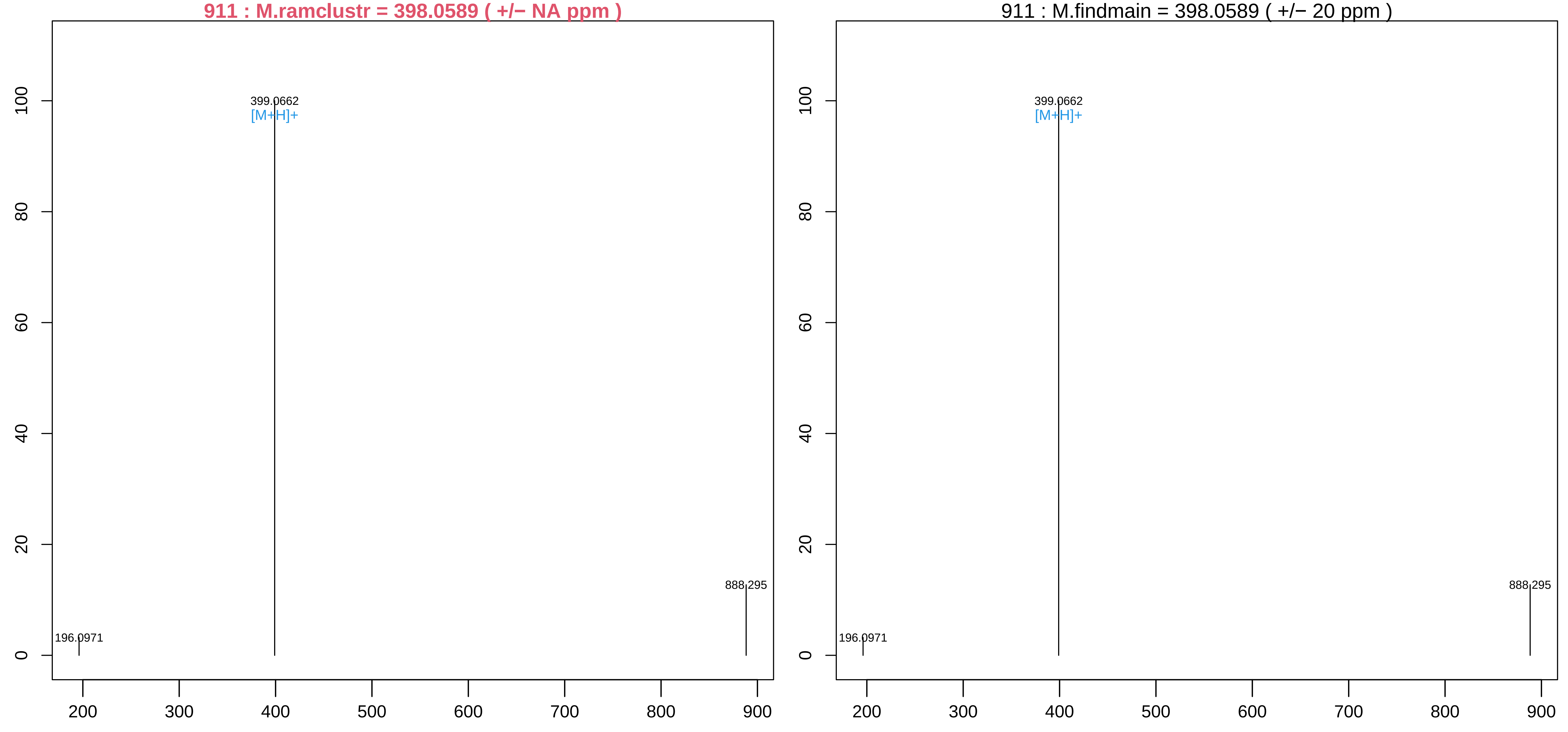
911 : M.ramclustr = 398.0589 (+/− NA ppm)
399.0662
[M+H]+
196.0971
888.295
911 : M.findmain = 398.0589 (+/− 20 ppm)
399.0662
[M+H]+
196.0971
888.295

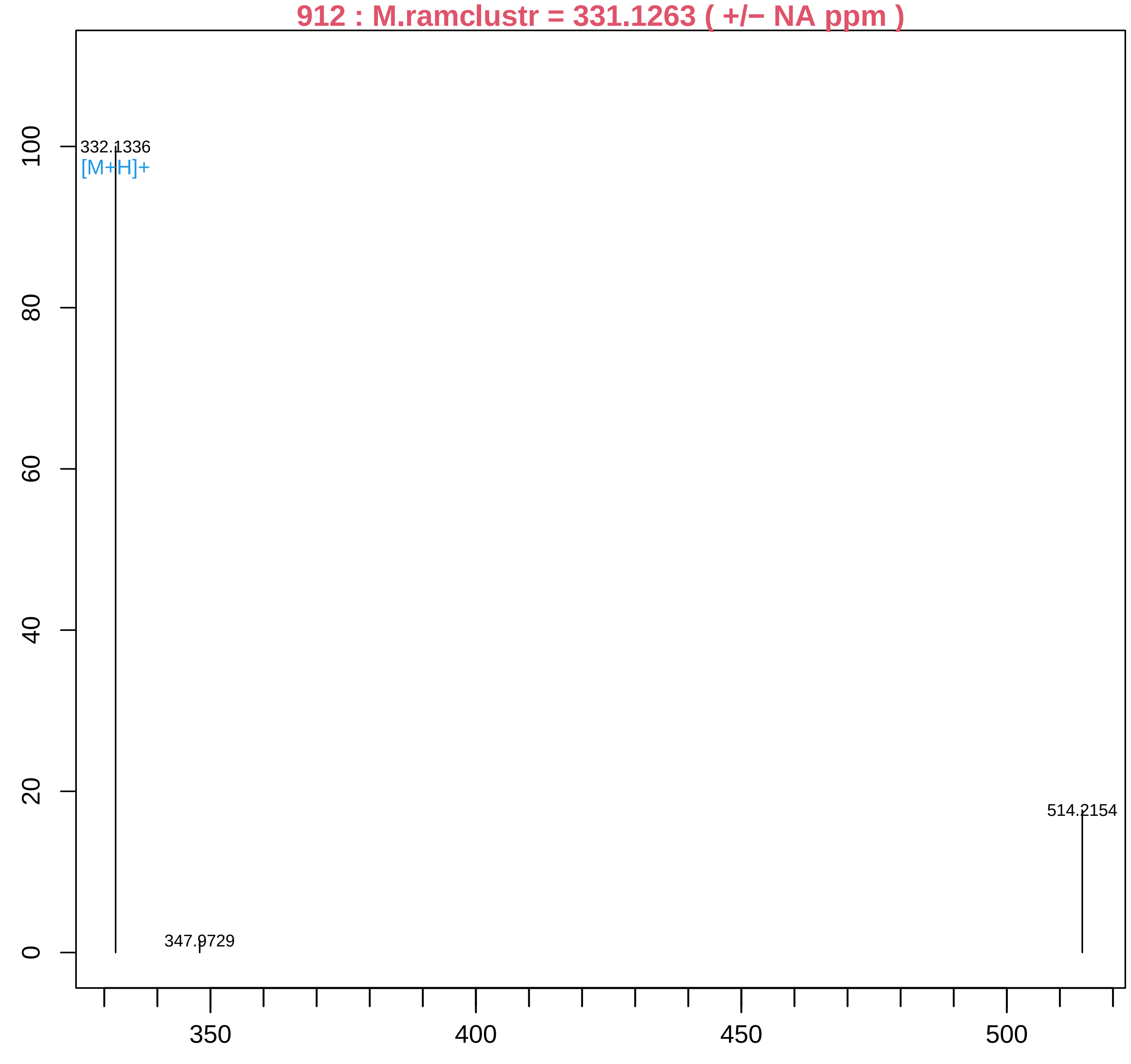
912 : M.ramclustr = 331.1263 (+/− NA ppm)
332.1336
[M+H]+
347.9729
514.2154
0
20
40
60
80
100
350
400
450
500

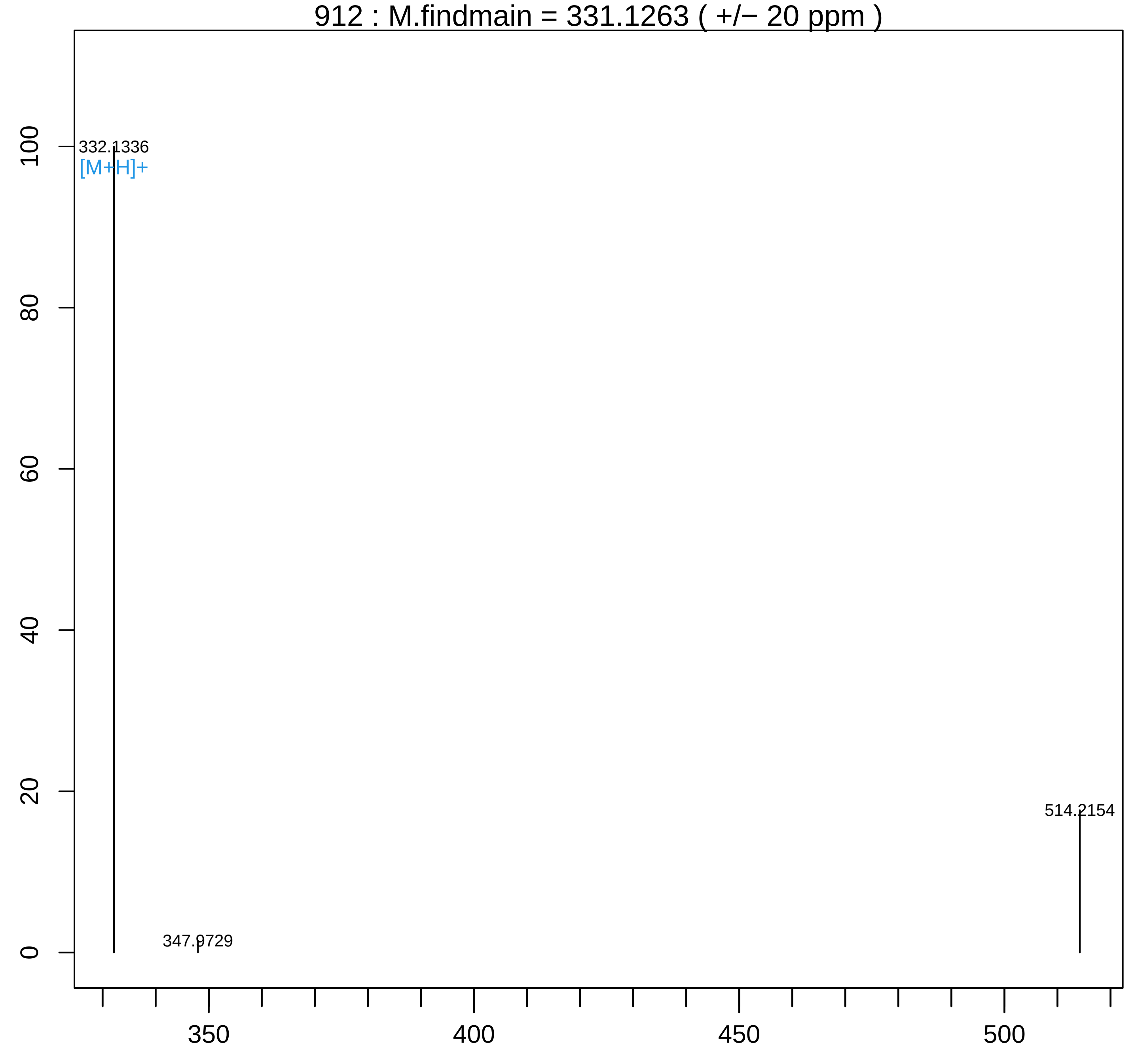
912 : M.findmain = 331.1263 (+/− 20 ppm)
332.1336
[M+H]+
347.9729
514.2154
0
20
40
60
80
100
350
400
450
500

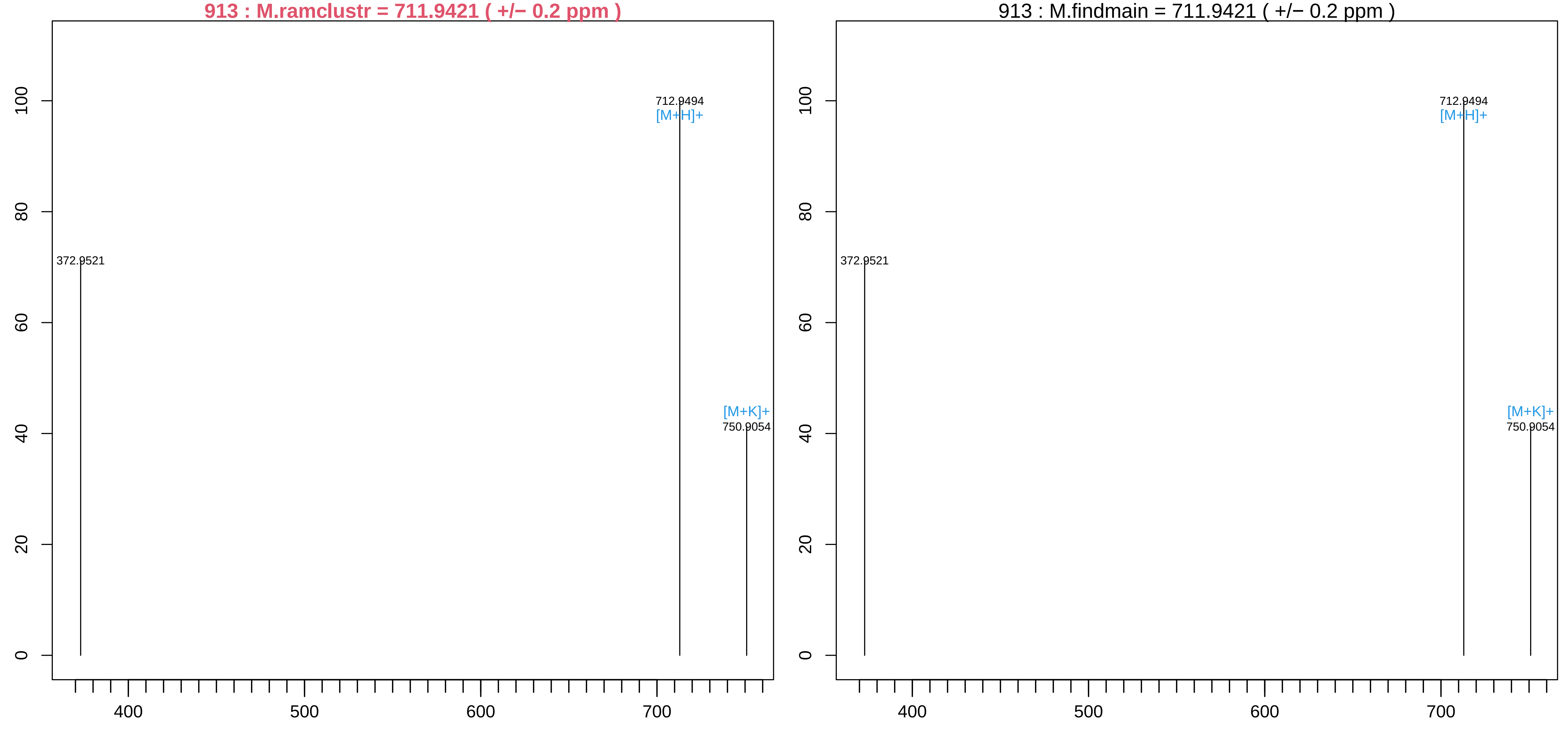
913 : M.ramclustr = 711.9421 (+/− 0.2 ppm)
712.9494
[M+H]+
372.9521
[M+K]+
750.9054
913 : M.findmain = 711.9421 (+/− 0.2 ppm)
712.9494
[M+H]+
372.9521
[M+K]+
750.9054

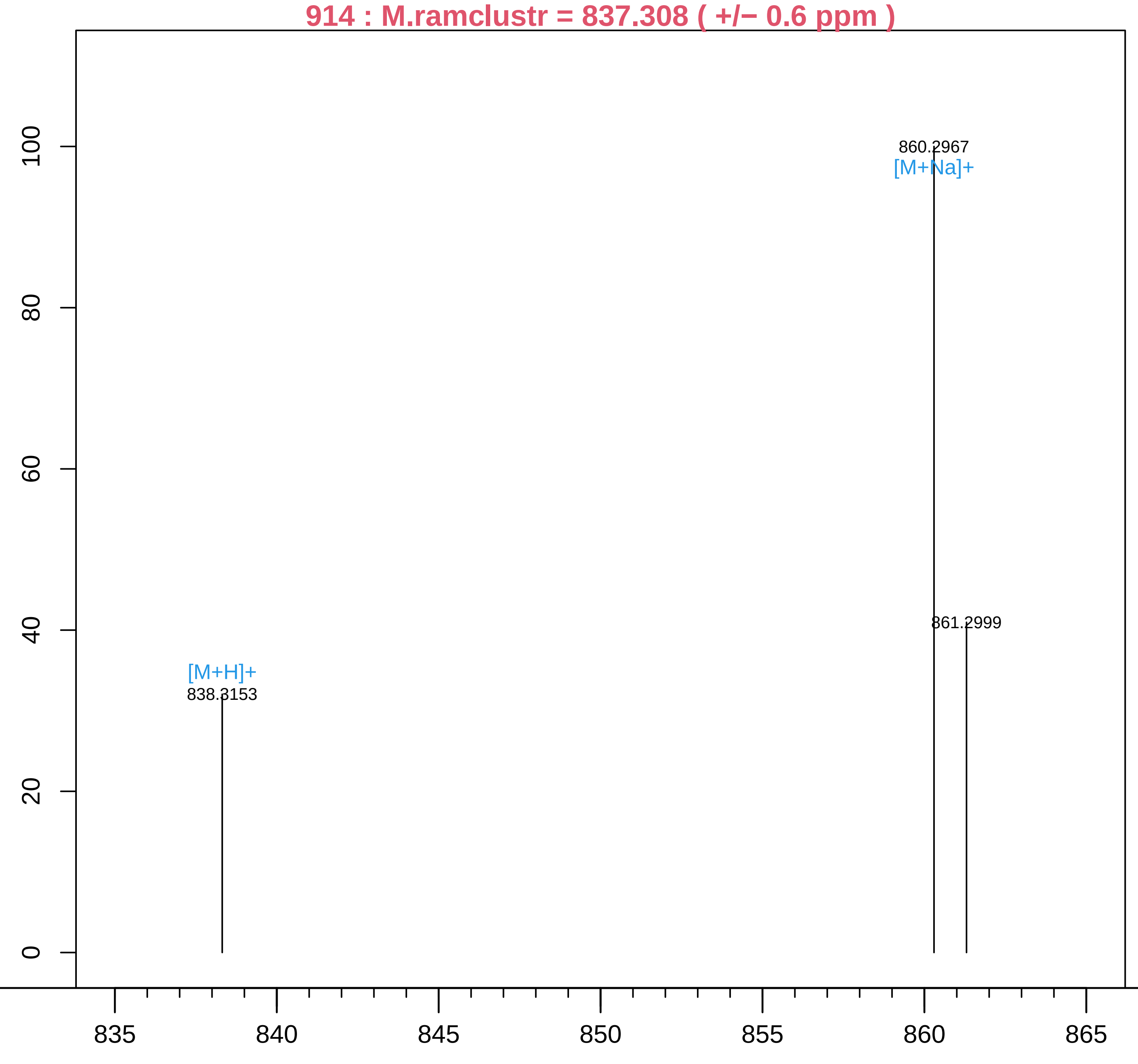
914 : M.ramclustr = 837.308 (+/− 0.6 ppm)
860.2967
[M+Na]+
861.2999
[M+H]+
838.3153
0
20
40
60
80
100
835
840
845
850
855
860
865

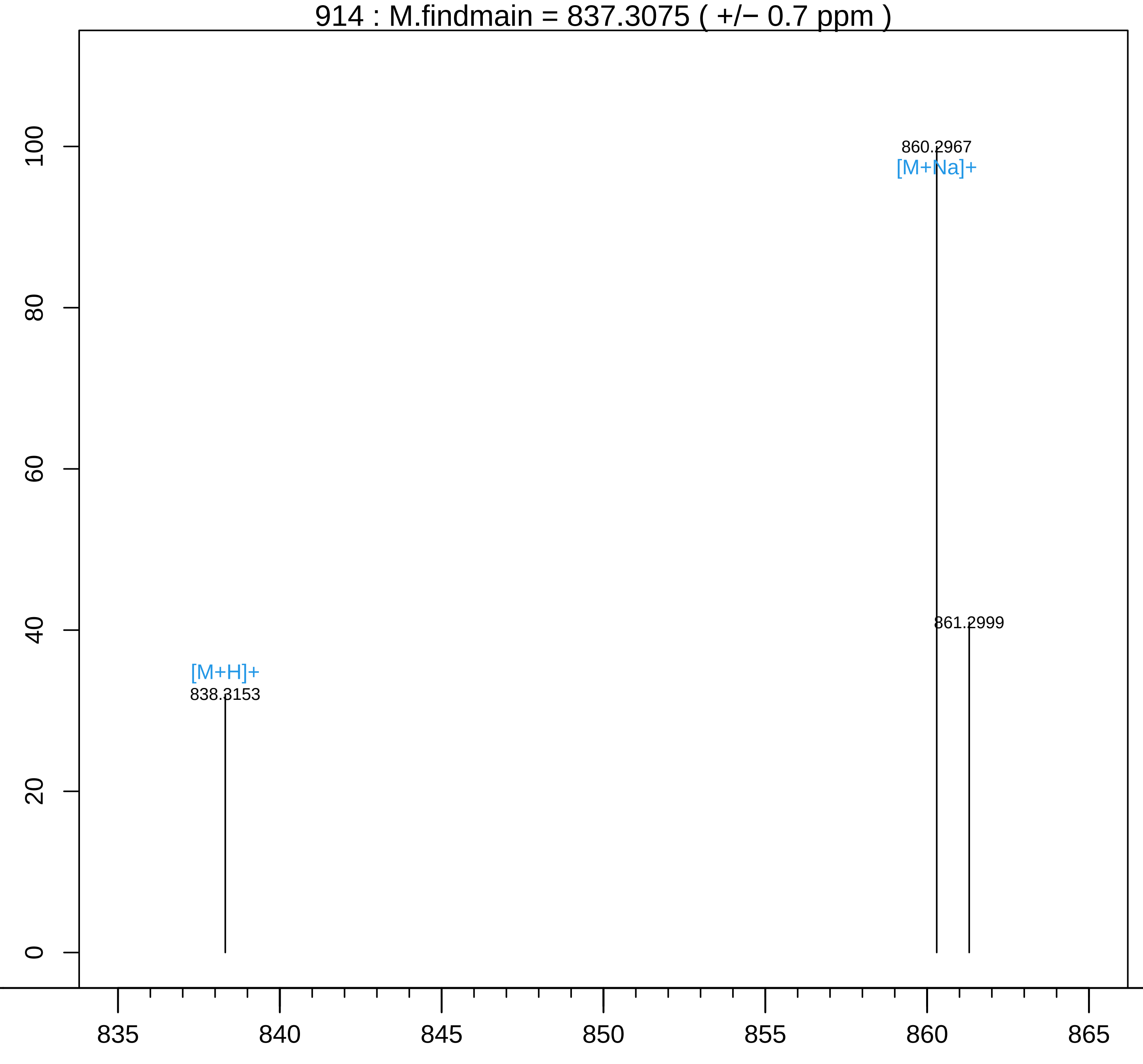
914 : M.findmain = 837.3075 (+/− 0.7 ppm)
860.2967
[M+Na]+
861.2999
[M+H]+
838.3153
0
20
40
60
80
100
835
840
845
850
855
860
865

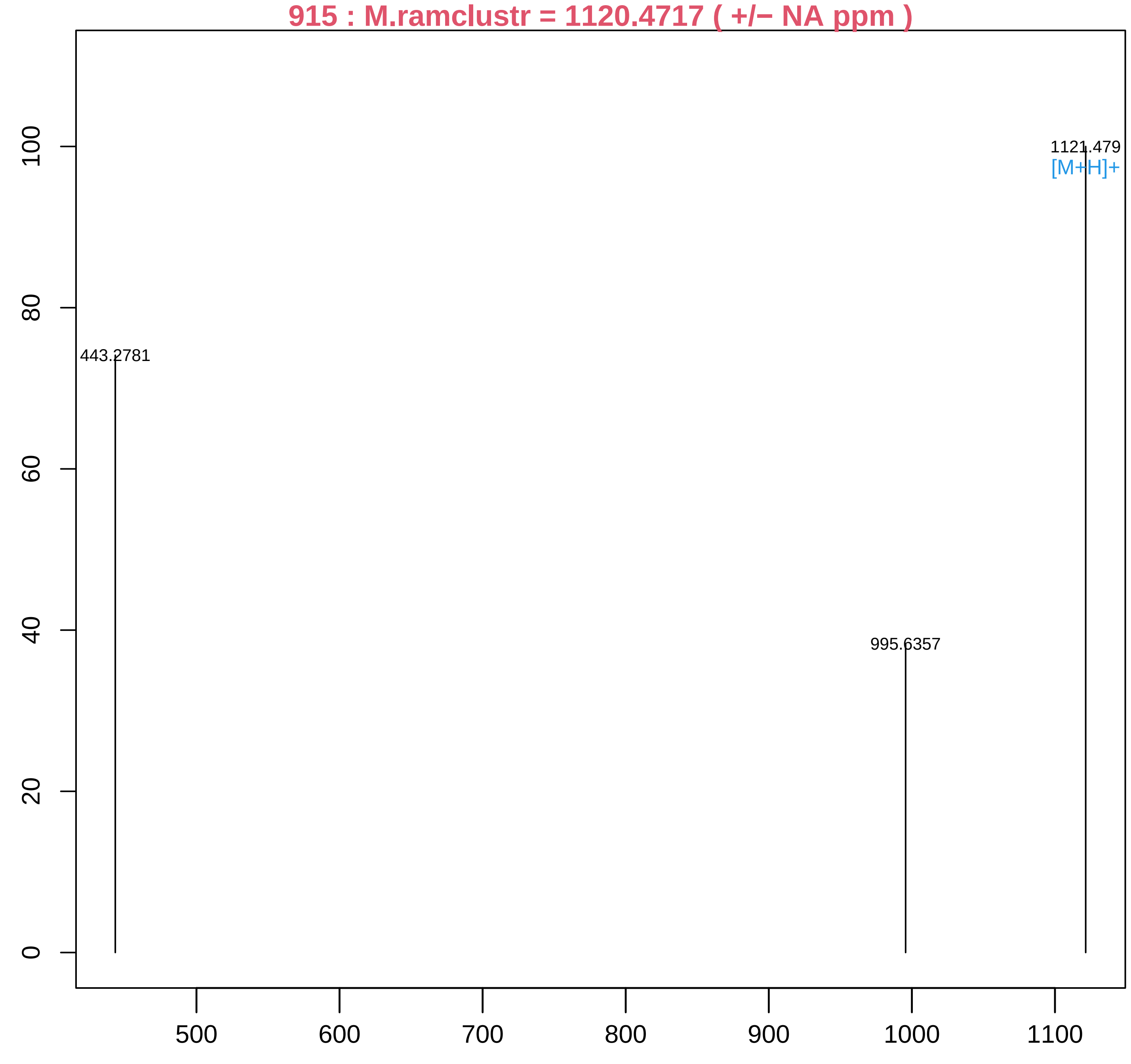

915 : M.ramclustr = 1120.4717 (+/− NA ppm)
1121.479
[M+H]+
443.2781
995.6357

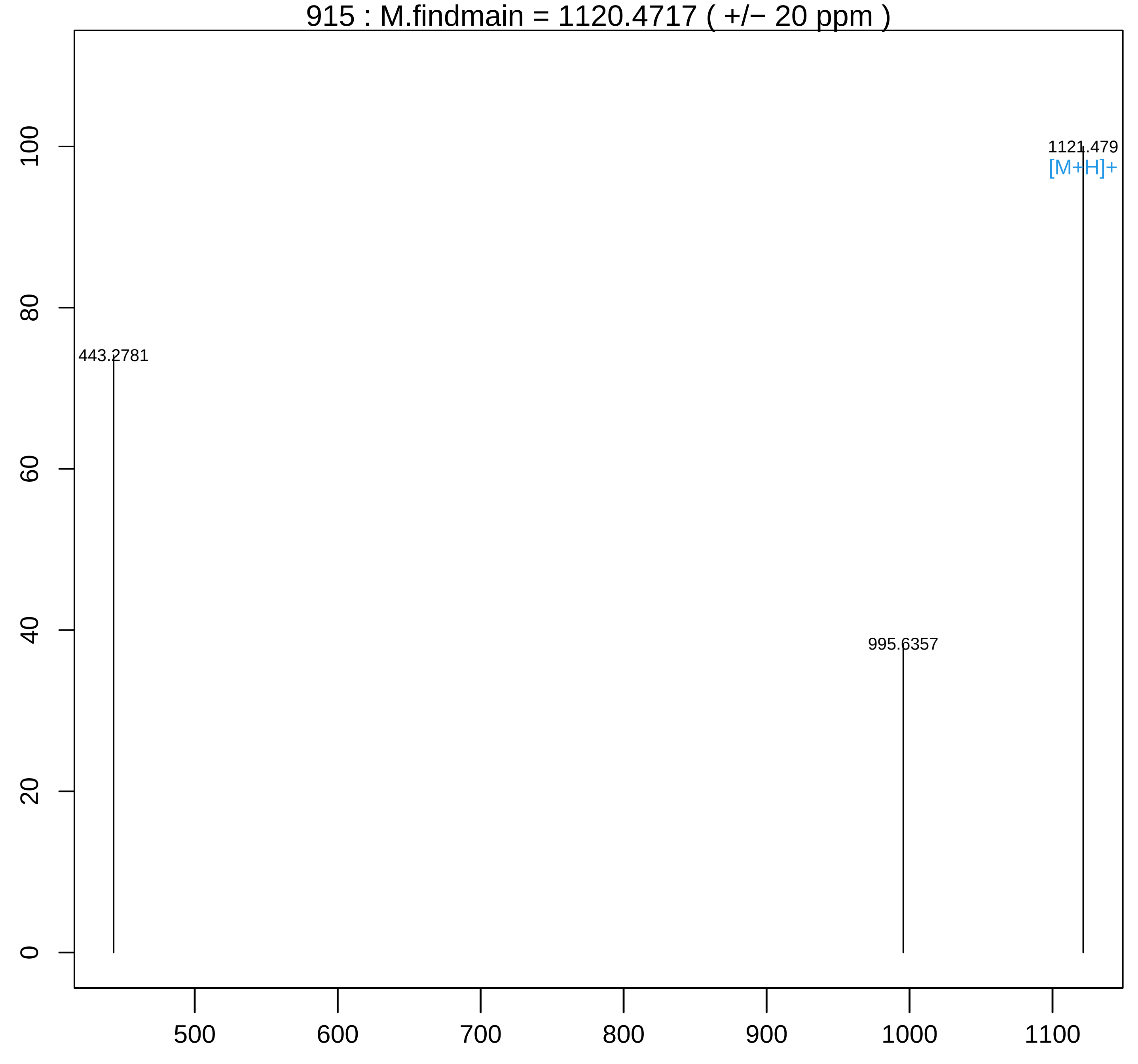

915 : M.findmain = 1120.4717 (+/− 20 ppm)
1121.479
[M+H]+
443.2781
995.6357

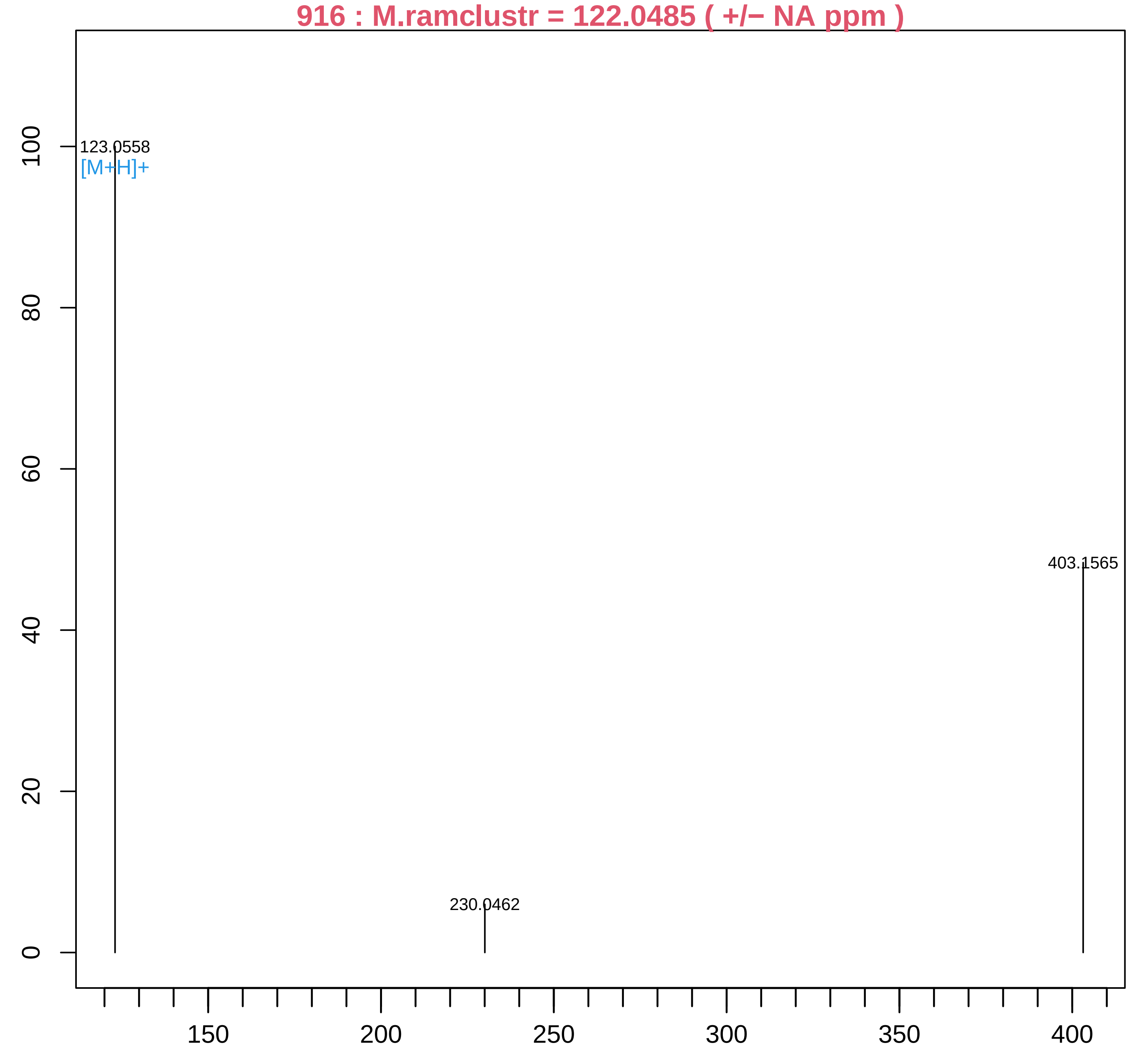
916 : M.ramclustr = 122.0485 (+/− NA ppm)
123.0558
[M+H]+
230.0462
403.1565
0
20
40
60
80
100
150
200
250
300
350
400
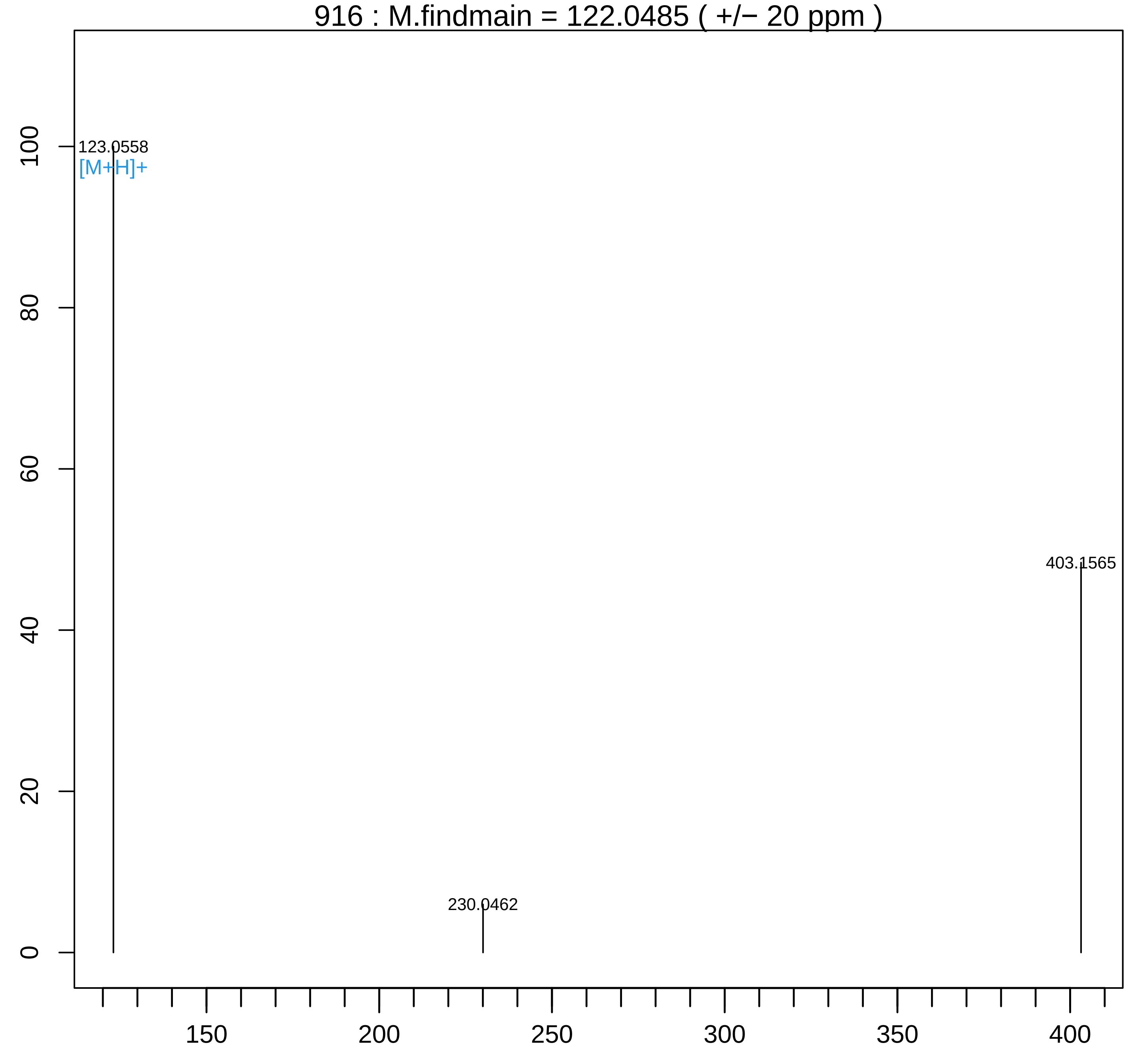
916 : M.findmain = 122.0485 (+/− 20 ppm)
123.0558
[M+H]+
230.0462
403.1565
0
20
40
60
80
100
150
200
250
300
350
400

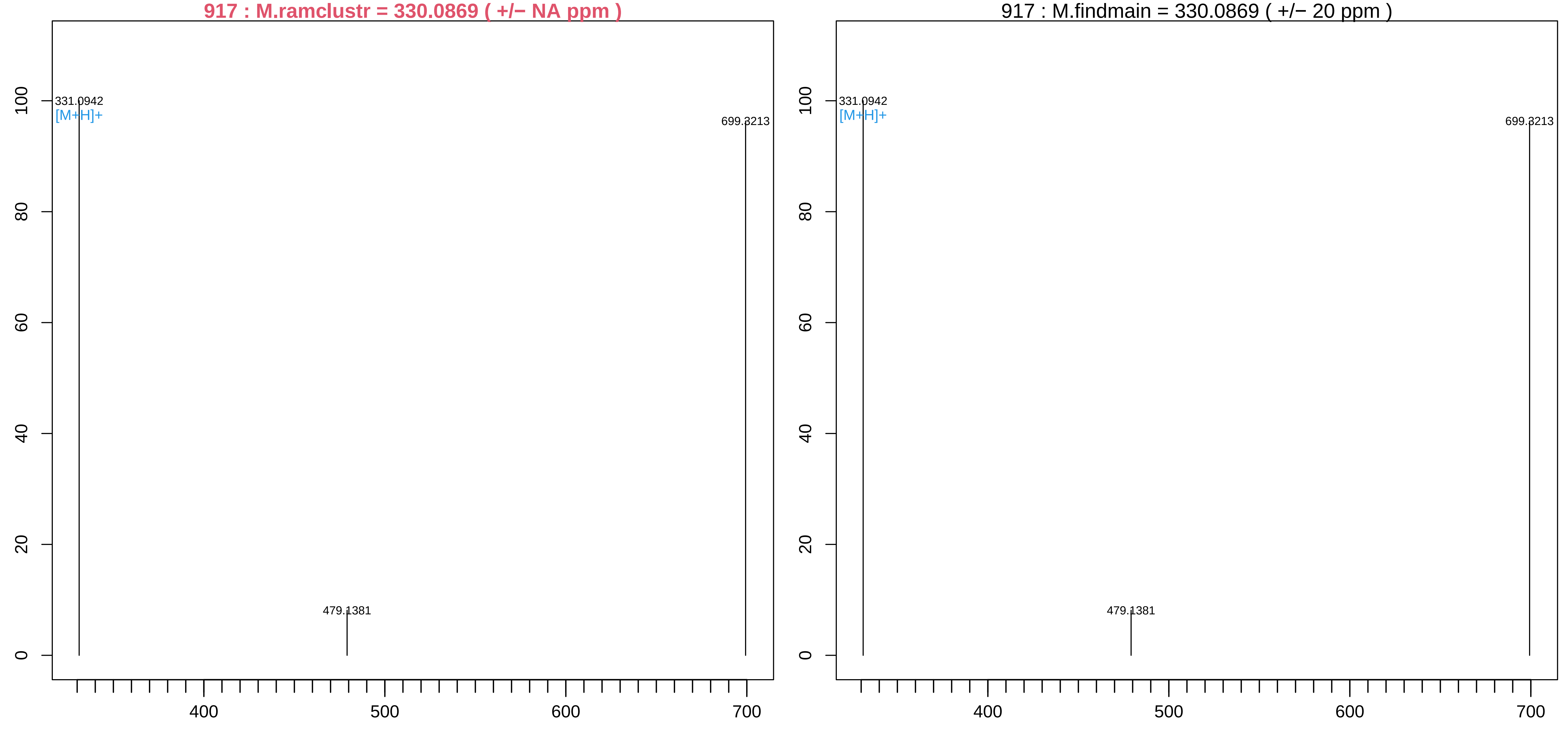
917 : M.ramclustr = 330.0869 (+/− NA ppm)
331.0942
[M+H]+
699.3213
479.1381
0
20
40
60
80
100
400
500
600
700
917 : M.findmain = 330.0869 (+/− 20 ppm)
331.0942
[M+H]+
699.3213
479.1381
0
20
40
60
80
100
400
500
600
700

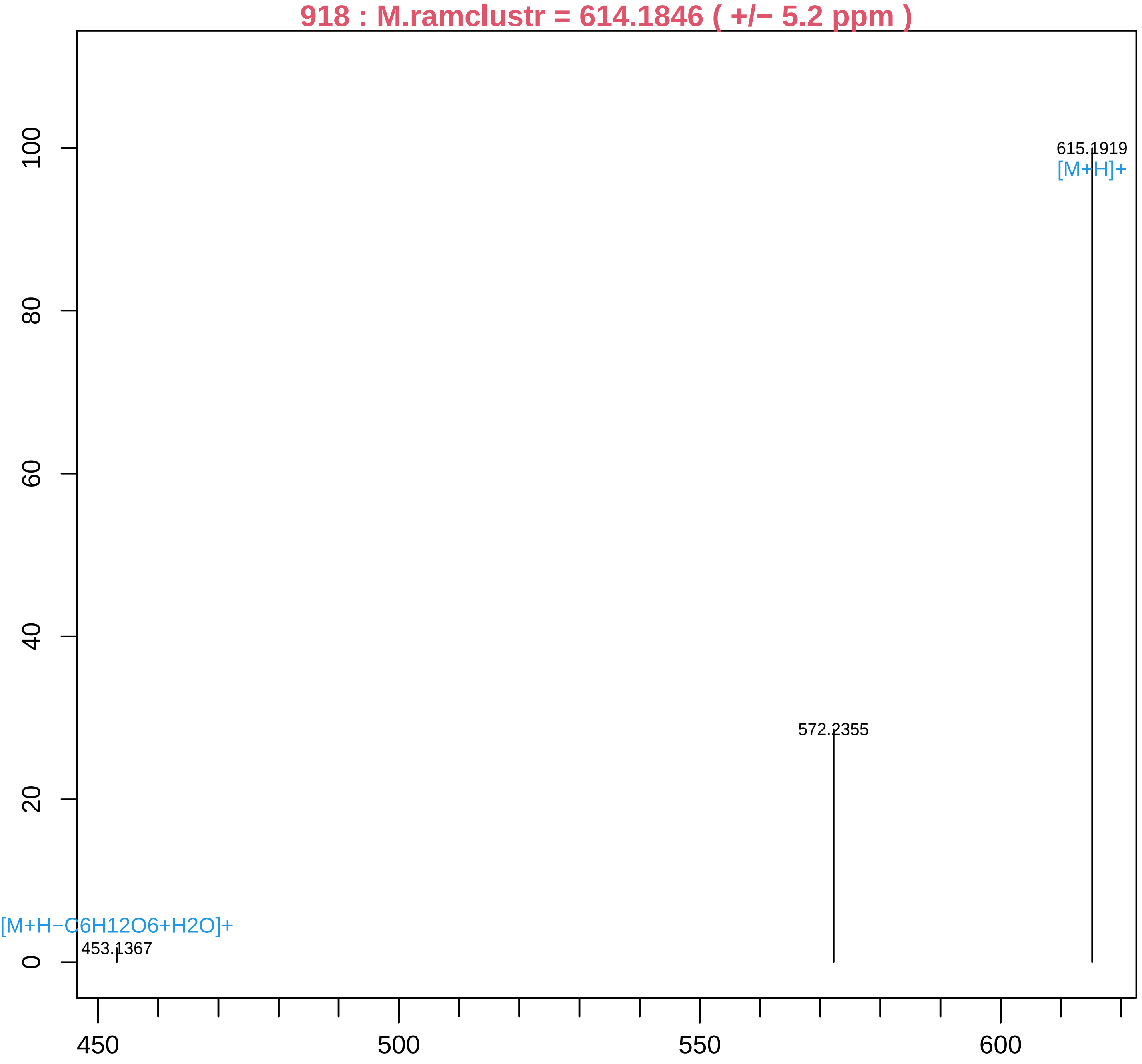
918 : M.ramclustr = 614.1846 (+/− 5.2 ppm)
615.1919
[M+H]+
572.2355
[M+H−C6H12O6+H2O]+
453.1367
0
20
40
60
80
100
450
500
550
600

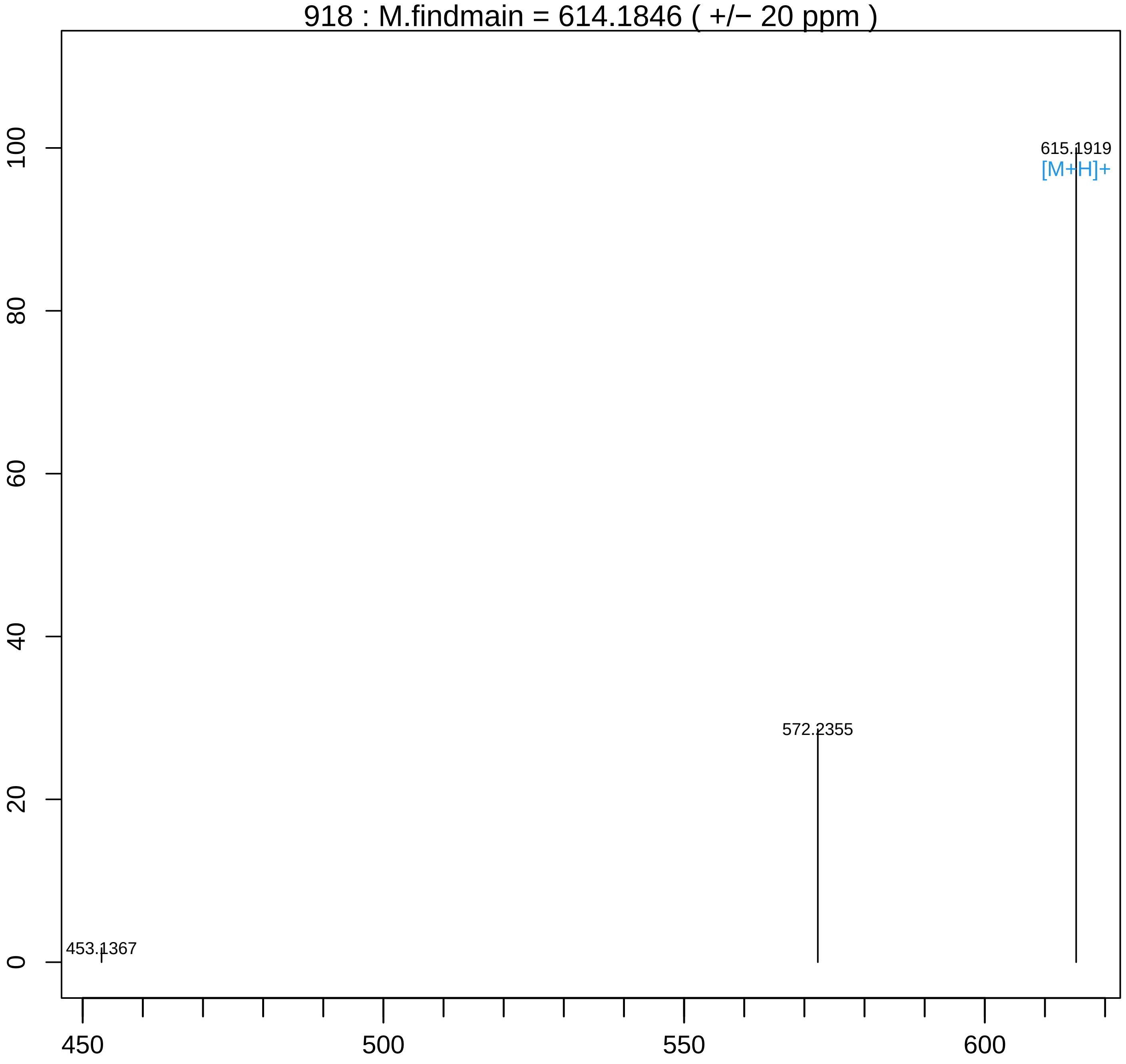
918 : M.findmain = 614.1846 (+/− 20 ppm)
615.1919
[M+H]+
572.2355
453.1367
0
20
40
60
80
100
450
500
550
600

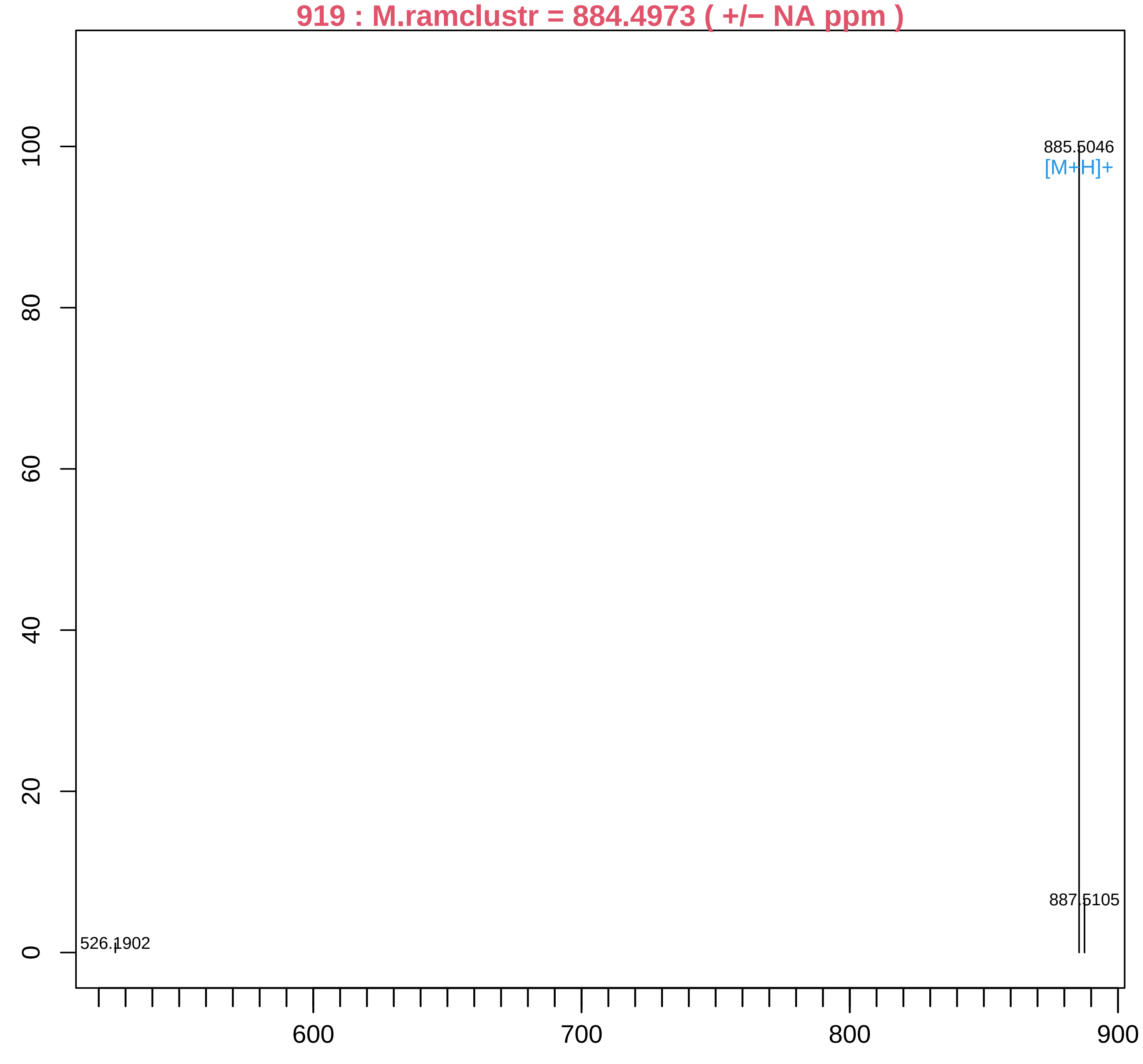
919 : M.ramclustr = 884.4973 (+/− NA ppm)
885.5046
[M+H]+
887.5105
526.1902
0
20
40
60
80
100
600
700
800
900

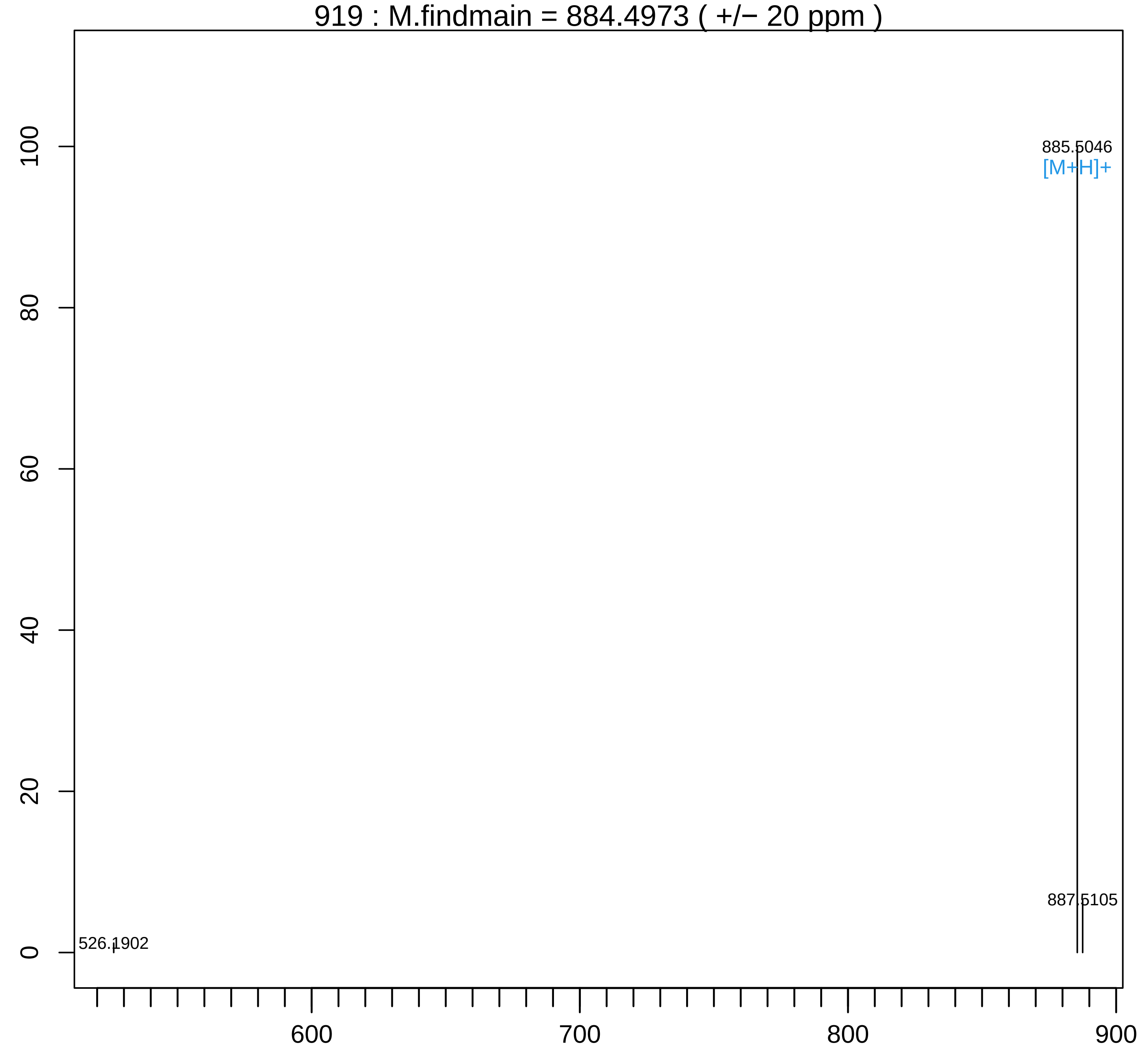
919 : M.findmain = 884.4973 (+/− 20 ppm)
885.5046
[M+H]+
887.5105
526.1902
0
20
40
60
80
100
600
700
800
900

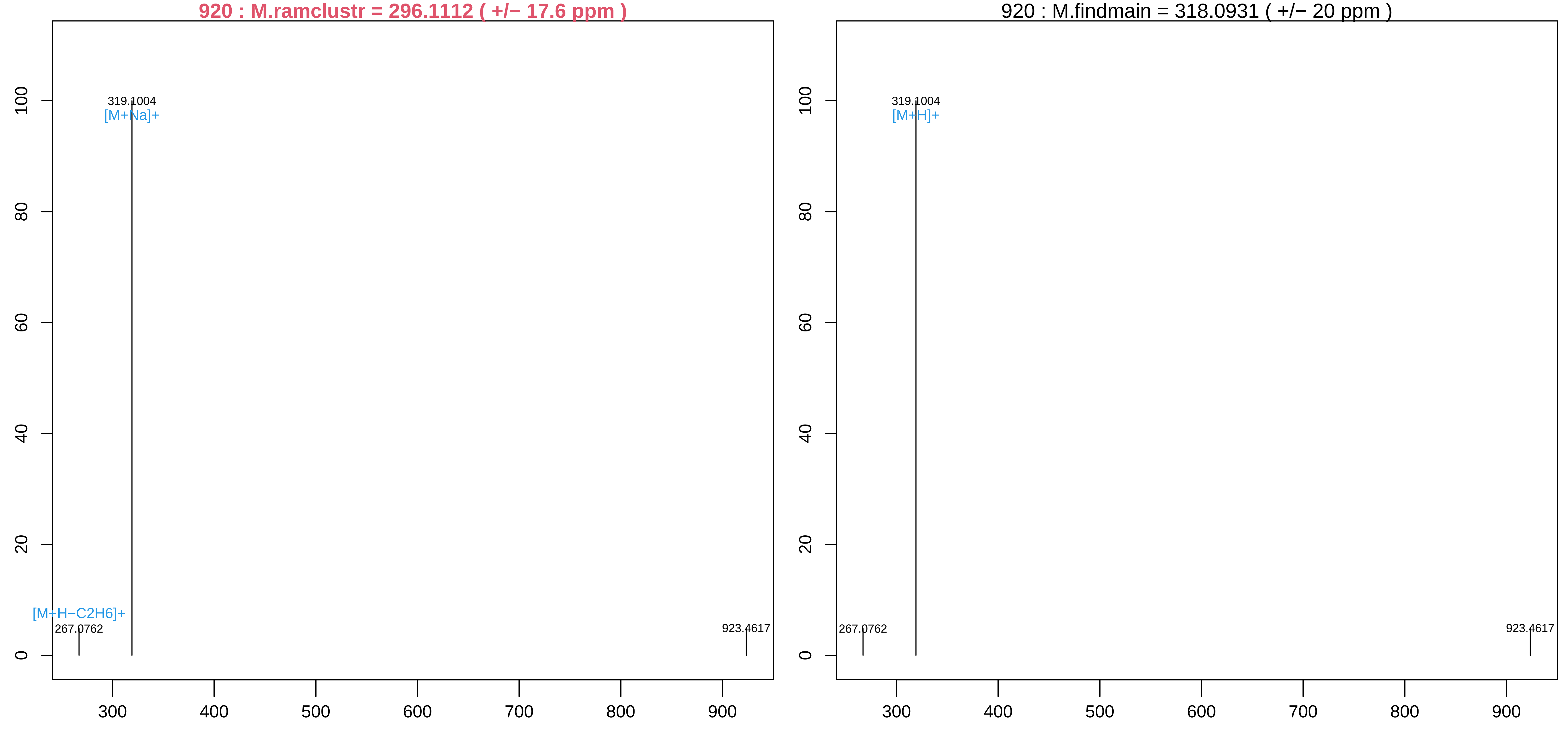

920 : M.ramclustr = 296.1112 (+/− 17.6 ppm)
319.1004
[M+Na]+
[M+H−C2H6]+
267.0762
923.4617
920 : M.findmain = 318.0931 (+/− 20 ppm)
319.1004
[M+H]+
267.0762
923.4617
0
20
40
60
80
100
300
400
500
600
700
800
900

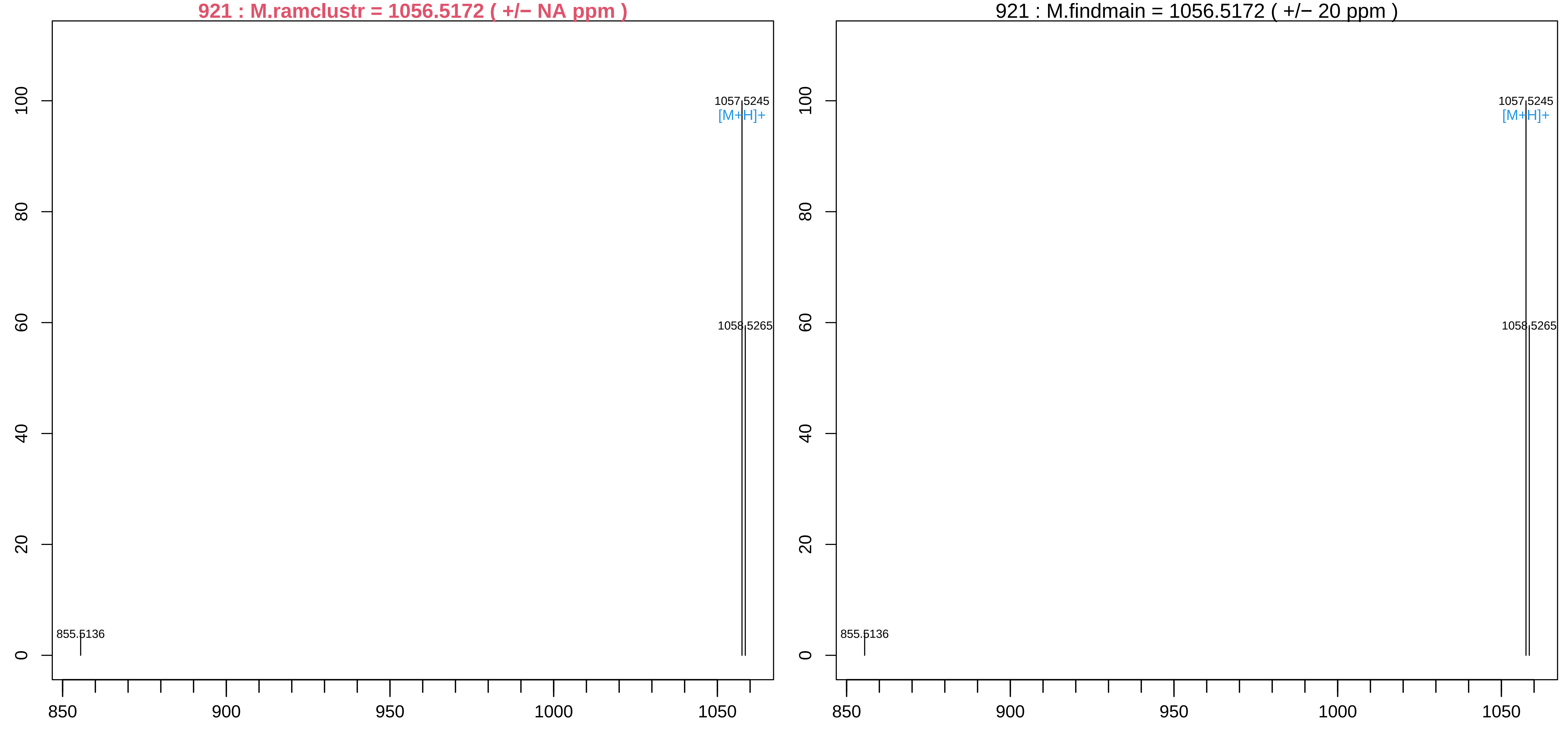
921 : M.ramclustr = 1056.5172 (+/− NA ppm)
1057.5245
[M+H]+
1058.5265
855.5136
0
20
40
60
80
100
850
900
950
1000
1050
921 : M.findmain = 1056.5172 (+/− 20 ppm)
1057.5245
[M+H]+
1058.5265
855.5136

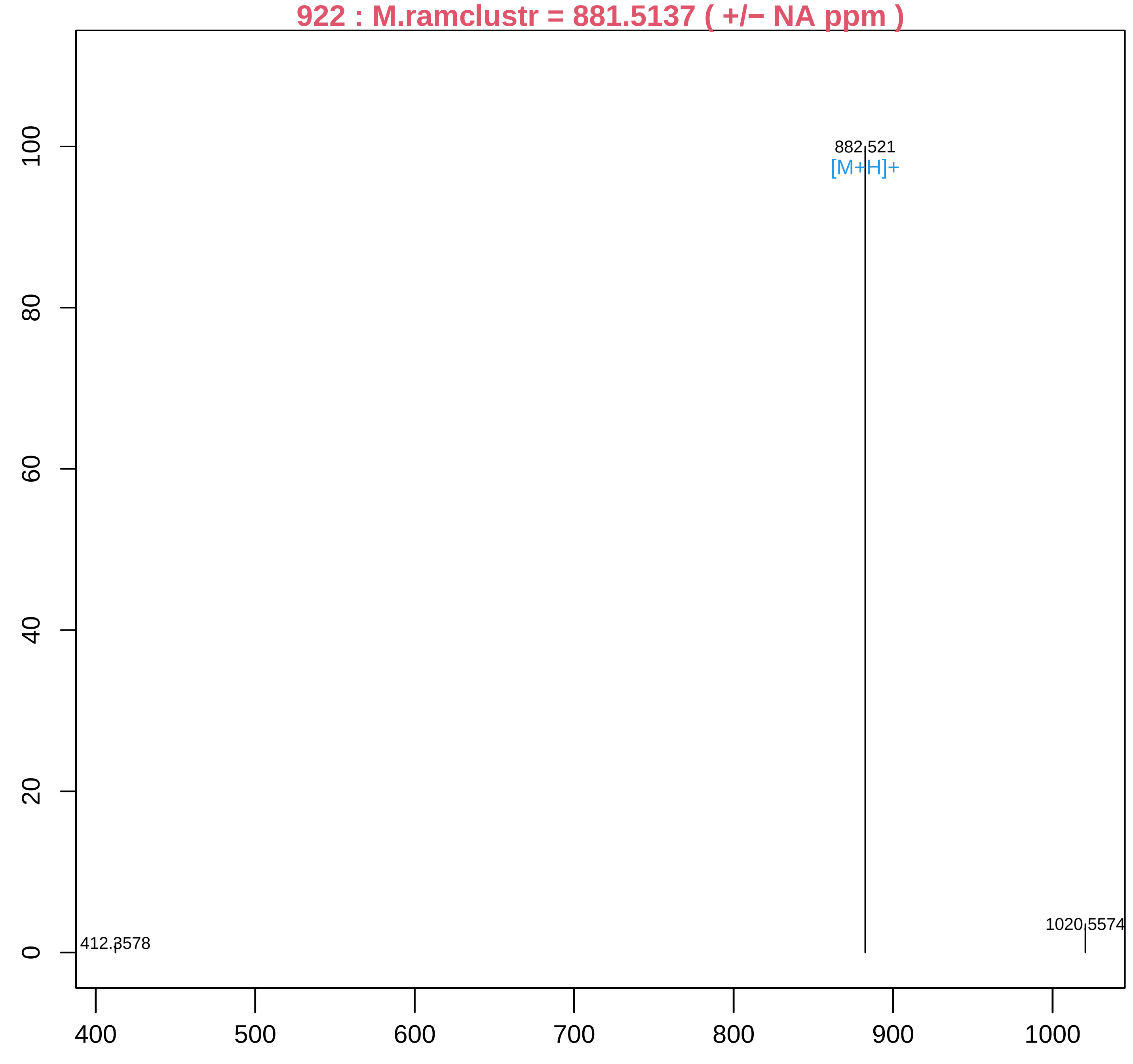
922 : M.ramclustr = 881.5137 (+/− NA ppm)
882.521
[M+H]+
412.3578
1020.5574
0
20
40
60
80
100
400
500
600
700
800
900
1000

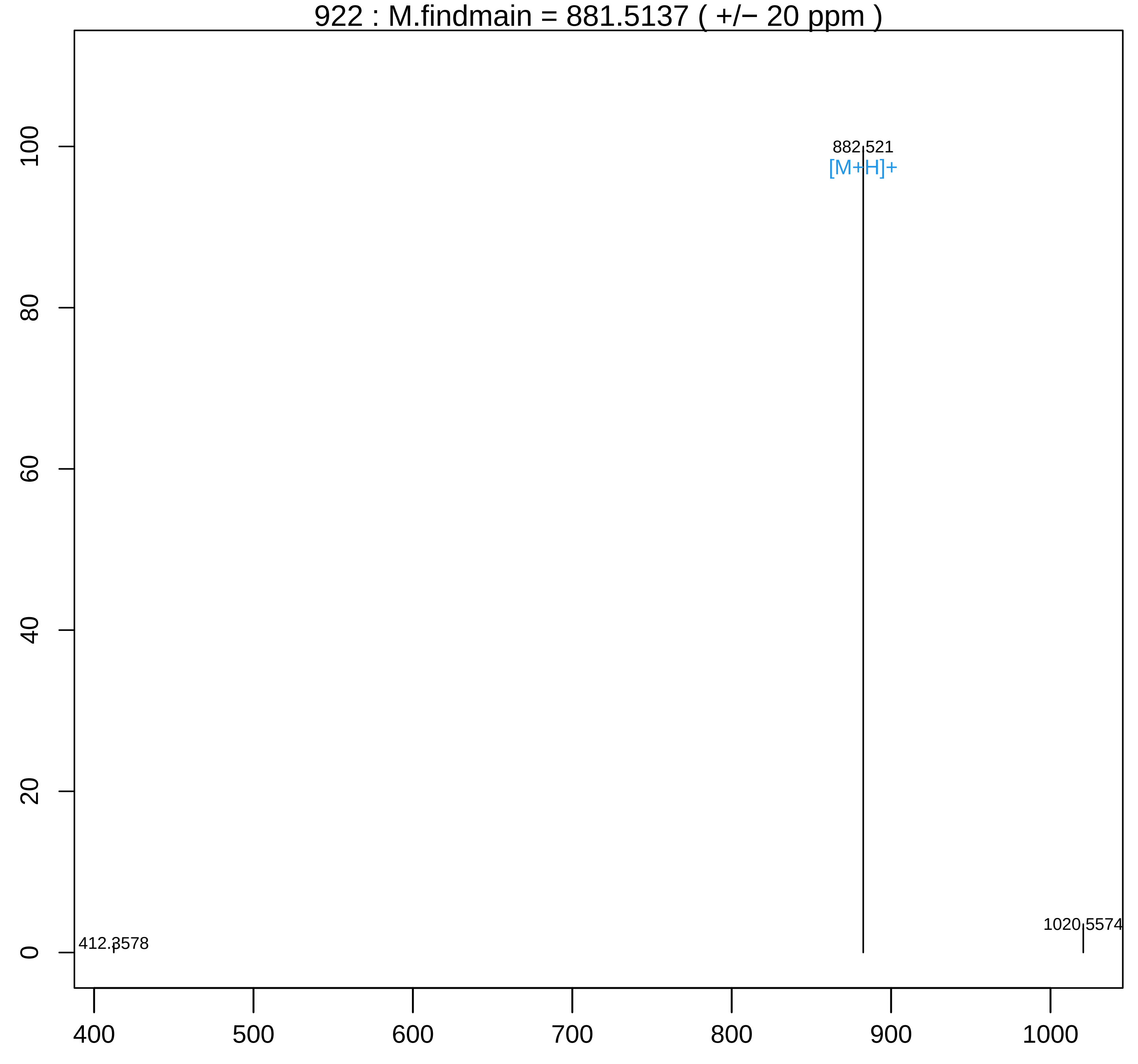
922 : M.findmain = 881.5137 (+/− 20 ppm)
882.521
[M+H]+
412.3578
1020.5574
0
20
40
60
80
100
400
500
600
700
800
900
1000

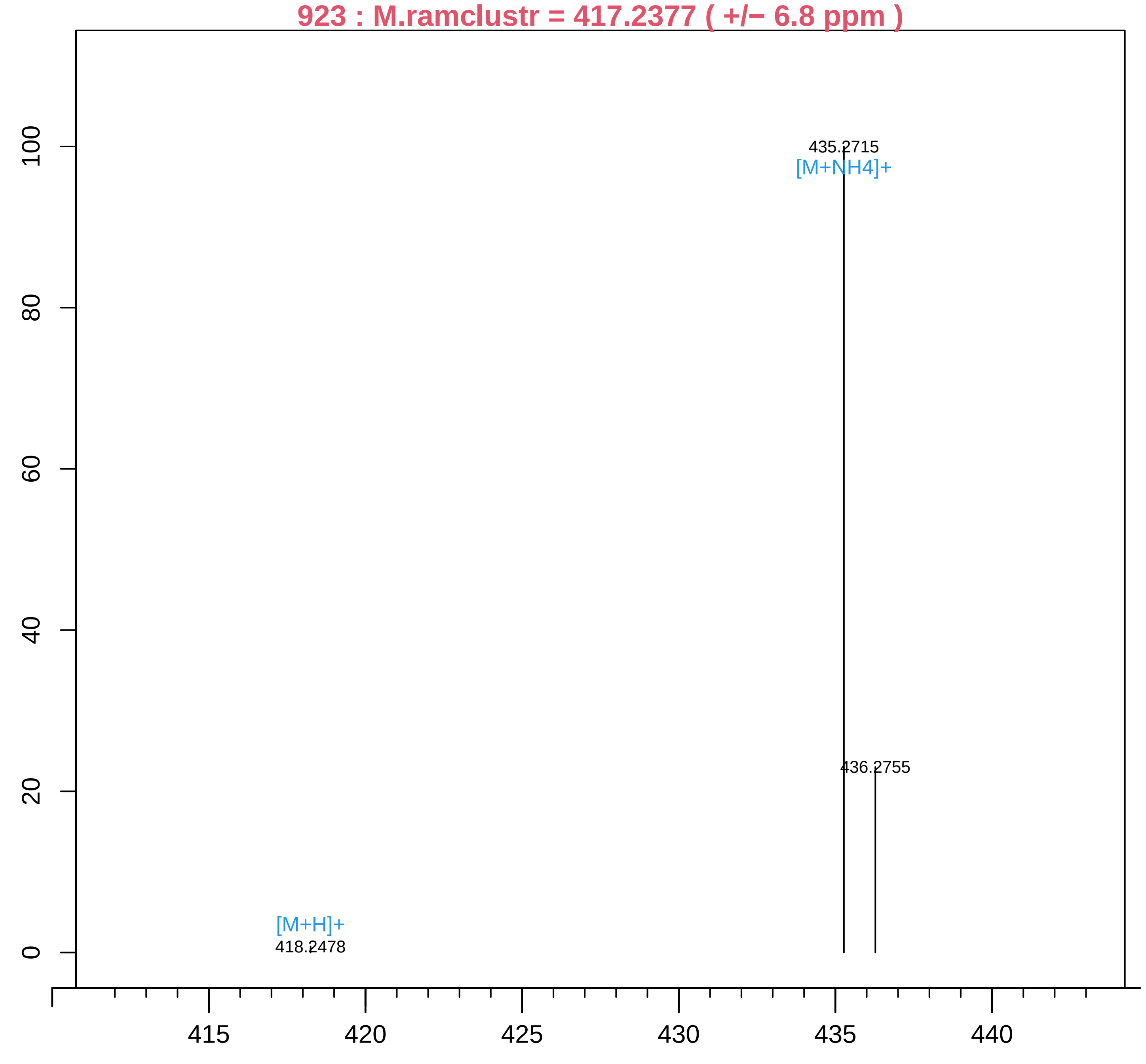

923 : M.ramclustr = 417.2377 (+/− 6.8 ppm)
435.2715
[M+NH4]+
436.2755
[M+H]+
418.2478
0
20
40
60
80
100
415
420
425
430
435
440

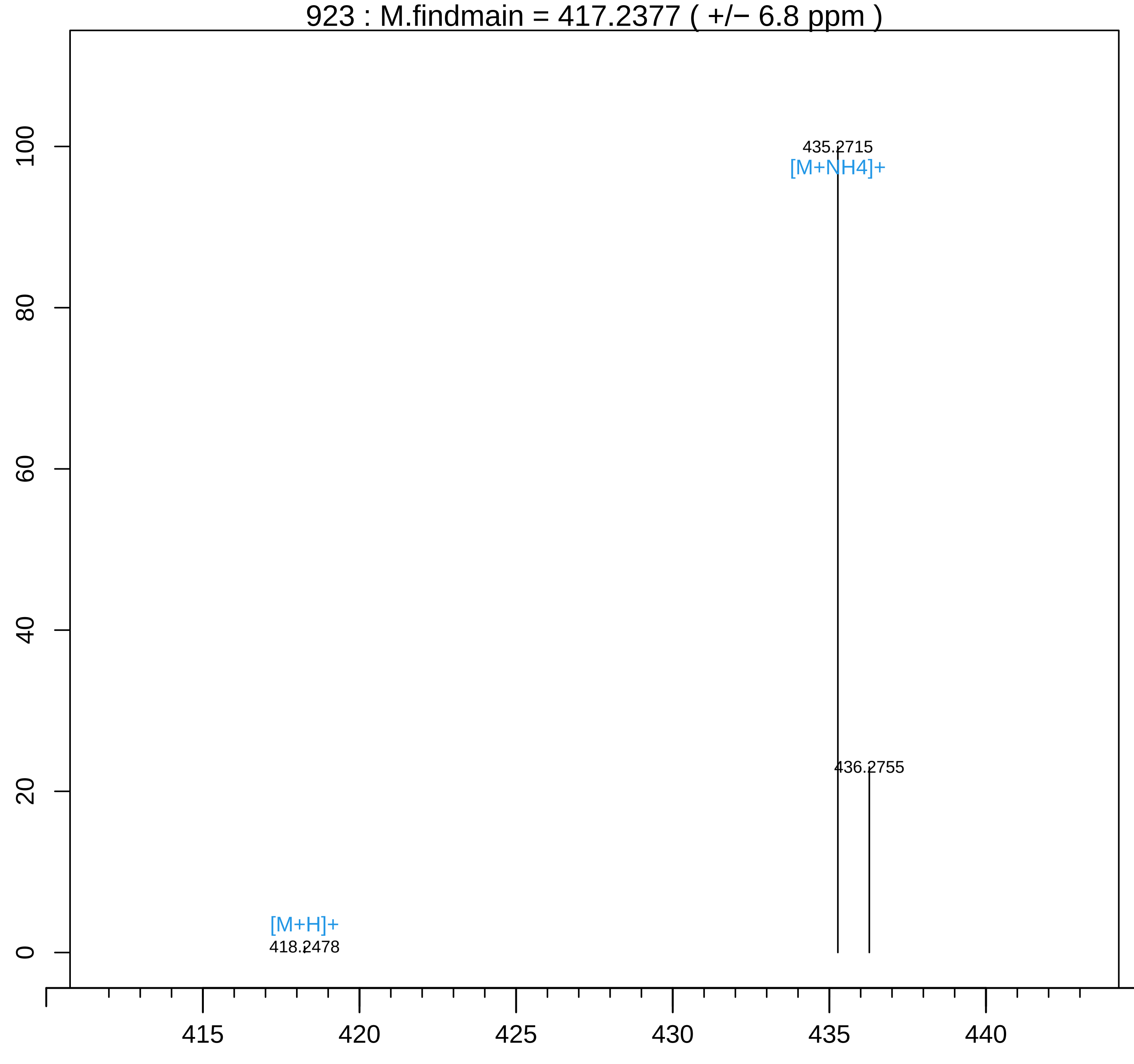

923 : M.findmain = 417.2377 (+/− 6.8 ppm)
435.2715
[M+NH4]+
436.2755
[M+H]+
418.2478
0
20
40
60
80
100
415
420
425
430
435
440

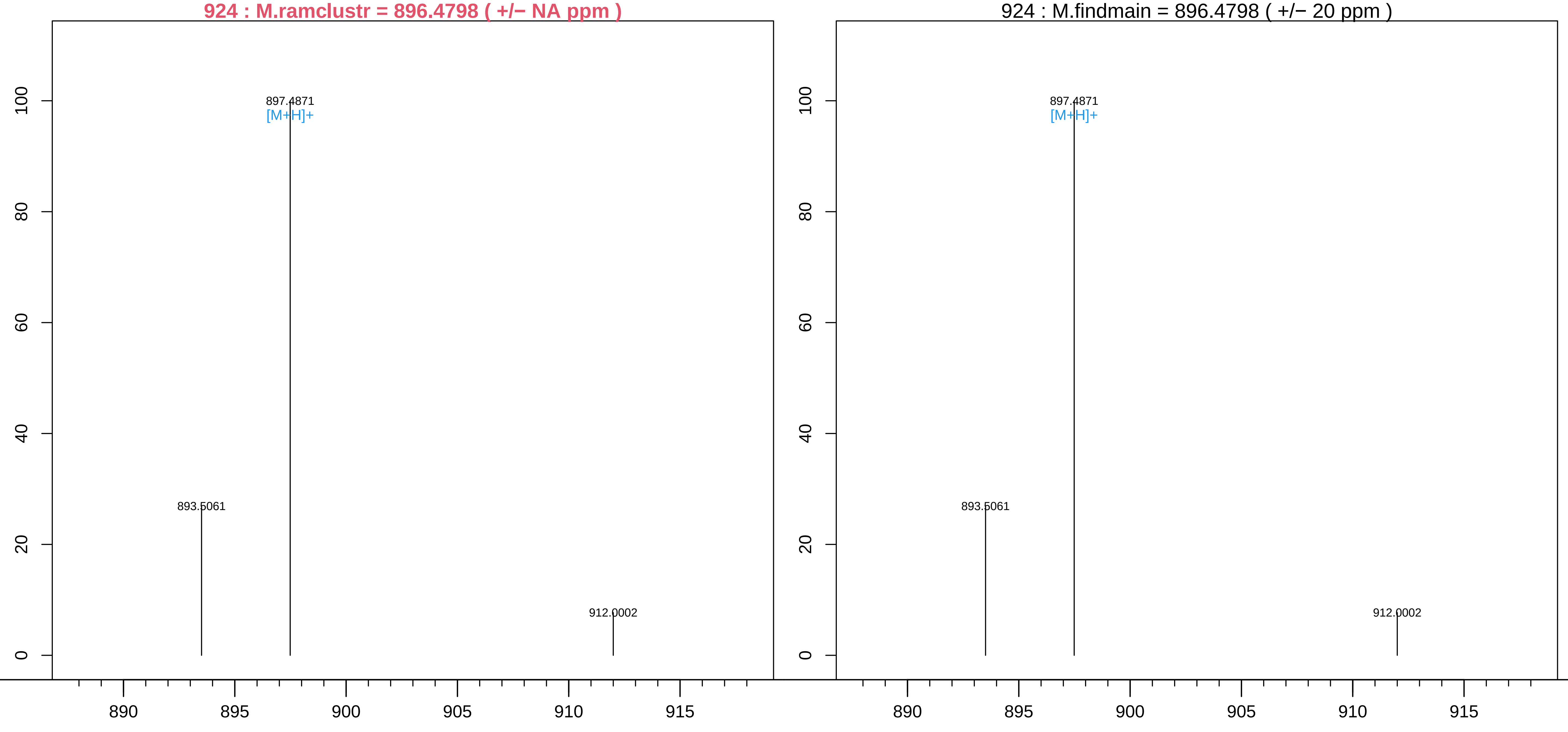
924 : M.ramclustr = 896.4798 (+/− NA ppm)
897.4871
[M+H]+
893.5061
912.0002
0
20
40
60
80
100
890
895
900
905
910
915
924 : M.findmain = 896.4798 (+/− 20 ppm)
897.4871
[M+H]+
893.5061
912.0002
0
20
40
60
80
100
890
895
900
905
910
915

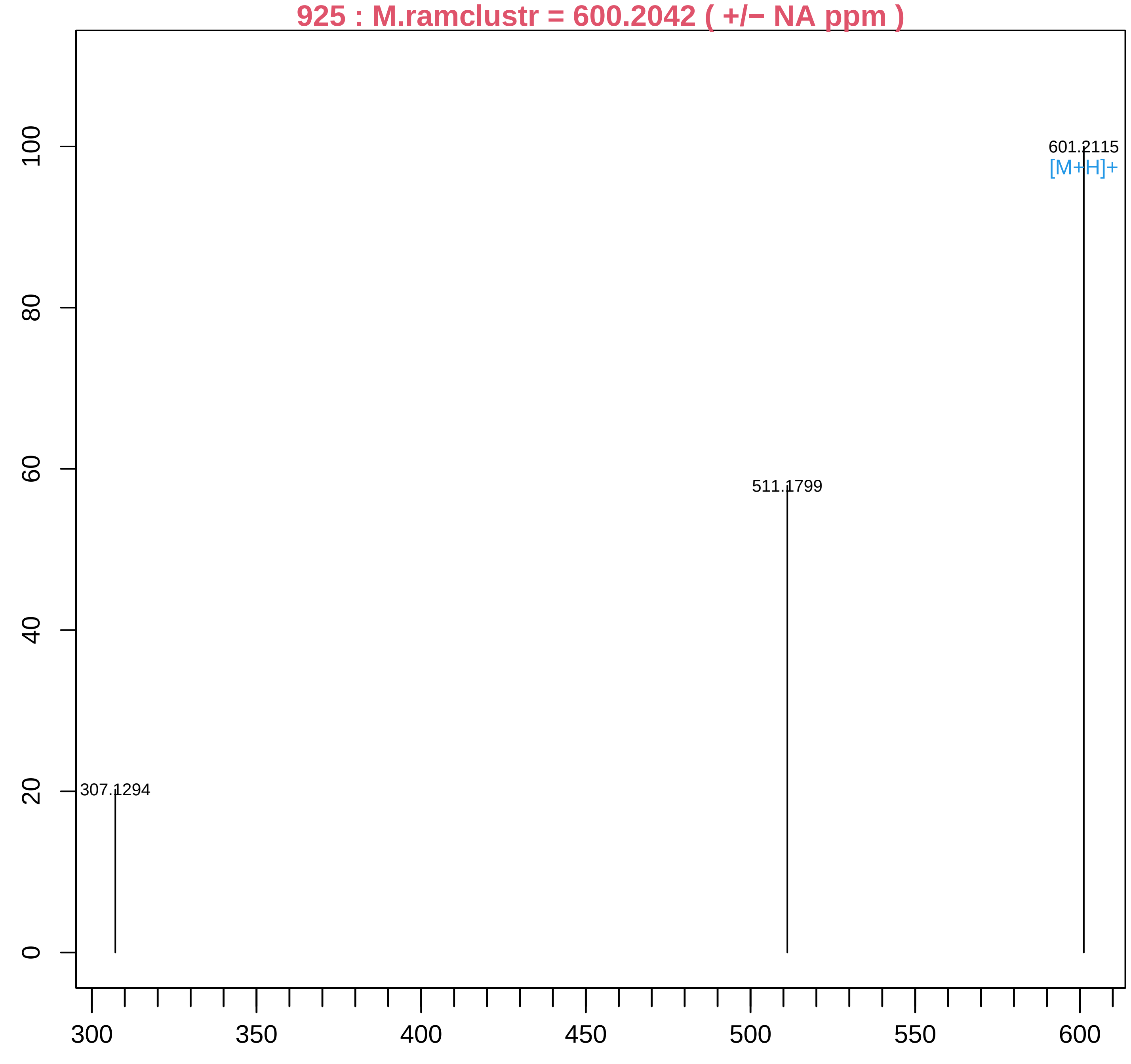
925 : M.ramclustr = 600.2042 (+/− NA ppm)
601.2115
[M+H]+
511.1799
307.1294
0
20
40
60
80
100
300
350
400
450
500
550
600

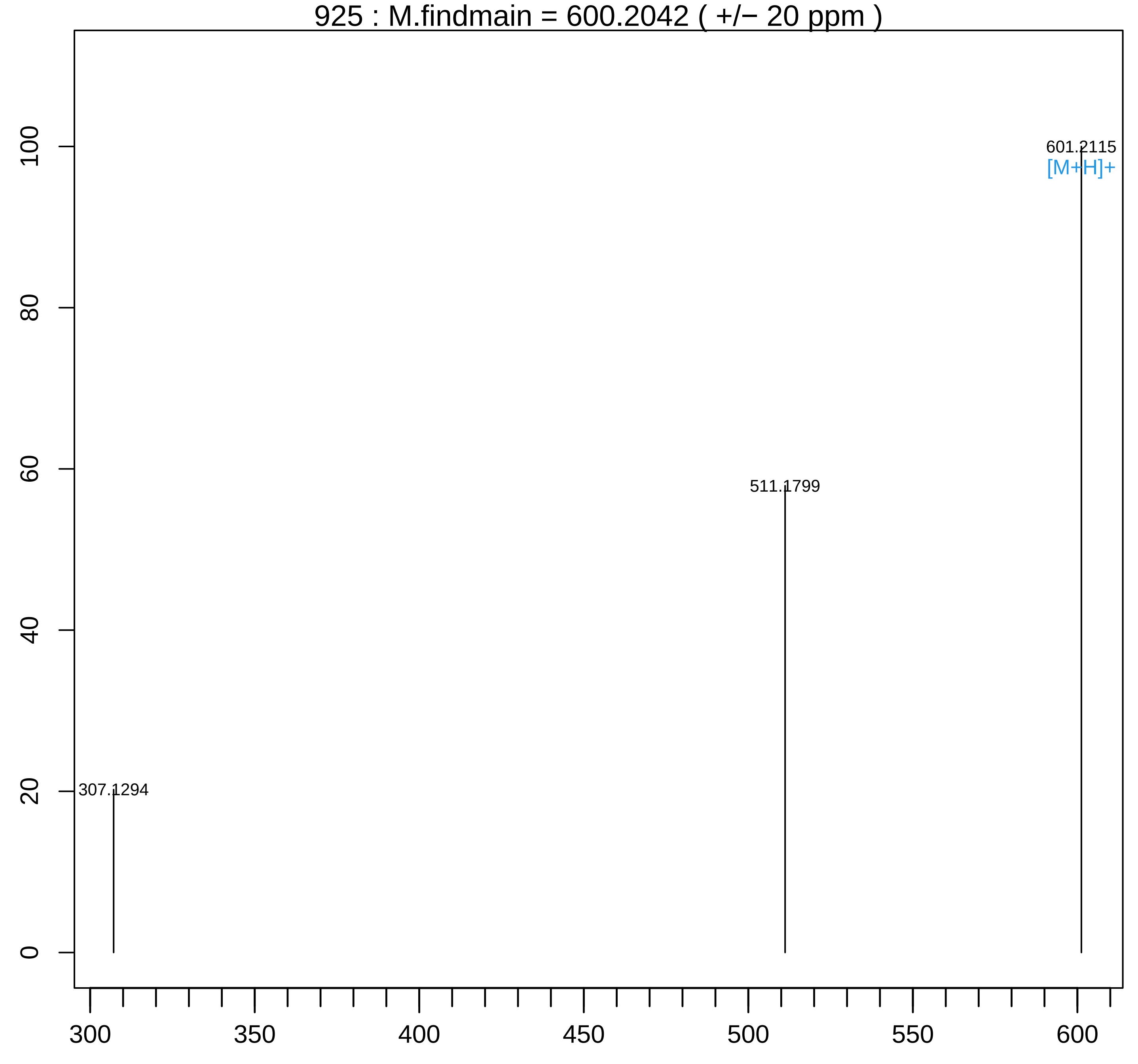
925 : M.findmain = 600.2042 (+/− 20 ppm)
601.2115
[M+H]+
511.1799
307.1294
0
20
40
60
80
100
300
350
400
450
500
550
600

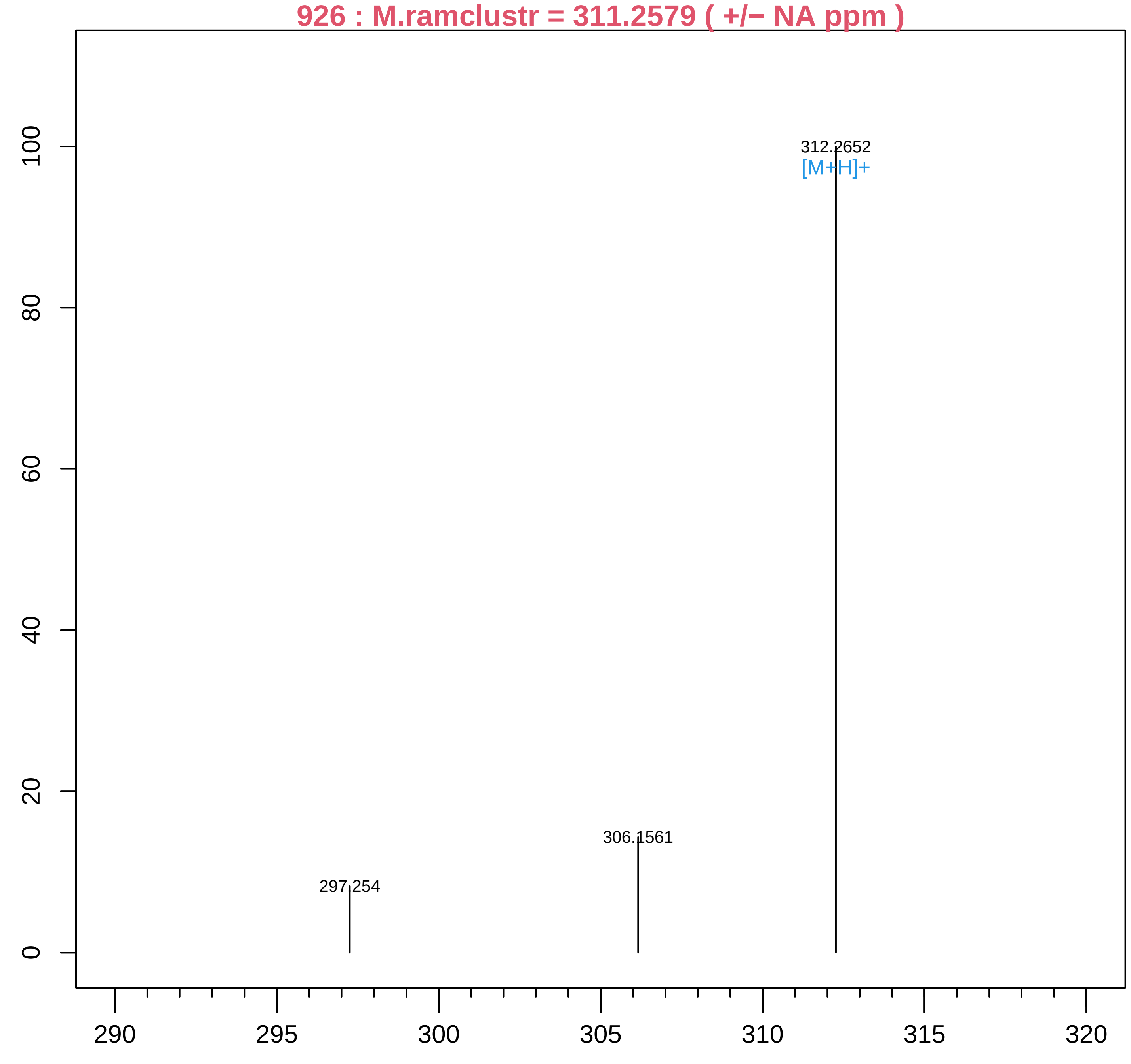
926 : M.ramclustr = 311.2579 (+/− NA ppm)
312.2652
[M+H]+
306.1561
297.254
0
20
40
60
80
100
290
295
300
305
310
315
320

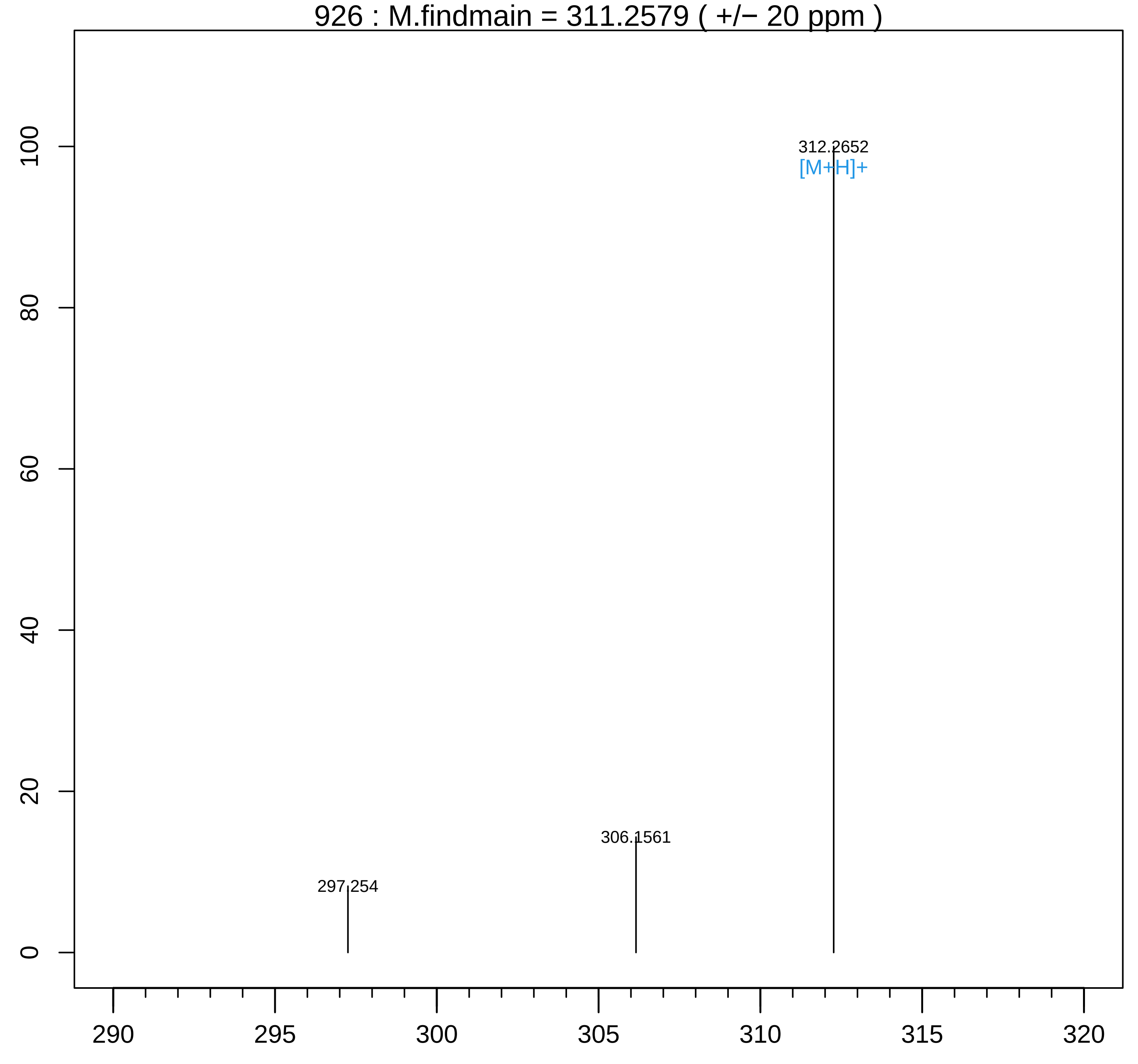
926 : M.findmain = 311.2579 (+/− 20 ppm)
312.2652
[M+H]+
306.1561
297.254
0
20
40
60
80
100
290
295
300
305
310
315
320

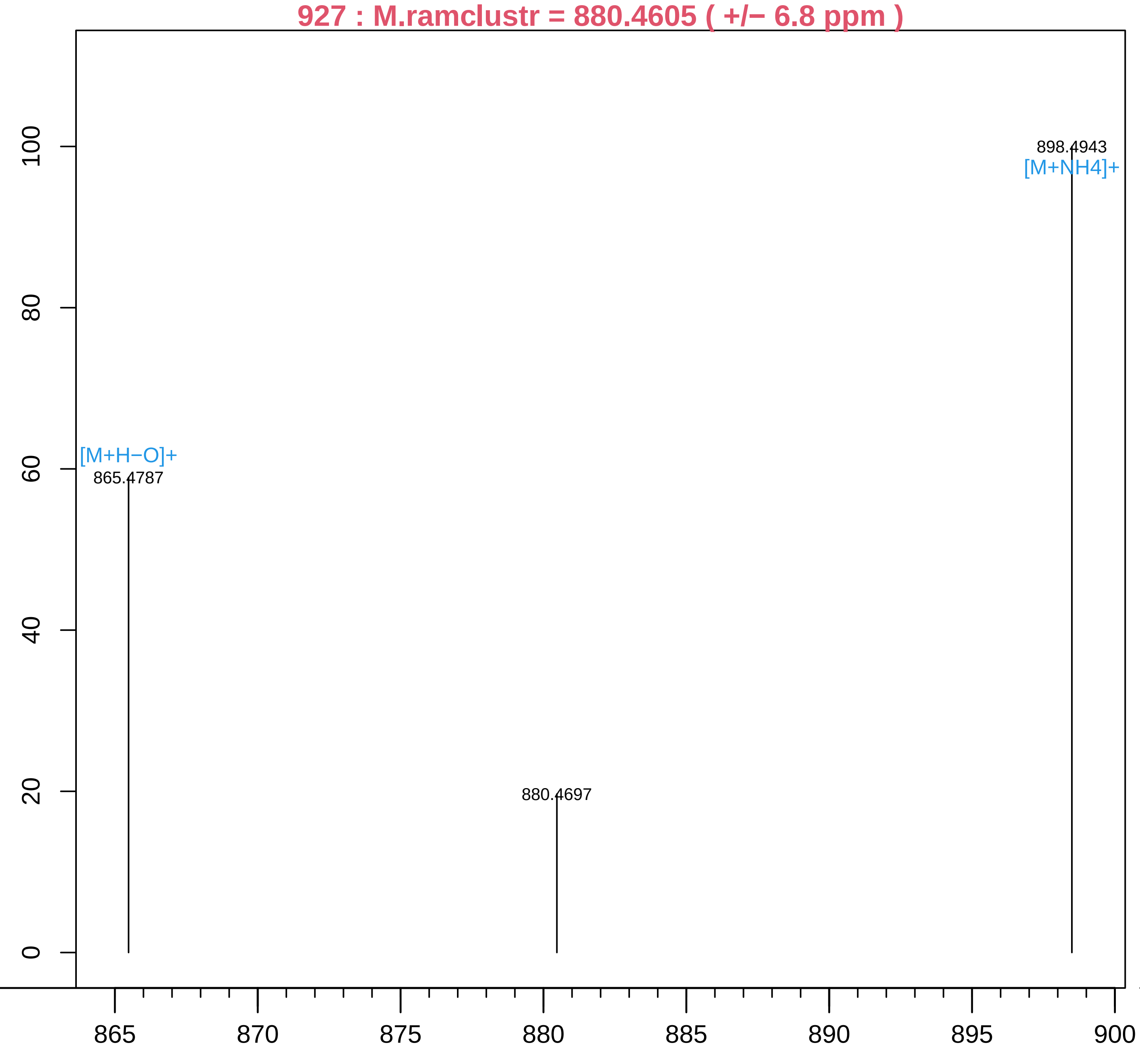
927 : M.ramclustr = 880.4605 (+/− 6.8 ppm)
898.4943
[M+NH4]+
[M+H−O]+
865.4787
880.4697
0
20
40
60
80
100
865
870
875
880
885
890
895
900

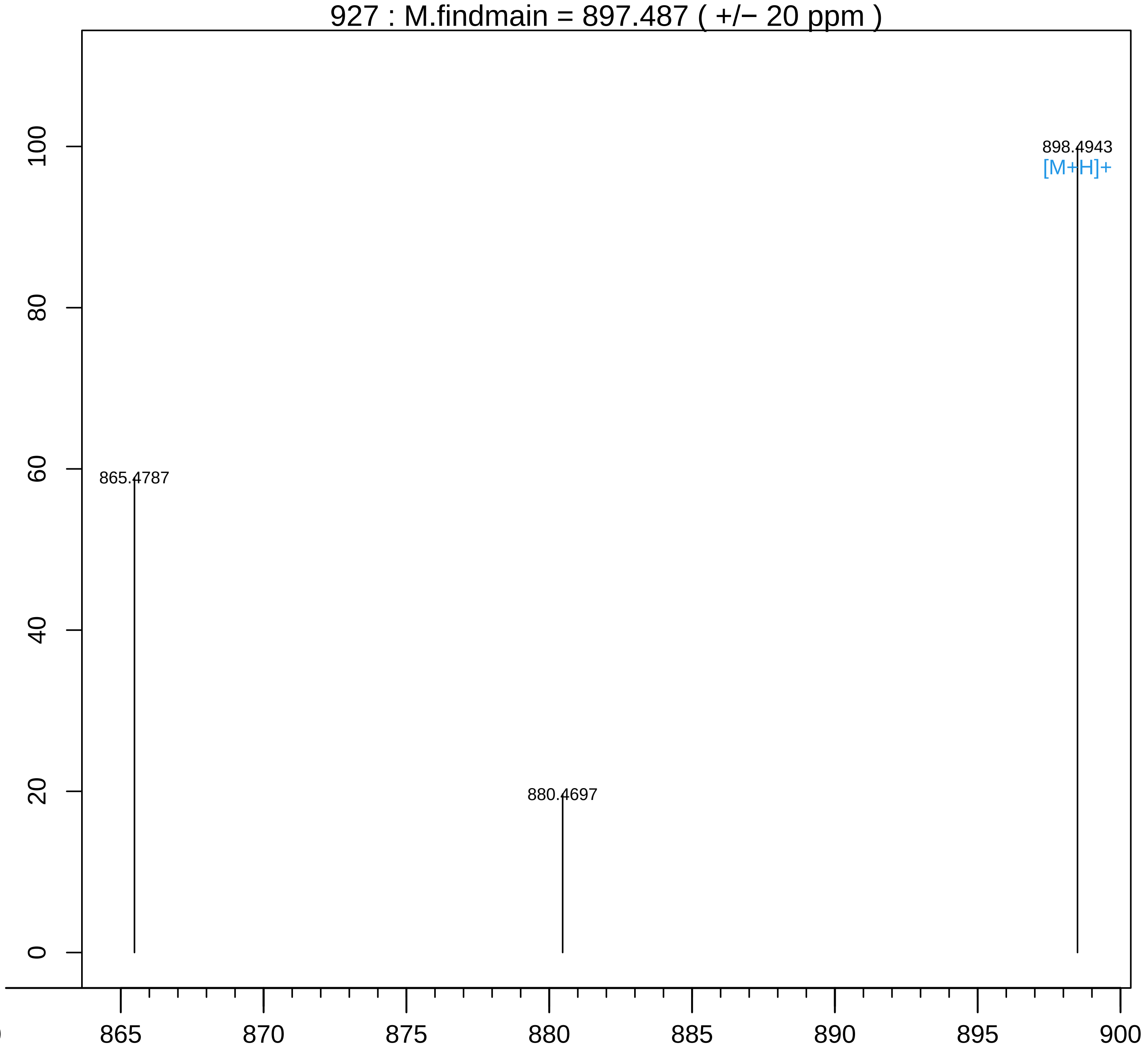
927 : M.findmain = 897.487 (+/− 20 ppm)
898.4943
[M+H]+
865.4787
880.4697
0
20
40
60
80
100
865
870
875
880
885
890
895
900

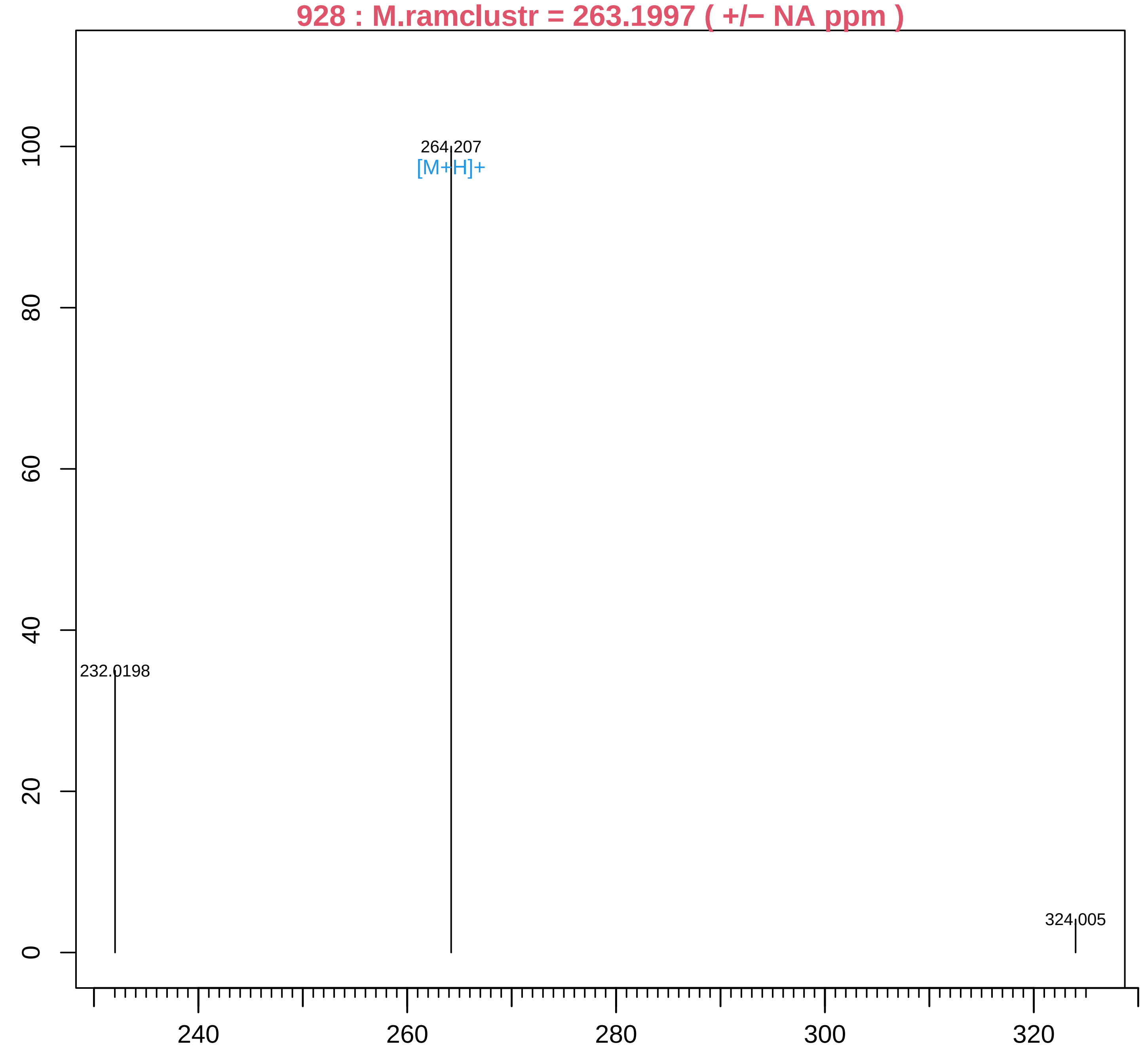

928 : M.ramclustr = 263.1997 (+/− NA ppm)
264.207
[M+H]+
232.0198
324.005
0
20
40
60
80
100
240
260
280
300
320

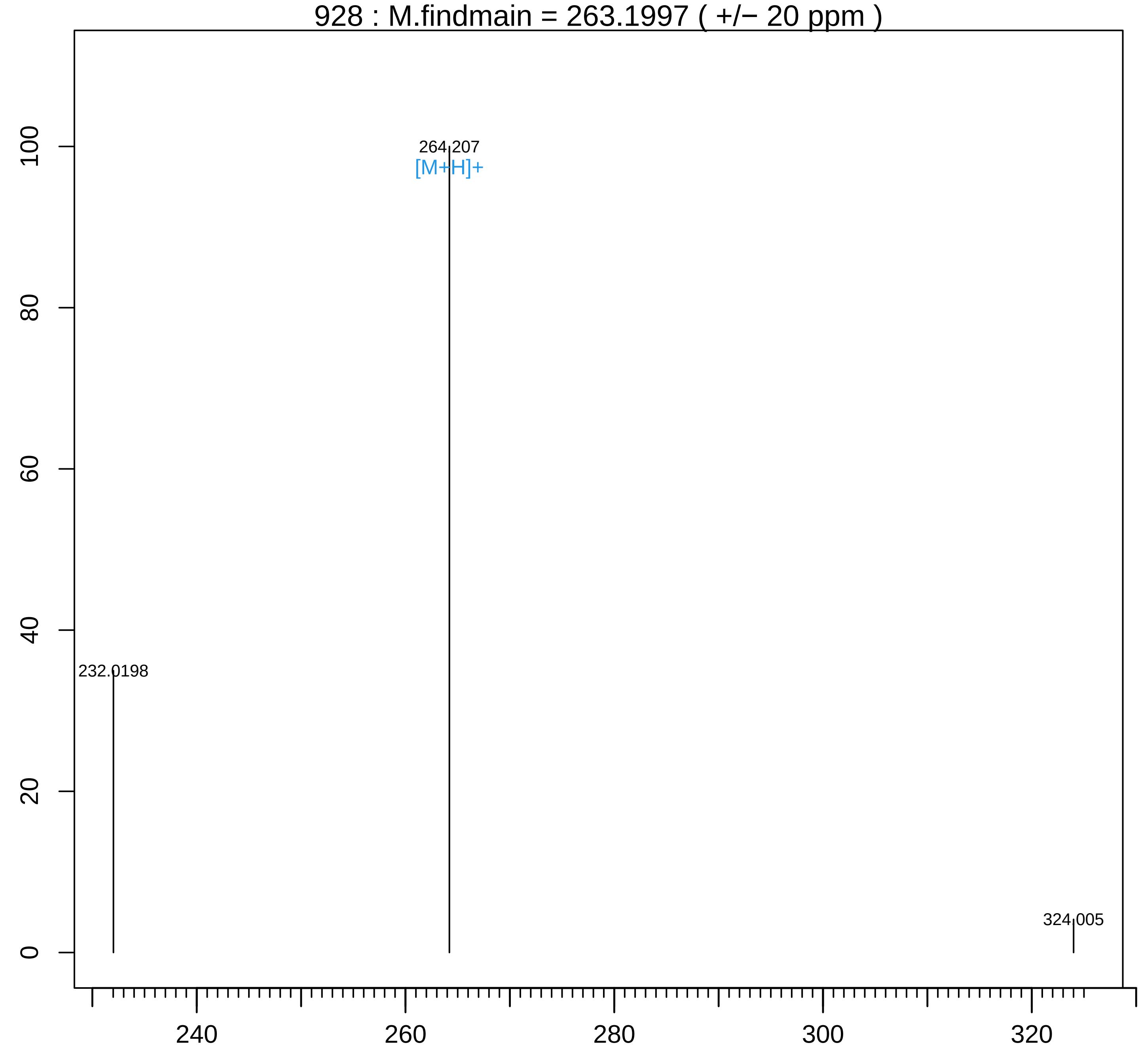

928 : M.findmain = 263.1997 (+/− 20 ppm)
264.207
[M+H]+
232.0198
324.005
0
20
40
60
80
100
240
260
280
300
320

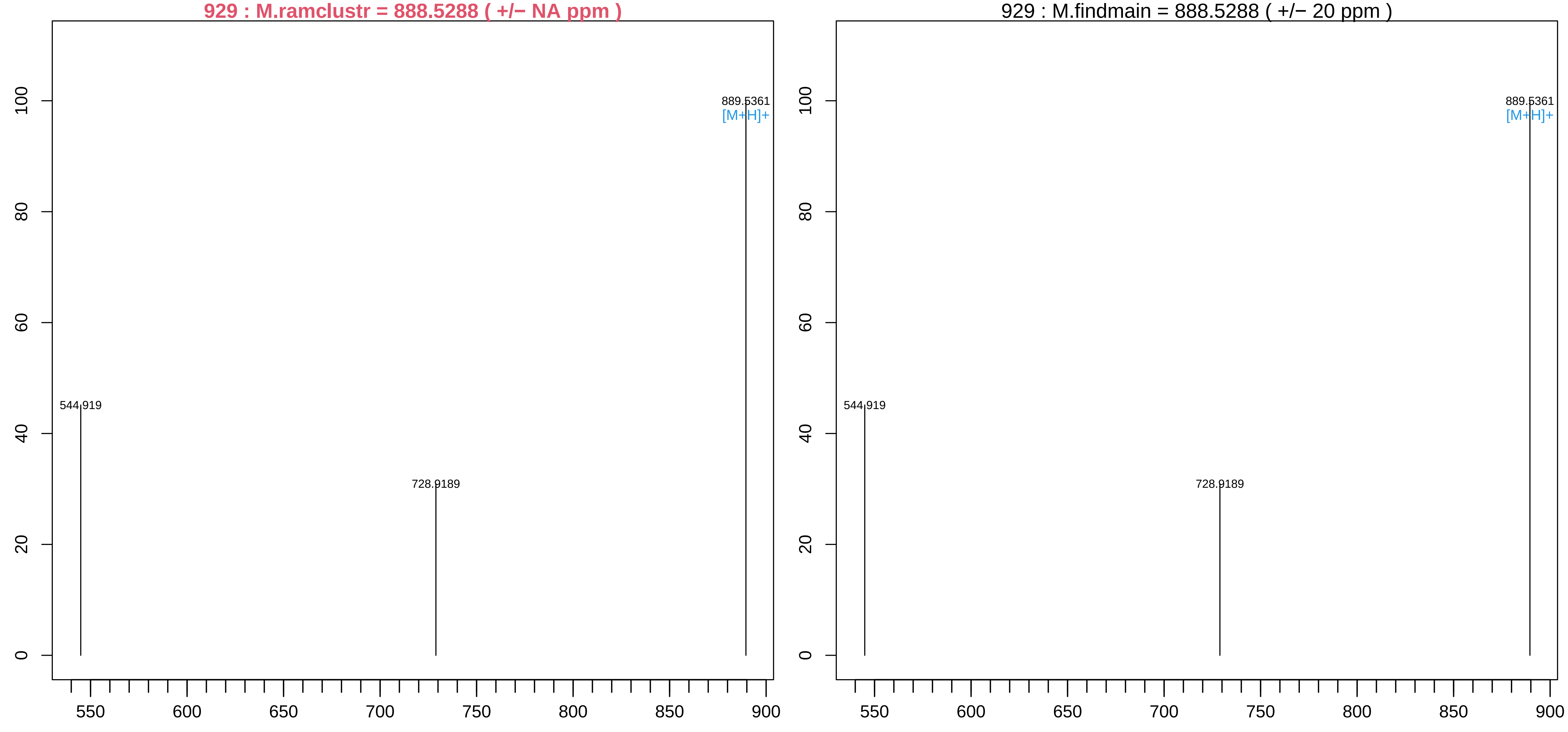
929 : M.ramclustr = 888.5288 (+/− NA ppm)
889.5361
[M+H]+
544.919
728.9189
0
20
40
60
80
100
550
600
650
700
750
800
850
900
929 : M.findmain = 888.5288 (+/− 20 ppm)
889.5361
[M+H]+
544.919
728.9189
0
20
40
60
80
100
550
600
650
700
750
800
850
900

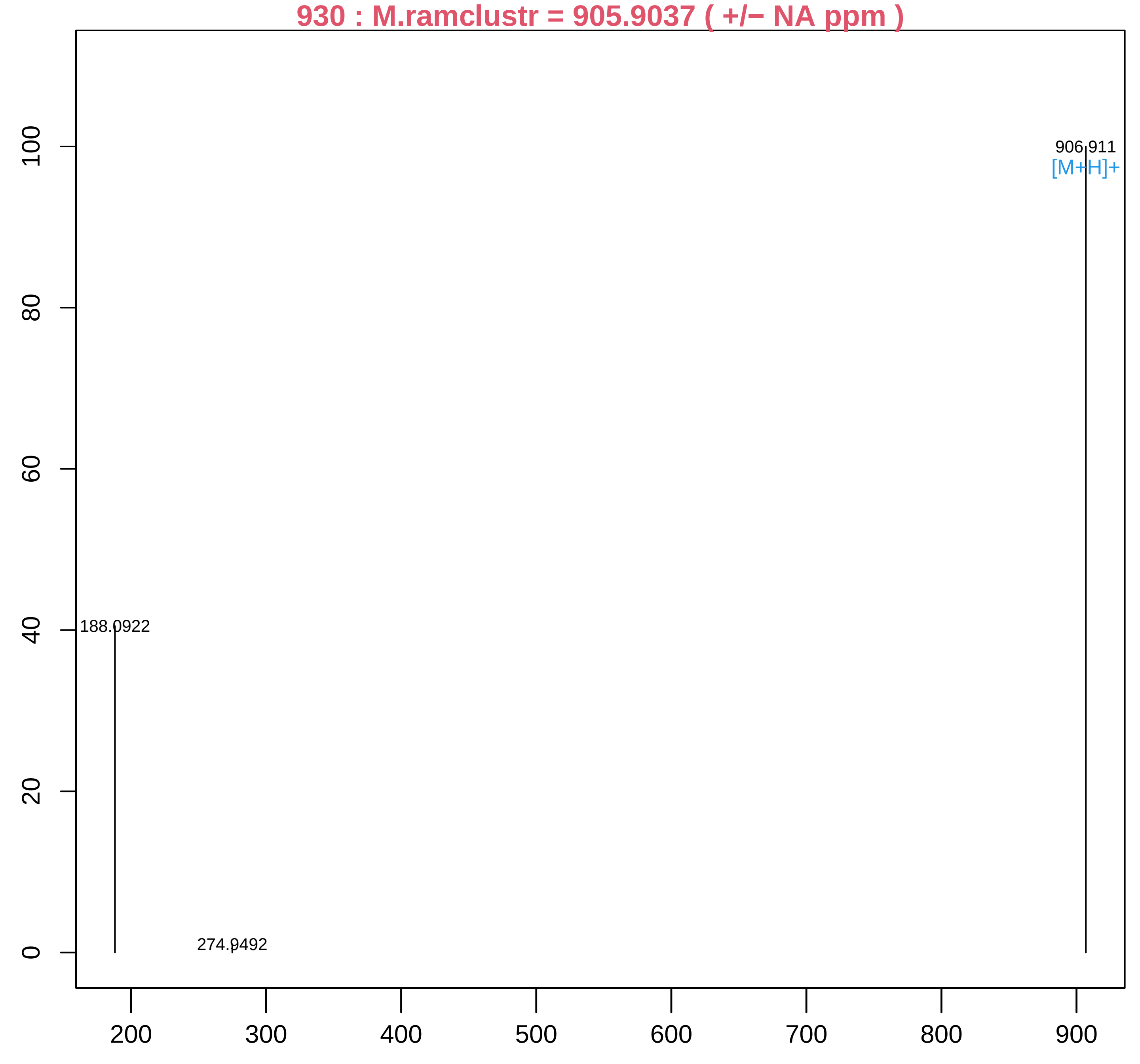
930 : M.ramclustr = 905.9037 (+/− NA ppm)
906.911
[M+H]+
188.0922
274.9492
0
20
40
60
80
100
200
300
400
500
600
700
800
900

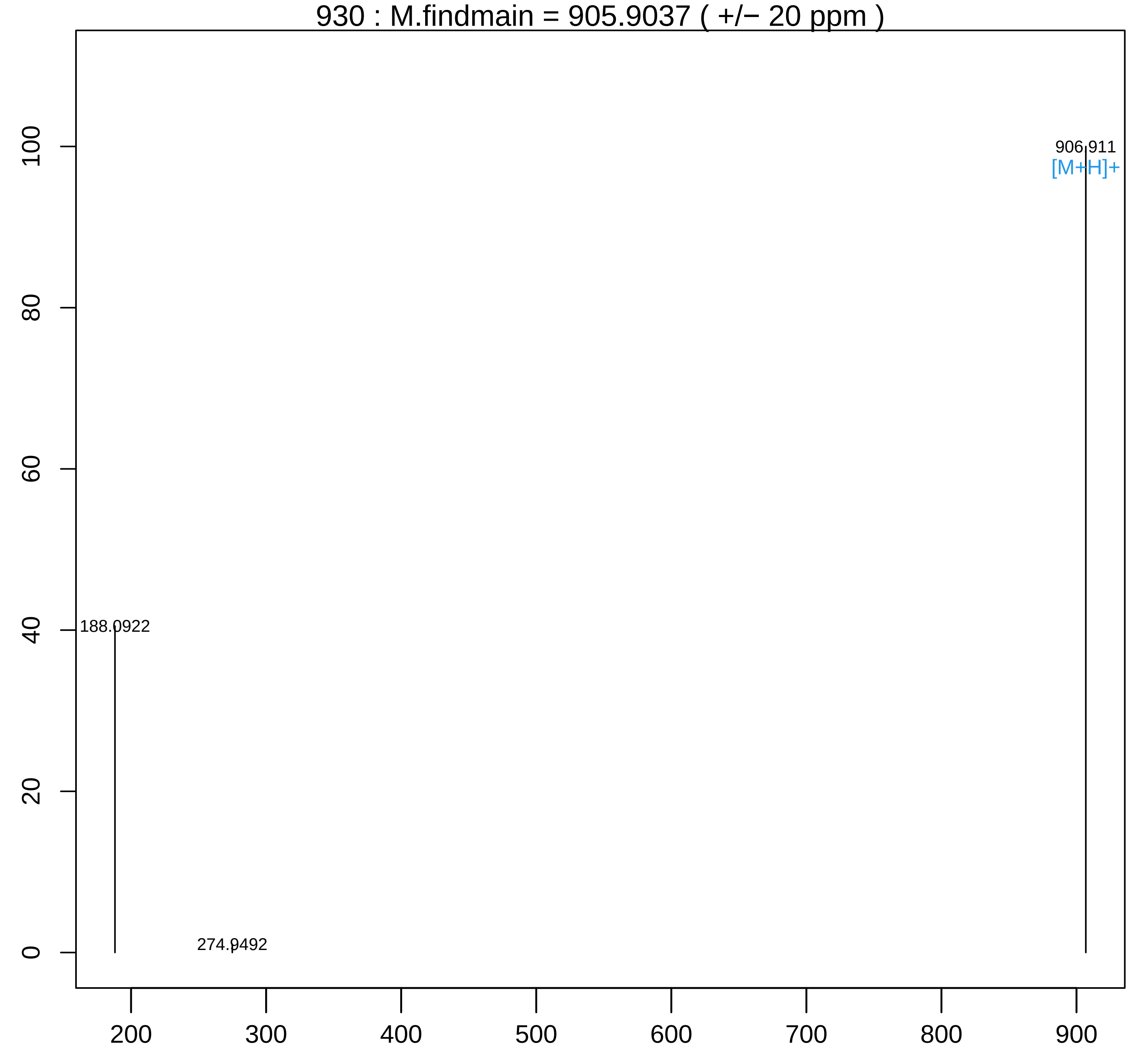
930 : M.findmain = 905.9037 (+/− 20 ppm)
906.911
[M+H]+
188.0922
274.9492
0
20
40
60
80
100
200
300
400
500
600
700
800
900

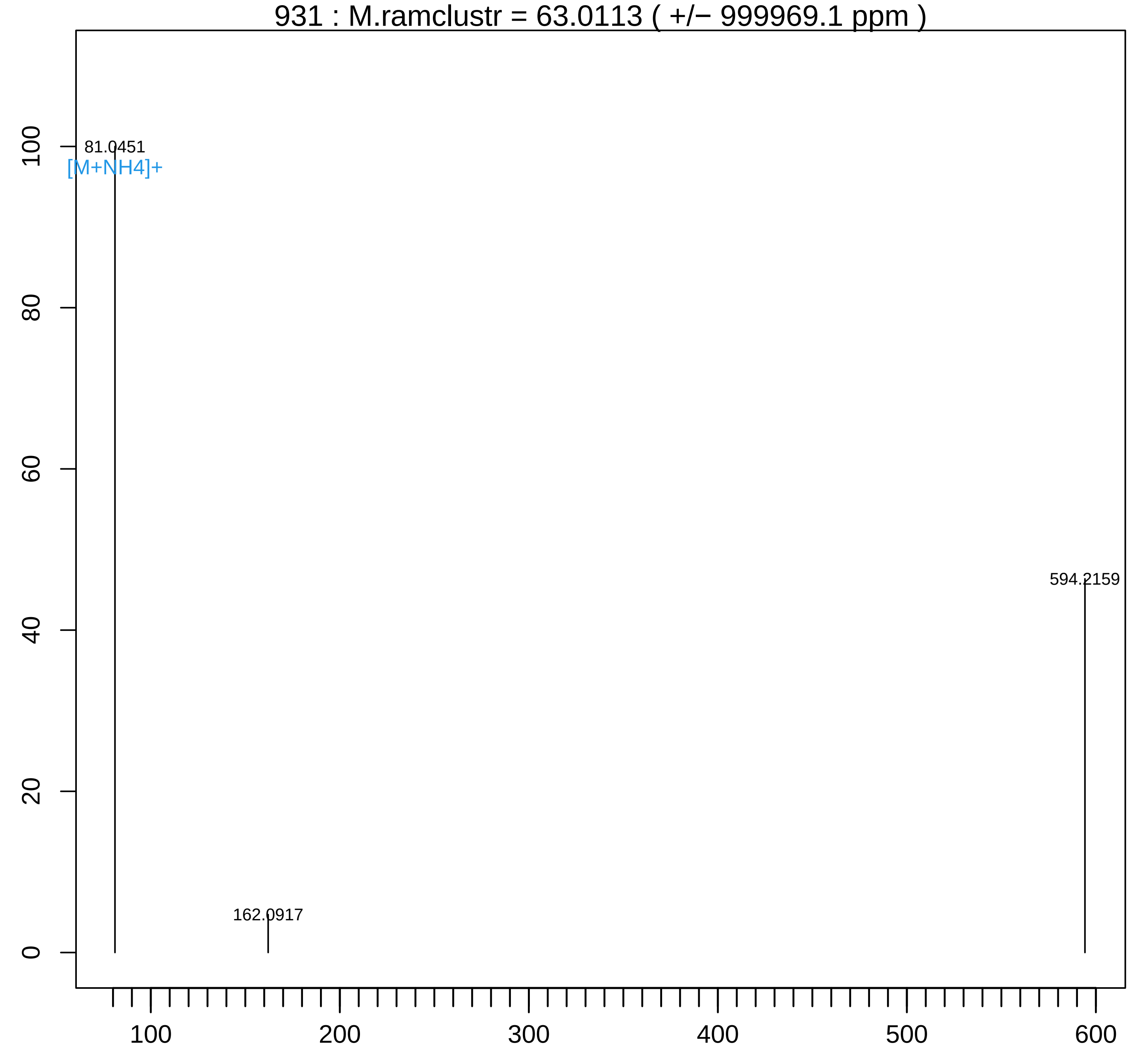

931 : M.ramclustr = 63.0113 (+/− 999969.1 ppm)
81.0451
[M+NH4]+
162.0917
594.2159
0
20
40
60
80
100
100
200
300
400
500
600

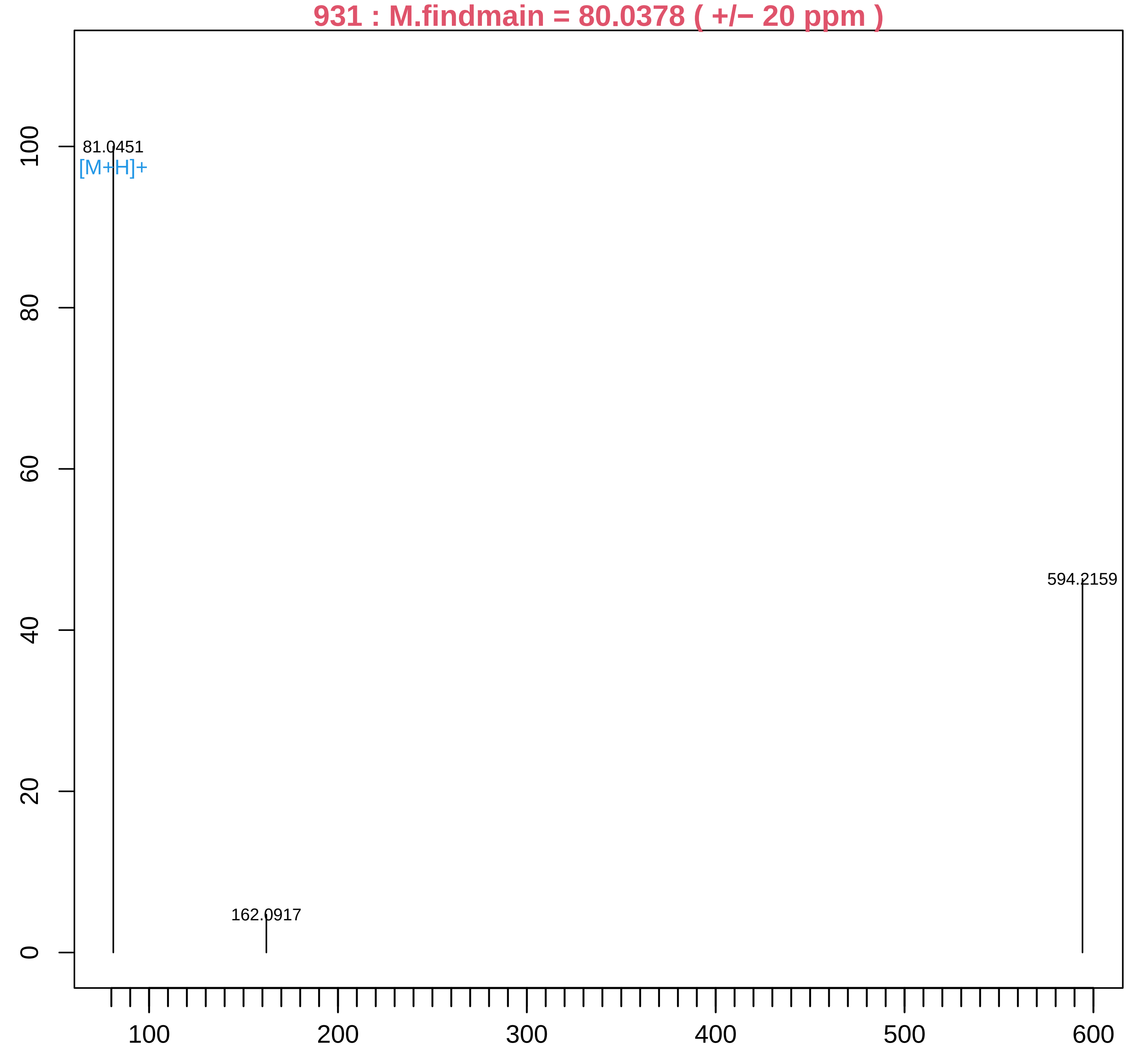

931 : M.findmain = 80.0378 (+/− 20 ppm)
81.0451
[M+H]+
162.0917
594.2159
0
20
40
60
80
100
100
200
300
400
500
600

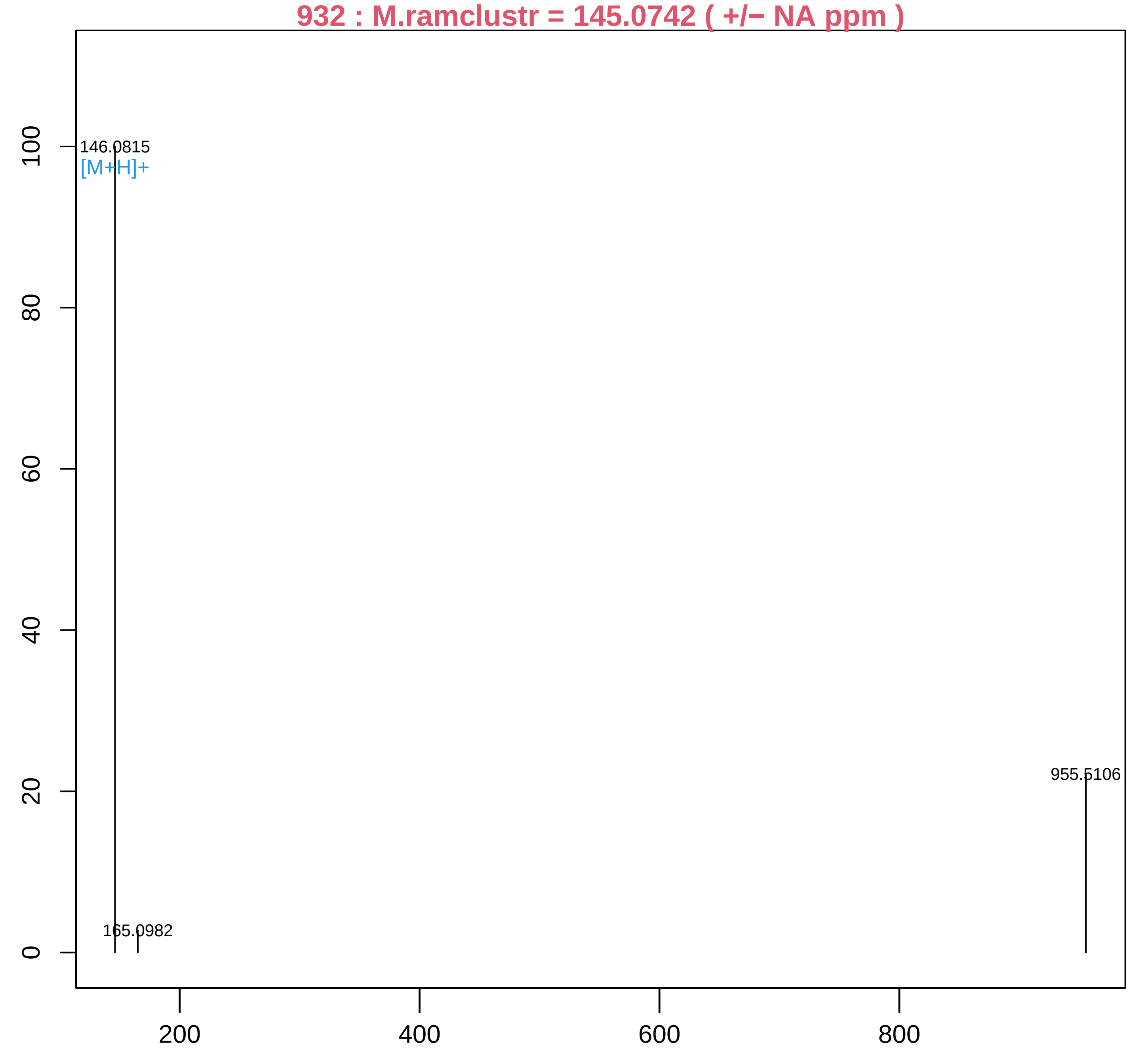

932 : M.ramclustr = 145.0742 (+/− NA ppm)
146.0815
[M+H]+
165.0982
955.5106
0
20
40
60
80
100
200
400
600
800

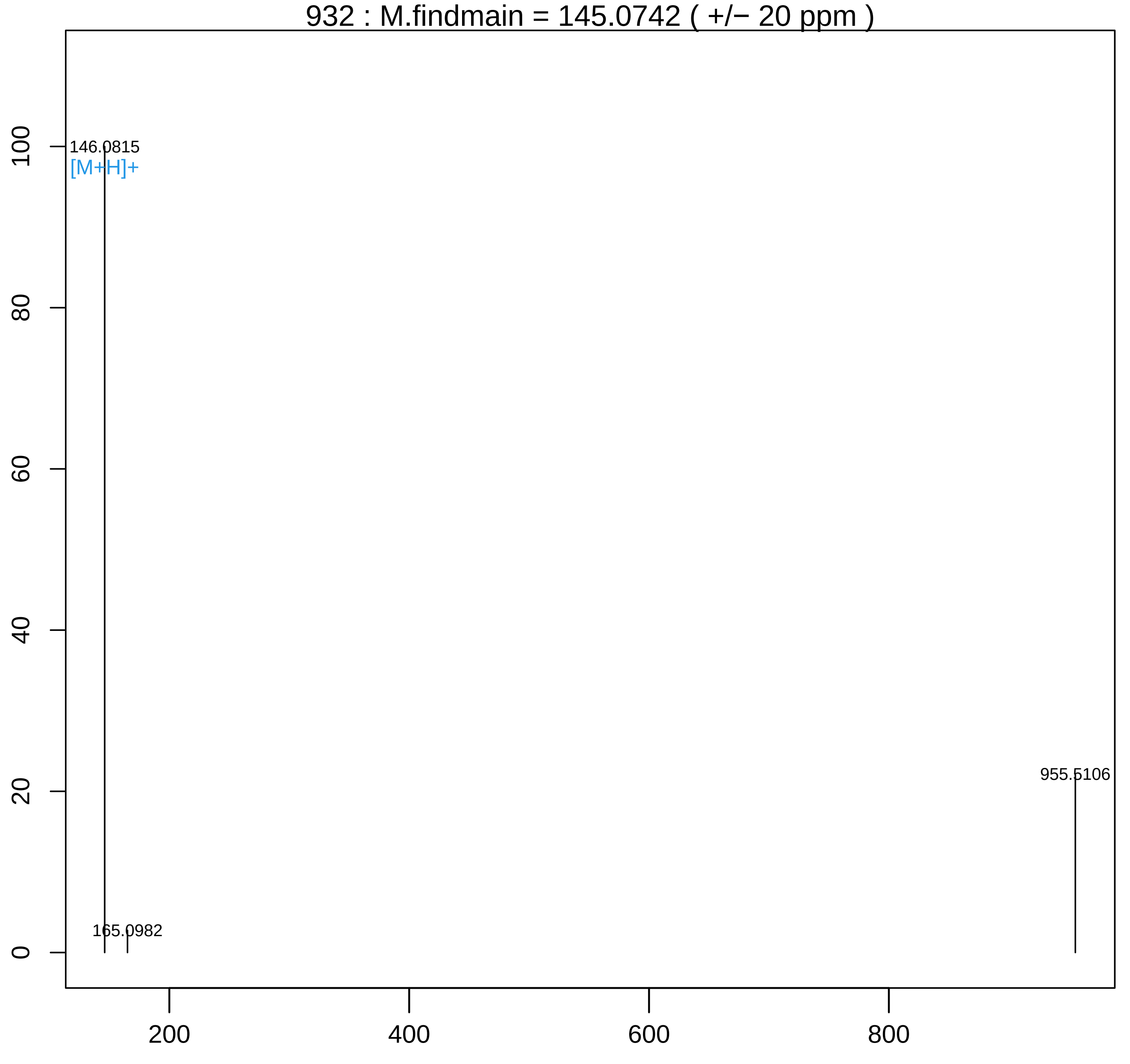

932 : M.findmain = 145.0742 (+/− 20 ppm)
146.0815
[M+H]+
165.0982
955.5106
0
20
40
60
80
100
200
400
600
800

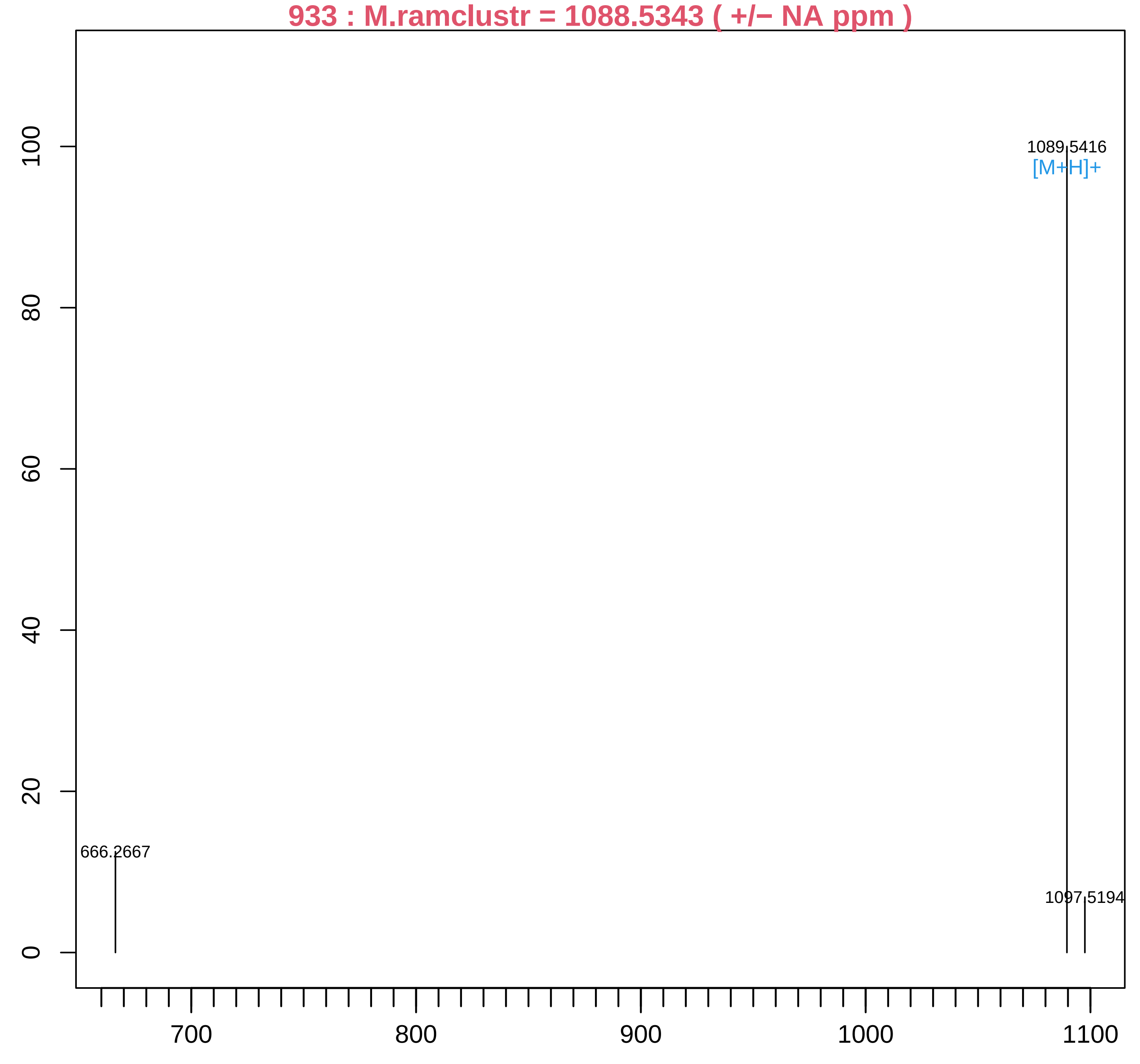

933 : M.ramclustr = 1088.5343 (+/− NA ppm)
1089.5416
[M+H]+
666.2667
1097.5194
0
20
40
60
80
100
700
800
900
1000
1100

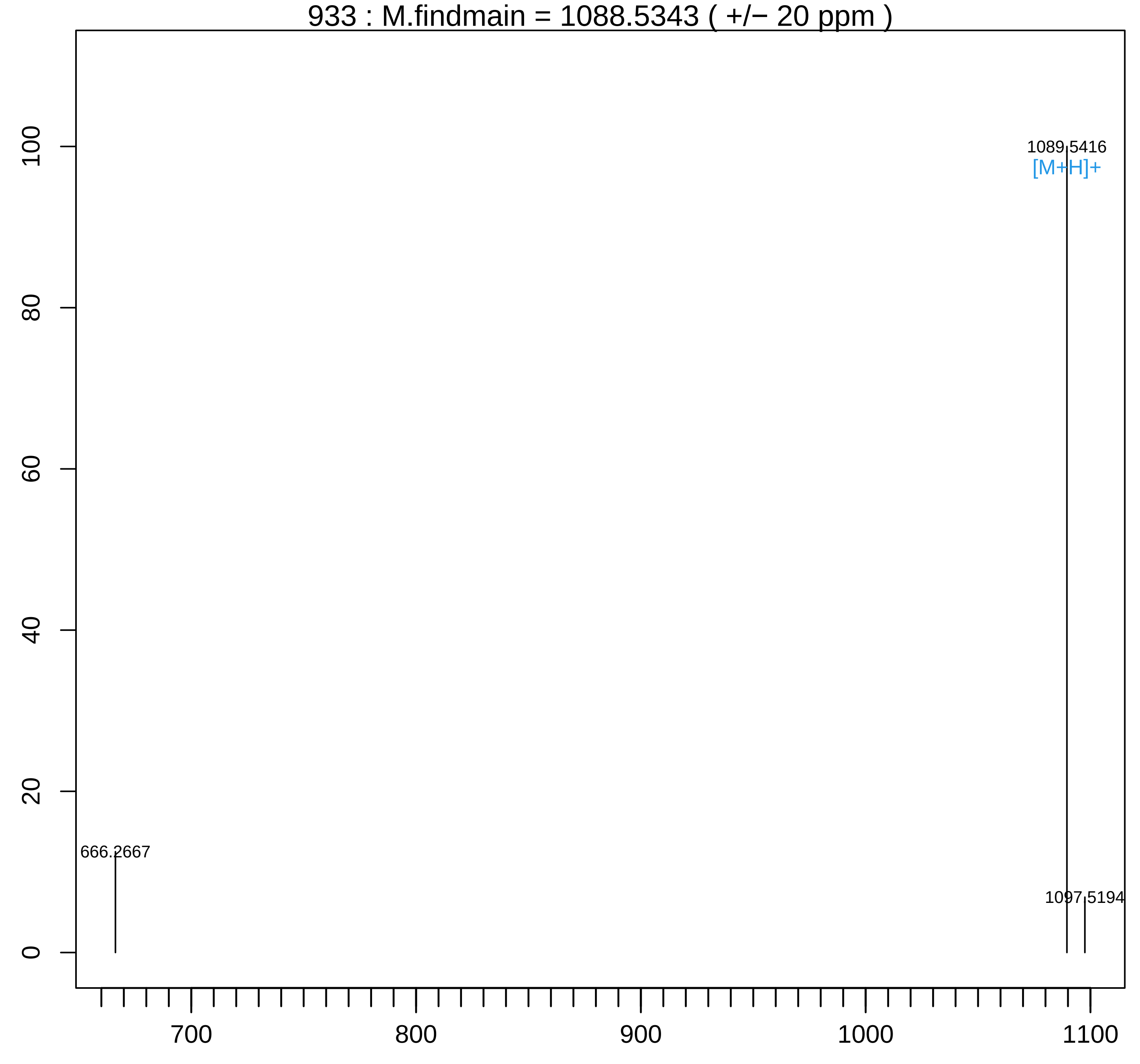

933 : M.findmain = 1088.5343 (+/− 20 ppm)
1089.5416
[M+H]+
666.2667
1097.5194
0
20
40
60
80
100
700
800
900
1000
1100

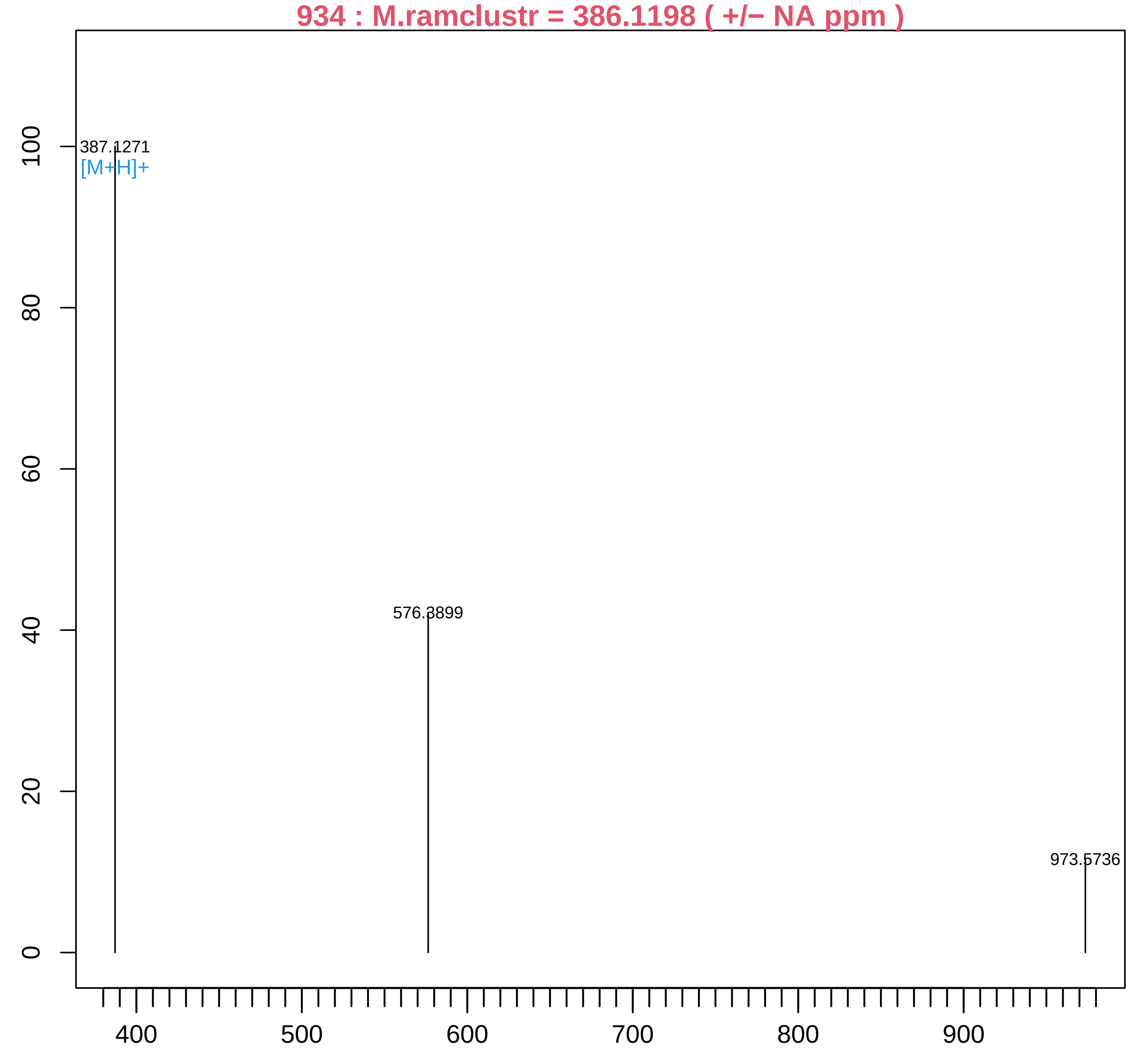
934 : M.ramclustr = 386.1198 (+/− NA ppm)
387.1271
[M+H]+
576.3899
973.5736
0
20
40
60
80
100
400
500
600
700
800
900
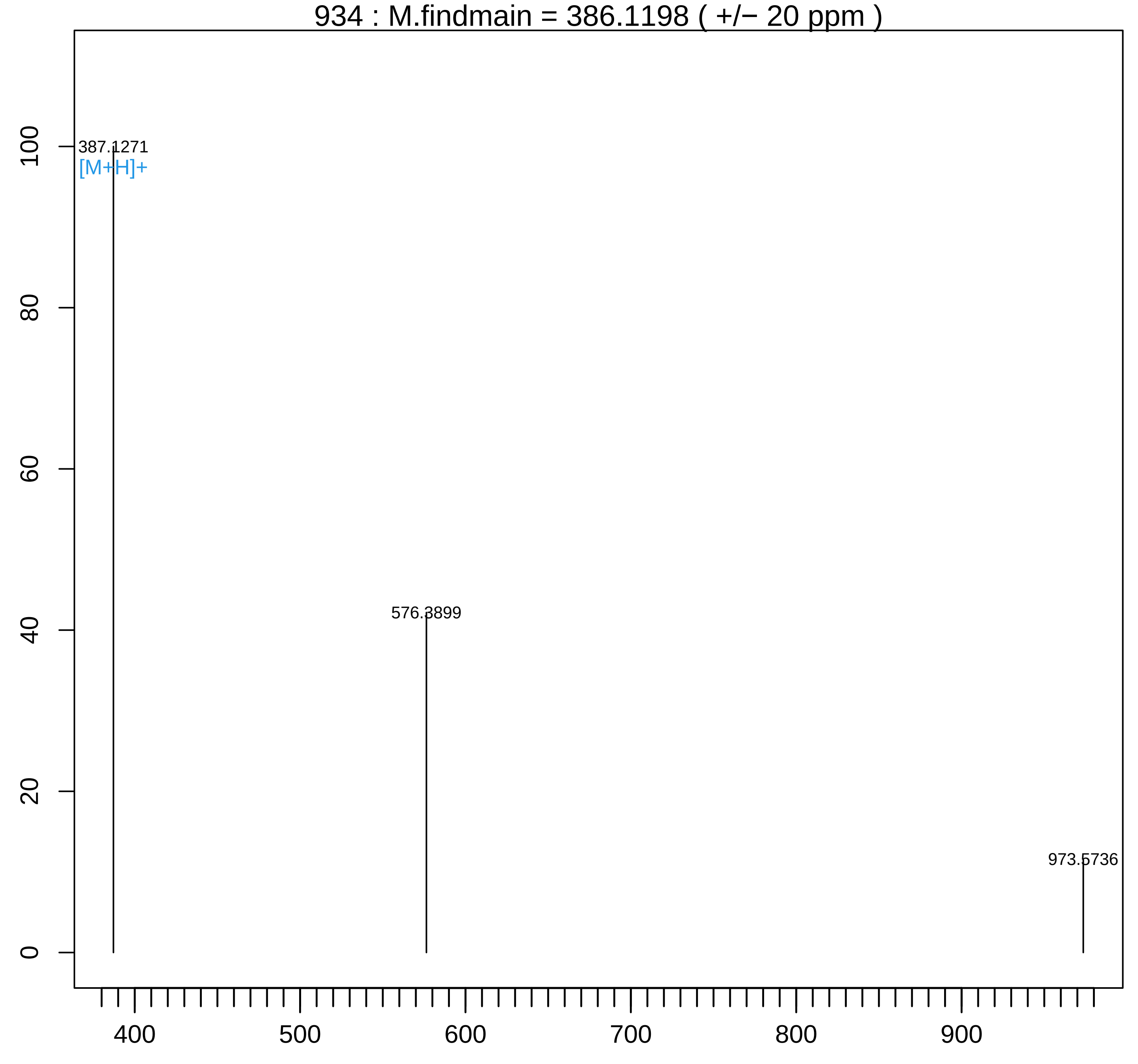
934 : M.findmain = 386.1198 (+/− 20 ppm)
387.1271
[M+H]+
576.3899
973.5736
0
20
40
60
80
100
400
500
600
700
800
900

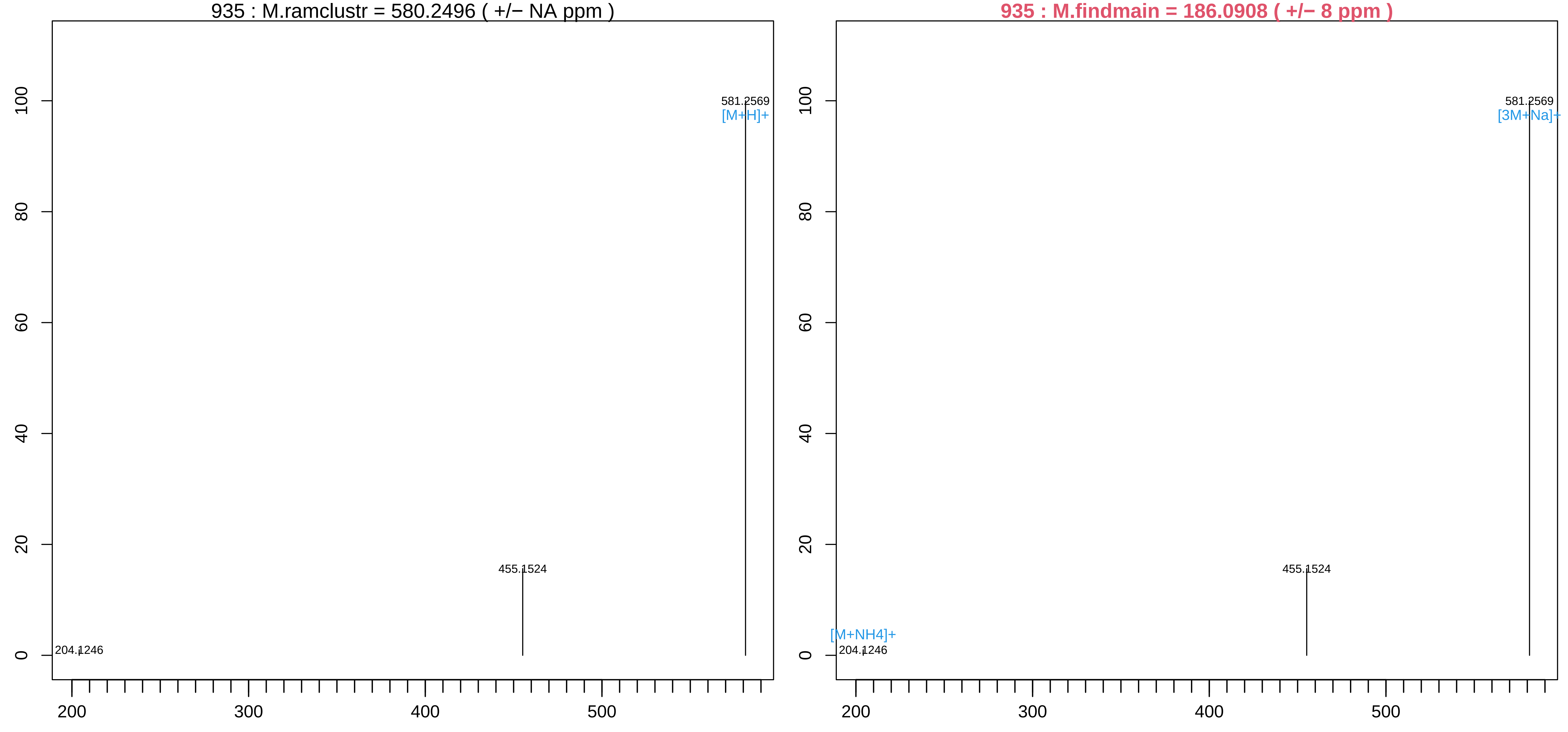

935 : M.ramclustr = 580.2496 (+/− NA ppm)
581.2569
[M+H]+
455.1524
204.1246
0
20
40
60
80
100
200
300
400
500
935 : M.findmain = 186.0908 (+/− 8 ppm)
581.2569
[3M+Na]+
455.1524
[M+NH4]+
204.1246
0
20
40
60
80
100
200
300
400
500

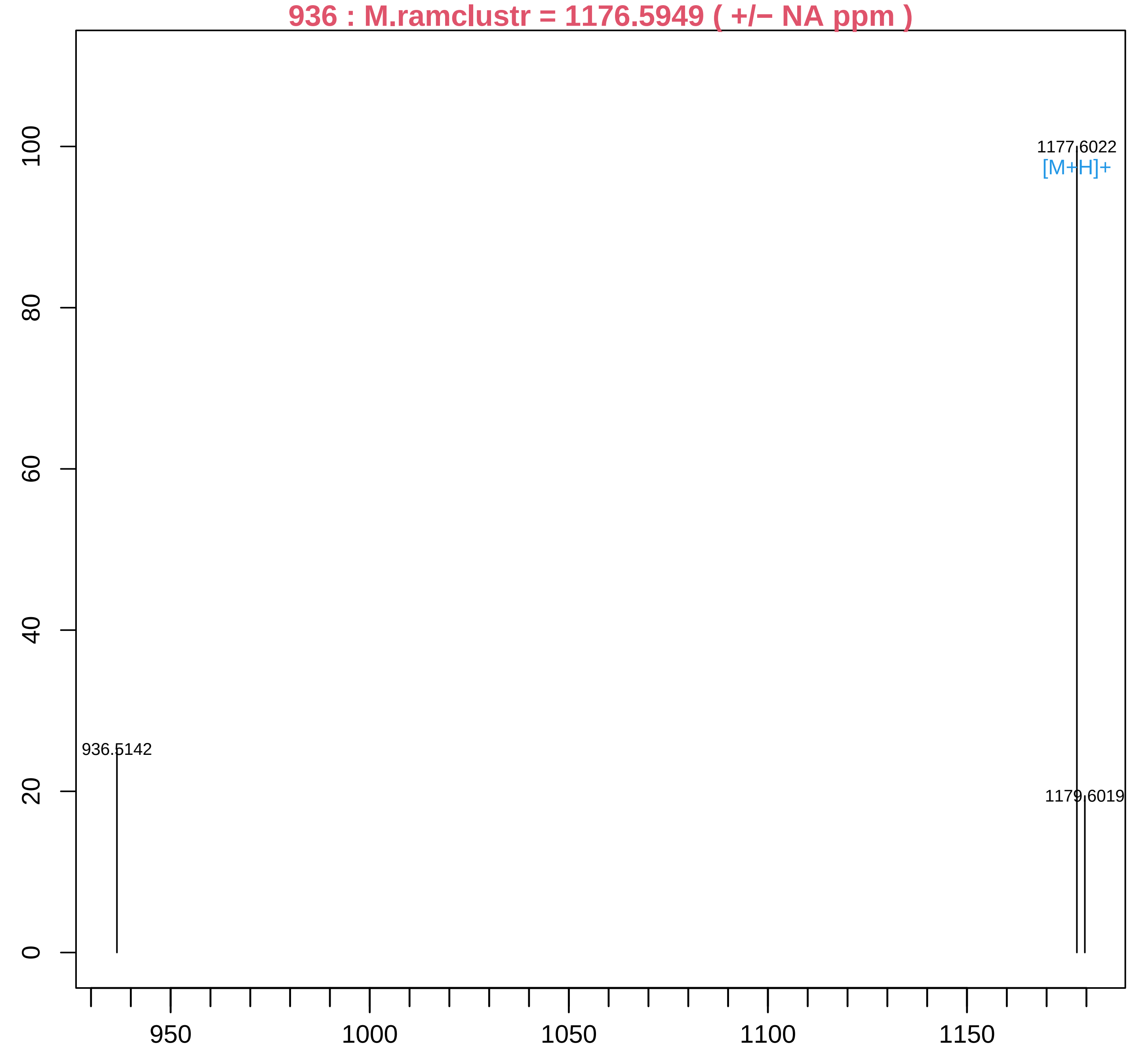
936 : M.ramclustr = 1176.5949 (+/− NA ppm)
1177.6022
[M+H]+
936.5142
1179.6019
0
20
40
60
80
100
950
1000
1050
1100
1150
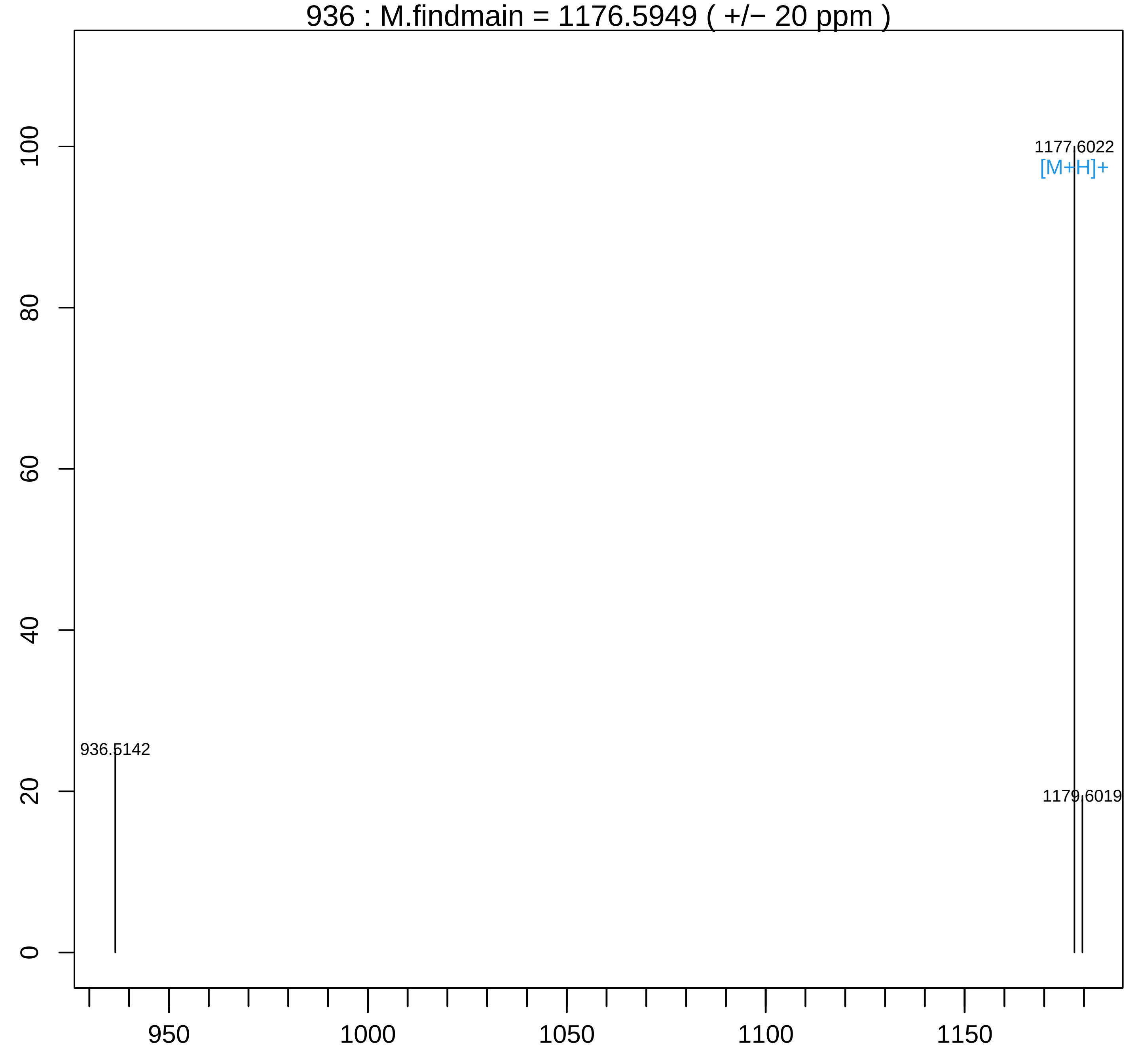
936 : M.findmain = 1176.5949 (+/− 20 ppm)
1177.6022
[M+H]+
936.5142
1179.6019
0
20
40
60
80
100
950
1000
1050
1100
1150

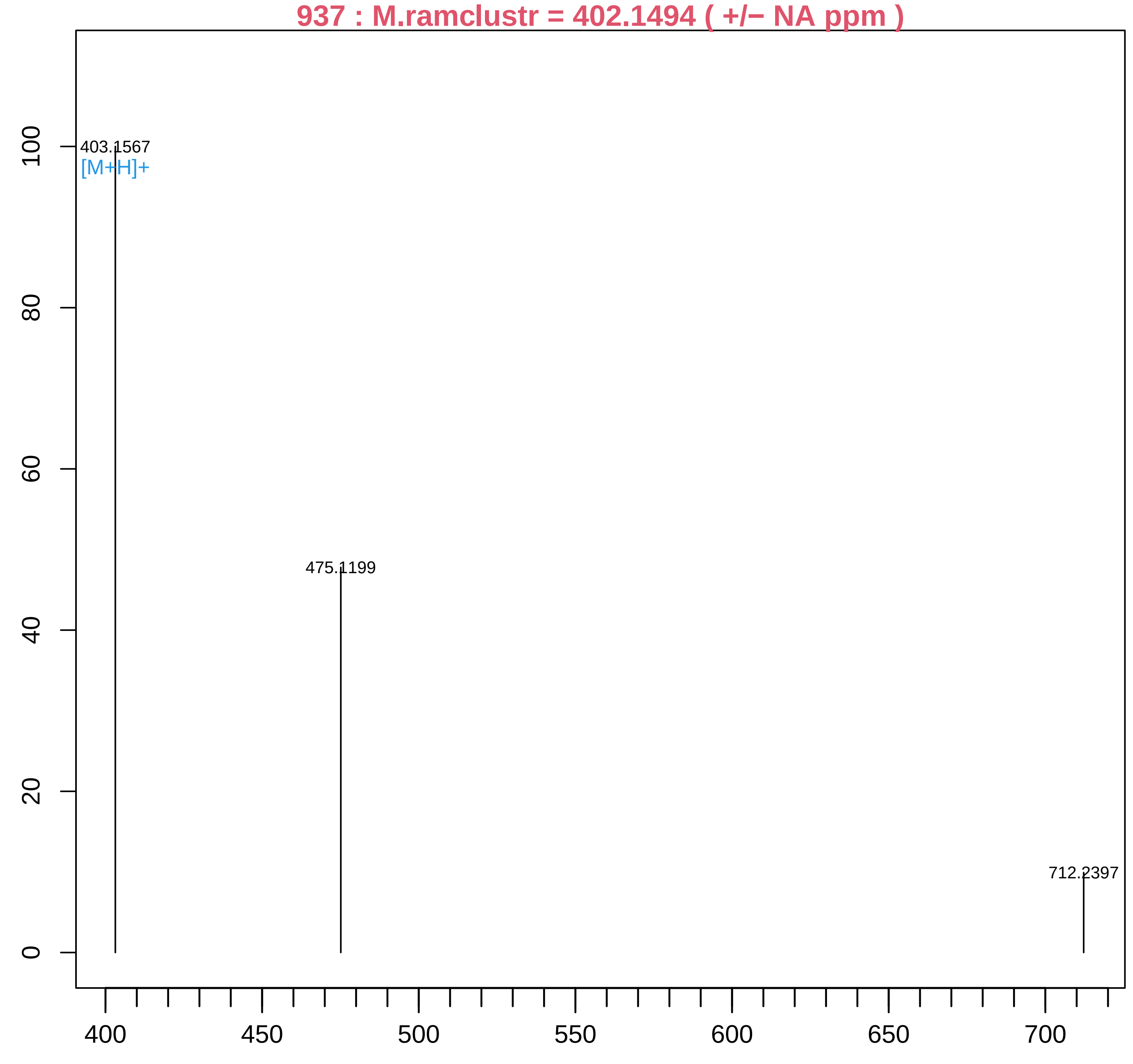

937 : M.ramclustr = 402.1494 (+/− NA ppm)
403.1567
[M+H]+
475.1199
712.2397
0
20
40
60
80
100
400
450
500
550
600
650
700

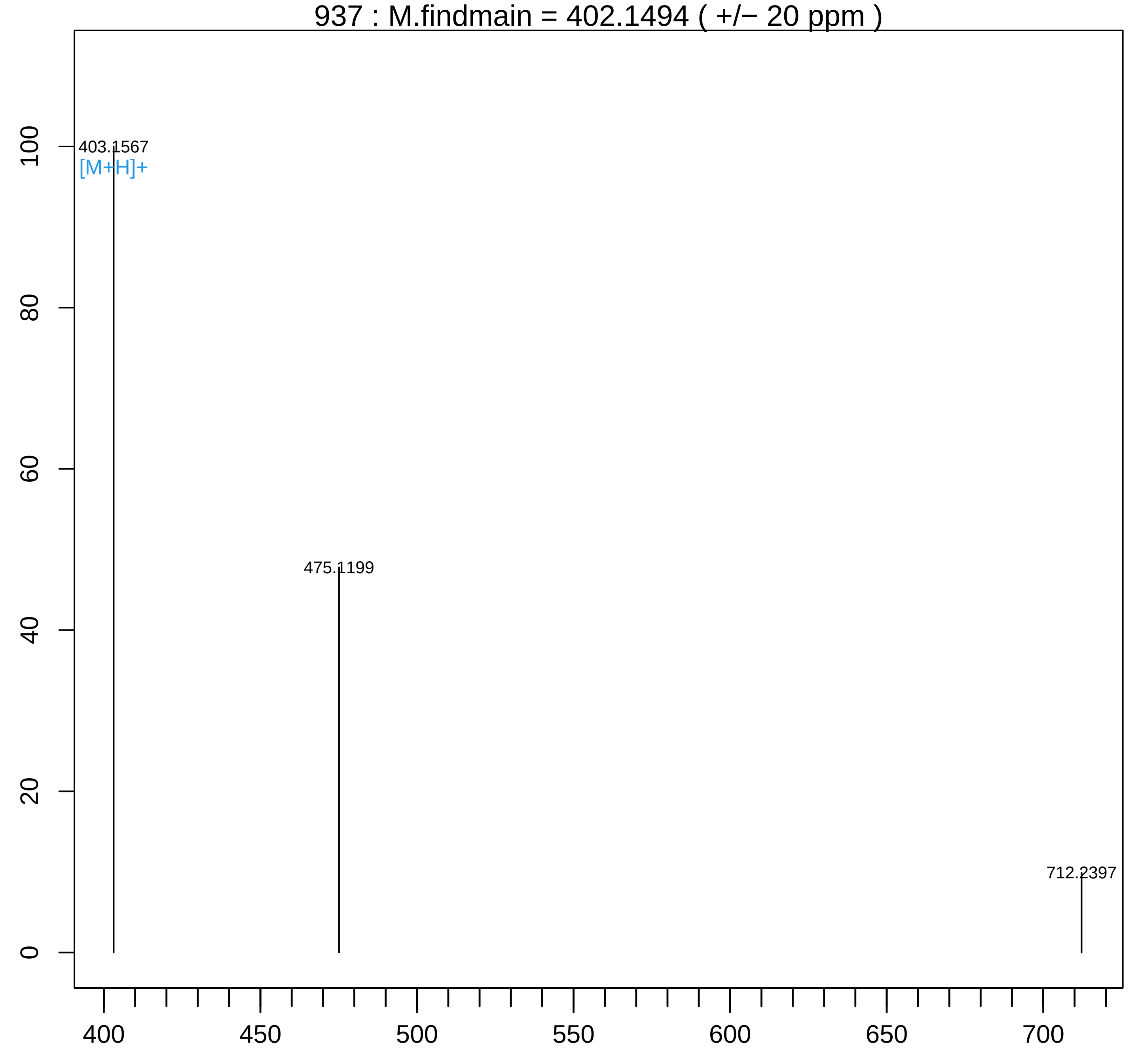

937 : M.findmain = 402.1494 (+/− 20 ppm)
403.1567
[M+H]+
475.1199
712.2397
0
20
40
60
80
100
400
450
500
550
600
650
700

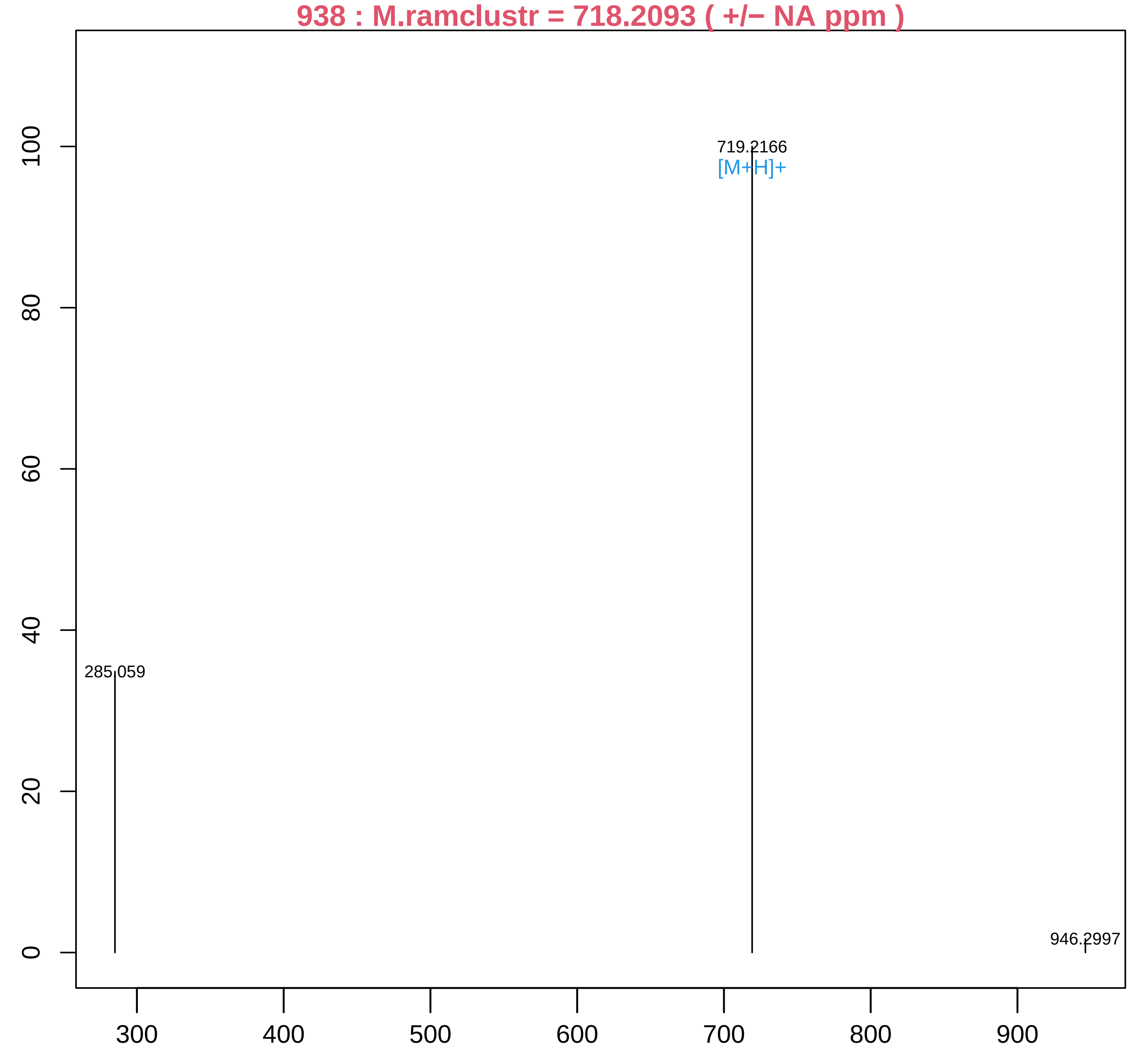
938 : M.ramclustr = 718.2093 (+/− NA ppm)
719.2166
[M+H]+
285.059
946.2997
0
20
40
60
80
100
300
400
500
600
700
800
900

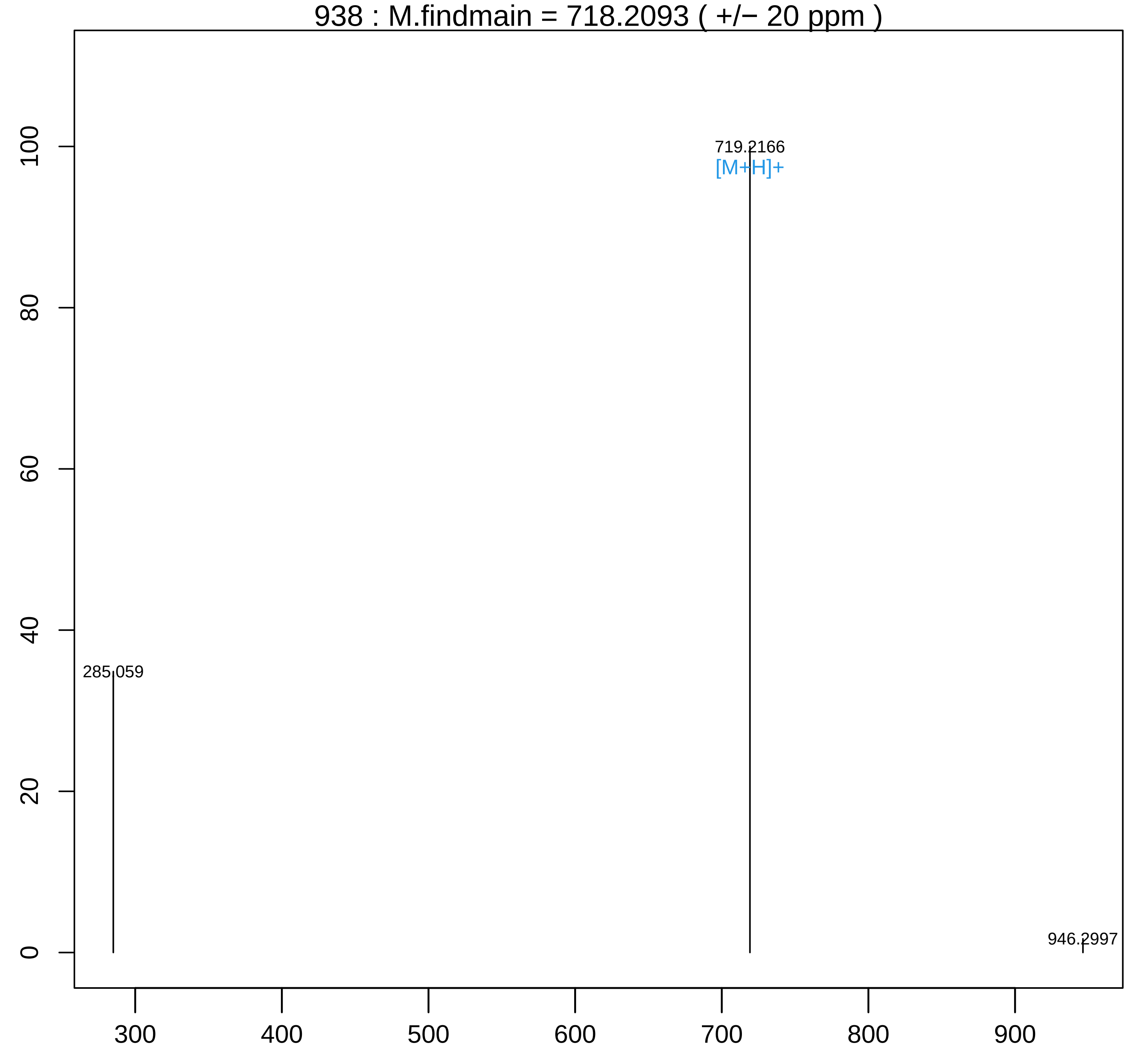
938 : M.findmain = 718.2093 (+/− 20 ppm)
719.2166
[M+H]+
285.059
946.2997
0
20
40
60
80
100
300
400
500
600
700
800
900

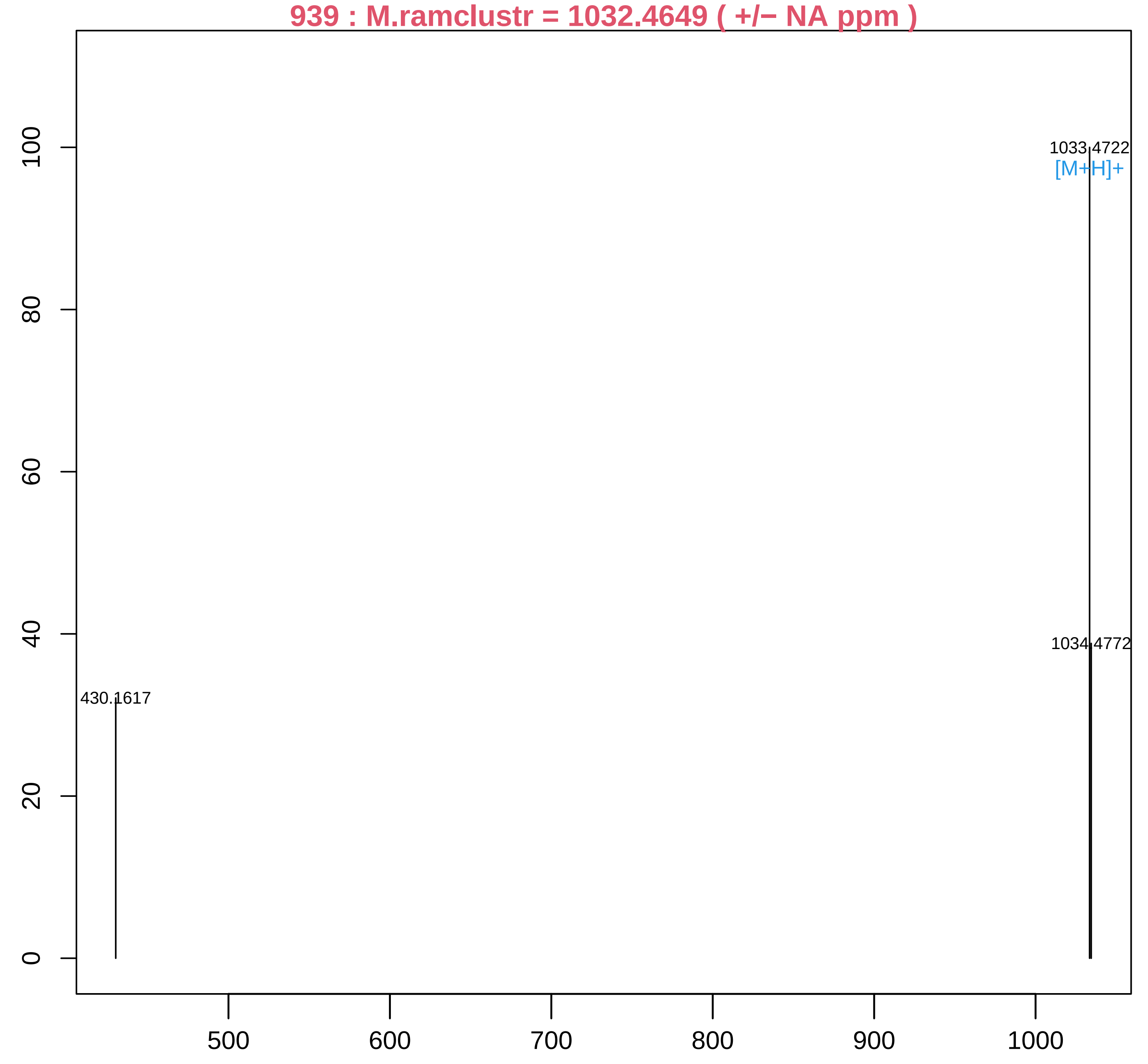
939 : M.ramclustr = 1032.4649 (+/− NA ppm)
1033.4722
[M+H]+
1034.4772
430.1617
0
20
40
60
80
100
500
600
700
800
900
1000

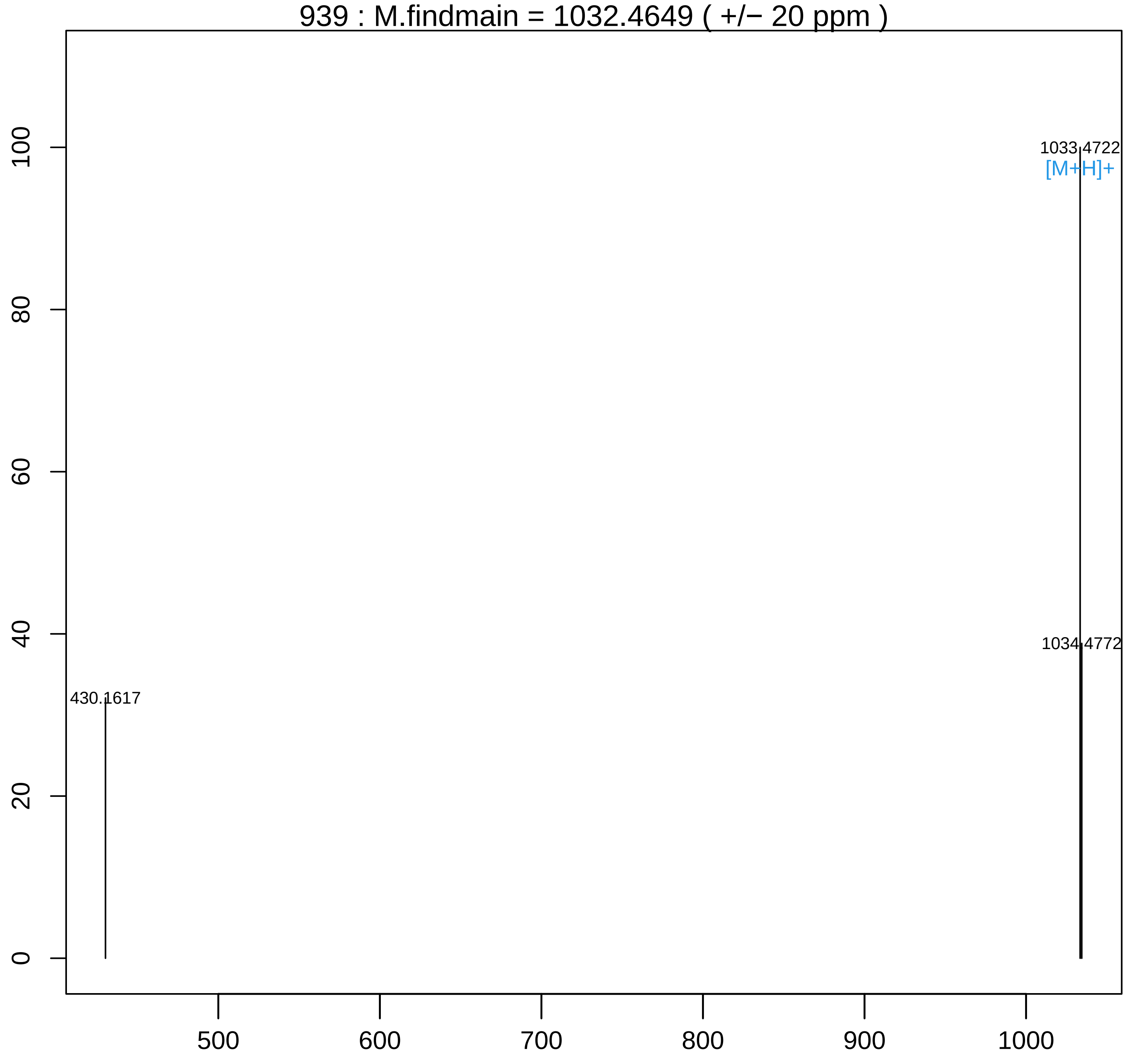
939 : M.findmain = 1032.4649 (+/− 20 ppm)
1033.4722
[M+H]+
1034.4772
430.1617
0
20
40
60
80
100
500
600
700
800
900
1000

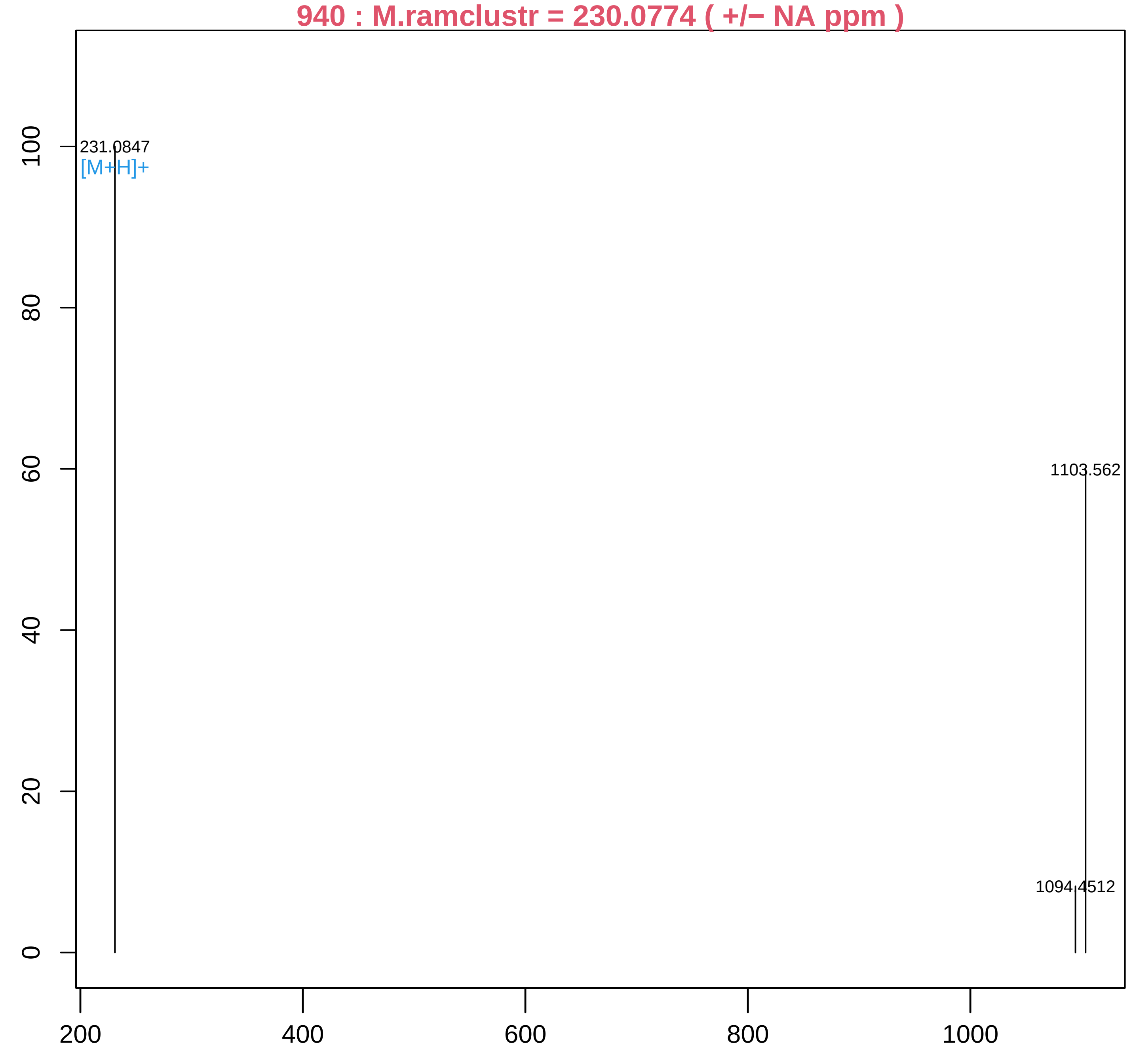
940 : M.ramclustr = 230.0774 (+/− NA ppm)
231.0847
[M+H]+
1103.562
1094.4512
0
20
40
60
80
100
200
400
600
800
1000

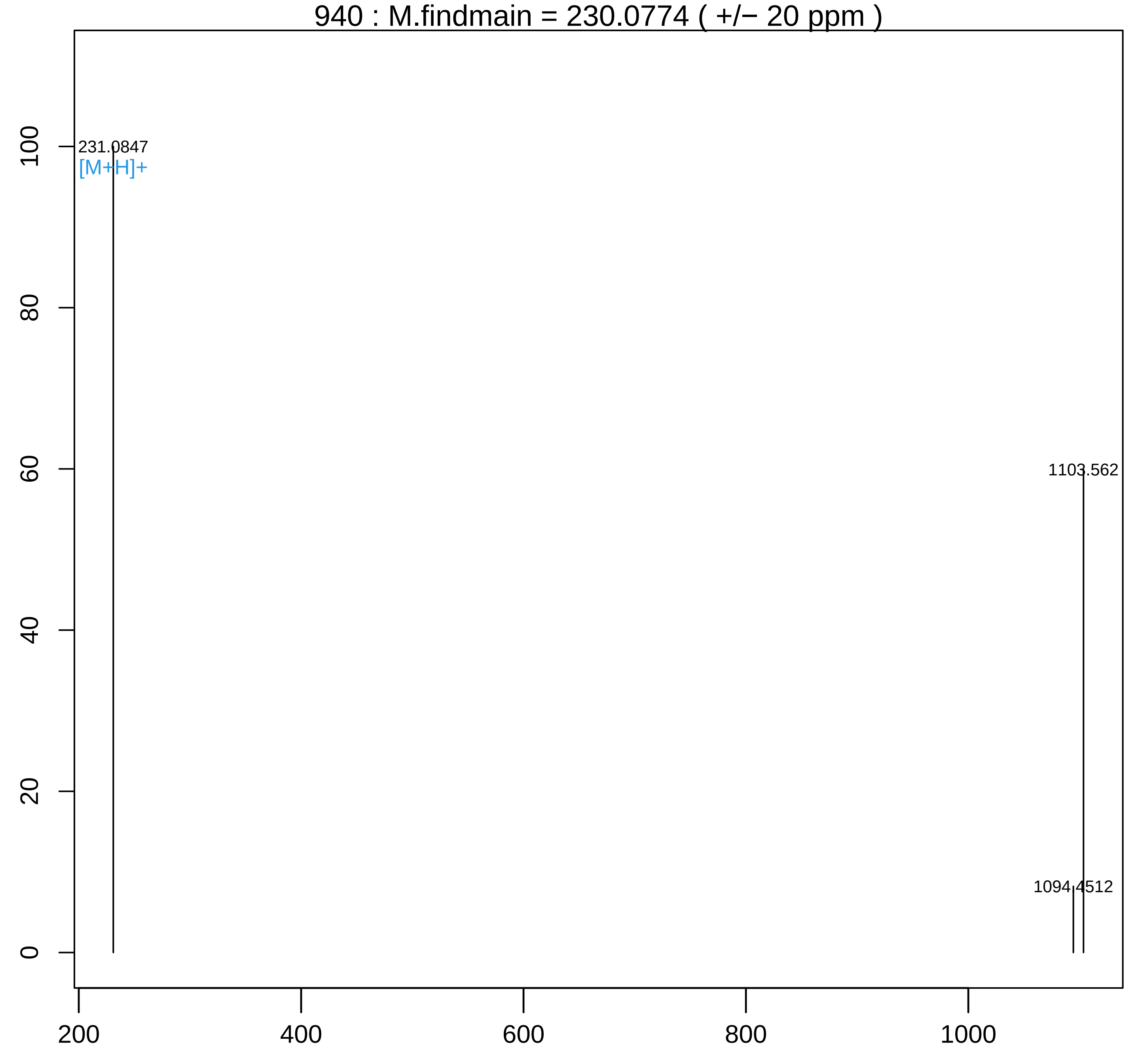
940 : M.findmain = 230.0774 (+/− 20 ppm)
231.0847
[M+H]+
1103.562
1094.4512
0
20
40
60
80
100
200
400
600
800
1000

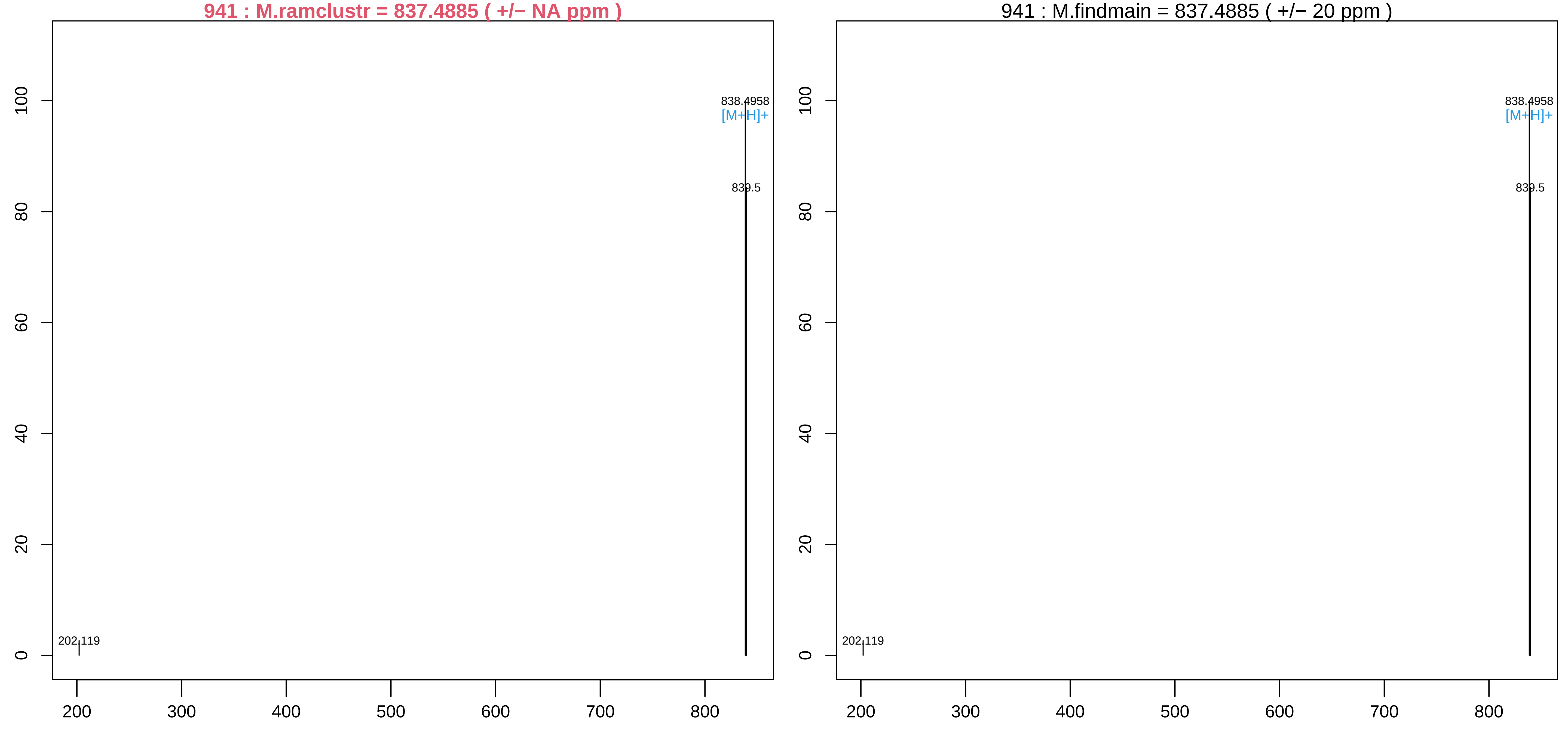
941 : M.ramclustr = 837.4885 (+/− NA ppm)
838.4958
[M+H]+
839.5
202.119
941 : M.findmain = 837.4885 (+/− 20 ppm)
838.4958
[M+H]+
839.5
202.119

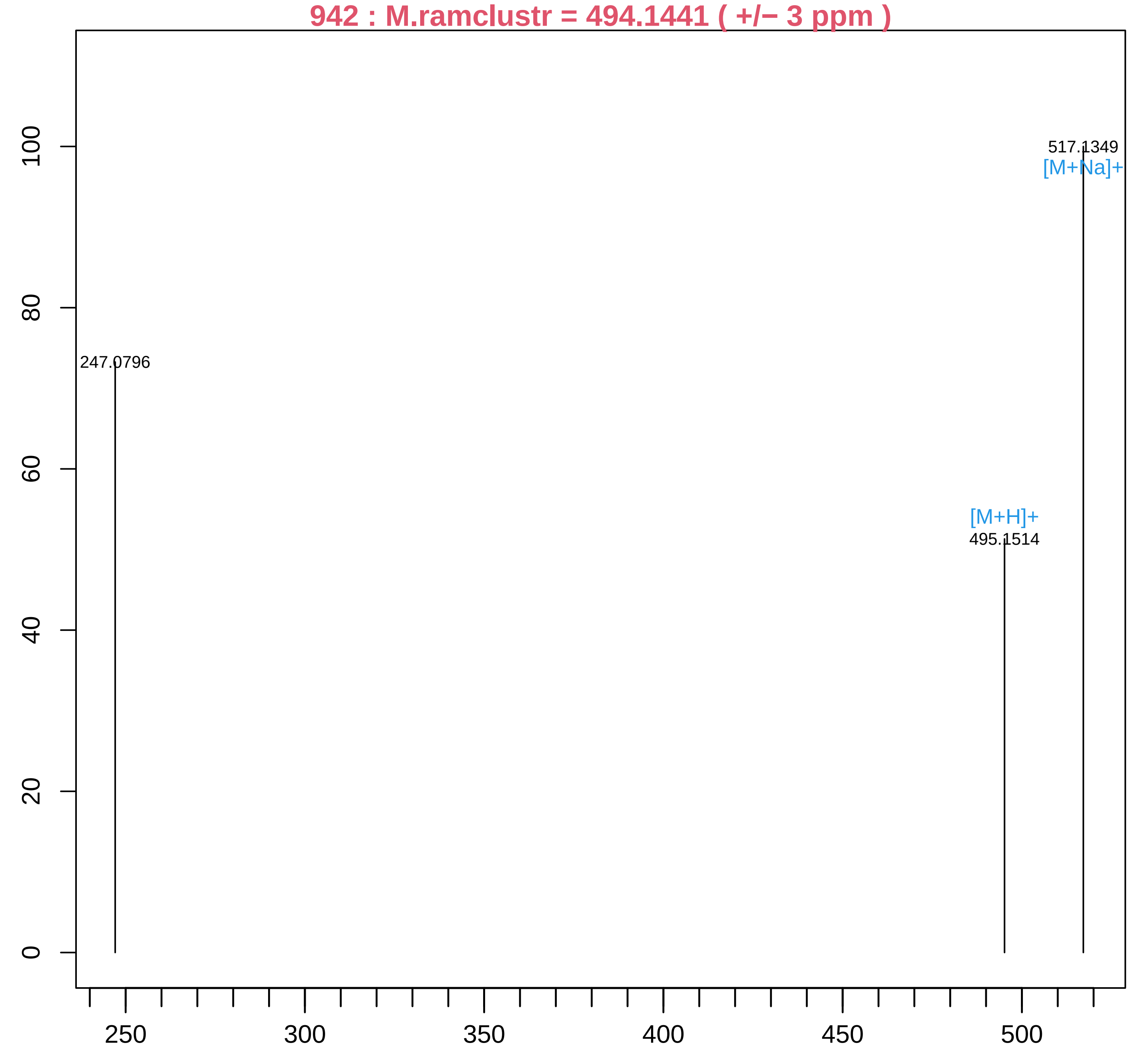
942 : M.ramclustr = 494.1441 (+/− 3 ppm)
517.1349
[M+Na]+
247.0796
[M+H]+
495.1514
0
20
40
60
80
100
250
300
350
400
450
500

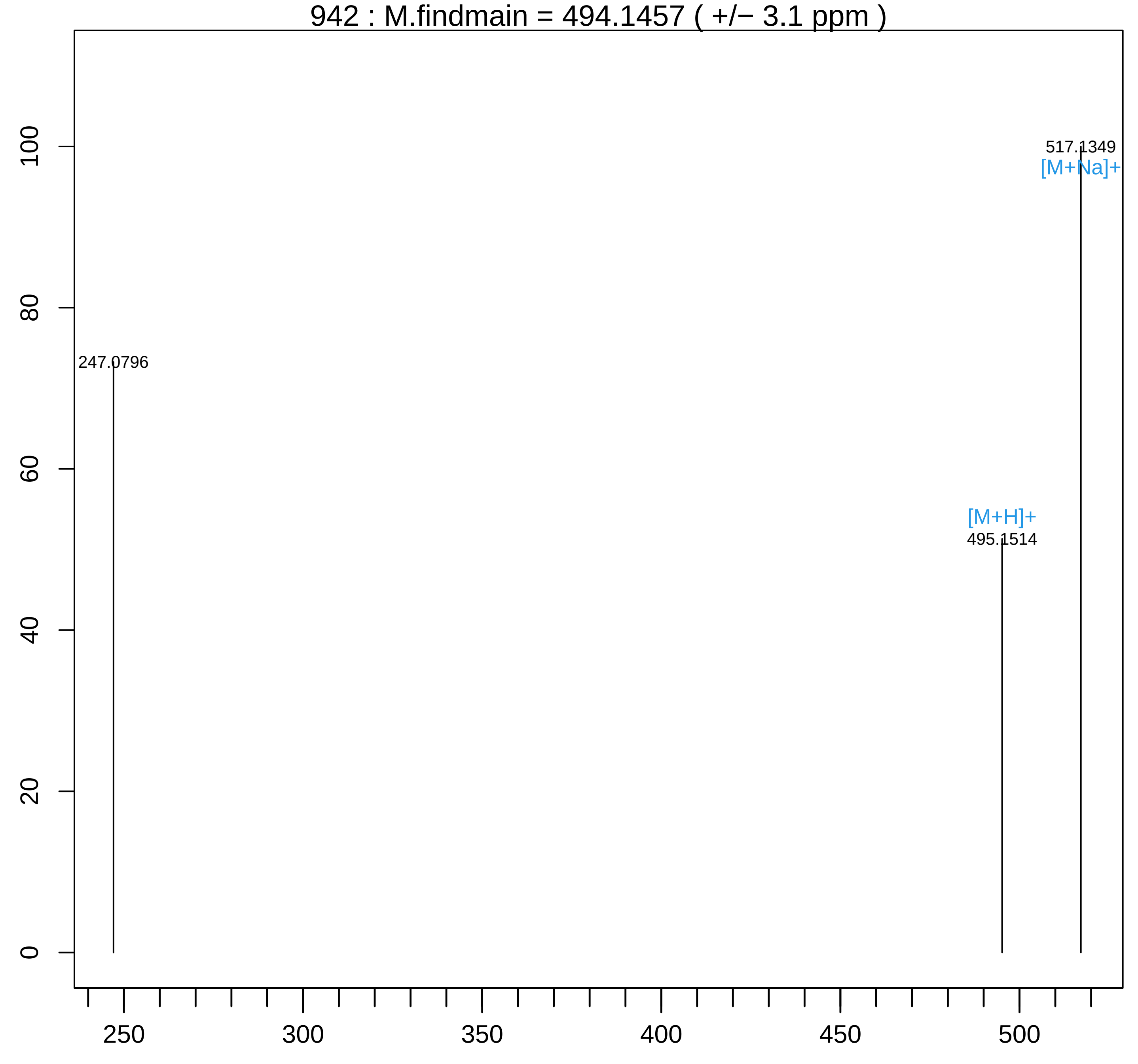
942 : M.findmain = 494.1457 (+/− 3.1 ppm)
517.1349
[M+Na]+
247.0796
[M+H]+
495.1514
0
20
40
60
80
100
250
300
350
400
450
500

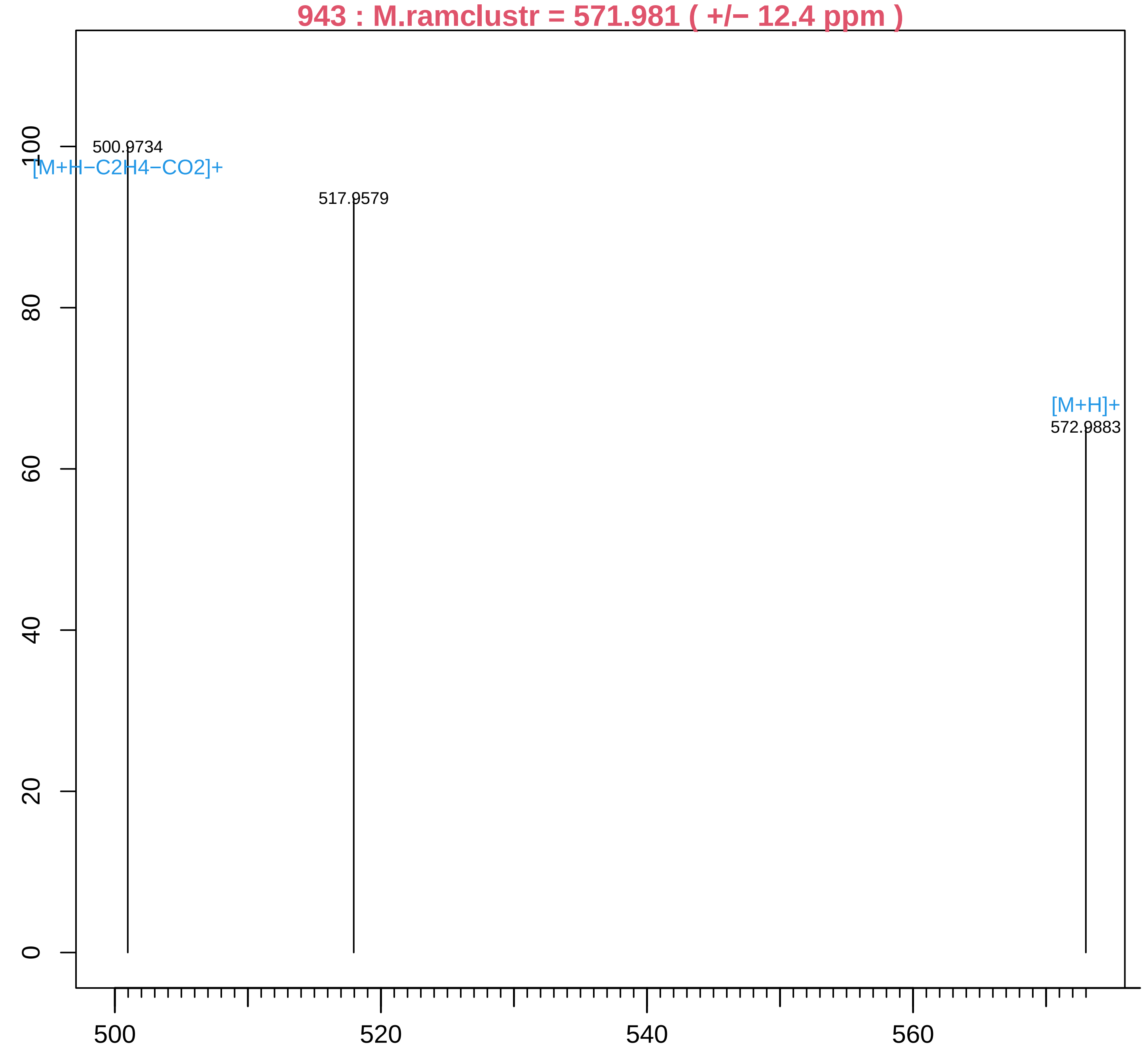
943 : M.ramclustr = 571.981 (+/− 12.4 ppm)
500.9734
[M+H−C2H4−CO2]+
517.9579
[M+H]+
572.9883
0
20
40
60
80
100
500
520
540
560

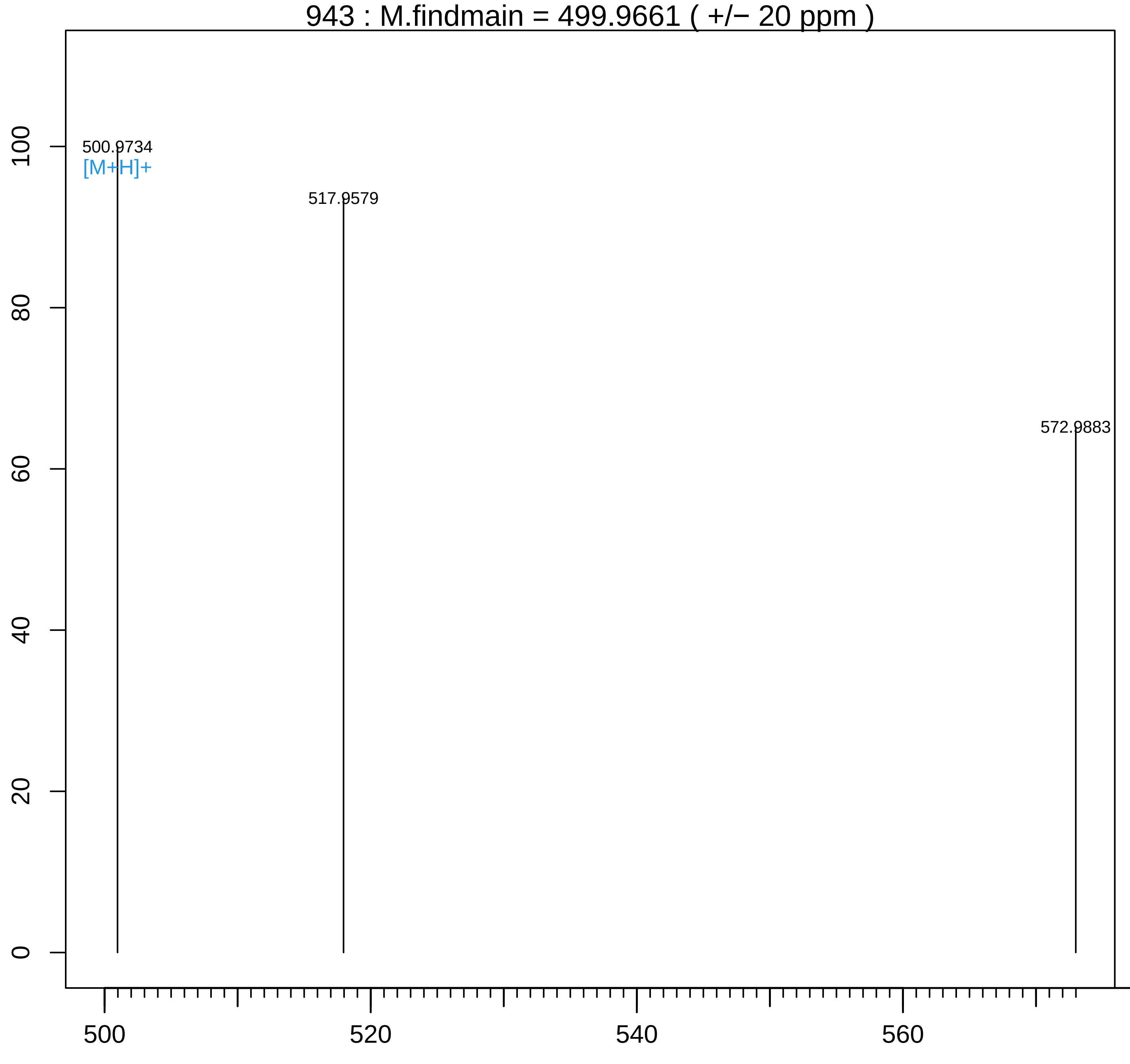
943 : M.findmain = 499.9661 (+/− 20 ppm)
500.9734
[M+H]+
517.9579
572.9883
0
20
40
60
80
100
500
520
540
560

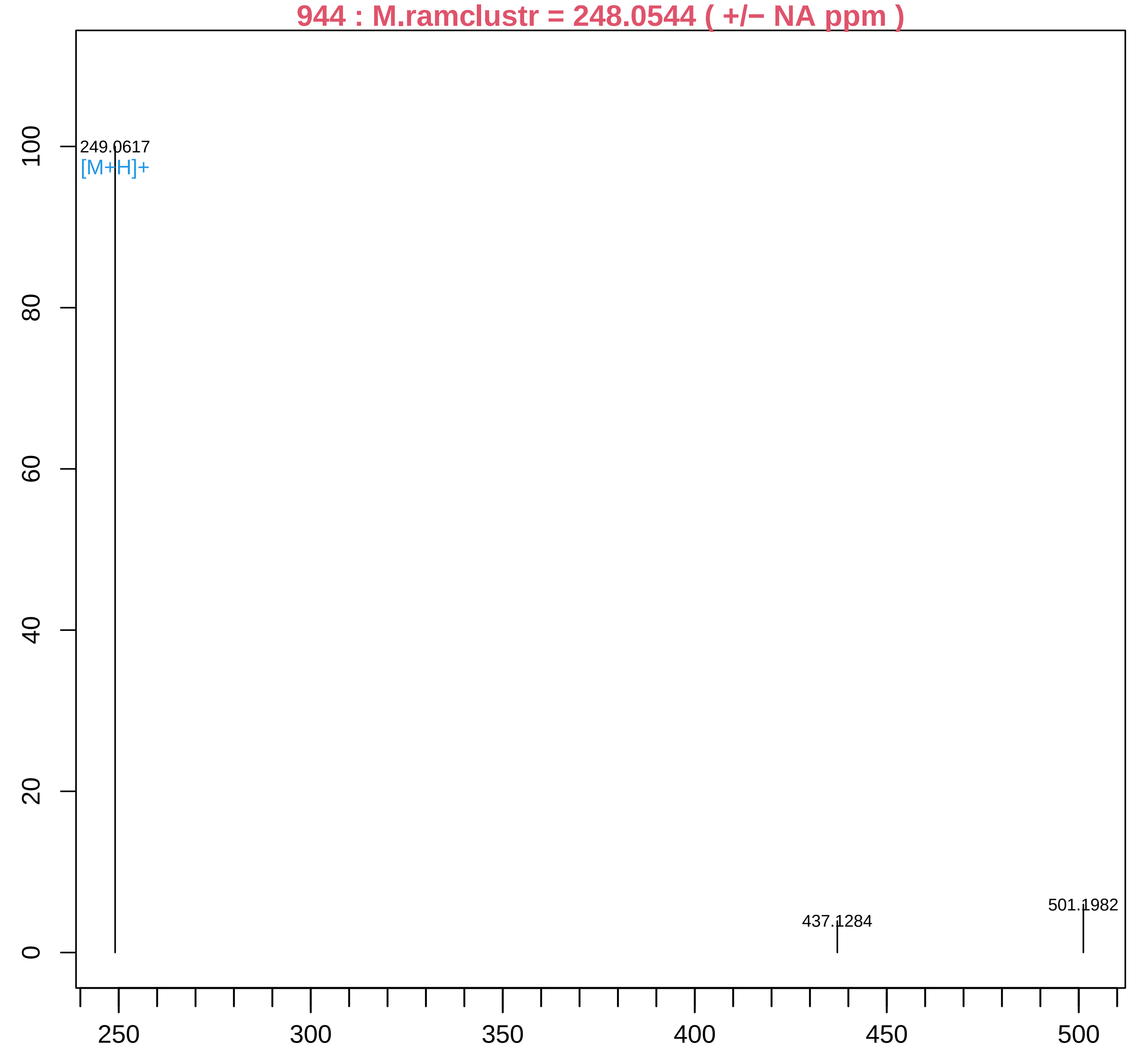
944 : M.ramclustr = 248.0544 (+/− NA ppm)
249.0617
[M+H]+
437.1284
501.1982
0
20
40
60
80
100
250
300
350
400
450
500

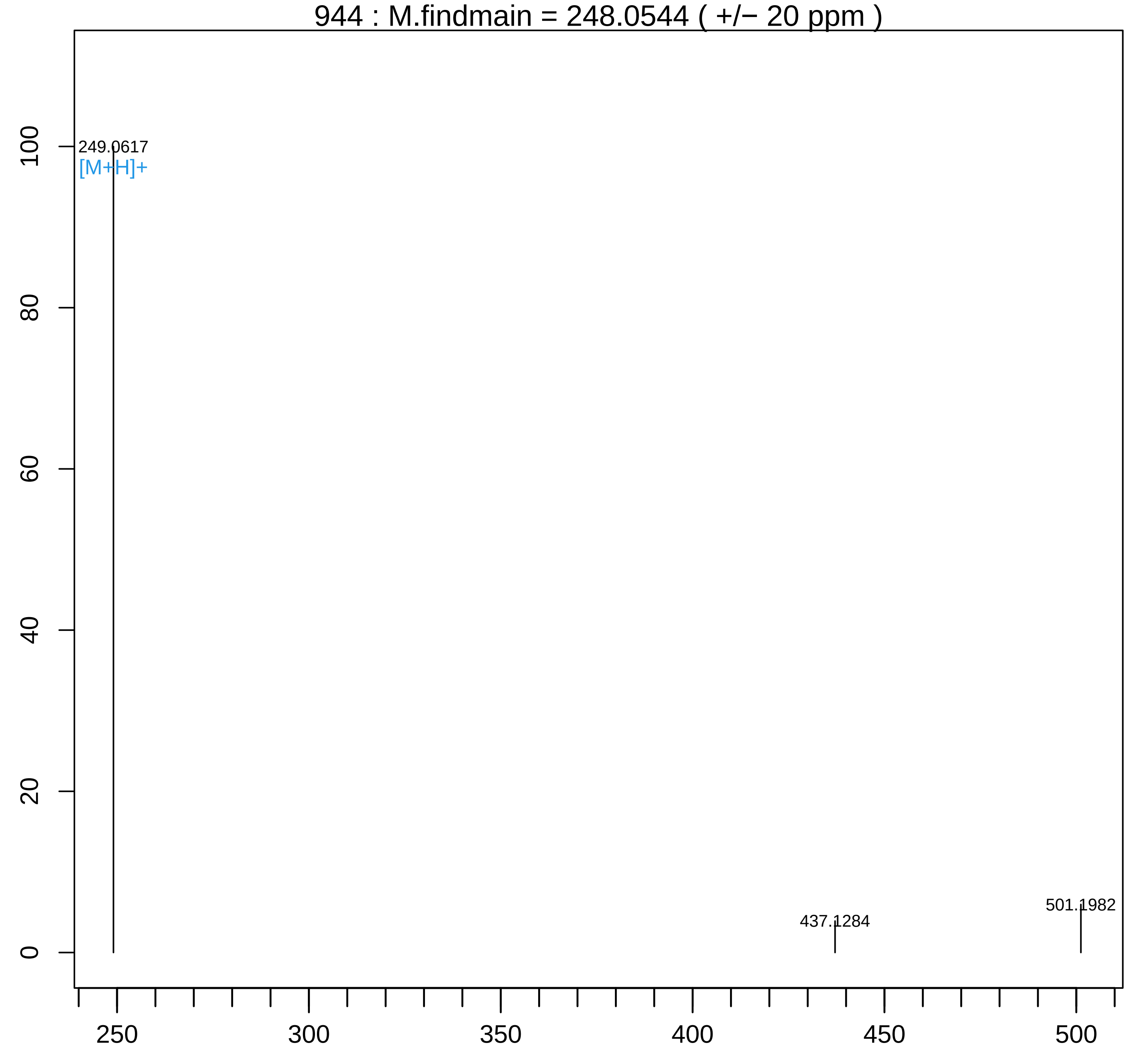
944 : M.findmain = 248.0544 (+/− 20 ppm)
249.0617
[M+H]+
437.1284
501.1982
0
20
40
60
80
100
250
300
350
400
450
500

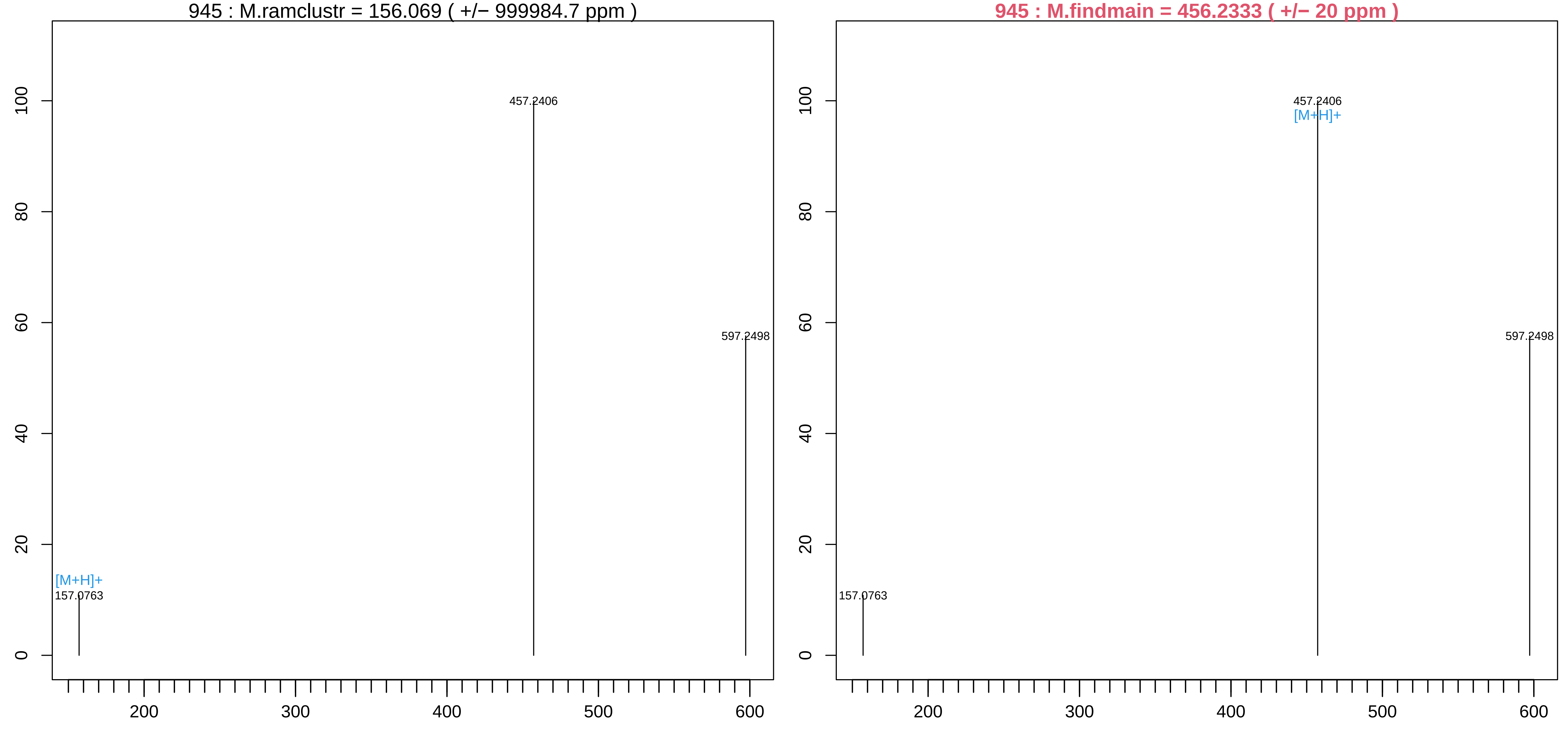
945 : M.ramclustr = 156.069 (+/− 999984.7 ppm)
457.2406
597.2498
[M+H]+
157.0763
0
20
40
60
80
100
200
300
400
500
600
945 : M.findmain = 456.2333 (+/− 20 ppm)
457.2406
[M+H]+
597.2498
157.0763
0
20
40
60
80
100
200
300
400
500
600

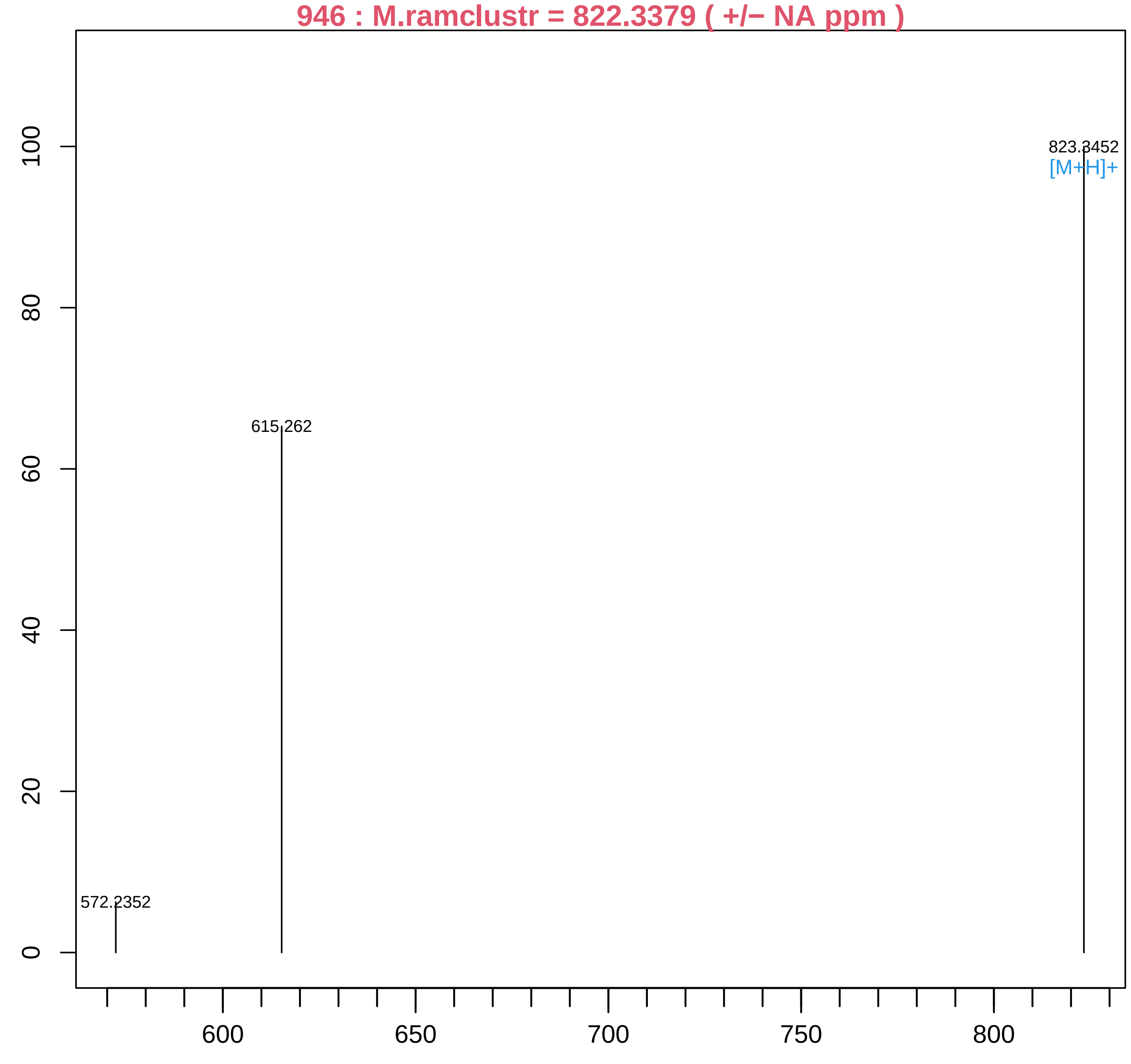
946 : M.ramclustr = 822.3379 (+/− NA ppm)
823.3452
[M+H]+
615.262
572.2352
0
20
40
60
80
100
600
650
700
750
800

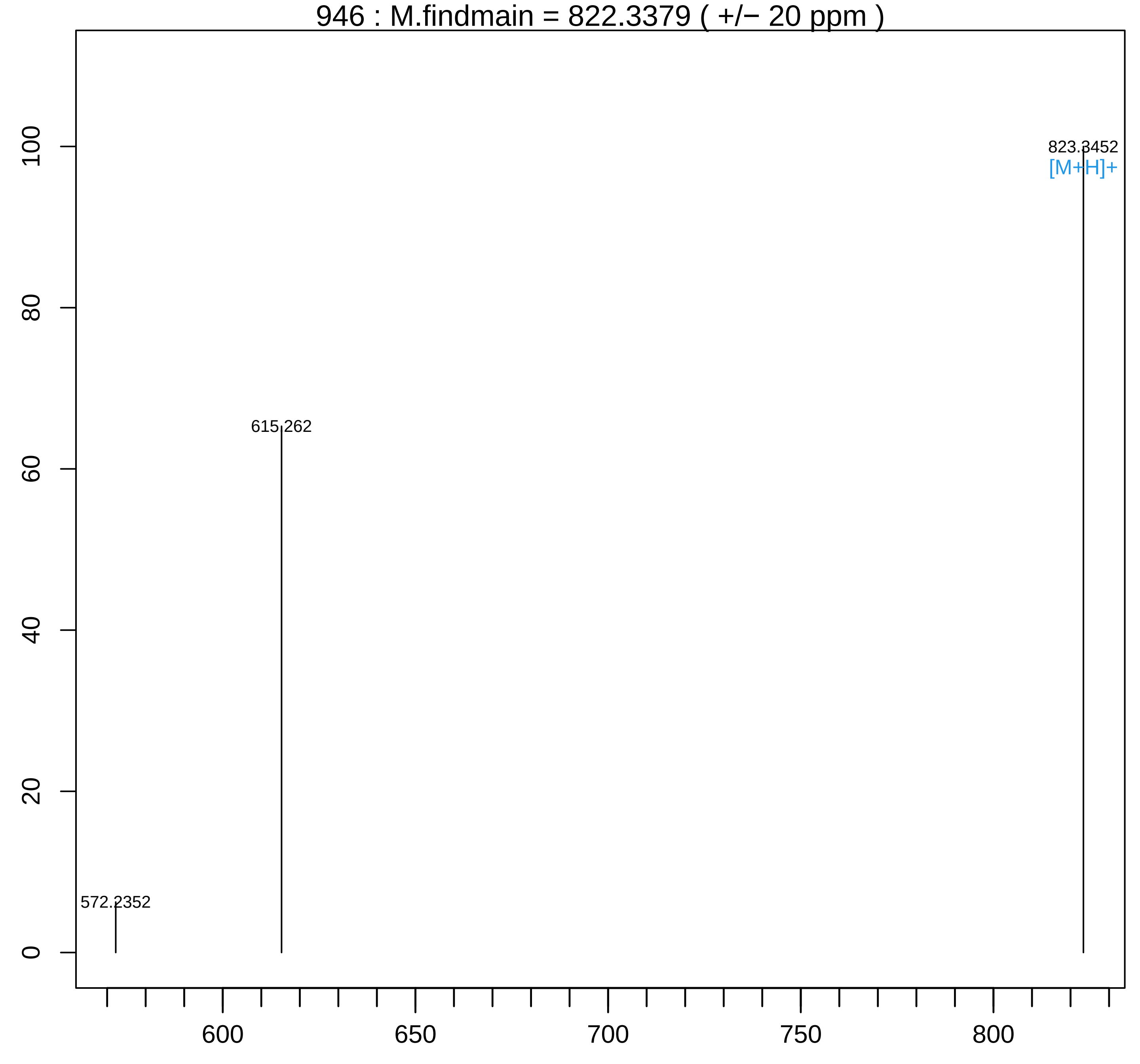
946 : M.findmain = 822.3379 (+/− 20 ppm)
823.3452
[M+H]+
615.262
572.2352
0
20
40
60
80
100
600
650
700
750
800

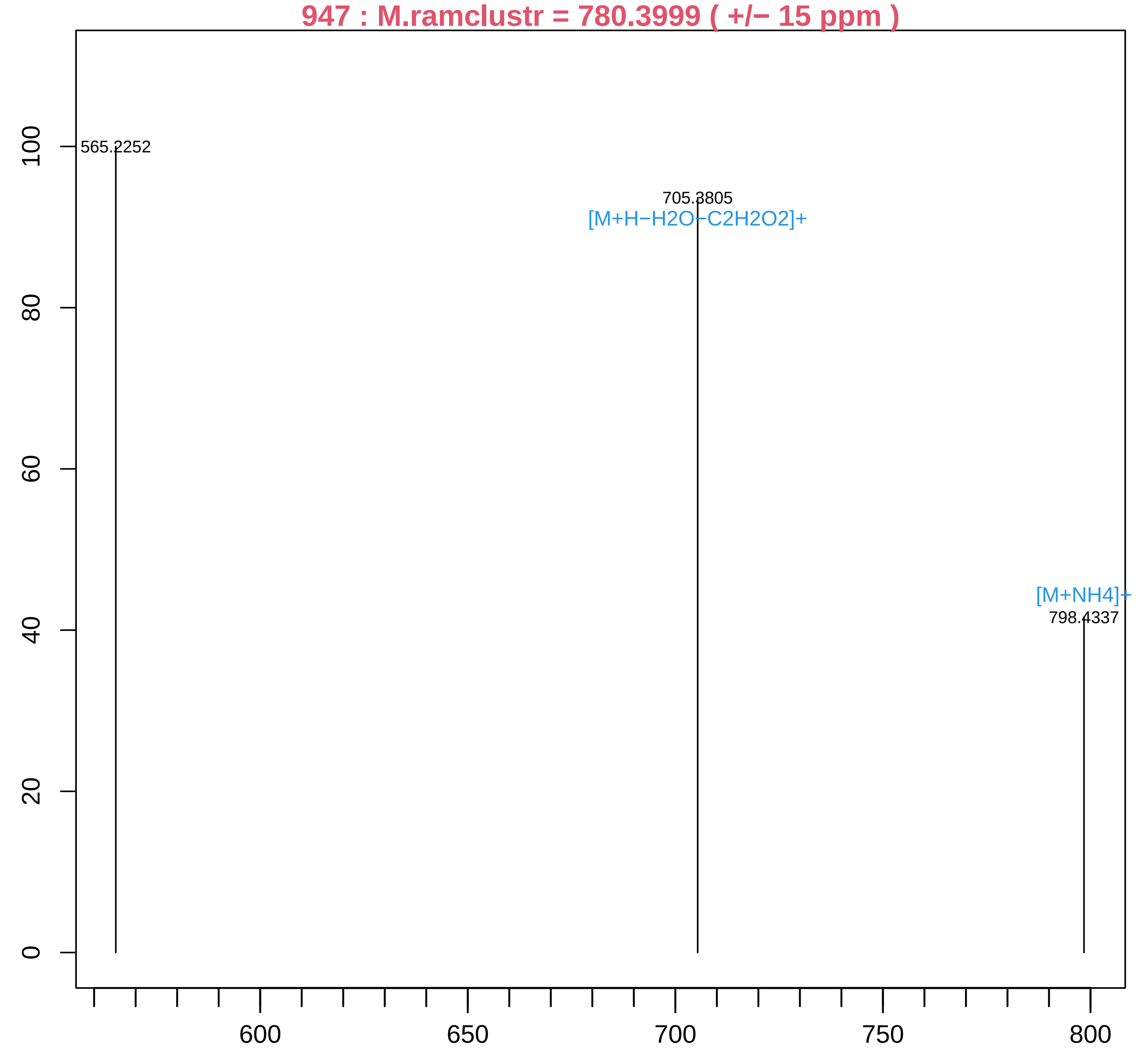

947 : M.ramclustr = 780.3999 (+/− 15 ppm)
565.2252
705.3805
[M+H−H2O−C2H2O2]+
[M+NH4]+
798.4337
0
20
40
60
80
100
600
650
700
750
800

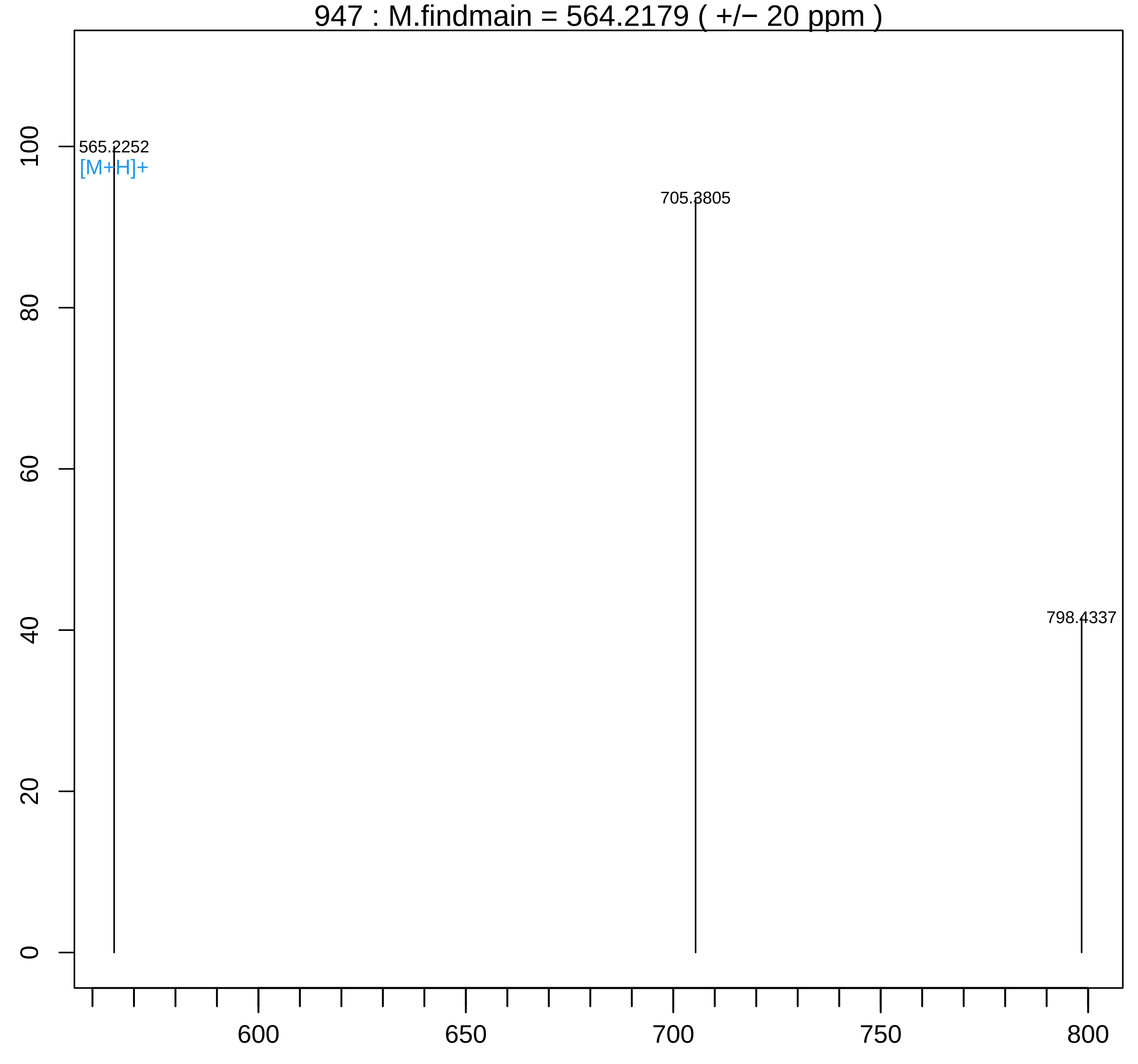

947 : M.findmain = 564.2179 (+/− 20 ppm)
565.2252
[M+H]+
705.3805
798.4337
0
20
40
60
80
100
600
650
700
750
800

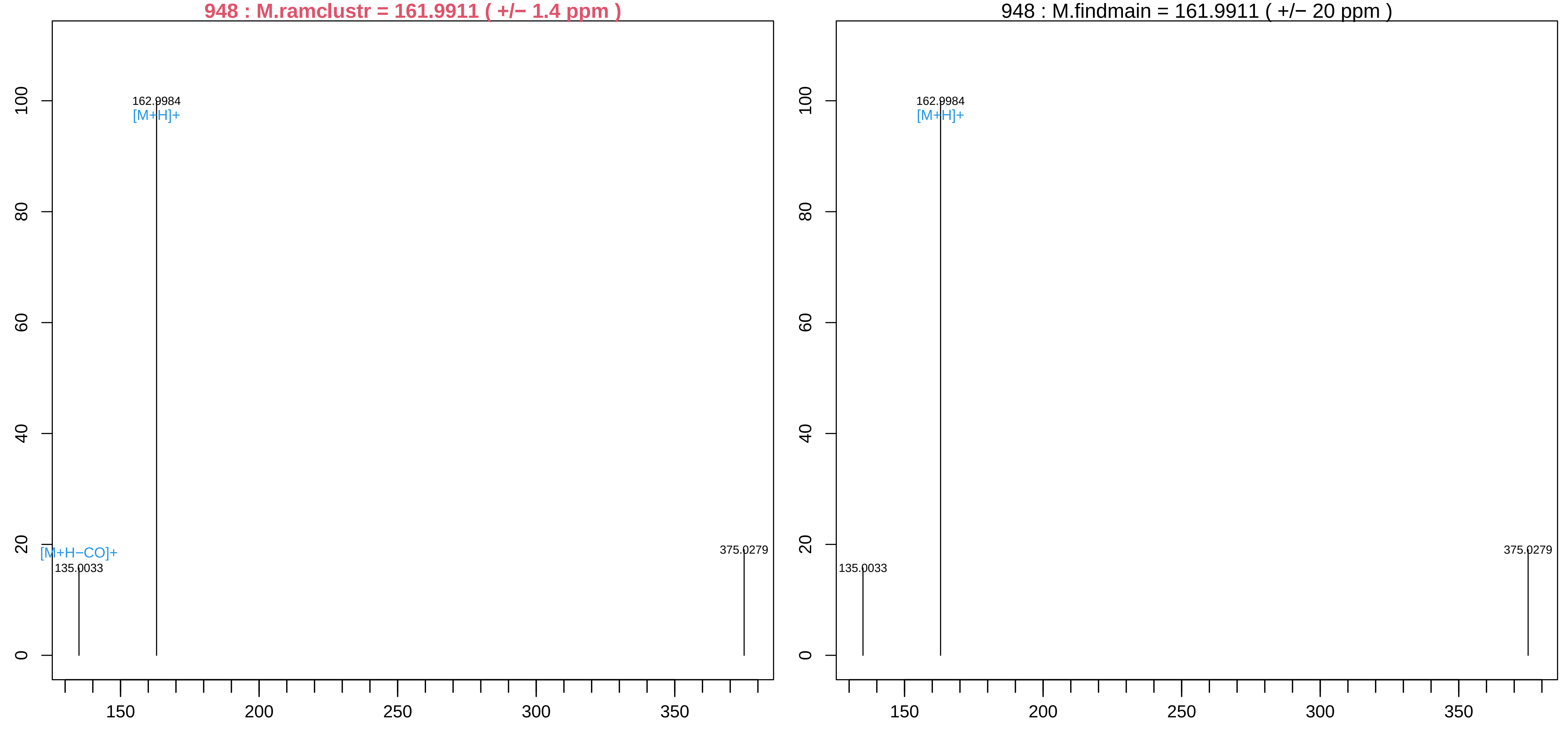
948 : M.ramclustr = 161.9911 (+/− 1.4 ppm)
162.9984
[M+H]+
[M+H−CO]+
135.0033
375.0279
0
20
40
60
80
100
150
200
250
300
350
948 : M.findmain = 161.9911 (+/− 20 ppm)
162.9984
[M+H]+
135.0033
375.0279
0
20
40
60
80
100
150
200
250
300
350

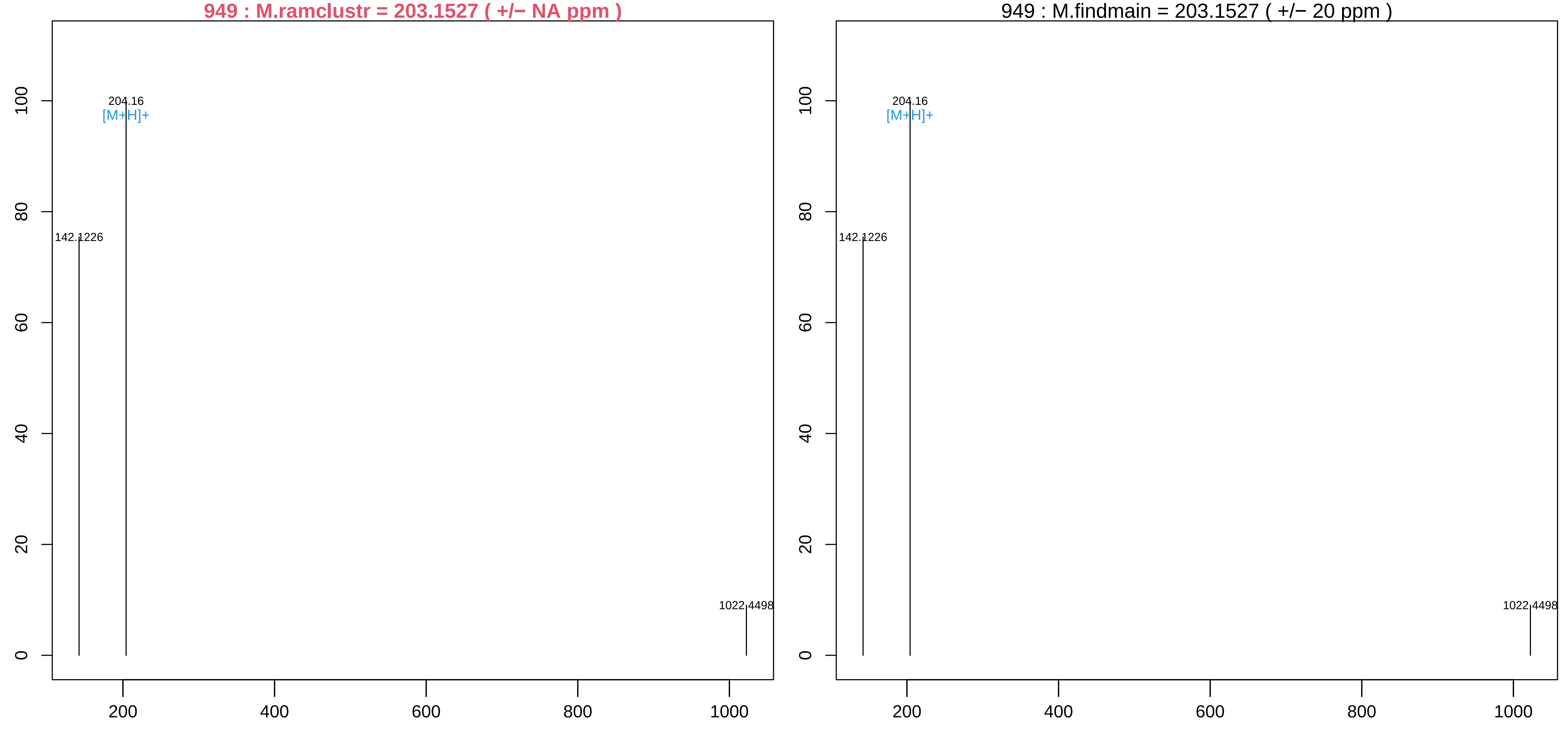

949 : M.ramclustr = 203.1527 (+/− NA ppm)
204.16
[M+H]+
142.1226
1022.4498
949 : M.findmain = 203.1527 (+/− 20 ppm)
204.16
[M+H]+
142.1226
1022.4498

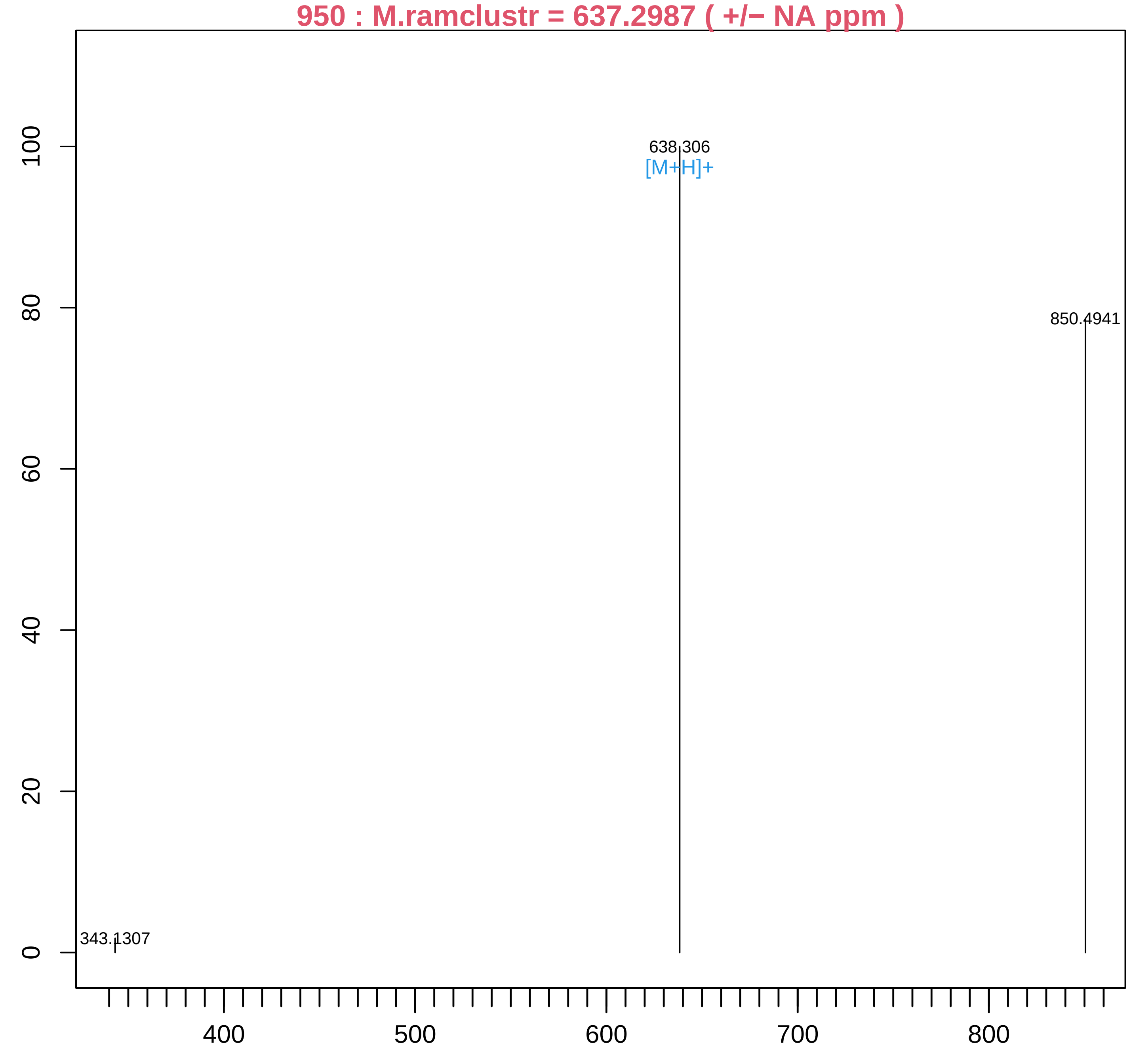
950 : M.ramclustr = 637.2987 (+/− NA ppm)
638.306
[M+H]+
850.4941
343.1307
0
20
40
60
80
100
400
500
600
700
800

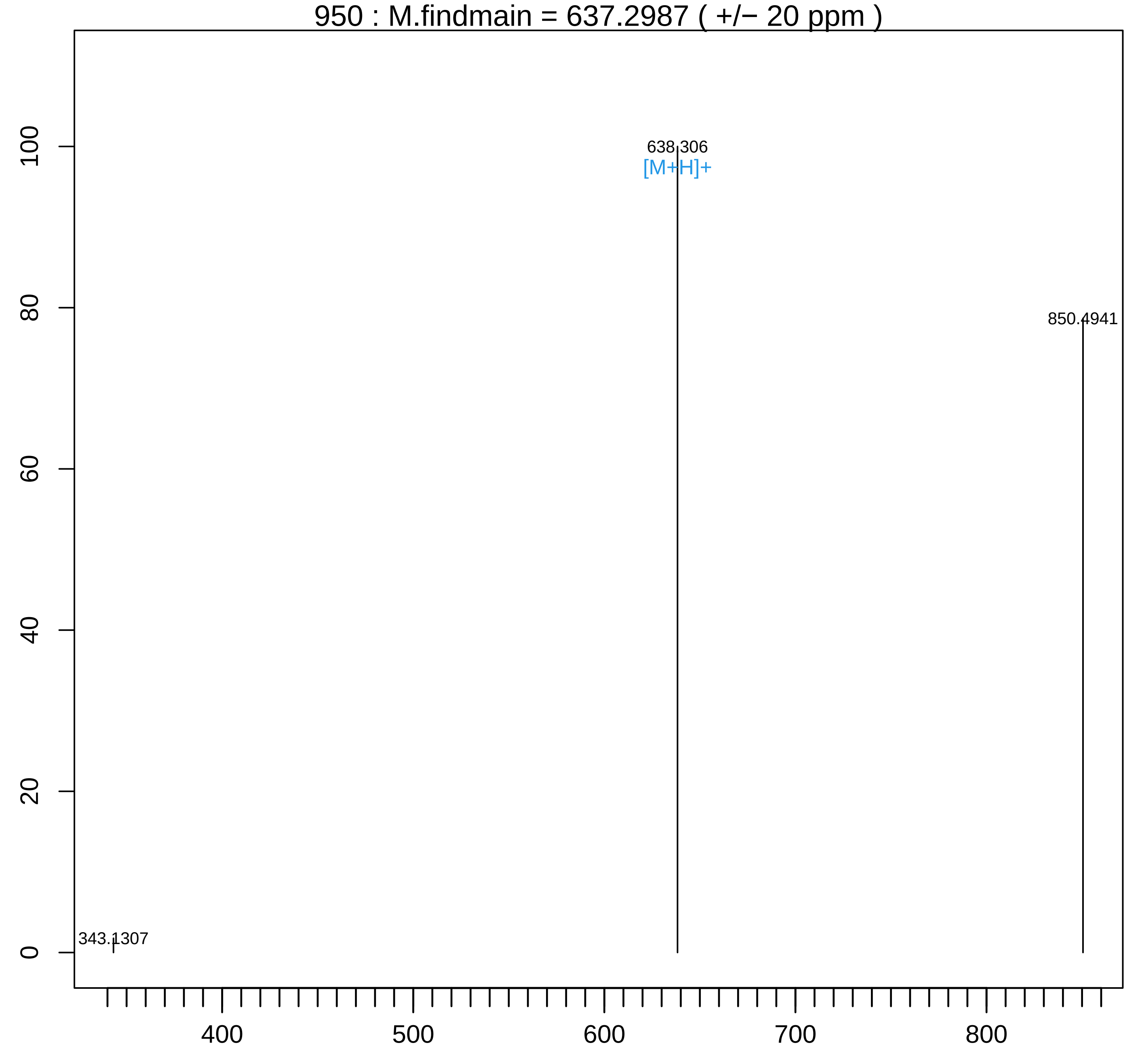
950 : M.findmain = 637.2987 (+/− 20 ppm)
638.306
[M+H]+
850.4941
343.1307
0
20
40
60
80
100
400
500
600
700
800

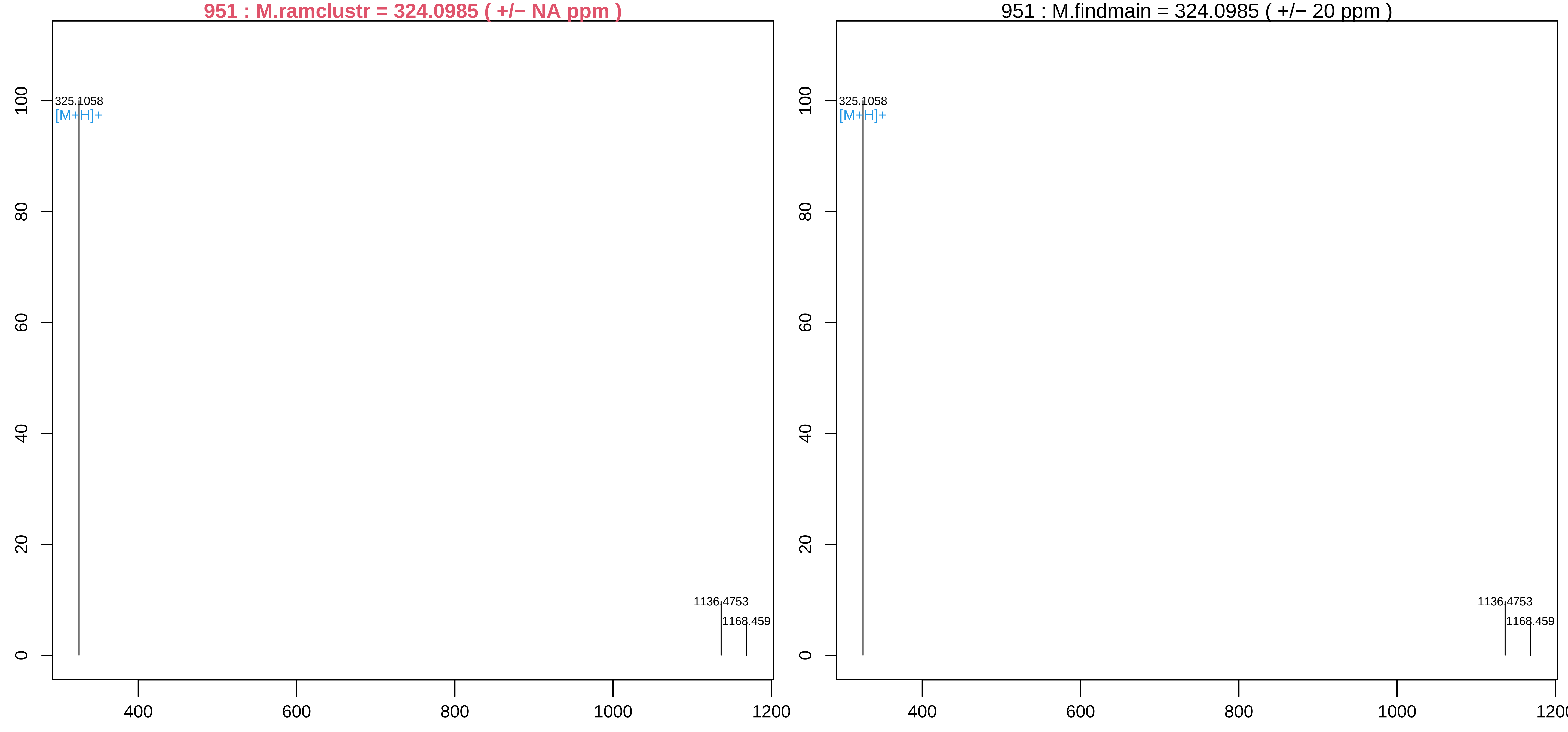

951 : M.ramclustr = 324.0985 (+/− NA ppm)
325.1058
[M+H]+
1136.4753
1168.459
951 : M.findmain = 324.0985 (+/− 20 ppm)
325.1058
[M+H]+
1136.4753
1168.459

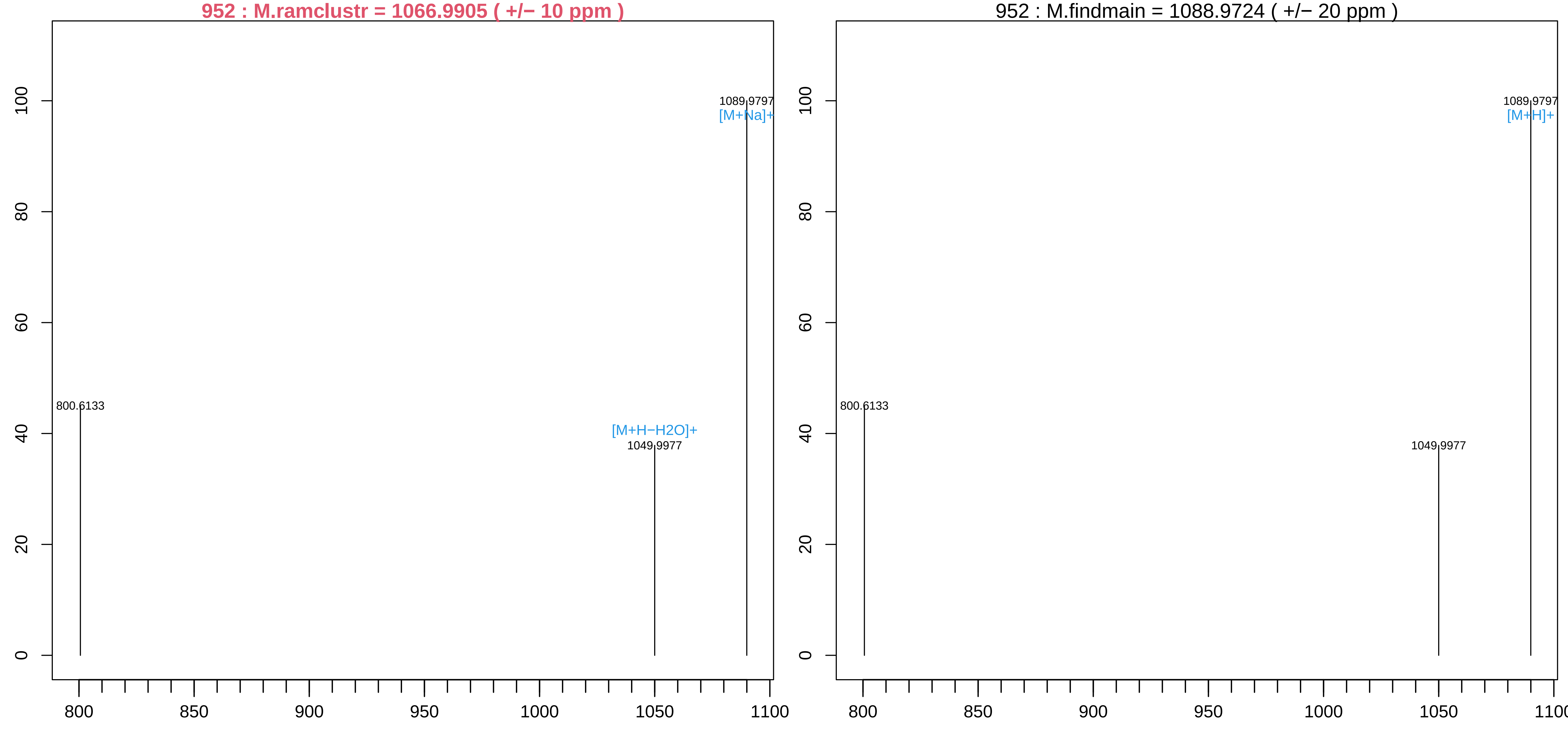
952 : M.ramclustr = 1066.9905 (+/− 10 ppm)
1089.9797
[M+Na]+
800.6133
[M+H−H2O]+
1049.9977
952 : M.findmain = 1088.9724 (+/− 20 ppm)
1089.9797
[M+H]+
800.6133
1049.9977

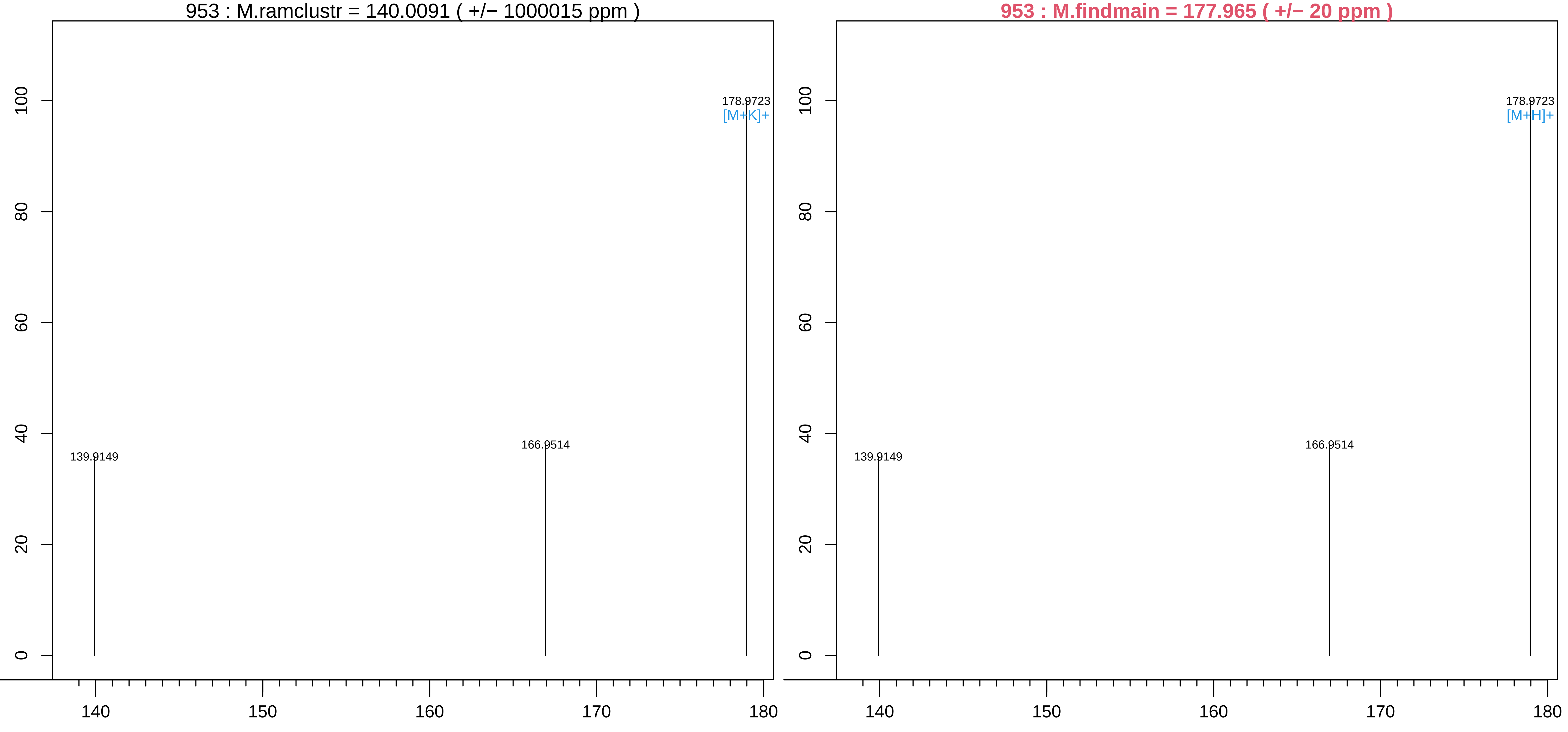

953 : M.ramclustr = 140.0091 (+/− 1000015 ppm)
178.9723
[M+K]+
139.9149
166.9514
953 : M.findmain = 177.965 (+/− 20 ppm)
178.9723
[M+H]+
139.9149
166.9514

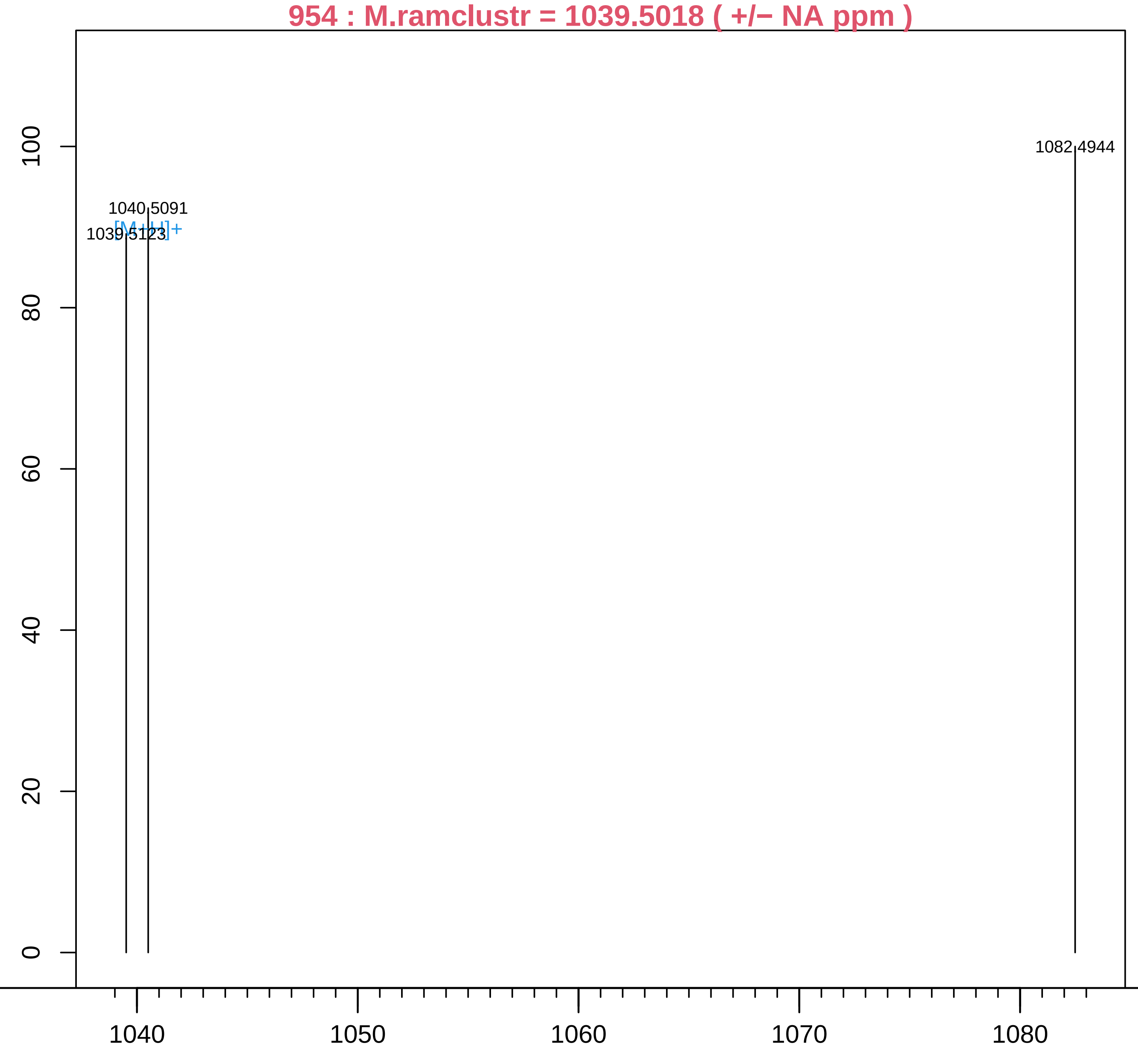
954 : M.ramclustr = 1039.5018 (+/− NA ppm)
1082.4944
1040.5091
[M+H]+
1039.5123
0
20
40
60
80
100
1040
1050
1060
1070
1080

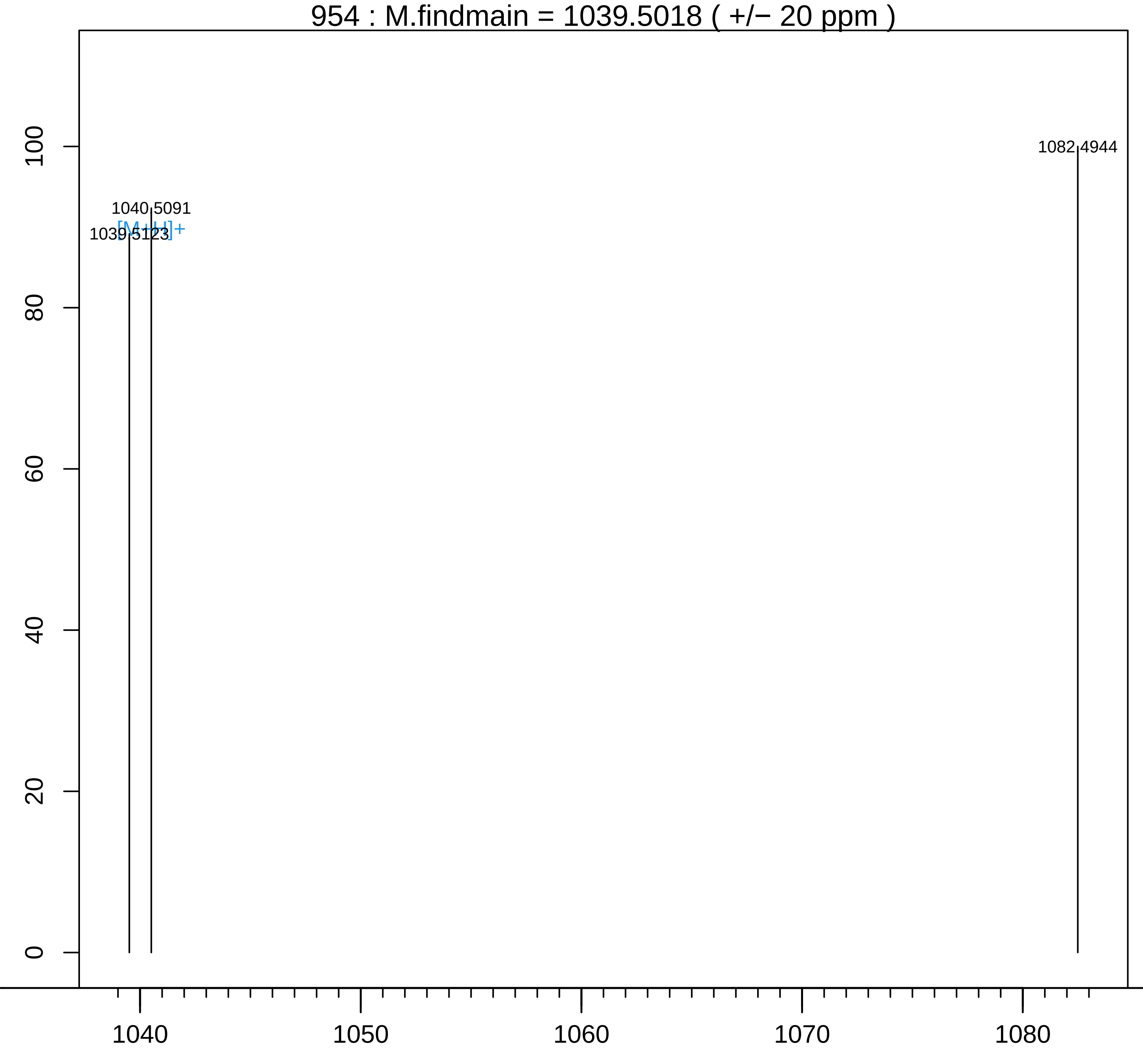
954 : M.findmain = 1039.5018 (+/− 20 ppm)
1082.4944
1040.5091
[M+H]+
1039.5123
0
20
40
60
80
100
1040
1050
1060
1070
1080

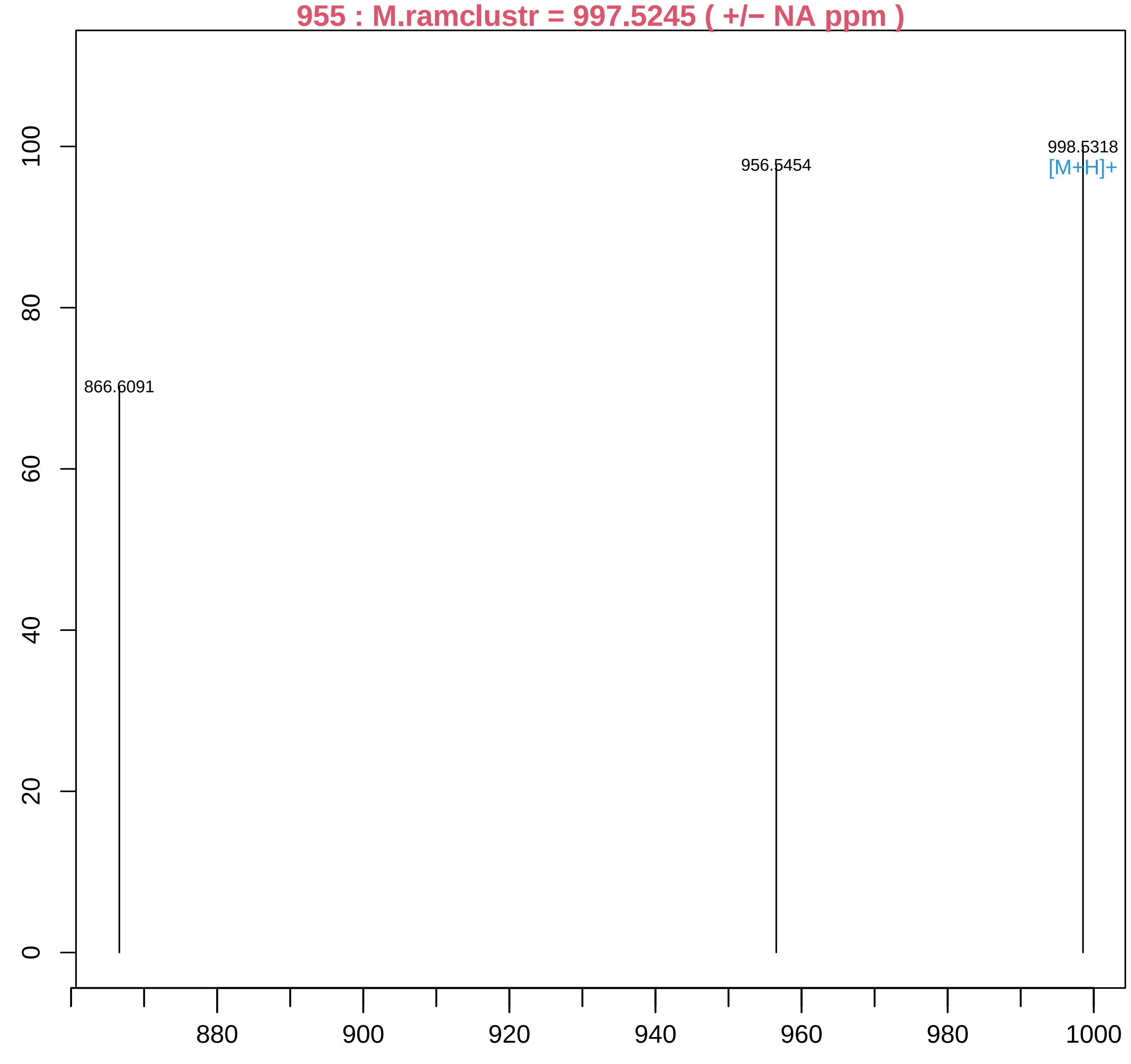
955 : M.ramclustr = 997.5245 (+/− NA ppm)
866.6091
956.5454
998.5318
[M+H]+
0
20
40
60
80
100
880
900
920
940
960
980
1000
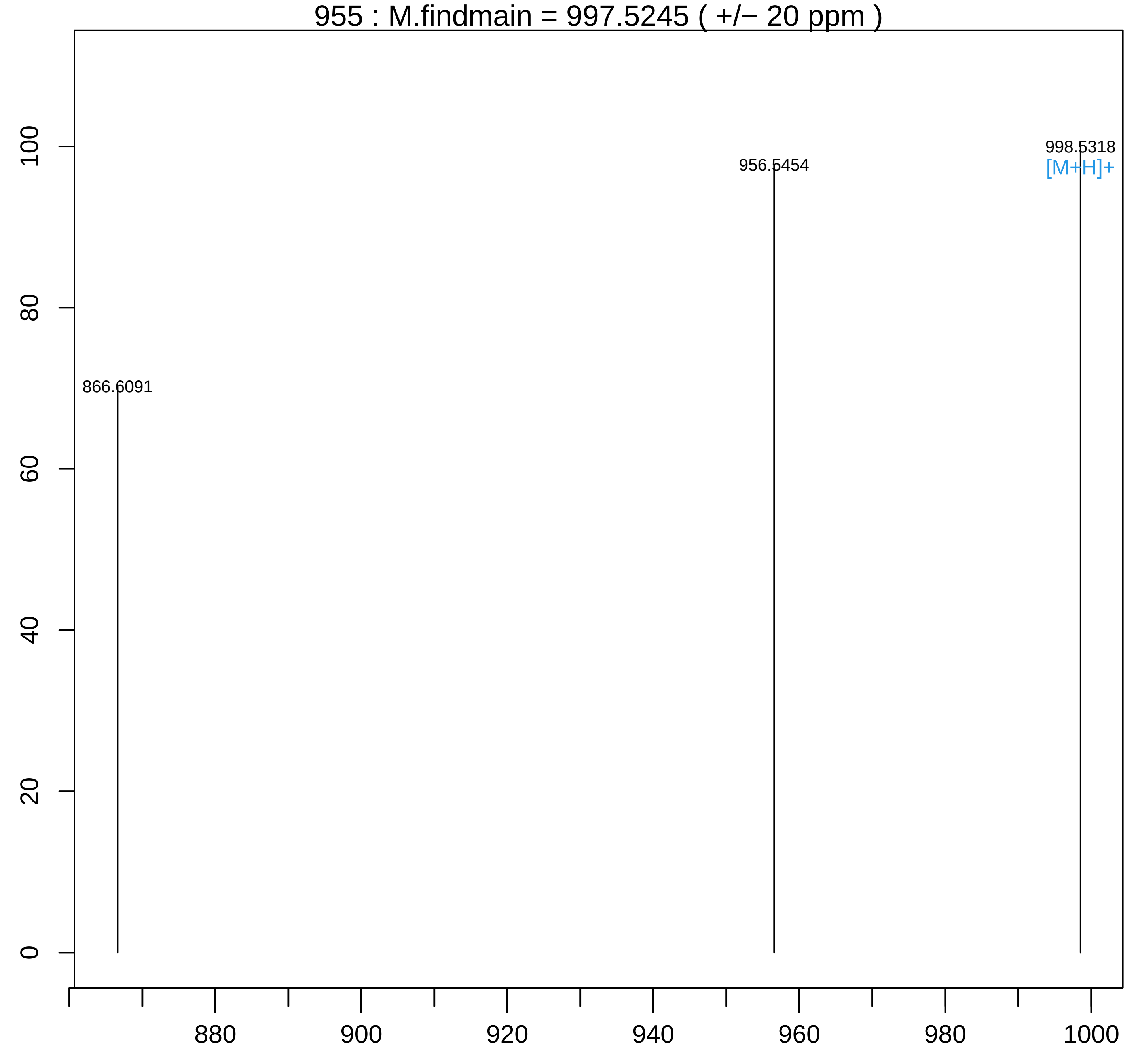
955 : M.findmain = 997.5245 (+/− 20 ppm)
866.6091
956.5454
998.5318
[M+H]+
0
20
40
60
80
100
880
900
920
940
960
980
1000

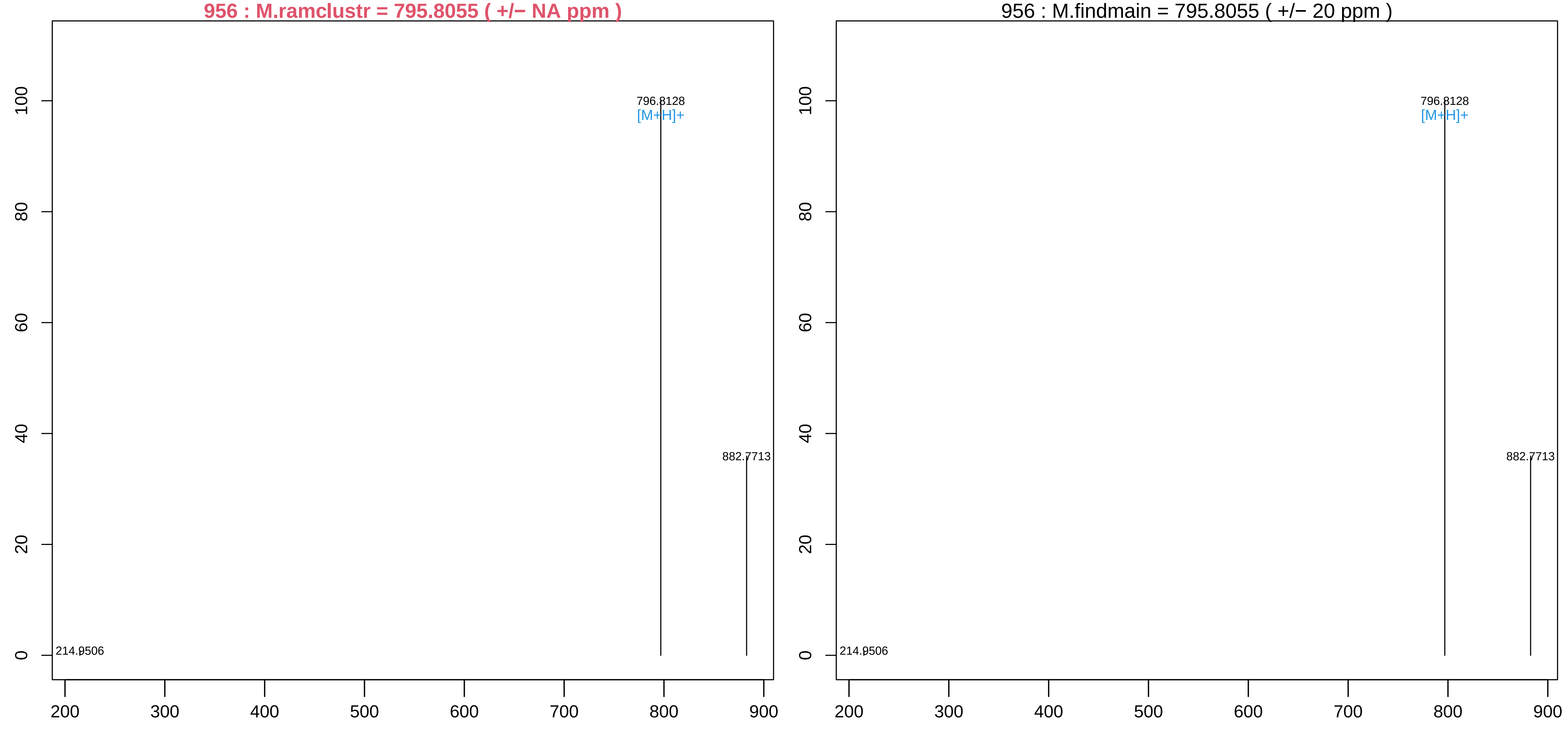
956 : M.ramclustr = 795.8055 (+/− NA ppm)
796.8128
[M+H]+
882.7713
214.9506
956 : M.findmain = 795.8055 (+/− 20 ppm)
796.8128
[M+H]+
882.7713
214.9506

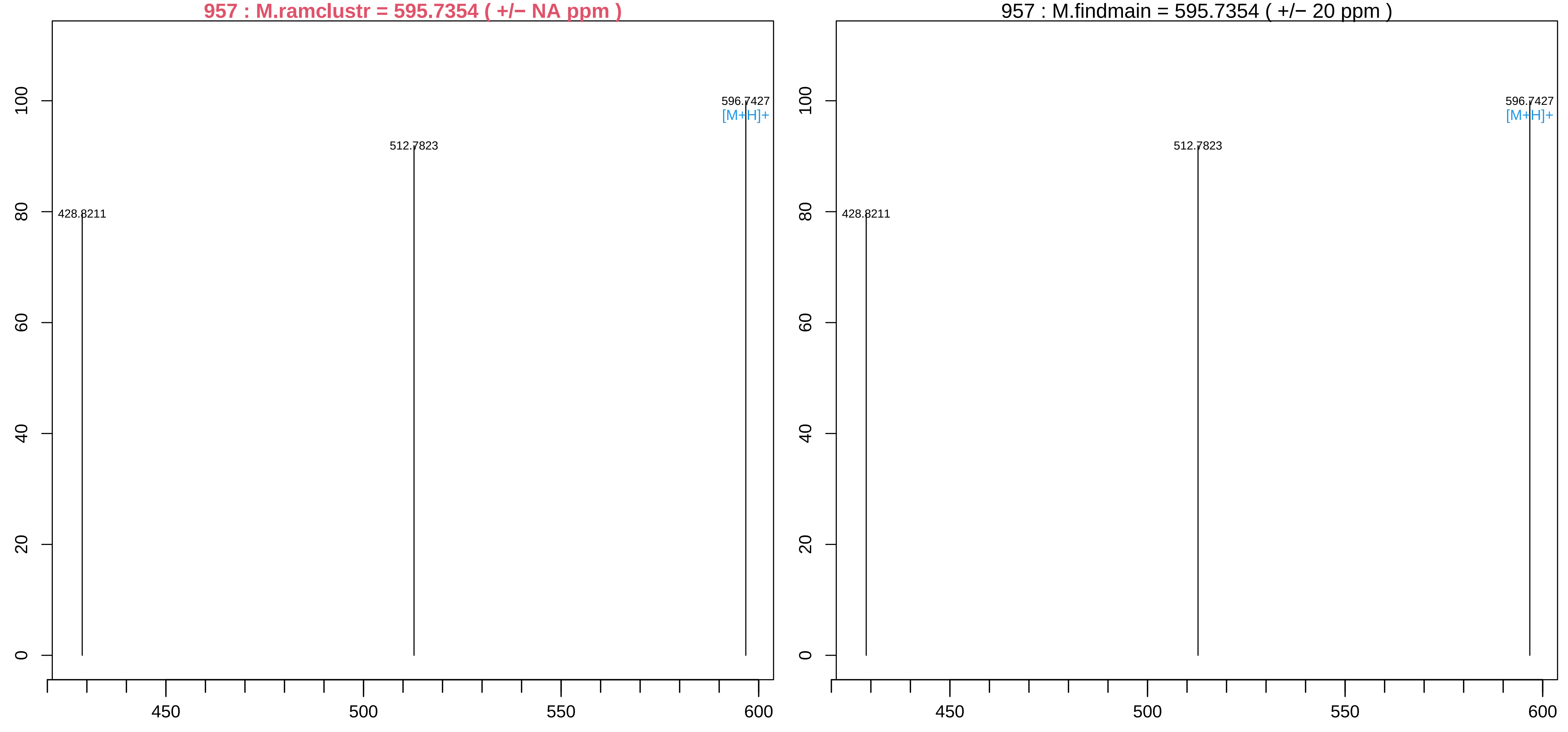

957 : M.ramclustr = 595.7354 (+/− NA ppm)
428.8211
512.7823
596.7427
[M+H]+
957 : M.findmain = 595.7354 (+/− 20 ppm)
428.8211
512.7823
596.7427
[M+H]+
0
20
40
60
80
100
450
500
550
600

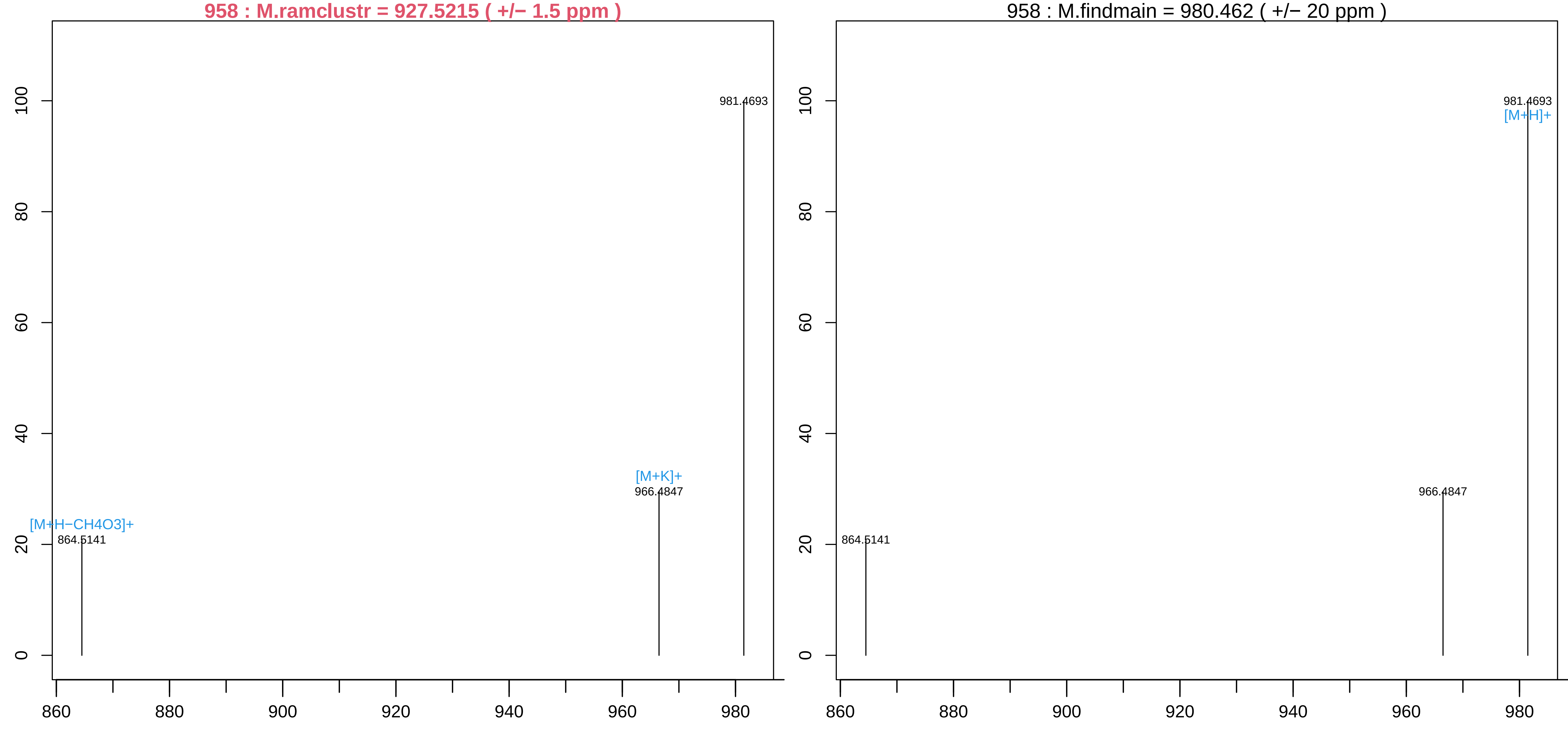
958 : M.ramclustr = 927.5215 (+/− 1.5 ppm)
981.4693
[M+K]+
966.4847
[M+H−CH4O3]+
864.5141
958 : M.findmain = 980.462 (+/− 20 ppm)
981.4693
[M+H]+
966.4847
864.5141
0
20
40
60
80
100
860
880
900
920
940
960
980

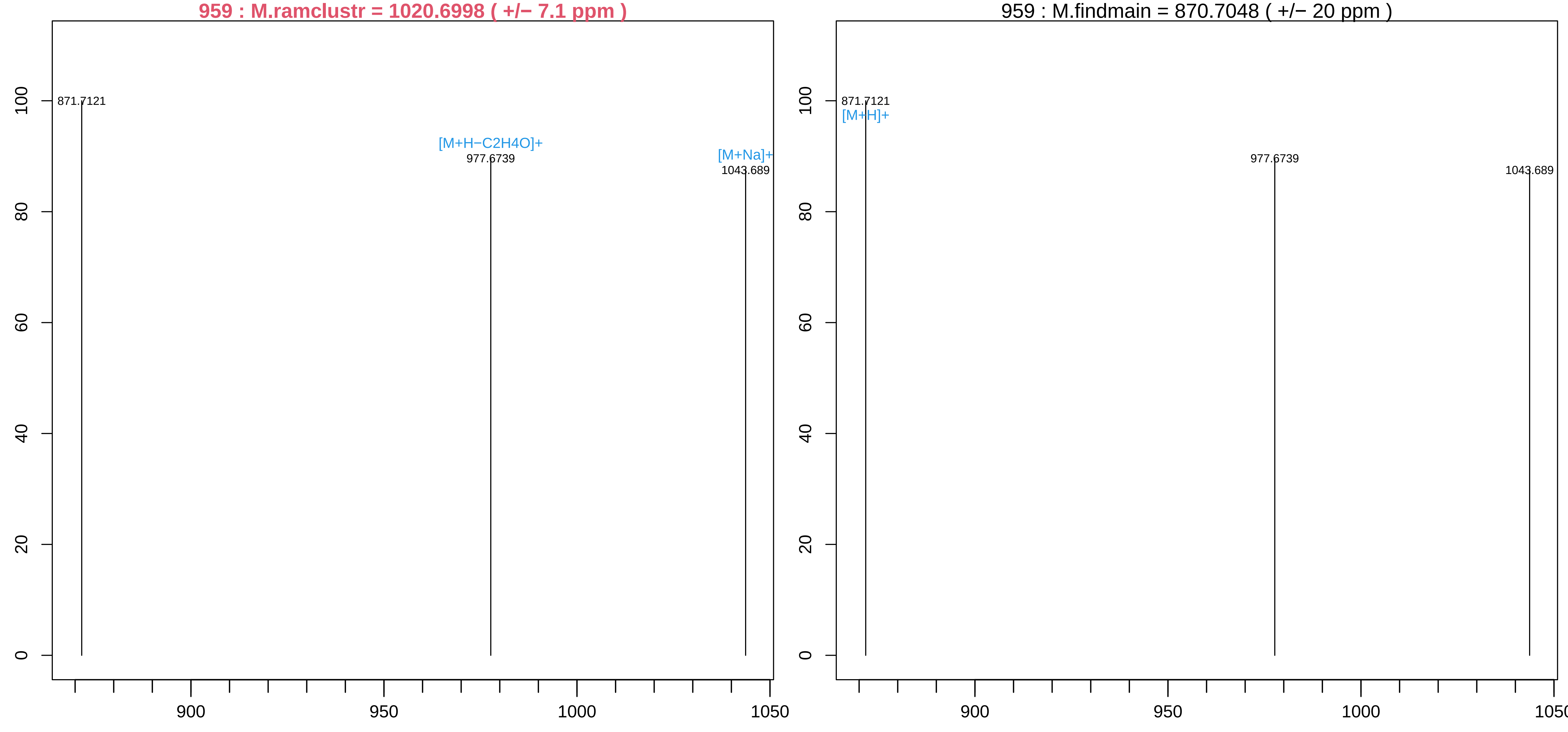
959 : M.ramclustr = 1020.6998 (+/− 7.1 ppm)
871.7121
[M+H−C2H4O]+
977.6739
[M+Na]+
1043.689
959 : M.findmain = 870.7048 (+/− 20 ppm)
871.7121
[M+H]+
977.6739
1043.689
0
20
40
60
80
100
900
950
1000
1050

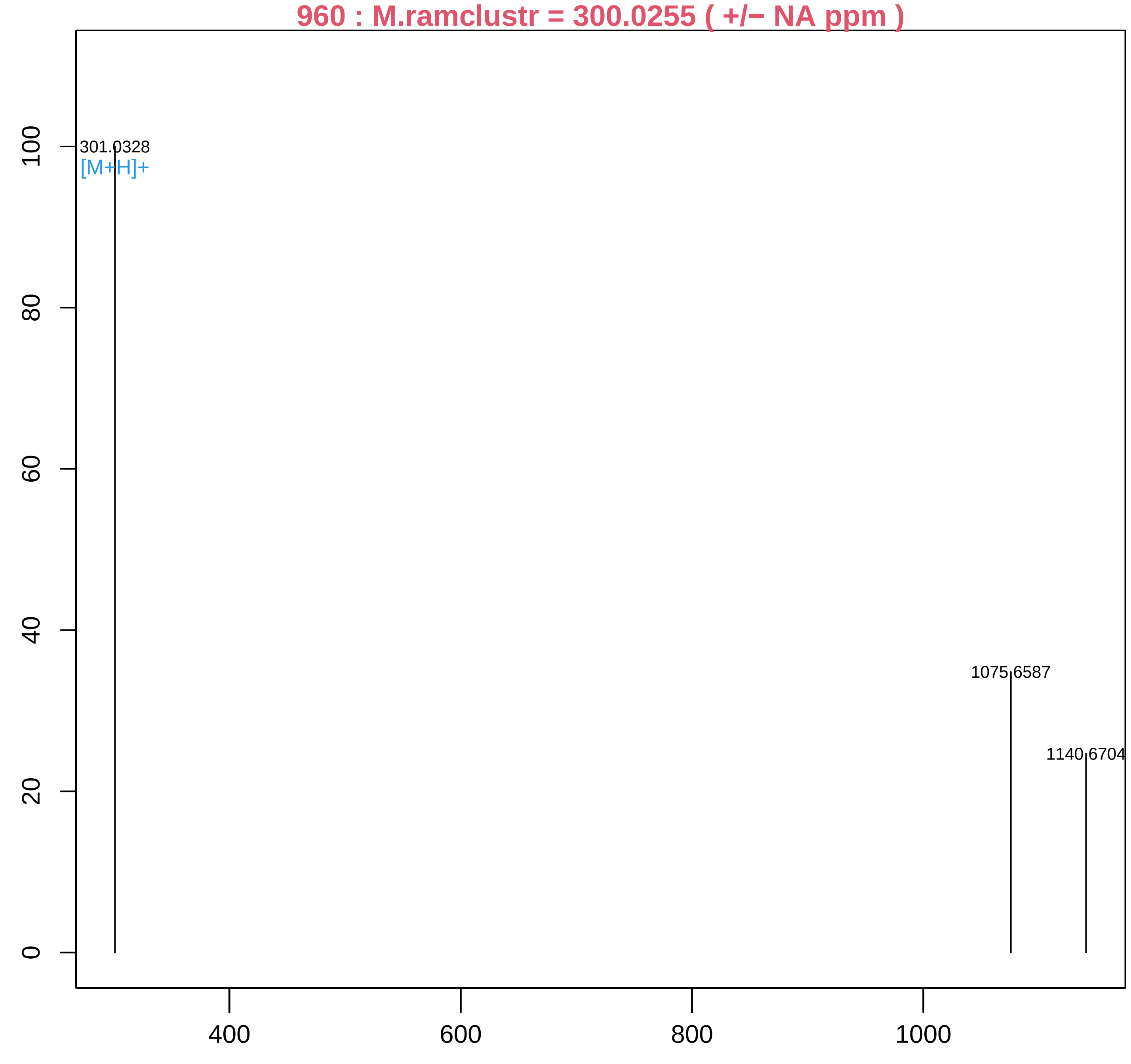

960 : M.ramclustr = 300.0255 (+/− NA ppm)
301.0328
[M+H]+
1075.6587
1140.6704
0
20
40
60
80
100
400
600
800
1000

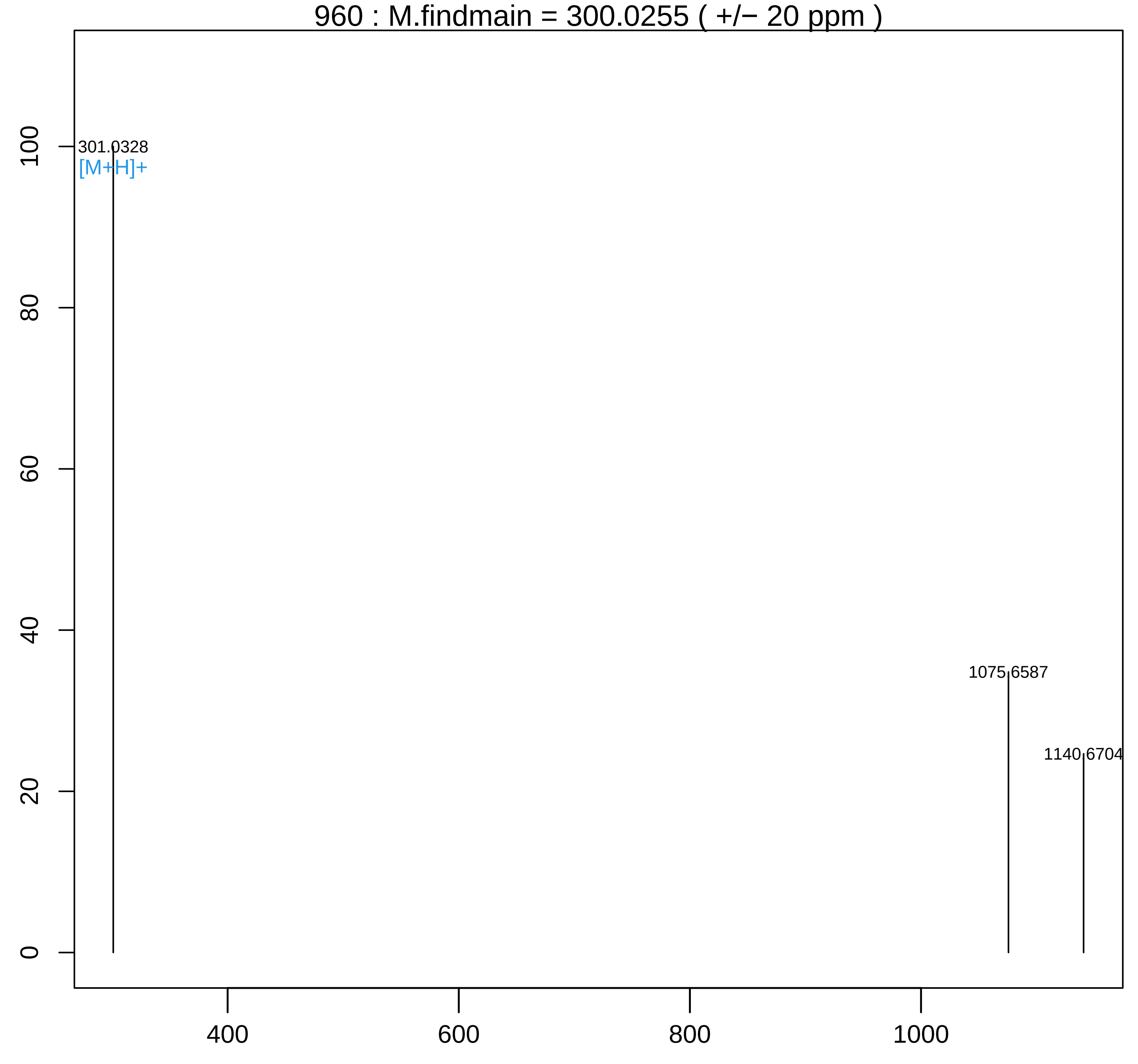

960 : M.findmain = 300.0255 (+/− 20 ppm)
301.0328
[M+H]+
1075.6587
1140.6704
0
20
40
60
80
100
400
600
800
1000

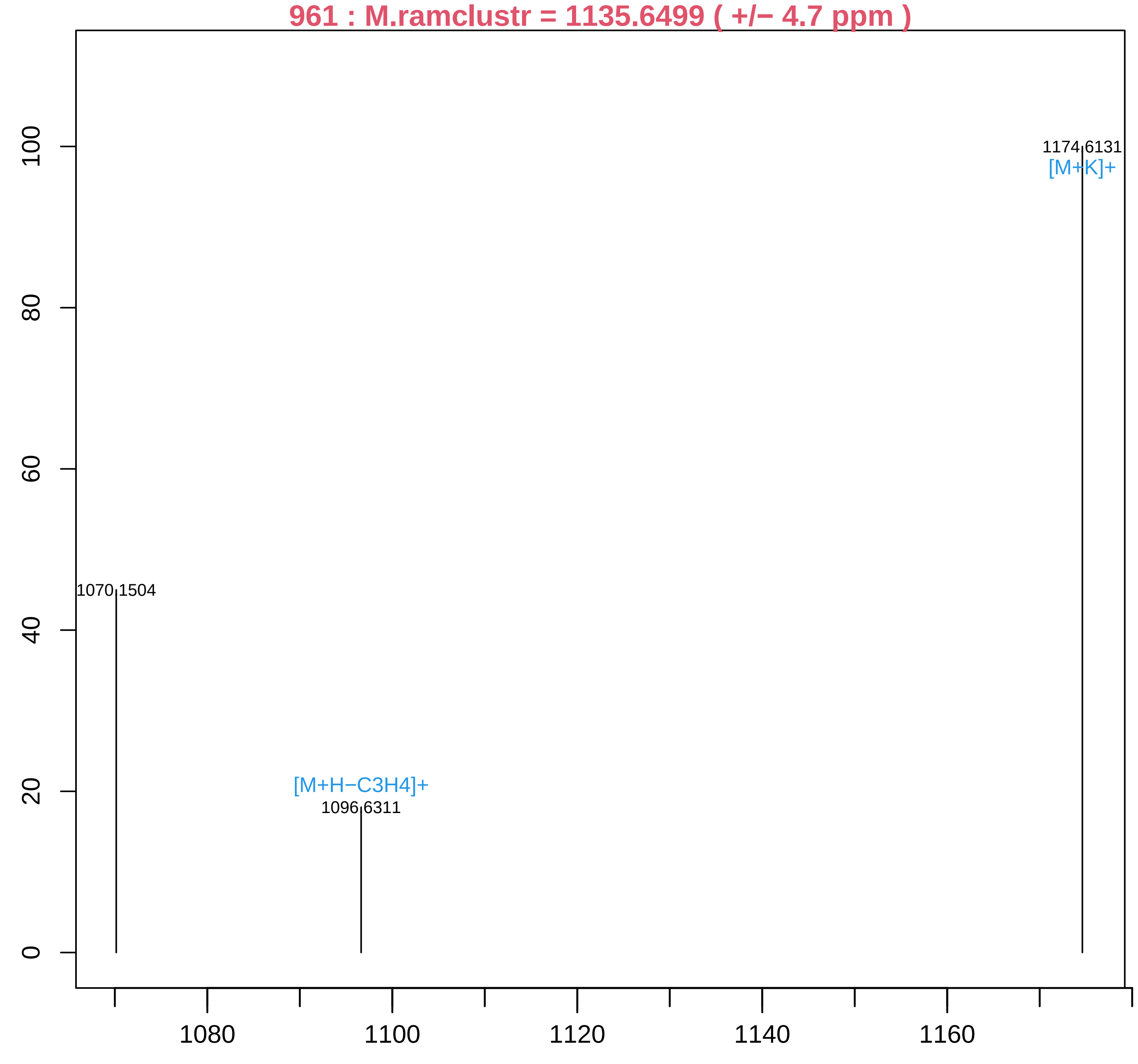
961 : M.ramclustr = 1135.6499 (+/− 4.7 ppm)
1174.6131
[M+K]+
1070.1504
[M+H−C3H4]+
1096.6311
0
20
40
60
80
100
1080
1100
1120
1140
1160

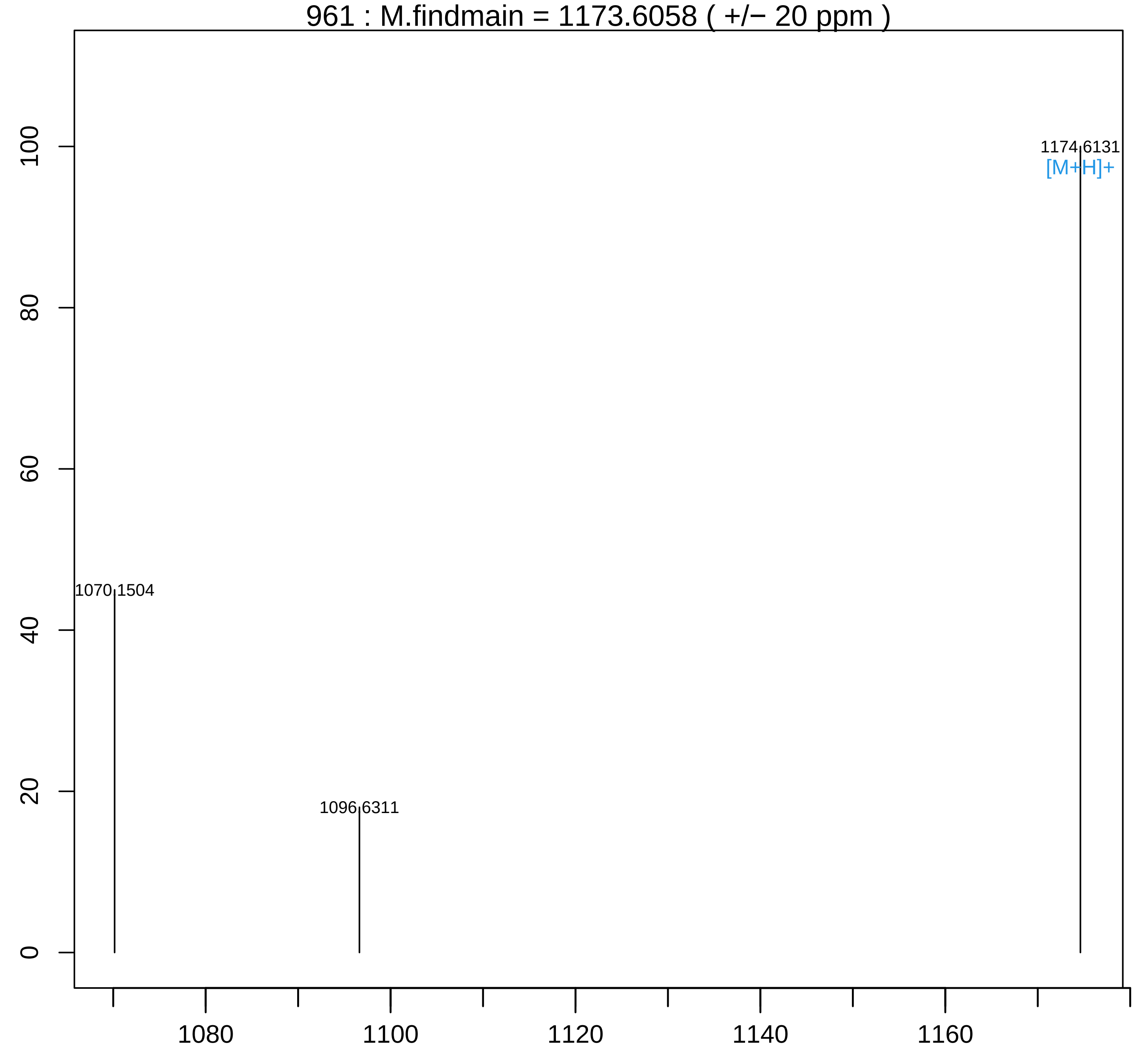
961 : M.findmain = 1173.6058 (+/− 20 ppm)
1174.6131
[M+H]+
1070.1504
1096.6311
0
20
40
60
80
100
1080
1100
1120
1140
1160

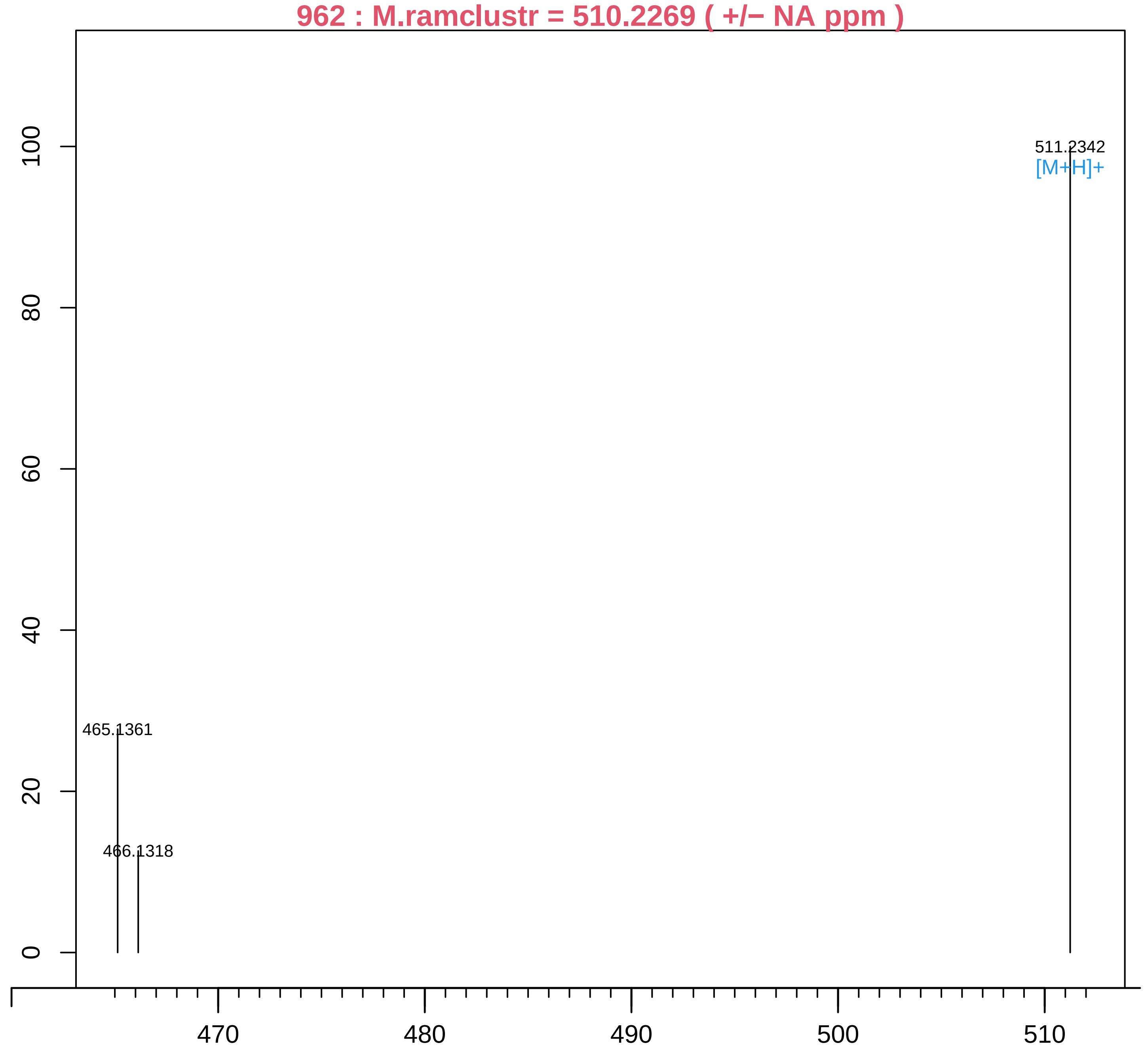
962 : M.ramclustr = 510.2269 (+/− NA ppm)
511.2342
[M+H]+
465.1361
466.1318
0
20
40
60
80
100
470
480
490
500
510

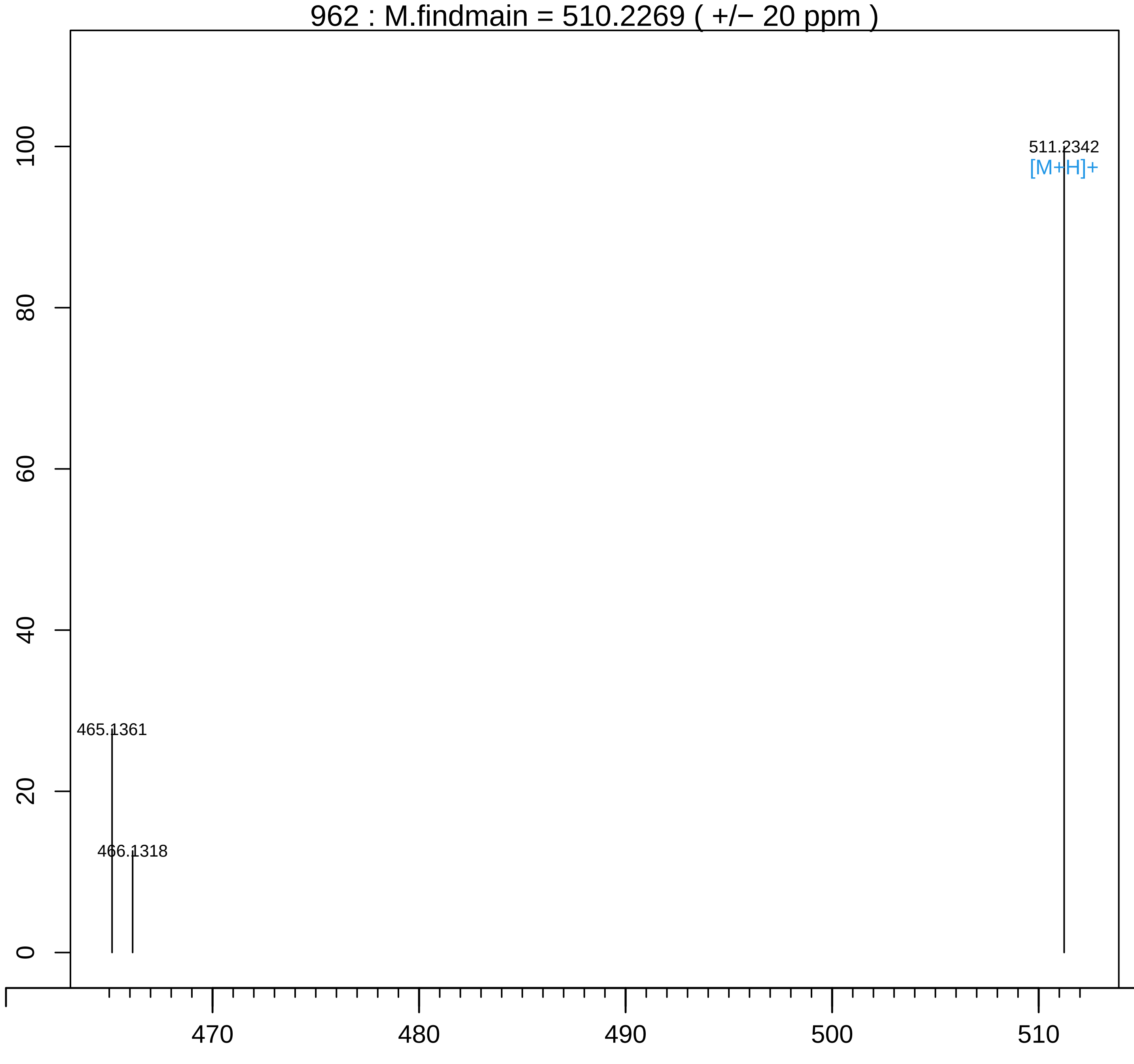
962 : M.findmain = 510.2269 (+/− 20 ppm)
511.2342
[M+H]+
465.1361
466.1318
0
20
40
60
80
100
470
480
490
500
510

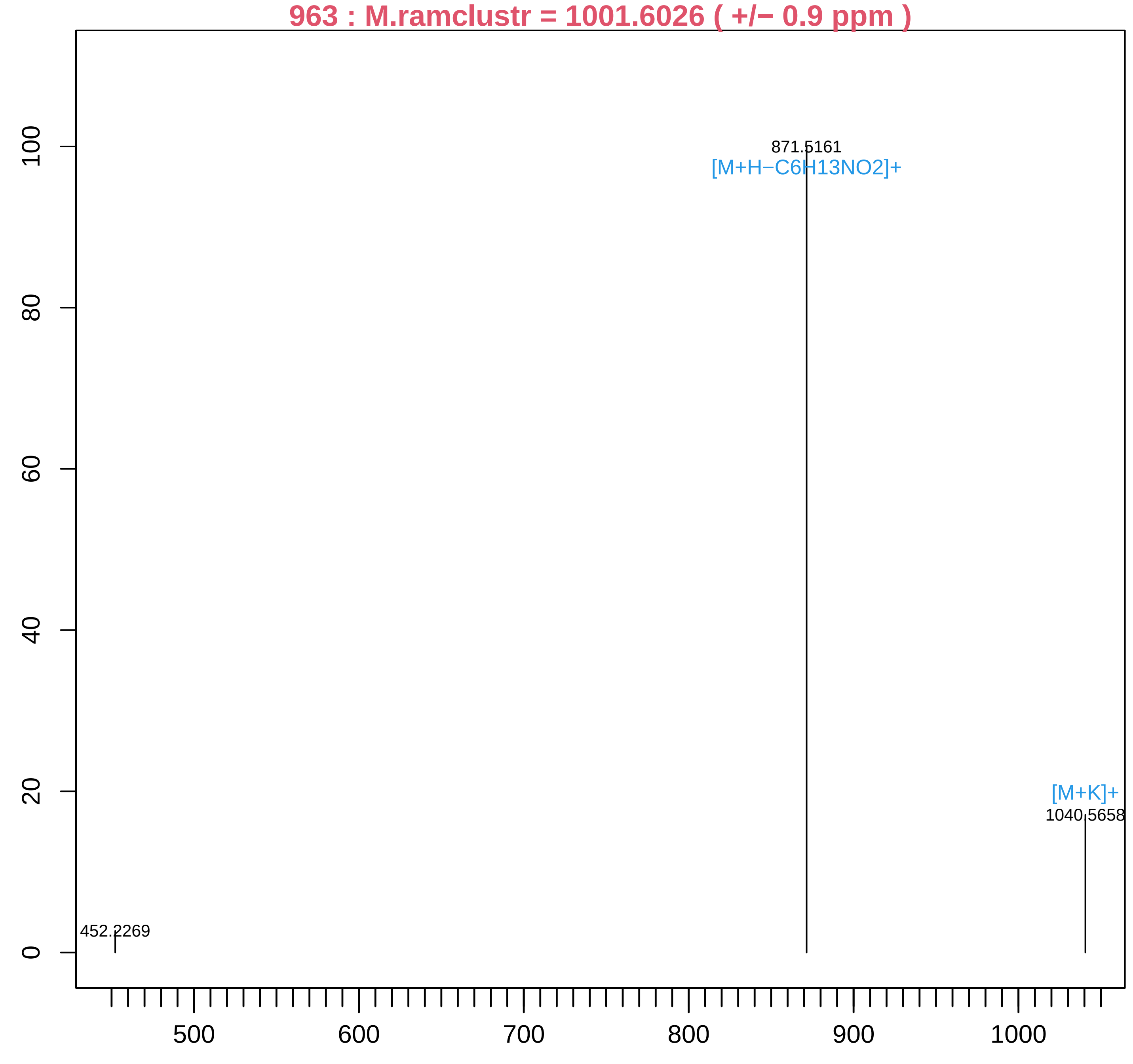
963 : M.ramclustr = 1001.6026 (+/− 0.9 ppm)
871.5161
[M+H−C6H13NO2]+
[M+K]+
1040.5658
452.2269
0
20
40
60
80
100
500
600
700
800
900
1000

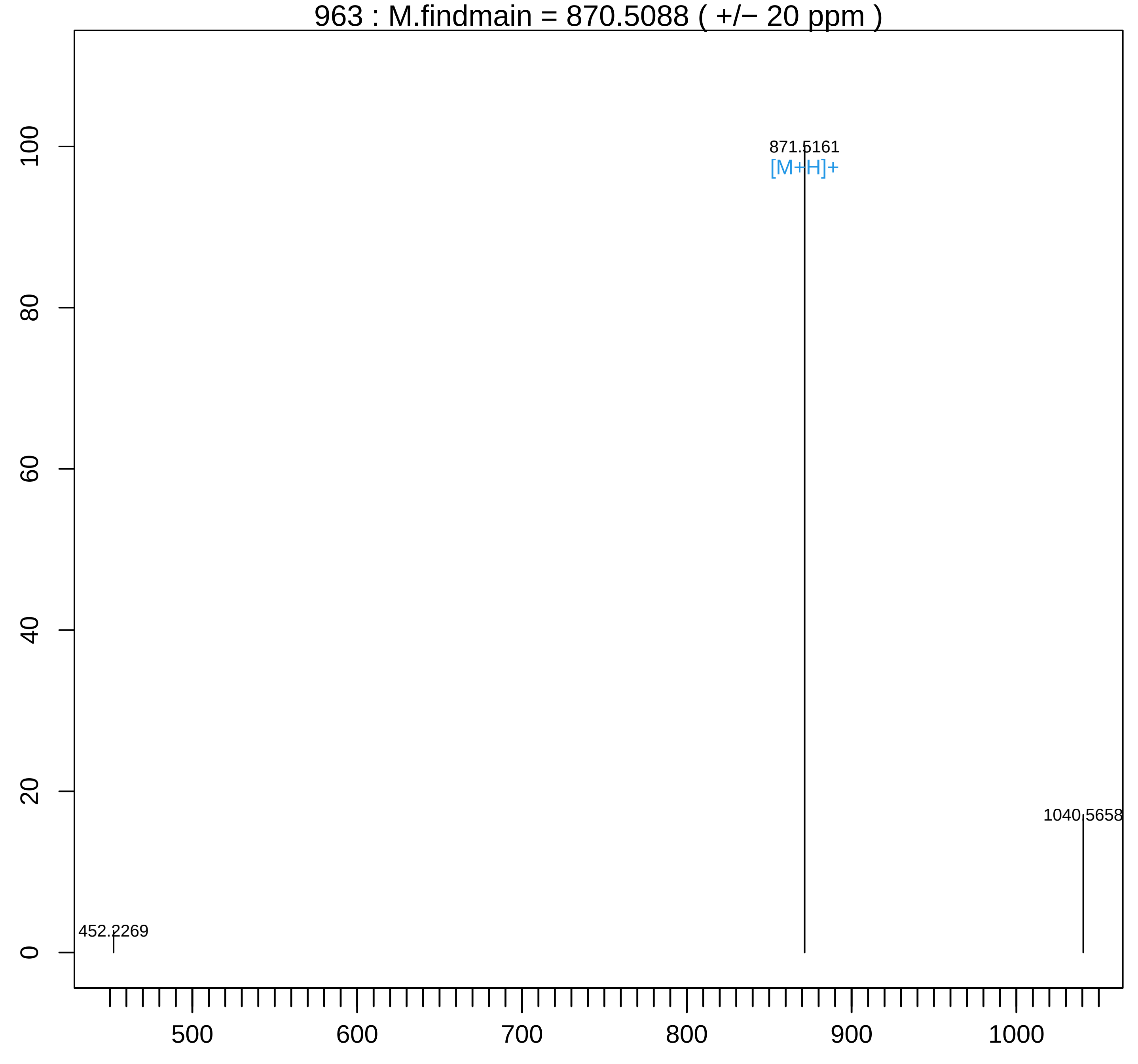
963 : M.findmain = 870.5088 (+/− 20 ppm)
871.5161
[M+H]+
1040.5658
452.2269
0
20
40
60
80
100
500
600
700
800
900
1000

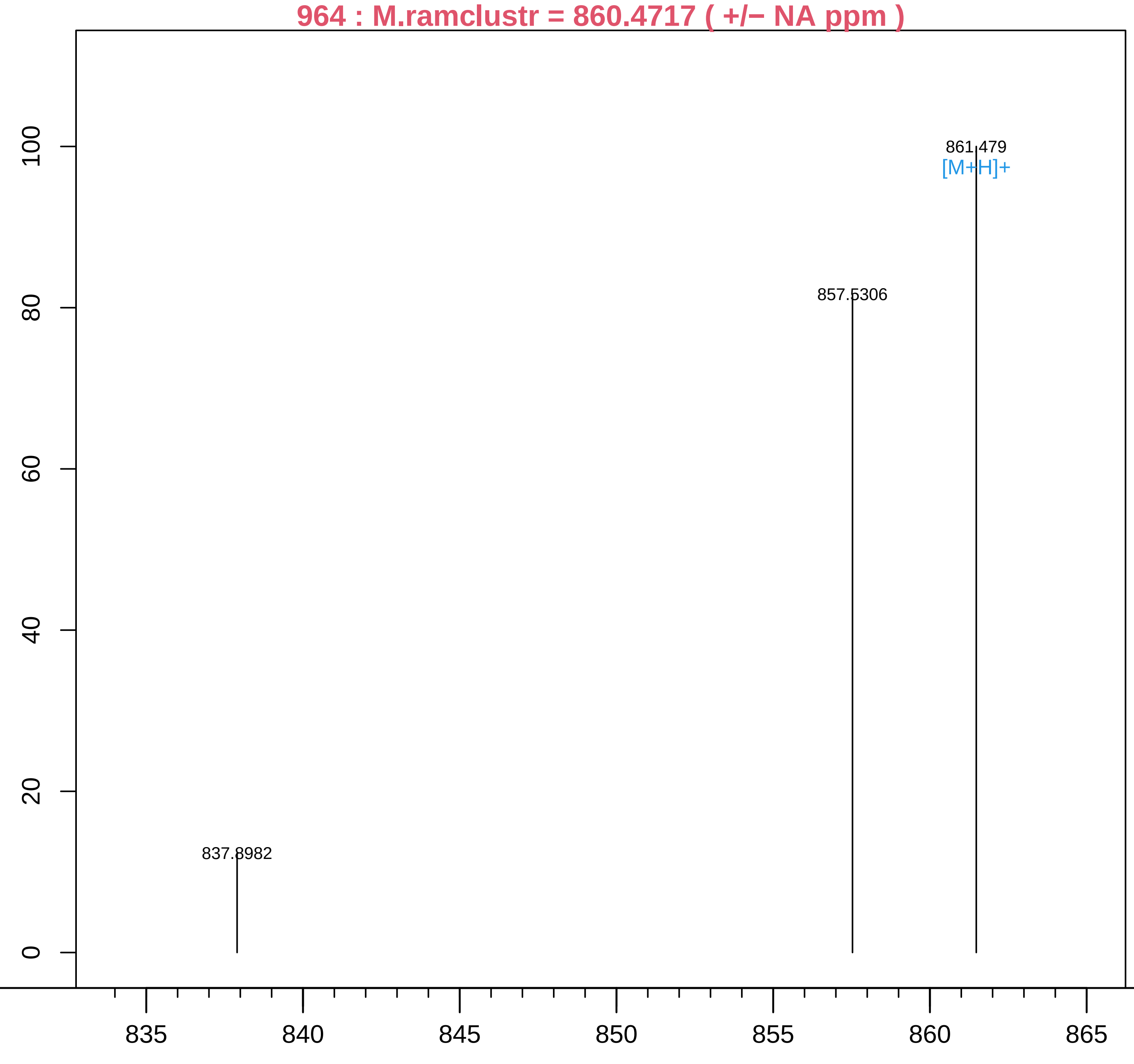
964 : M.ramclustr = 860.4717 (+/− NA ppm)
861.479
[M+H]+
857.5306
837.8982
0
20
40
60
80
100
835
840
845
850
855
860
865

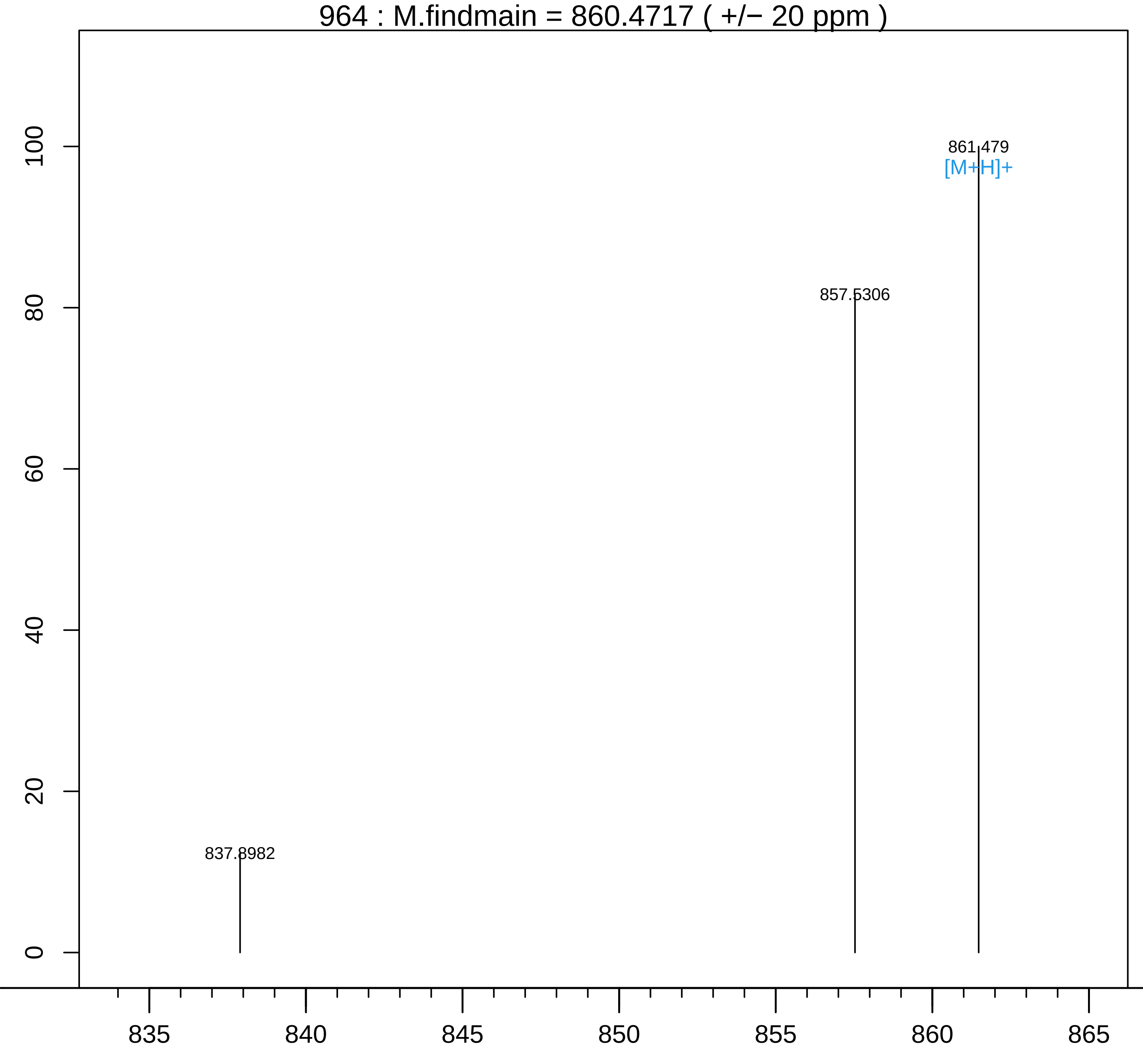
964 : M.findmain = 860.4717 (+/− 20 ppm)
861.479
[M+H]+
857.5306
837.8982
0
20
40
60
80
100
835
840
845
850
855
860
865

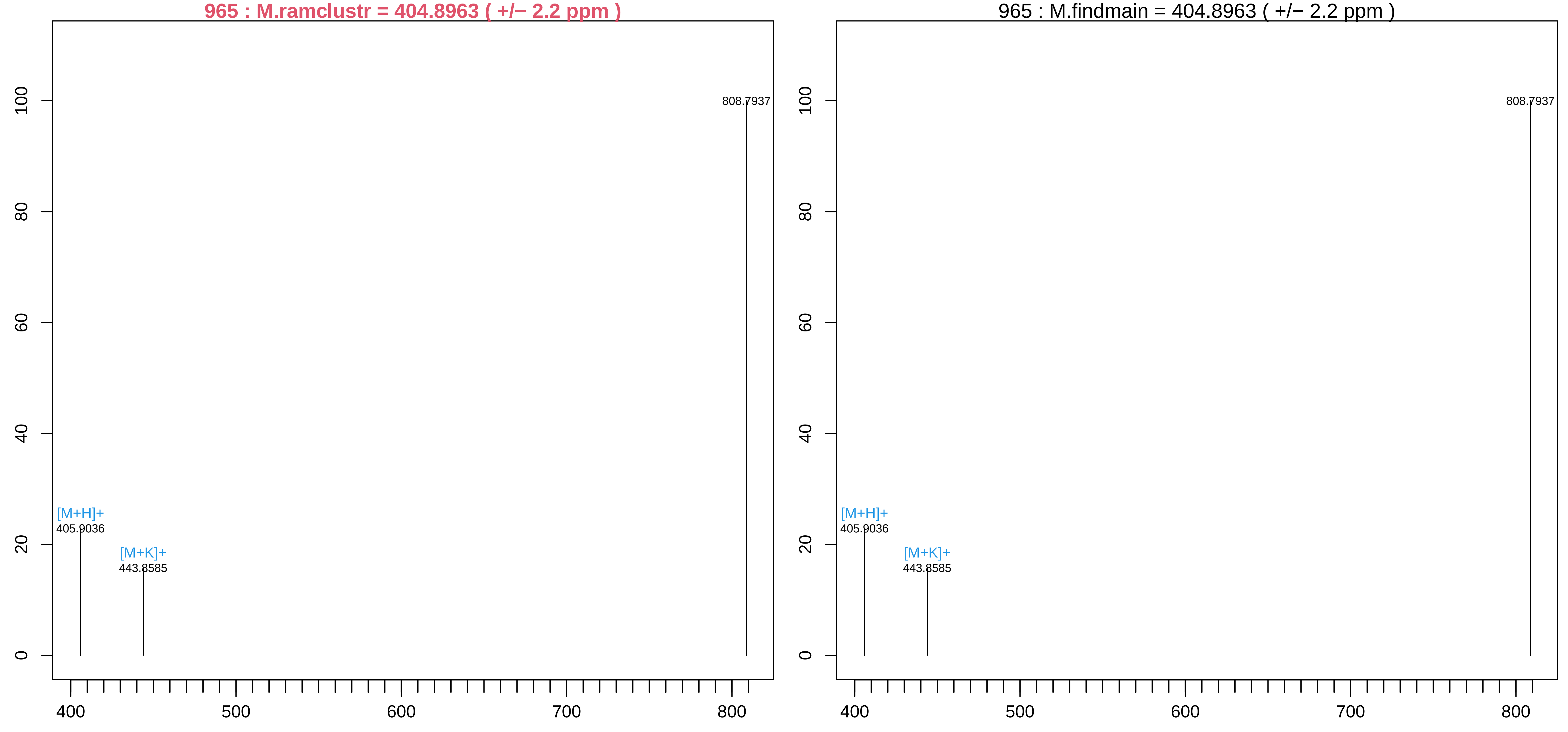
965 : M.ramclustr = 404.8963 (+/− 2.2 ppm)
965 : M.findmain = 404.8963 (+/− 2.2 ppm)
808.7937
[M+H]+
405.9036
[M+K]+
443.8585
0
20
40
60
80
100
400
500
600
700
800

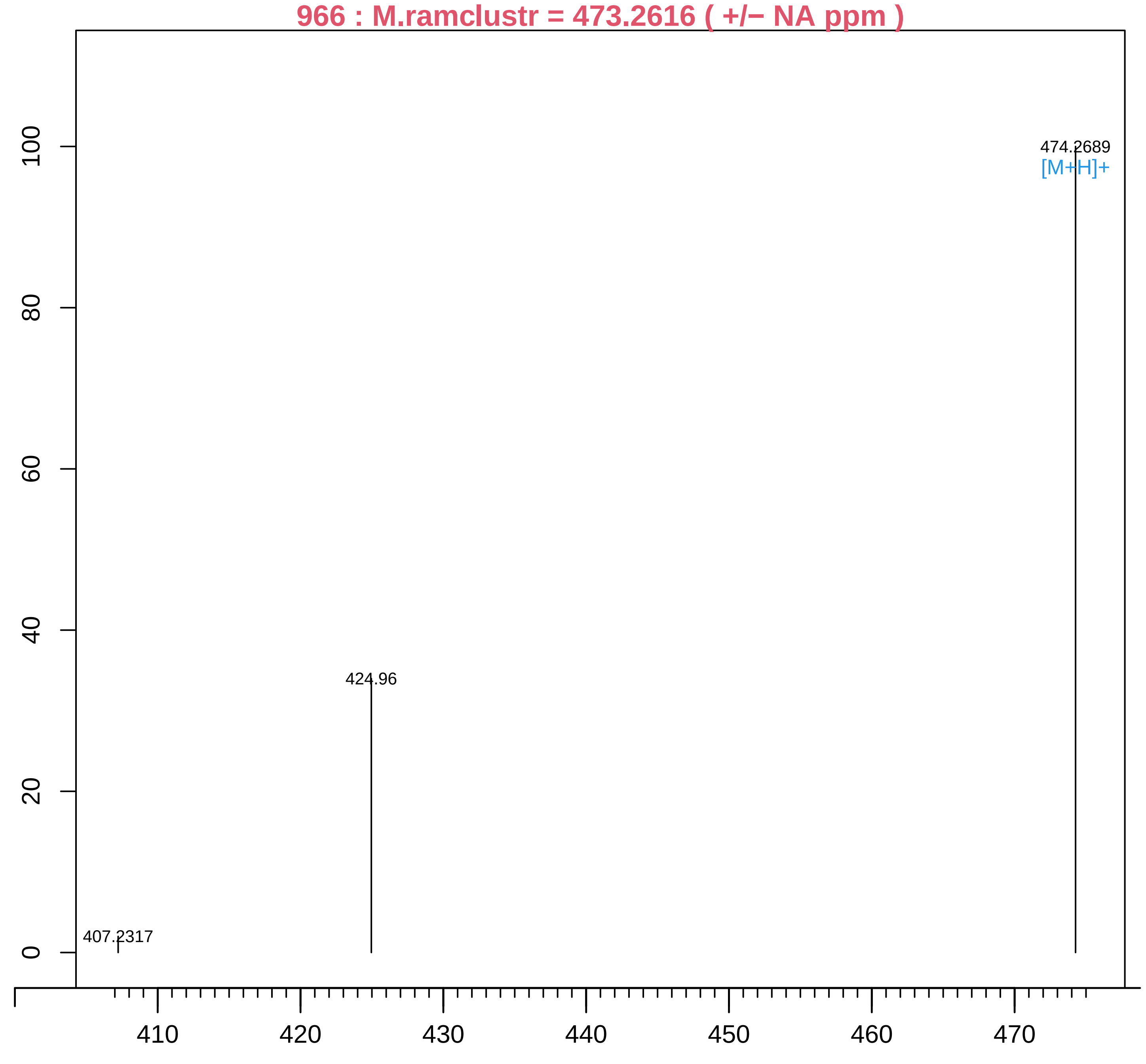
966 : M.ramclustr = 473.2616 (+/− NA ppm)
474.2689
[M+H]+
424.96
407.2317
0
20
40
60
80
100
410
420
430
440
450
460
470

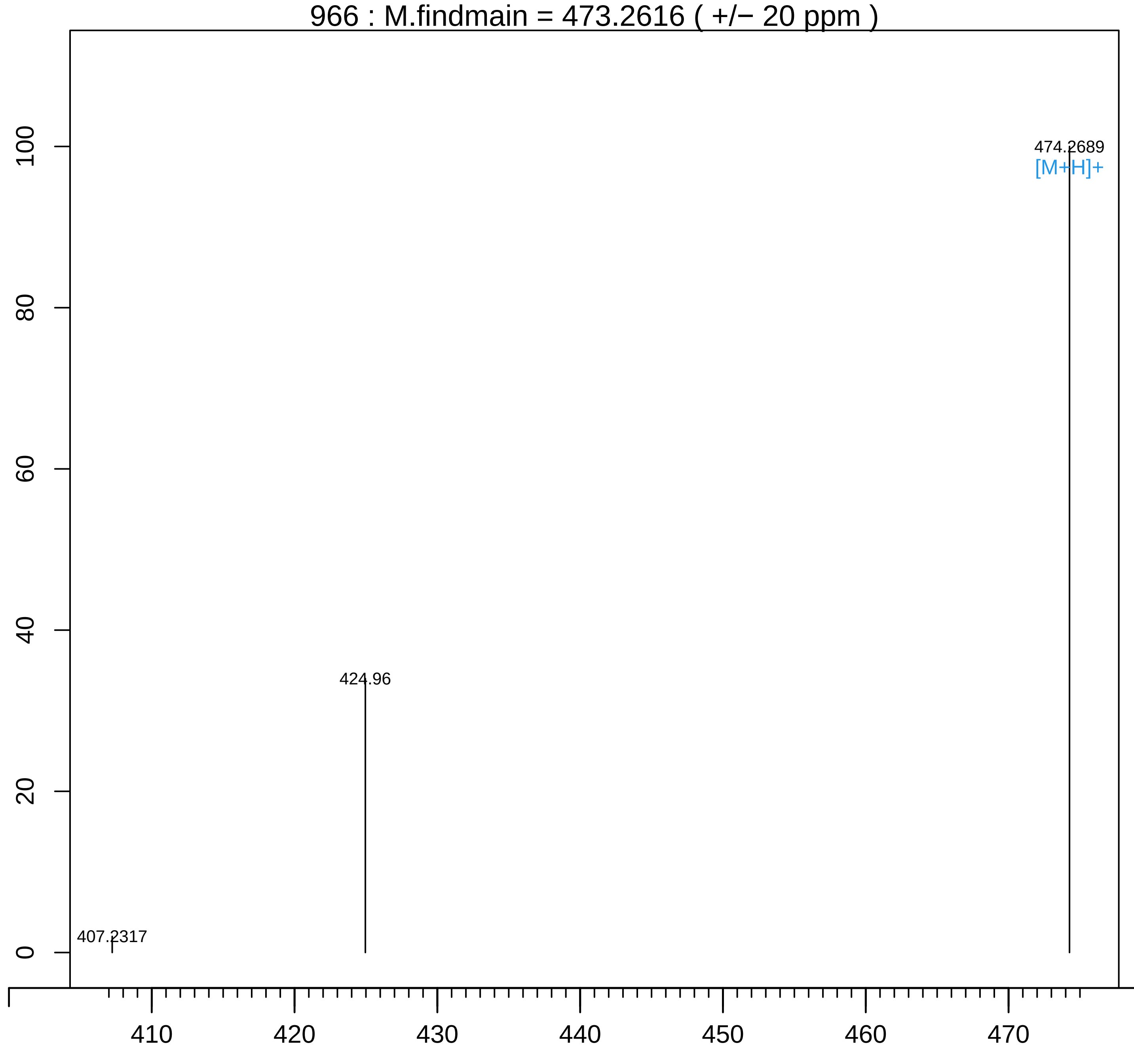
966 : M.findmain = 473.2616 (+/− 20 ppm)
474.2689
[M+H]+
424.96
407.2317
0
20
40
60
80
100
410
420
430
440
450
460
470

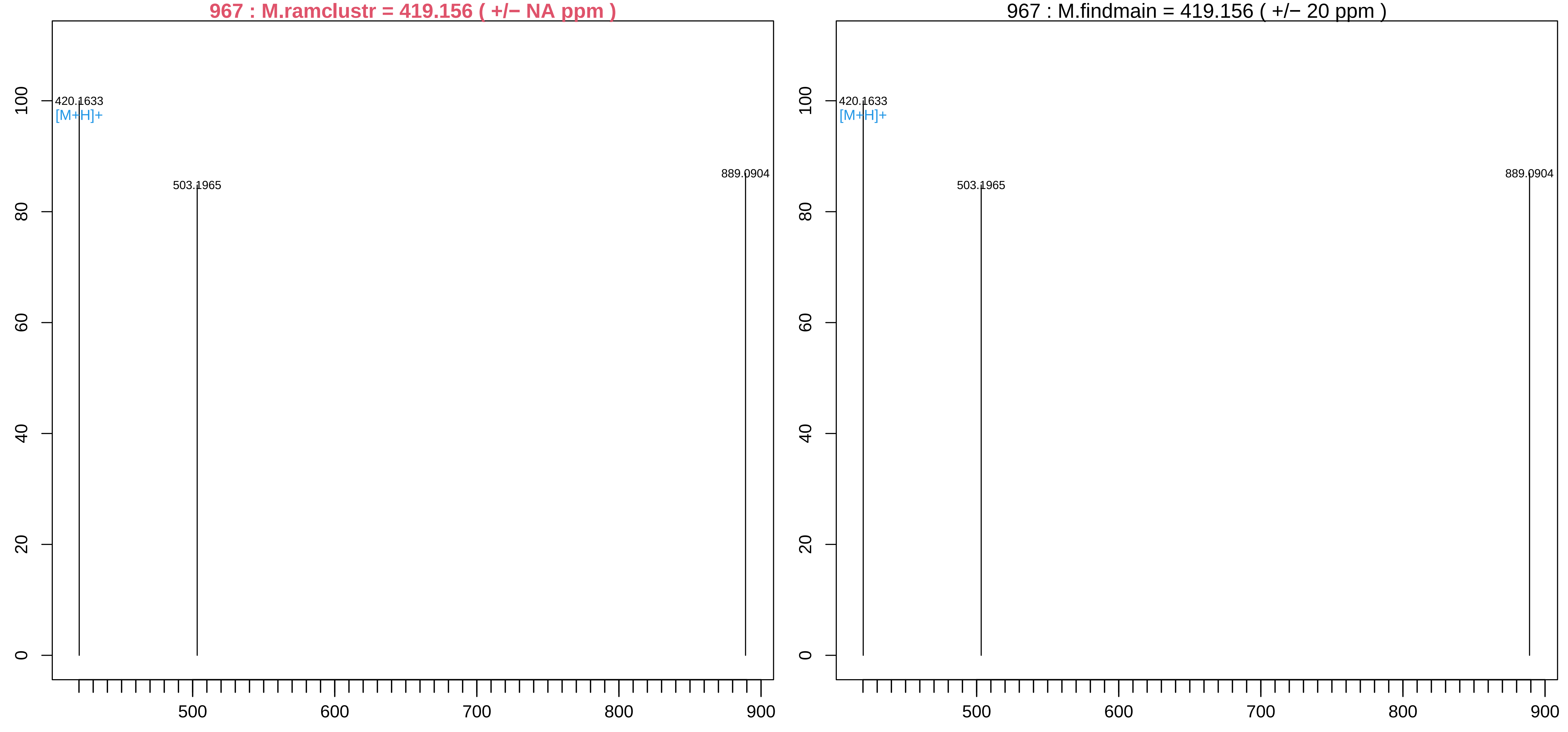

967 : M.ramclustr = 419.156 (+/− NA ppm)
420.1633
[M+H]+
503.1965
889.0904
0
20
40
60
80
100
500
600
700
800
900
967 : M.findmain = 419.156 (+/− 20 ppm)
420.1633
[M+H]+
503.1965
889.0904
0
20
40
60
80
100
500
600
700
800
900

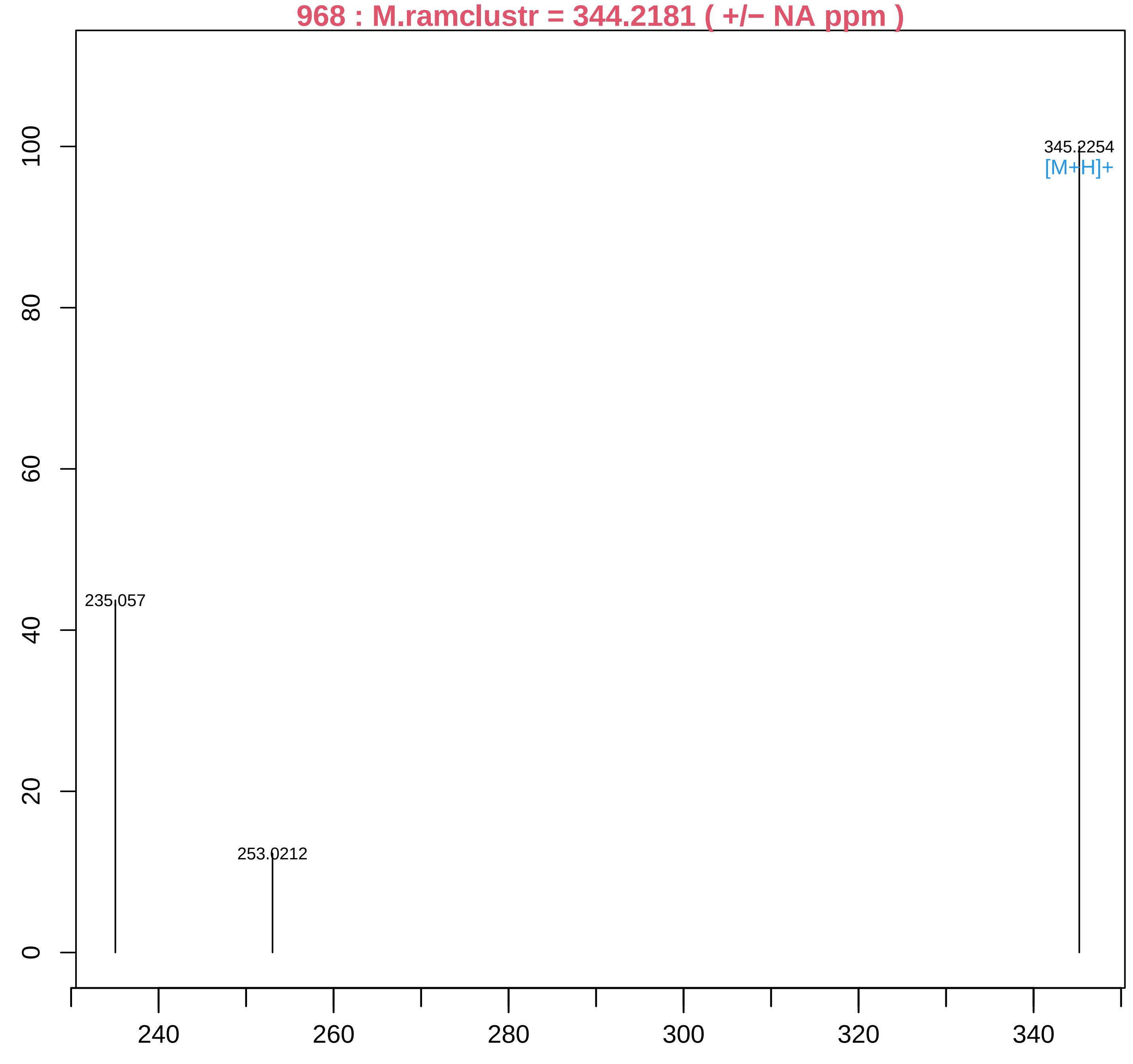
968 : M.ramclustr = 344.2181 (+/− NA ppm)
345.2254
[M+H]+
235.057
253.0212
0
20
40
60
80
100
240
260
280
300
320
340

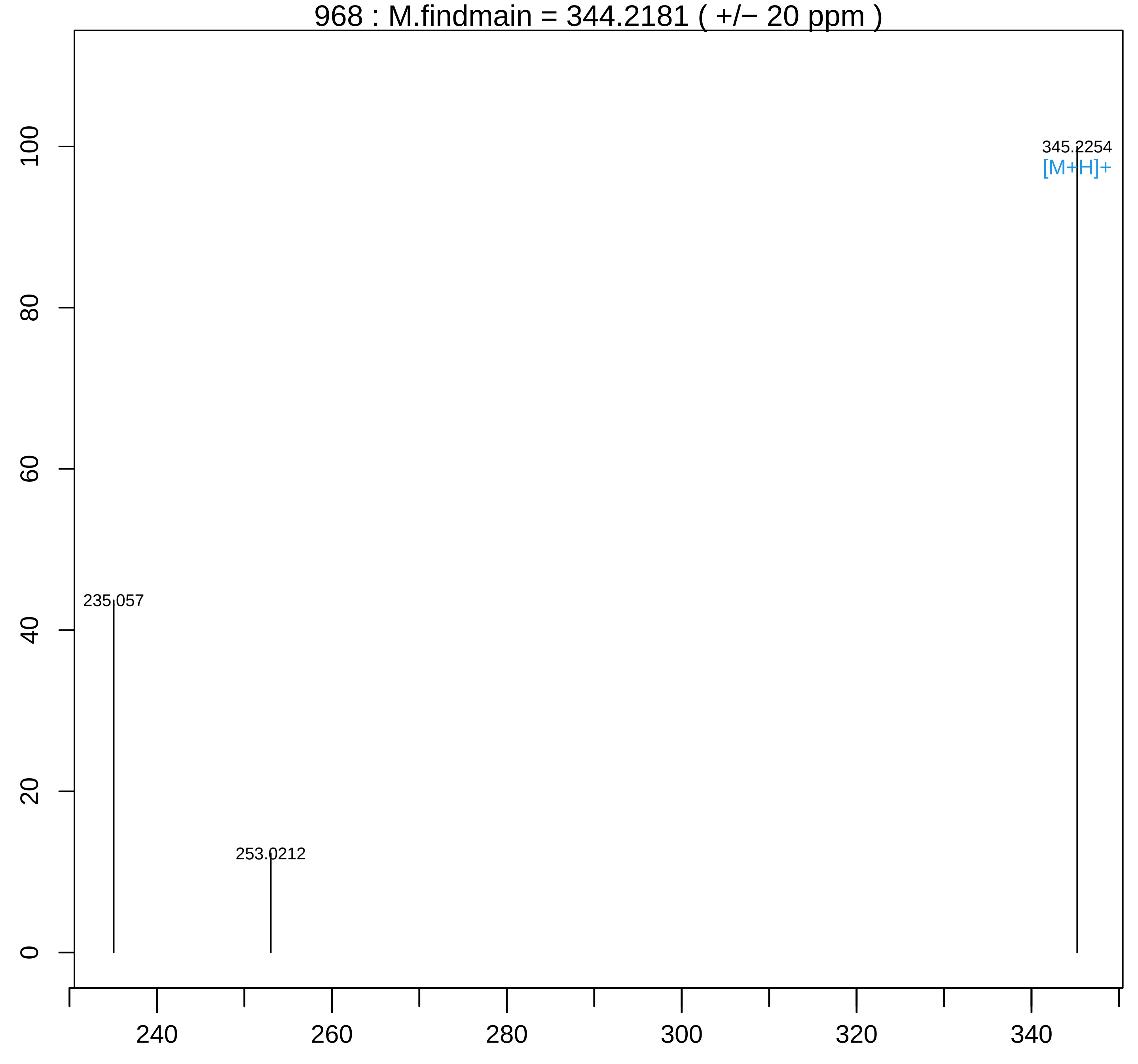
968 : M.findmain = 344.2181 (+/− 20 ppm)
345.2254
[M+H]+
235.057
253.0212
0
20
40
60
80
100
240
260
280
300
320
340

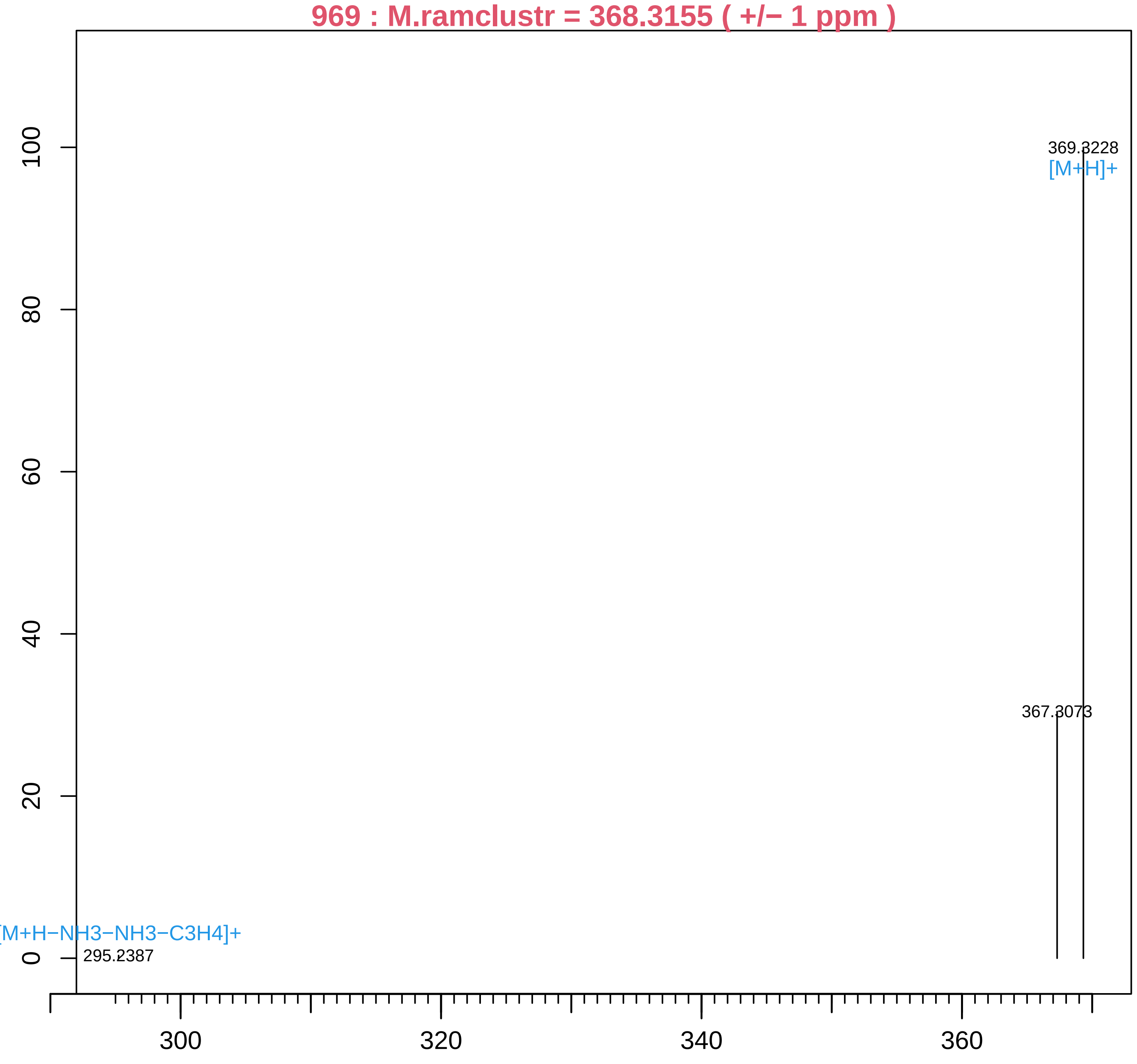
969 : M.ramclustr = 368.3155 (+/− 1 ppm)
369.3228
[M+H]+
367.3073
[M+H−NH3−NH3−C3H4]+
295.2387
0
20
40
60
80
100
300
320
340
360

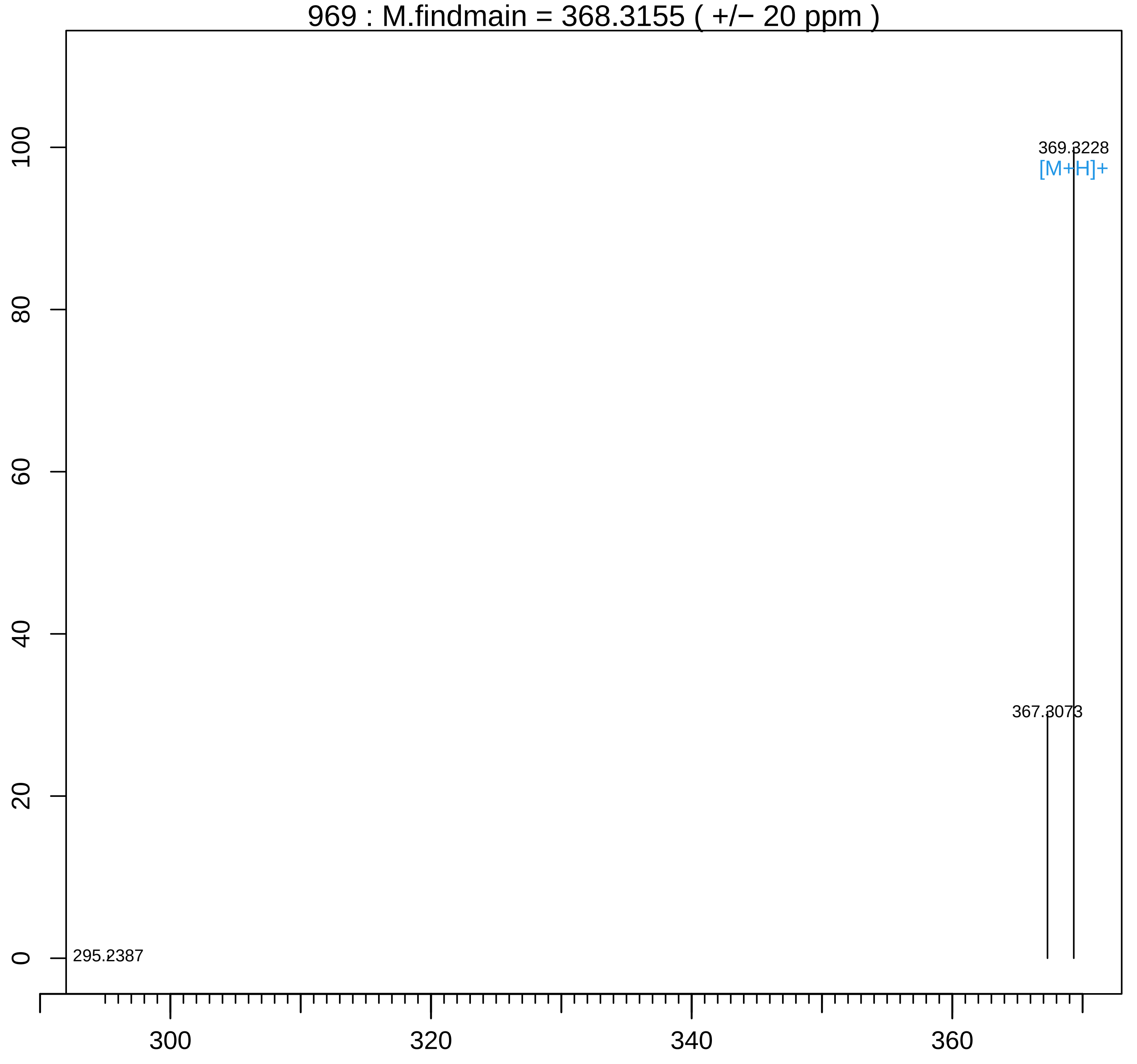
969 : M.findmain = 368.3155 (+/− 20 ppm)
369.3228
[M+H]+
367.3073
295.2387
0
20
40
60
80
100
300
320
340
360

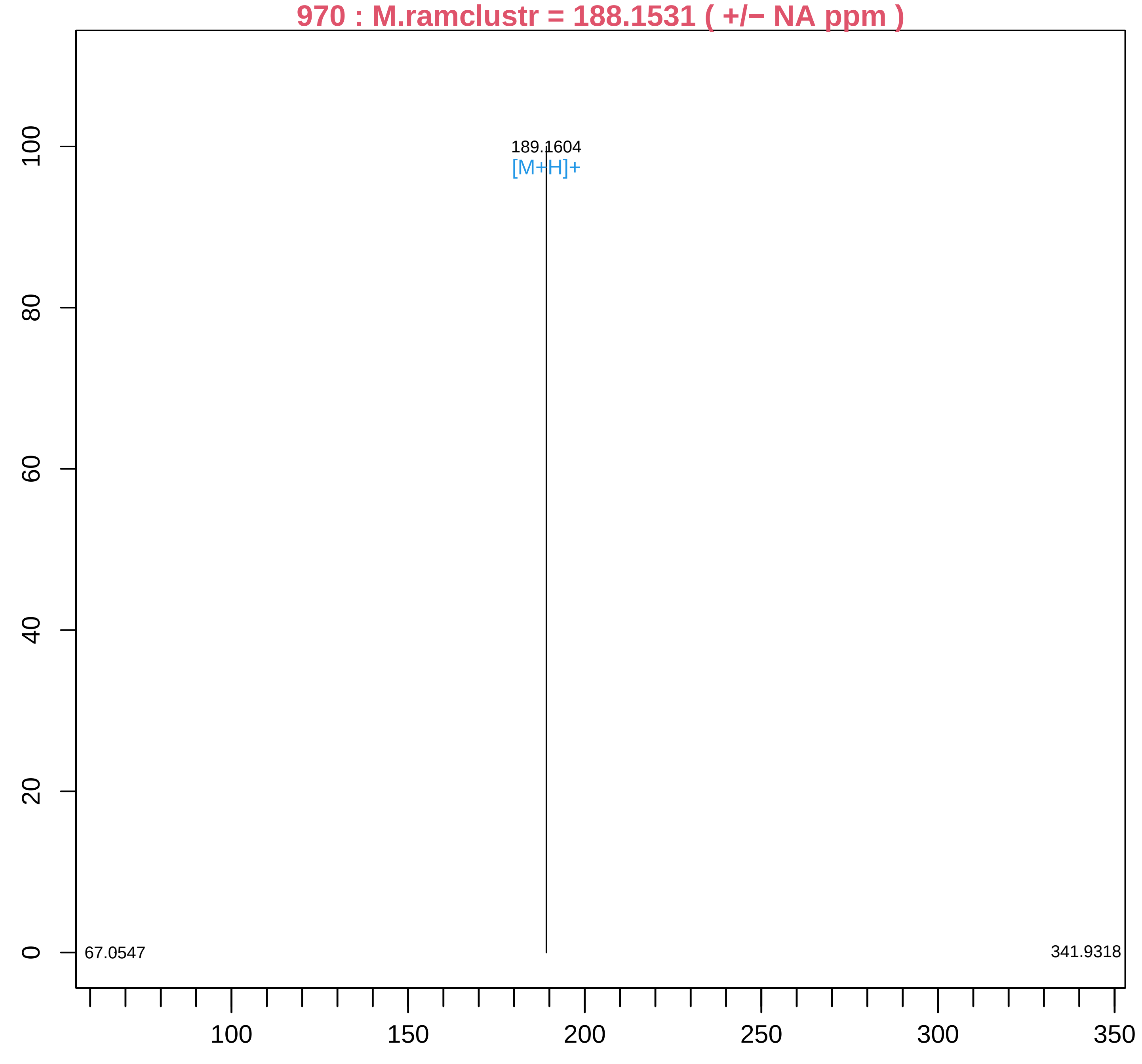

970 : M.ramclustr = 188.1531 (+/− NA ppm)
189.1604
[M+H]+
67.0547
341.9318
0
20
40
60
80
100
100
150
200
250
300
350

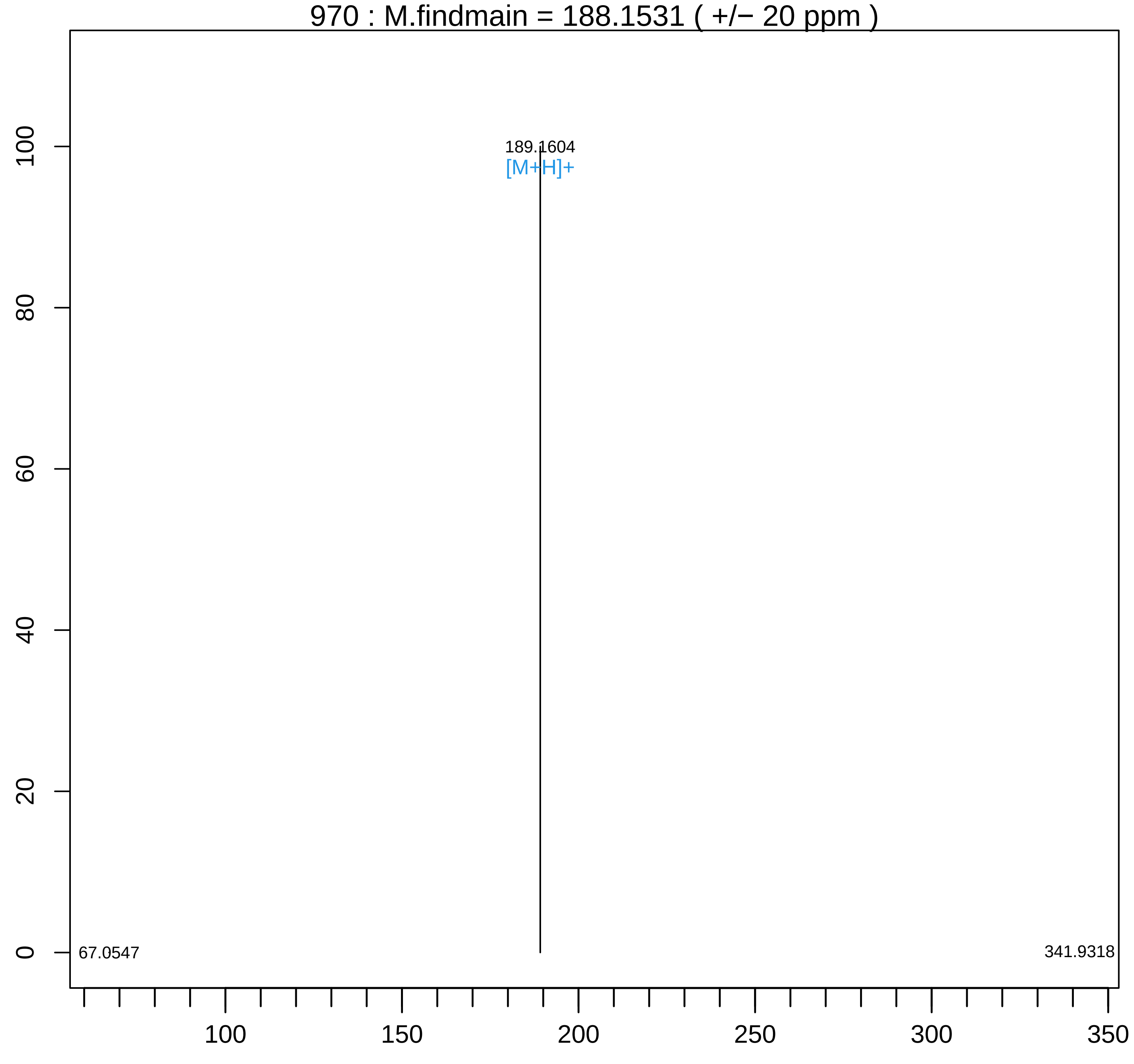

970 : M.findmain = 188.1531 (+/− 20 ppm)
189.1604
[M+H]+
67.0547
341.9318
0
20
40
60
80
100
100
150
200
250
300
350

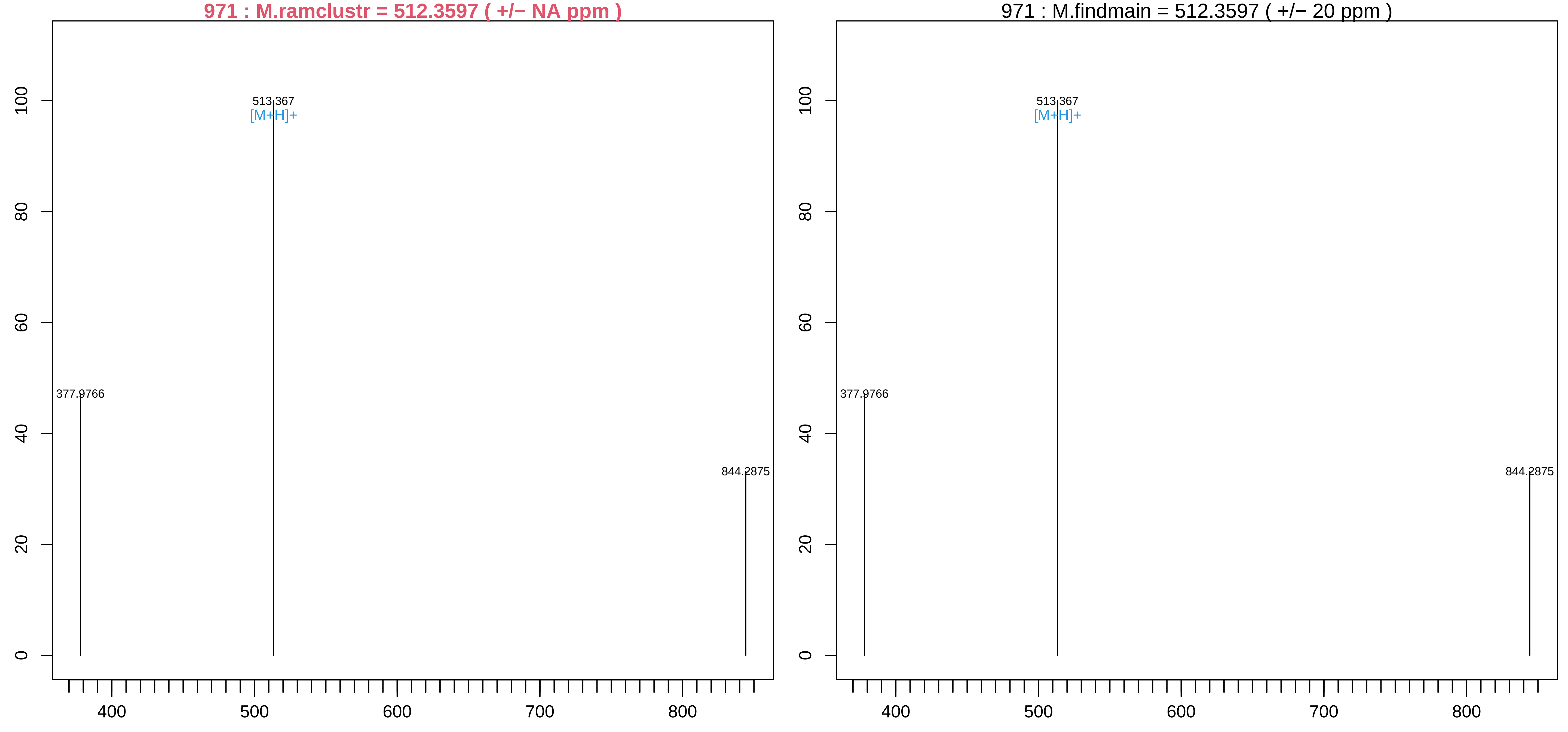
971 : M.ramclustr = 512.3597 (+/− NA ppm)
513.367
[M+H]+
377.9766
844.2875
971 : M.findmain = 512.3597 (+/− 20 ppm)
513.367
[M+H]+
377.9766
844.2875
0
20
40
60
80
100
400
500
600
700
800

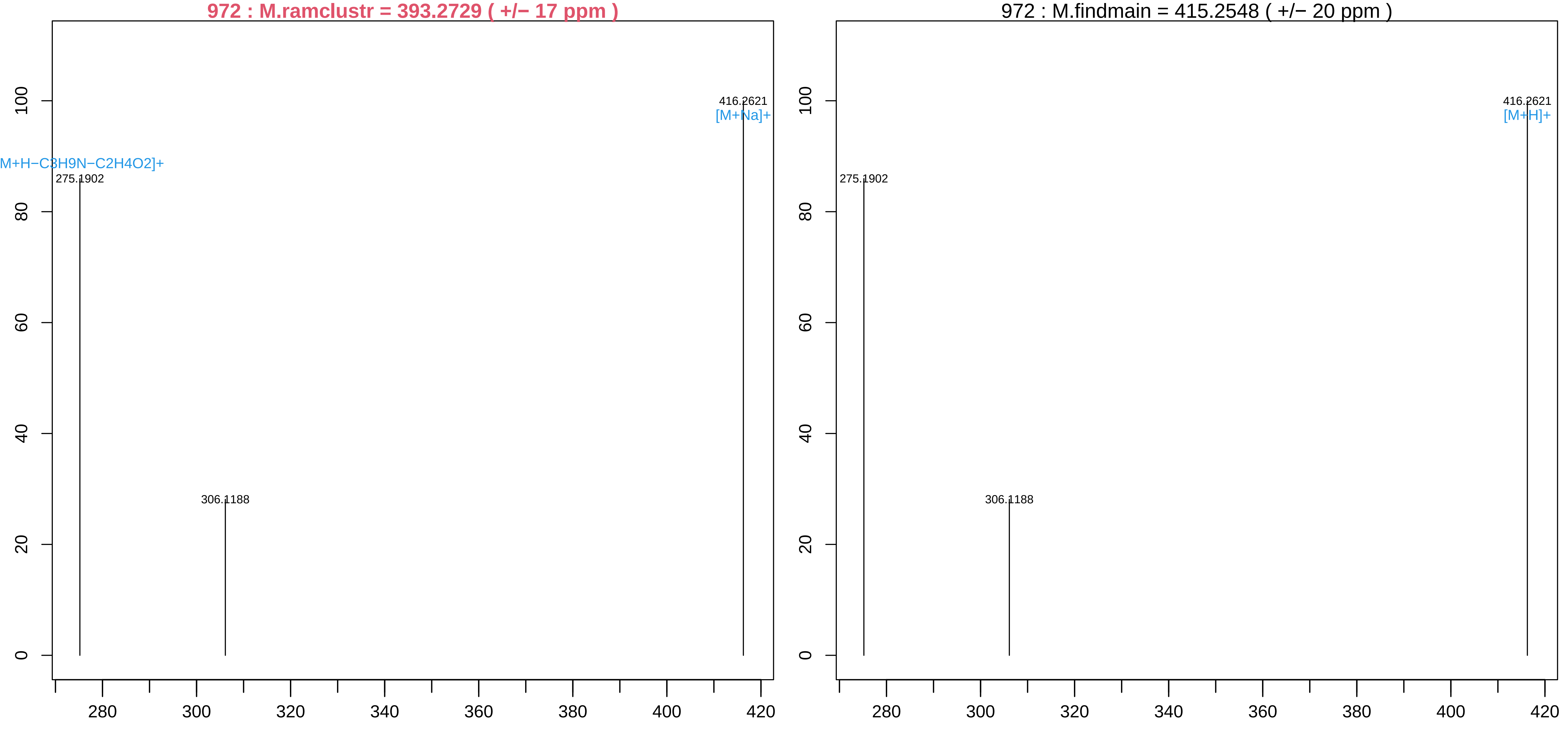

972 : M.ramclustr = 393.2729 (+/− 17 ppm)
416.2621
[M+Na]+
M+H−C3H9N−C2H4O2]+
275.1902
306.1188
0
20
40
60
80
100
280
300
320
340
360
380
400
420
972 : M.findmain = 415.2548 (+/− 20 ppm)
416.2621
[M+H]+
275.1902
306.1188
0
20
40
60
80
100
280
300
320
340
360
380
400
420

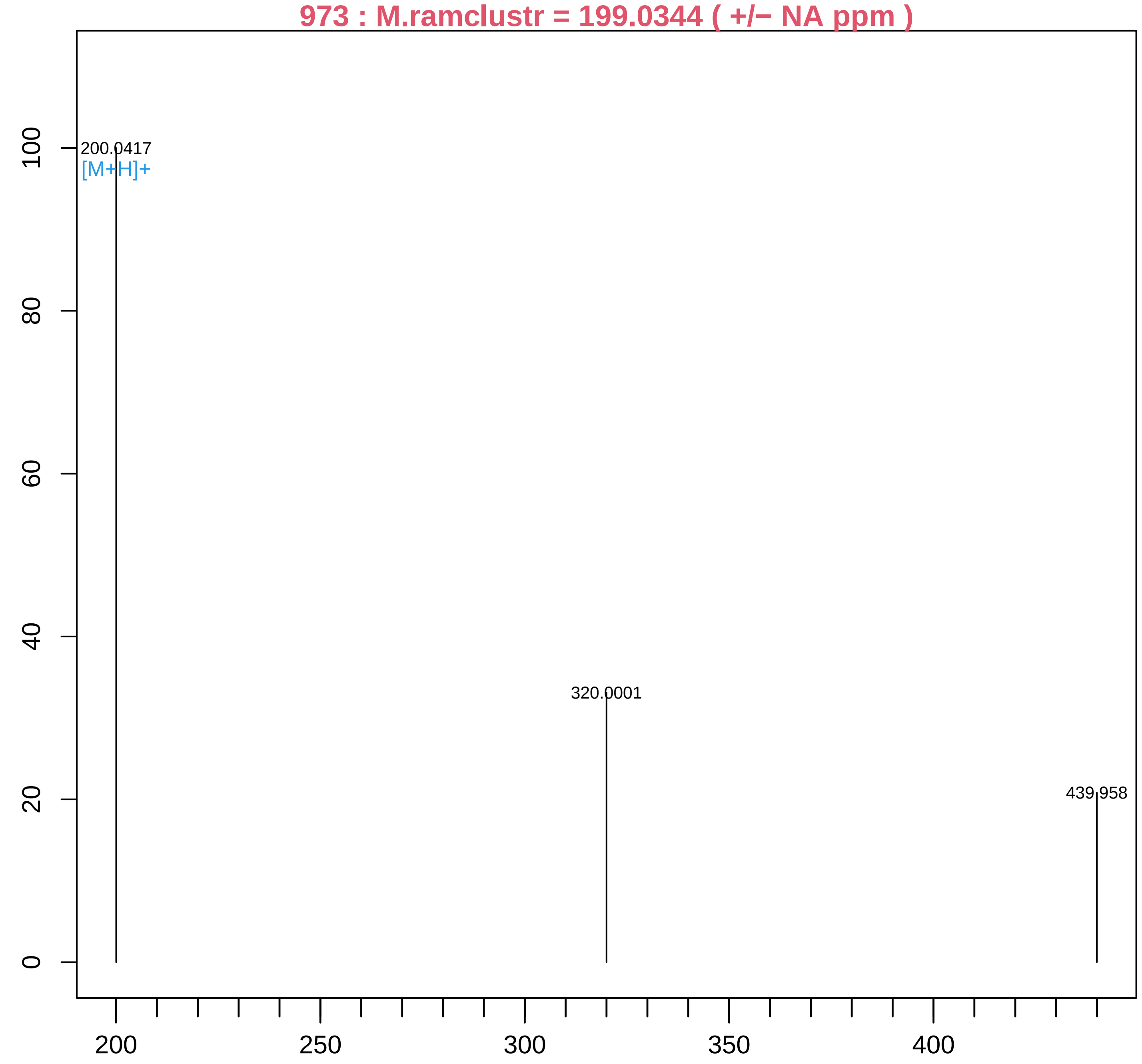
973 : M.ramclustr = 199.0344 (+/− NA ppm)
200.0417
[M+H]+
320.0001
439.958
0
20
40
60
80
100
200
250
300
350
400

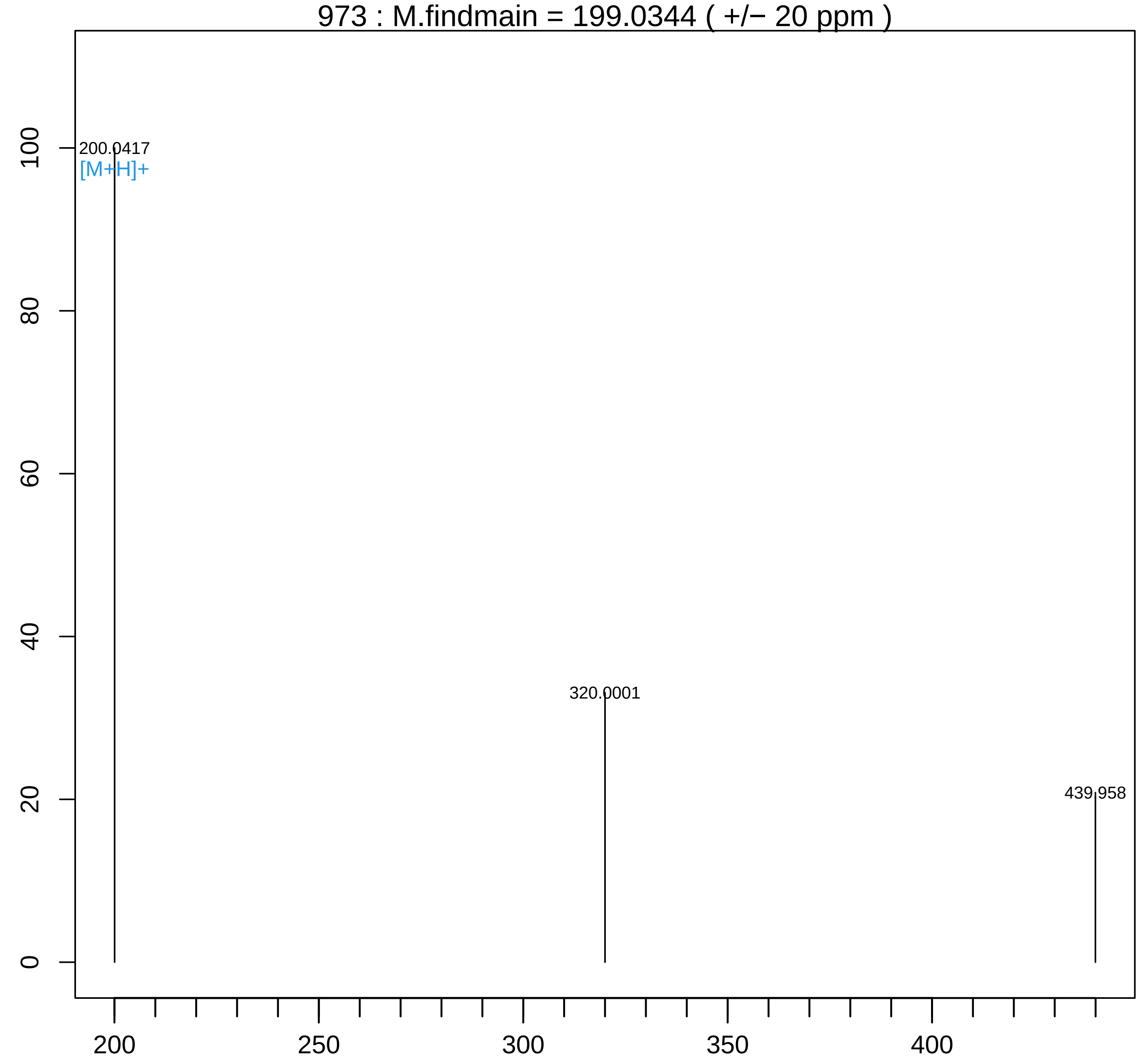
973 : M.findmain = 199.0344 (+/− 20 ppm)
200.0417
[M+H]+
320.0001
439.958
0
20
40
60
80
100
200
250
300
350
400

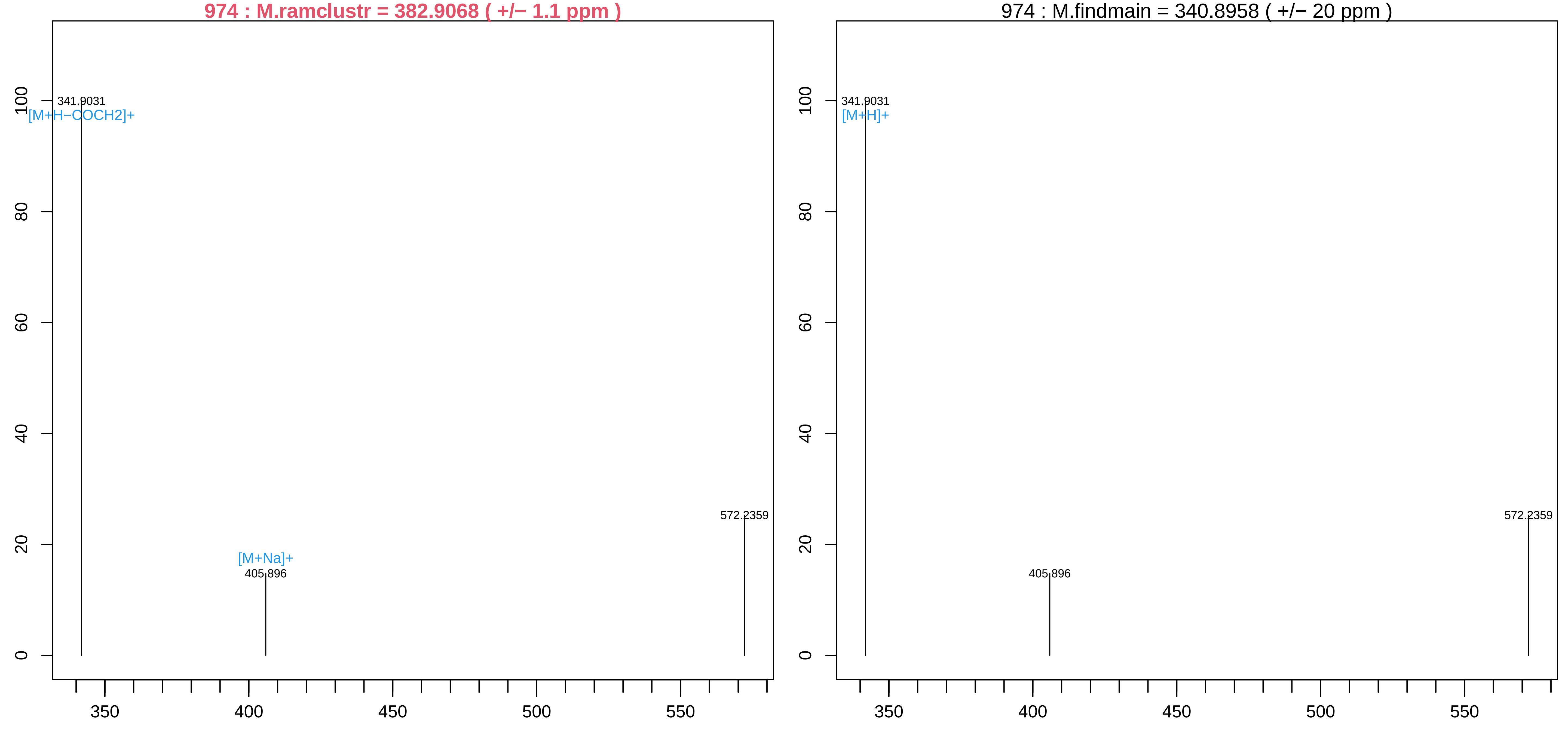
974 : M.ramclustr = 382.9068 (+/− 1.1 ppm)
341.9031
[M+H−COCH2]+
[M+Na]+
405.896
572.2359
974 : M.findmain = 340.8958 (+/− 20 ppm)
341.9031
[M+H]+
405.896
572.2359

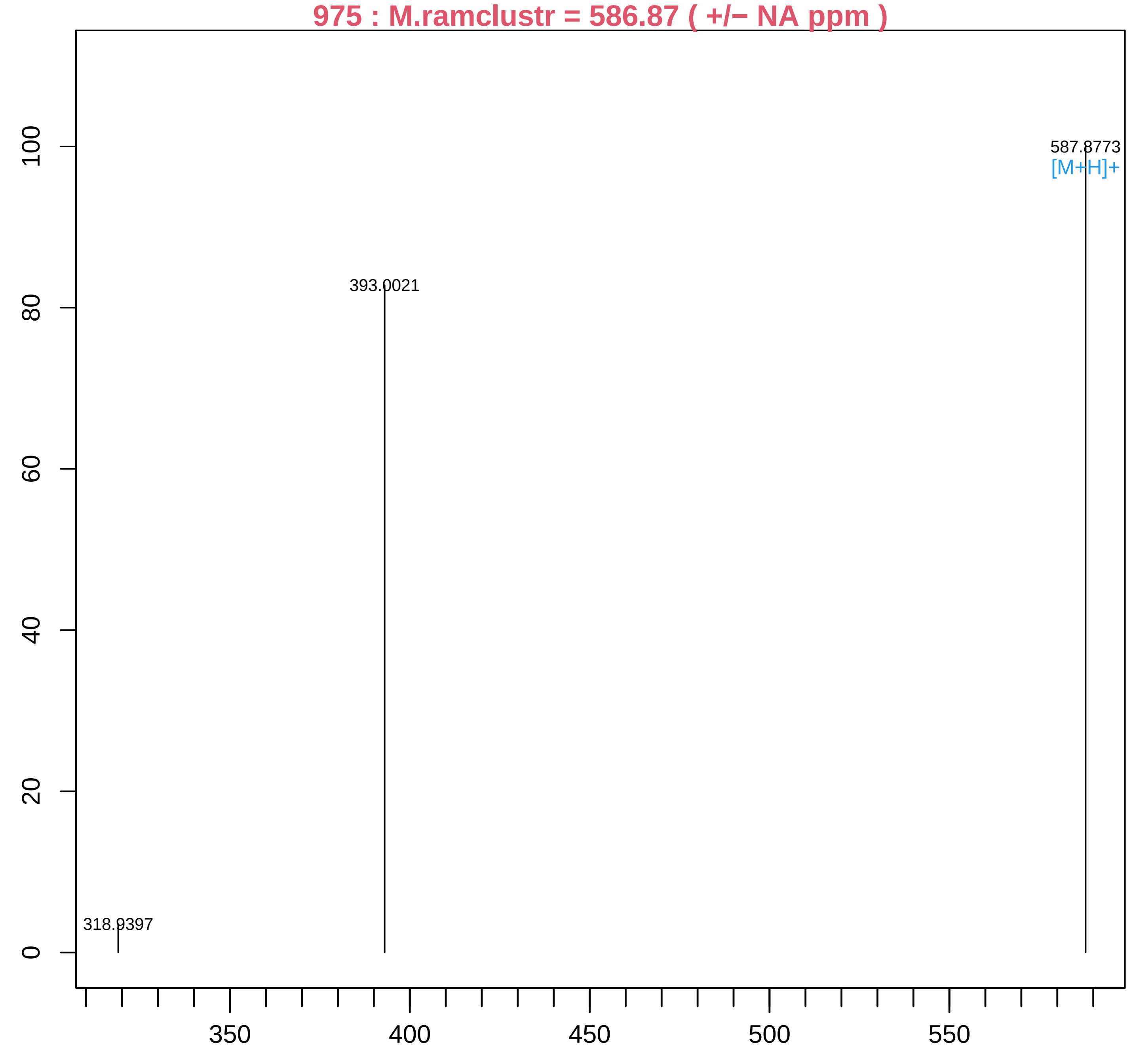

975 : M.ramclustr = 586.87 (+/− NA ppm)
587.8773
[M+H]+
393.0021
318.9397
0
20
40
60
80
100
350
400
450
500
550

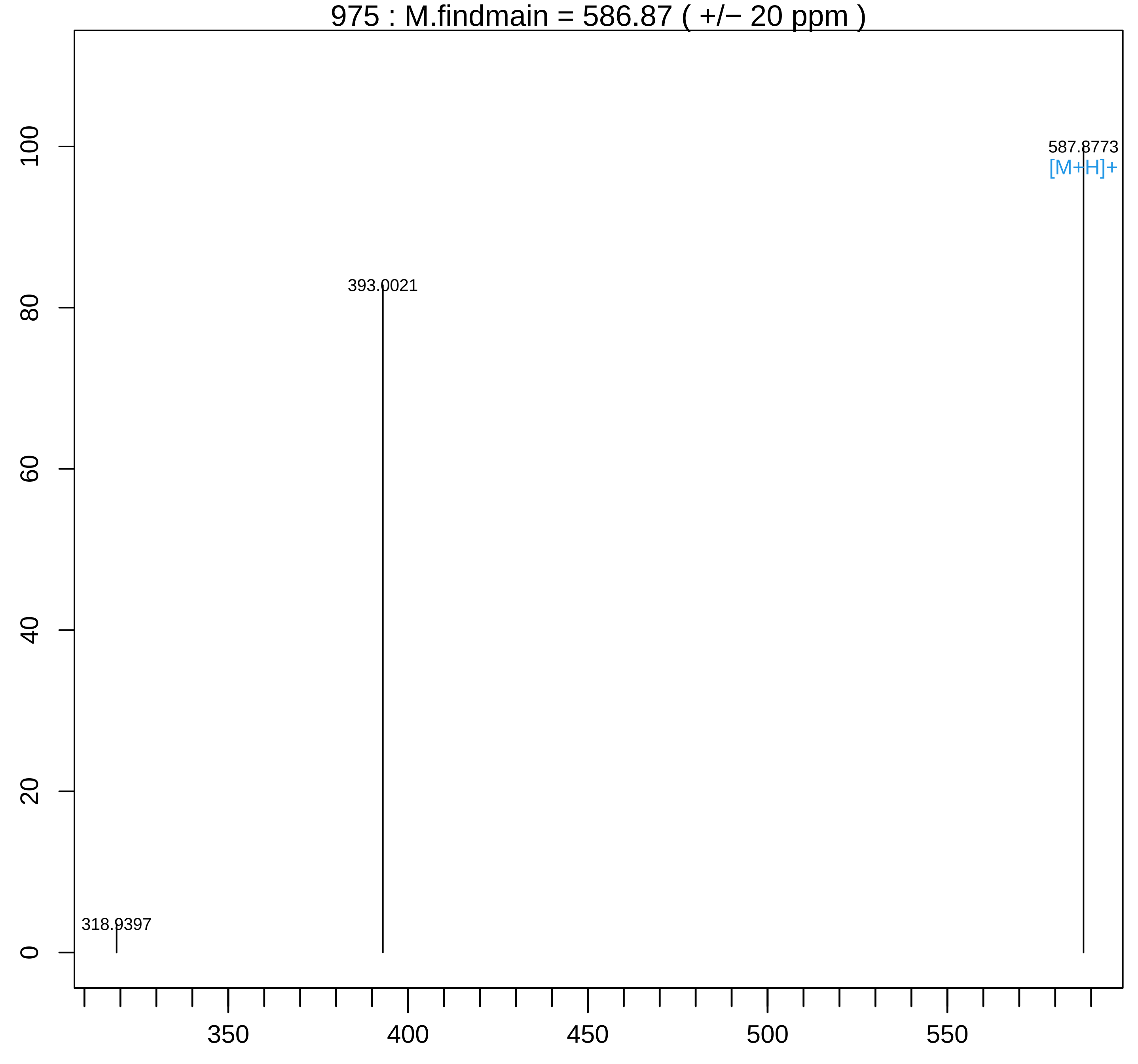

975 : M.findmain = 586.87 (+/− 20 ppm)
587.8773
[M+H]+
393.0021
318.9397
0
20
40
60
80
100
350
400
450
500
550

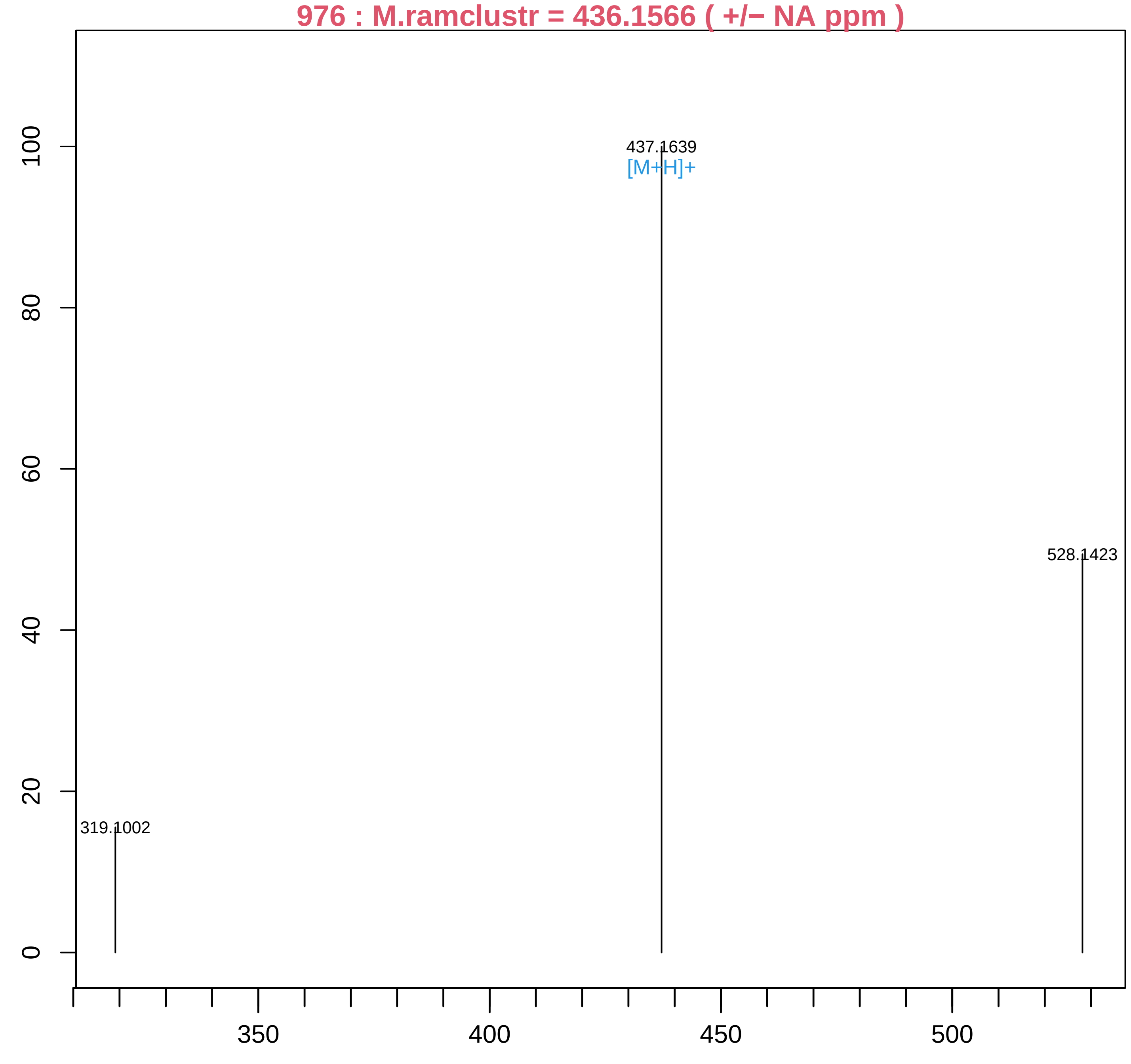
976 : M.ramclustr = 436.1566 (+/− NA ppm)
437.1639
[M+H]+
528.1423
319.1002
0
20
40
60
80
100
350
400
450
500

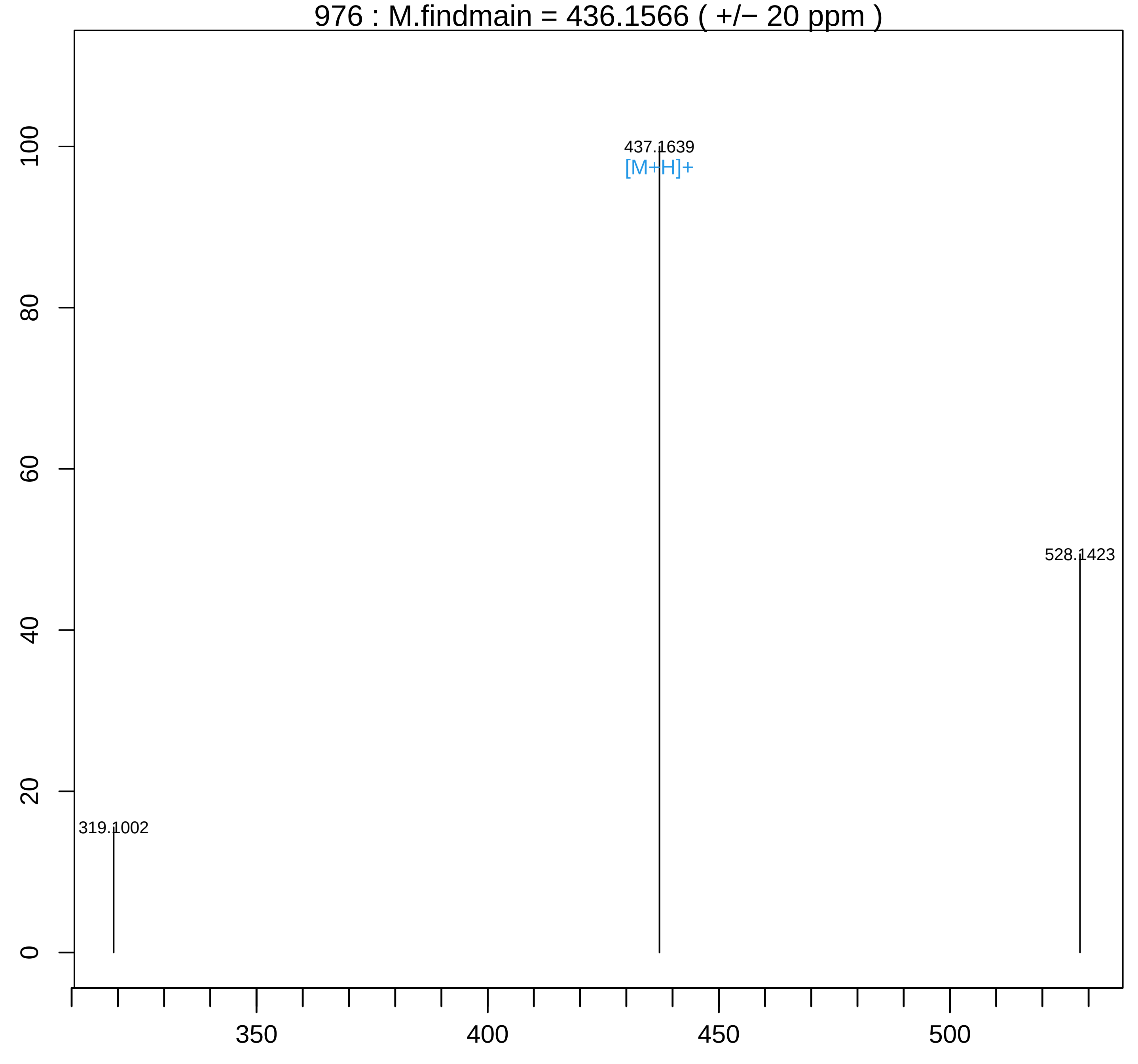
976 : M.findmain = 436.1566 (+/− 20 ppm)
437.1639
[M+H]+
528.1423
319.1002
0
20
40
60
80
100
350
400
450
500

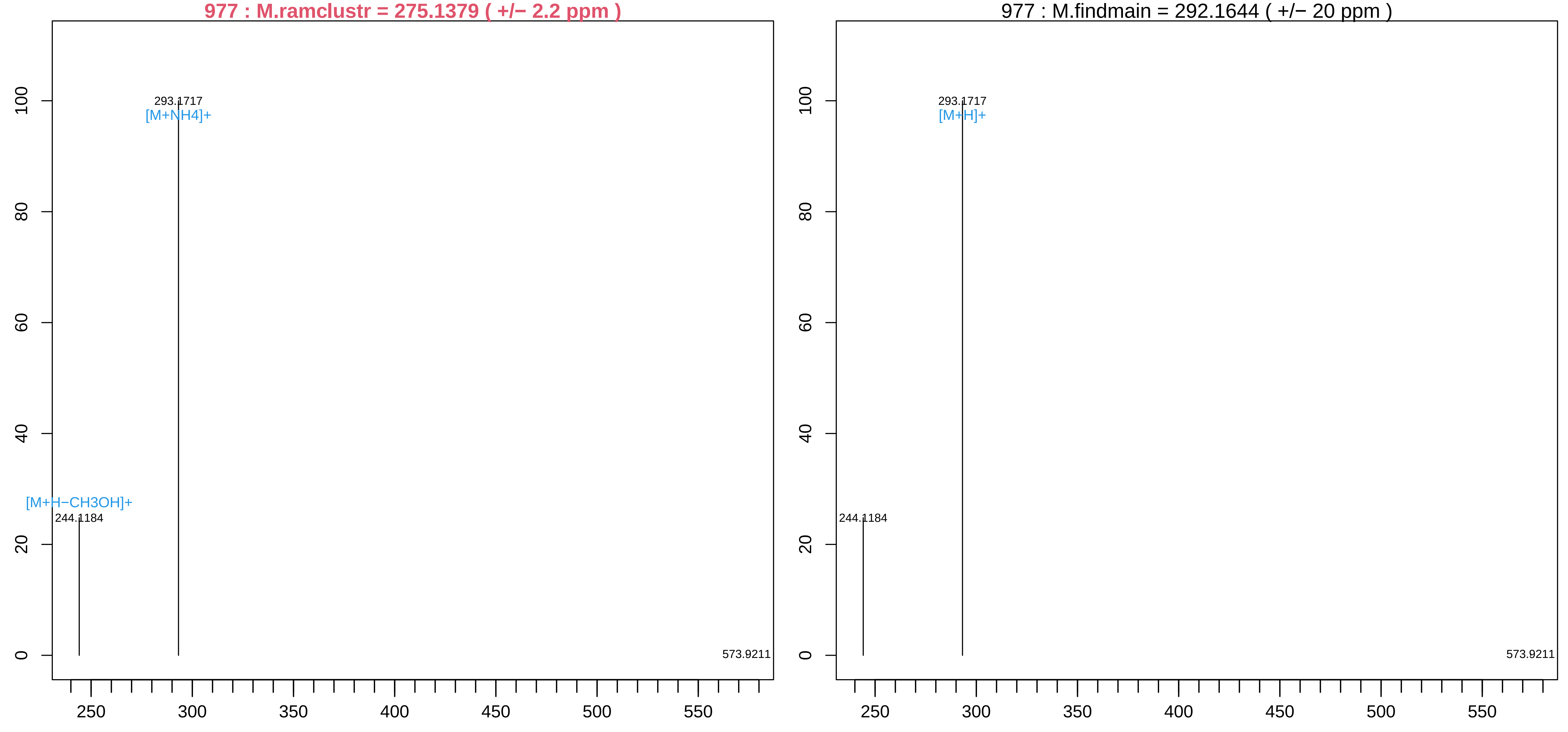

977 : M.ramclustr = 275.1379 (+/− 2.2 ppm)
293.1717
[M+NH4]+
[M+H−CH3OH]+
244.1184
573.9211
977 : M.findmain = 292.1644 (+/− 20 ppm)
293.1717
[M+H]+
244.1184
573.9211
0
20
40
60
80
100
250
300
350
400
450
500
550

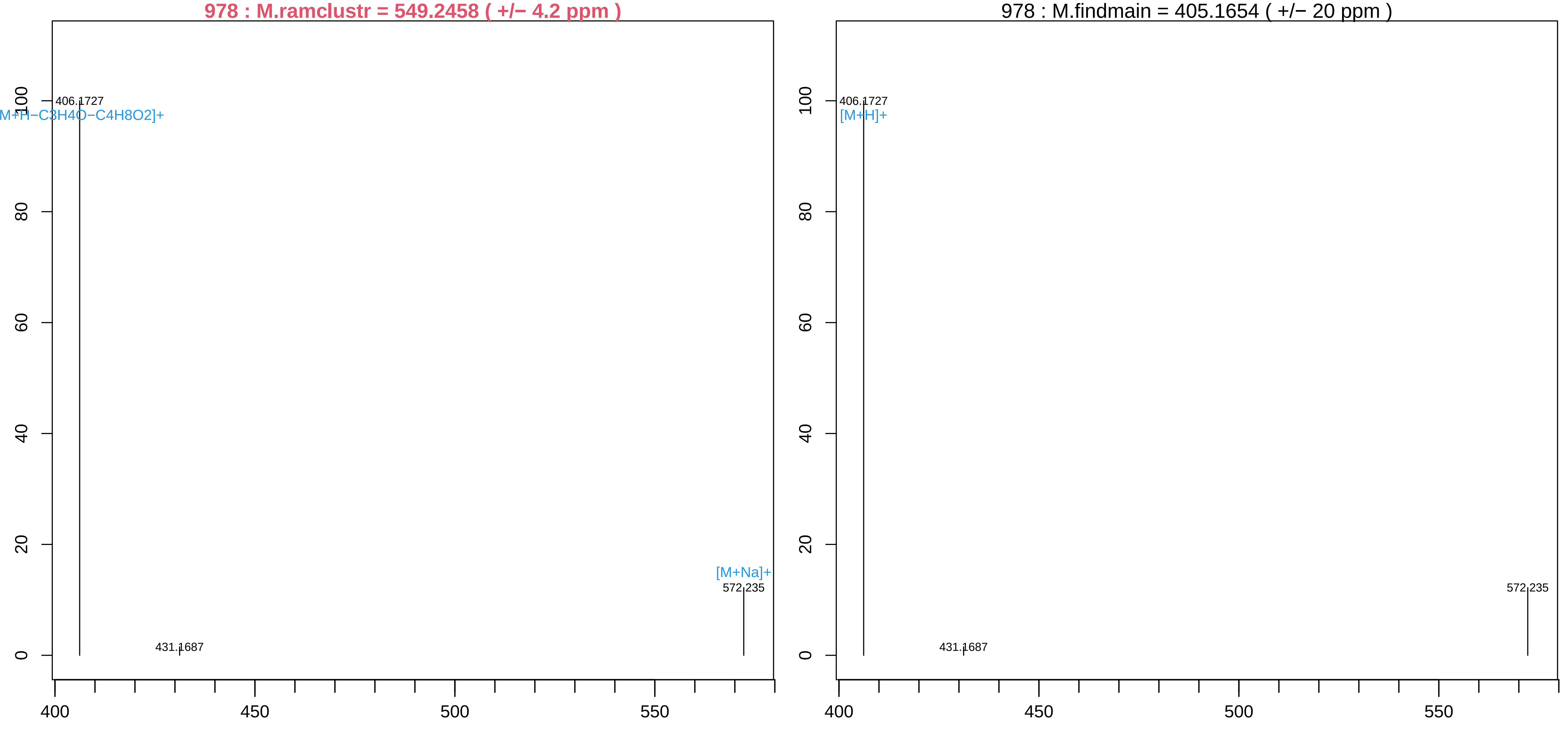
978 : M.ramclustr = 549.2458 (+/− 4.2 ppm)
406.1727
[M+H−C3H4O−C4H8O2]+
431.1687
[M+Na]+
572.235
978 : M.findmain = 405.1654 (+/− 20 ppm)
406.1727
[M+H]+
431.1687
572.235
0
20
40
60
80
100
400
450
500
550

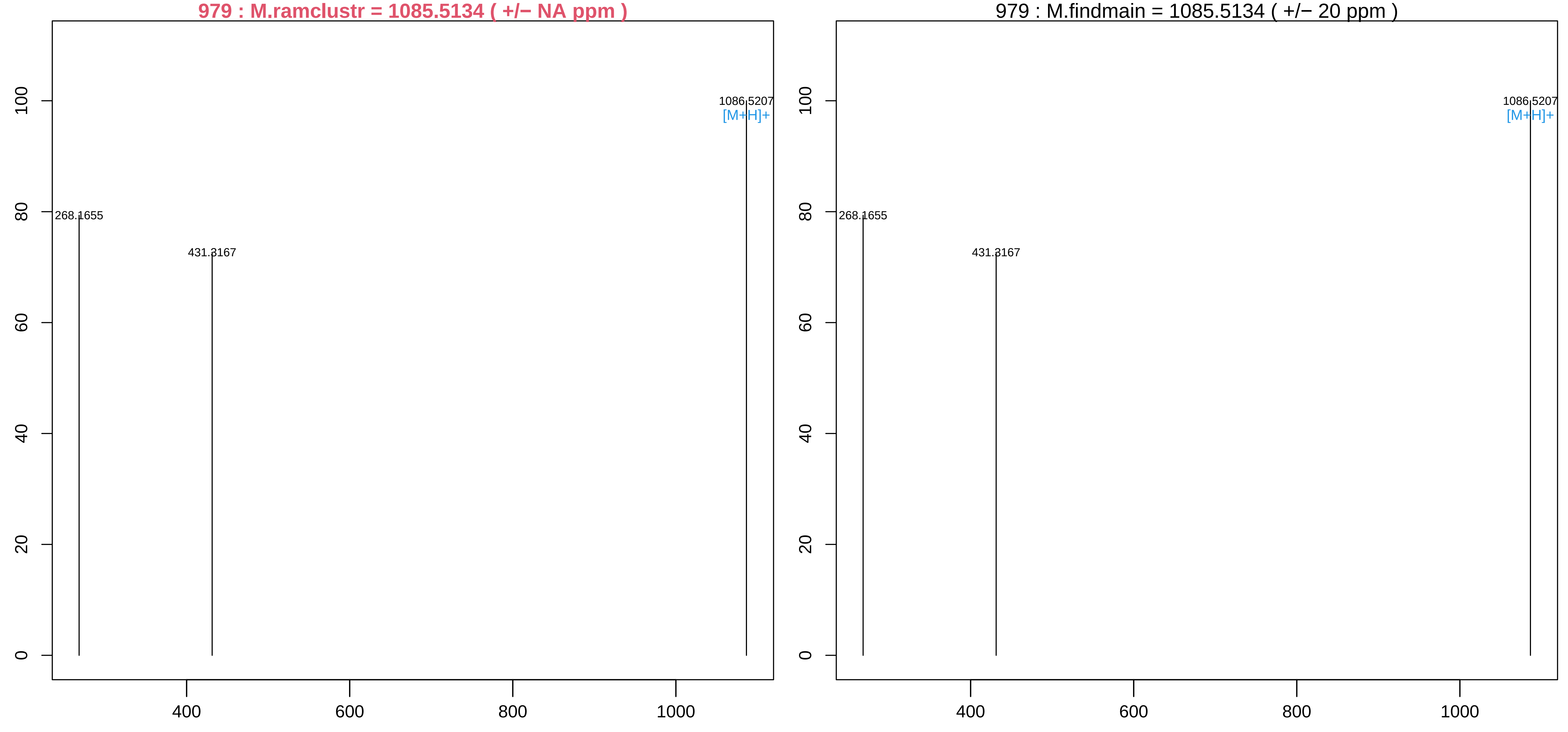
979 : M.ramclustr = 1085.5134 (+/− NA ppm)
979 : M.findmain = 1085.5134 (+/− 20 ppm)
1086.5207
[M+H]+
268.1655
431.3167
0
20
40
60
80
100
400
600
800
1000

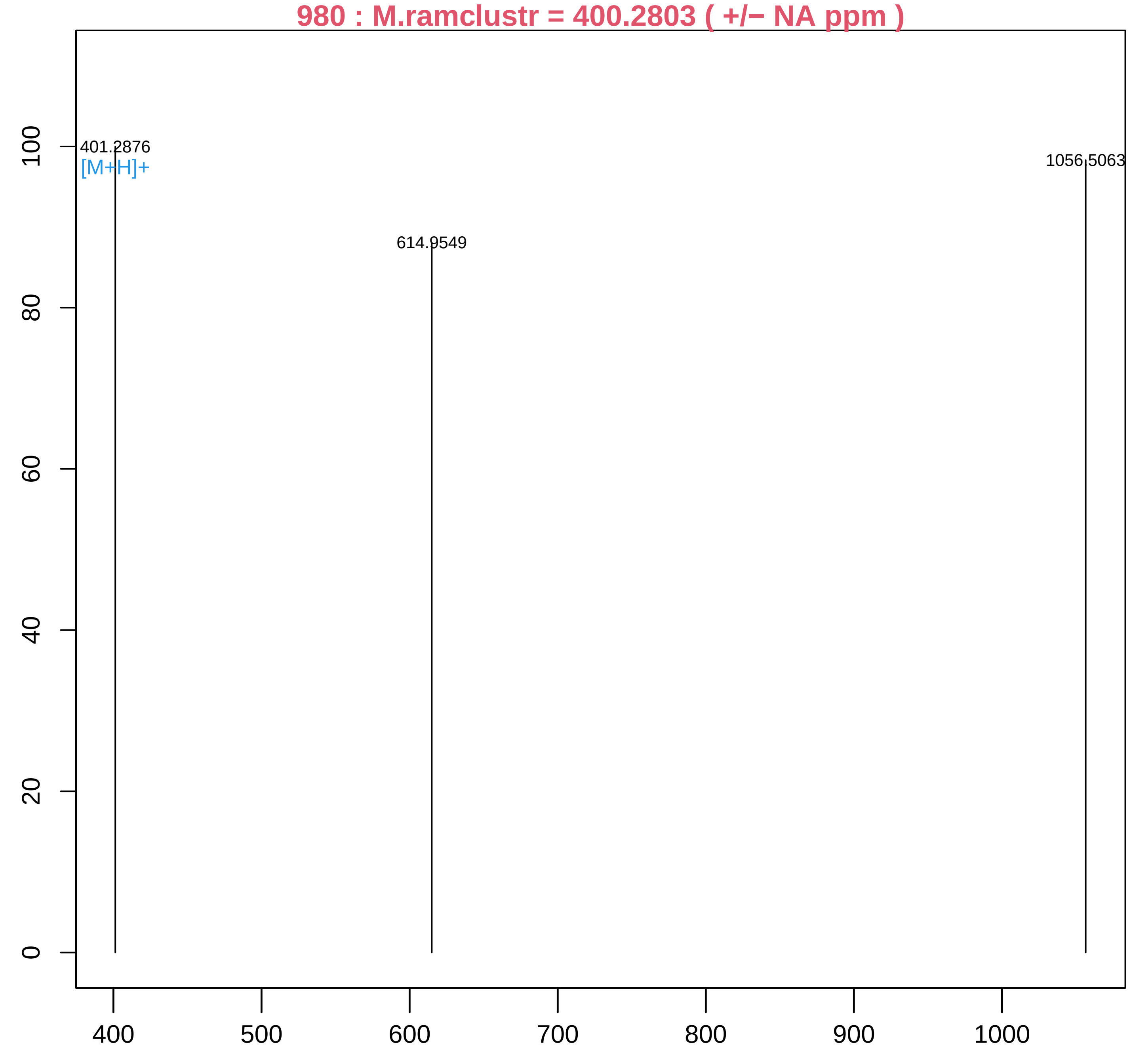
980 : M.ramclustr = 400.2803 (+/− NA ppm)
401.2876
[M+H]+
614.9549
1056.5063
0
20
40
60
80
100
400
500
600
700
800
900
1000

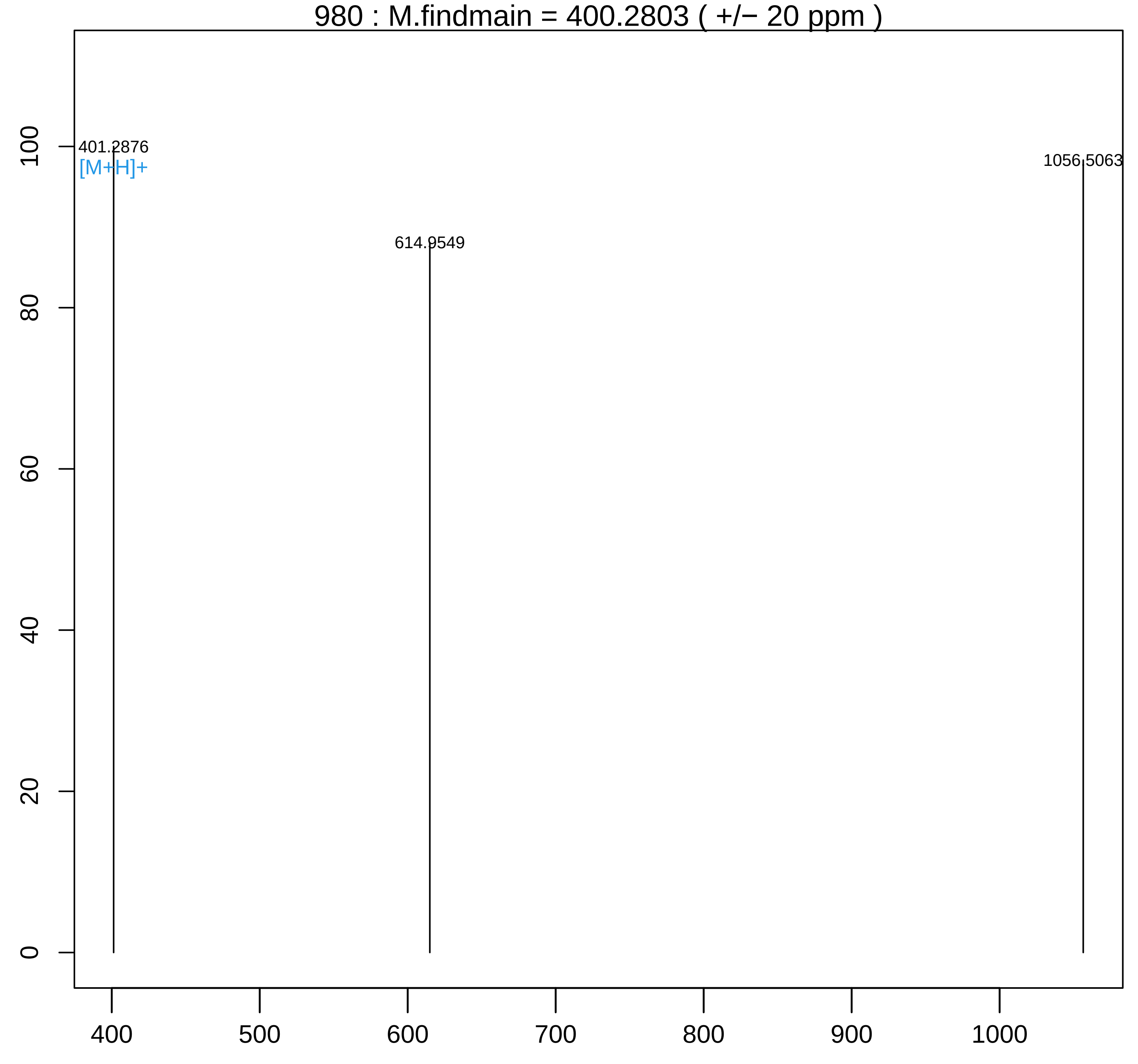
980 : M.findmain = 400.2803 (+/− 20 ppm)
401.2876
[M+H]+
614.9549
1056.5063
0
20
40
60
80
100
400
500
600
700
800
900
1000

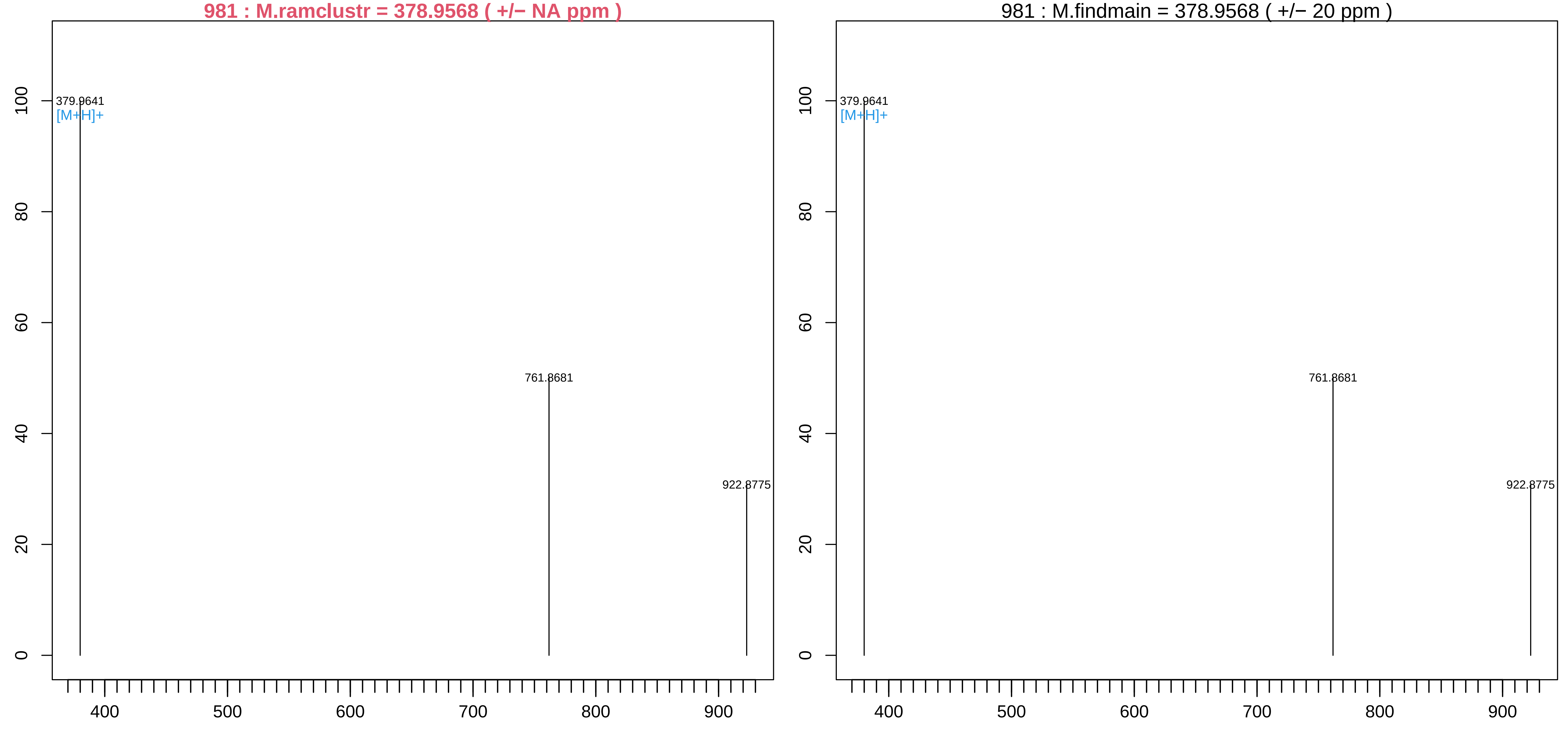
981 : M.ramclustr = 378.9568 (+/− NA ppm)
379.9641
[M+H]+
761.8681
922.8775
981 : M.findmain = 378.9568 (+/− 20 ppm)
379.9641
[M+H]+
761.8681
922.8775
0
20
40
60
80
100
400
500
600
700
800
900

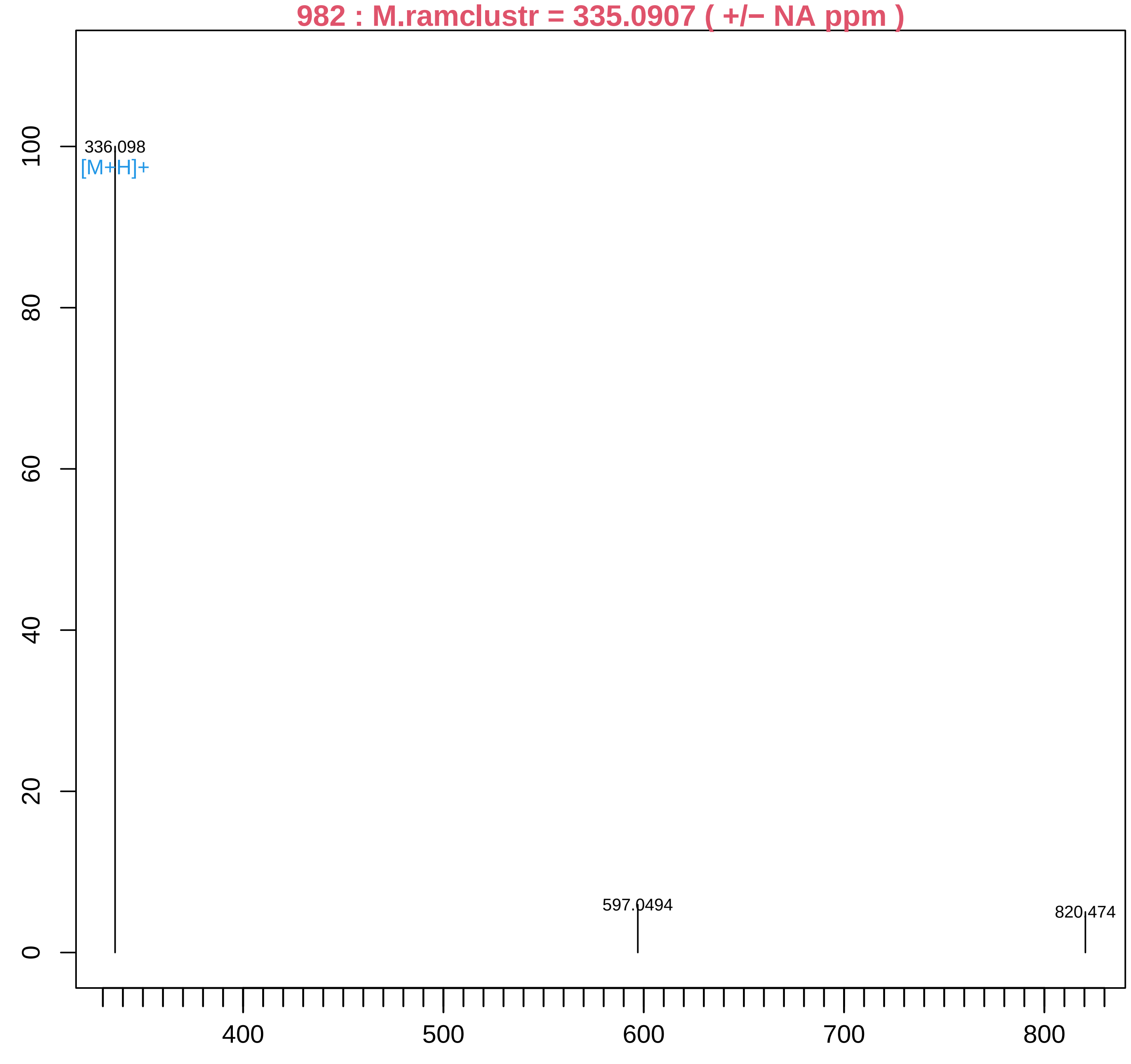
982 : M.ramclustr = 335.0907 (+/− NA ppm)
336.098
[M+H]+
597.0494
820.474
0
20
40
60
80
100
400
500
600
700
800

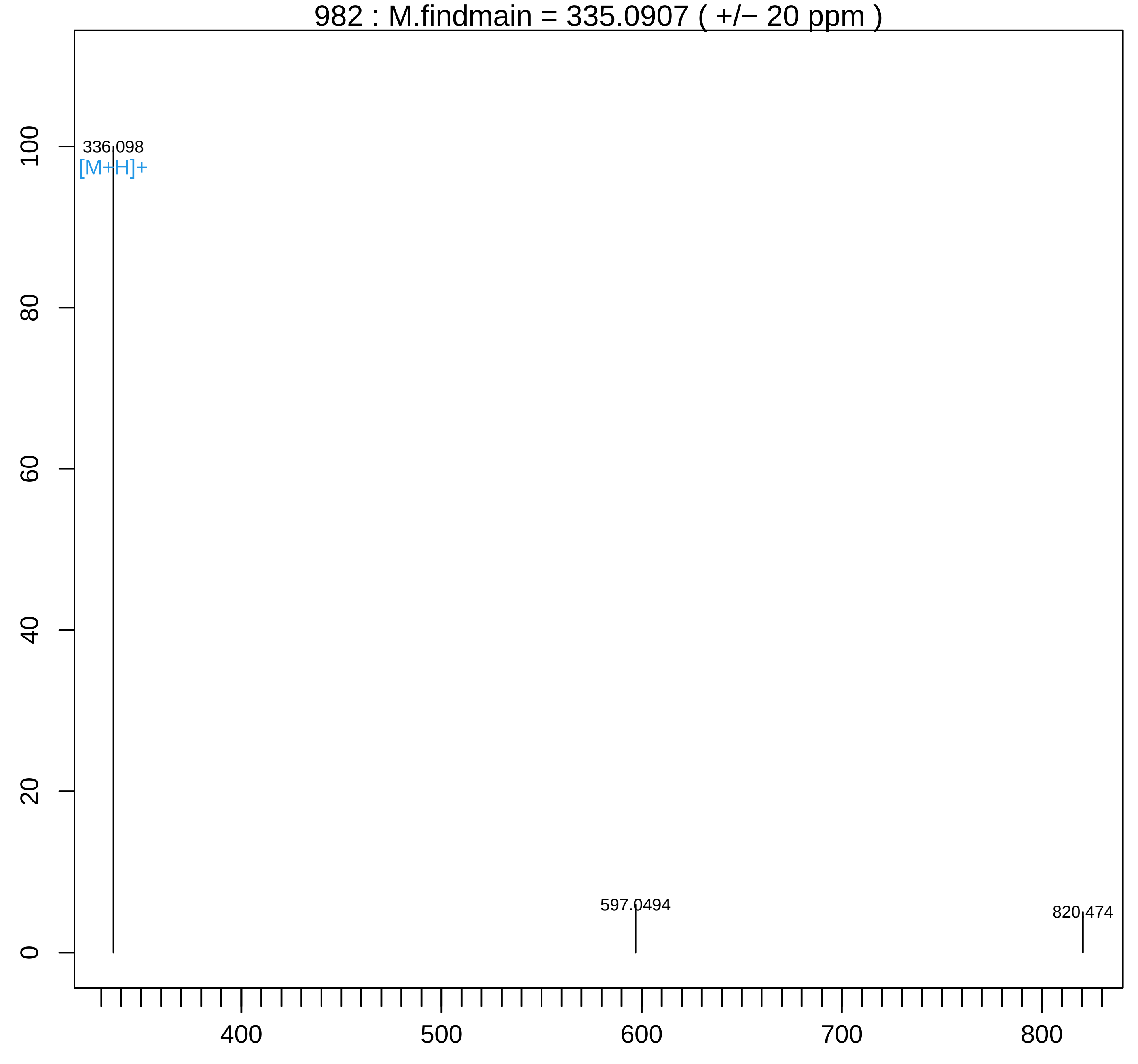
982 : M.findmain = 335.0907 (+/− 20 ppm)
336.098
[M+H]+
597.0494
820.474
0
20
40
60
80
100
400
500
600
700
800

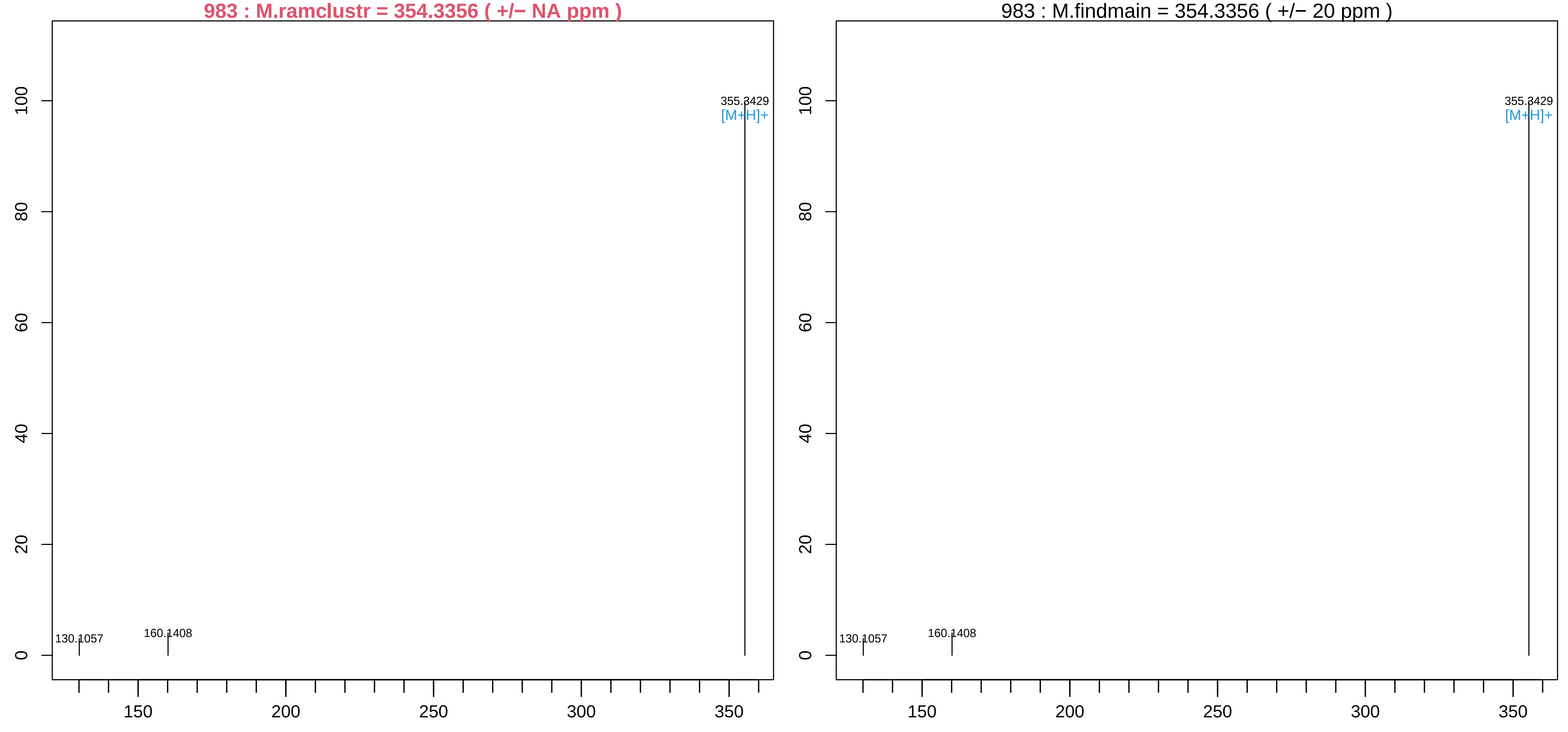
983 : M.ramclustr = 354.3356 (+/− NA ppm)
355.3429
[M+H]+
130.1057
160.1408
100
80
60
40
20
0
150
200
250
300
350
983 : M.findmain = 354.3356 (+/− 20 ppm)
355.3429
[M+H]+
130.1057
160.1408
100
80
60
40
20
0
150
200
250
300
350

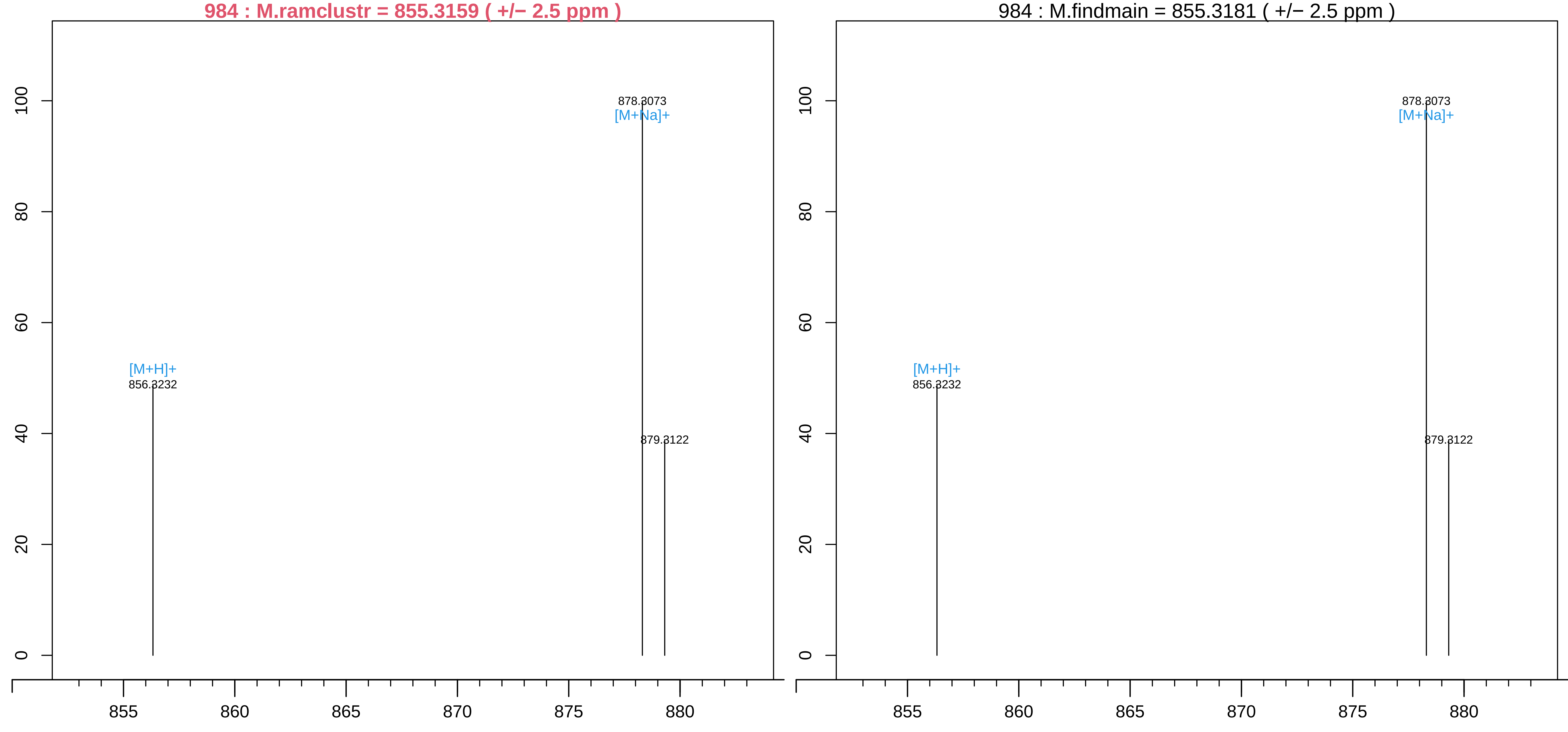
984 : M.ramclustr = 855.3159 (+/− 2.5 ppm)
984 : M.findmain = 855.3181 (+/− 2.5 ppm)
878.3073
[M+Na]+
[M+H]+
856.3232
879.3122
0
20
40
60
80
100
855
860
865
870
875
880

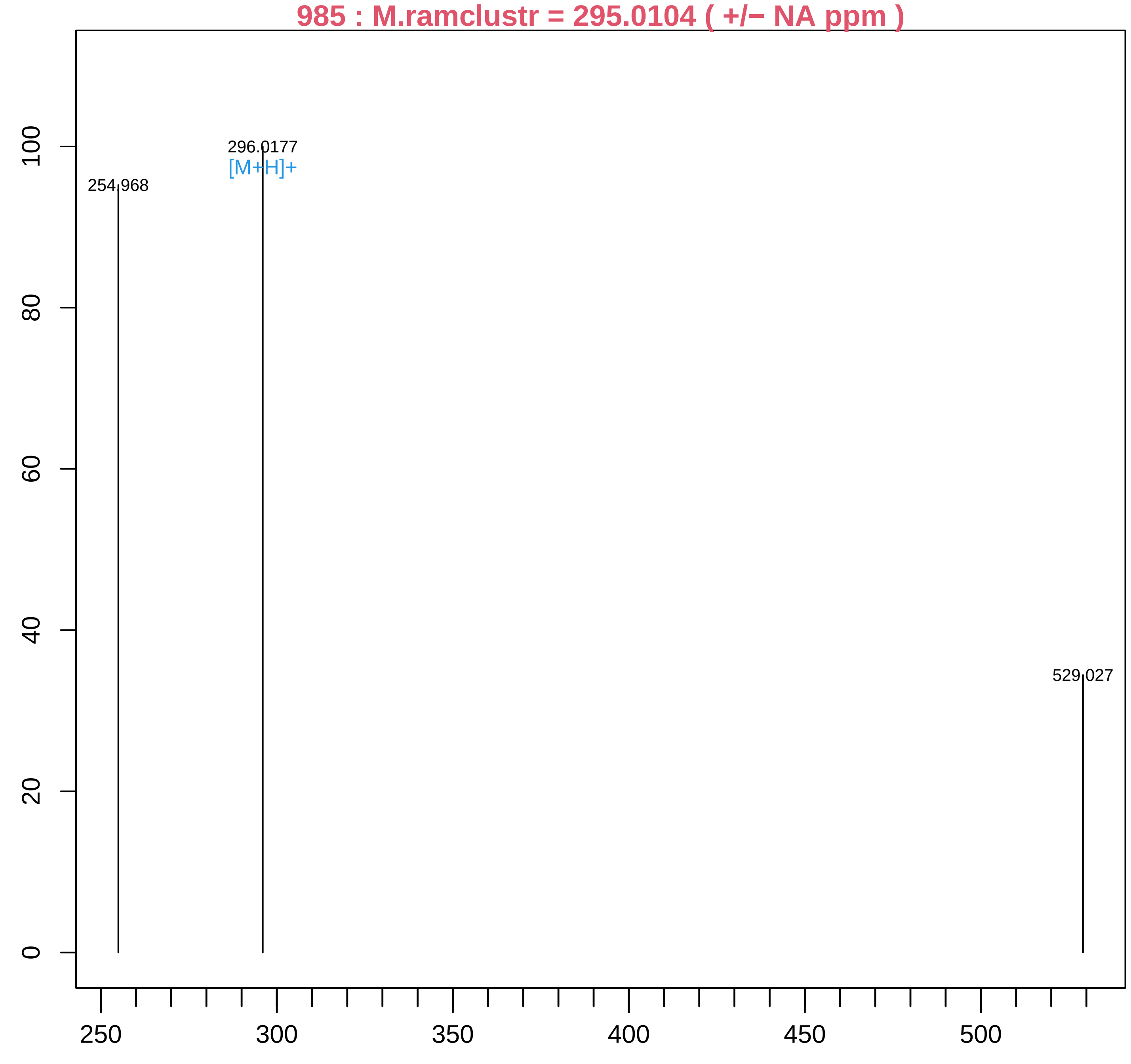
985 : M.ramclustr = 295.0104 (+/− NA ppm)
296.0177
[M+H]+
254.968
529.027
0
20
40
60
80
100
250
300
350
400
450
500

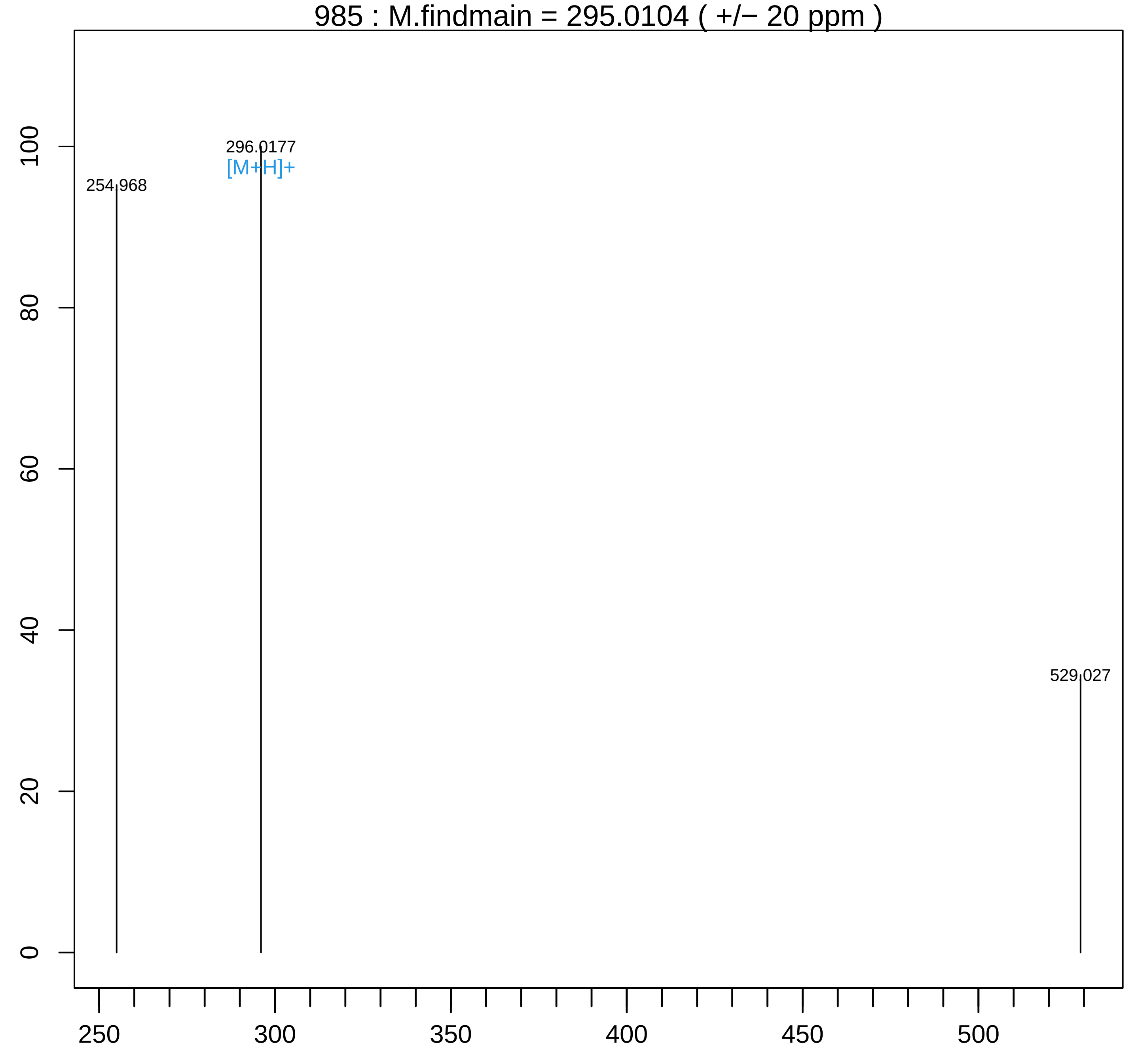
985 : M.findmain = 295.0104 (+/− 20 ppm)
296.0177
[M+H]+
254.968
529.027
0
20
40
60
80
100
250
300
350
400
450
500

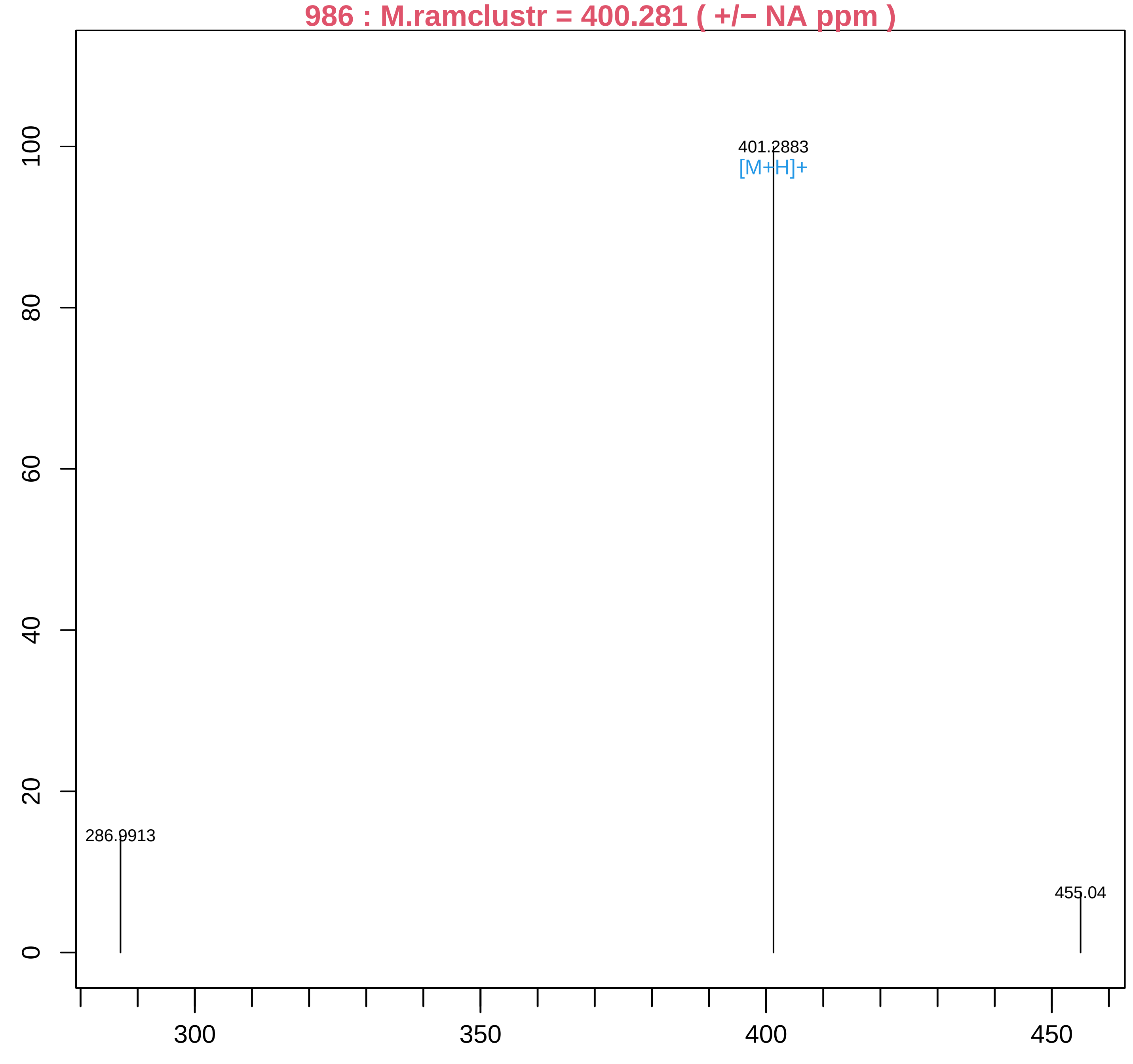
986 : M.ramclustr = 400.281 (+/− NA ppm)
401.2883
[M+H]+
286.9913
455.04
0
20
40
60
80
100
300
350
400
450

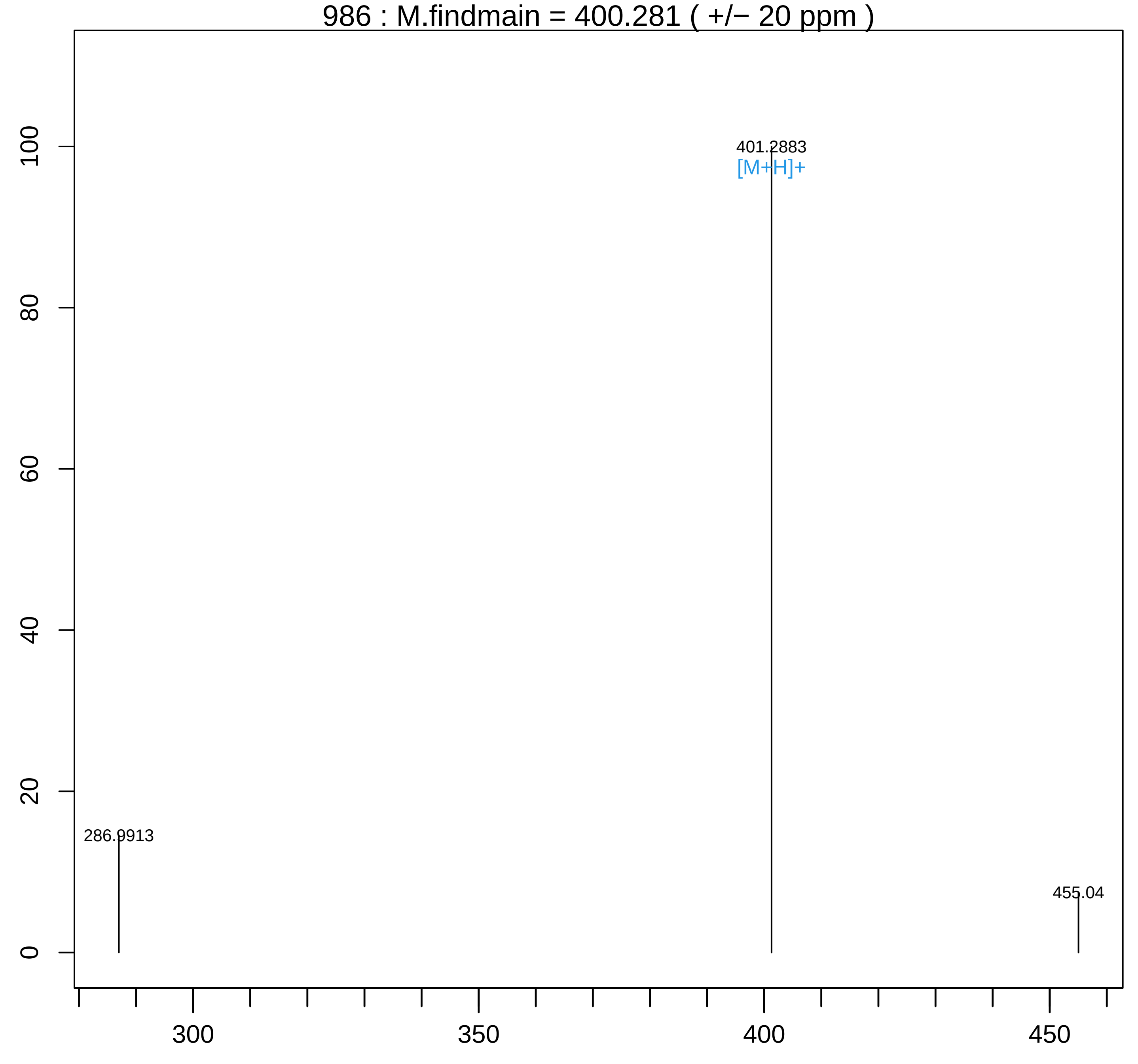
986 : M.findmain = 400.281 (+/− 20 ppm)
401.2883
[M+H]+
286.9913
455.04
0
20
40
60
80
100
300
350
400
450

987 : M.ramclustr = 297.1223 (+/− NA ppm)

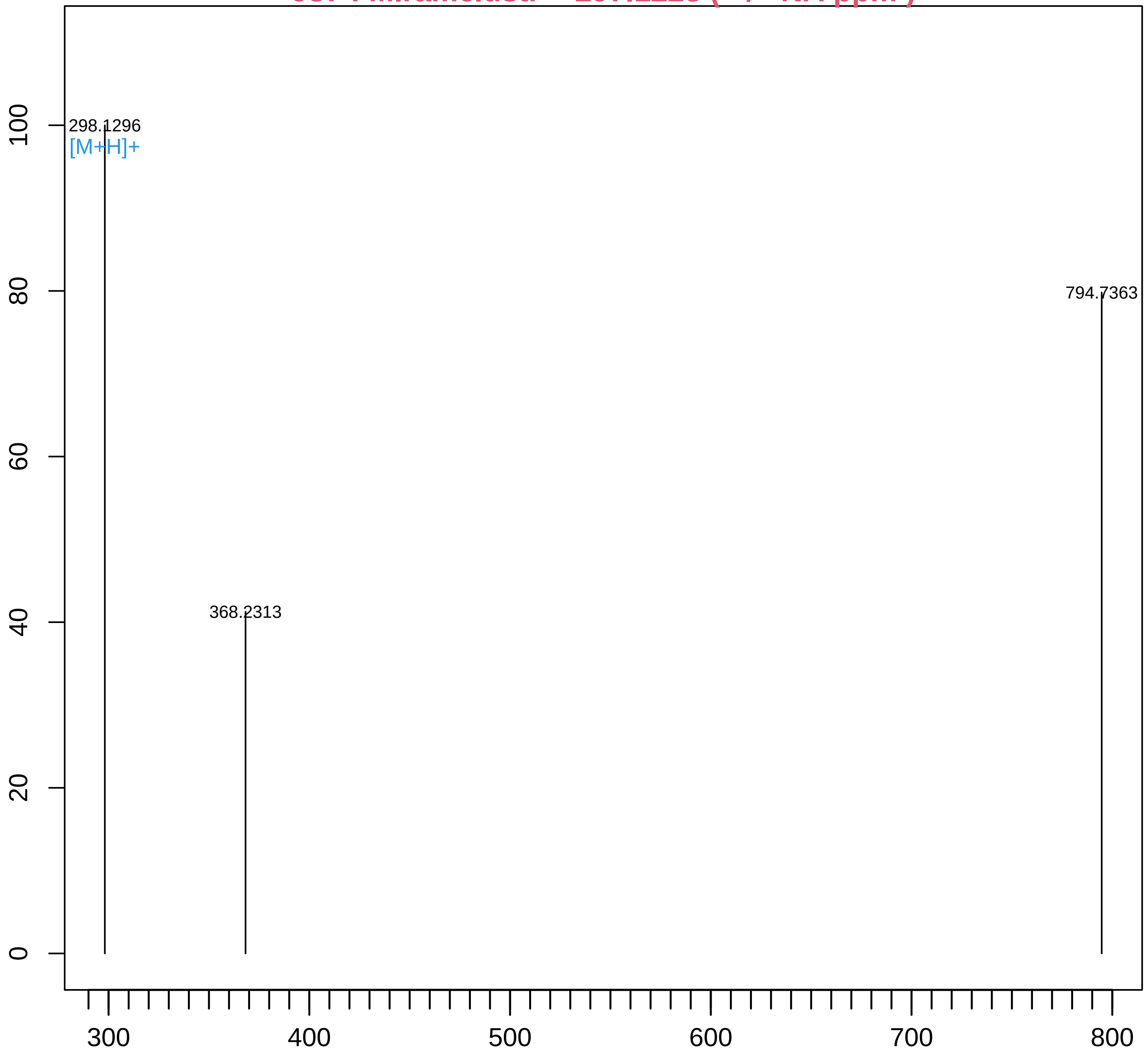

987 : M.findmain = 297.1223 (+/− 20 ppm)

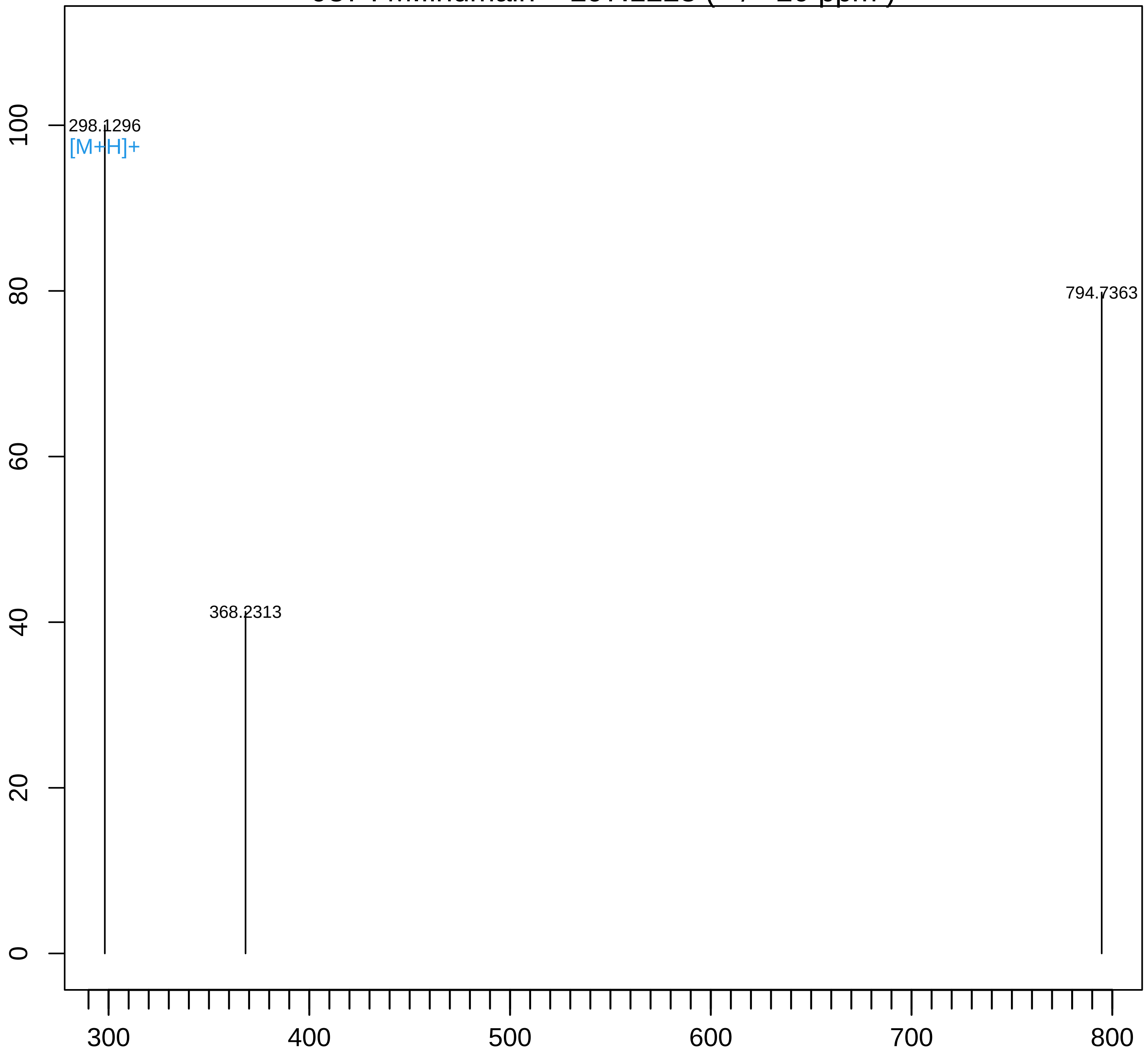

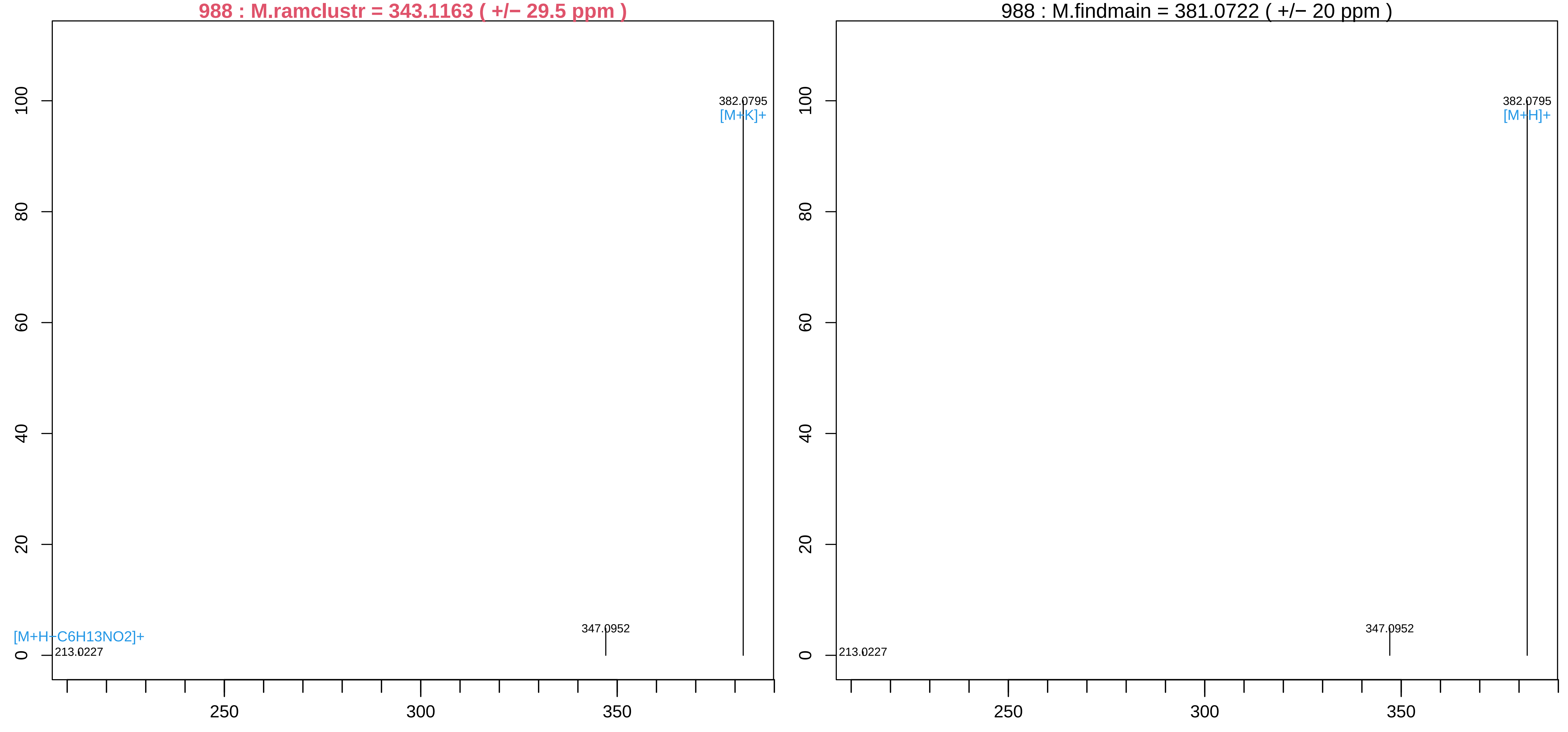

988 : M.ramclustr = 343.1163 (+/− 29.5 ppm)
382.0795
[M+K]+
347.0952
[M+H−C6H13NO2]+
213.0227
988 : M.findmain = 381.0722 (+/− 20 ppm)
382.0795
[M+H]+
347.0952
213.0227

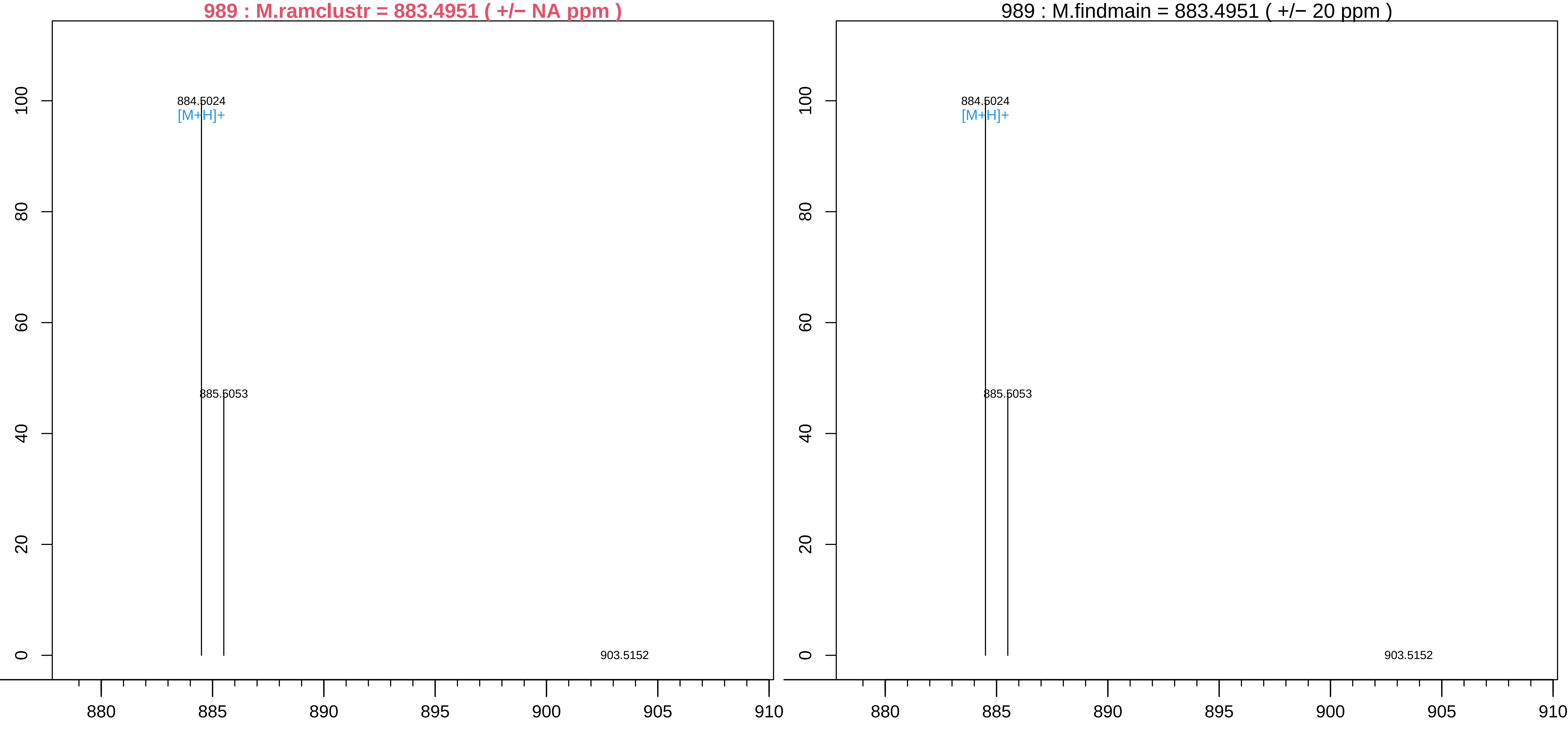

989 : M.ramclustr = 883.4951 (+/− NA ppm)
884.5024
[M+H]+
885.5053
903.5152
0
20
40
60
80
100
880
885
890
895
900
905
910
989 : M.findmain = 883.4951 (+/− 20 ppm)
884.5024
[M+H]+
885.5053
903.5152
0
20
40
60
80
100
880
885
890
895
900
905
910

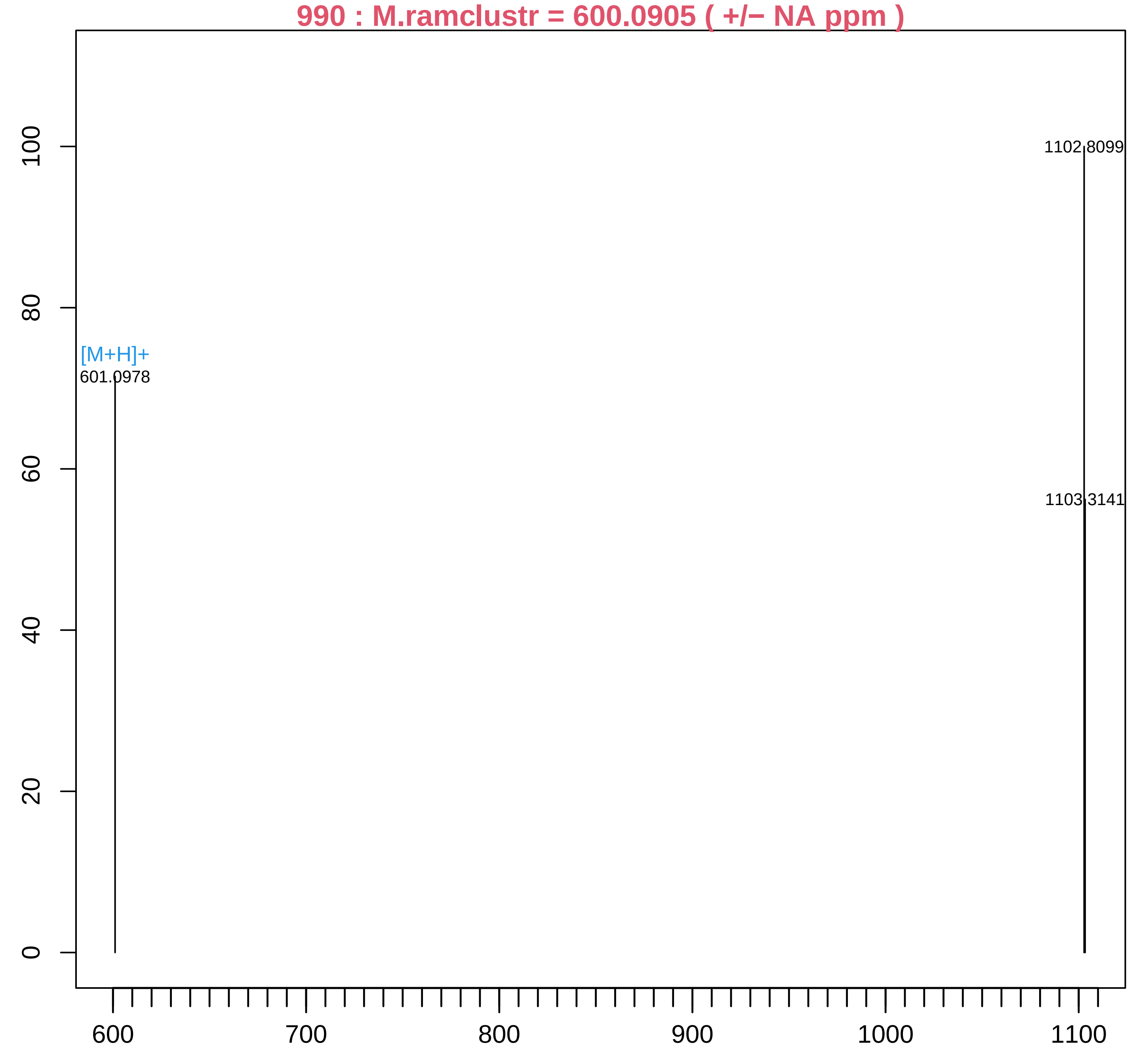
990 : M.ramclustr = 600.0905 (+/− NA ppm)
[M+H]+
601.0978
1102.8099
1103.3141
0
20
40
60
80
100
600
700
800
900
1000
1100

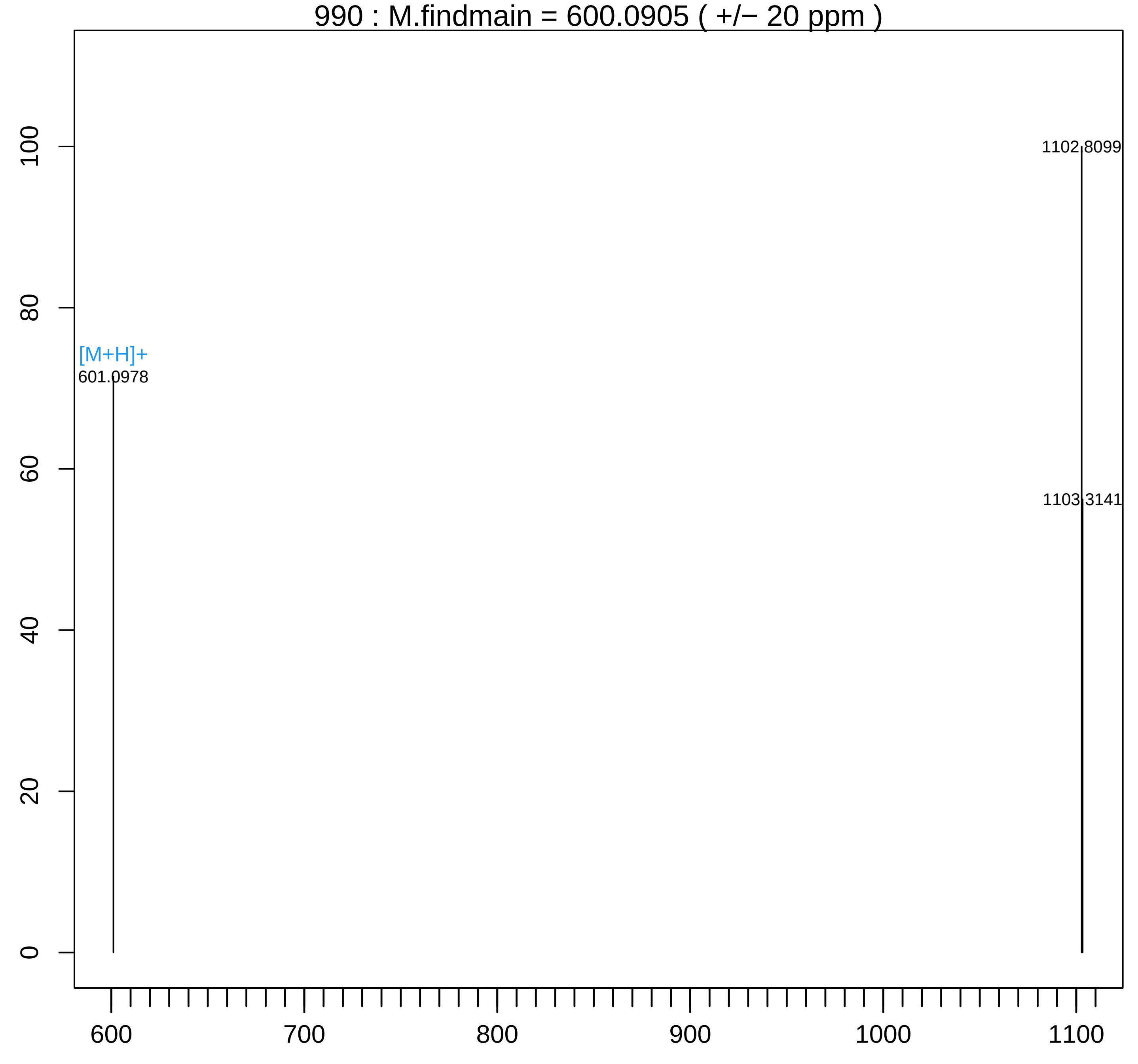
990 : M.findmain = 600.0905 (+/− 20 ppm)
[M+H]+
601.0978
1102.8099
1103.3141
0
20
40
60
80
100
600
700
800
900
1000
1100

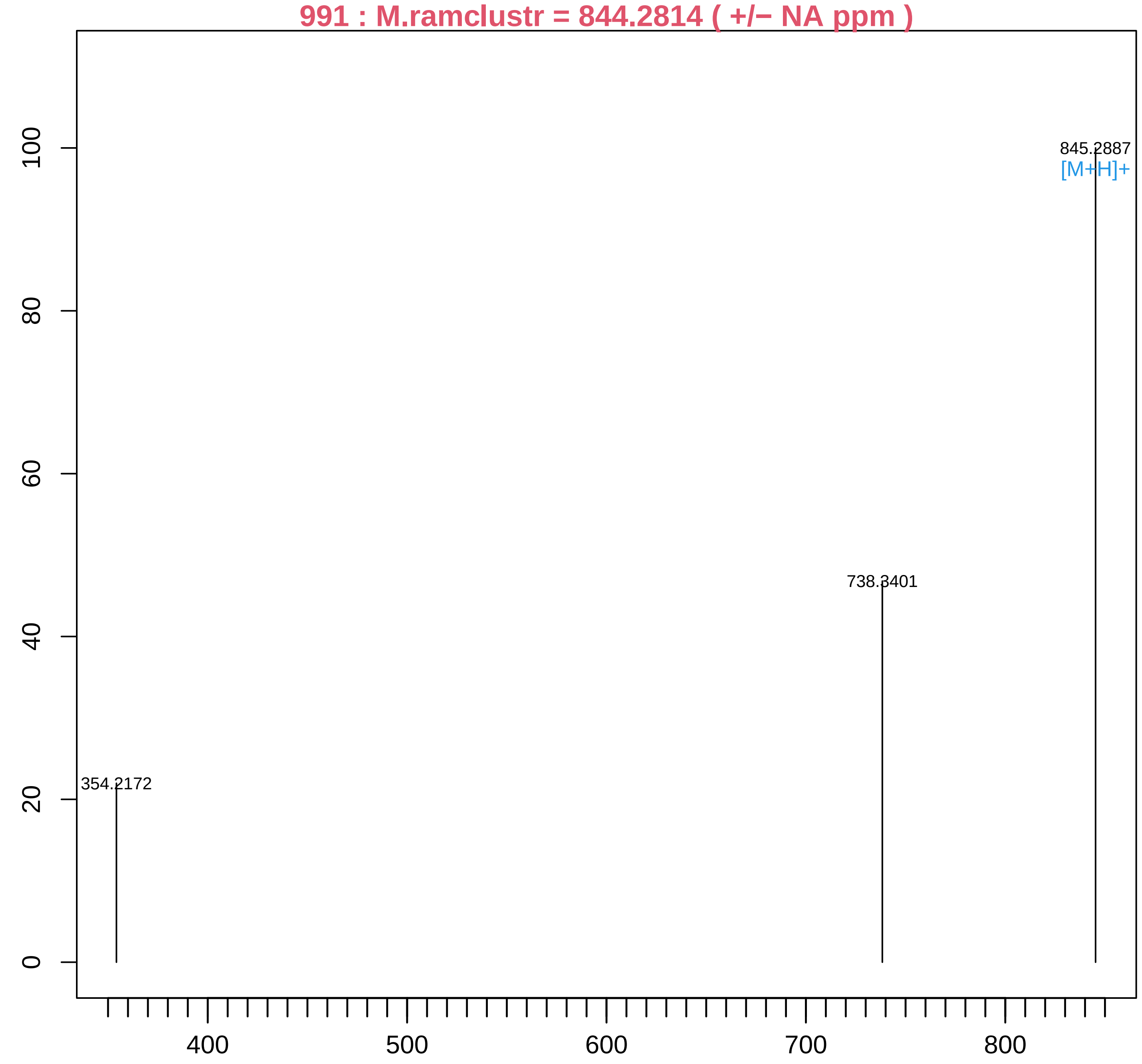
991 : M.ramclustr = 844.2814 (+/− NA ppm)
845.2887
[M+H]+
738.3401
354.2172
0
20
40
60
80
100
400
500
600
700
800

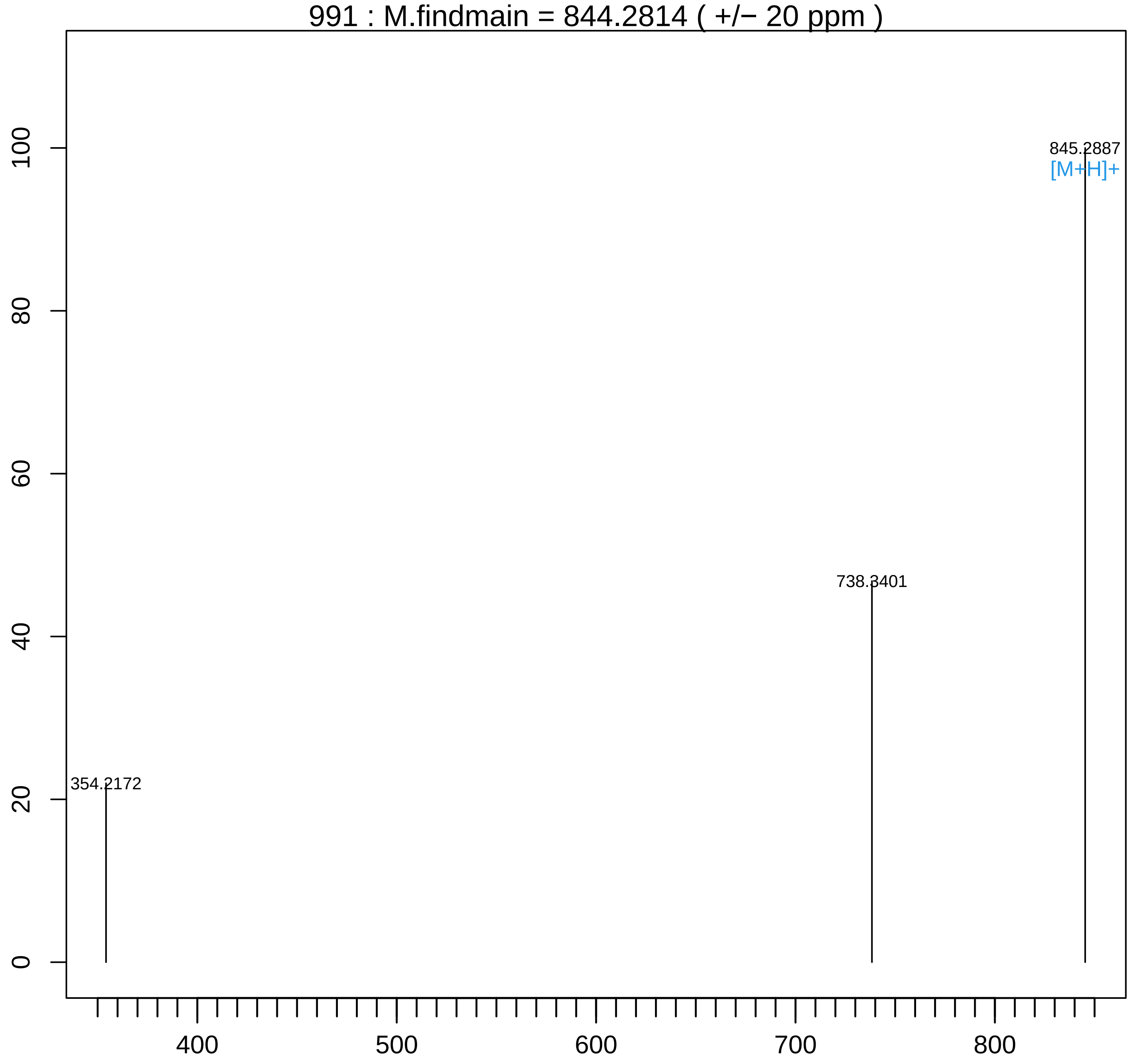
991 : M.findmain = 844.2814 (+/− 20 ppm)
845.2887
[M+H]+
738.3401
354.2172
0
20
40
60
80
100
400
500
600
700
800

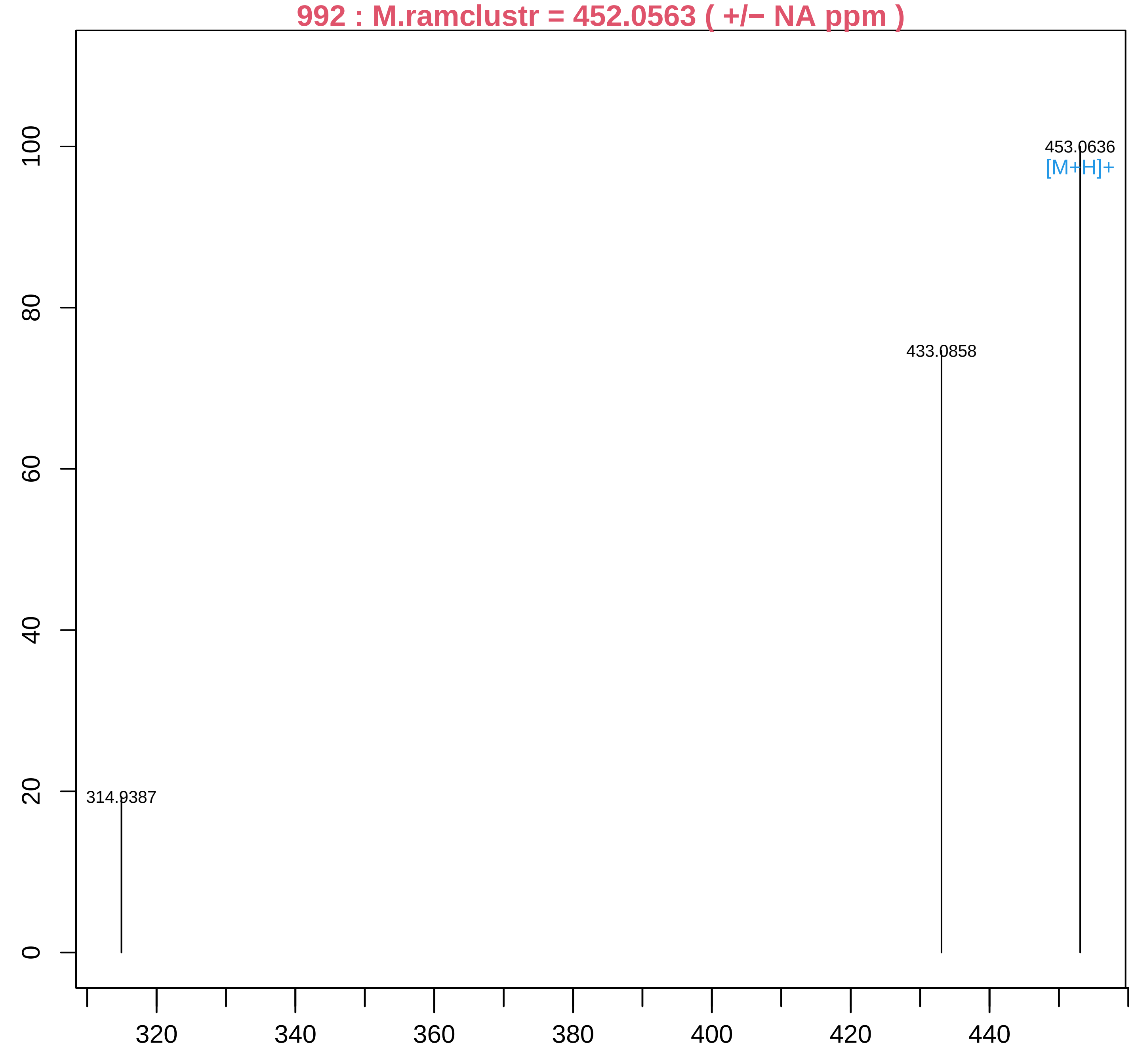

992 : M.ramclustr = 452.0563 (+/− NA ppm)
453.0636
[M+H]+
433.0858
314.9387
0
20
40
60
80
100
320
340
360
380
400
420
440

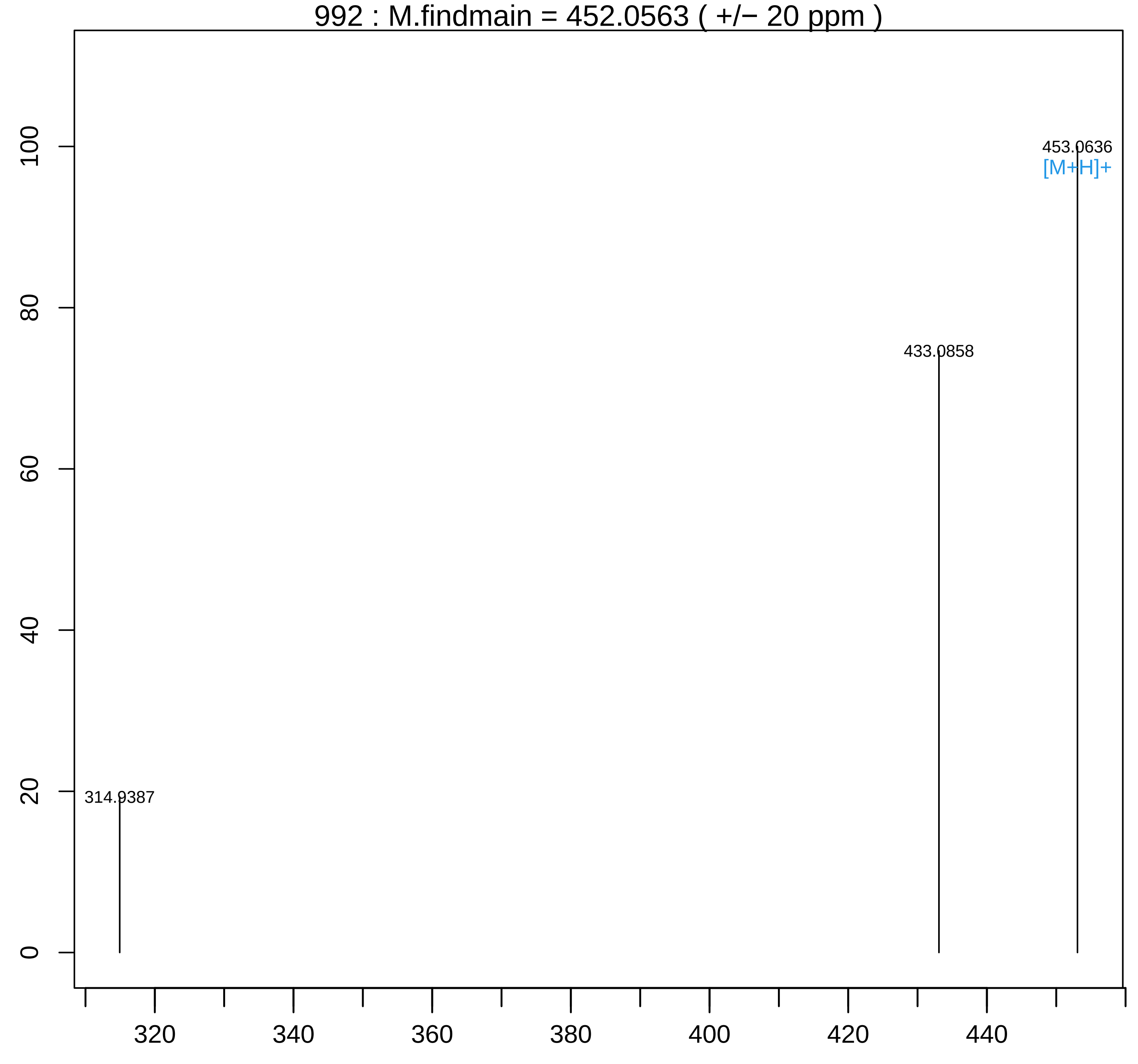

992 : M.findmain = 452.0563 (+/− 20 ppm)
453.0636
[M+H]+
433.0858
314.9387
0
20
40
60
80
100
320
340
360
380
400
420
440

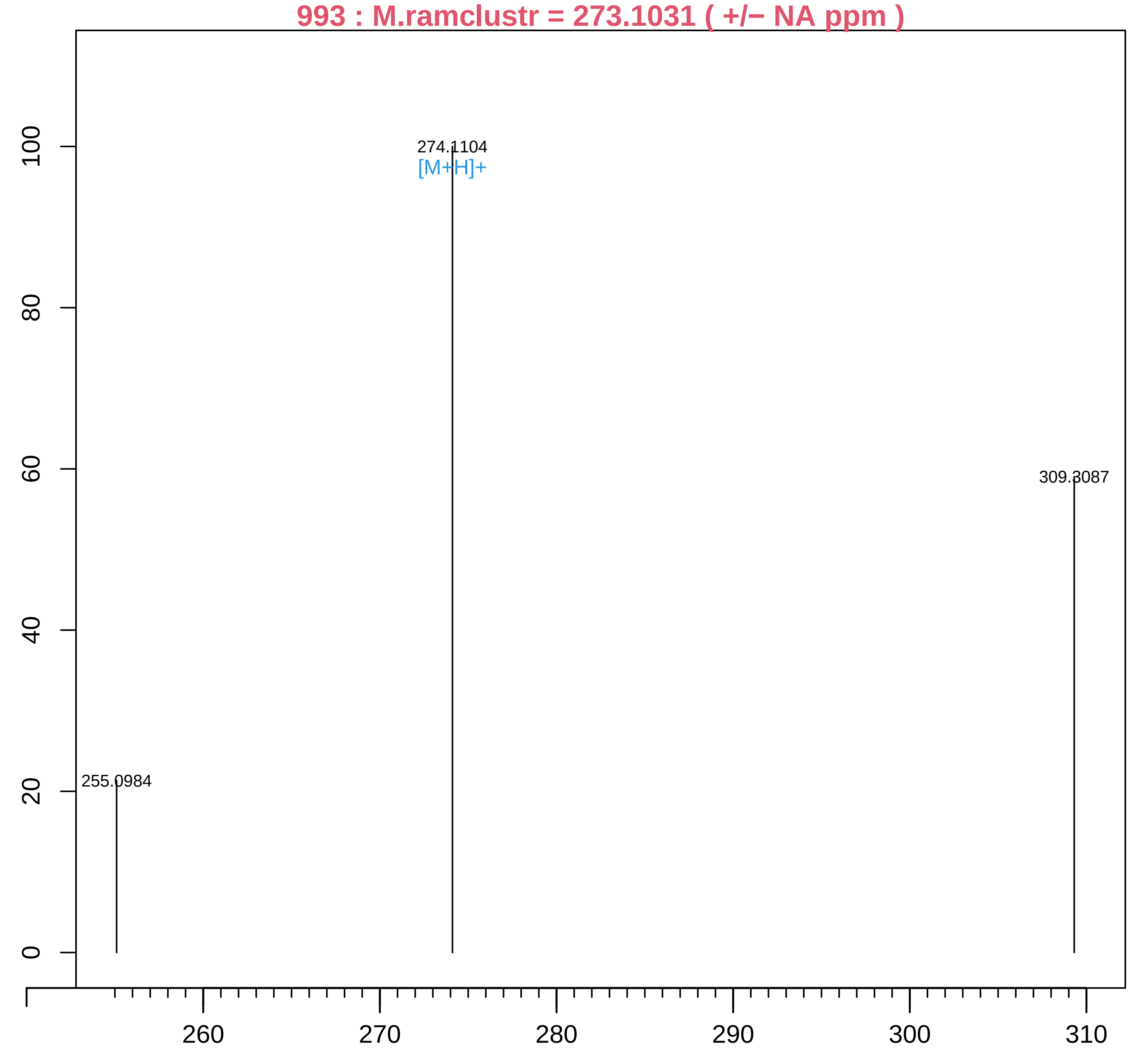
993 : M.ramclustr = 273.1031 (+/− NA ppm)
274.1104
[M+H]+
309.3087
255.0984
0
20
40
60
80
100
260
270
280
290
300
310
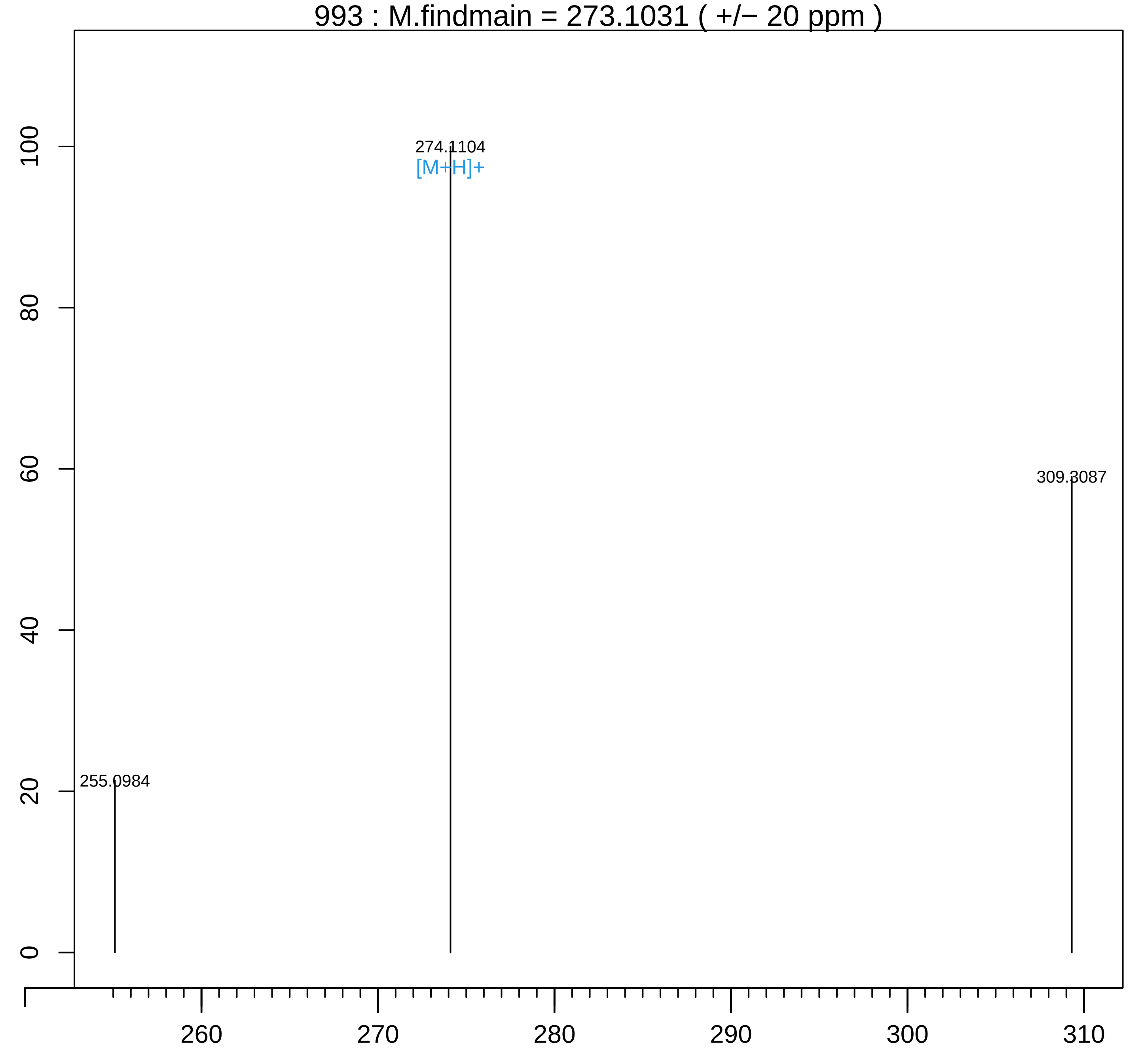
993 : M.findmain = 273.1031 (+/− 20 ppm)
274.1104
[M+H]+
309.3087
255.0984
0
20
40
60
80
100
260
270
280
290
300
310

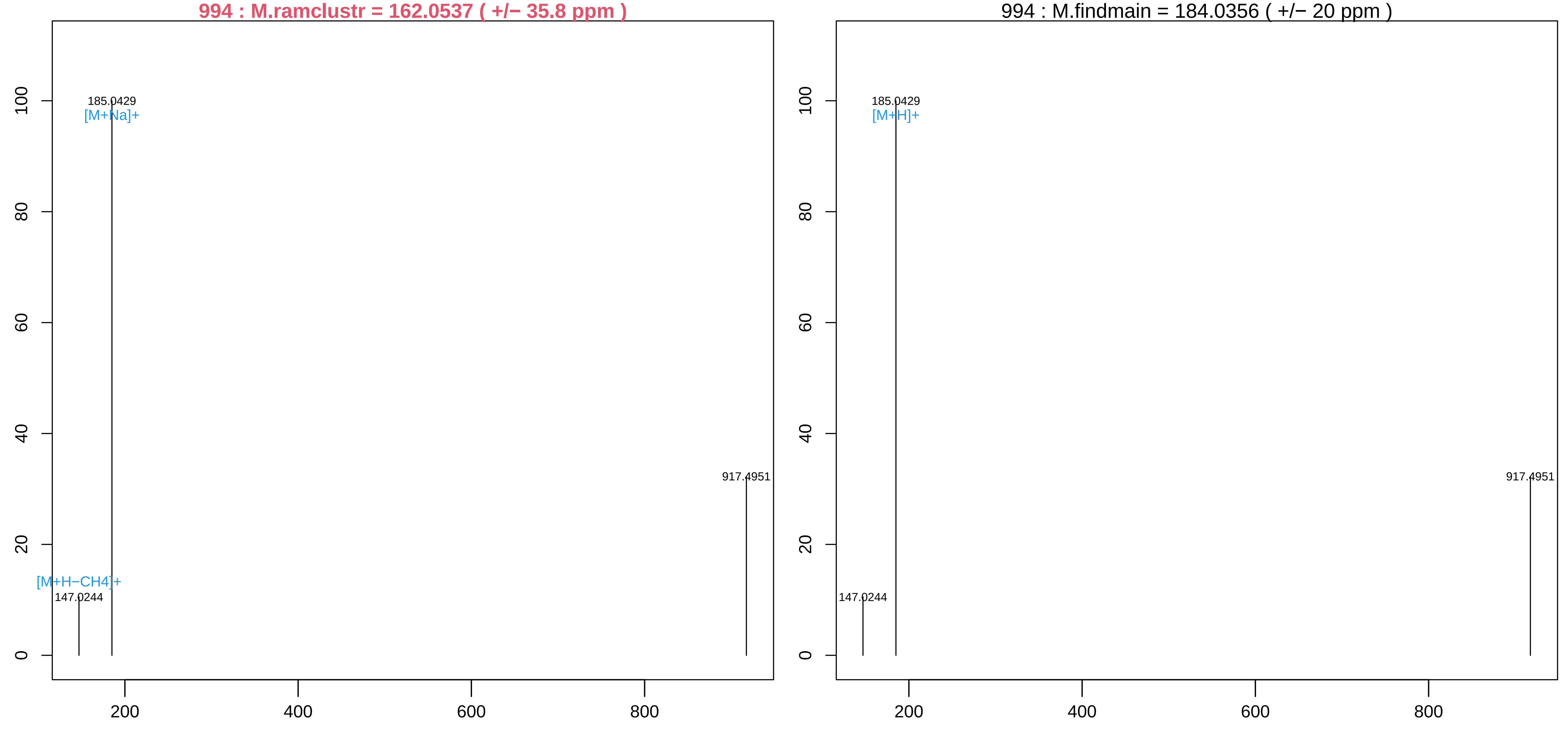
994 : M.ramclustr = 162.0537 (+/− 35.8 ppm)
185.0429
[M+Na]+
[M+H−CH4]+
147.0244
917.4951
0
20
40
60
80
100
200
400
600
800
994 : M.findmain = 184.0356 (+/− 20 ppm)
185.0429
[M+H]+
147.0244
917.4951
0
20
40
60
80
100
200
400
600
800

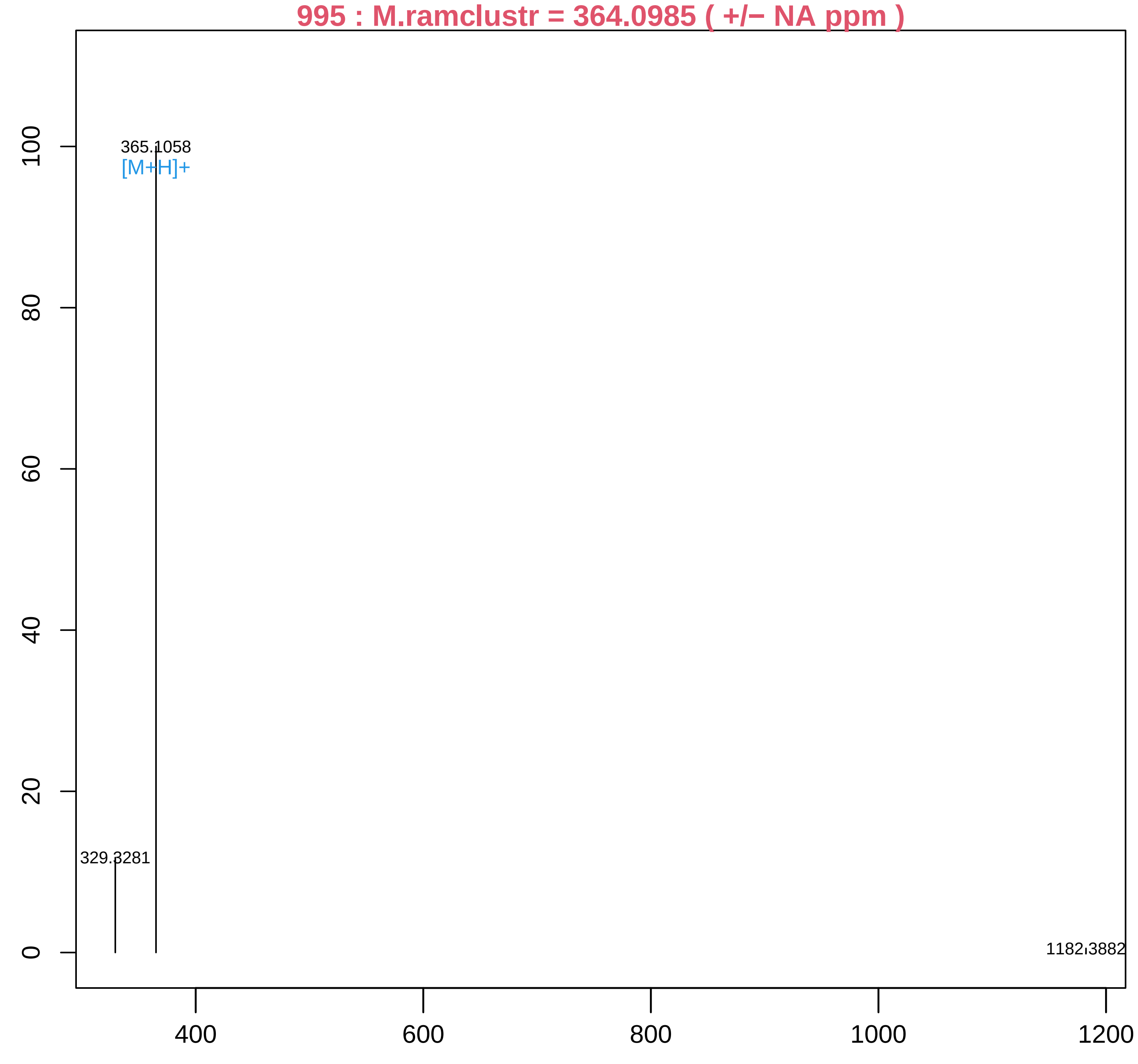
995 : M.ramclustr = 364.0985 (+/− NA ppm)
365.1058
[M+H]+
329.3281
1182.3882
0
20
40
60
80
100
400
600
800
1000
1200

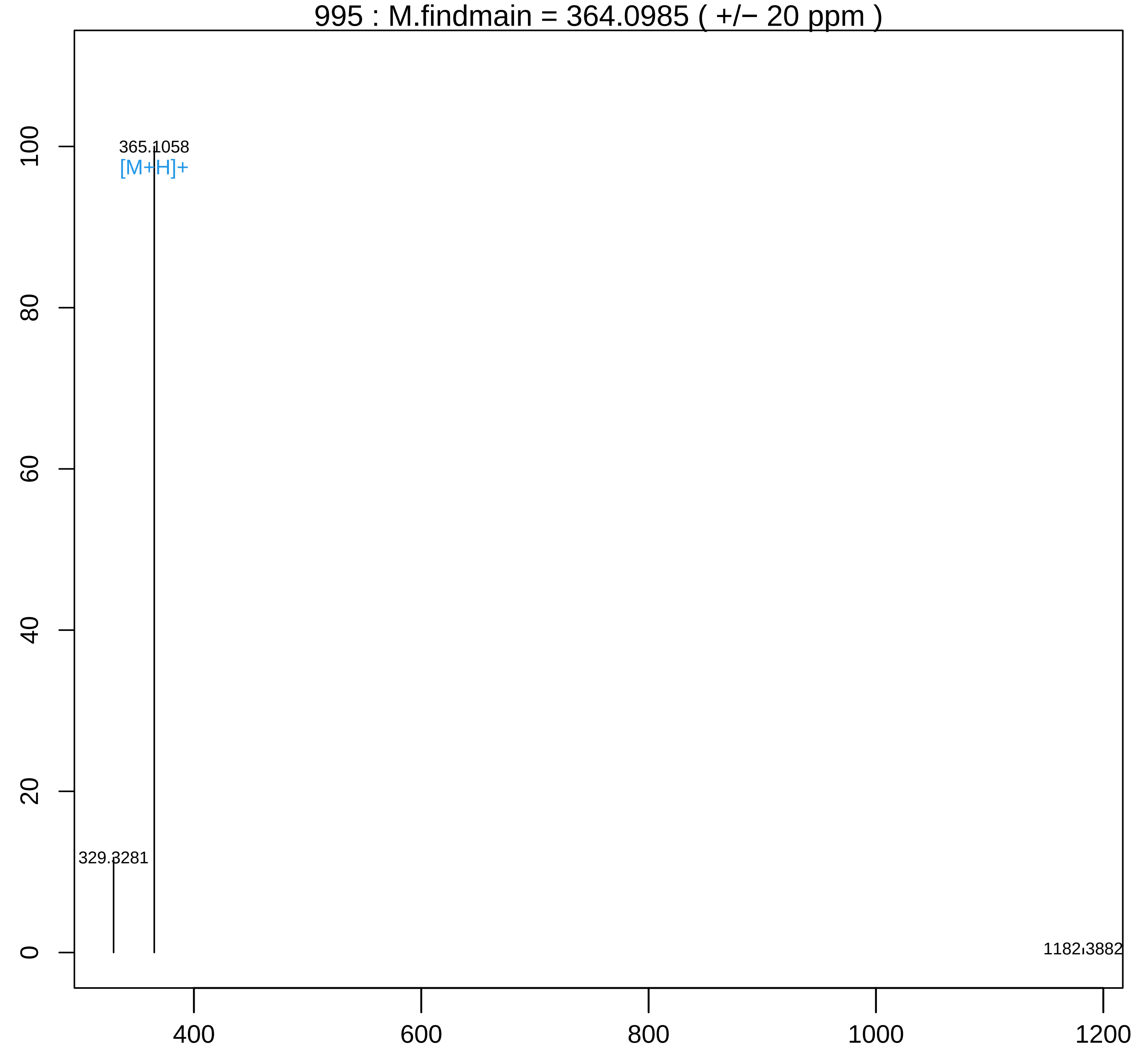
995 : M.findmain = 364.0985 (+/− 20 ppm)
365.1058
[M+H]+
329.3281
1182.3882
0
20
40
60
80
100
400
600
800
1000
1200

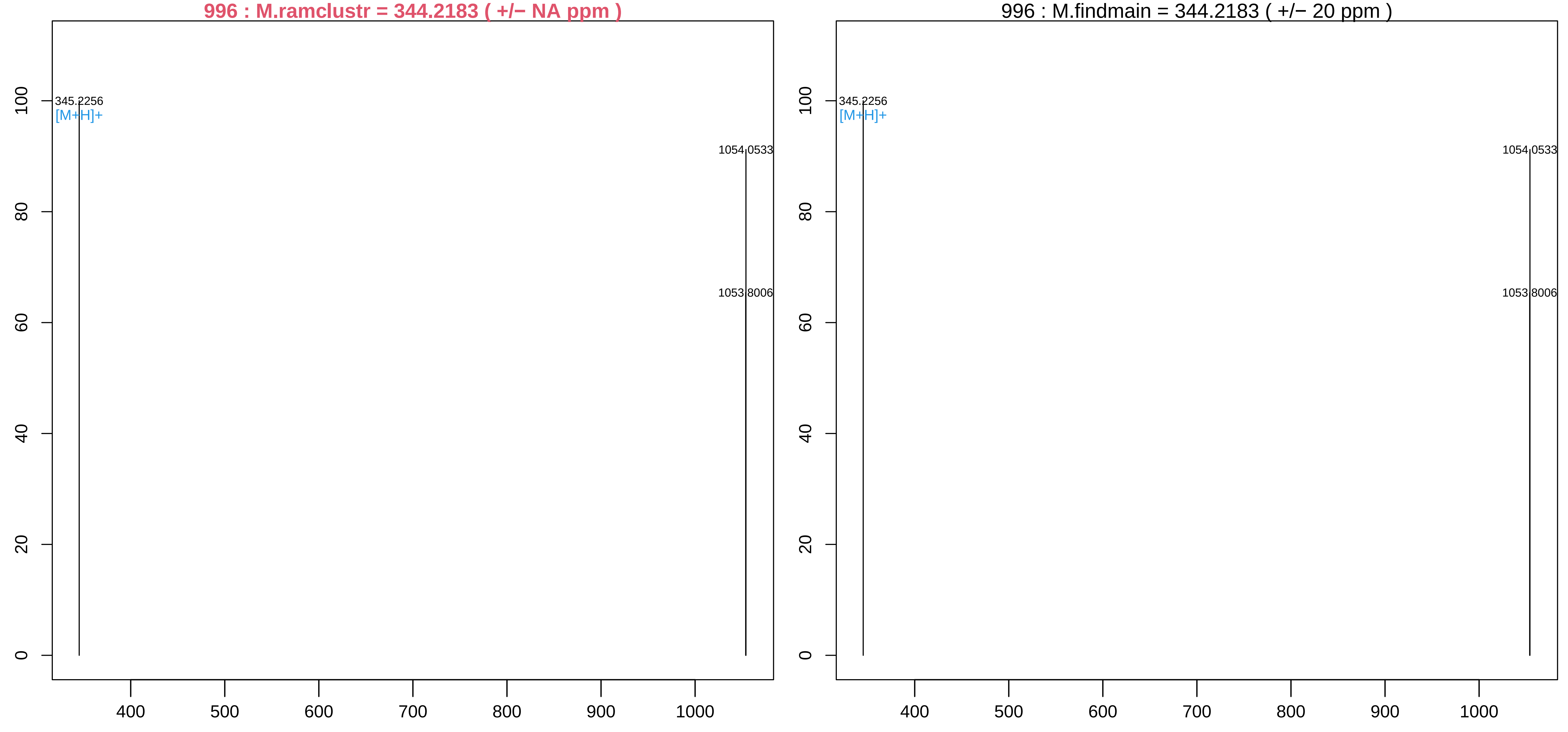
996 : M.ramclustr = 344.2183 (+/− NA ppm)
345.2256
[M+H]+
1054.0533
1053.8006
0
20
40
60
80
100
400
500
600
700
800
900
1000
996 : M.findmain = 344.2183 (+/− 20 ppm)
345.2256
[M+H]+
1054.0533
1053.8006
0
20
40
60
80
100
400
500
600
700
800
900
1000

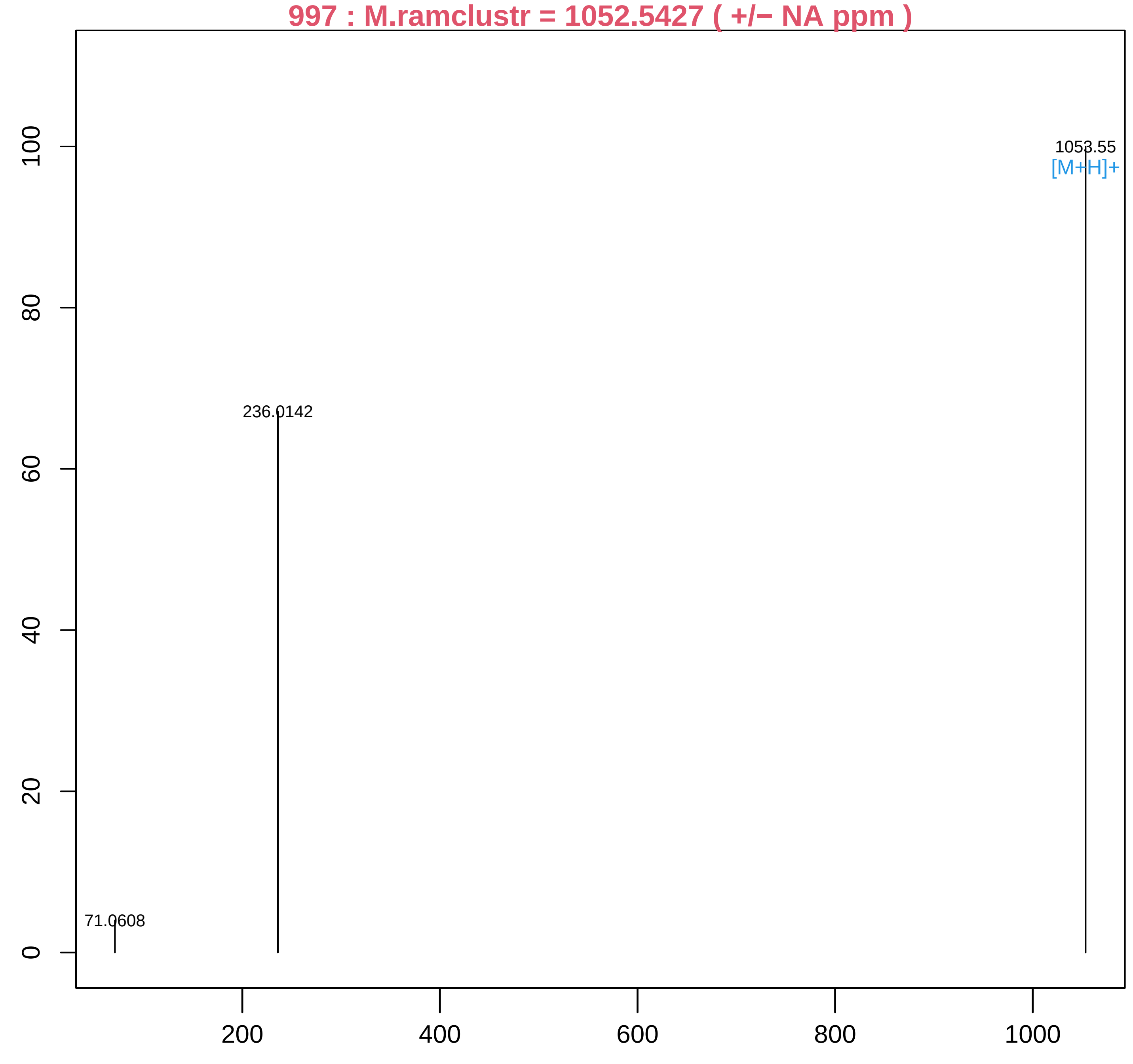
997 : M.ramclustr = 1052.5427 (+/− NA ppm)
1053.55
[M+H]+
236.0142
71.0608
0
20
40
60
80
100
200
400
600
800
1000

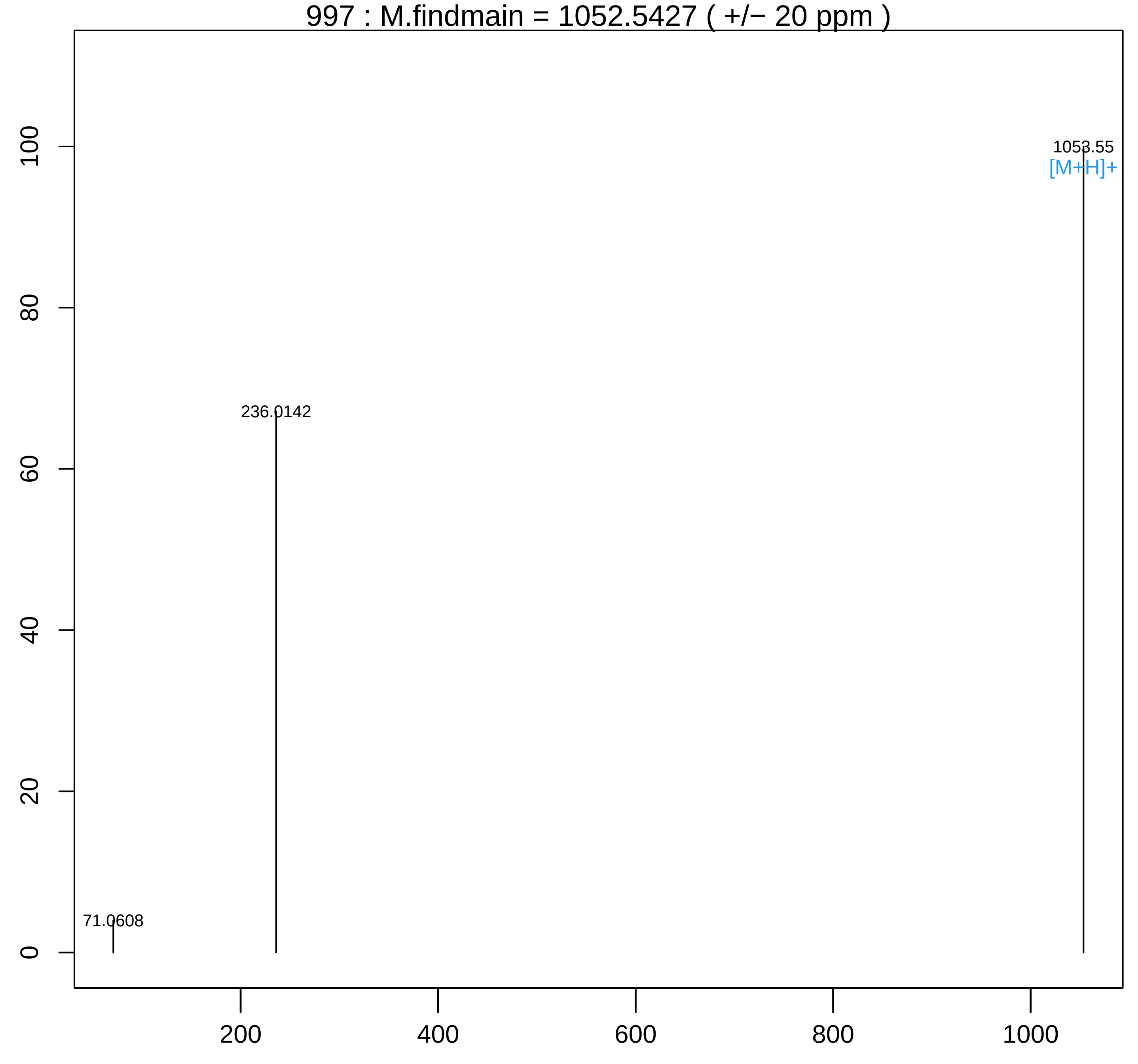
997 : M.findmain = 1052.5427 (+/− 20 ppm)
1053.55
[M+H]+
236.0142
71.0608
0
20
40
60
80
100
200
400
600
800
1000

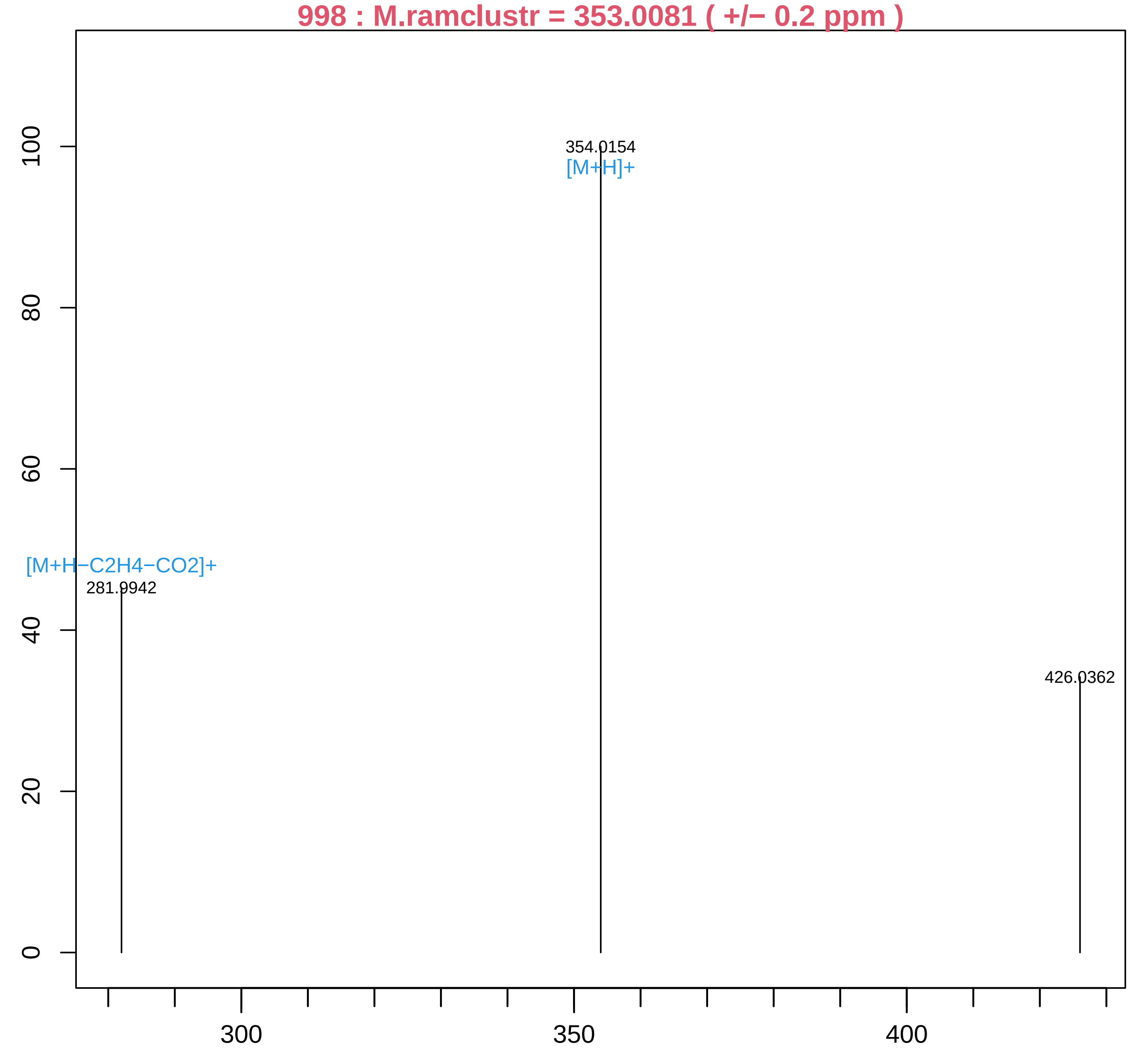

998 : M.ramclustr = 353.0081 (+/− 0.2 ppm)
354.0154
[M+H]+
[M+H−C2H4−CO2]+
281.9942
426.0362
0
20
40
60
80
100
300
350
400

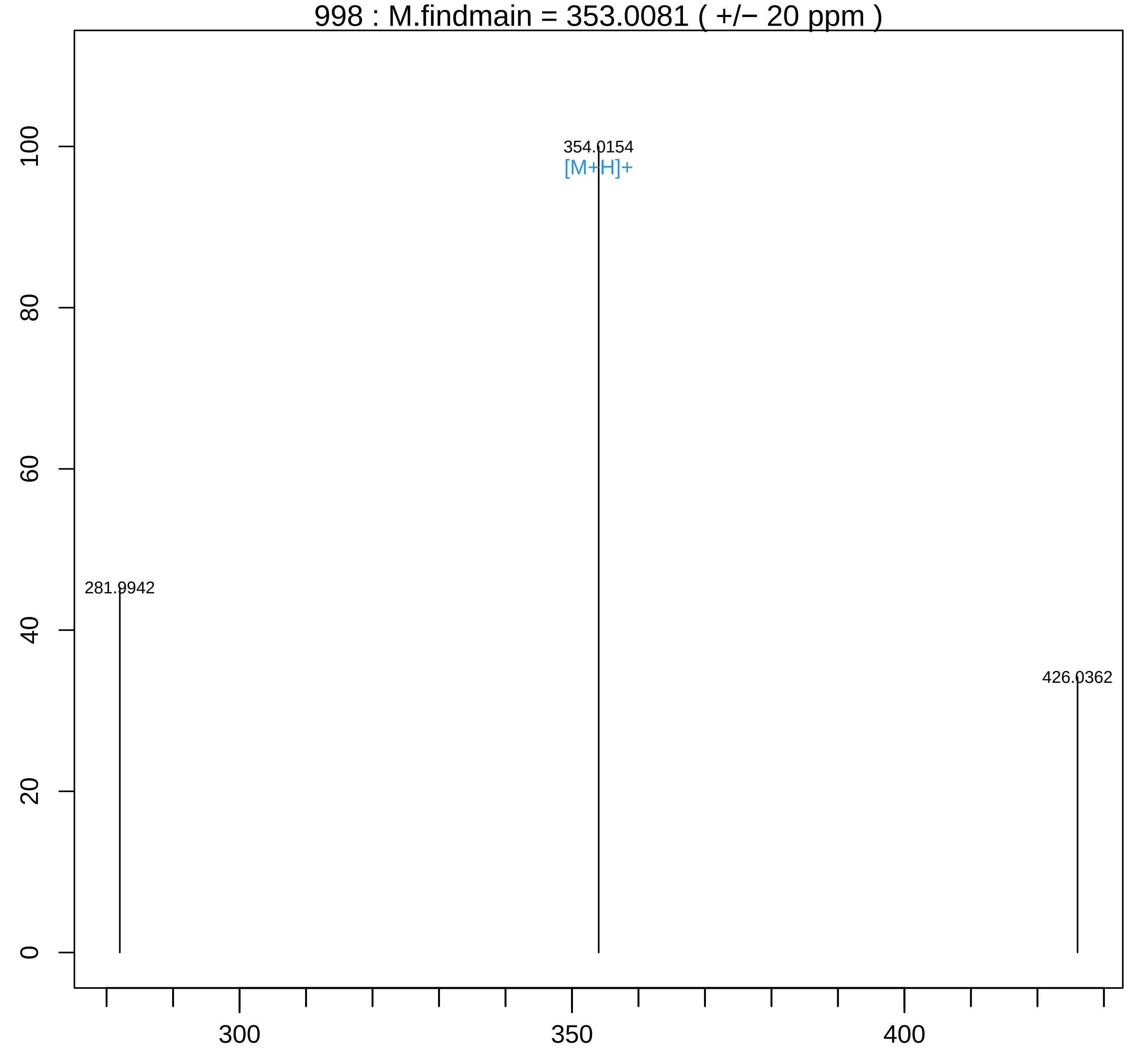

998 : M.findmain = 353.0081 (+/− 20 ppm)
354.0154
[M+H]+
281.9942
426.0362
0
20
40
60
80
100
300
350
400

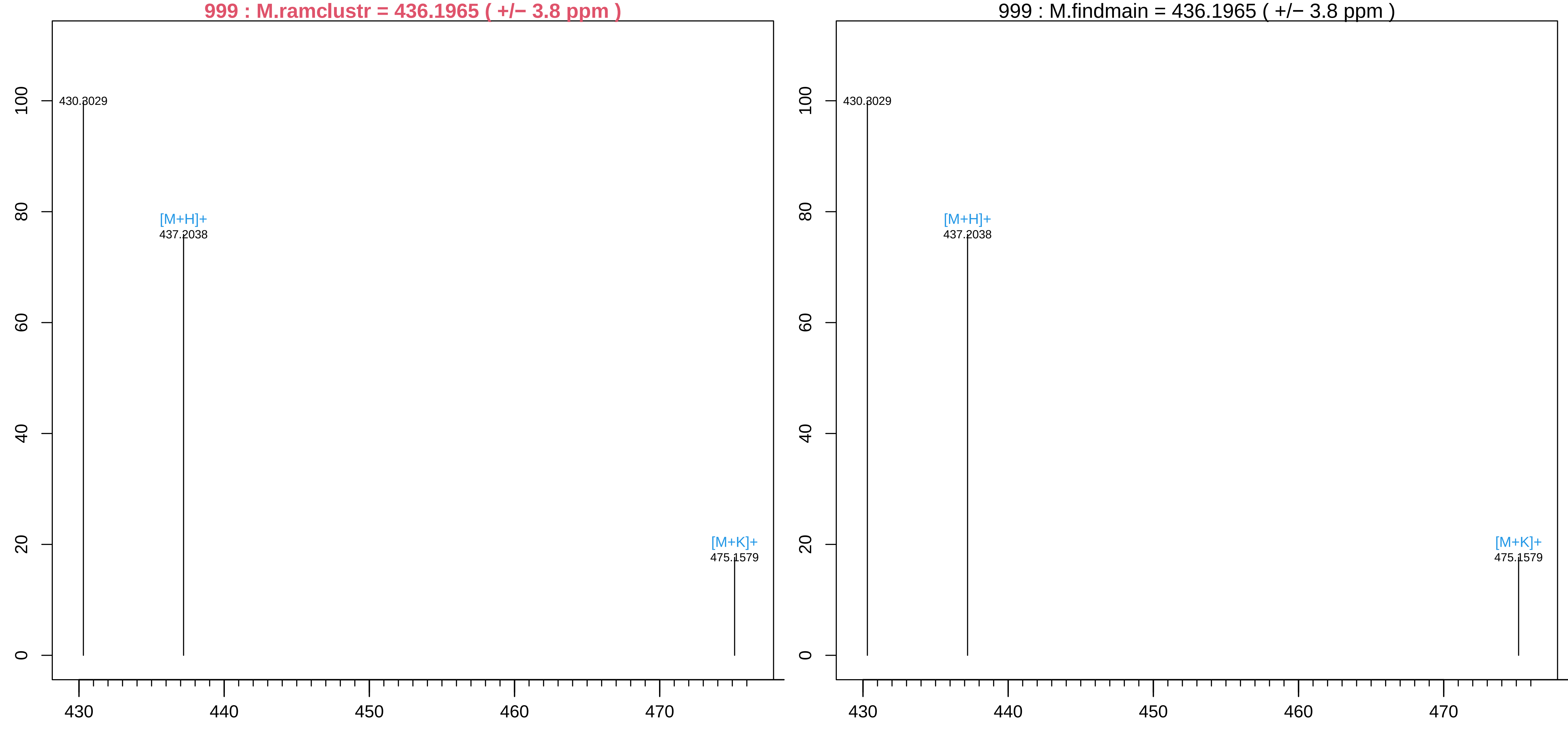
999 : M.ramclustr = 436.1965 (+/− 3.8 ppm)
430.3029
[M+H]+
437.2038
[M+K]+
475.1579
999 : M.findmain = 436.1965 (+/− 3.8 ppm)
430.3029
[M+H]+
437.2038
[M+K]+
475.1579

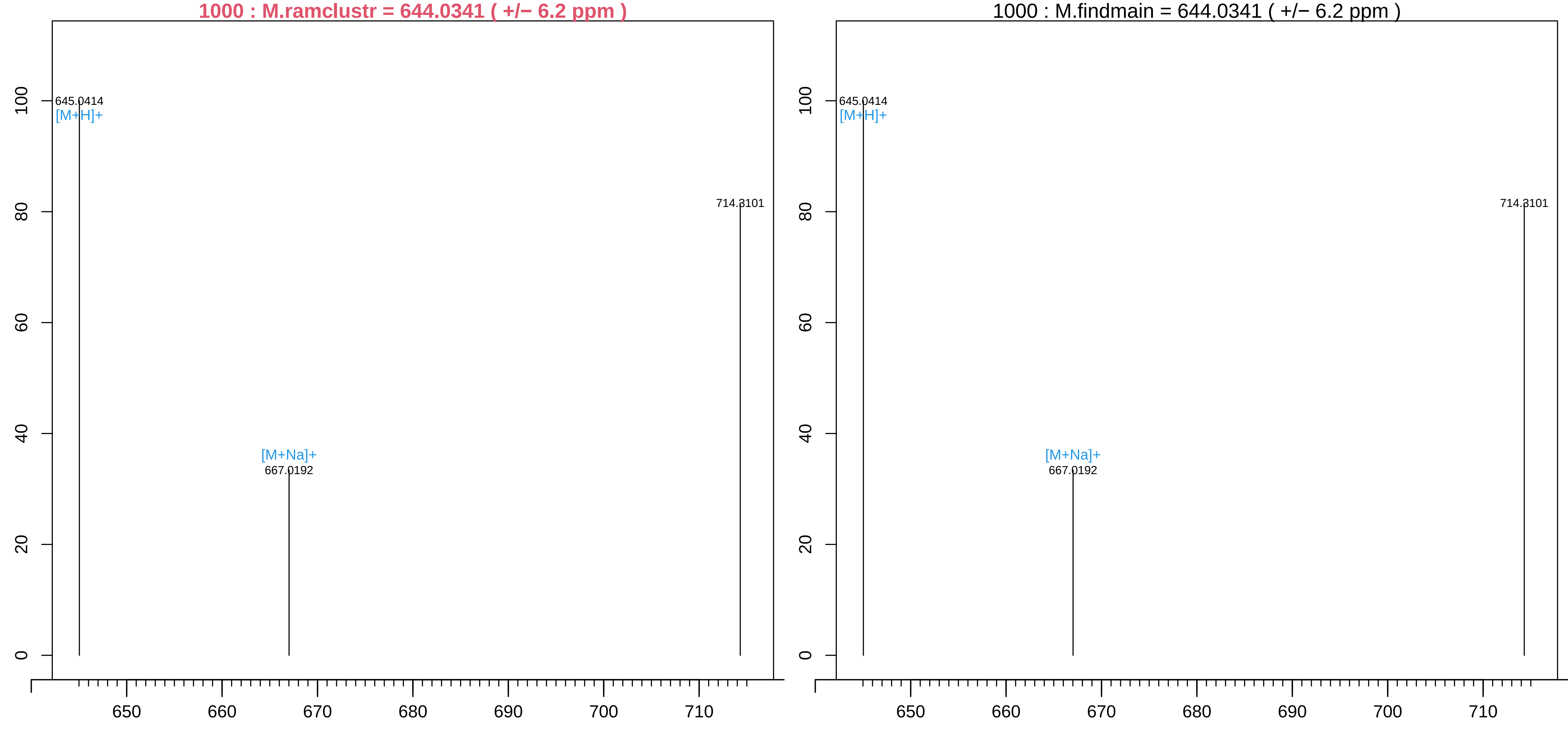
1000 : M.ramclustr = 644.0341 (+/− 6.2 ppm)
1000 : M.findmain = 644.0341 (+/− 6.2 ppm)
645.0414
[M+H]+
[M+Na]+
667.0192
714.3101
0
20
40
60
80
100
650
660
670
680
690
700
710

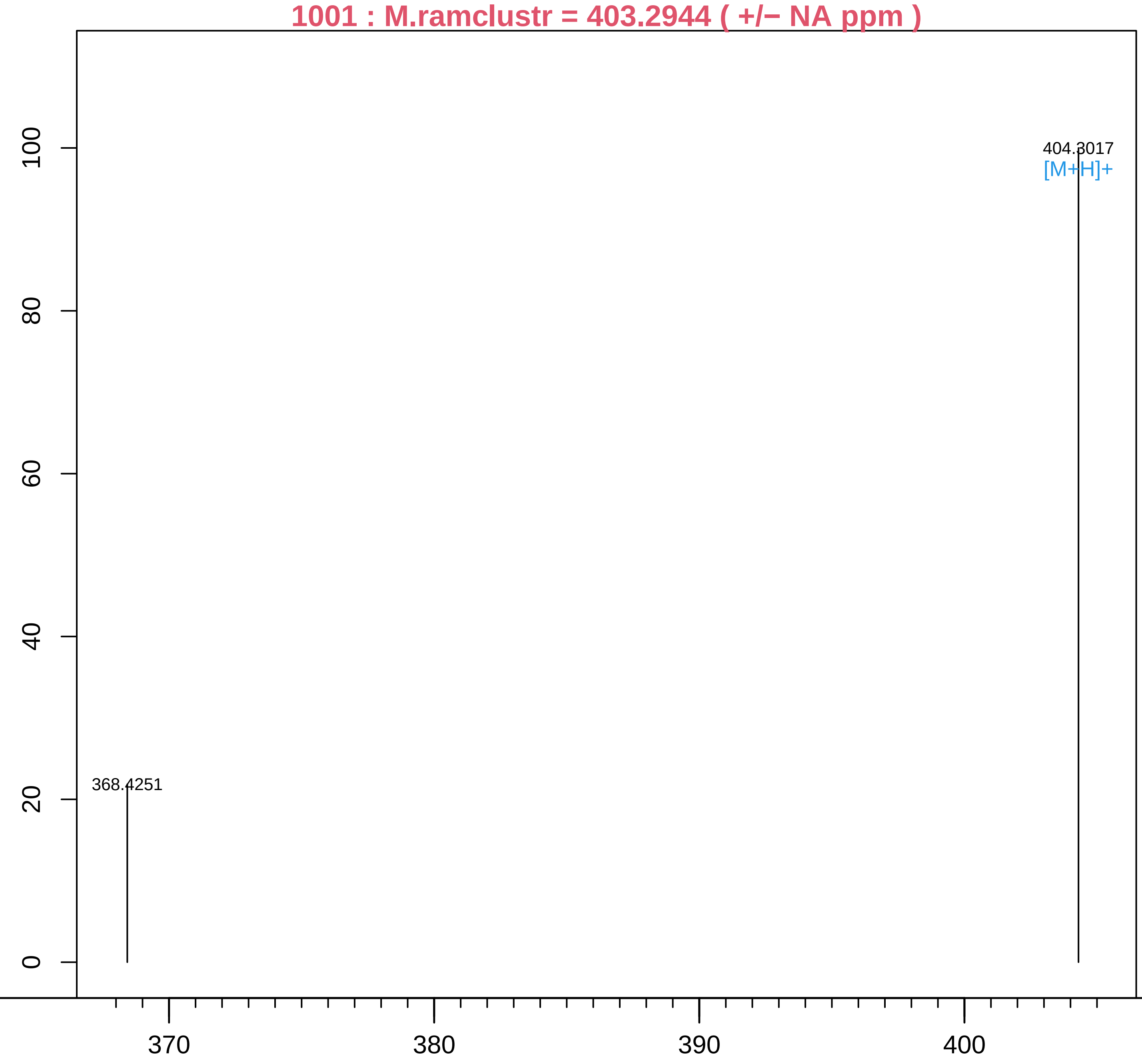

1001 : M.ramclustr = 403.2944 (+/− NA ppm)
404.3017
[M+H]+
368.4251
0
20
40
60
80
100
370
380
390
400

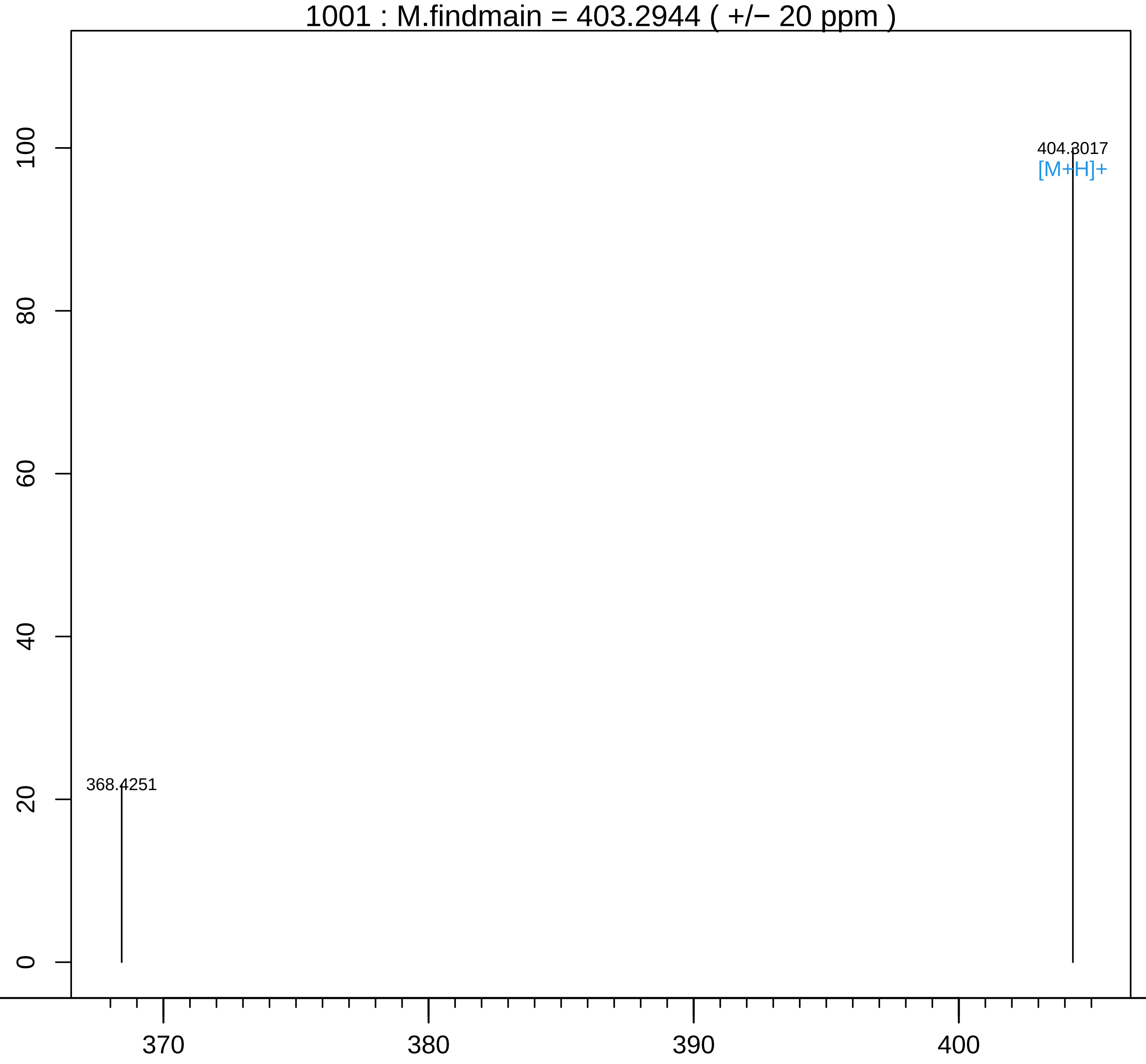

1001 : M.findmain = 403.2944 (+/− 20 ppm)
404.3017
[M+H]+
368.4251
0
20
40
60
80
100
370
380
390
400

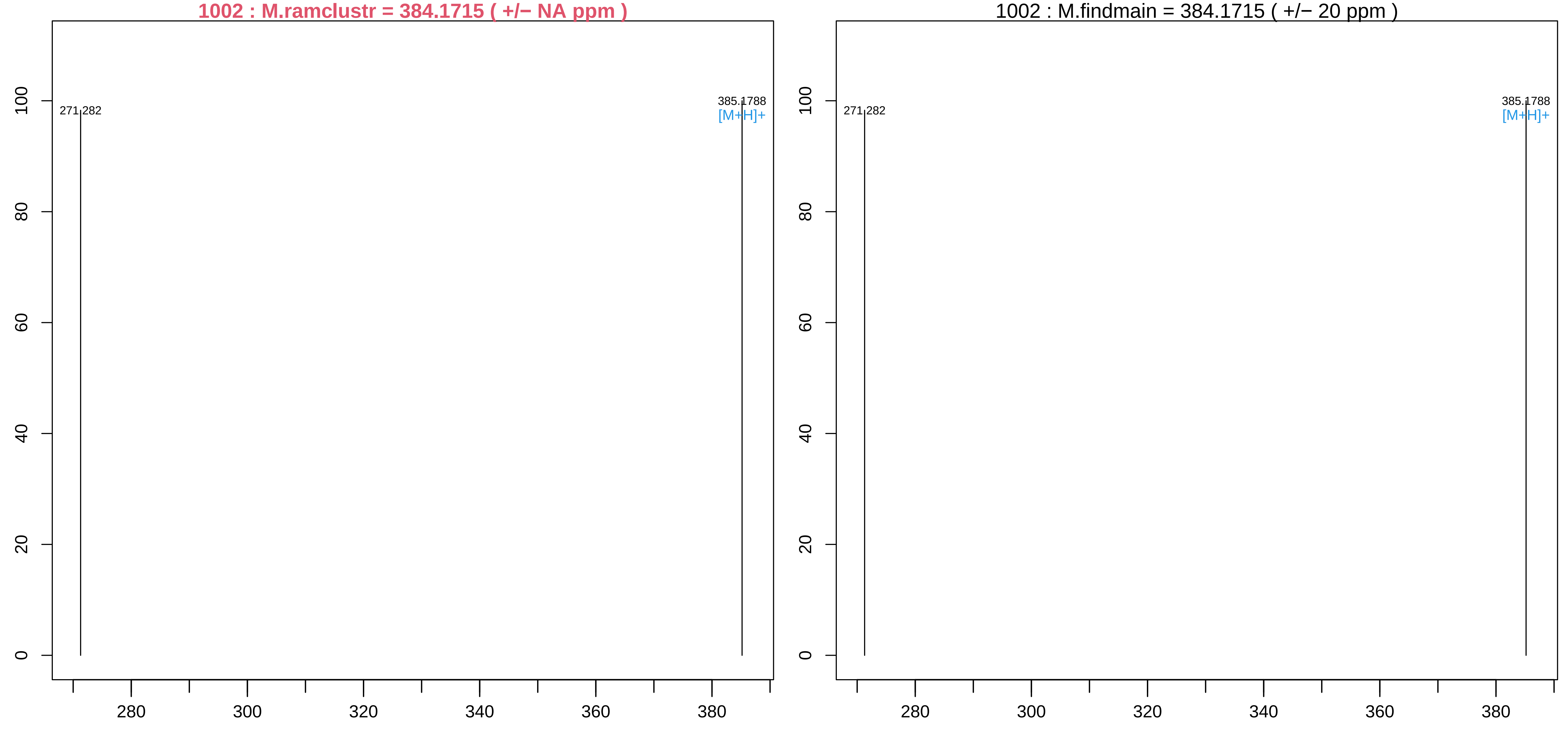
1002 : M.ramclustr = 384.1715 (+/− NA ppm)
1002 : M.findmain = 384.1715 (+/− 20 ppm)
271.282
385.1788
[M+H]+
0
20
40
60
80
100
280
300
320
340
360
380

1003 : M.ramclustr = 374.0209 (+/− NA ppm)

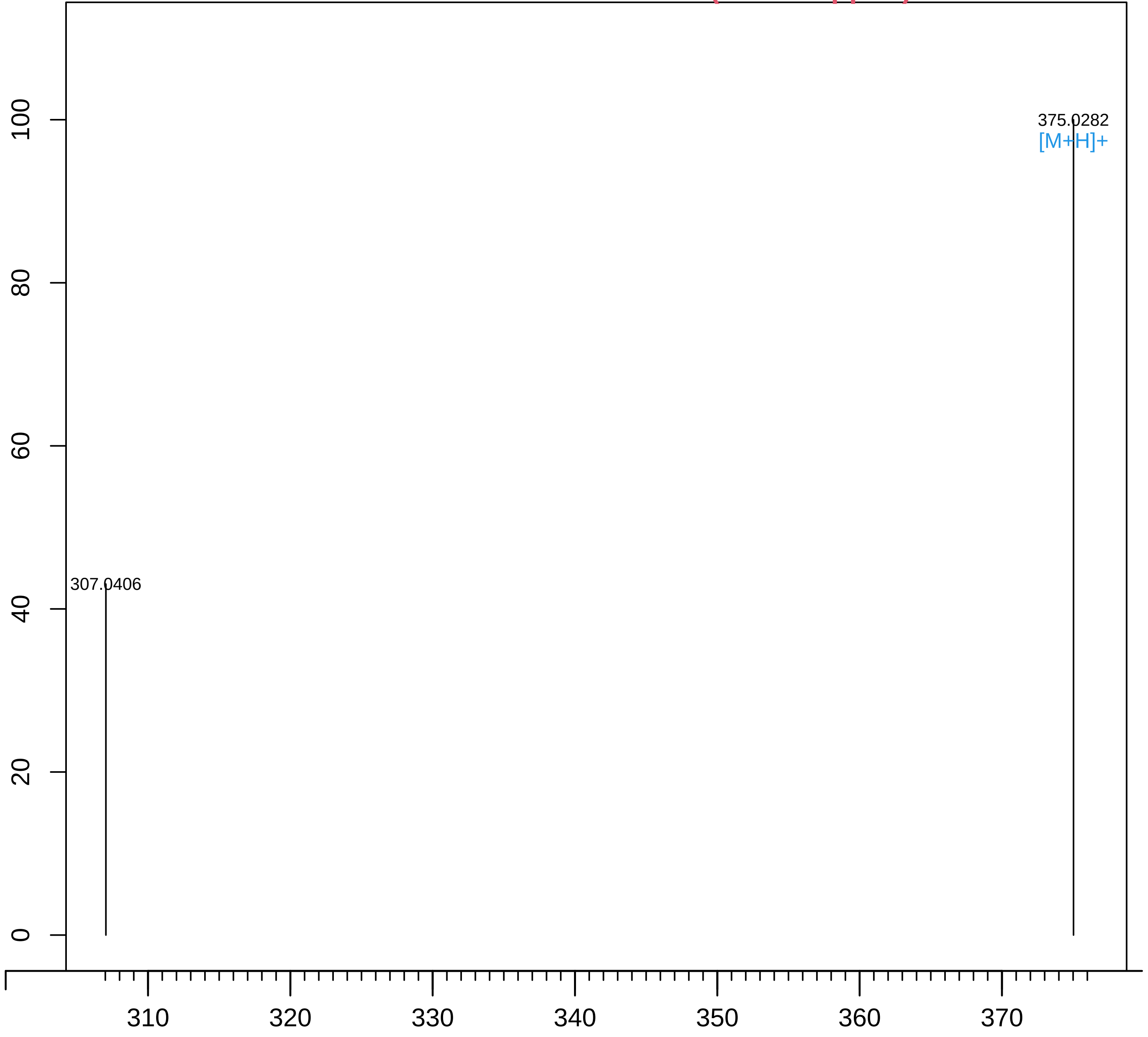

1003 : M.findmain = 374.0209 (+/− 20 ppm)

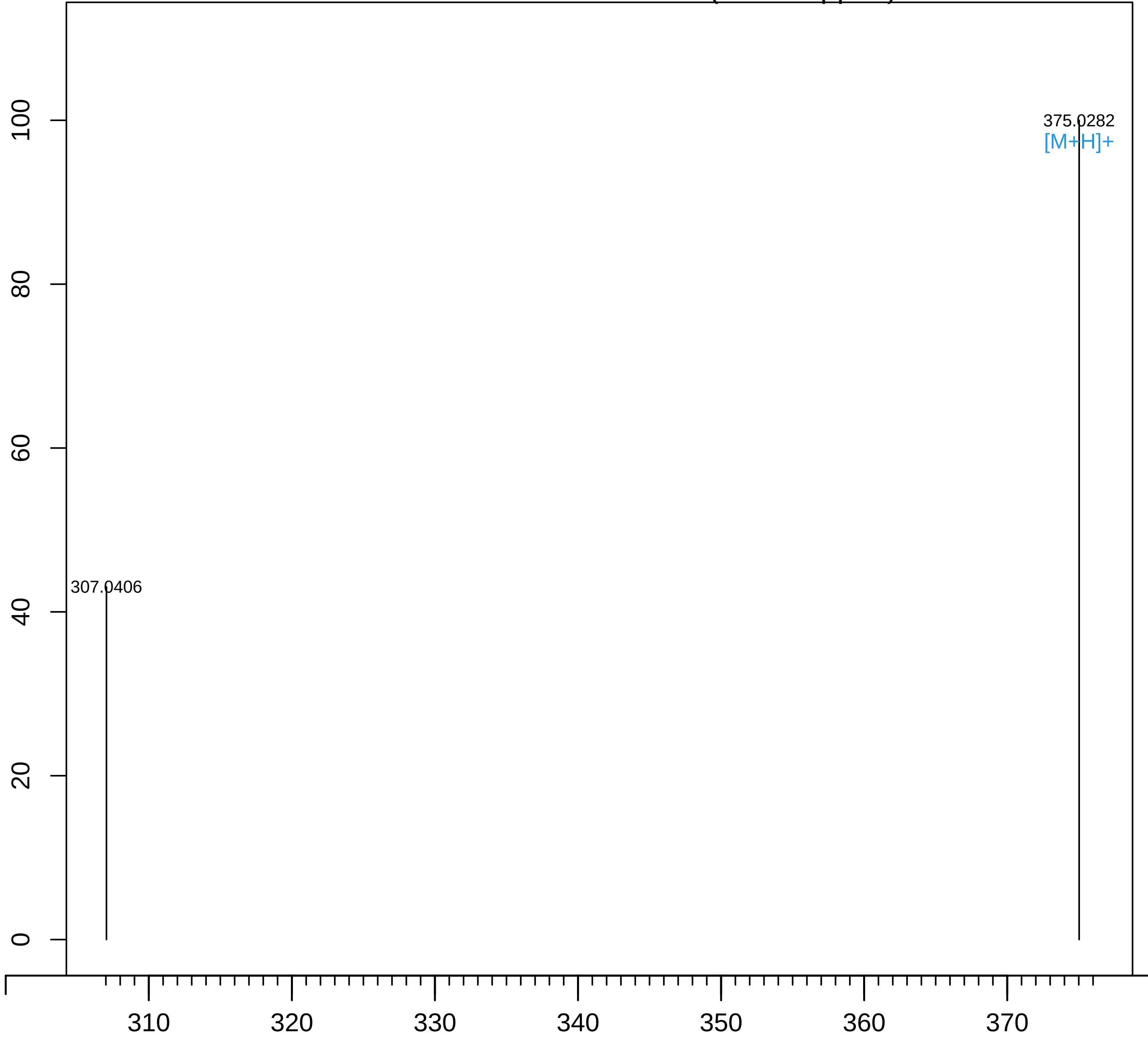

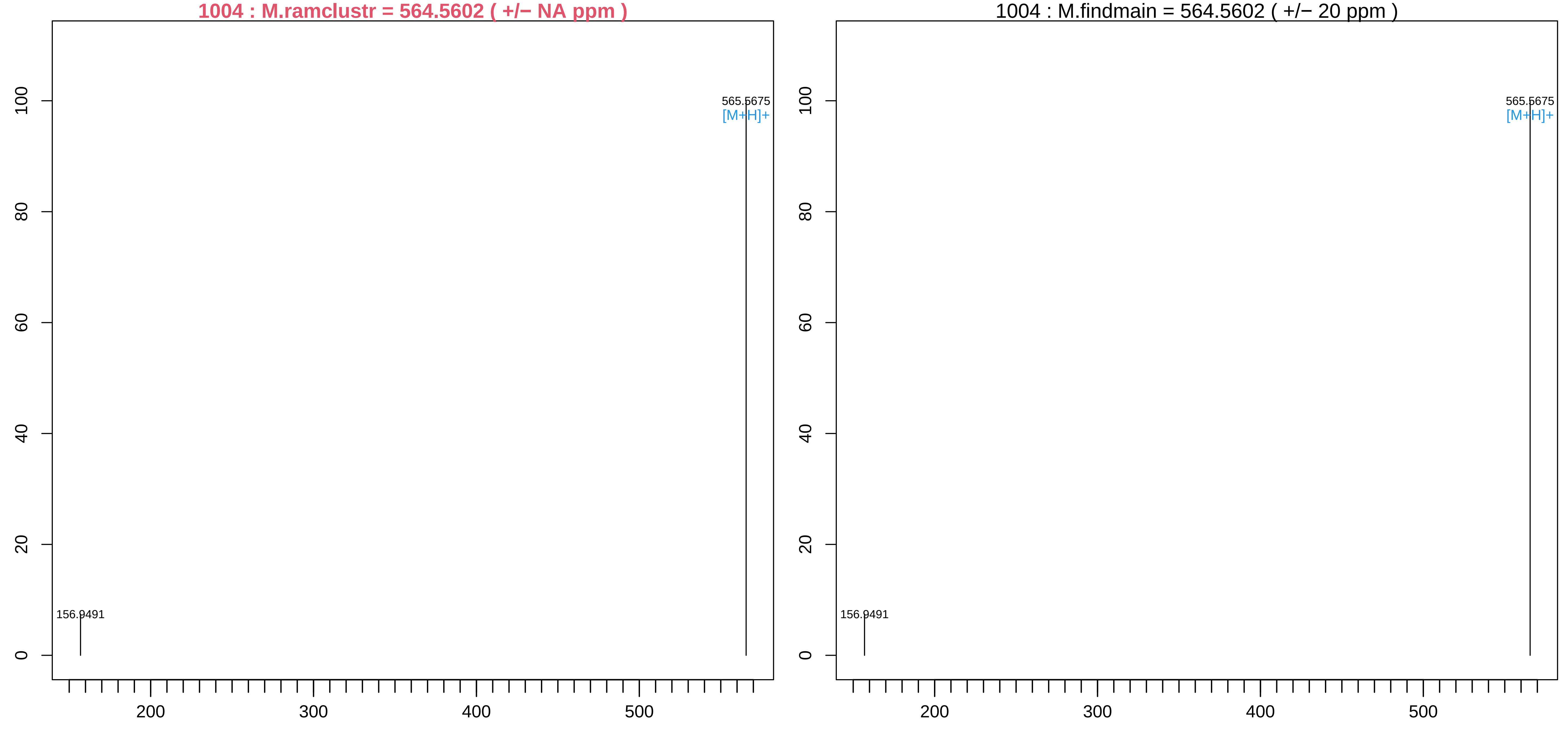
1004 : M.ramclustr = 564.5602 (+/− NA ppm)
565.5675
[M+H]+
156.9491
0
20
40
60
80
100
200
300
400
500
1004 : M.findmain = 564.5602 (+/− 20 ppm)
565.5675
[M+H]+
156.9491
0
20
40
60
80
100
200
300
400
500

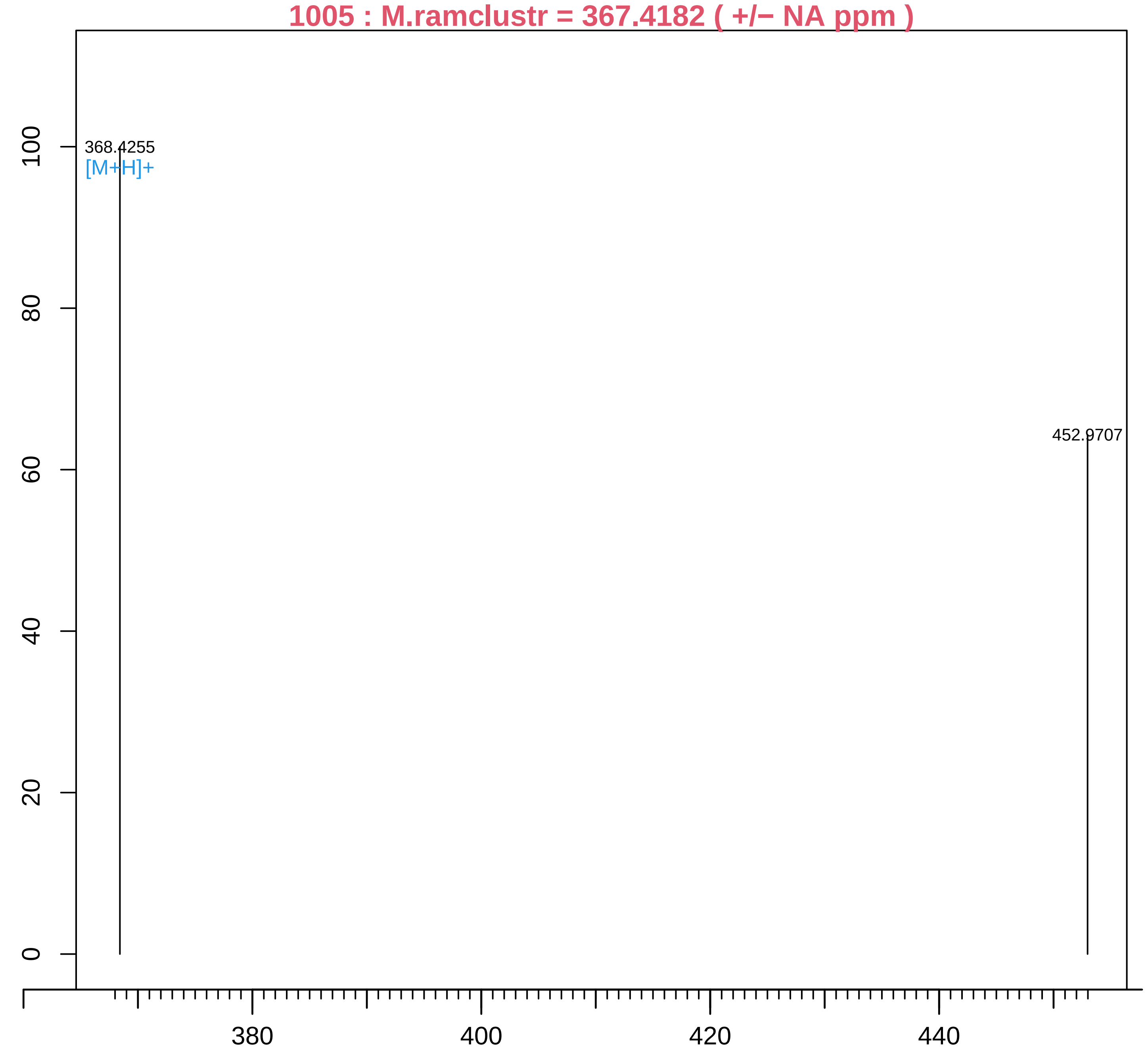
1005 : M.ramclustr = 367.4182 (+/− NA ppm)
368.4255
[M+H]+
452.9707
0
20
40
60
80
100
380
400
420
440

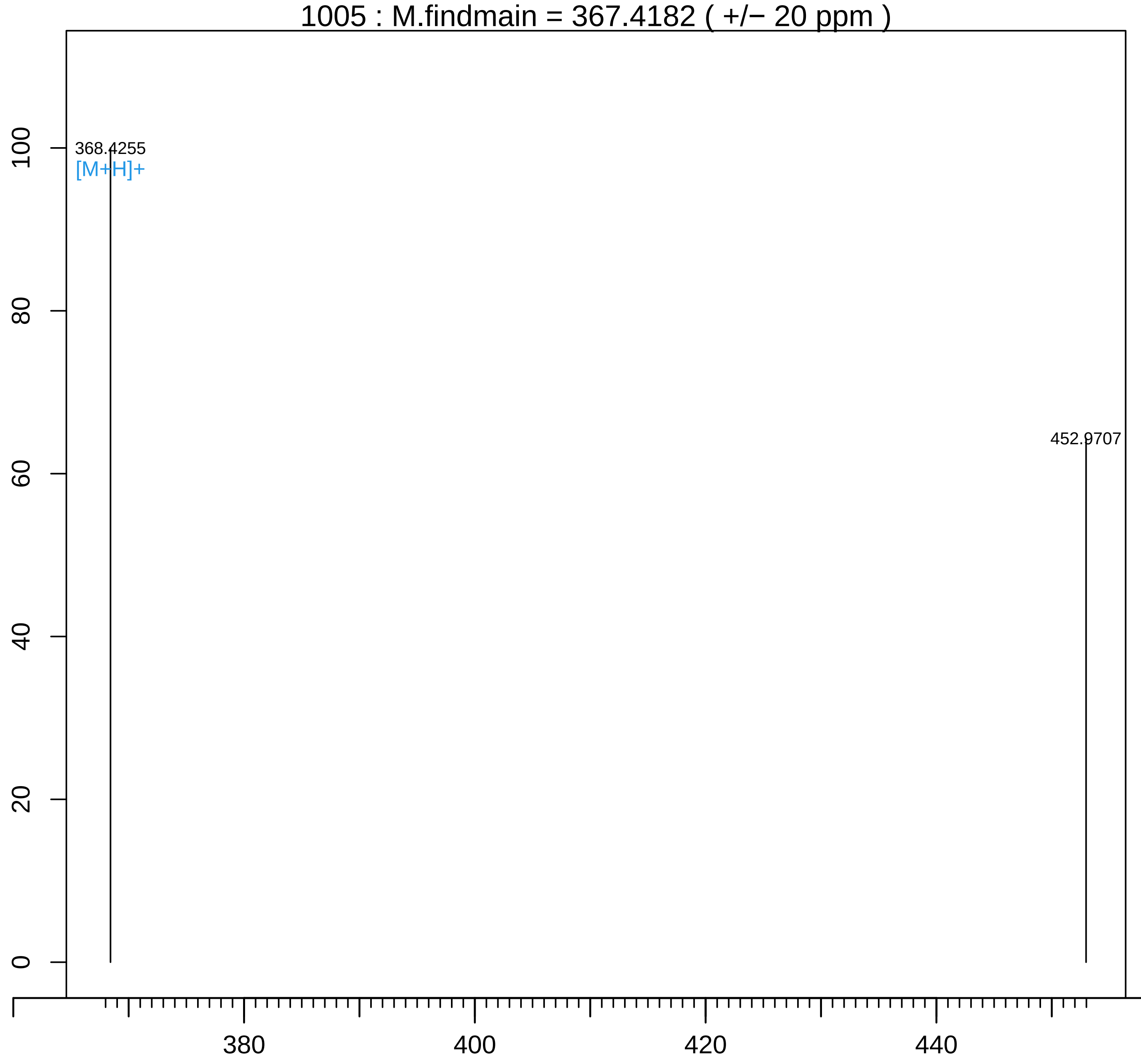
1005 : M.findmain = 367.4182 (+/− 20 ppm)
368.4255
[M+H]+
452.9707
0
20
40
60
80
100
380
400
420
440

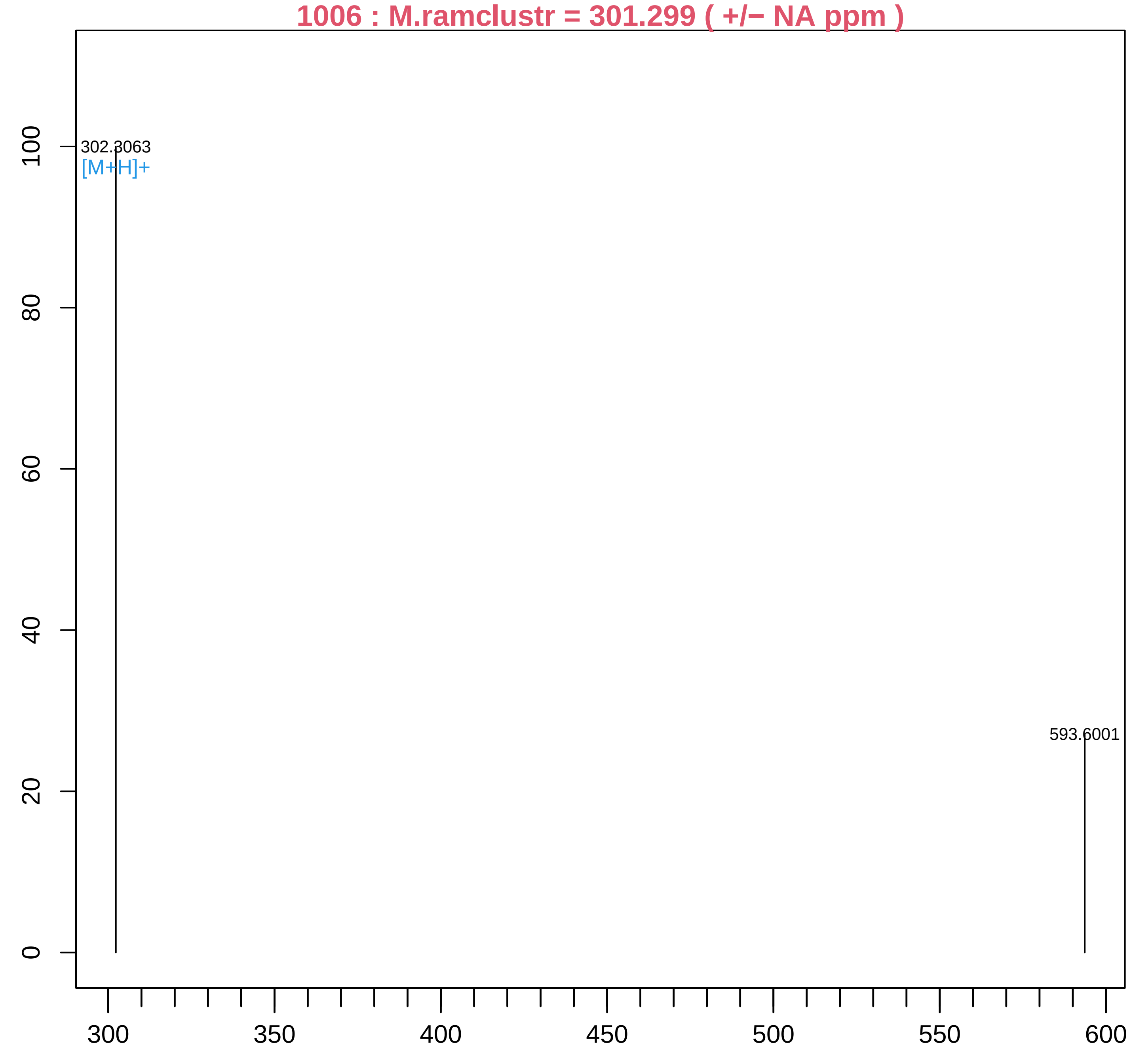
1006 : M.ramclustr = 301.299 (+/− NA ppm)
302.3063
[M+H]+
593.6001
0
20
40
60
80
100
300
350
400
450
500
550
600

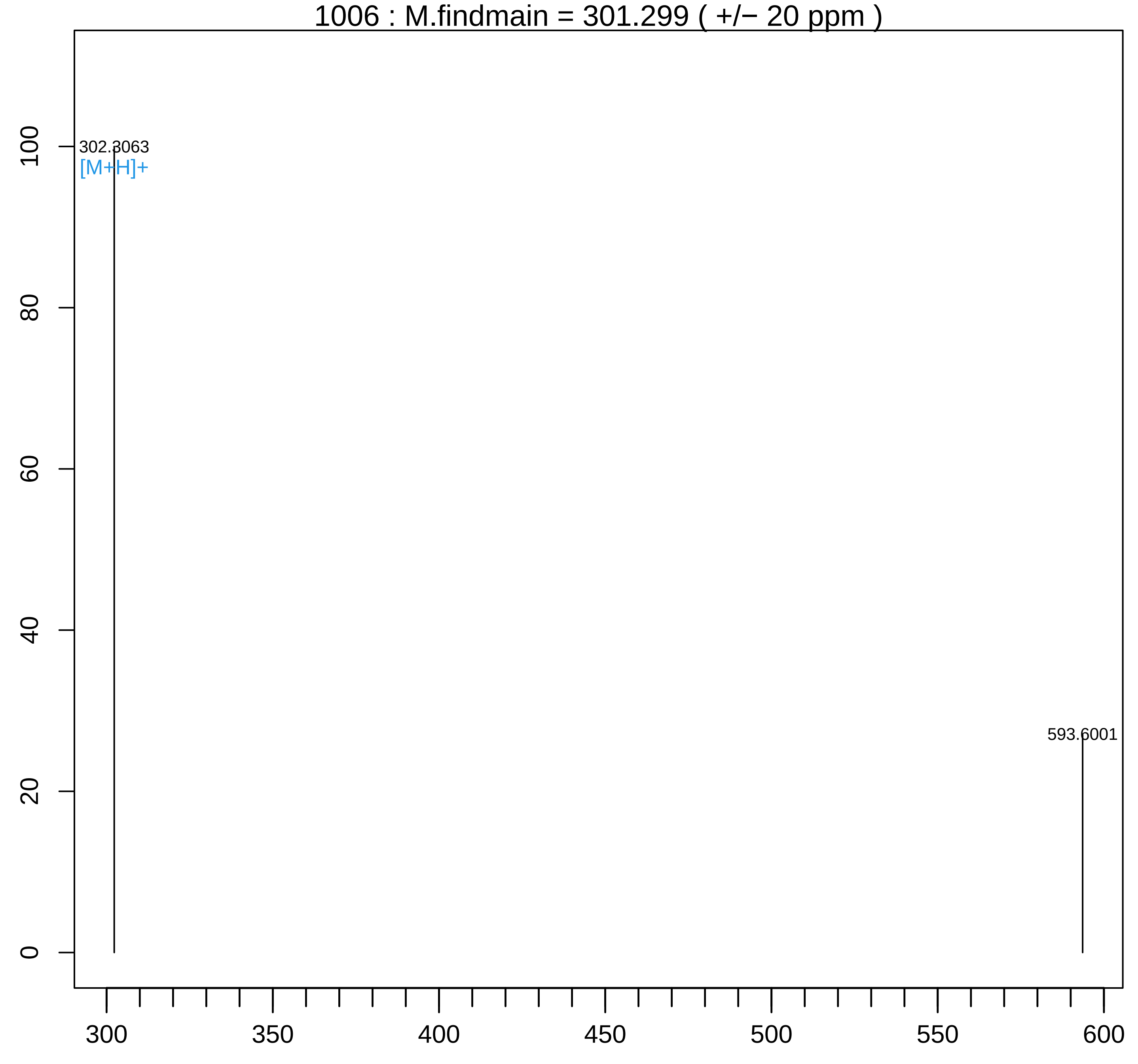
1006 : M.findmain = 301.299 (+/− 20 ppm)
302.3063
[M+H]+
593.6001
0
20
40
60
80
100
300
350
400
450
500
550
600

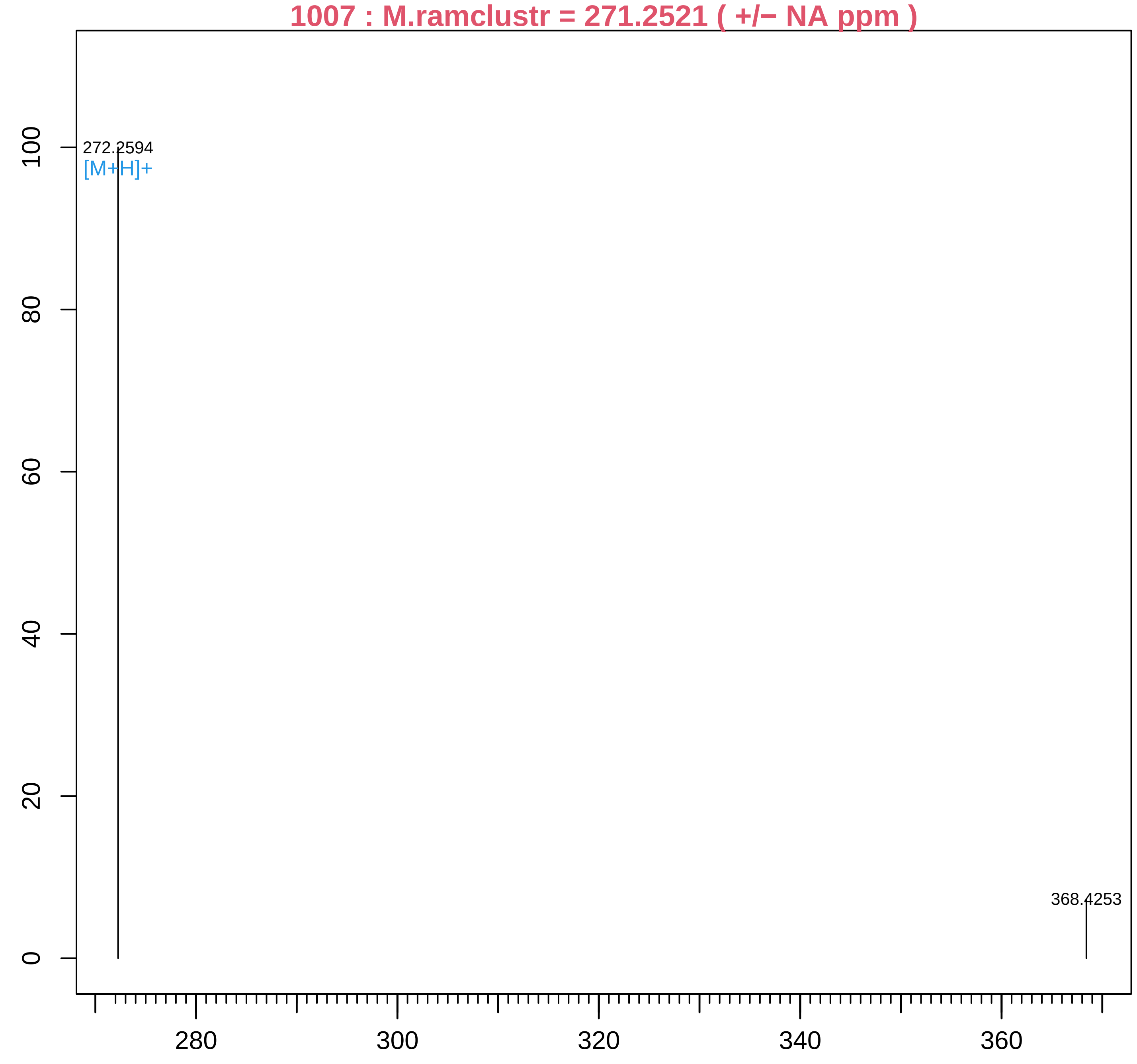

1007 : M.ramclustr = 271.2521 (+/− NA ppm)
272.2594
[M+H]+
368.4253
0
20
40
60
80
100
280
300
320
340
360

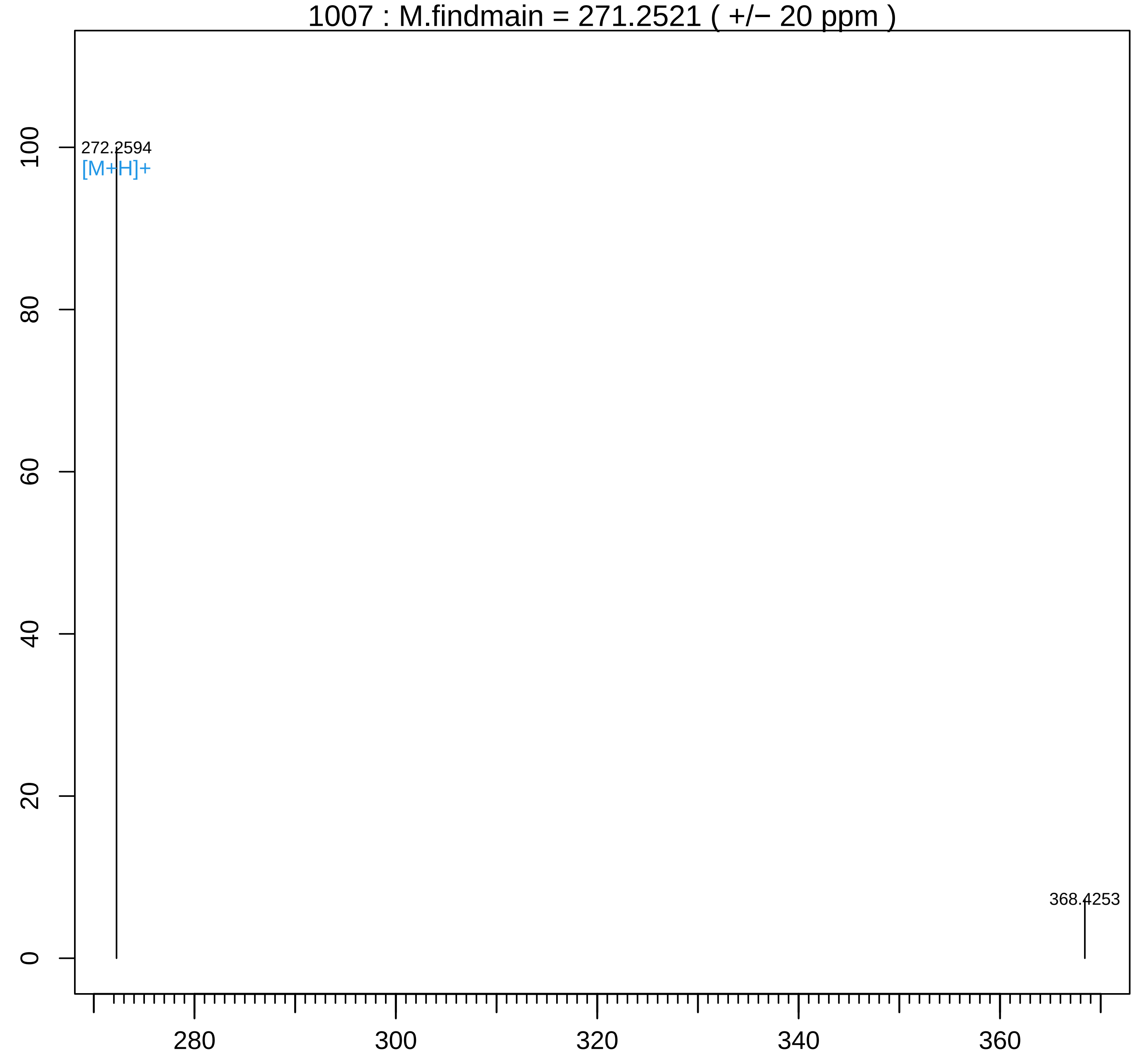

1007 : M.findmain = 271.2521 (+/− 20 ppm)
272.2594
[M+H]+
368.4253
0
20
40
60
80
100
280
300
320
340
360

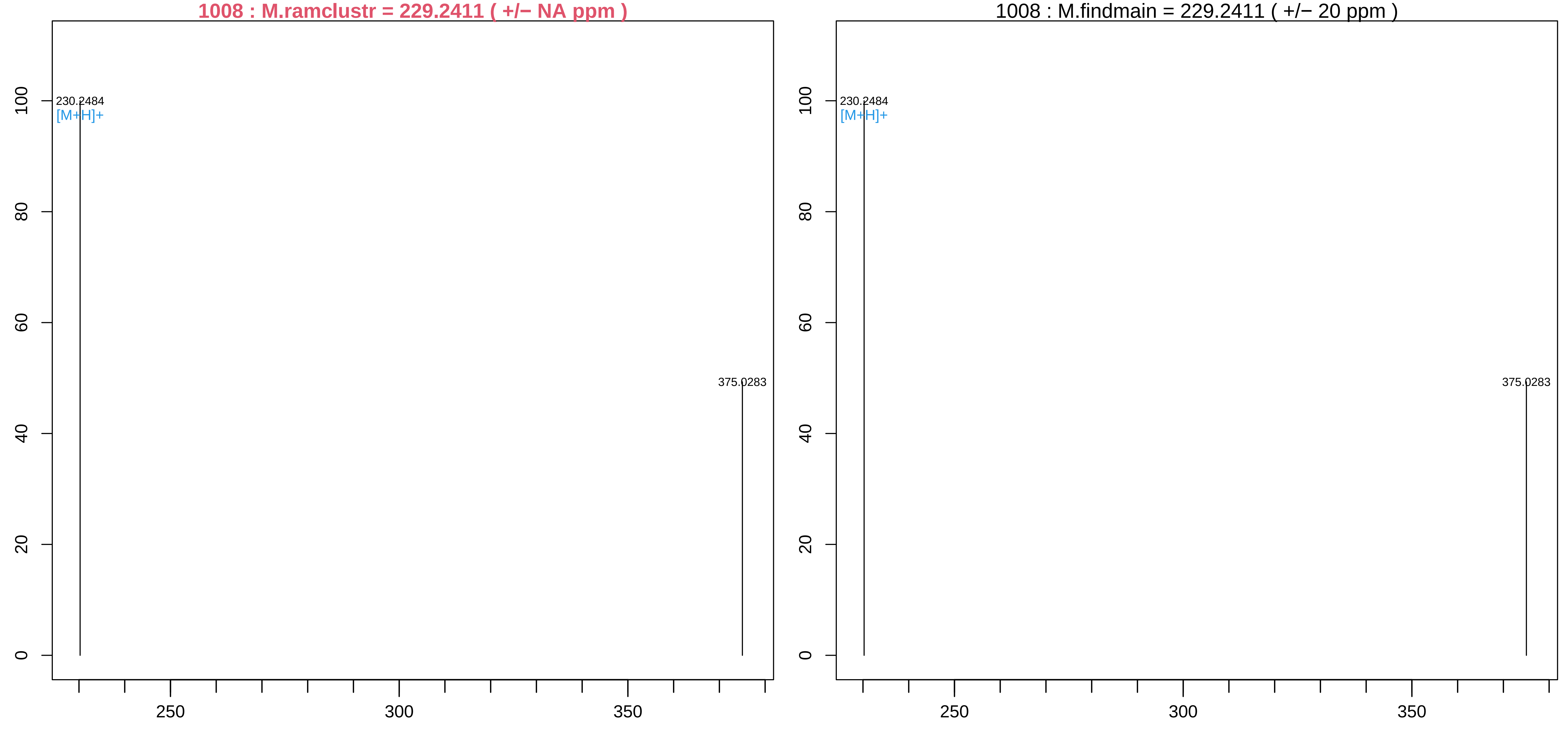

1008 : M.ramclustr = 229.2411 (+/− NA ppm)
230.2484
[M+H]+
375.0283
0
20
40
60
80
100
250
300
350
1008 : M.findmain = 229.2411 (+/− 20 ppm)
230.2484
[M+H]+
375.0283
0
20
40
60
80
100
250
300
350

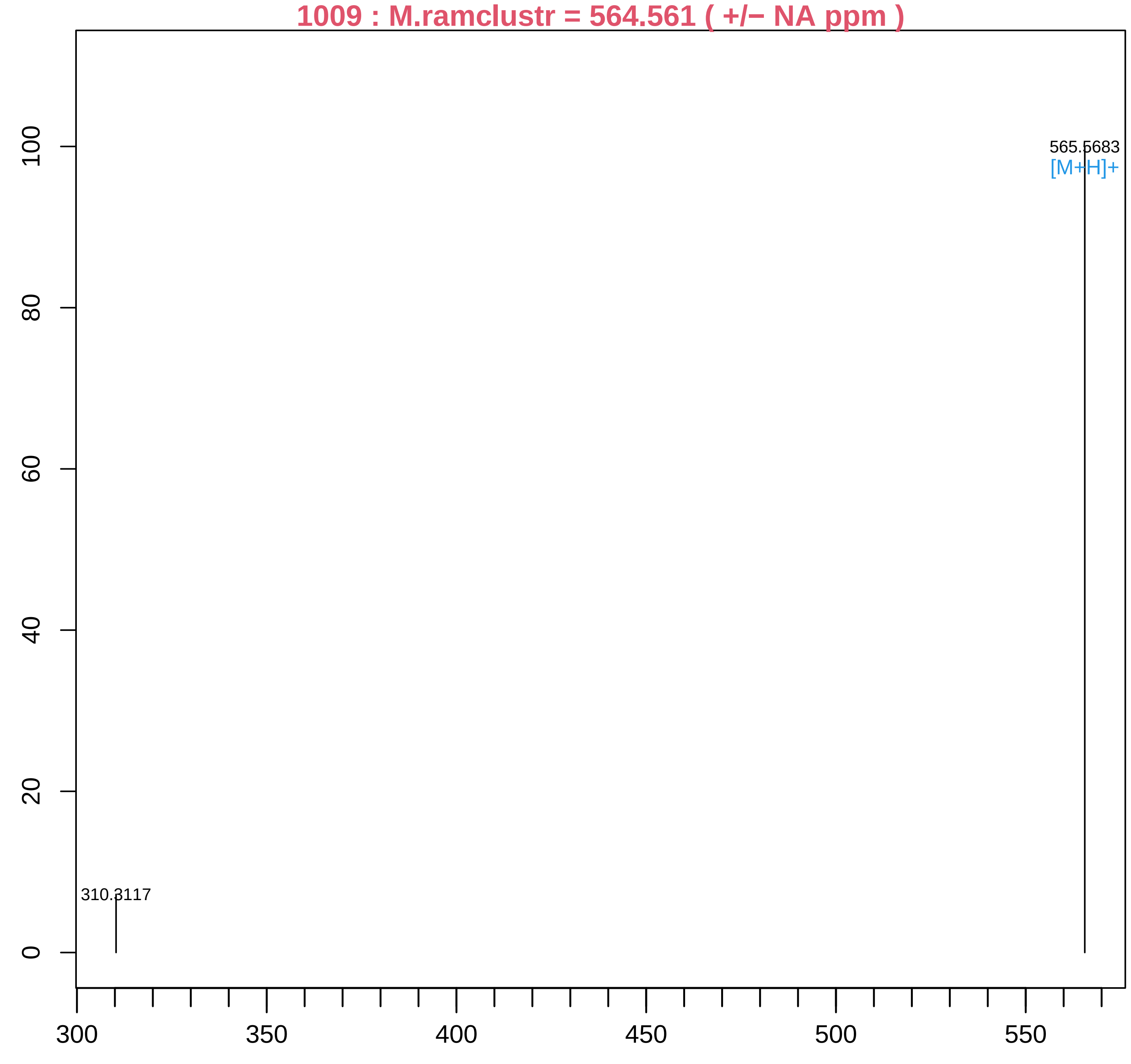

1009 : M.ramclustr = 564.561 (+/− NA ppm)
565.5683
[M+H]+
310.3117
0
20
40
60
80
100
300
350
400
450
500
550

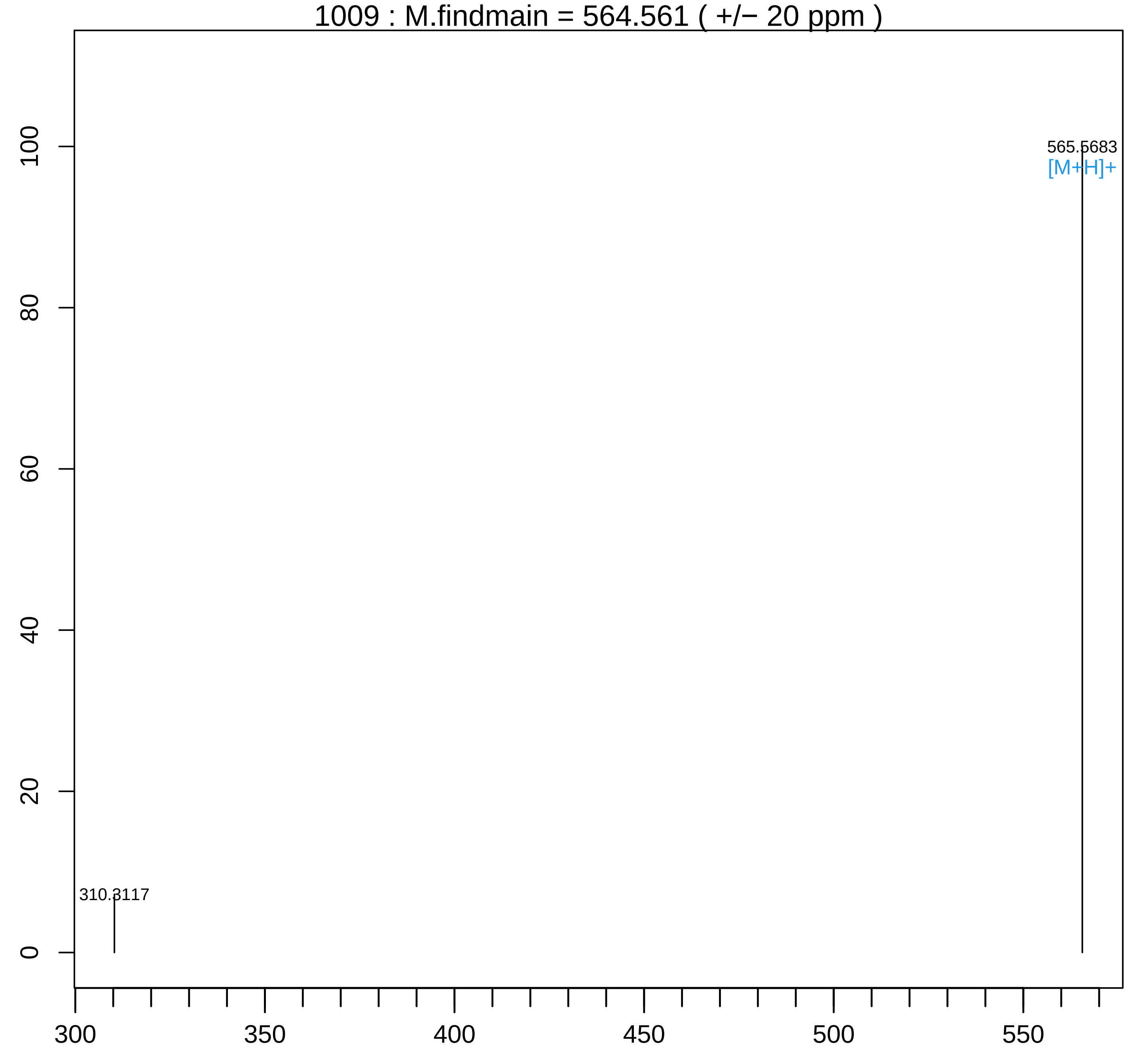

1009 : M.findmain = 564.561 (+/− 20 ppm)
565.5683
[M+H]+
310.3117
0
20
40
60
80
100
300
350
400
450
500
550

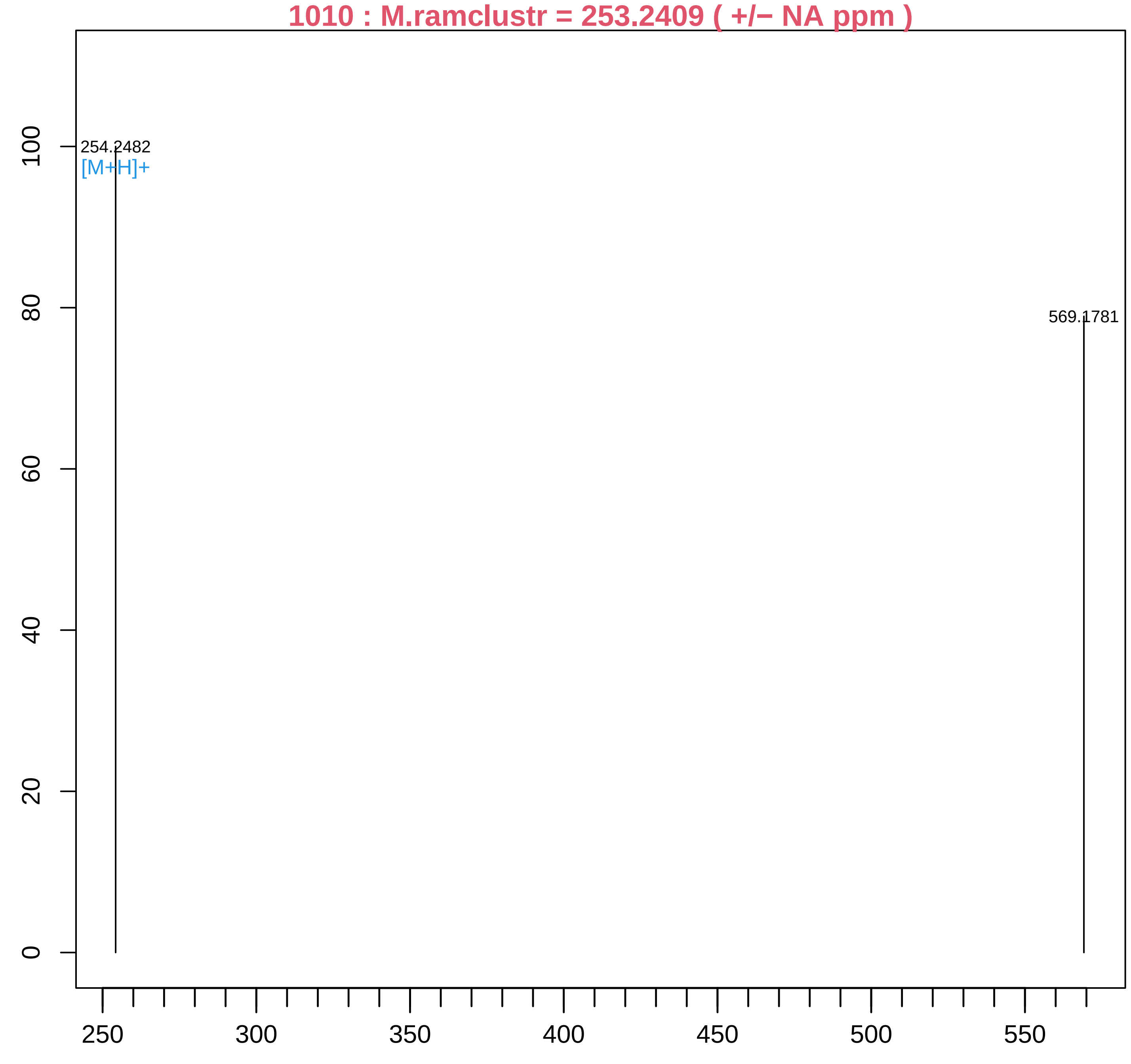
1010 : M.ramclustr = 253.2409 (+/− NA ppm)
254.2482
[M+H]+
569.1781
0
20
40
60
80
100
250
300
350
400
450
500
550

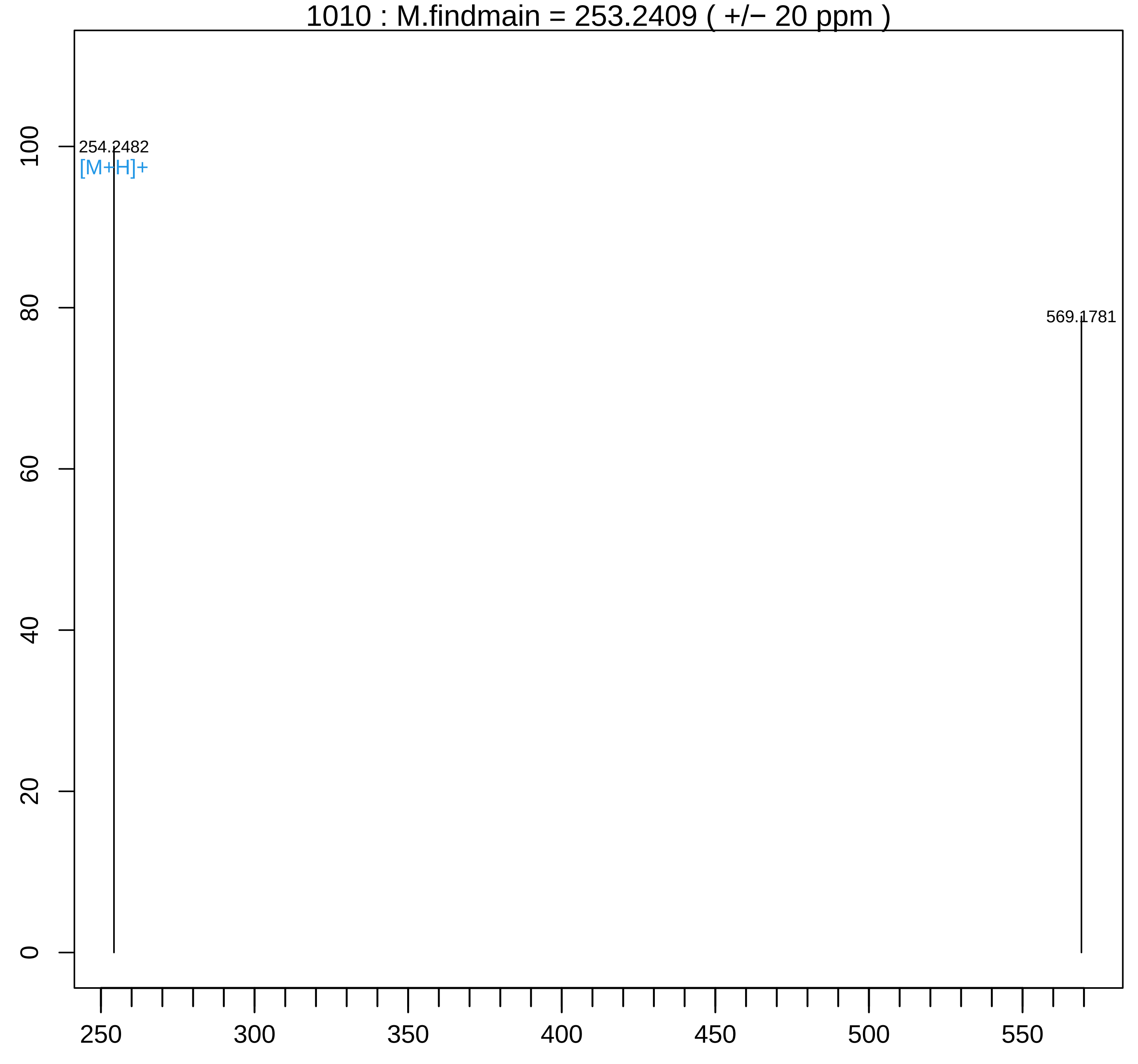
1010 : M.findmain = 253.2409 (+/− 20 ppm)
254.2482
[M+H]+
569.1781
0
20
40
60
80
100
250
300
350
400
450
500
550

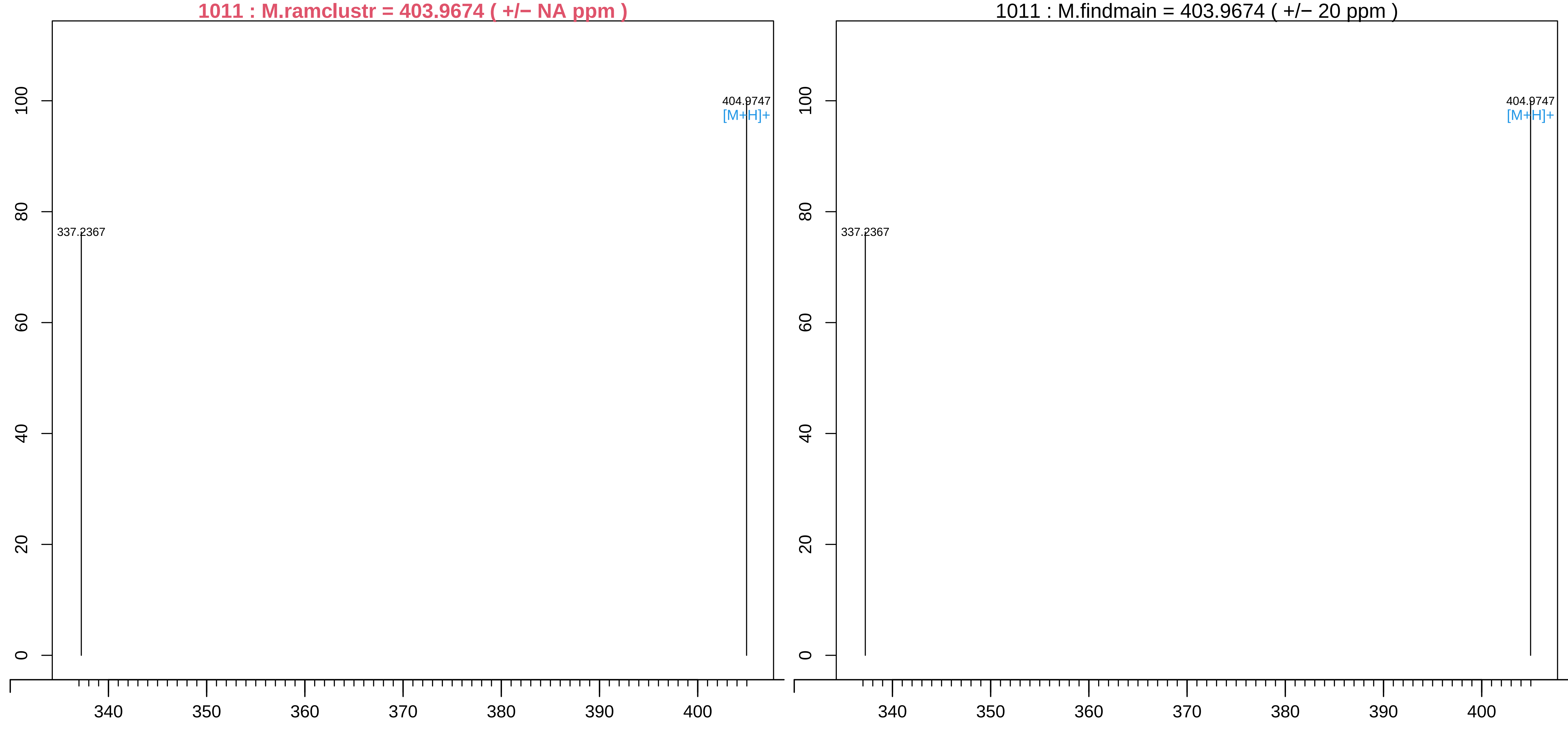

1011 : M.ramclustr = 403.9674 (+/− NA ppm)
404.9747
[M+H]+
337.2367
1011 : M.findmain = 403.9674 (+/− 20 ppm)
404.9747
[M+H]+
337.2367

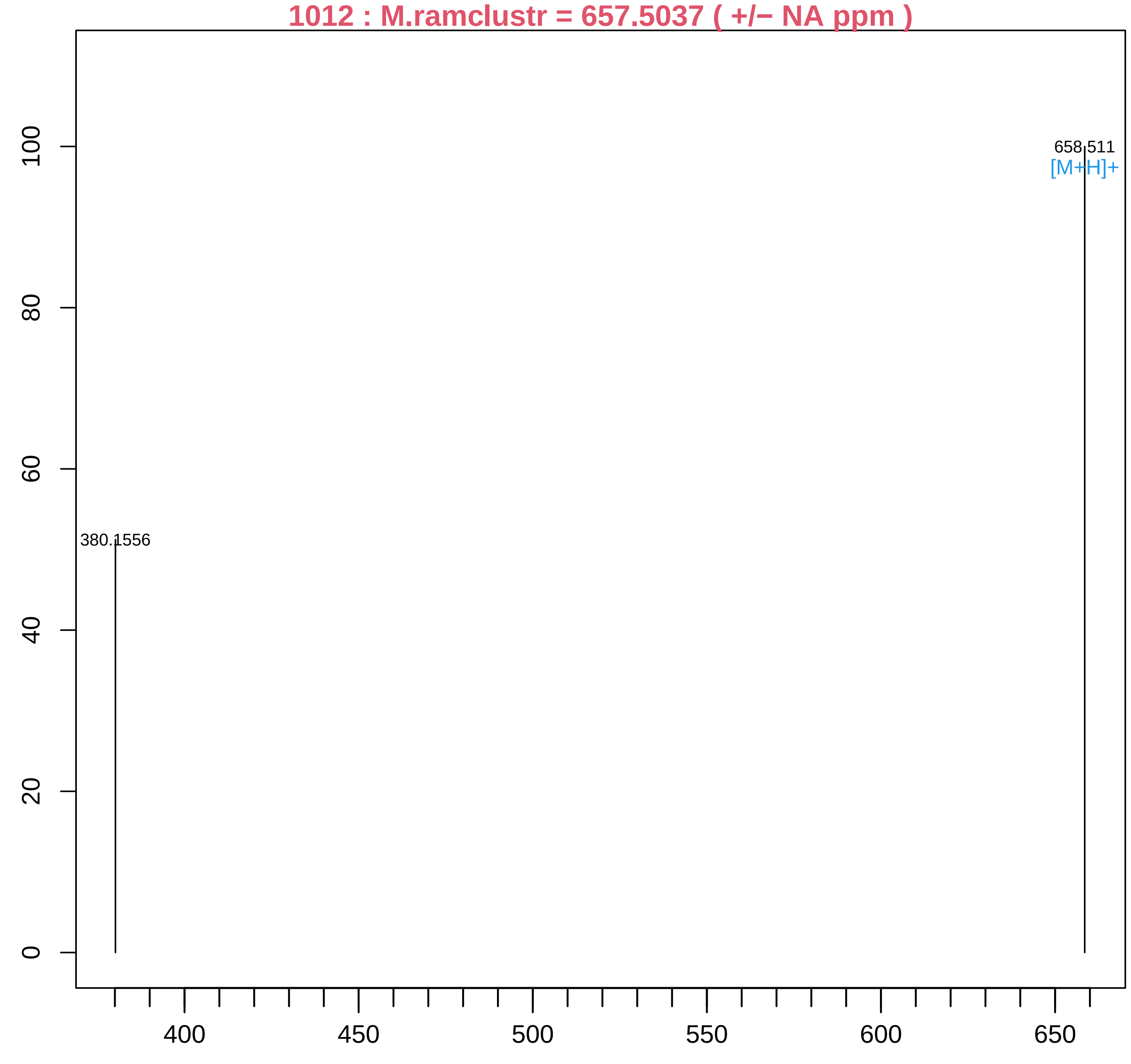
1012 : M.ramclustr = 657.5037 (+/− NA ppm)
658.511
[M+H]+
380.1556
0
20
40
60
80
100
400
450
500
550
600
650

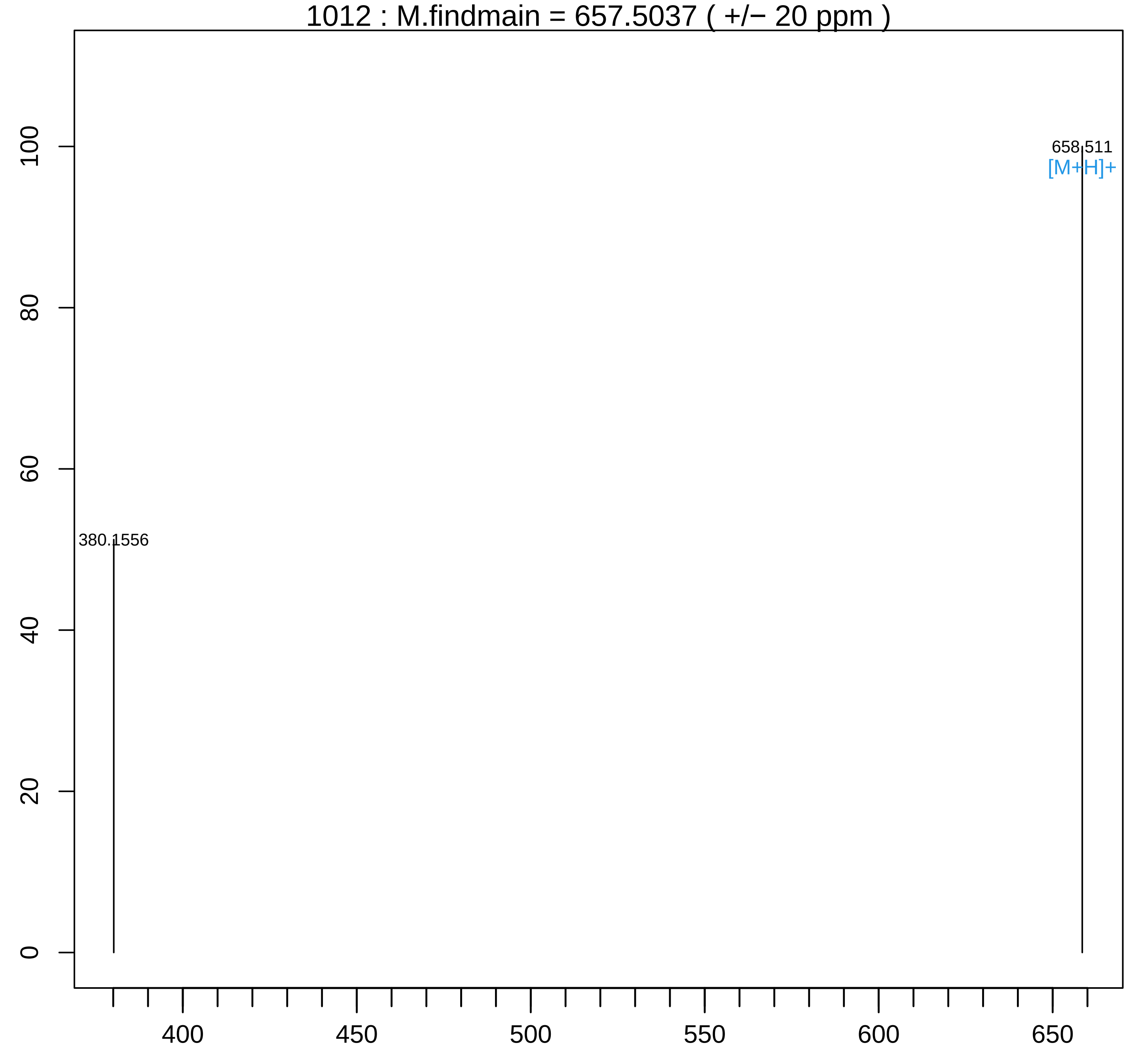
1012 : M.findmain = 657.5037 (+/− 20 ppm)
658.511
[M+H]+
380.1556
0
20
40
60
80
100
400
450
500
550
600
650

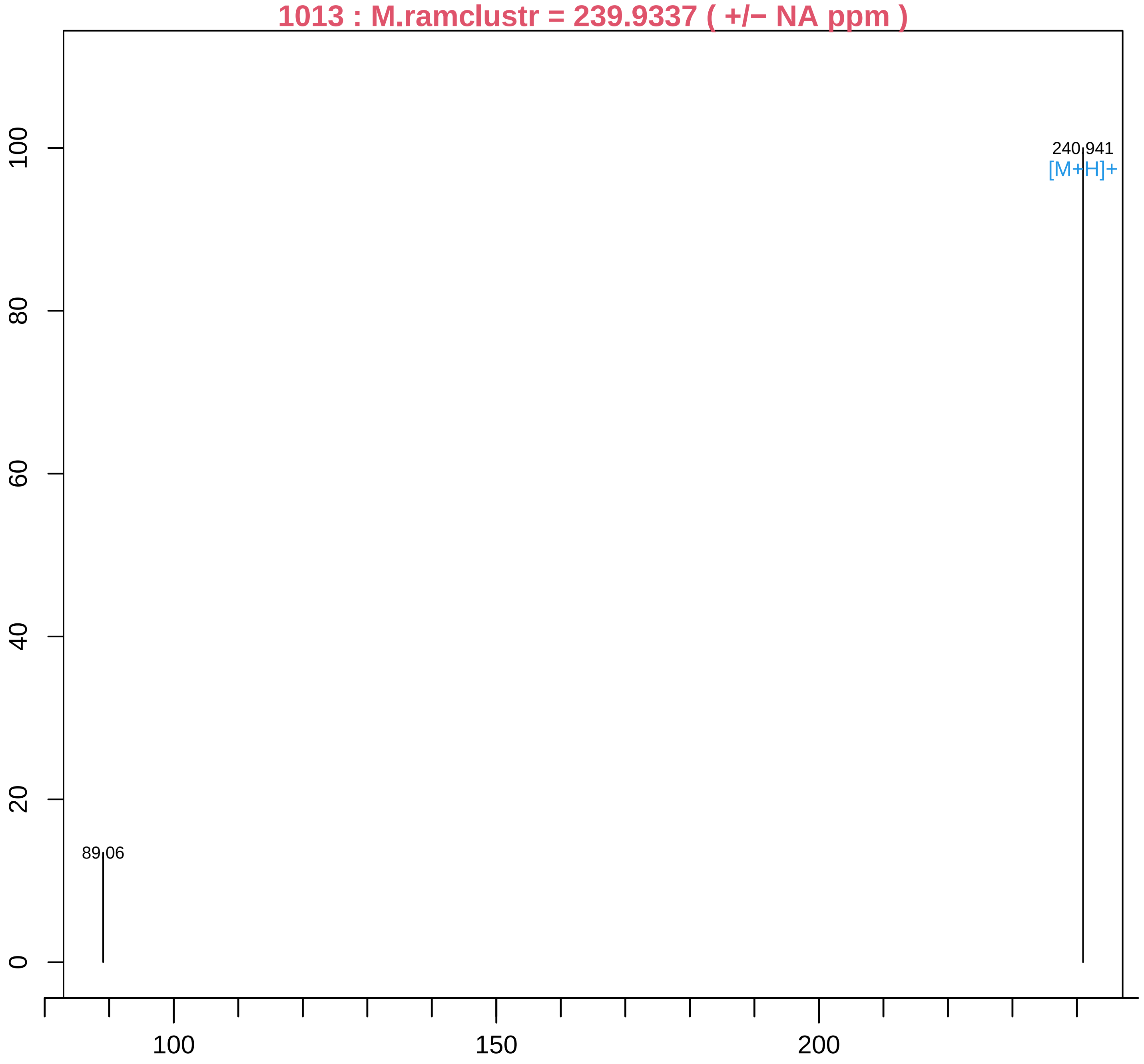
1013 : M.ramclustr = 239.9337 (+/− NA ppm)
240.941
[M+H]+
89.06
0
20
40
60
80
100
100
150
200

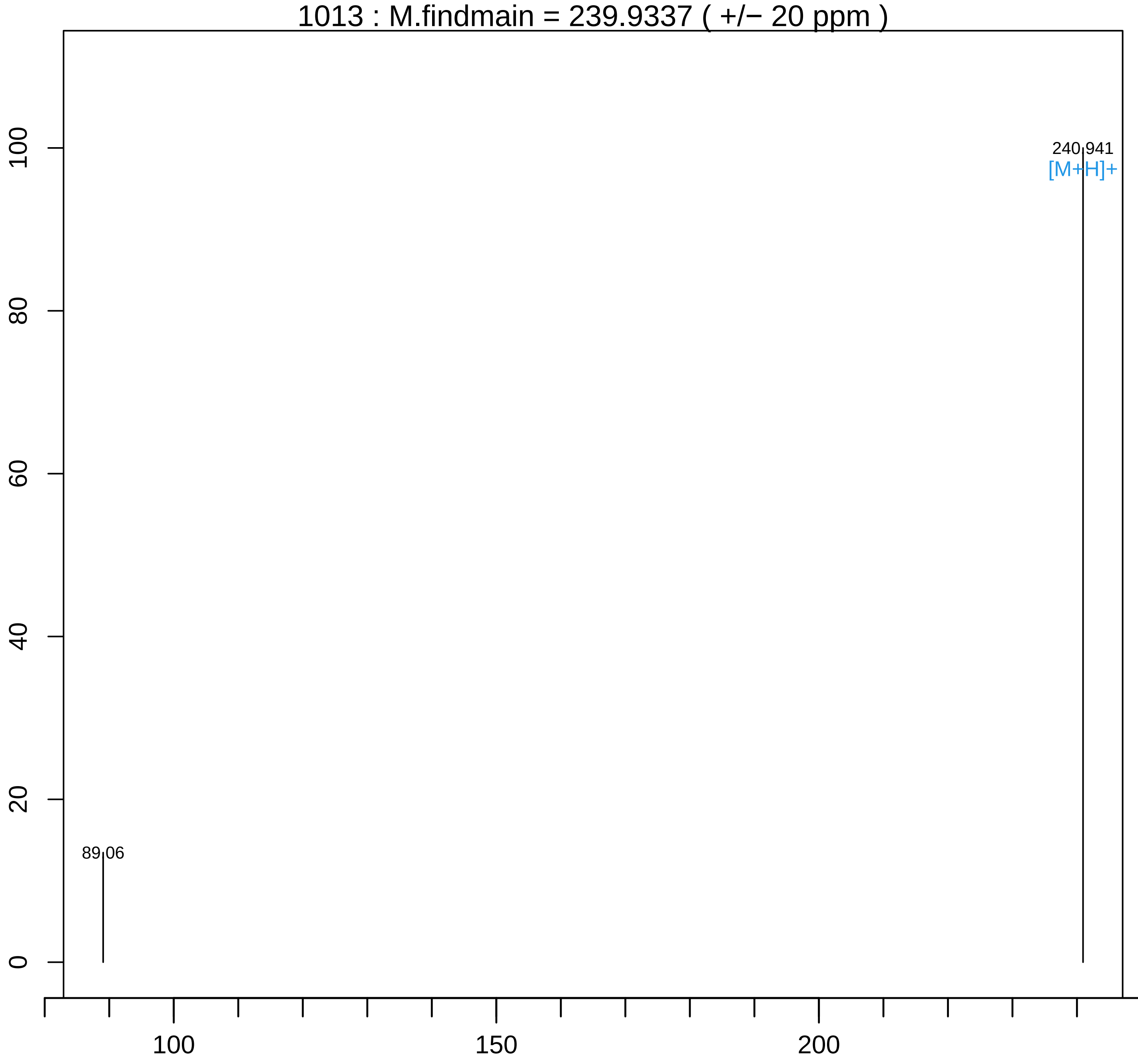
1013 : M.findmain = 239.9337 (+/− 20 ppm)
240.941
[M+H]+
89.06
0
20
40
60
80
100
100
150
200

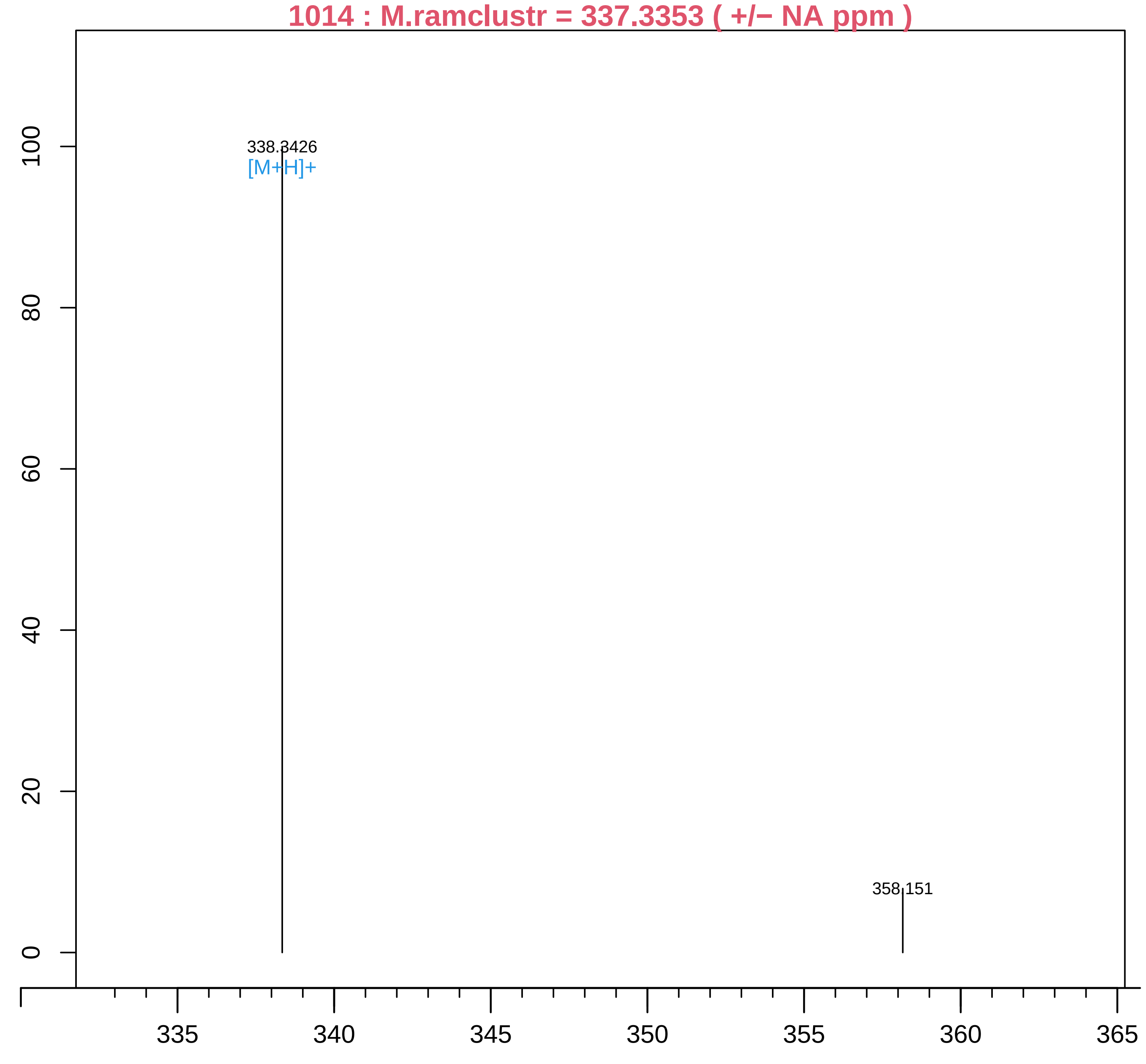
1014 : M.ramclustr = 337.3353 (+/− NA ppm)
338.3426
[M+H]+
358.151
0
20
40
60
80
100
335
340
345
350
355
360
365

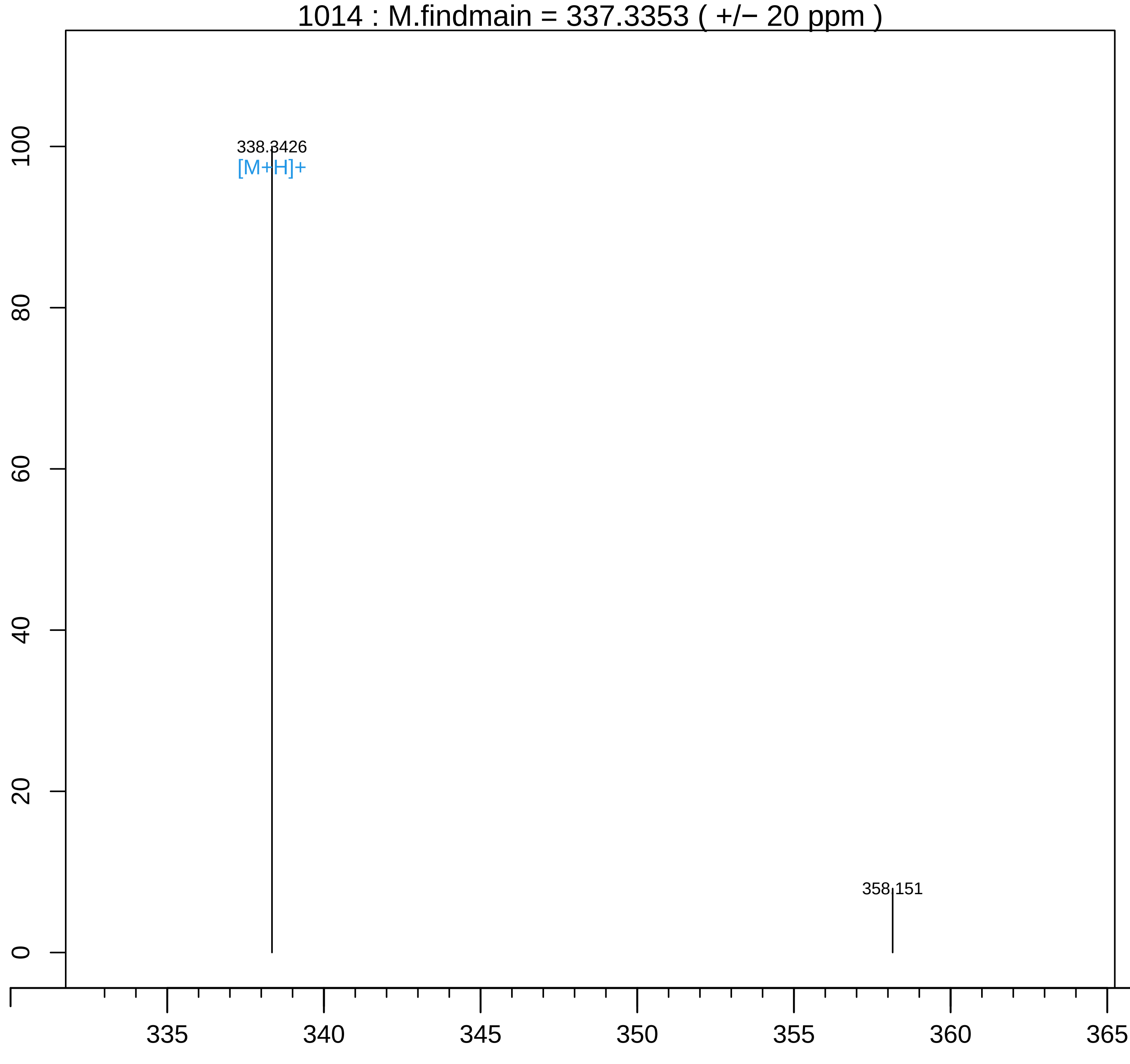
1014 : M.findmain = 337.3353 (+/− 20 ppm)
338.3426
[M+H]+
358.151
0
20
40
60
80
100
335
340
345
350
355
360
365

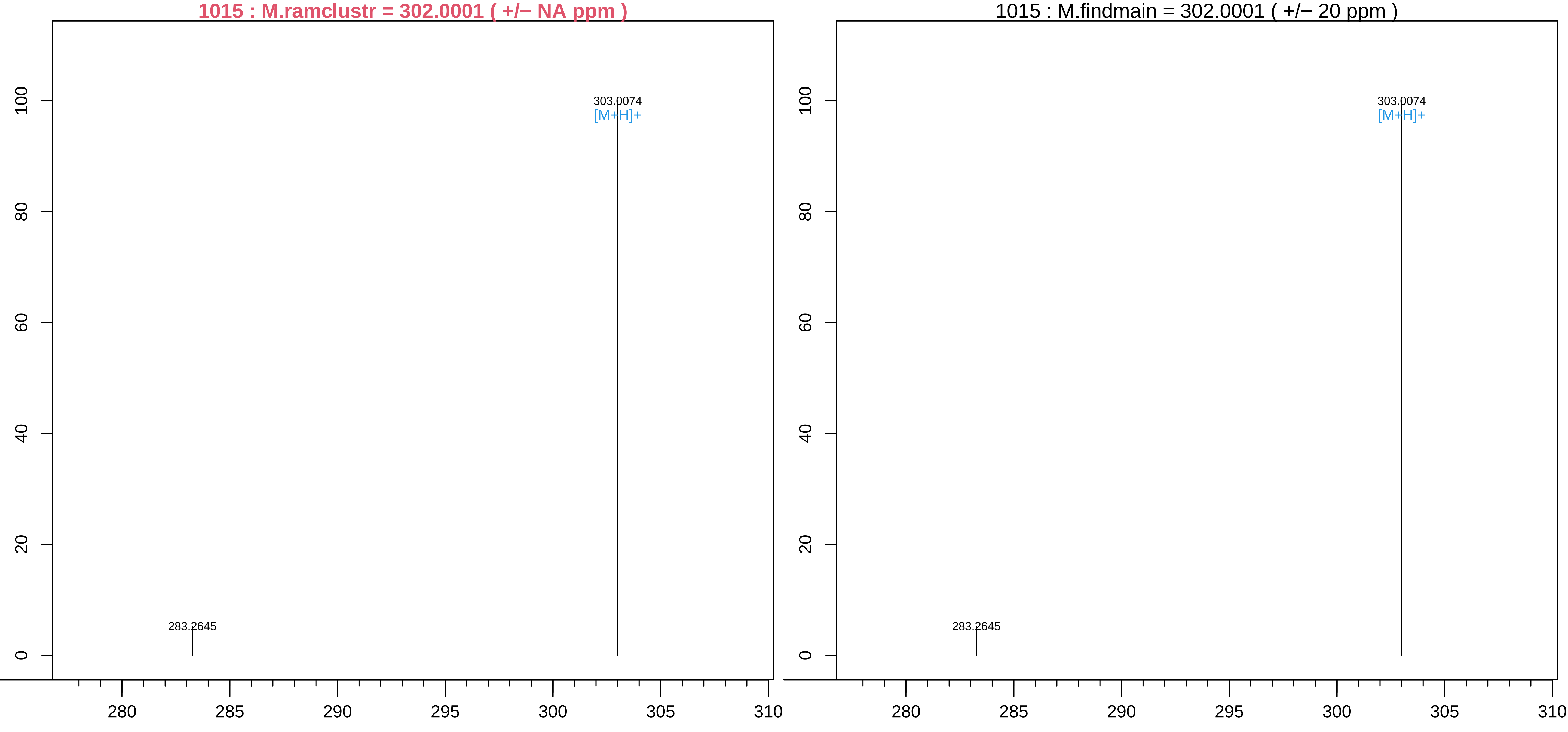
1015 : M.ramclustr = 302.0001 (+/− NA ppm)
303.0074
[M+H]+
283.2645
1015 : M.findmain = 302.0001 (+/− 20 ppm)
303.0074
[M+H]+
283.2645
0
20
40
60
80
100
280
285
290
295
300
305
310

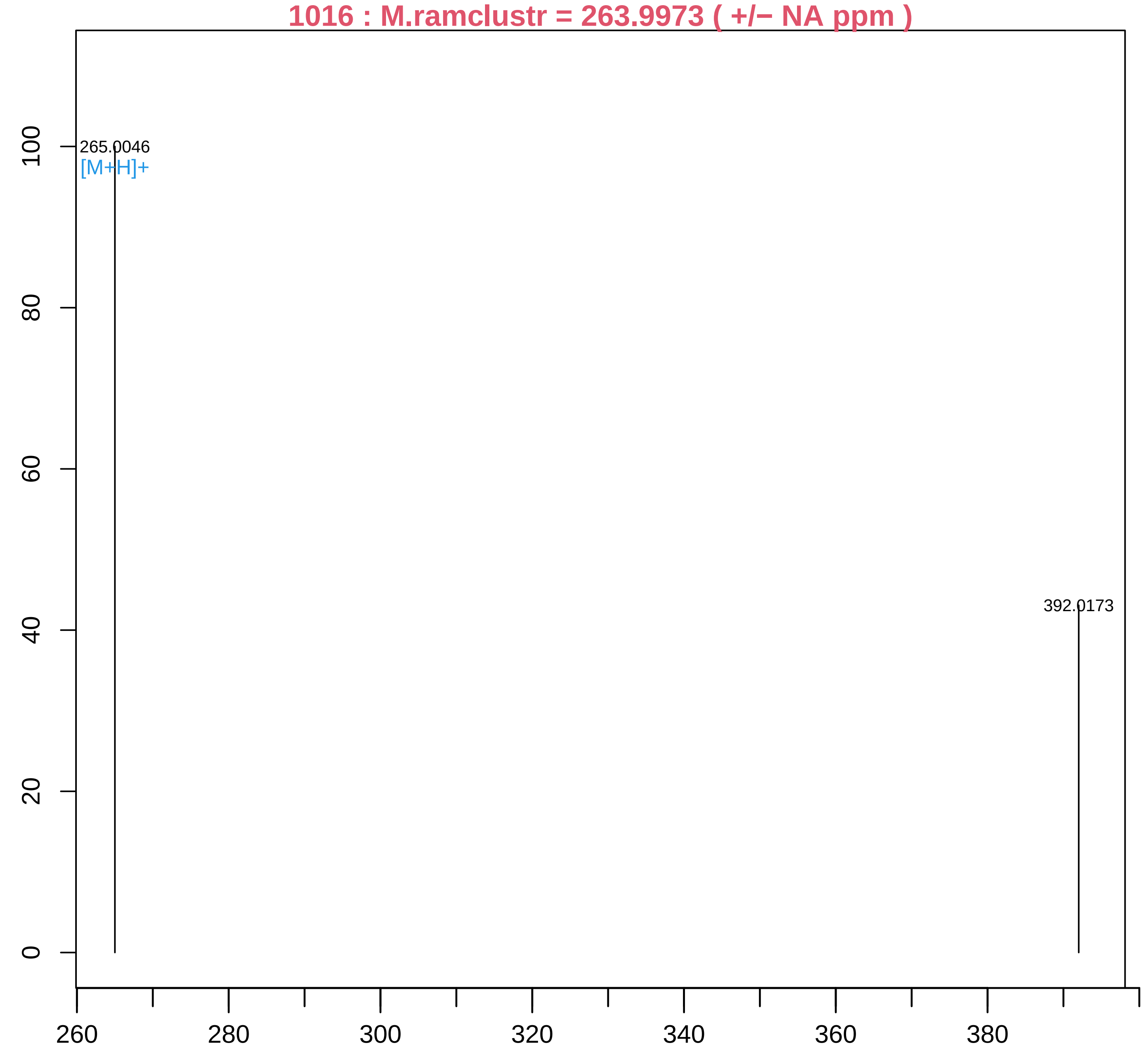
1016 : M.ramclustr = 263.9973 (+/− NA ppm)
265.0046
[M+H]+
392.0173
0
20
40
60
80
100
260
280
300
320
340
360
380

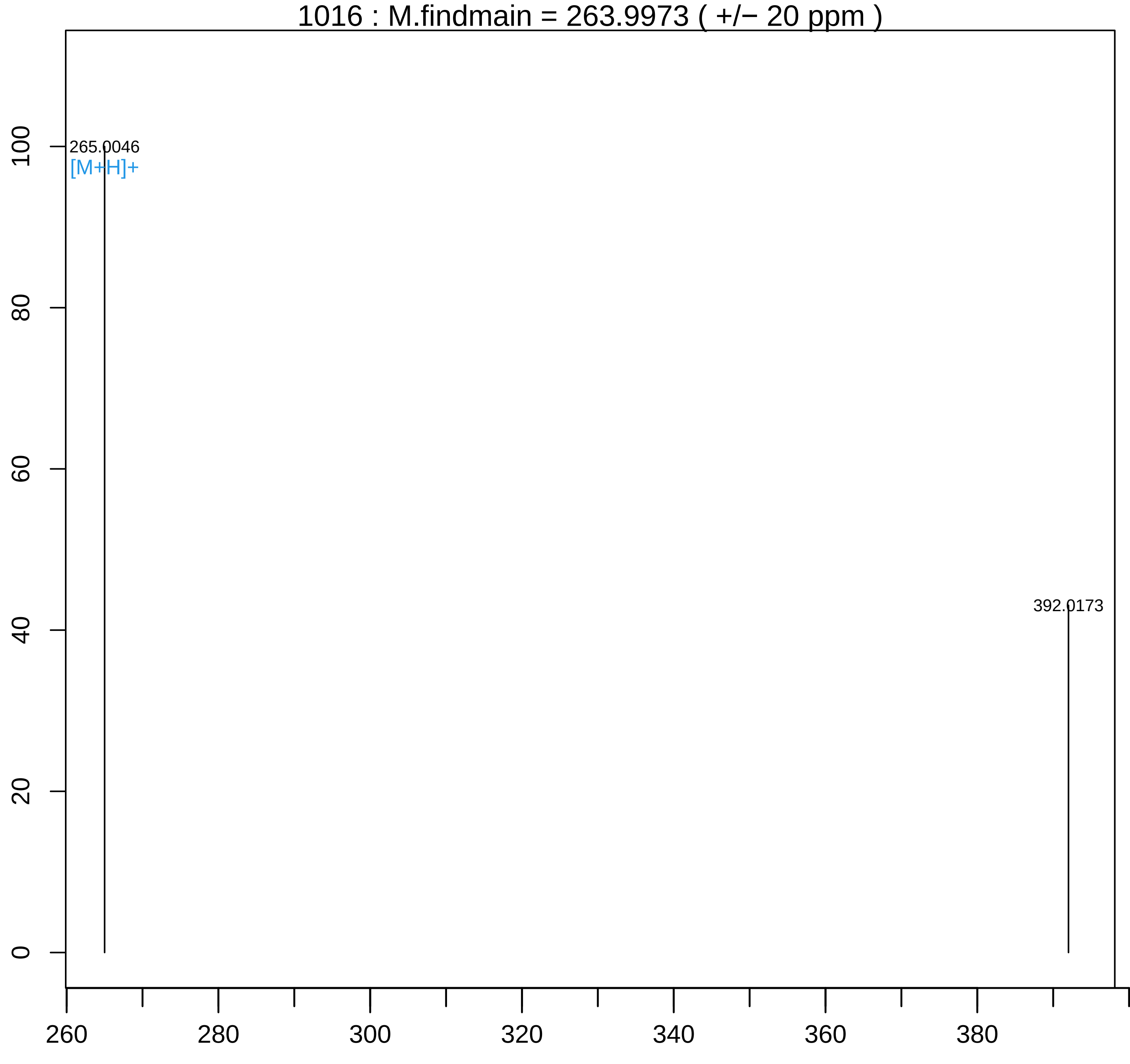
1016 : M.findmain = 263.9973 (+/− 20 ppm)
265.0046
[M+H]+
392.0173
0
20
40
60
80
100
260
280
300
320
340
360
380

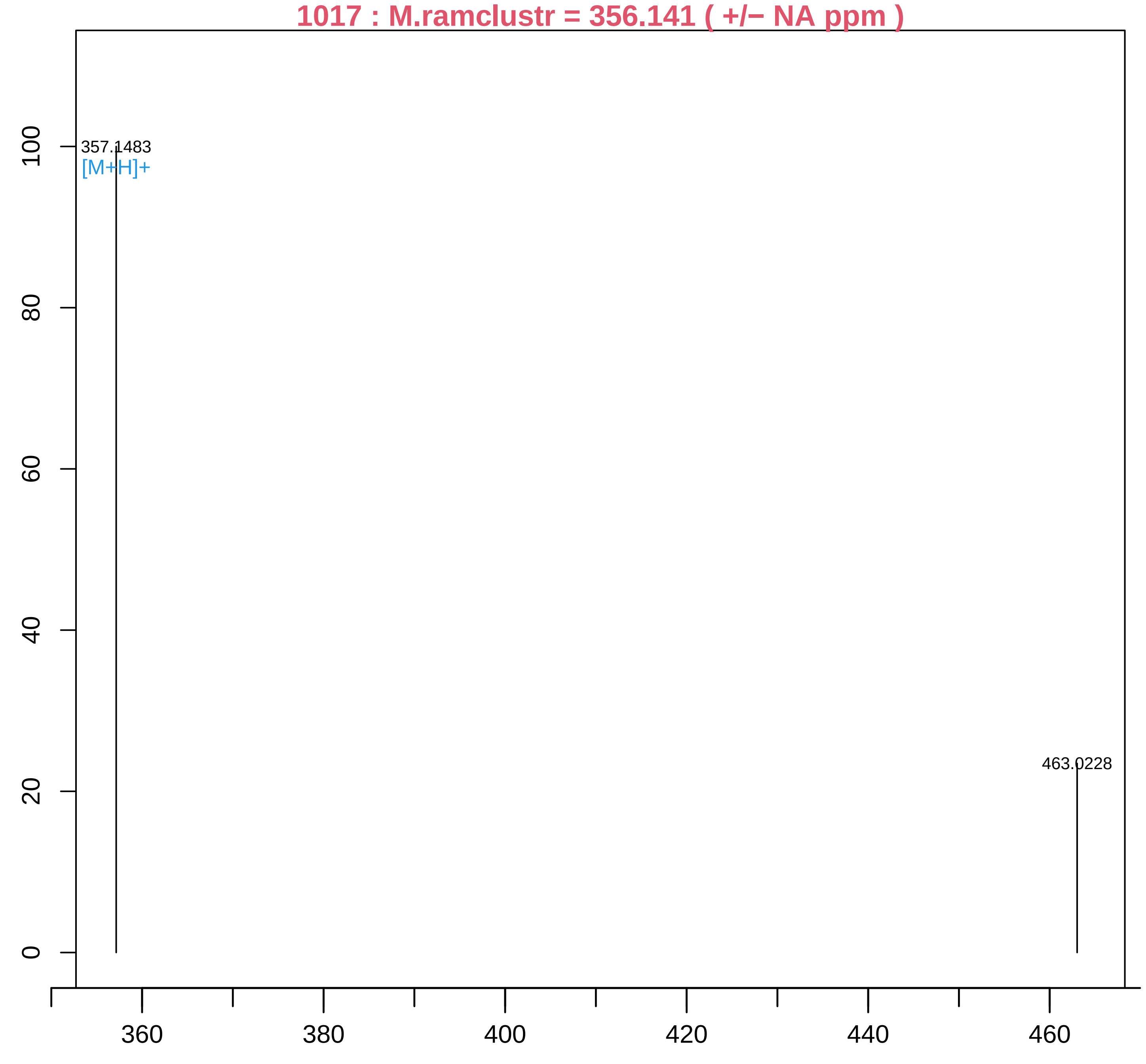

1017 : M.ramclustr = 356.141 (+/− NA ppm)
357.1483
[M+H]+
463.0228
0
20
40
60
80
100
360
380
400
420
440
460

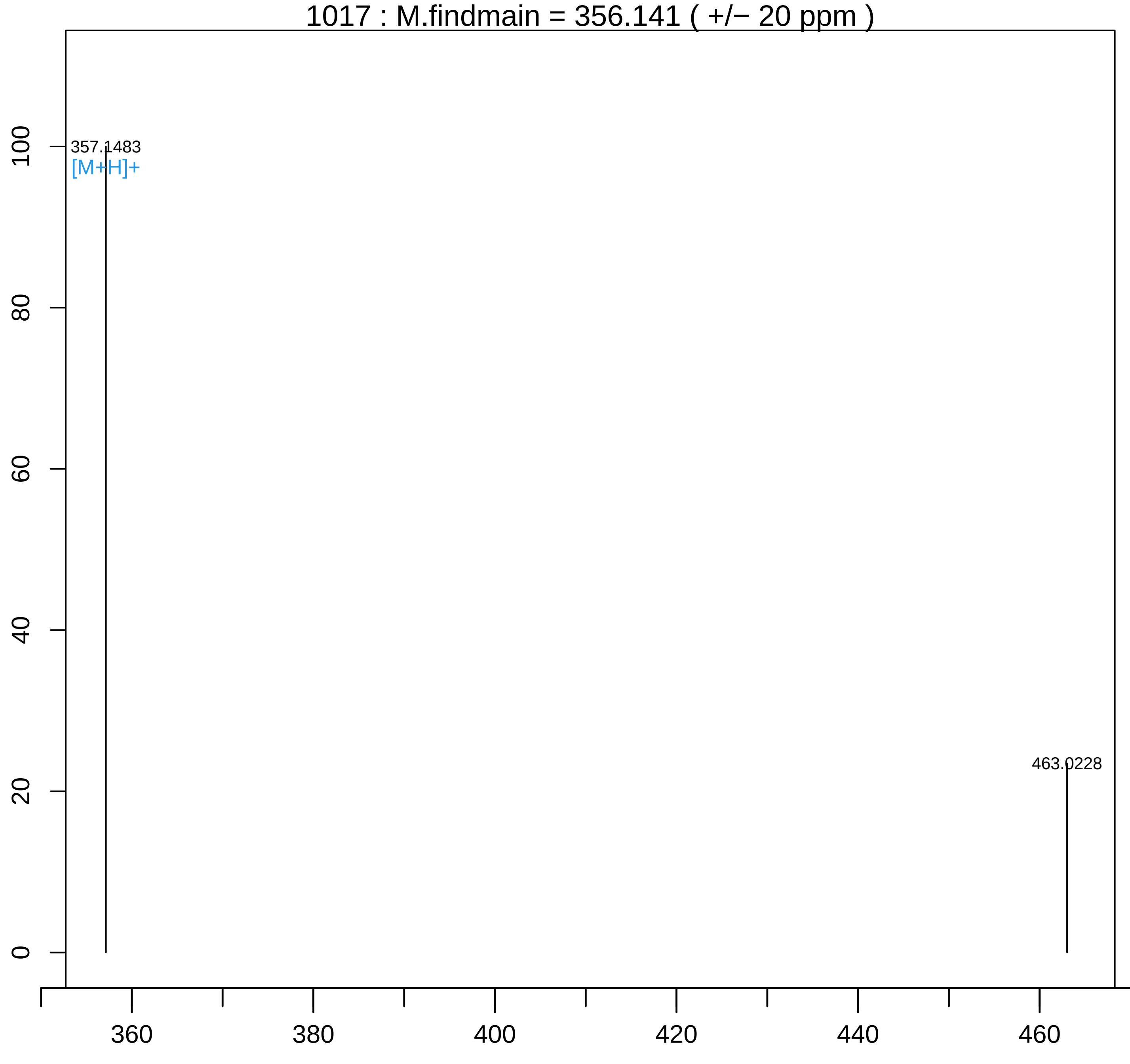

1017 : M.findmain = 356.141 (+/− 20 ppm)
357.1483
[M+H]+
463.0228
0
20
40
60
80
100
360
380
400
420
440
460

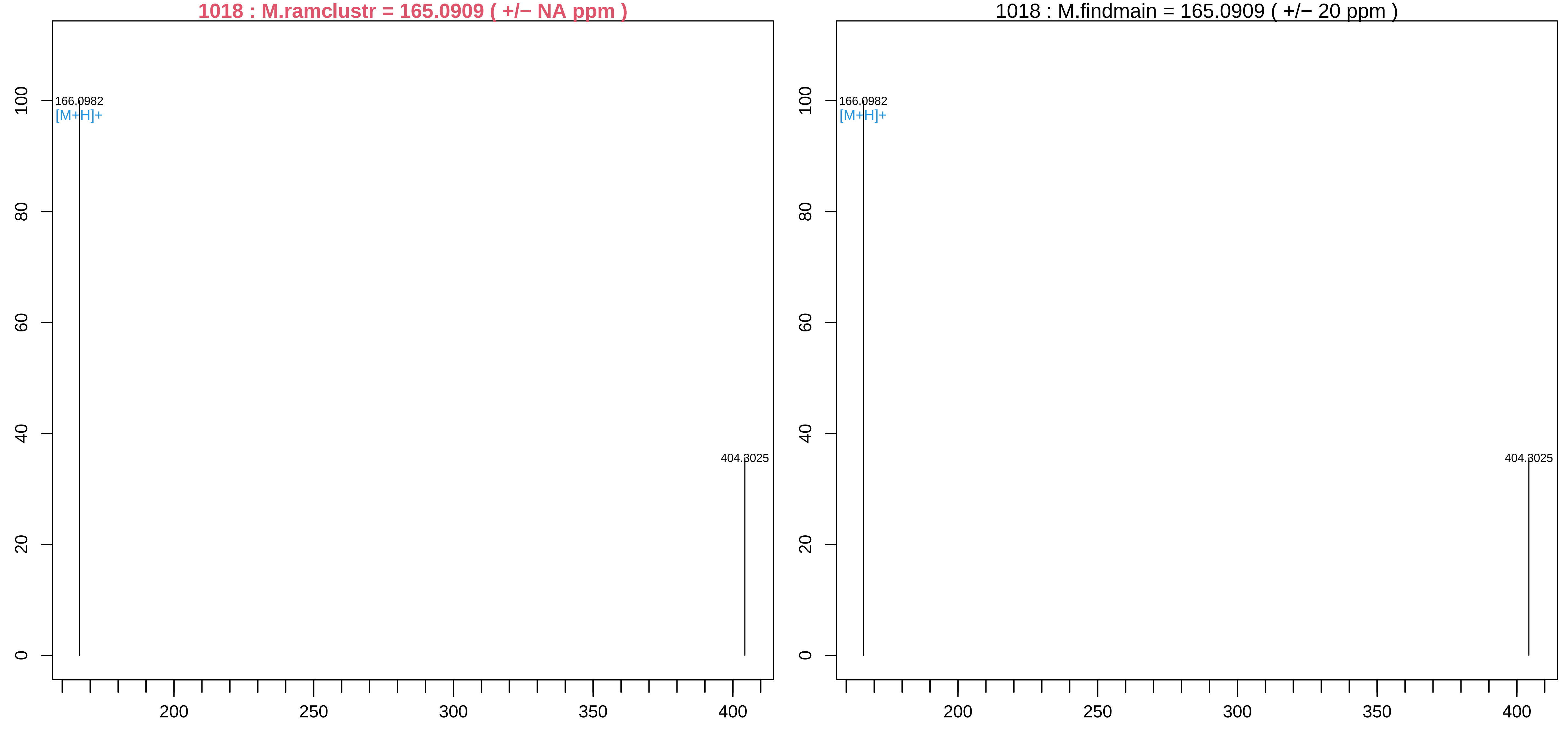

1018 : M.ramclustr = 165.0909 (+/− NA ppm)
166.0982
[M+H]+
404.3025
0
20
40
60
80
100
200
250
300
350
400
1018 : M.findmain = 165.0909 (+/− 20 ppm)
166.0982
[M+H]+
404.3025
0
20
40
60
80
100
200
250
300
350
400

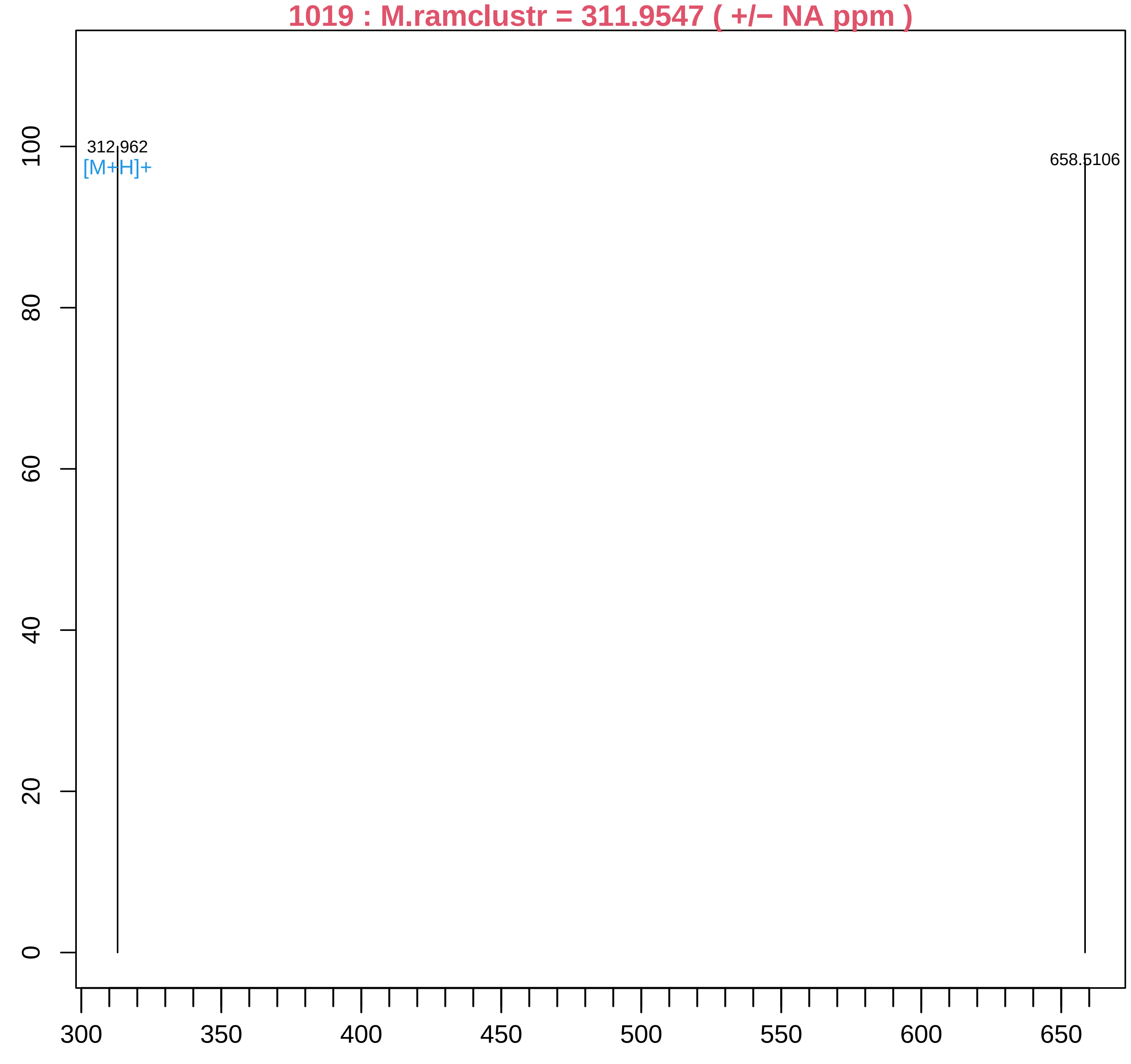
1019 : M.ramclustr = 311.9547 (+/− NA ppm)
312.962
[M+H]+
658.5106
0
20
40
60
80
100
300
350
400
450
500
550
600
650

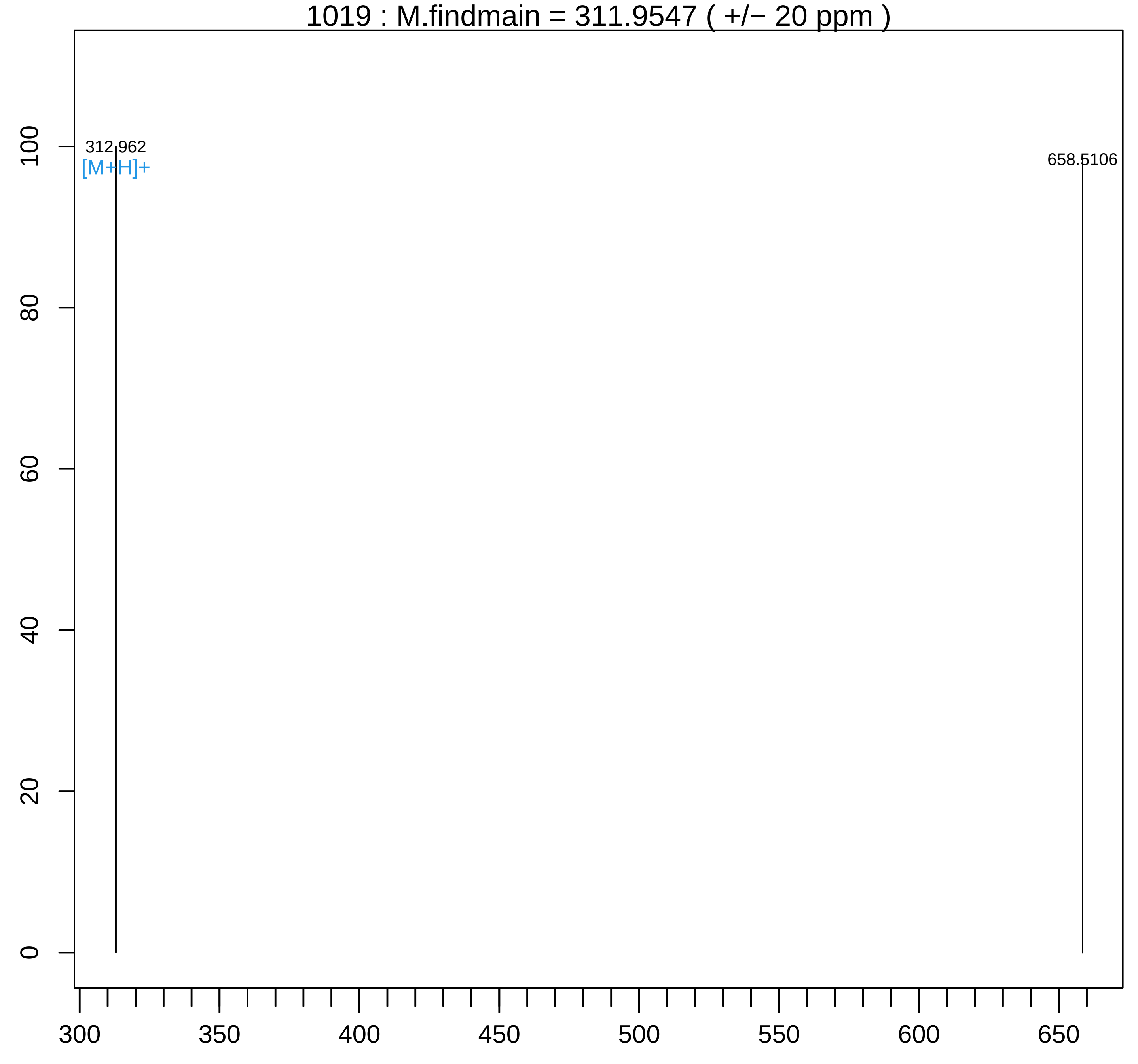
1019 : M.findmain = 311.9547 (+/− 20 ppm)
312.962
[M+H]+
658.5106
0
20
40
60
80
100
300
350
400
450
500
550
600
650

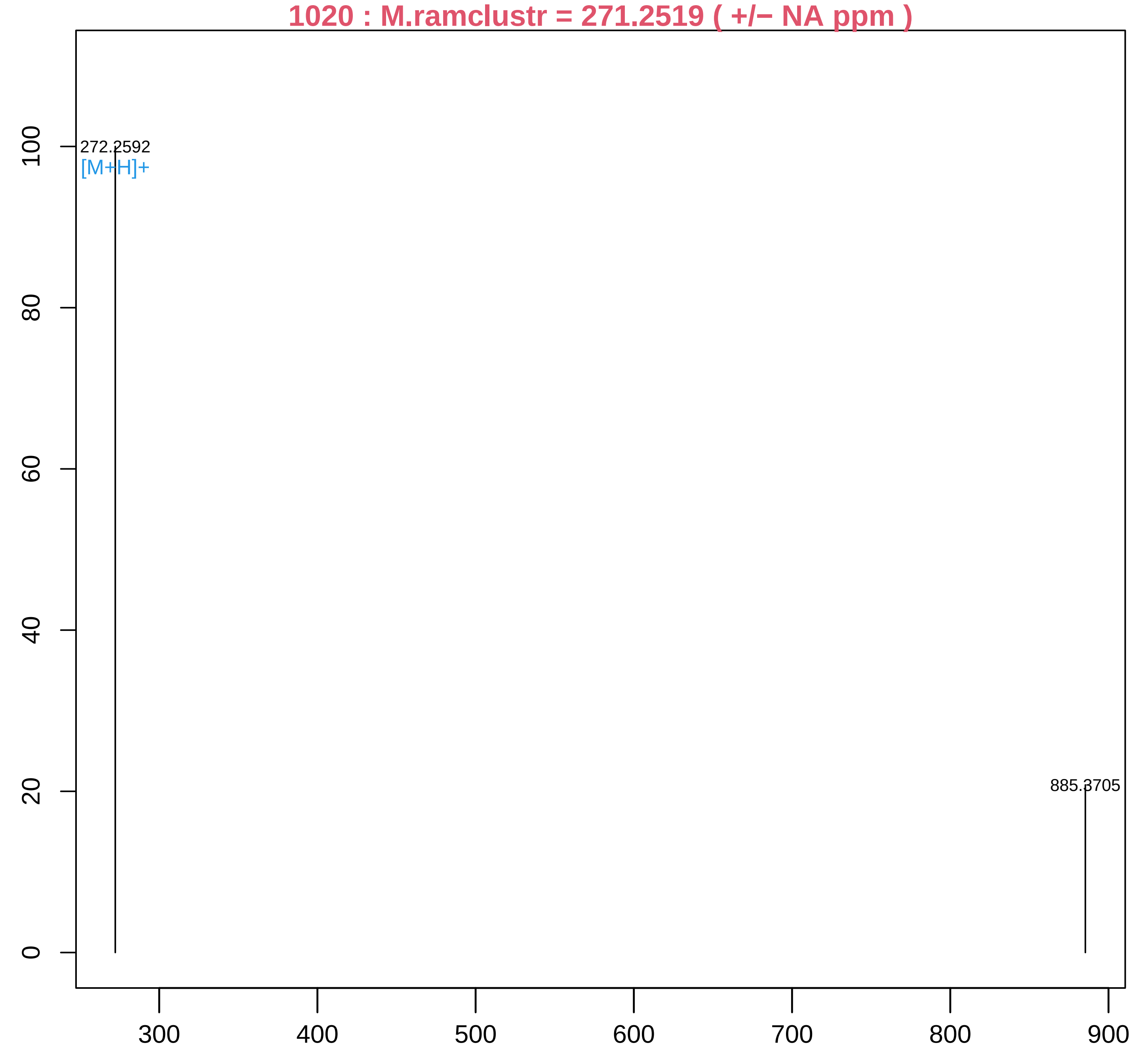
1020 : M.ramclustr = 271.2519 (+/− NA ppm)
272.2592
[M+H]+
885.3705
0
20
40
60
80
100
300
400
500
600
700
800
900
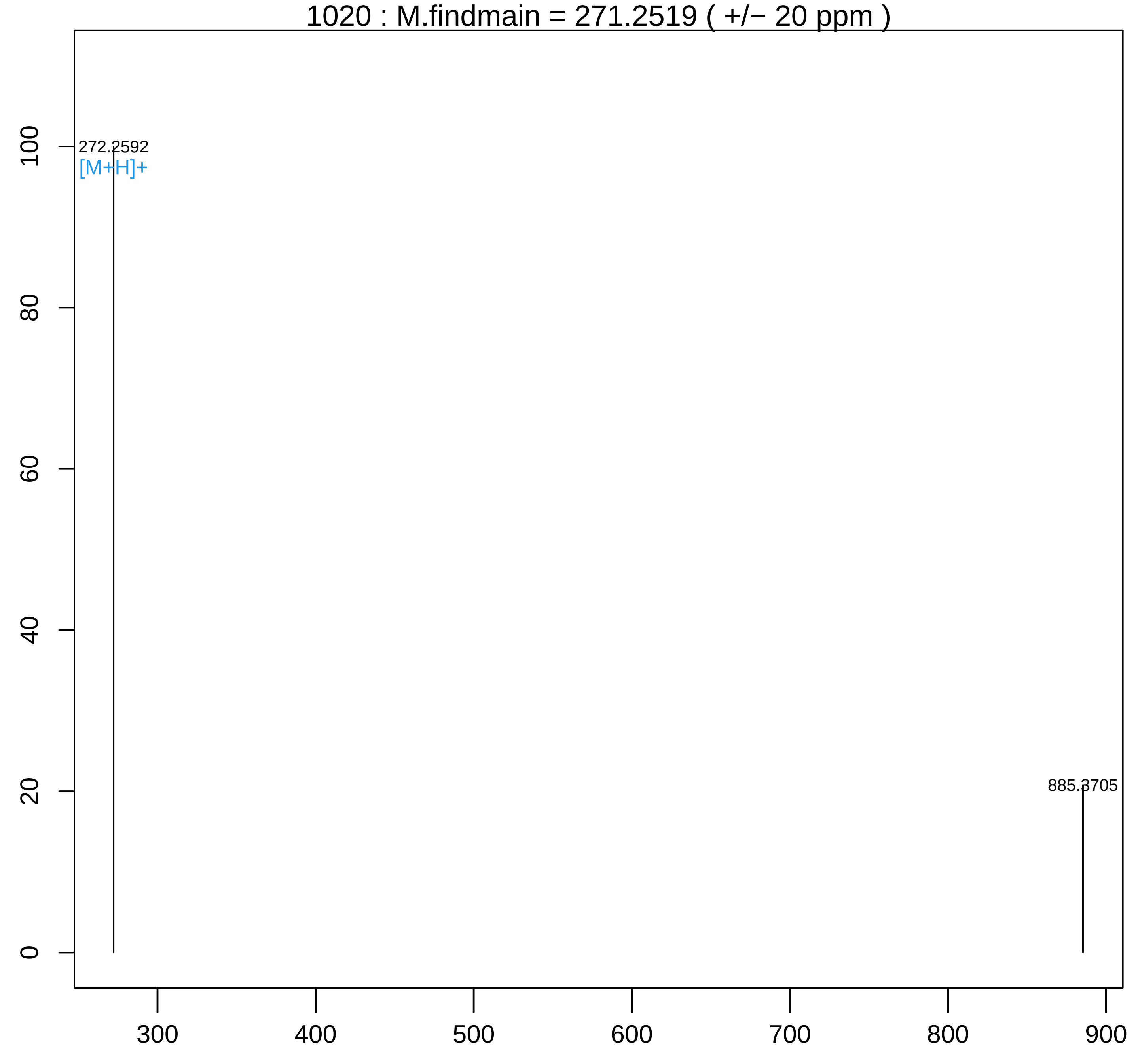
1020 : M.findmain = 271.2519 (+/− 20 ppm)
272.2592
[M+H]+
885.3705
0
20
40
60
80
100
300
400
500
600
700
800
900

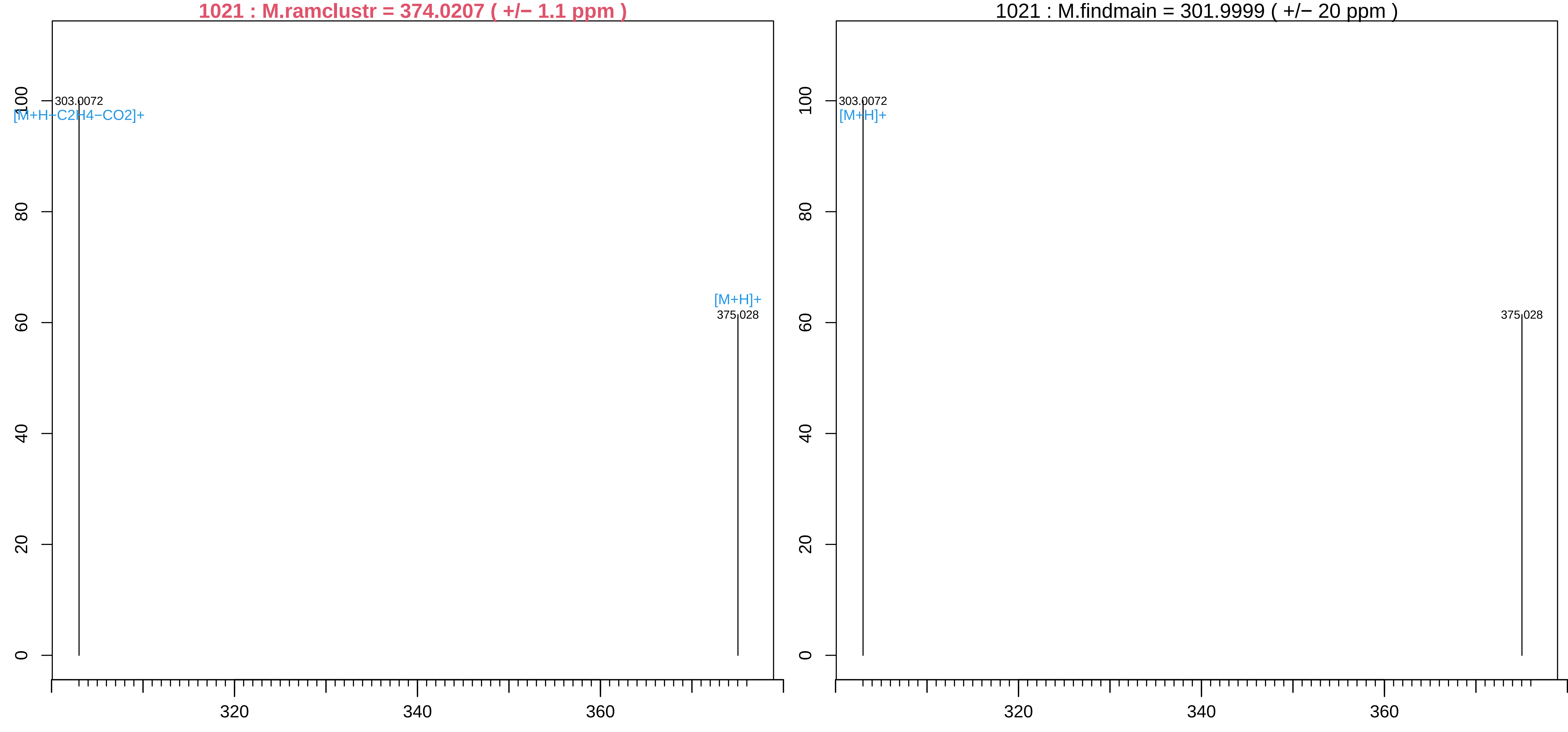
1021 : M.ramclustr = 374.0207 (+/− 1.1 ppm)
303.0072
[M+H−C2H4−CO2]+
[M+H]+
375.028
1021 : M.findmain = 301.9999 (+/− 20 ppm)
303.0072
[M+H]+
375.028
0
20
40
60
80
100
320
340
360

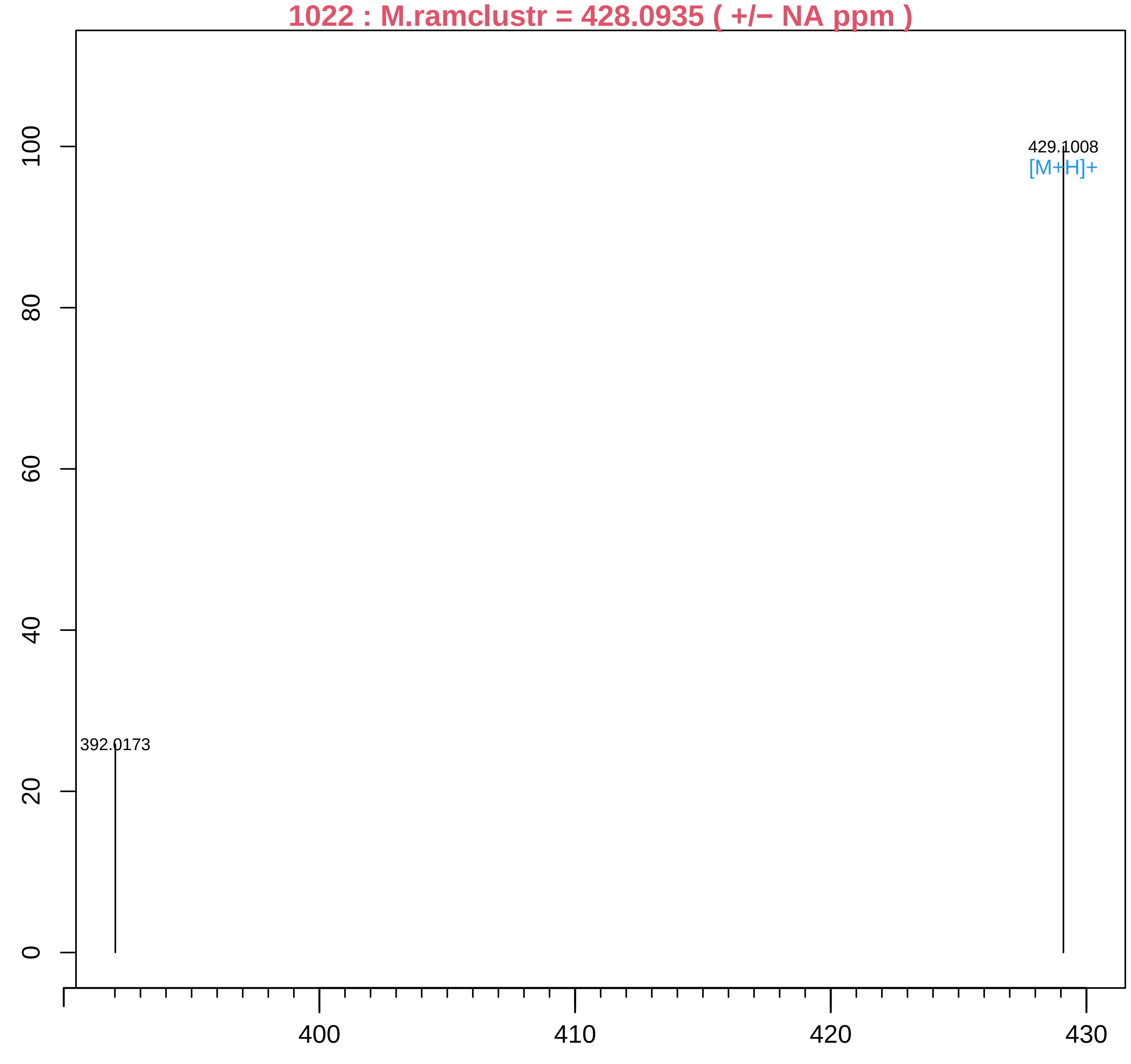
1022 : M.ramclustr = 428.0935 (+/− NA ppm)
429.1008
[M+H]+
392.0173
0
20
40
60
80
100
400
410
420
430
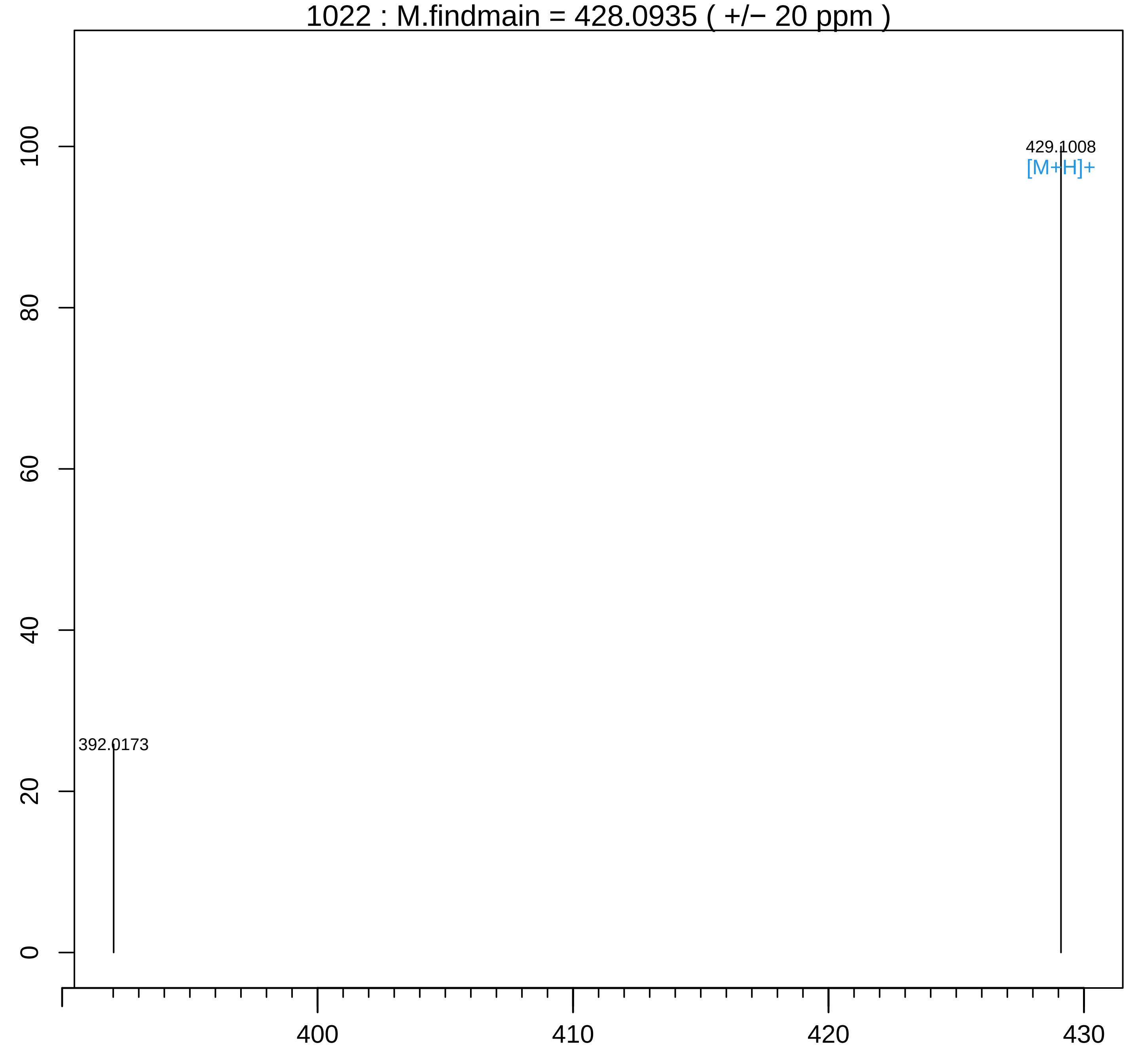
1022 : M.findmain = 428.0935 (+/− 20 ppm)
429.1008
[M+H]+
392.0173
0
20
40
60
80
100
400
410
420
430

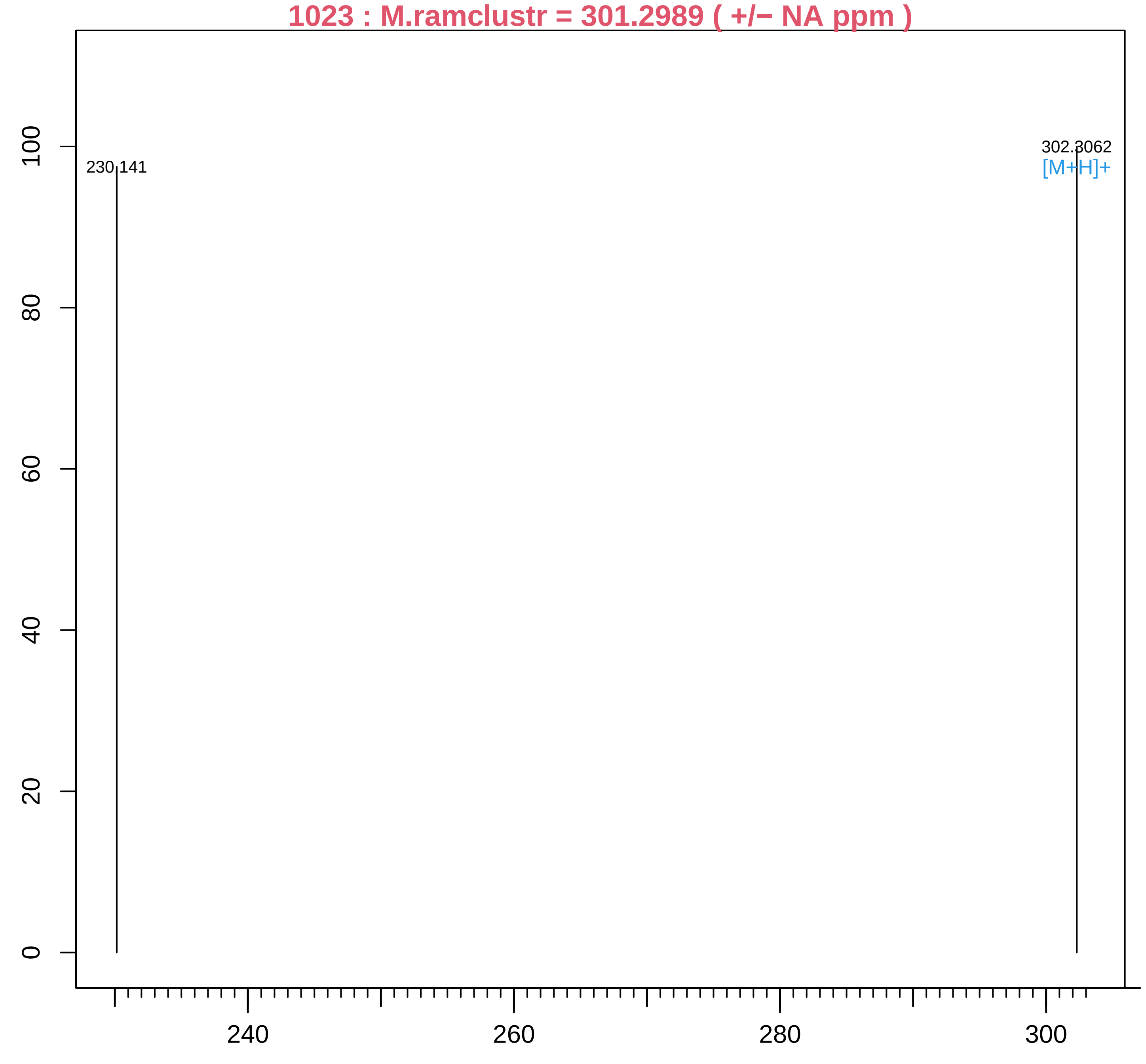
1023 : M.ramclustr = 301.2989 (+/− NA ppm)
230.141
302.3062
[M+H]+
0
20
40
60
80
100
240
260
280
300

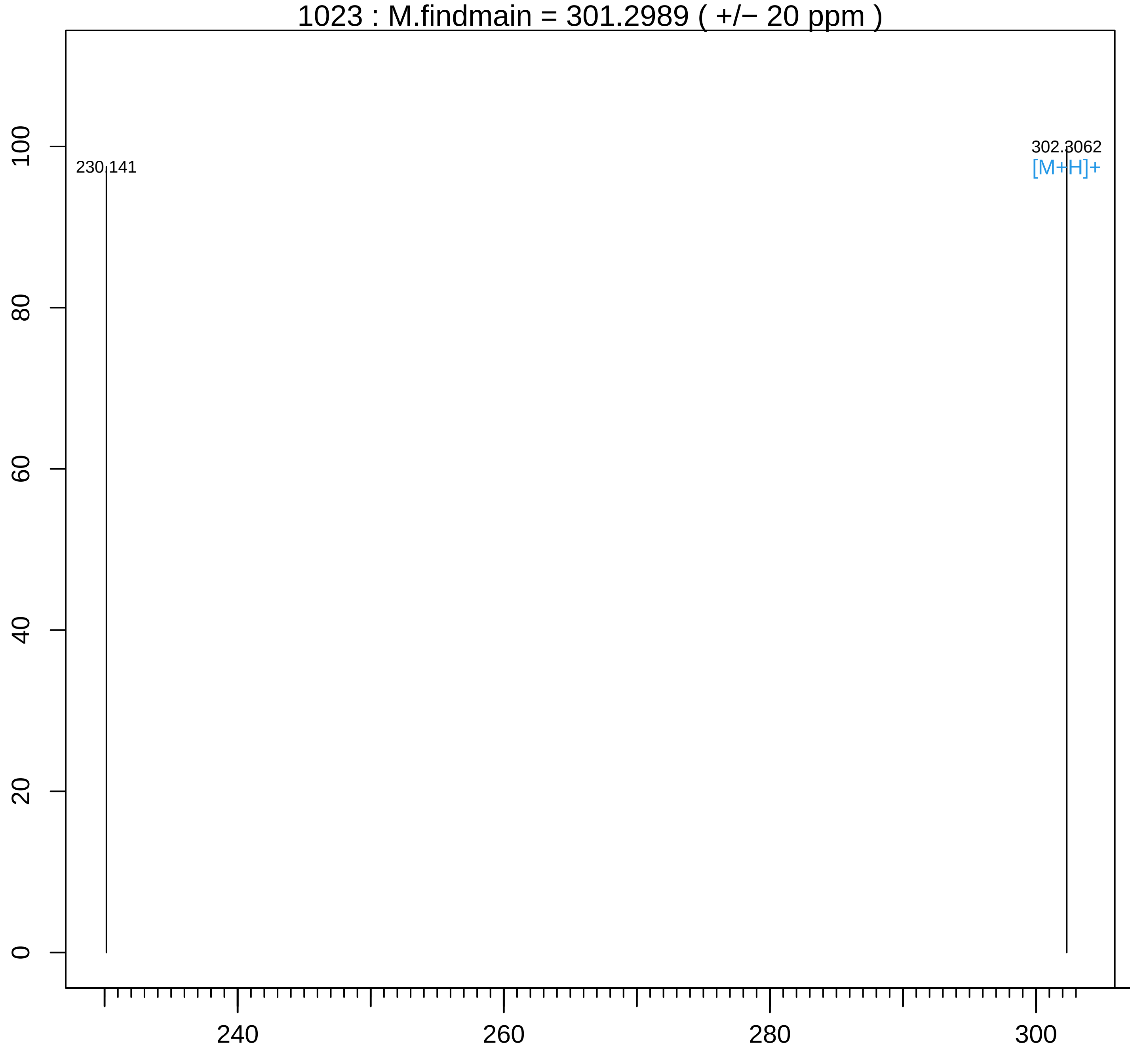
1023 : M.findmain = 301.2989 (+/− 20 ppm)
230.141
302.3062
[M+H]+
0
20
40
60
80
100
240
260
280
300

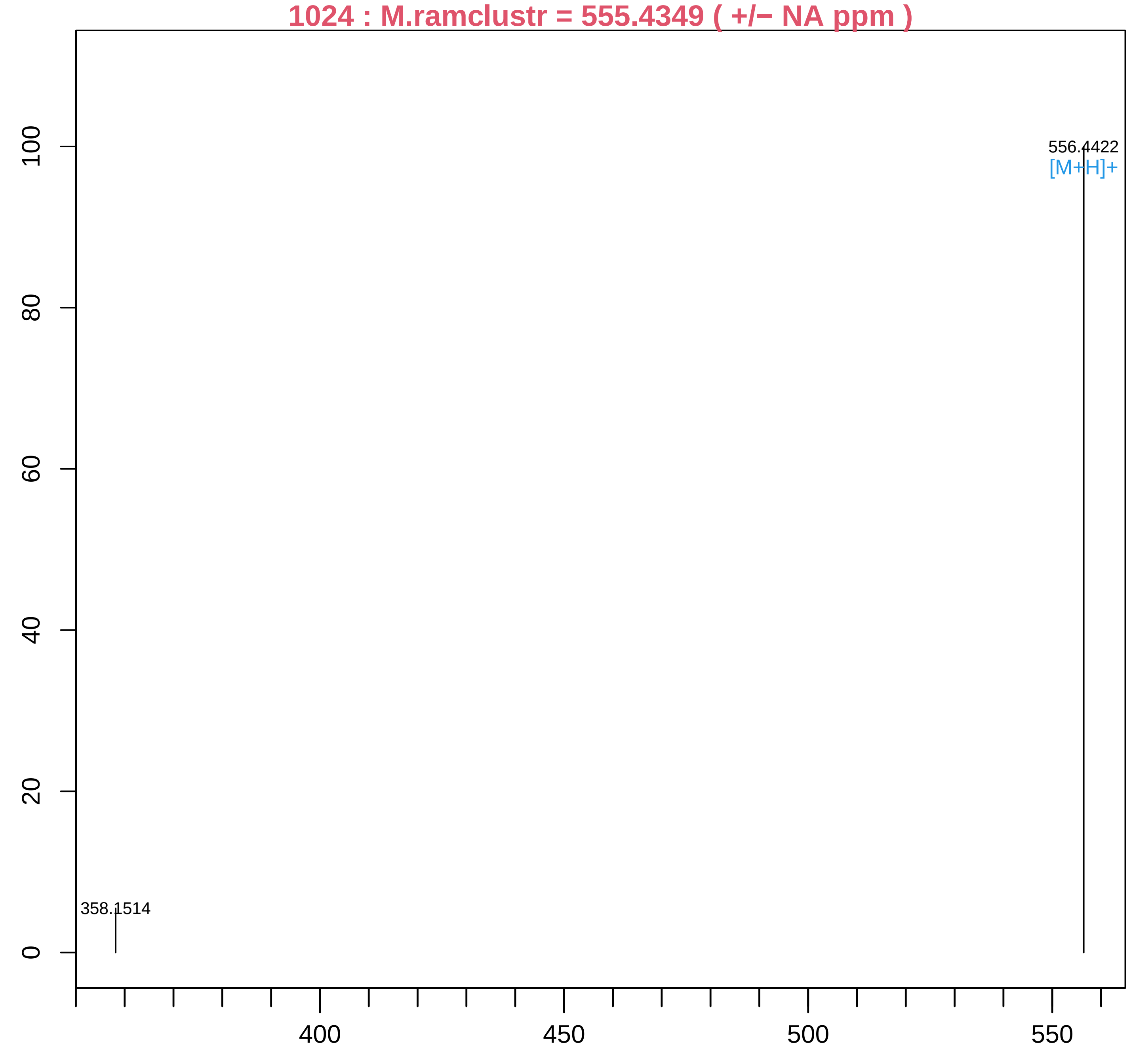

1024 : M.ramclustr = 555.4349 (+/− NA ppm)
556.4422
[M+H]+
358.1514
0
20
40
60
80
100
400
450
500
550

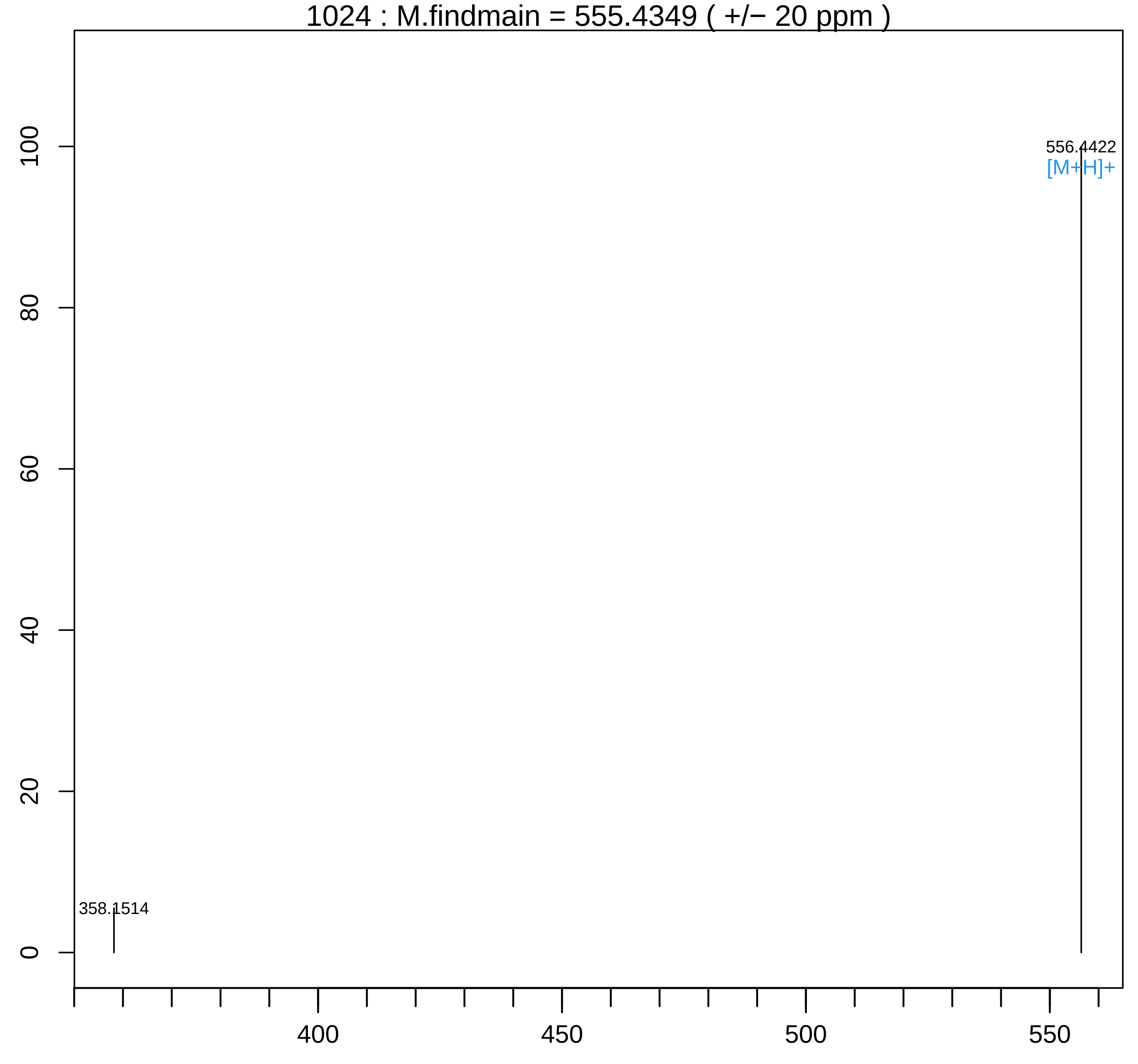

1024 : M.findmain = 555.4349 (+/− 20 ppm)
556.4422
[M+H]+
358.1514
0
20
40
60
80
100
400
450
500
550

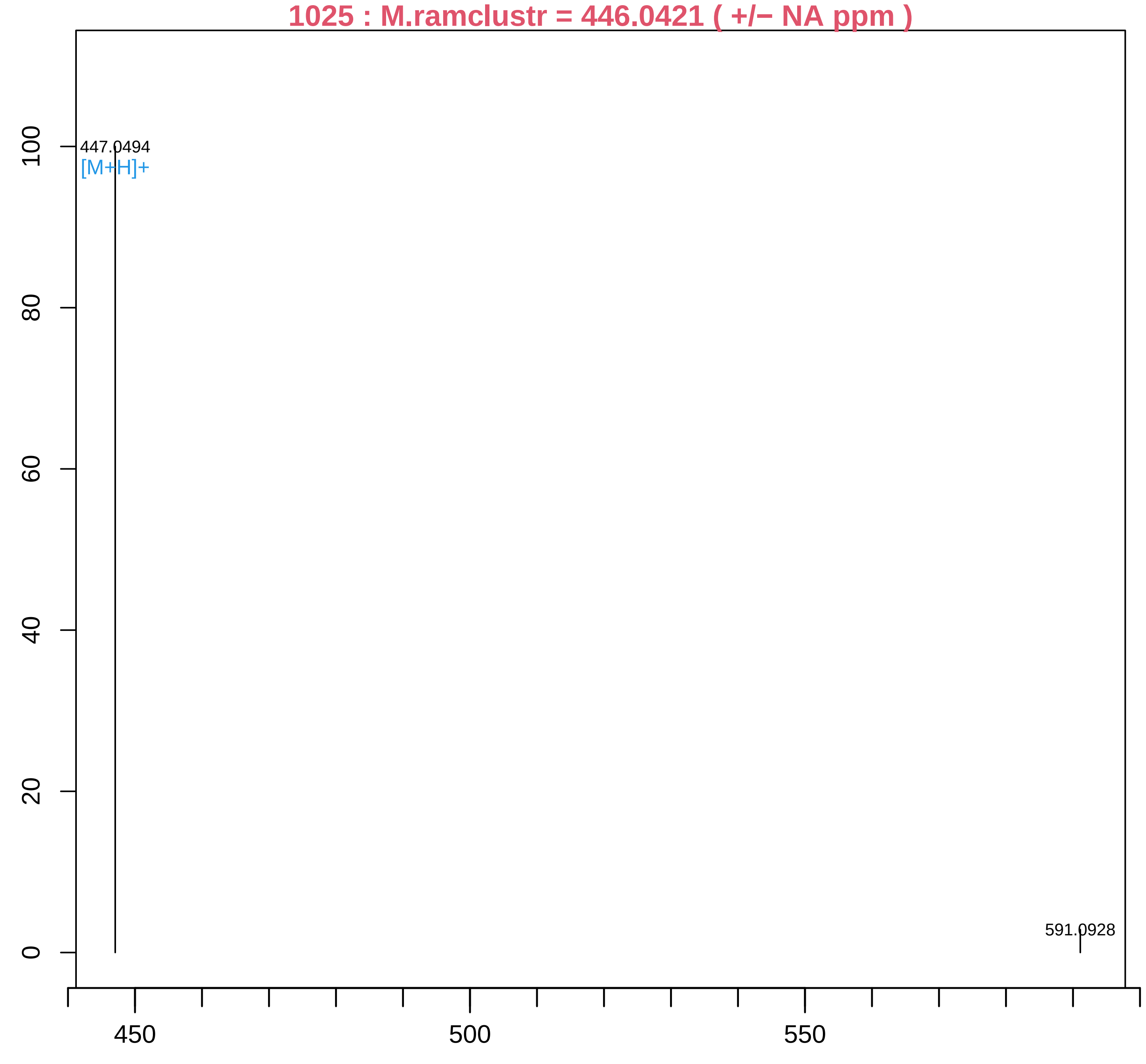

1025 : M.ramclustr = 446.0421 (+/− NA ppm)
447.0494
[M+H]+
591.0928
0
20
40
60
80
100
450
500
550

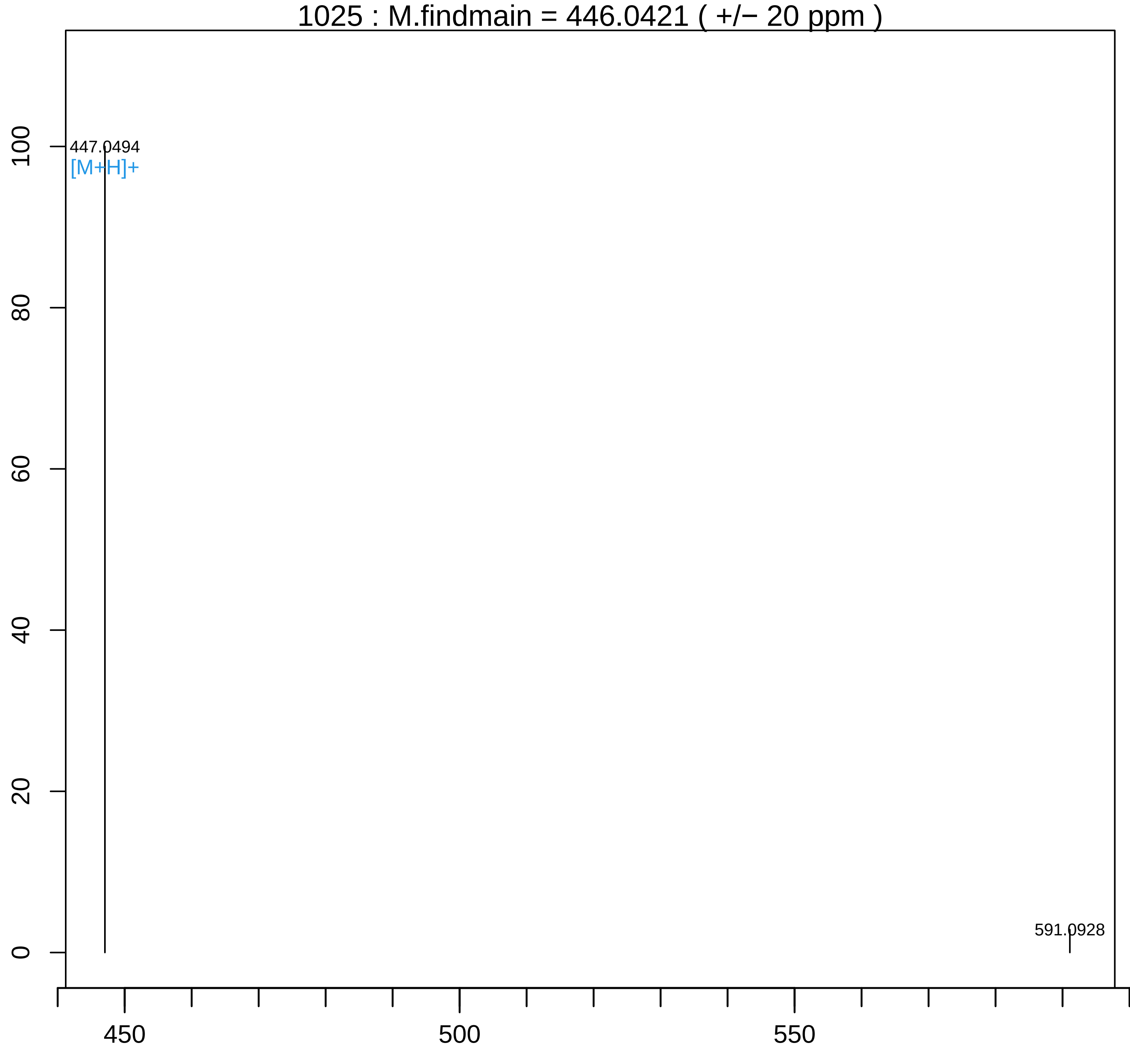

1025 : M.findmain = 446.0421 (+/− 20 ppm)
447.0494
[M+H]+
591.0928
0
20
40
60
80
100
450
500
550

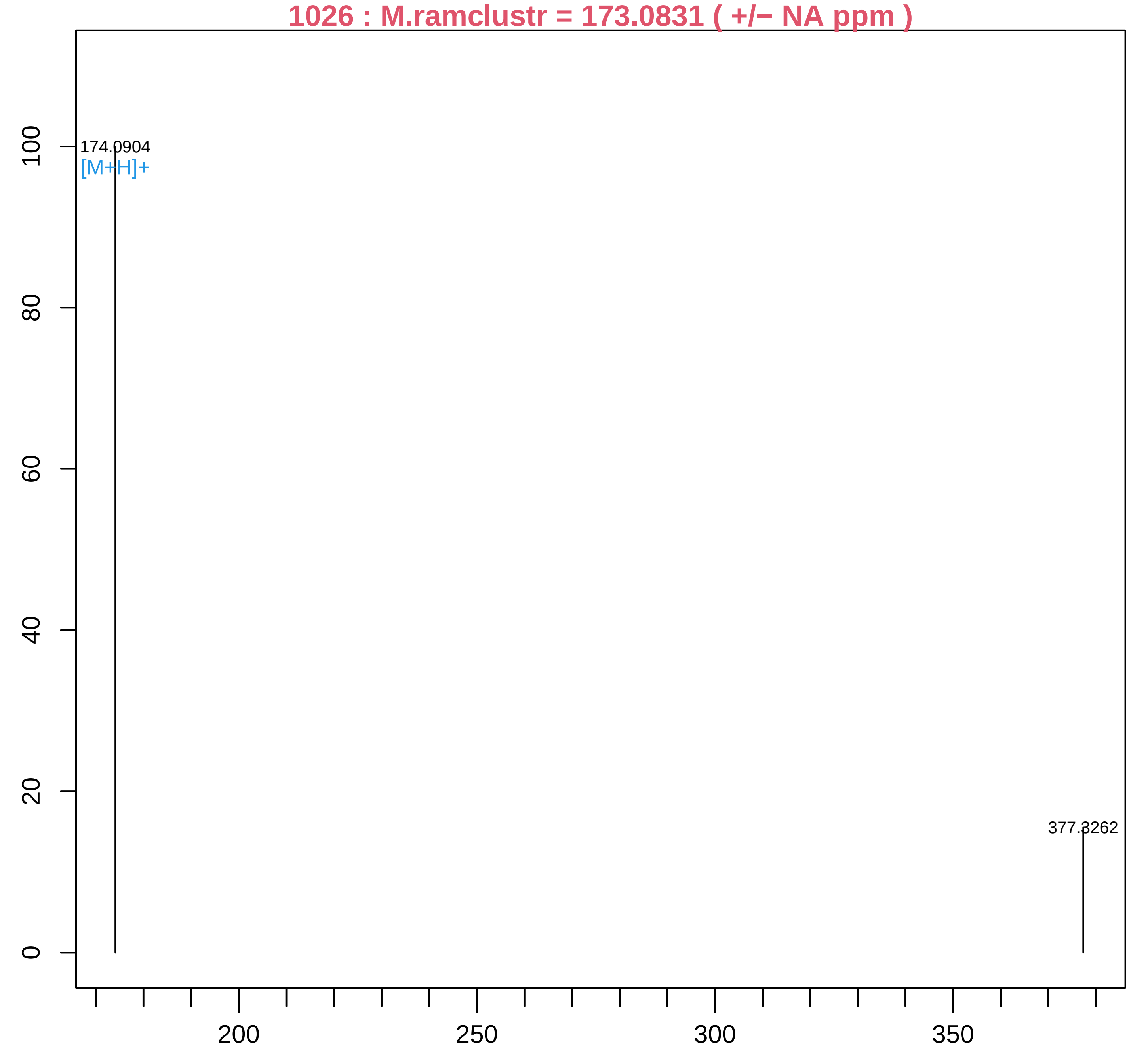
1026 : M.ramclustr = 173.0831 (+/− NA ppm)
174.0904
[M+H]+
377.3262
0
20
40
60
80
100
200
250
300
350

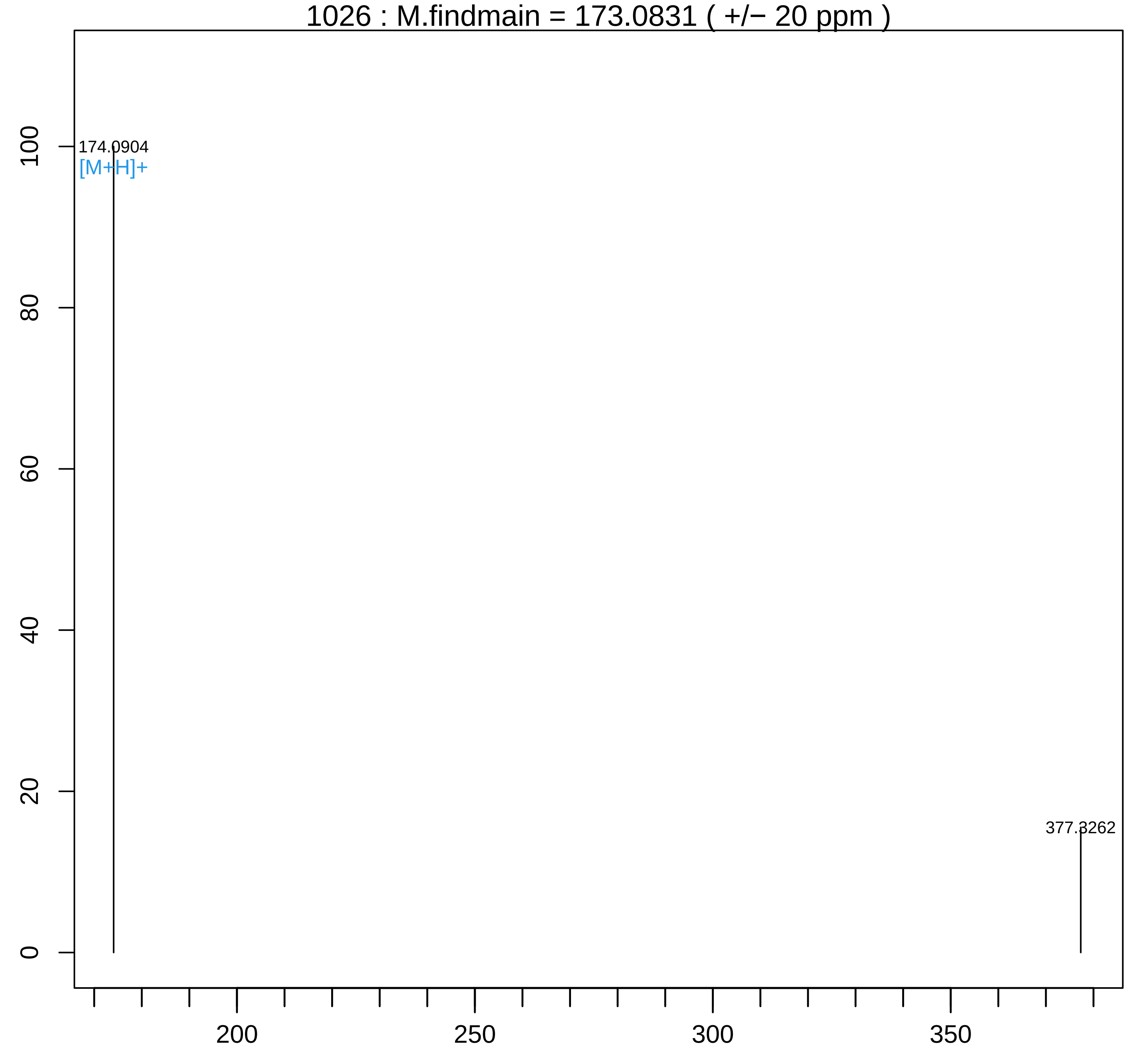
1026 : M.findmain = 173.0831 (+/− 20 ppm)
174.0904
[M+H]+
377.3262
0
20
40
60
80
100
200
250
300
350

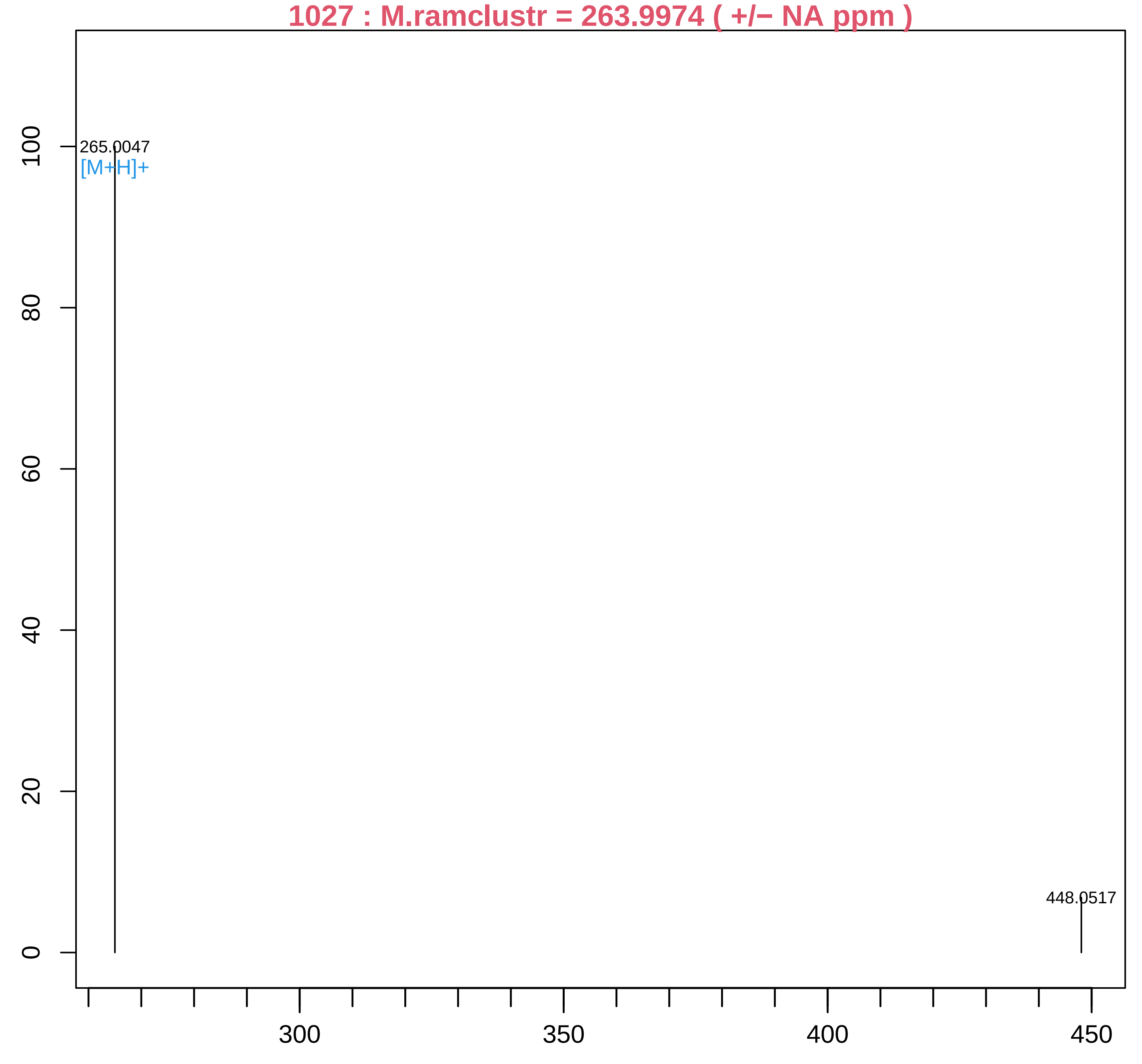
1027 : M.ramclustr = 263.9974 (+/− NA ppm)
265.0047
[M+H]+
448.0517
0
20
40
60
80
100
300
350
400
450

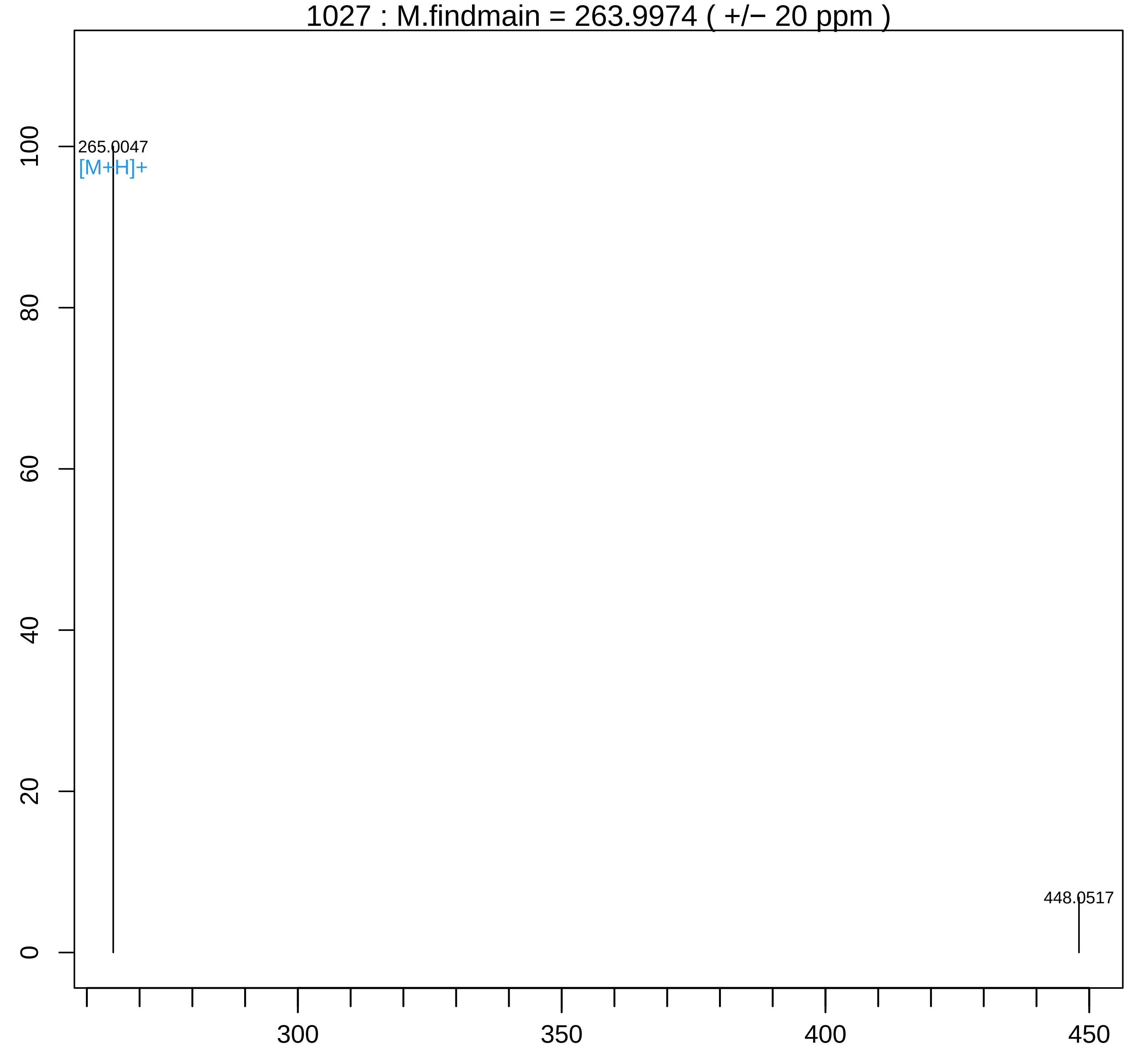
1027 : M.findmain = 263.9974 (+/− 20 ppm)
265.0047
[M+H]+
448.0517
0
20
40
60
80
100
300
350
400
450

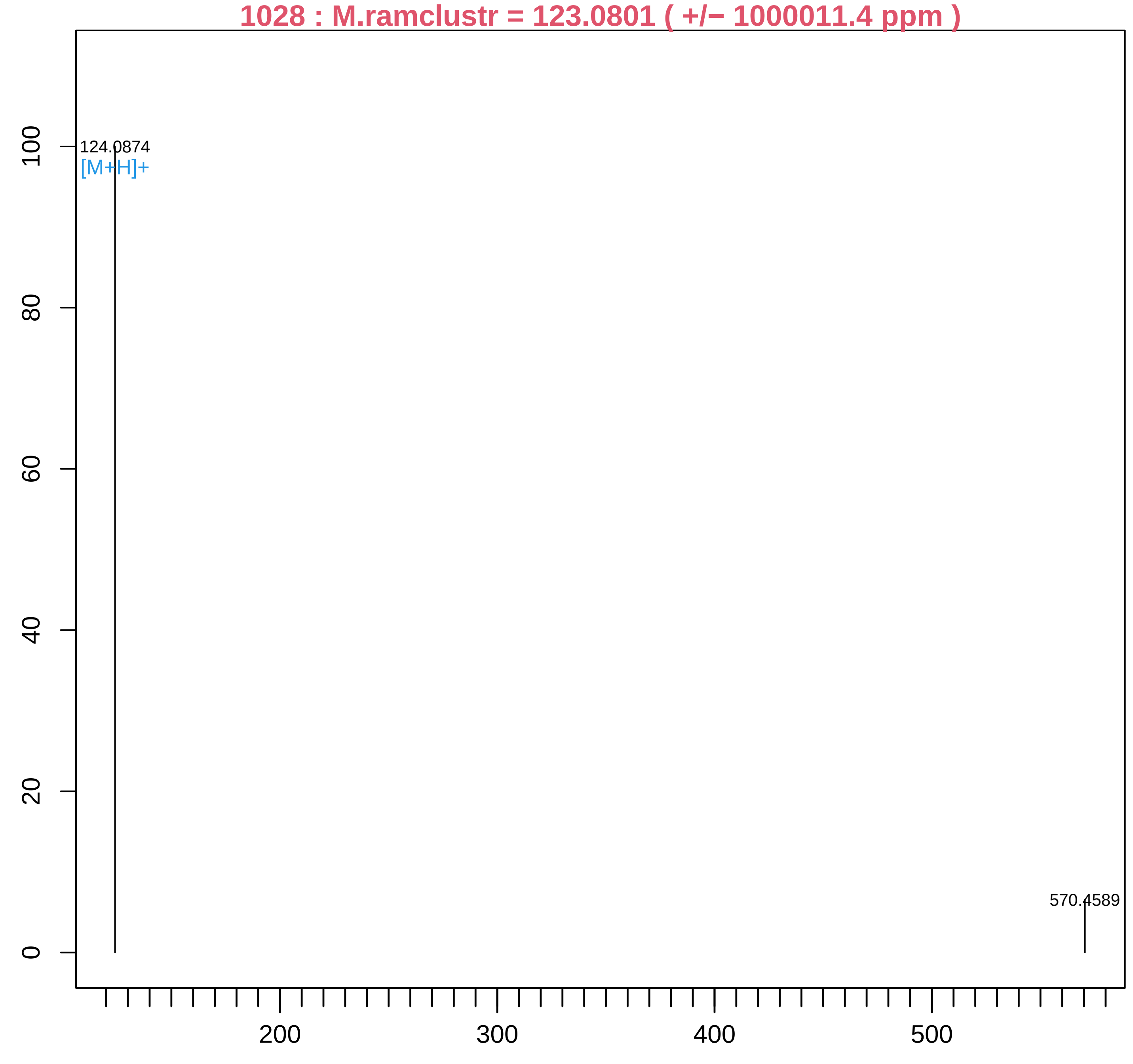
1028 : M.ramclustr = 123.0801 (+/− 1000011.4 ppm)
124.0874
[M+H]+
570.4589
0
20
40
60
80
100
200
300
400
500

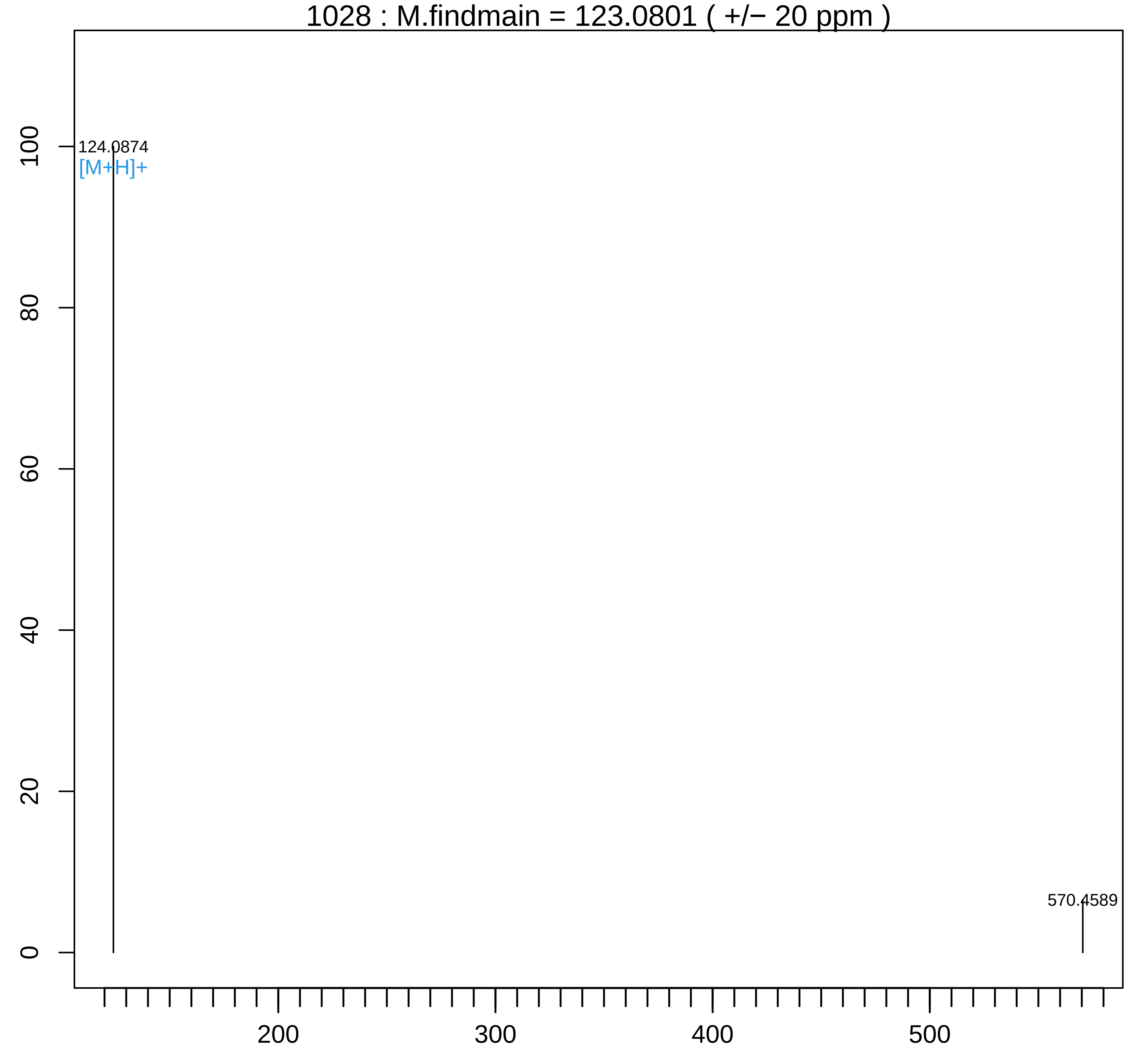
1028 : M.findmain = 123.0801 (+/− 20 ppm)
124.0874
[M+H]+
570.4589
0
20
40
60
80
100
200
300
400
500

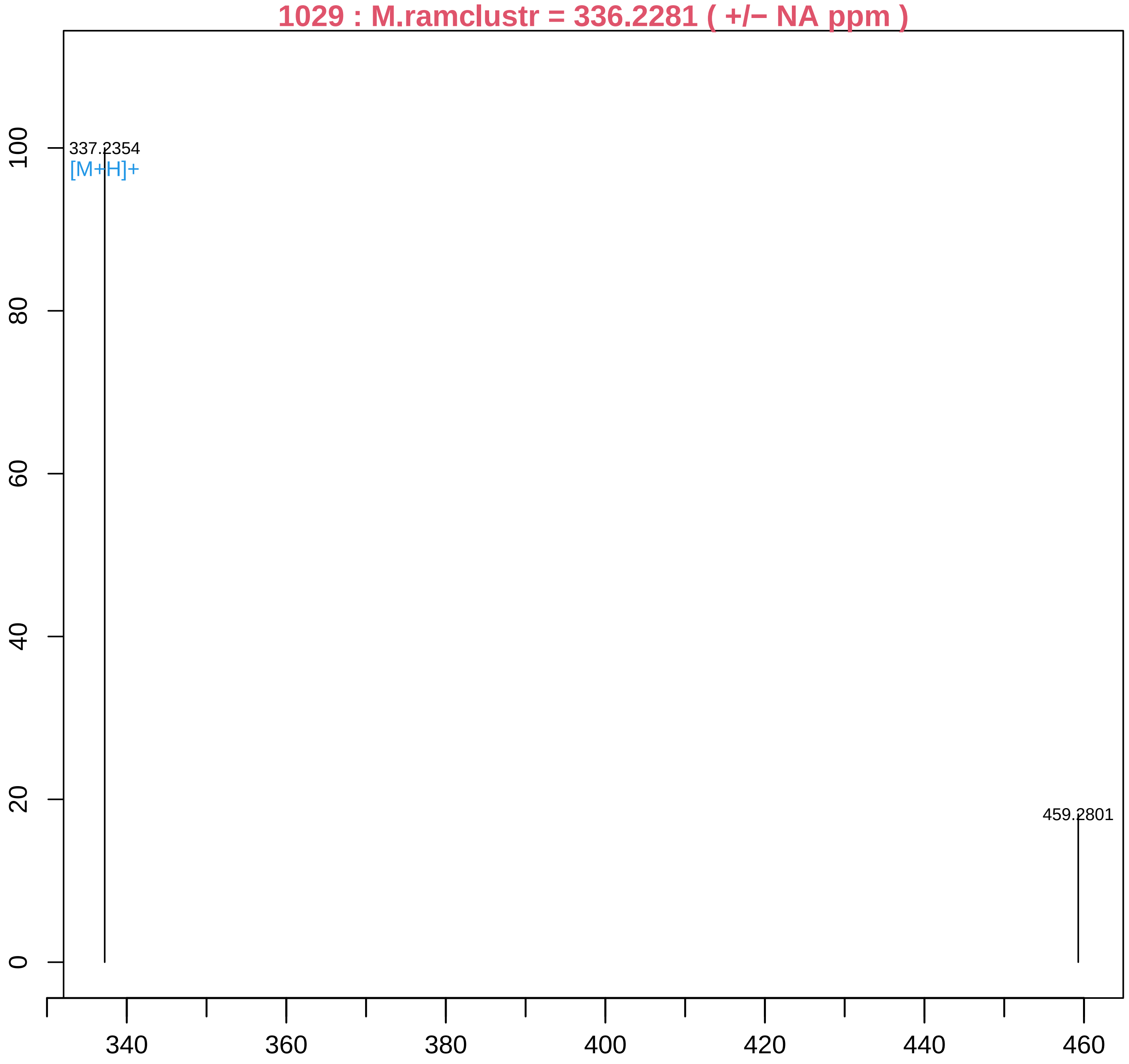
1029 : M.ramclustr = 336.2281 (+/− NA ppm)
337.2354
[M+H]+
459.2801
0
20
40
60
80
100
340
360
380
400
420
440
460

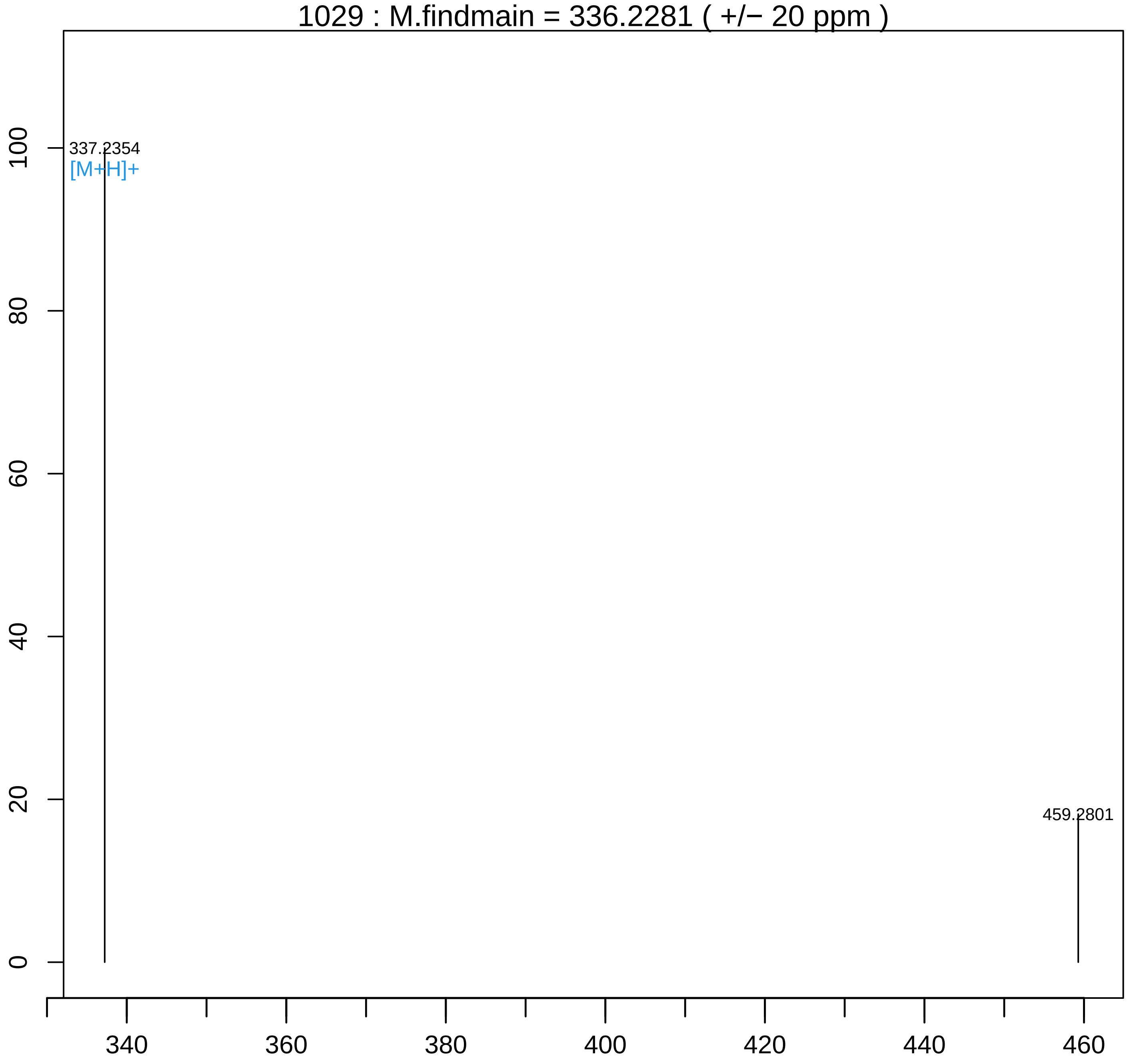
1029 : M.findmain = 336.2281 (+/− 20 ppm)
337.2354
[M+H]+
459.2801
0
20
40
60
80
100
340
360
380
400
420
440
460

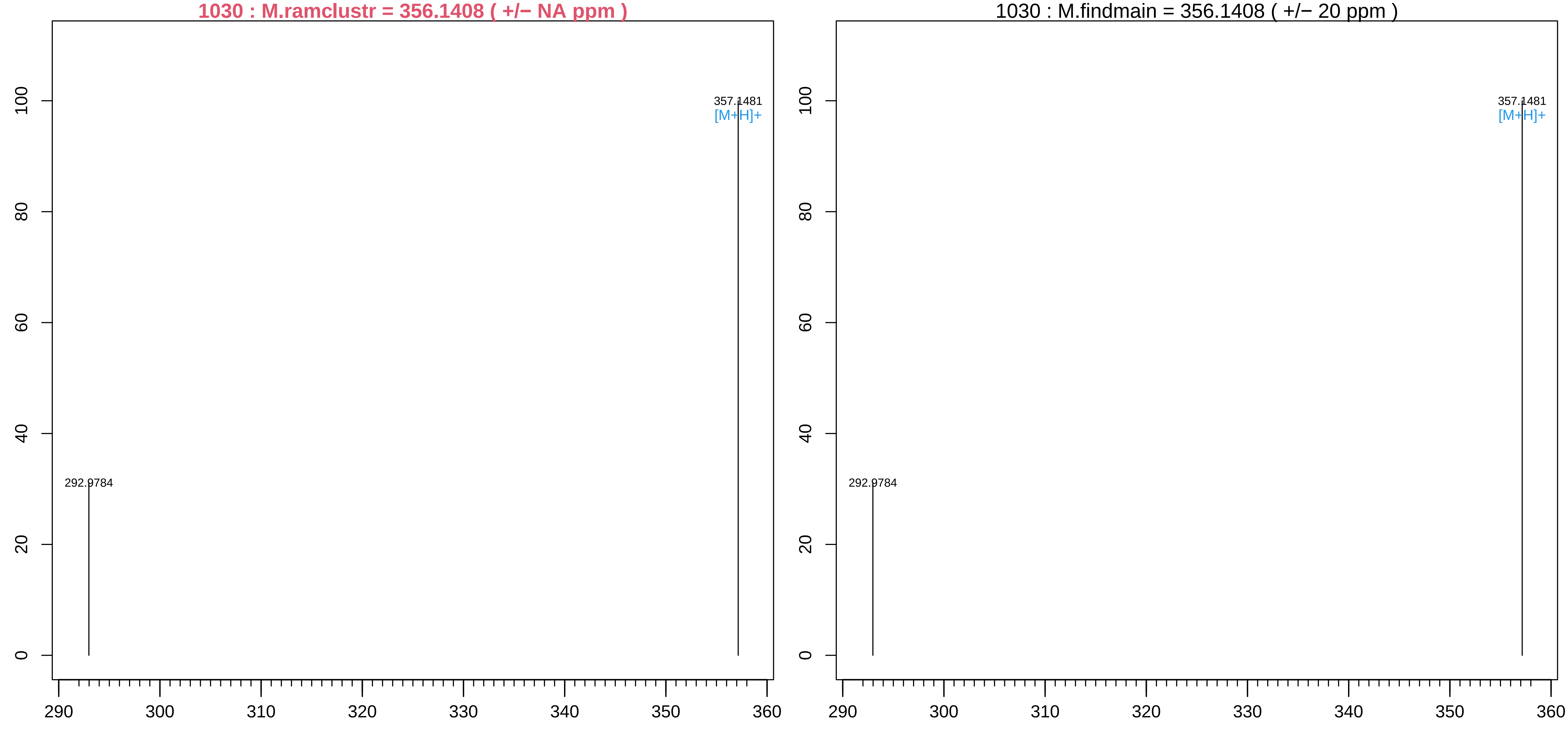
1030 : M.ramclustr = 356.1408 (+/− NA ppm)
357.1481
[M+H]+
292.9784
1030 : M.findmain = 356.1408 (+/− 20 ppm)
357.1481
[M+H]+
292.9784
0
20
40
60
80
100
290
300
310
320
330
340
350
360

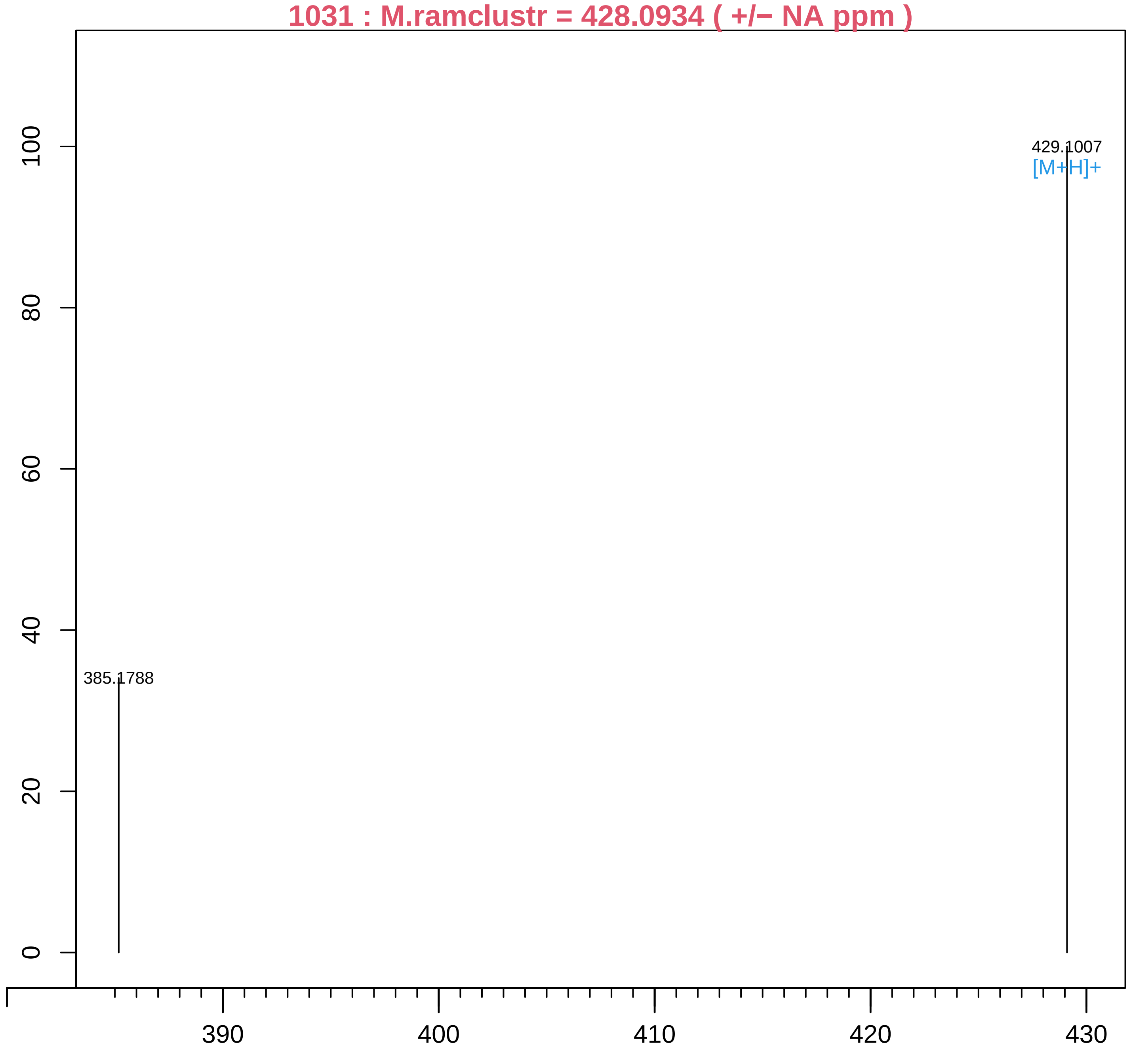

1031 : M.ramclustr = 428.0934 (+/− NA ppm)
429.1007
[M+H]+
385.1788
0
20
40
60
80
100
390
400
410
420
430

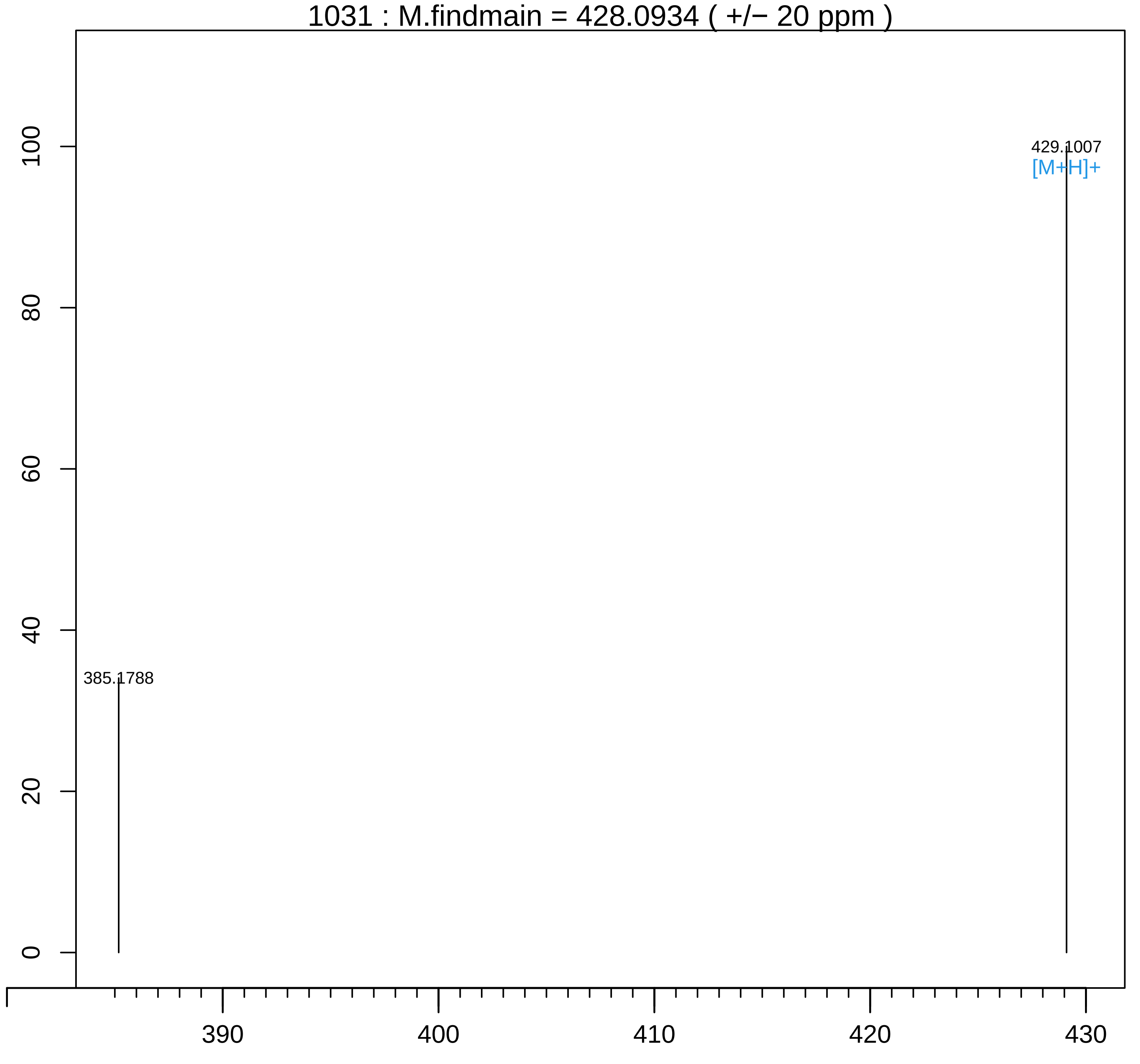

1031 : M.findmain = 428.0934 (+/− 20 ppm)
429.1007
[M+H]+
385.1788
0
20
40
60
80
100
390
400
410
420
430

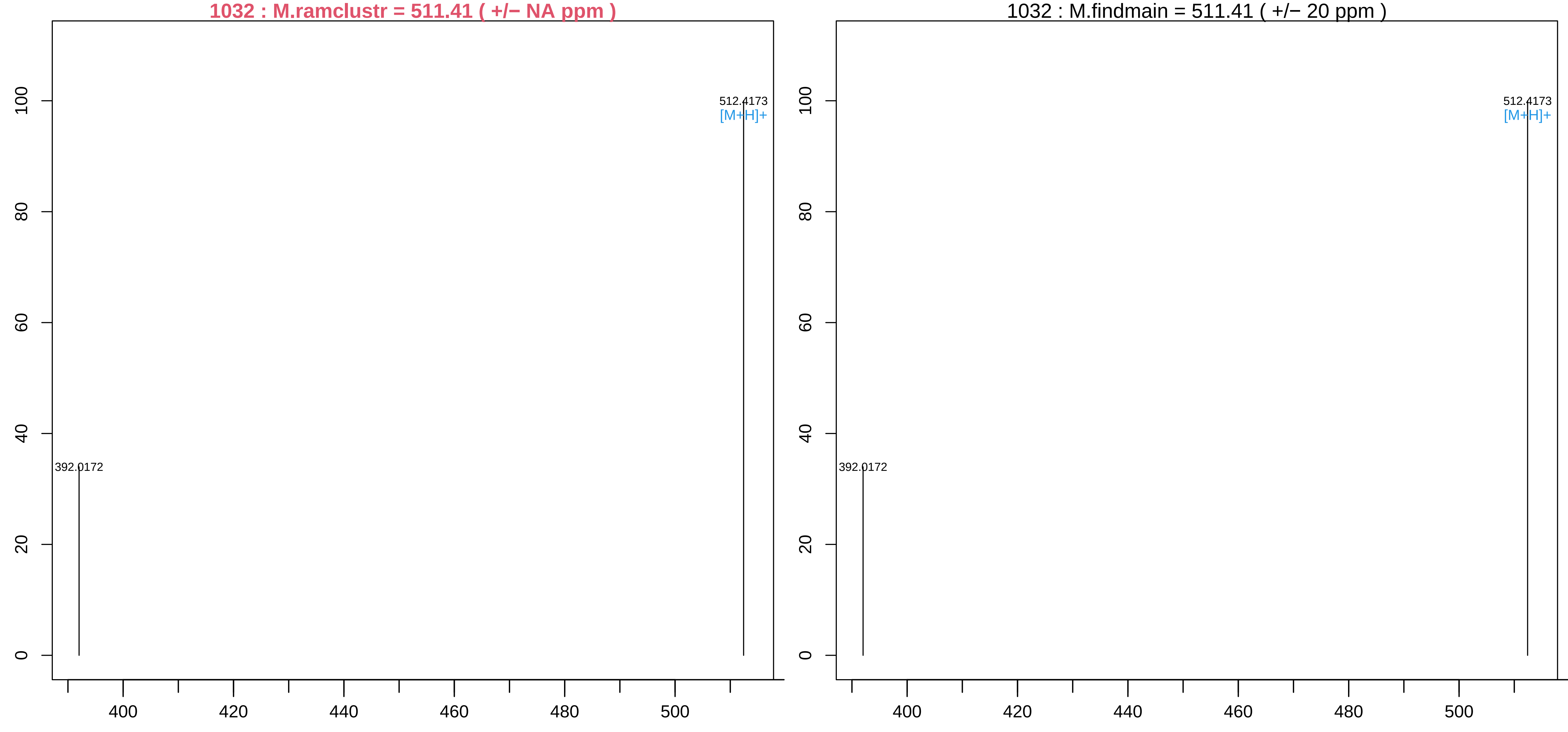

1032 : M.ramclustr = 511.41 (+/− NA ppm)
512.4173
[M+H]+
392.0172
1032 : M.findmain = 511.41 (+/− 20 ppm)
512.4173
[M+H]+
392.0172

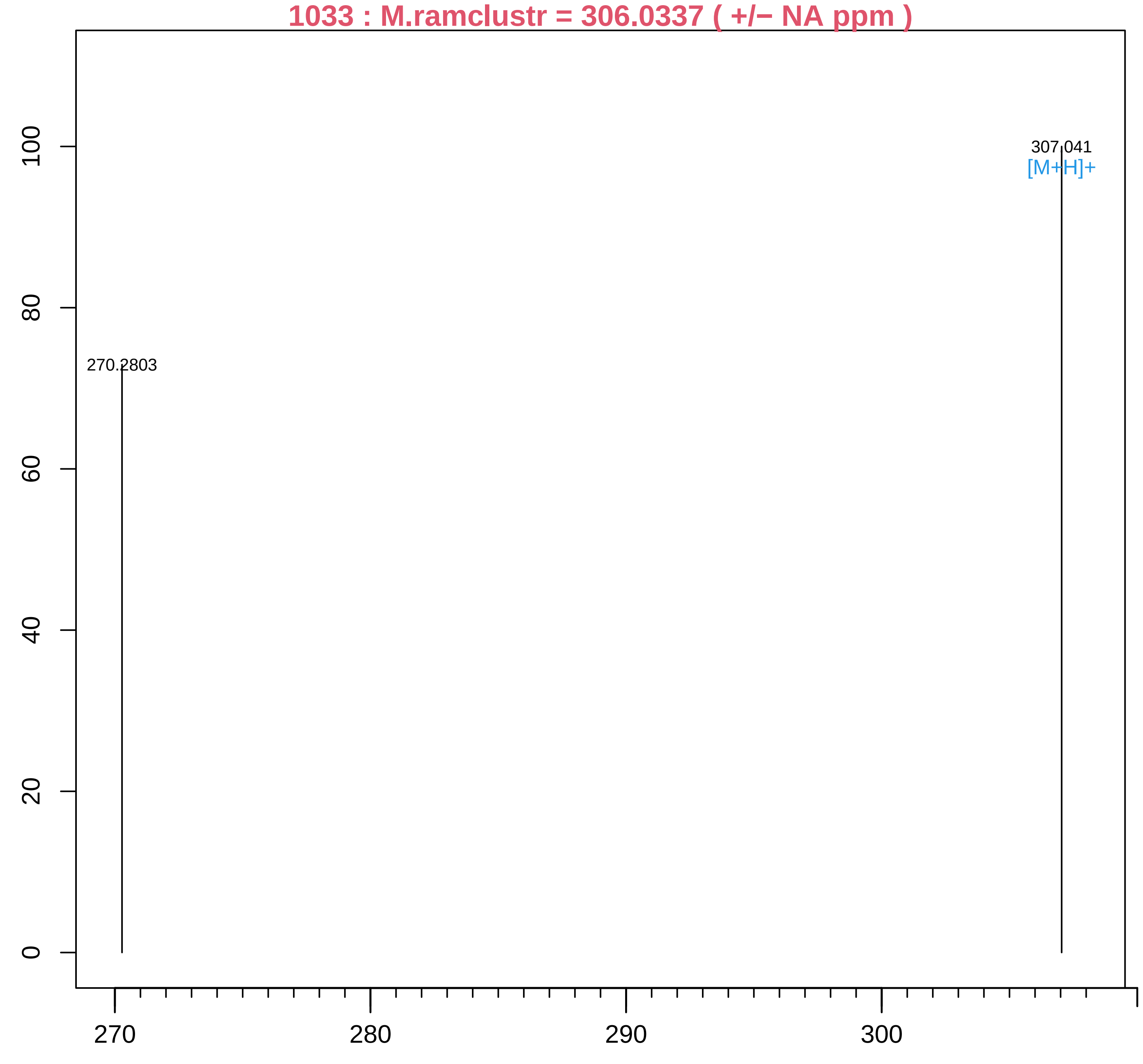
1033 : M.ramclustr = 306.0337 (+/− NA ppm)
307.041
[M+H]+
270.2803
0
20
40
60
80
100
270
280
290
300

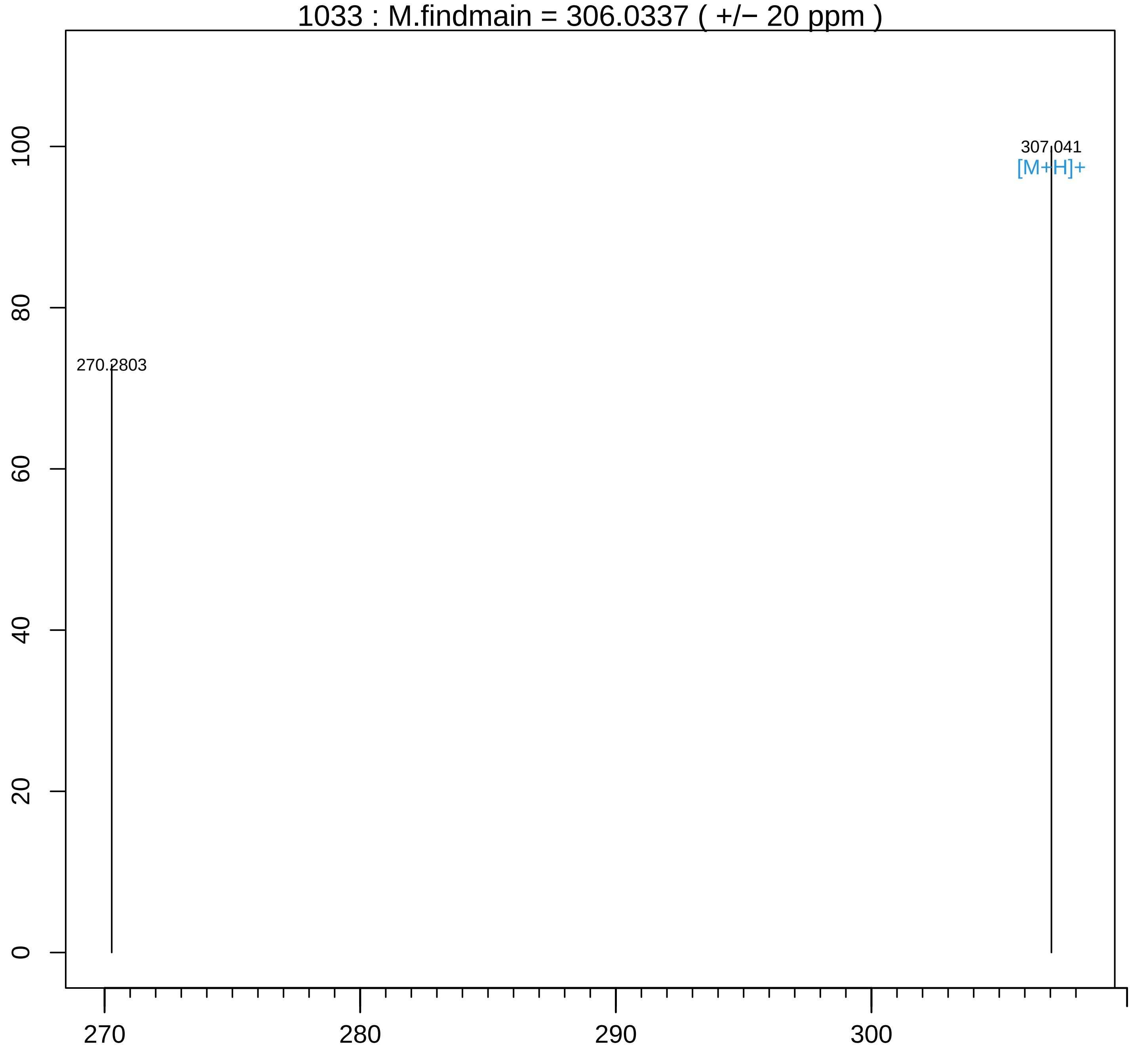
1033 : M.findmain = 306.0337 (+/− 20 ppm)
307.041
[M+H]+
270.2803
0
20
40
60
80
100
270
280
290
300

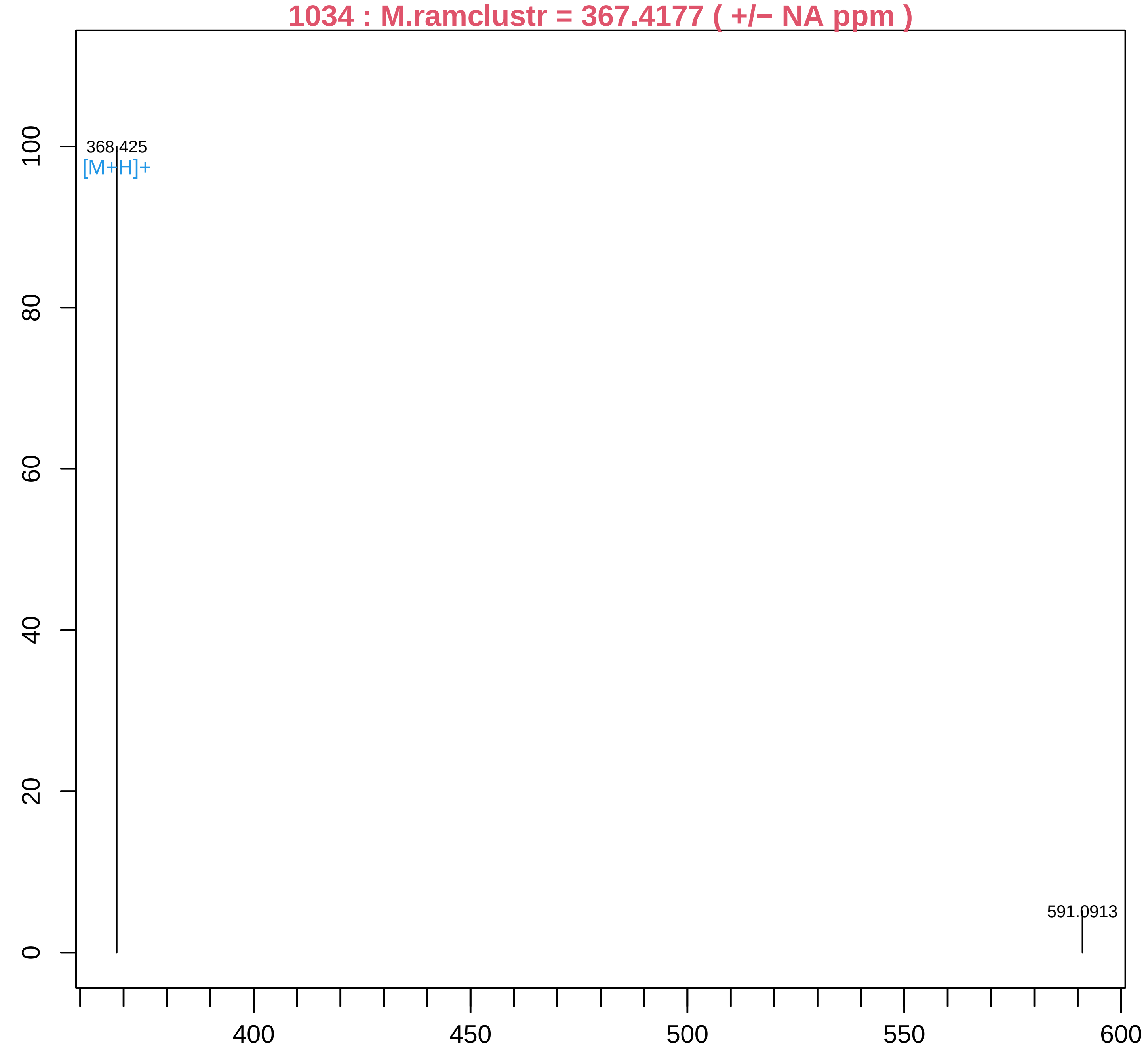

1034 : M.ramclustr = 367.4177 (+/− NA ppm)
368.425
[M+H]+
591.0913
0
20
40
60
80
100
400
450
500
550
600

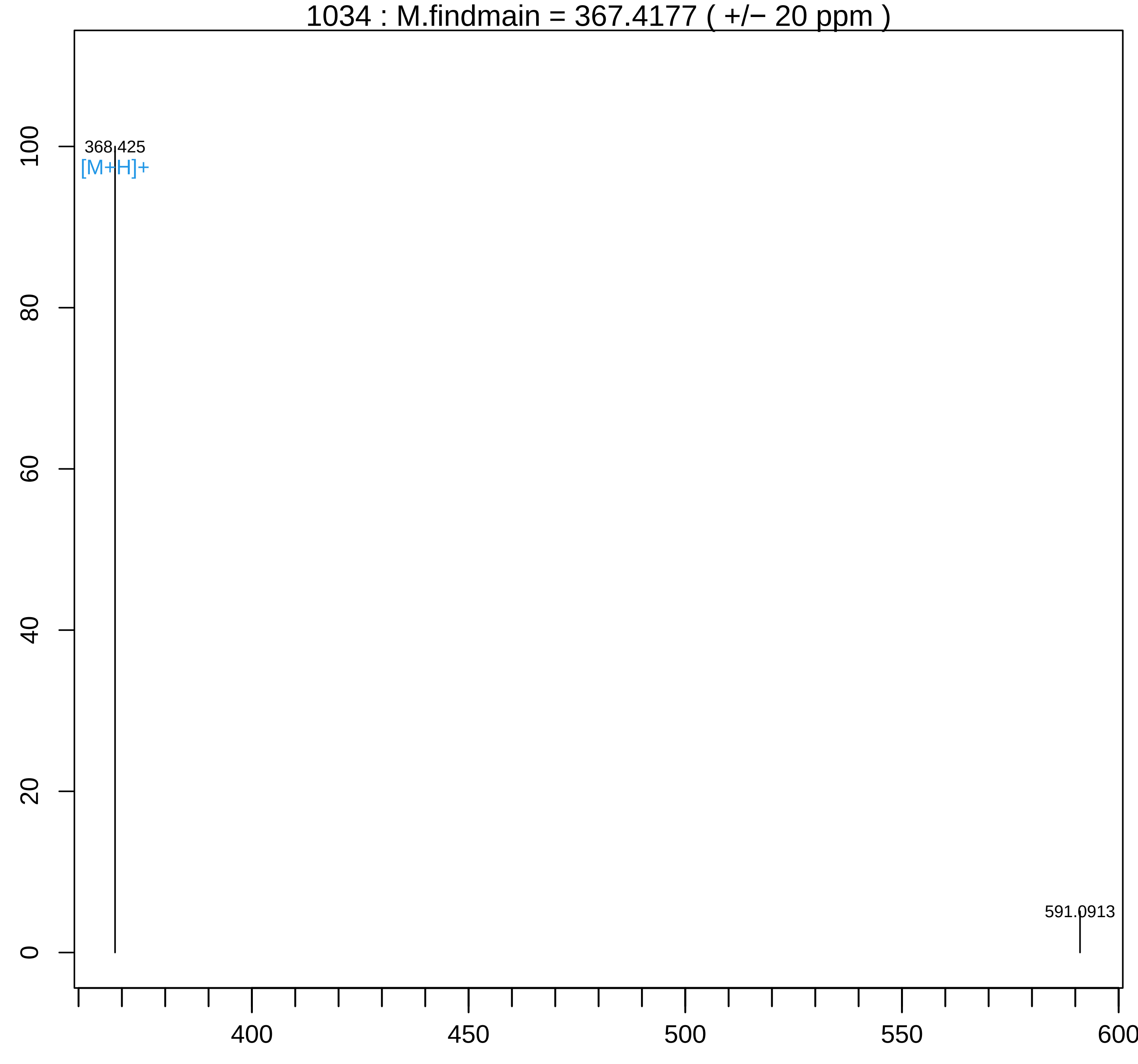

1034 : M.findmain = 367.4177 (+/− 20 ppm)
368.425
[M+H]+
591.0913
0
20
40
60
80
100
400
450
500
550
600

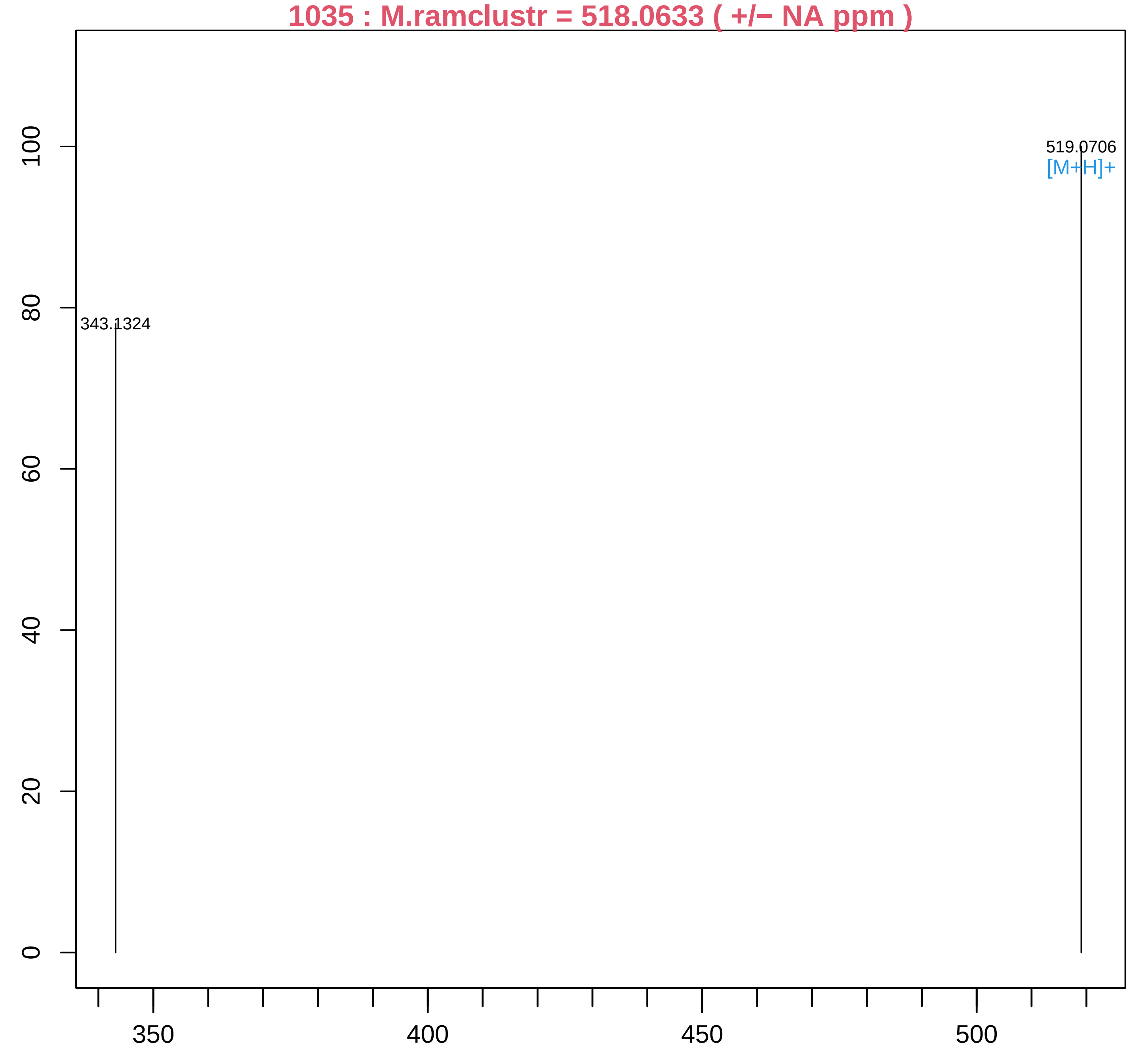
1035 : M.ramclustr = 518.0633 (+/− NA ppm)
519.0706
[M+H]+
343.1324
0
20
40
60
80
100
350
400
450
500

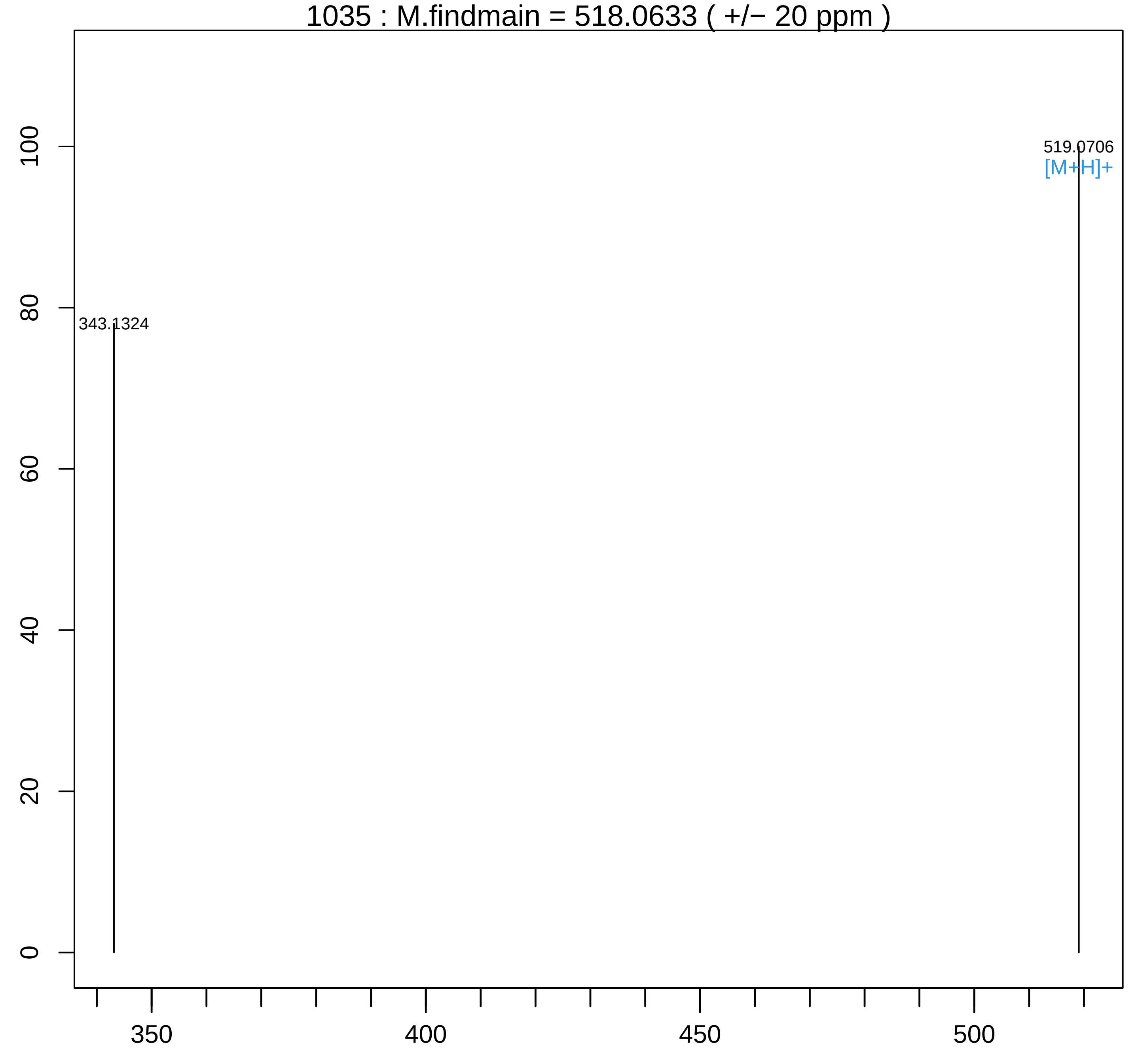
1035 : M.findmain = 518.0633 (+/− 20 ppm)
519.0706
[M+H]+
343.1324
0
20
40
60
80
100
350
400
450
500

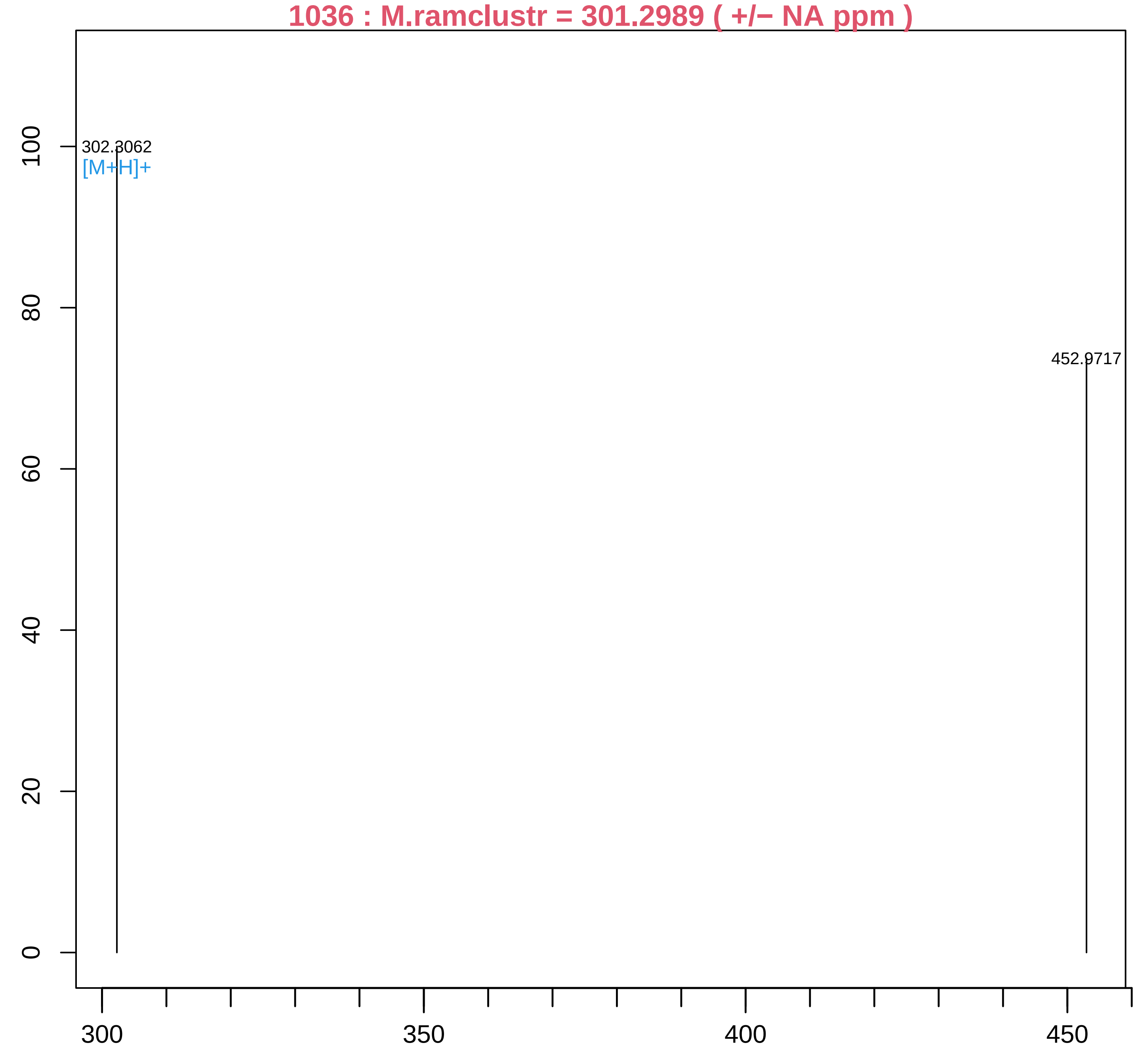
1036 : M.ramclustr = 301.2989 (+/− NA ppm)
302.3062
[M+H]+
452.9717
0
20
40
60
80
100
300
350
400
450

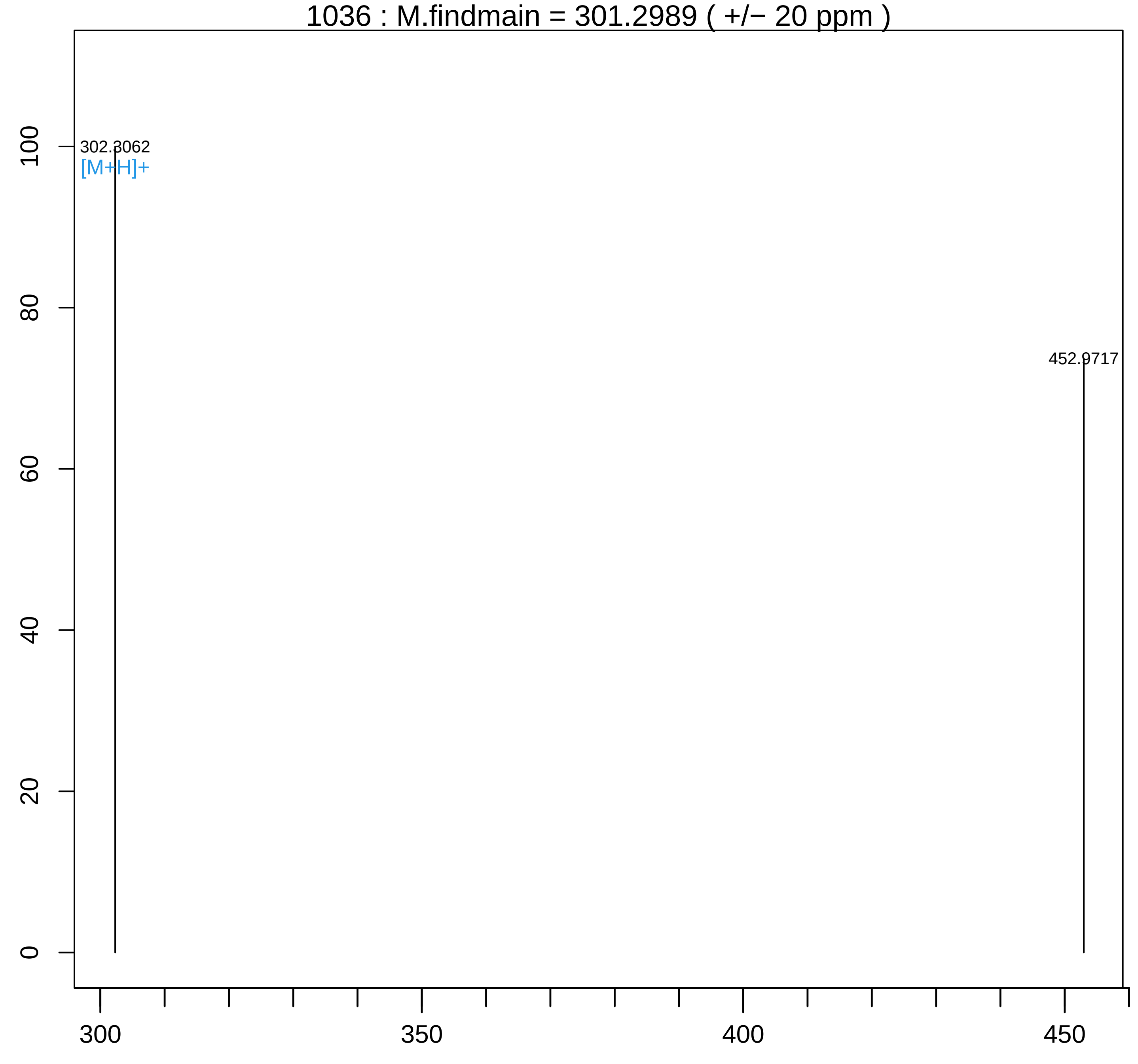
1036 : M.findmain = 301.2989 (+/− 20 ppm)
302.3062
[M+H]+
452.9717
0
20
40
60
80
100
300
350
400
450

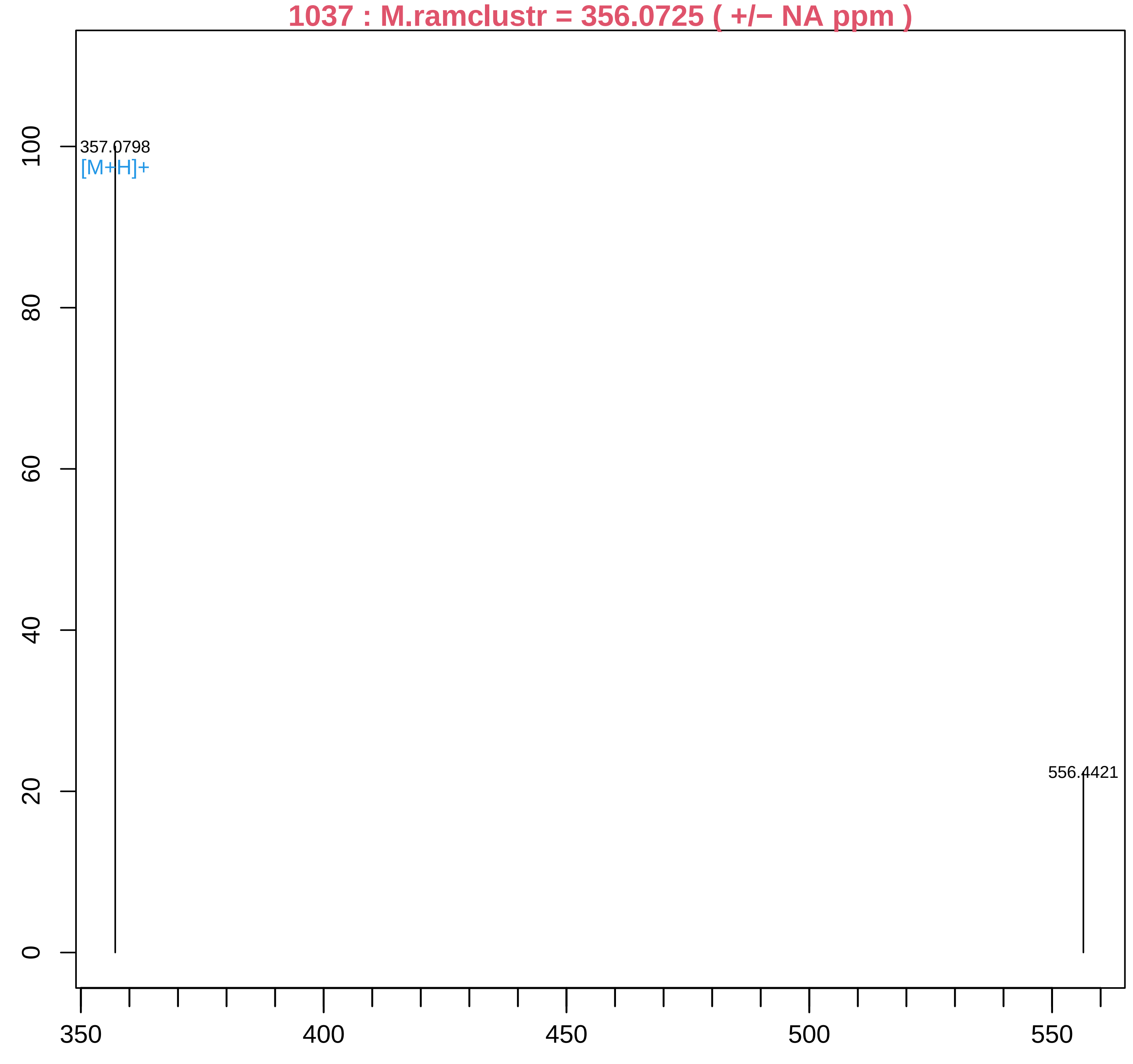
1037 : M.ramclustr = 356.0725 (+/− NA ppm)
357.0798
[M+H]+
556.4421
0
20
40
60
80
100
350
400
450
500
550

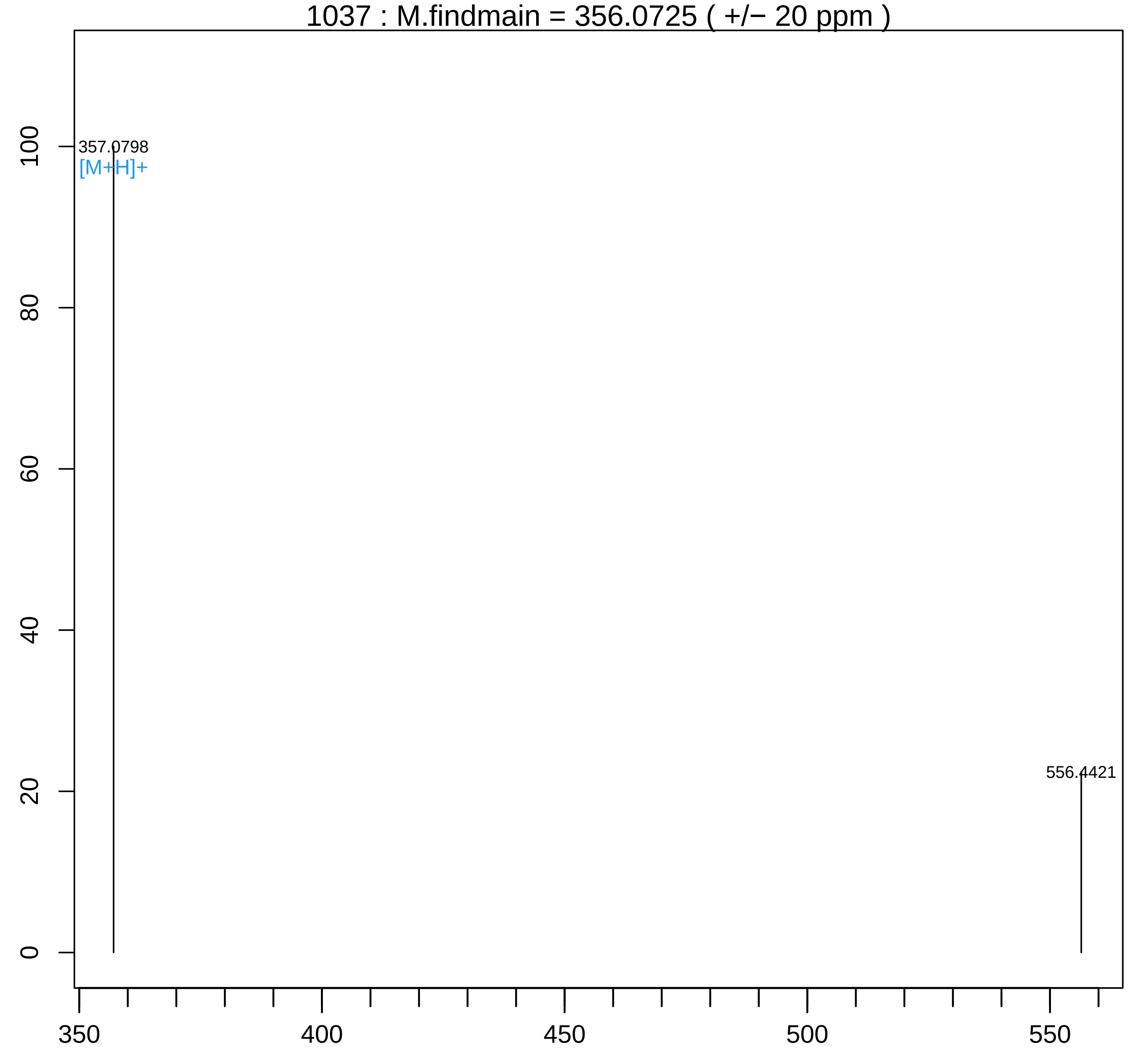
1037 : M.findmain = 356.0725 (+/− 20 ppm)
357.0798
[M+H]+
556.4421
0
20
40
60
80
100
350
400
450
500
550

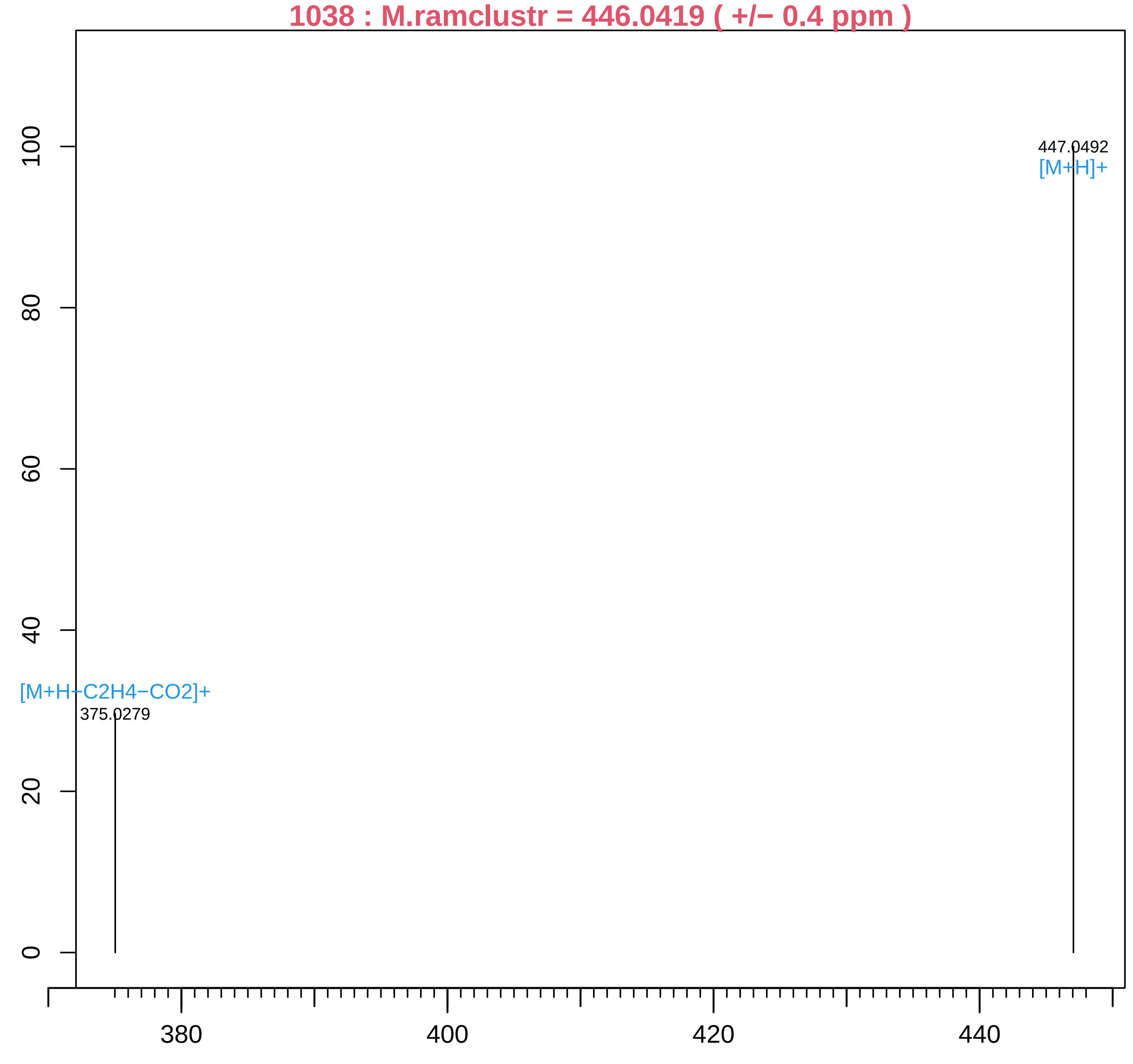
1038 : M.ramclustr = 446.0419 (+/− 0.4 ppm)
447.0492
[M+H]+
[M+H−C2H4−CO2]+
375.0279
0
20
40
60
80
100
380
400
420
440

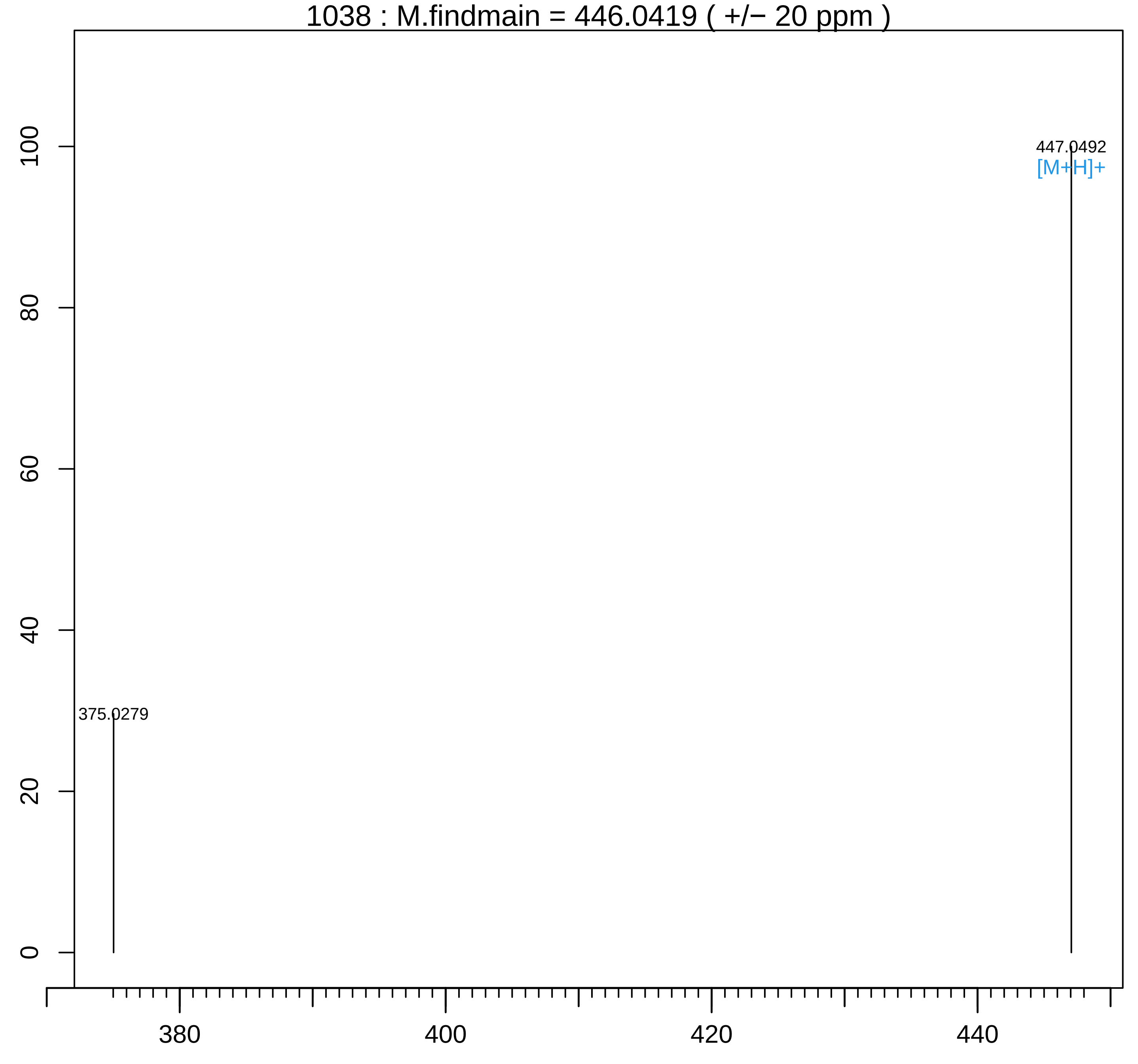
1038 : M.findmain = 446.0419 (+/− 20 ppm)
447.0492
[M+H]+
375.0279
0
20
40
60
80
100
380
400
420
440

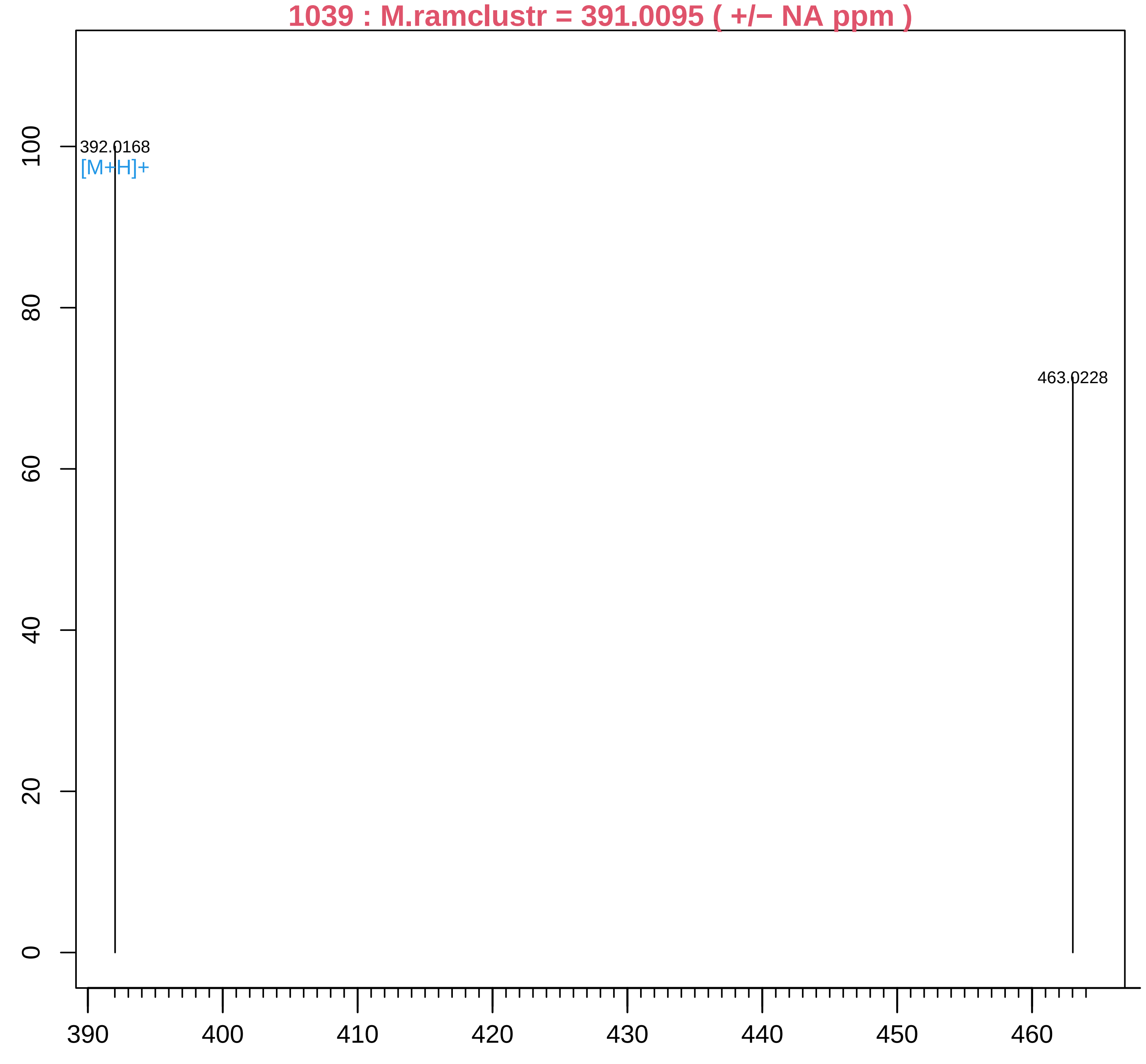
1039 : M.ramclustr = 391.0095 (+/− NA ppm)
392.0168
[M+H]+
463.0228
0
20
40
60
80
100
390
400
410
420
430
440
450
460

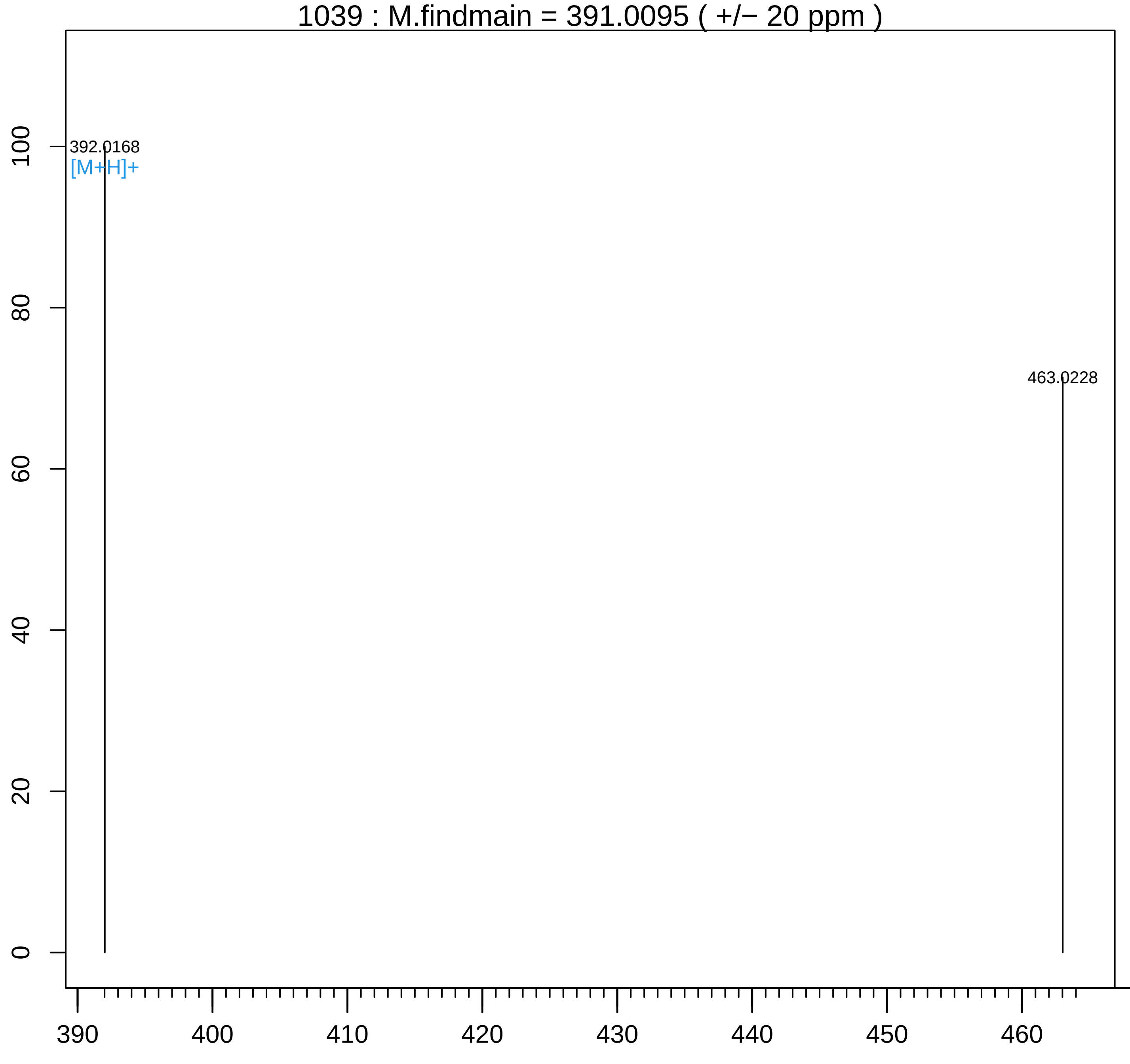
1039 : M.findmain = 391.0095 (+/− 20 ppm)
392.0168
[M+H]+
463.0228
0
20
40
60
80
100
390
400
410
420
430
440
450
460

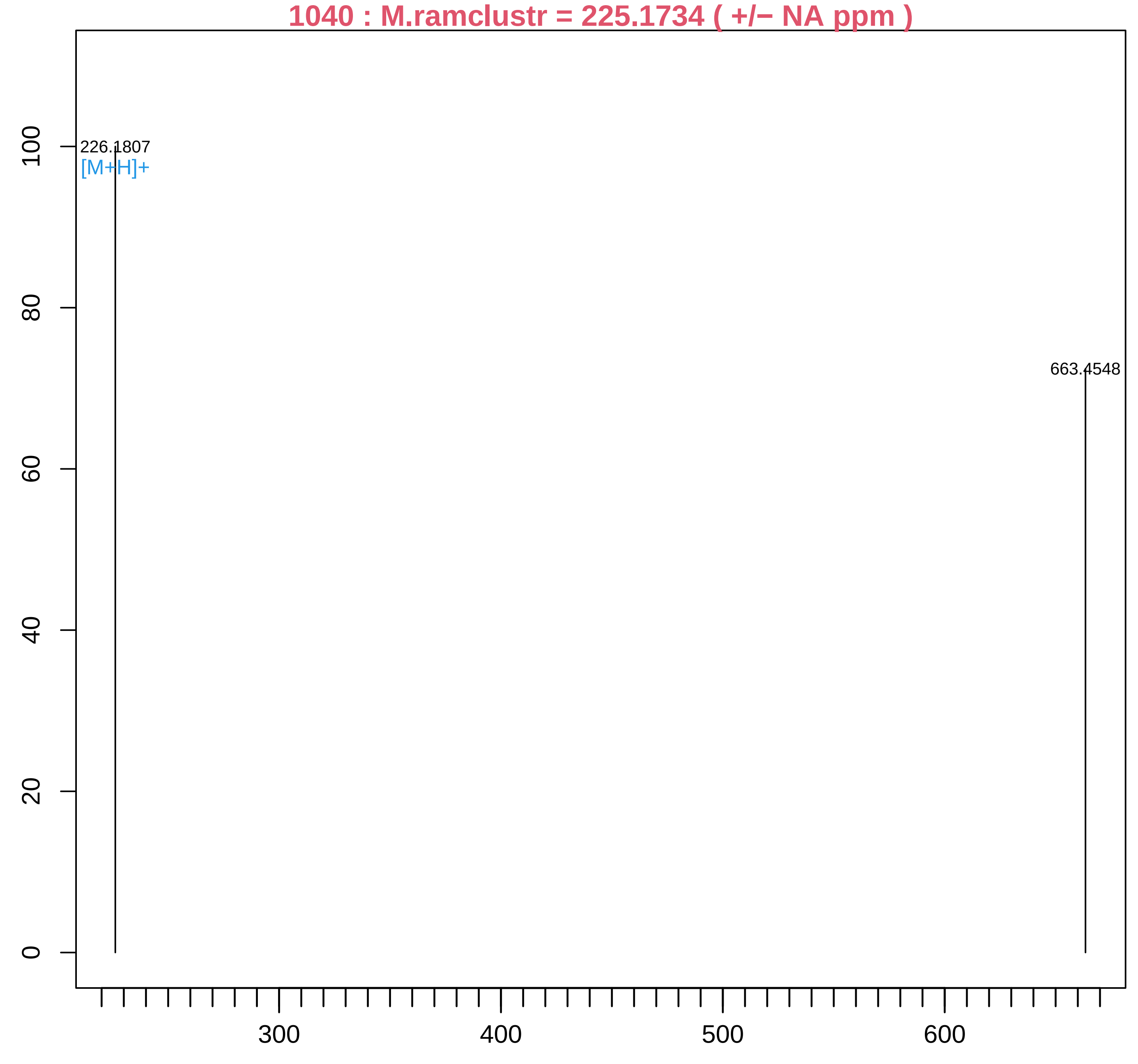
1040 : M.ramclustr = 225.1734 (+/− NA ppm)
226.1807
[M+H]+
663.4548
0
20
40
60
80
100
300
400
500
600

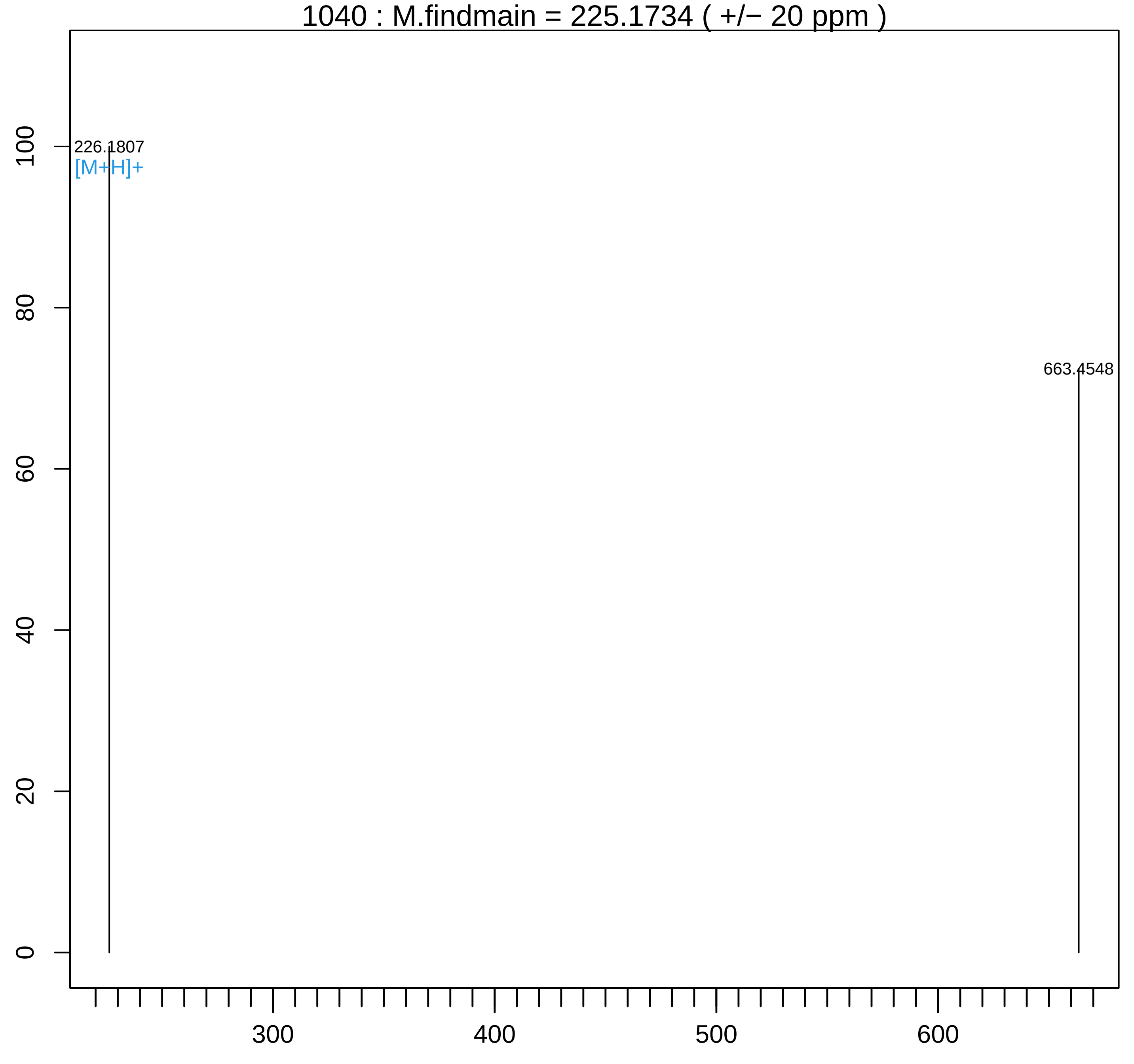
1040 : M.findmain = 225.1734 (+/− 20 ppm)
226.1807
[M+H]+
663.4548
0
20
40
60
80
100
300
400
500
600

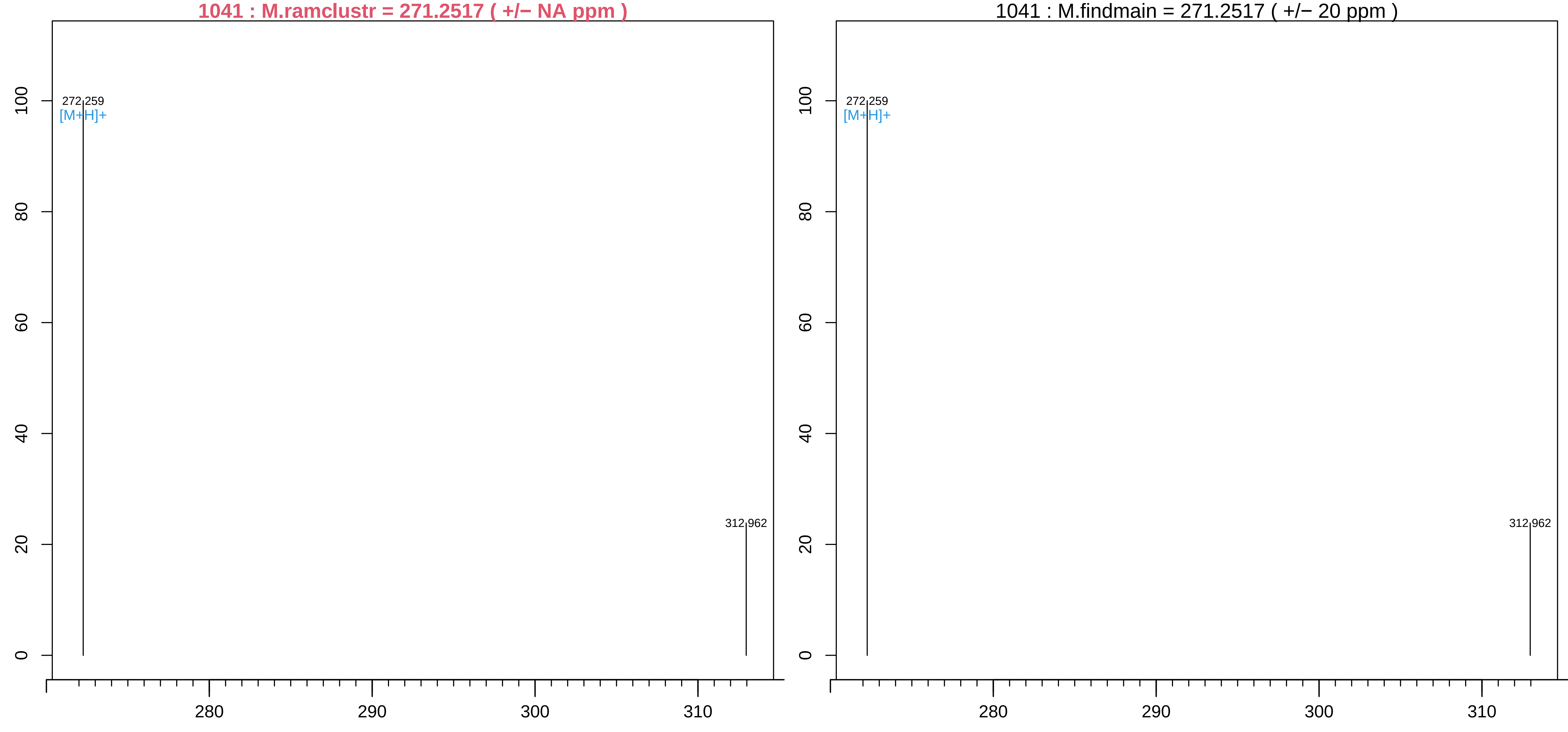
1041 : M.ramclustr = 271.2517 (+/− NA ppm)
272.259
[M+H]+
312.962
0
20
40
60
80
100
280
290
300
310
1041 : M.findmain = 271.2517 (+/− 20 ppm)
272.259
[M+H]+
312.962
0
20
40
60
80
100
280
290
300
310

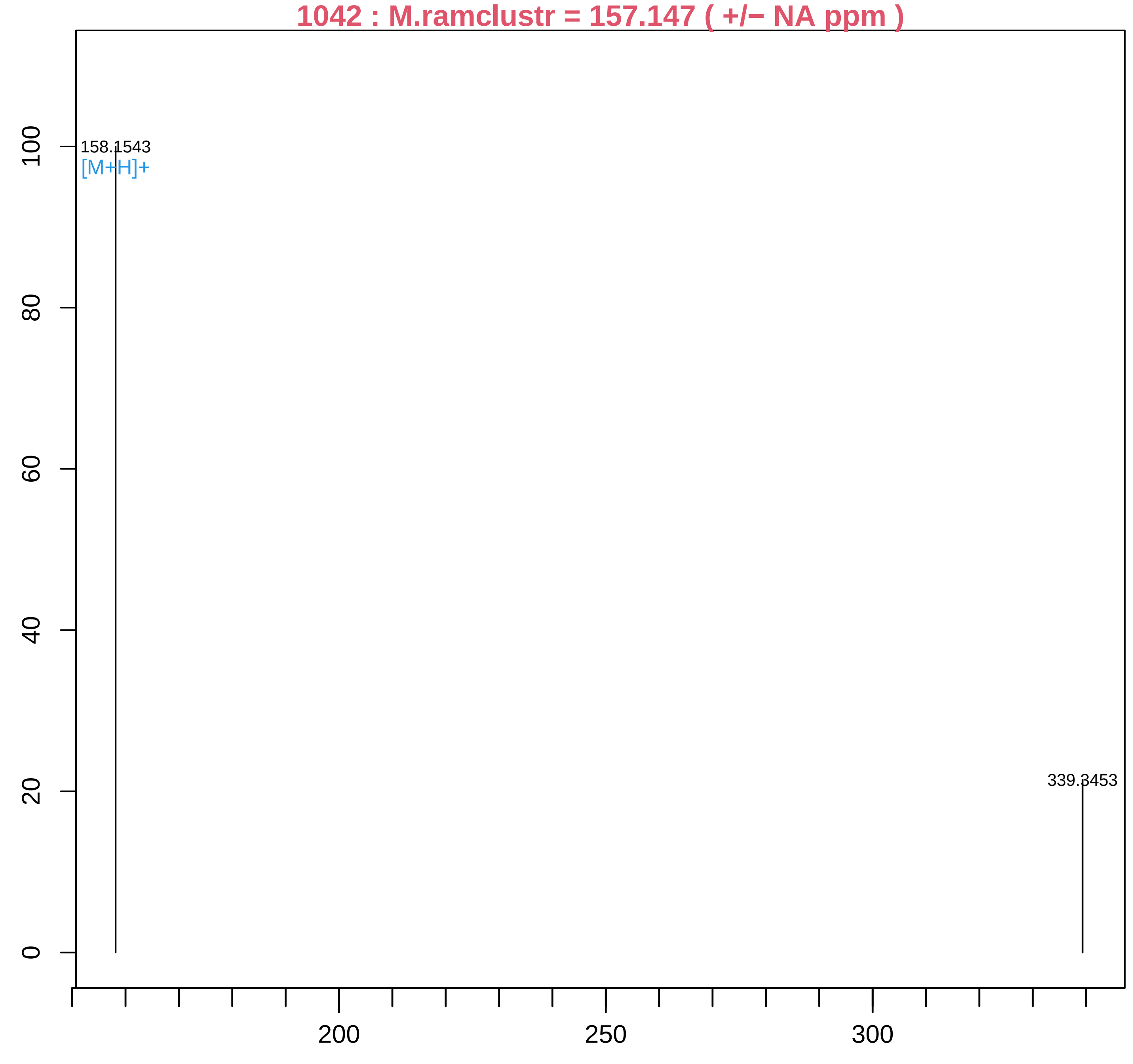
1042 : M.ramclustr = 157.147 (+/− NA ppm)
158.1543
[M+H]+
339.3453
0
20
40
60
80
100
200
250
300

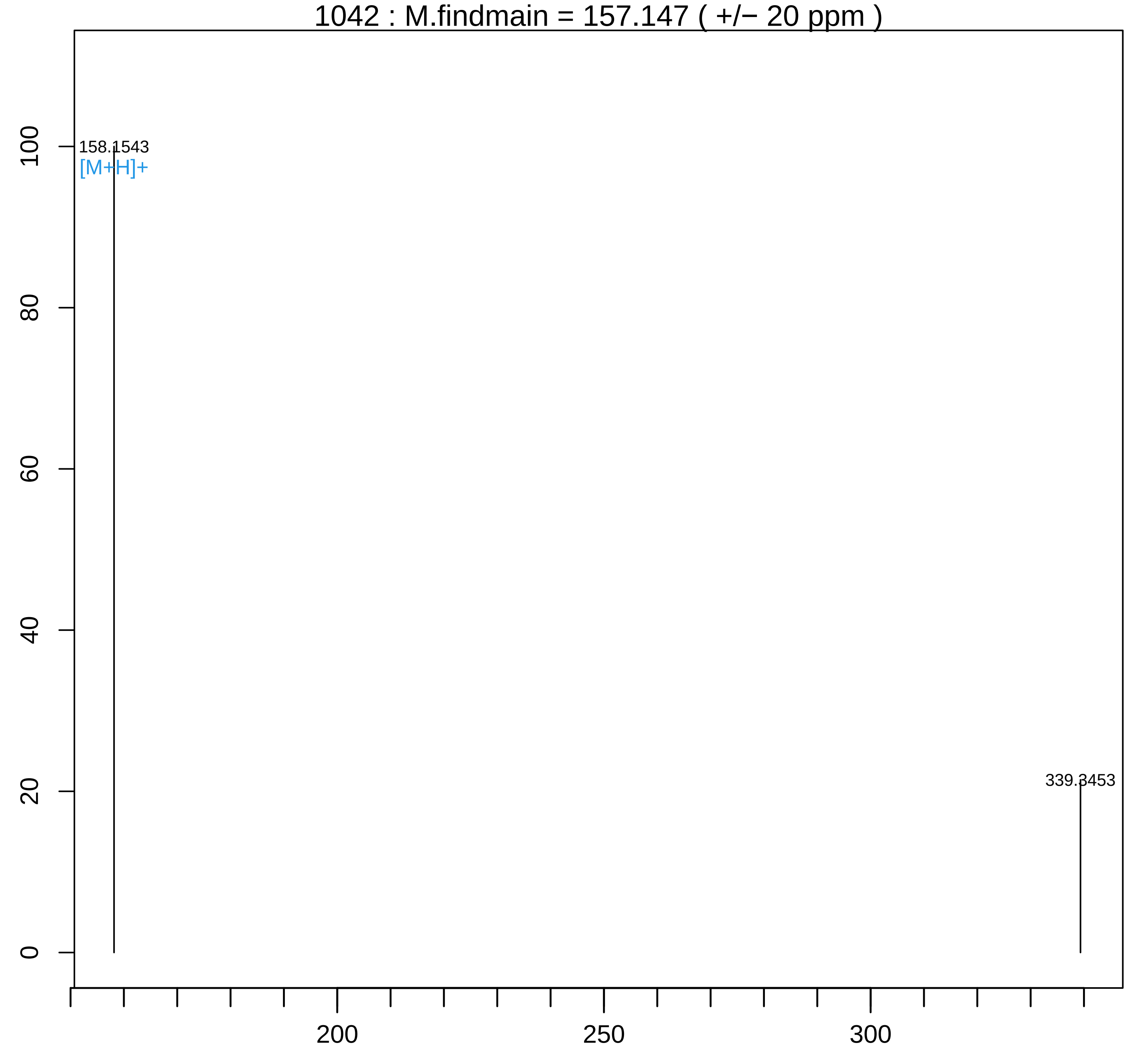
1042 : M.findmain = 157.147 (+/− 20 ppm)
158.1543
[M+H]+
339.3453
0
20
40
60
80
100
200
250
300

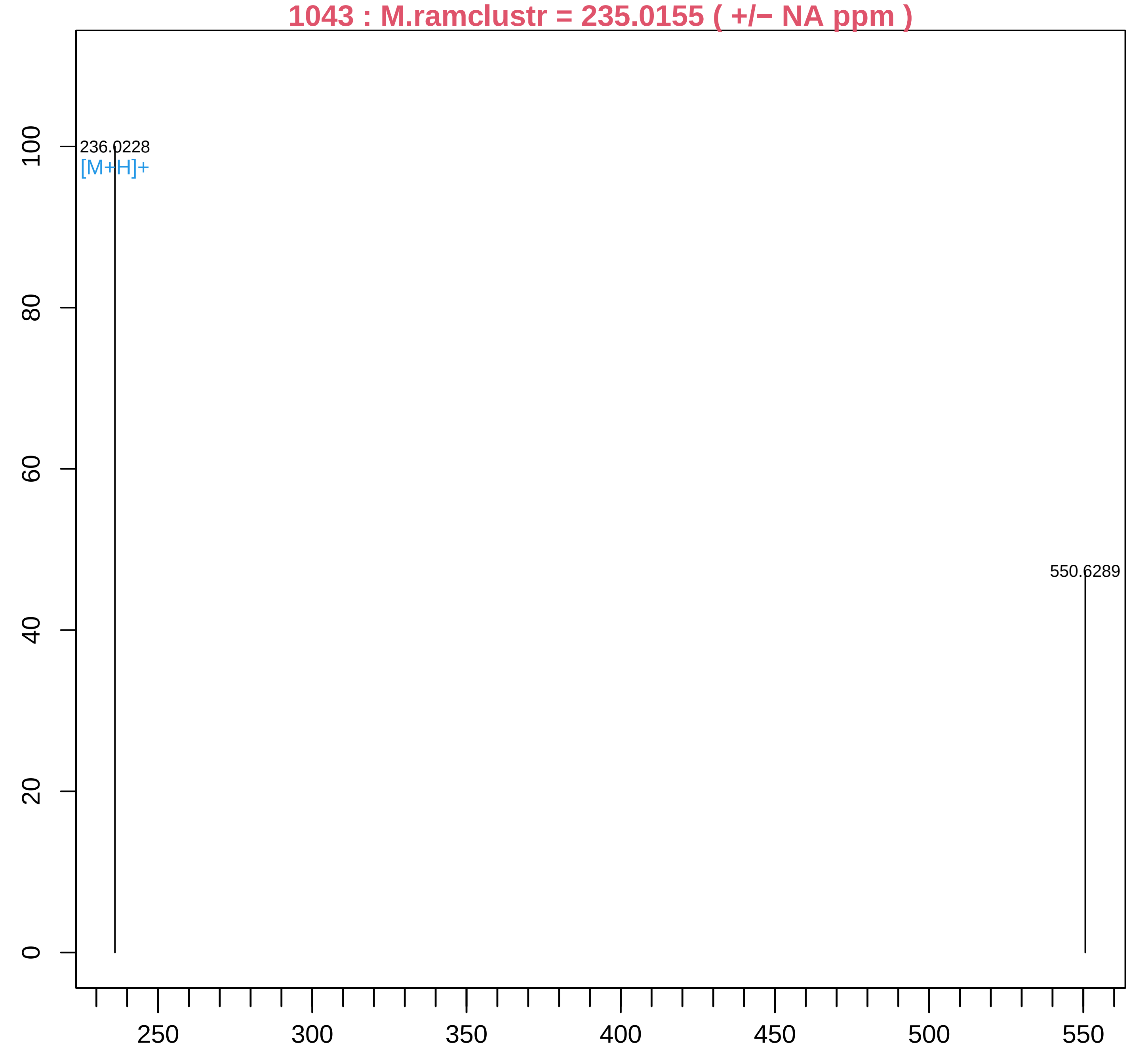

1043 : M.ramclustr = 235.0155 (+/− NA ppm)
236.0228
[M+H]+
550.6289
0
20
40
60
80
100
250
300
350
400
450
500
550

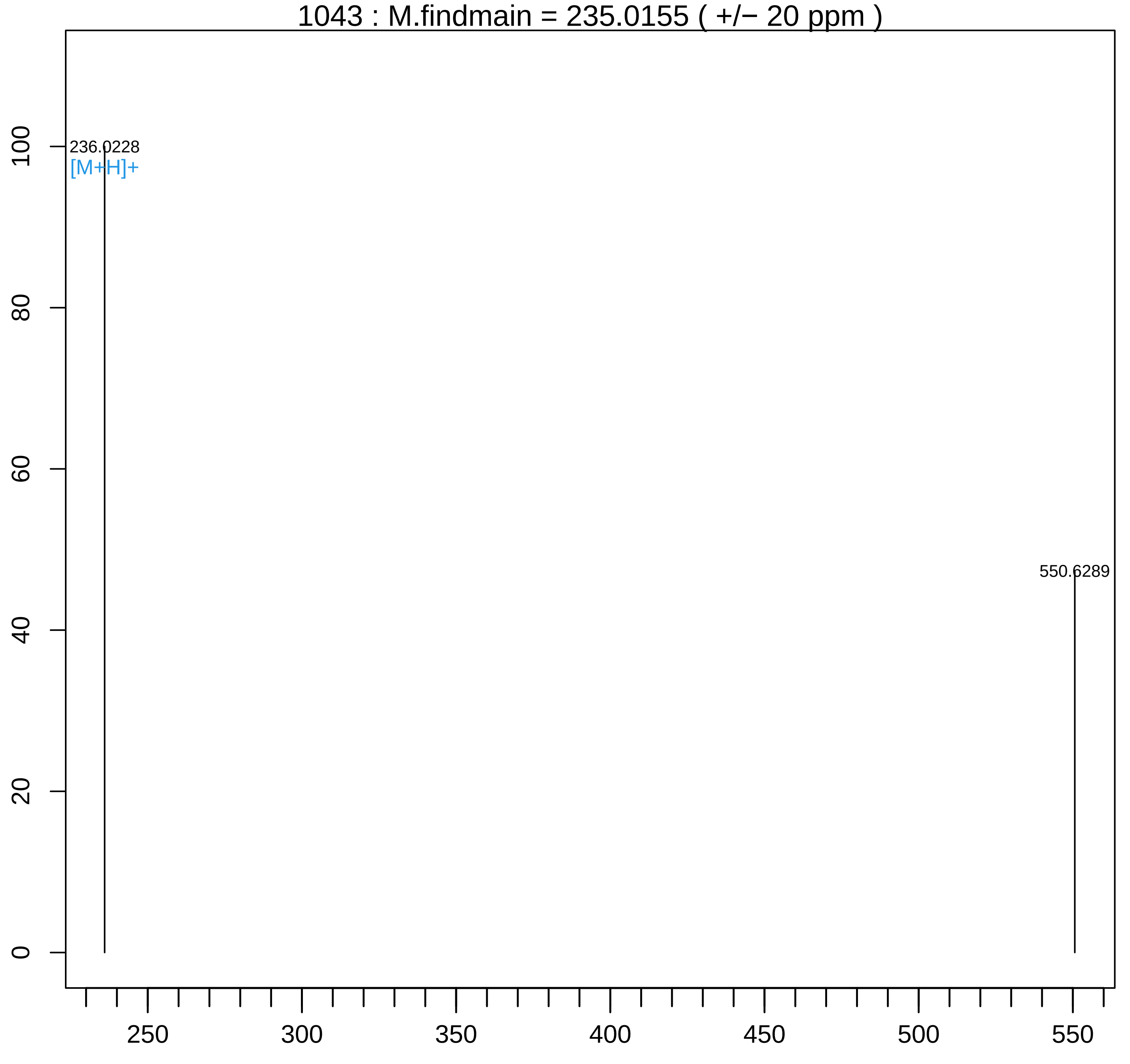

1043 : M.findmain = 235.0155 (+/− 20 ppm)
236.0228
[M+H]+
550.6289
0
20
40
60
80
100
250
300
350
400
450
500
550

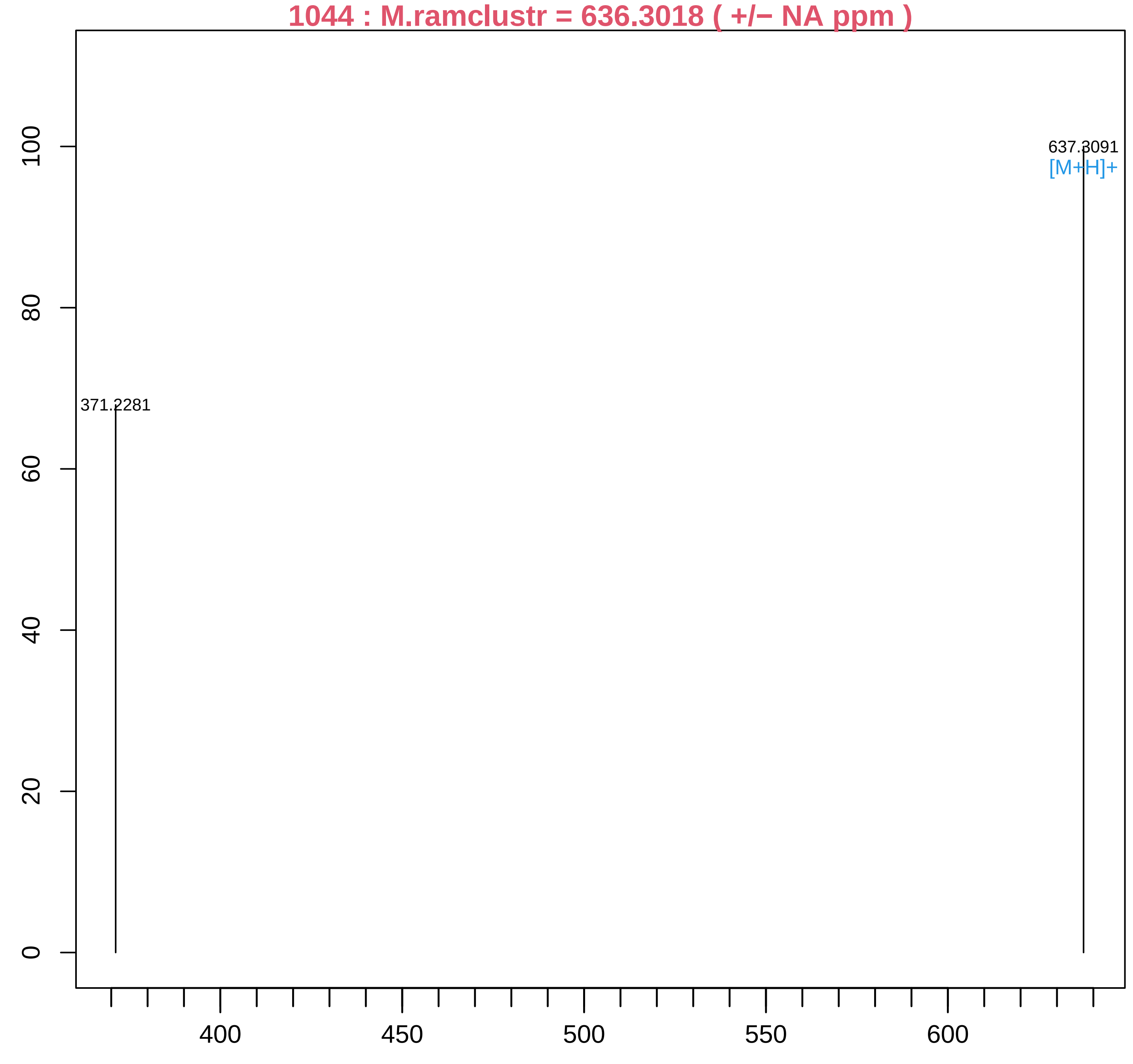

1044 : M.ramclustr = 636.3018 (+/− NA ppm)
637.3091
[M+H]+
371.2281
0
20
40
60
80
100
400
450
500
550
600

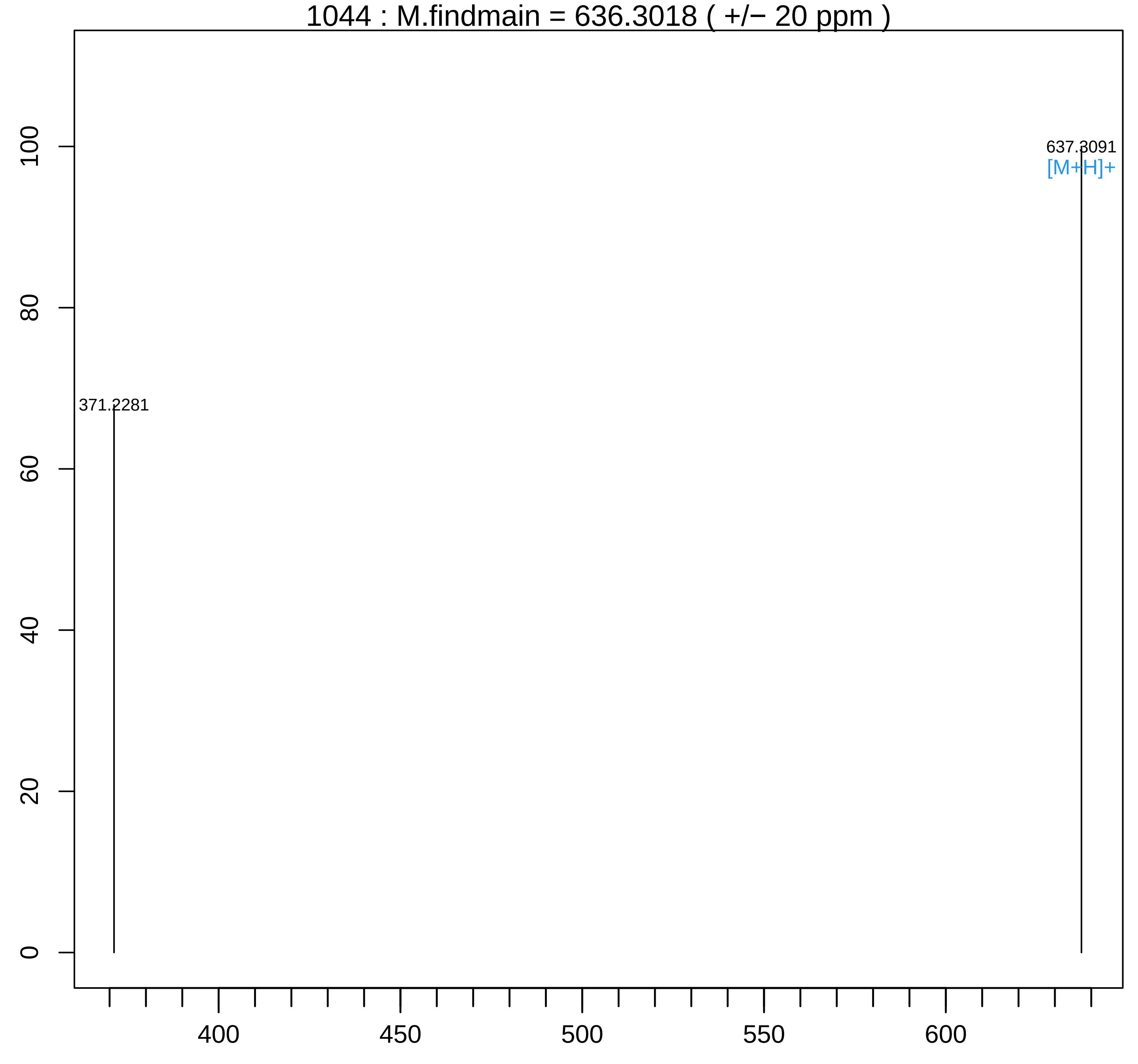

1044 : M.findmain = 636.3018 (+/− 20 ppm)
637.3091
[M+H]+
371.2281
0
20
40
60
80
100
400
450
500
550
600

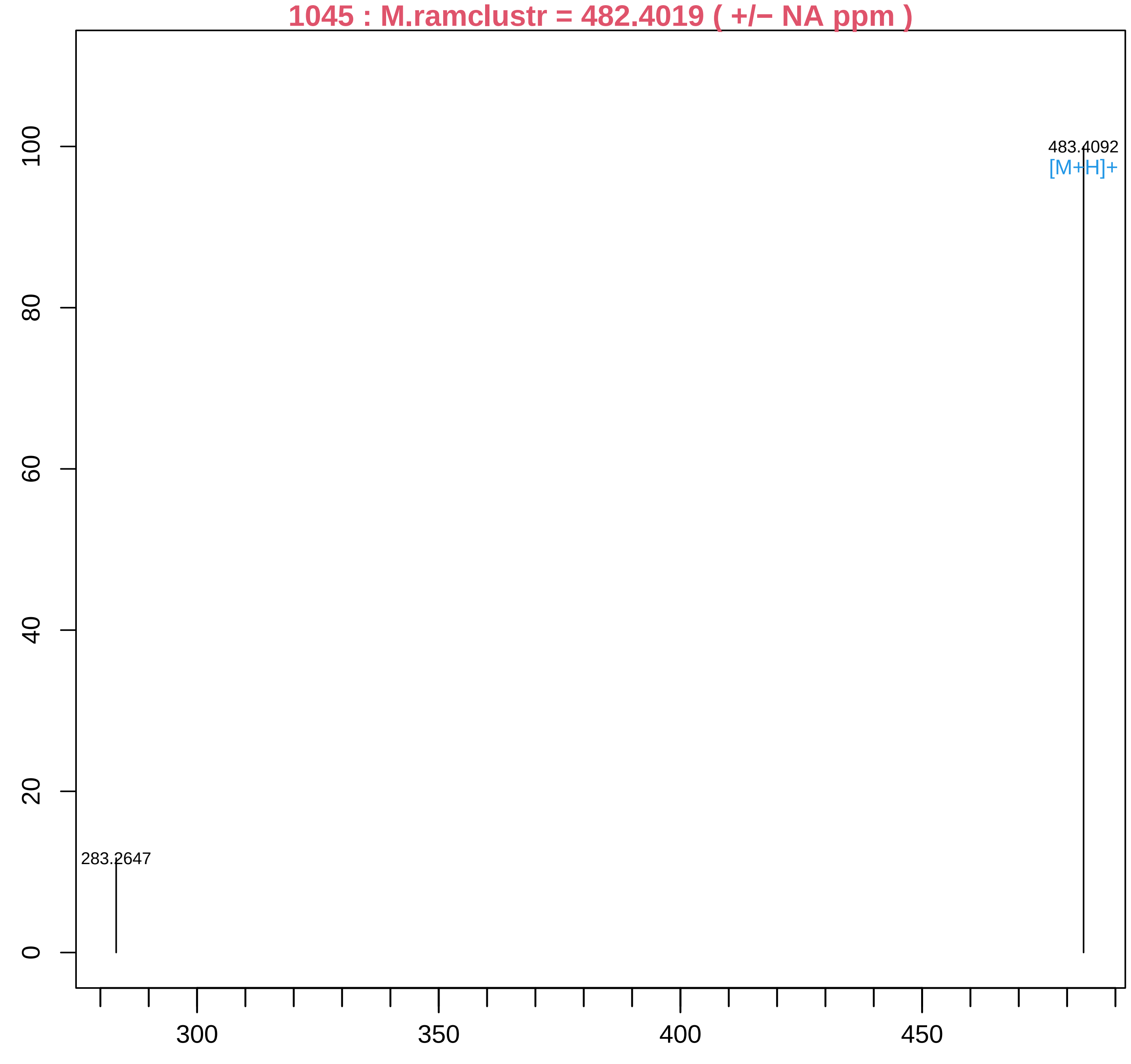

1045 : M.ramclustr = 482.4019 (+/− NA ppm)
483.4092
[M+H]+
283.2647
0
20
40
60
80
100
300
350
400
450

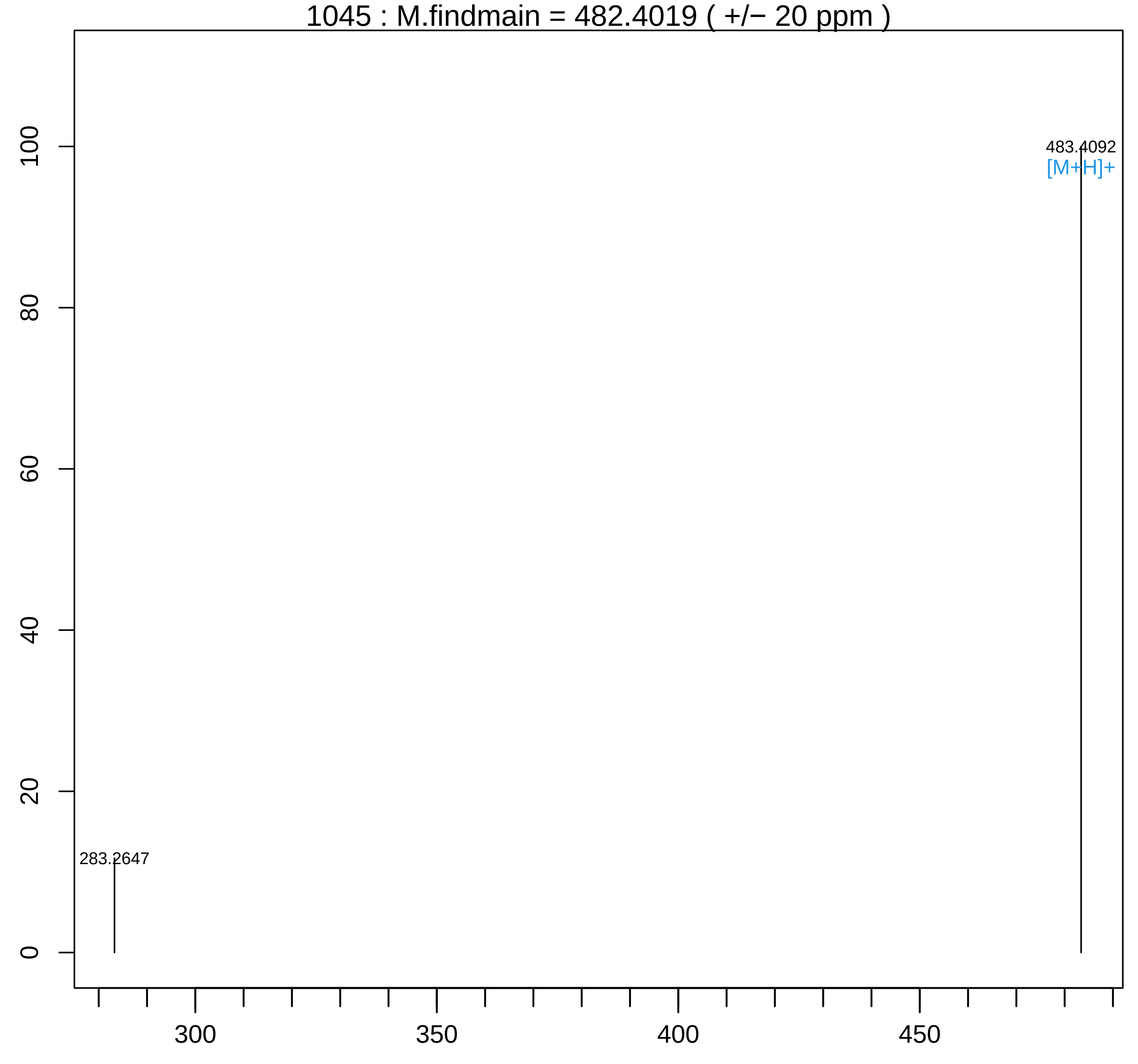

1045 : M.findmain = 482.4019 (+/− 20 ppm)
483.4092
[M+H]+
283.2647
0
20
40
60
80
100
300
350
400
450

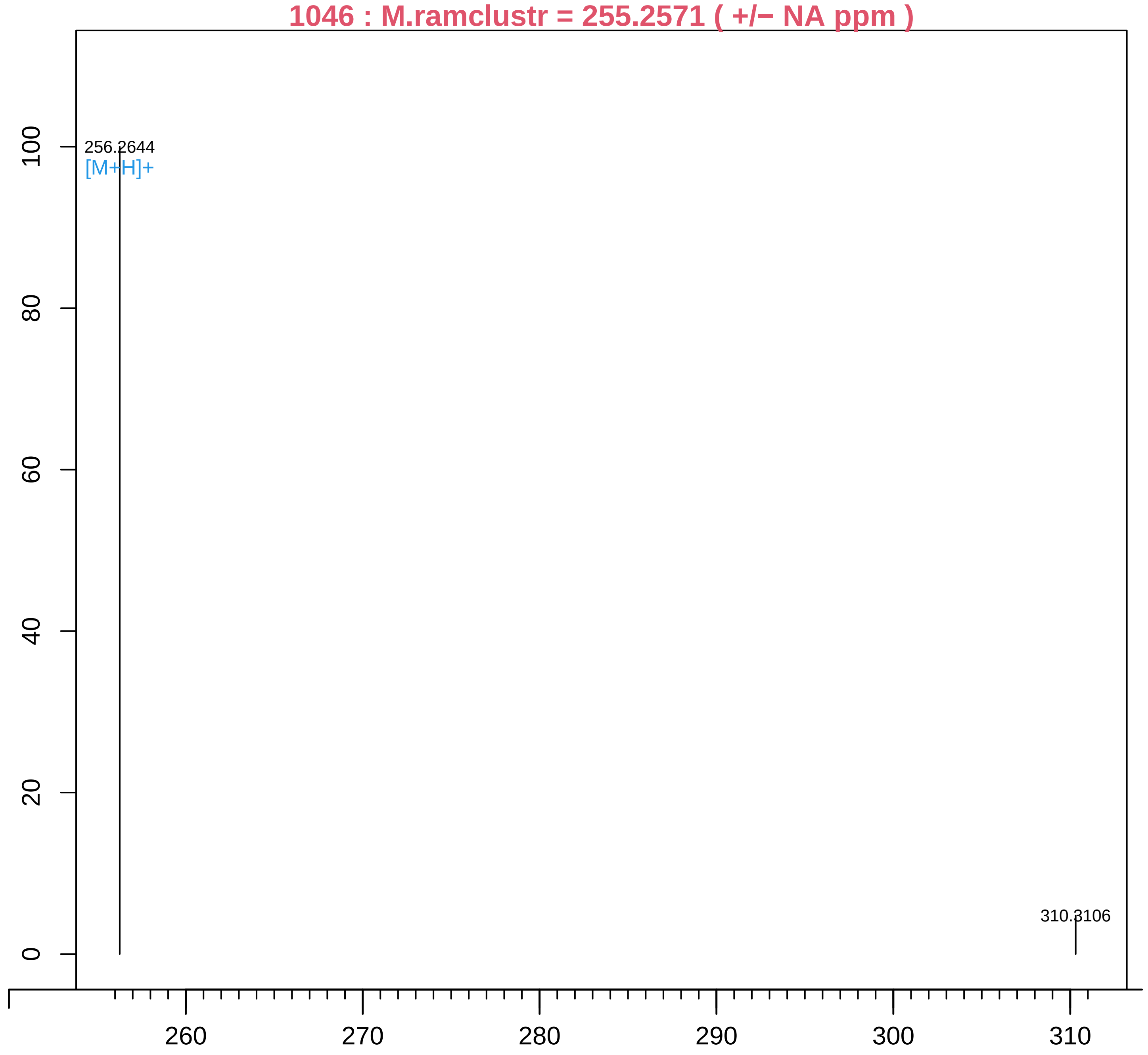
1046 : M.ramclustr = 255.2571 (+/− NA ppm)
256.2644
[M+H]+
310.3106
0
20
40
60
80
100
260
270
280
290
300
310

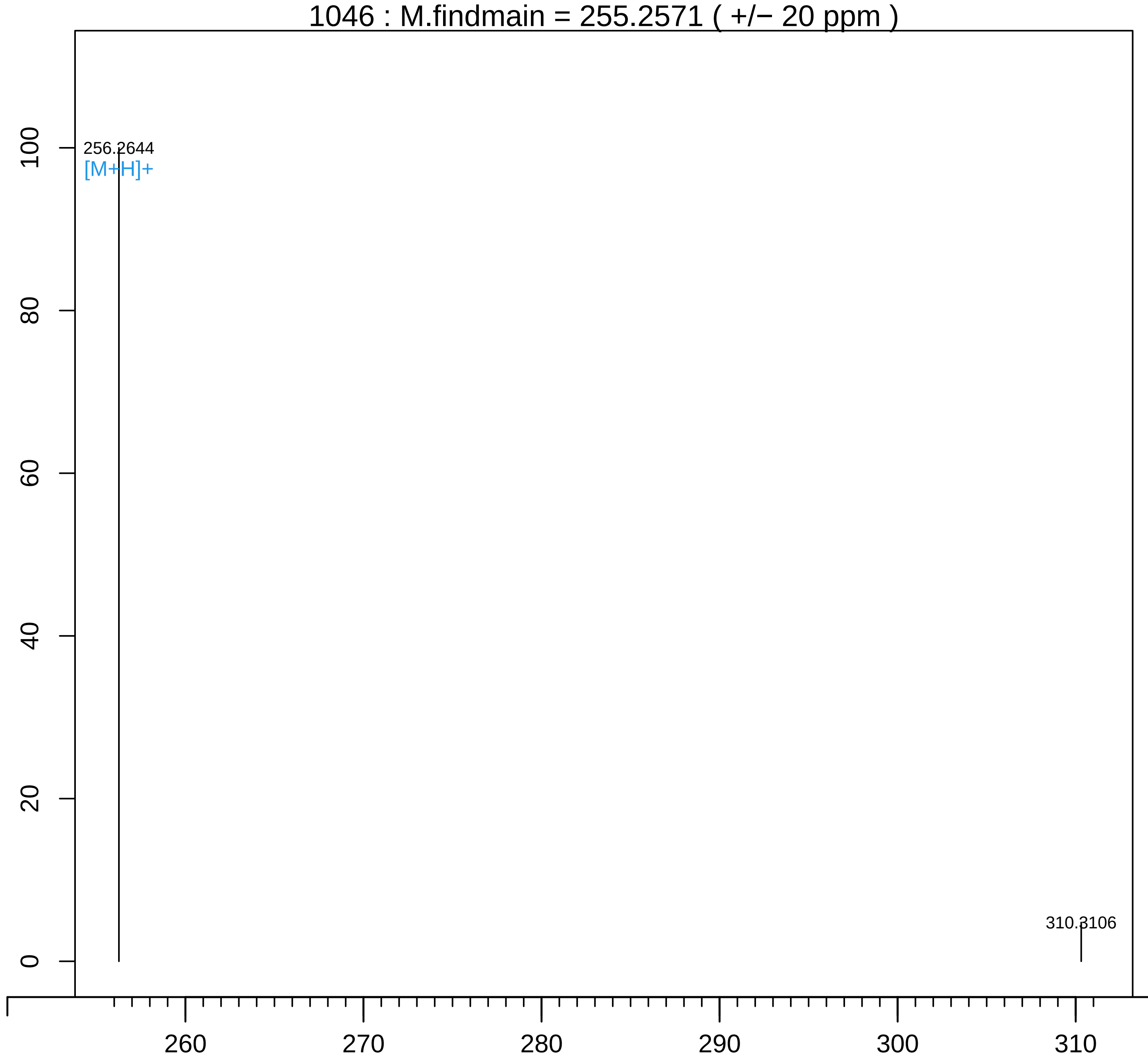
1046 : M.findmain = 255.2571 (+/− 20 ppm)
256.2644
[M+H]+
310.3106
0
20
40
60
80
100
260
270
280
290
300
310

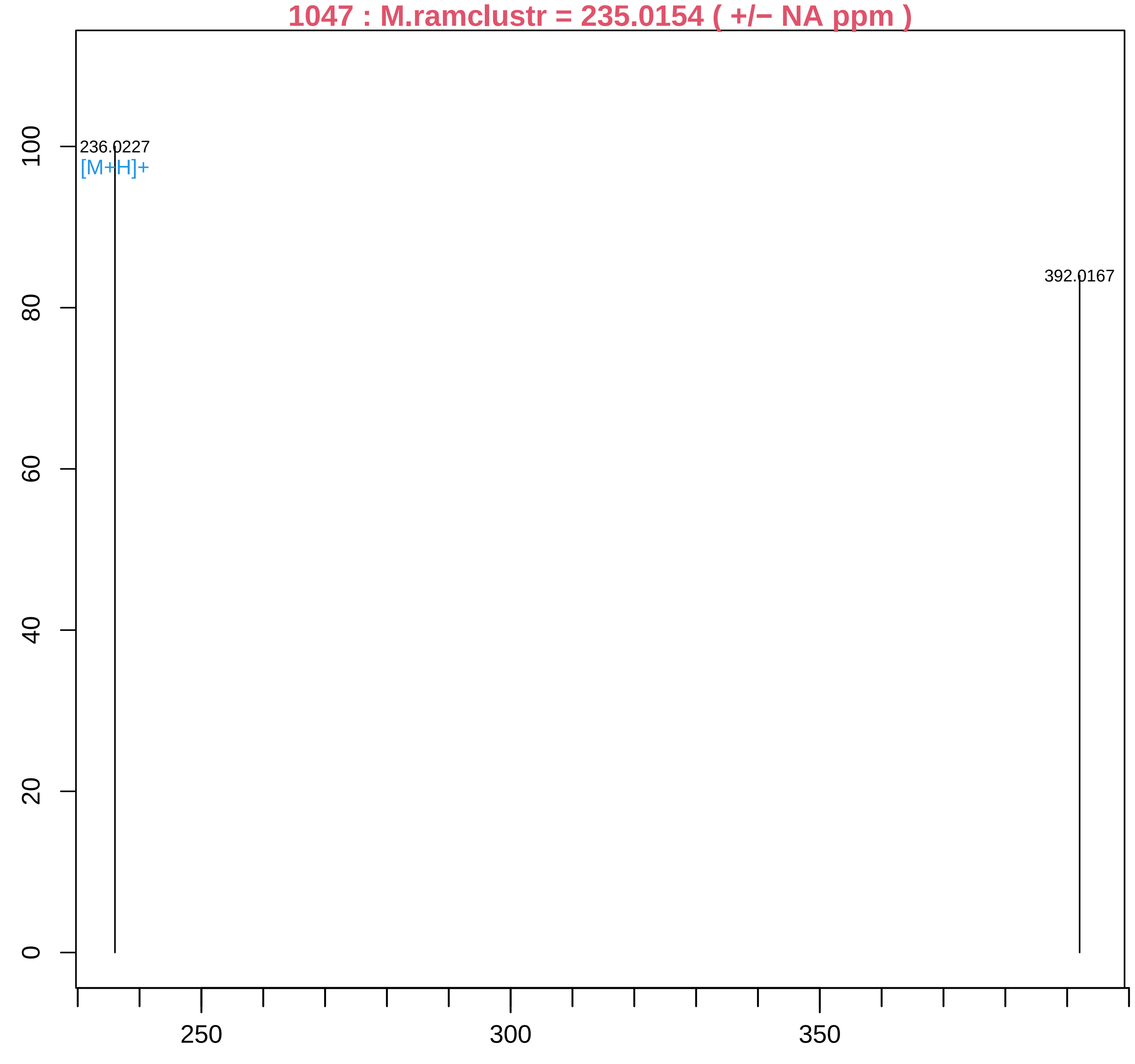
1047 : M.ramclustr = 235.0154 (+/− NA ppm)
236.0227
[M+H]+
392.0167
0
20
40
60
80
100
250
300
350

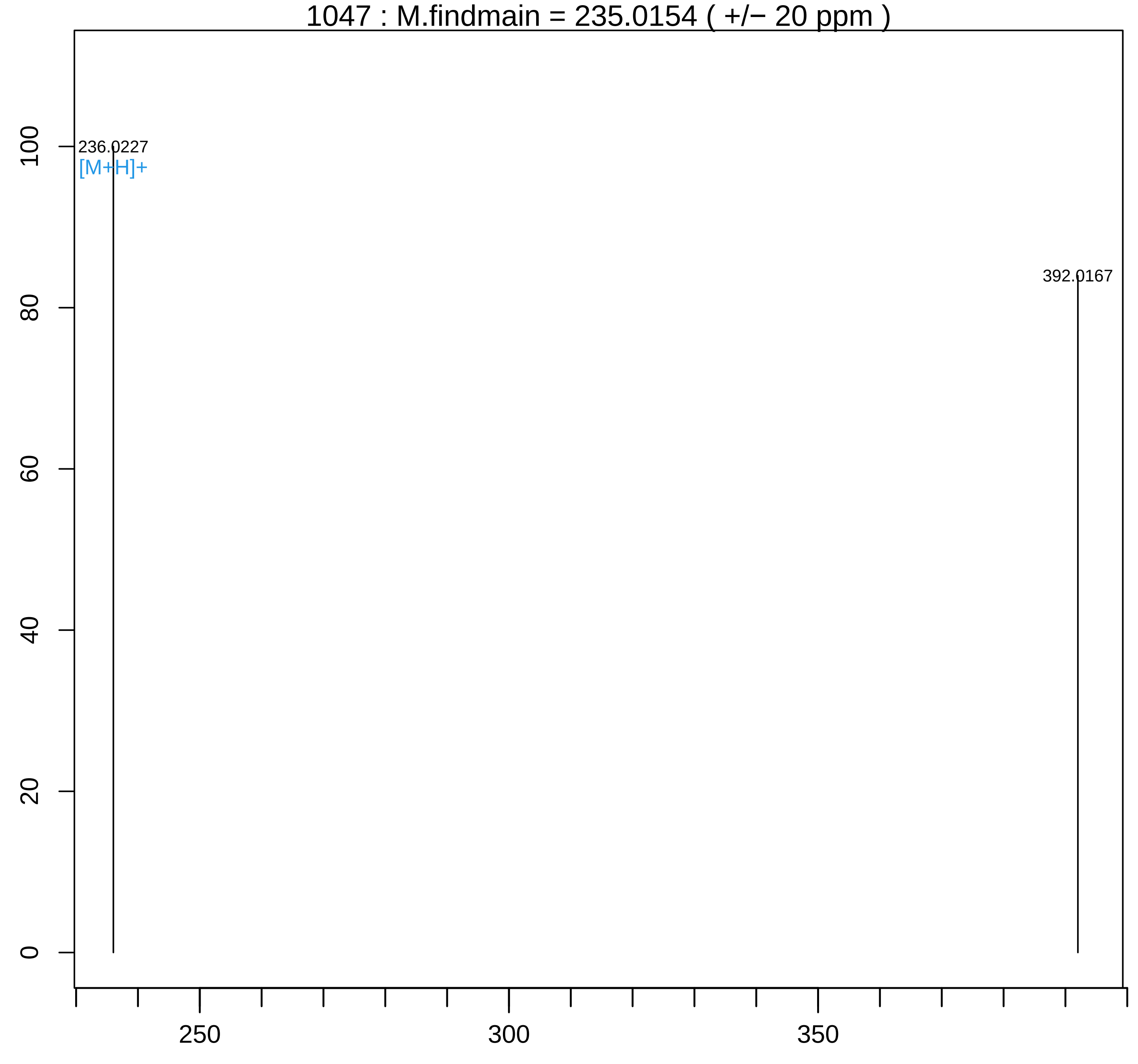
1047 : M.findmain = 235.0154 (+/− 20 ppm)
236.0227
[M+H]+
392.0167
0
20
40
60
80
100
250
300
350

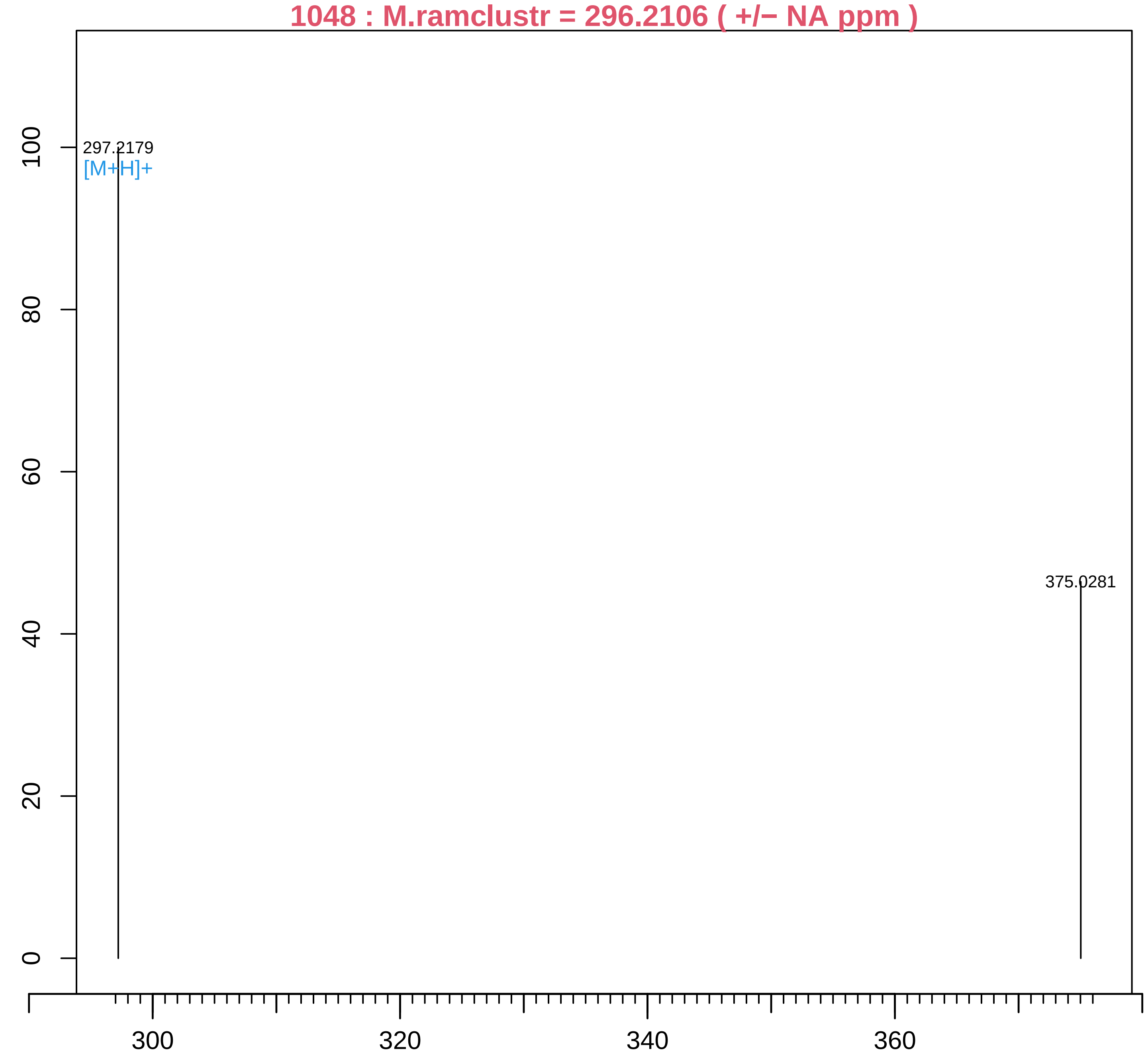
1048 : M.ramclustr = 296.2106 (+/− NA ppm)
297.2179
[M+H]+
375.0281
0
20
40
60
80
100
300
320
340
360

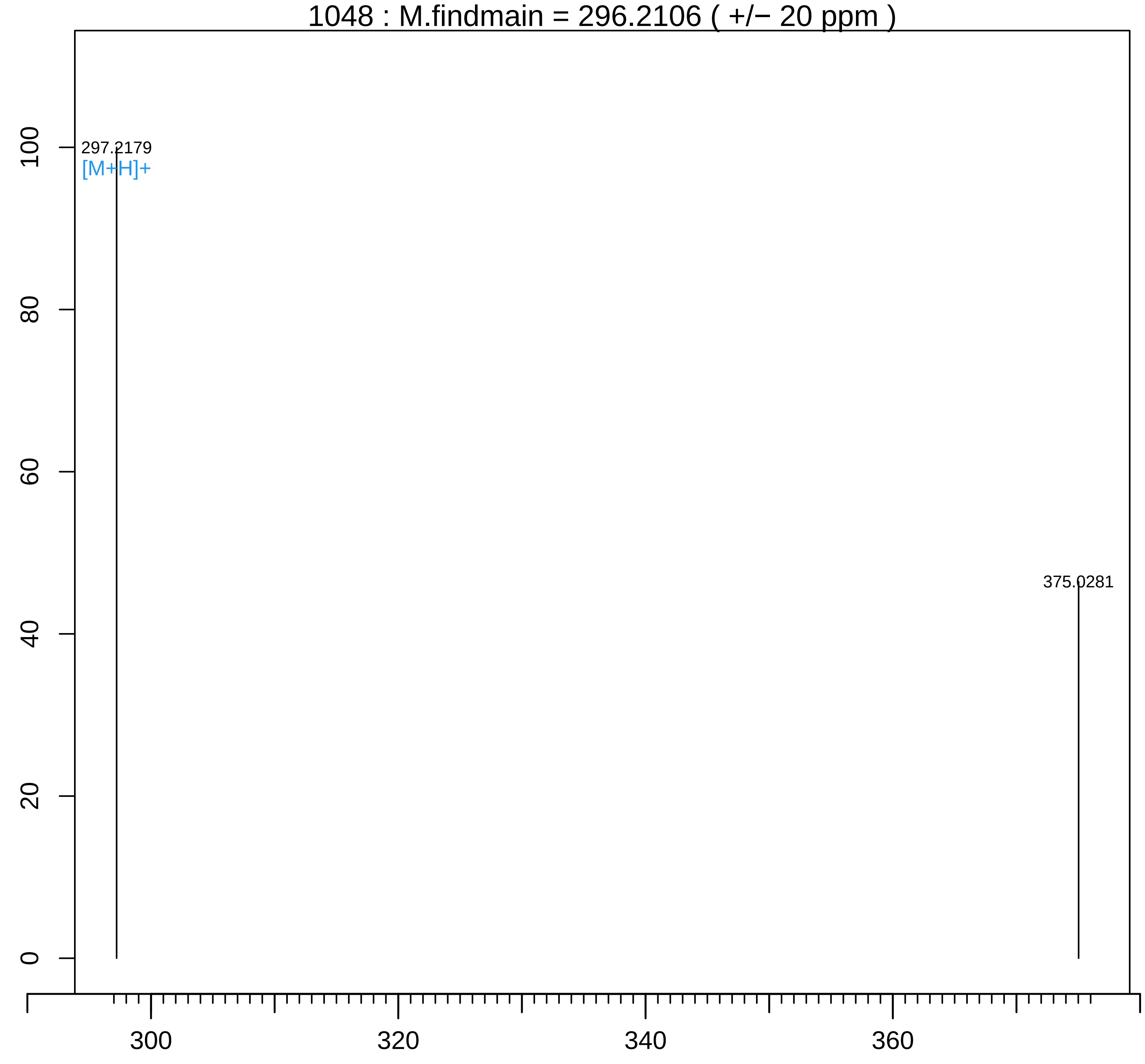
1048 : M.findmain = 296.2106 (+/− 20 ppm)
297.2179
[M+H]+
375.0281
0
20
40
60
80
100
300
320
340
360

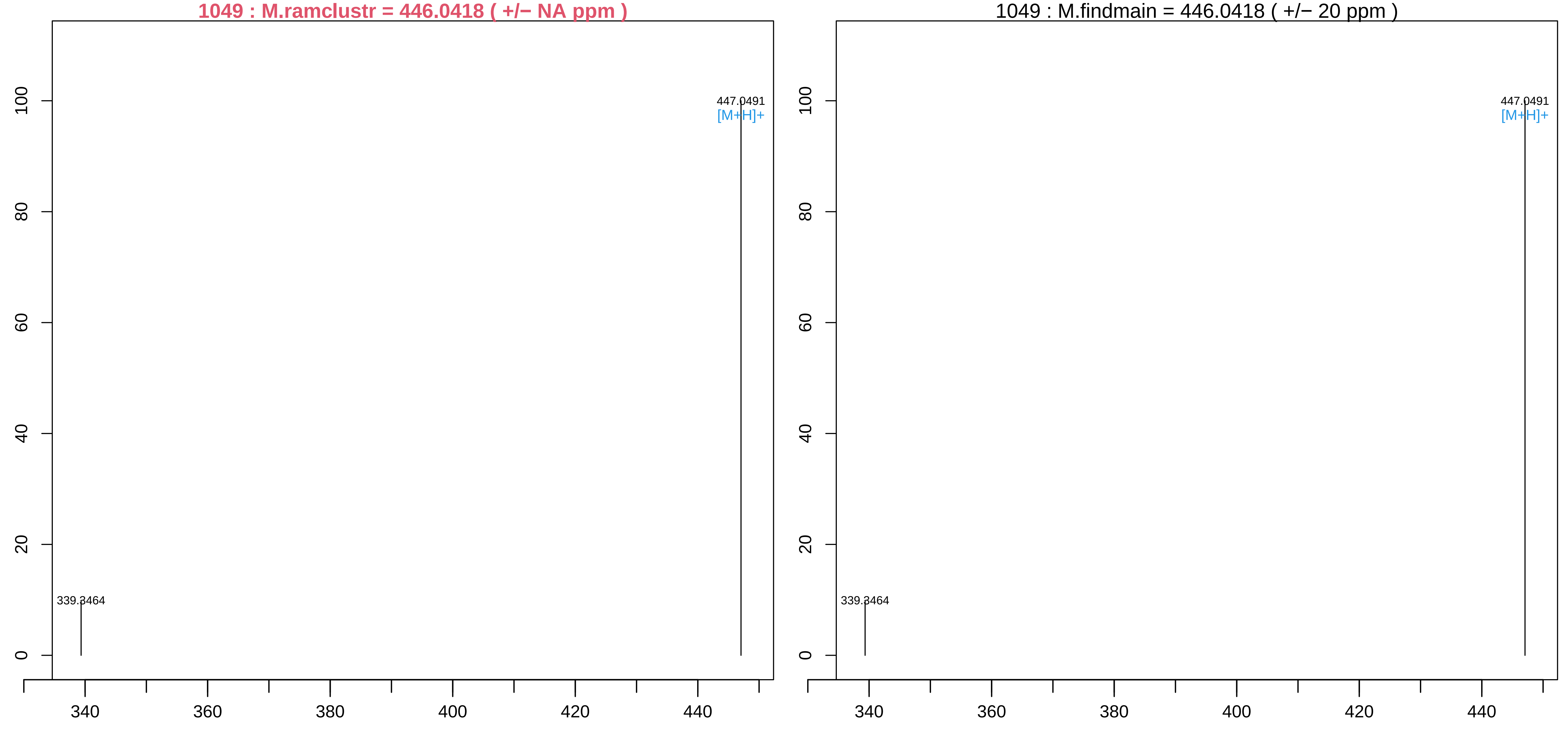
1049 : M.ramclustr = 446.0418 (+/− NA ppm)
447.0491
[M+H]+
339.3464
1049 : M.findmain = 446.0418 (+/− 20 ppm)
447.0491
[M+H]+
339.3464

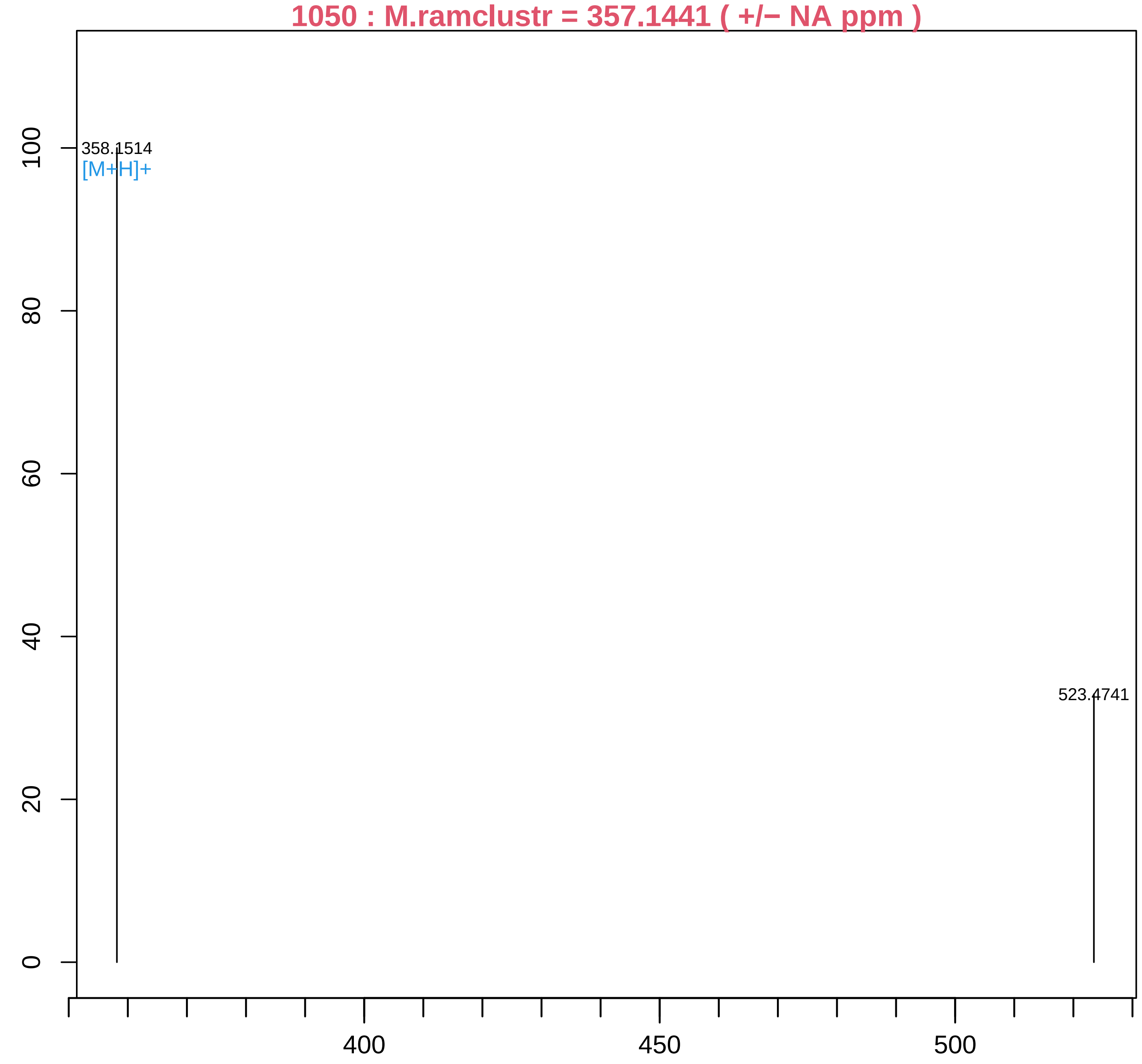

1050 : M.ramclustr = 357.1441 (+/− NA ppm)
358.1514
[M+H]+
523.4741
0
20
40
60
80
100
400
450
500

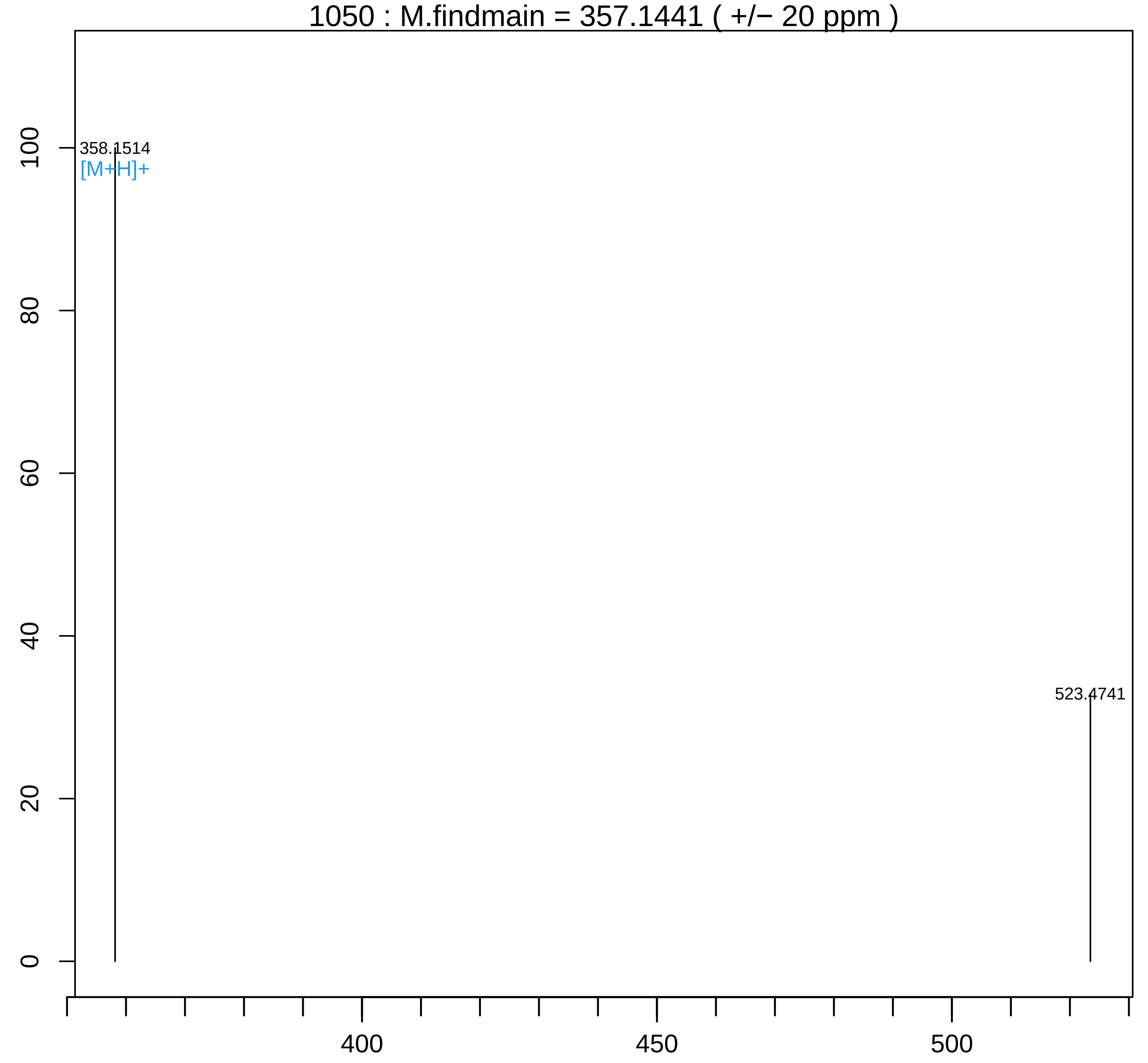

1050 : M.findmain = 357.1441 (+/− 20 ppm)
358.1514
[M+H]+
523.4741
0
20
40
60
80
100
400
450
500

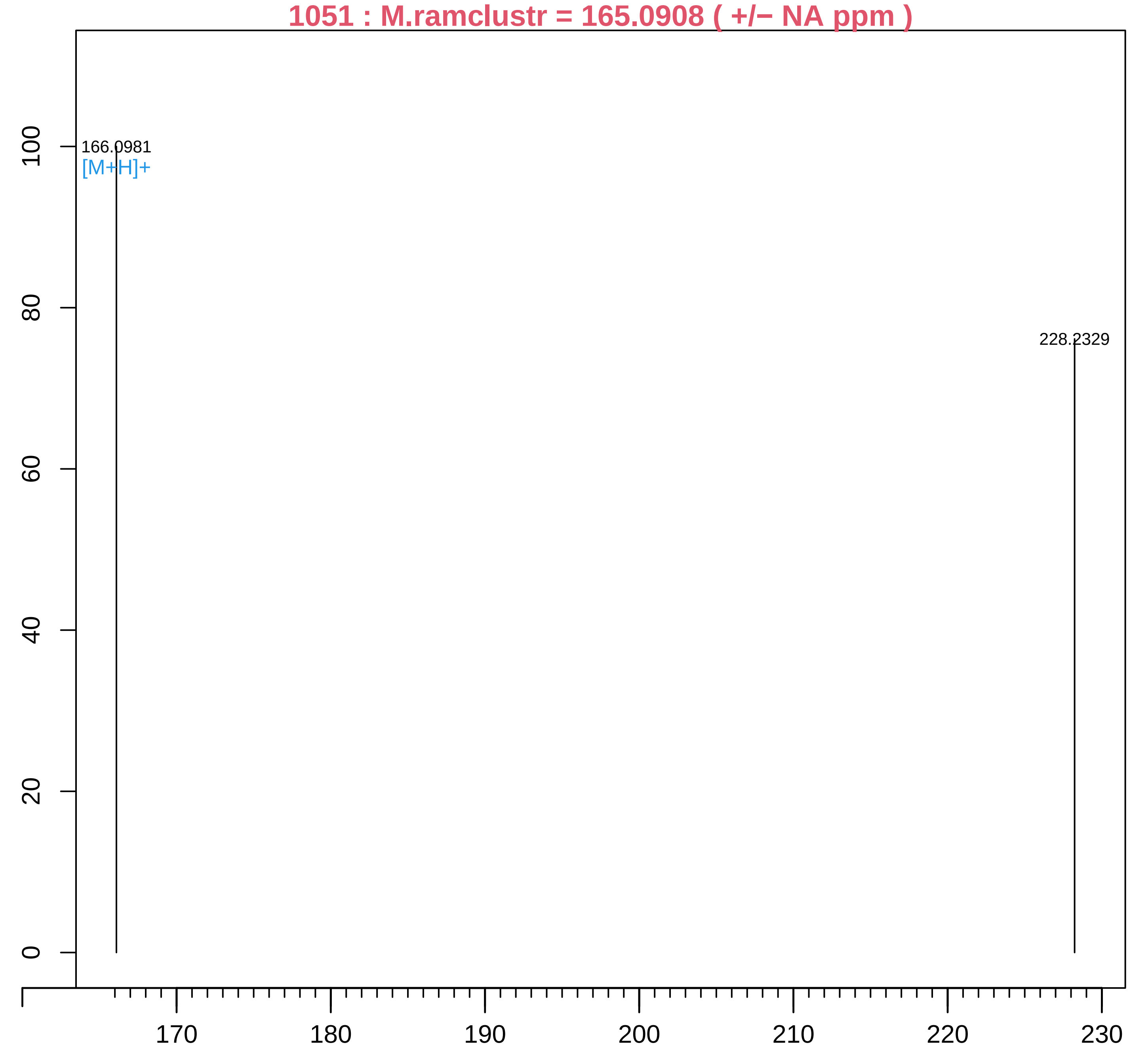
1051 : M.ramclustr = 165.0908 (+/− NA ppm)
166.0981
[M+H]+
228.2329
0
20
40
60
80
100
170
180
190
200
210
220
230

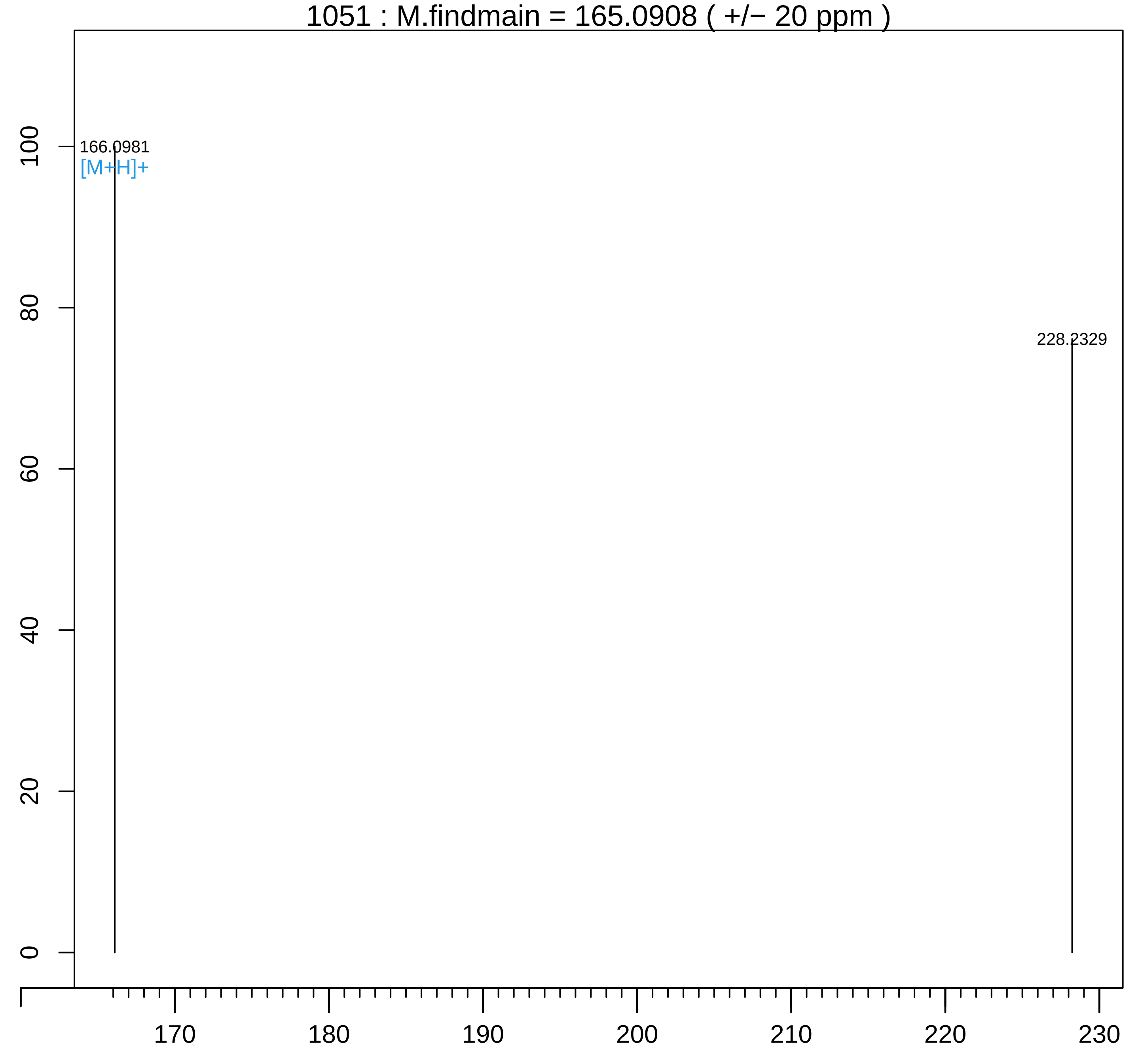
1051 : M.findmain = 165.0908 (+/− 20 ppm)
166.0981
[M+H]+
228.2329
0
20
40
60
80
100
170
180
190
200
210
220
230

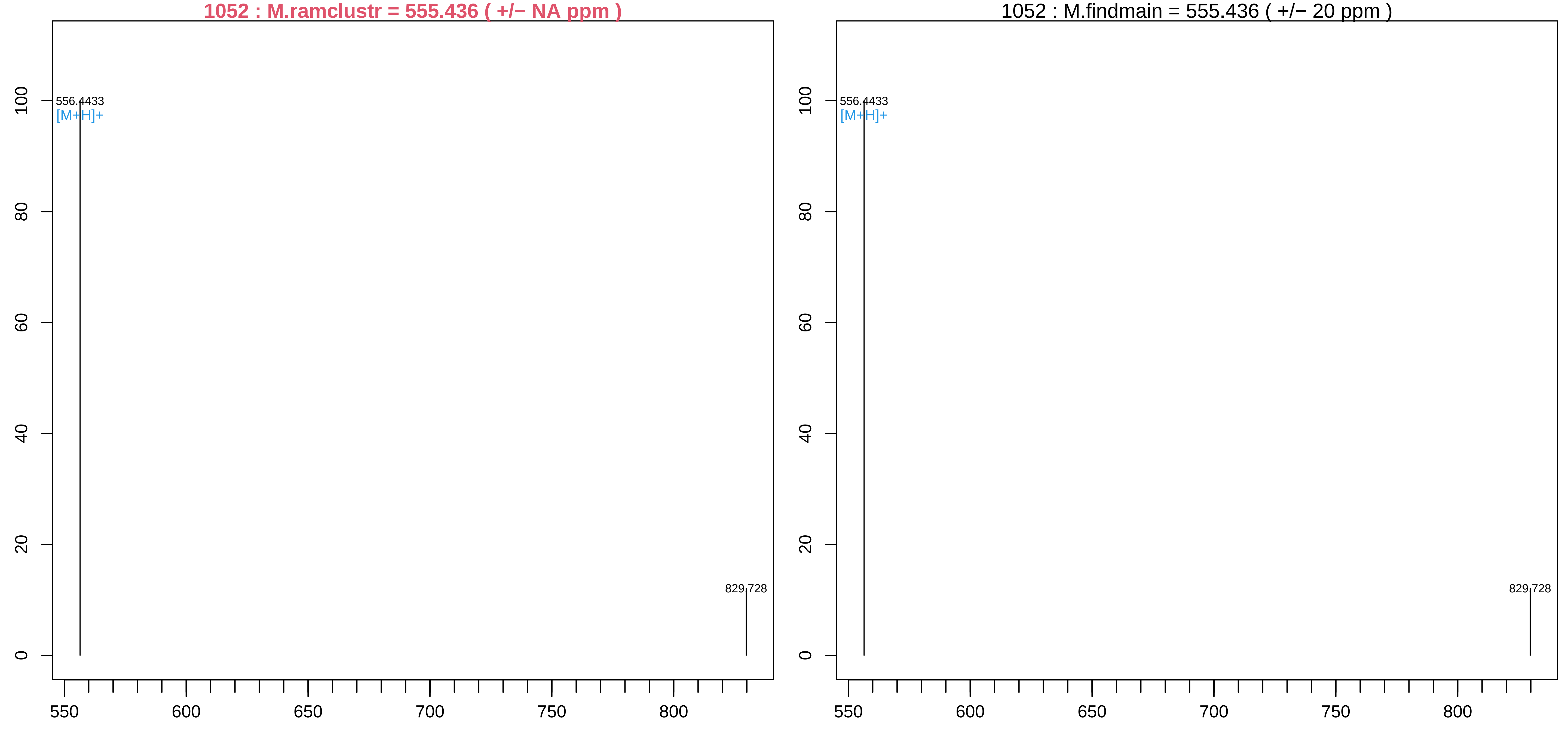

1052 : M.ramclustr = 555.436 (+/− NA ppm)
556.4433
[M+H]+
829.728
1052 : M.findmain = 555.436 (+/− 20 ppm)
556.4433
[M+H]+
829.728
0
20
40
60
80
100
550
600
650
700
750
800

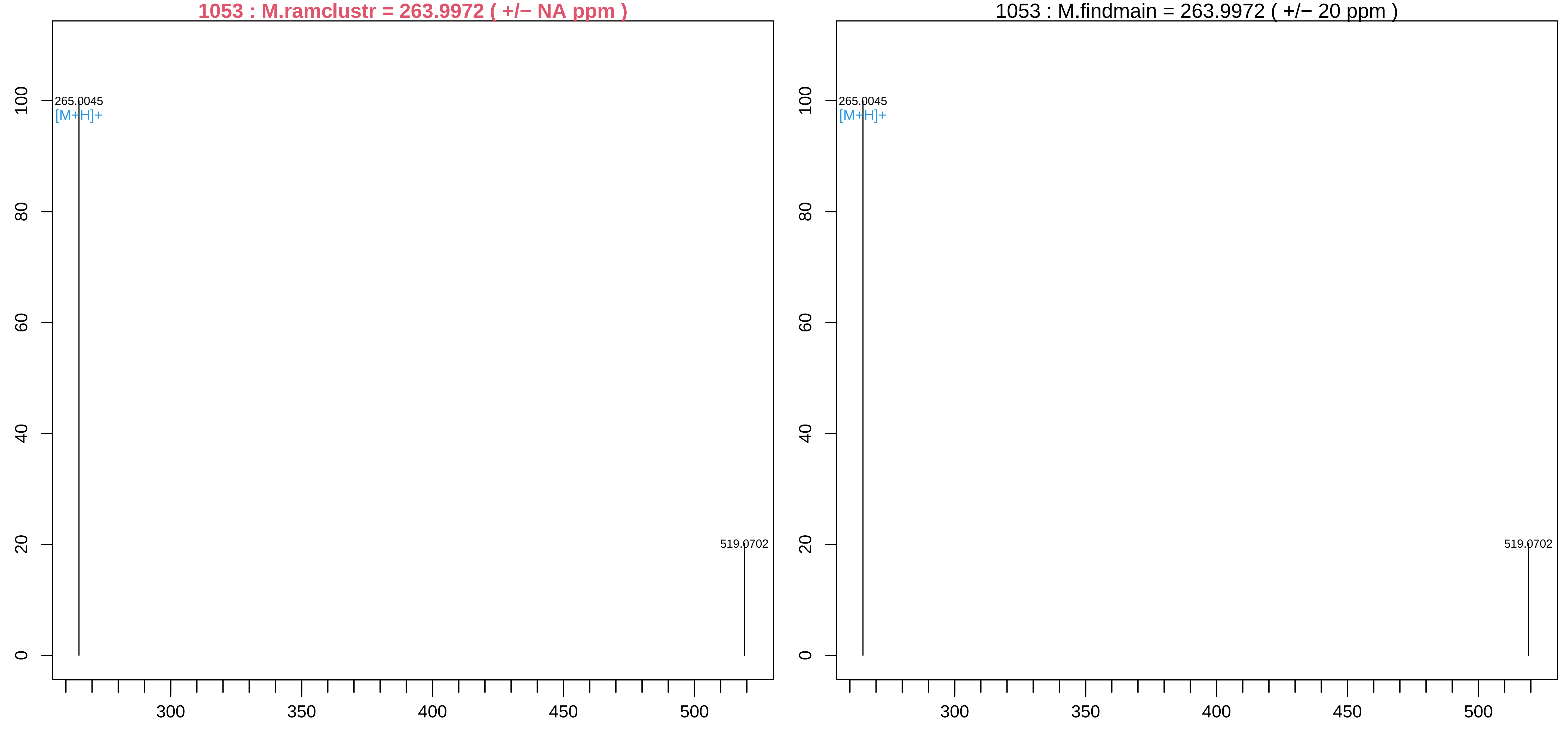
1053 : M.ramclustr = 263.9972 (+/− NA ppm)
265.0045
[M+H]+
519.0702
1053 : M.findmain = 263.9972 (+/− 20 ppm)
265.0045
[M+H]+
519.0702
0
20
40
60
80
100
300
350
400
450
500

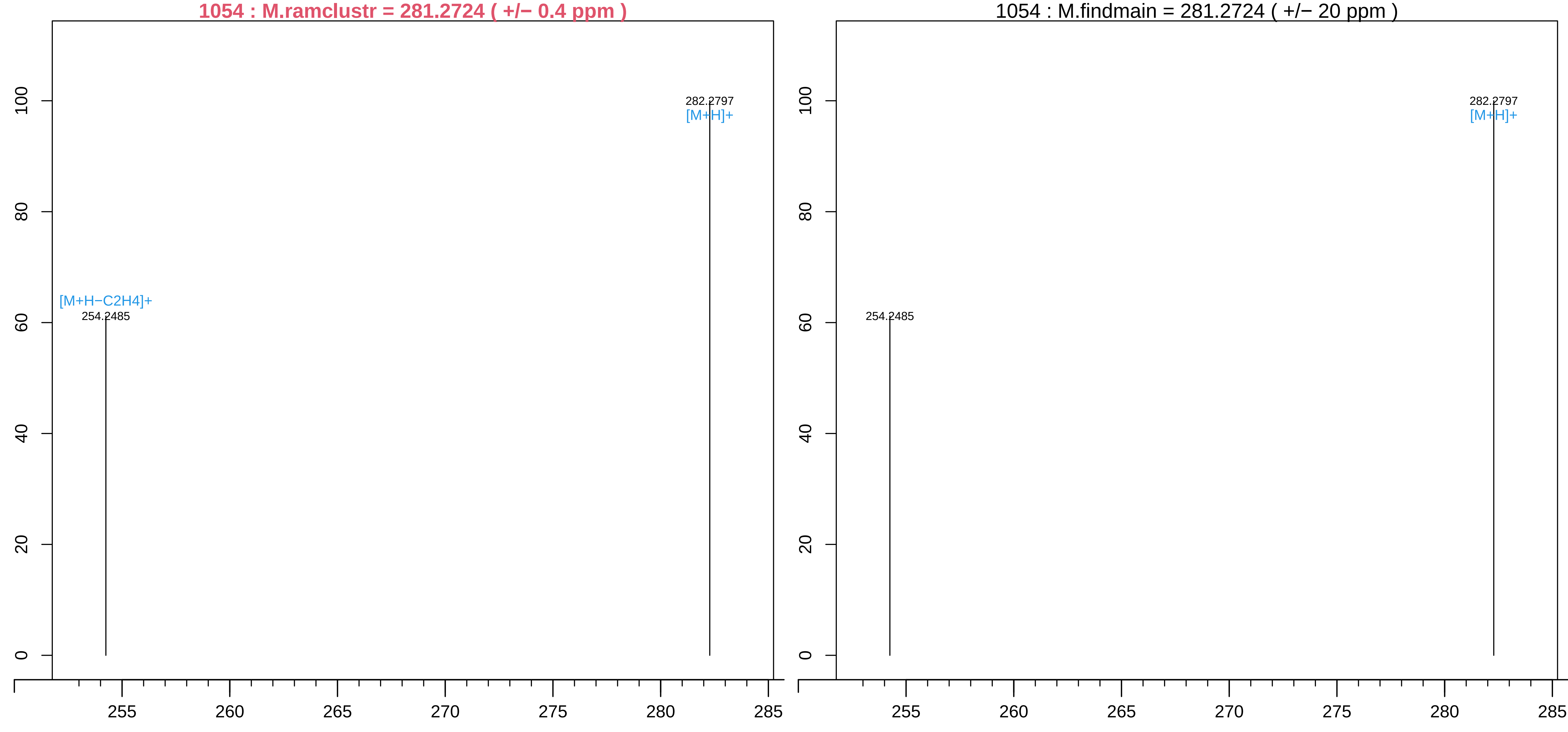
1054 : M.ramclustr = 281.2724 (+/− 0.4 ppm)
[M+H−C2H4]+
254.2485
282.2797
[M+H]+
1054 : M.findmain = 281.2724 (+/− 20 ppm)
254.2485
282.2797
[M+H]+
0
20
40
60
80
100
255
260
265
270
275
280
285

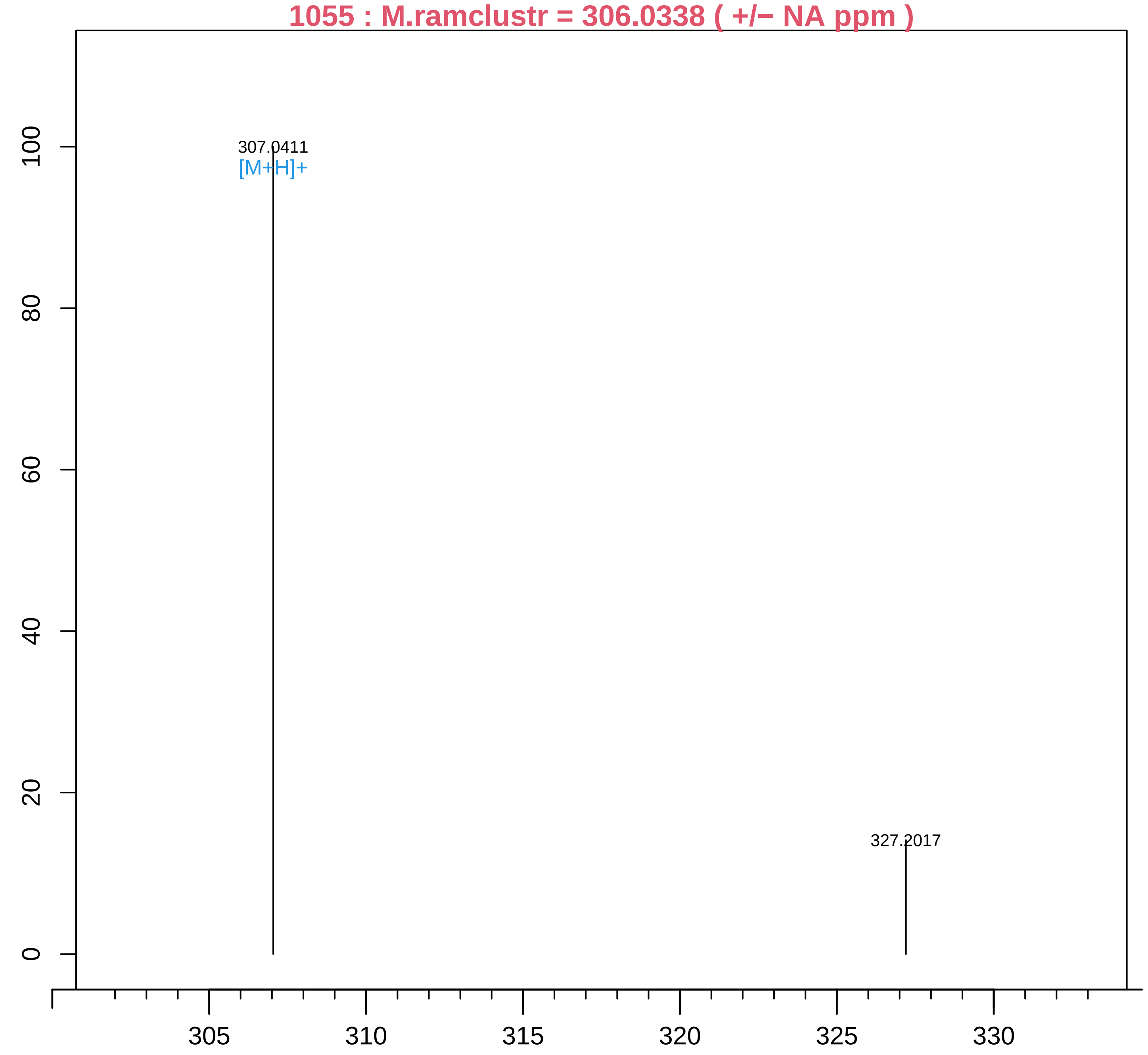
1055 : M.ramclustr = 306.0338 (+/− NA ppm)
307.0411
[M+H]+
327.2017
0
20
40
60
80
100
305
310
315
320
325
330

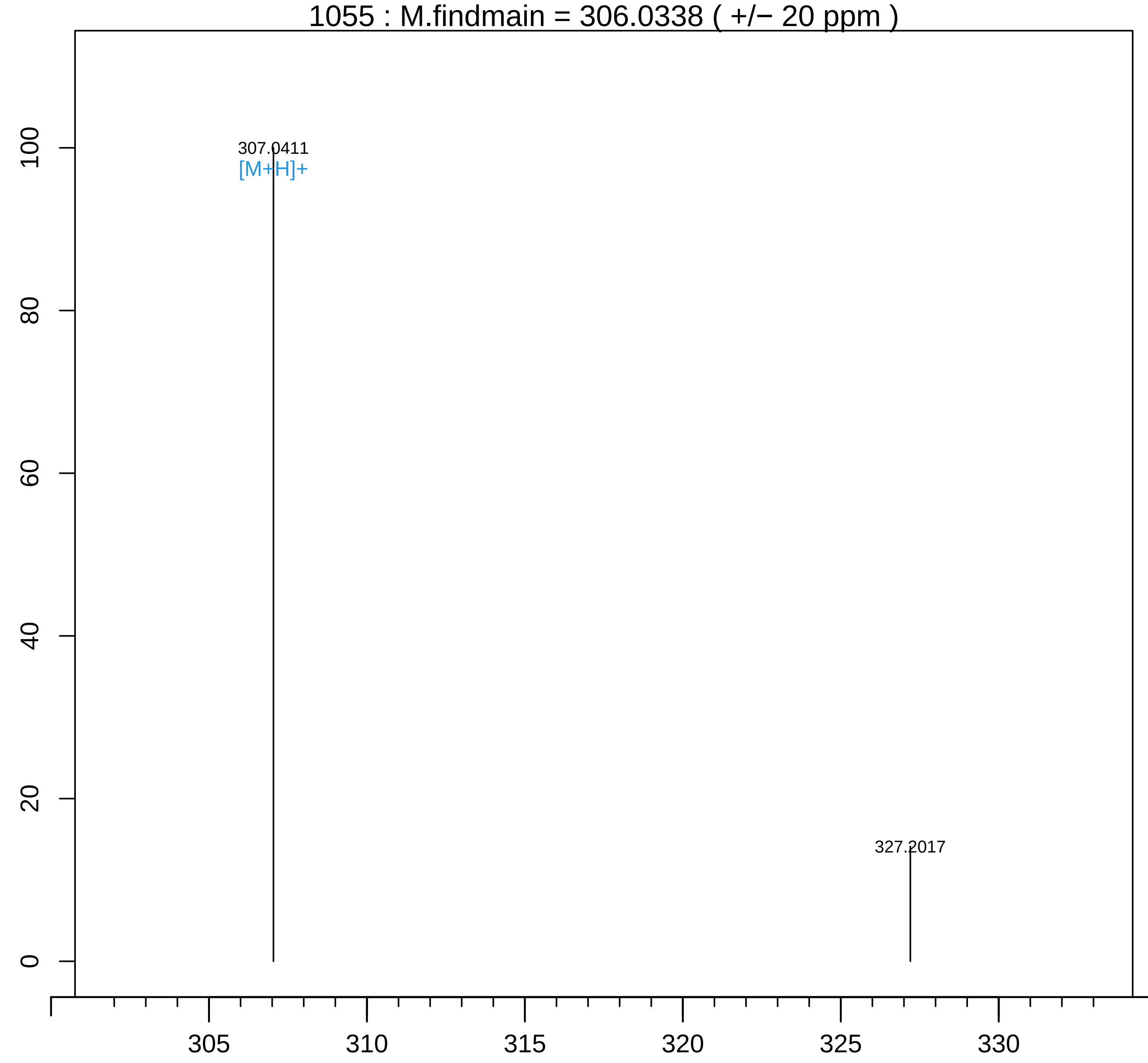
1055 : M.findmain = 306.0338 (+/− 20 ppm)
307.0411
[M+H]+
327.2017
0
20
40
60
80
100
305
310
315
320
325
330

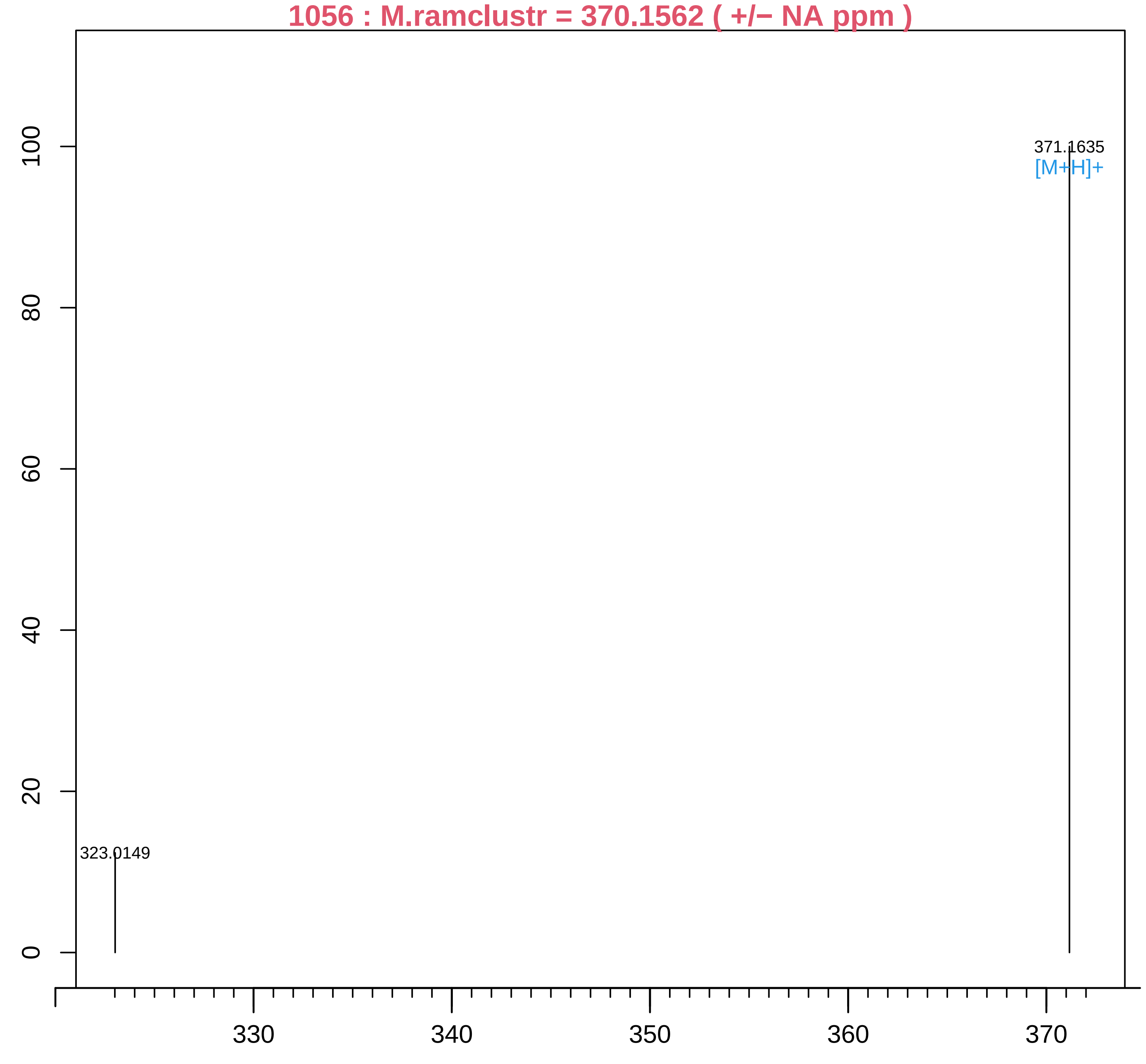
1056 : M.ramclustr = 370.1562 (+/− NA ppm)
371.1635
[M+H]+
323.0149
0
20
40
60
80
100
330
340
350
360
370

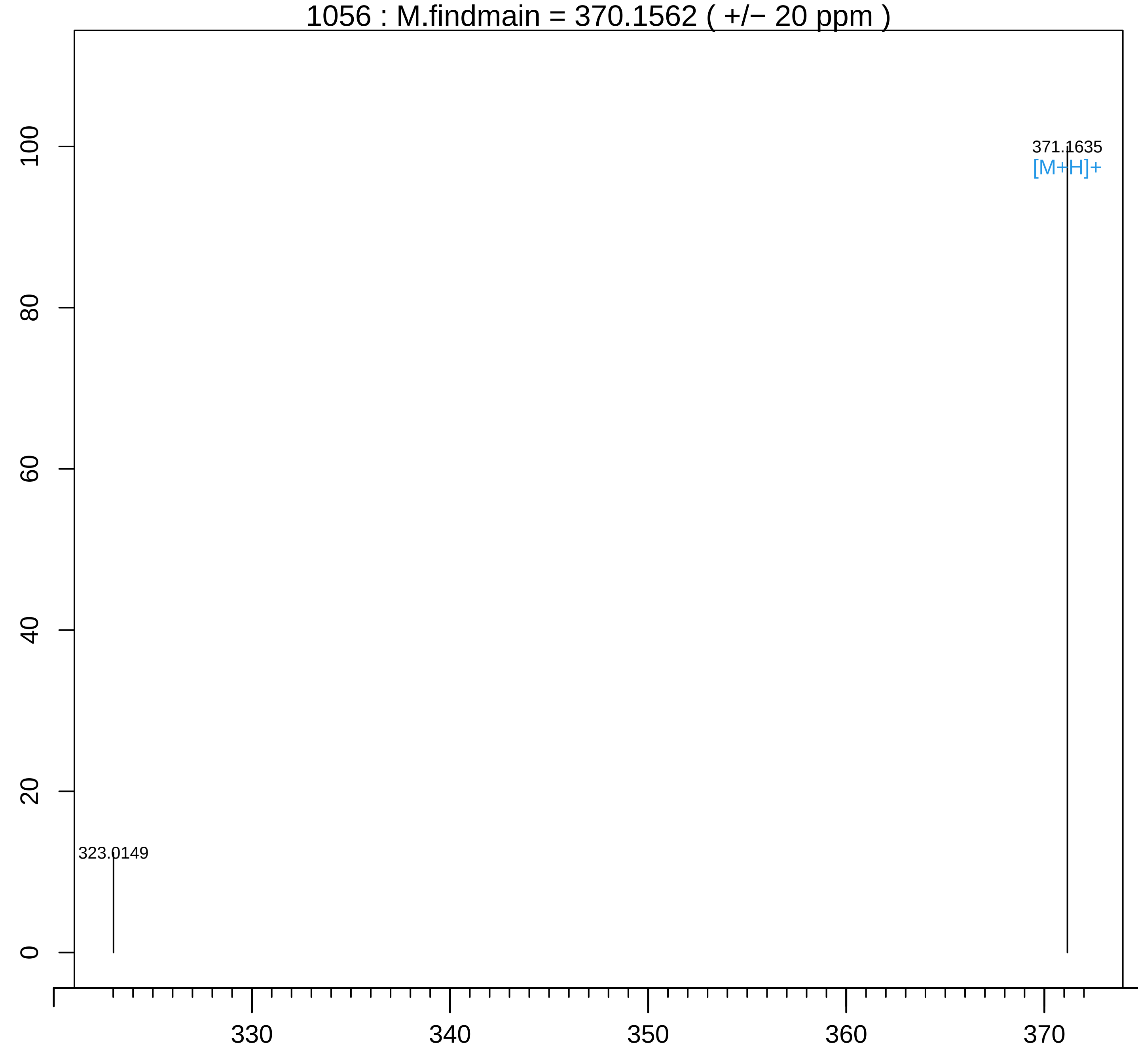
1056 : M.findmain = 370.1562 (+/− 20 ppm)
371.1635
[M+H]+
323.0149
0
20
40
60
80
100
330
340
350
360
370

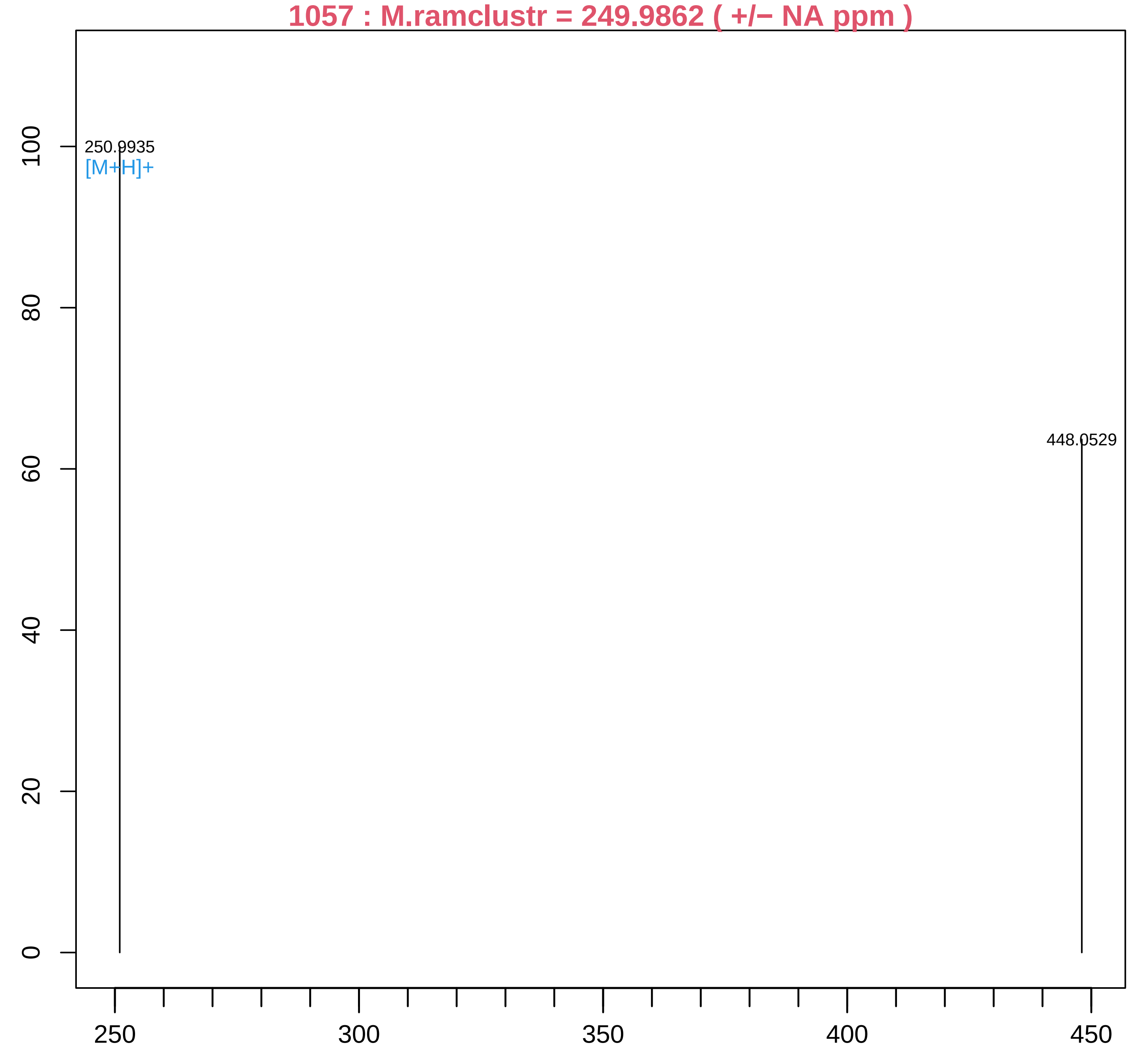

1057 : M.ramclustr = 249.9862 (+/− NA ppm)
250.9935
[M+H]+
448.0529
0
20
40
60
80
100
250
300
350
400
450

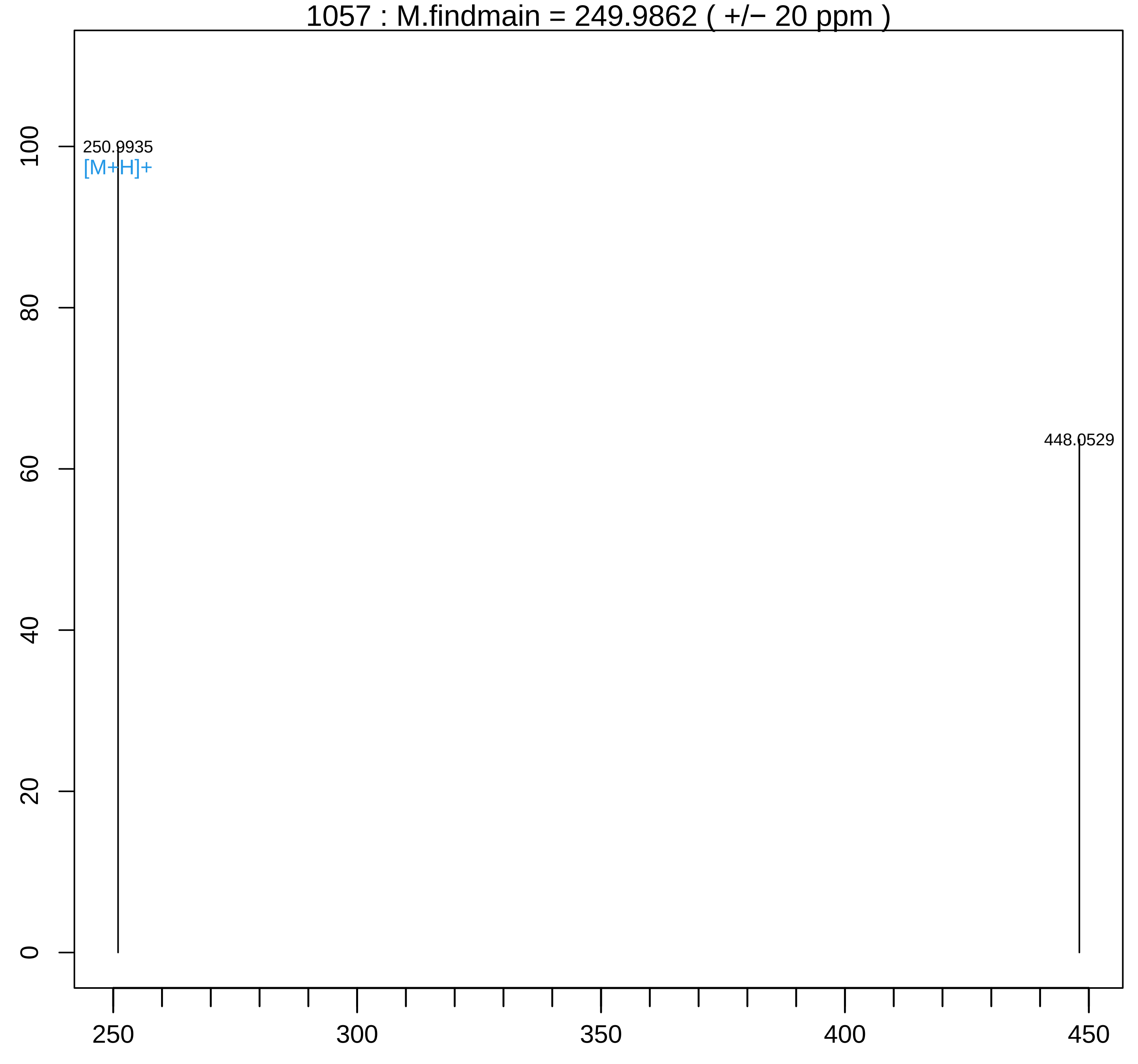

1057 : M.findmain = 249.9862 (+/− 20 ppm)
250.9935
[M+H]+
448.0529
0
20
40
60
80
100
250
300
350
400
450

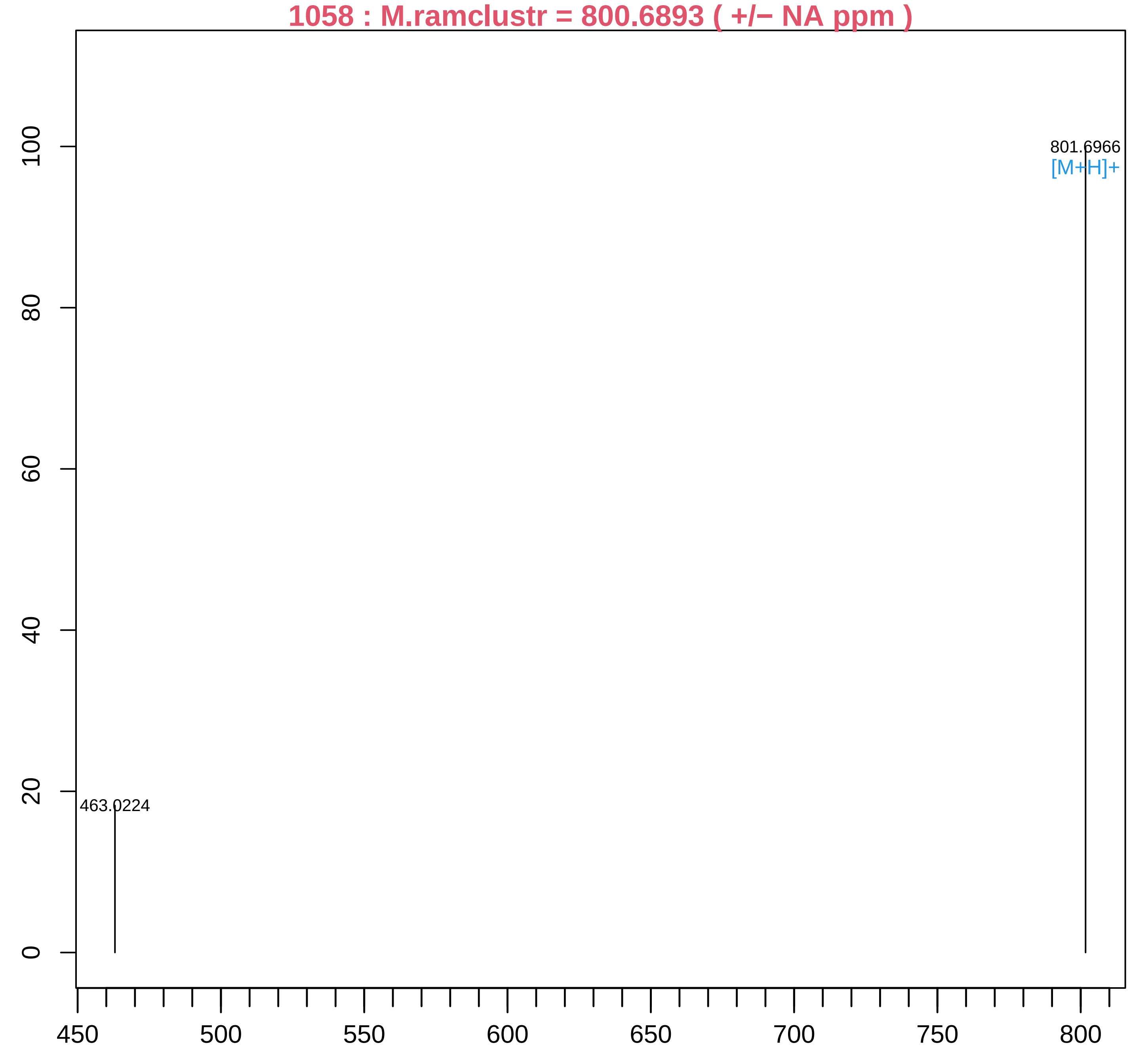
1058 : M.ramclustr = 800.6893 (+/− NA ppm)
801.6966
[M+H]+
463.0224
0
20
40
60
80
100
450
500
550
600
650
700
750
800

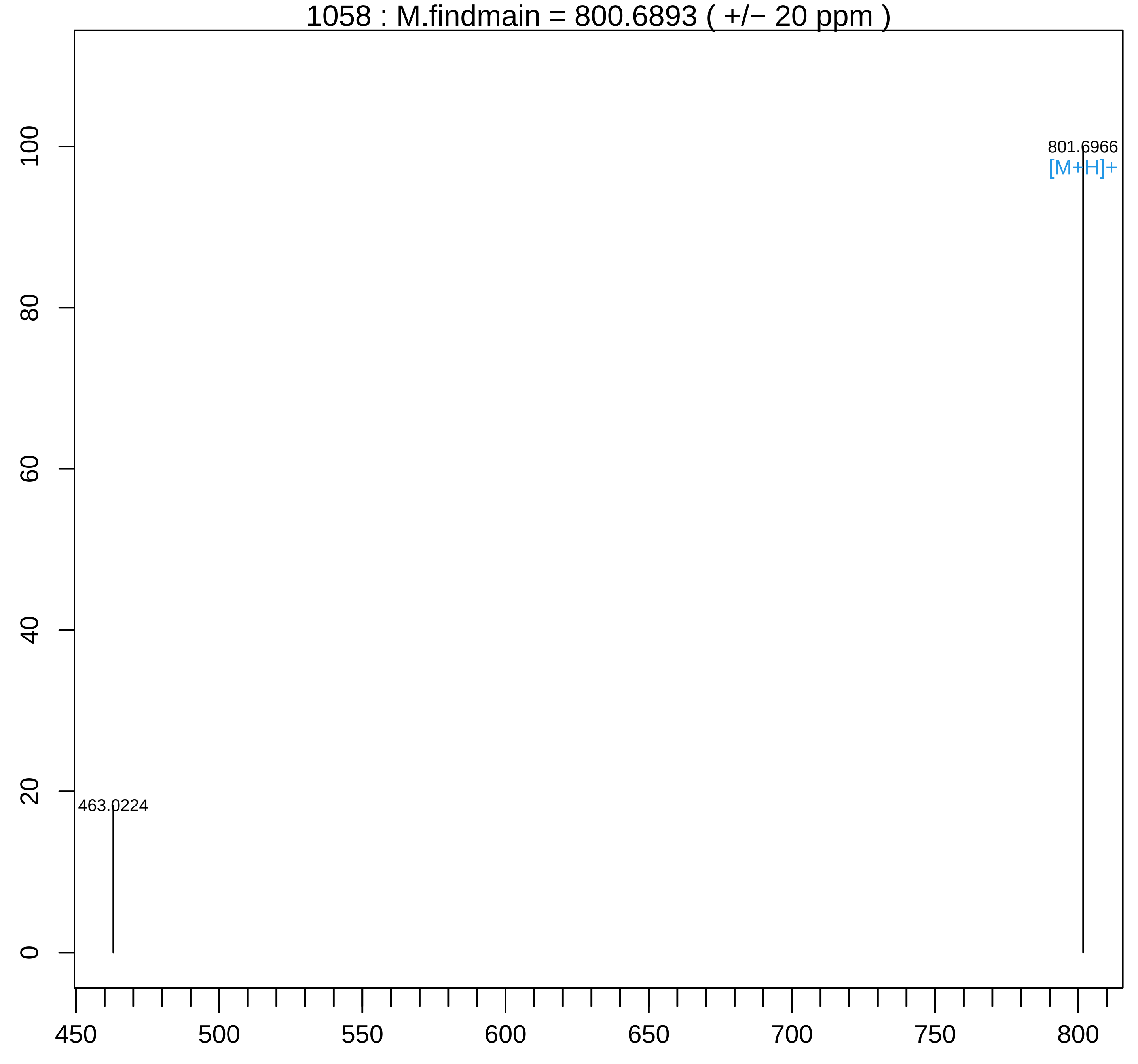
1058 : M.findmain = 800.6893 (+/− 20 ppm)
801.6966
[M+H]+
463.0224
0
20
40
60
80
100
450
500
550
600
650
700
750
800

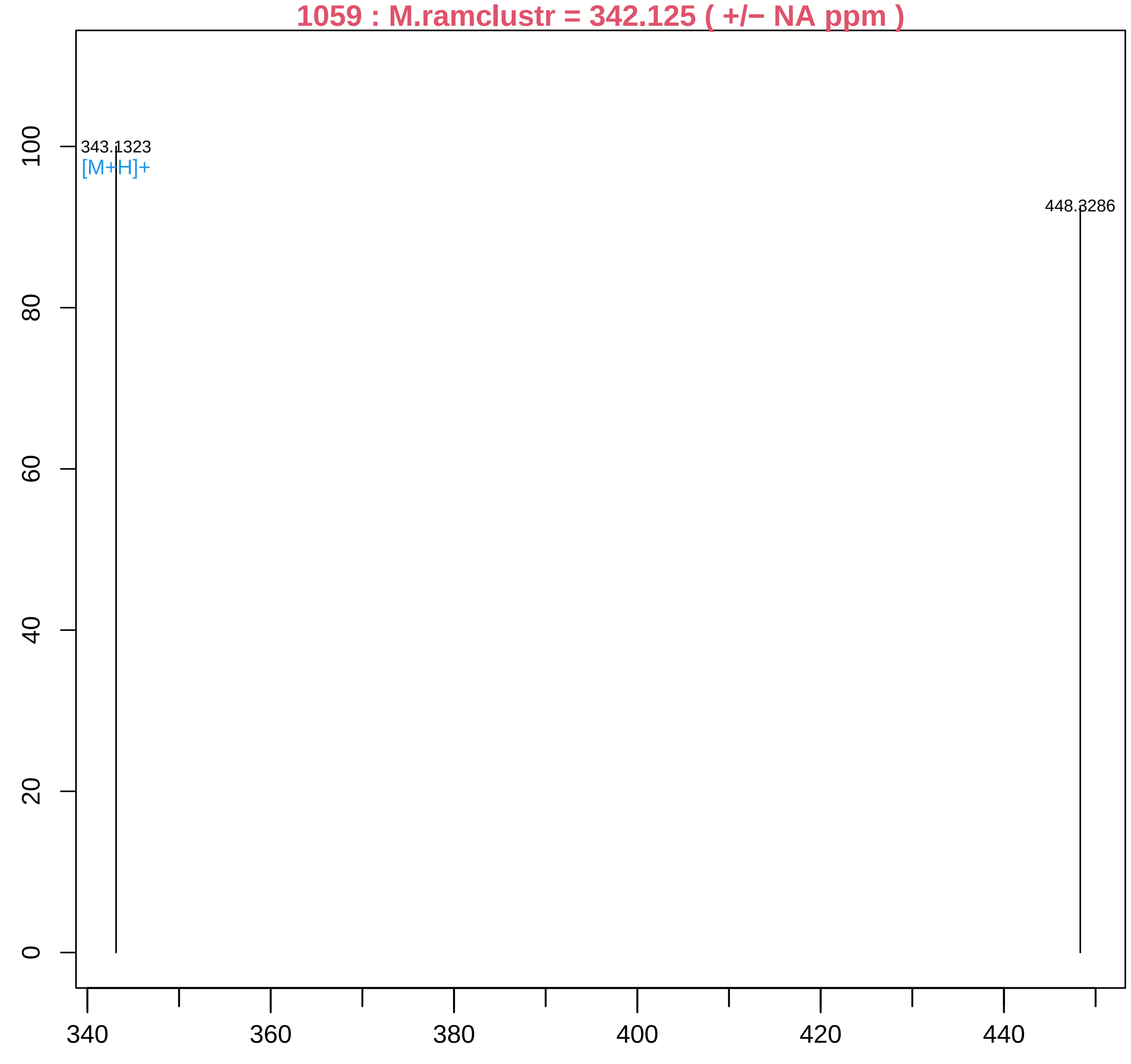
1059 : M.ramclustr = 342.125 (+/− NA ppm)
343.1323
[M+H]+
448.3286
0
20
40
60
80
100
340
360
380
400
420
440

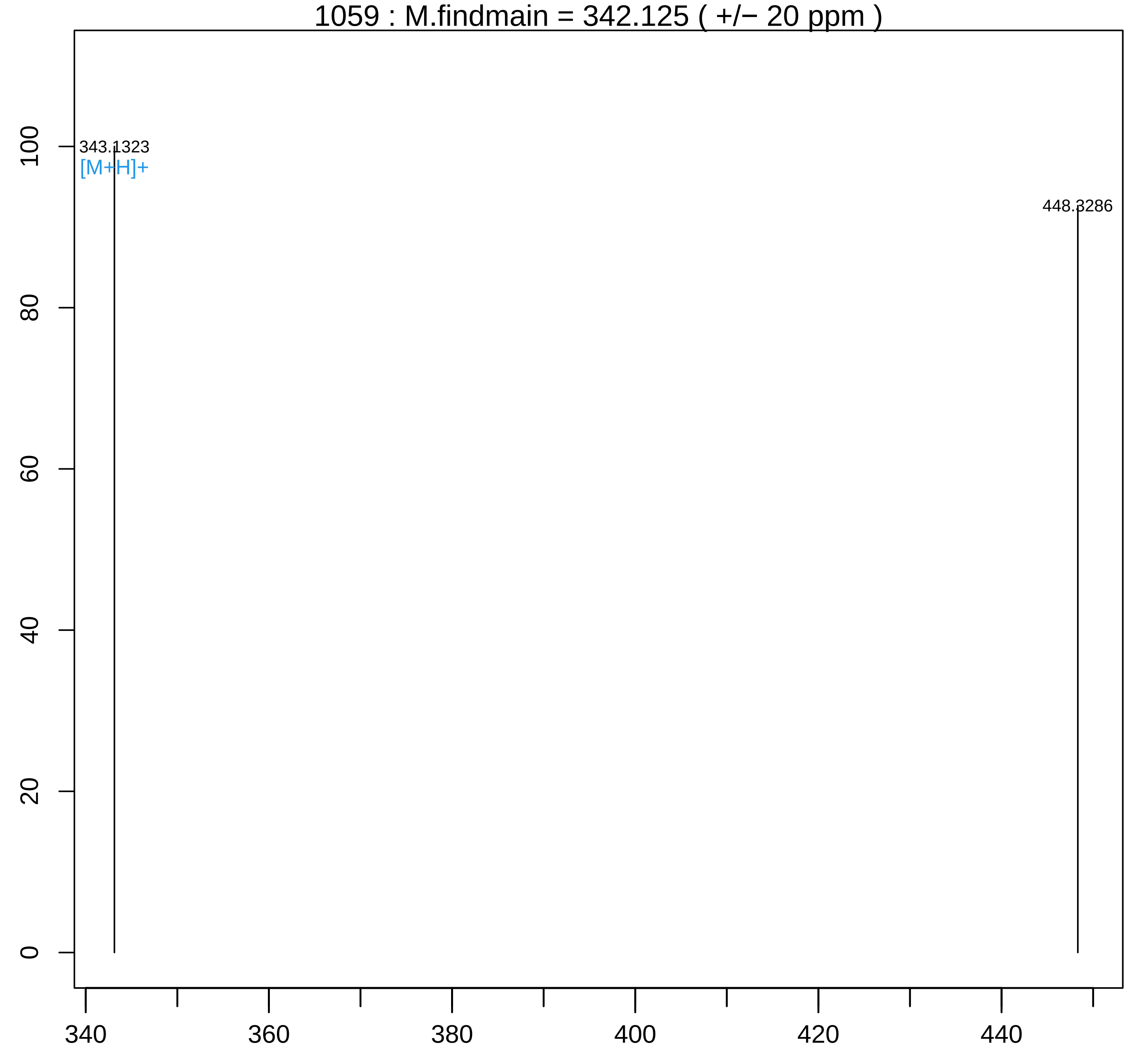
1059 : M.findmain = 342.125 (+/− 20 ppm)
343.1323
[M+H]+
448.3286
0
20
40
60
80
100
340
360
380
400
420
440

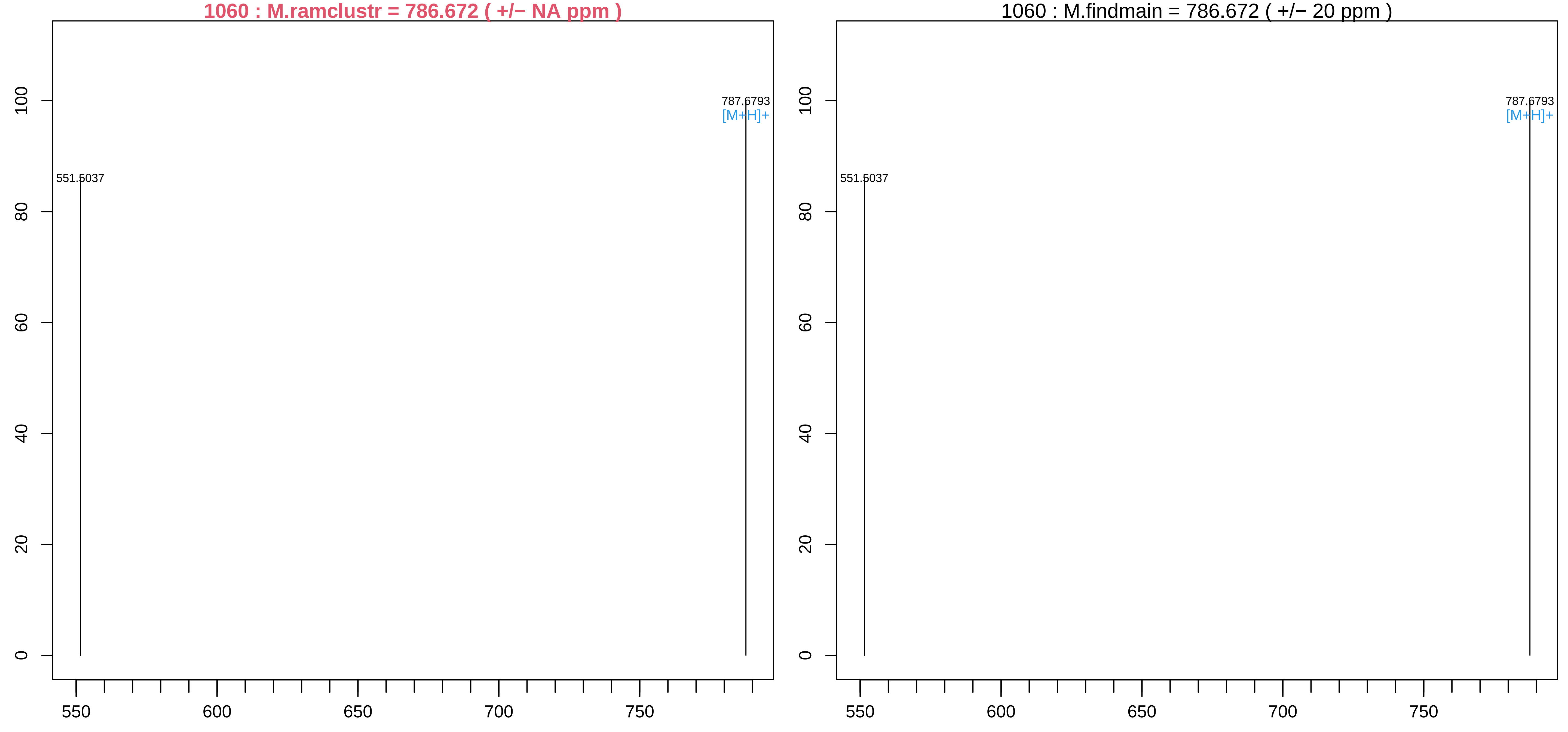

1060 : M.ramclustr = 786.672 (+/− NA ppm)
1060 : M.findmain = 786.672 (+/− 20 ppm)
787.6793
[M+H]+
551.5037
0
20
40
60
80
100
550
600
650
700
750

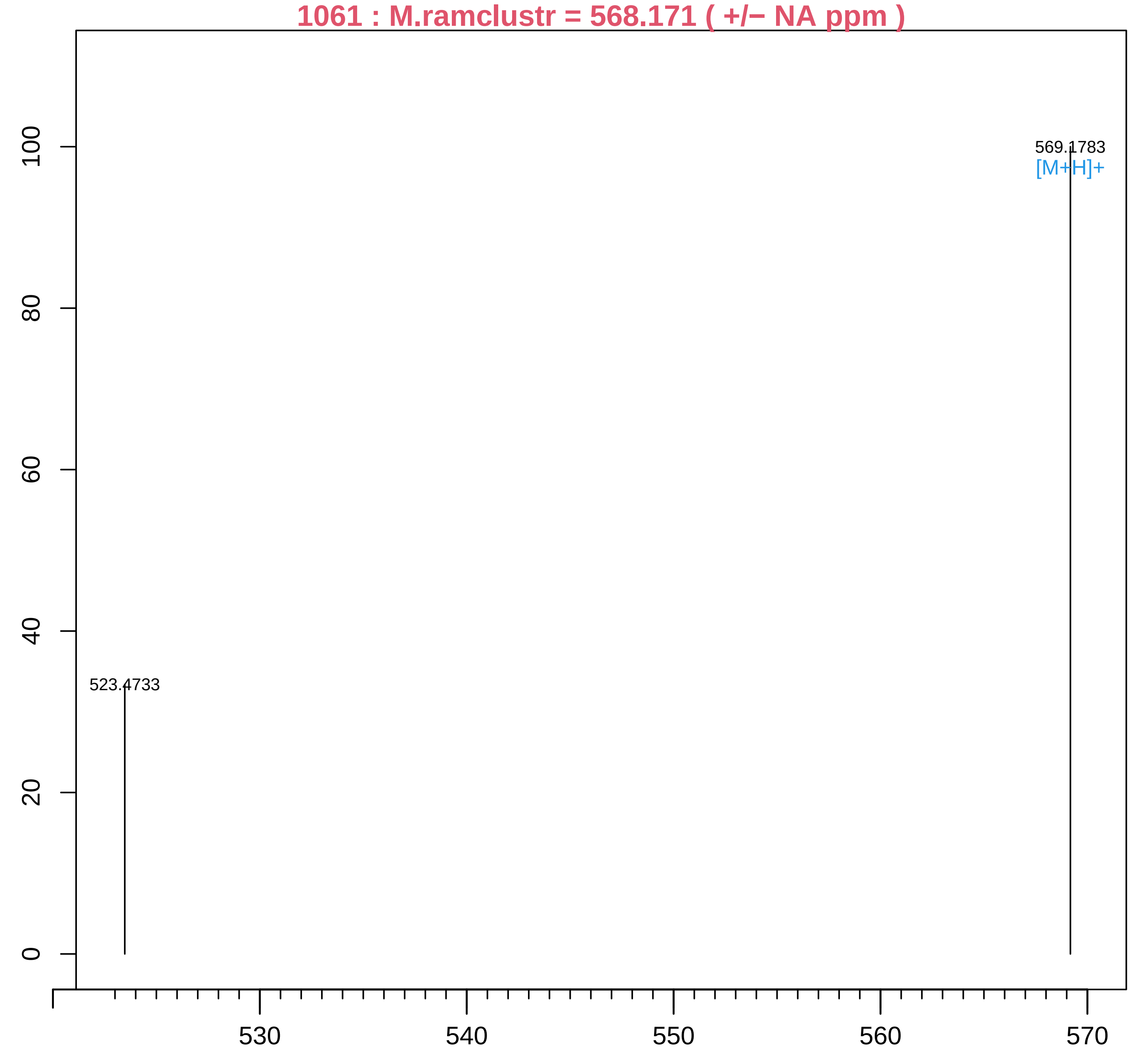
1061 : M.ramclustr = 568.171 (+/− NA ppm)
569.1783
[M+H]+
523.4733
0
20
40
60
80
100
530
540
550
560
570

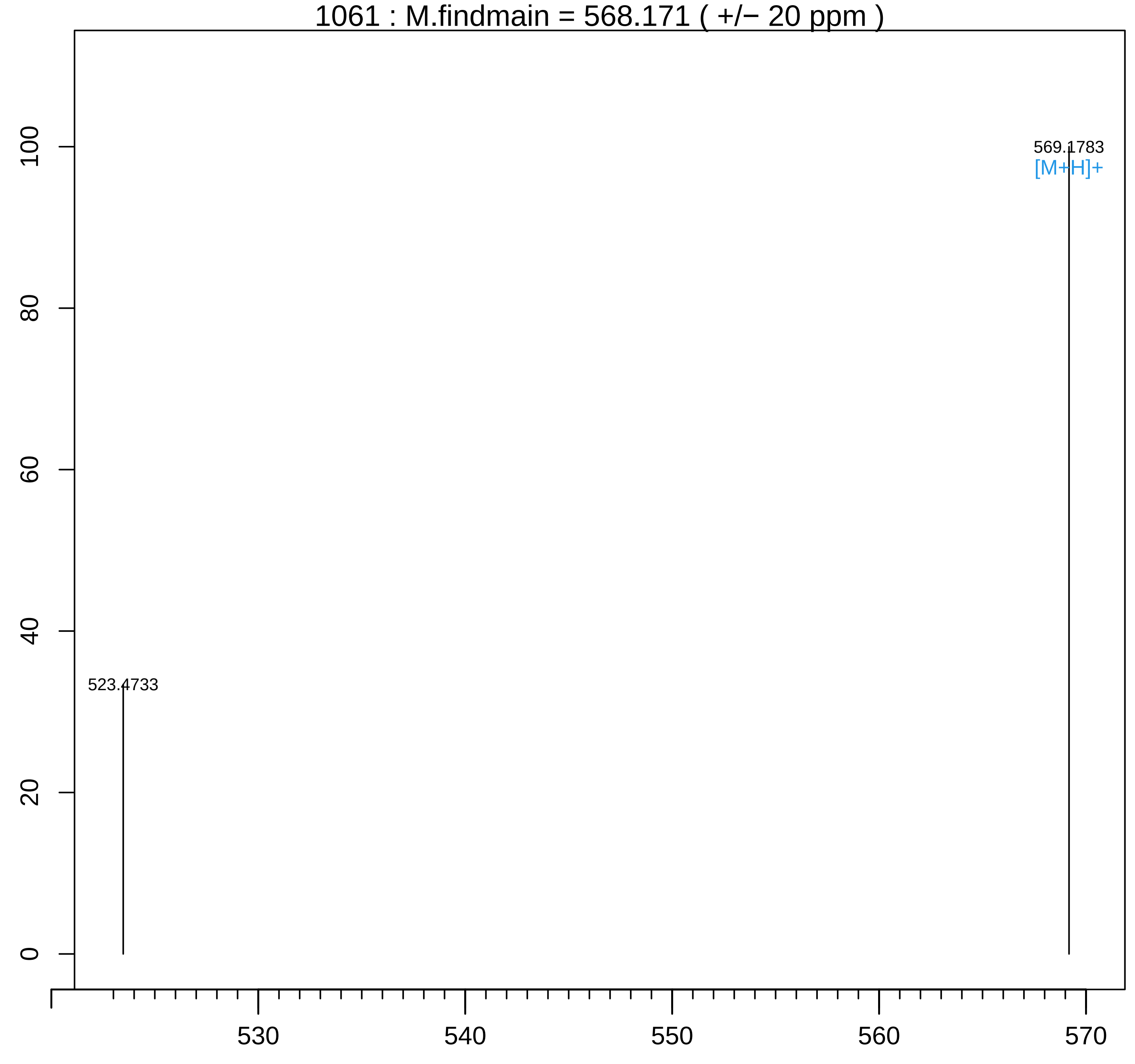
1061 : M.findmain = 568.171 (+/− 20 ppm)
569.1783
[M+H]+
523.4733
0
20
40
60
80
100
530
540
550
560
570

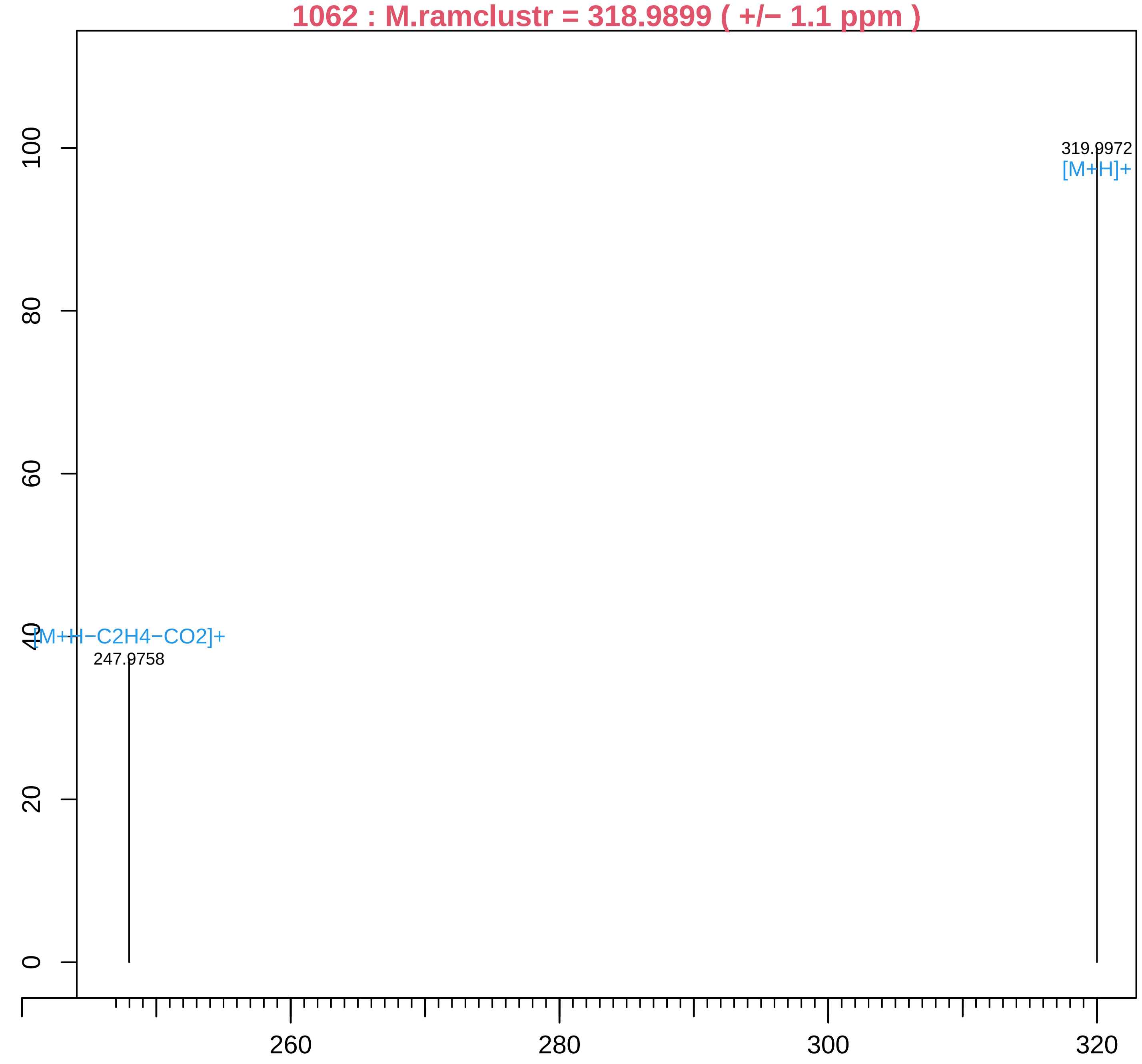

1062 : M.ramclustr = 318.9899 (+/− 1.1 ppm)
319.9972
[M+H]+
[M+H−C2H4−CO2]+
247.9758
0
20
40
60
80
100
260
280
300
320

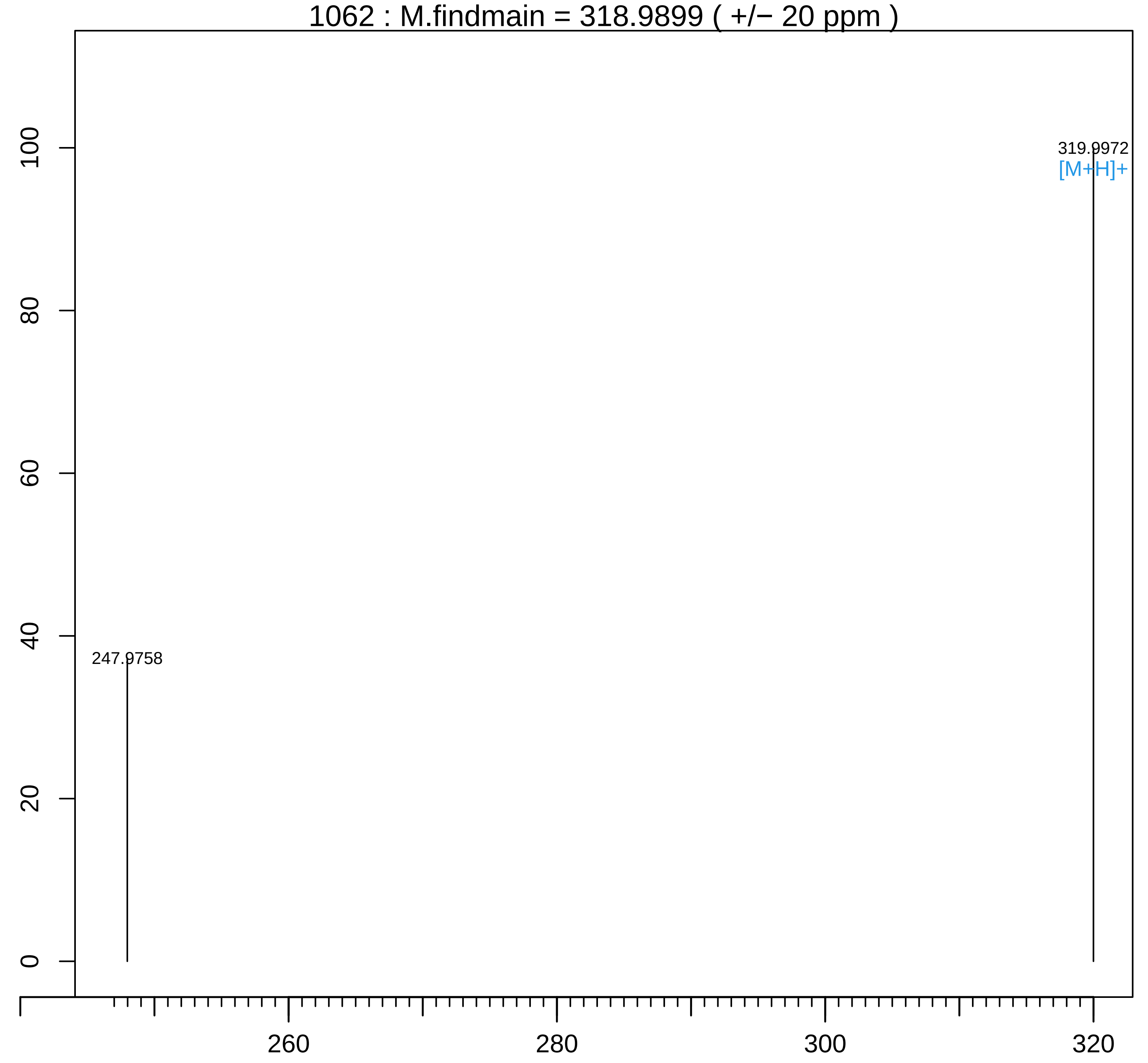

1062 : M.findmain = 318.9899 (+/− 20 ppm)
319.9972
[M+H]+
247.9758
0
20
40
60
80
100
260
280
300
320

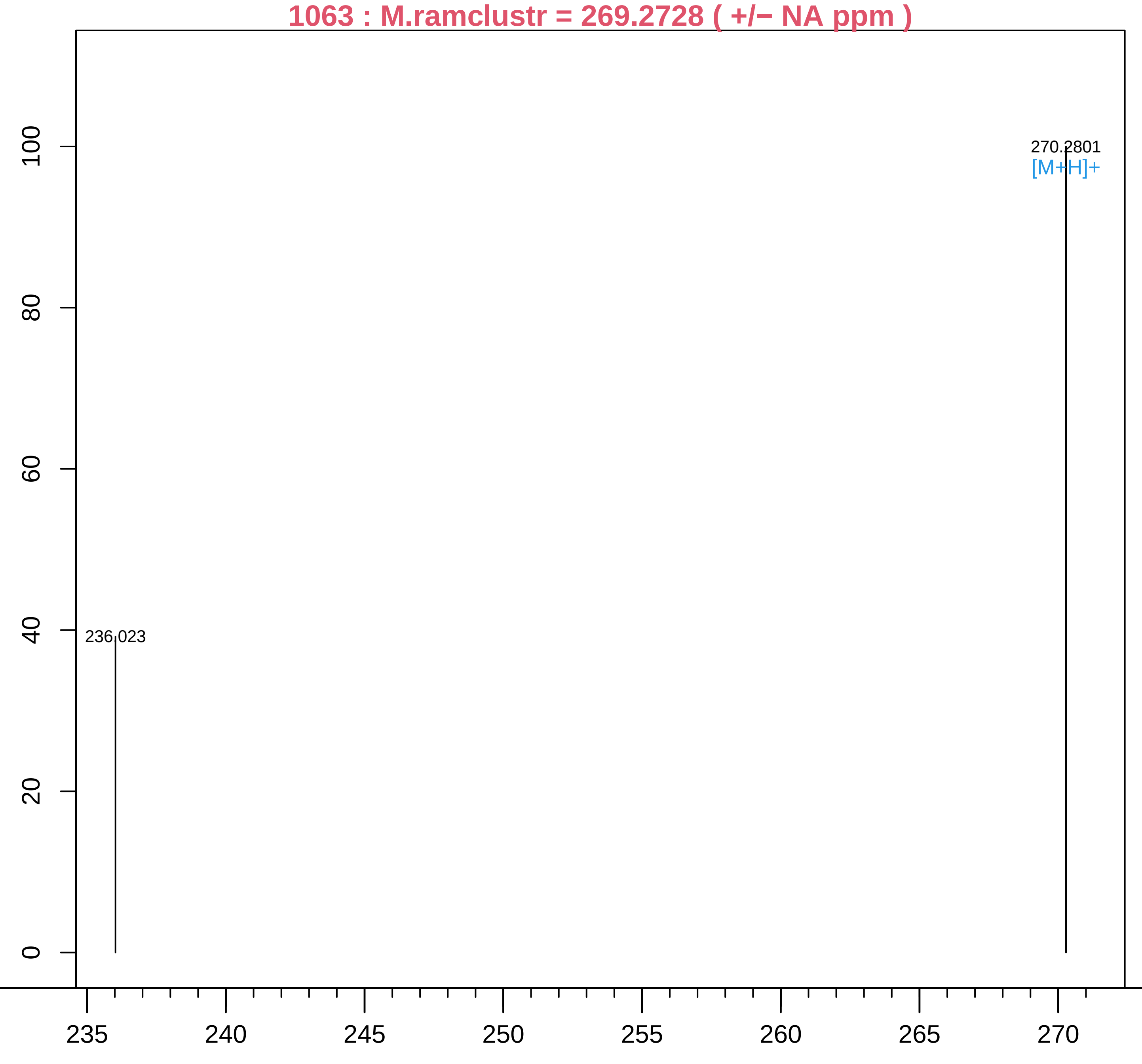
1063 : M.ramclustr = 269.2728 (+/− NA ppm)
270.2801
[M+H]+
236.023
0
20
40
60
80
100
235
240
245
250
255
260
265
270

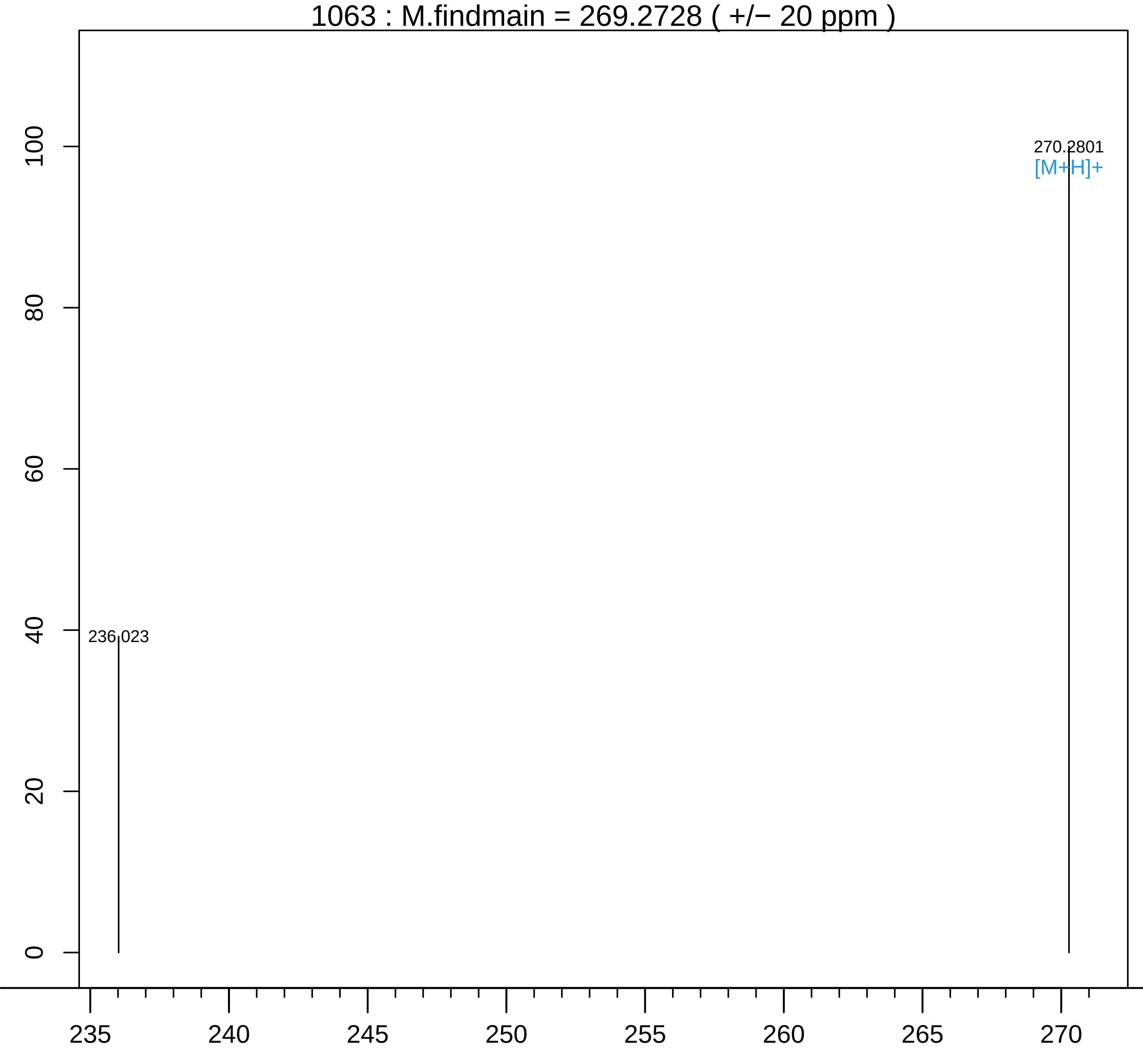
1063 : M.findmain = 269.2728 (+/− 20 ppm)
270.2801
[M+H]+
236.023
0
20
40
60
80
100
235
240
245
250
255
260
265
270

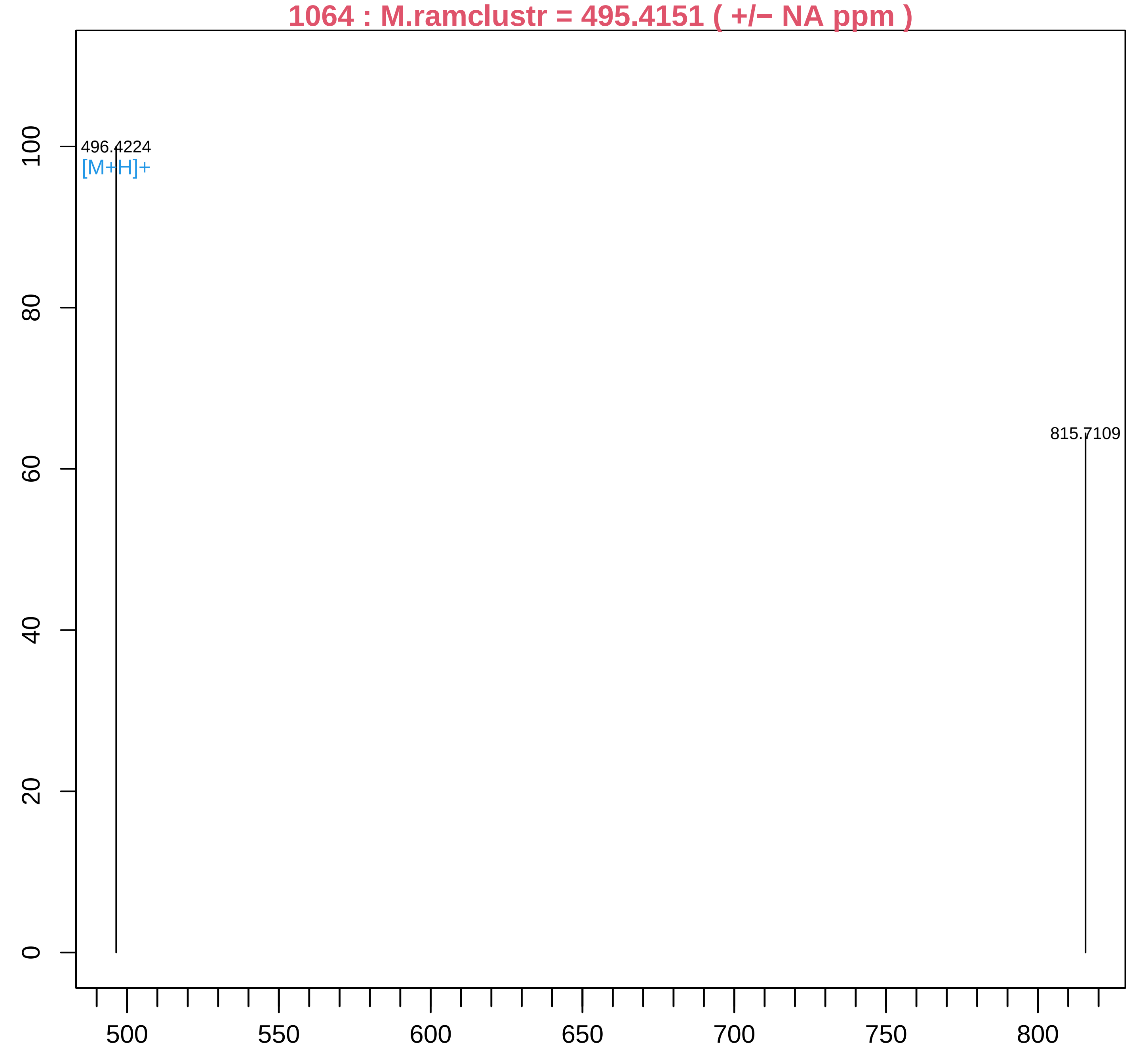
1064 : M.ramclustr = 495.4151 (+/− NA ppm)
496.4224
[M+H]+
815.7109
0
20
40
60
80
100
500
550
600
650
700
750
800

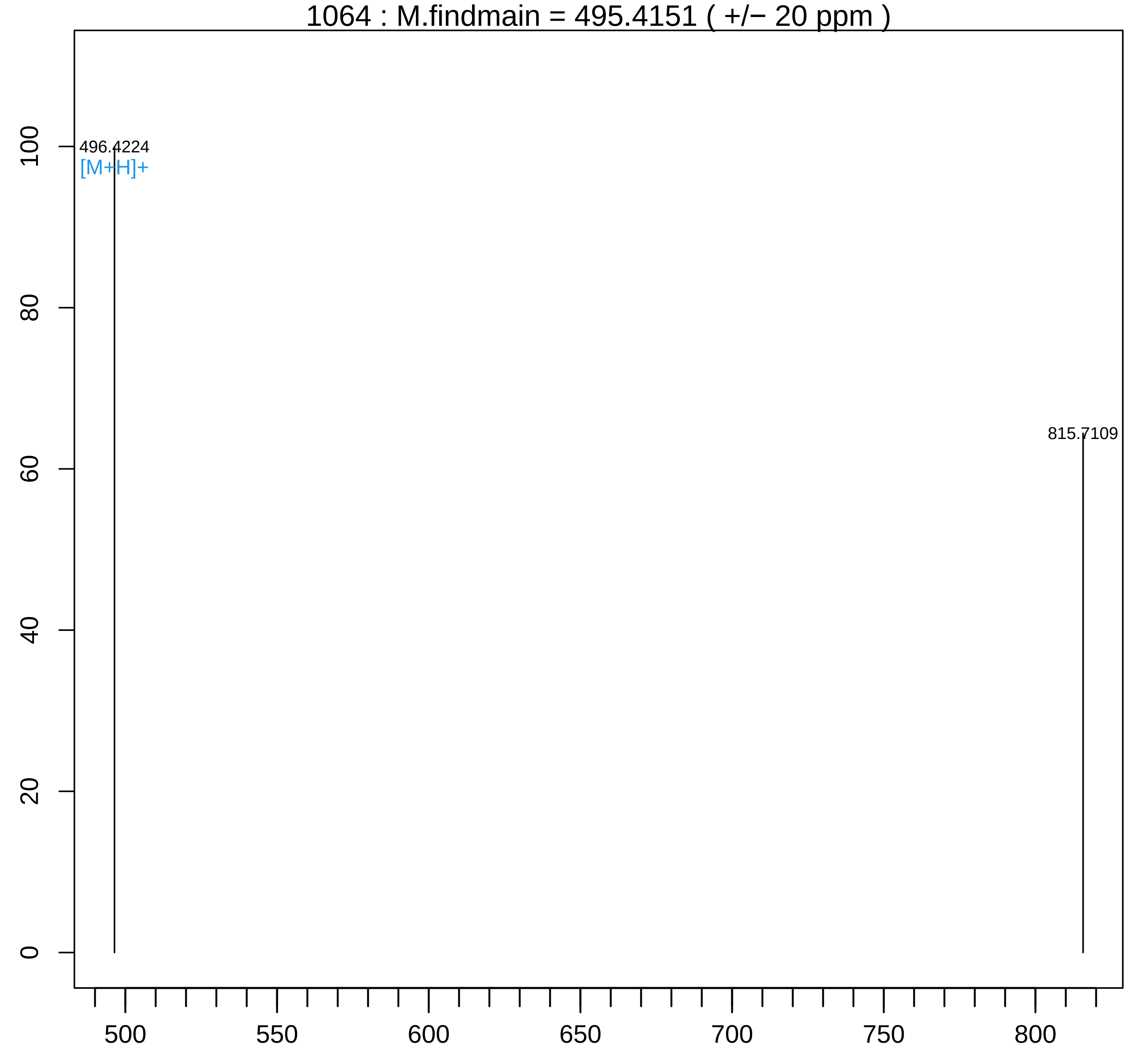
1064 : M.findmain = 495.4151 (+/− 20 ppm)
496.4224
[M+H]+
815.7109
0
20
40
60
80
100
500
550
600
650
700
750
800

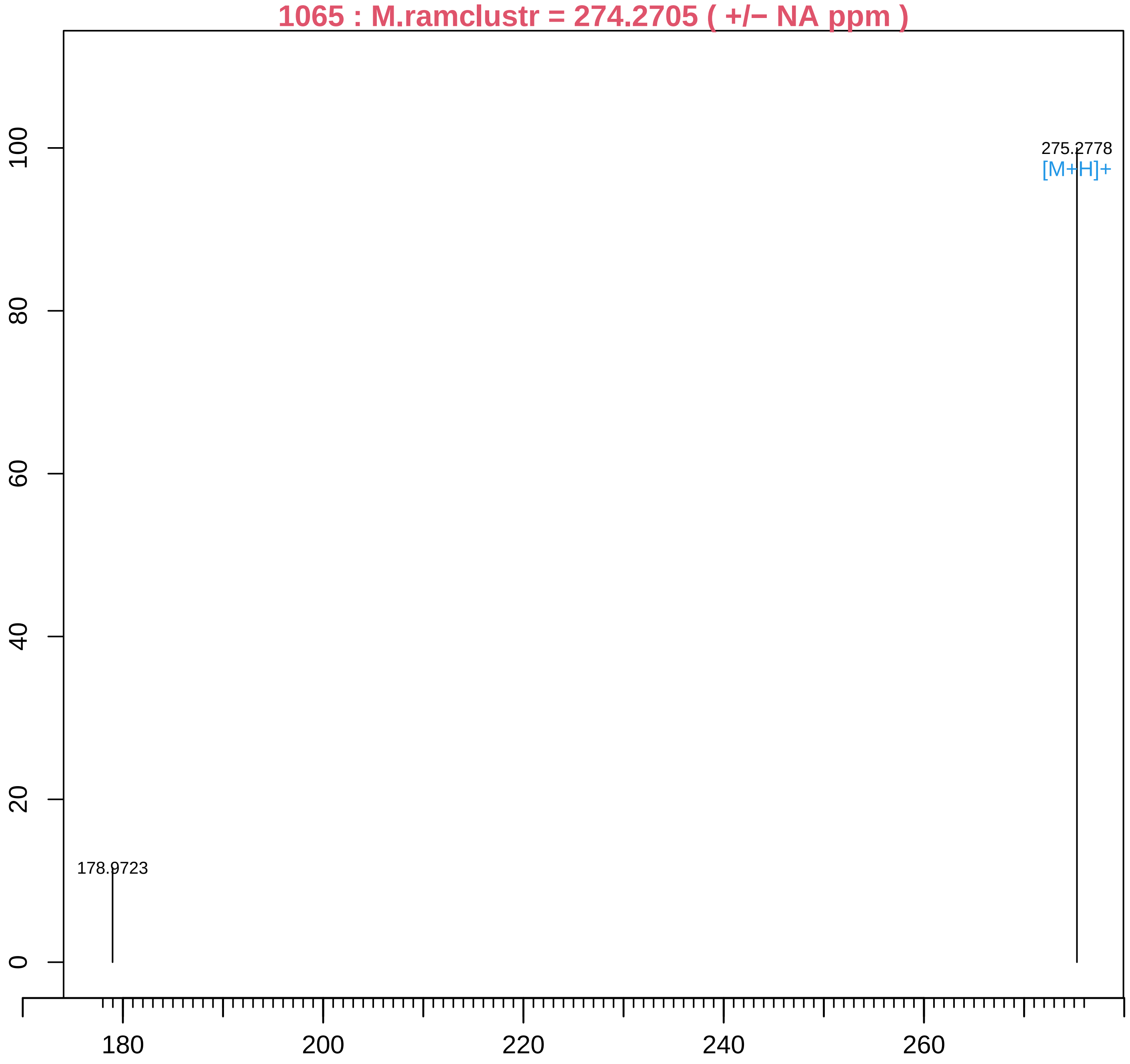

1065 : M.ramclustr = 274.2705 (+/− NA ppm)
275.2778
[M+H]+
178.9723
0
20
40
60
80
100
180
200
220
240
260

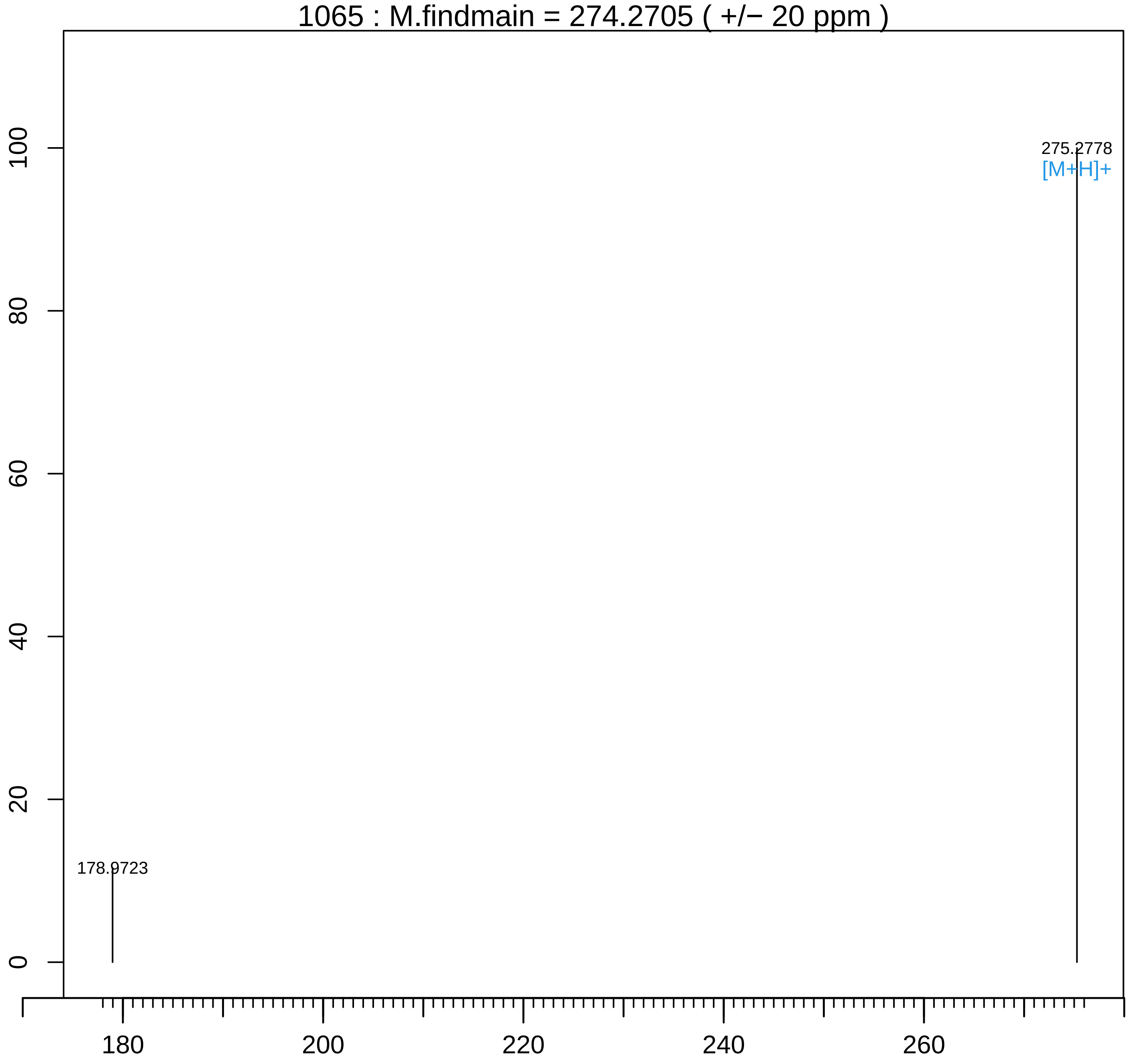

1065 : M.findmain = 274.2705 (+/− 20 ppm)
275.2778
[M+H]+
178.9723
0
20
40
60
80
100
180
200
220
240
260

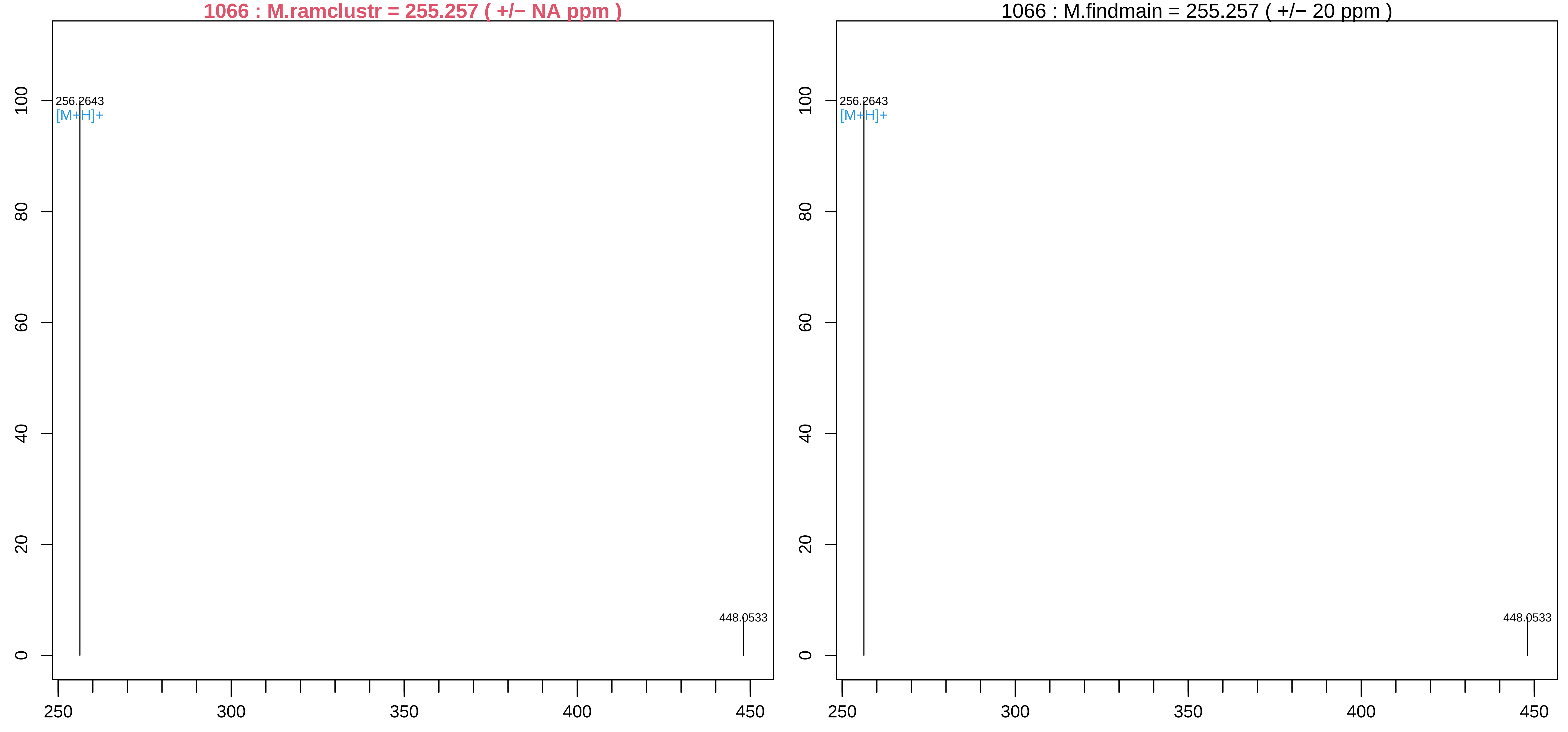
1066 : M.ramclustr = 255.257 (+/− NA ppm)
256.2643
[M+H]+
448.0533
0
20
40
60
80
100
250
300
350
400
450
1066 : M.findmain = 255.257 (+/− 20 ppm)
256.2643
[M+H]+
448.0533
0
20
40
60
80
100
250
300
350
400
450

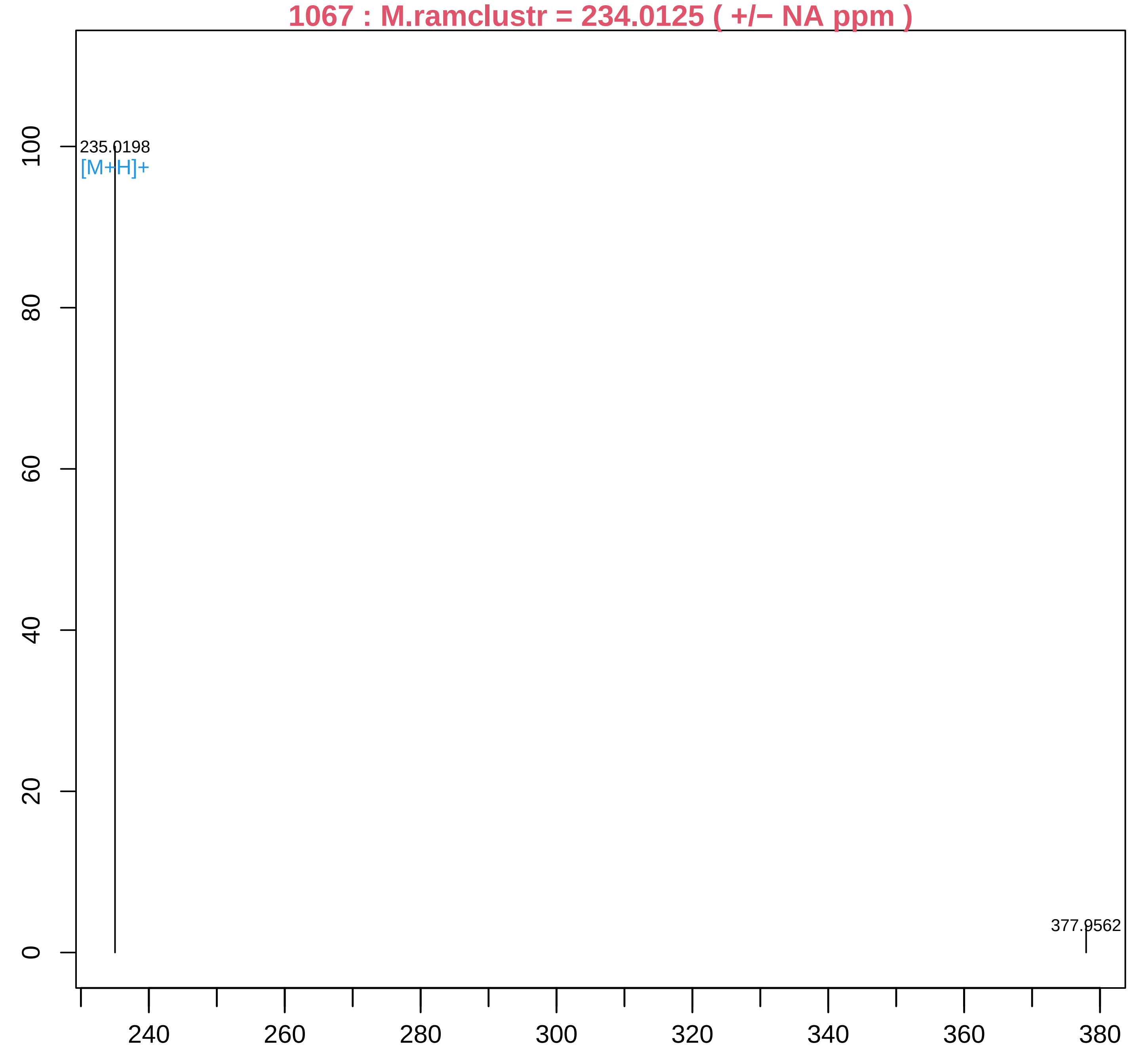

1067 : M.ramclustr = 234.0125 (+/− NA ppm)
235.0198
[M+H]+
377.9562
0
20
40
60
80
100
240
260
280
300
320
340
360
380

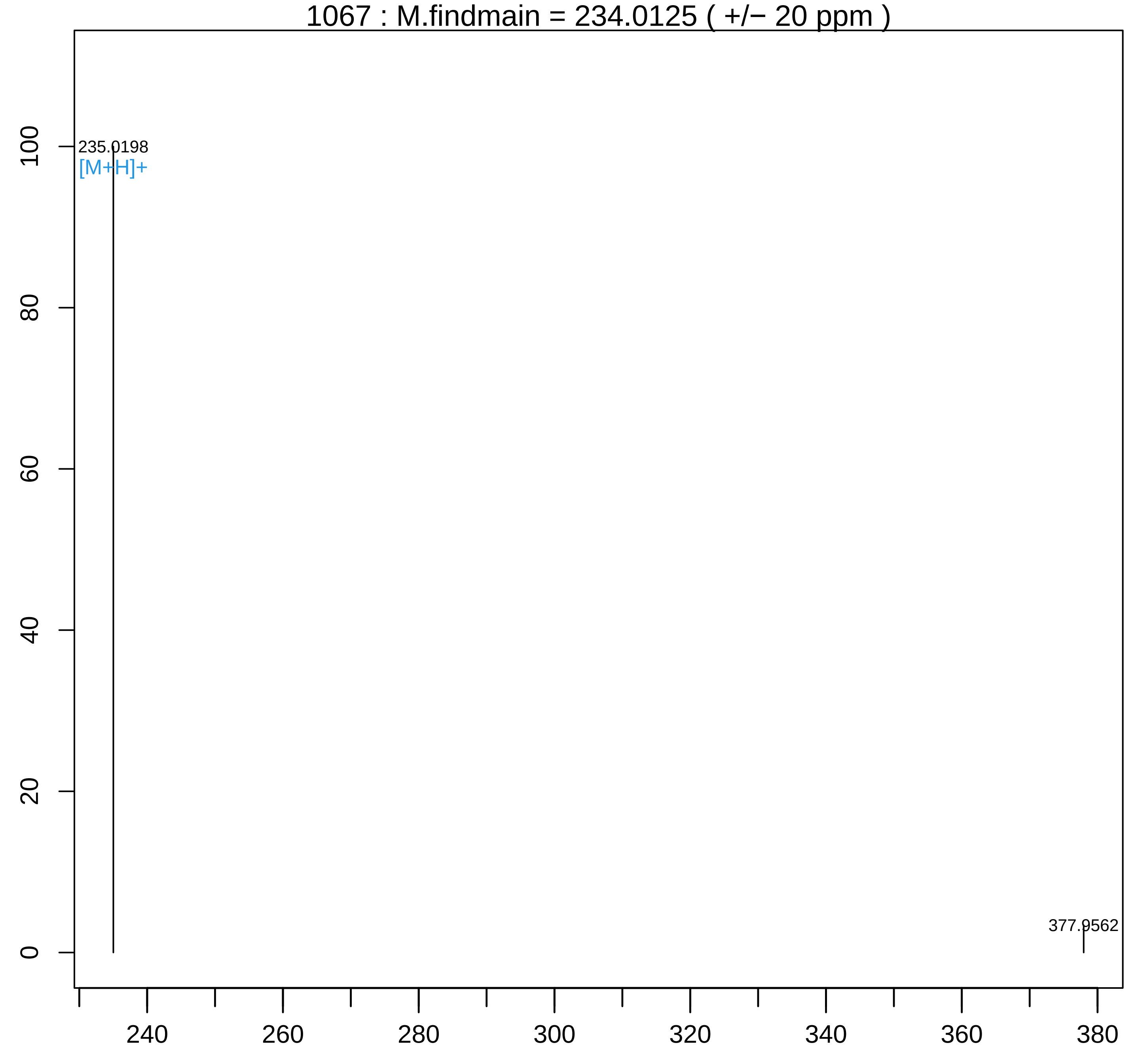

1067 : M.findmain = 234.0125 (+/− 20 ppm)
235.0198
[M+H]+
377.9562
0
20
40
60
80
100
240
260
280
300
320
340
360
380

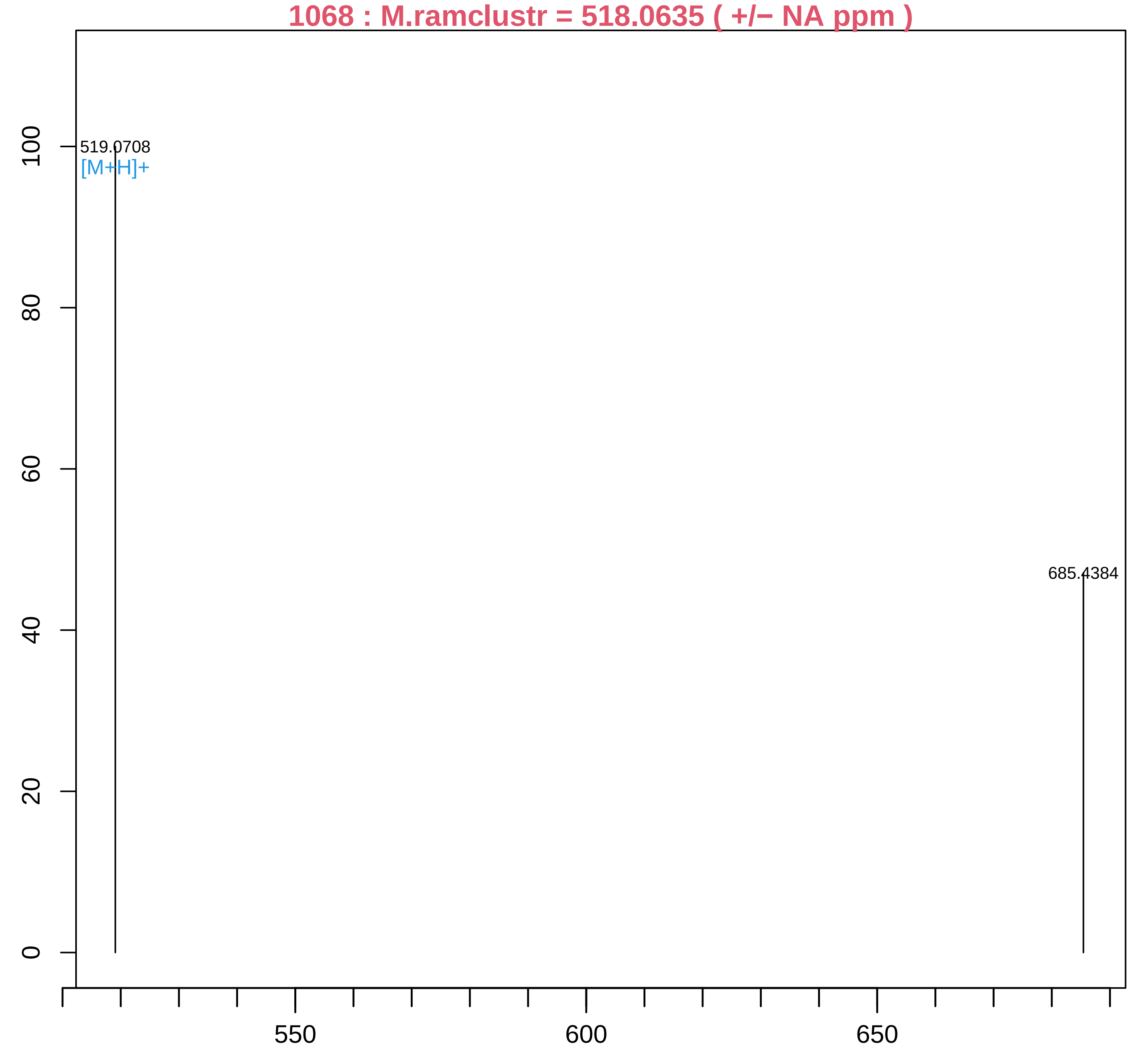
1068 : M.ramclustr = 518.0635 (+/− NA ppm)
519.0708
[M+H]+
685.4384
0
20
40
60
80
100
550
600
650

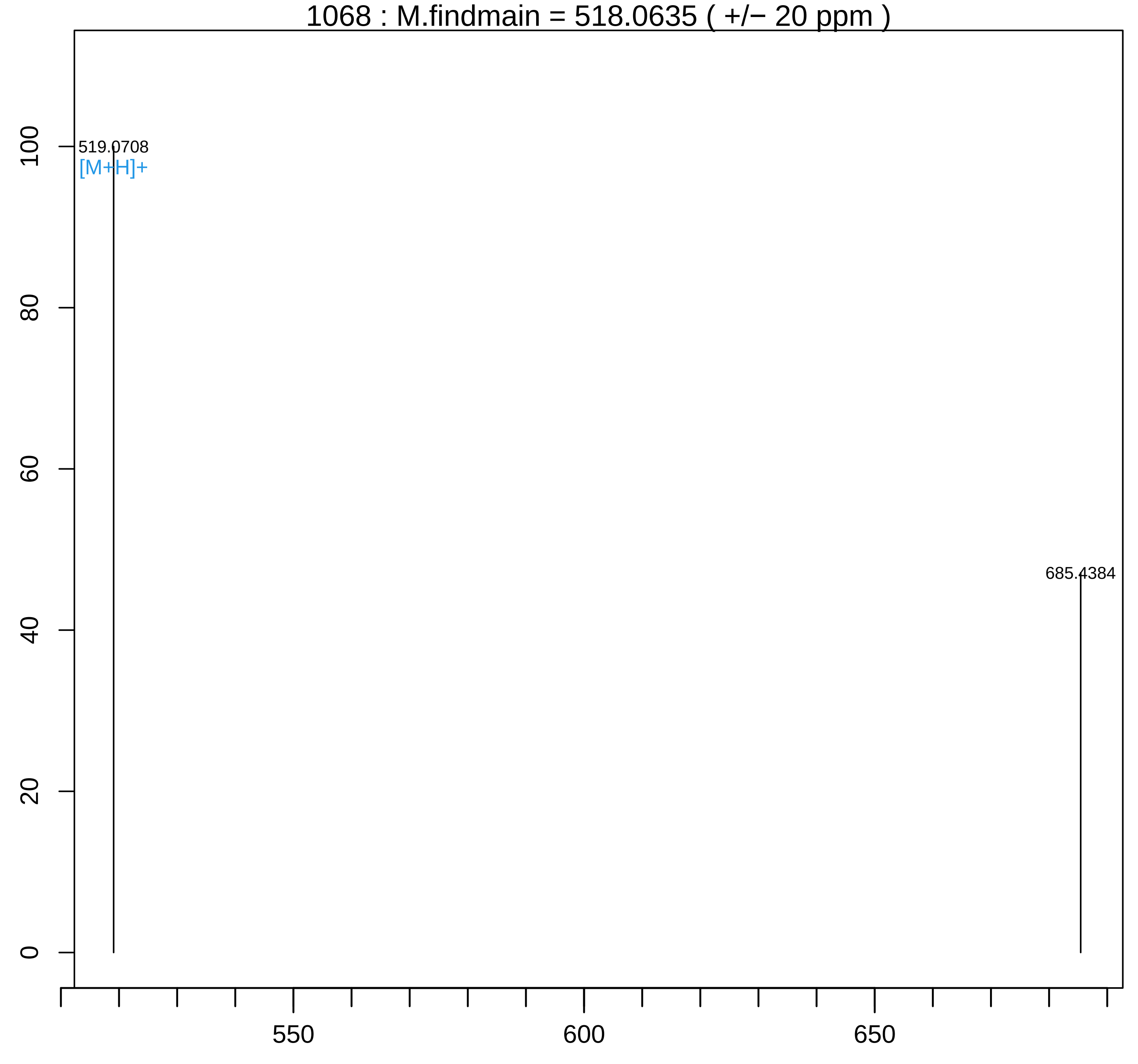
1068 : M.findmain = 518.0635 (+/− 20 ppm)
519.0708
[M+H]+
685.4384
0
20
40
60
80
100
550
600
650

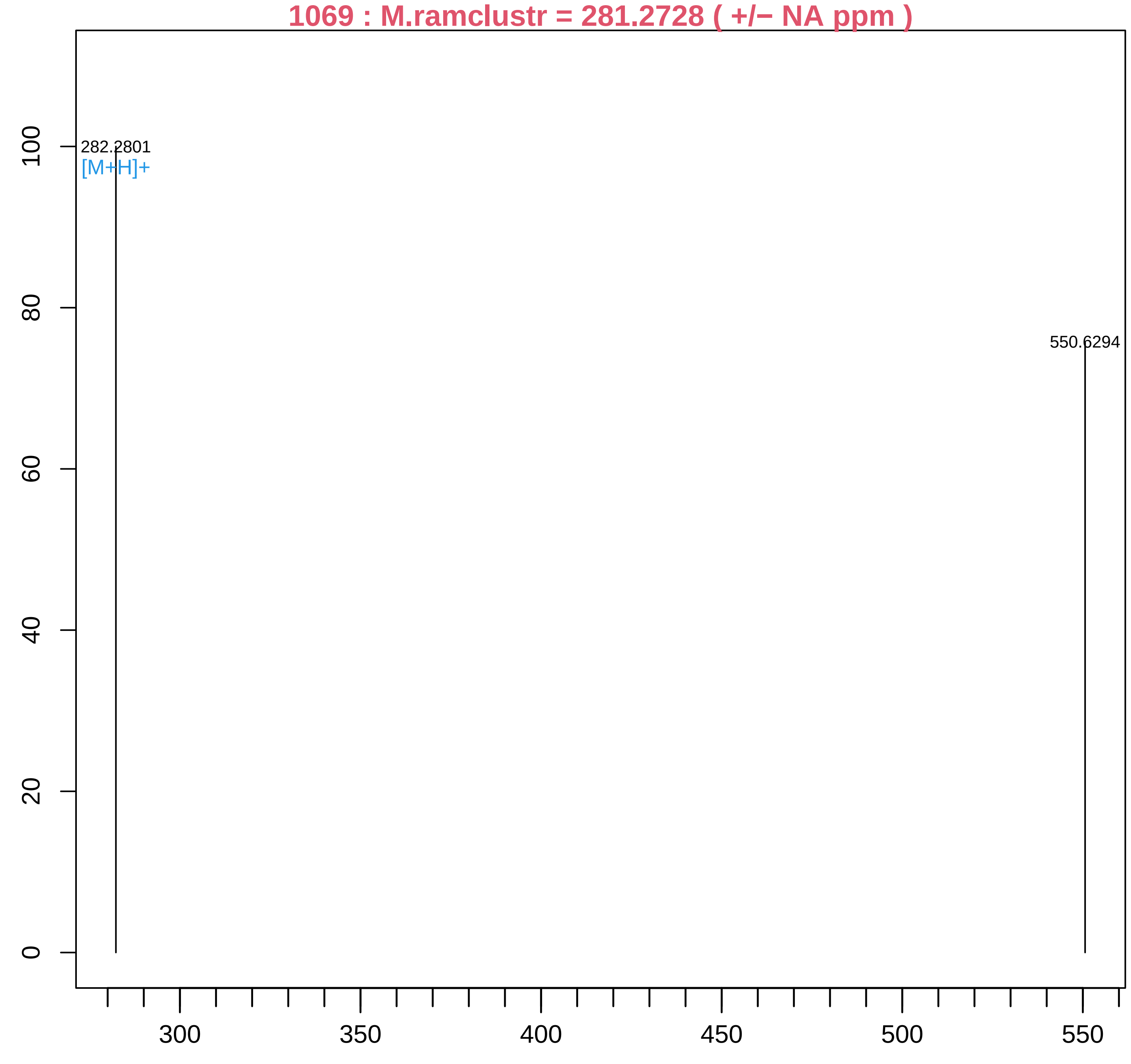

1069 : M.ramclustr = 281.2728 (+/− NA ppm)
282.2801
[M+H]+
550.6294
0
20
40
60
80
100
300
350
400
450
500
550

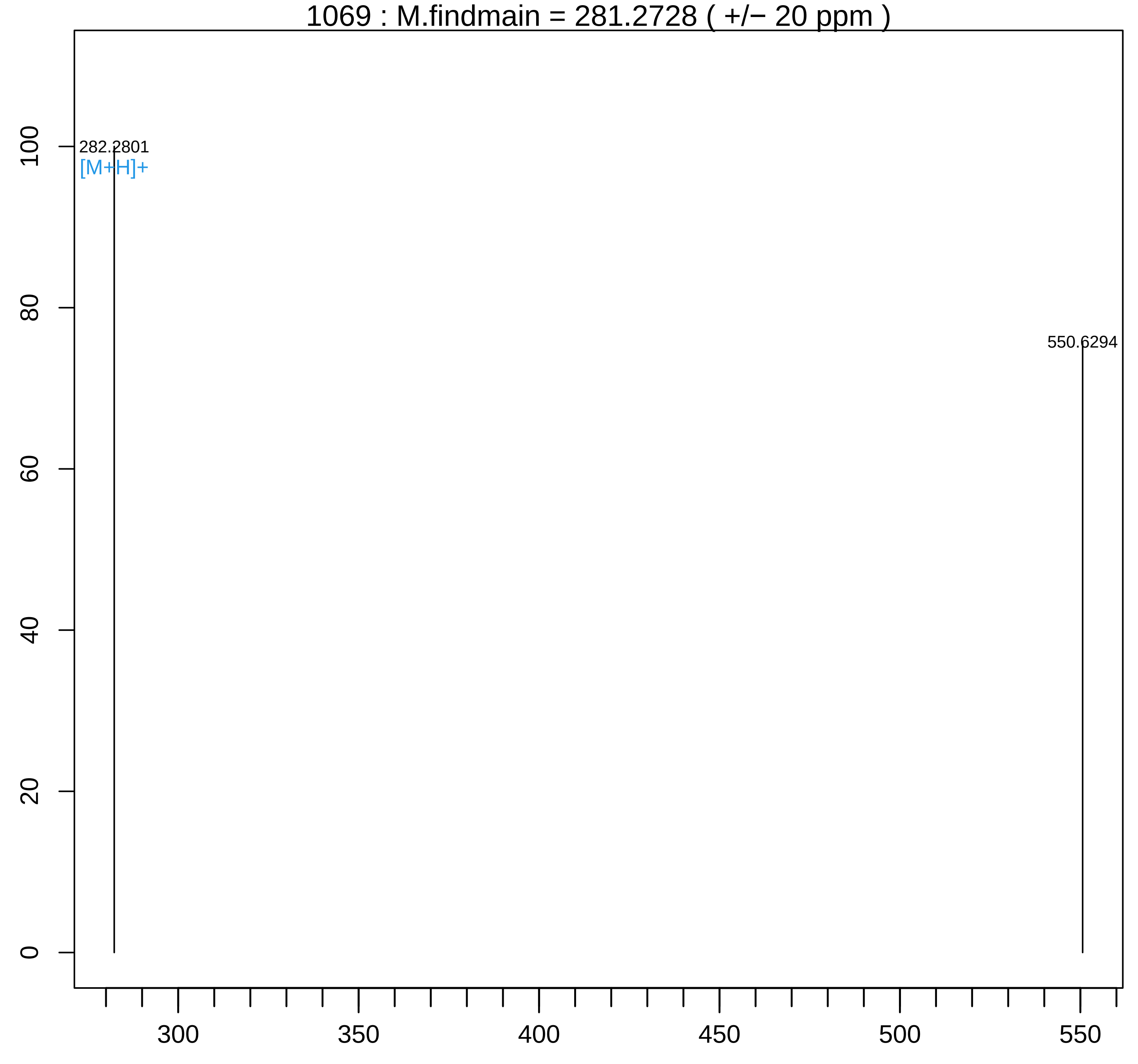

1069 : M.findmain = 281.2728 (+/− 20 ppm)
282.2801
[M+H]+
550.6294
0
20
40
60
80
100
300
350
400
450
500
550

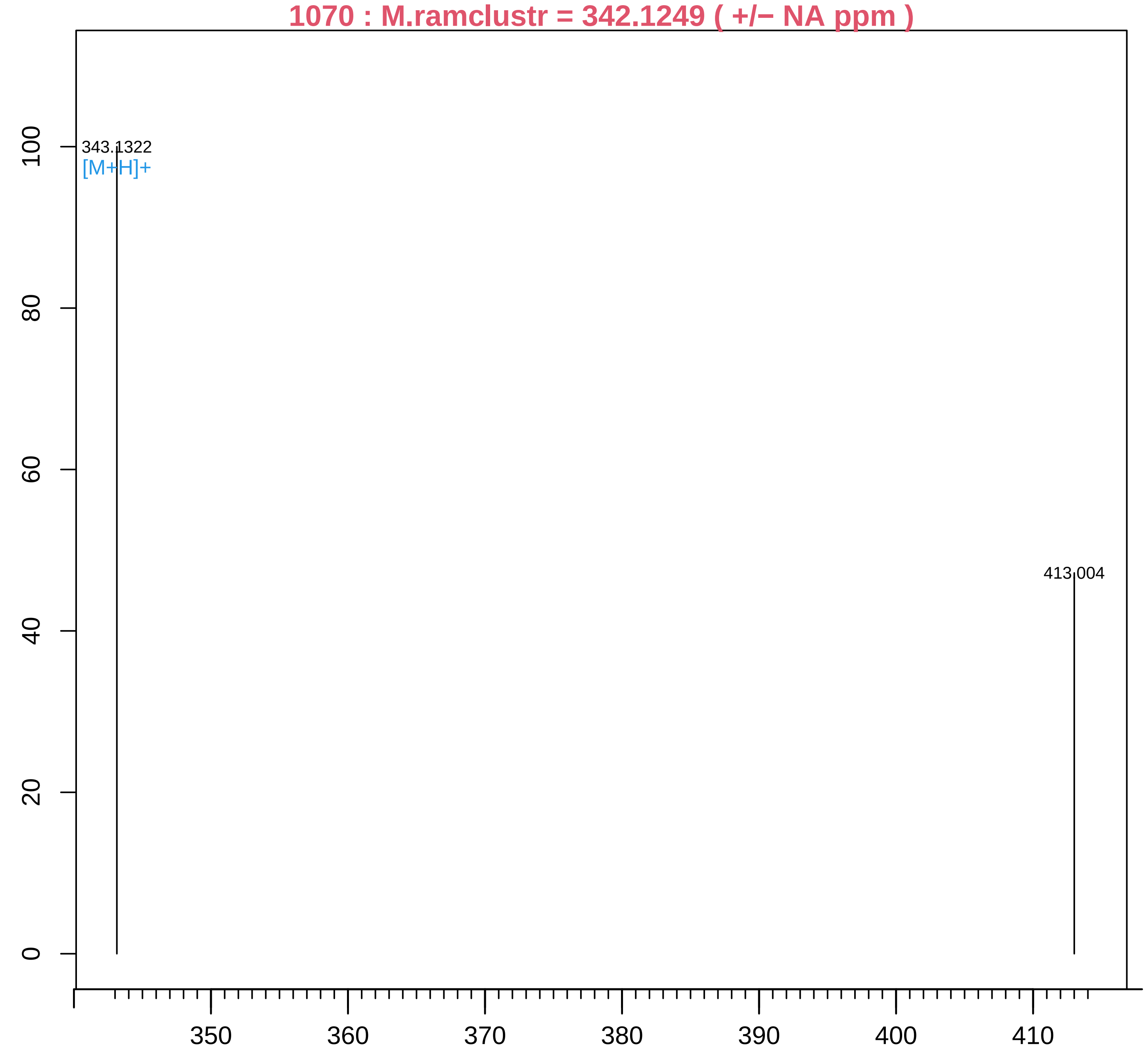
1070 : M.ramclustr = 342.1249 (+/− NA ppm)
343.1322
[M+H]+
413.004
0
20
40
60
80
100
350
360
370
380
390
400
410

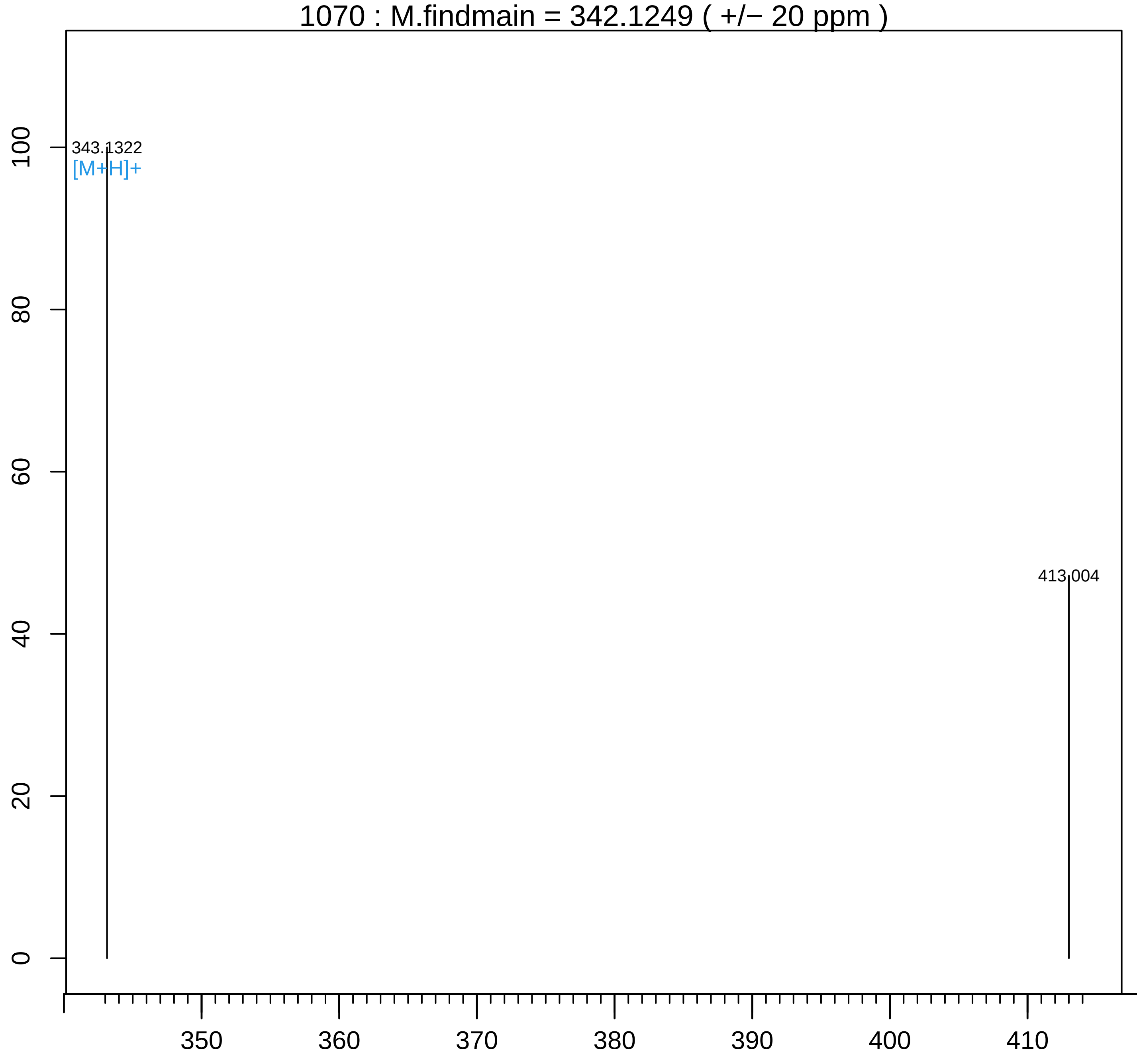
1070 : M.findmain = 342.1249 (+/− 20 ppm)
343.1322
[M+H]+
413.004
0
20
40
60
80
100
350
360
370
380
390
400
410

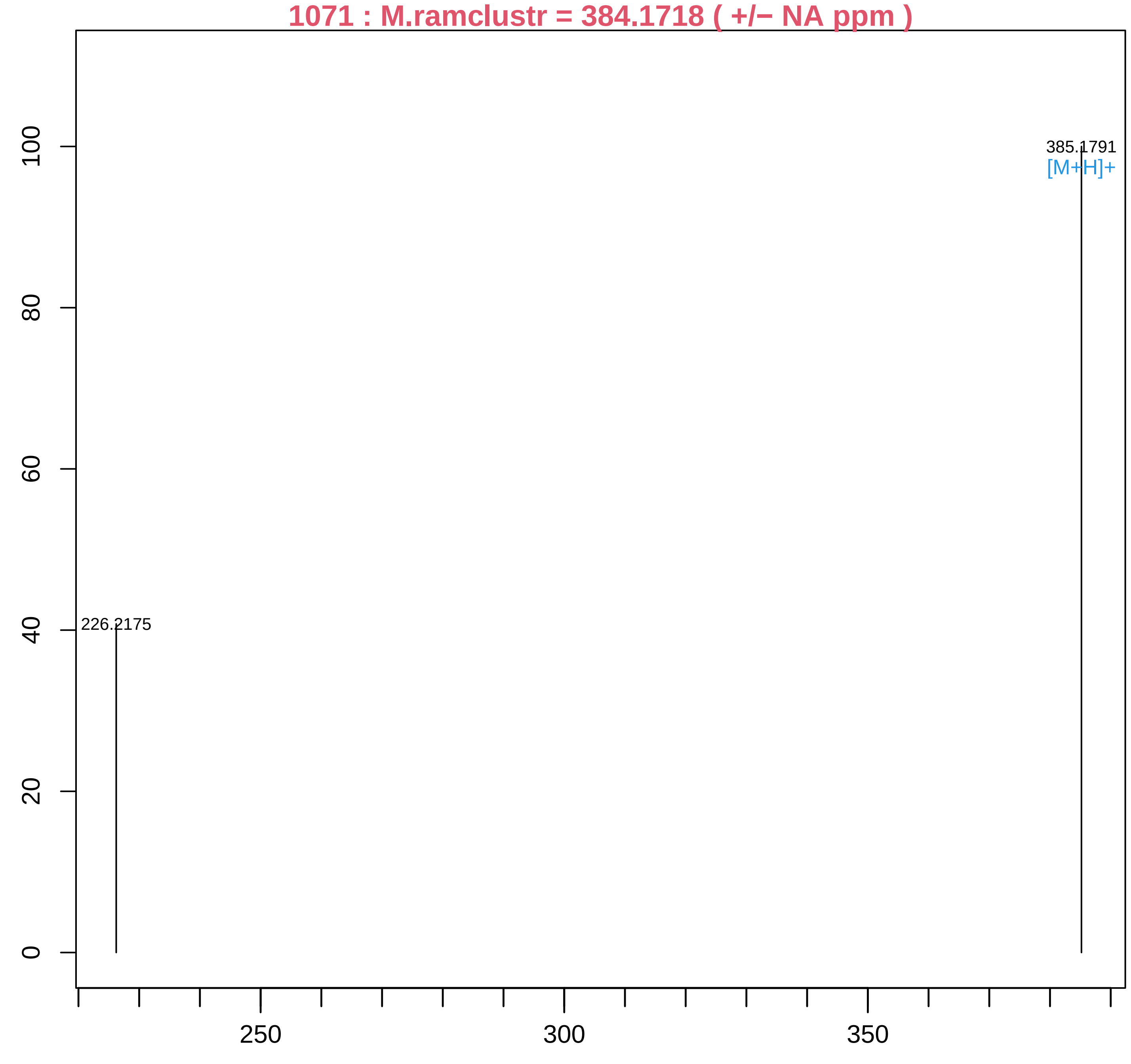
1071 : M.ramclustr = 384.1718 (+/− NA ppm)
385.1791
[M+H]+
226.2175
0
20
40
60
80
100
250
300
350

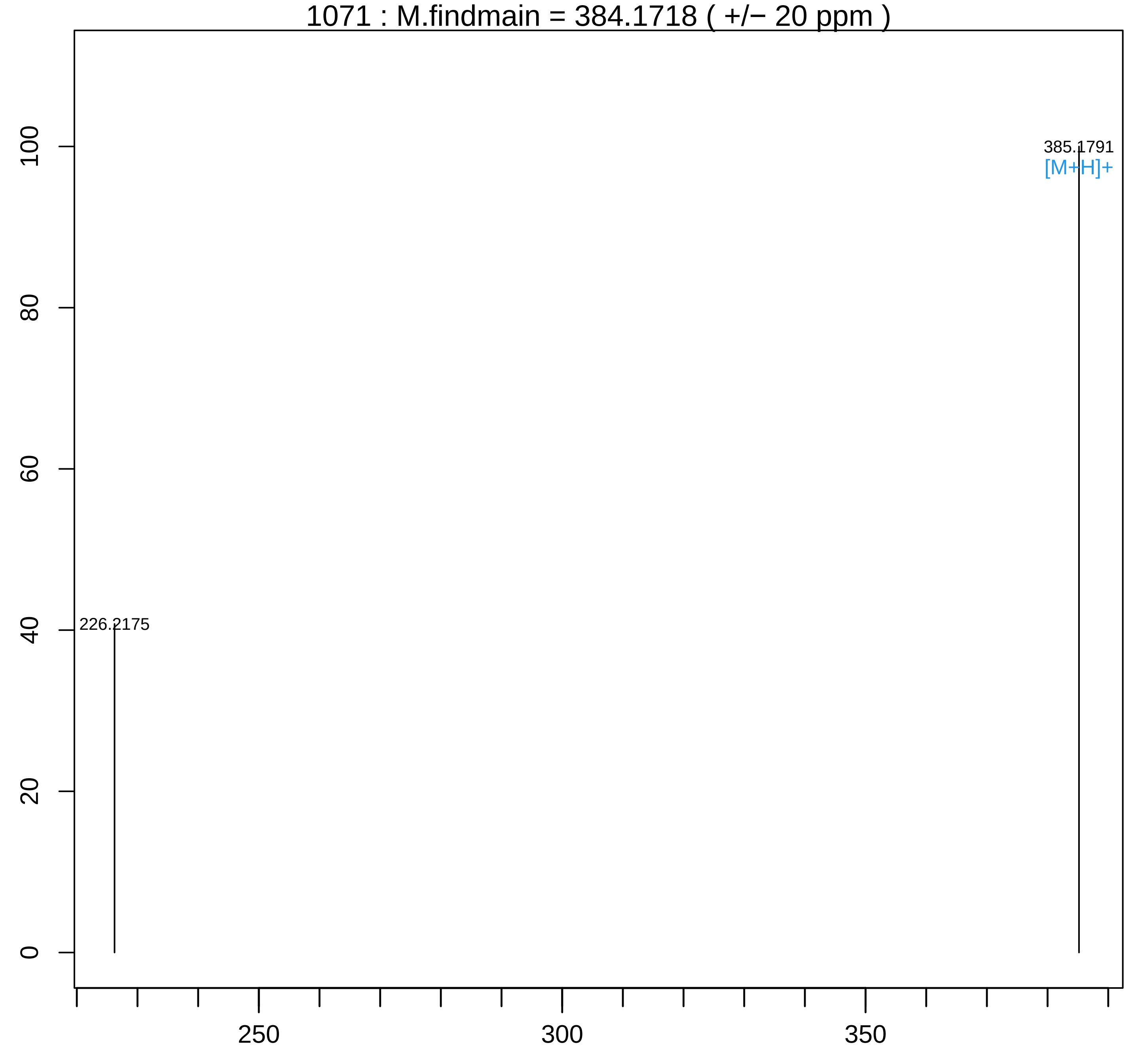
1071 : M.findmain = 384.1718 (+/− 20 ppm)
385.1791
[M+H]+
226.2175
0
20
40
60
80
100
250
300
350

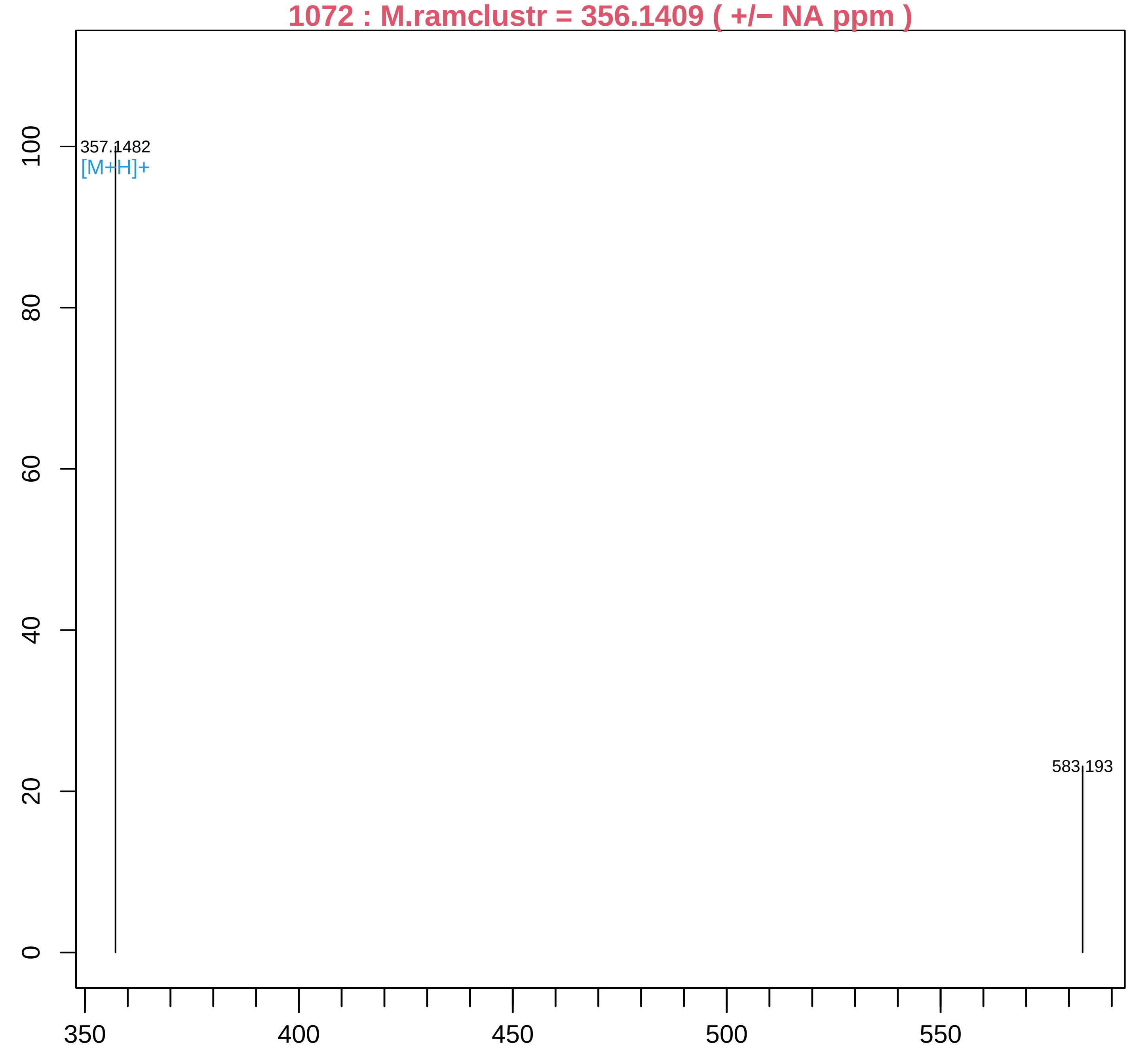
1072 : M.ramclustr = 356.1409 (+/− NA ppm)
357.1482
[M+H]+
583.193
0
20
40
60
80
100
350
400
450
500
550

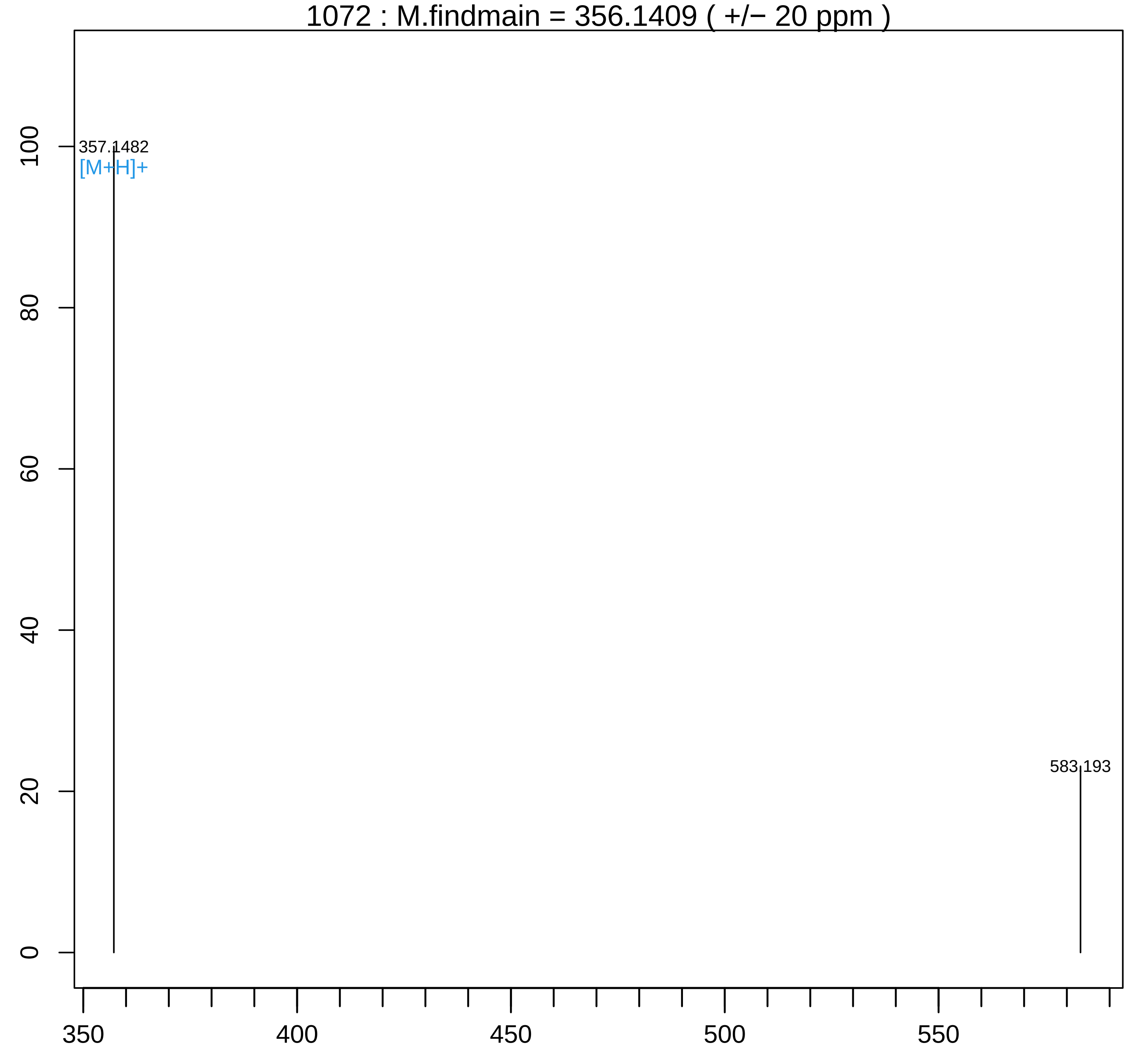
1072 : M.findmain = 356.1409 (+/− 20 ppm)
357.1482
[M+H]+
583.193
0
20
40
60
80
100
350
400
450
500
550

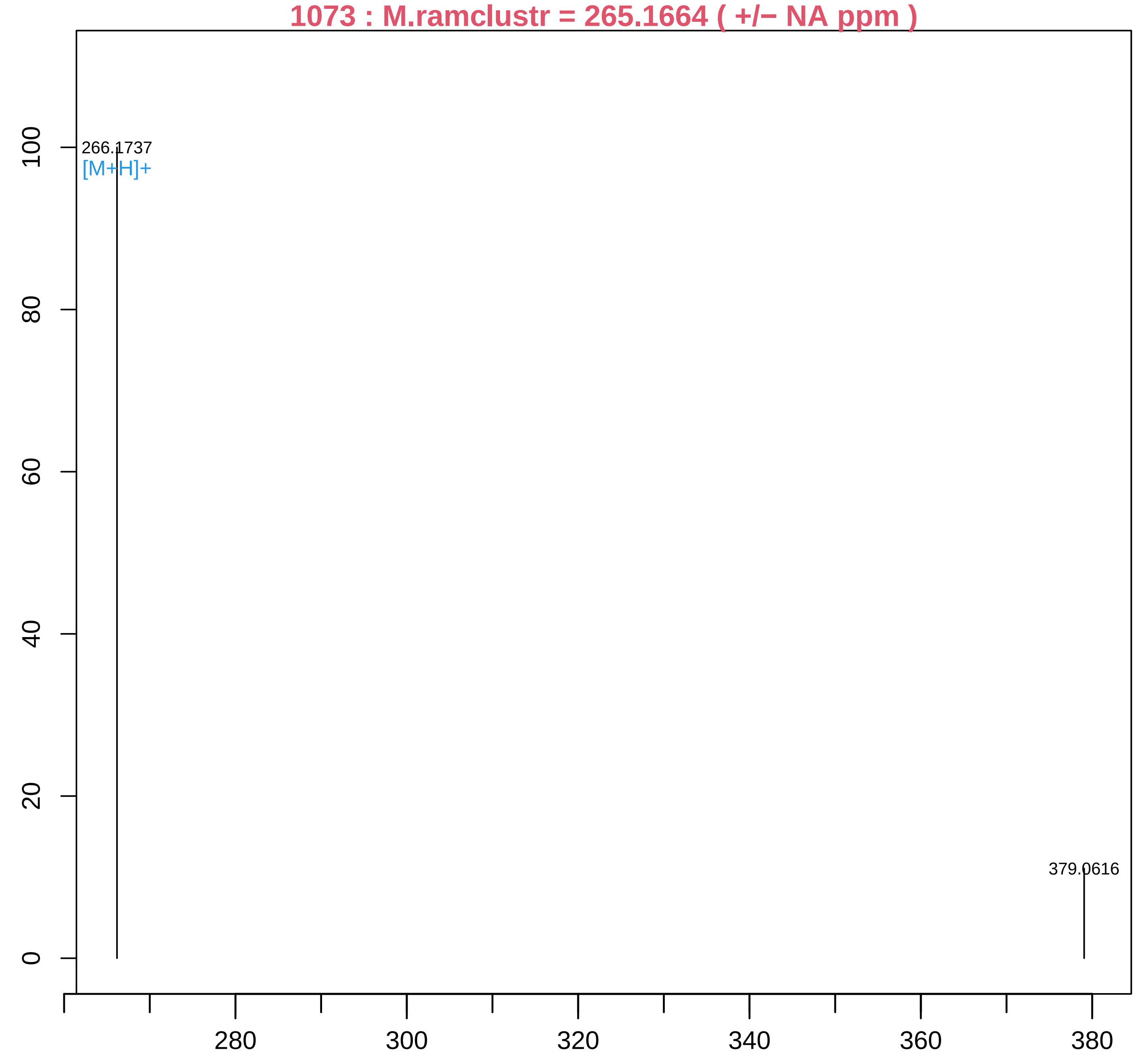
1073 : M.ramclustr = 265.1664 (+/− NA ppm)
266.1737
[M+H]+
379.0616
0
20
40
60
80
100
280
300
320
340
360
380

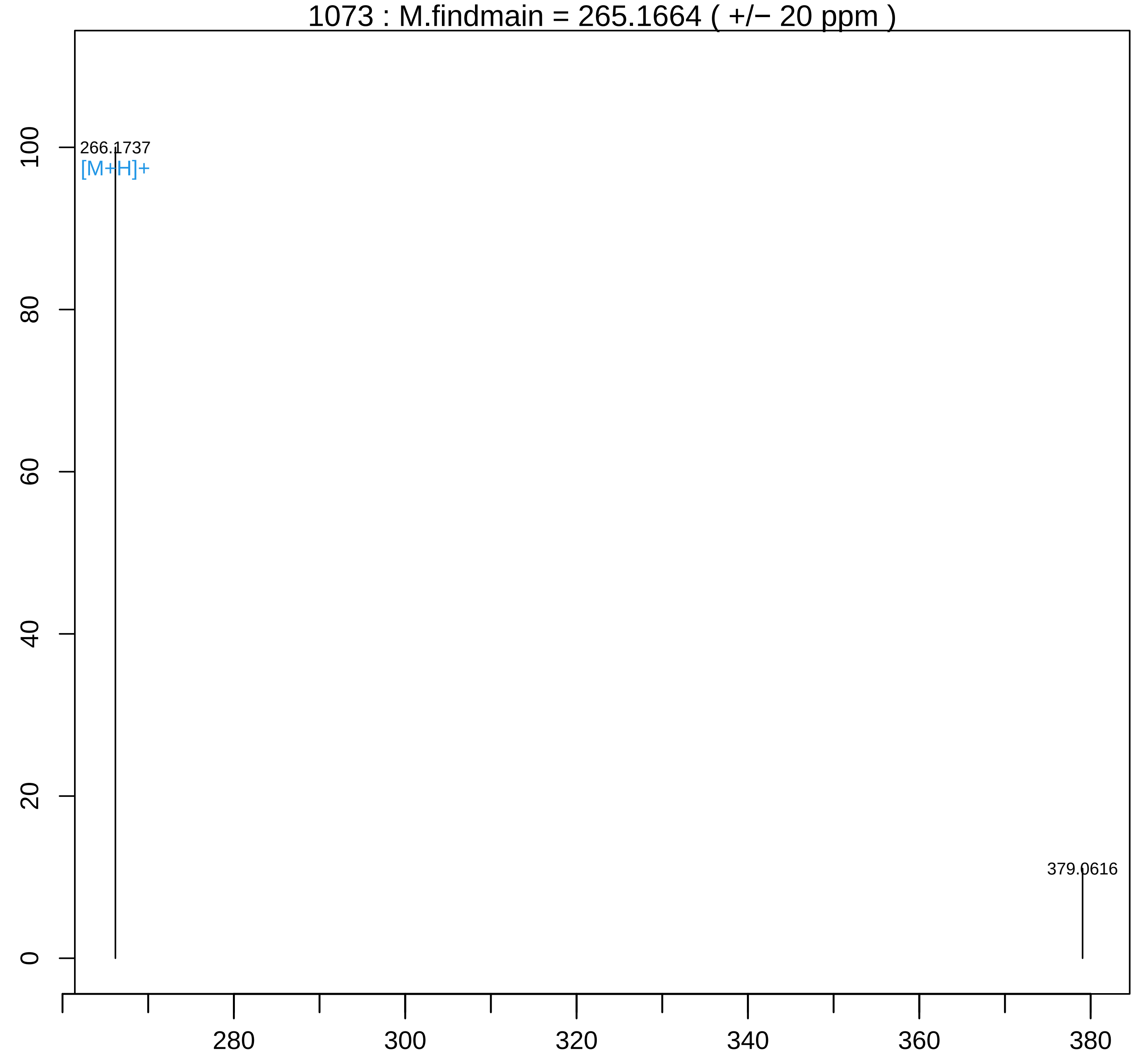
1073 : M.findmain = 265.1664 (+/− 20 ppm)
266.1737
[M+H]+
379.0616
0
20
40
60
80
100
280
300
320
340
360
380

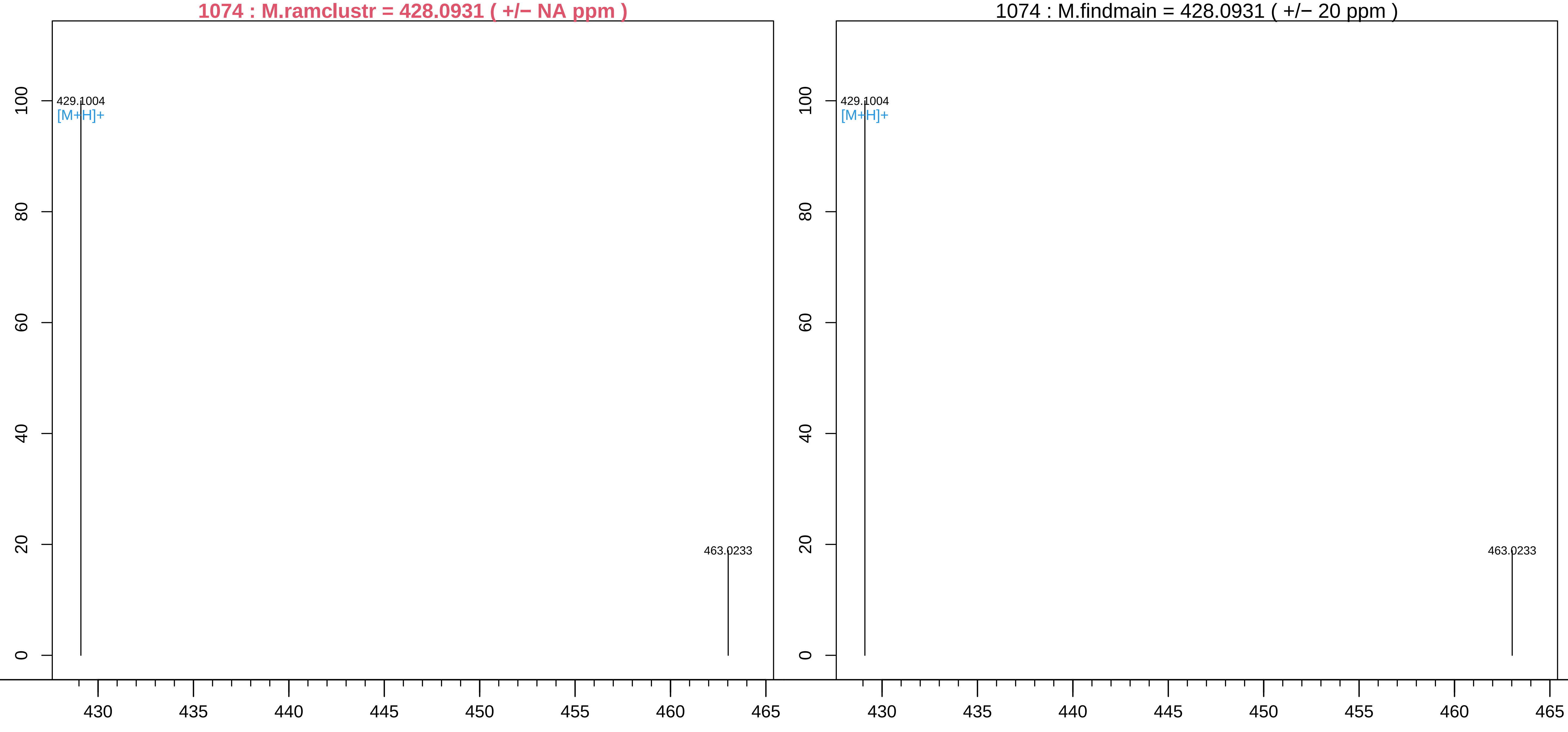

1074 : M.ramclustr = 428.0931 (+/− NA ppm)
429.1004
[M+H]+
463.0233
1074 : M.findmain = 428.0931 (+/− 20 ppm)
429.1004
[M+H]+
463.0233

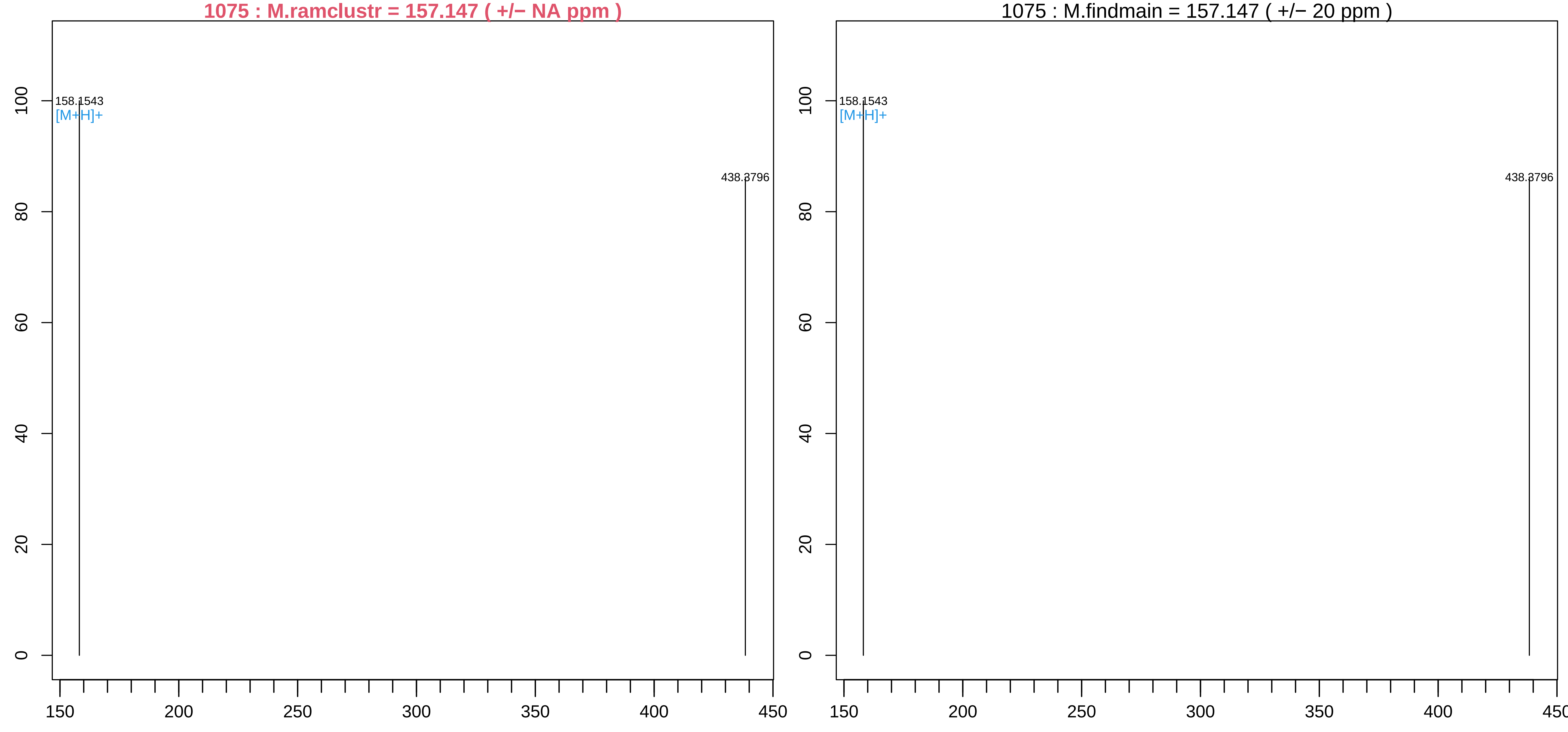
1075 : M.ramclustr = 157.147 (+/− NA ppm)
158.1543
[M+H]+
438.3796
1075 : M.findmain = 157.147 (+/− 20 ppm)
158.1543
[M+H]+
438.3796
0
20
40
60
80
100
150
200
250
300
350
400
450

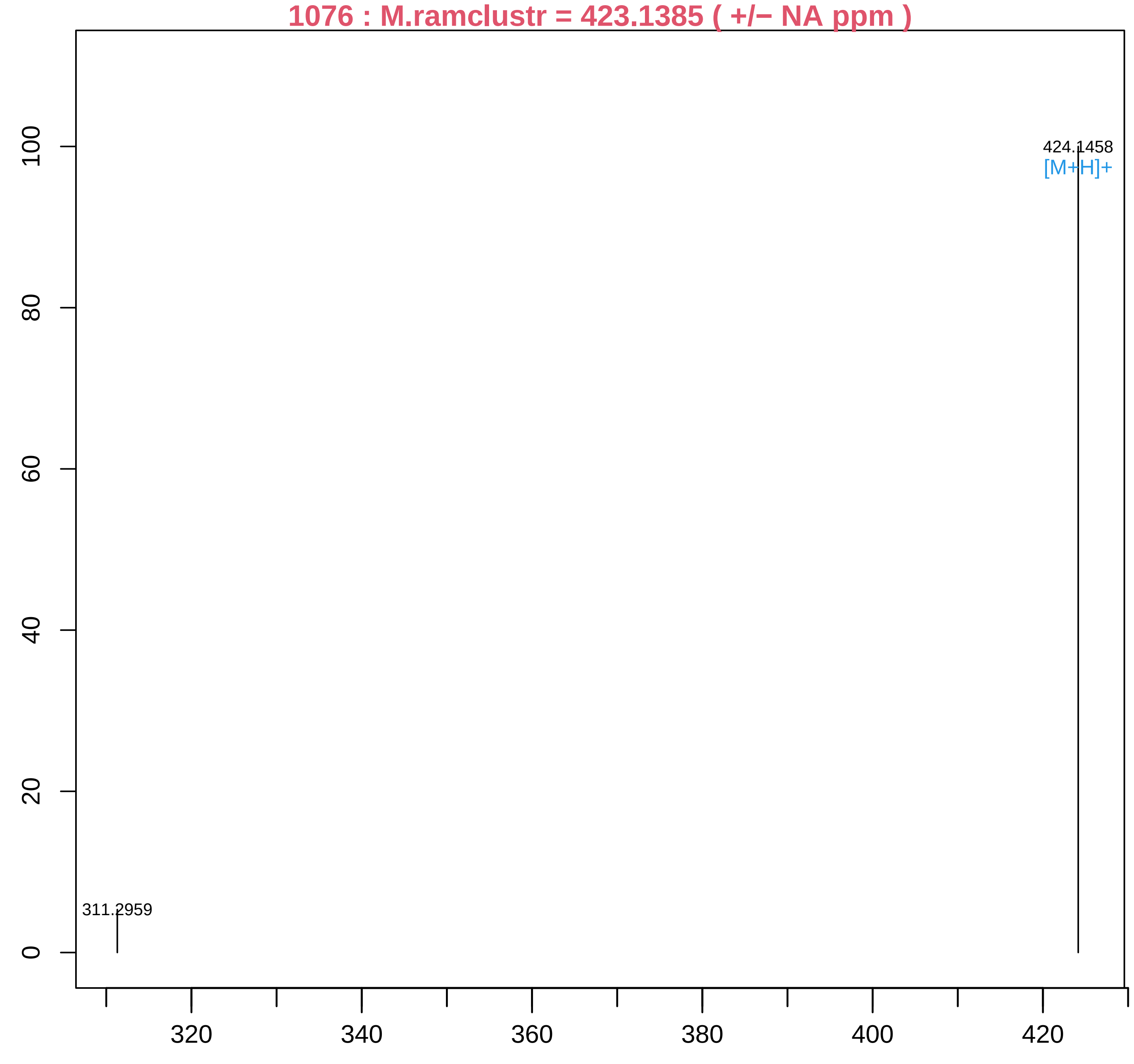

1076 : M.ramclustr = 423.1385 (+/− NA ppm)
424.1458
[M+H]+
311.2959
0
20
40
60
80
100
320
340
360
380
400
420

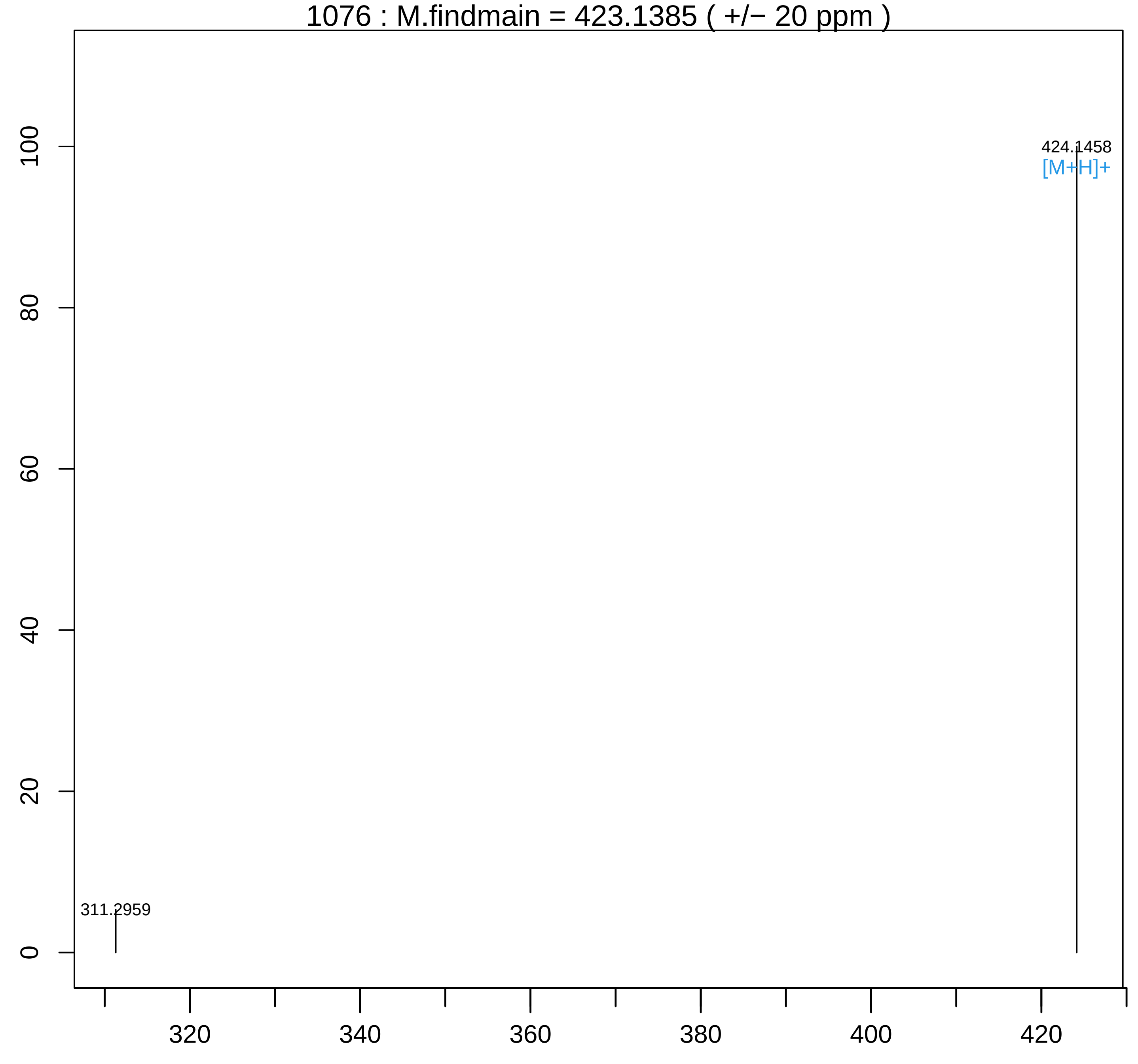

1076 : M.findmain = 423.1385 (+/− 20 ppm)
424.1458
[M+H]+
311.2959
0
20
40
60
80
100
320
340
360
380
400
420

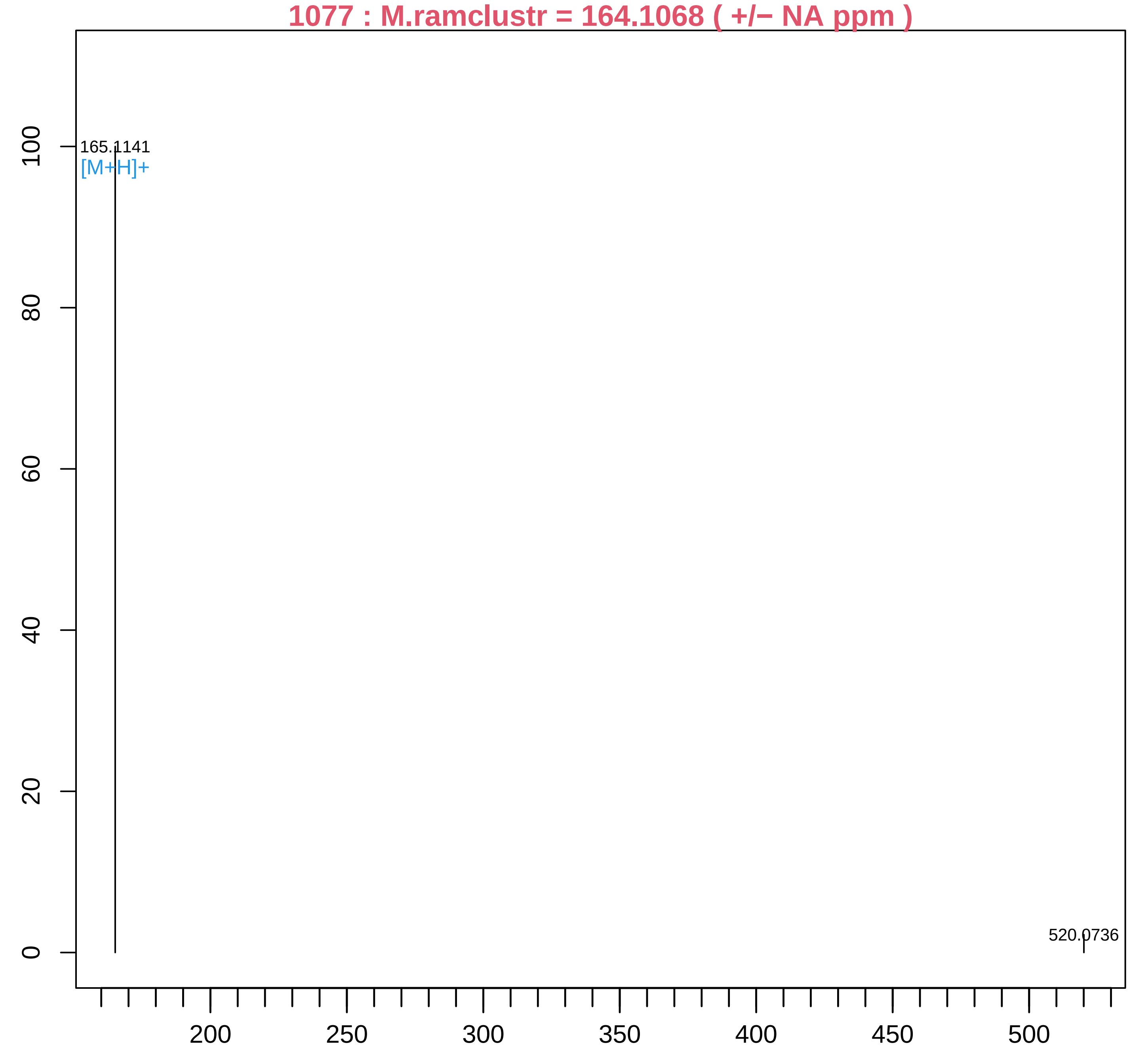
1077 : M.ramclustr = 164.1068 (+/− NA ppm)
165.1141
[M+H]+
520.0736
0
20
40
60
80
100
200
250
300
350
400
450
500

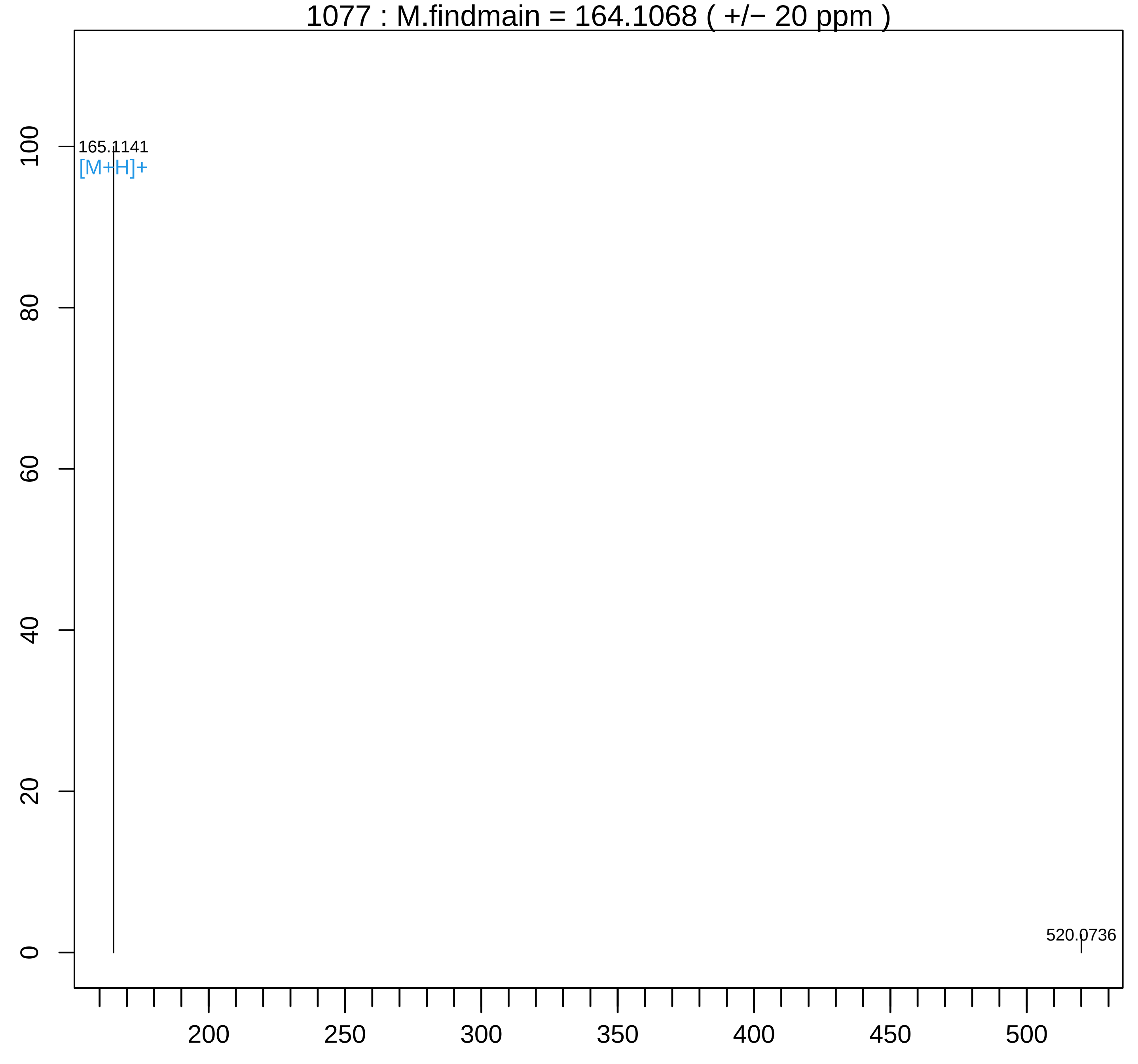
1077 : M.findmain = 164.1068 (+/− 20 ppm)
165.1141
[M+H]+
520.0736
0
20
40
60
80
100
200
250
300
350
400
450
500

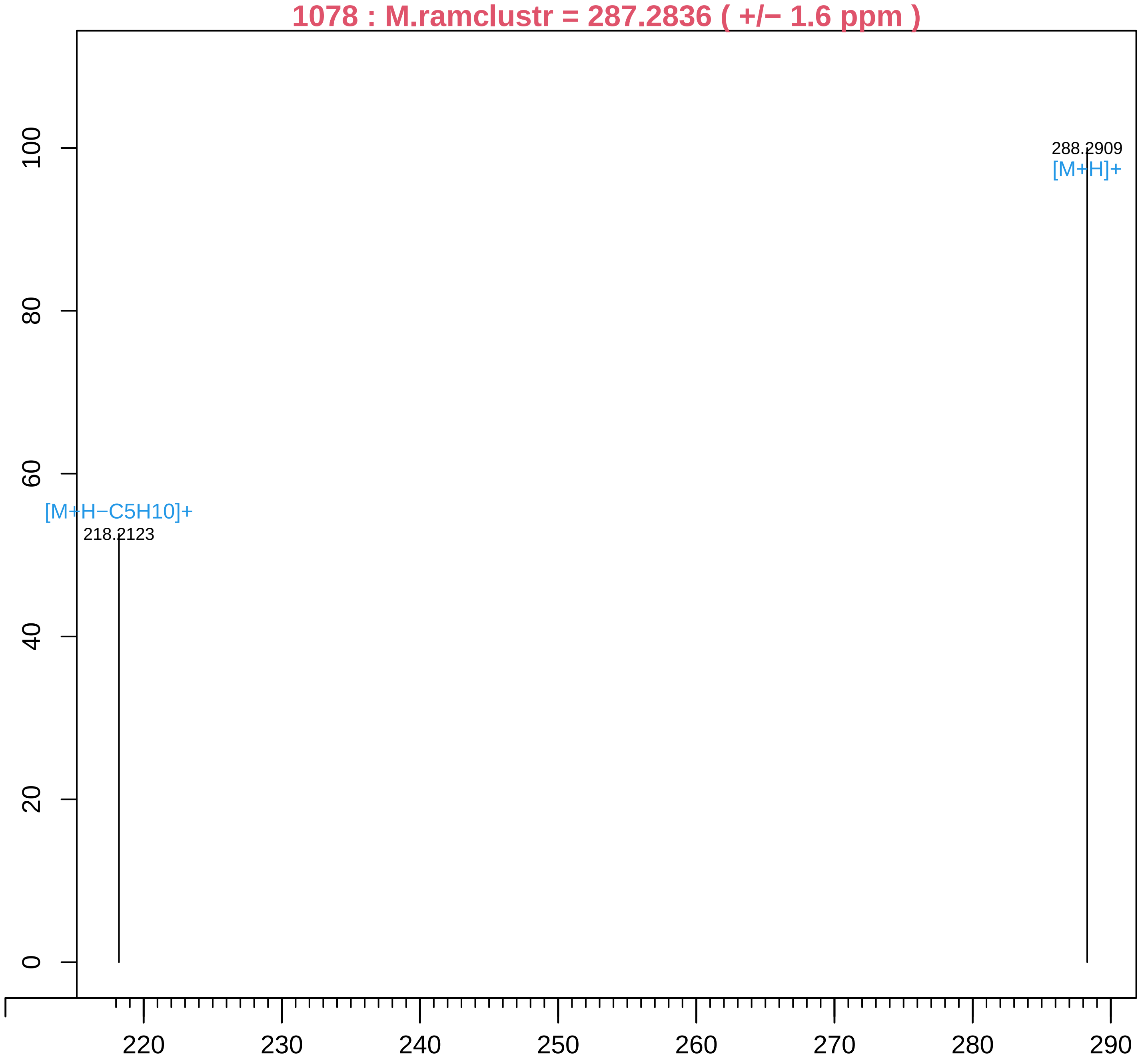

1078 : M.ramclustr = 287.2836 (+/− 1.6 ppm)
288.2909
[M+H]+
[M+H−C5H10]+
218.2123
0
20
40
60
80
100
220
230
240
250
260
270
280
290

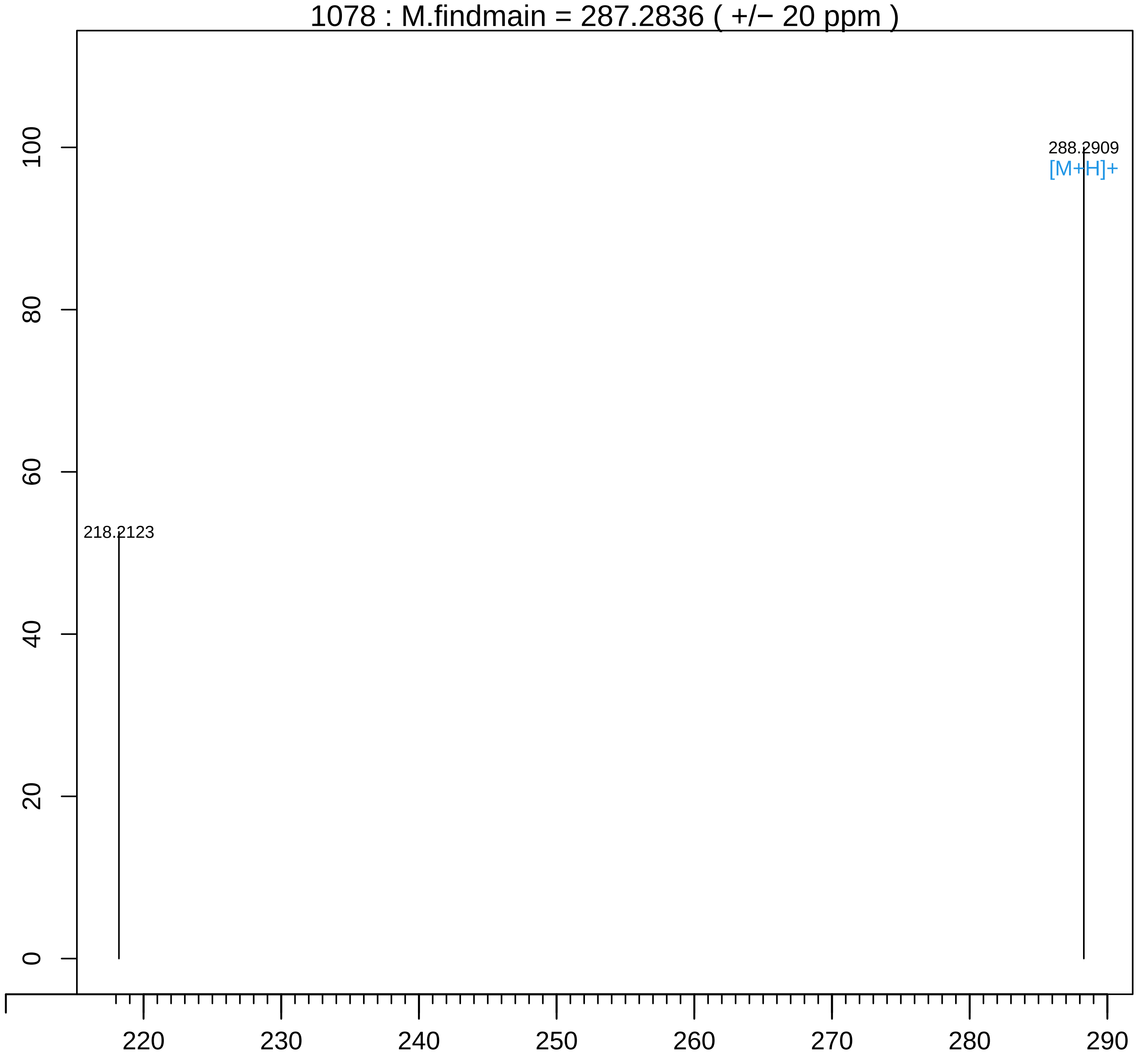

1078 : M.findmain = 287.2836 (+/− 20 ppm)
288.2909
[M+H]+
218.2123
0
20
40
60
80
100
220
230
240
250
260
270
280
290

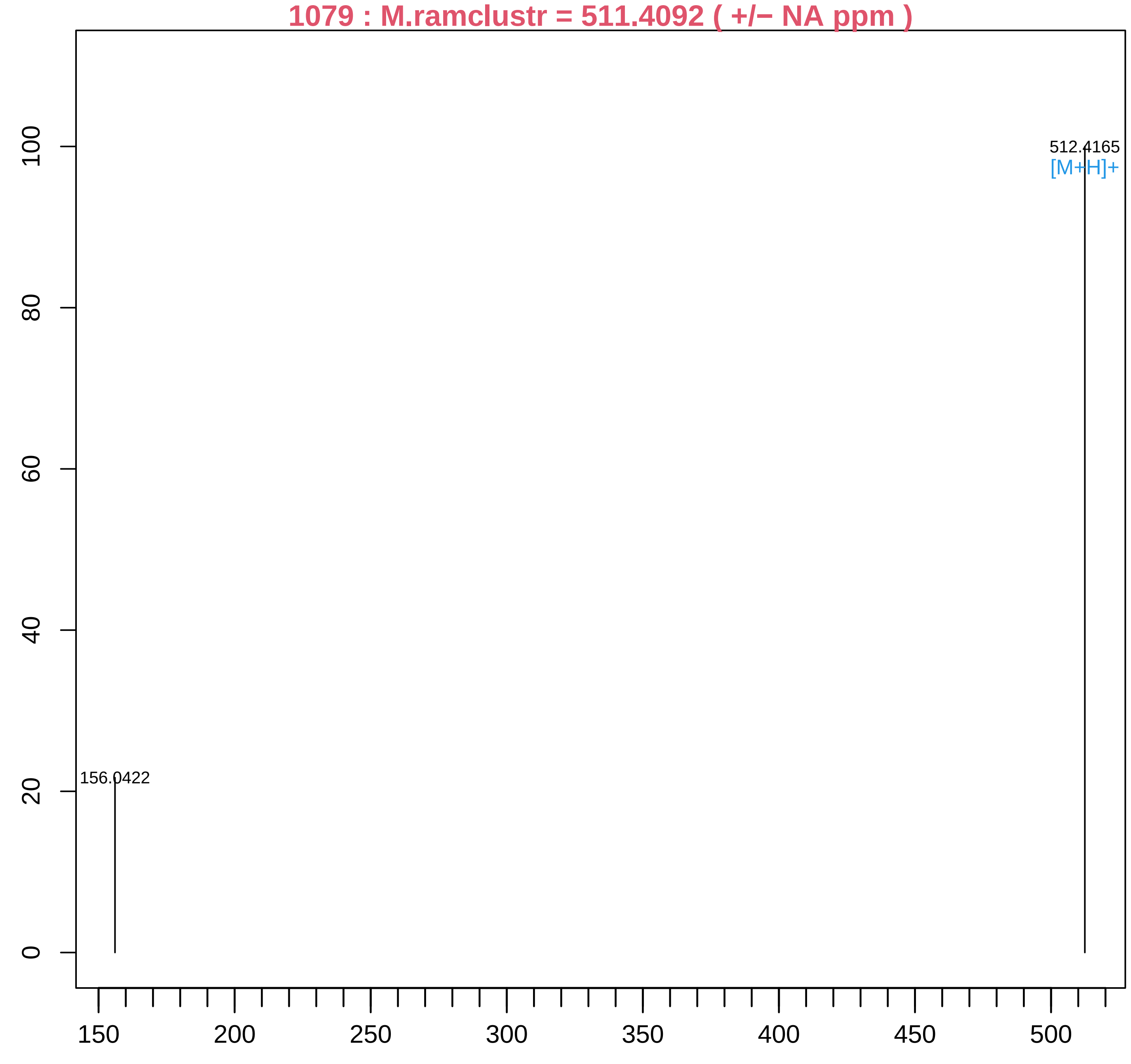

1079 : M.ramclustr = 511.4092 (+/− NA ppm)
512.4165
[M+H]+
156.0422
0
20
40
60
80
100
150
200
250
300
350
400
450
500

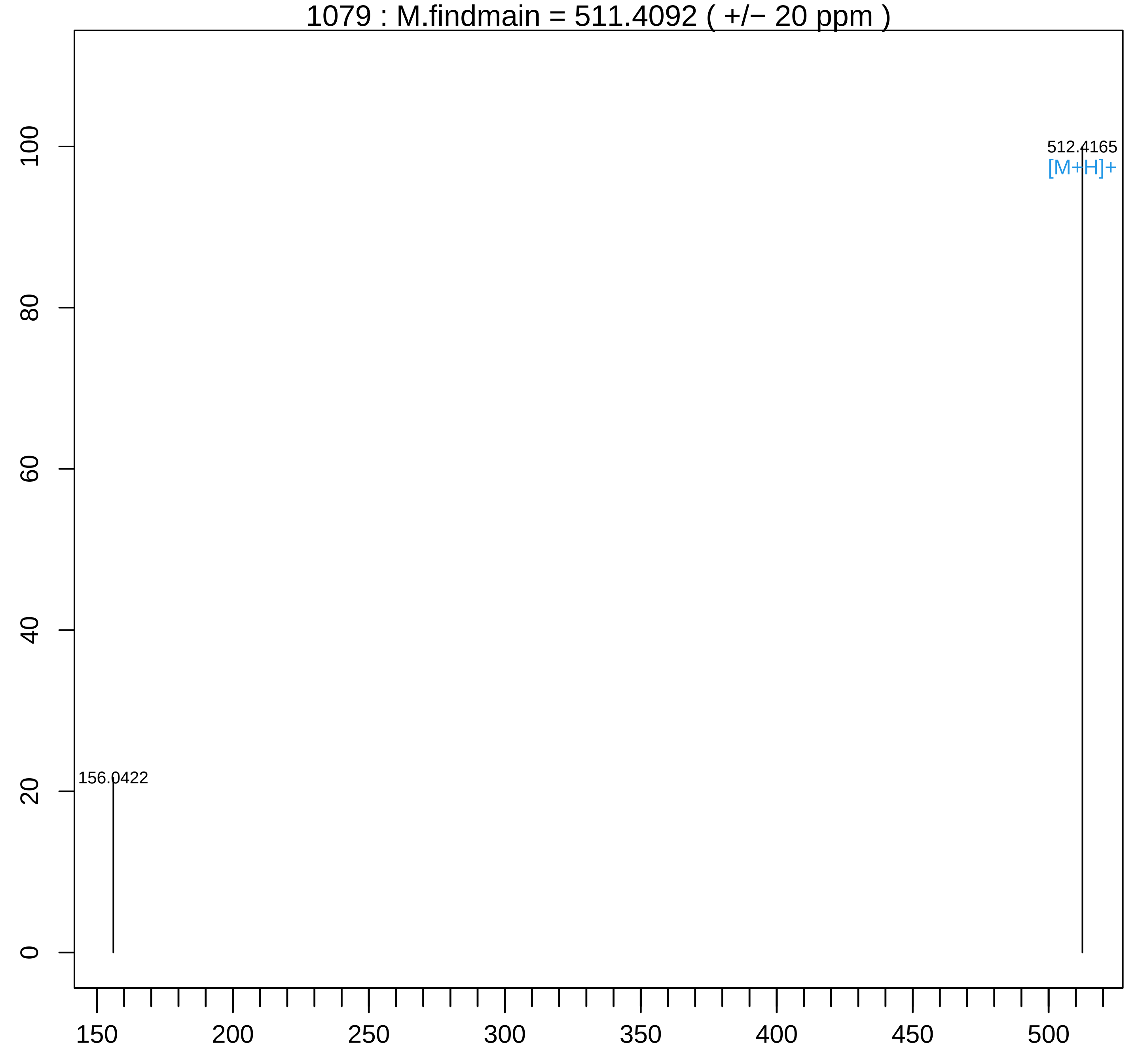

1079 : M.findmain = 511.4092 (+/− 20 ppm)
512.4165
[M+H]+
156.0422
0
20
40
60
80
100
150
200
250
300
350
400
450
500

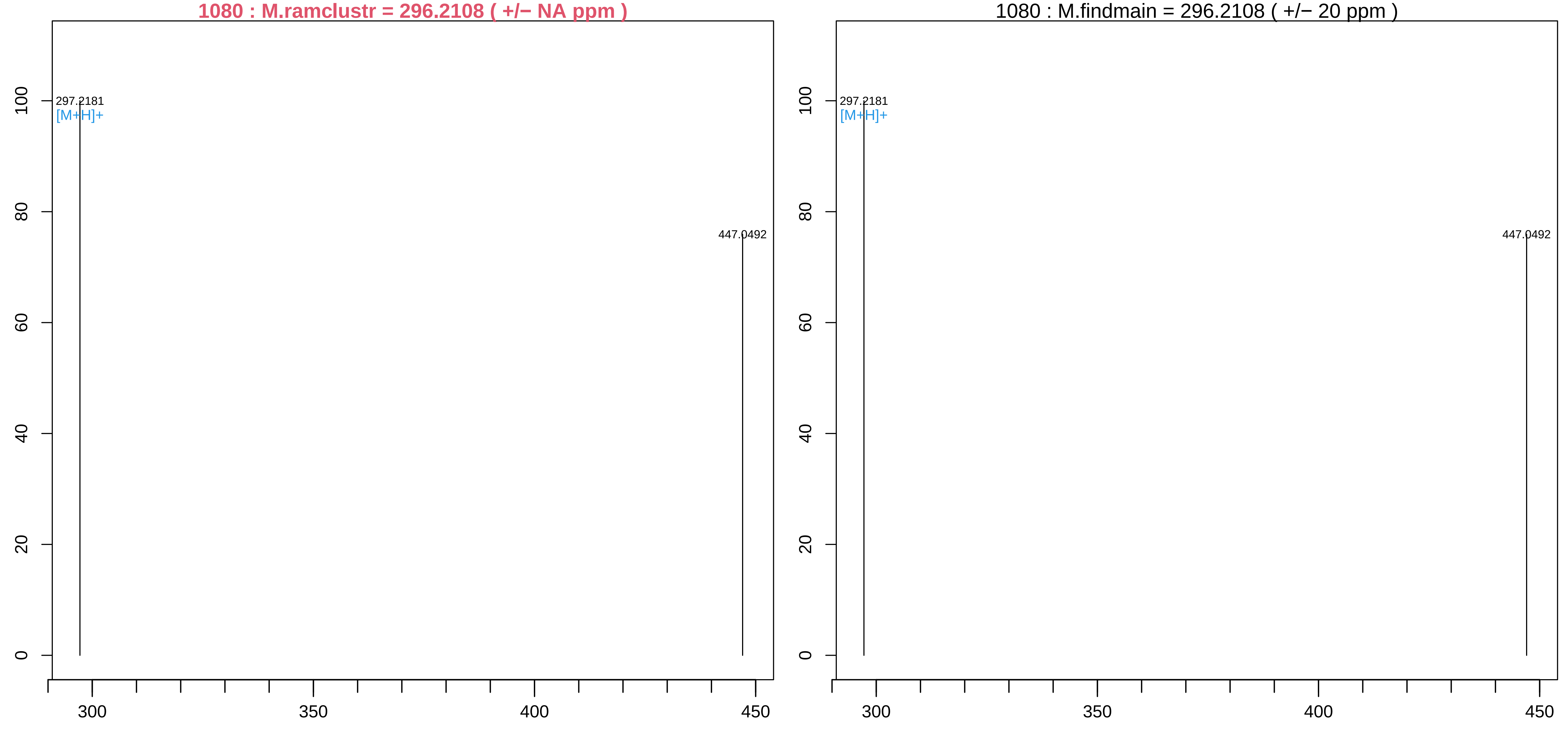
1080 : M.ramclustr = 296.2108 (+/− NA ppm)
297.2181
[M+H]+
447.0492
1080 : M.findmain = 296.2108 (+/− 20 ppm)
297.2181
[M+H]+
447.0492
0
20
40
60
80
100
300
350
400
450

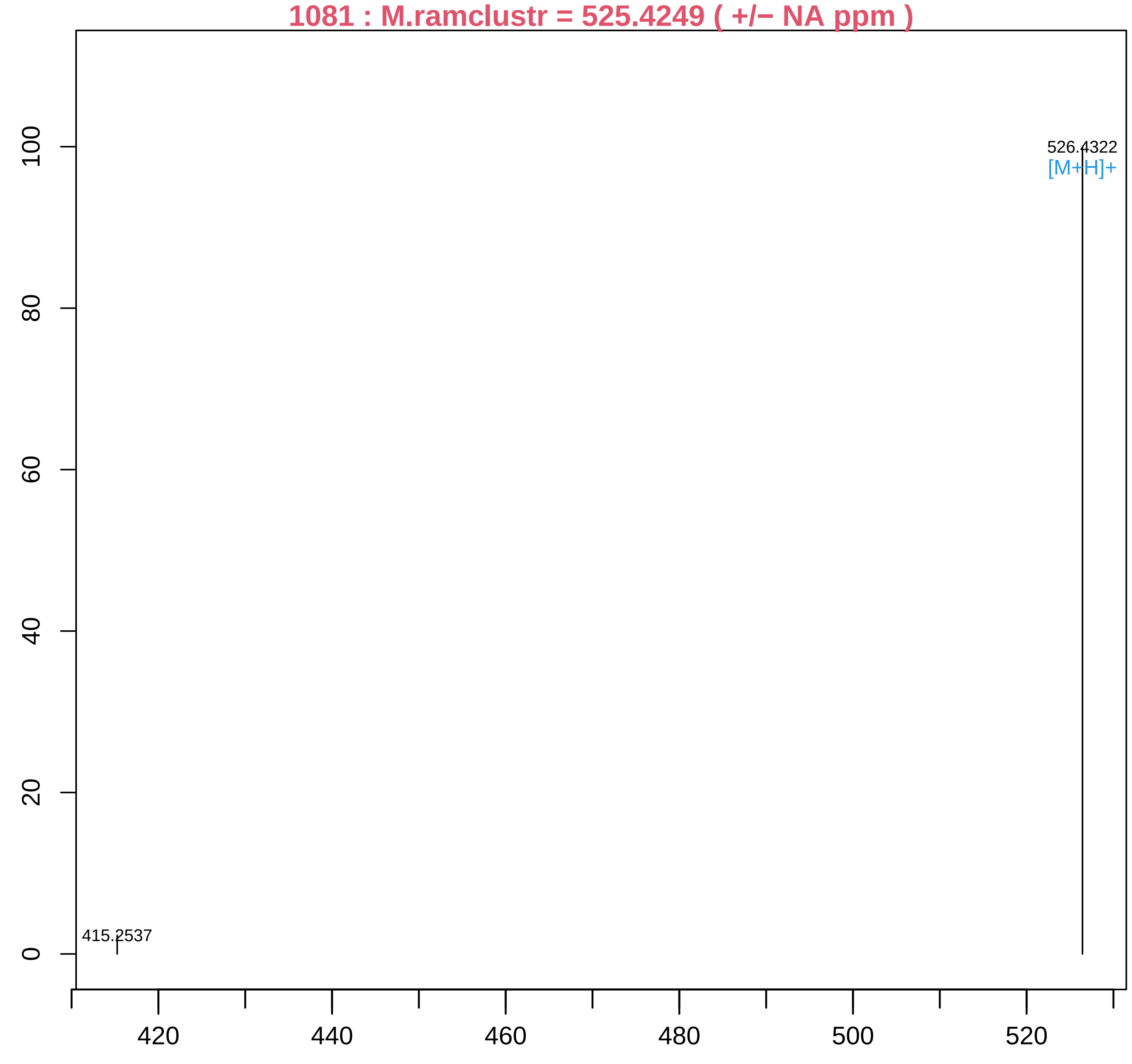
1081 : M.ramclustr = 525.4249 (+/− NA ppm)
526.4322
[M+H]+
415.2537
0
20
40
60
80
100
420
440
460
480
500
520

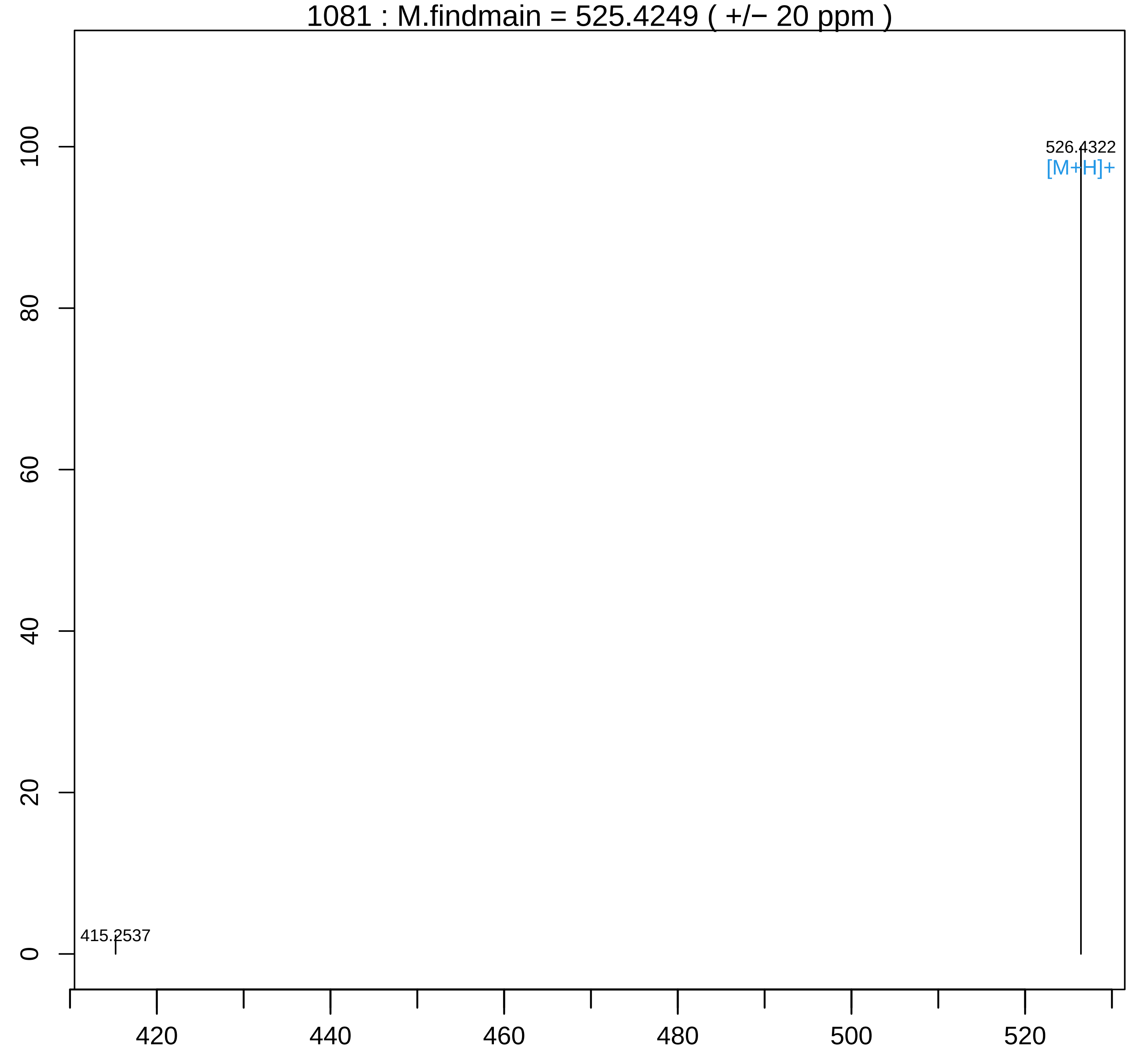
1081 : M.findmain = 525.4249 (+/− 20 ppm)
526.4322
[M+H]+
415.2537
0
20
40
60
80
100
420
440
460
480
500
520

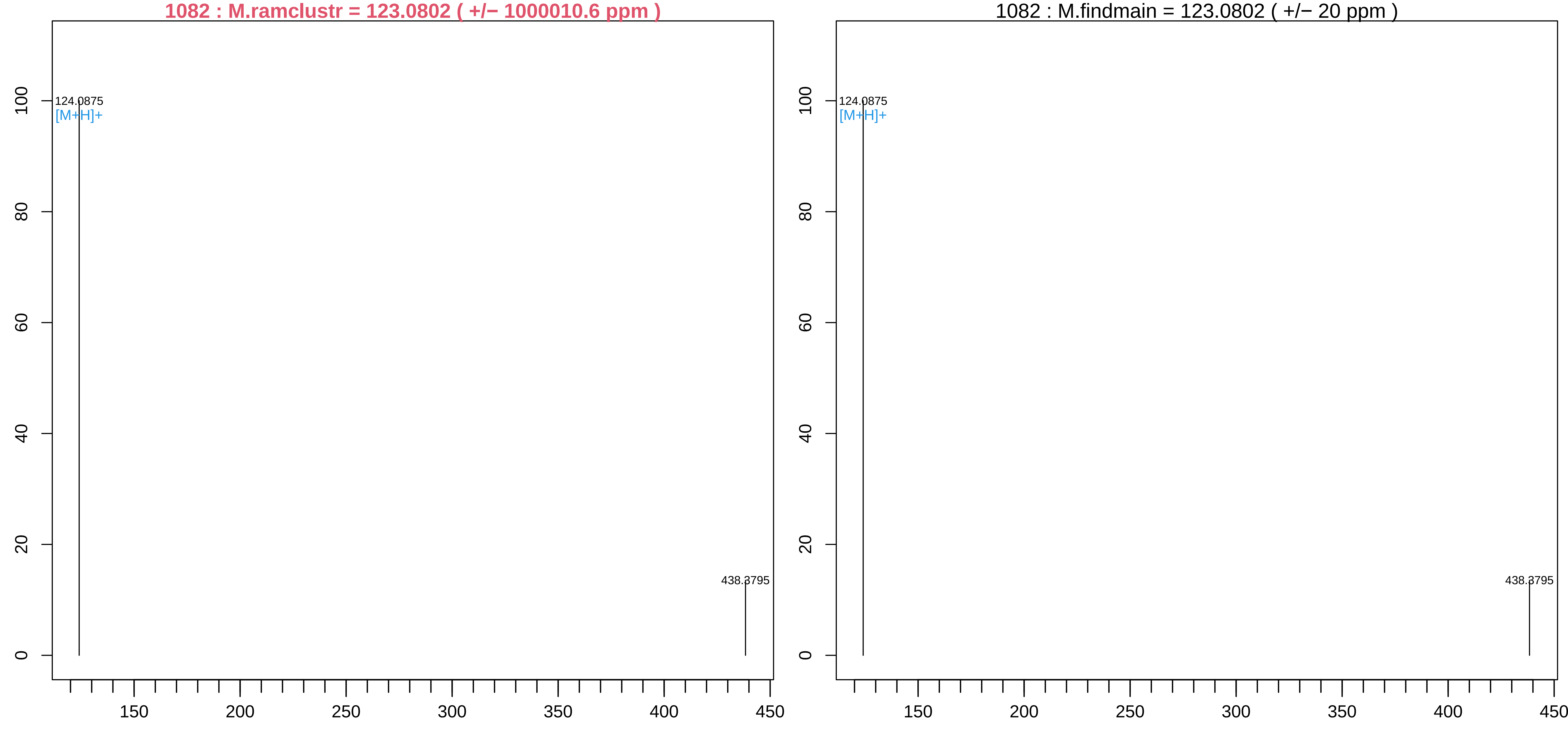

1082 : M.ramclustr = 123.0802 (+/− 1000010.6 ppm)
124.0875
[M+H]+
438.3795
0
20
40
60
80
100
150
200
250
300
350
400
450
1082 : M.findmain = 123.0802 (+/− 20 ppm)
124.0875
[M+H]+
438.3795

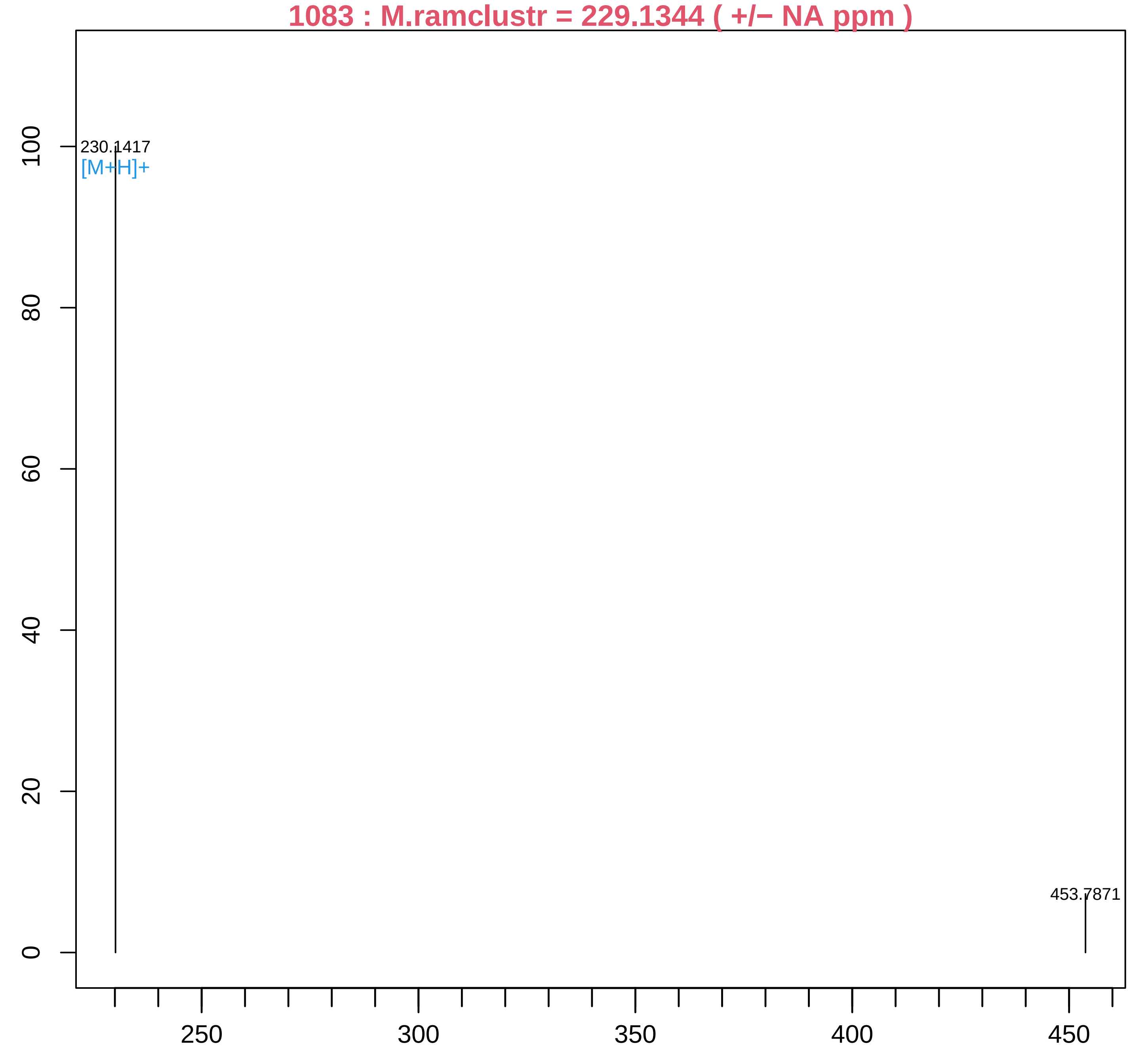
1083 : M.ramclustr = 229.1344 (+/− NA ppm)
230.1417
[M+H]+
453.7871
0
20
40
60
80
100
250
300
350
400
450

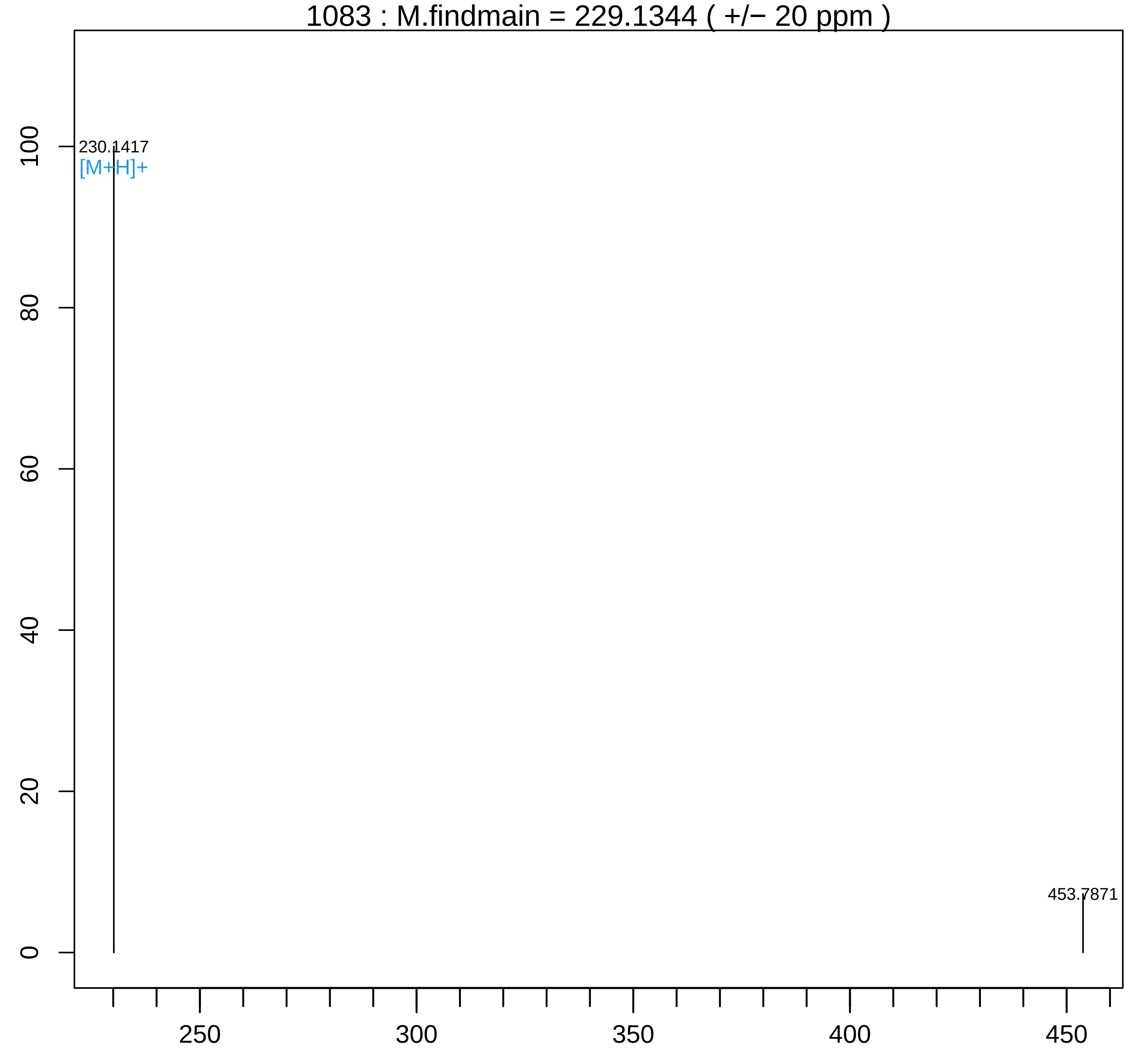
1083 : M.findmain = 229.1344 (+/− 20 ppm)
230.1417
[M+H]+
453.7871
0
20
40
60
80
100
250
300
350
400
450

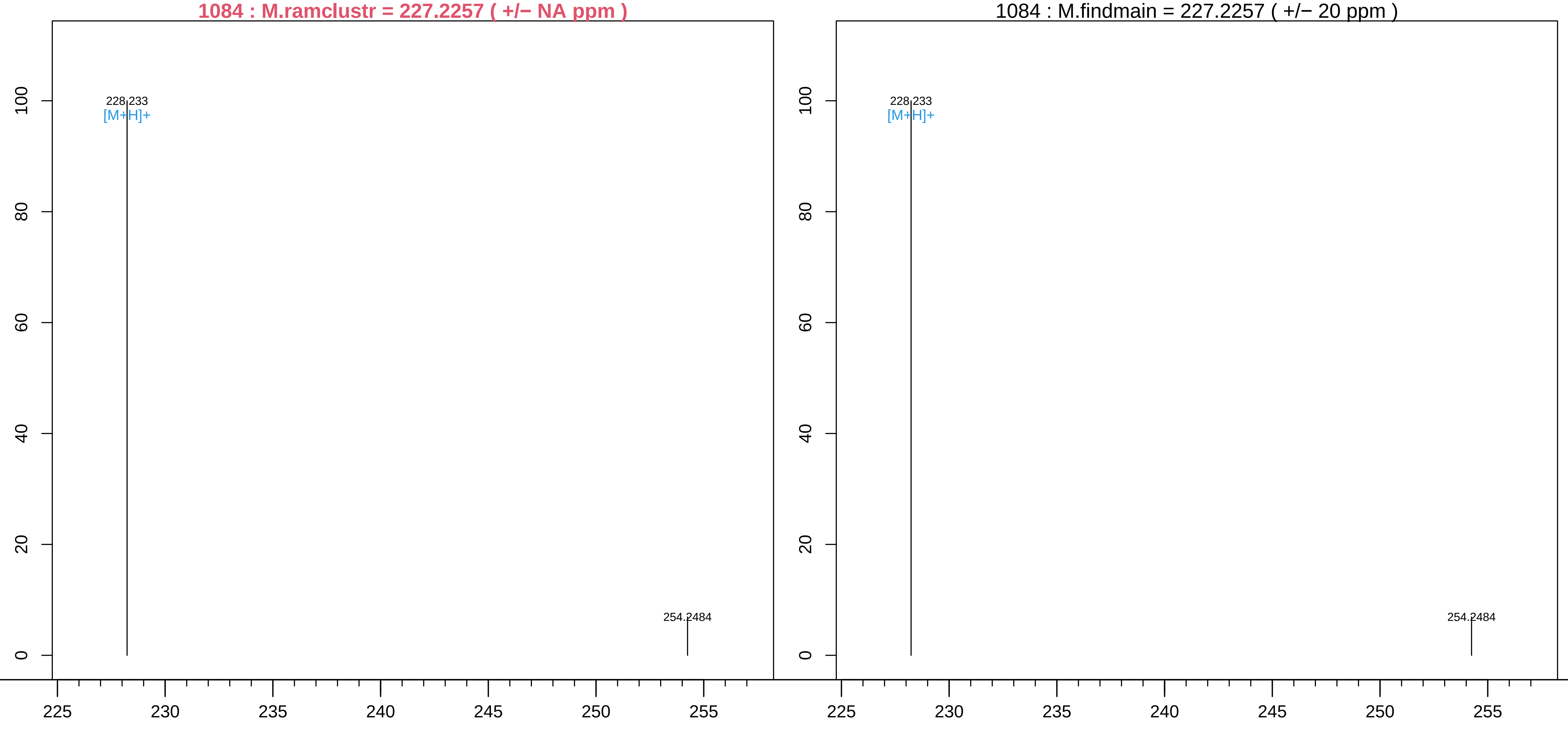

1084 : M.ramclustr = 227.2257 (+/− NA ppm)
1084 : M.findmain = 227.2257 (+/− 20 ppm)
228.233
[M+H]+
254.2484
0
20
40
60
80
100
225
230
235
240
245
250
255

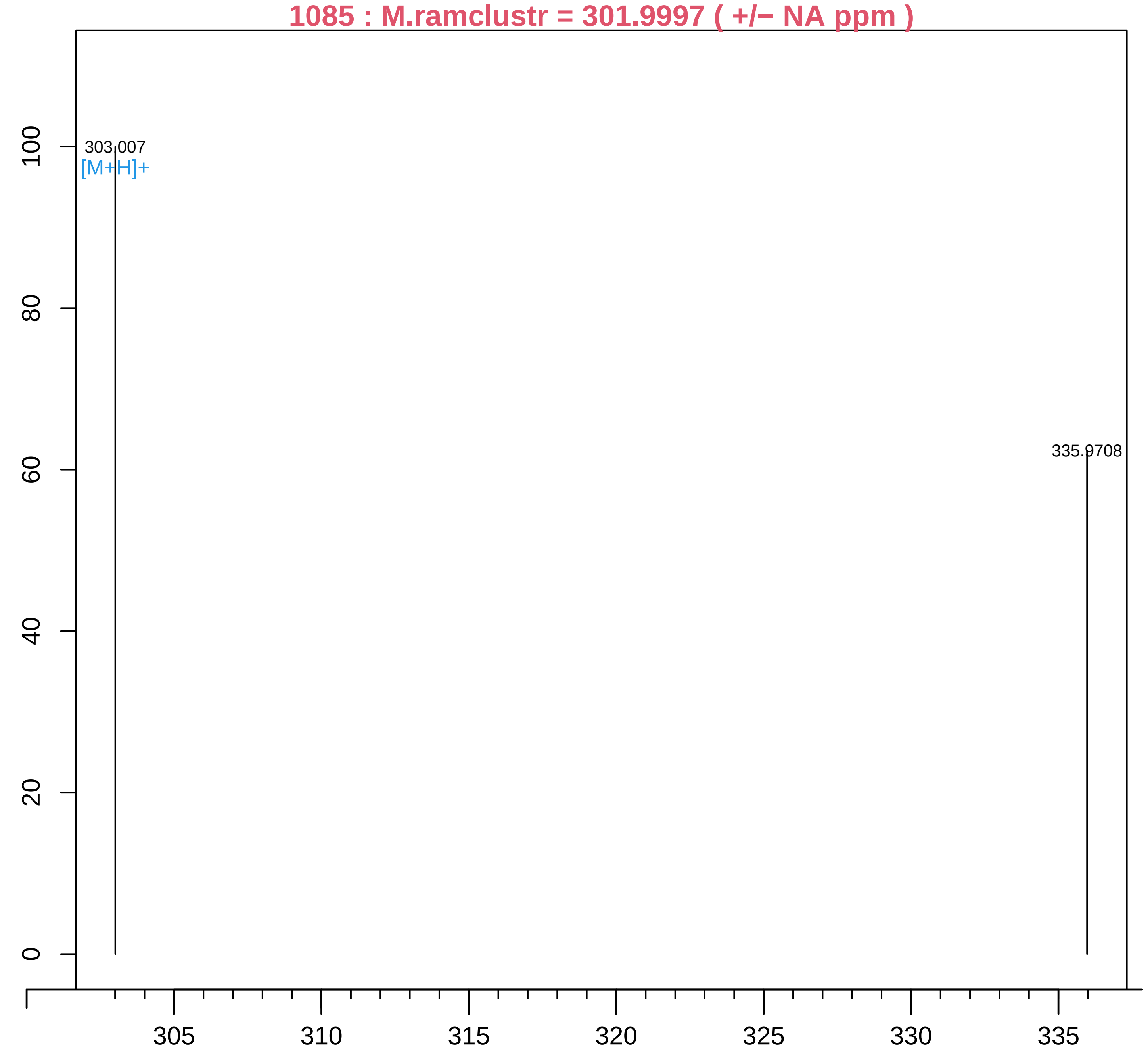
1085 : M.ramclustr = 301.9997 (+/− NA ppm)
303.007
[M+H]+
335.9708
0
20
40
60
80
100
305
310
315
320
325
330
335

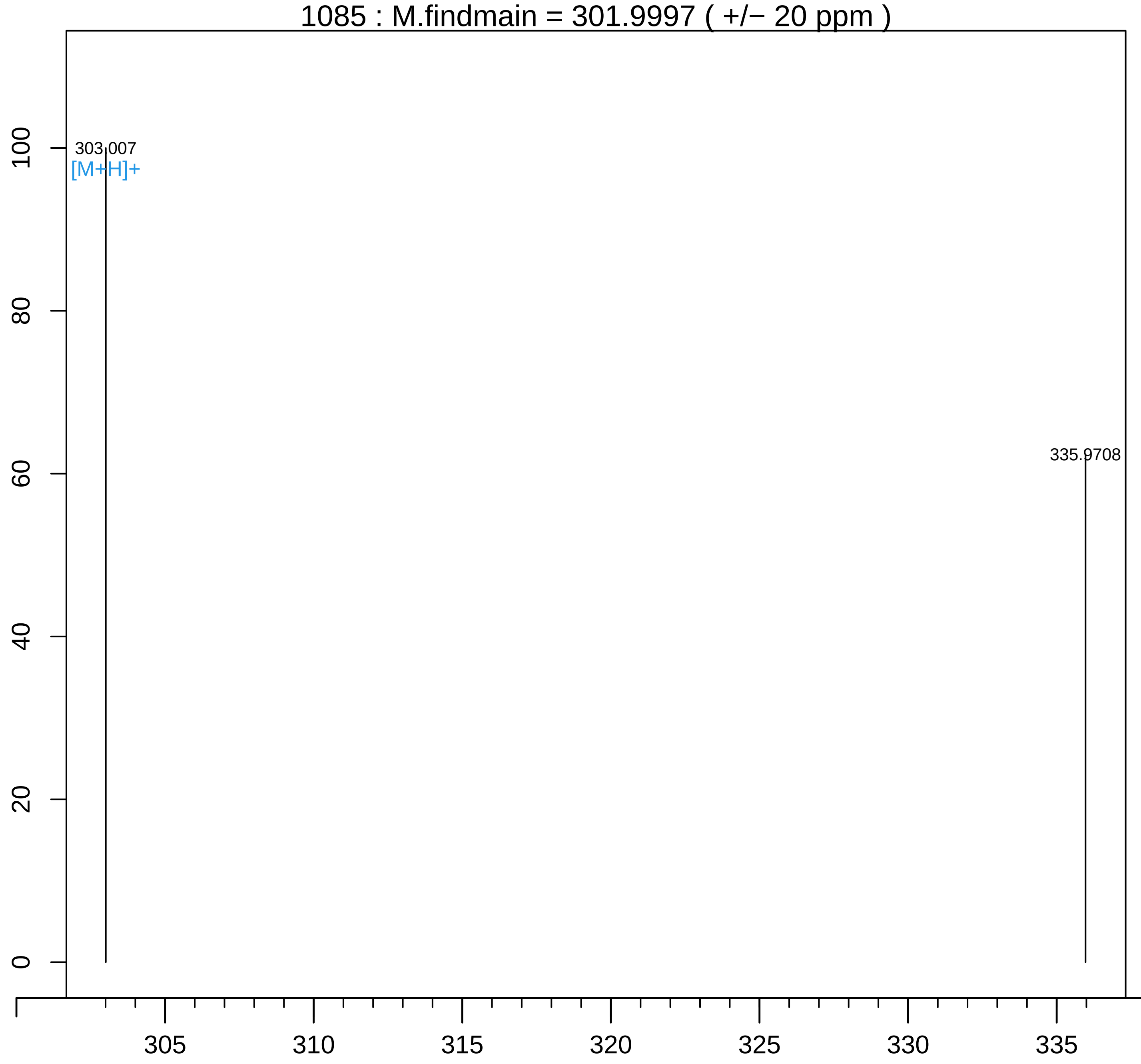
1085 : M.findmain = 301.9997 (+/− 20 ppm)
303.007
[M+H]+
335.9708
0
20
40
60
80
100
305
310
315
320
325
330
335

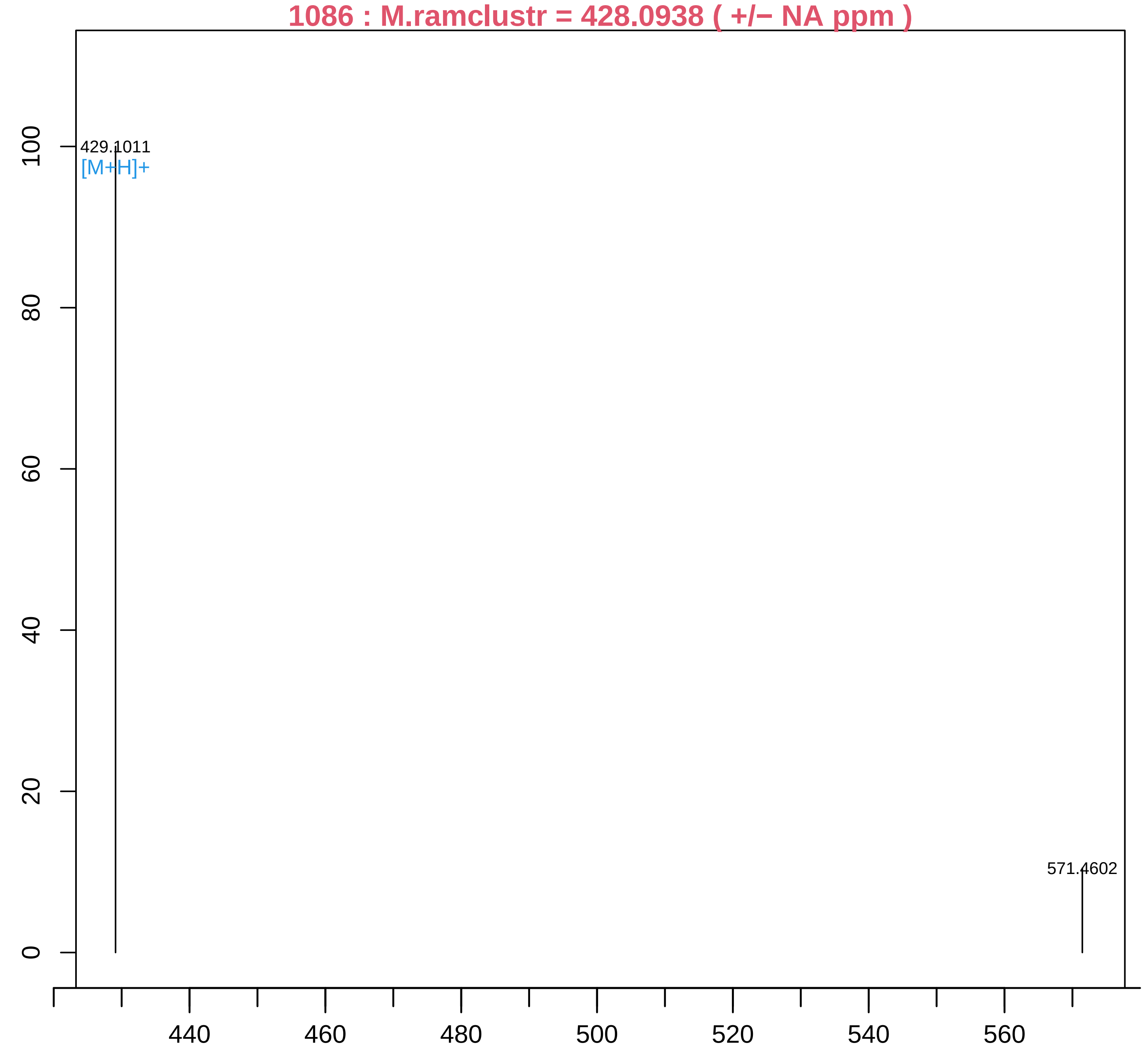
1086 : M.ramclustr = 428.0938 (+/− NA ppm)
429.1011
[M+H]+
571.4602
0
20
40
60
80
100
440
460
480
500
520
540
560

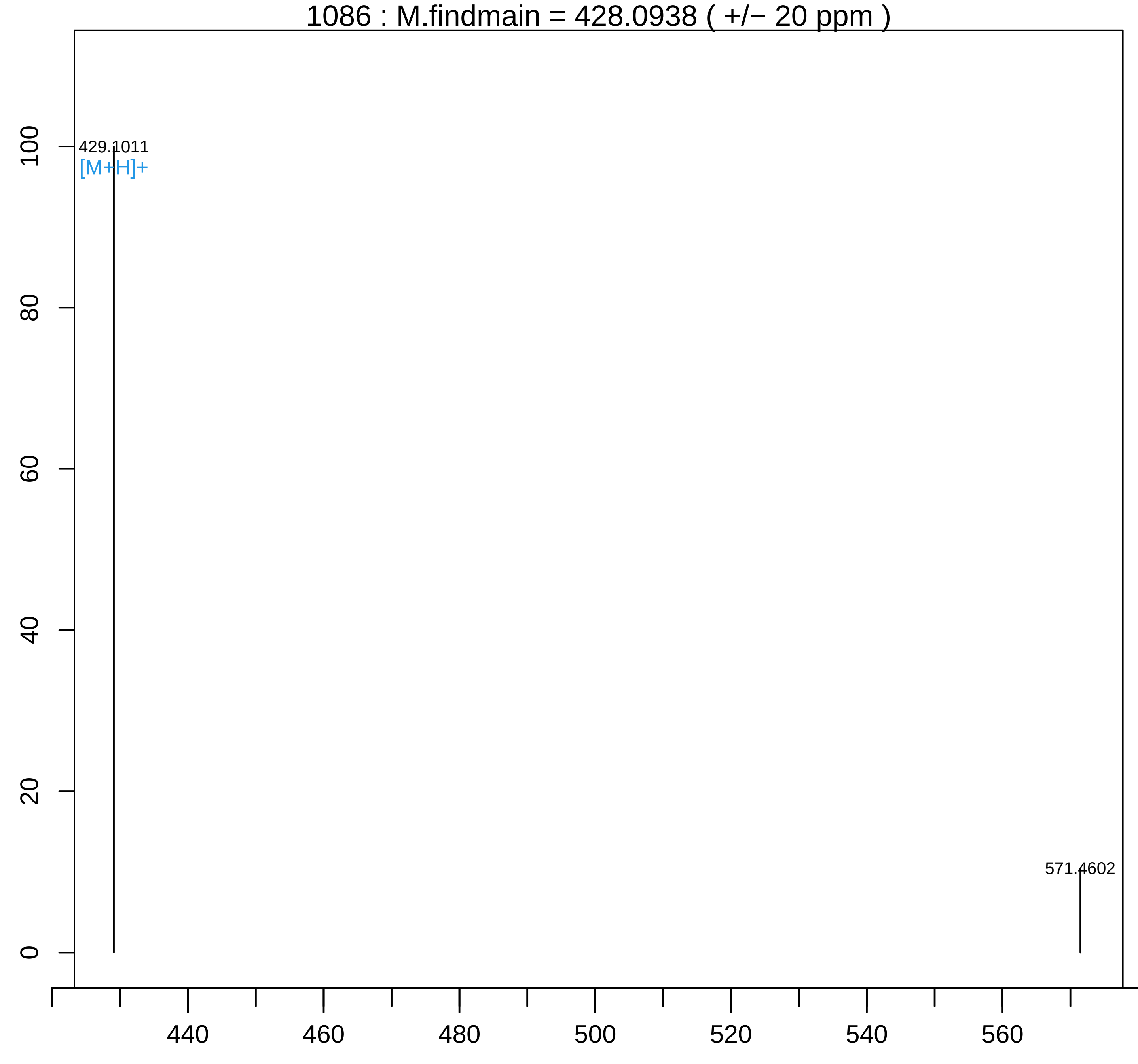
1086 : M.findmain = 428.0938 (+/− 20 ppm)
429.1011
[M+H]+
571.4602
0
20
40
60
80
100
440
460
480
500
520
540
560

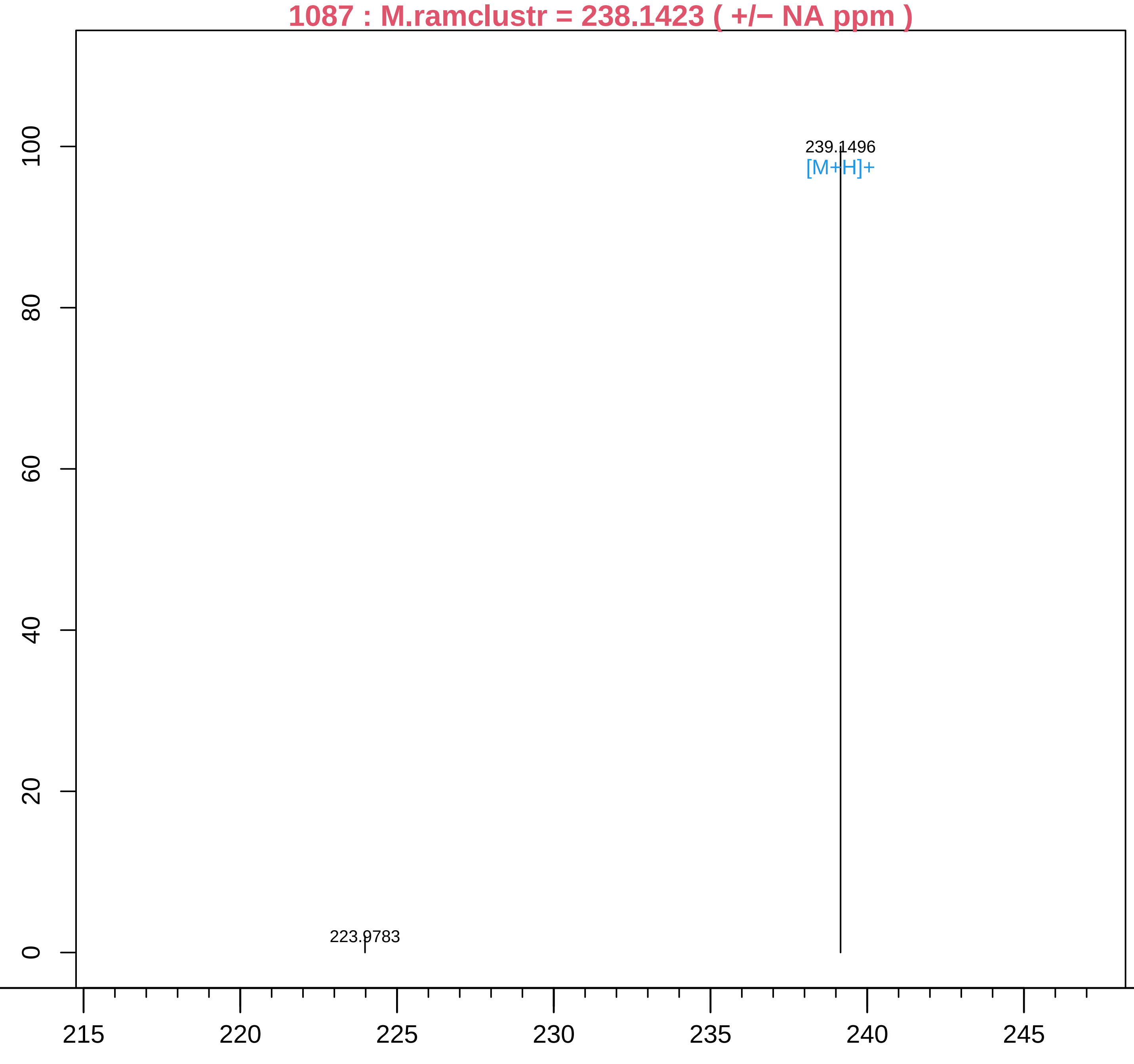
1087 : M.ramclustr = 238.1423 (+/− NA ppm)
239.1496
[M+H]+
223.9783
0
20
40
60
80
100
215
220
225
230
235
240
245

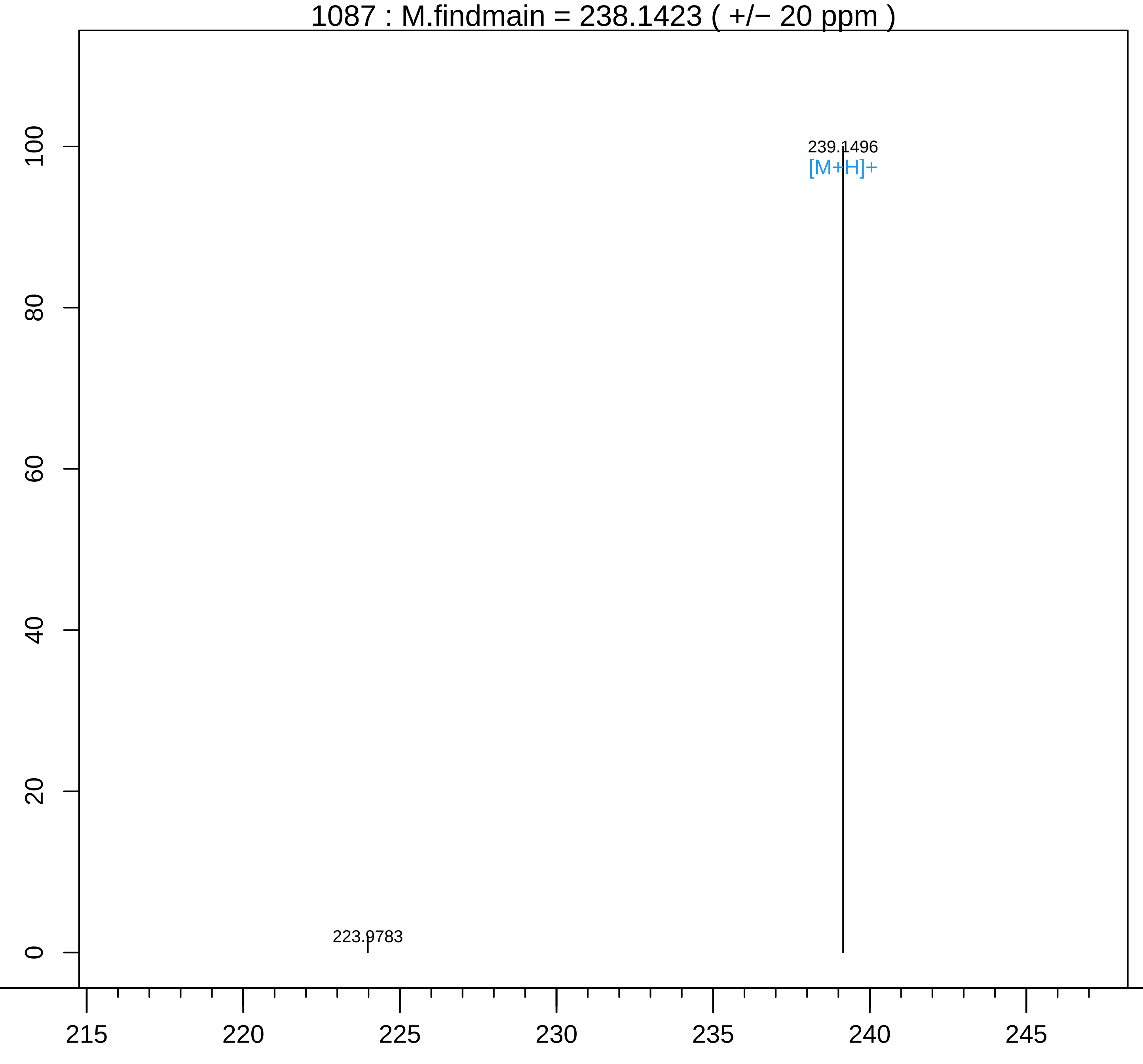
1087 : M.findmain = 238.1423 (+/− 20 ppm)
239.1496
[M+H]+
223.9783
0
20
40
60
80
100
215
220
225
230
235
240
245

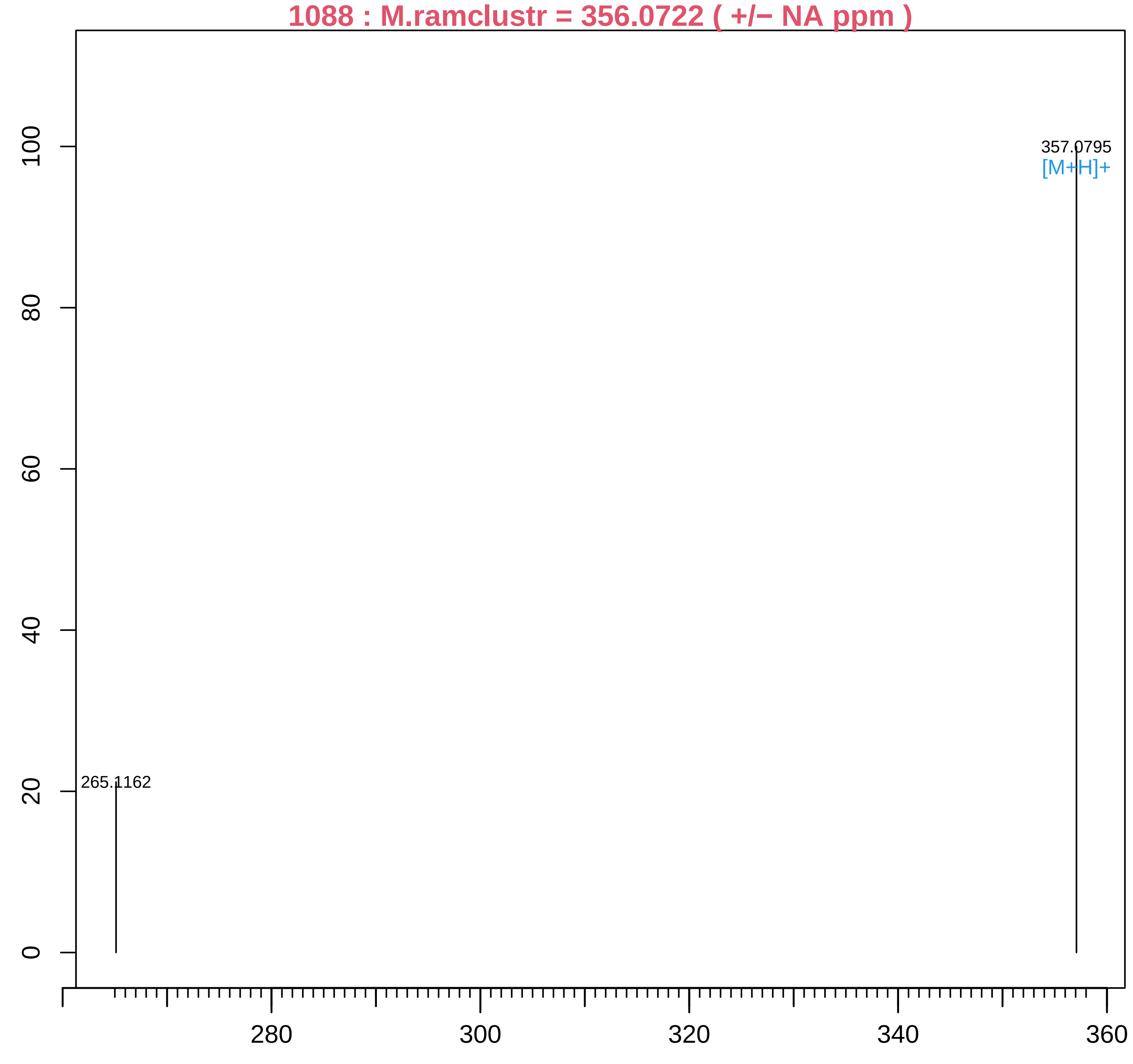
1088 : M.ramclustr = 356.0722 (+/− NA ppm)
357.0795
[M+H]+
265.1162
0
20
40
60
80
100
280
300
320
340
360

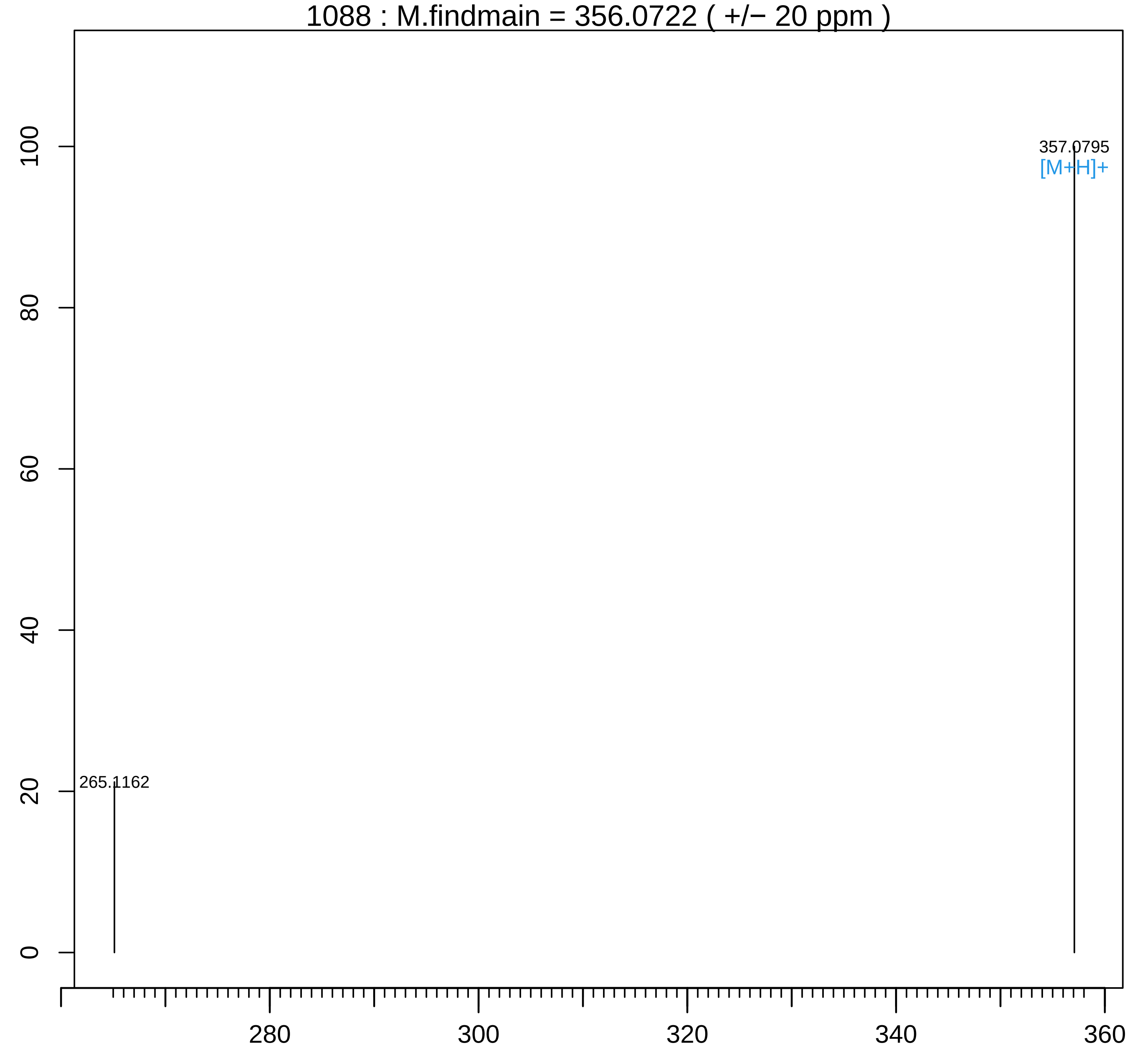
1088 : M.findmain = 356.0722 (+/− 20 ppm)
357.0795
[M+H]+
265.1162
0
20
40
60
80
100
280
300
320
340
360

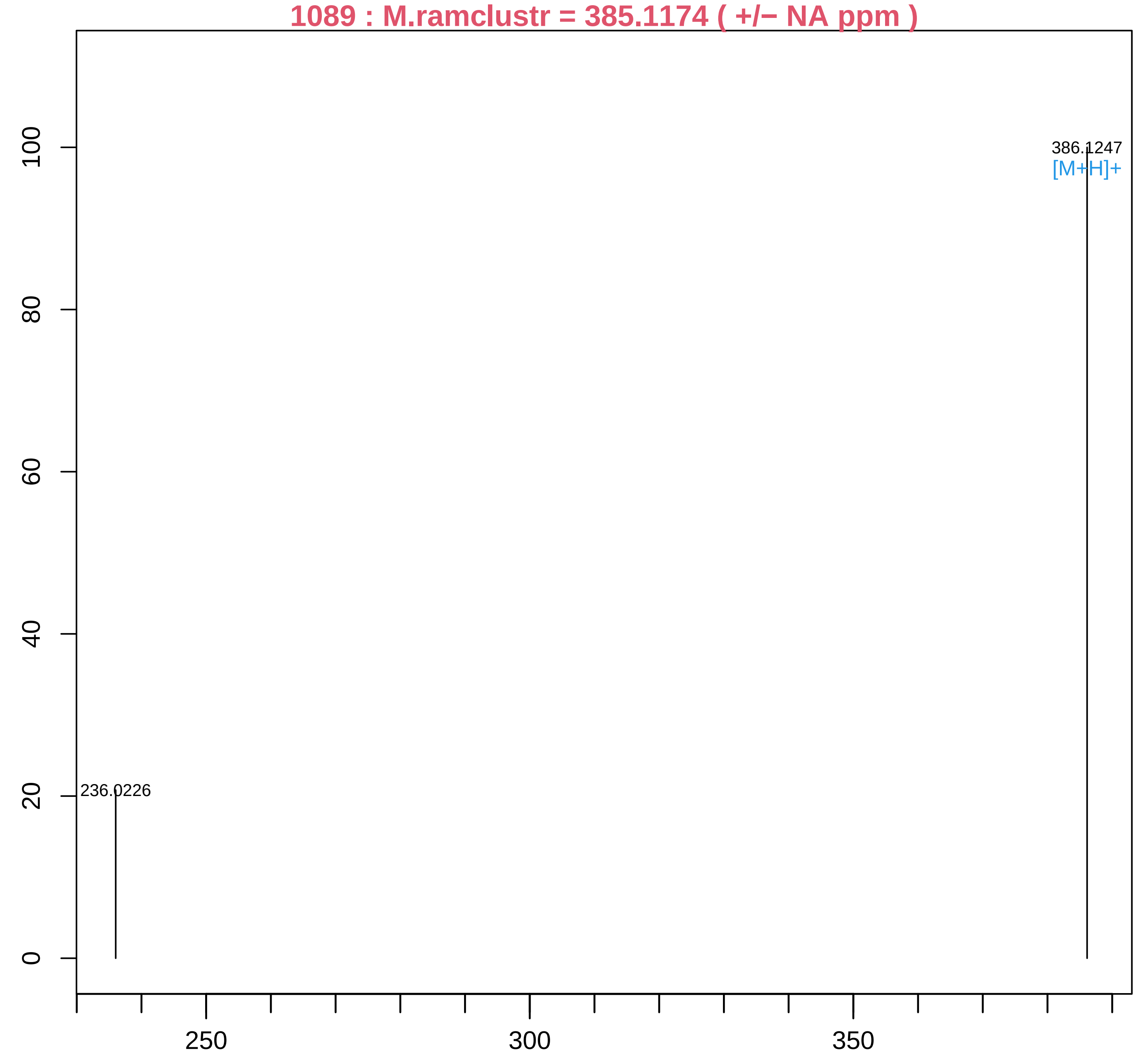

1089 : M.ramclustr = 385.1174 (+/− NA ppm)
386.1247
[M+H]+
236.0226
0
20
40
60
80
100
250
300
350

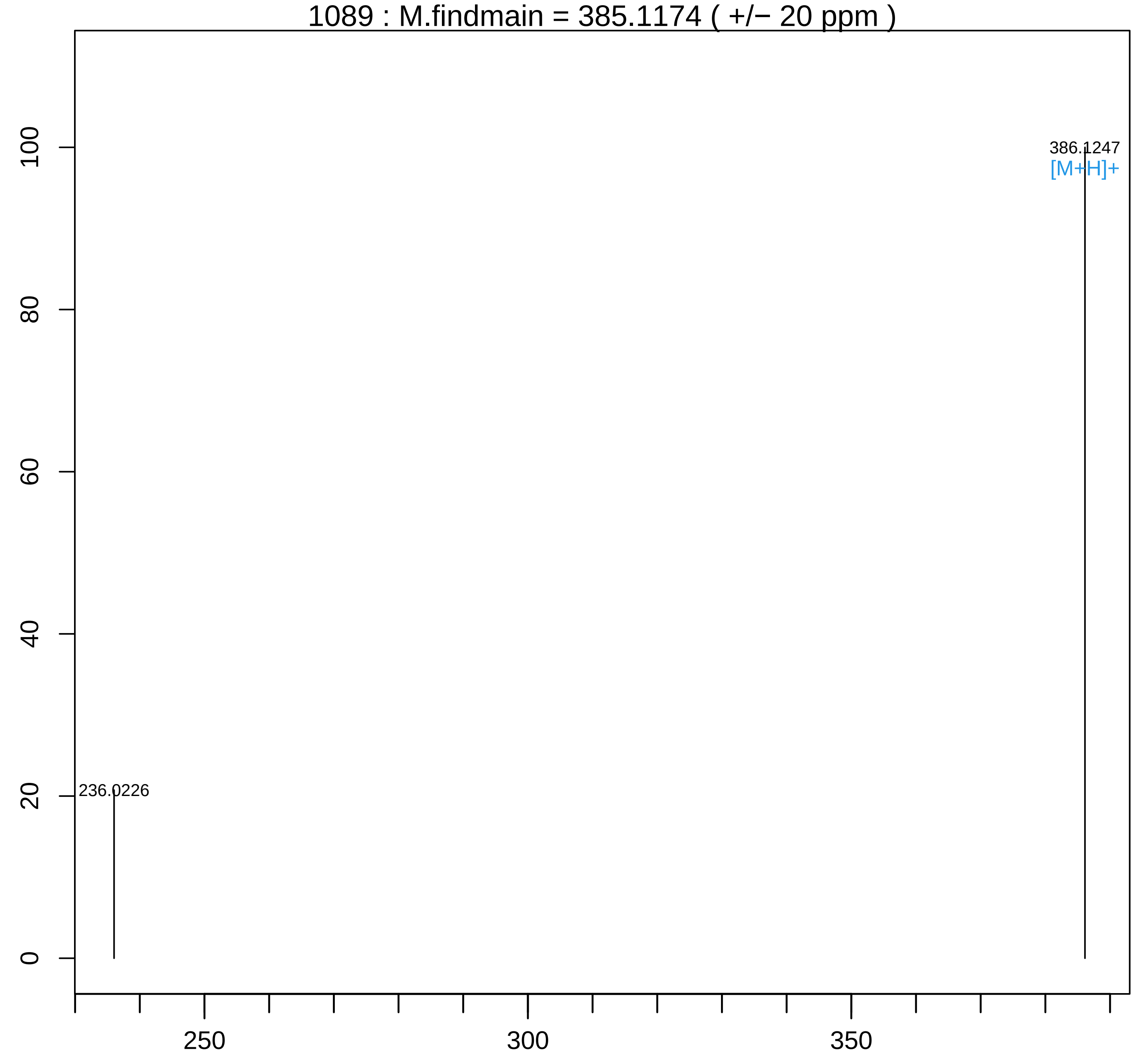

1089 : M.findmain = 385.1174 (+/− 20 ppm)
386.1247
[M+H]+
236.0226
0
20
40
60
80
100
250
300
350

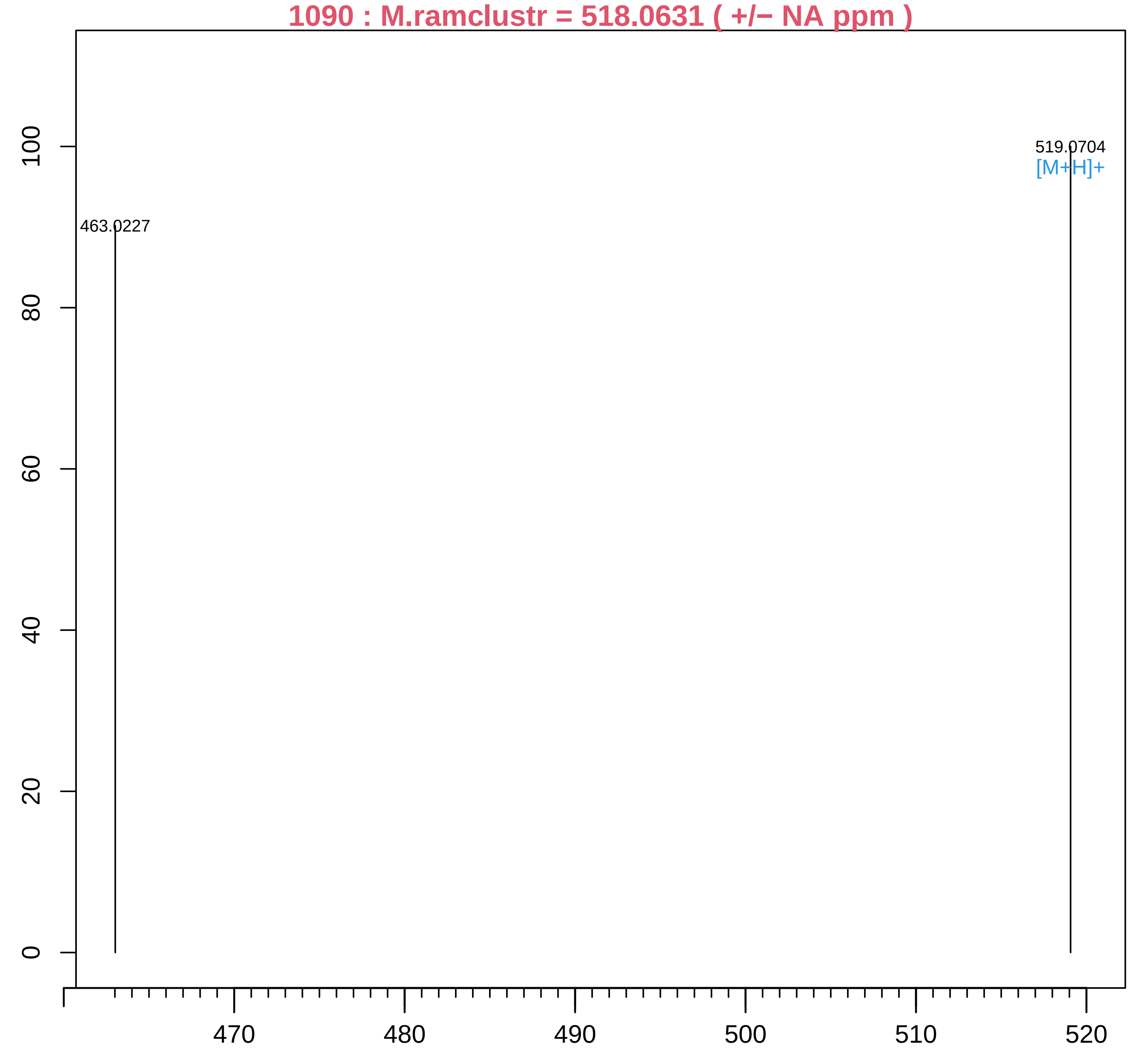

1090 : M.ramclustr = 518.0631 (+/− NA ppm)
463.0227
519.0704
[M+H]+
0
20
40
60
80
100
470
480
490
500
510
520

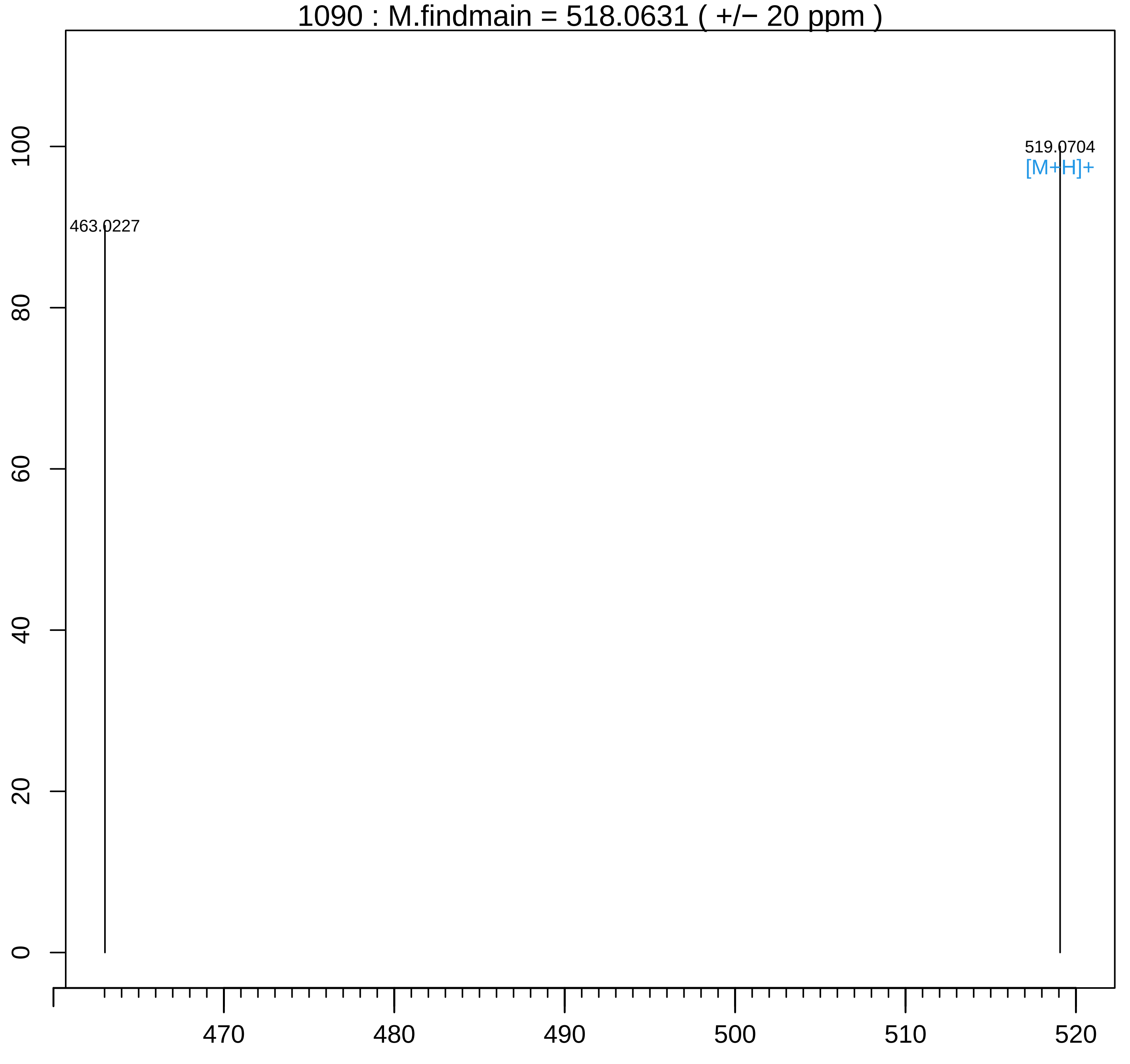

1090 : M.findmain = 518.0631 (+/− 20 ppm)
463.0227
519.0704
[M+H]+
0
20
40
60
80
100
470
480
490
500
510
520

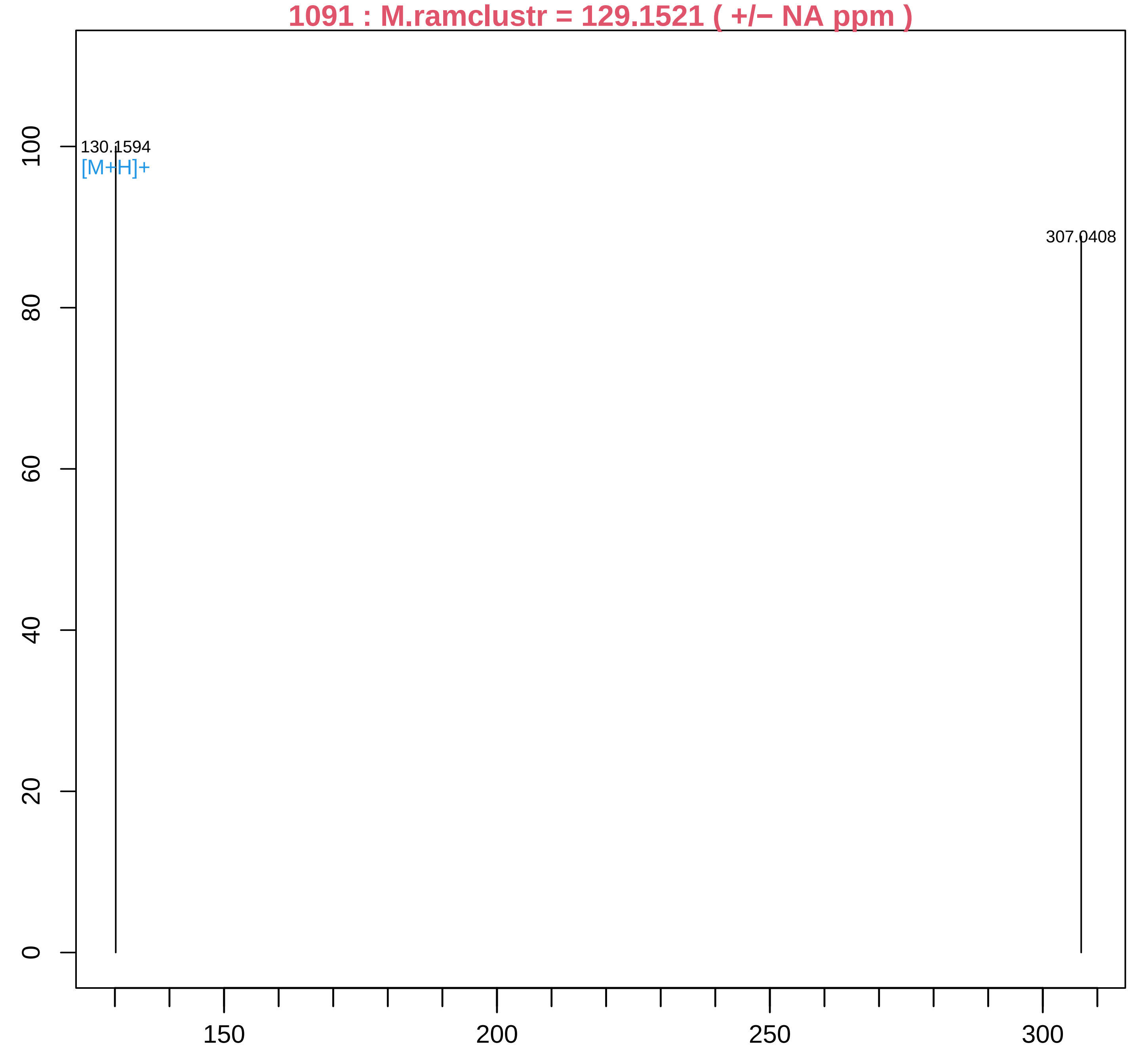

1091 : M.ramclustr = 129.1521 (+/− NA ppm)
130.1594
[M+H]+
307.0408
0
20
40
60
80
100
150
200
250
300

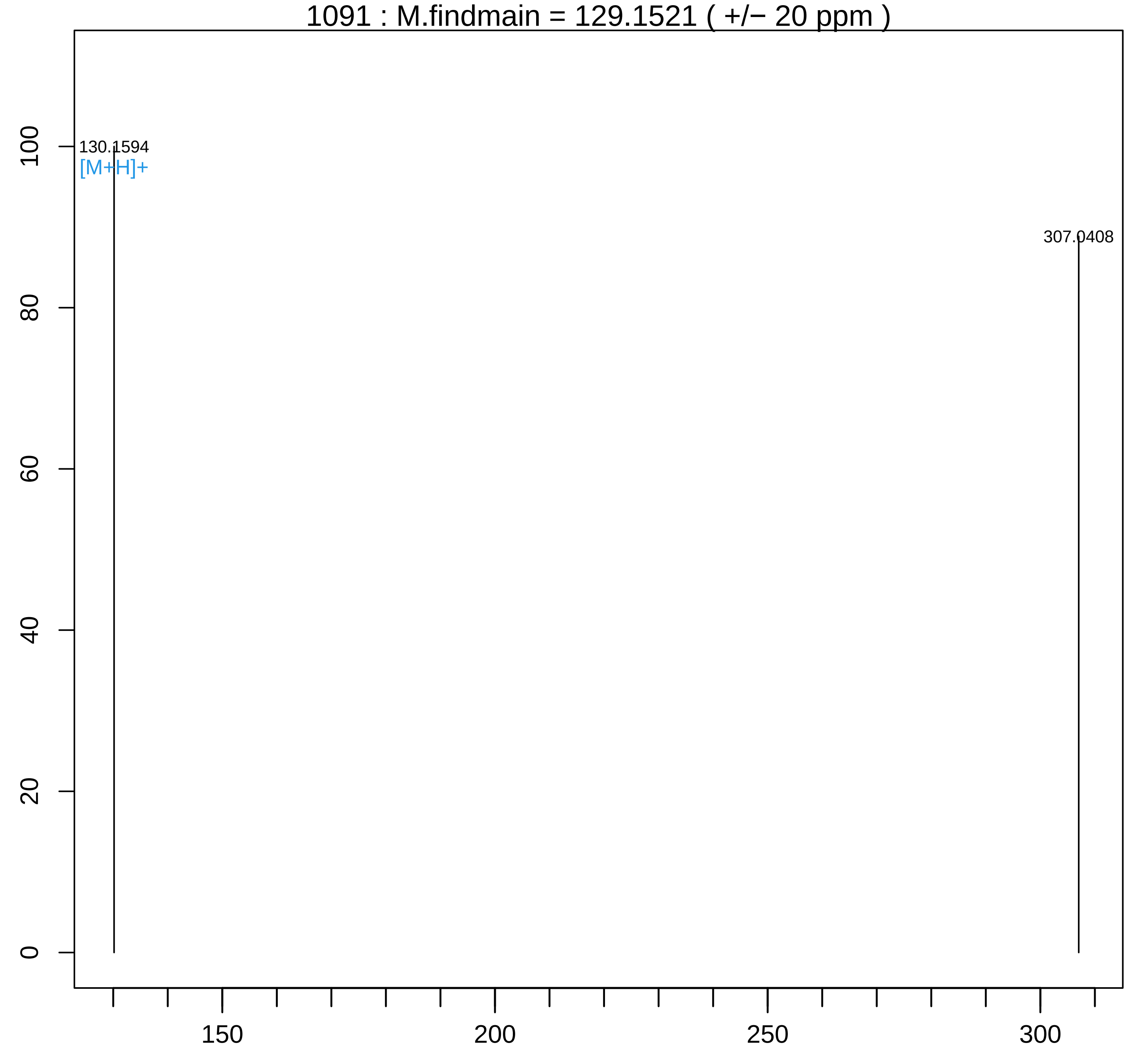

1091 : M.findmain = 129.1521 (+/− 20 ppm)
130.1594
[M+H]+
307.0408
0
20
40
60
80
100
150
200
250
300

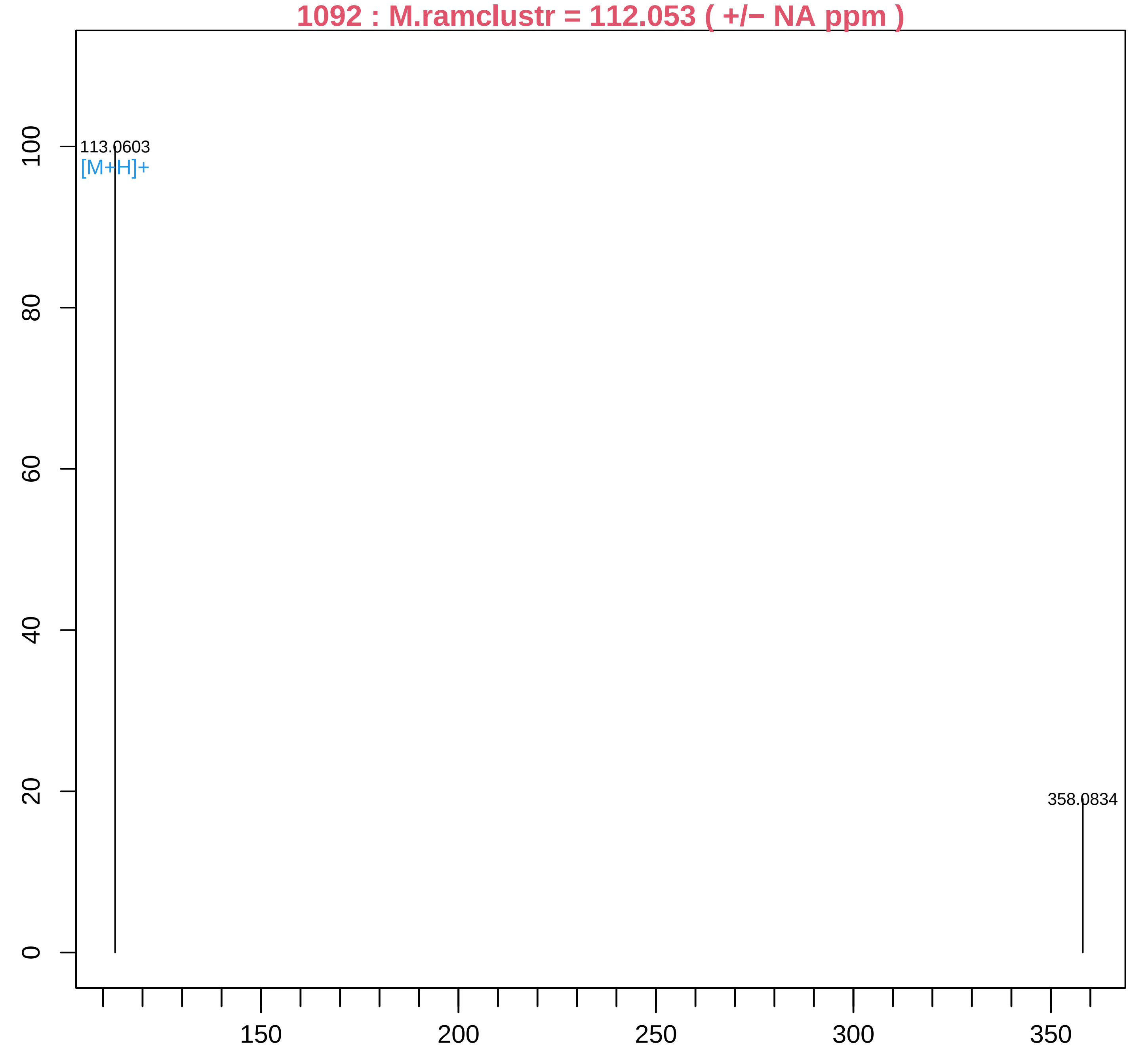
1092 : M.ramclustr = 112.053 (+/− NA ppm)
113.0603
[M+H]+
358.0834
0
20
40
60
80
100
150
200
250
300
350

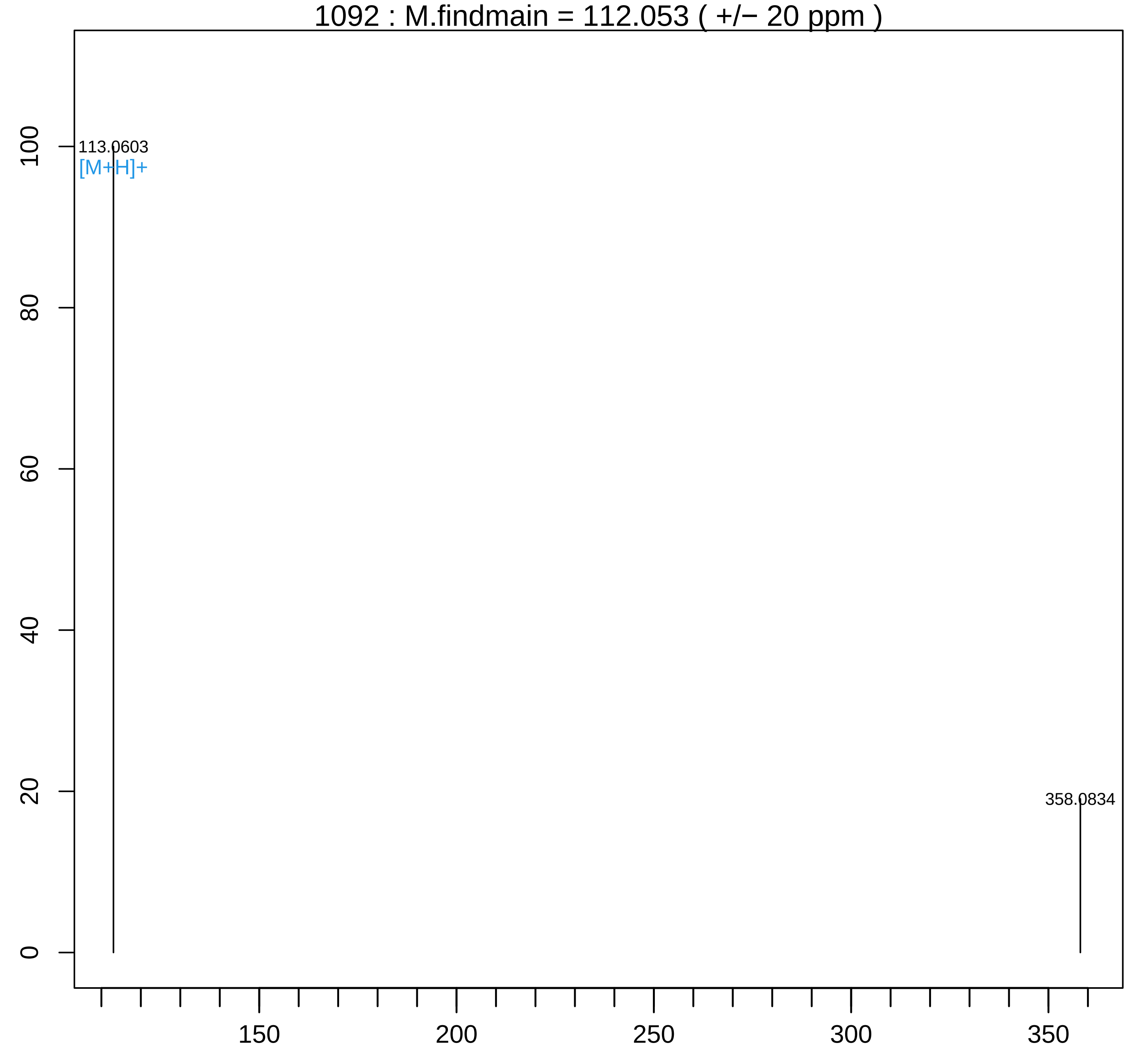
1092 : M.findmain = 112.053 (+/− 20 ppm)
113.0603
[M+H]+
358.0834
0
20
40
60
80
100
150
200
250
300
350

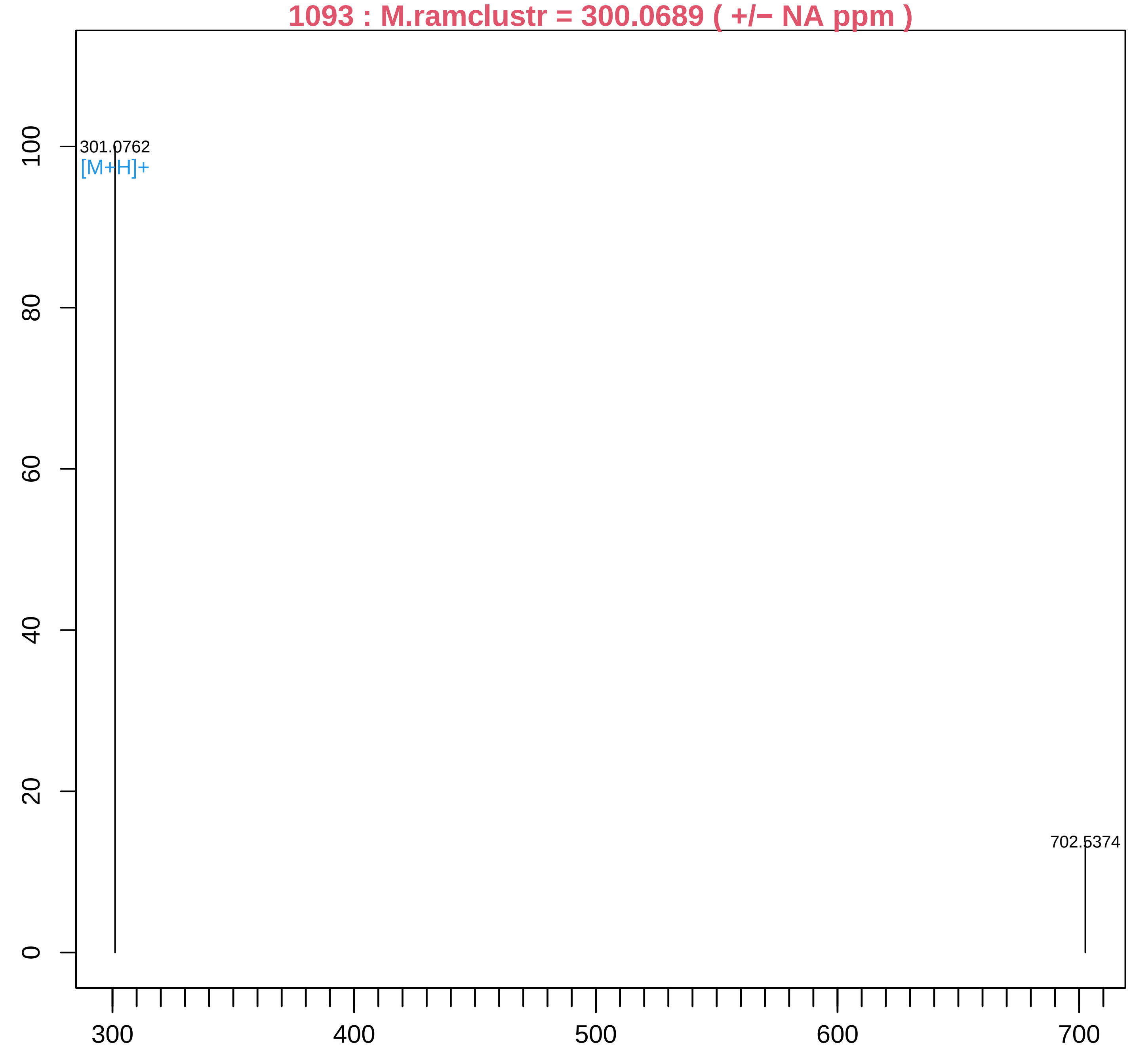
1093 : M.ramclustr = 300.0689 (+/− NA ppm)
301.0762
[M+H]+
702.5374
0
20
40
60
80
100
300
400
500
600
700

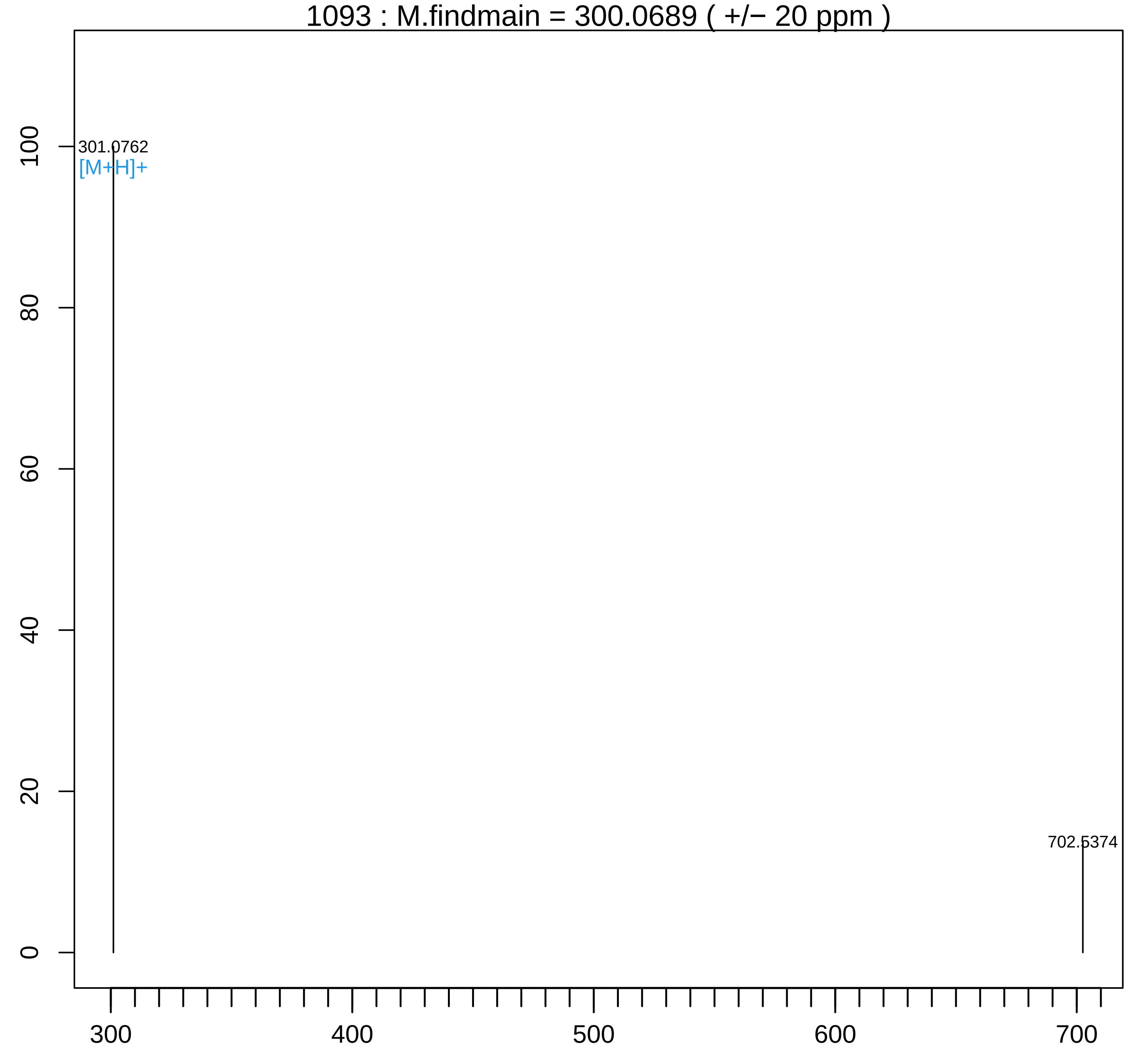
1093 : M.findmain = 300.0689 (+/− 20 ppm)
301.0762
[M+H]+
702.5374
0
20
40
60
80
100
300
400
500
600
700

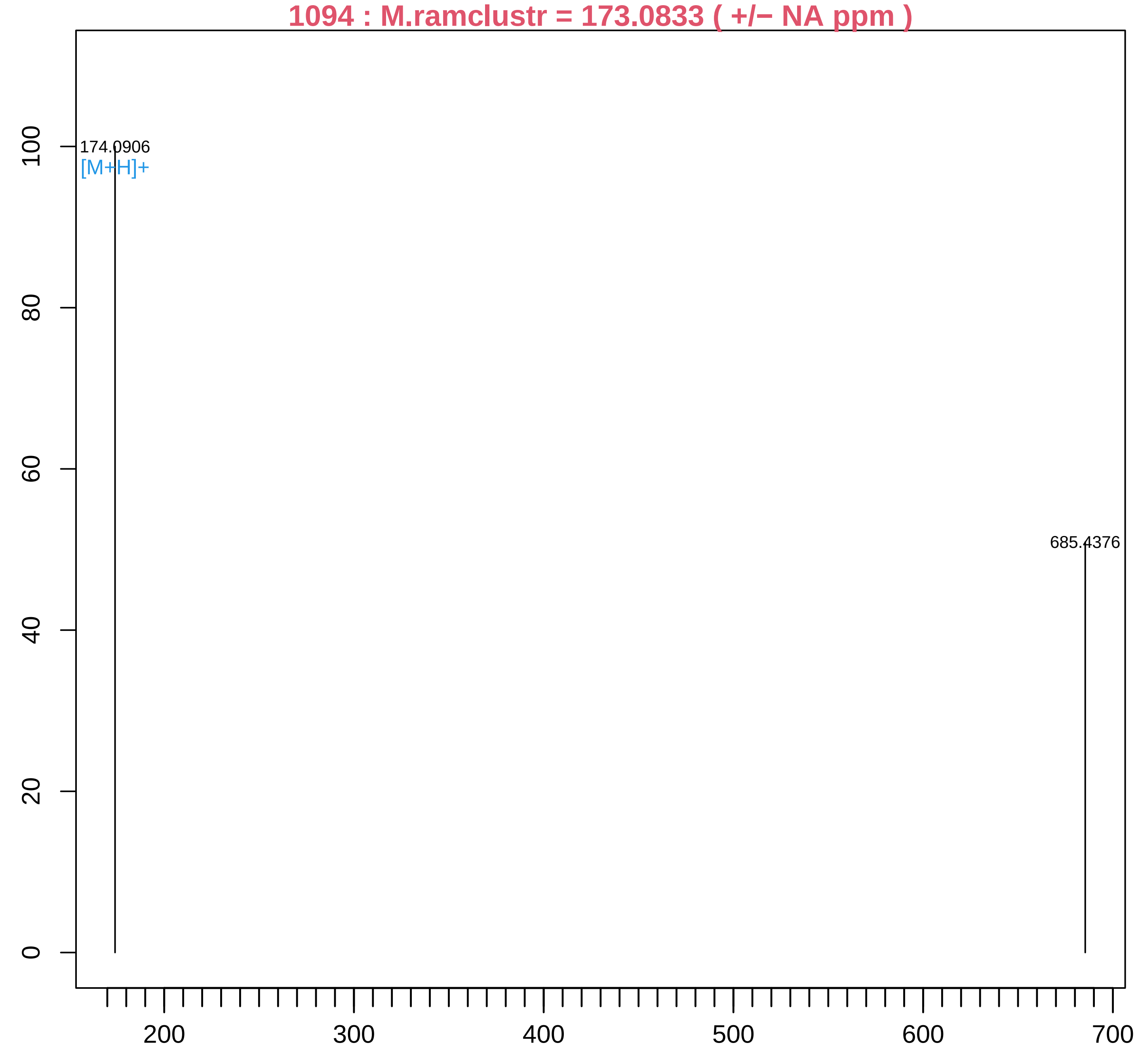
1094 : M.ramclustr = 173.0833 (+/− NA ppm)
174.0906
[M+H]+
685.4376
0
20
40
60
80
100
200
300
400
500
600
700

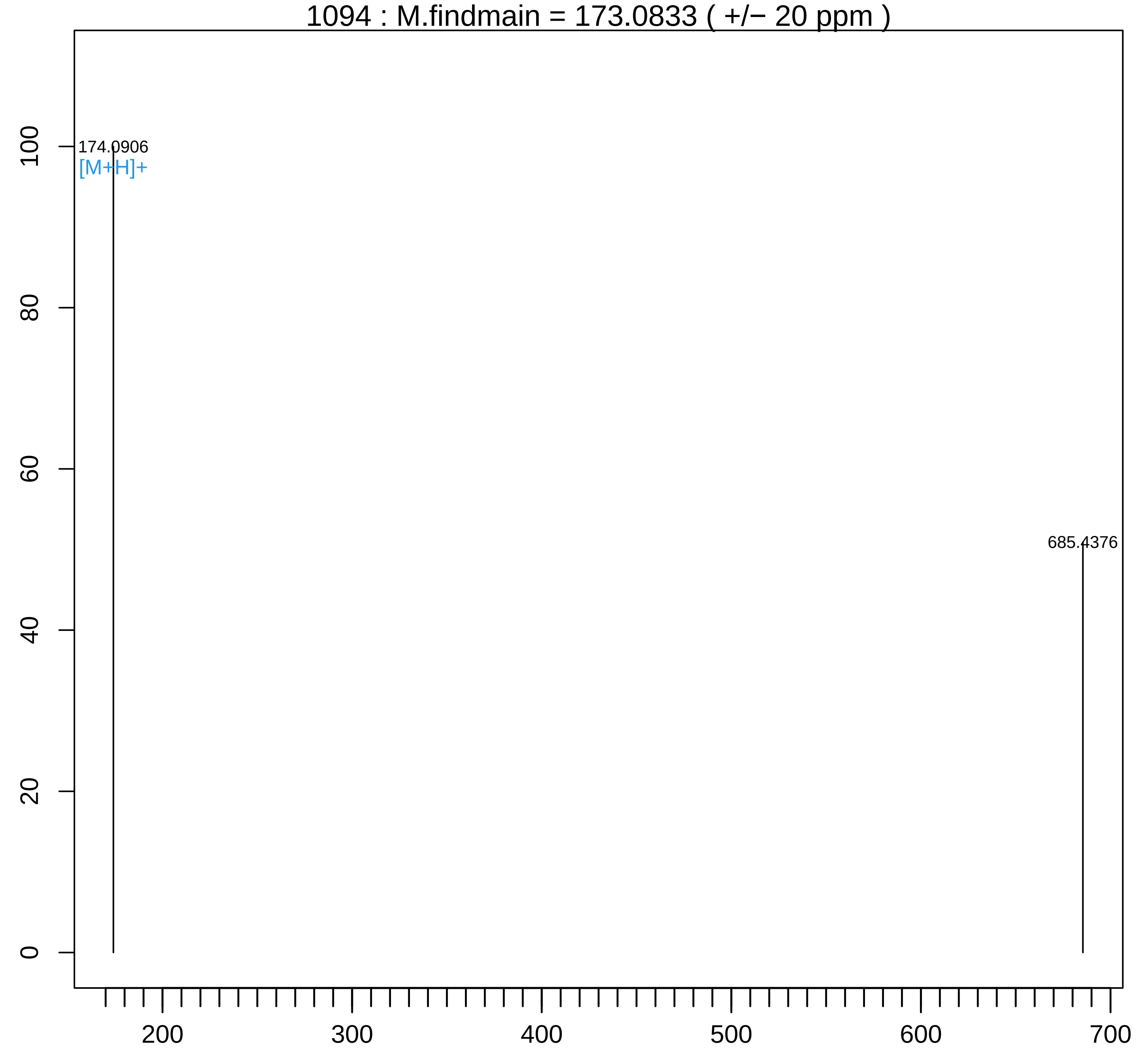
1094 : M.findmain = 173.0833 (+/− 20 ppm)
174.0906
[M+H]+
685.4376
0
20
40
60
80
100
200
300
400
500
600
700

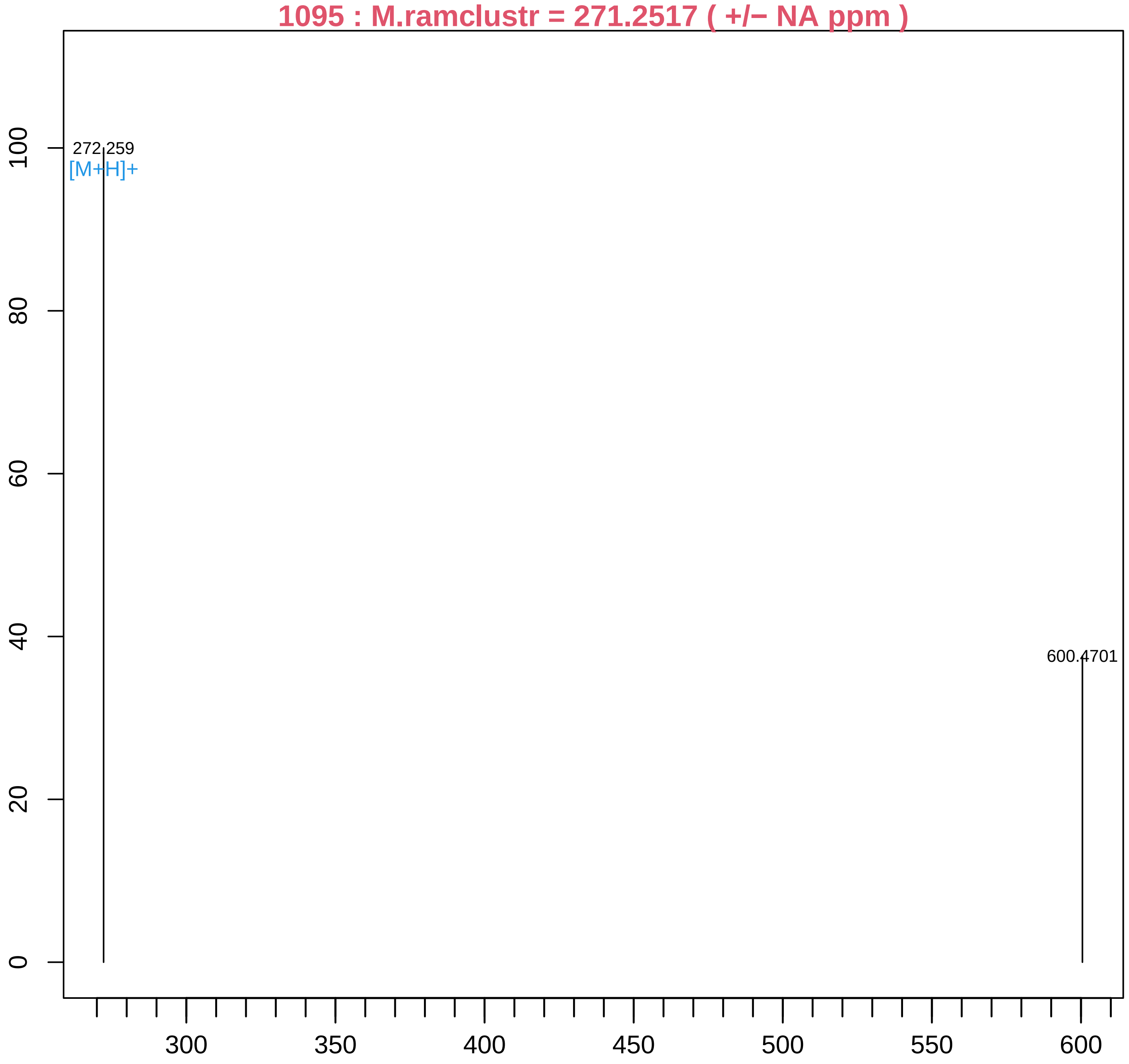
1095 : M.ramclustr = 271.2517 (+/− NA ppm)
272.259
[M+H]+
600.4701
0
20
40
60
80
100
300
350
400
450
500
550
600

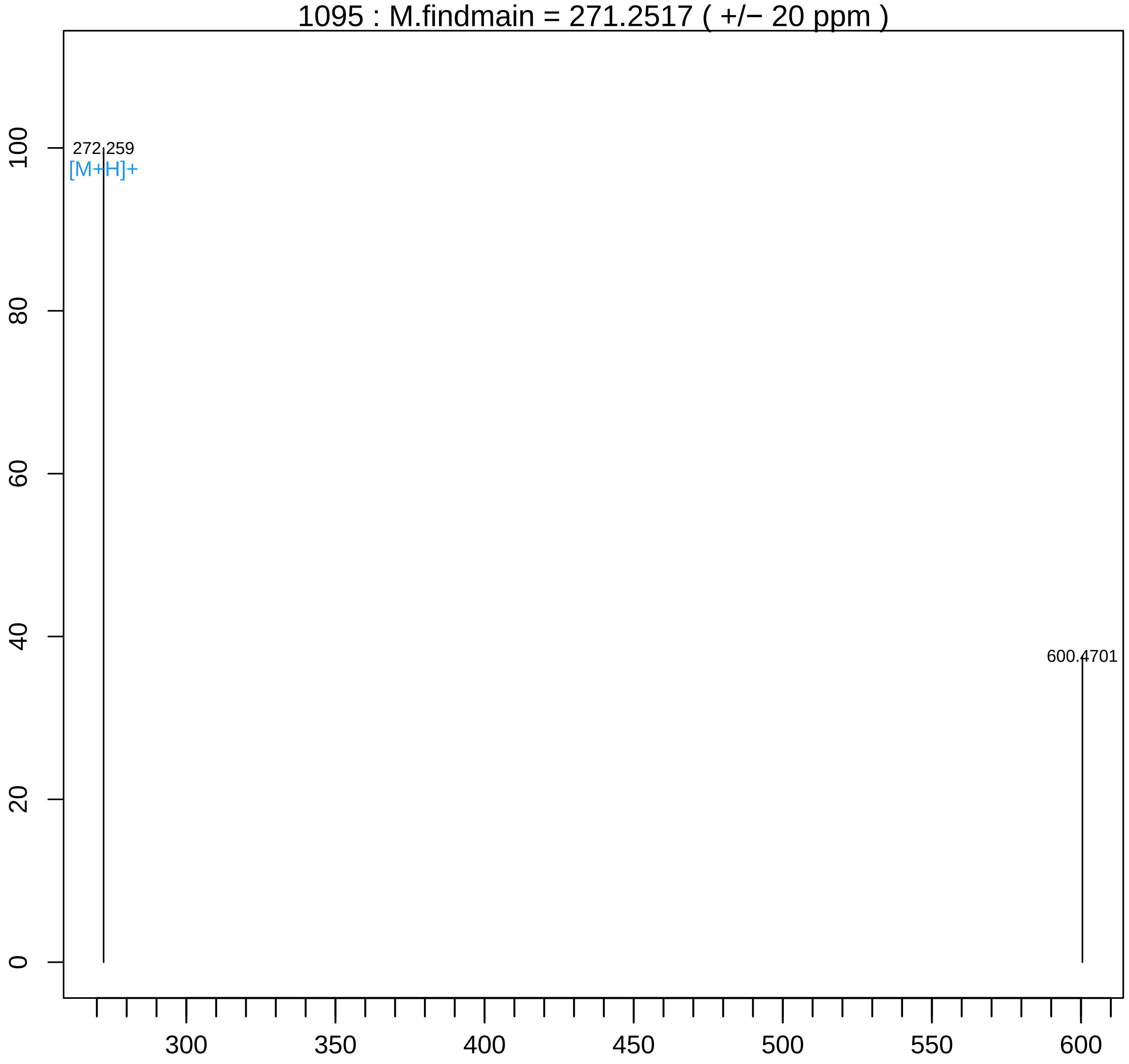
1095 : M.findmain = 271.2517 (+/− 20 ppm)
272.259
[M+H]+
600.4701
0
20
40
60
80
100
300
350
400
450
500
550
600

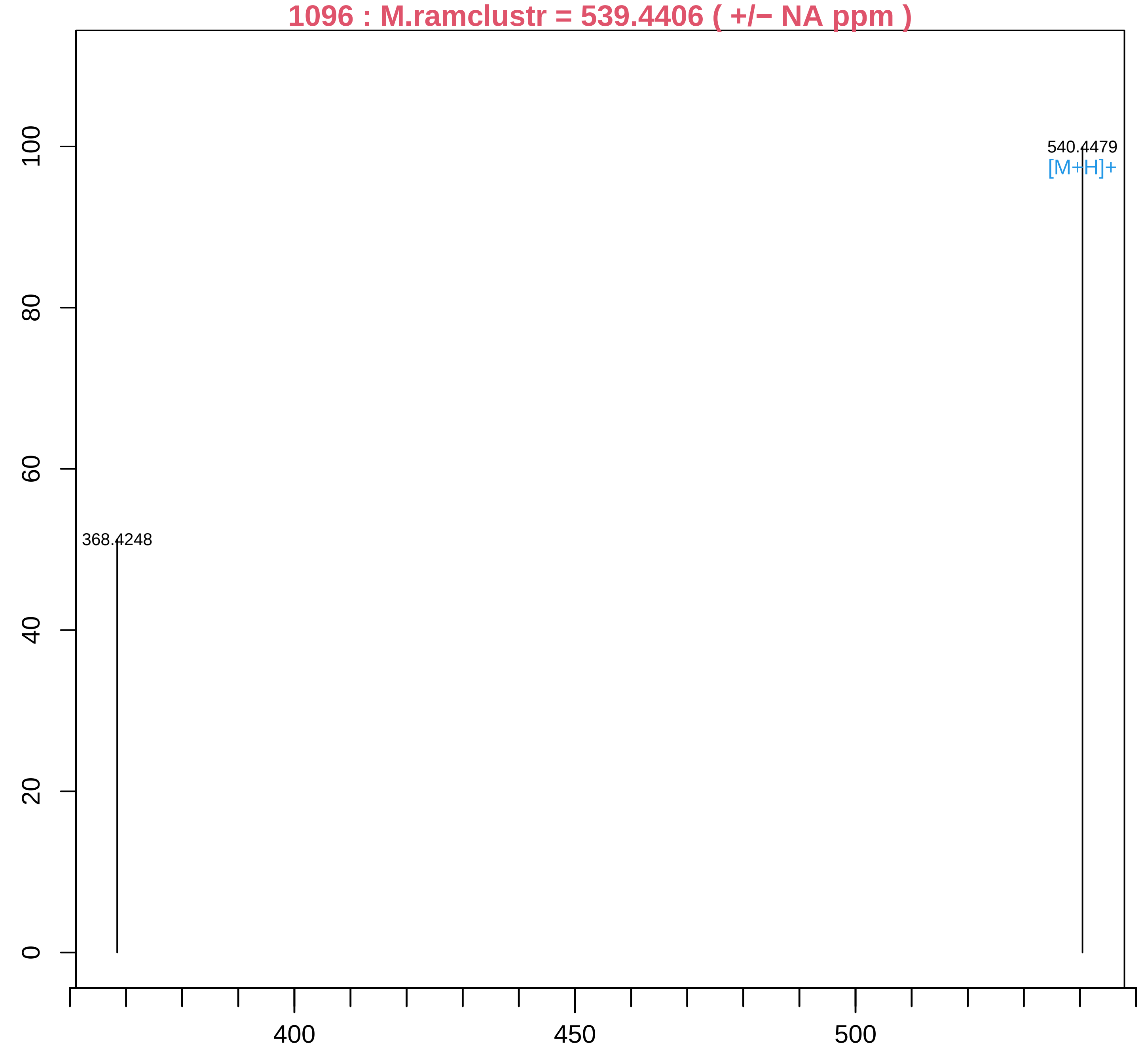
1096 : M.ramclustr = 539.4406 (+/− NA ppm)
540.4479
[M+H]+
368.4248
0
20
40
60
80
100
400
450
500

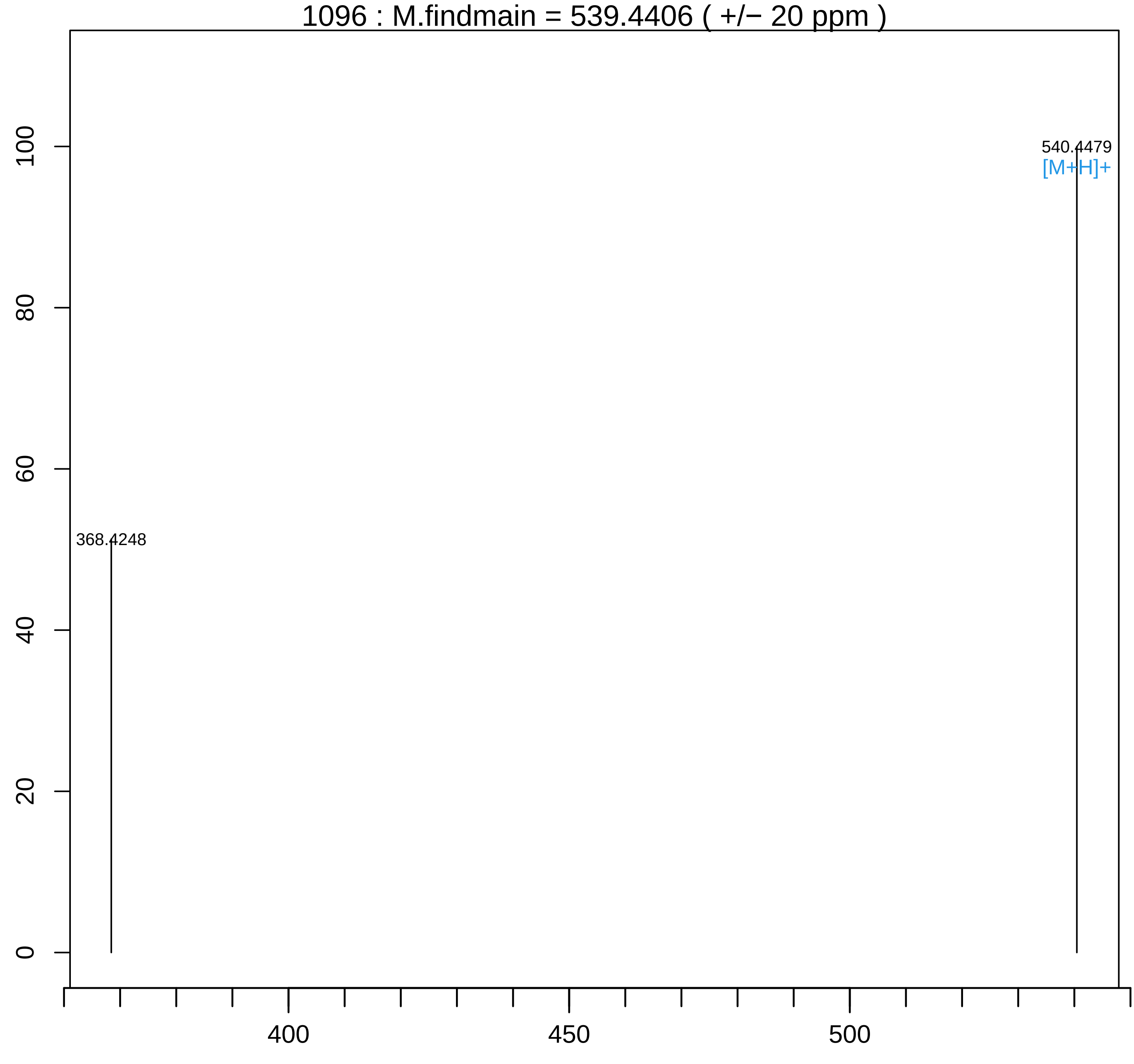
1096 : M.findmain = 539.4406 (+/− 20 ppm)
540.4479
[M+H]+
368.4248
0
20
40
60
80
100
400
450
500

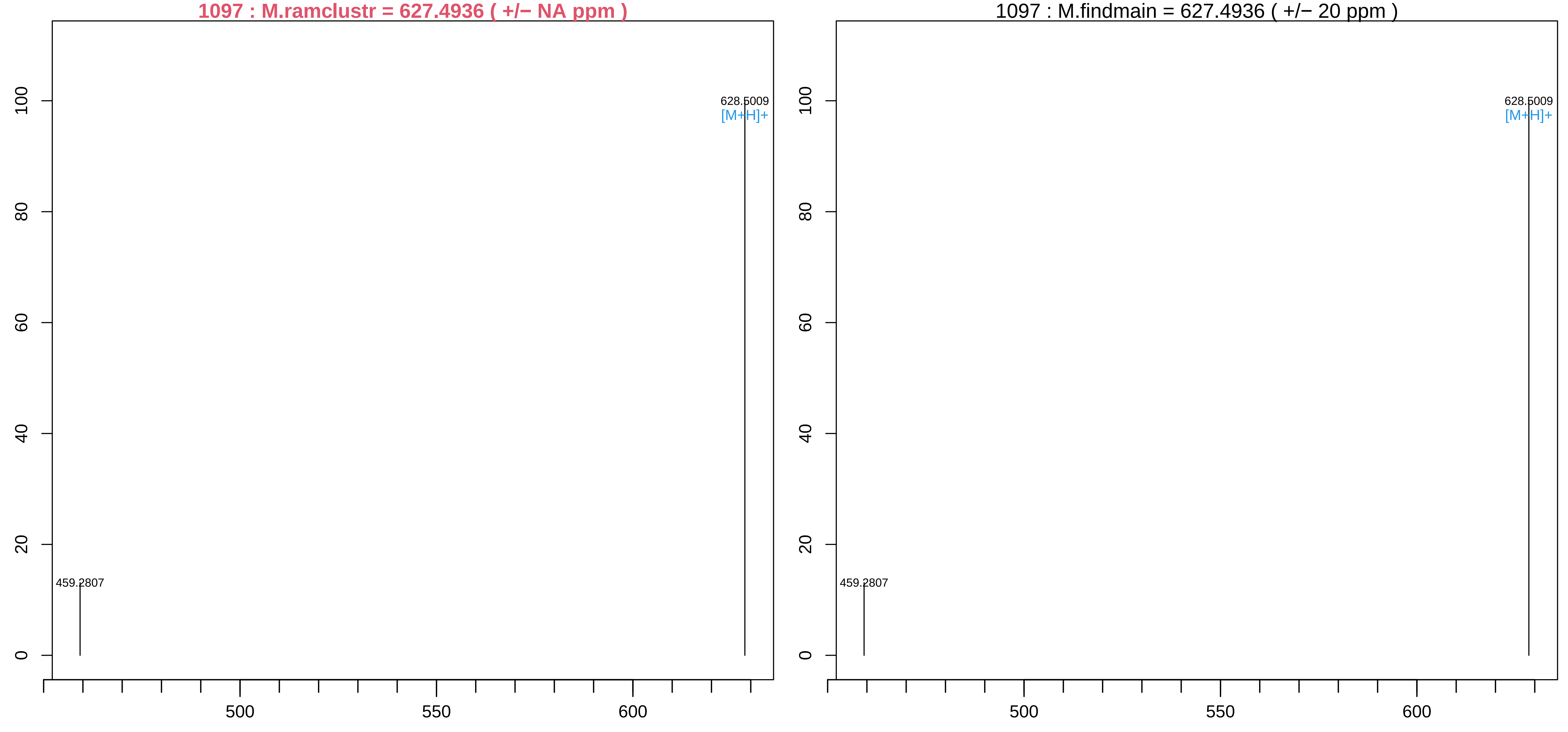
1097 : M.ramclustr = 627.4936 (+/− NA ppm)
628.5009
[M+H]+
459.2807
1097 : M.findmain = 627.4936 (+/− 20 ppm)
628.5009
[M+H]+
459.2807
0
20
40
60
80
100
500
550
600

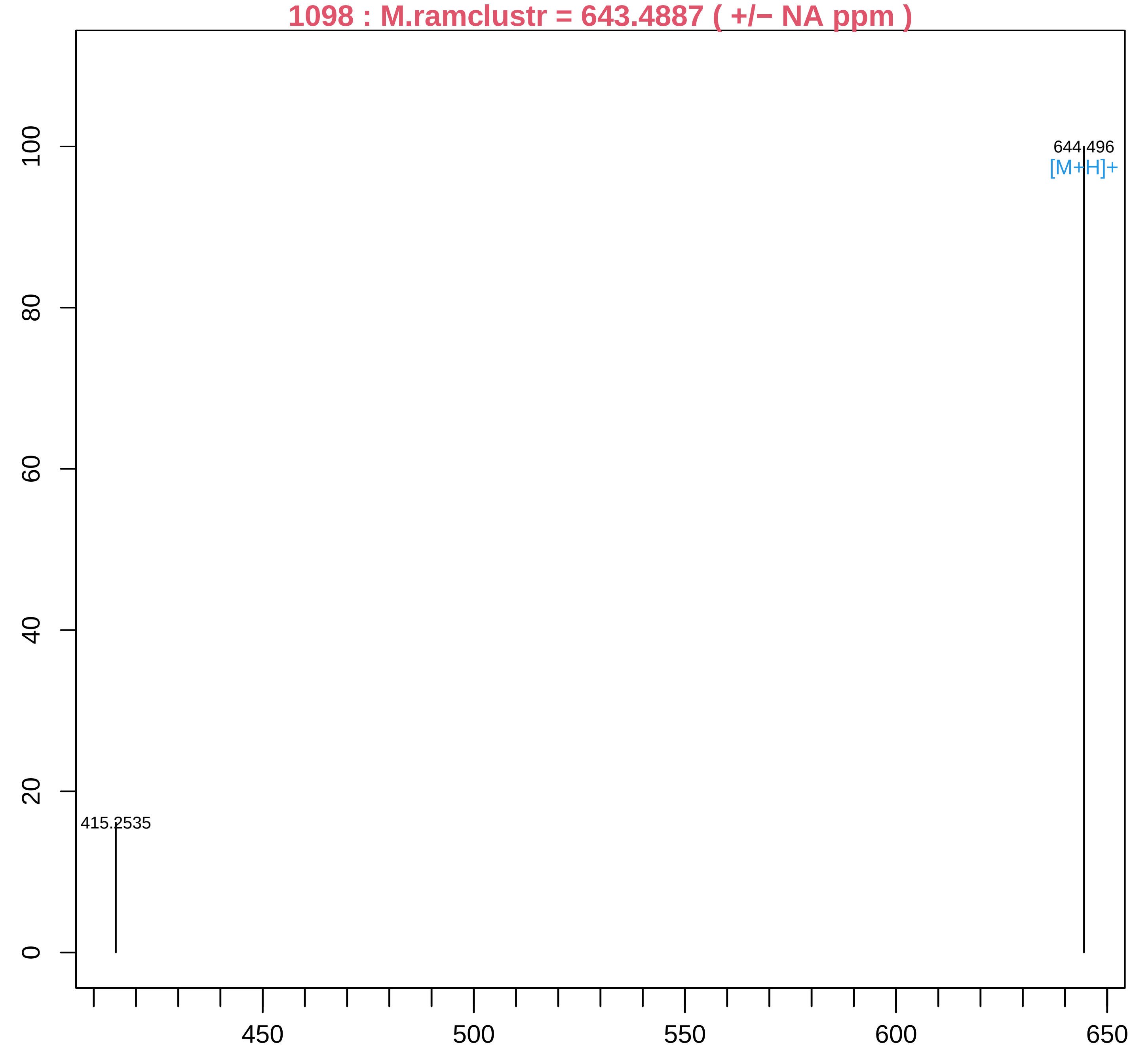

1098 : M.ramclustr = 643.4887 (+/− NA ppm)
644.496
[M+H]+
415.2535
0
20
40
60
80
100
450
500
550
600
650

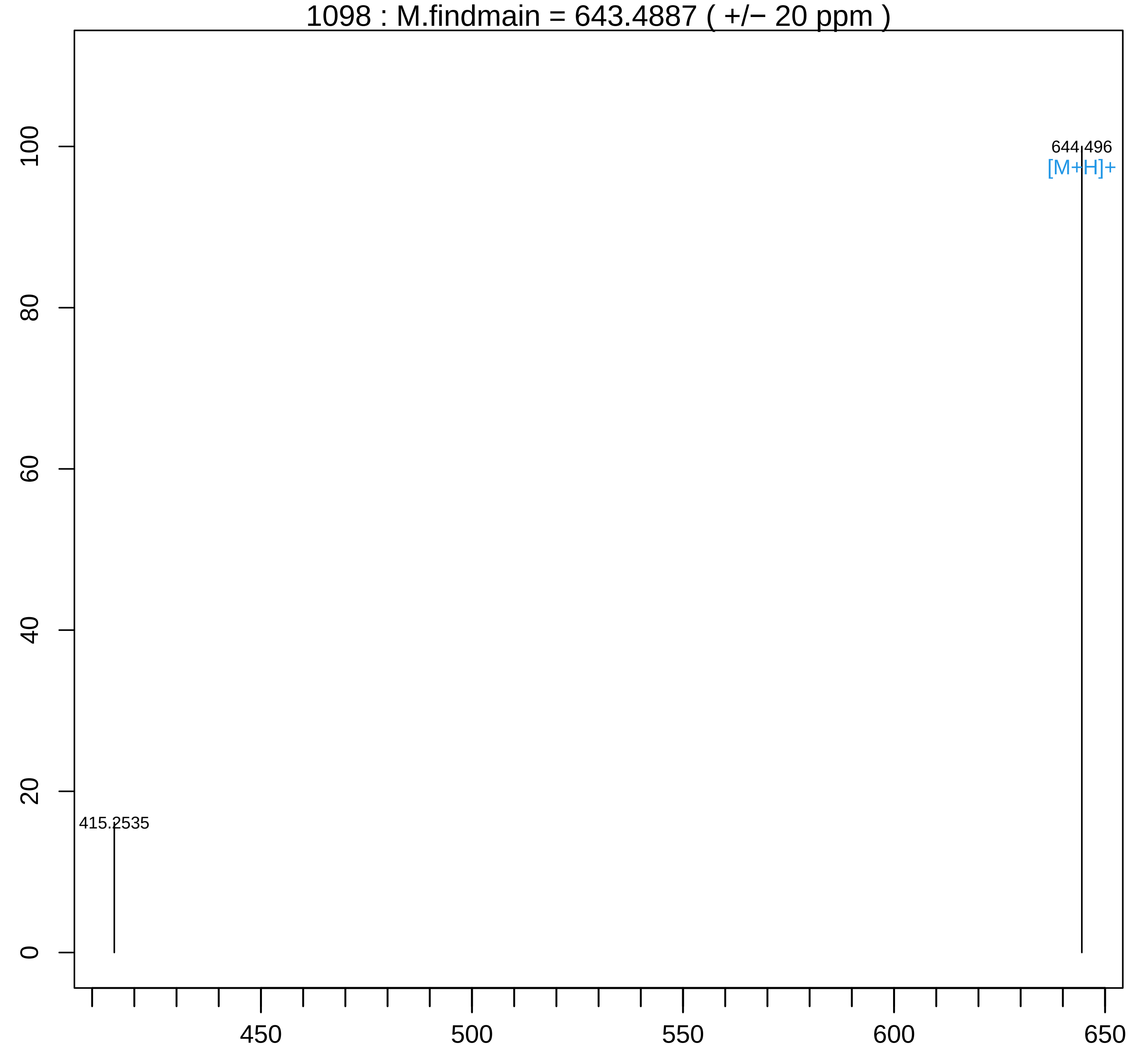

1098 : M.findmain = 643.4887 (+/− 20 ppm)
644.496
[M+H]+
415.2535
0
20
40
60
80
100
450
500
550
600
650

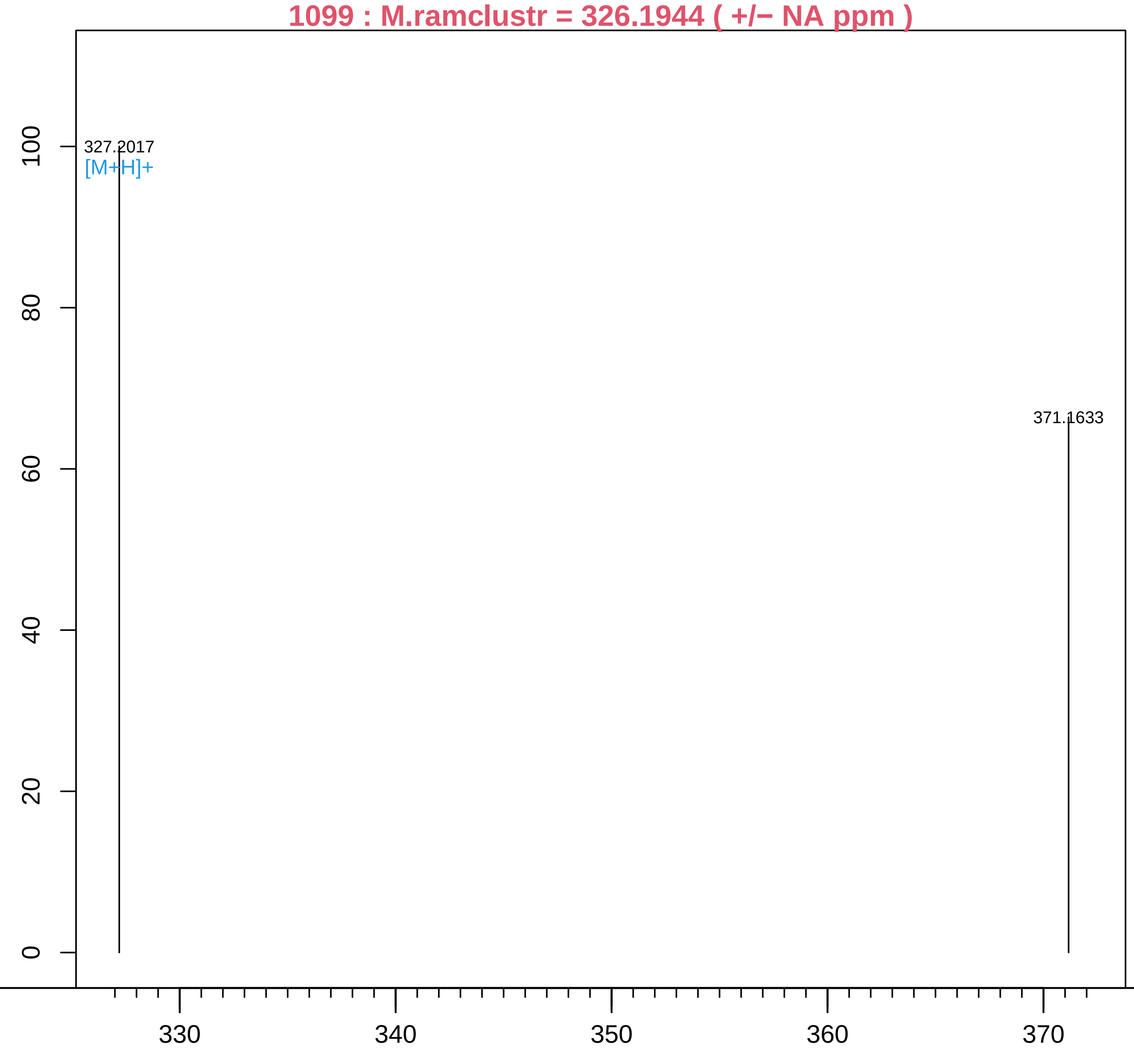
1099 : M.ramclustr = 326.1944 (+/− NA ppm)
327.2017
[M+H]+
371.1633
0
20
40
60
80
100
330
340
350
360
370

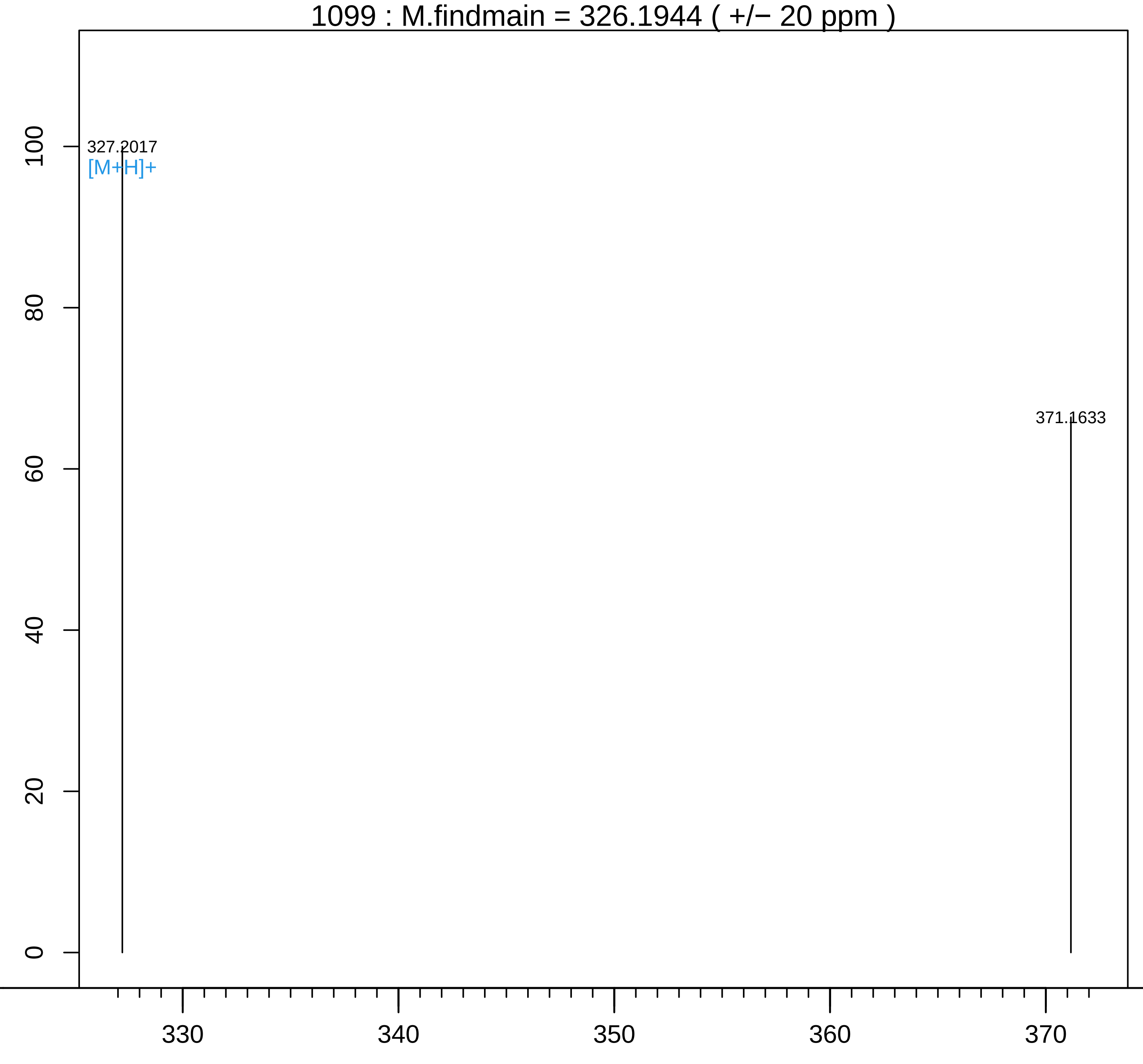
1099 : M.findmain = 326.1944 (+/− 20 ppm)
327.2017
[M+H]+
371.1633
0
20
40
60
80
100
330
340
350
360
370

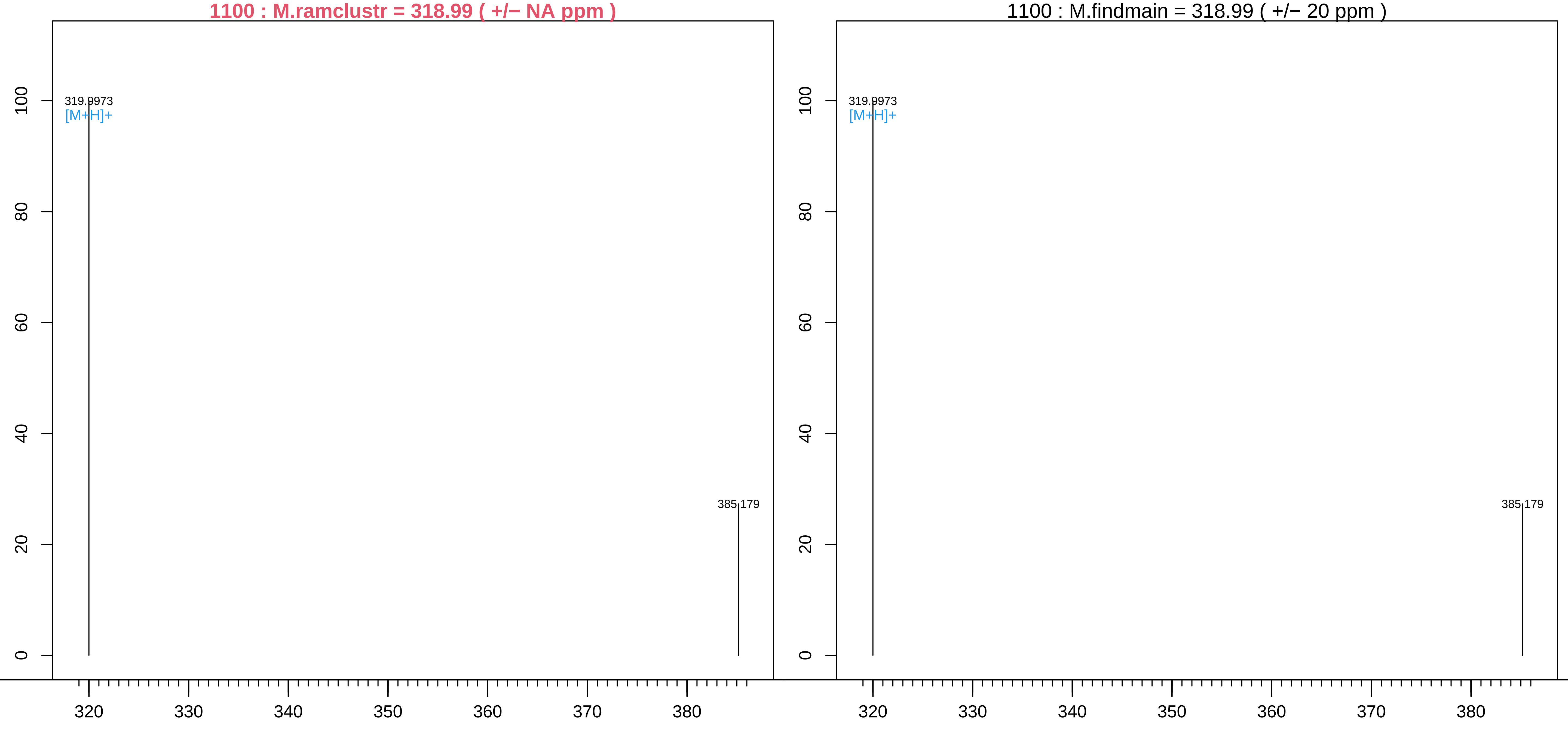
1100 : M.ramclustr = 318.99 (+/− NA ppm)
1100 : M.findmain = 318.99 (+/− 20 ppm)
319.9973
[M+H]+
385.179
319.9973
[M+H]+
385.179
0
20
40
60
80
100
320
330
340
350
360
370
380

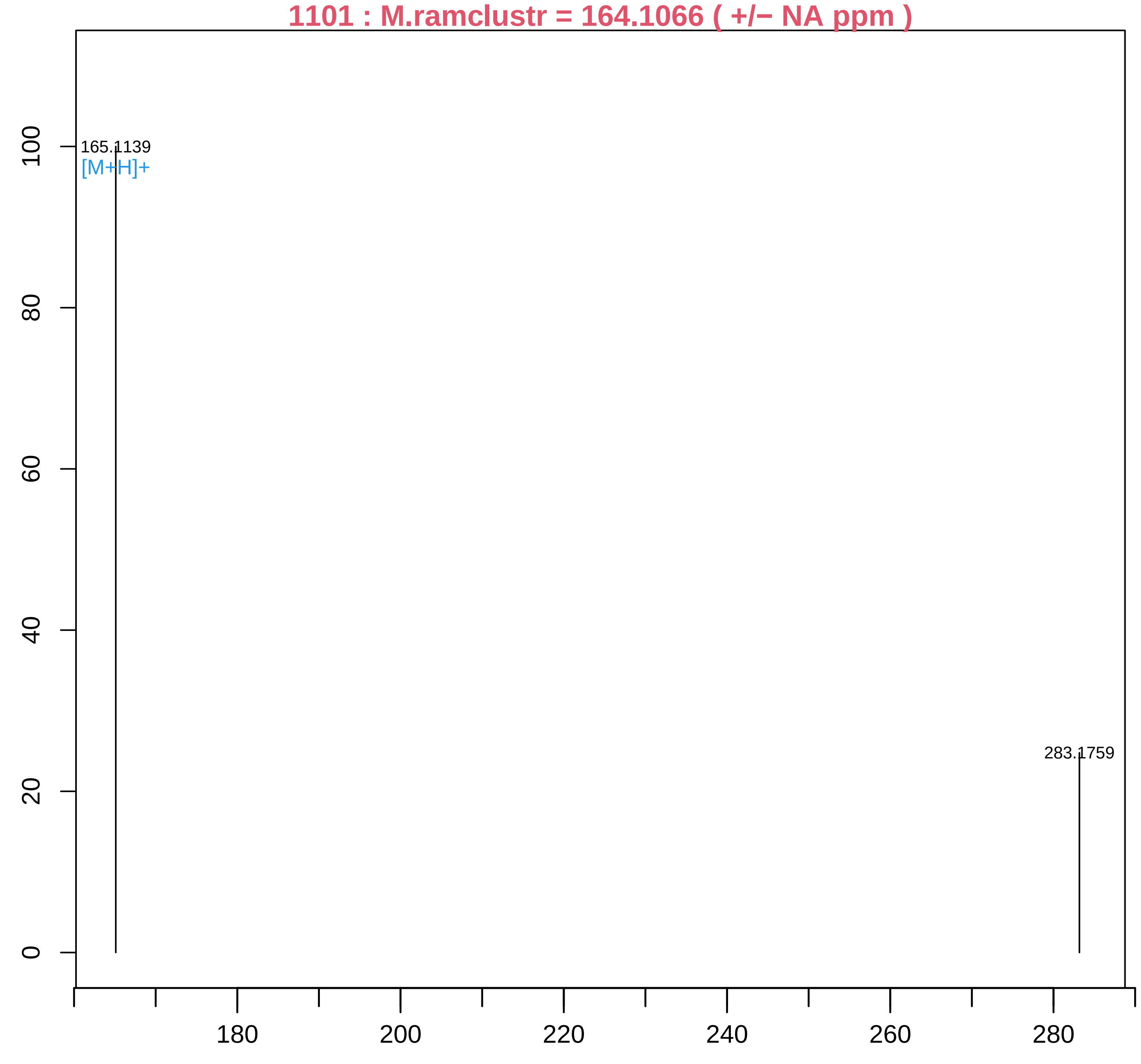

1101 : M.ramclustr = 164.1066 (+/− NA ppm)
165.1139
[M+H]+
283.1759
0
20
40
60
80
100
180
200
220
240
260
280

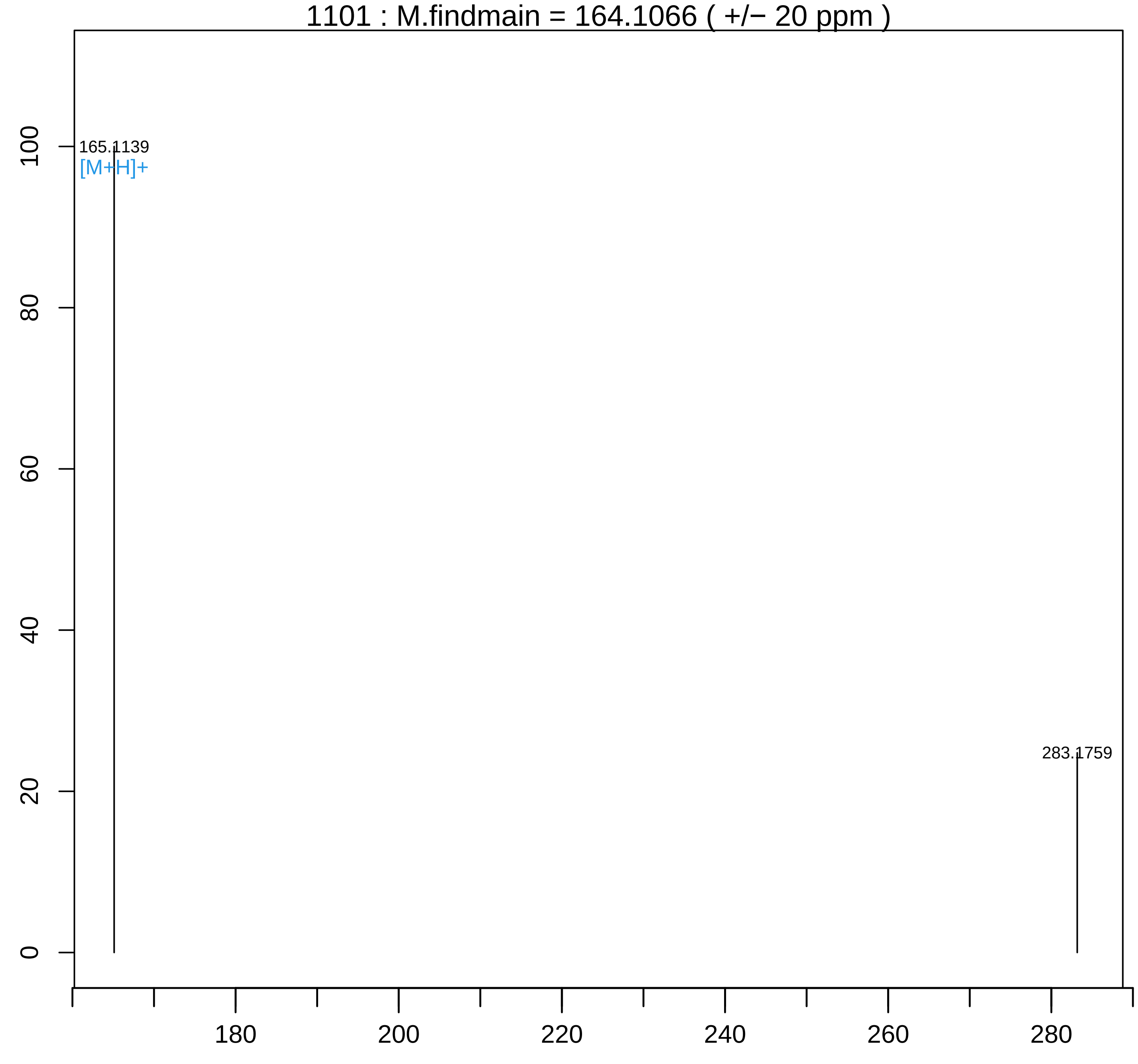

1101 : M.findmain = 164.1066 (+/− 20 ppm)
165.1139
[M+H]+
283.1759
0
20
40
60
80
100
180
200
220
240
260
280

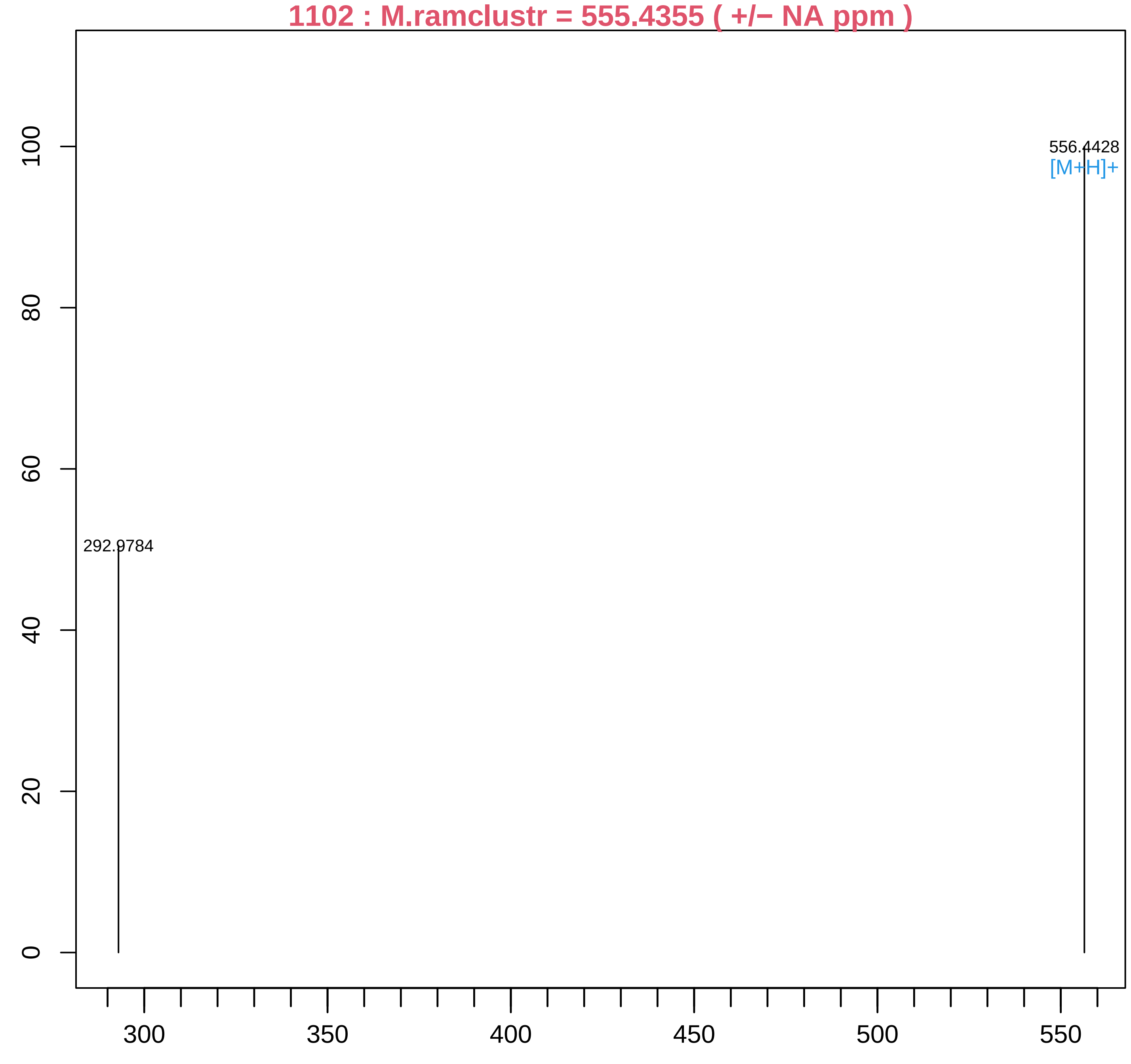
1102 : M.ramclustr = 555.4355 (+/− NA ppm)
556.4428
[M+H]+
292.9784
0
20
40
60
80
100
300
350
400
450
500
550

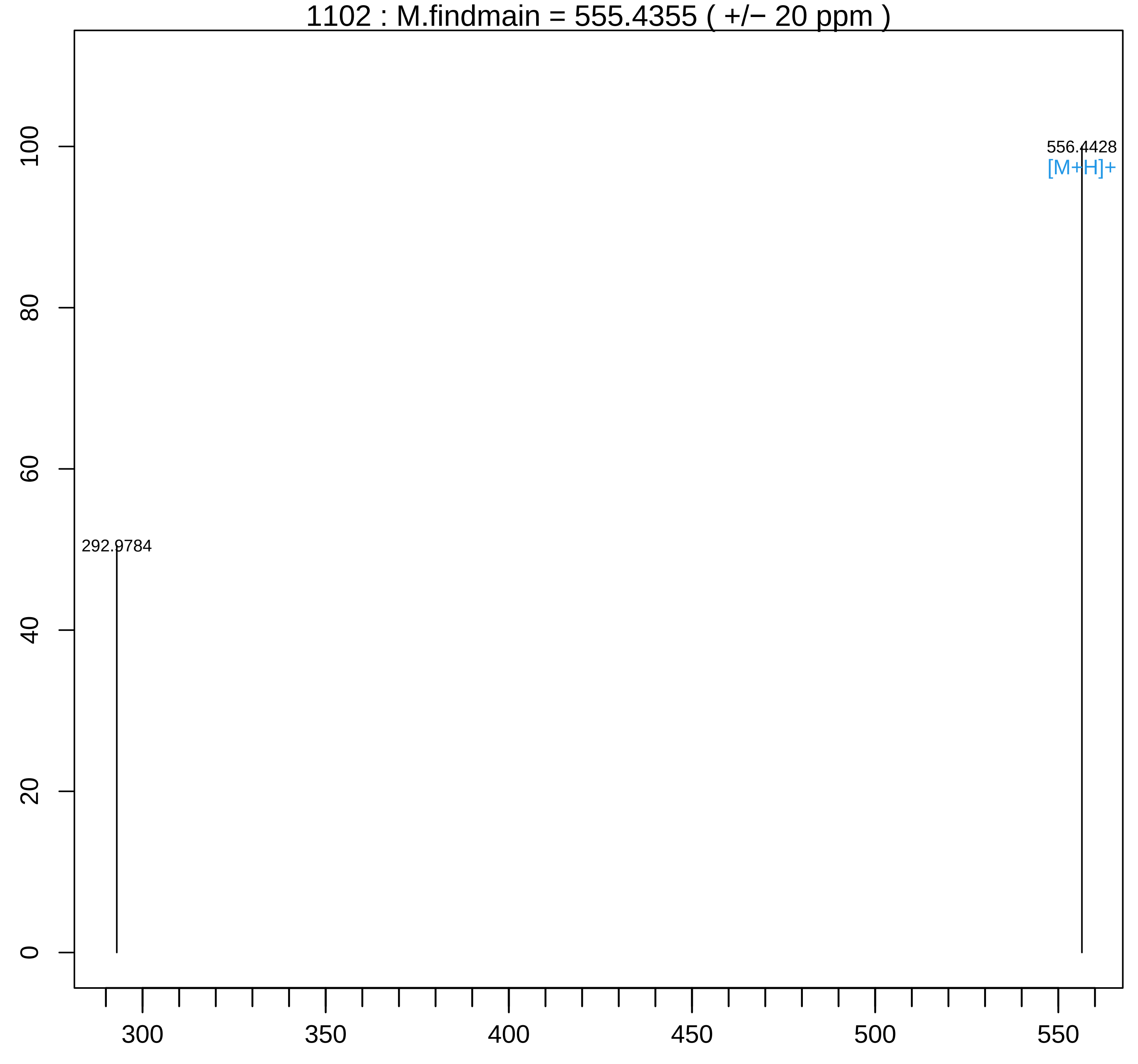
1102 : M.findmain = 555.4355 (+/− 20 ppm)
556.4428
[M+H]+
292.9784
0
20
40
60
80
100
300
350
400
450
500
550

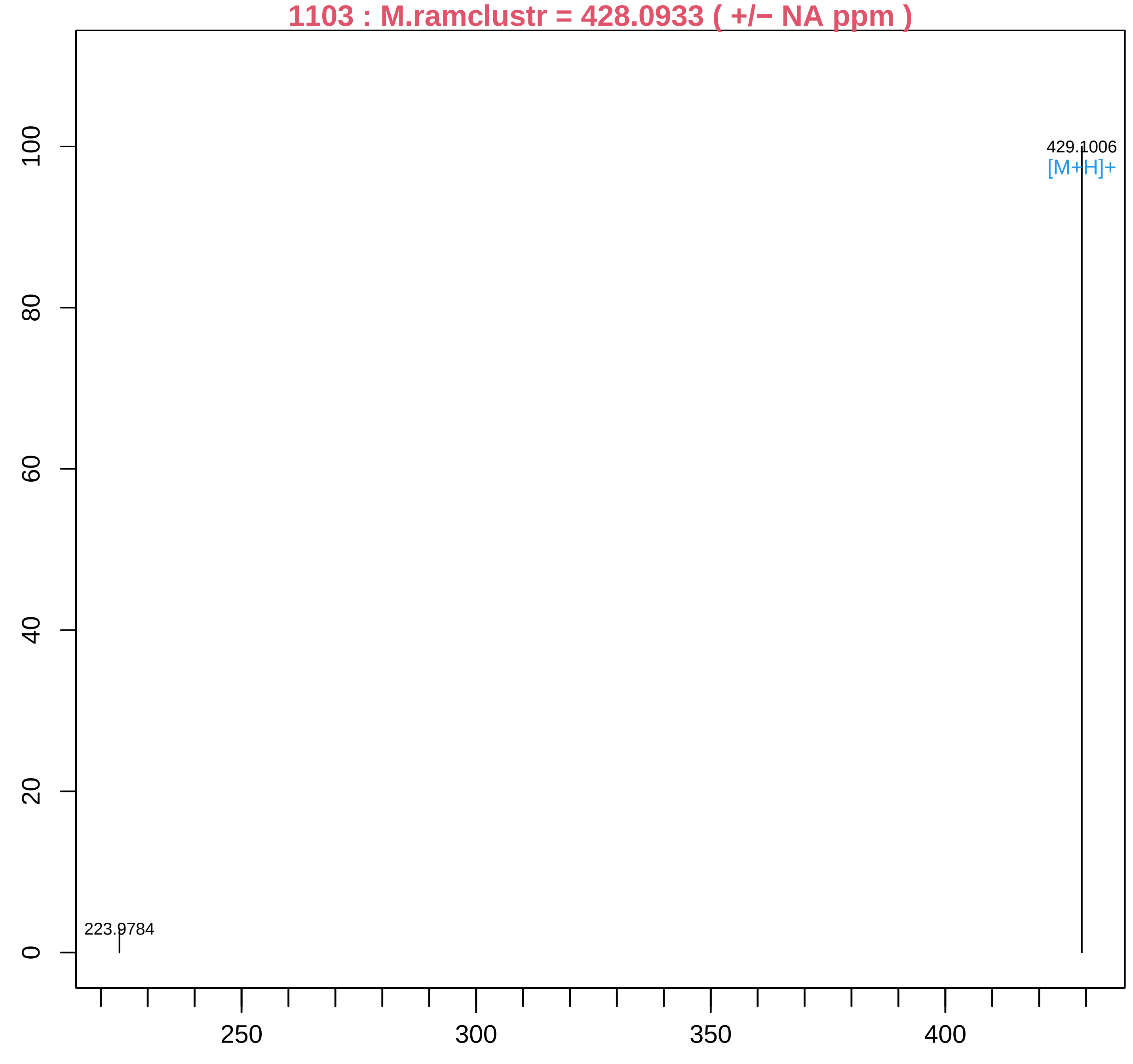
1103 : M.ramclustr = 428.0933 (+/− NA ppm)
429.1006
[M+H]+
223.9784
0
20
40
60
80
100
250
300
350
400

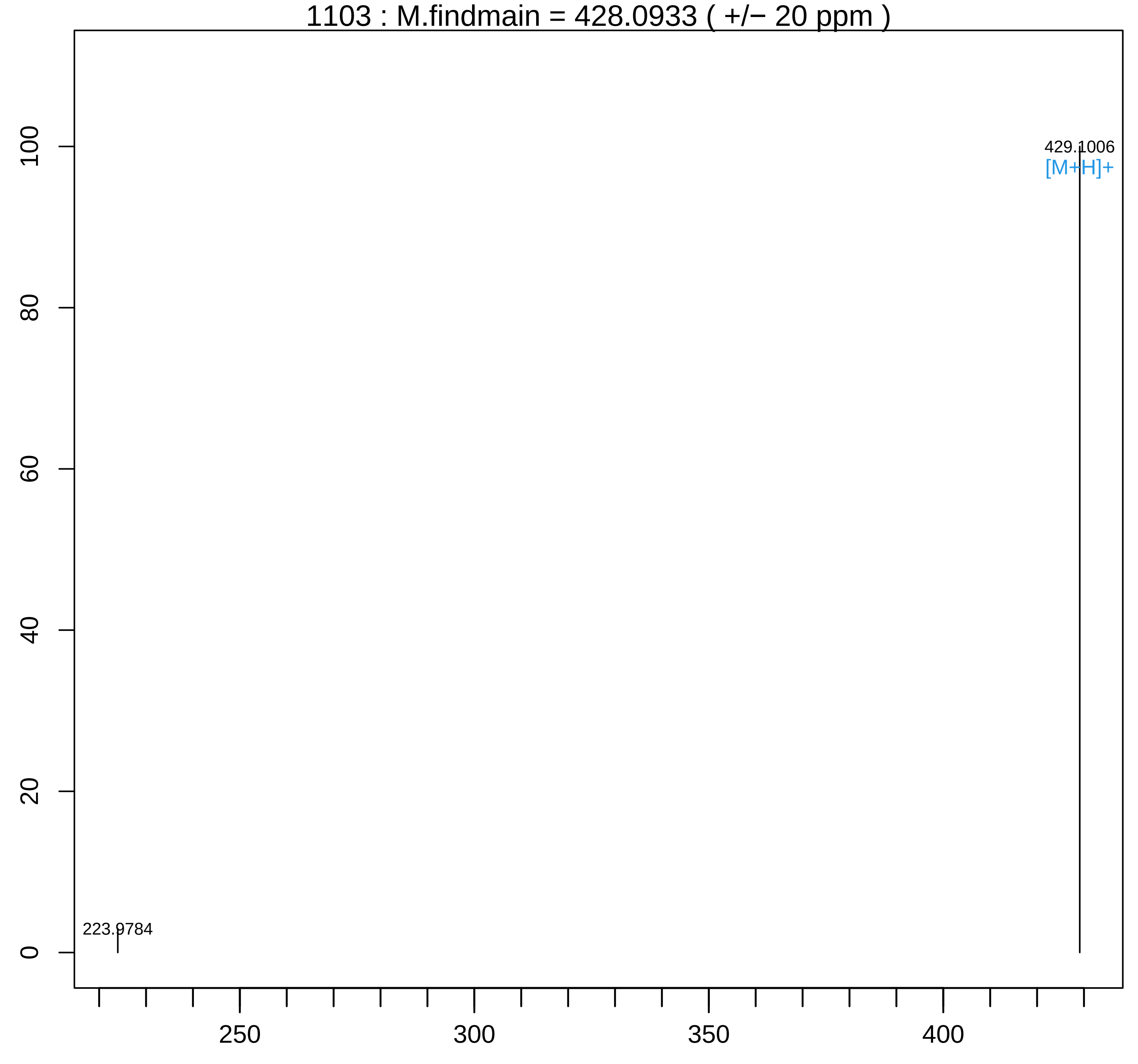
1103 : M.findmain = 428.0933 (+/− 20 ppm)
429.1006
[M+H]+
223.9784
0
20
40
60
80
100
250
300
350
400

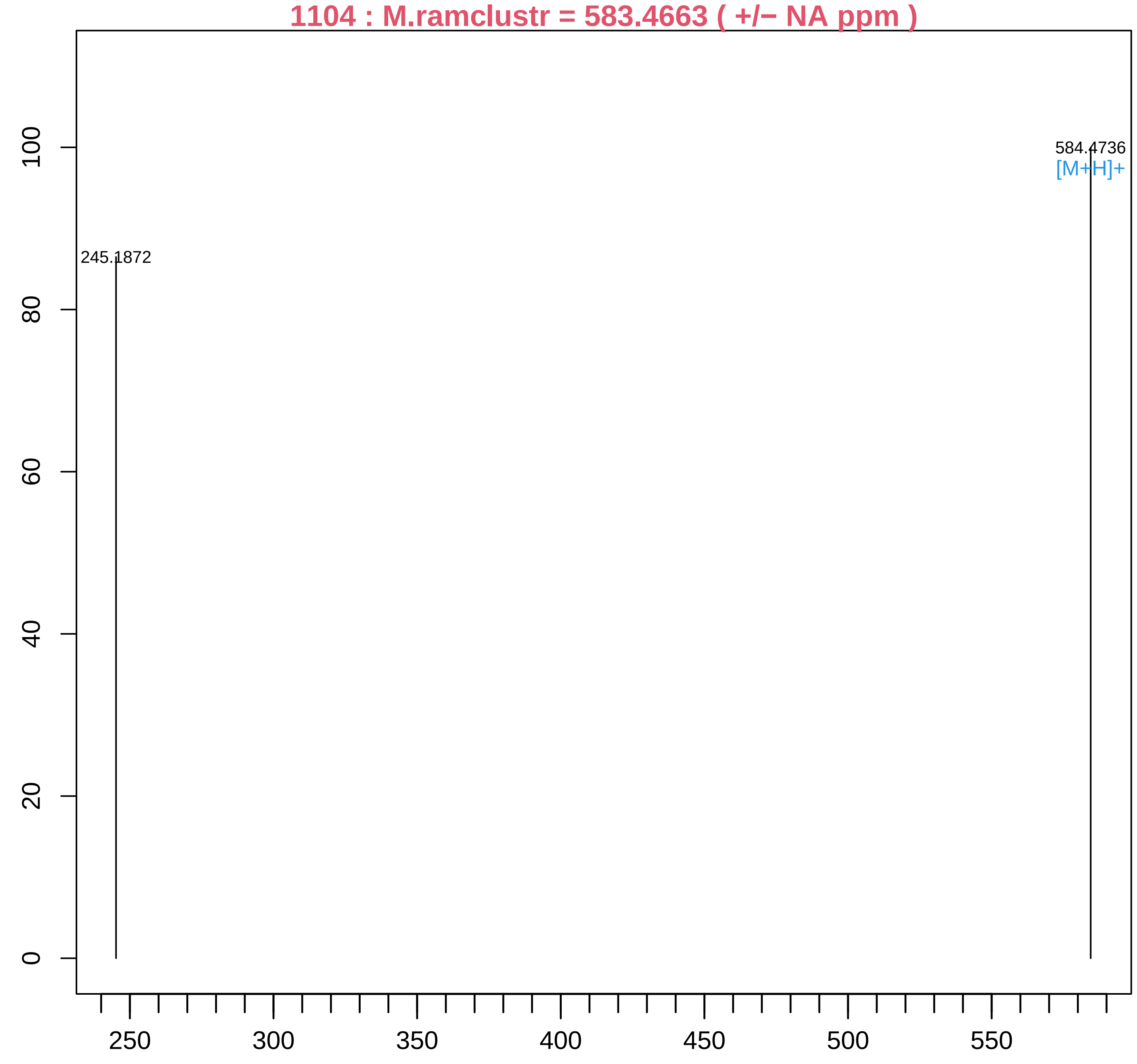

1104 : M.ramclustr = 583.4663 (+/− NA ppm)
584.4736
[M+H]+
245.1872
0
20
40
60
80
100
250
300
350
400
450
500
550

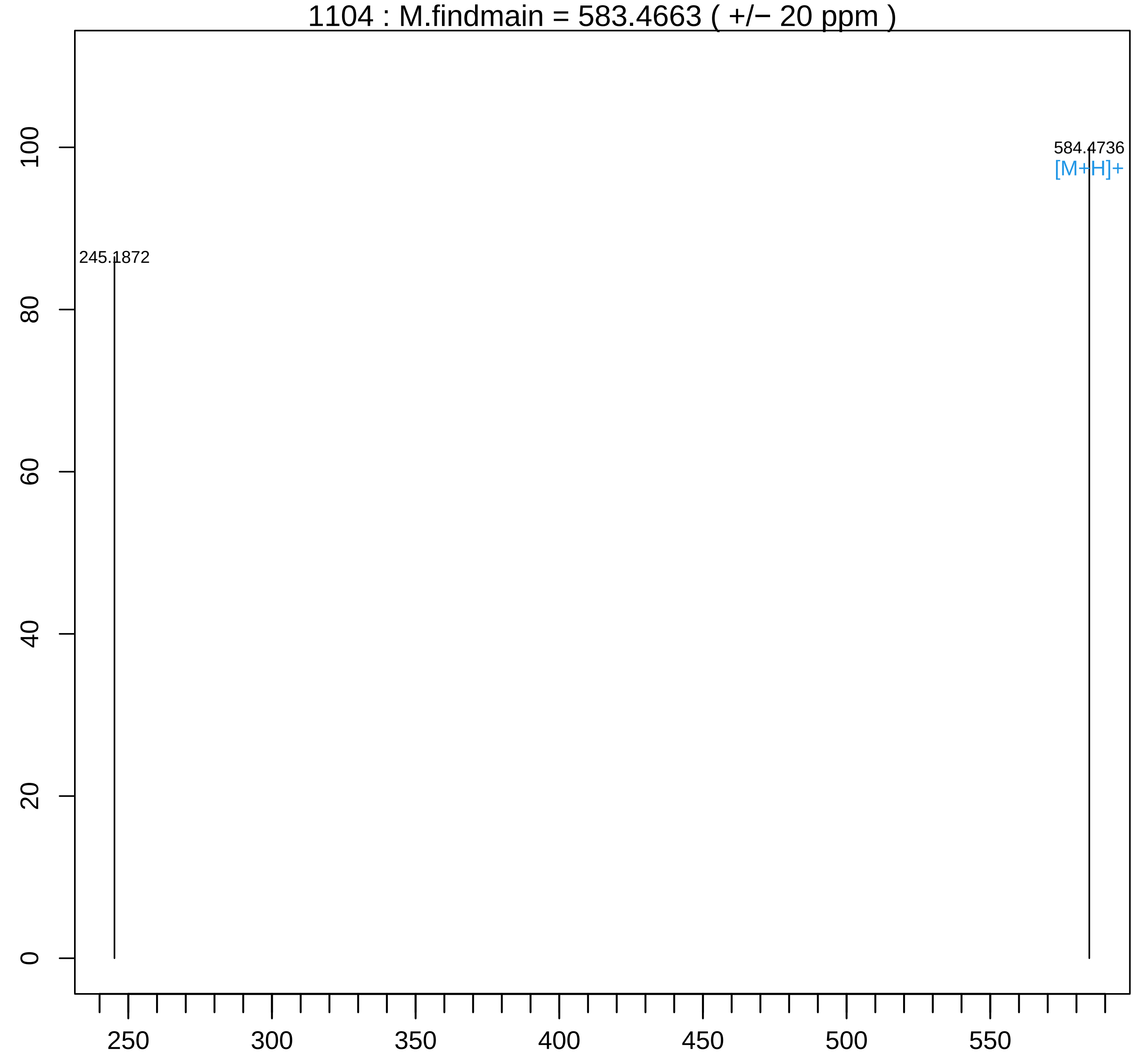

1104 : M.findmain = 583.4663 (+/− 20 ppm)
584.4736
[M+H]+
245.1872
0
20
40
60
80
100
250
300
350
400
450
500
550

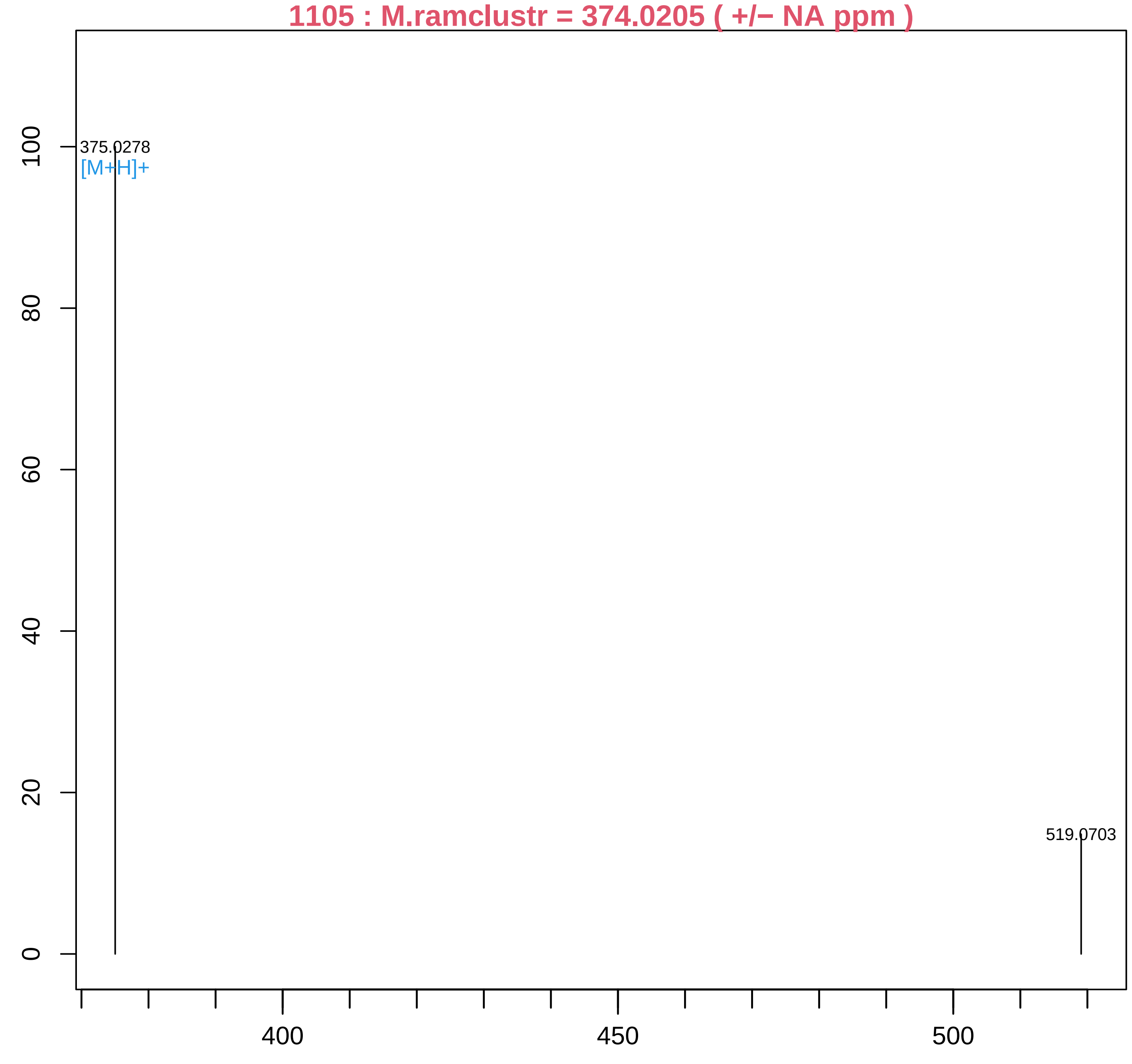
1105 : M.ramclustr = 374.0205 (+/− NA ppm)
375.0278
[M+H]+
519.0703
0
20
40
60
80
100
400
450
500

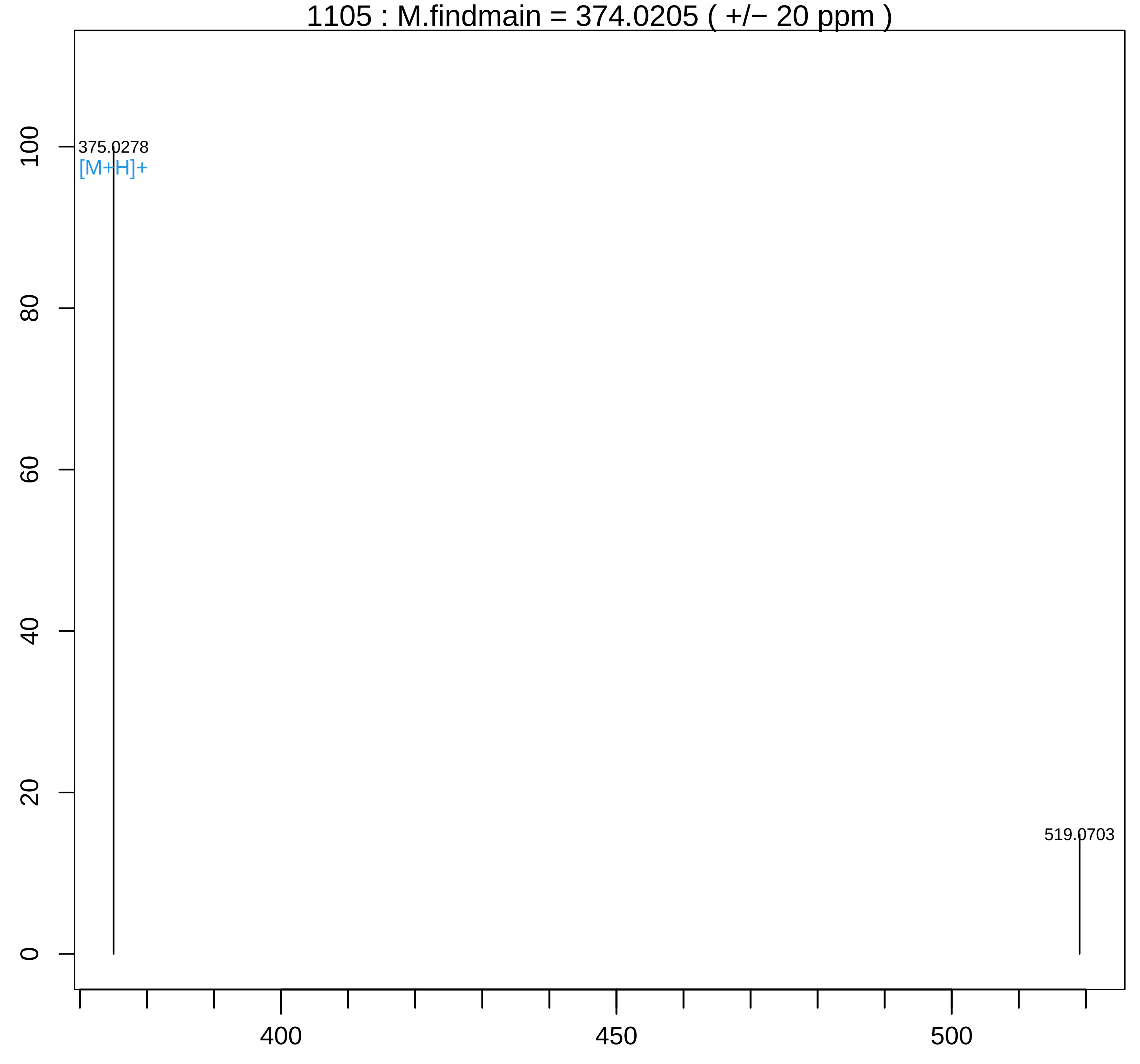
1105 : M.findmain = 374.0205 (+/− 20 ppm)
375.0278
[M+H]+
519.0703
0
20
40
60
80
100
400
450
500

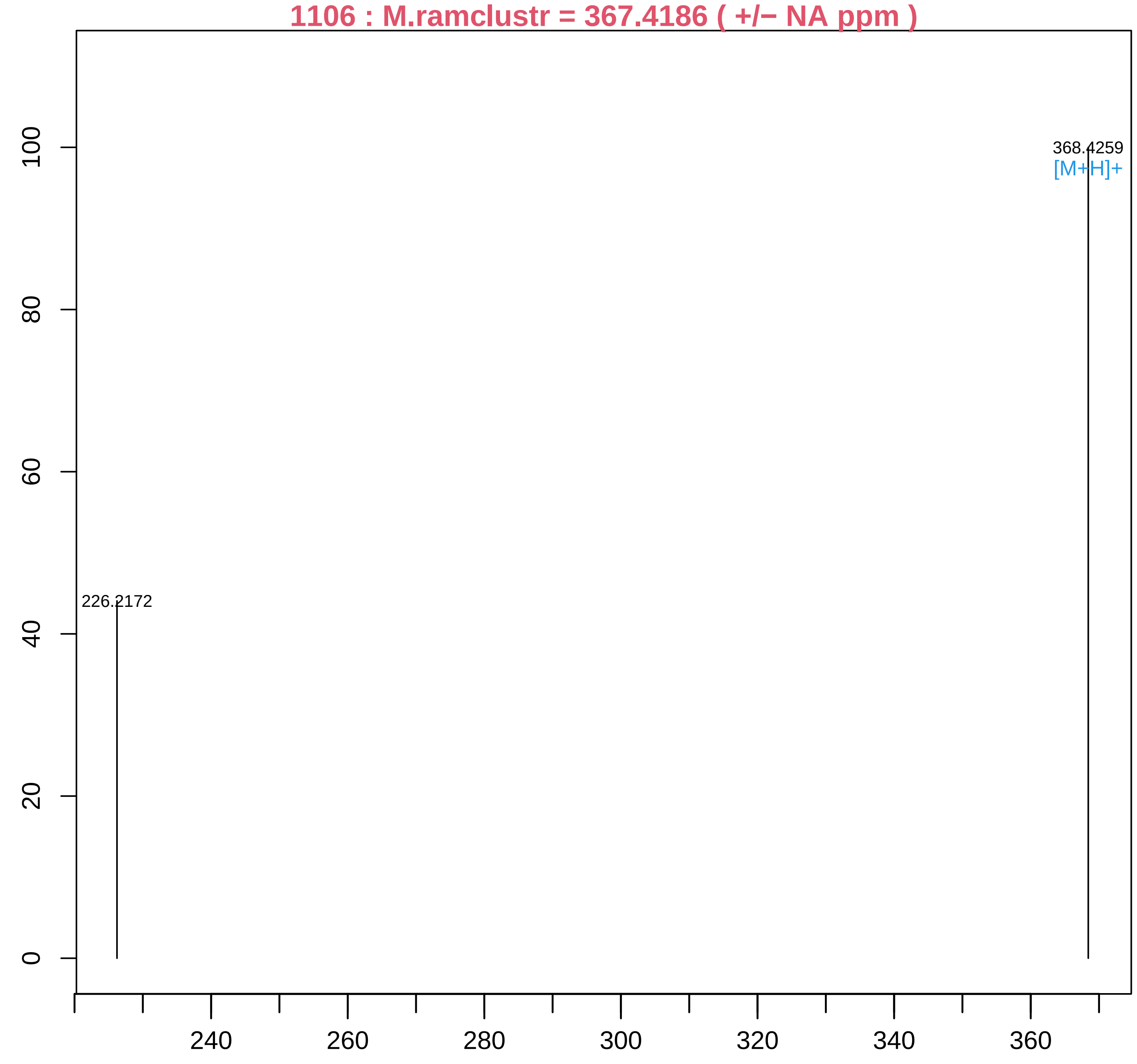
1106 : M.ramclustr = 367.4186 (+/− NA ppm)
368.4259
[M+H]+
226.2172
0
20
40
60
80
100
240
260
280
300
320
340
360

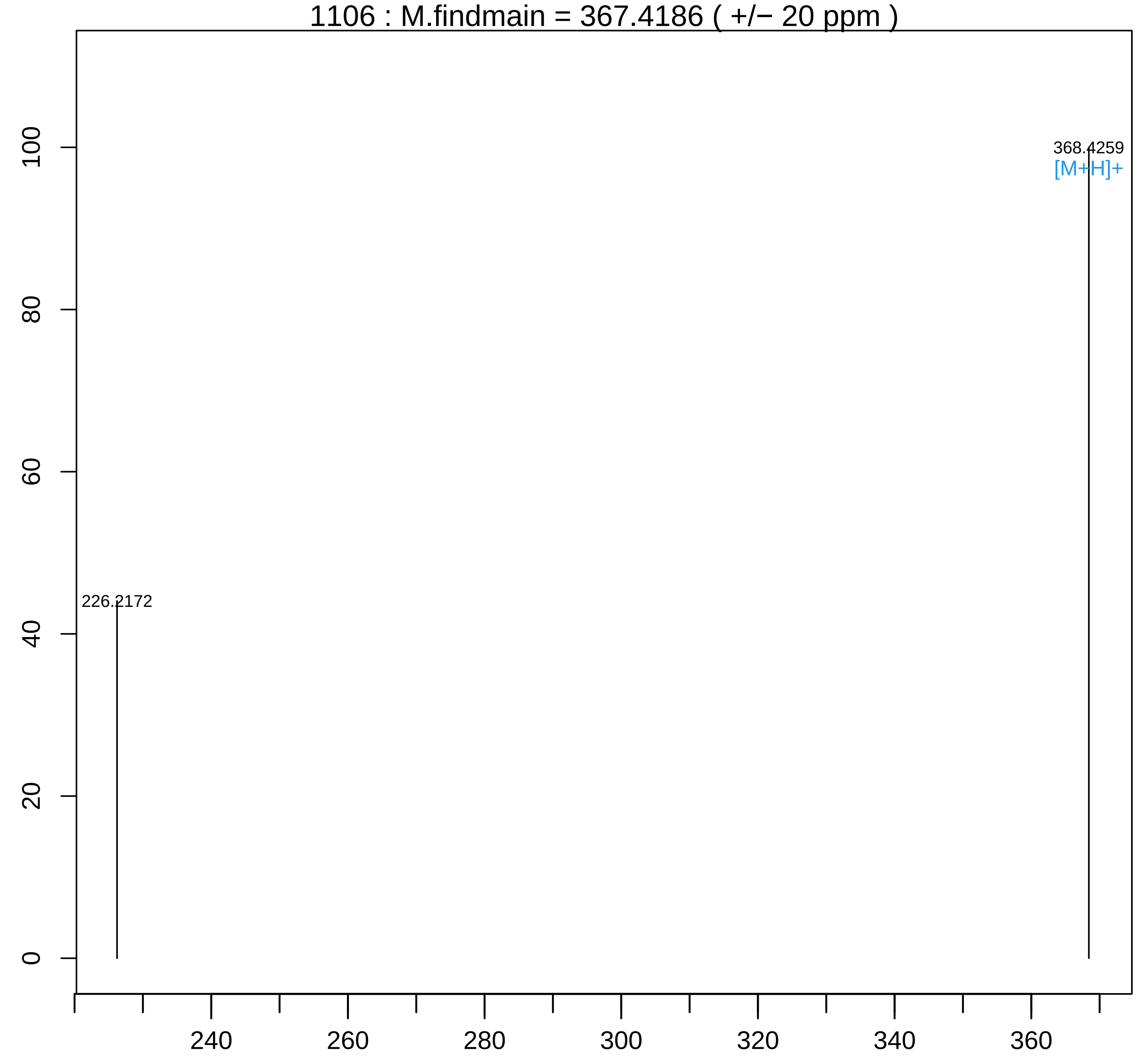
1106 : M.findmain = 367.4186 (+/− 20 ppm)
368.4259
[M+H]+
226.2172
0
20
40
60
80
100
240
260
280
300
320
340
360

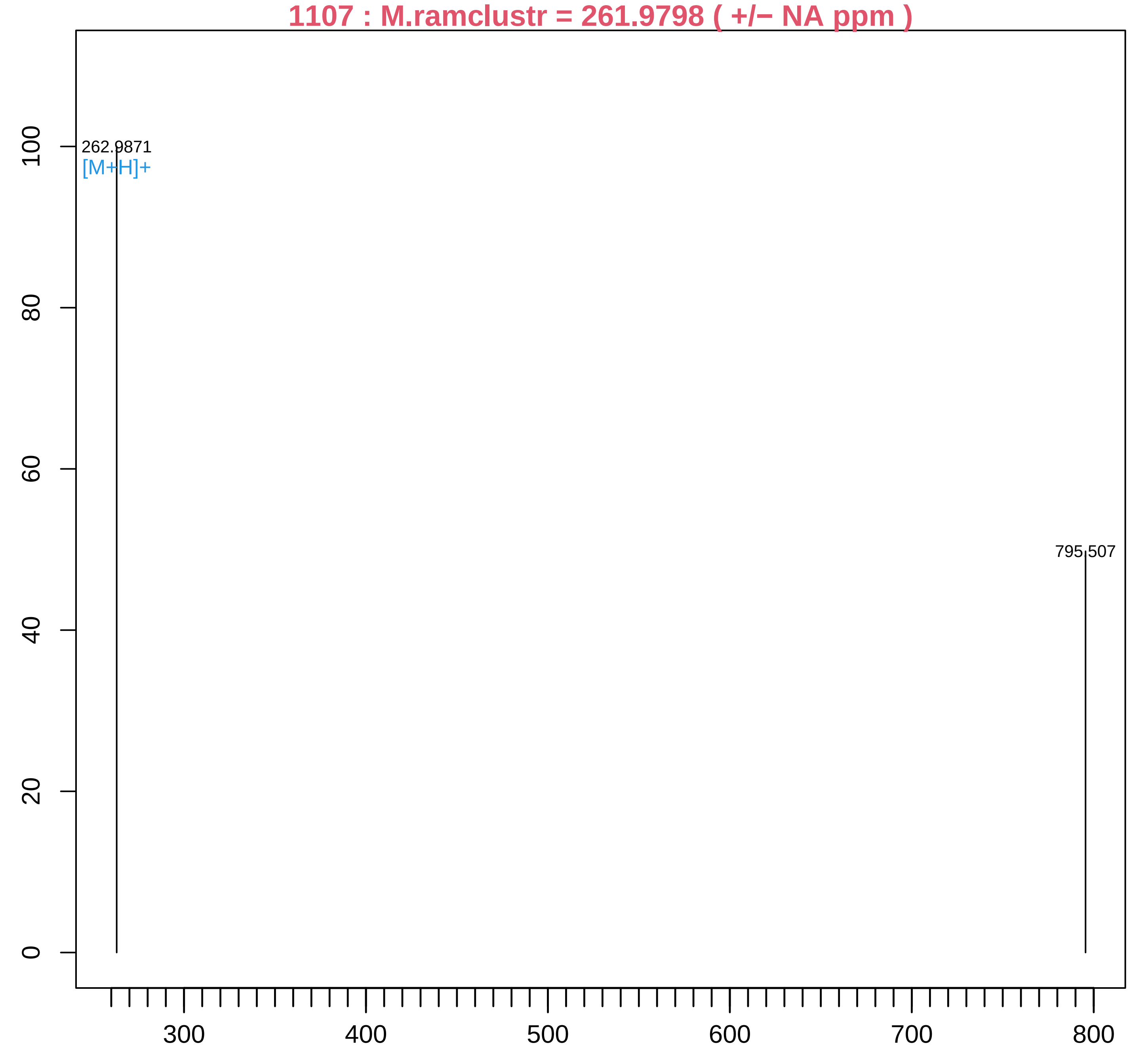
1107 : M.ramclustr = 261.9798 (+/− NA ppm)
262.9871
[M+H]+
795.507
0
20
40
60
80
100
300
400
500
600
700
800

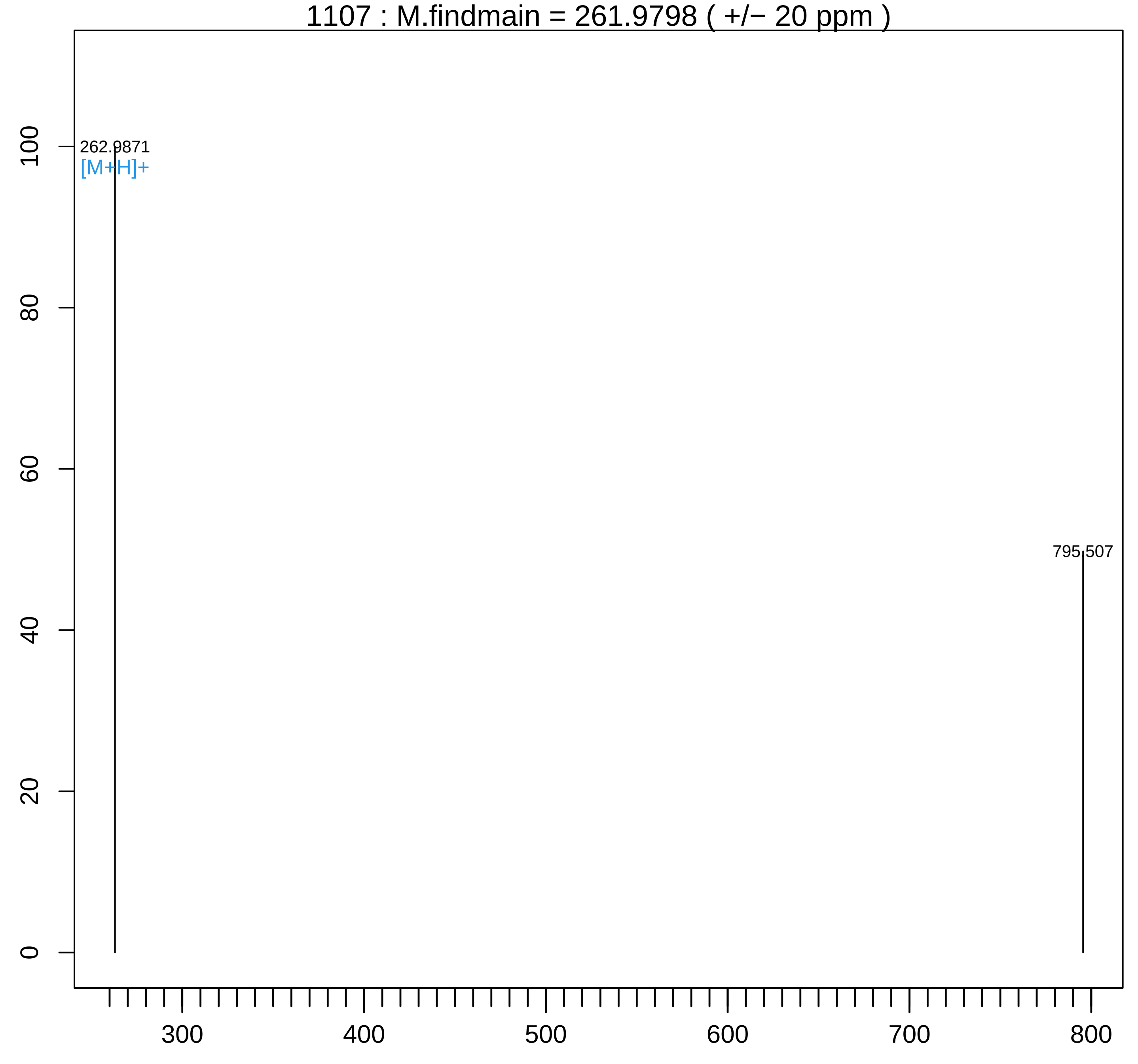
1107 : M.findmain = 261.9798 (+/− 20 ppm)
262.9871
[M+H]+
795.507
0
20
40
60
80
100
300
400
500
600
700
800

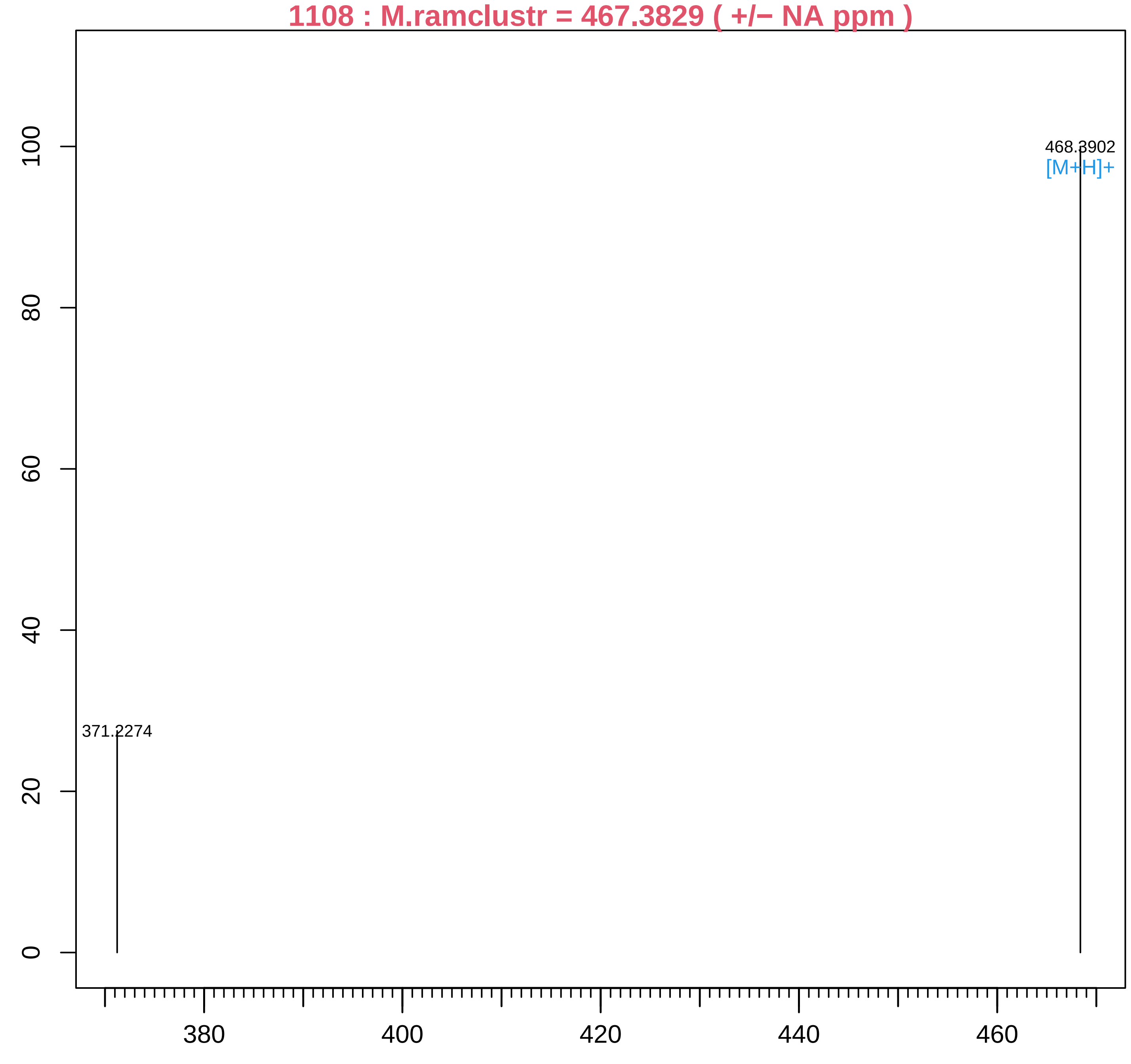
1108 : M.ramclustr = 467.3829 (+/− NA ppm)
468.3902
[M+H]+
371.2274
0
20
40
60
80
100
380
400
420
440
460

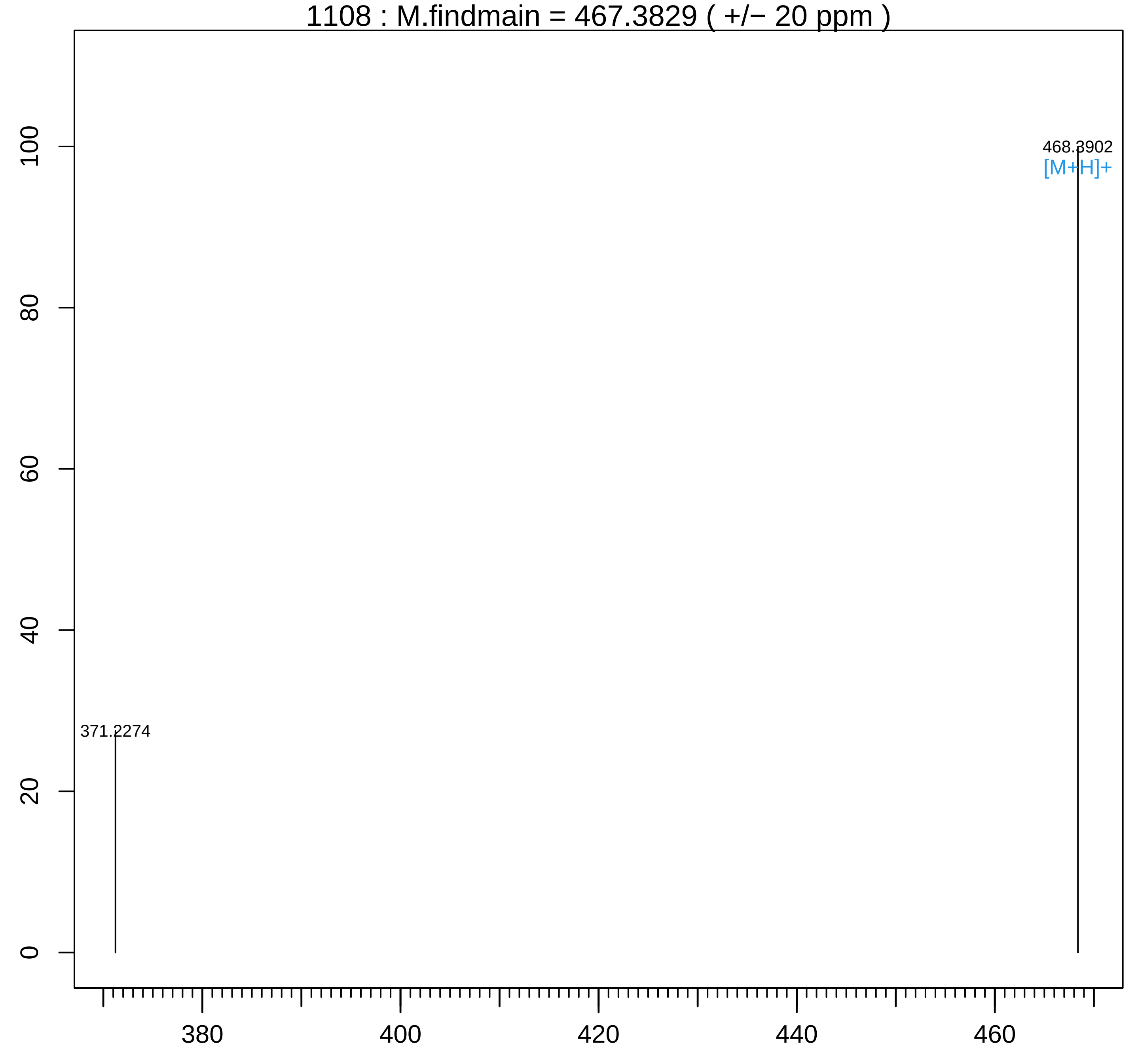
1108 : M.findmain = 467.3829 (+/− 20 ppm)
468.3902
[M+H]+
371.2274
0
20
40
60
80
100
380
400
420
440
460

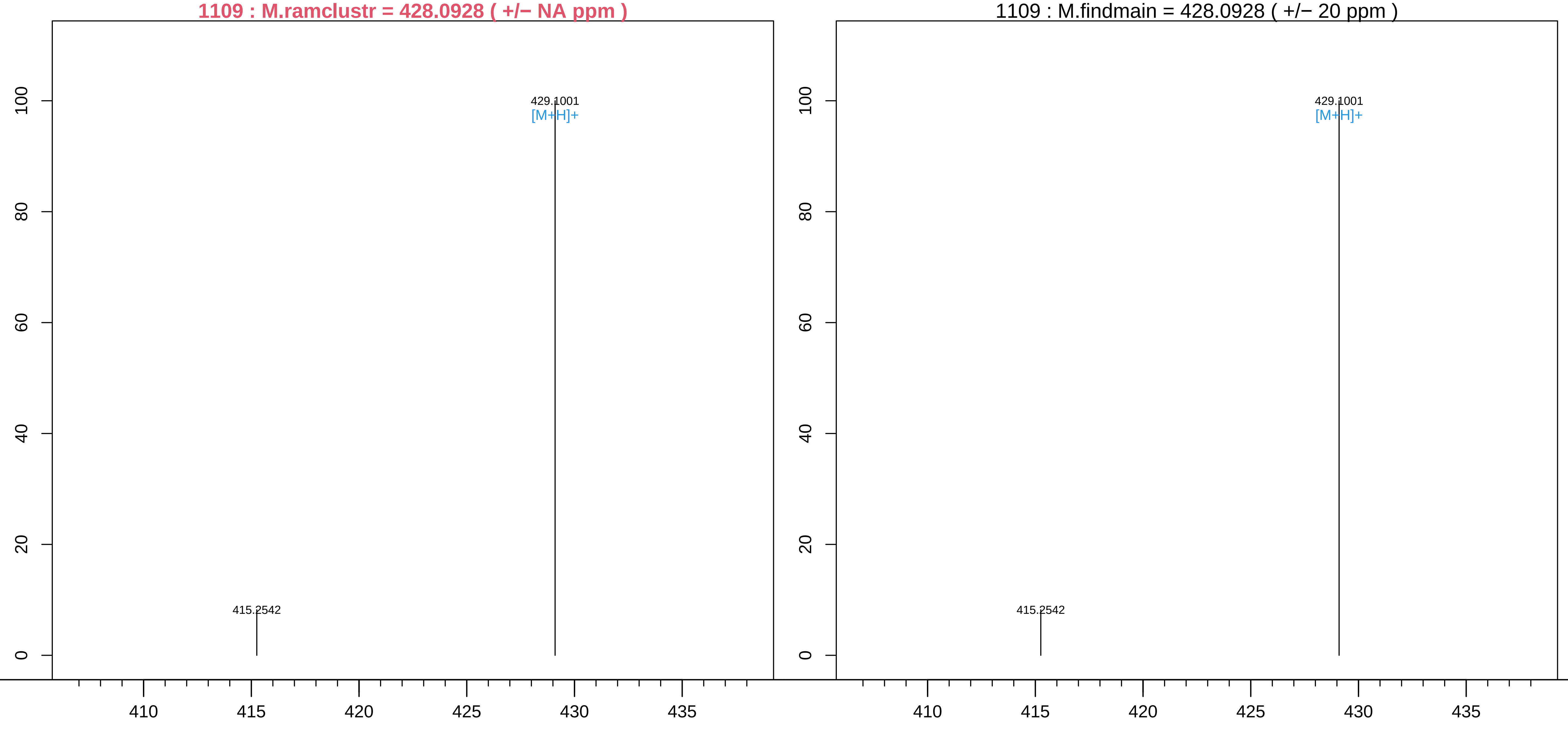
1109 : M.ramclustr = 428.0928 (+/− NA ppm)
1109 : M.findmain = 428.0928 (+/− 20 ppm)
429.1001
[M+H]+
415.2542
429.1001
[M+H]+
415.2542
0
20
40
60
80
100
410
415
420
425
430
435

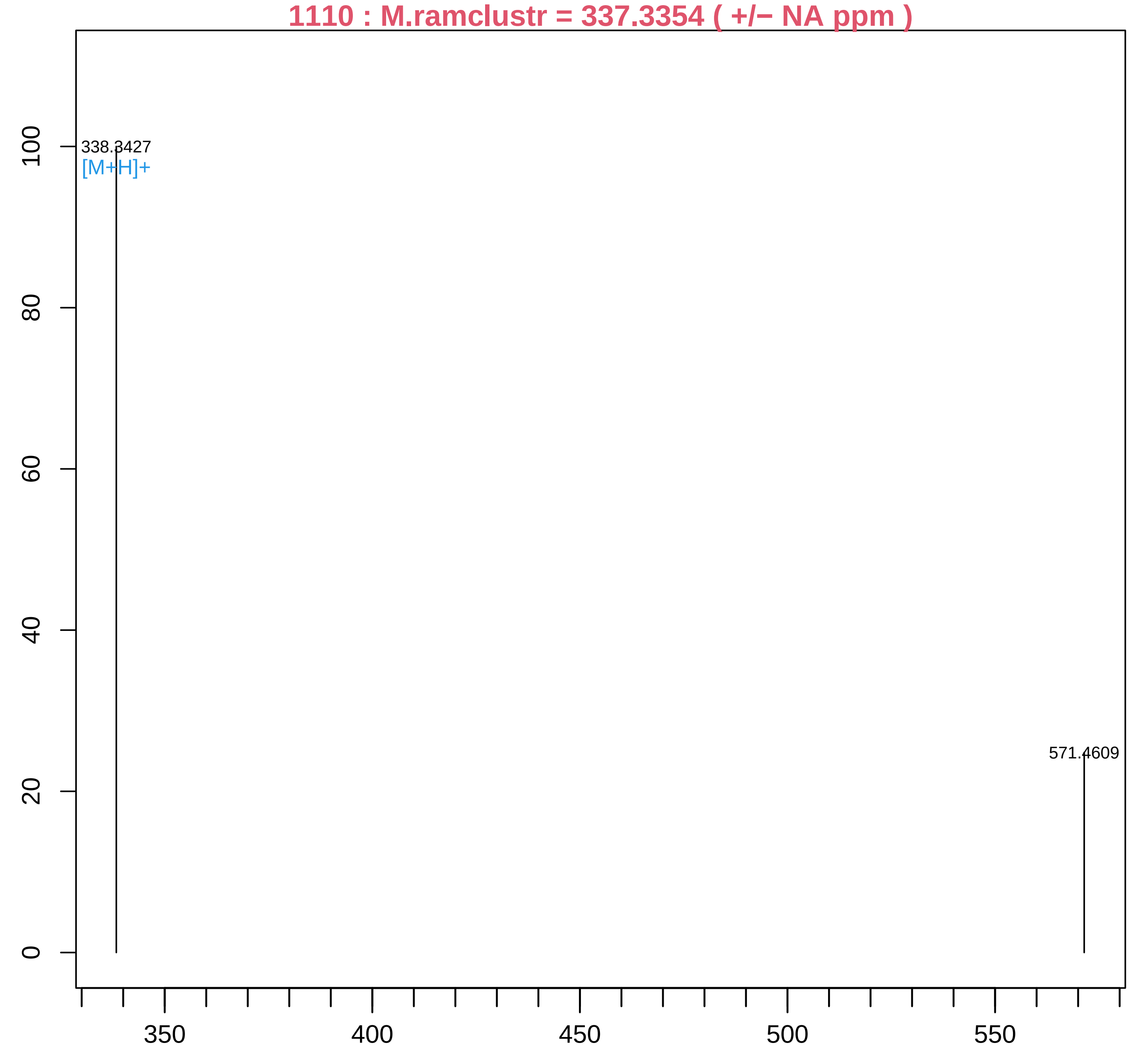
1110 : M.ramclustr = 337.3354 (+/− NA ppm)
338.3427
[M+H]+
571.4609
0
20
40
60
80
100
350
400
450
500
550

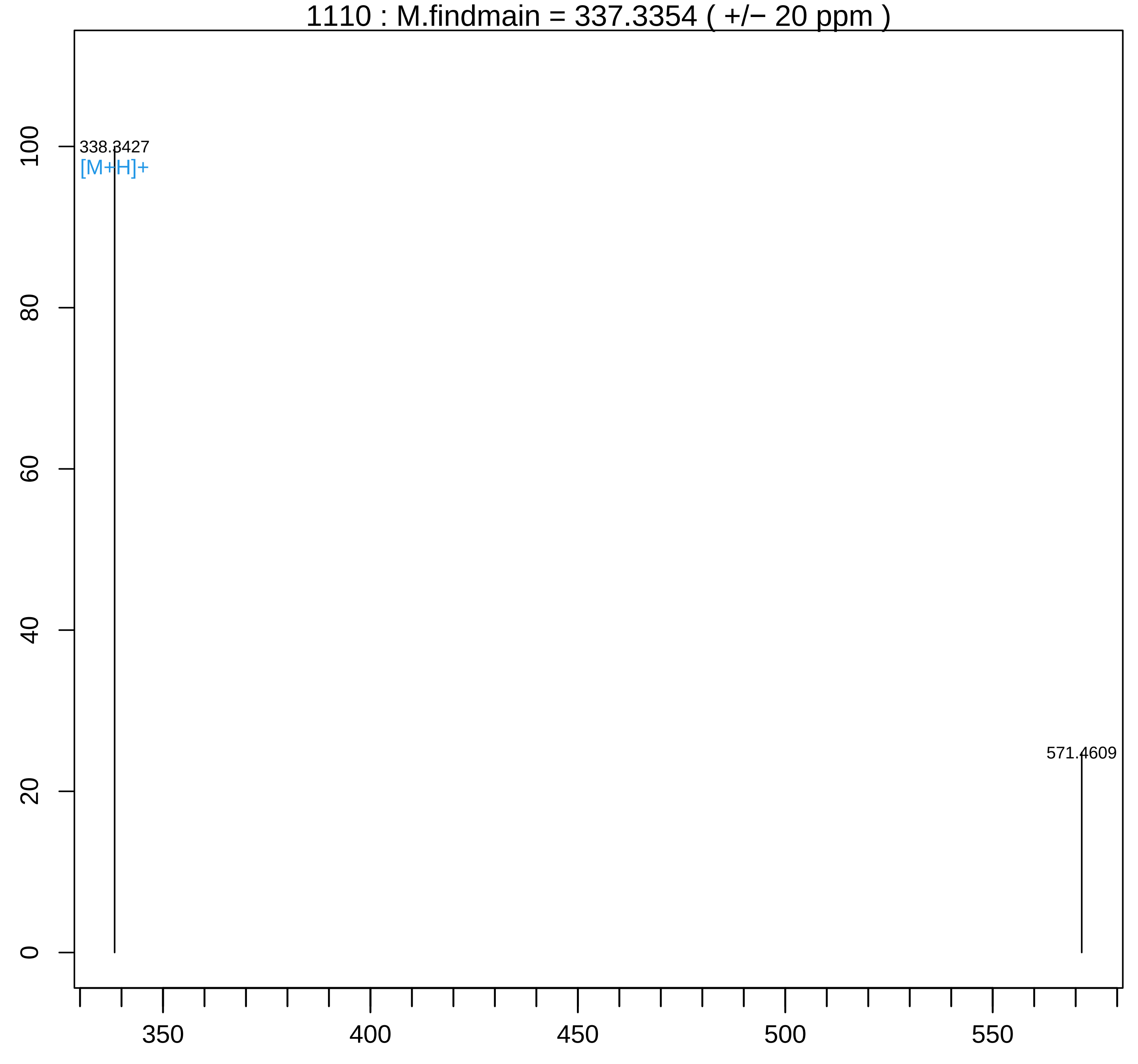
1110 : M.findmain = 337.3354 (+/− 20 ppm)
338.3427
[M+H]+
571.4609
0
20
40
60
80
100
350
400
450
500
550

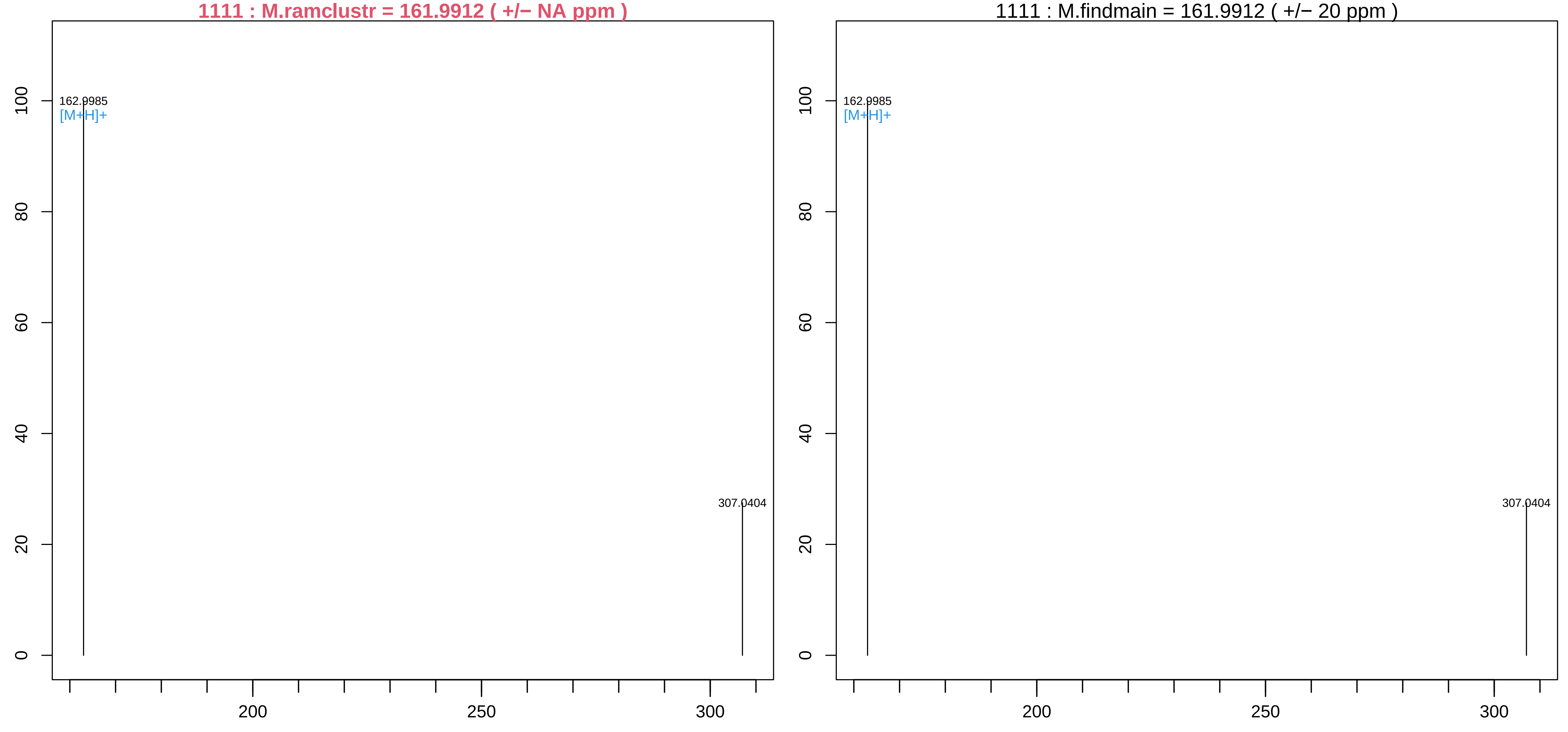
1111 : M.ramclustr = 161.9912 (+/− NA ppm)
162.9985
[M+H]+
307.0404
1111 : M.findmain = 161.9912 (+/− 20 ppm)
162.9985
[M+H]+
307.0404
0
20
40
60
80
100
200
250
300

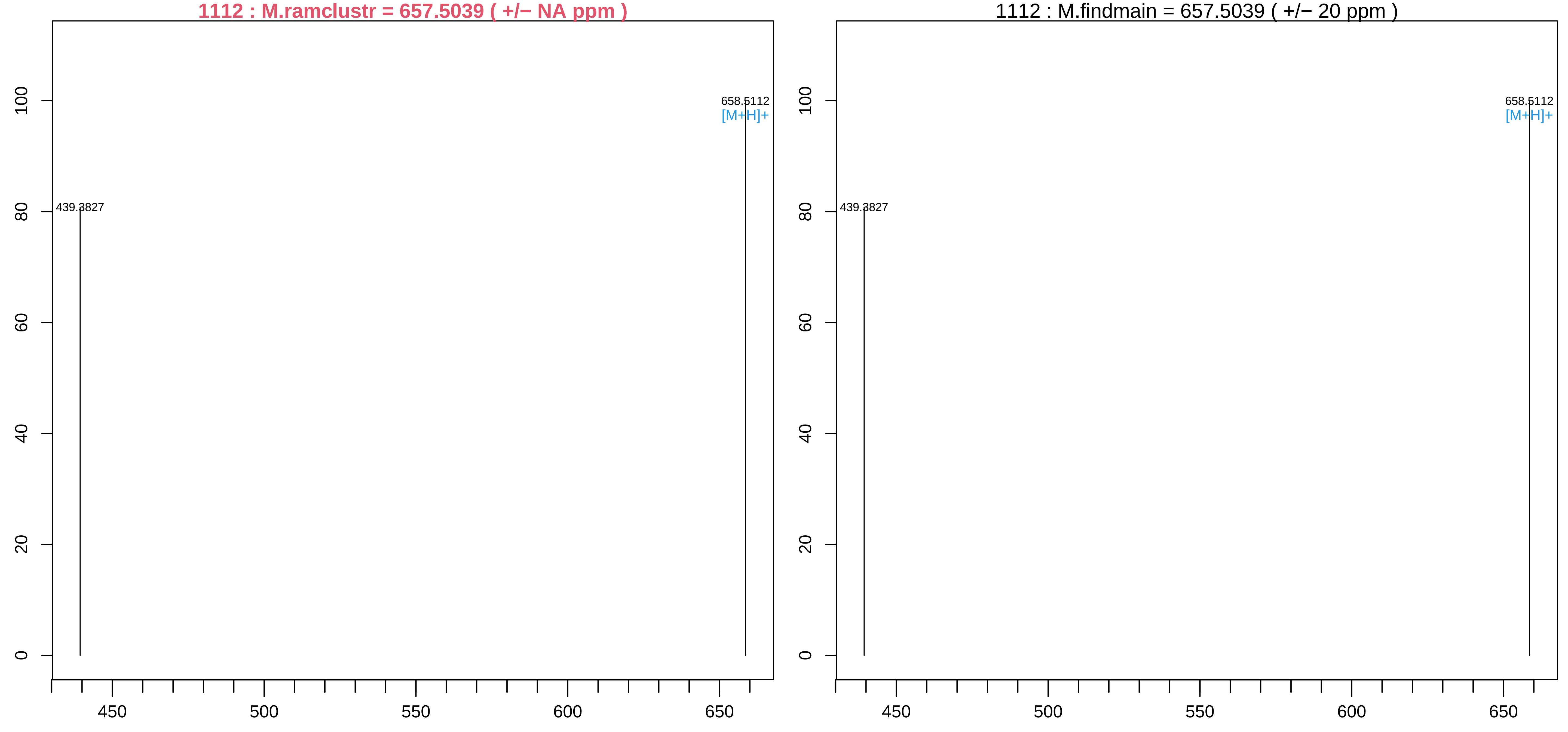
1112 : M.ramclustr = 657.5039 (+/− NA ppm)
658.5112
[M+H]+
439.3827
100
80
60
40
20
0
450
500
550
600
650
1112 : M.findmain = 657.5039 (+/− 20 ppm)
658.5112
[M+H]+
439.3827
100
80
60
40
20
0
450
500
550
600
650

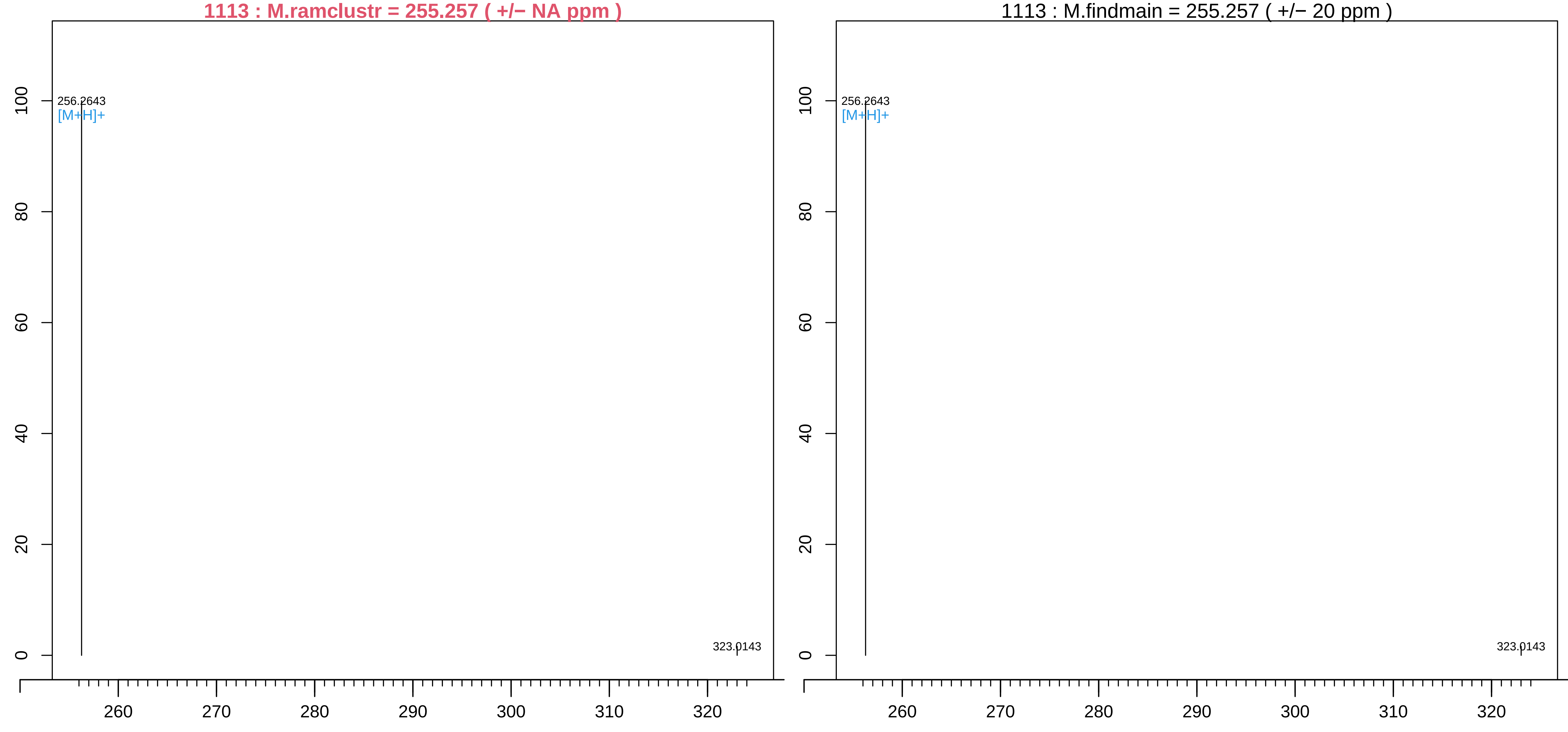
1113 : M.ramclustr = 255.257 (+/− NA ppm)
256.2643
[M+H]+
323.0143
1113 : M.findmain = 255.257 (+/− 20 ppm)
256.2643
[M+H]+
323.0143

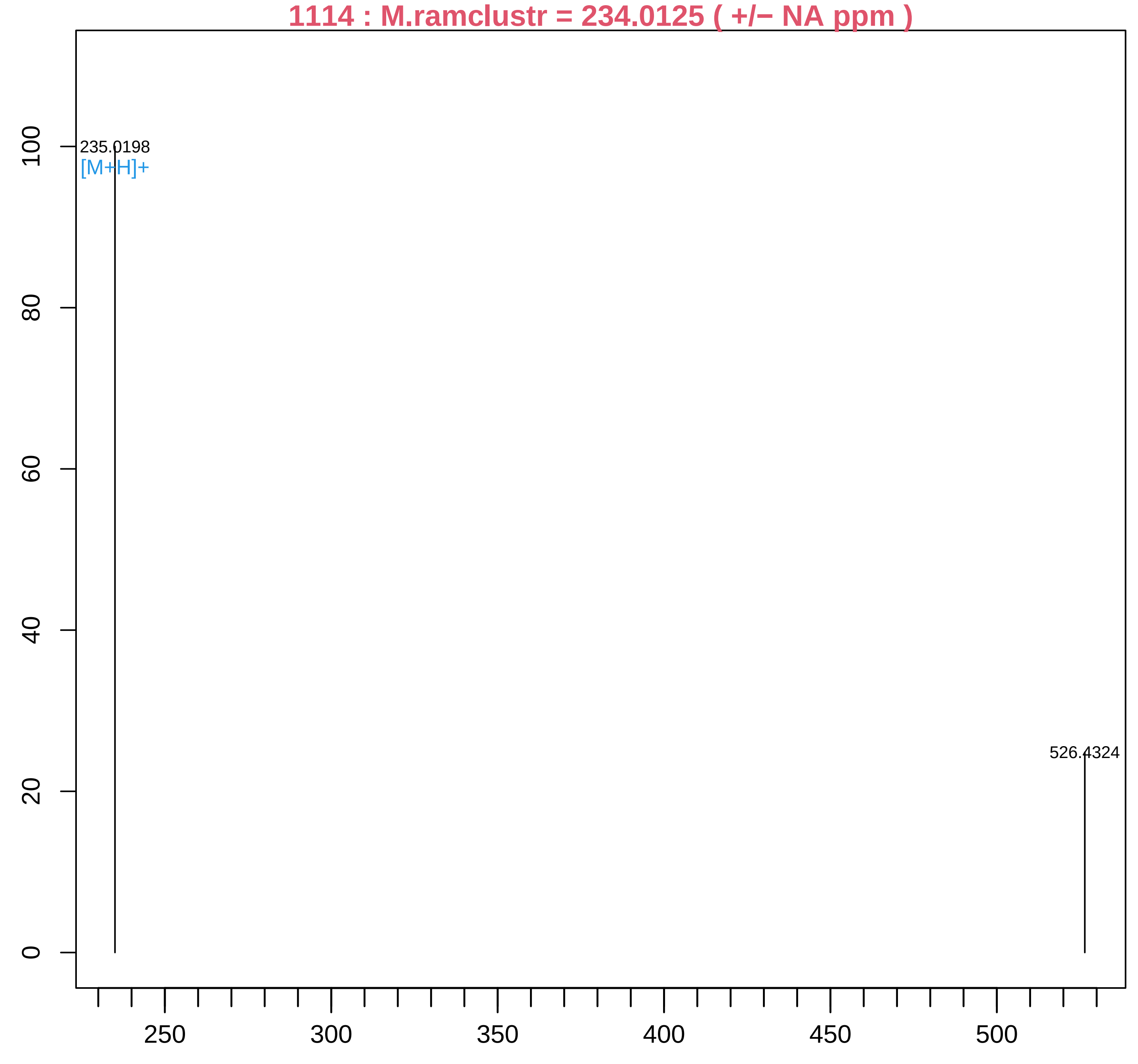
1114 : M.ramclustr = 234.0125 (+/− NA ppm)
235.0198
[M+H]+
526.4324
0
20
40
60
80
100
250
300
350
400
450
500

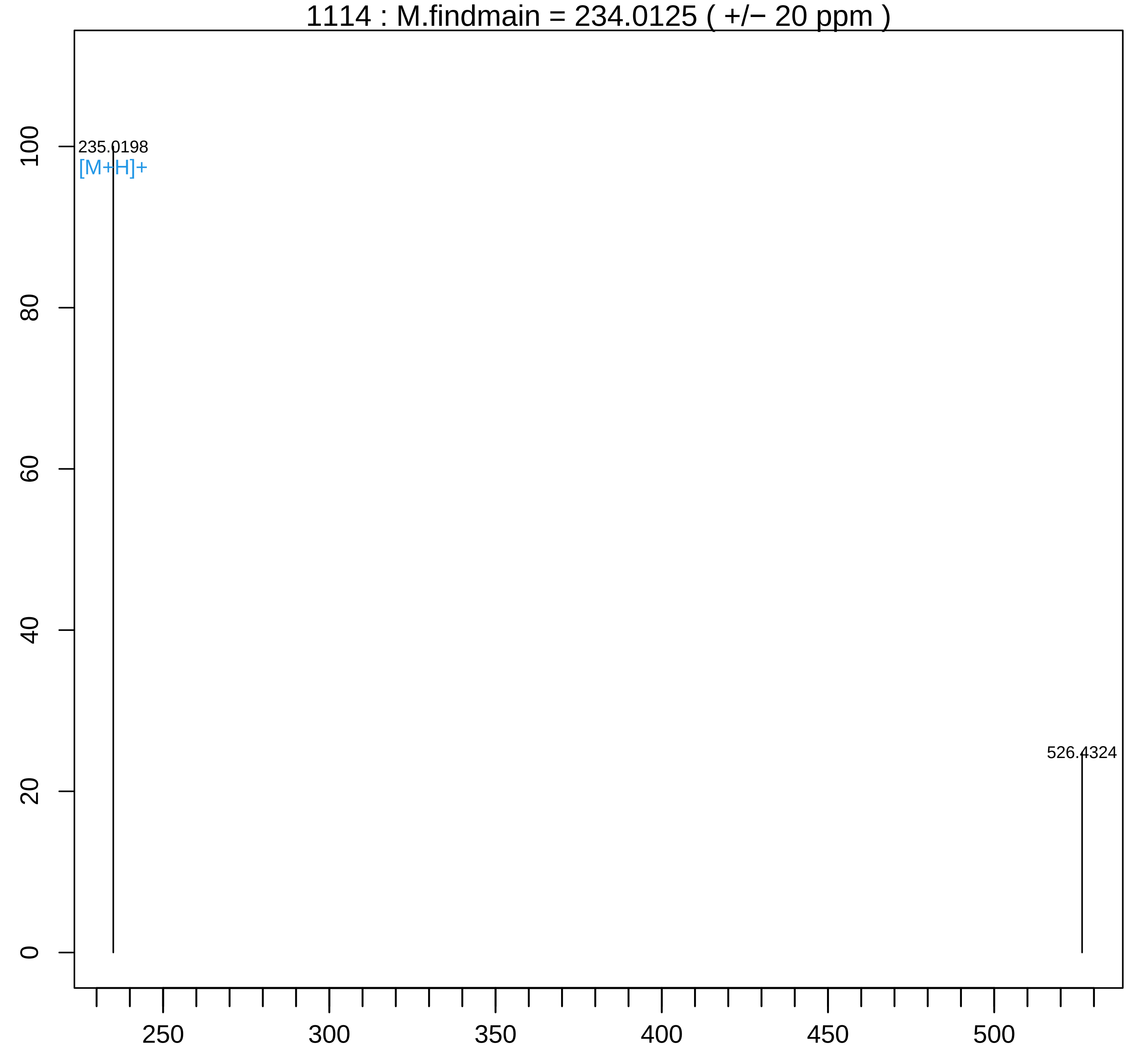
1114 : M.findmain = 234.0125 (+/− 20 ppm)
235.0198
[M+H]+
526.4324
0
20
40
60
80
100
250
300
350
400
450
500

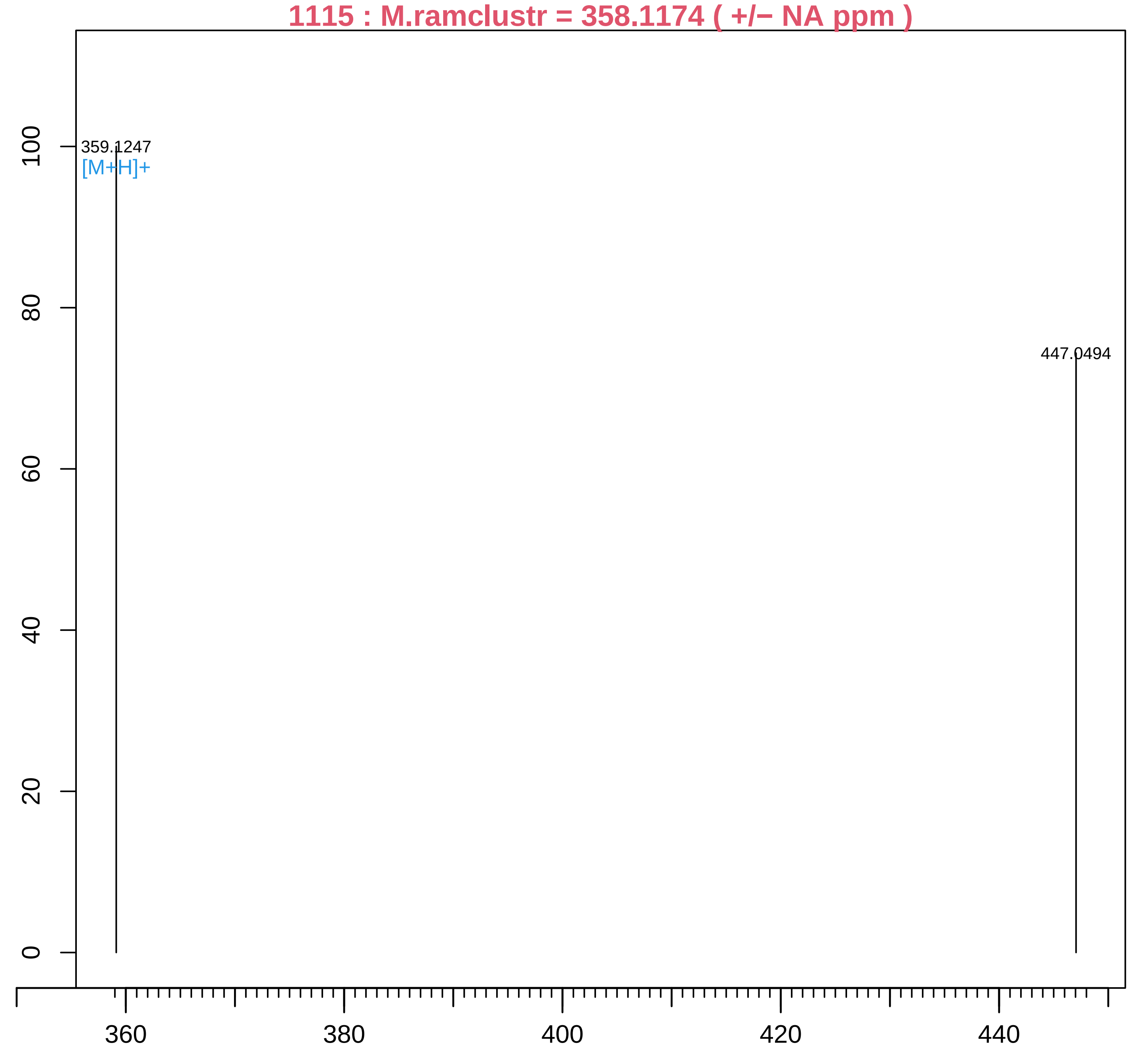

1115 : M.ramclustr = 358.1174 (+/− NA ppm)
359.1247
[M+H]+
447.0494
0
20
40
60
80
100
360
380
400
420
440

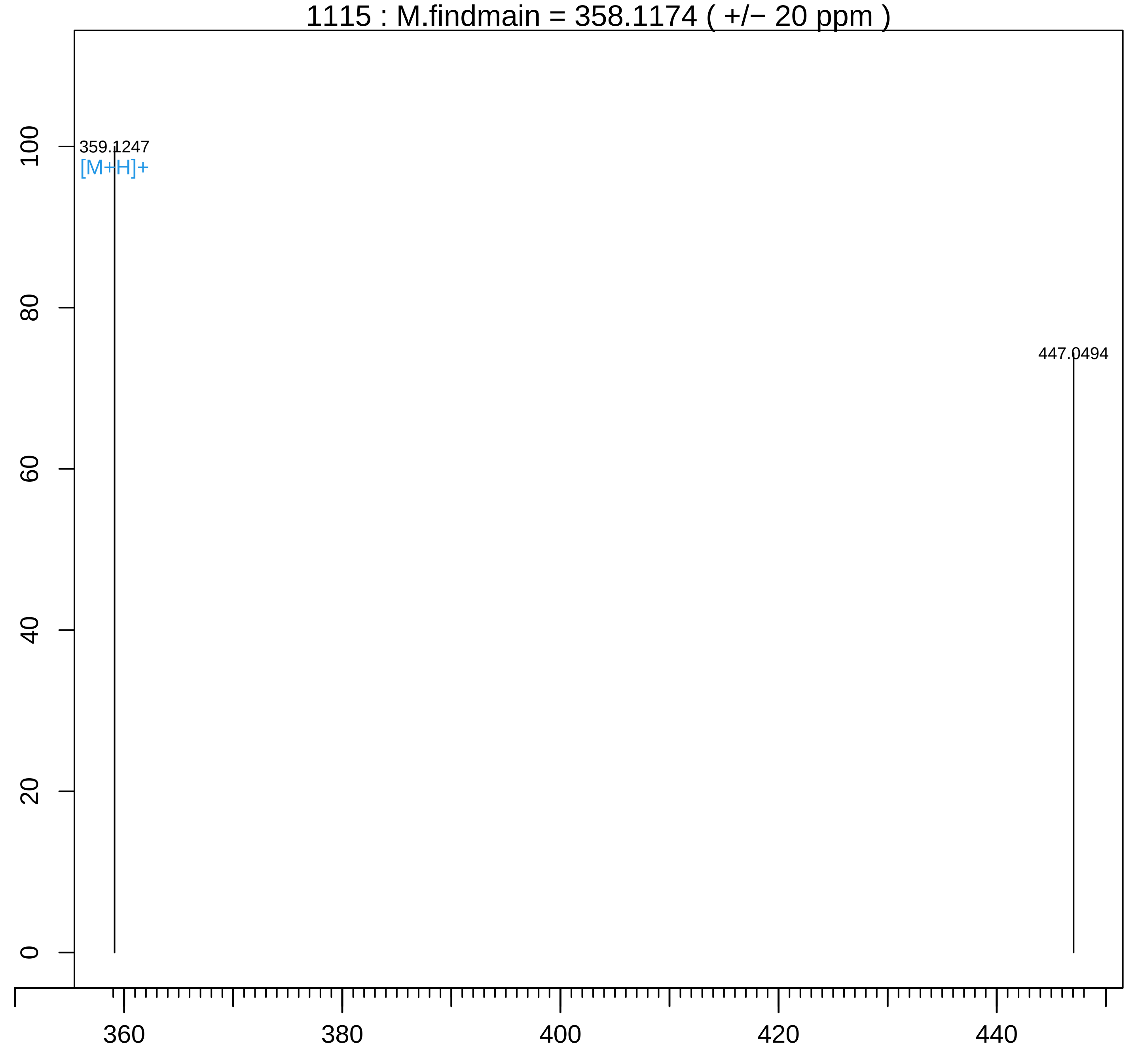

1115 : M.findmain = 358.1174 (+/− 20 ppm)
359.1247
[M+H]+
447.0494
0
20
40
60
80
100
360
380
400
420
440

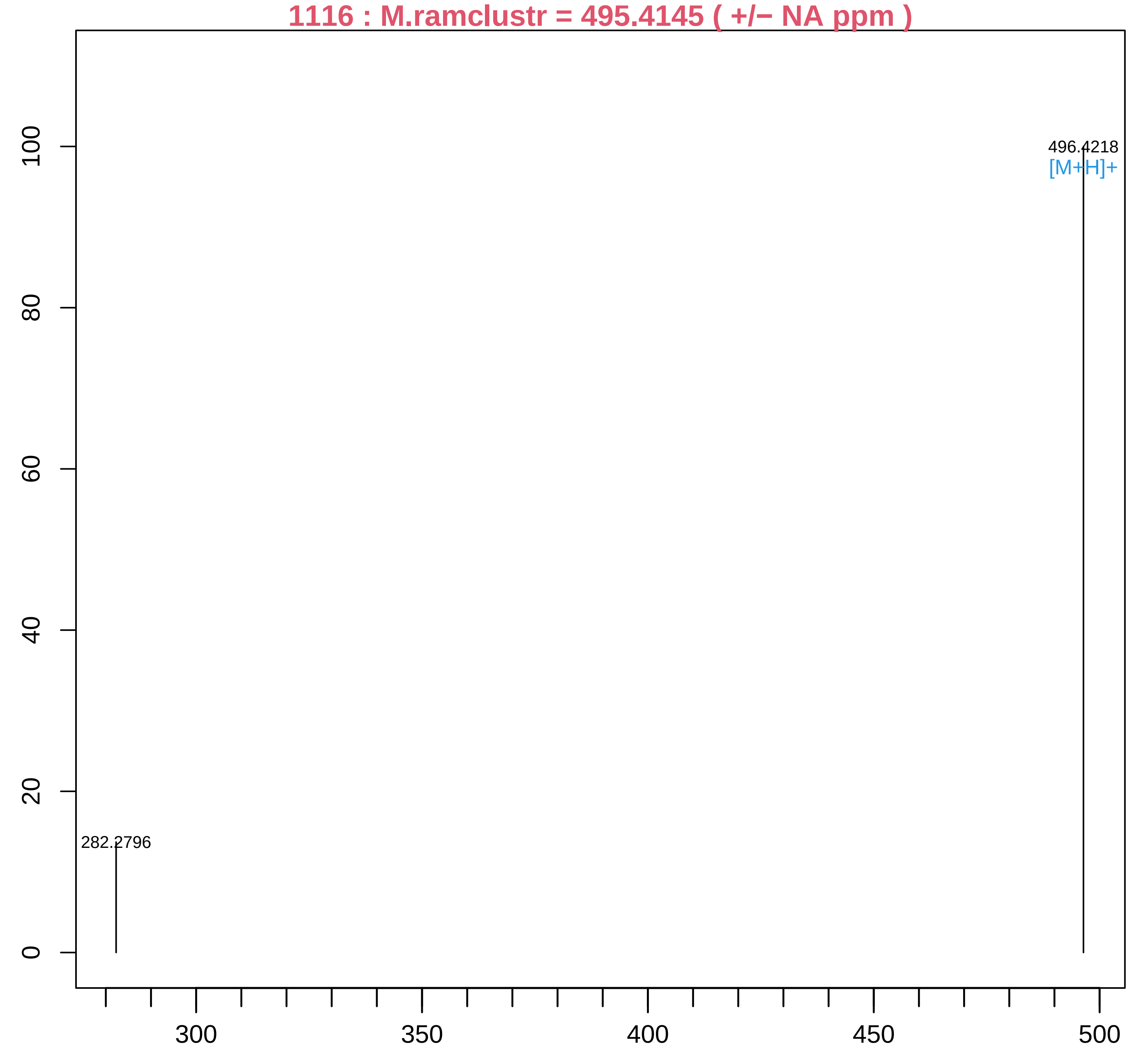
1116 : M.ramclustr = 495.4145 (+/− NA ppm)
496.4218
[M+H]+
282.2796
0
20
40
60
80
100
300
350
400
450
500

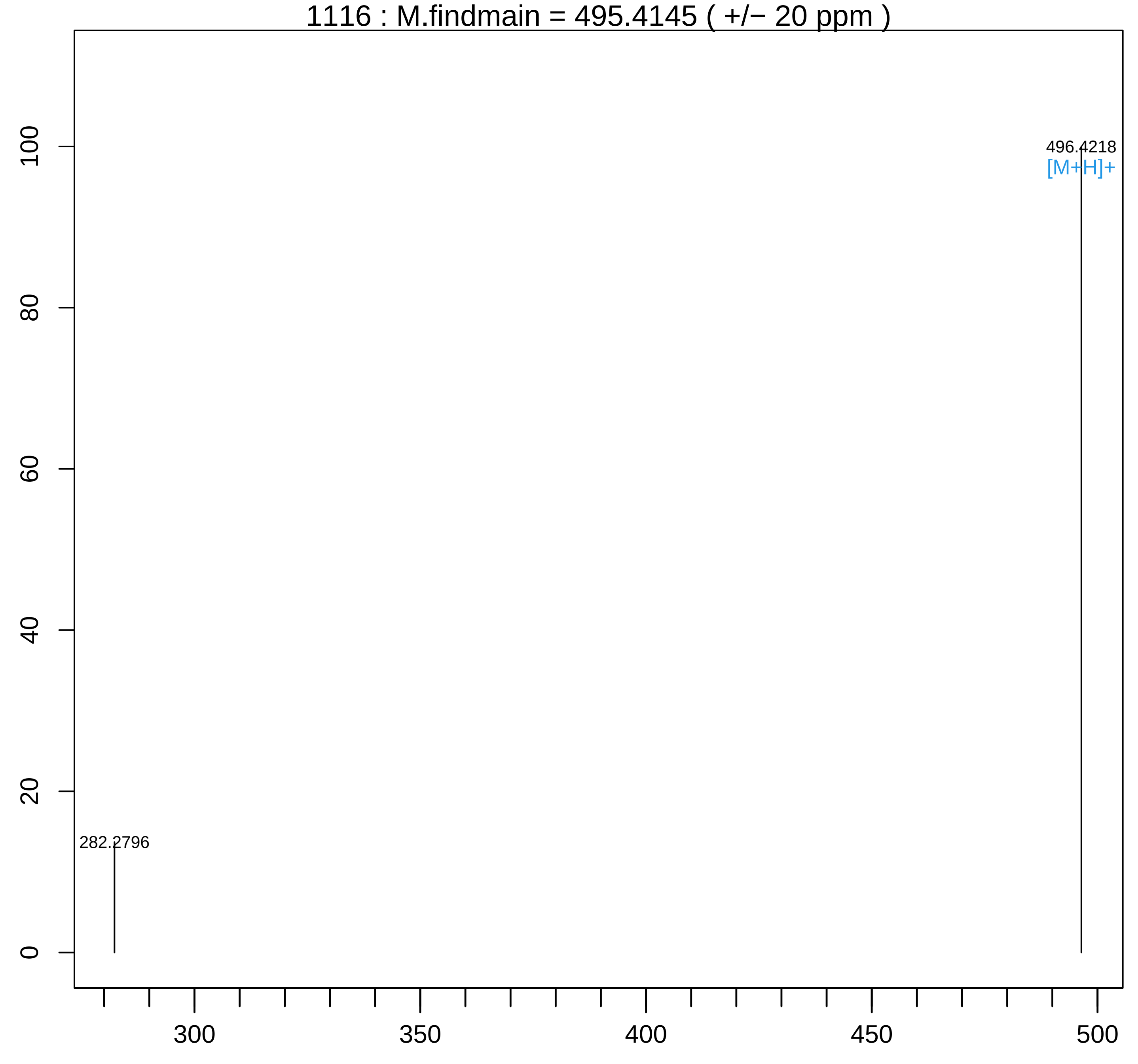
1116 : M.findmain = 495.4145 (+/− 20 ppm)
496.4218
[M+H]+
282.2796
0
20
40
60
80
100
300
350
400
450
500

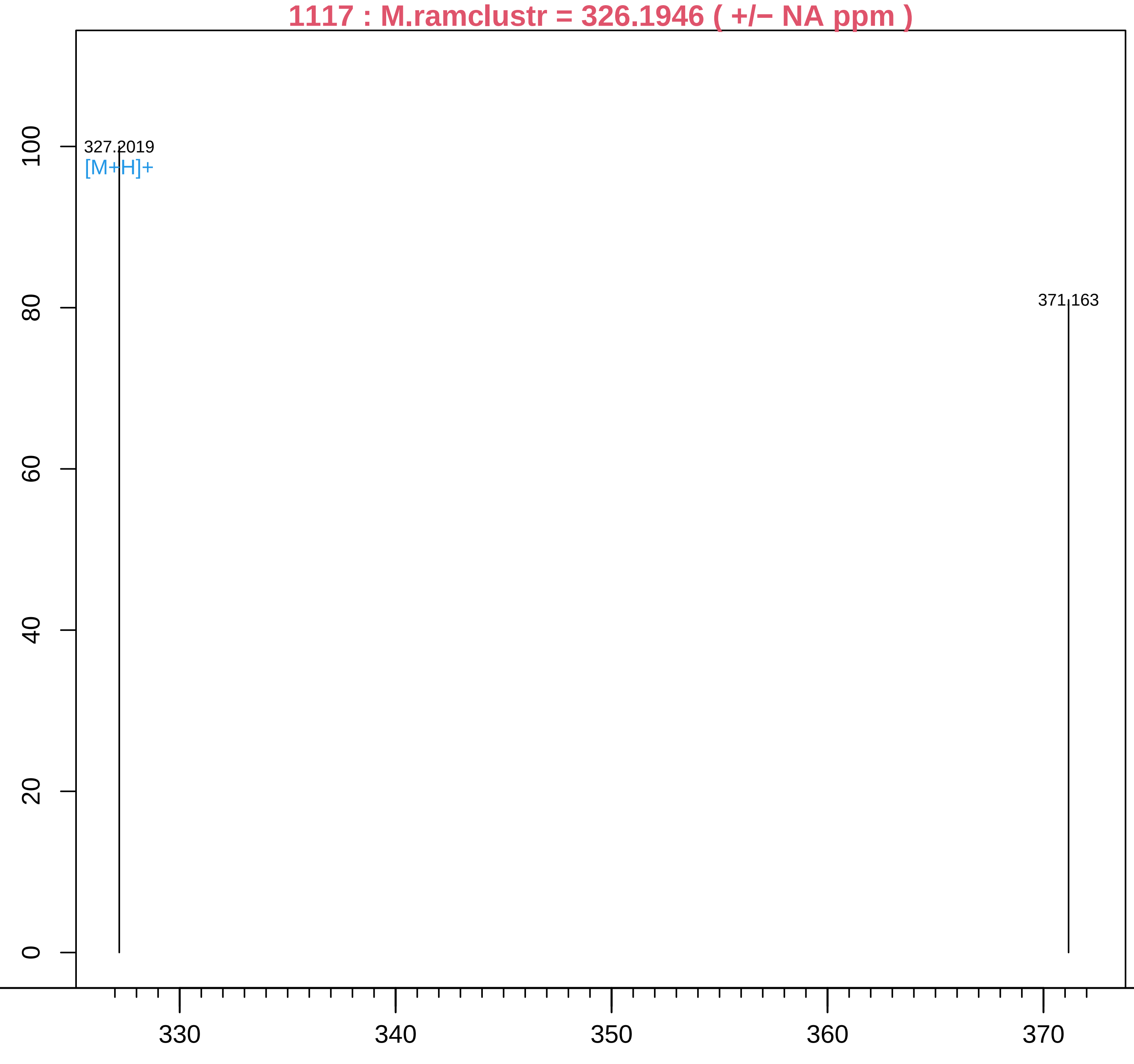
1117 : M.ramclustr = 326.1946 (+/− NA ppm)
327.2019
[M+H]+
371.163
0
20
40
60
80
100
330
340
350
360
370

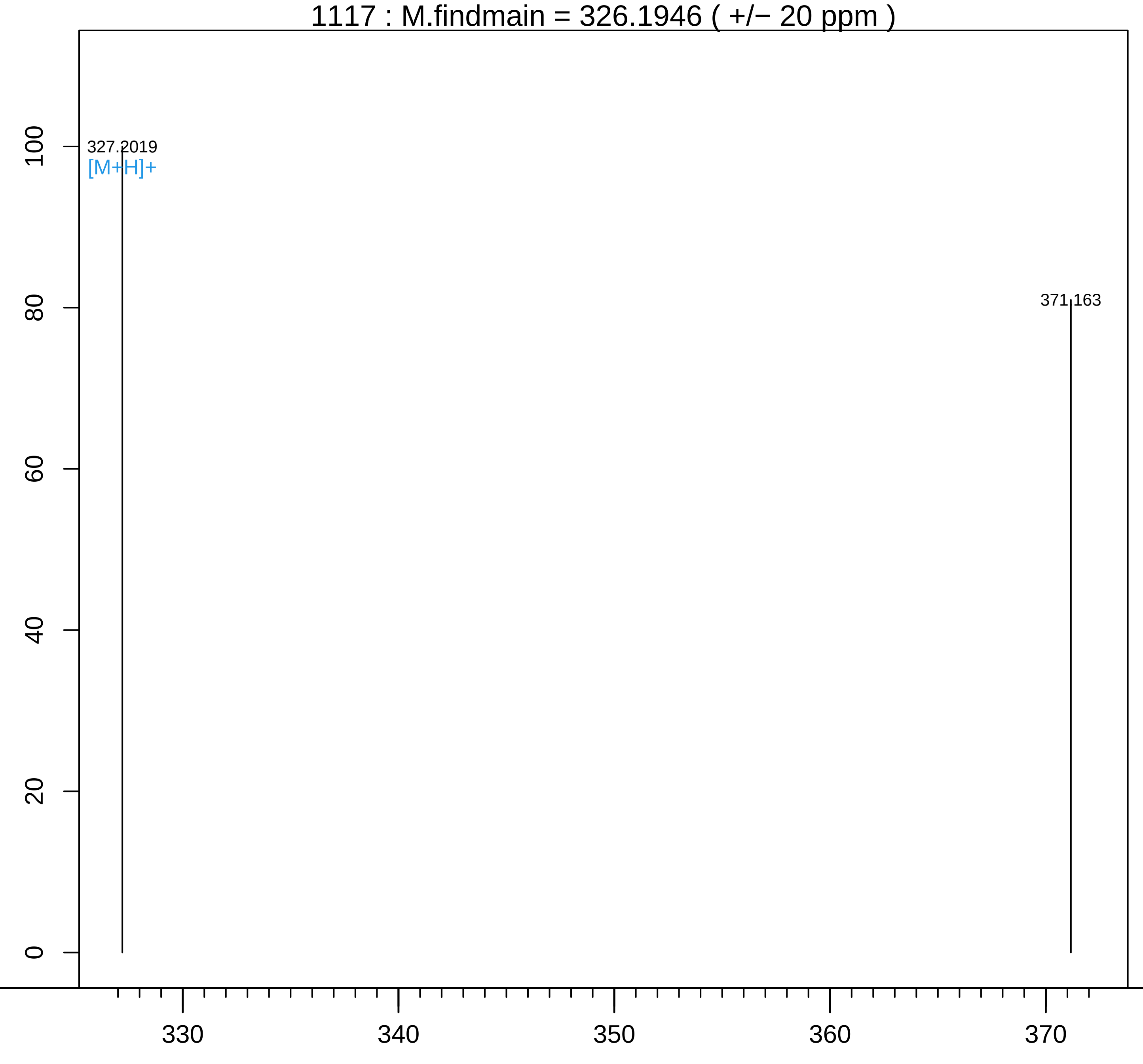
1117 : M.findmain = 326.1946 (+/− 20 ppm)
327.2019
[M+H]+
371.163
0
20
40
60
80
100
330
340
350
360
370

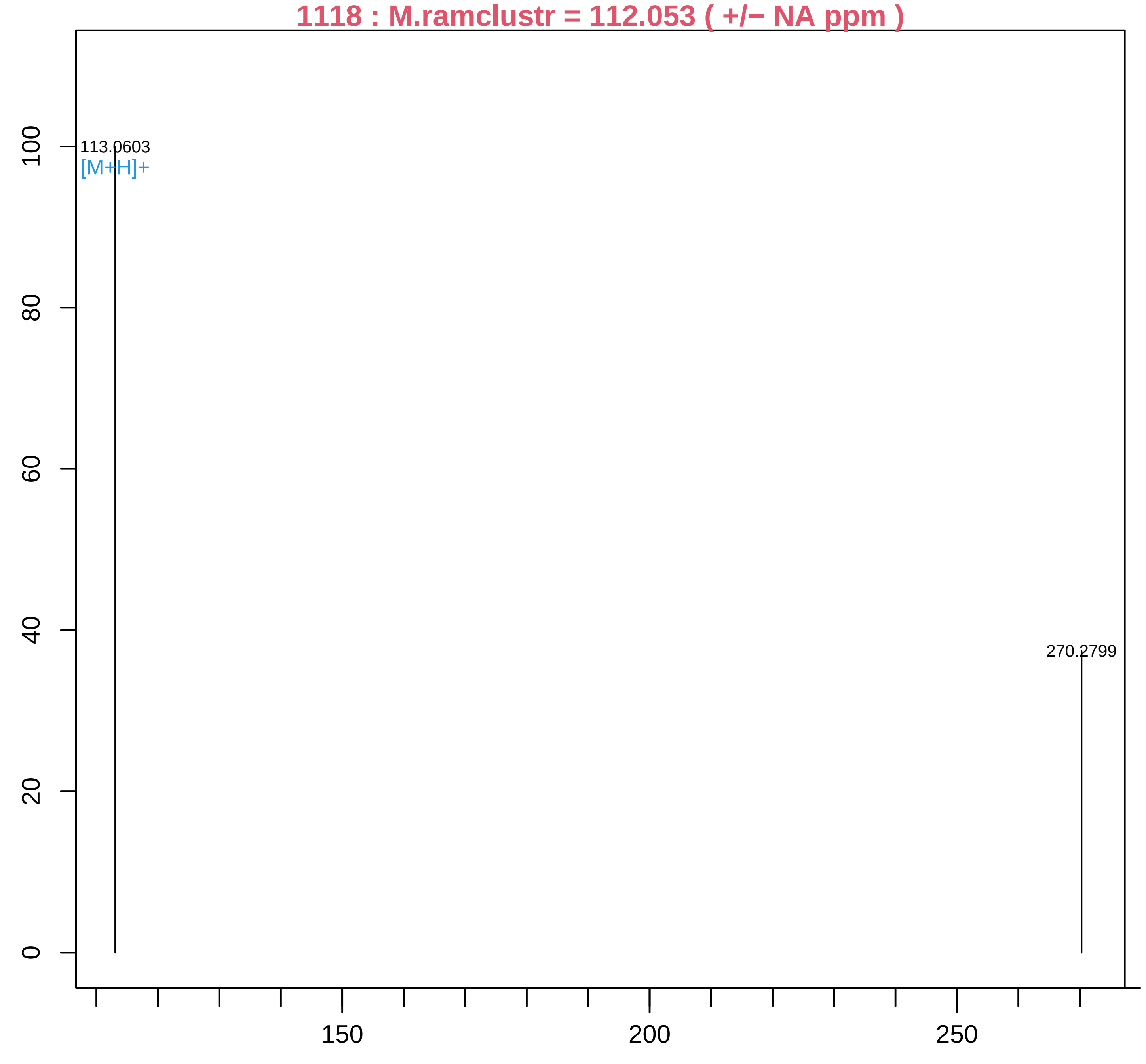
1118 : M.ramclustr = 112.053 (+/− NA ppm)
113.0603
[M+H]+
270.2799
0
20
40
60
80
100
150
200
250

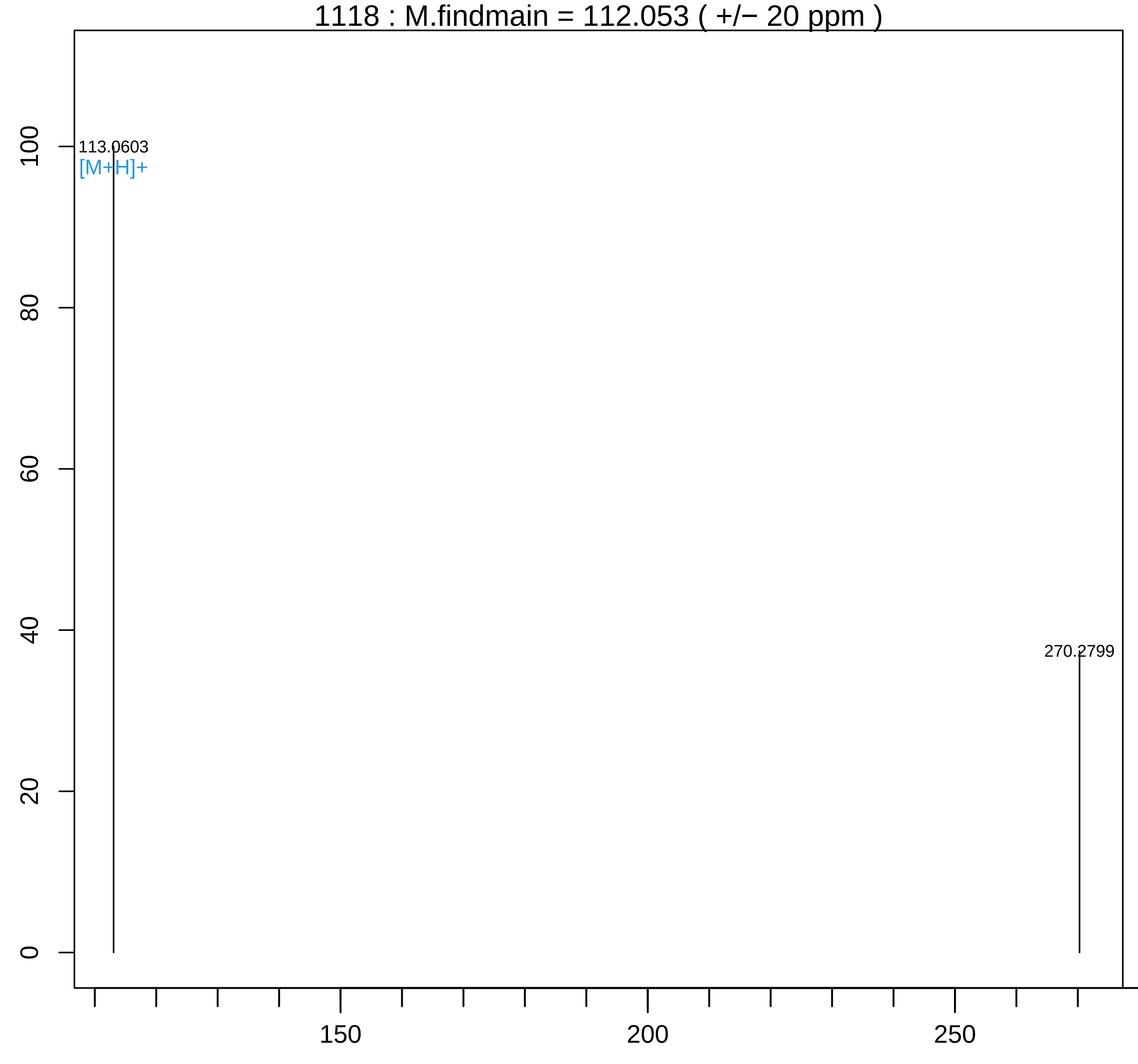
1118 : M.findmain = 112.053 (+/− 20 ppm)
113.0603
[M+H]+
270.2799
0
20
40
60
80
100
150
200
250

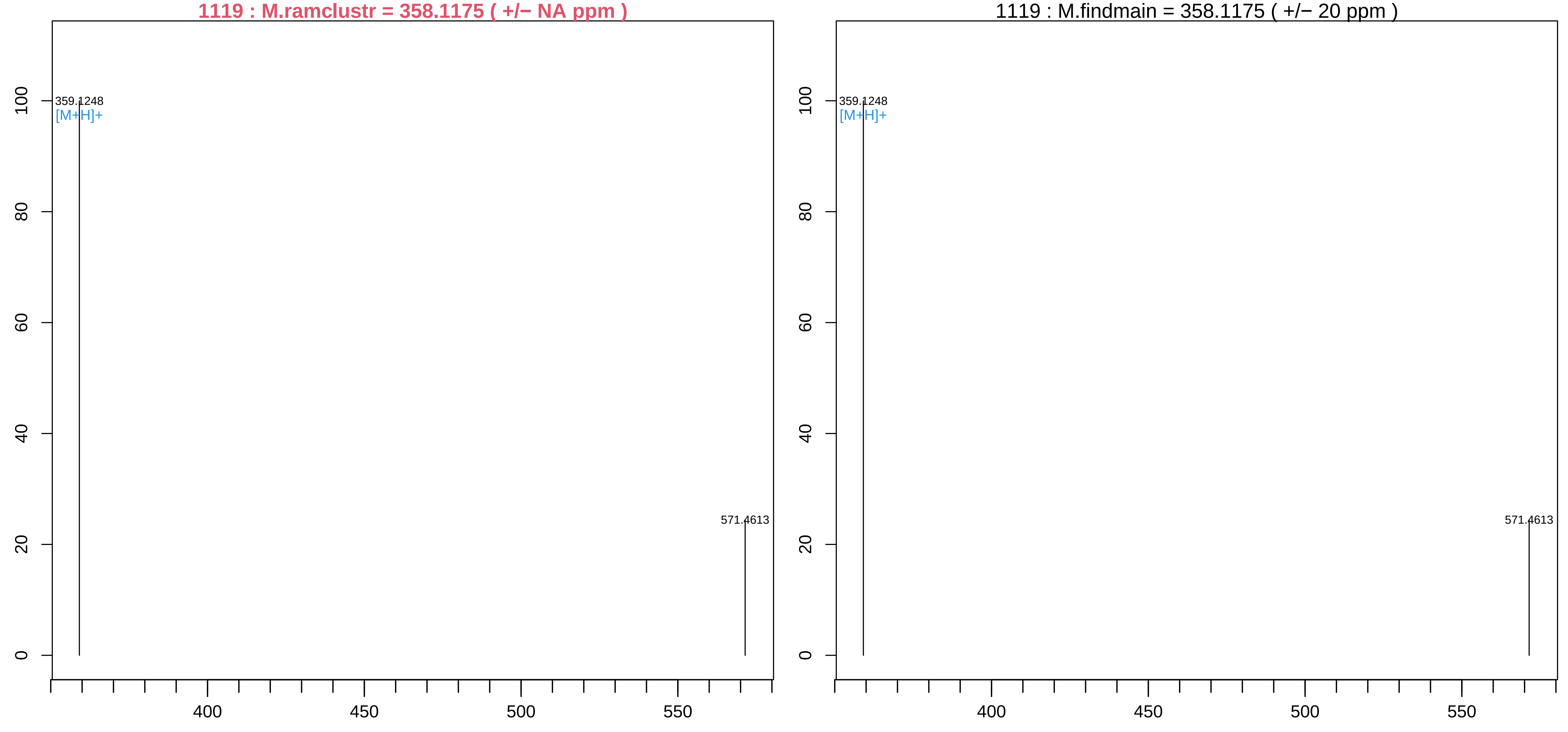

1119 : M.ramclustr = 358.1175 (+/− NA ppm)
1119 : M.findmain = 358.1175 (+/− 20 ppm)
359.1248
[M+H]+
571.4613
0
20
40
60
80
100
400
450
500
550

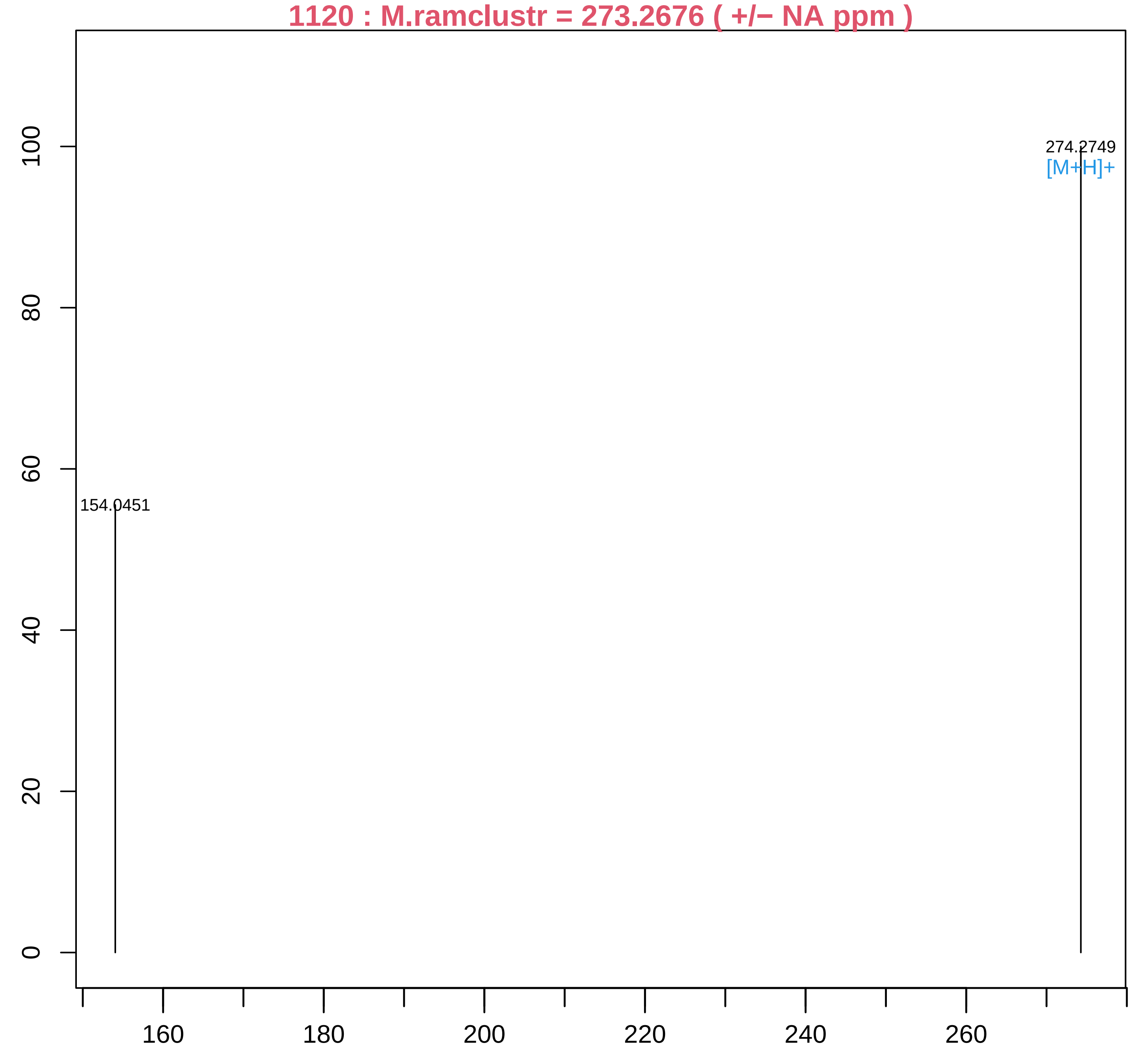
1120 : M.ramclustr = 273.2676 (+/− NA ppm)
274.2749
[M+H]+
154.0451
0
20
40
60
80
100
160
180
200
220
240
260

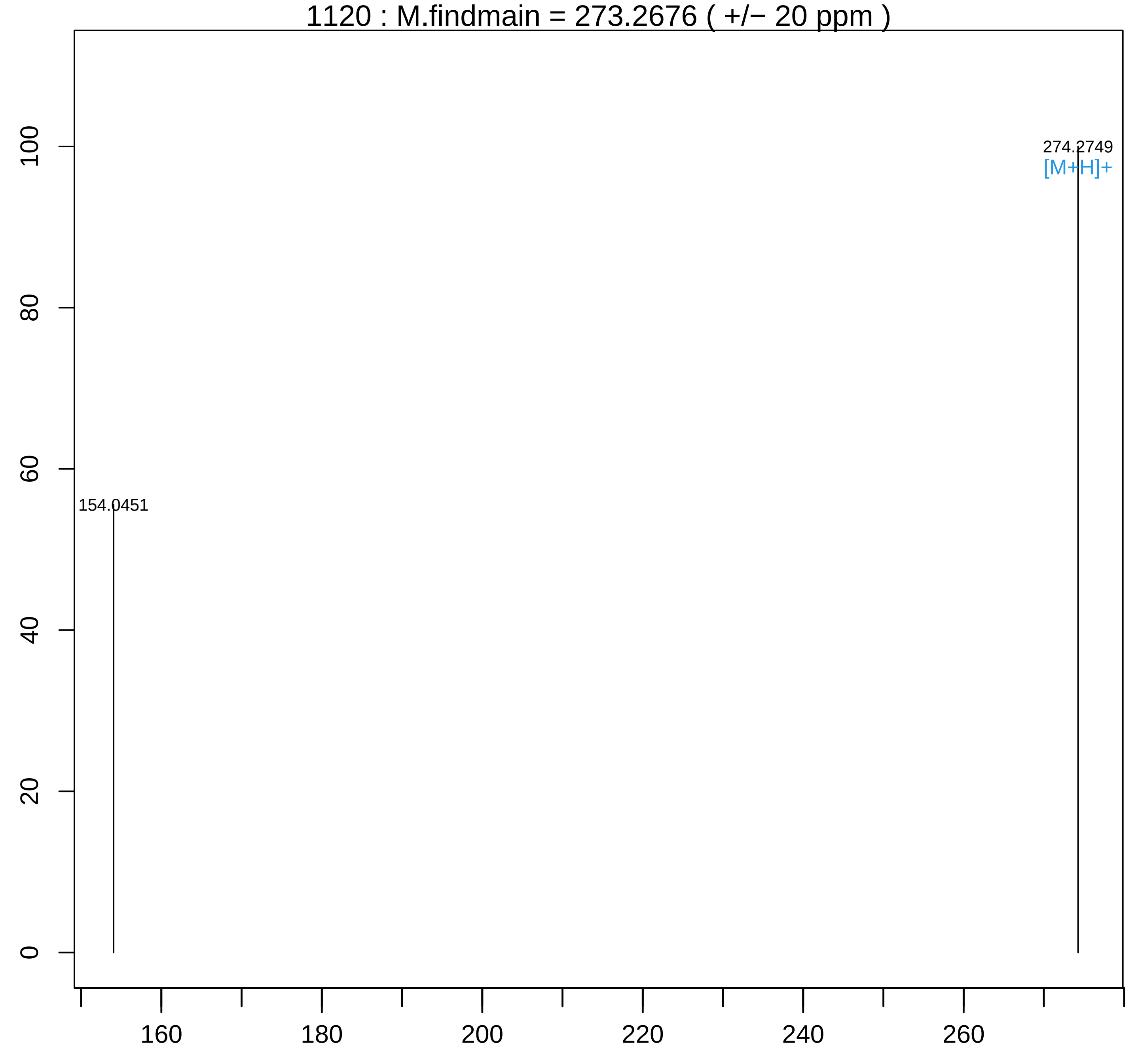
1120 : M.findmain = 273.2676 (+/− 20 ppm)
274.2749
[M+H]+
154.0451
0
20
40
60
80
100
160
180
200
220
240
260

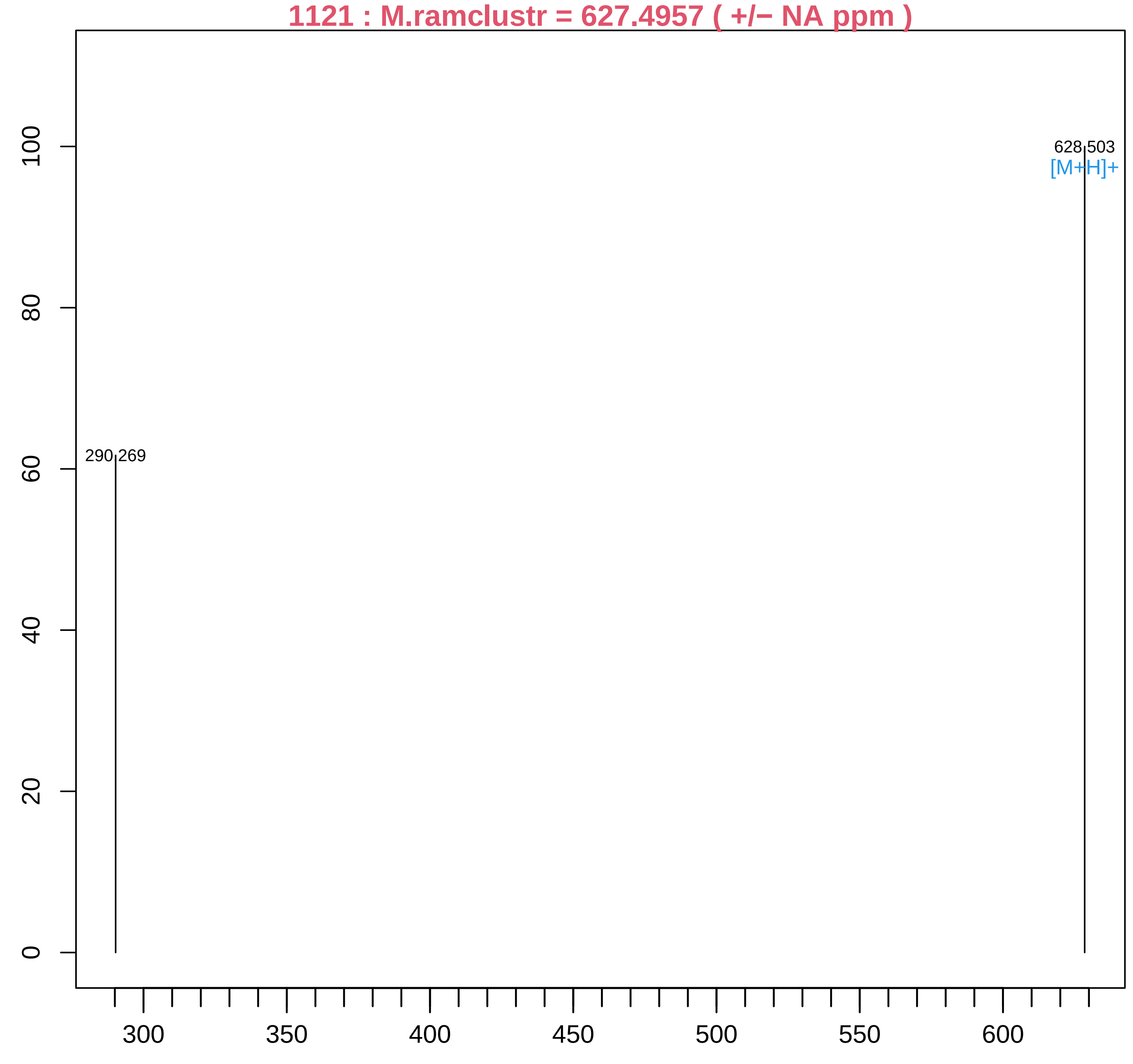
1121 : M.ramclustr = 627.4957 (+/− NA ppm)
628.503
[M+H]+
290.269
0
20
40
60
80
100
300
350
400
450
500
550
600

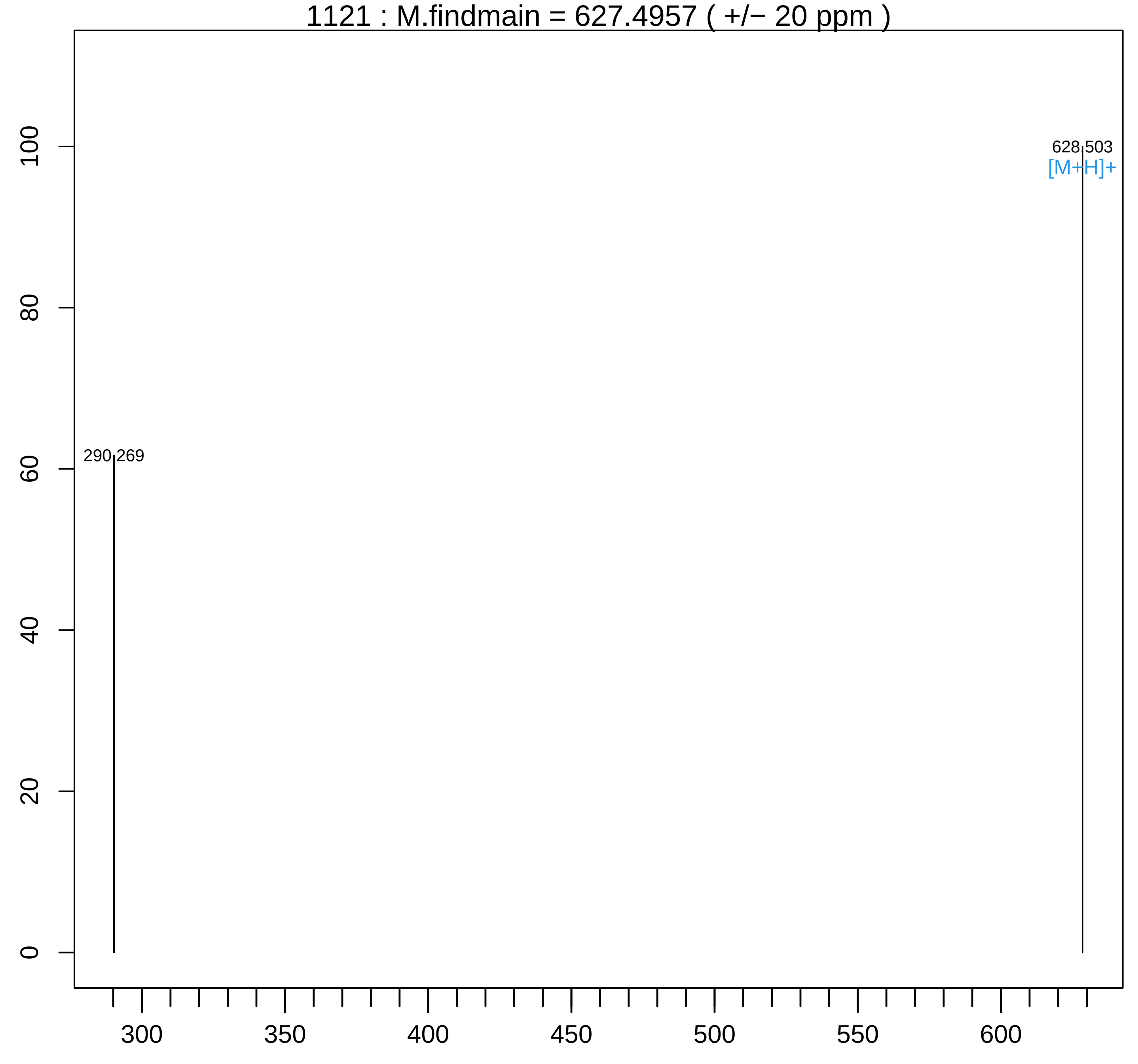
1121 : M.findmain = 627.4957 (+/− 20 ppm)
628.503
[M+H]+
290.269
0
20
40
60
80
100
300
350
400
450
500
550
600

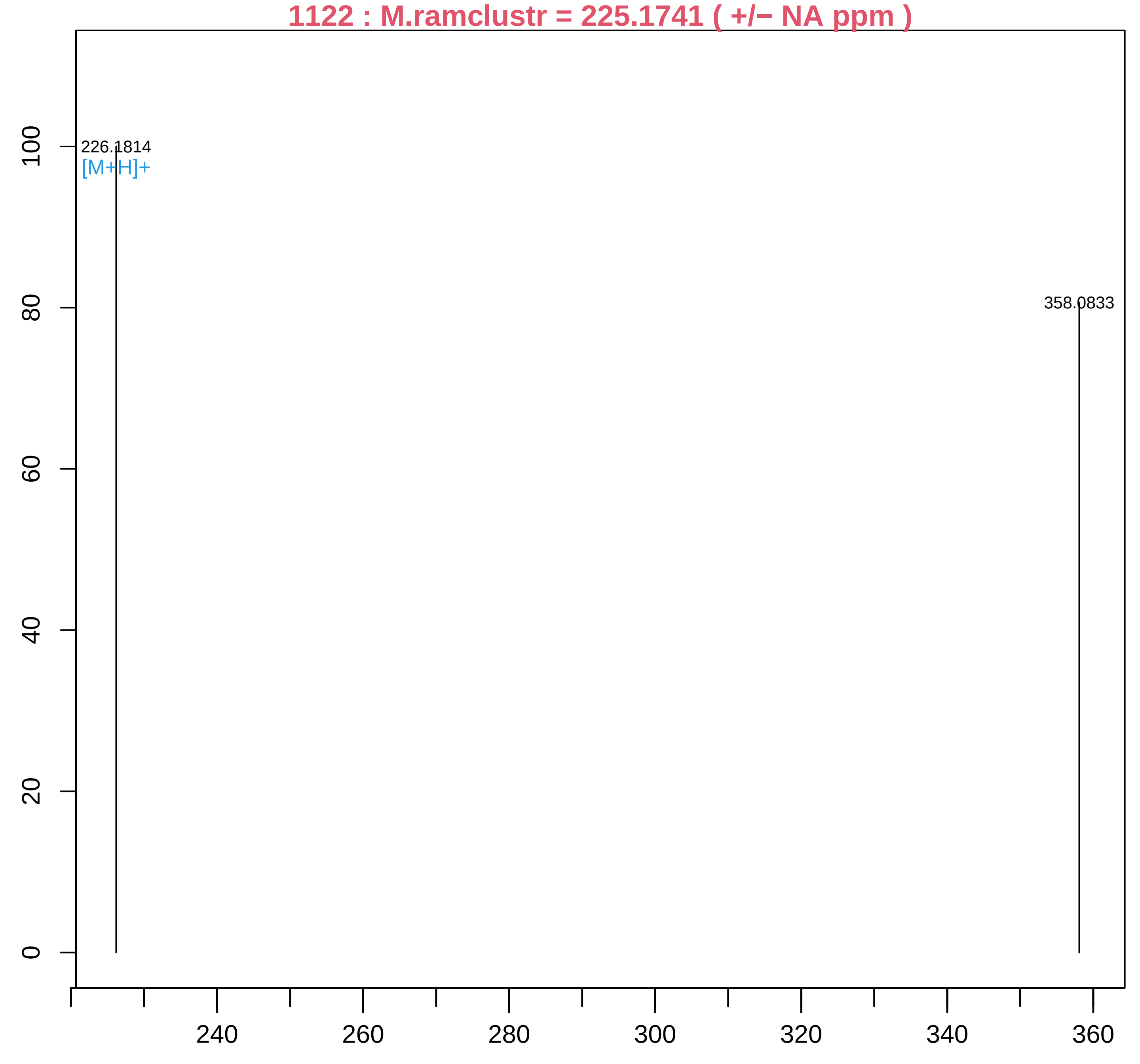
1122 : M.ramclustr = 225.1741 (+/− NA ppm)
226.1814
[M+H]+
358.0833
0
20
40
60
80
100
240
260
280
300
320
340
360

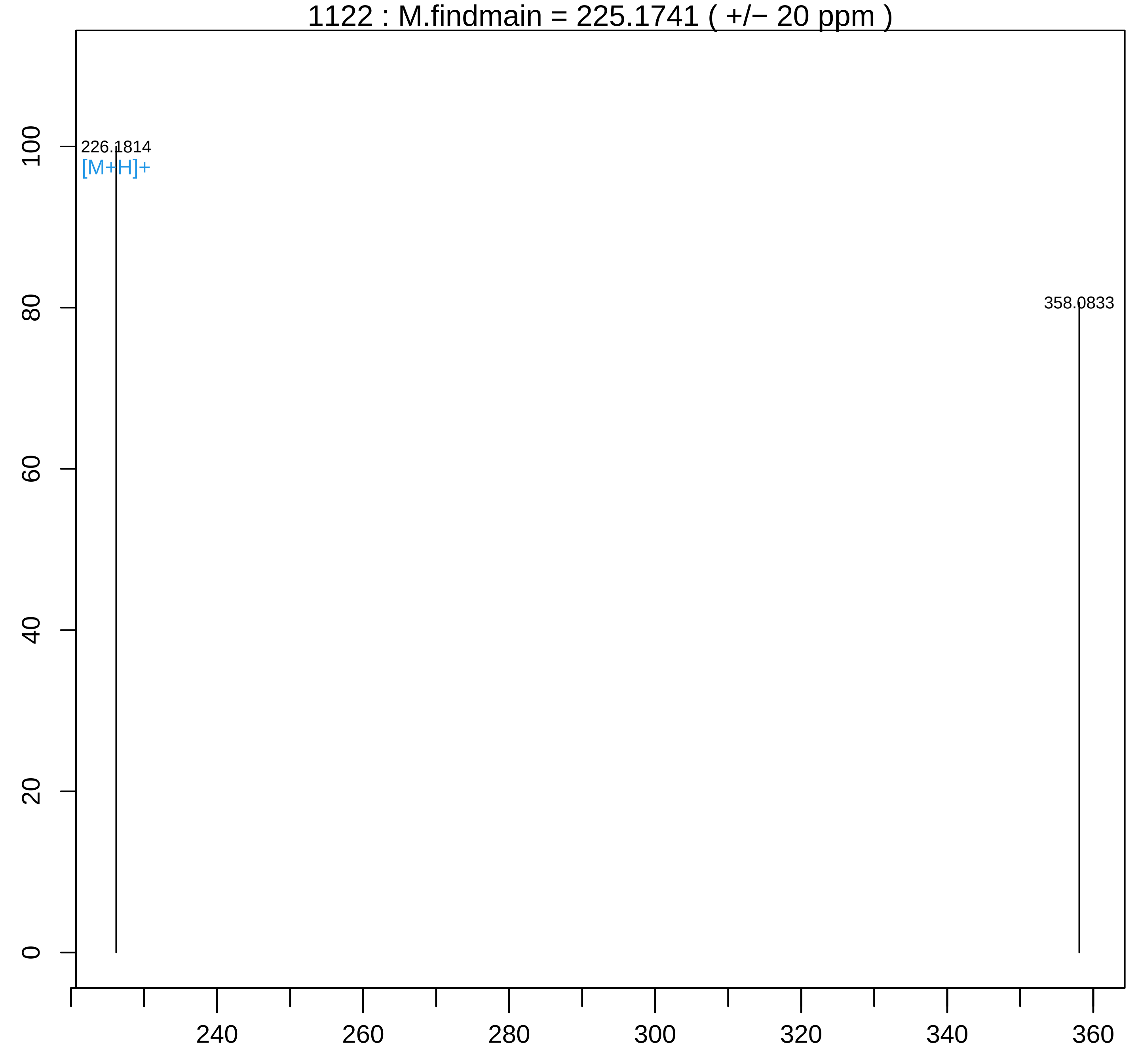
1122 : M.findmain = 225.1741 (+/− 20 ppm)
226.1814
[M+H]+
358.0833
0
20
40
60
80
100
240
260
280
300
320
340
360

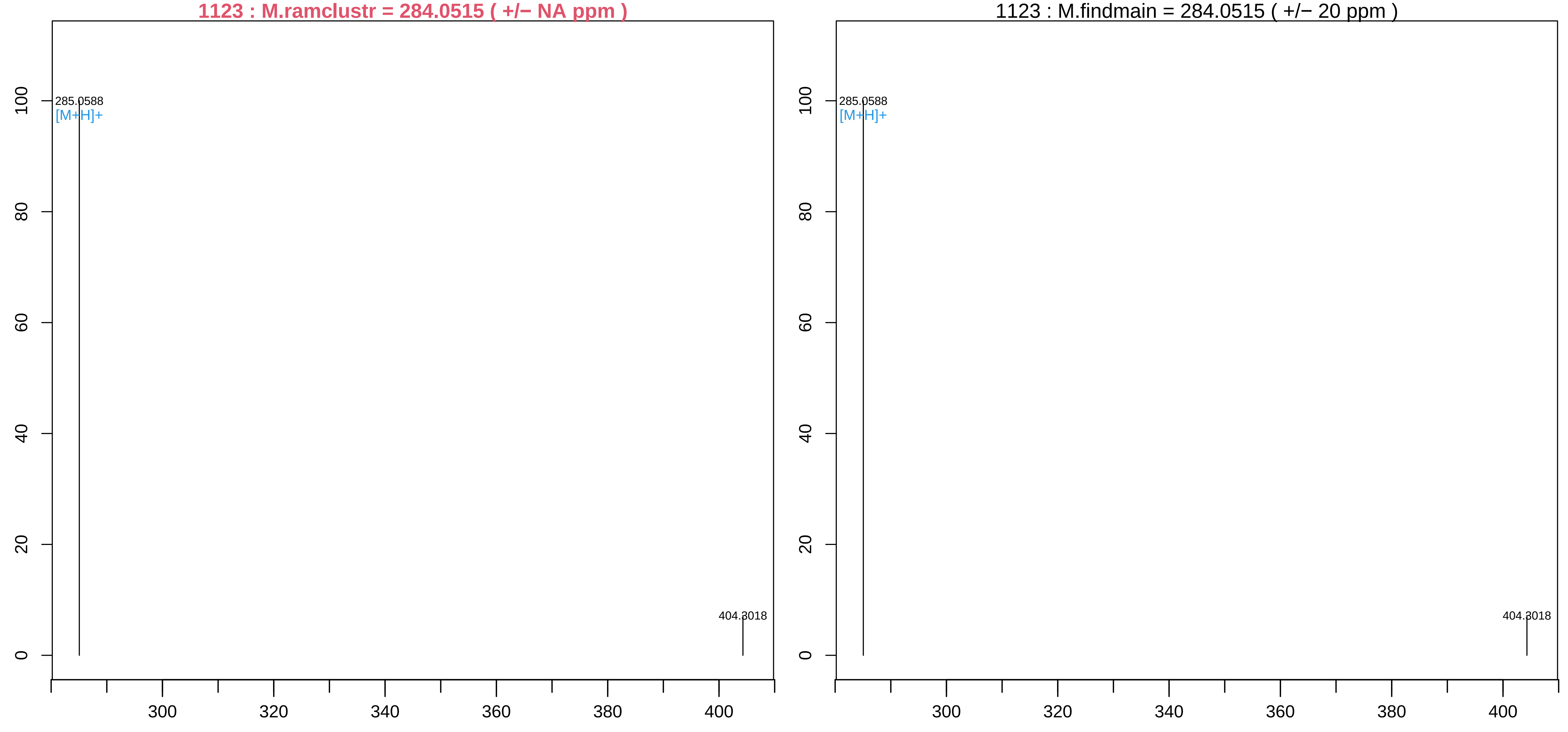
1123 : M.ramclustr = 284.0515 (+/− NA ppm)
285.0588
[M+H]+
404.3018
0
20
40
60
80
100
300
320
340
360
380
400
1123 : M.findmain = 284.0515 (+/− 20 ppm)
285.0588
[M+H]+
404.3018
0
20
40
60
80
100
300
320
340
360
380
400

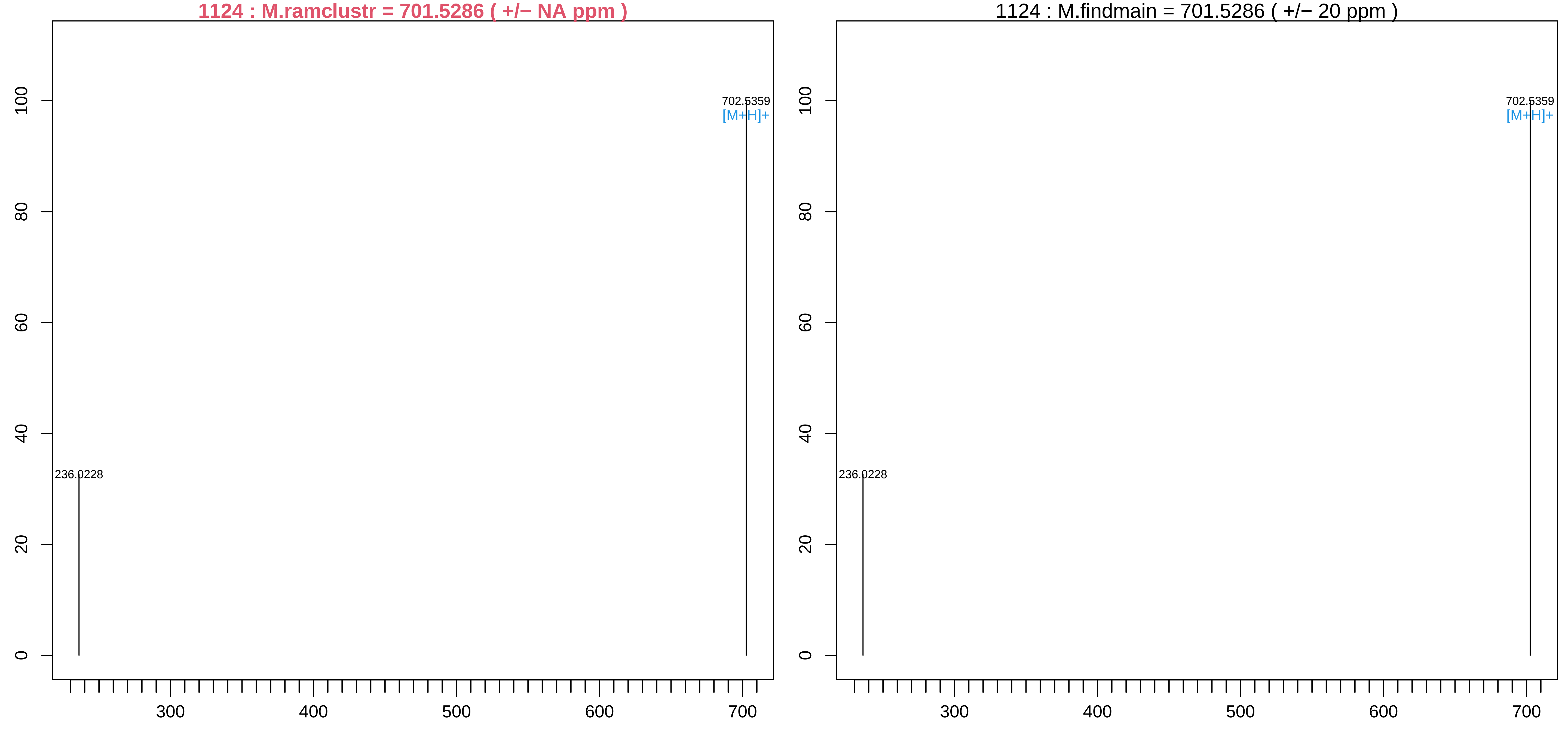
1124 : M.ramclustr = 701.5286 (+/− NA ppm)
702.5359
[M+H]+
236.0228
0
20
40
60
80
100
300
400
500
600
700
1124 : M.findmain = 701.5286 (+/− 20 ppm)
702.5359
[M+H]+
236.0228
0
20
40
60
80
100
300
400
500
600
700

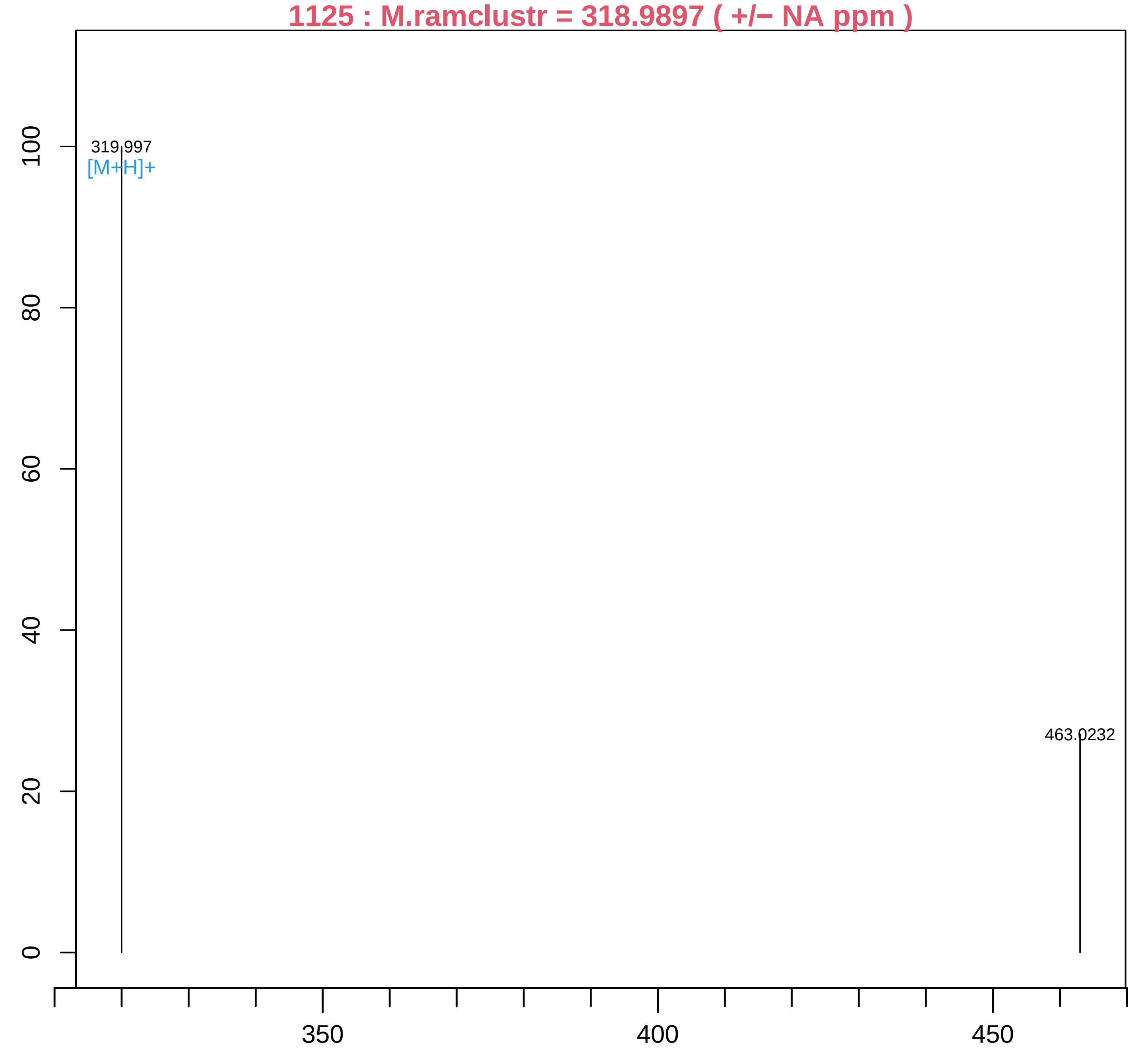
1125 : M.ramclustr = 318.9897 (+/− NA ppm)
319.997
[M+H]+
463.0232
0
20
40
60
80
100
350
400
450

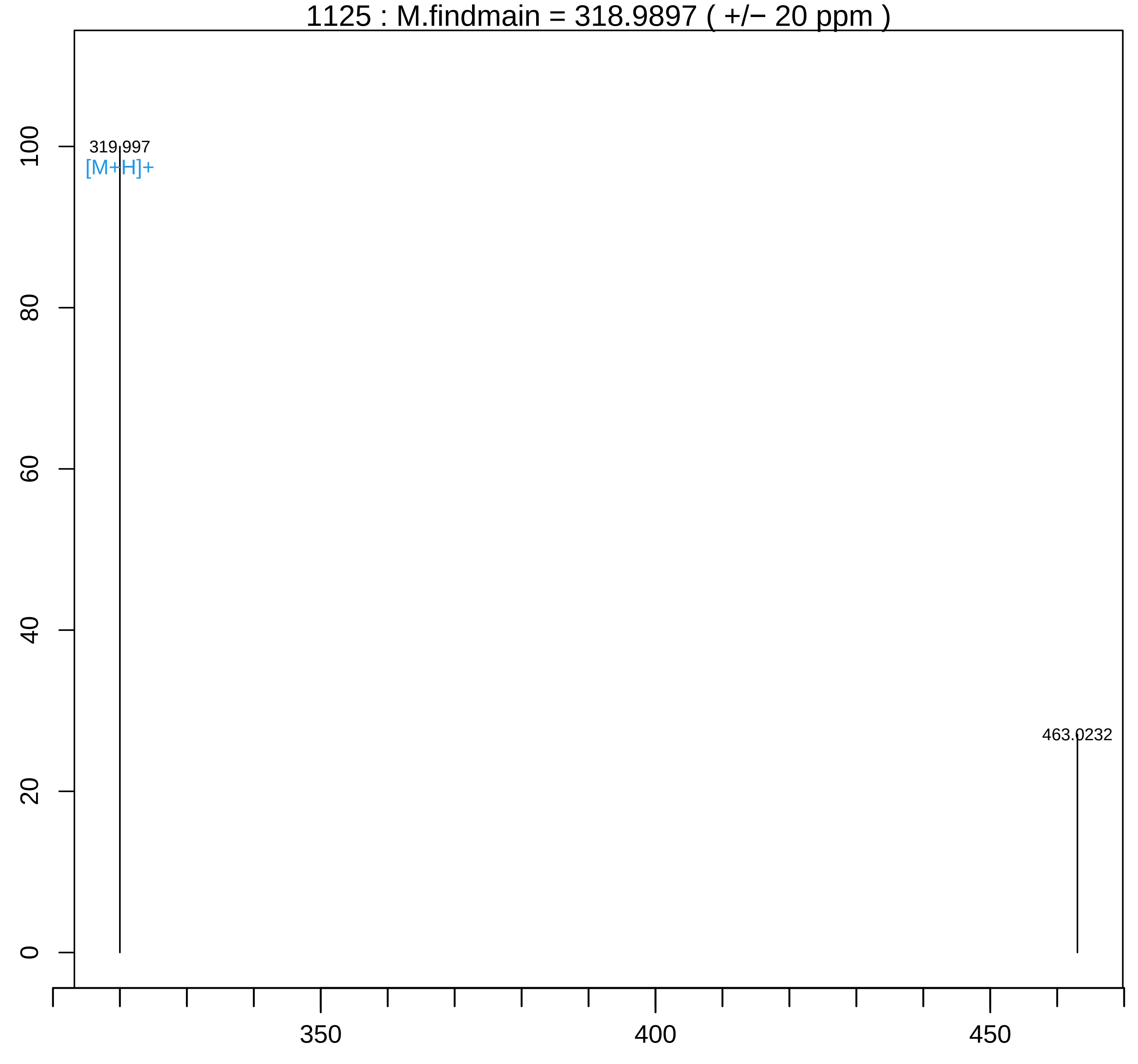
1125 : M.findmain = 318.9897 (+/− 20 ppm)
319.997
[M+H]+
463.0232
0
20
40
60
80
100
350
400
450

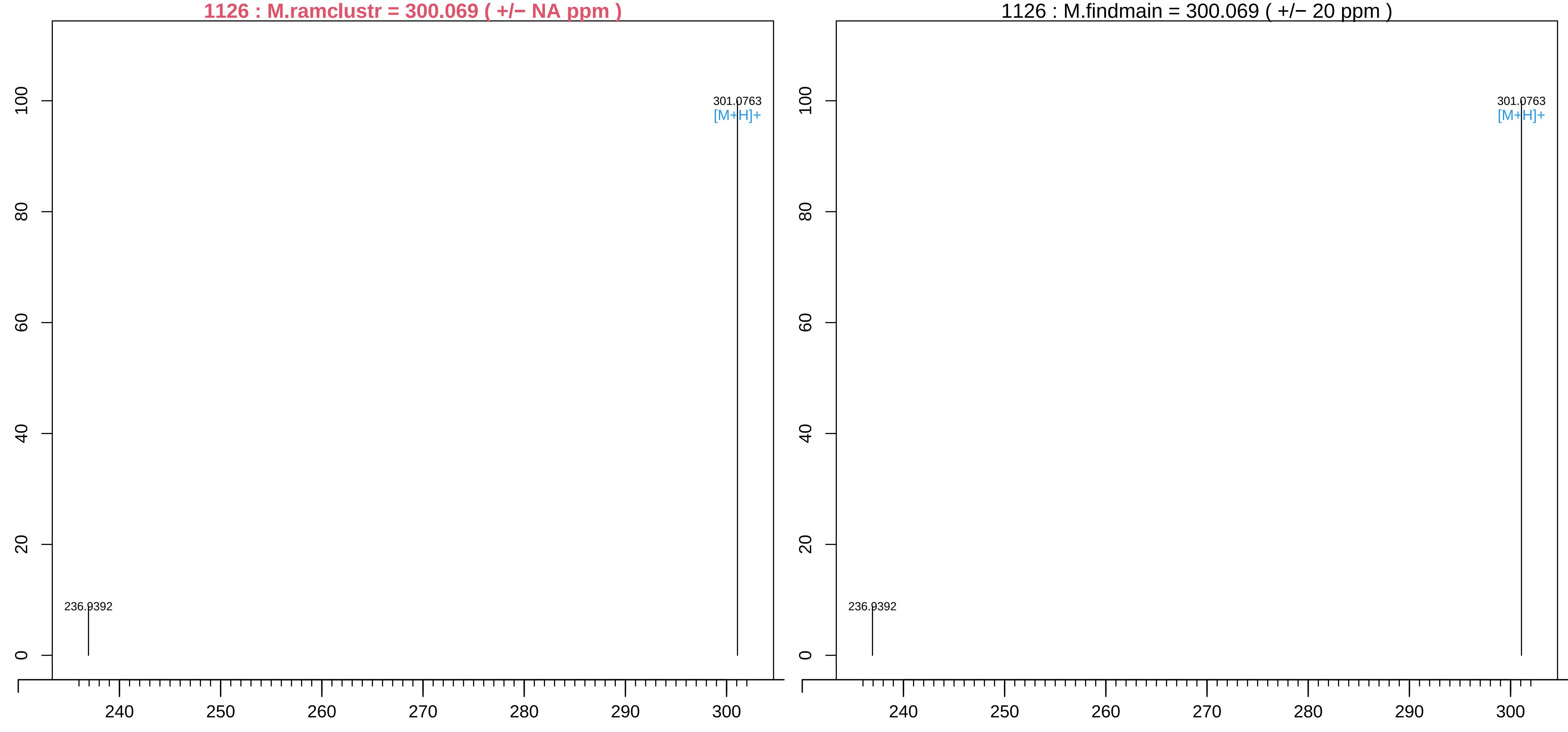
1126 : M.ramclustr = 300.069 (+/− NA ppm)
301.0763
[M+H]+
236.9392
1126 : M.findmain = 300.069 (+/− 20 ppm)
301.0763
[M+H]+
236.9392
0
20
40
60
80
100
240
250
260
270
280
290
300

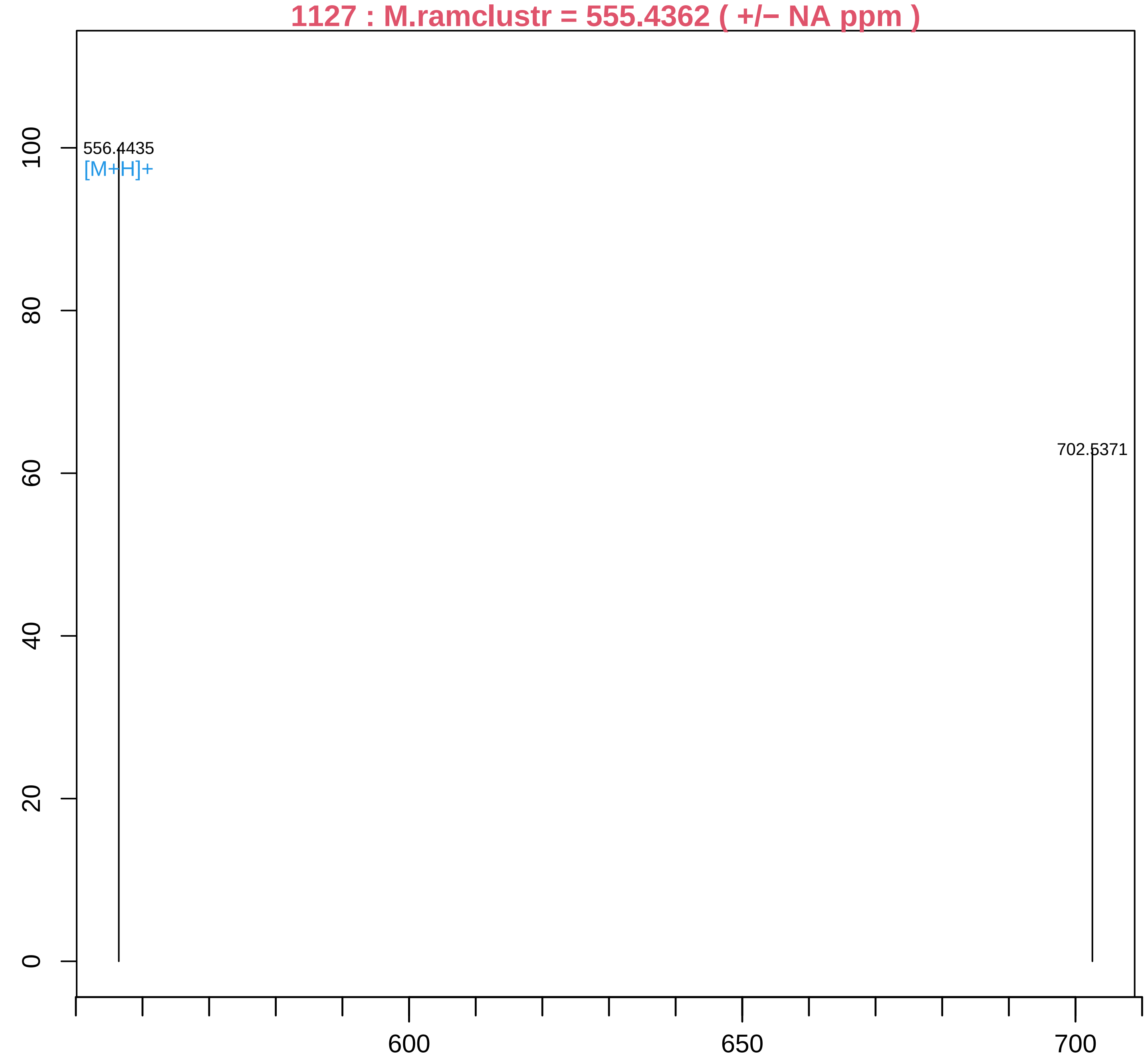
1127 : M.ramclustr = 555.4362 (+/− NA ppm)
556.4435
[M+H]+
702.5371
0
20
40
60
80
100
600
650
700

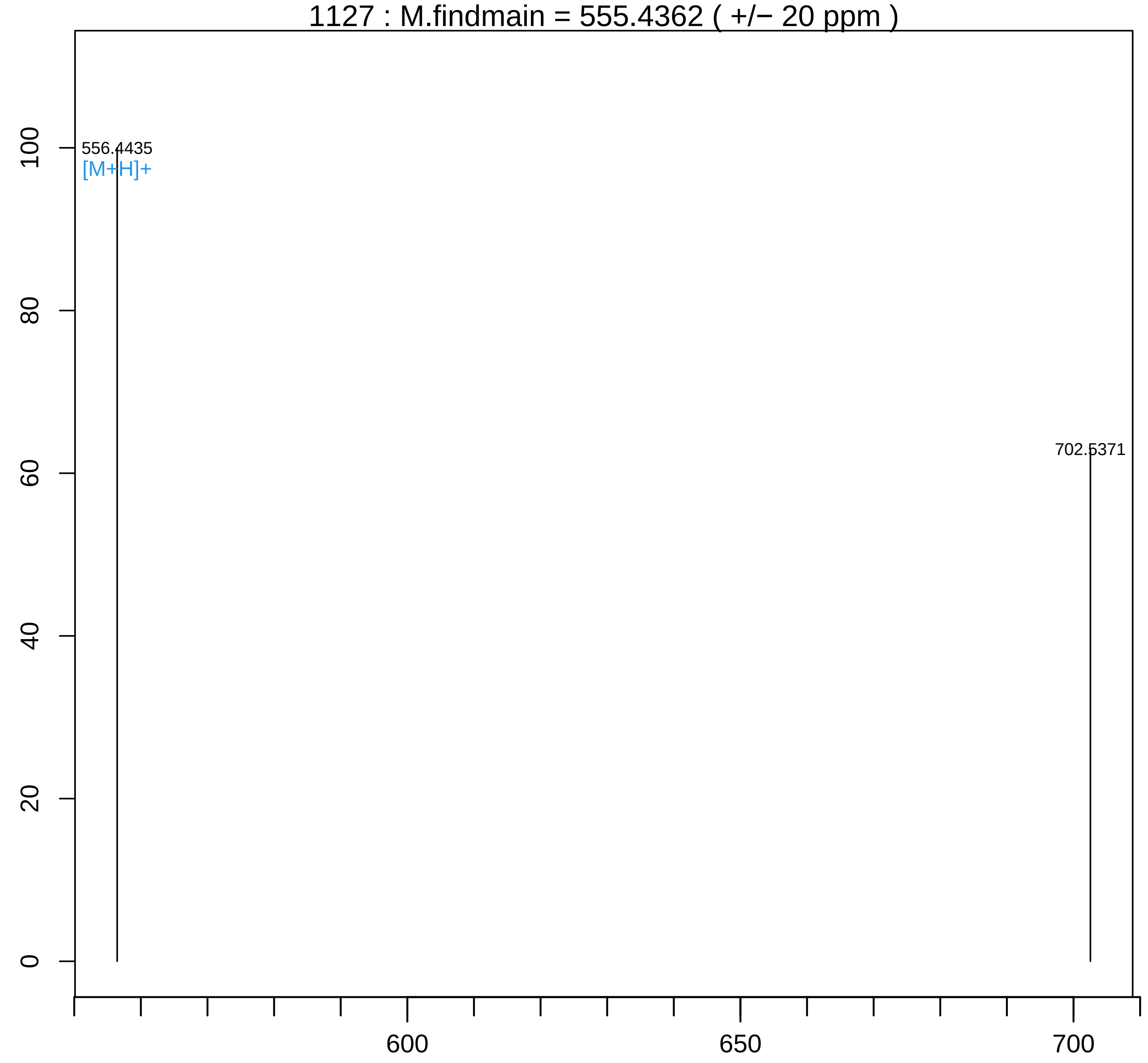
1127 : M.findmain = 555.4362 (+/− 20 ppm)
556.4435
[M+H]+
702.5371
0
20
40
60
80
100
600
650
700

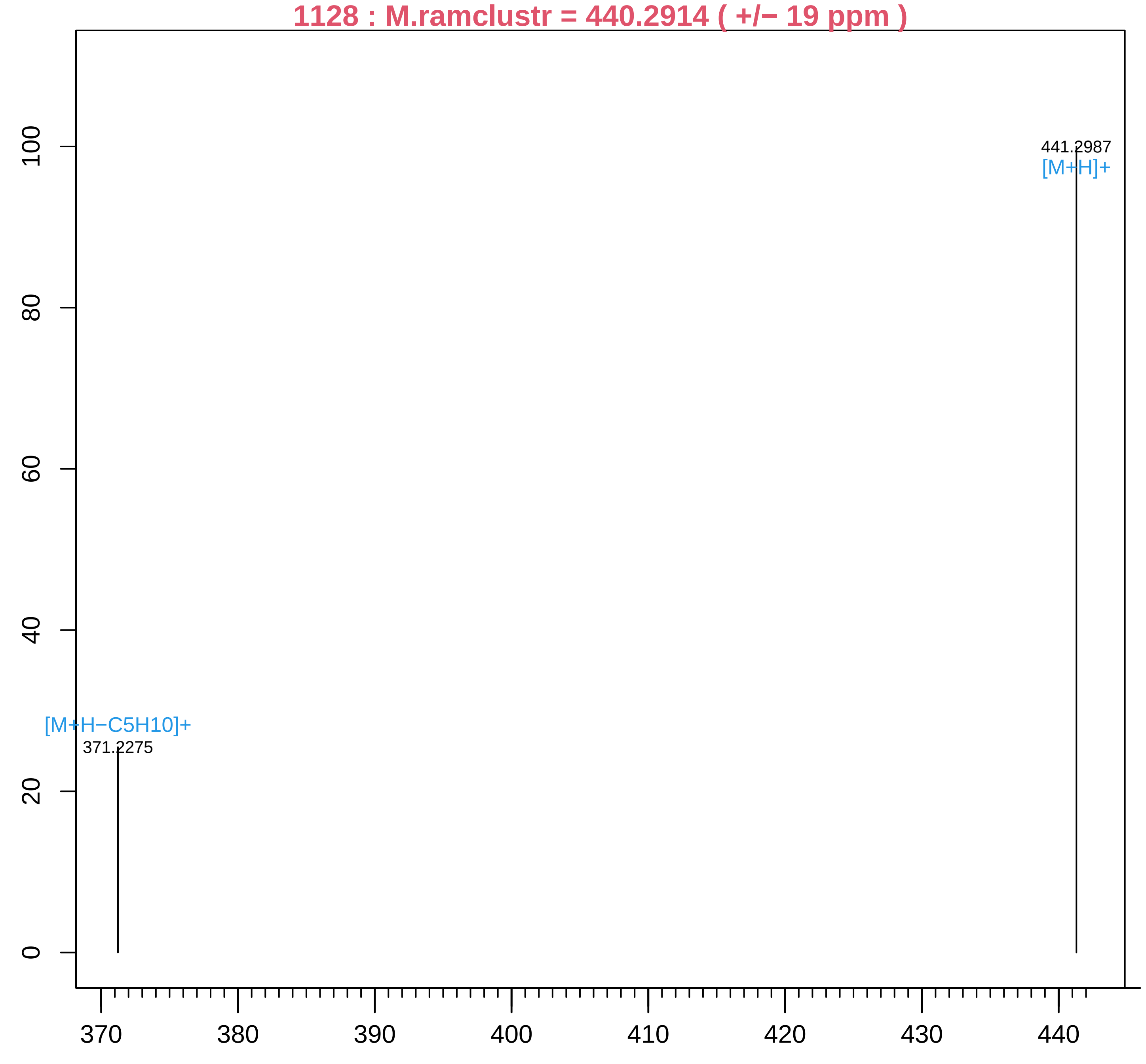
1128 : M.ramclustr = 440.2914 (+/− 19 ppm)
441.2987
[M+H]+
[M+H−C5H10]+
371.2275

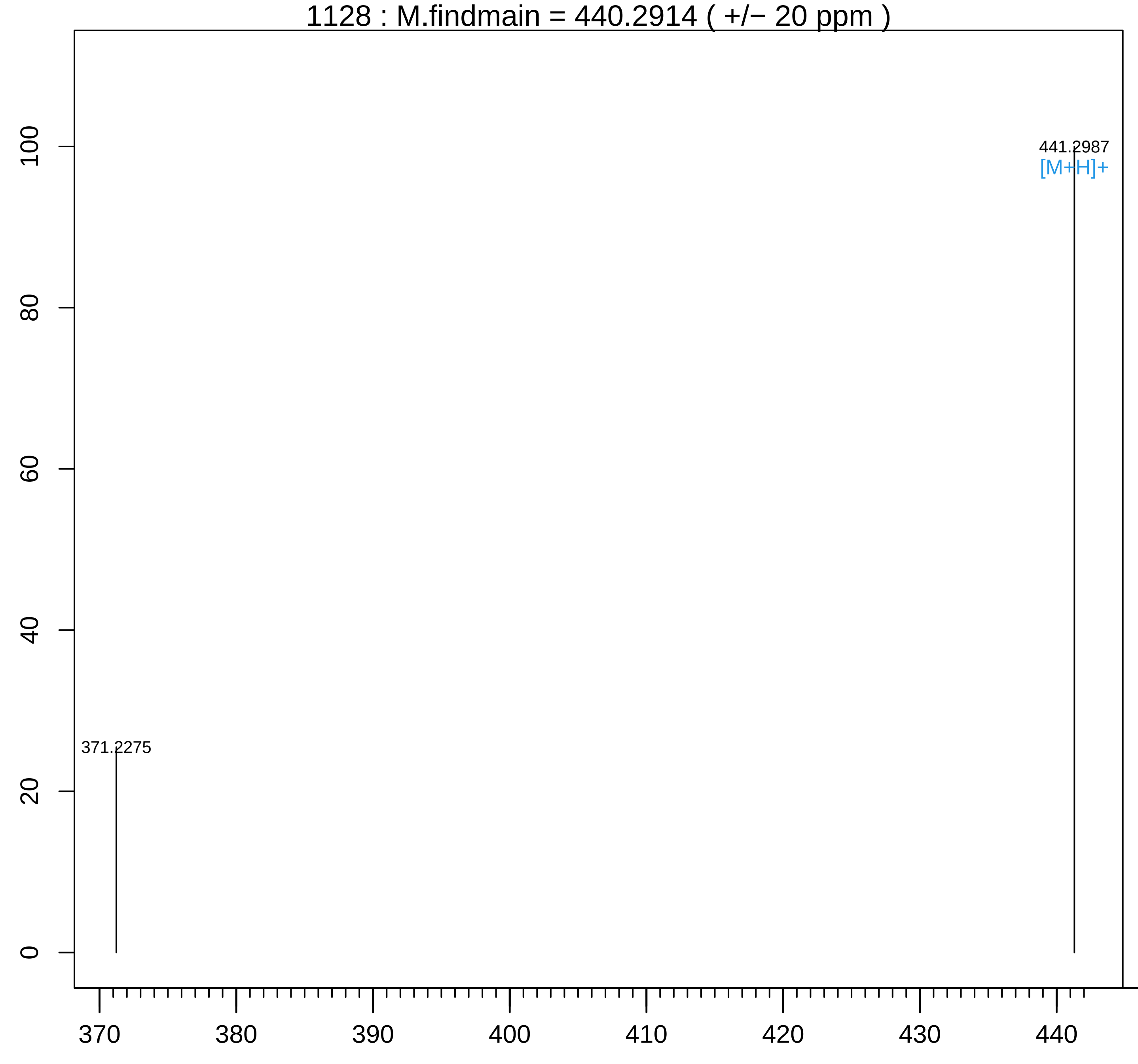
1128 : M.findmain = 440.2914 (+/− 20 ppm)
441.2987
[M+H]+
371.2275

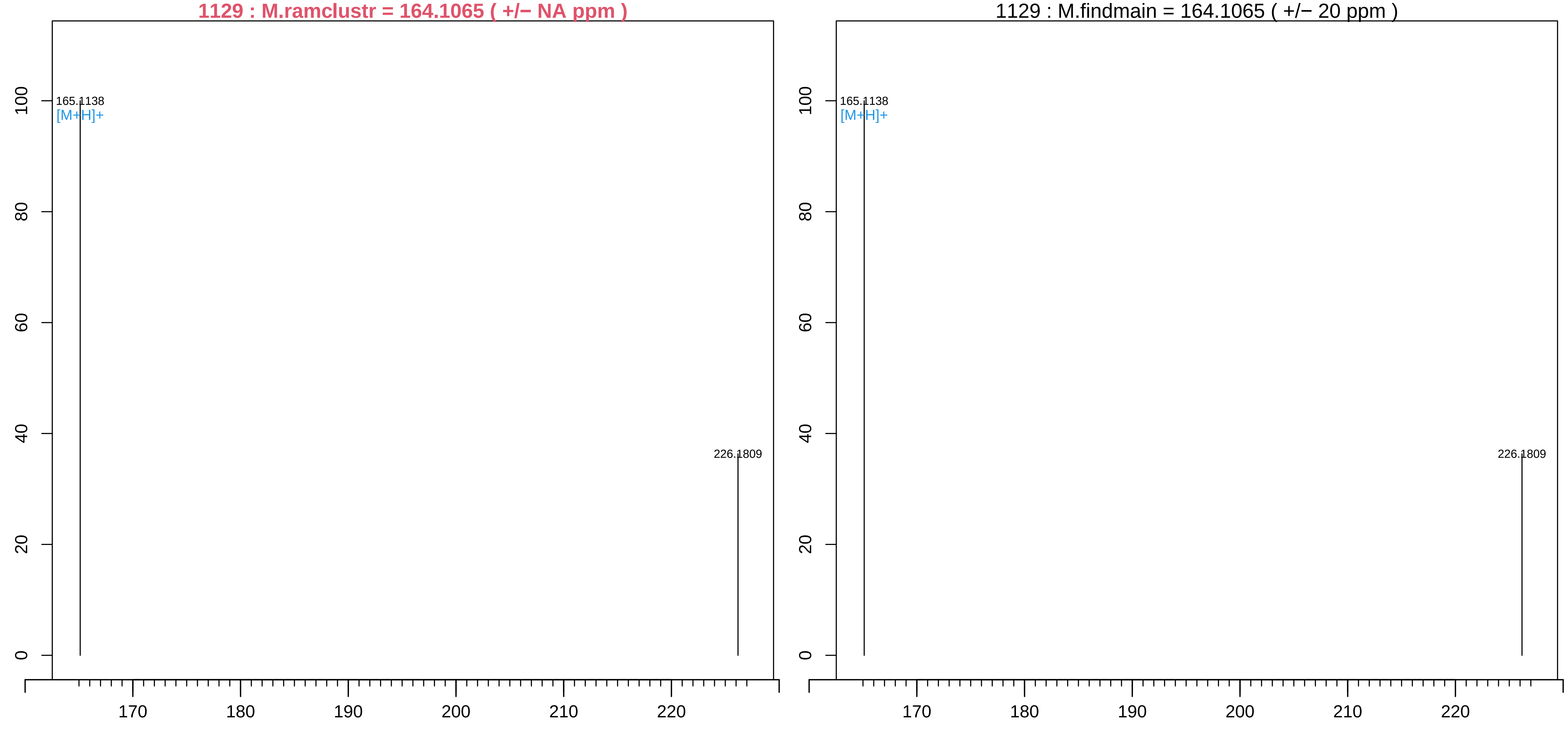
1129 : M.ramclustr = 164.1065 (+/− NA ppm)
165.1138
[M+H]+
226.1809
1129 : M.findmain = 164.1065 (+/− 20 ppm)
165.1138
[M+H]+
226.1809
0
20
40
60
80
100
170
180
190
200
210
220

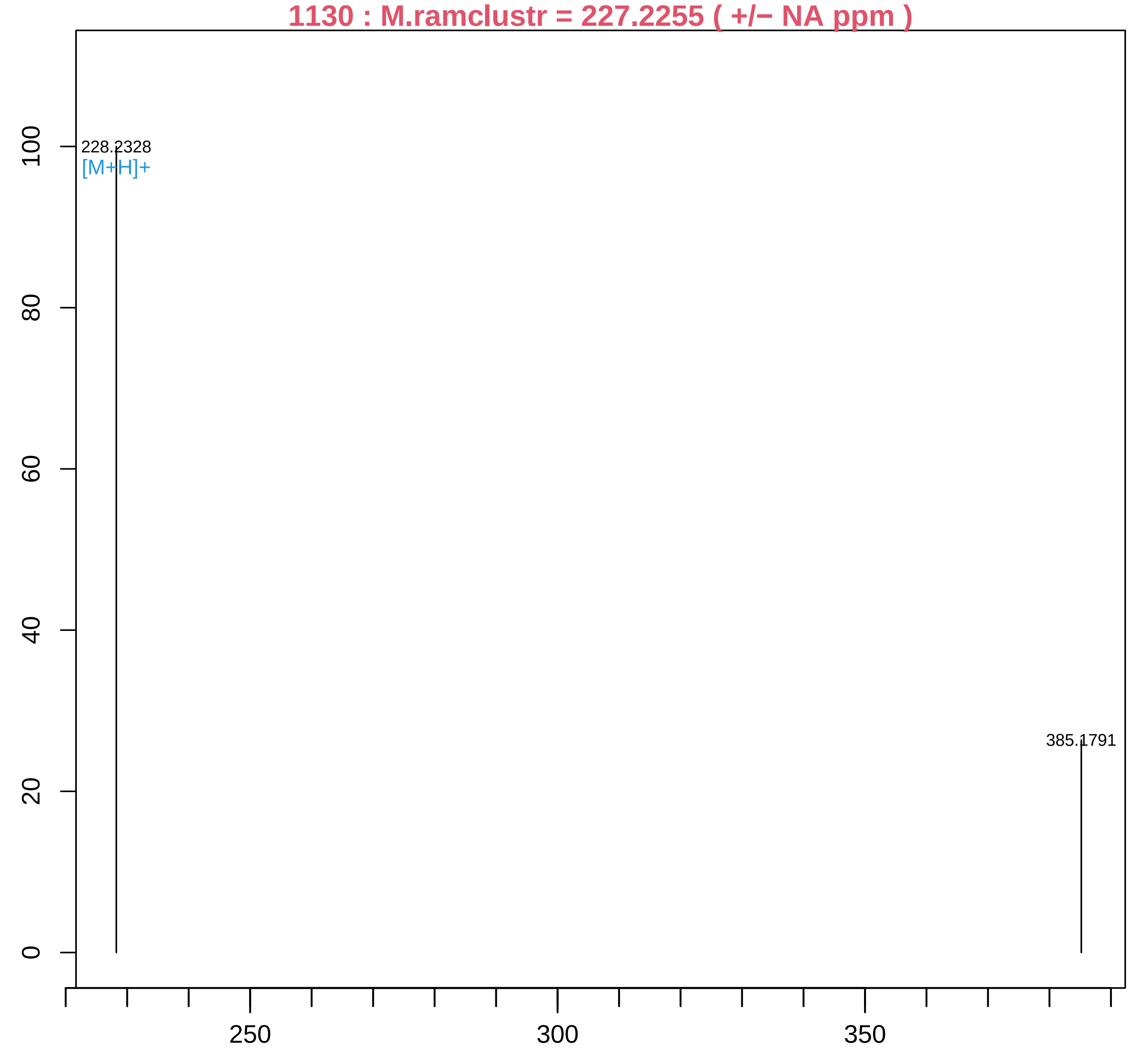
1130 : M.ramclustr = 227.2255 (+/− NA ppm)
228.2328
[M+H]+
385.1791
0
20
40
60
80
100
250
300
350

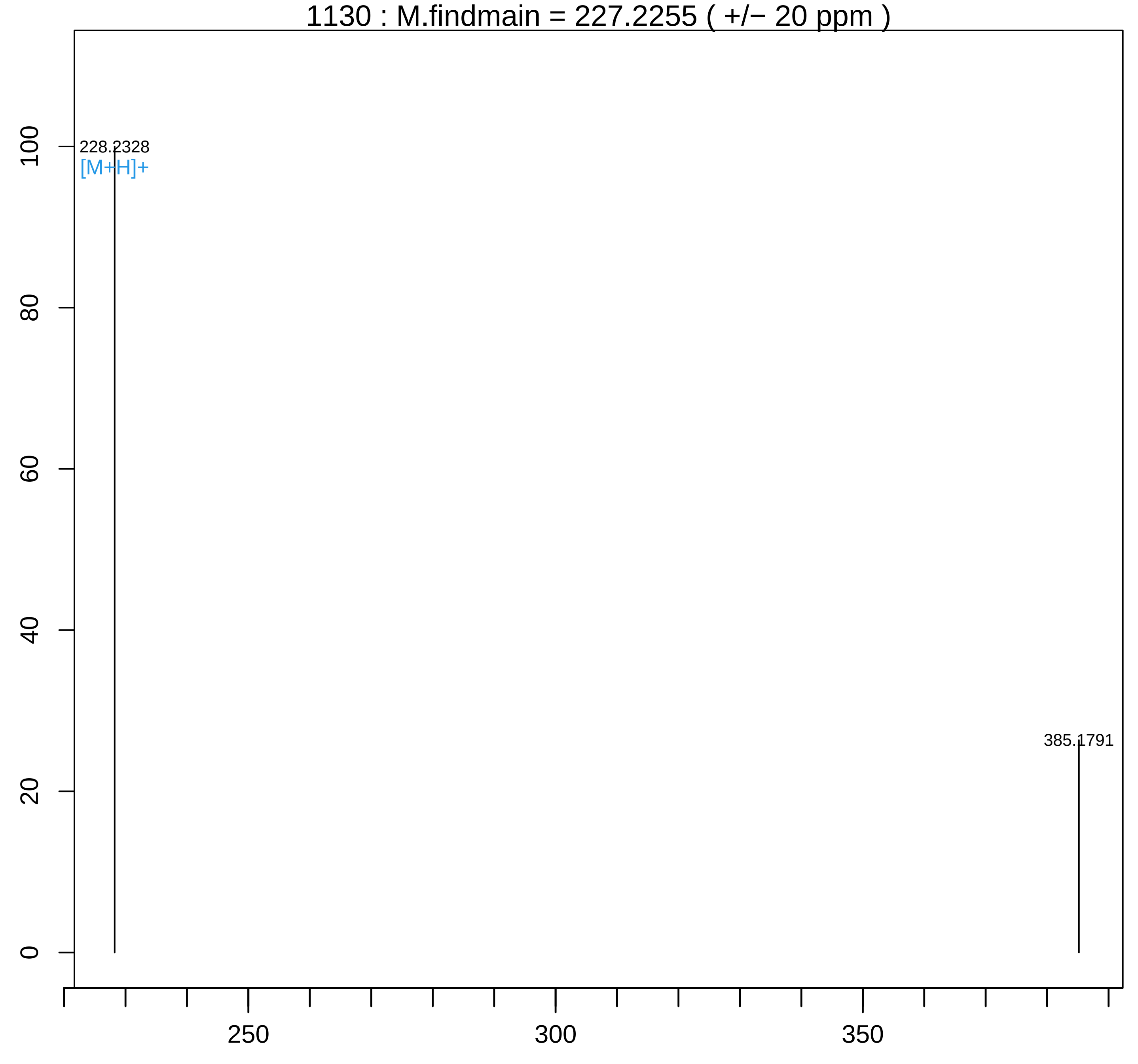
1130 : M.findmain = 227.2255 (+/− 20 ppm)
228.2328
[M+H]+
385.1791
0
20
40
60
80
100
250
300
350

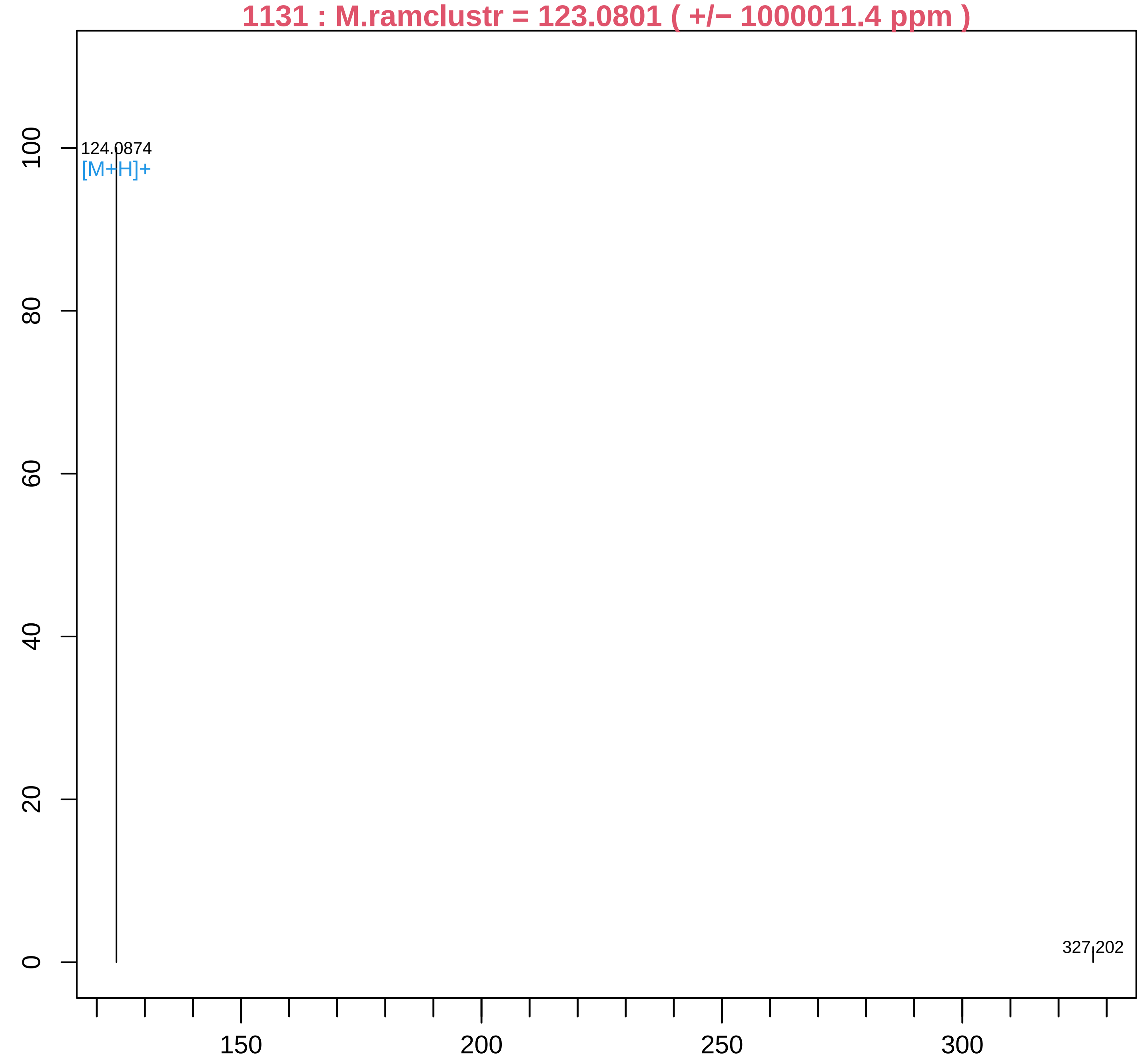
1131 : M.ramclustr = 123.0801 (+/− 1000011.4 ppm)
124.0874
[M+H]+
327.202
0
20
40
60
80
100
150
200
250
300

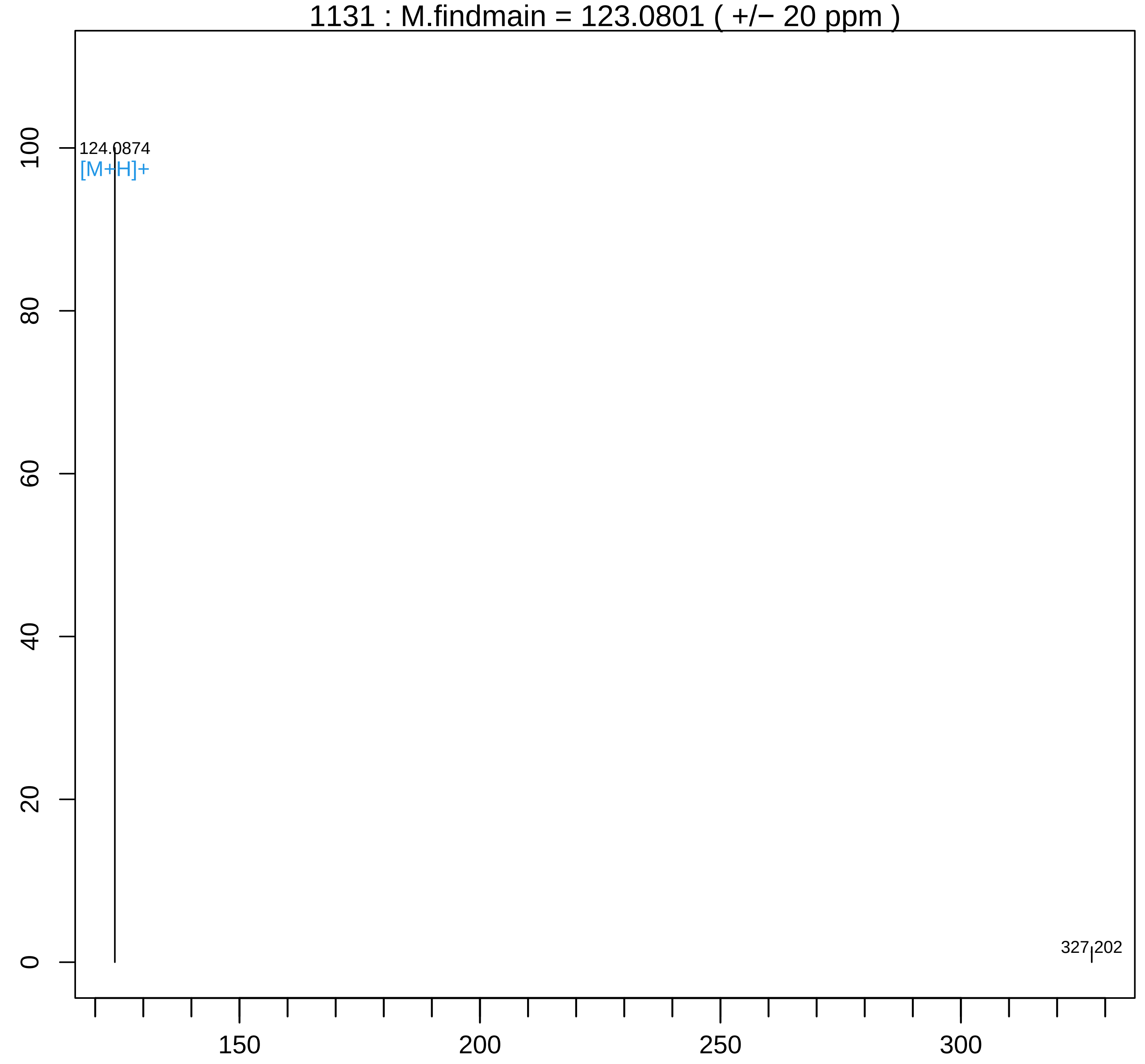
1131 : M.findmain = 123.0801 (+/− 20 ppm)
124.0874
[M+H]+
327.202
0
20
40
60
80
100
150
200
250
300

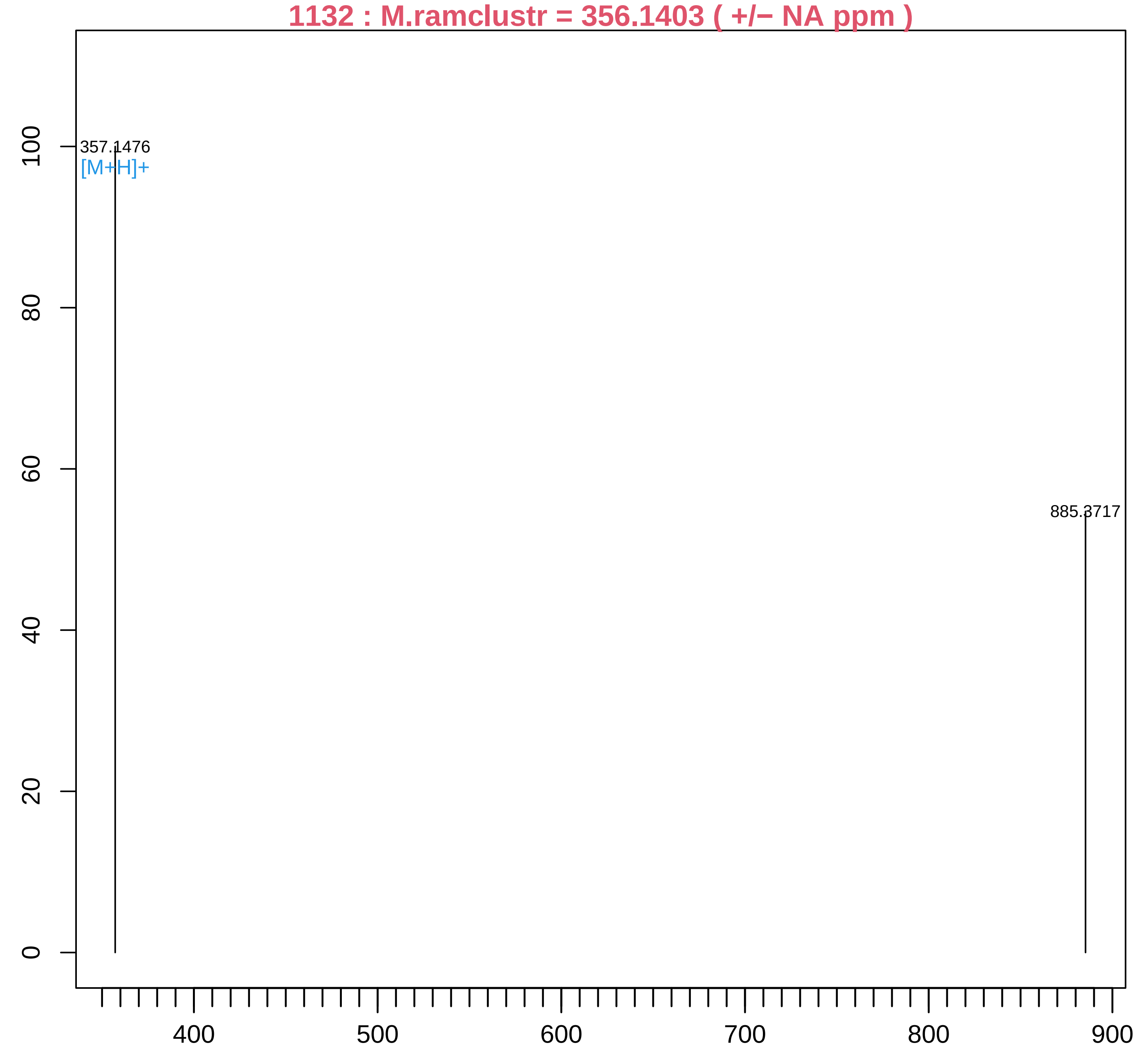
1132 : M.ramclustr = 356.1403 (+/− NA ppm)
357.1476
[M+H]+
885.3717
0
20
40
60
80
100
400
500
600
700
800
900

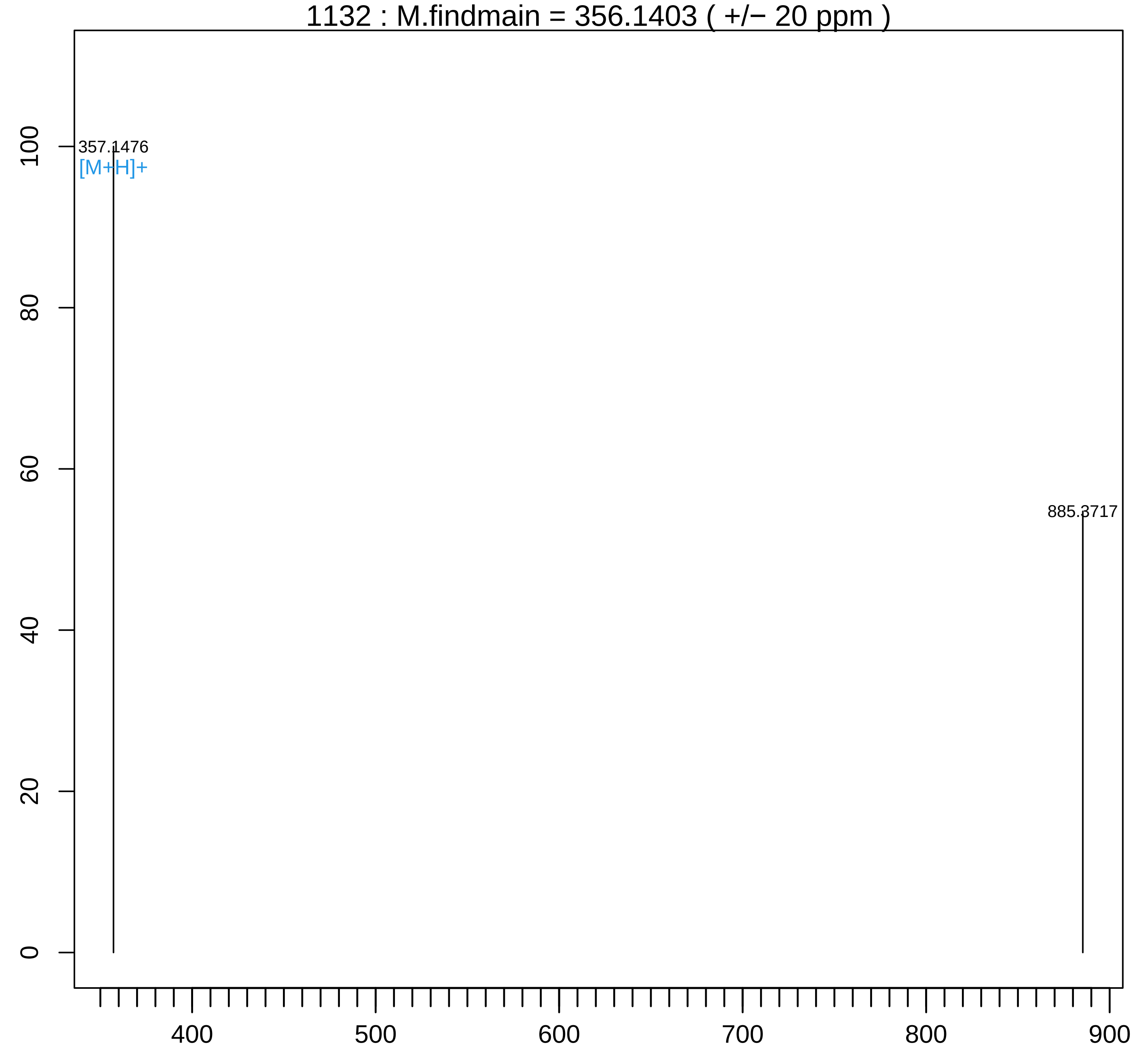
1132 : M.findmain = 356.1403 (+/− 20 ppm)
357.1476
[M+H]+
885.3717
0
20
40
60
80
100
400
500
600
700
800
900

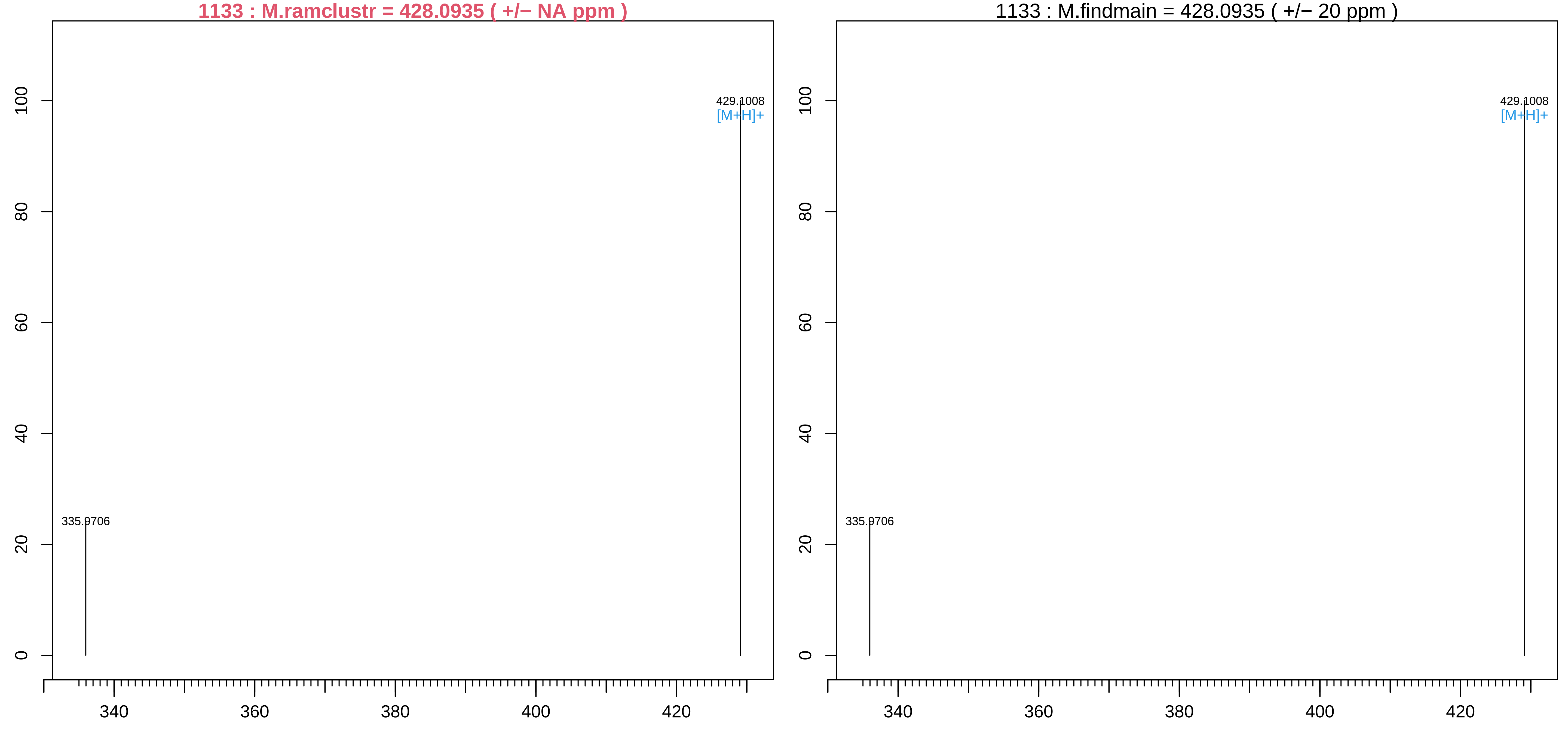

1133 : M.ramclustr = 428.0935 (+/− NA ppm)
429.1008
[M+H]+
335.9706
0
20
40
60
80
100
340
360
380
400
420
1133 : M.findmain = 428.0935 (+/− 20 ppm)
429.1008
[M+H]+
335.9706
0
20
40
60
80
100
340
360
380
400
420

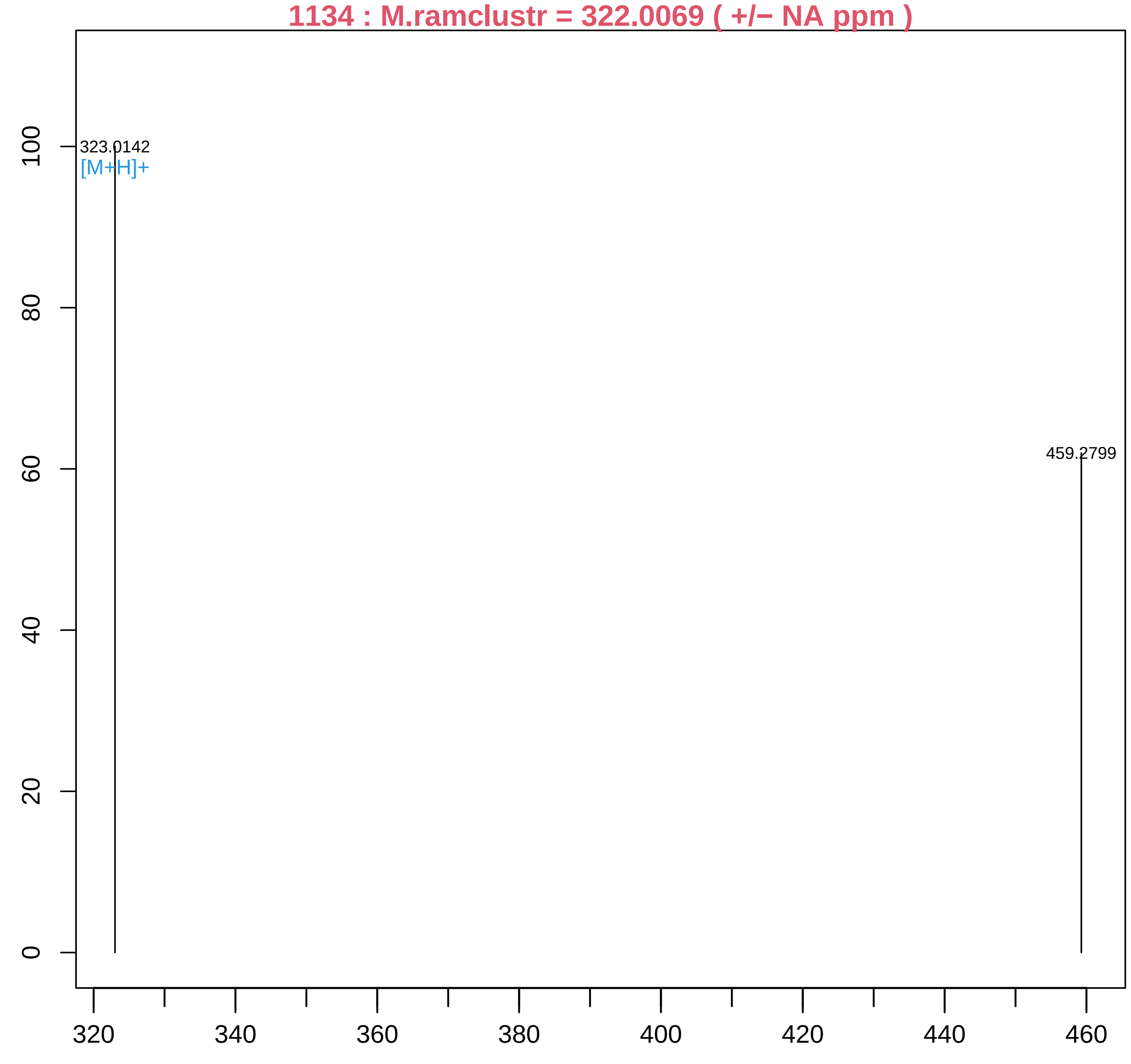
1134 : M.ramclustr = 322.0069 (+/− NA ppm)
323.0142
[M+H]+
459.2799
0
20
40
60
80
100
320
340
360
380
400
420
440
460

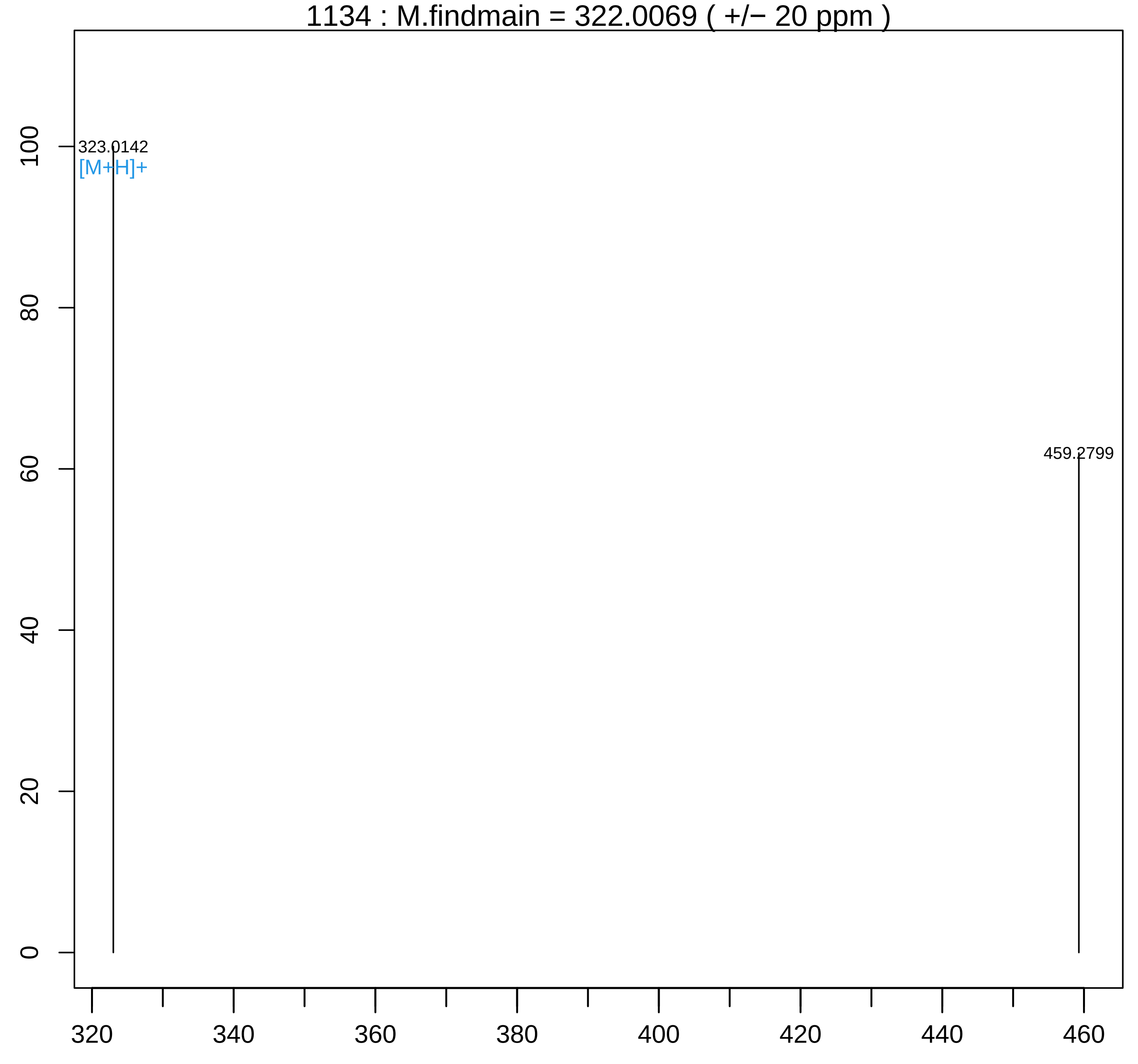
1134 : M.findmain = 322.0069 (+/− 20 ppm)
323.0142
[M+H]+
459.2799
0
20
40
60
80
100
320
340
360
380
400
420
440
460

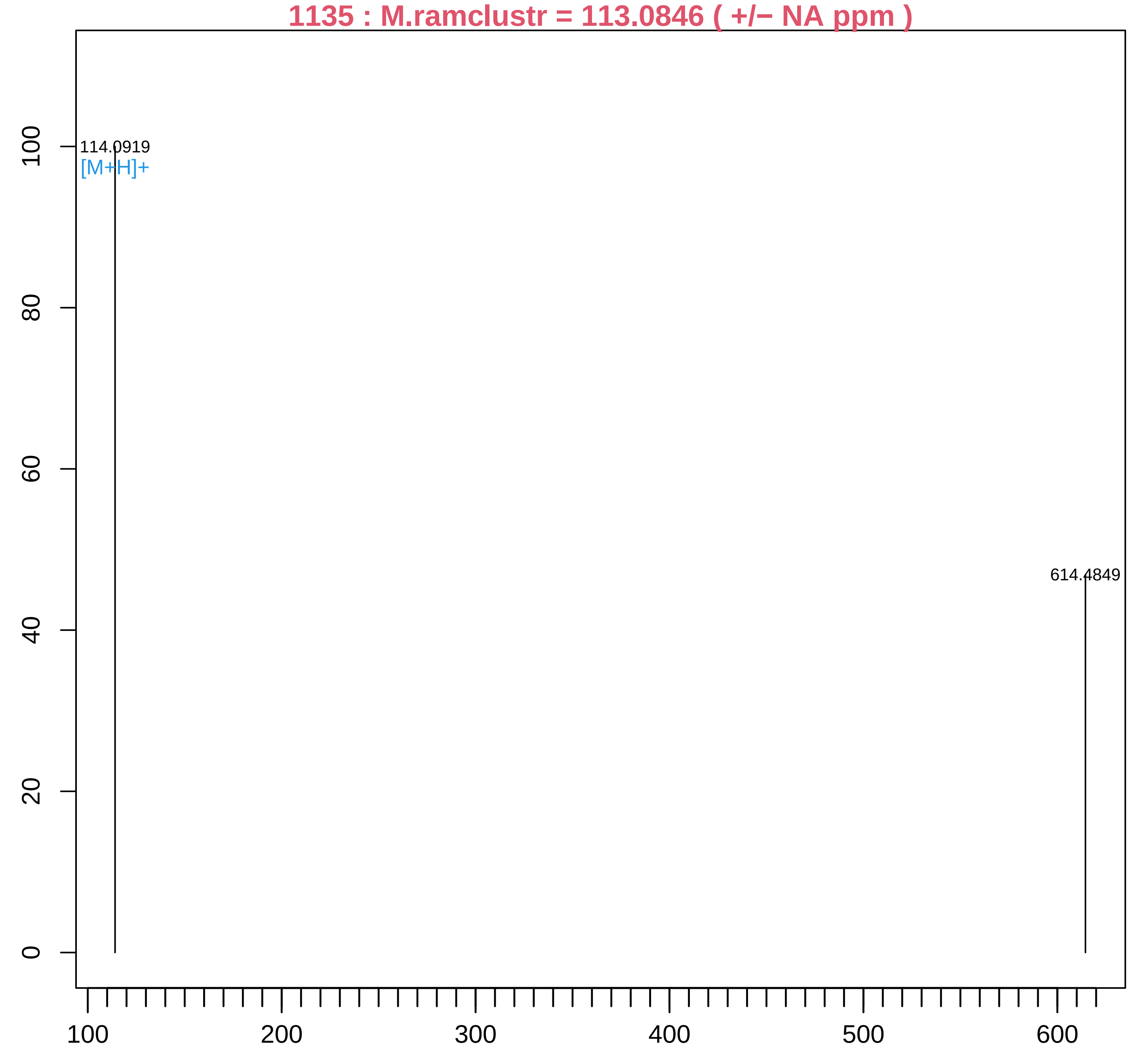

1135 : M.ramclustr = 113.0846 (+/− NA ppm)
114.0919
[M+H]+
614.4849
0
20
40
60
80
100
100
200
300
400
500
600

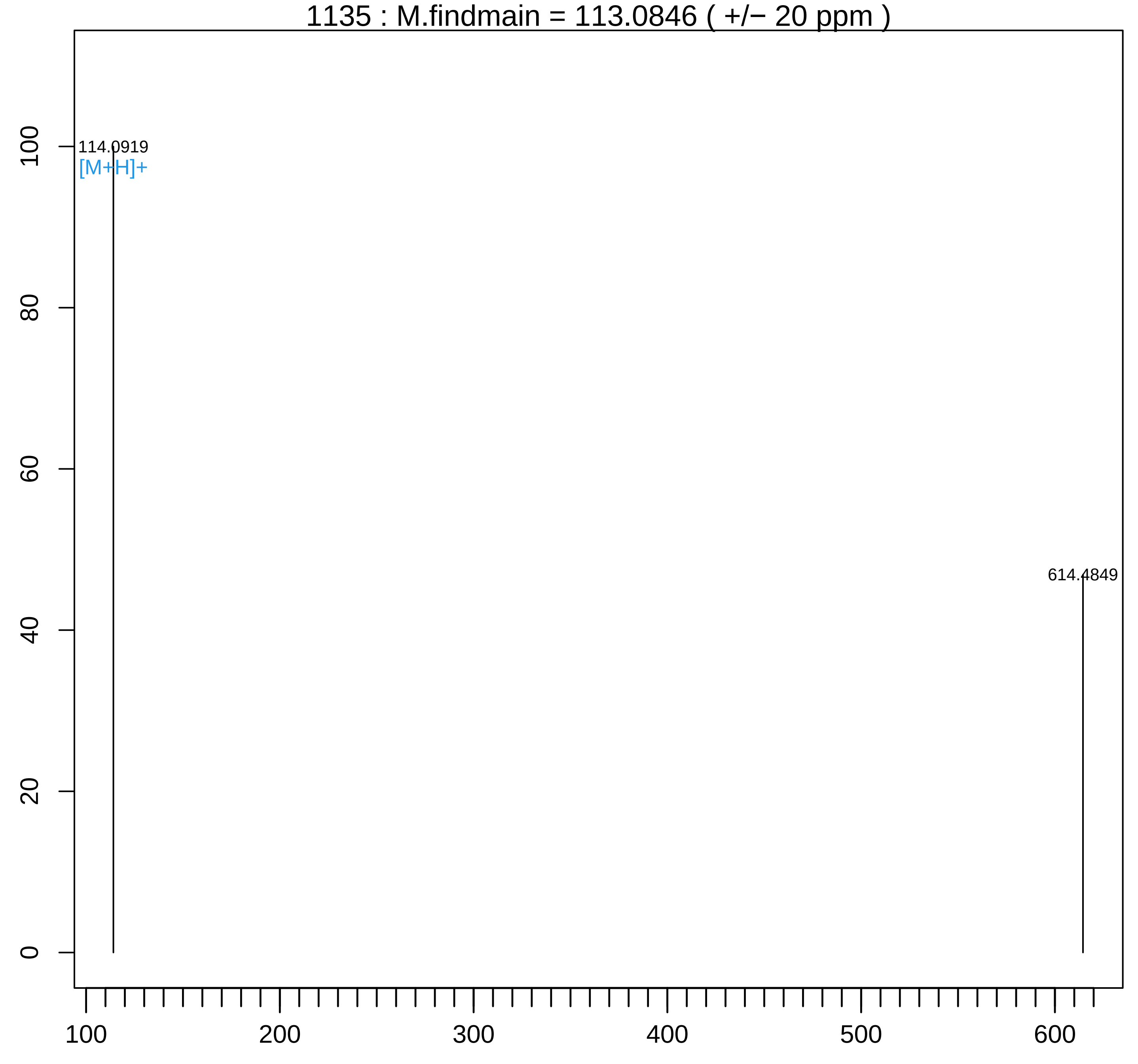

1135 : M.findmain = 113.0846 (+/− 20 ppm)
114.0919
[M+H]+
614.4849
0
20
40
60
80
100
100
200
300
400
500
600

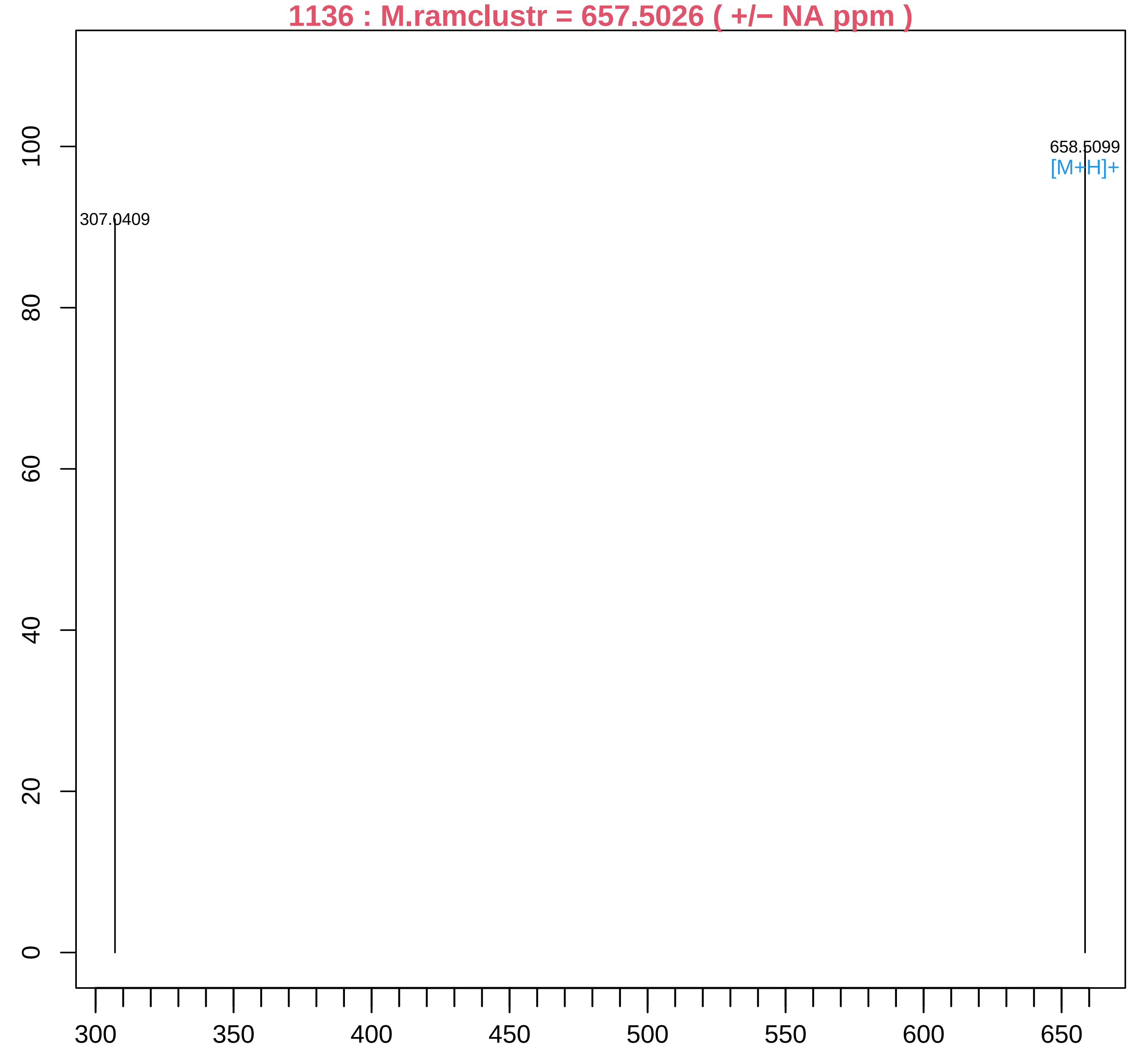
1136 : M.ramclustr = 657.5026 (+/− NA ppm)
658.5099
[M+H]+
307.0409
0
20
40
60
80
100
300
350
400
450
500
550
600
650

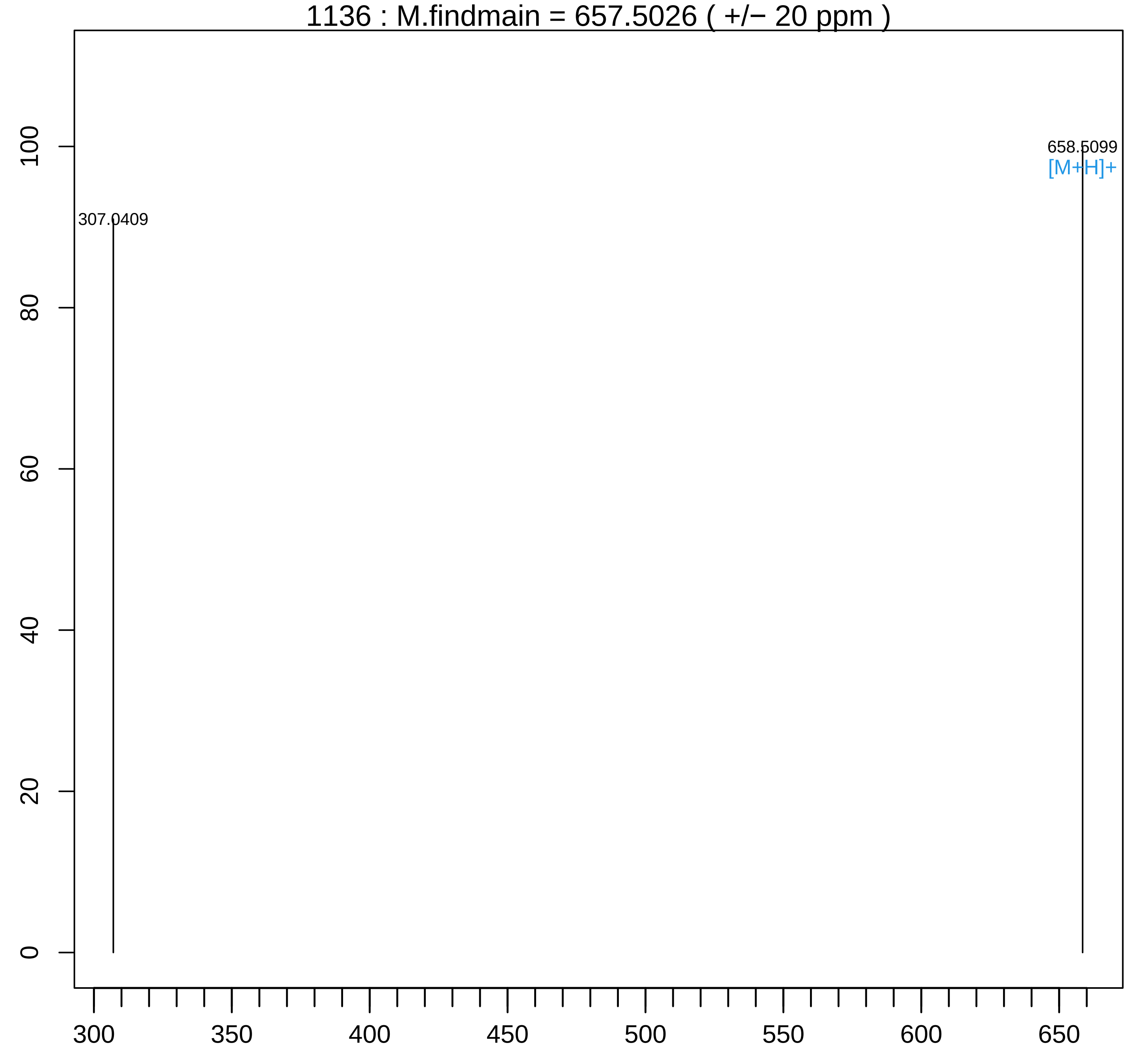
1136 : M.findmain = 657.5026 (+/− 20 ppm)
658.5099
[M+H]+
307.0409
0
20
40
60
80
100
300
350
400
450
500
550
600
650

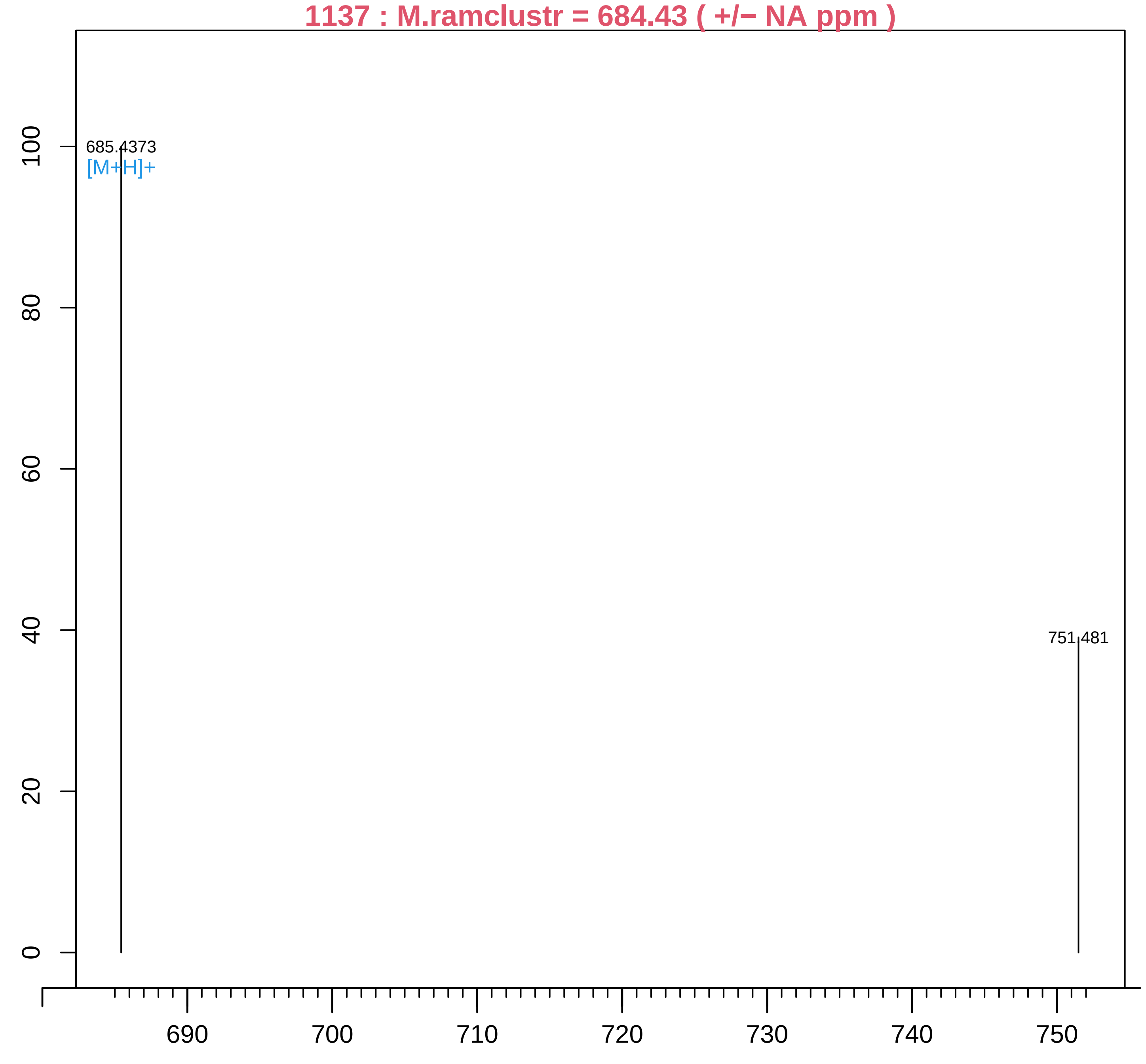
1137 : M.ramclustr = 684.43 (+/− NA ppm)
685.4373
[M+H]+
751.481
0
20
40
60
80
100
690
700
710
720
730
740
750

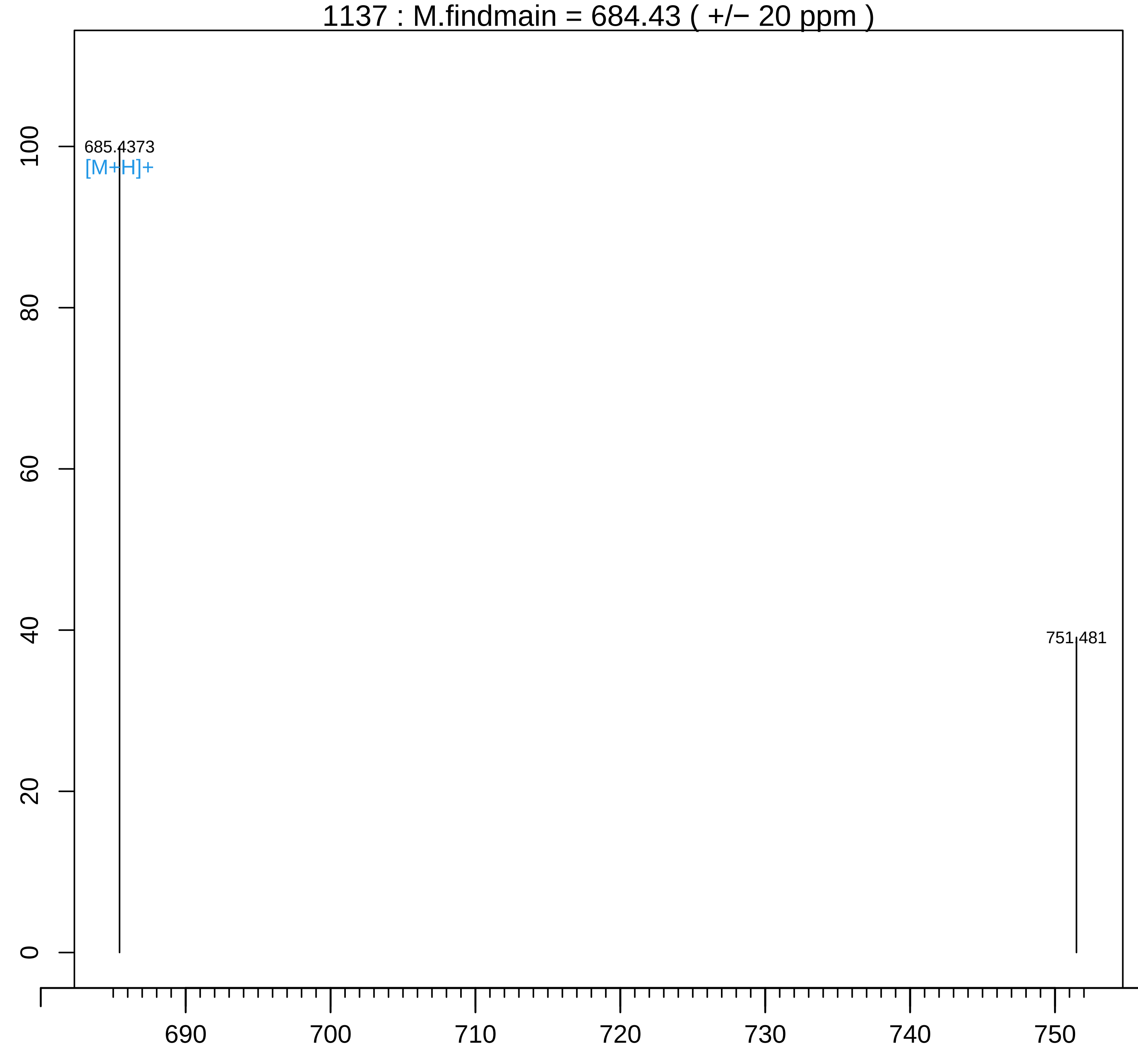
1137 : M.findmain = 684.43 (+/− 20 ppm)
685.4373
[M+H]+
751.481
0
20
40
60
80
100
690
700
710
720
730
740
750

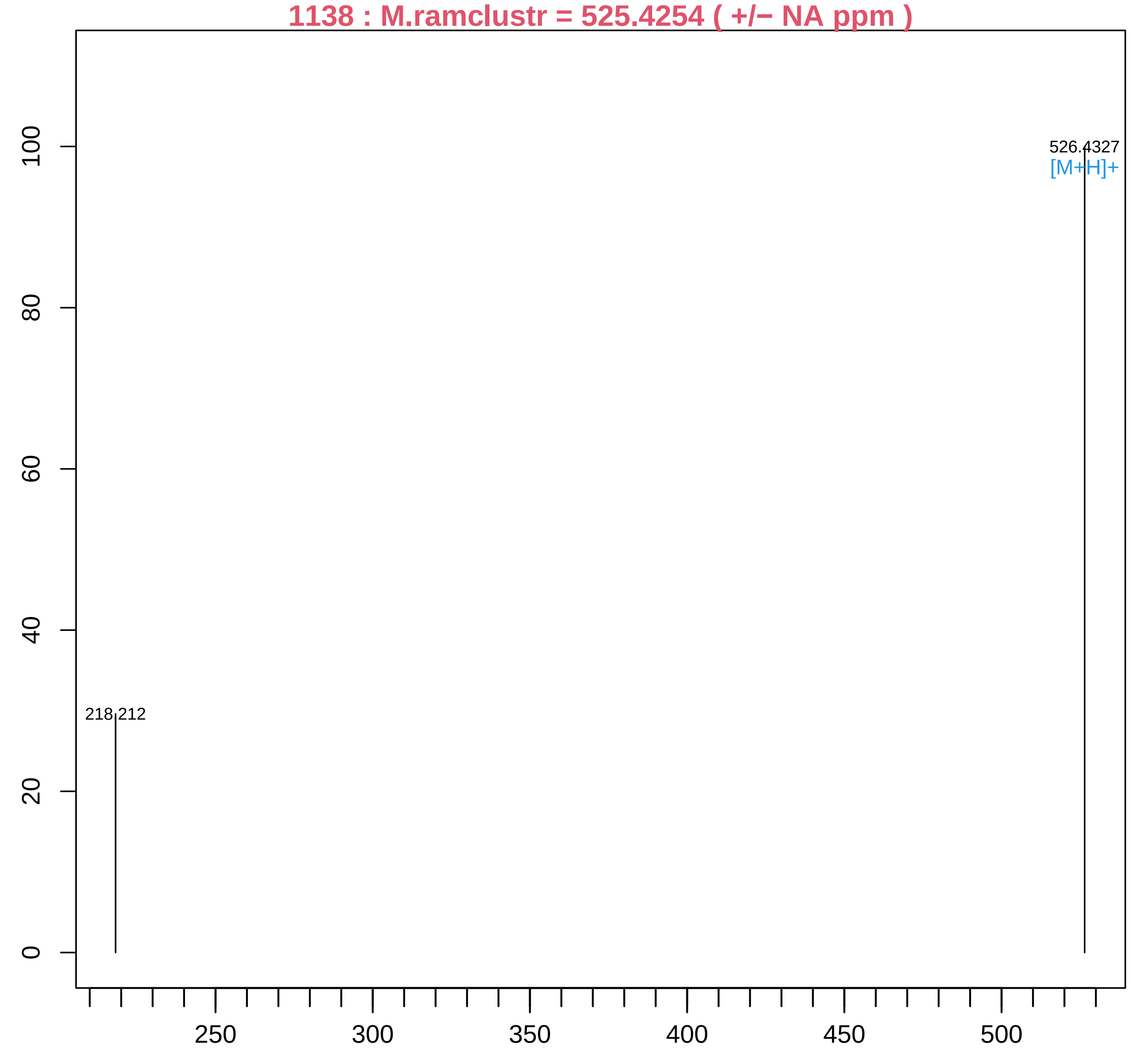
1138 : M.ramclustr = 525.4254 (+/− NA ppm)
526.4327
[M+H]+
218.212
0
20
40
60
80
100
250
300
350
400
450
500

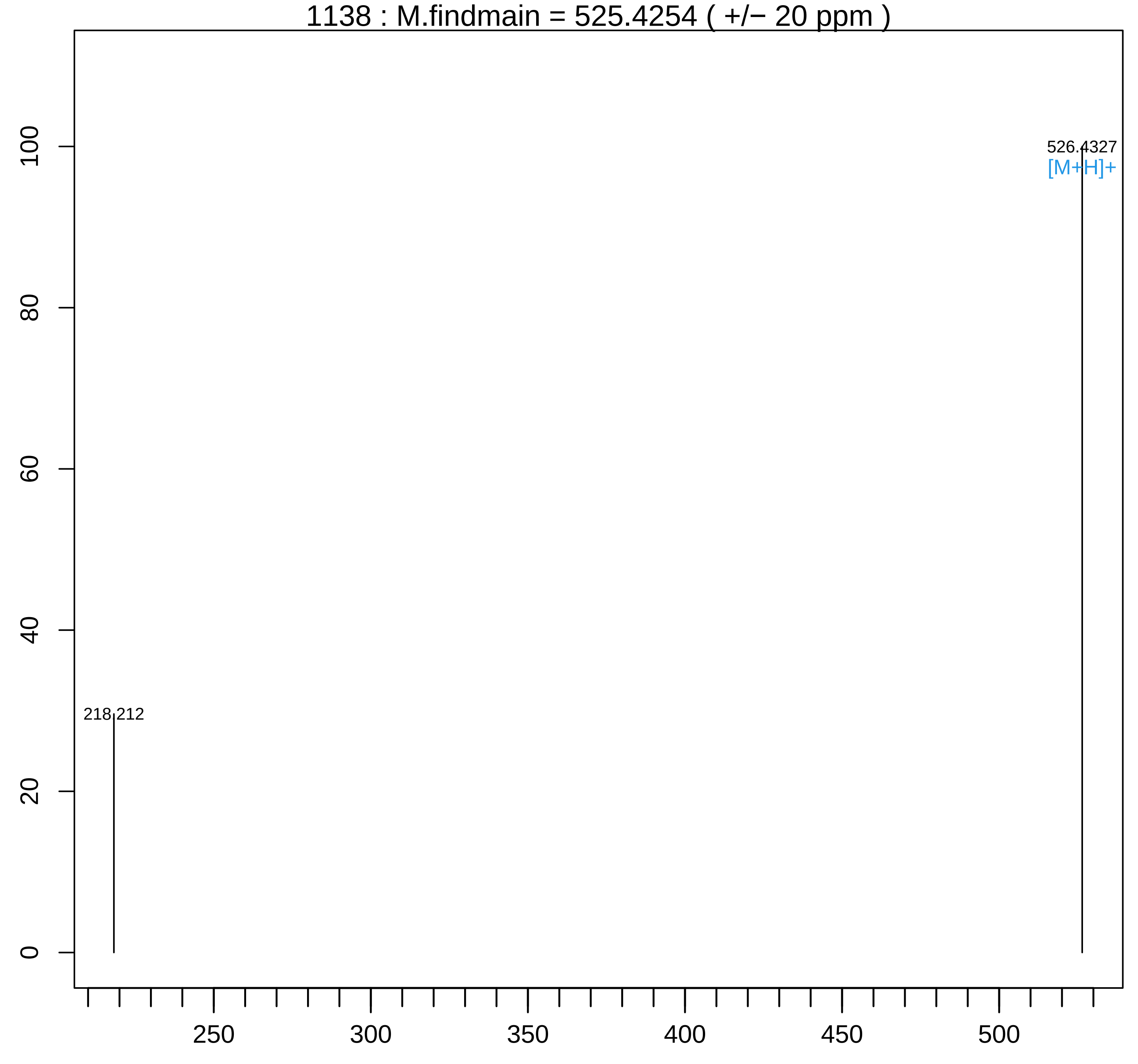
1138 : M.findmain = 525.4254 (+/− 20 ppm)
526.4327
[M+H]+
218.212
0
20
40
60
80
100
250
300
350
400
450
500

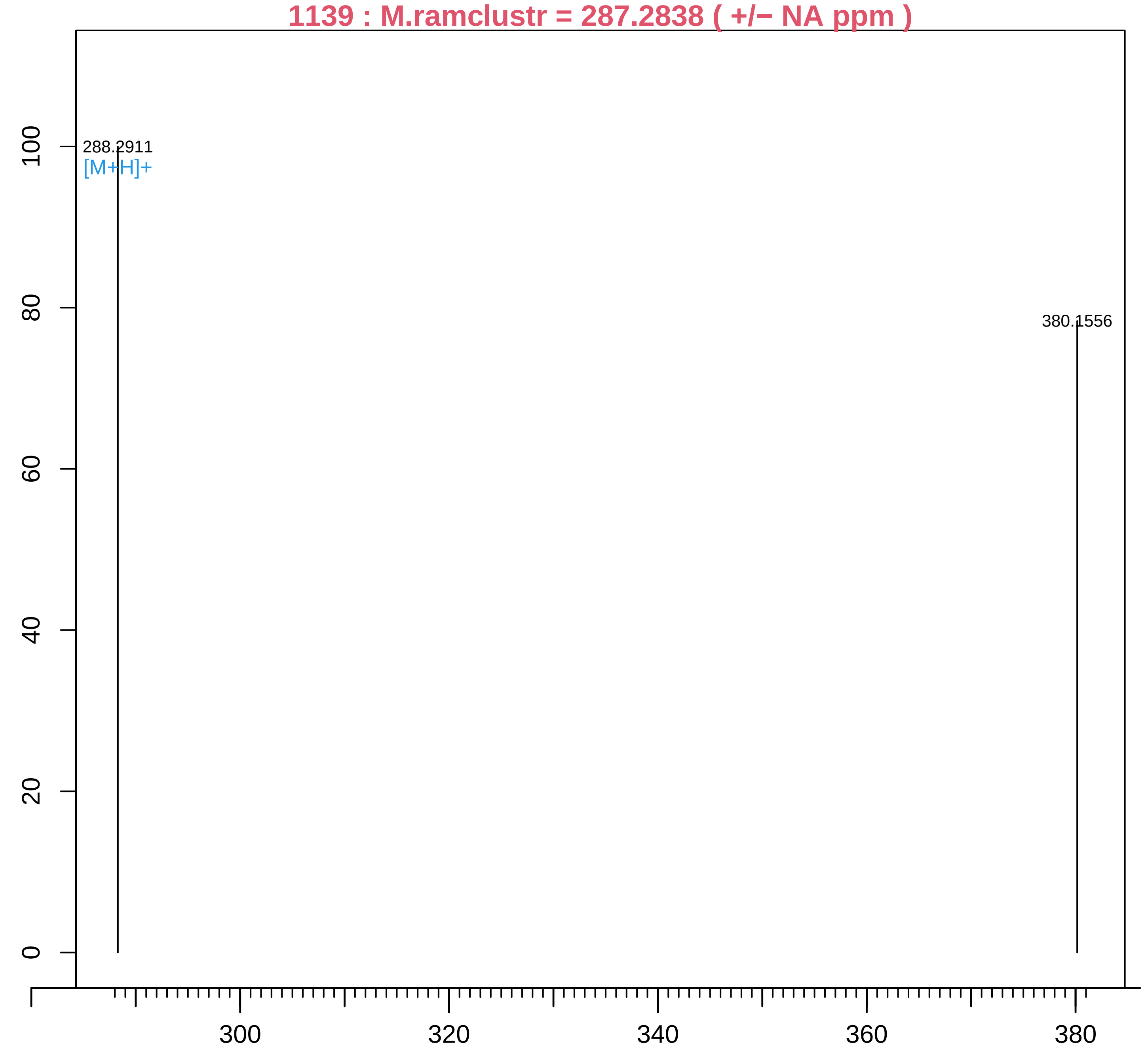
1139 : M.ramclustr = 287.2838 (+/− NA ppm)
288.2911
[M+H]+
380.1556
0
20
40
60
80
100
300
320
340
360
380

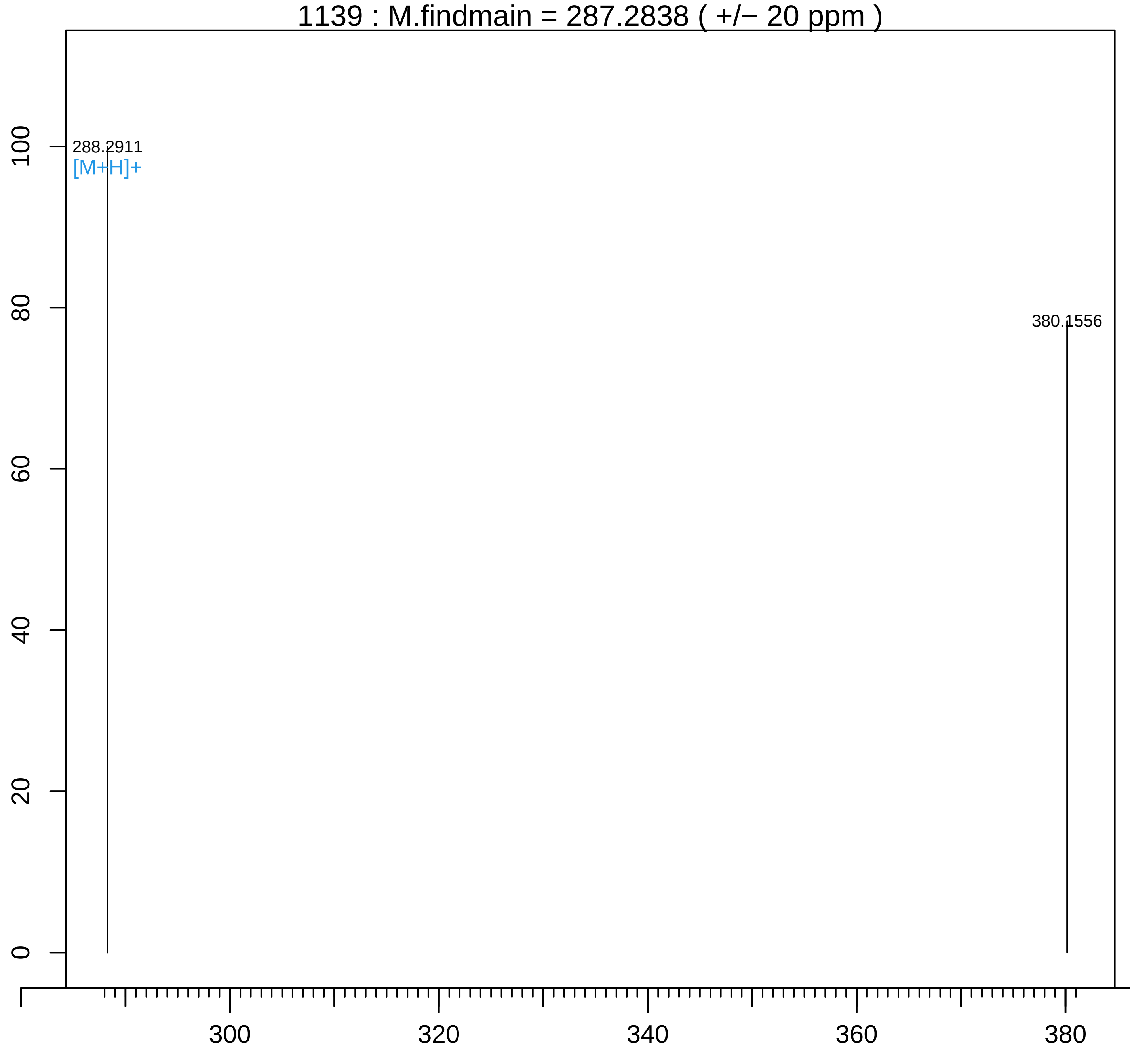
1139 : M.findmain = 287.2838 (+/− 20 ppm)
288.2911
[M+H]+
380.1556
0
20
40
60
80
100
300
320
340
360
380

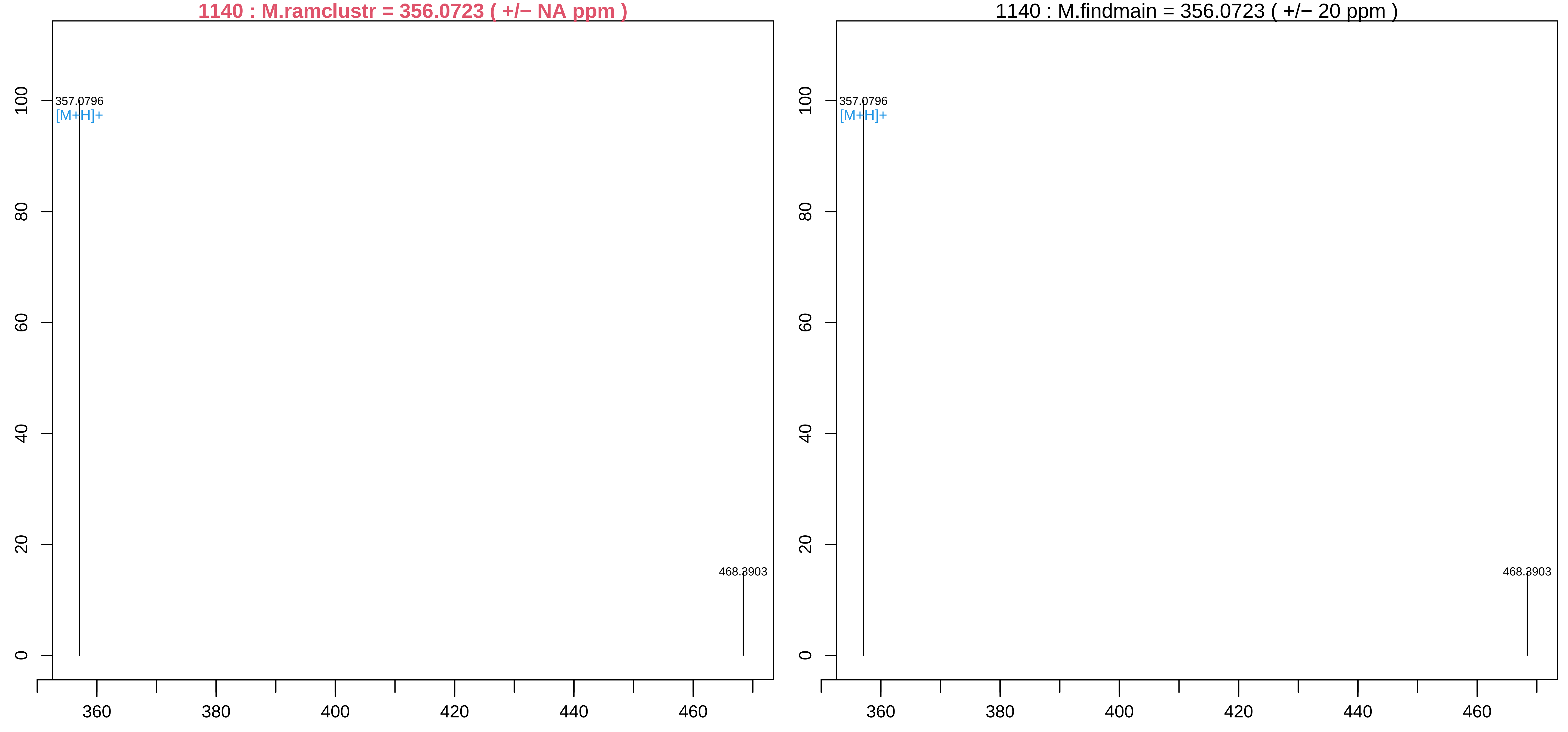
1140 : M.ramclustr = 356.0723 (+/− NA ppm)
357.0796
[M+H]+
468.3903
1140 : M.findmain = 356.0723 (+/− 20 ppm)
357.0796
[M+H]+
468.3903
0
20
40
60
80
100
360
380
400
420
440
460

1141 : M.ramclustr = 173.0831 (+/− NA ppm)

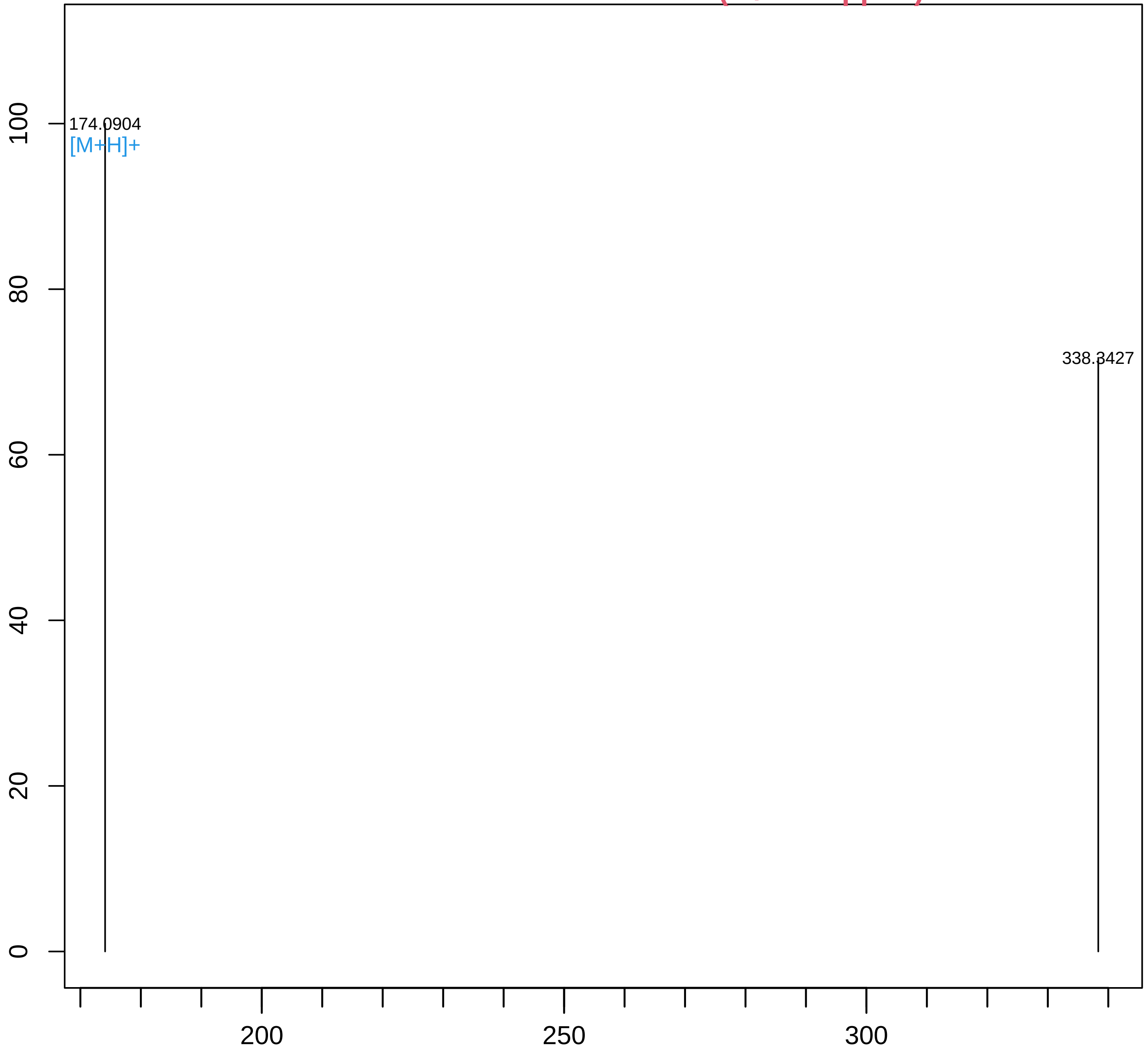

1141 : M.findmain = 173.0831 (+/− 20 ppm)

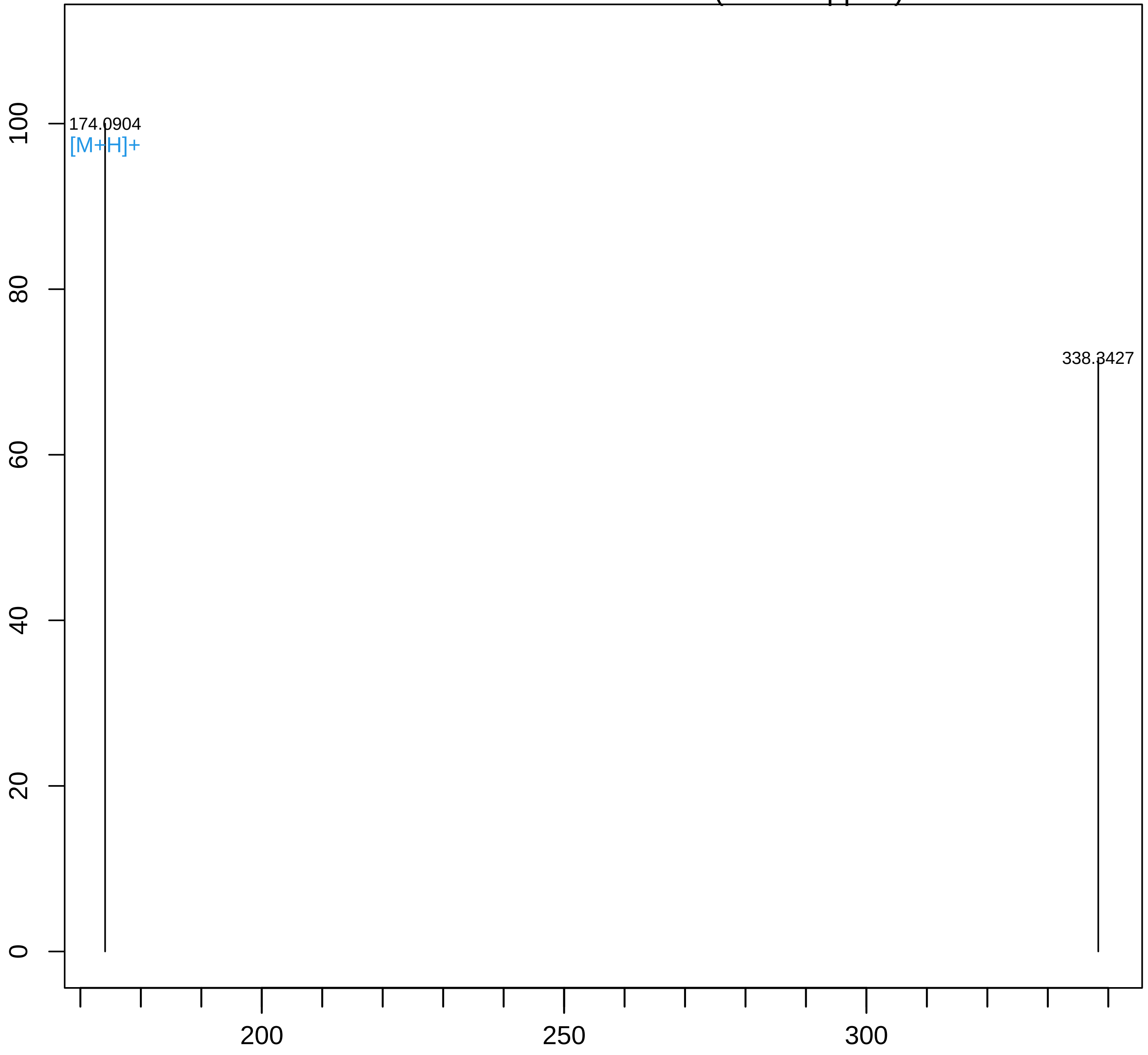

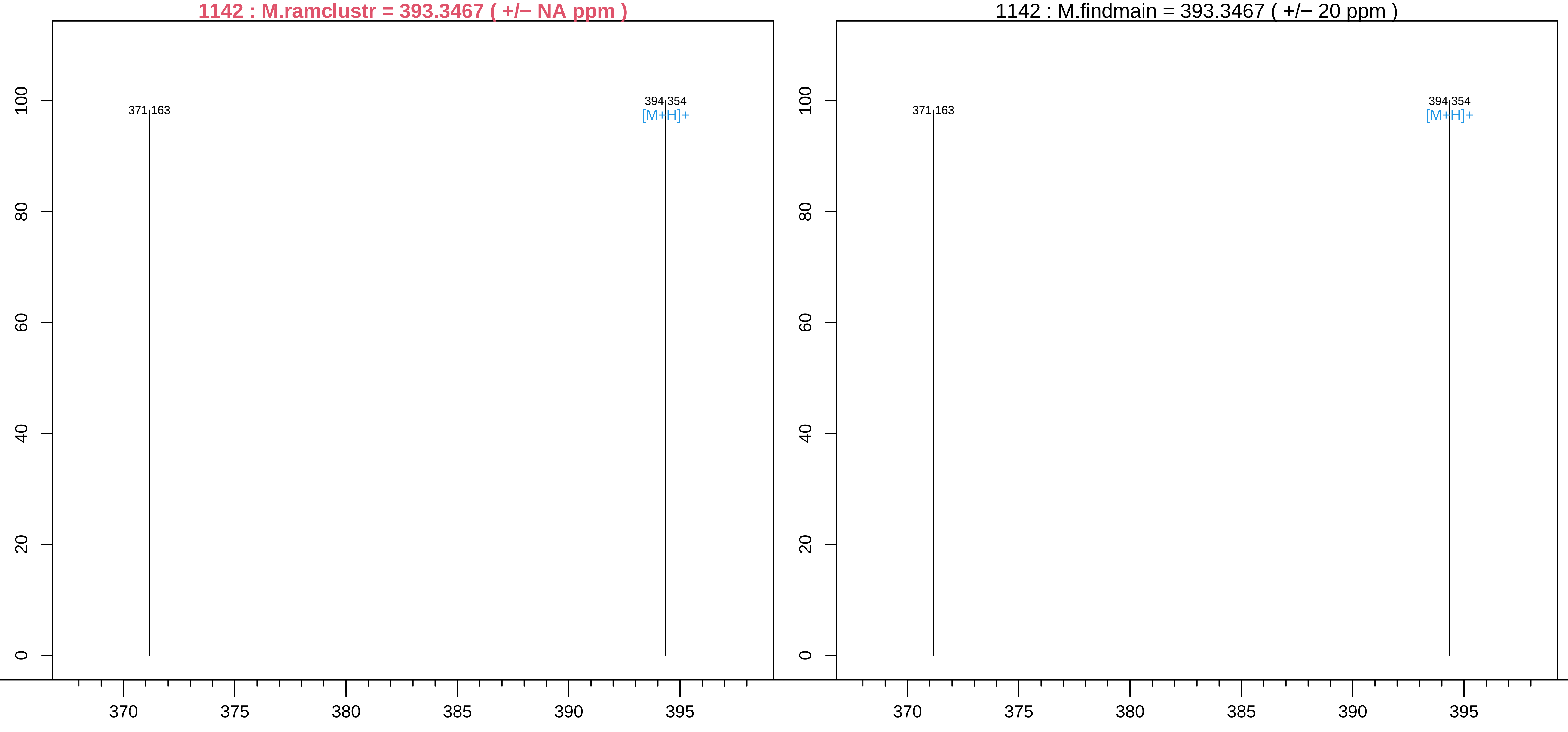
1142 : M.ramclustr = 393.3467 (+/− NA ppm)
1142 : M.findmain = 393.3467 (+/− 20 ppm)
371.163
394.354
[M+H]+
0
20
40
60
80
100
370
375
380
385
390
395

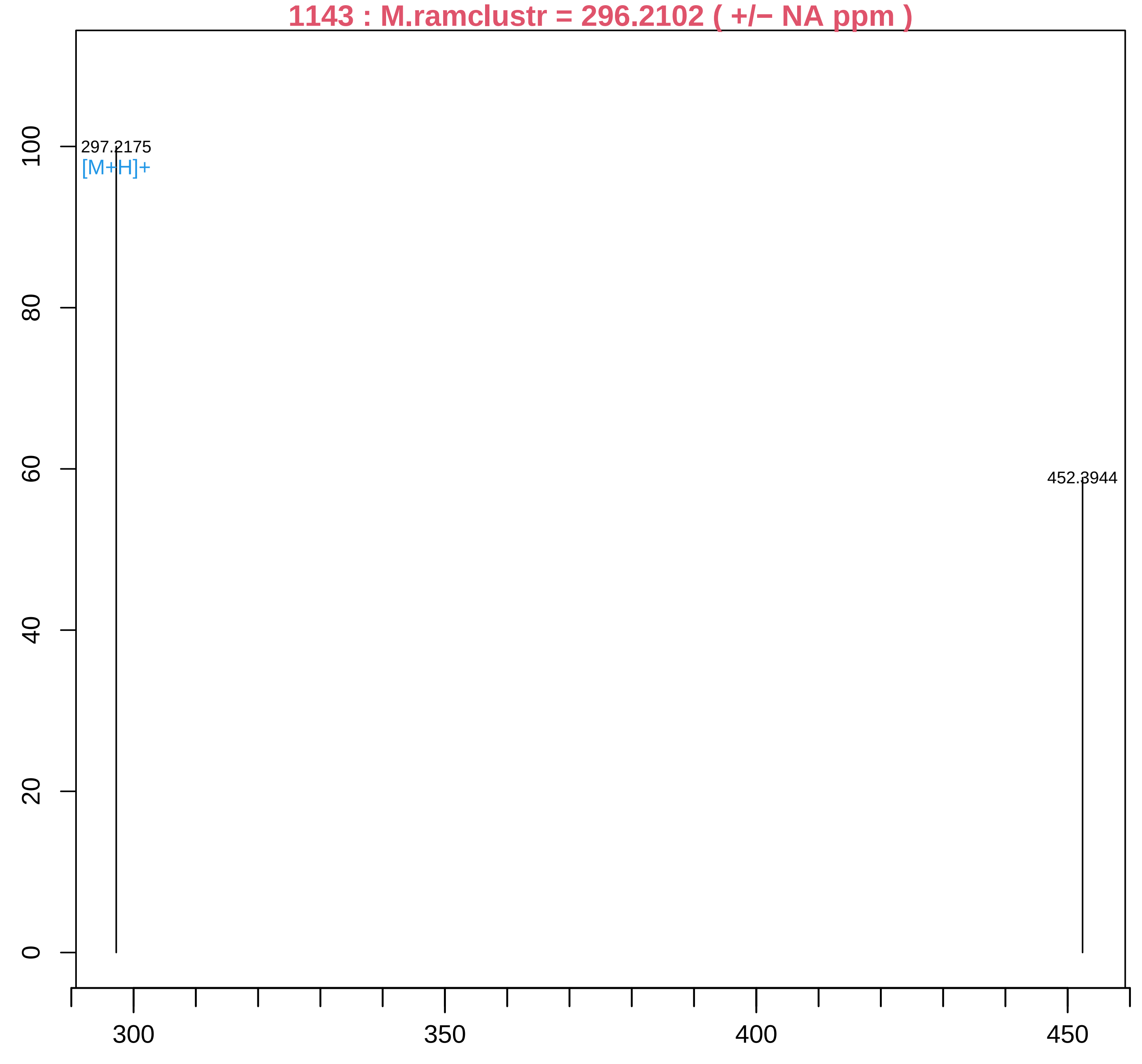
1143 : M.ramclustr = 296.2102 (+/− NA ppm)
297.2175
[M+H]+
452.3944
0
20
40
60
80
100
300
350
400
450

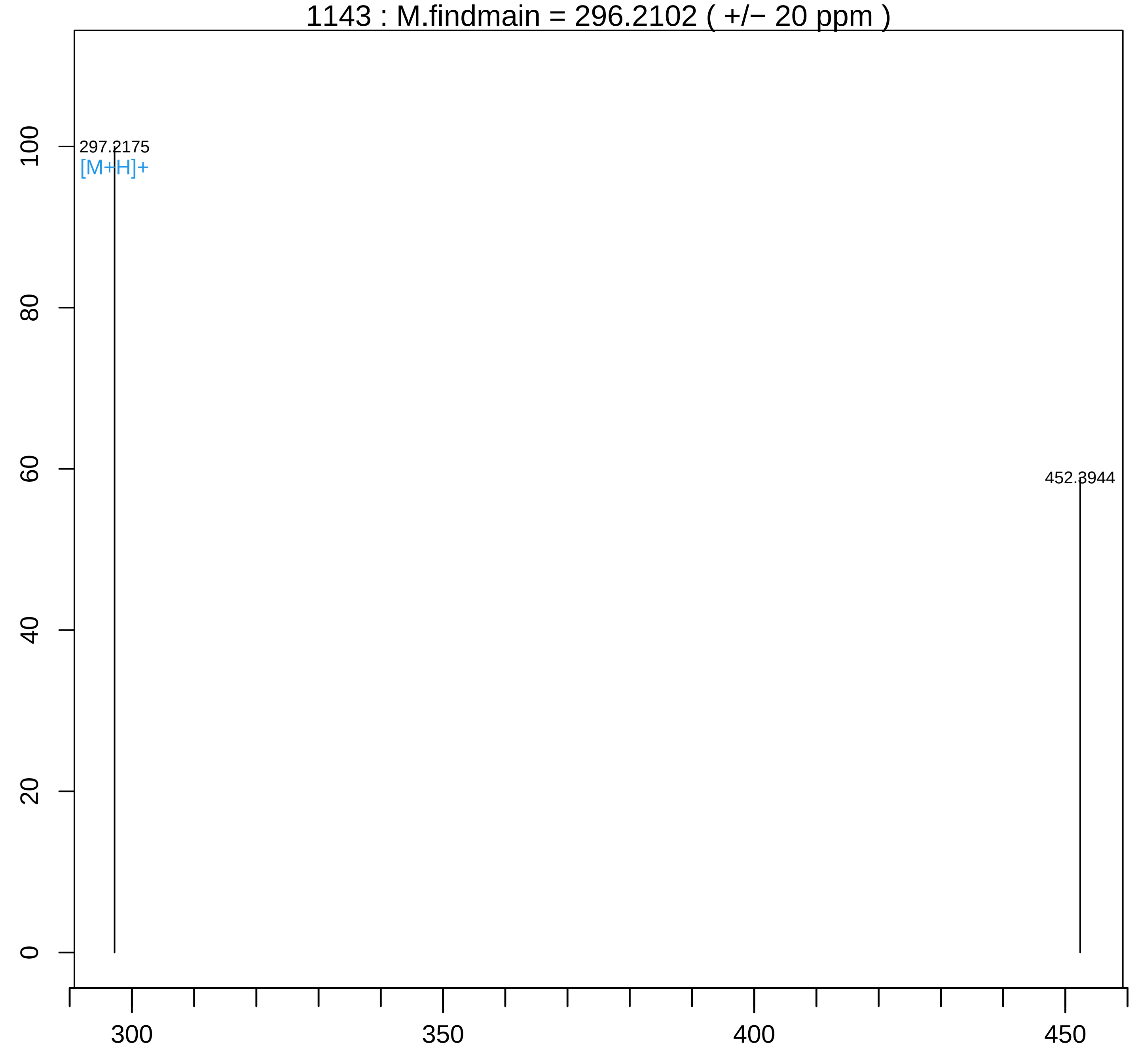
1143 : M.findmain = 296.2102 (+/− 20 ppm)
297.2175
[M+H]+
452.3944
0
20
40
60
80
100
300
350
400
450

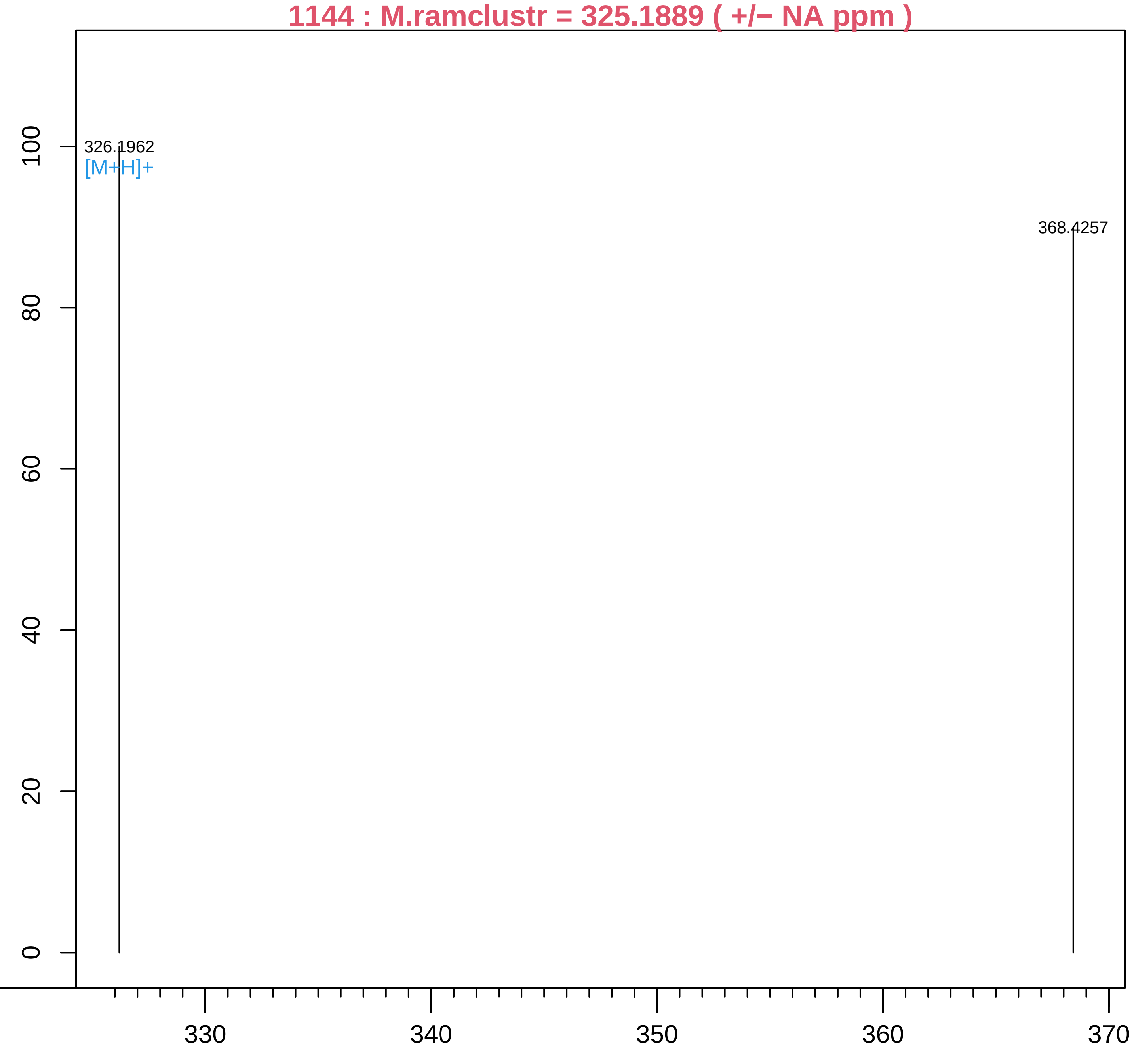

1144 : M.ramclustr = 325.1889 (+/− NA ppm)
326.1962
[M+H]+
368.4257
0
20
40
60
80
100
330
340
350
360
370

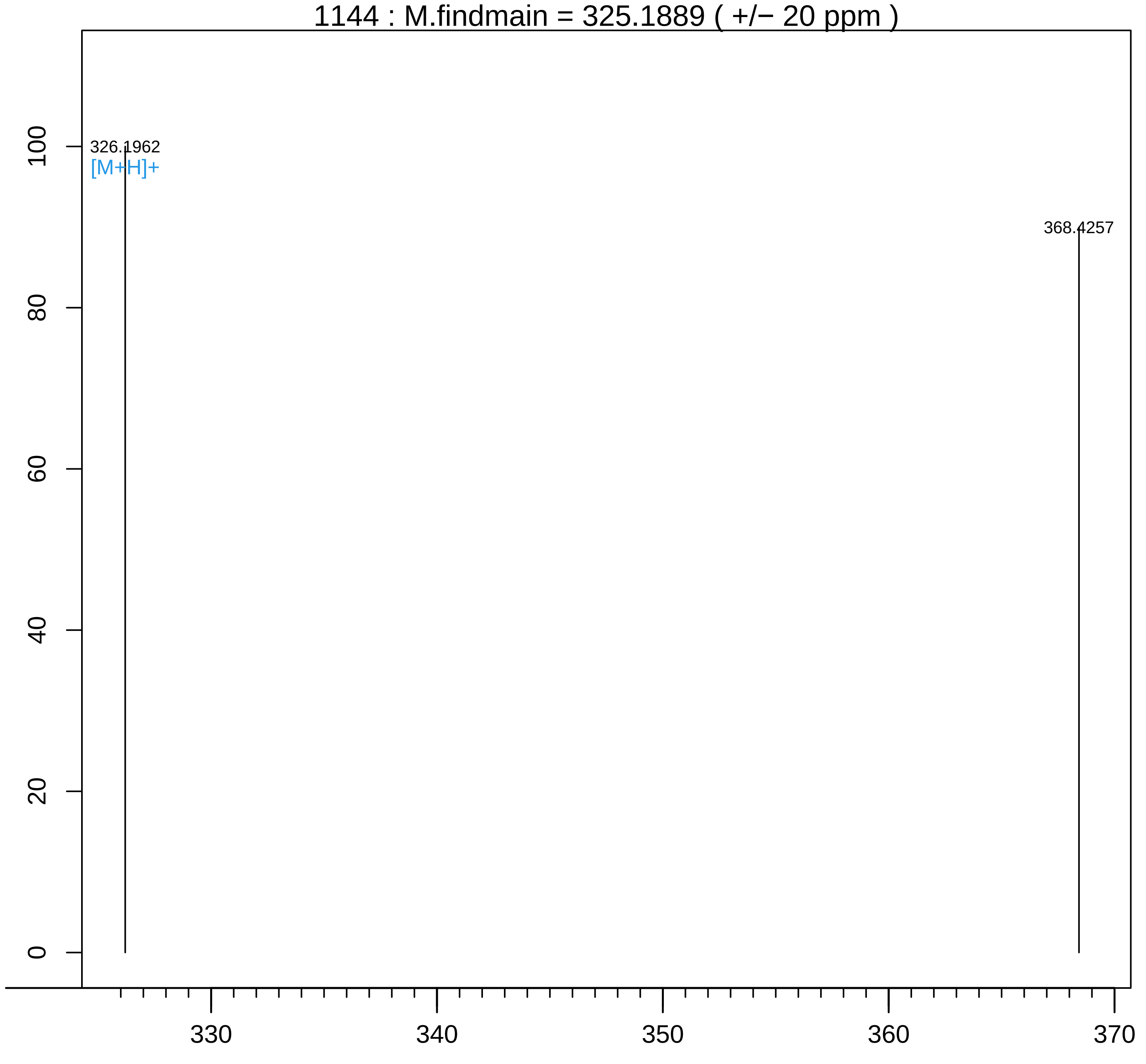

1144 : M.findmain = 325.1889 (+/− 20 ppm)
326.1962
[M+H]+
368.4257
0
20
40
60
80
100
330
340
350
360
370

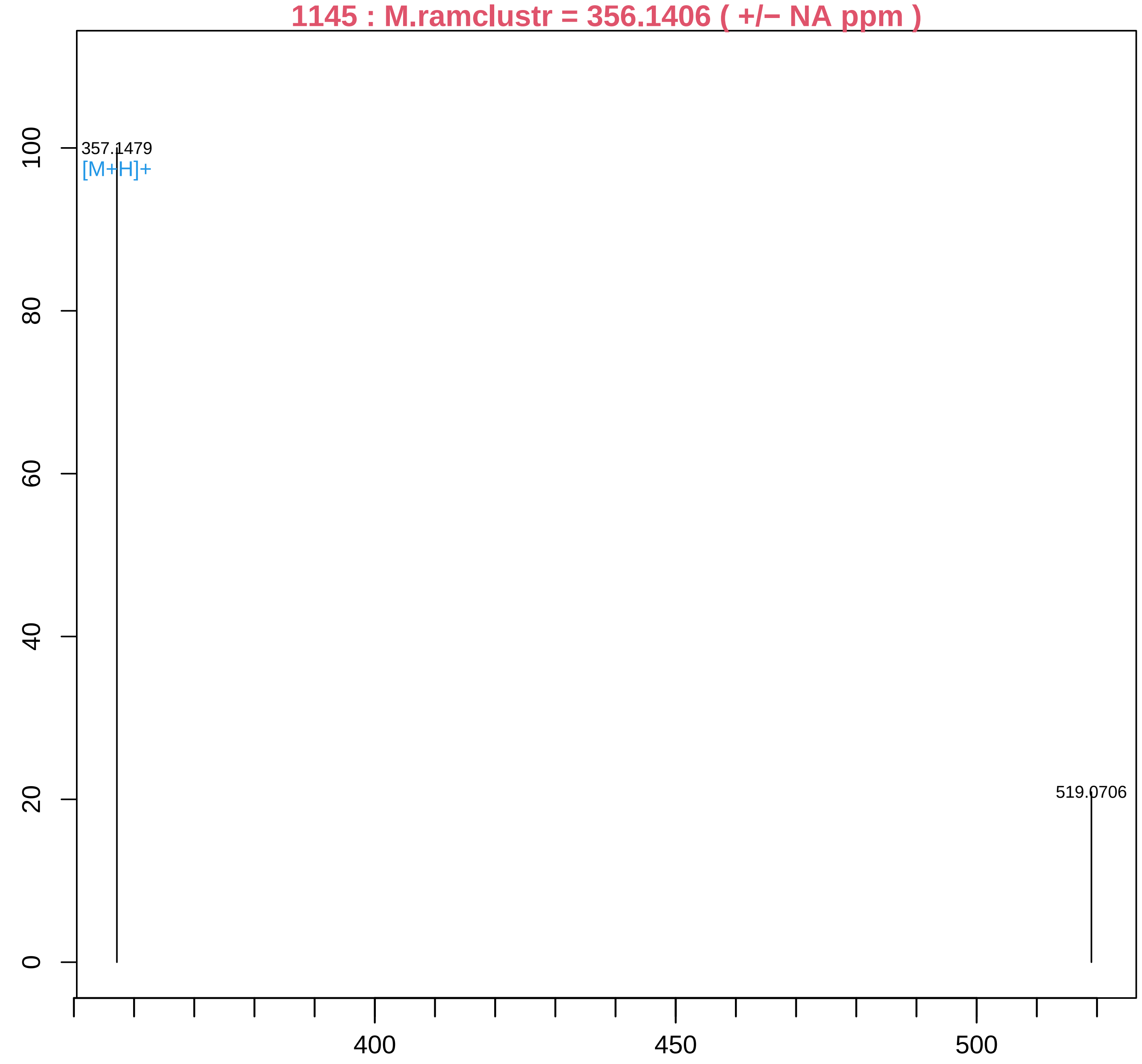

1145 : M.ramclustr = 356.1406 (+/− NA ppm)
357.1479
[M+H]+
519.0706
0
20
40
60
80
100
400
450
500

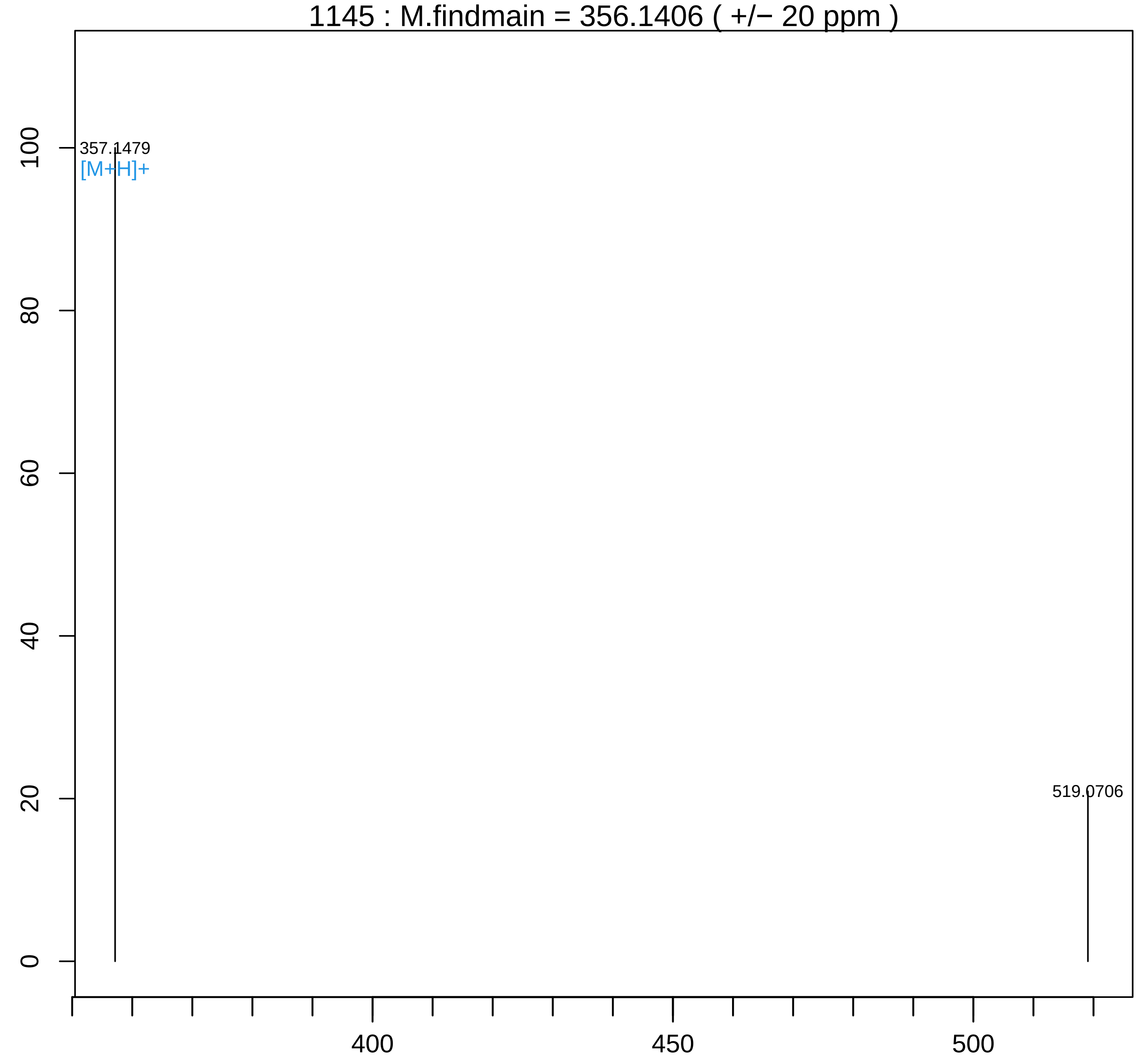

1145 : M.findmain = 356.1406 (+/− 20 ppm)
357.1479
[M+H]+
519.0706
0
20
40
60
80
100
400
450
500

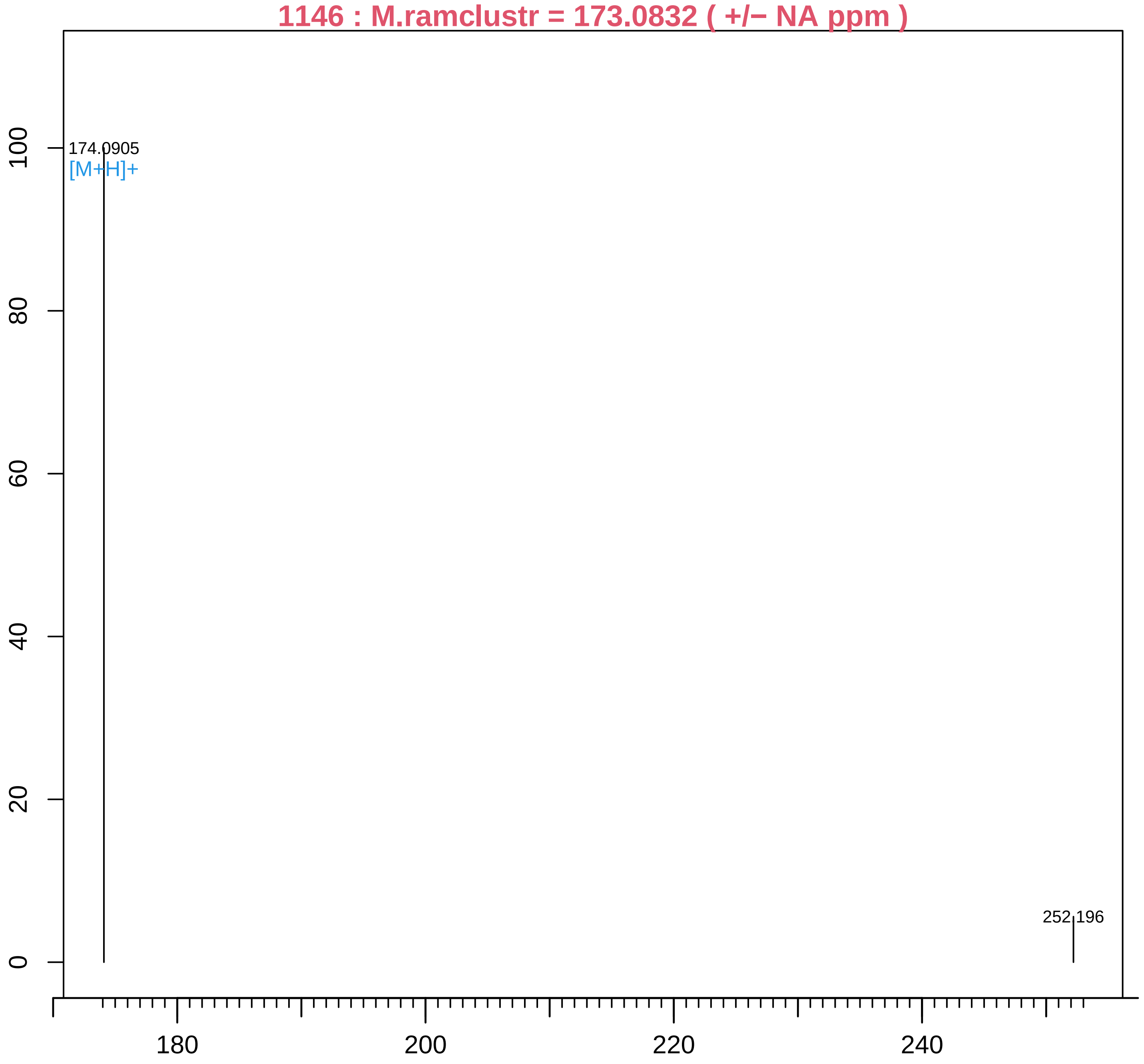
1146 : M.ramclustr = 173.0832 (+/− NA ppm)
174.0905
[M+H]+
252.196
0
20
40
60
80
100
180
200
220
240

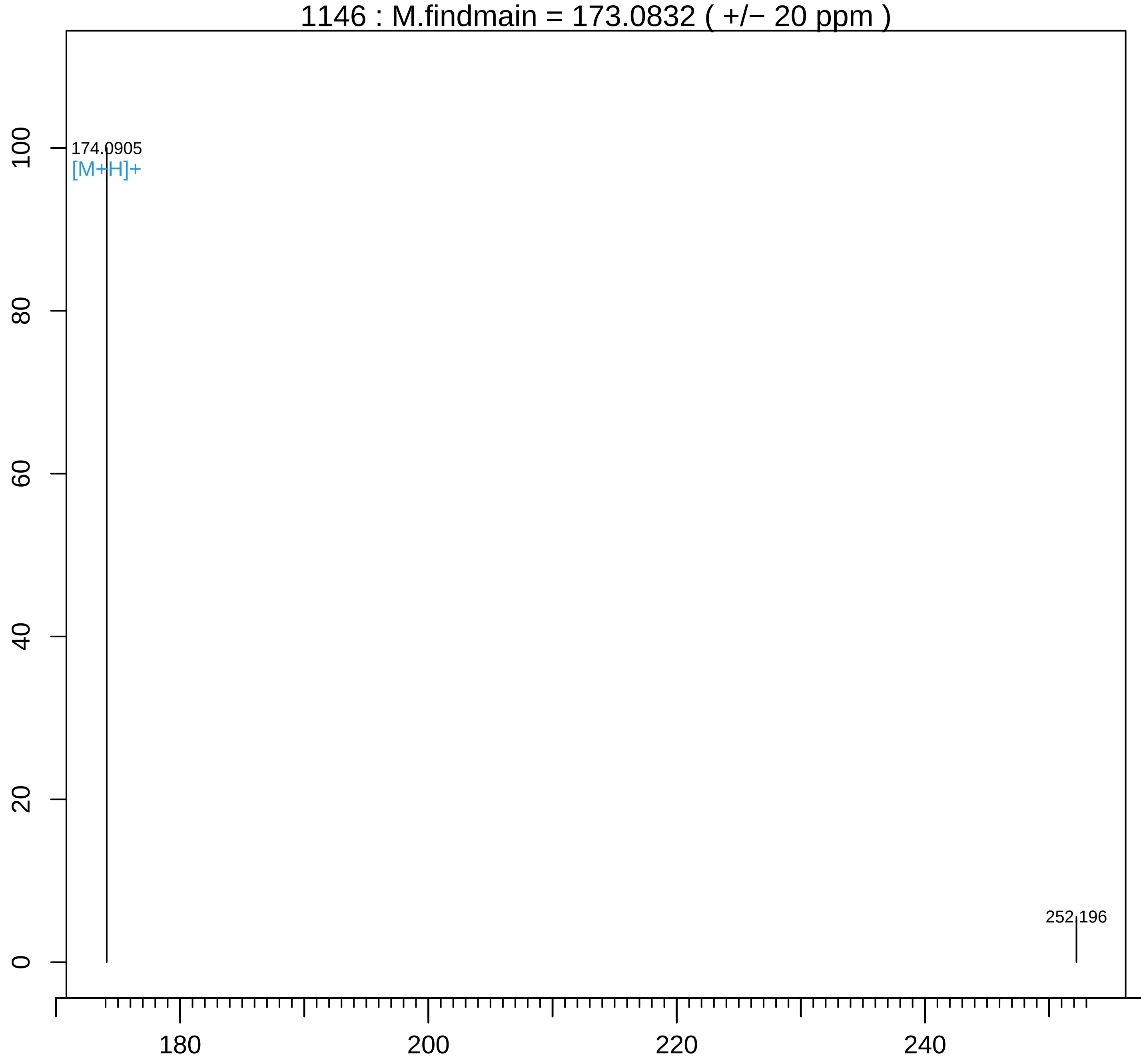
1146 : M.findmain = 173.0832 (+/− 20 ppm)
174.0905
[M+H]+
252.196
0
20
40
60
80
100
180
200
220
240

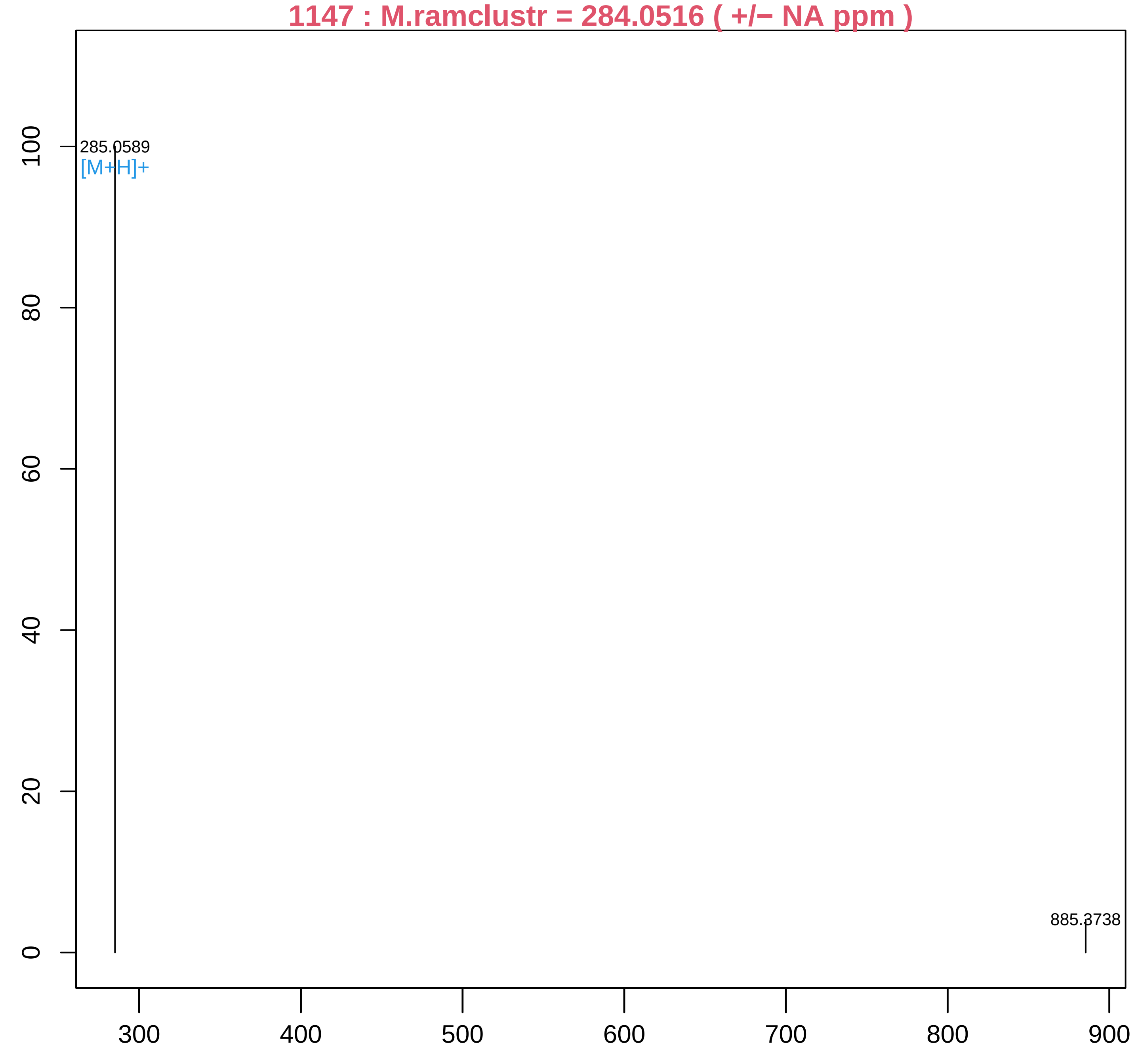
1147 : M.ramclustr = 284.0516 (+/− NA ppm)
285.0589
[M+H]+
885.3738
0
20
40
60
80
100
300
400
500
600
700
800
900

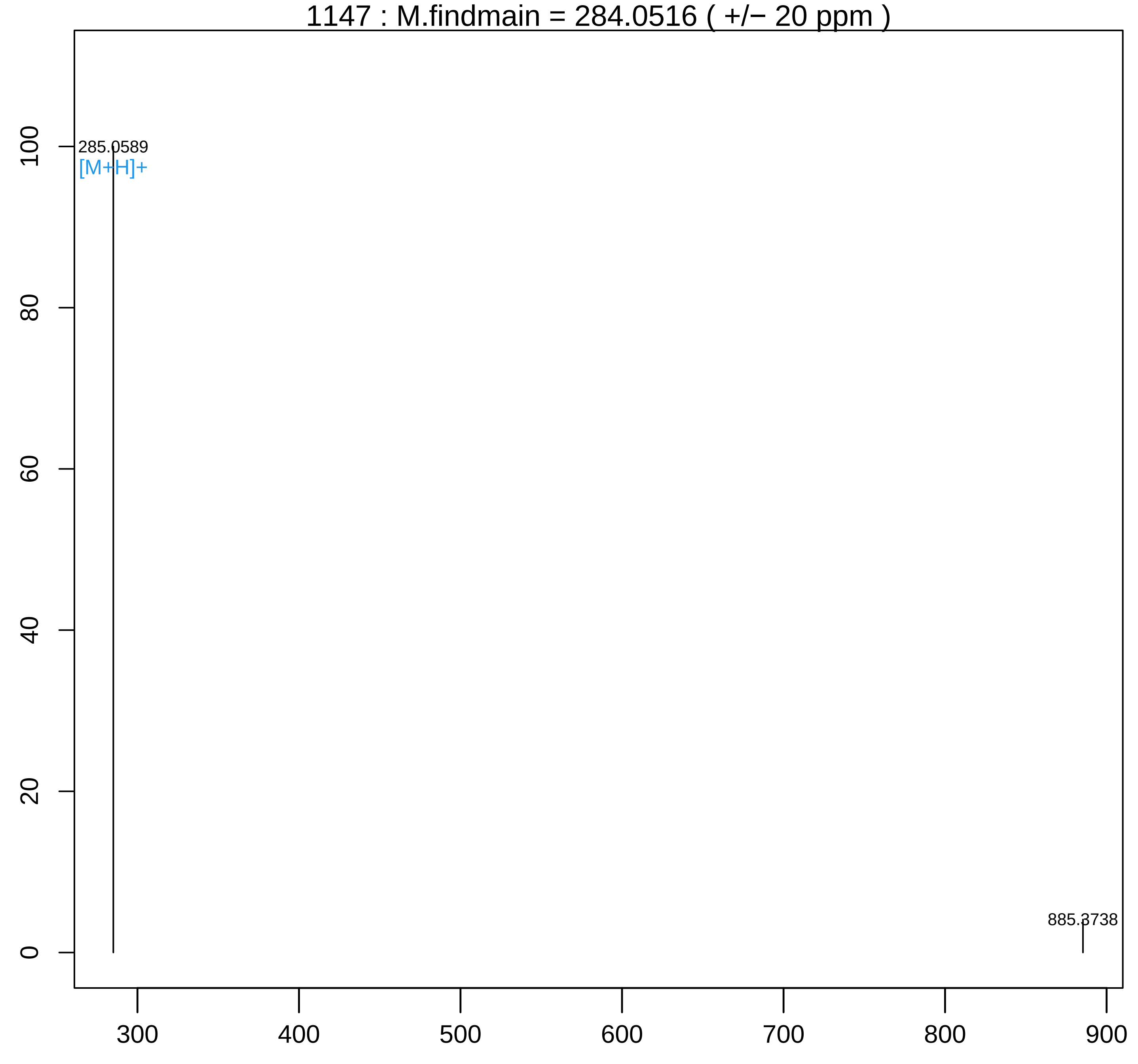
1147 : M.findmain = 284.0516 (+/− 20 ppm)
285.0589
[M+H]+
885.3738
0
20
40
60
80
100
300
400
500
600
700
800
900

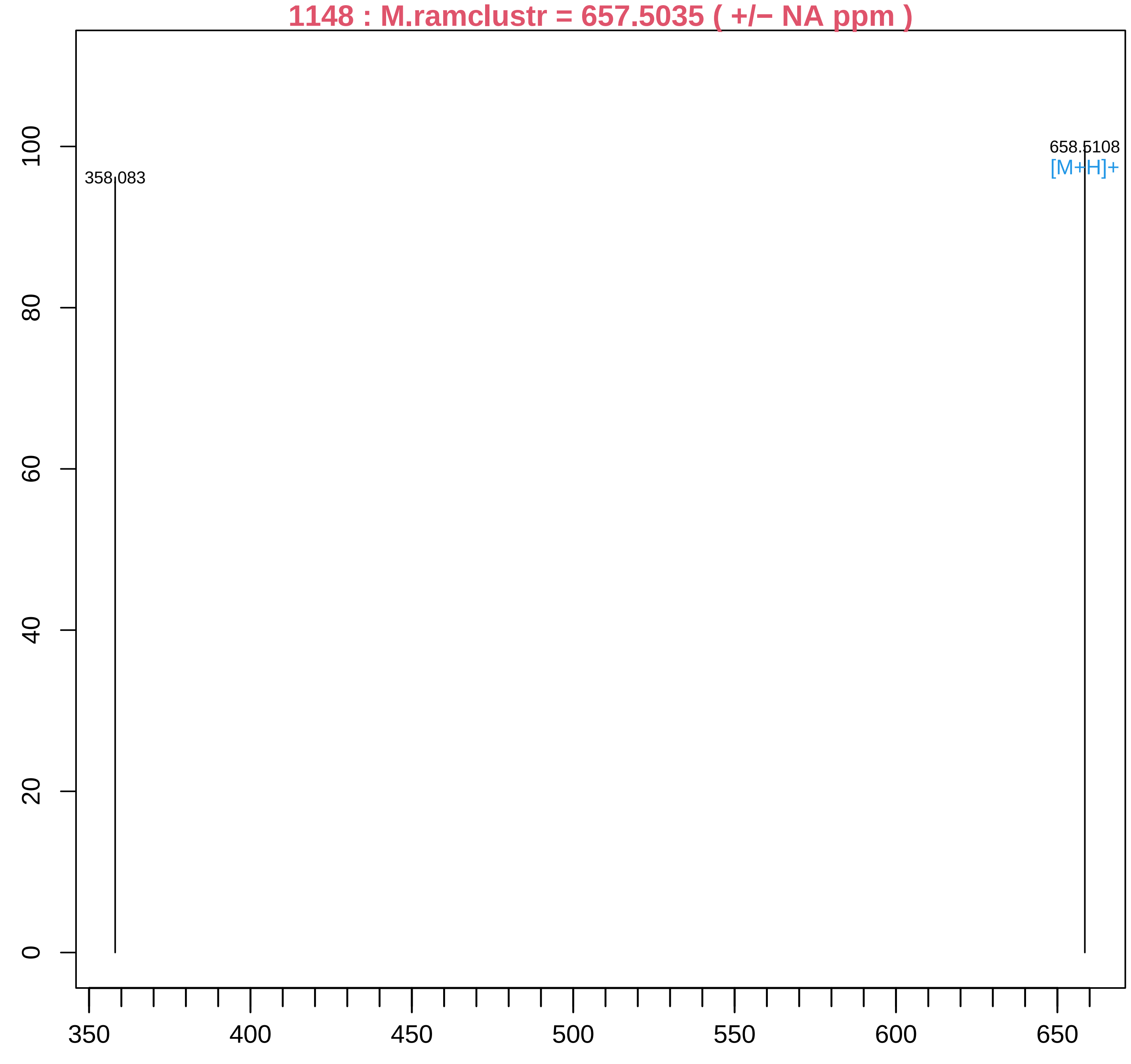

1148 : M.ramclustr = 657.5035 (+/− NA ppm)
358.083
658.5108
[M+H]+
0
20
40
60
80
100
350
400
450
500
550
600
650

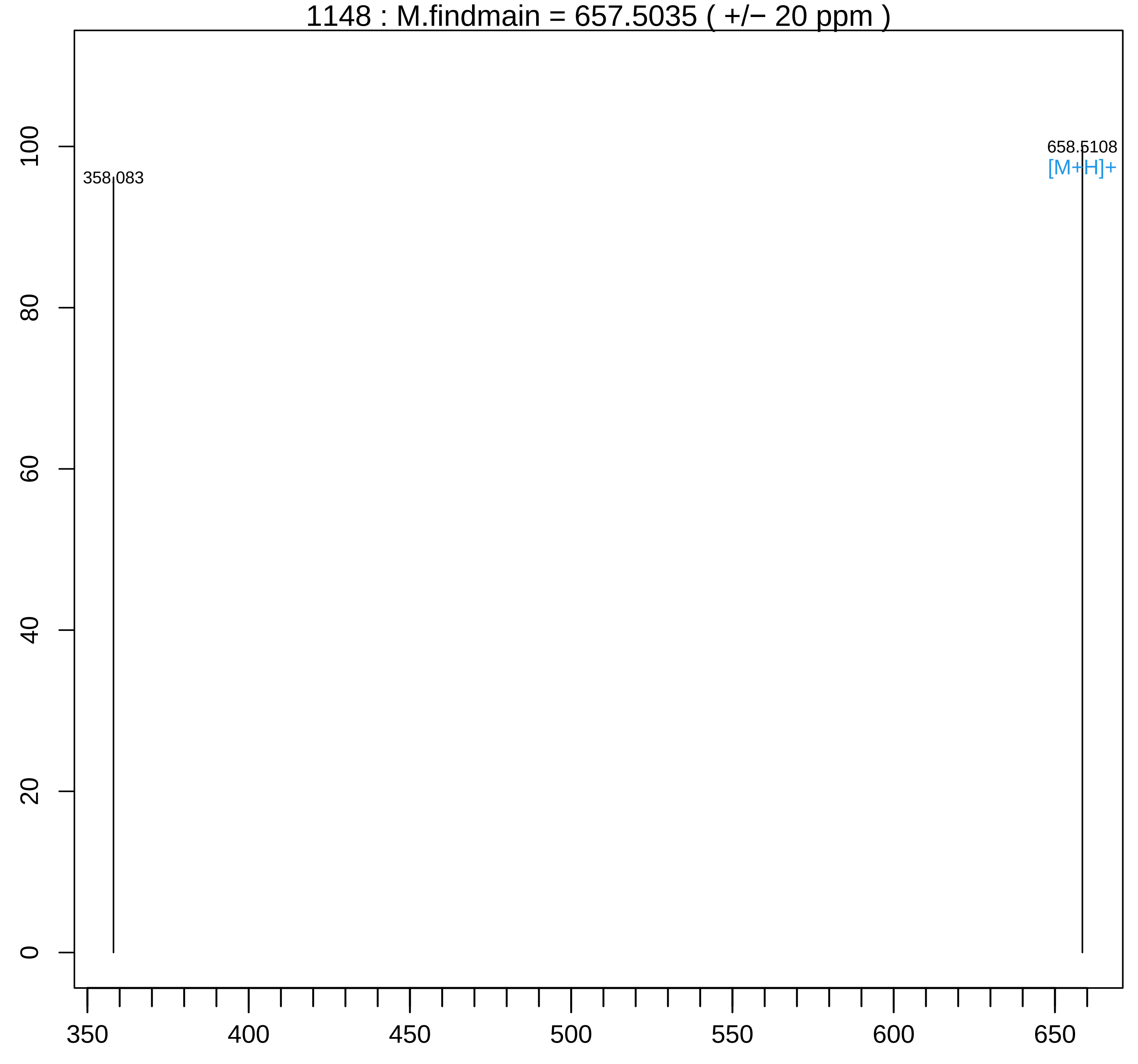

1148 : M.findmain = 657.5035 (+/− 20 ppm)
358.083
658.5108
[M+H]+
0
20
40
60
80
100
350
400
450
500
550
600
650

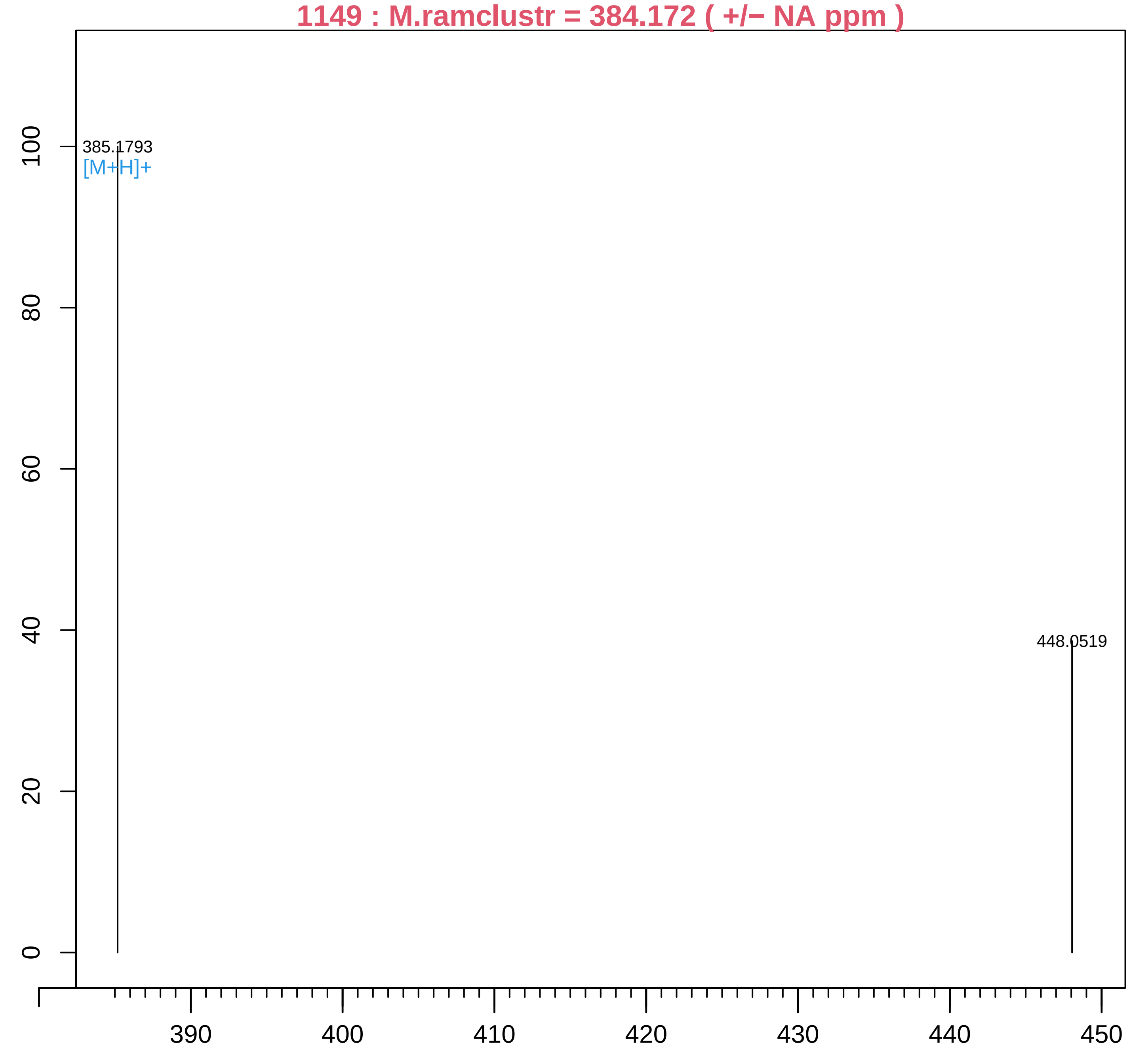
1149 : M.ramclustr = 384.172 (+/− NA ppm)
385.1793
[M+H]+
448.0519
0
20
40
60
80
100
390
400
410
420
430
440
450

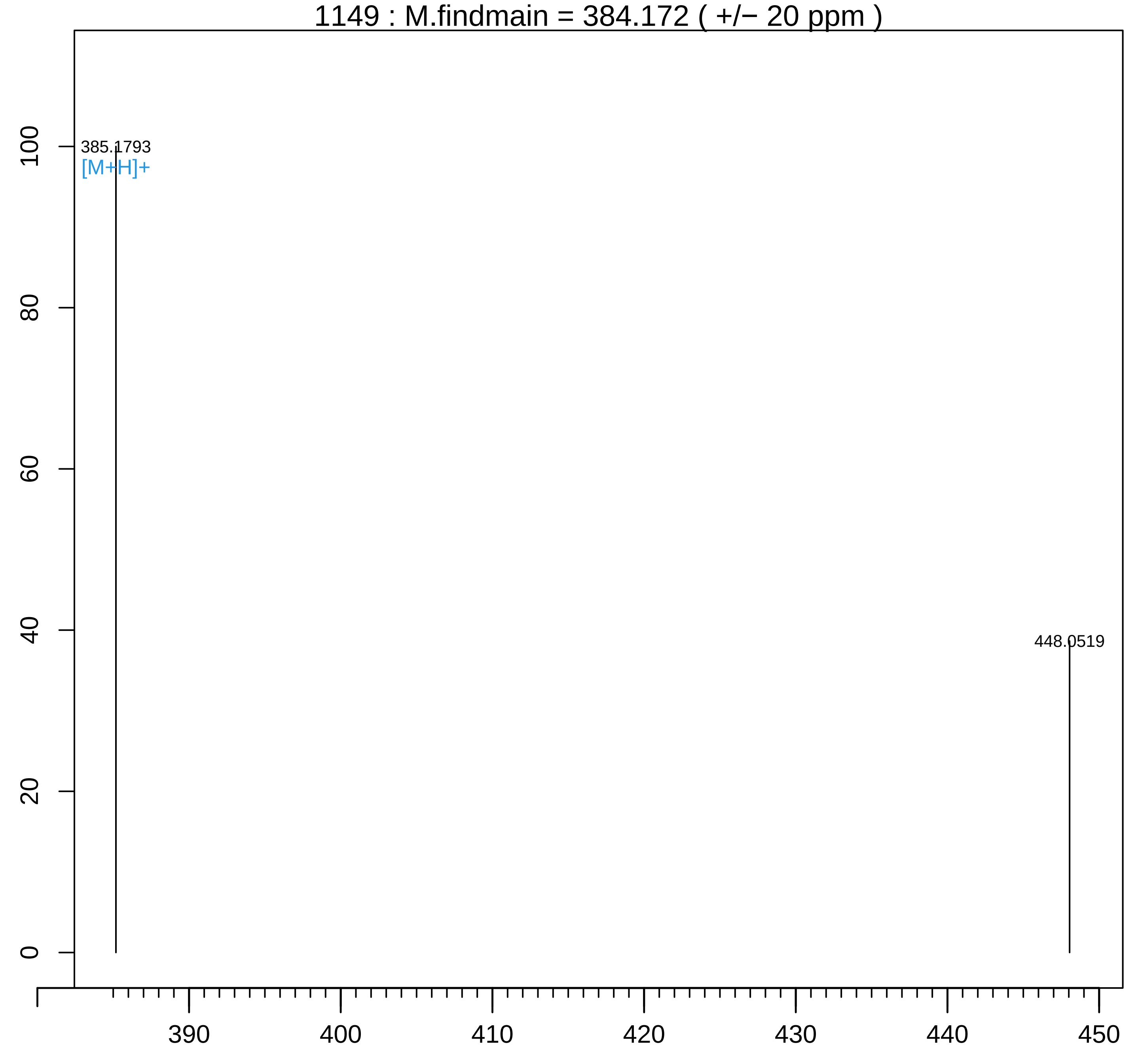
1149 : M.findmain = 384.172 (+/− 20 ppm)
385.1793
[M+H]+
448.0519
0
20
40
60
80
100
390
400
410
420
430
440
450

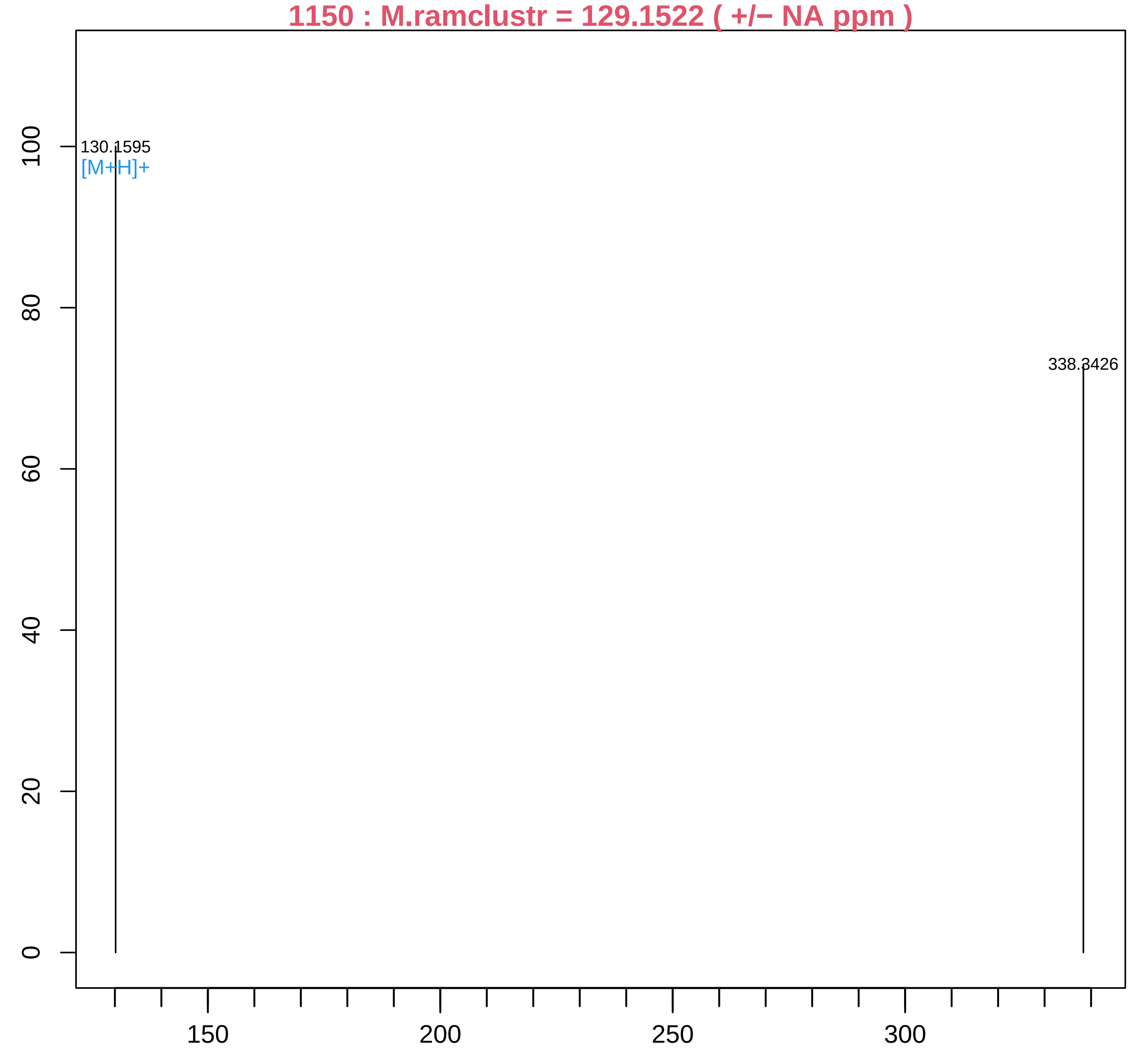
1150 : M.ramclustr = 129.1522 (+/− NA ppm)
130.1595
[M+H]+
338.3426
0
20
40
60
80
100
150
200
250
300

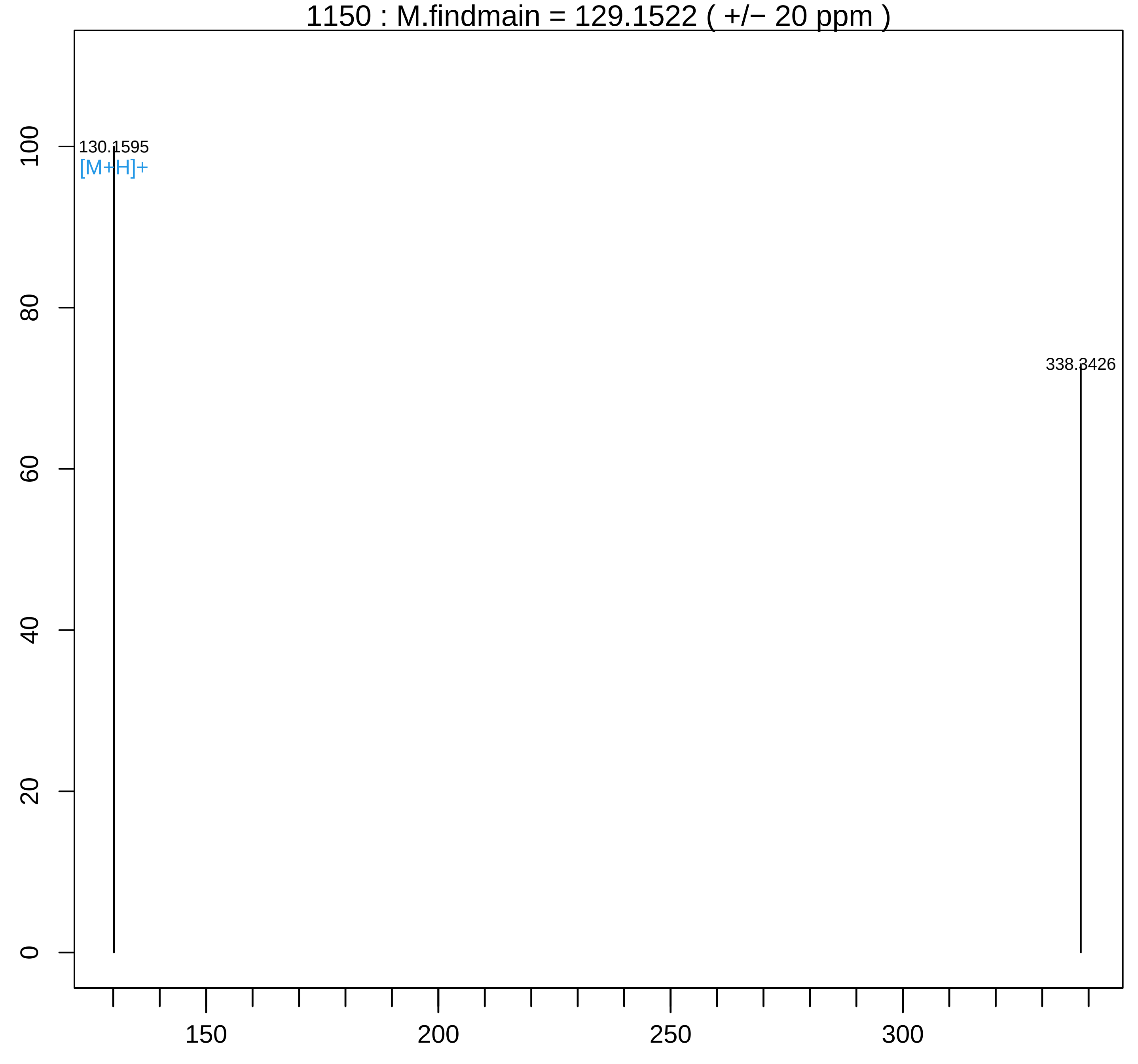
1150 : M.findmain = 129.1522 (+/− 20 ppm)
130.1595
[M+H]+
338.3426
0
20
40
60
80
100
150
200
250
300

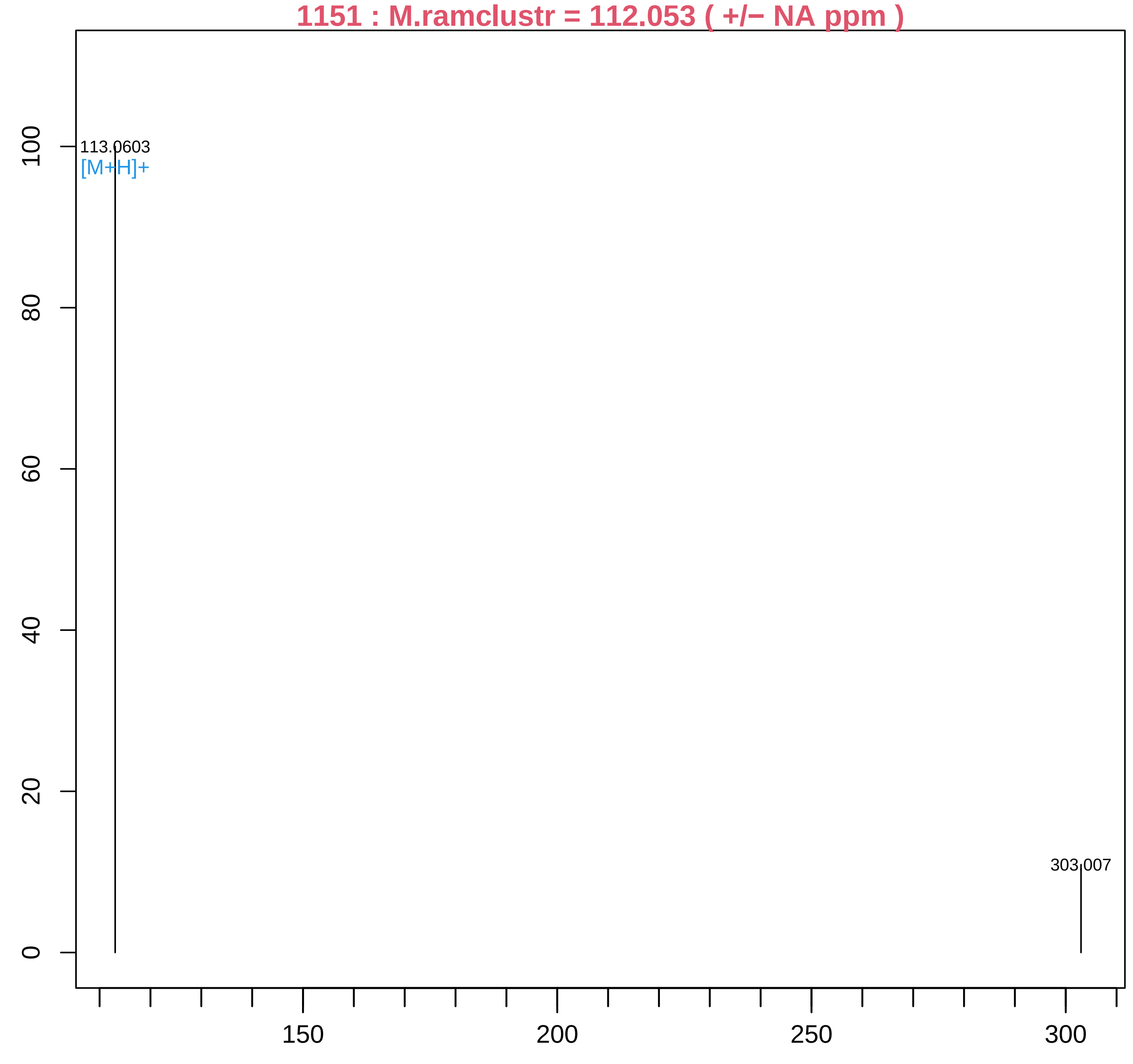
1151 : M.ramclustr = 112.053 (+/− NA ppm)
113.0603
[M+H]+
303.007
0
20
40
60
80
100
150
200
250
300
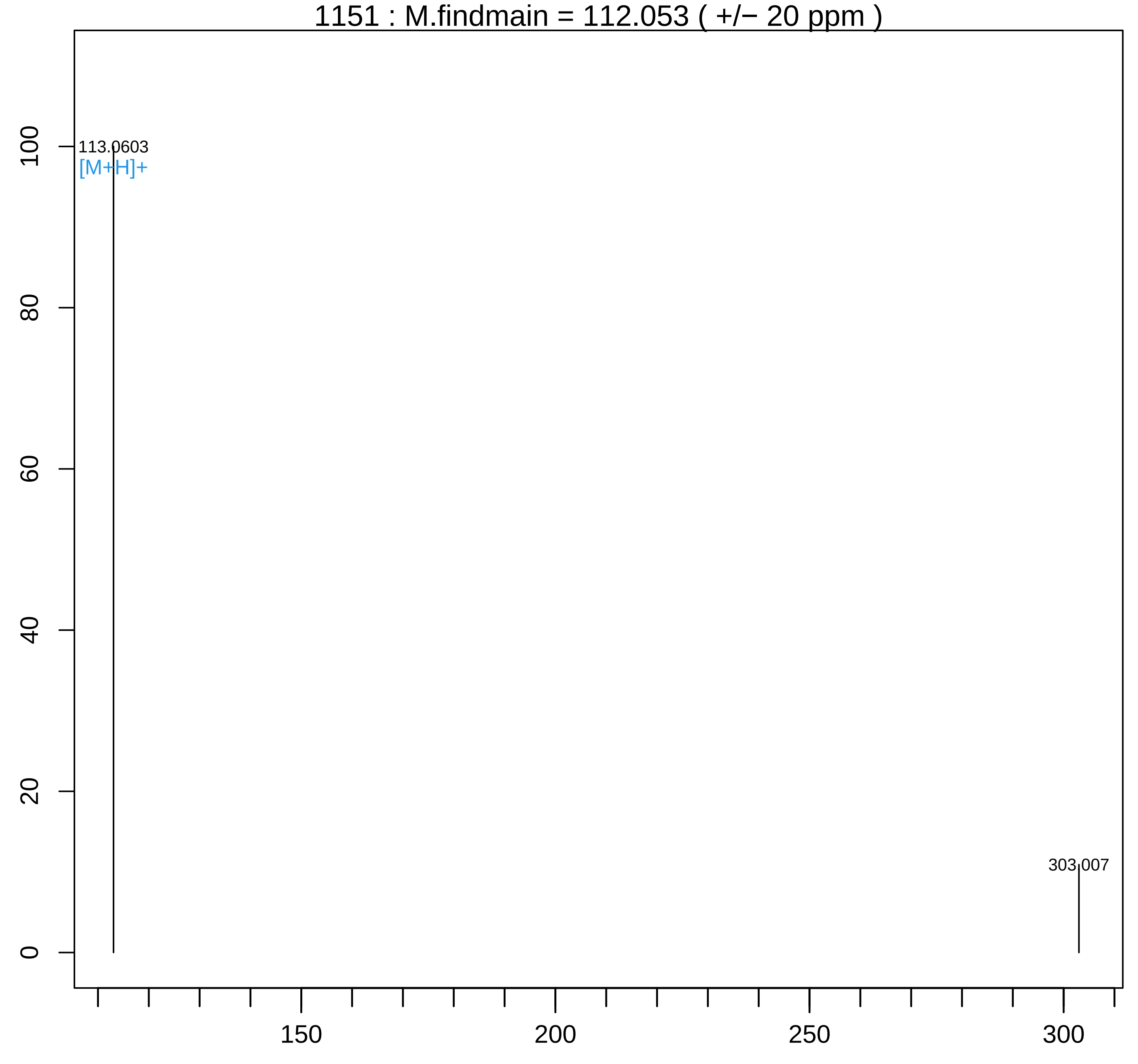
1151 : M.findmain = 112.053 (+/− 20 ppm)
113.0603
[M+H]+
303.007
0
20
40
60
80
100
150
200
250
300

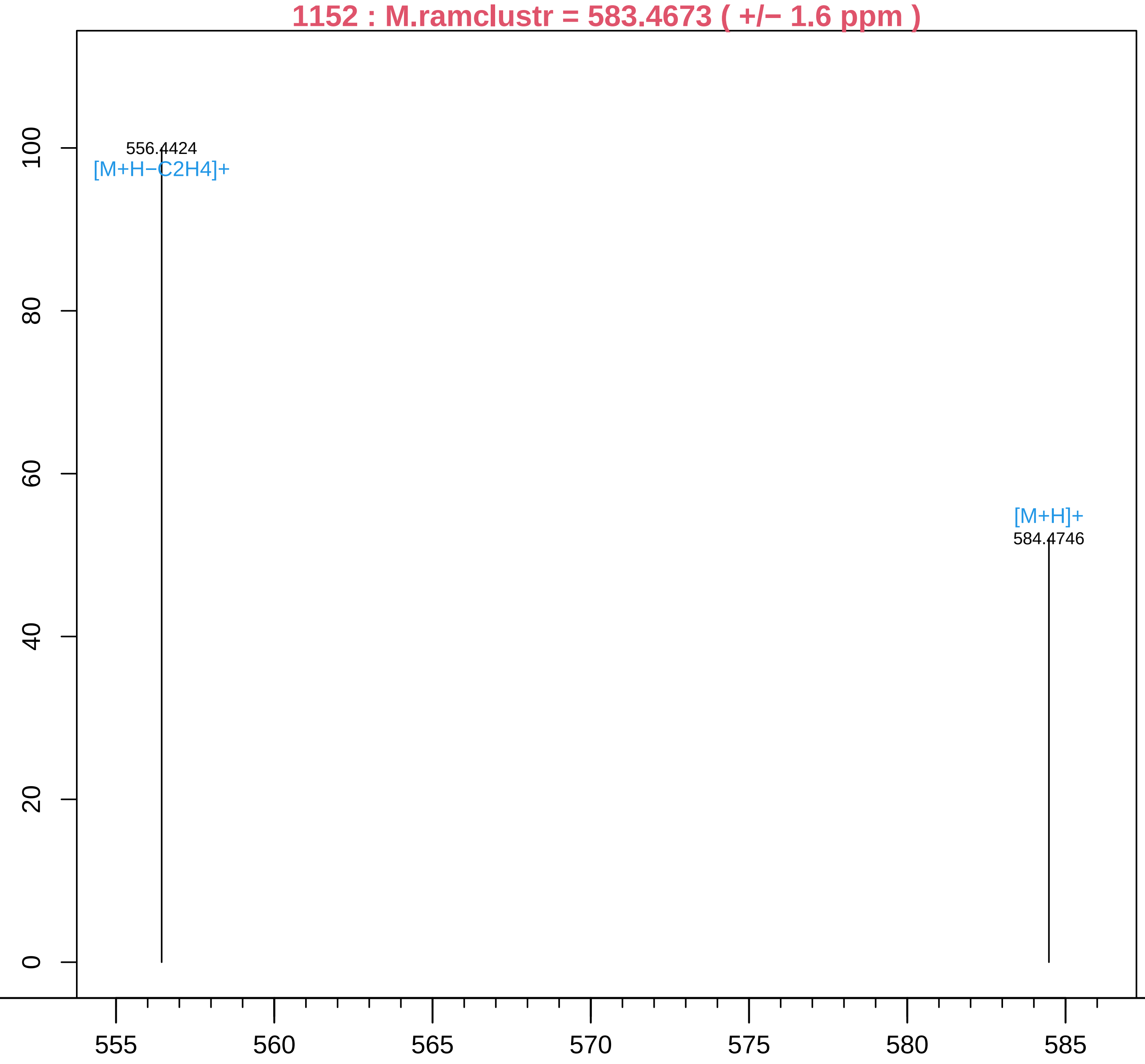
1152 : M.ramclustr = 583.4673 (+/− 1.6 ppm)
556.4424
[M+H−C2H4]+
[M+H]+
584.4746
0
20
40
60
80
100
555
560
565
570
575
580
585

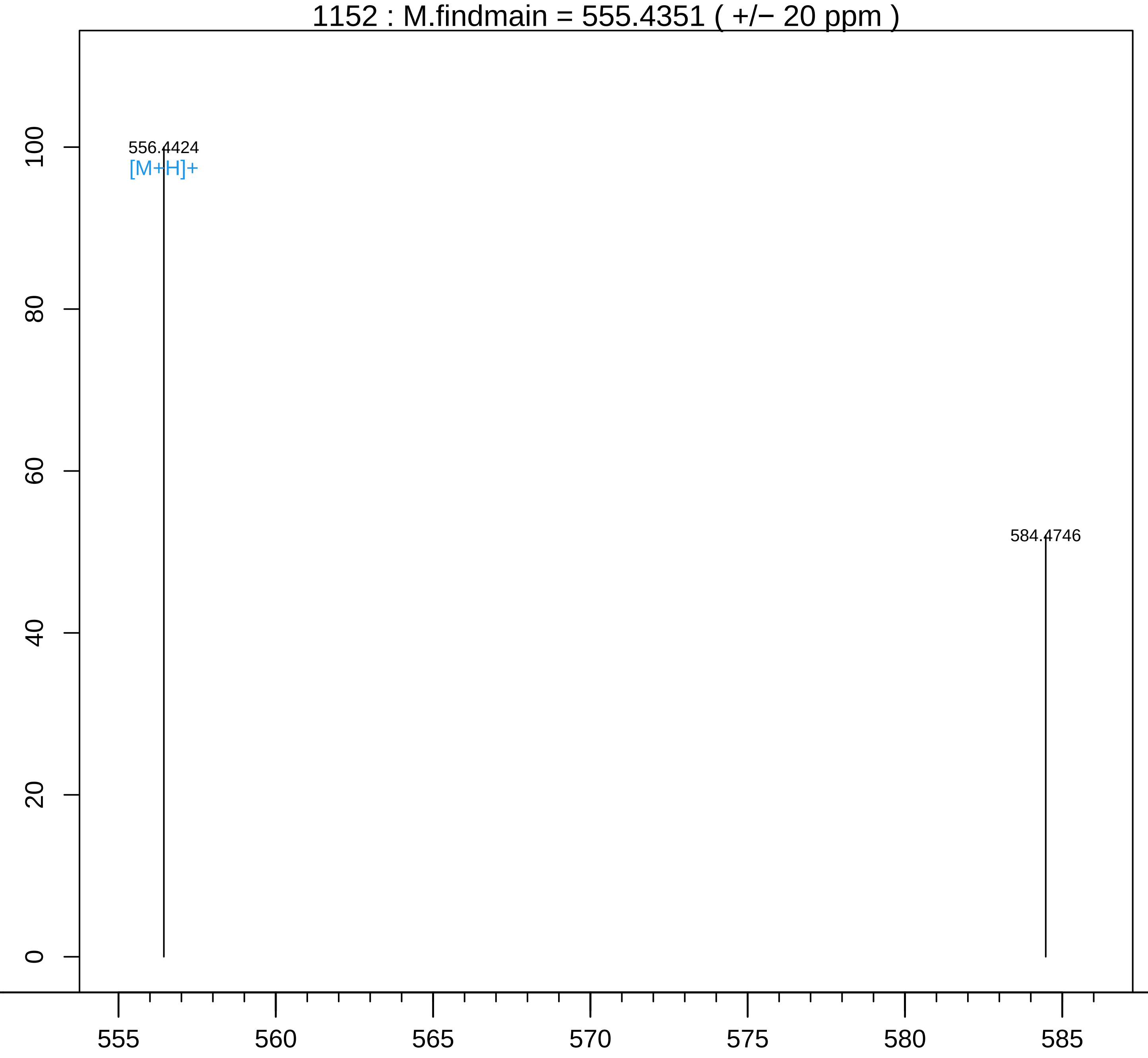
1152 : M.findmain = 555.4351 (+/− 20 ppm)
556.4424
[M+H]+
584.4746
0
20
40
60
80
100
555
560
565
570
575
580
585

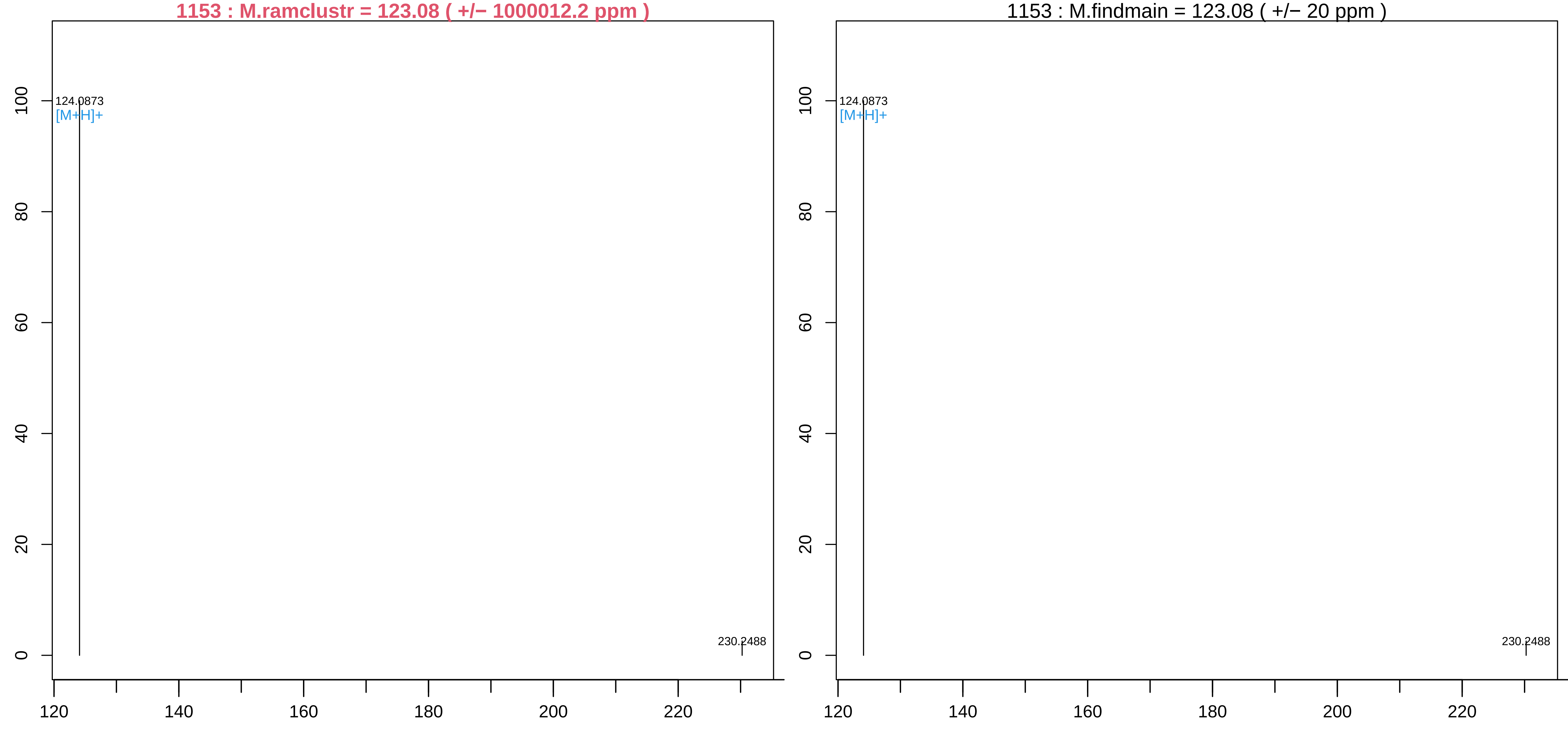
1153 : M.ramclustr = 123.08 (+/− 1000012.2 ppm)
124.0873
[M+H]+
230.2488
0
20
40
60
80
100
120
140
160
180
200
220
1153 : M.findmain = 123.08 (+/− 20 ppm)
124.0873
[M+H]+
230.2488
0
20
40
60
80
100
120
140
160
180
200
220

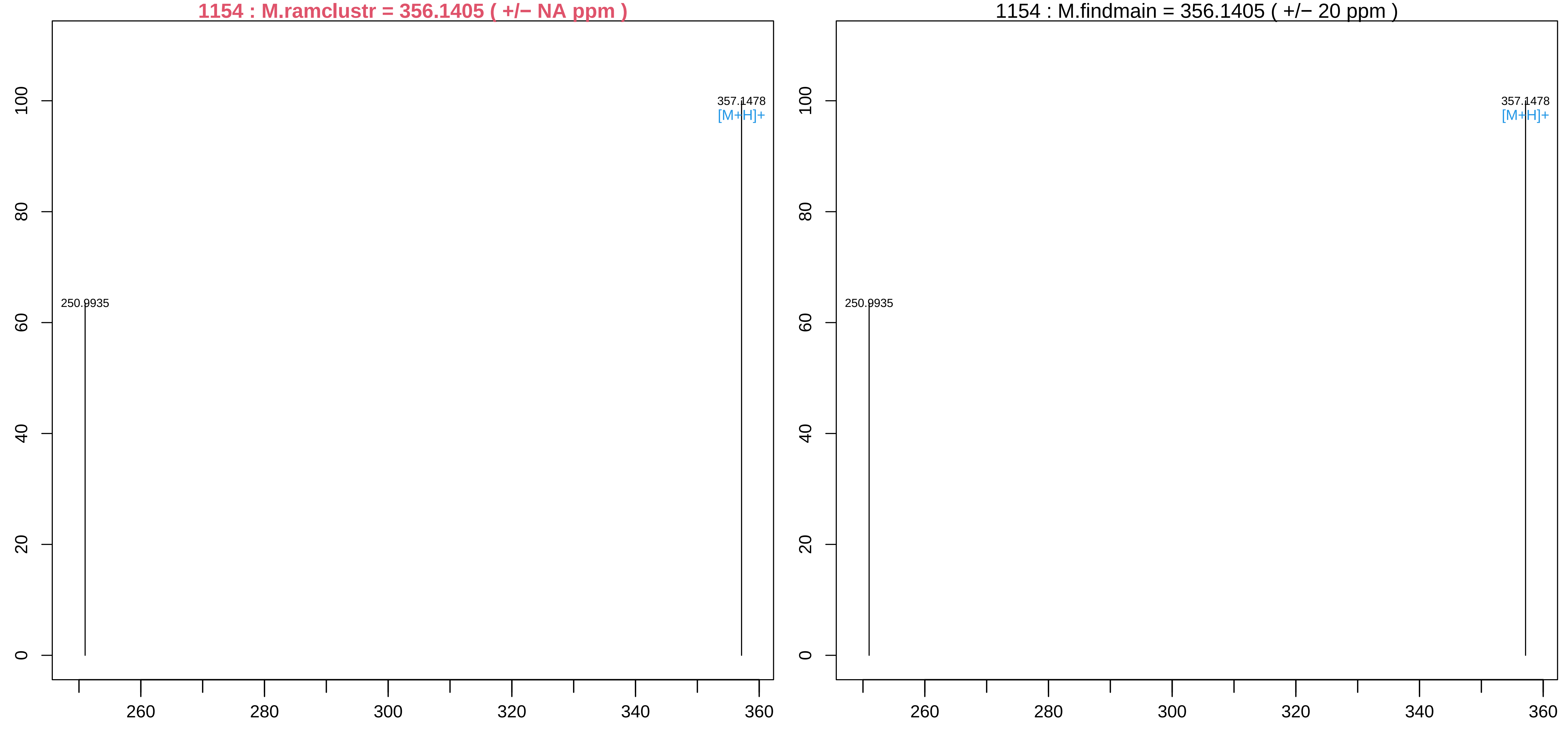
1154 : M.ramclustr = 356.1405 (+/− NA ppm)
357.1478
[M+H]+
250.9935
1154 : M.findmain = 356.1405 (+/− 20 ppm)
357.1478
[M+H]+
250.9935
0
20
40
60
80
100
260
280
300
320
340
360

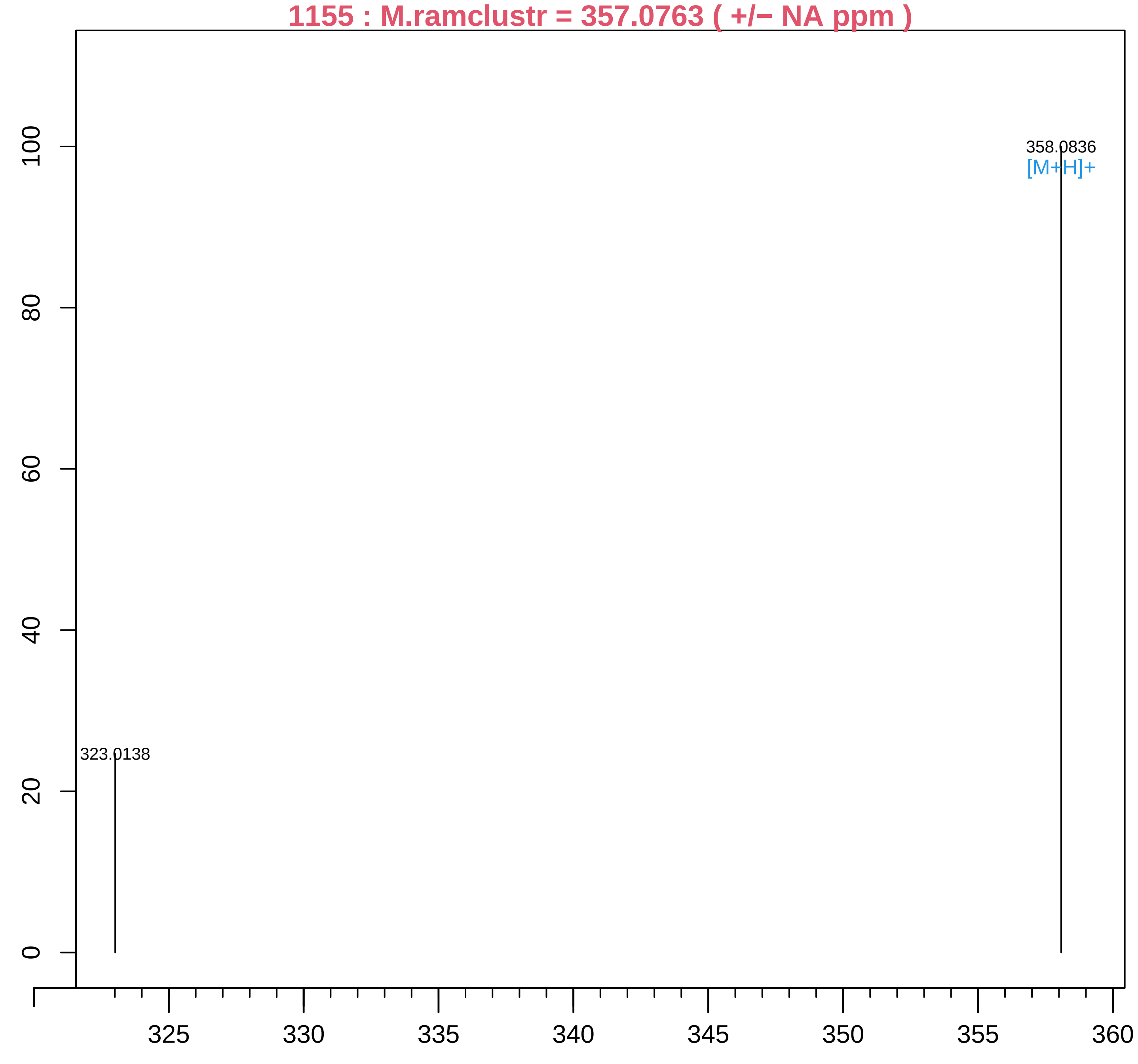

1155 : M.ramclustr = 357.0763 (+/− NA ppm)
358.0836
[M+H]+
323.0138
0
20
40
60
80
100
325
330
335
340
345
350
355
360

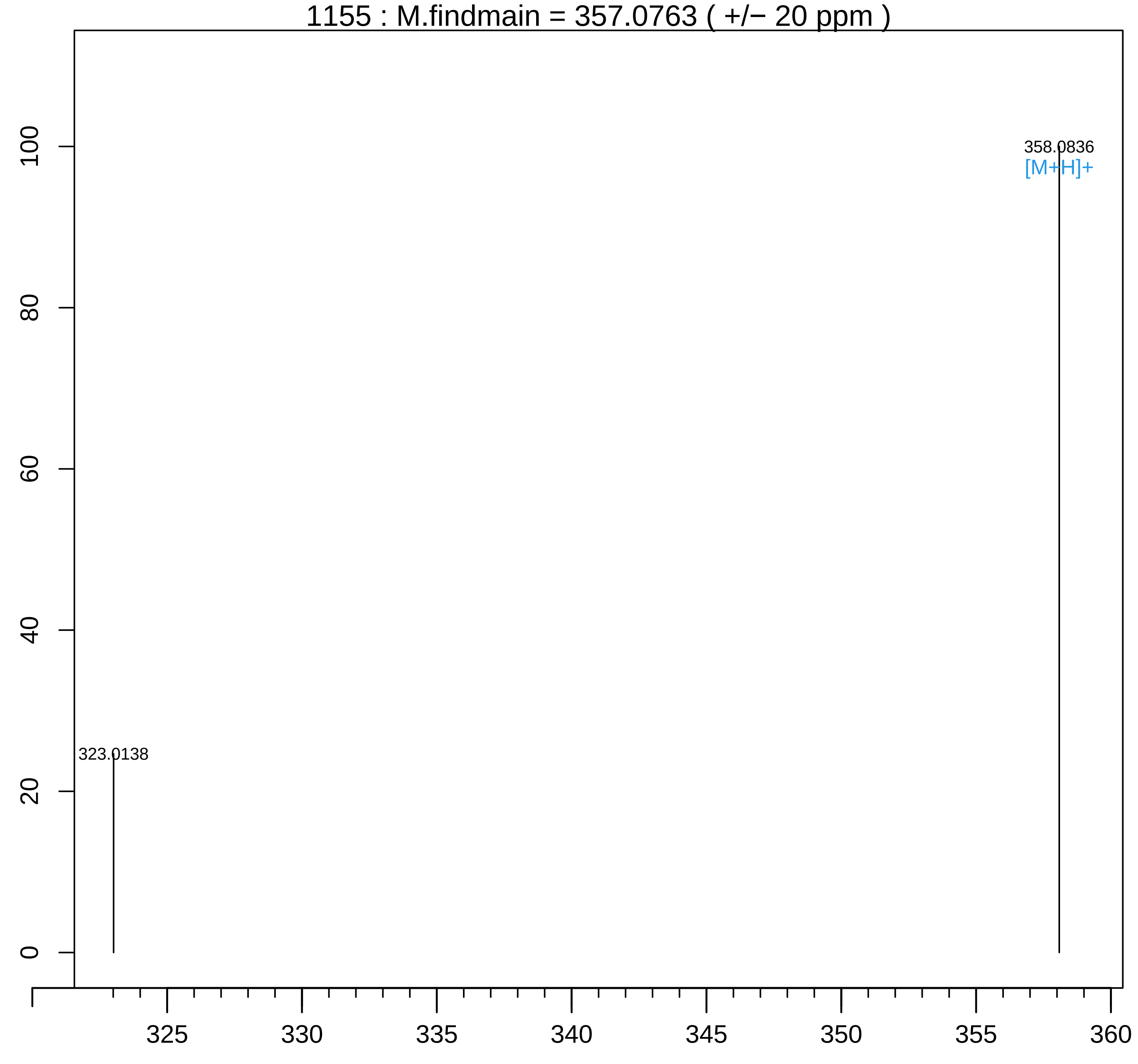

1155 : M.findmain = 357.0763 (+/− 20 ppm)
358.0836
[M+H]+
323.0138
0
20
40
60
80
100
325
330
335
340
345
350
355
360

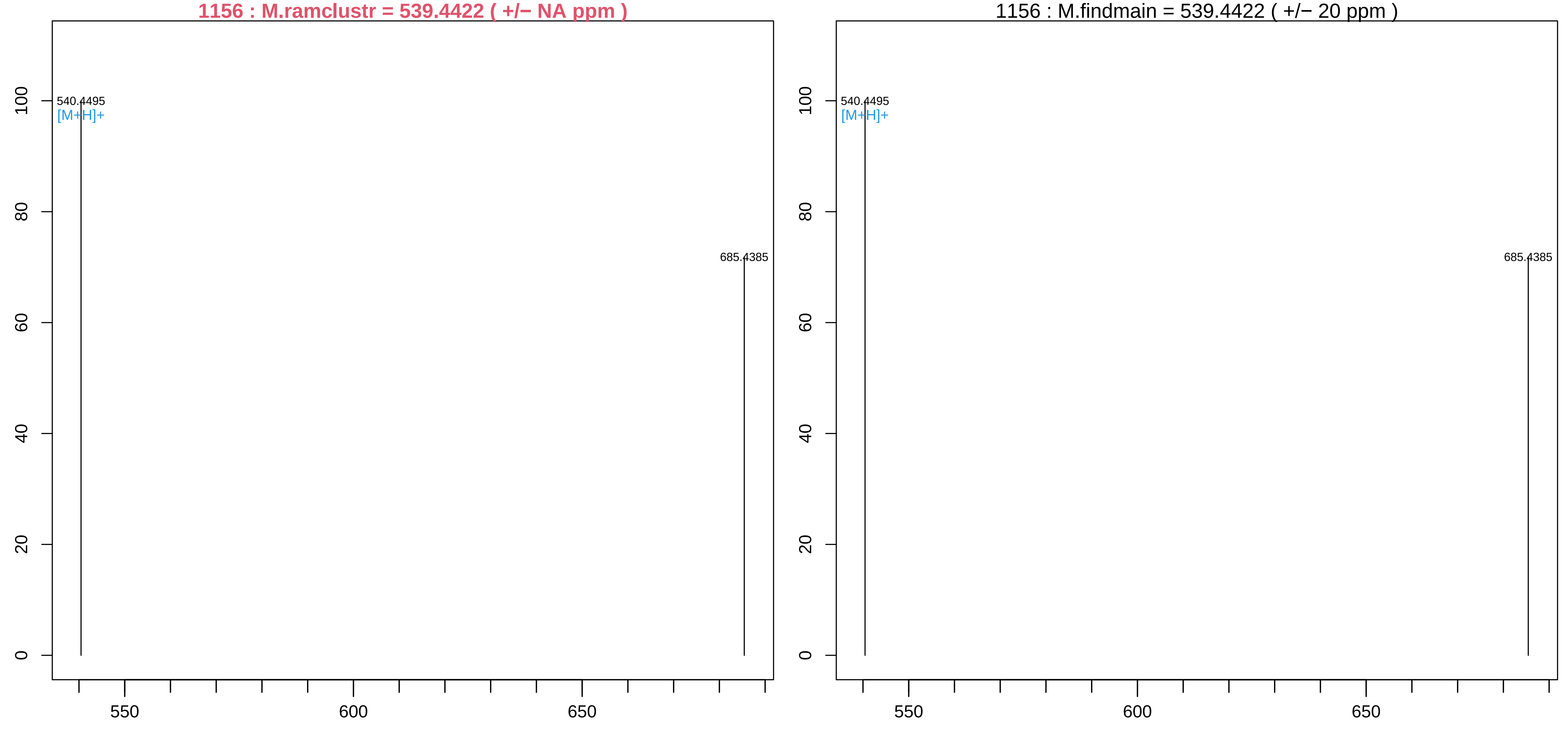

1156 : M.ramclustr = 539.4422 (+/− NA ppm)
540.4495
[M+H]+
685.4385
0
20
40
60
80
100
550
600
650
1156 : M.findmain = 539.4422 (+/− 20 ppm)
540.4495
[M+H]+
685.4385
0
20
40
60
80
100
550
600
650

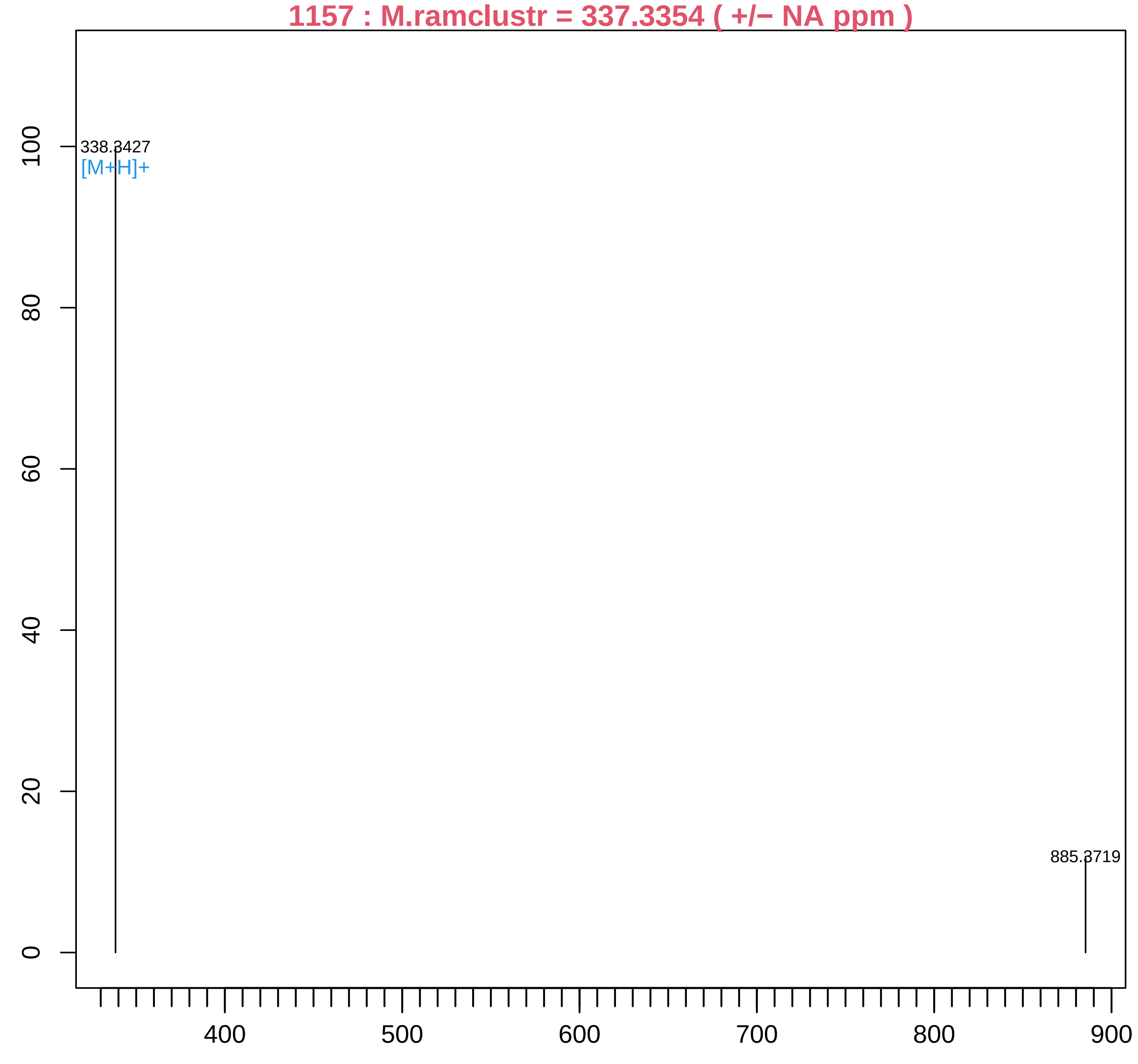
1157 : M.ramclustr = 337.3354 (+/− NA ppm)
338.3427
[M+H]+
885.3719
0
20
40
60
80
100
400
500
600
700
800
900

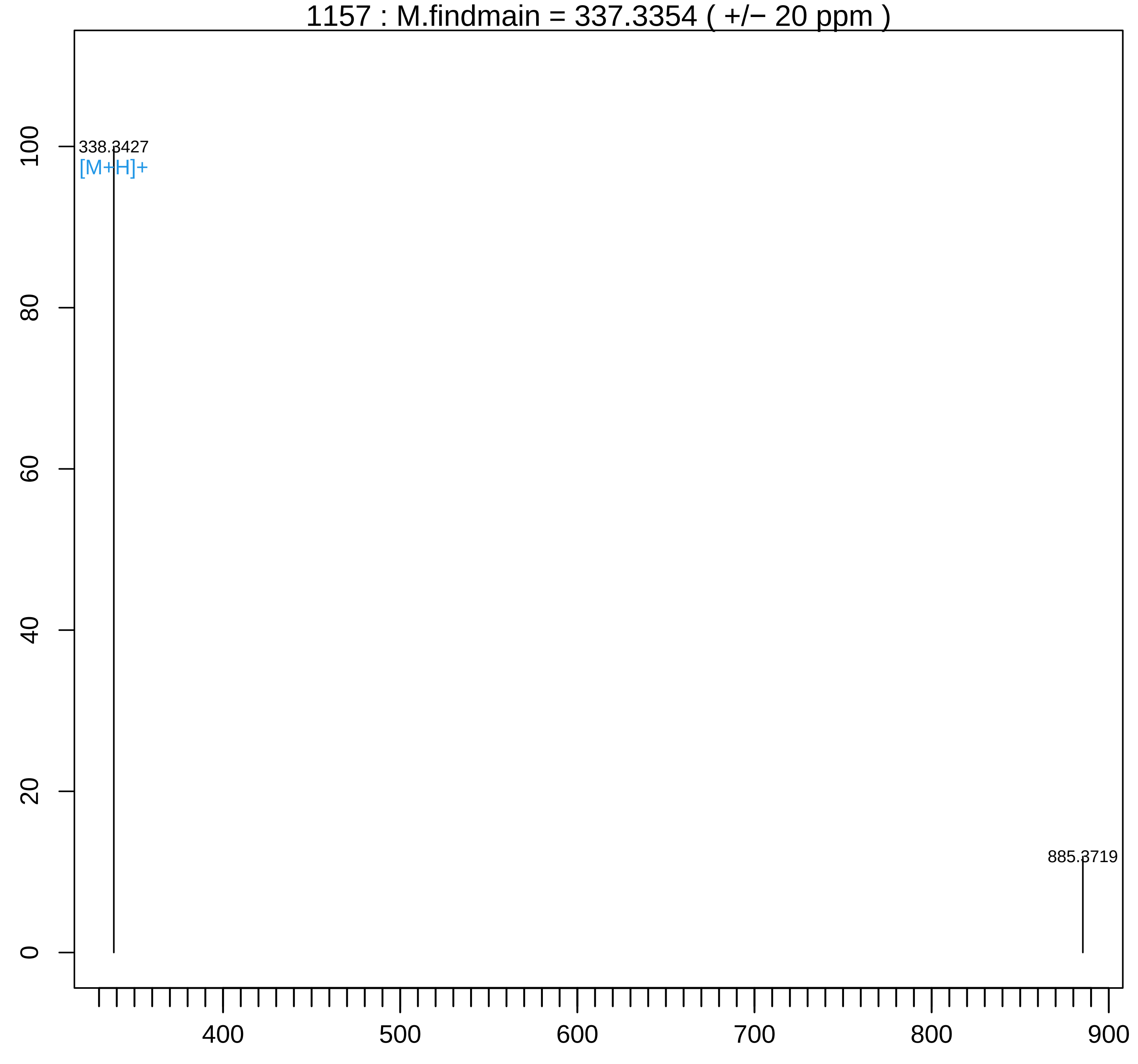
1157 : M.findmain = 337.3354 (+/− 20 ppm)
338.3427
[M+H]+
885.3719
0
20
40
60
80
100
400
500
600
700
800
900

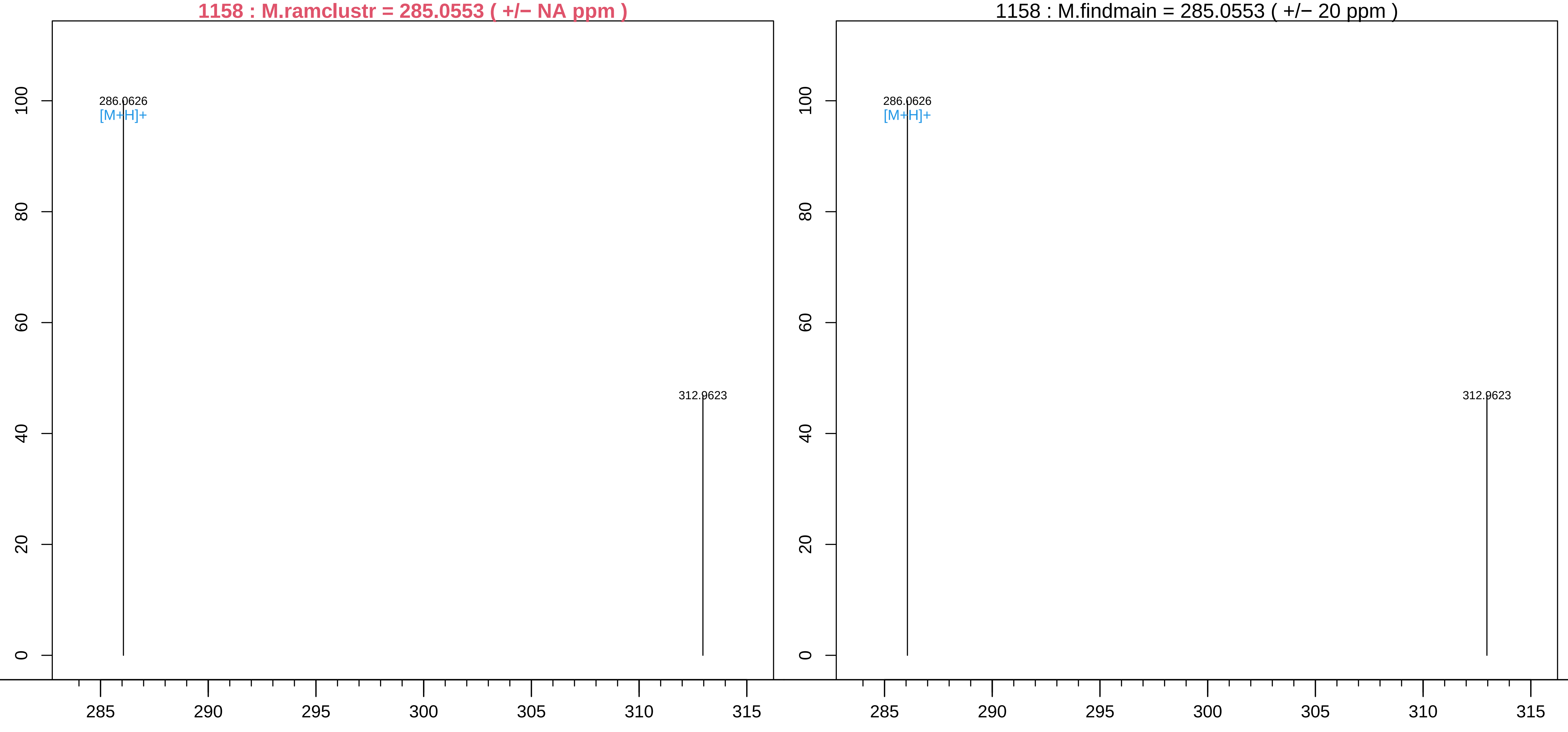

1158 : M.ramclustr = 285.0553 (+/− NA ppm)
286.0626
[M+H]+
312.9623
0
20
40
60
80
100
285
290
295
300
305
310
315
1158 : M.findmain = 285.0553 (+/− 20 ppm)
286.0626
[M+H]+
312.9623
0
20
40
60
80
100
285
290
295
300
305
310
315

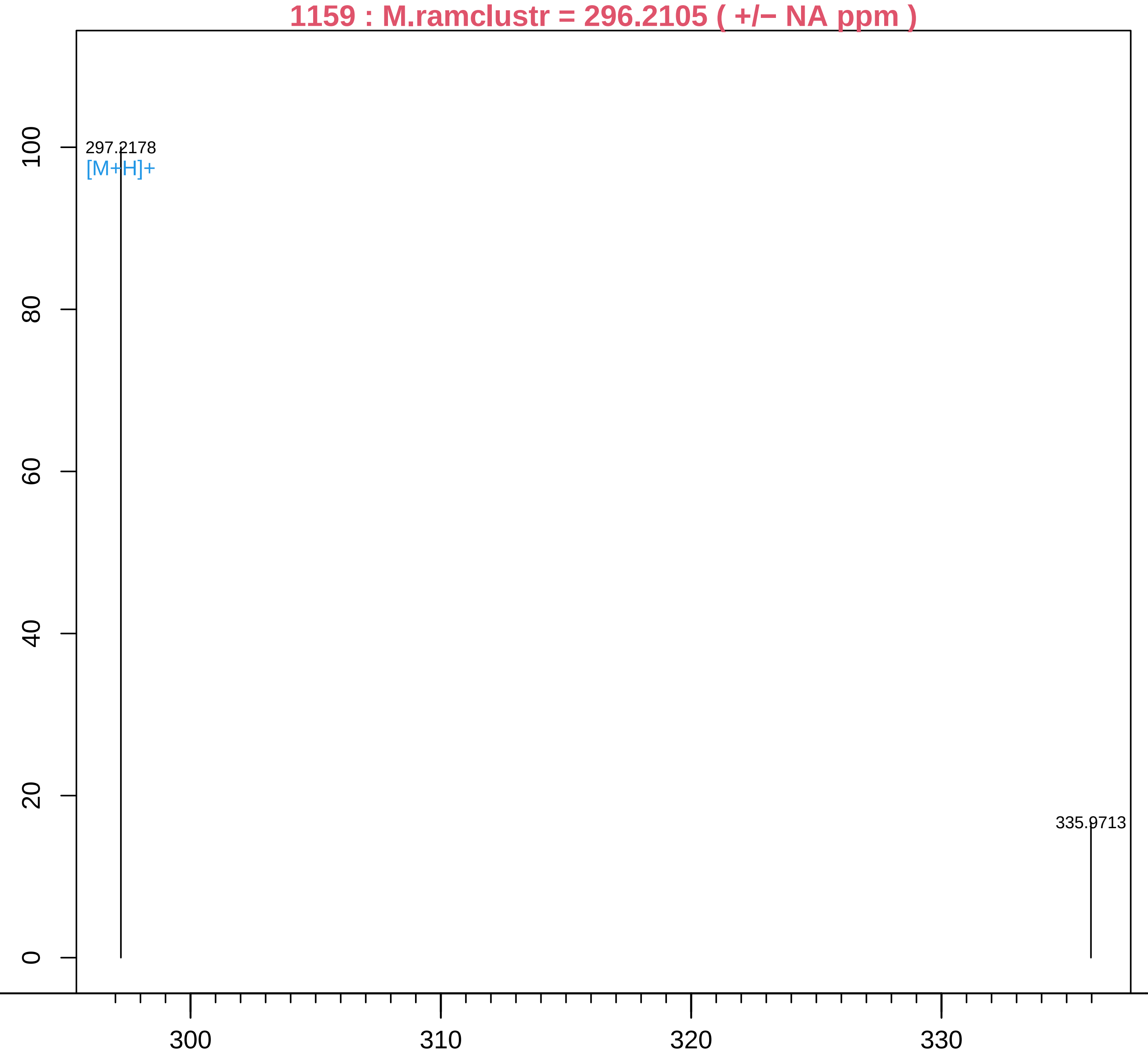
1159 : M.ramclustr = 296.2105 (+/− NA ppm)
297.2178
[M+H]+
335.9713
0
20
40
60
80
100
300
310
320
330

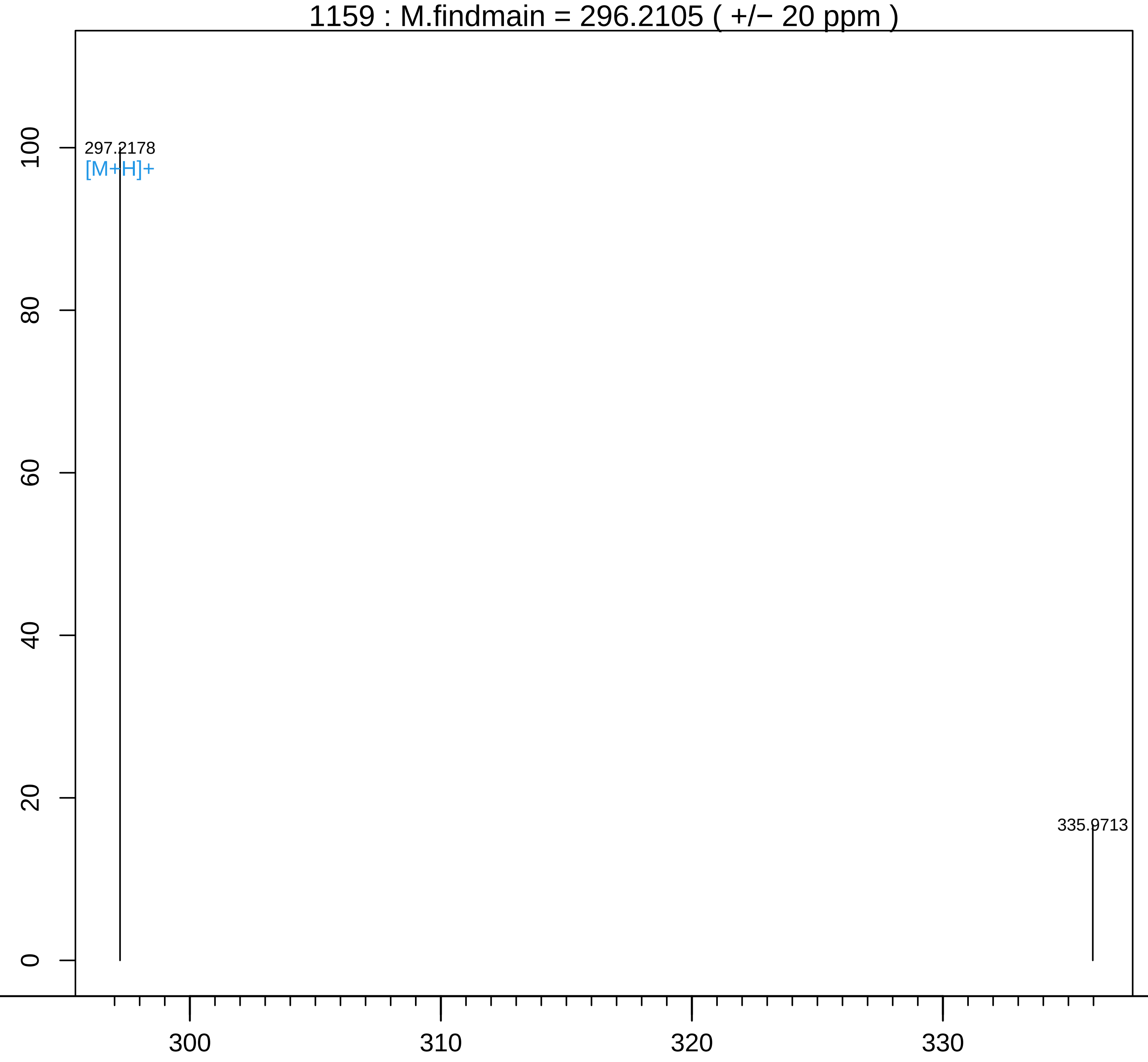
1159 : M.findmain = 296.2105 (+/− 20 ppm)
297.2178
[M+H]+
335.9713
0
20
40
60
80
100
300
310
320
330

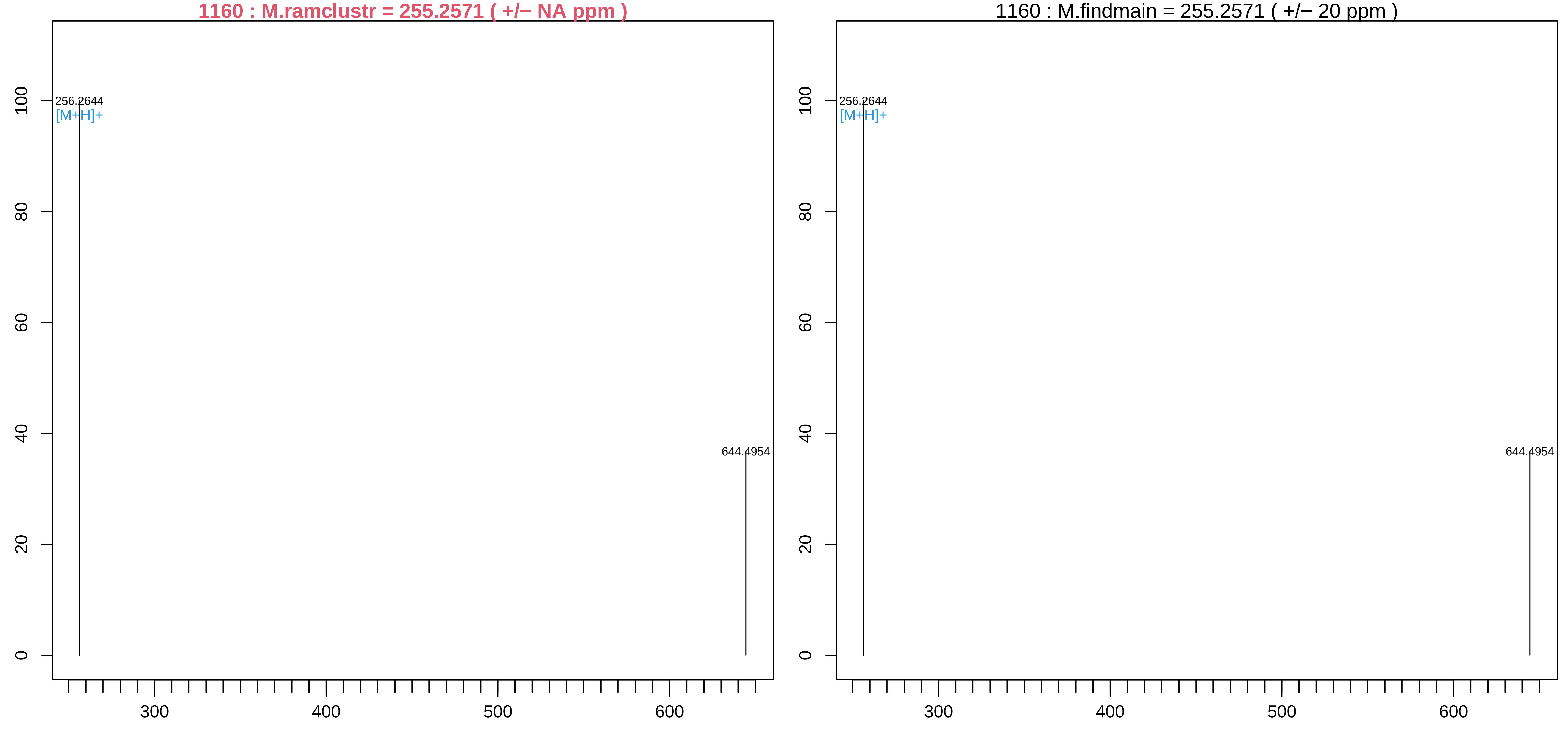
1160 : M.ramclustr = 255.2571 (+/− NA ppm)
256.2644
[M+H]+
644.4954
1160 : M.findmain = 255.2571 (+/− 20 ppm)
256.2644
[M+H]+
644.4954

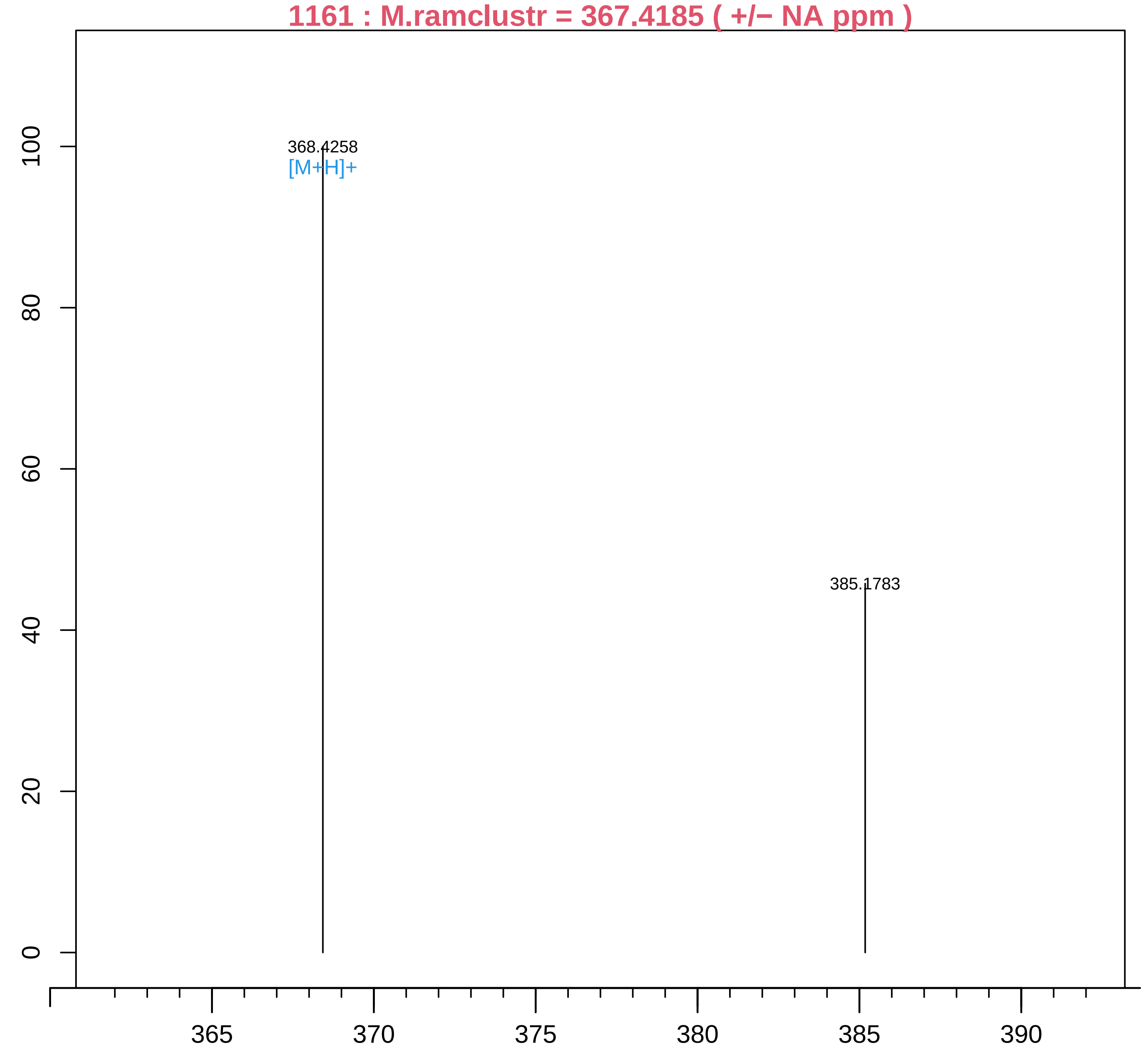
1161 : M.ramclustr = 367.4185 (+/− NA ppm)
368.4258
[M+H]+
385.1783
0
20
40
60
80
100
365
370
375
380
385
390

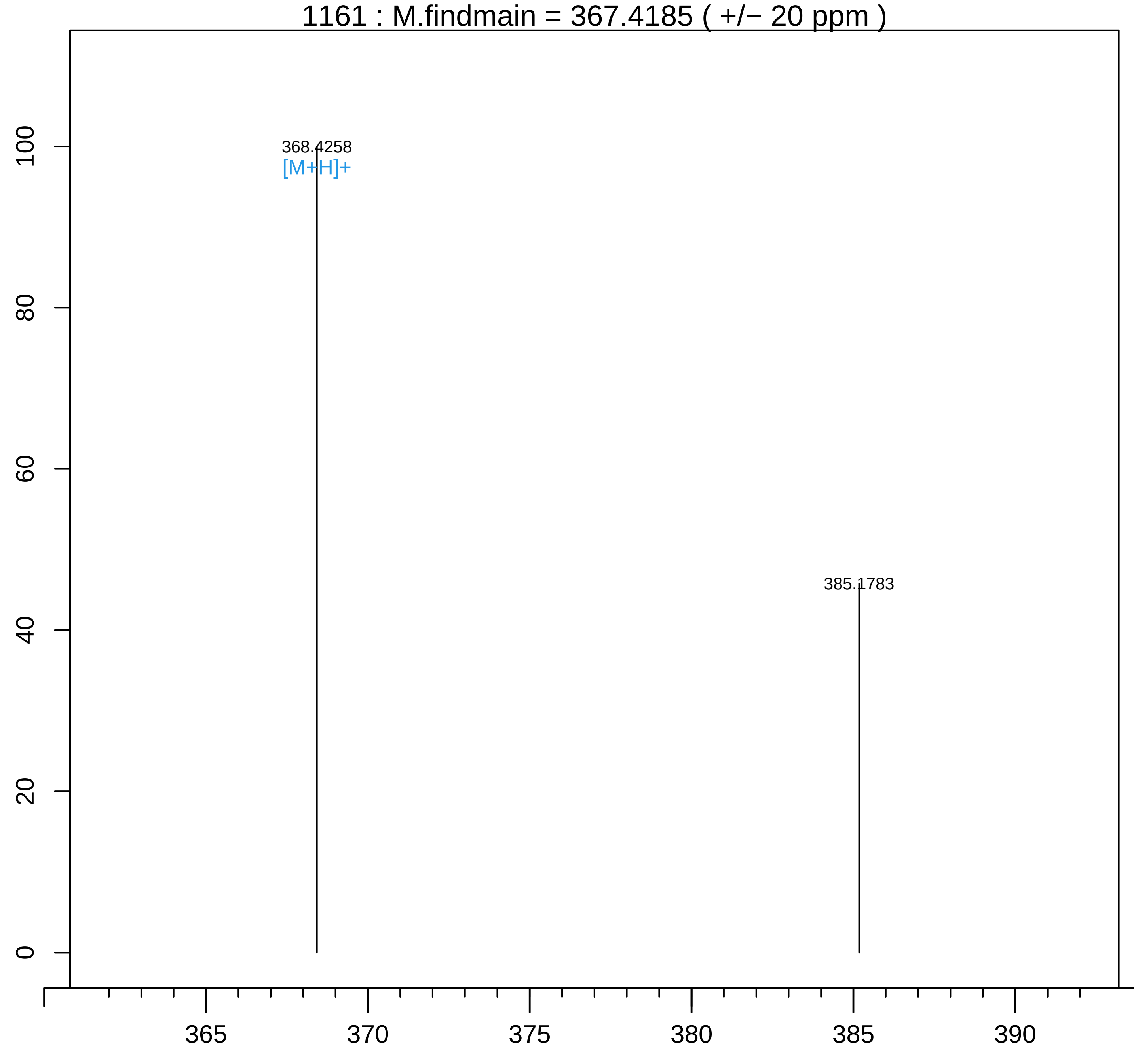
1161 : M.findmain = 367.4185 (+/− 20 ppm)
368.4258
[M+H]+
385.1783
0
20
40
60
80
100
365
370
375
380
385
390

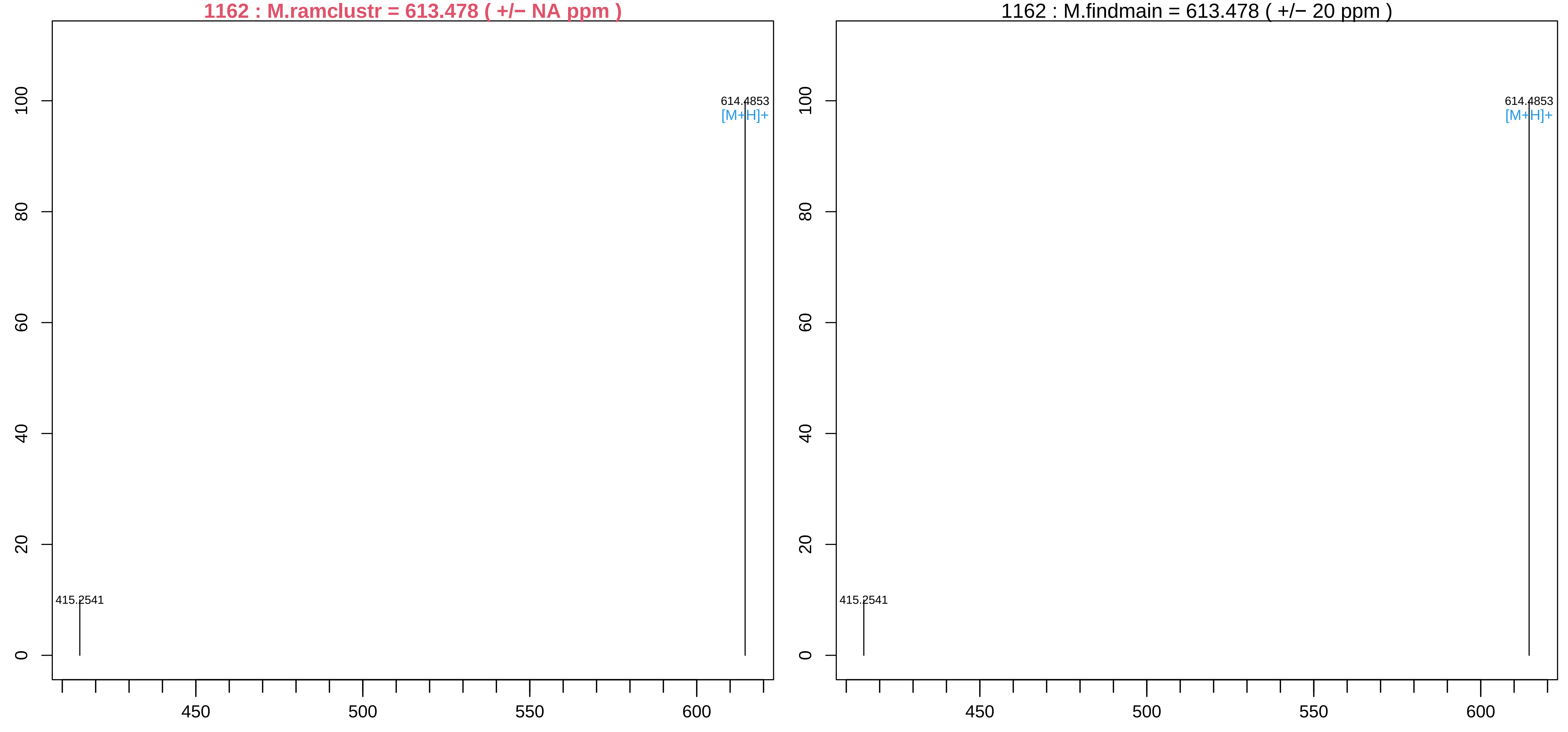
1162 : M.ramclustr = 613.478 (+/− NA ppm)
1162 : M.findmain = 613.478 (+/− 20 ppm)
614.4853
[M+H]+
415.2541
0
20
40
60
80
100
450
500
550
600

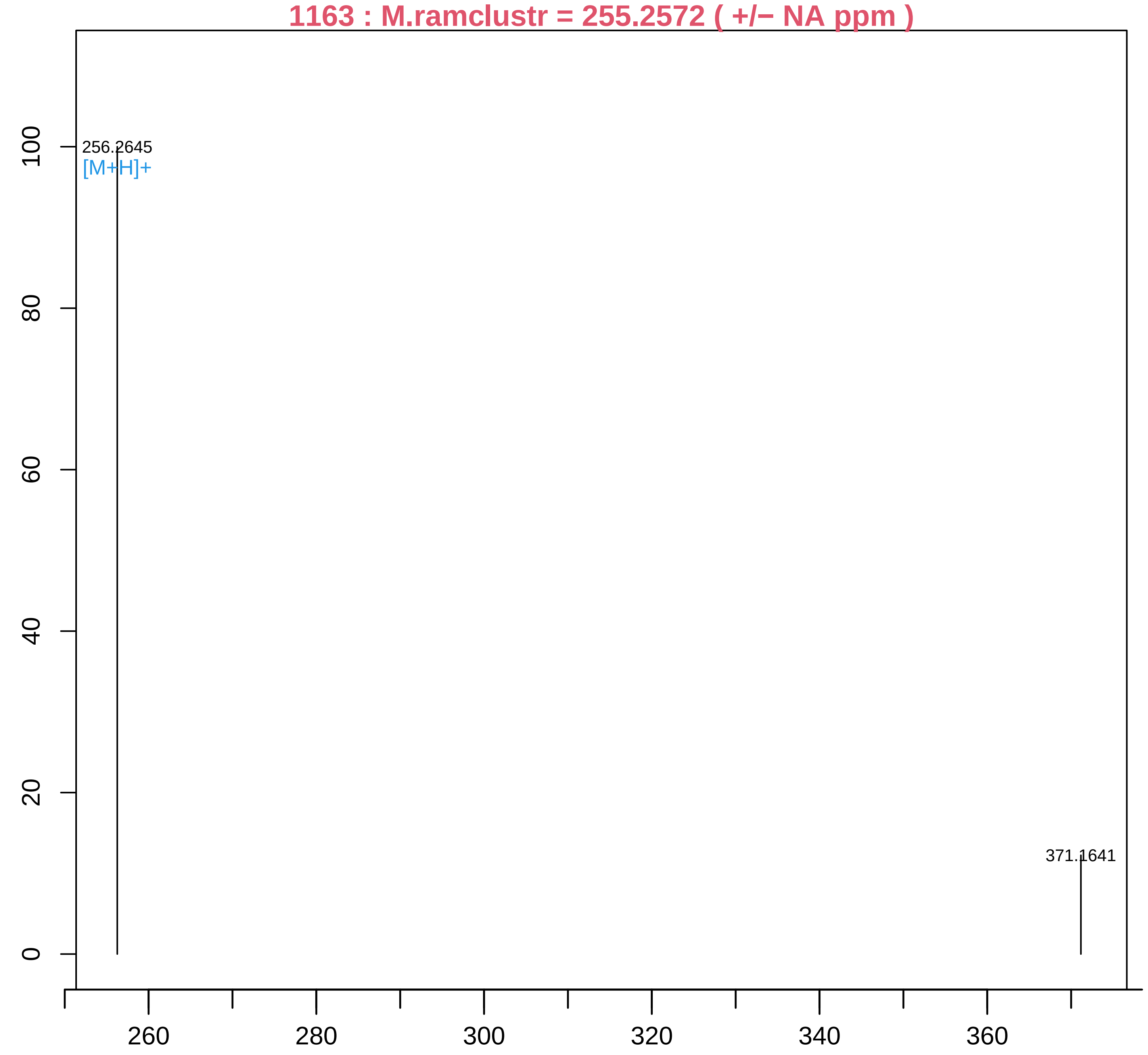
1163 : M.ramclustr = 255.2572 (+/− NA ppm)
256.2645
[M+H]+
371.1641
0
20
40
60
80
100
260
280
300
320
340
360

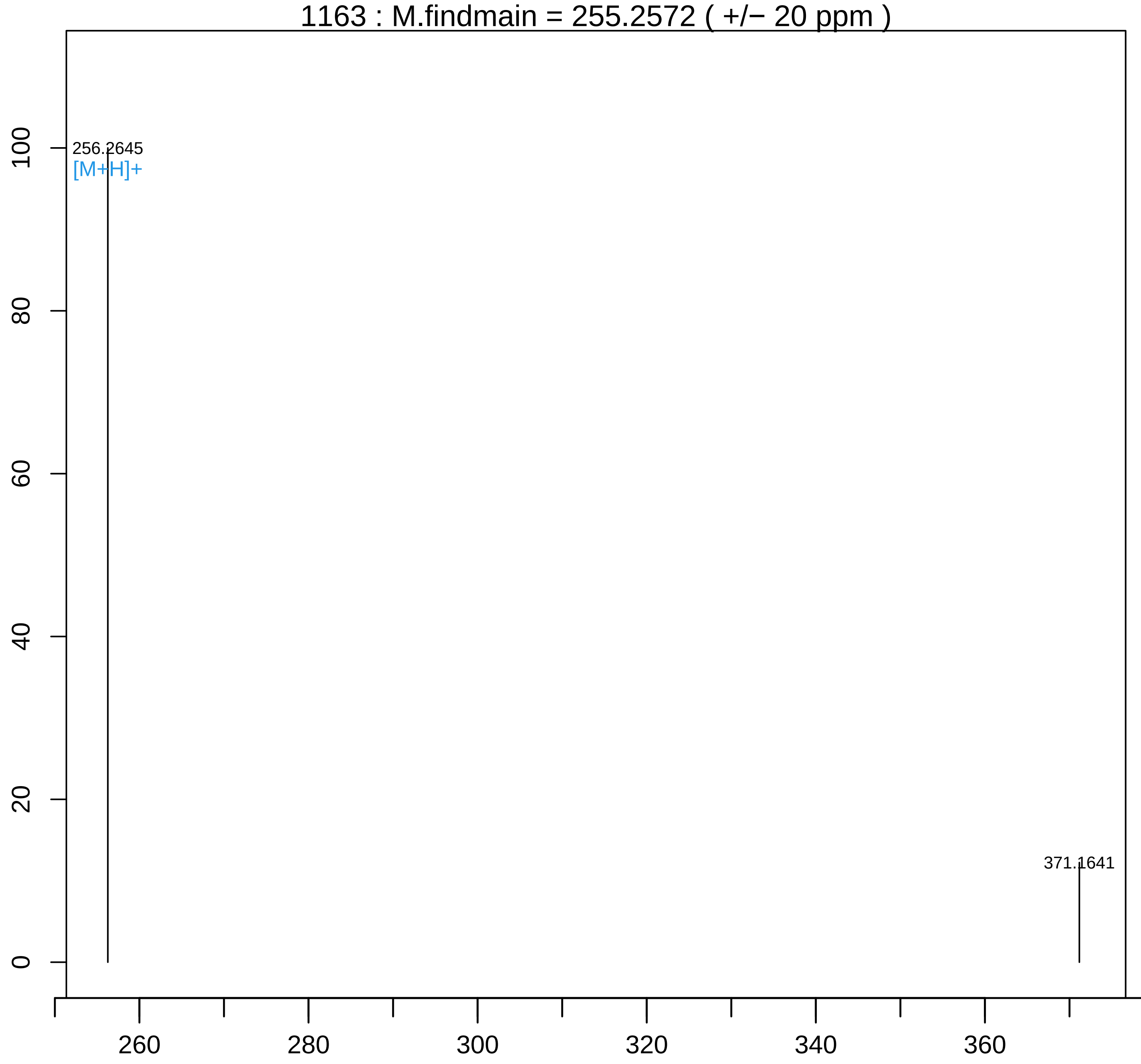
1163 : M.findmain = 255.2572 (+/− 20 ppm)
256.2645
[M+H]+
371.1641
0
20
40
60
80
100
260
280
300
320
340
360

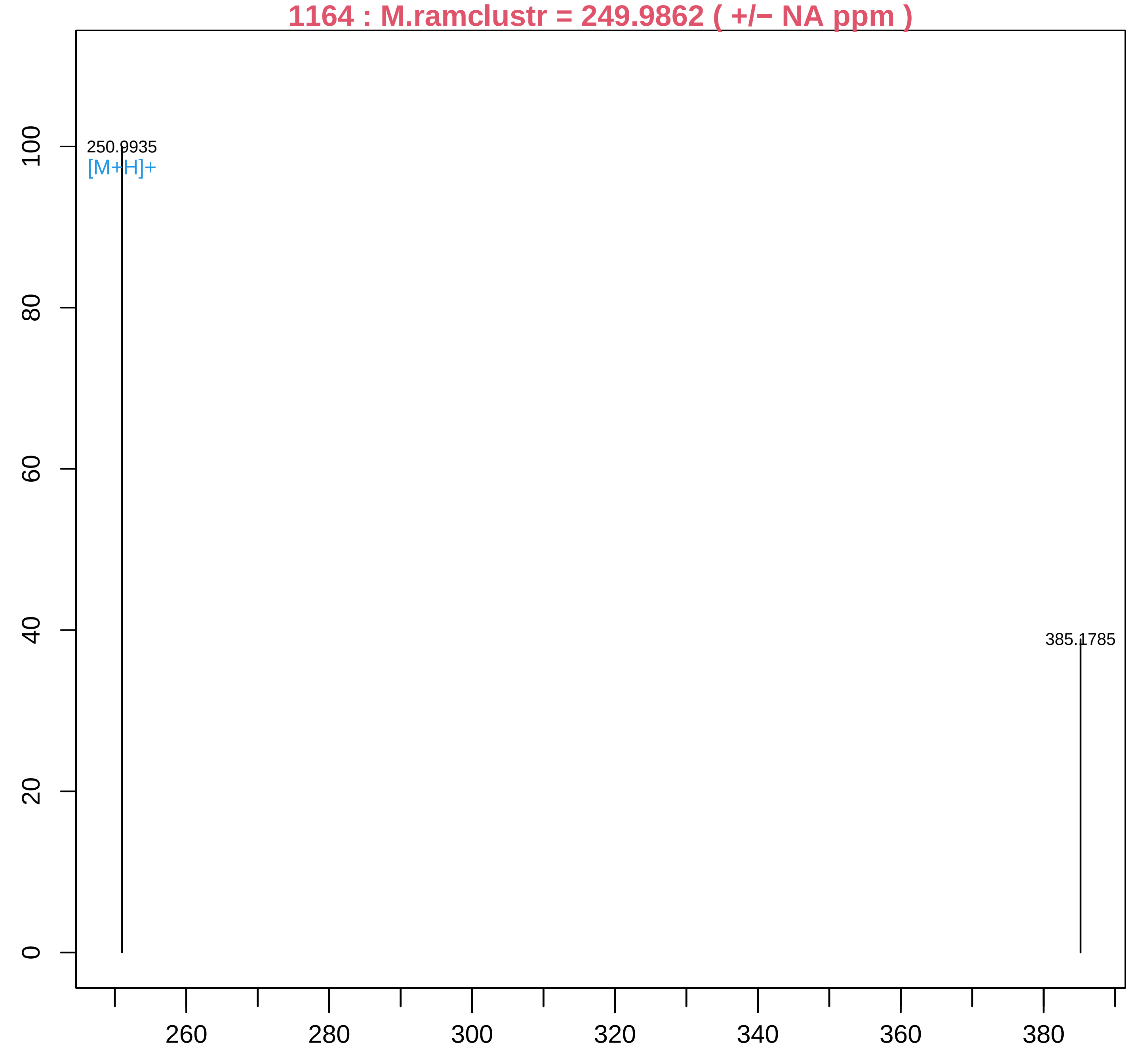
1164 : M.ramclustr = 249.9862 (+/− NA ppm)
250.9935
[M+H]+
385.1785
0
20
40
60
80
100
260
280
300
320
340
360
380

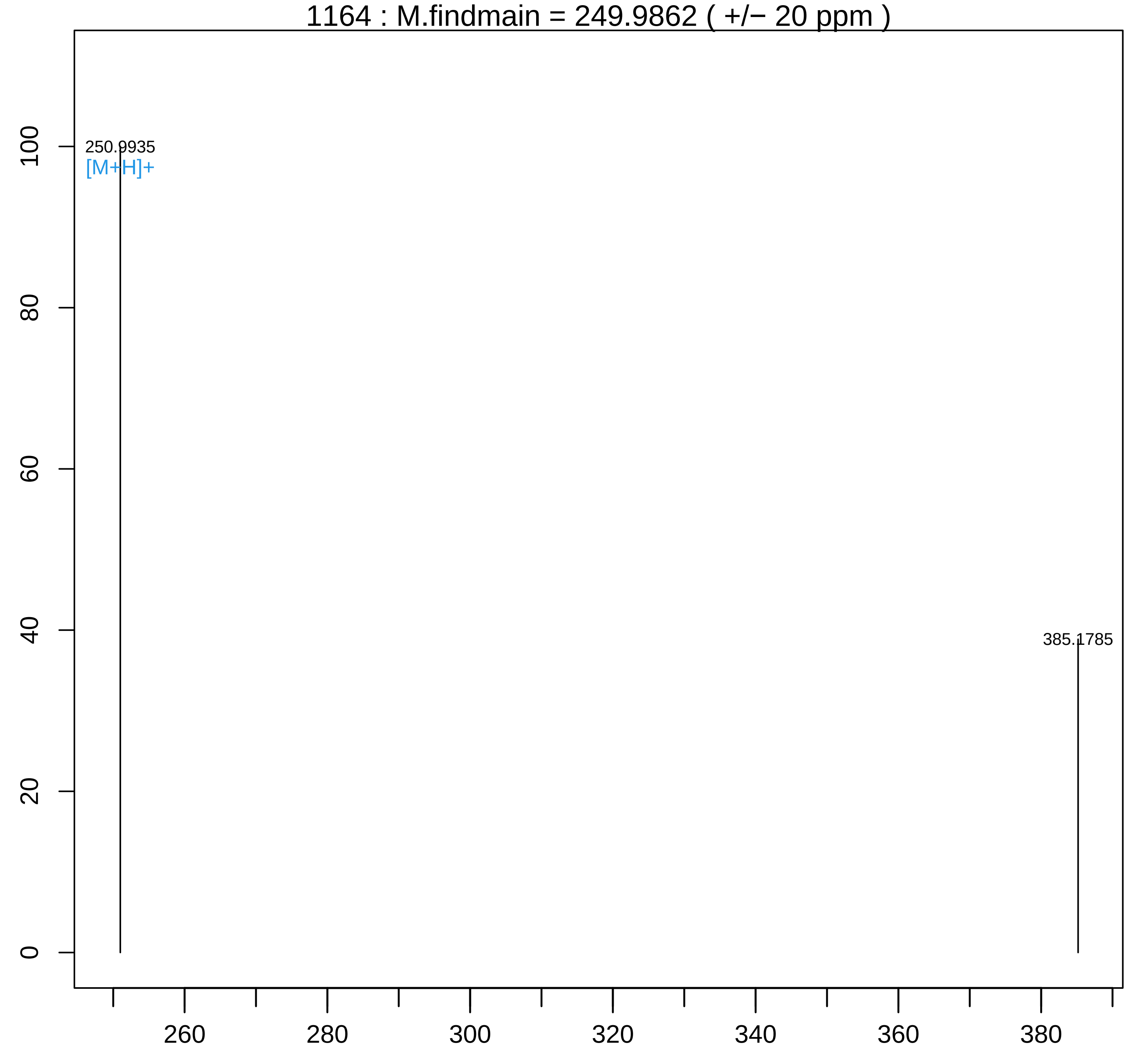
1164 : M.findmain = 249.9862 (+/− 20 ppm)
250.9935
[M+H]+
385.1785
0
20
40
60
80
100
260
280
300
320
340
360
380

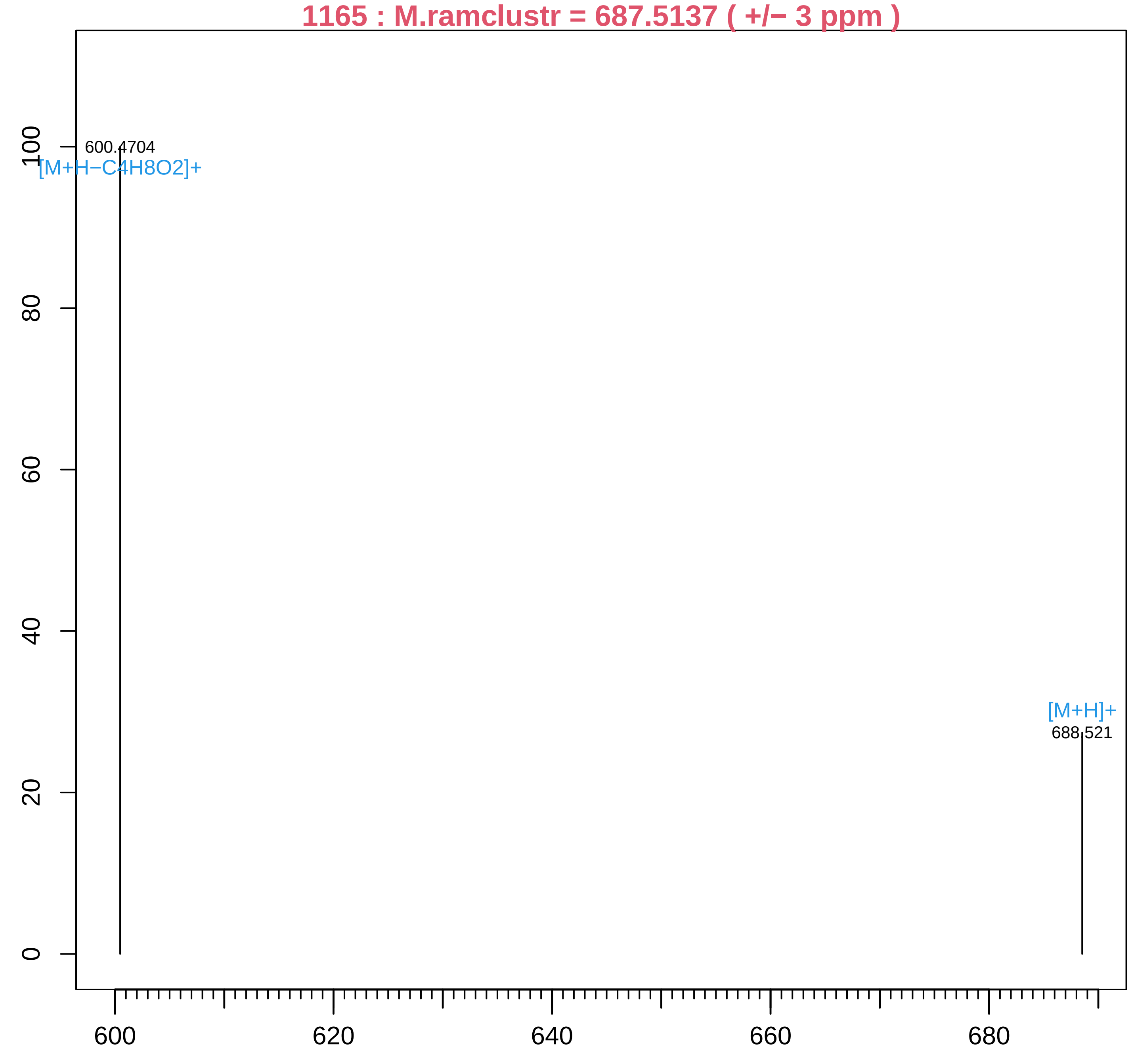
1165 : M.ramclustr = 687.5137 (+/− 3 ppm)
600.4704
[M+H−C4H8O2]+
[M+H]+
688.521
0
20
40
60
80
100
600
620
640
660
680

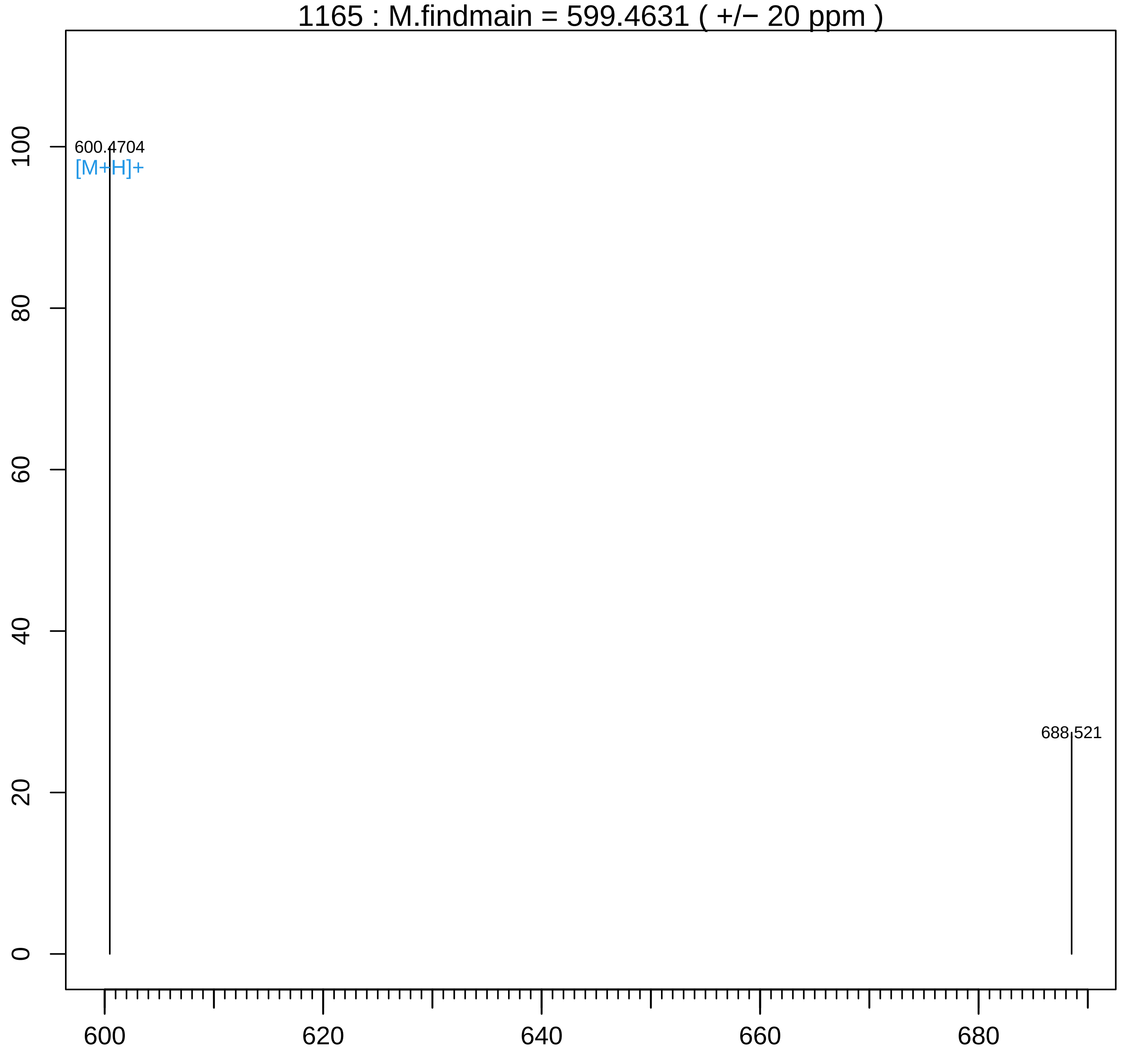
1165 : M.findmain = 599.4631 (+/− 20 ppm)
600.4704
[M+H]+
688.521
0
20
40
60
80
100
600
620
640
660
680

1166 : M.ramclustr = 284.0516 (+/− NA ppm)

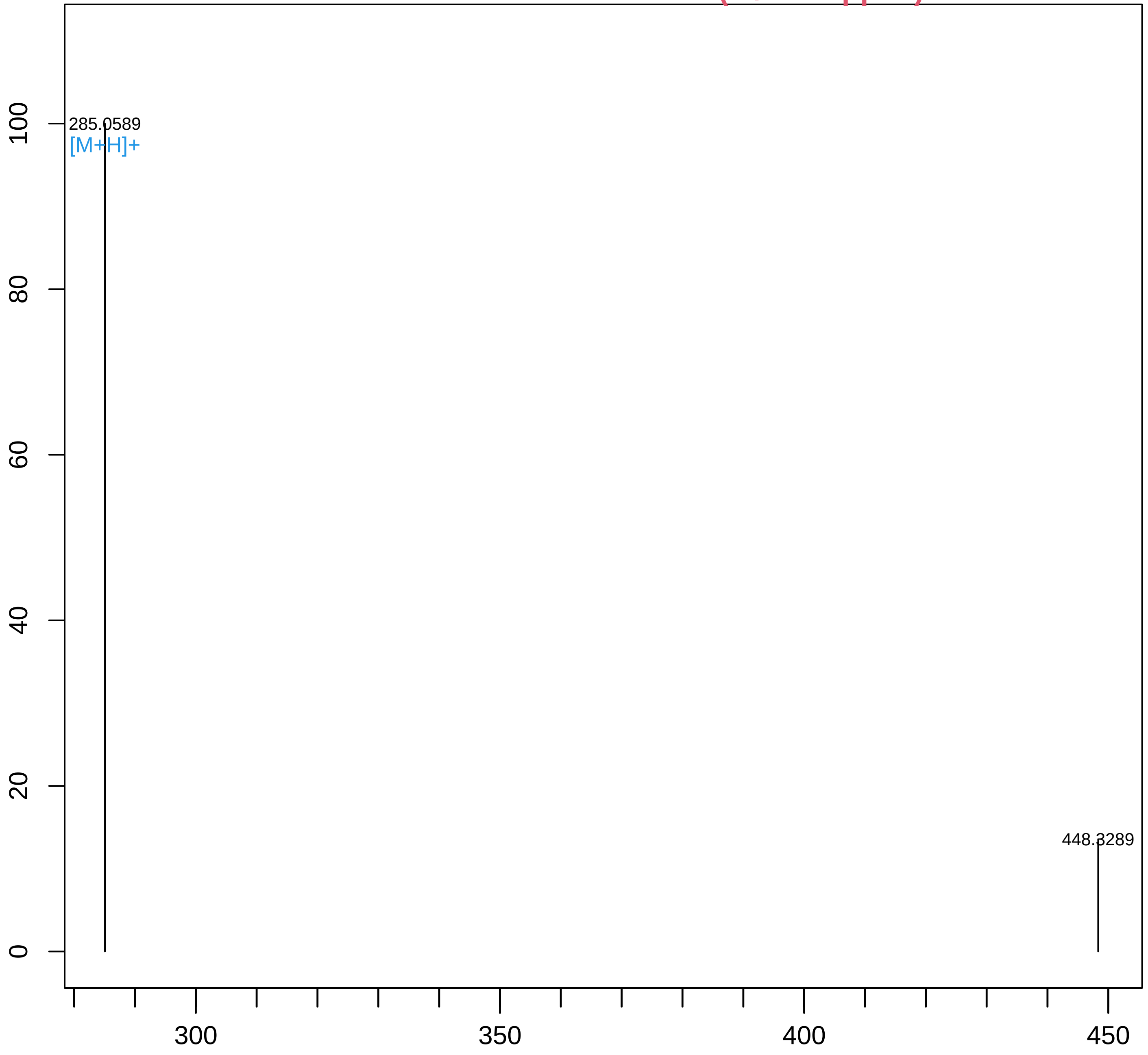

1166 : M.findmain = 284.0516 (+/− 20 ppm)

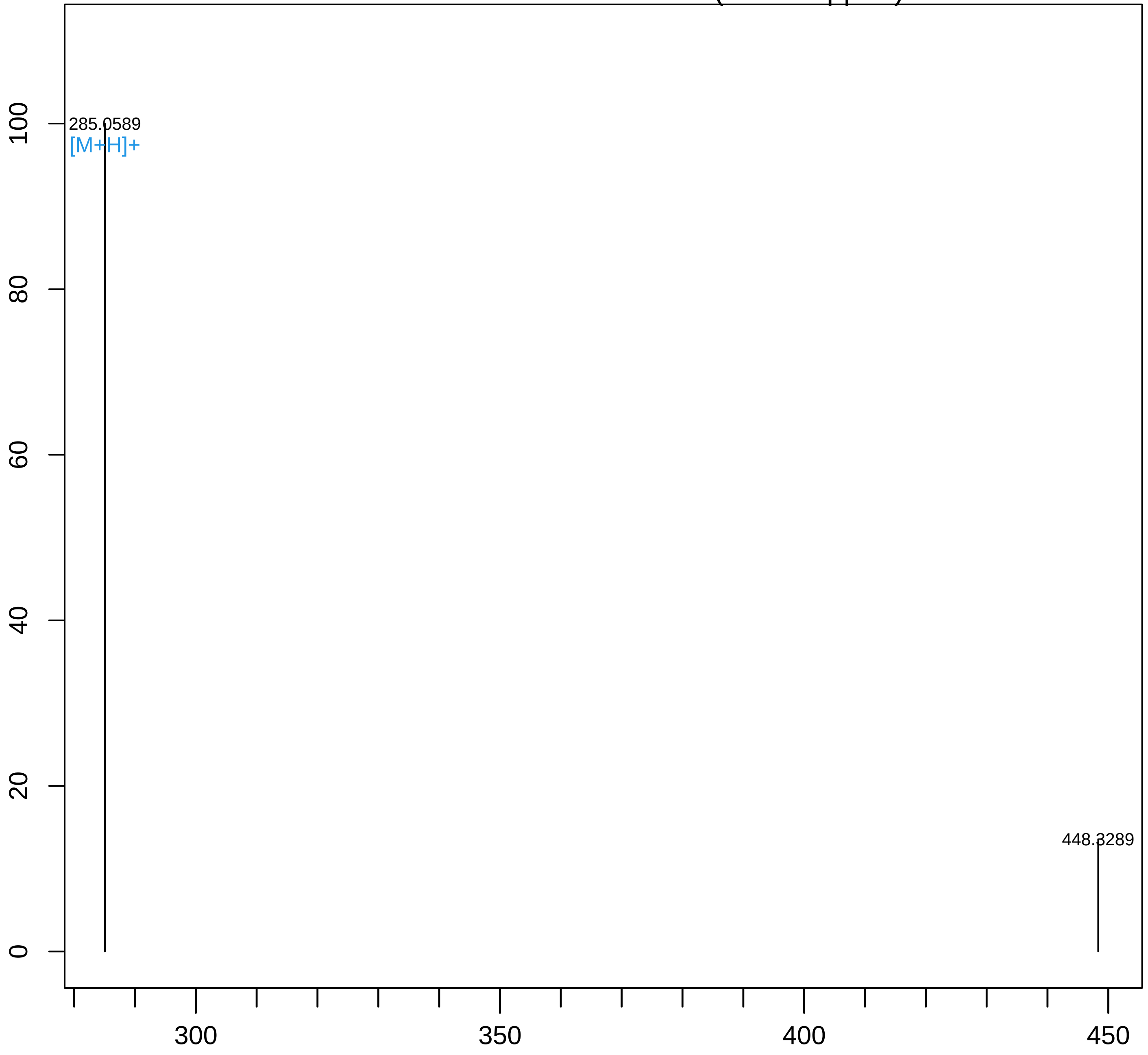

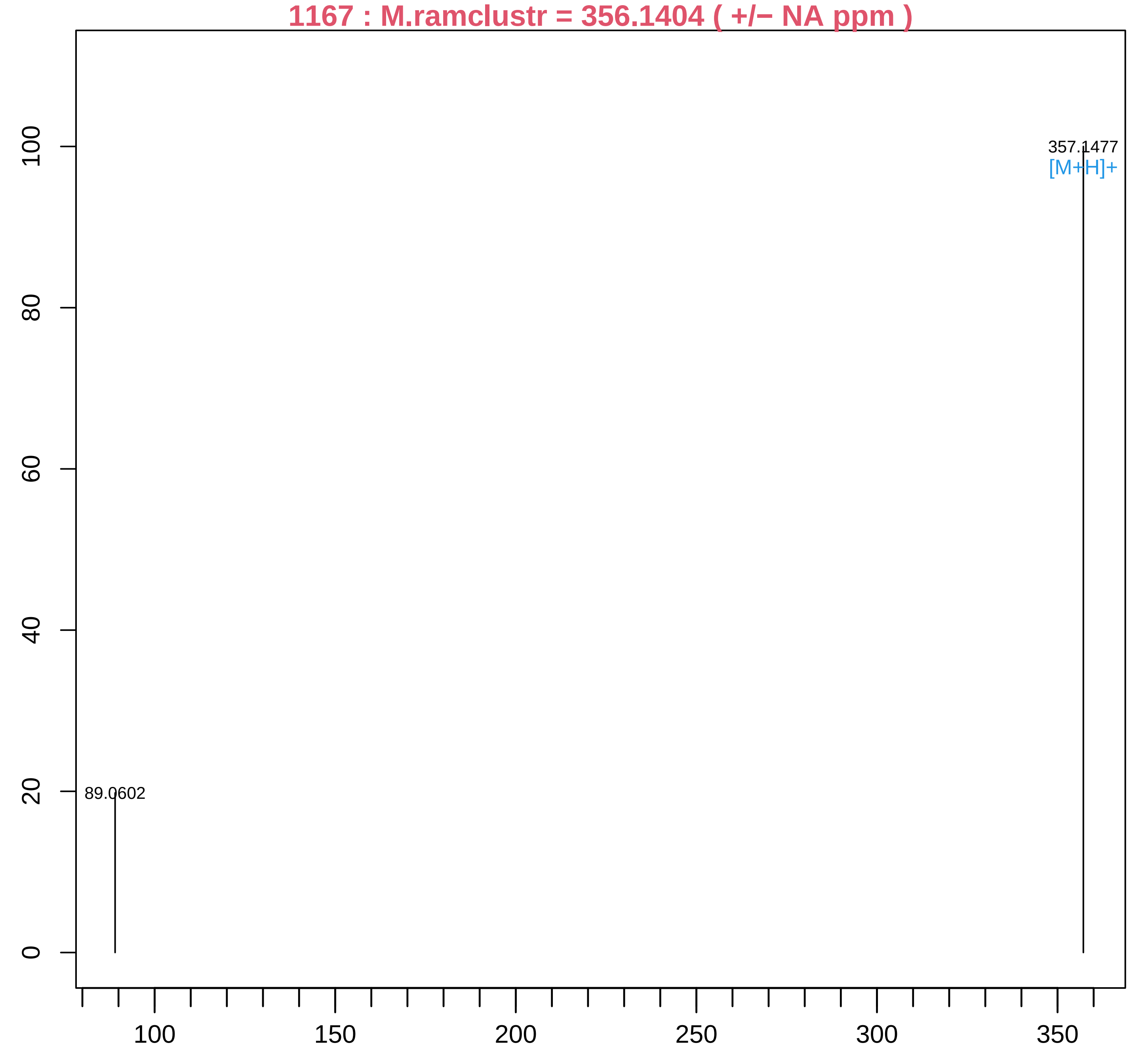

1167 : M.ramclustr = 356.1404 (+/− NA ppm)
357.1477
[M+H]+
89.0602

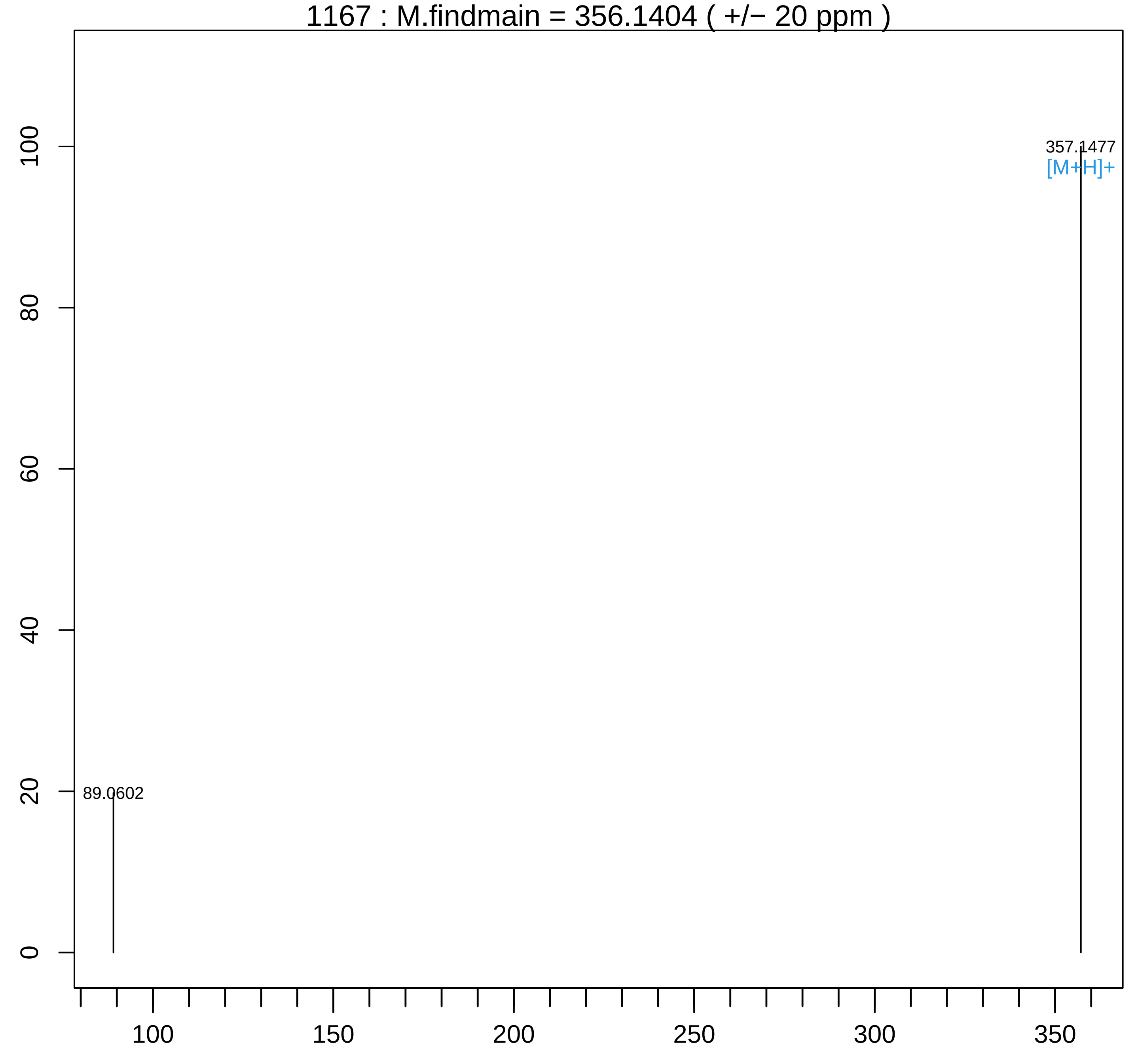

1167 : M.findmain = 356.1404 (+/− 20 ppm)
357.1477
[M+H]+
89.0602

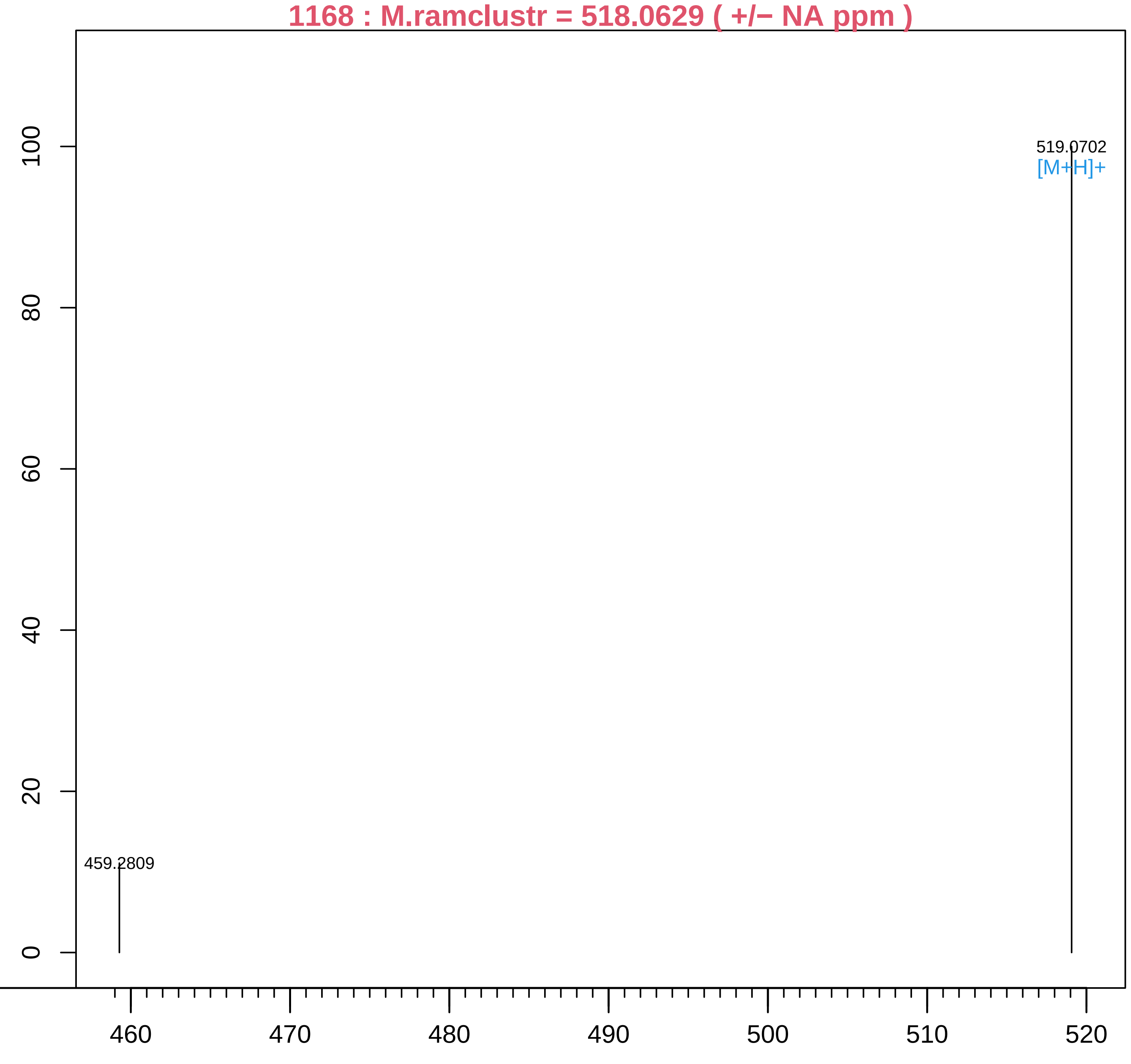
1168 : M.ramclustr = 518.0629 (+/− NA ppm)
519.0702
[M+H]+
459.2809
0
20
40
60
80
100
460
470
480
490
500
510
520

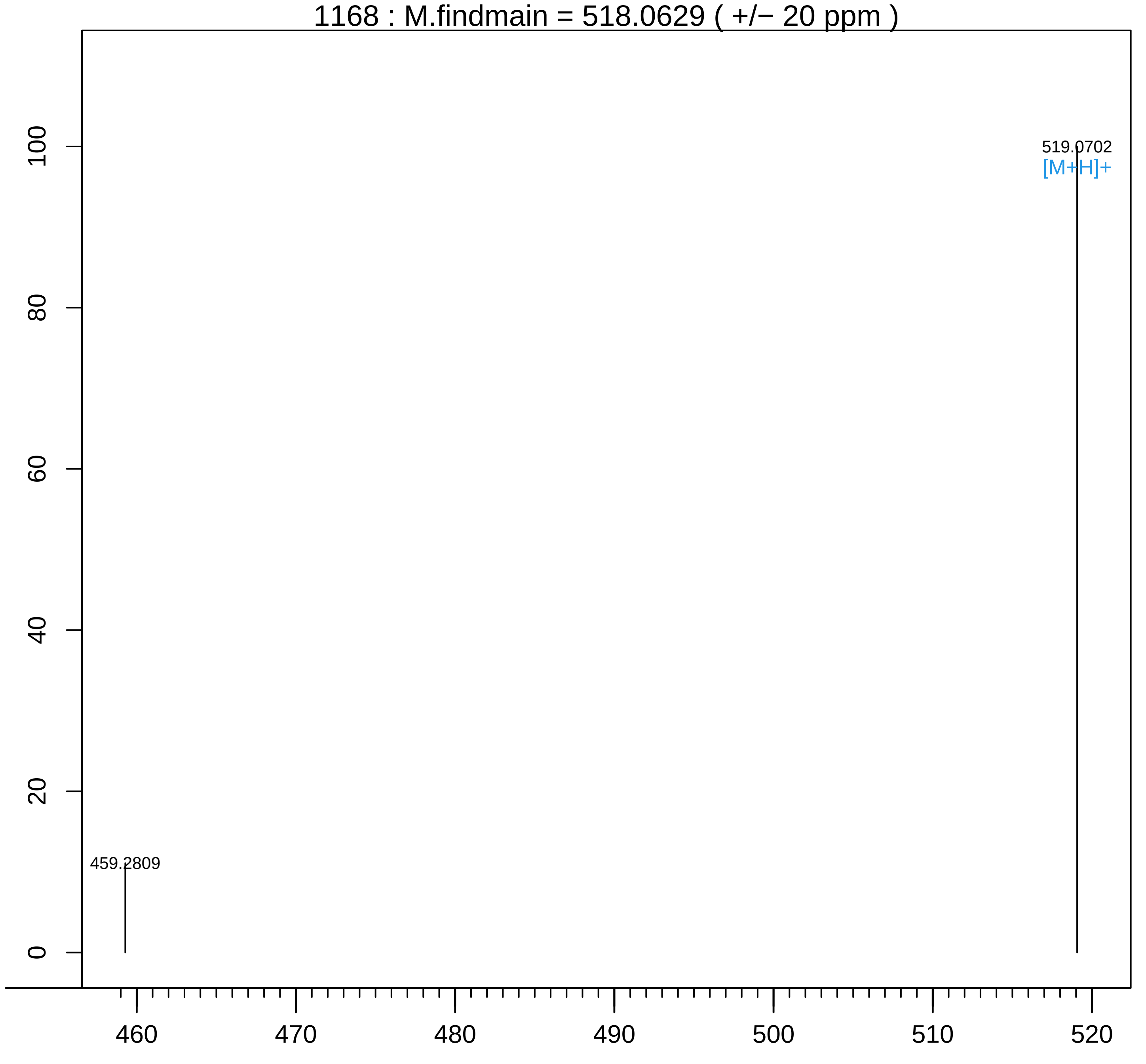
1168 : M.findmain = 518.0629 (+/− 20 ppm)
519.0702
[M+H]+
459.2809
0
20
40
60
80
100
460
470
480
490
500
510
520

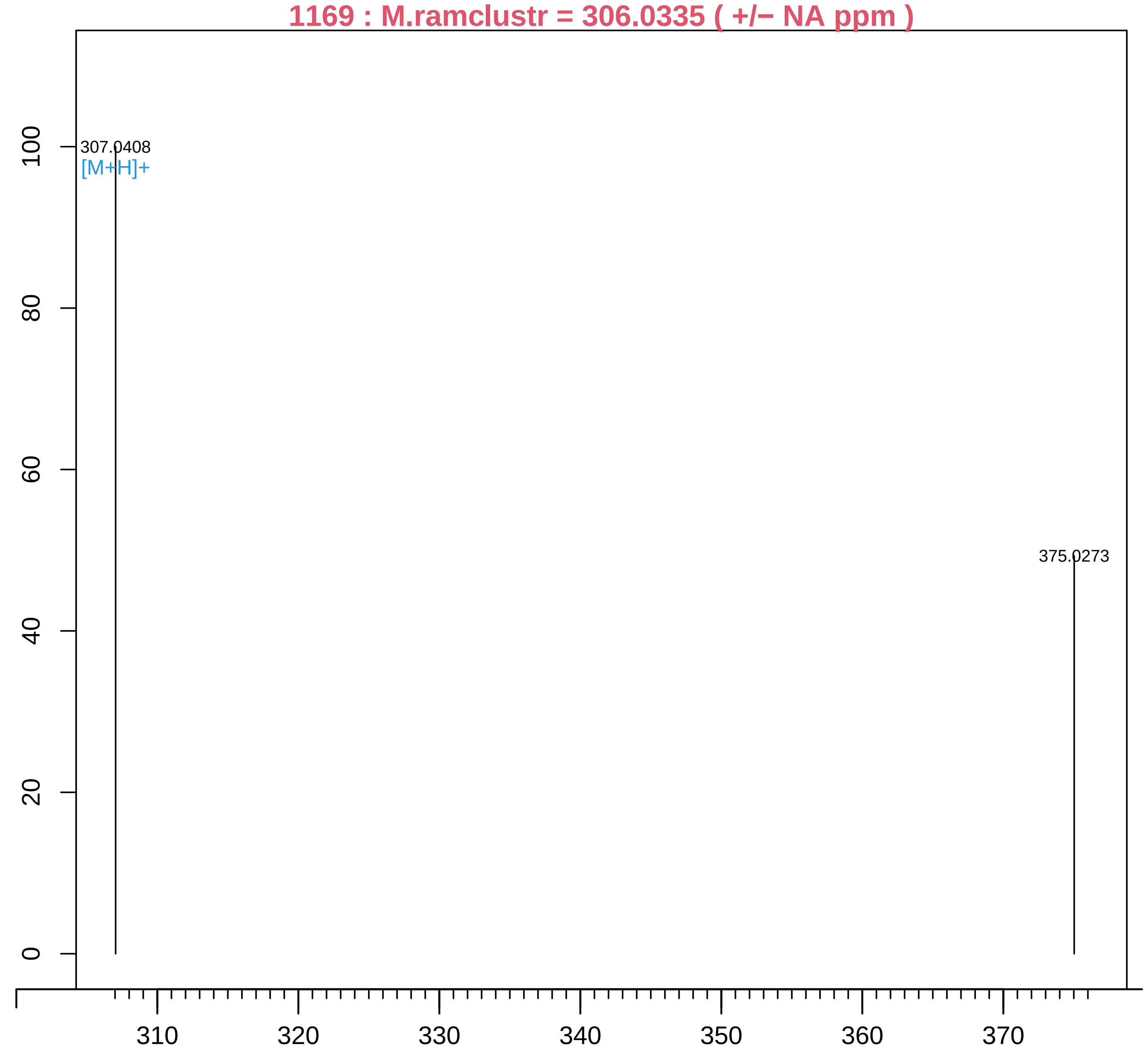
1169 : M.ramclustr = 306.0335 (+/− NA ppm)
307.0408
[M+H]+
375.0273
0
20
40
60
80
100
310
320
330
340
350
360
370

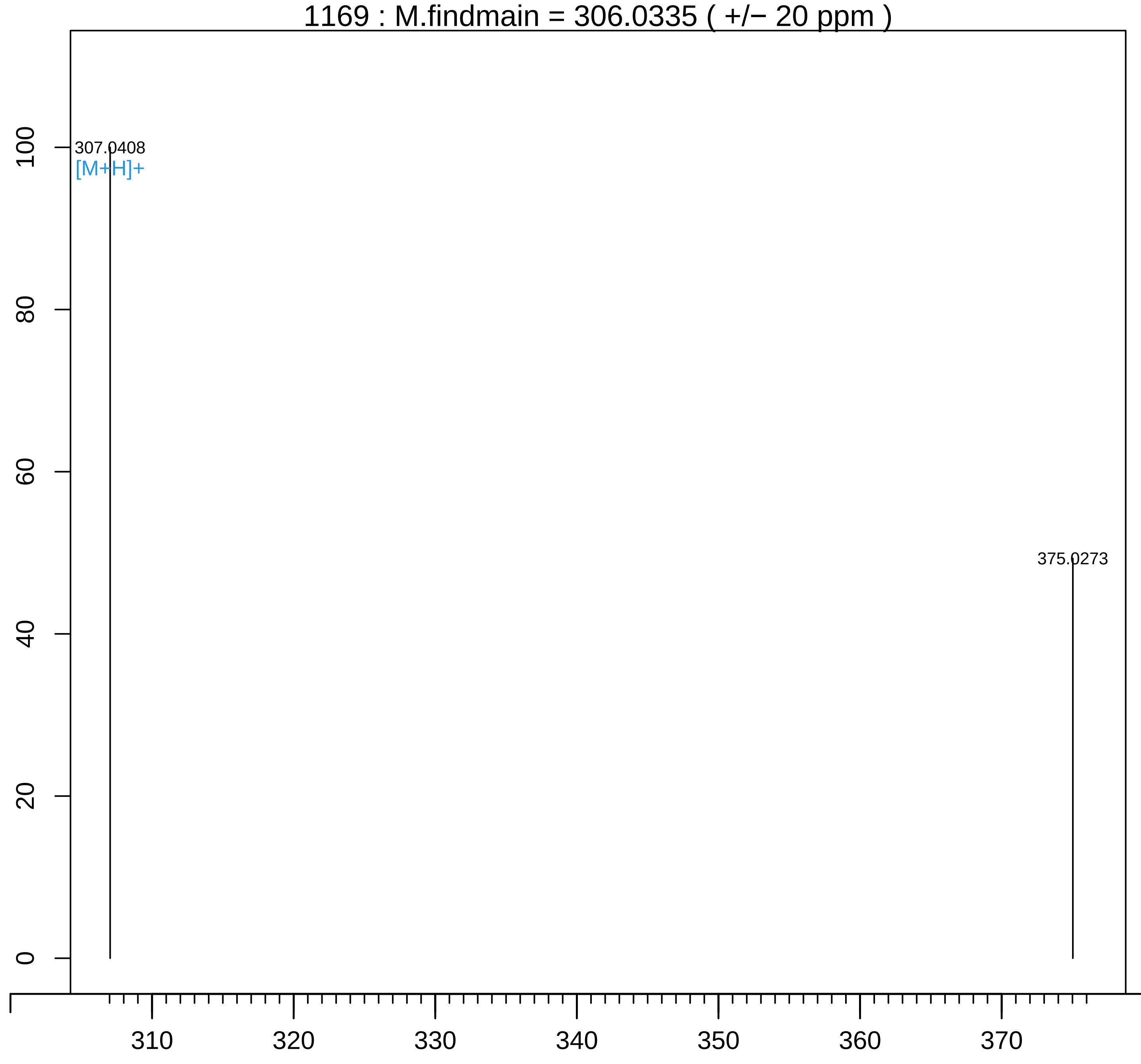
1169 : M.findmain = 306.0335 (+/− 20 ppm)
307.0408
[M+H]+
375.0273
0
20
40
60
80
100
310
320
330
340
350
360
370

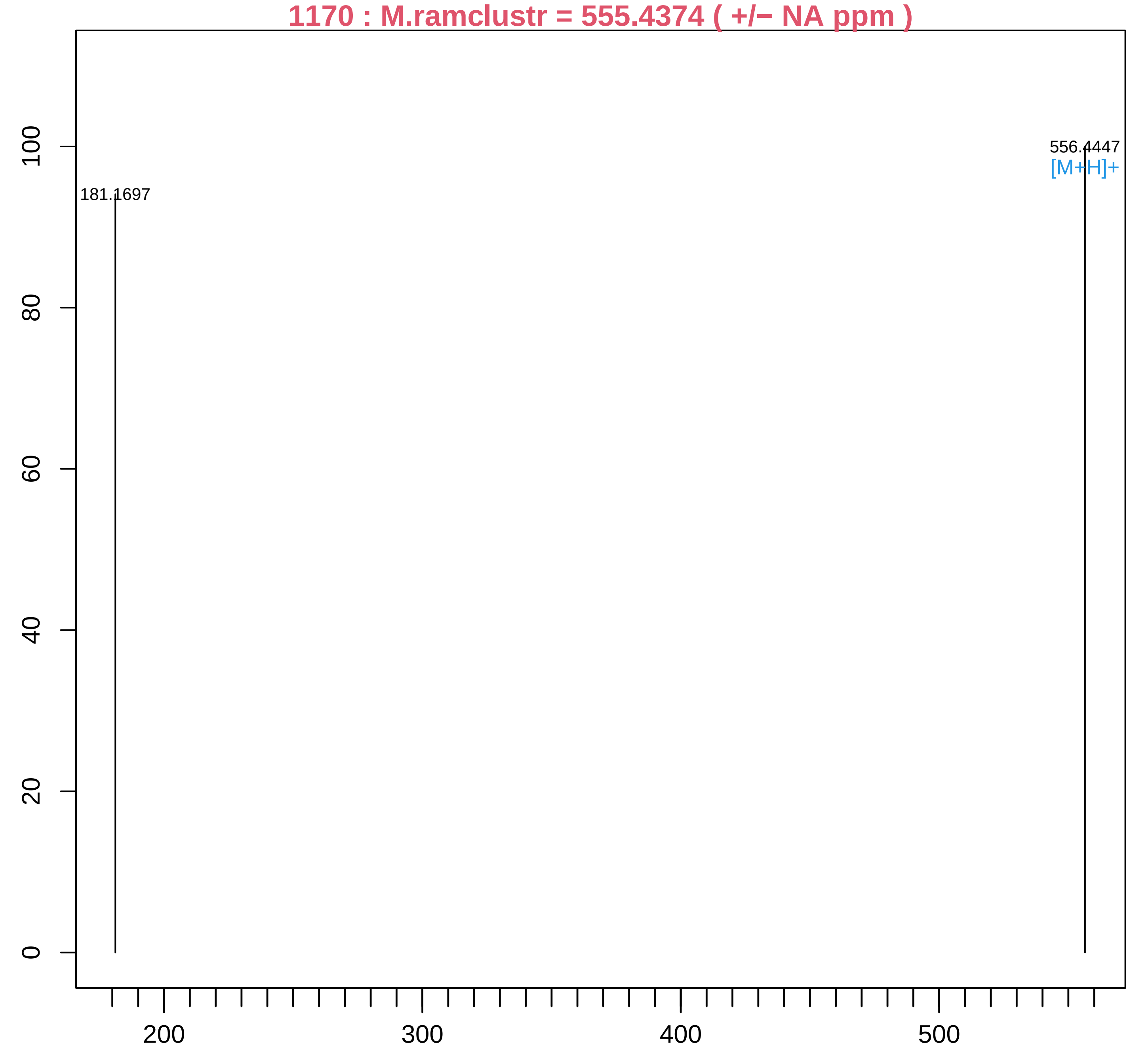
1170 : M.ramclustr = 555.4374 (+/− NA ppm)
181.1697
556.4447
[M+H]+
0
20
40
60
80
100
200
300
400
500

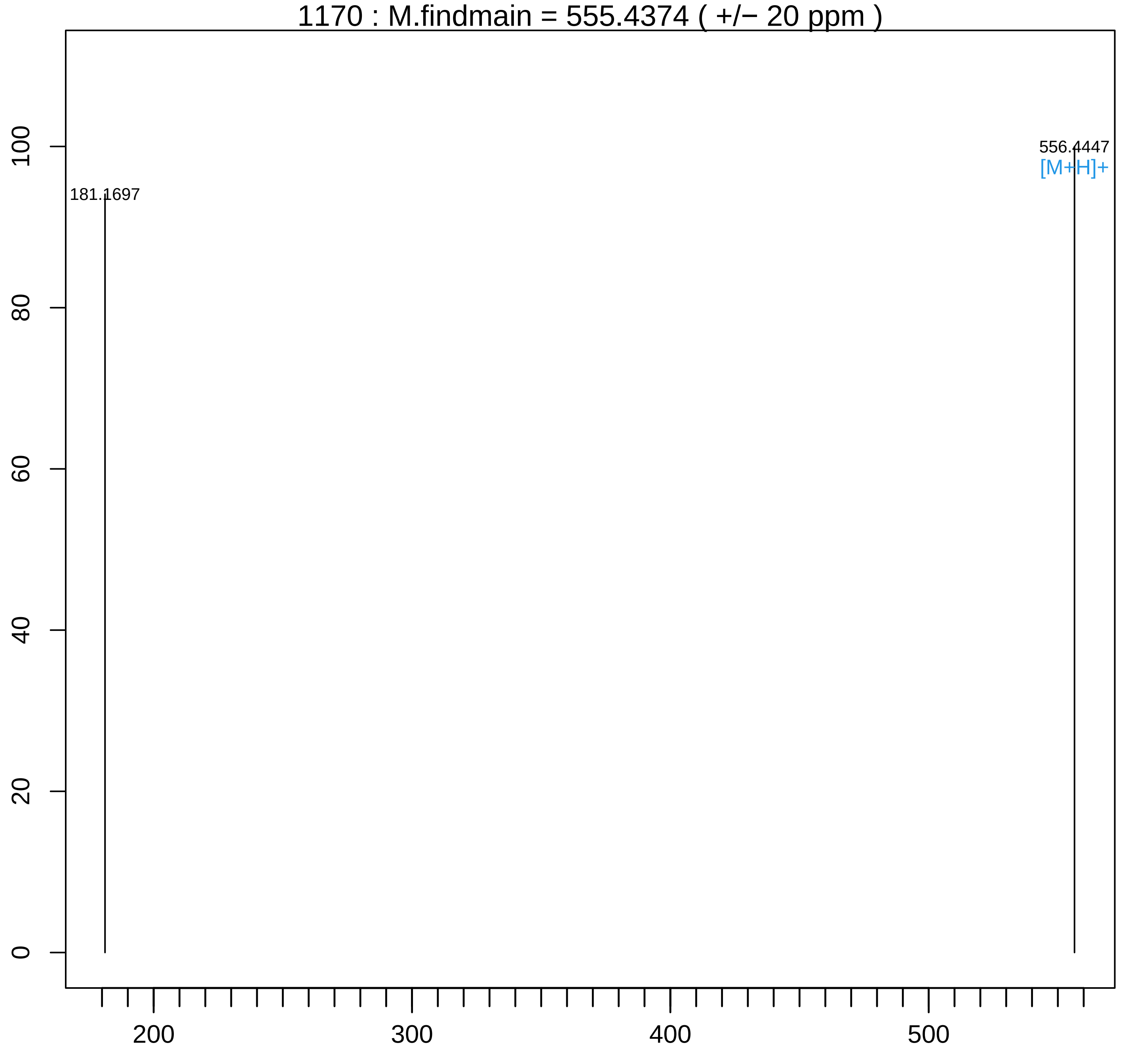
1170 : M.findmain = 555.4374 (+/− 20 ppm)
181.1697
556.4447
[M+H]+
0
20
40
60
80
100
200
300
400
500

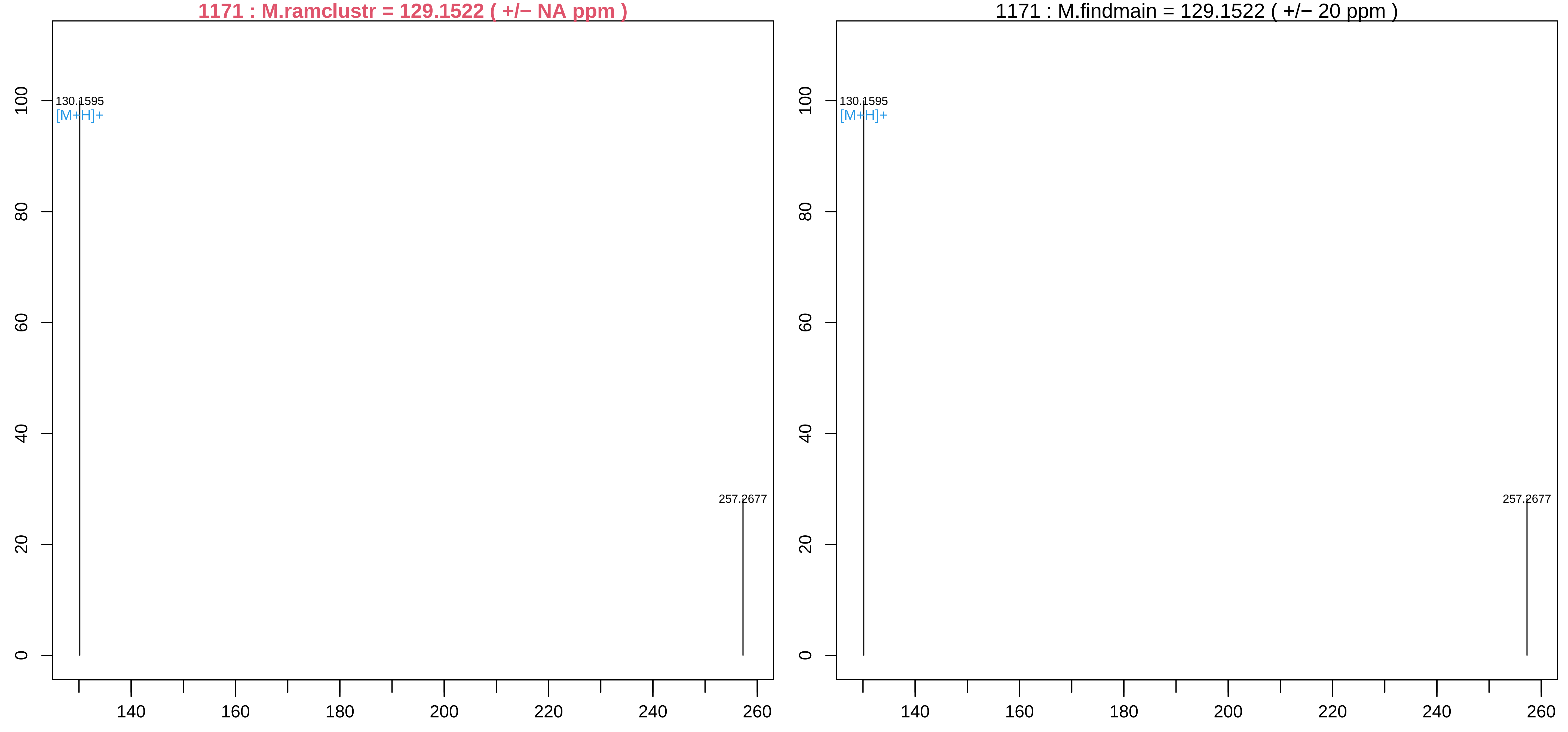
1171 : M.ramclustr = 129.1522 (+/− NA ppm)
130.1595
[M+H]+
257.2677
0
20
40
60
80
100
140
160
180
200
220
240
260
1171 : M.findmain = 129.1522 (+/− 20 ppm)
130.1595
[M+H]+
257.2677

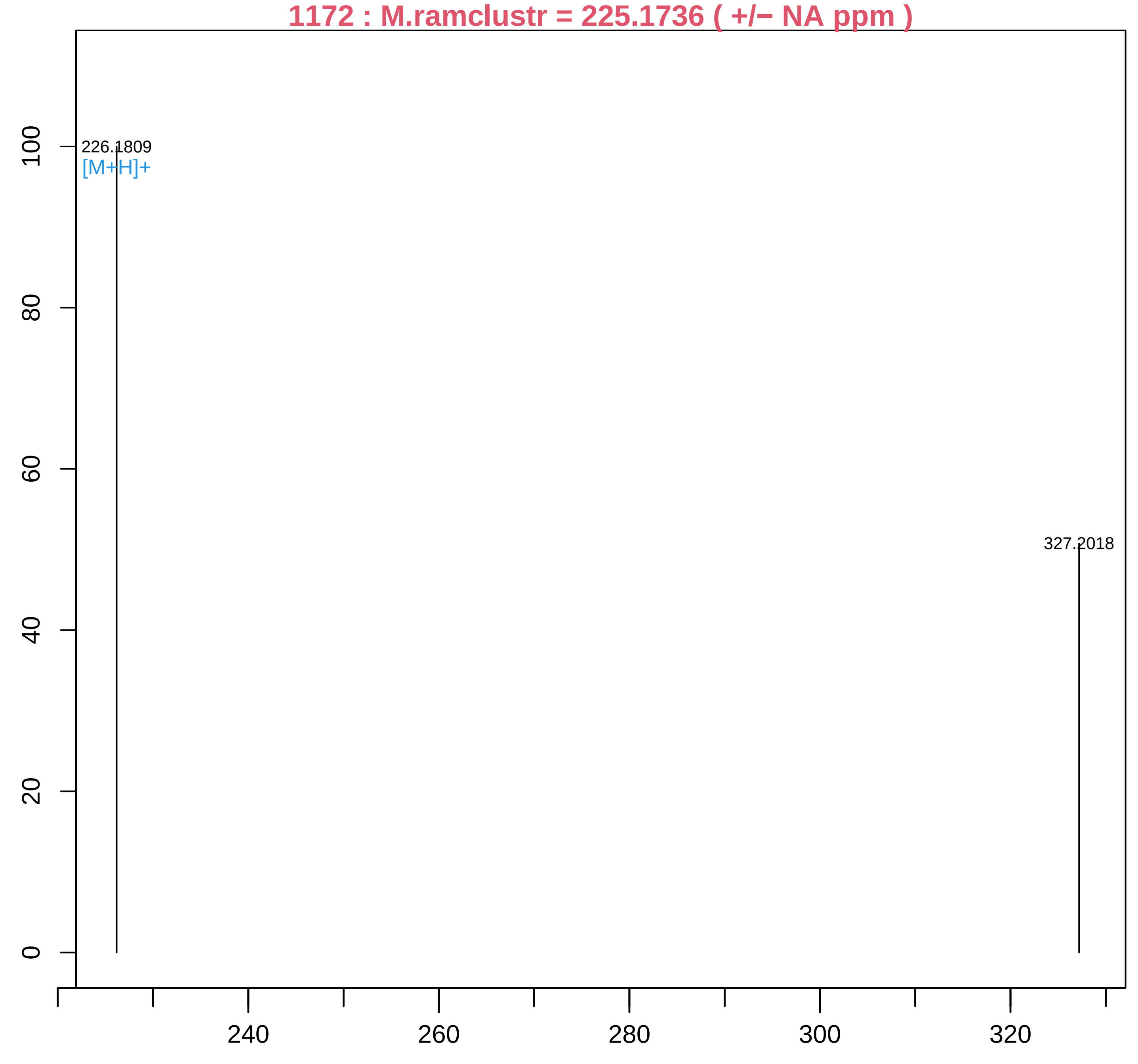

1172 : M.ramclustr = 225.1736 (+/− NA ppm)
226.1809
[M+H]+
327.2018
0
20
40
60
80
100
240
260
280
300
320

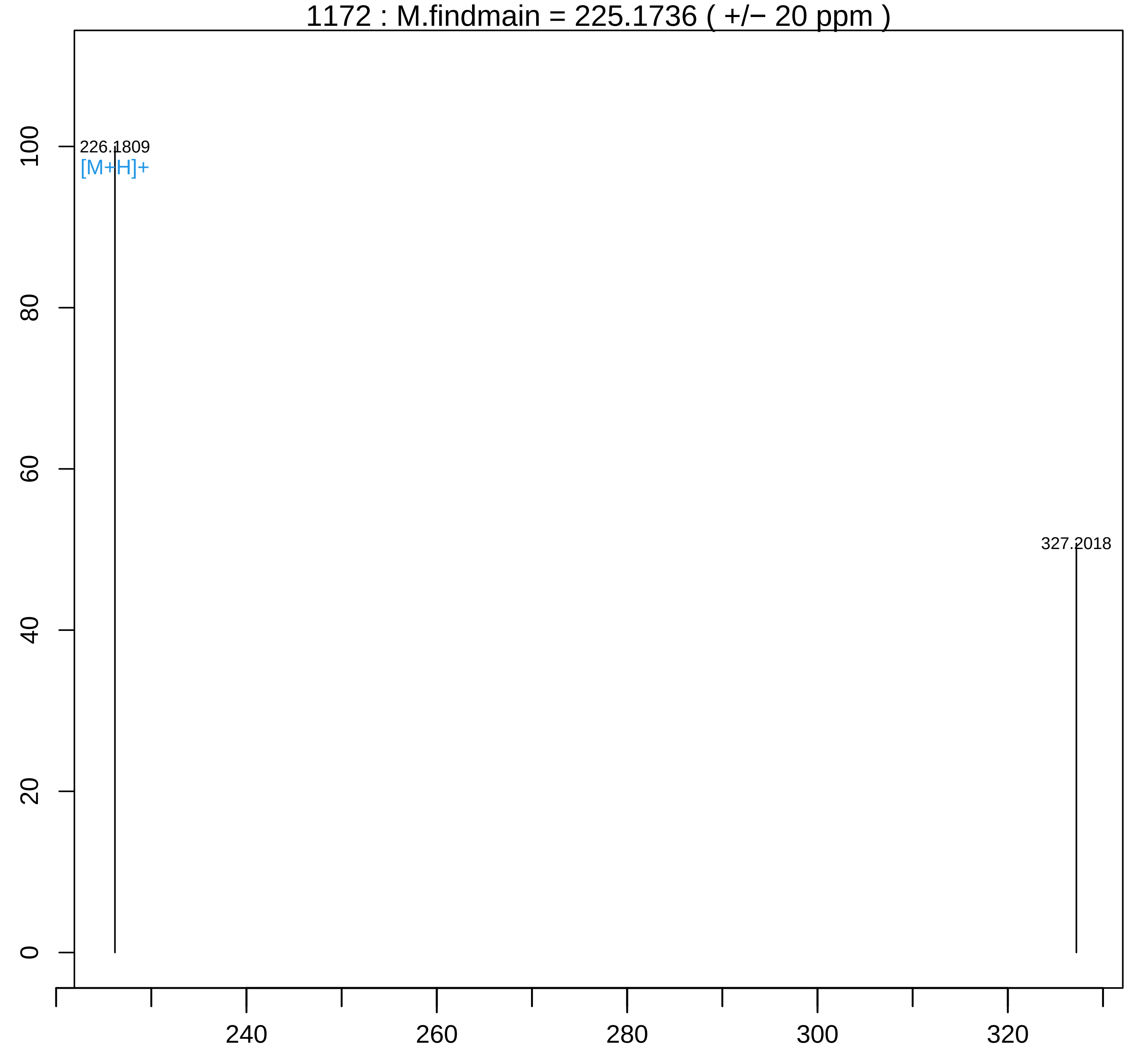

1172 : M.findmain = 225.1736 (+/− 20 ppm)
226.1809
[M+H]+
327.2018
0
20
40
60
80
100
240
260
280
300
320

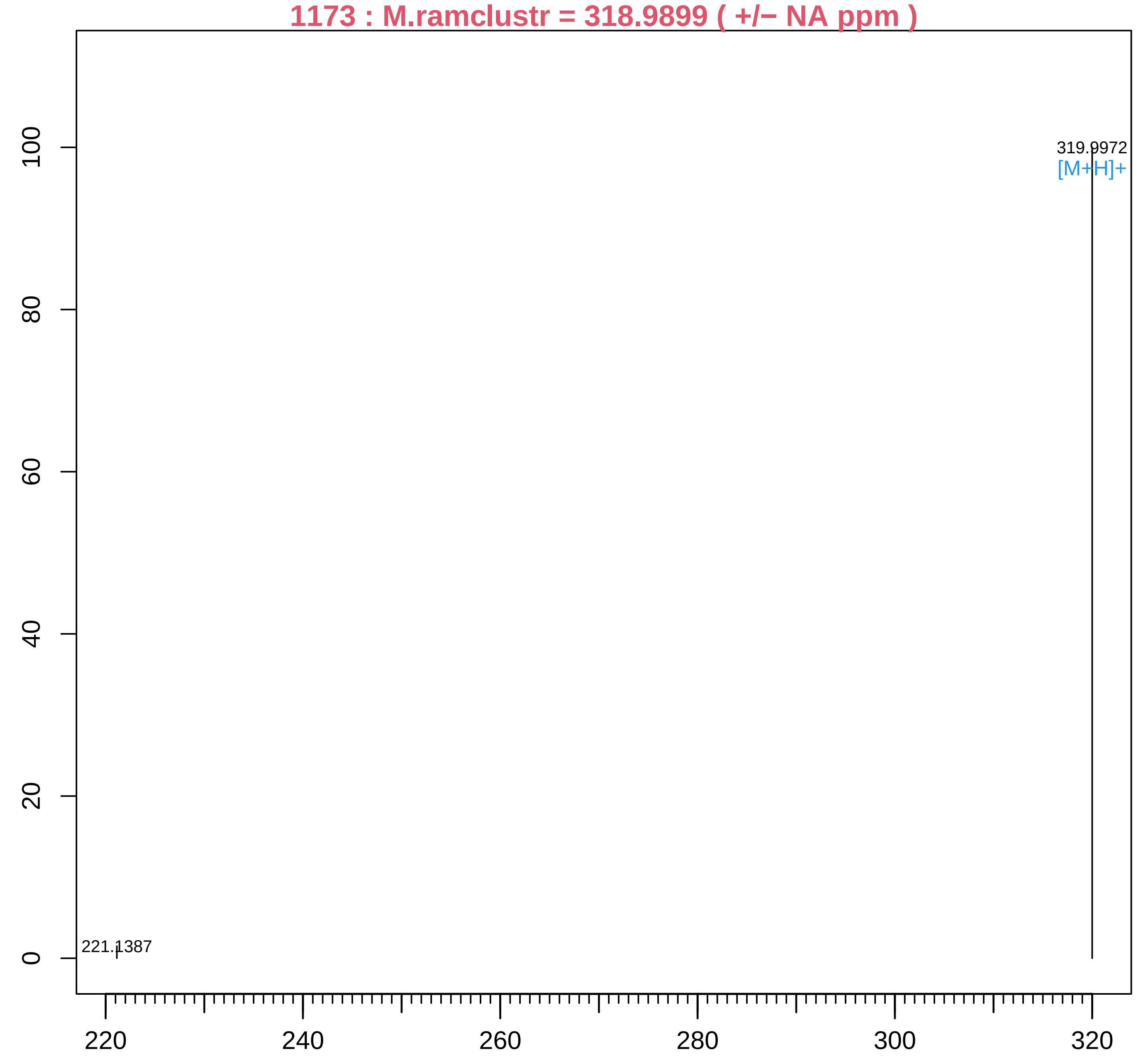
1173 : M.ramclustr = 318.9899 (+/− NA ppm)
319.9972
[M+H]+
221.1387
0
20
40
60
80
100
220
240
260
280
300
320

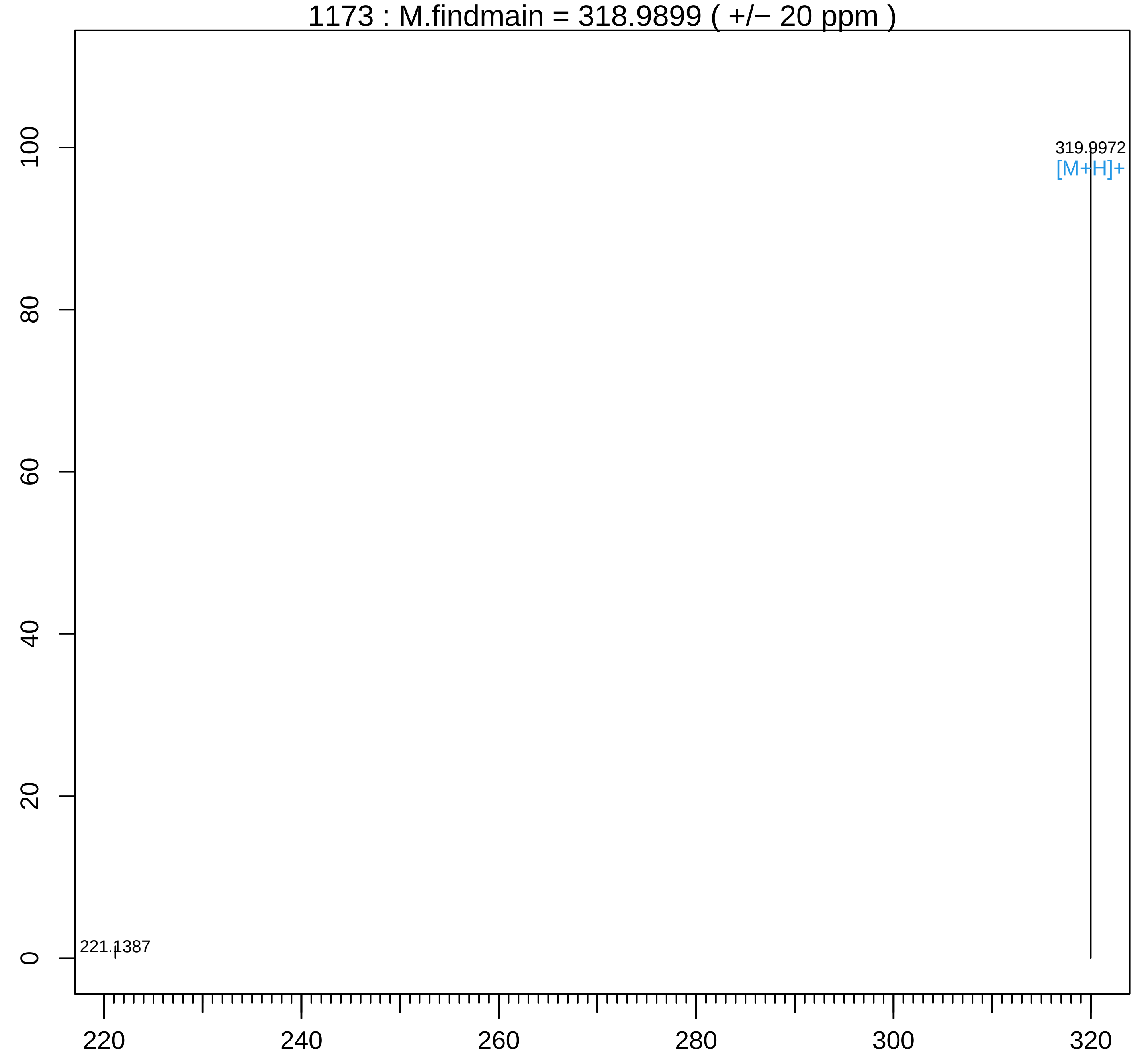
1173 : M.findmain = 318.9899 (+/− 20 ppm)
319.9972
[M+H]+
221.1387
0
20
40
60
80
100
220
240
260
280
300
320

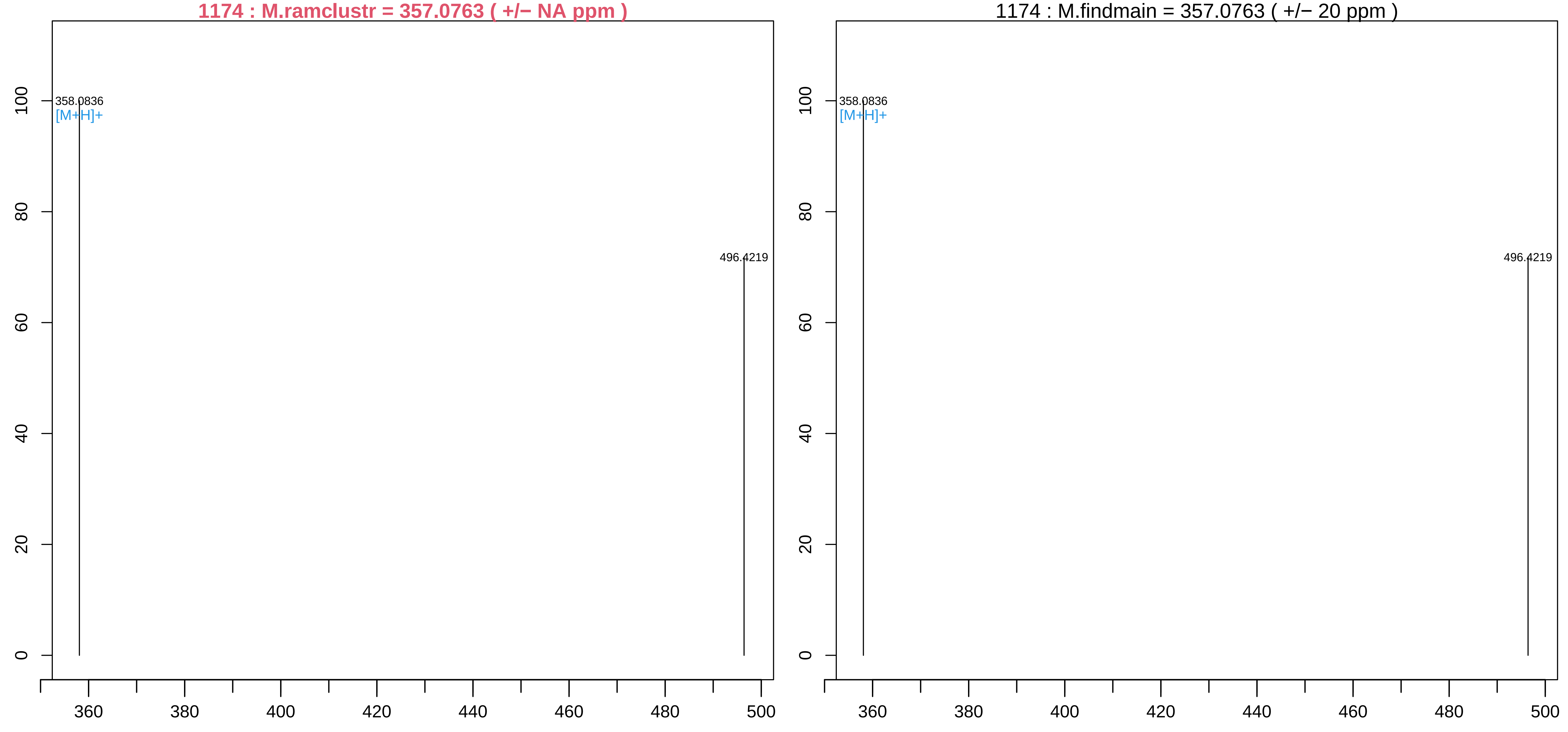

1174 : M.ramclustr = 357.0763 (+/− NA ppm)
358.0836
[M+H]+
496.4219
1174 : M.findmain = 357.0763 (+/− 20 ppm)
358.0836
[M+H]+
496.4219

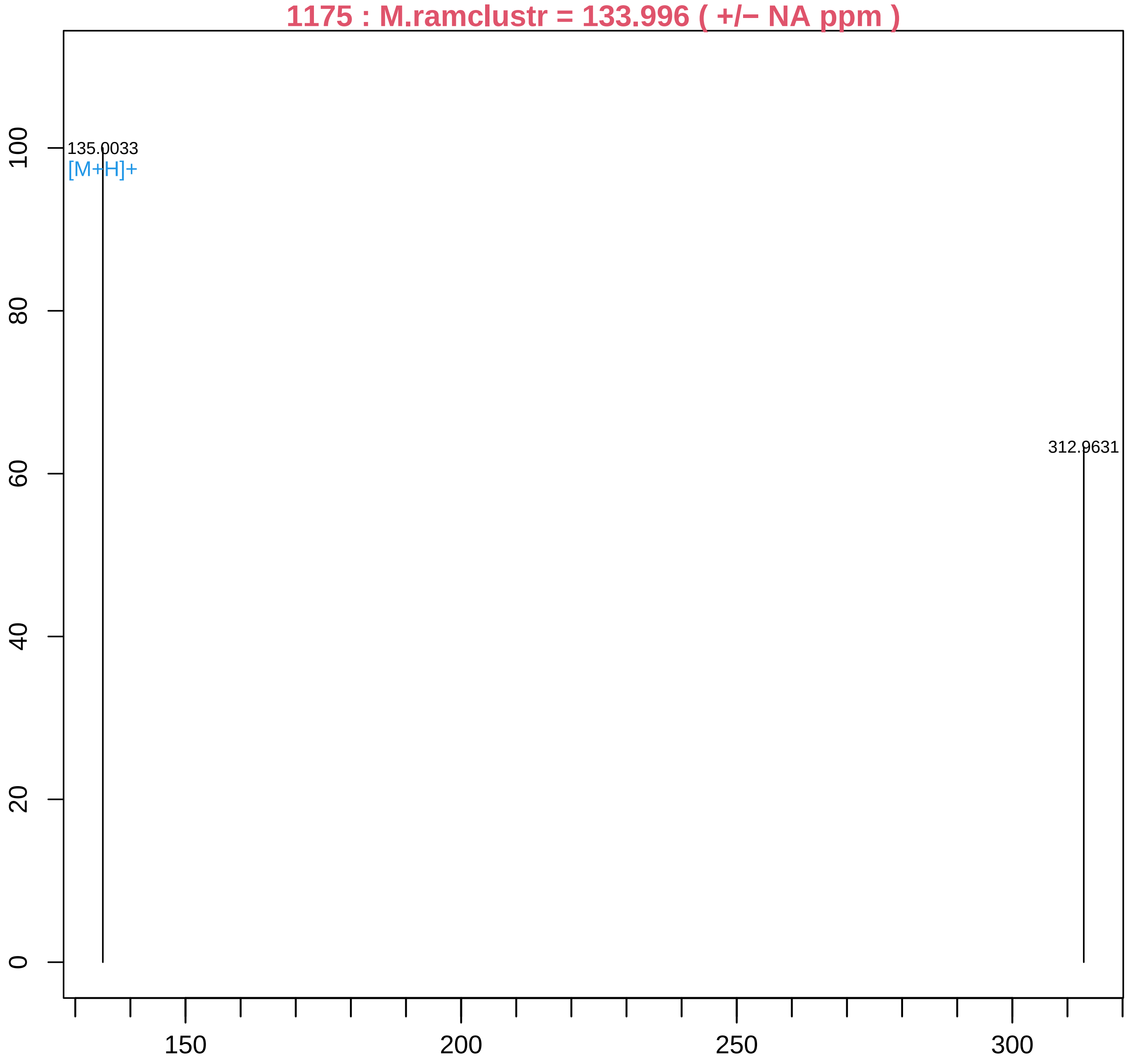
1175 : M.ramclustr = 133.996 (+/− NA ppm)
135.0033
[M+H]+
312.9631
0
20
40
60
80
100
150
200
250
300

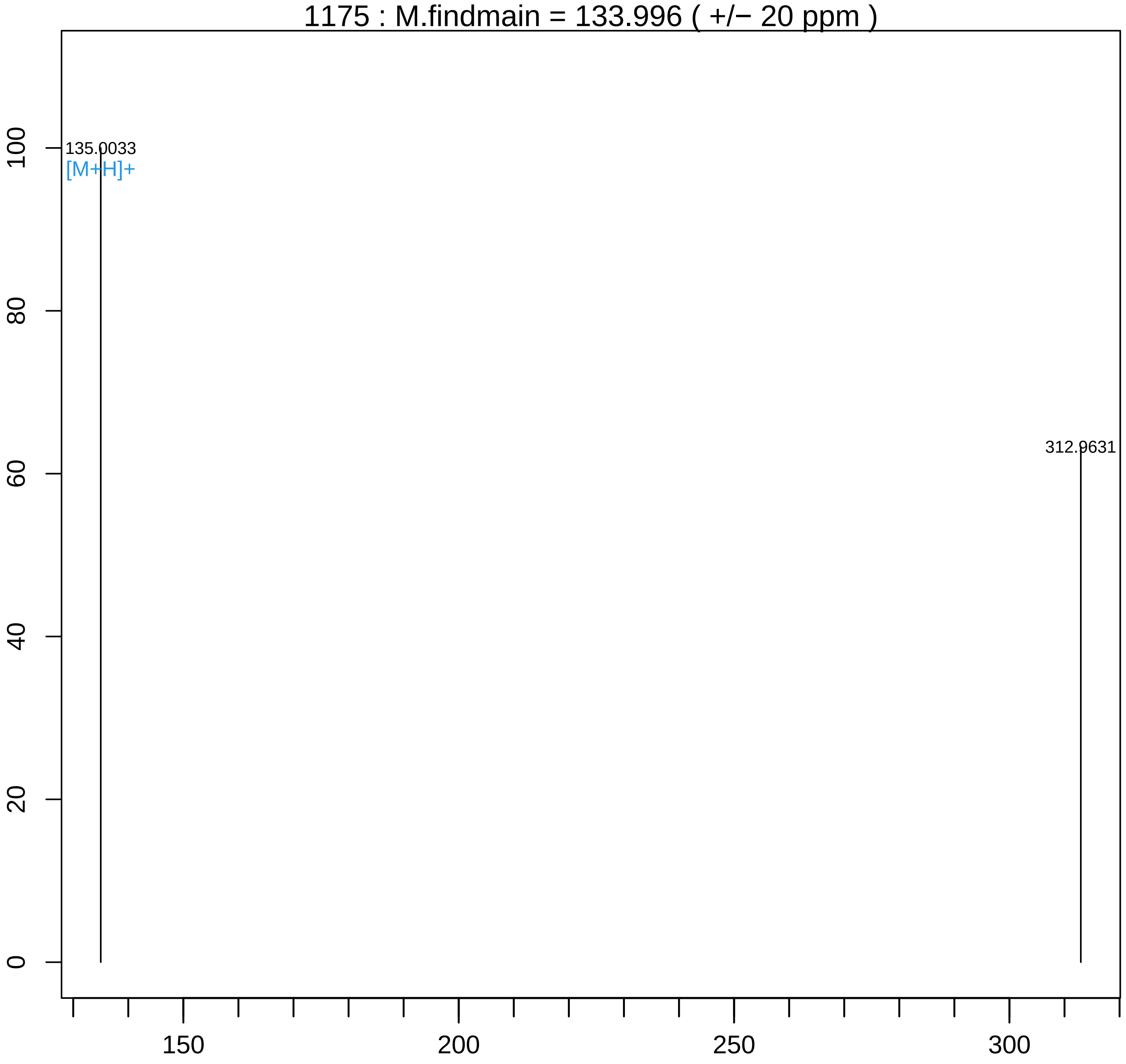
1175 : M.findmain = 133.996 (+/− 20 ppm)
135.0033
[M+H]+
312.9631
0
20
40
60
80
100
150
200
250
300

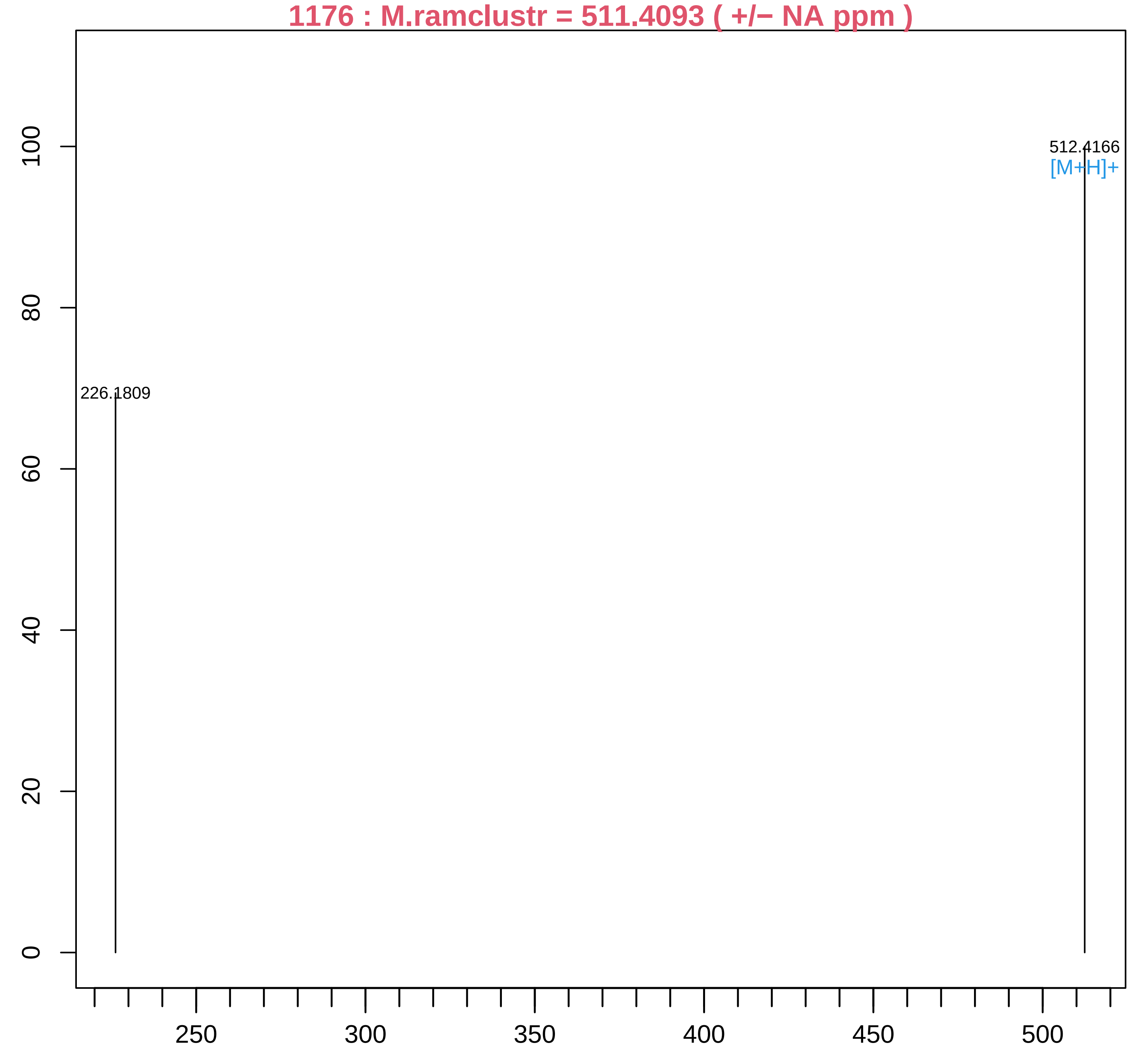
1176 : M.ramclustr = 511.4093 (+/− NA ppm)
512.4166
[M+H]+
226.1809

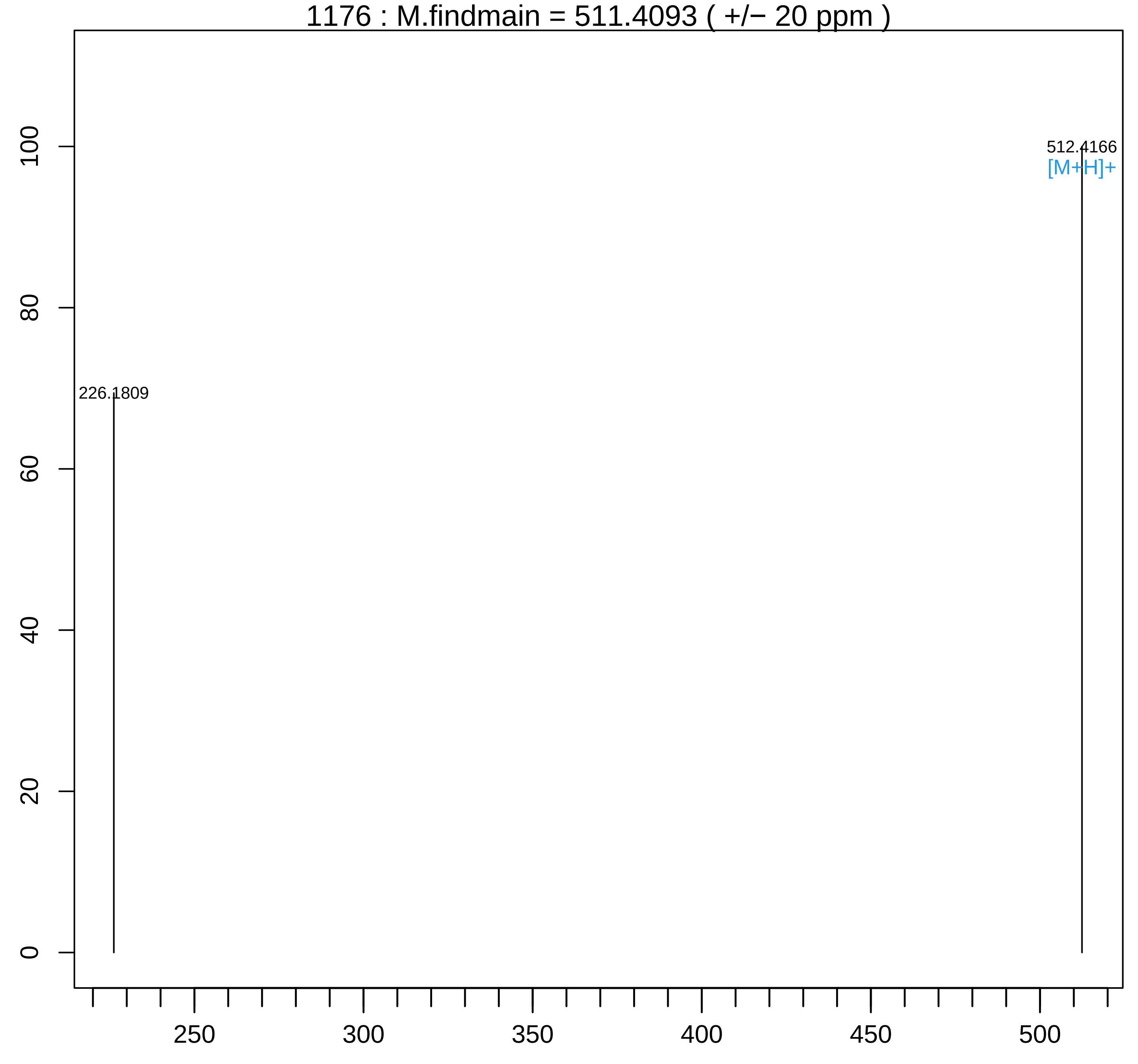
1176 : M.findmain = 511.4093 (+/− 20 ppm)
512.4166
[M+H]+
226.1809

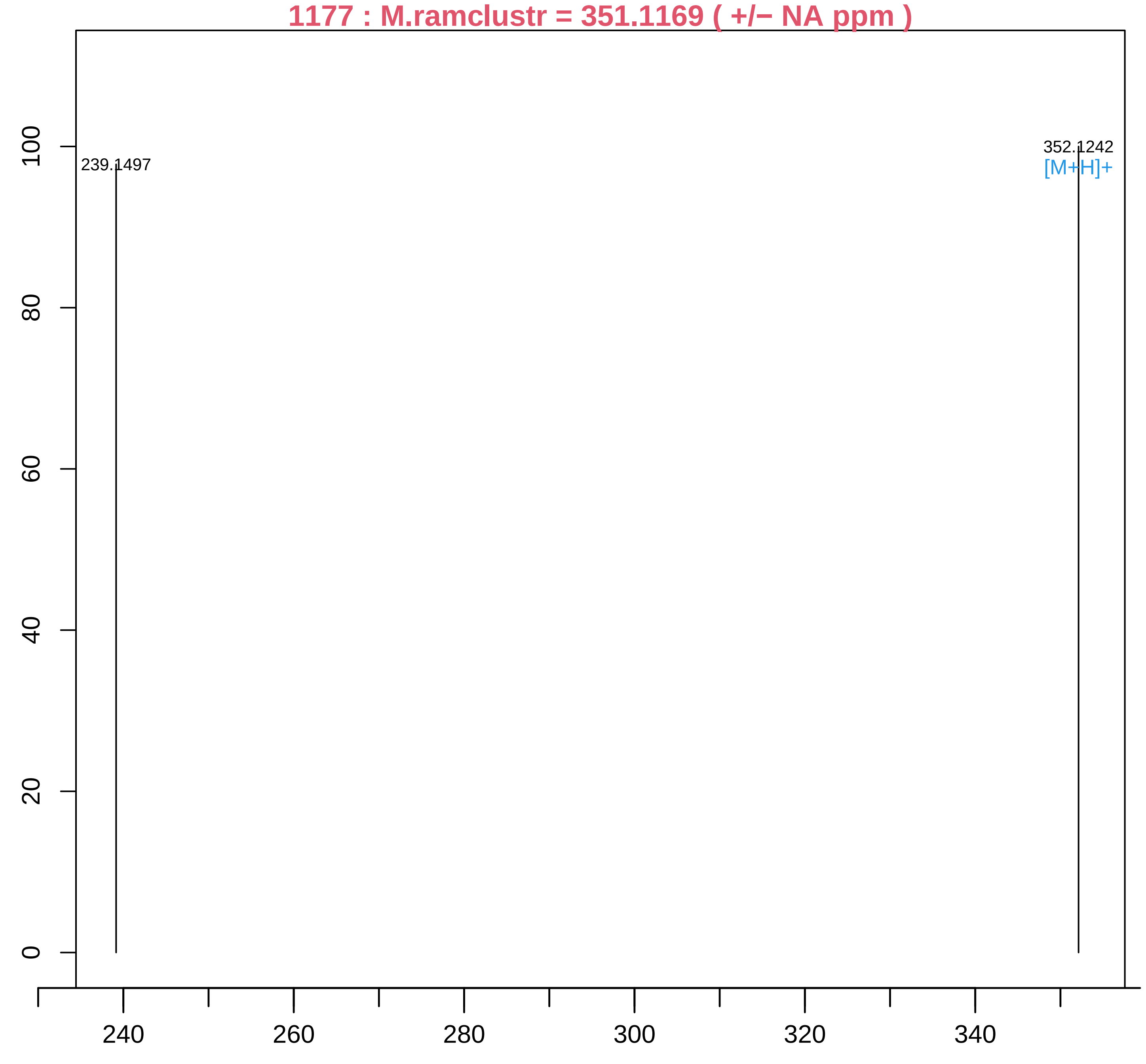

1177 : M.ramclustr = 351.1169 (+/− NA ppm)
239.1497
352.1242
[M+H]+
0
20
40
60
80
100
240
260
280
300
320
340

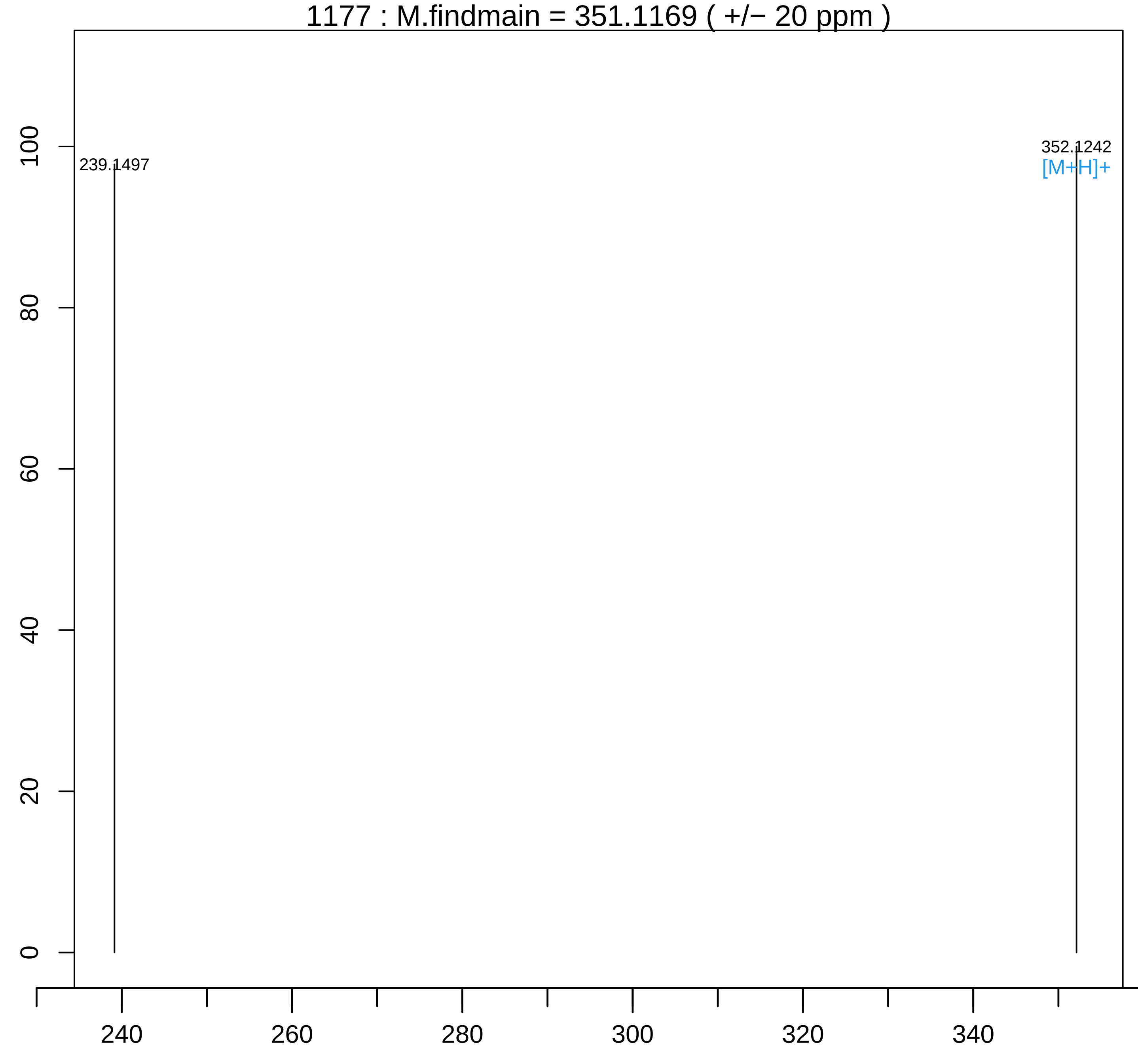

1177 : M.findmain = 351.1169 (+/− 20 ppm)
239.1497
352.1242
[M+H]+
0
20
40
60
80
100
240
260
280
300
320
340

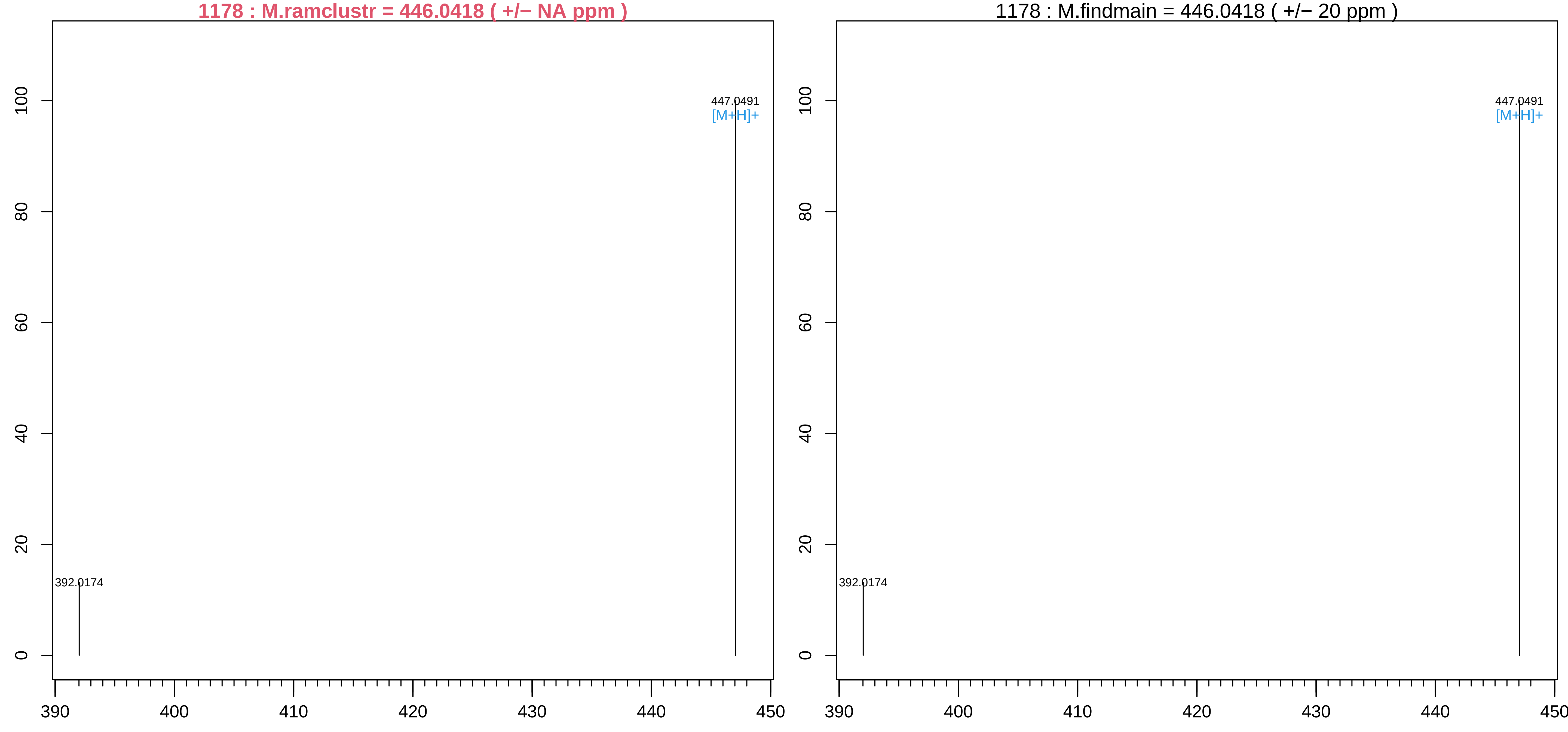
1178 : M.ramclustr = 446.0418 (+/− NA ppm)
447.0491
[M+H]+
392.0174
1178 : M.findmain = 446.0418 (+/− 20 ppm)
447.0491
[M+H]+
392.0174
0
20
40
60
80
100
390
400
410
420
430
440
450

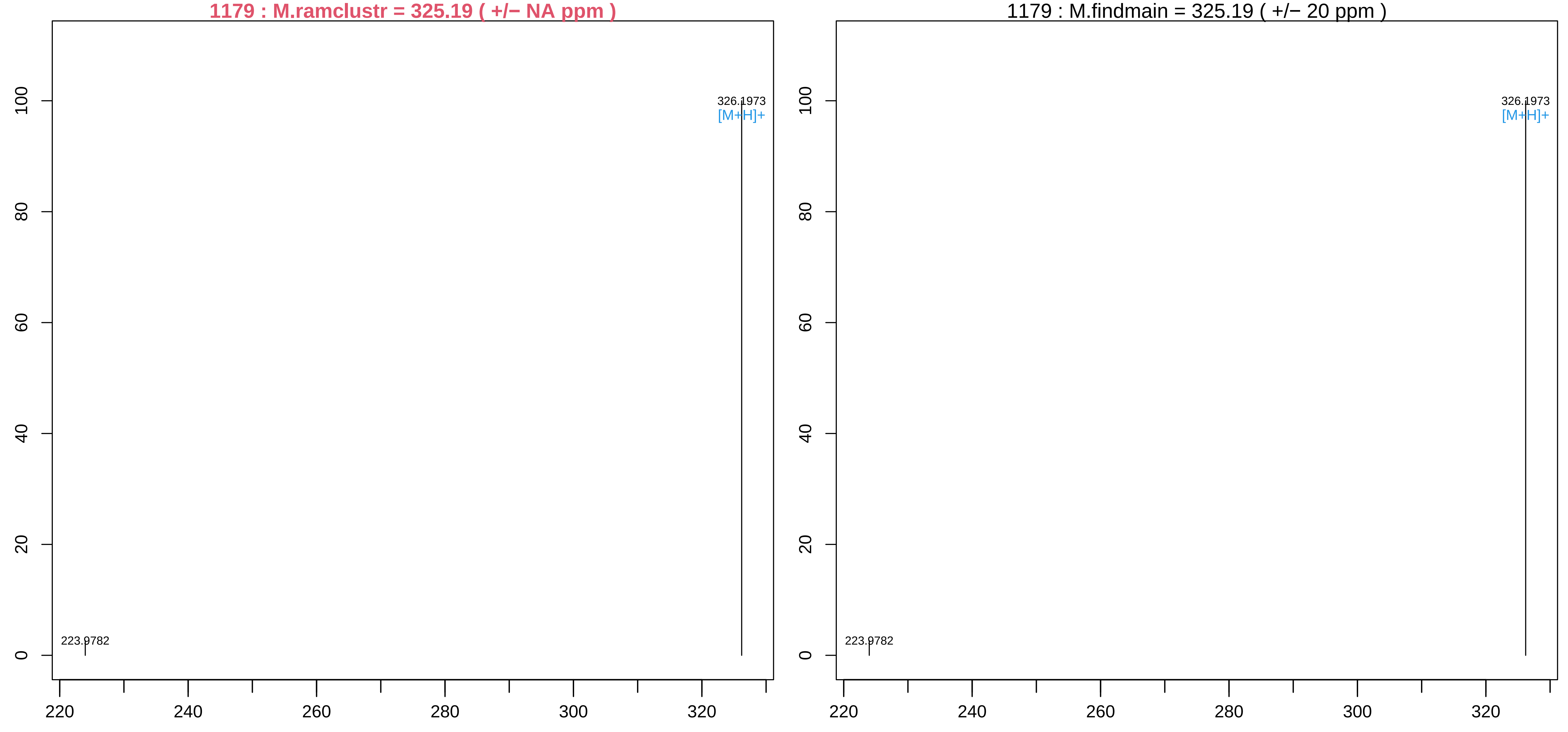

1179 : M.ramclustr = 325.19 (+/− NA ppm)
1179 : M.findmain = 325.19 (+/− 20 ppm)
326.1973
[M+H]+
223.9782
326.1973
[M+H]+
223.9782
0
20
40
60
80
100
220
240
260
280
300
320

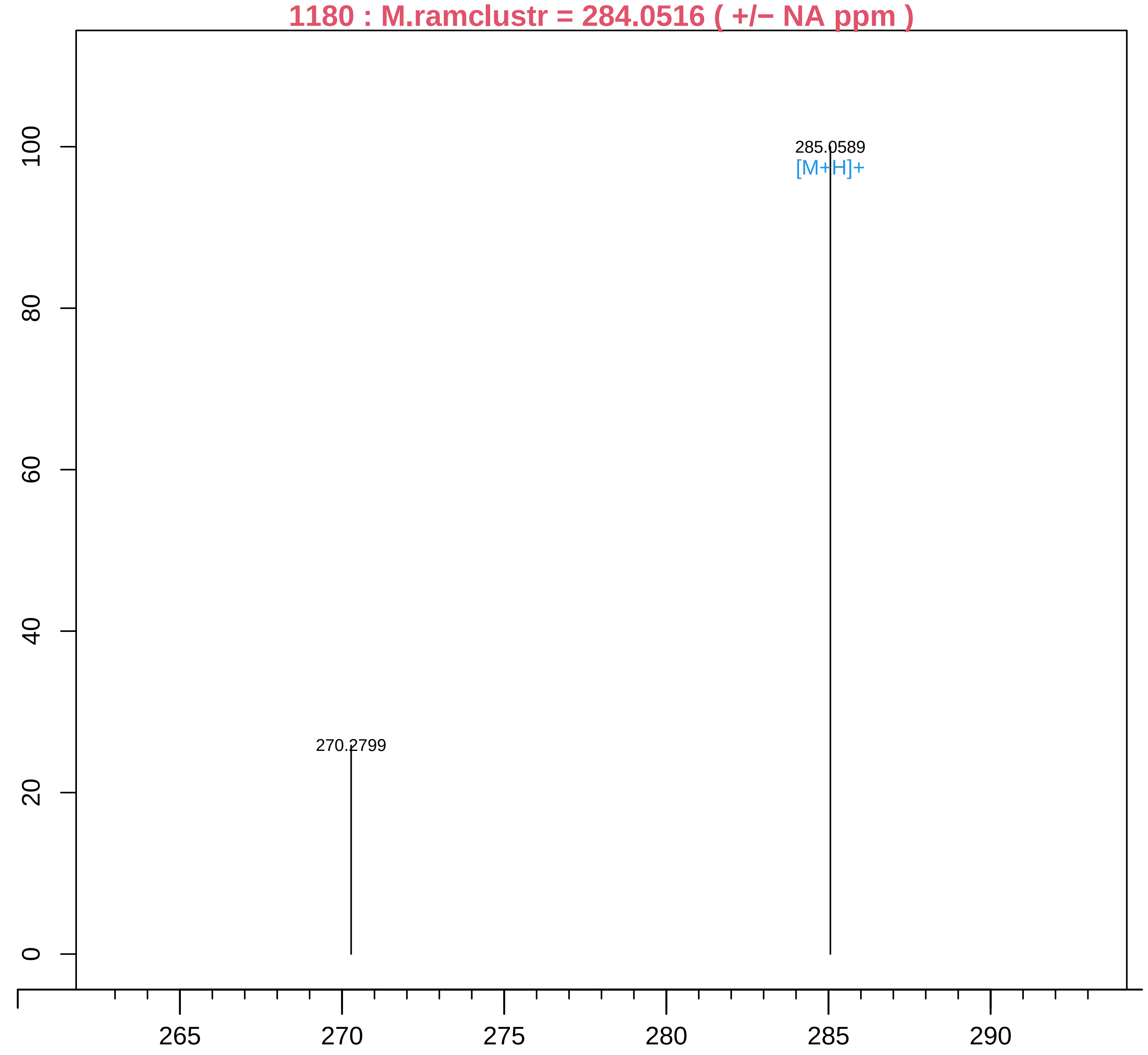
1180 : M.ramclustr = 284.0516 (+/− NA ppm)
285.0589
[M+H]+
270.2799
0
20
40
60
80
100
265
270
275
280
285
290

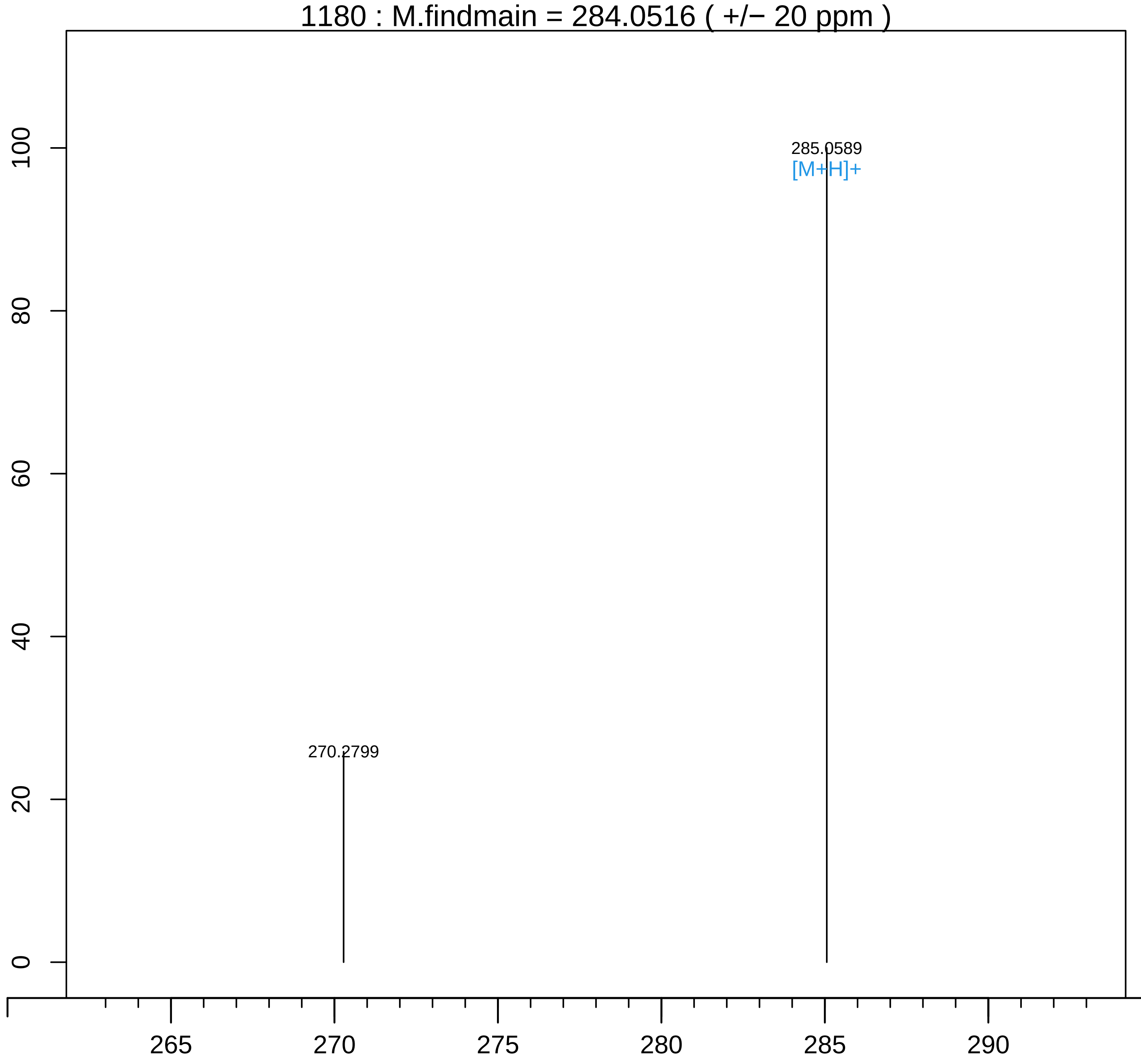
1180 : M.findmain = 284.0516 (+/− 20 ppm)
285.0589
[M+H]+
270.2799
0
20
40
60
80
100
265
270
275
280
285
290

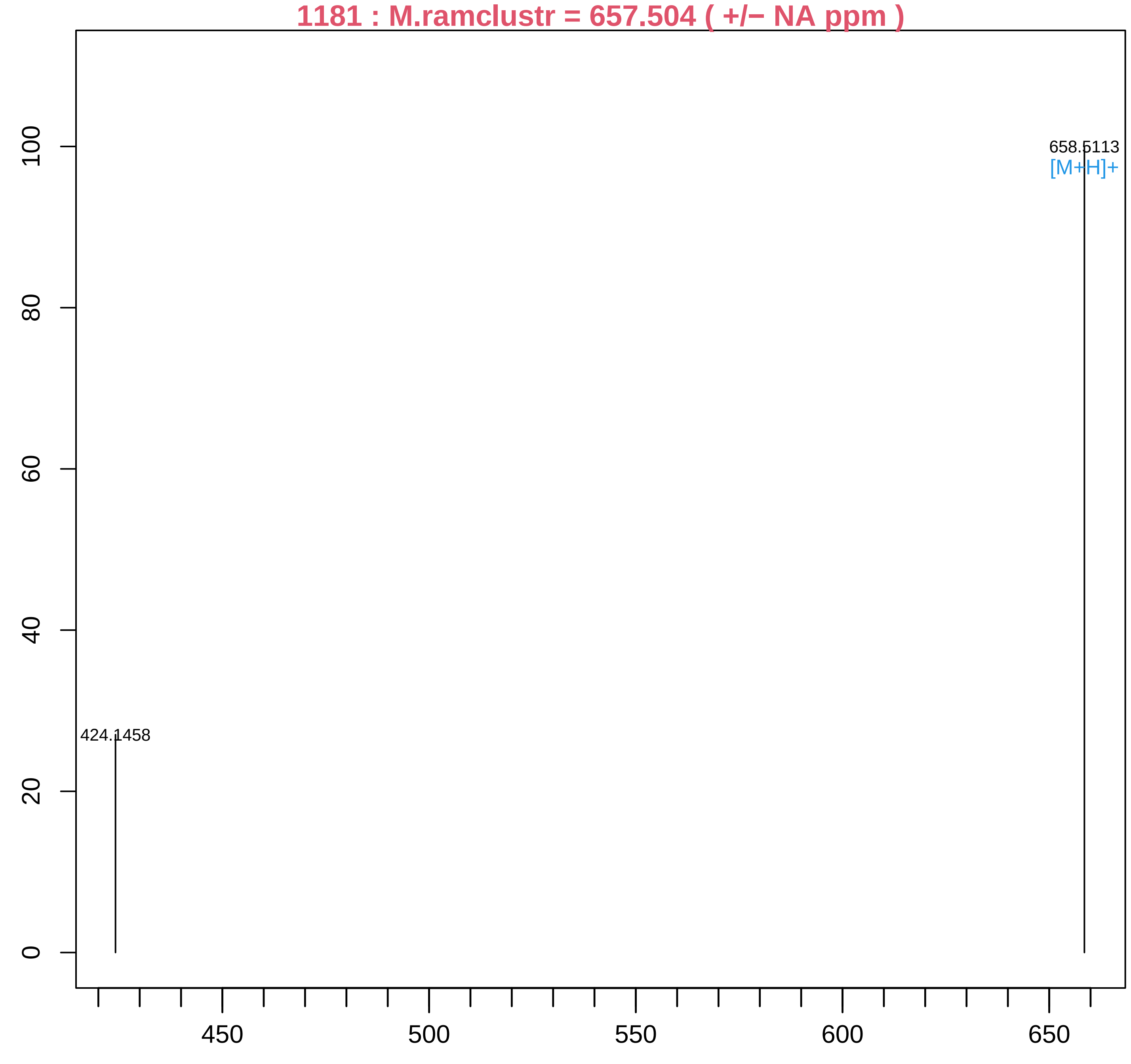
1181 : M.ramclustr = 657.504 (+/− NA ppm)
658.5113
[M+H]+
424.1458
0
20
40
60
80
100
450
500
550
600
650

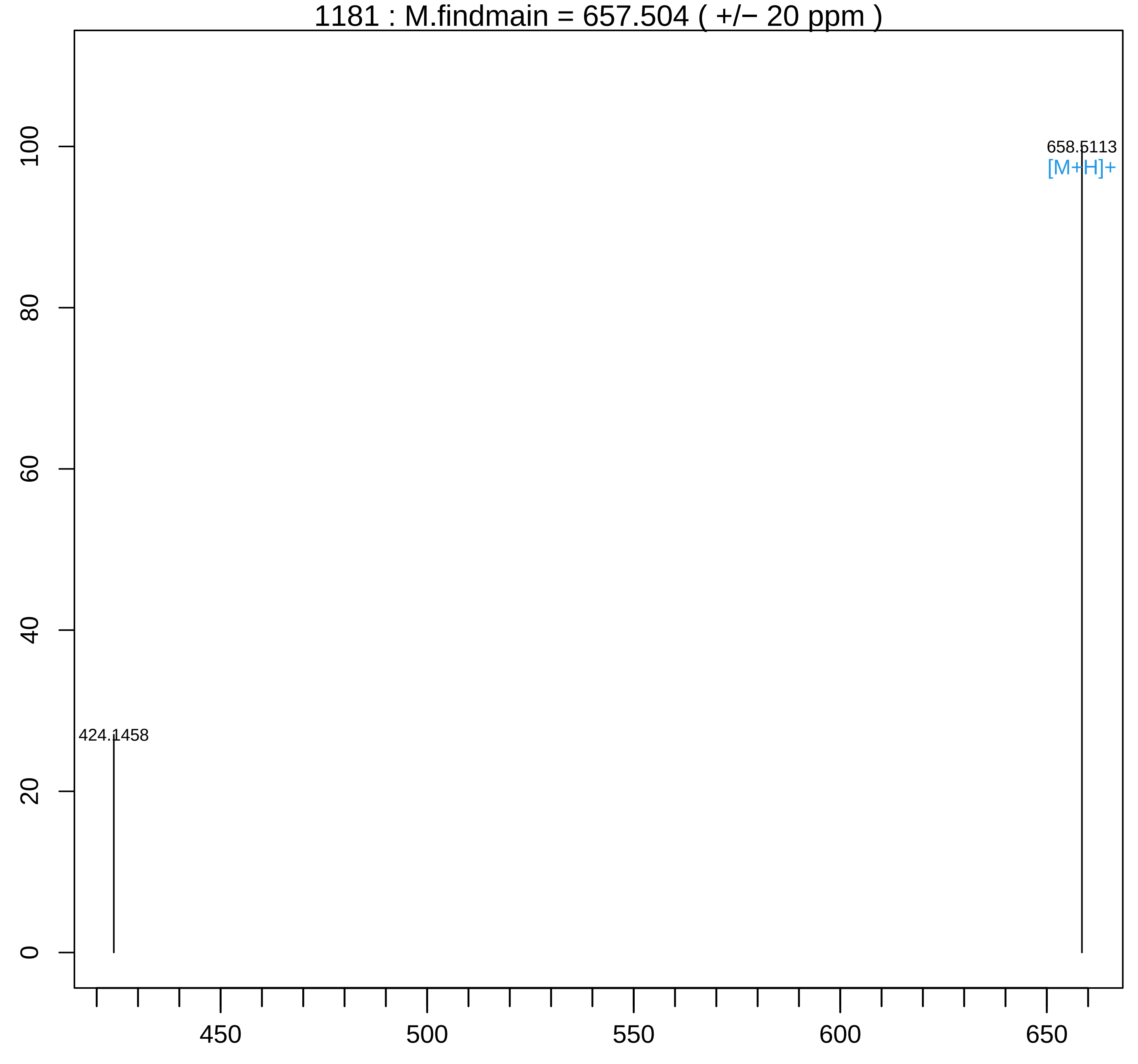
1181 : M.findmain = 657.504 (+/− 20 ppm)
658.5113
[M+H]+
424.1458
0
20
40
60
80
100
450
500
550
600
650

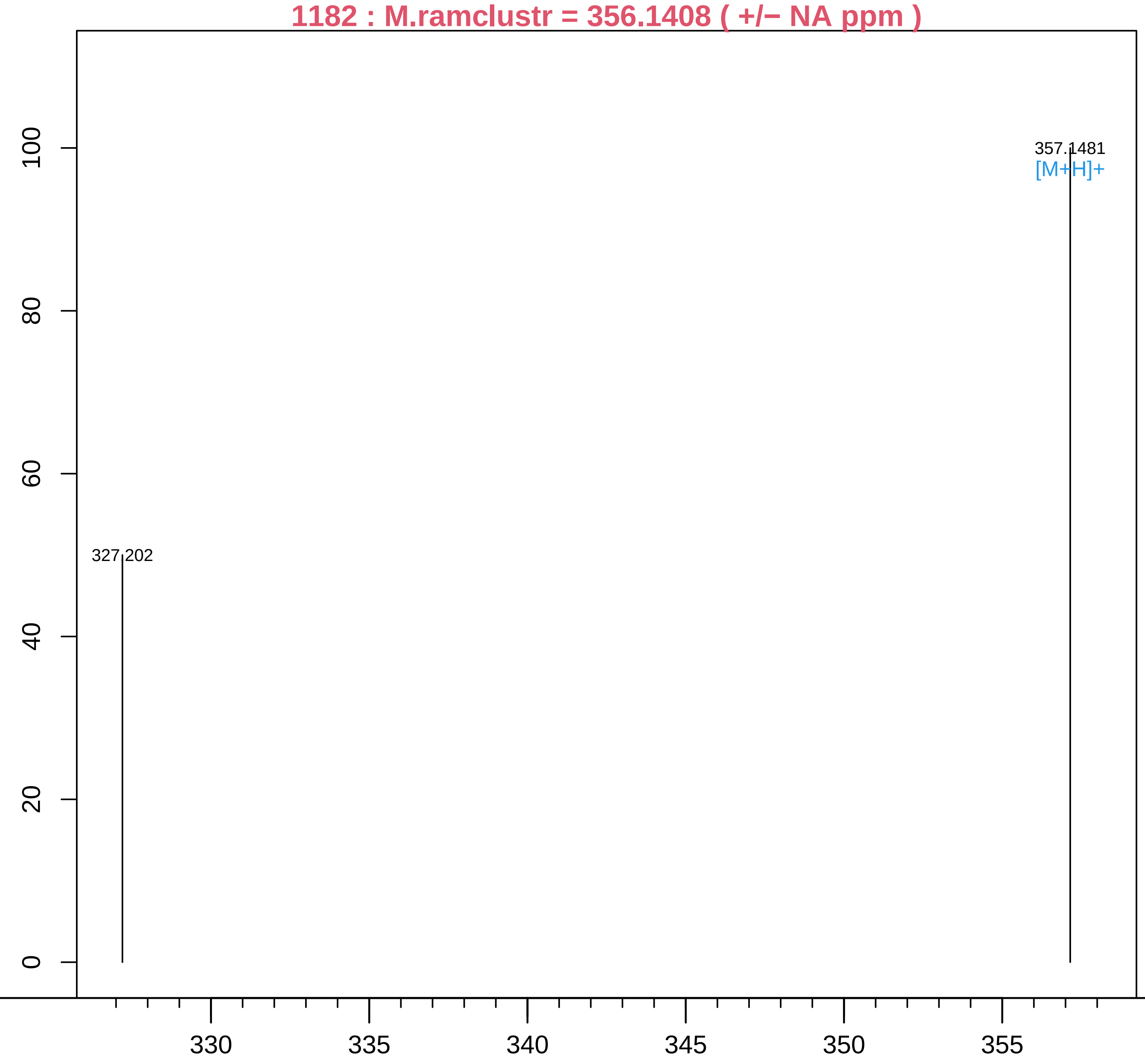
1182 : M.ramclustr = 356.1408 (+/− NA ppm)
357.1481
[M+H]+
327.202
0
20
40
60
80
100
330
335
340
345
350
355

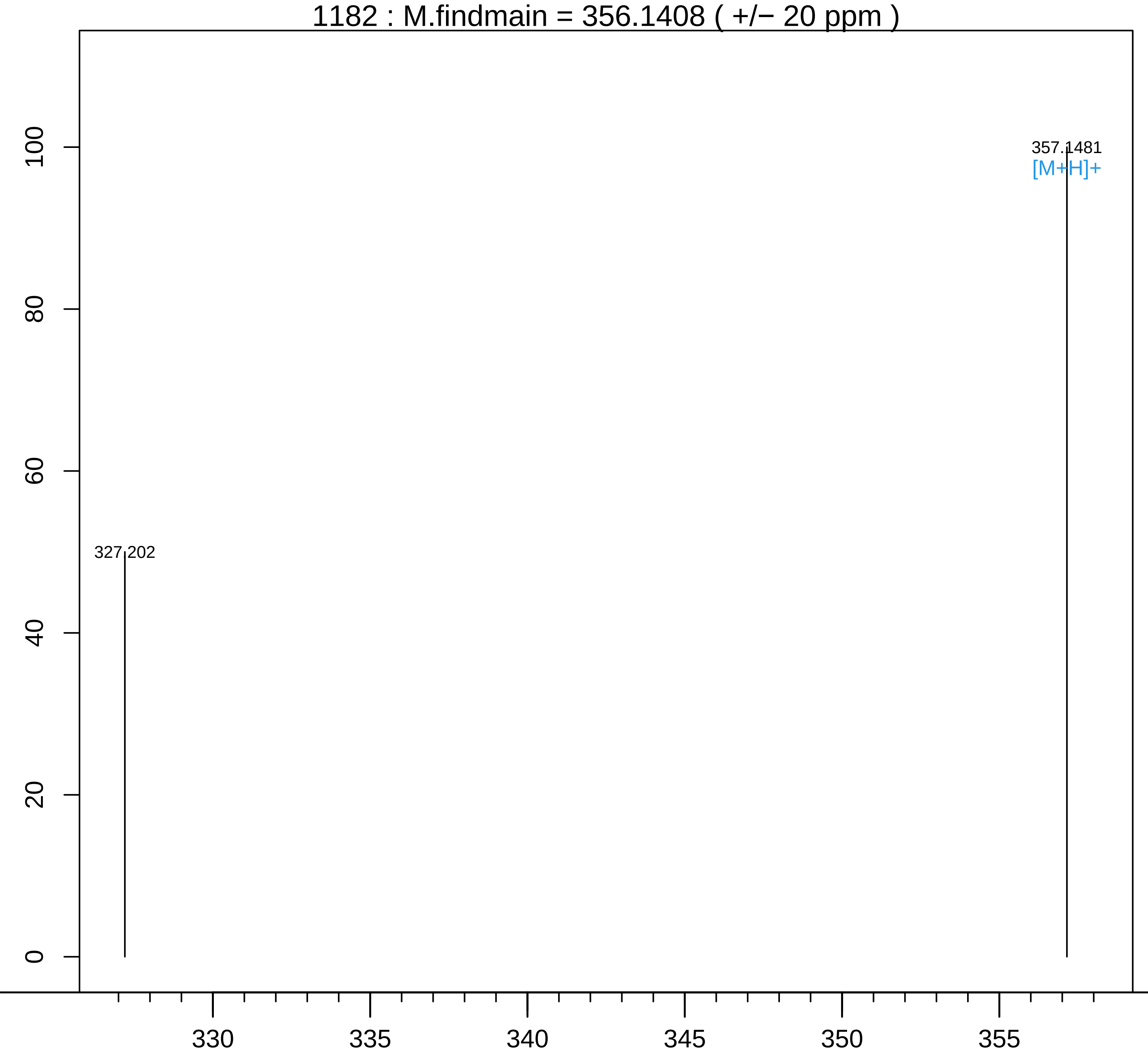
1182 : M.findmain = 356.1408 (+/− 20 ppm)
357.1481
[M+H]+
327.202
0
20
40
60
80
100
330
335
340
345
350
355

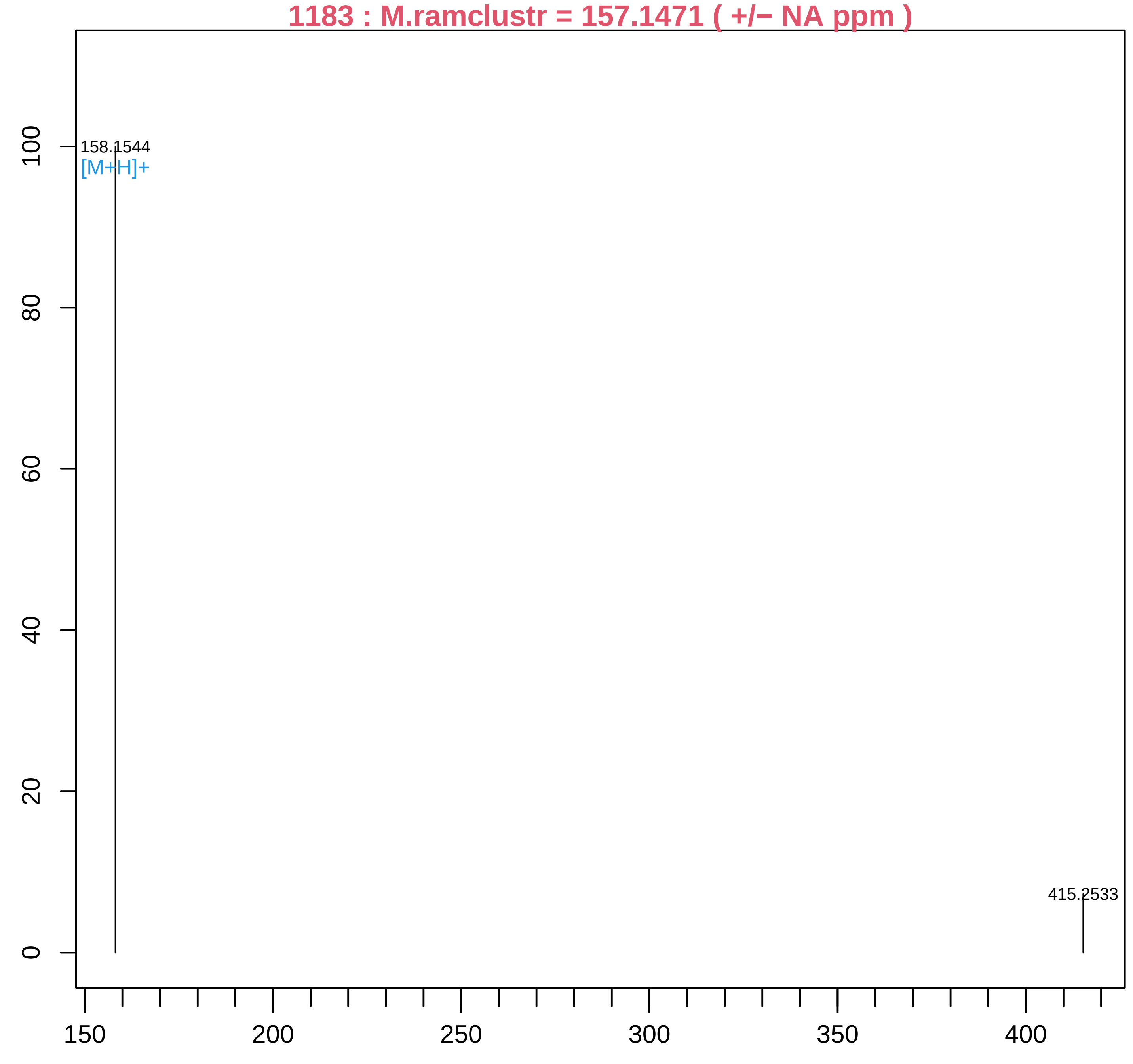

1183 : M.ramclustr = 157.1471 (+/− NA ppm)
158.1544
[M+H]+
415.2533
0
20
40
60
80
100
150
200
250
300
350
400

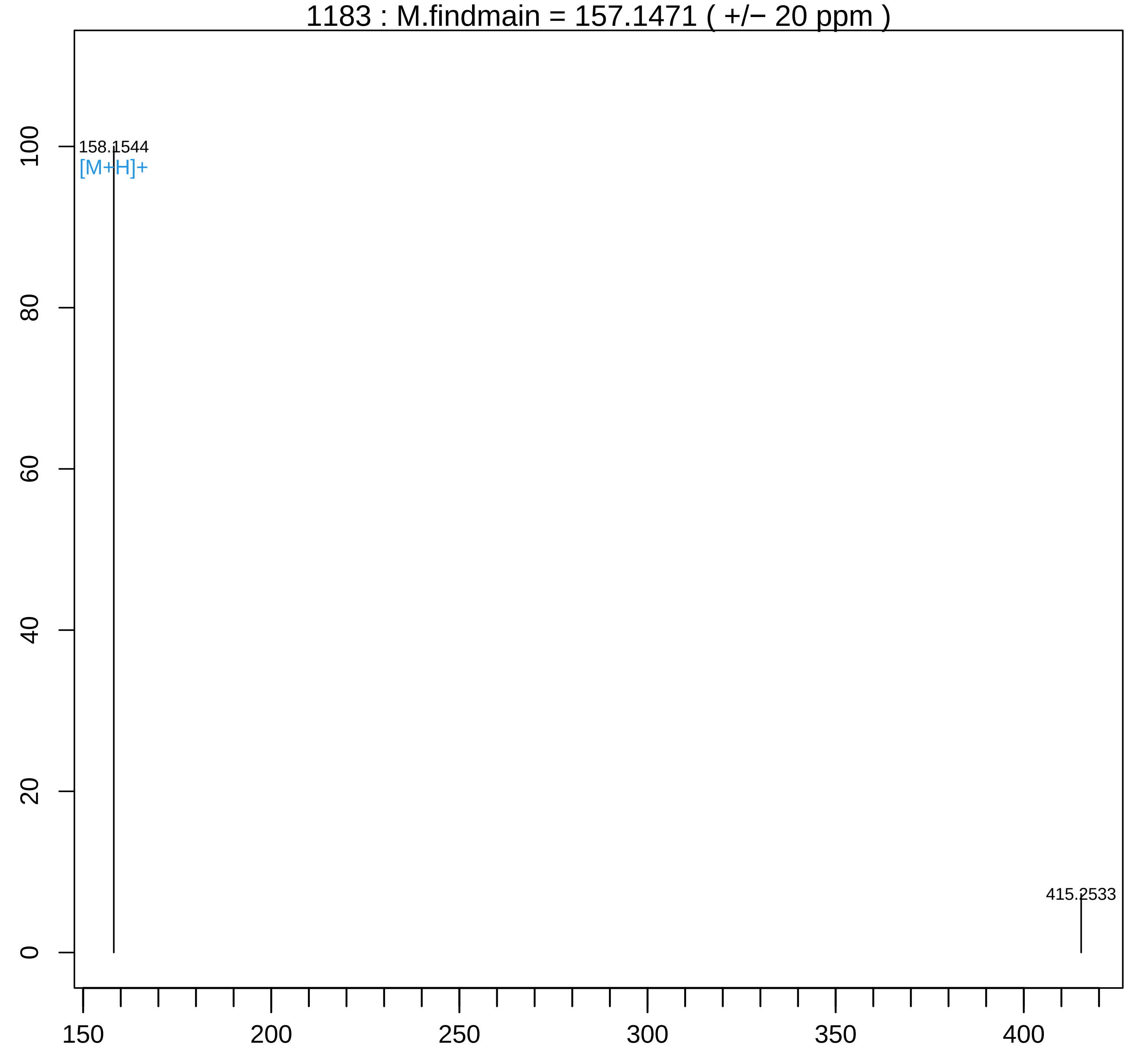

1183 : M.findmain = 157.1471 (+/− 20 ppm)
158.1544
[M+H]+
415.2533
0
20
40
60
80
100
150
200
250
300
350
400

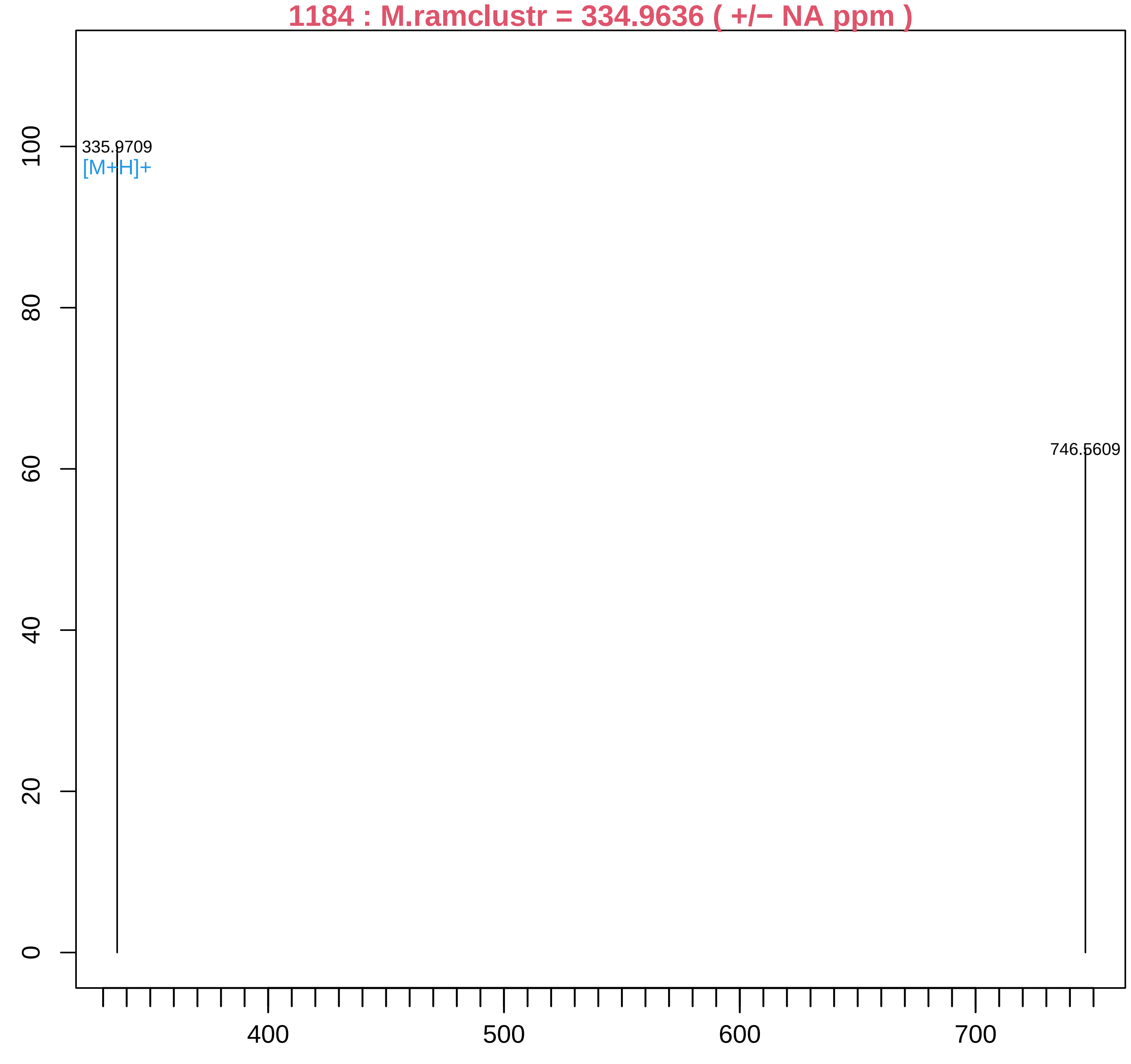

1184 : M.ramclustr = 334.9636 (+/− NA ppm)
335.9709
[M+H]+
746.5609
0
20
40
60
80
100
400
500
600
700

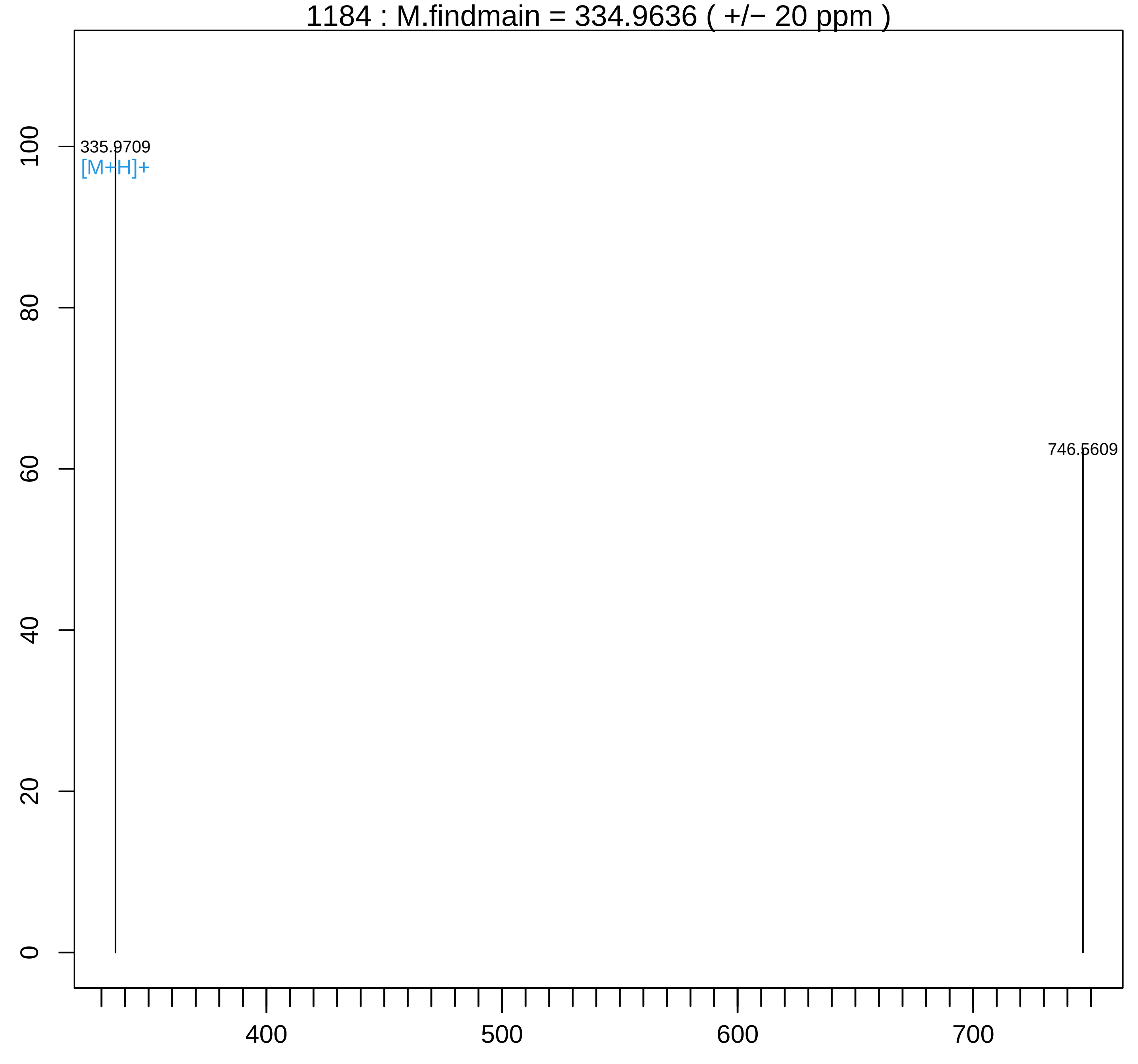

1184 : M.findmain = 334.9636 (+/− 20 ppm)
335.9709
[M+H]+
746.5609
0
20
40
60
80
100
400
500
600
700

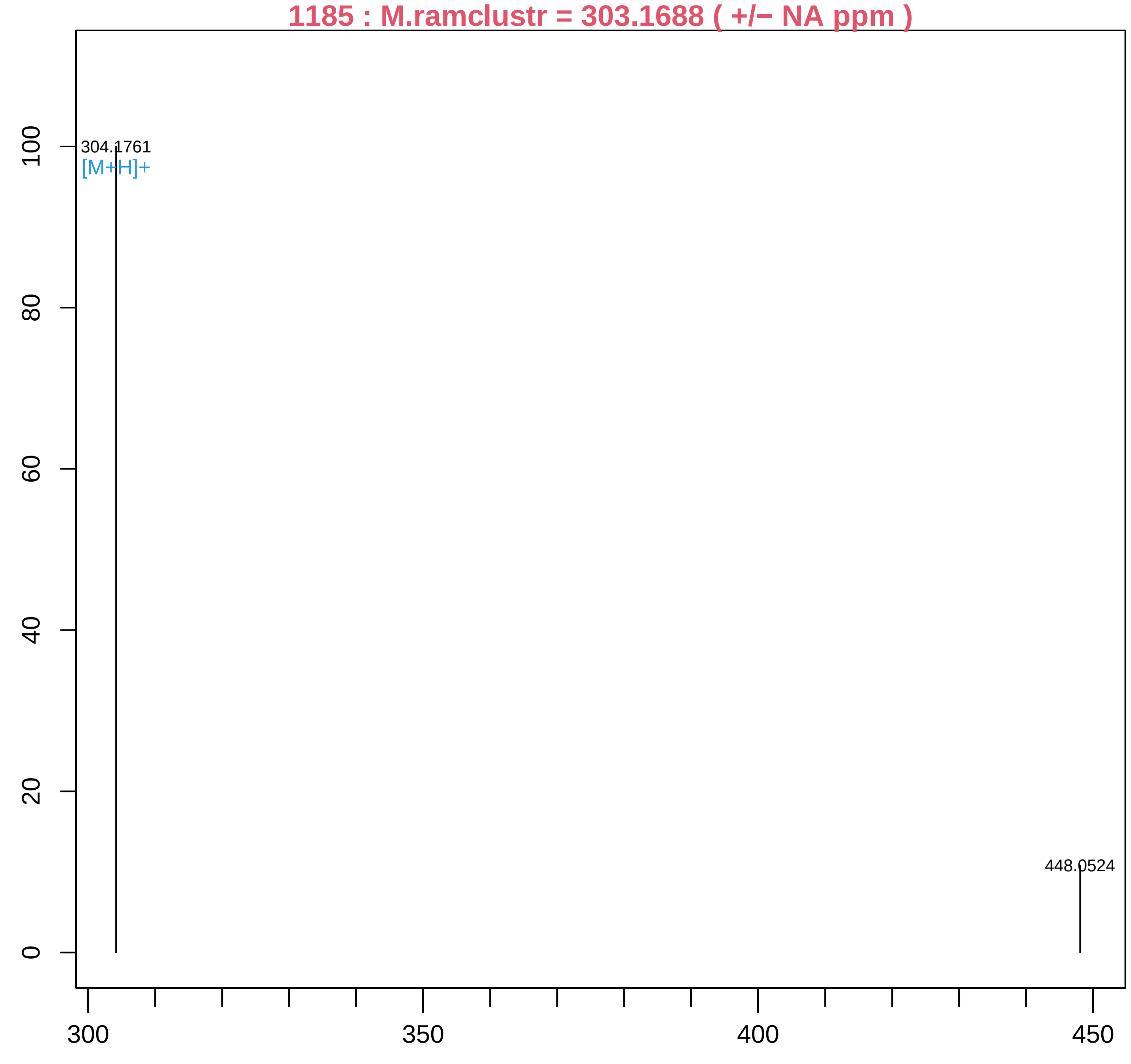
1185 : M.ramclustr = 303.1688 (+/− NA ppm)
304.1761
[M+H]+
448.0524
0
20
40
60
80
100
300
350
400
450

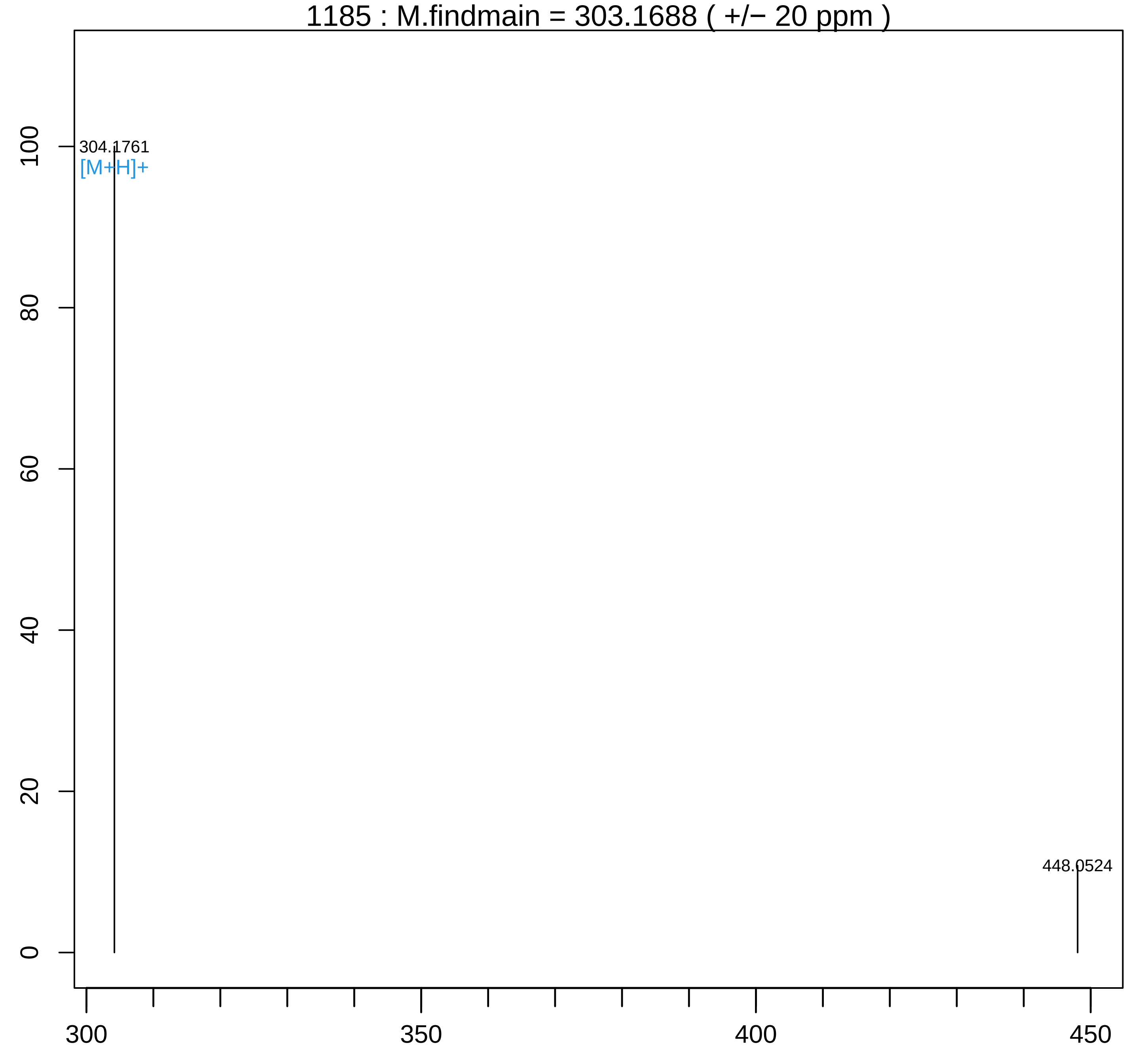
1185 : M.findmain = 303.1688 (+/− 20 ppm)
304.1761
[M+H]+
448.0524
0
20
40
60
80
100
300
350
400
450

1186 : M.ramclustr = 356.0725 (+/− NA ppm)

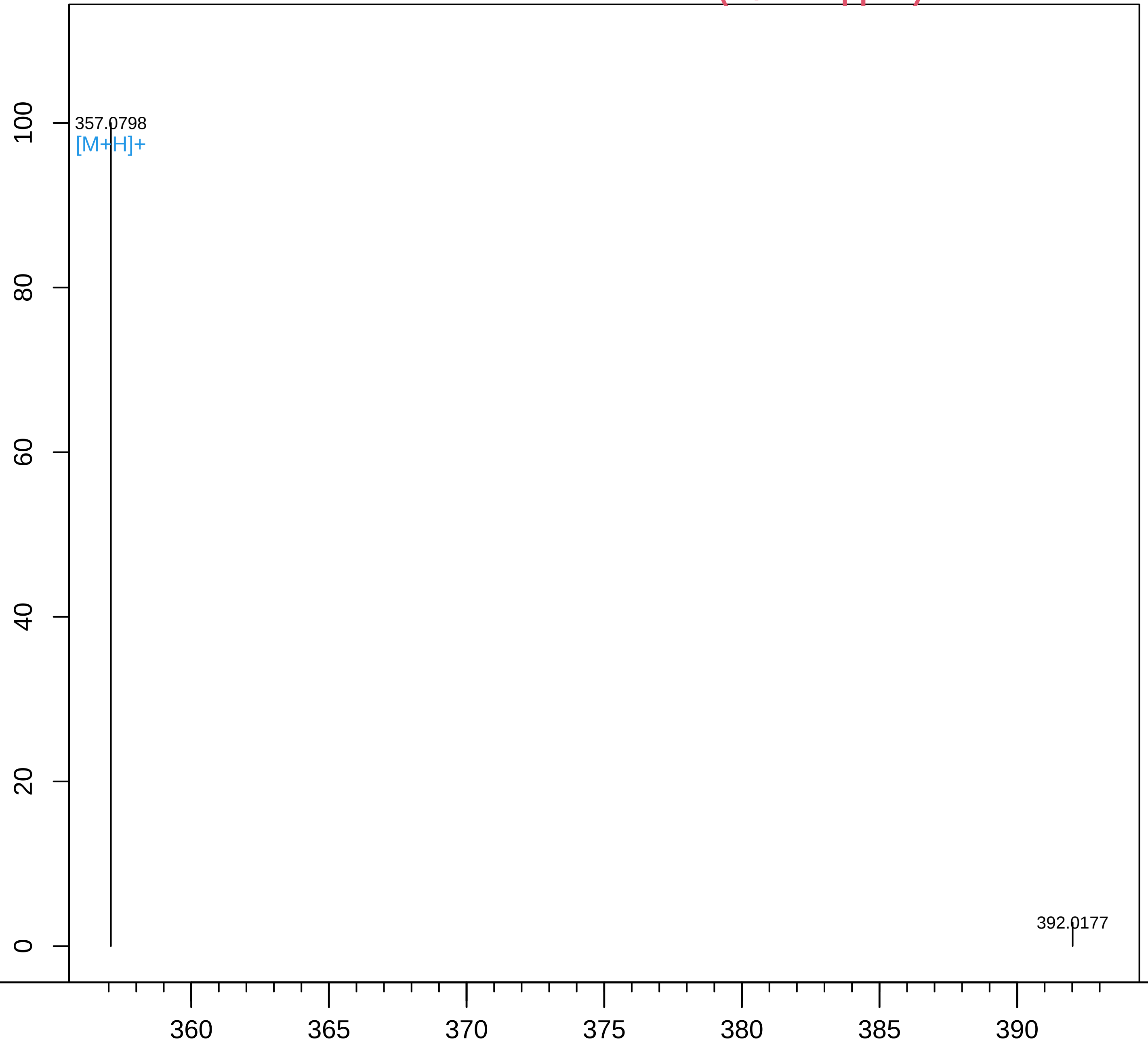

1186 : M.findmain = 356.0725 (+/− 20 ppm)

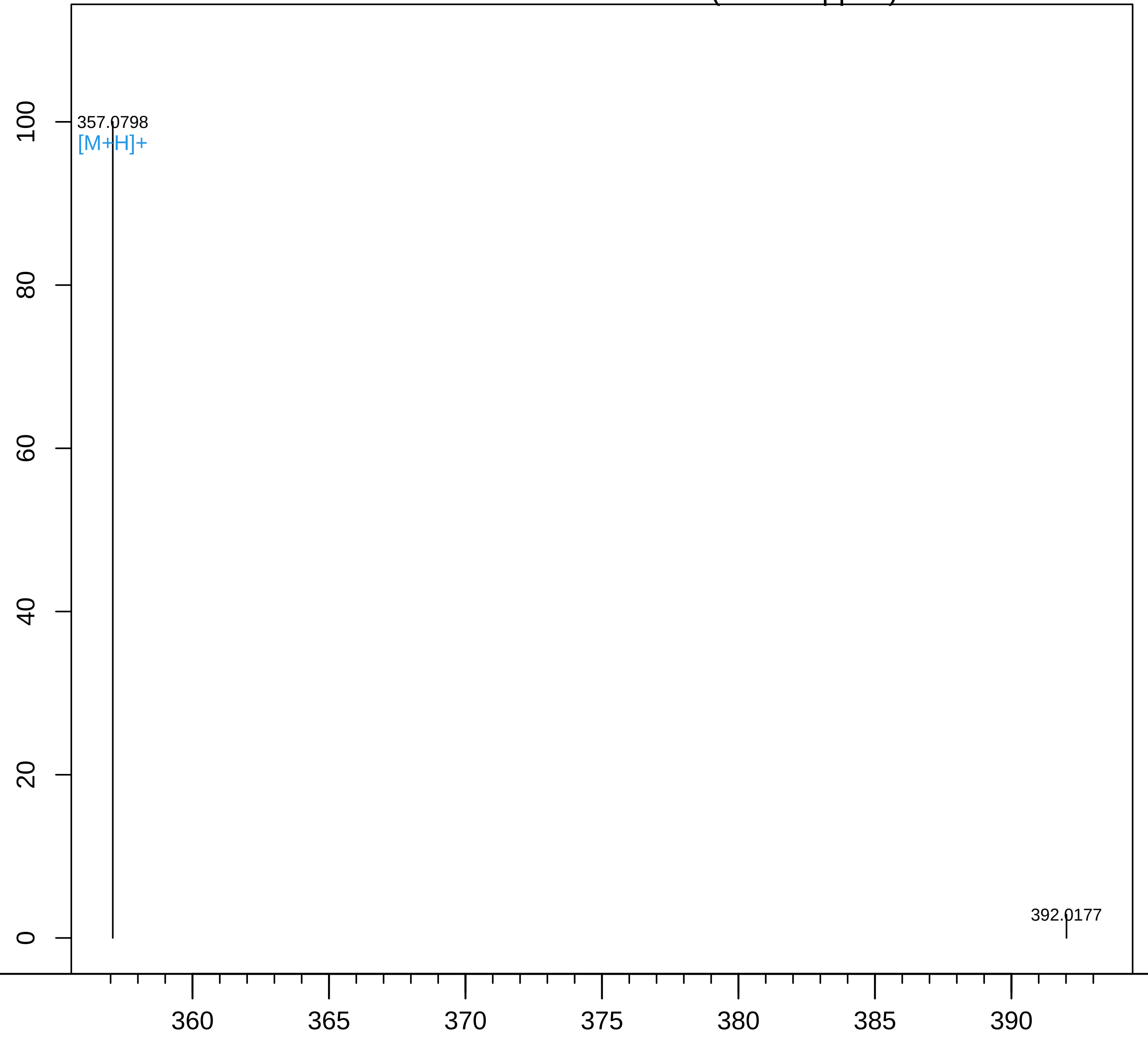

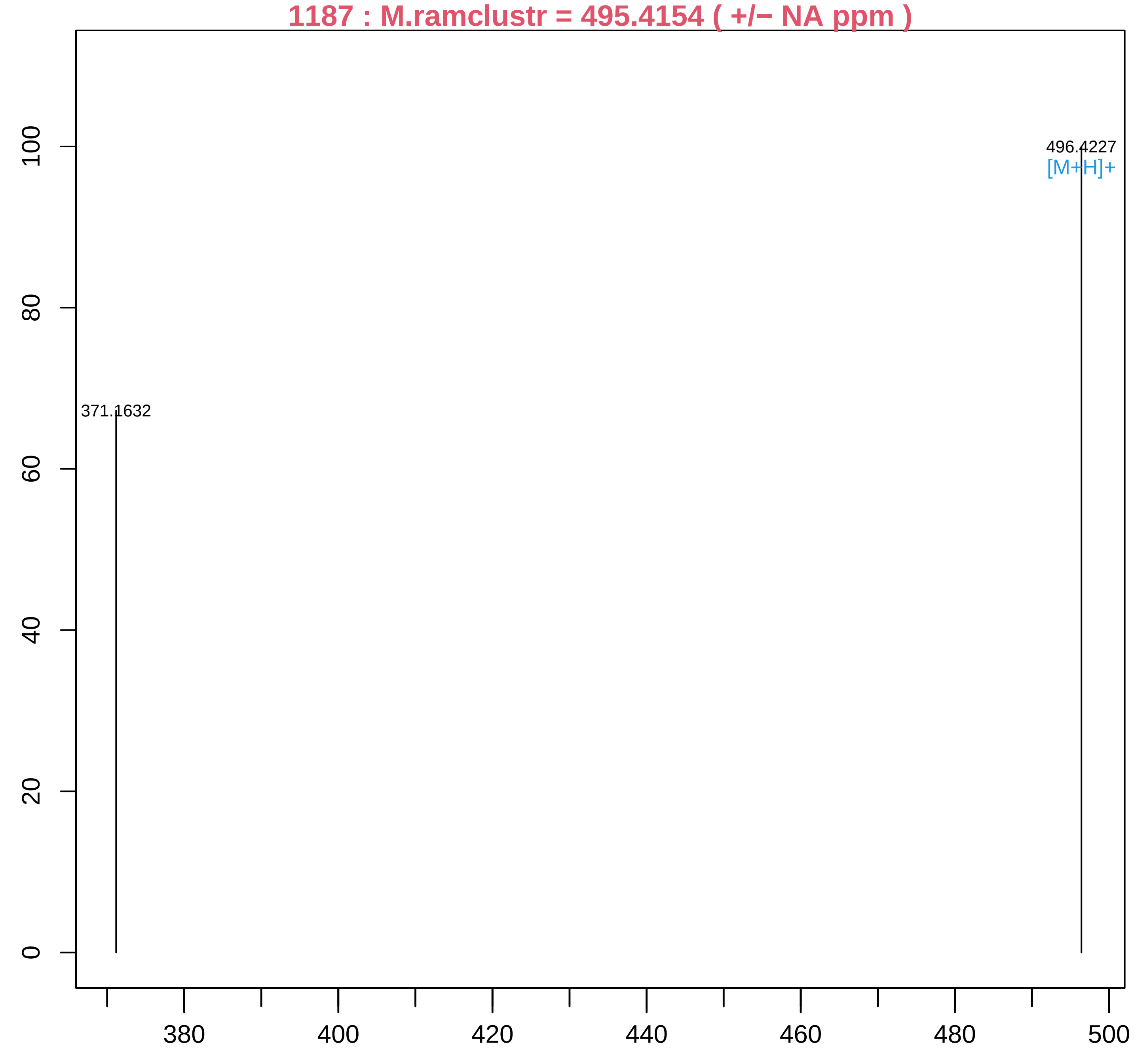
1187 : M.ramclustr = 495.4154 (+/− NA ppm)
496.4227
[M+H]+
371.1632
0
20
40
60
80
100
380
400
420
440
460
480
500

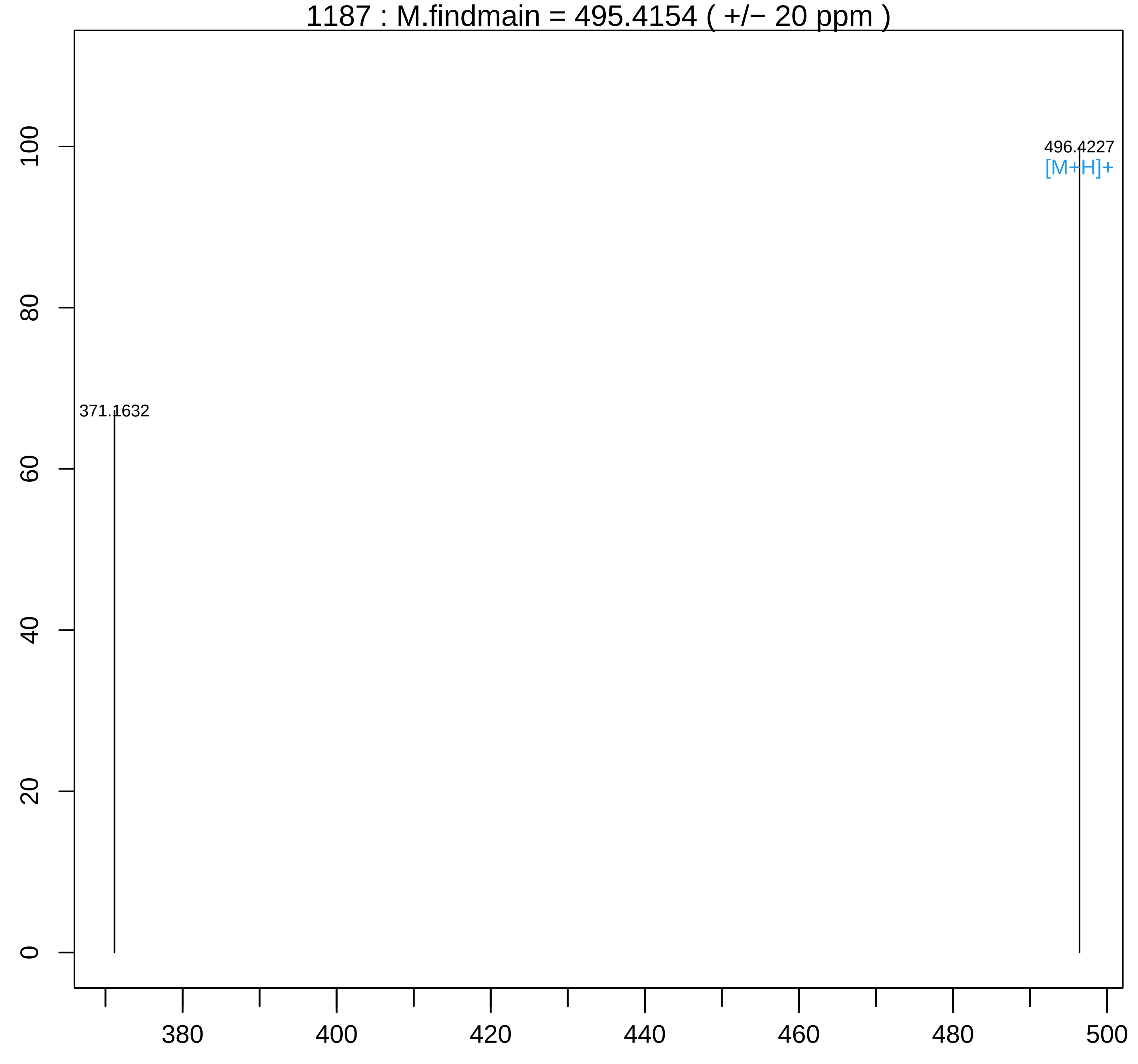
1187 : M.findmain = 495.4154 (+/− 20 ppm)
496.4227
[M+H]+
371.1632
0
20
40
60
80
100
380
400
420
440
460
480
500

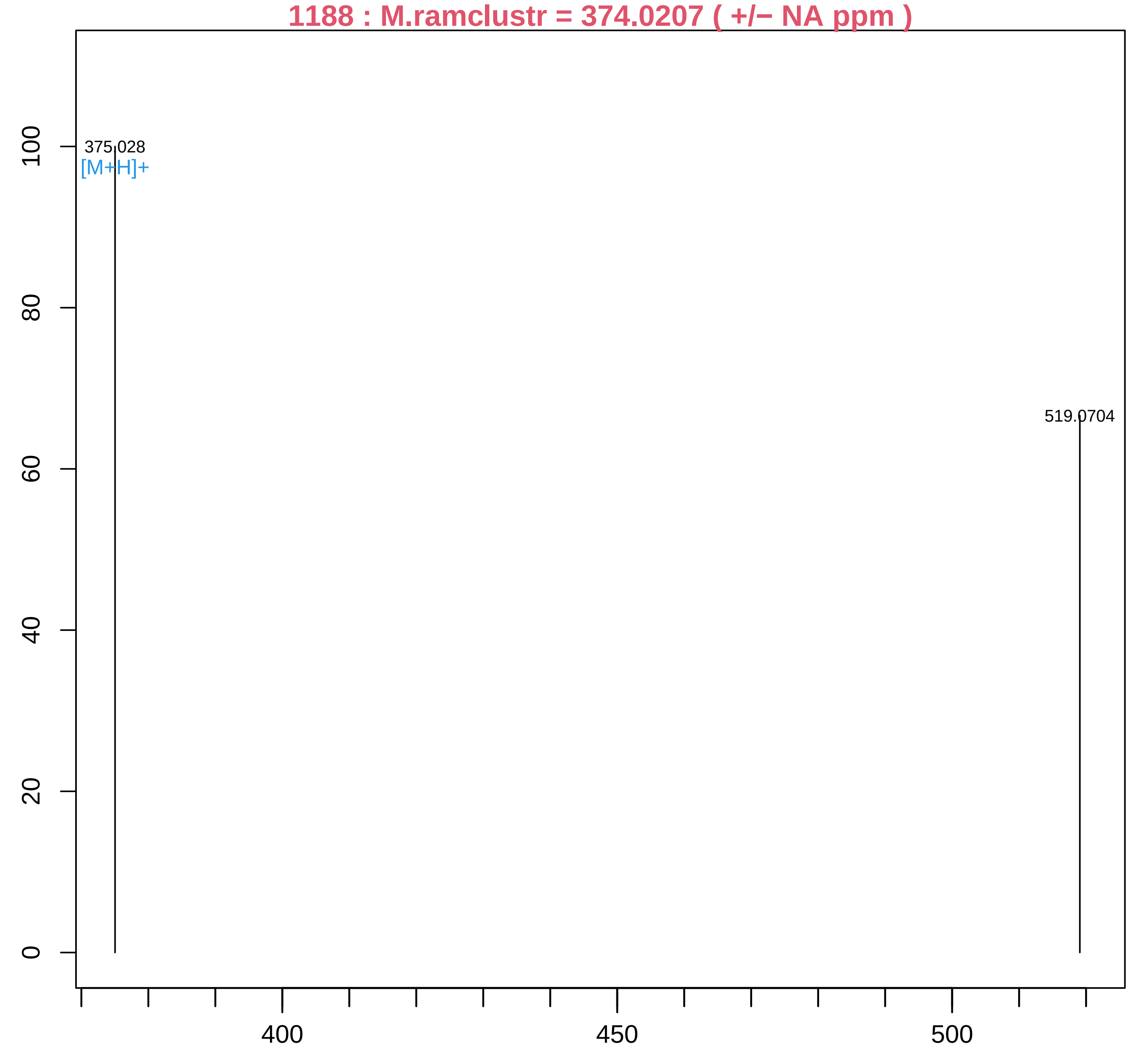
1188 : M.ramclustr = 374.0207 (+/− NA ppm)
375.028
[M+H]+
519.0704
0
20
40
60
80
100
400
450
500

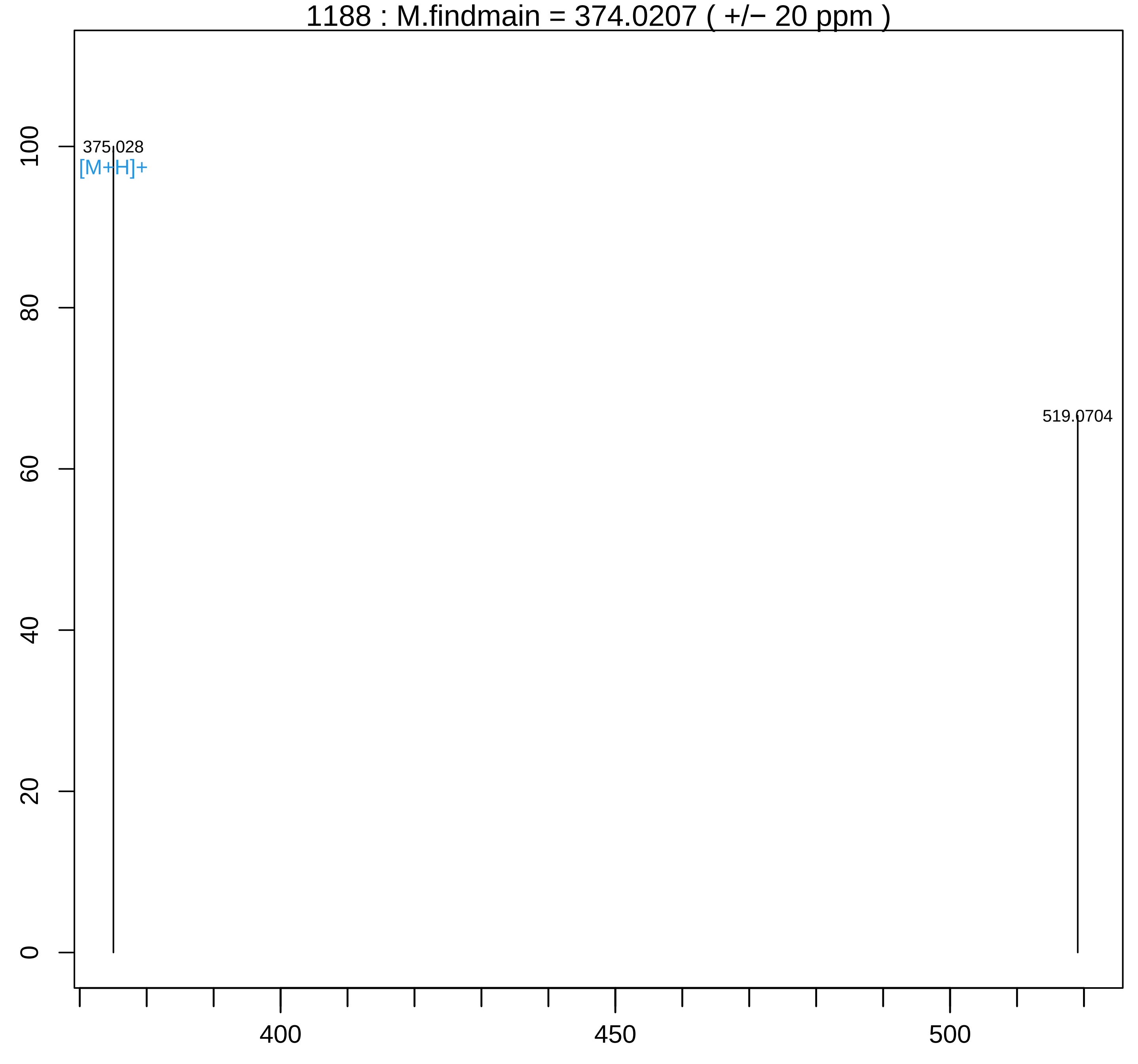
1188 : M.findmain = 374.0207 (+/− 20 ppm)
375.028
[M+H]+
519.0704
0
20
40
60
80
100
400
450
500

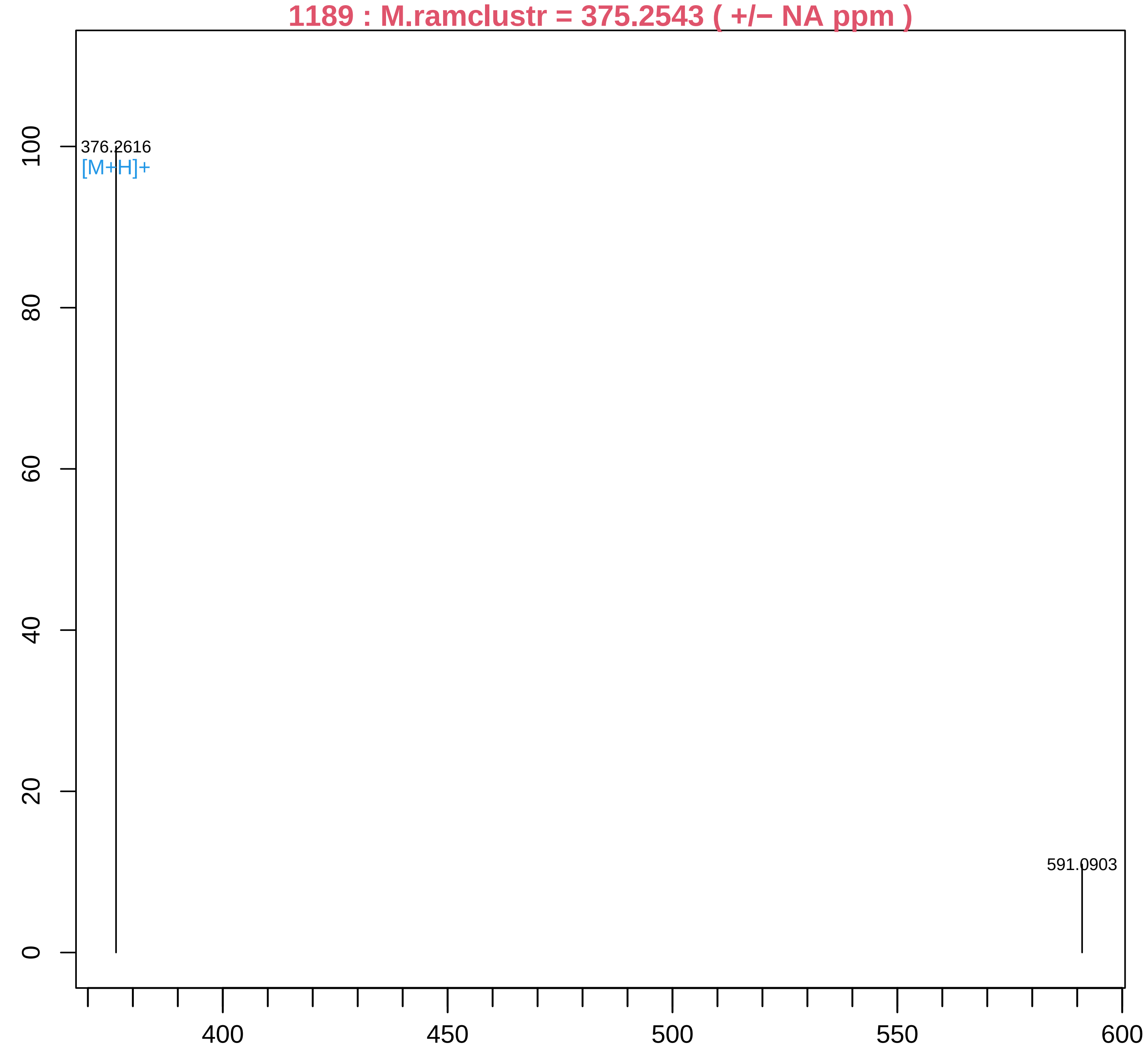
1189 : M.ramclustr = 375.2543 (+/− NA ppm)
376.2616
[M+H]+
591.0903
0
20
40
60
80
100
400
450
500
550
600

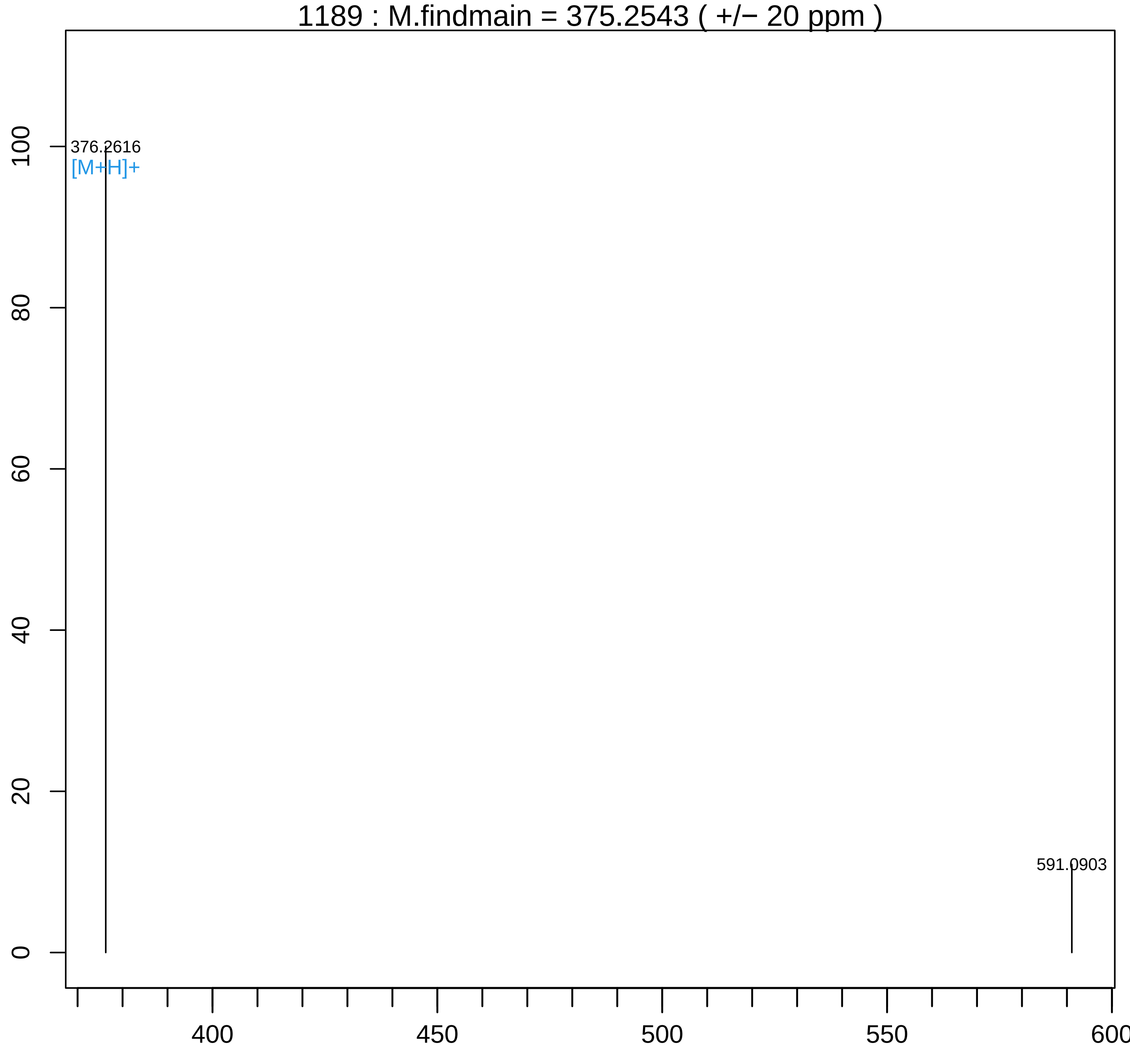
1189 : M.findmain = 375.2543 (+/− 20 ppm)
376.2616
[M+H]+
591.0903
0
20
40
60
80
100
400
450
500
550
600

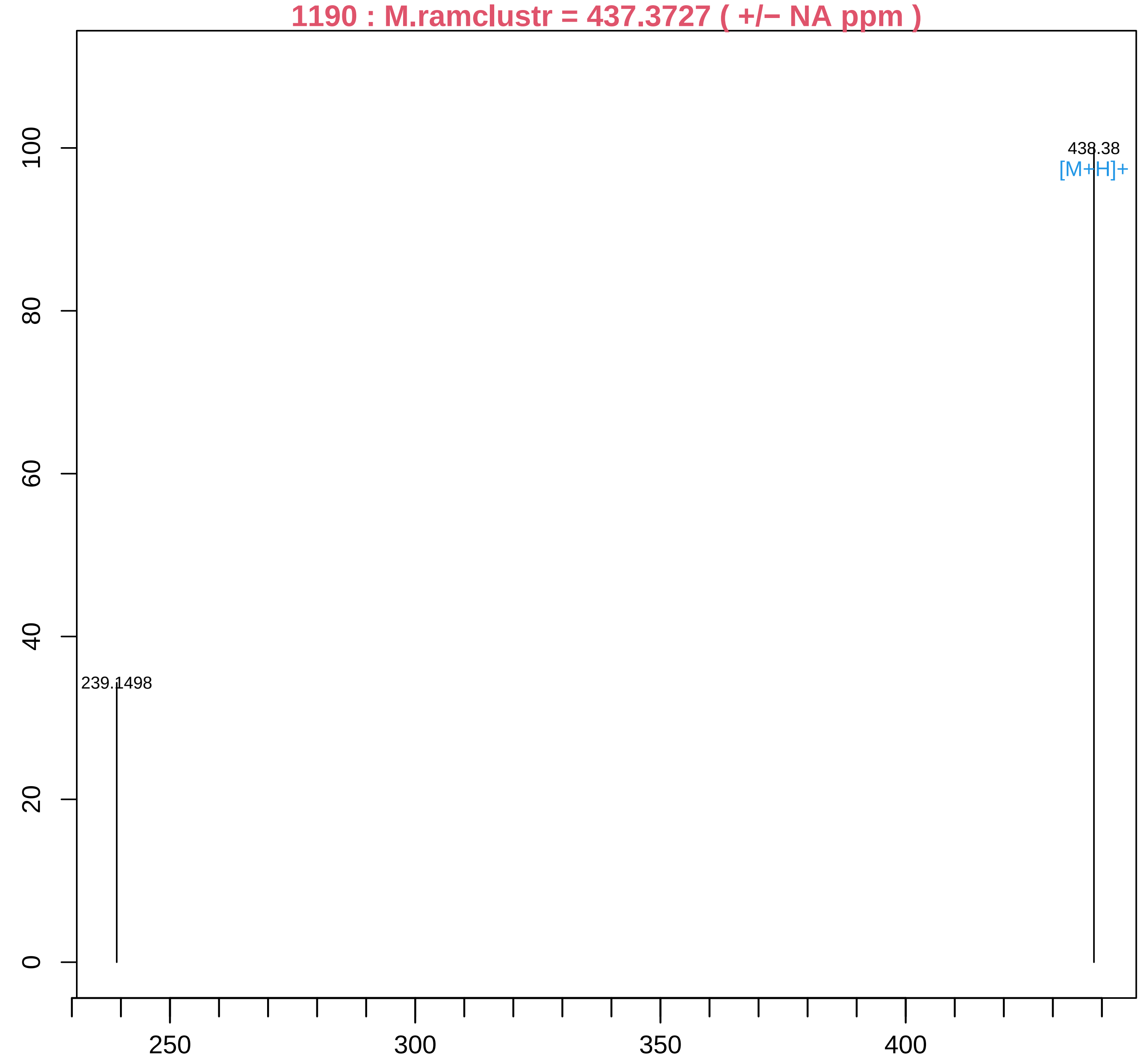

1190 : M.ramclustr = 437.3727 (+/− NA ppm)
438.38
[M+H]+
239.1498
0
20
40
60
80
100
250
300
350
400

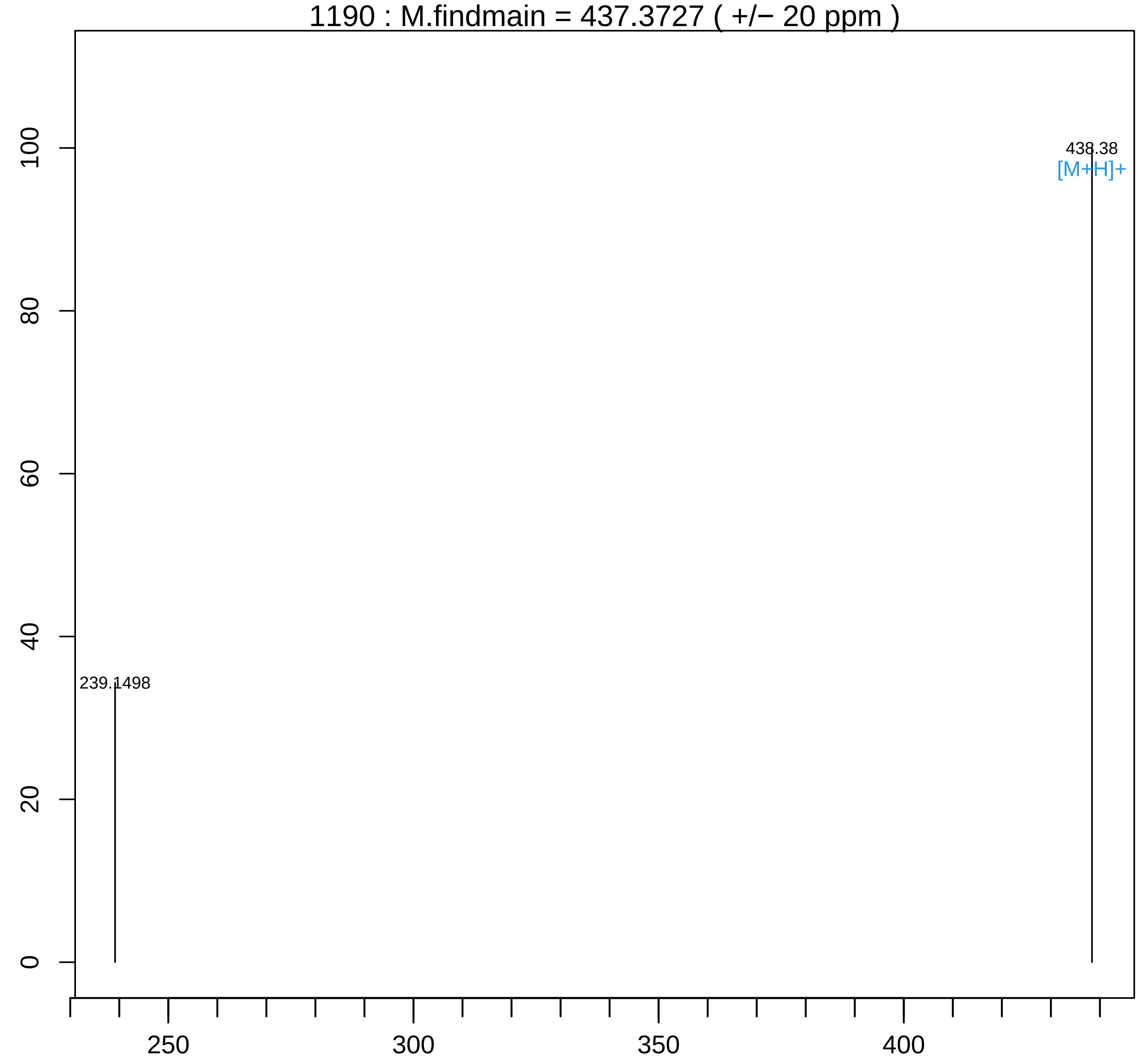

1190 : M.findmain = 437.3727 (+/− 20 ppm)
438.38
[M+H]+
239.1498
0
20
40
60
80
100
250
300
350
400

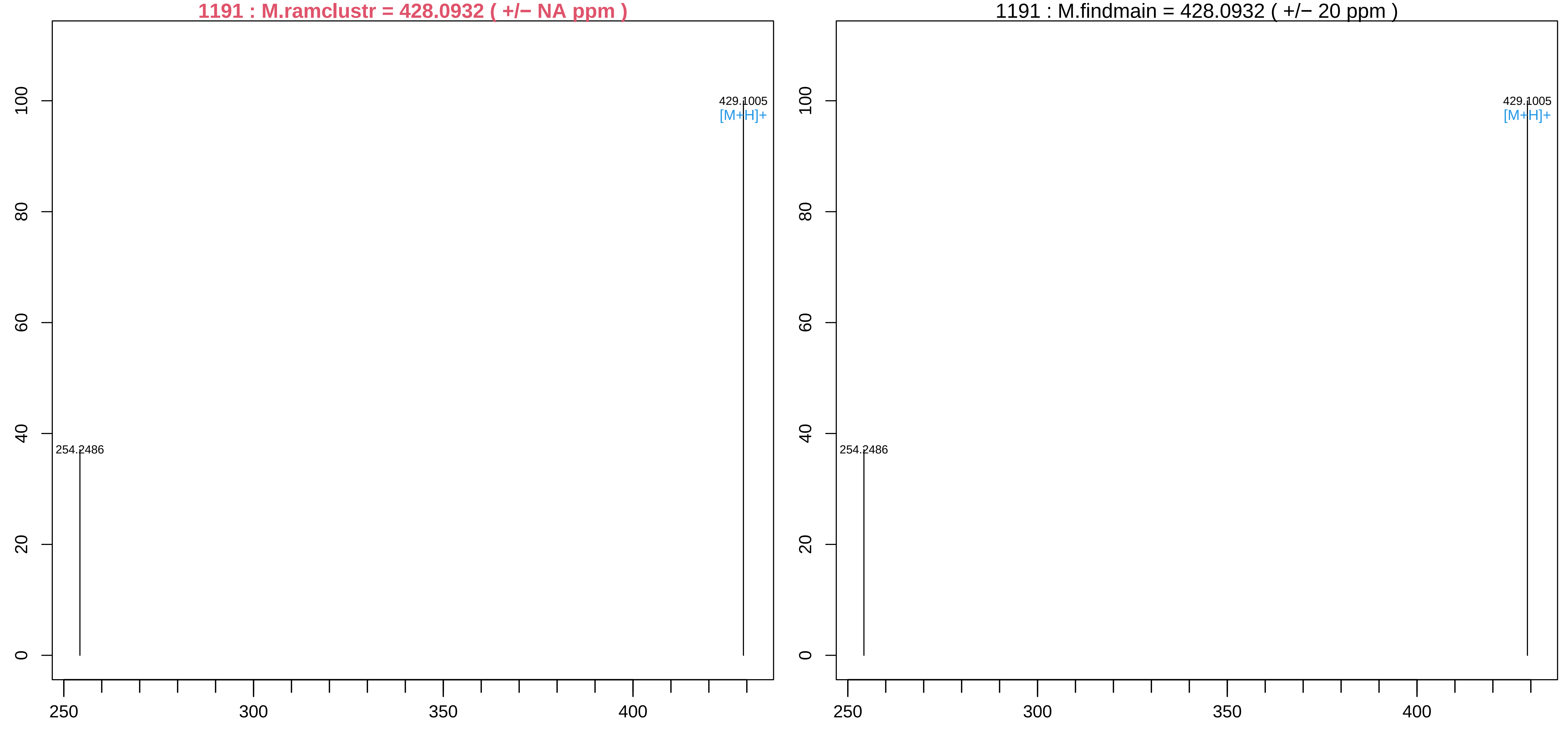

1191 : M.ramclustr = 428.0932 (+/− NA ppm)
429.1005
[M+H]+
254.2486
1191 : M.findmain = 428.0932 (+/− 20 ppm)
429.1005
[M+H]+
254.2486
0
20
40
60
80
100
250
300
350
400

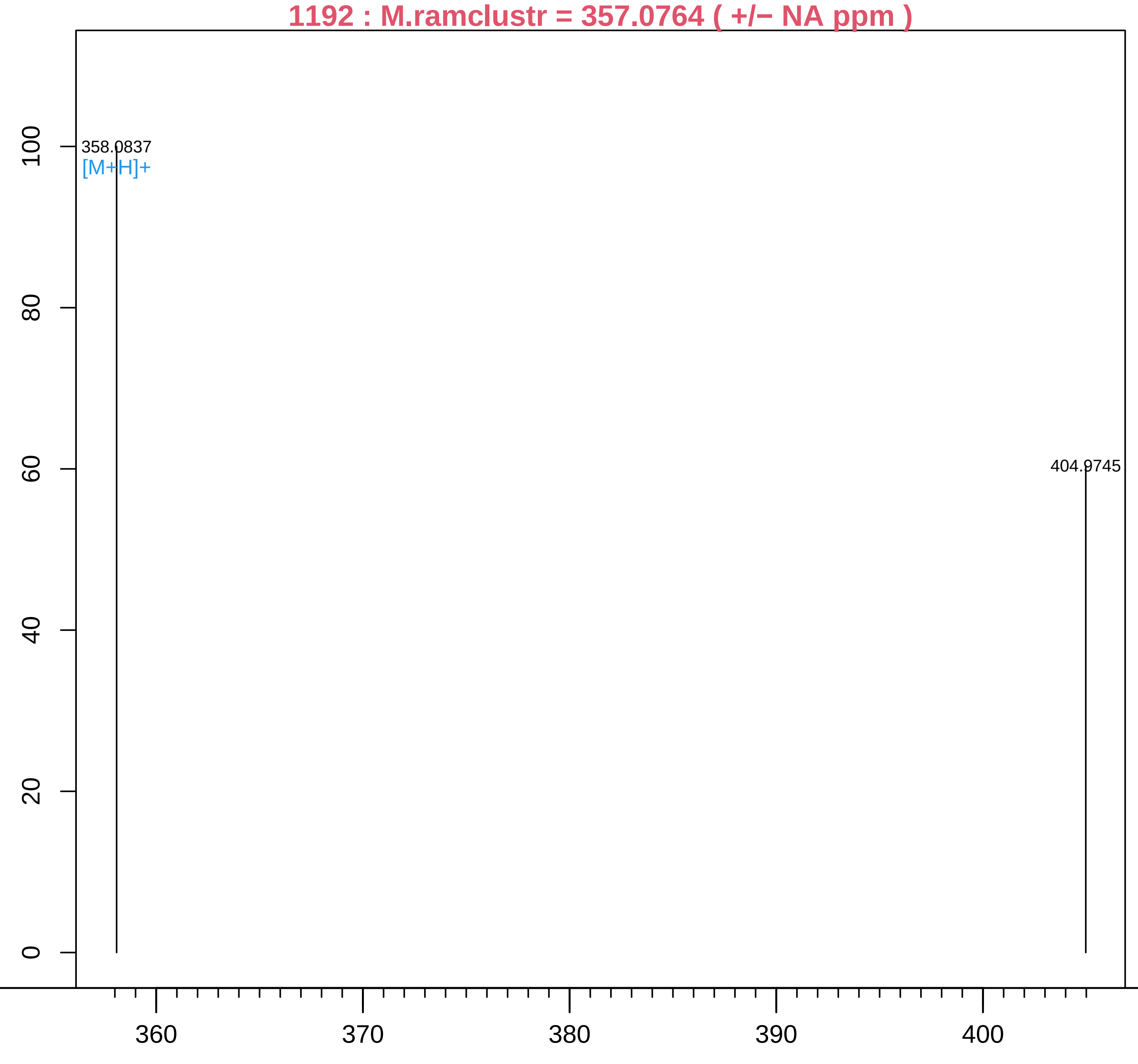
1192 : M.ramclustr = 357.0764 (+/− NA ppm)
358.0837
[M+H]+
404.9745
0
20
40
60
80
100
360
370
380
390
400

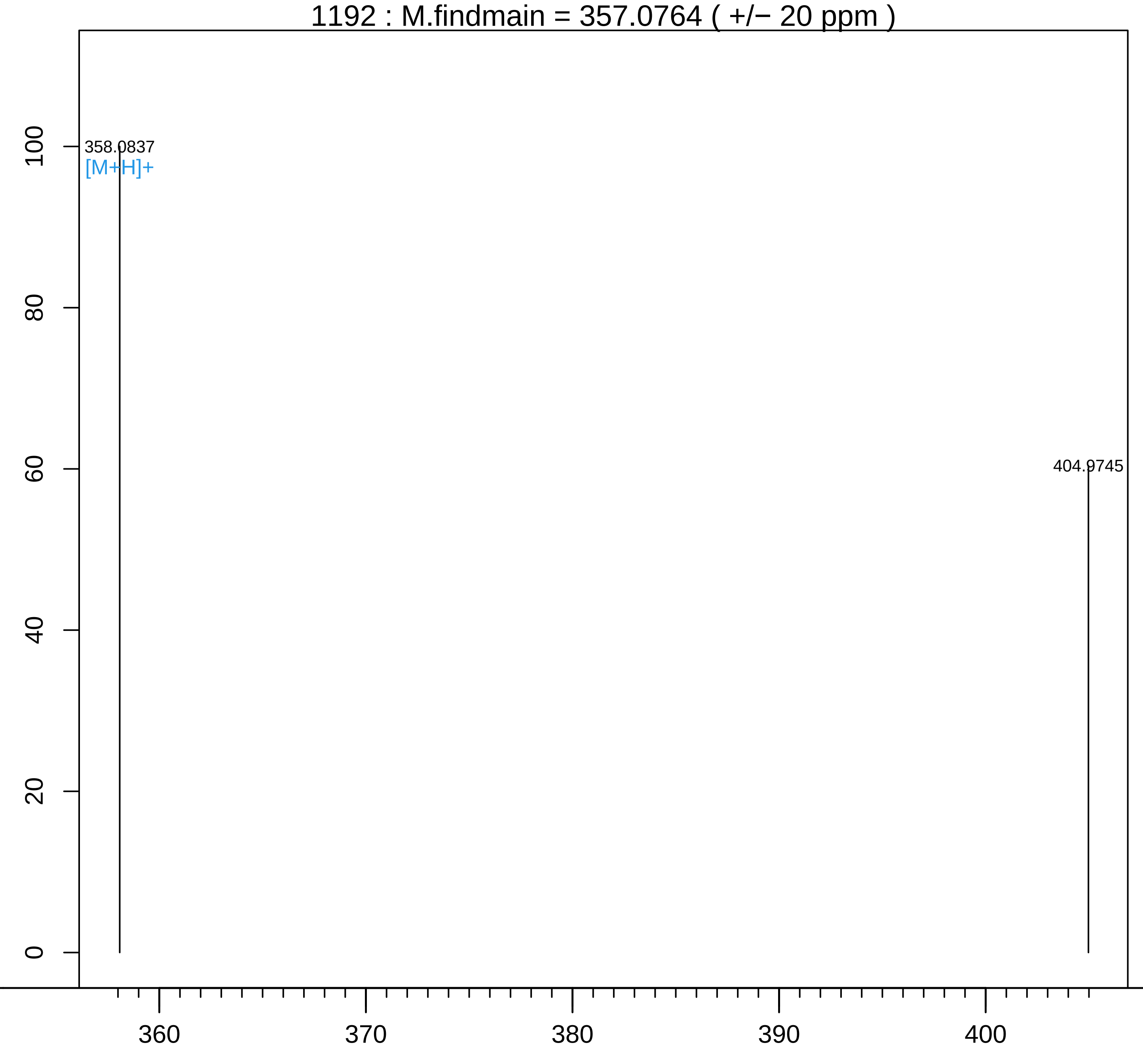
1192 : M.findmain = 357.0764 (+/− 20 ppm)
358.0837
[M+H]+
404.9745
0
20
40
60
80
100
360
370
380
390
400

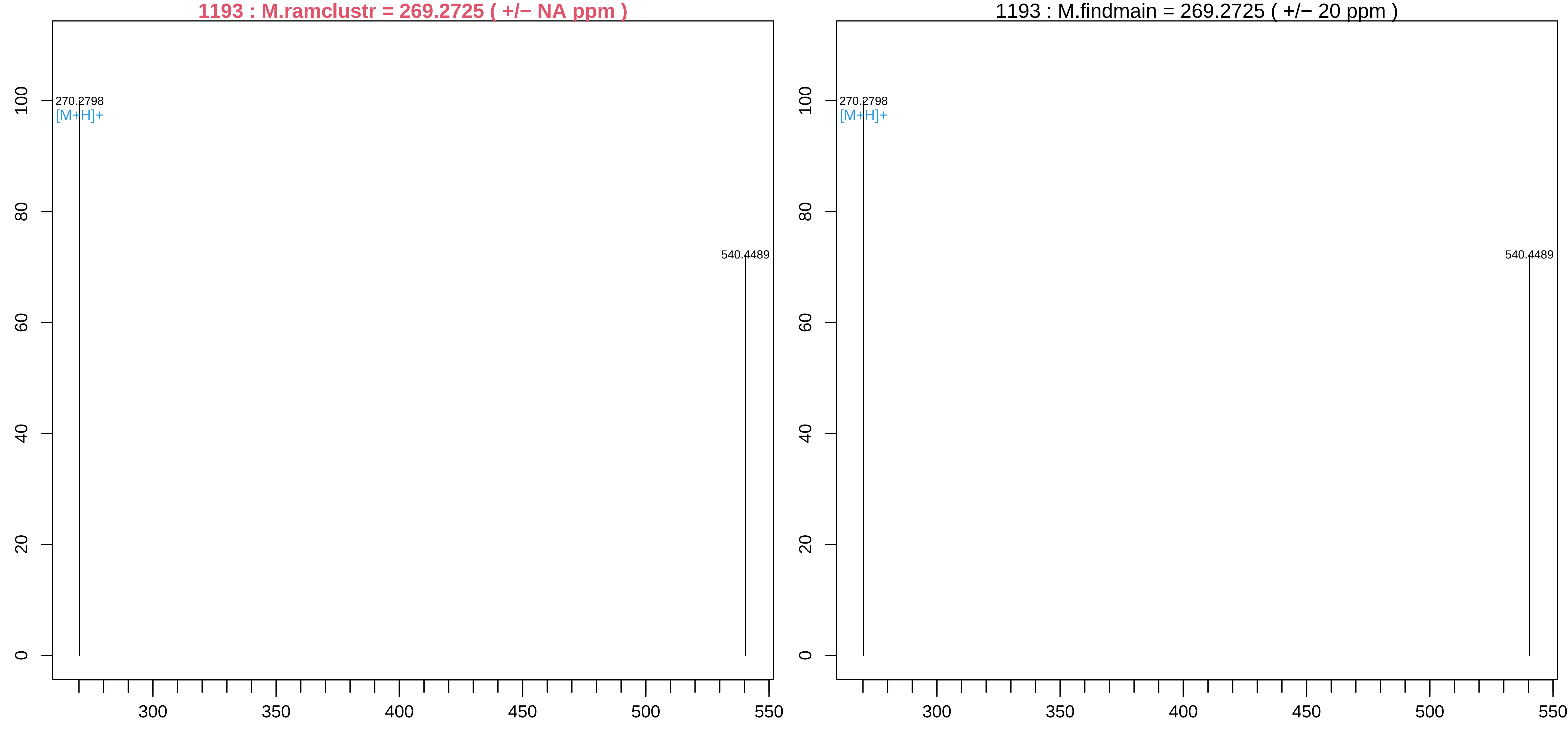

1193 : M.ramclustr = 269.2725 (+/− NA ppm)
270.2798
[M+H]+
540.4489
0
20
40
60
80
100
300
350
400
450
500
550
1193 : M.findmain = 269.2725 (+/− 20 ppm)
270.2798
[M+H]+
540.4489
0
20
40
60
80
100
300
350
400
450
500
550

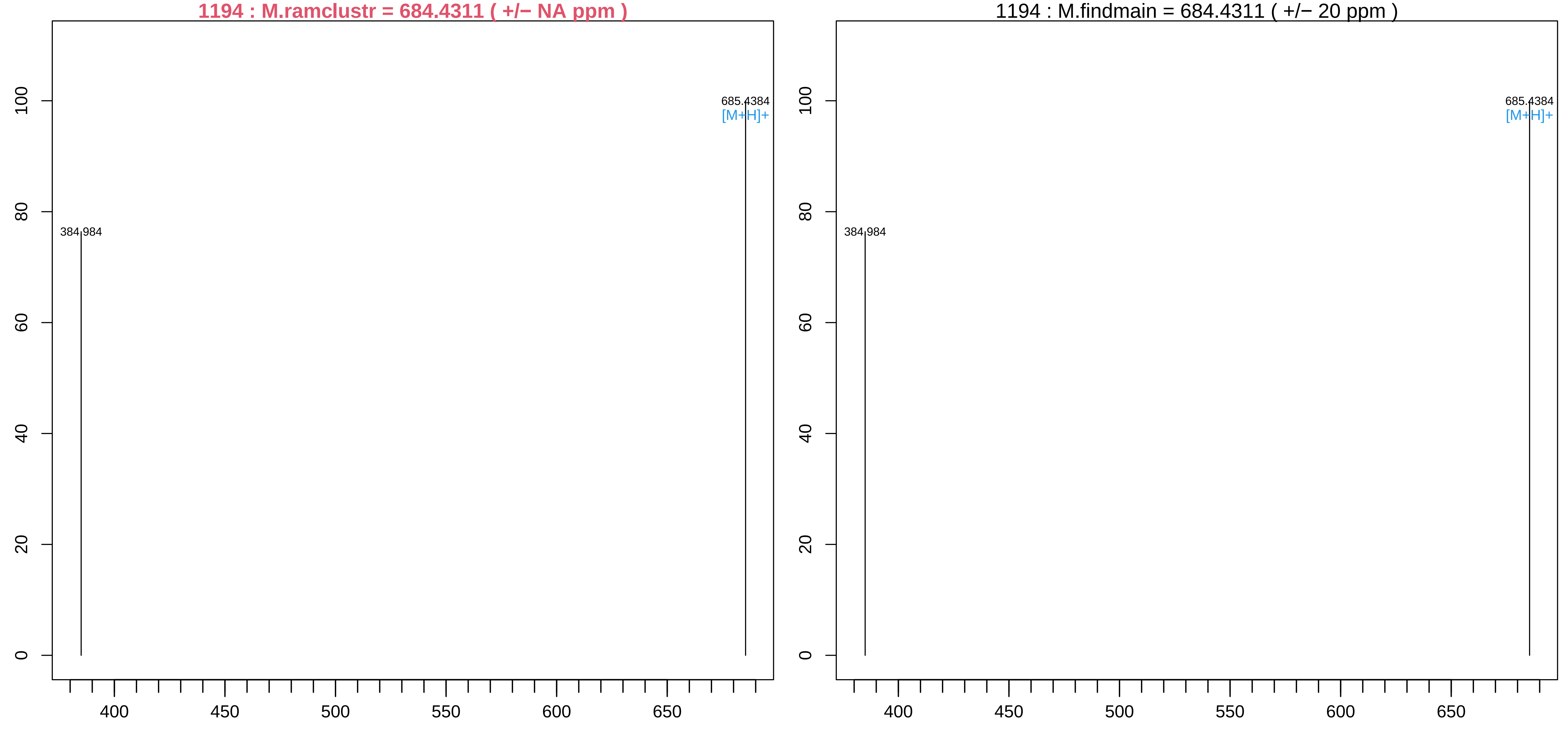
1194 : M.ramclustr = 684.4311 (+/− NA ppm)
1194 : M.findmain = 684.4311 (+/− 20 ppm)
685.4384
[M+H]+
384.984
685.4384
[M+H]+
384.984
0
20
40
60
80
100
400
450
500
550
600
650

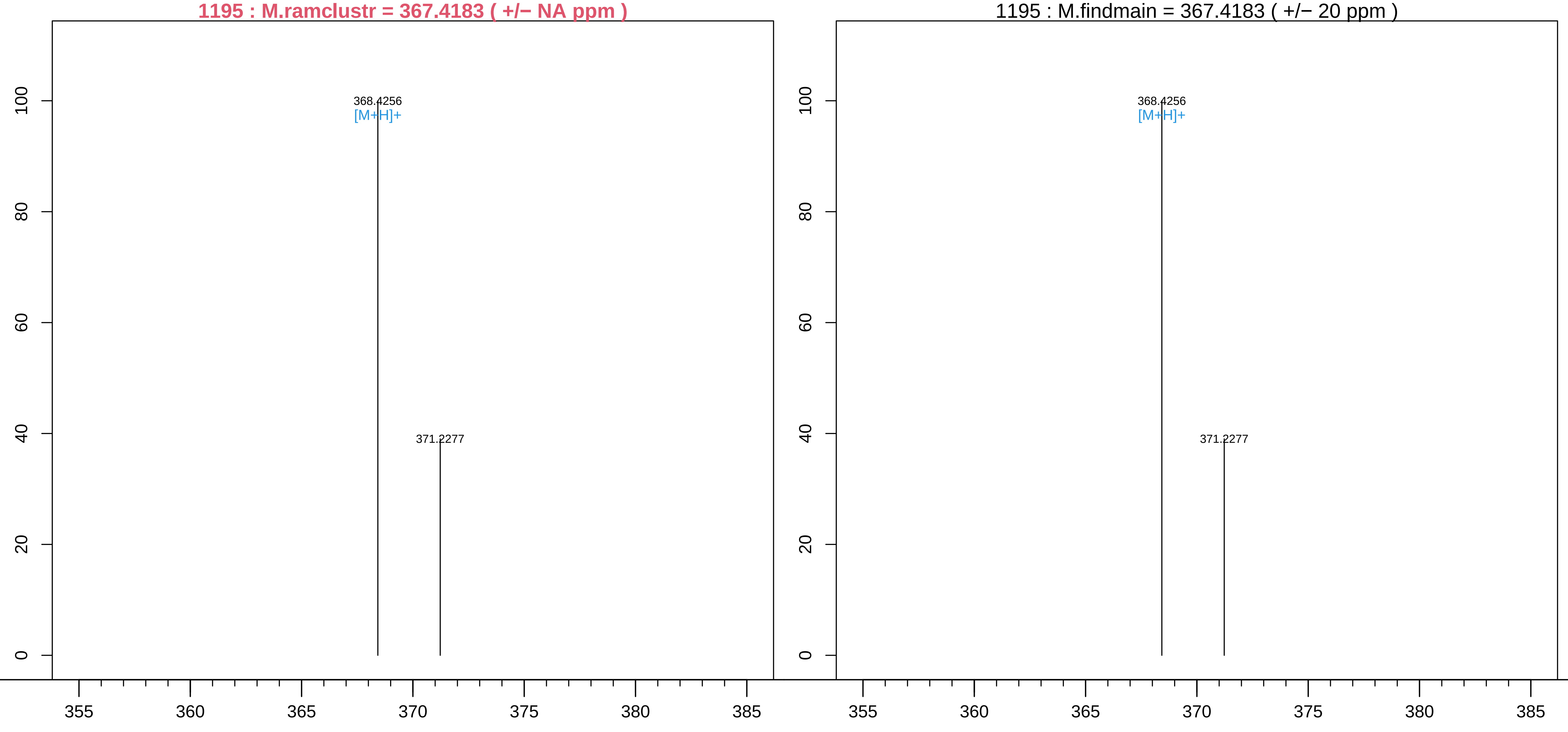
1195 : M.ramclustr = 367.4183 (+/− NA ppm)
368.4256
[M+H]+
371.2277
0
20
40
60
80
100
355
360
365
370
375
380
385
1195 : M.findmain = 367.4183 (+/− 20 ppm)
368.4256
[M+H]+
371.2277
0
20
40
60
80
100
355
360
365
370
375
380
385

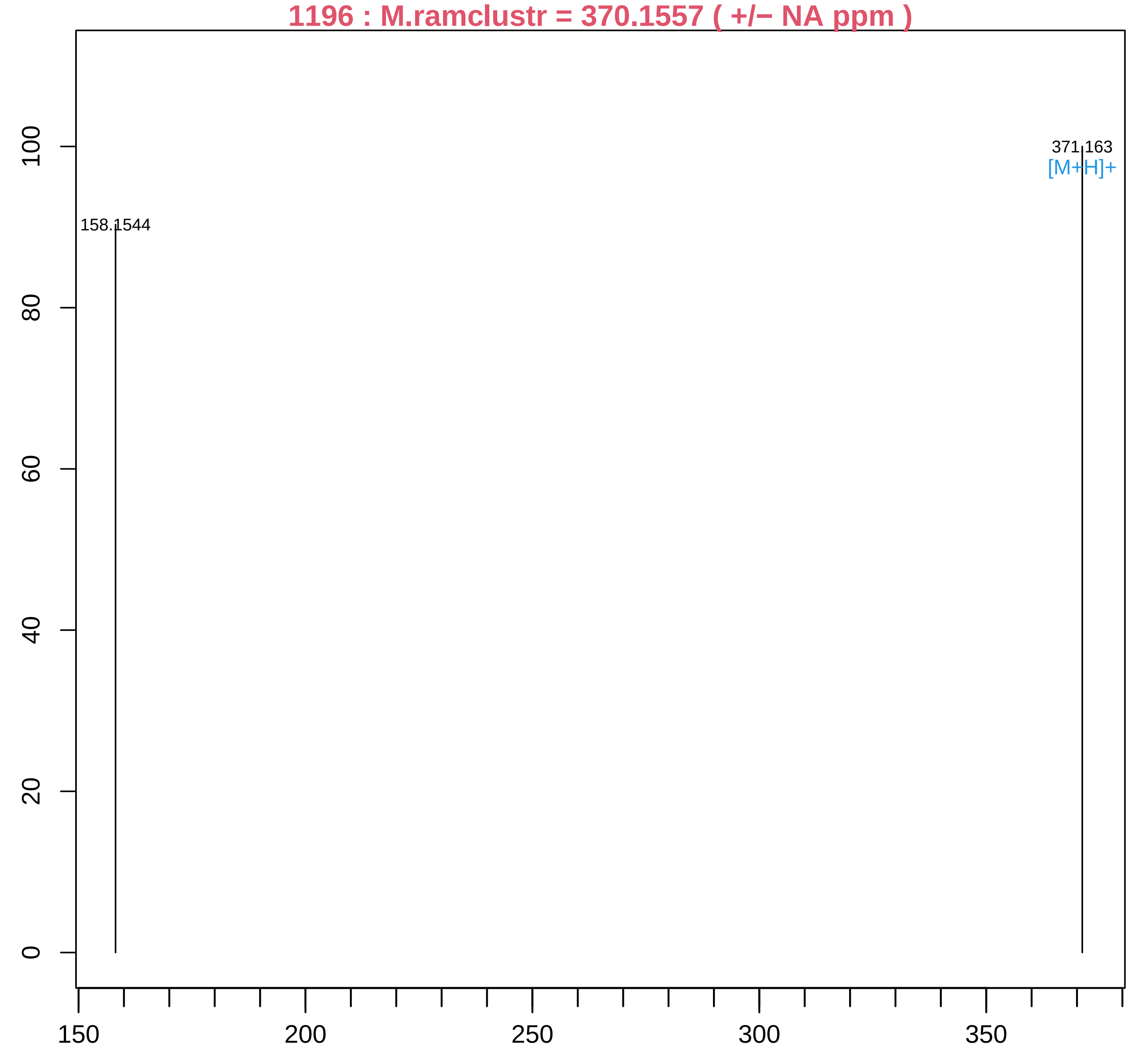
1196 : M.ramclustr = 370.1557 (+/− NA ppm)
158.1544
371.163
[M+H]+
0
20
40
60
80
100
150
200
250
300
350

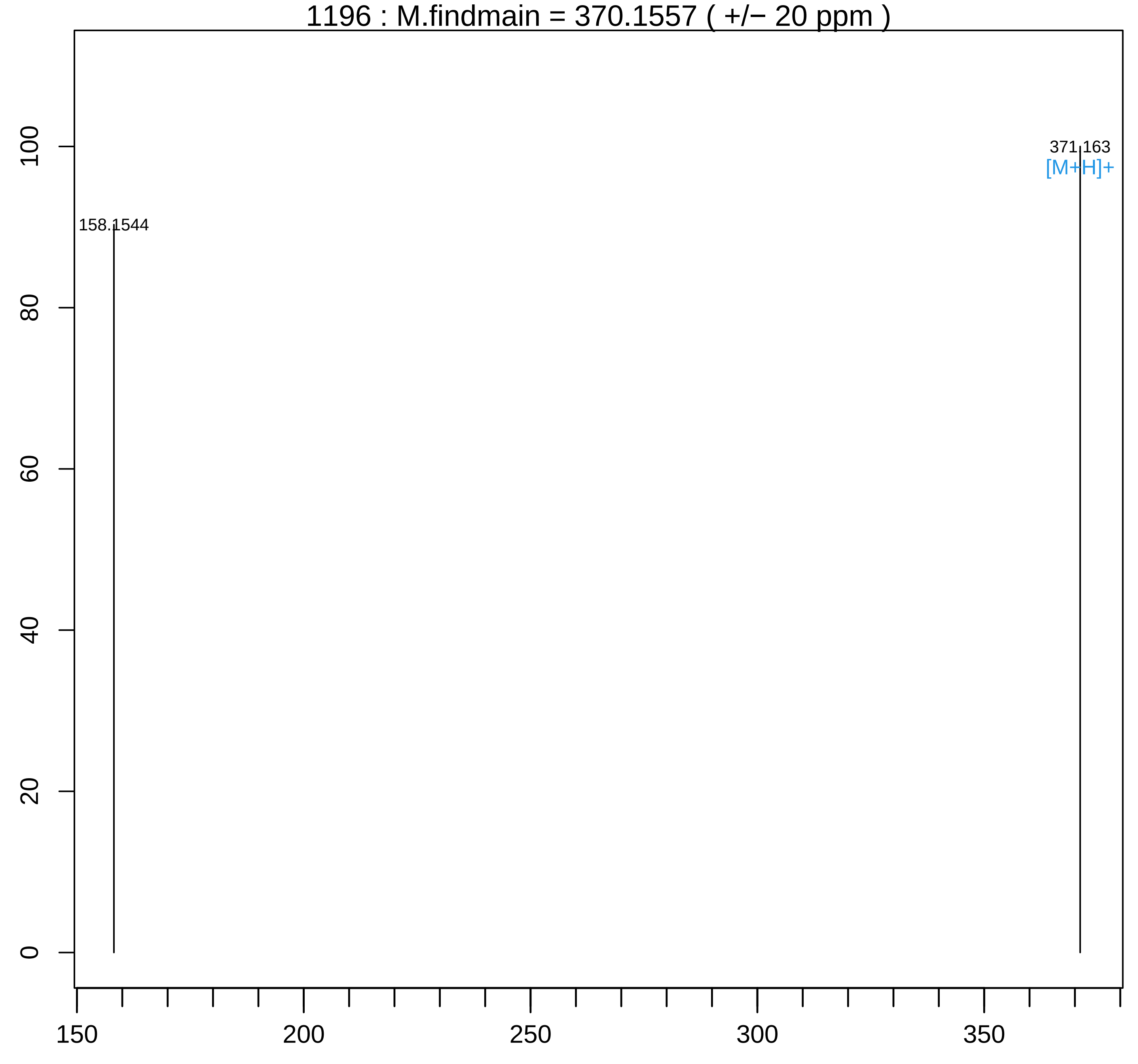
1196 : M.findmain = 370.1557 (+/− 20 ppm)
158.1544
371.163
[M+H]+
0
20
40
60
80
100
150
200
250
300
350

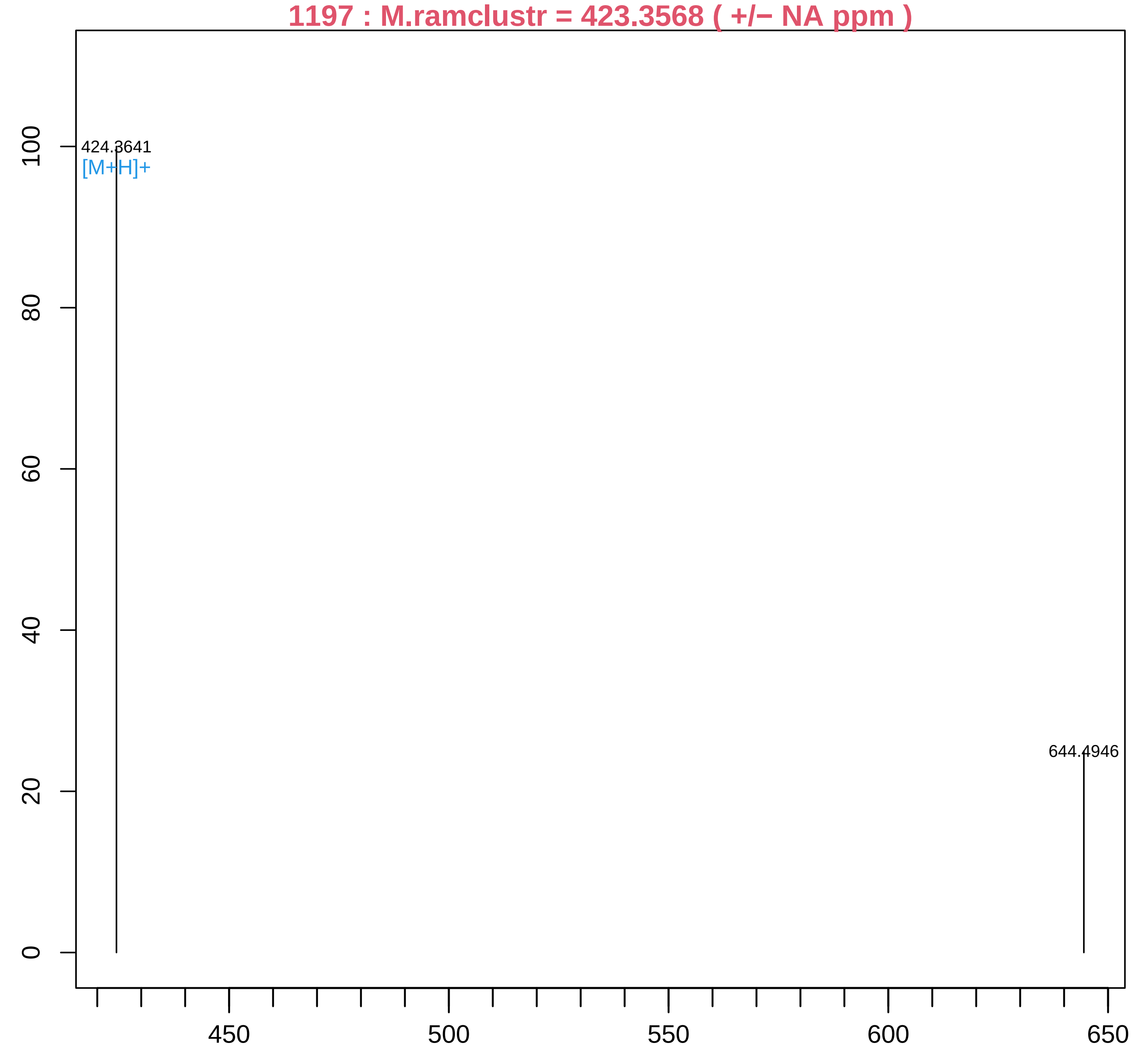
1197 : M.ramclustr = 423.3568 (+/− NA ppm)
424.3641
[M+H]+
644.4946
0
20
40
60
80
100
450
500
550
600
650

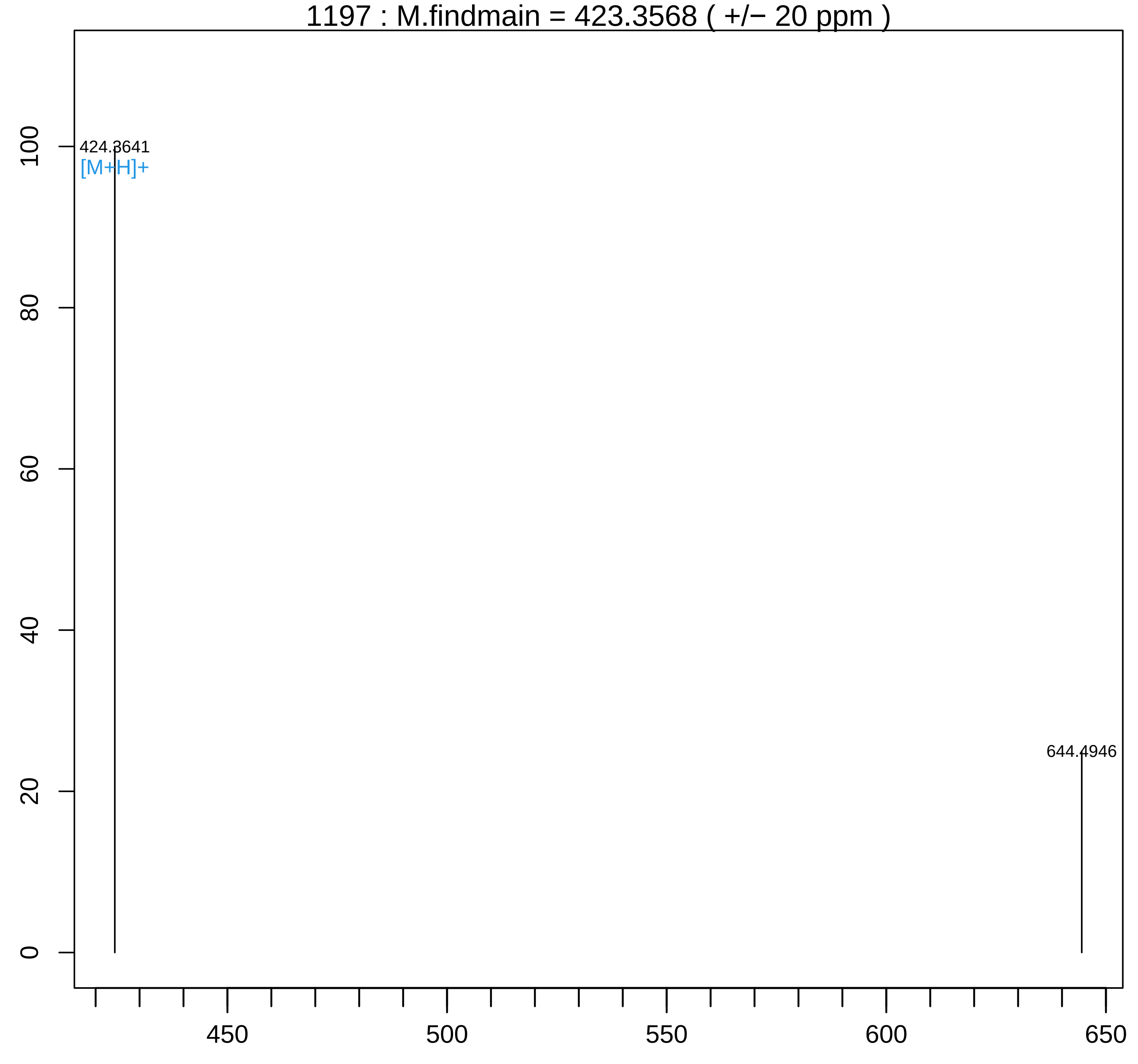
1197 : M.findmain = 423.3568 (+/− 20 ppm)
424.3641
[M+H]+
644.4946
0
20
40
60
80
100
450
500
550
600
650

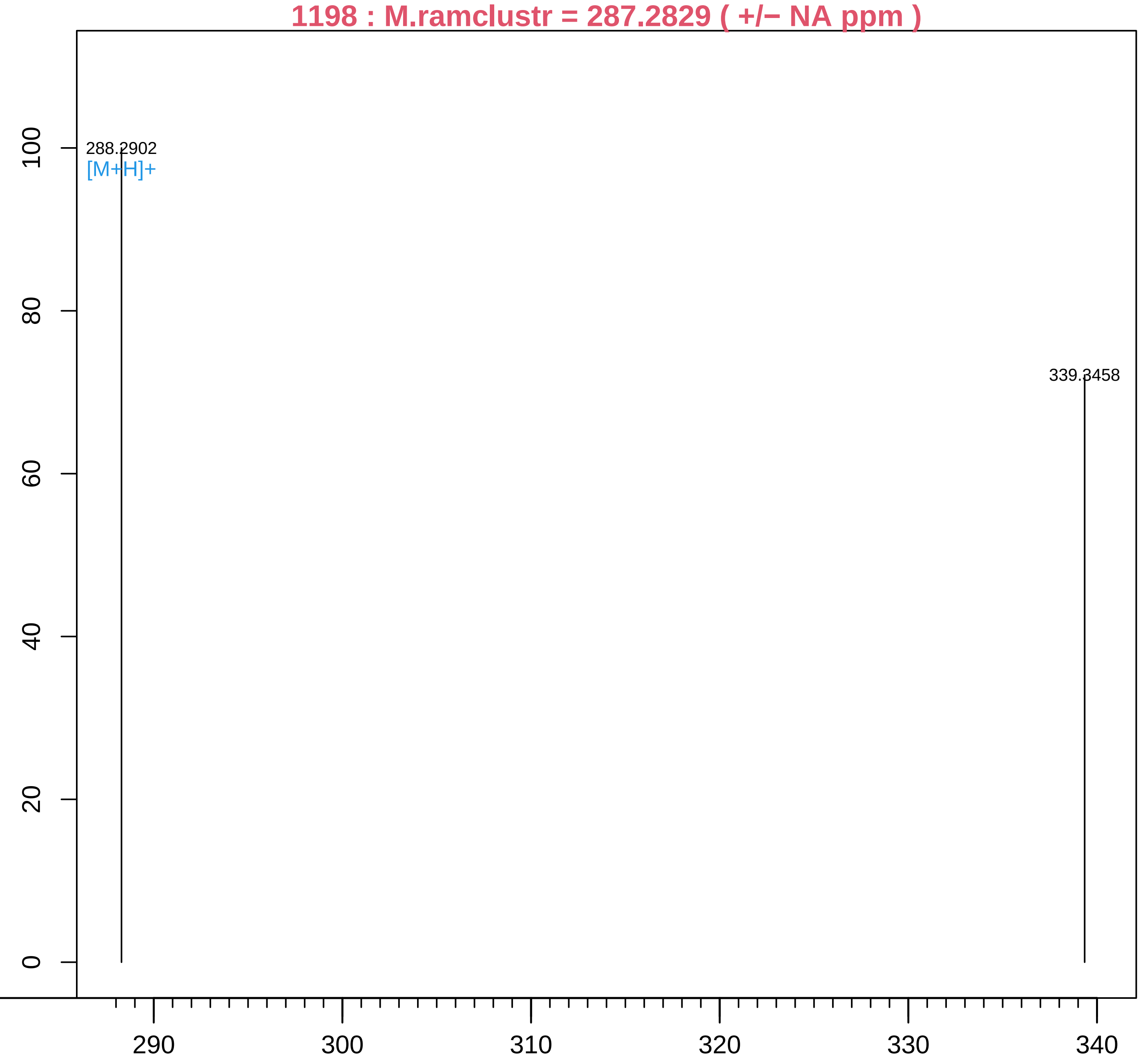

1198 : M.ramclustr = 287.2829 (+/− NA ppm)
288.2902
[M+H]+
339.3458
0
20
40
60
80
100
290
300
310
320
330
340

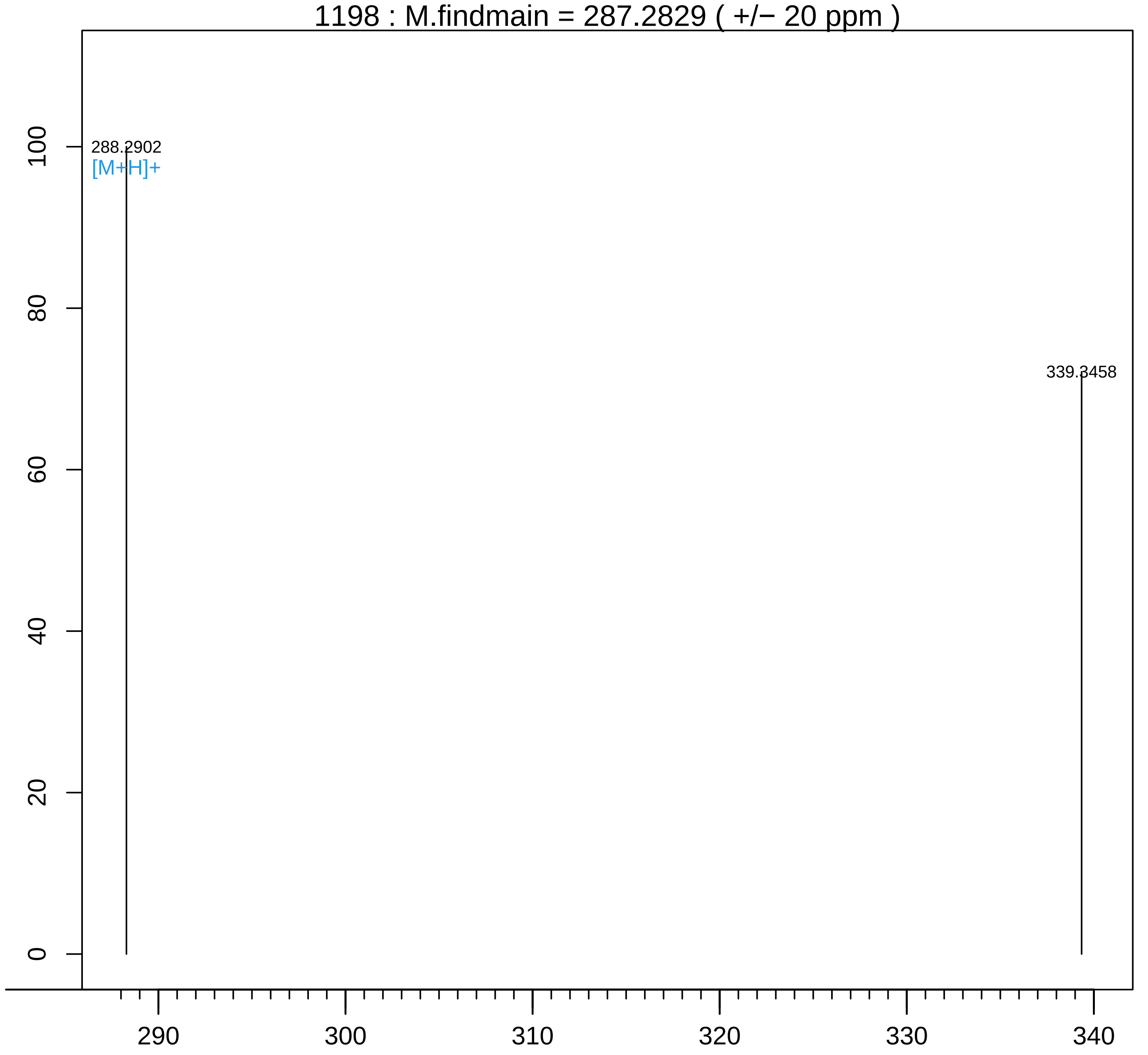

1198 : M.findmain = 287.2829 (+/− 20 ppm)
288.2902
[M+H]+
339.3458
0
20
40
60
80
100
290
300
310
320
330
340

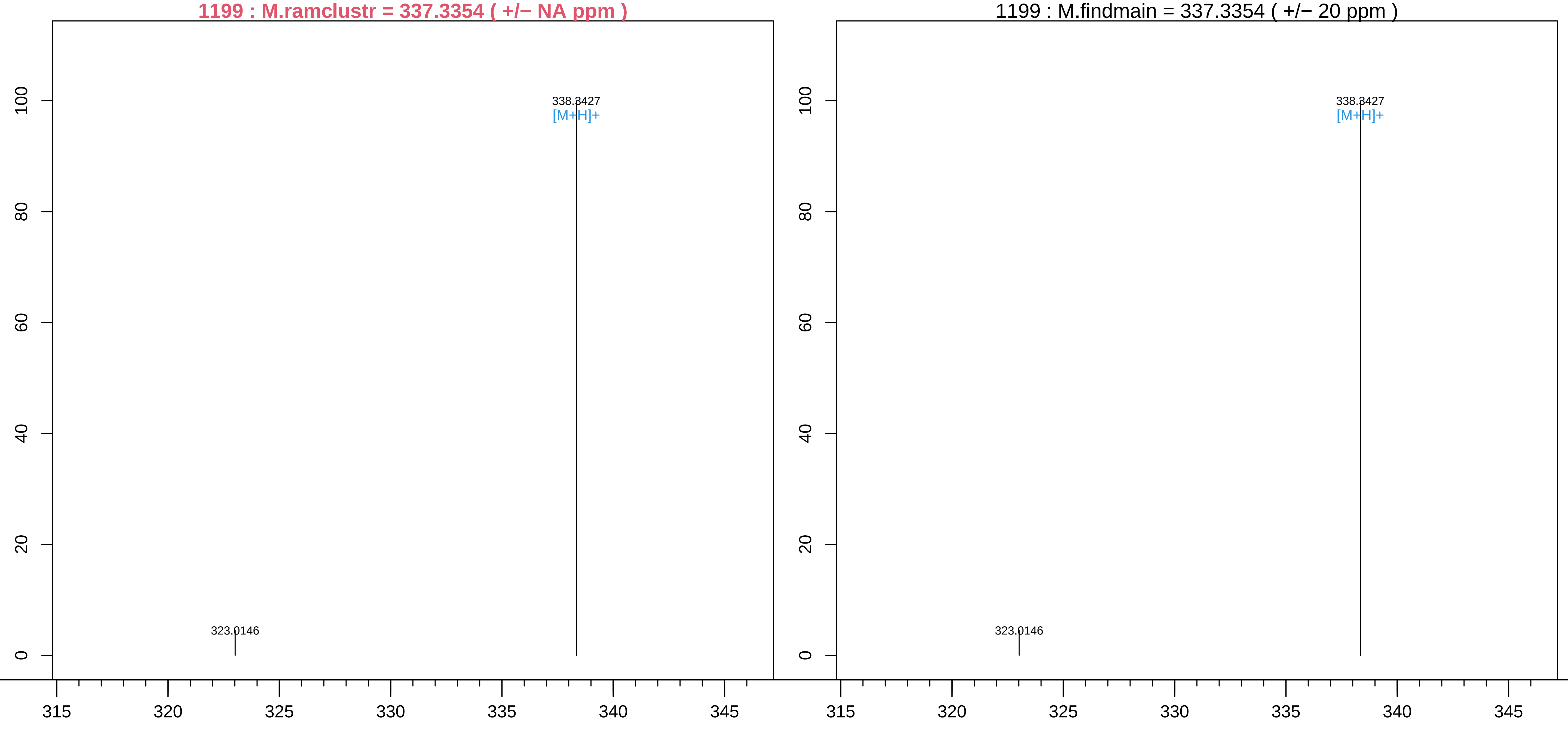
1199 : M.ramclustr = 337.3354 (+/− NA ppm)
338.3427
[M+H]+
323.0146
1199 : M.findmain = 337.3354 (+/− 20 ppm)
338.3427
[M+H]+
323.0146

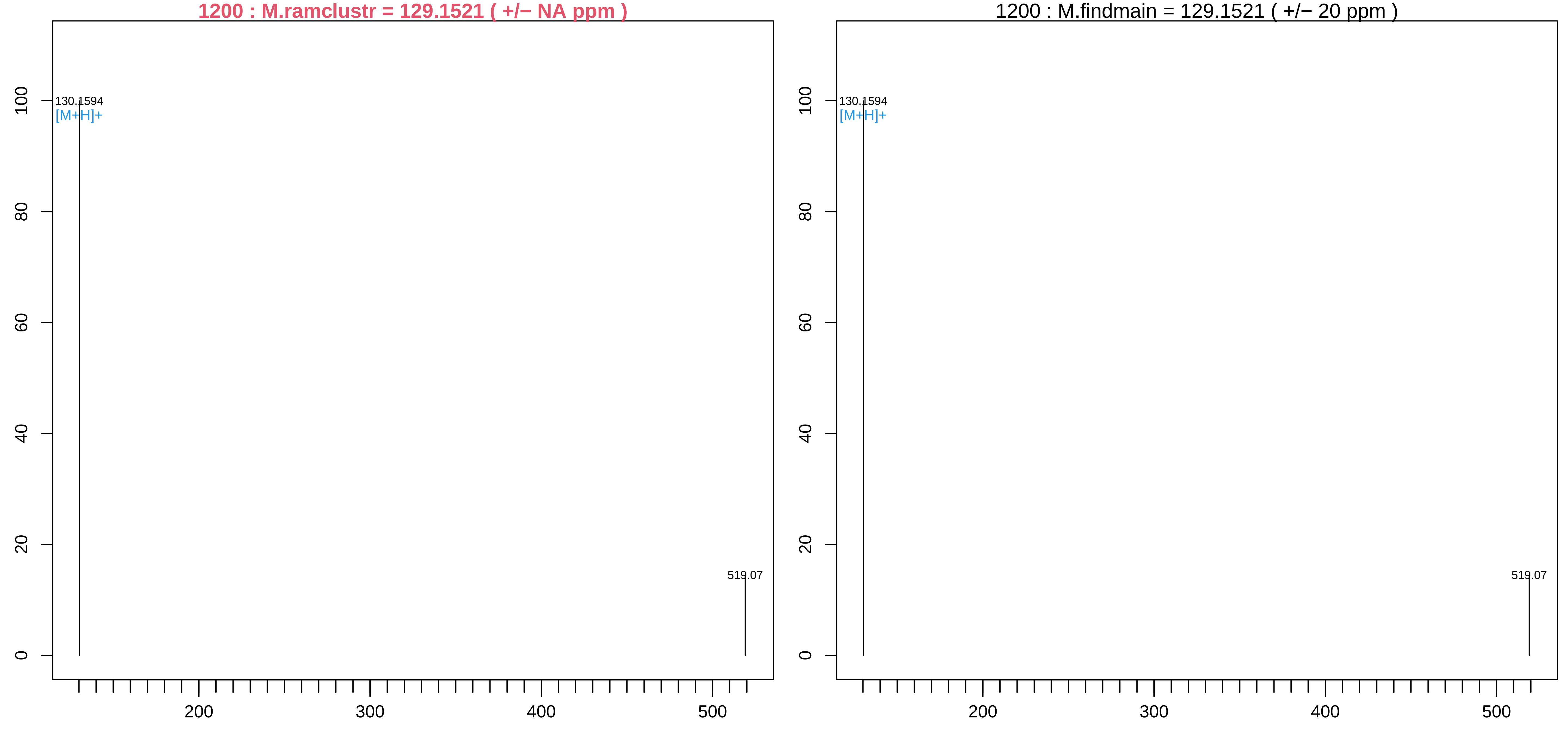
1200 : M.ramclustr = 129.1521 (+/− NA ppm)
130.1594
[M+H]+
519.07
1200 : M.findmain = 129.1521 (+/− 20 ppm)
130.1594
[M+H]+
519.07
0
20
40
60
80
100
200
300
400
500

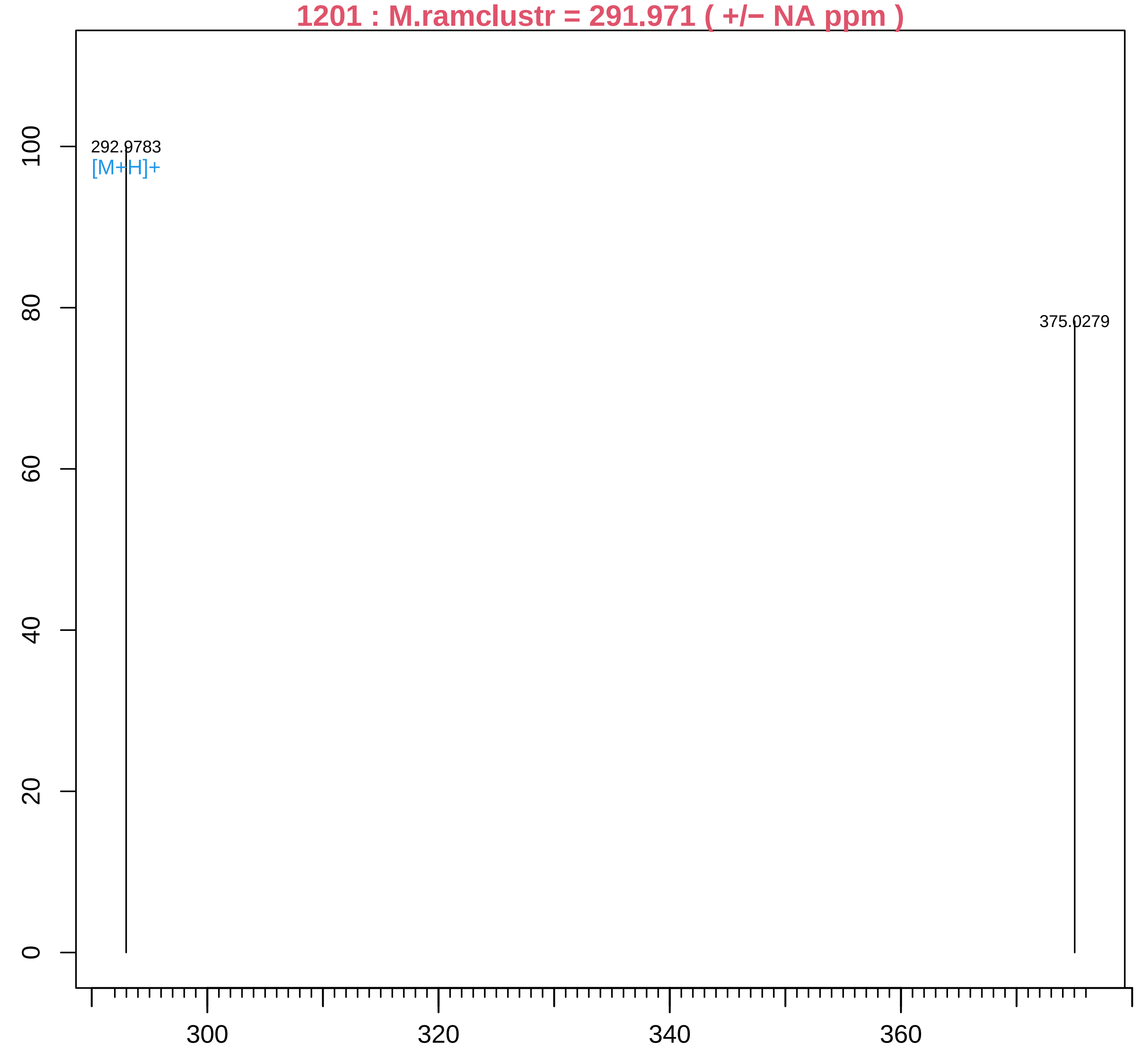
1201 : M.ramclustr = 291.971 (+/− NA ppm)
292.9783
[M+H]+
375.0279

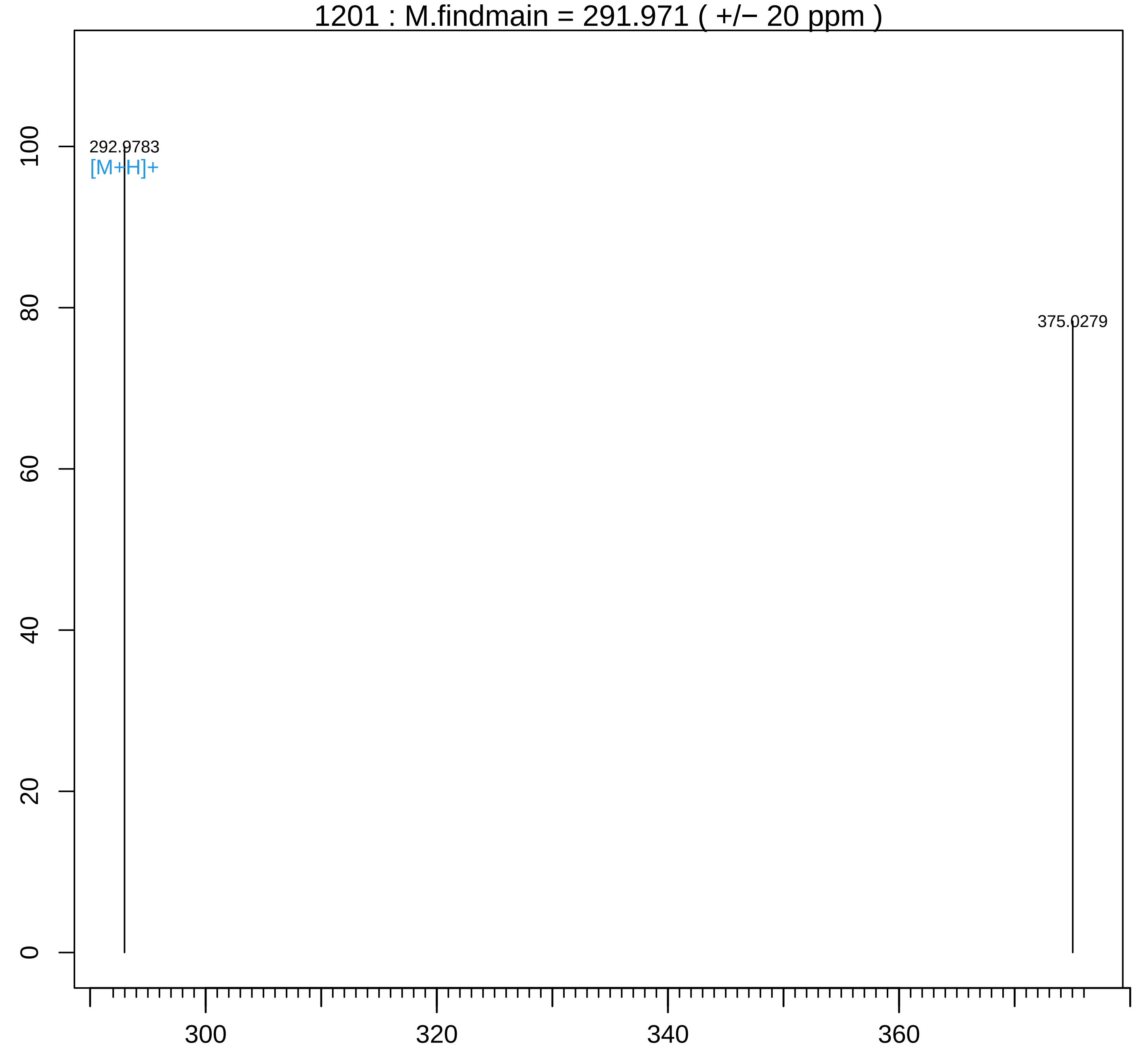
1201 : M.findmain = 291.971 (+/− 20 ppm)
292.9783
[M+H]+
375.0279

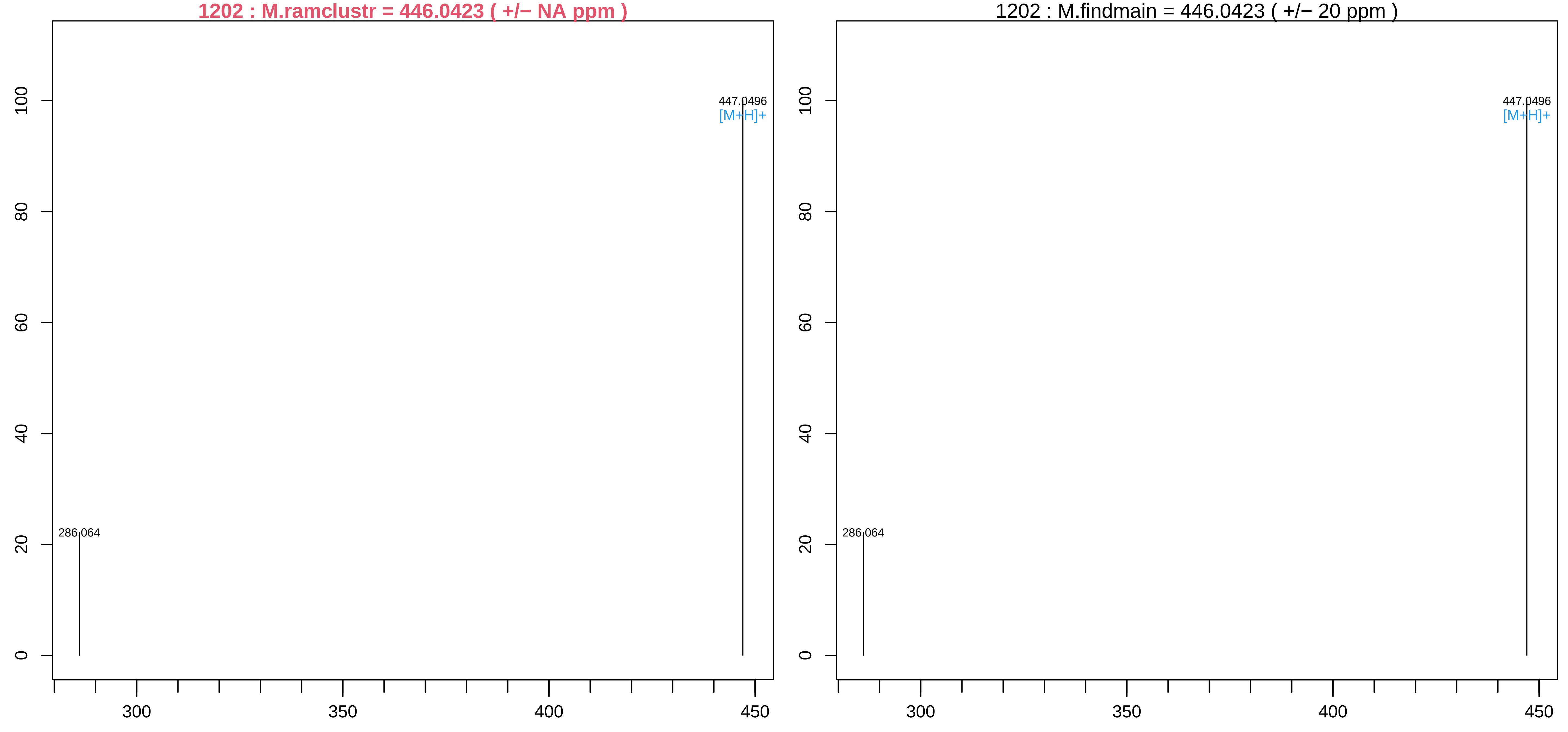

1202 : M.ramclustr = 446.0423 (+/− NA ppm)
447.0496
[M+H]+
286.064
1202 : M.findmain = 446.0423 (+/− 20 ppm)
447.0496
[M+H]+
286.064
0
20
40
60
80
100
300
350
400
450

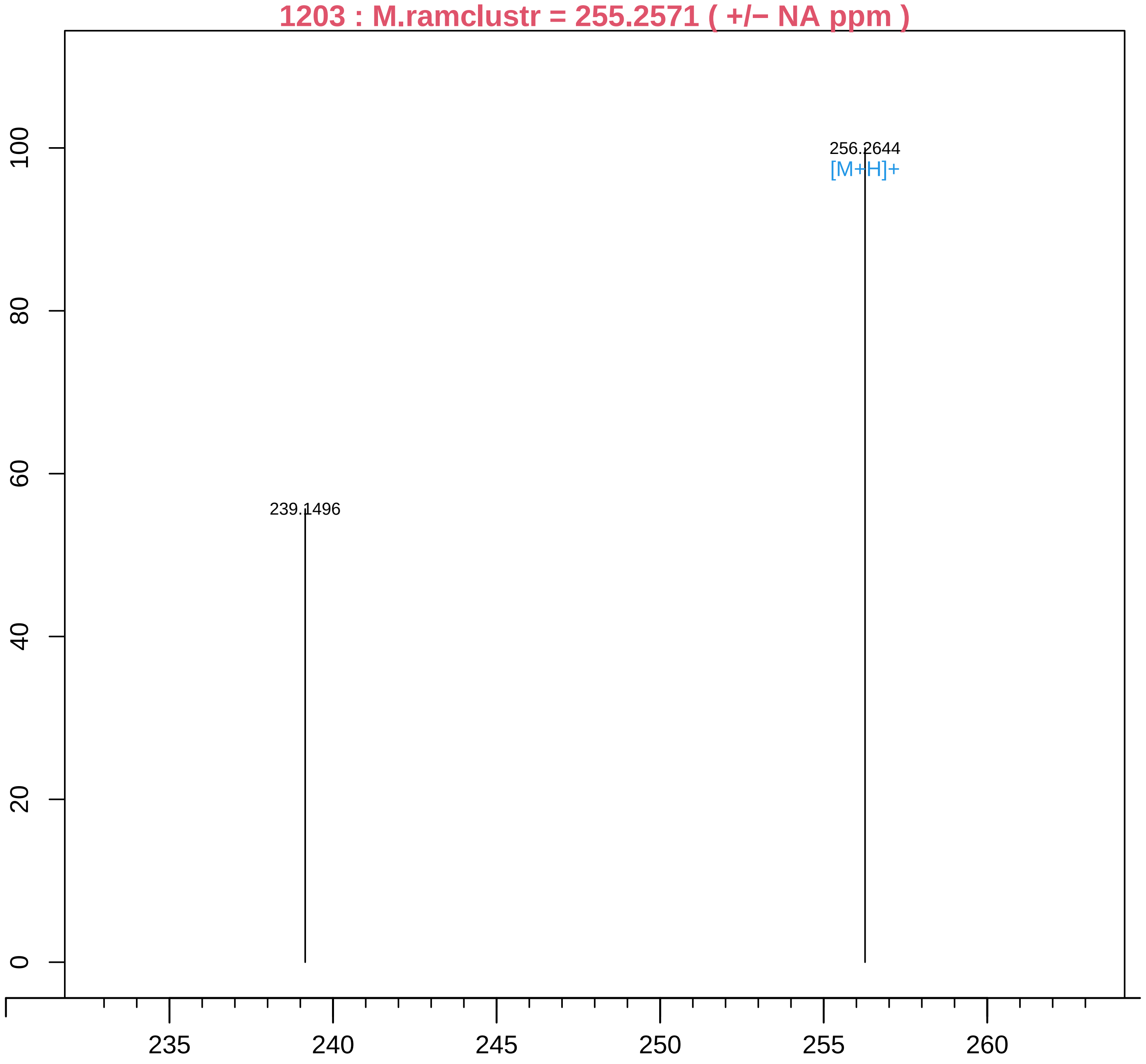
1203 : M.ramclustr = 255.2571 (+/− NA ppm)
256.2644
[M+H]+
239.1496
0
20
40
60
80
100
235
240
245
250
255
260

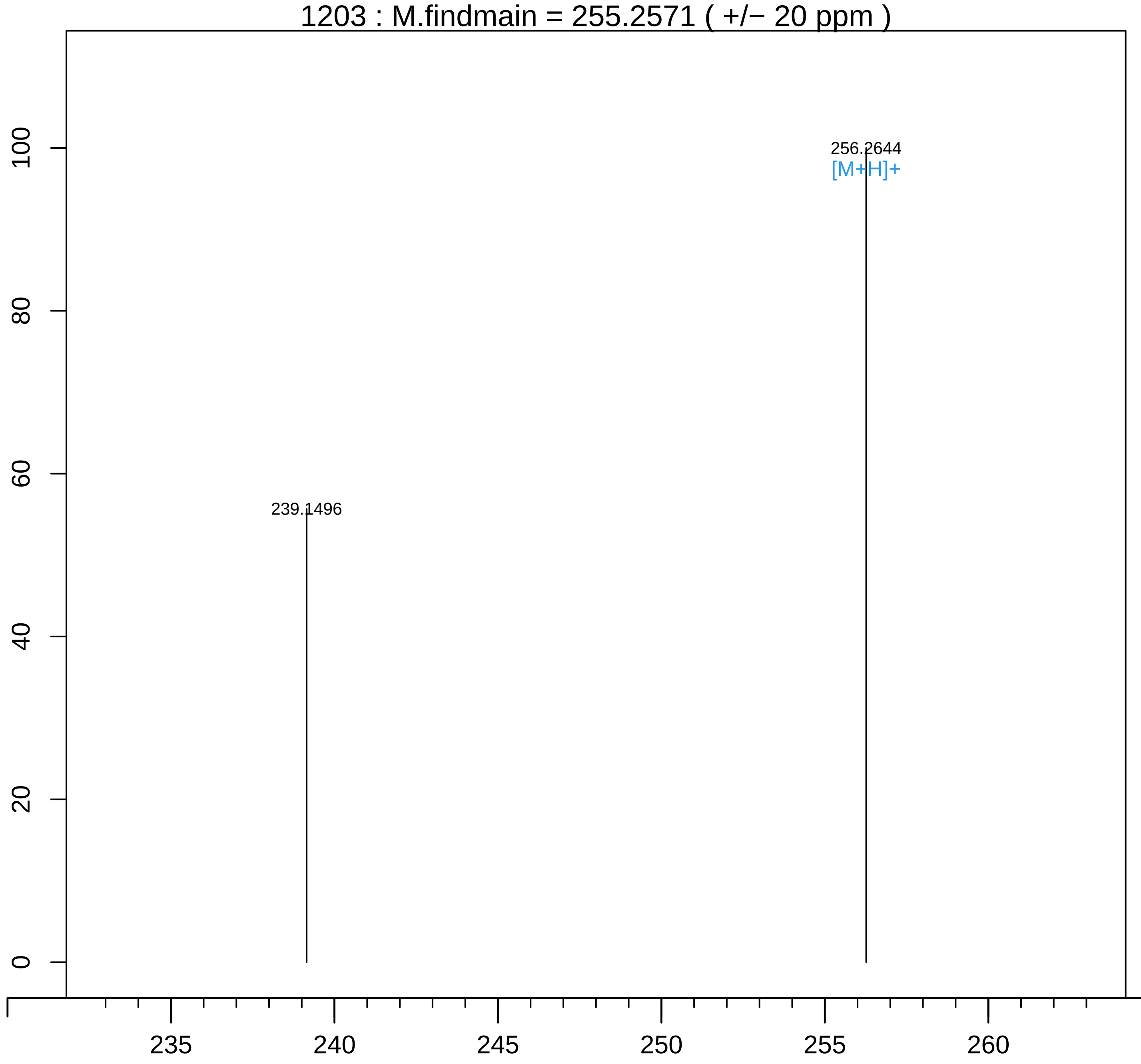
1203 : M.findmain = 255.2571 (+/− 20 ppm)
256.2644
[M+H]+
239.1496
0
20
40
60
80
100
235
240
245
250
255
260

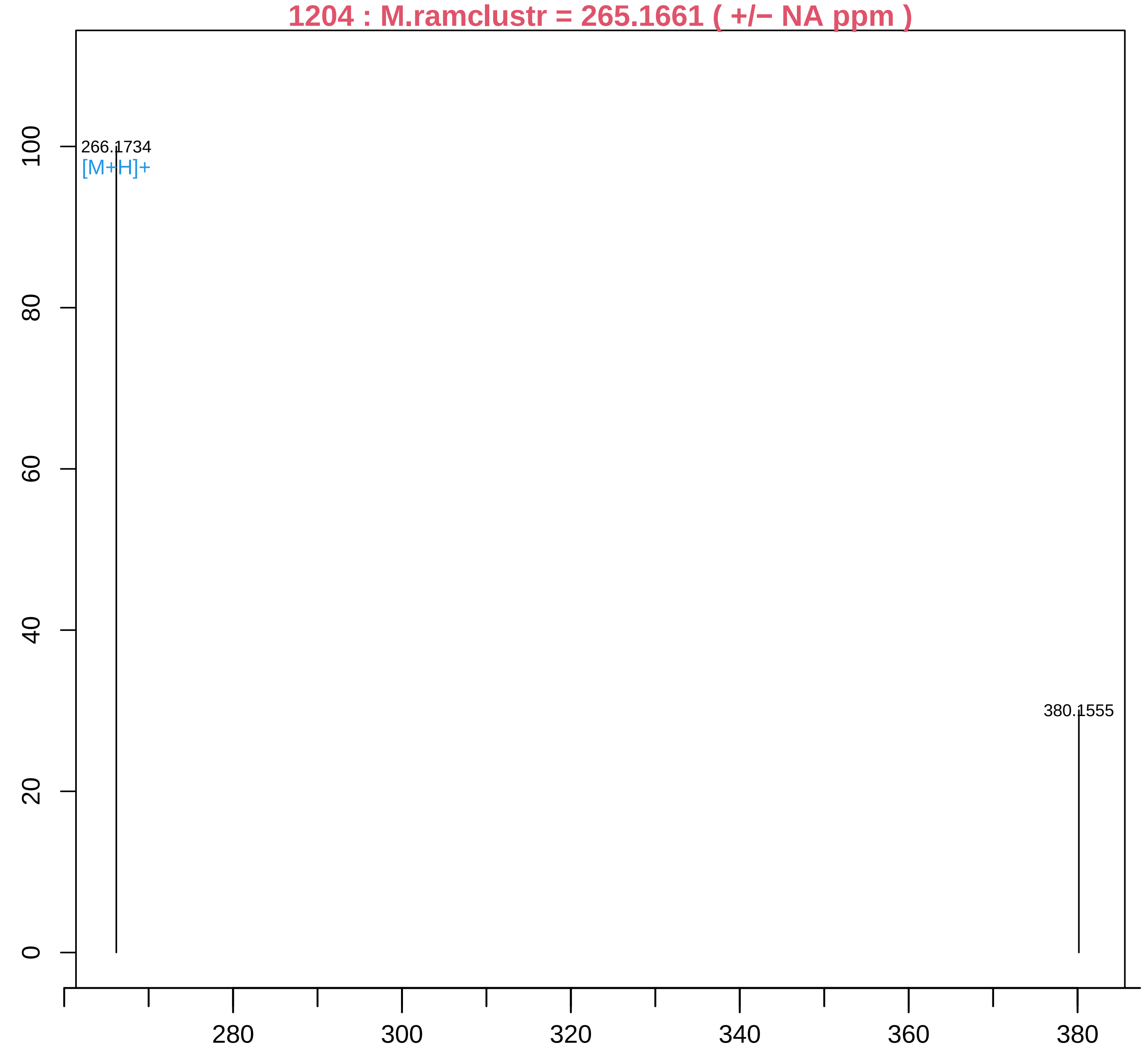
1204 : M.ramclustr = 265.1661 (+/− NA ppm)
266.1734
[M+H]+
380.1555
0
20
40
60
80
100
280
300
320
340
360
380

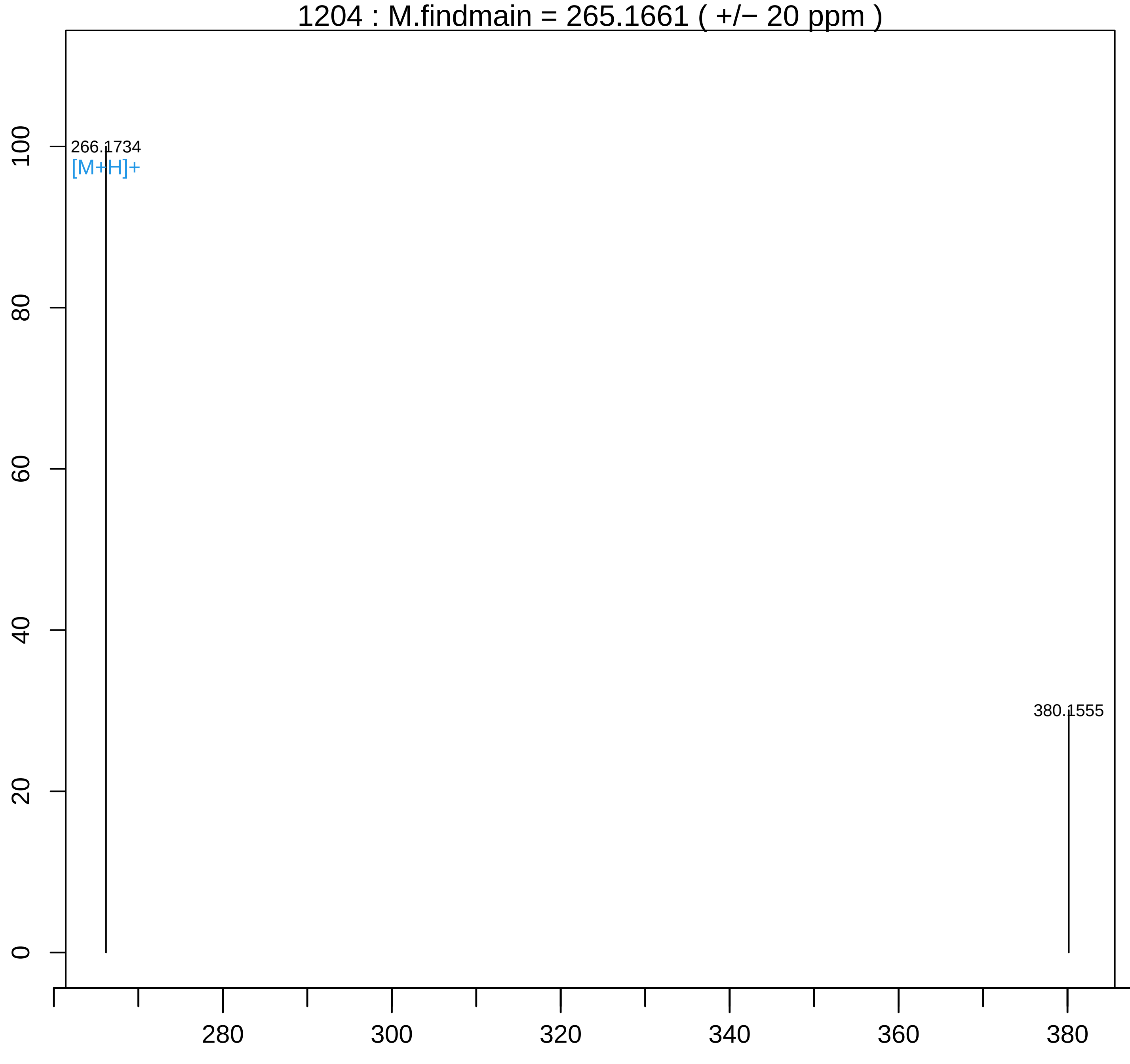
1204 : M.findmain = 265.1661 (+/− 20 ppm)
266.1734
[M+H]+
380.1555
0
20
40
60
80
100
280
300
320
340
360
380

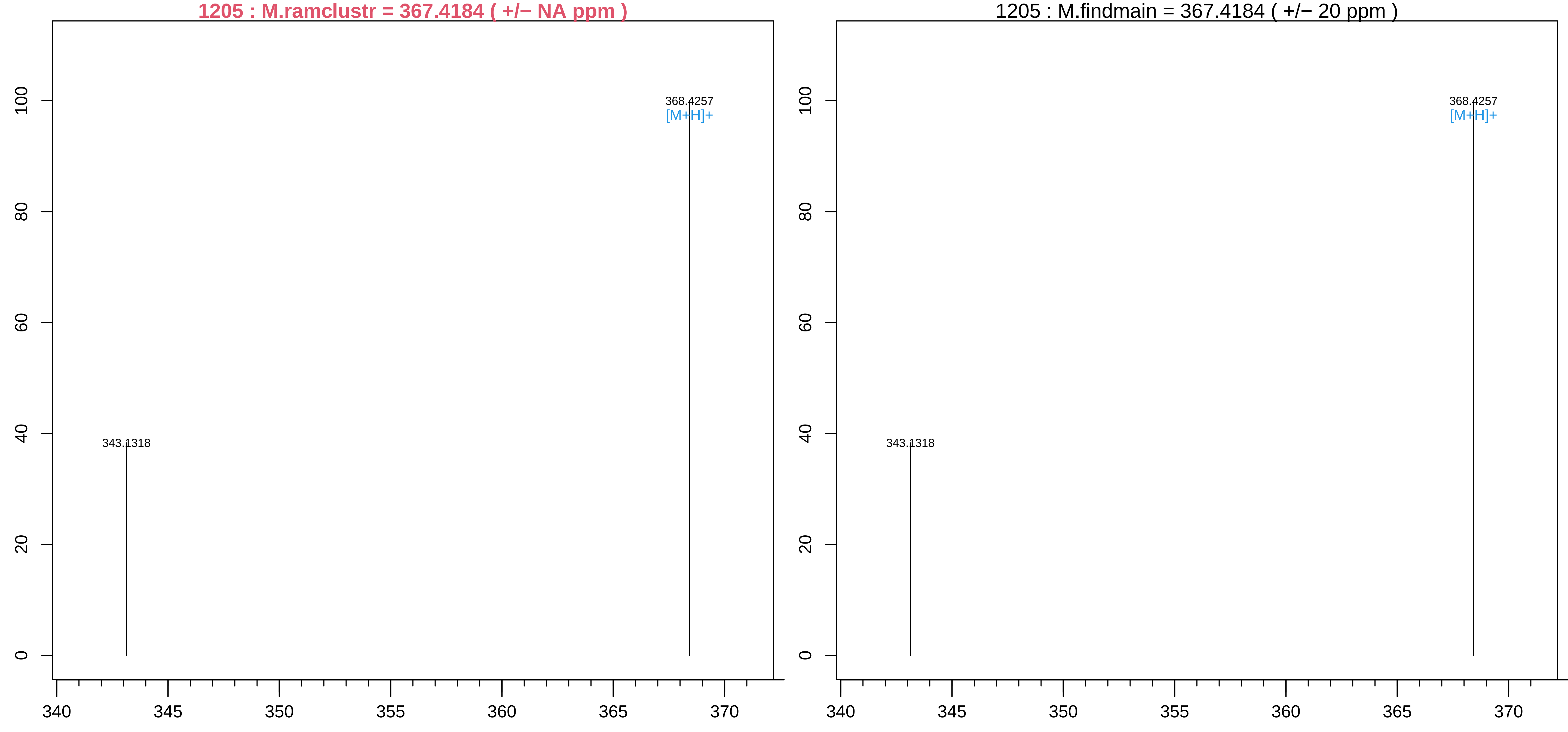
1205 : M.ramclustr = 367.4184 (+/− NA ppm)
1205 : M.findmain = 367.4184 (+/− 20 ppm)
368.4257
[M+H]+
343.1318
0
20
40
60
80
100
340
345
350
355
360
365
370

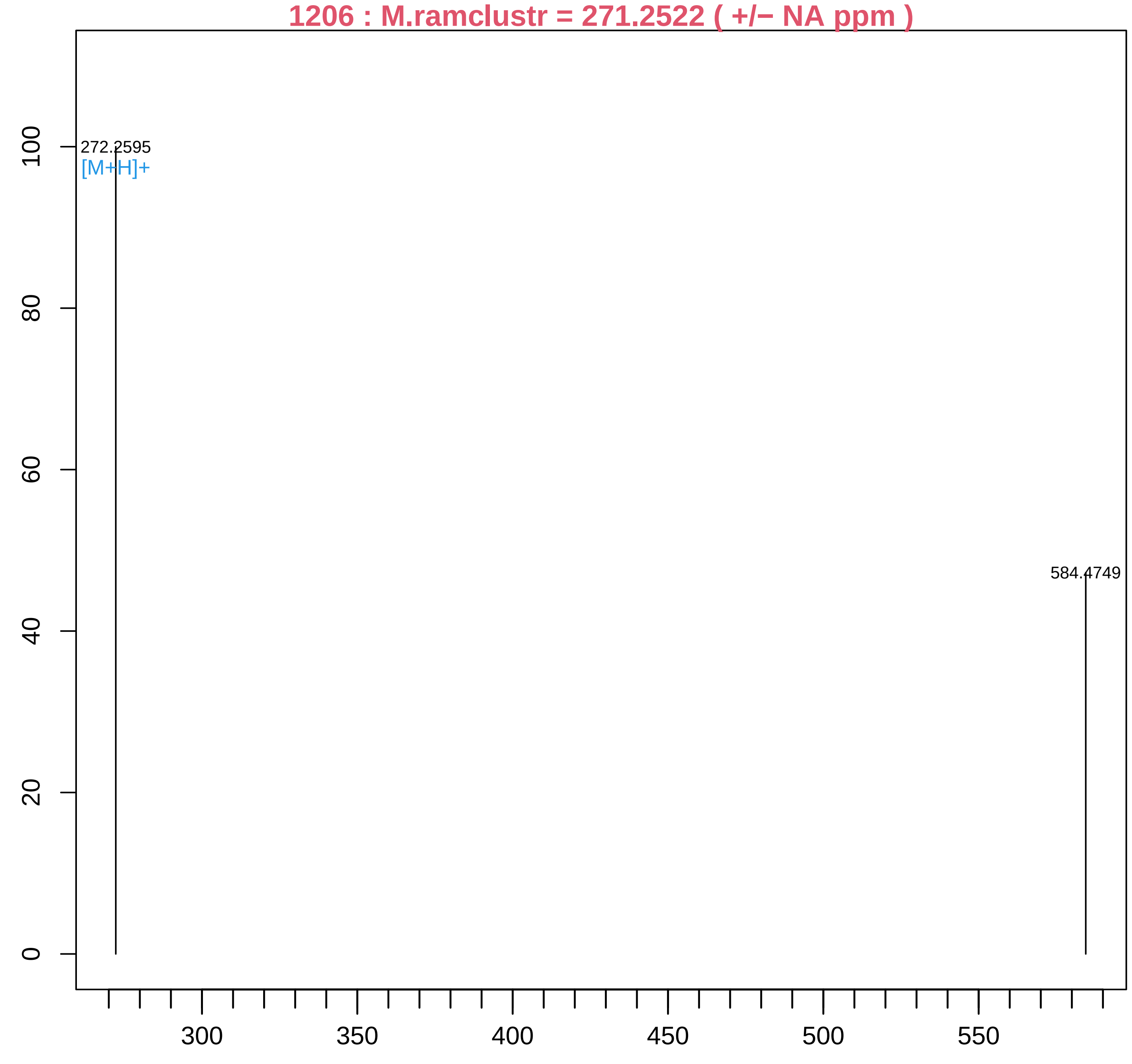

1206 : M.ramclustr = 271.2522 (+/− NA ppm)
272.2595
[M+H]+
584.4749
0
20
40
60
80
100
300
350
400
450
500
550

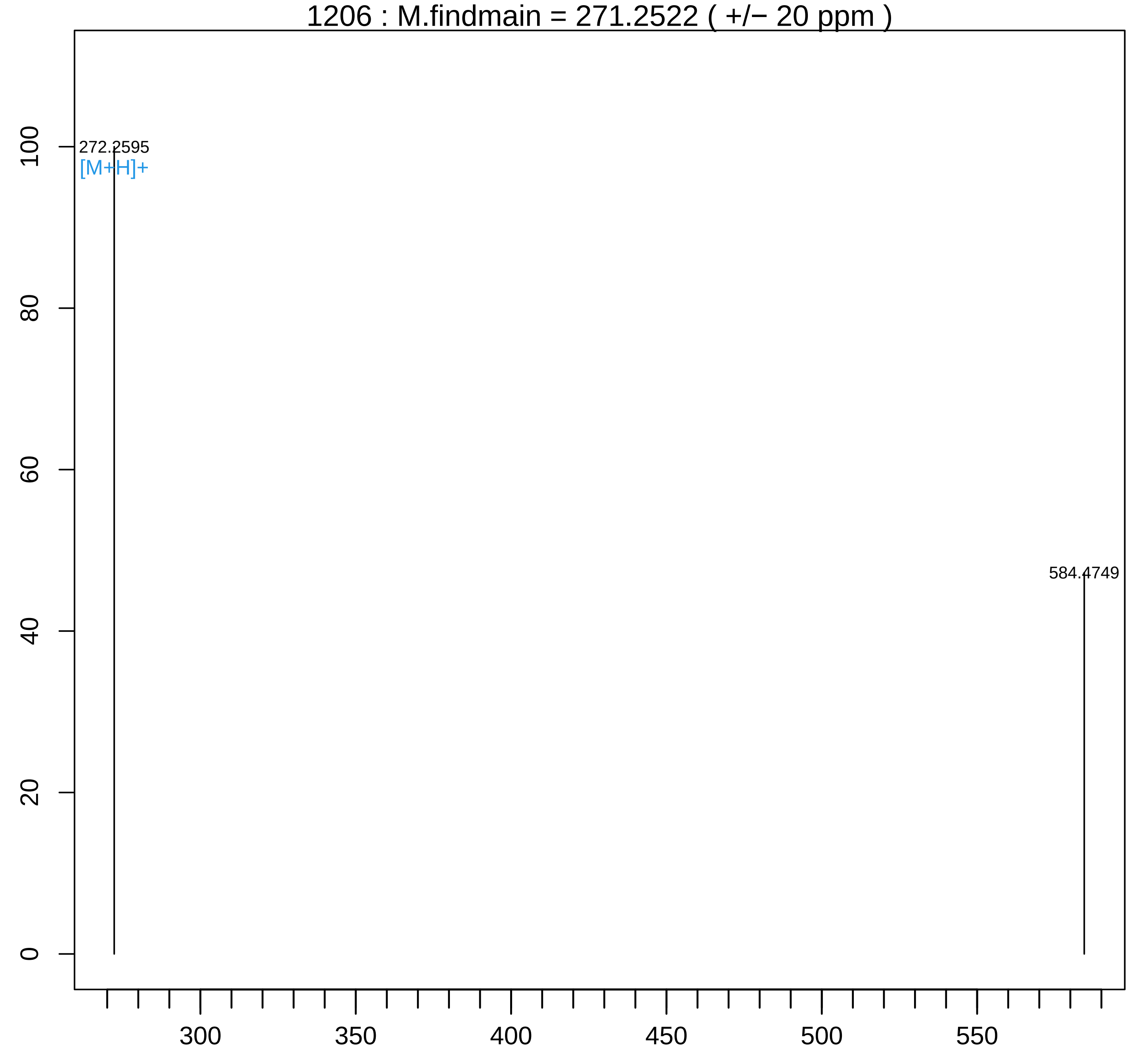

1206 : M.findmain = 271.2522 (+/− 20 ppm)
272.2595
[M+H]+
584.4749
0
20
40
60
80
100
300
350
400
450
500
550

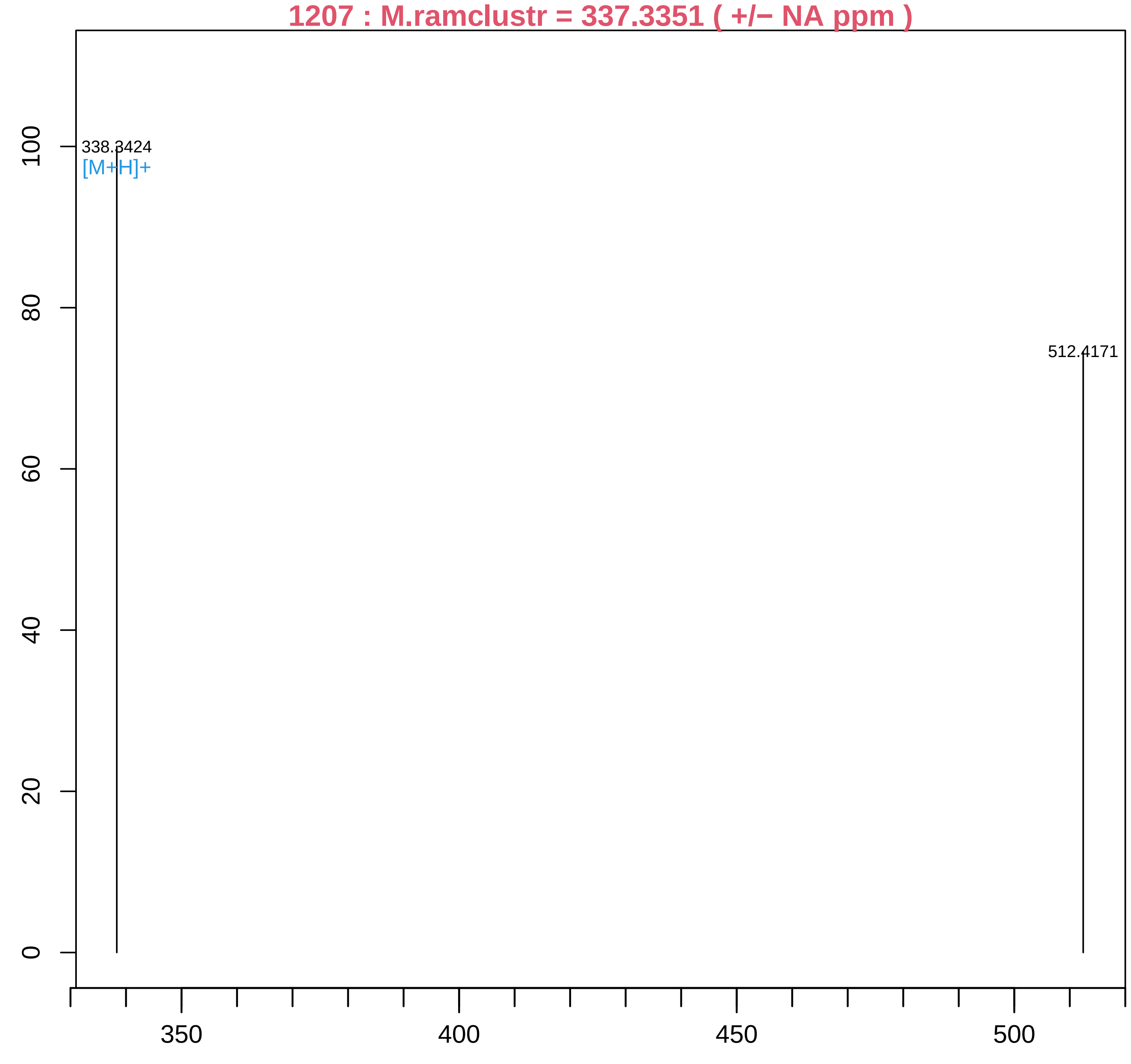
1207 : M.ramclustr = 337.3351 (+/− NA ppm)
338.3424
[M+H]+
512.4171
0
20
40
60
80
100
350
400
450
500

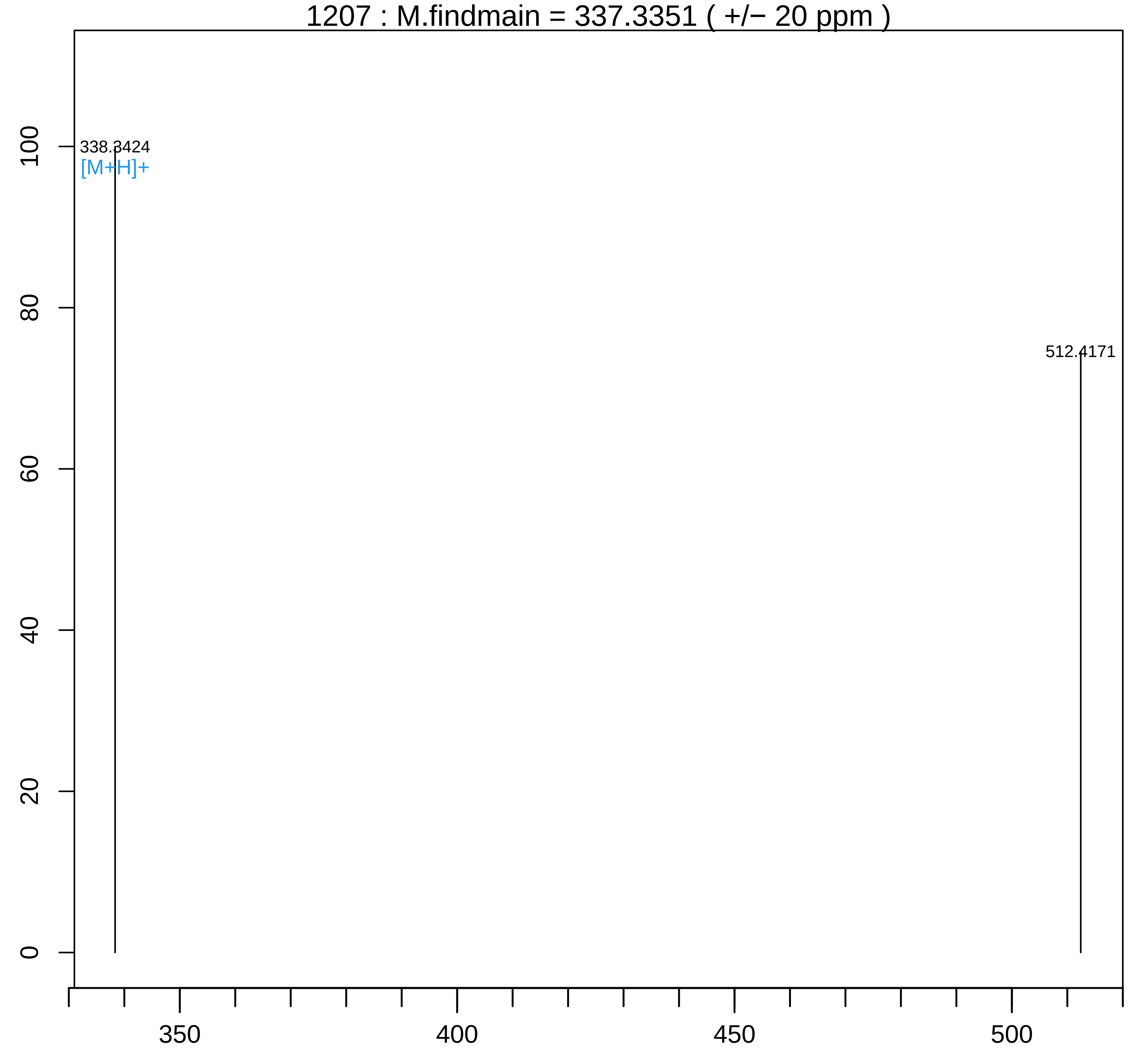
1207 : M.findmain = 337.3351 (+/− 20 ppm)
338.3424
[M+H]+
512.4171
0
20
40
60
80
100
350
400
450
500

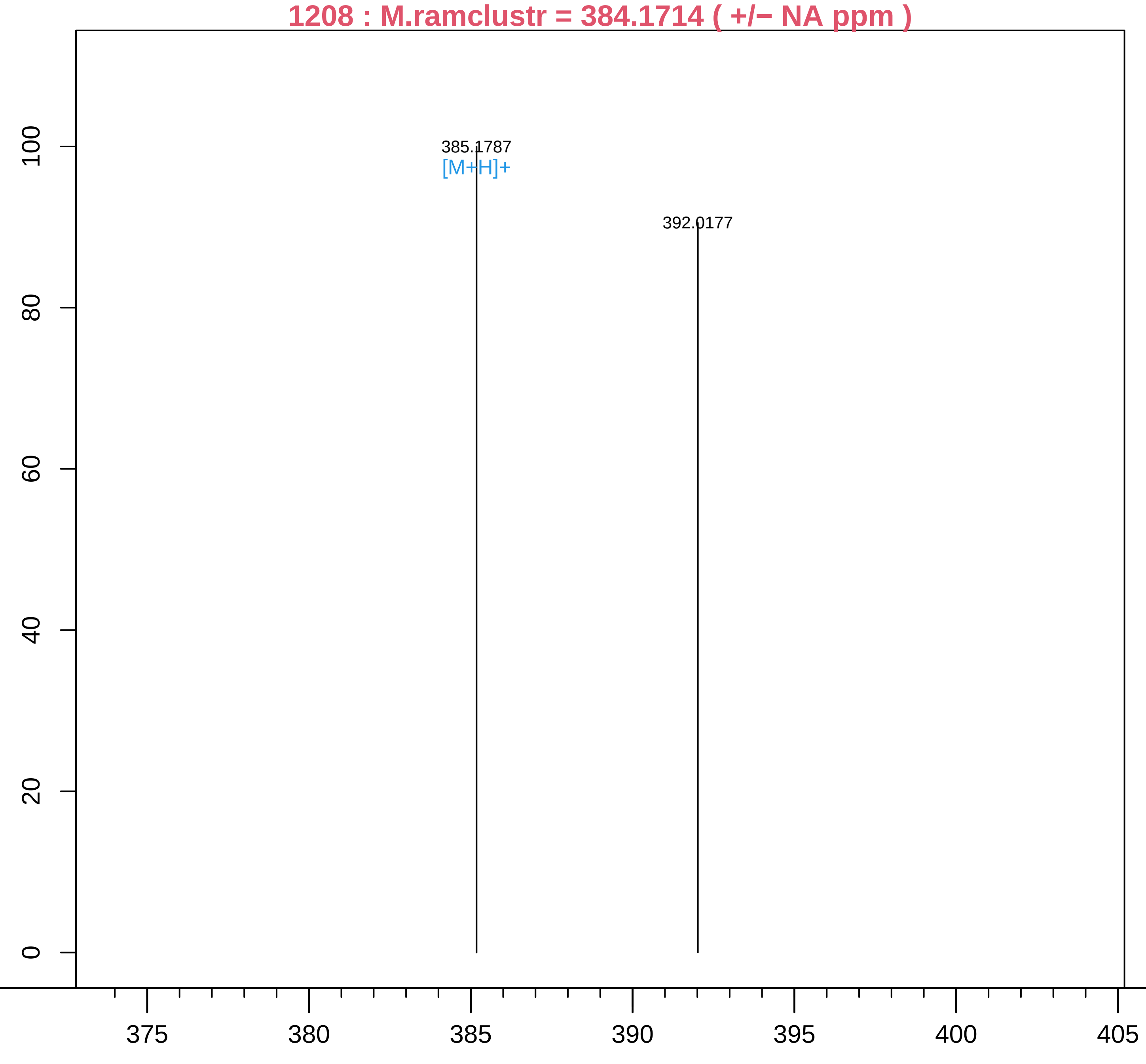
1208 : M.ramclustr = 384.1714 (+/− NA ppm)
385.1787
[M+H]+
392.0177
0
20
40
60
80
100
375
380
385
390
395
400
405

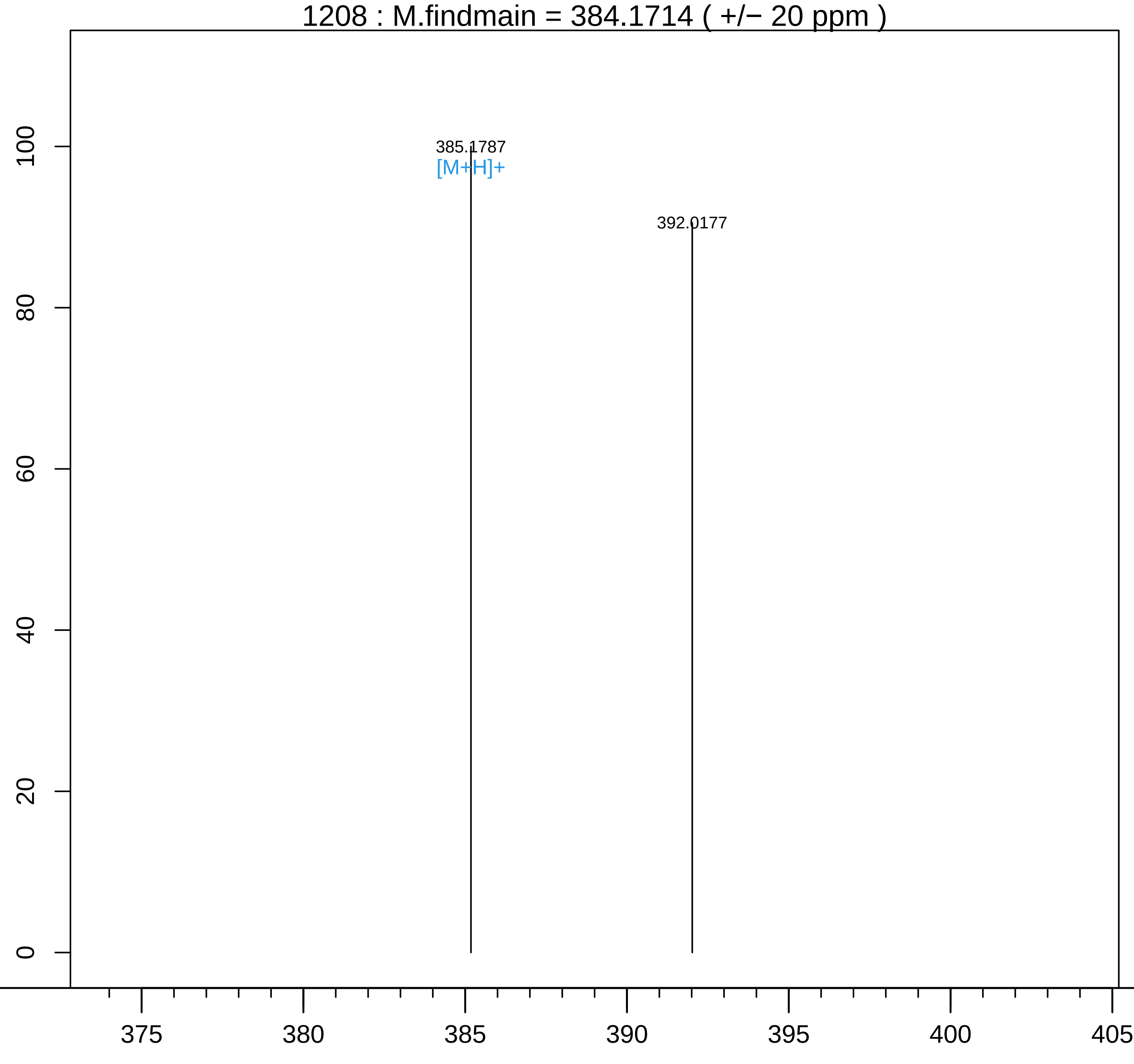
1208 : M.findmain = 384.1714 (+/− 20 ppm)
385.1787
[M+H]+
392.0177
0
20
40
60
80
100
375
380
385
390
395
400
405

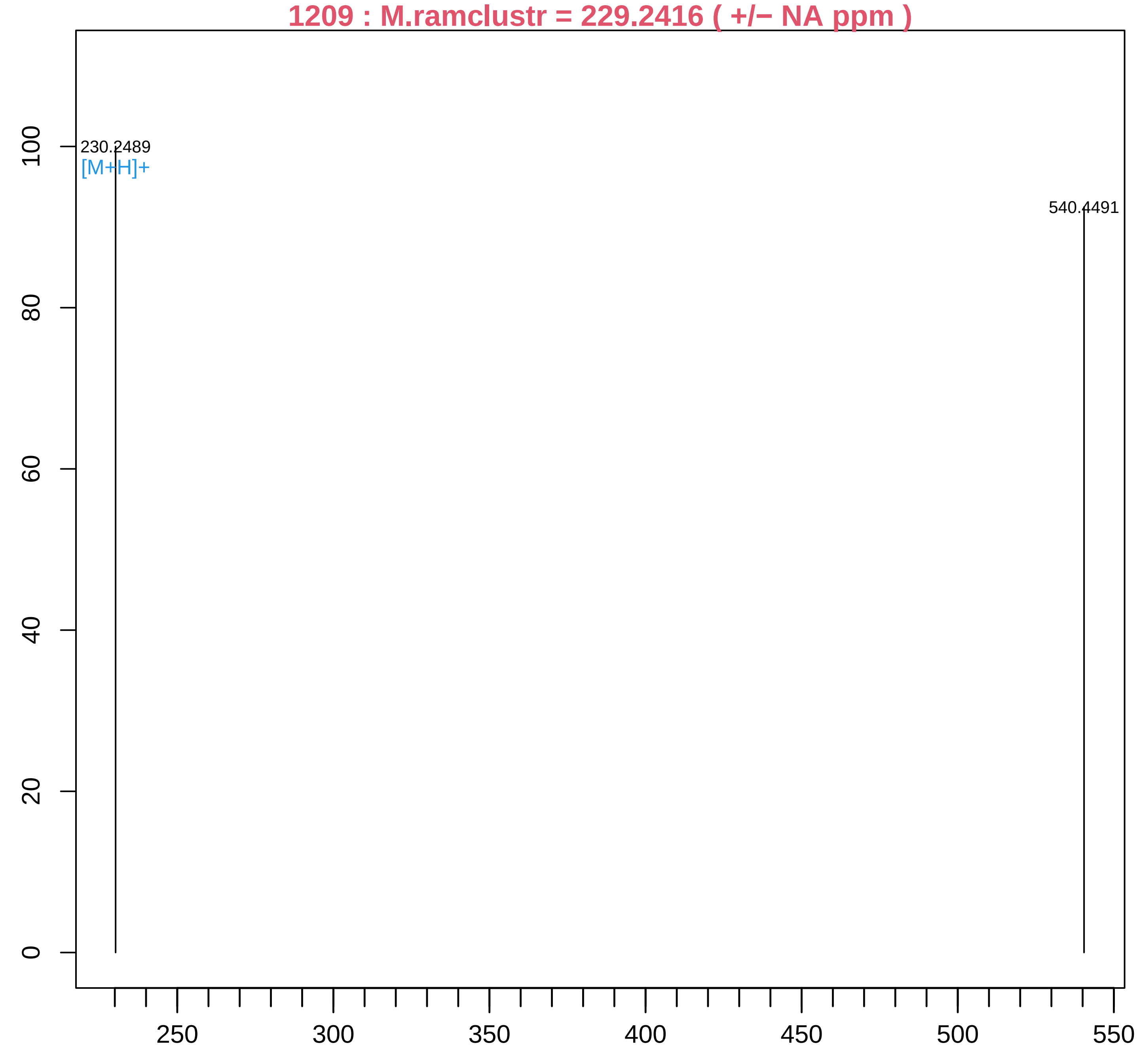
1209 : M.ramclustr = 229.2416 (+/− NA ppm)
230.2489
[M+H]+
540.4491
0
20
40
60
80
100
250
300
350
400
450
500
550

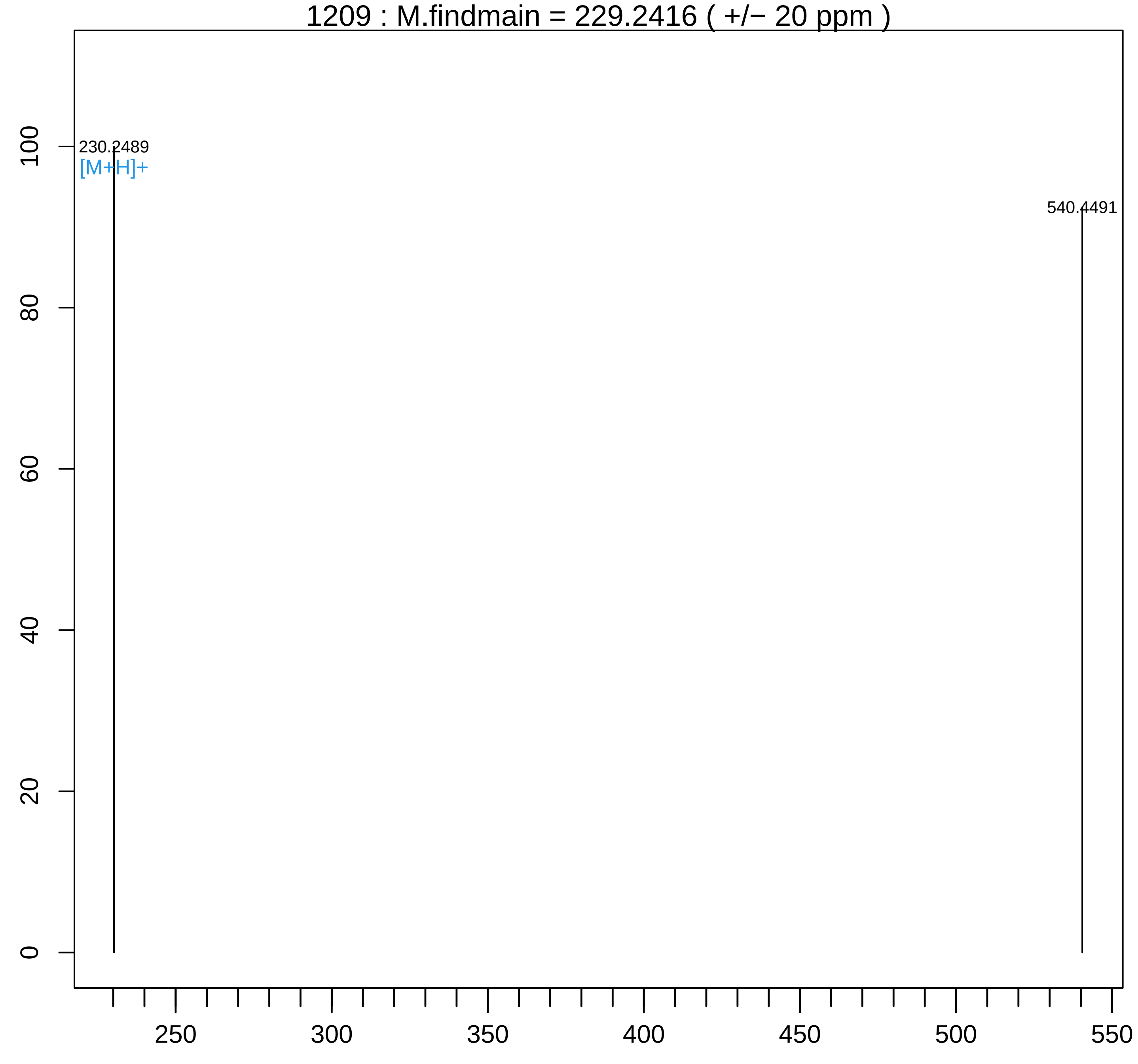
1209 : M.findmain = 229.2416 (+/− 20 ppm)
230.2489
[M+H]+
540.4491
0
20
40
60
80
100
250
300
350
400
450
500
550

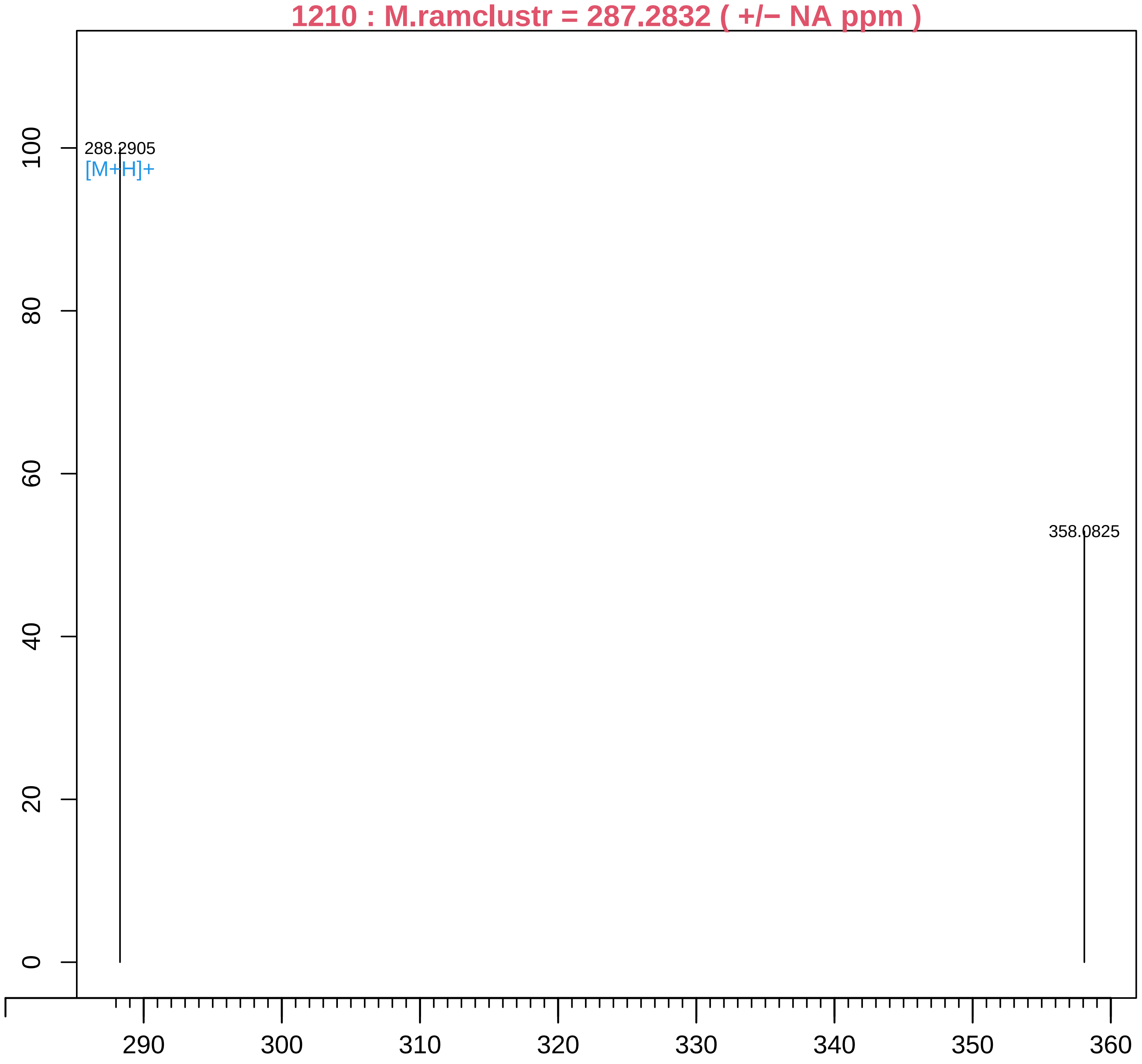
1210 : M.ramclustr = 287.2832 (+/− NA ppm)
288.2905
[M+H]+
358.0825
0
20
40
60
80
100
290
300
310
320
330
340
350
360

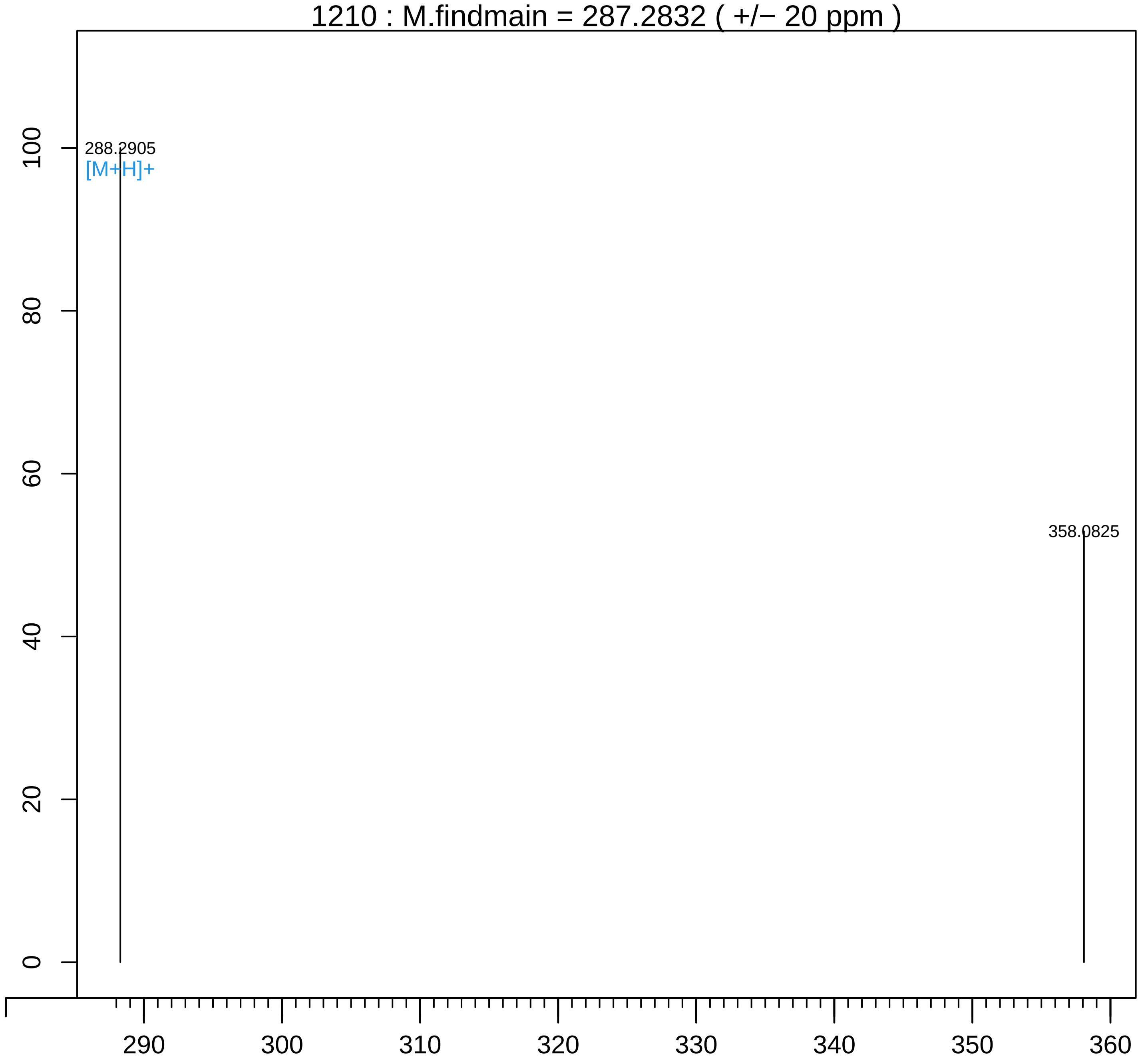
1210 : M.findmain = 287.2832 (+/− 20 ppm)
288.2905
[M+H]+
358.0825
0
20
40
60
80
100
290
300
310
320
330
340
350
360

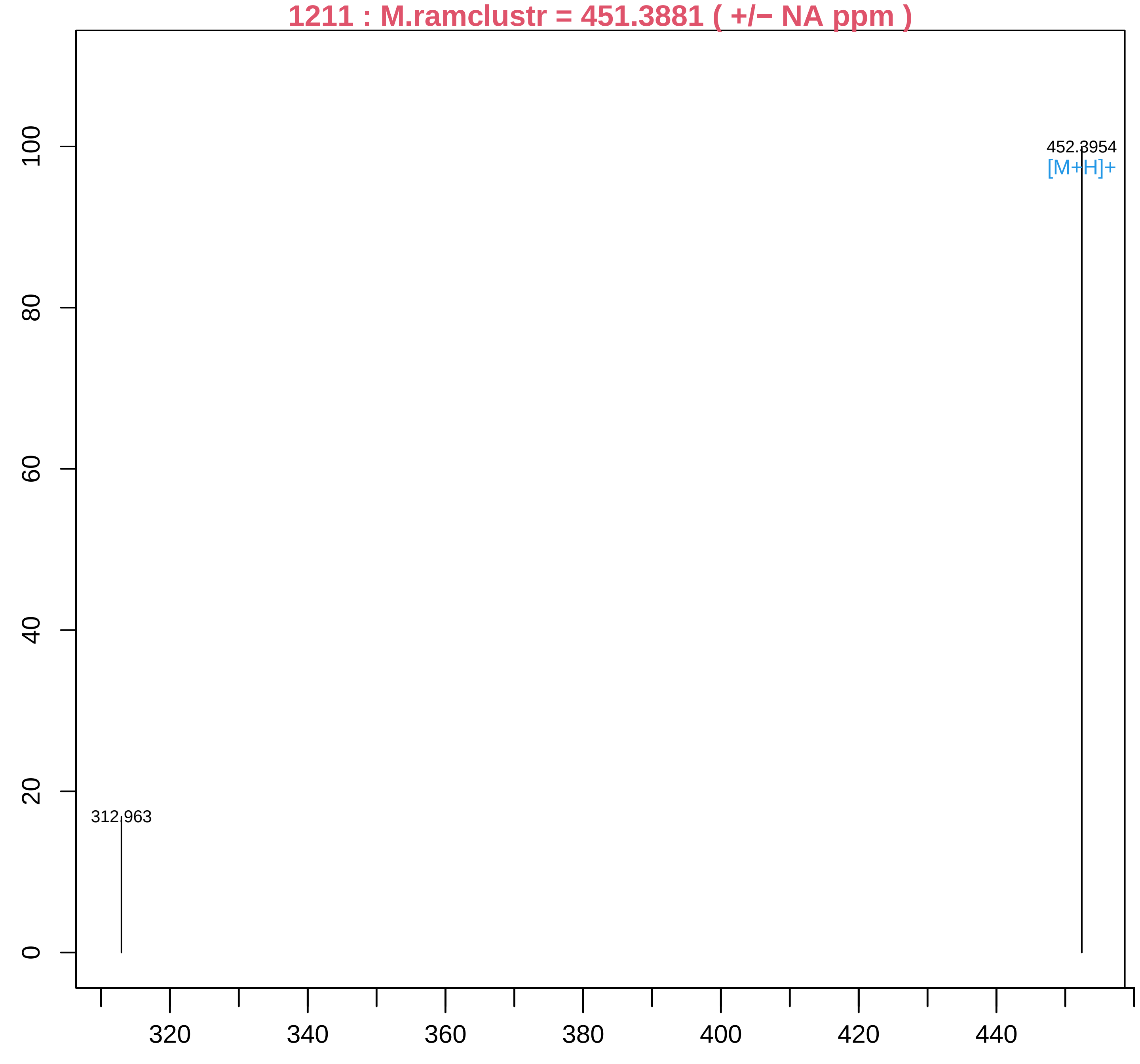
1211 : M.ramclustr = 451.3881 (+/− NA ppm)
452.3954
[M+H]+
312.963
0
20
40
60
80
100
320
340
360
380
400
420
440

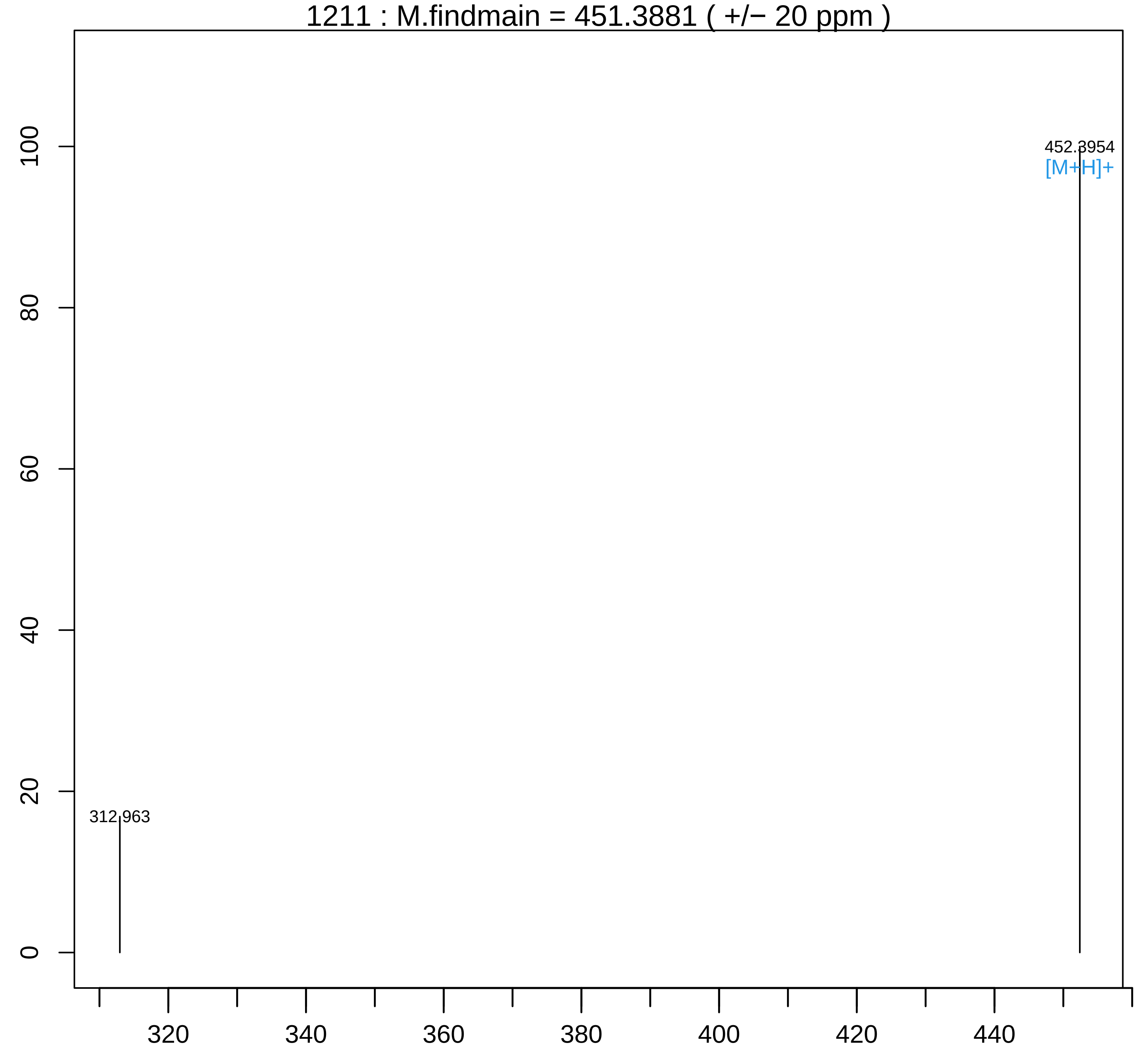
1211 : M.findmain = 451.3881 (+/− 20 ppm)
452.3954
[M+H]+
312.963
0
20
40
60
80
100
320
340
360
380
400
420
440

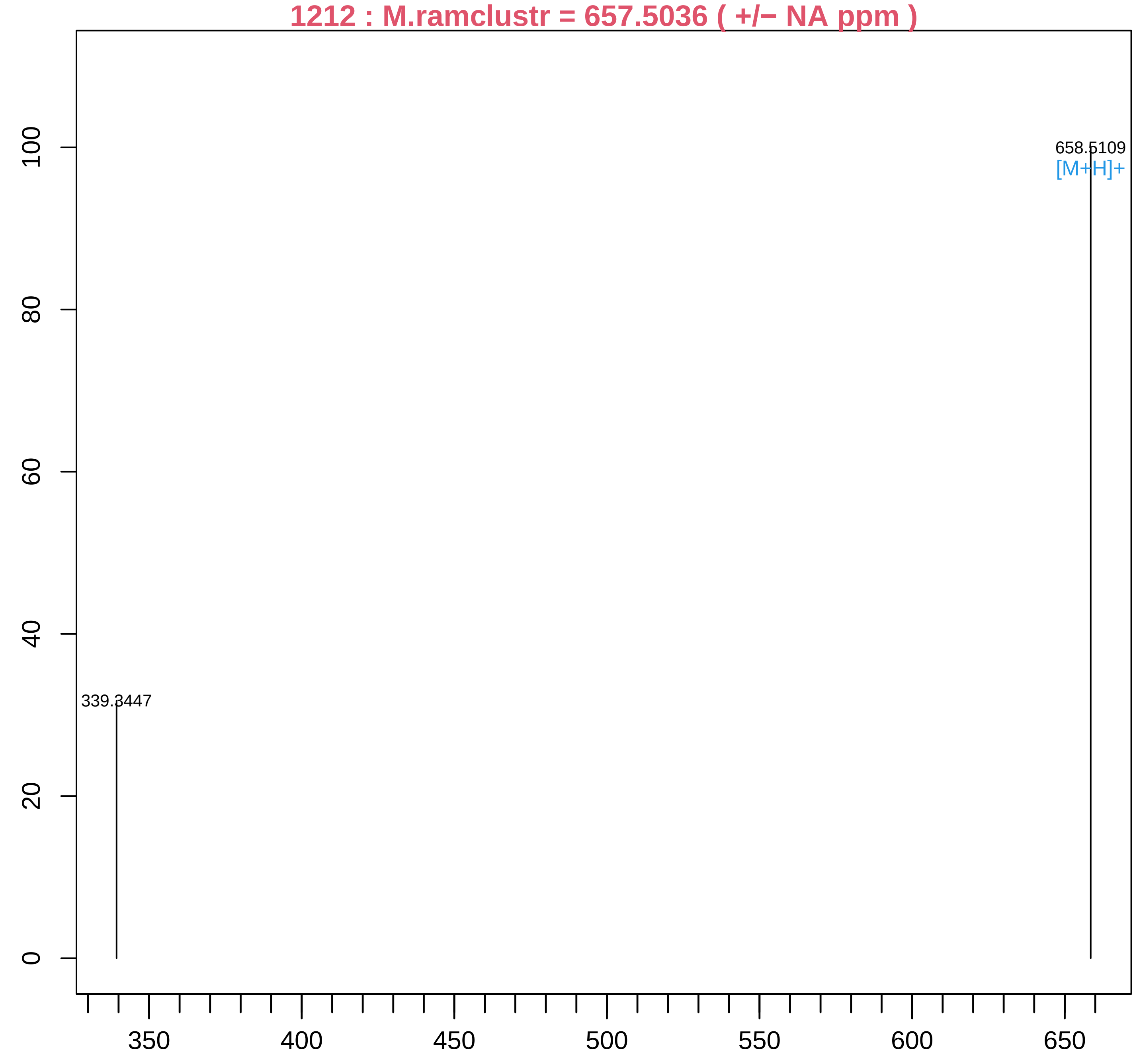
1212 : M.ramclustr = 657.5036 (+/− NA ppm)
658.5109
[M+H]+
339.3447
0
20
40
60
80
100
350
400
450
500
550
600
650

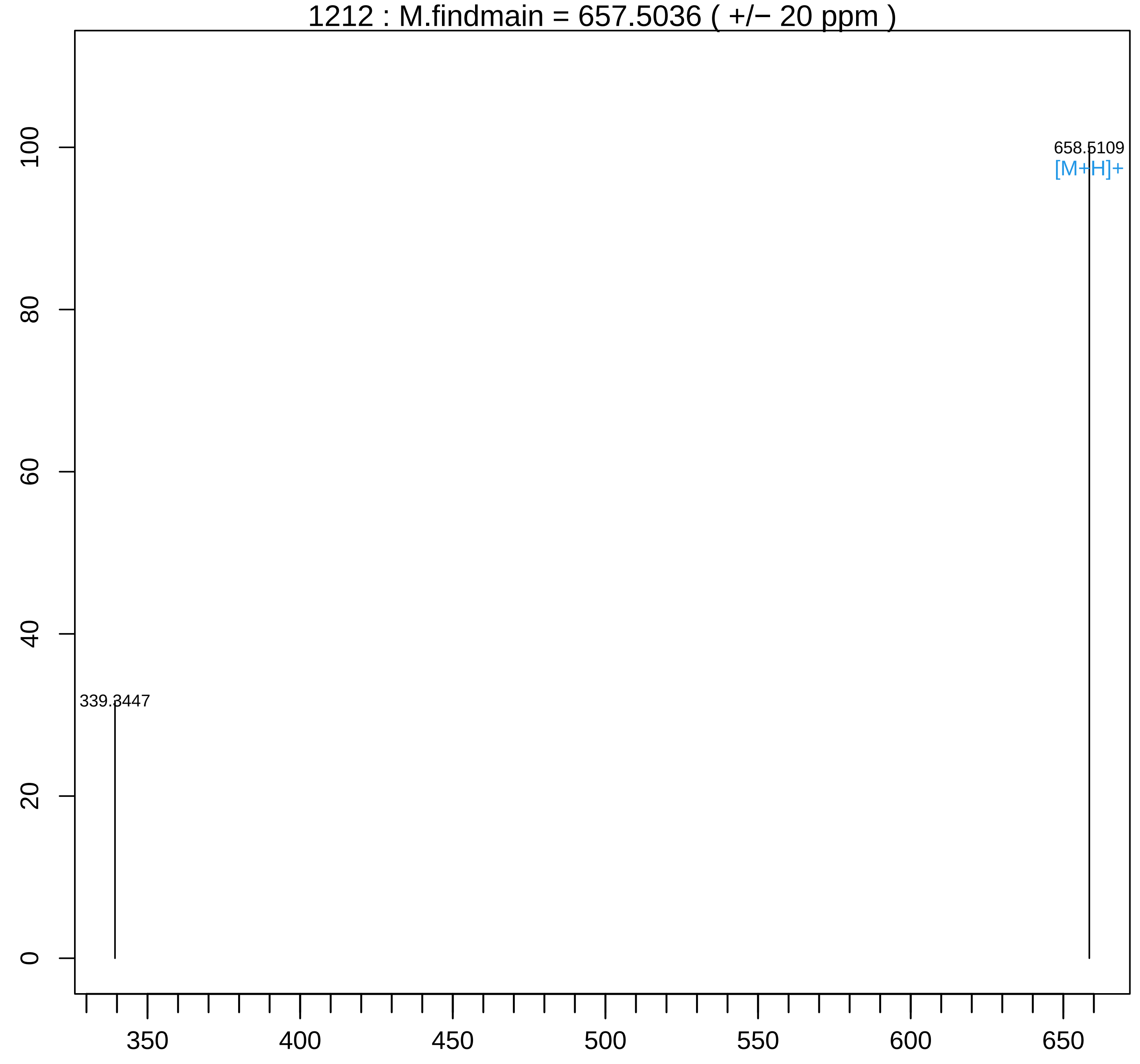
1212 : M.findmain = 657.5036 (+/− 20 ppm)
658.5109
[M+H]+
339.3447
0
20
40
60
80
100
350
400
450
500
550
600
650

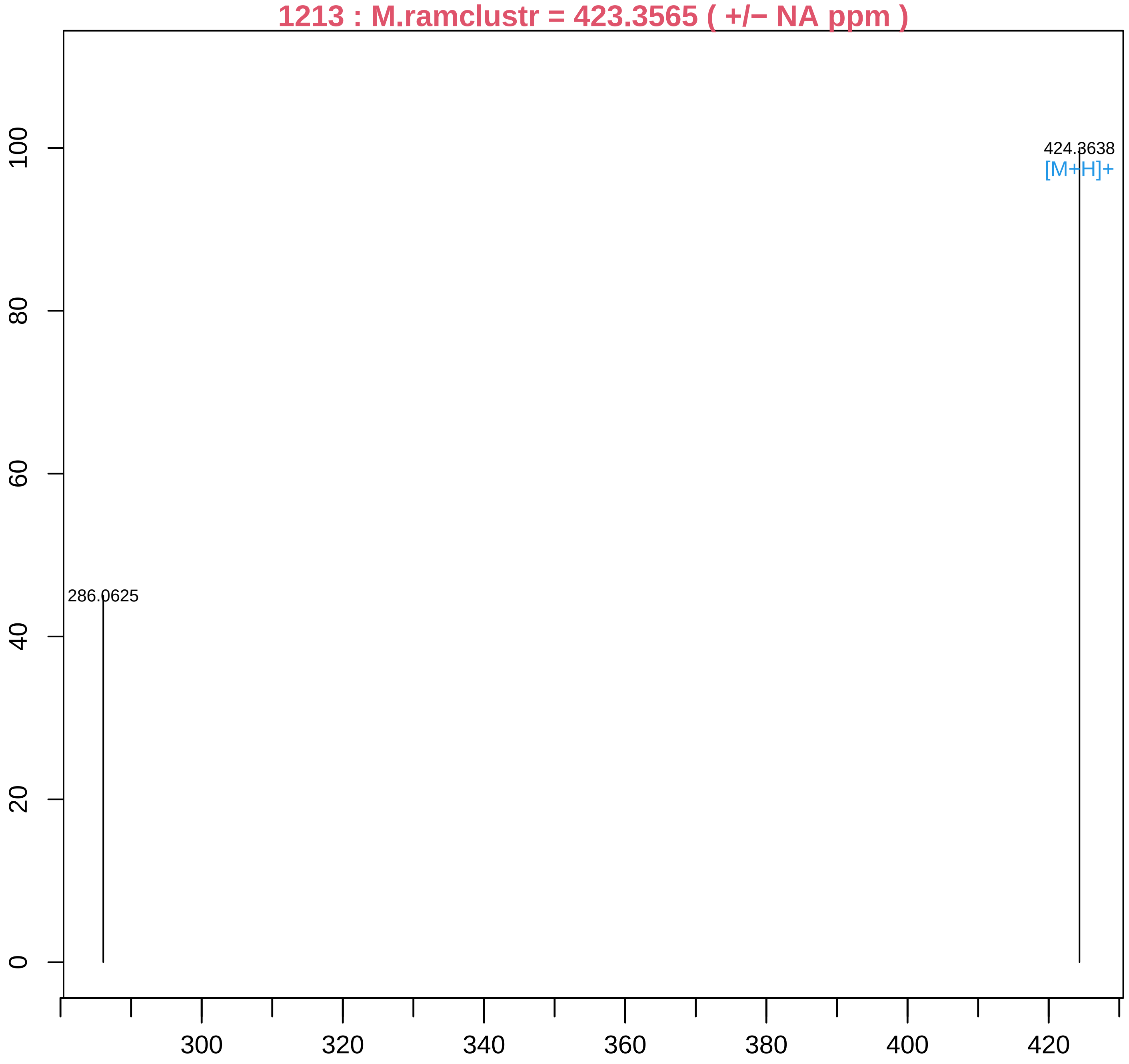
1213 : M.ramclustr = 423.3565 (+/− NA ppm)
424.3638
[M+H]+
286.0625
0
20
40
60
80
100
300
320
340
360
380
400
420

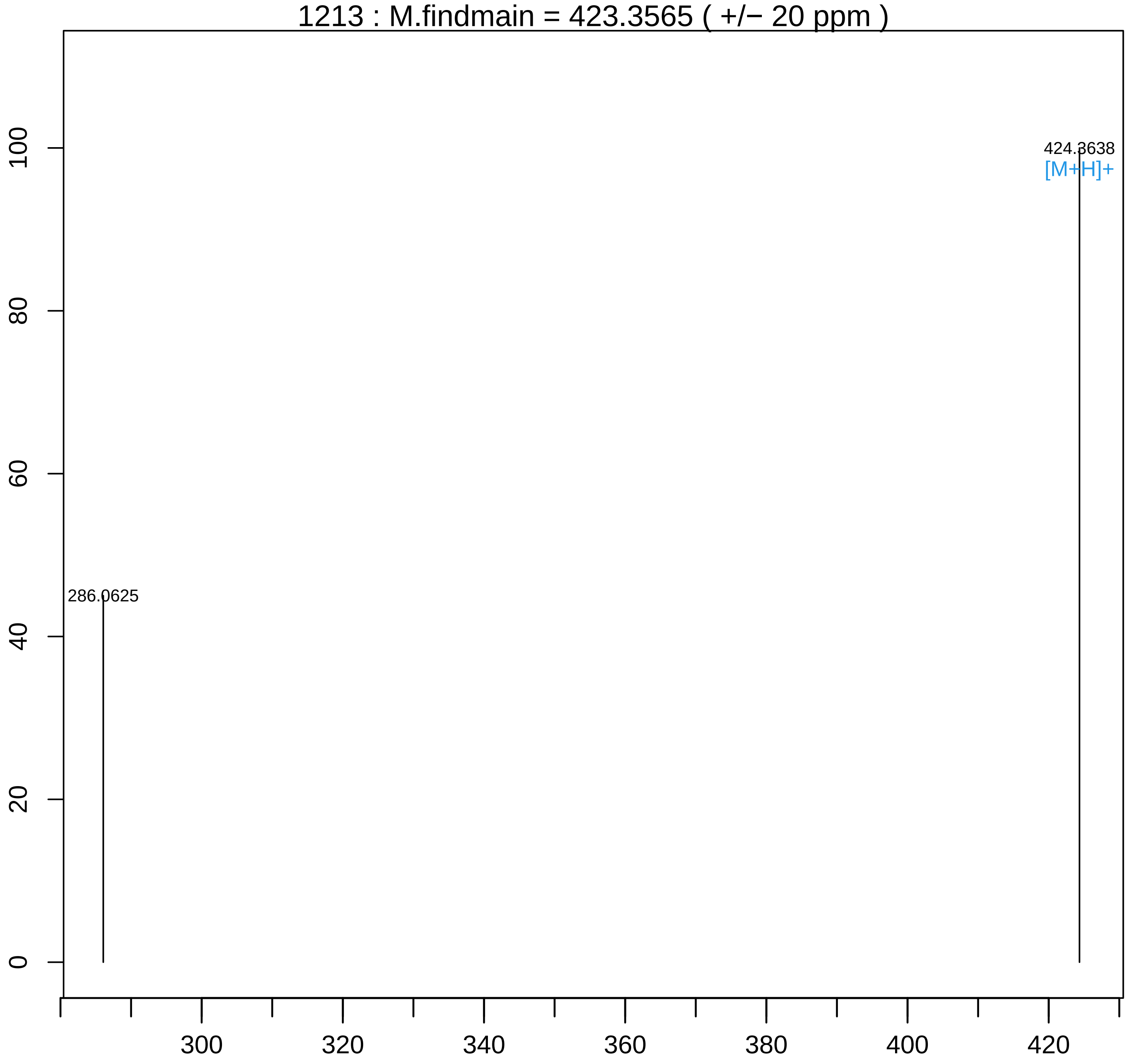
1213 : M.findmain = 423.3565 (+/− 20 ppm)
424.3638
[M+H]+
286.0625
0
20
40
60
80
100
300
320
340
360
380
400
420

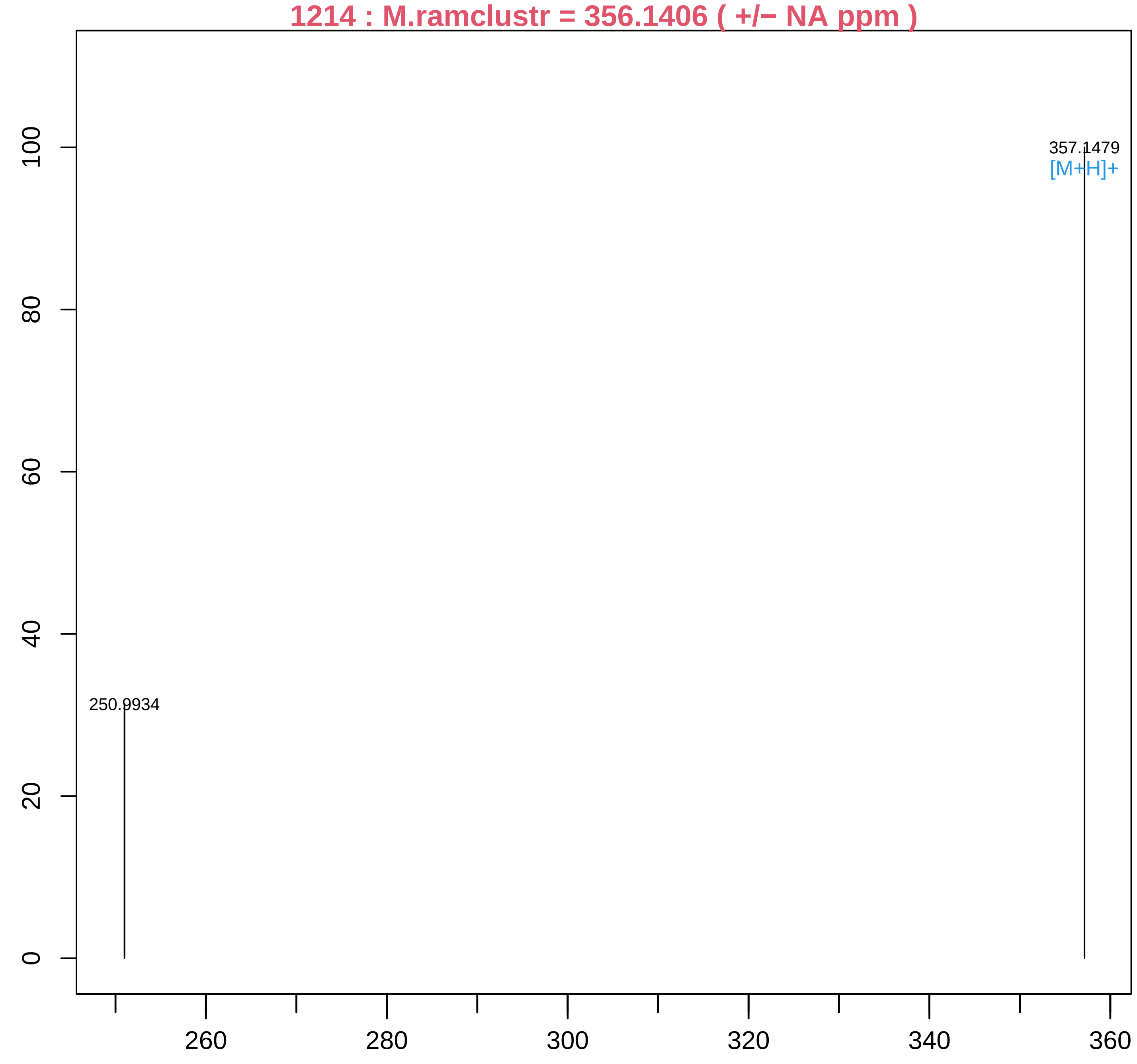
1214 : M.ramclustr = 356.1406 (+/− NA ppm)
357.1479
[M+H]+
250.9934
0
20
40
60
80
100
260
280
300
320
340
360

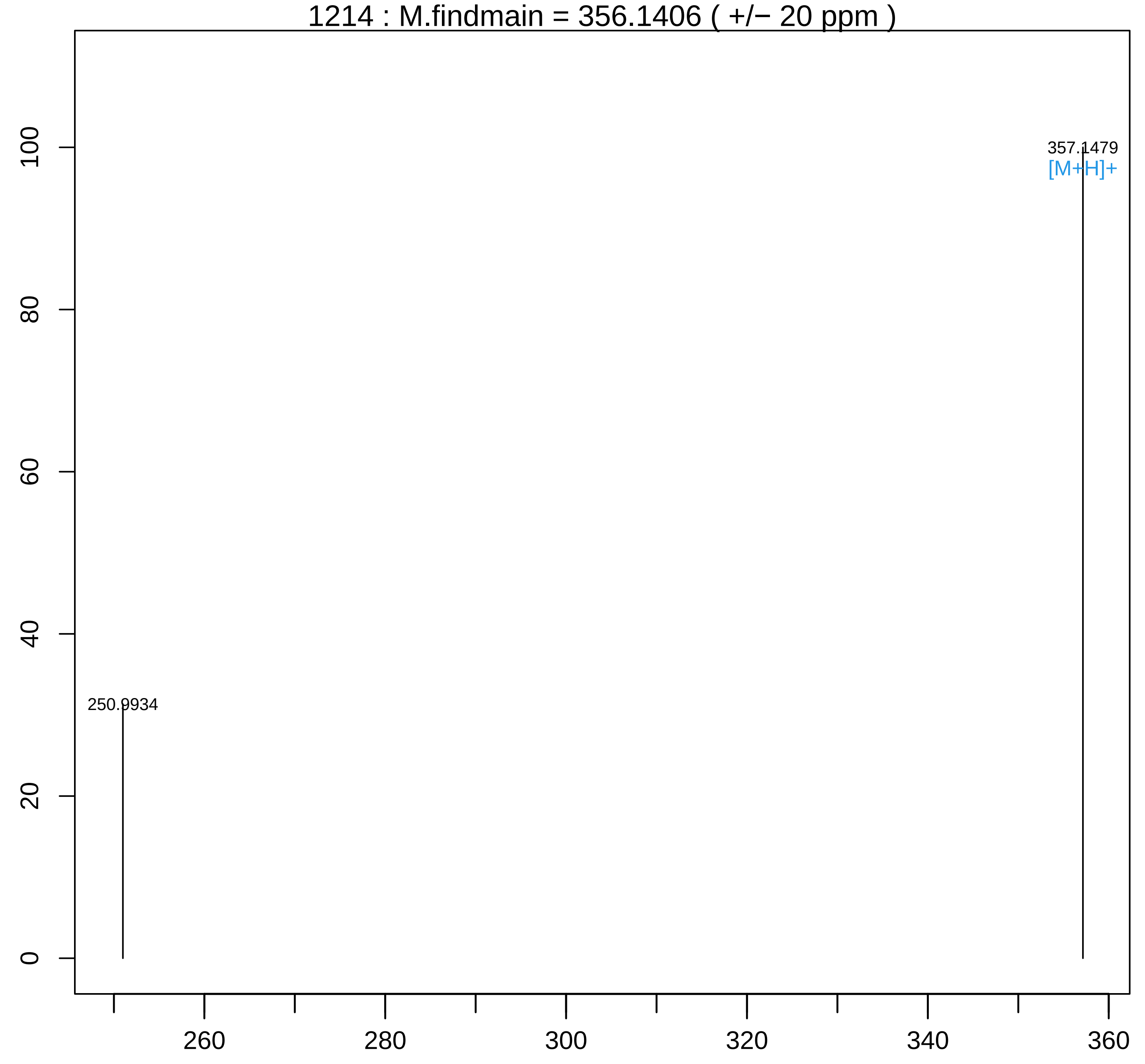
1214 : M.findmain = 356.1406 (+/− 20 ppm)
357.1479
[M+H]+
250.9934
0
20
40
60
80
100
260
280
300
320
340
360

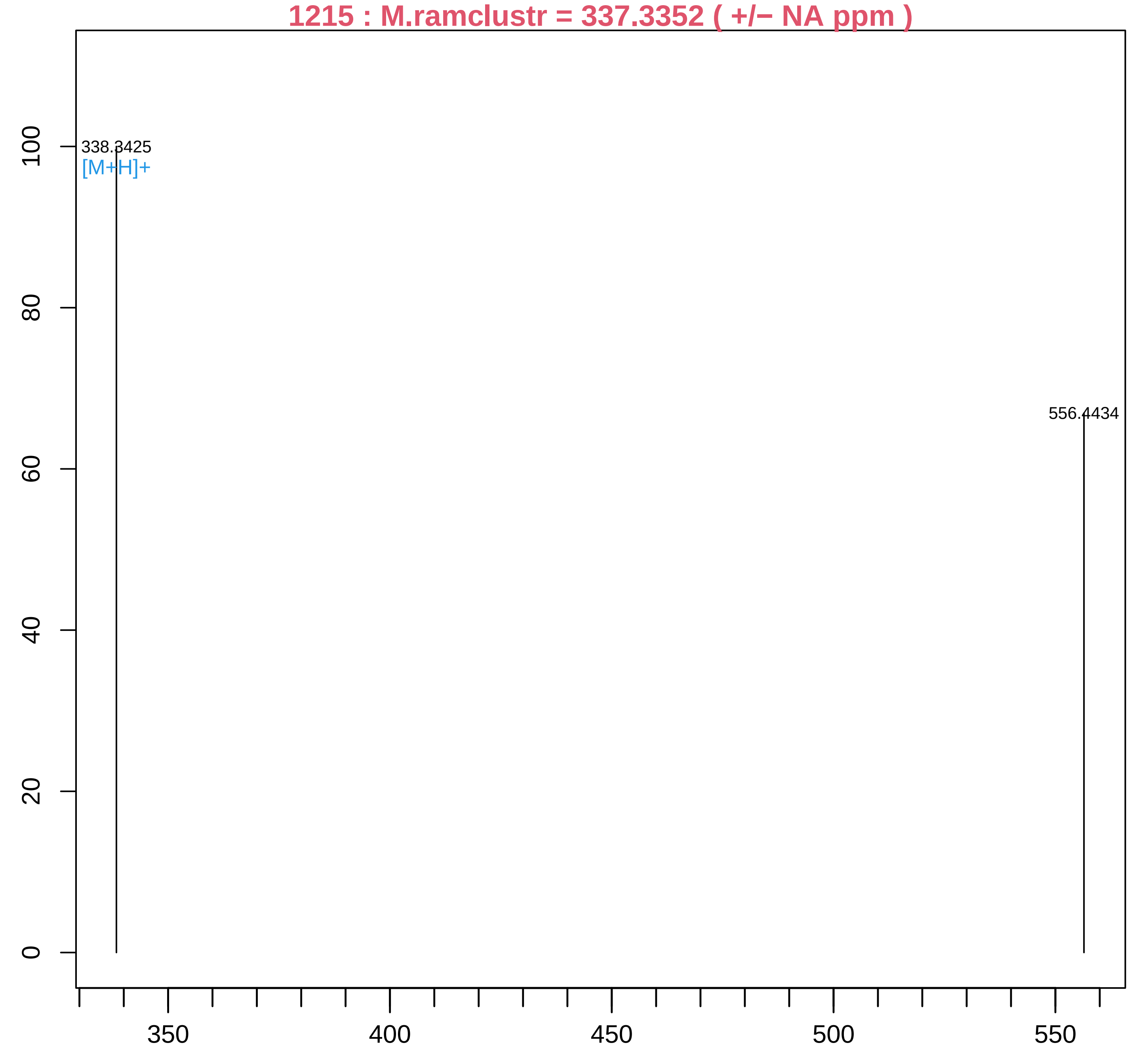
1215 : M.ramclustr = 337.3352 (+/− NA ppm)
338.3425
[M+H]+
556.4434
0
20
40
60
80
100
350
400
450
500
550

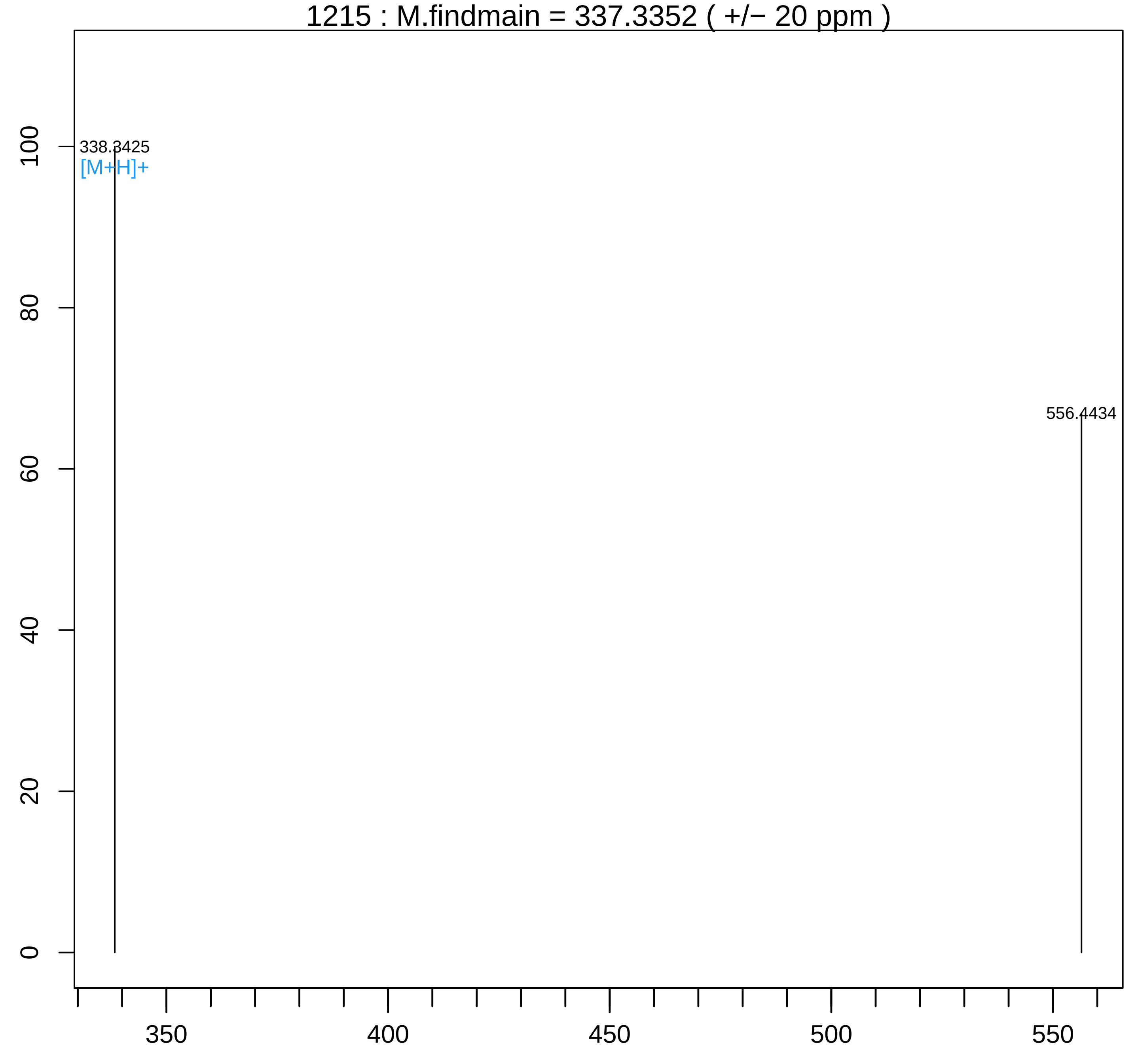
1215 : M.findmain = 337.3352 (+/− 20 ppm)
338.3425
[M+H]+
556.4434
0
20
40
60
80
100
350
400
450
500
550

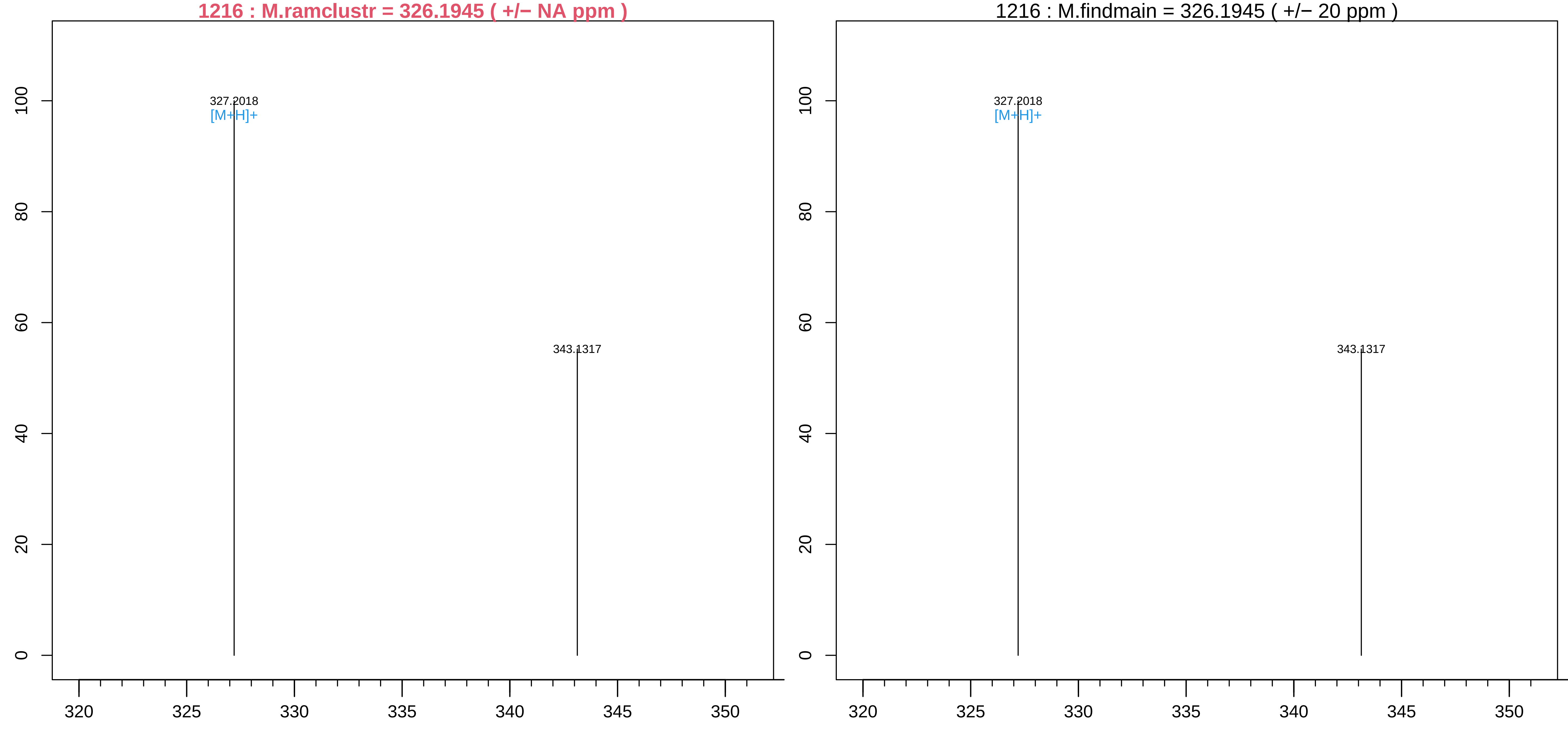
1216 : M.ramclustr = 326.1945 (+/− NA ppm)
1216 : M.findmain = 326.1945 (+/− 20 ppm)
327.2018
[M+H]+
343.1317
0
20
40
60
80
100
320
325
330
335
340
345
350

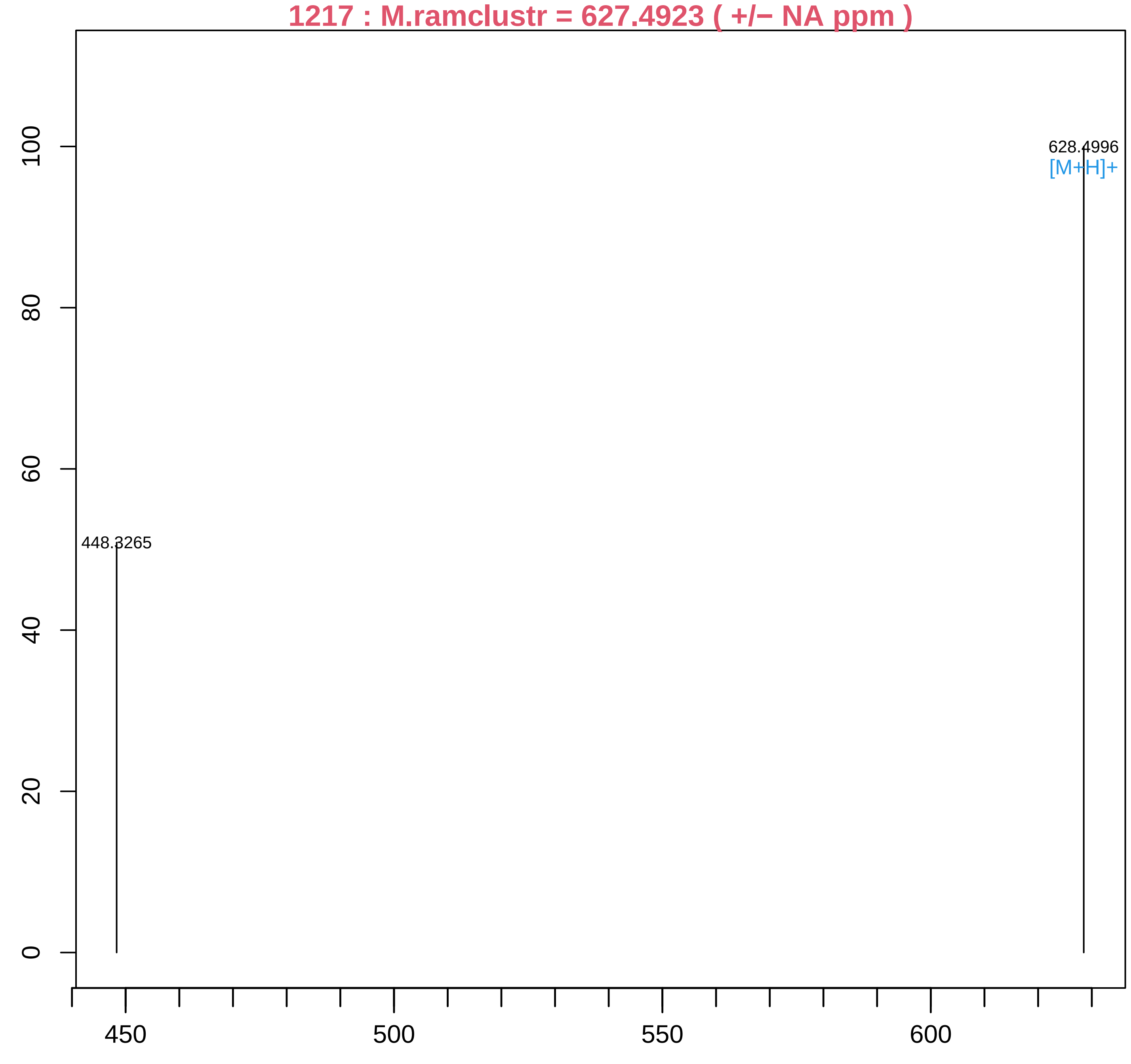
1217 : M.ramclustr = 627.4923 (+/− NA ppm)
628.4996
[M+H]+
448.3265
0
20
40
60
80
100
450
500
550
600

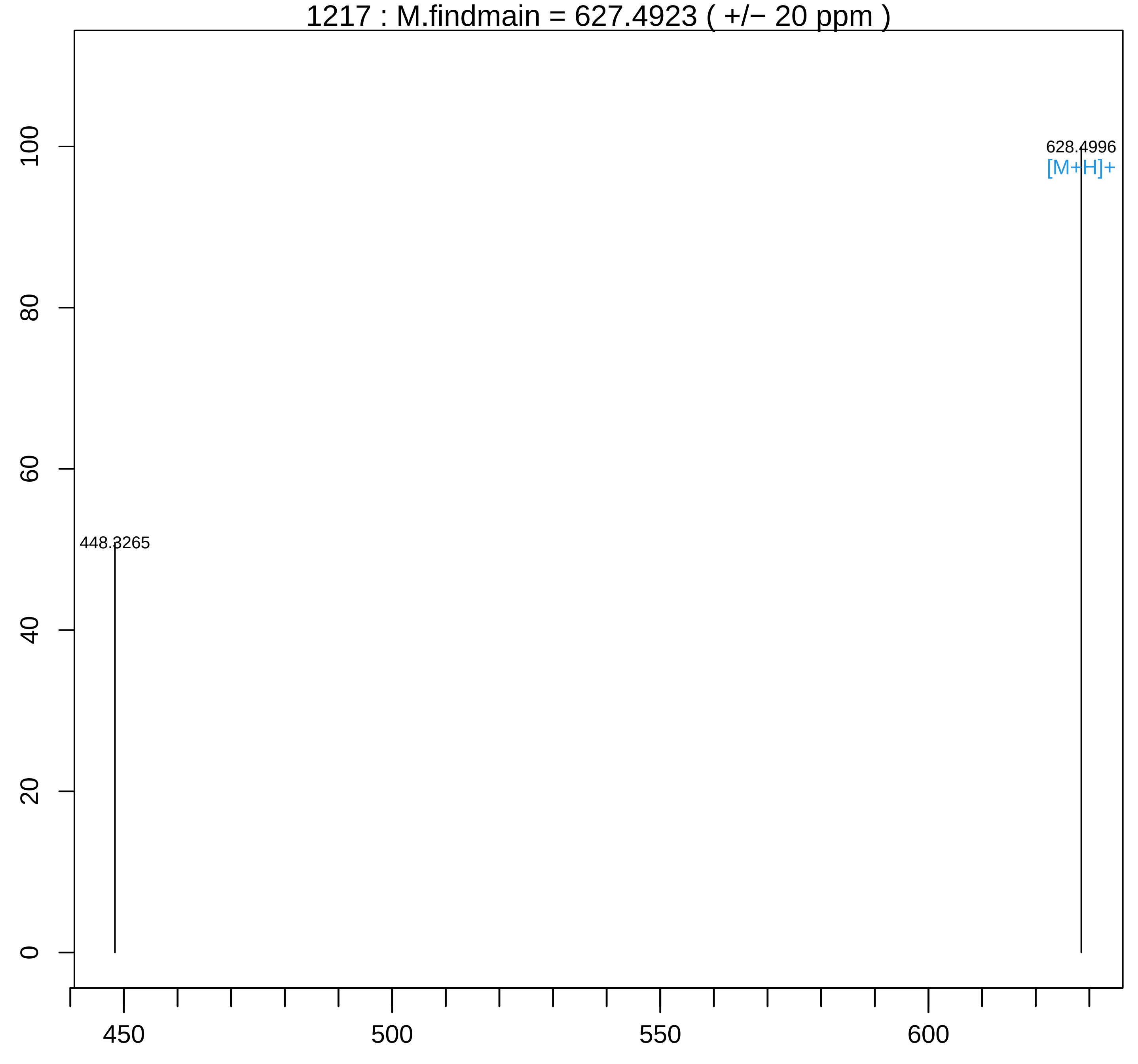
1217 : M.findmain = 627.4923 (+/− 20 ppm)
628.4996
[M+H]+
448.3265
0
20
40
60
80
100
450
500
550
600

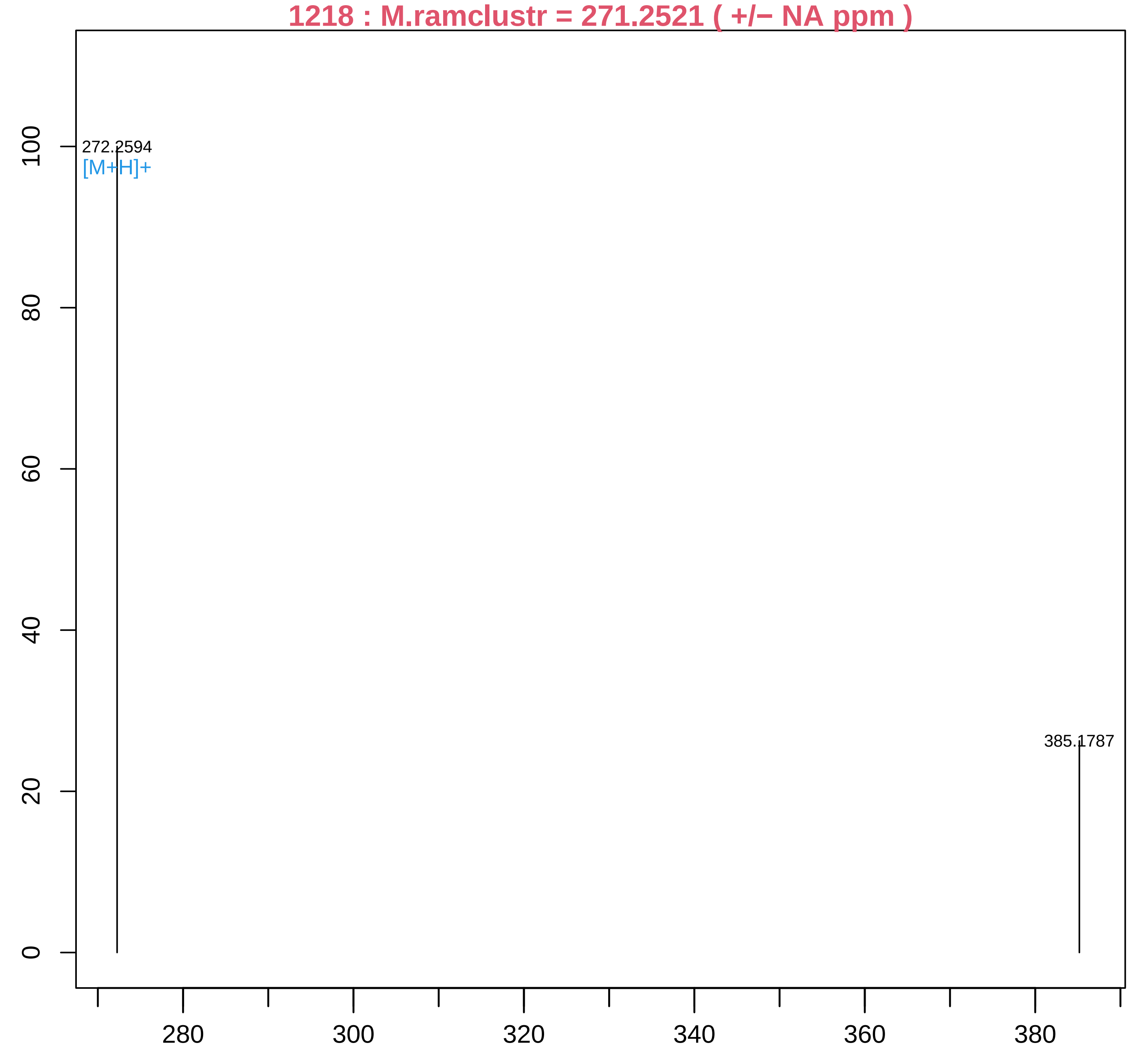

1218 : M.ramclustr = 271.2521 (+/− NA ppm)
272.2594
[M+H]+
385.1787
0
20
40
60
80
100
280
300
320
340
360
380

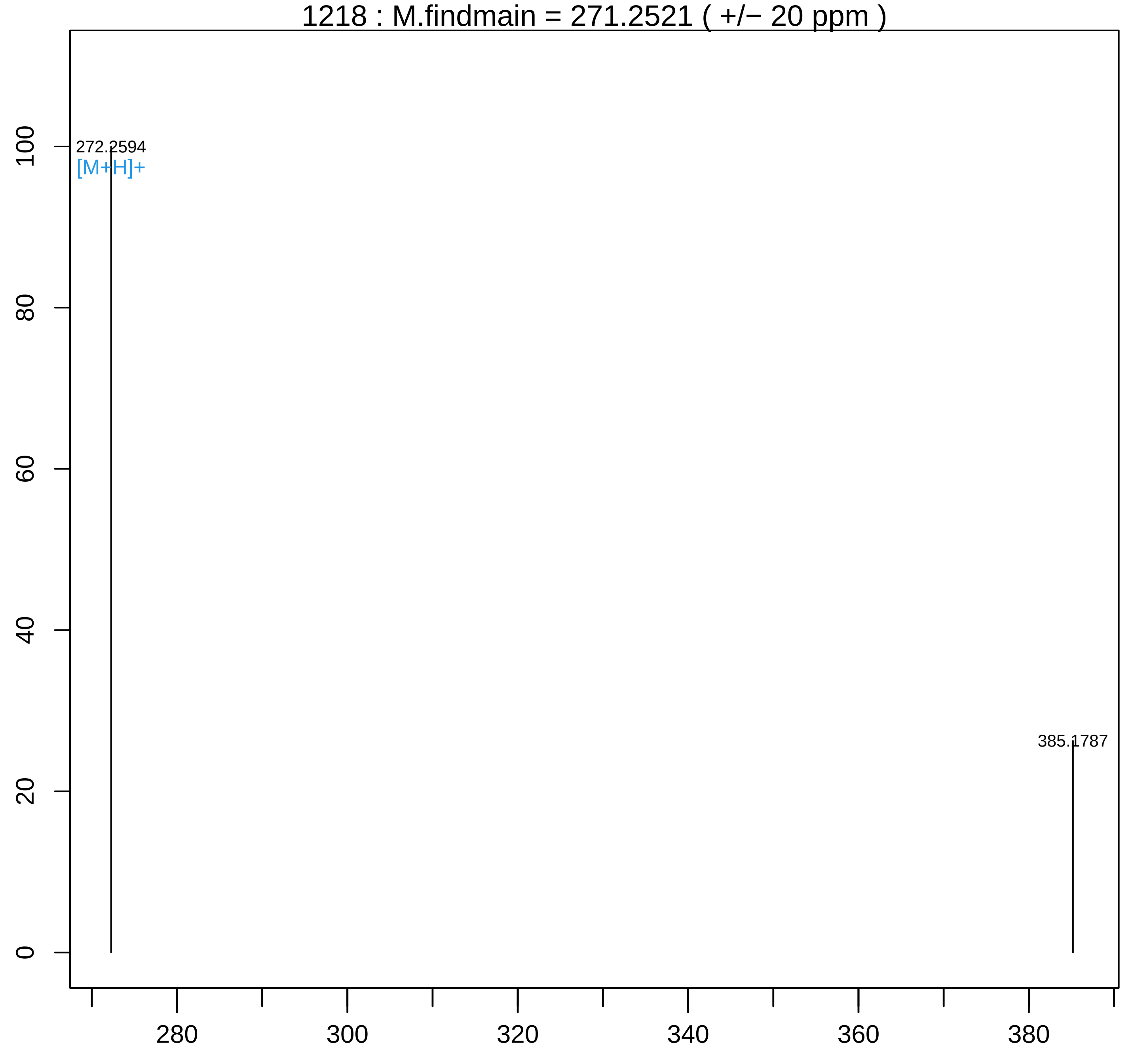

1218 : M.findmain = 271.2521 (+/− 20 ppm)
272.2594
[M+H]+
385.1787
0
20
40
60
80
100
280
300
320
340
360
380

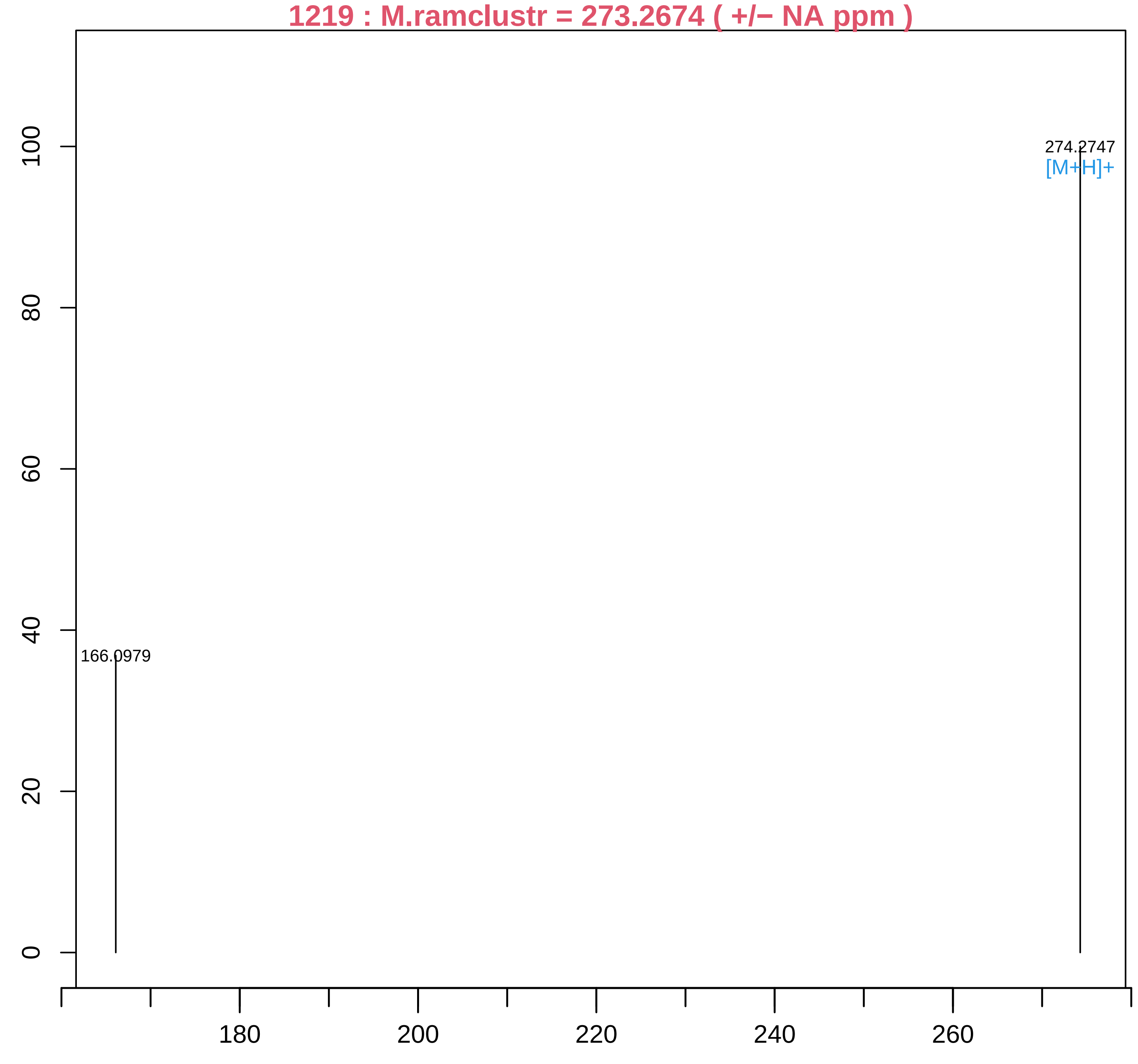
1219 : M.ramclustr = 273.2674 (+/− NA ppm)
274.2747
[M+H]+
166.0979
0
20
40
60
80
100
180
200
220
240
260

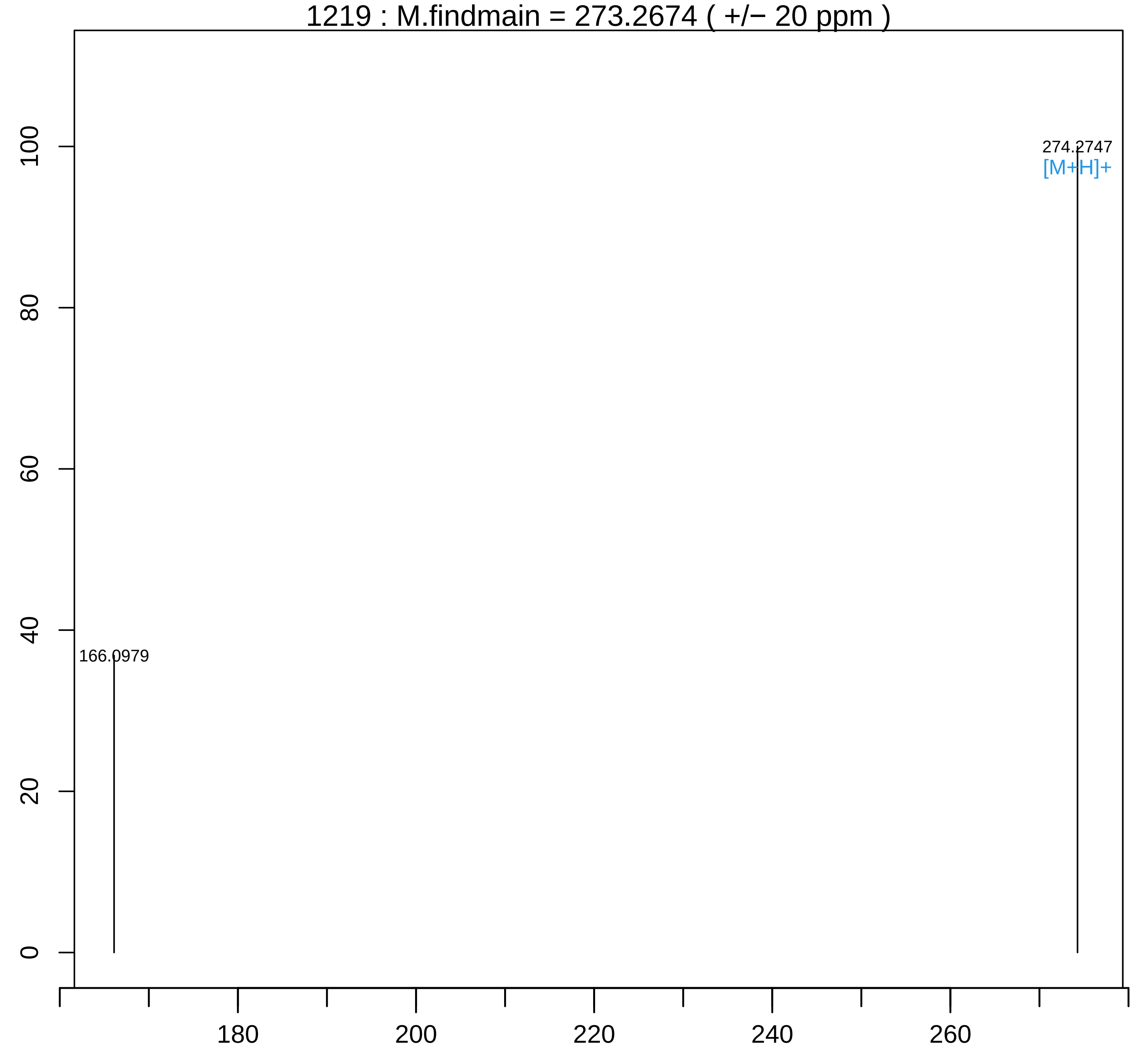
1219 : M.findmain = 273.2674 (+/− 20 ppm)
274.2747
[M+H]+
166.0979
0
20
40
60
80
100
180
200
220
240
260

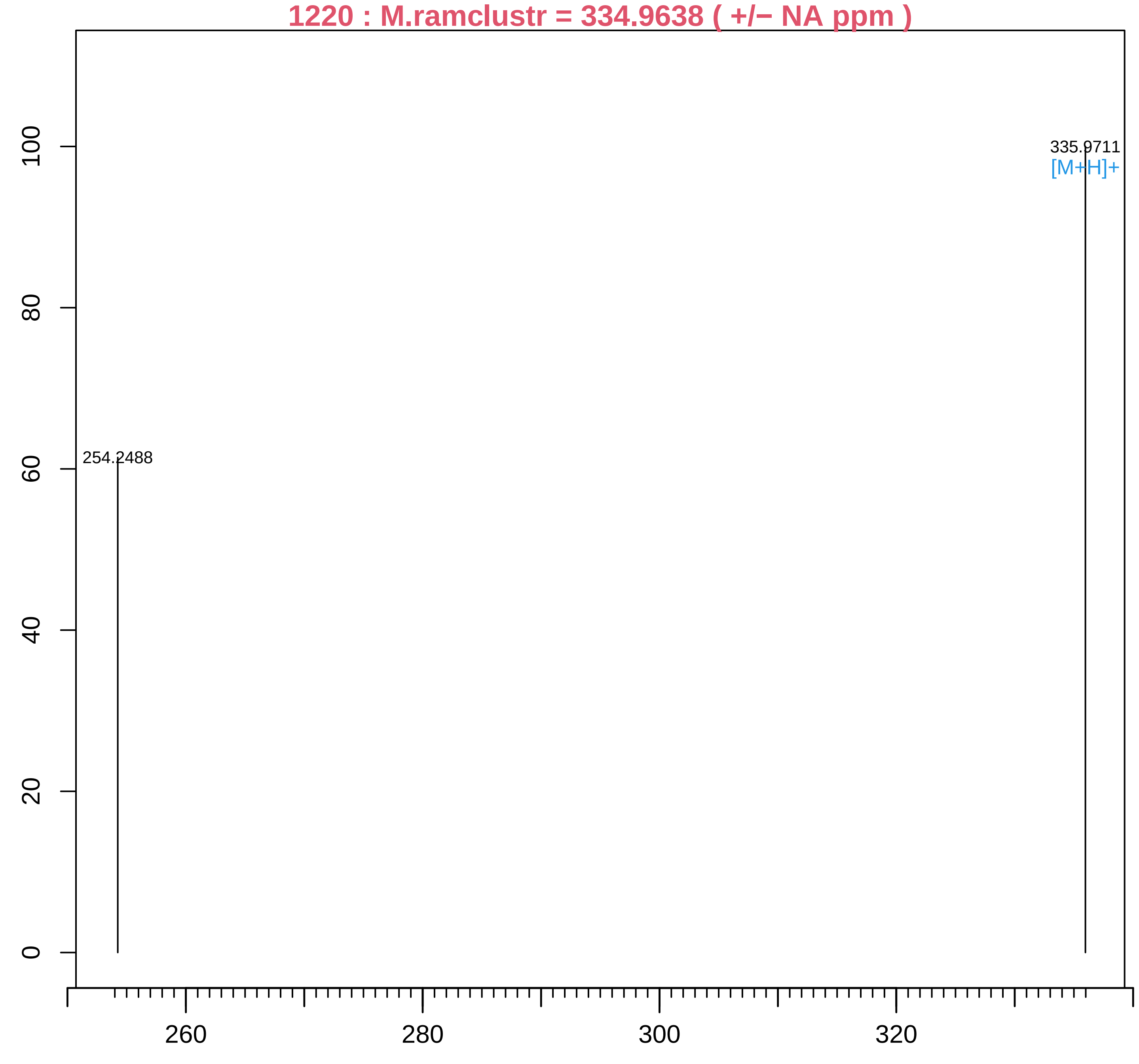

1220 : M.ramclustr = 334.9638 (+/− NA ppm)
335.9711
[M+H]+
254.2488
0
20
40
60
80
100
260
280
300
320

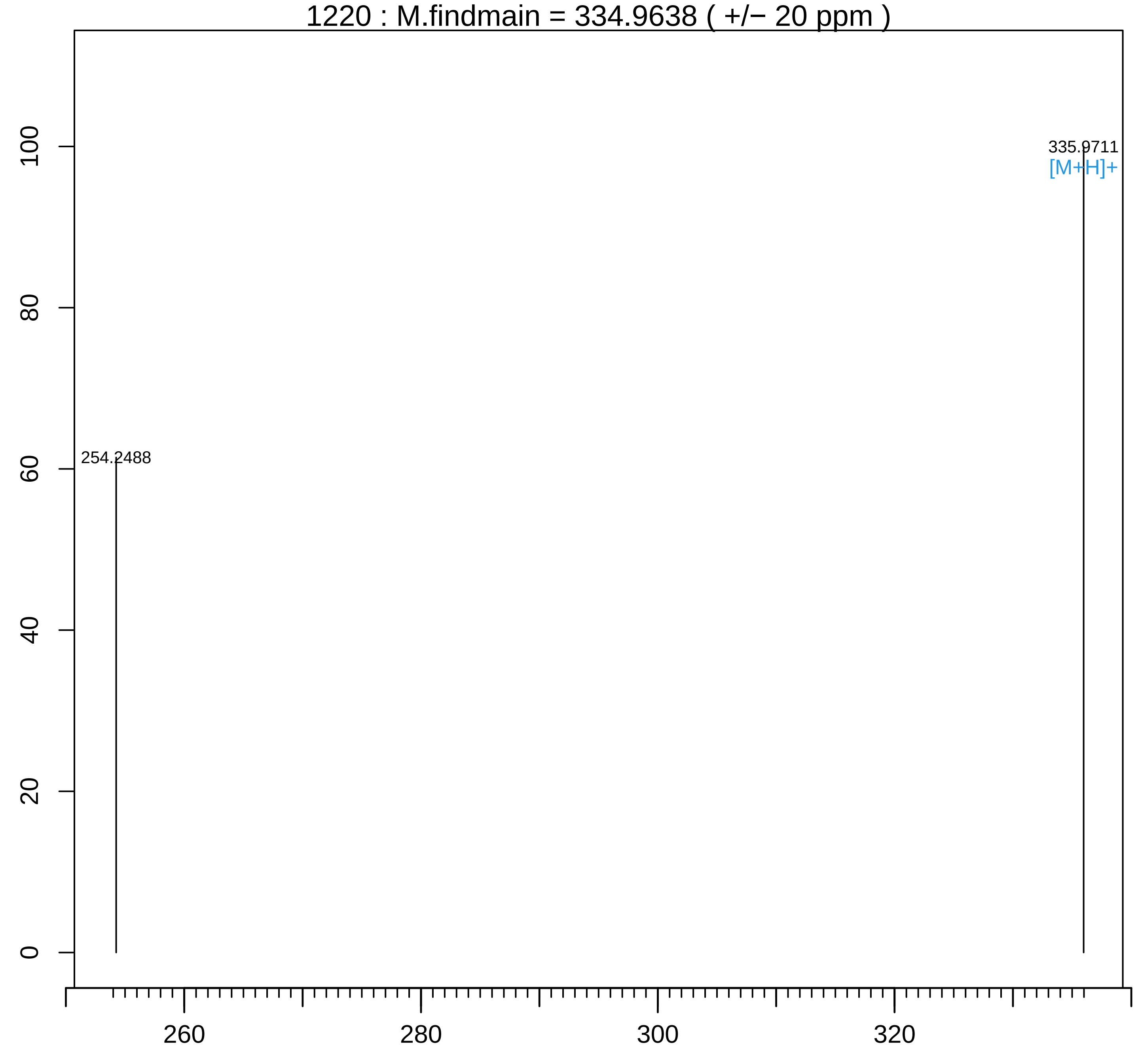

1220 : M.findmain = 334.9638 (+/− 20 ppm)
335.9711
[M+H]+
254.2488
0
20
40
60
80
100
260
280
300
320

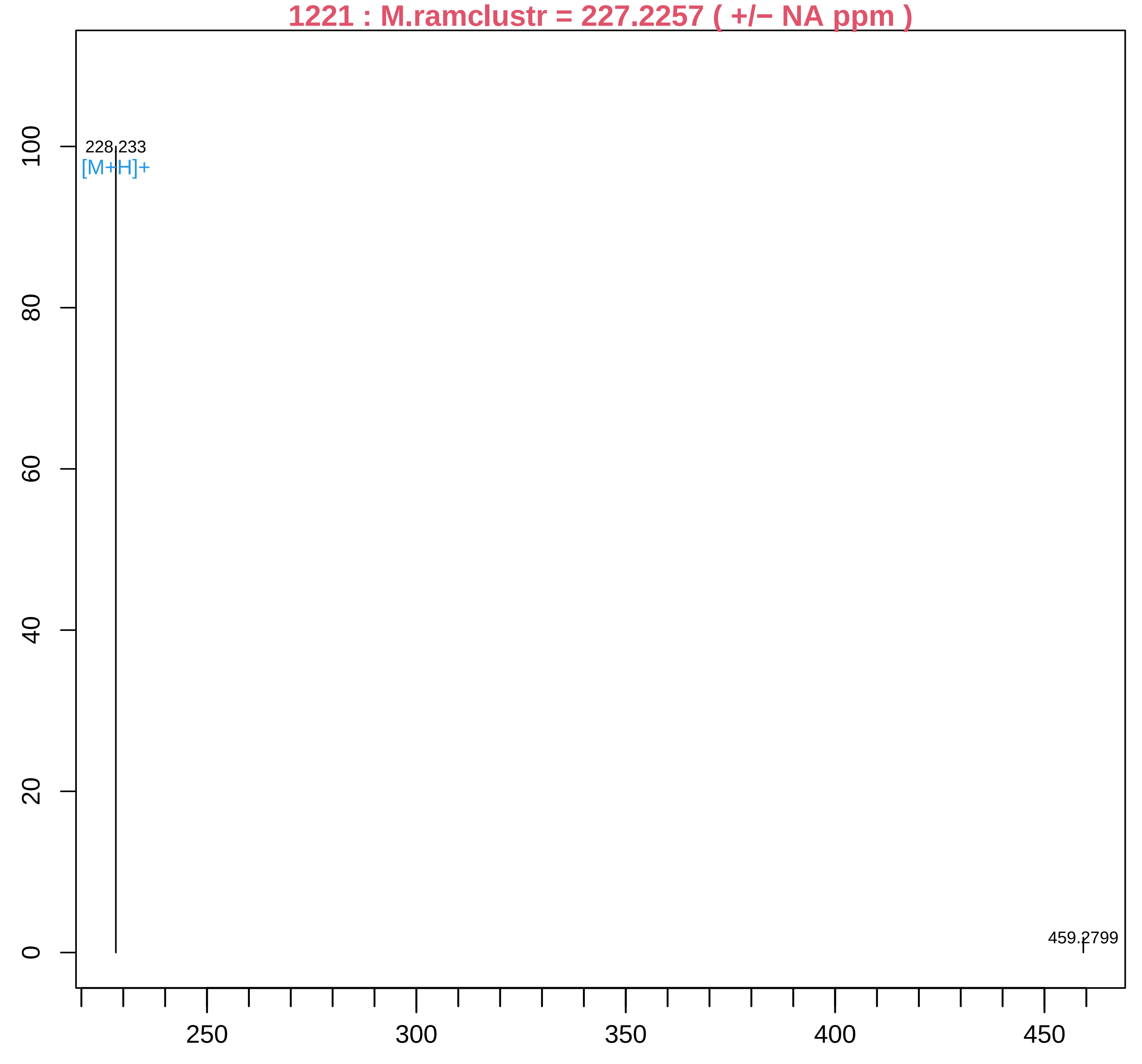
1221 : M.ramclustr = 227.2257 (+/− NA ppm)
228.233
[M+H]+
459.2799
0
20
40
60
80
100
250
300
350
400
450

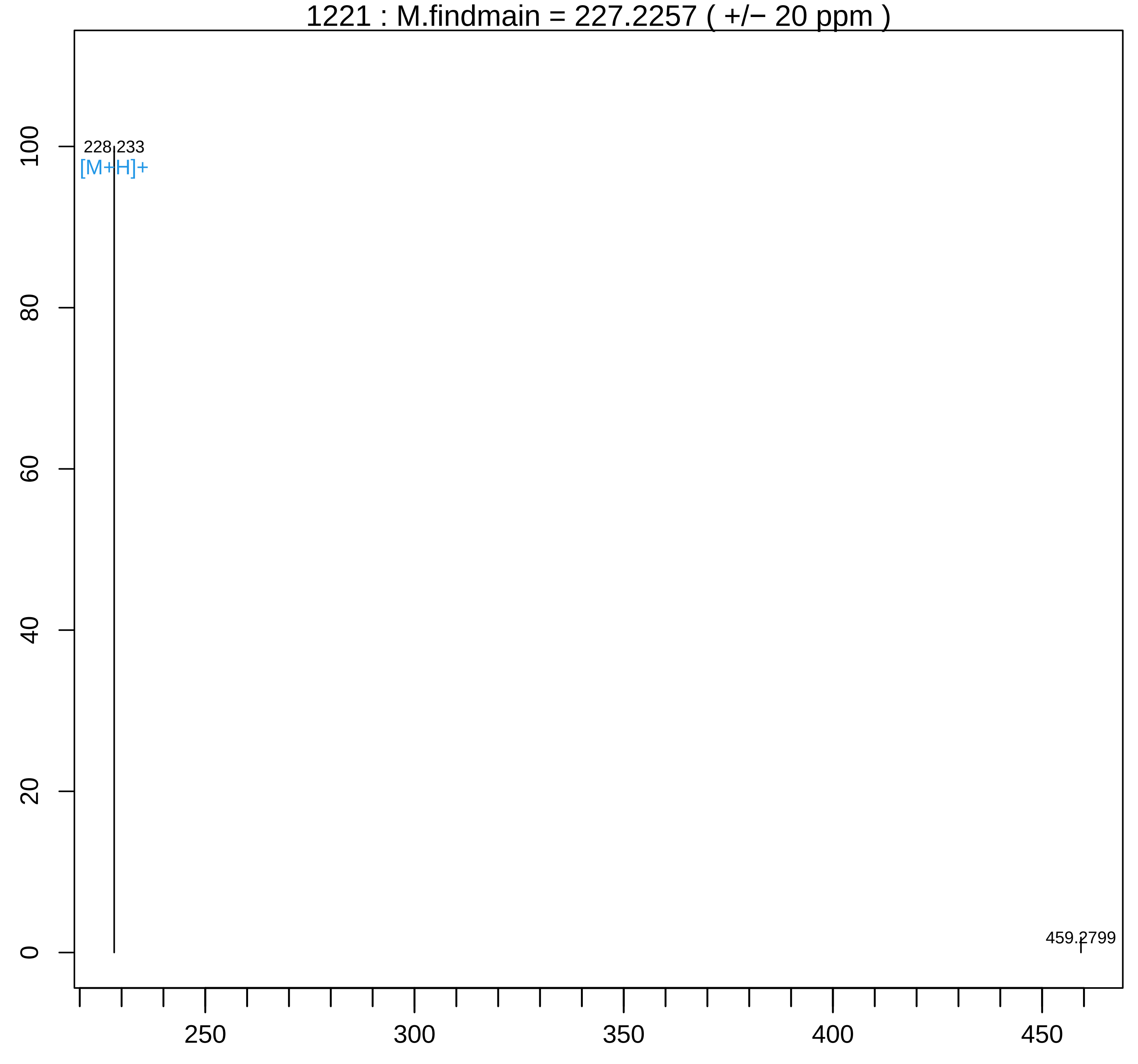
1221 : M.findmain = 227.2257 (+/− 20 ppm)
228.233
[M+H]+
459.2799
0
20
40
60
80
100
250
300
350
400
450

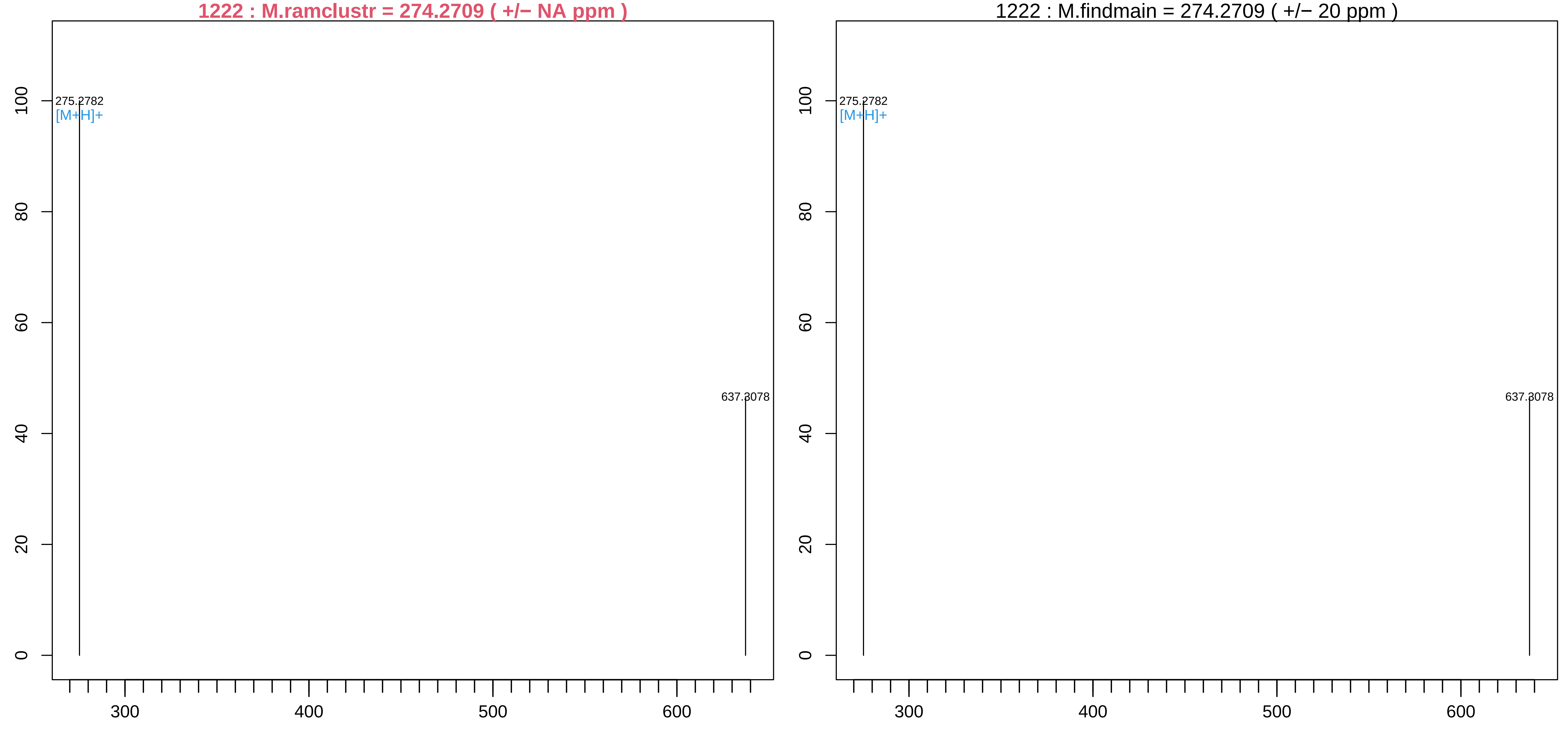
1222 : M.ramclustr = 274.2709 (+/− NA ppm)
275.2782
[M+H]+
637.3078
1222 : M.findmain = 274.2709 (+/− 20 ppm)
275.2782
[M+H]+
637.3078
0
20
40
60
80
100
300
400
500
600

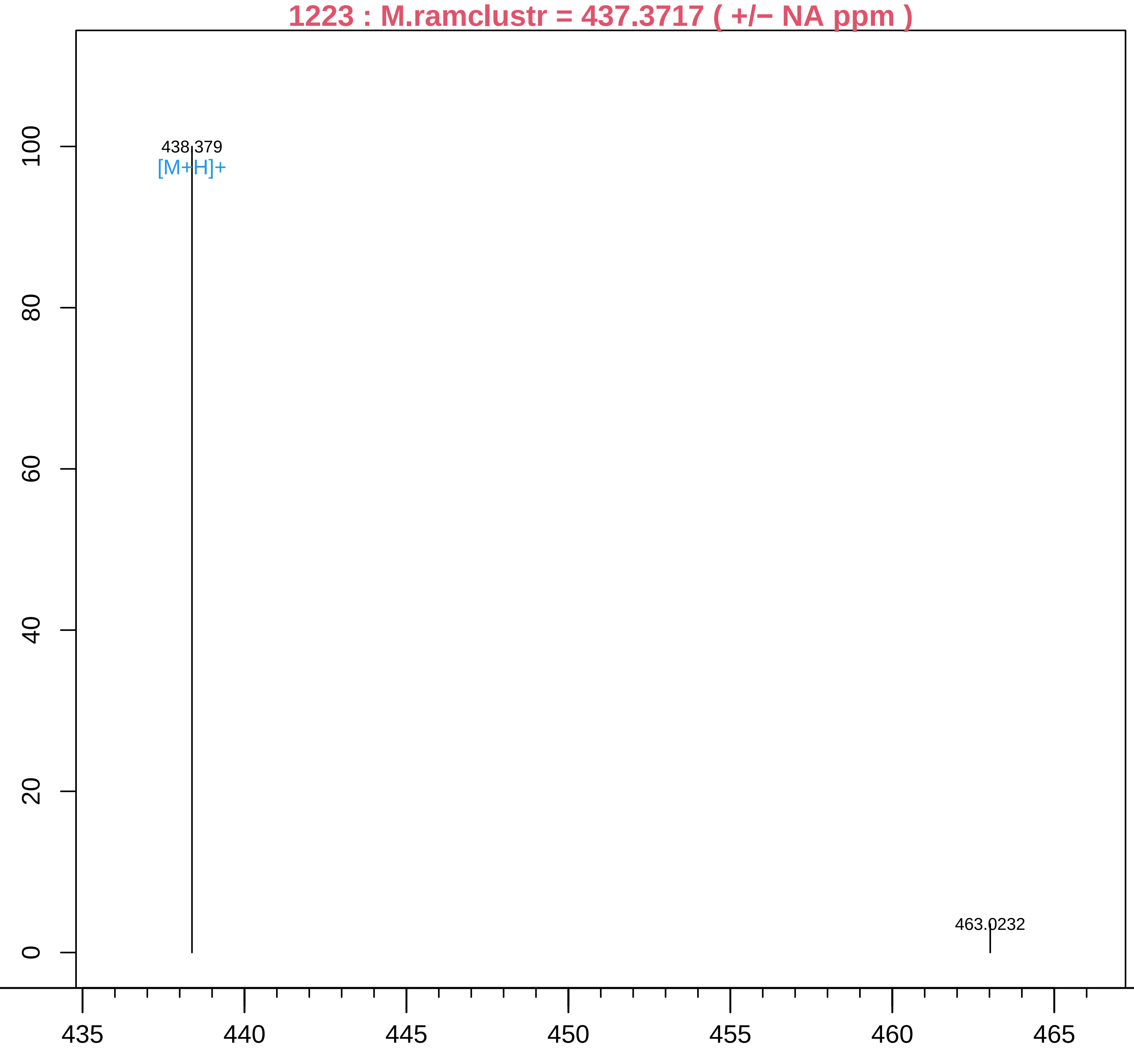

1223 : M.ramclustr = 437.3717 (+/− NA ppm)
438.379
[M+H]+
463.0232
0
20
40
60
80
100
435
440
445
450
455
460
465

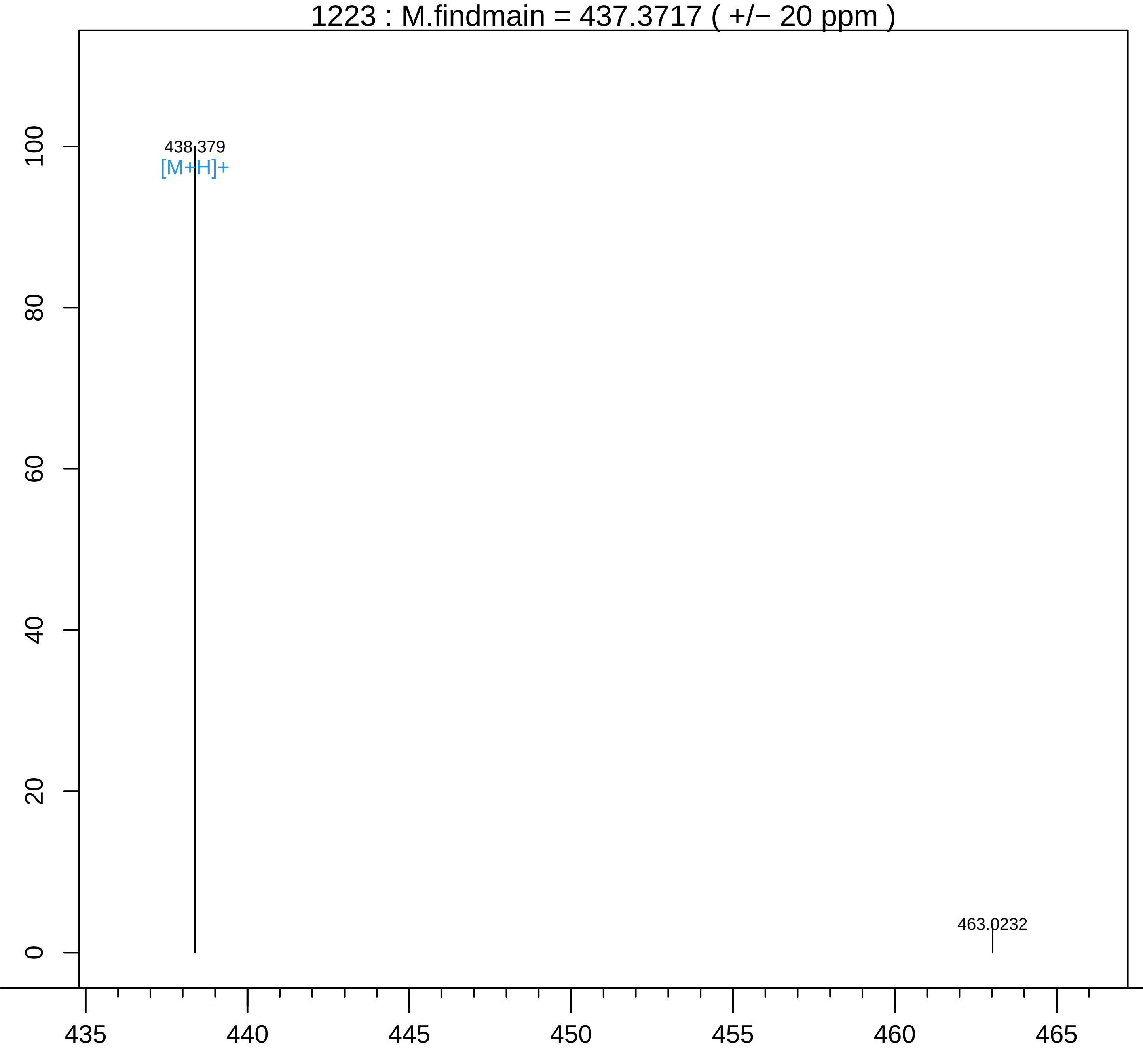

1223 : M.findmain = 437.3717 (+/− 20 ppm)
438.379
[M+H]+
463.0232
0
20
40
60
80
100
435
440
445
450
455
460
465

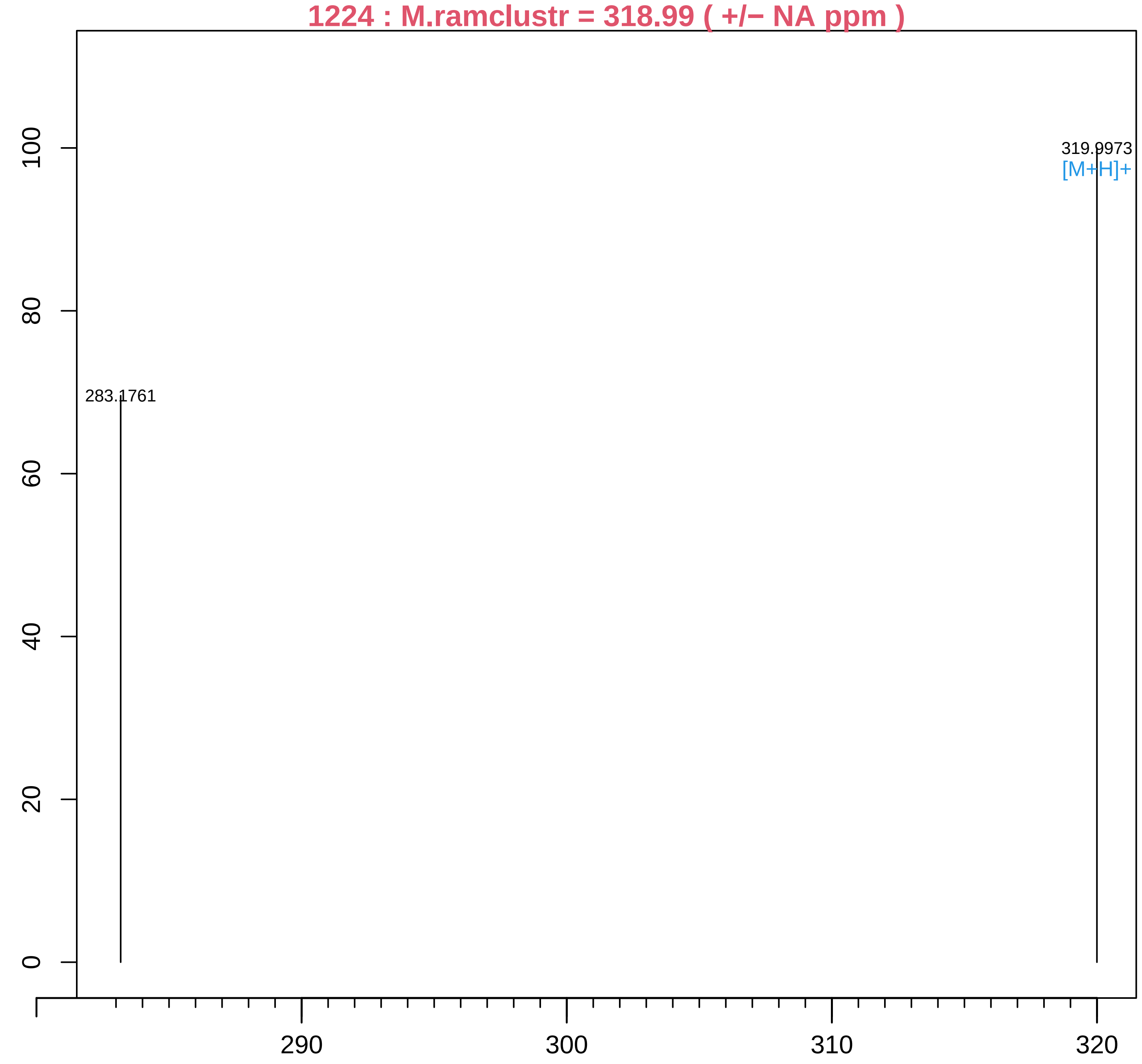
1224 : M.ramclustr = 318.99 (+/− NA ppm)
319.9973
[M+H]+
283.1761
0
20
40
60
80
100
290
300
310
320

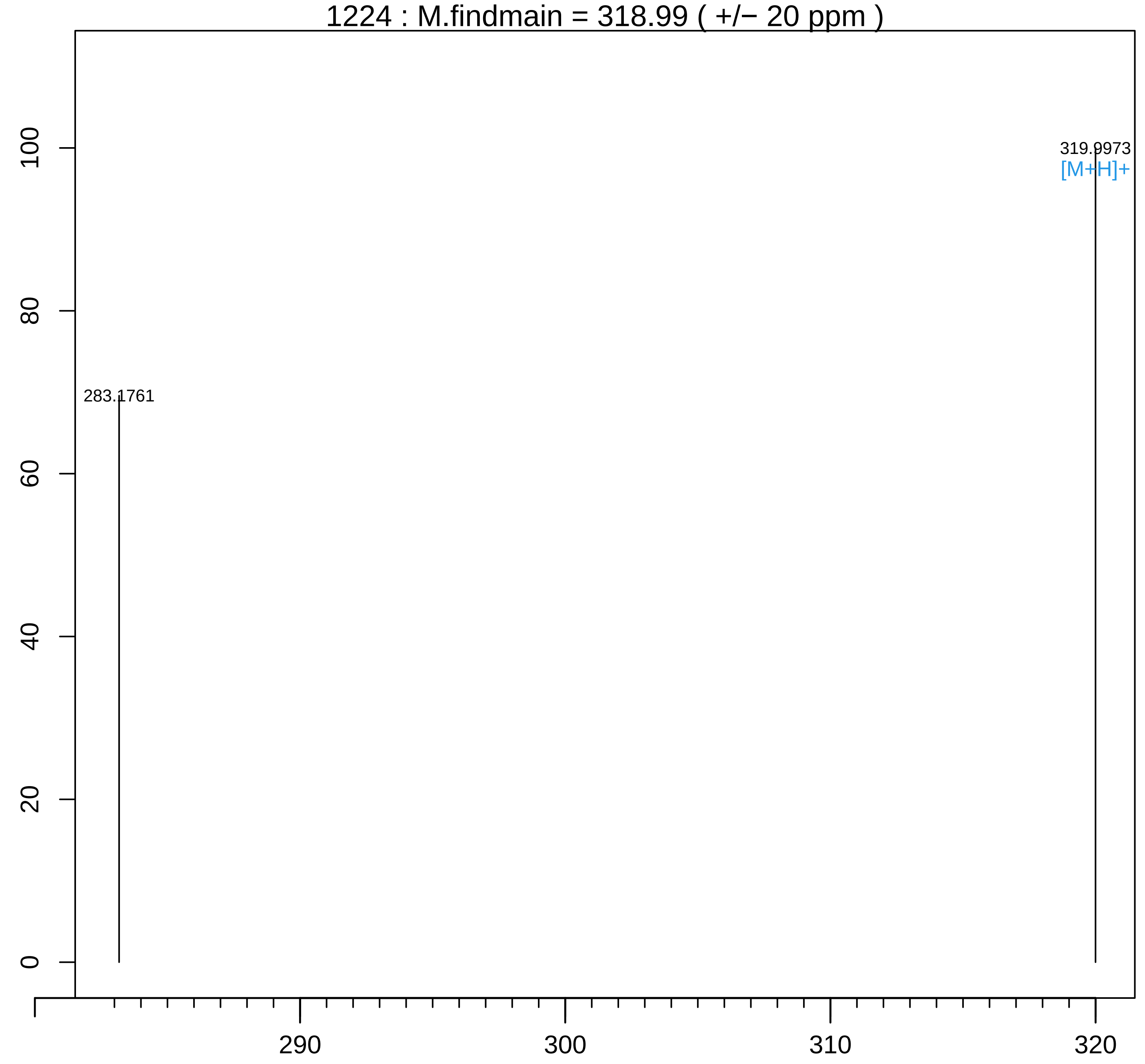
1224 : M.findmain = 318.99 (+/− 20 ppm)
319.9973
[M+H]+
283.1761
0
20
40
60
80
100
290
300
310
320

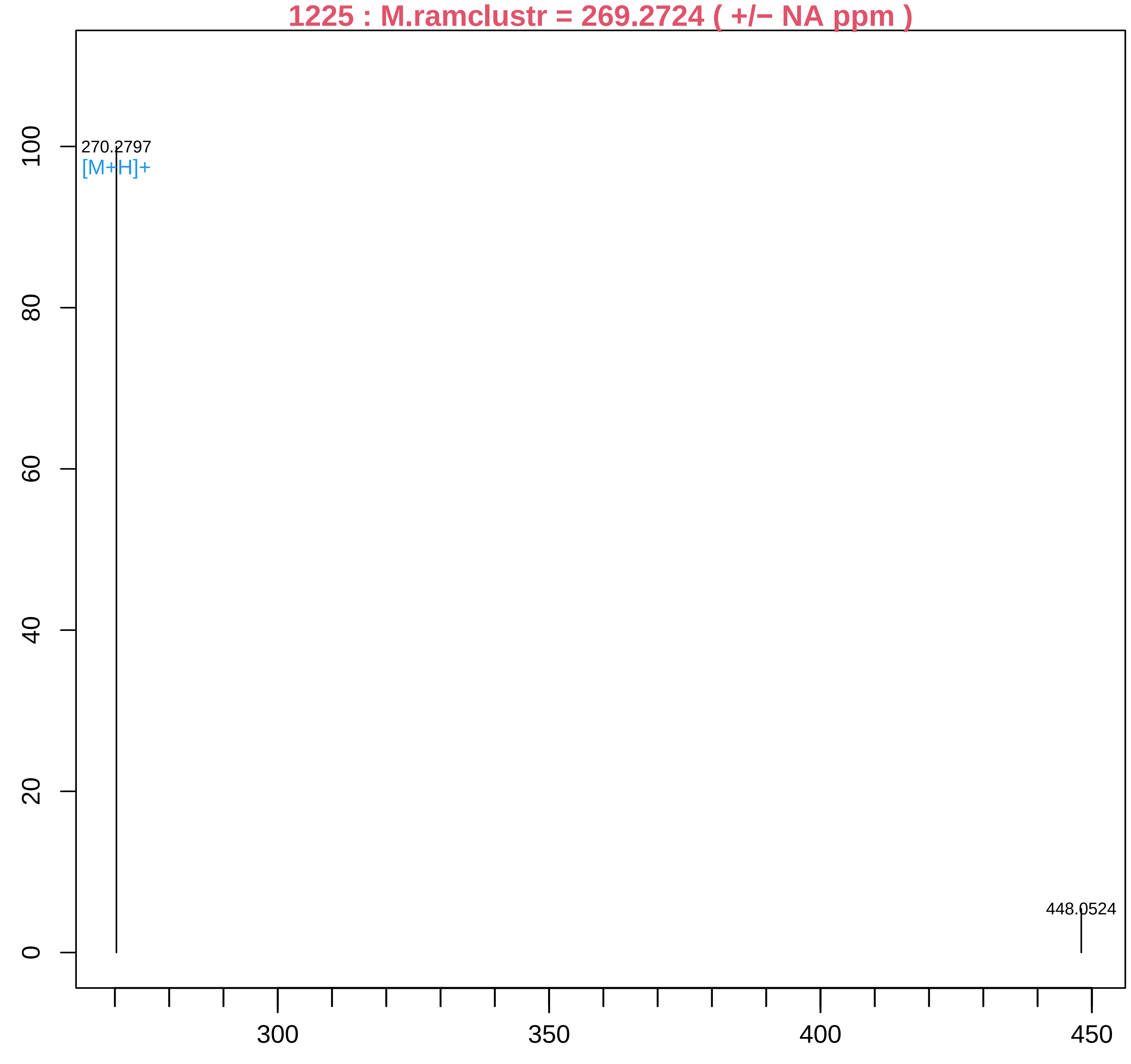
1225 : M.ramclustr = 269.2724 (+/− NA ppm)
270.2797
[M+H]+
448.0524
0
20
40
60
80
100
300
350
400
450

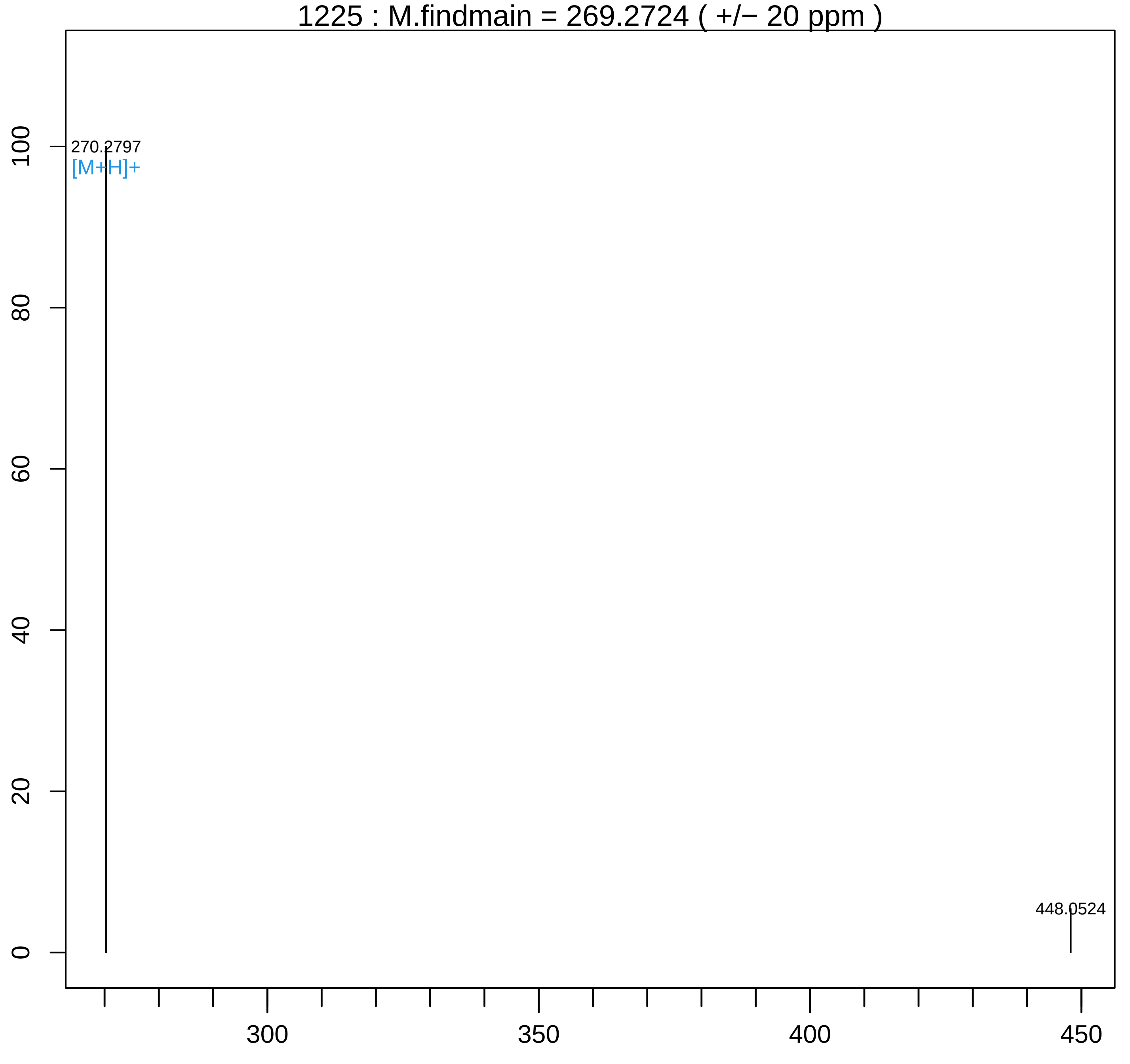
1225 : M.findmain = 269.2724 (+/− 20 ppm)
270.2797
[M+H]+
448.0524
0
20
40
60
80
100
300
350
400
450

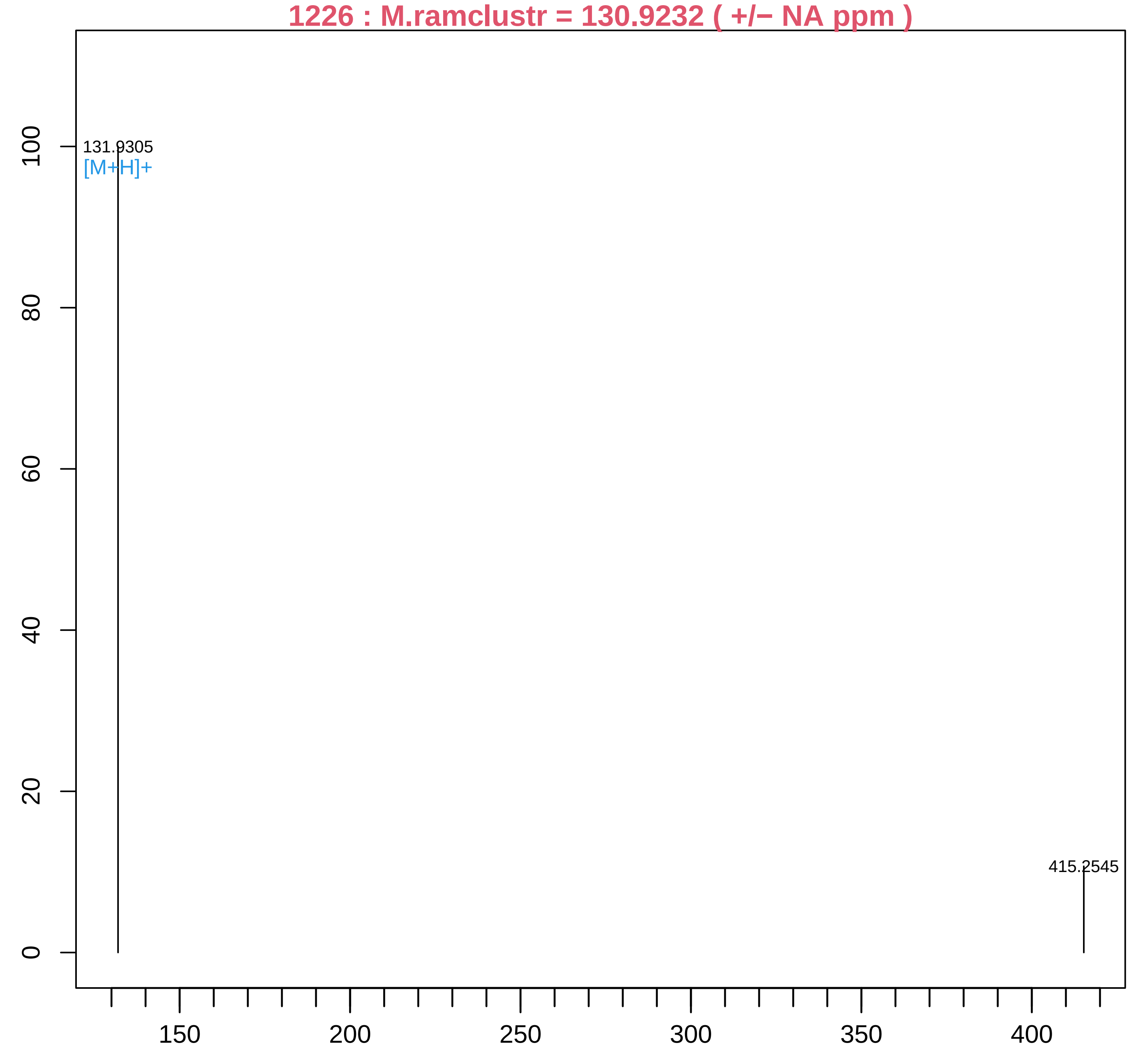
1226 : M.ramclustr = 130.9232 (+/− NA ppm)
131.9305
[M+H]+
415.2545

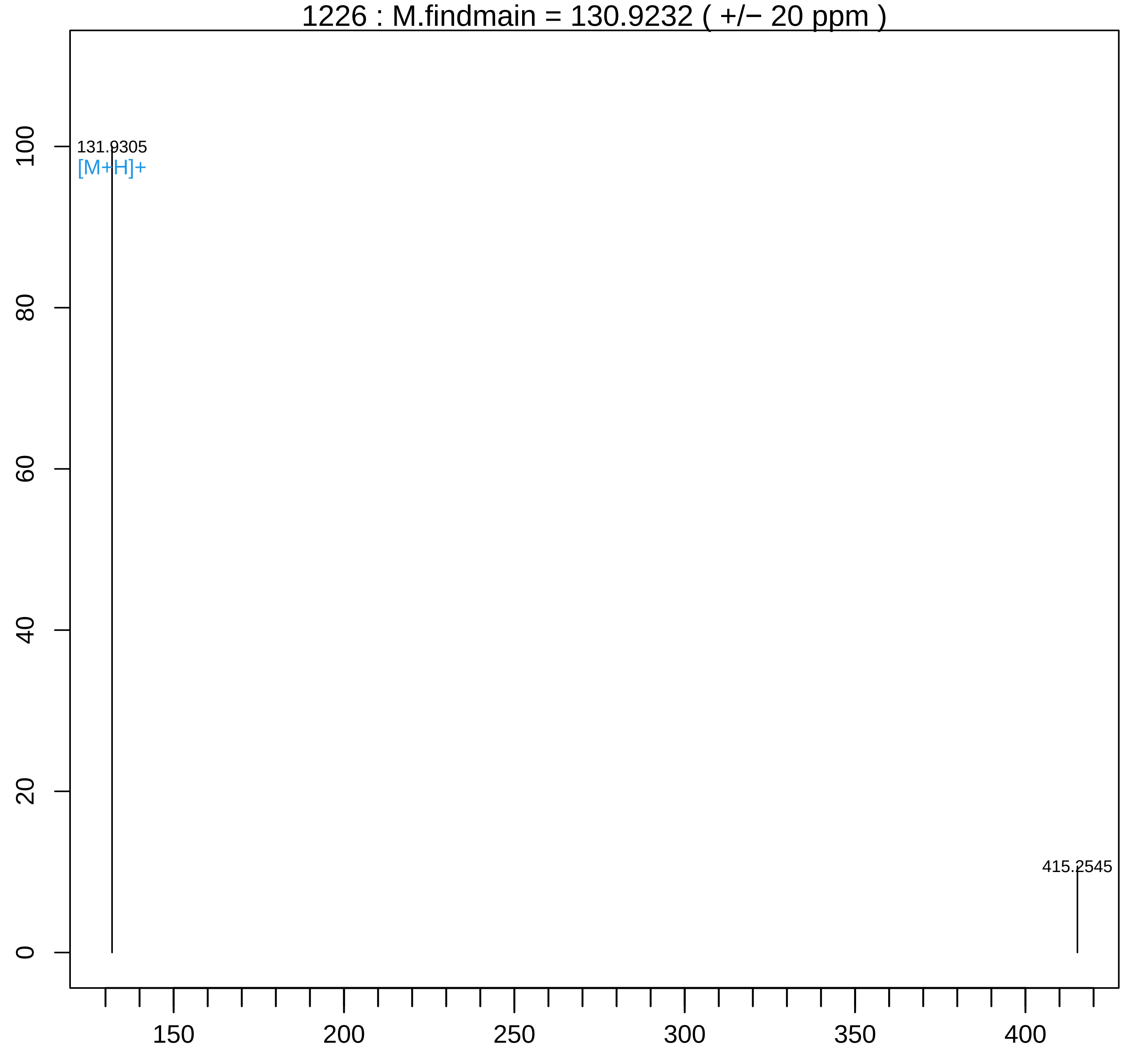
1226 : M.findmain = 130.9232 (+/− 20 ppm)
131.9305
[M+H]+
415.2545

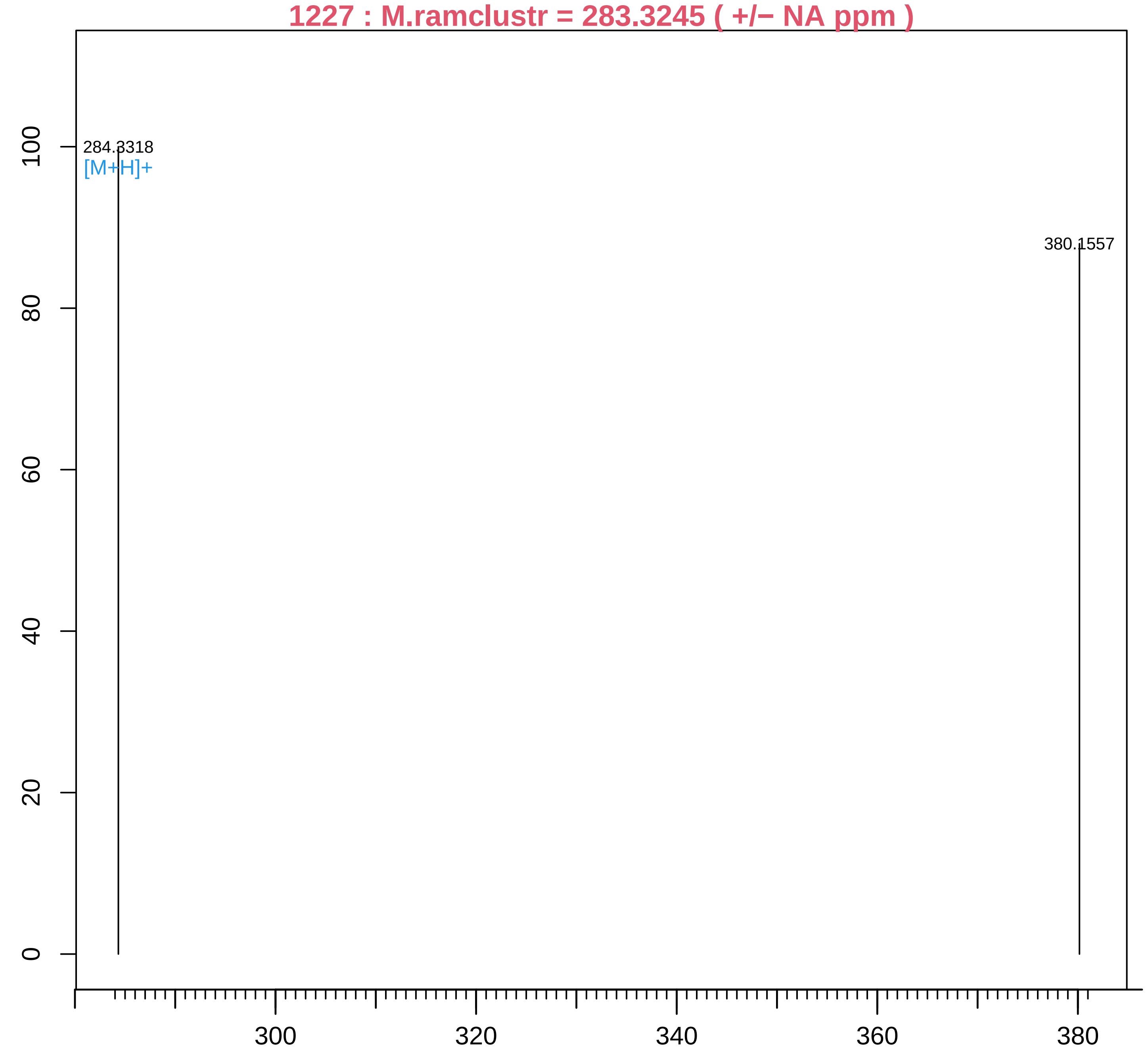
1227 : M.ramclustr = 283.3245 (+/− NA ppm)
284.3318
[M+H]+
380.1557
0
20
40
60
80
100
300
320
340
360
380

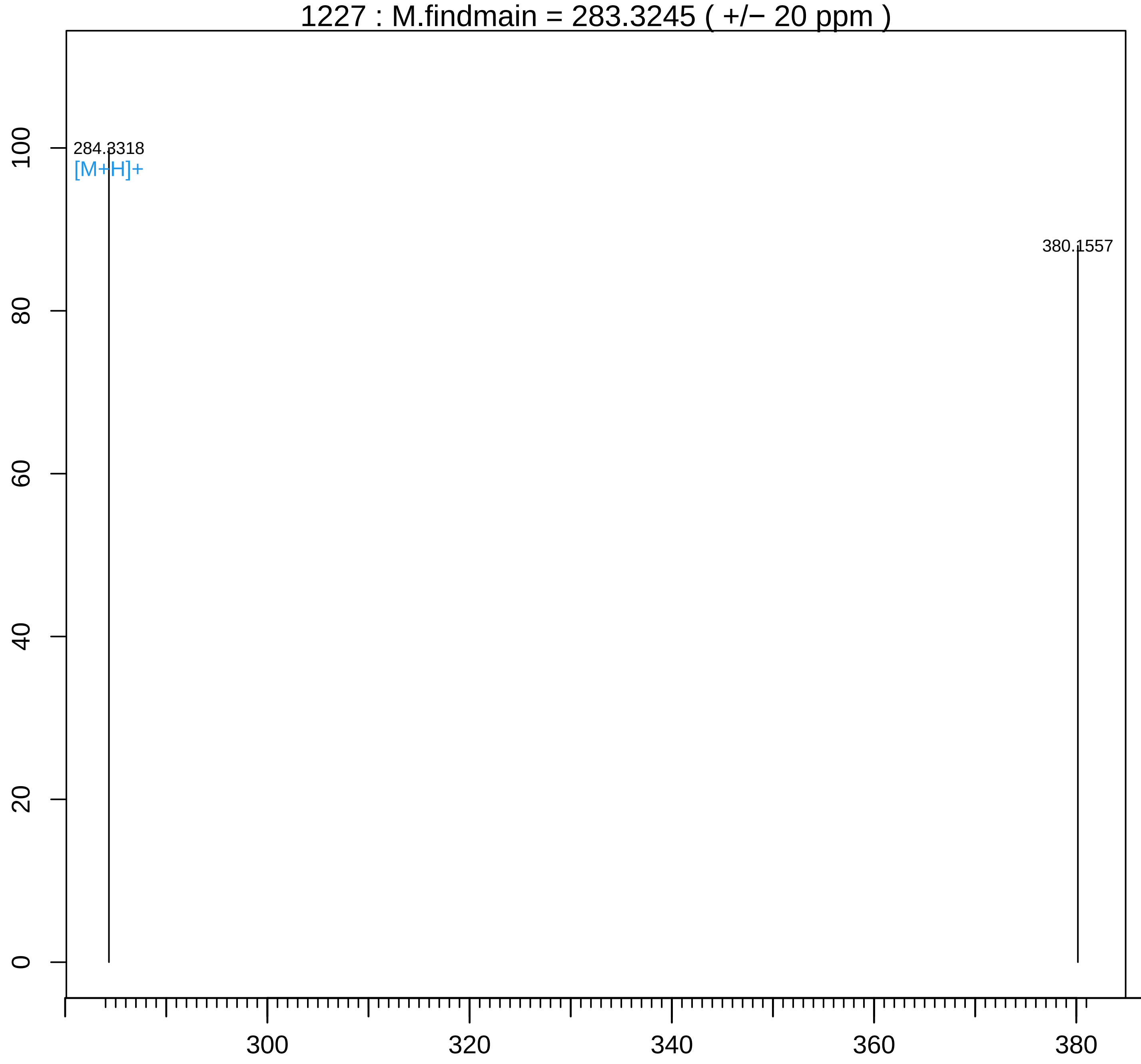
1227 : M.findmain = 283.3245 (+/− 20 ppm)
284.3318
[M+H]+
380.1557
0
20
40
60
80
100
300
320
340
360
380

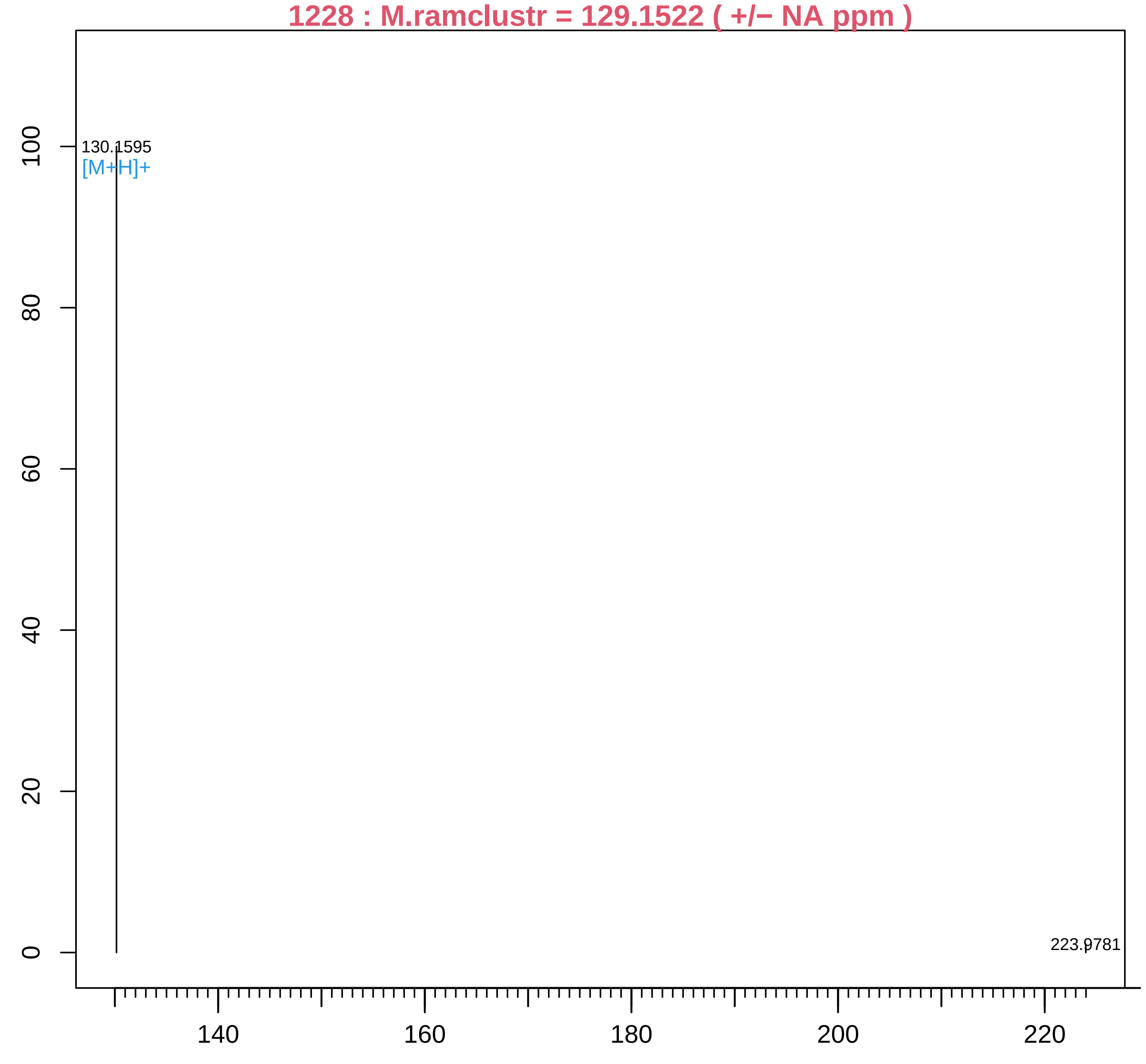
1228 : M.ramclustr = 129.1522 (+/− NA ppm)
130.1595
[M+H]+
223.9781
0
20
40
60
80
100
140
160
180
200
220

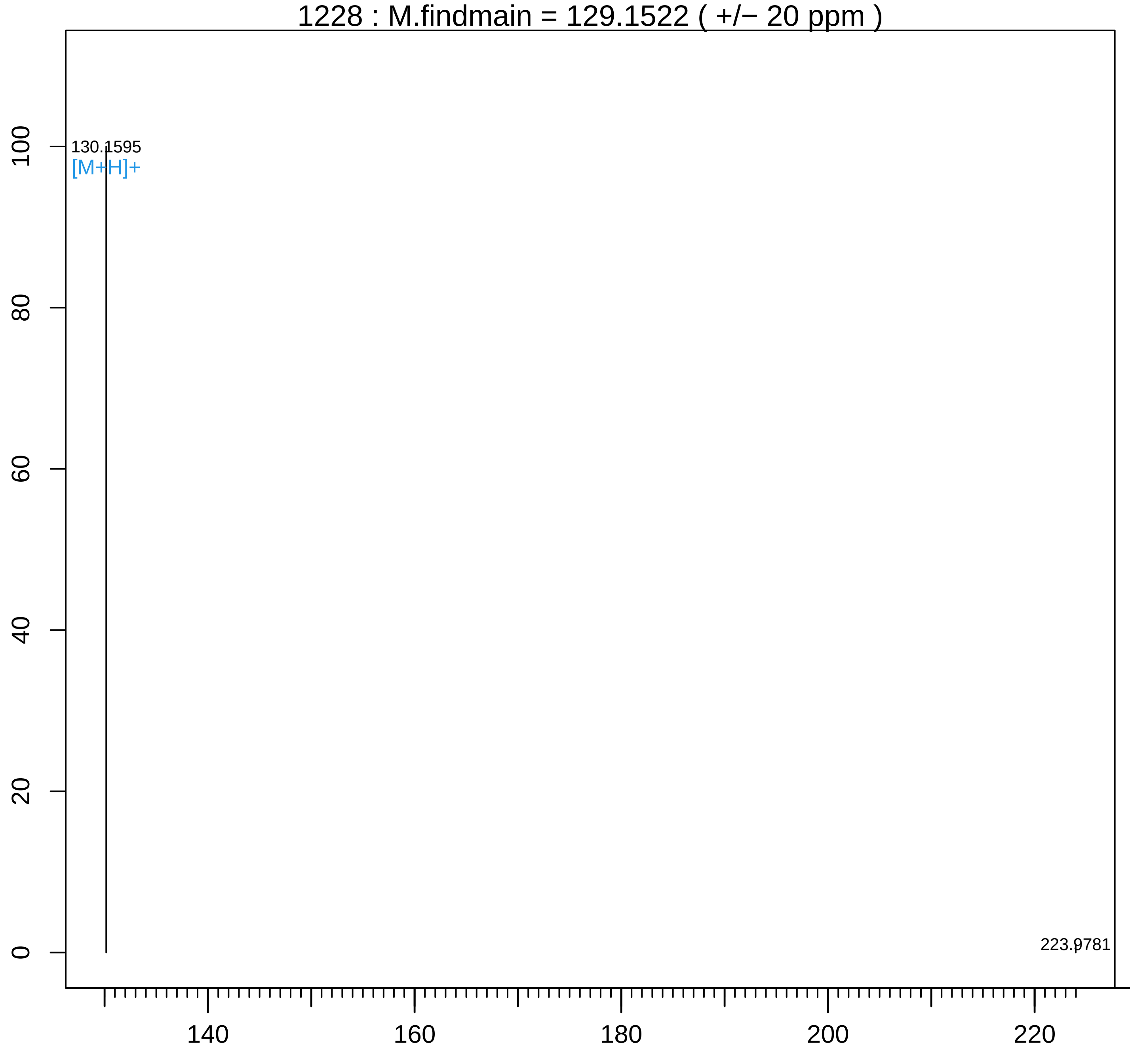
1228 : M.findmain = 129.1522 (+/− 20 ppm)
130.1595
[M+H]+
223.9781
0
20
40
60
80
100
140
160
180
200
220

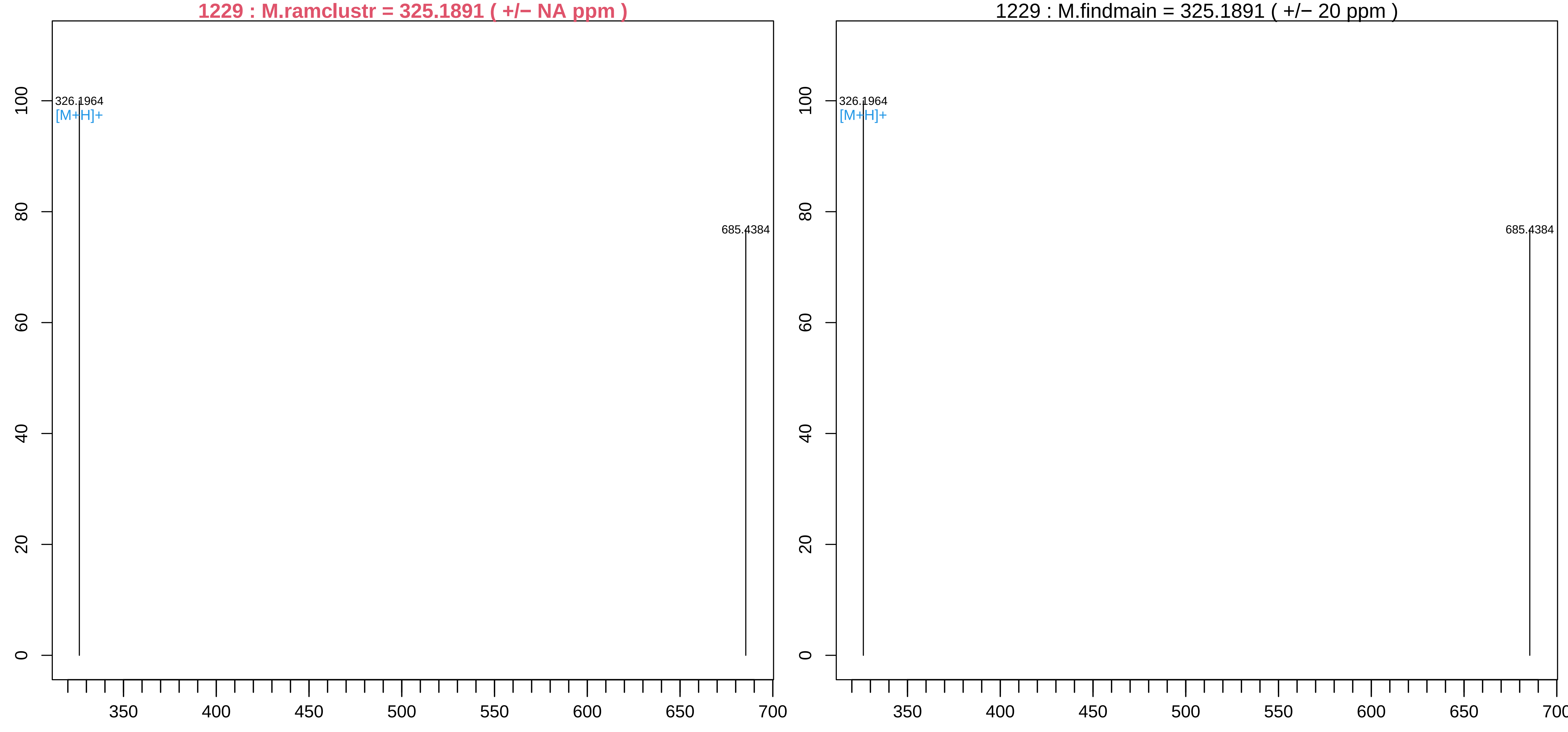
1229 : M.ramclustr = 325.1891 (+/− NA ppm)
326.1964
[M+H]+
685.4384
1229 : M.findmain = 325.1891 (+/− 20 ppm)
326.1964
[M+H]+
685.4384

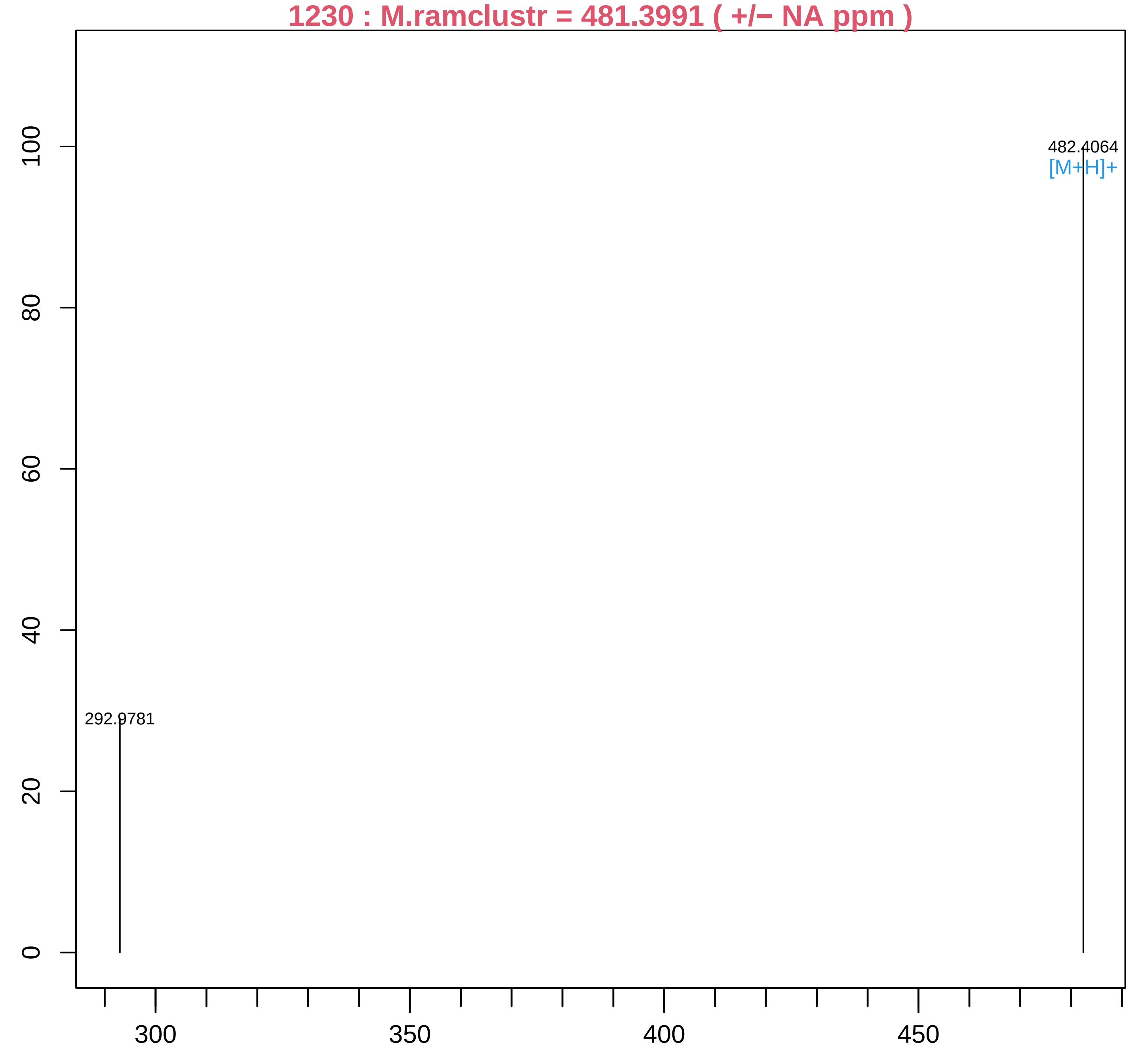
1230 : M.ramclustr = 481.3991 (+/− NA ppm)
482.4064
[M+H]+
292.9781
0
20
40
60
80
100
300
350
400
450

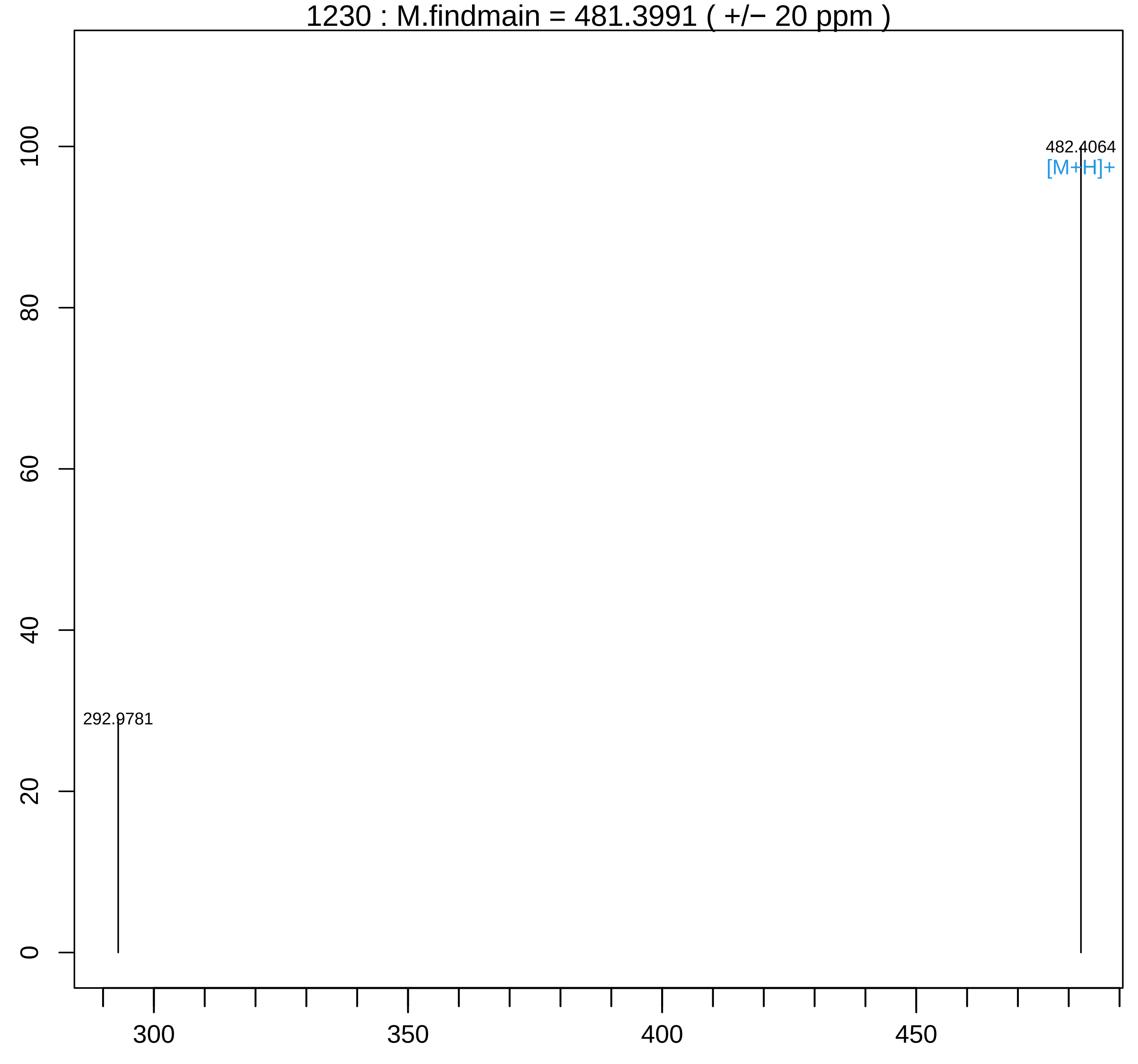
1230 : M.findmain = 481.3991 (+/− 20 ppm)
482.4064
[M+H]+
292.9781
0
20
40
60
80
100
300
350
400
450

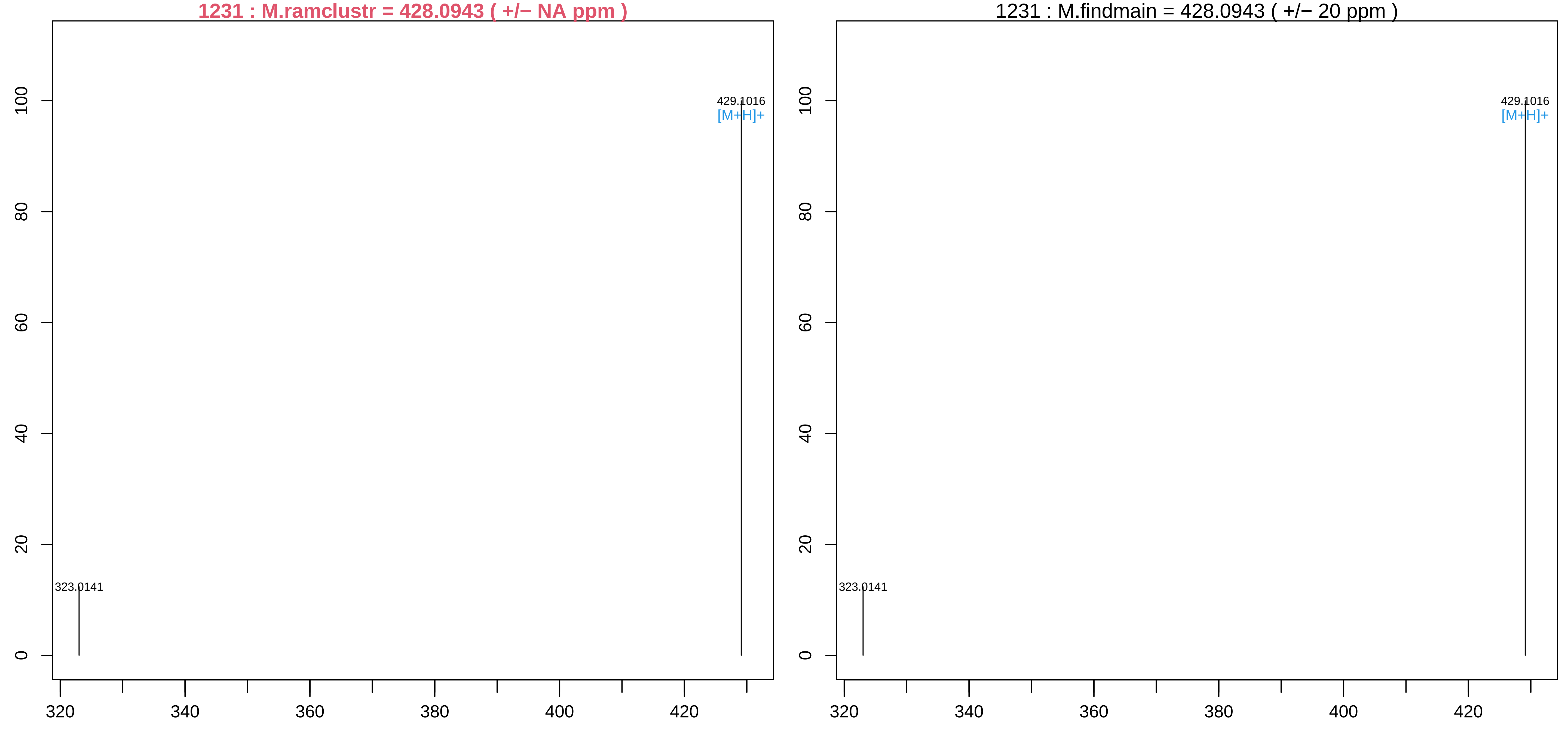

1231 : M.ramclustr = 428.0943 (+/− NA ppm)
429.1016
[M+H]+
323.0141
1231 : M.findmain = 428.0943 (+/− 20 ppm)
429.1016
[M+H]+
323.0141

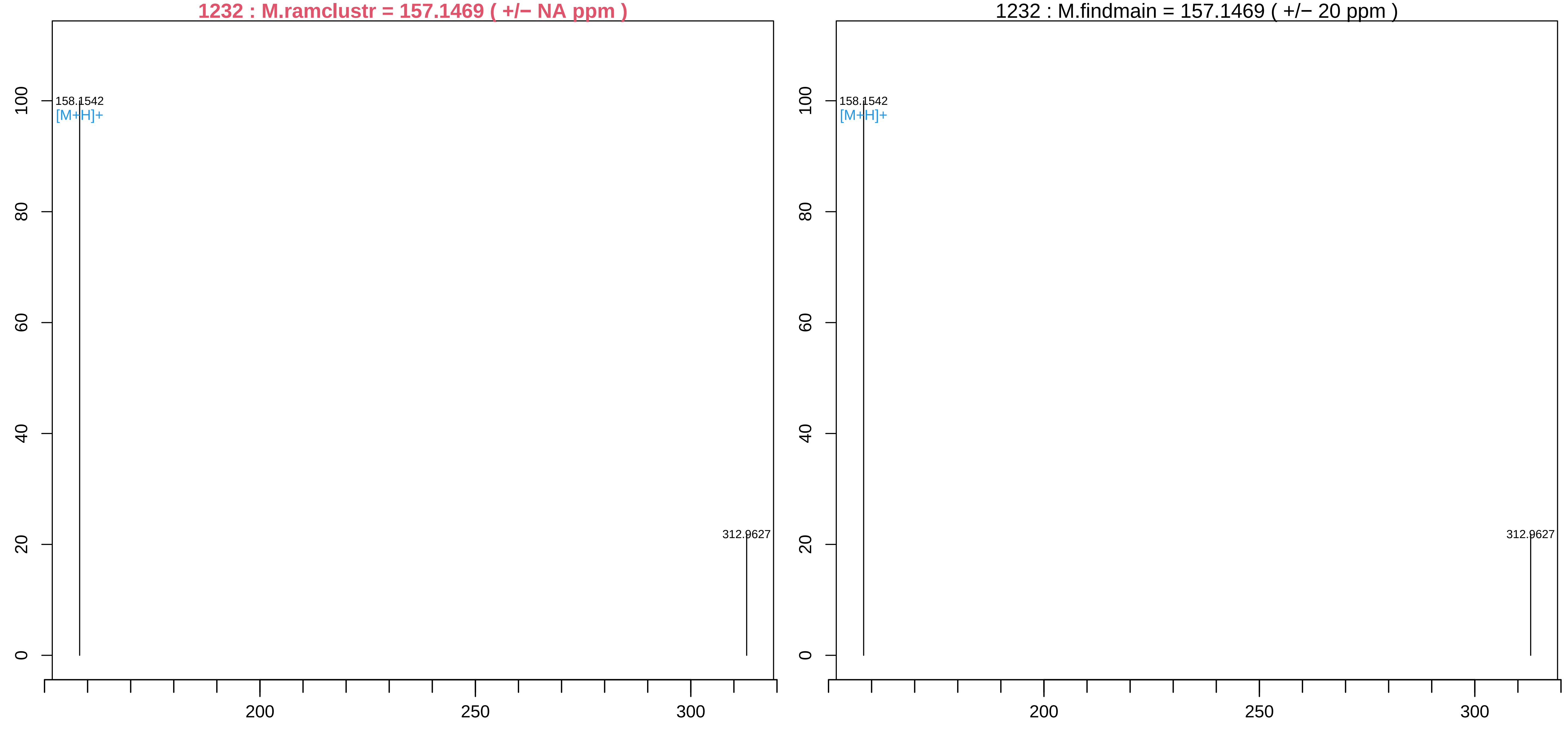

1232 : M.ramclustr = 157.1469 (+/− NA ppm)
158.1542
[M+H]+
312.9627
0
20
40
60
80
100
200
250
300
1232 : M.findmain = 157.1469 (+/− 20 ppm)
158.1542
[M+H]+
312.9627
0
20
40
60
80
100
200
250
300

1233 : M.ramclustr = 271.2522 (+/− NA ppm)

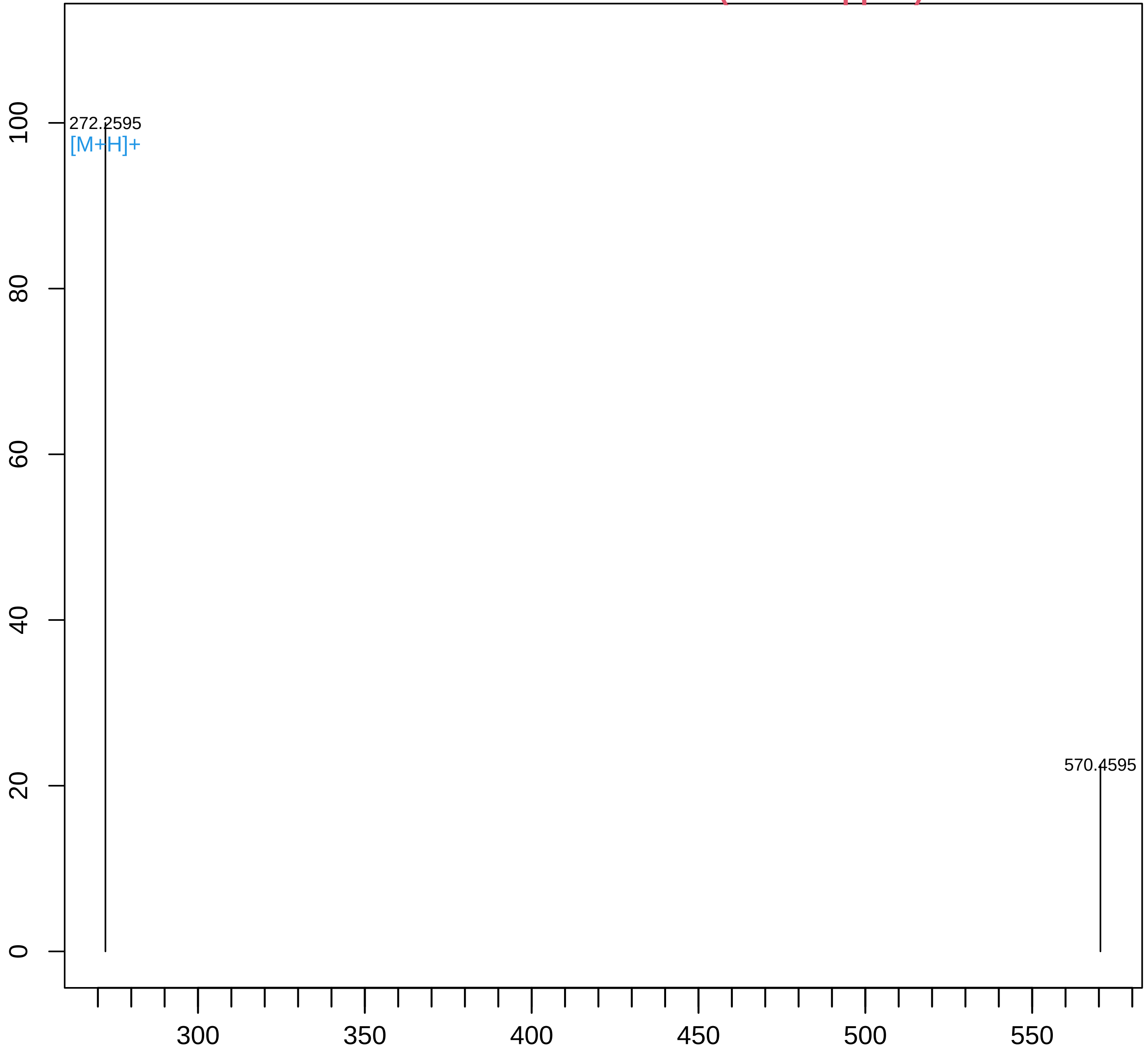

1233 : M.findmain = 271.2522 (+/− 20 ppm)

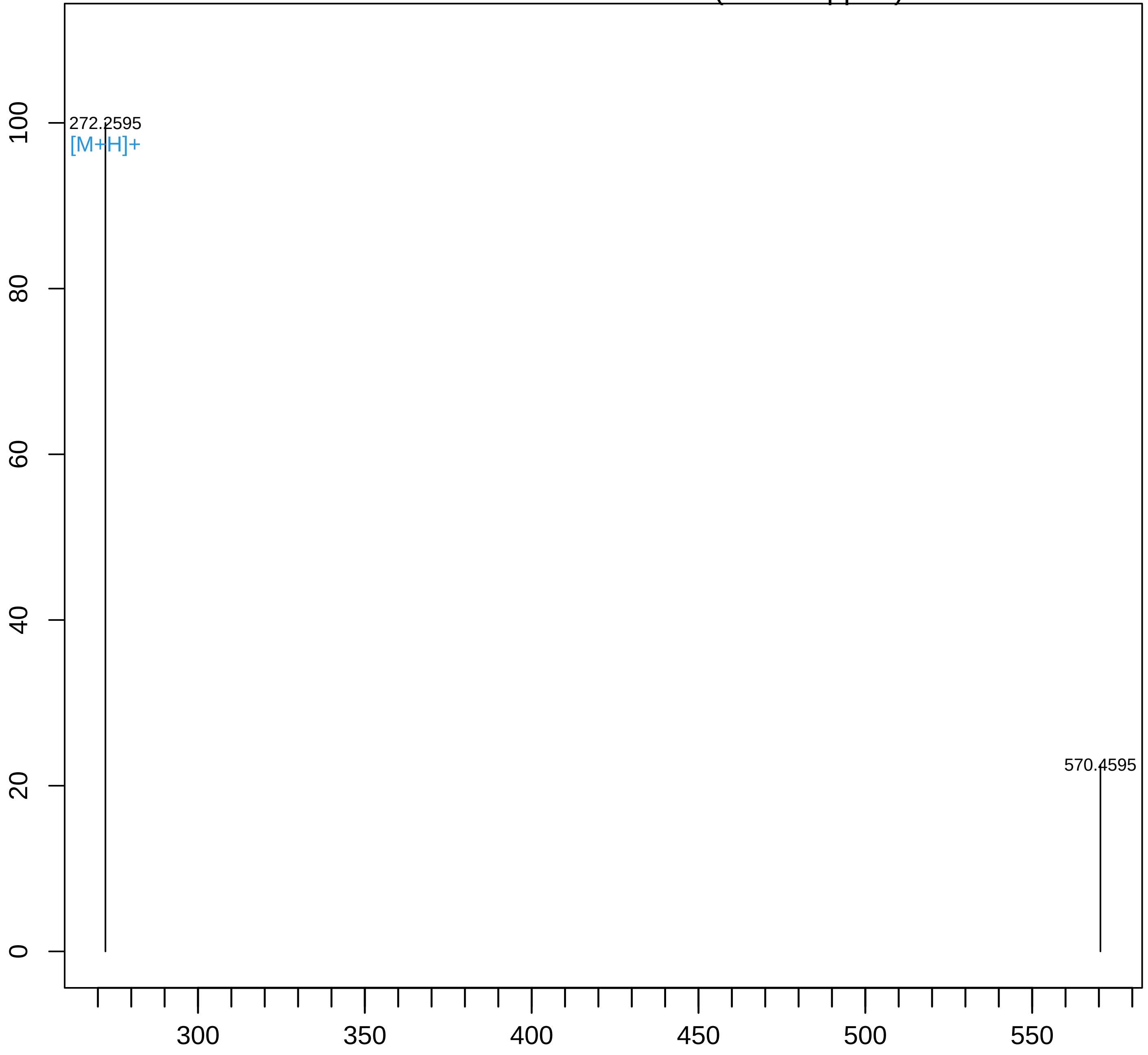

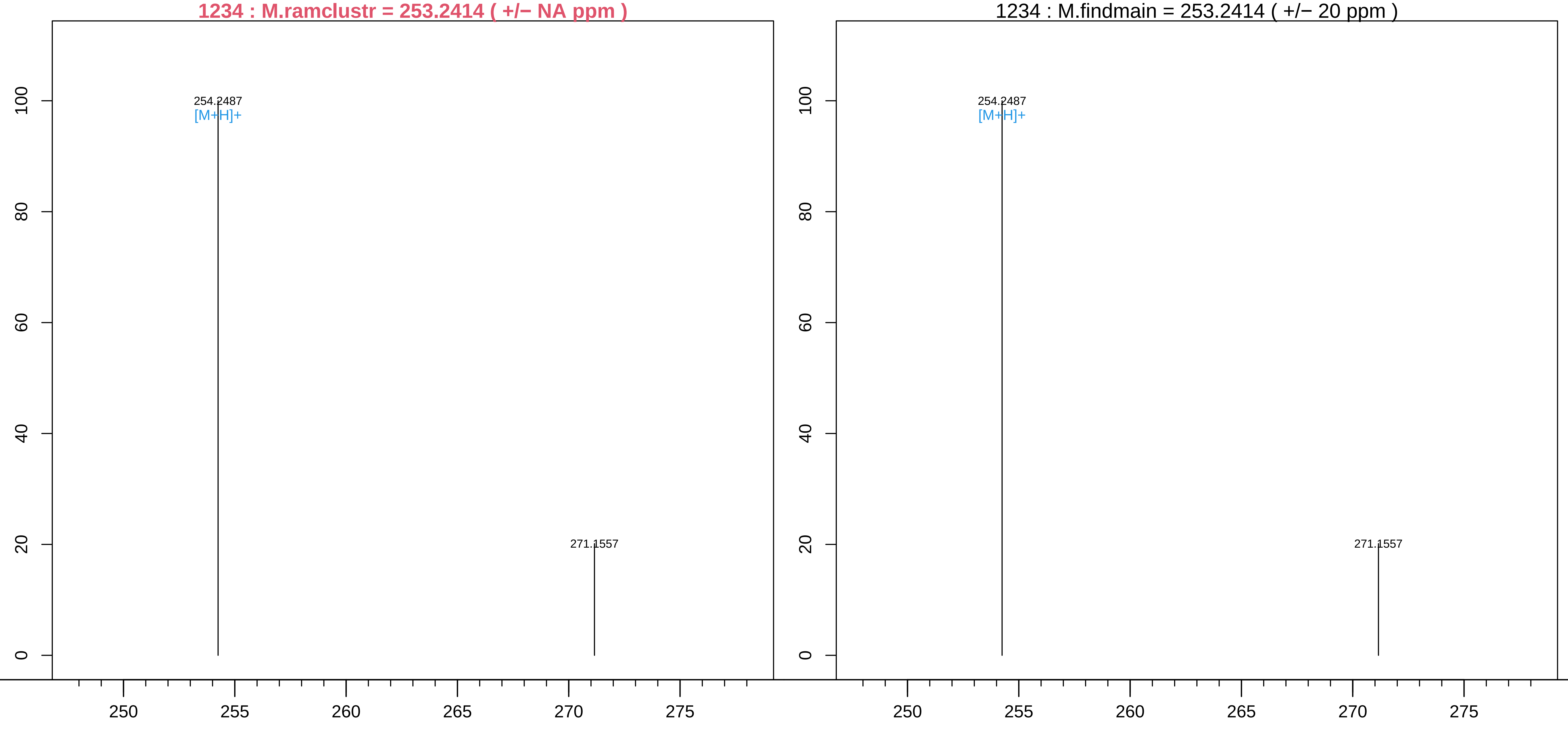
1234 : M.ramclustr = 253.2414 (+/− NA ppm)
254.2487
[M+H]+
271.1557
1234 : M.findmain = 253.2414 (+/− 20 ppm)
254.2487
[M+H]+
271.1557
0
20
40
60
80
100
250
255
260
265
270
275

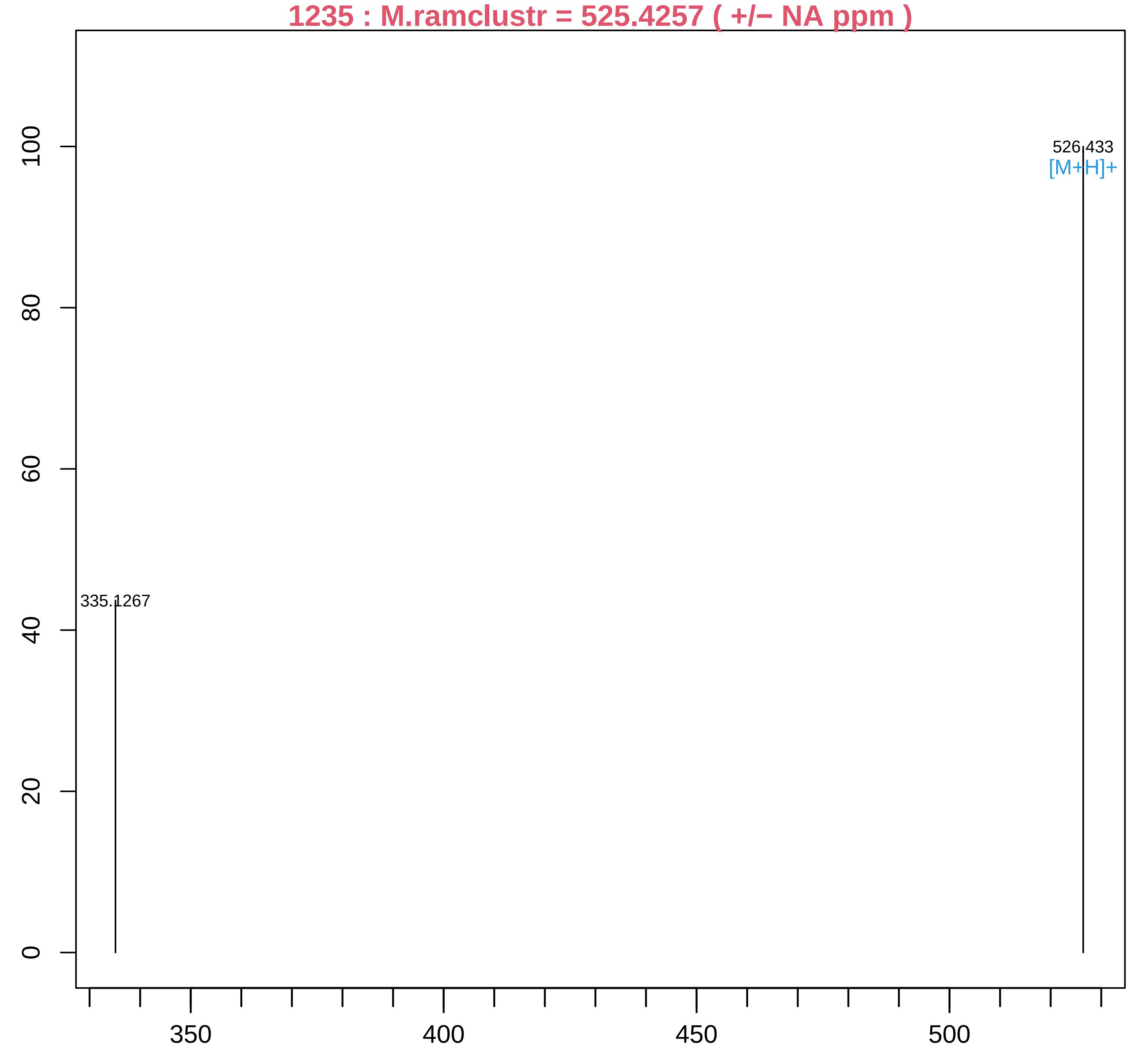

1235 : M.ramclustr = 525.4257 (+/− NA ppm)
526.433
[M+H]+
335.1267
0
20
40
60
80
100
350
400
450
500

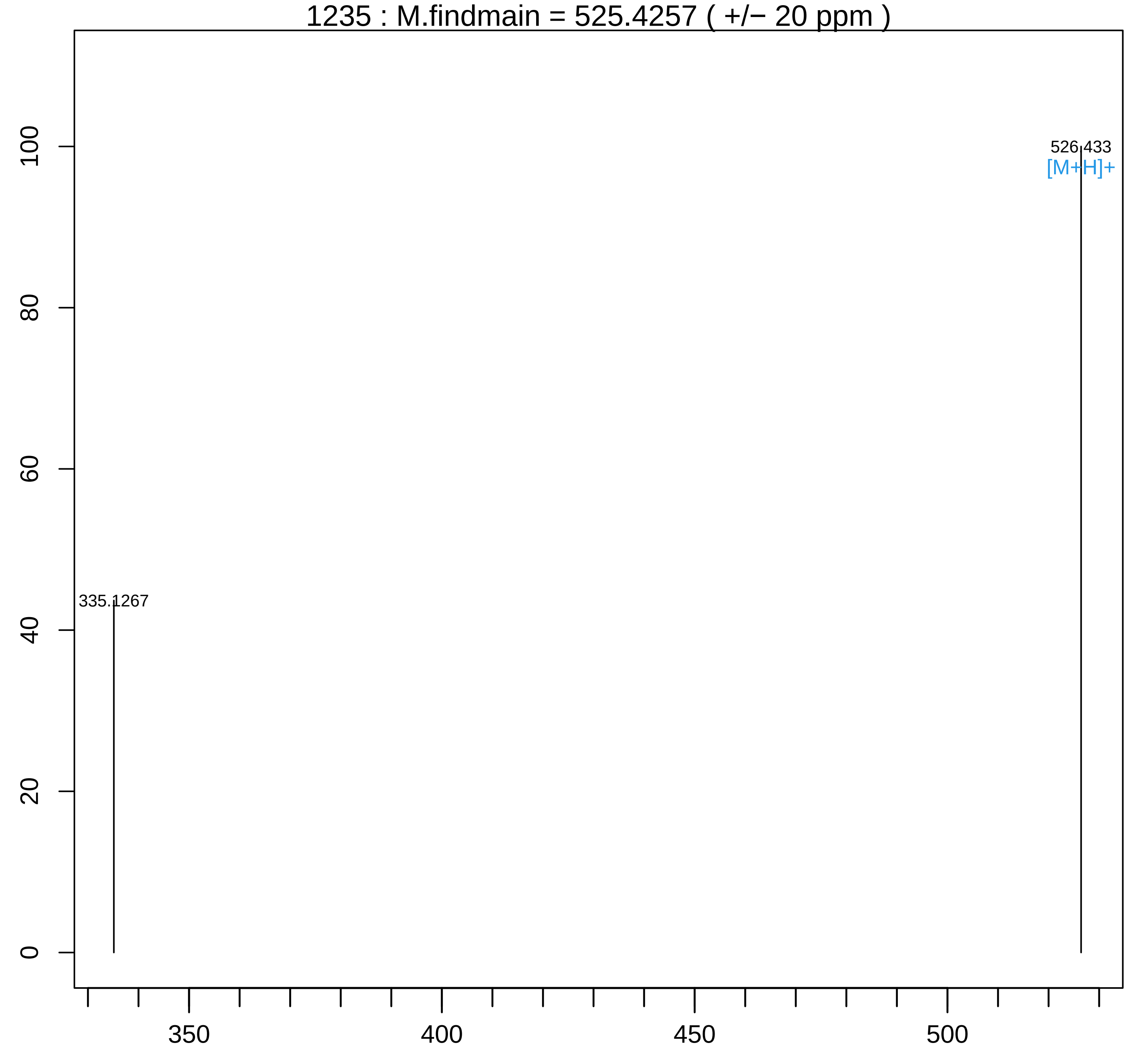

1235 : M.findmain = 525.4257 (+/− 20 ppm)
526.433
[M+H]+
335.1267
0
20
40
60
80
100
350
400
450
500

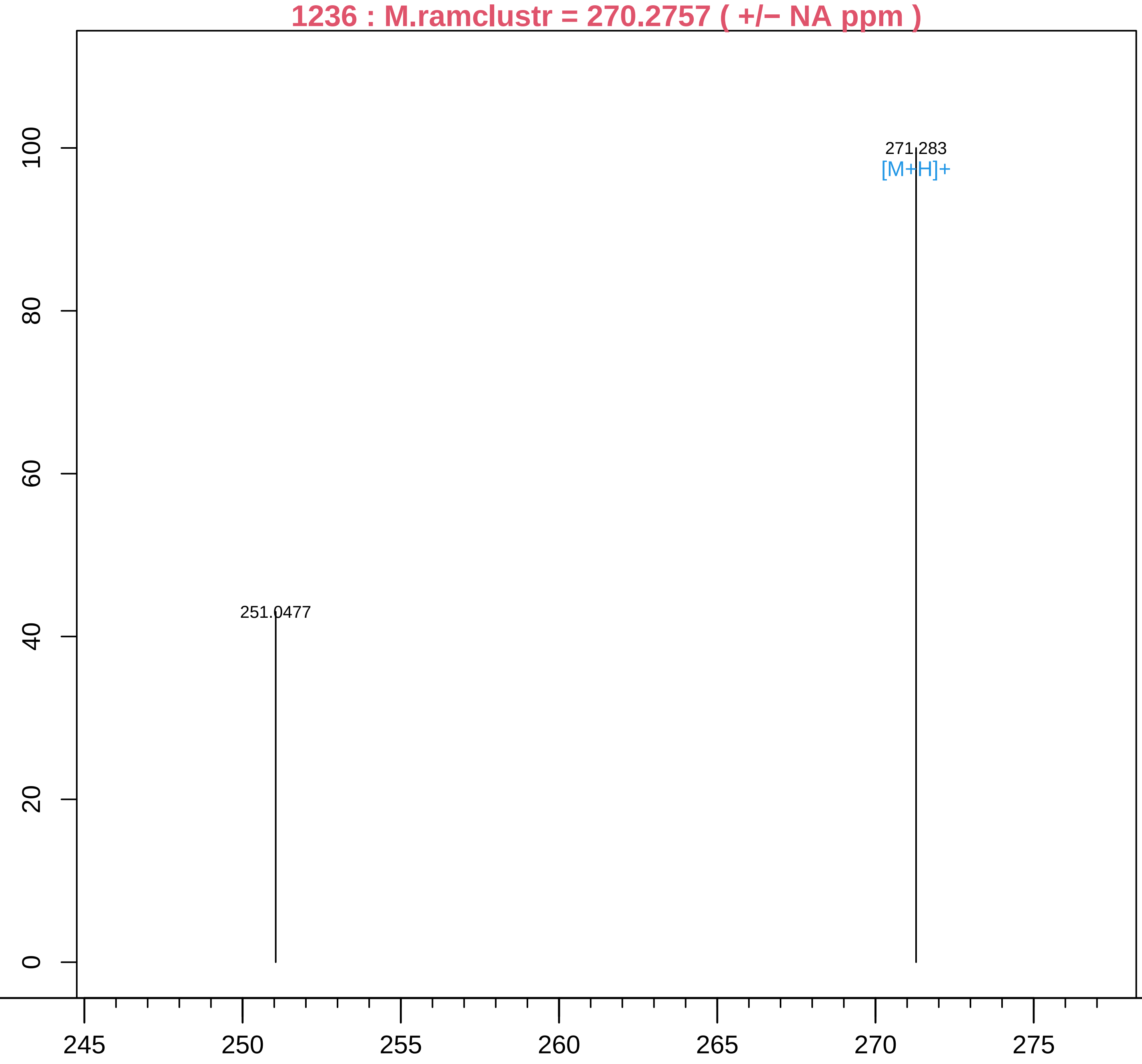
1236 : M.ramclustr = 270.2757 (+/− NA ppm)
271.283
[M+H]+
251.0477
0
20
40
60
80
100
245
250
255
260
265
270
275

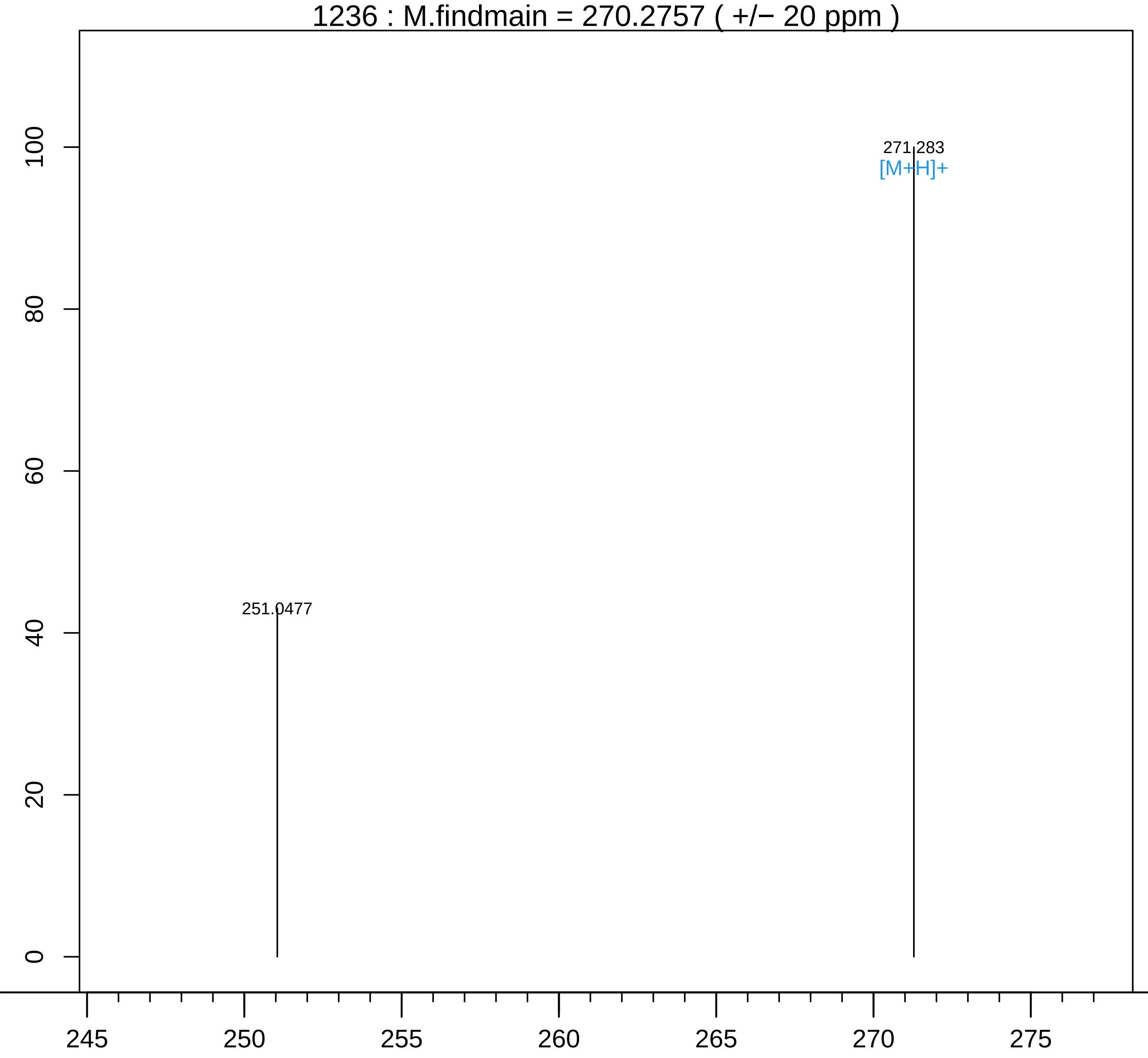
1236 : M.findmain = 270.2757 (+/− 20 ppm)
271.283
[M+H]+
251.0477
0
20
40
60
80
100
245
250
255
260
265
270
275

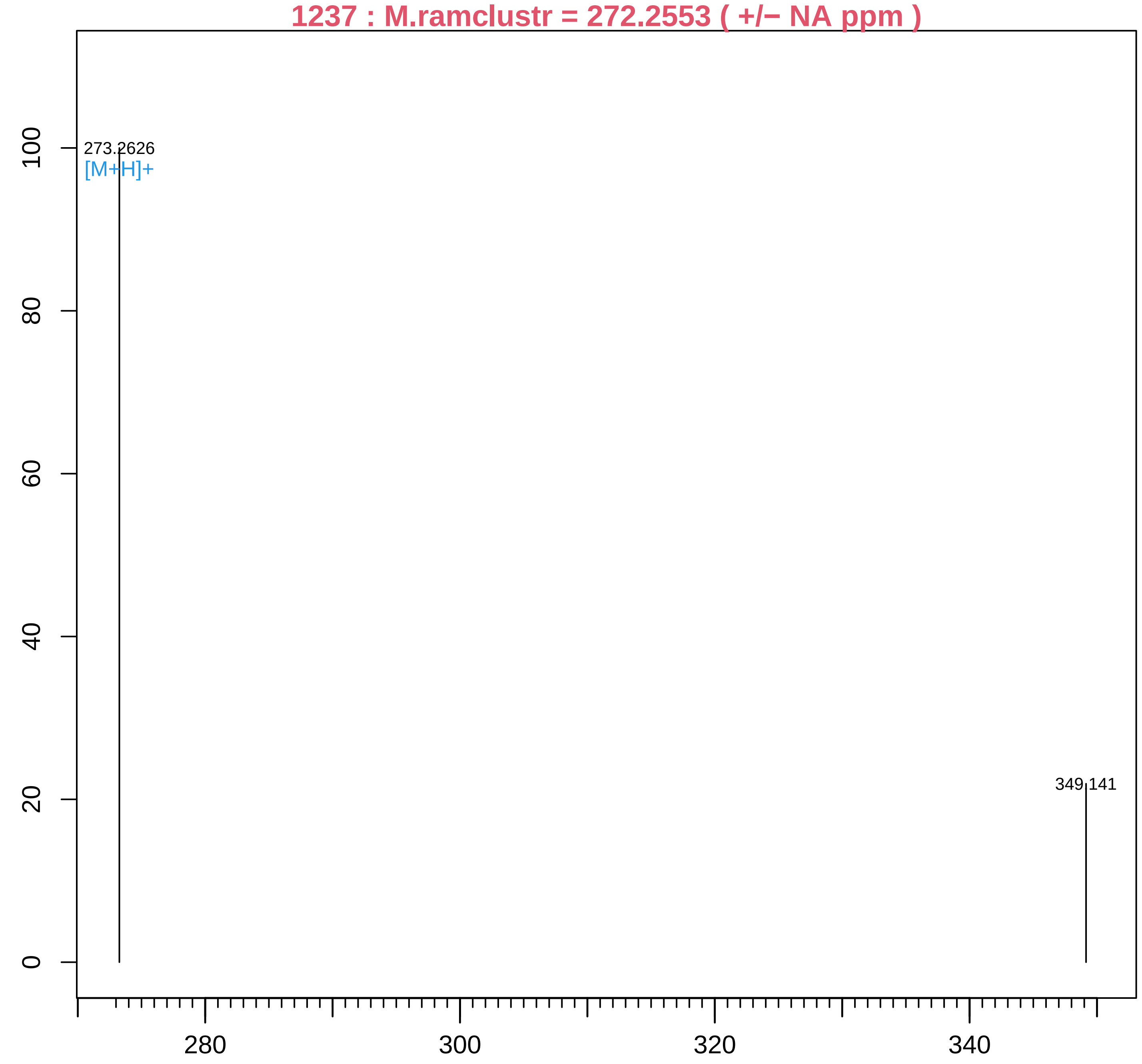
1237 : M.ramclustr = 272.2553 (+/− NA ppm)
273.2626
[M+H]+
349.141
0
20
40
60
80
100
280
300
320
340

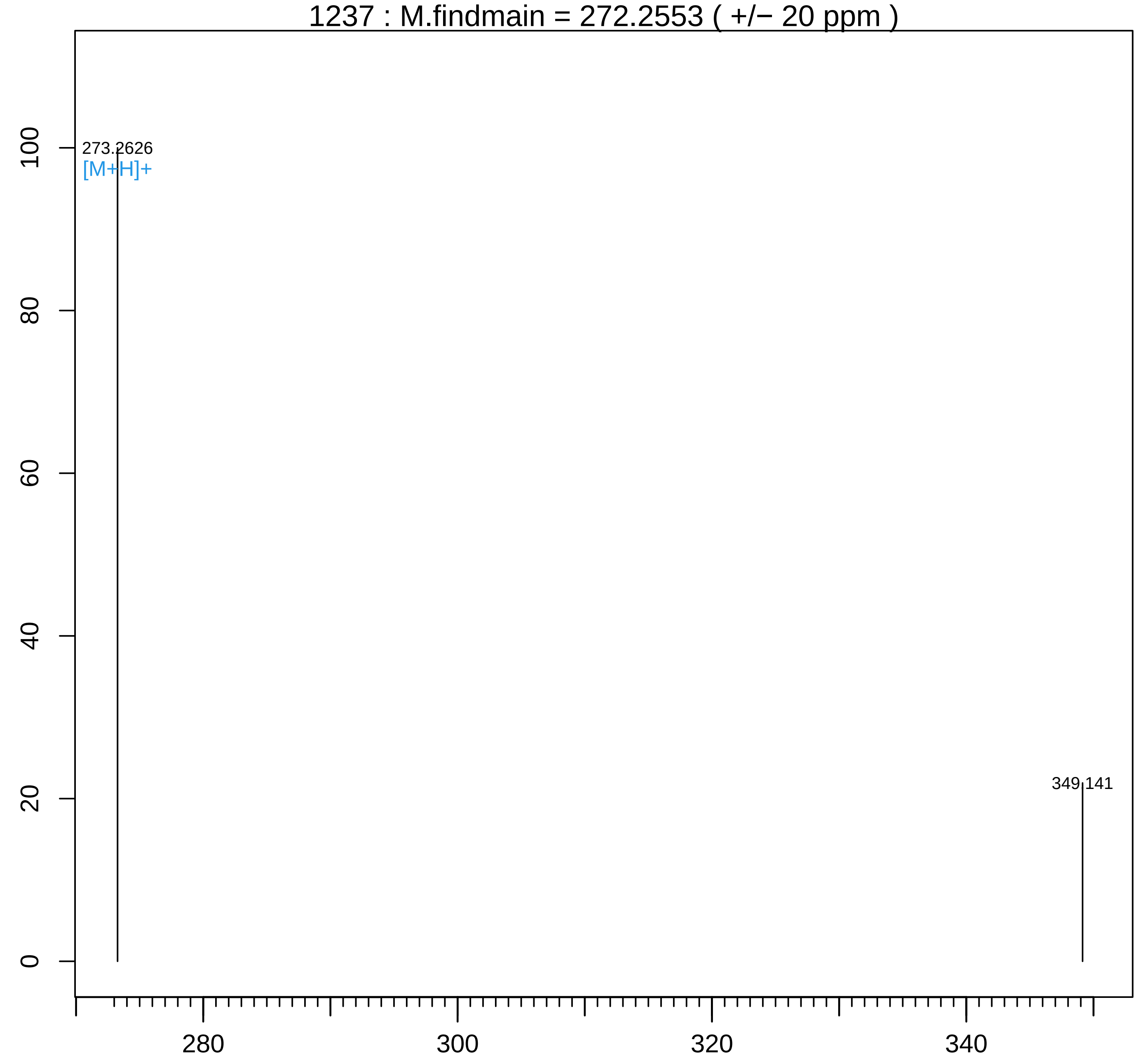
1237 : M.findmain = 272.2553 (+/− 20 ppm)
273.2626
[M+H]+
349.141
0
20
40
60
80
100
280
300
320
340

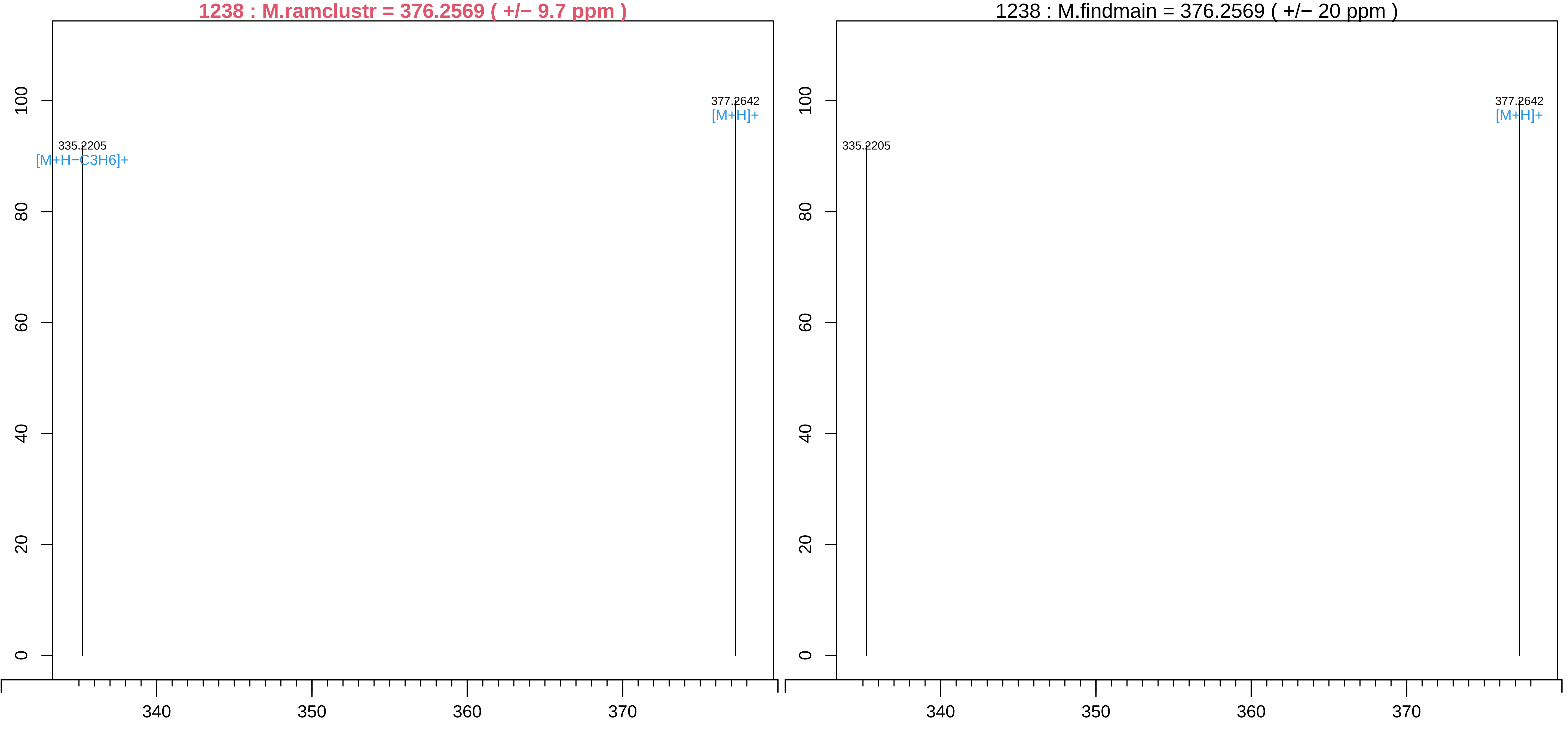

1238 : M.ramclustr = 376.2569 (+/− 9.7 ppm)
335.2205
[M+H−C3H6]+
377.2642
[M+H]+
1238 : M.findmain = 376.2569 (+/− 20 ppm)
335.2205
377.2642
[M+H]+
0
20
40
60
80
100
340
350
360
370

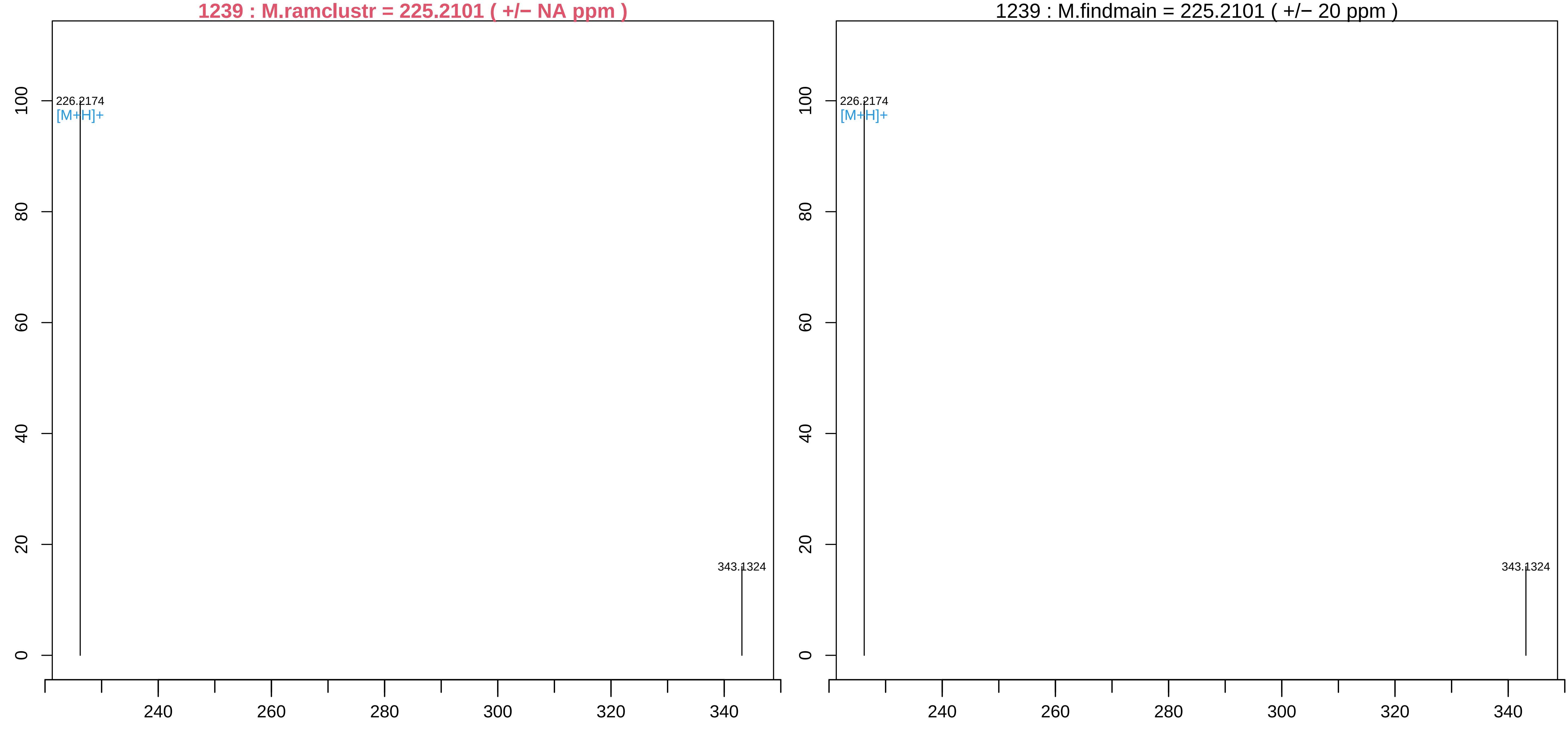
1239 : M.ramclustr = 225.2101 (+/− NA ppm)
226.2174
[M+H]+
343.1324
0
20
40
60
80
100
240
260
280
300
320
340
1239 : M.findmain = 225.2101 (+/− 20 ppm)
226.2174
[M+H]+
343.1324
0
20
40
60
80
100
240
260
280
300
320
340

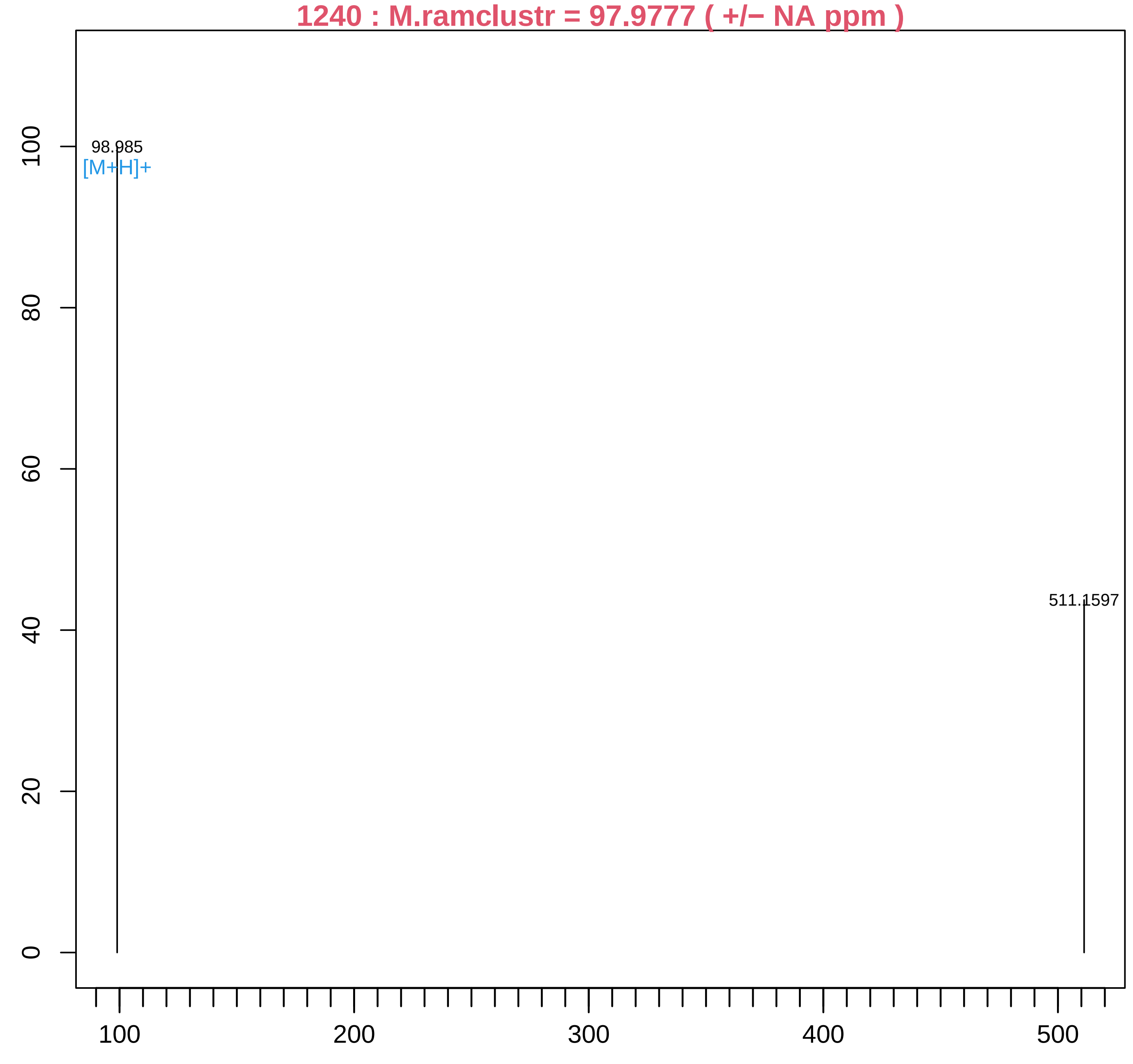

1240 : M.ramclustr = 97.9777 (+/− NA ppm)
98.985
[M+H]+
511.1597
0
20
40
60
80
100
100
200
300
400
500

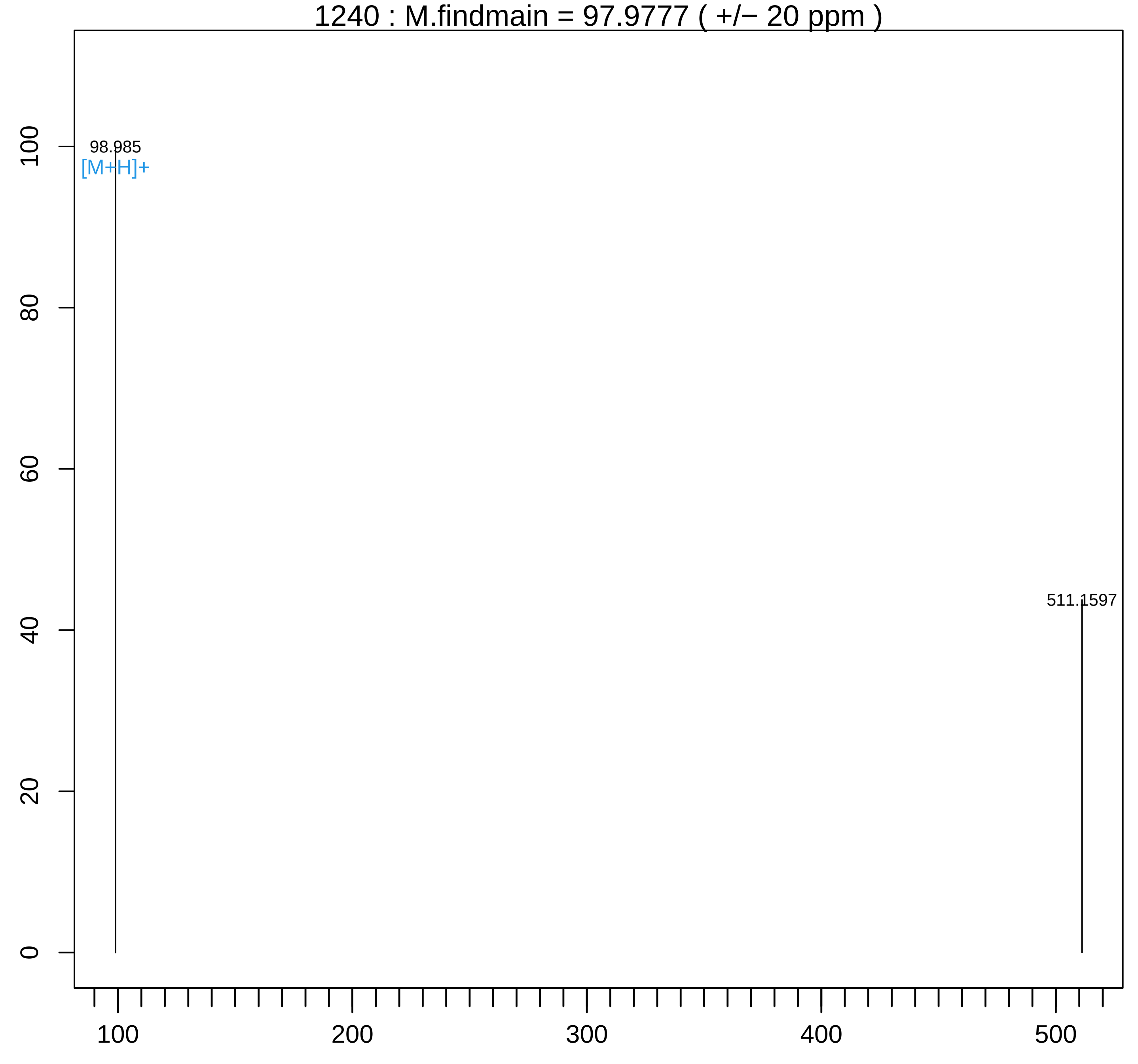

1240 : M.findmain = 97.9777 (+/− 20 ppm)
98.985
[M+H]+
511.1597
0
20
40
60
80
100
100
200
300
400
500

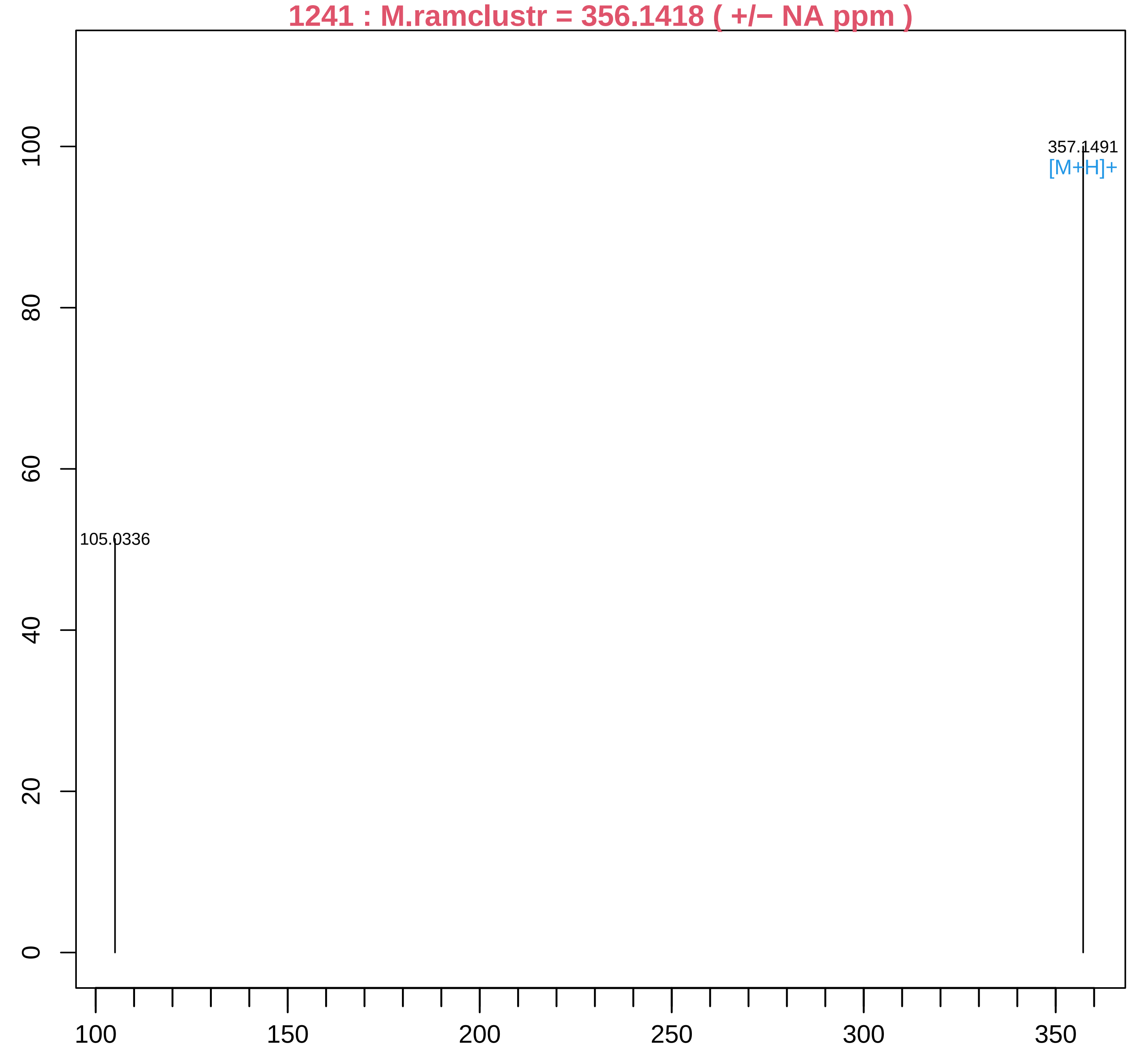
1241 : M.ramclustr = 356.1418 (+/− NA ppm)
357.1491
[M+H]+
105.0336
0
20
40
60
80
100
100
150
200
250
300
350

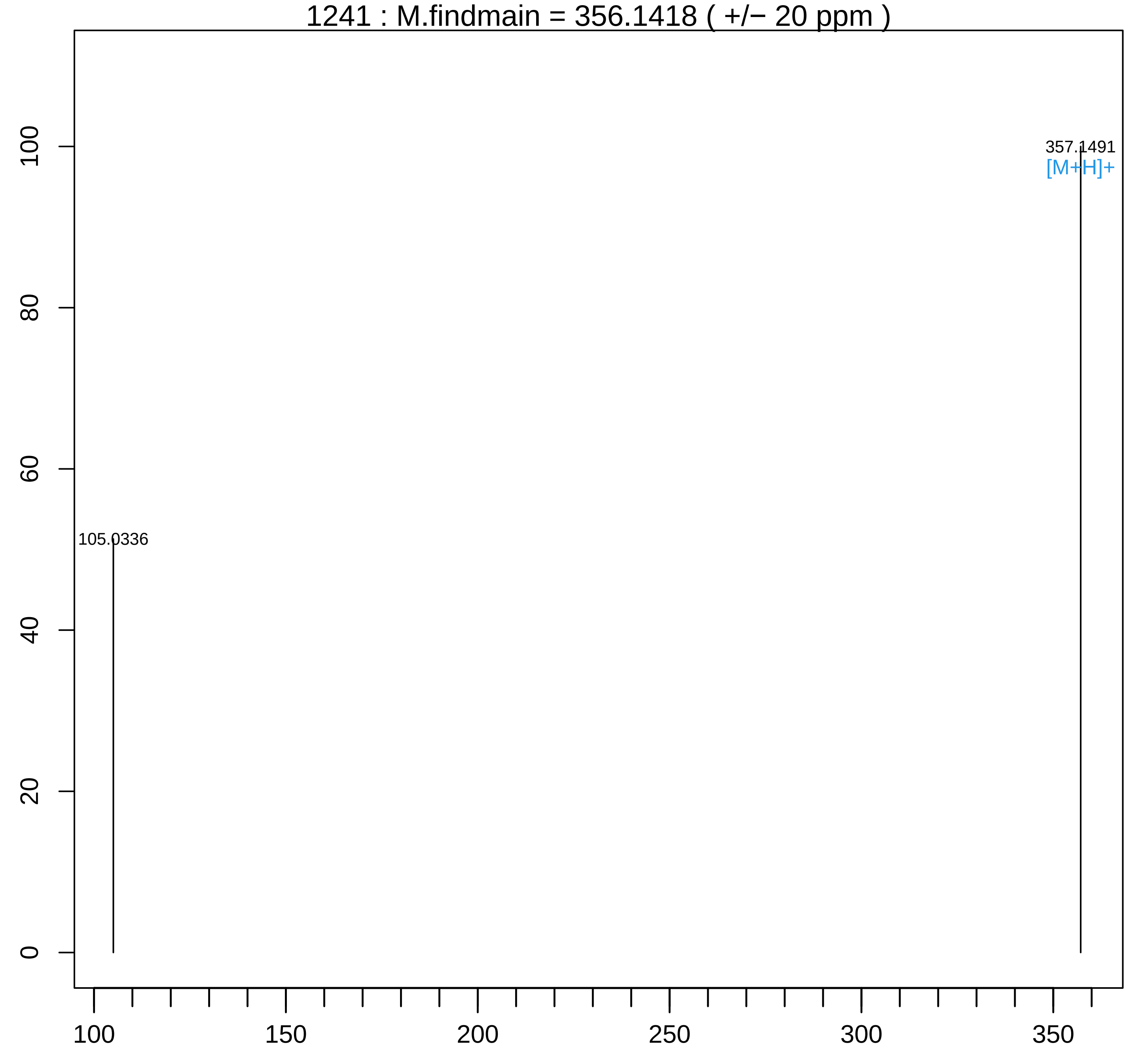
1241 : M.findmain = 356.1418 (+/− 20 ppm)
357.1491
[M+H]+
105.0336
0
20
40
60
80
100
100
150
200
250
300
350

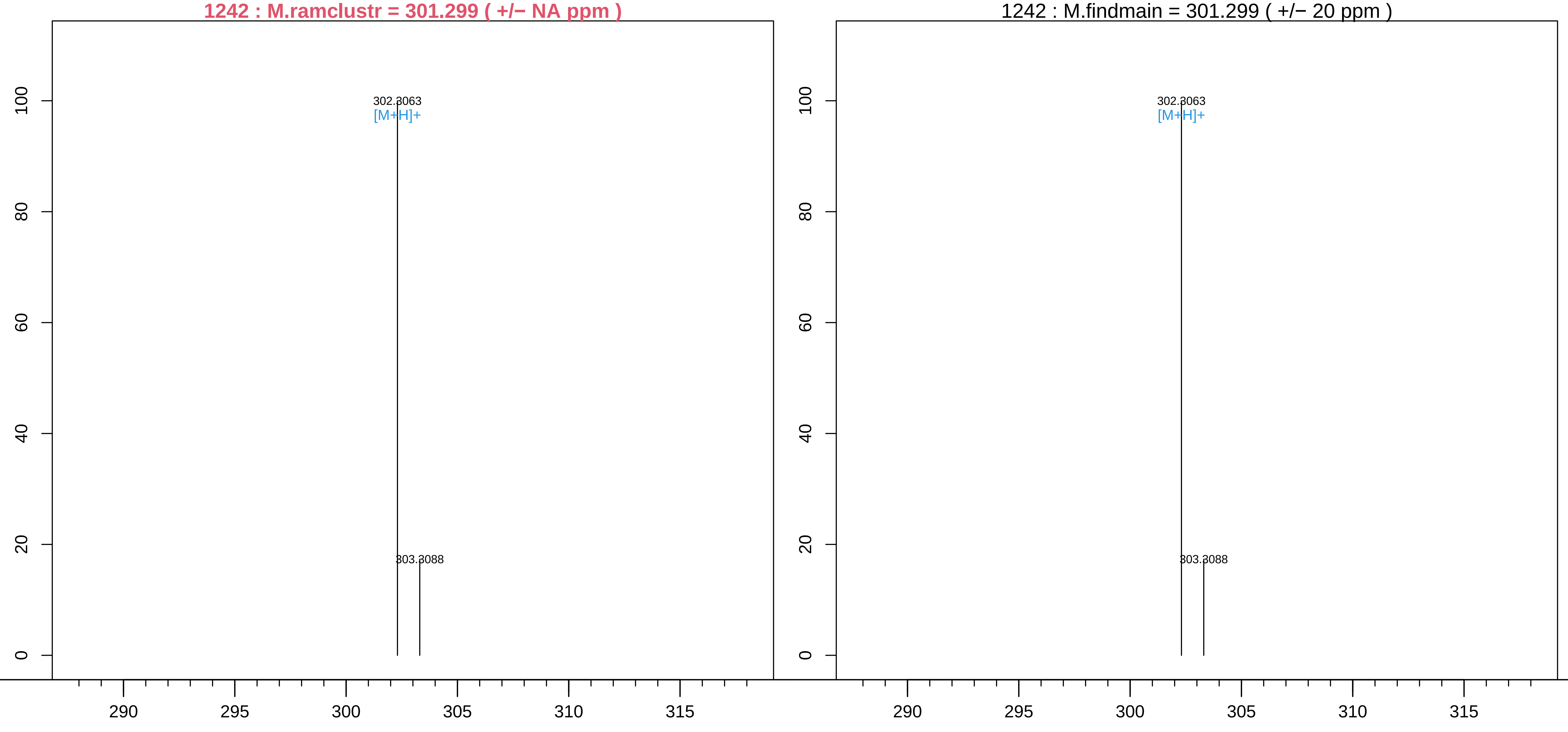
1242 : M.ramclustr = 301.299 (+/− NA ppm)
302.3063
[M+H]+
303.3088
1242 : M.findmain = 301.299 (+/− 20 ppm)
302.3063
[M+H]+
303.3088
0
20
40
60
80
100
290
295
300
305
310
315

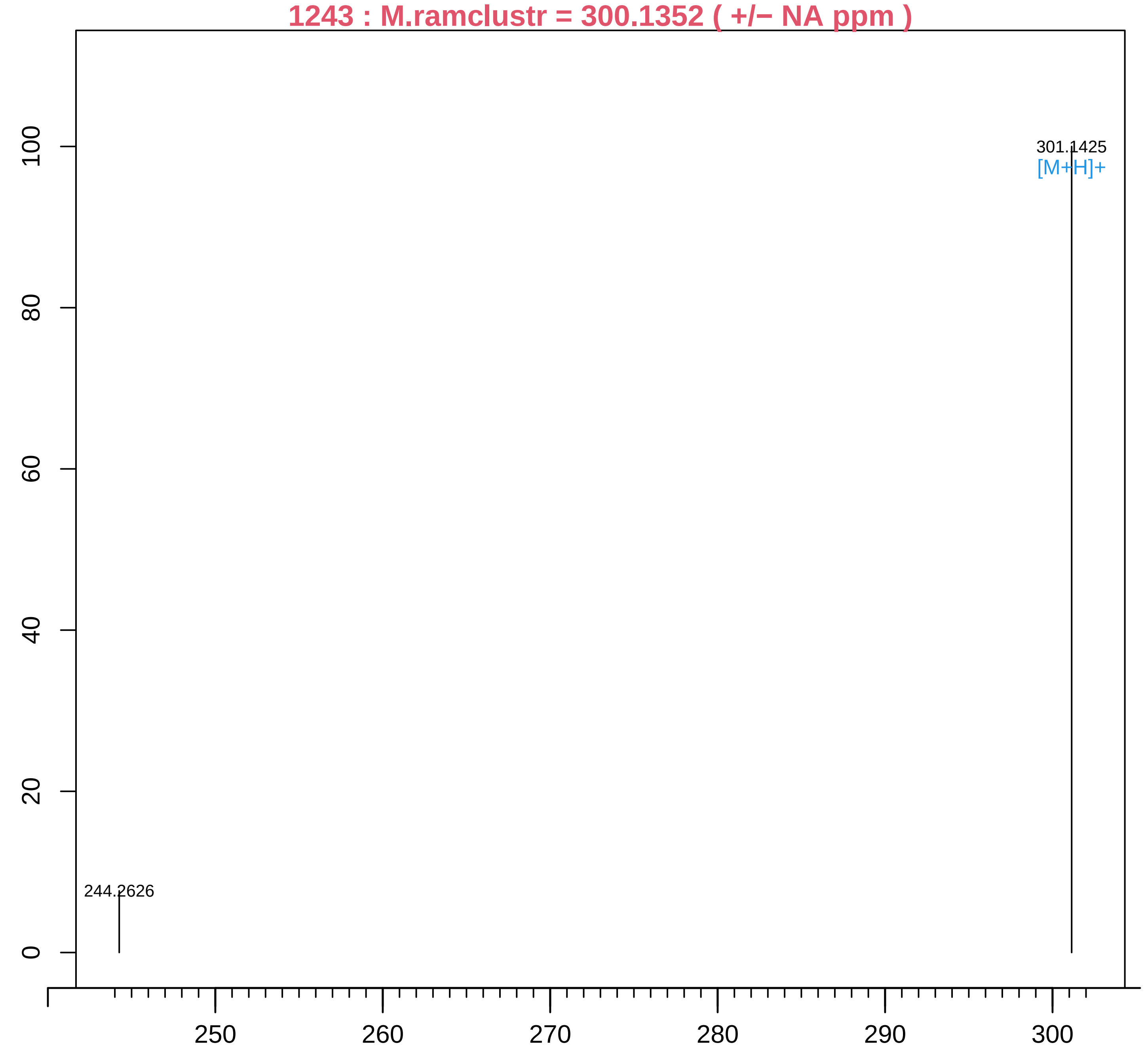
1243 : M.ramclustr = 300.1352 (+/− NA ppm)
301.1425
[M+H]+
244.2626
0
20
40
60
80
100
250
260
270
280
290
300

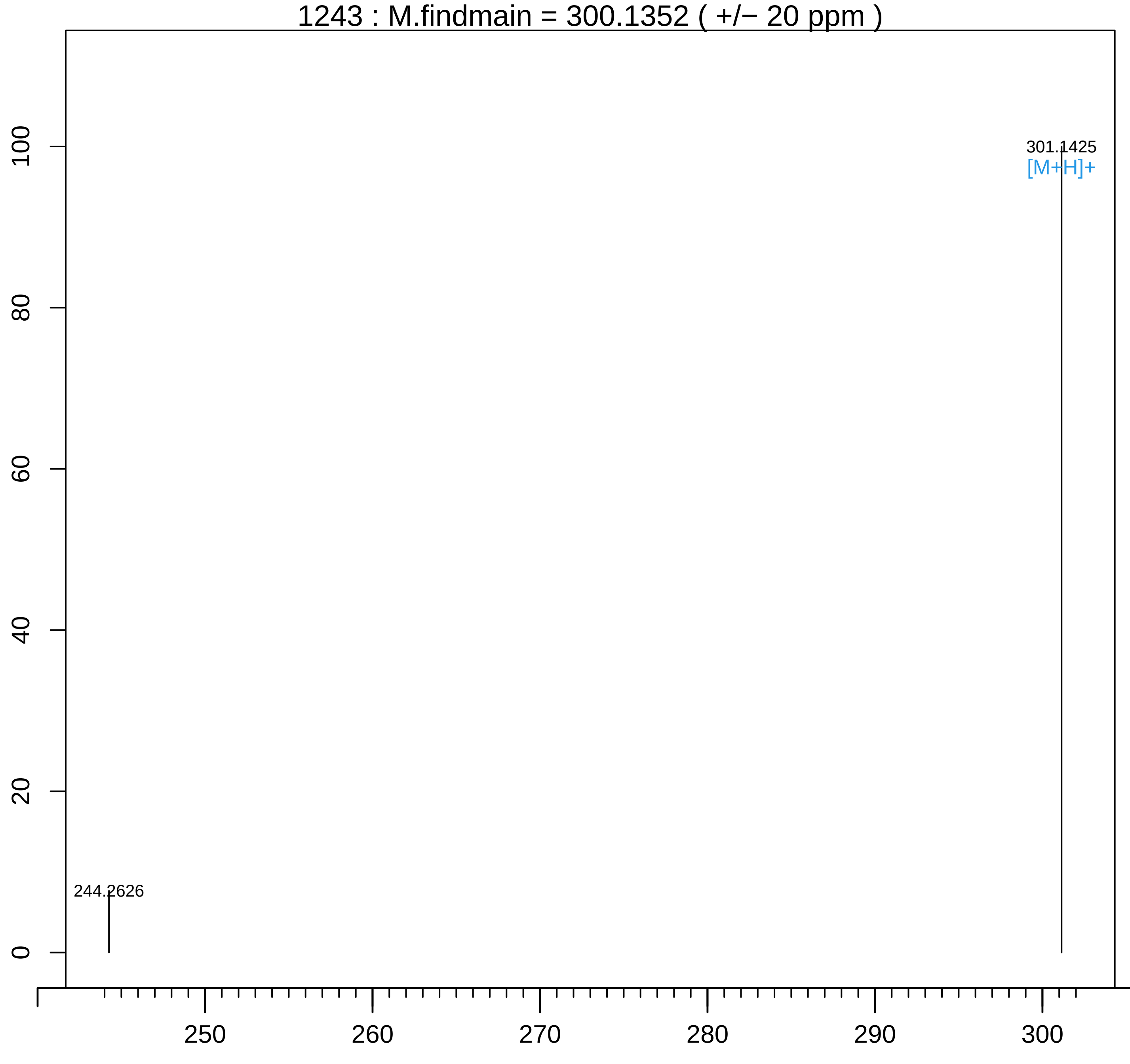
1243 : M.findmain = 300.1352 (+/− 20 ppm)
301.1425
[M+H]+
244.2626
0
20
40
60
80
100
250
260
270
280
290
300

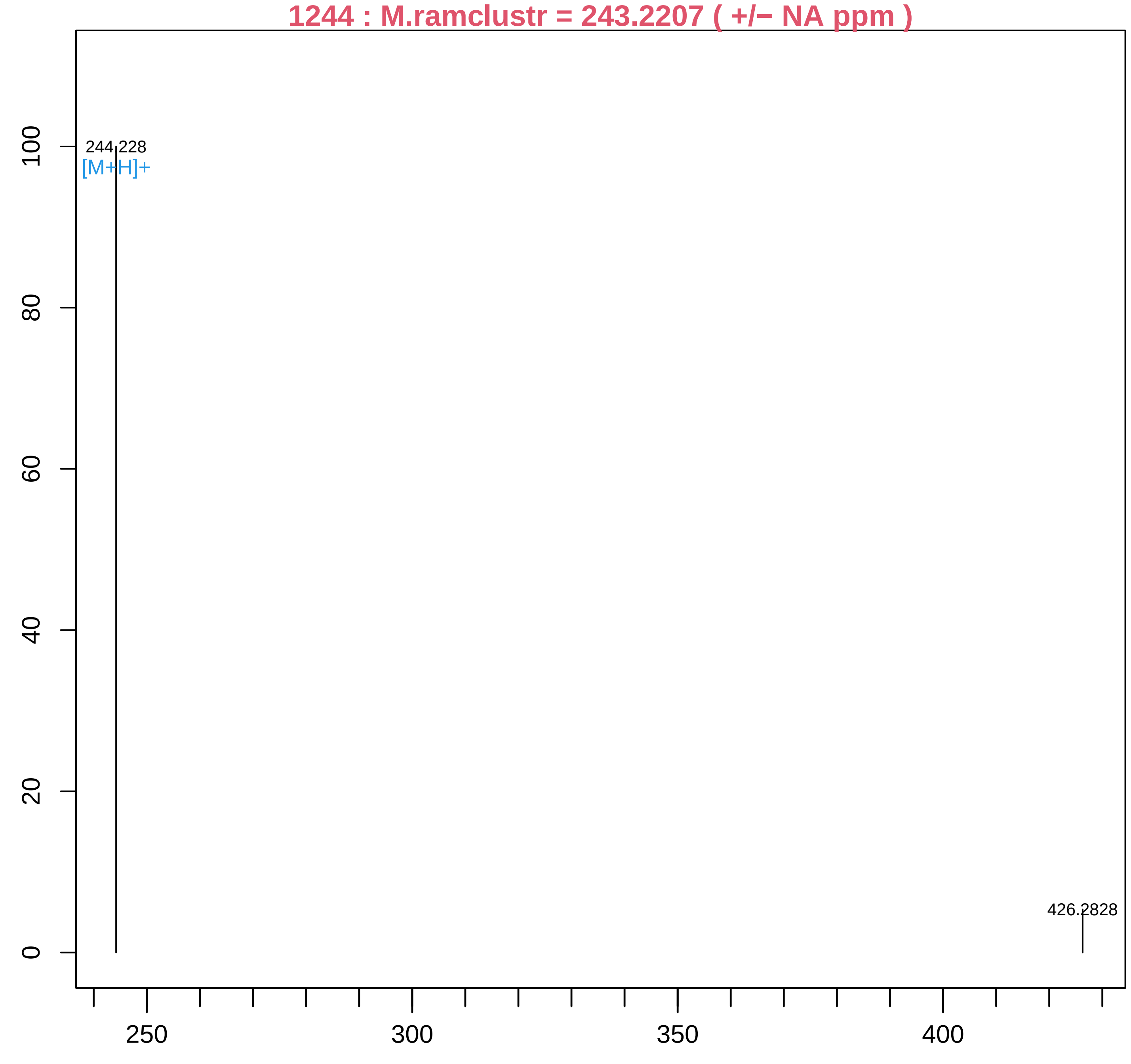
1244 : M.ramclustr = 243.2207 (+/− NA ppm)
244.228
[M+H]+
426.2828
0
20
40
60
80
100
250
300
350
400

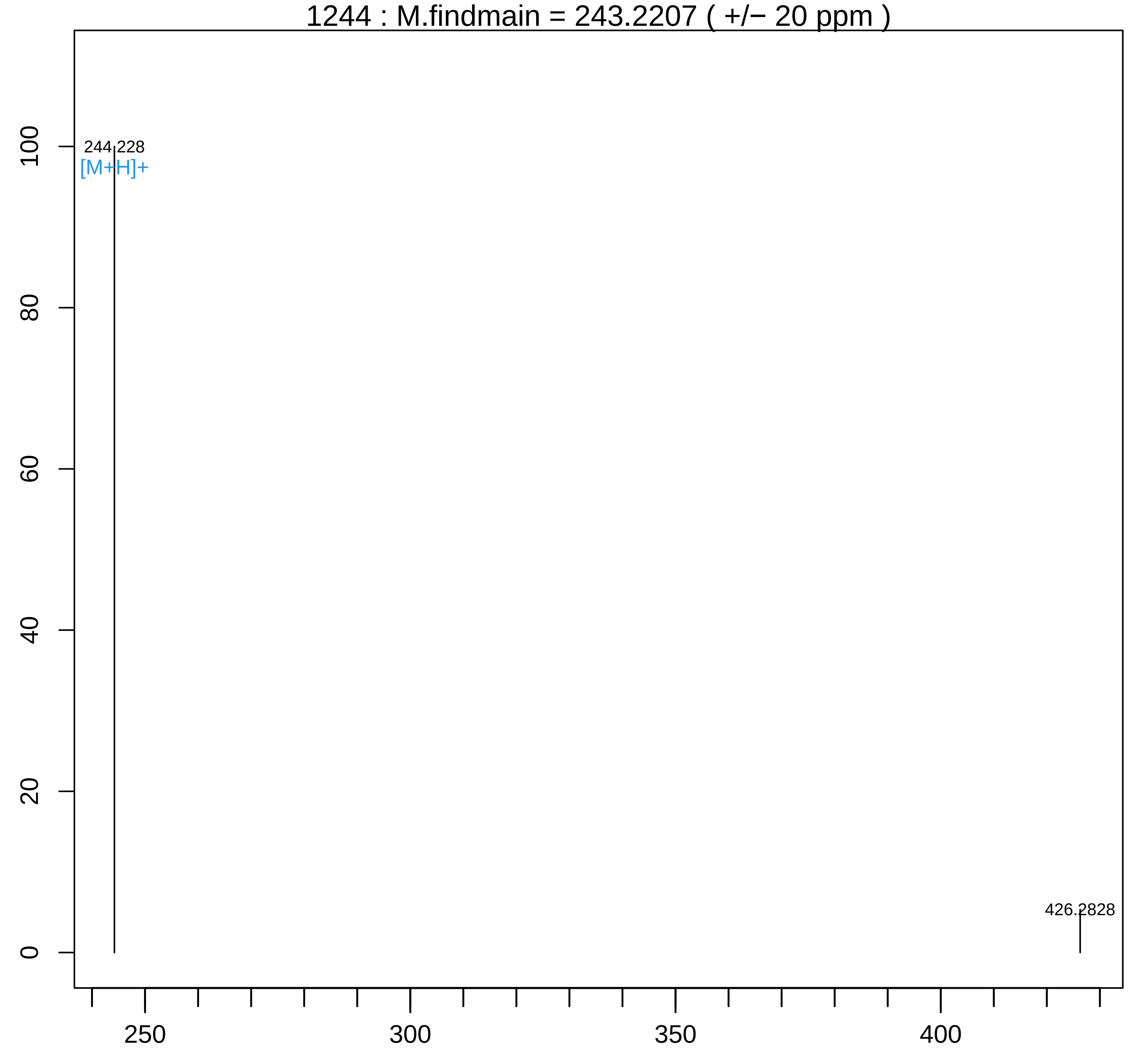
1244 : M.findmain = 243.2207 (+/− 20 ppm)
244.228
[M+H]+
426.2828
0
20
40
60
80
100
250
300
350
400

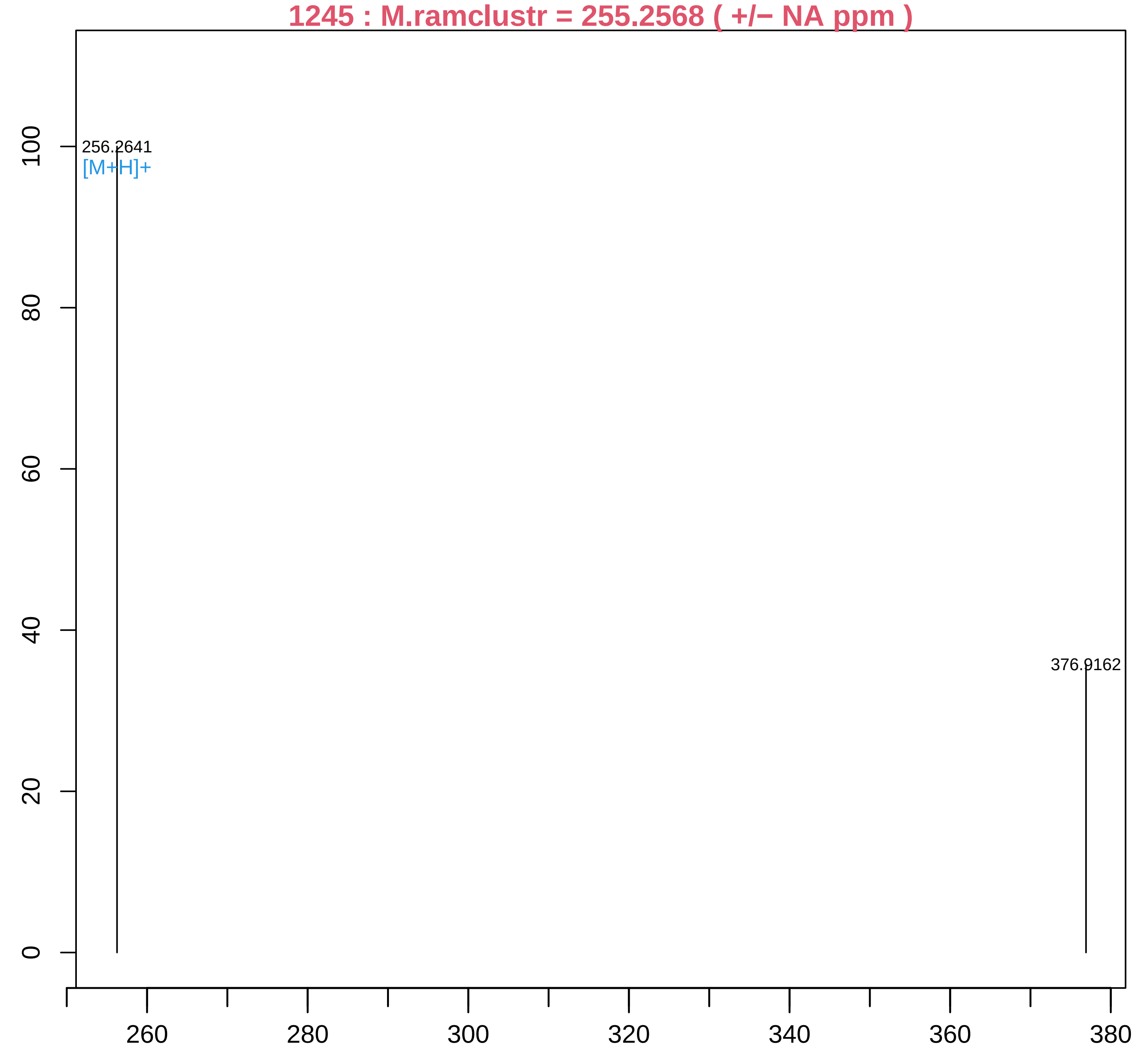
1245 : M.ramclustr = 255.2568 (+/− NA ppm)
256.2641
[M+H]+
376.9162
0
20
40
60
80
100
260
280
300
320
340
360
380

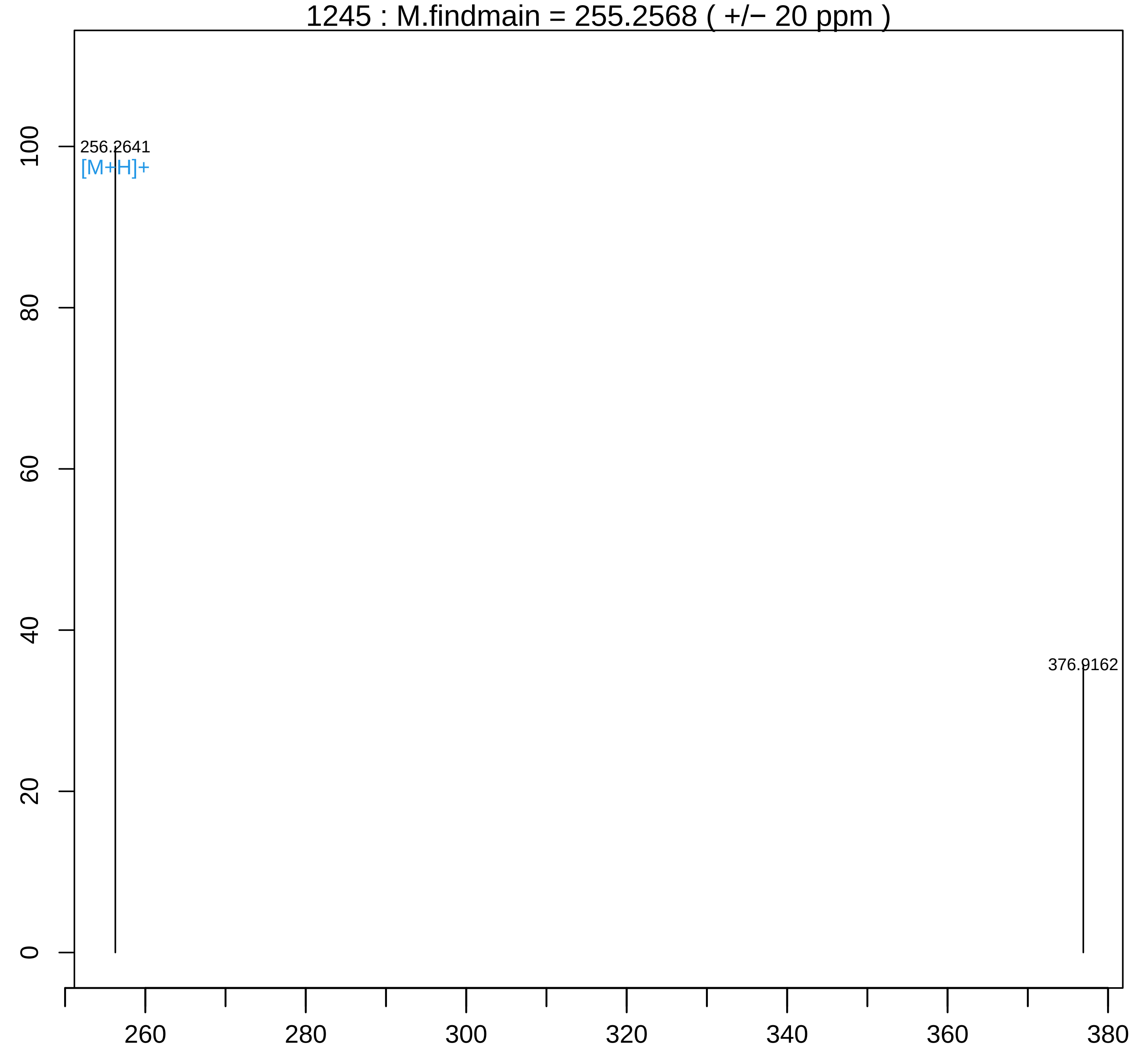
1245 : M.findmain = 255.2568 (+/− 20 ppm)
256.2641
[M+H]+
376.9162
0
20
40
60
80
100
260
280
300
320
340
360
380

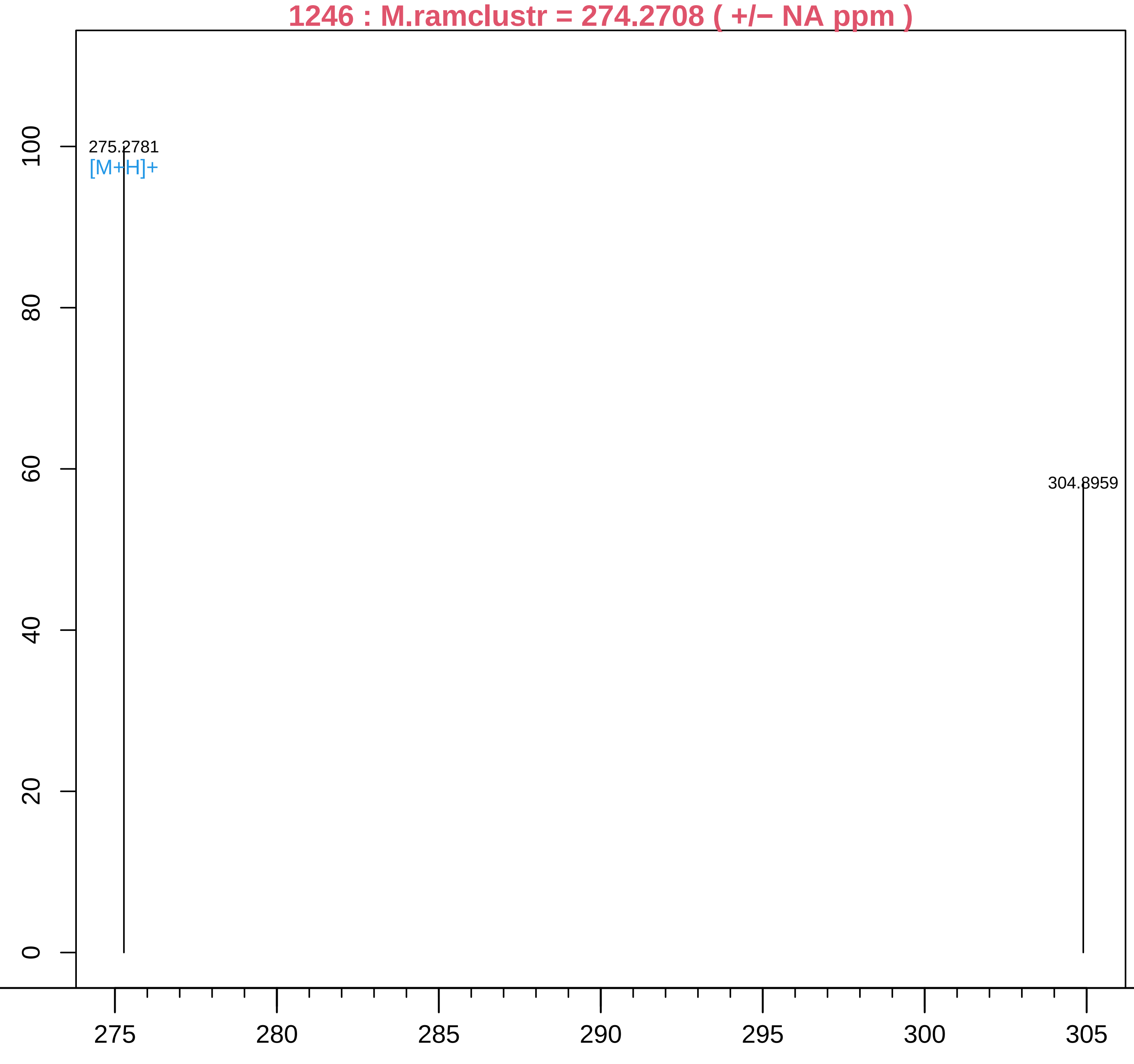
1246 : M.ramclustr = 274.2708 (+/− NA ppm)
275.2781
[M+H]+
304.8959
0
20
40
60
80
100
275
280
285
290
295
300
305

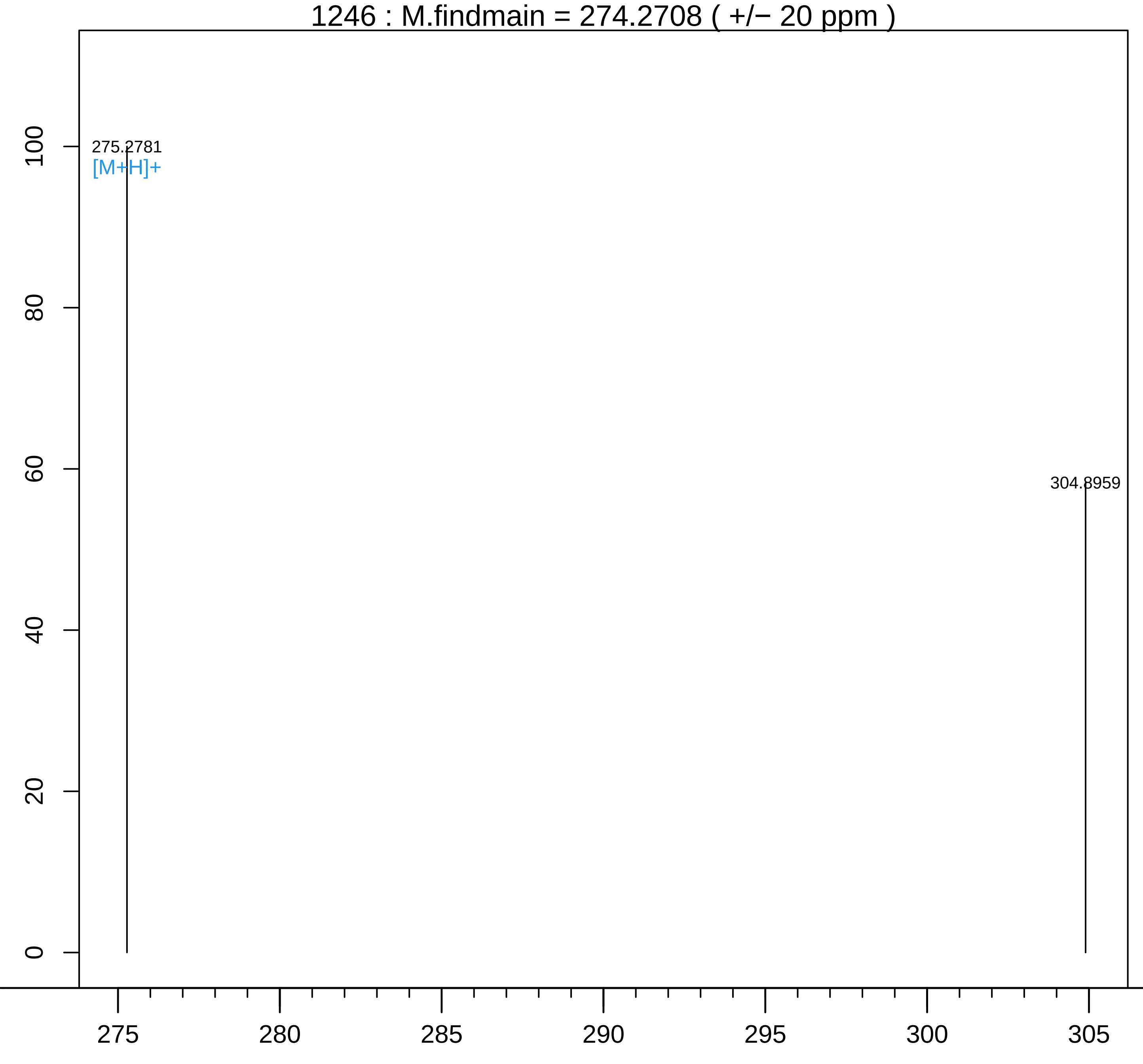
1246 : M.findmain = 274.2708 (+/− 20 ppm)
275.2781
[M+H]+
304.8959
0
20
40
60
80
100
275
280
285
290
295
300
305

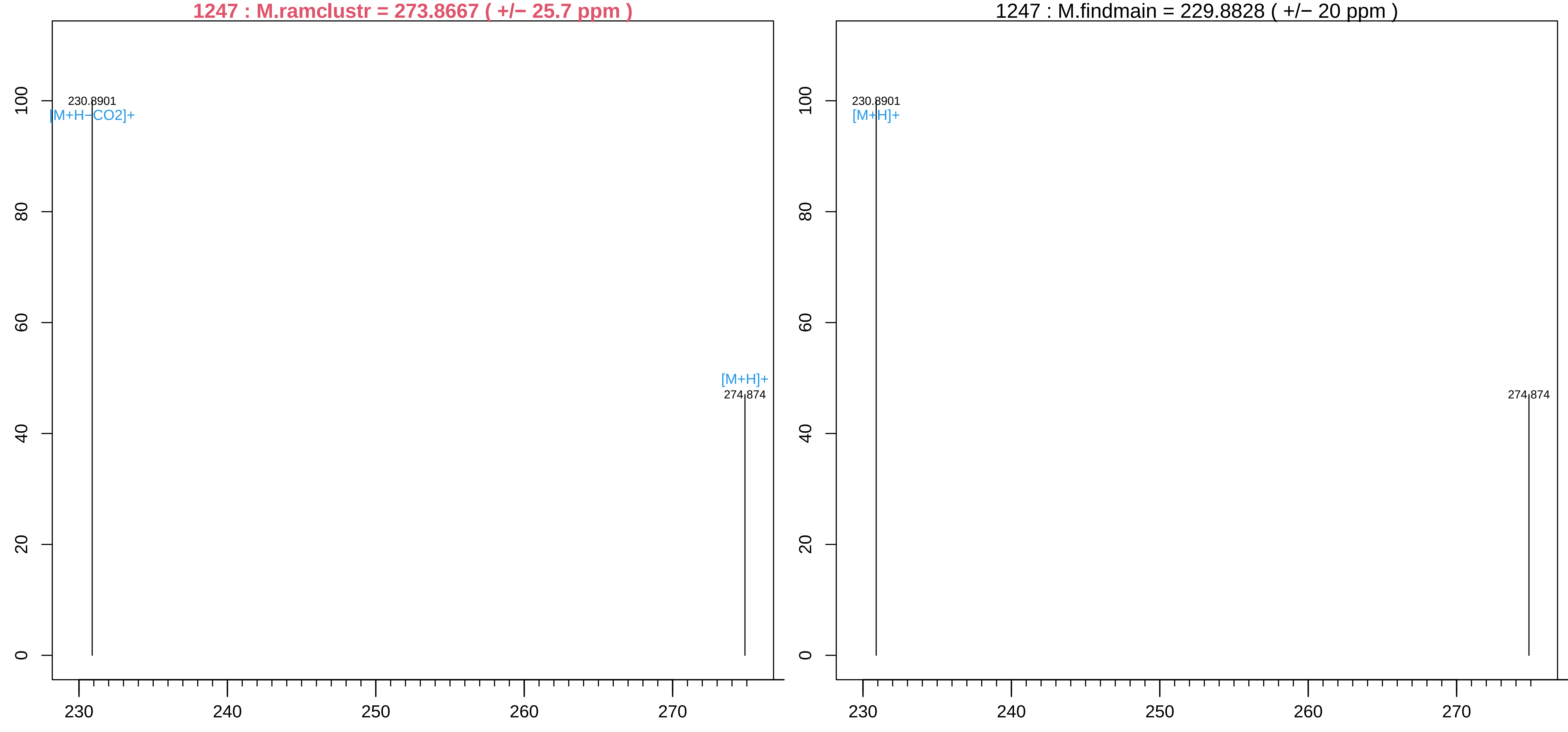

1247 : M.ramclustr = 273.8667 (+/− 25.7 ppm)
230.8901
[M+H−CO2]+
[M+H]+
274.874
1247 : M.findmain = 229.8828 (+/− 20 ppm)
230.8901
[M+H]+
274.874

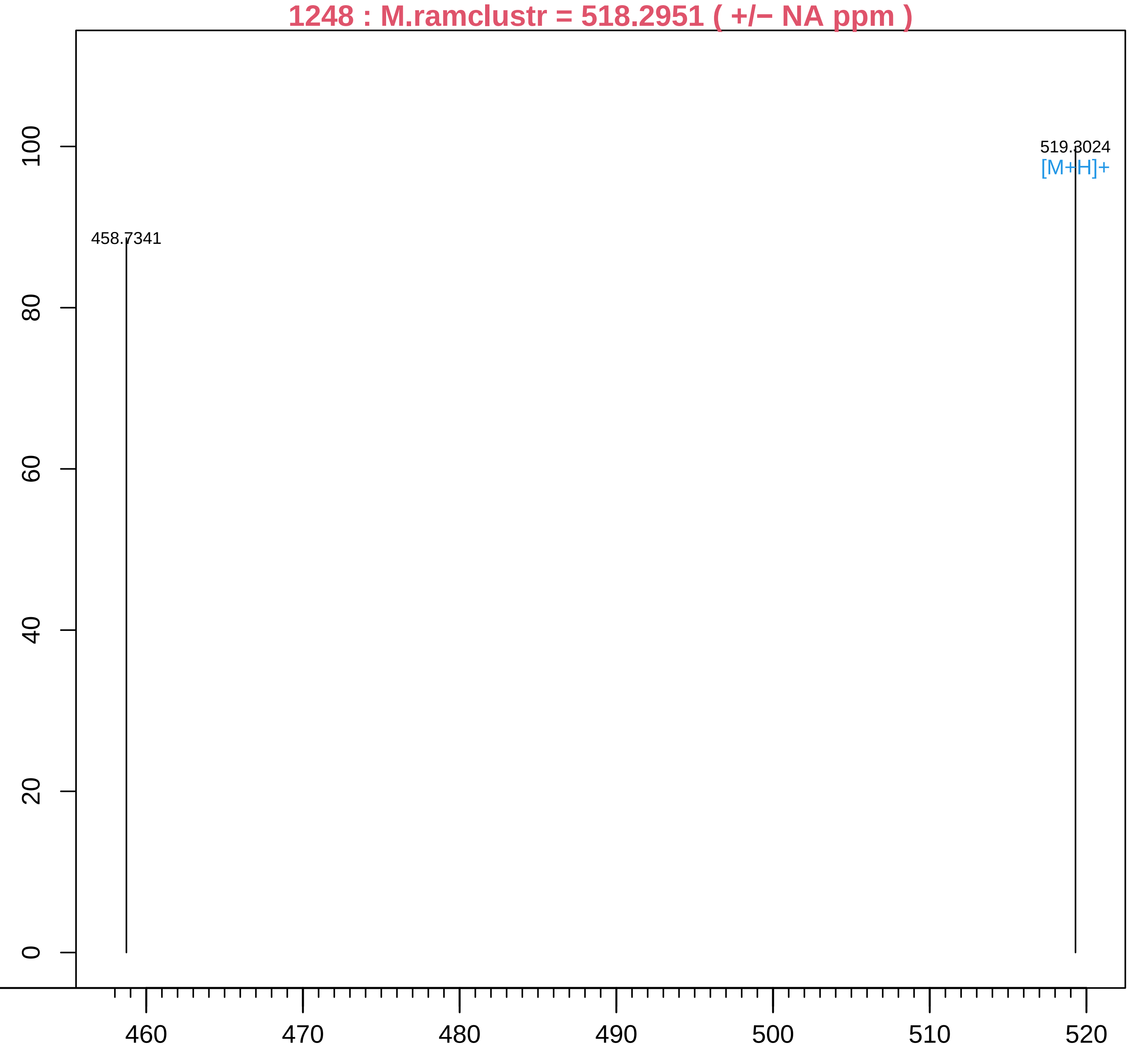

1248 : M.ramclustr = 518.2951 (+/− NA ppm)
519.3024
[M+H]+
458.7341
0
20
40
60
80
100
460
470
480
490
500
510
520

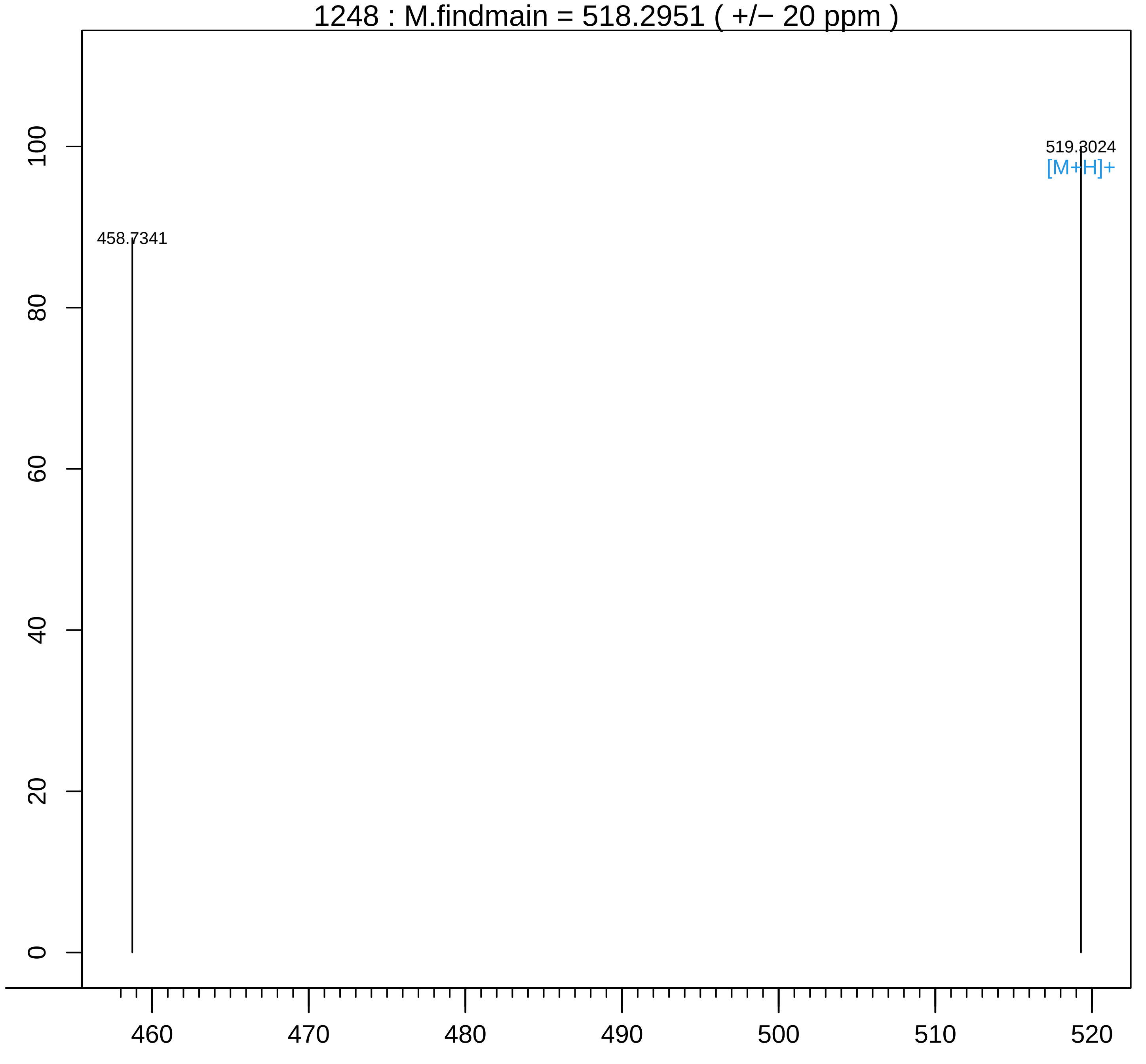

1248 : M.findmain = 518.2951 (+/− 20 ppm)
519.3024
[M+H]+
458.7341
0
20
40
60
80
100
460
470
480
490
500
510
520

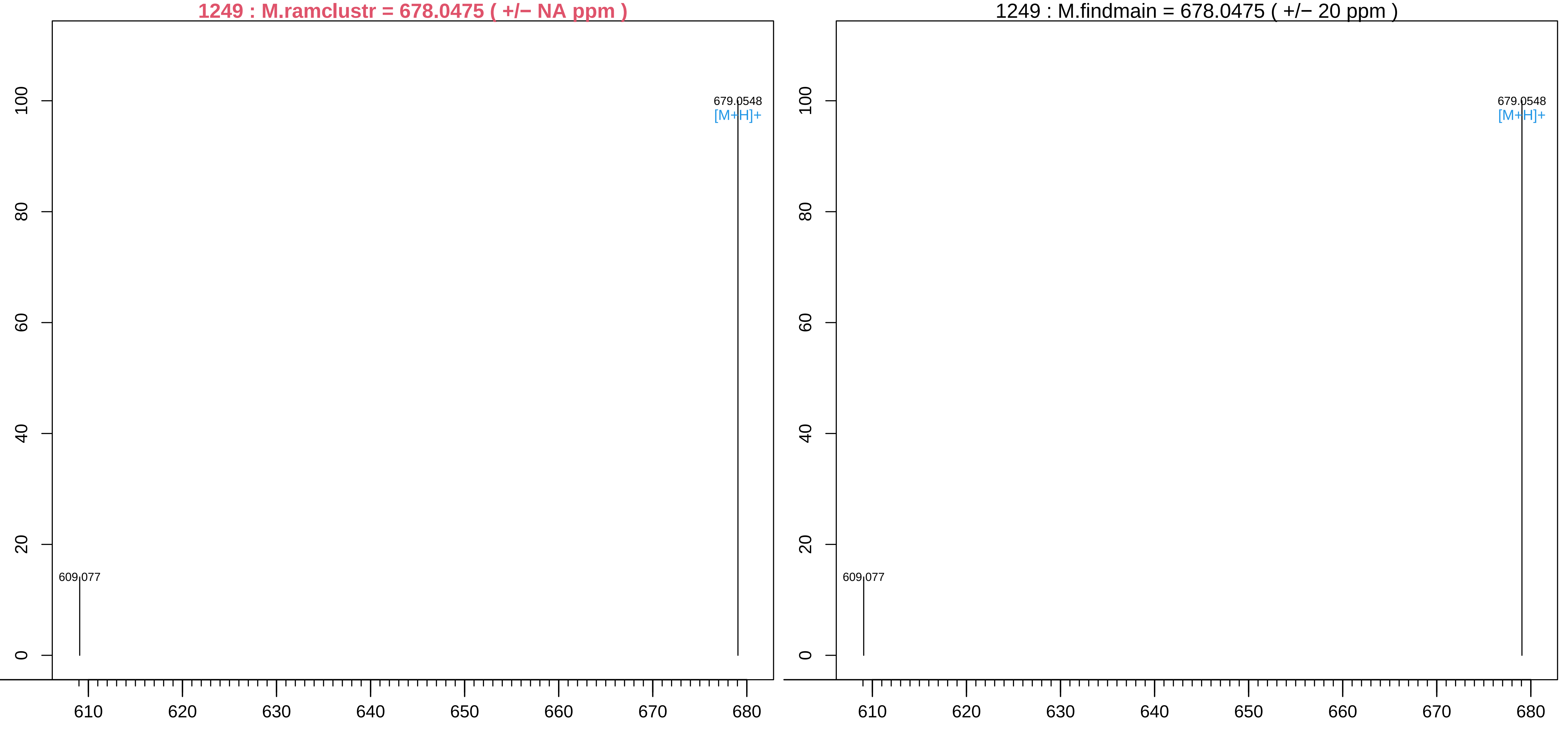

1249 : M.ramclustr = 678.0475 (+/− NA ppm)
679.0548
[M+H]+
609.077
1249 : M.findmain = 678.0475 (+/− 20 ppm)
679.0548
[M+H]+
609.077
0
20
40
60
80
100
610
620
630
640
650
660
670
680

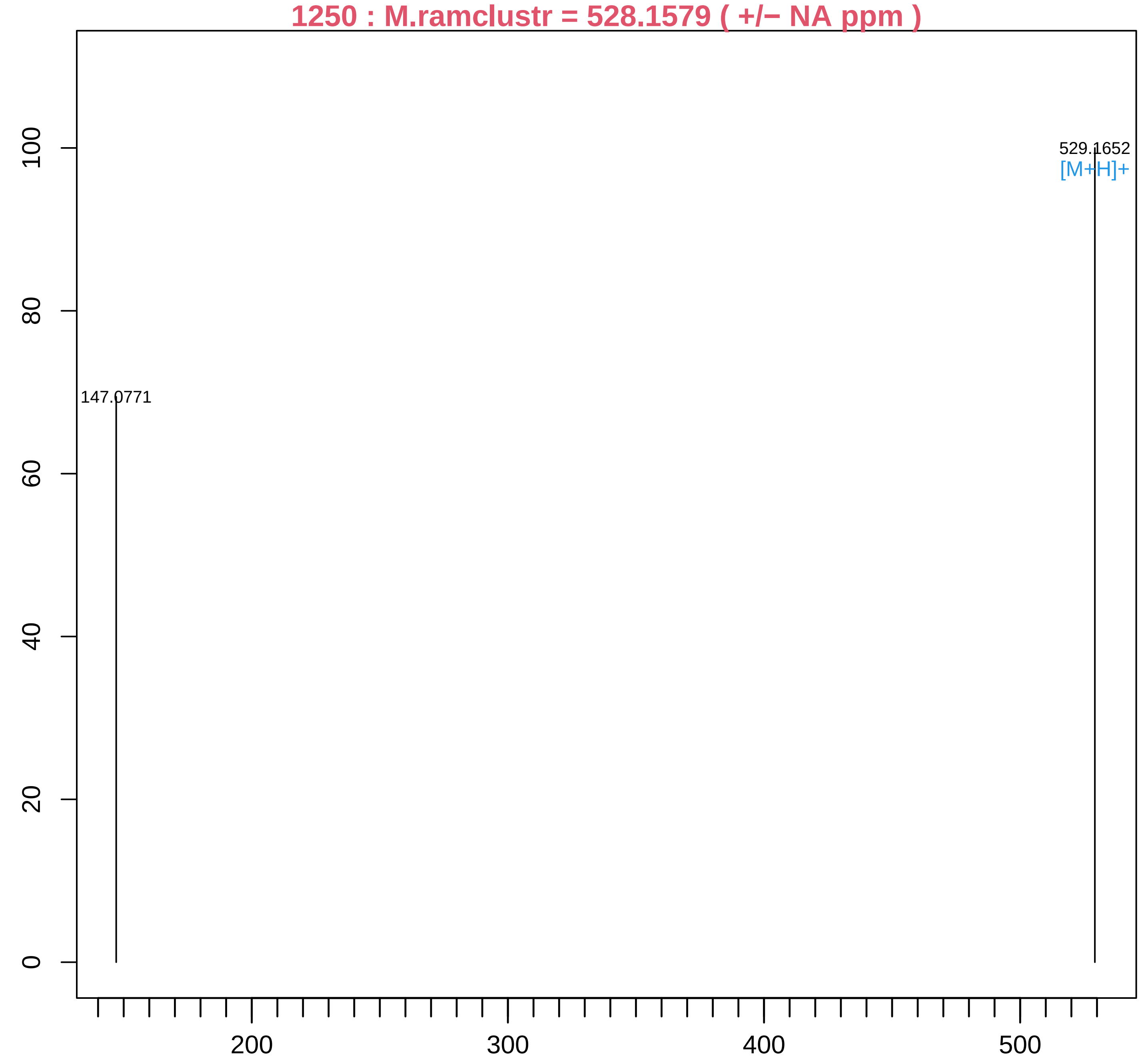
1250 : M.ramclustr = 528.1579 (+/− NA ppm)
529.1652
[M+H]+
147.0771
0
20
40
60
80
100
200
300
400
500

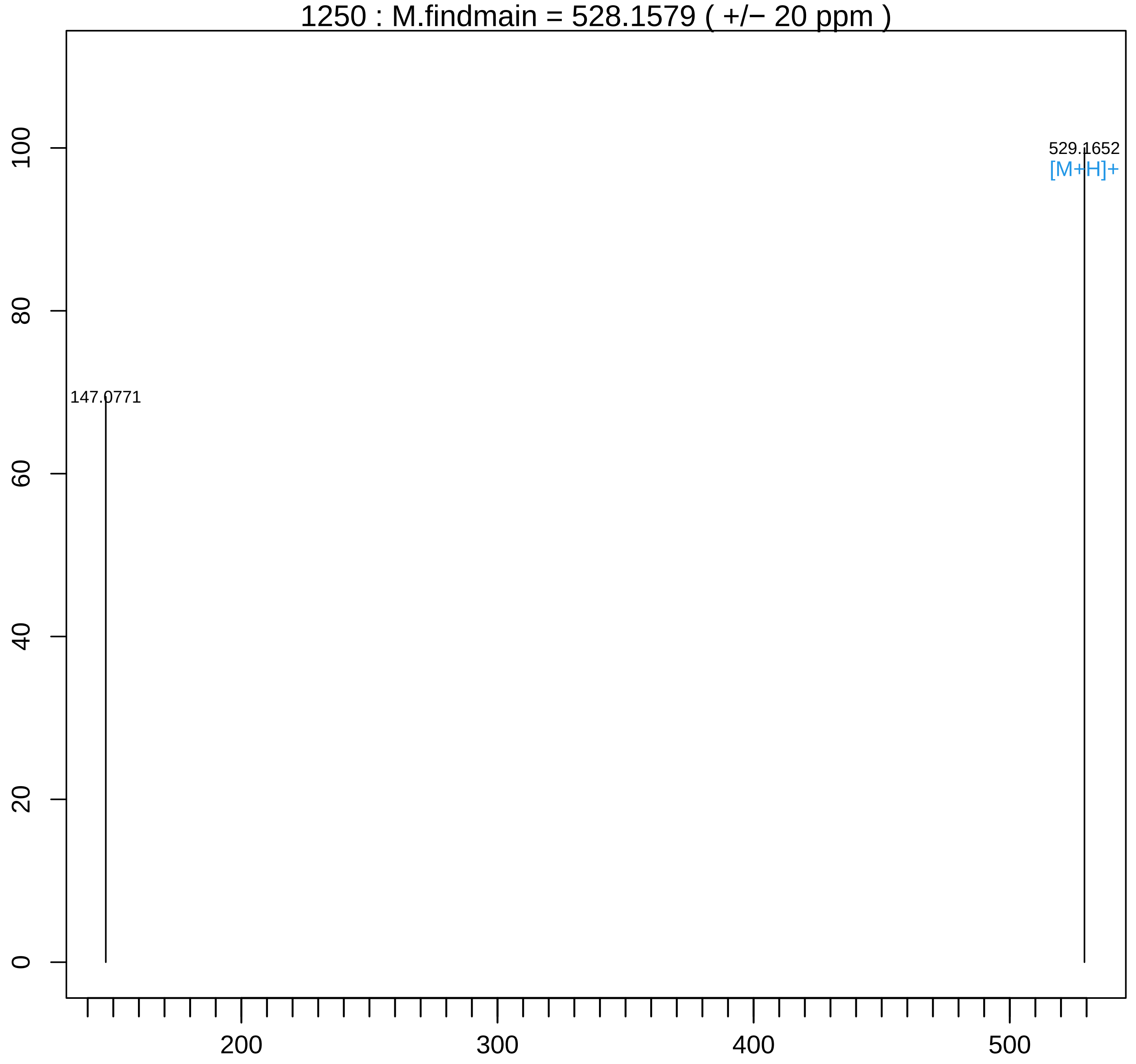
1250 : M.findmain = 528.1579 (+/− 20 ppm)
529.1652
[M+H]+
147.0771
0
20
40
60
80
100
200
300
400
500

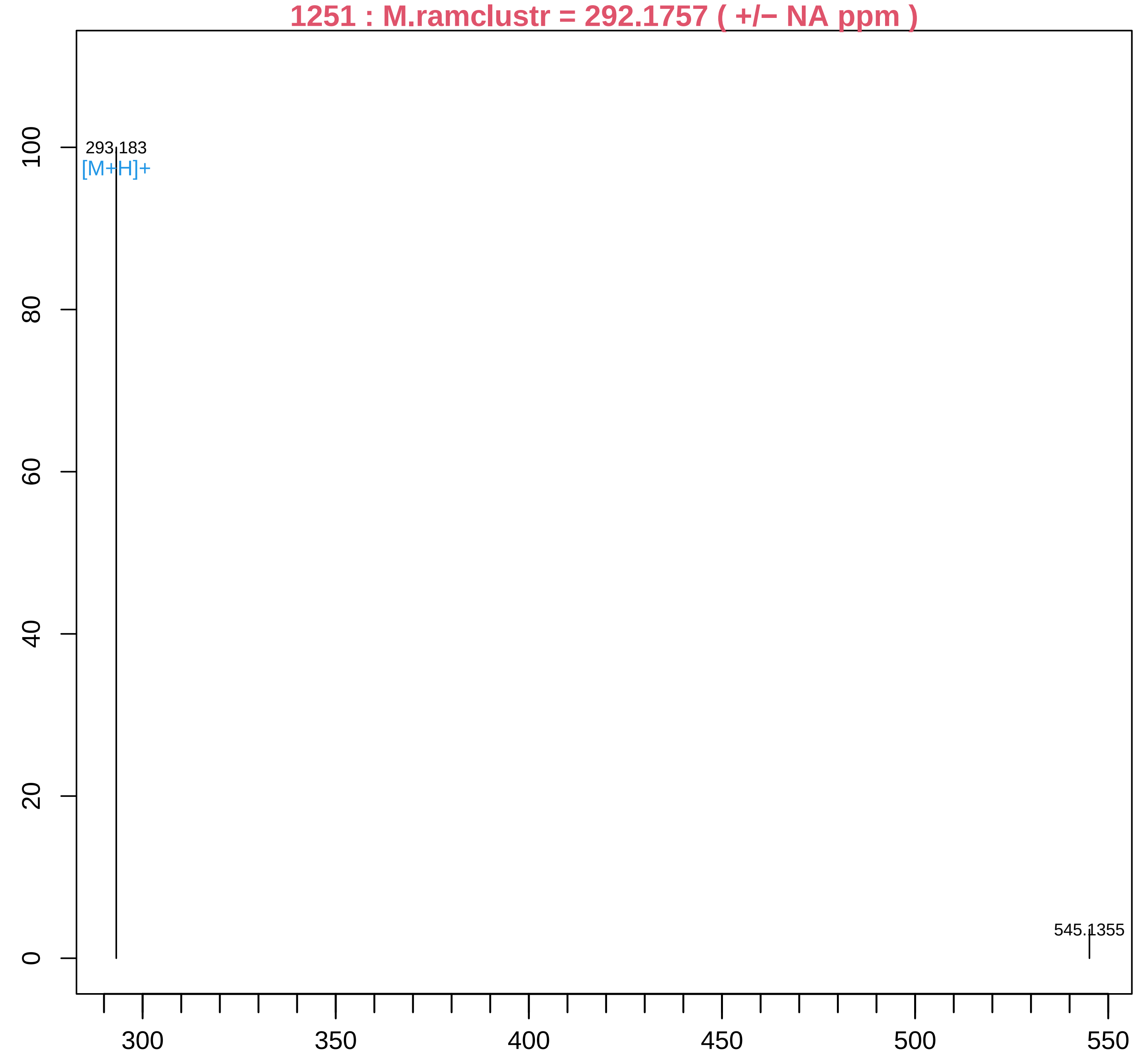
1251 : M.ramclustr = 292.1757 (+/− NA ppm)
293.183
[M+H]+
545.1355
0
20
40
60
80
100
300
350
400
450
500
550

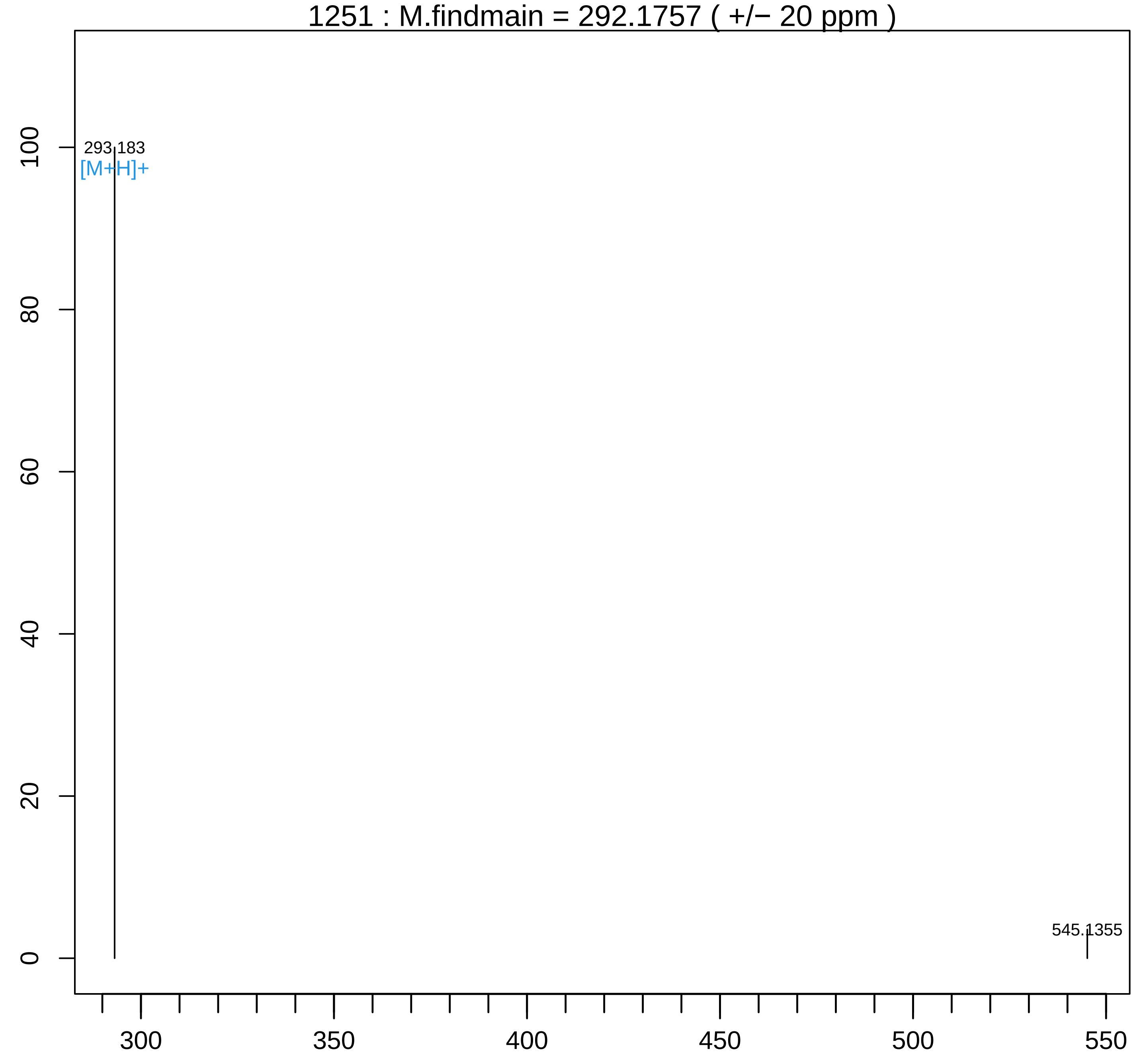
1251 : M.findmain = 292.1757 (+/− 20 ppm)
293.183
[M+H]+
545.1355
0
20
40
60
80
100
300
350
400
450
500
550

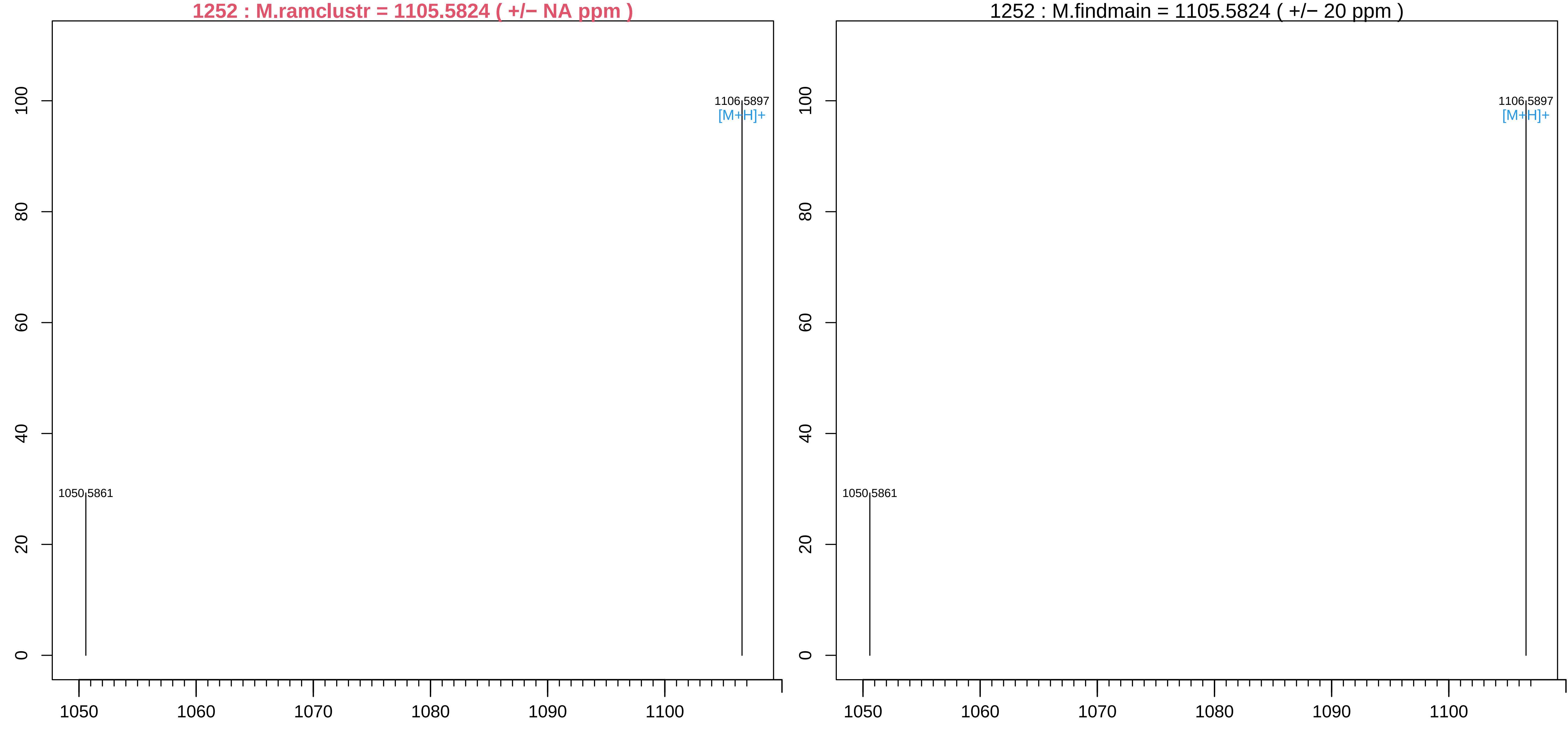
1252 : M.ramclustr = 1105.5824 (+/− NA ppm)
1252 : M.findmain = 1105.5824 (+/− 20 ppm)
1106.5897
[M+H]+
1050.5861
0
20
40
60
80
100
1050
1060
1070
1080
1090
1100

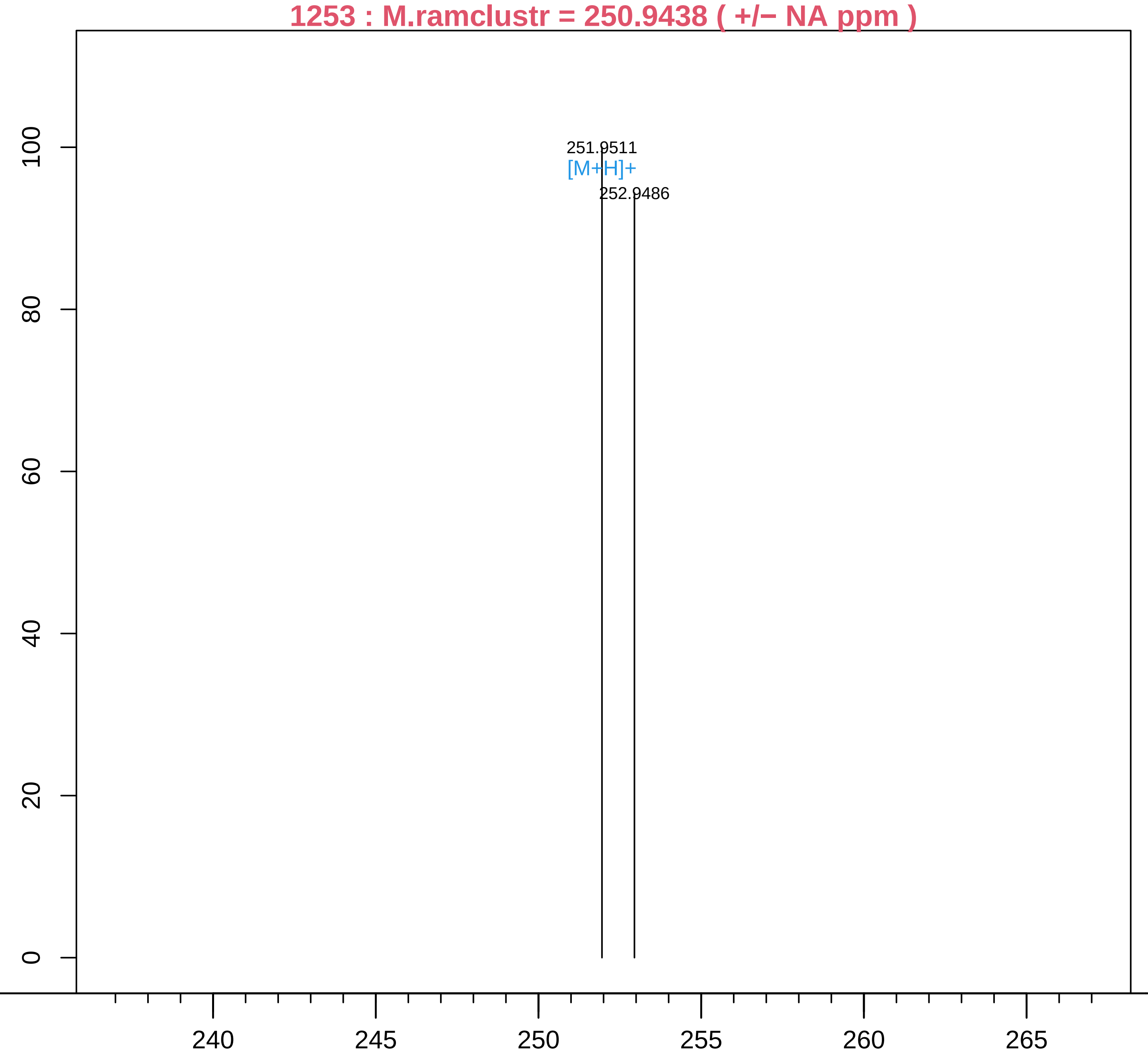

1253 : M.ramclustr = 250.9438 (+/− NA ppm)
251.9511
[M+H]+
252.9486
0
20
40
60
80
100
240
245
250
255
260
265

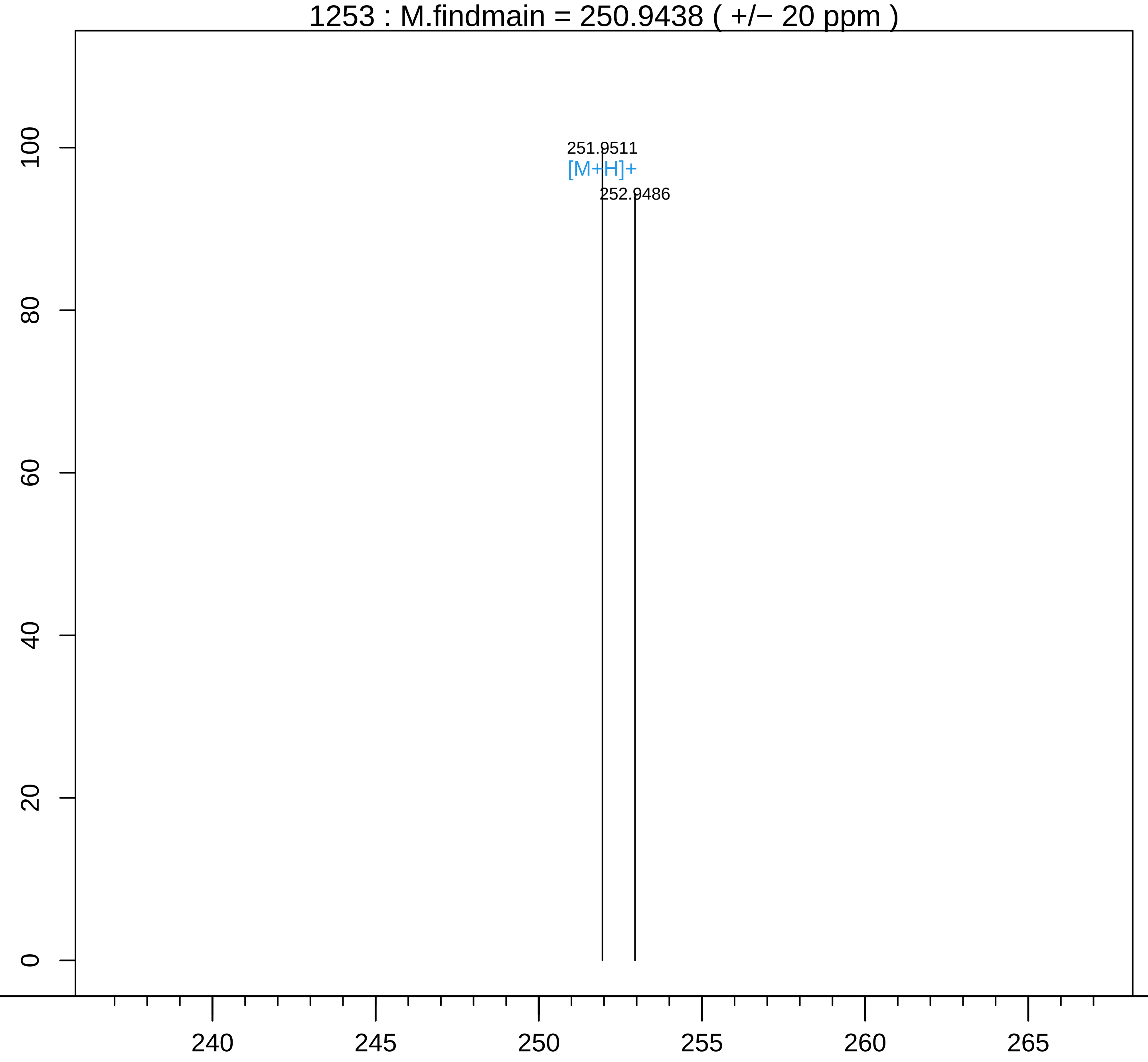

1253 : M.findmain = 250.9438 (+/− 20 ppm)
251.9511
[M+H]+
252.9486
0
20
40
60
80
100
240
245
250
255
260
265

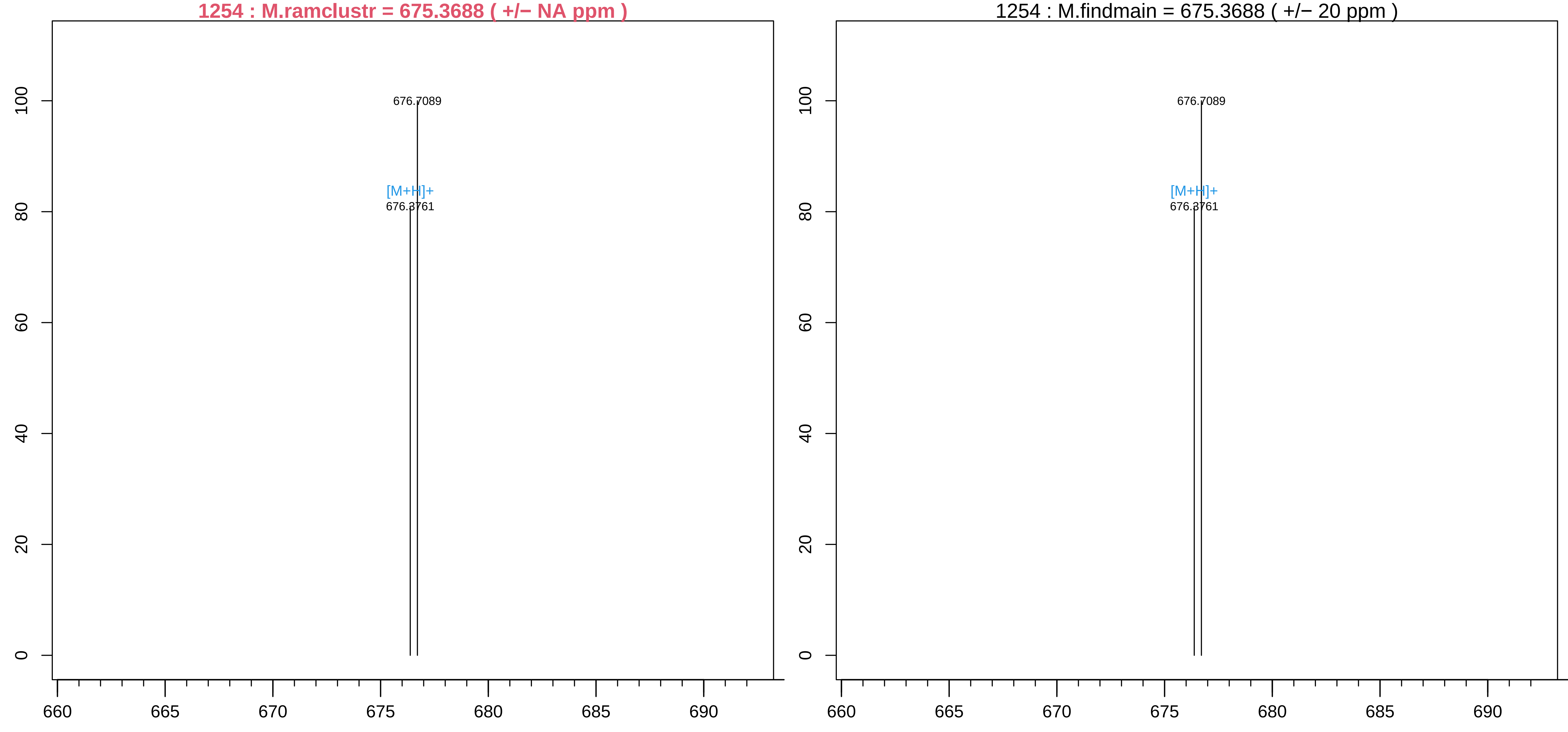

1254 : M.ramclustr = 675.3688 (+/− NA ppm)
676.7089
[M+H]+
676.3761
1254 : M.findmain = 675.3688 (+/− 20 ppm)
676.7089
[M+H]+
676.3761

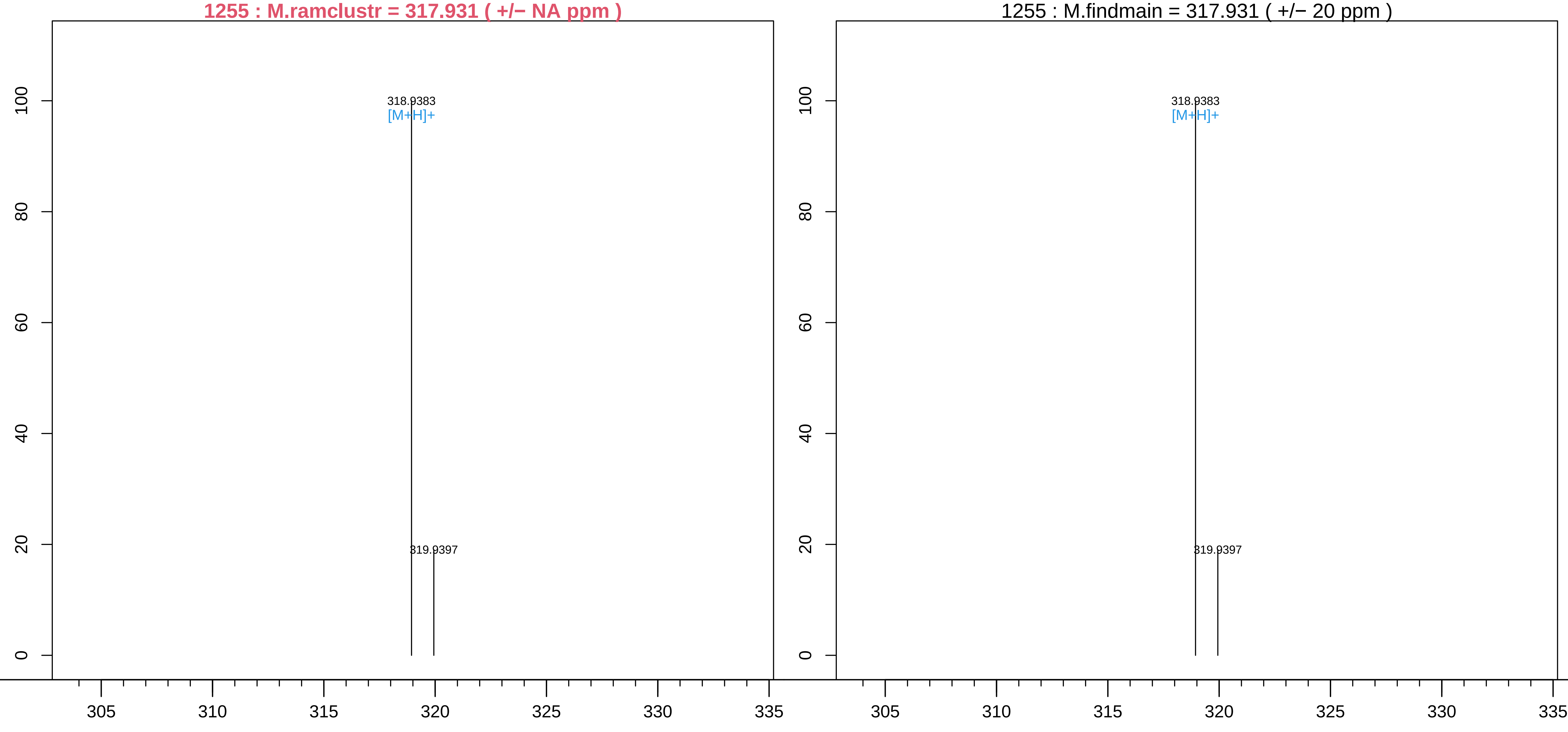

1255 : M.ramclustr = 317.931 (+/− NA ppm)
318.9383
[M+H]+
319.9397
1255 : M.findmain = 317.931 (+/− 20 ppm)
318.9383
[M+H]+
319.9397
0
20
40
60
80
100
305
310
315
320
325
330
335

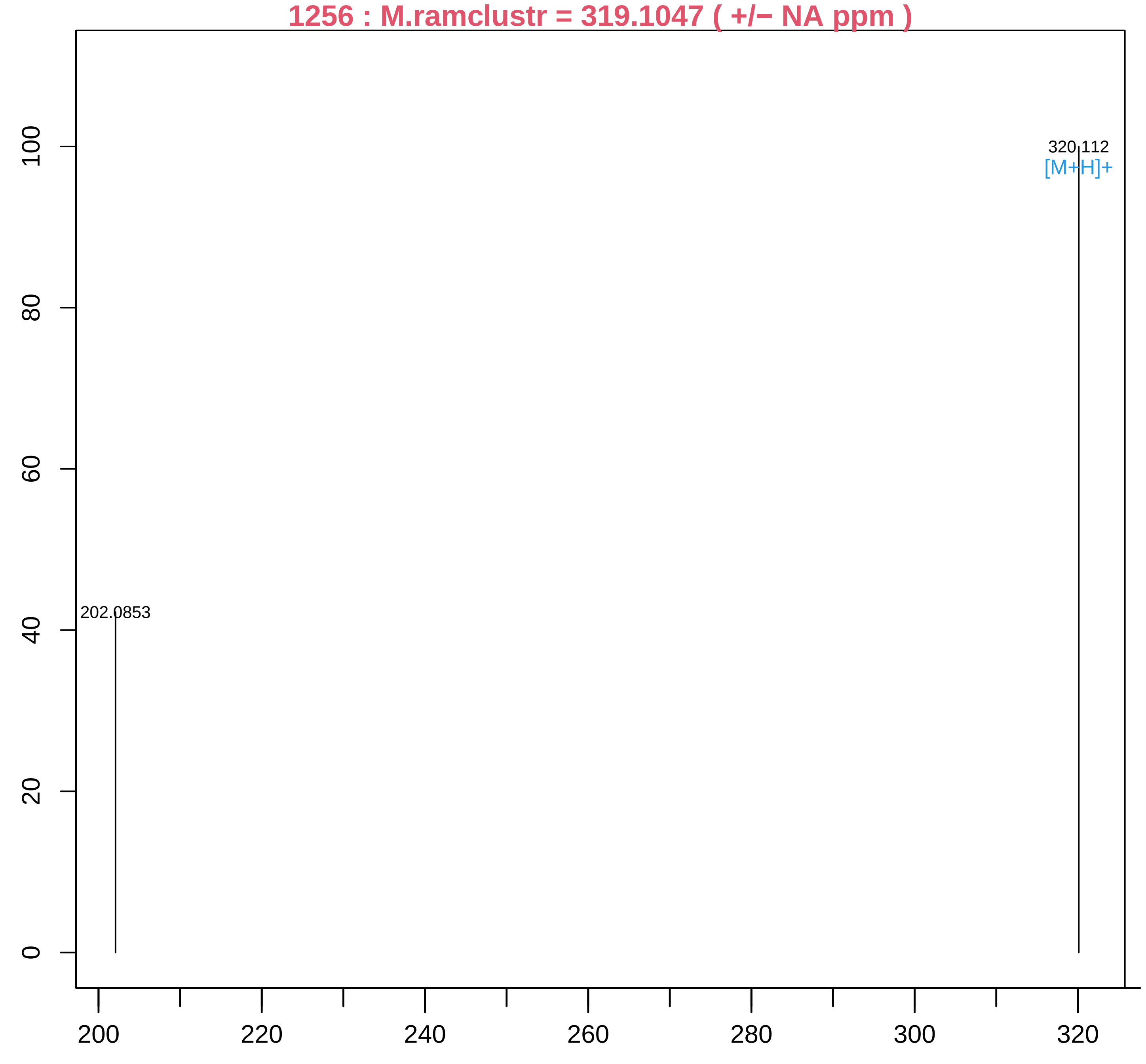
1256 : M.ramclustr = 319.1047 (+/− NA ppm)
320.112
[M+H]+
202.0853
0
20
40
60
80
100
200
220
240
260
280
300
320

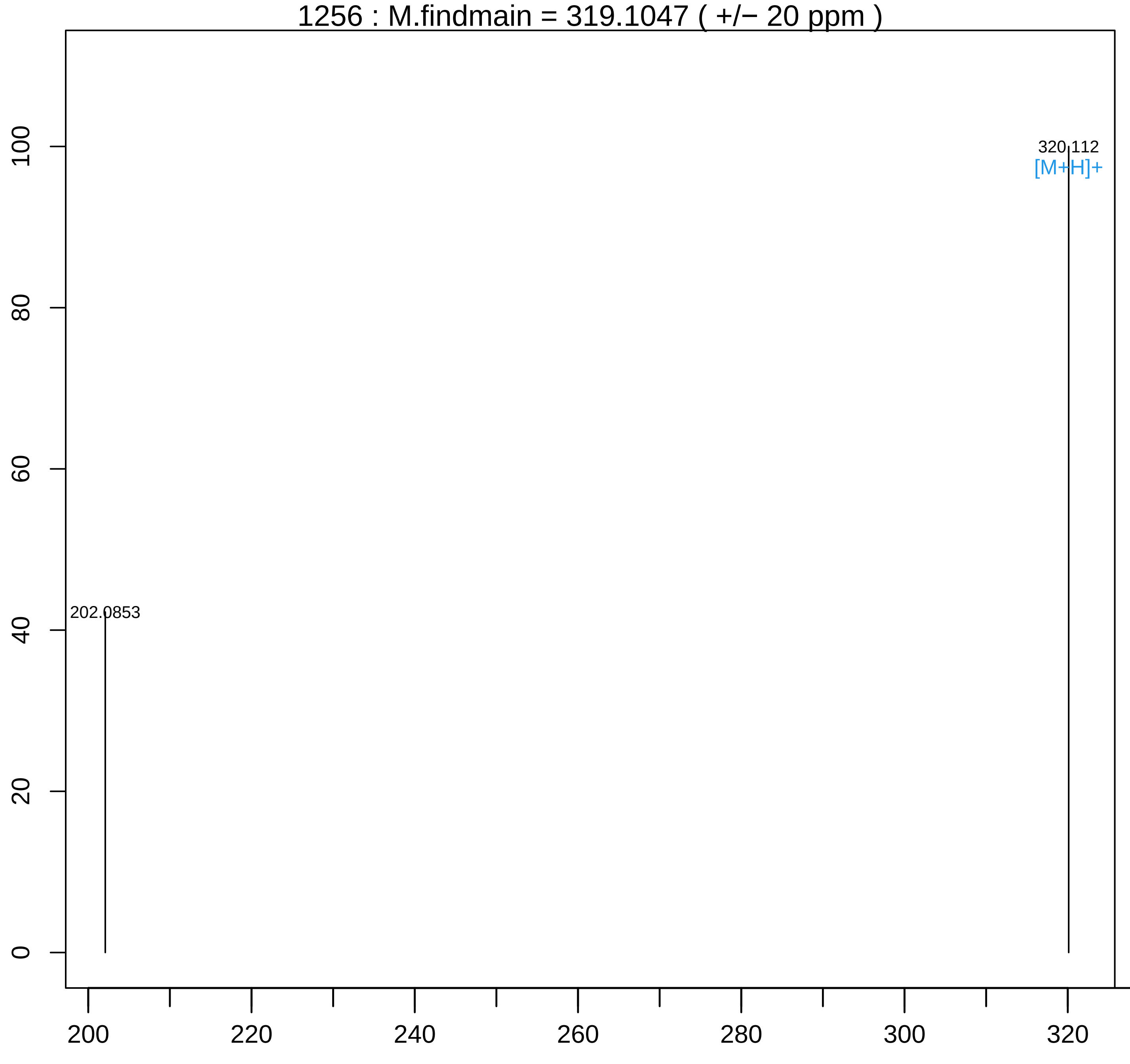
1256 : M.findmain = 319.1047 (+/− 20 ppm)
320.112
[M+H]+
202.0853
0
20
40
60
80
100
200
220
240
260
280
300
320

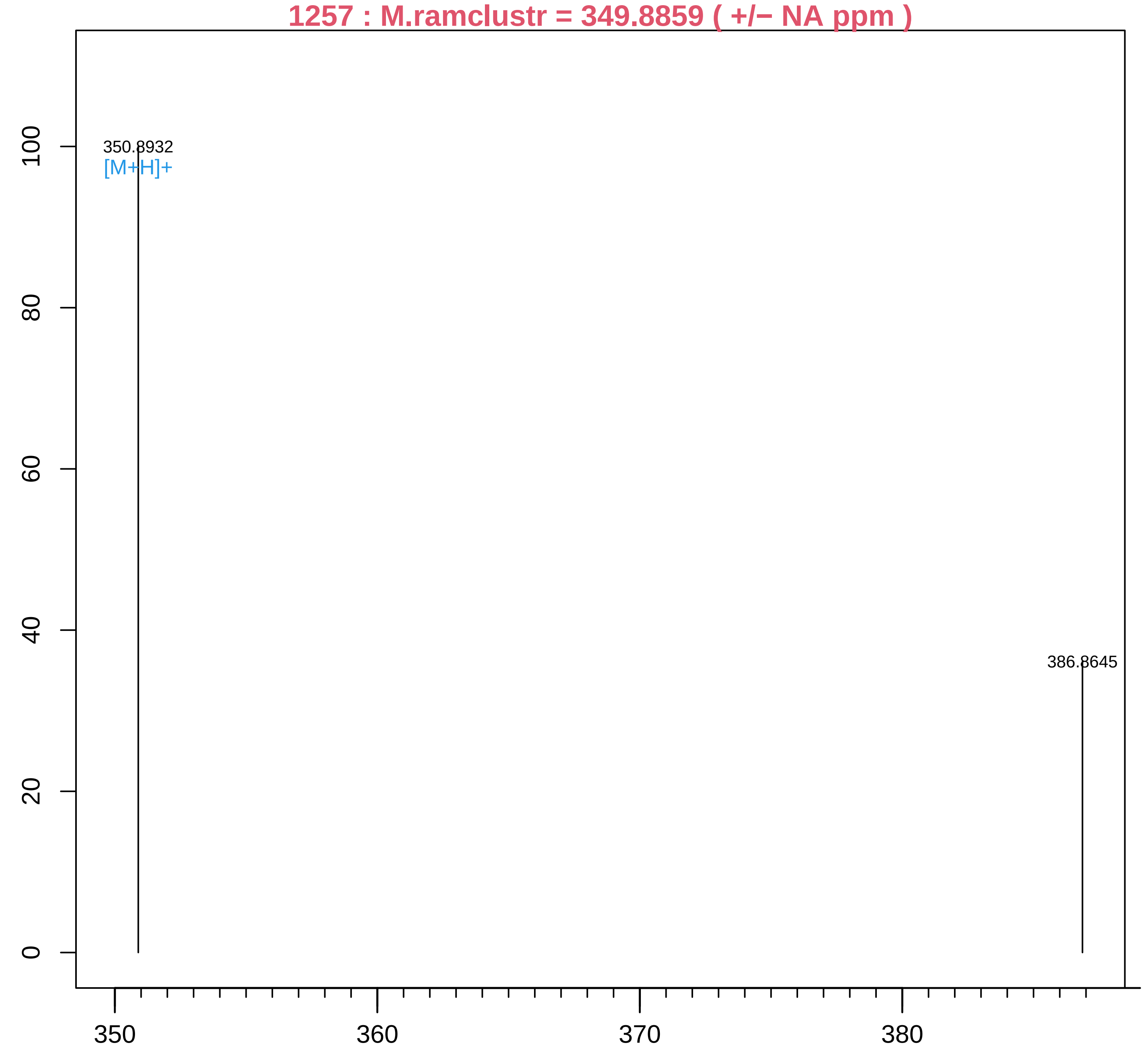

1257 : M.ramclustr = 349.8859 (+/− NA ppm)
350.8932
[M+H]+
386.8645
0
20
40
60
80
100
350
360
370
380

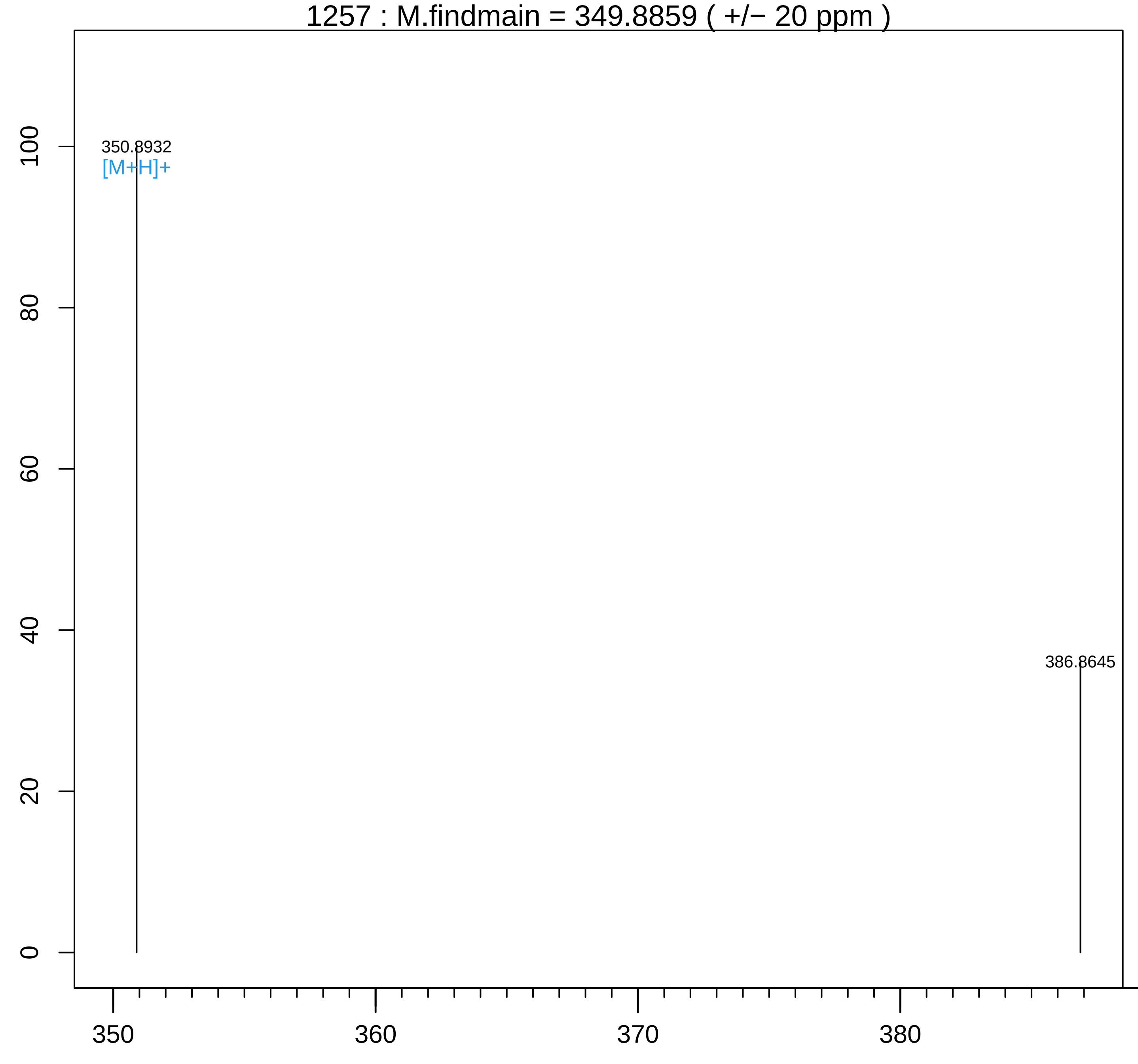

1257 : M.findmain = 349.8859 (+/− 20 ppm)
350.8932
[M+H]+
386.8645
0
20
40
60
80
100
350
360
370
380

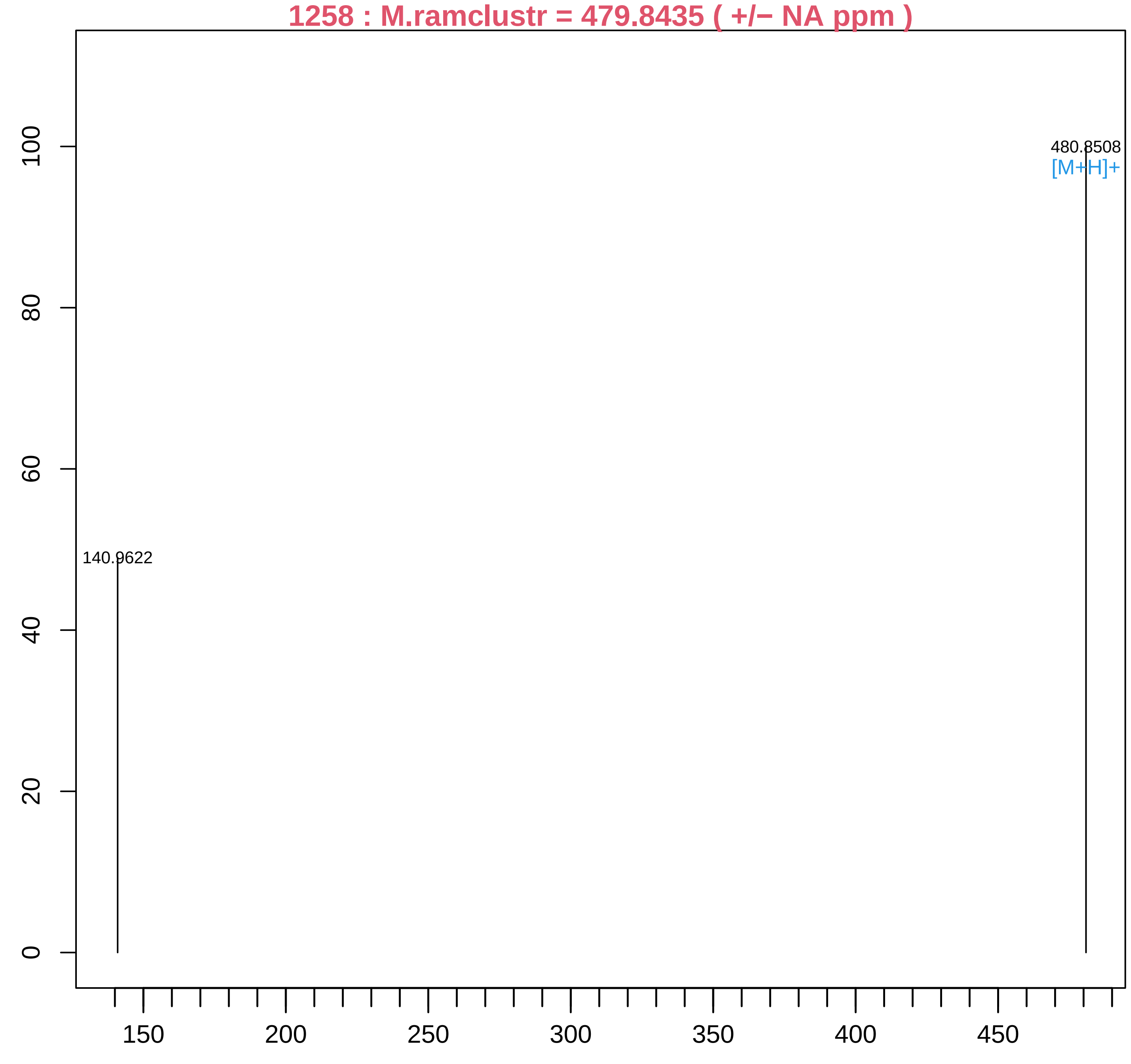
1258 : M.ramclustr = 479.8435 (+/− NA ppm)
480.8508
[M+H]+
140.9622
0
20
40
60
80
100
150
200
250
300
350
400
450

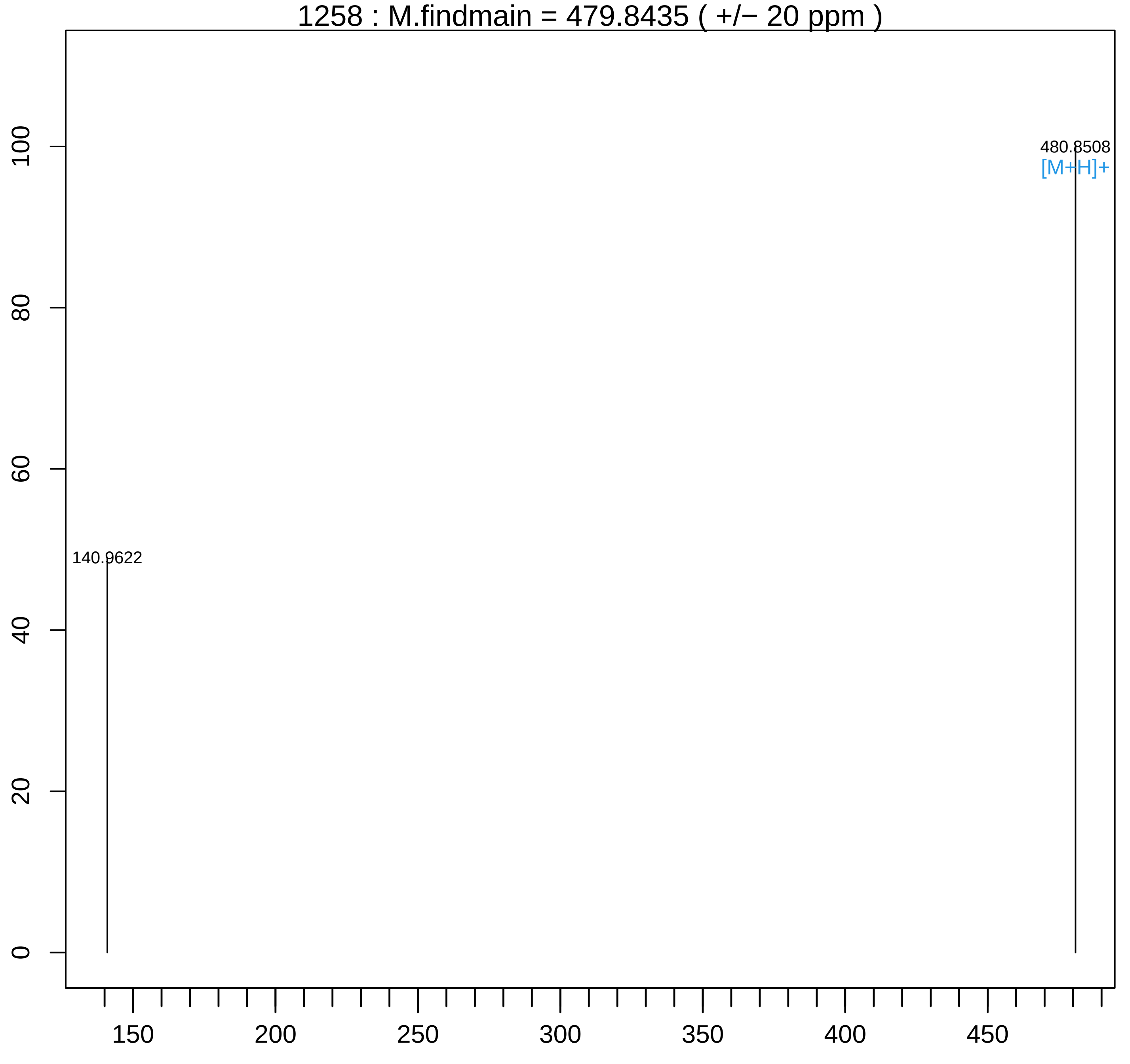
1258 : M.findmain = 479.8435 (+/− 20 ppm)
480.8508
[M+H]+
140.9622
0
20
40
60
80
100
150
200
250
300
350
400
450

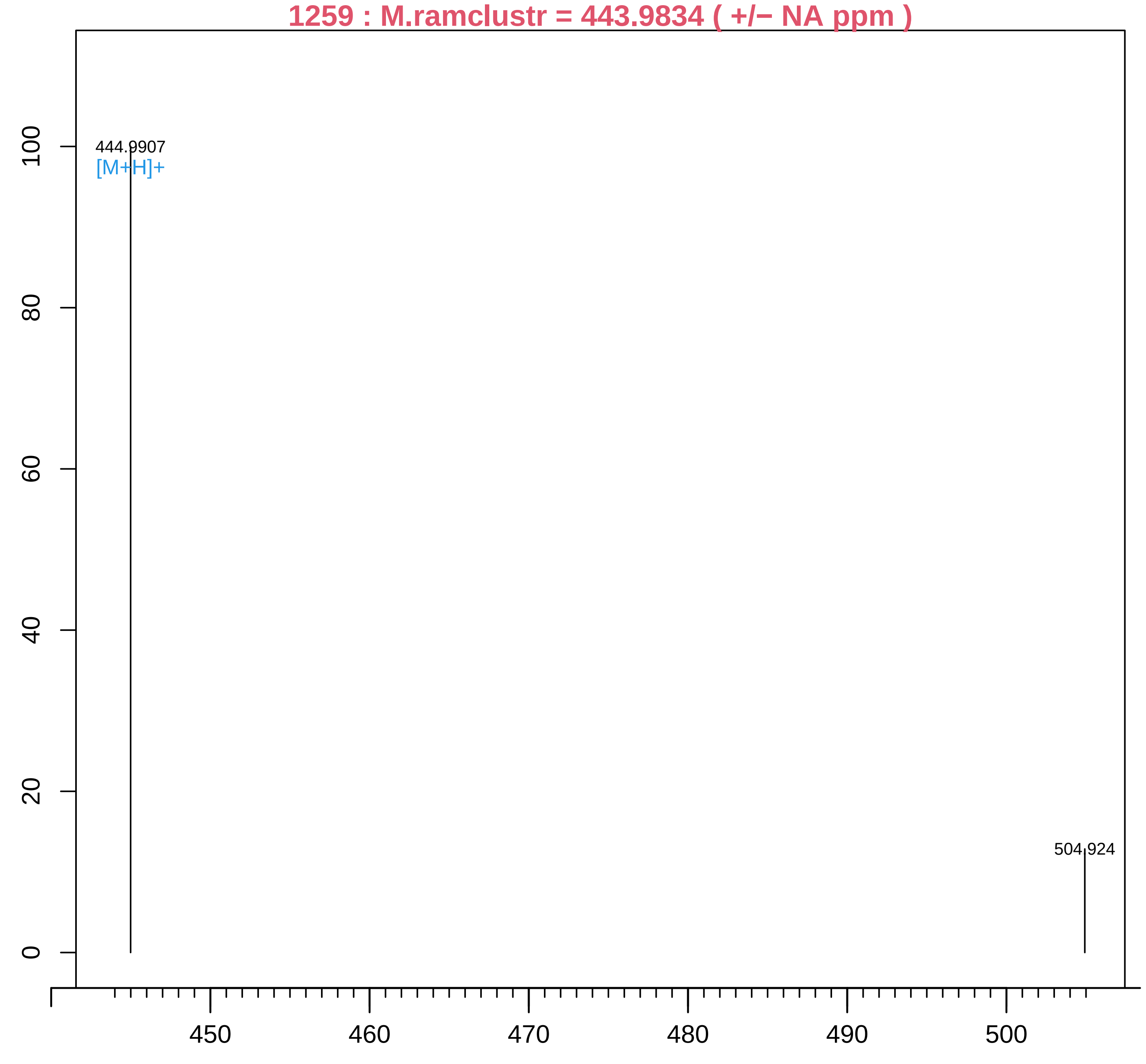

1259 : M.ramclustr = 443.9834 (+/− NA ppm)
444.9907
[M+H]+
504.924
0
20
40
60
80
100
450
460
470
480
490
500

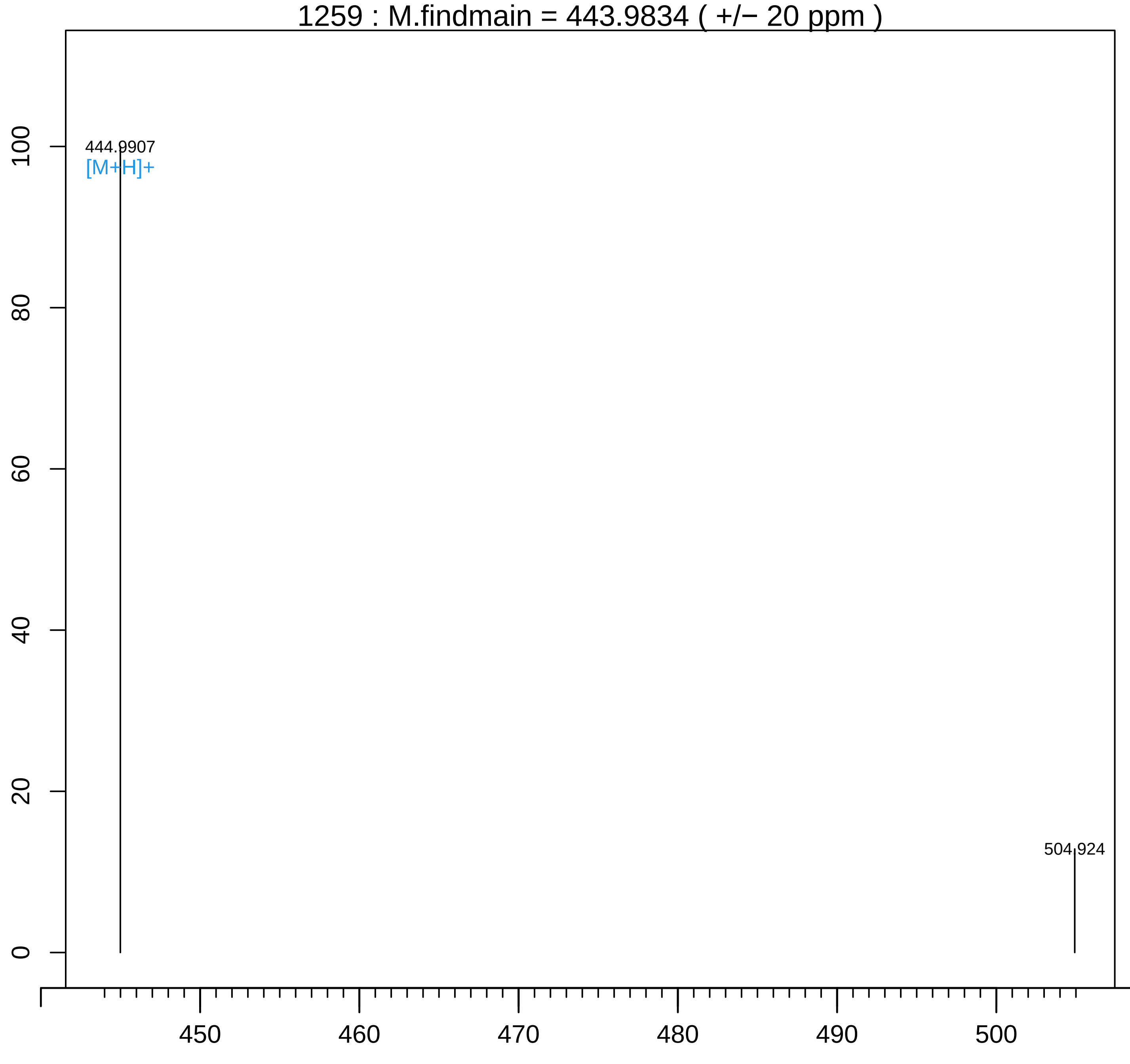

1259 : M.findmain = 443.9834 (+/− 20 ppm)
444.9907
[M+H]+
504.924
0
20
40
60
80
100
450
460
470
480
490
500

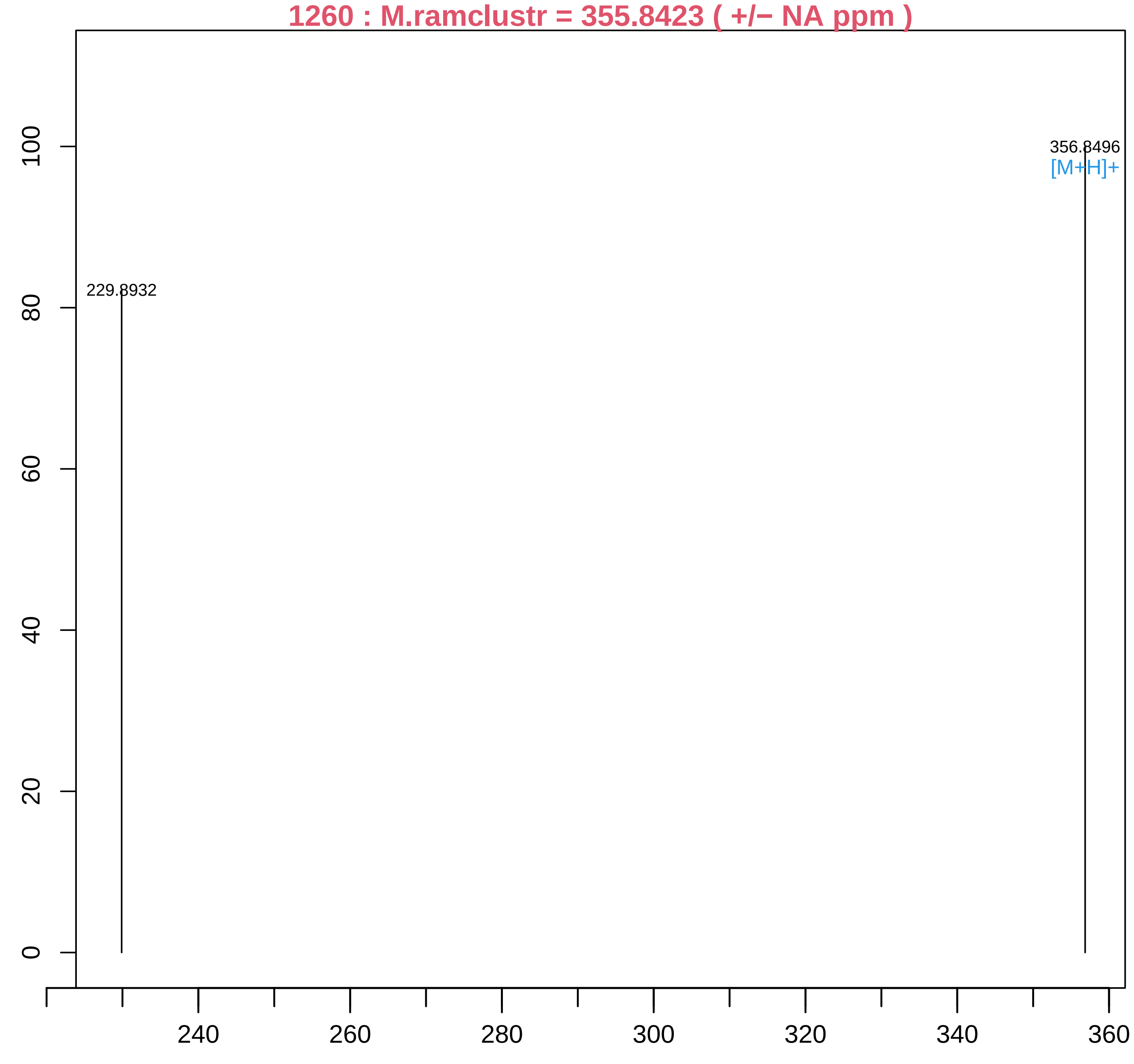
1260 : M.ramclustr = 355.8423 (+/− NA ppm)
356.8496
[M+H]+
229.8932
0
20
40
60
80
100
240
260
280
300
320
340
360

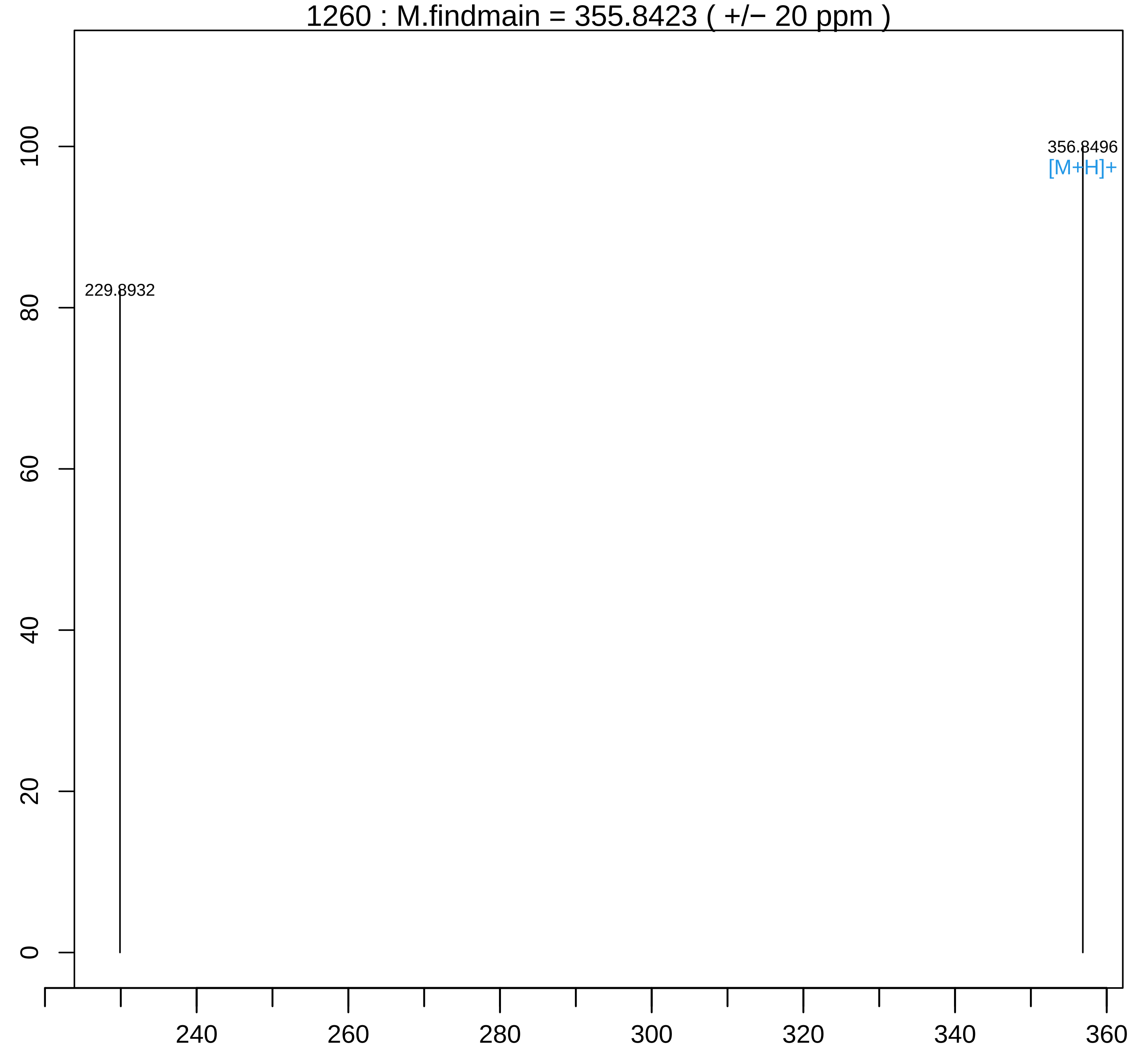
1260 : M.findmain = 355.8423 (+/− 20 ppm)
356.8496
[M+H]+
229.8932
0
20
40
60
80
100
240
260
280
300
320
340
360

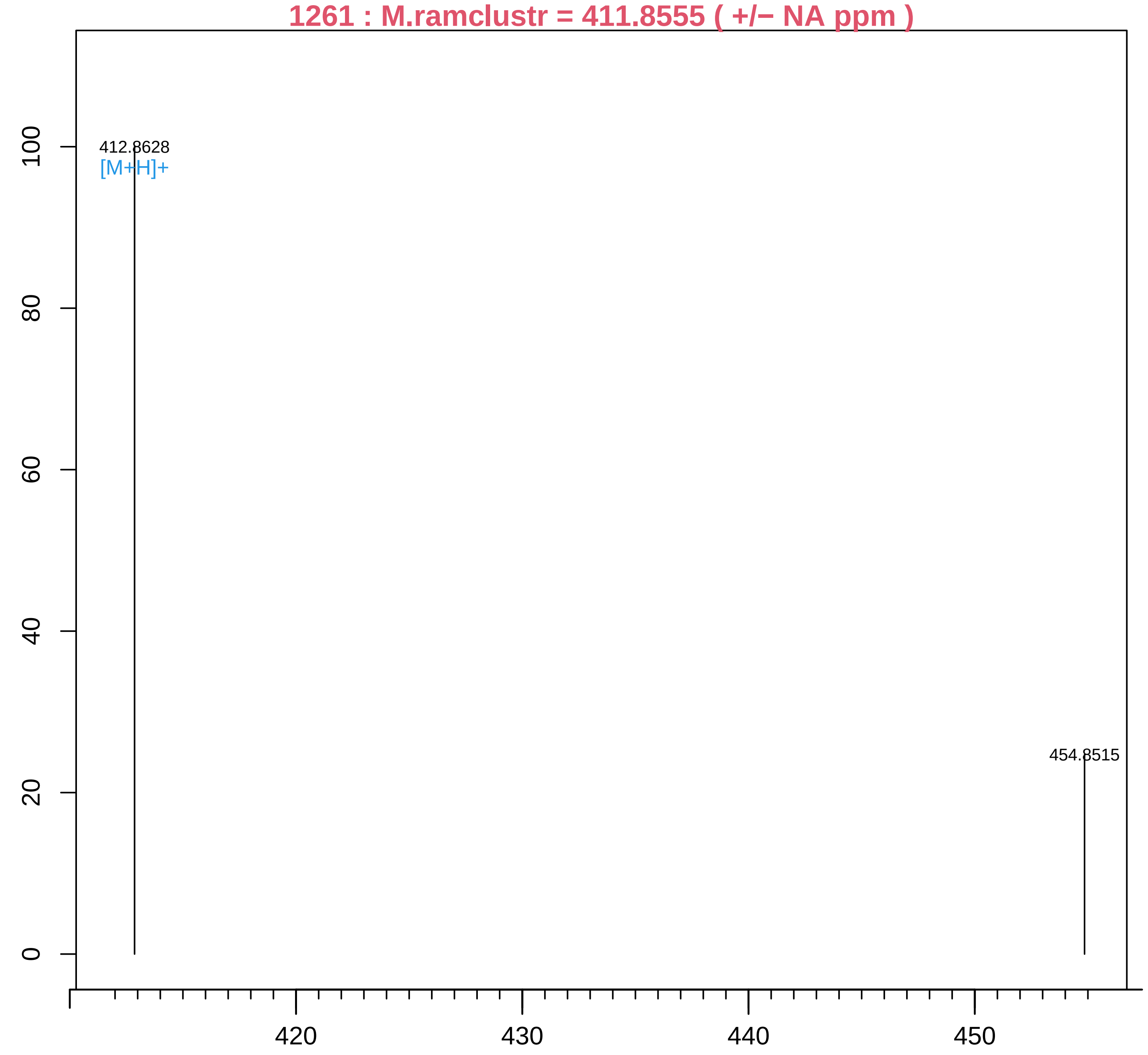

1261 : M.ramclustr = 411.8555 (+/− NA ppm)
412.8628
[M+H]+
454.8515
0
20
40
60
80
100
420
430
440
450

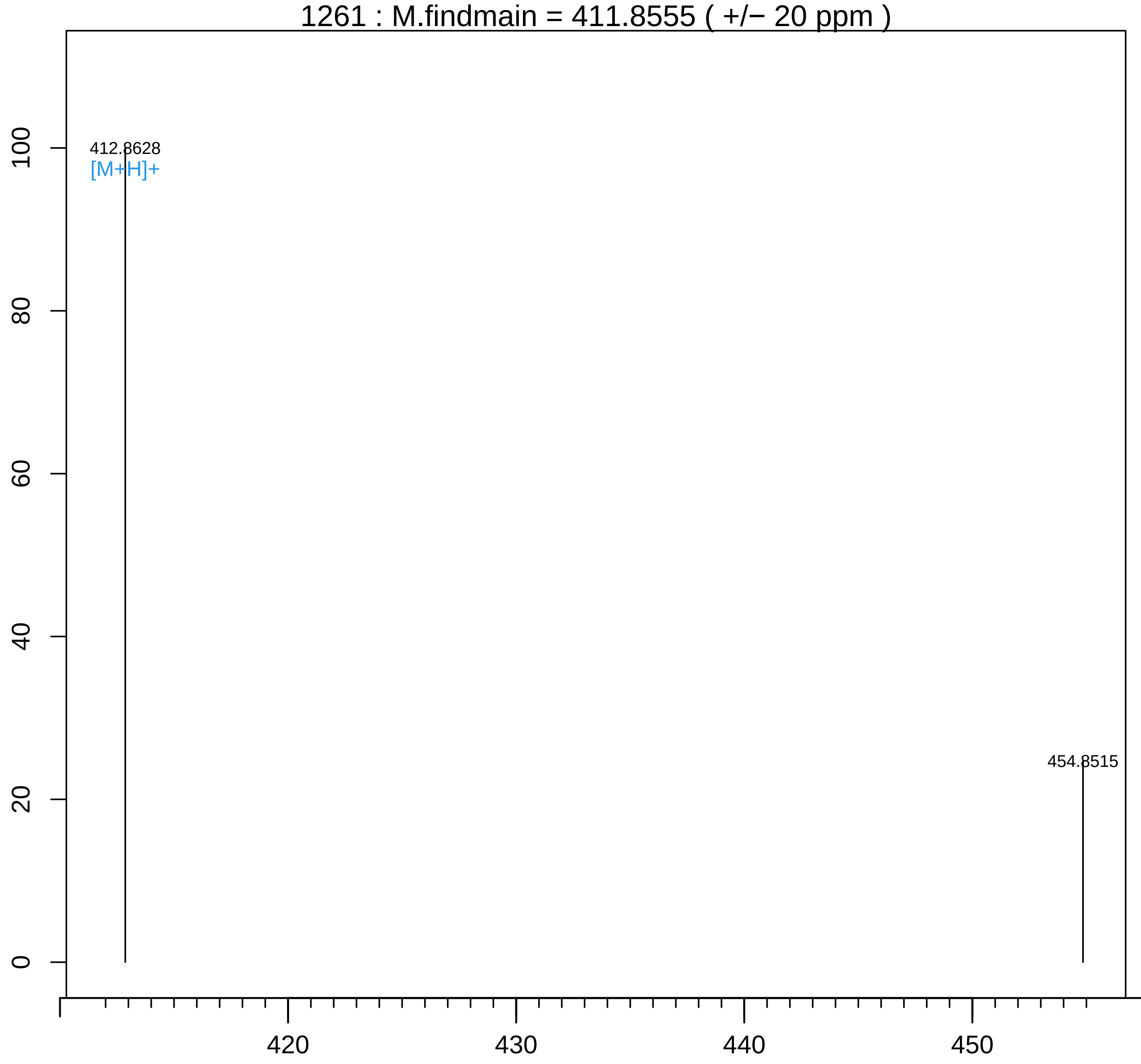

1261 : M.findmain = 411.8555 (+/− 20 ppm)
412.8628
[M+H]+
454.8515
0
20
40
60
80
100
420
430
440
450

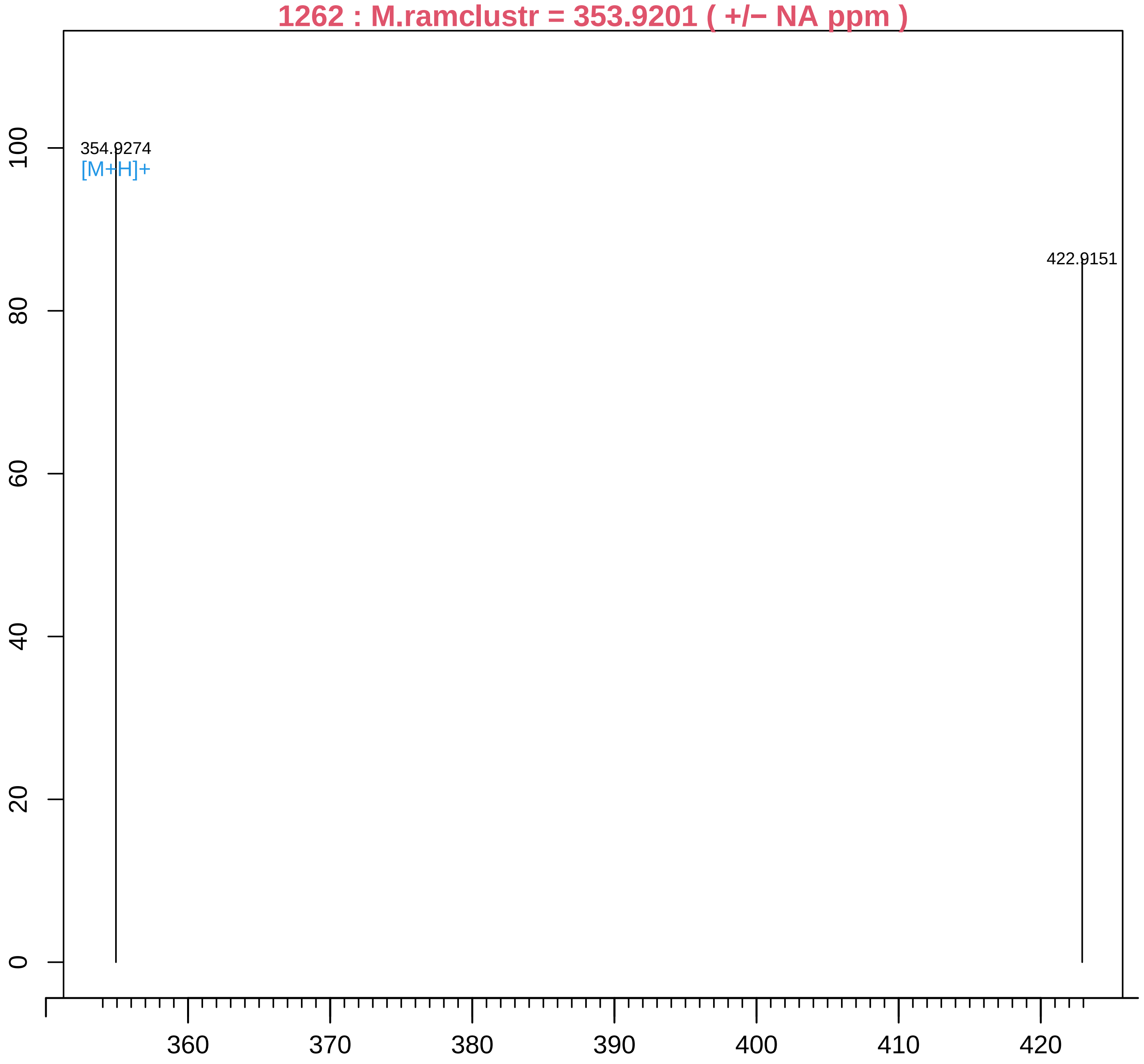

1262 : M.ramclustr = 353.9201 (+/− NA ppm)
354.9274
[M+H]+
422.9151
0
20
40
60
80
100
360
370
380
390
400
410
420

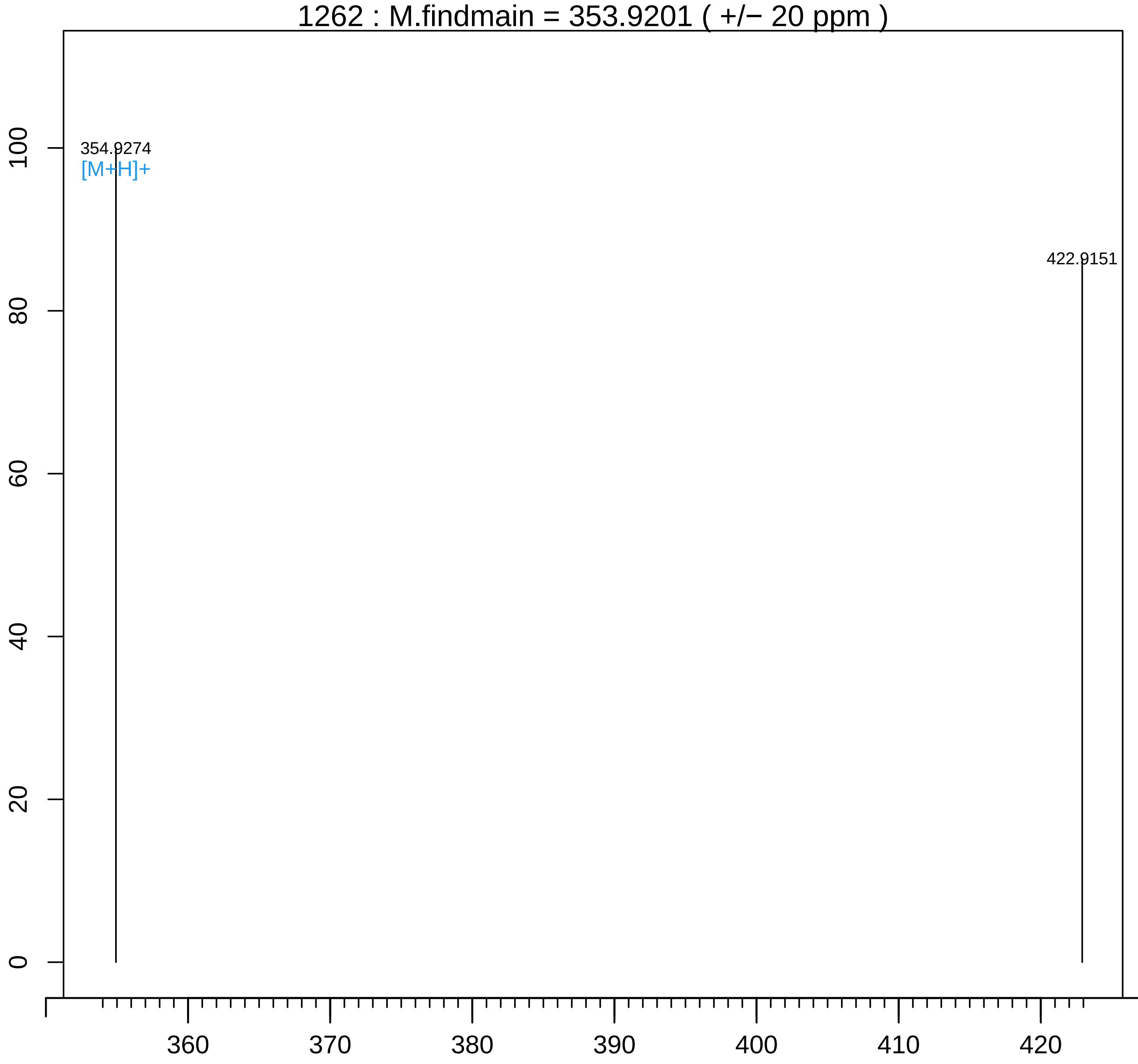

1262 : M.findmain = 353.9201 (+/− 20 ppm)
354.9274
[M+H]+
422.9151
0
20
40
60
80
100
360
370
380
390
400
410
420

1263 : M.ramclustr = 145.1586 (+/− NA ppm)

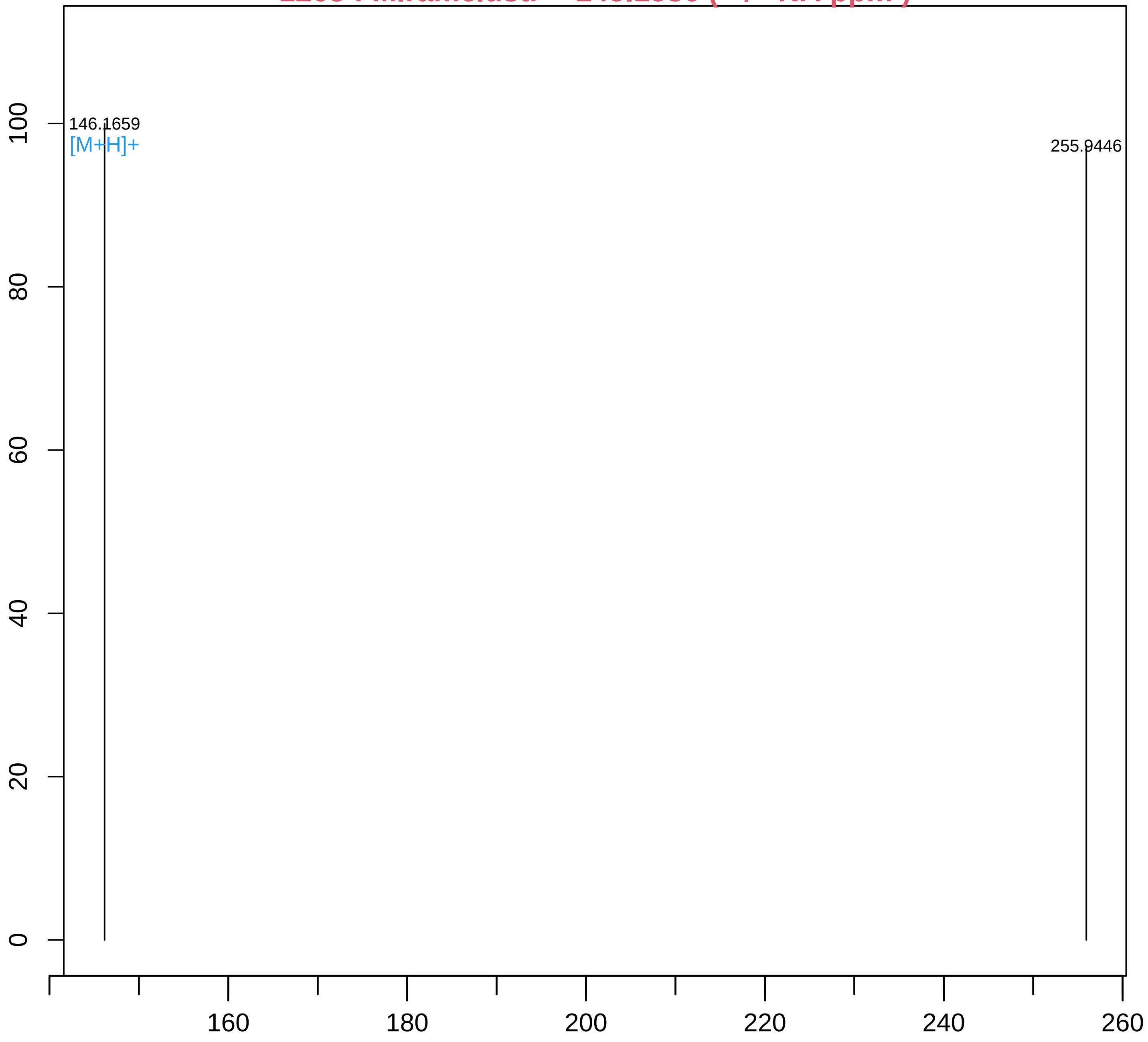

1263 : M.findmain = 145.1586 (+/− 20 ppm)

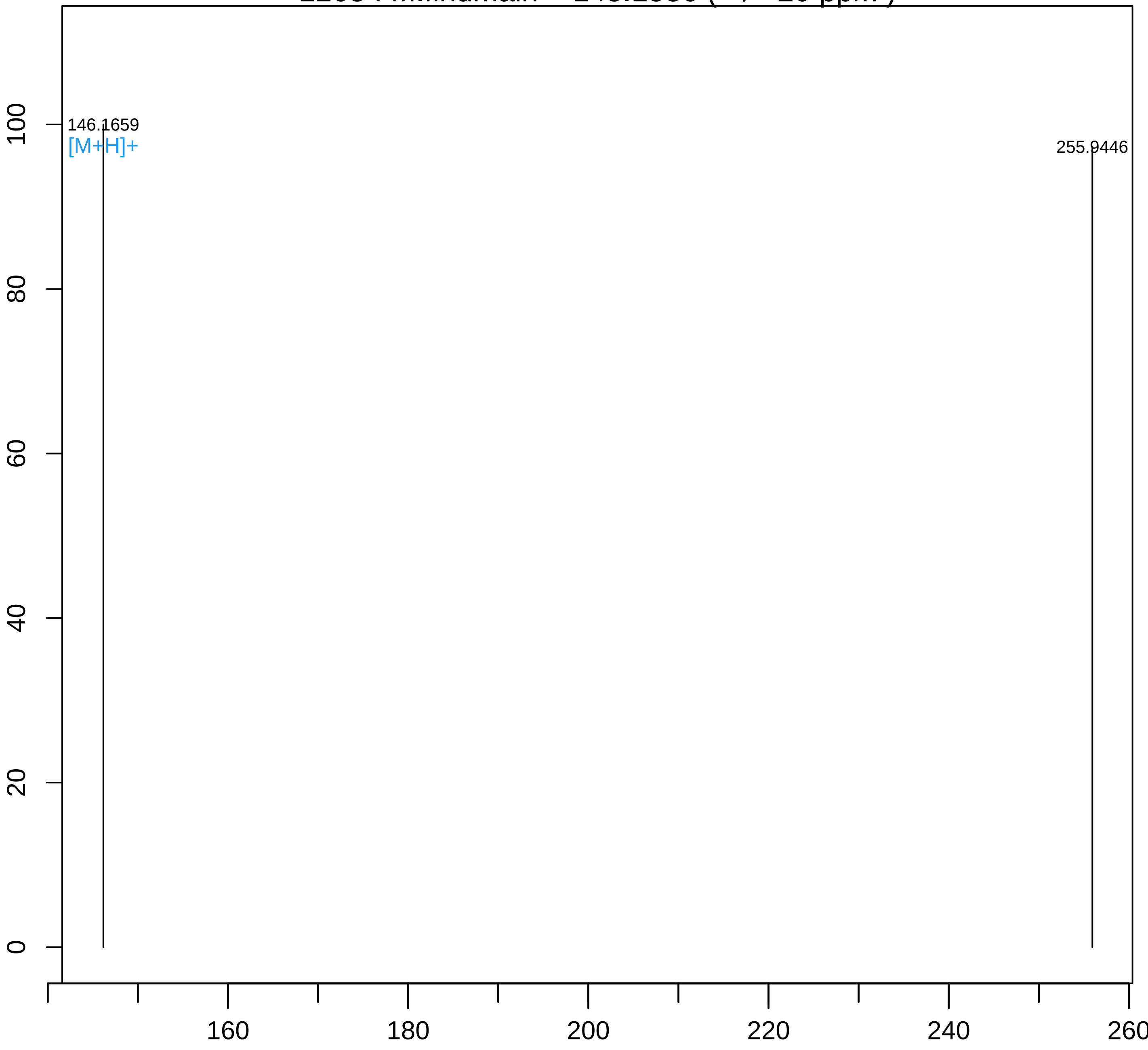

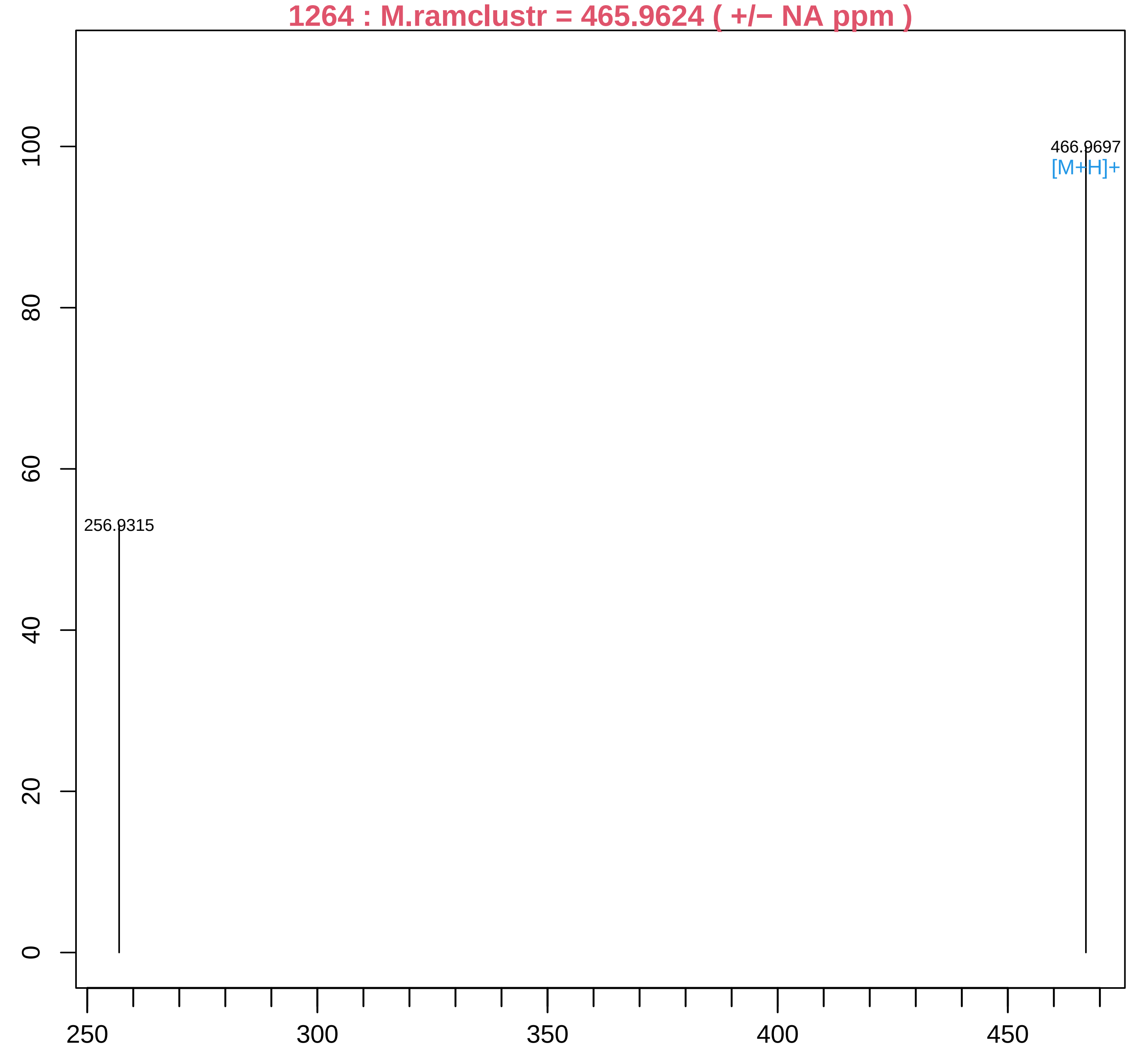
1264 : M.ramclustr = 465.9624 (+/− NA ppm)
466.9697
[M+H]+
256.9315
0
20
40
60
80
100
250
300
350
400
450

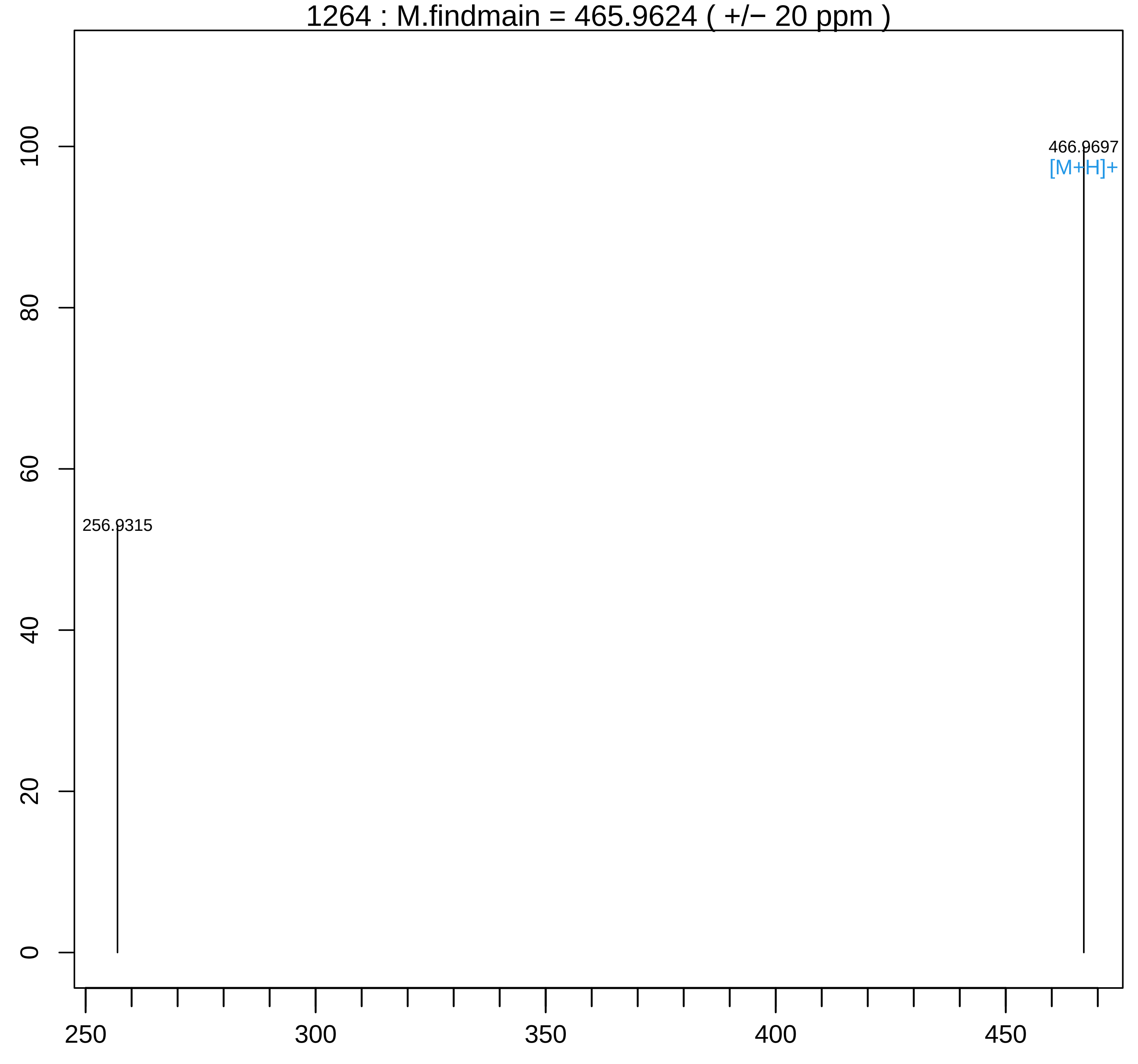
1264 : M.findmain = 465.9624 (+/− 20 ppm)
466.9697
[M+H]+
256.9315
0
20
40
60
80
100
250
300
350
400
450

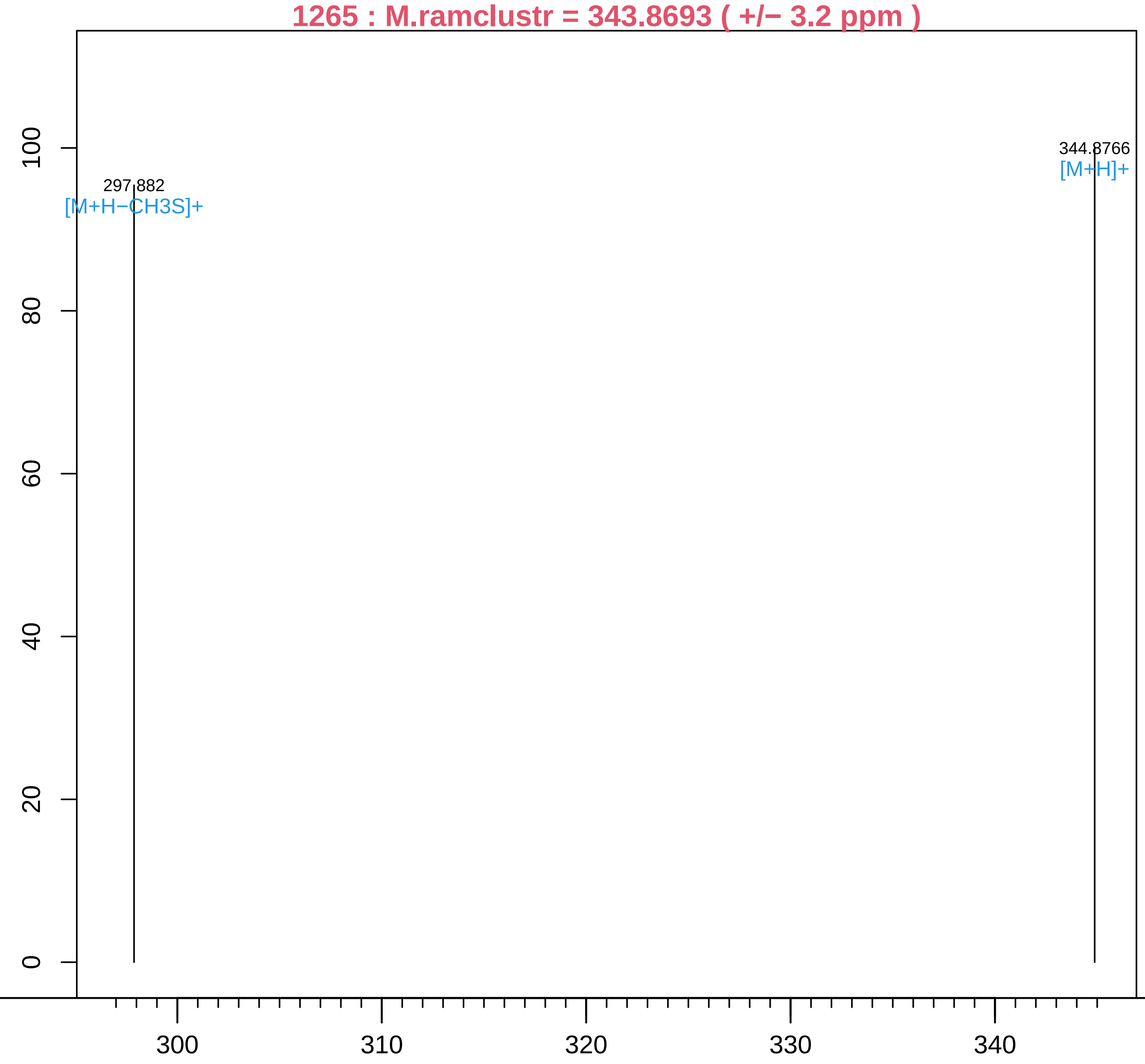

1265 : M.ramclustr = 343.8693 (+/− 3.2 ppm)
297.882
[M+H−CH3S]+
344.8766
[M+H]+
0
20
40
60
80
100
300
310
320
330
340

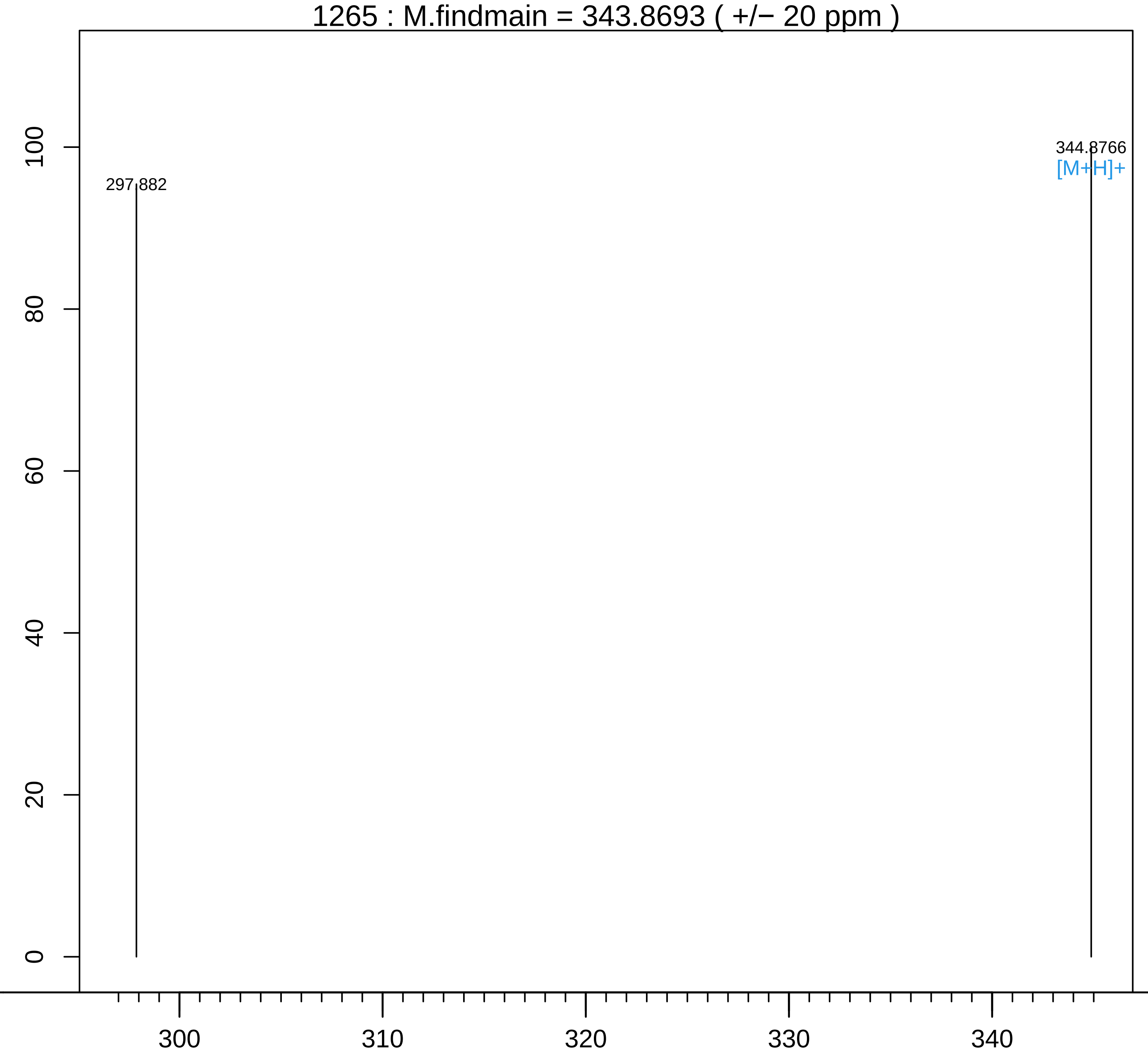

1265 : M.findmain = 343.8693 (+/− 20 ppm)
297.882
344.8766
[M+H]+
0
20
40
60
80
100
300
310
320
330
340

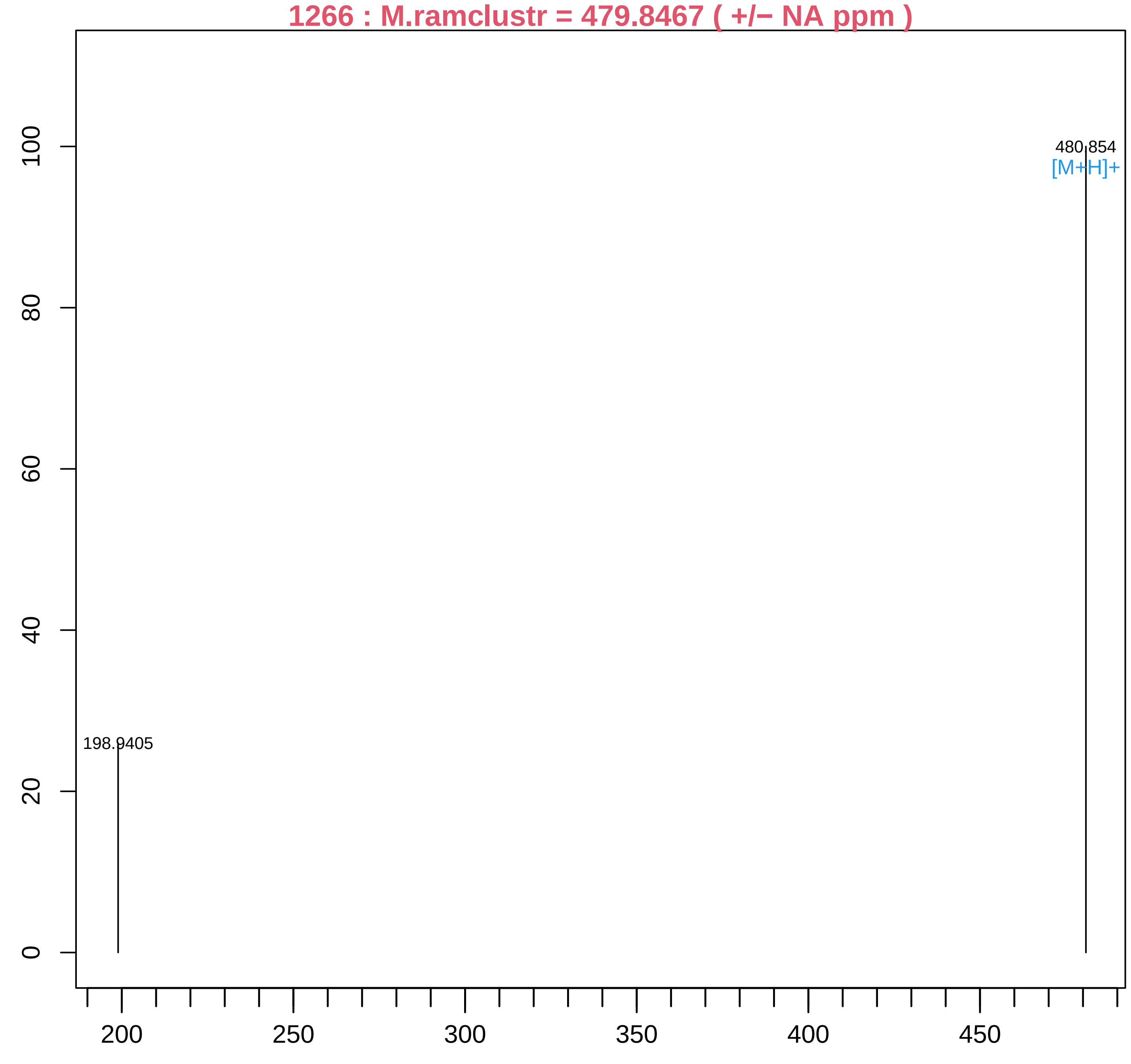
1266 : M.ramclustr = 479.8467 (+/− NA ppm)
480.854
[M+H]+
198.9405
0
20
40
60
80
100
200
250
300
350
400
450

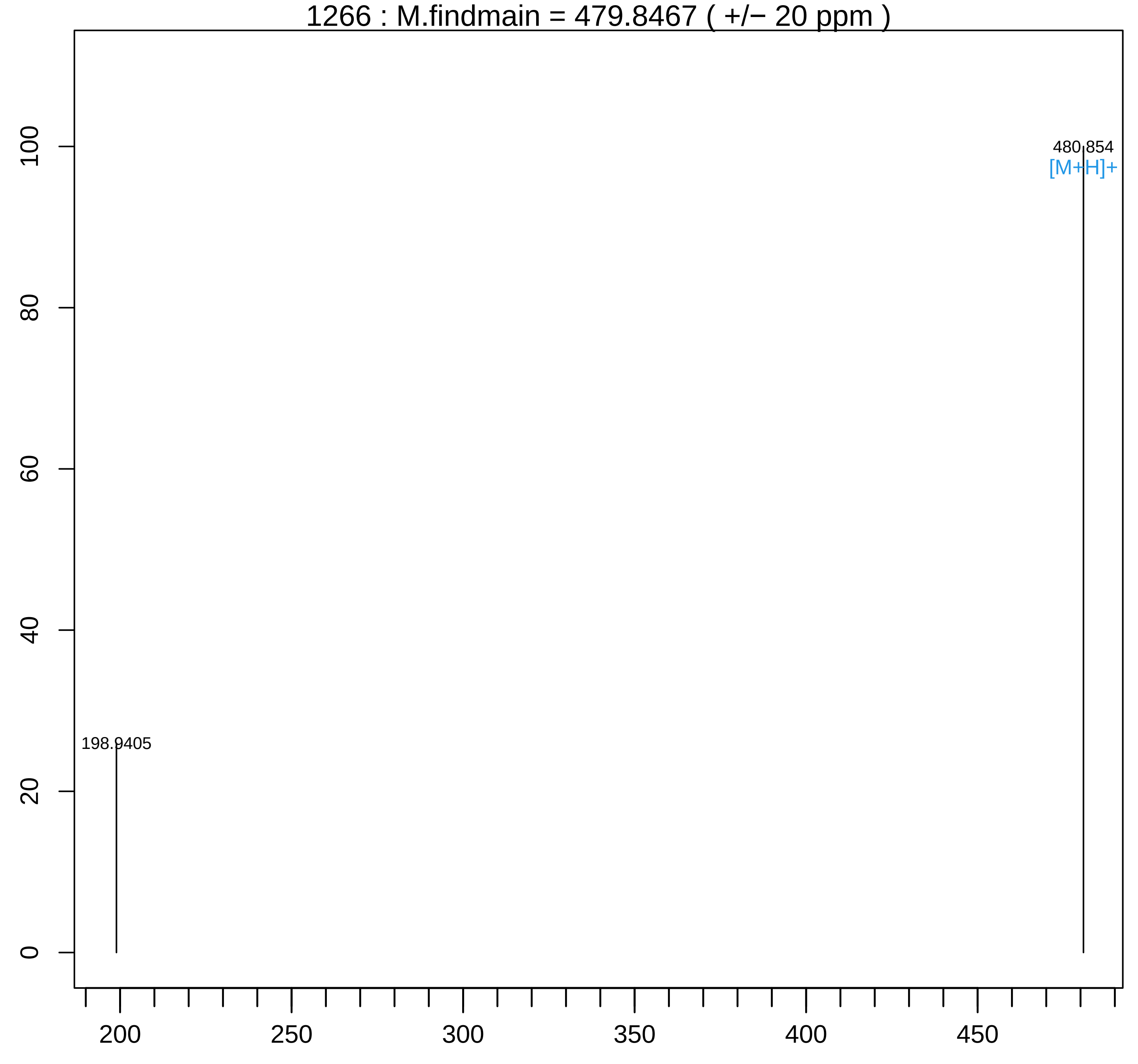
1266 : M.findmain = 479.8467 (+/− 20 ppm)
480.854
[M+H]+
198.9405
0
20
40
60
80
100
200
250
300
350
400
450

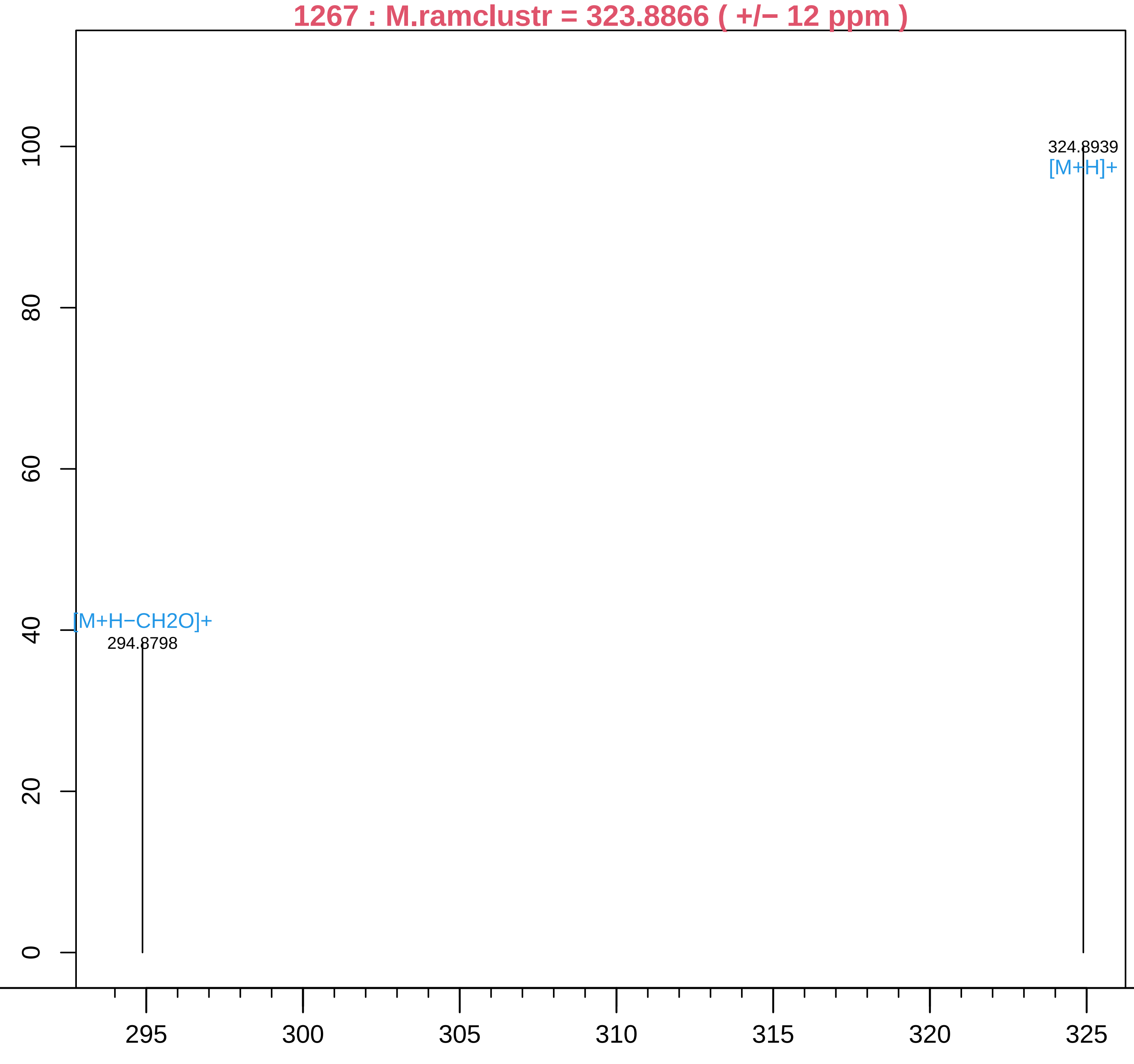

1267 : M.ramclustr = 323.8866 (+/− 12 ppm)
324.8939
[M+H]+
[M+H−CH2O]+
294.8798
0
20
40
60
80
100
295
300
305
310
315
320
325

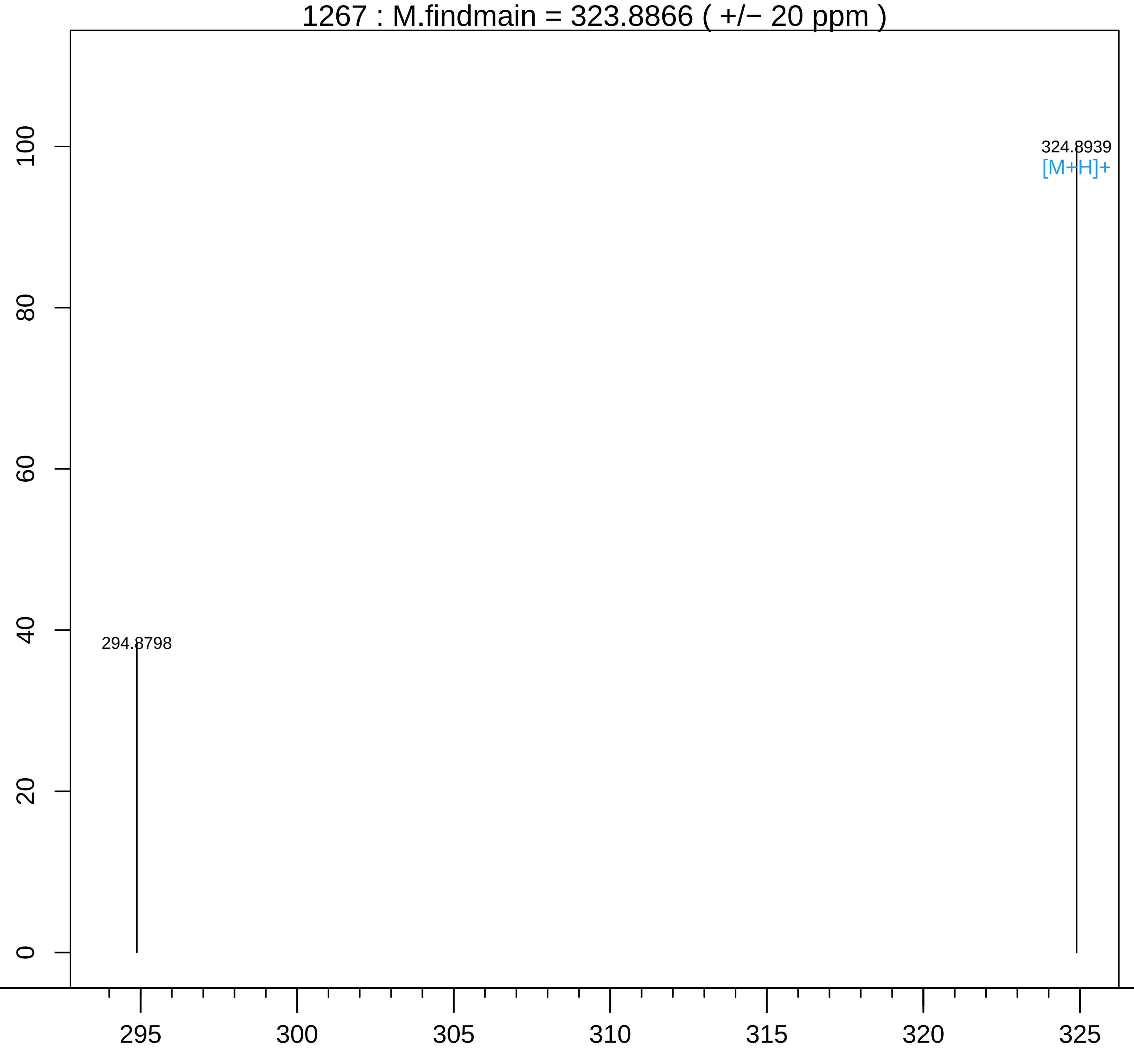

1267 : M.findmain = 323.8866 (+/− 20 ppm)
324.8939
[M+H]+
294.8798
0
20
40
60
80
100
295
300
305
310
315
320
325

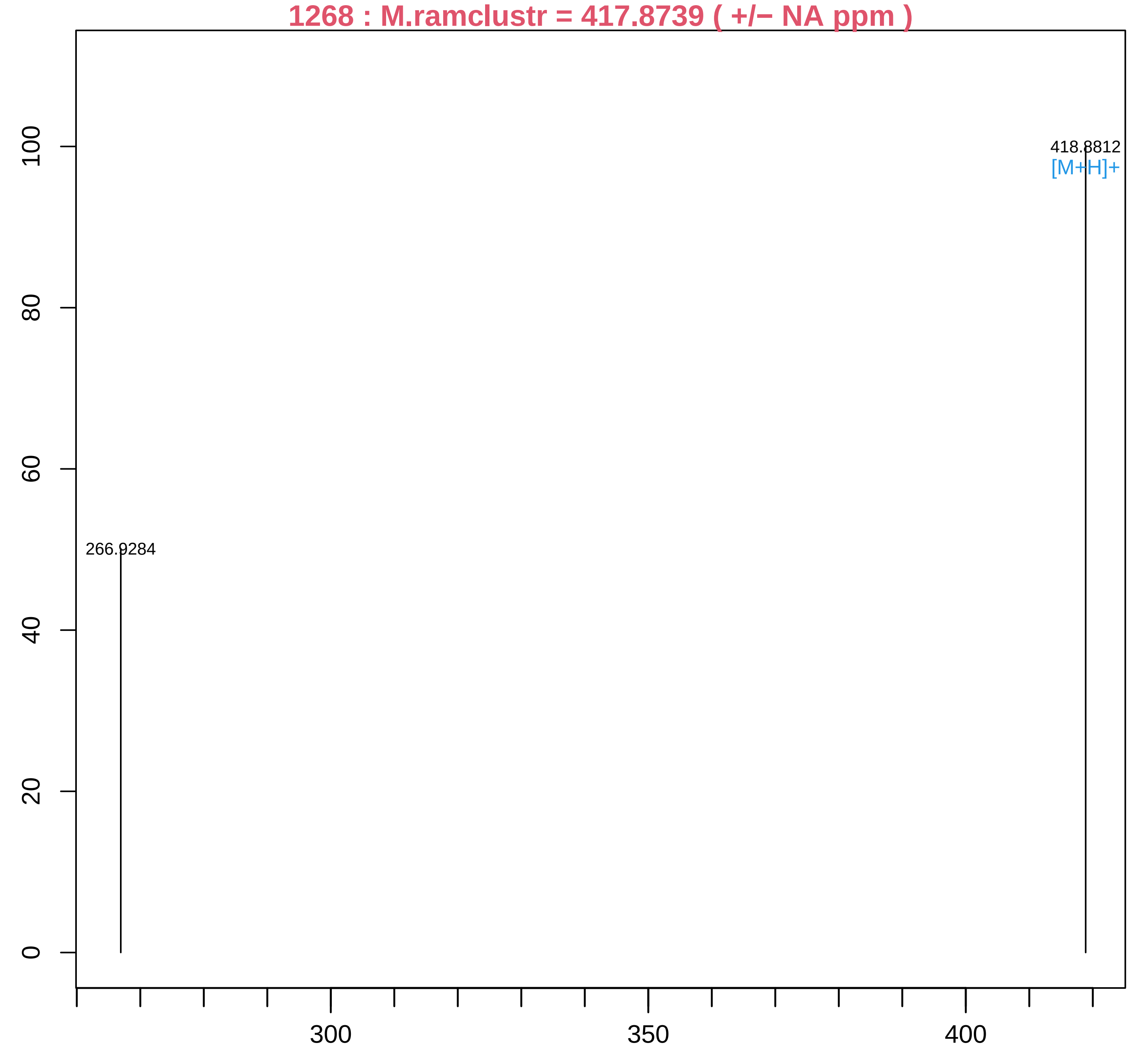
1268 : M.ramclustr = 417.8739 (+/− NA ppm)
418.8812
[M+H]+
266.9284
0
20
40
60
80
100
300
350
400

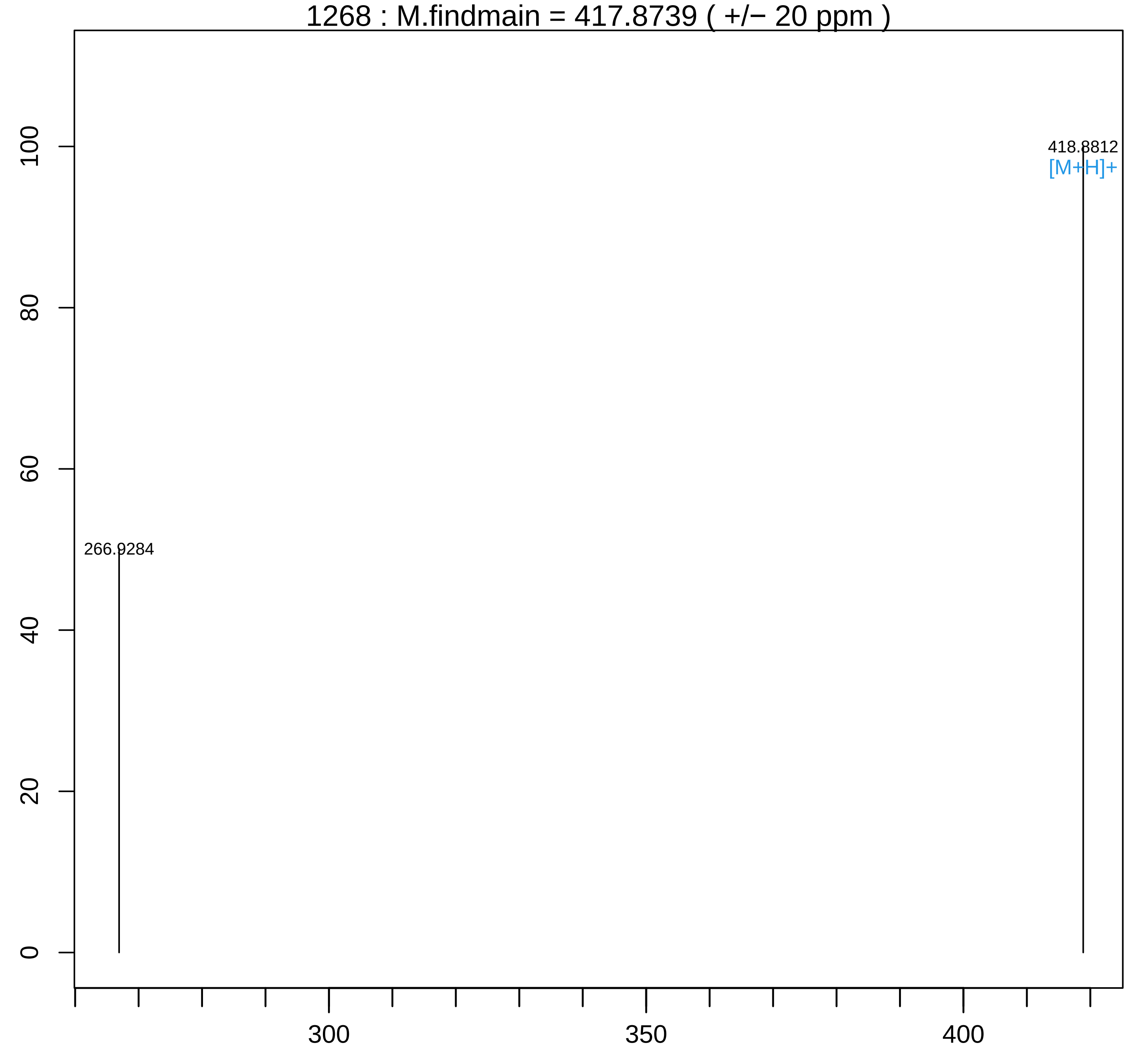
1268 : M.findmain = 417.8739 (+/− 20 ppm)
418.8812
[M+H]+
266.9284
0
20
40
60
80
100
300
350
400

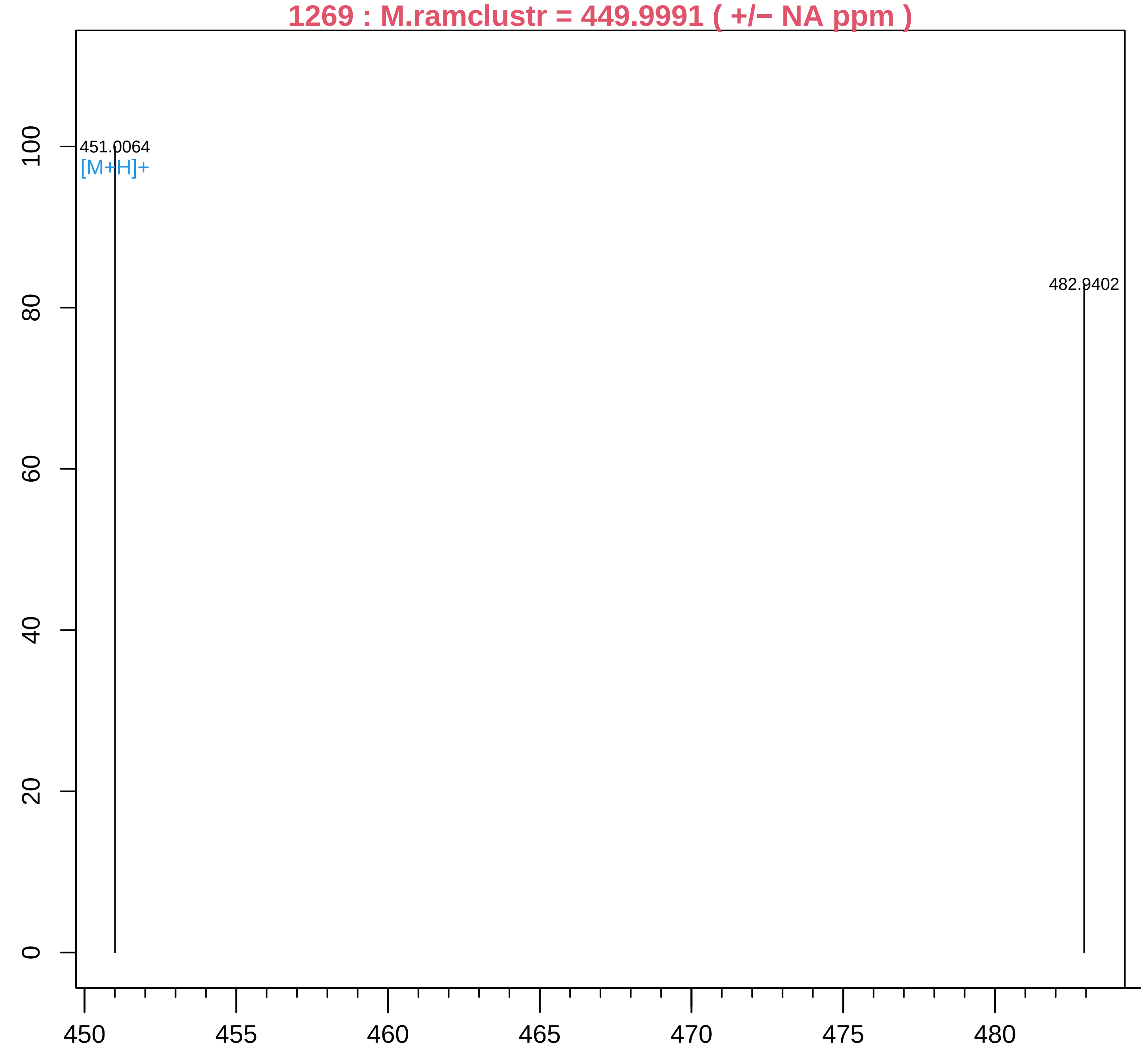
1269 : M.ramclustr = 449.9991 (+/− NA ppm)
451.0064
[M+H]+
482.9402
0
20
40
60
80
100
450
455
460
465
470
475
480

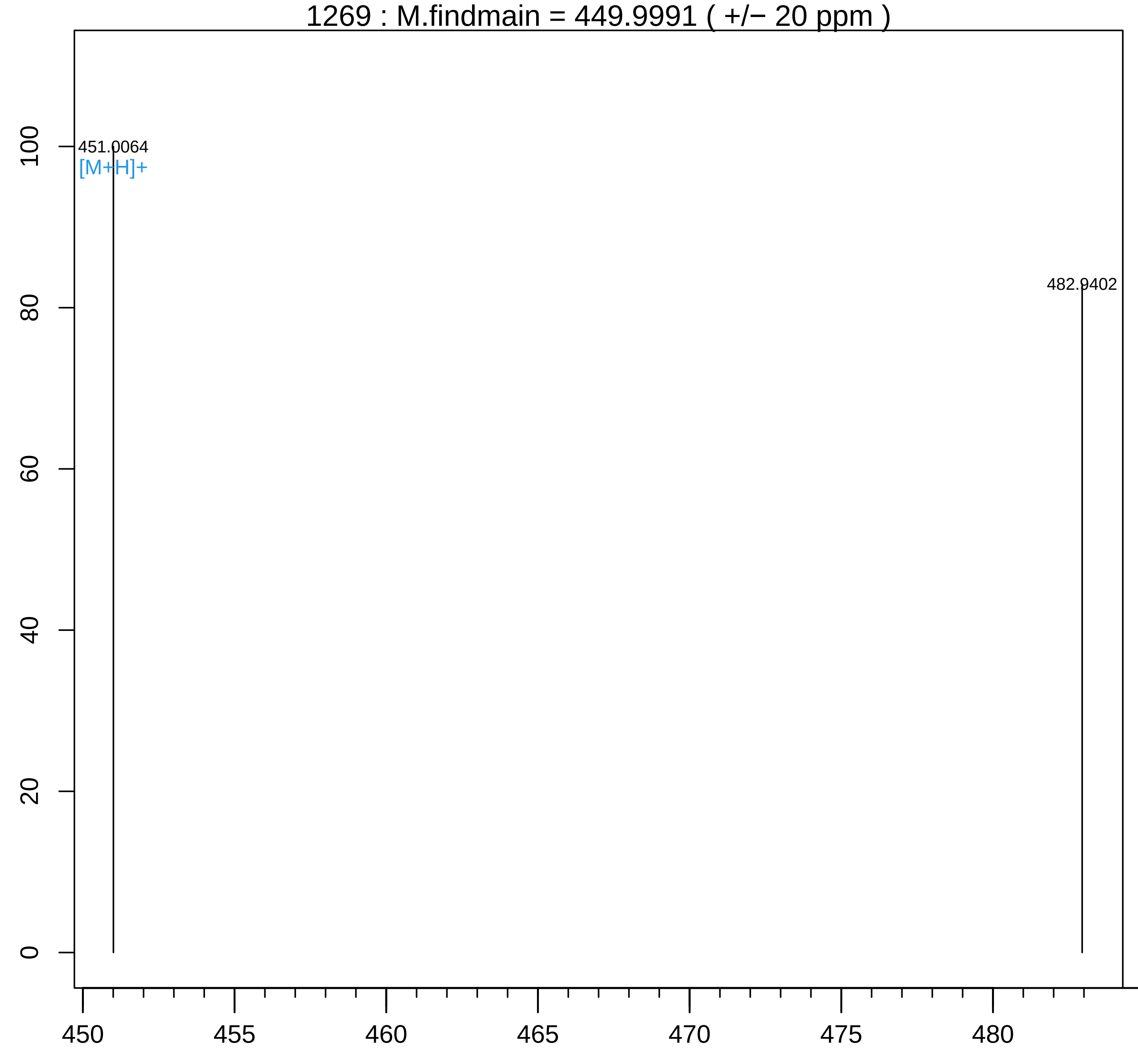
1269 : M.findmain = 449.9991 (+/− 20 ppm)
451.0064
[M+H]+
482.9402
0
20
40
60
80
100
450
455
460
465
470
475
480

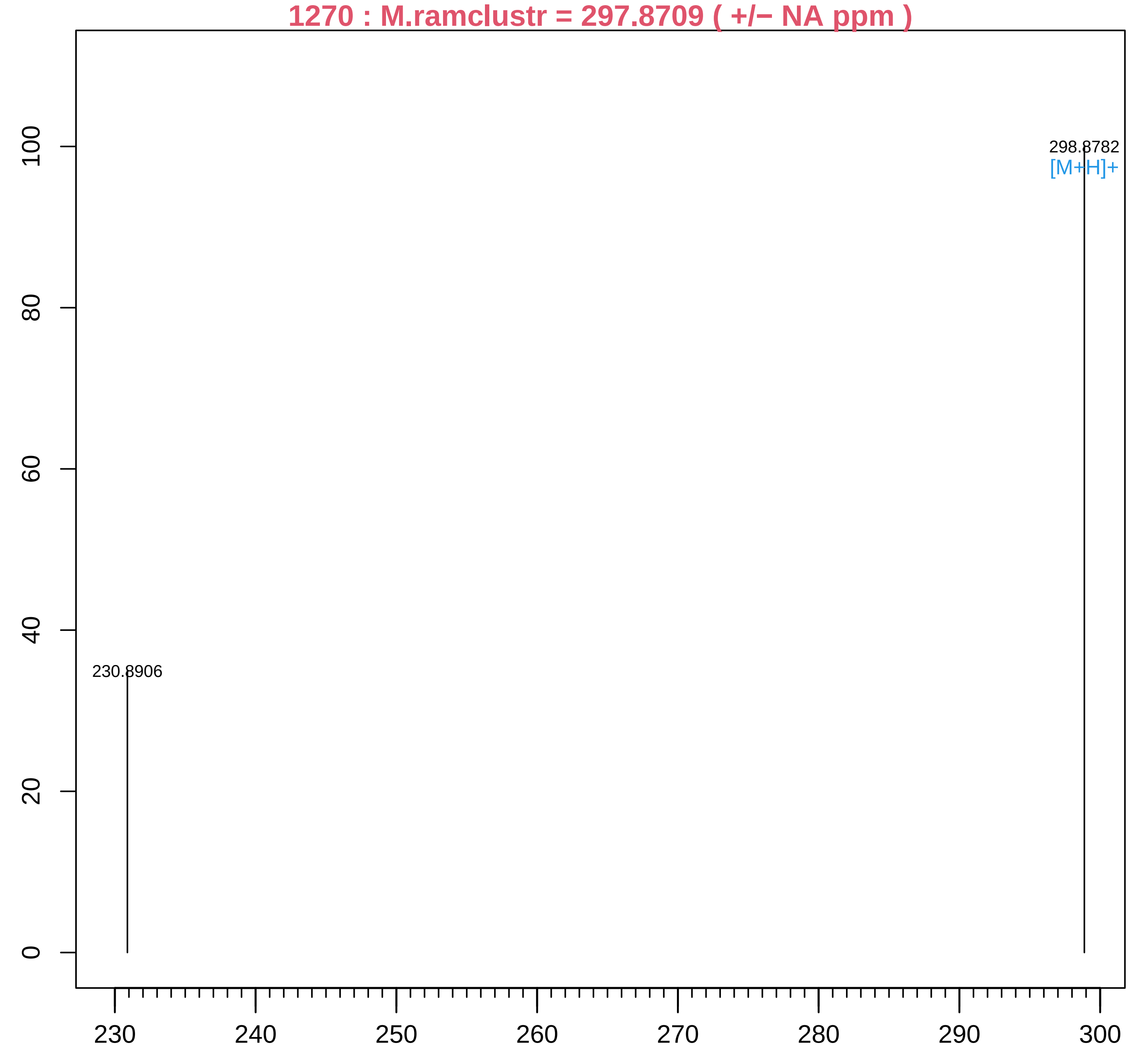
1270 : M.ramclustr = 297.8709 (+/− NA ppm)
298.8782
[M+H]+
230.8906

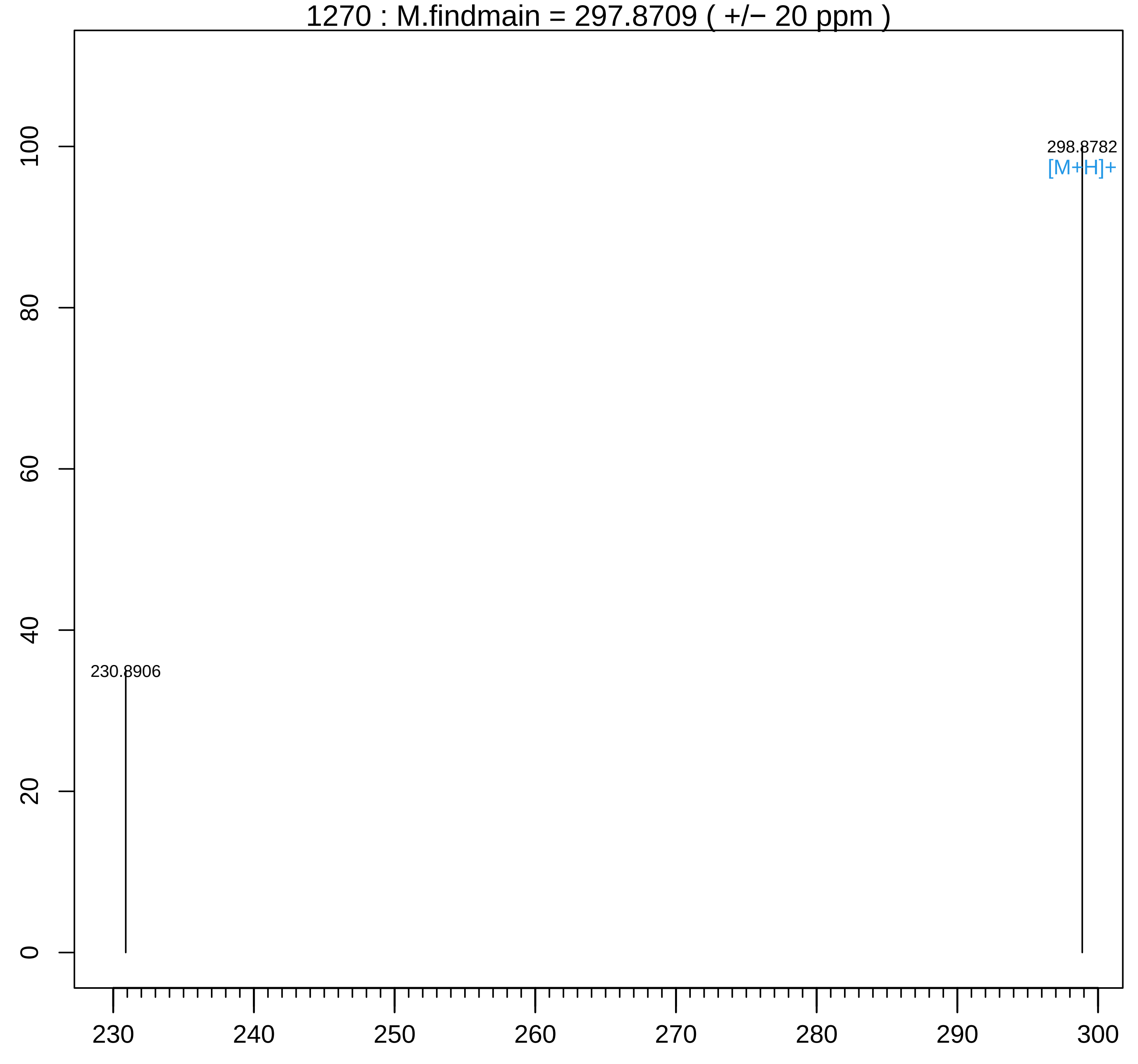
1270 : M.findmain = 297.8709 (+/− 20 ppm)
298.8782
[M+H]+
230.8906

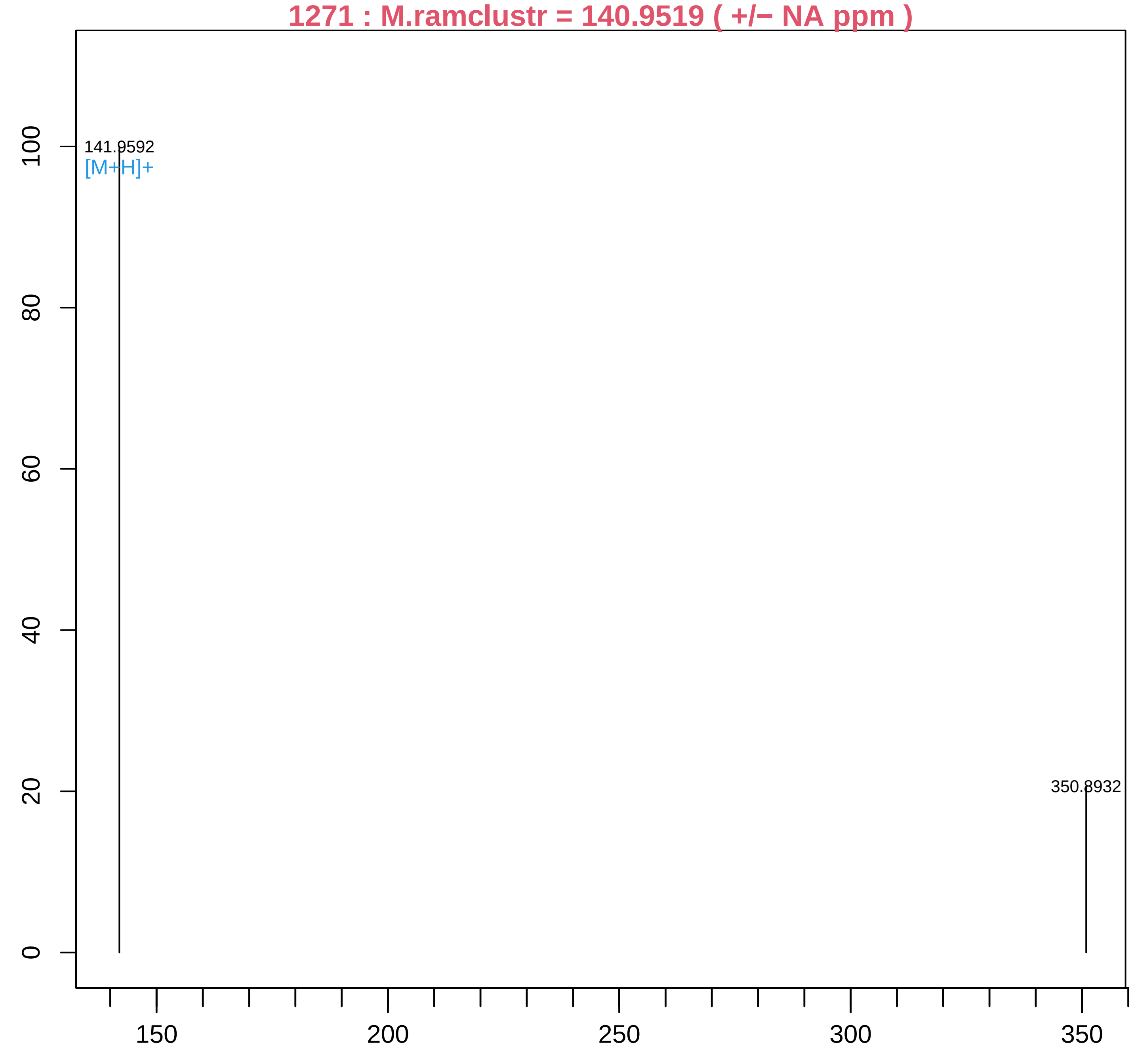
1271 : M.ramclustr = 140.9519 (+/− NA ppm)
141.9592
[M+H]+
350.8932
0
20
40
60
80
100
150
200
250
300
350

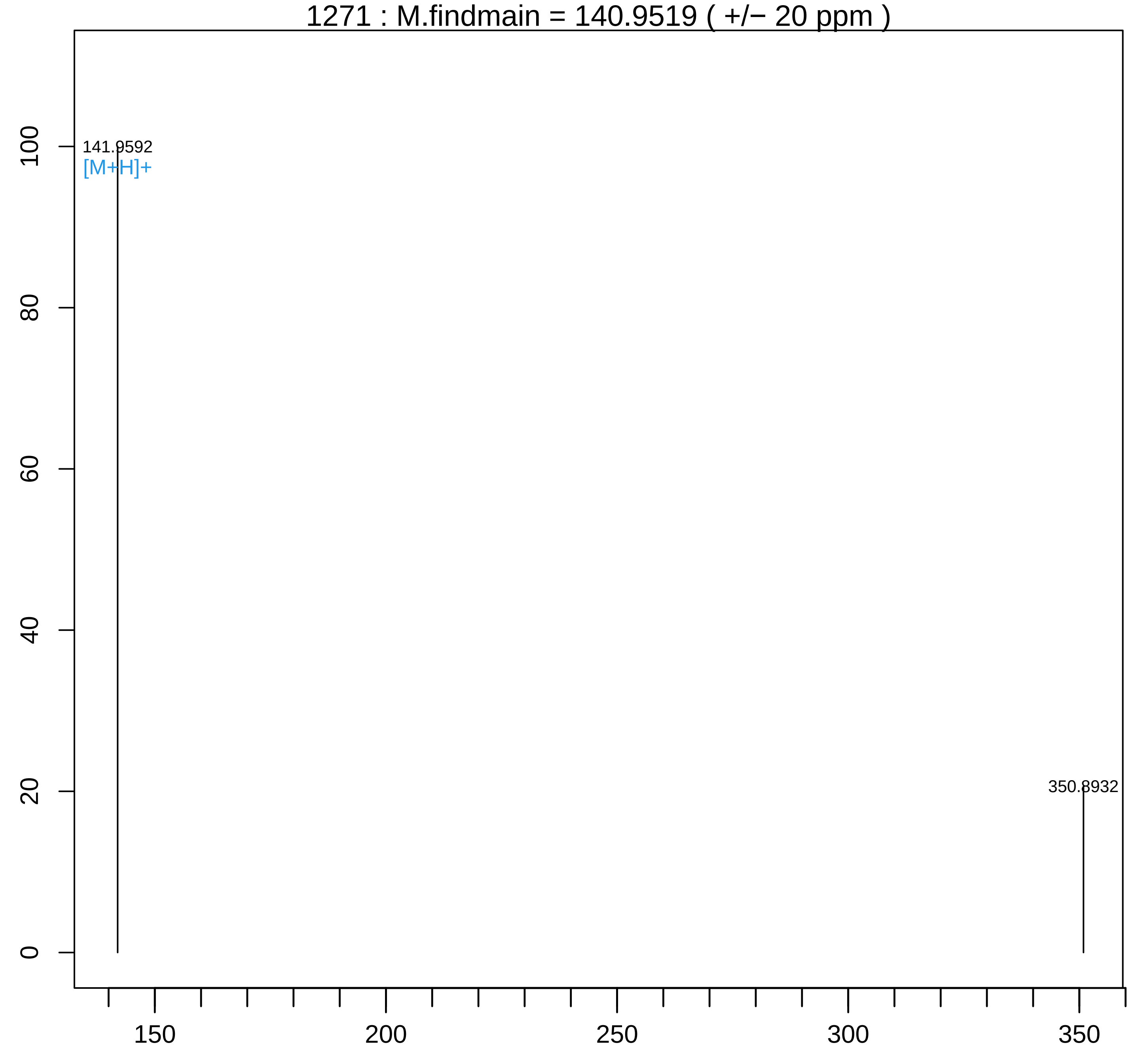
1271 : M.findmain = 140.9519 (+/− 20 ppm)
141.9592
[M+H]+
350.8932
0
20
40
60
80
100
150
200
250
300
350

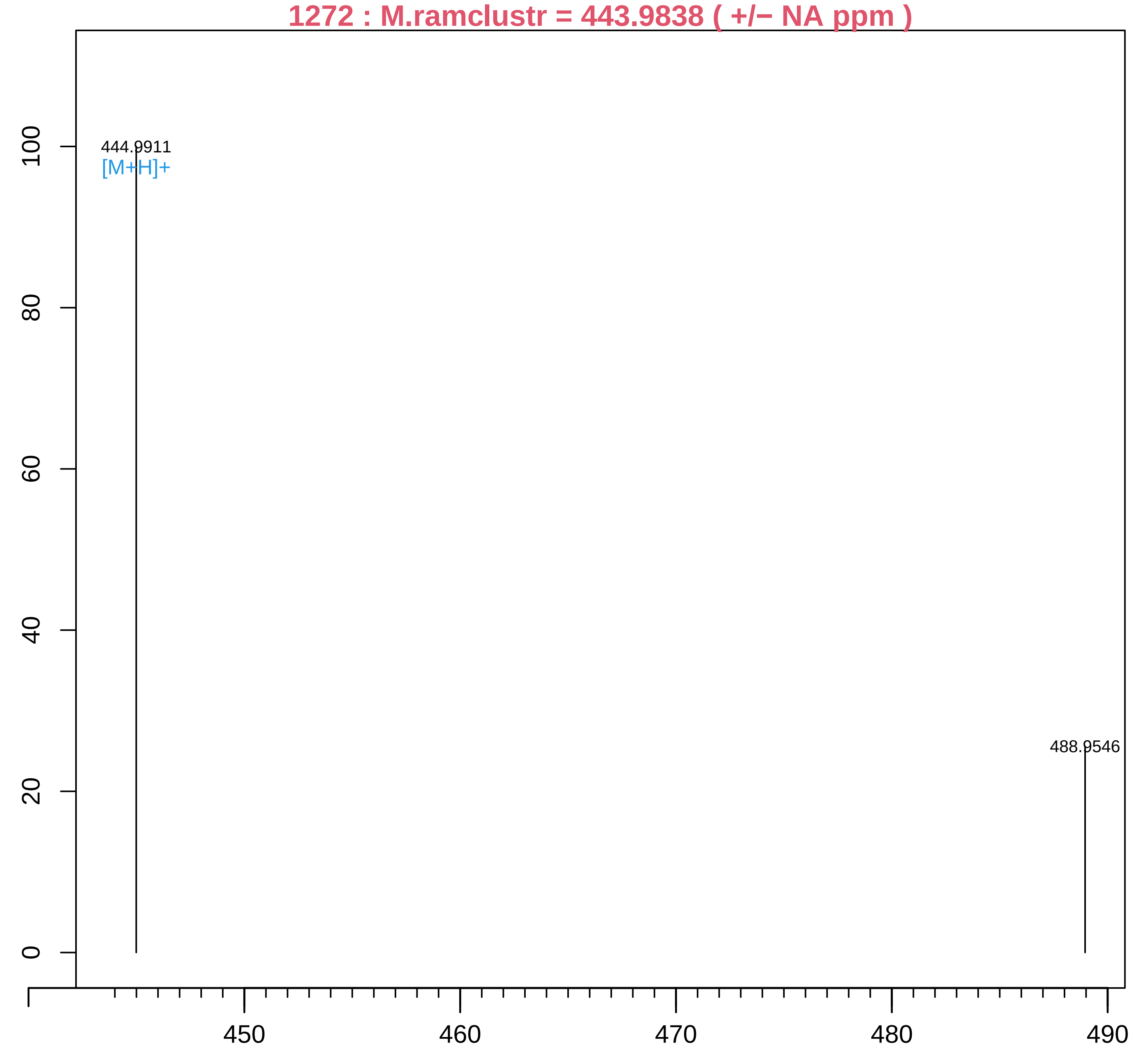
1272 : M.ramclustr = 443.9838 (+/− NA ppm)
444.9911
[M+H]+
488.9546
0
20
40
60
80
100
450
460
470
480
490

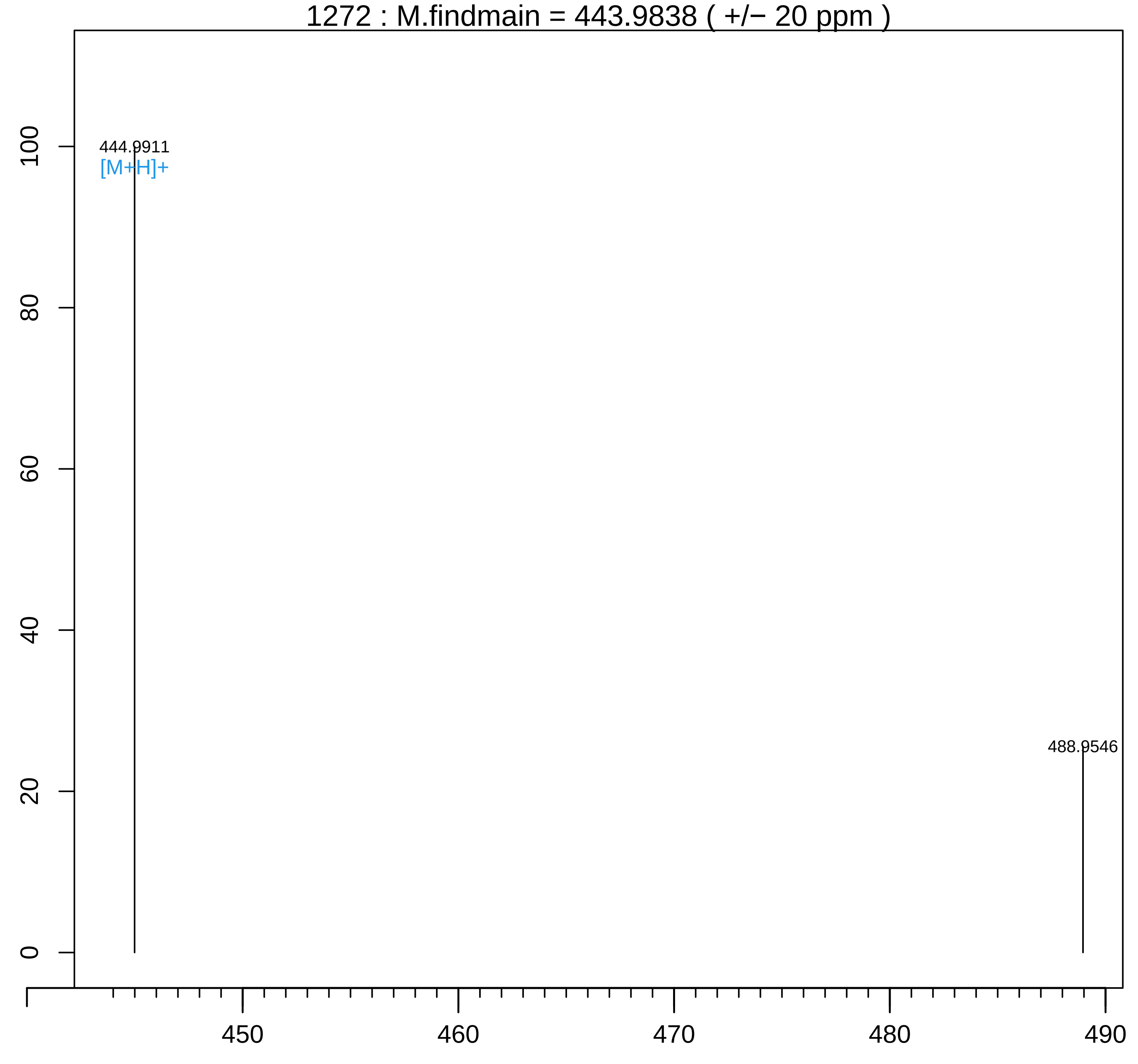
1272 : M.findmain = 443.9838 (+/− 20 ppm)
444.9911
[M+H]+
488.9546
0
20
40
60
80
100
450
460
470
480
490

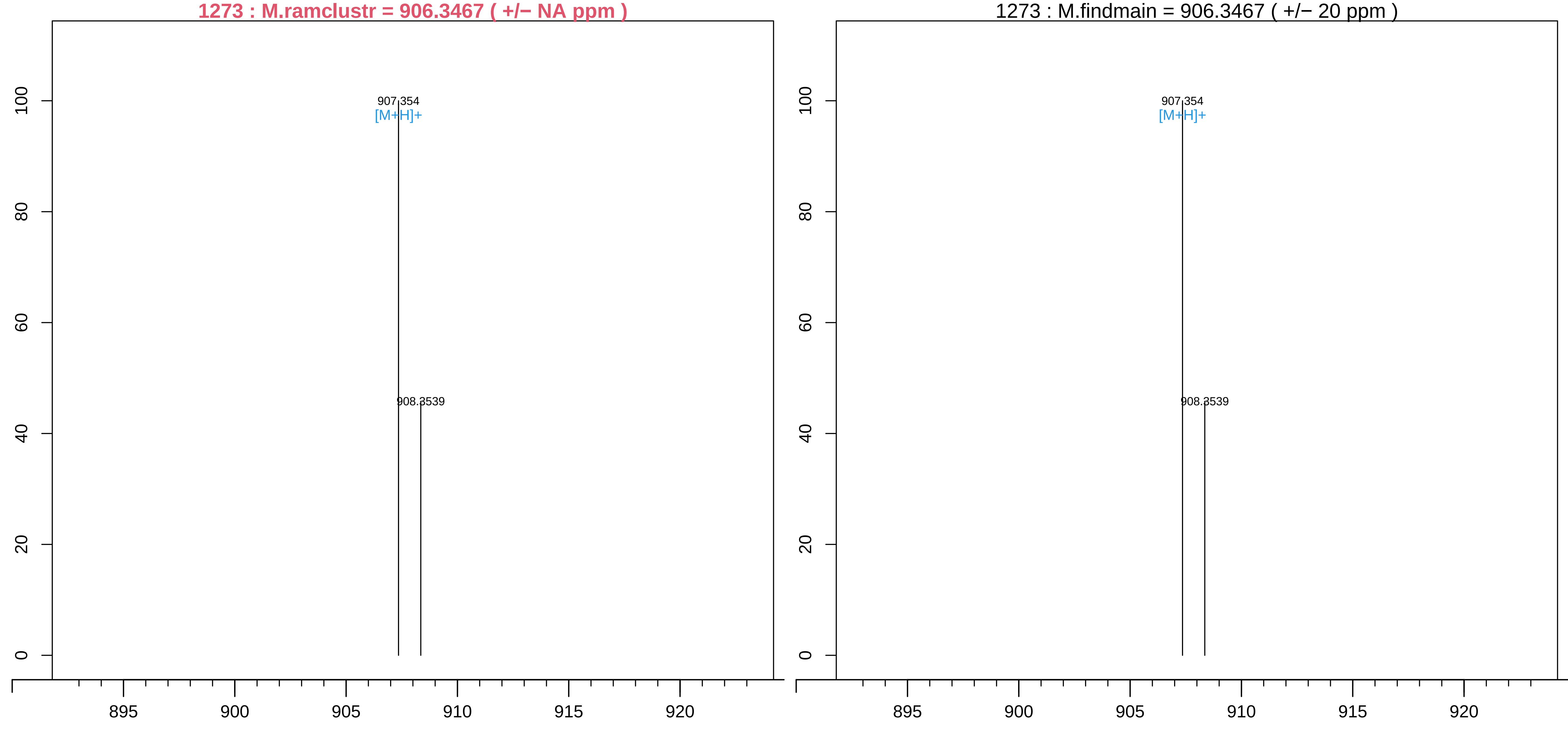
1273 : M.ramclustr = 906.3467 (+/− NA ppm)
907.354
[M+H]+
908.3539
1273 : M.findmain = 906.3467 (+/− 20 ppm)
907.354
[M+H]+
908.3539

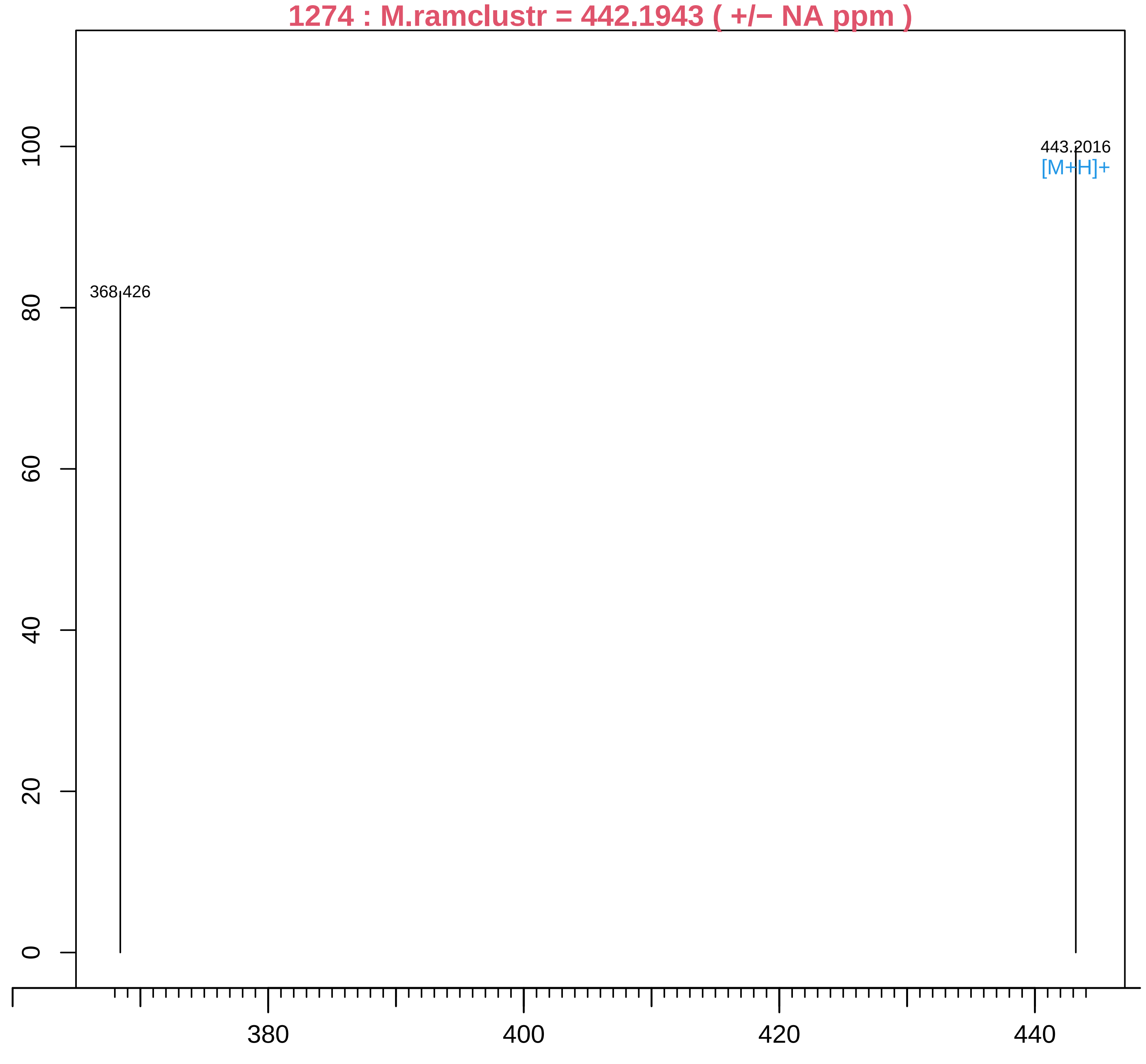
1274 : M.ramclustr = 442.1943 (+/− NA ppm)
443.2016
[M+H]+
368.426
0
20
40
60
80
100
380
400
420
440

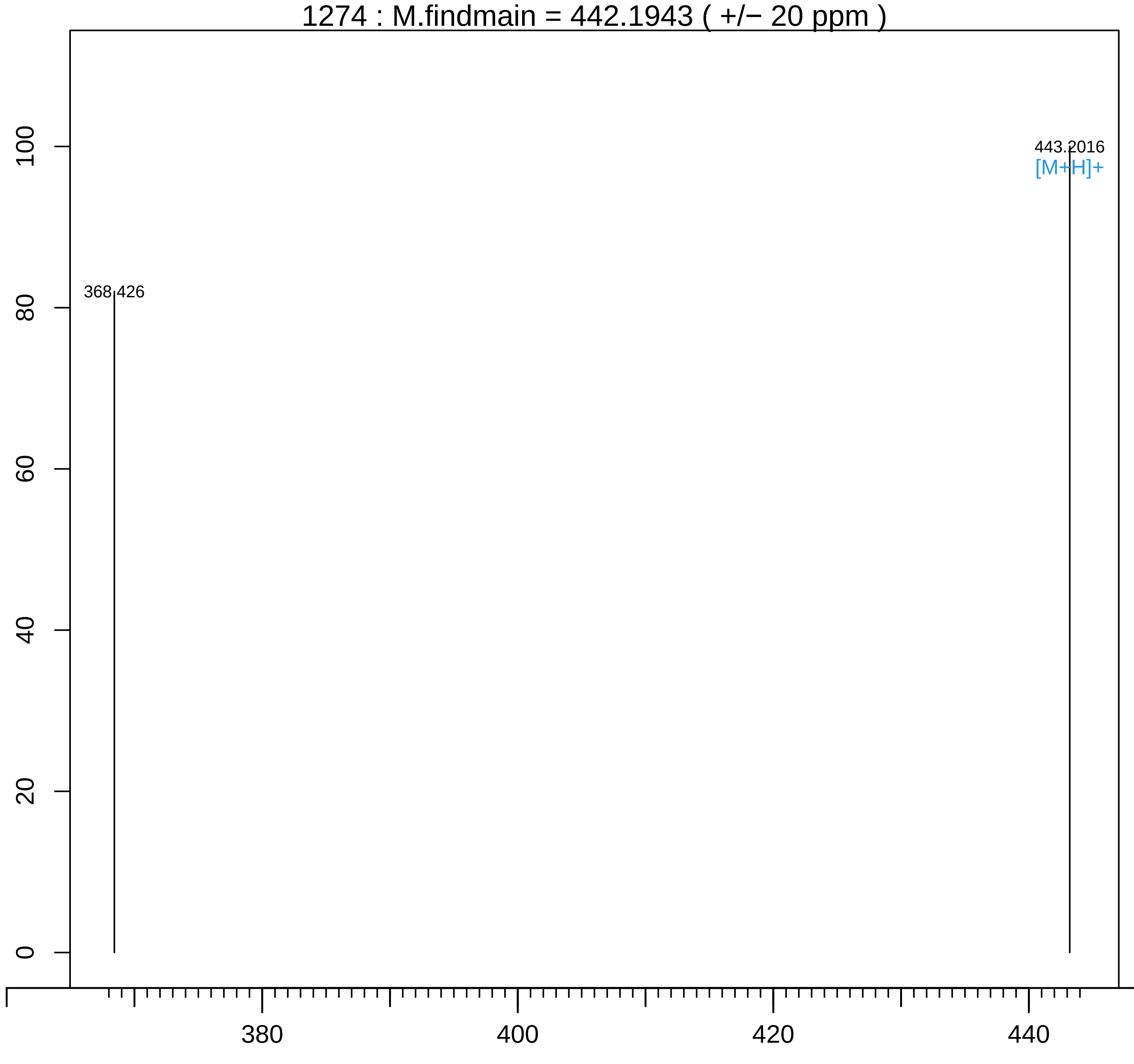
1274 : M.findmain = 442.1943 (+/− 20 ppm)
443.2016
[M+H]+
368.426
0
20
40
60
80
100
380
400
420
440

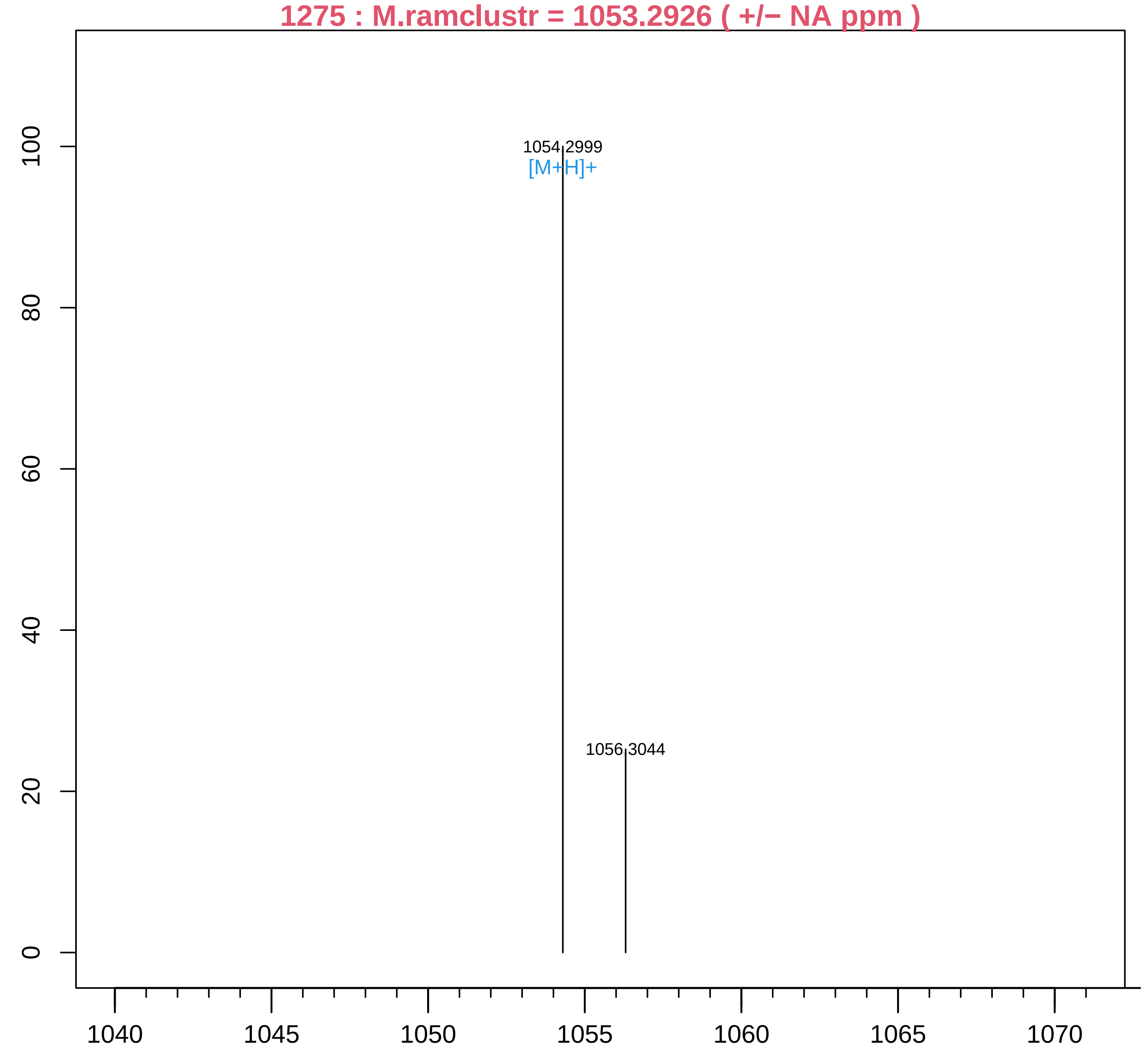
1275 : M.ramclustr = 1053.2926 (+/− NA ppm)
1054.2999
[M+H]+
1056.3044
0
20
40
60
80
100
1040
1045
1050
1055
1060
1065
1070

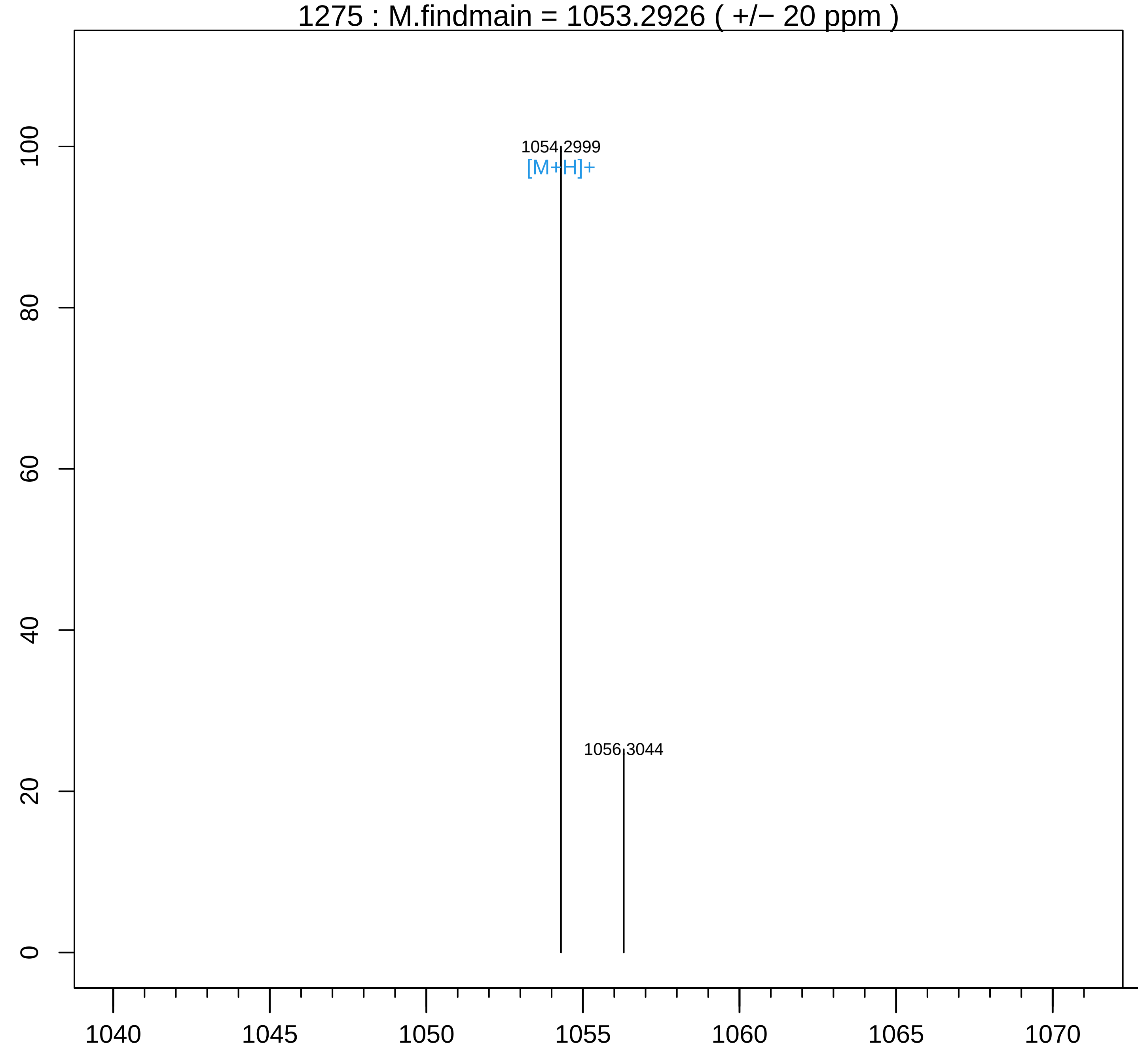
1275 : M.findmain = 1053.2926 (+/− 20 ppm)
1054.2999
[M+H]+
1056.3044
0
20
40
60
80
100
1040
1045
1050
1055
1060
1065
1070

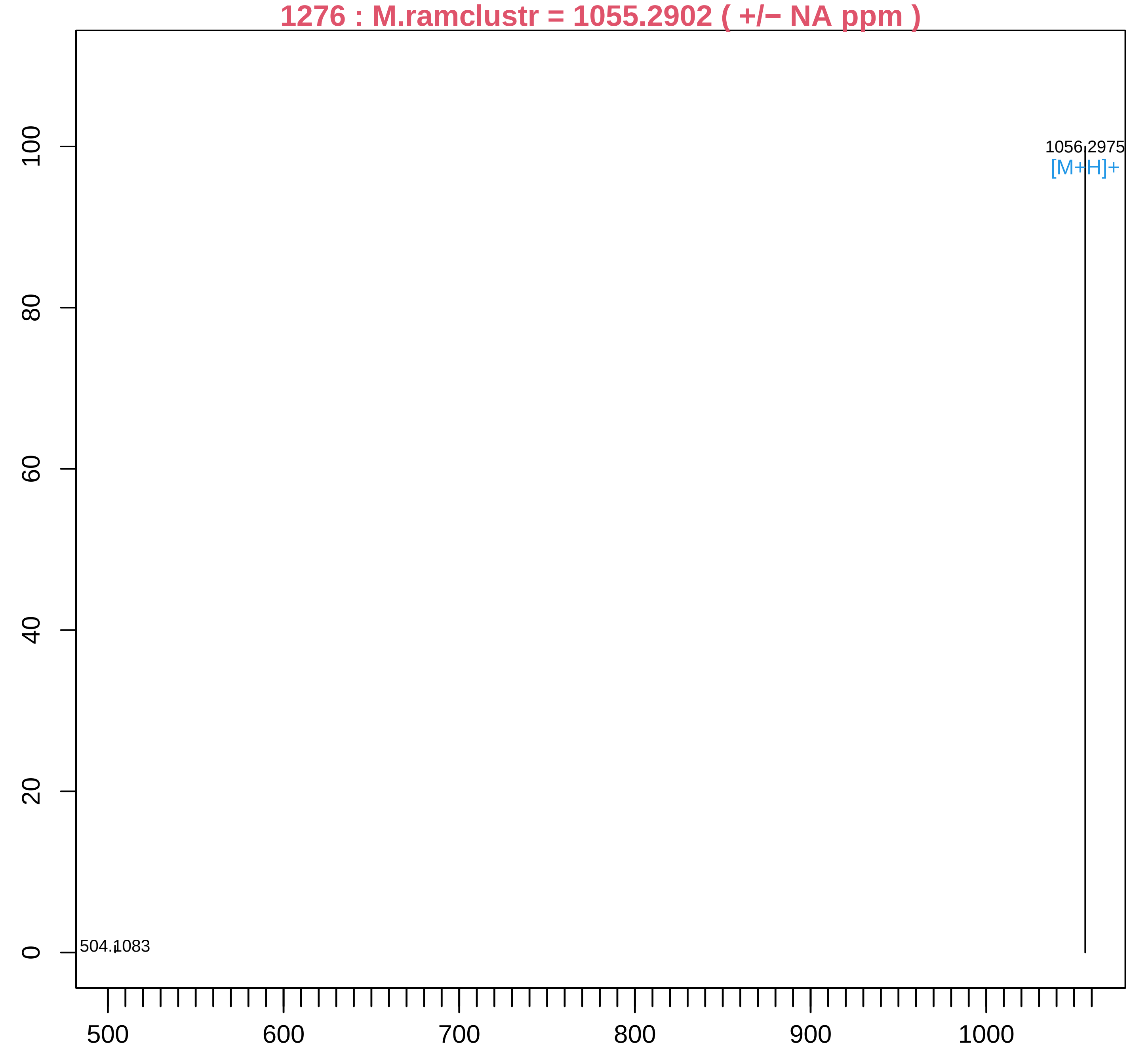
1276 : M.ramclustr = 1055.2902 (+/− NA ppm)
100
80
60
40
20
0
1056.2975
[M+H]+
504.1083
500
600
700
800
900
1000

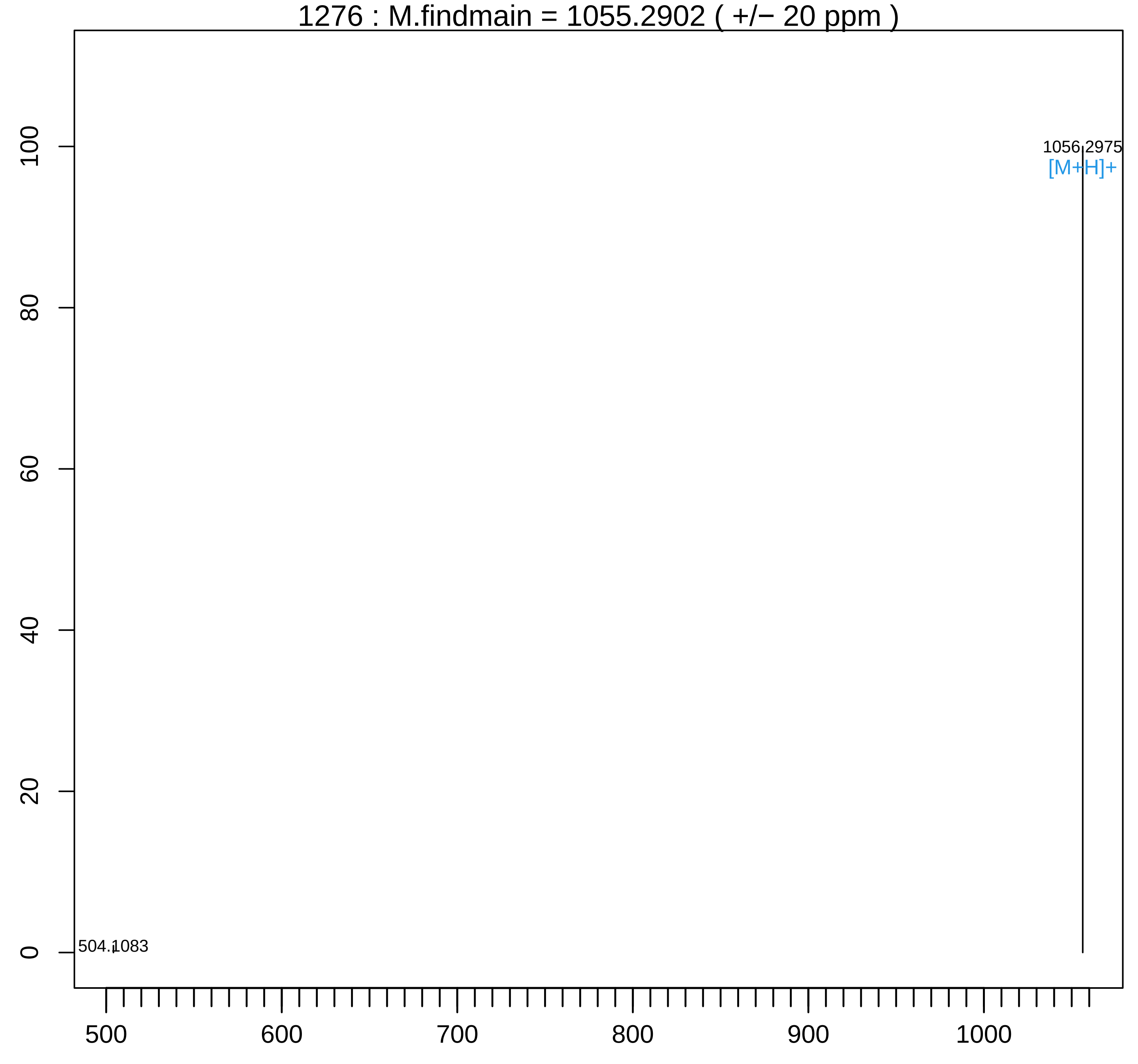
1276 : M.findmain = 1055.2902 (+/− 20 ppm)
100
80
60
40
20
0
1056.2975
[M+H]+
504.1083
500
600
700
800
900
1000

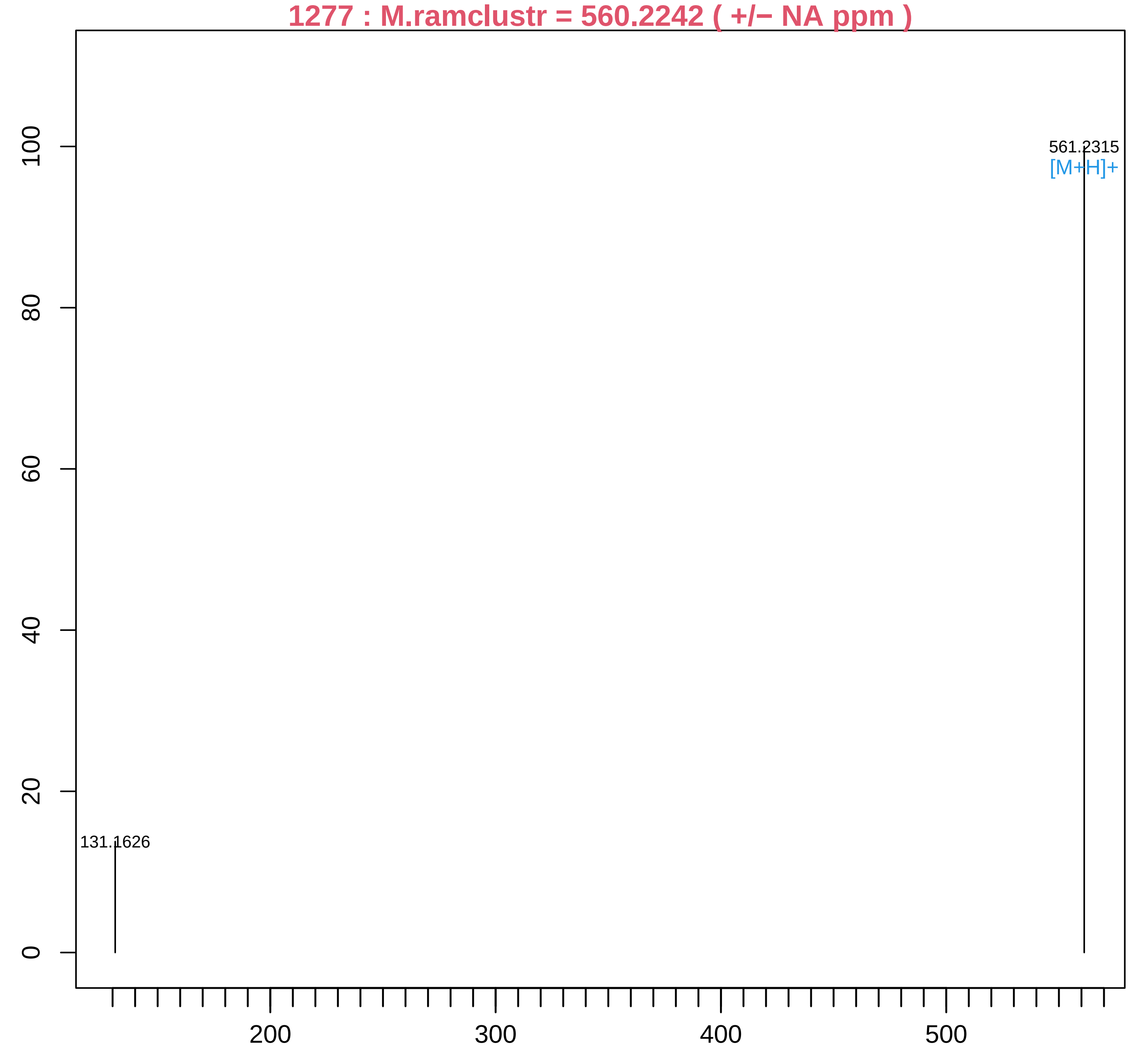
1277 : M.ramclustr = 560.2242 (+/− NA ppm)
561.2315
[M+H]+
131.1626
0
20
40
60
80
100
200
300
400
500

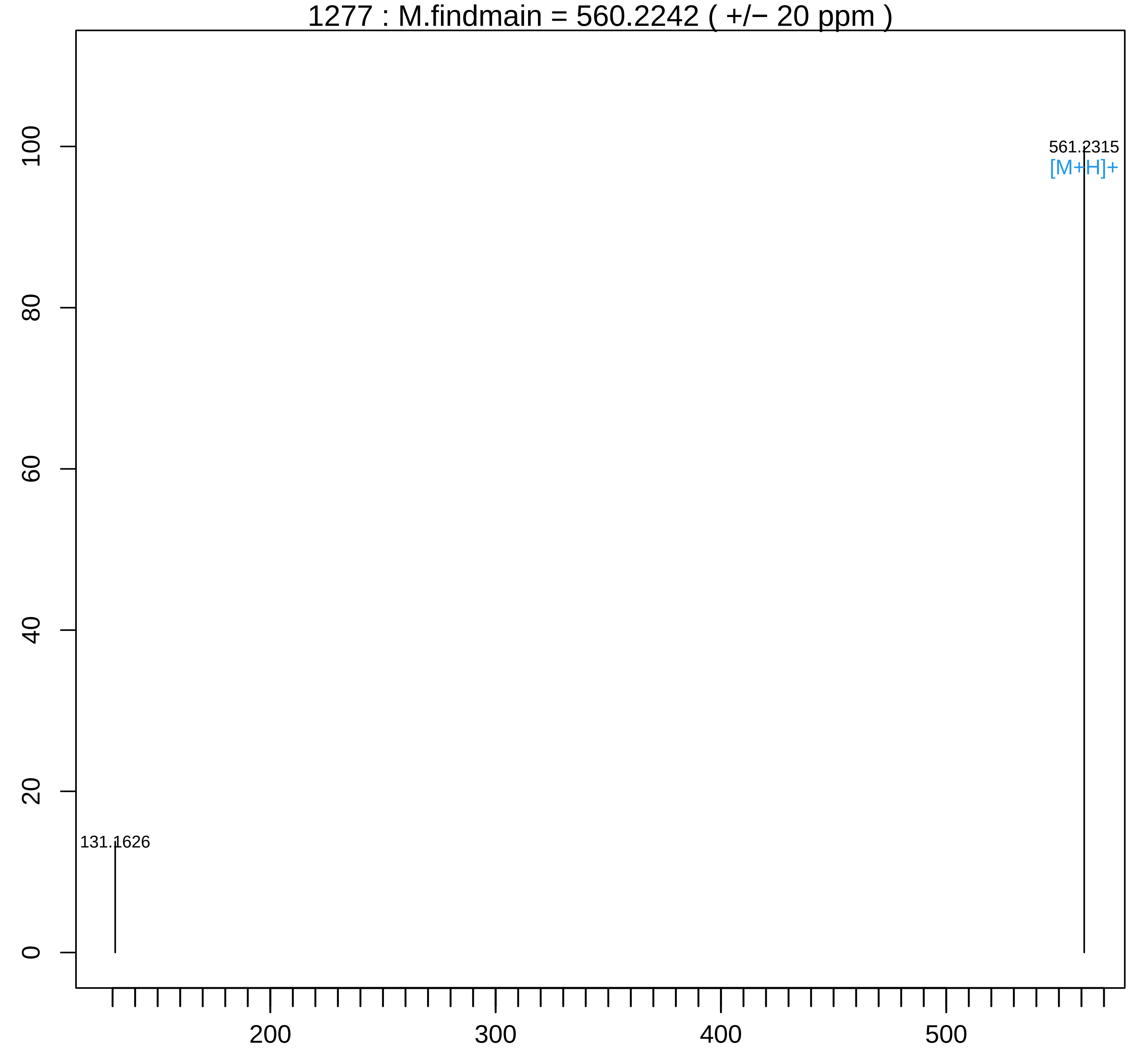
1277 : M.findmain = 560.2242 (+/− 20 ppm)
561.2315
[M+H]+
131.1626
0
20
40
60
80
100
200
300
400
500

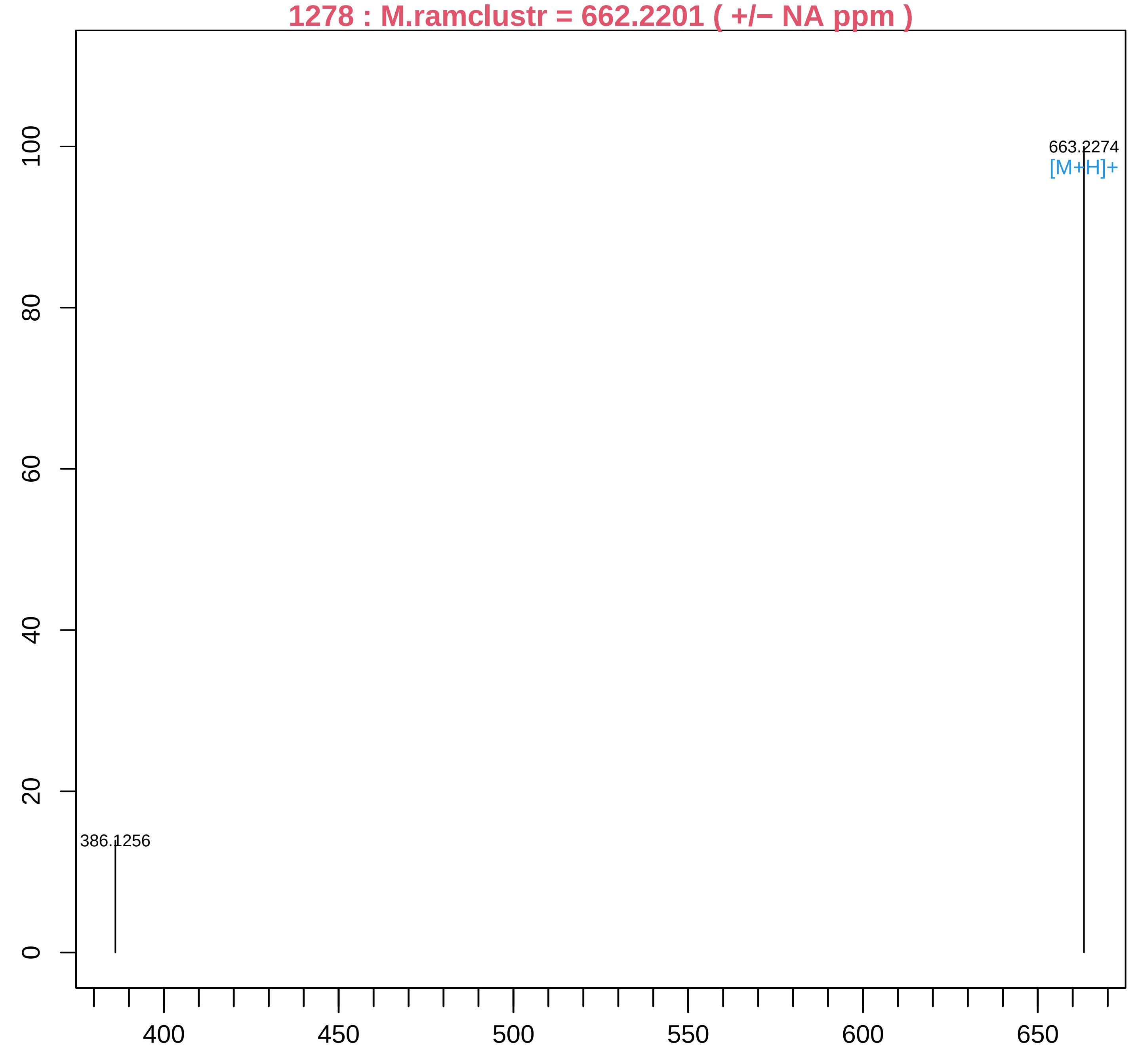
1278 : M.ramclustr = 662.2201 (+/− NA ppm)
663.2274
[M+H]+
386.1256
0
20
40
60
80
100
400
450
500
550
600
650

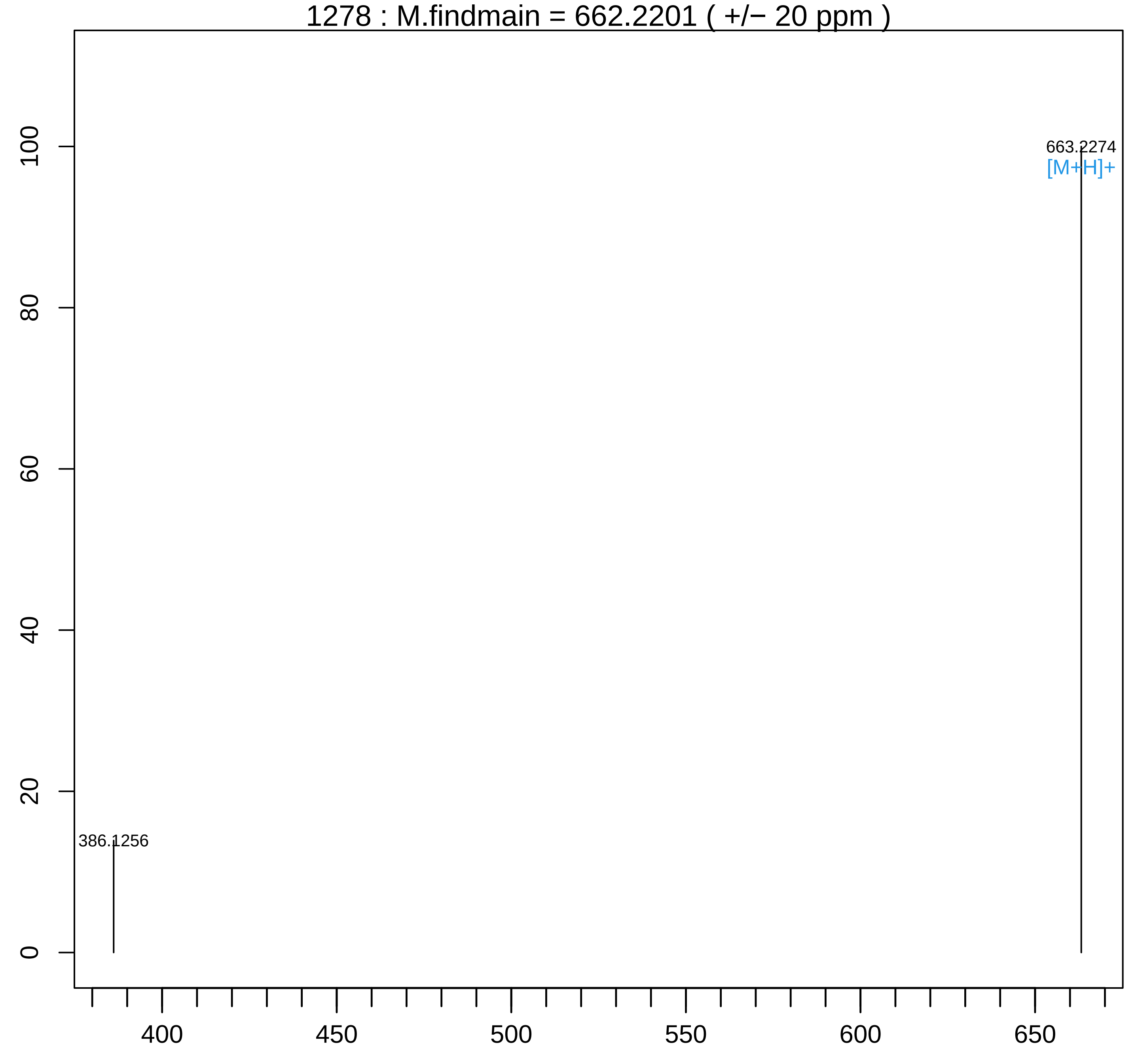
1278 : M.findmain = 662.2201 (+/− 20 ppm)
663.2274
[M+H]+
386.1256
0
20
40
60
80
100
400
450
500
550
600
650

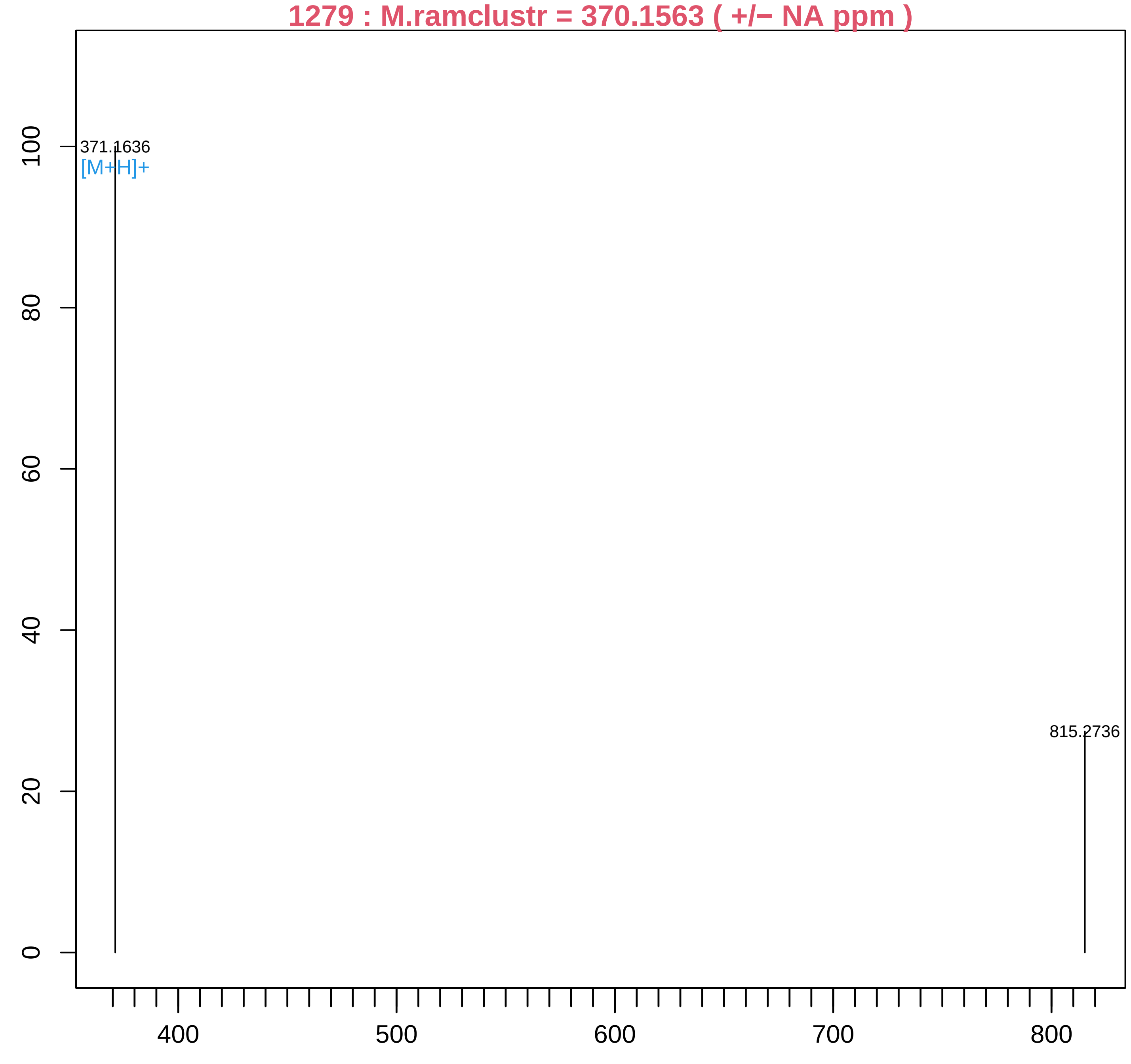
1279 : M.ramclustr = 370.1563 (+/− NA ppm)
371.1636
[M+H]+
815.2736
0
20
40
60
80
100
400
500
600
700
800

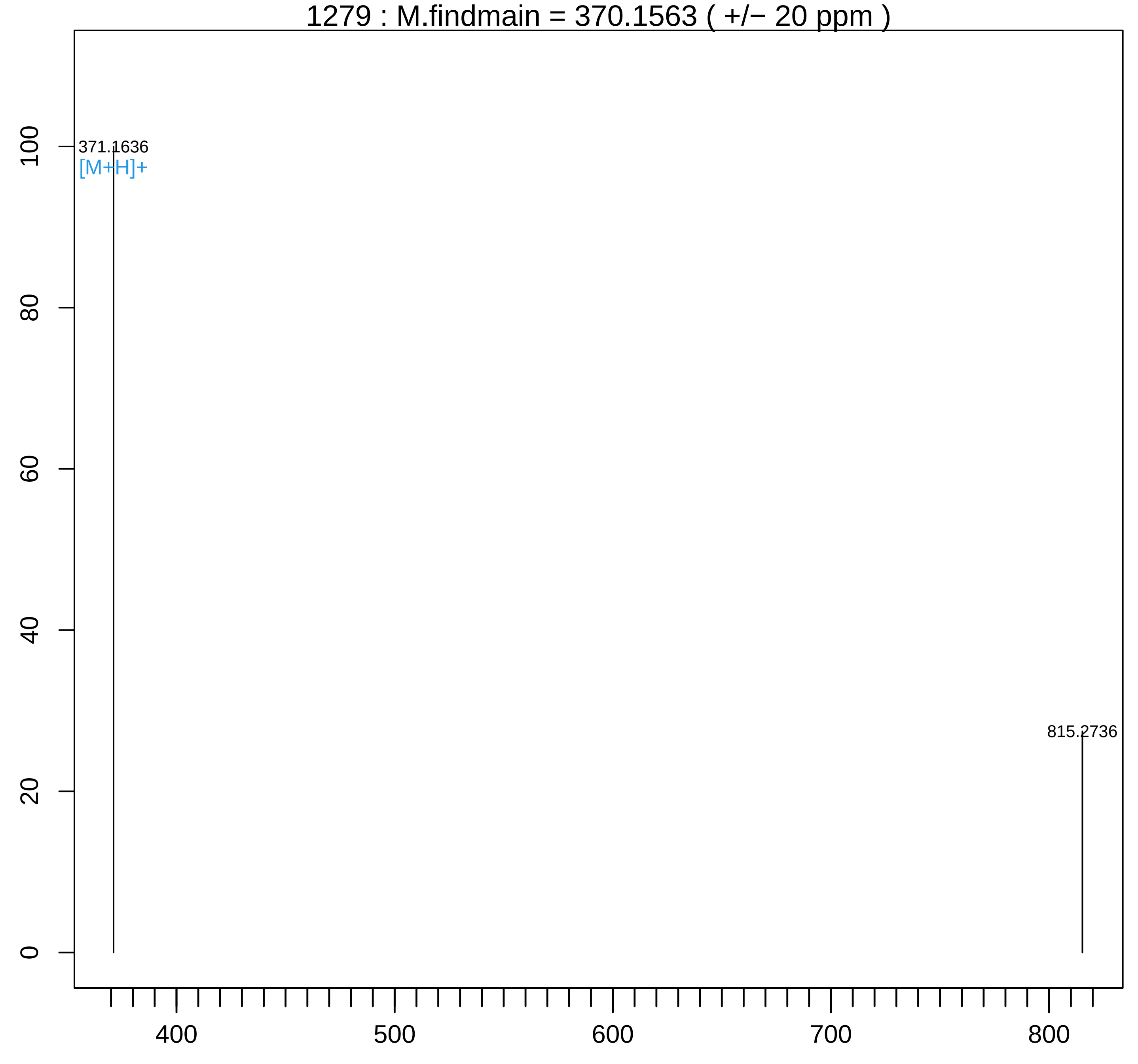
1279 : M.findmain = 370.1563 (+/− 20 ppm)
371.1636
[M+H]+
815.2736
0
20
40
60
80
100
400
500
600
700
800

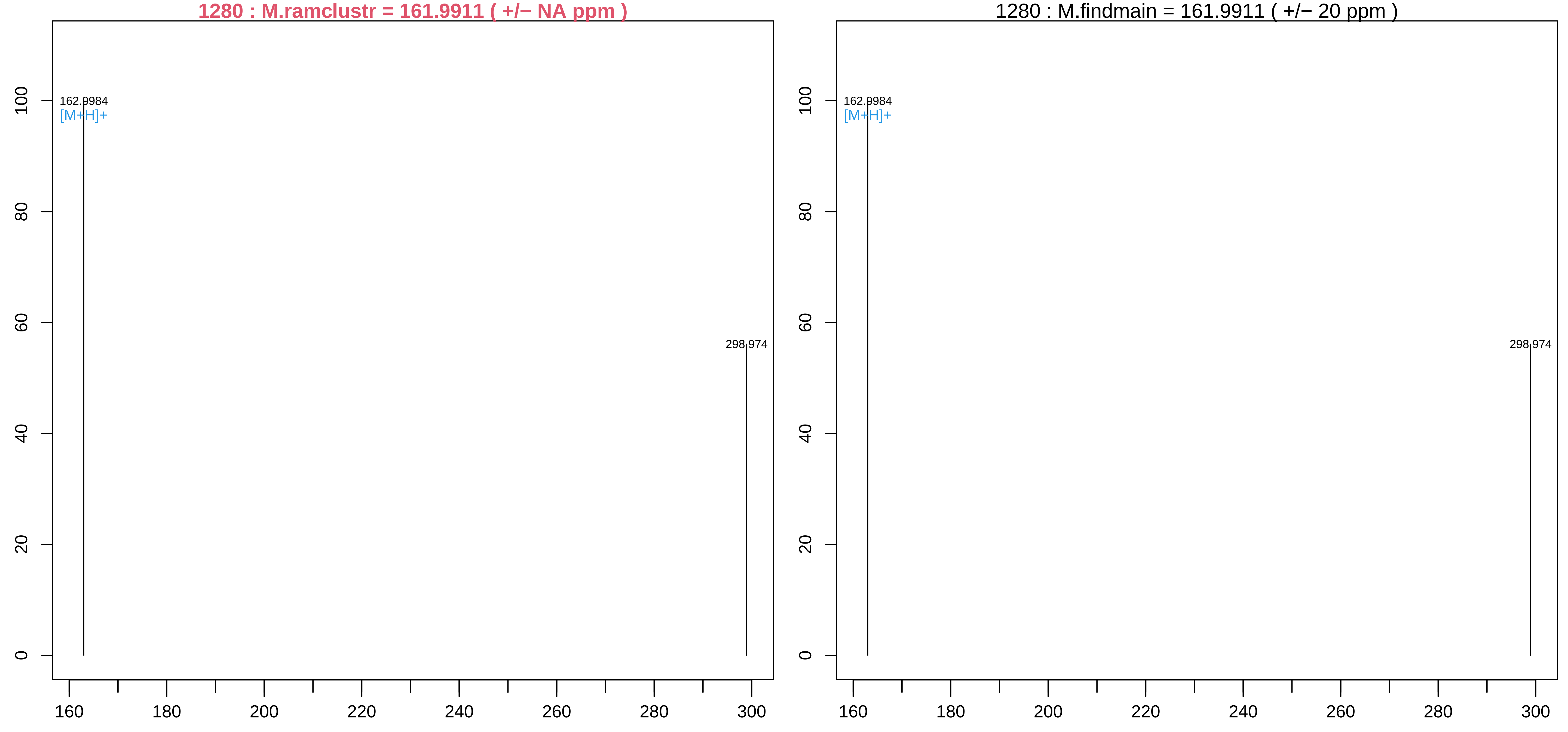
1280 : M.ramclustr = 161.9911 (+/− NA ppm)
162.9984
[M+H]+
298.974
0
20
40
60
80
100
160
180
200
220
240
260
280
300
1280 : M.findmain = 161.9911 (+/− 20 ppm)
162.9984
[M+H]+
298.974
0
20
40
60
80
100
160
180
200
220
240
260
280
300

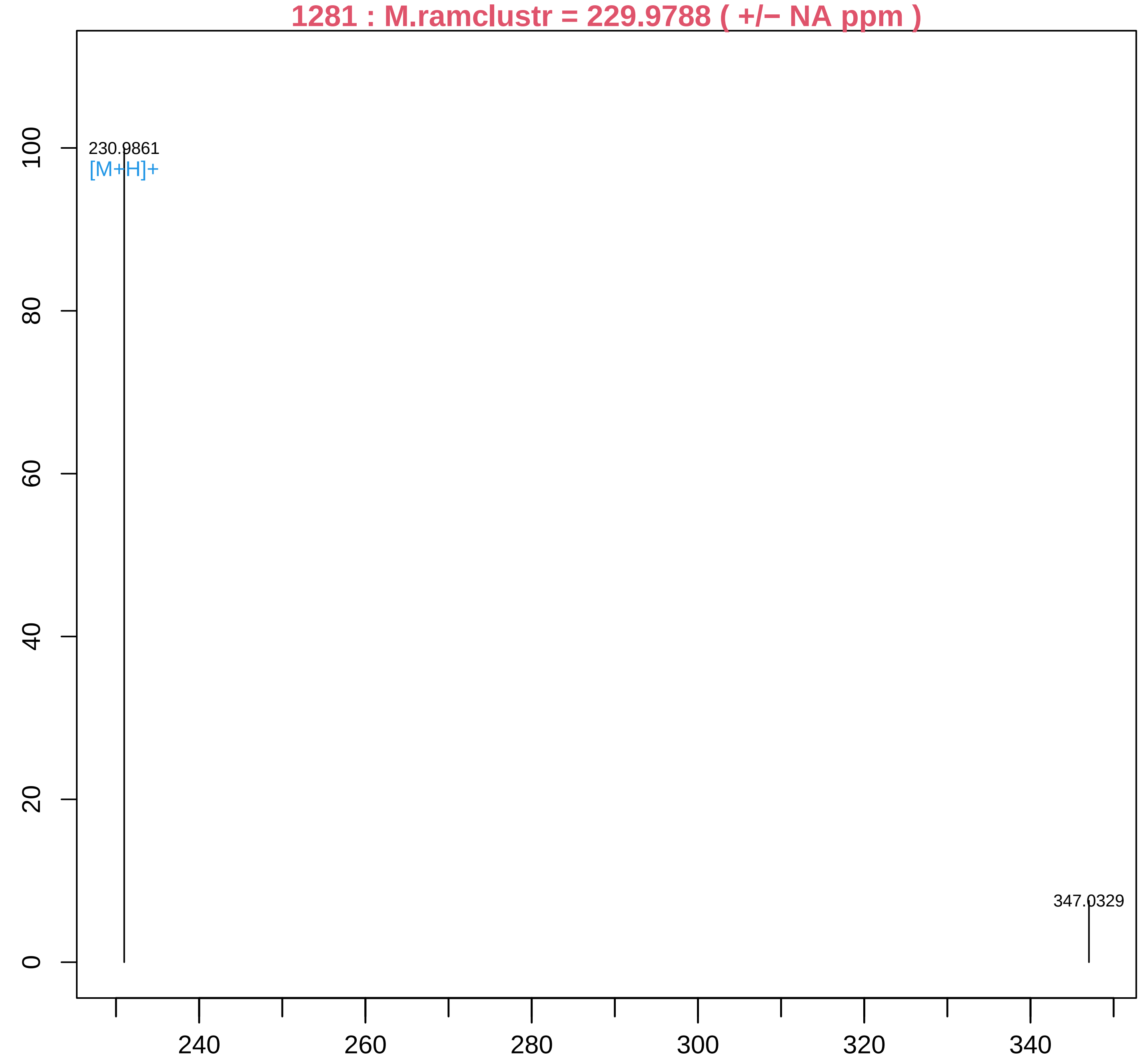
1281 : M.ramclustr = 229.9788 (+/− NA ppm)
230.9861
[M+H]+
347.0329
0
20
40
60
80
100
240
260
280
300
320
340

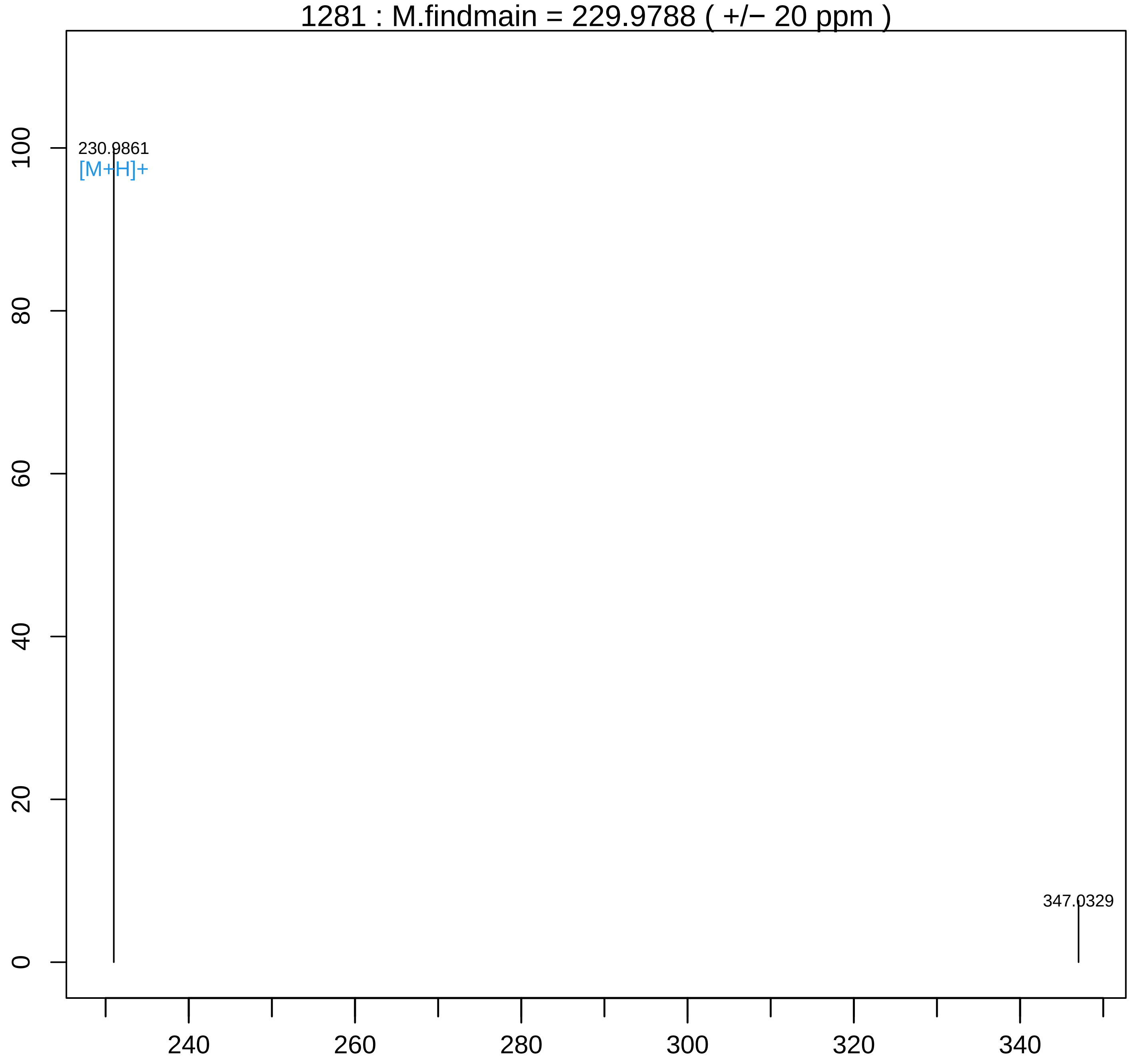
1281 : M.findmain = 229.9788 (+/− 20 ppm)
230.9861
[M+H]+
347.0329
0
20
40
60
80
100
240
260
280
300
320
340

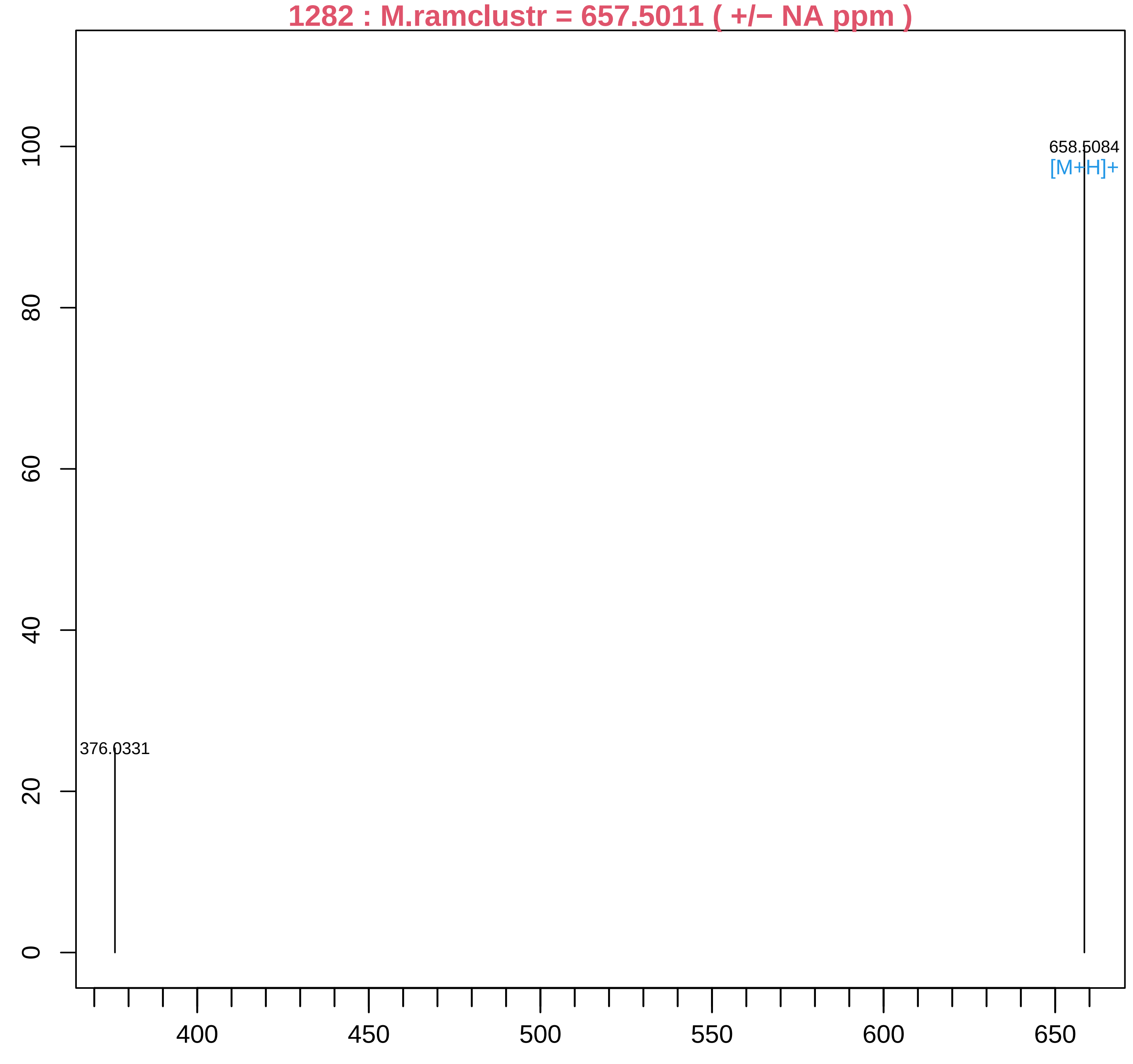

1282 : M.ramclustr = 657.5011 (+/− NA ppm)
658.5084
[M+H]+
376.0331

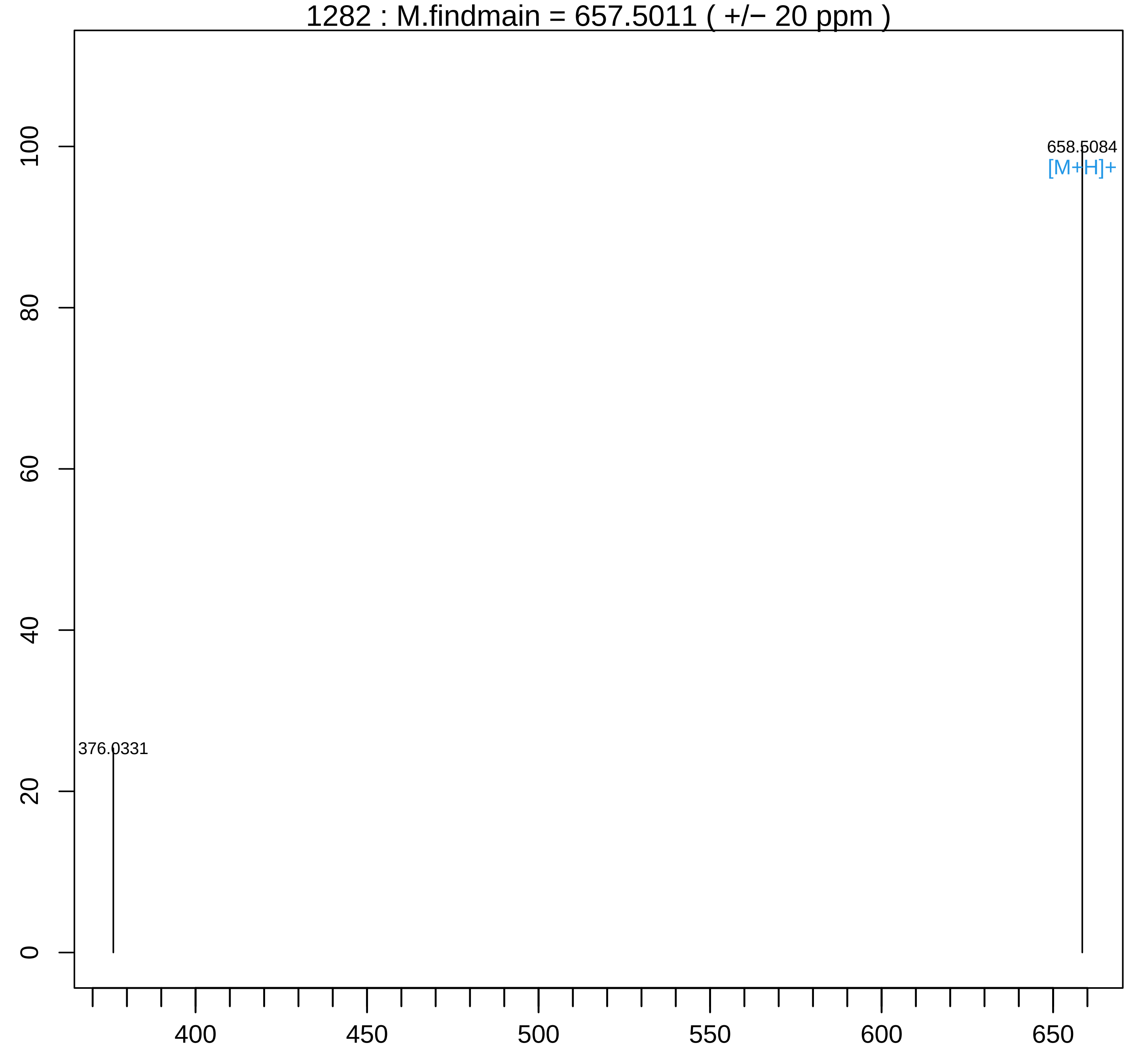

1282 : M.findmain = 657.5011 (+/− 20 ppm)
658.5084
[M+H]+
376.0331

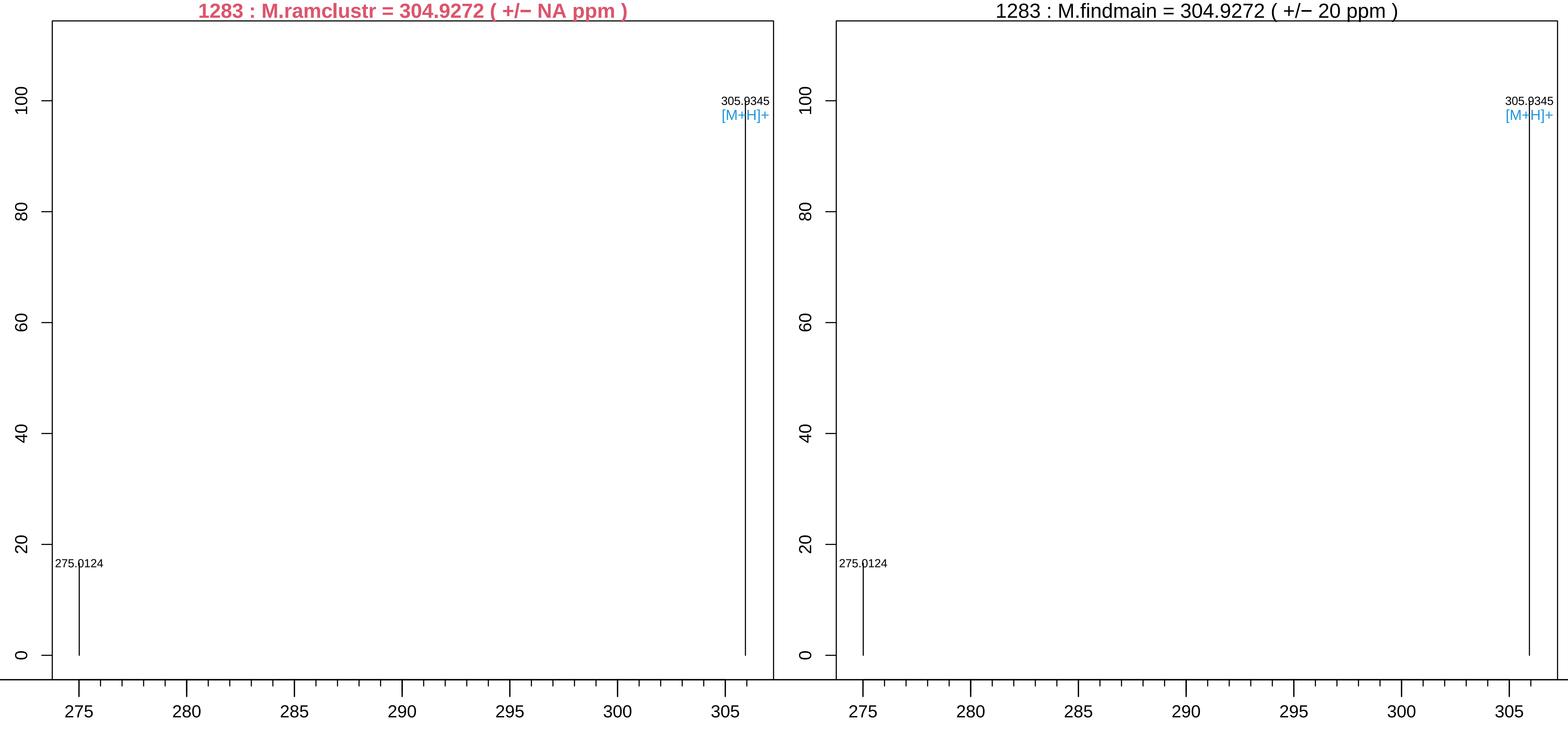

1283 : M.ramclustr = 304.9272 (+/− NA ppm)
305.9345
[M+H]+
275.0124
1283 : M.findmain = 304.9272 (+/− 20 ppm)
305.9345
[M+H]+
275.0124

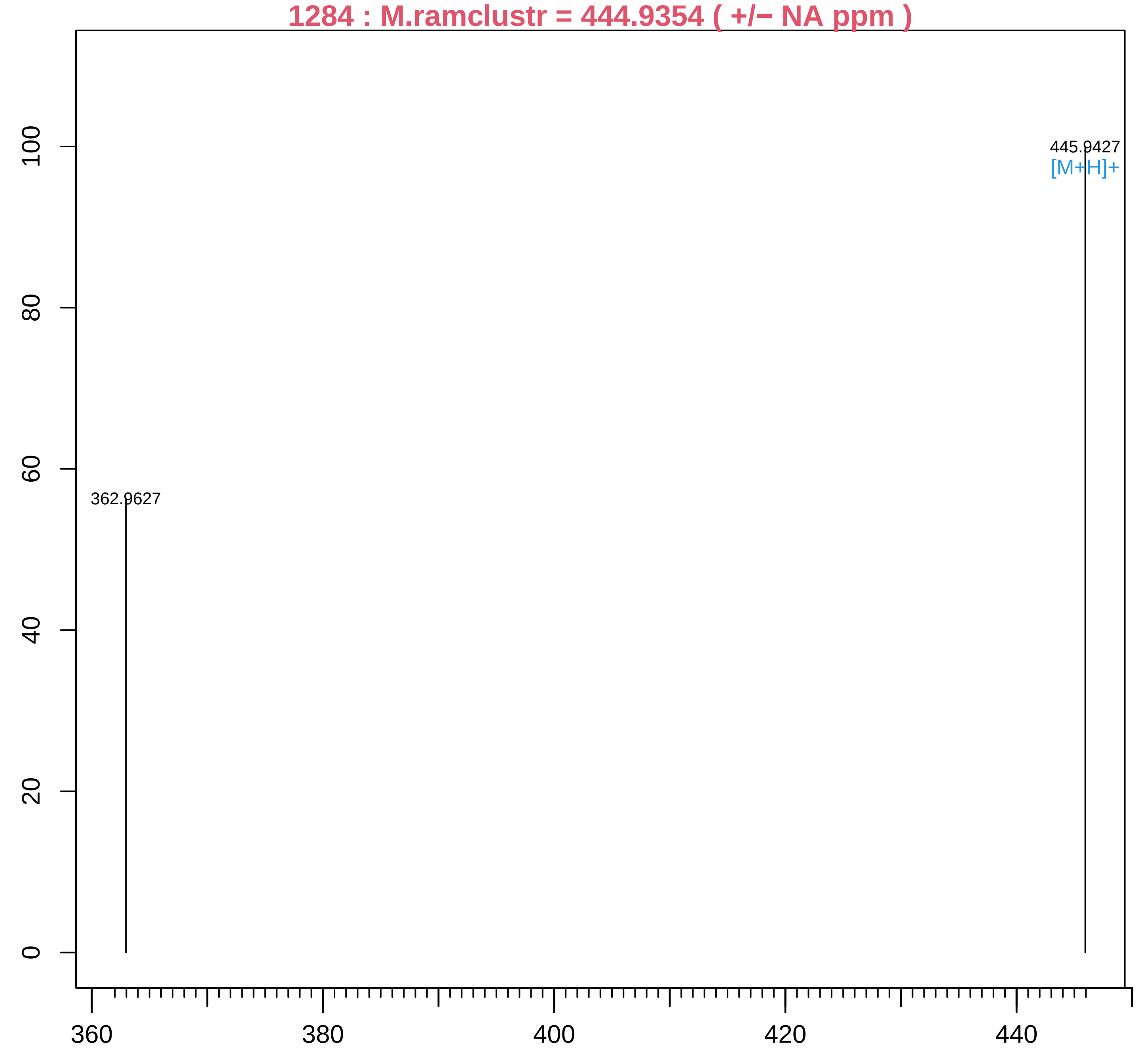

1284 : M.ramclustr = 444.9354 (+/− NA ppm)
445.9427
[M+H]+
362.9627
0
20
40
60
80
100
360
380
400
420
440

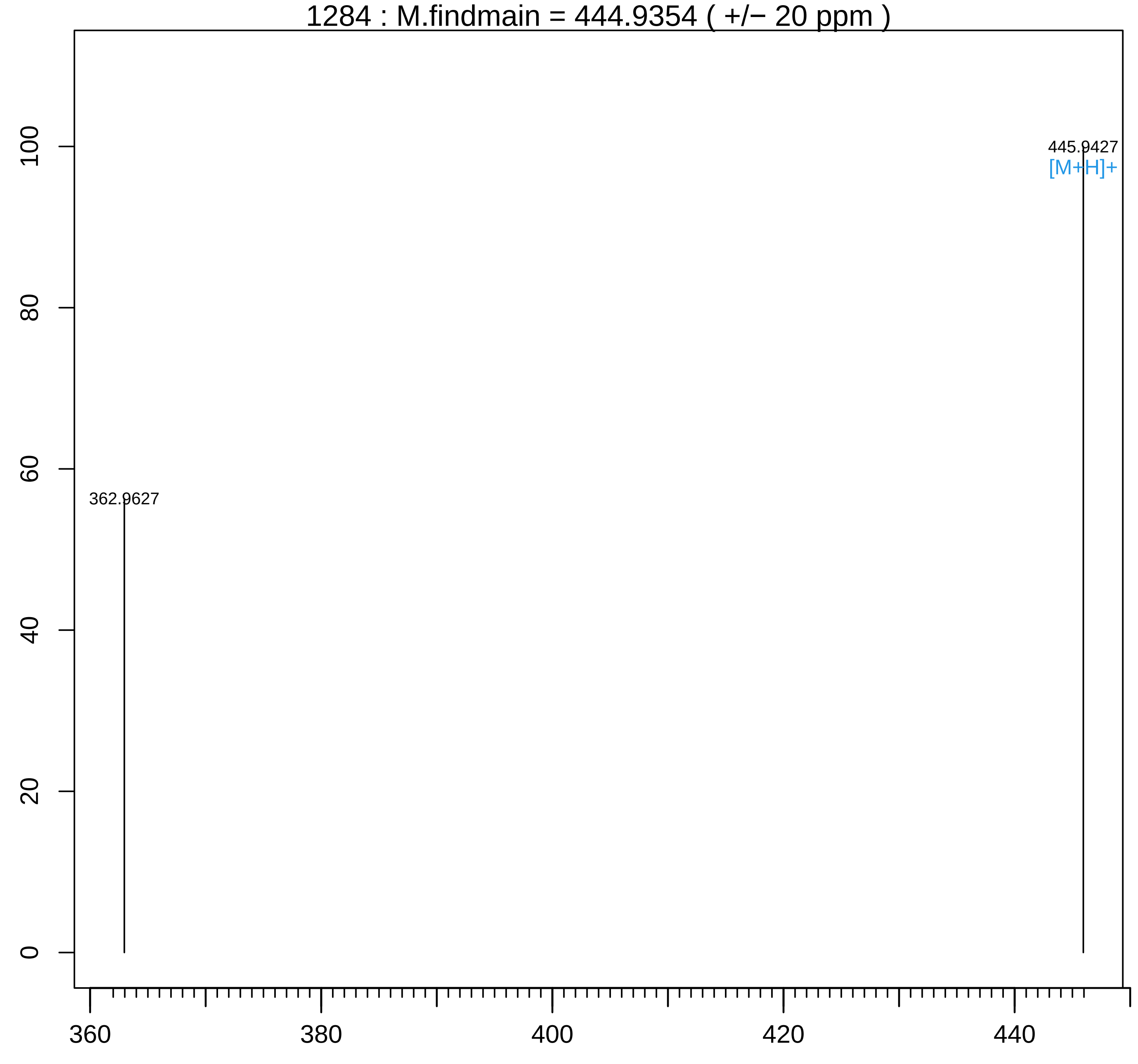

1284 : M.findmain = 444.9354 (+/− 20 ppm)
445.9427
[M+H]+
362.9627
0
20
40
60
80
100
360
380
400
420
440

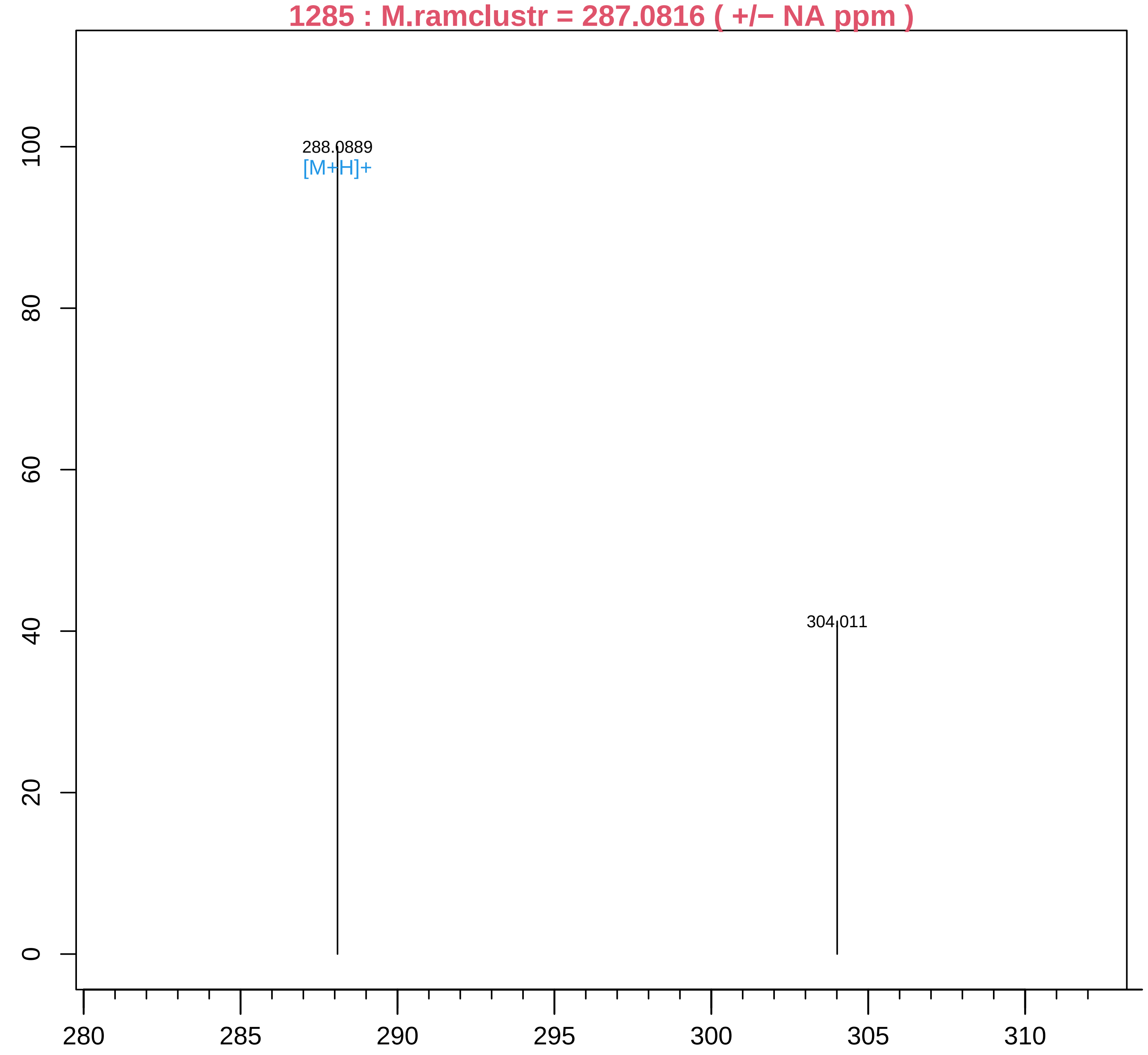
1285 : M.ramclustr = 287.0816 (+/− NA ppm)
288.0889
[M+H]+
304.011
0
20
40
60
80
100
280
285
290
295
300
305
310

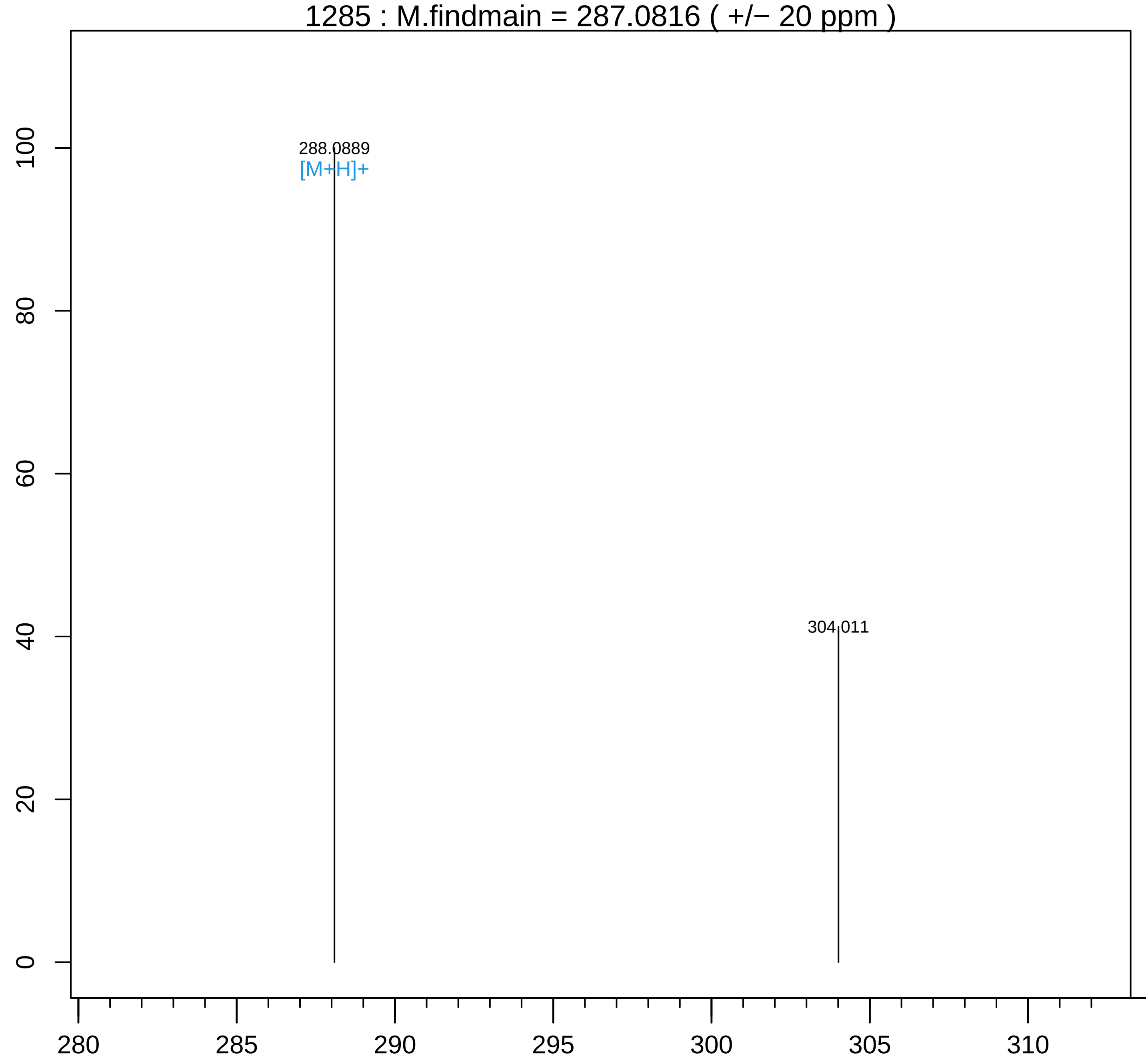
1285 : M.findmain = 287.0816 (+/− 20 ppm)
288.0889
[M+H]+
304.011
0
20
40
60
80
100
280
285
290
295
300
305
310

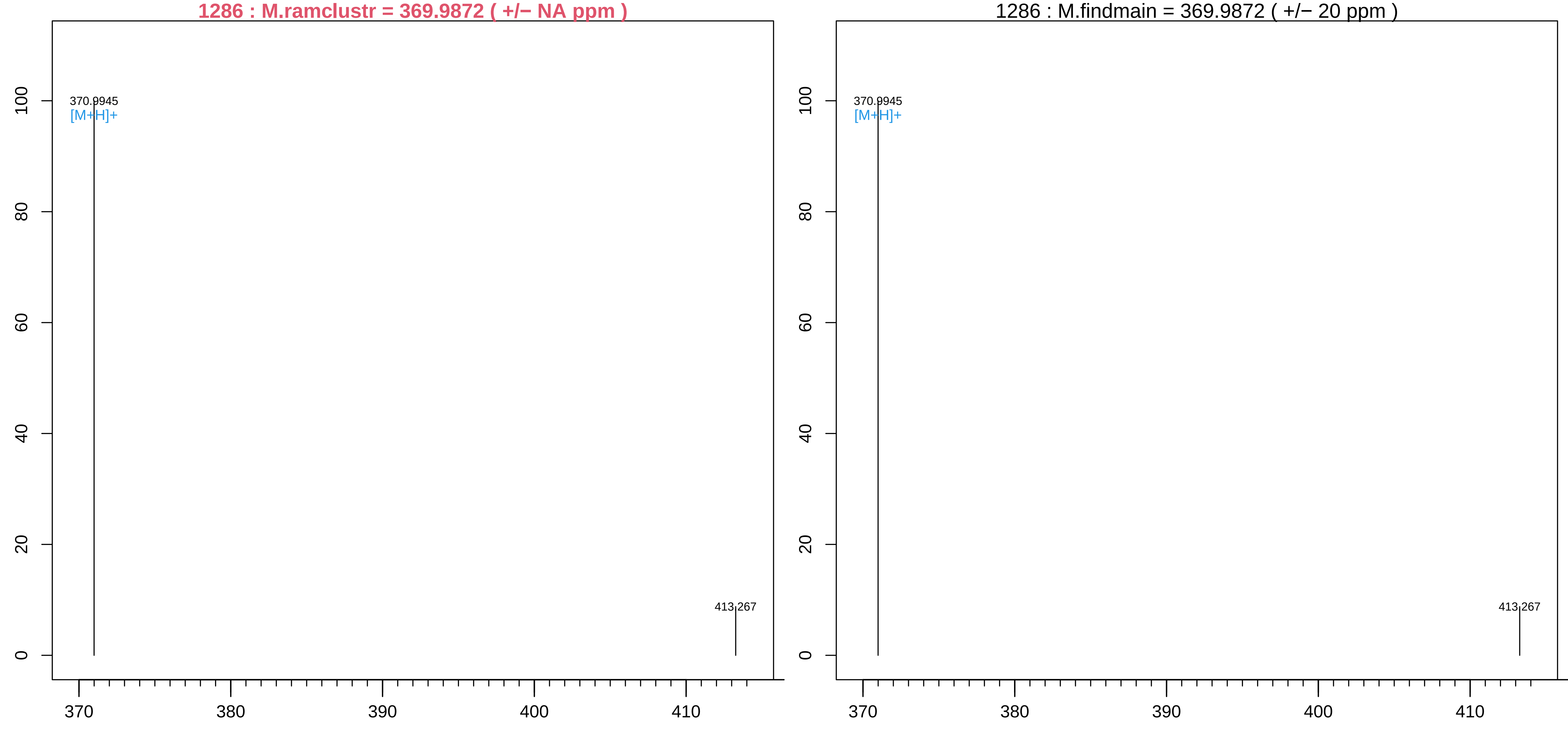
1286 : M.ramclustr = 369.9872 (+/− NA ppm)
370.9945
[M+H]+
413.267
1286 : M.findmain = 369.9872 (+/− 20 ppm)
370.9945
[M+H]+
413.267
0
20
40
60
80
100
370
380
390
400
410

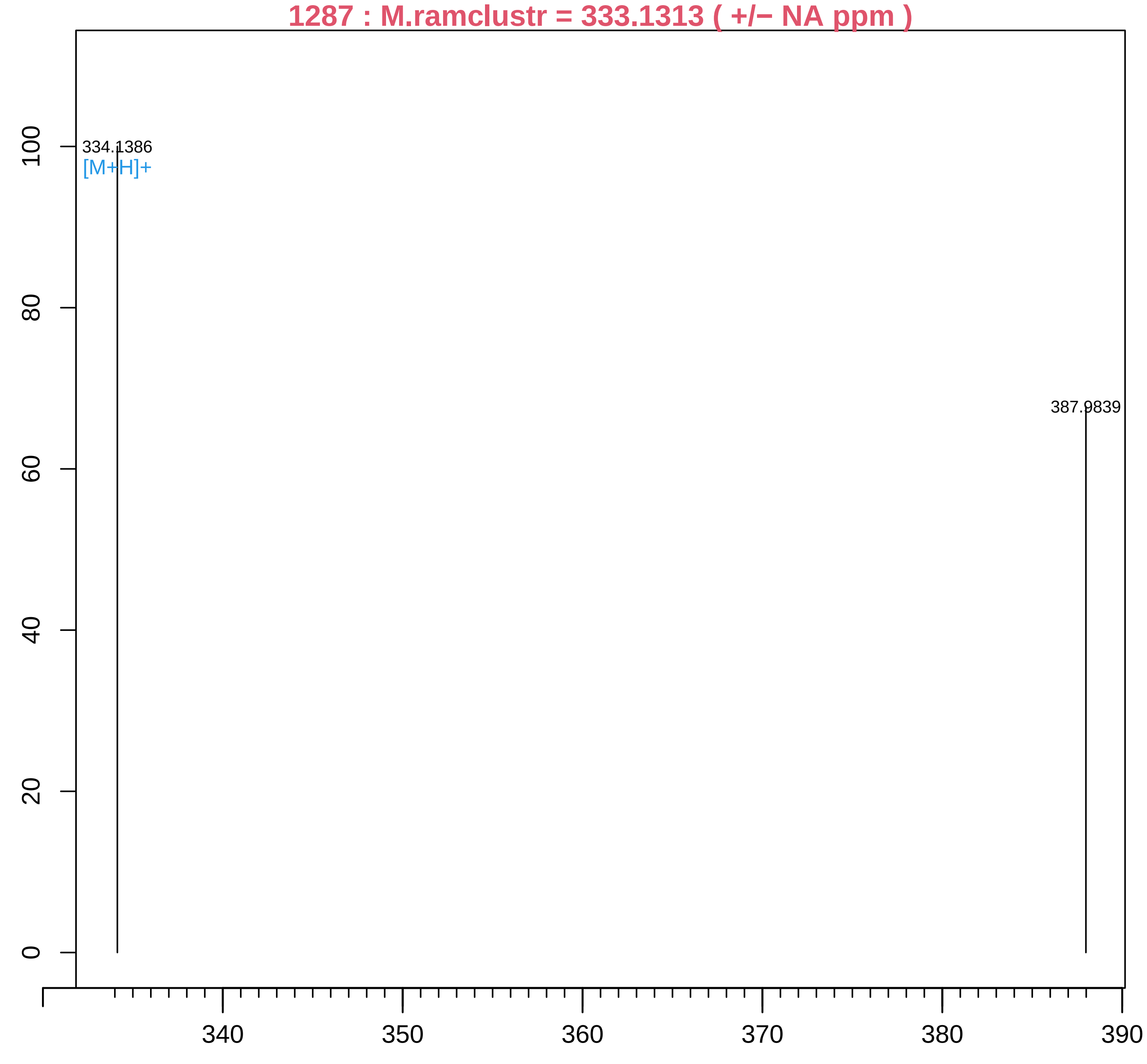
1287 : M.ramclustr = 333.1313 (+/− NA ppm)
334.1386
[M+H]+
387.9839
0
20
40
60
80
100
340
350
360
370
380
390

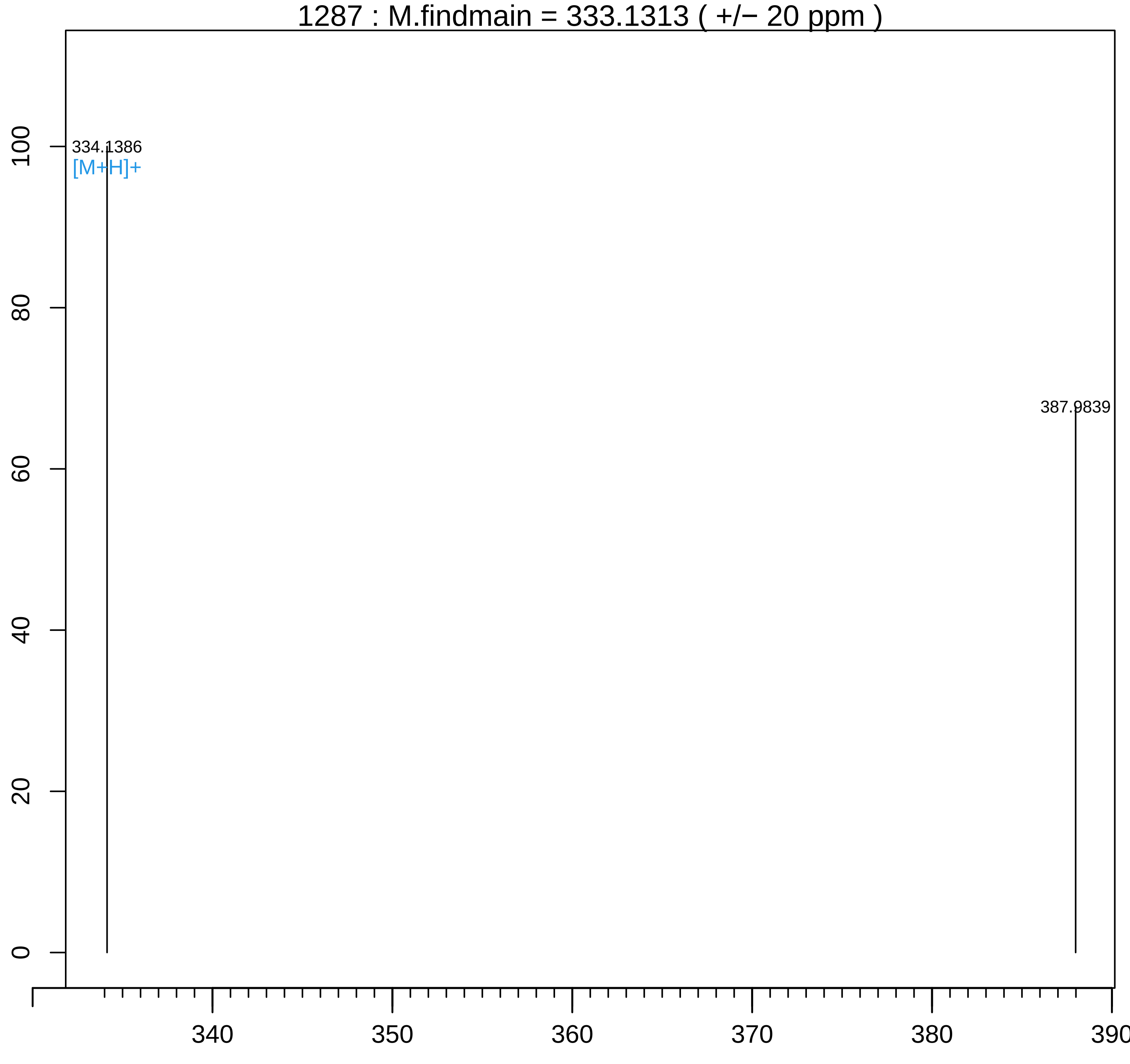
1287 : M.findmain = 333.1313 (+/− 20 ppm)
334.1386
[M+H]+
387.9839
0
20
40
60
80
100
340
350
360
370
380
390

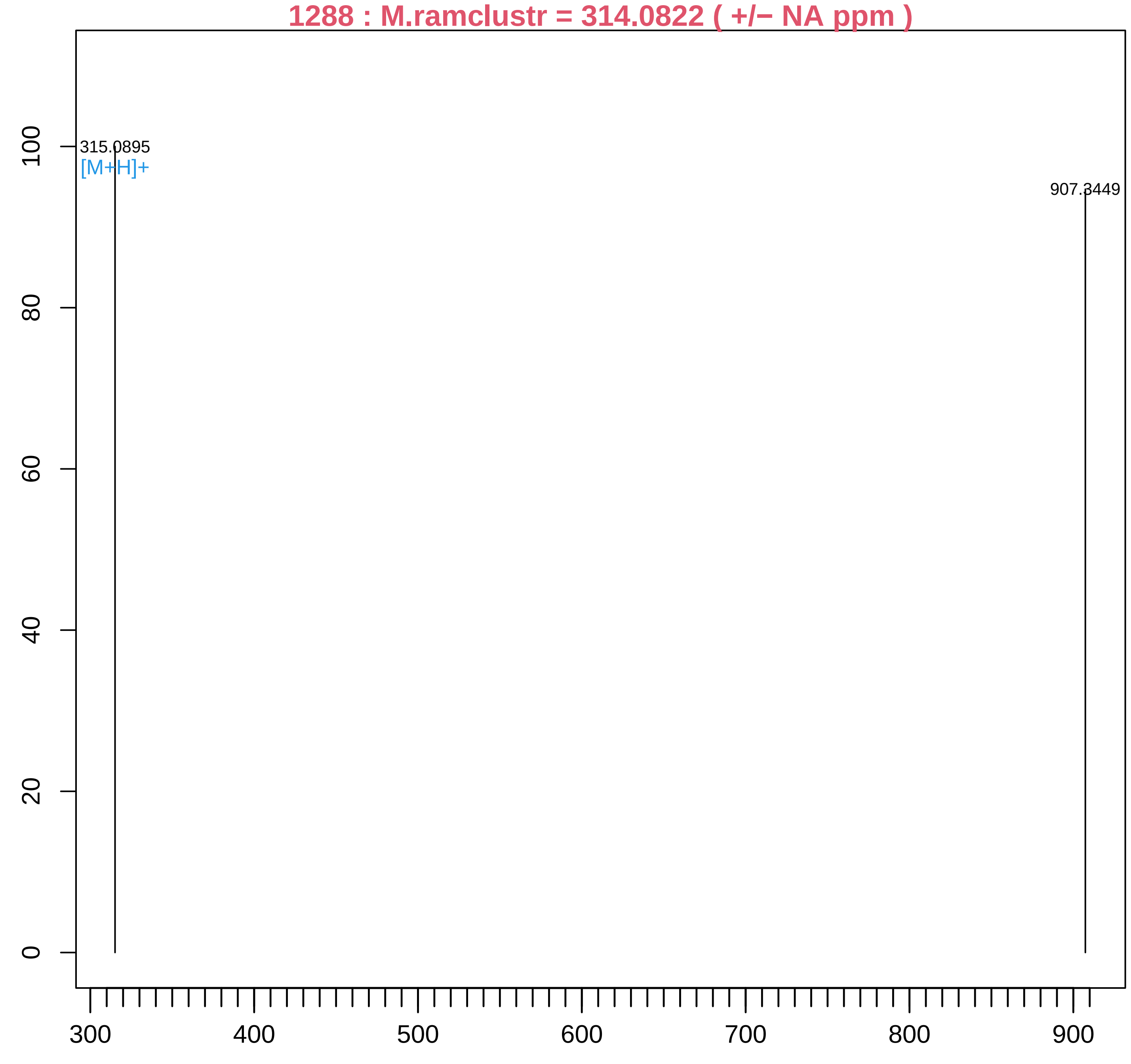
1288 : M.ramclustr = 314.0822 (+/− NA ppm)
315.0895
[M+H]+
907.3449
0
20
40
60
80
100
300
400
500
600
700
800
900

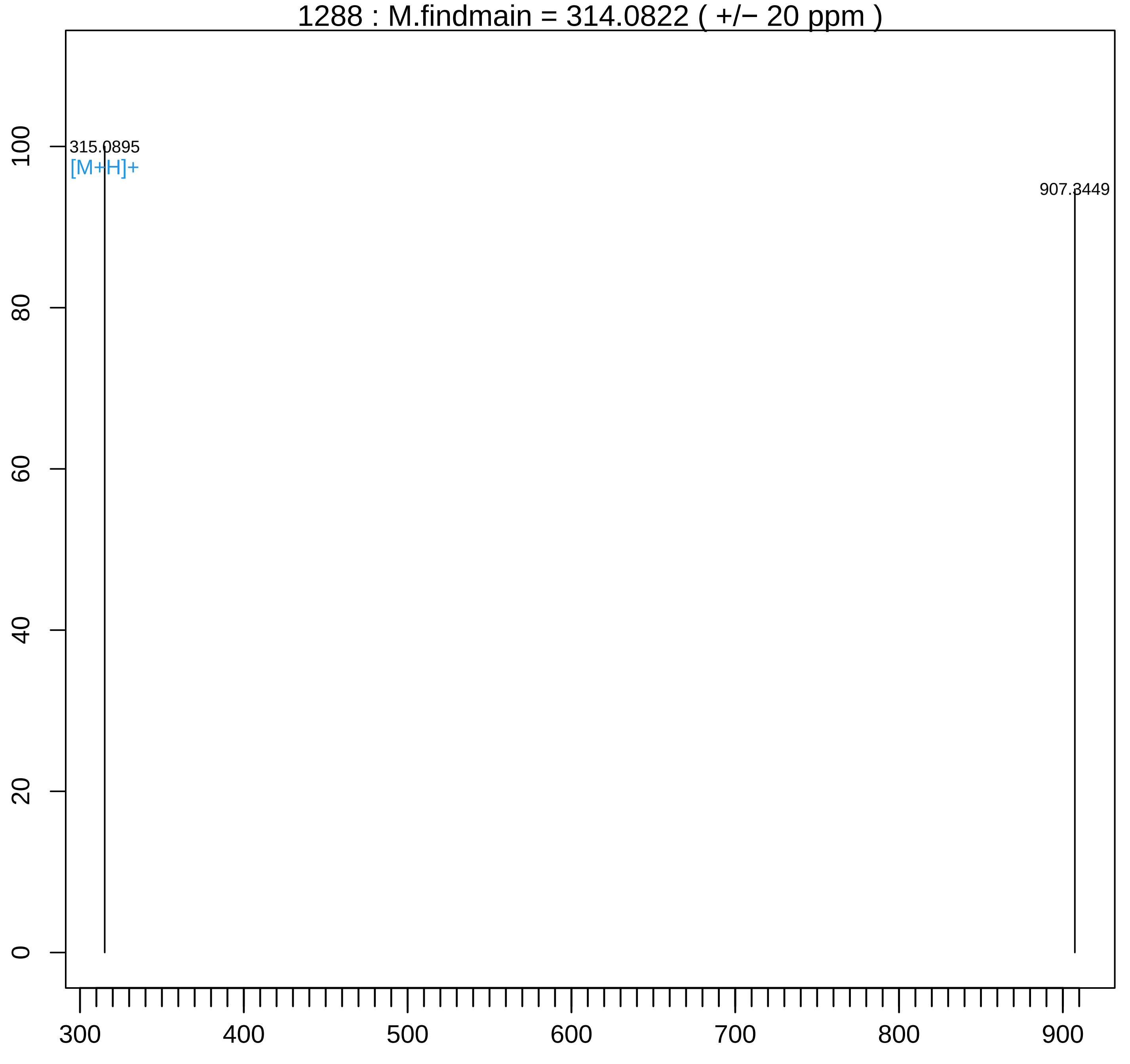
1288 : M.findmain = 314.0822 (+/− 20 ppm)
315.0895
[M+H]+
907.3449
0
20
40
60
80
100
300
400
500
600
700
800
900

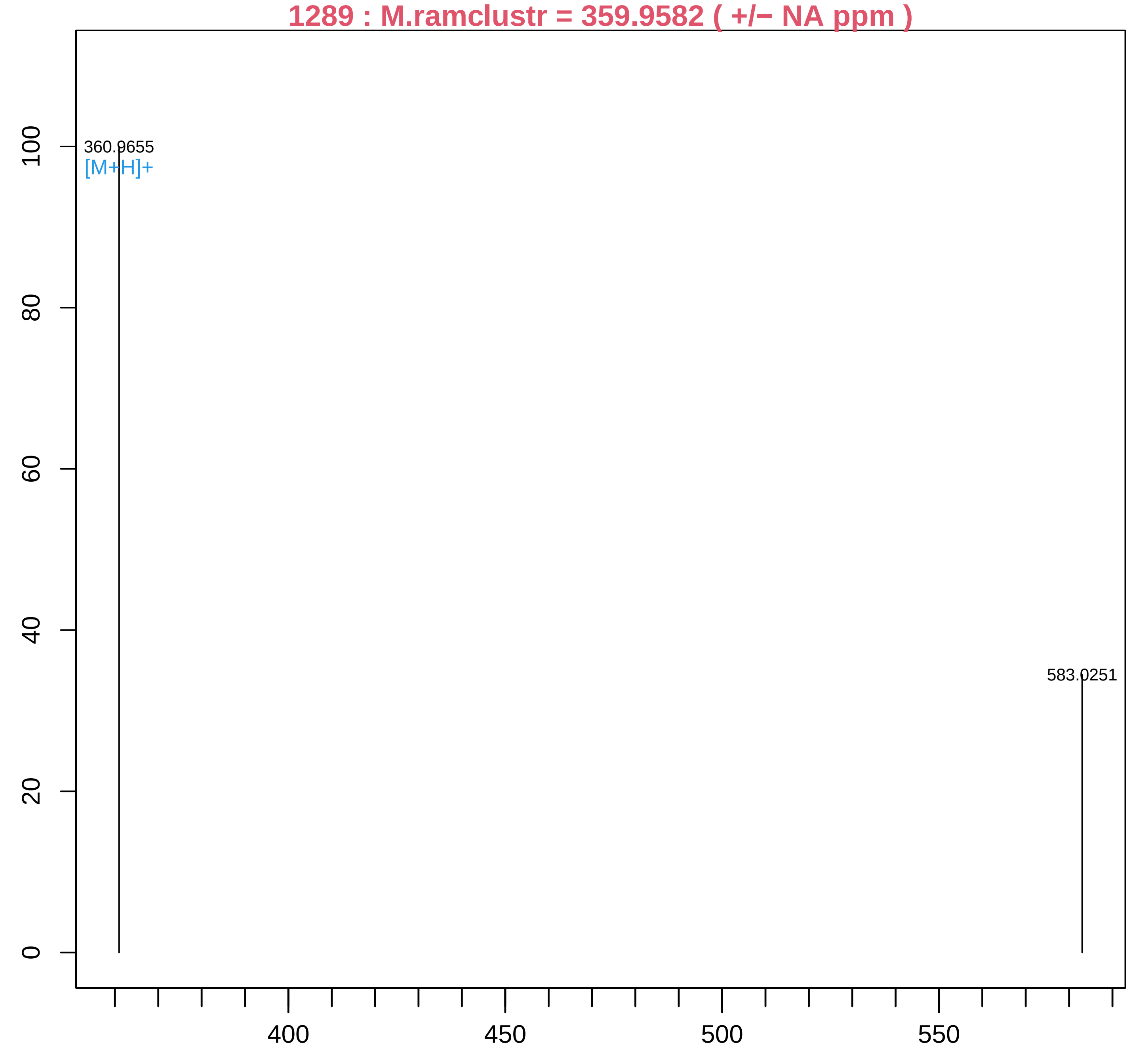
1289 : M.ramclustr = 359.9582 (+/− NA ppm)
360.9655
[M+H]+
583.0251
0
20
40
60
80
100
400
450
500
550

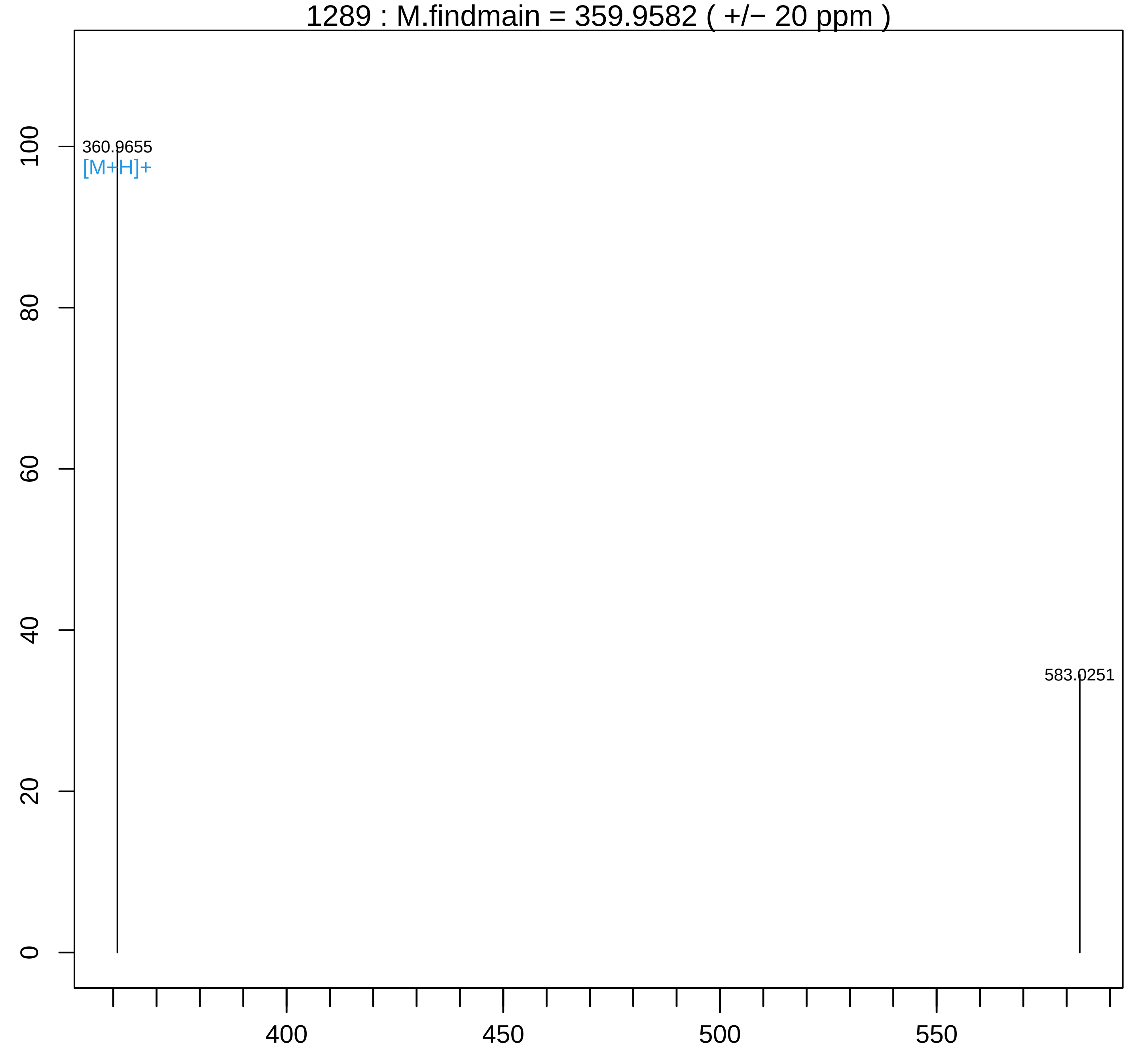
1289 : M.findmain = 359.9582 (+/− 20 ppm)
360.9655
[M+H]+
583.0251
0
20
40
60
80
100
400
450
500
550

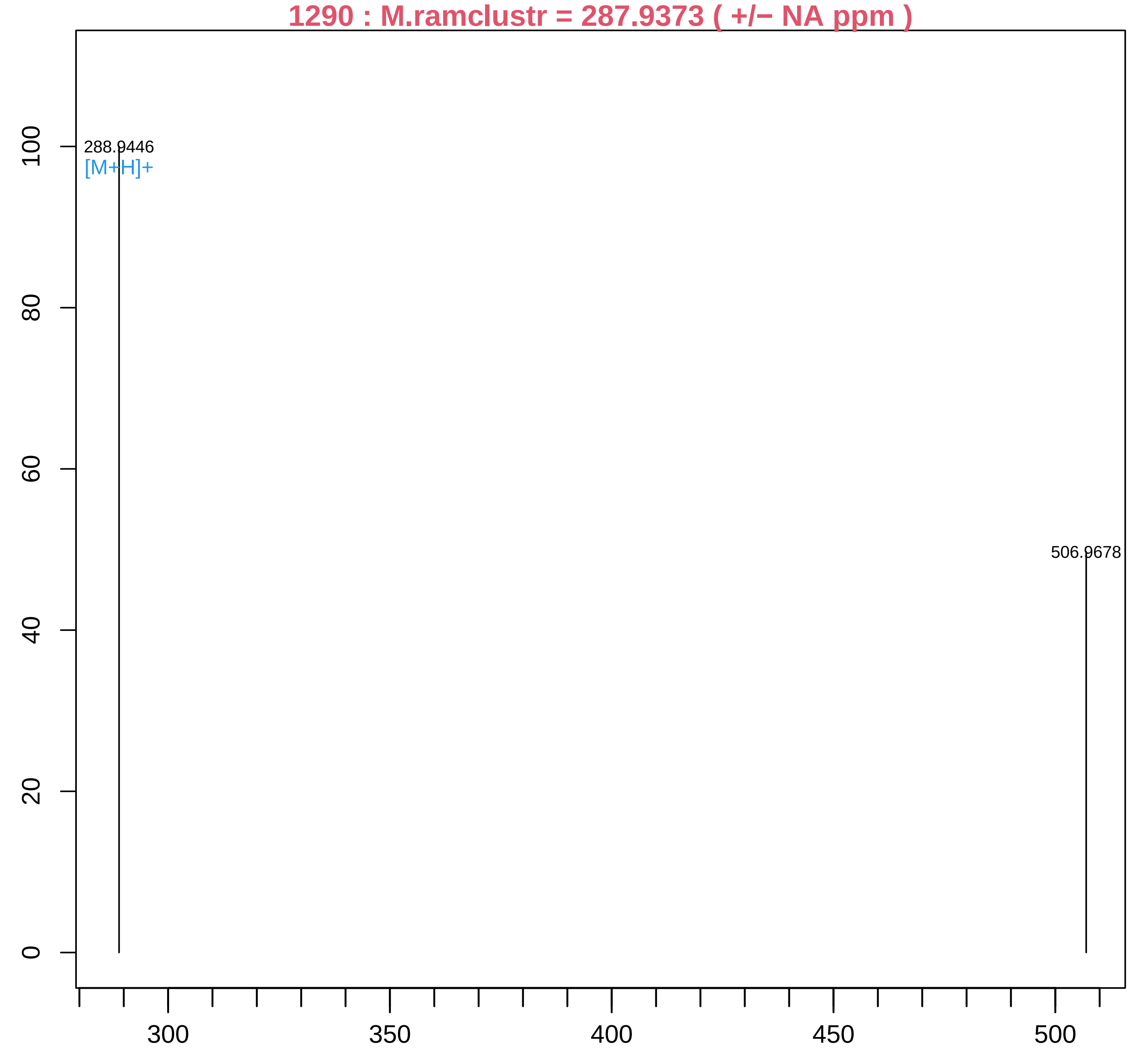
1290 : M.ramclustr = 287.9373 (+/− NA ppm)
288.9446
[M+H]+
506.9678
0
20
40
60
80
100
300
350
400
450
500

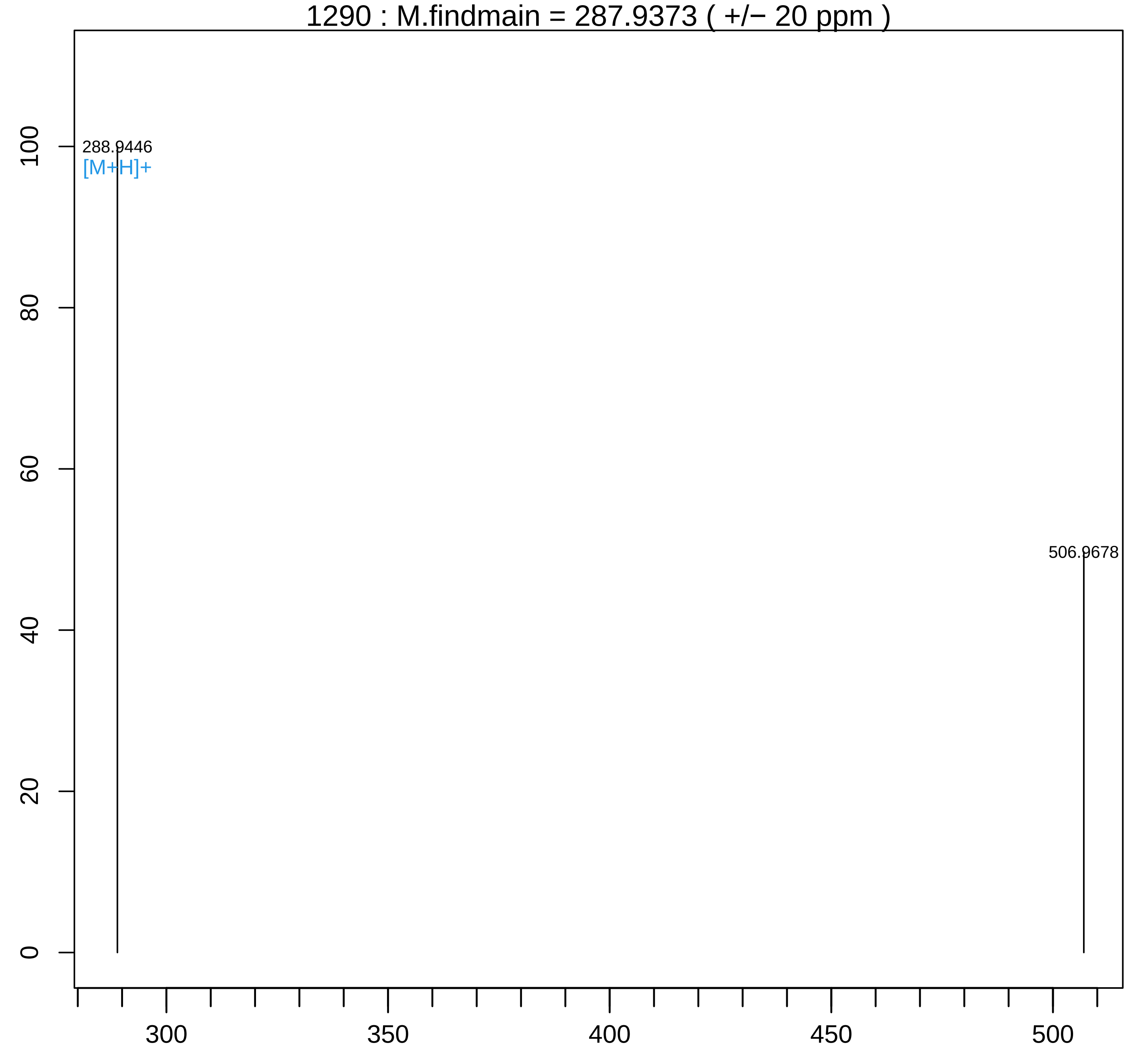
1290 : M.findmain = 287.9373 (+/− 20 ppm)
288.9446
[M+H]+
506.9678
0
20
40
60
80
100
300
350
400
450
500

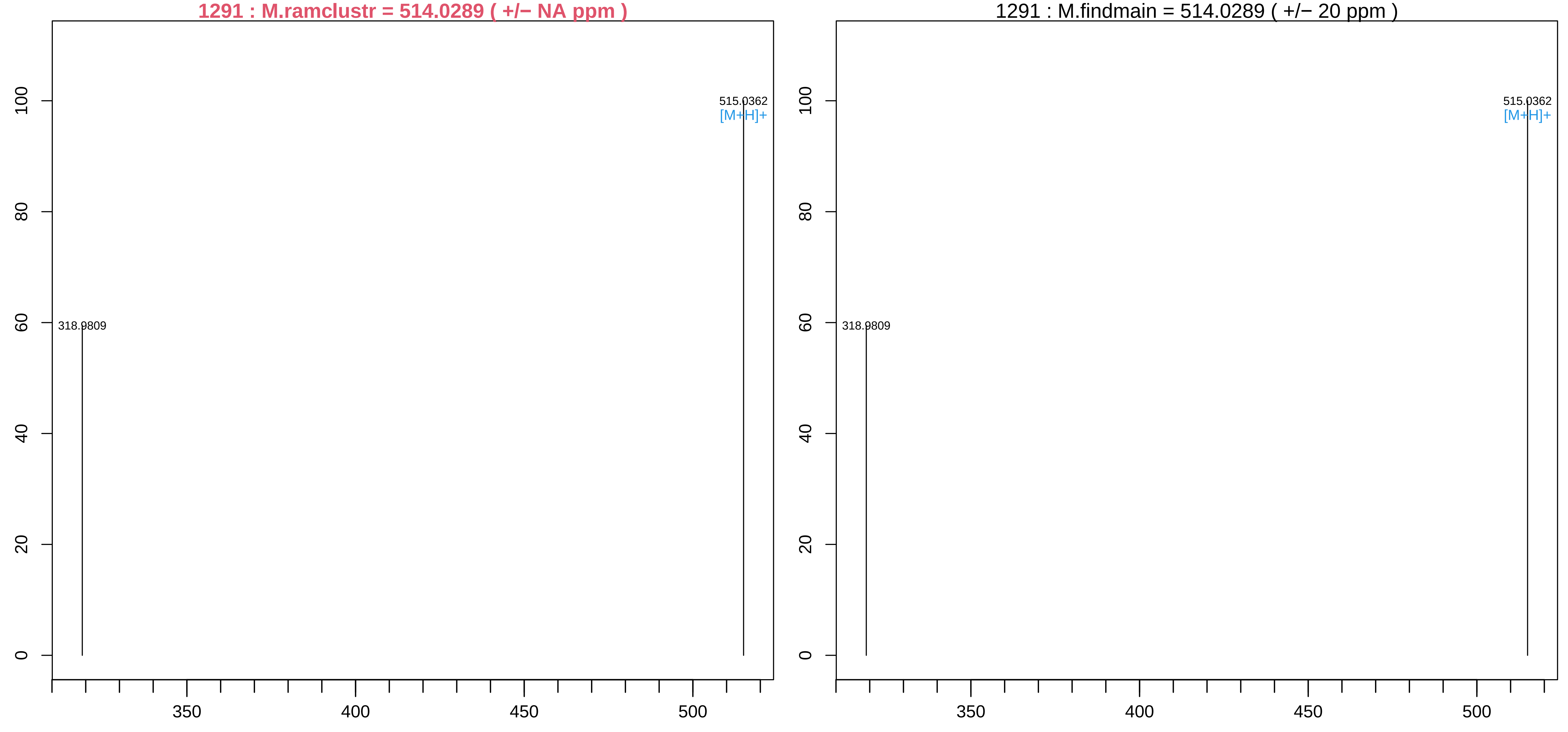
1291 : M.ramclustr = 514.0289 (+/− NA ppm)
515.0362
[M+H]+
318.9809
1291 : M.findmain = 514.0289 (+/− 20 ppm)
515.0362
[M+H]+
318.9809

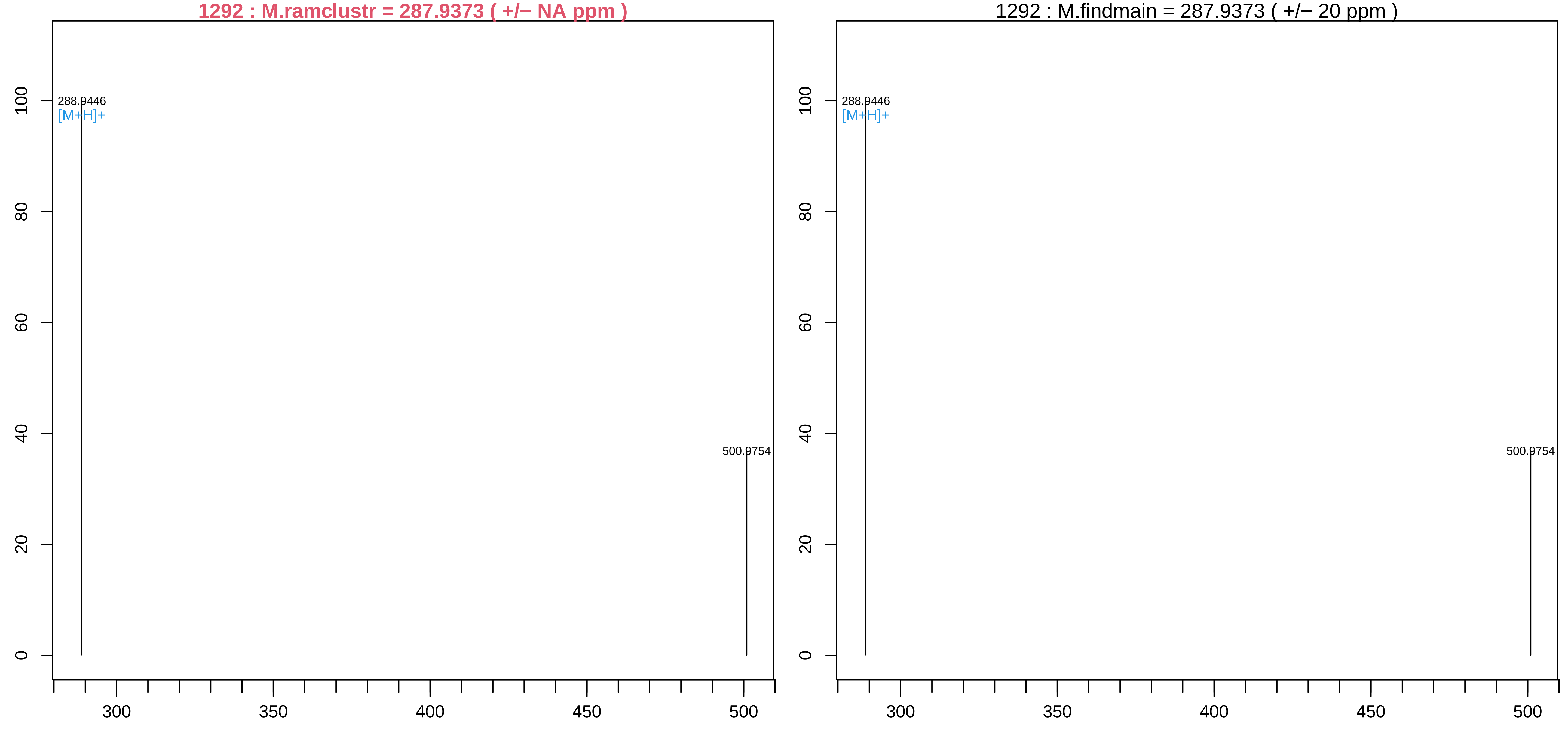
1292 : M.ramclustr = 287.9373 (+/− NA ppm)
288.9446
[M+H]+
500.9754
1292 : M.findmain = 287.9373 (+/− 20 ppm)
288.9446
[M+H]+
500.9754
0
20
40
60
80
100
300
350
400
450
500

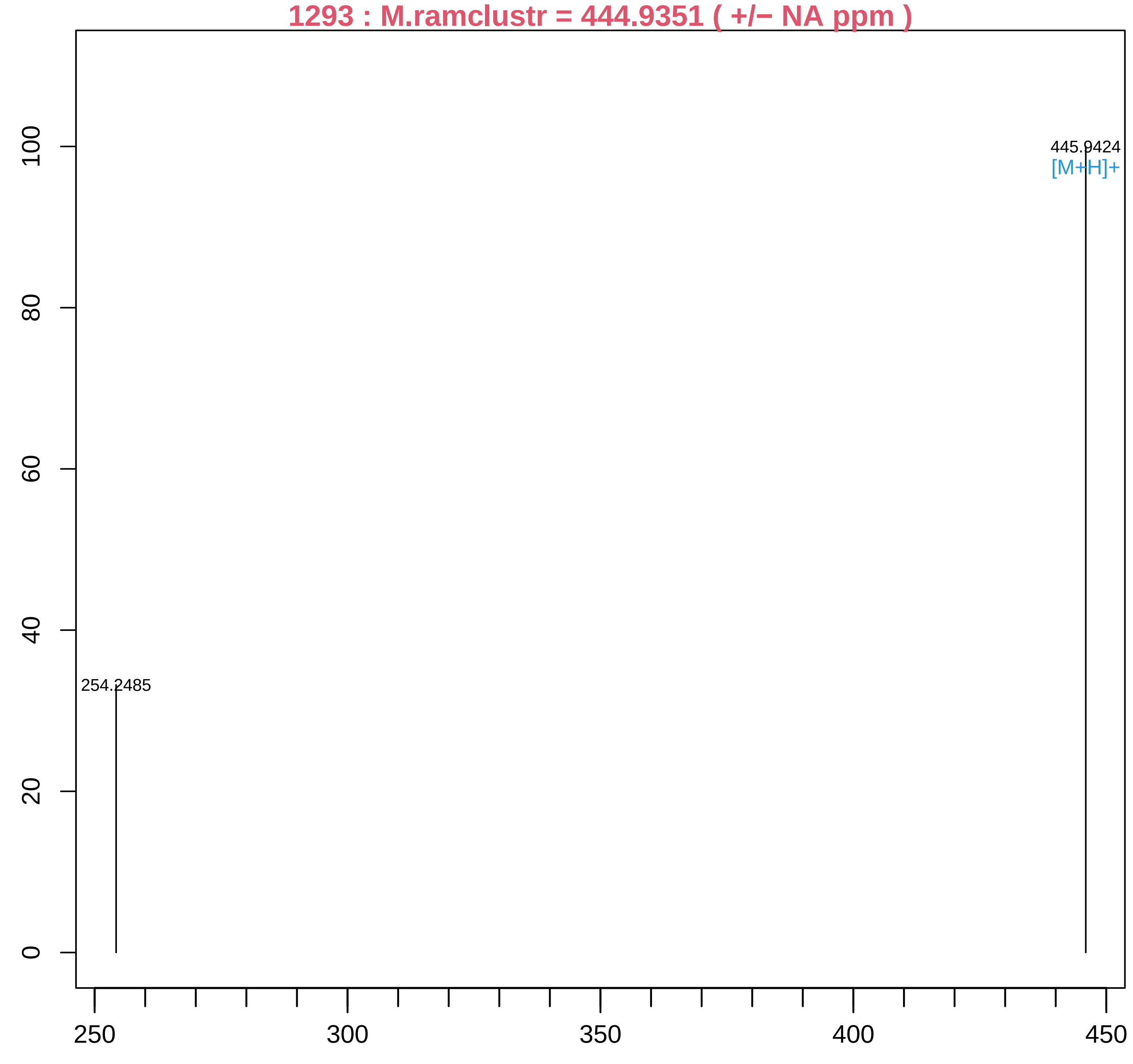

1293 : M.ramclustr = 444.9351 (+/− NA ppm)
445.9424
[M+H]+
254.2485

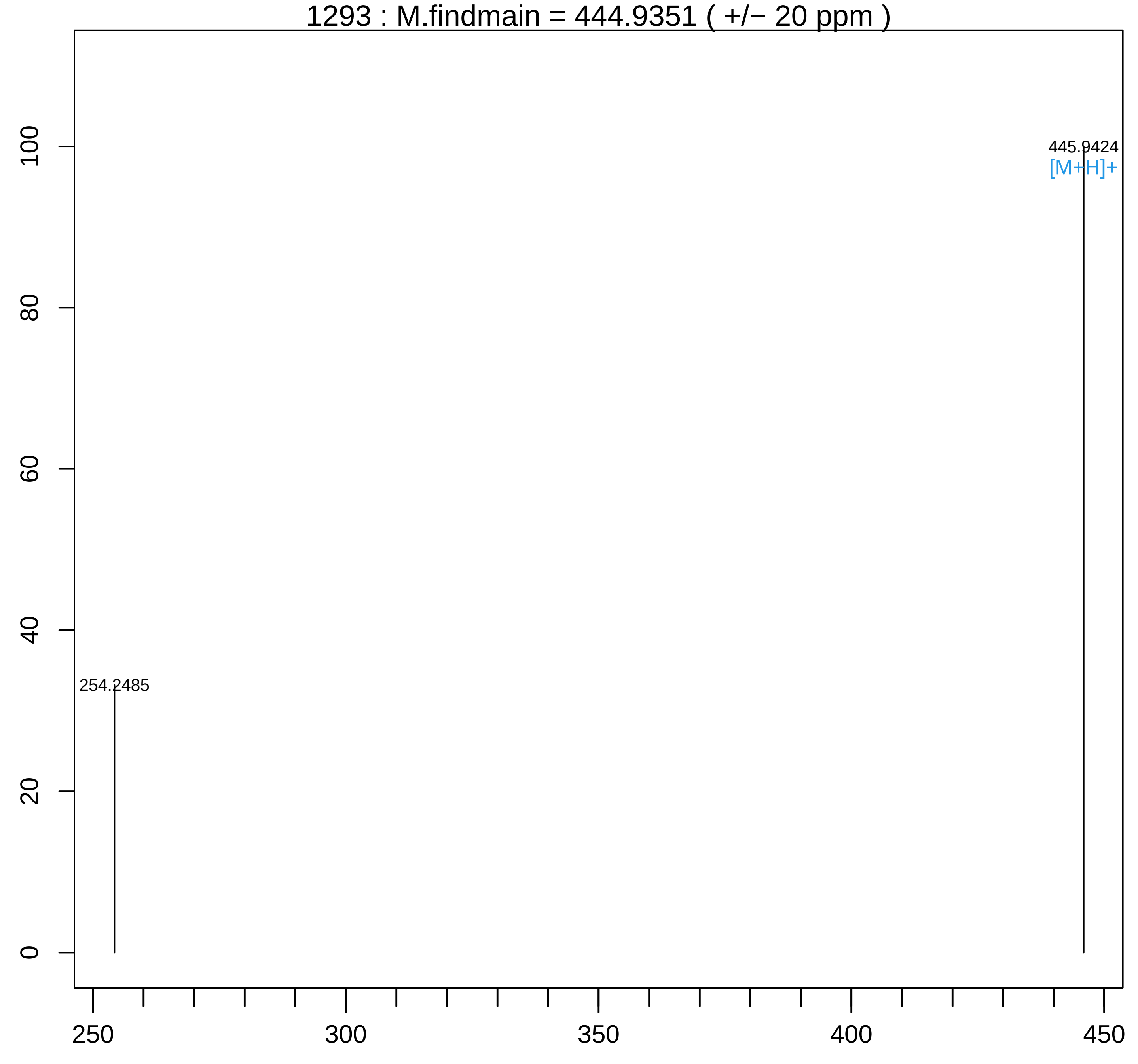

1293 : M.findmain = 444.9351 (+/− 20 ppm)
445.9424
[M+H]+
254.2485

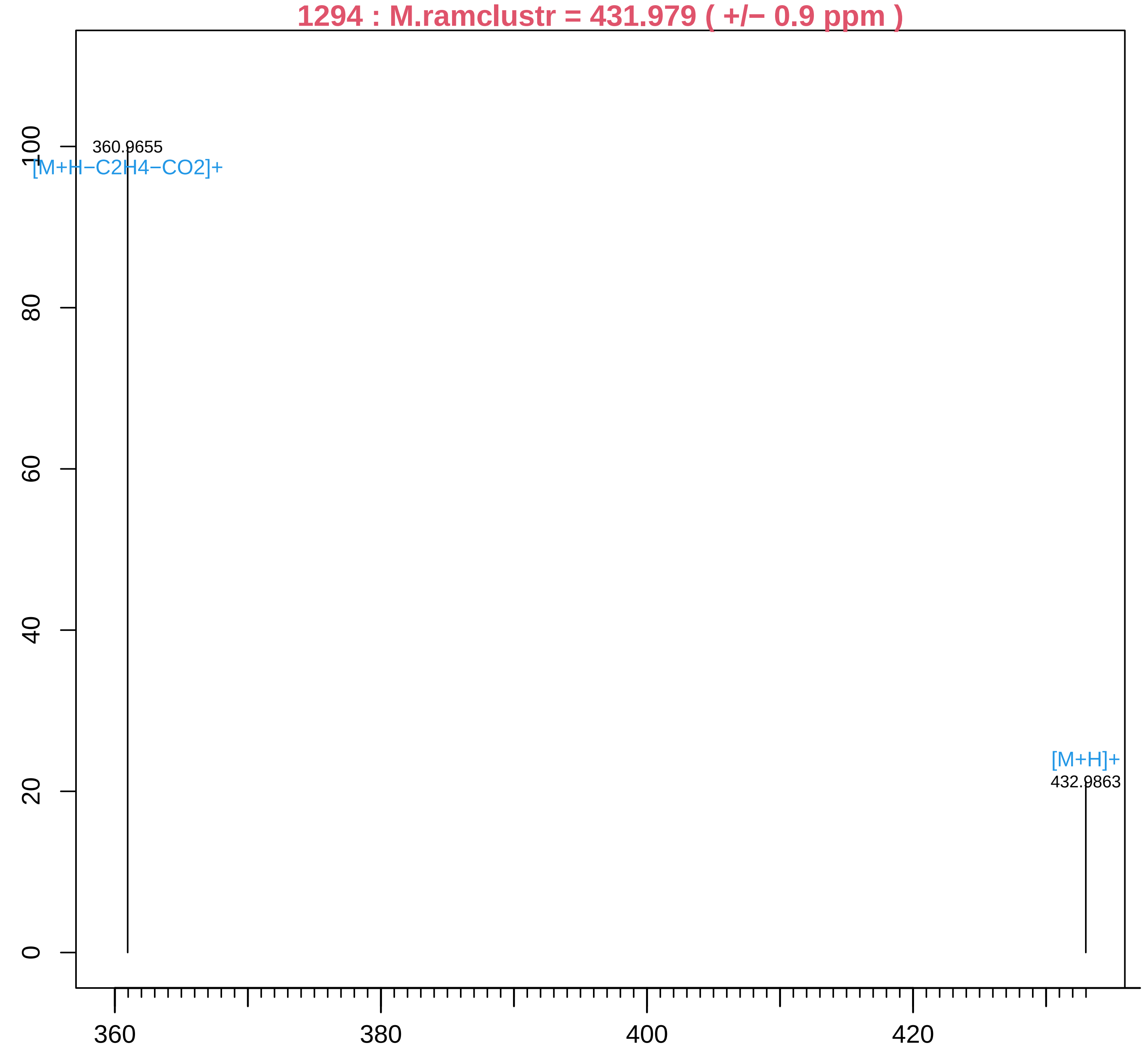

1294 : M.ramclustr = 431.979 (+/− 0.9 ppm)
360.9655
[M+H−C2H4−CO2]+
[M+H]+
432.9863
0
20
40
60
80
100
360
380
400
420

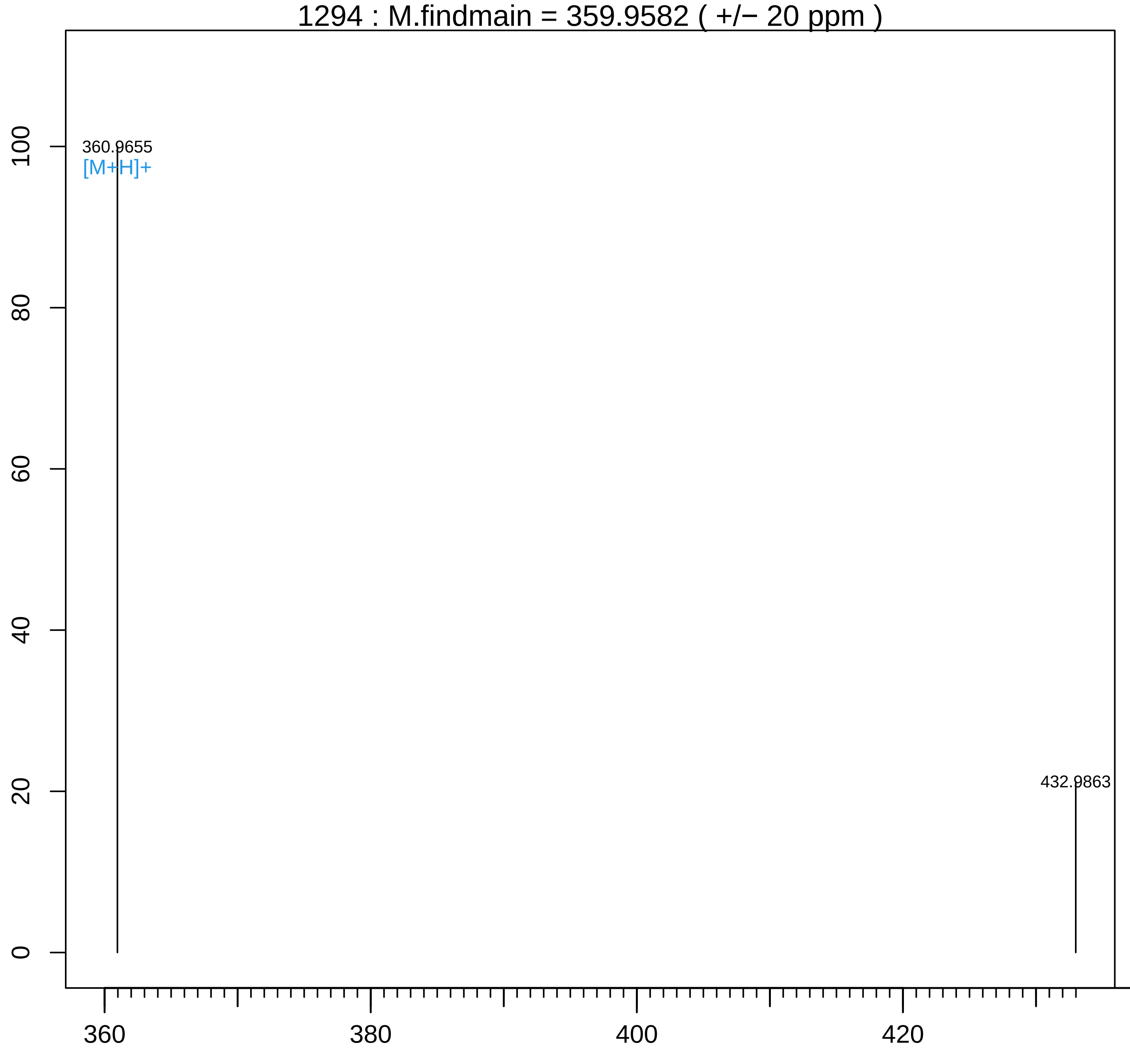

1294 : M.findmain = 359.9582 (+/− 20 ppm)
360.9655
[M+H]+
432.9863
0
20
40
60
80
100
360
380
400
420

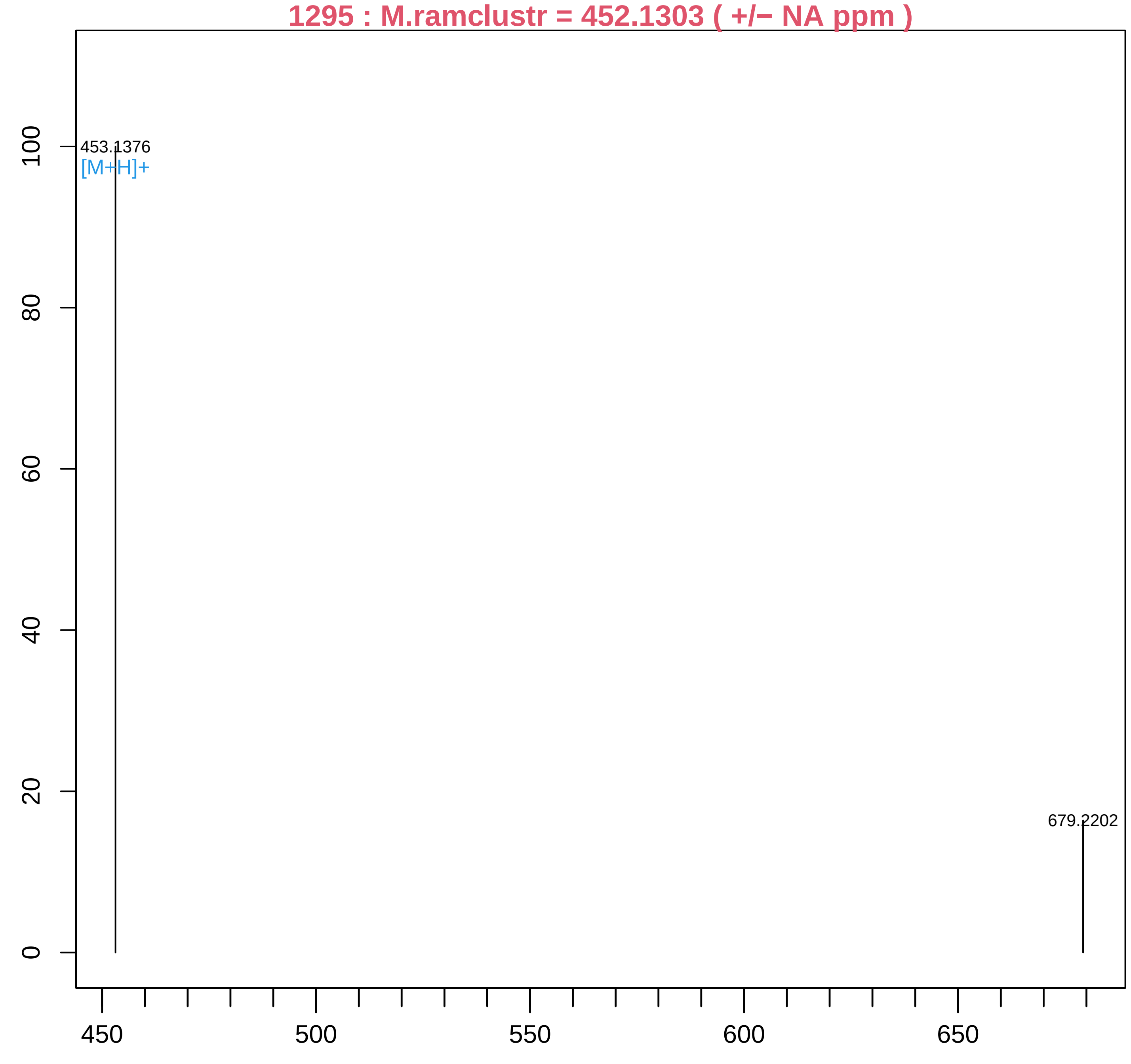
1295 : M.ramclustr = 452.1303 (+/− NA ppm)
453.1376
[M+H]+
679.2202
0
20
40
60
80
100
450
500
550
600
650

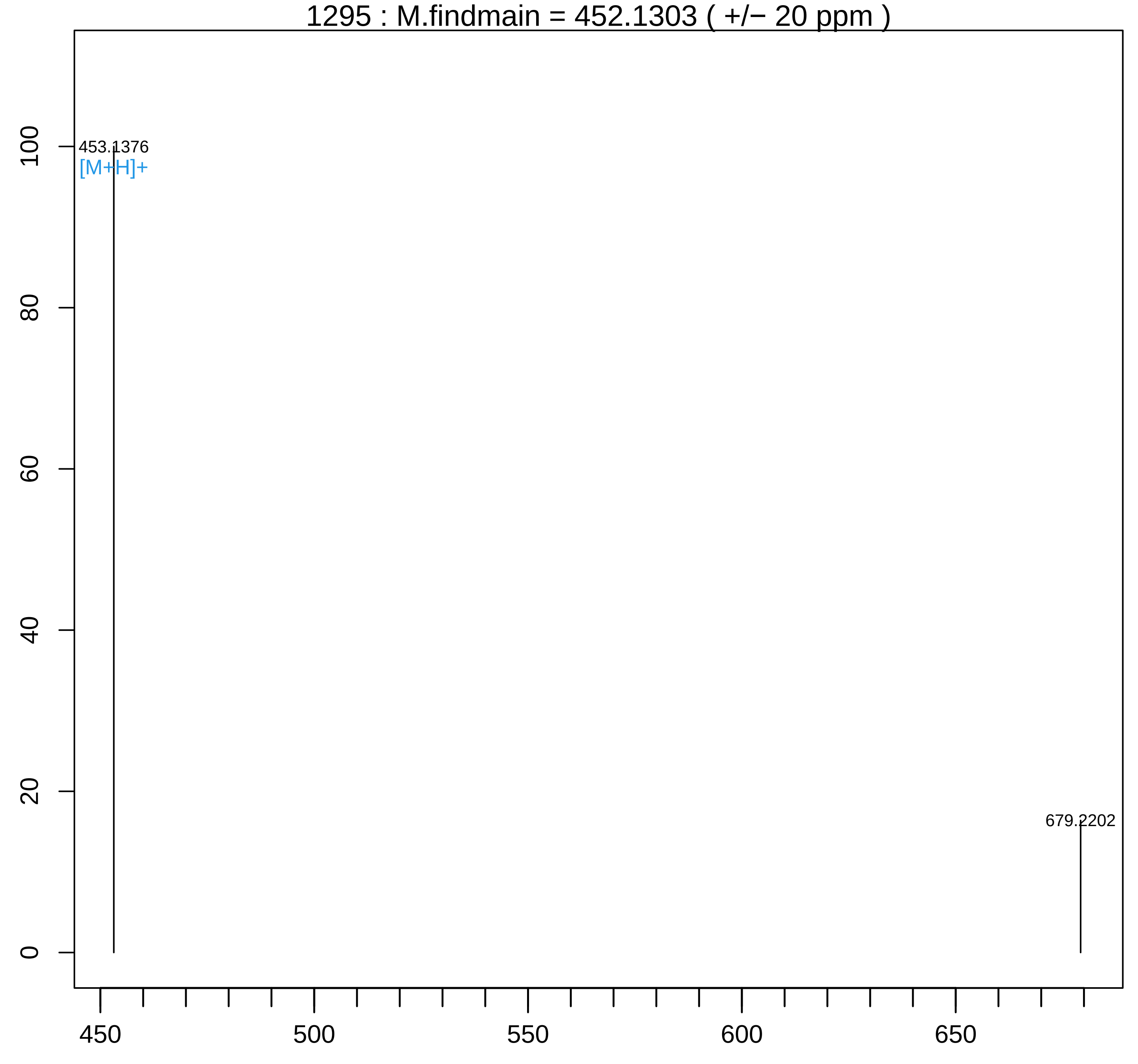
1295 : M.findmain = 452.1303 (+/− 20 ppm)
453.1376
[M+H]+
679.2202
0
20
40
60
80
100
450
500
550
600
650

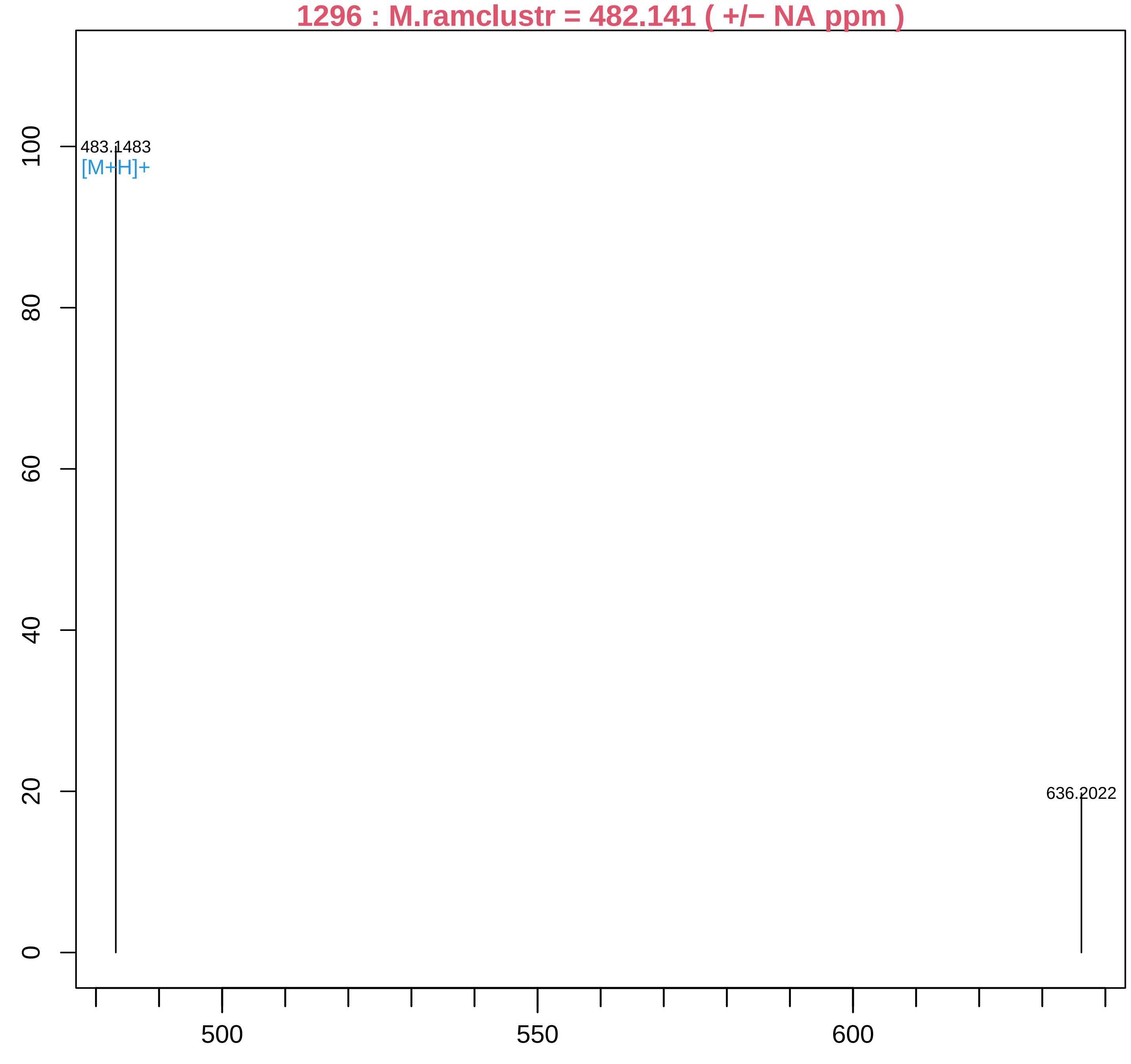
1296 : M.ramclustr = 482.141 (+/− NA ppm)
483.1483
[M+H]+
636.2022
0
20
40
60
80
100
500
550
600

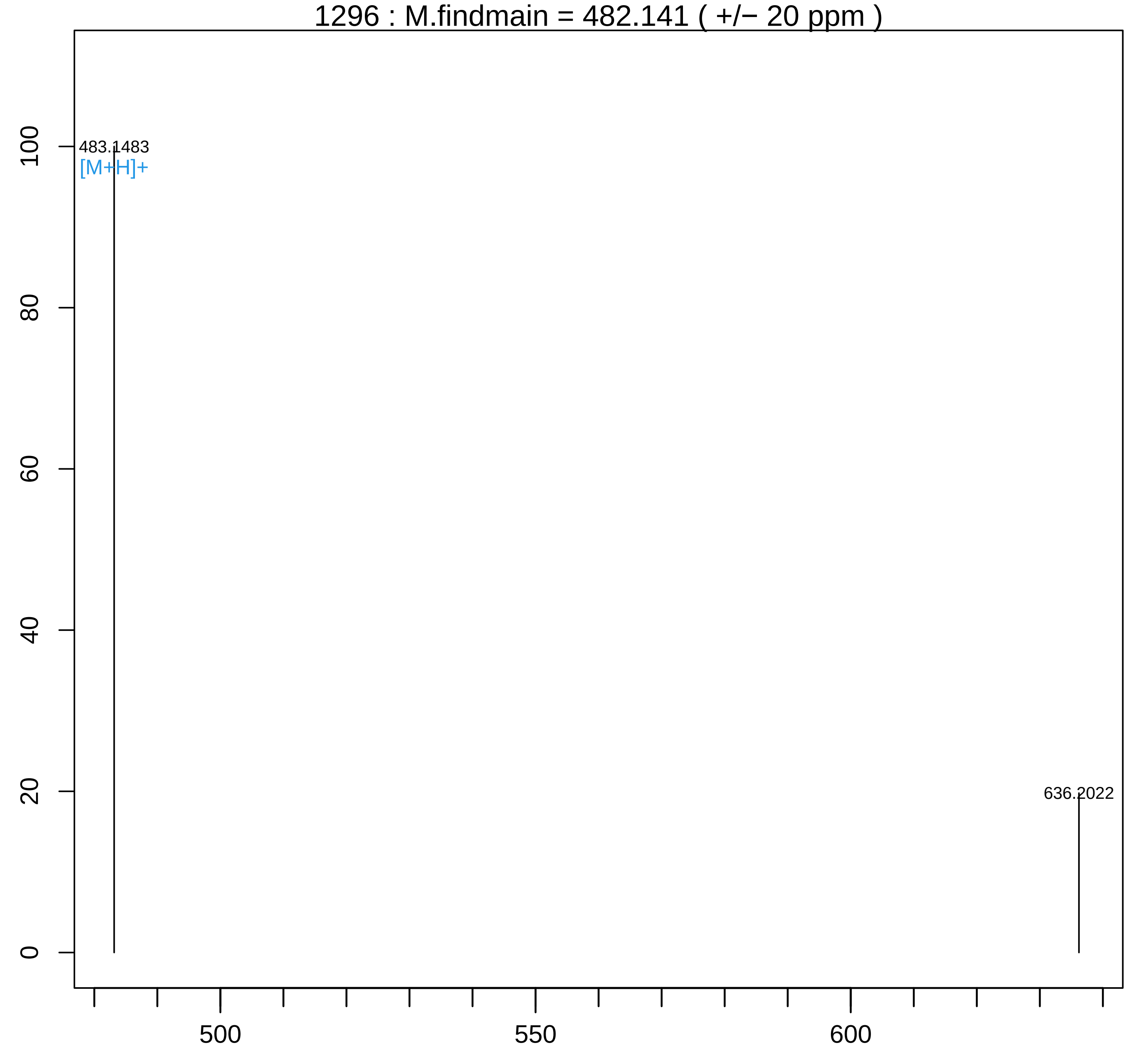
1296 : M.findmain = 482.141 (+/− 20 ppm)
483.1483
[M+H]+
636.2022
0
20
40
60
80
100
500
550
600

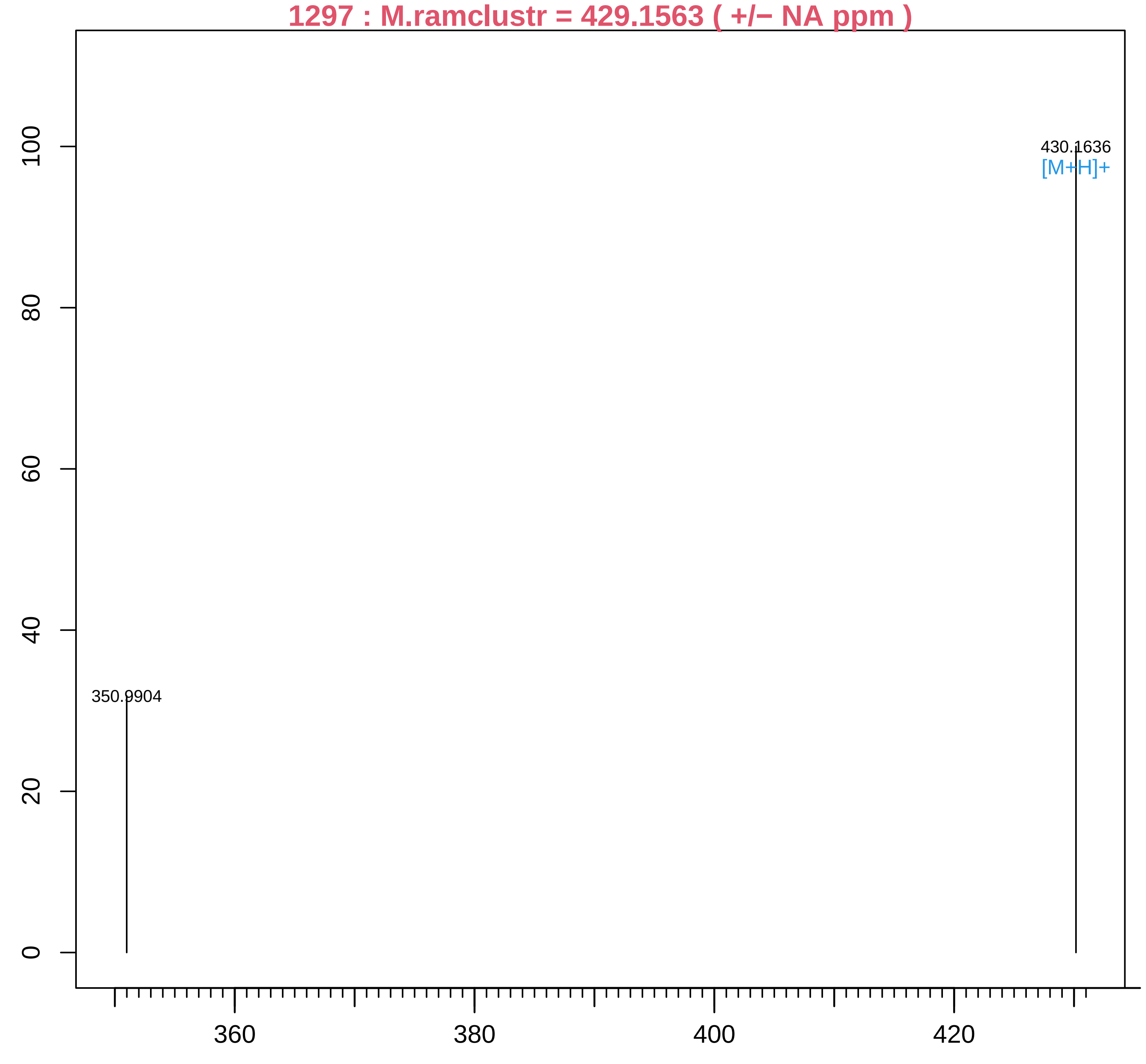
1297 : M.ramclustr = 429.1563 (+/− NA ppm)
430.1636
[M+H]+
350.9904
0
20
40
60
80
100
360
380
400
420

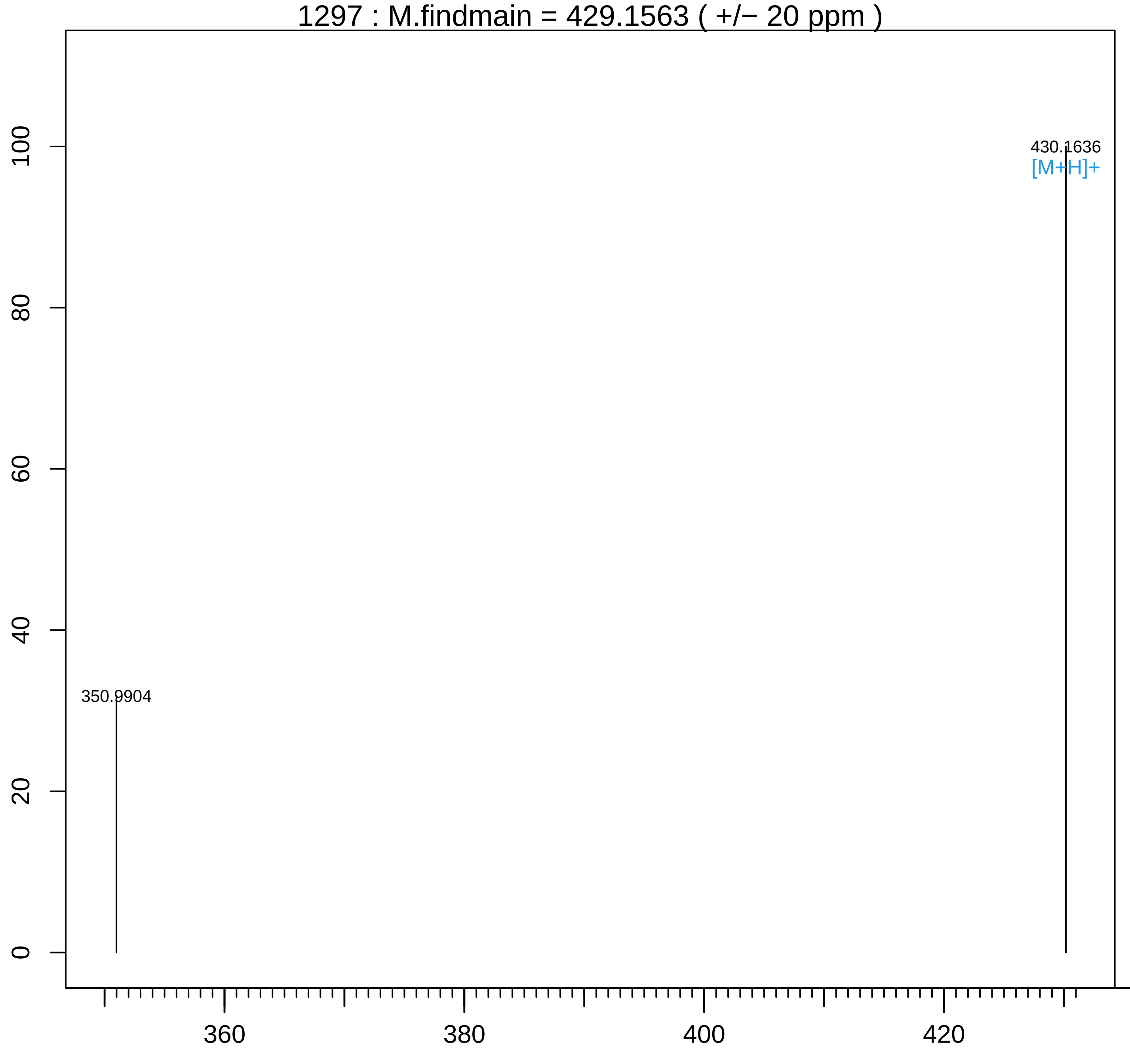
1297 : M.findmain = 429.1563 (+/− 20 ppm)
430.1636
[M+H]+
350.9904
0
20
40
60
80
100
360
380
400
420

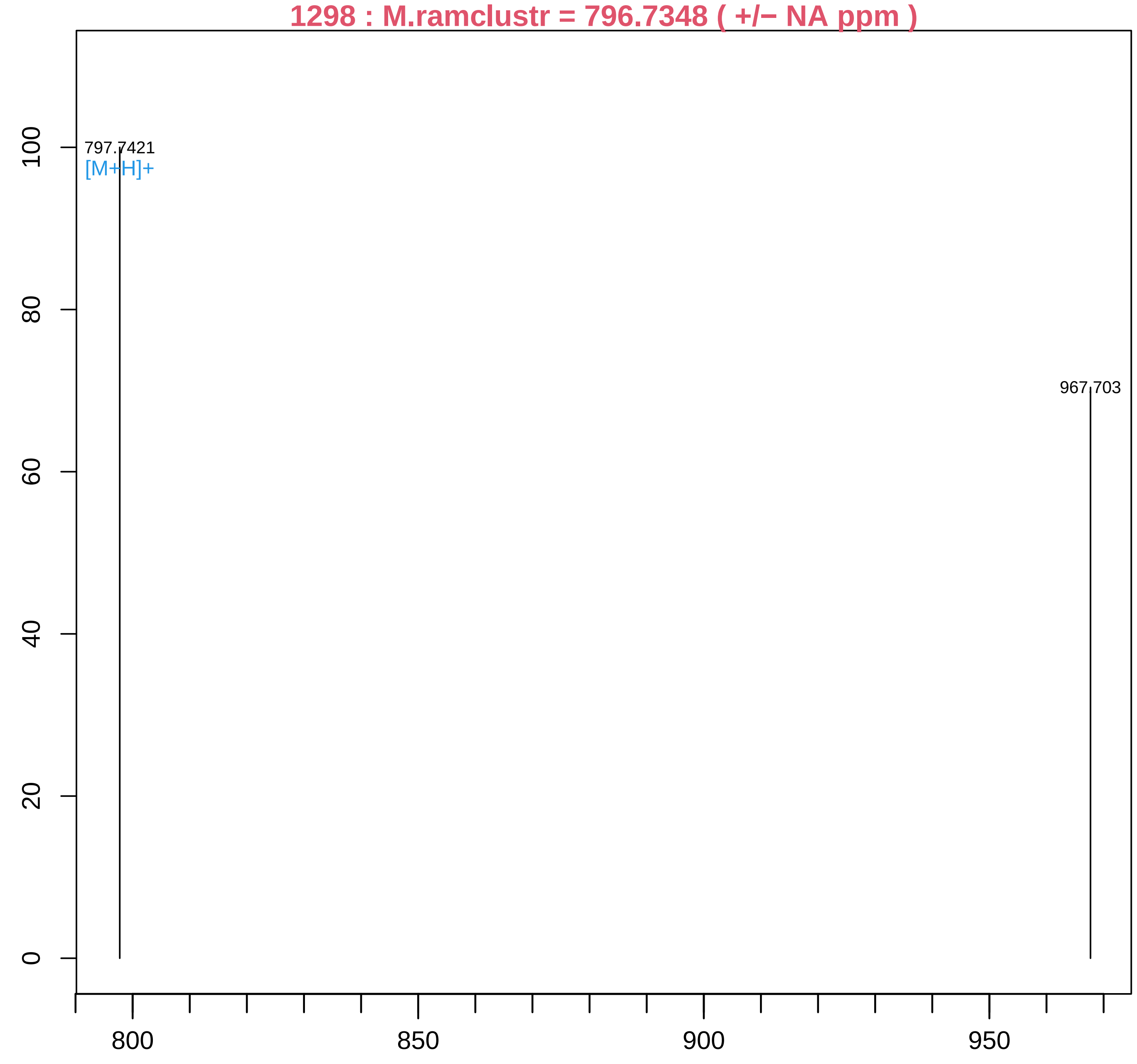
1298 : M.ramclustr = 796.7348 (+/− NA ppm)
797.7421
[M+H]+
967.703
0
20
40
60
80
100
800
850
900
950

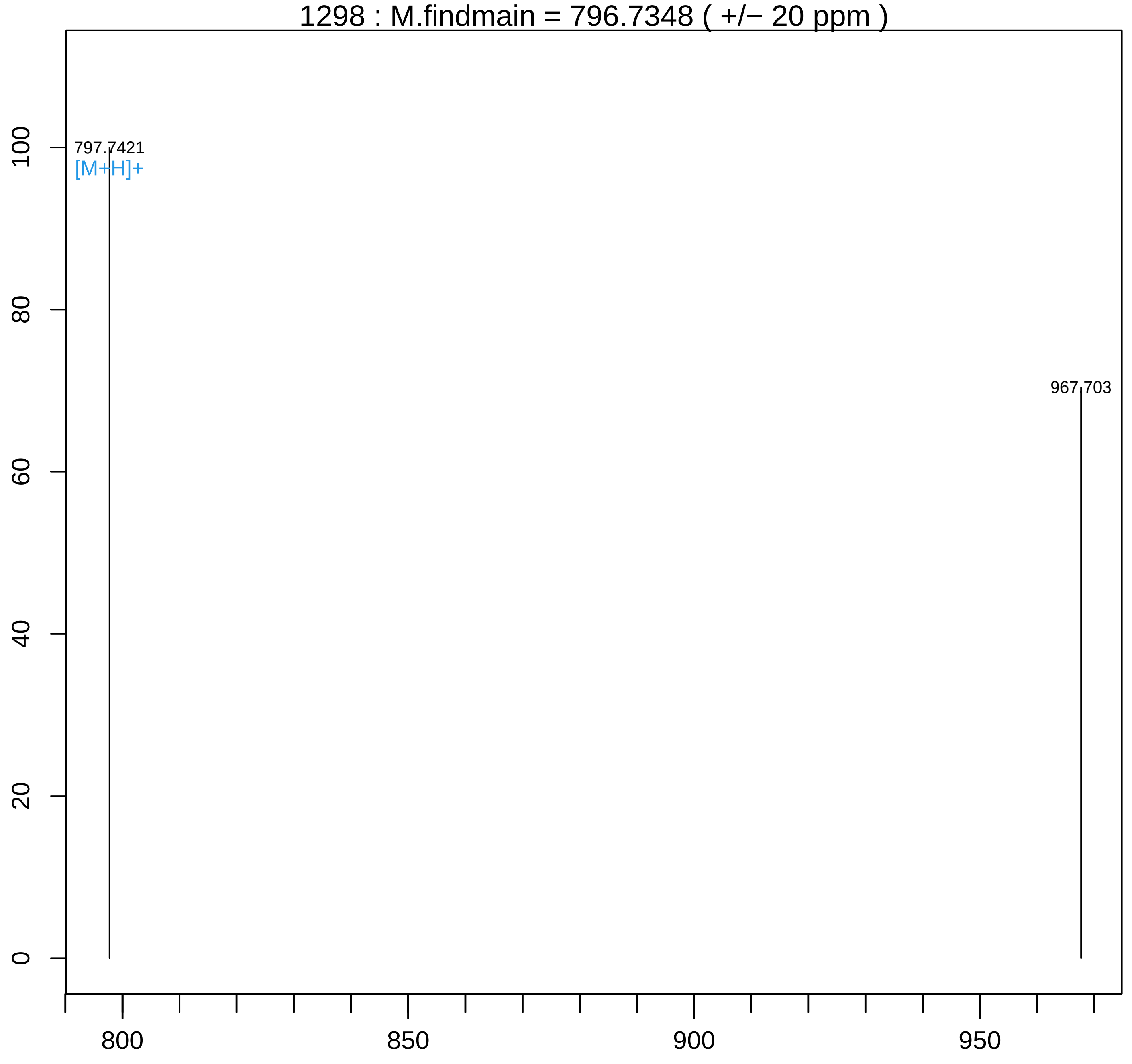
1298 : M.findmain = 796.7348 (+/− 20 ppm)
797.7421
[M+H]+
967.703
0
20
40
60
80
100
800
850
900
950

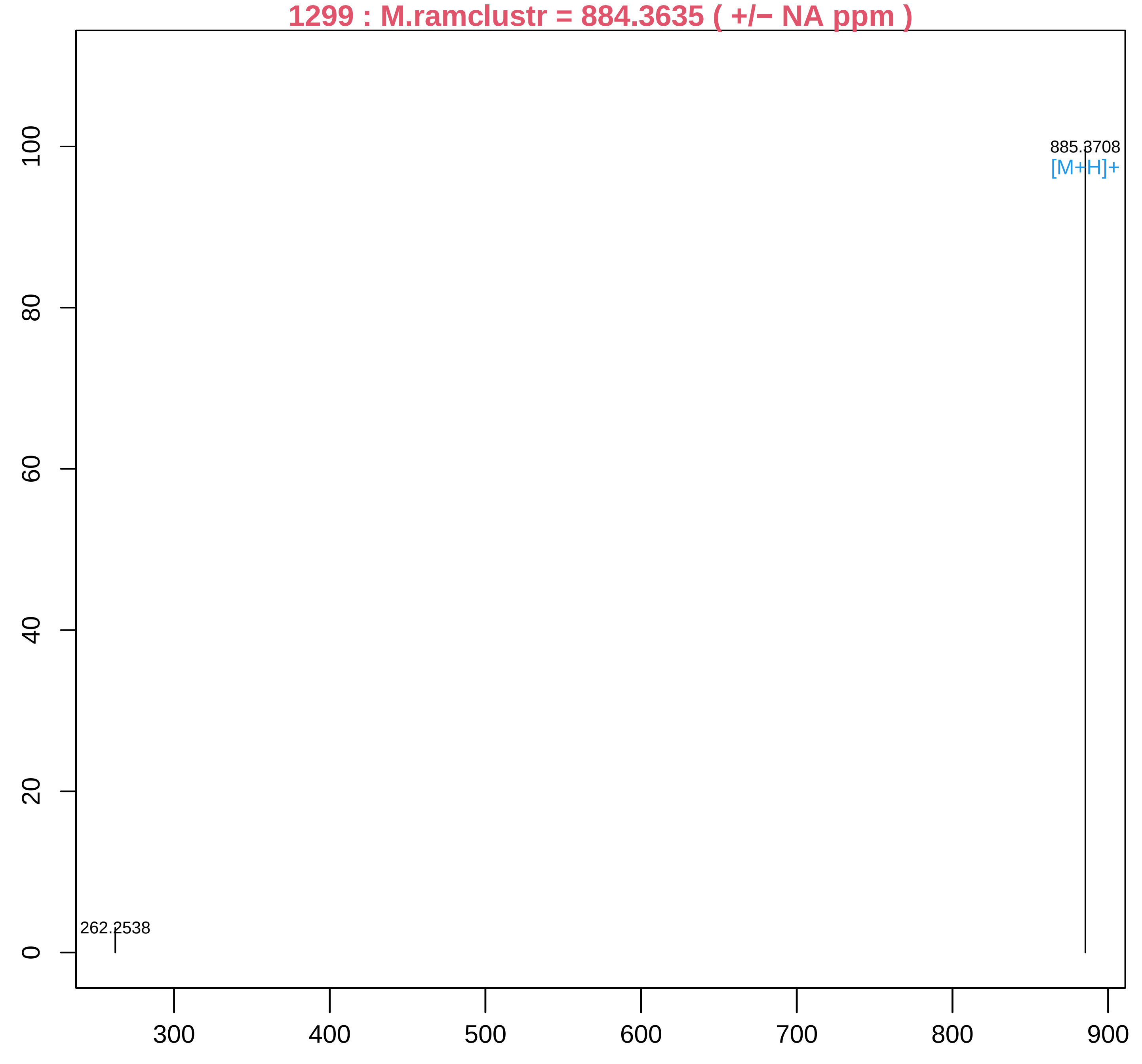
1299 : M.ramclustr = 884.3635 (+/− NA ppm)
885.3708
[M+H]+
262.2538
0
20
40
60
80
100
300
400
500
600
700
800
900

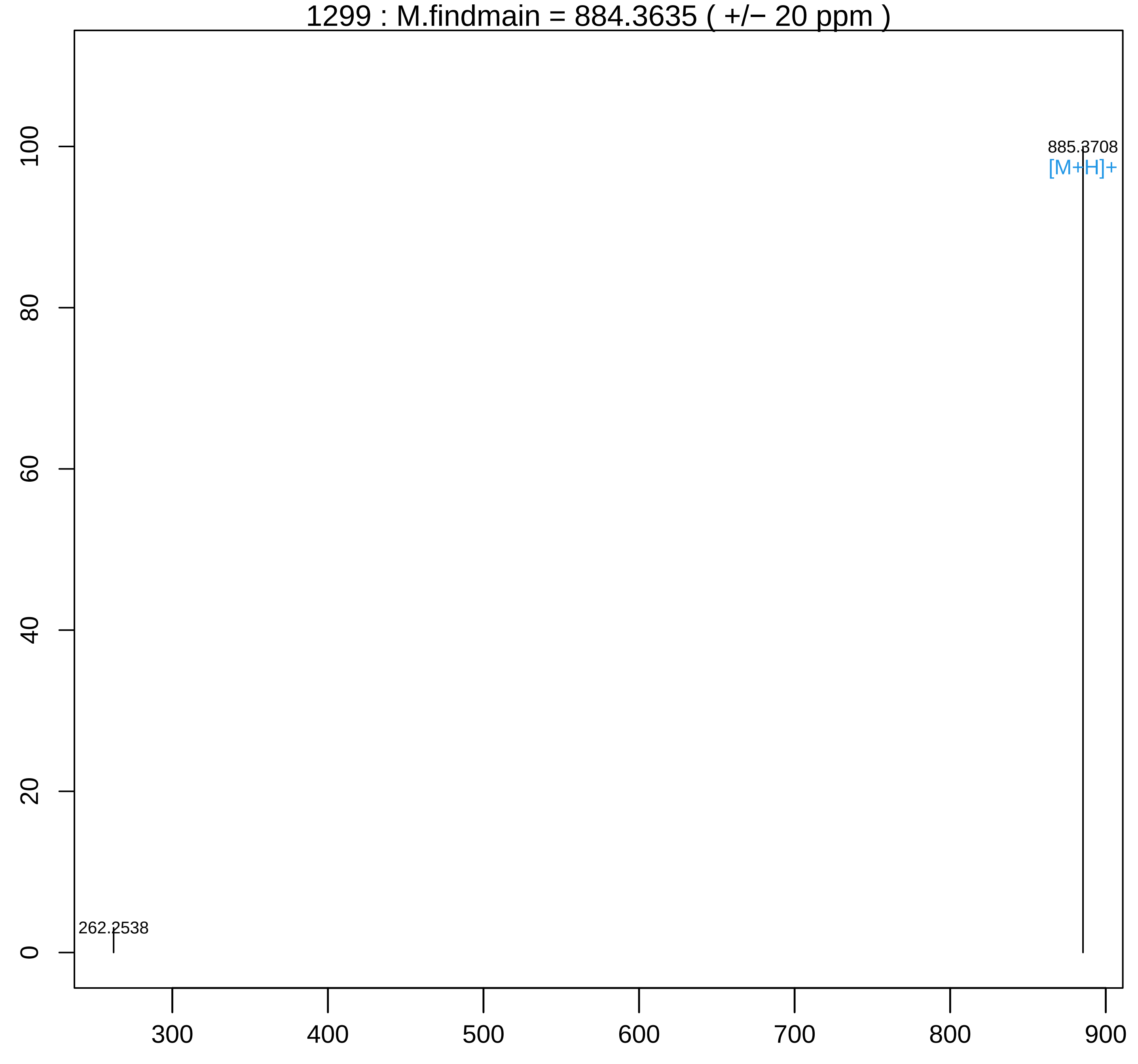
1299 : M.findmain = 884.3635 (+/− 20 ppm)
885.3708
[M+H]+
262.2538
0
20
40
60
80
100
300
400
500
600
700
800
900

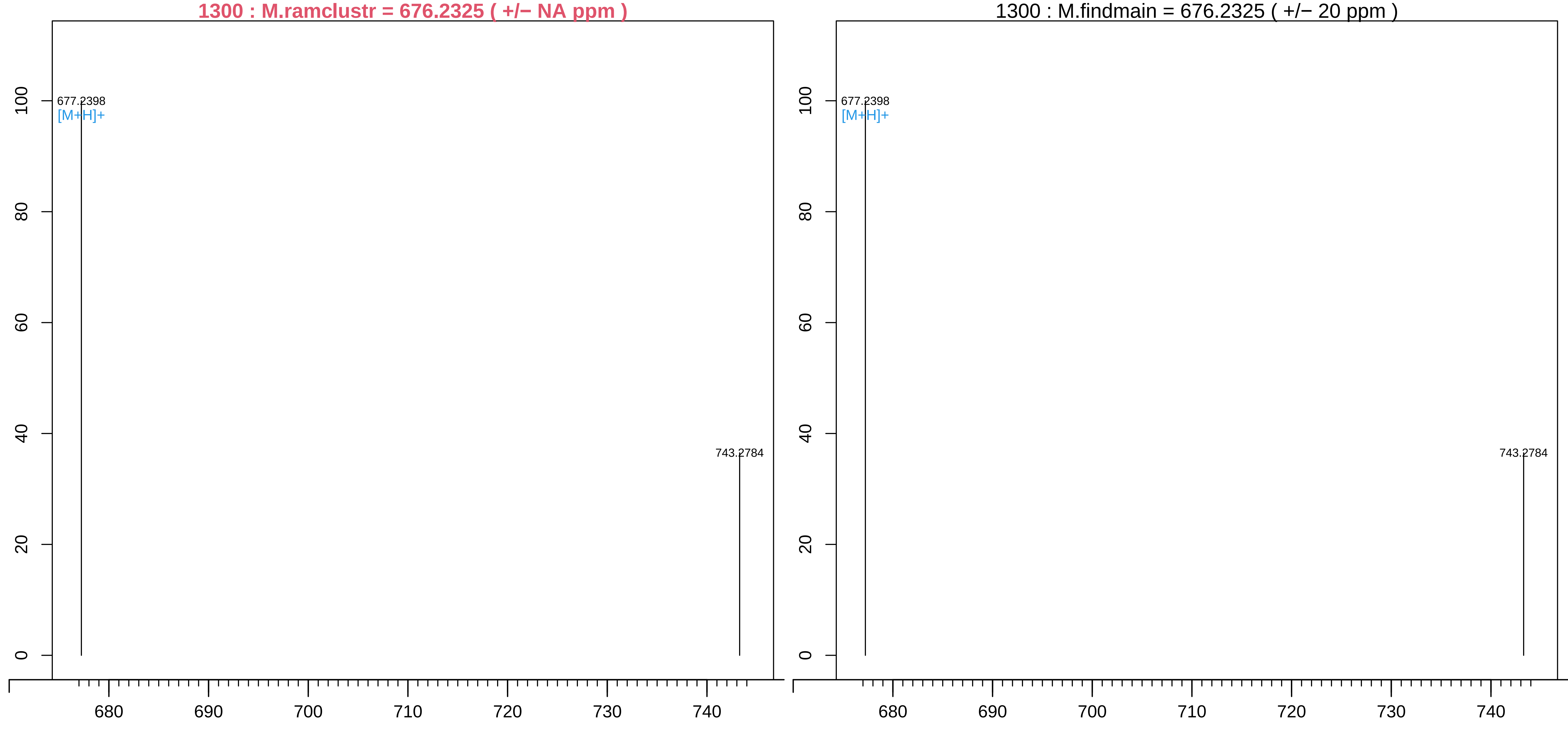
1300 : M.ramclustr = 676.2325 (+/− NA ppm)
1300 : M.findmain = 676.2325 (+/− 20 ppm)
677.2398
[M+H]+
743.2784
0
20
40
60
80
100
680
690
700
710
720
730
740

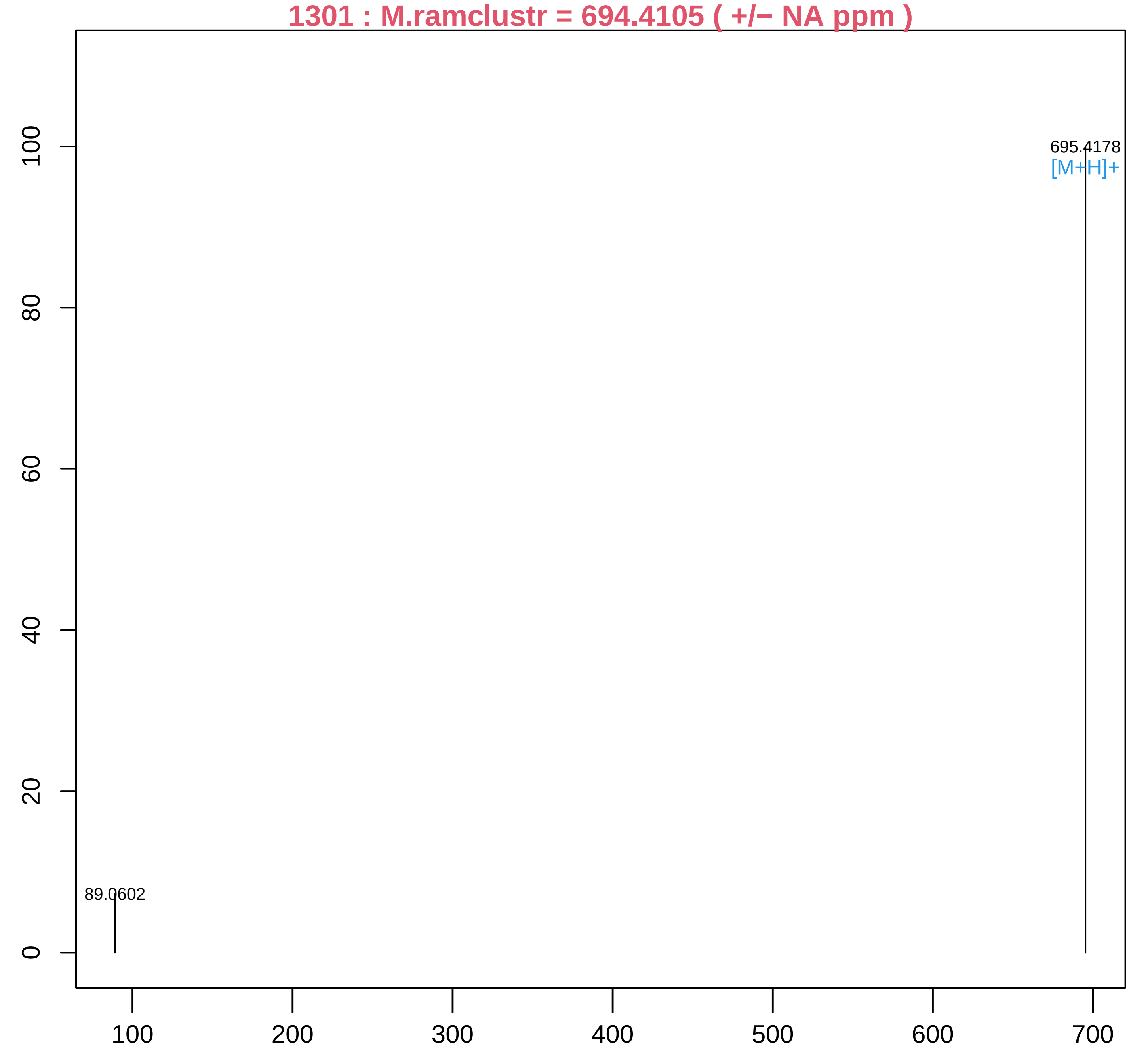
1301 : M.ramclustr = 694.4105 (+/− NA ppm)
695.4178
[M+H]+
89.0602
0
20
40
60
80
100
100
200
300
400
500
600
700

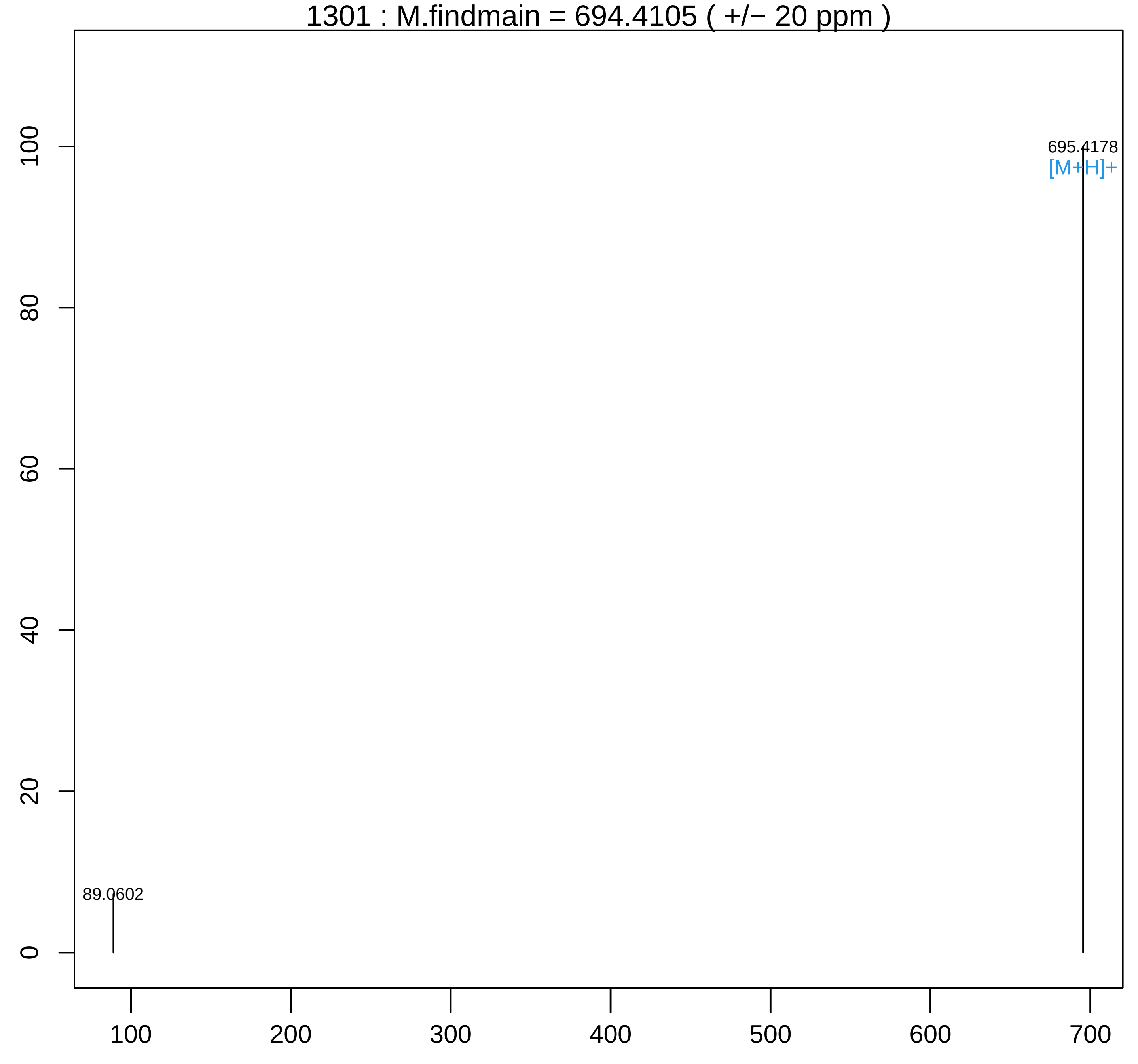
1301 : M.findmain = 694.4105 (+/− 20 ppm)
695.4178
[M+H]+
89.0602
0
20
40
60
80
100
100
200
300
400
500
600
700

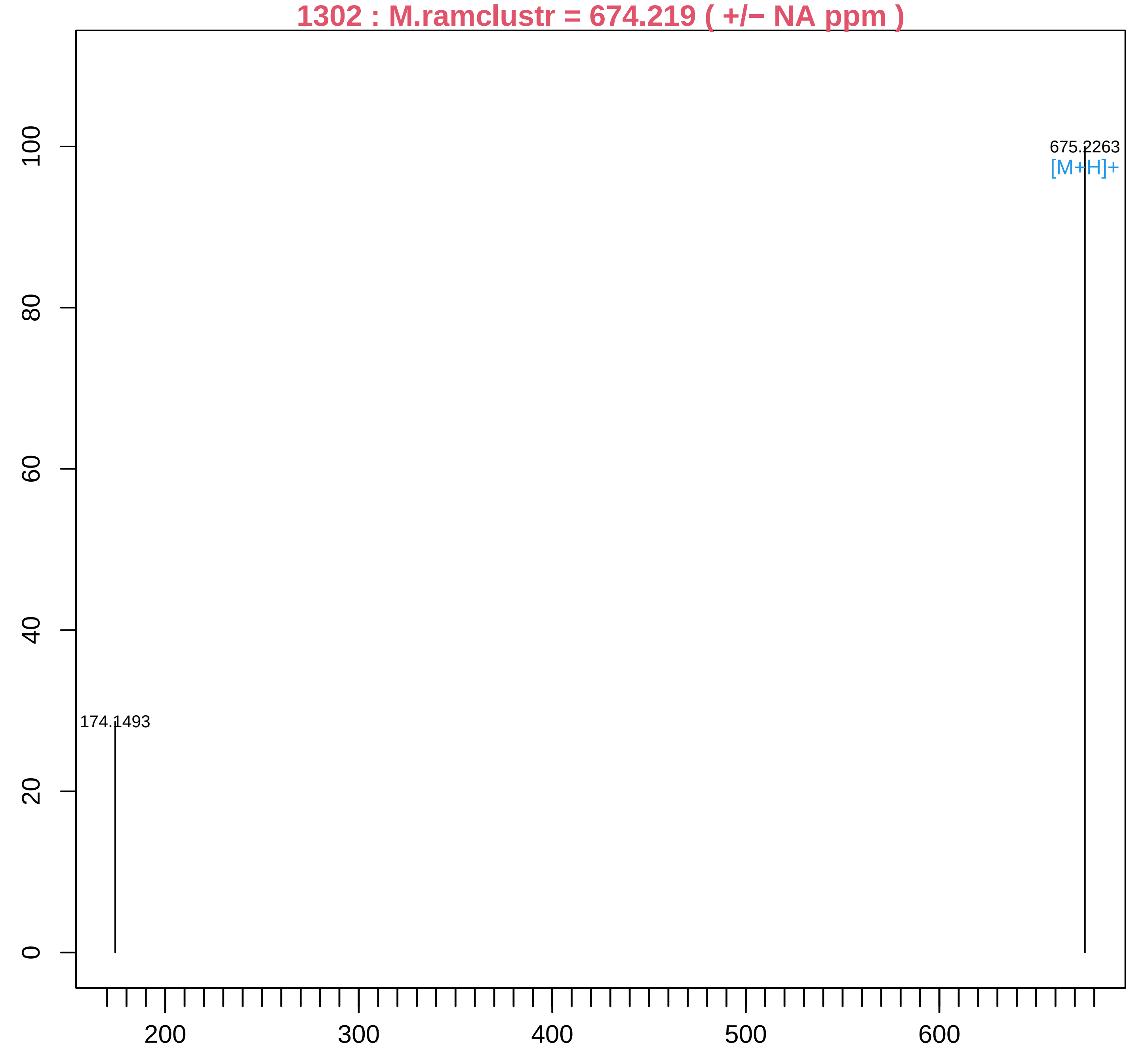

1302 : M.ramclustr = 674.219 (+/− NA ppm)
675.2263
[M+H]+
174.1493
0
20
40
60
80
100
200
300
400
500
600

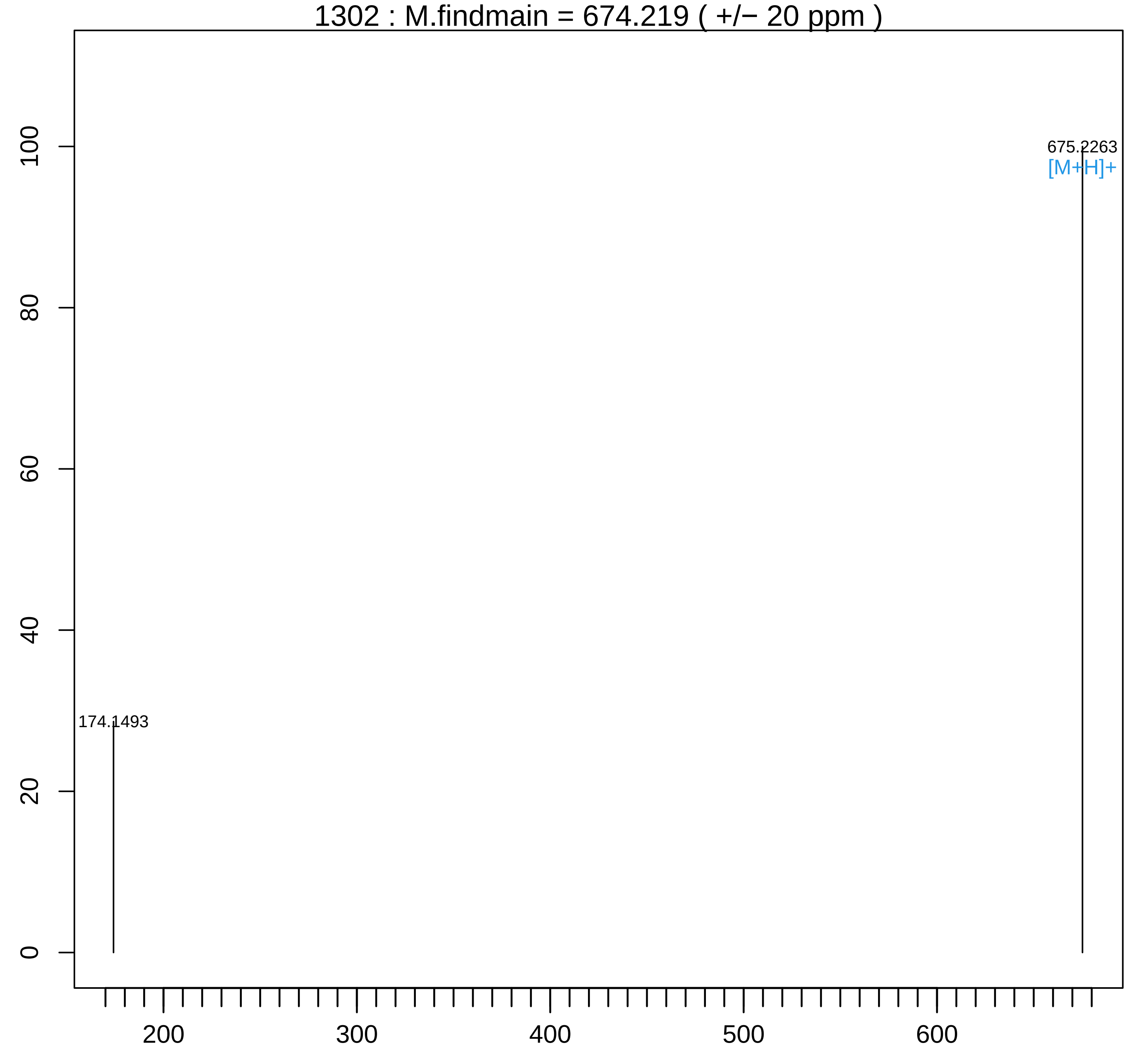

1302 : M.findmain = 674.219 (+/− 20 ppm)
675.2263
[M+H]+
174.1493
0
20
40
60
80
100
200
300
400
500
600

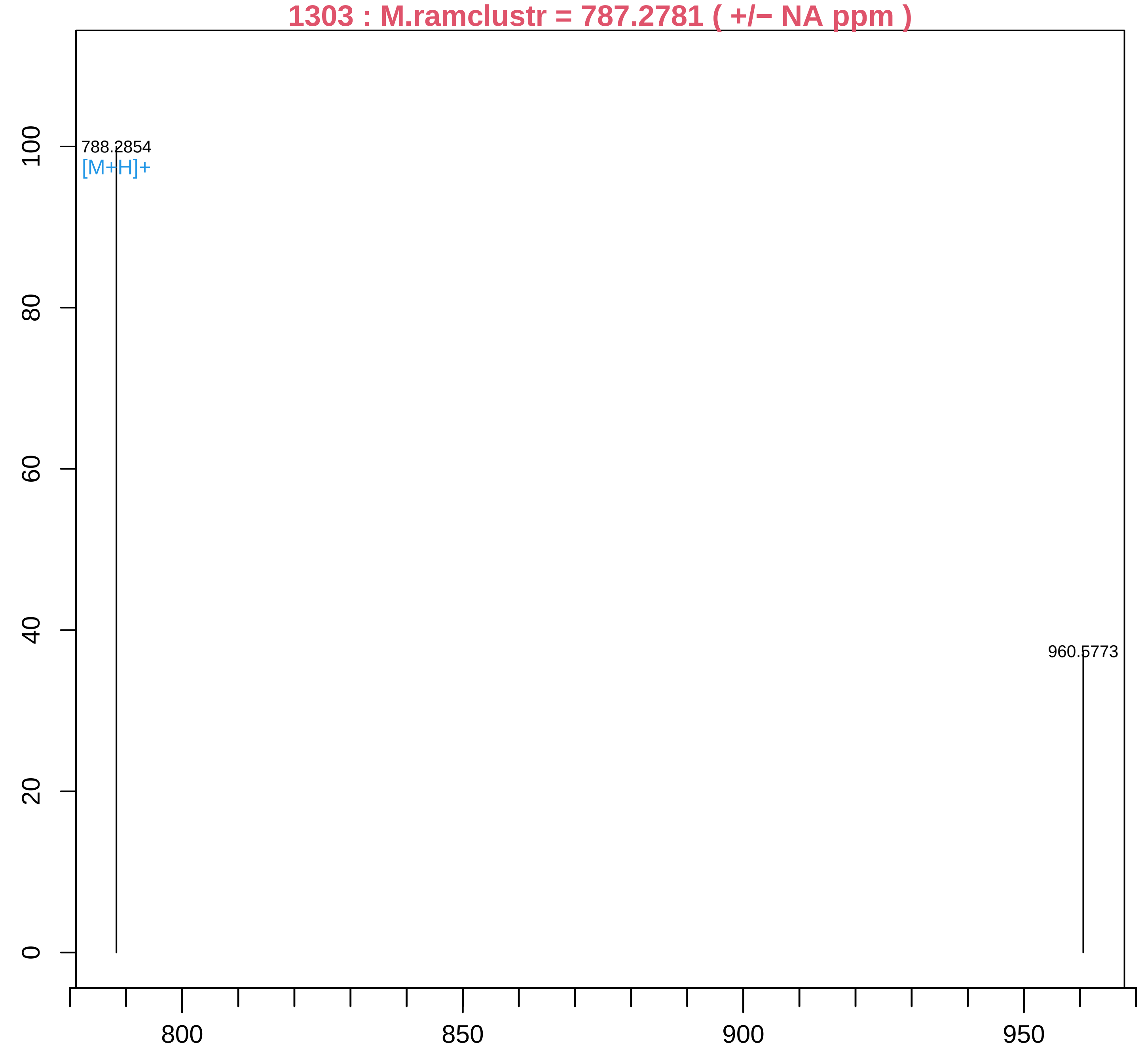

1303 : M.ramclustr = 787.2781 (+/− NA ppm)
788.2854
[M+H]+
960.5773
0
20
40
60
80
100
800
850
900
950

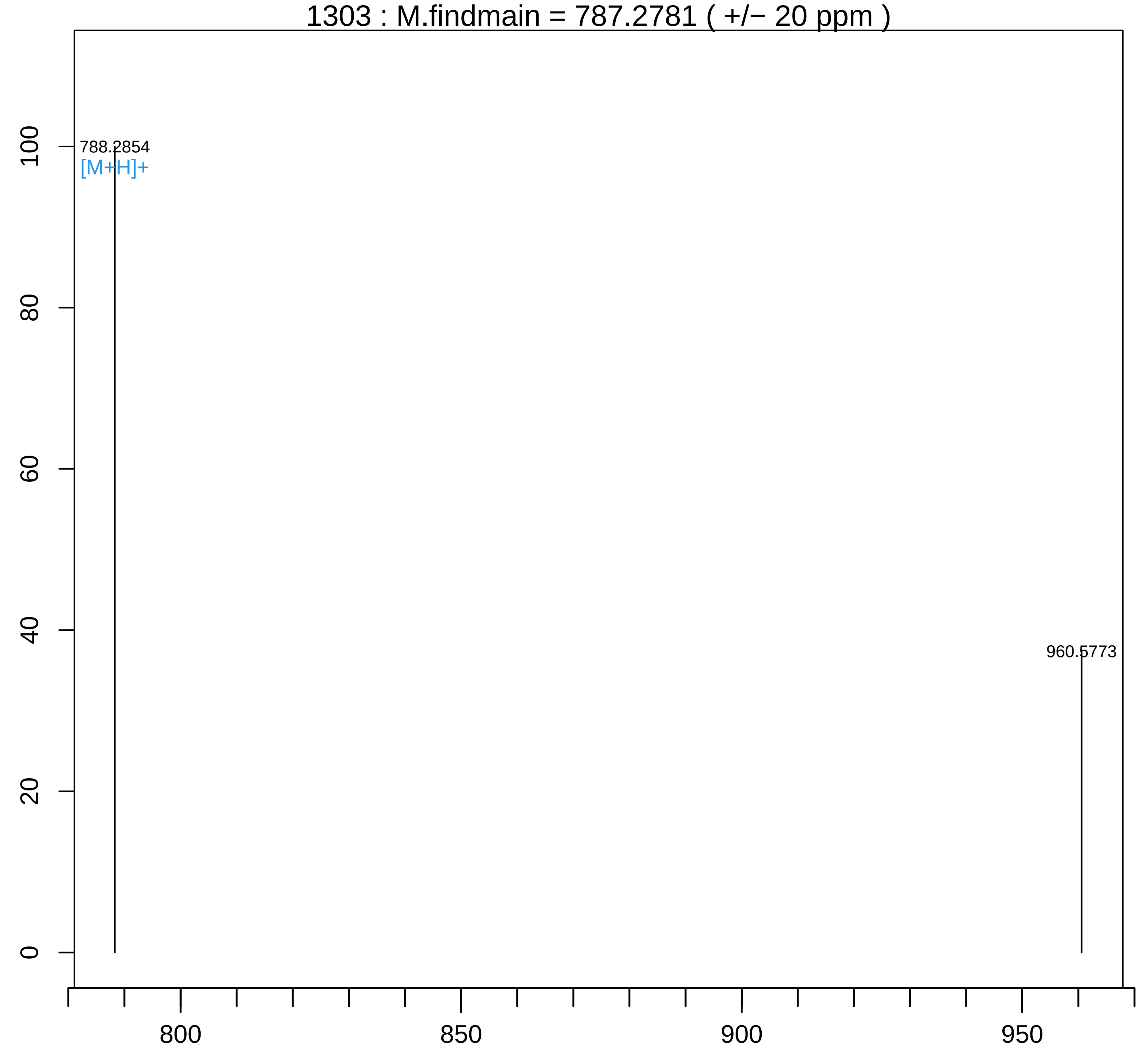

1303 : M.findmain = 787.2781 (+/− 20 ppm)
788.2854
[M+H]+
960.5773
0
20
40
60
80
100
800
850
900
950

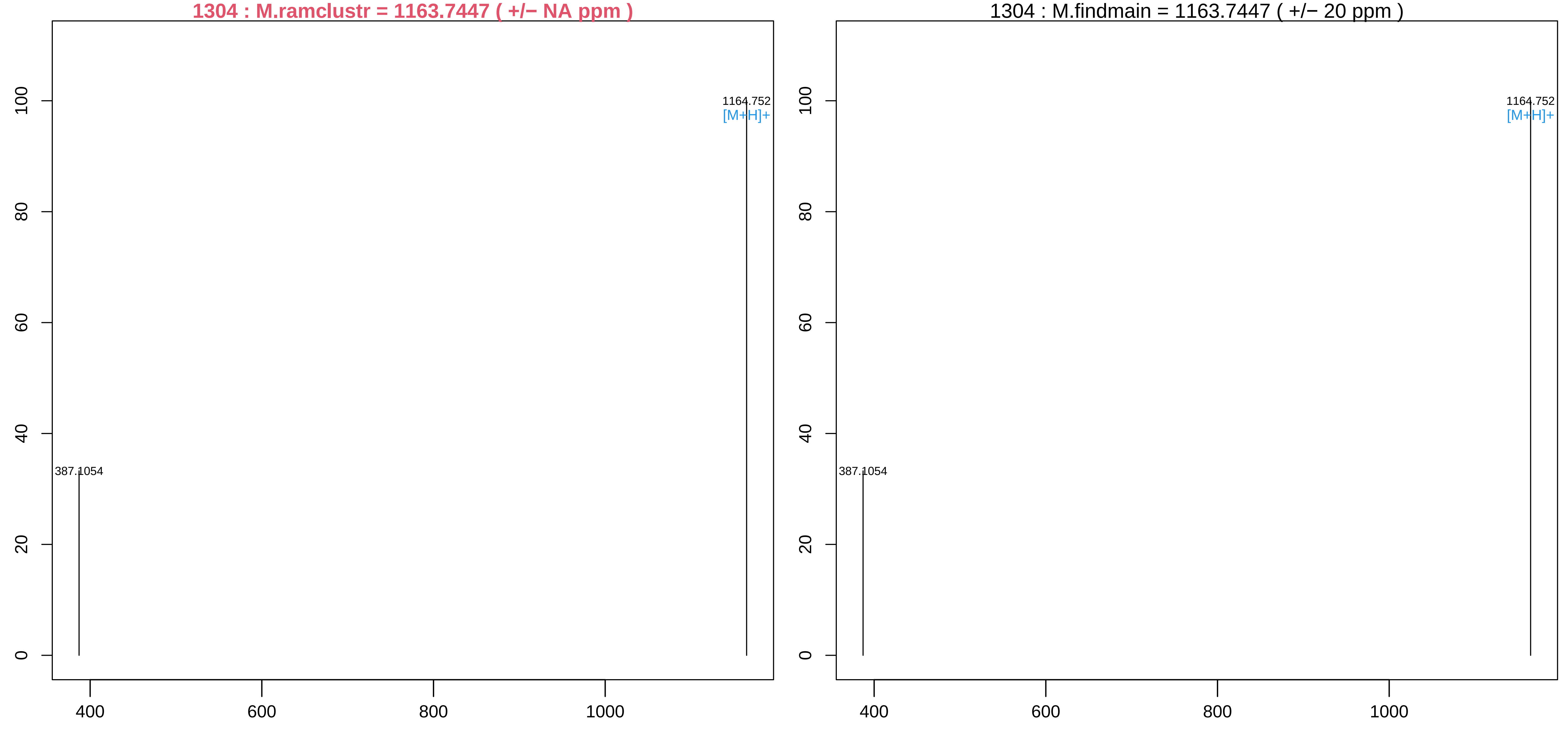

1304 : M.ramclustr = 1163.7447 (+/− NA ppm)
1164.752
[M+H]+
387.1054
1304 : M.findmain = 1163.7447 (+/− 20 ppm)
1164.752
[M+H]+
387.1054
0
20
40
60
80
100
400
600
800
1000

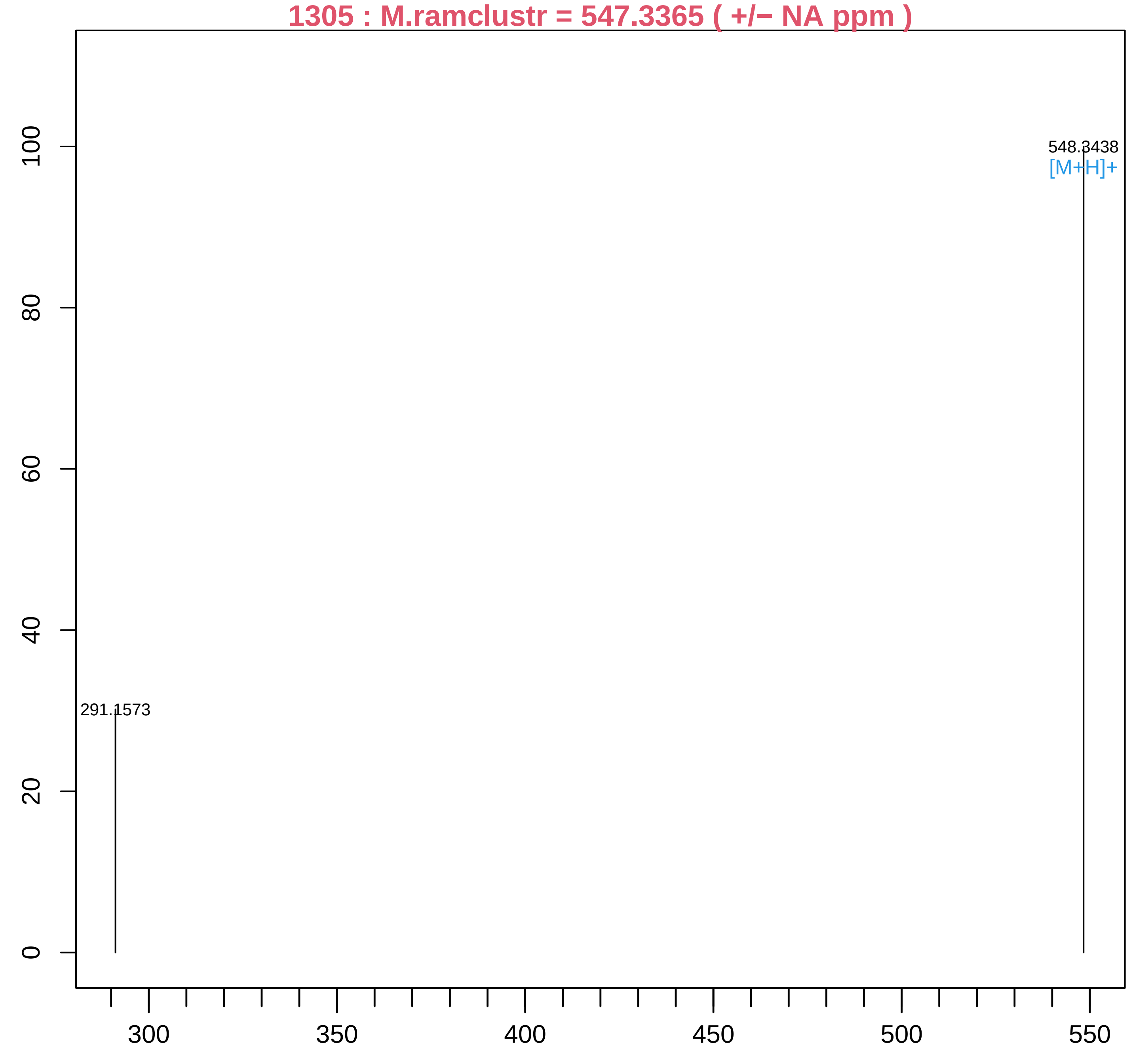

1305 : M.ramclustr = 547.3365 (+/− NA ppm)
548.3438
[M+H]+
291.1573
0
20
40
60
80
100
300
350
400
450
500
550

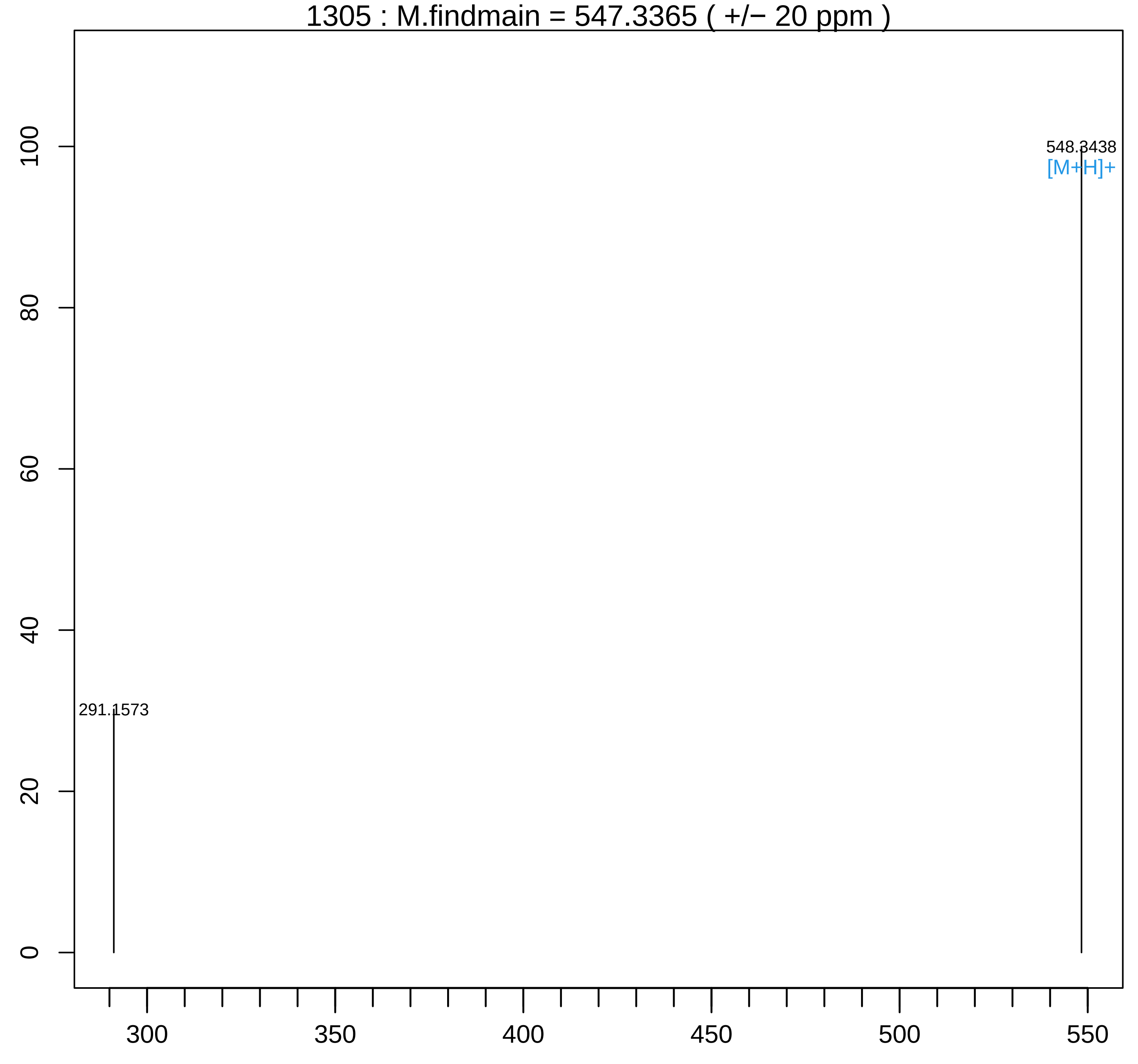

1305 : M.findmain = 547.3365 (+/− 20 ppm)
548.3438
[M+H]+
291.1573
0
20
40
60
80
100
300
350
400
450
500
550

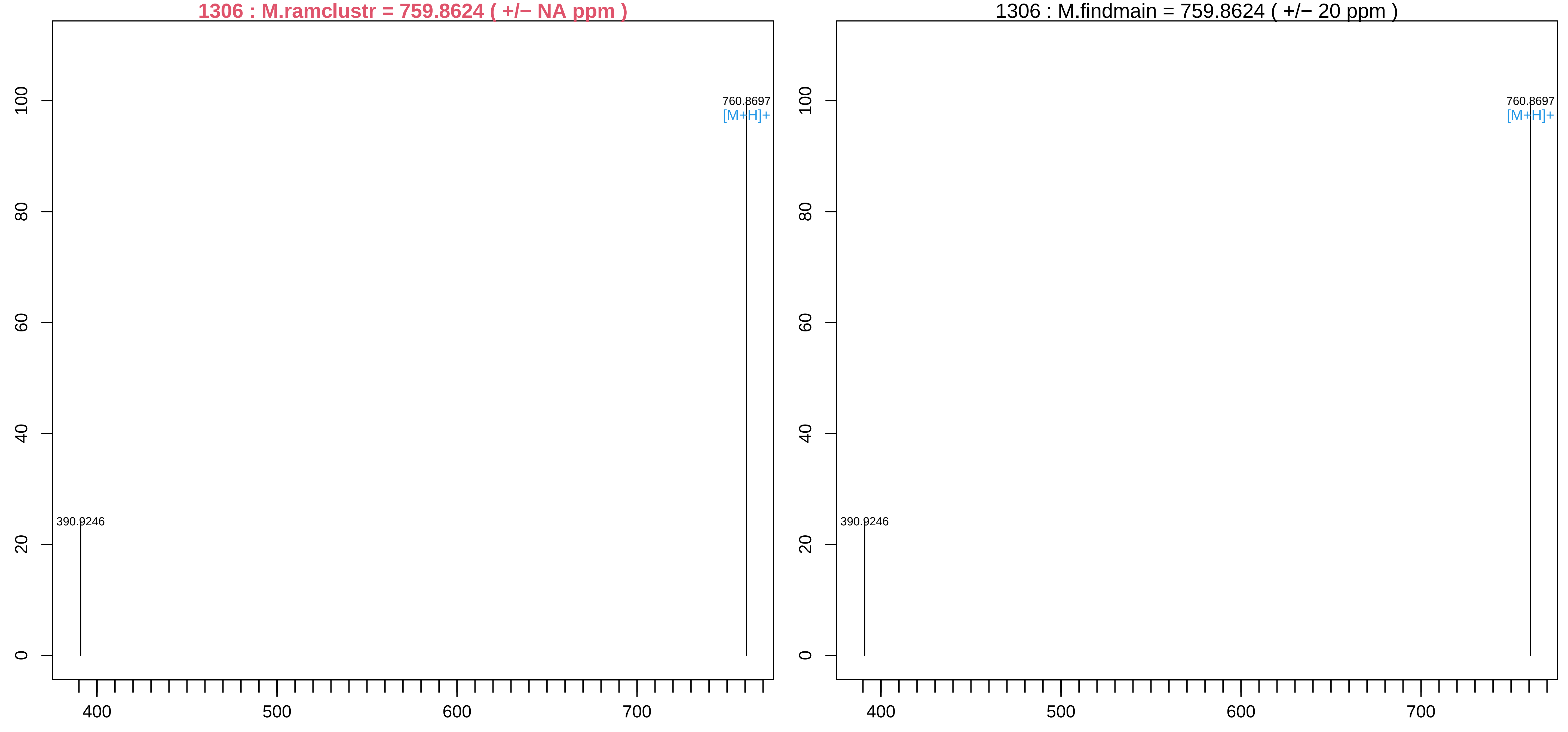
1306 : M.ramclustr = 759.8624 (+/− NA ppm)
760.8697
[M+H]+
390.9246
1306 : M.findmain = 759.8624 (+/− 20 ppm)
760.8697
[M+H]+
390.9246
0
20
40
60
80
100
400
500
600
700

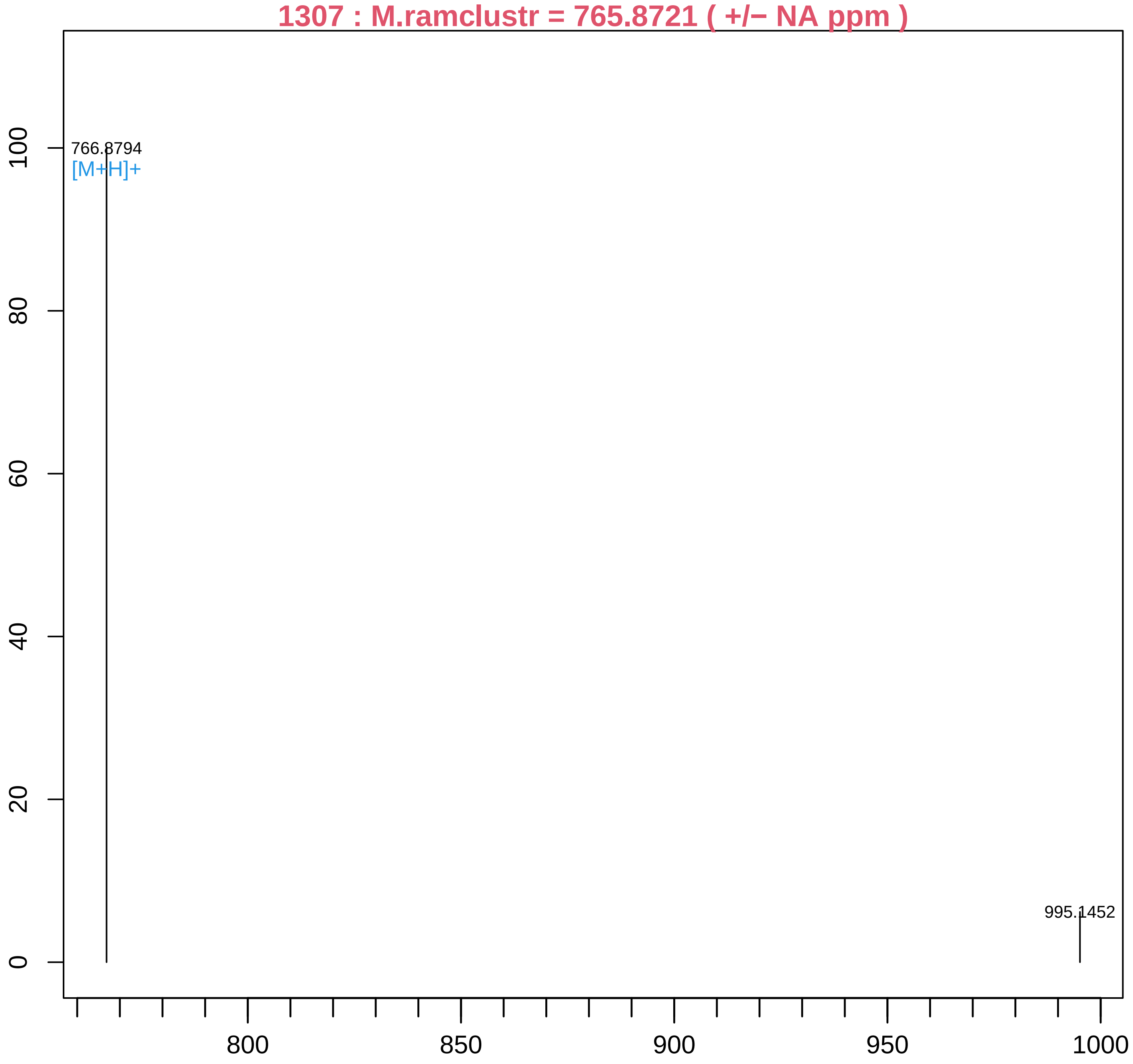
1307 : M.ramclustr = 765.8721 (+/− NA ppm)
766.8794
[M+H]+
995.1452
0
20
40
60
80
100
800
850
900
950
1000

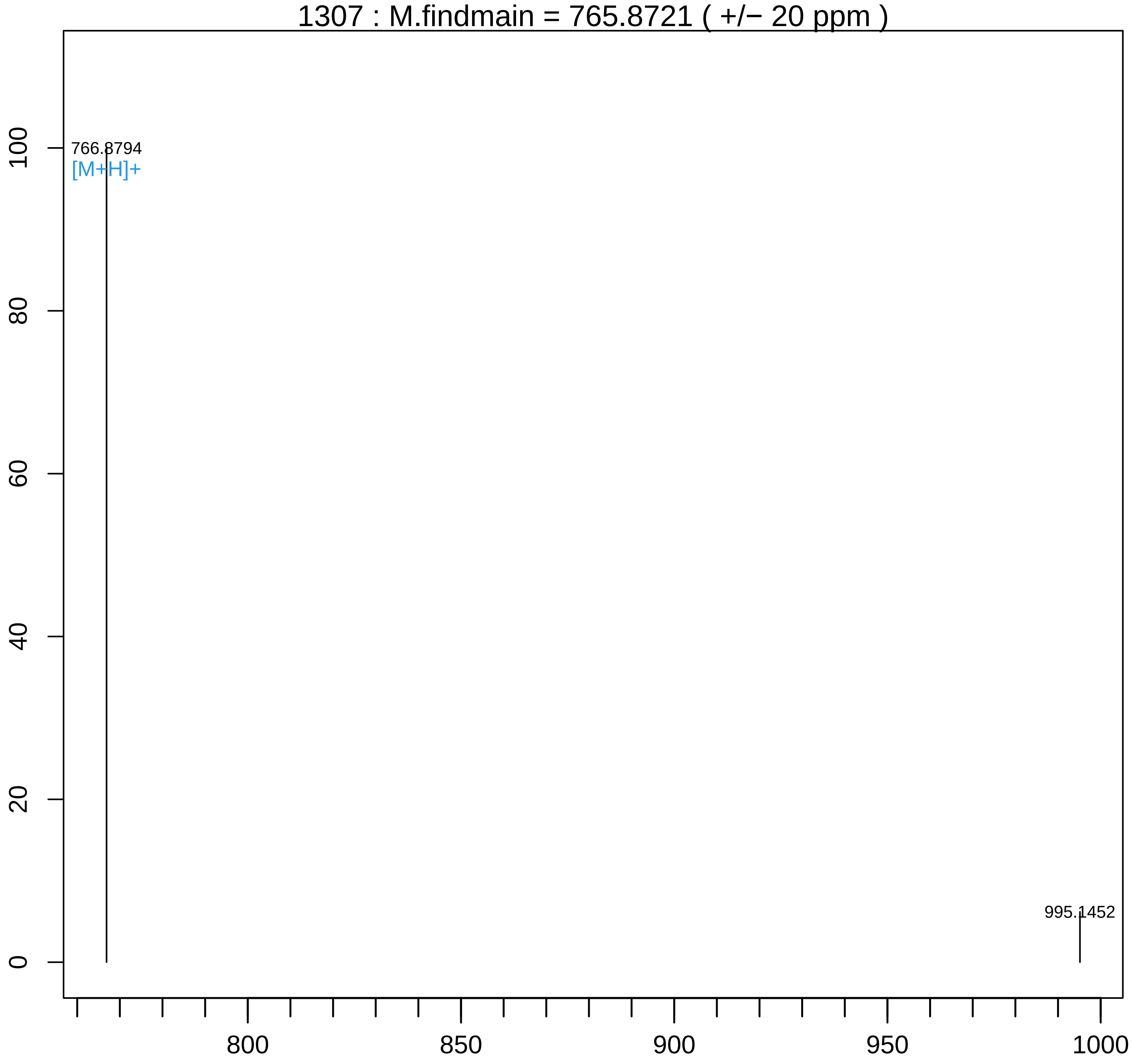
1307 : M.findmain = 765.8721 (+/− 20 ppm)
766.8794
[M+H]+
995.1452
0
20
40
60
80
100
800
850
900
950
1000

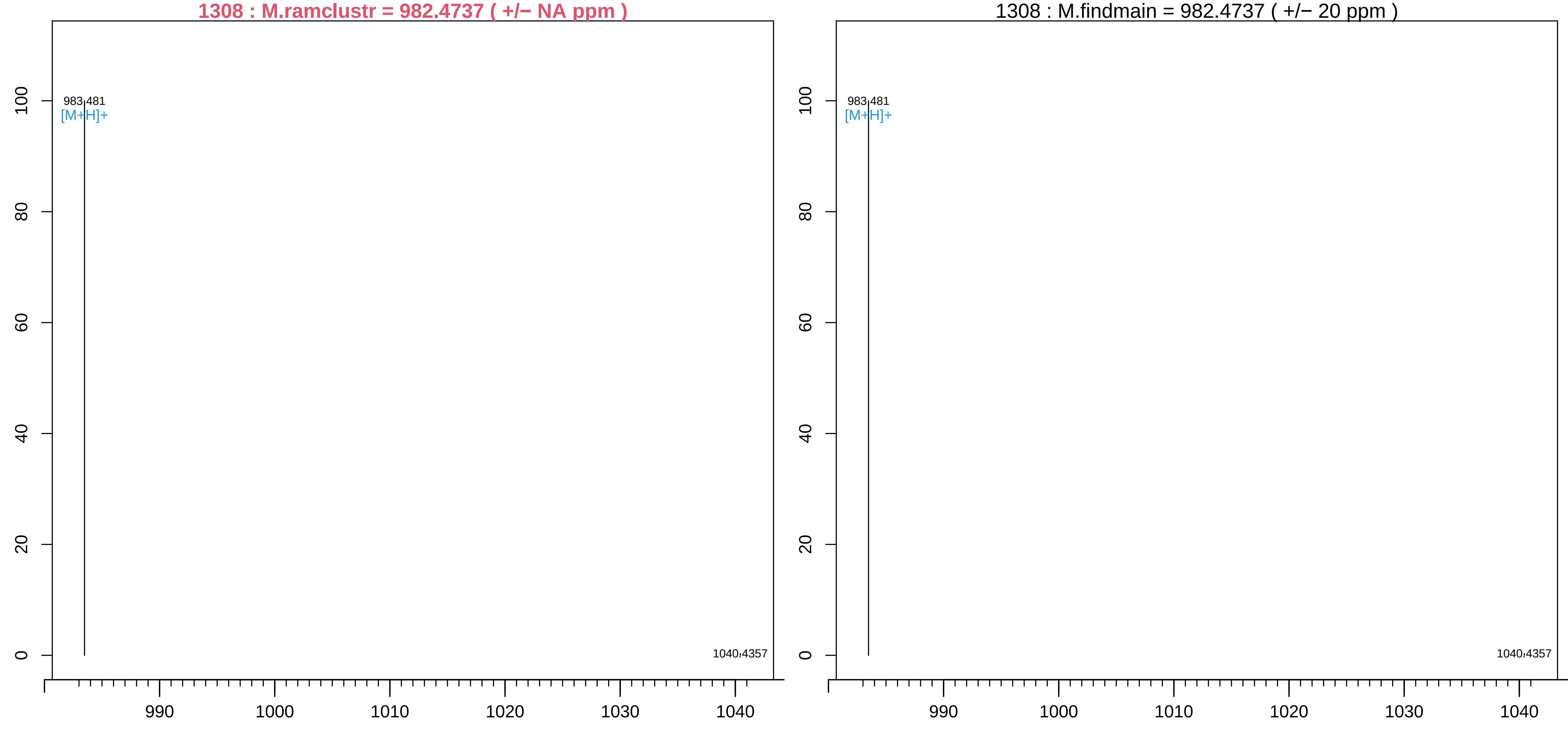
1308 : M.ramclustr = 982.4737 (+/− NA ppm)
983.481
[M+H]+
1040.4357
1308 : M.findmain = 982.4737 (+/− 20 ppm)
983.481
[M+H]+
1040.4357
0
20
40
60
80
100
990
1000
1010
1020
1030
1040

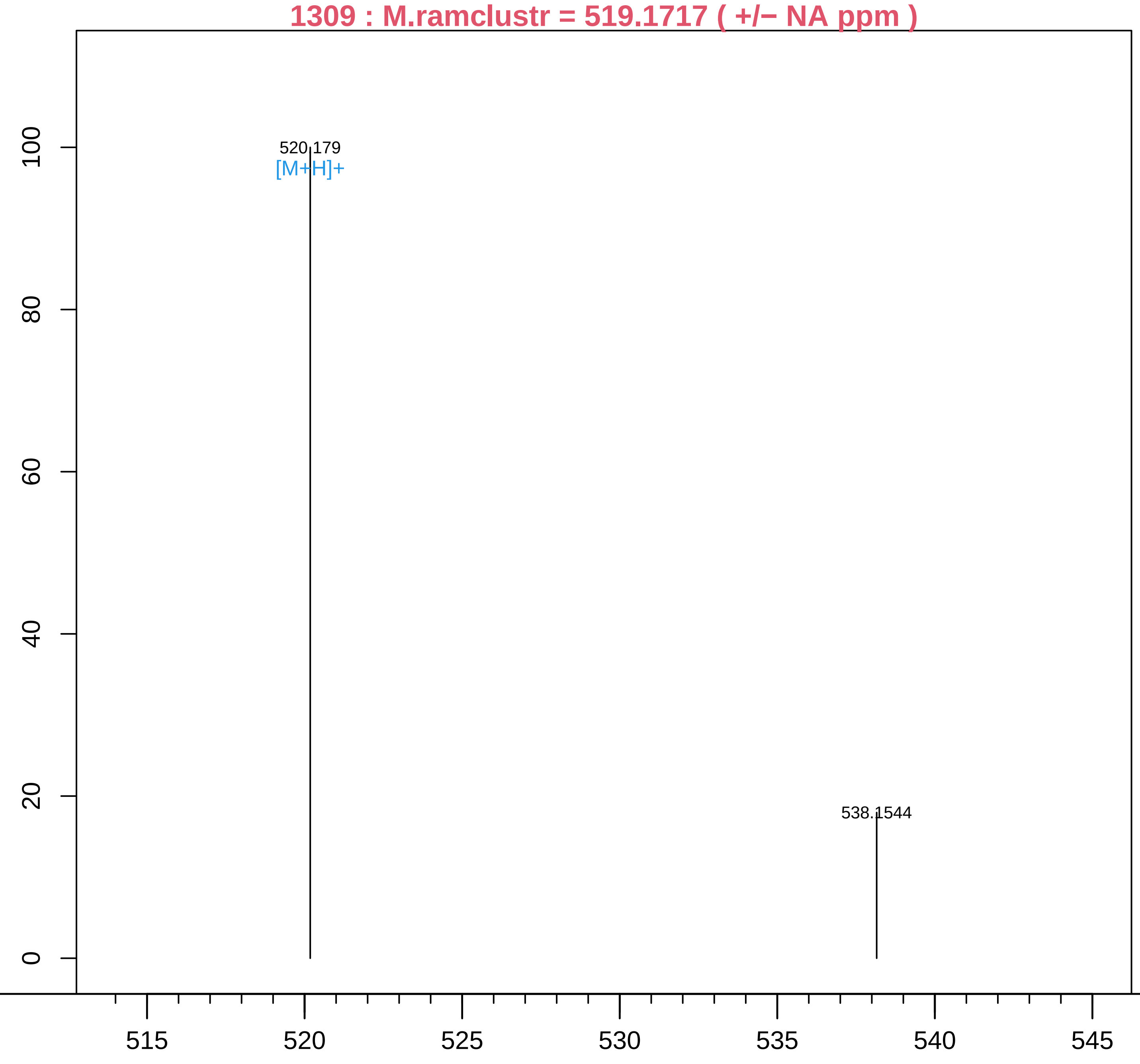
1309 : M.ramclustr = 519.1717 (+/− NA ppm)
520.179
[M+H]+
538.1544
0
20
40
60
80
100
515
520
525
530
535
540
545

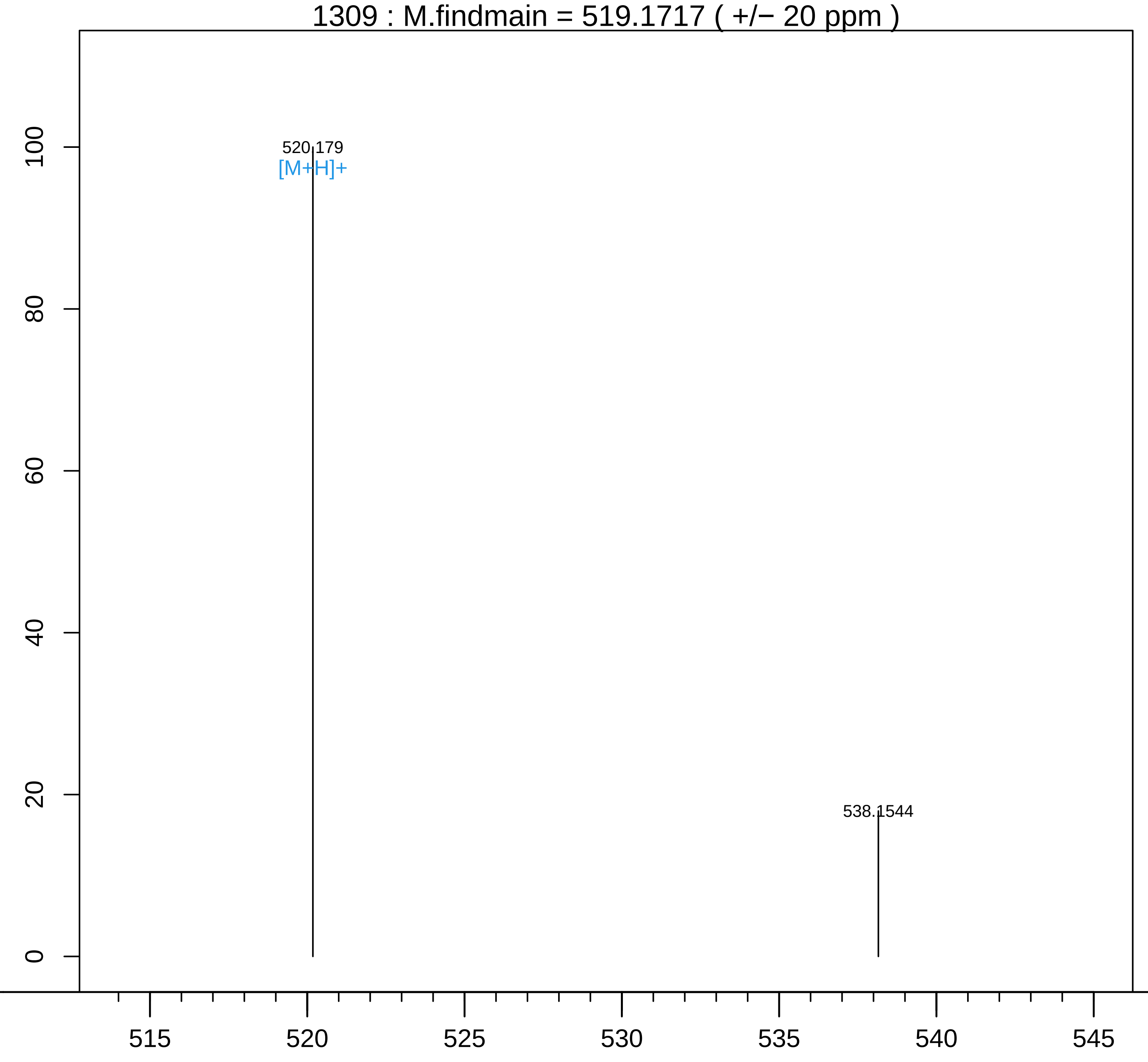
1309 : M.findmain = 519.1717 (+/− 20 ppm)
520.179
[M+H]+
538.1544
0
20
40
60
80
100
515
520
525
530
535
540
545

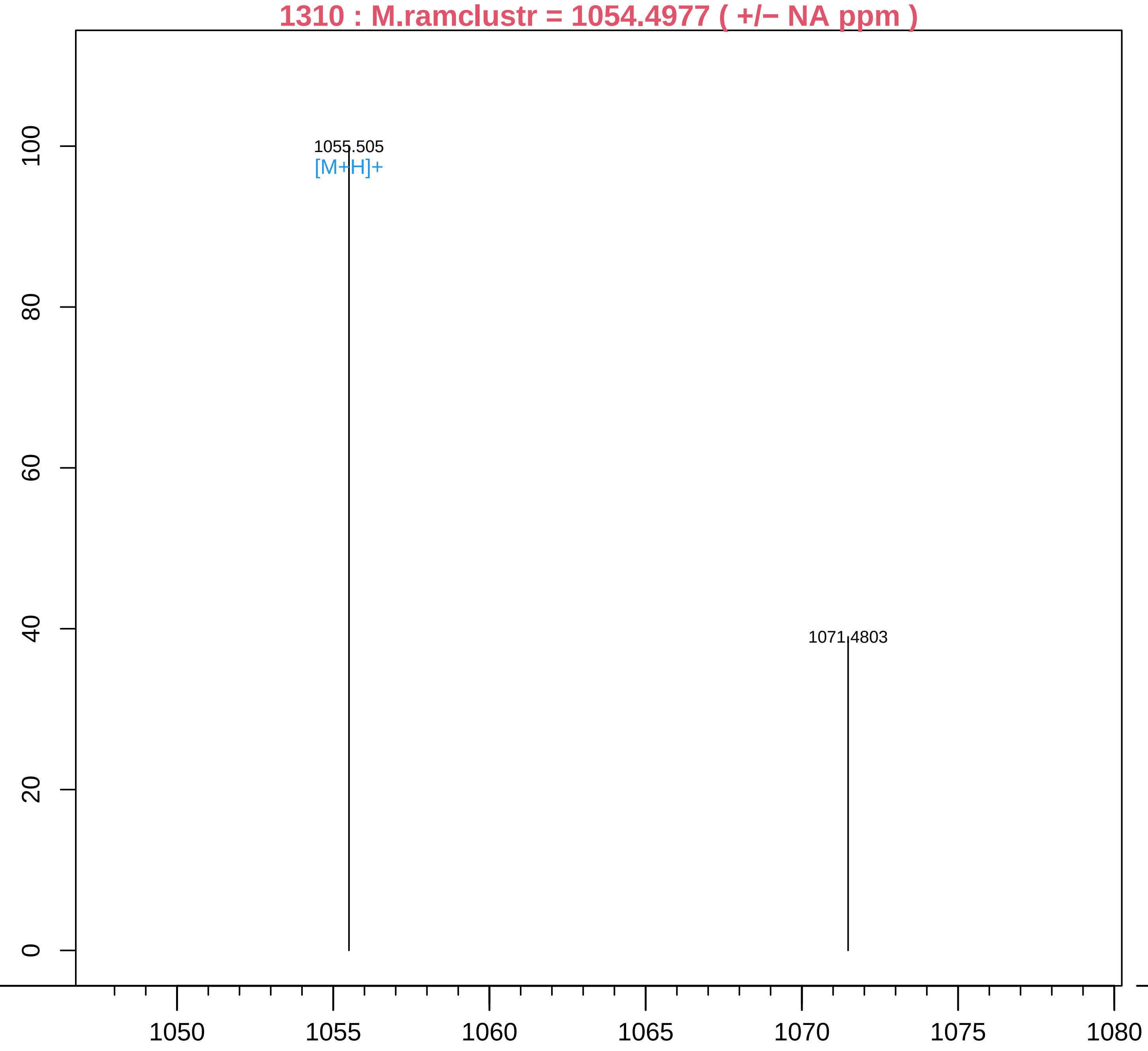

1310 : M.ramclustr = 1054.4977 (+/− NA ppm)
1055.505
[M+H]+
1071.4803
0
20
40
60
80
100
1050
1055
1060
1065
1070
1075
1080

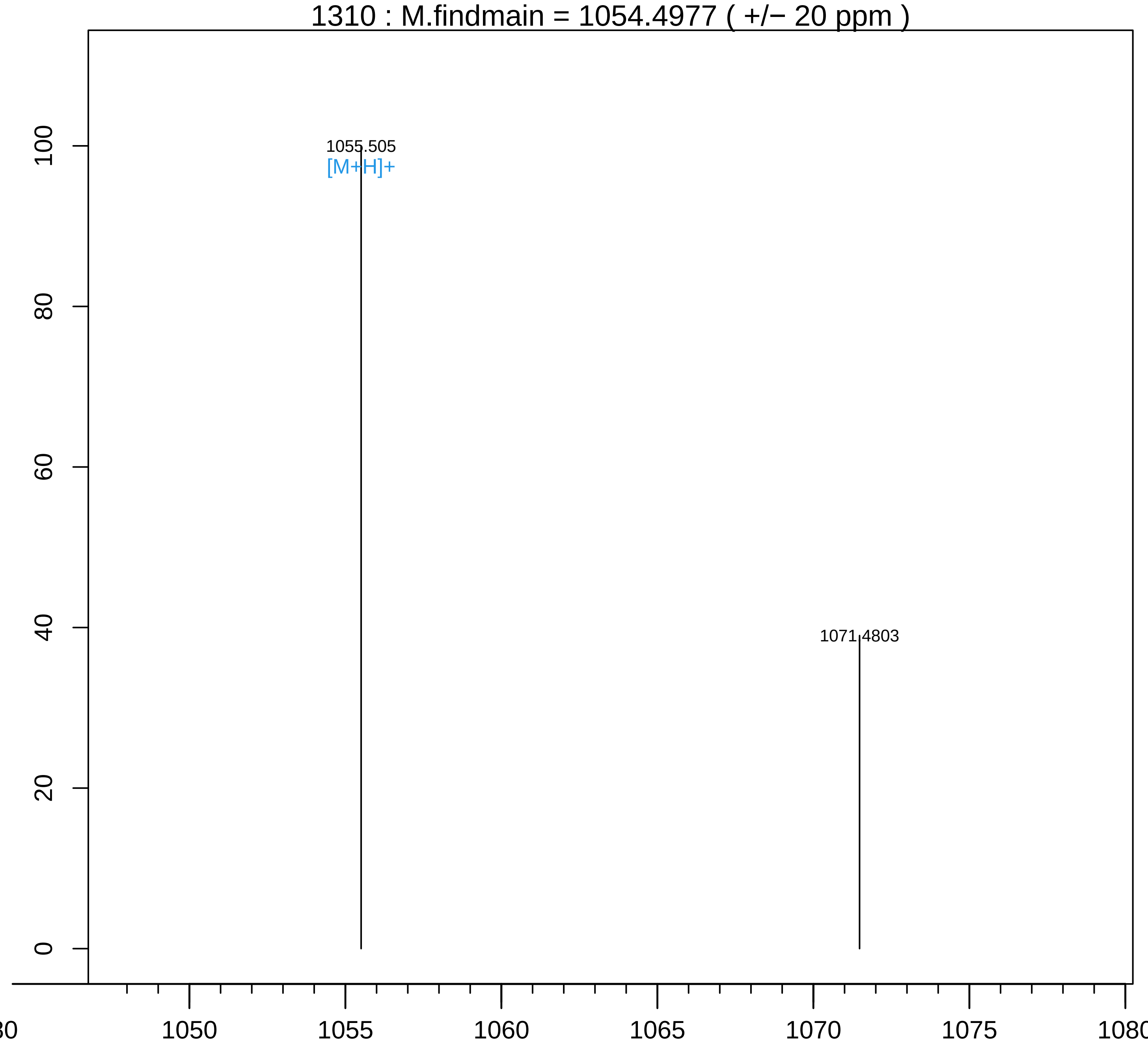

1310 : M.findmain = 1054.4977 (+/− 20 ppm)
1055.505
[M+H]+
1071.4803
0
20
40
60
80
100
1050
1055
1060
1065
1070
1075
1080

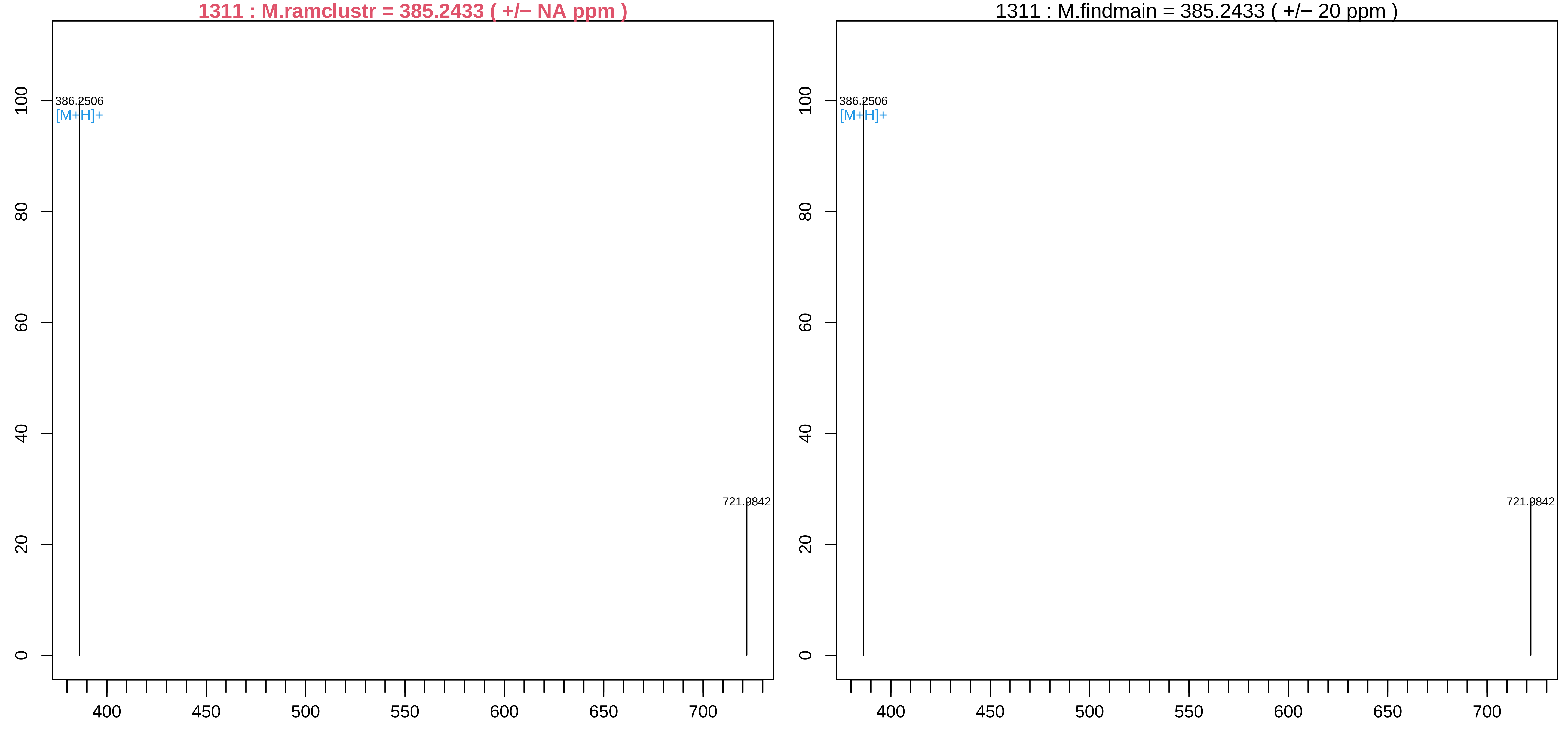
1311 : M.ramclustr = 385.2433 (+/− NA ppm)
386.2506
[M+H]+
721.9842
1311 : M.findmain = 385.2433 (+/− 20 ppm)
386.2506
[M+H]+
721.9842
0
20
40
60
80
100
400
450
500
550
600
650
700

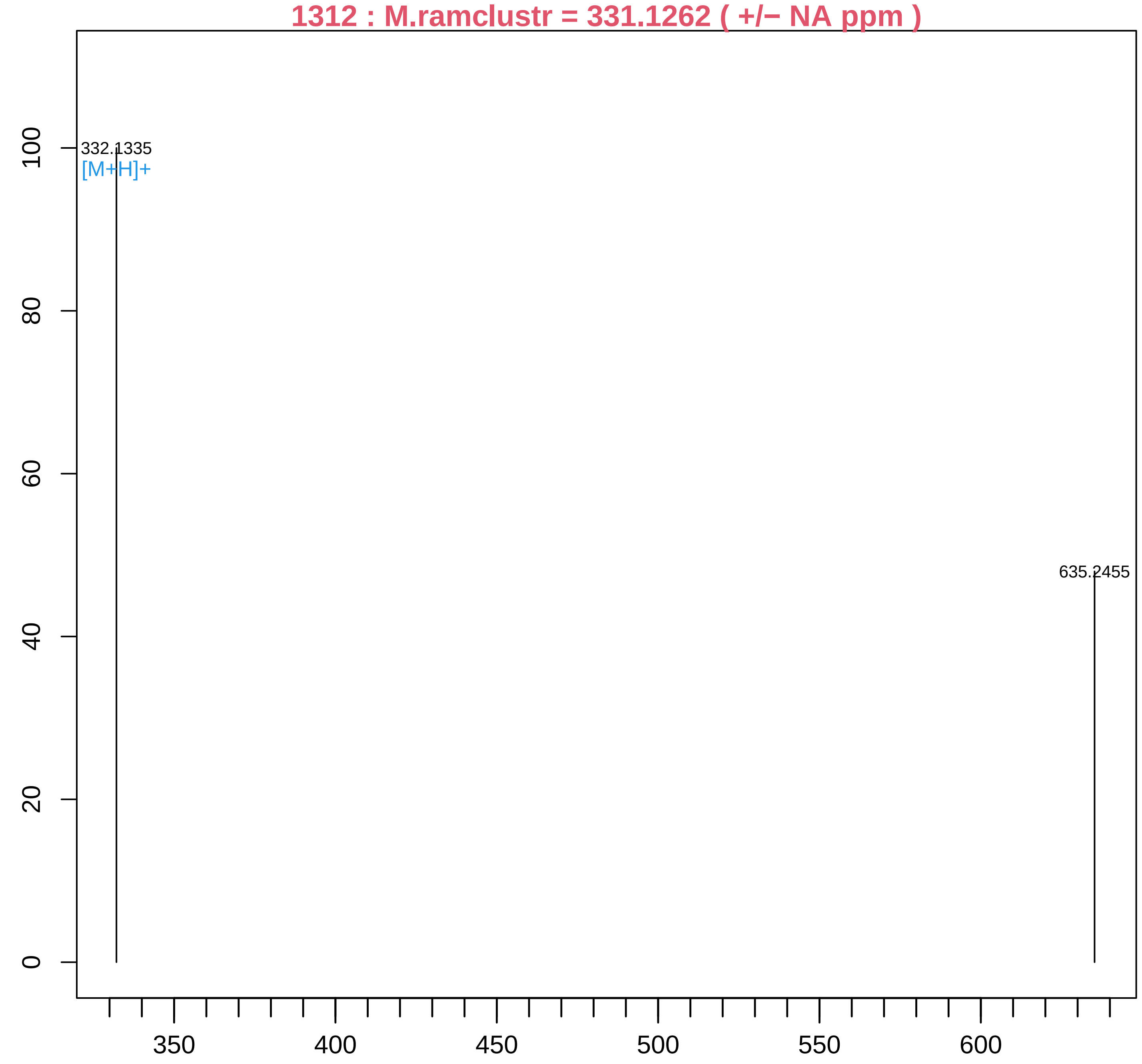
1312 : M.ramclustr = 331.1262 (+/− NA ppm)
332.1335
[M+H]+
635.2455
0
20
40
60
80
100
350
400
450
500
550
600

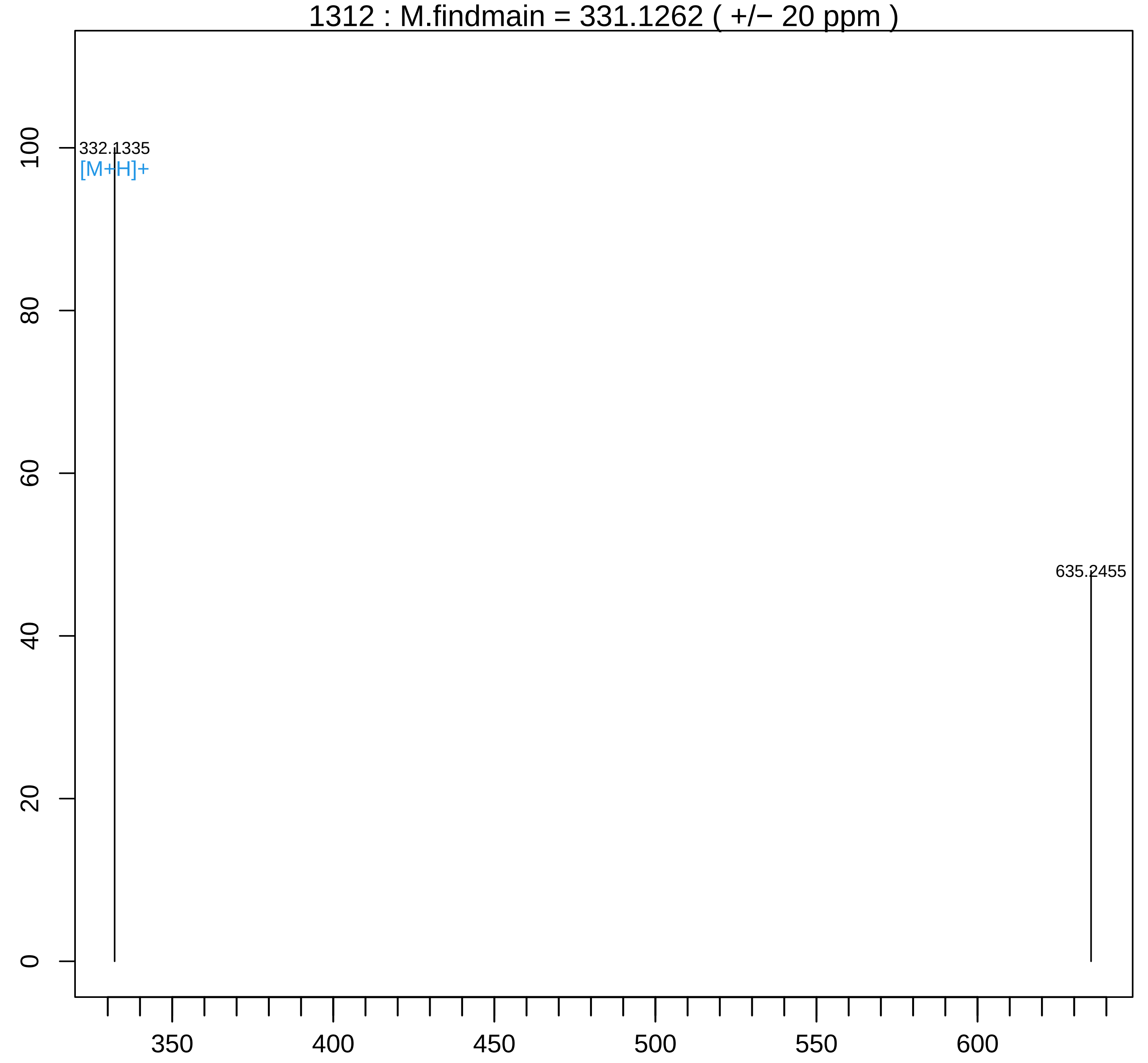
1312 : M.findmain = 331.1262 (+/− 20 ppm)
332.1335
[M+H]+
635.2455
0
20
40
60
80
100
350
400
450
500
550
600

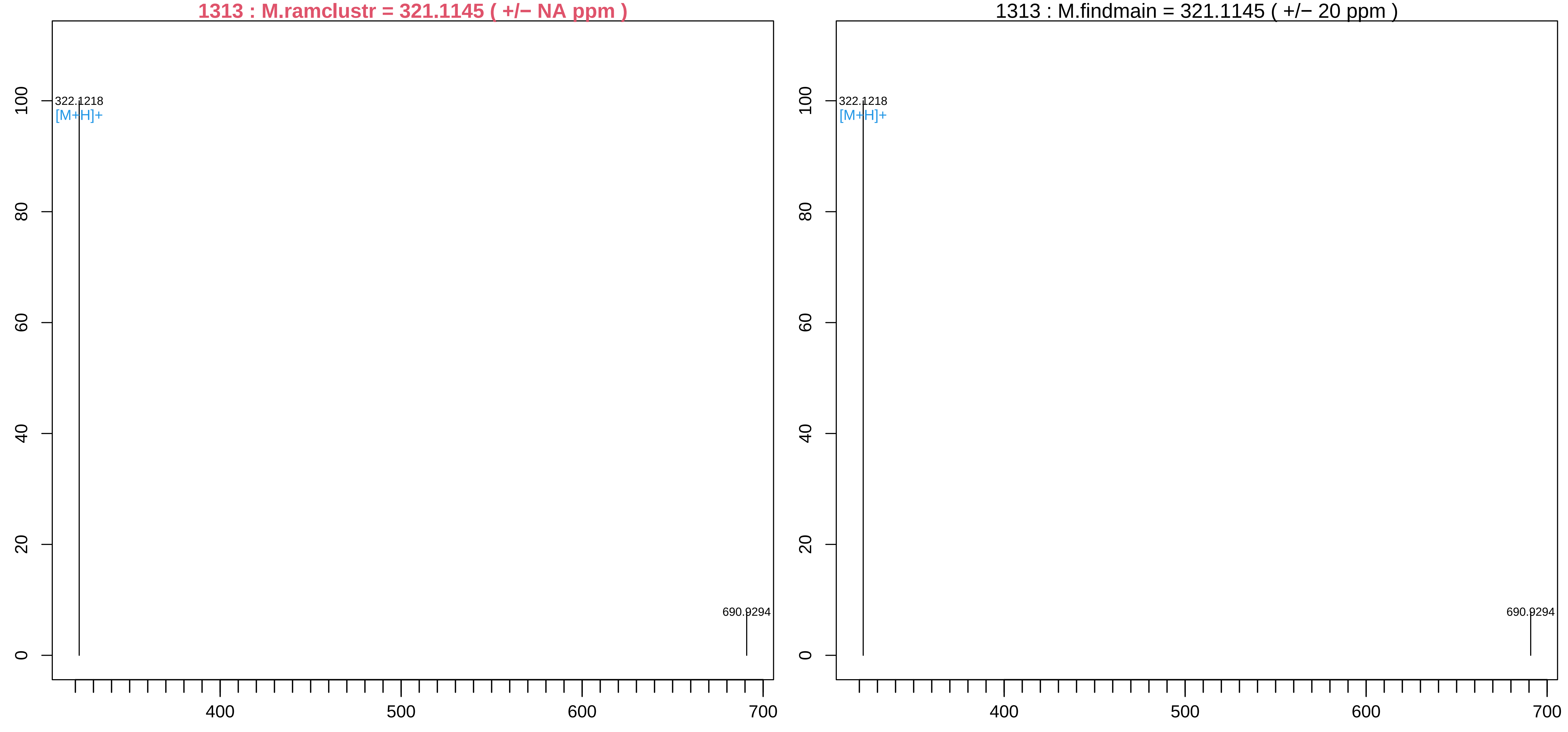

1313 : M.ramclustr = 321.1145 (+/− NA ppm)
322.1218
[M+H]+
690.9294
1313 : M.findmain = 321.1145 (+/− 20 ppm)
322.1218
[M+H]+
690.9294
0
20
40
60
80
100
400
500
600
700

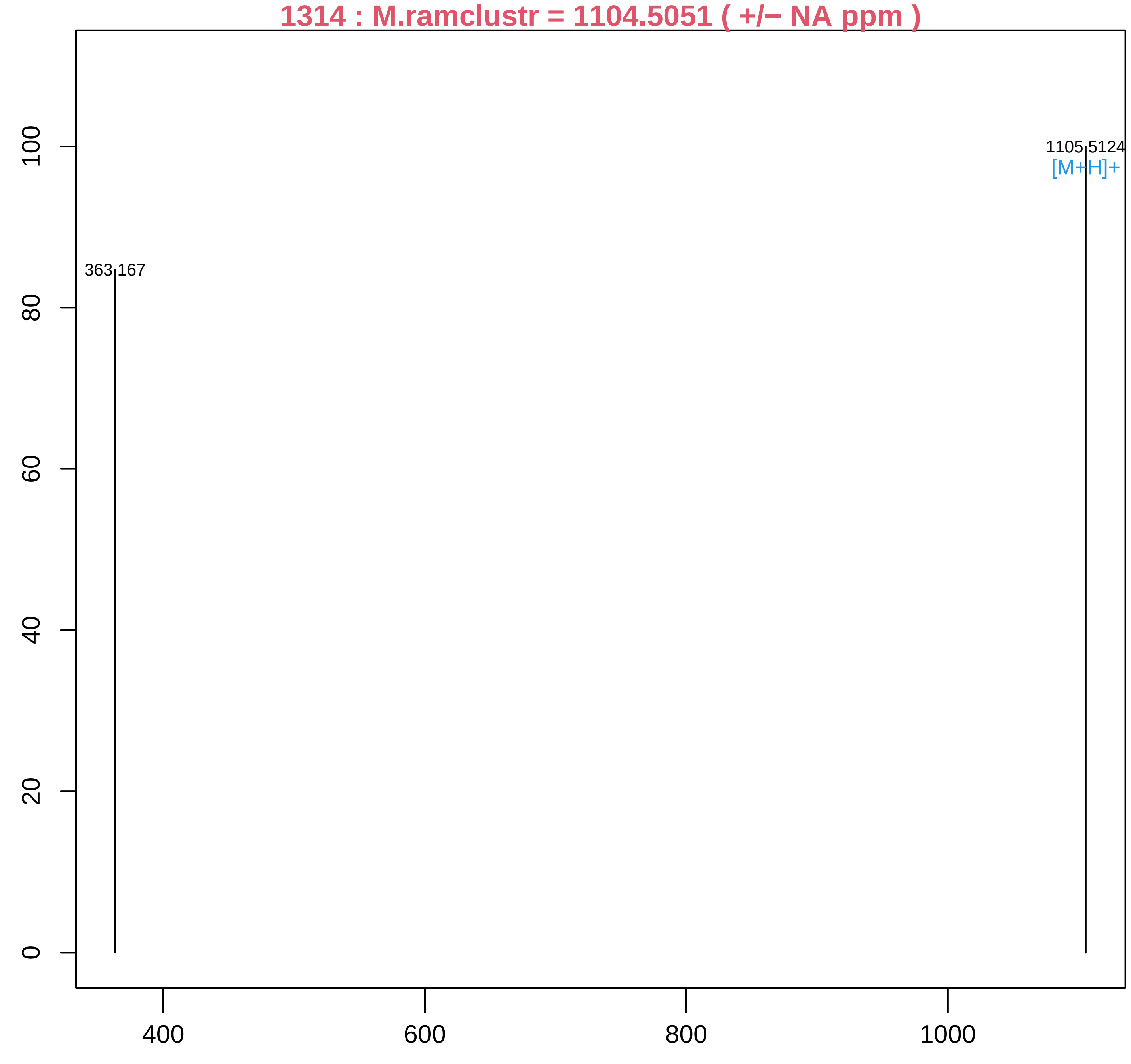

1314 : M.ramclustr = 1104.5051 (+/− NA ppm)
1105.5124
[M+H]+
363.167
0
20
40
60
80
100
400
600
800
1000

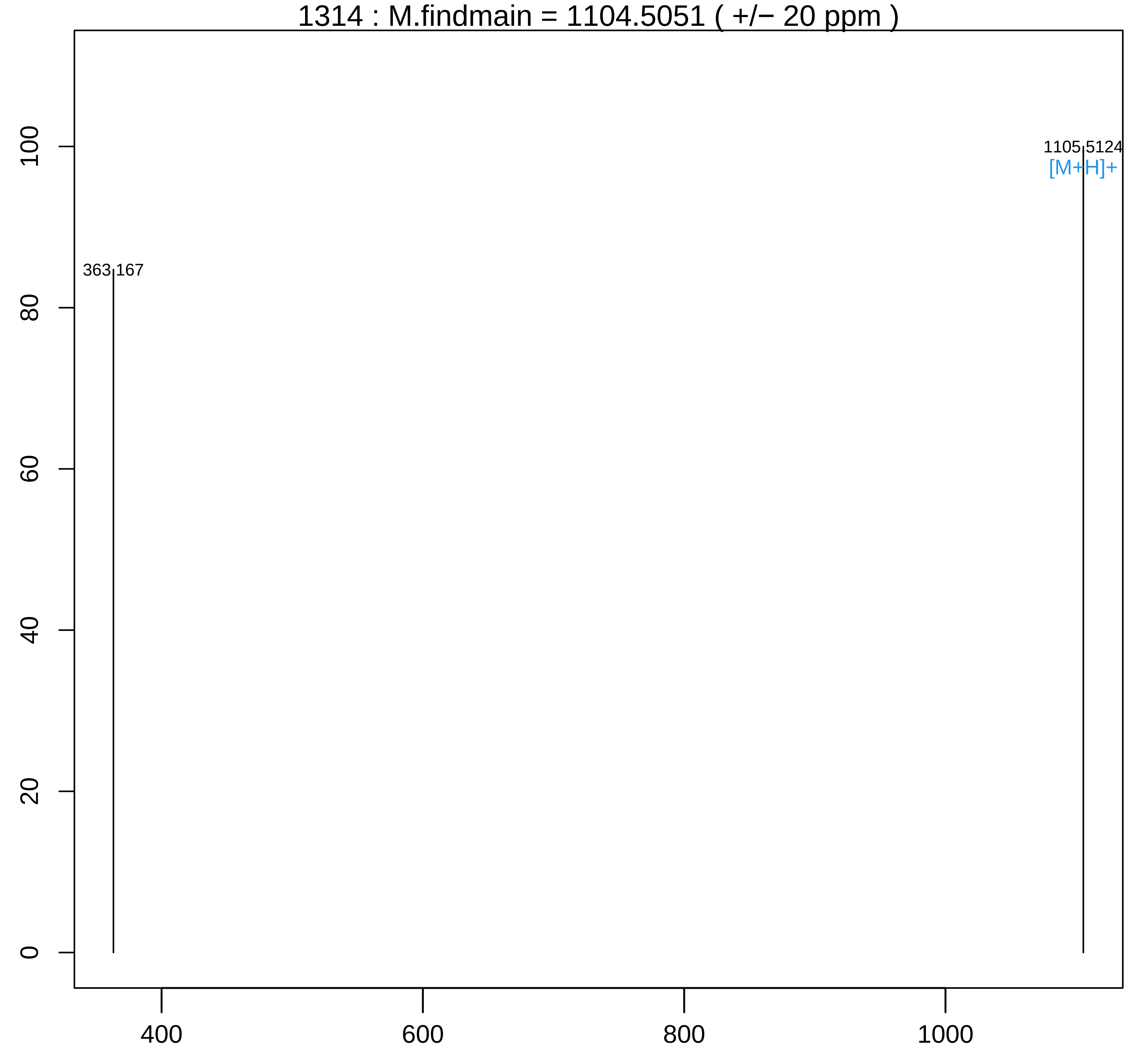

1314 : M.findmain = 1104.5051 (+/− 20 ppm)
1105.5124
[M+H]+
363.167
0
20
40
60
80
100
400
600
800
1000

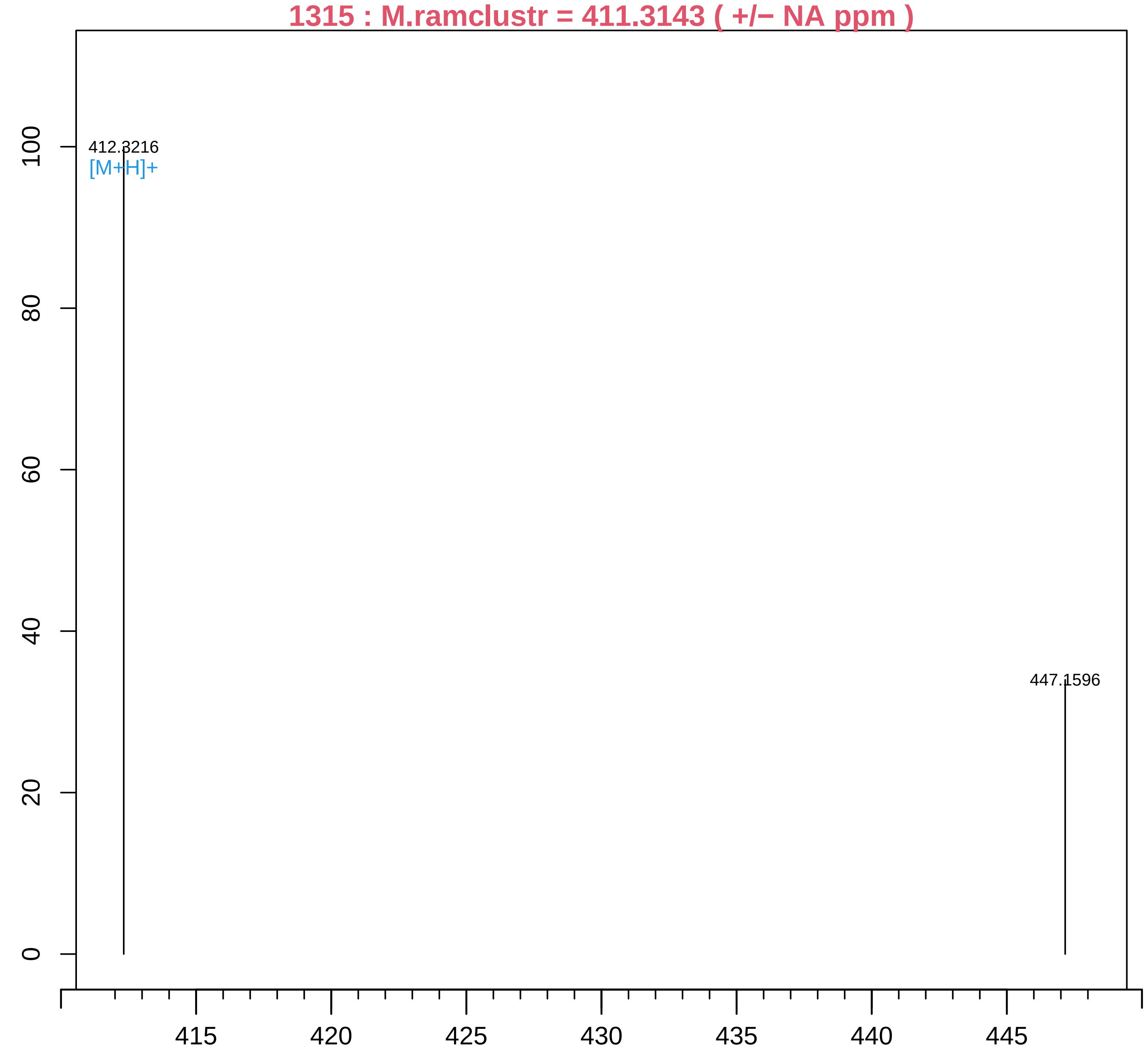
1315 : M.ramclustr = 411.3143 (+/− NA ppm)
412.3216
[M+H]+
447.1596
0
20
40
60
80
100
415
420
425
430
435
440
445

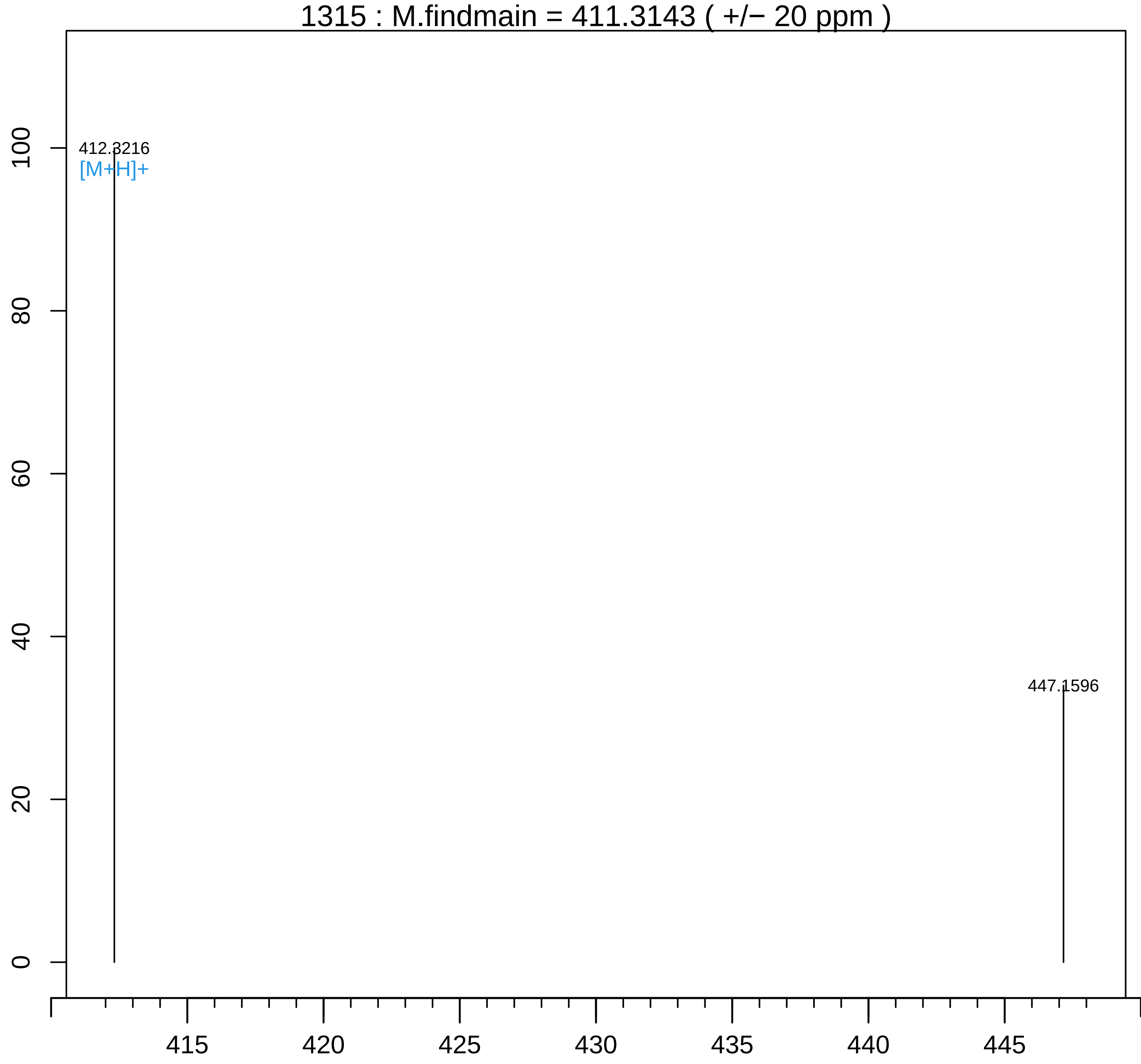
1315 : M.findmain = 411.3143 (+/− 20 ppm)
412.3216
[M+H]+
447.1596
0
20
40
60
80
100
415
420
425
430
435
440
445

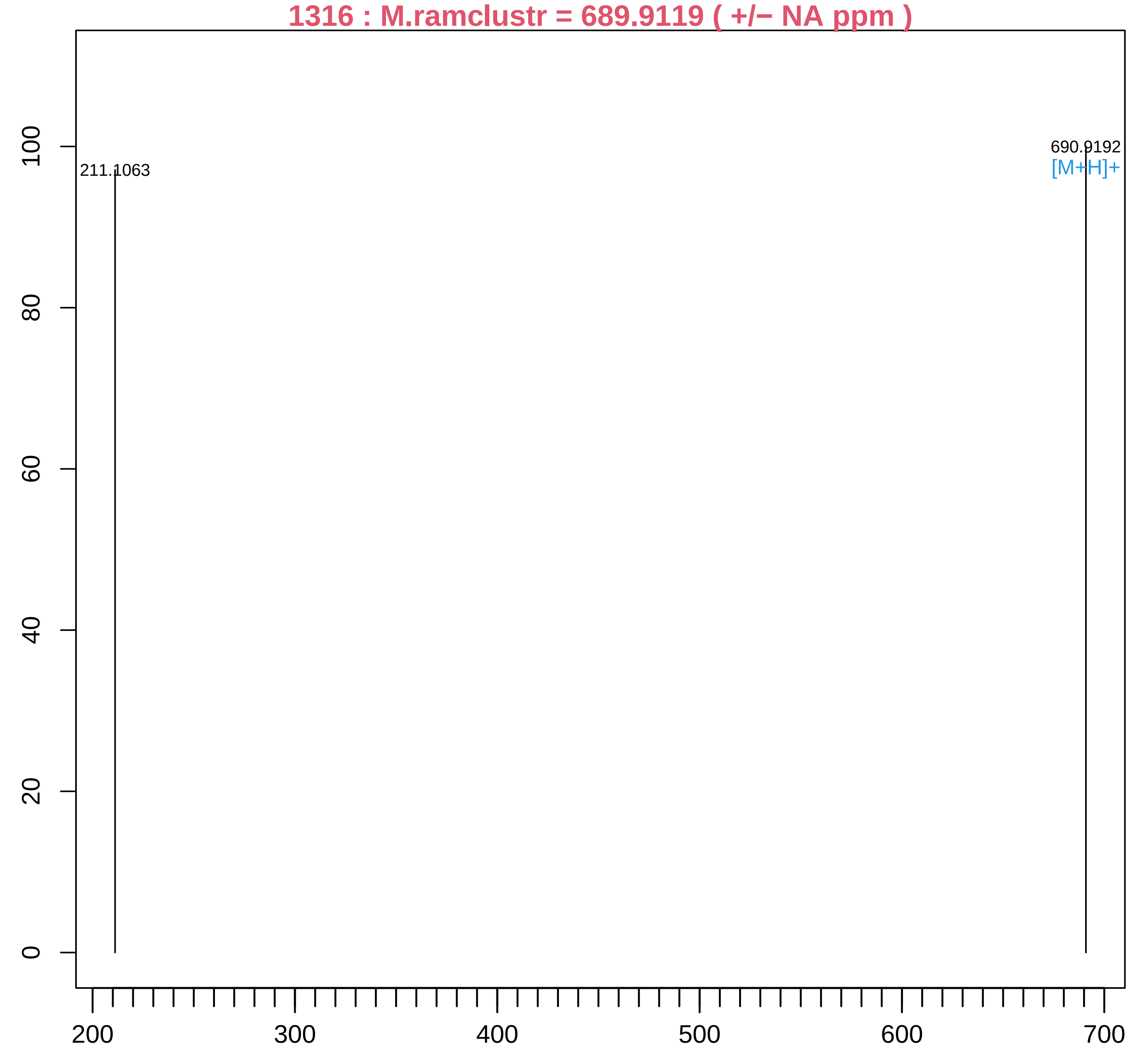

1316 : M.ramclustr = 689.9119 (+/− NA ppm)
211.1063
690.9192
[M+H]+
0
20
40
60
80
100
200
300
400
500
600
700

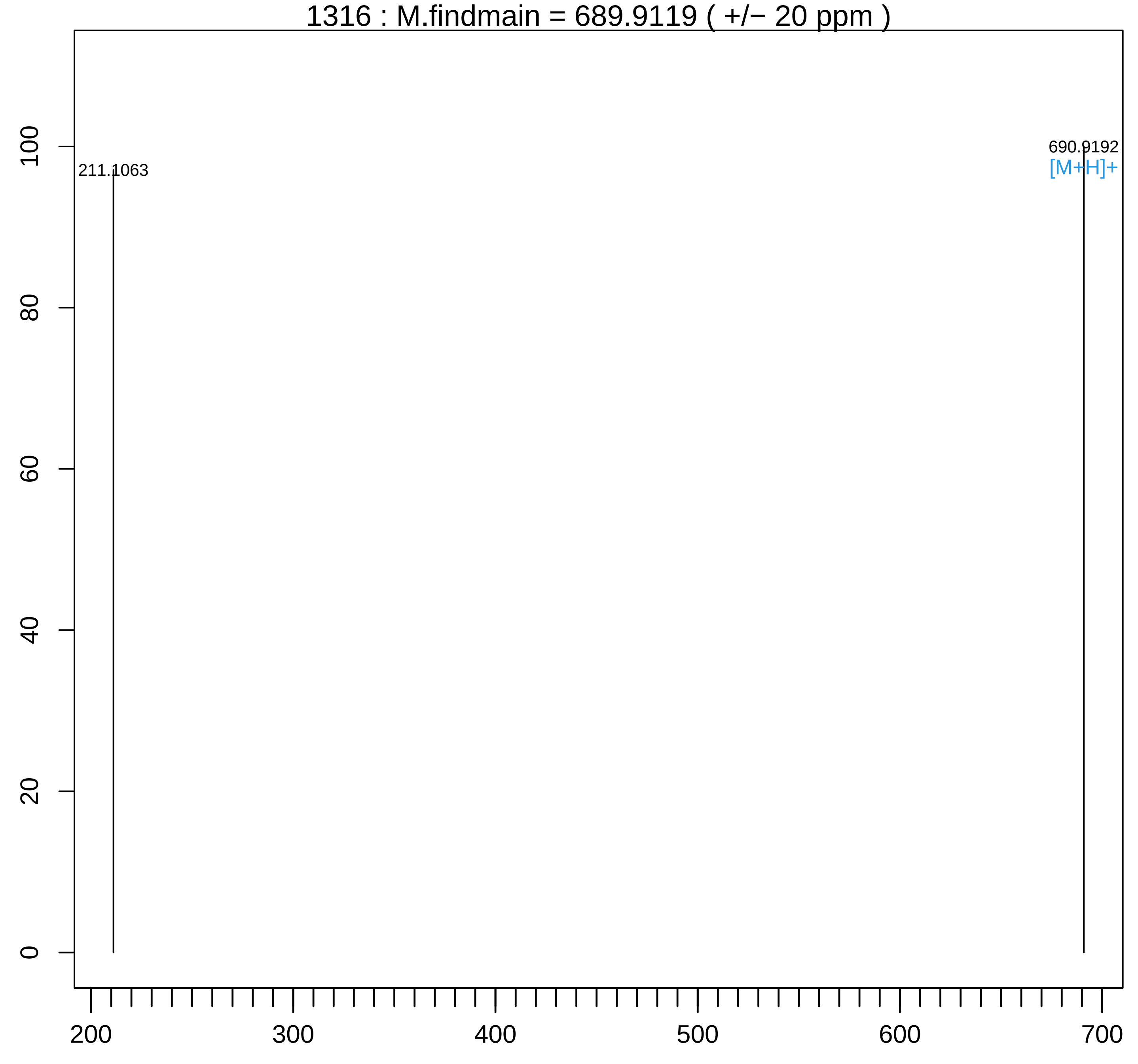

1316 : M.findmain = 689.9119 (+/− 20 ppm)
211.1063
690.9192
[M+H]+
0
20
40
60
80
100
200
300
400
500
600
700

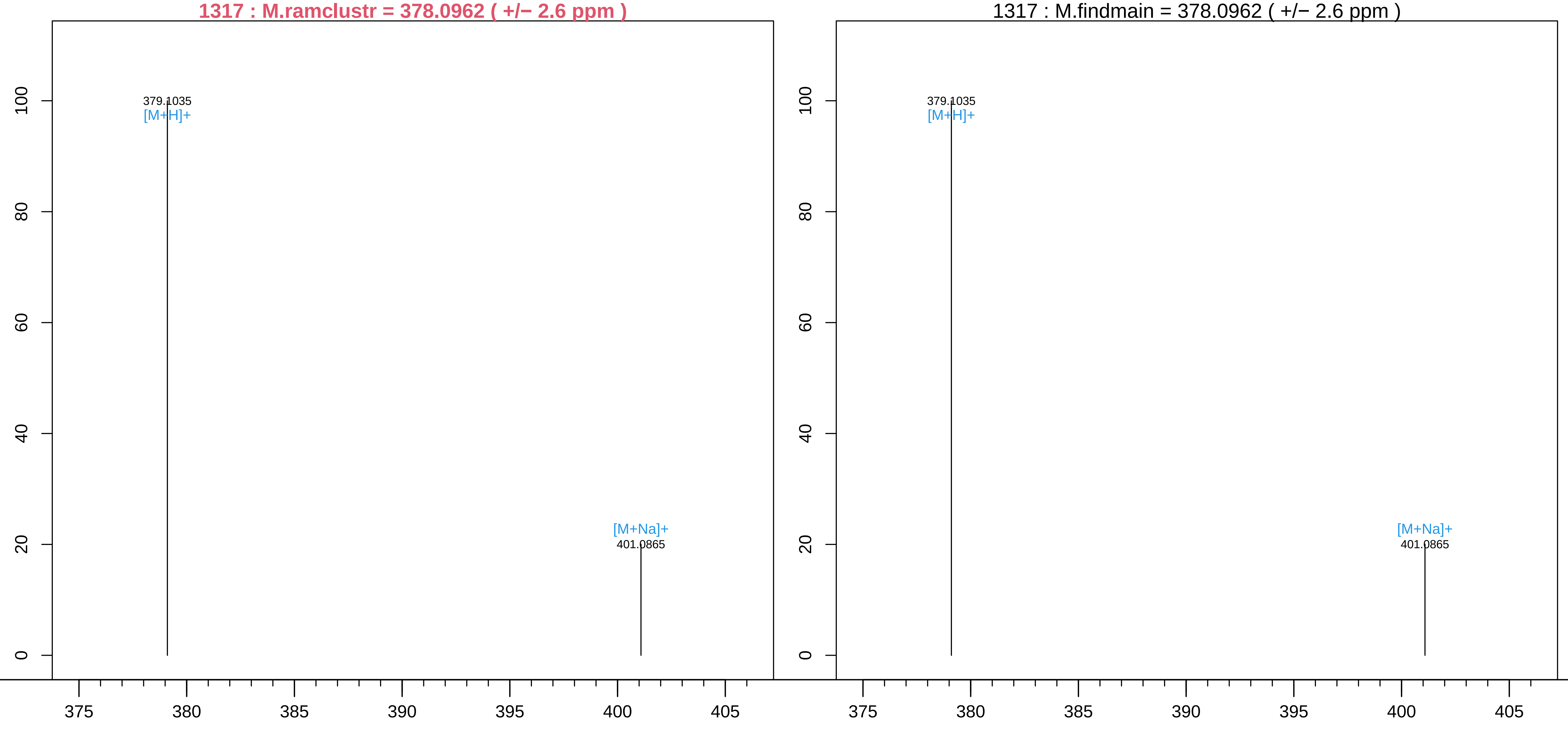

1317 : M.ramclustr = 378.0962 (+/− 2.6 ppm)
379.1035
[M+H]+
[M+Na]+
401.0865
1317 : M.findmain = 378.0962 (+/− 2.6 ppm)
379.1035
[M+H]+
[M+Na]+
401.0865

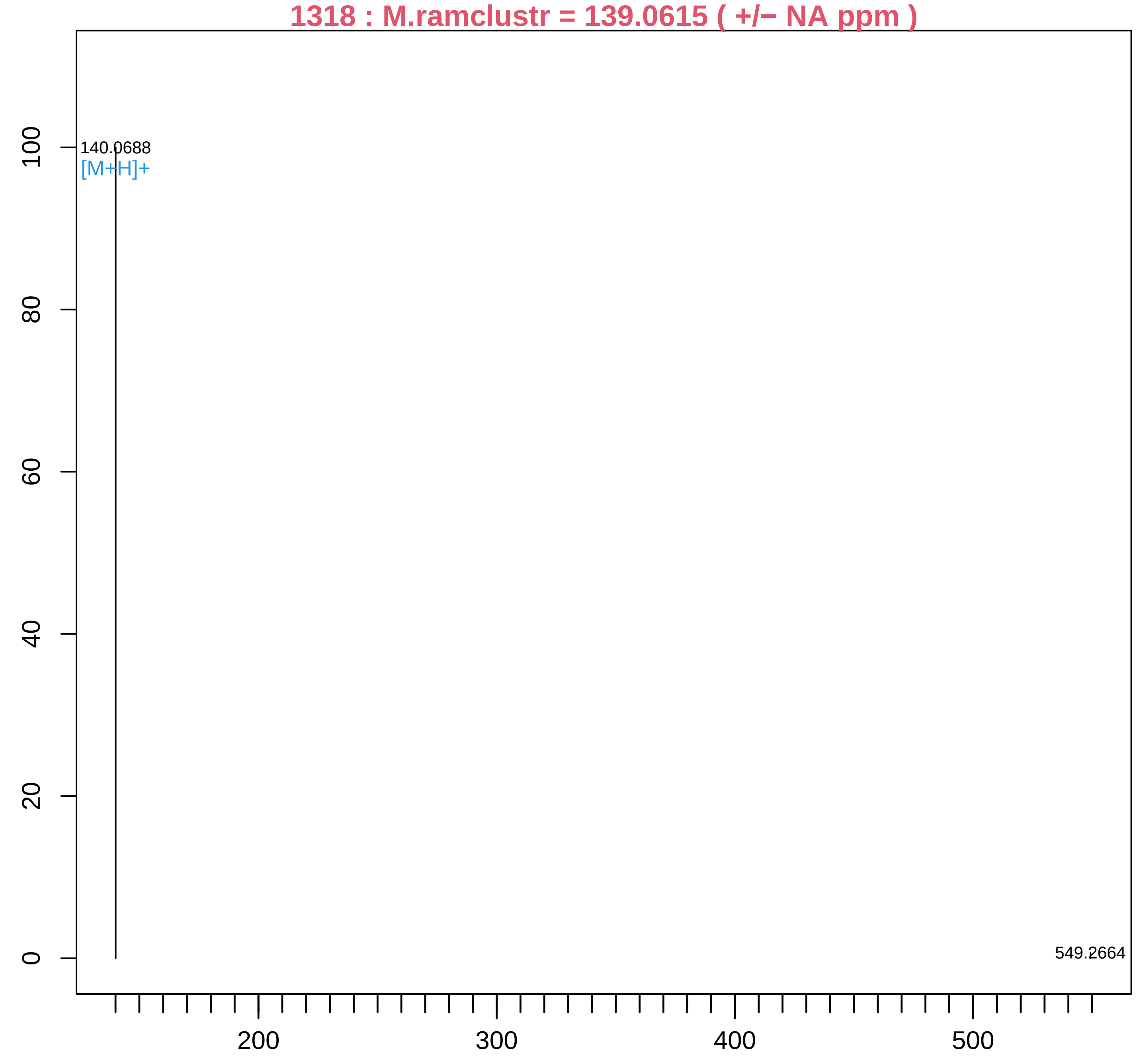
1318 : M.ramclustr = 139.0615 (+/− NA ppm)
140.0688
[M+H]+
549.2664
0
20
40
60
80
100
200
300
400
500

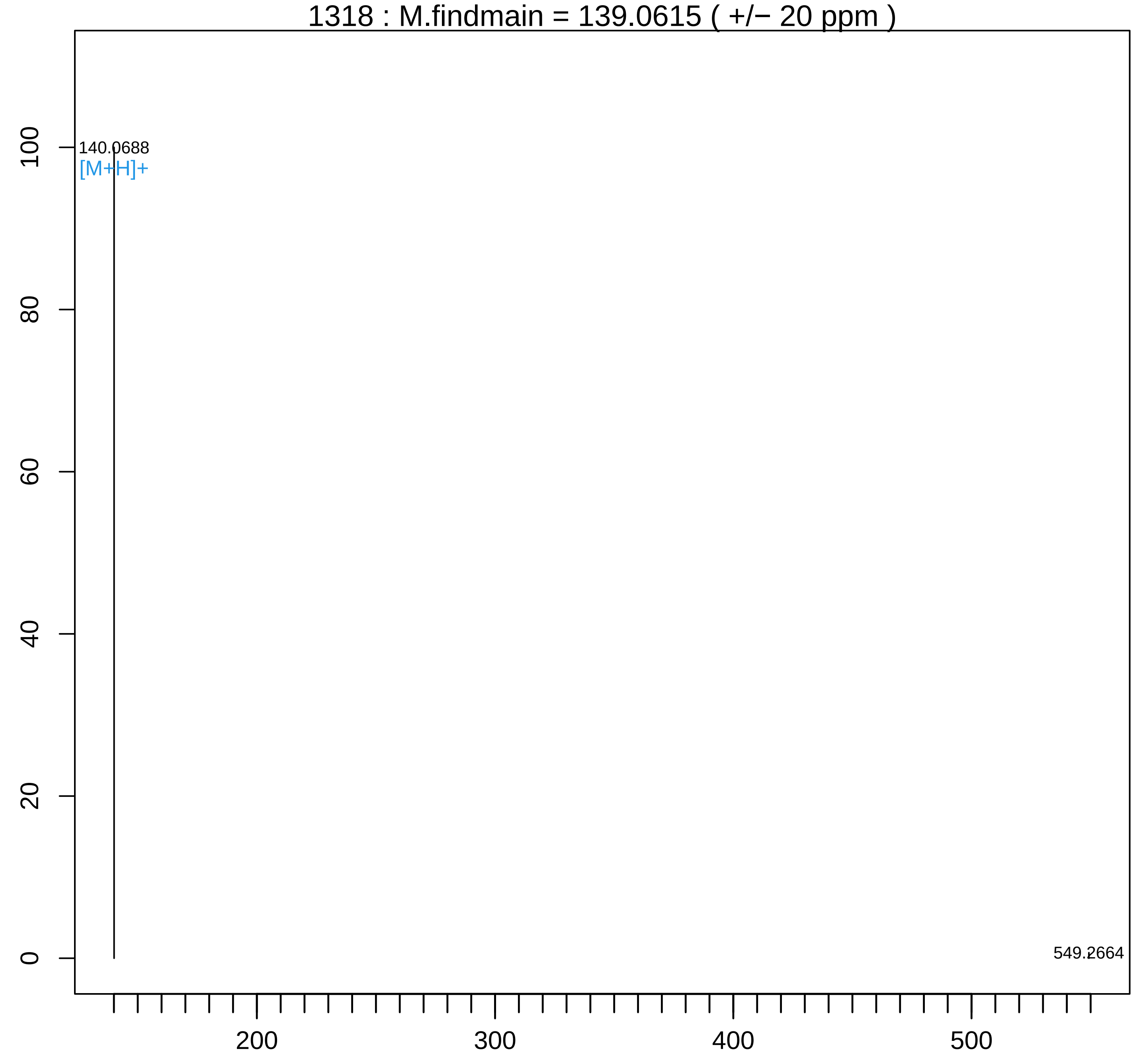
1318 : M.findmain = 139.0615 (+/− 20 ppm)
140.0688
[M+H]+
549.2664
0
20
40
60
80
100
200
300
400
500

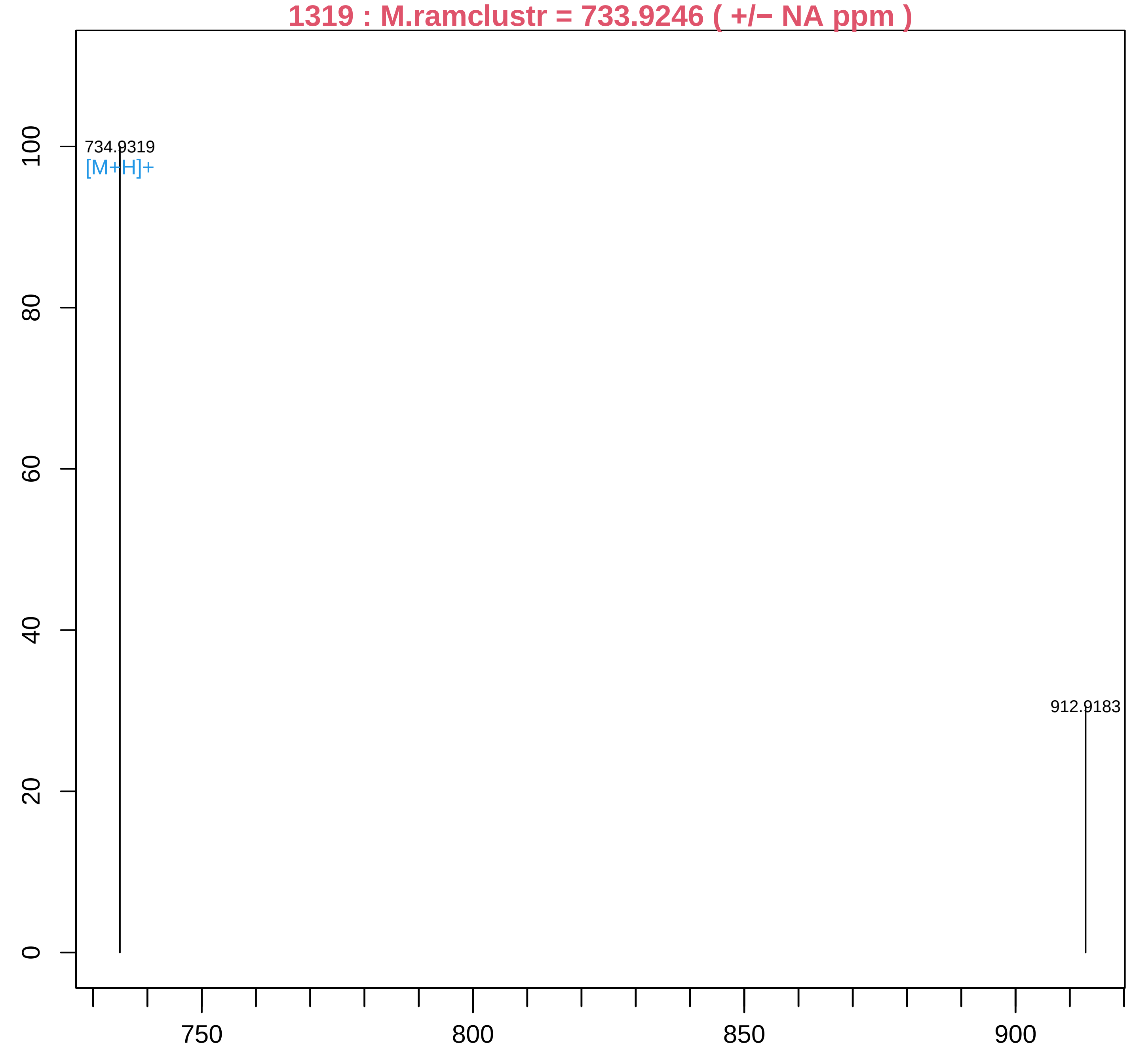

1319 : M.ramclustr = 733.9246 (+/− NA ppm)
734.9319
[M+H]+
912.9183
0
20
40
60
80
100
750
800
850
900

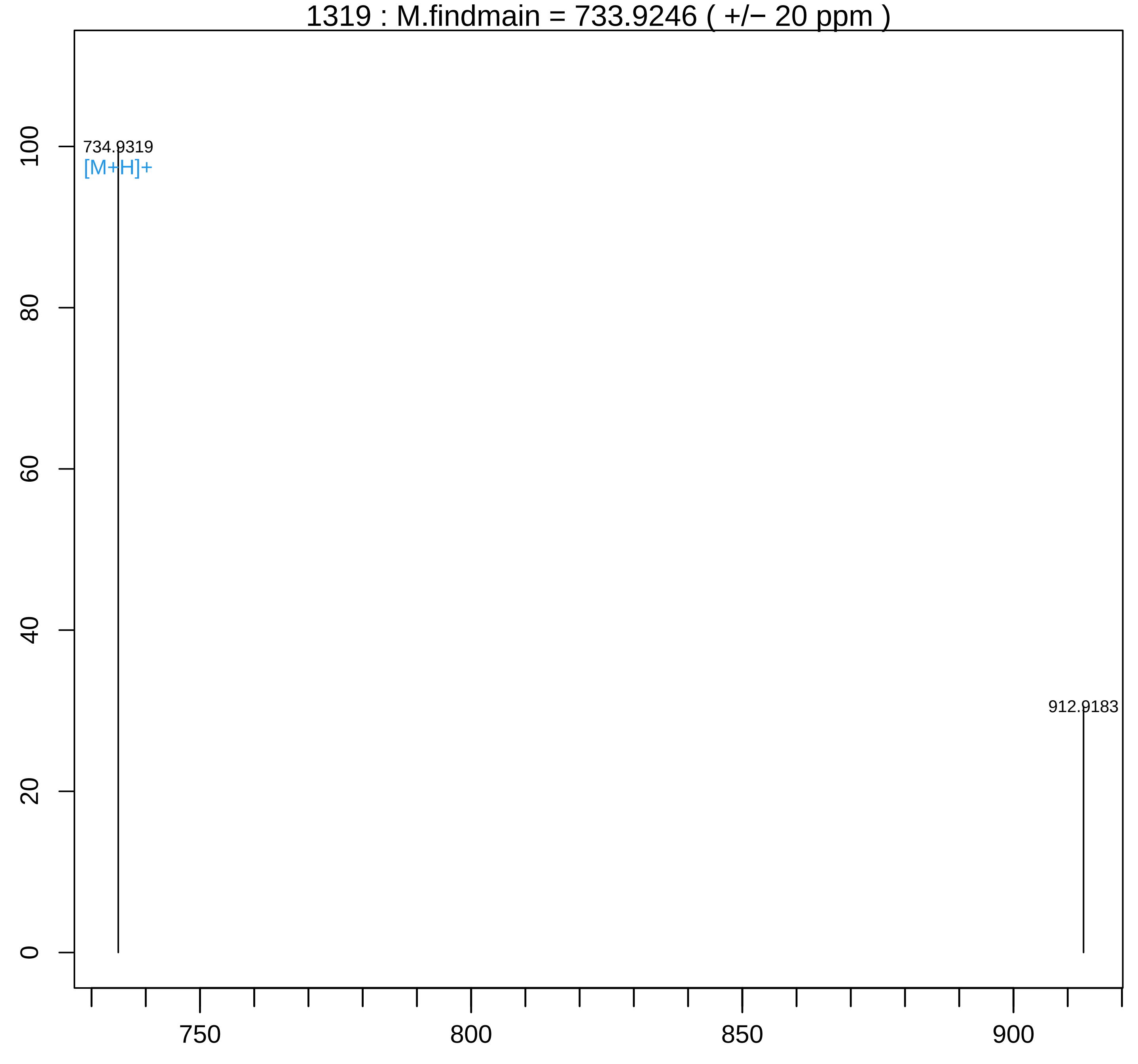

1319 : M.findmain = 733.9246 (+/− 20 ppm)
734.9319
[M+H]+
912.9183
0
20
40
60
80
100
750
800
850
900

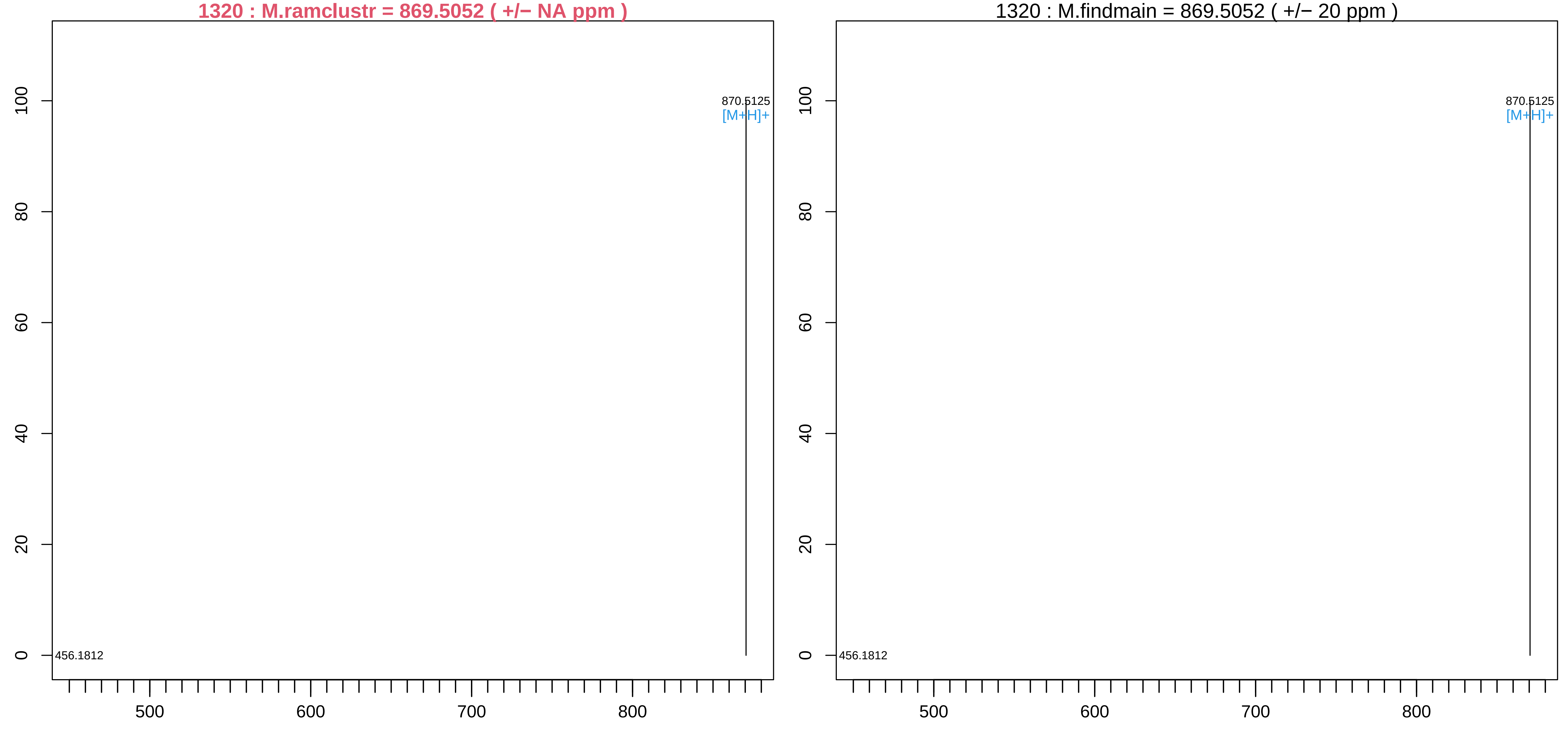
1320 : M.ramclustr = 869.5052 (+/− NA ppm)
870.5125
[M+H]+
456.1812
1320 : M.findmain = 869.5052 (+/− 20 ppm)
870.5125
[M+H]+
456.1812
0
20
40
60
80
100
500
600
700
800

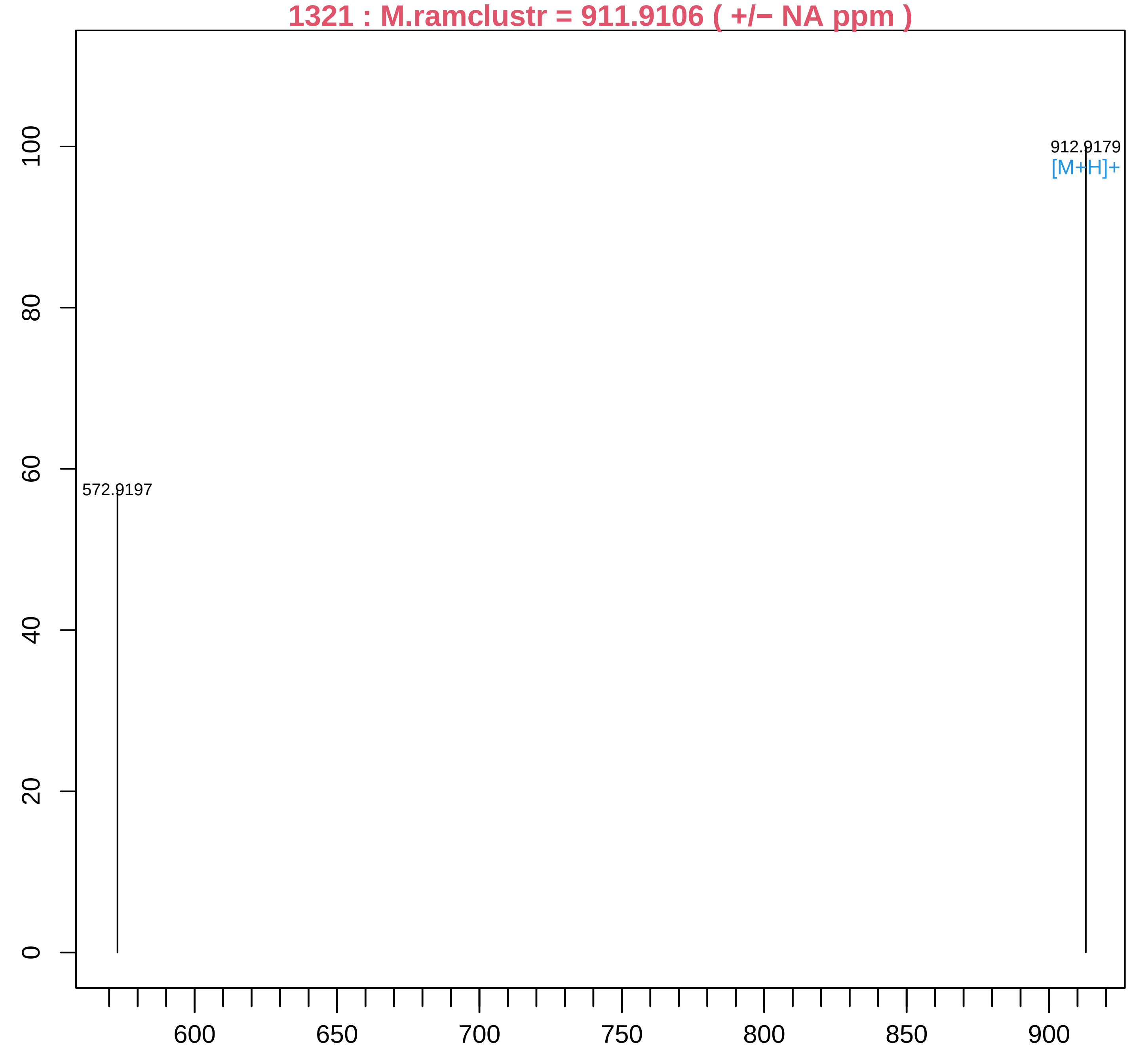
1321 : M.ramclustr = 911.9106 (+/− NA ppm)
912.9179
[M+H]+
572.9197
0
20
40
60
80
100
600
650
700
750
800
850
900

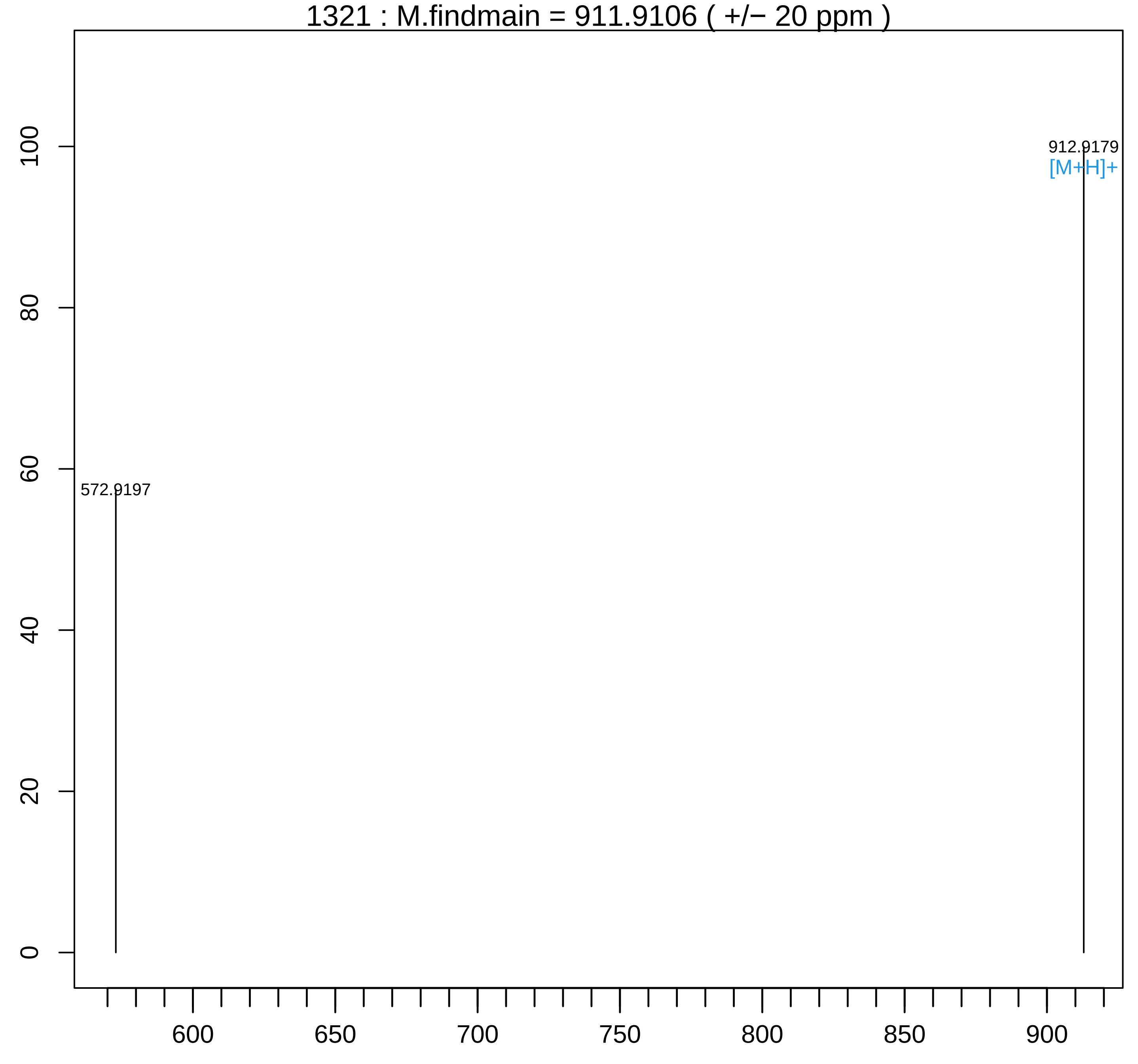
1321 : M.findmain = 911.9106 (+/− 20 ppm)
912.9179
[M+H]+
572.9197
0
20
40
60
80
100
600
650
700
750
800
850
900

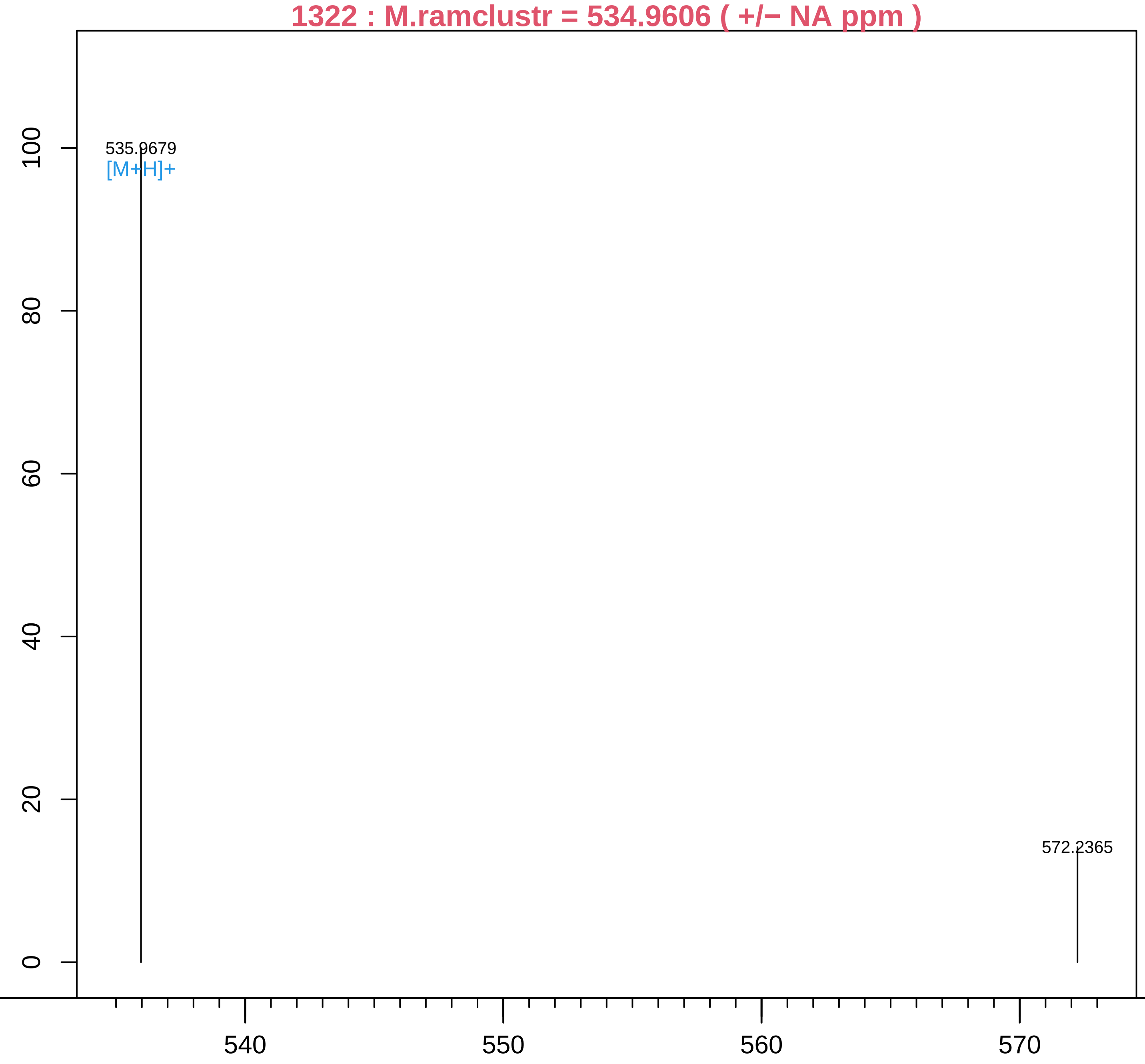
1322 : M.ramclustr = 534.9606 (+/− NA ppm)
535.9679
[M+H]+
572.2365
0
20
40
60
80
100
540
550
560
570

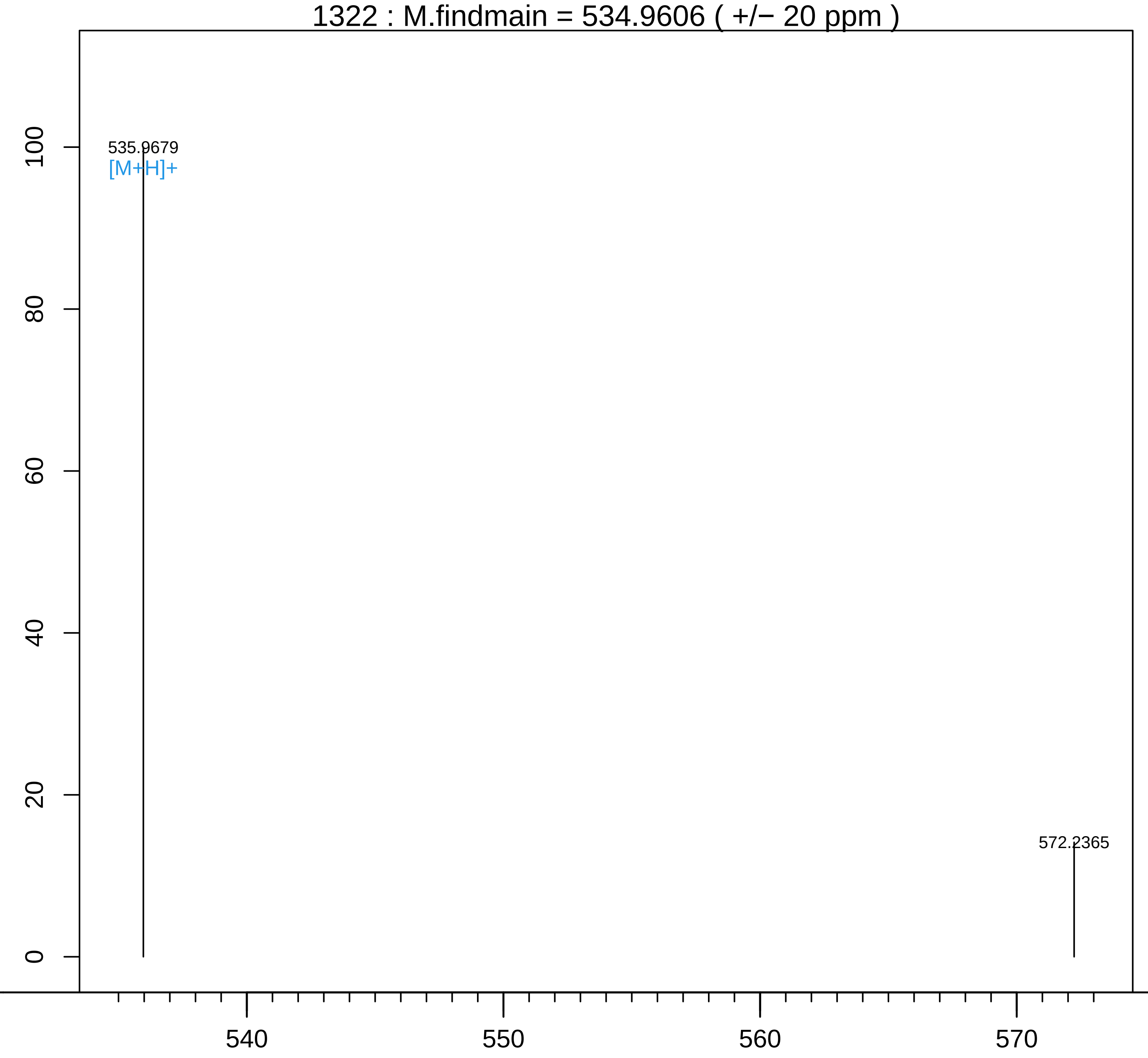
1322 : M.findmain = 534.9606 (+/− 20 ppm)
535.9679
[M+H]+
572.2365
0
20
40
60
80
100
540
550
560
570

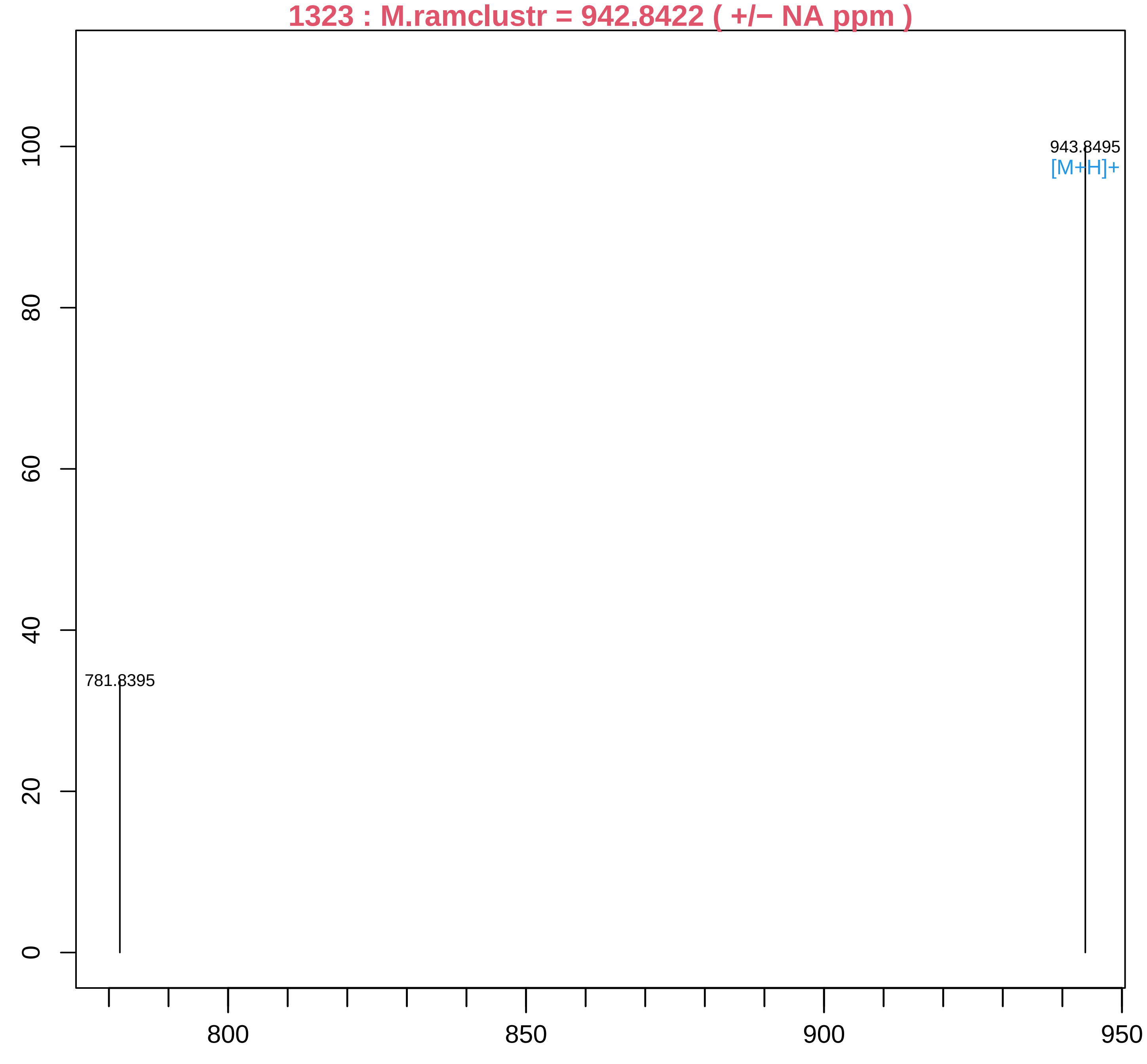
1323 : M.ramclustr = 942.8422 (+/− NA ppm)
943.8495
[M+H]+
781.8395
0
20
40
60
80
100
800
850
900
950

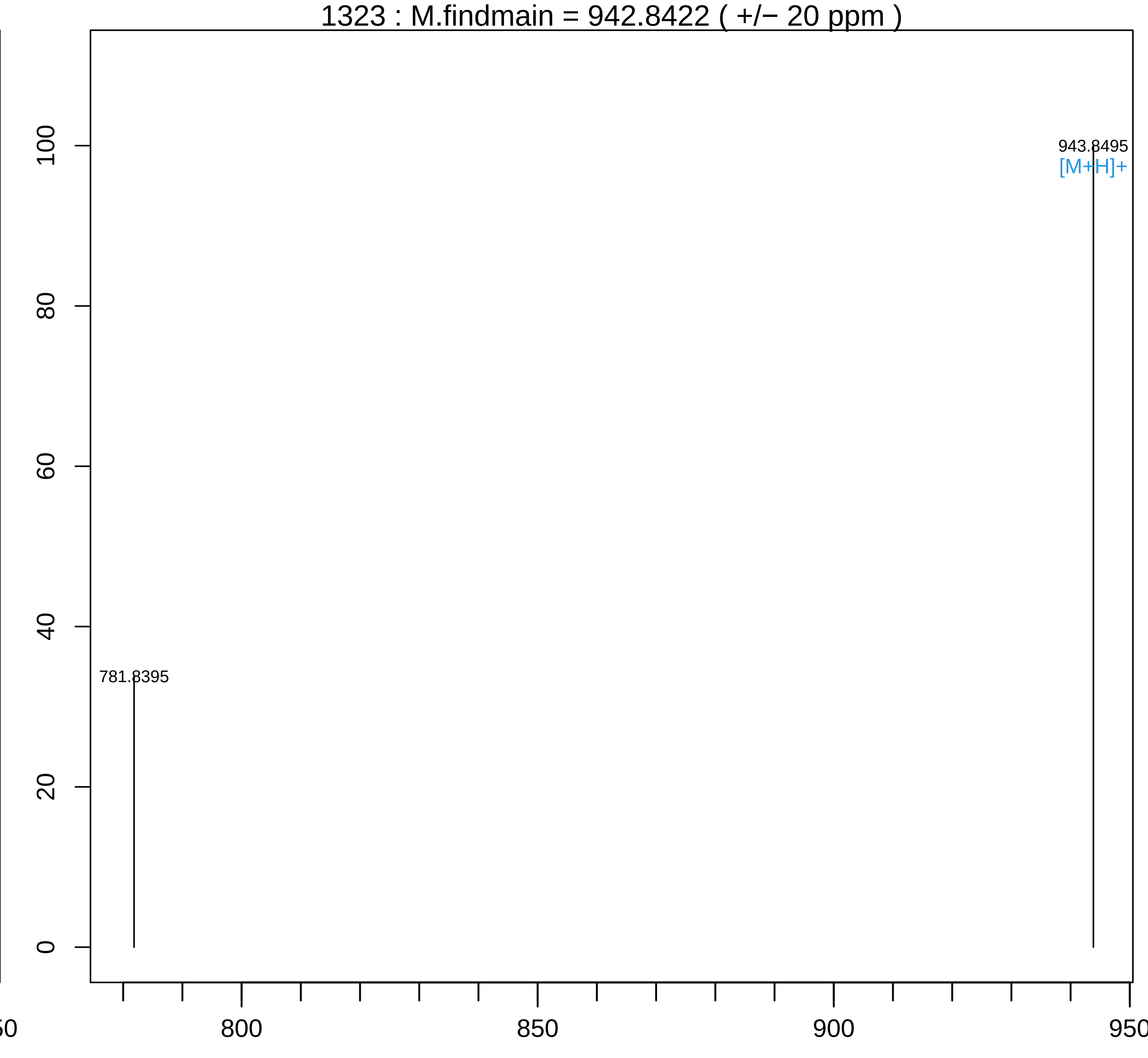
1323 : M.findmain = 942.8422 (+/− 20 ppm)
943.8495
[M+H]+
781.8395
0
20
40
60
80
100
800
850
900
950

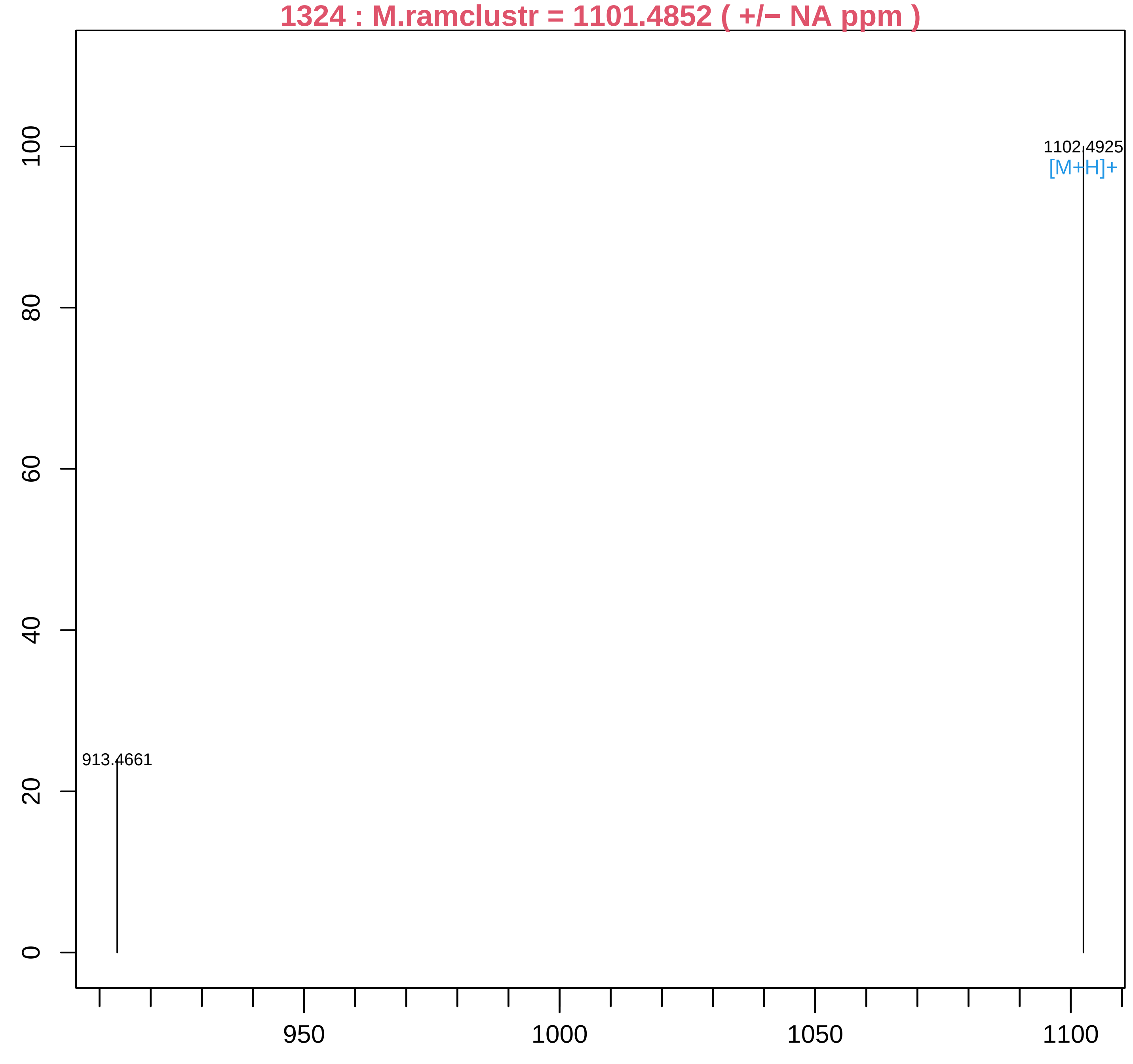
1324 : M.ramclustr = 1101.4852 (+/− NA ppm)
1102.4925
[M+H]+
913.4661
0
20
40
60
80
100
950
1000
1050
1100

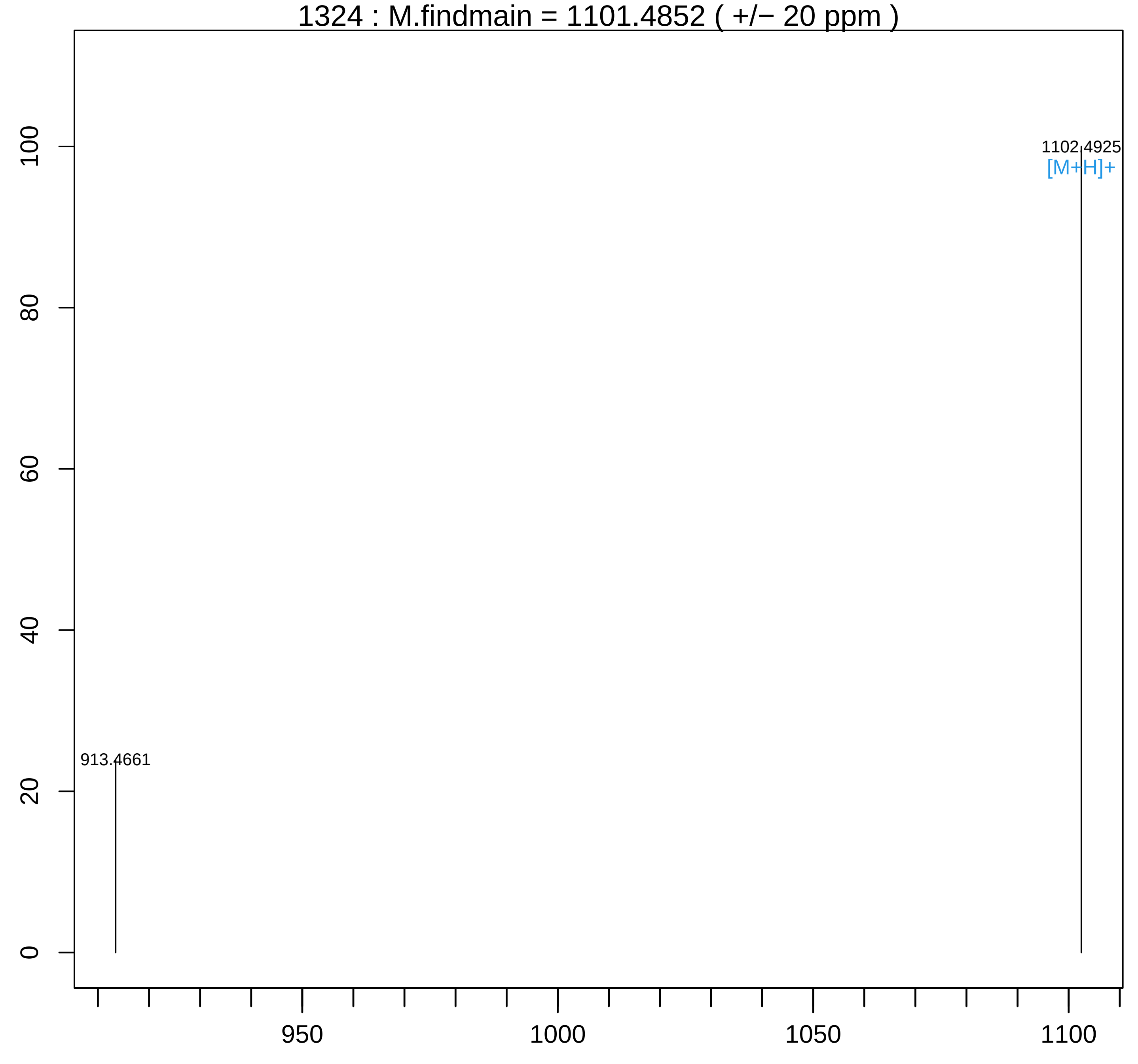
1324 : M.findmain = 1101.4852 (+/− 20 ppm)
1102.4925
[M+H]+
913.4661
0
20
40
60
80
100
950
1000
1050
1100

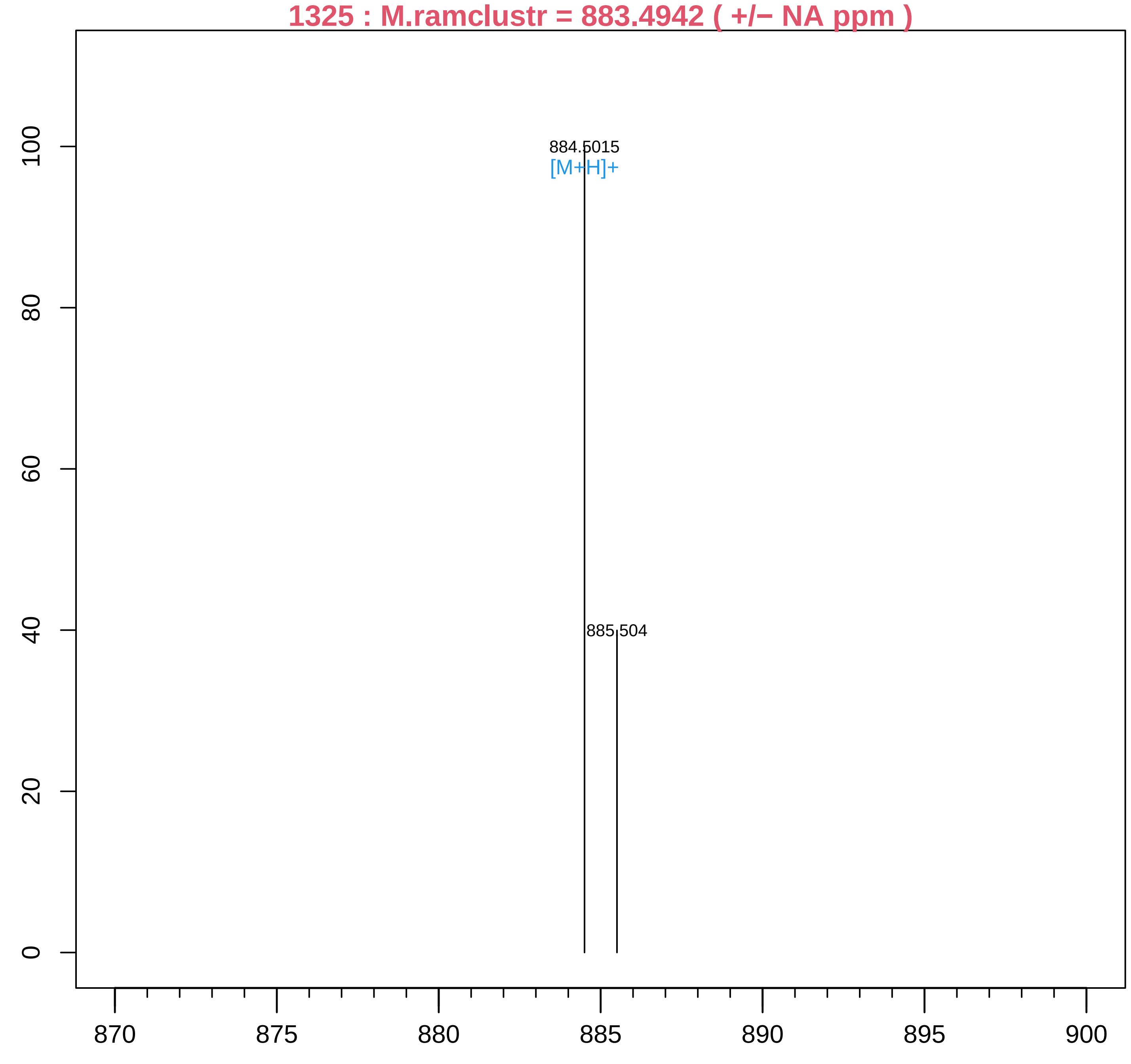
1325 : M.ramclustr = 883.4942 (+/− NA ppm)
884.5015
[M+H]+
885.504
0
20
40
60
80
100
870
875
880
885
890
895
900

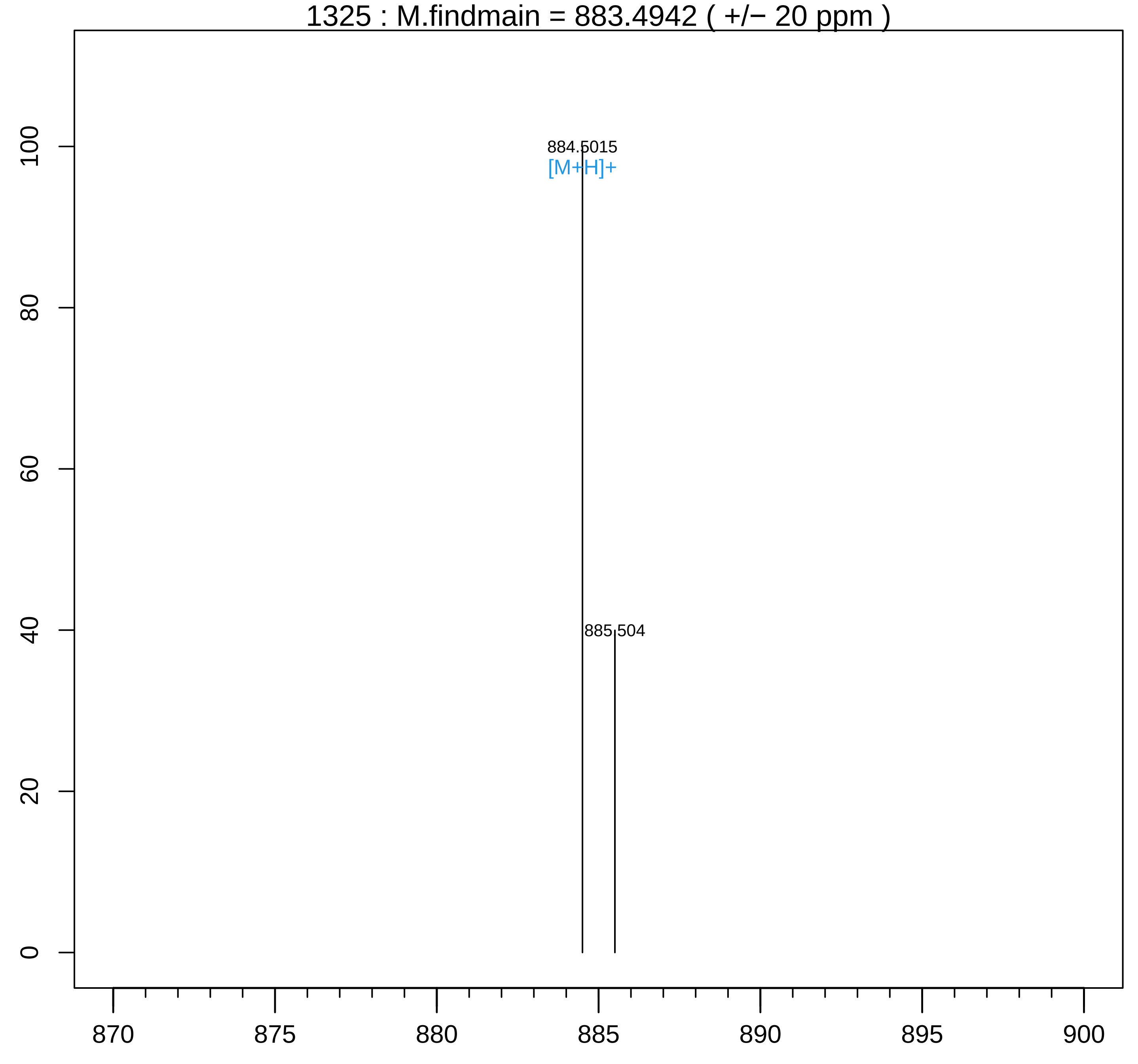
1325 : M.findmain = 883.4942 (+/− 20 ppm)
884.5015
[M+H]+
885.504
0
20
40
60
80
100
870
875
880
885
890
895
900

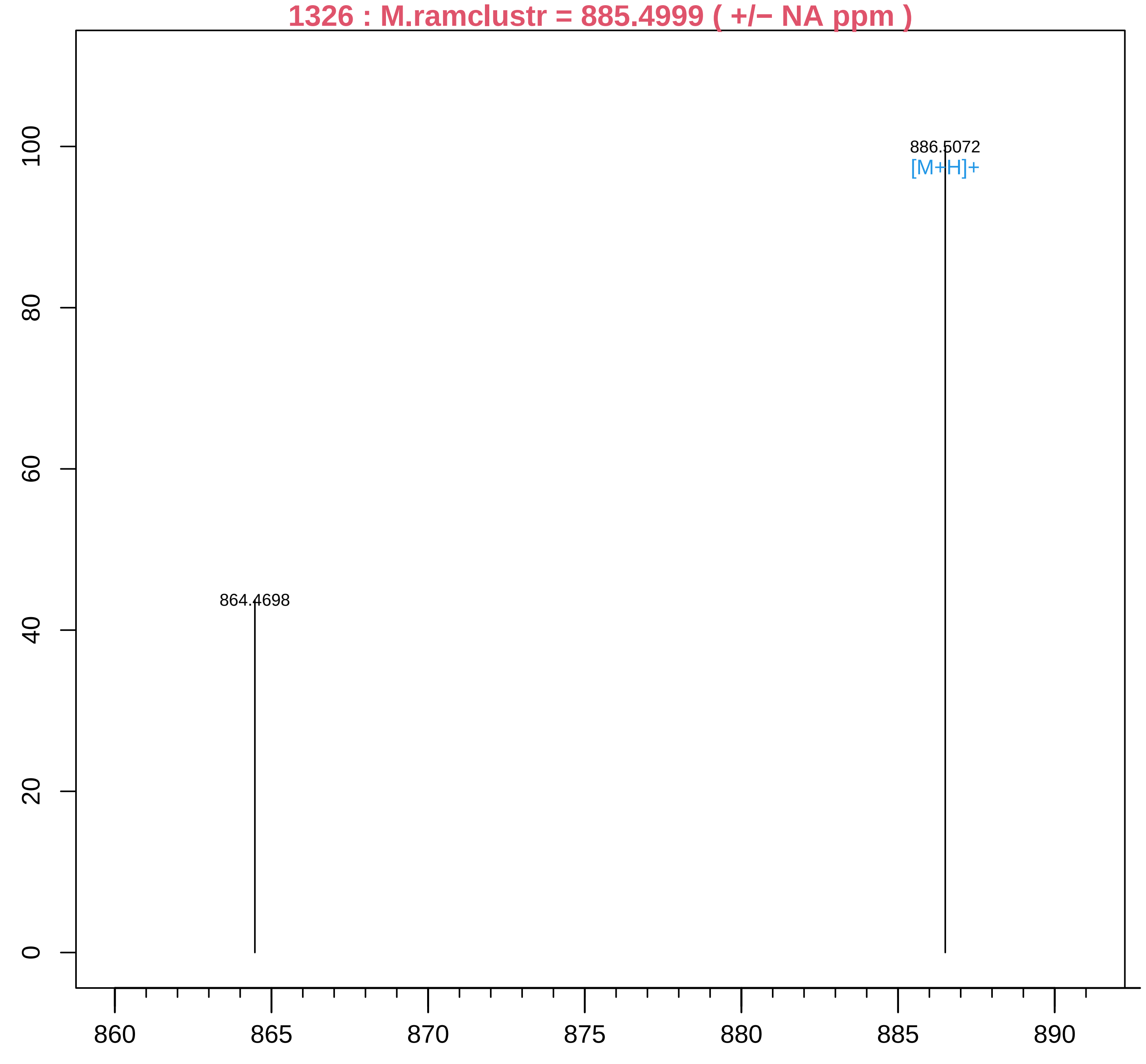

1326 : M.ramclustr = 885.4999 (+/− NA ppm)
886.5072
[M+H]+
864.4698
0
20
40
60
80
100
860
865
870
875
880
885
890

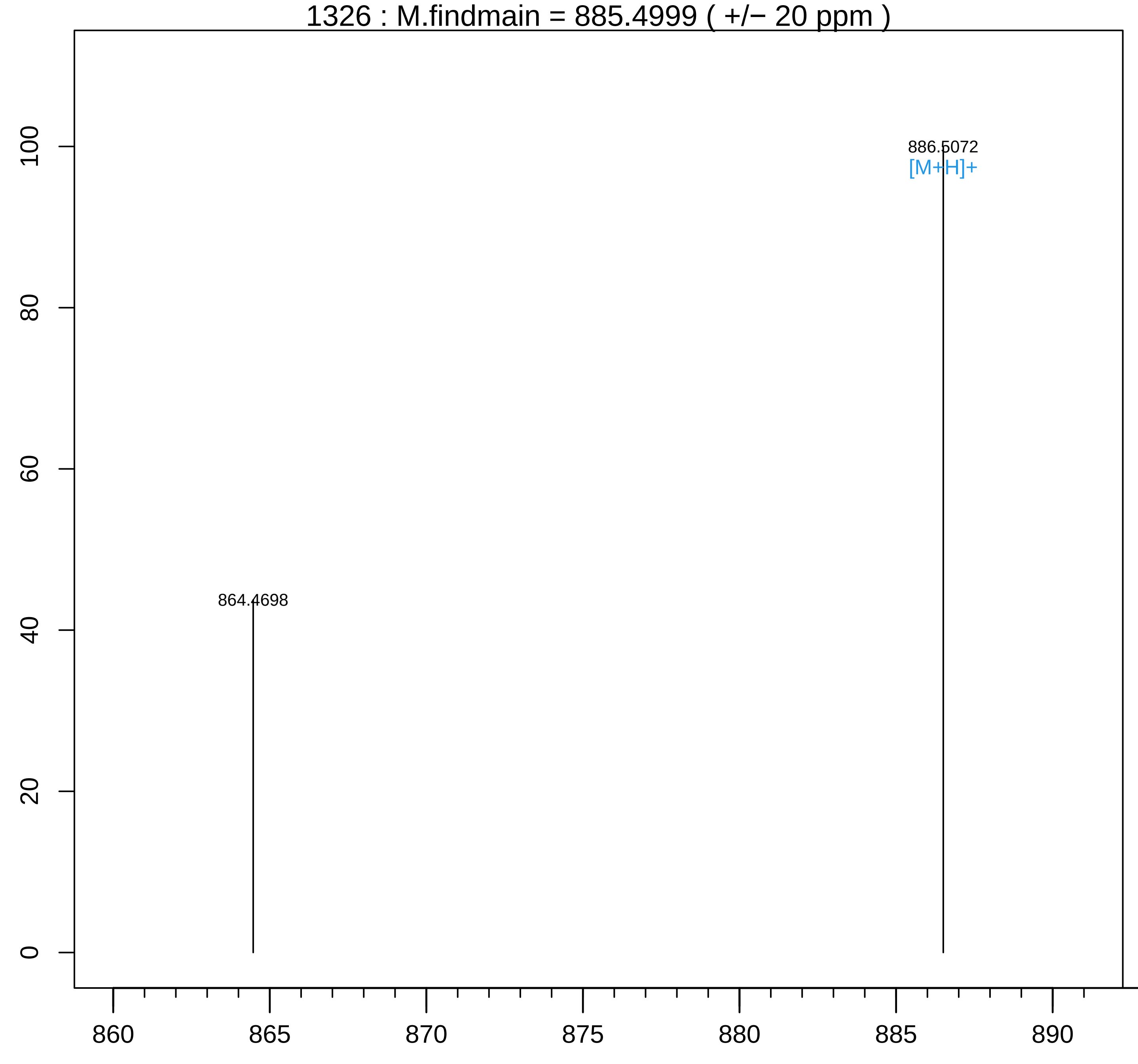

1326 : M.findmain = 885.4999 (+/− 20 ppm)
886.5072
[M+H]+
864.4698
0
20
40
60
80
100
860
865
870
875
880
885
890

1327 : M.ramclustr = 1089.8958 (+/− NA ppm)

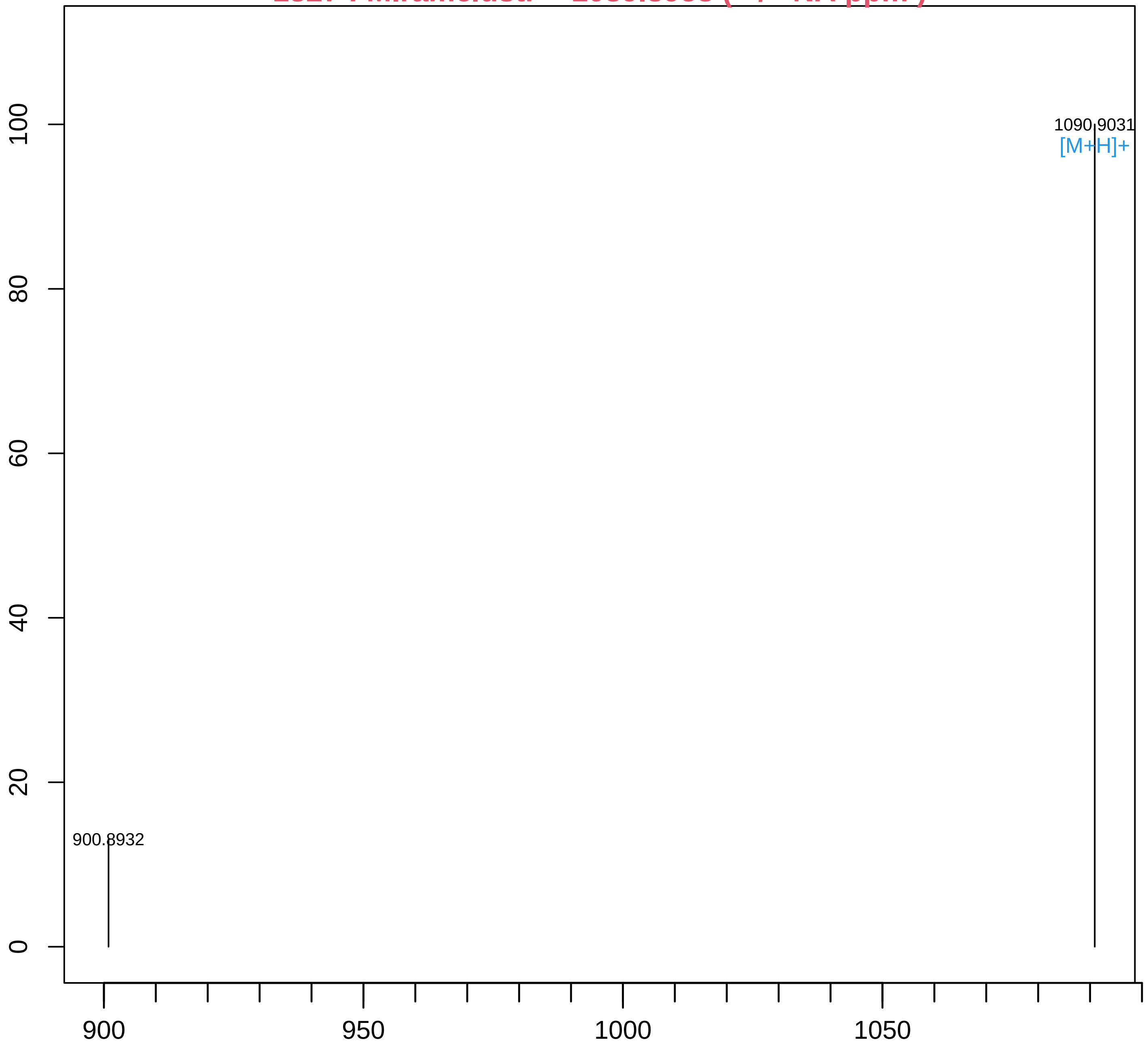

1327 : M.findmain = 1089.8958 (+/− 20 ppm)

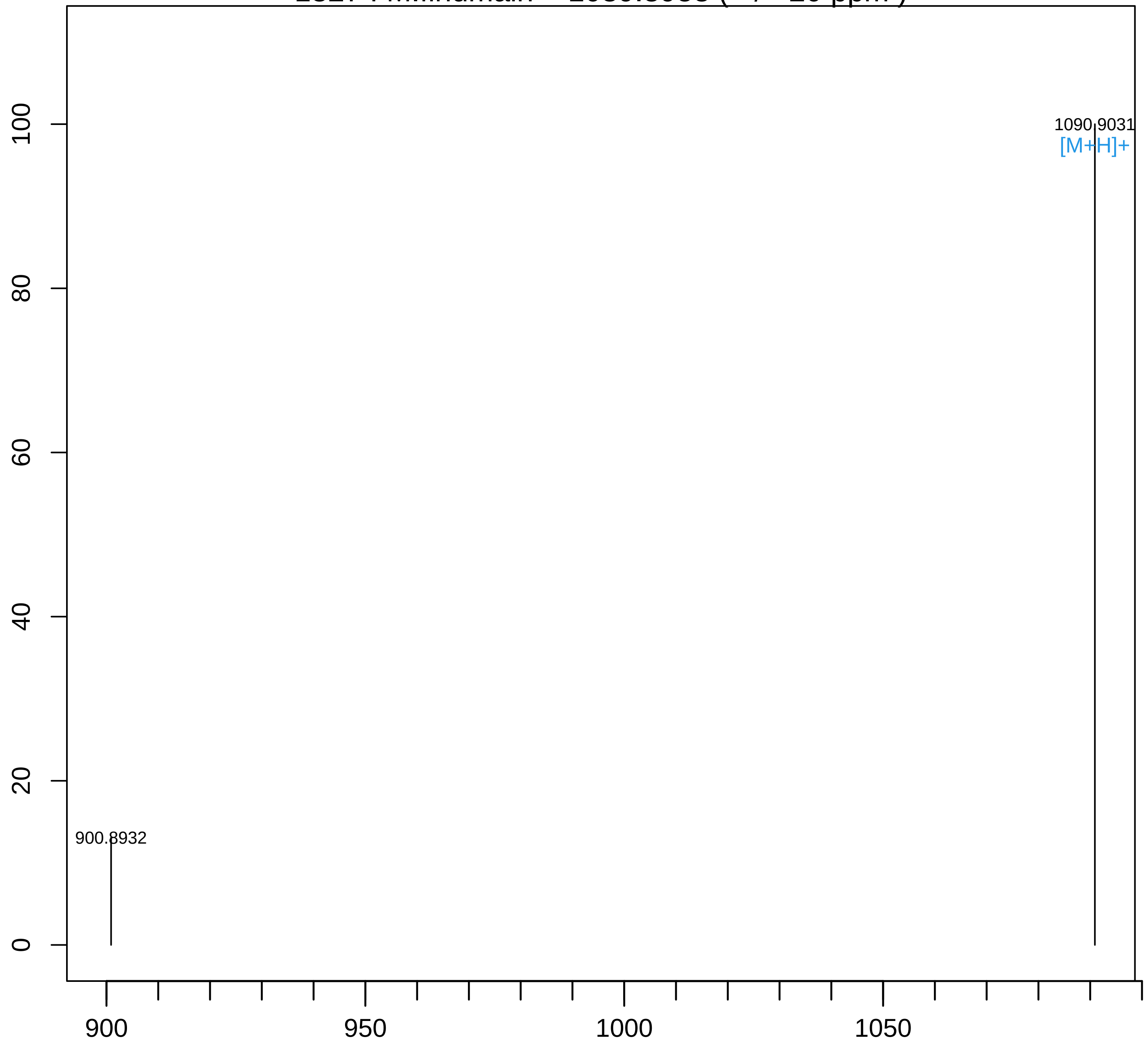

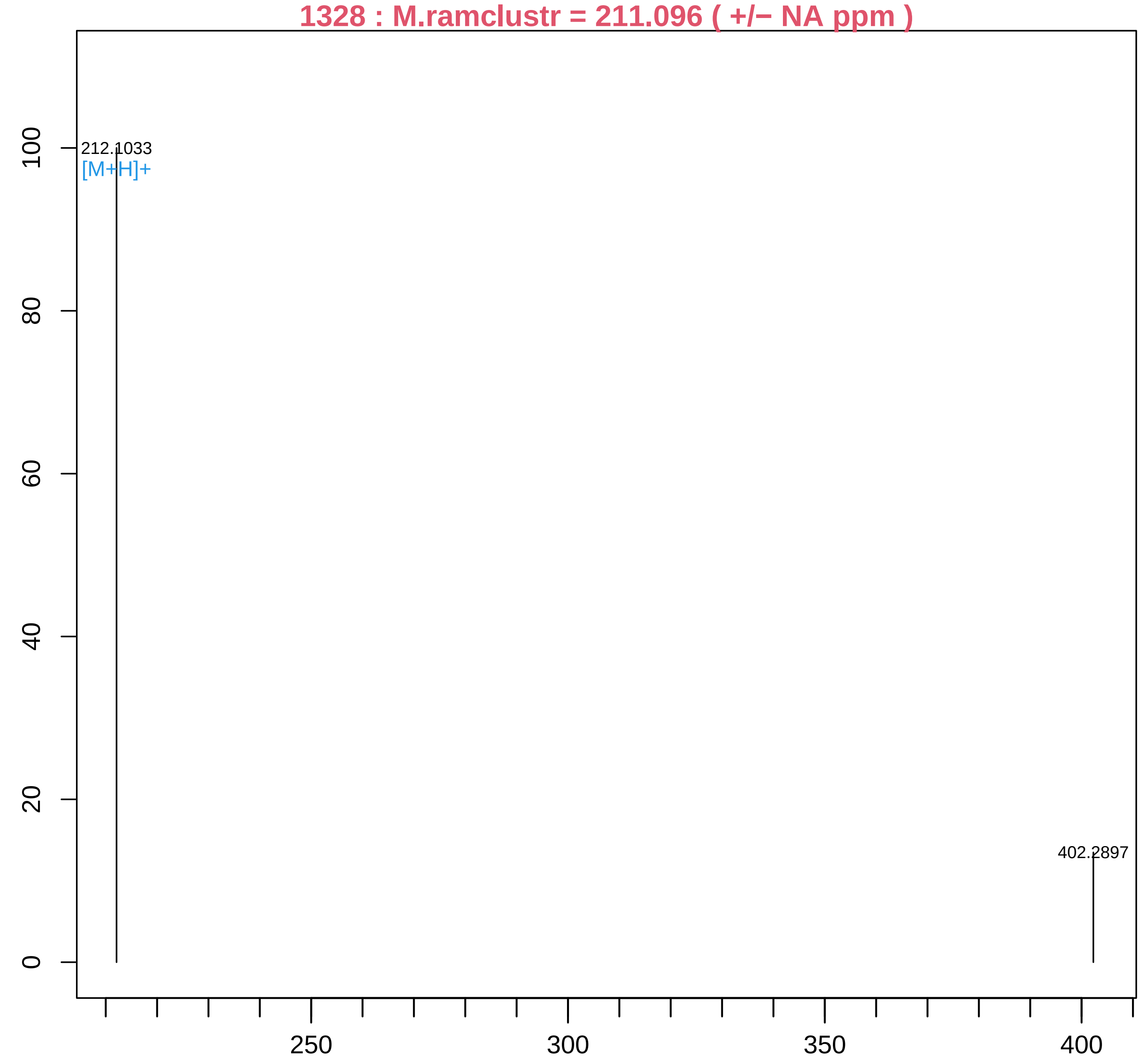
1328 : M.ramclustr = 211.096 (+/− NA ppm)
212.1033
[M+H]+
402.2897
0
20
40
60
80
100
250
300
350
400

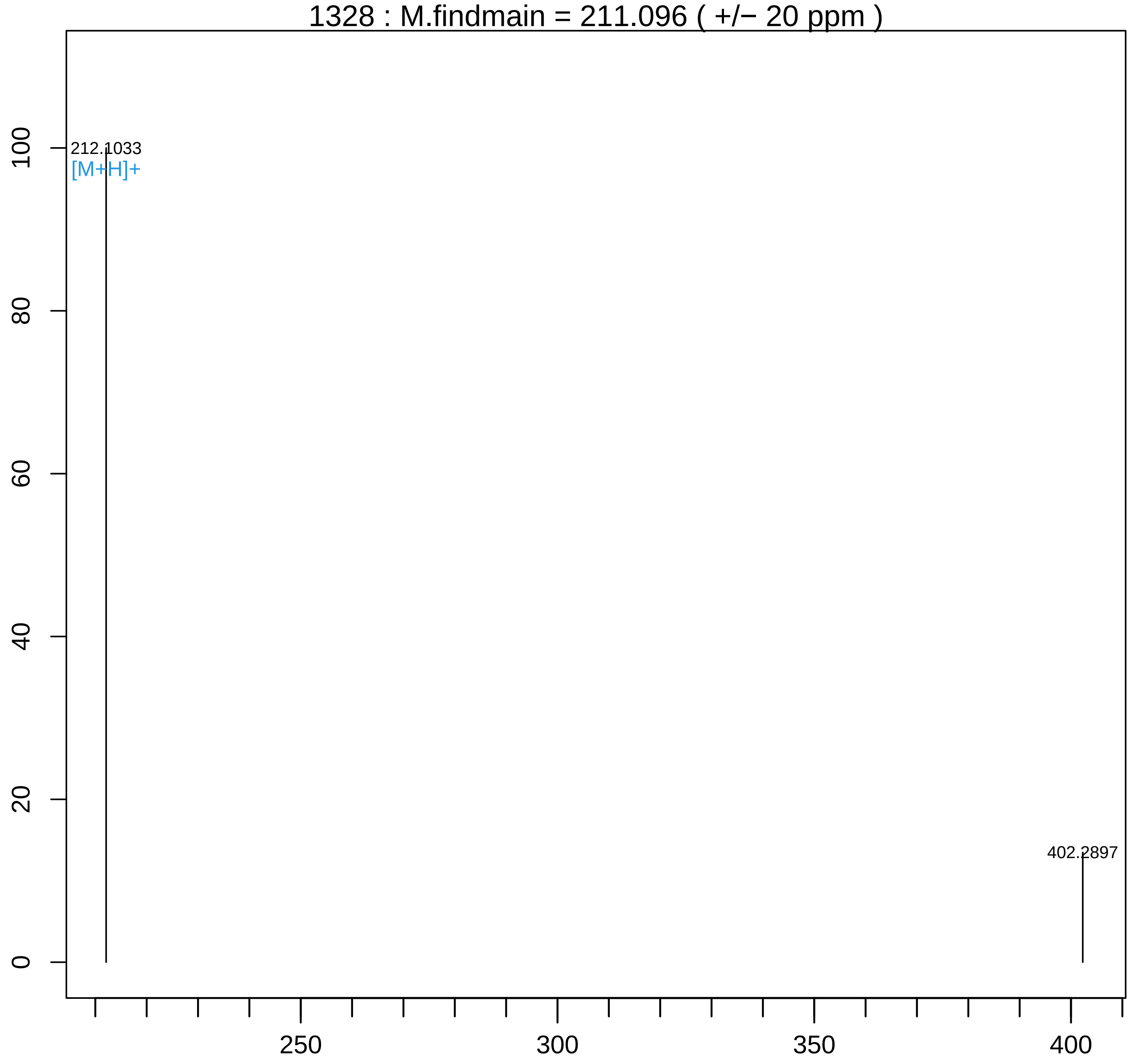
1328 : M.findmain = 211.096 (+/− 20 ppm)
212.1033
[M+H]+
402.2897
0
20
40
60
80
100
250
300
350
400

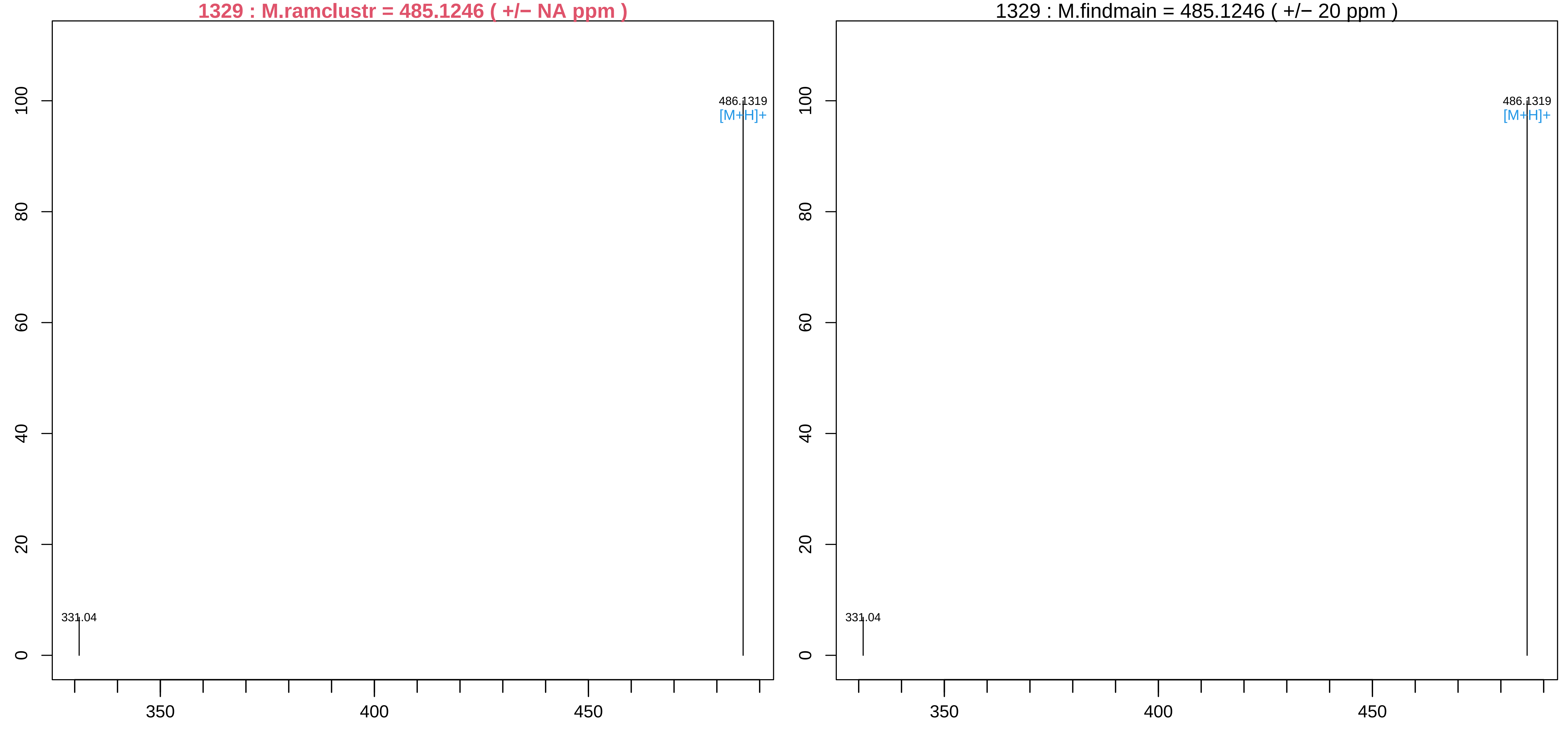
1329 : M.ramclustr = 485.1246 (+/− NA ppm)
486.1319
[M+H]+
331.04
0
20
40
60
80
100
350
400
450
1329 : M.findmain = 485.1246 (+/− 20 ppm)
486.1319
[M+H]+
331.04
0
20
40
60
80
100
350
400
450

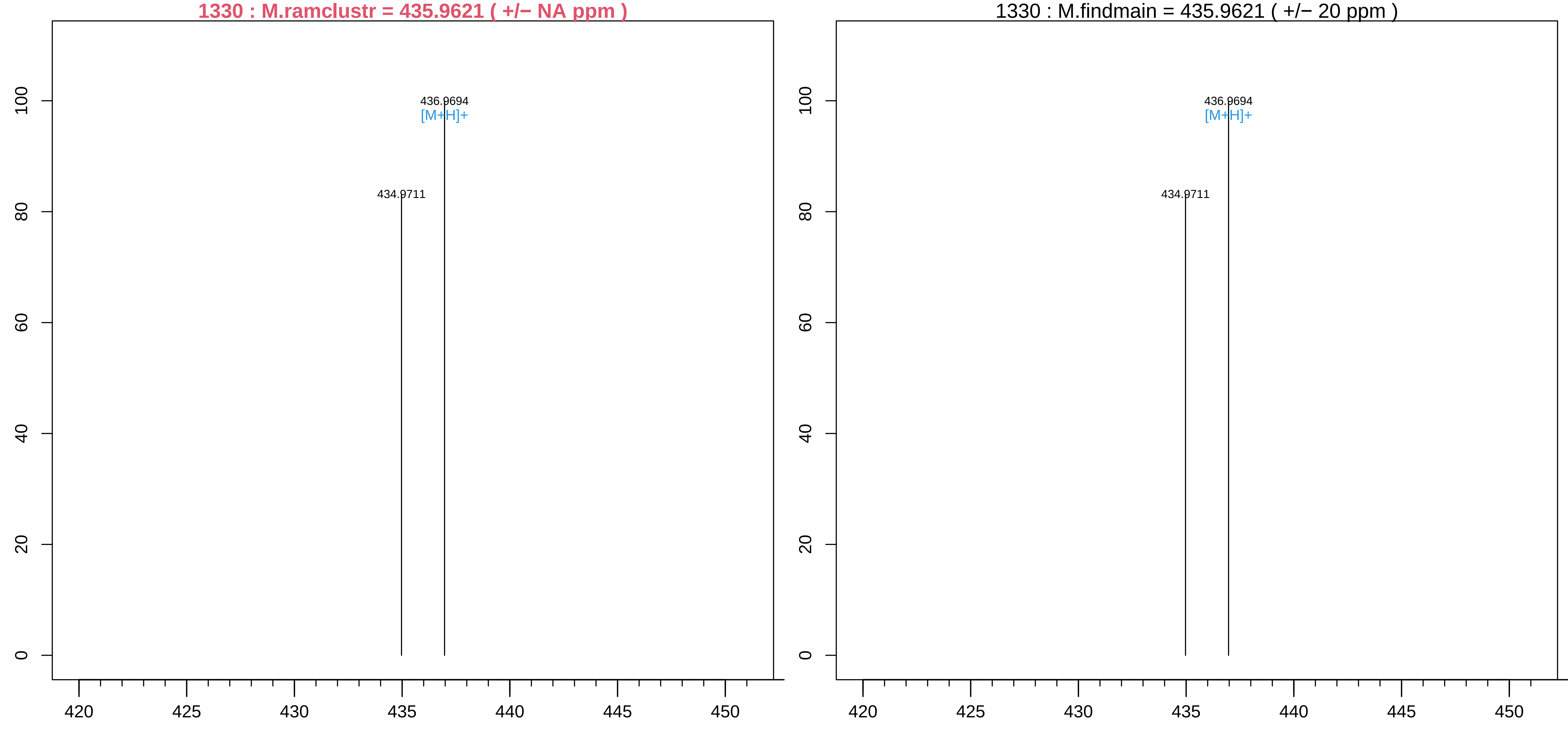
1330 : M.ramclustr = 435.9621 (+/− NA ppm)
436.9694
[M+H]+
434.9711
1330 : M.findmain = 435.9621 (+/− 20 ppm)
436.9694
[M+H]+
434.9711
0
20
40
60
80
100
420
425
430
435
440
445
450

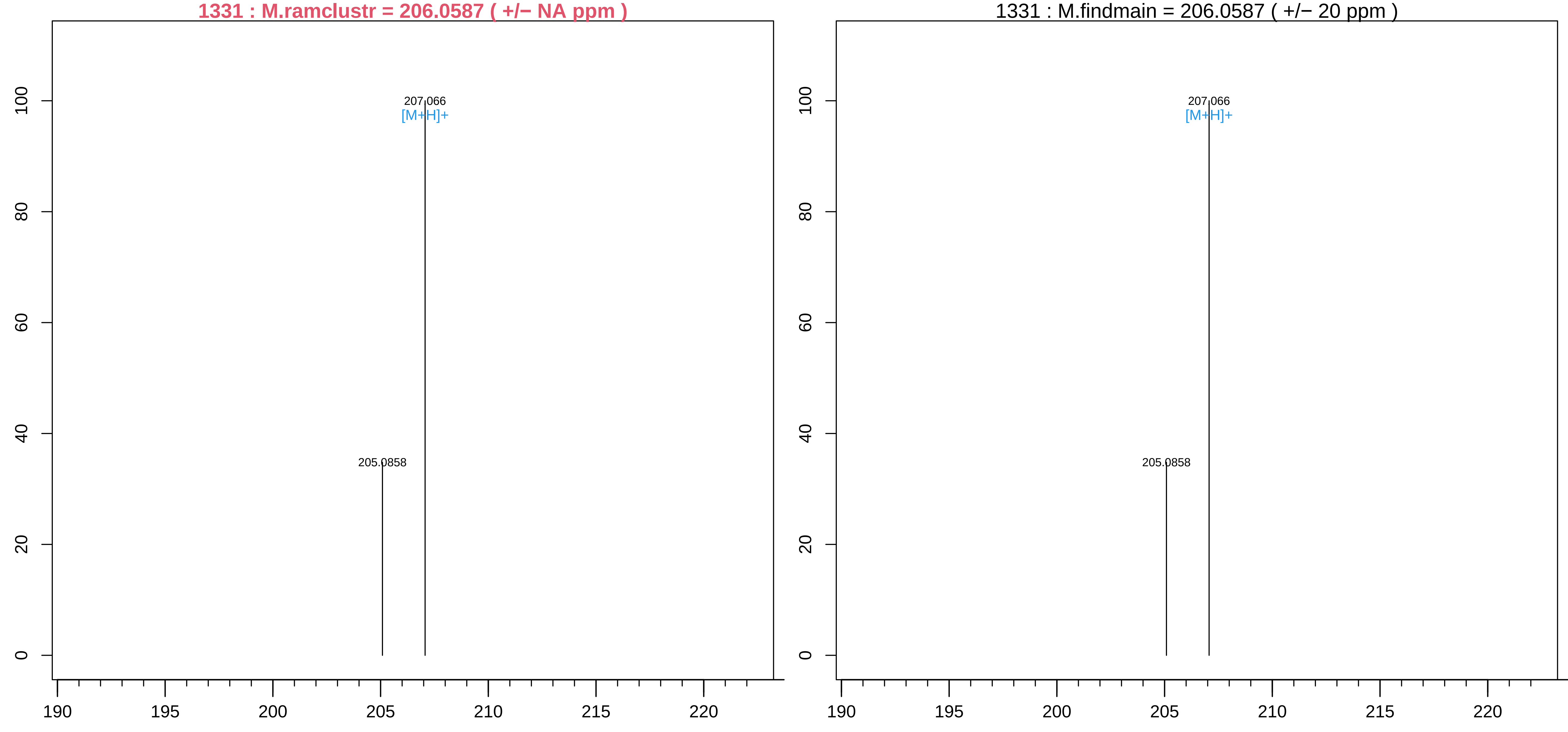

1331 : M.ramclustr = 206.0587 (+/− NA ppm)
207.066
[M+H]+
205.0858
1331 : M.findmain = 206.0587 (+/− 20 ppm)
207.066
[M+H]+
205.0858

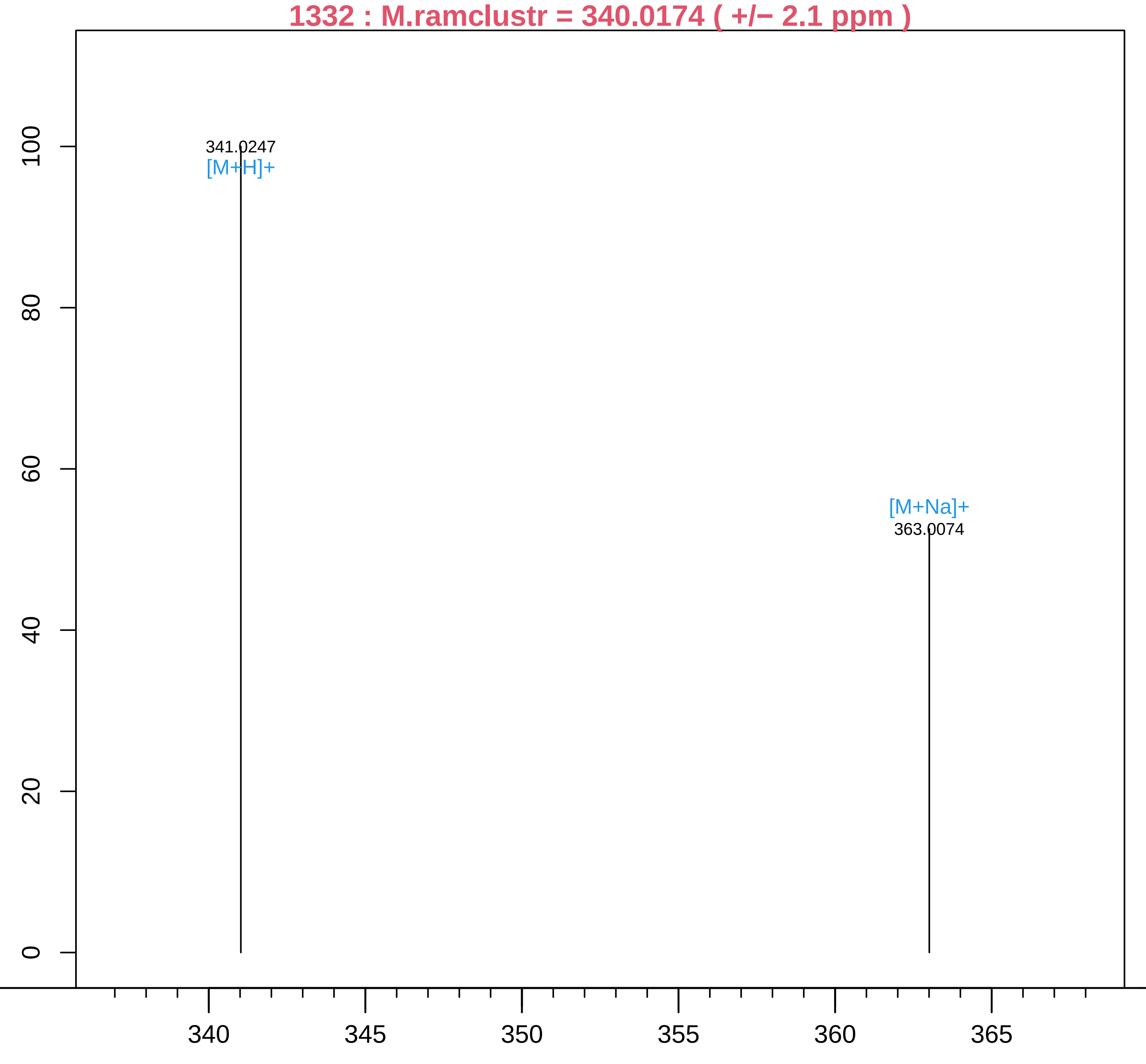
1332 : M.ramclustr = 340.0174 (+/− 2.1 ppm)
341.0247
[M+H]+
[M+Na]+
363.0074
0
20
40
60
80
100
340
345
350
355
360
365

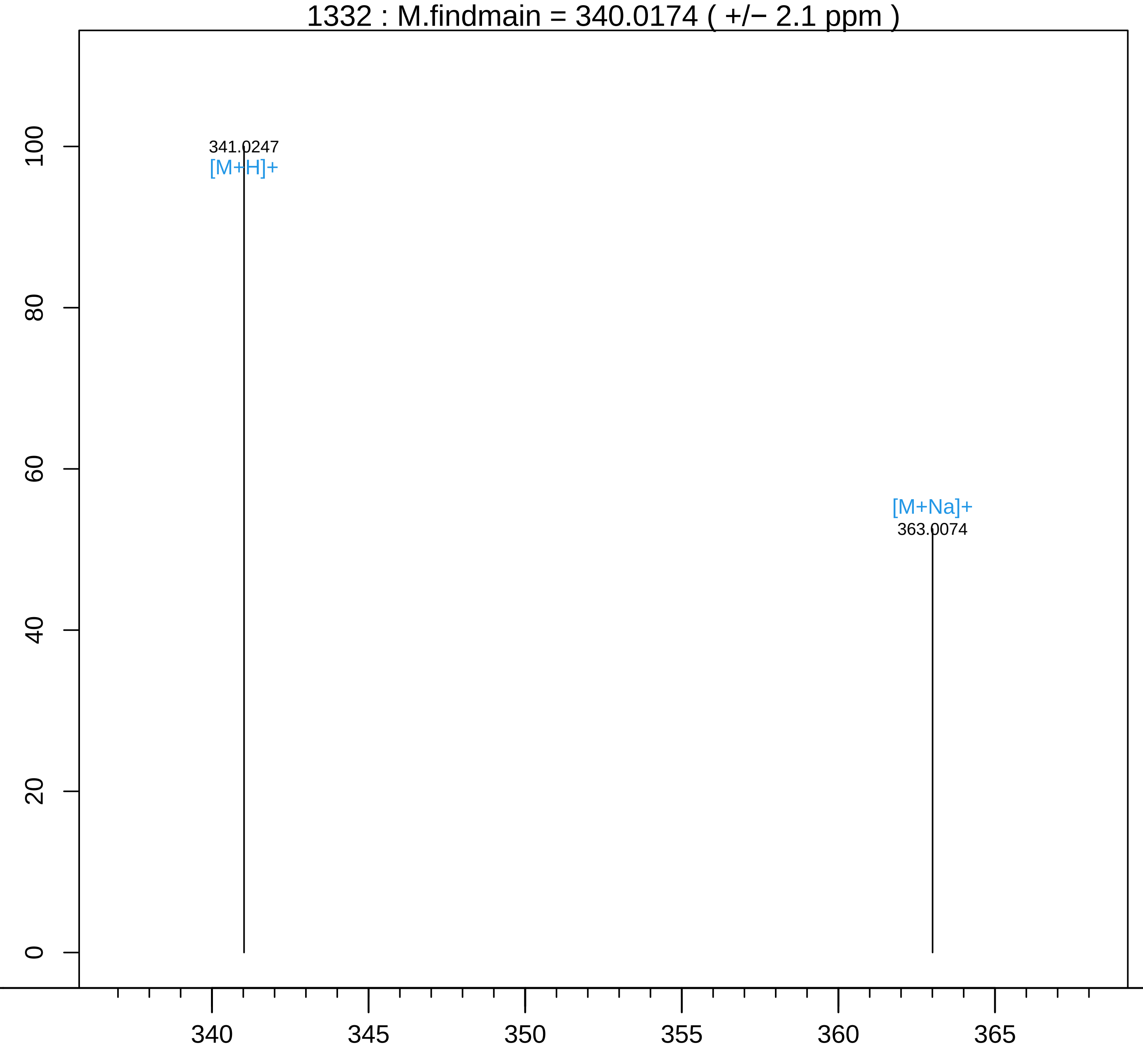
1332 : M.findmain = 340.0174 (+/− 2.1 ppm)
341.0247
[M+H]+
[M+Na]+
363.0074
0
20
40
60
80
100
340
345
350
355
360
365

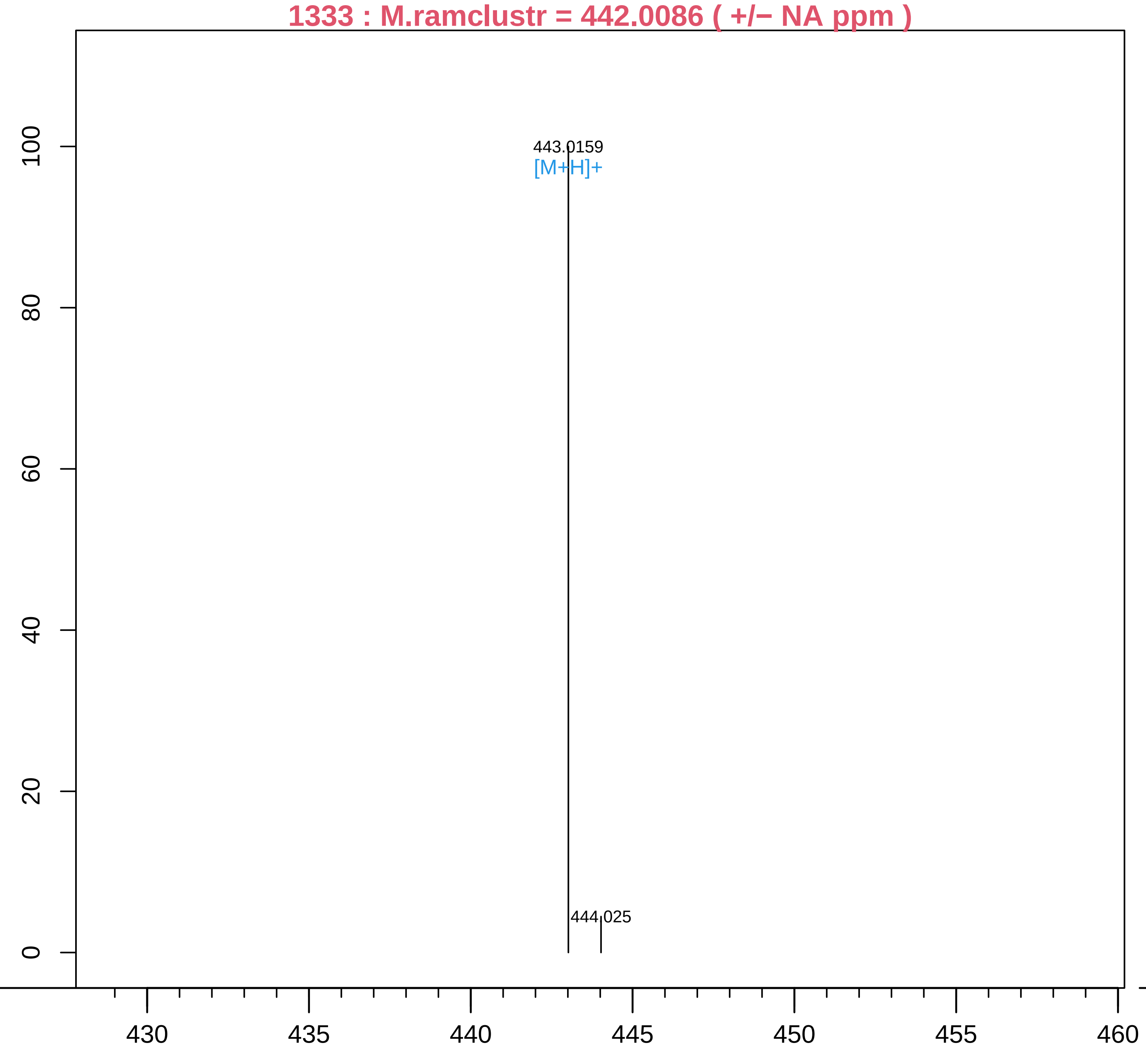

1333 : M.ramclustr = 442.0086 (+/− NA ppm)
443.0159
[M+H]+
444.025
0
20
40
60
80
100
430
435
440
445
450
455
460

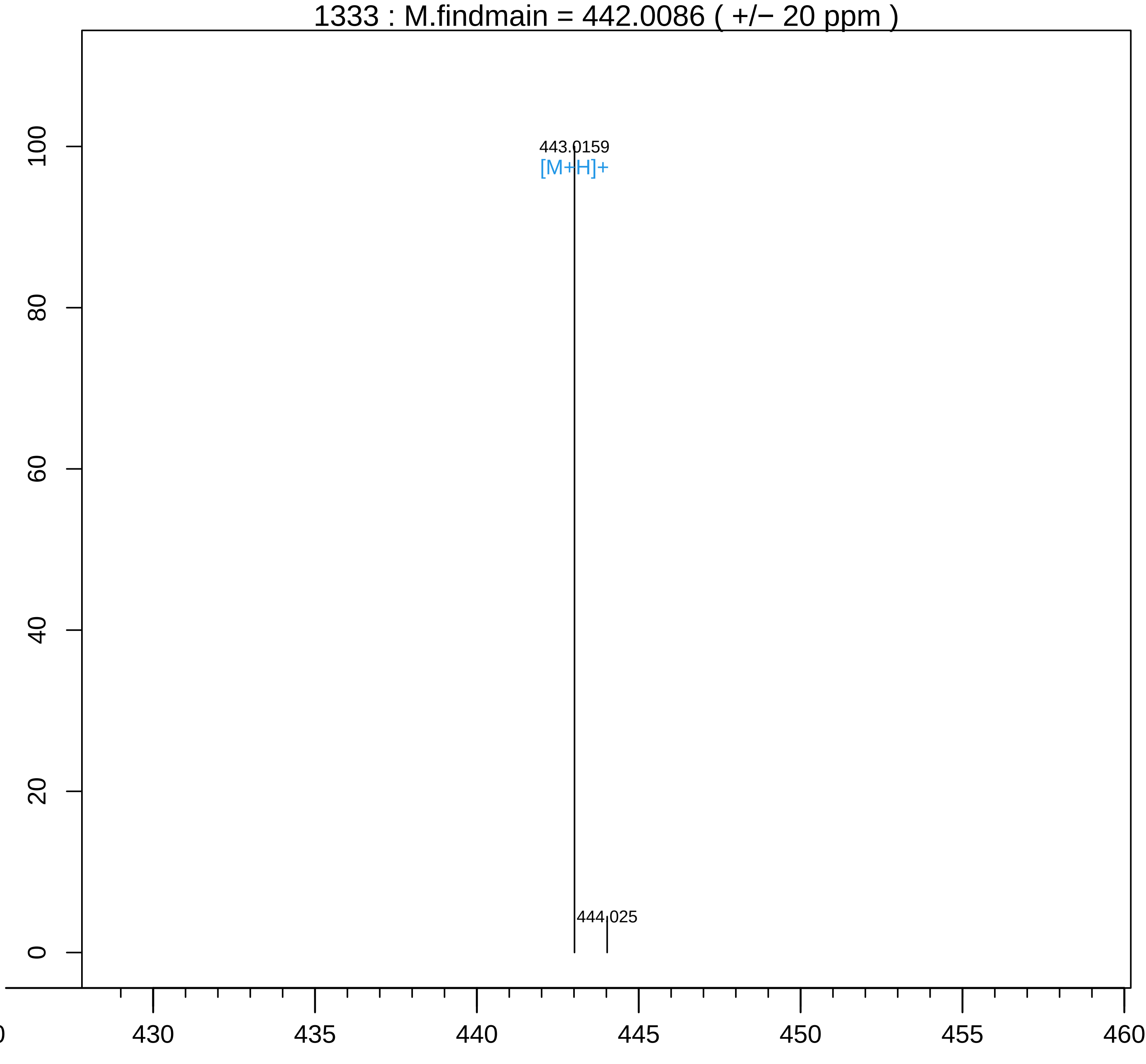

1333 : M.findmain = 442.0086 (+/− 20 ppm)
443.0159
[M+H]+
444.025
0
20
40
60
80
100
430
435
440
445
450
455
460

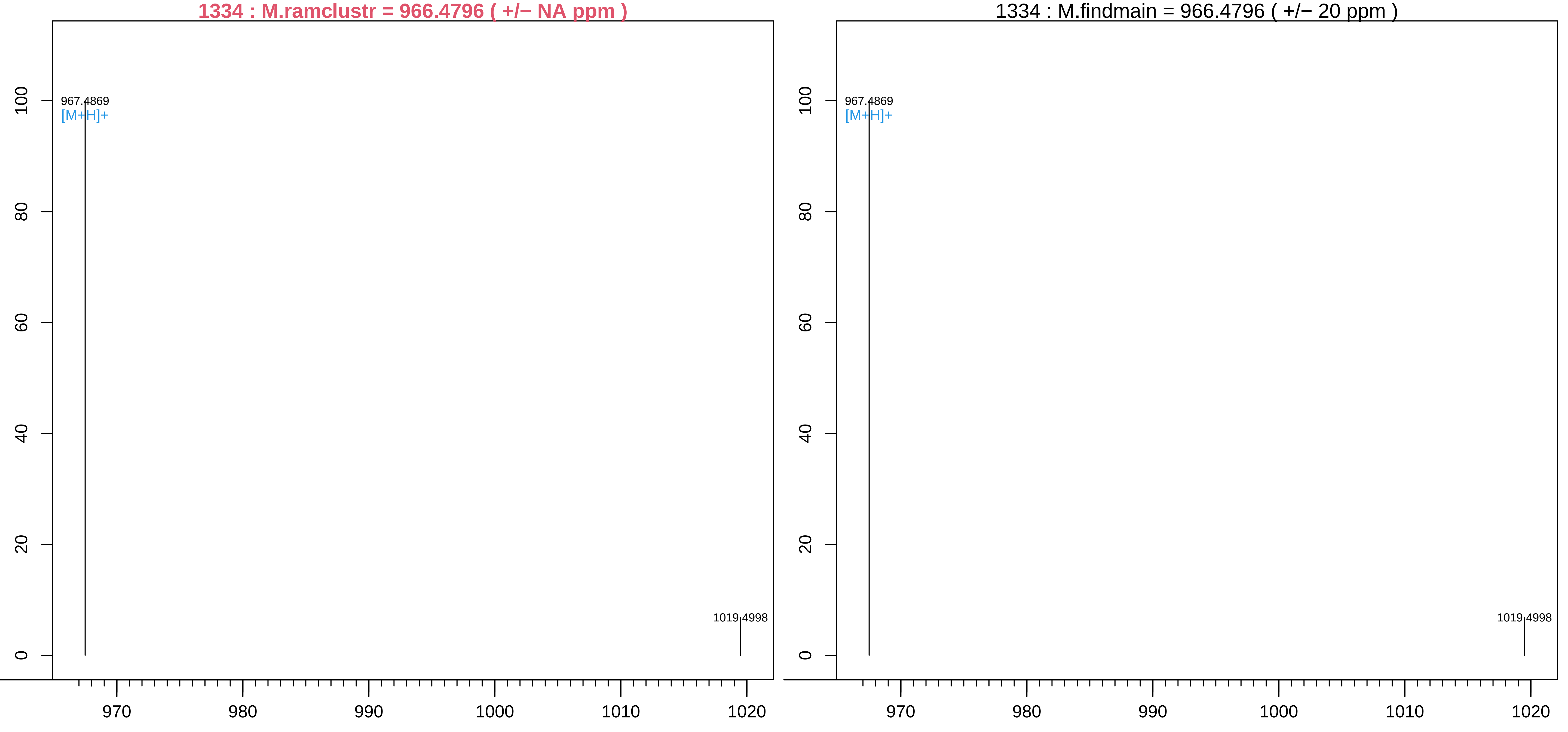

1334 : M.ramclustr = 966.4796 (+/− NA ppm)
967.4869
[M+H]+
1019.4998
1334 : M.findmain = 966.4796 (+/− 20 ppm)
967.4869
[M+H]+
1019.4998

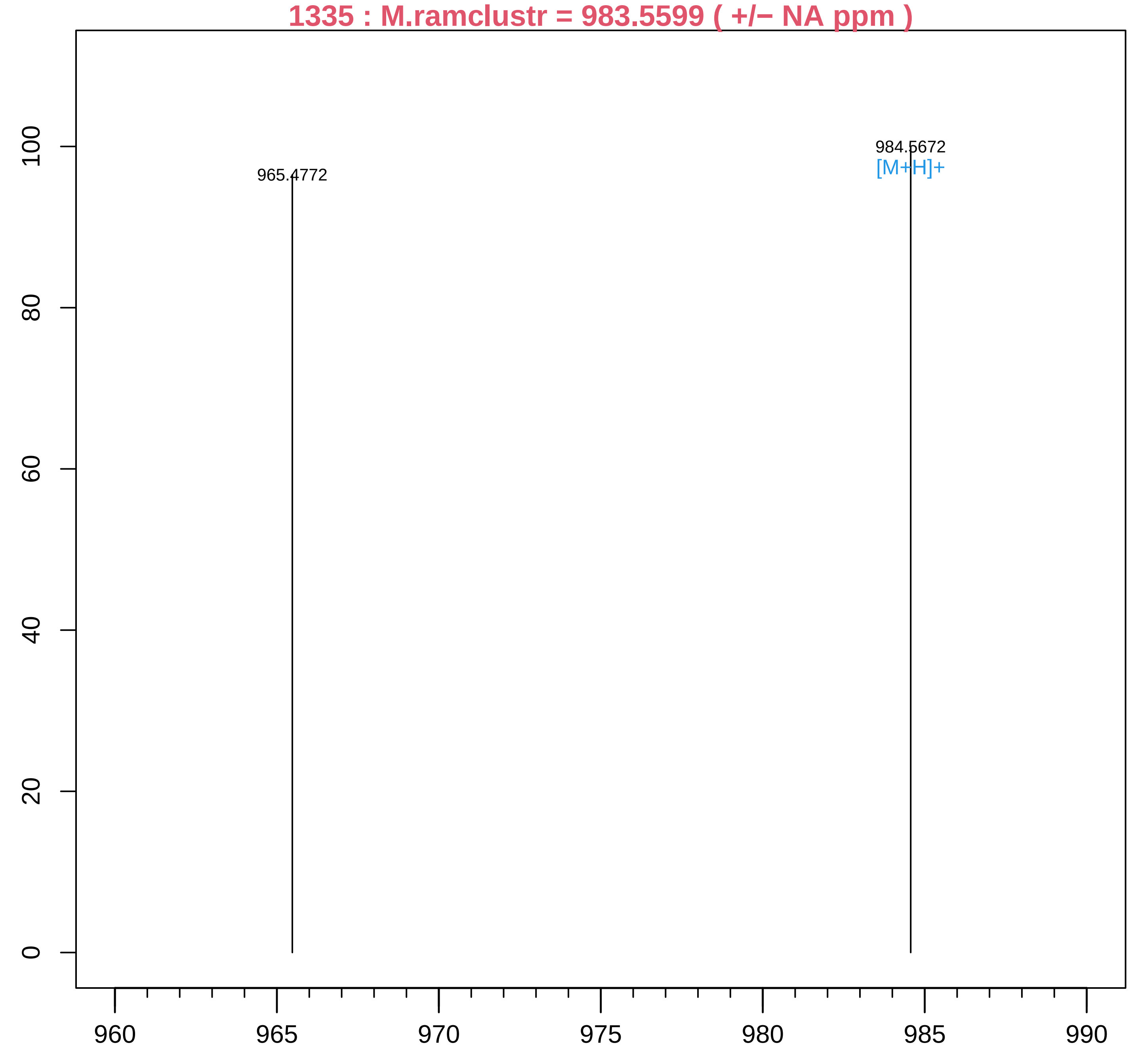
1335 : M.ramclustr = 983.5599 (+/− NA ppm)
965.4772
984.5672
[M+H]+
0
20
40
60
80
100
960
965
970
975
980
985
990

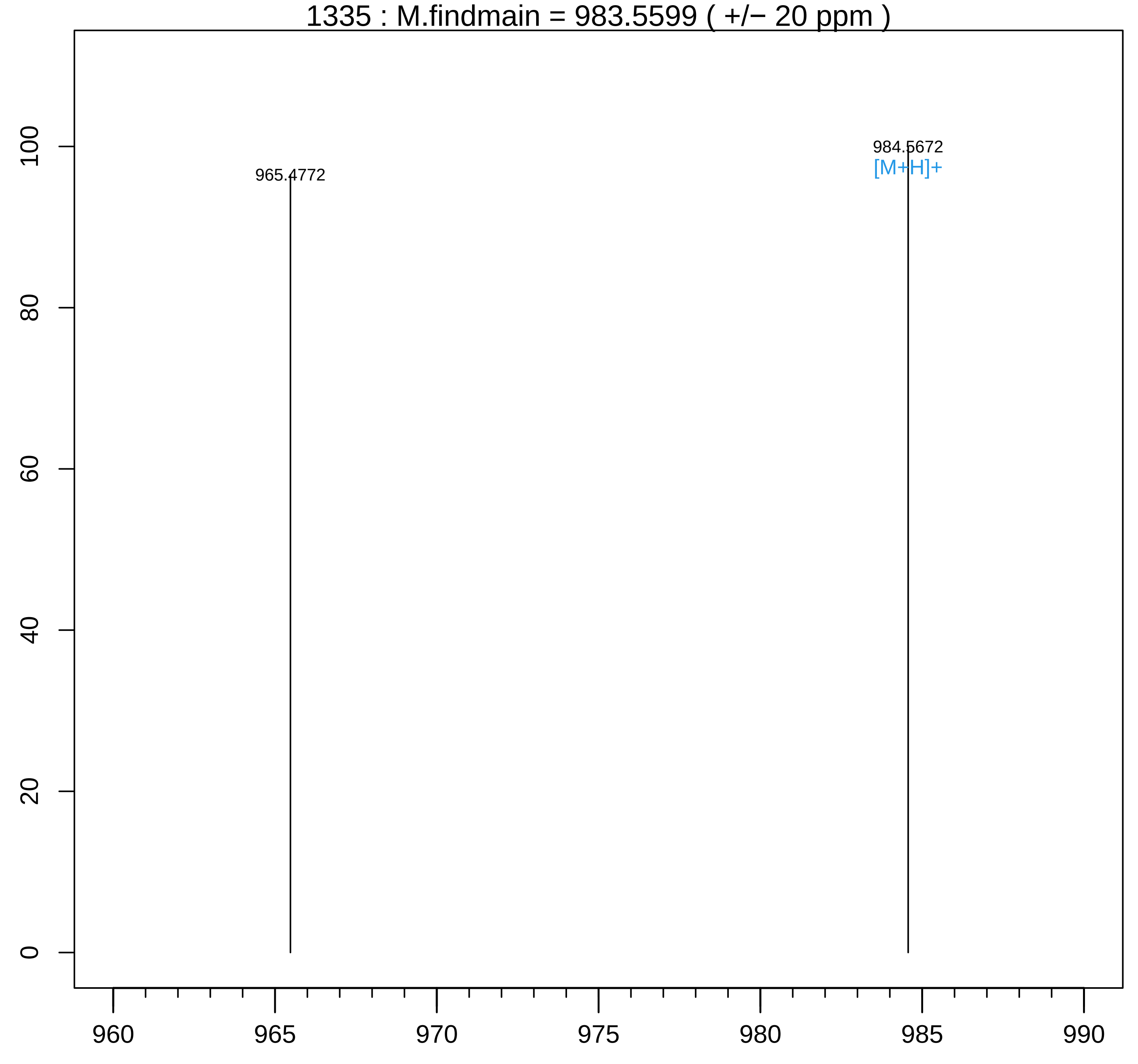
1335 : M.findmain = 983.5599 (+/− 20 ppm)
965.4772
984.5672
[M+H]+
0
20
40
60
80
100
960
965
970
975
980
985
990

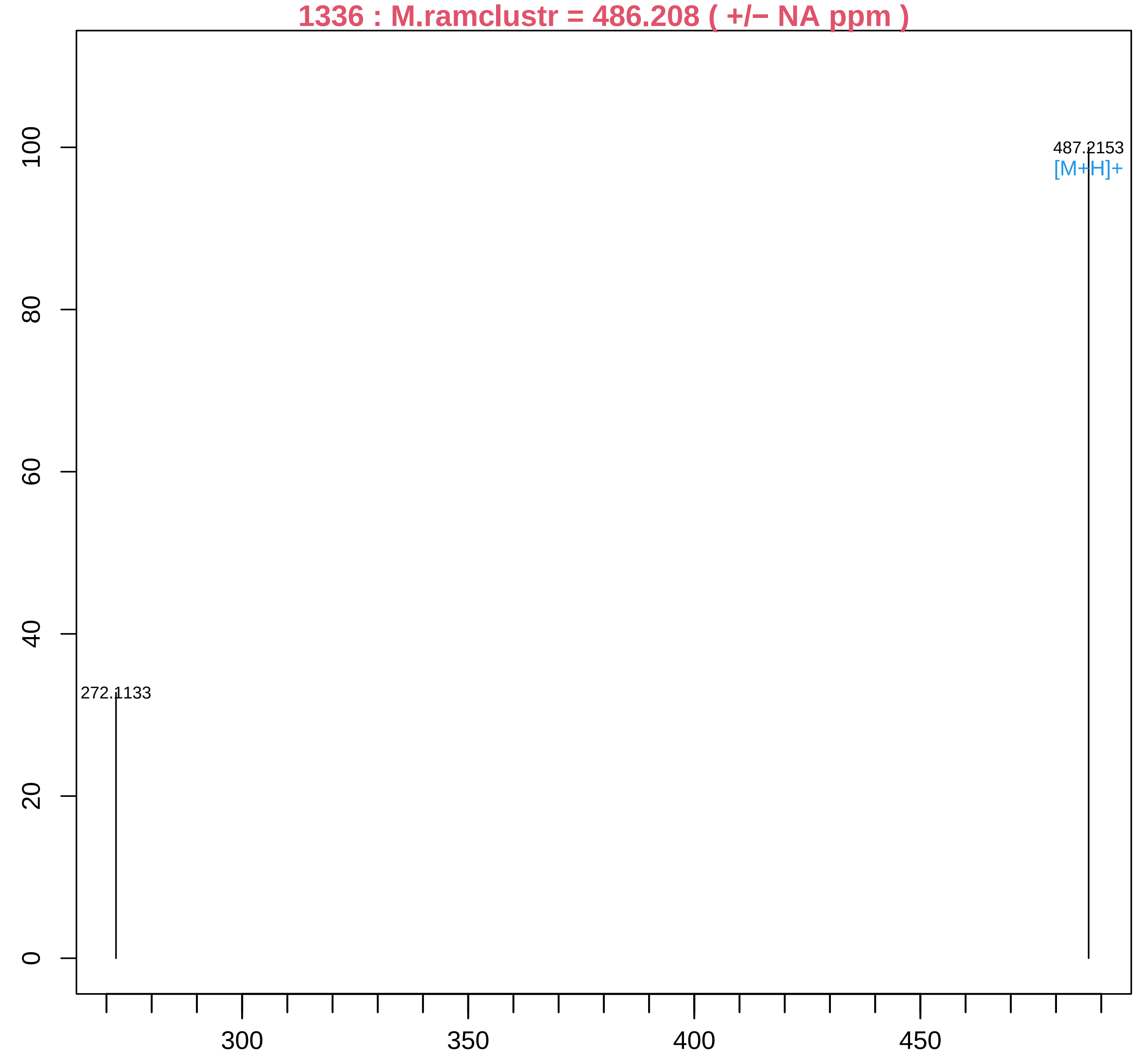

1336 : M.ramclustr = 486.208 (+/− NA ppm)
487.2153
[M+H]+
272.1133
0
20
40
60
80
100
300
350
400
450

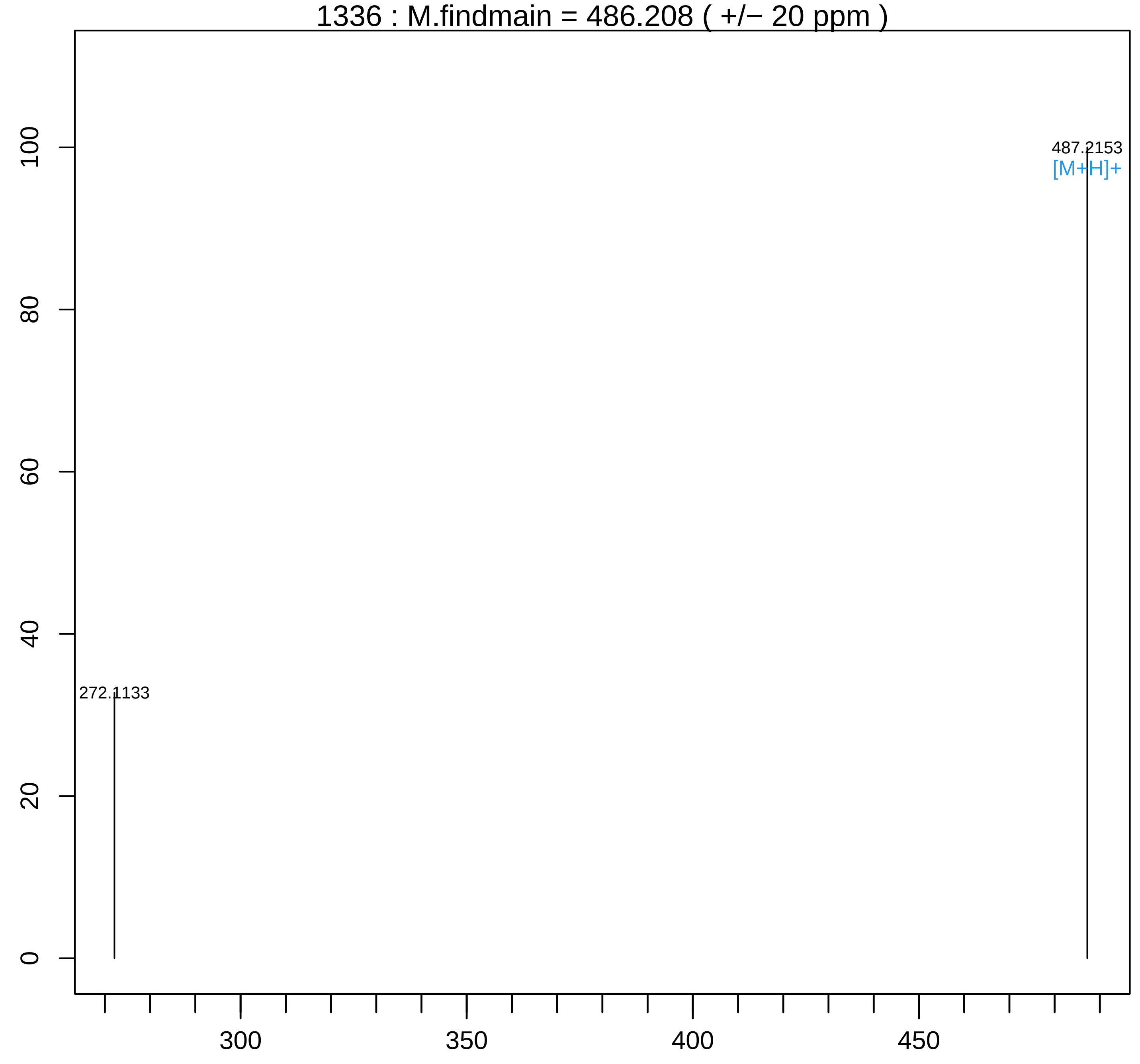

1336 : M.findmain = 486.208 (+/− 20 ppm)
487.2153
[M+H]+
272.1133
0
20
40
60
80
100
300
350
400
450

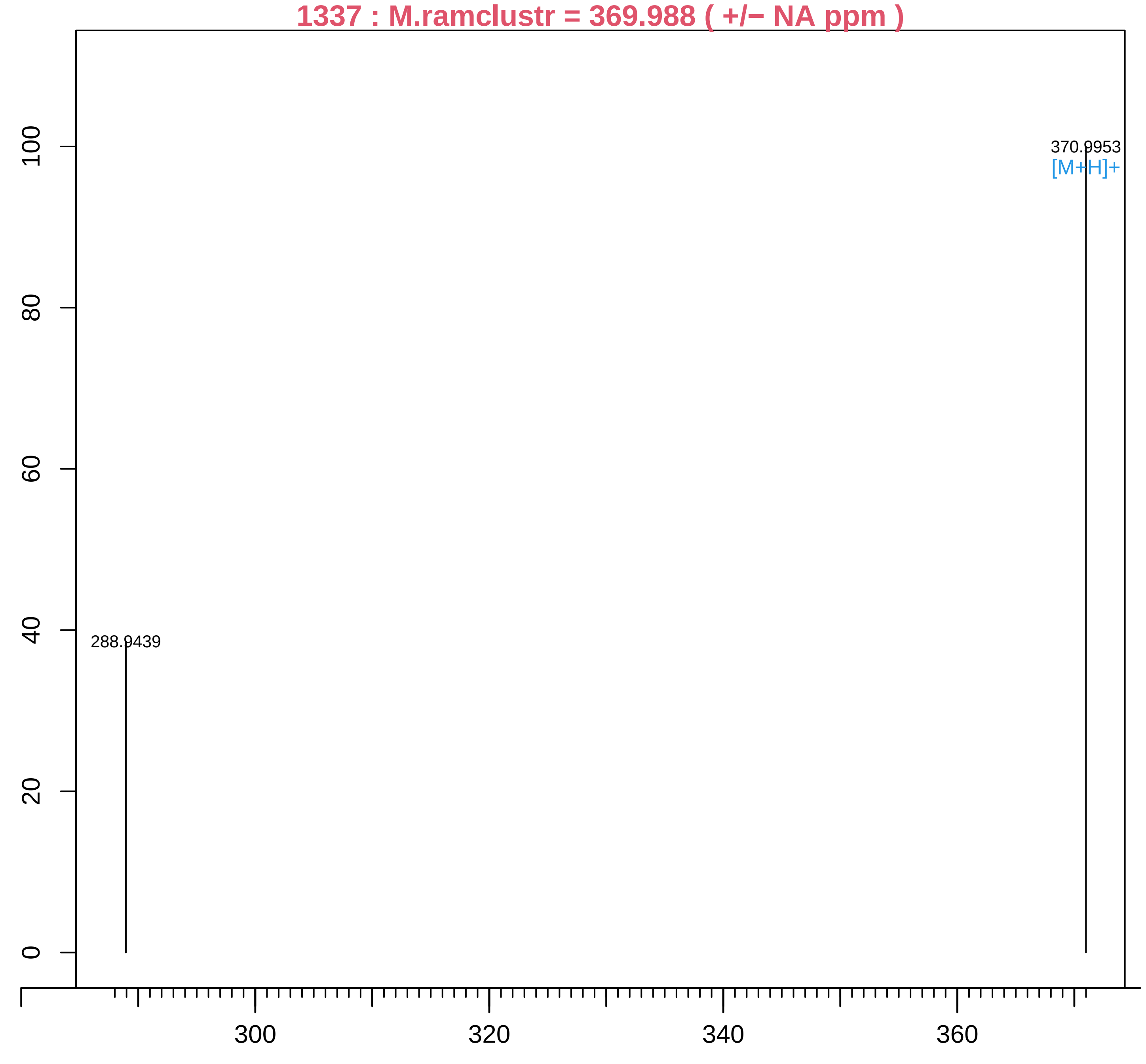
1337 : M.ramclustr = 369.988 (+/− NA ppm)
100
80
60
40
20
0
370.9953
[M+H]+
288.9439
300
320
340
360

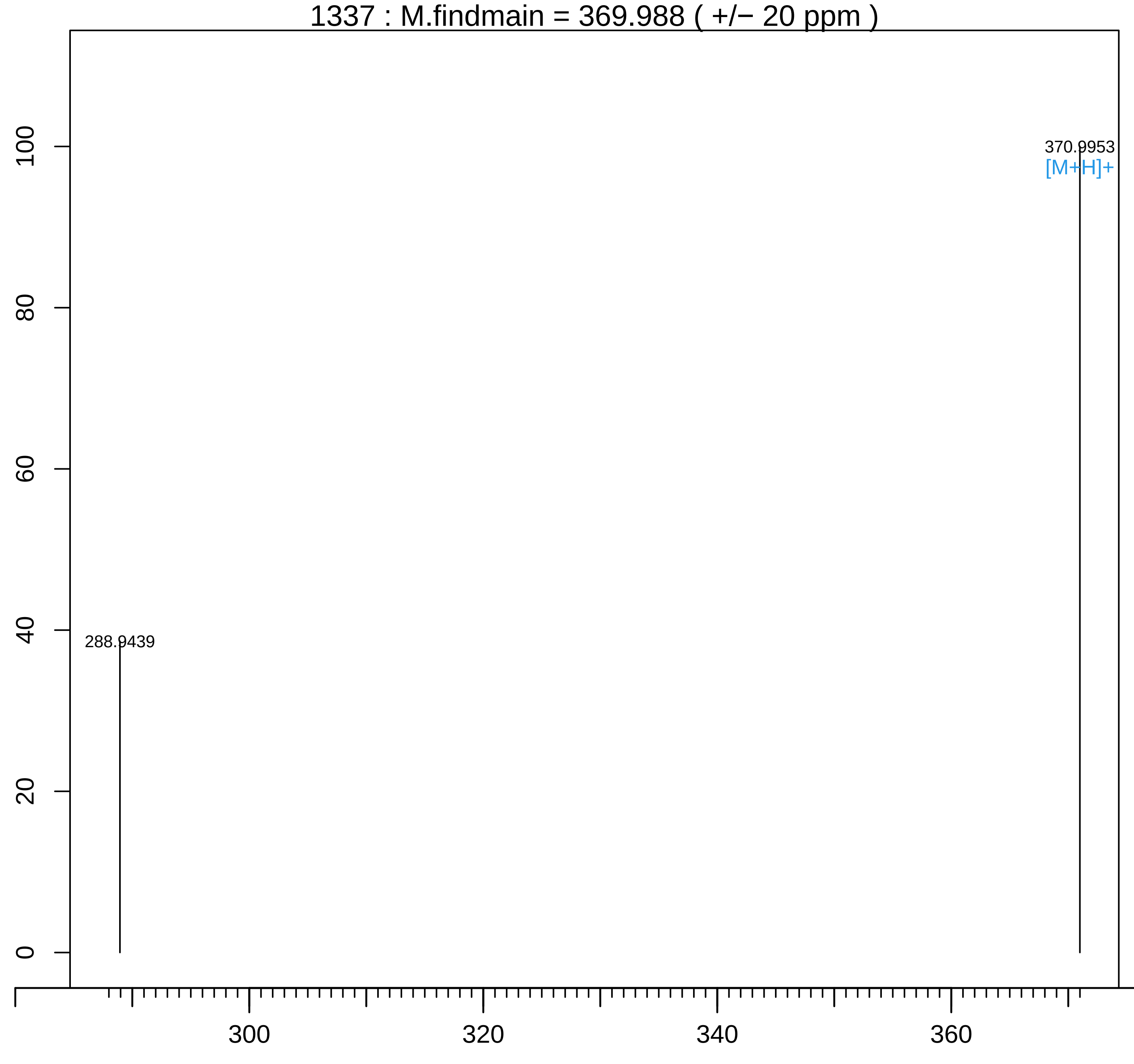
1337 : M.findmain = 369.988 (+/− 20 ppm)
100
80
60
40
20
0
370.9953
[M+H]+
288.9439
300
320
340
360

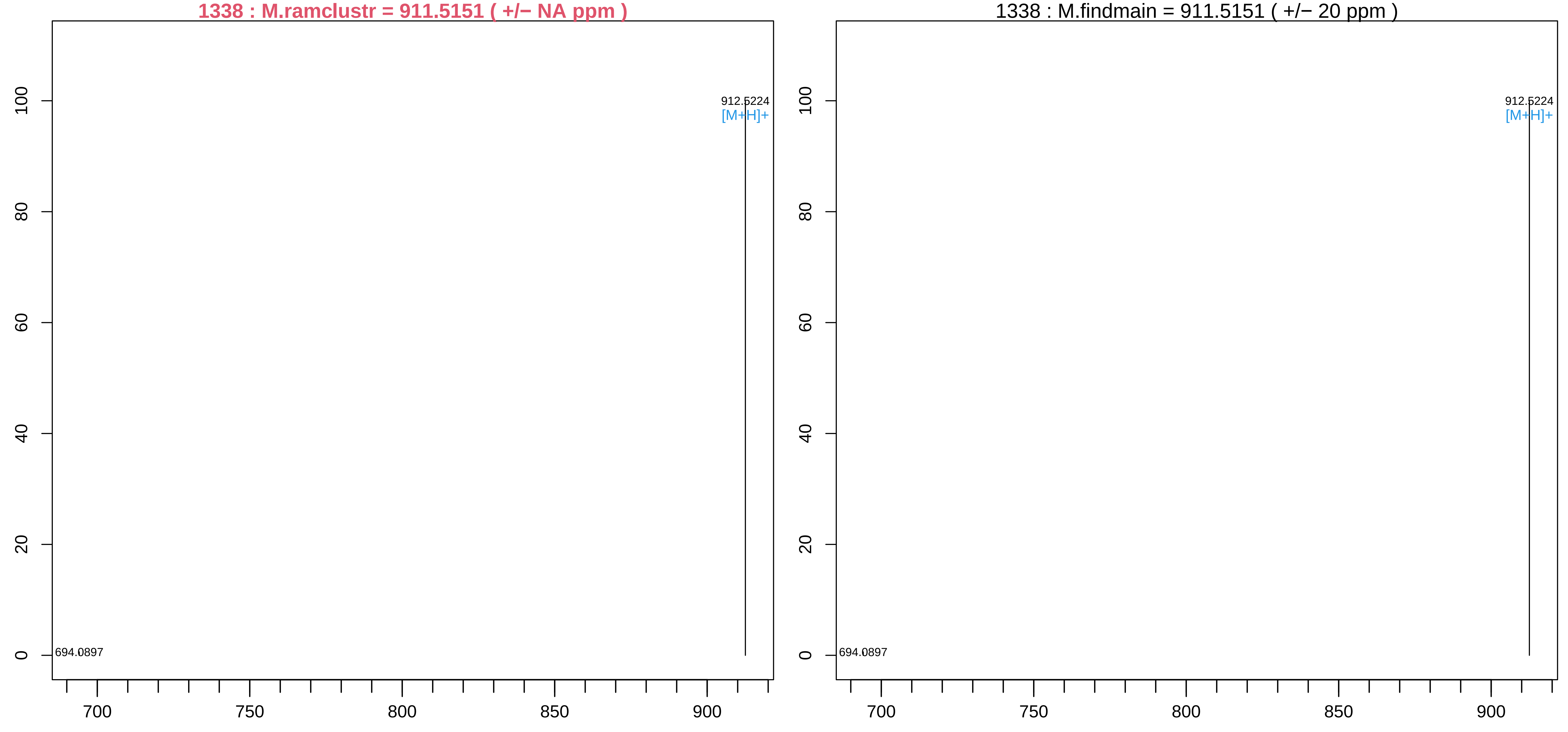

1338 : M.ramclustr = 911.5151 (+/− NA ppm)
912.5224
[M+H]+
694.0897
1338 : M.findmain = 911.5151 (+/− 20 ppm)
912.5224
[M+H]+
694.0897

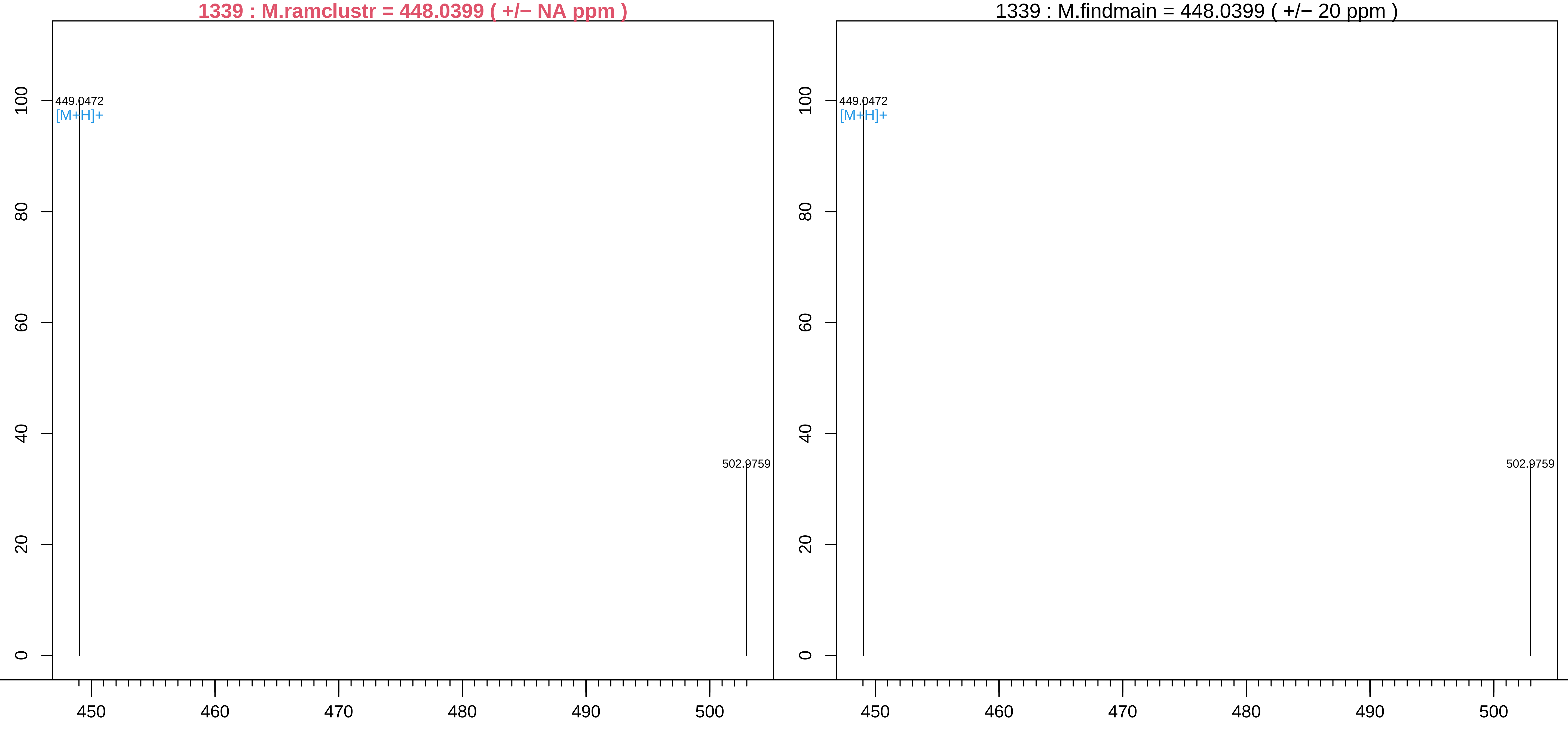
1339 : M.ramclustr = 448.0399 (+/− NA ppm)
449.0472
[M+H]+
502.9759
1339 : M.findmain = 448.0399 (+/− 20 ppm)
449.0472
[M+H]+
502.9759
0
20
40
60
80
100
450
460
470
480
490
500

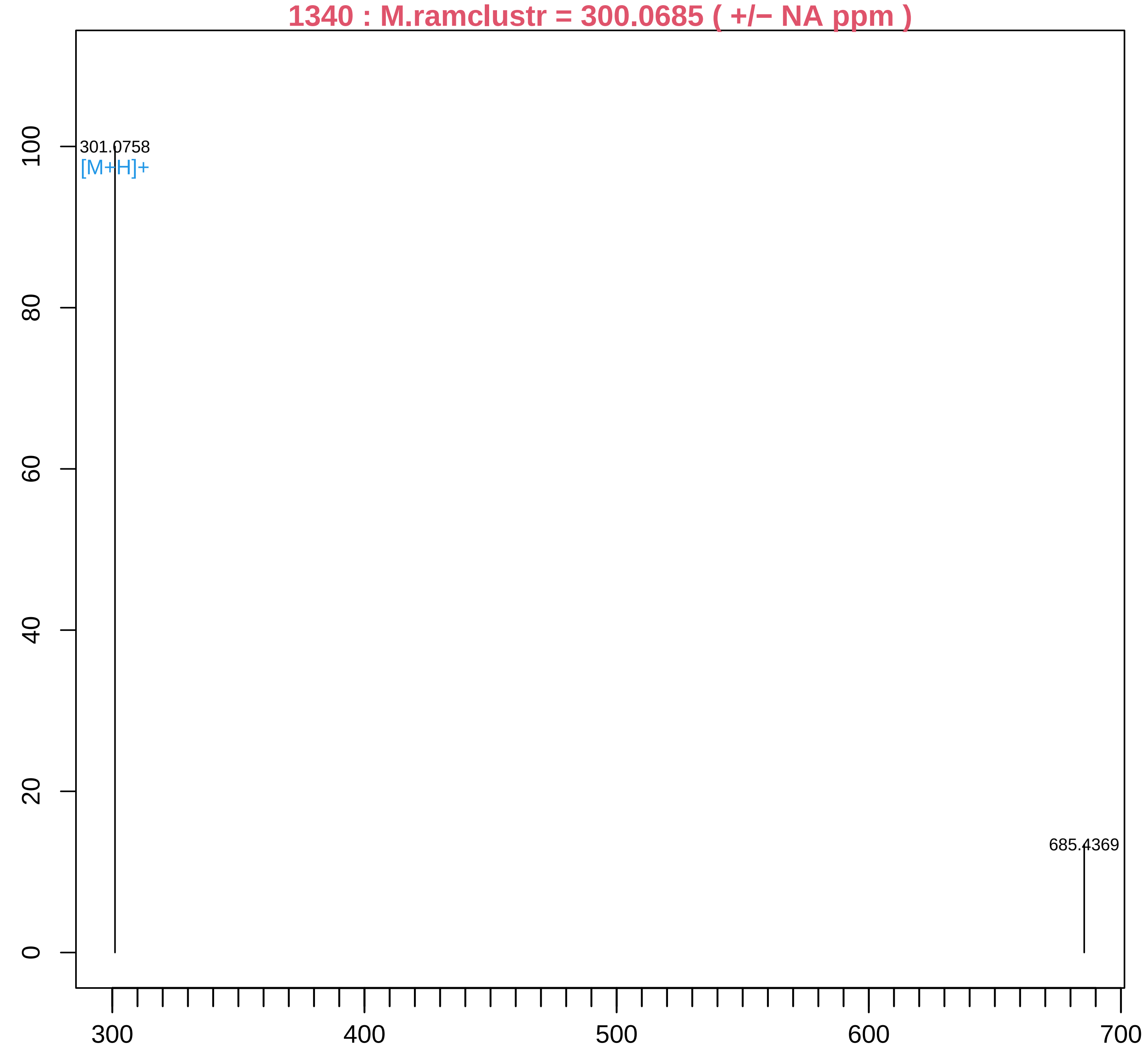
1340 : M.ramclustr = 300.0685 (+/− NA ppm)
301.0758
[M+H]+
685.4369
0
20
40
60
80
100
300
400
500
600
700

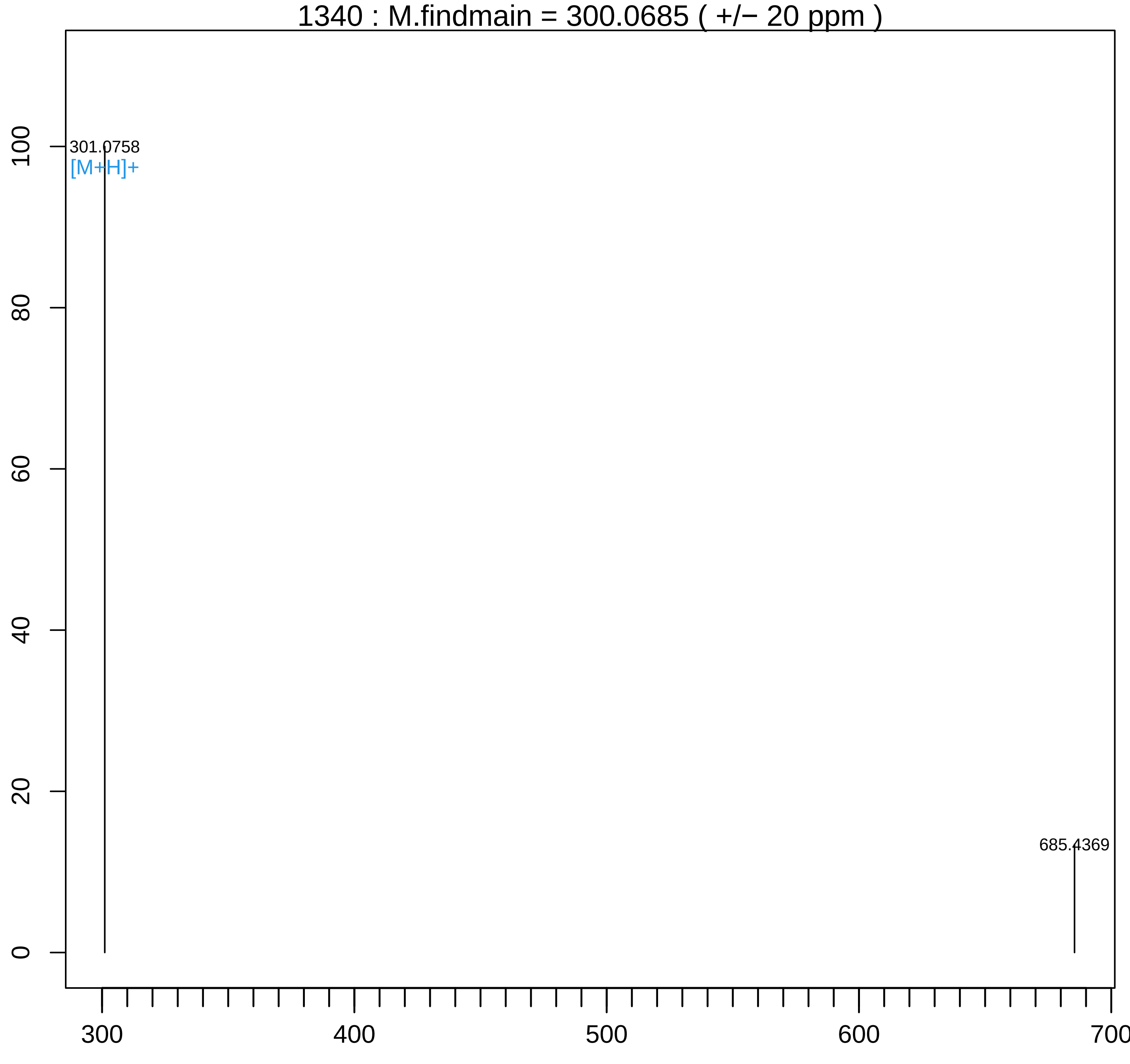
1340 : M.findmain = 300.0685 (+/− 20 ppm)
301.0758
[M+H]+
685.4369
0
20
40
60
80
100
300
400
500
600
700

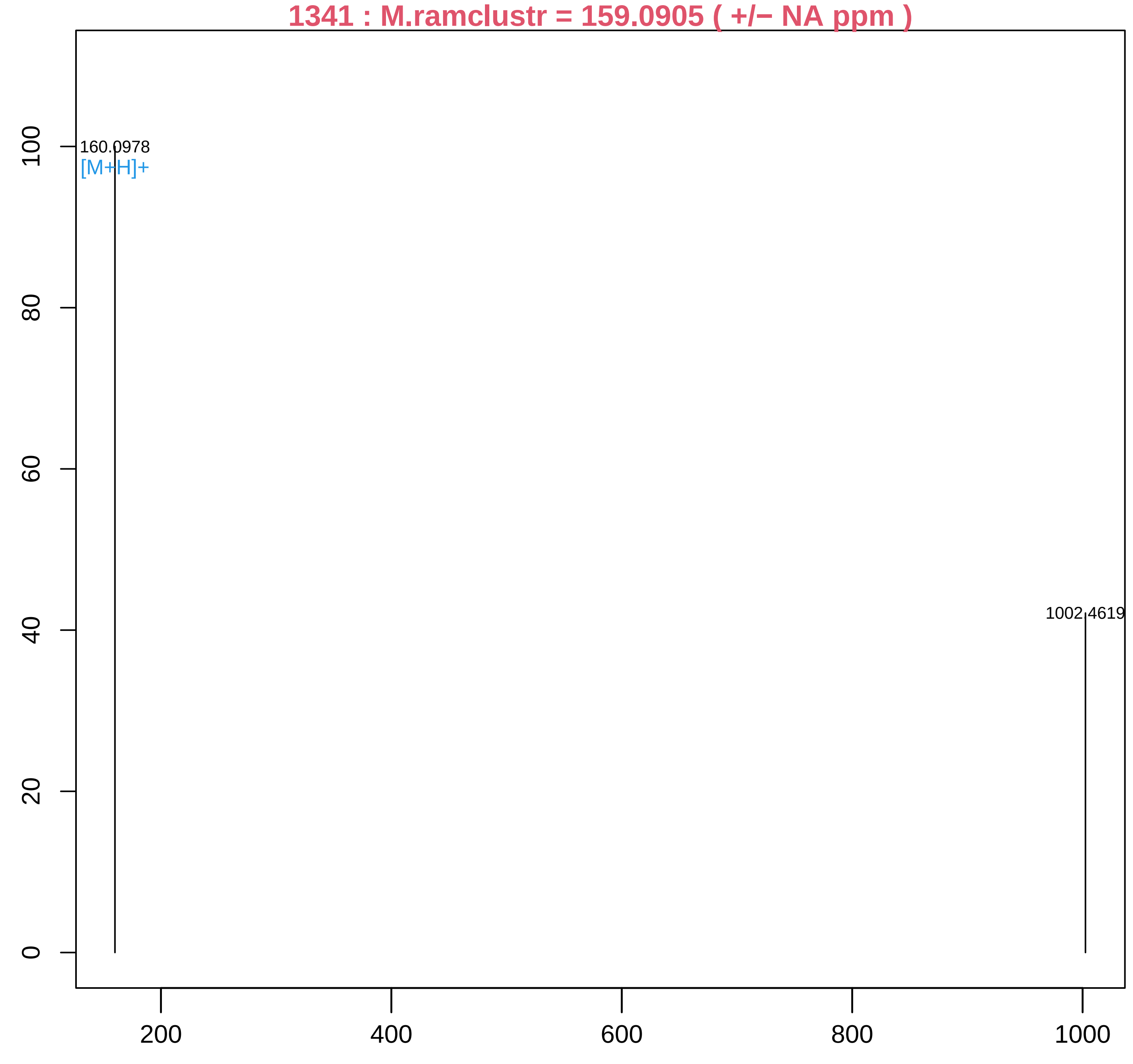
1341 : M.ramclustr = 159.0905 (+/− NA ppm)
160.0978
[M+H]+
1002.4619
0
20
40
60
80
100
200
400
600
800
1000

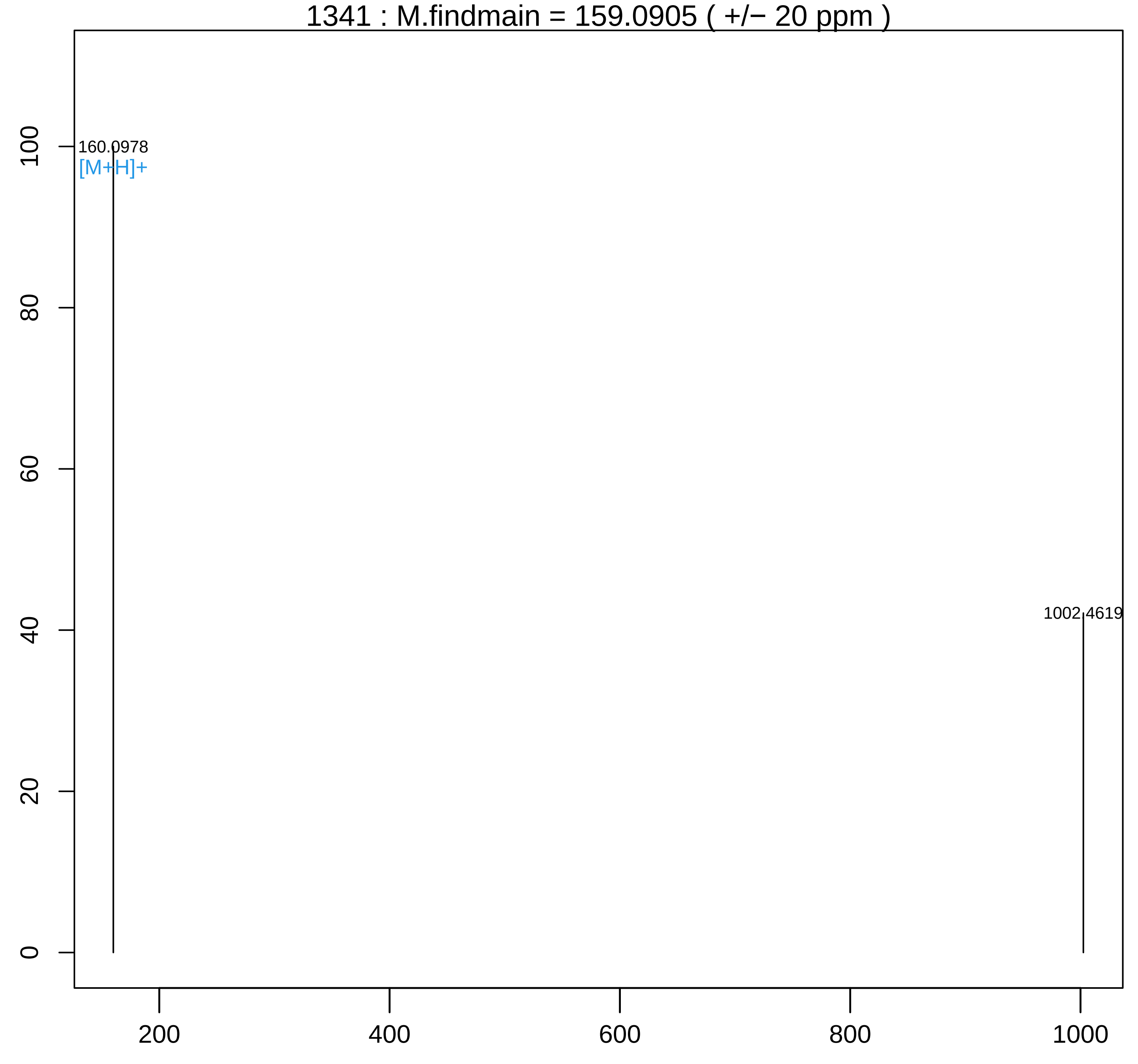
1341 : M.findmain = 159.0905 (+/− 20 ppm)
160.0978
[M+H]+
1002.4619
0
20
40
60
80
100
200
400
600
800
1000

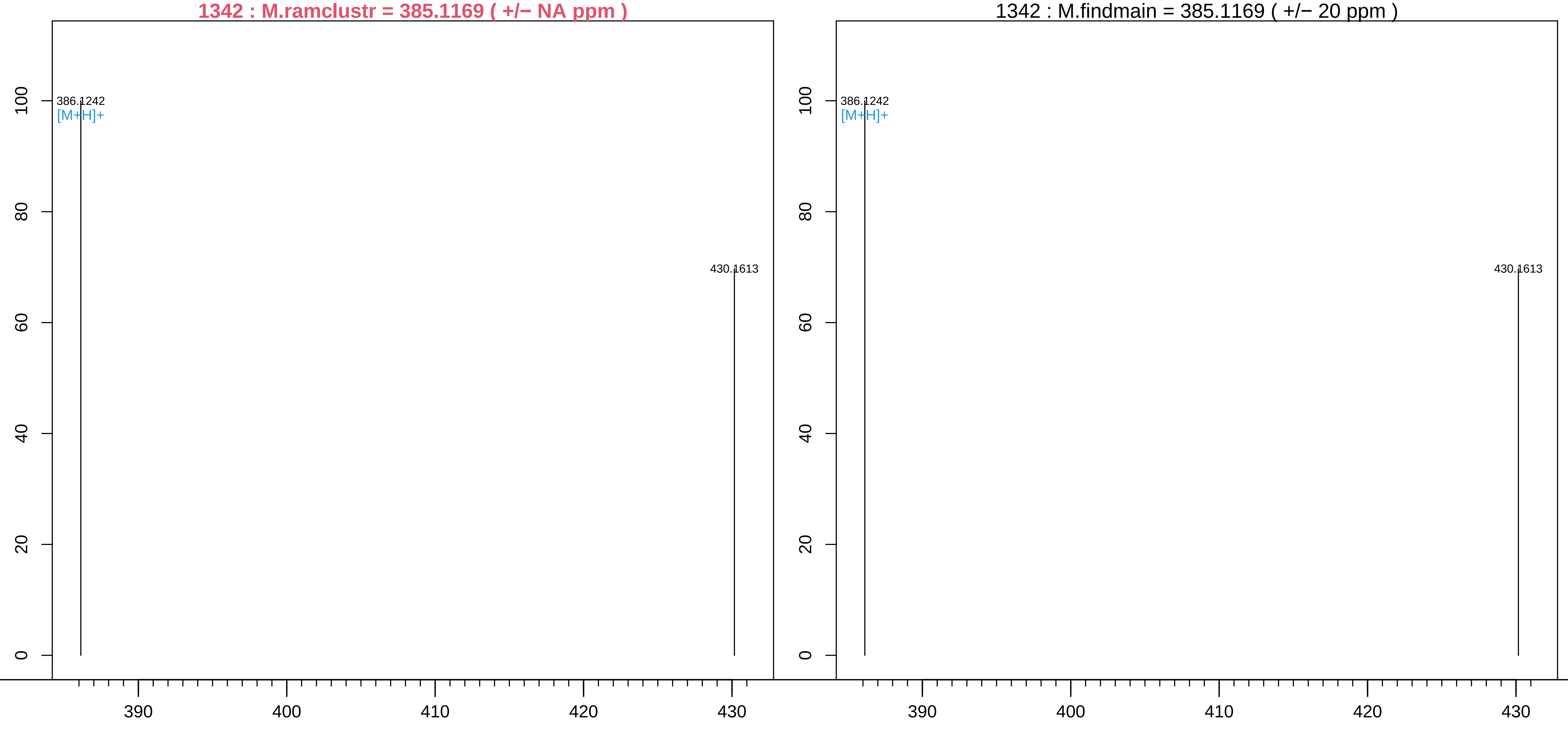

1342 : M.ramclustr = 385.1169 (+/− NA ppm)
386.1242
[M+H]+
430.1613
1342 : M.findmain = 385.1169 (+/− 20 ppm)
386.1242
[M+H]+
430.1613
0
20
40
60
80
100
390
400
410
420
430

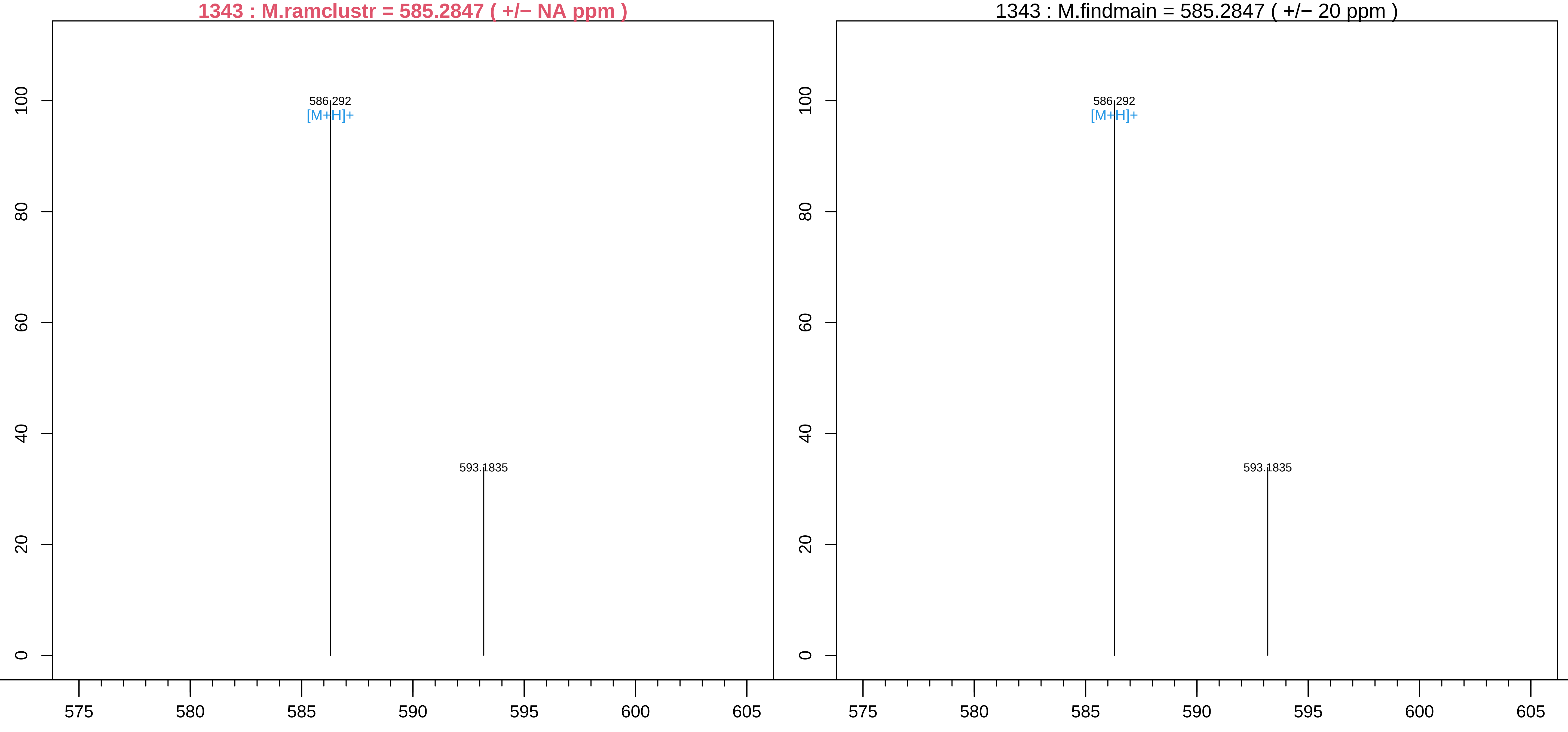

1343 : M.ramclustr = 585.2847 (+/− NA ppm)
586.292
[M+H]+
593.1835
1343 : M.findmain = 585.2847 (+/− 20 ppm)
586.292
[M+H]+
593.1835
0
20
40
60
80
100
575
580
585
590
595
600
605

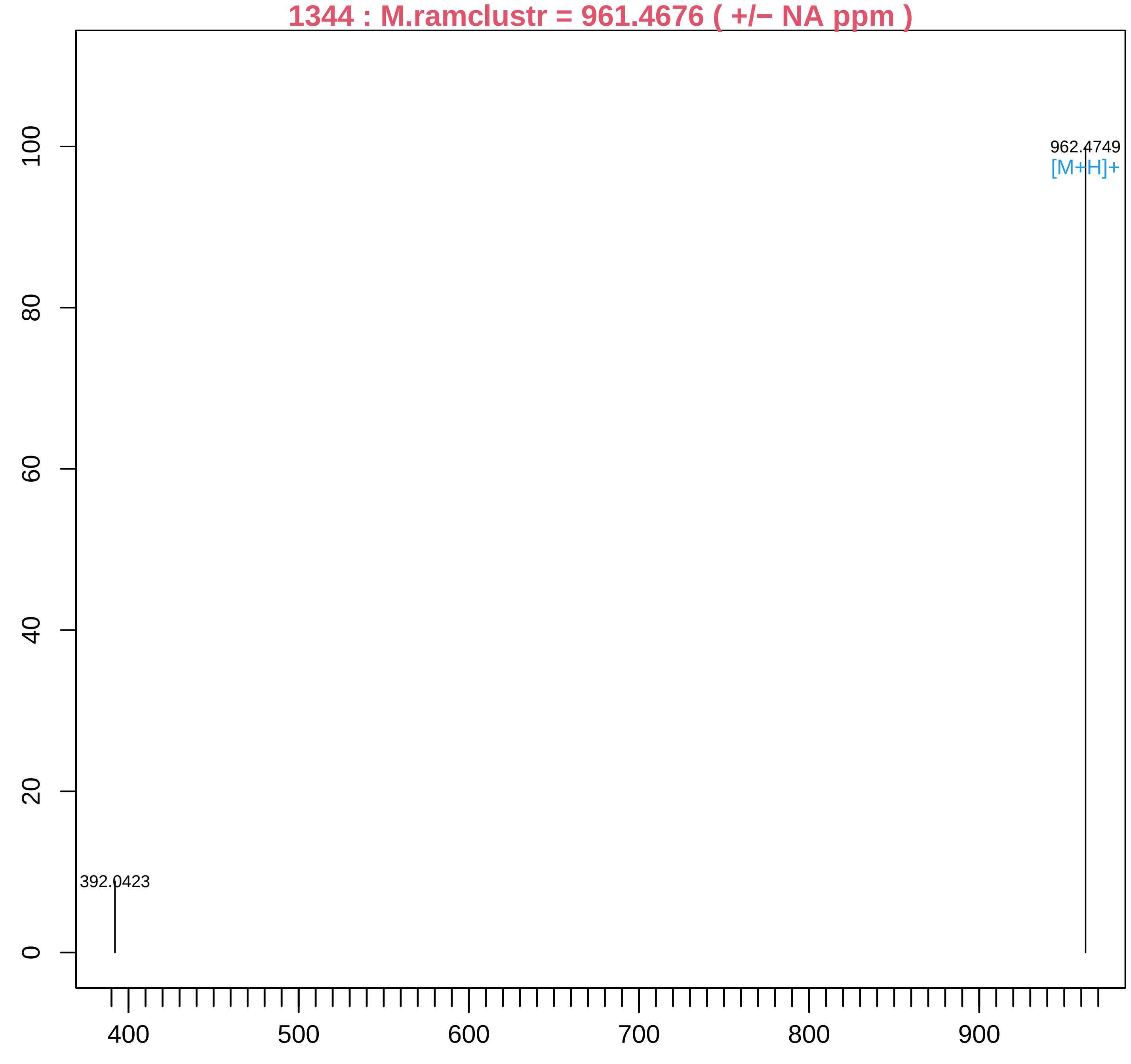
1344 : M.ramclustr = 961.4676 (+/− NA ppm)
962.4749
[M+H]+
392.0423
0
20
40
60
80
100
400
500
600
700
800
900

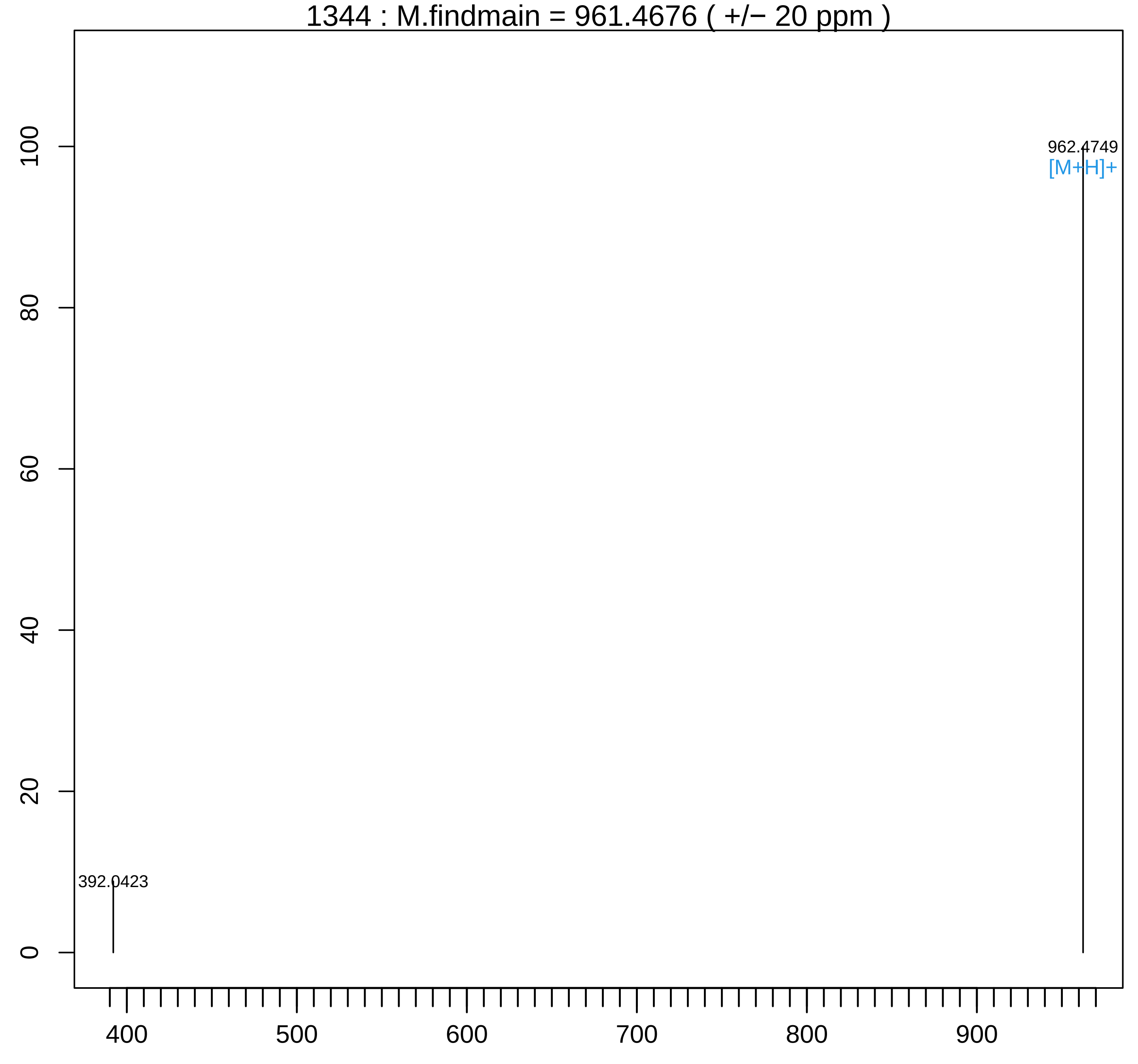
1344 : M.findmain = 961.4676 (+/− 20 ppm)
962.4749
[M+H]+
392.0423
0
20
40
60
80
100
400
500
600
700
800
900

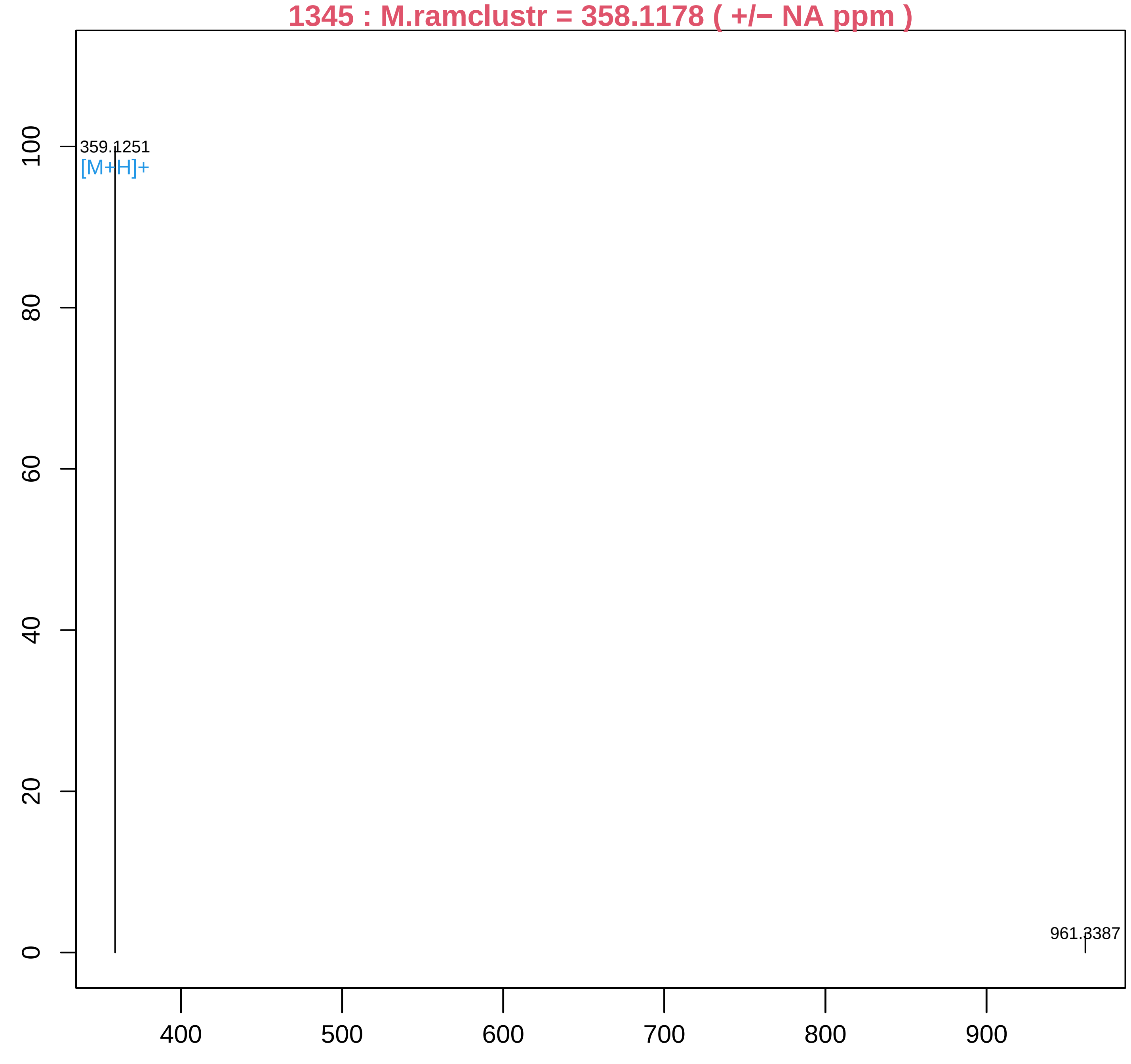
1345 : M.ramclustr = 358.1178 (+/− NA ppm)
359.1251
[M+H]+
961.3387
0
20
40
60
80
100
400
500
600
700
800
900

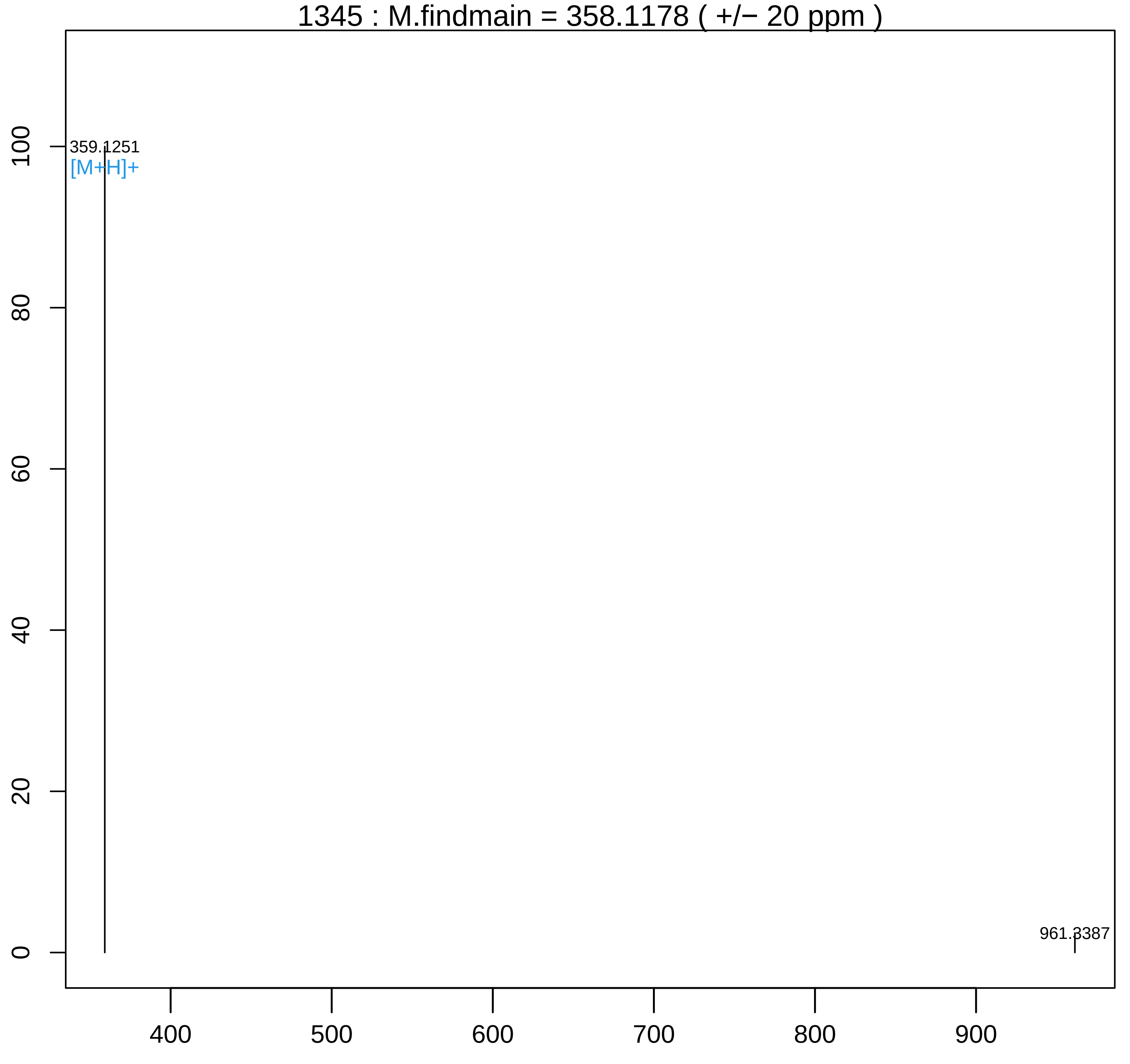
1345 : M.findmain = 358.1178 (+/− 20 ppm)
359.1251
[M+H]+
961.3387
0
20
40
60
80
100
400
500
600
700
800
900

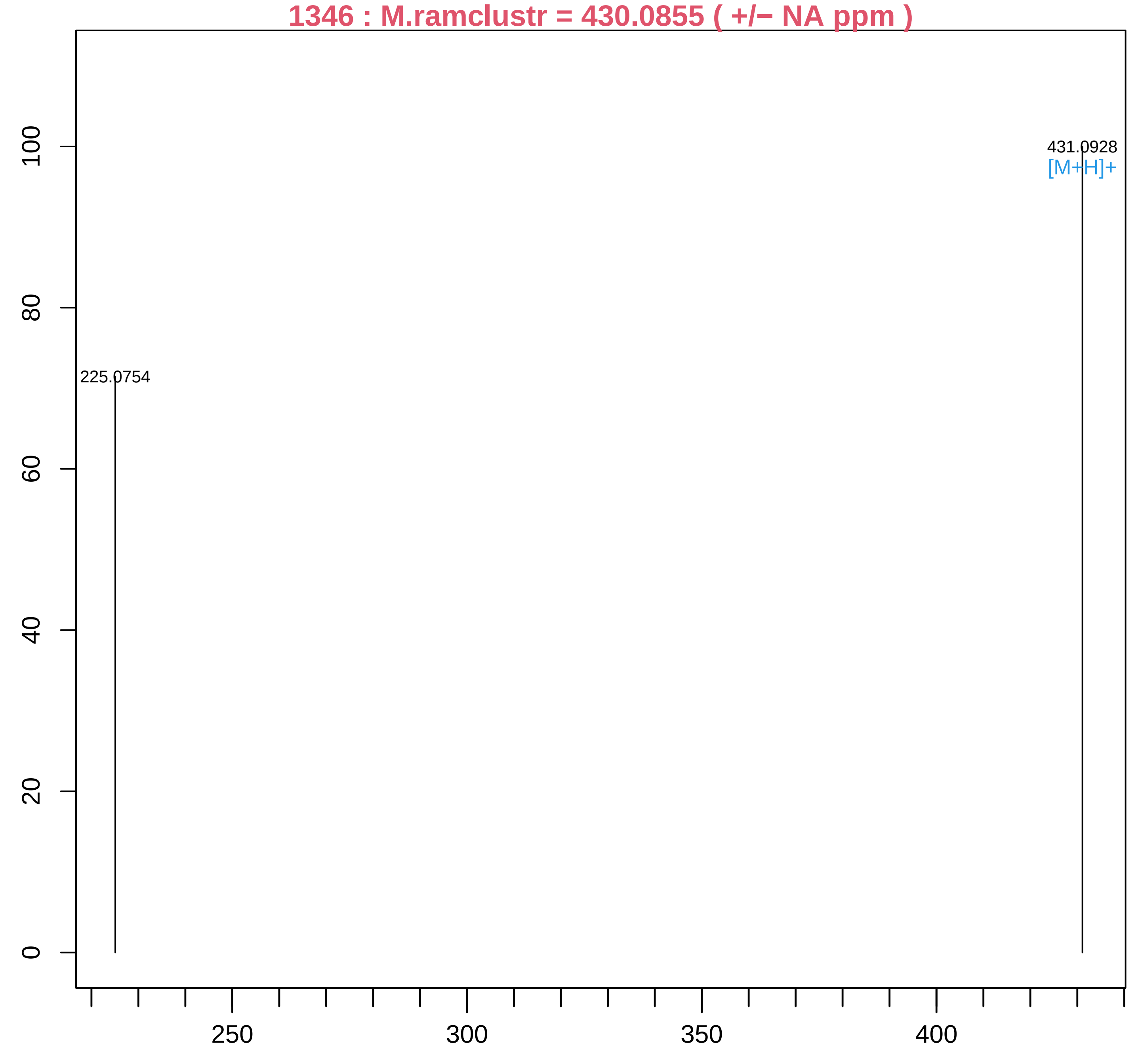
1346 : M.ramclustr = 430.0855 (+/− NA ppm)
431.0928
[M+H]+
225.0754

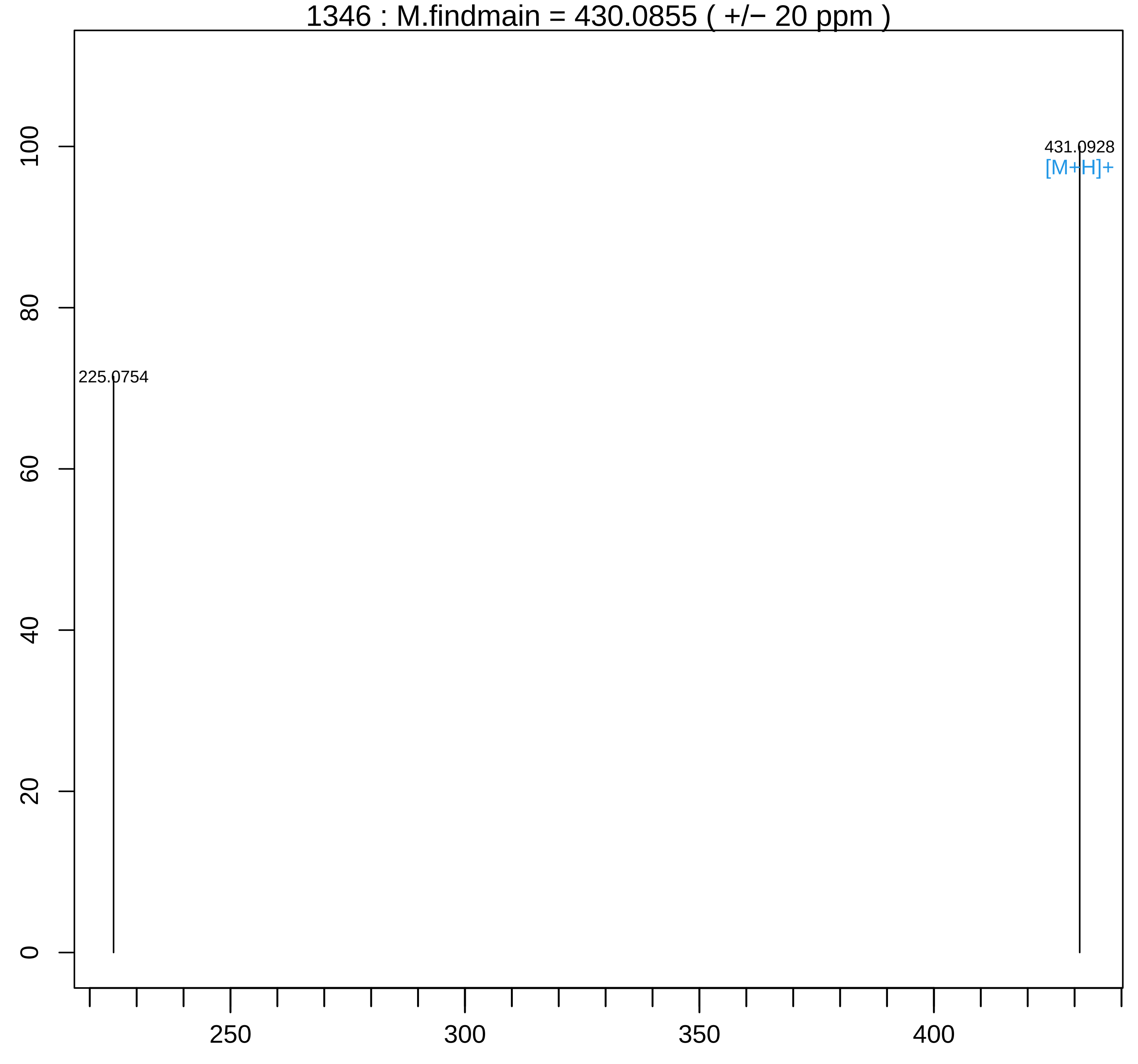
1346 : M.findmain = 430.0855 (+/− 20 ppm)
431.0928
[M+H]+
225.0754

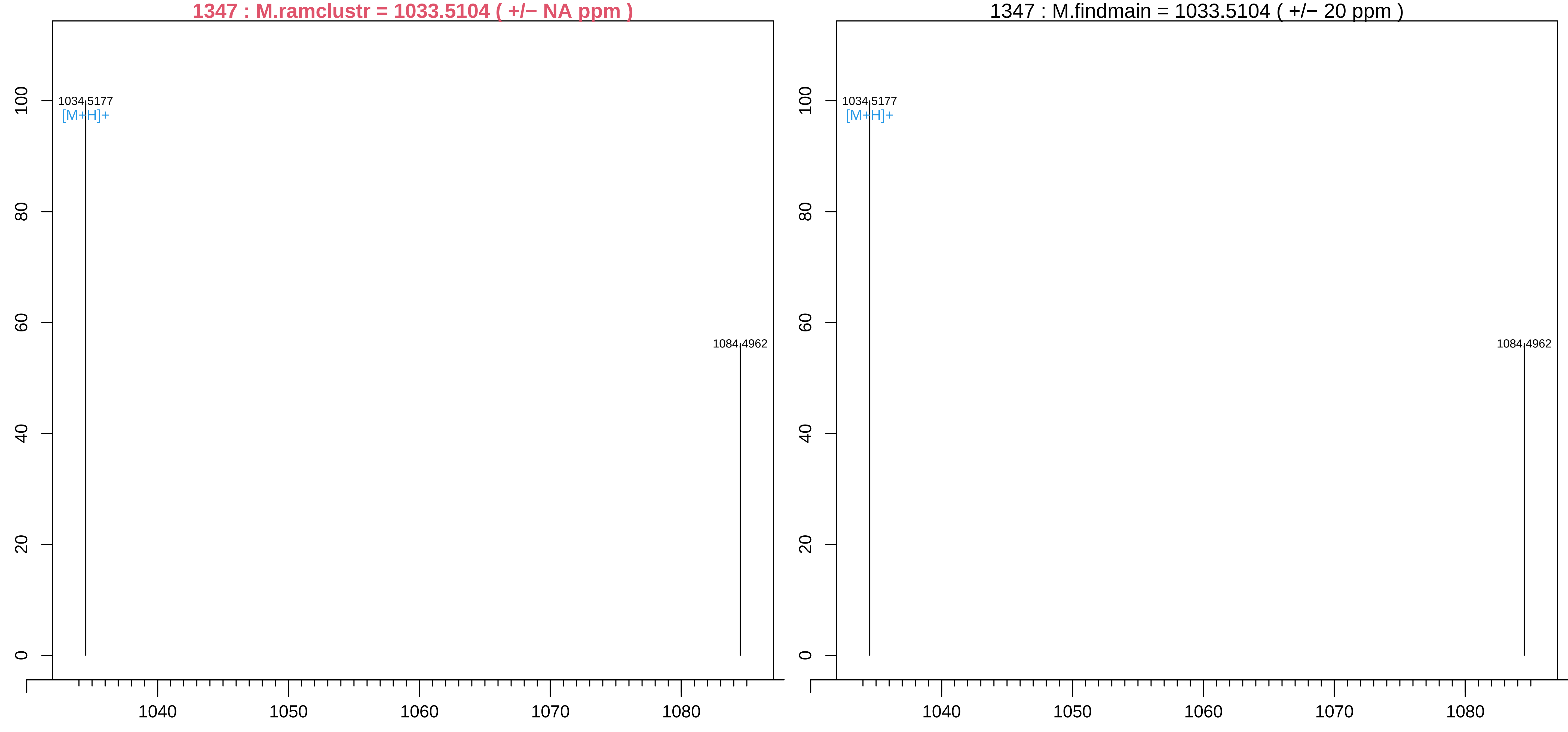
1347 : M.ramclustr = 1033.5104 (+/− NA ppm)
1034.5177
[M+H]+
1084.4962
0
20
40
60
80
100
1040
1050
1060
1070
1080
1347 : M.findmain = 1033.5104 (+/− 20 ppm)
1034.5177
[M+H]+
1084.4962
0
20
40
60
80
100
1040
1050
1060
1070
1080

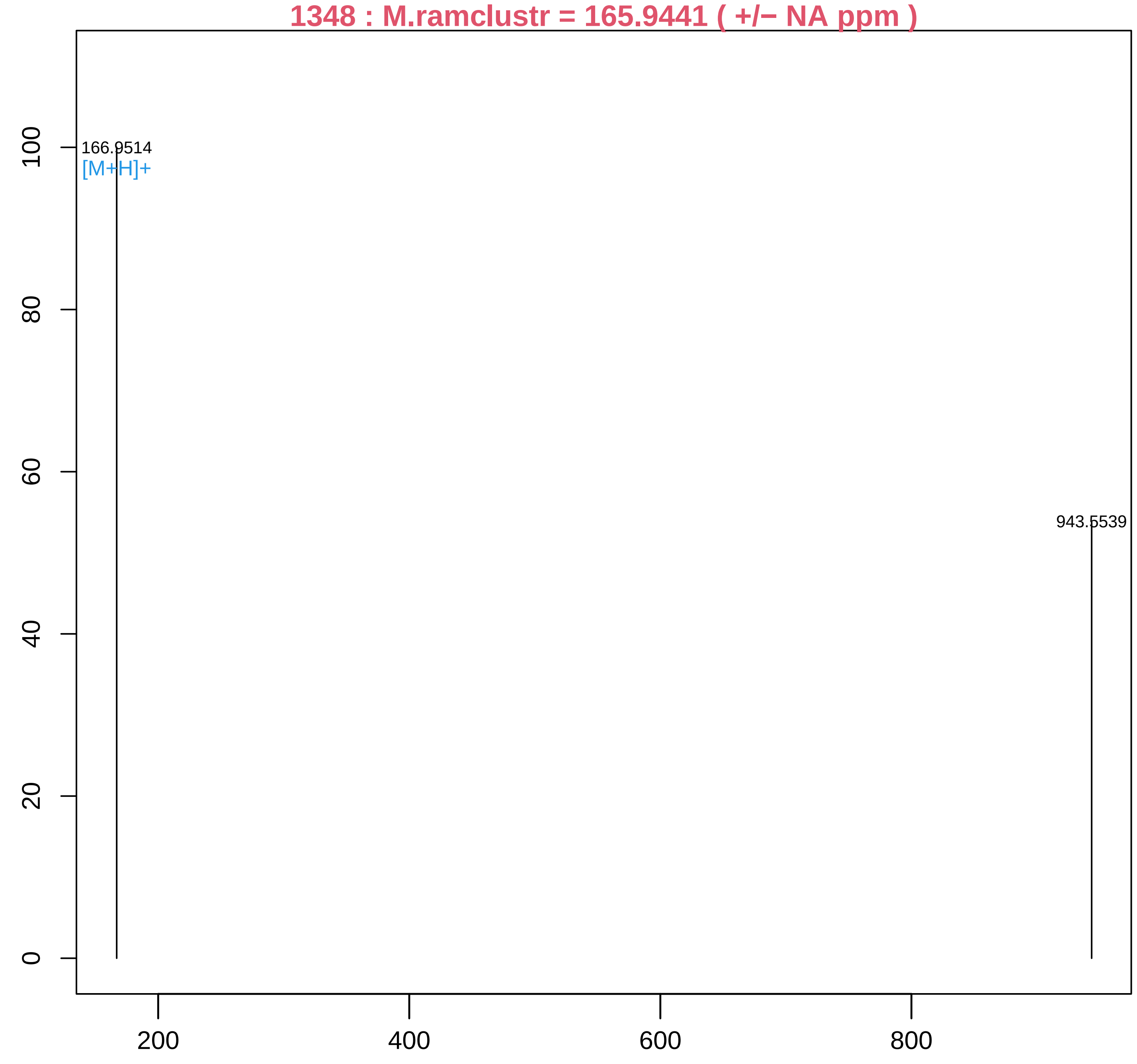
1348 : M.ramclustr = 165.9441 (+/− NA ppm)
166.9514
[M+H]+
943.5539
0
20
40
60
80
100
200
400
600
800

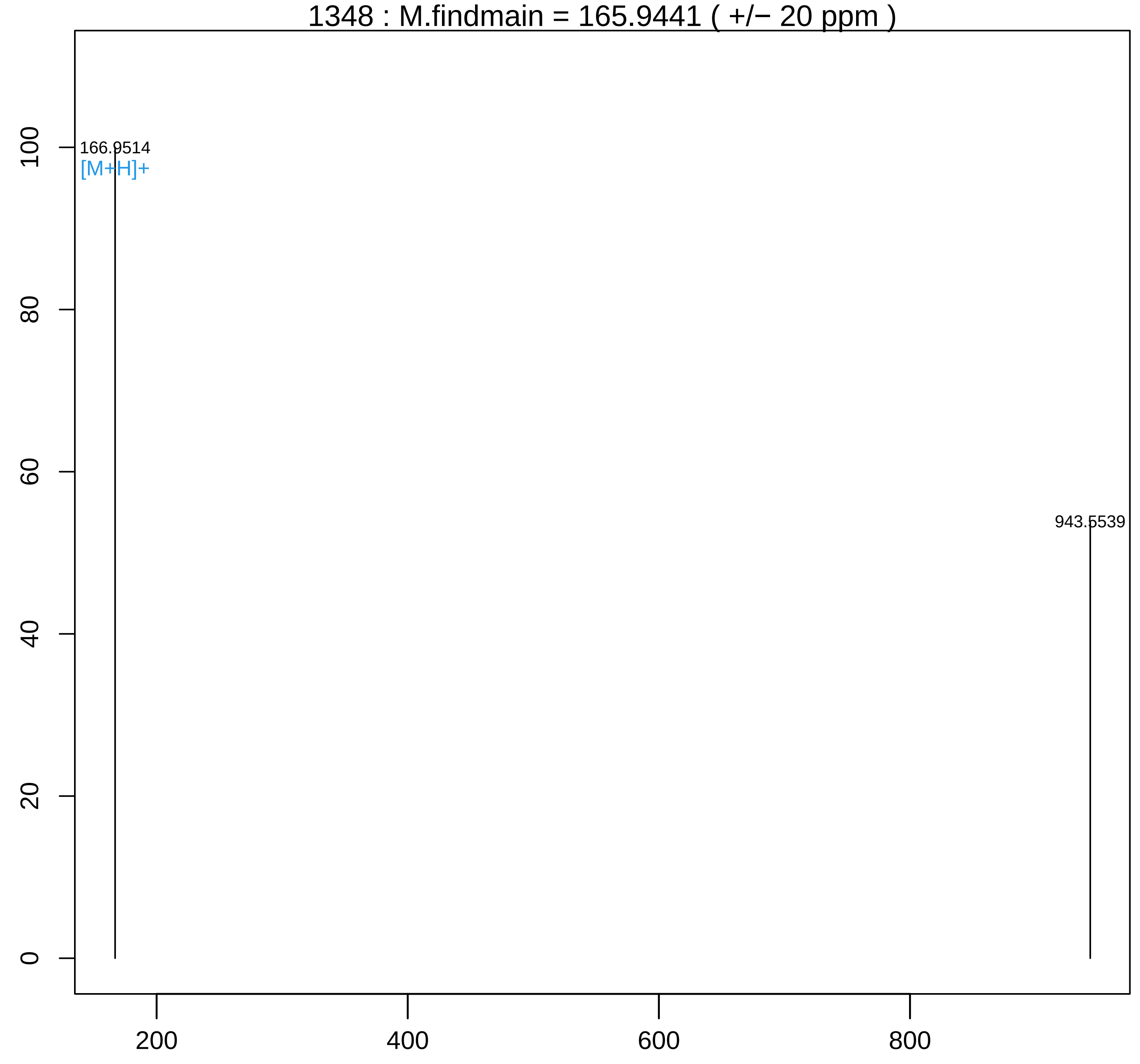
1348 : M.findmain = 165.9441 (+/− 20 ppm)
166.9514
[M+H]+
943.5539
0
20
40
60
80
100
200
400
600
800

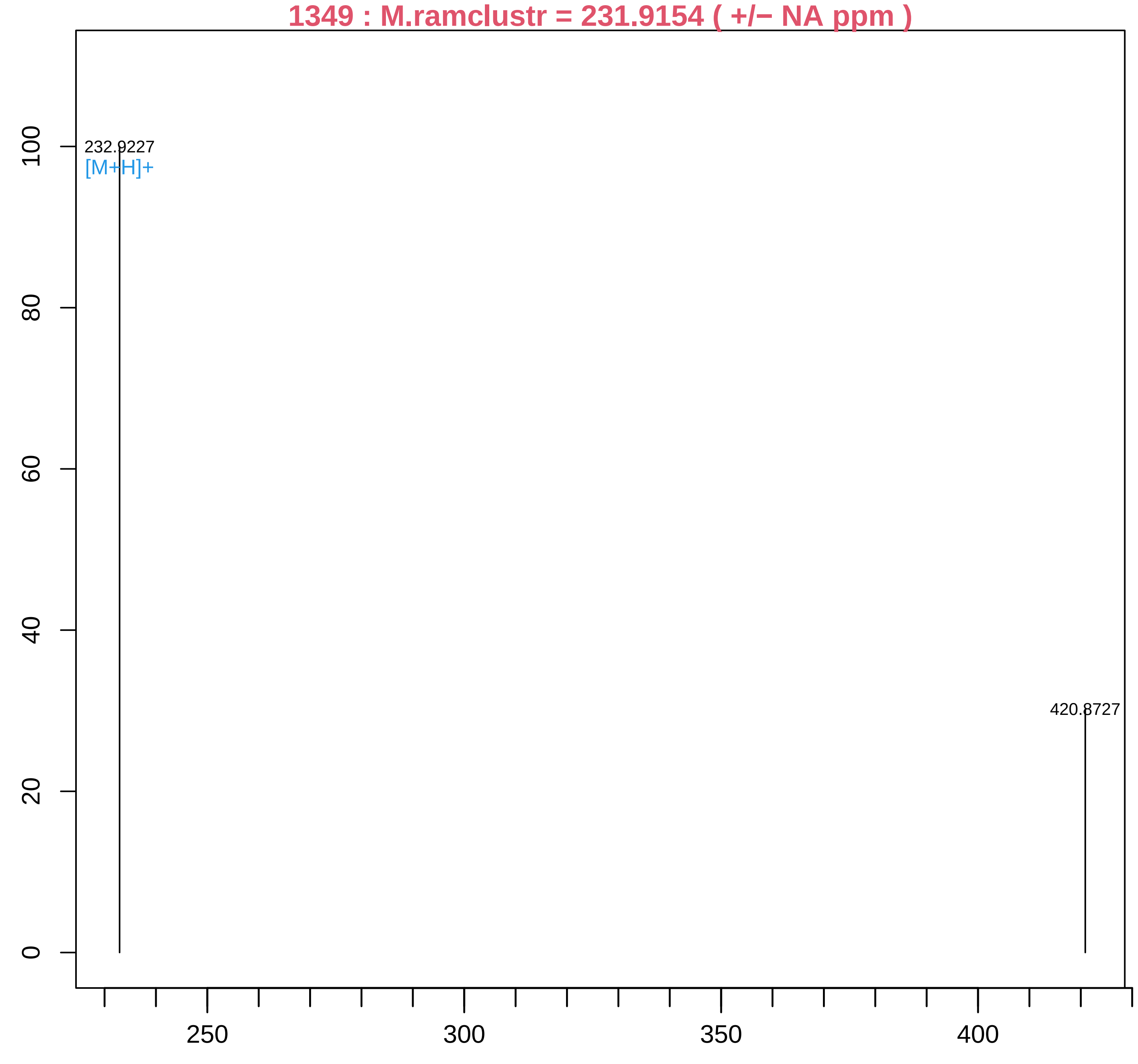
1349 : M.ramclustr = 231.9154 (+/− NA ppm)
232.9227
[M+H]+
420.8727
0
20
40
60
80
100
250
300
350
400

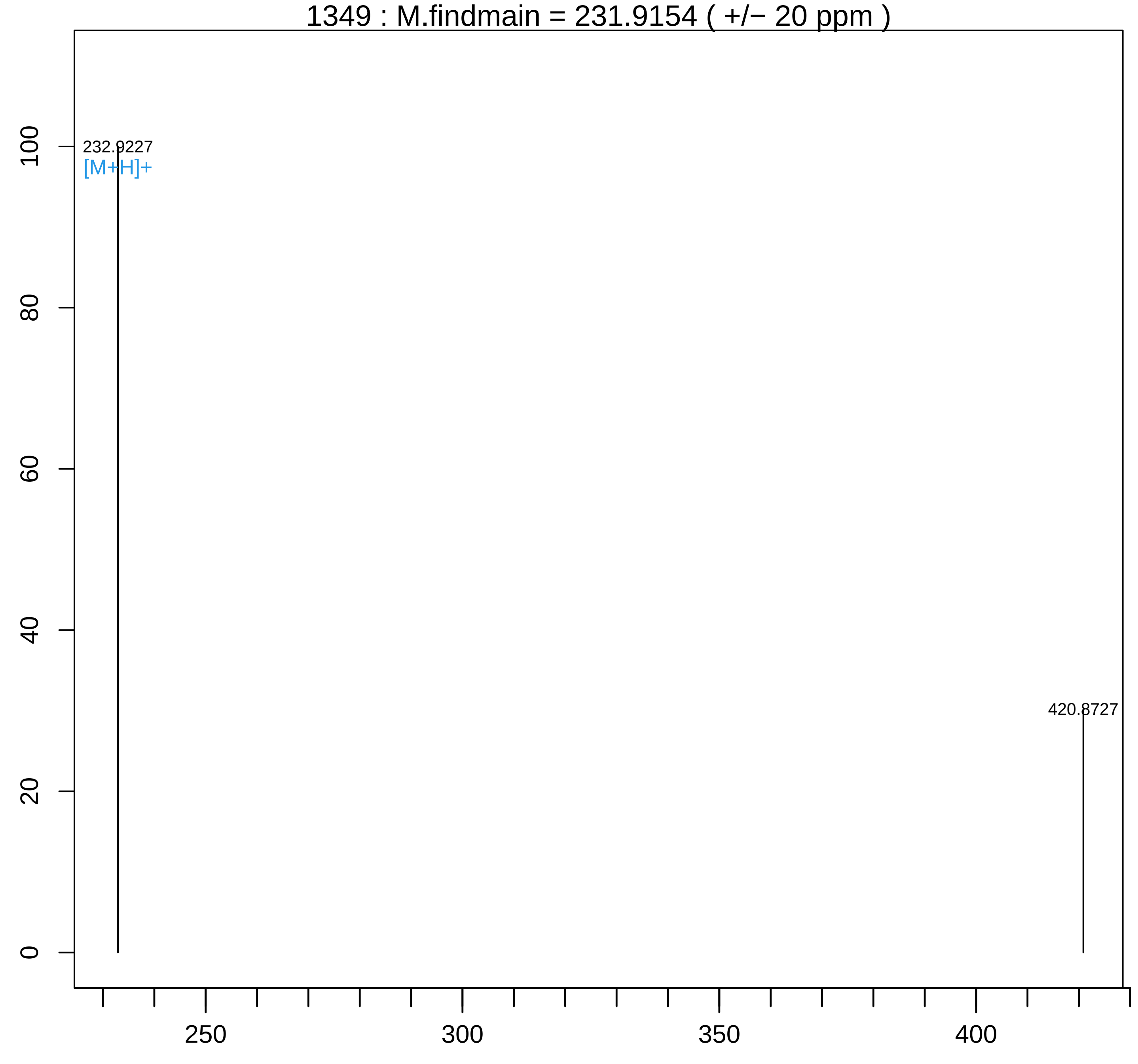
1349 : M.findmain = 231.9154 (+/− 20 ppm)
232.9227
[M+H]+
420.8727
0
20
40
60
80
100
250
300
350
400

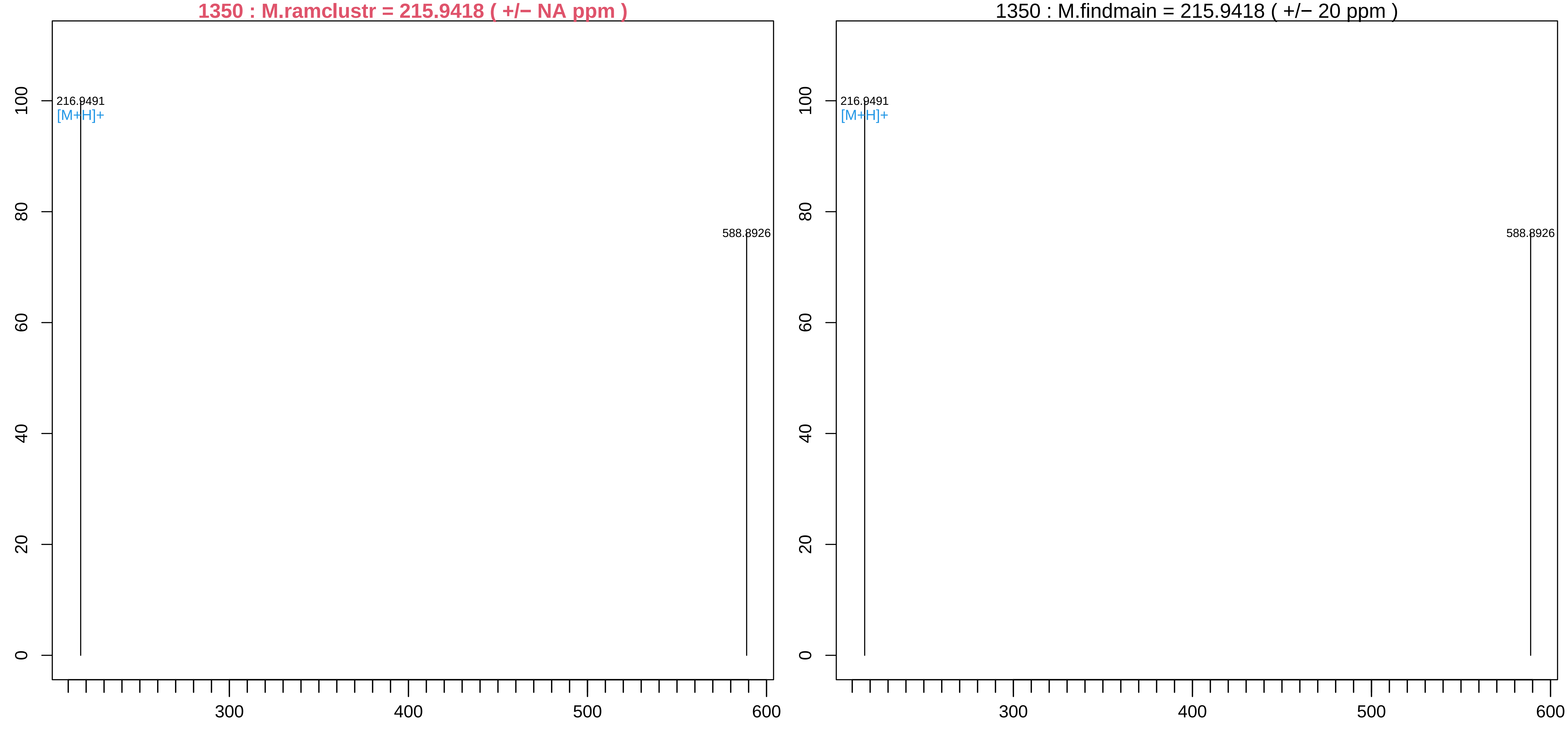

1350 : M.ramclustr = 215.9418 (+/− NA ppm)
216.9491
[M+H]+
588.8926
1350 : M.findmain = 215.9418 (+/− 20 ppm)
216.9491
[M+H]+
588.8926
0
20
40
60
80
100
300
400
500
600

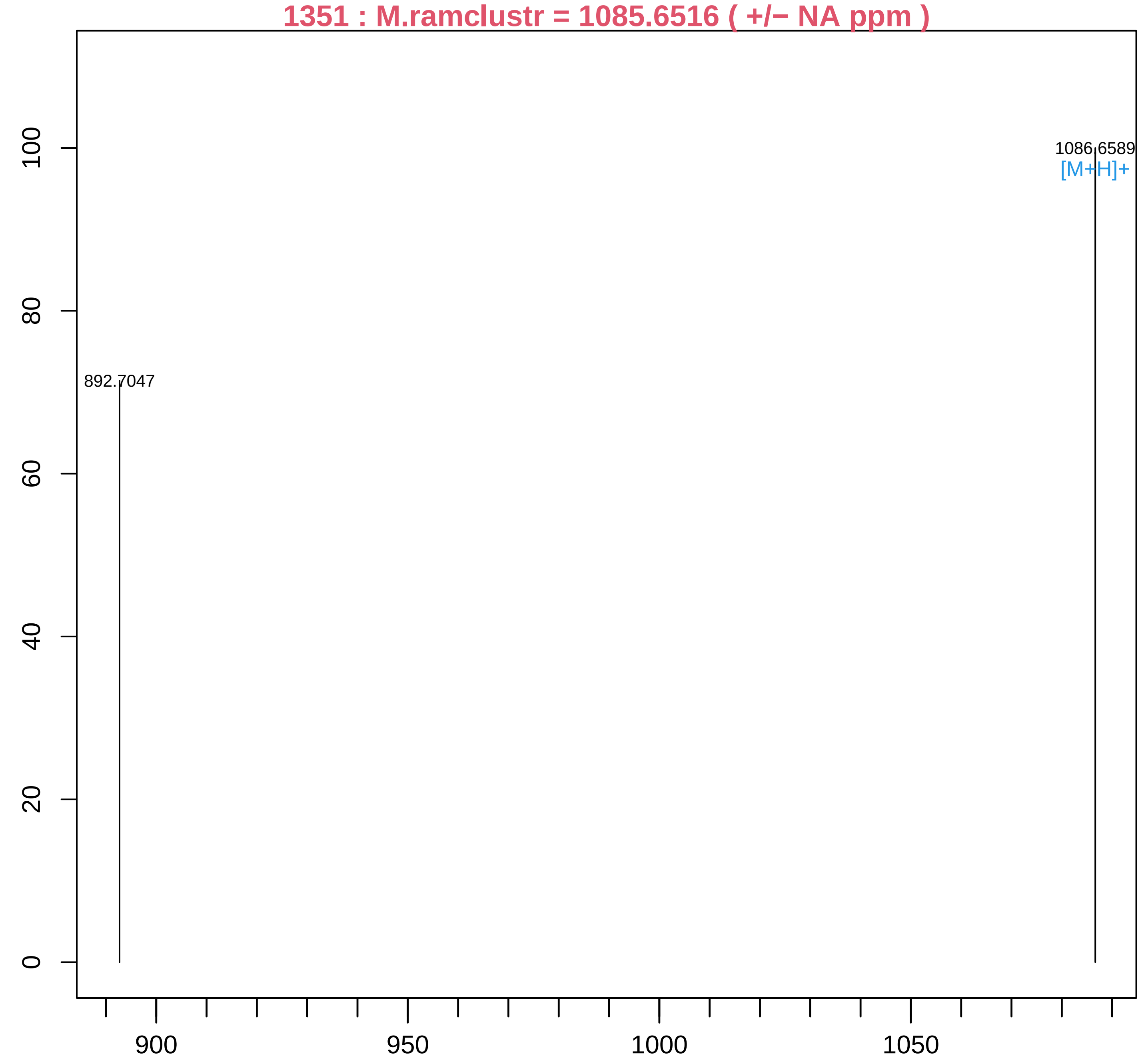
1351 : M.ramclustr = 1085.6516 (+/− NA ppm)
1086.6589
[M+H]+
892.7047
0
20
40
60
80
100
900
950
1000
1050

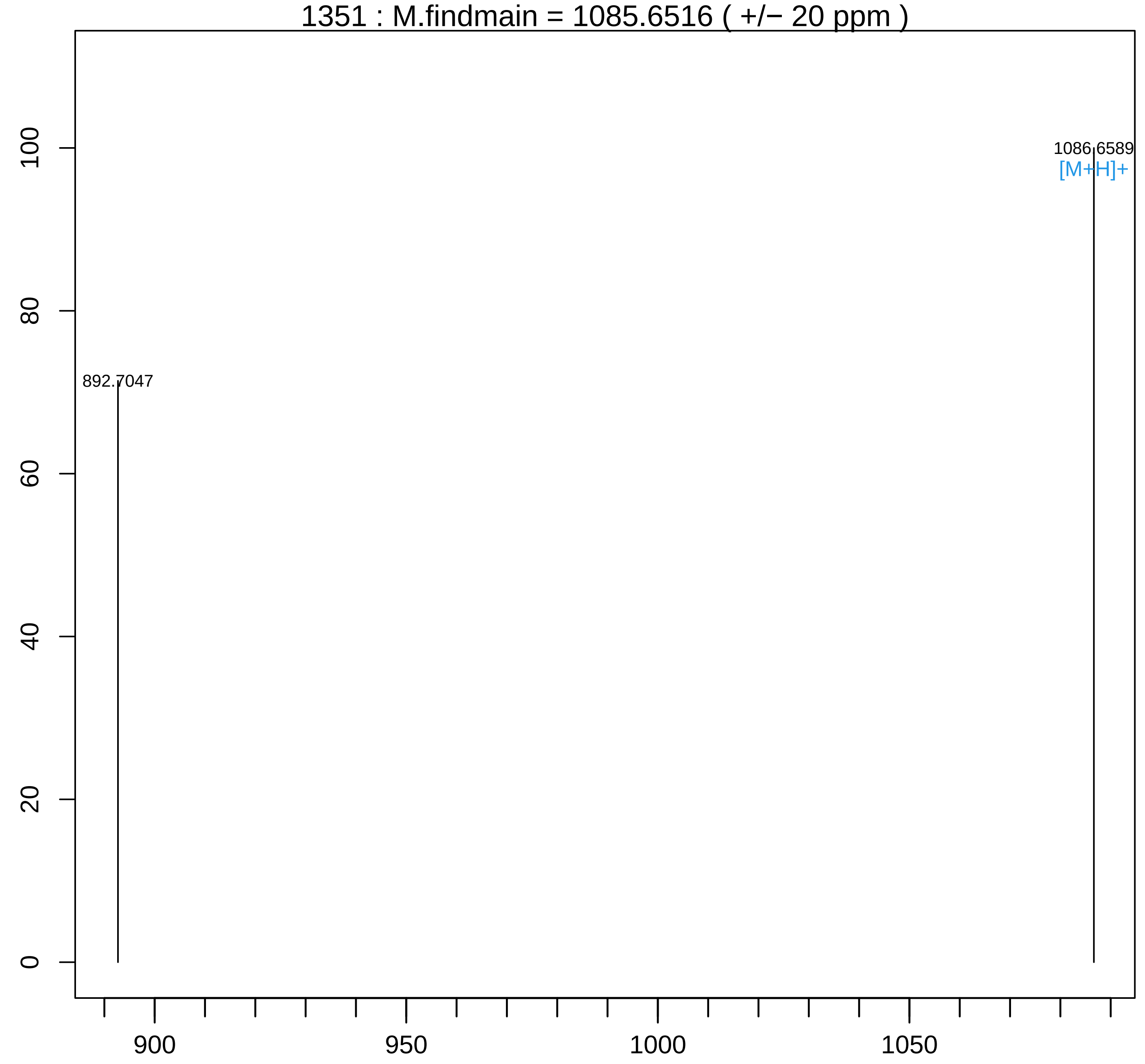
1351 : M.findmain = 1085.6516 (+/− 20 ppm)
1086.6589
[M+H]+
892.7047
0
20
40
60
80
100
900
950
1000
1050

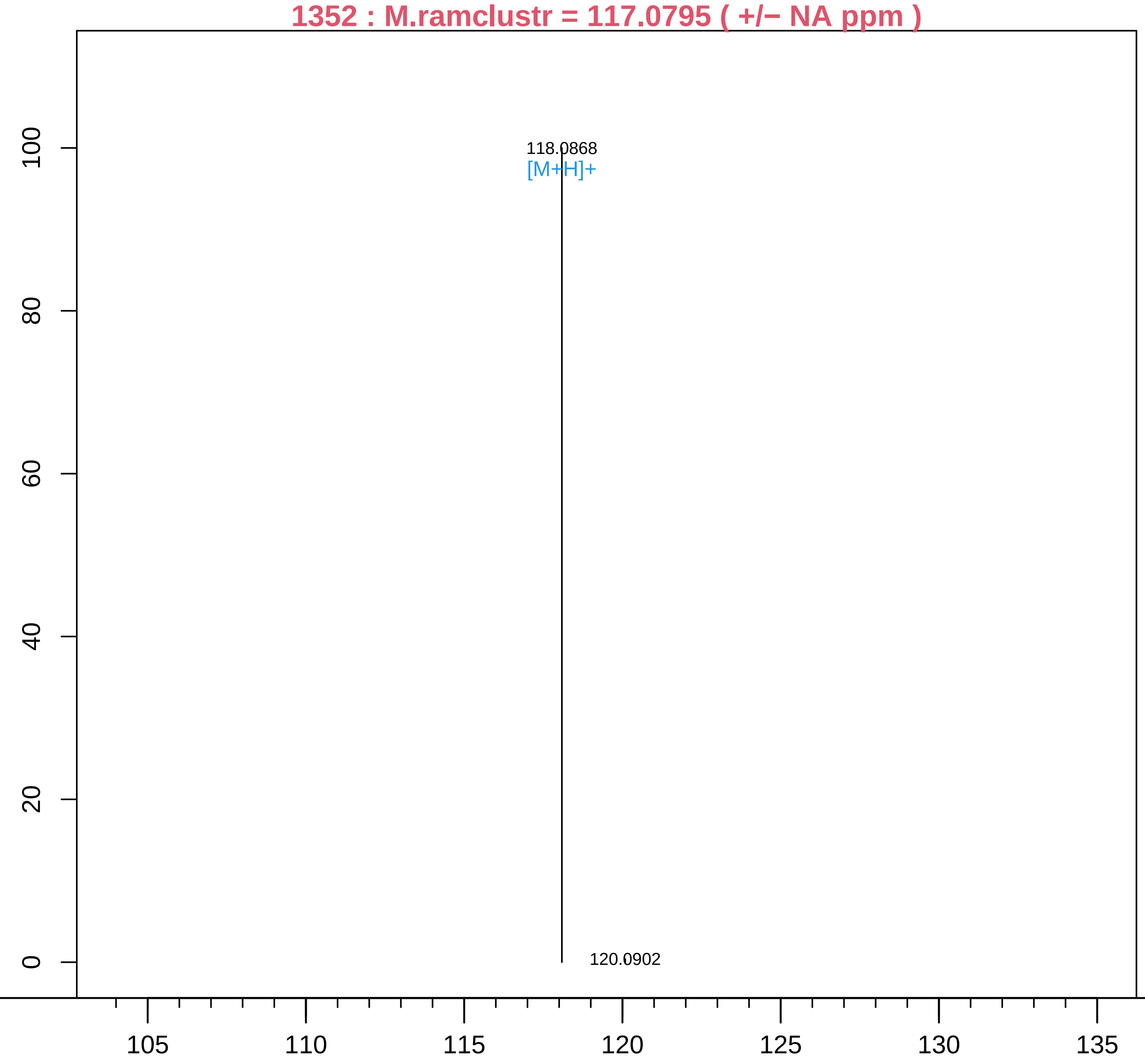

1352 : M.ramclustr = 117.0795 (+/− NA ppm)
118.0868
[M+H]+
120.0902
0
20
40
60
80
100
105
110
115
120
125
130
135

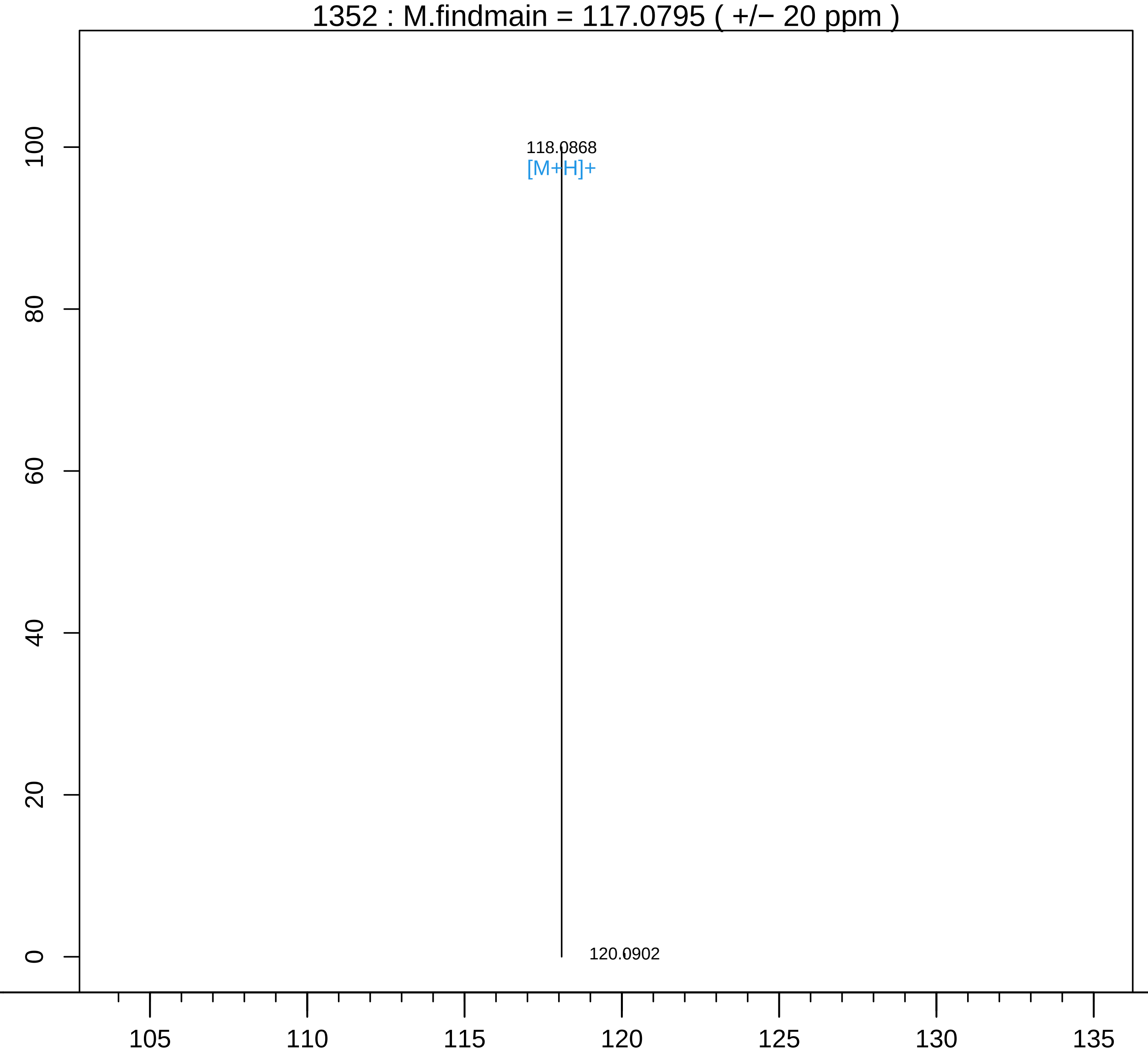

1352 : M.findmain = 117.0795 (+/− 20 ppm)
118.0868
[M+H]+
120.0902
0
20
40
60
80
100
105
110
115
120
125
130
135

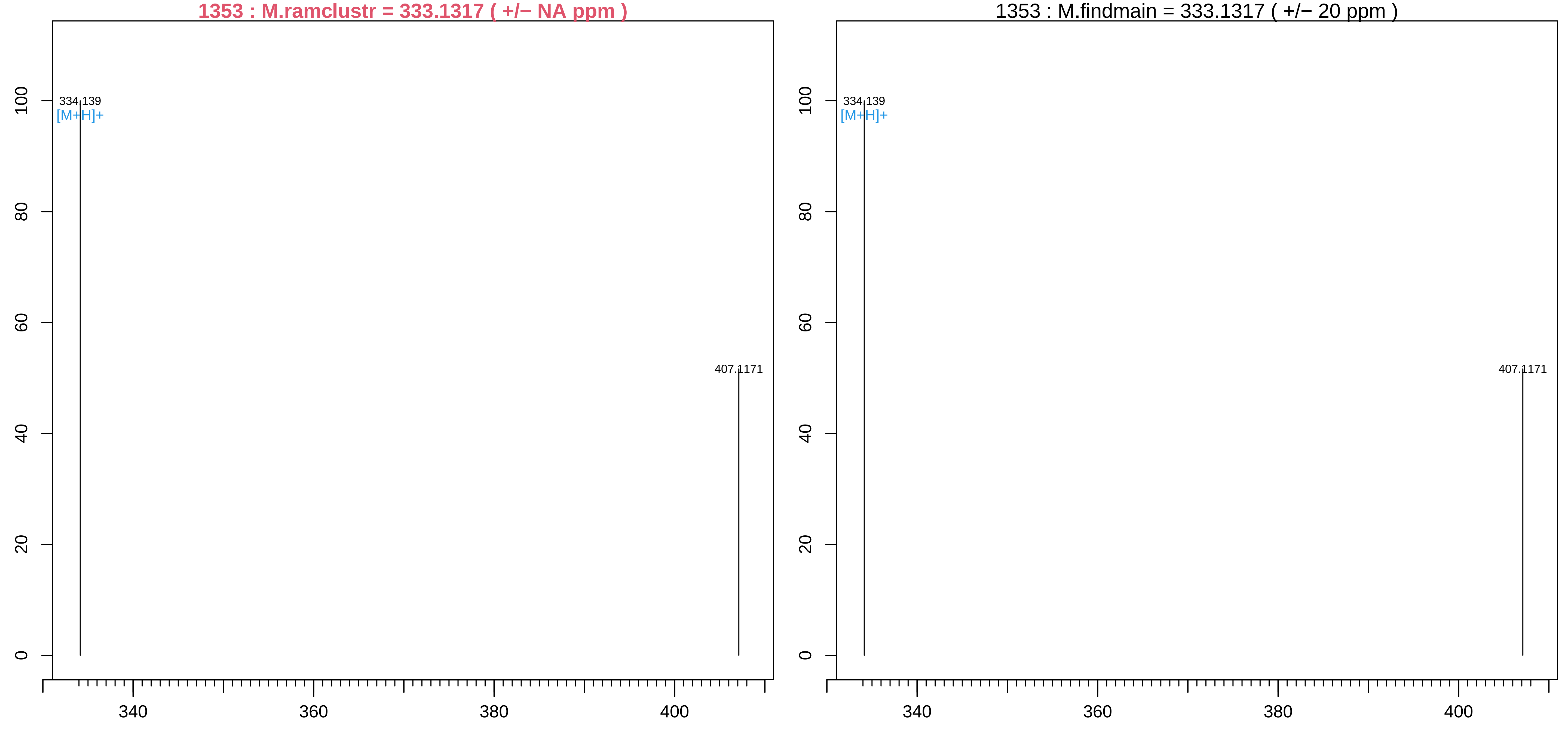

1353 : M.ramclustr = 333.1317 (+/− NA ppm)
1353 : M.findmain = 333.1317 (+/− 20 ppm)
334.139
[M+H]+
407.1171
0
20
40
60
80
100
340
360
380
400

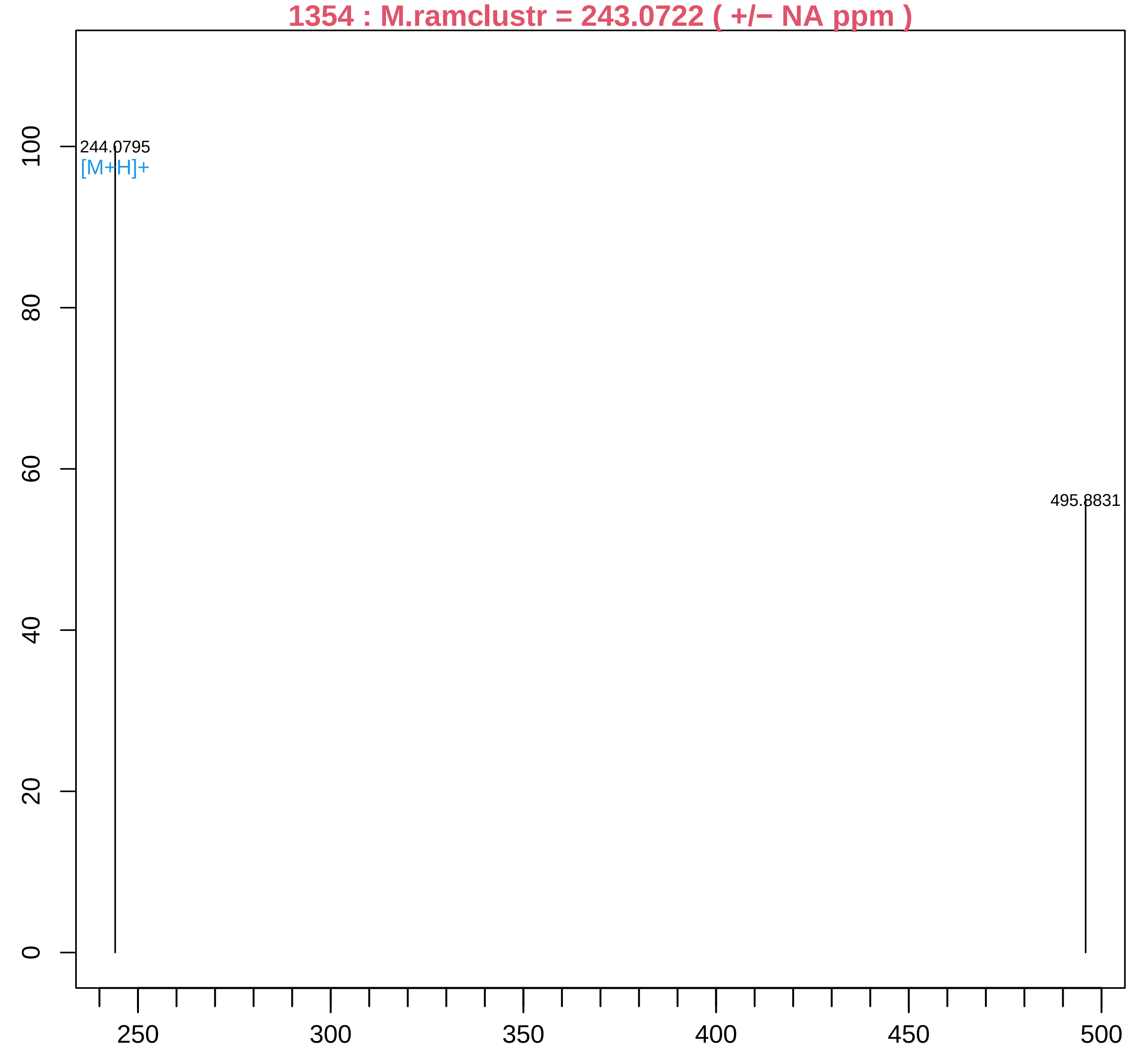
1354 : M.ramclustr = 243.0722 (+/− NA ppm)
244.0795
[M+H]+
495.8831
0
20
40
60
80
100
250
300
350
400
450
500

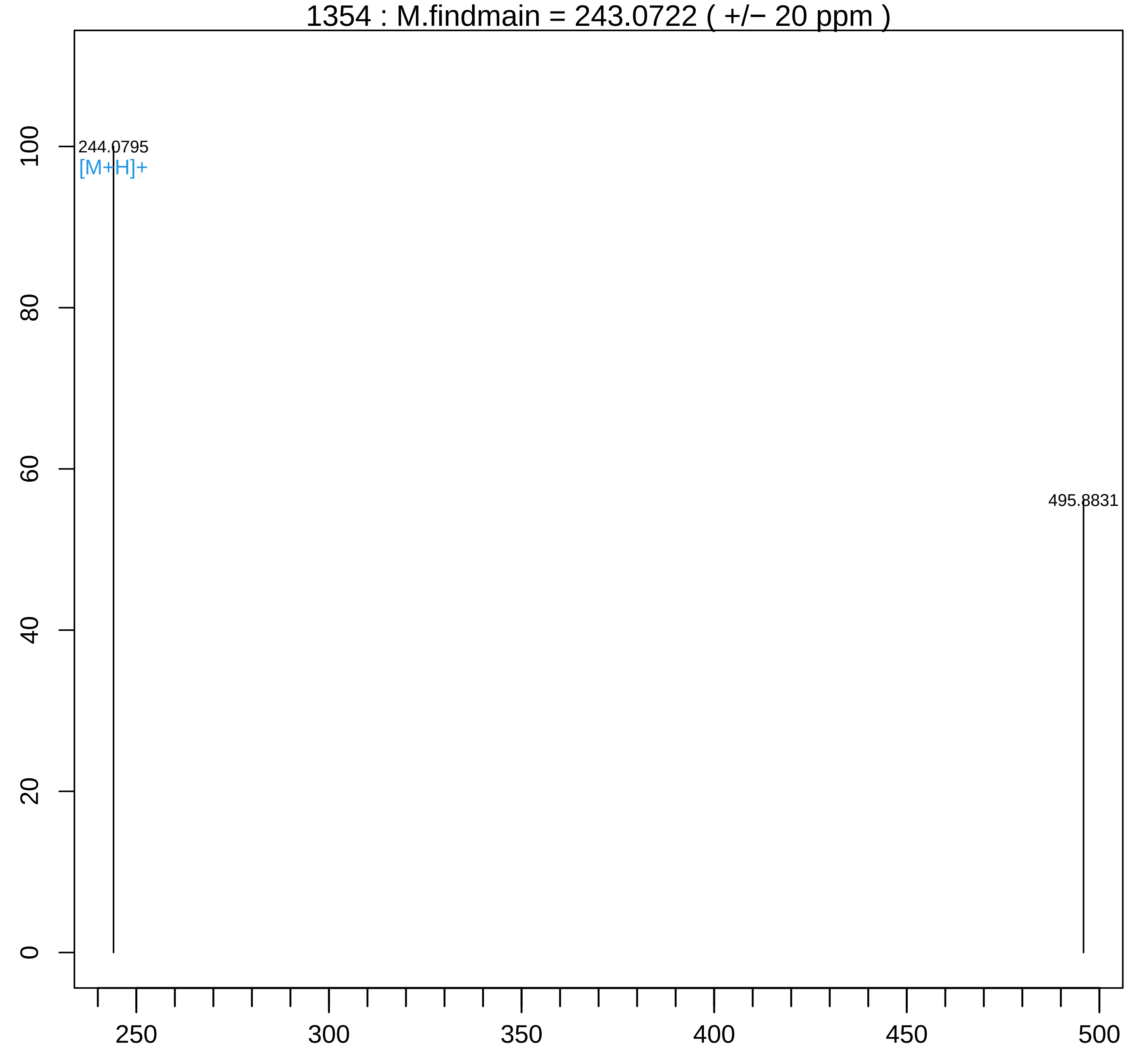
1354 : M.findmain = 243.0722 (+/− 20 ppm)
244.0795
[M+H]+
495.8831
0
20
40
60
80
100
250
300
350
400
450
500

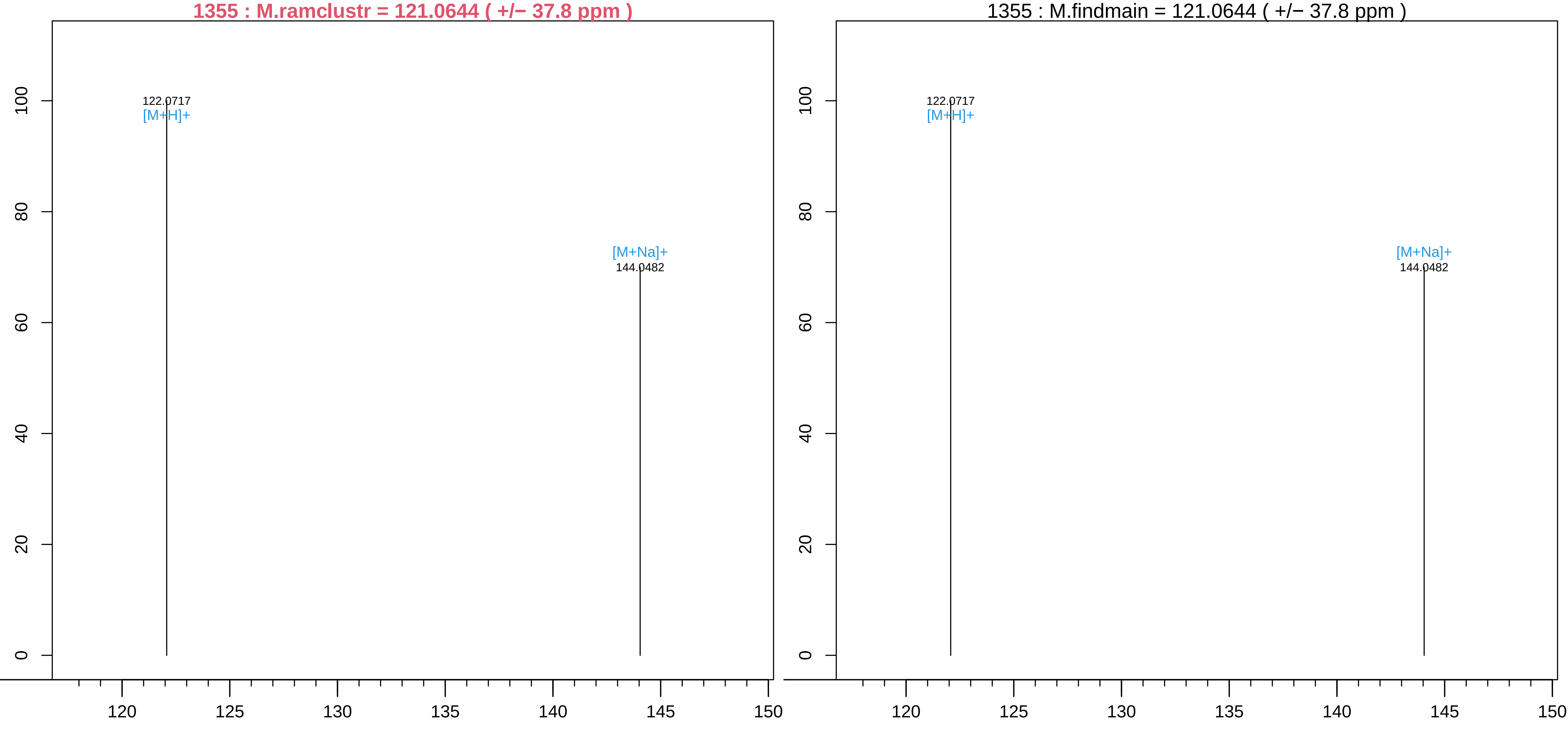
1355 : M.ramclustr = 121.0644 (+/− 37.8 ppm)
122.0717
[M+H]+
[M+Na]+
144.0482
1355 : M.findmain = 121.0644 (+/− 37.8 ppm)
122.0717
[M+H]+
[M+Na]+
144.0482
0
20
40
60
80
100
120
125
130
135
140
145
150

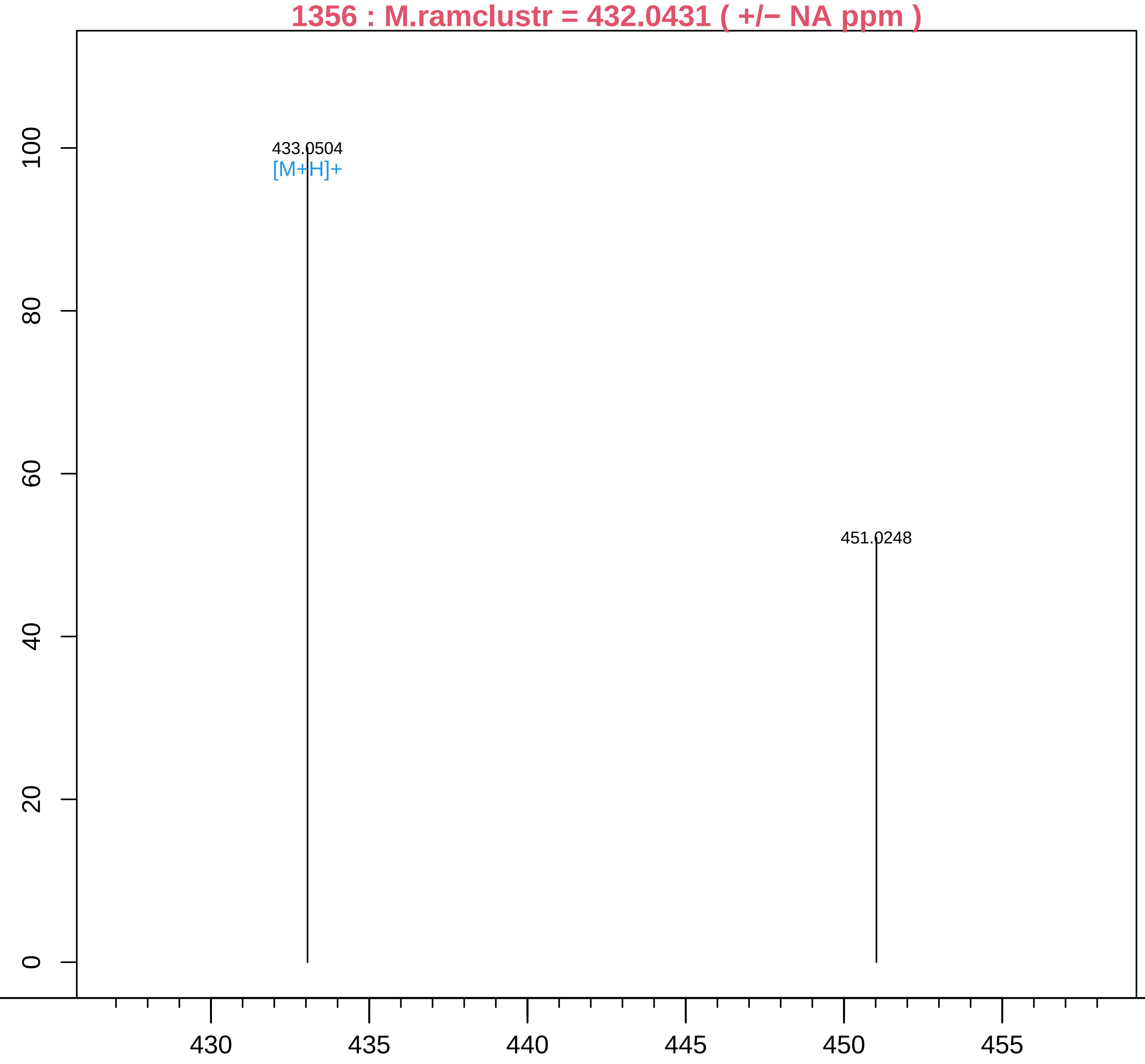
1356 : M.ramclustr = 432.0431 (+/− NA ppm)
433.0504
[M+H]+
451.0248
0
20
40
60
80
100
430
435
440
445
450
455

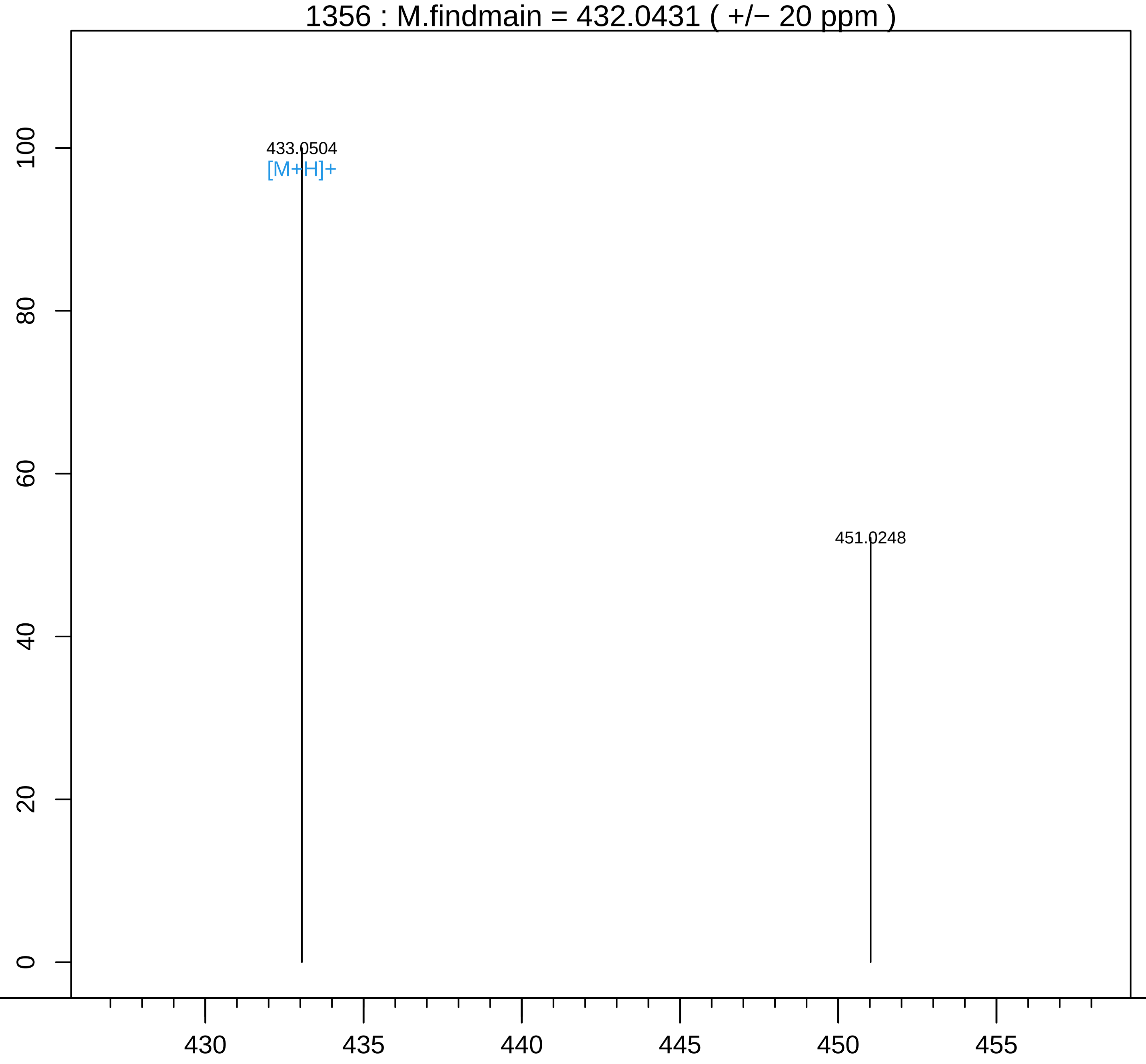
1356 : M.findmain = 432.0431 (+/− 20 ppm)
433.0504
[M+H]+
451.0248
0
20
40
60
80
100
430
435
440
445
450
455

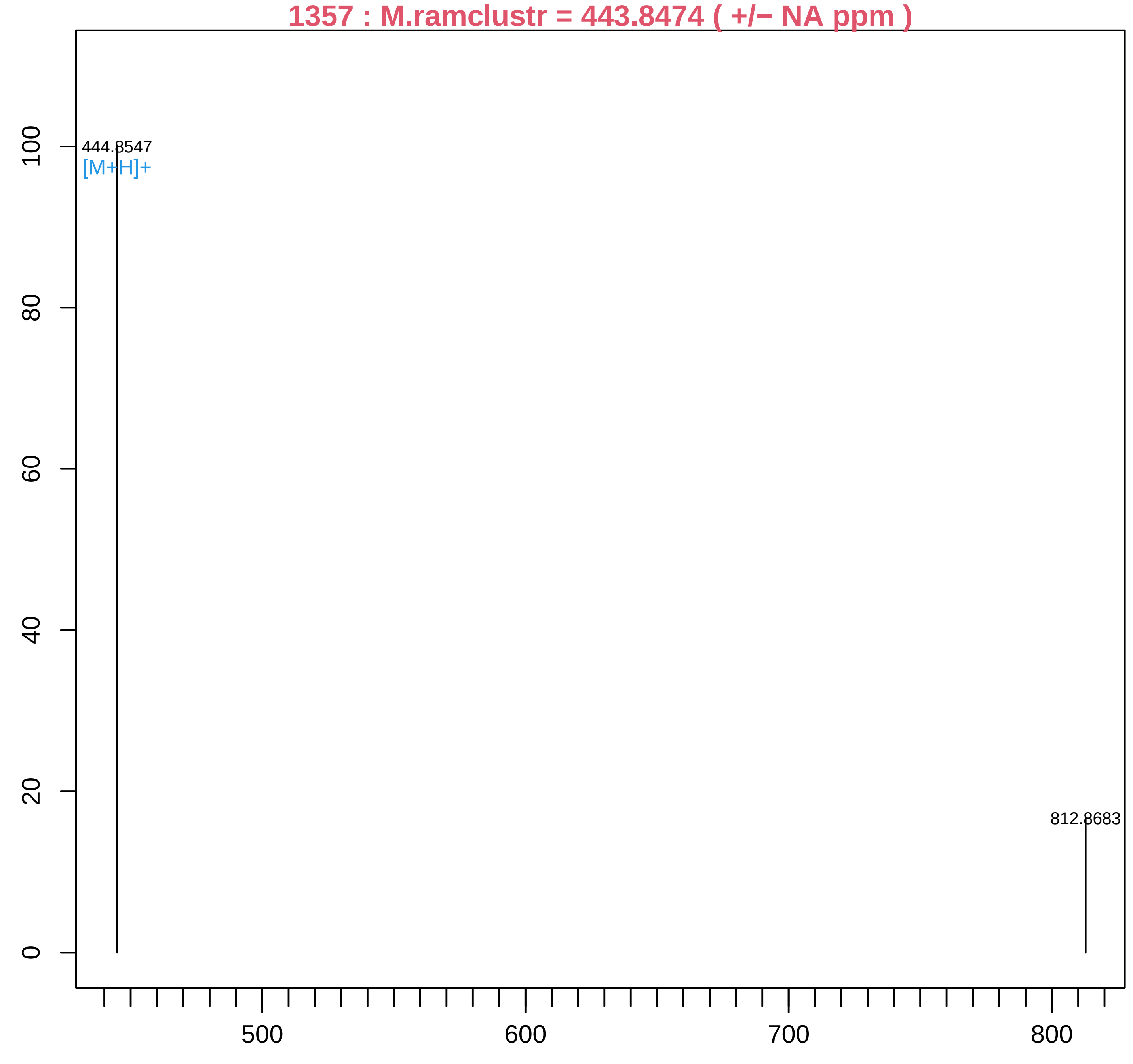
1357 : M.ramclustr = 443.8474 (+/− NA ppm)
444.8547
[M+H]+
812.8683
0
20
40
60
80
100
500
600
700
800

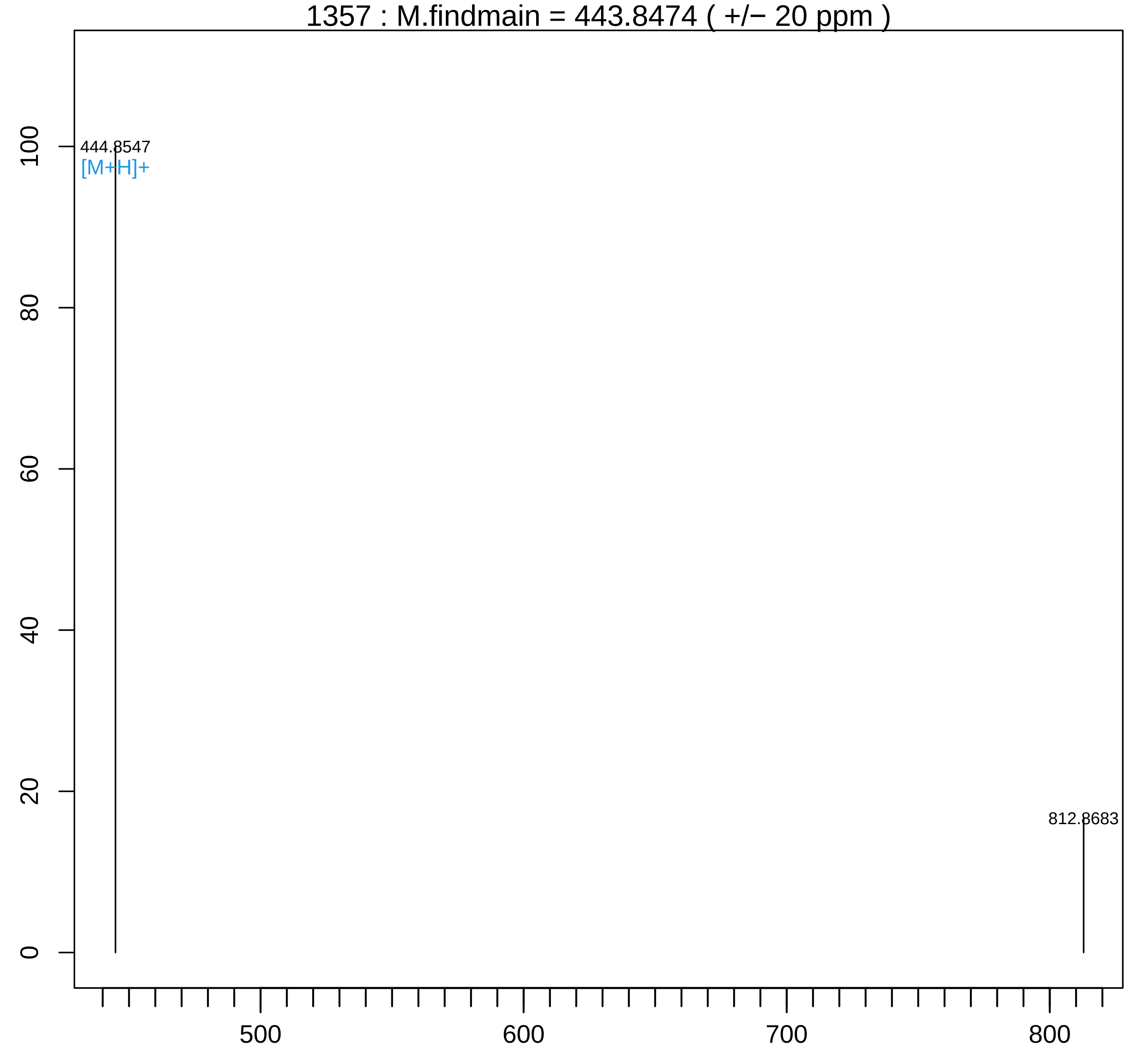
1357 : M.findmain = 443.8474 (+/− 20 ppm)
444.8547
[M+H]+
812.8683
0
20
40
60
80
100
500
600
700
800

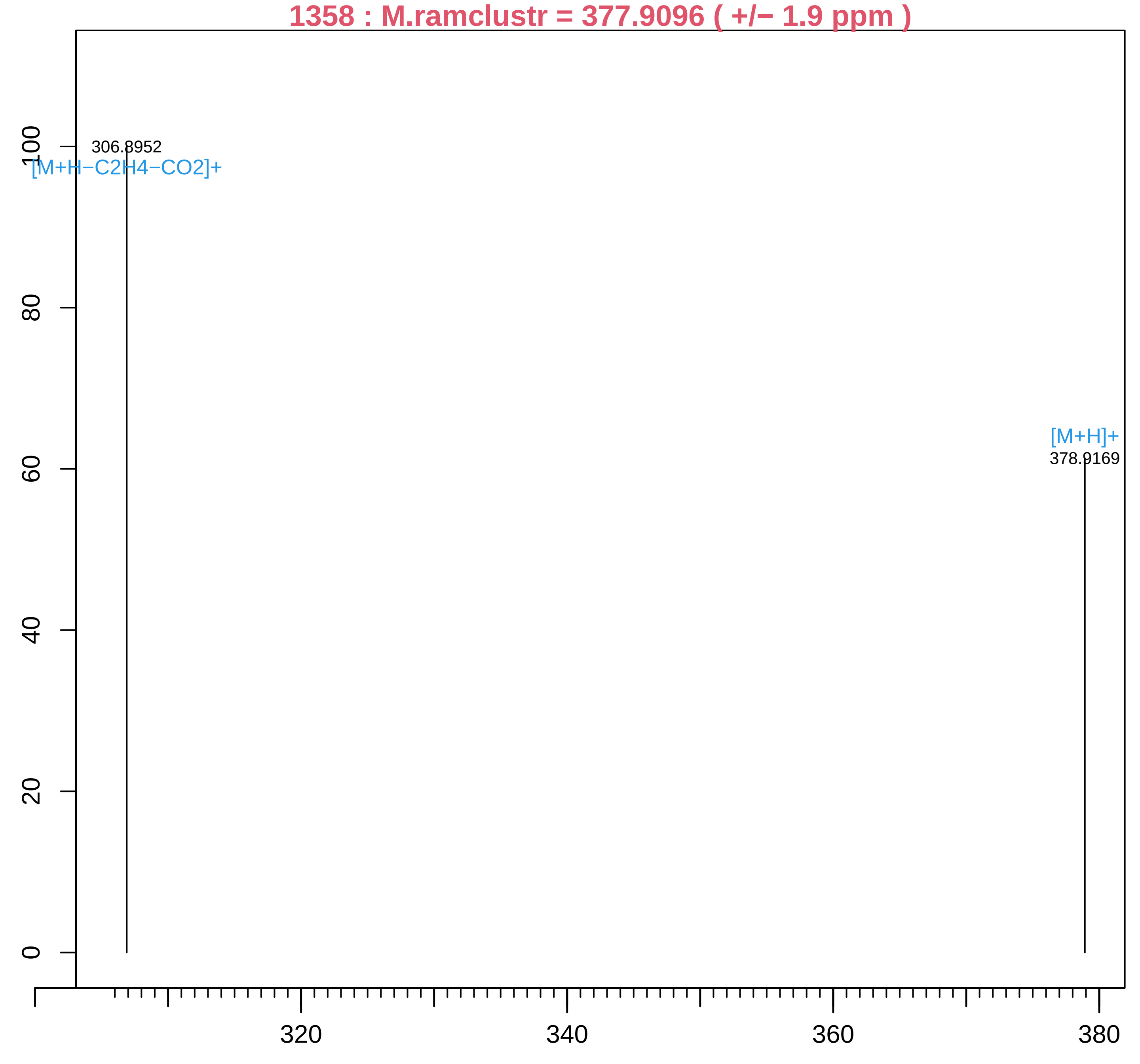
1358 : M.ramclustr = 377.9096 (+/− 1.9 ppm)
306.8952
[M+H−C2H4−CO2]+
[M+H]+
378.9169
0
20
40
60
80
100
320
340
360
380
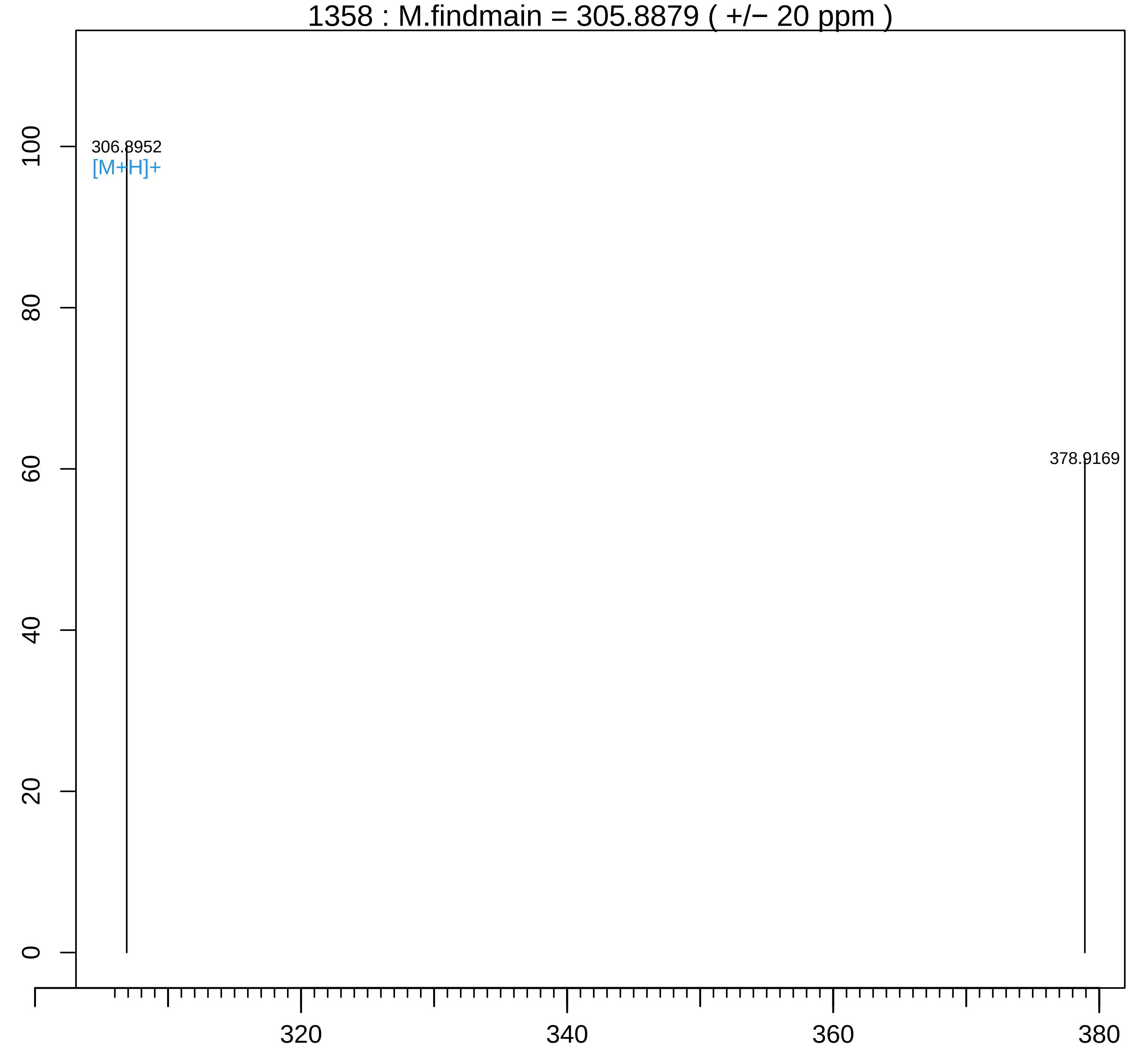
1358 : M.findmain = 305.8879 (+/− 20 ppm)
306.8952
[M+H]+
378.9169
0
20
40
60
80
100
320
340
360
380

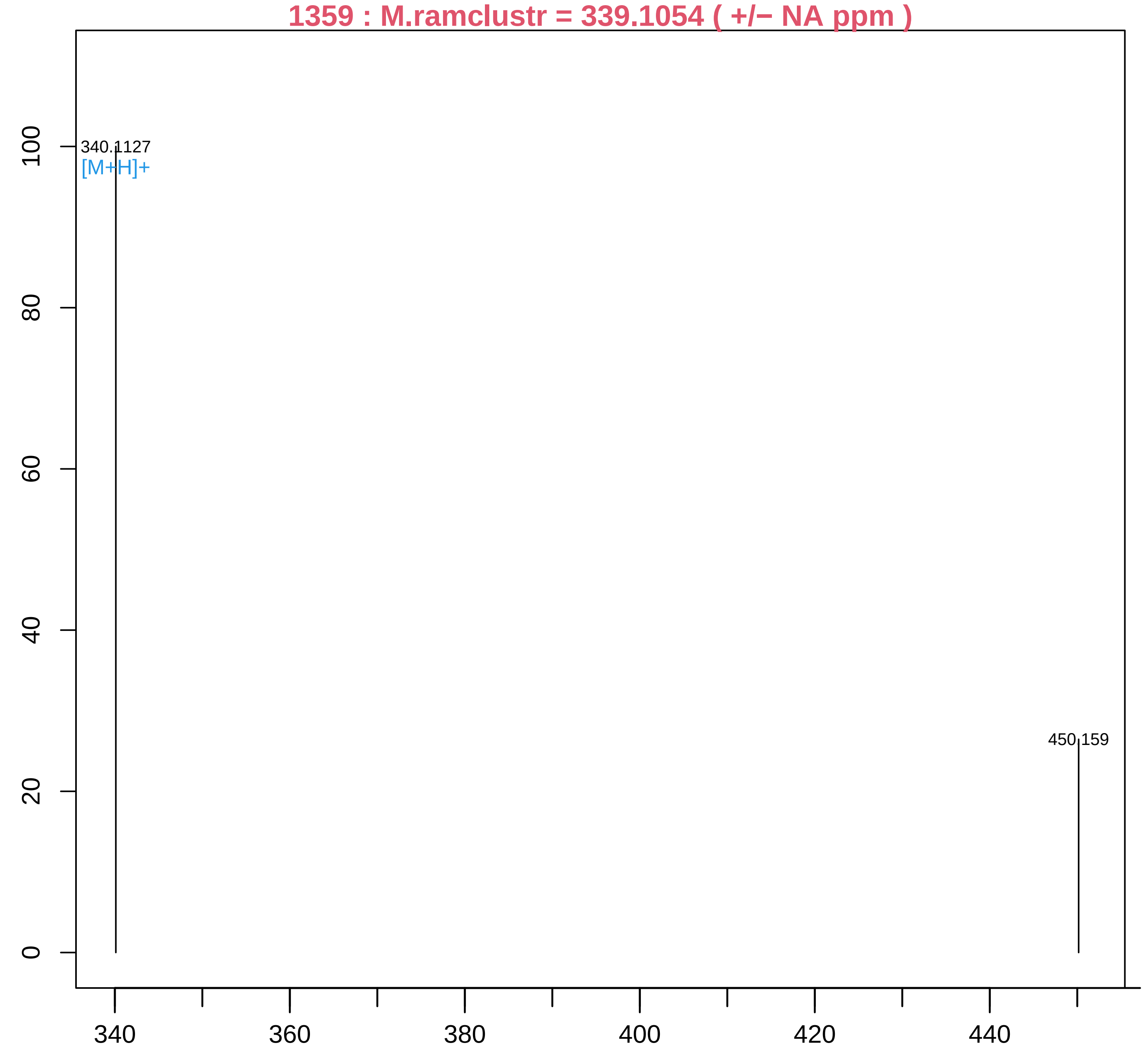

1359 : M.ramclustr = 339.1054 (+/− NA ppm)
340.1127
[M+H]+
450.159
0
20
40
60
80
100
340
360
380
400
420
440

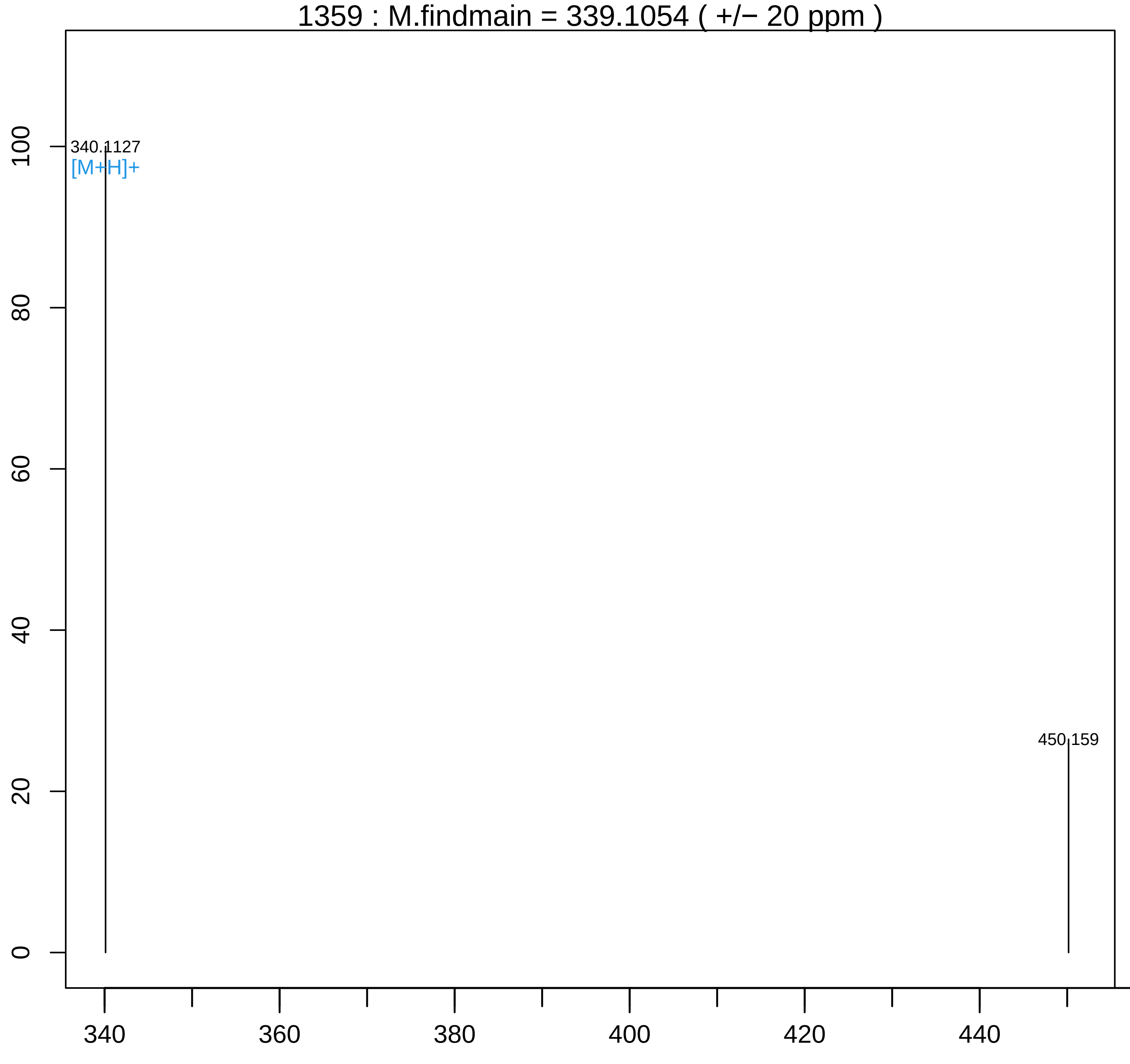

1359 : M.findmain = 339.1054 (+/− 20 ppm)
340.1127
[M+H]+
450.159
0
20
40
60
80
100
340
360
380
400
420
440

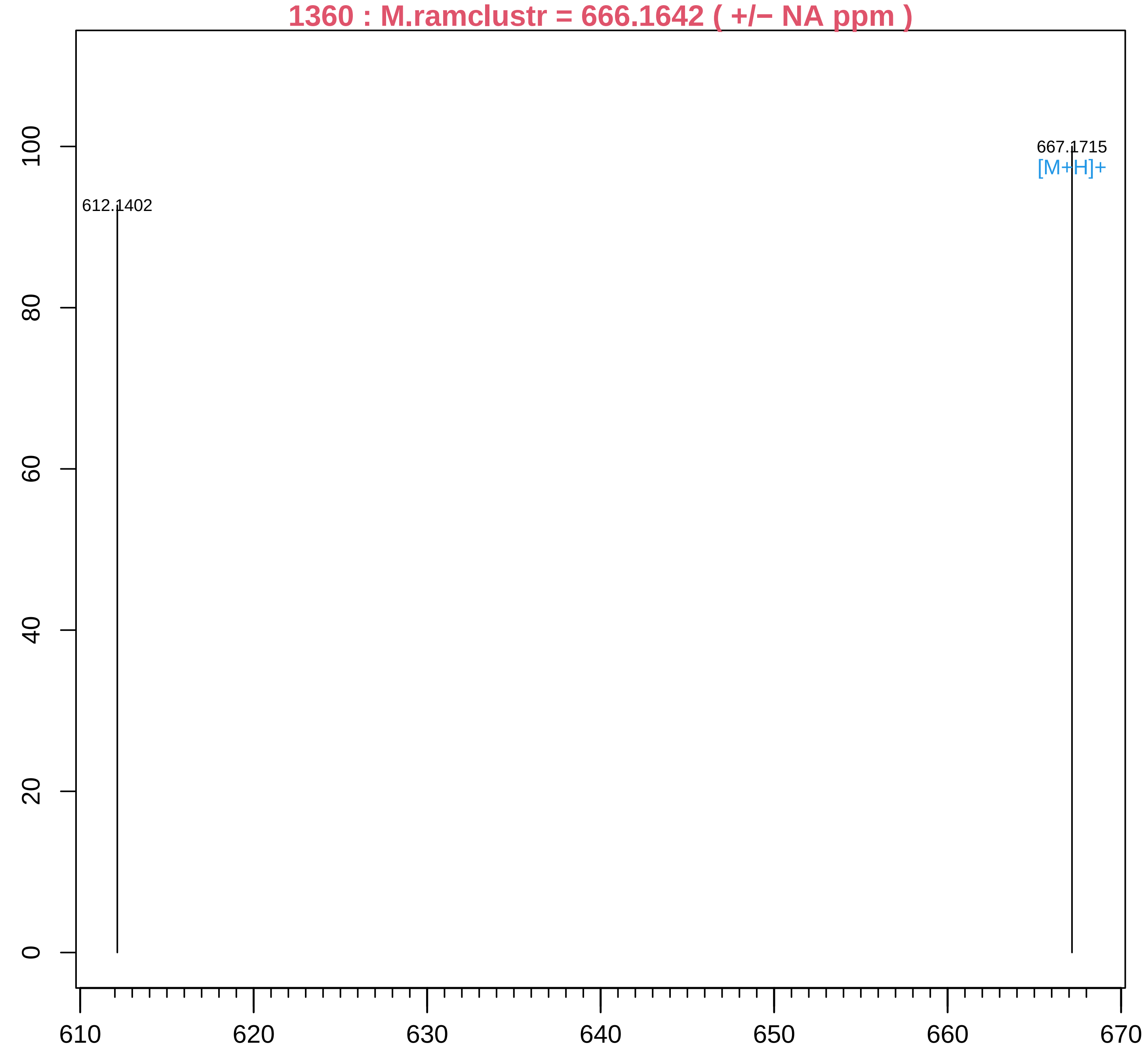
1360 : M.ramclustr = 666.1642 (+/− NA ppm)
612.1402
667.1715
[M+H]+
0
20
40
60
80
100
610
620
630
640
650
660
670

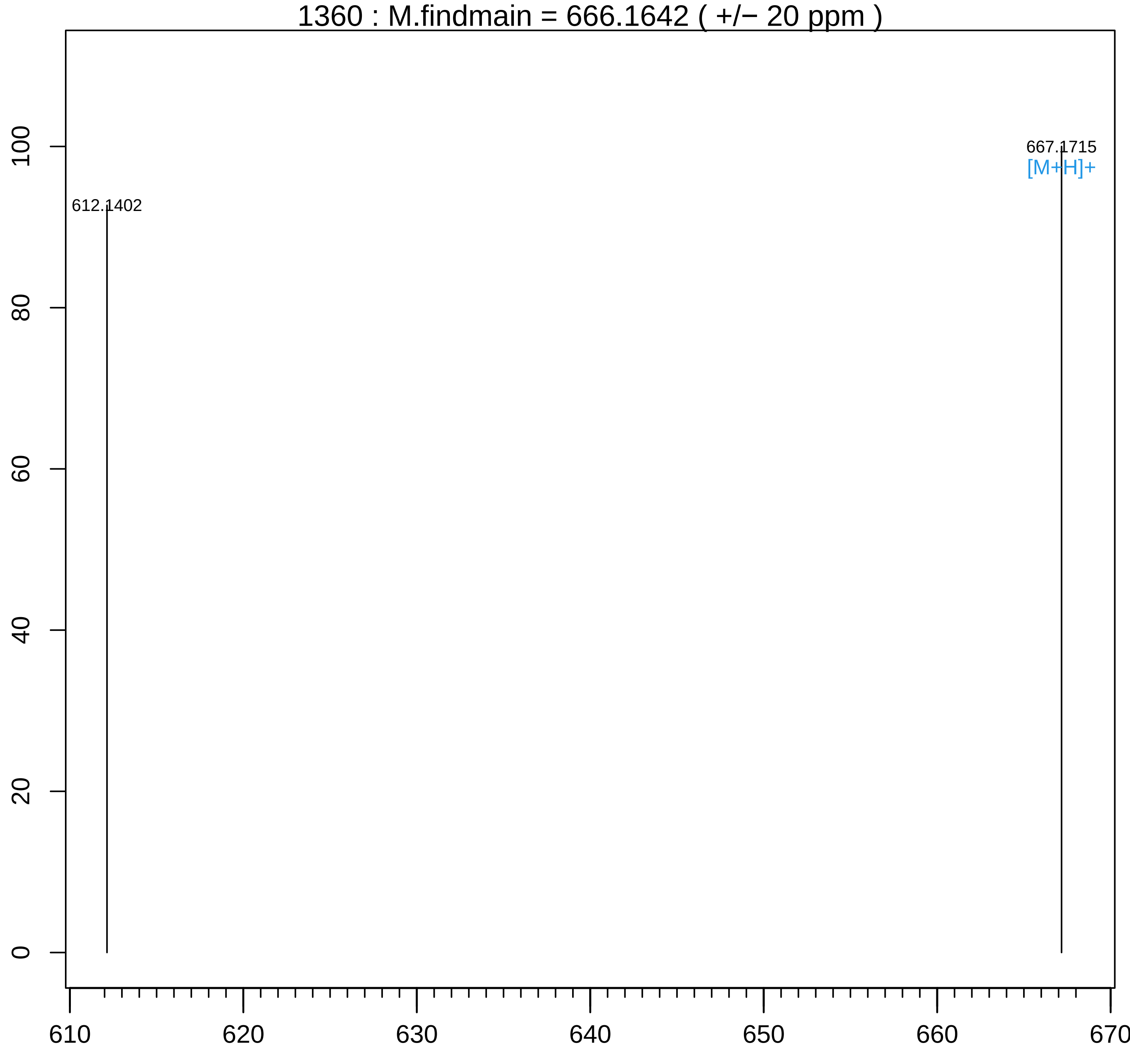
1360 : M.findmain = 666.1642 (+/− 20 ppm)
612.1402
667.1715
[M+H]+
0
20
40
60
80
100
610
620
630
640
650
660
670

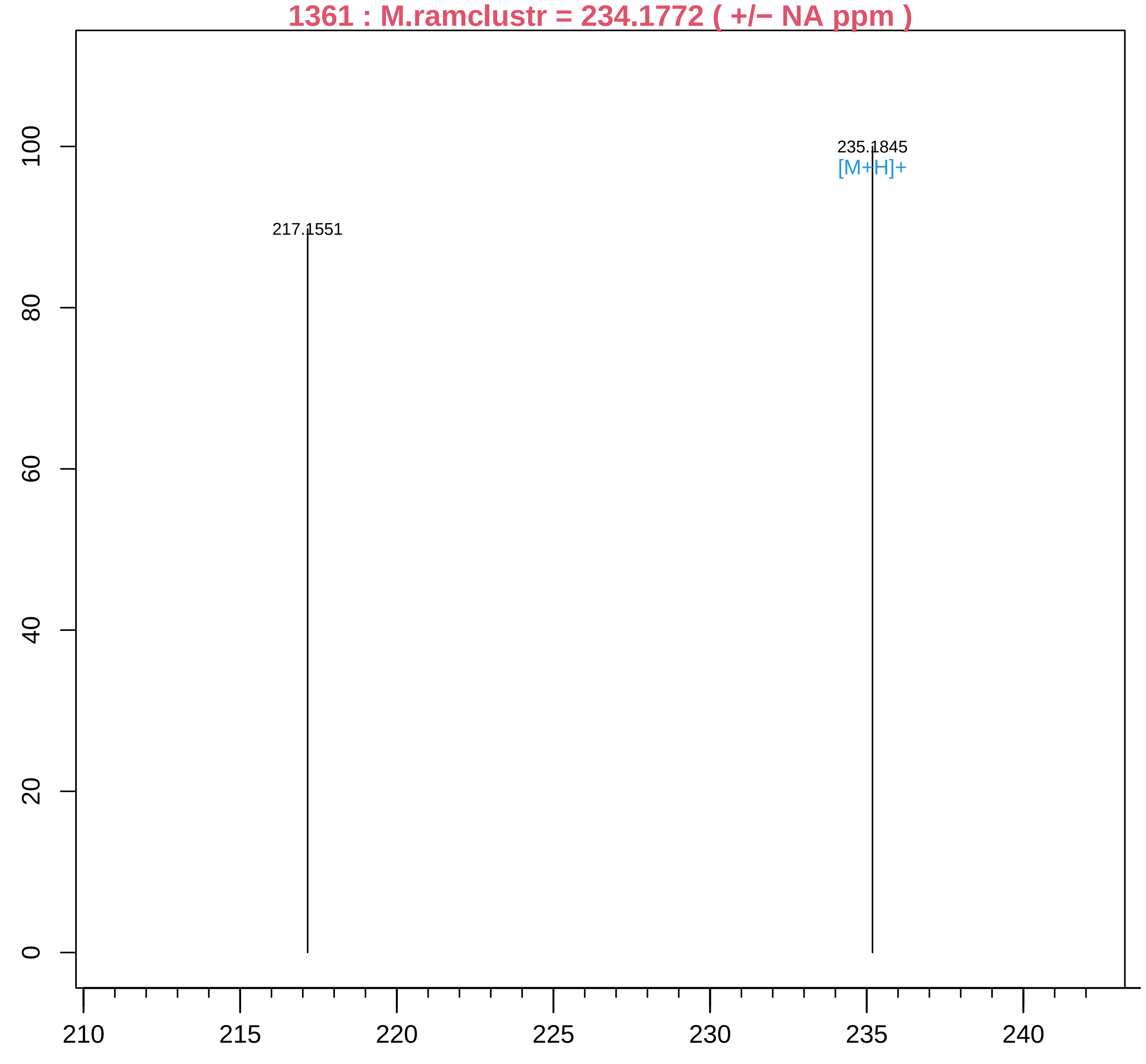
1361 : M.ramclustr = 234.1772 (+/− NA ppm)
235.1845
[M+H]+
217.1551
0
20
40
60
80
100
210
215
220
225
230
235
240

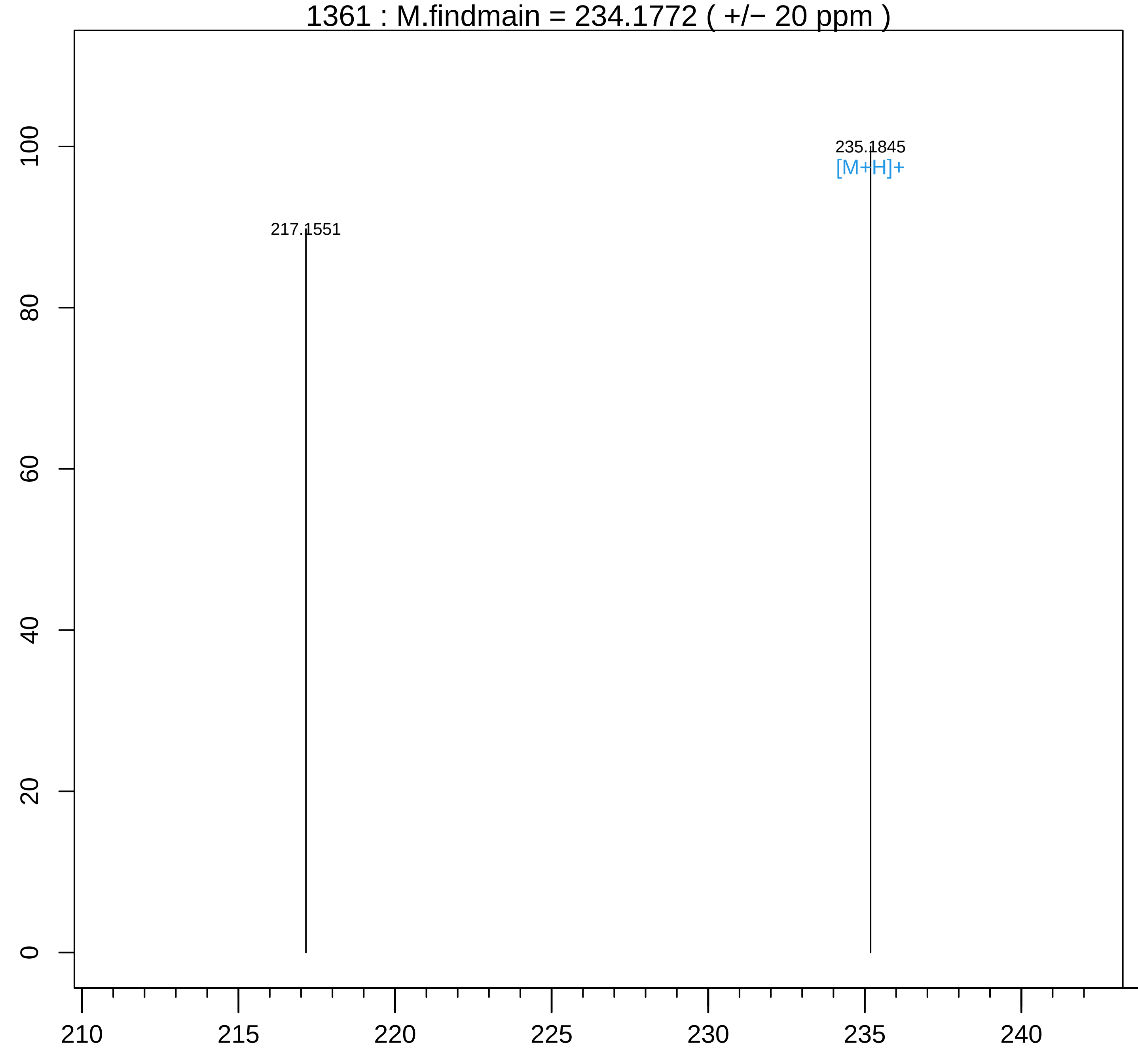
1361 : M.findmain = 234.1772 (+/− 20 ppm)
235.1845
[M+H]+
217.1551
0
20
40
60
80
100
210
215
220
225
230
235
240

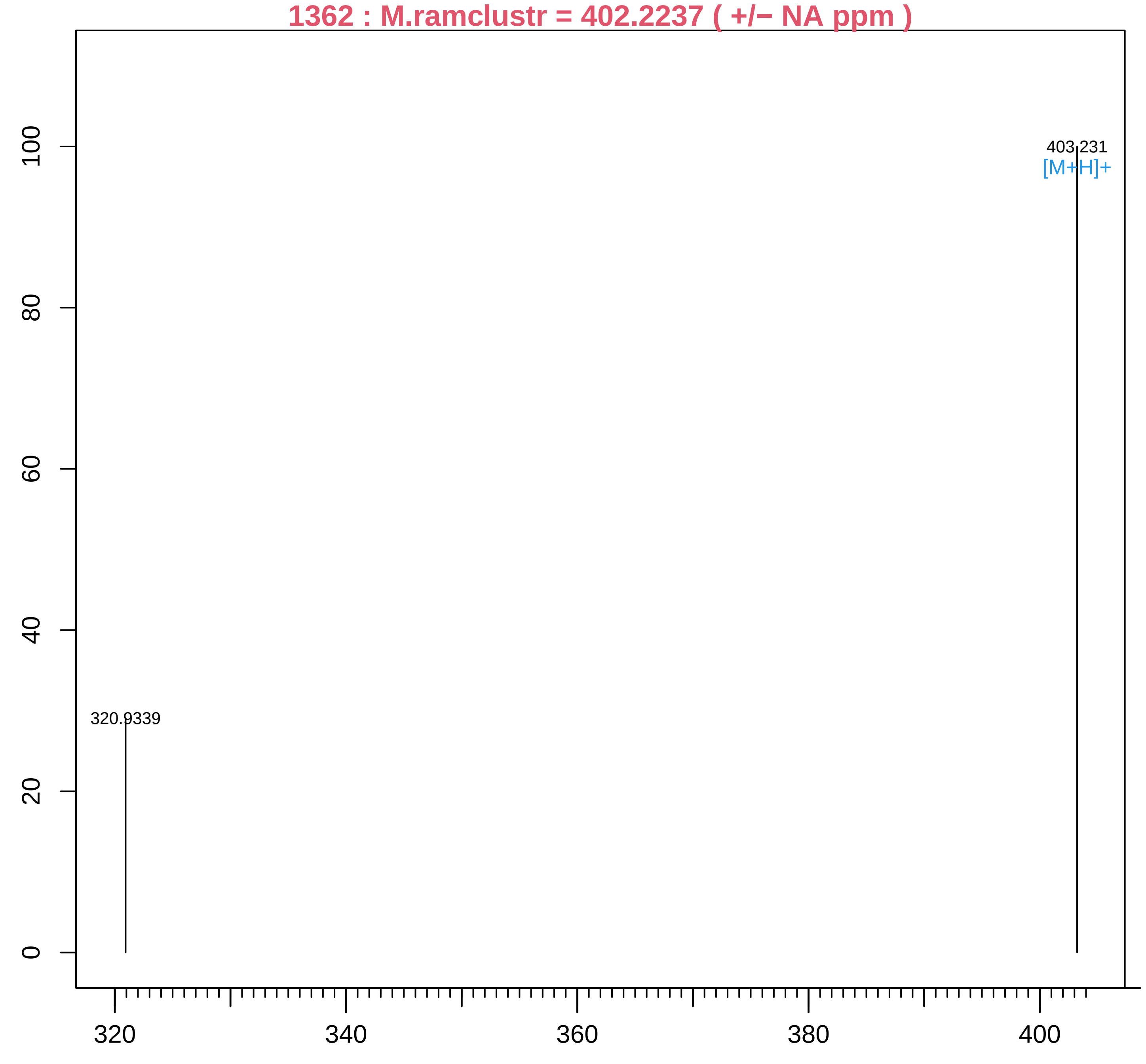
1362 : M.ramclustr = 402.2237 (+/− NA ppm)
403.231
[M+H]+
320.9339
0
20
40
60
80
100
320
340
360
380
400

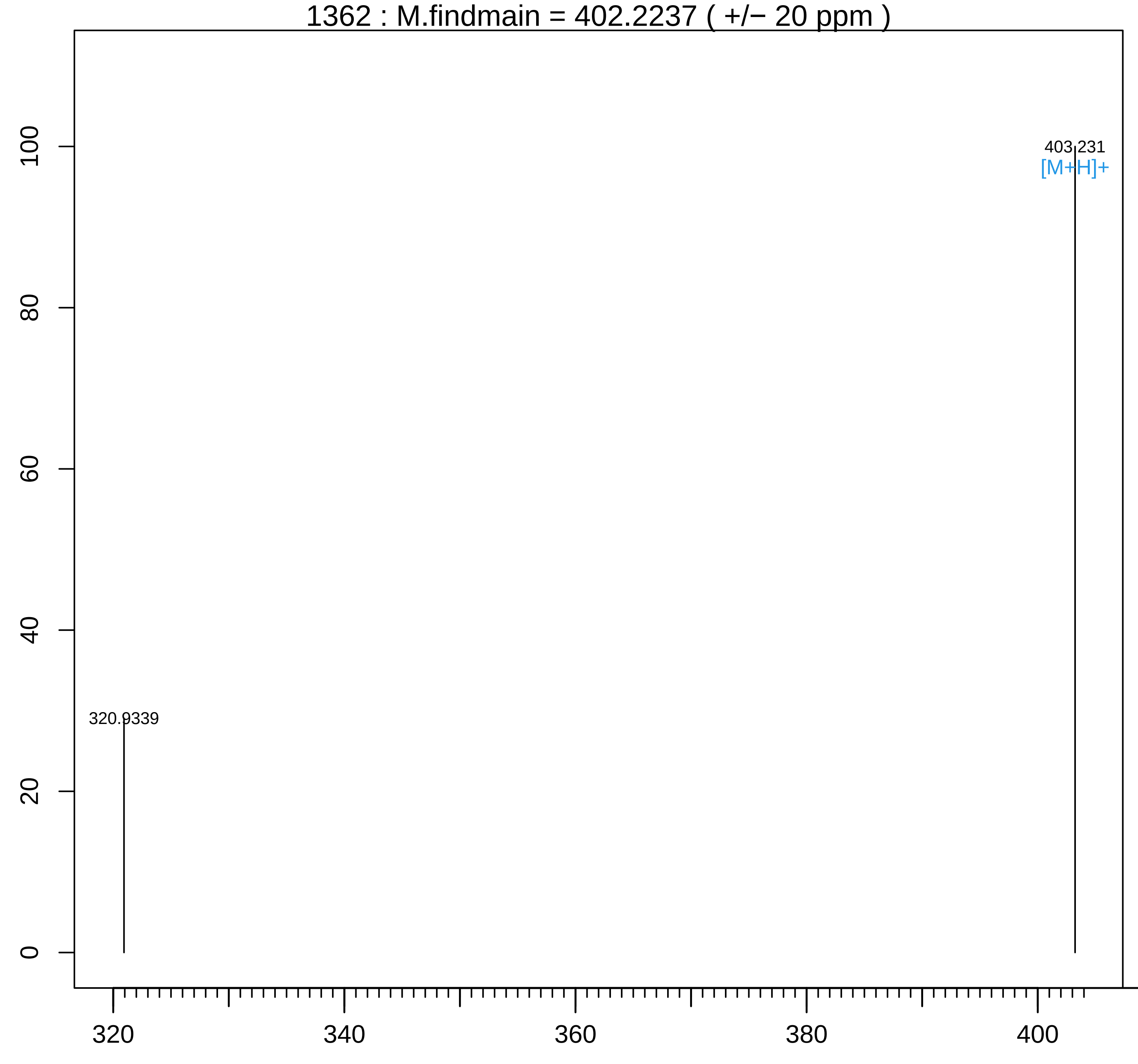
1362 : M.findmain = 402.2237 (+/− 20 ppm)
403.231
[M+H]+
320.9339
0
20
40
60
80
100
320
340
360
380
400

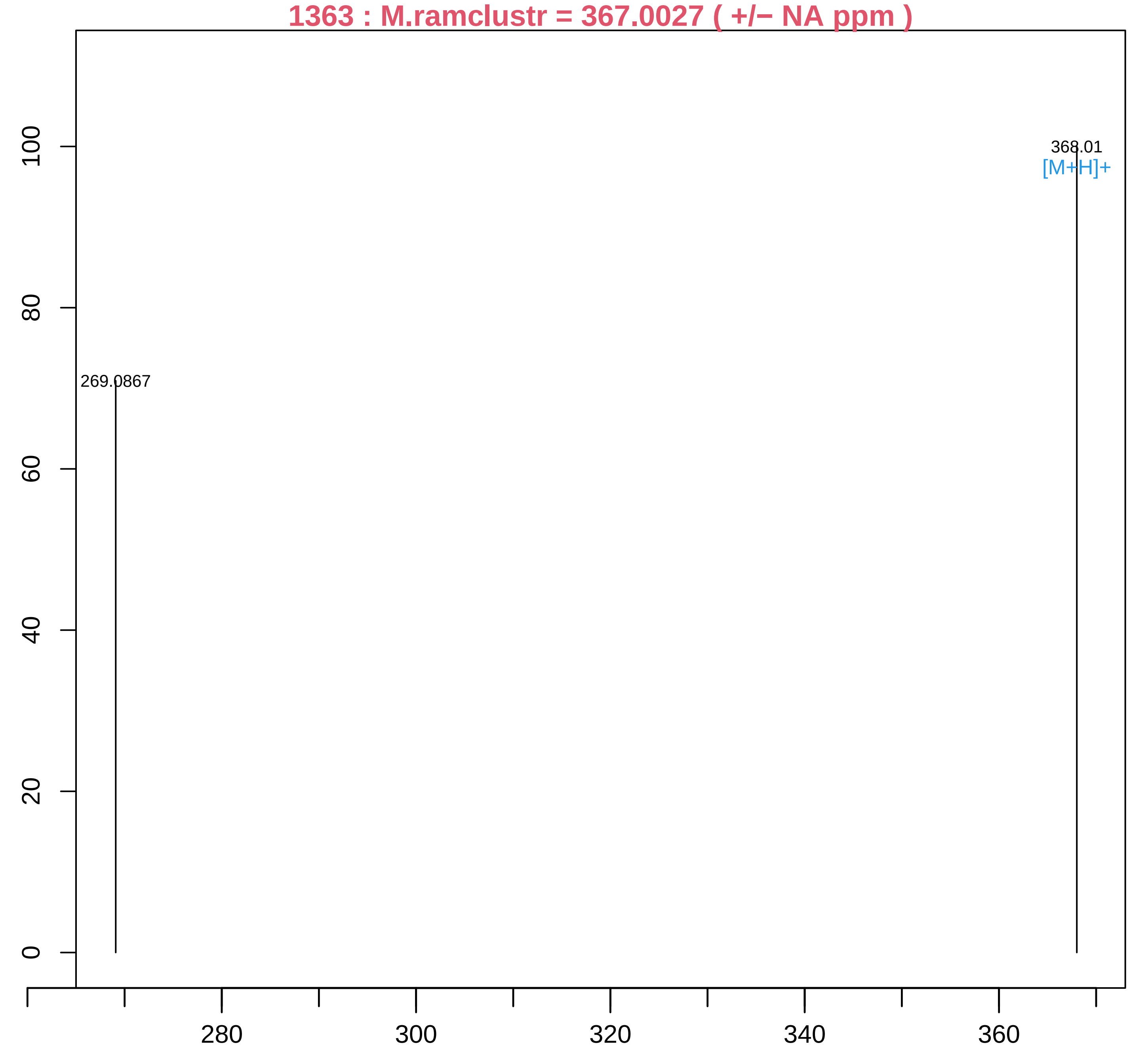
1363 : M.ramclustr = 367.0027 (+/− NA ppm)
269.0867
368.01
[M+H]+
0
20
40
60
80
100
280
300
320
340
360

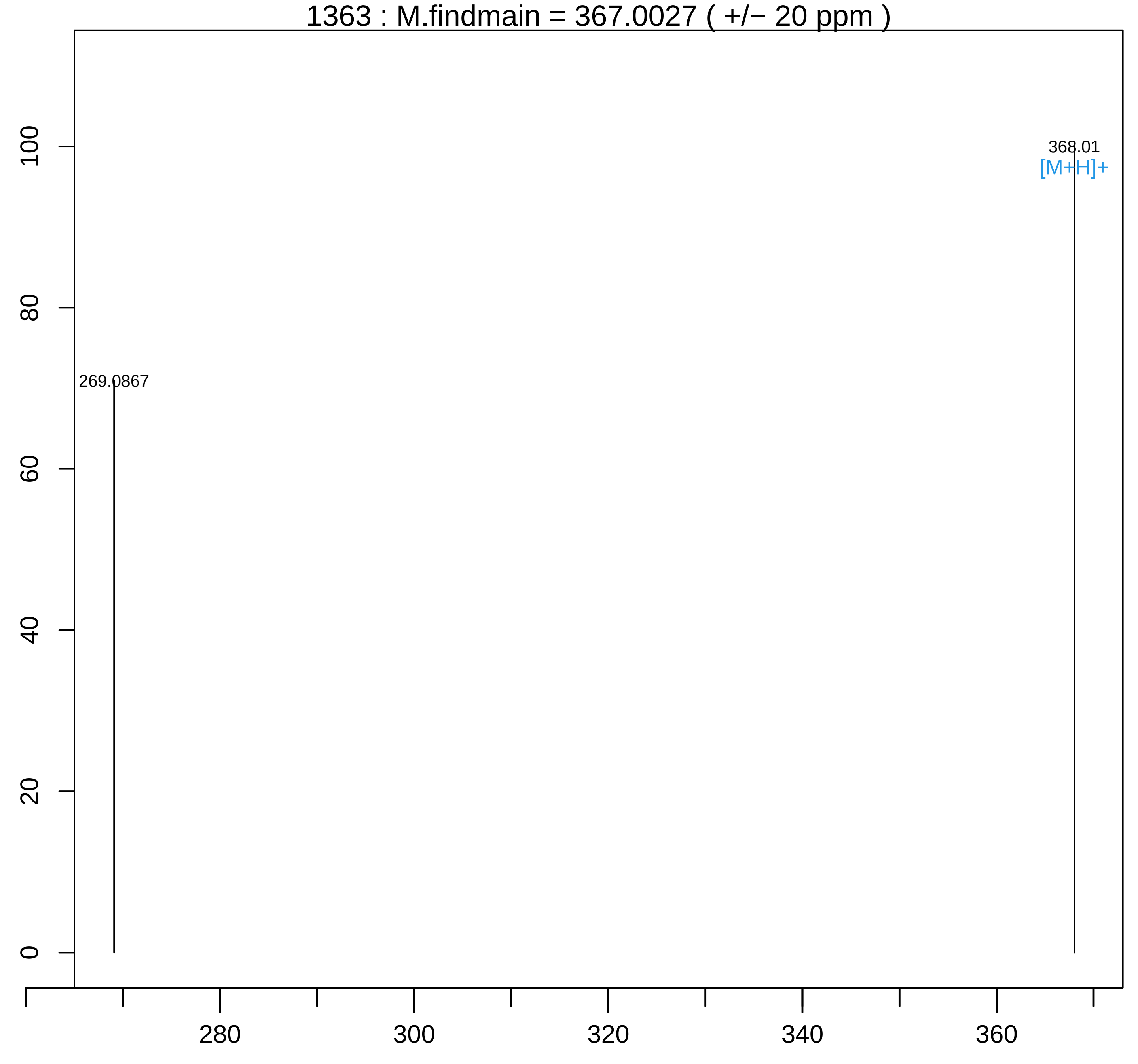
1363 : M.findmain = 367.0027 (+/− 20 ppm)
269.0867
368.01
[M+H]+
0
20
40
60
80
100
280
300
320
340
360

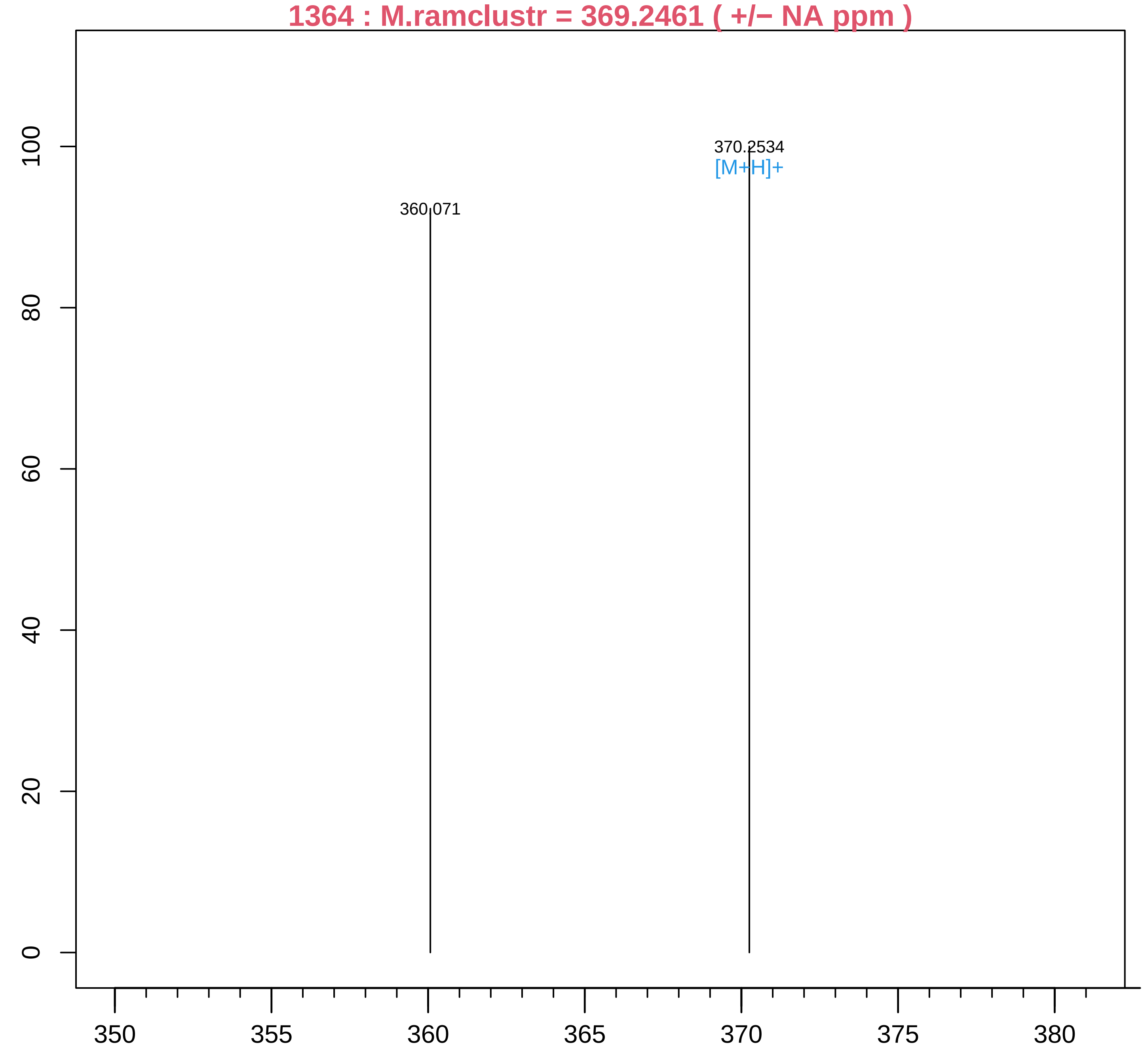
1364 : M.ramclustr = 369.2461 (+/− NA ppm)
370.2534
[M+H]+
360.071
0
20
40
60
80
100
350
355
360
365
370
375
380

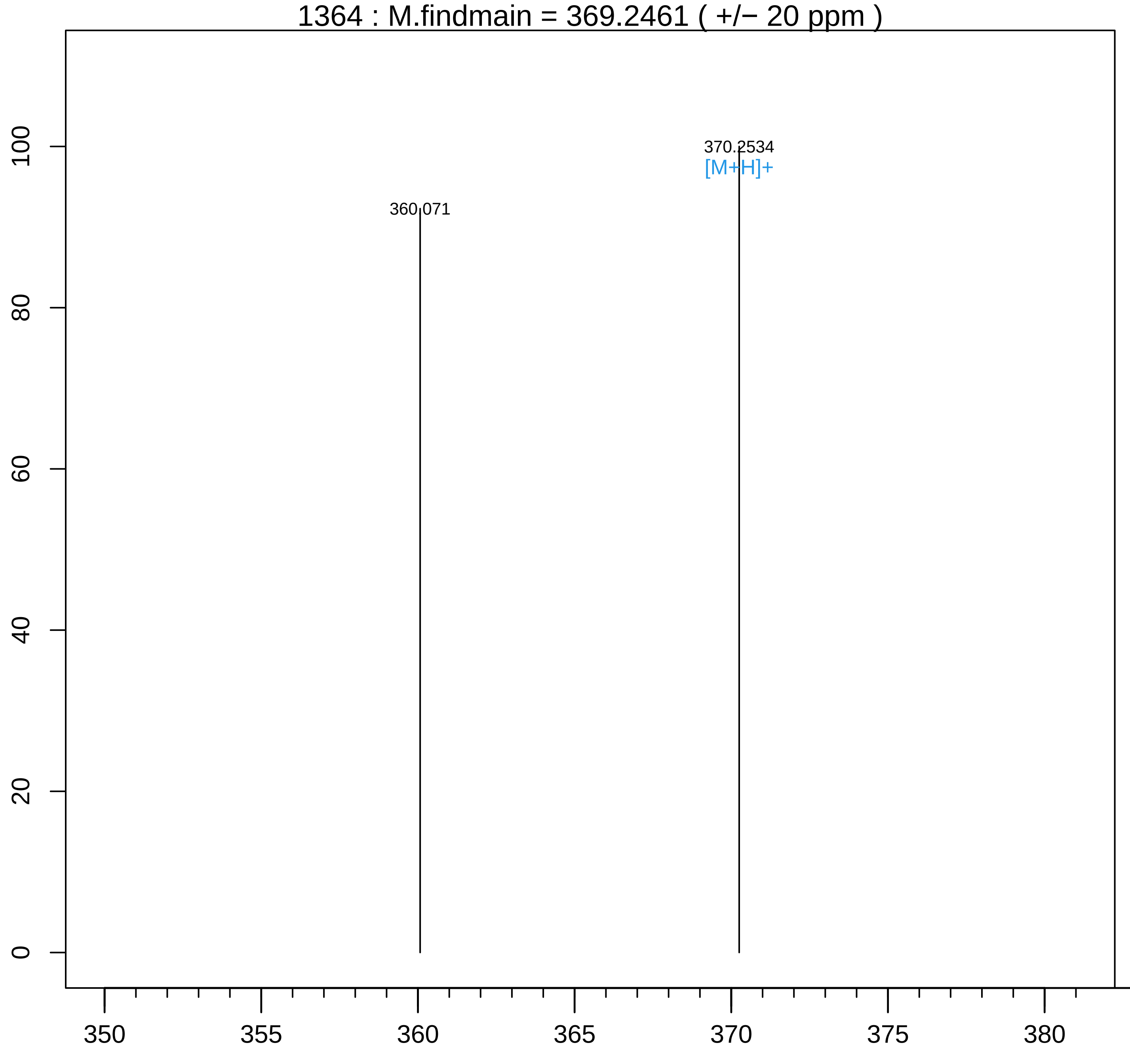
1364 : M.findmain = 369.2461 (+/− 20 ppm)
370.2534
[M+H]+
360.071
0
20
40
60
80
100
350
355
360
365
370
375
380

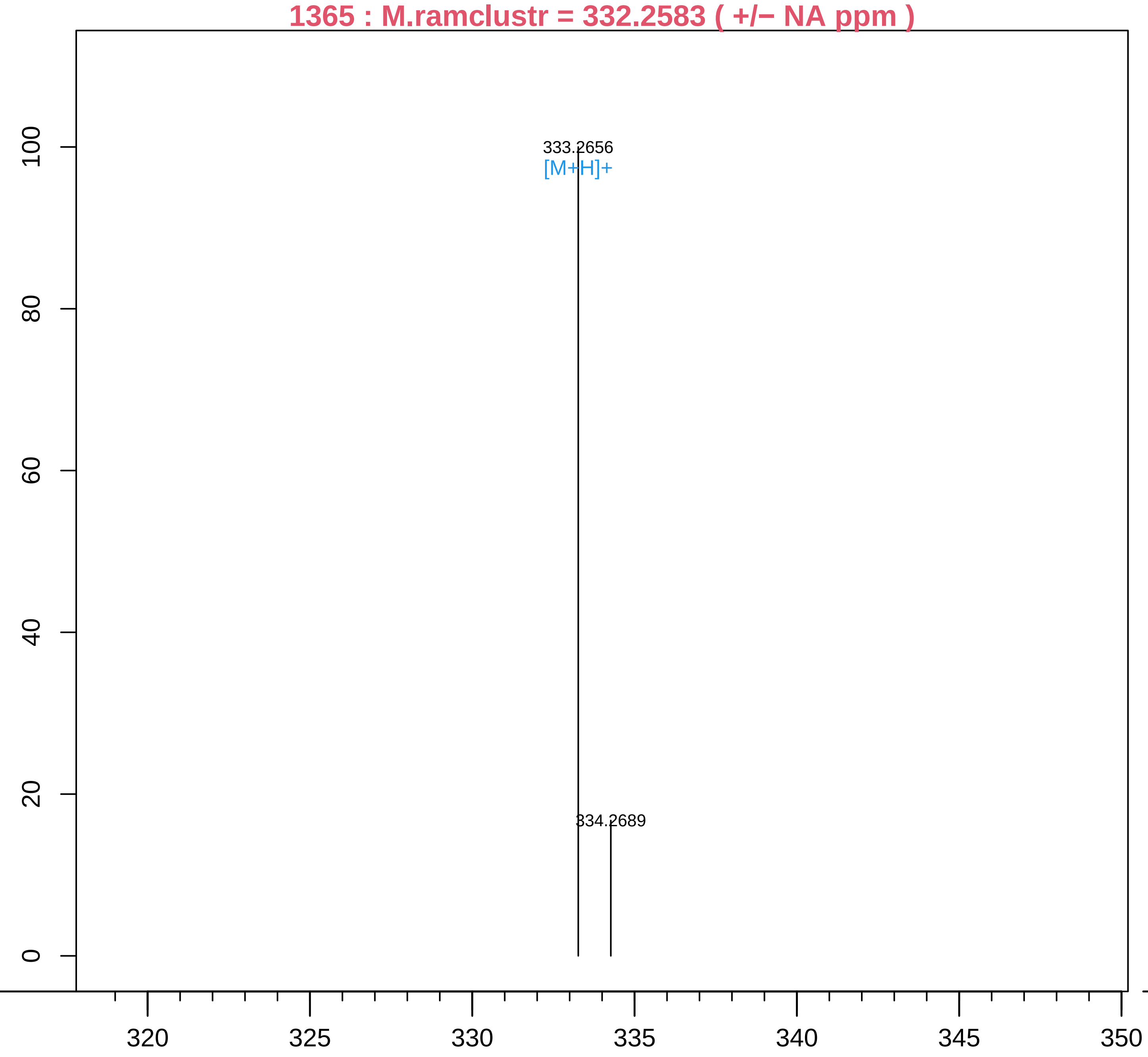

1365 : M.ramclustr = 332.2583 (+/− NA ppm)
333.2656
[M+H]+
334.2689
0
20
40
60
80
100
320
325
330
335
340
345
350

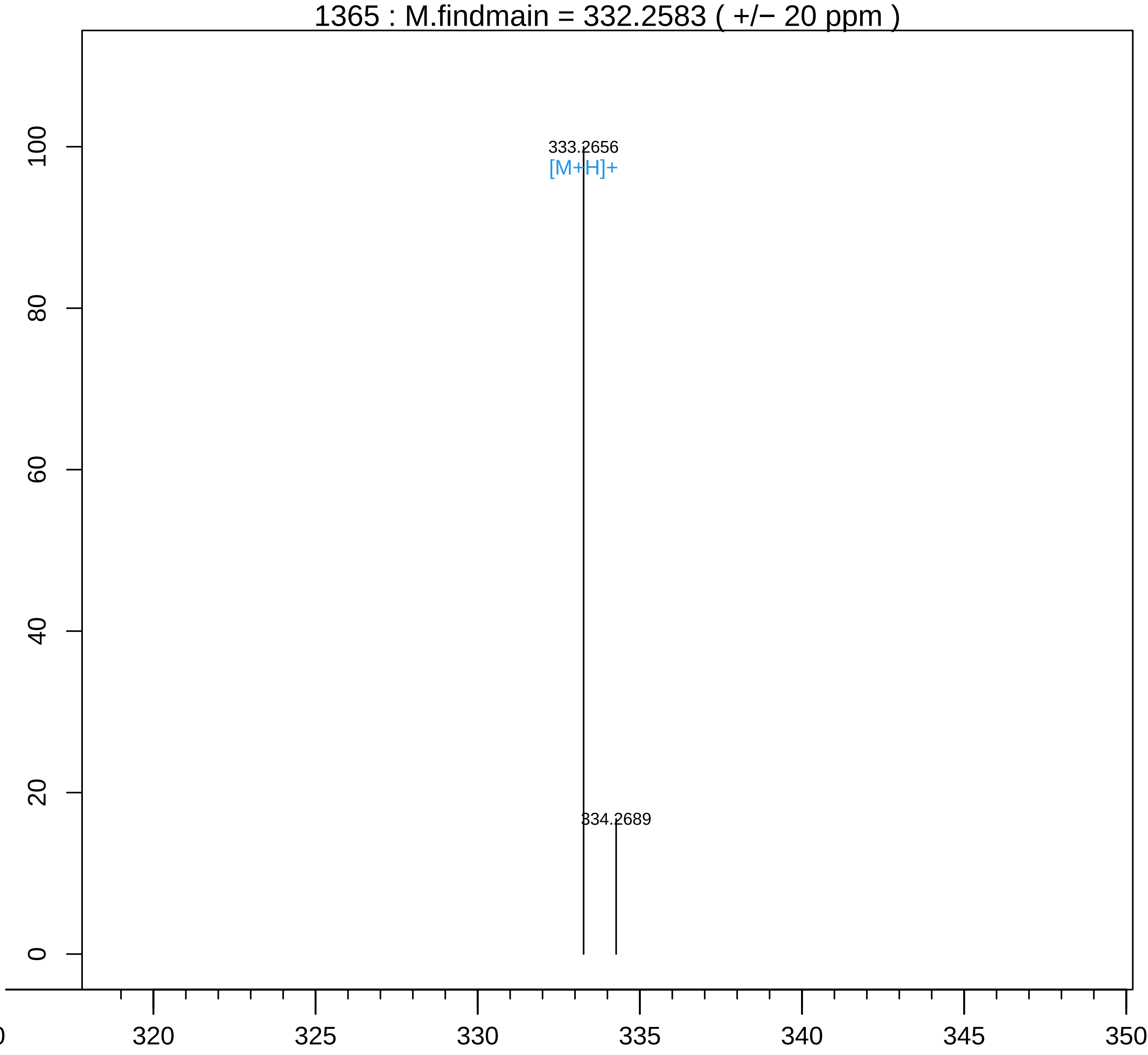

1365 : M.findmain = 332.2583 (+/− 20 ppm)
333.2656
[M+H]+
334.2689
0
20
40
60
80
100
320
325
330
335
340
345
350

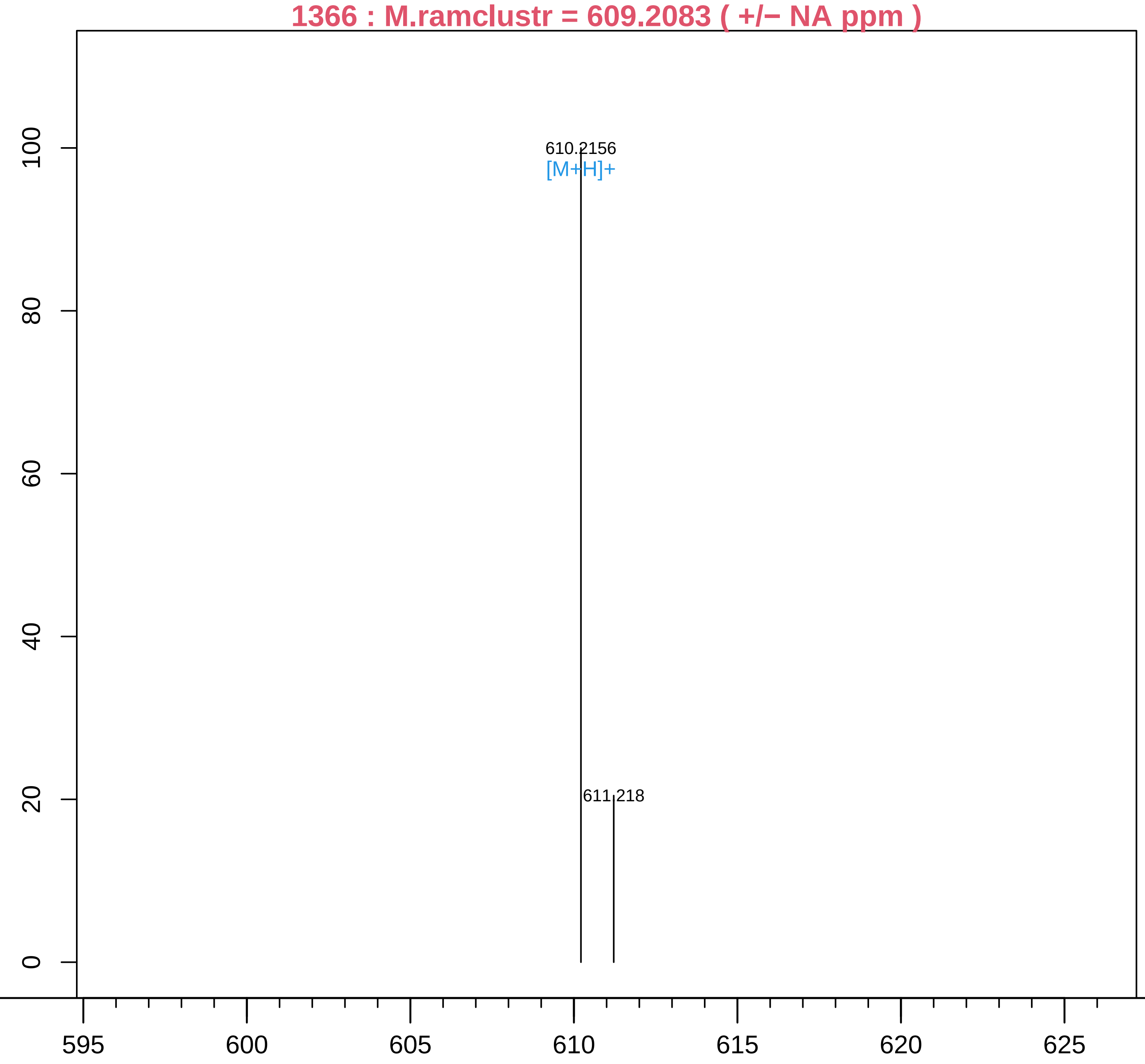
1366 : M.ramclustr = 609.2083 (+/− NA ppm)
610.2156
[M+H]+
611.218
0
20
40
60
80
100
595
600
605
610
615
620
625

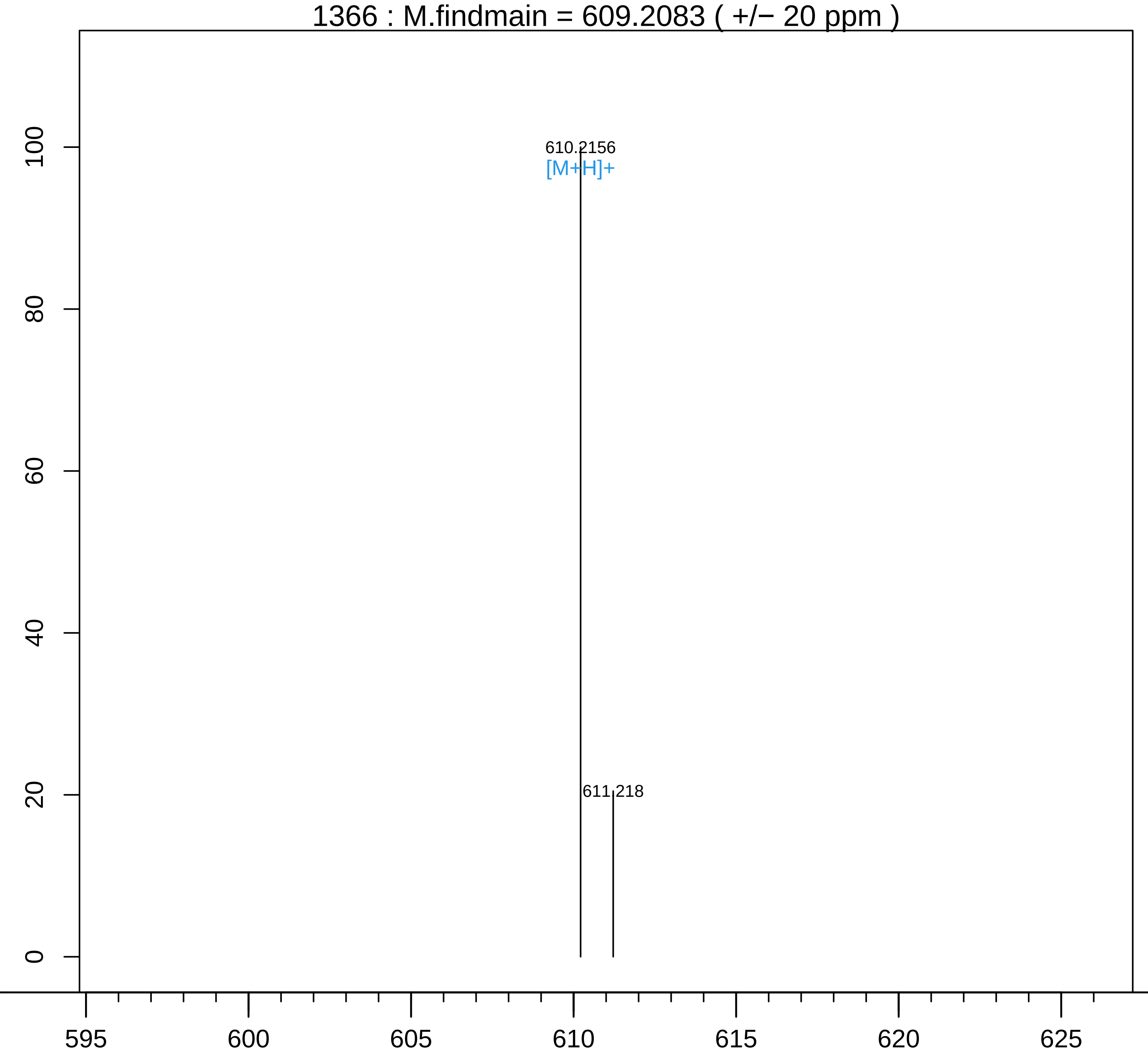
1366 : M.findmain = 609.2083 (+/− 20 ppm)
610.2156
[M+H]+
611.218
0
20
40
60
80
100
595
600
605
610
615
620
625

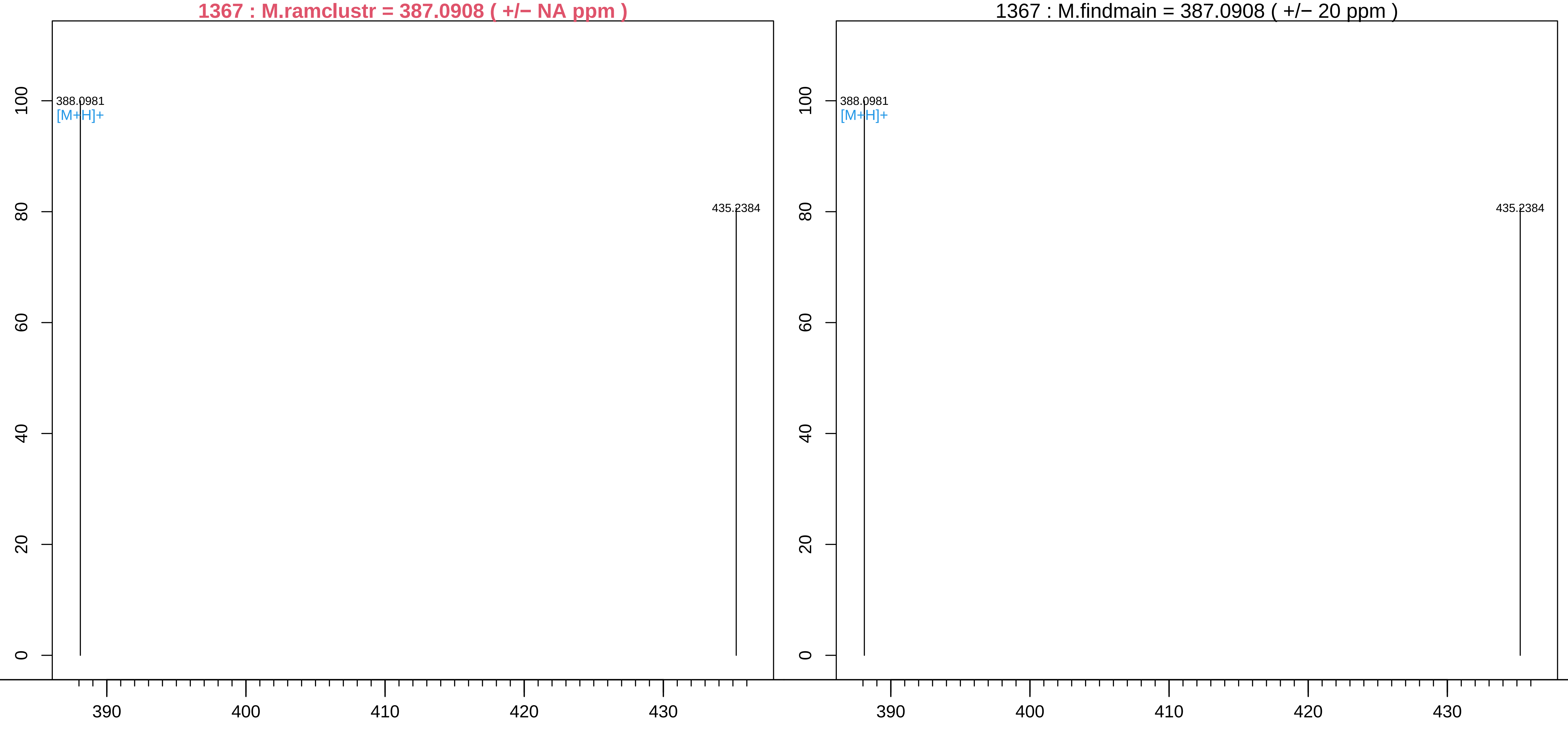
1367 : M.ramclustr = 387.0908 (+/− NA ppm)
388.0981
[M+H]+
435.2384
1367 : M.findmain = 387.0908 (+/− 20 ppm)
388.0981
[M+H]+
435.2384

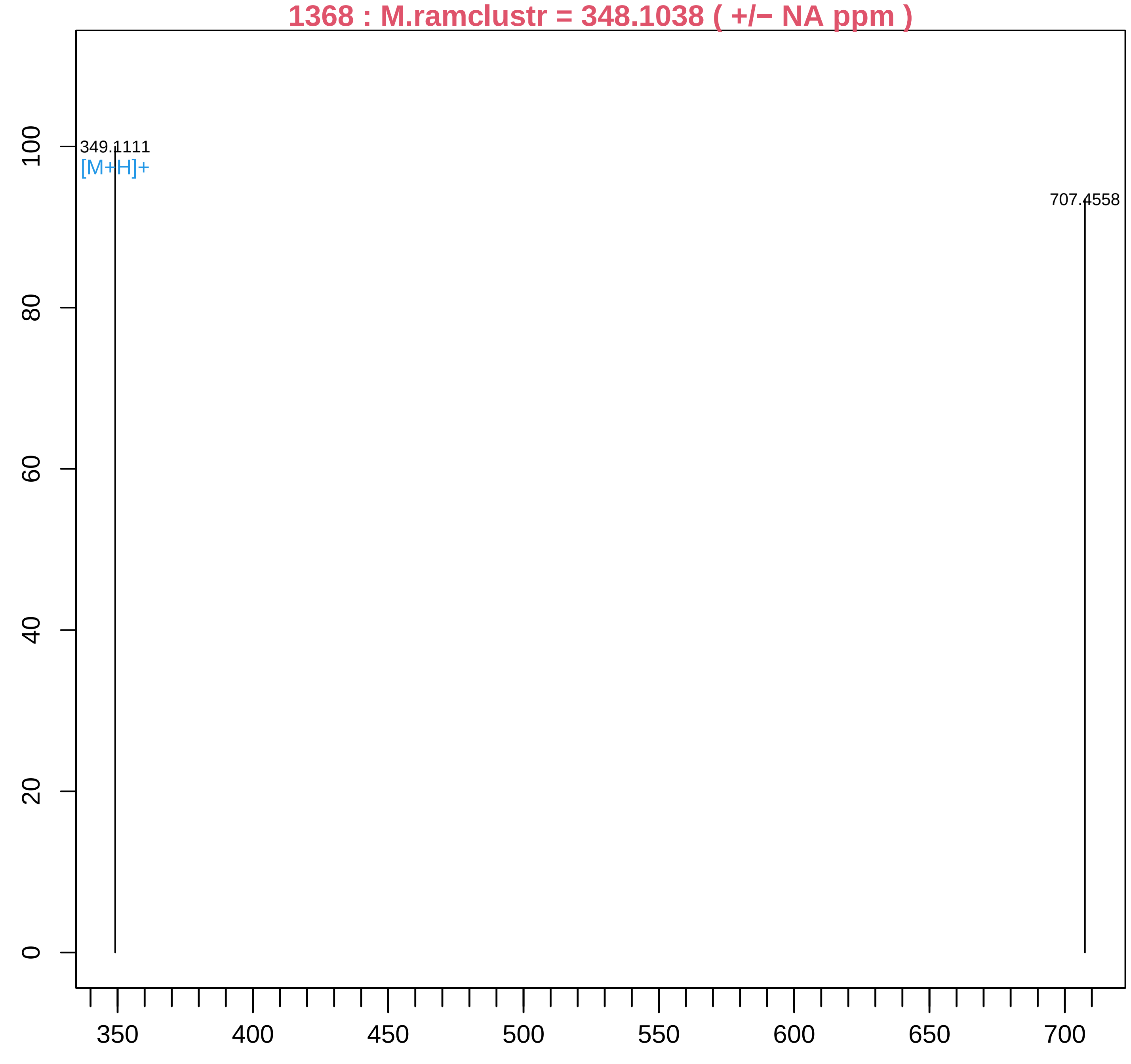
1368 : M.ramclustr = 348.1038 (+/− NA ppm)
349.1111
[M+H]+
707.4558
0
20
40
60
80
100
350
400
450
500
550
600
650
700

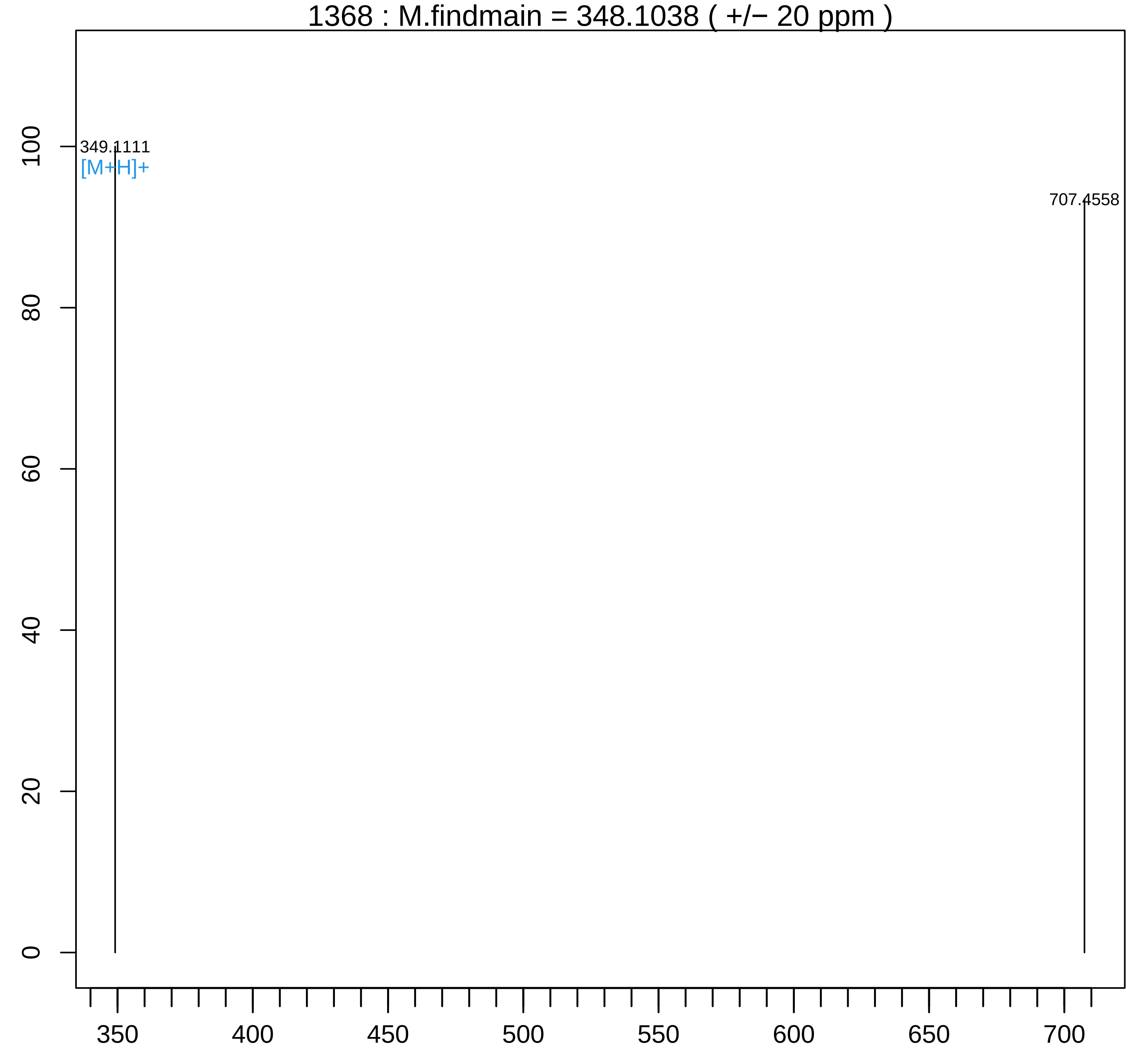
1368 : M.findmain = 348.1038 (+/− 20 ppm)
349.1111
[M+H]+
707.4558
0
20
40
60
80
100
350
400
450
500
550
600
650
700

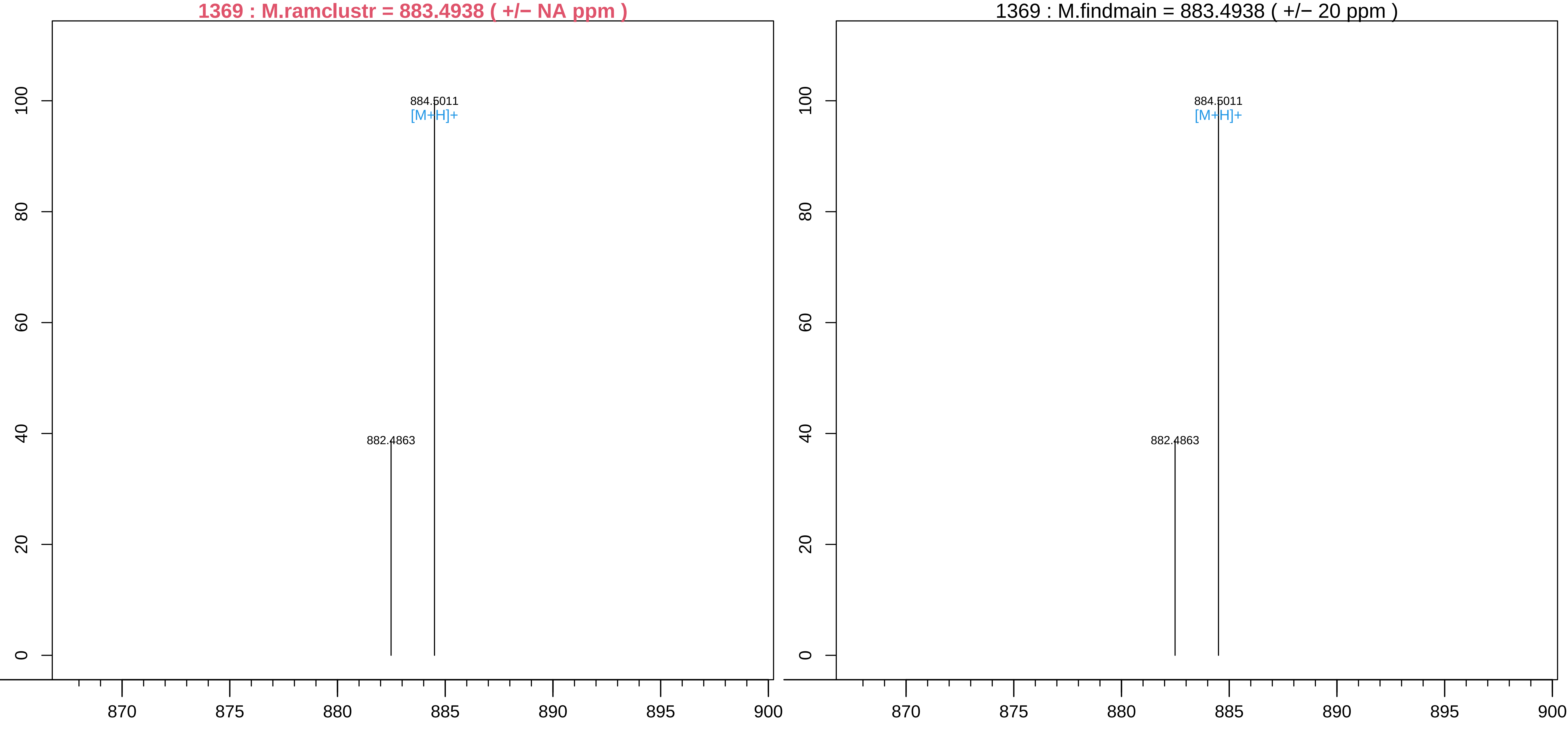
1369 : M.ramclustr = 883.4938 (+/− NA ppm)
884.5011
[M+H]+
882.4863
1369 : M.findmain = 883.4938 (+/− 20 ppm)
884.5011
[M+H]+
882.4863

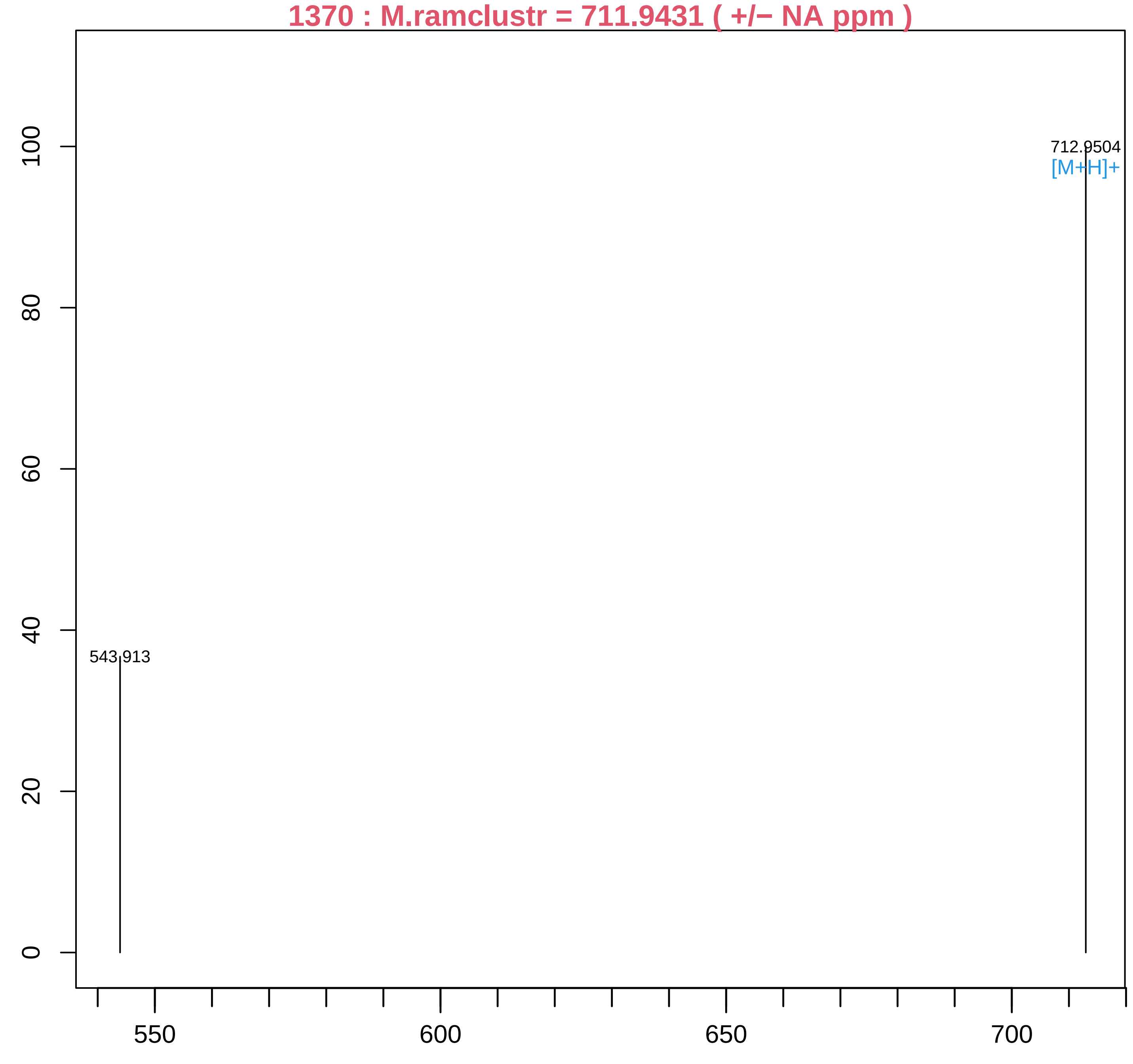

1370 : M.ramclustr = 711.9431 (+/− NA ppm)
712.9504
[M+H]+
543.913
0
20
40
60
80
100
550
600
650
700

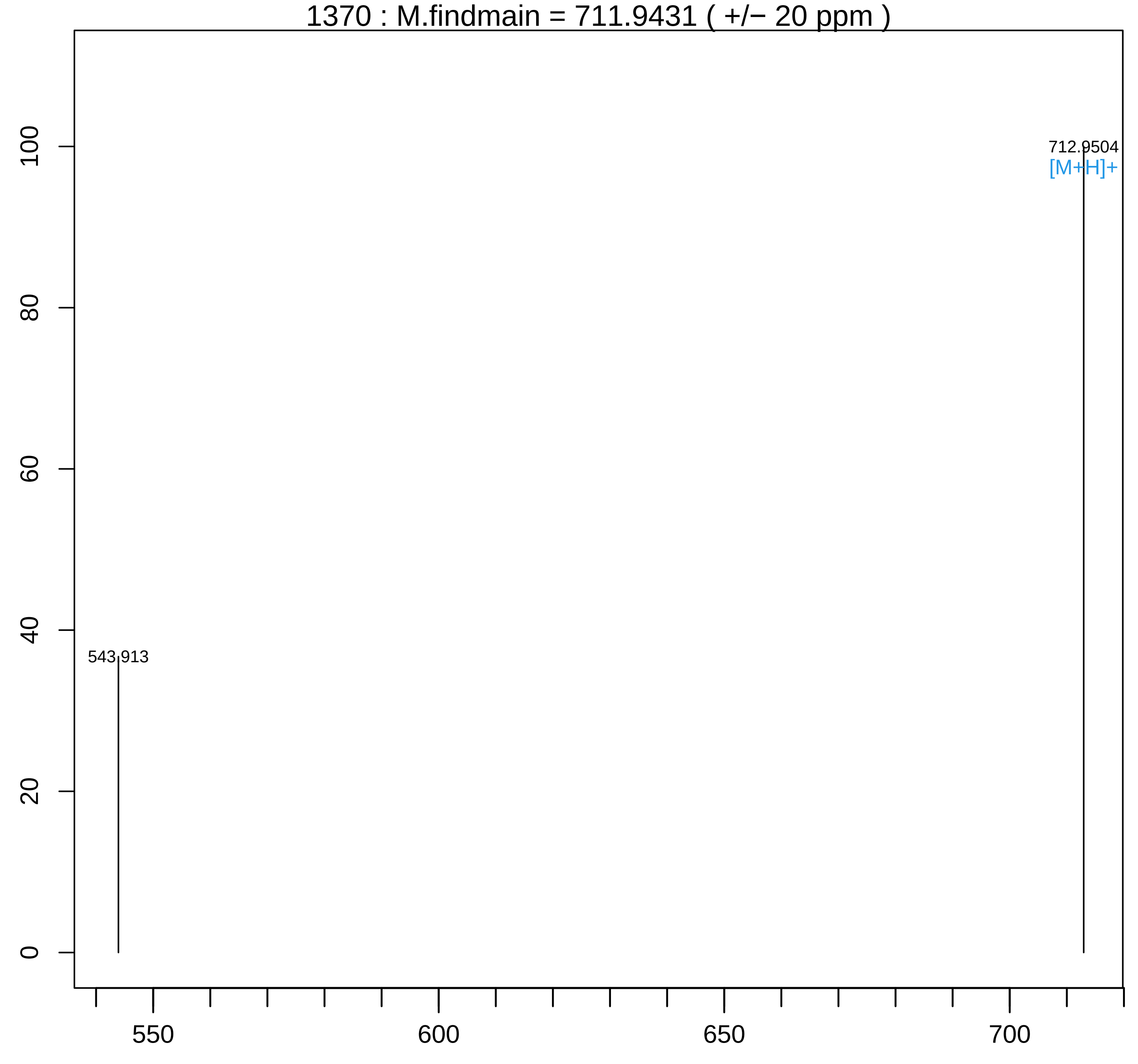

1370 : M.findmain = 711.9431 (+/− 20 ppm)
712.9504
[M+H]+
543.913
0
20
40
60
80
100
550
600
650
700

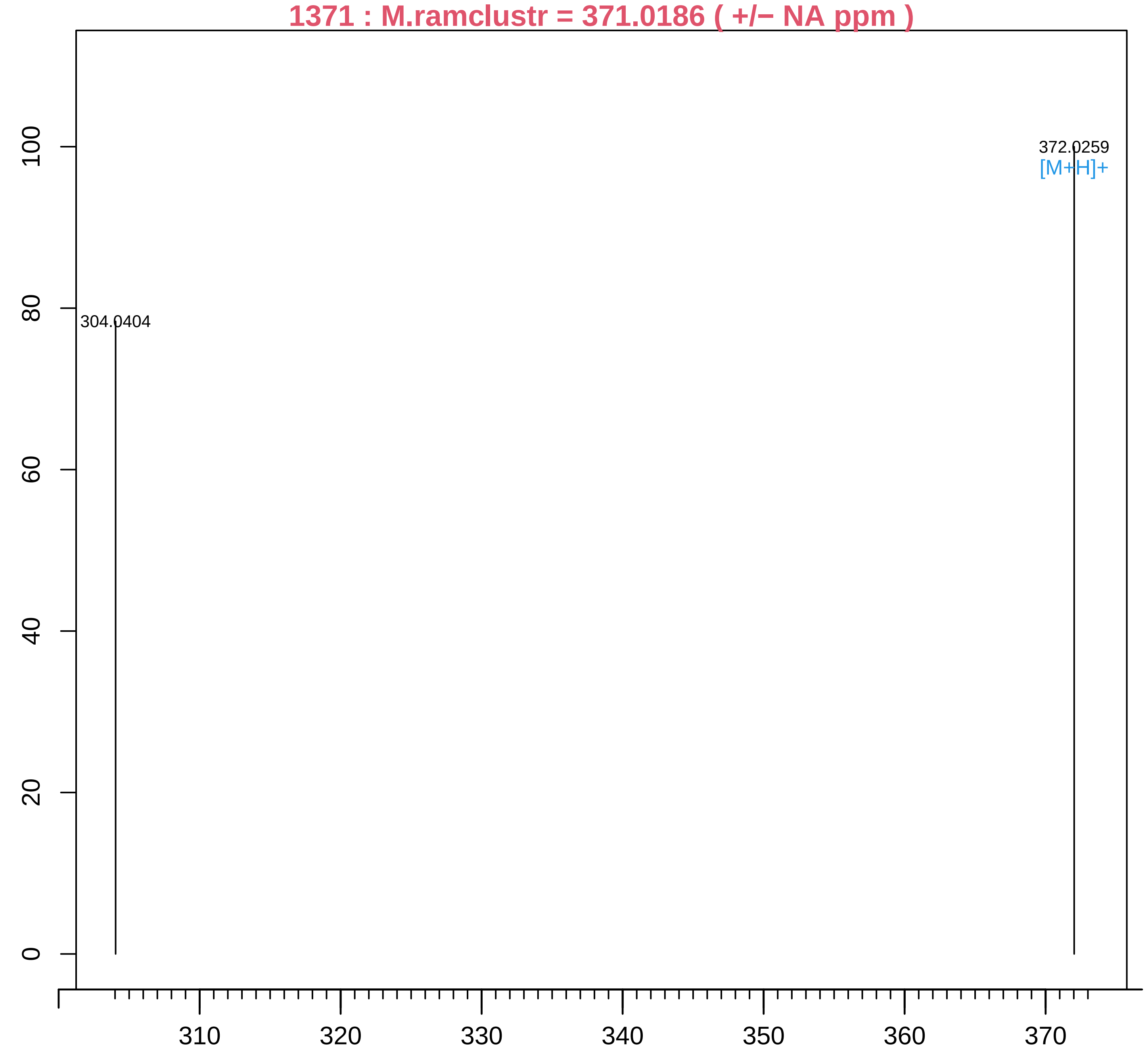
1371 : M.ramclustr = 371.0186 (+/− NA ppm)
304.0404
372.0259
[M+H]+
0
20
40
60
80
100
310
320
330
340
350
360
370

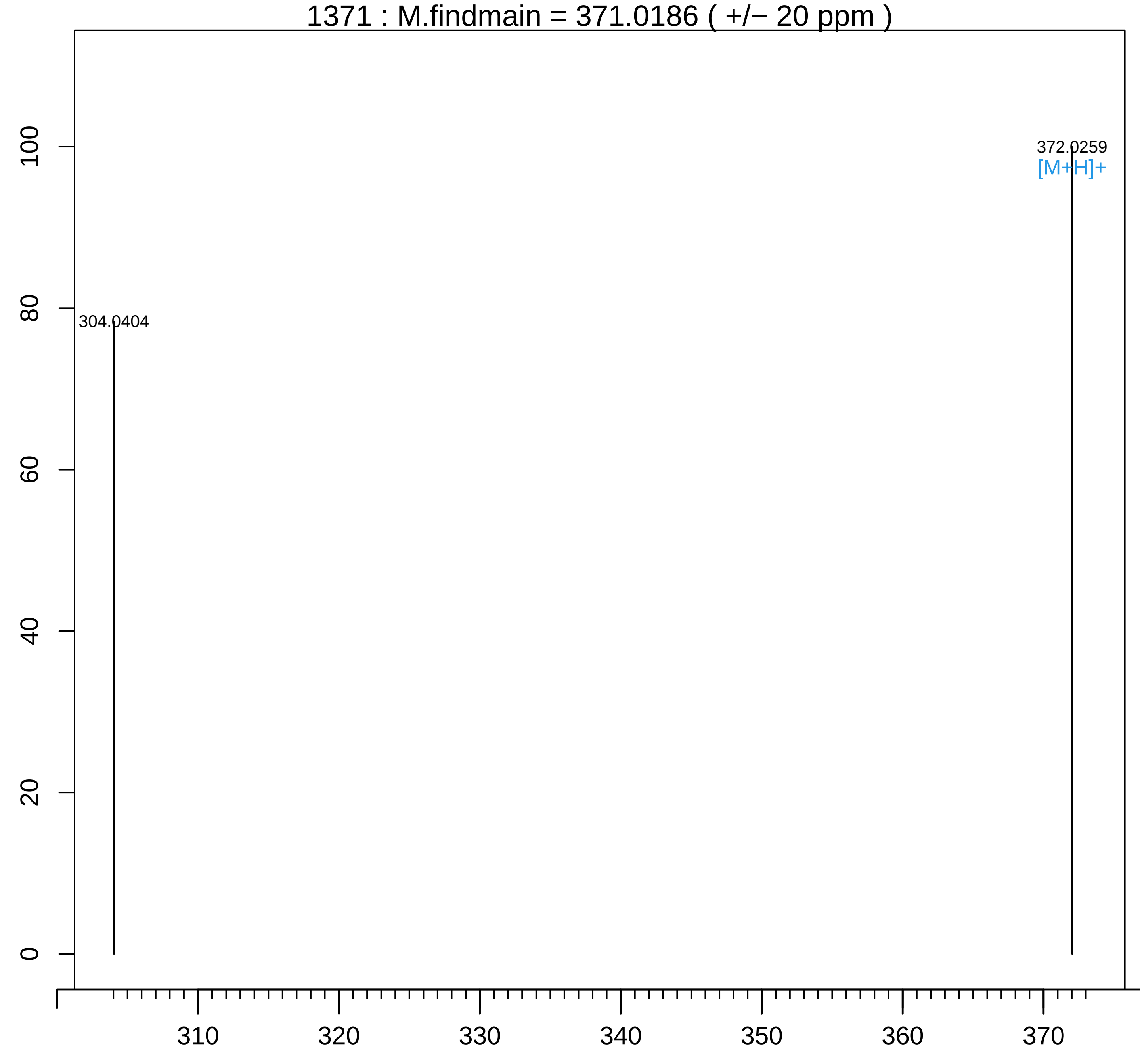
1371 : M.findmain = 371.0186 (+/− 20 ppm)
304.0404
372.0259
[M+H]+
0
20
40
60
80
100
310
320
330
340
350
360
370

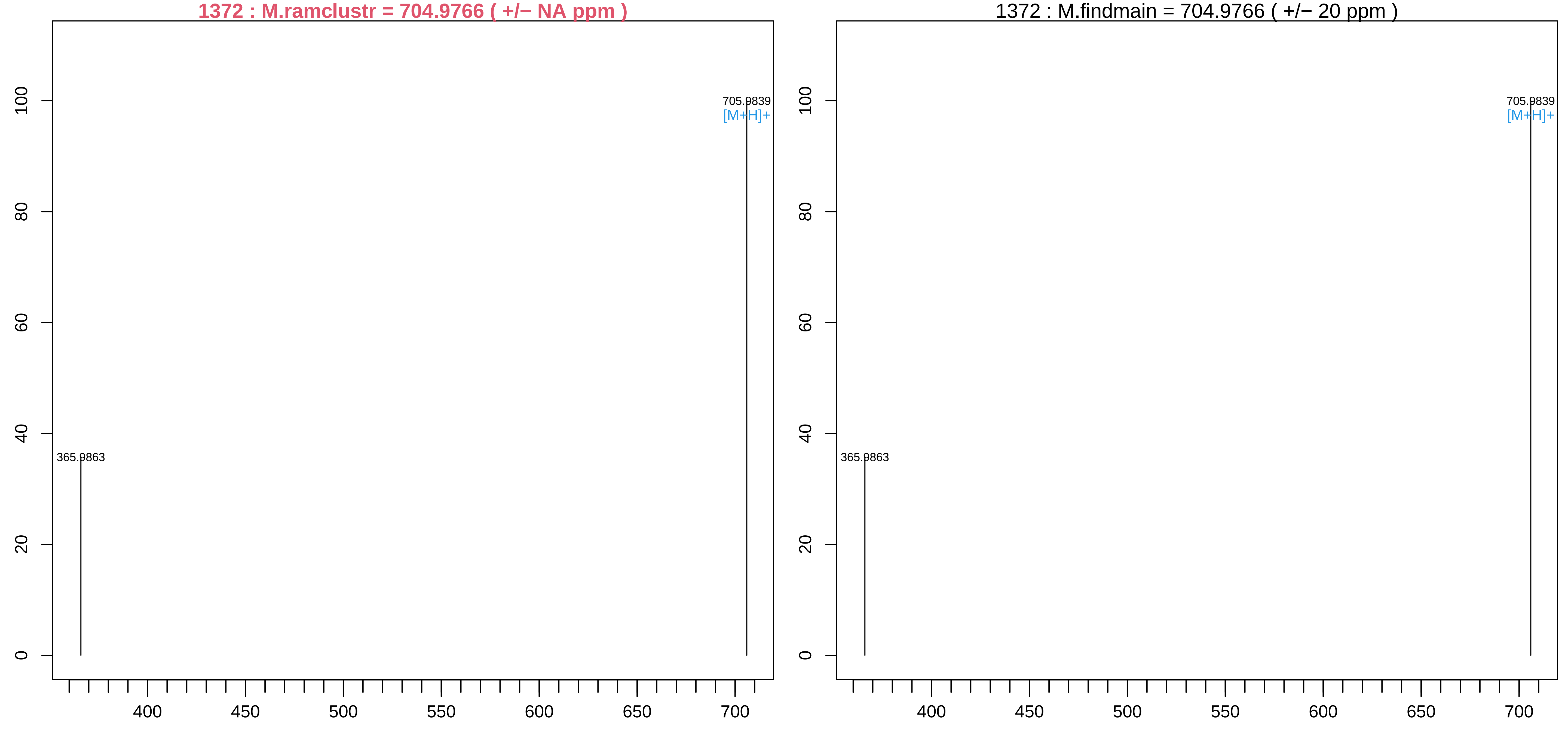

1372 : M.ramclustr = 704.9766 (+/− NA ppm)
705.9839
[M+H]+
365.9863
1372 : M.findmain = 704.9766 (+/− 20 ppm)
705.9839
[M+H]+
365.9863
0
20
40
60
80
100
400
450
500
550
600
650
700

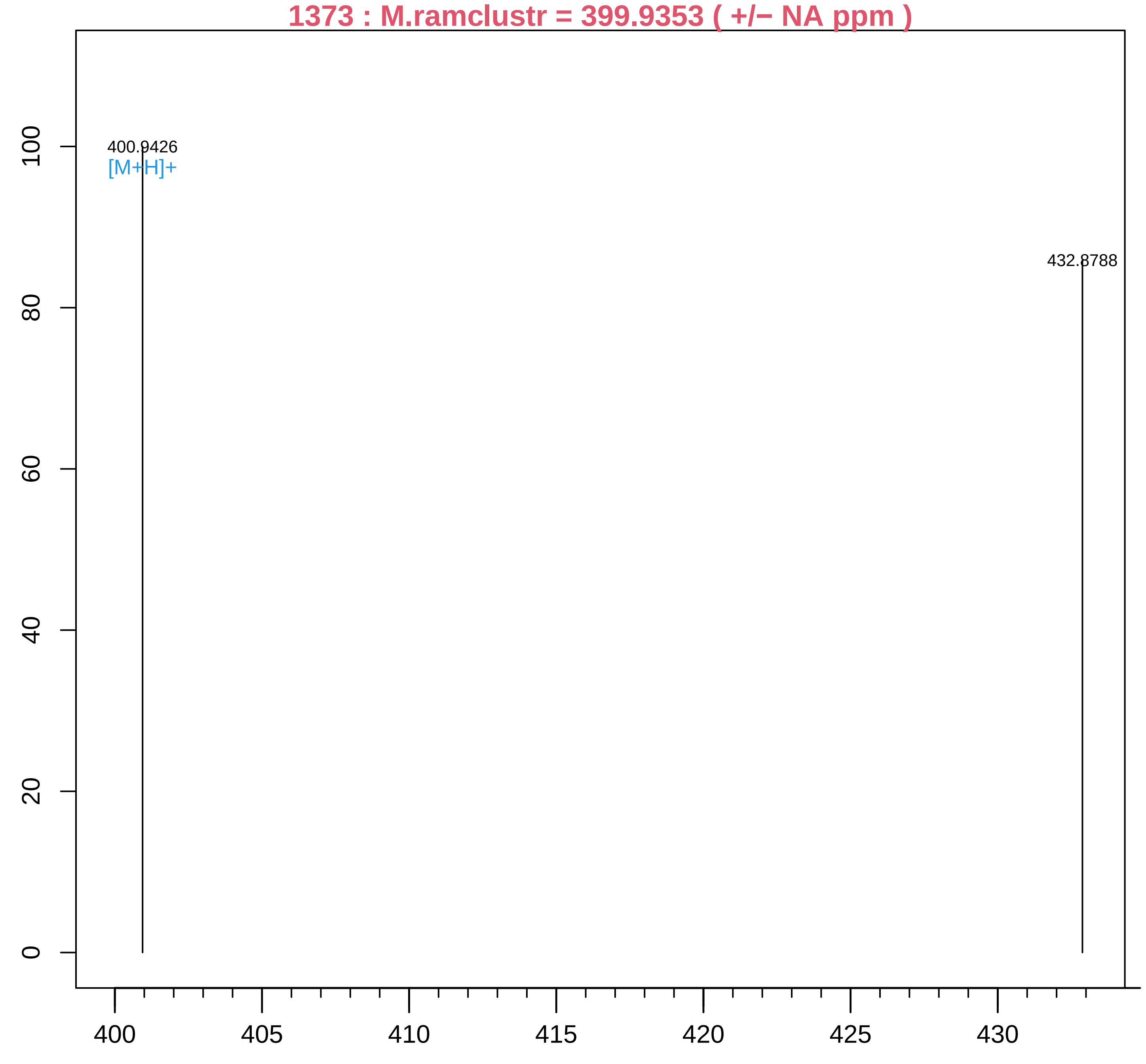
1373 : M.ramclustr = 399.9353 (+/− NA ppm)
400.9426
[M+H]+
432.8788
0
20
40
60
80
100
400
405
410
415
420
425
430

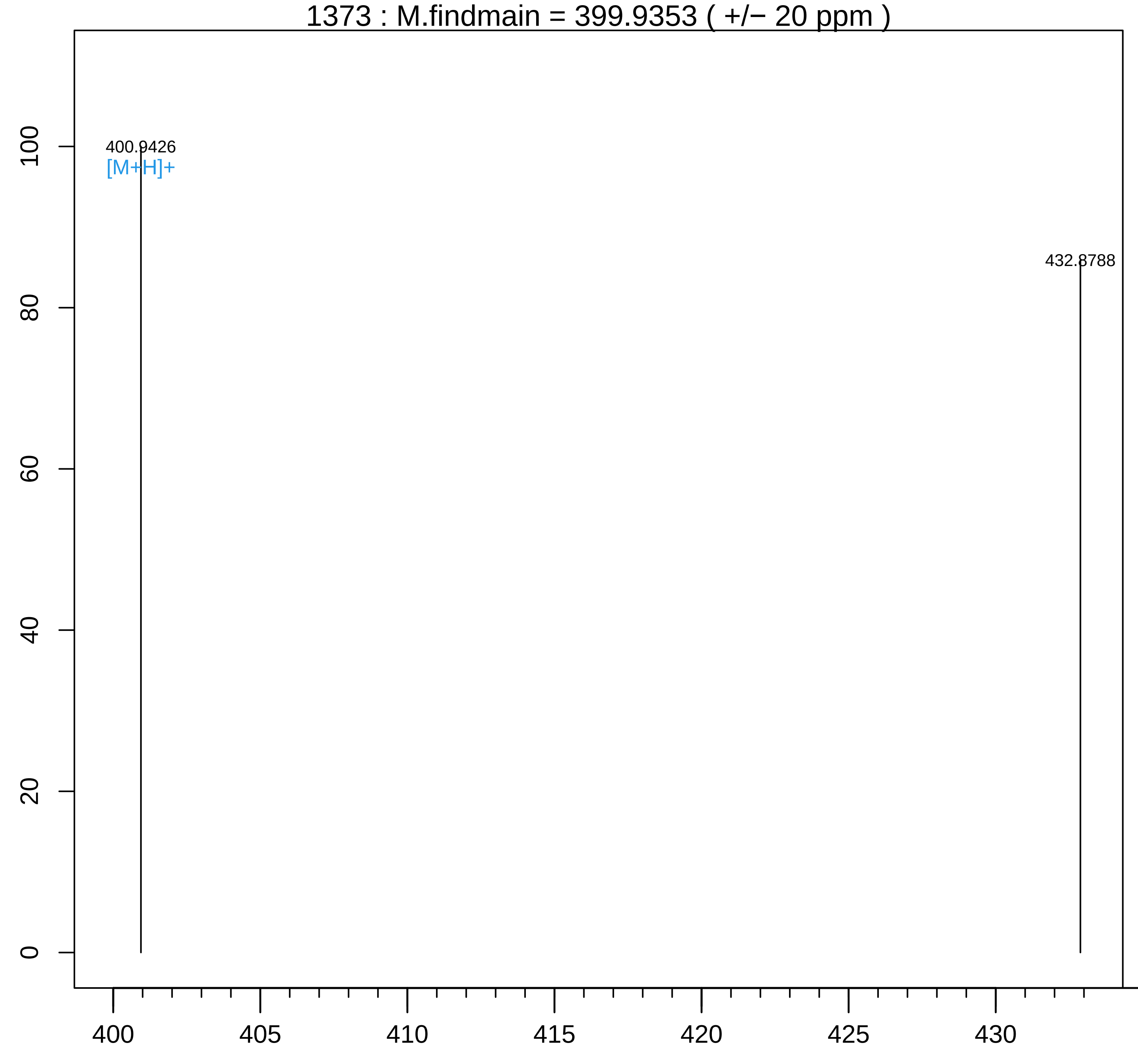
1373 : M.findmain = 399.9353 (+/− 20 ppm)
400.9426
[M+H]+
432.8788
0
20
40
60
80
100
400
405
410
415
420
425
430

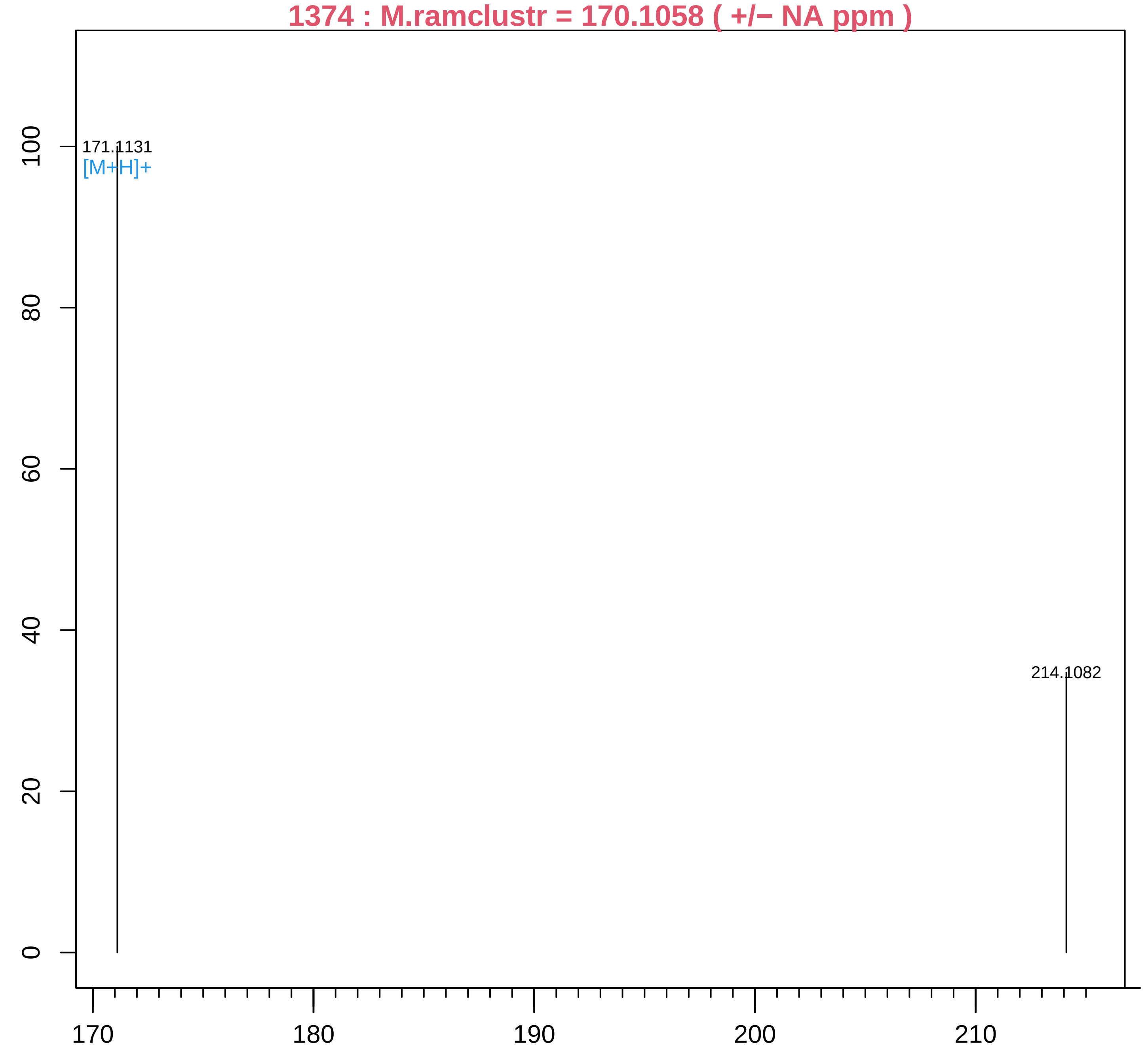
1374 : M.ramclustr = 170.1058 (+/− NA ppm)
171.1131
[M+H]+
214.1082
0
20
40
60
80
100
170
180
190
200
210

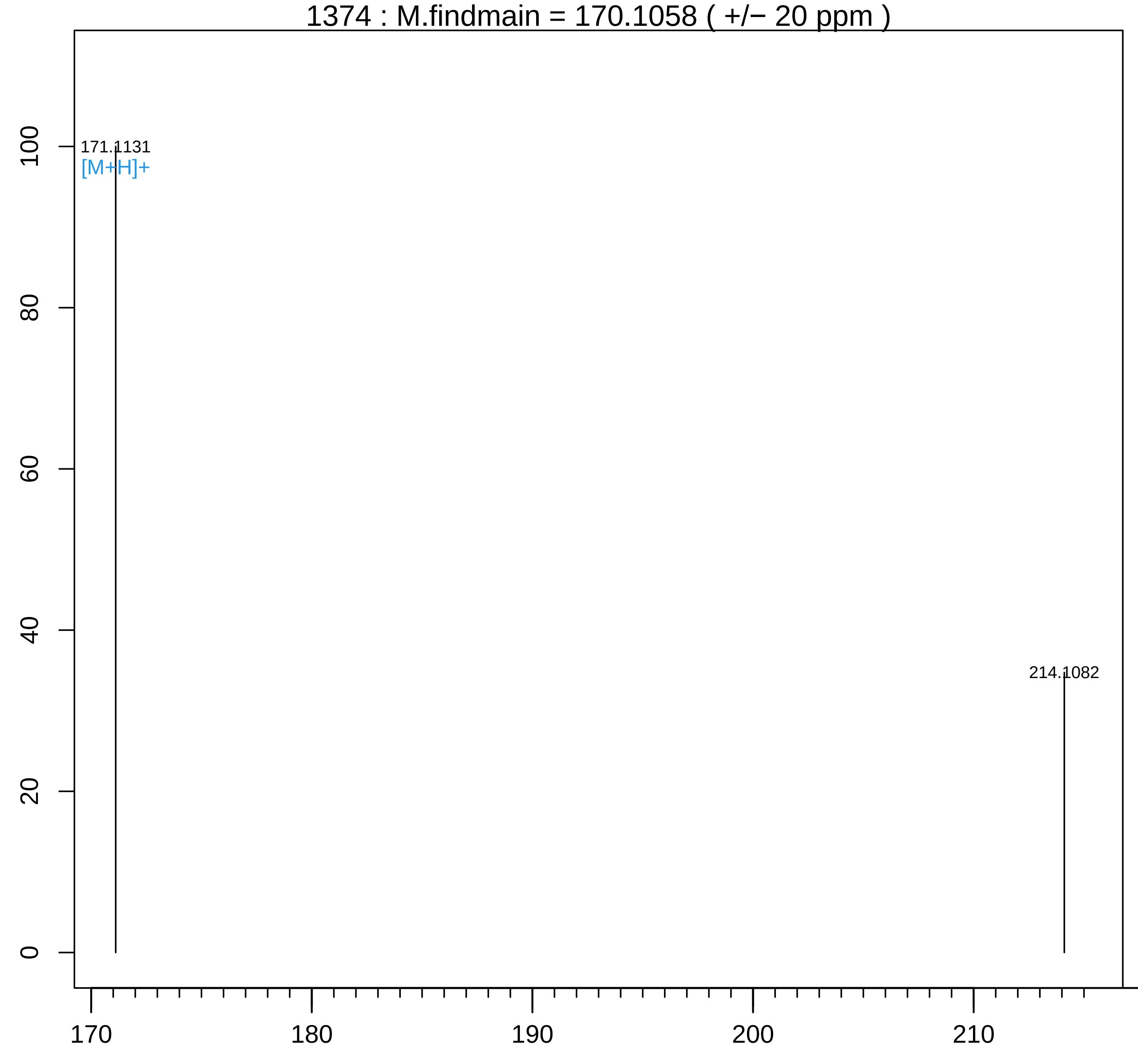
1374 : M.findmain = 170.1058 (+/− 20 ppm)
171.1131
[M+H]+
214.1082
0
20
40
60
80
100
170
180
190
200
210

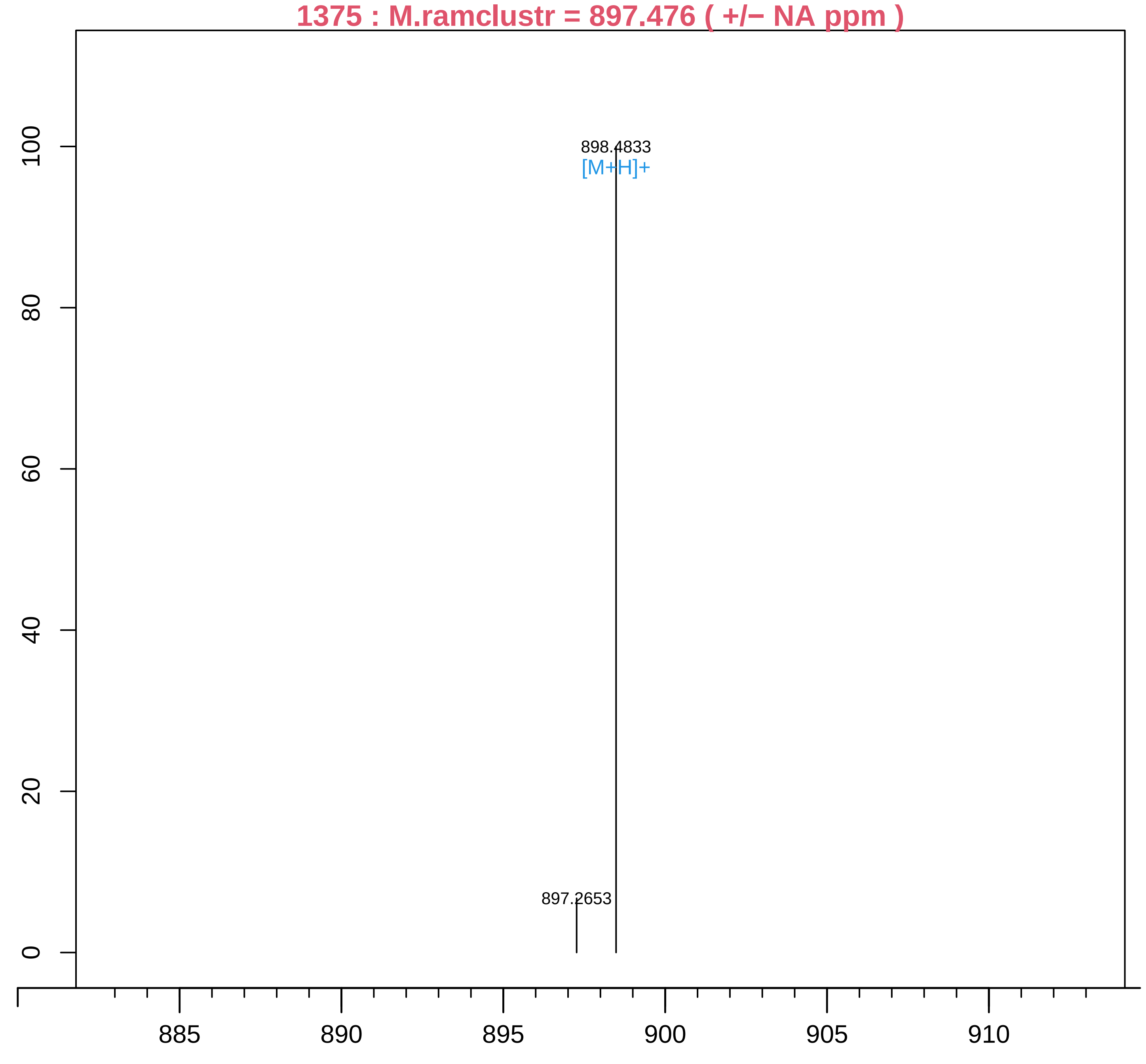
1375 : M.ramclustr = 897.476 (+/− NA ppm)
898.4833
[M+H]+
897.2653
0
20
40
60
80
100
885
890
895
900
905
910

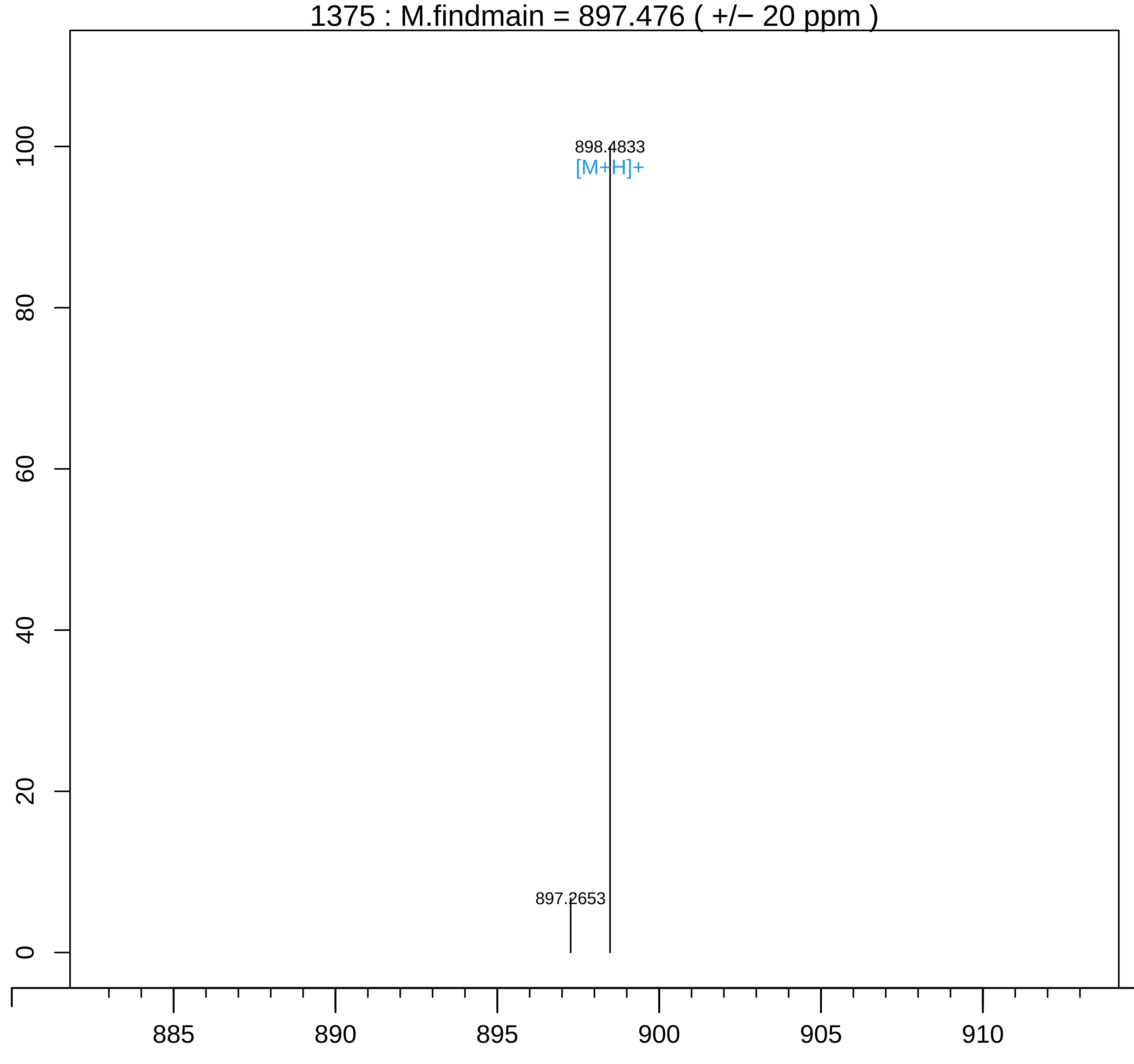
1375 : M.findmain = 897.476 (+/− 20 ppm)
898.4833
[M+H]+
897.2653
0
20
40
60
80
100
885
890
895
900
905
910

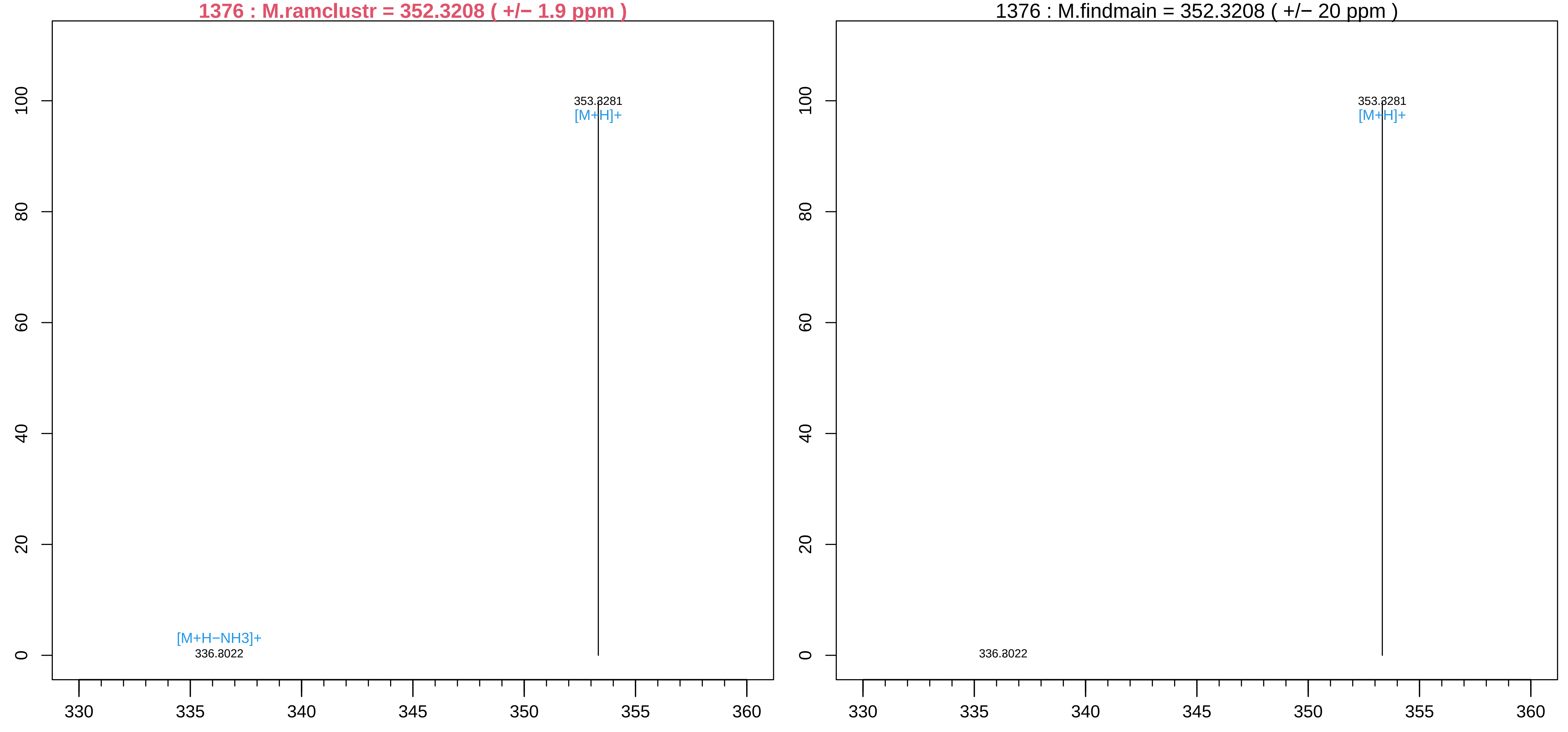
1376 : M.ramclustr = 352.3208 (+/− 1.9 ppm)
353.3281
[M+H]+
[M+H−NH3]+
336.8022
0
20
40
60
80
100
330
335
340
345
350
355
360
1376 : M.findmain = 352.3208 (+/− 20 ppm)
353.3281
[M+H]+
336.8022
0
20
40
60
80
100
330
335
340
345
350
355
360

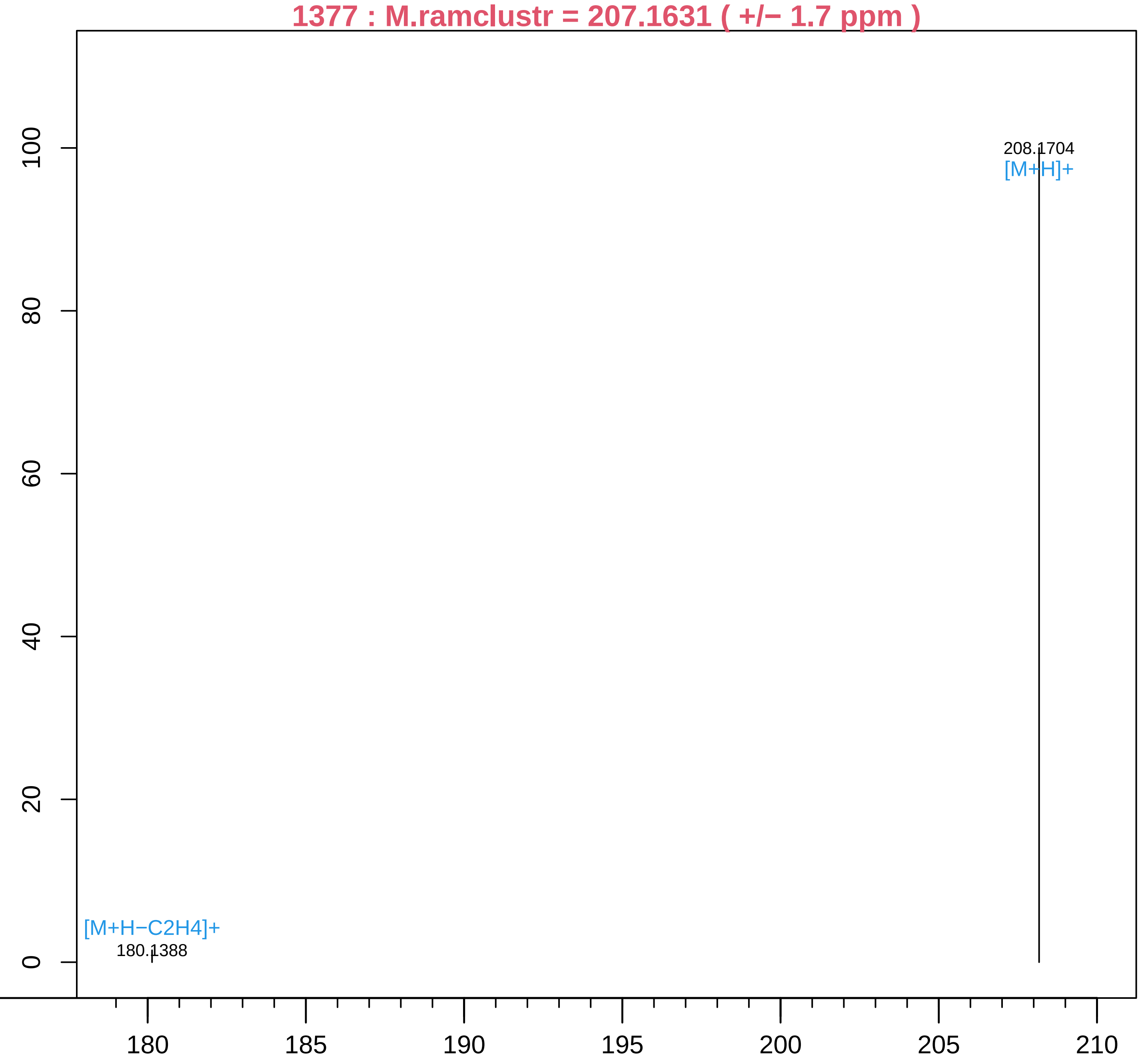
1377 : M.ramclustr = 207.1631 (+/− 1.7 ppm)
208.1704
[M+H]+
[M+H−C2H4]+
180.1388
0
20
40
60
80
100
180
185
190
195
200
205
210

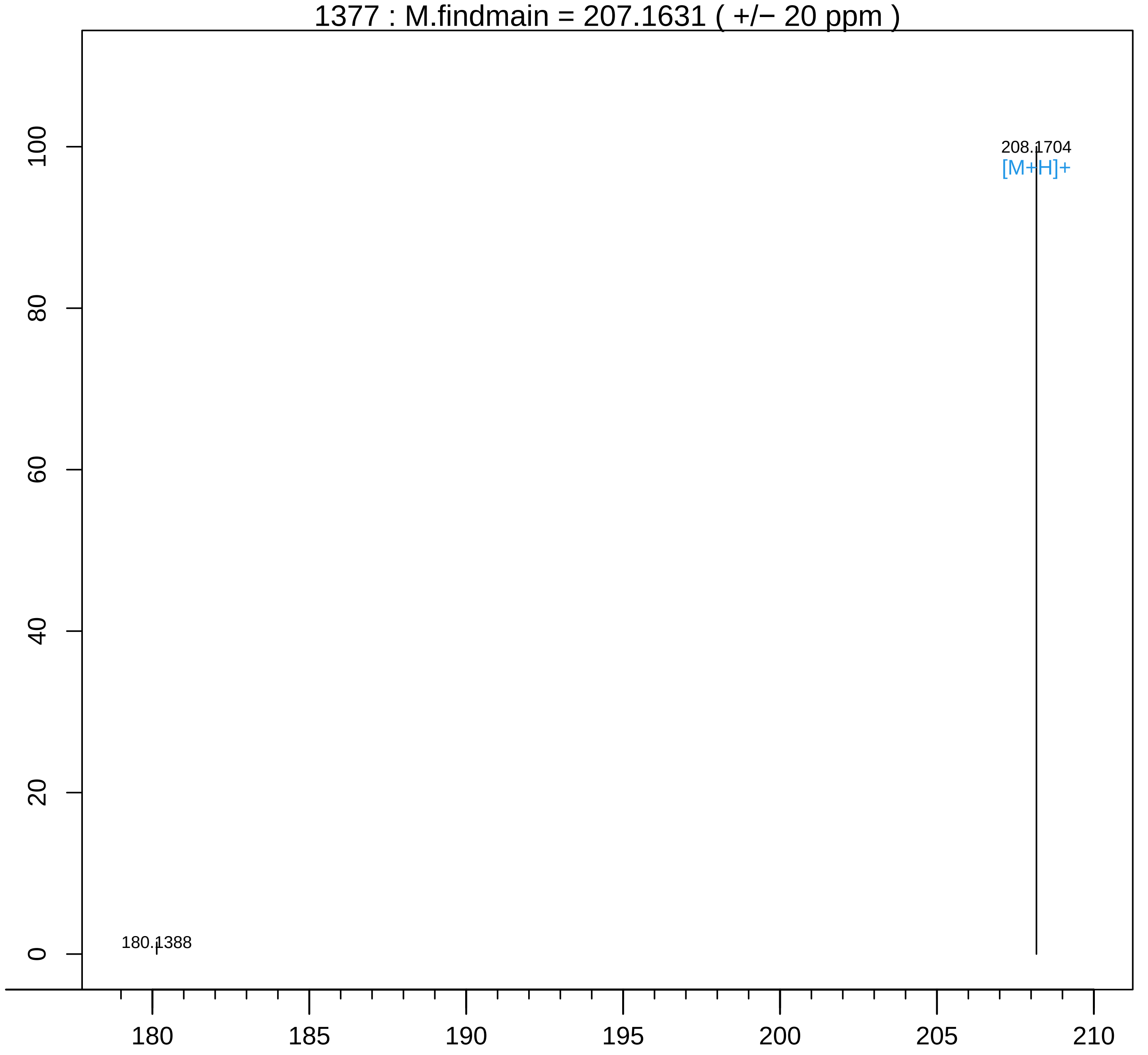
1377 : M.findmain = 207.1631 (+/− 20 ppm)
208.1704
[M+H]+
180.1388
0
20
40
60
80
100
180
185
190
195
200
205
210

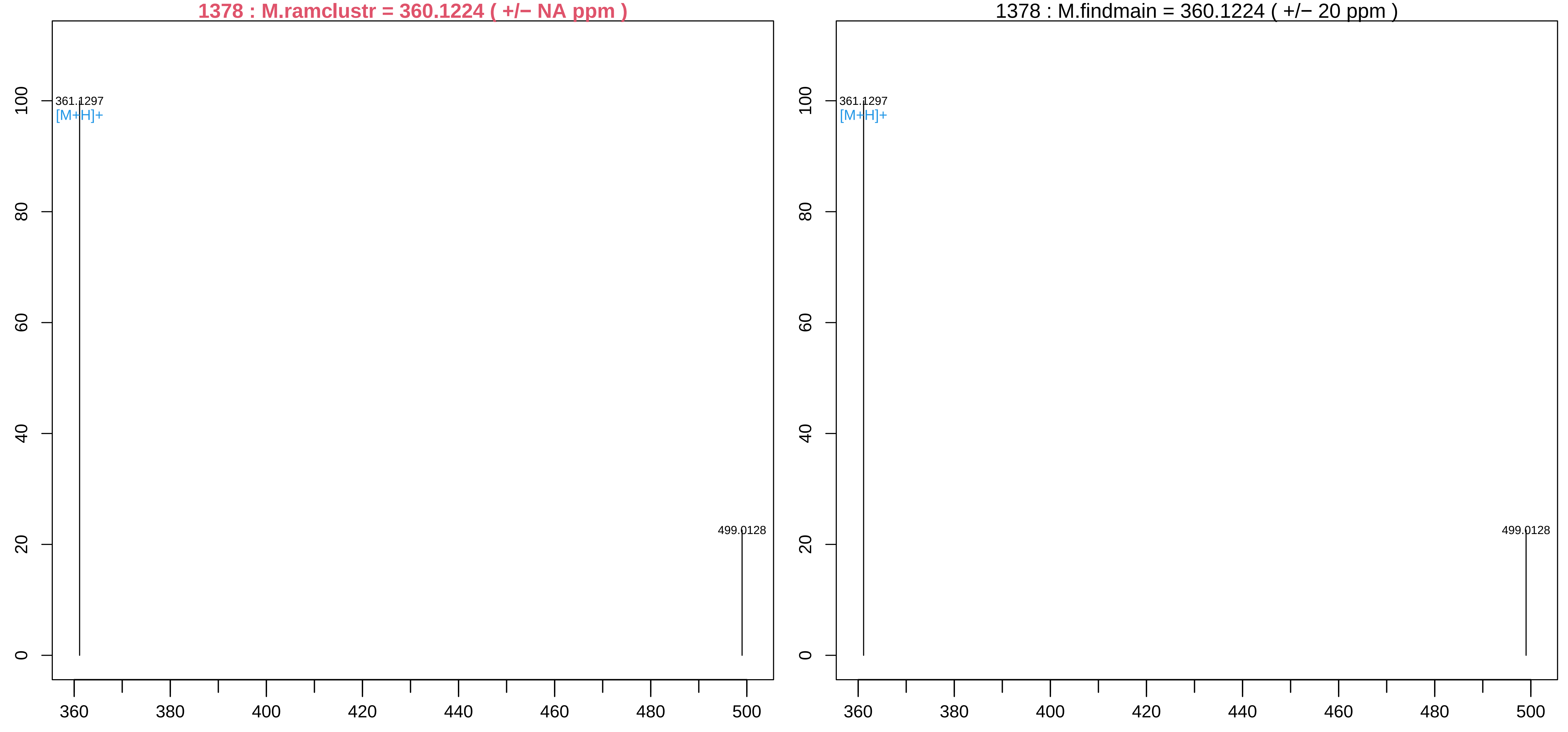

1378 : M.ramclustr = 360.1224 (+/− NA ppm)
361.1297
[M+H]+
499.0128
1378 : M.findmain = 360.1224 (+/− 20 ppm)
361.1297
[M+H]+
499.0128
0
20
40
60
80
100
360
380
400
420
440
460
480
500

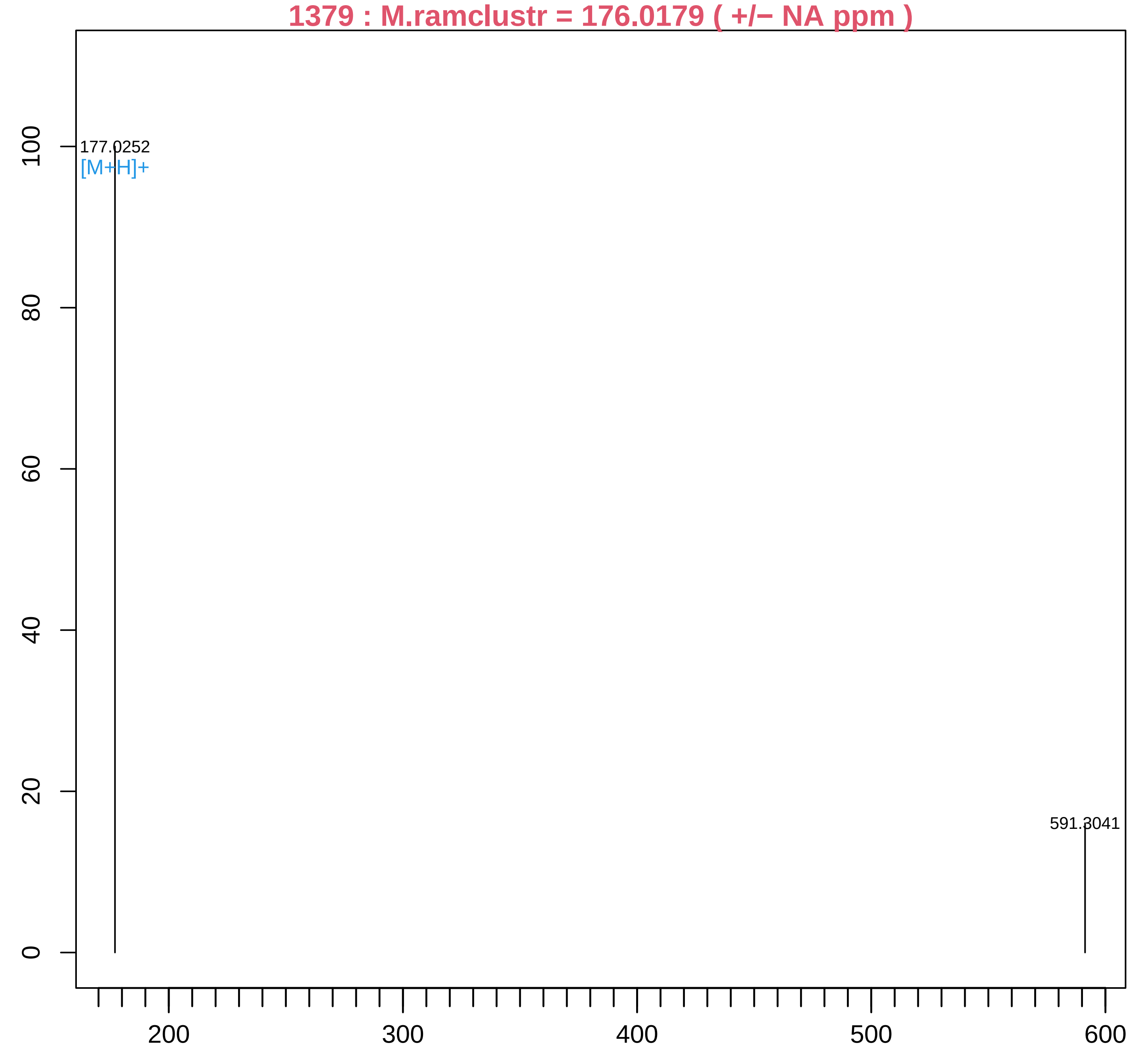
1379 : M.ramclustr = 176.0179 (+/− NA ppm)
177.0252
[M+H]+
591.3041
0
20
40
60
80
100
200
300
400
500
600

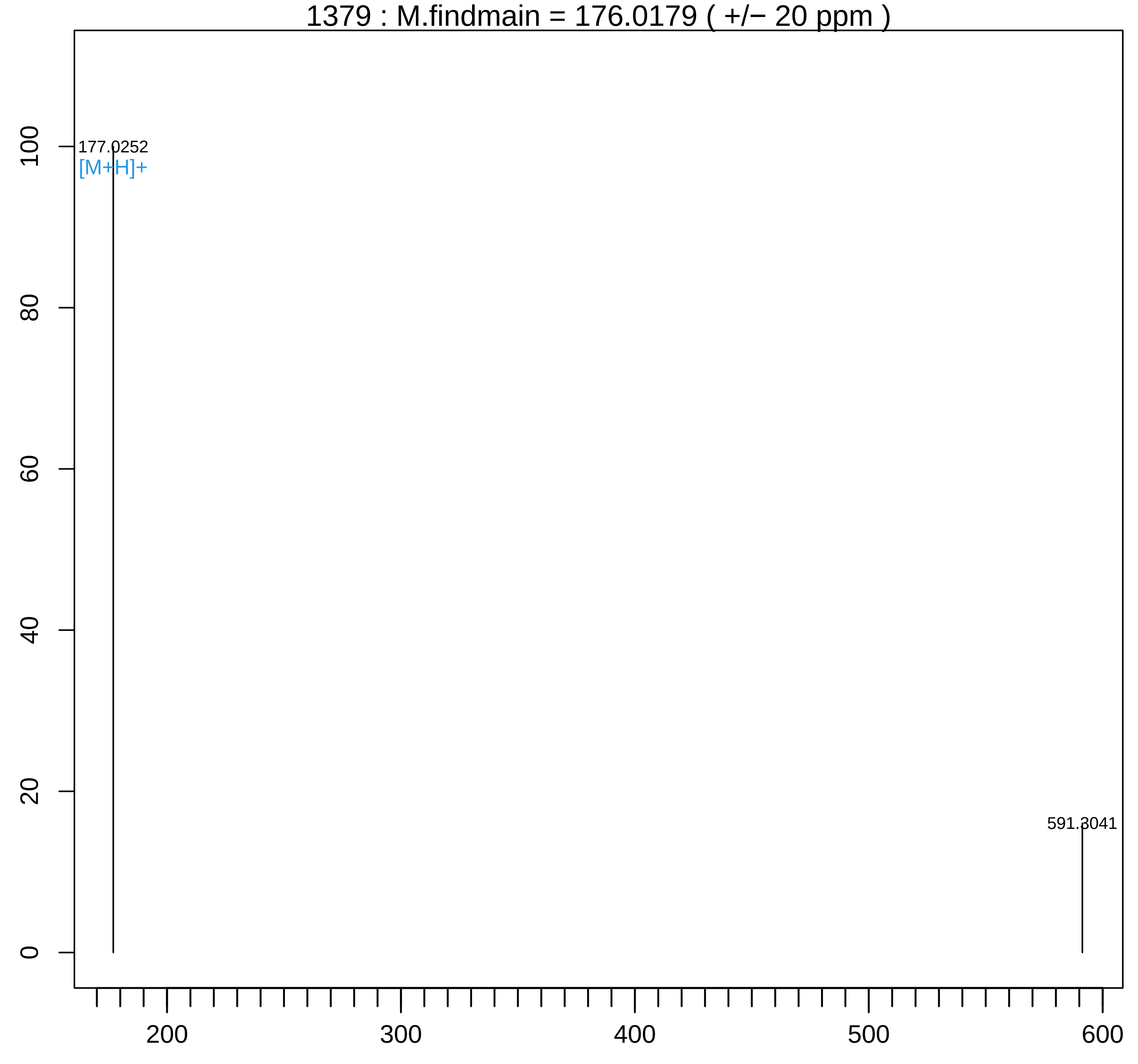
1379 : M.findmain = 176.0179 (+/− 20 ppm)
177.0252
[M+H]+
591.3041
0
20
40
60
80
100
200
300
400
500
600

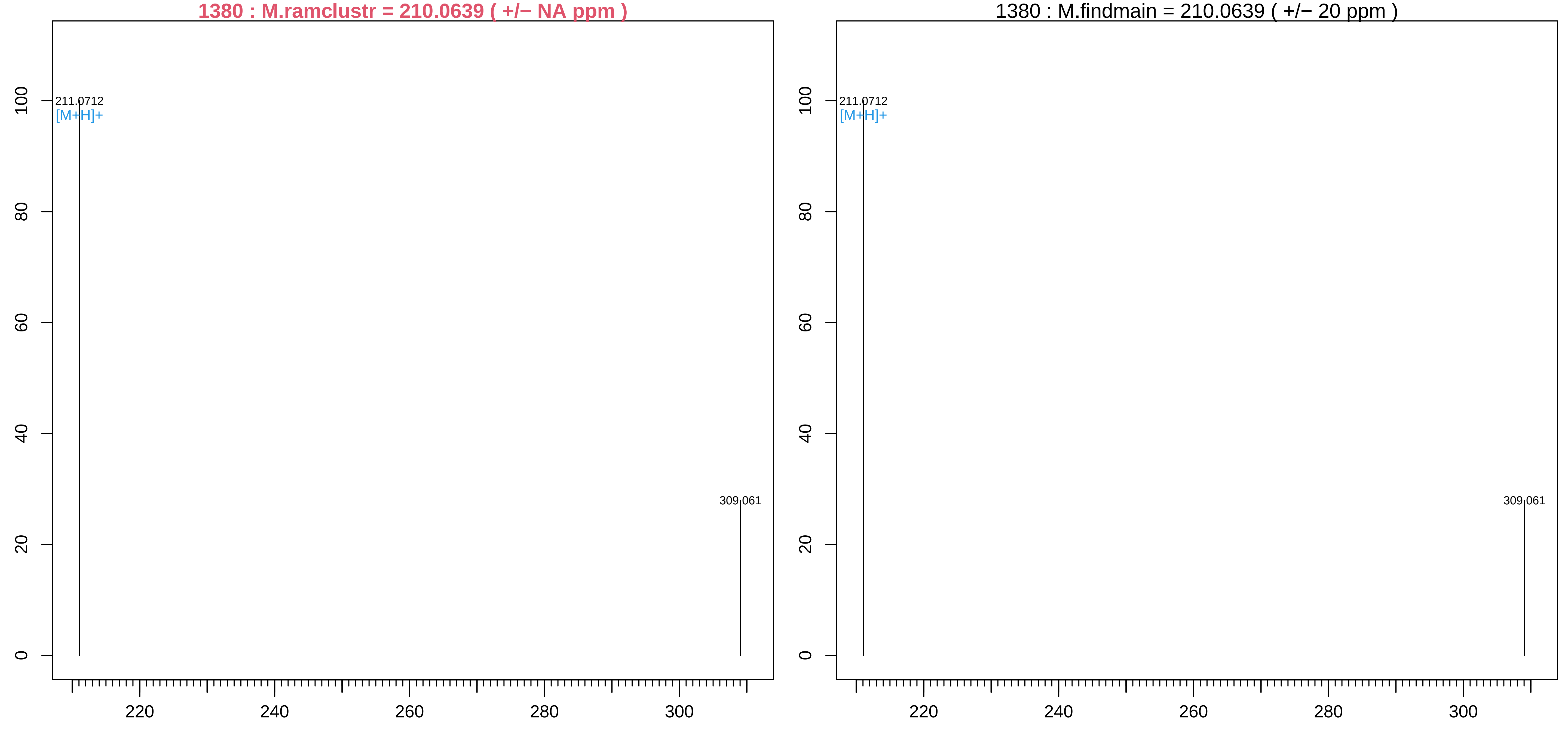

1380 : M.ramclustr = 210.0639 (+/− NA ppm)
1380 : M.findmain = 210.0639 (+/− 20 ppm)
211.0712
[M+H]+
309.061
0
20
40
60
80
100
220
240
260
280
300

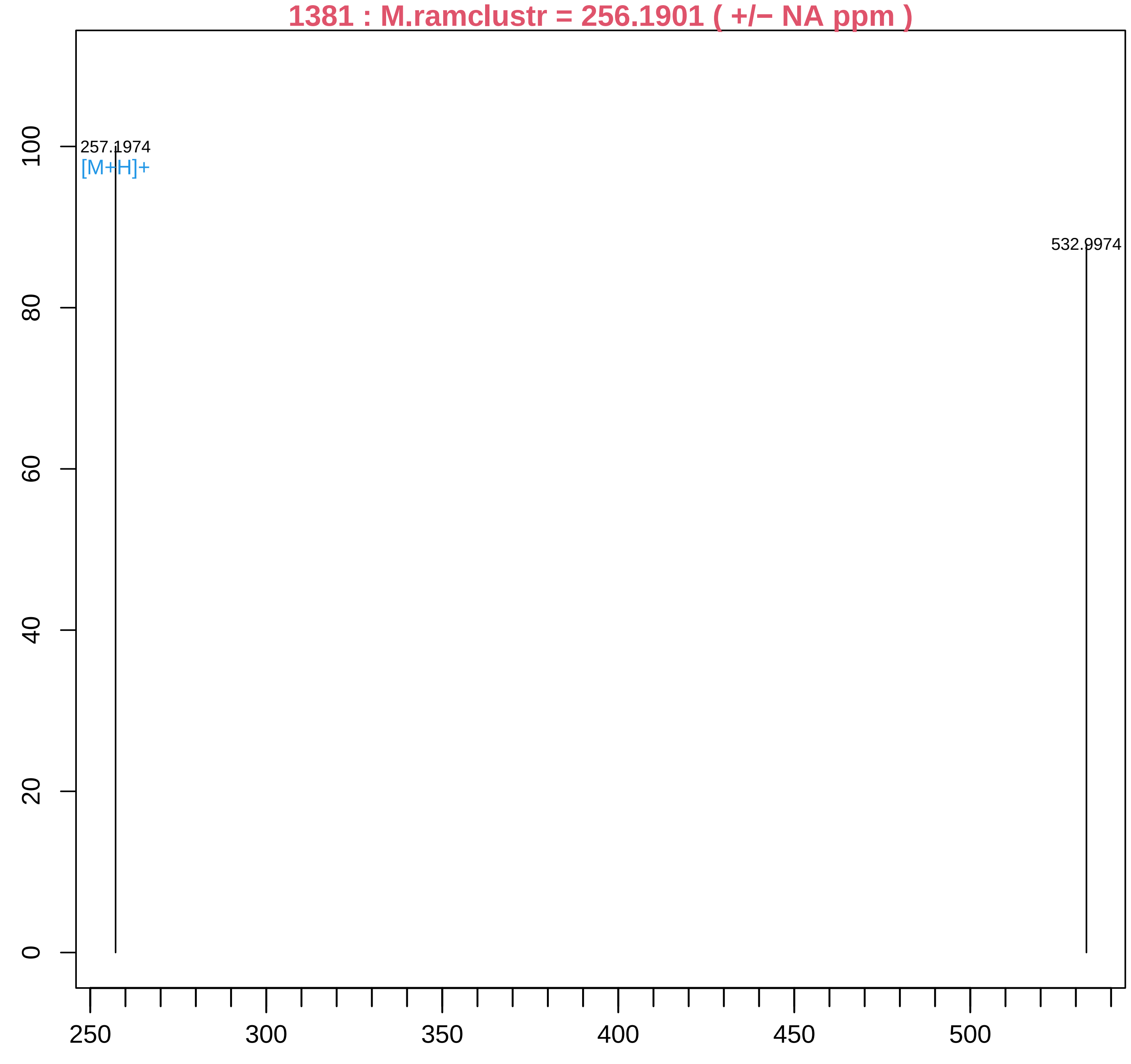
1381 : M.ramclustr = 256.1901 (+/− NA ppm)
257.1974
[M+H]+
532.9974
0
20
40
60
80
100
250
300
350
400
450
500

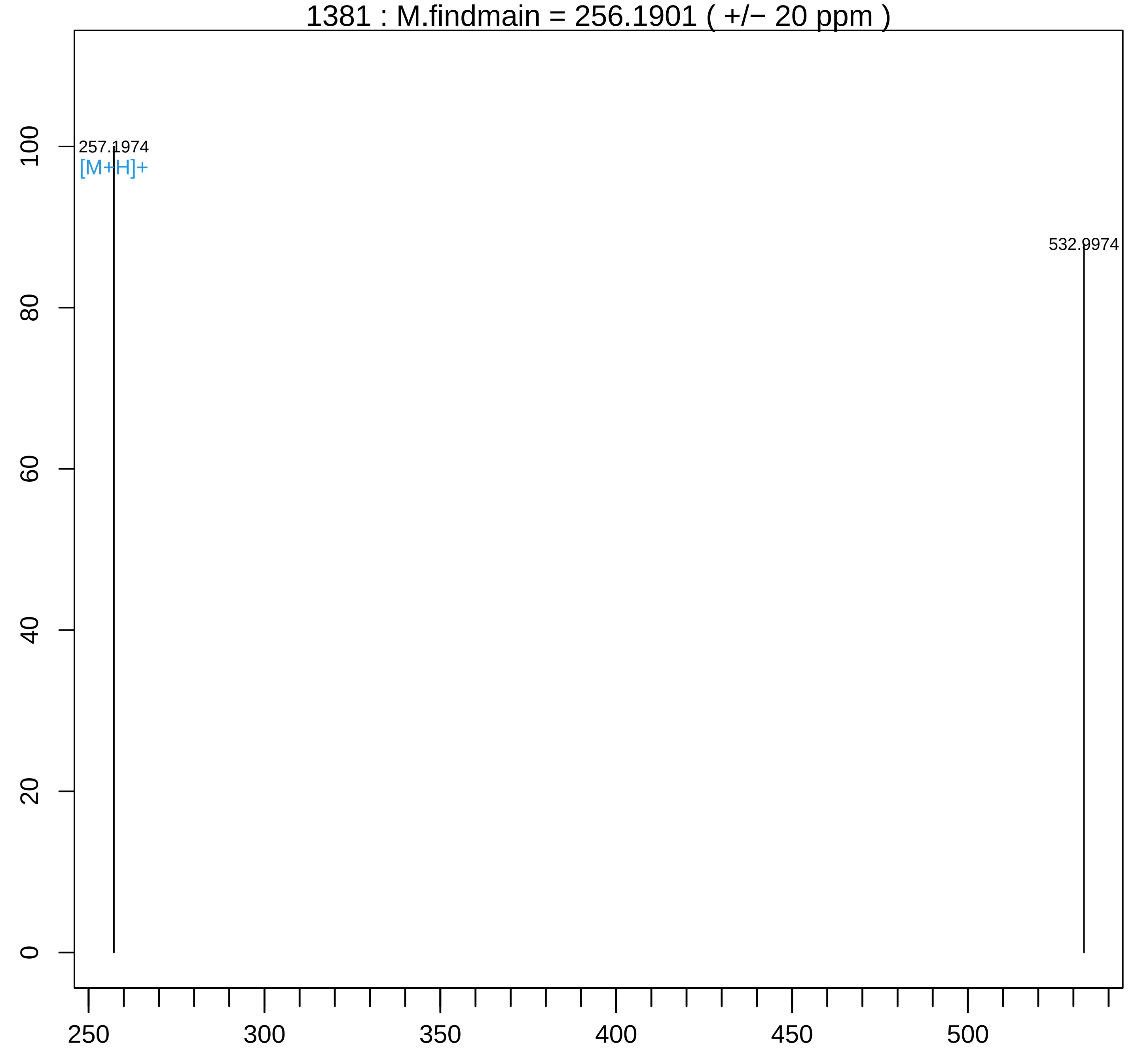
1381 : M.findmain = 256.1901 (+/− 20 ppm)
257.1974
[M+H]+
532.9974
0
20
40
60
80
100
250
300
350
400
450
500

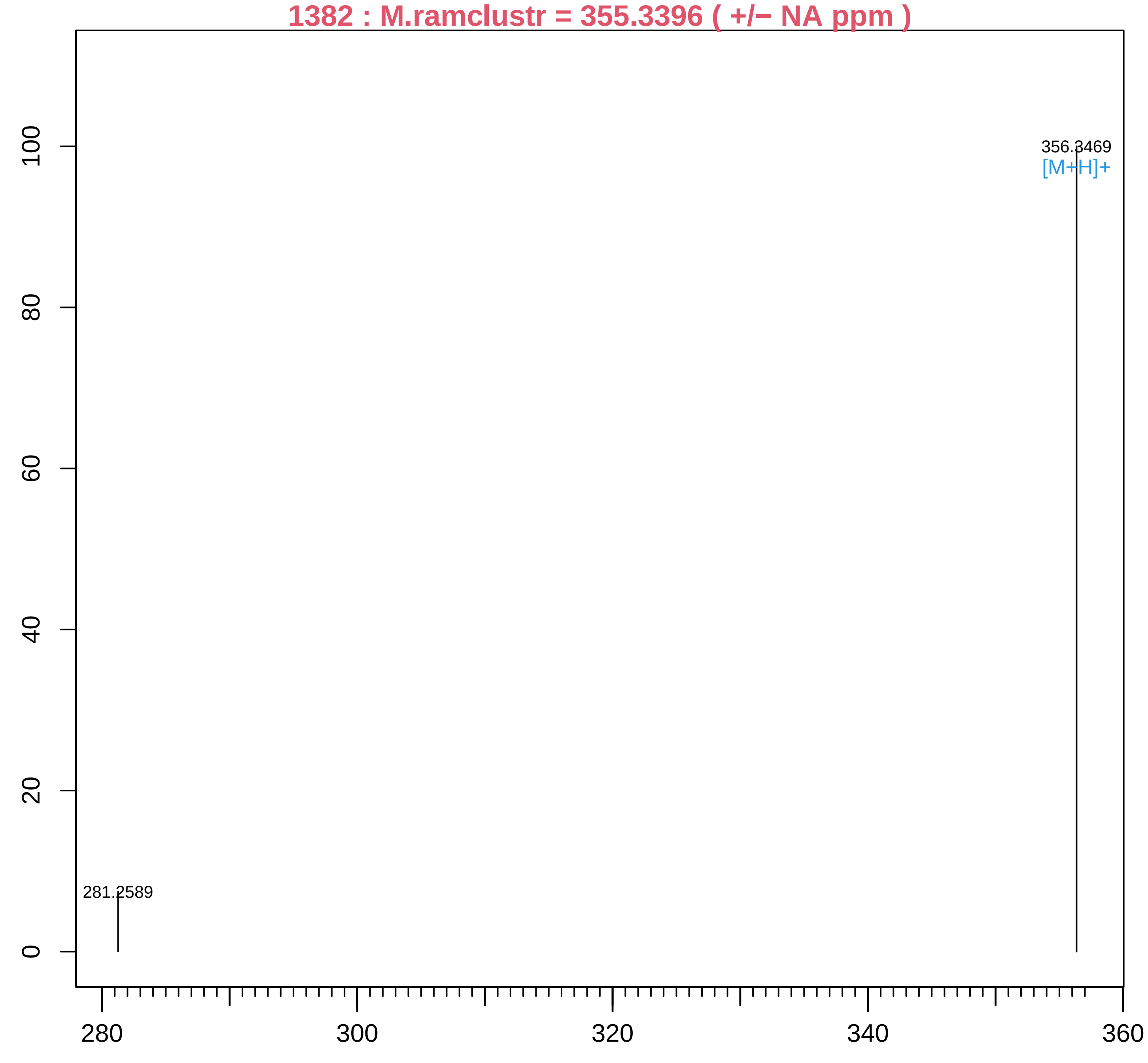
1382 : M.ramclustr = 355.3396 (+/− NA ppm)
356.3469
[M+H]+
281.2589
0
20
40
60
80
100
280
300
320
340
360

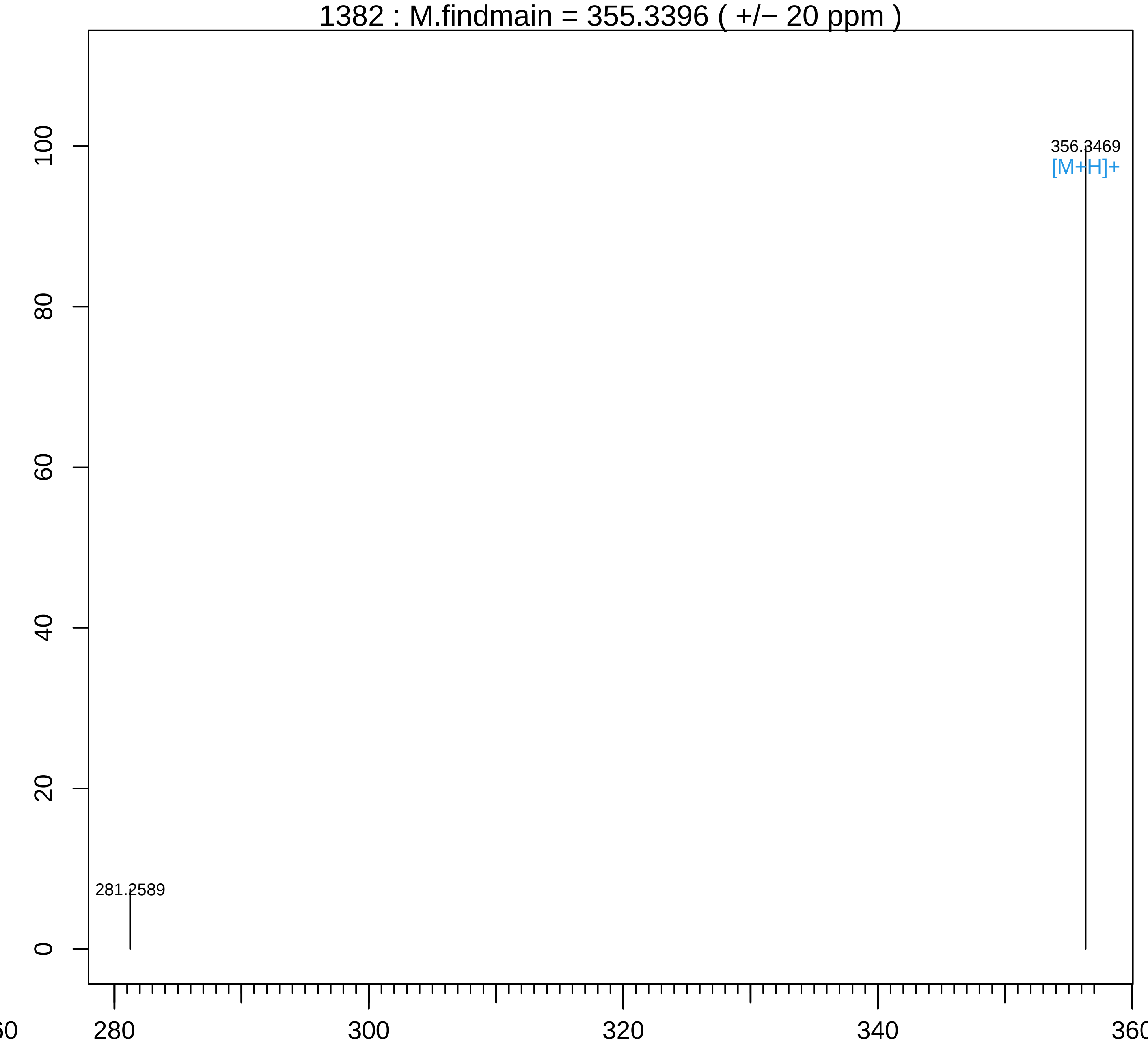
1382 : M.findmain = 355.3396 (+/− 20 ppm)
356.3469
[M+H]+
281.2589
0
20
40
60
80
100
280
300
320
340
360

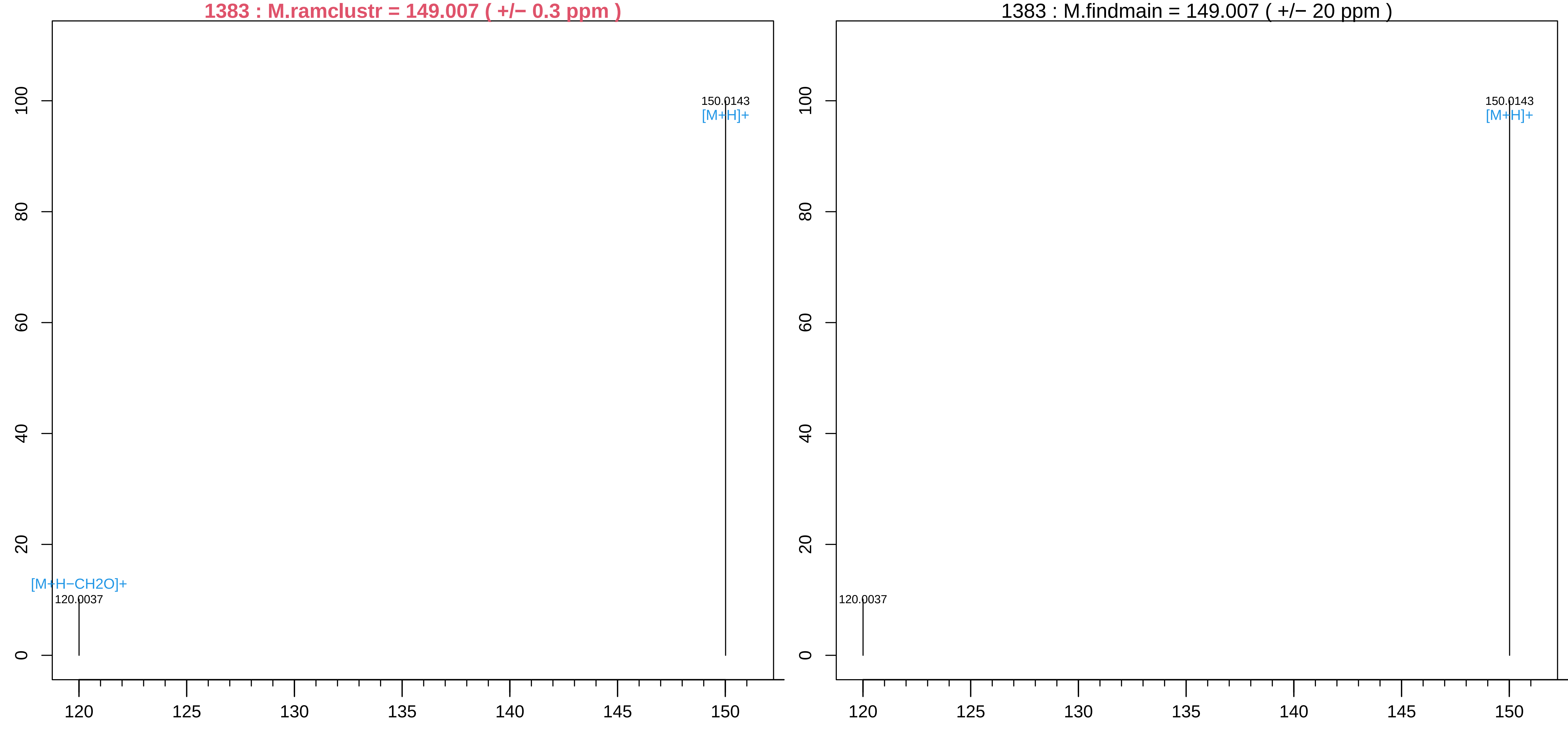

1383 : M.ramclustr = 149.007 (+/− 0.3 ppm)
150.0143
[M+H]+
[M+H−CH2O]+
120.0037
0
20
40
60
80
100
120
125
130
135
140
145
150
1383 : M.findmain = 149.007 (+/− 20 ppm)
150.0143
[M+H]+
120.0037
0
20
40
60
80
100
120
125
130
135
140
145
150

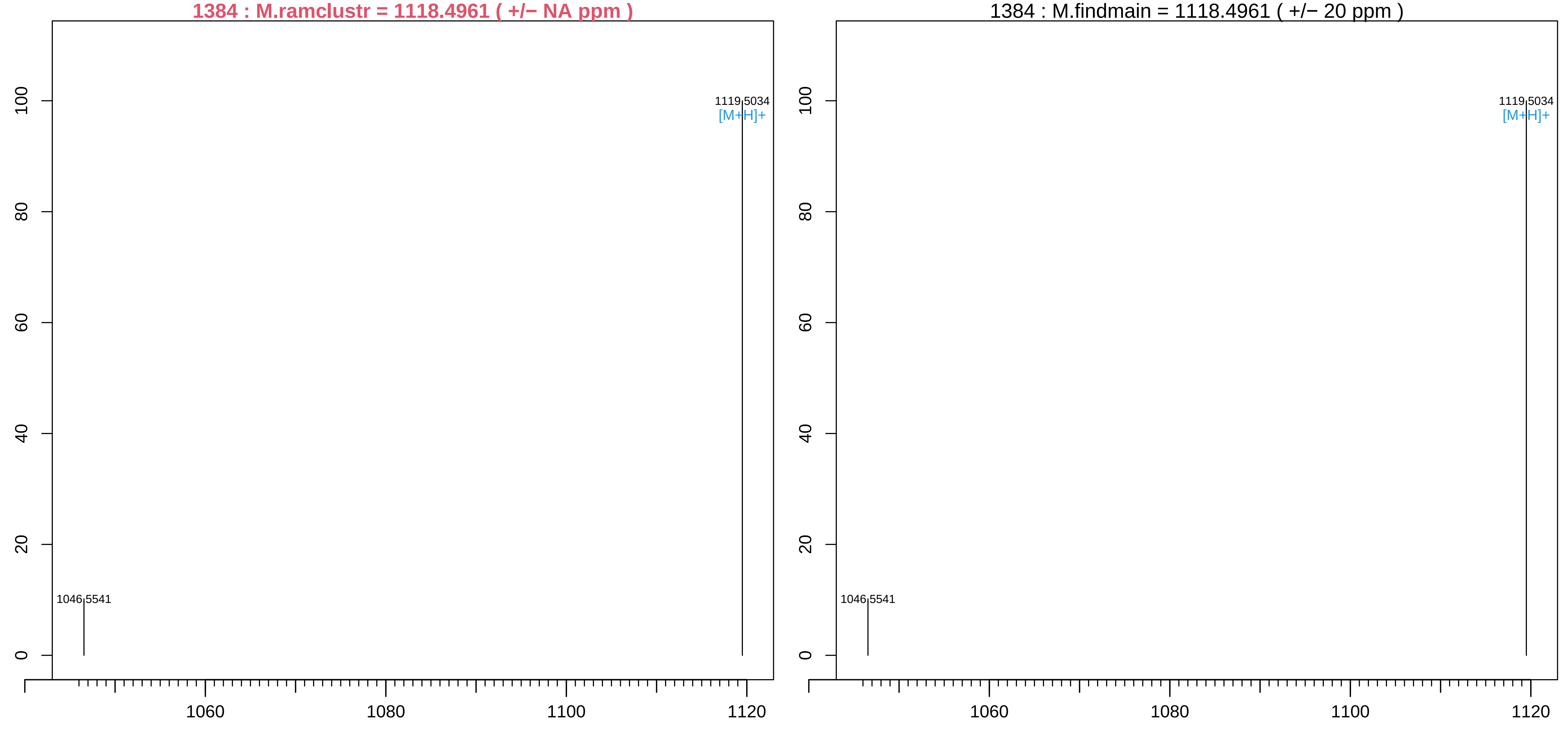
1384 : M.ramclustr = 1118.4961 (+/− NA ppm)
1119.5034
[M+H]+
1046.5541
1384 : M.findmain = 1118.4961 (+/− 20 ppm)
1119.5034
[M+H]+
1046.5541
0
20
40
60
80
100
1060
1080
1100
1120

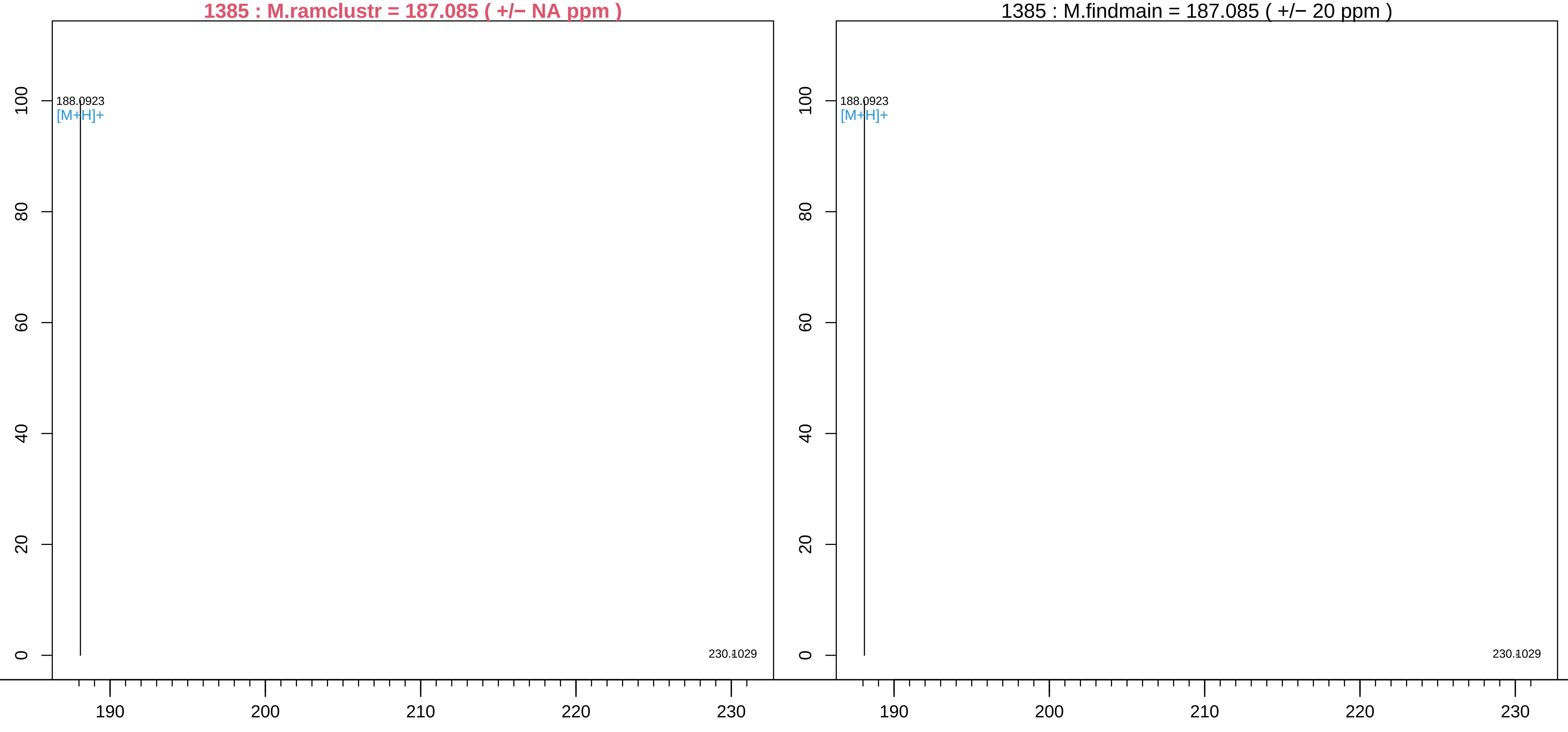
1385 : M.ramclustr = 187.085 (+/− NA ppm)
188.0923
[M+H]+
230.1029
1385 : M.findmain = 187.085 (+/− 20 ppm)
188.0923
[M+H]+
230.1029
0
20
40
60
80
100
190
200
210
220
230

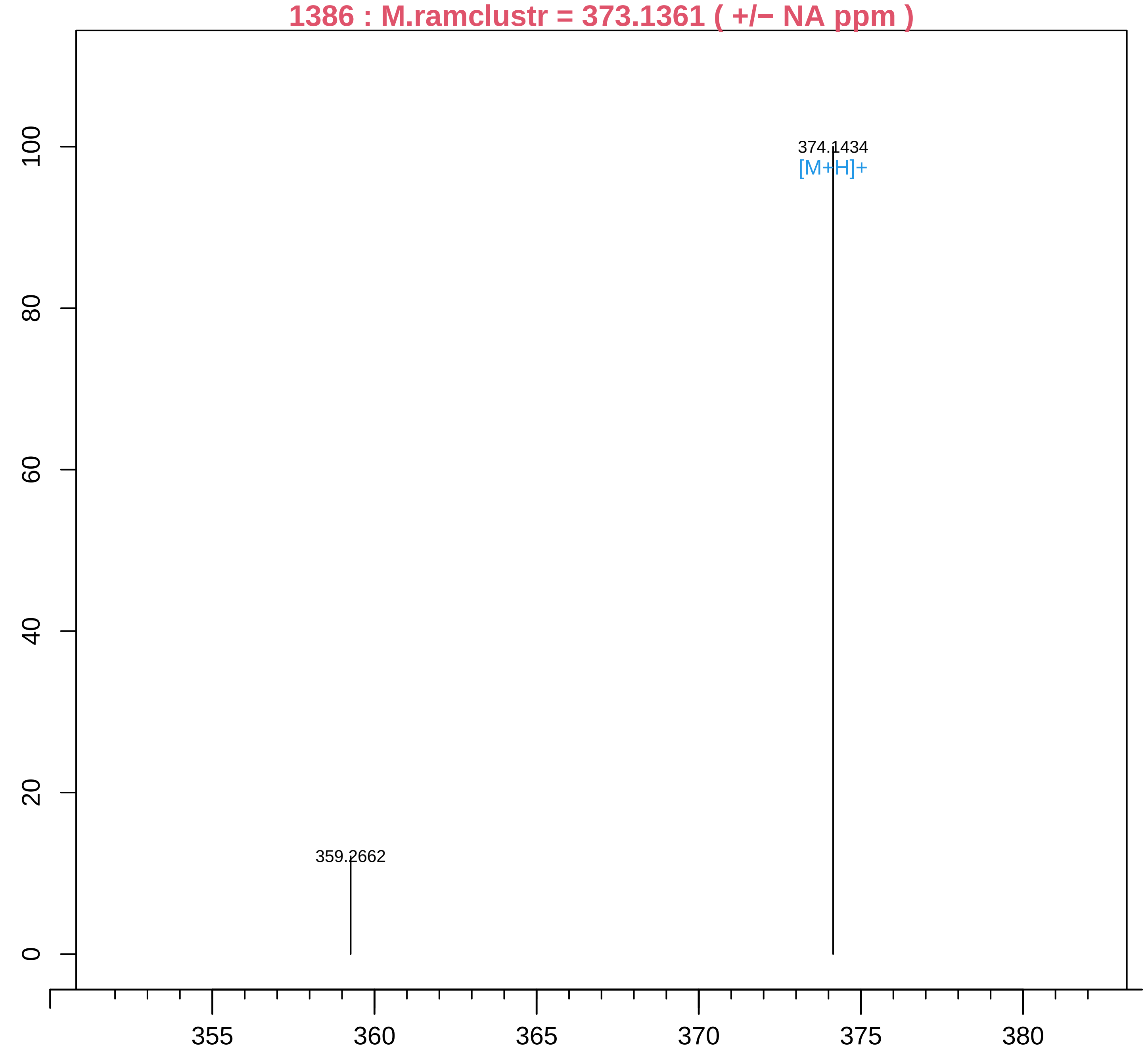
1386 : M.ramclustr = 373.1361 (+/− NA ppm)
374.1434
[M+H]+
359.2662
0
20
40
60
80
100
355
360
365
370
375
380

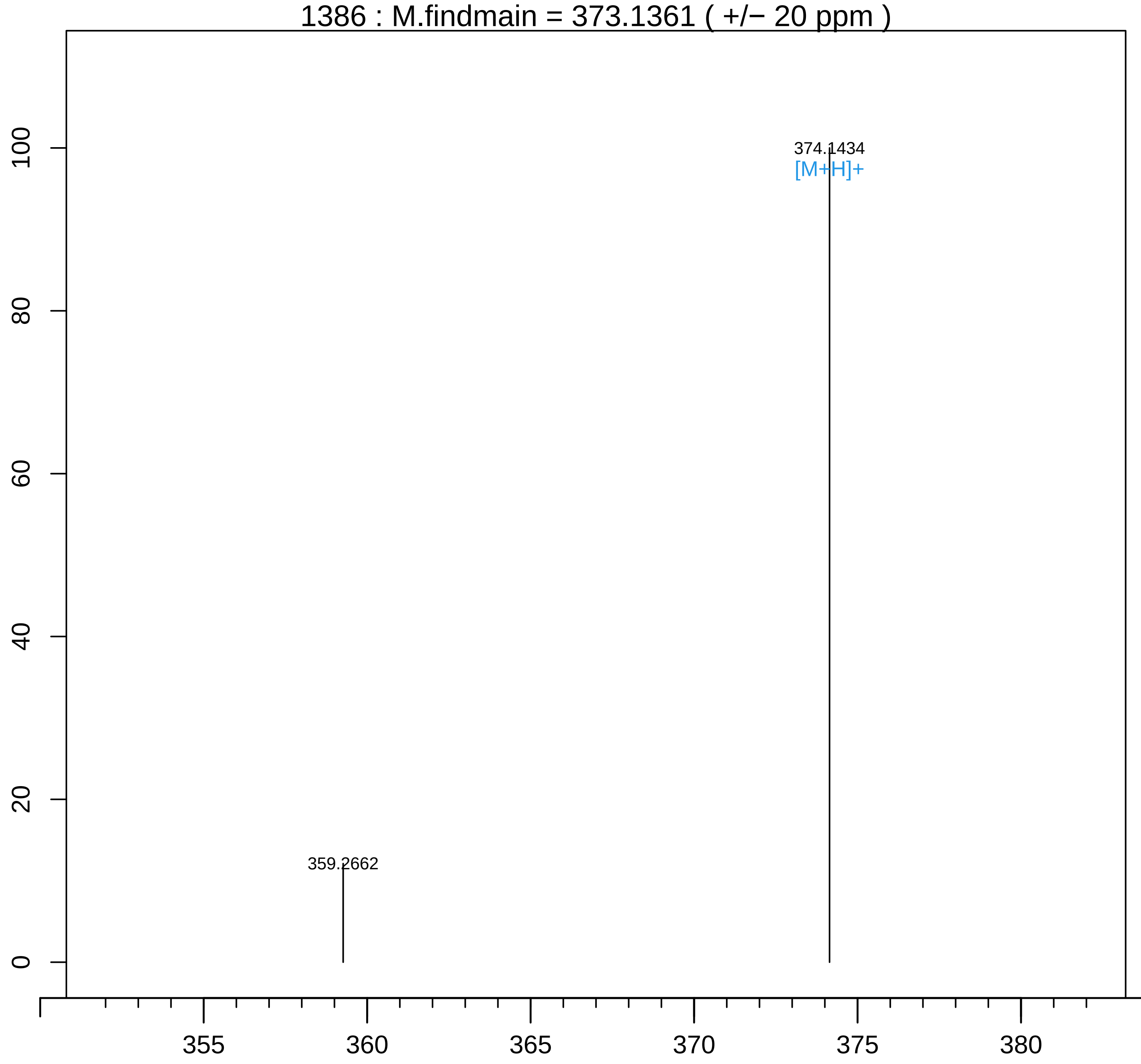
1386 : M.findmain = 373.1361 (+/− 20 ppm)
374.1434
[M+H]+
359.2662
0
20
40
60
80
100
355
360
365
370
375
380

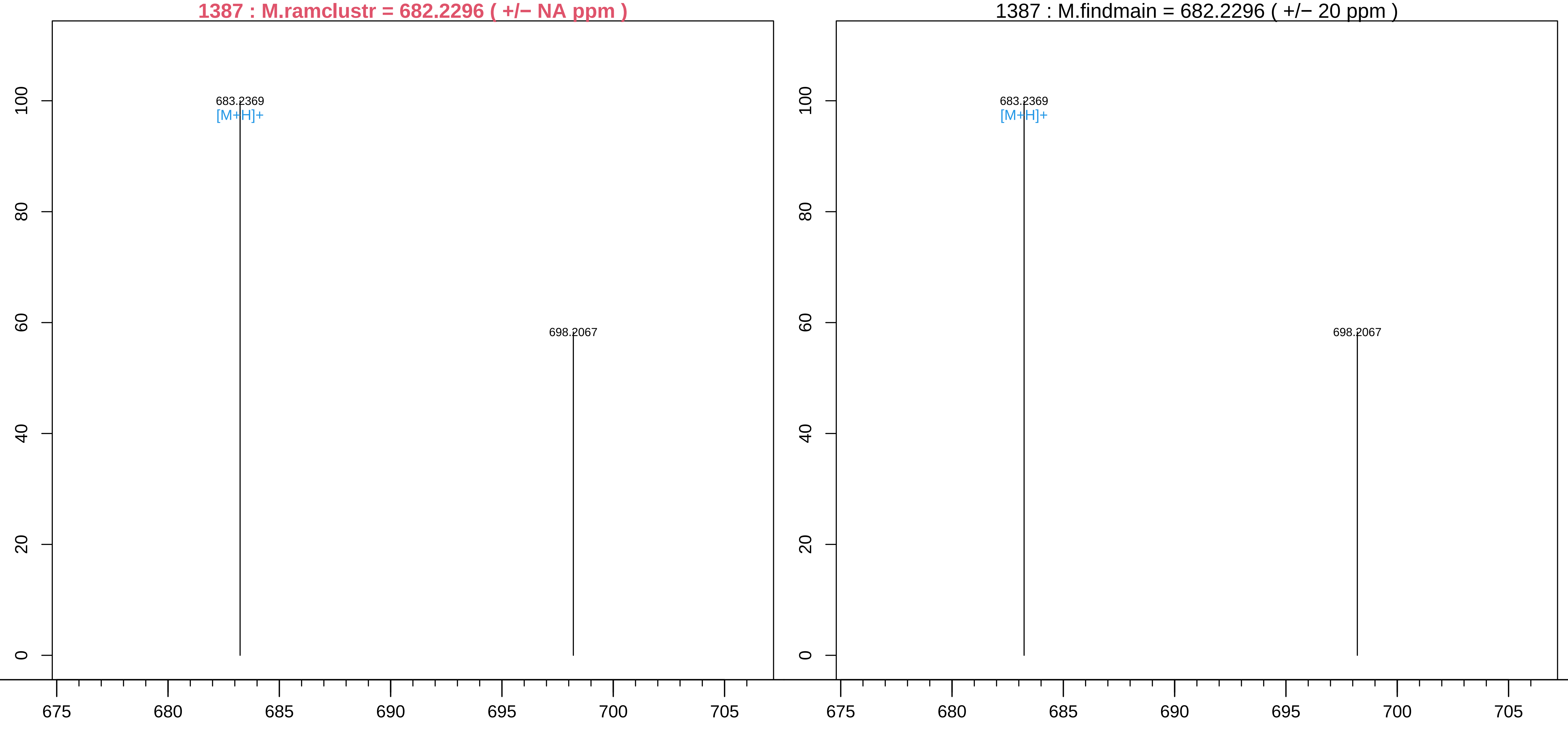
1387 : M.ramclustr = 682.2296 (+/− NA ppm)
683.2369
[M+H]+
698.2067
1387 : M.findmain = 682.2296 (+/− 20 ppm)
683.2369
[M+H]+
698.2067
0
20
40
60
80
100
675
680
685
690
695
700
705

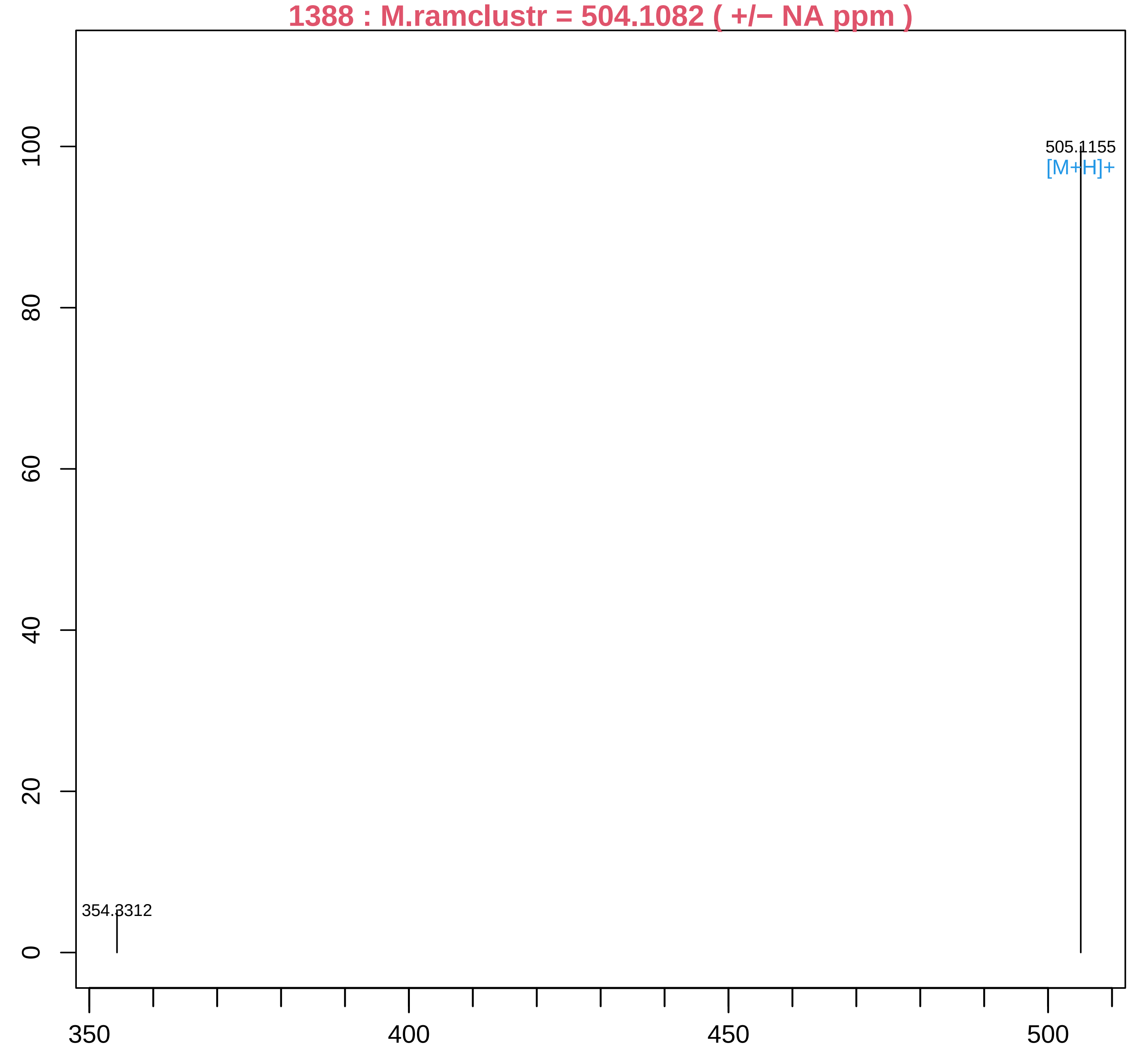
1388 : M.ramclustr = 504.1082 (+/− NA ppm)
505.1155
[M+H]+
354.3312
0
20
40
60
80
100
350
400
450
500

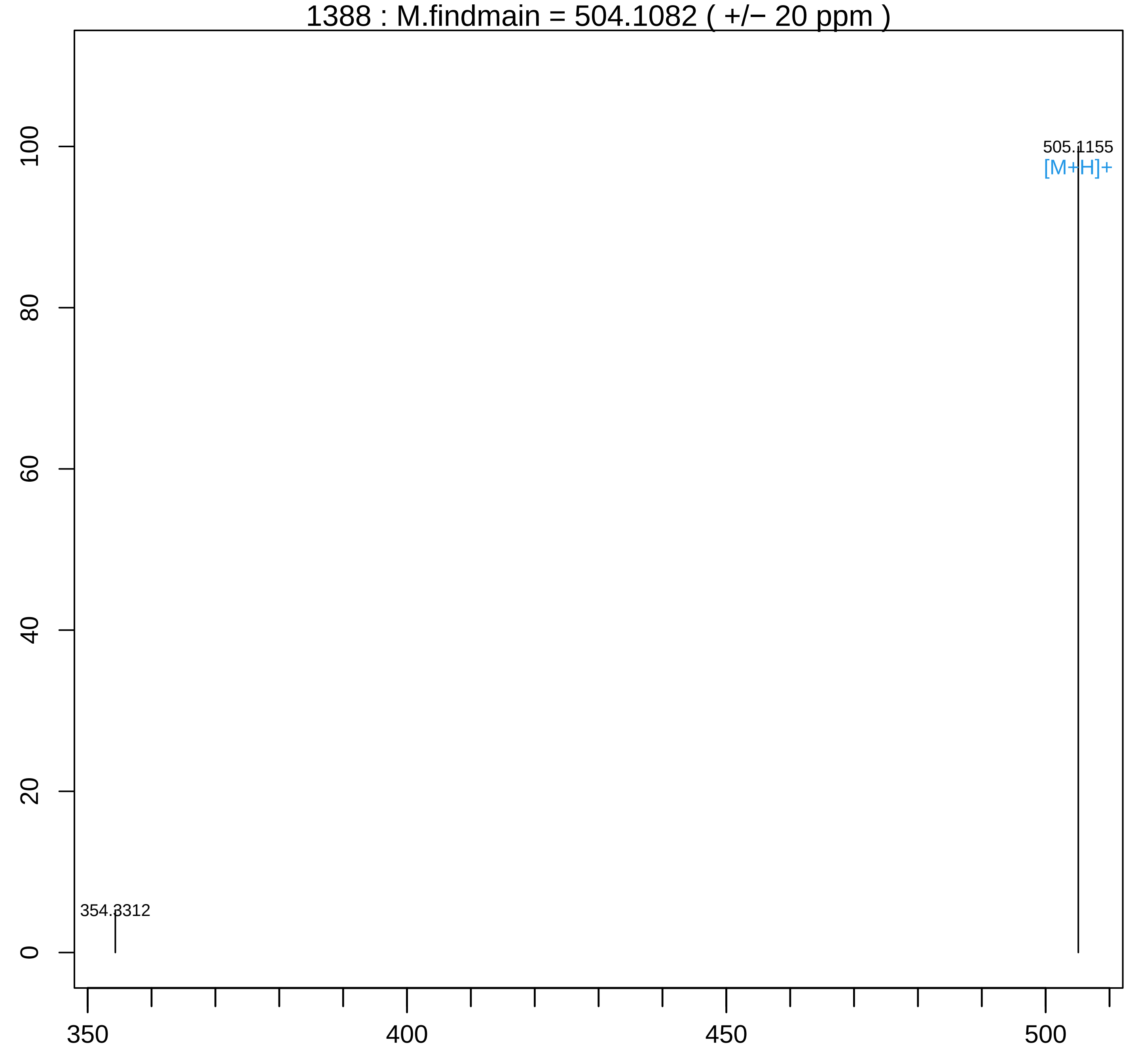
1388 : M.findmain = 504.1082 (+/− 20 ppm)
505.1155
[M+H]+
354.3312
0
20
40
60
80
100
350
400
450
500

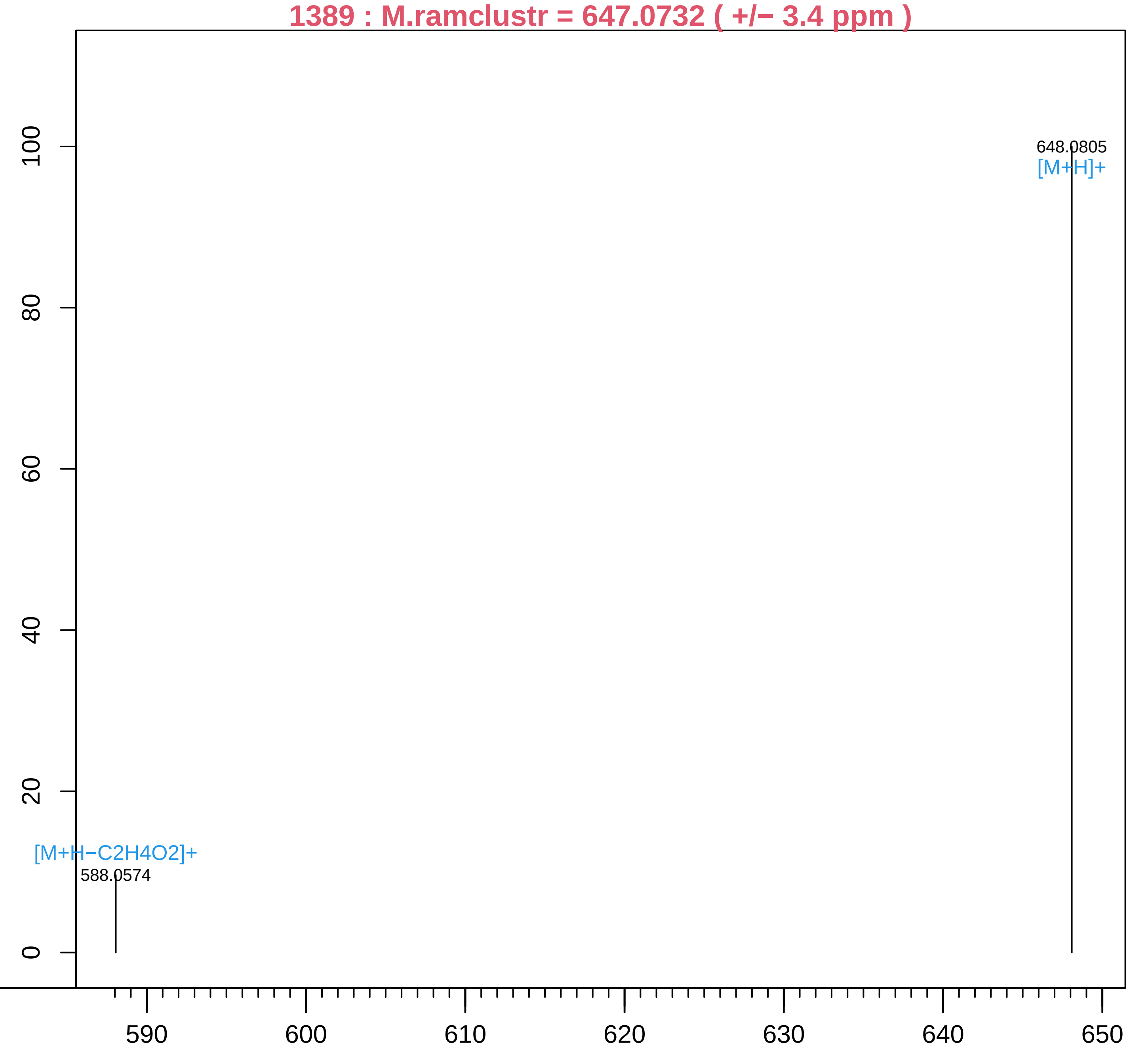
1389 : M.ramclustr = 647.0732 (+/− 3.4 ppm)
648.0805
[M+H]+
[M+H−C2H4O2]+
588.0574
0
20
40
60
80
100
590
600
610
620
630
640
650

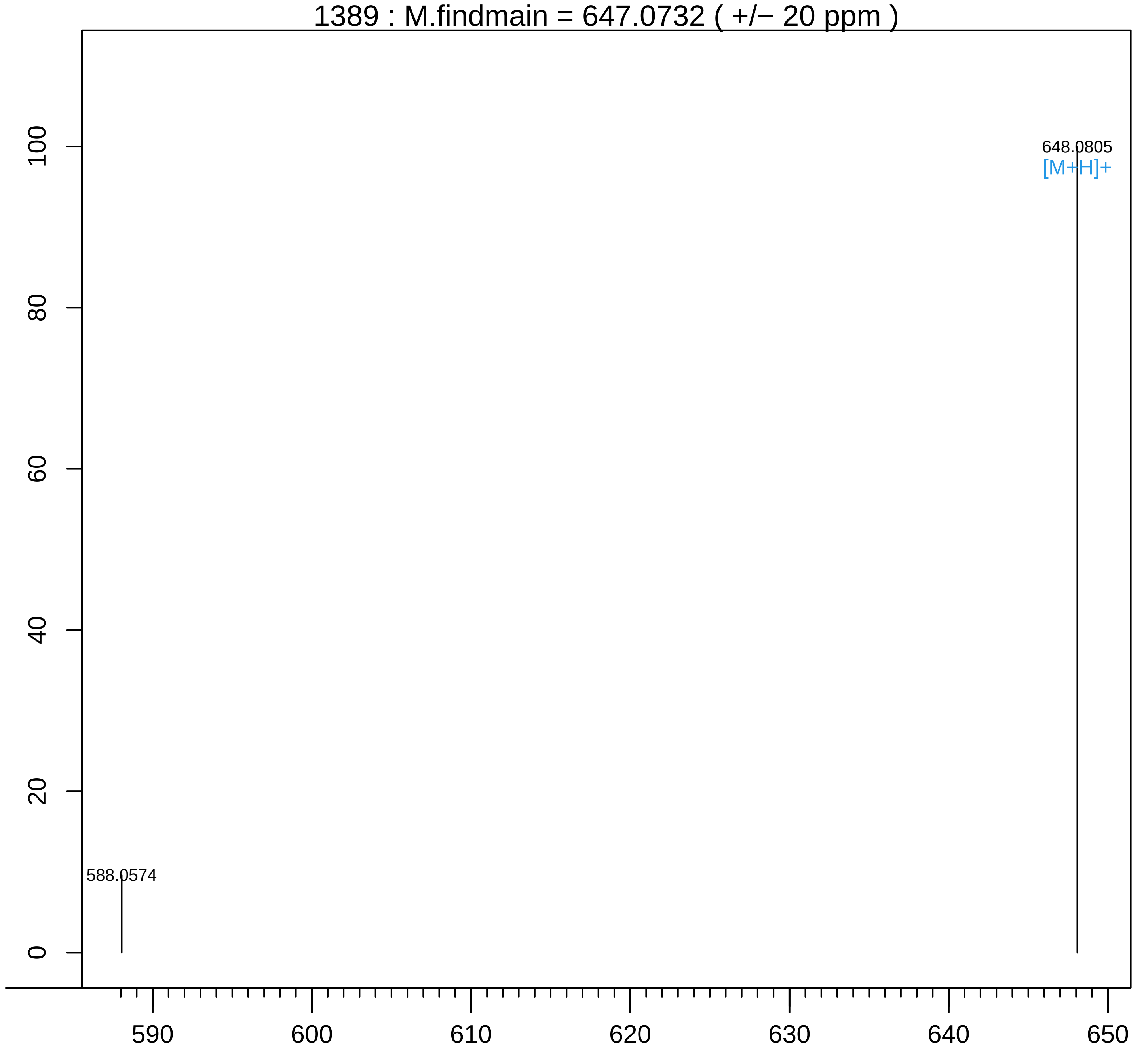
1389 : M.findmain = 647.0732 (+/− 20 ppm)
648.0805
[M+H]+
588.0574
0
20
40
60
80
100
590
600
610
620
630
640
650

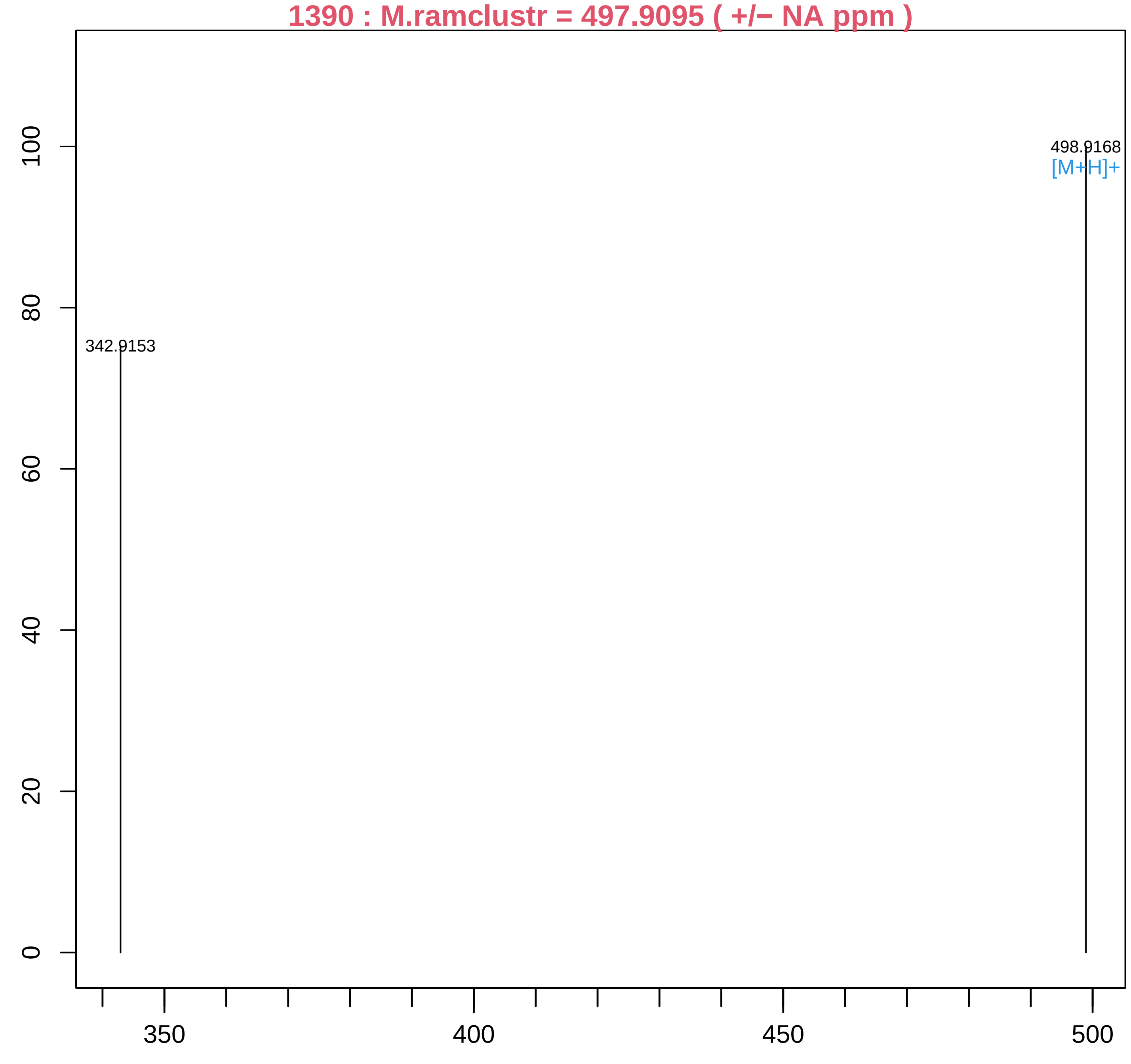

1390 : M.ramclustr = 497.9095 (+/− NA ppm)
342.9153
498.9168
[M+H]+

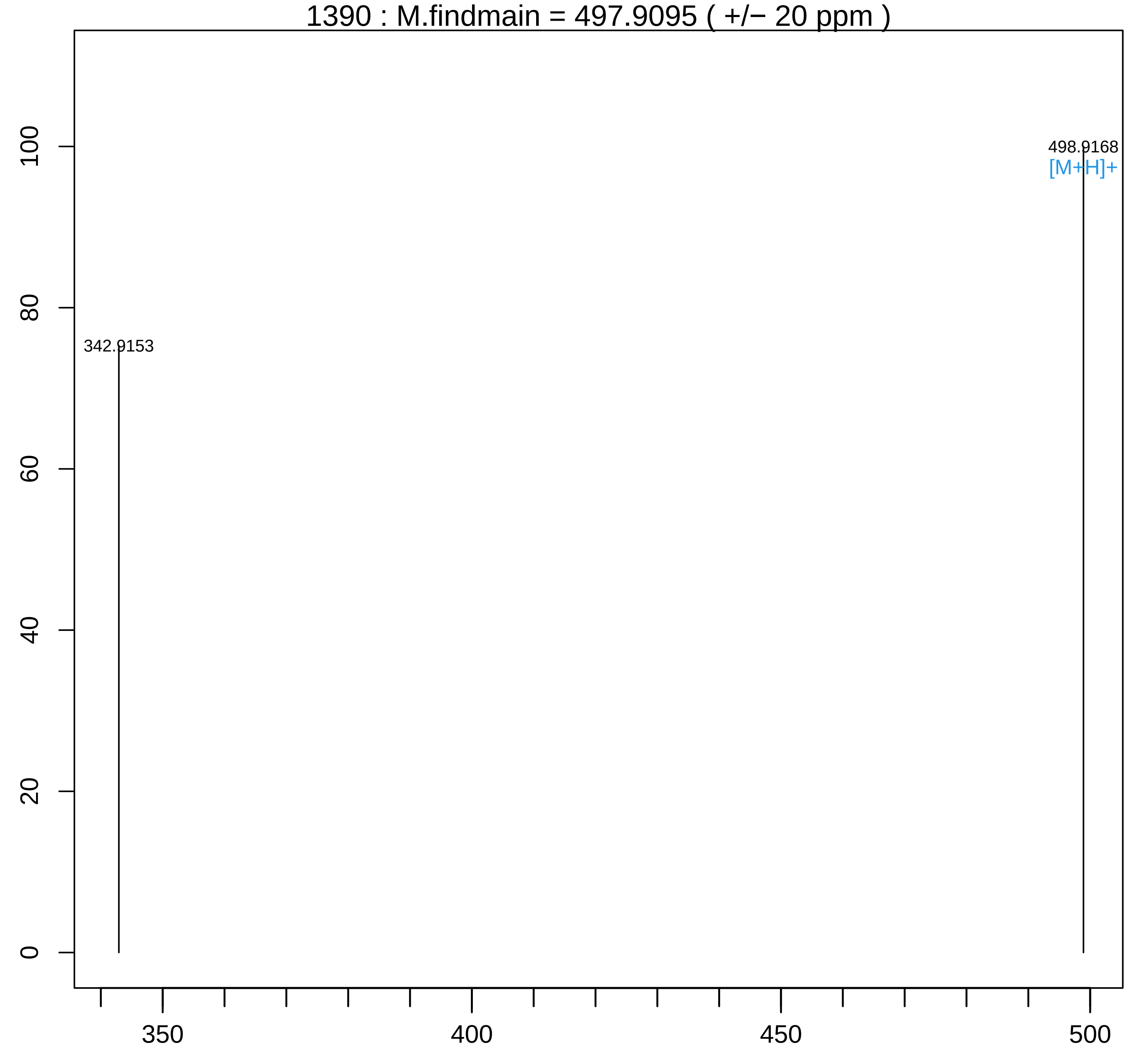

1390 : M.findmain = 497.9095 (+/− 20 ppm)
342.9153
498.9168
[M+H]+

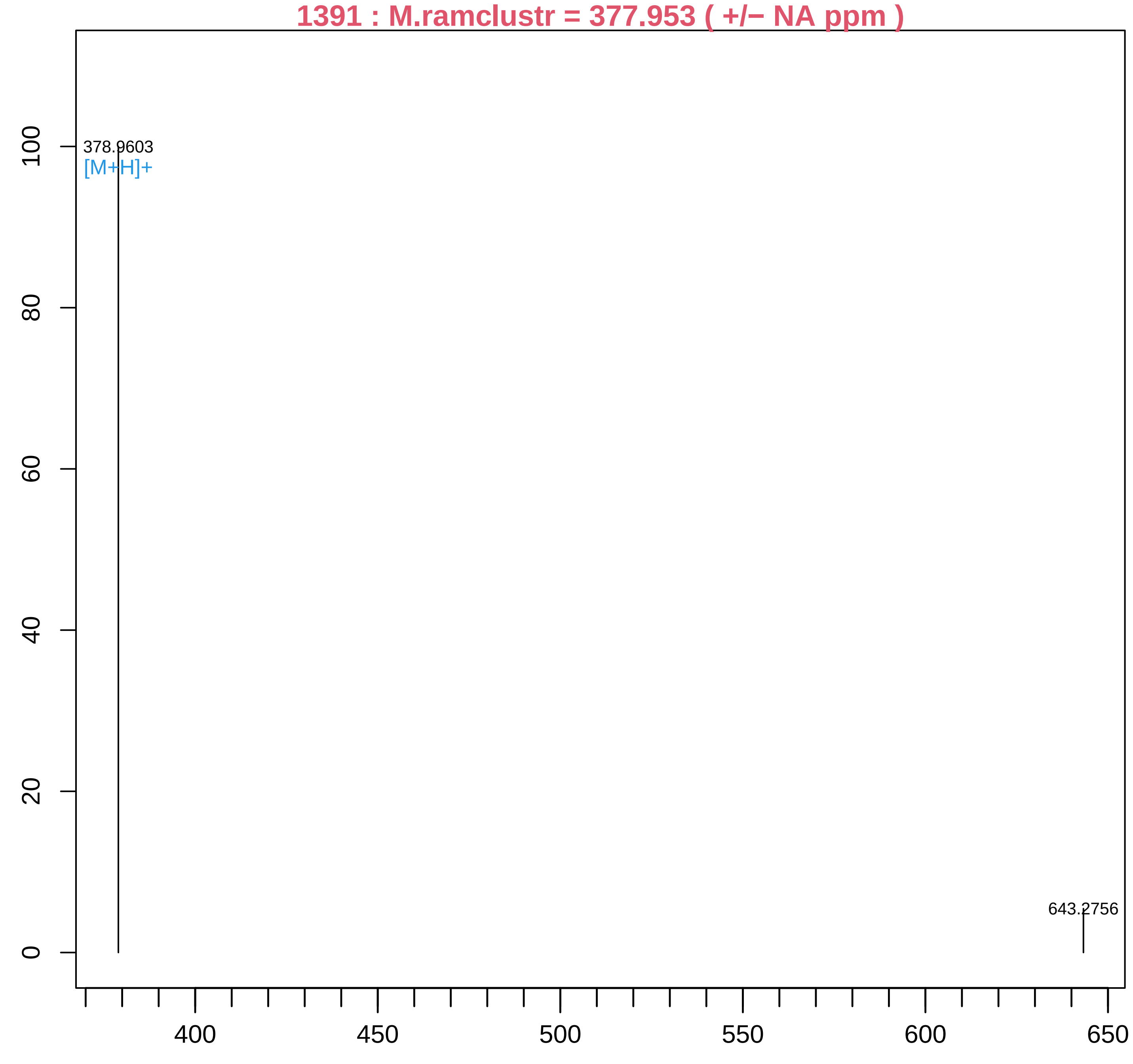
1391 : M.ramclustr = 377.953 (+/− NA ppm)
378.9603
[M+H]+
643.2756
0
20
40
60
80
100
400
450
500
550
600
650

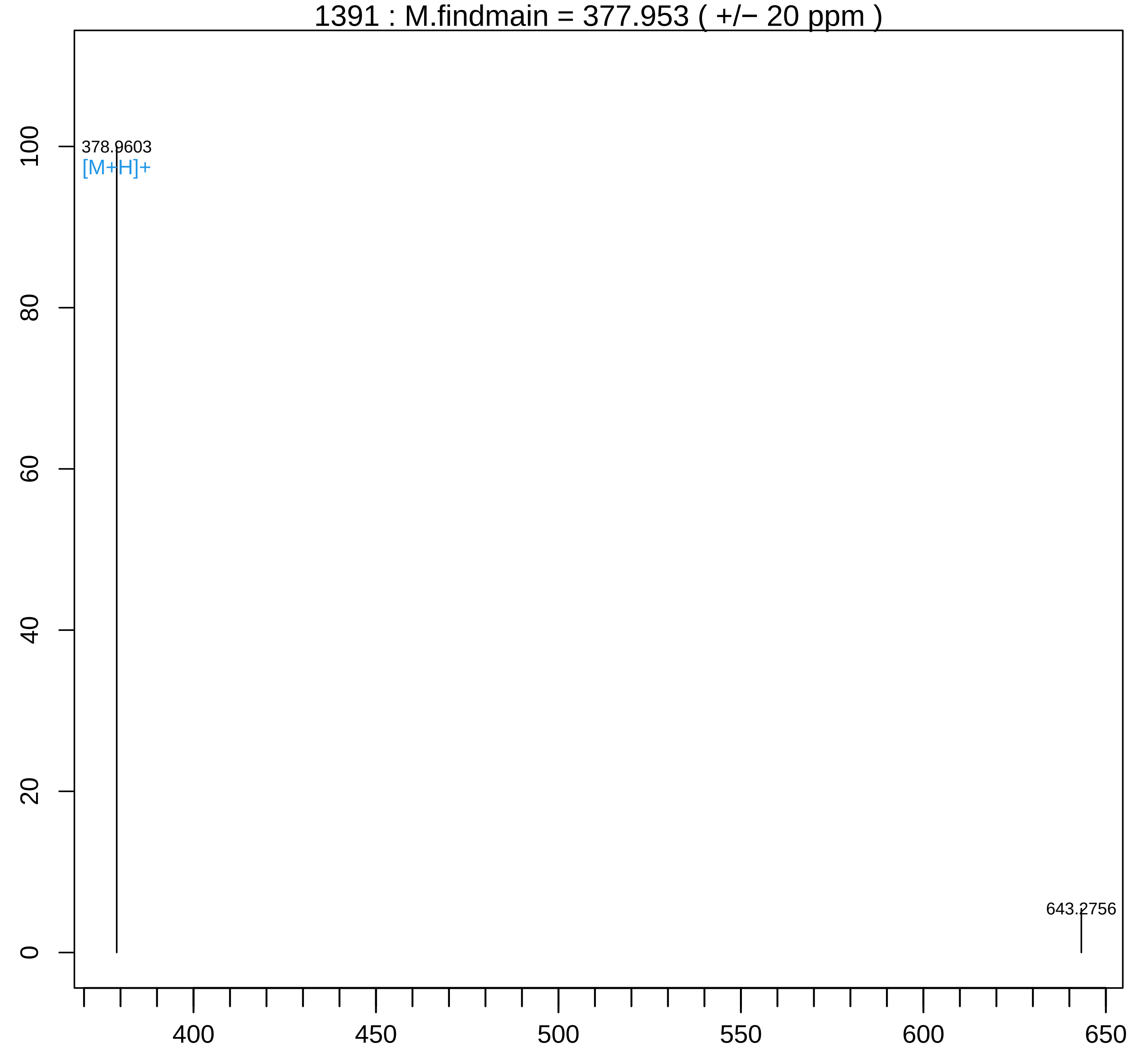
1391 : M.findmain = 377.953 (+/− 20 ppm)
378.9603
[M+H]+
643.2756
0
20
40
60
80
100
400
450
500
550
600
650

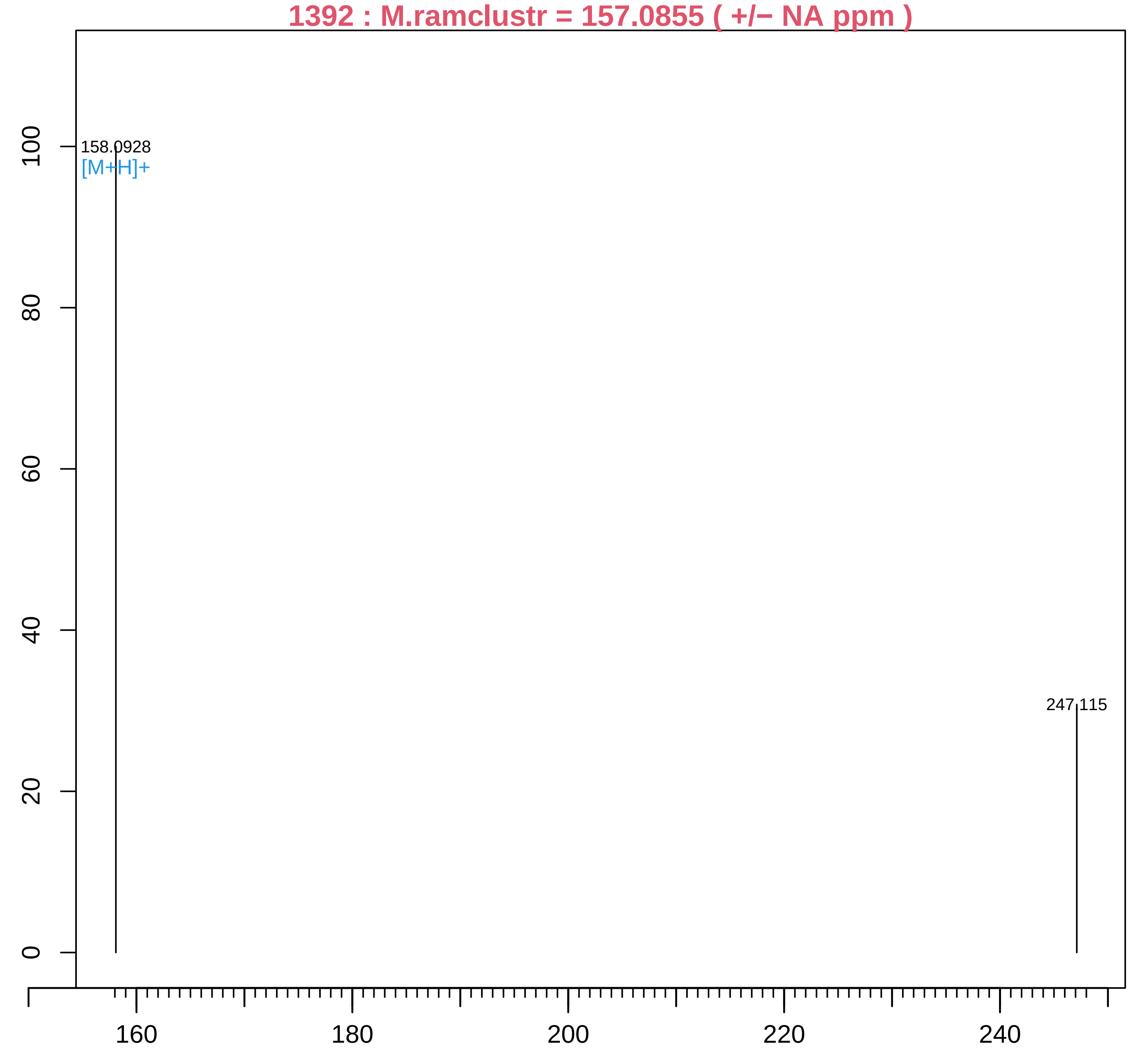
1392 : M.ramclustr = 157.0855 (+/− NA ppm)
158.0928
[M+H]+
247.115
0
20
40
60
80
100
160
180
200
220
240

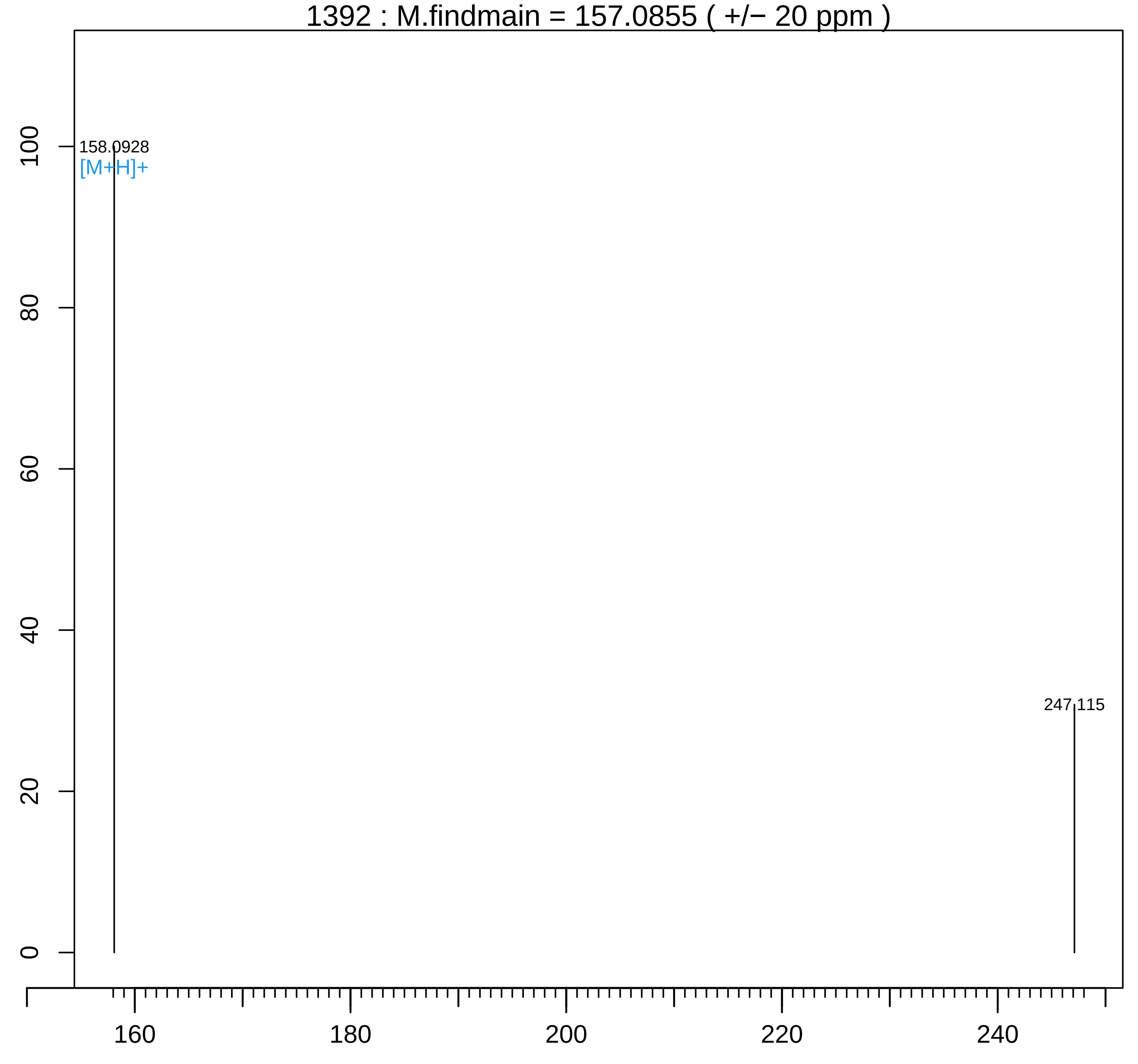
1392 : M.findmain = 157.0855 (+/− 20 ppm)
158.0928
[M+H]+
247.115
0
20
40
60
80
100
160
180
200
220
240

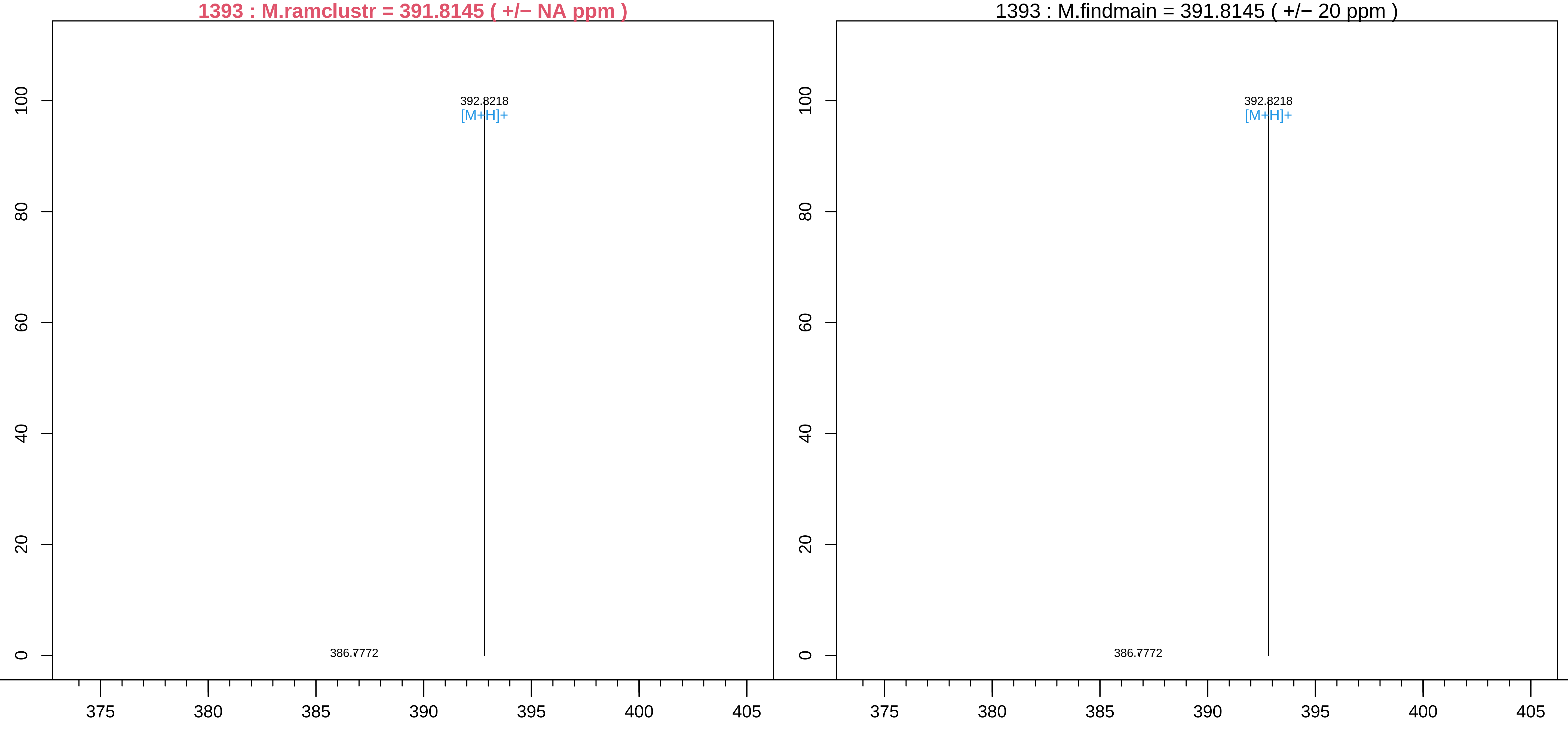
1393 : M.ramclustr = 391.8145 (+/− NA ppm)
392.8218
[M+H]+
386.7772
1393 : M.findmain = 391.8145 (+/− 20 ppm)
392.8218
[M+H]+
386.7772
0
20
40
60
80
100
375
380
385
390
395
400
405

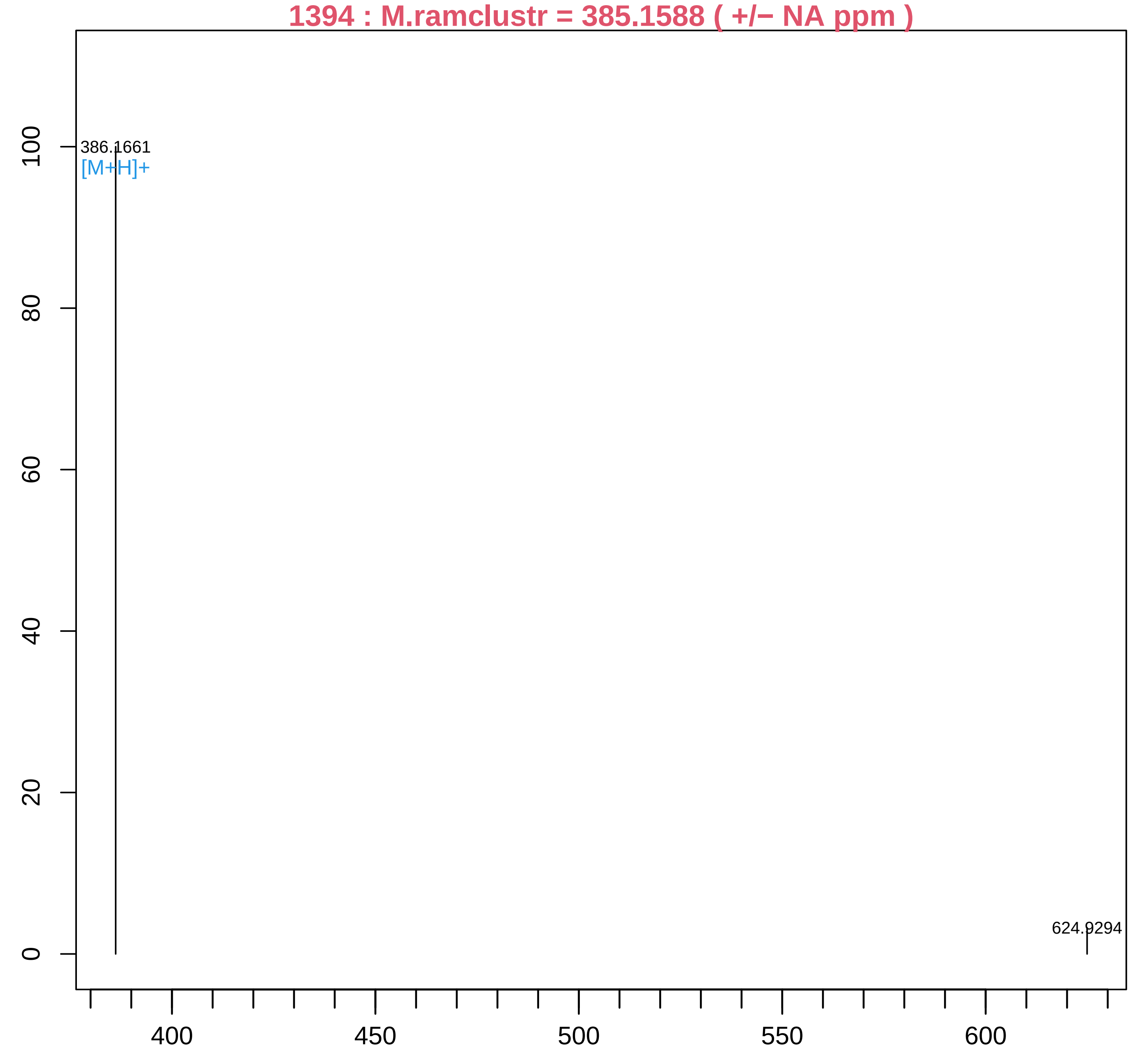
1394 : M.ramclustr = 385.1588 (+/− NA ppm)
386.1661
[M+H]+
624.9294
0
20
40
60
80
100
400
450
500
550
600

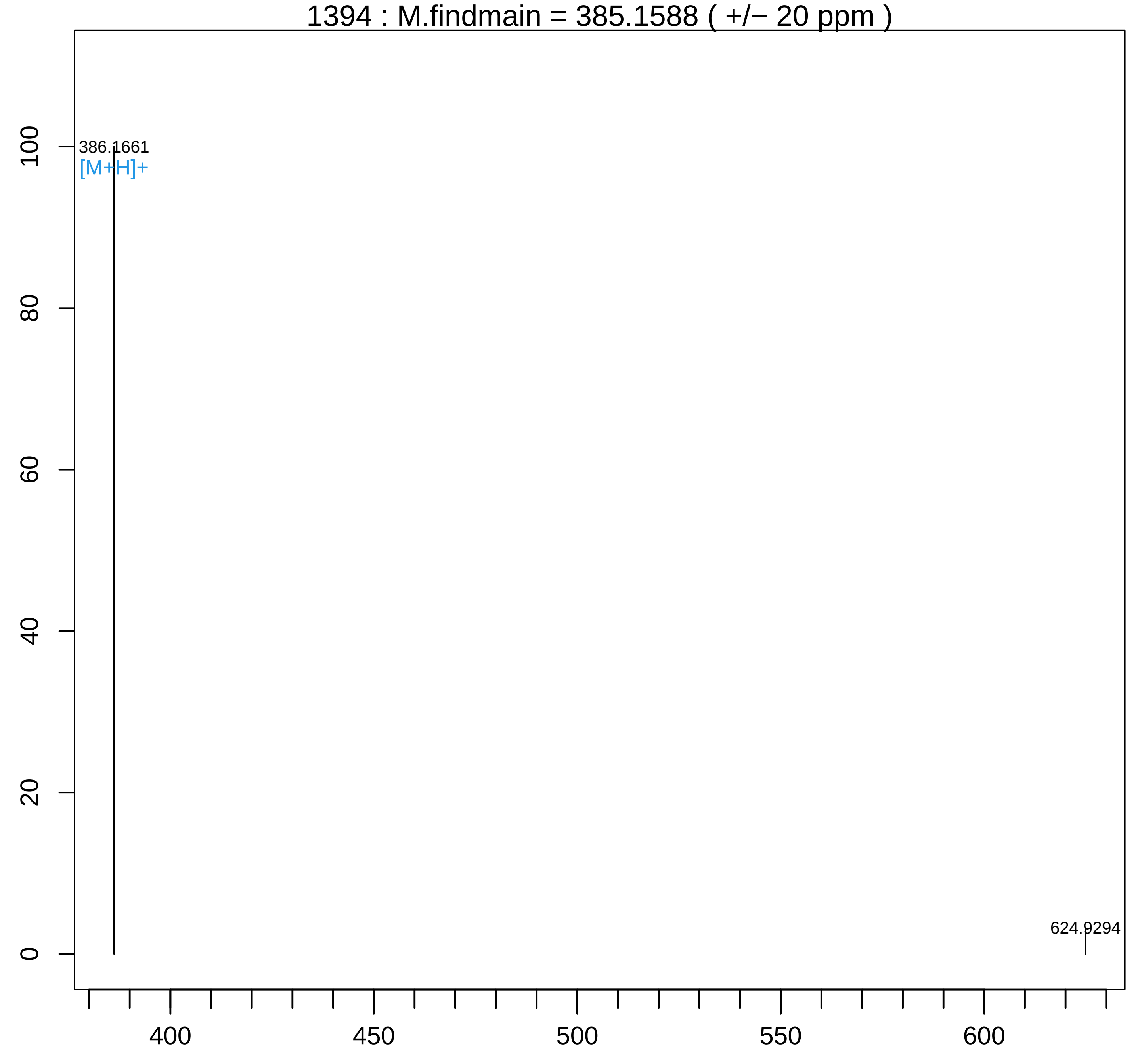
1394 : M.findmain = 385.1588 (+/− 20 ppm)
386.1661
[M+H]+
624.9294
0
20
40
60
80
100
400
450
500
550
600

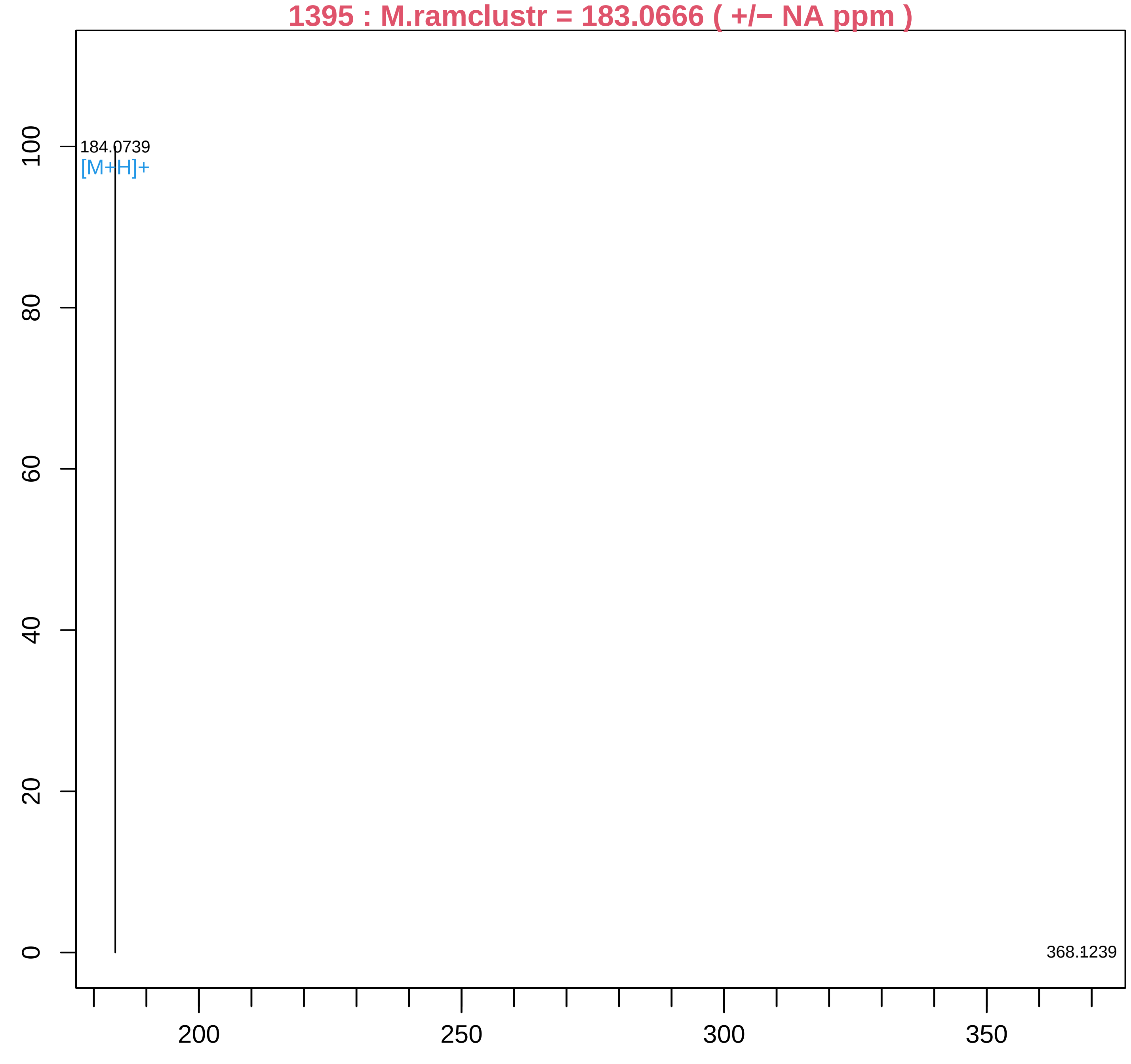
1395 : M.ramclustr = 183.0666 (+/− NA ppm)
184.0739
[M+H]+
368.1239
0
20
40
60
80
100
200
250
300
350

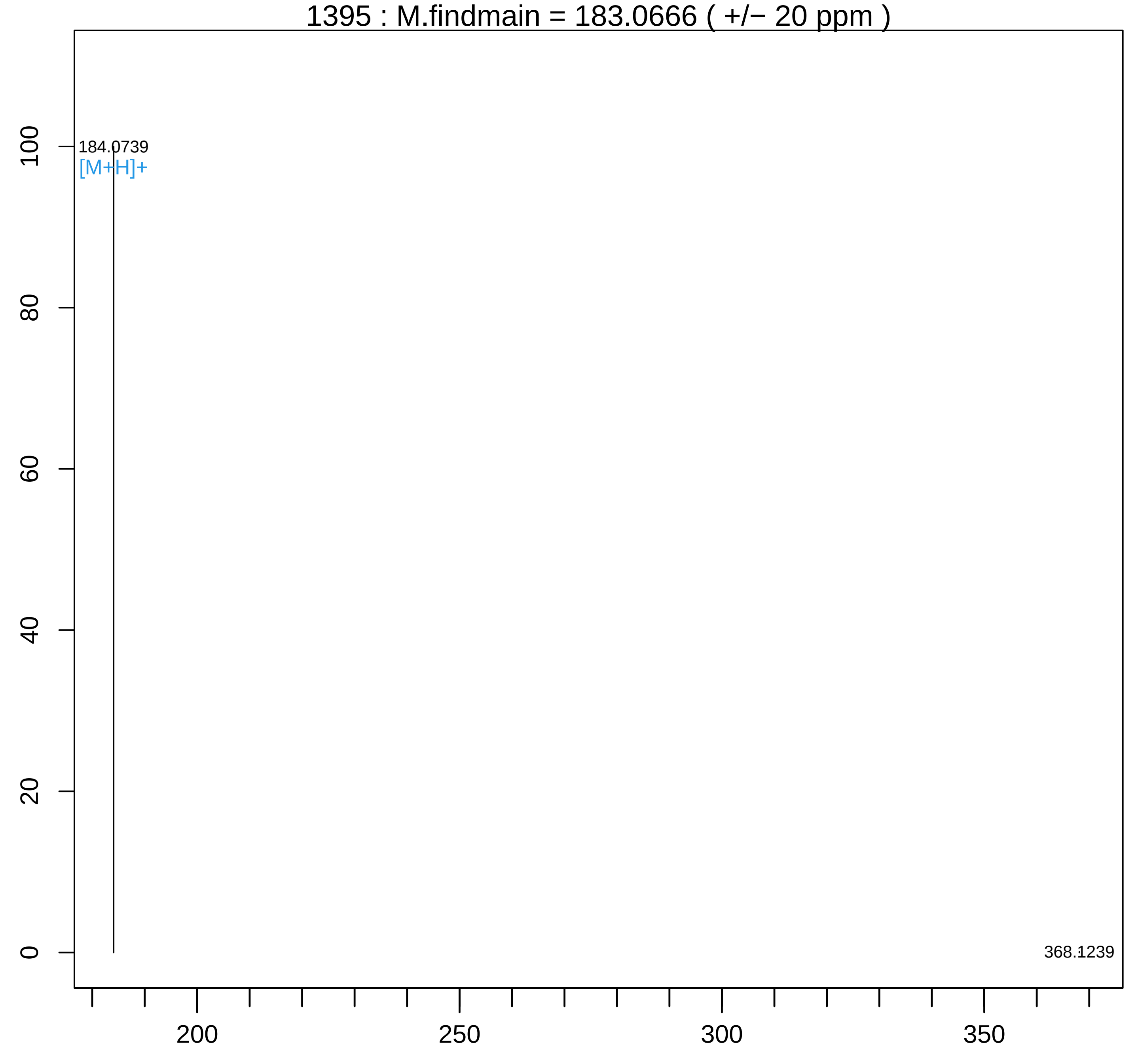
1395 : M.findmain = 183.0666 (+/− 20 ppm)
184.0739
[M+H]+
368.1239
0
20
40
60
80
100
200
250
300
350

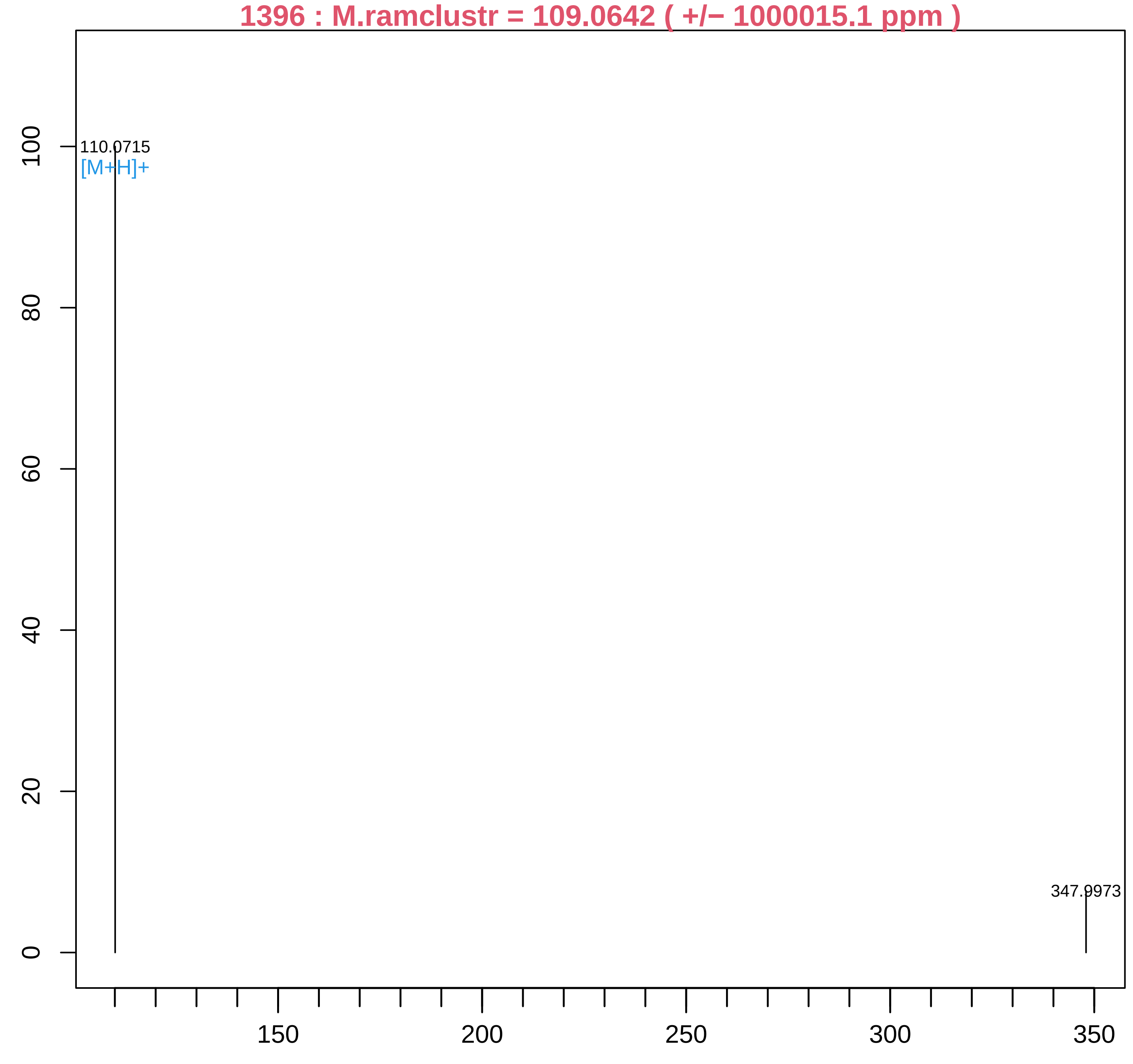
1396 : M.ramclustr = 109.0642 (+/− 1000015.1 ppm)
110.0715
[M+H]+
347.9973
0
20
40
60
80
100
150
200
250
300
350

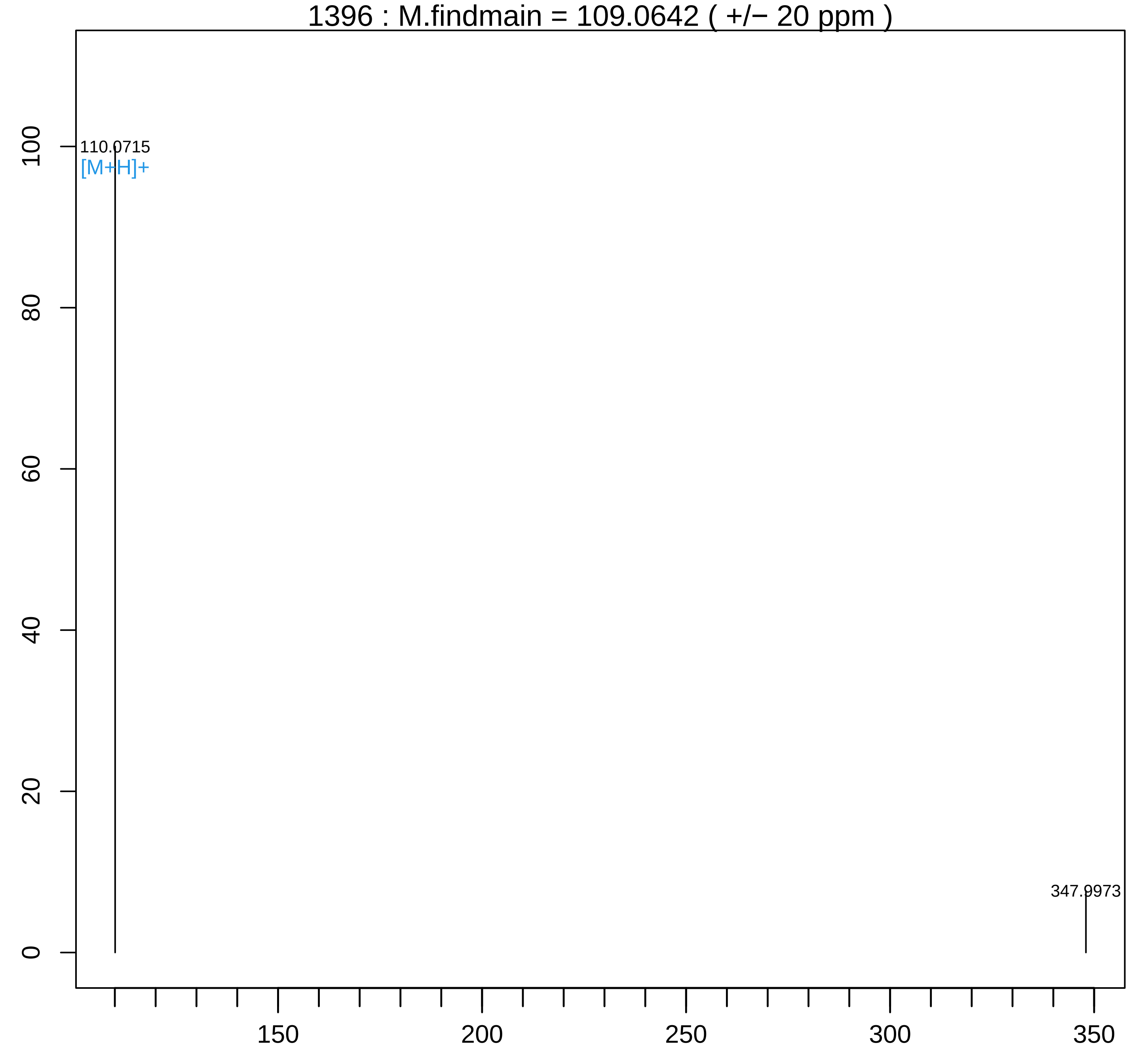
1396 : M.findmain = 109.0642 (+/− 20 ppm)
110.0715
[M+H]+
347.9973
0
20
40
60
80
100
150
200
250
300
350

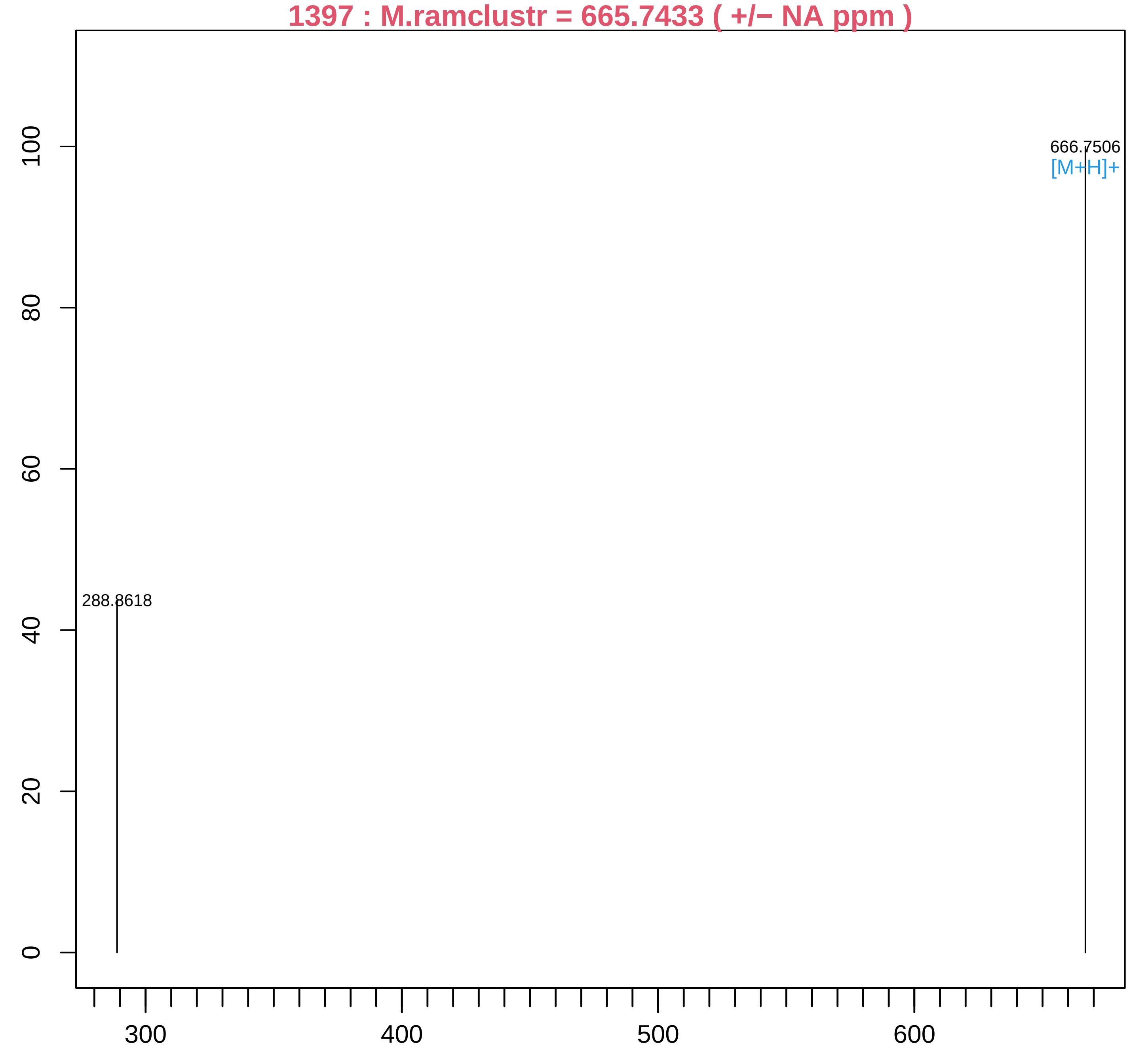

1397 : M.ramclustr = 665.7433 (+/− NA ppm)
666.7506
[M+H]+
288.8618
0
20
40
60
80
100
300
400
500
600

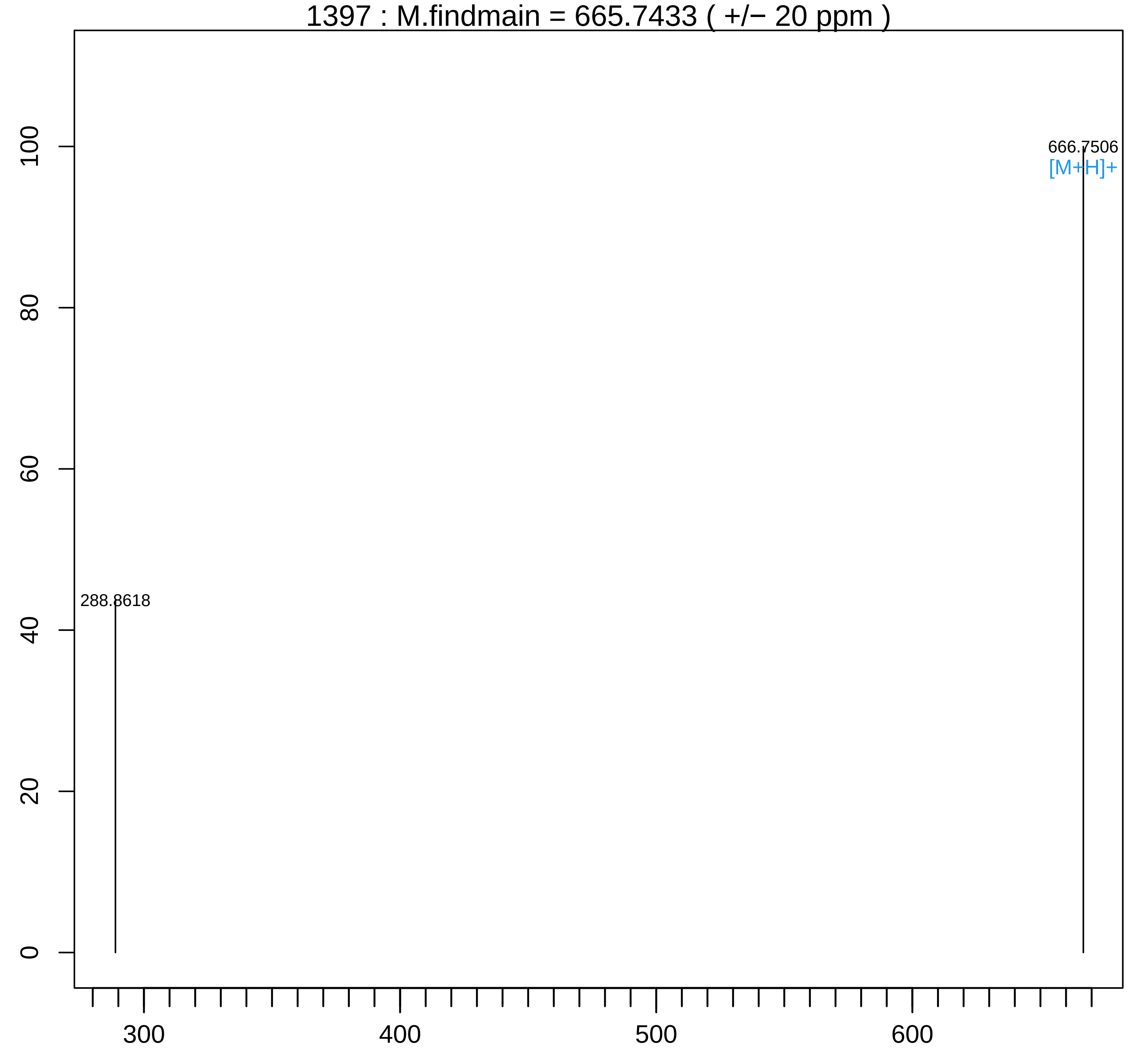

1397 : M.findmain = 665.7433 (+/− 20 ppm)
666.7506
[M+H]+
288.8618
0
20
40
60
80
100
300
400
500
600

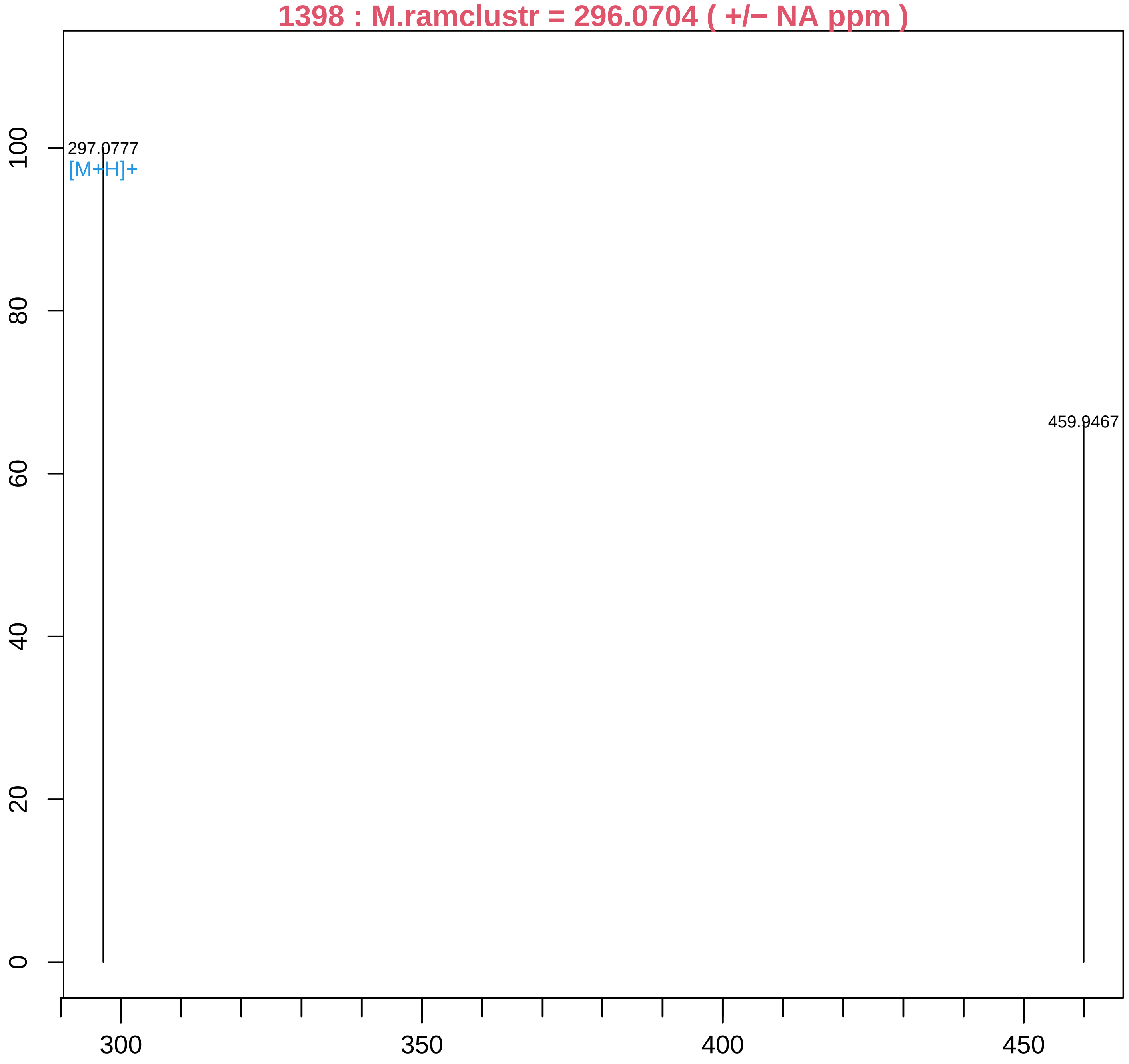
1398 : M.ramclustr = 296.0704 (+/− NA ppm)
297.0777
[M+H]+
459.9467
0
20
40
60
80
100
300
350
400
450

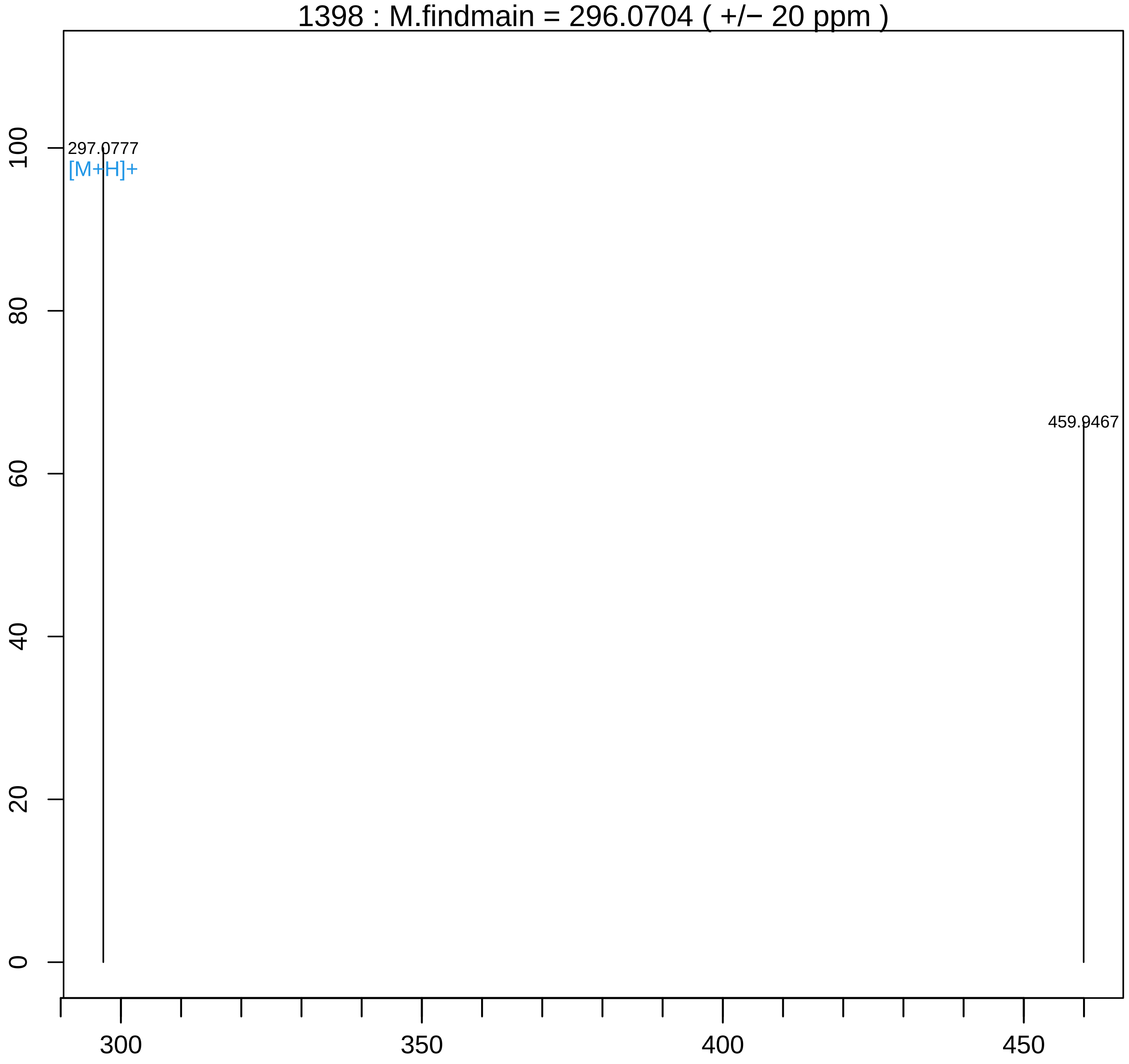
1398 : M.findmain = 296.0704 (+/− 20 ppm)
297.0777
[M+H]+
459.9467
0
20
40
60
80
100
300
350
400
450

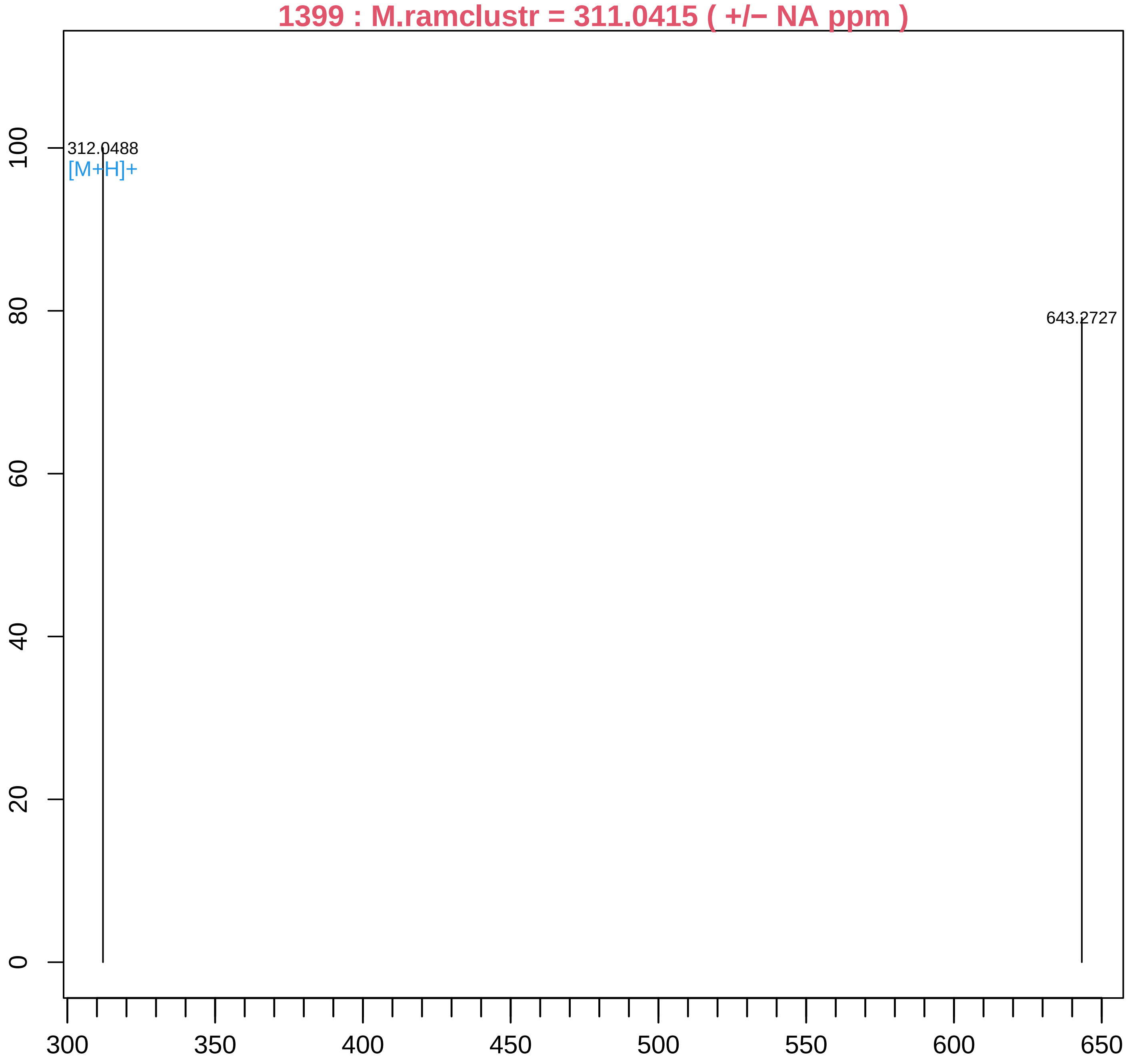

1399 : M.ramclustr = 311.0415 (+/− NA ppm)
312.0488
[M+H]+
643.2727
0
20
40
60
80
100
300
350
400
450
500
550
600
650

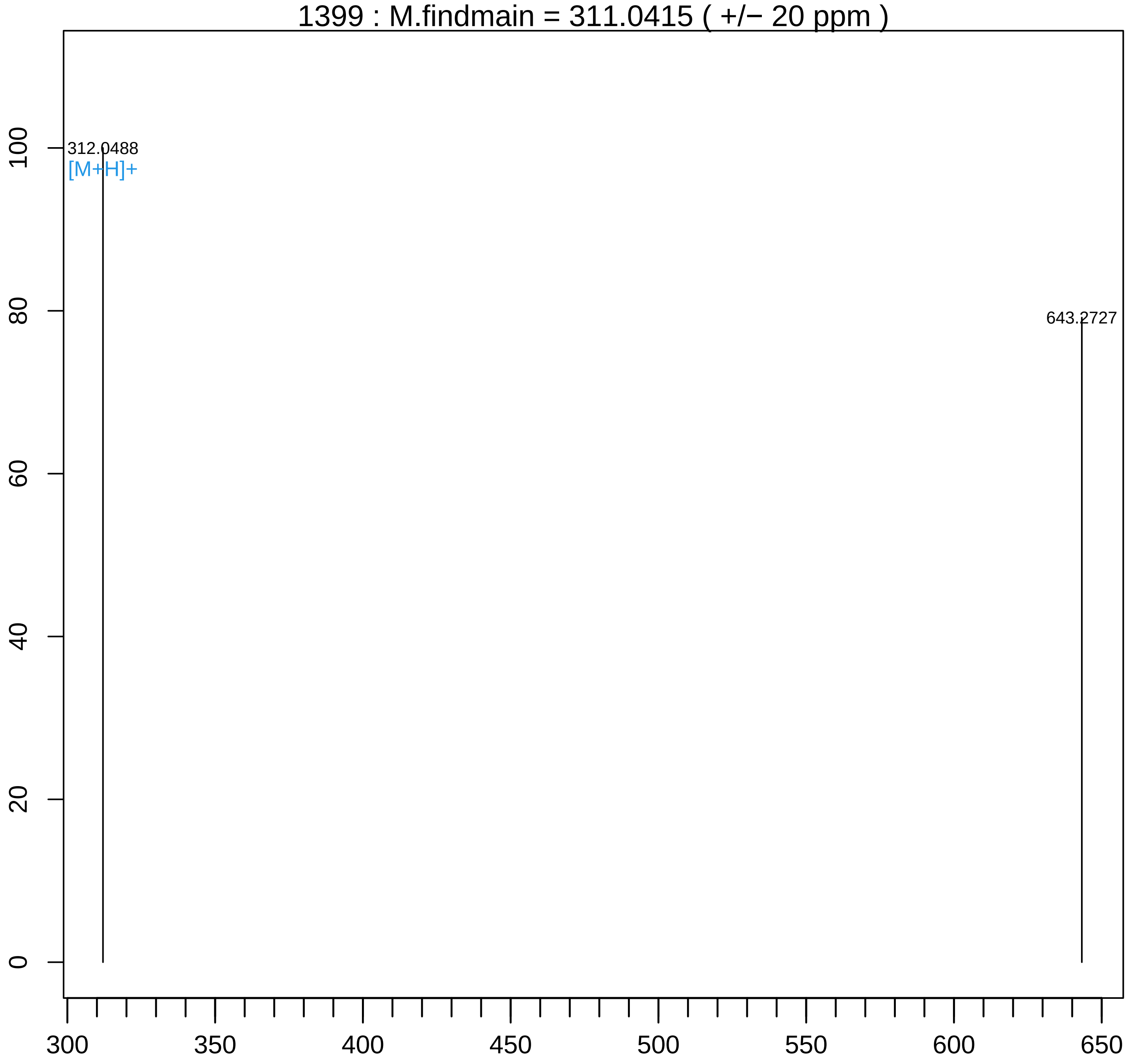

1399 : M.findmain = 311.0415 (+/− 20 ppm)
312.0488
[M+H]+
643.2727
0
20
40
60
80
100
300
350
400
450
500
550
600
650

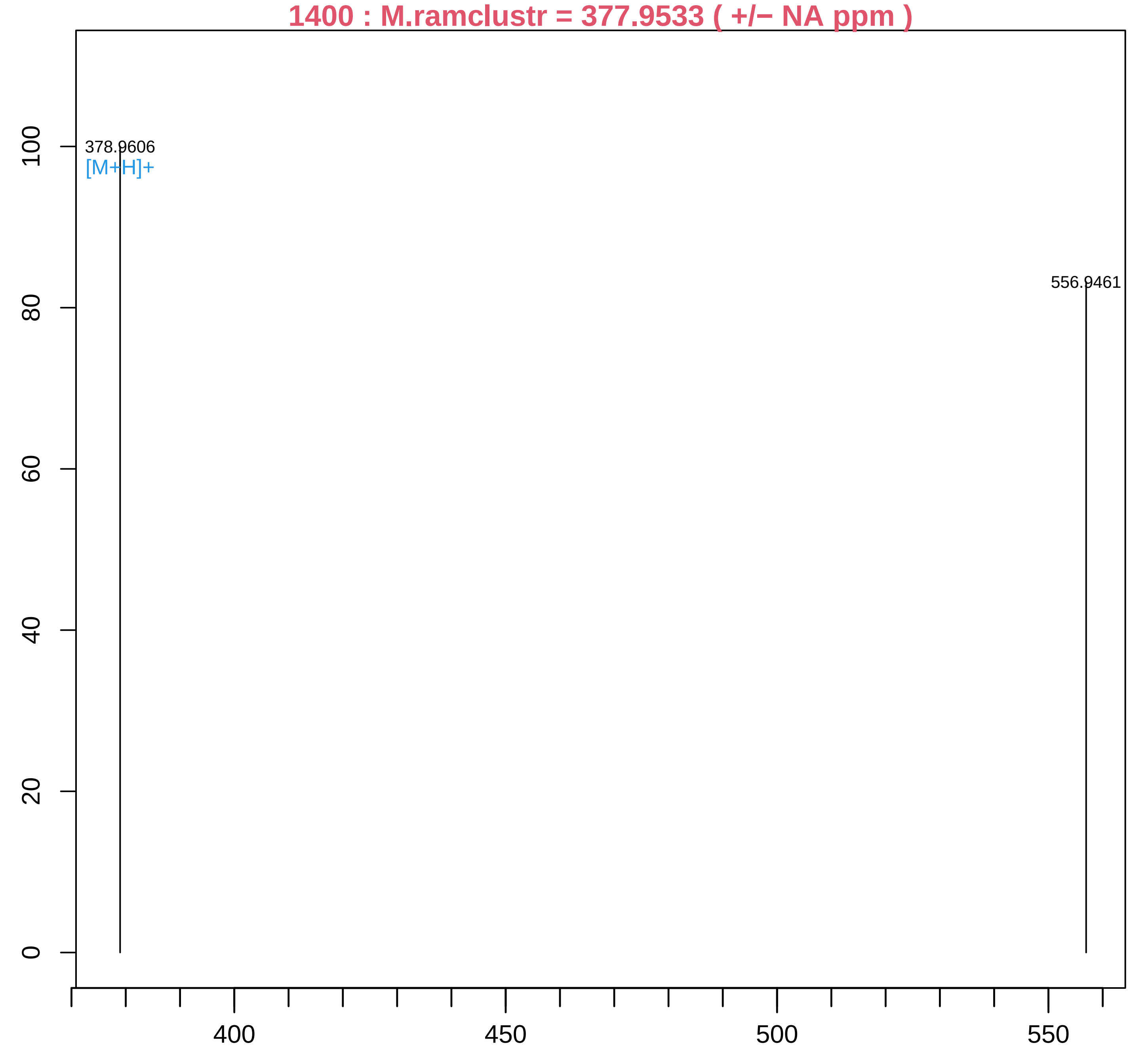
1400 : M.ramclustr = 377.9533 (+/− NA ppm)
378.9606
[M+H]+
556.9461
0
20
40
60
80
100
400
450
500
550

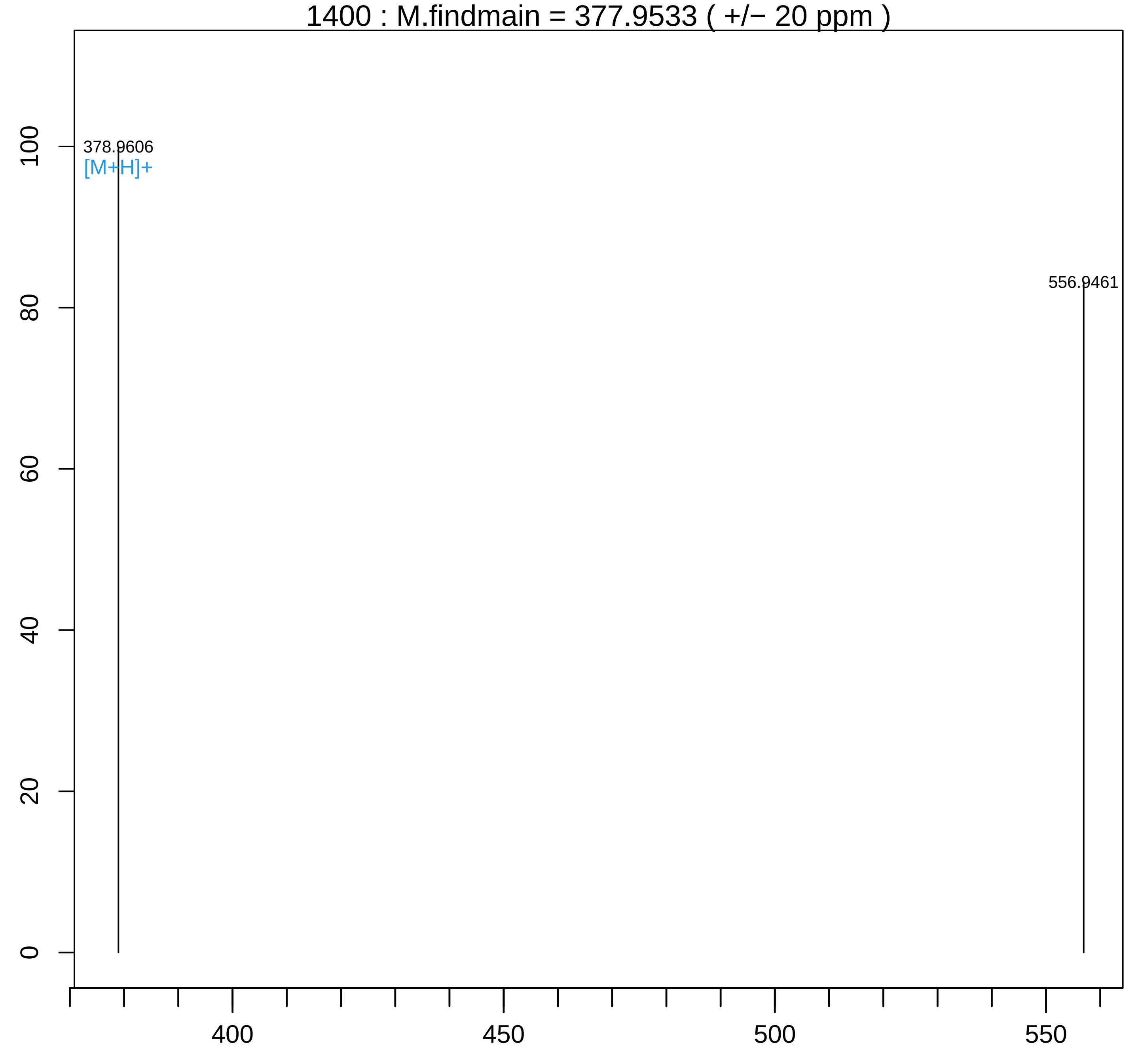
1400 : M.findmain = 377.9533 (+/− 20 ppm)
378.9606
[M+H]+
556.9461
0
20
40
60
80
100
400
450
500
550

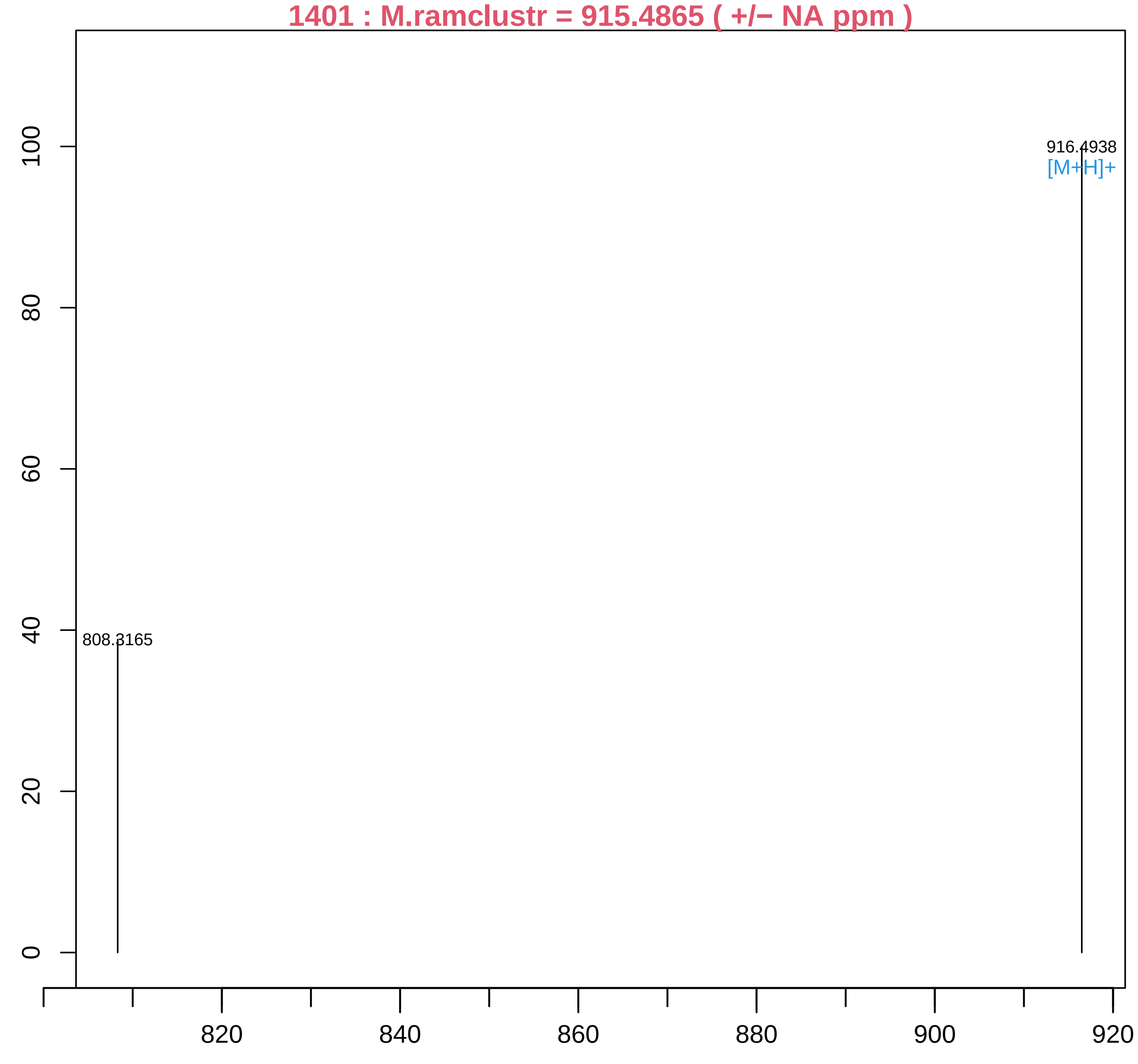
1401 : M.ramclustr = 915.4865 (+/− NA ppm)
916.4938
[M+H]+
808.3165
0
20
40
60
80
100
820
840
860
880
900
920

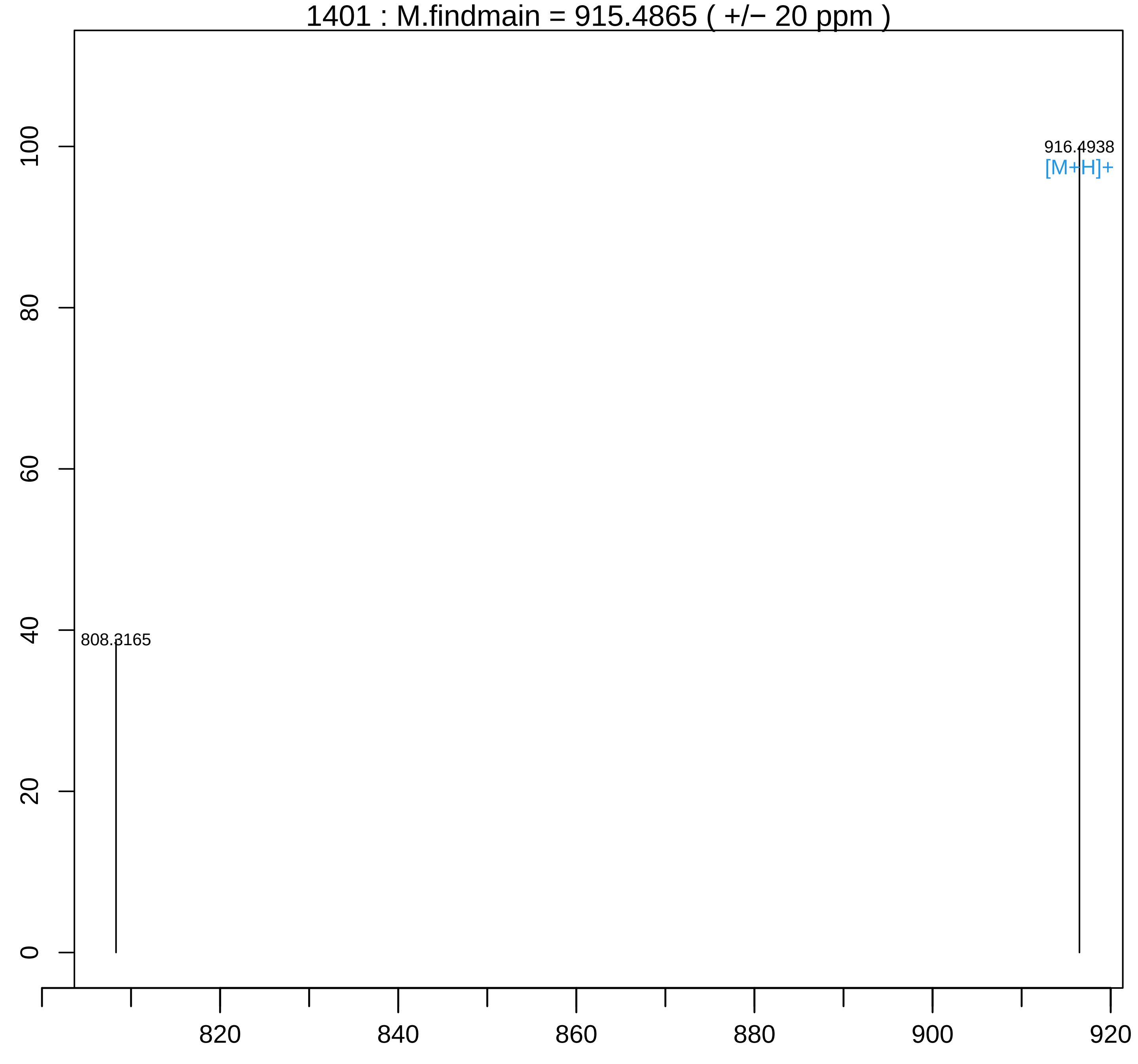
1401 : M.findmain = 915.4865 (+/− 20 ppm)
916.4938
[M+H]+
808.3165
0
20
40
60
80
100
820
840
860
880
900
920

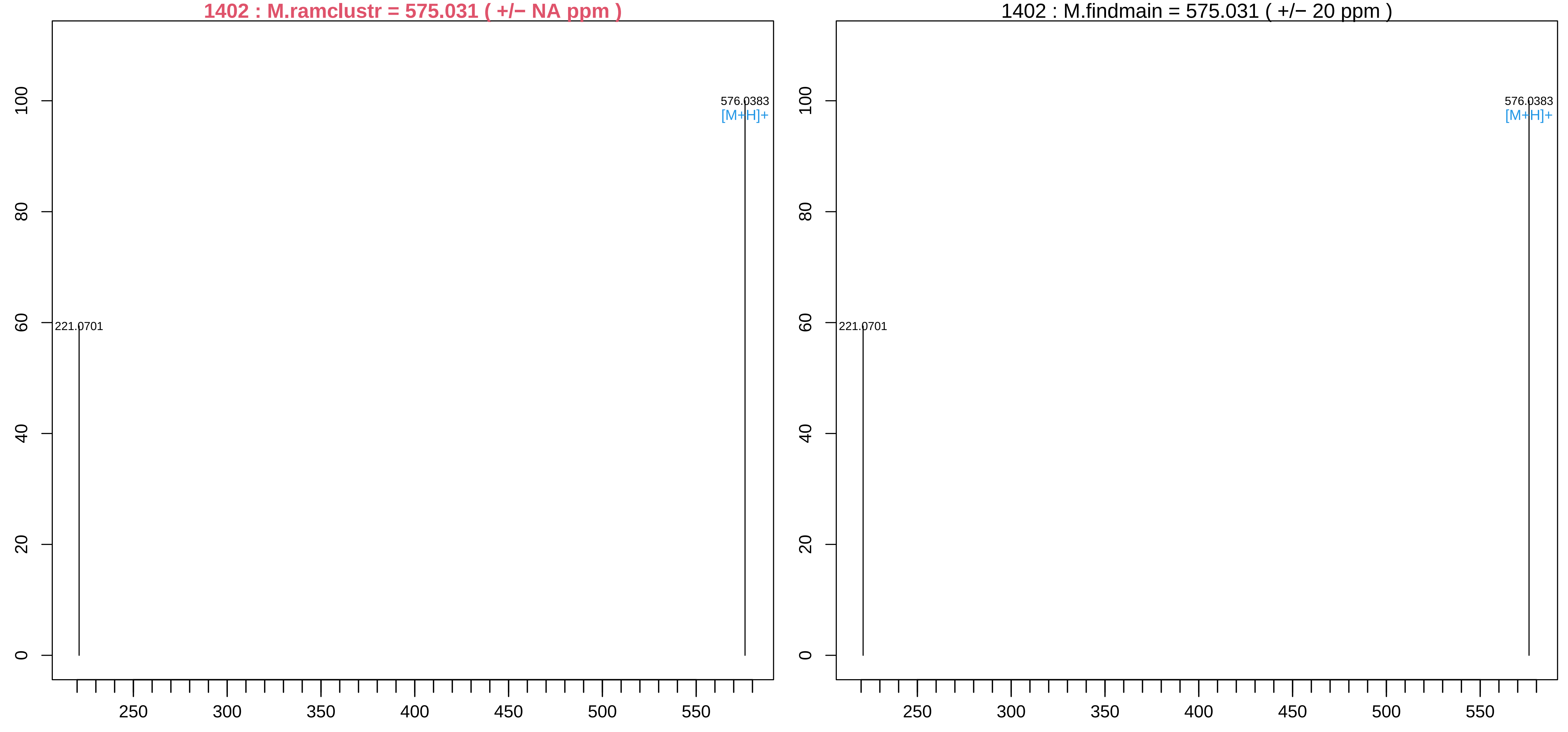

1402 : M.ramclustr = 575.031 (+/− NA ppm)
576.0383
[M+H]+
221.0701
0
20
40
60
80
100
250
300
350
400
450
500
550
1402 : M.findmain = 575.031 (+/− 20 ppm)
576.0383
[M+H]+
221.0701
0
20
40
60
80
100
250
300
350
400
450
500
550

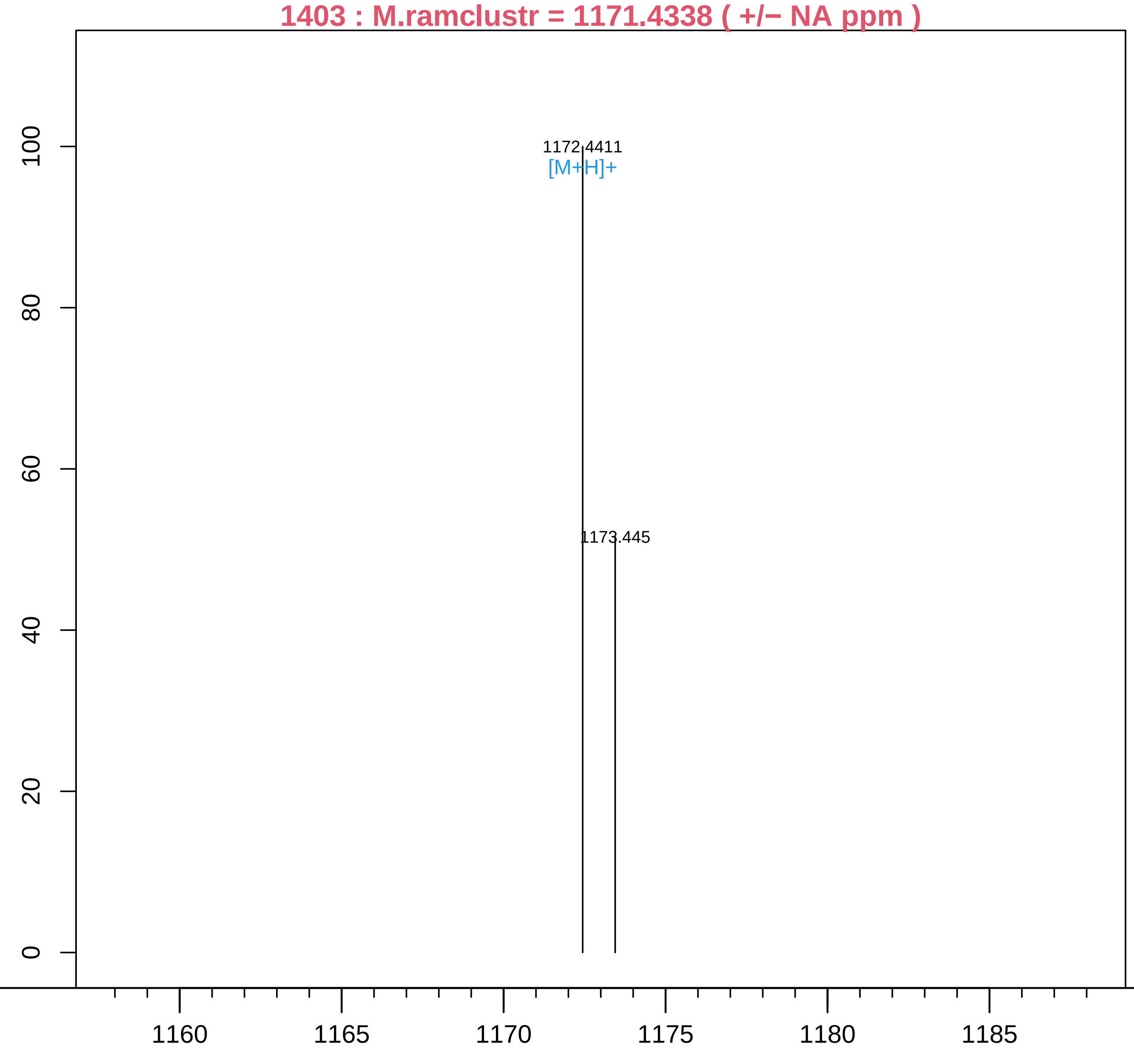
1403 : M.ramclustr = 1171.4338 (+/− NA ppm)
1172.4411
[M+H]+
1173.445
0
20
40
60
80
100
1160
1165
1170
1175
1180
1185

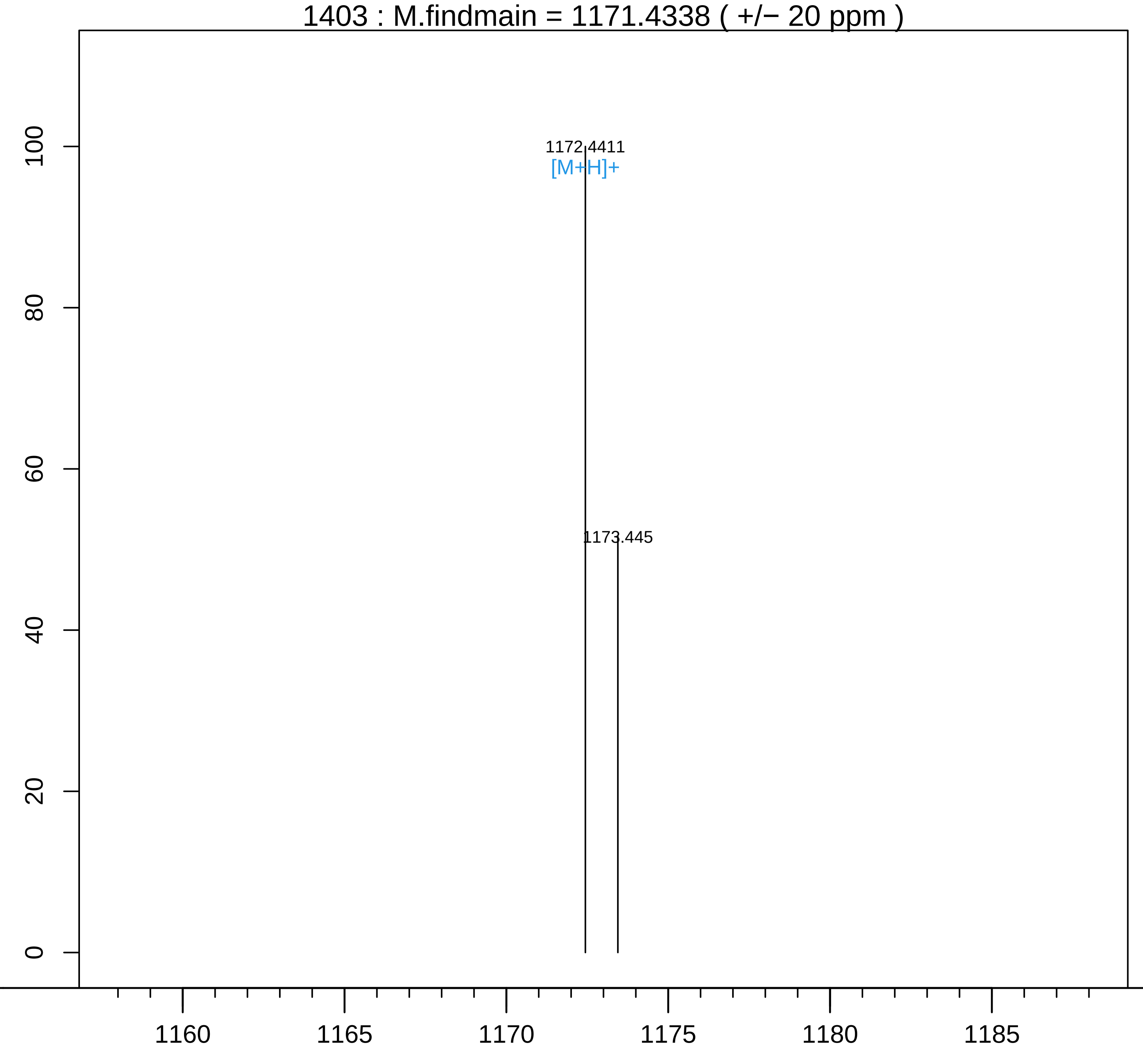
1403 : M.findmain = 1171.4338 (+/− 20 ppm)
1172.4411
[M+H]+
1173.445
0
20
40
60
80
100
1160
1165
1170
1175
1180
1185

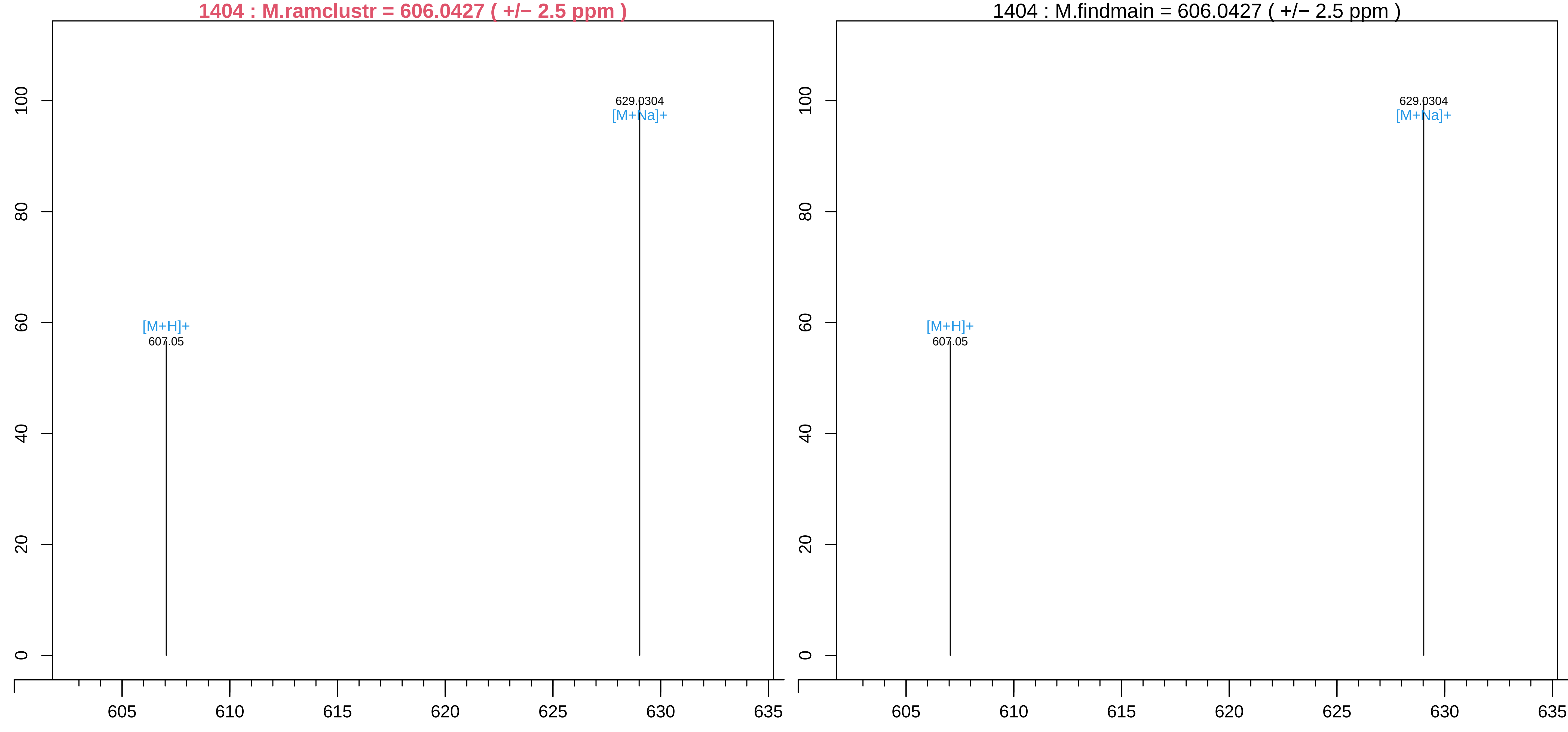

1404 : M.ramclustr = 606.0427 (+/− 2.5 ppm)
629.0304
[M+Na]+
[M+H]+
607.05
1404 : M.findmain = 606.0427 (+/− 2.5 ppm)
629.0304
[M+Na]+
[M+H]+
607.05
