# Supplementary material for: Plant necrotrophic bacterial disease resistance phenotypes, QTL, and metabolites identified through integrated genetic mapping and metabolomics in Solanum species
Source: Front Plant Sci. 2024 Mar 5;15:1336513. doi: 10.3389/fpls.2024.1336513 (PMC10949924; doi:10.3389/fpls.2024.1336513)
Supplement: Supplementary file 3 [file DataSheet_3.pdf]

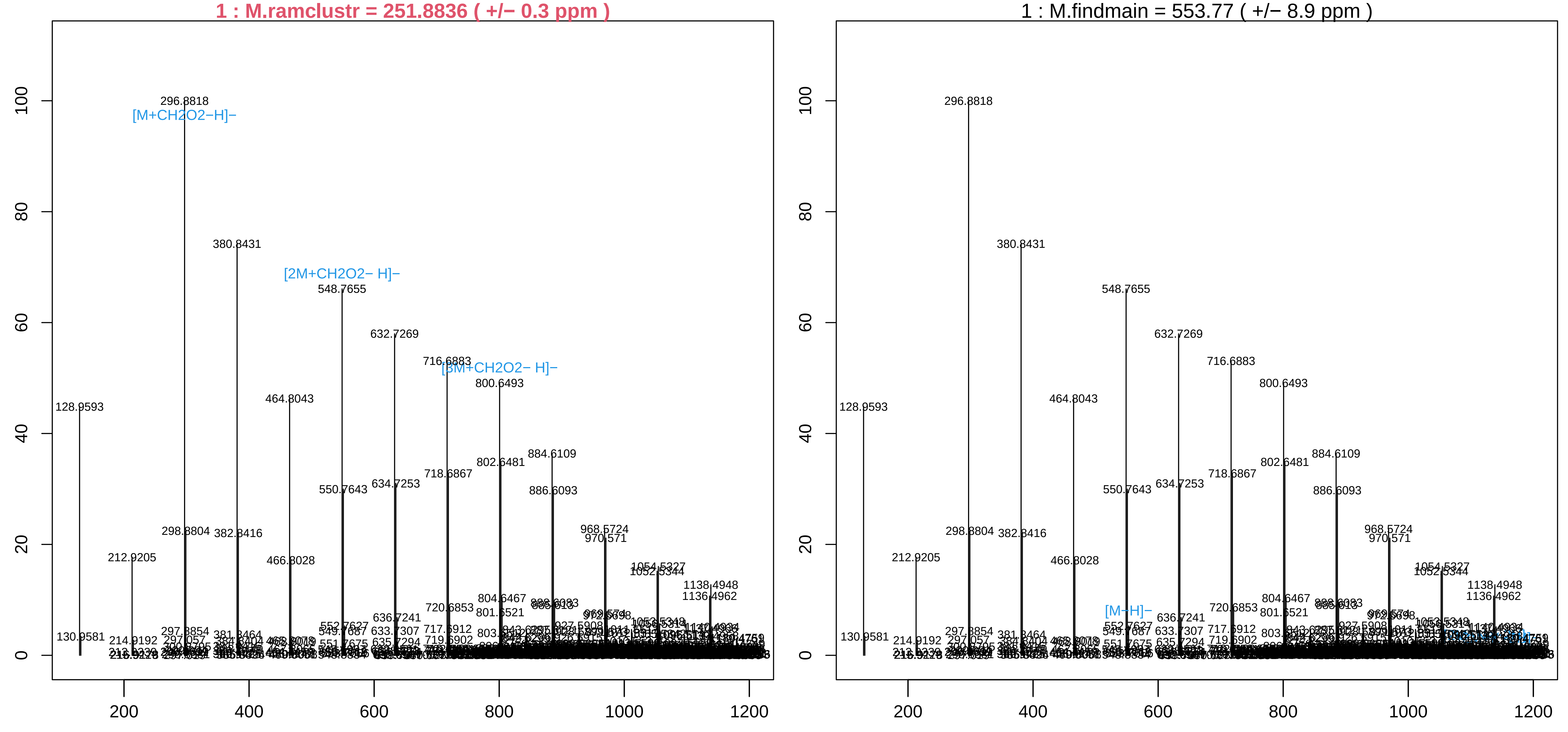
1 : M.ramclustr = 251.8836 (+/− 0.3 ppm)
[M+CH2O2−H]−
[2M+CH2O2− H]−
[3M+CH2O2− H]−
296.8818
380.8431
548.7655
632.7269
716.6883
800.6493
464.8043
128.9593
884.6109
802.6481
718.6867
550.7643
634.7253
886.6093
298.8804
382.8416
466.8028
212.9205
968.5724
970.571
1054.5327
1052.5344
1138.4948
1136.4962
804.6467
801.6521
720.6853
636.7241
130.9581
214.9192
1 : M.findmain = 553.77 (+/− 8.9 ppm)
[M−H]−
0
20
40
60
80
100
200
400
600
800
1000
1200

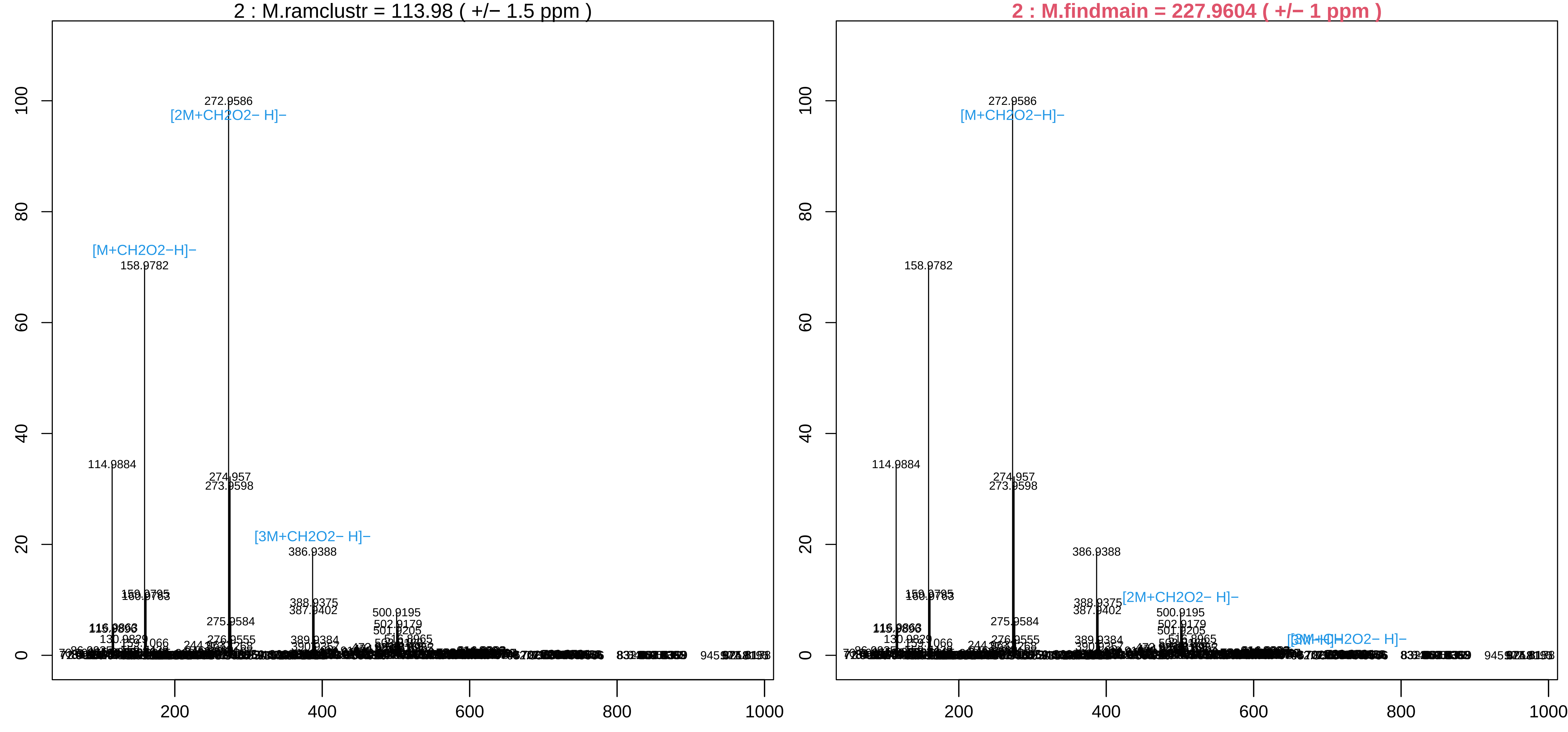
2 : M.ramclustr = 113.98 (+/− 1.5 ppm)
272.9586
[2M+CH2O2− H]−
[M+CH2O2−H]−
158.9782
114.9884
274.957
273.9598
[3M+CH2O2− H]−
386.9388
388.9375
387.9402
500.9195
502.9179
501.9205
275.9584
276.9555
389.9384
2 : M.findmain = 227.9604 (+/− 1 ppm)
272.9586
[M+CH2O2−H]−
158.9782
114.9884
274.957
273.9598
386.9388
[2M+CH2O2− H]−
388.9375
387.9402
500.9195
502.9179
501.9205
275.9584
276.9555
389.9384
0
20
40
60
80
100
200
400
600
800
1000

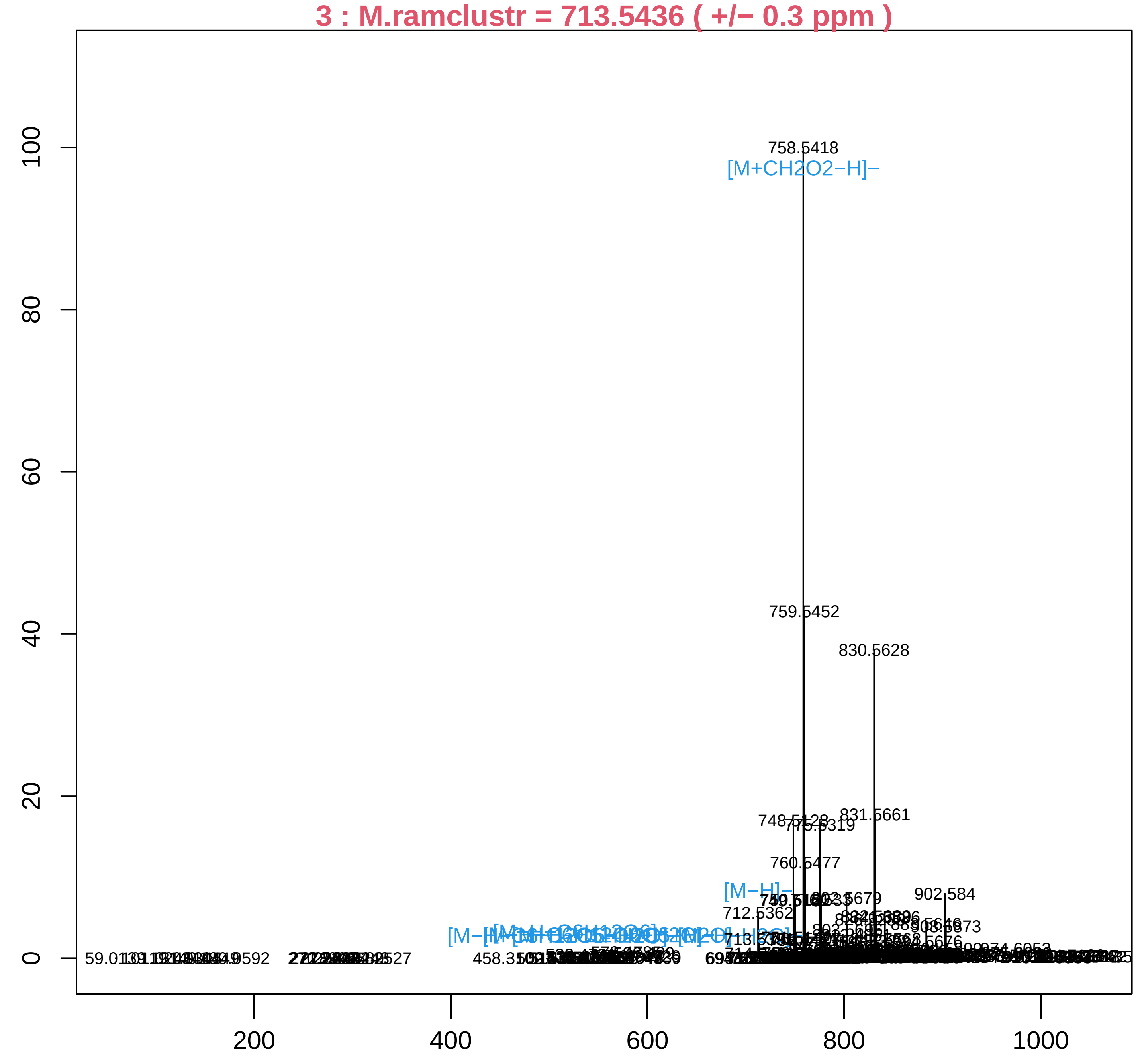
3 : M.ramclustr = 713.5436 (+/− 0.3 ppm)
758.5418
[M+CH2O2−H]−
759.5452
830.5628
748.5128
831.5661
760.5477
[M−H]−
902.584
712.5362
100
80
60
40
20
0
200
400
600
800
1000

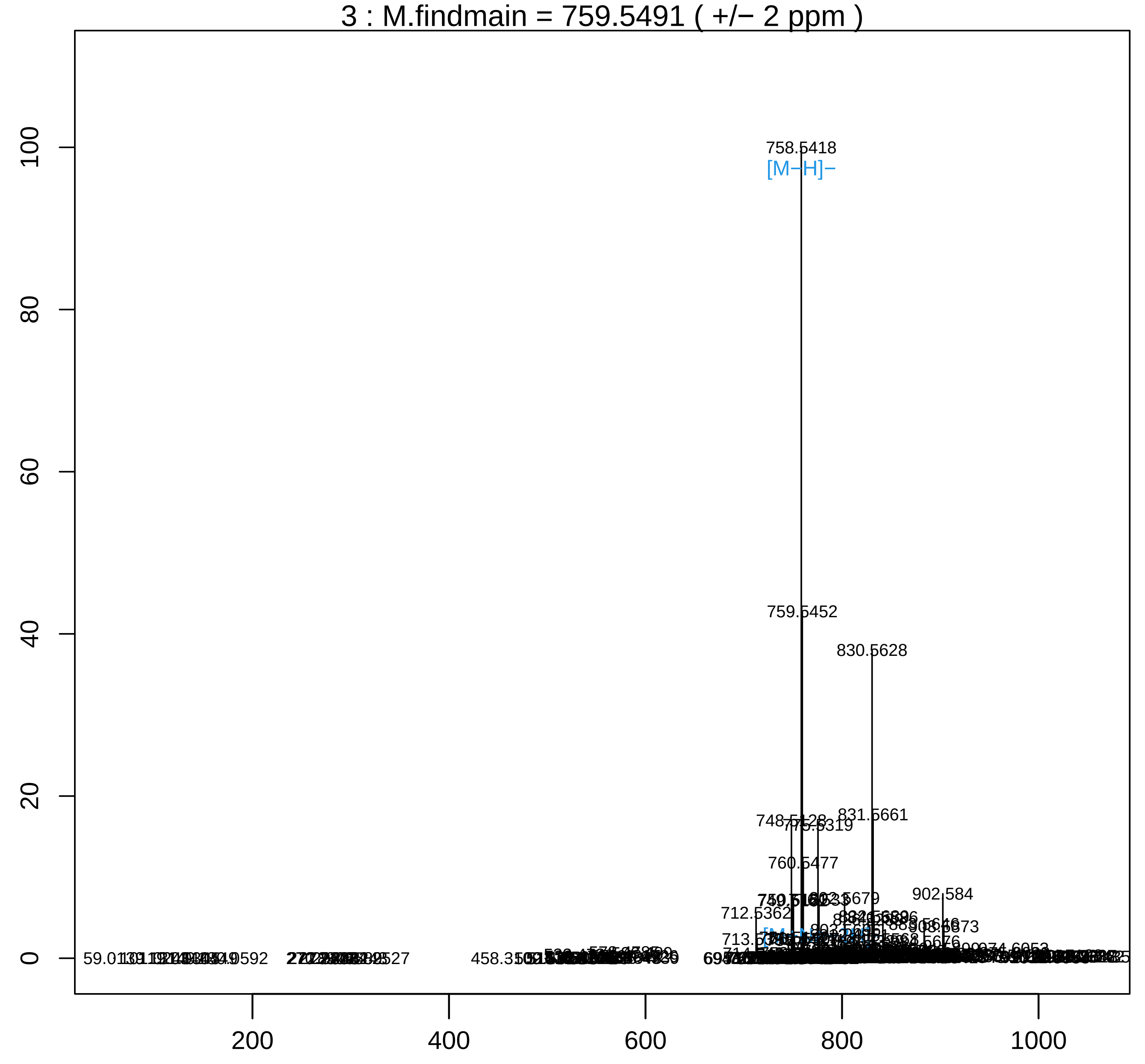
3 : M.findmain = 759.5491 (+/− 2 ppm)
758.5418
[M−H]−
759.5452
830.5628
748.5128
831.5661
760.5477
902.584
712.5362
100
80
60
40
20
0
200
400
600
800
1000

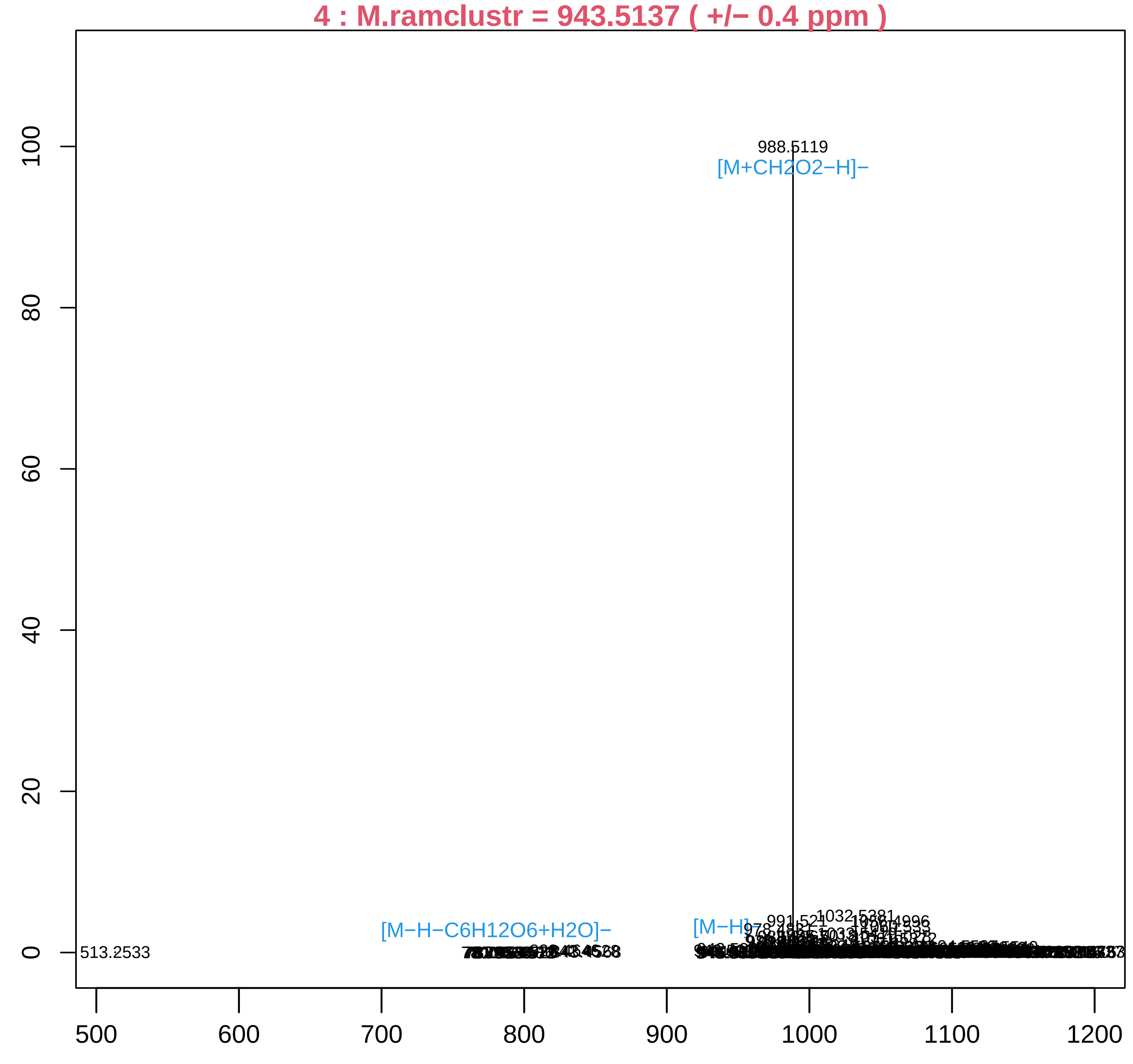
4 : M.ramclustr = 943.5137 (+/− 0.4 ppm)
988.5119
[M+CH2O2−H]−
[M−H−C6H12O6+H2O]−
[M−H]−
513.2533
0
20
40
60
80
100
500
600
700
800
900
1000
1100
1200

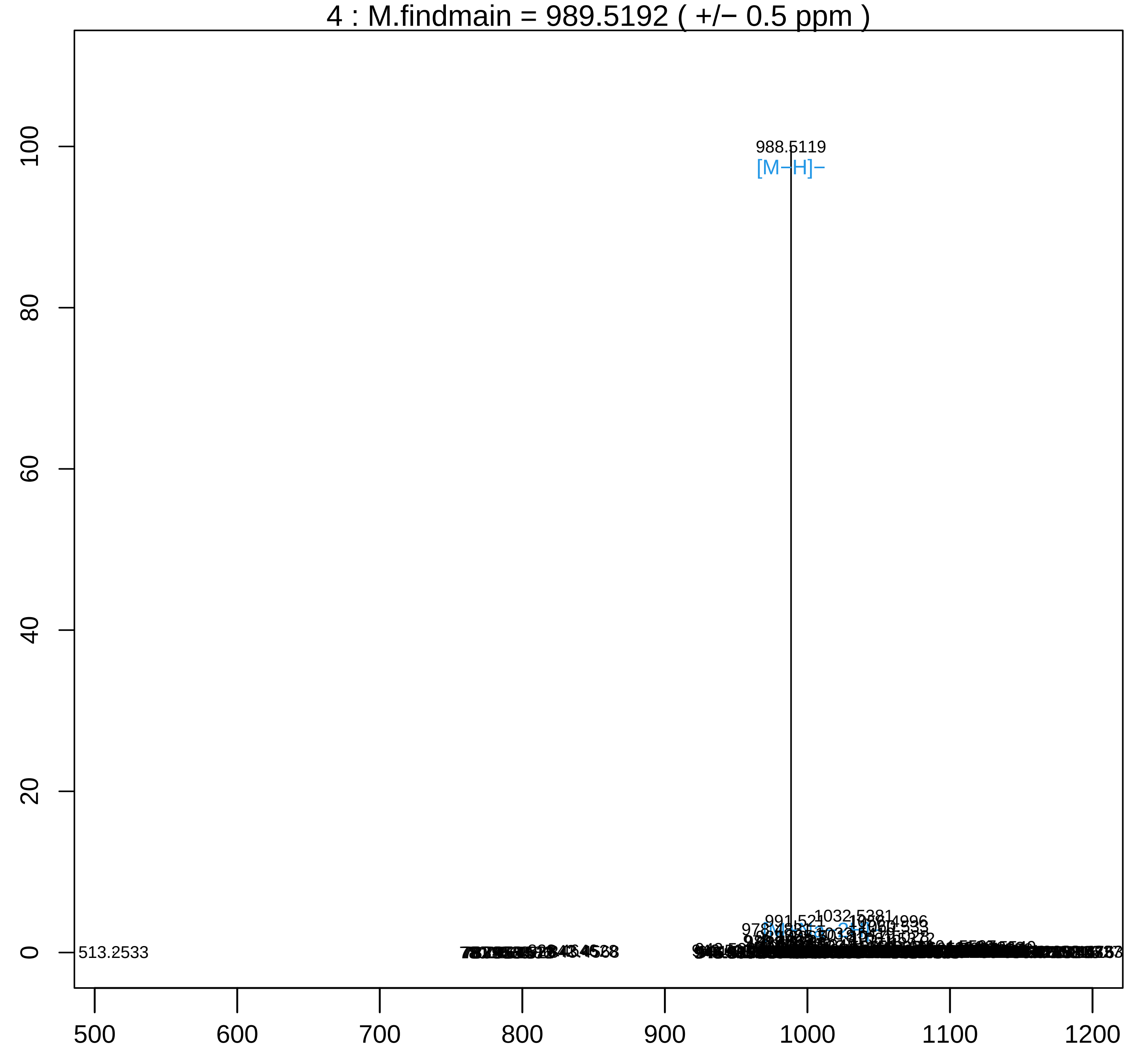
4 : M.findmain = 989.5192 (+/− 0.5 ppm)
988.5119
[M−H]−
513.2533
0
20
40
60
80
100
500
600
700
800
900
1000
1100
1200

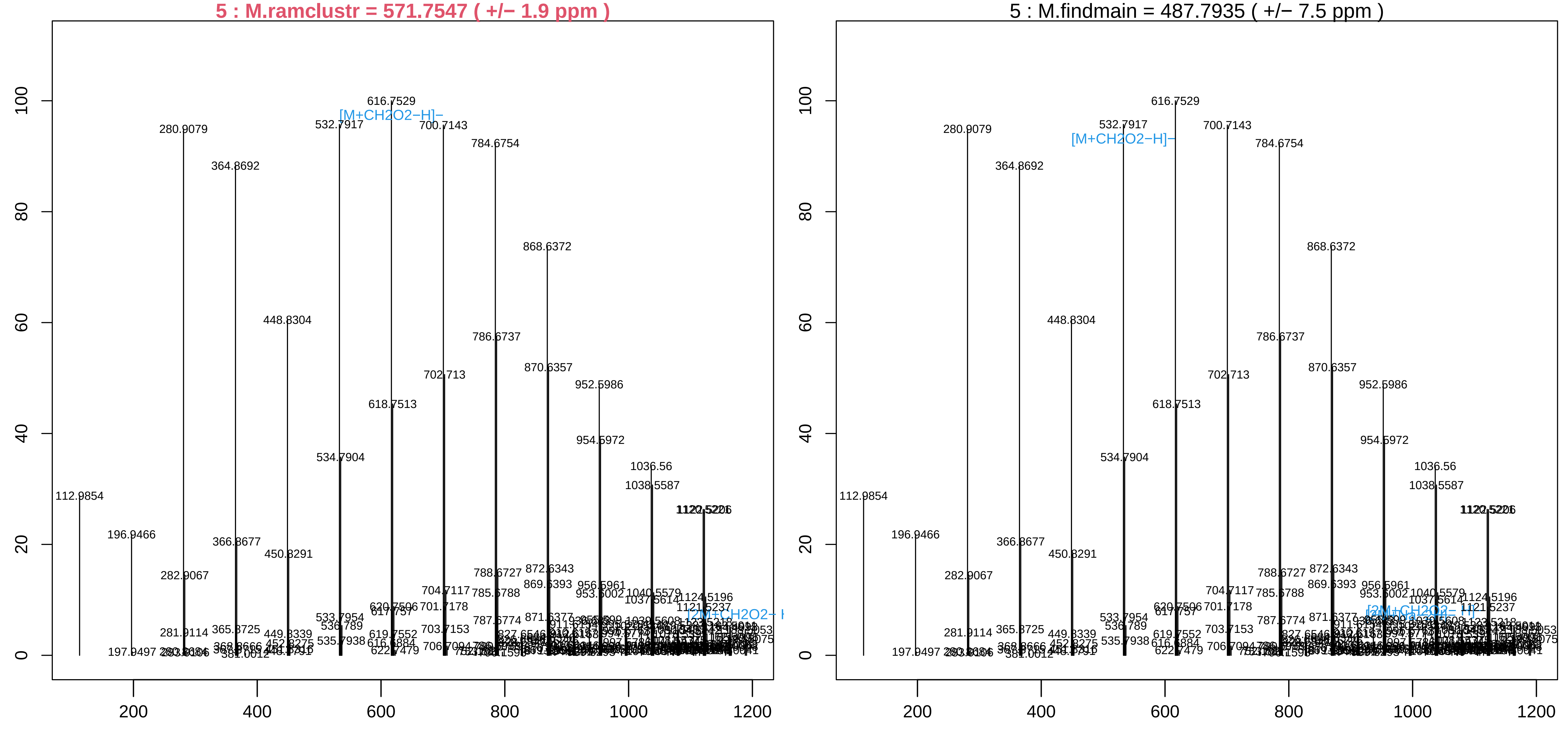

5 : M.ramclustr = 571.7547 (+/− 1.9 ppm)
5 : M.findmain = 487.7935 (+/− 7.5 ppm)
[M+CH2O2−H]−
616.7529
532.7917
700.7143
280.9079
784.6754
364.8692
868.6372
448.8304
786.6737
702.713
618.7513
870.6357
952.5986
954.6972
534.7904
1036.56
1038.5587
112.9854
196.9466
366.8677
450.8291
282.9067
788.6727
872.6343
704.7117
785.5788
869.5393
956.5961
953.5002
1040.5579
1124.5196
1121.5237
620.7506
617.737
701.7178
533.7954
536.789
787.6774
871.6377
281.9114
365.3725
449.3339
619.7552
703.7153
535.7938
616.6884
622.7479
197.9497
381.0012
0
20
40
60
80
100
200
400
600
800
1000
1200

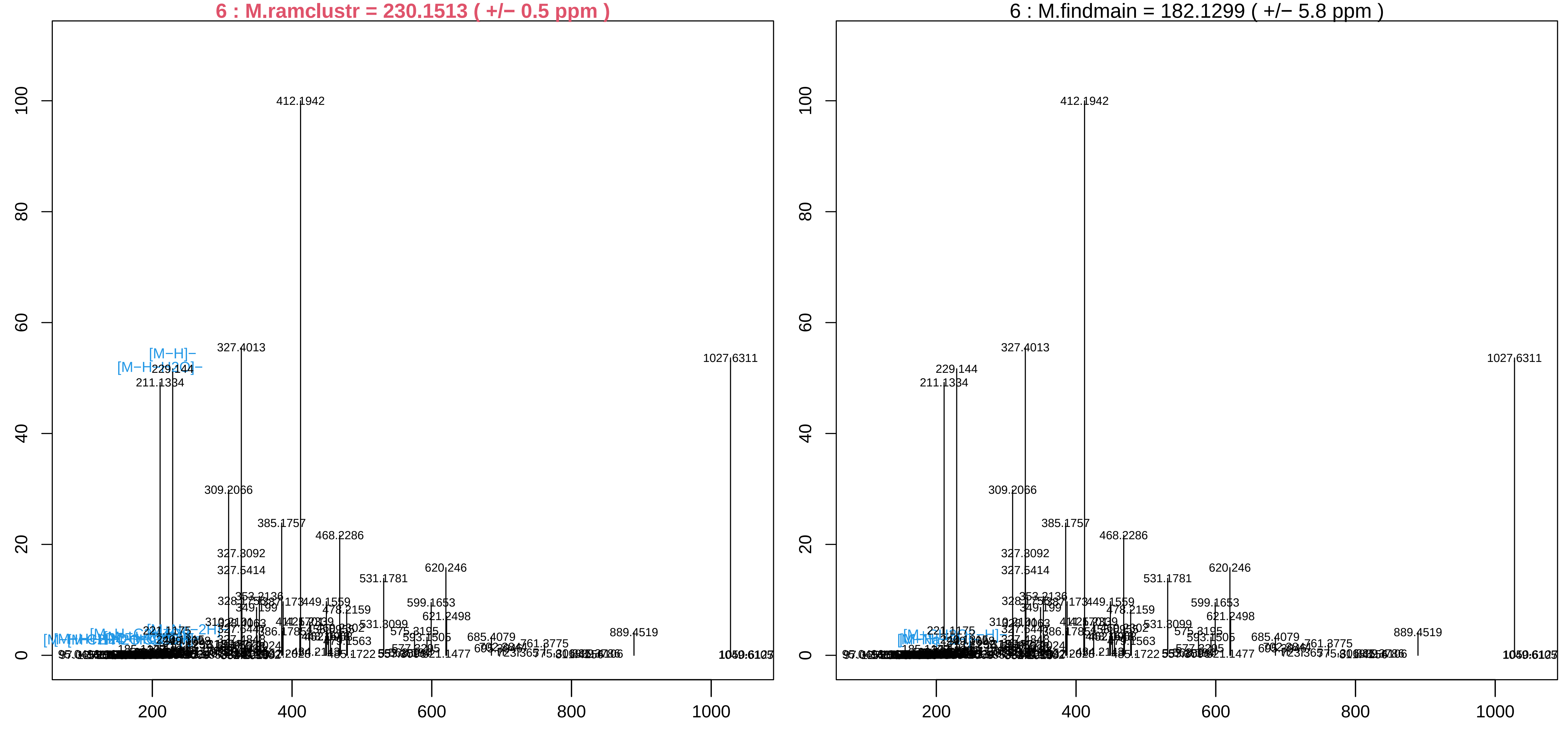

6 : M.ramclustr = 230.1513 (+/− 0.5 ppm)
6 : M.findmain = 182.1299 (+/− 5.8 ppm)
412.1942
327.4013
1027.6311
229.144
211.1334
[M−H]−
[M−H−H2O]−
309.2066
385.1757
468.2286
327.3092
327.5414
620.246
531.1781
599.1653
621.2498
889.4519
685.4079
761.3775
449.1559
478.2159
531.3099
593.1505
0
20
40
60
80
100
200
400
600
800
1000

7 : M.ramclustr = 867.4974 (+/− 0.5 ppm)

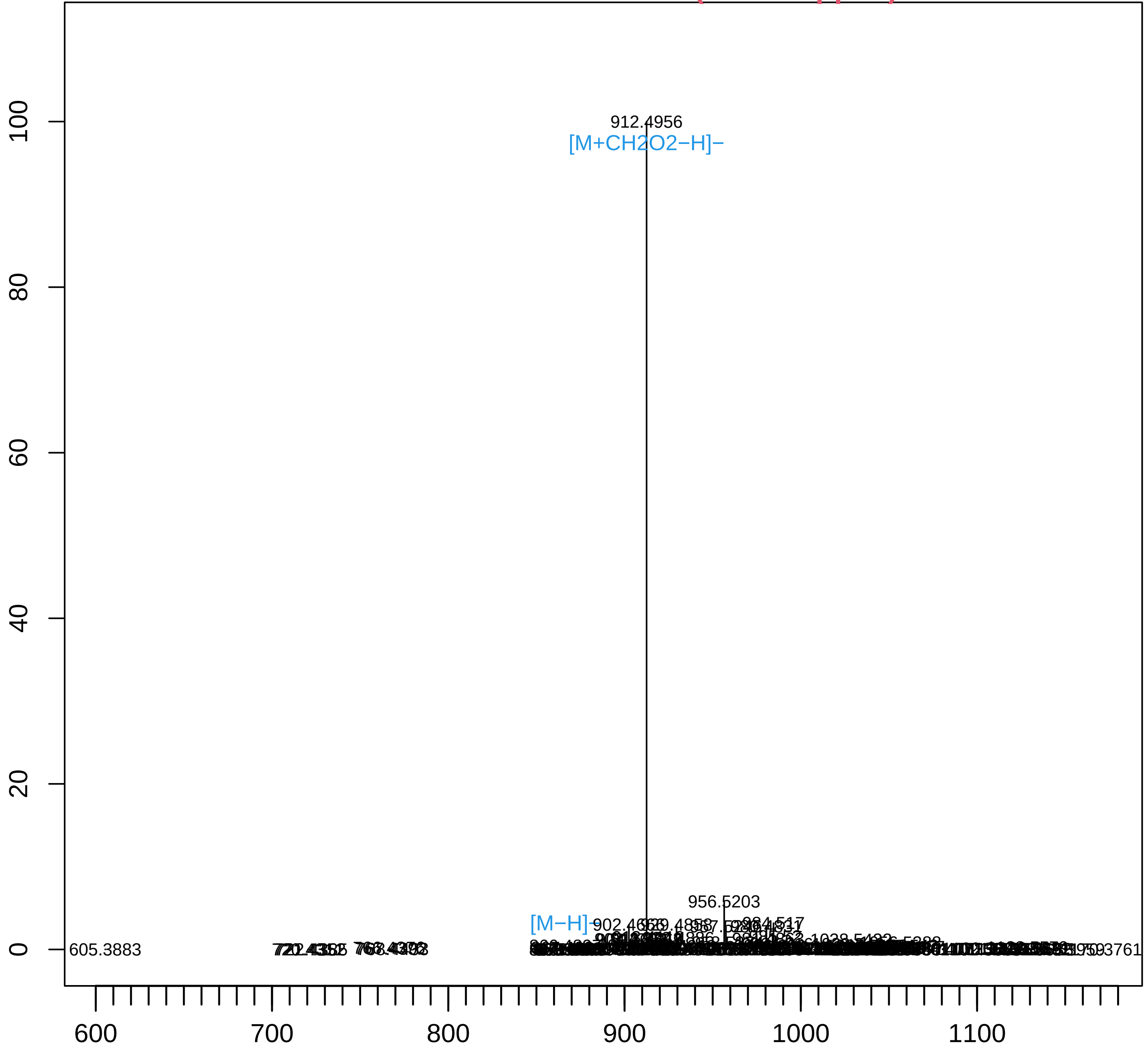

7 : M.findmain = 913.5029 (+/− 1.1 ppm)

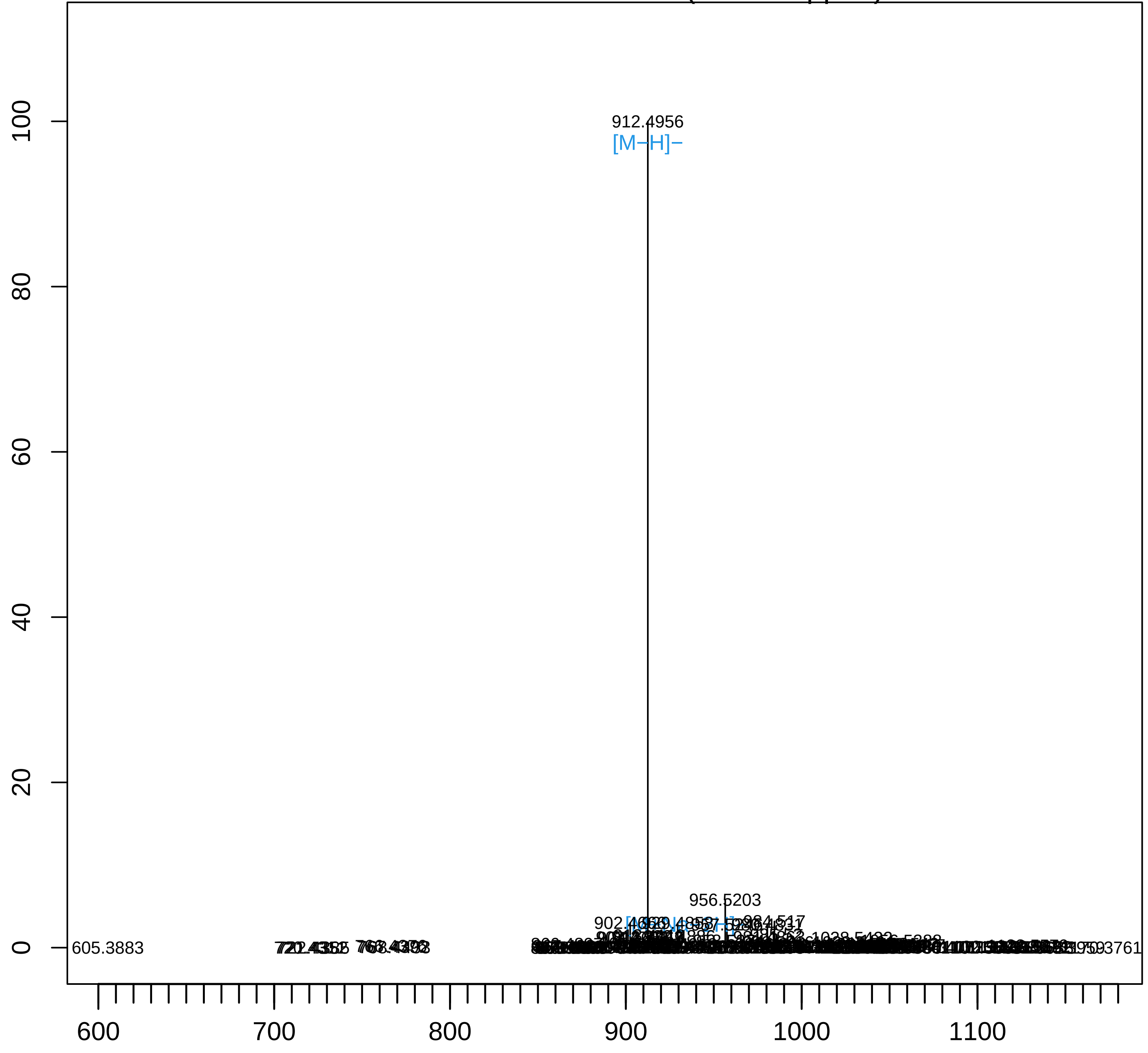

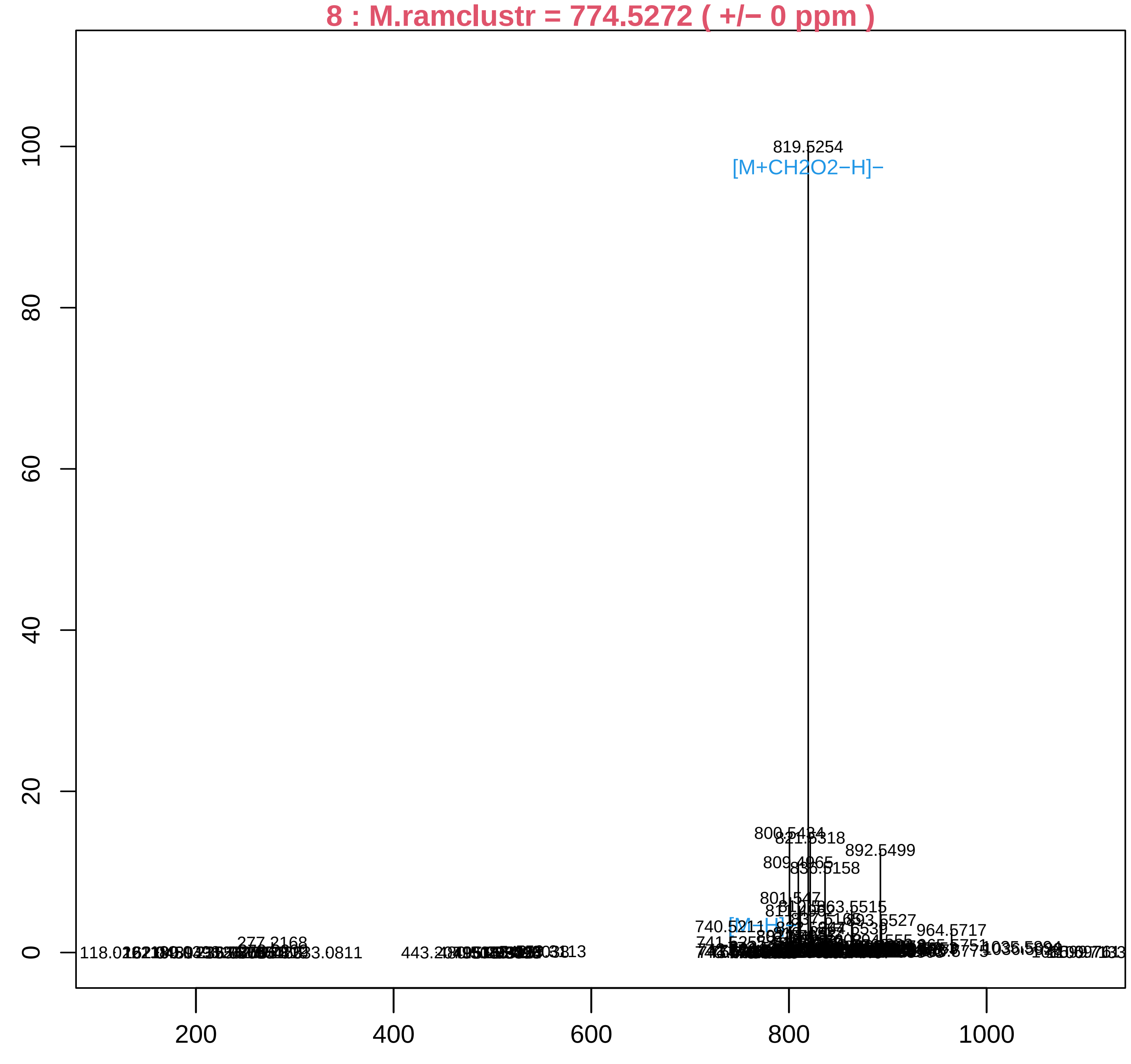
8 : M.ramclustr = 774.5272 (+/− 0 ppm)
819.5254
[M+CH2O2−H]−
800.5434
892.5499
809.4965
836.5158
964.5717
740.5211
277.2168
118.0
443.2
0
20
40
60
80
100
200
400
600
800
1000

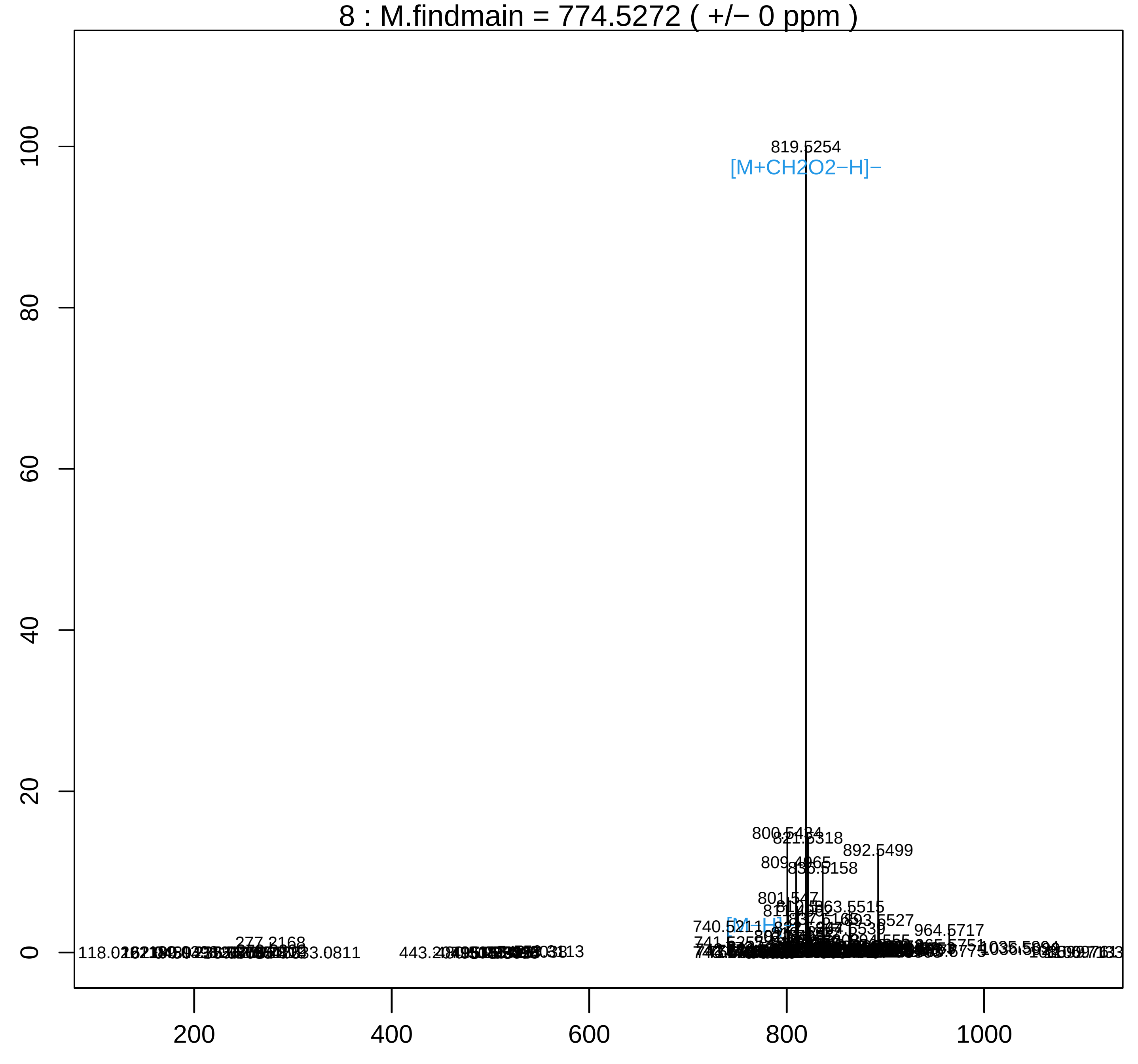
8 : M.findmain = 774.5272 (+/− 0 ppm)
819.5254
[M+CH2O2−H]−
800.5434
892.5499
809.4965
836.5158
964.5717
740.5211
277.2168
118.0
443.2
0
20
40
60
80
100
200
400
600
800
1000

9 : M.ramclustr = 683.4657 (+/− 3.7 ppm)

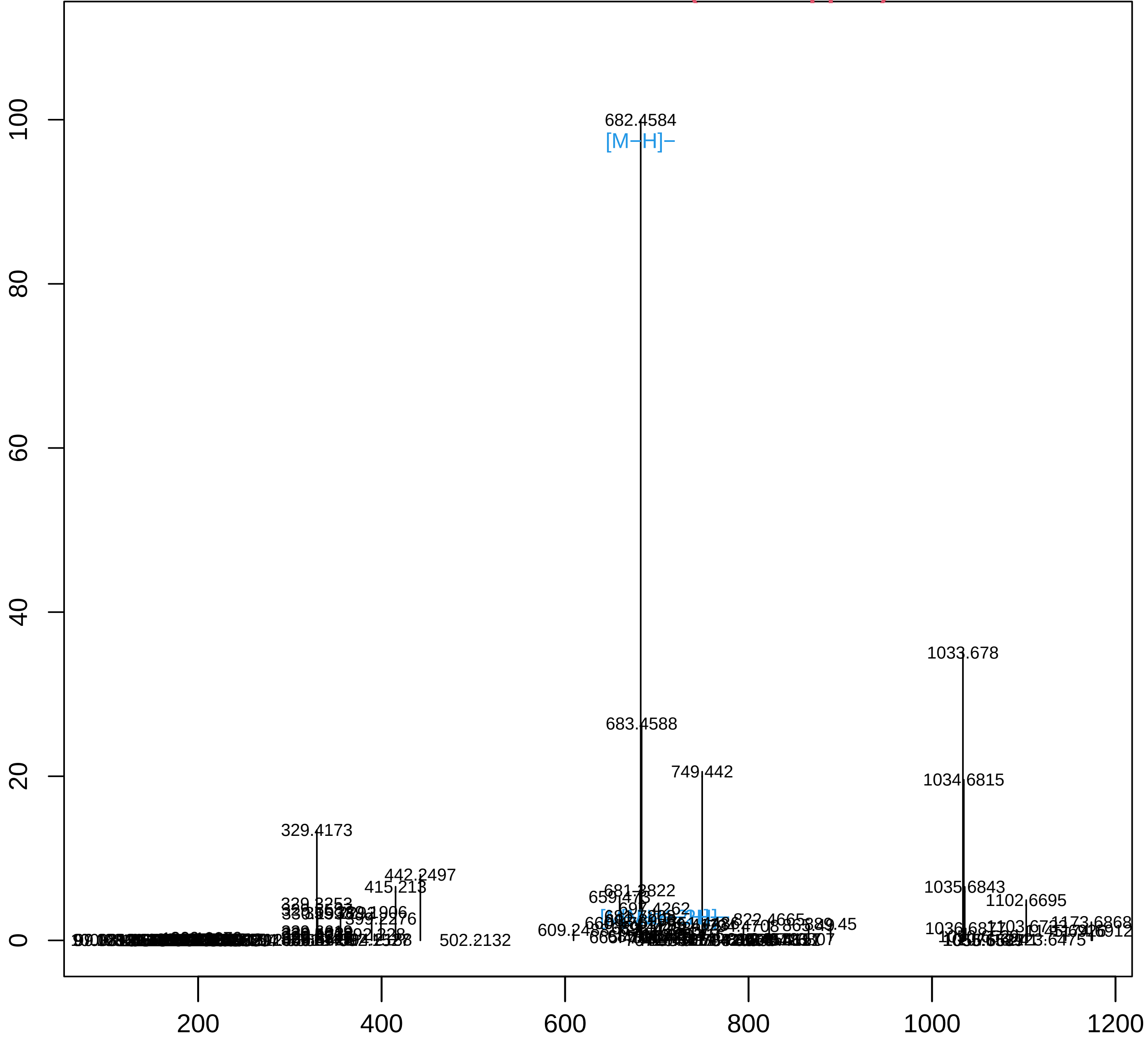

9 : M.findmain = 683.4657 (+/− 3.7 ppm)

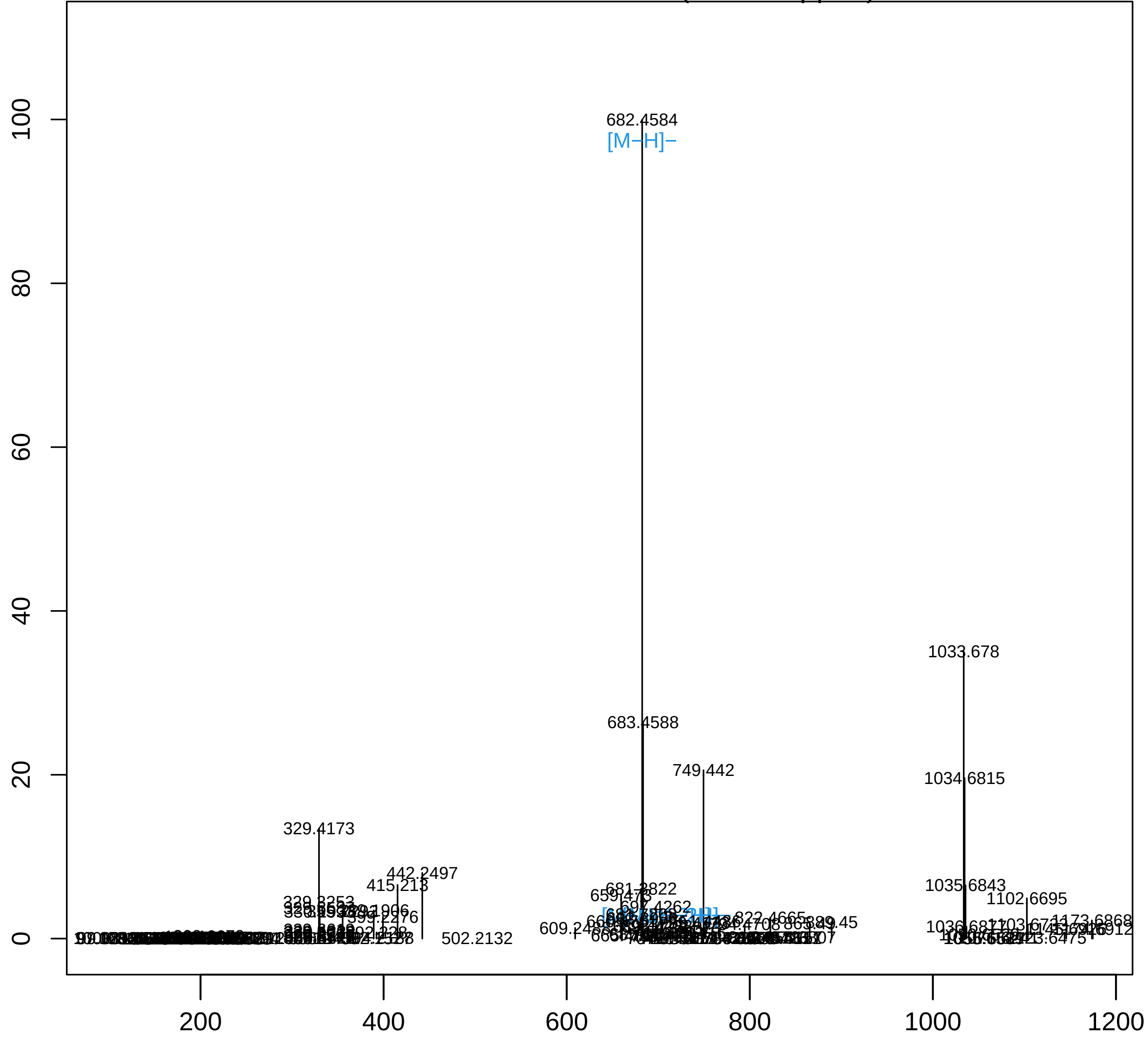

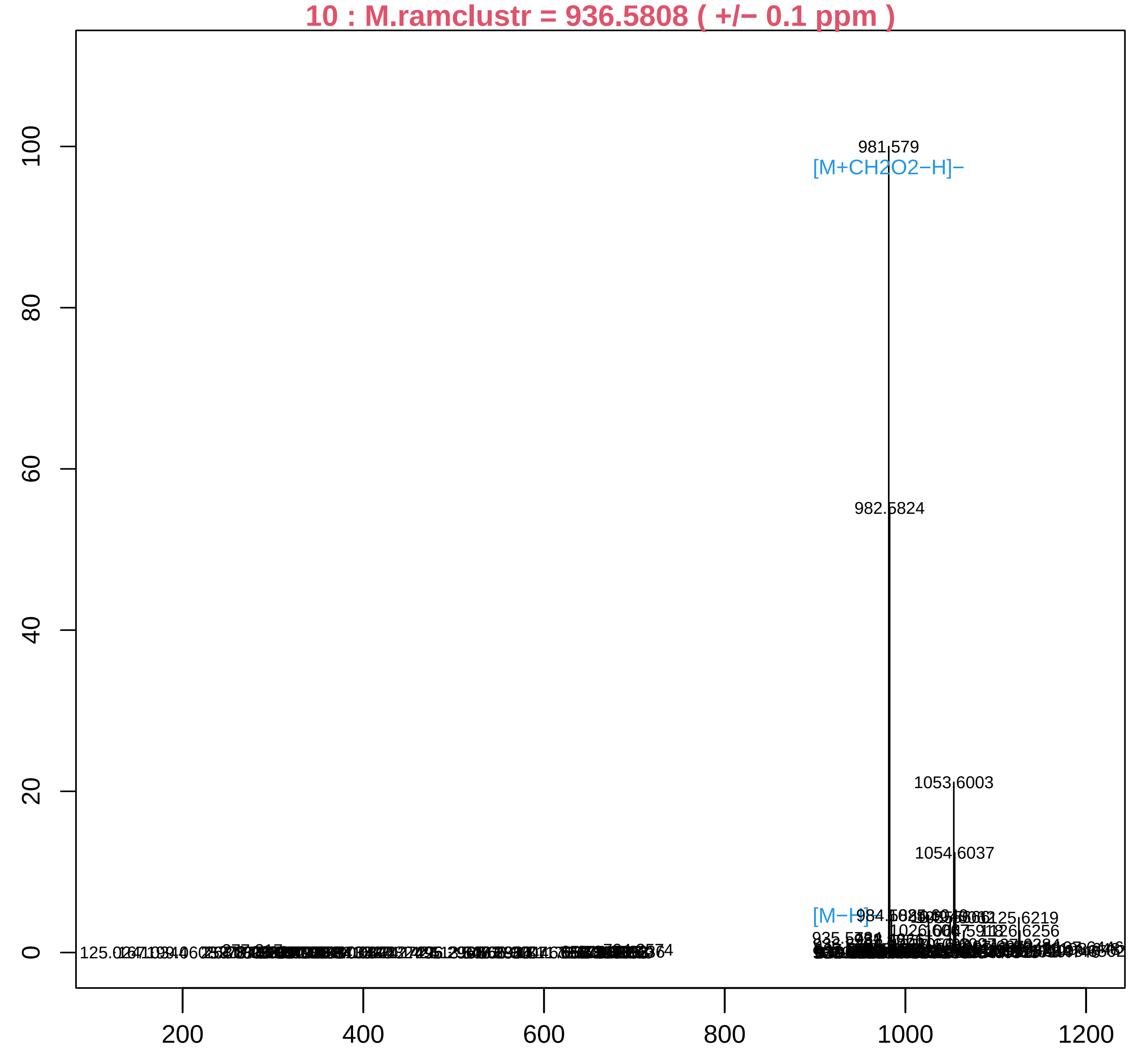

10 : M.ramclustr = 936.5808 (+/− 0.1 ppm)
981.579
[M+CH2O2−H]−
982.5824
1053.6003
1054.6037
[M−H]−

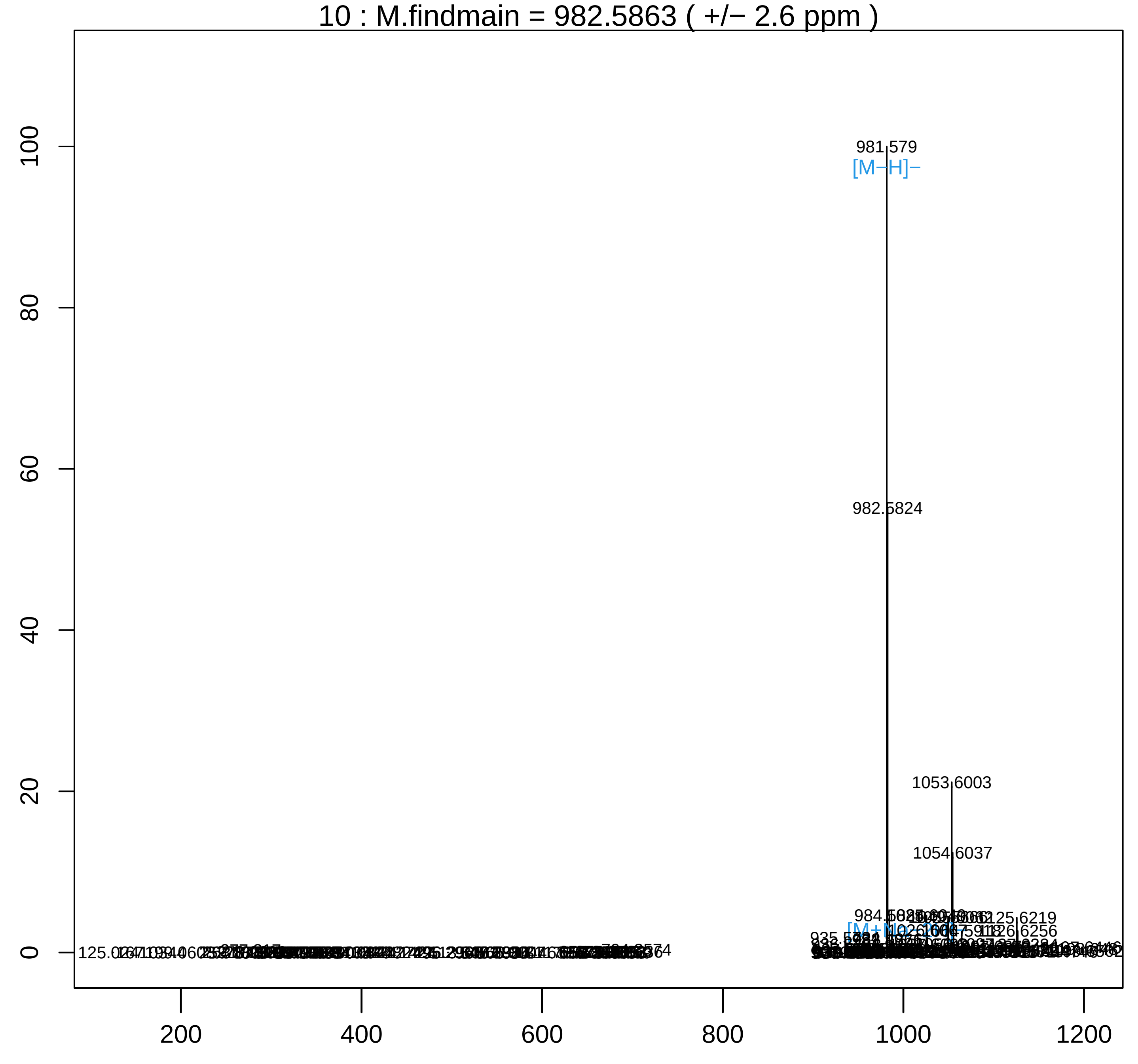

10 : M.findmain = 982.5863 (+/− 2.6 ppm)
981.579
[M−H]−
982.5824
1053.6003
1054.6037

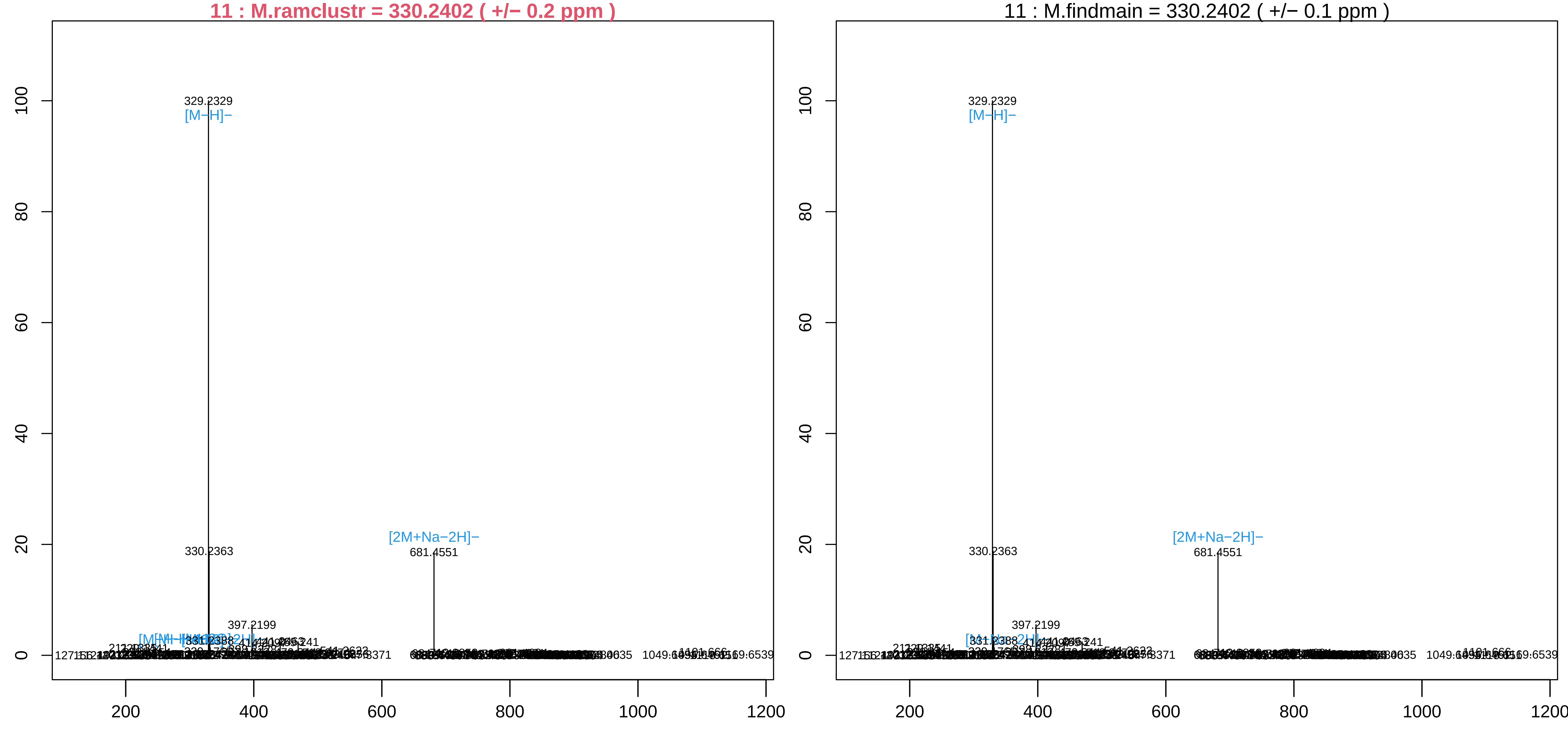
11 : M.ramclustr = 330.2402 (+/− 0.2 ppm)
329.2329
[M−H]−
330.2363
[2M+Na−2H]−
681.4551
397.2199
11 : M.findmain = 330.2402 (+/− 0.1 ppm)
329.2329
[M−H]−
330.2363
[2M+Na−2H]−
681.4551
397.2199
0
20
40
60
80
100
200
400
600
800
1000
1200

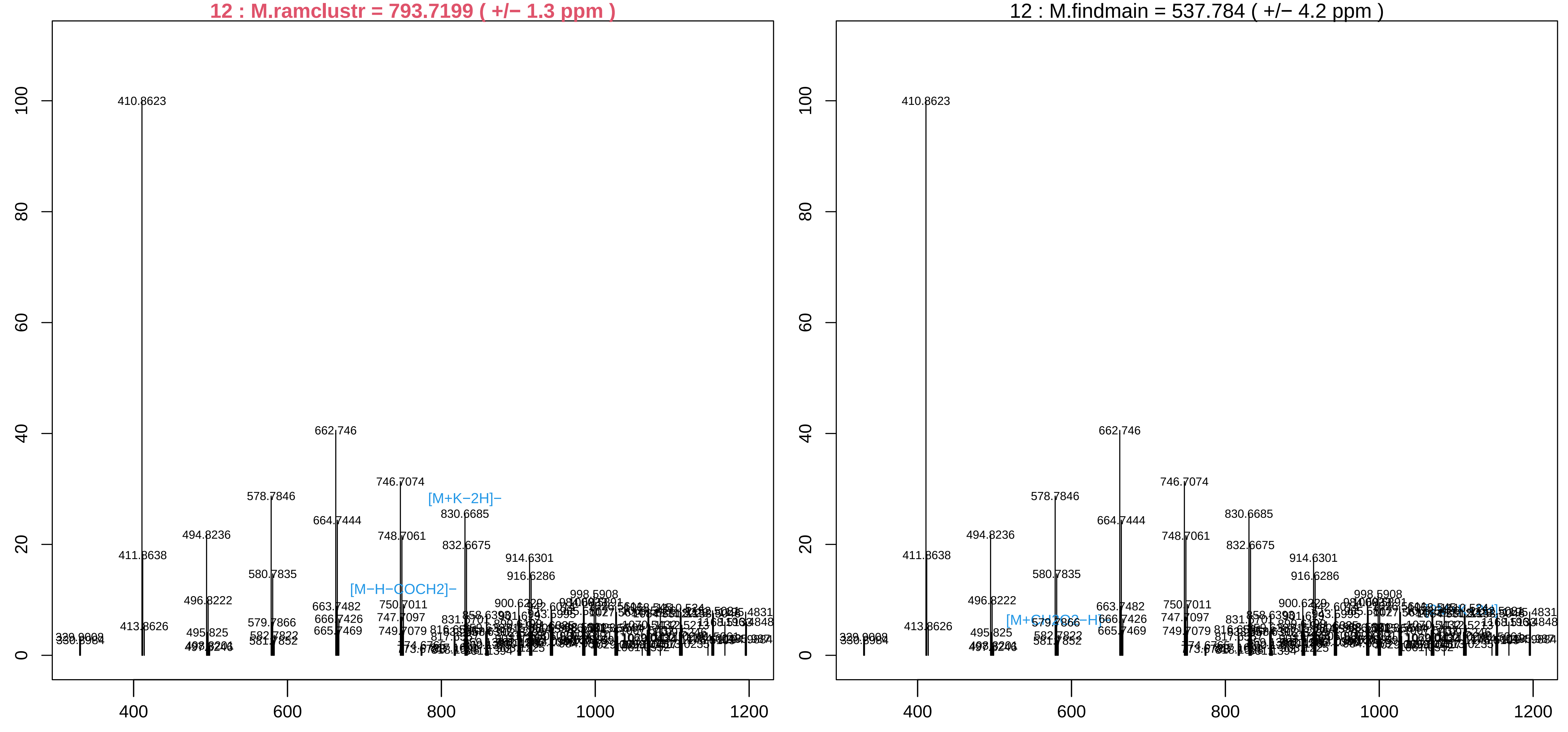
12 : M.ramclustr = 793.7199 (+/− 1.3 ppm)
12 : M.findmain = 537.784 (+/− 4.2 ppm)
410.8623
411.8638
413.8626
494.8236
496.8222
495.825
578.7846
580.7835
579.7866
582.7822
581.7852
662.746
664.7444
663.7482
666.7426
665.7469
746.7074
748.7061
750.7011
747.7097
749.7079
830.6685
832.6675
914.6301
916.6286
900.6229
998.6908
329.9009
330.8984
[M+K−2H]−
[M−H−COCH2]−
0
20
40
60
80
100
400
600
800
1000
1200

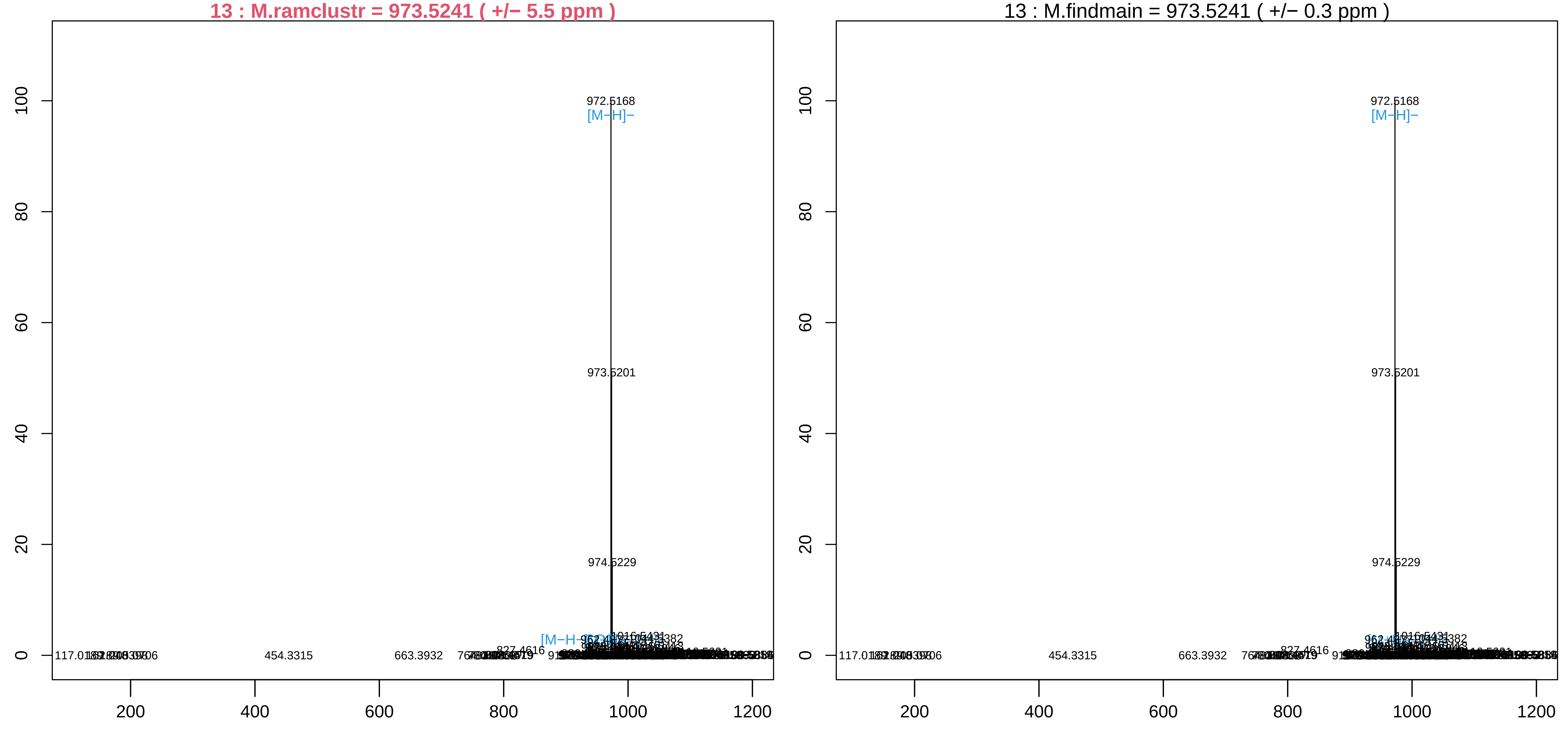

13 : M.ramclustr = 973.5241 (+/− 5.5 ppm)
13 : M.findmain = 973.5241 (+/− 0.3 ppm)
972.5168
[M−H]−
973.5201
974.5229
827.4616
117.0182
454.3315
663.3932
0
20
40
60
80
100
200
400
600
800
1000
1200

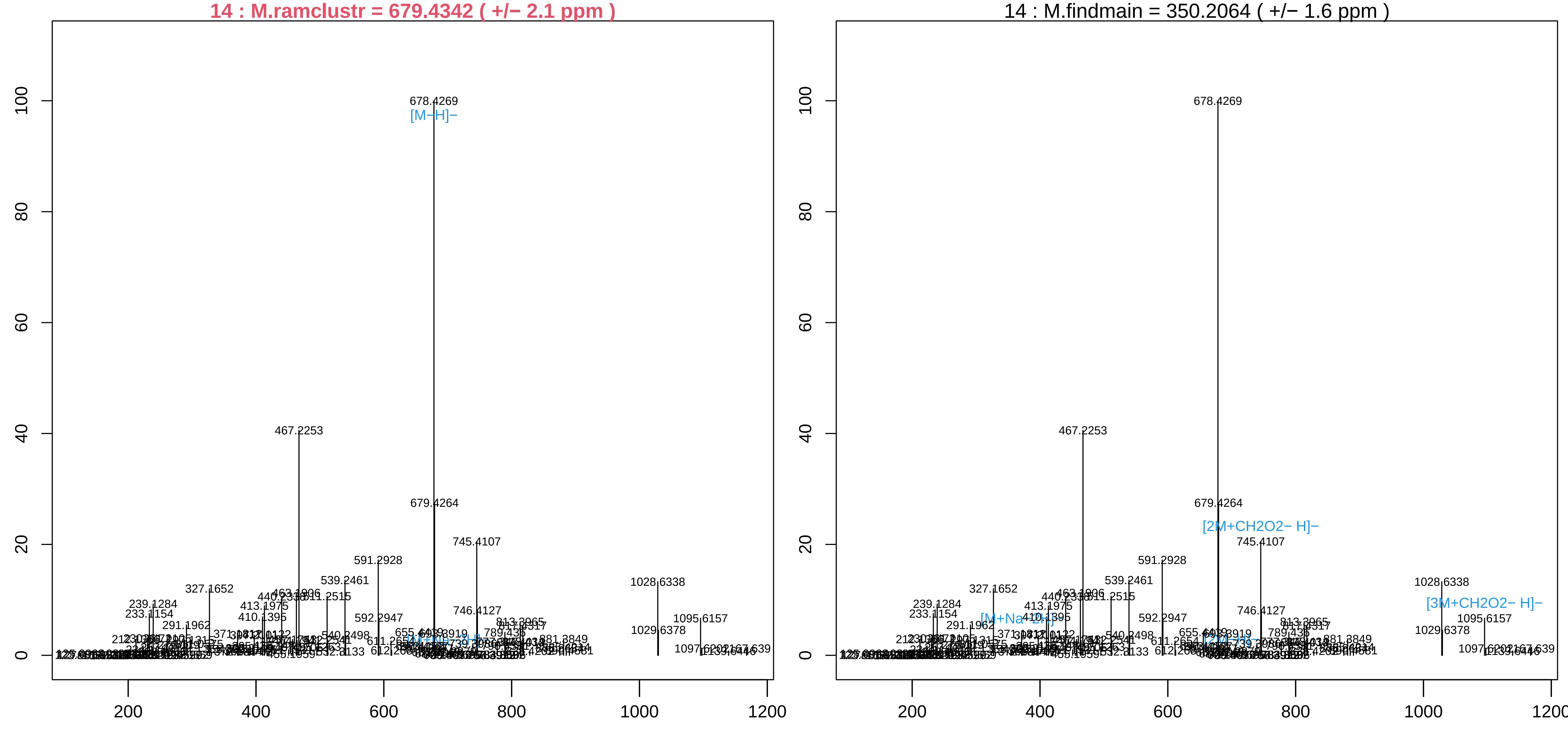
14 : M.ramclustr = 679.4342 (+/− 2.1 ppm)
678.4269
[M−H]−
467.2253
679.4264
745.4107
591.2928
539.2461
327.1652
1028.6338
239.1284
233.1154
413.1975
410.1395
511.2515
592.2947
746.4127
813.3965
817.4317
1095.6157
1029.6378
291.1962
789.436
881.3849
540.2498
14 : M.findmain = 350.2064 (+/− 1.6 ppm)
678.4269
467.2253
679.4264
[2M+CH2O2− H]−
745.4107
591.2928
539.2461
327.1652
1028.6338
[3M+CH2O2− H]−
239.1284
233.1154
413.1975
410.1395
511.2515
592.2947
746.4127
813.3965
817.4317
1095.6157
1029.6378
291.1962
789.436
881.3849
540.2498
0
20
40
60
80
100
200
400
600
800
1000
1200

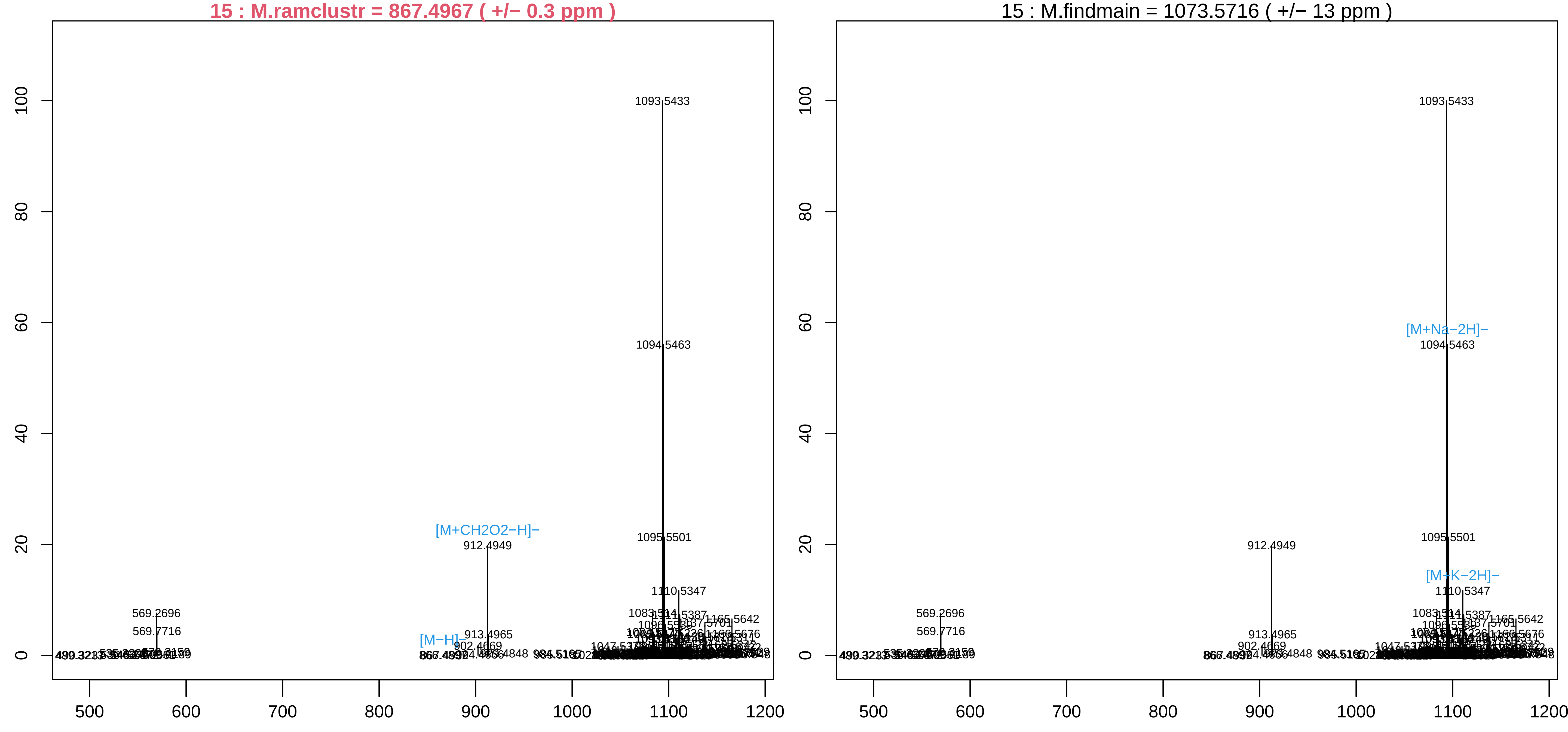

15 : M.ramclustr = 867.4967 (+/− 0.3 ppm)
1093.5433
1094.5463
[M+CH2O2−H]−
912.4949
1095.5501
1110.5347
569.2696
569.7716
1083.5414
1165.5642
[M−H]−
913.4965
902.4669
984.5165
1047.5378
499.3233
866.4992
0
20
40
60
80
100
500
600
700
800
900
1000
1100
1200
15 : M.findmain = 1073.5716 (+/− 13 ppm)
1093.5433
[M+Na−2H]−
1094.5463
912.4949
1095.5501
[M+K−2H]−
1110.5347
569.2696
569.7716
1083.5414
1165.5642
913.4965
902.4669
984.5165
1047.5378
499.3233
866.4992
0
20
40
60
80
100
500
600
700
800
900
1000
1100
1200

16 : M.ramclustr = 746.4958 (+/− 0.2 ppm)

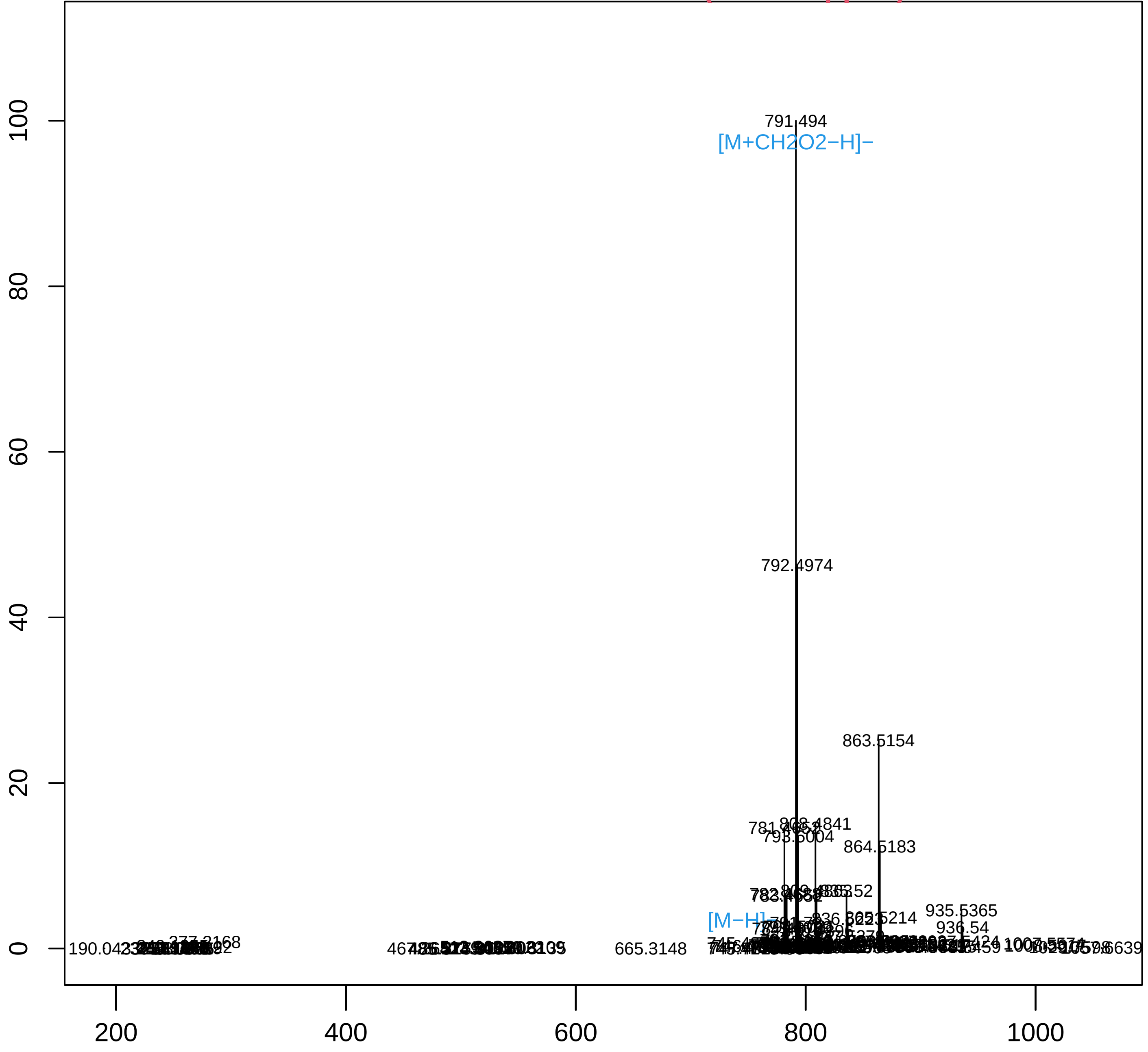

16 : M.findmain = 747.4992 (+/− 0.1 ppm)

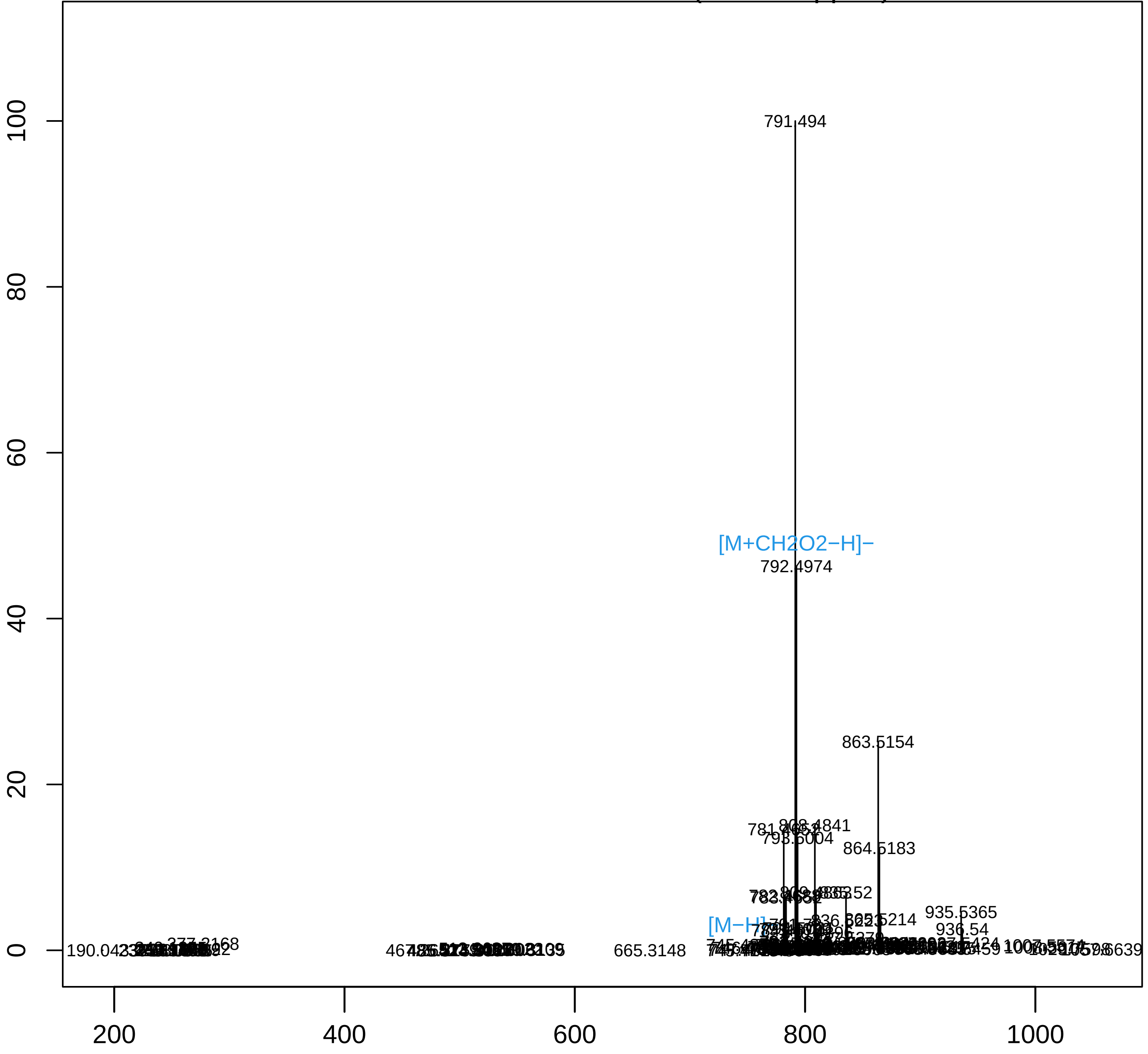

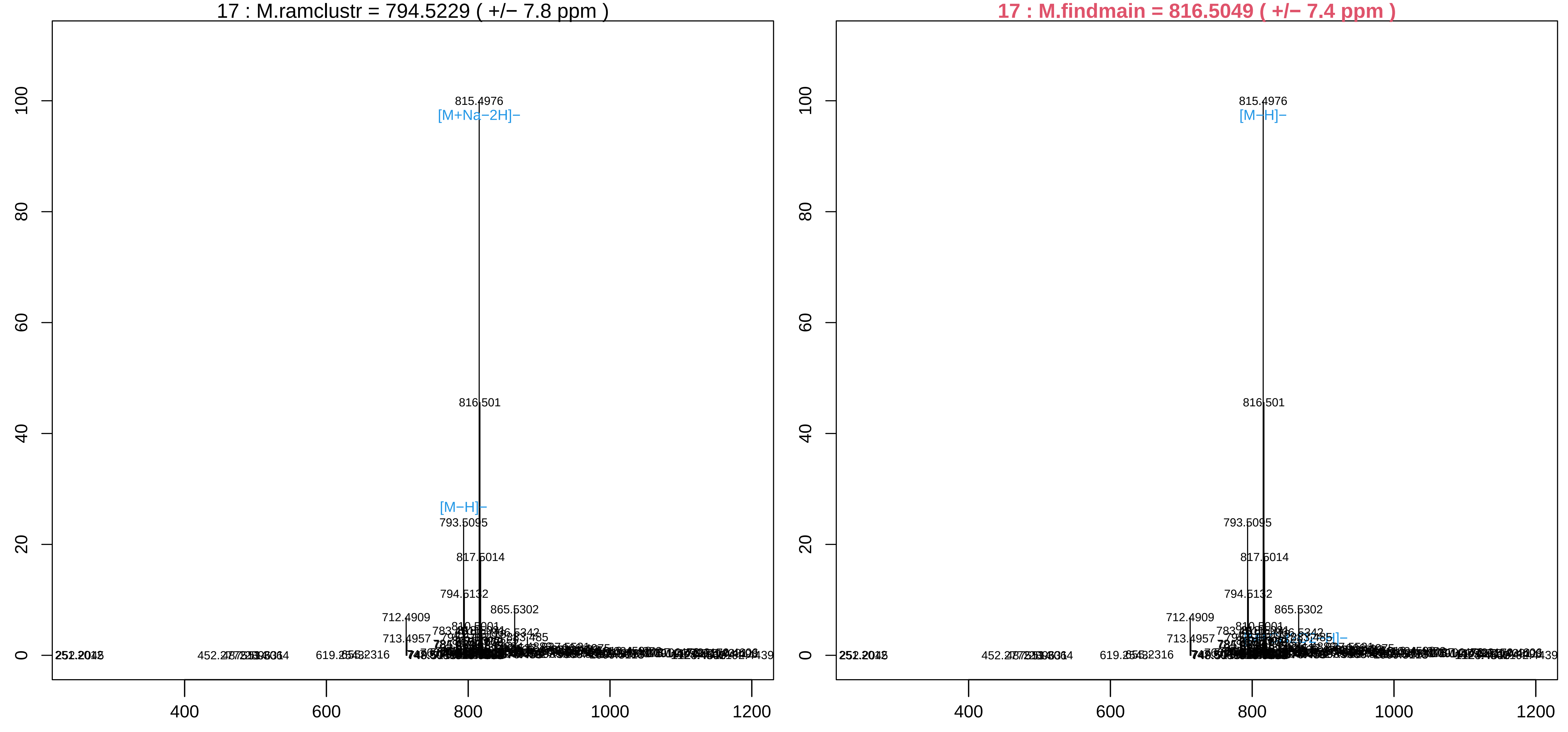
17 : M.ramclustr = 794.5229 (+/− 7.8 ppm)
815.4976
[M+Na−2H]−
816.501
[M−H]−
793.5095
817.5014
794.5132
865.5302
712.4909
713.4957
17 : M.findmain = 816.5049 (+/− 7.4 ppm)
815.4976
[M−H]−
816.501
793.5095
817.5014
794.5132
865.5302
712.4909
713.4957

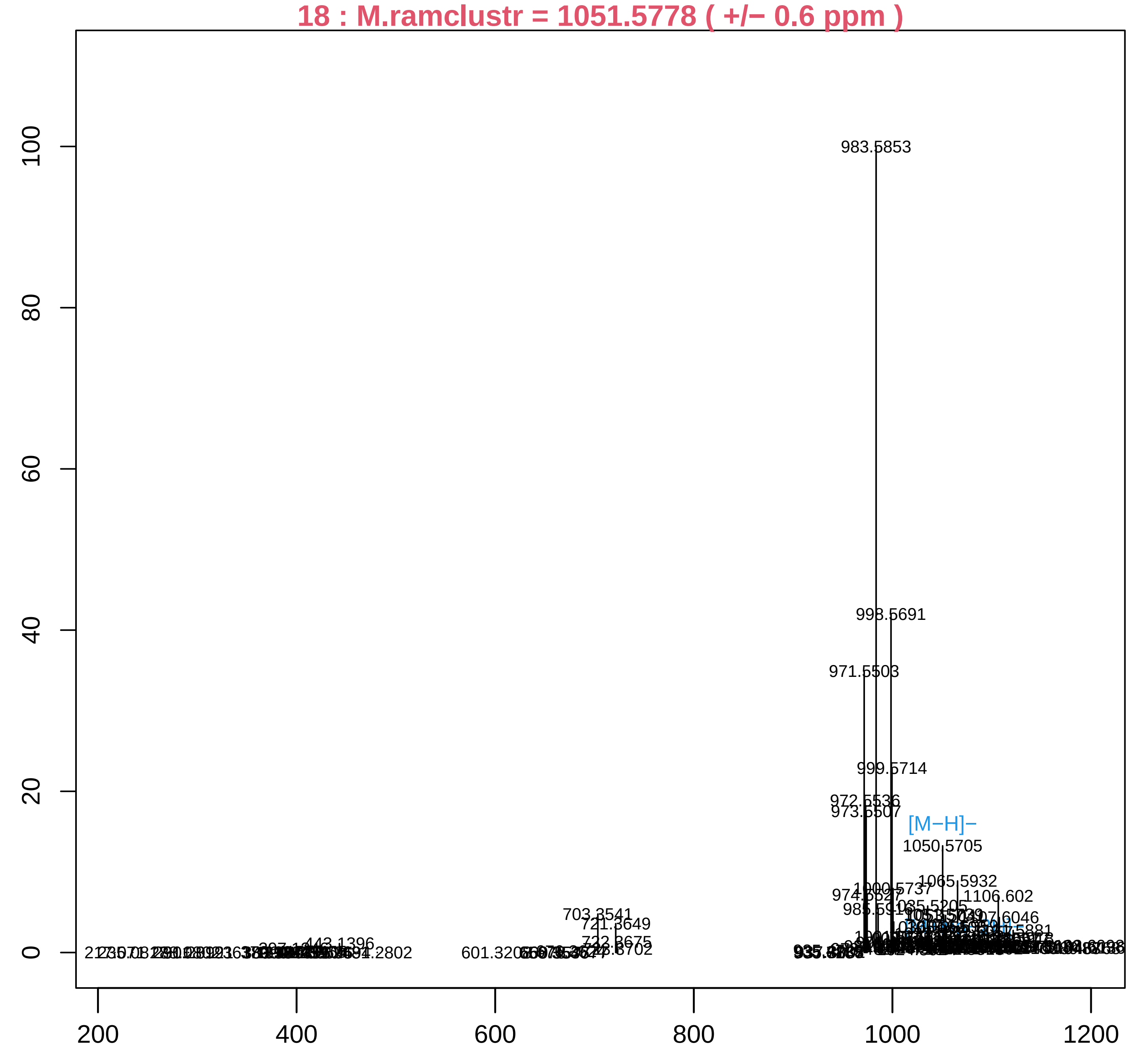
18 : M.ramclustr = 1051.5778 (+/− 0.6 ppm)
983.5853
998.5691
971.5503
999.5714
972.5536
973.5507
[M−H]−
1050.5705
1065.5932
1106.602
703.3541
721.3649
722.3675
443.1396
601.3206
935.8361
0
20
40
60
80
100
200
400
600
800
1000
1200

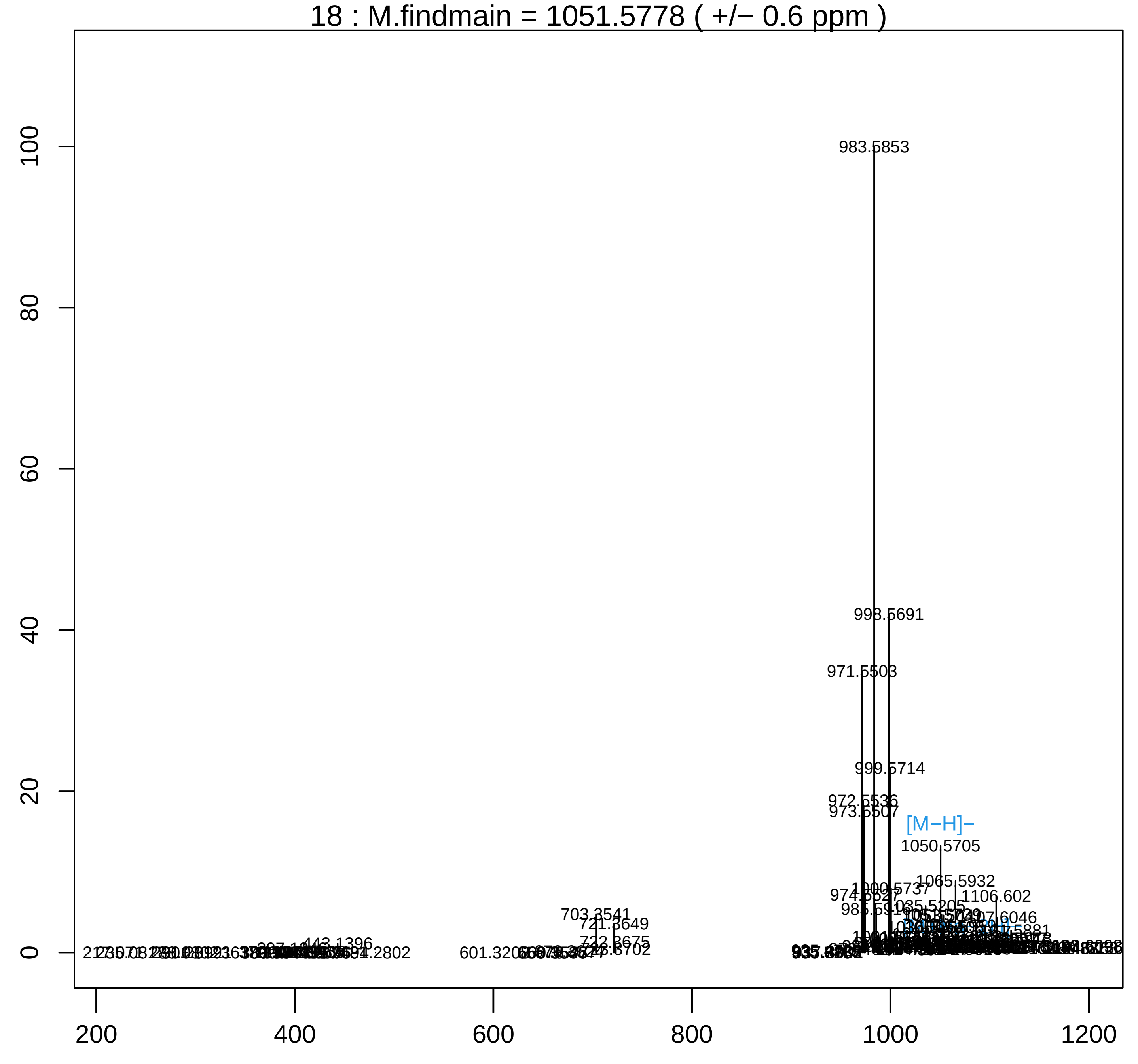
18 : M.findmain = 1051.5778 (+/− 0.6 ppm)
983.5853
998.5691
971.5503
999.5714
972.5536
973.5507
[M−H]−
1050.5705
1065.5932
1106.602
703.3541
721.3649
722.3675
443.1396
601.3206
935.8361
0
20
40
60
80
100
200
400
600
800
1000
1200

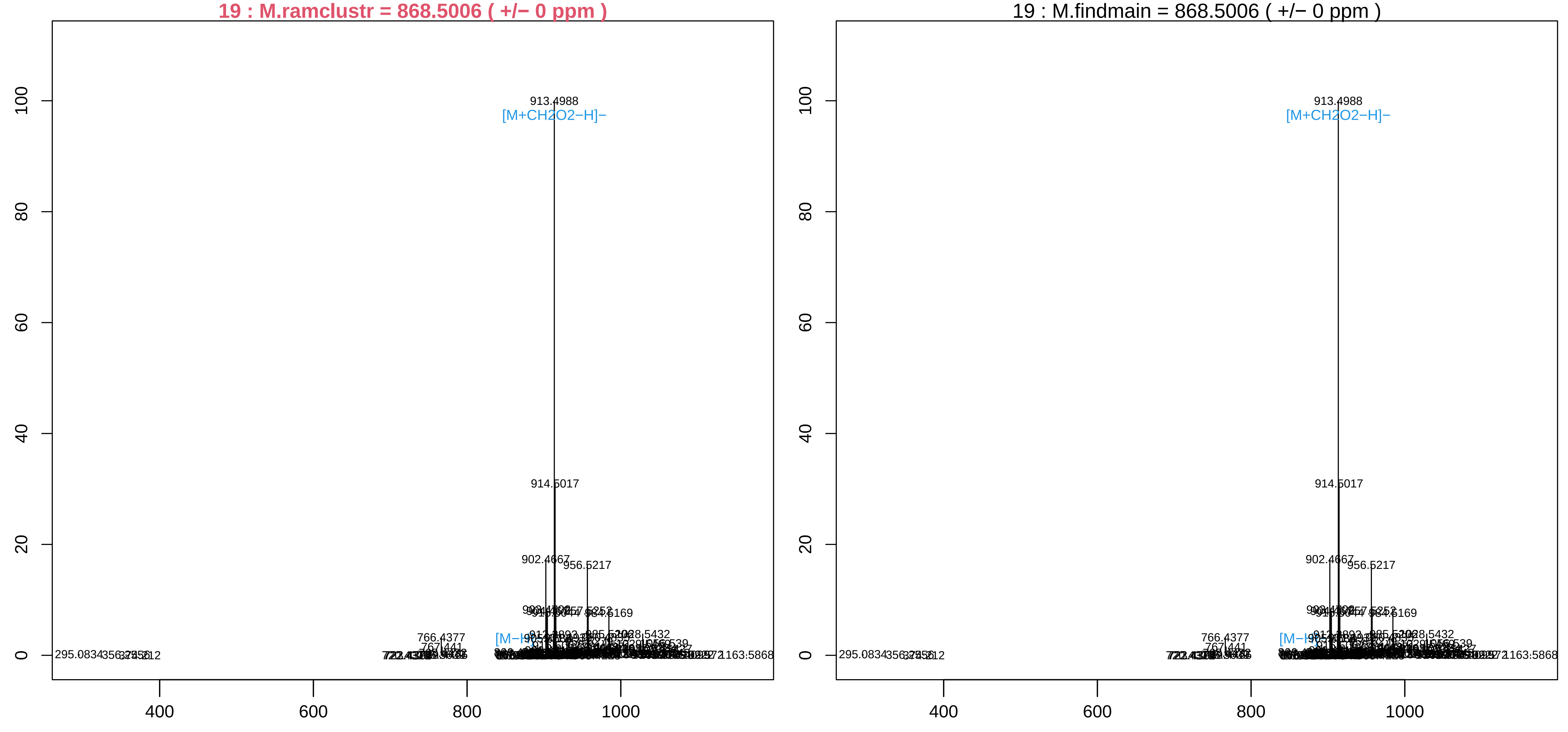

19 : M.ramclustr = 868.5006 (+/− 0 ppm)
913.4988
[M+CH2O2−H]−
914.5017
902.4667
956.5217
984.5169
1028.5432
766.4377
295.0834
1163.5868
19 : M.findmain = 868.5006 (+/− 0 ppm)
913.4988
[M+CH2O2−H]−
914.5017
902.4667
956.5217
984.5169
1028.5432
766.4377
295.0834
1163.5868

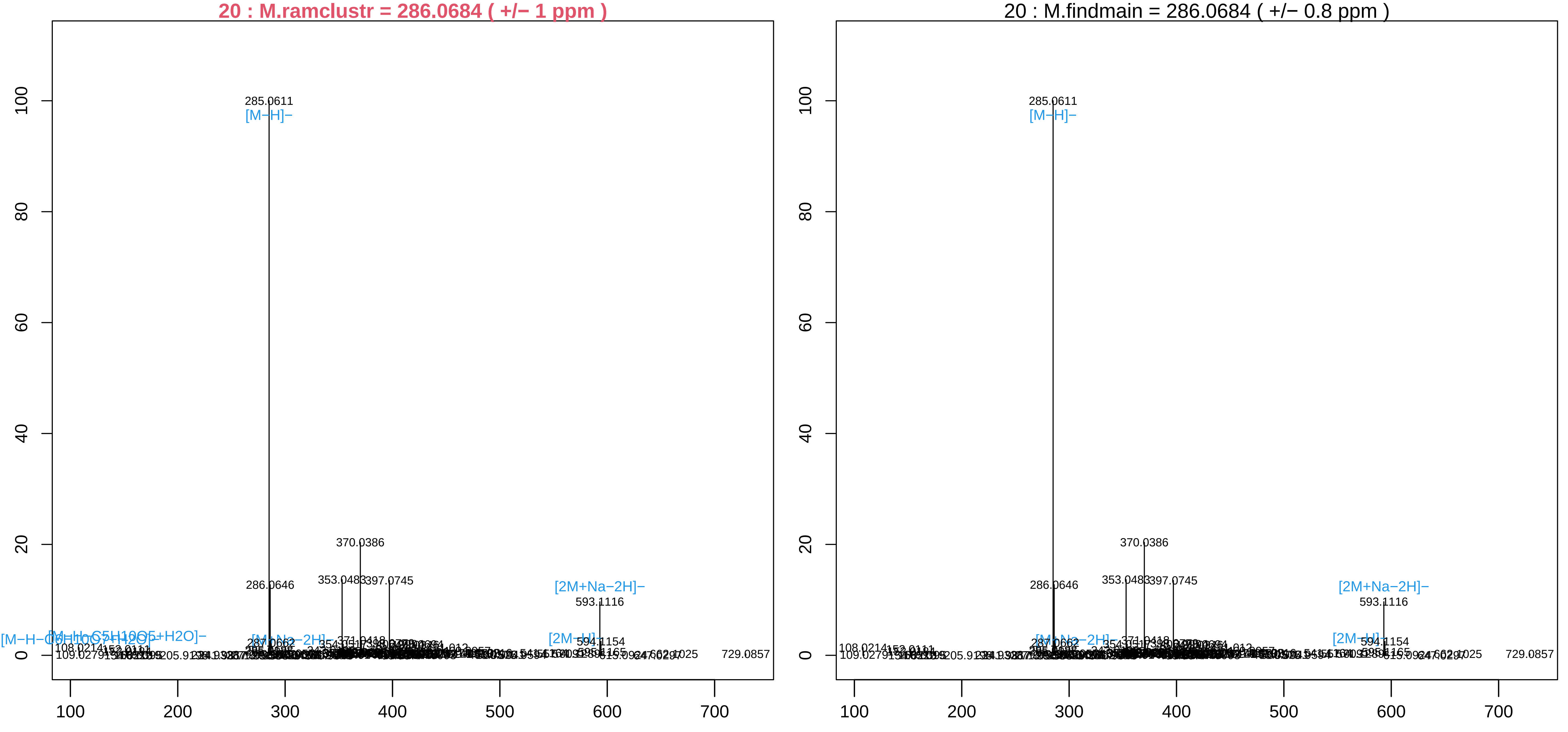
20 : M.ramclustr = 286.0684 (+/− 1 ppm)
20 : M.findmain = 286.0684 (+/− 0.8 ppm)
285.0611
[M−H]−
370.0386
353.0483
397.0745
286.0646
[2M+Na−2H]−
593.1116
[2M−H]−
594.1154
729.0857
100
80
60
40
20
0
100
200
300
400
500
600
700

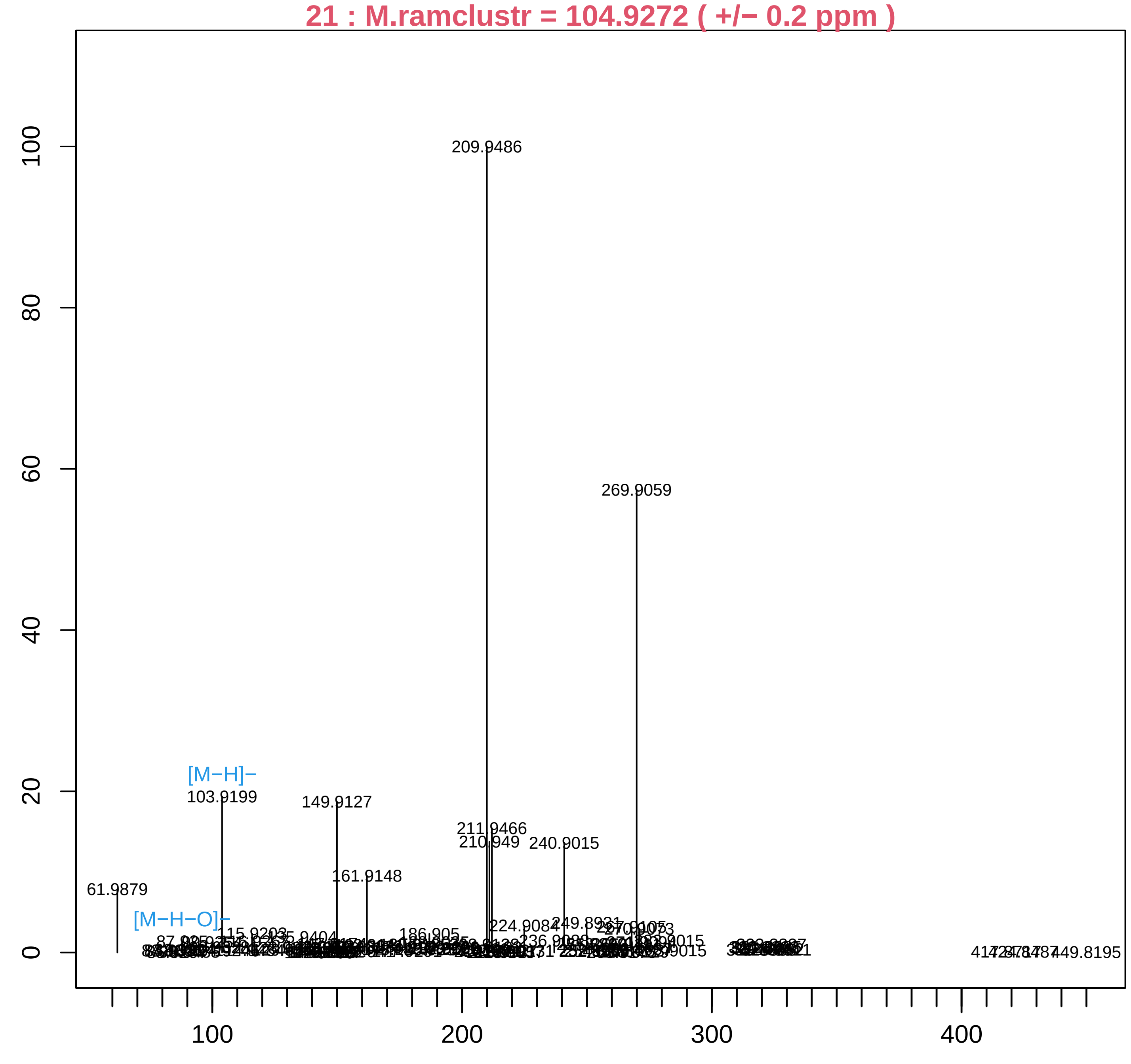
21 : M.ramclustr = 104.9272 (+/− 0.2 ppm)
209.9486
269.9059
[M−H]−
103.9199
149.9127
211.9466
210.949
240.9015
61.9879
161.9148
[M−H−O]−
224.9084
249.8931
186.905
449.8195
0
20
40
60
80
100
100
200
300
400

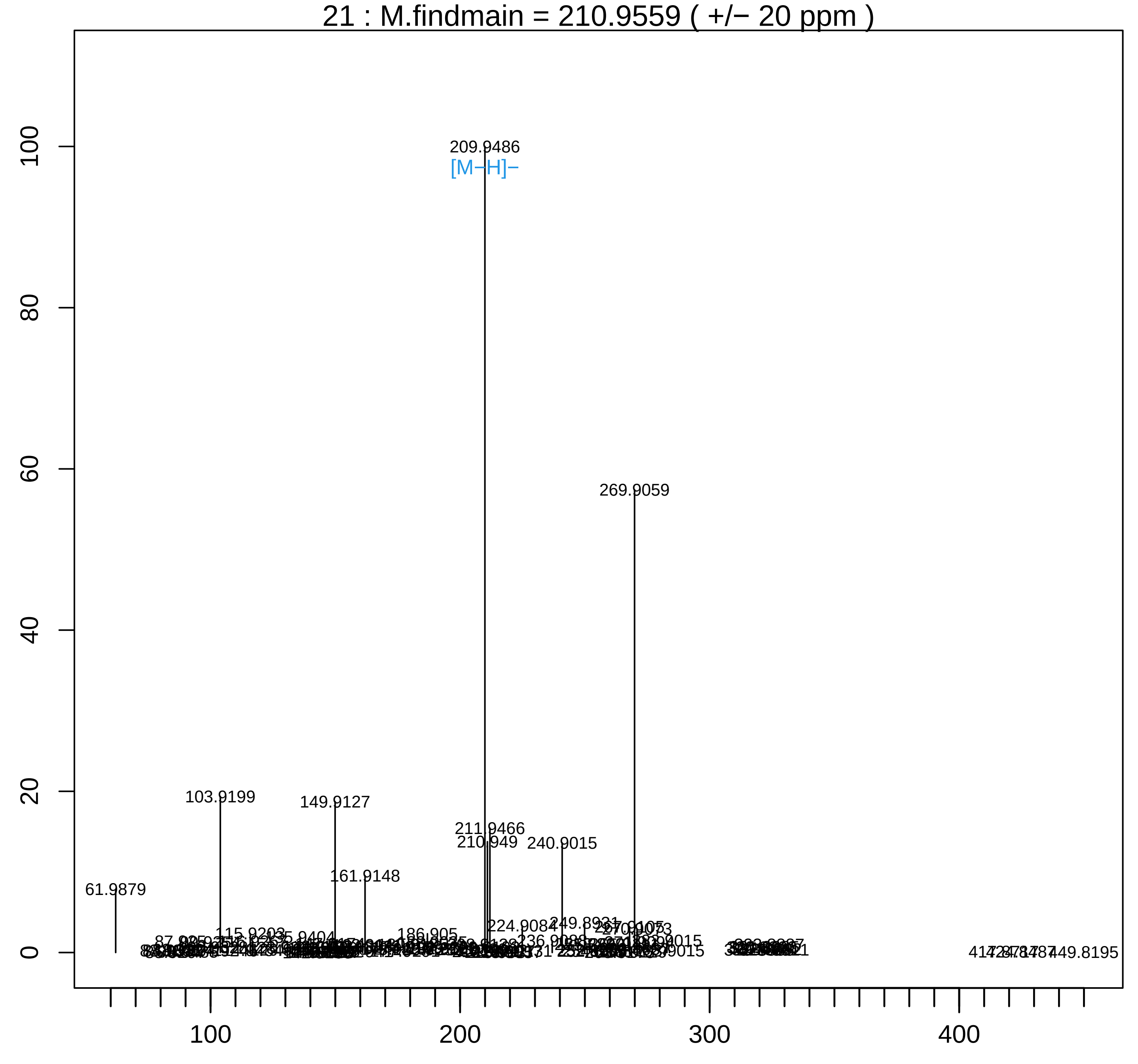
21 : M.findmain = 210.9559 (+/− 20 ppm)
209.9486
[M−H]−
269.9059
103.9199
149.9127
211.9466
210.949
240.9015
61.9879
161.9148
224.9084
249.8931
186.905
449.8195
0
20
40
60
80
100
100
200
300
400

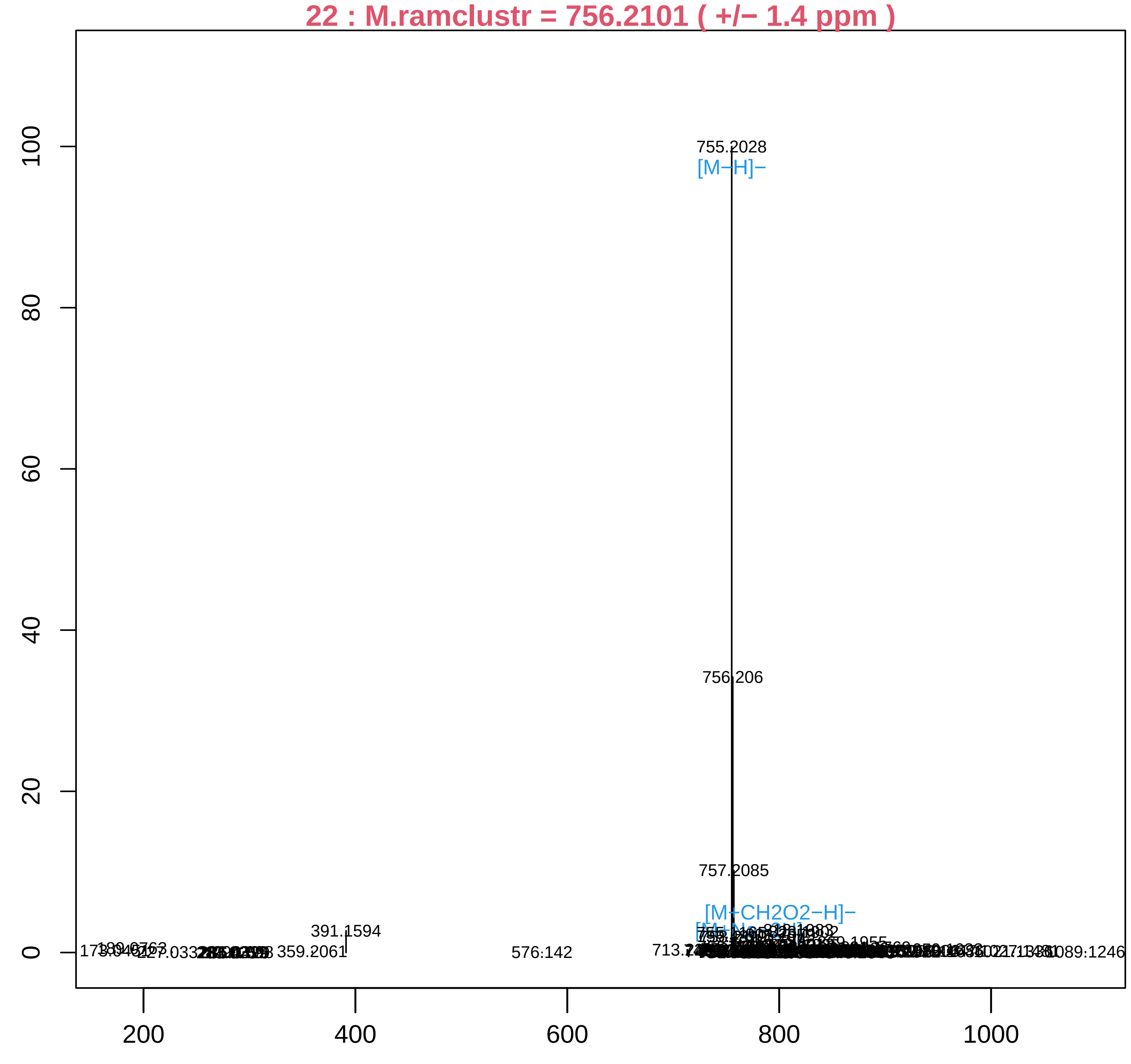
22 : M.ramclustr = 756.2101 (+/− 1.4 ppm)
755.2028
[M−H]−
756.206
757.2085
[M+CH2O2−H]−
391.1594
359.2061
576.142
1089.1246
0
20
40
60
80
100
200
400
600
800
1000

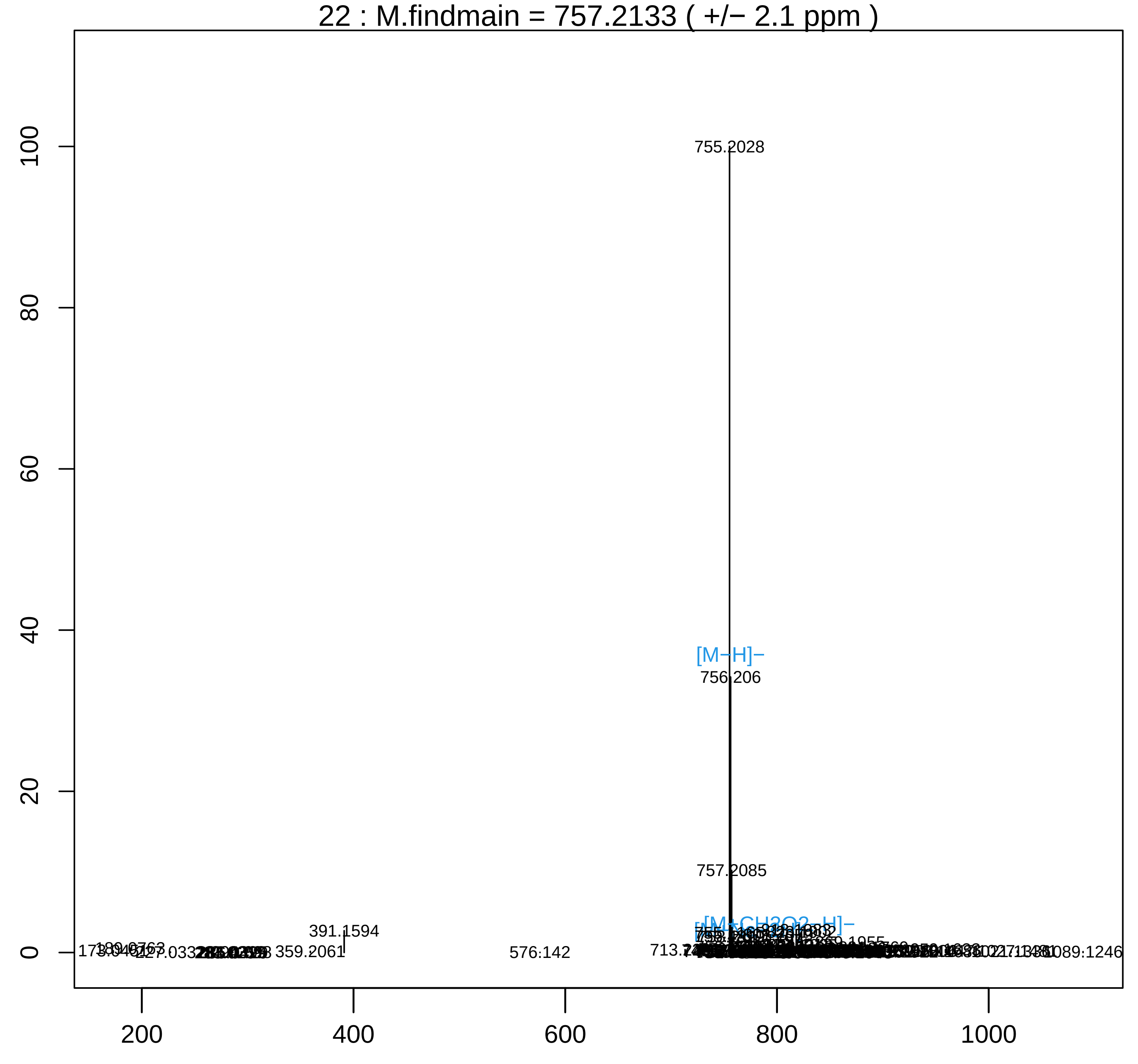
22 : M.findmain = 757.2133 (+/− 2.1 ppm)
755.2028
[M−H]−
756.206
757.2085
391.1594
359.2061
576.142
1089.1246
0
20
40
60
80
100
200
400
600
800
1000

23 : M.ramclustr = 316.0789 (+/− 2.5 ppm)

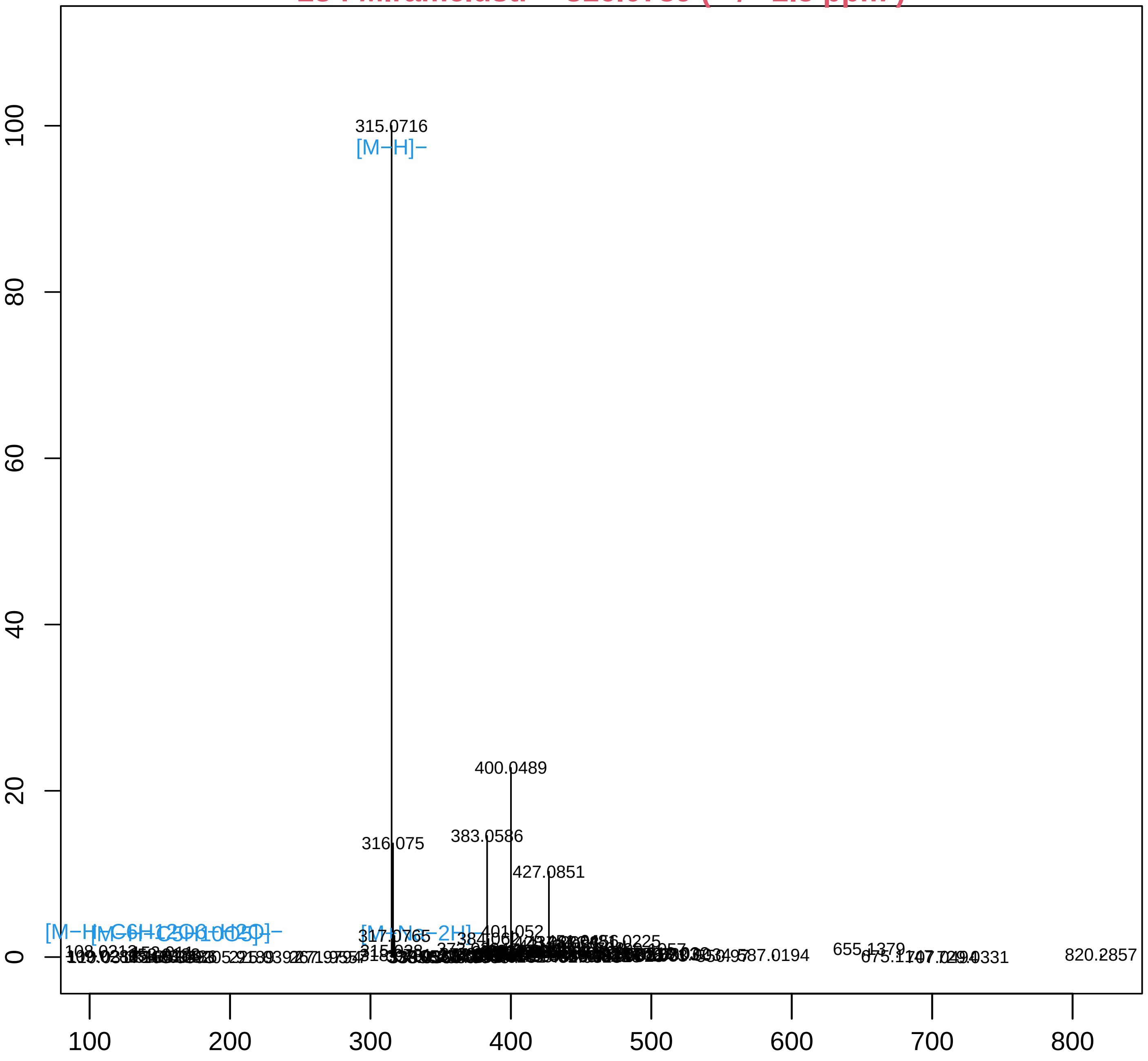

23 : M.findmain = 316.0789 (+/− 2.5 ppm)

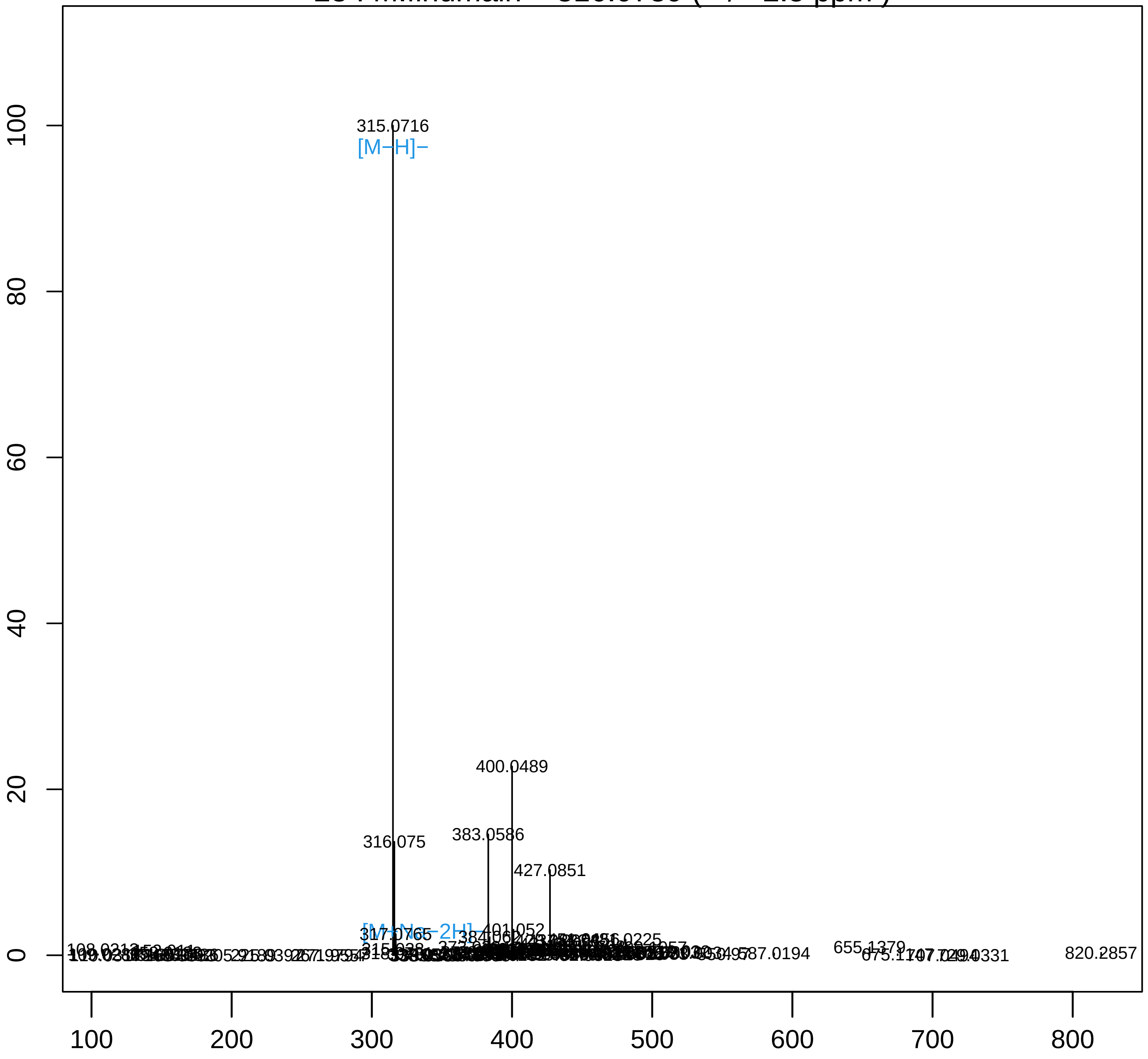

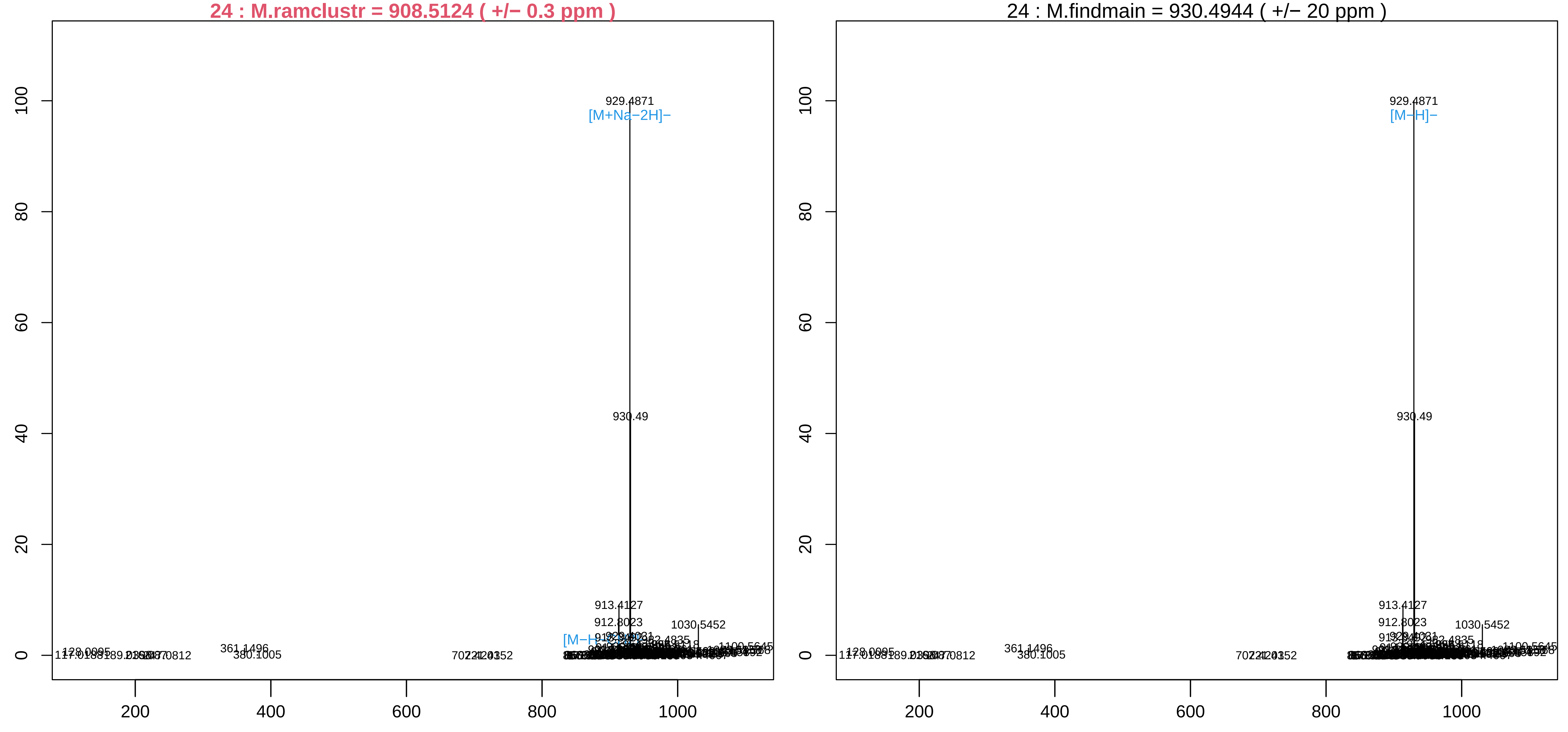

24 : M.ramclustr = 908.5124 (+/− 0.3 ppm)
929.4871
[M+Na−2H]−
930.49
913.4127
912.8023
1030.5452
361.1496
380.1005
128.0095
100
80
60
40
20
0
200
400
600
800
1000
24 : M.findmain = 930.4944 (+/− 20 ppm)
929.4871
[M−H]−
930.49
913.4127
912.8023
1030.5452
361.1496
380.1005
128.0095
100
80
60
40
20
0
200
400
600
800
1000

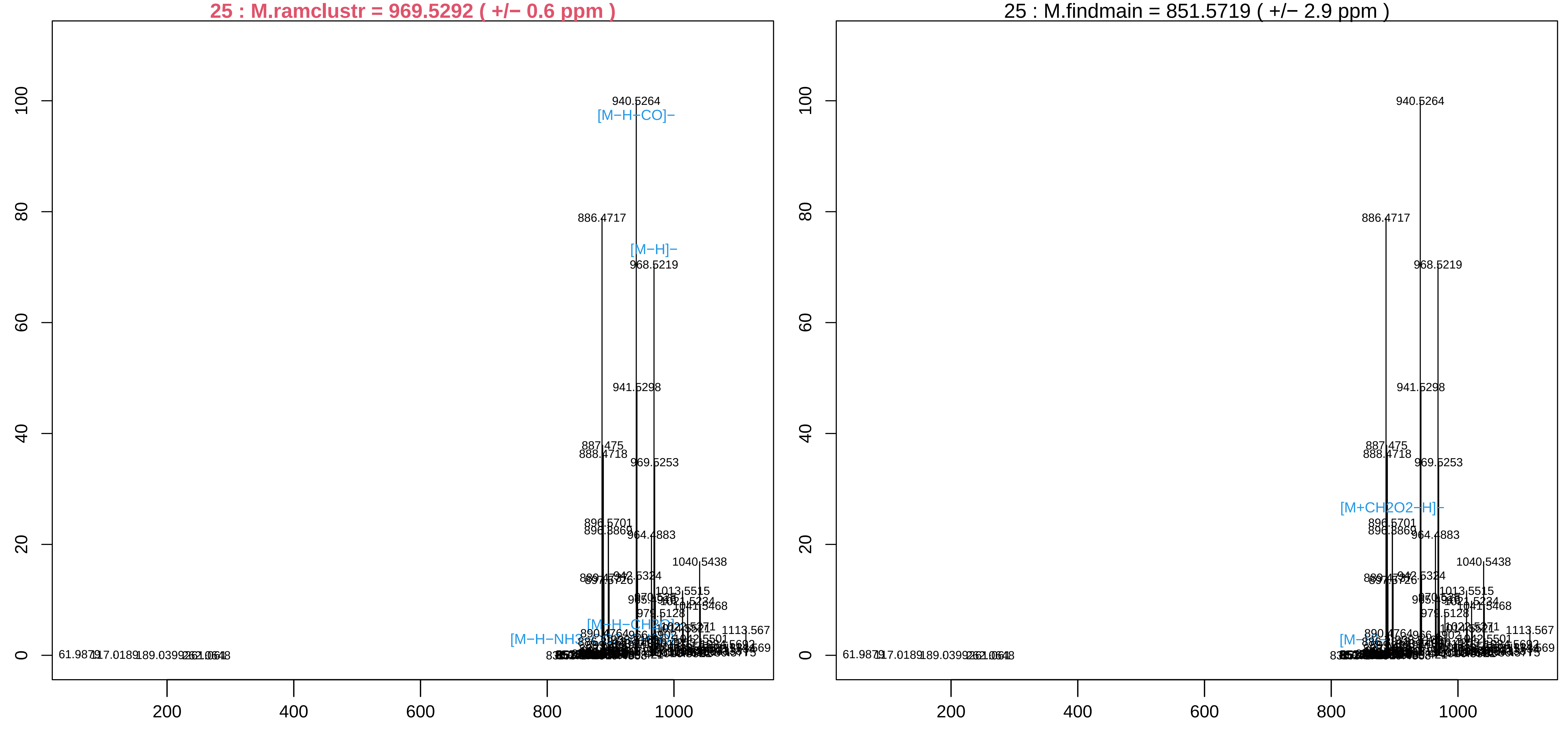

25 : M.ramclustr = 969.5292 (+/− 0.6 ppm)
940.5264
[M−H−CO]−
886.4717
[M−H]−
968.5219
941.5298
887.475
888.4718
969.5253
896.5701
964.4883
1040.5438
942.5324
1013.5515
1041.5468
1113.567
61.9879
117.0189
189.0399
0
20
40
60
80
100
200
400
600
800
1000
25 : M.findmain = 851.5719 (+/− 2.9 ppm)
940.5264
886.4717
968.5219
941.5298
887.475
888.4718
969.5253
[M+CH2O2−H]−
896.5701
964.4883
1040.5438
942.5324
1013.5515
1041.5468
1113.567
61.9879
117.0189
189.0399
0
20
40
60
80
100
200
400
600
800
1000

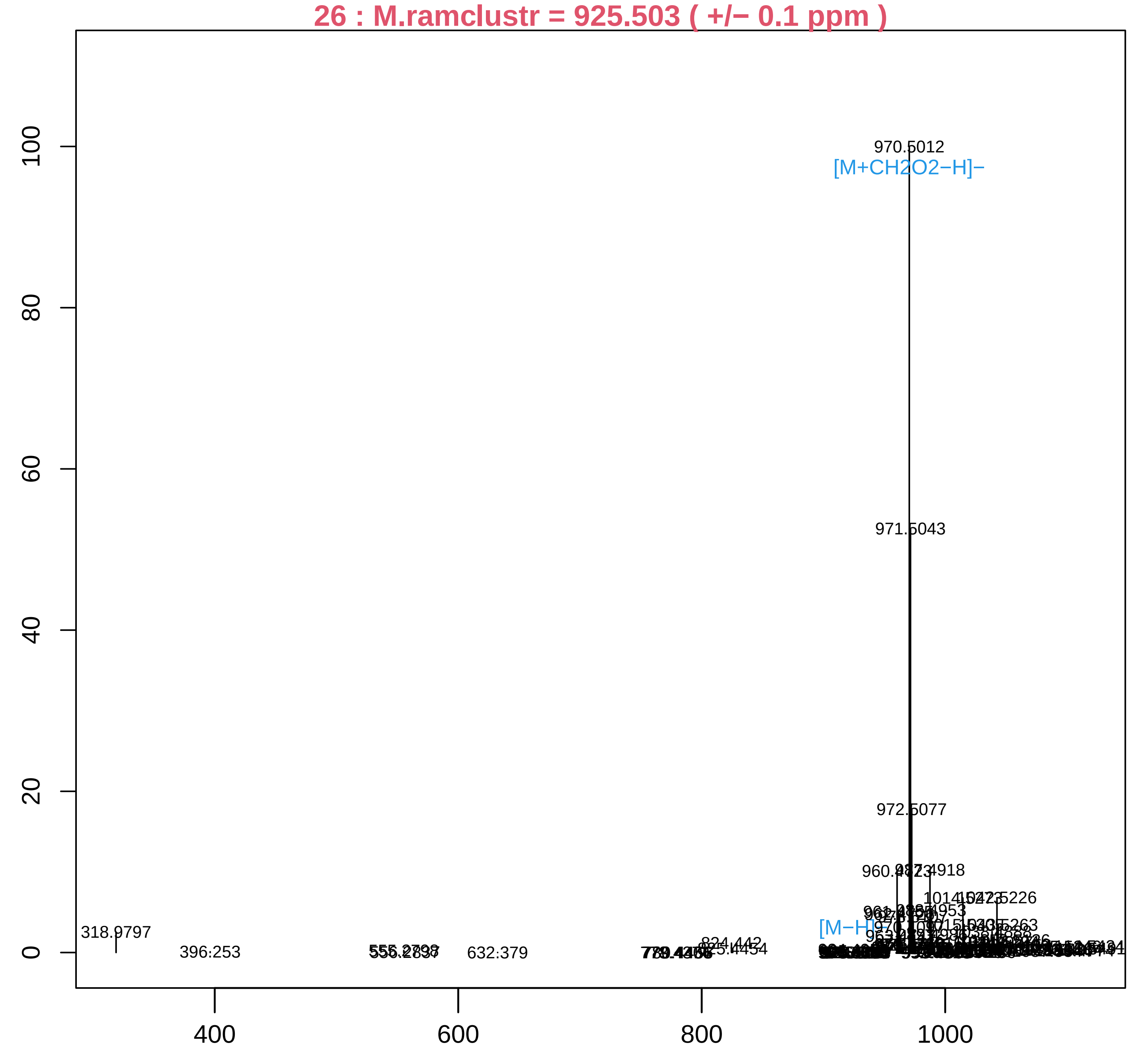
26 : M.ramclustr = 925.503 (+/− 0.1 ppm)
970.5012
[M+CH2O2−H]−
971.5043
972.5077
[M−H]−
318.9797
396:253
632:379
824.442
0
20
40
60
80
100
400
600
800
1000

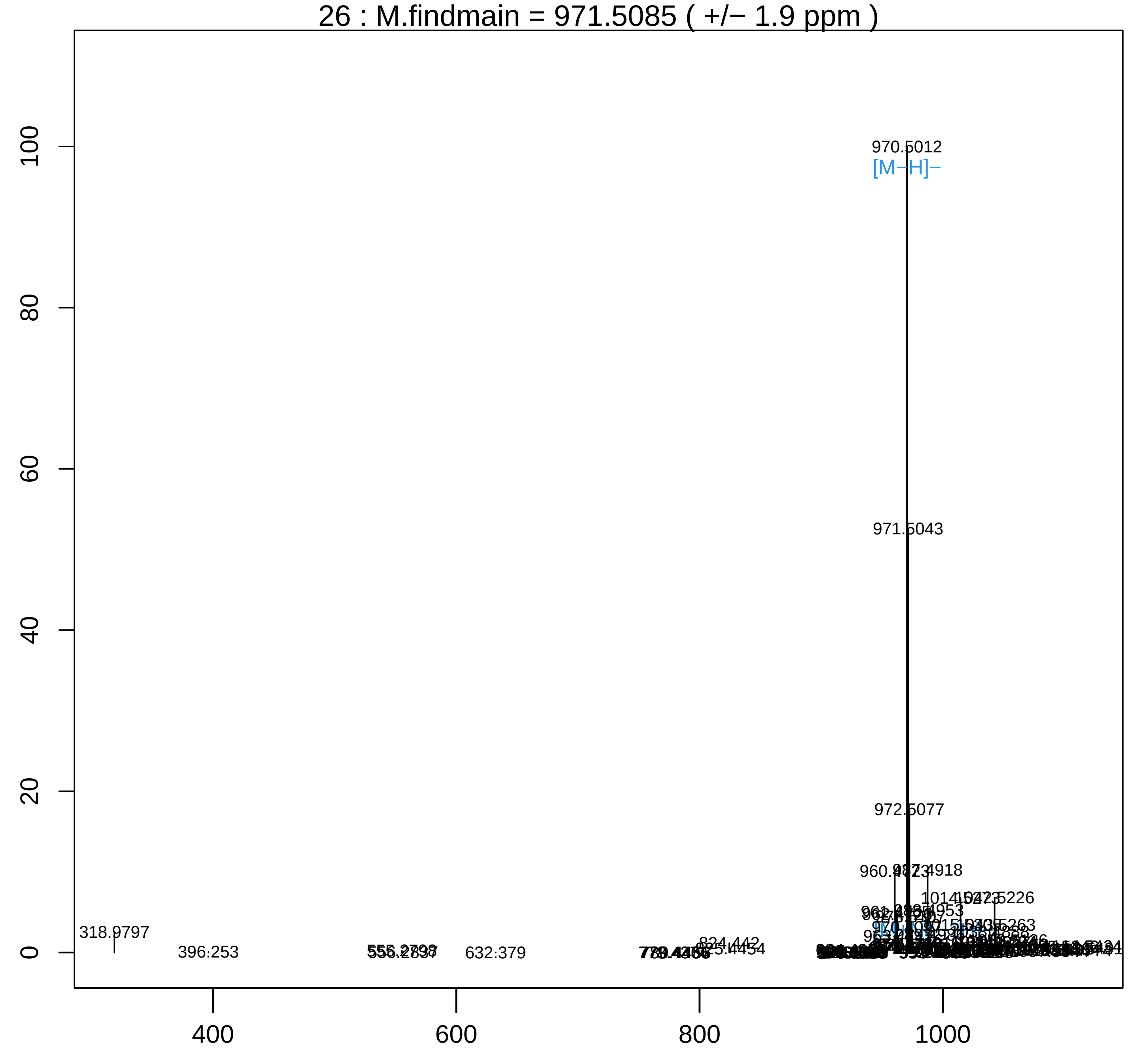
26 : M.findmain = 971.5085 (+/− 1.9 ppm)
970.5012
[M−H]−
971.5043
972.5077
318.9797
396:253
632:379
824.442
0
20
40
60
80
100
400
600
800
1000

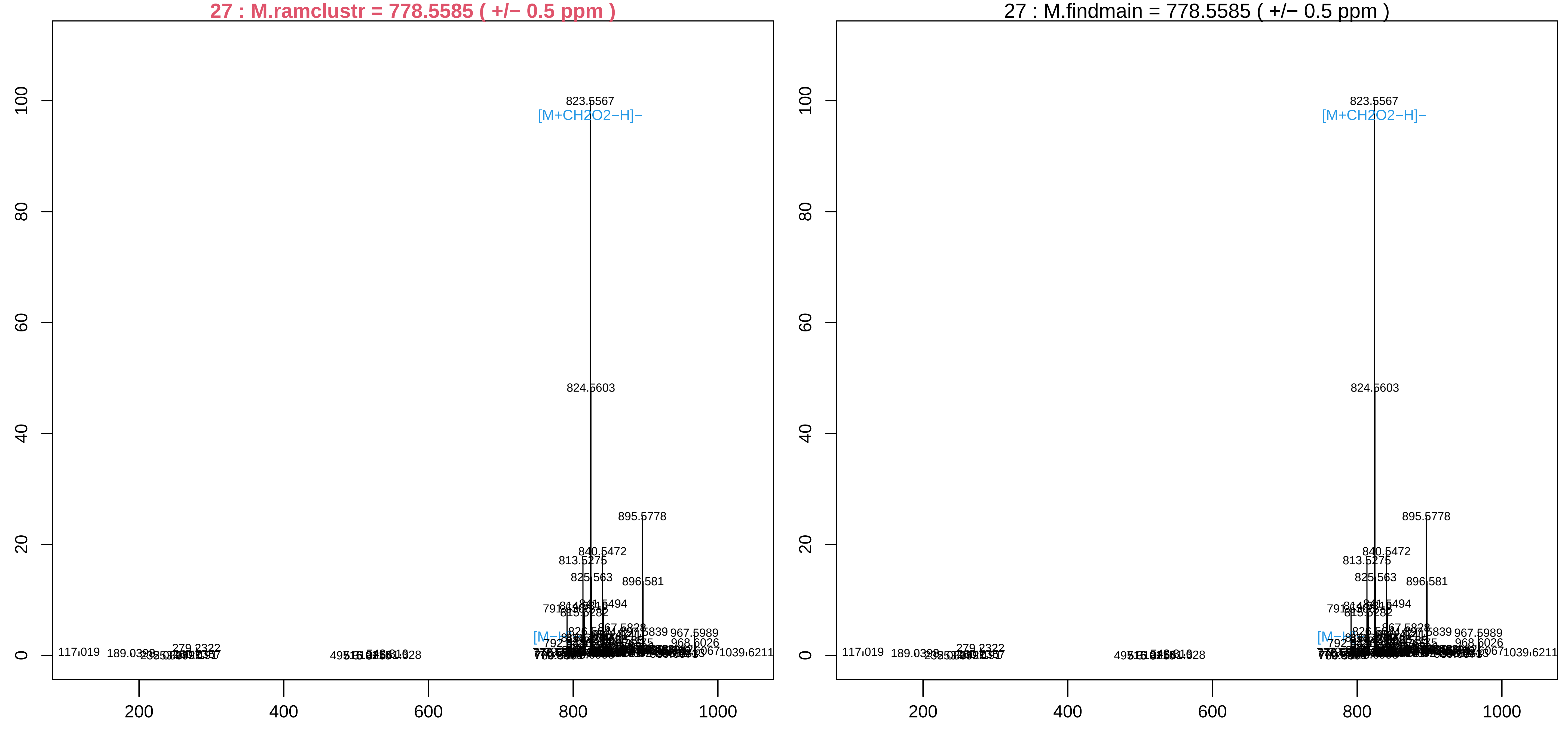

27 : M.ramclustr = 778.5585 (+/− 0.5 ppm)
27 : M.findmain = 778.5585 (+/− 0.5 ppm)
823.5567
[M+CH2O2−H]−
824.5603
895.5778
840.5472
813.5275
825.563
896.581
841.5494
967.5989
968.6026
279.2322
117.019
189.0398
1039.6211
0
20
40
60
80
100
200
400
600
800
1000

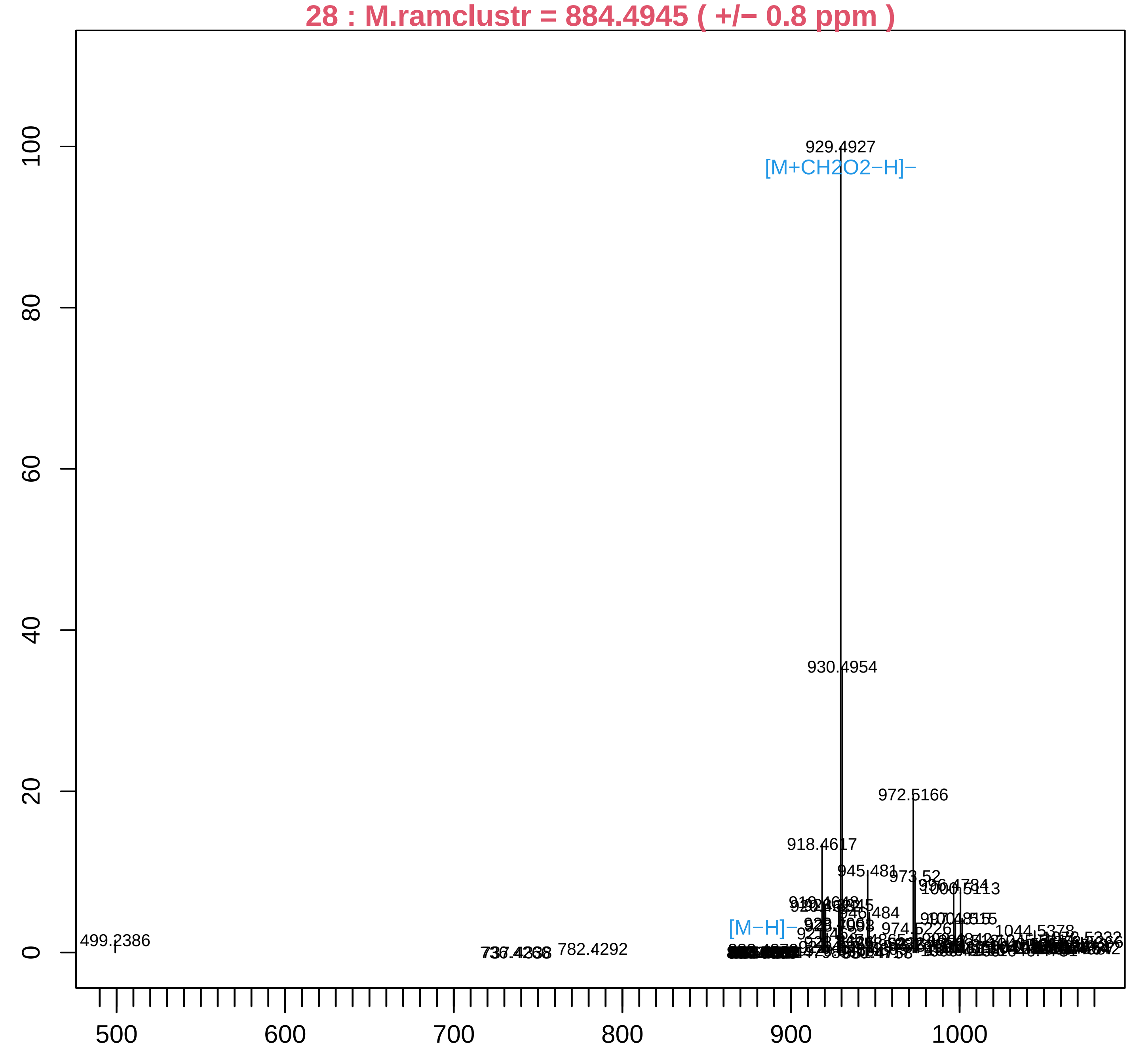
28 : M.ramclustr = 884.4945 (+/− 0.8 ppm)
929.4927
[M+CH2O2−H]−
930.4954
972.5166
918.4617
945.481
[M−H]−
499.2386
782.4292
0
20
40
60
80
100
500
600
700
800
900
1000

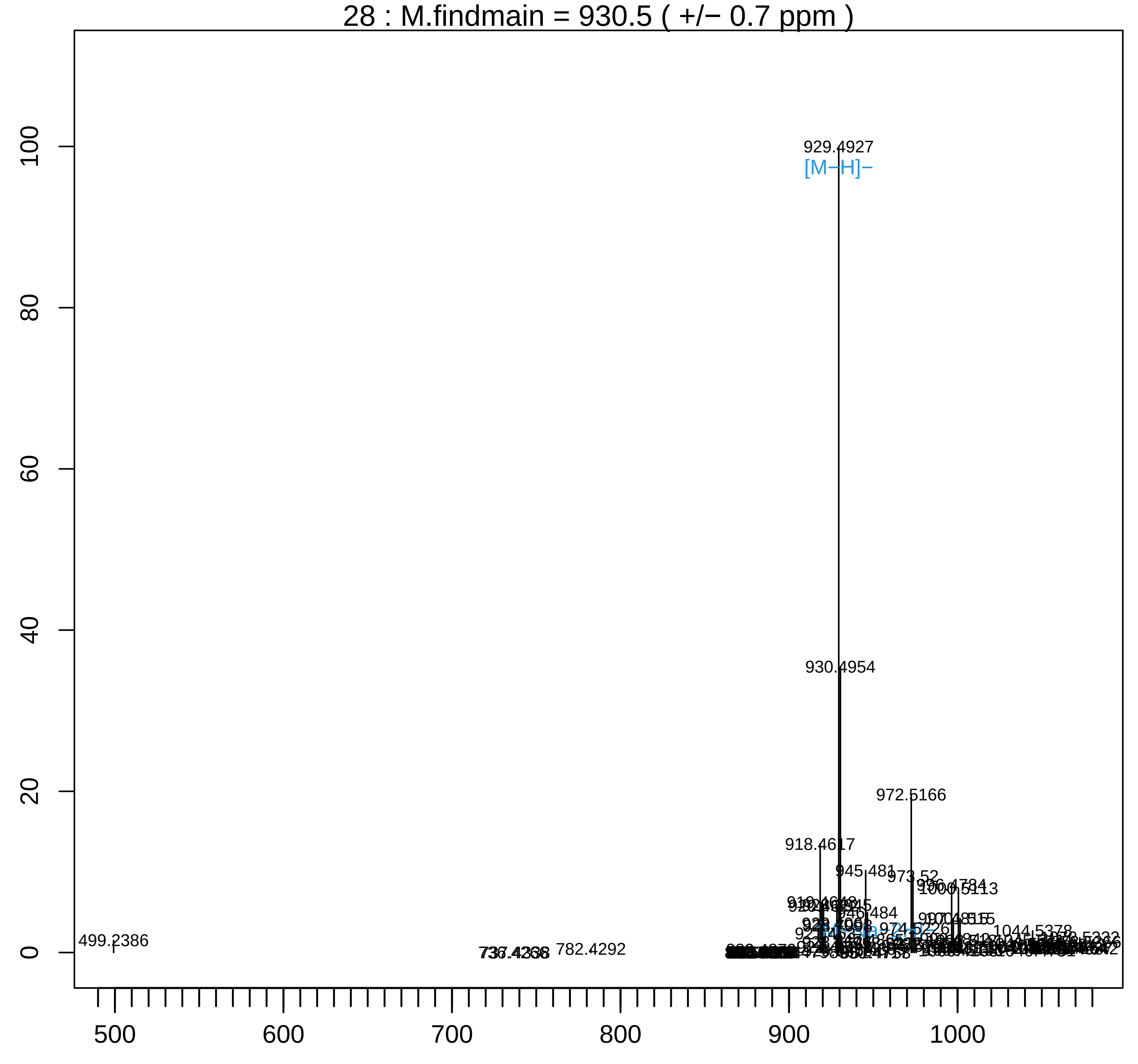
28 : M.findmain = 930.5 (+/− 0.7 ppm)
929.4927
[M−H]−
930.4954
972.5166
918.4617
945.481
499.2386
782.4292
0
20
40
60
80
100
500
600
700
800
900
1000

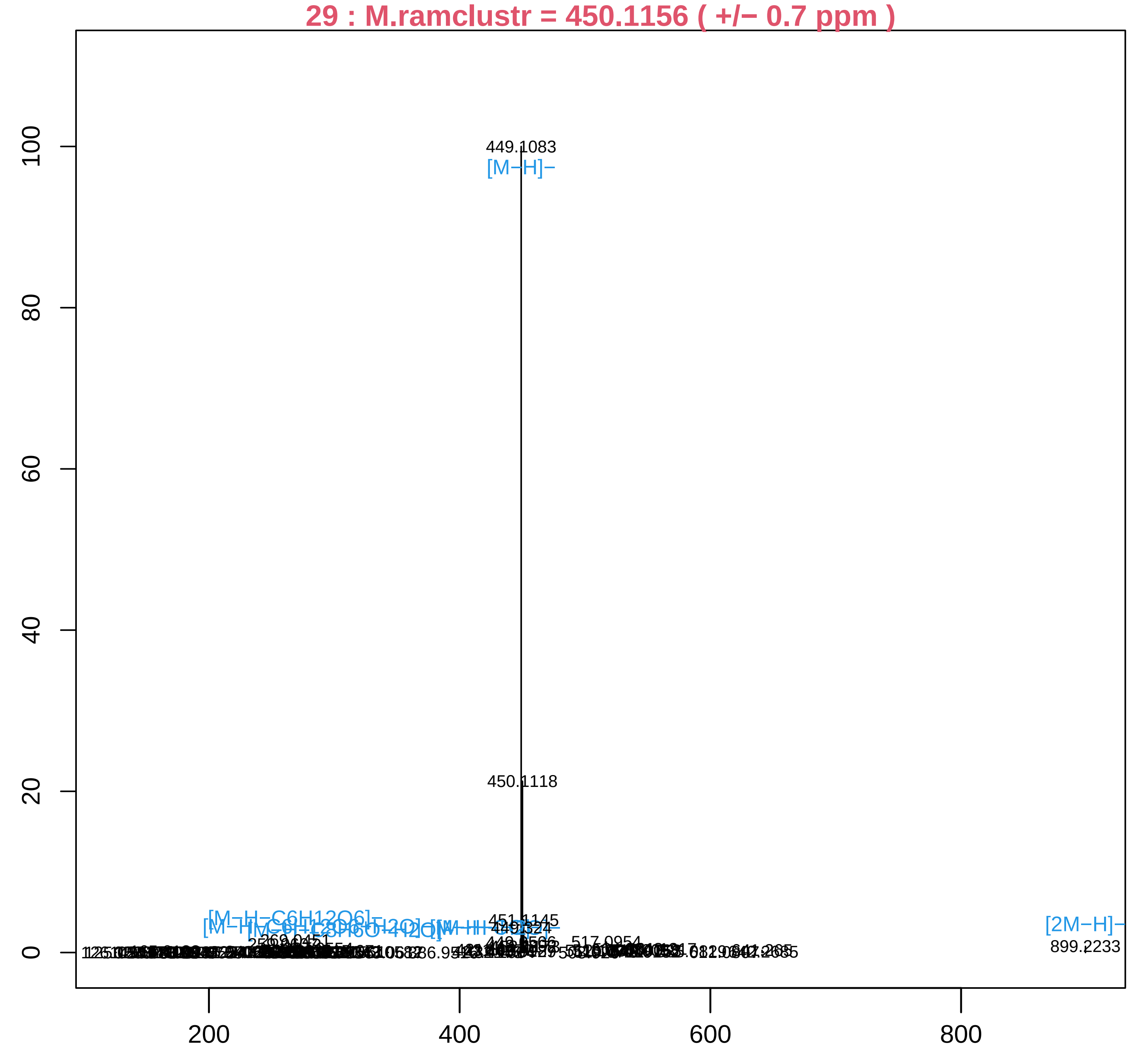
29 : M.ramclustr = 450.1156 (+/− 0.7 ppm)
449.1083
[M−H]−
450.1118
451.1145
517.0954
[2M−H]−
899.2233
0
20
40
60
80
100
200
400
600
800

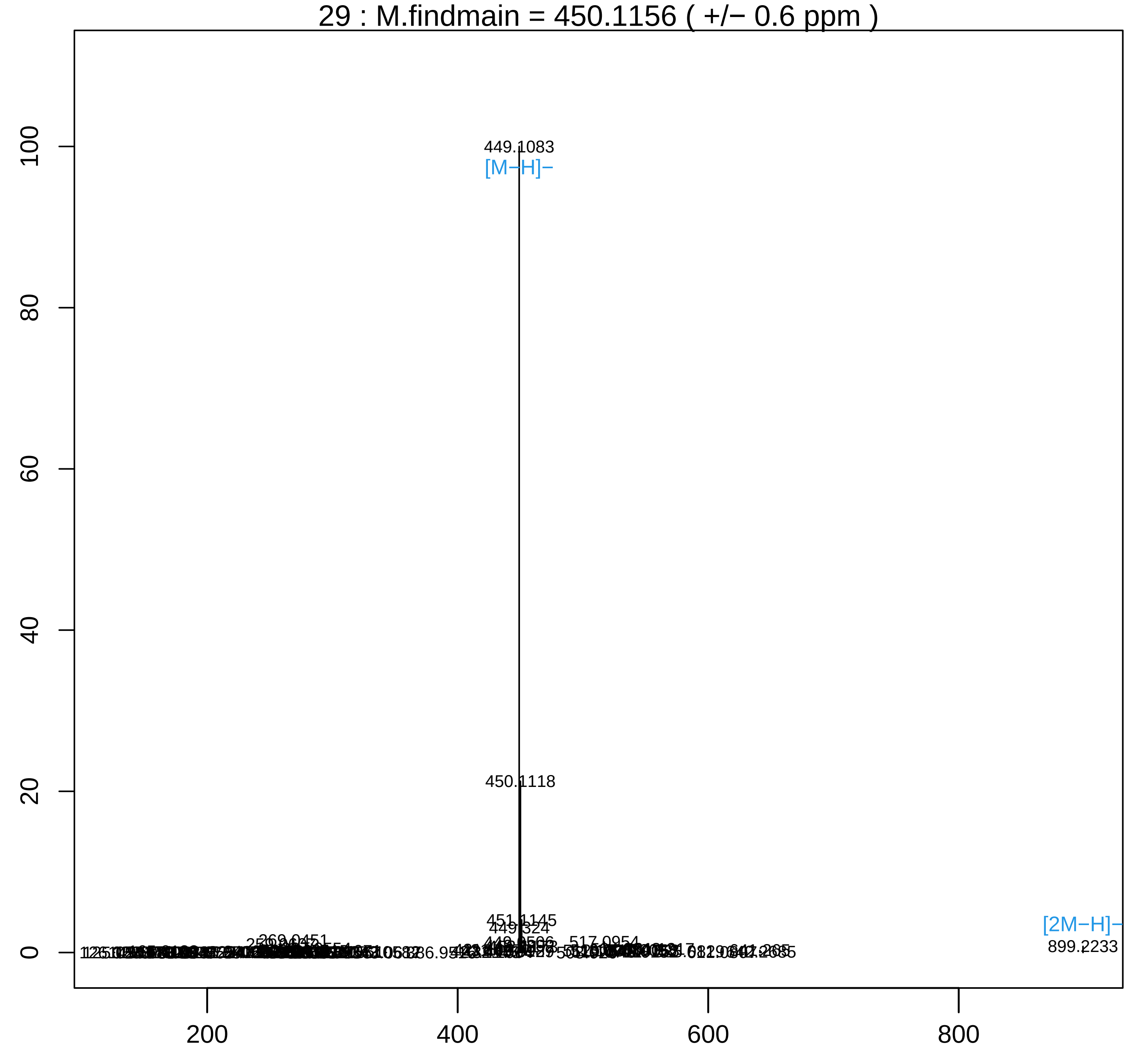
29 : M.findmain = 450.1156 (+/− 0.6 ppm)
449.1083
[M−H]−
450.1118
451.1145
269.0451
517.0954
[2M−H]−
899.2233
0
20
40
60
80
100
200
400
600
800

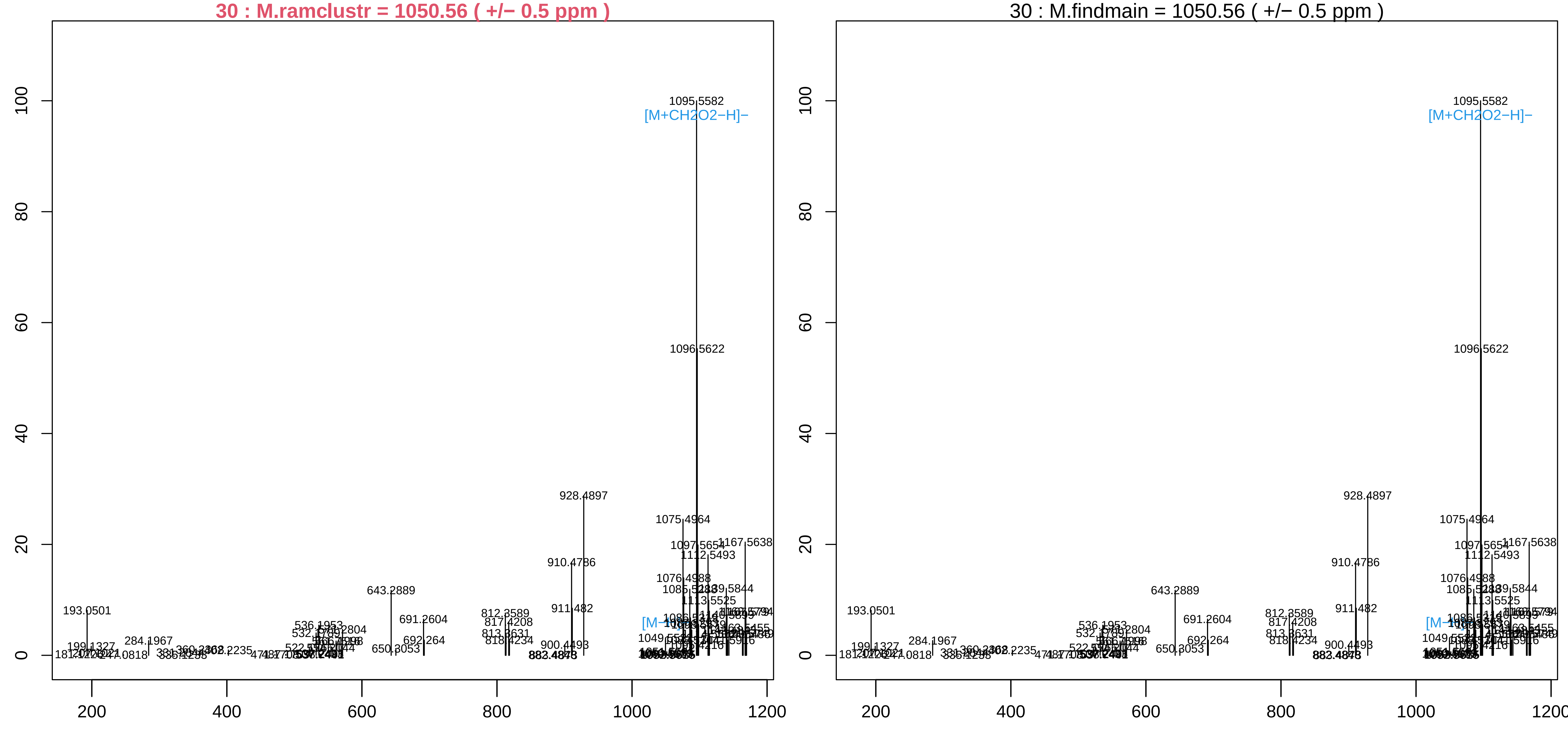

30 : M.ramclustr = 1050.56 (+/− 0.5 ppm)
30 : M.findmain = 1050.56 (+/− 0.5 ppm)
1095.5582
[M+CH2O2−H]−
1096.5622
928.4897
1075.4964
1097.5654
1167.5638
1112.5493
910.4786
1076.4988
643.2889
193.0501
911.482
812.3589
817.4208
691.2604
536.1953
813.3631
818.4234
692.264
284.1967
650.3053
900.4493
199.1327
882.4878
0
20
40
60
80
100
200
400
600
800
1000
1200

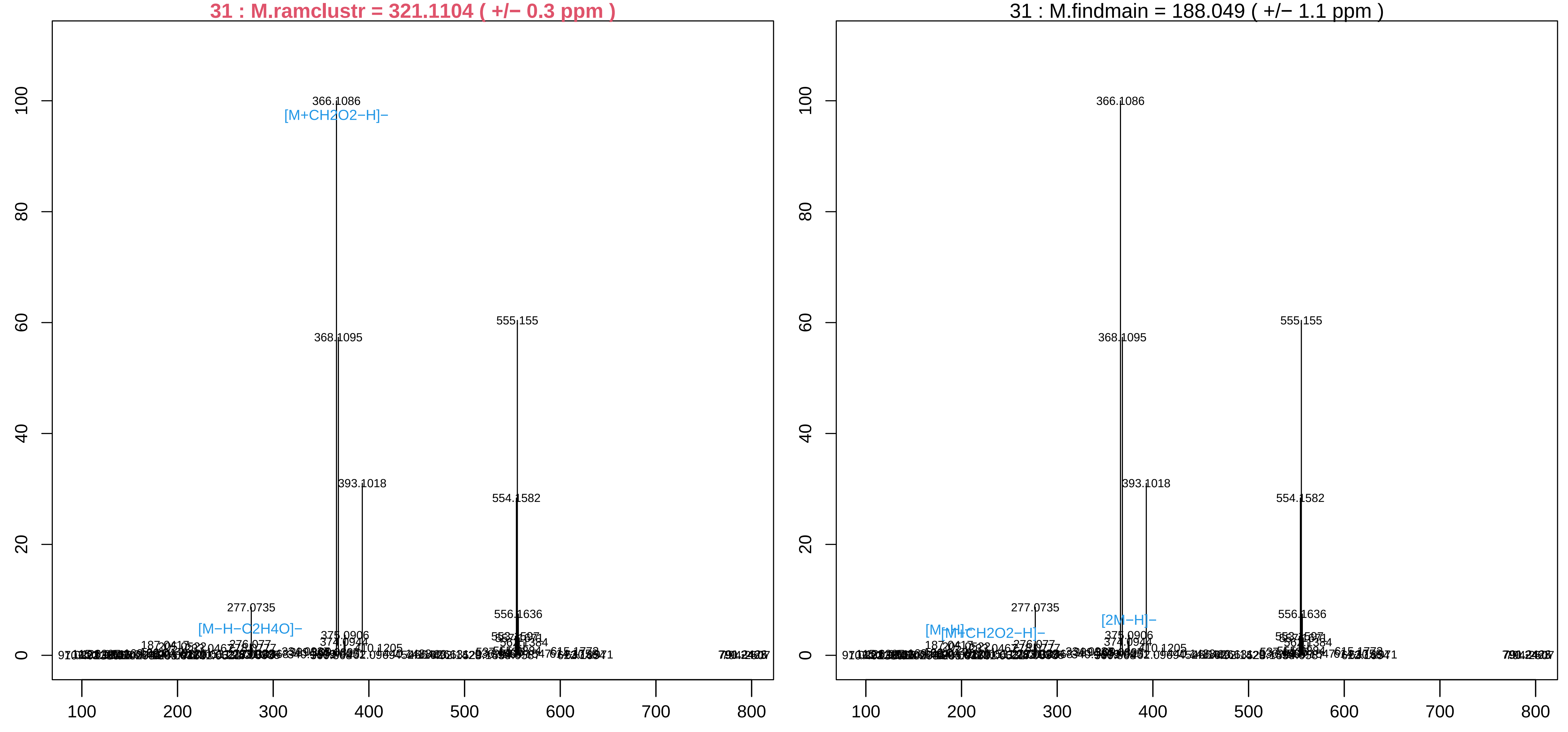
31 : M.ramclustr = 321.1104 (+/− 0.3 ppm)
31 : M.findmain = 188.049 (+/− 1.1 ppm)
366.1086
[M+CH2O2−H]−
368.1095
393.1018
555.155
554.1582
556.1636
277.0735
[M−H−C2H4O]−
375.0906
374.0944
410.1205
[M−H]−
[M+CH2O2−H]−
[2M−H]−
0
20
40
60
80
100
100
200
300
400
500
600
700
800

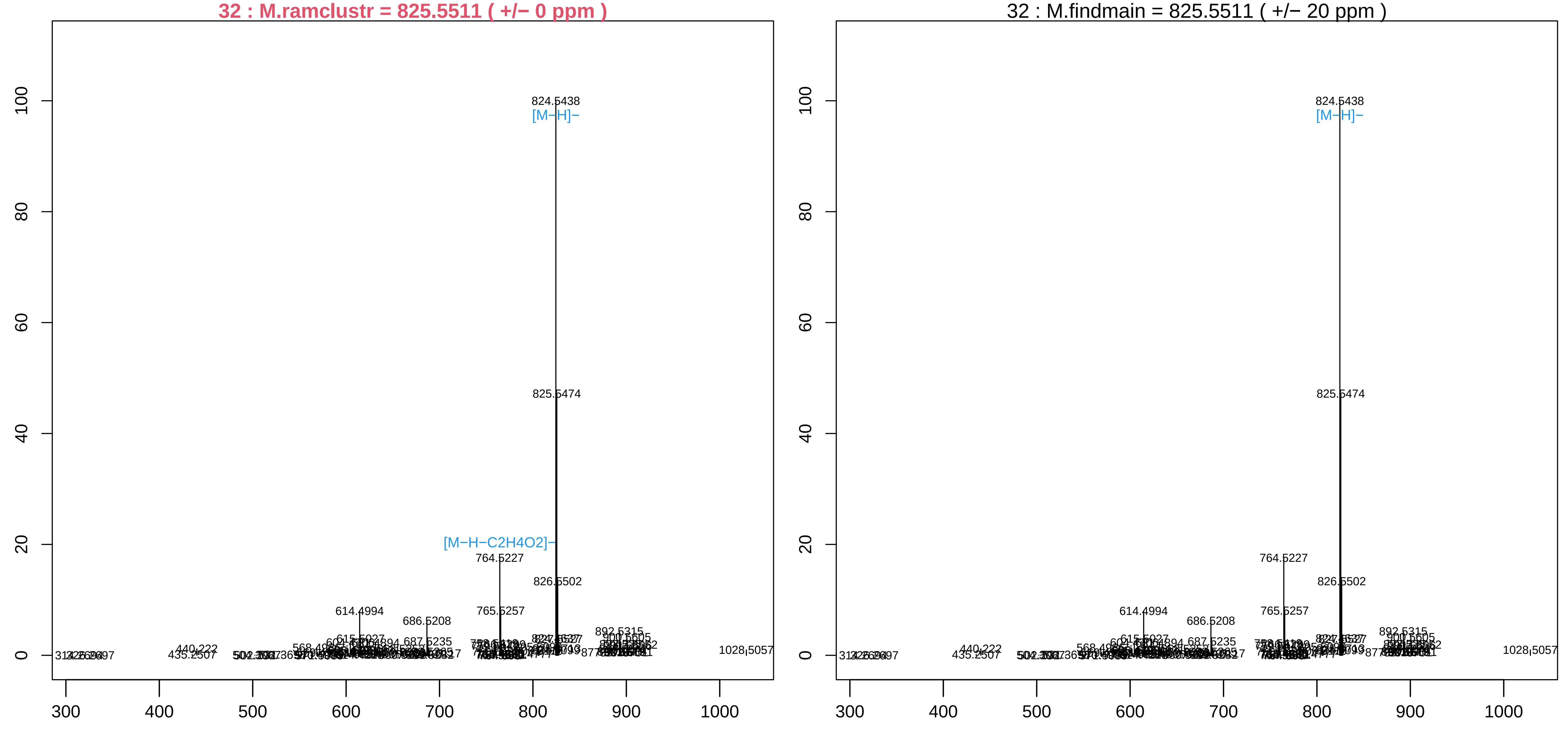

32 : M.ramclustr = 825.5511 (+/− 0 ppm)
824.5438
[M−H]−
825.5474
[M−H−C2H4O2]−
764.5227
826.5502
614.4994
765.5257
686.5208
892.5315
1028.5057
435.2507
440.222
32 : M.findmain = 825.5511 (+/− 20 ppm)

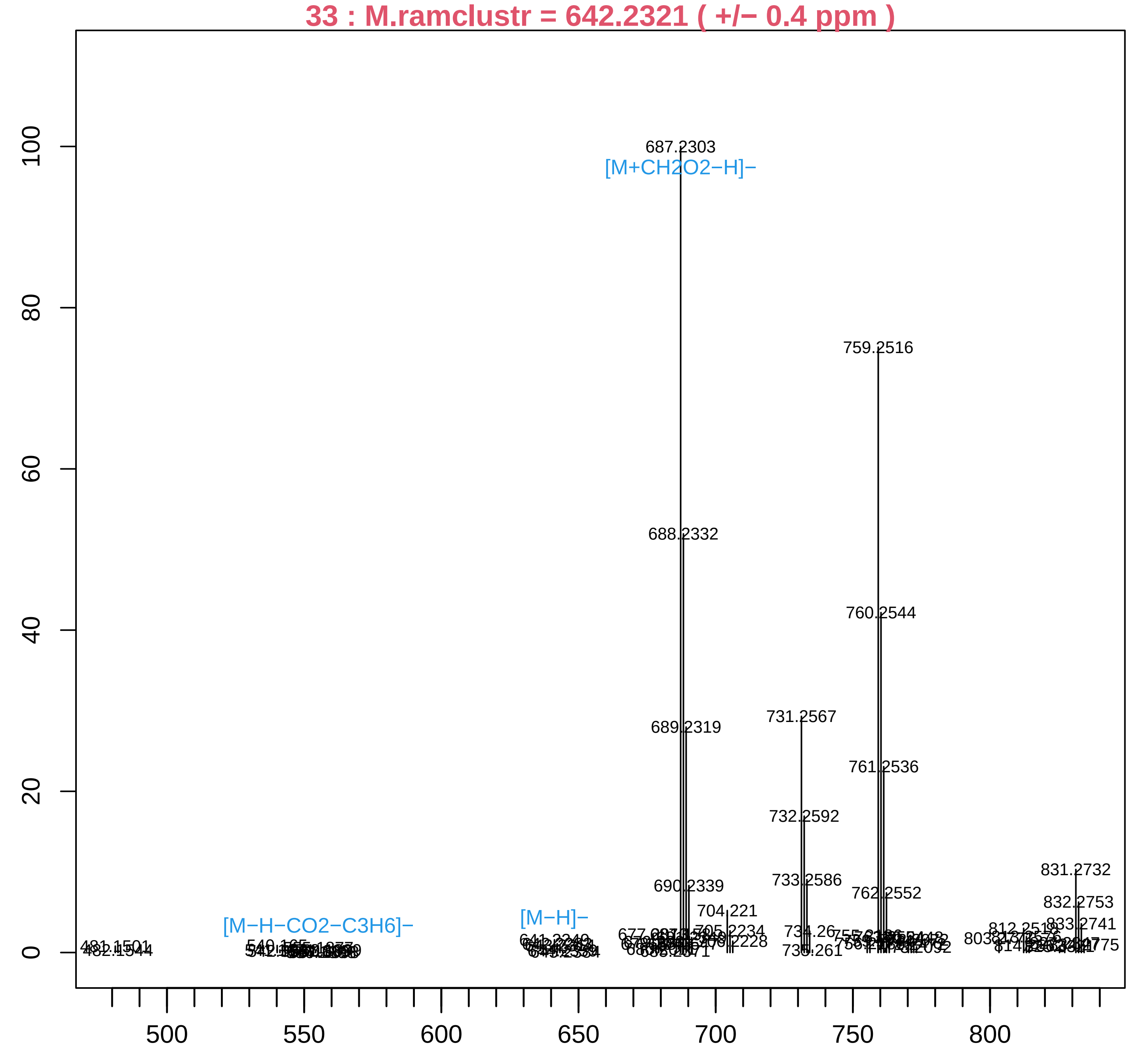
33 : M.ramclustr = 642.2321 (+/− 0.4 ppm)
100
80
60
40
20
0
500
550
600
650
700
750
800
687.2303
[M+CH2O2−H]−
759.2516
688.2332
760.2544
689.2319
731.2567
761.2536
732.2592
831.2732
690.2339
733.2586
762.2552
704.221
832.2753
705.2234
734.26
833.2741
[M−H]−
[M−H−CO2−C3H6]−
481.1501
482.1544
736.261

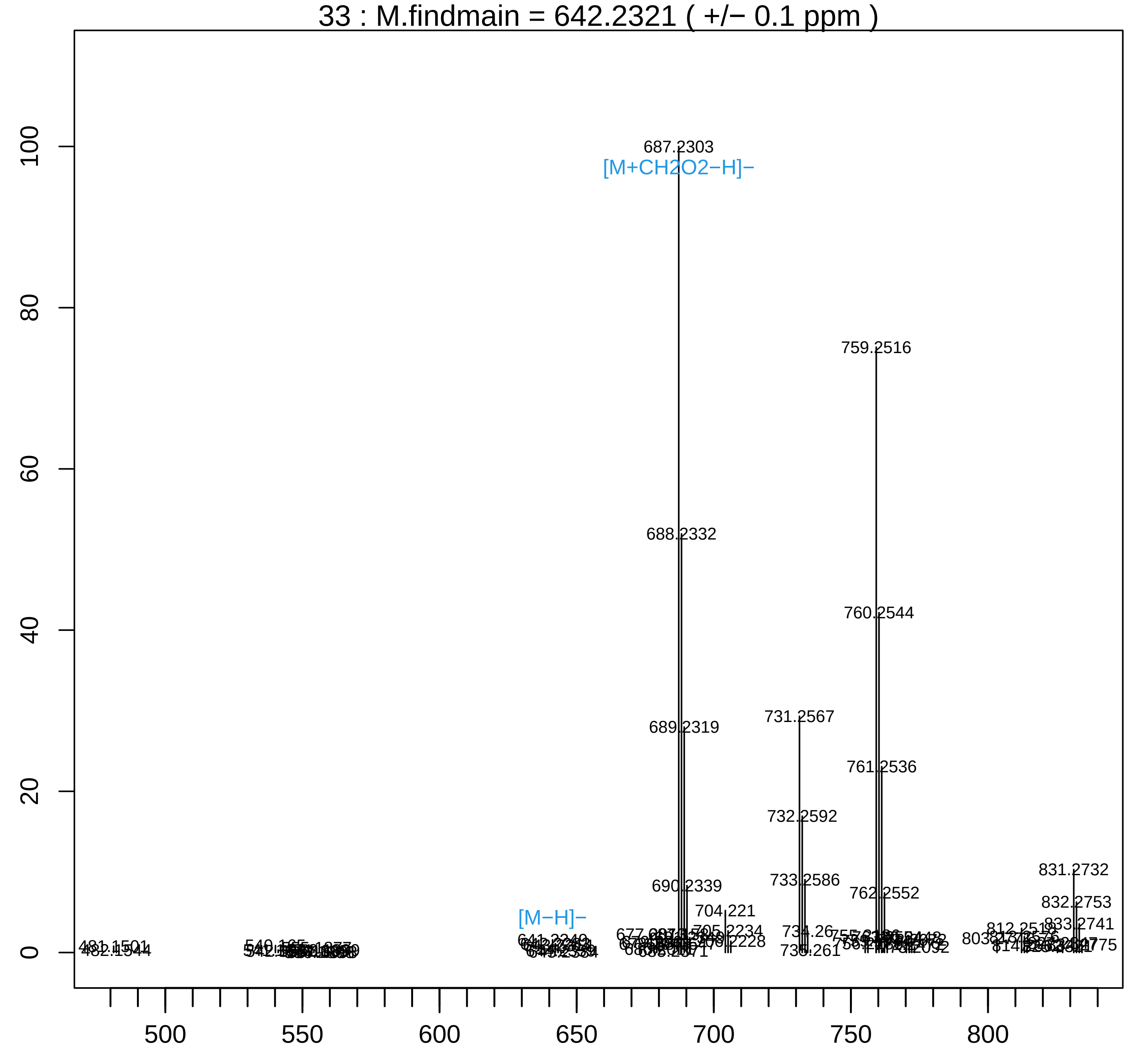
33 : M.findmain = 642.2321 (+/− 0.1 ppm)
100
80
60
40
20
0
500
550
600
650
700
750
800
687.2303
[M+CH2O2−H]−
759.2516
688.2332
760.2544
689.2319
731.2567
761.2536
732.2592
831.2732
690.2339
733.2586
762.2552
704.221
832.2753
705.2234
734.26
833.2741
[M−H]−
481.1501
482.1544
736.261

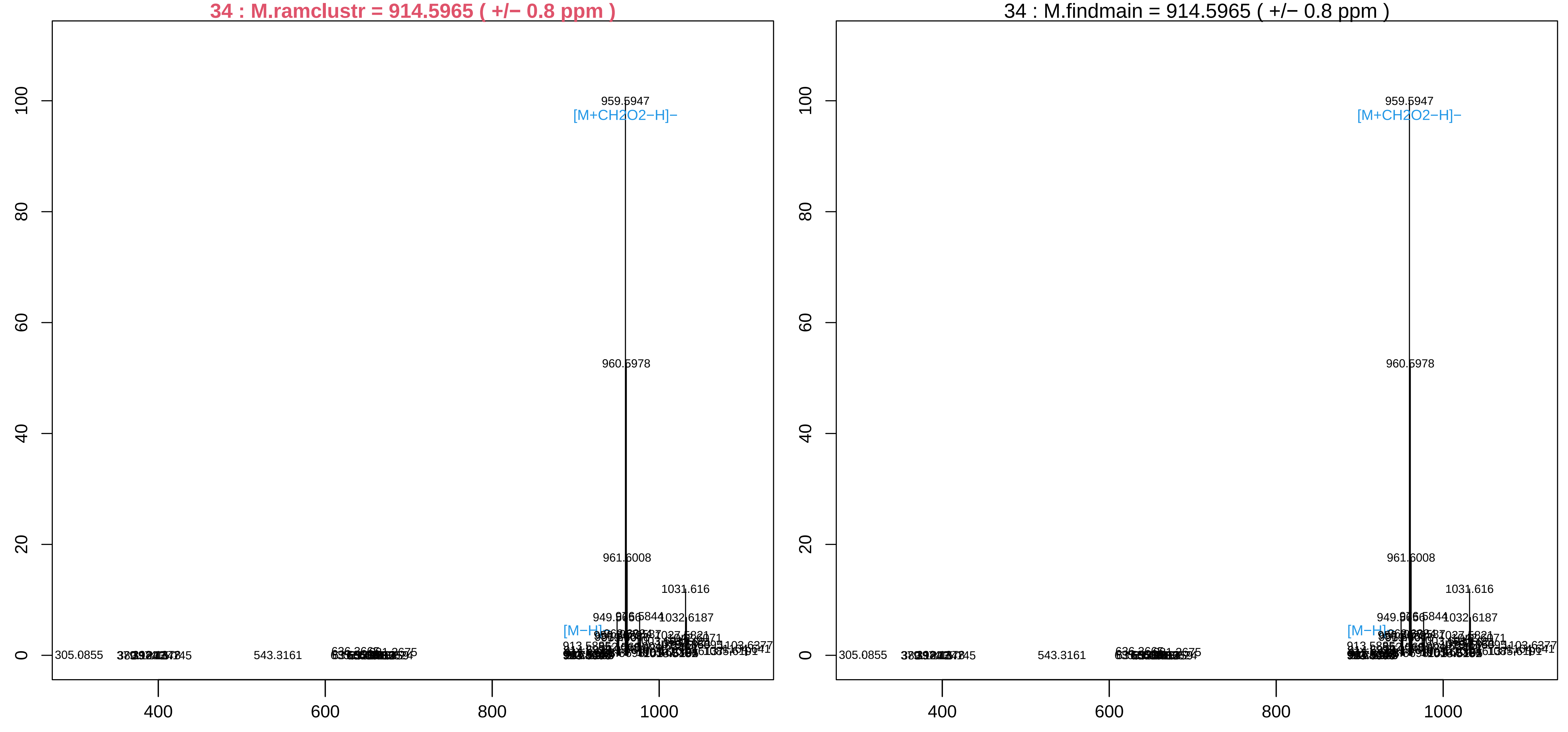
34 : M.ramclustr = 914.5965 (+/− 0.8 ppm)
959.5947
[M+CH2O2−H]−
960.5978
961.6008
1031.616
949.5656
1032.6187
[M−H]−
305.0855
543.3161
0
20
40
60
80
100
400
600
800
1000
34 : M.findmain = 914.5965 (+/− 0.8 ppm)
959.5947
[M+CH2O2−H]−
960.5978
961.6008
1031.616
949.5656
1032.6187
[M−H]−
305.0855
543.3161
0
20
40
60
80
100
400
600
800
1000

35 : M.ramclustr = 280.2399 (+/− 3.1 ppm)

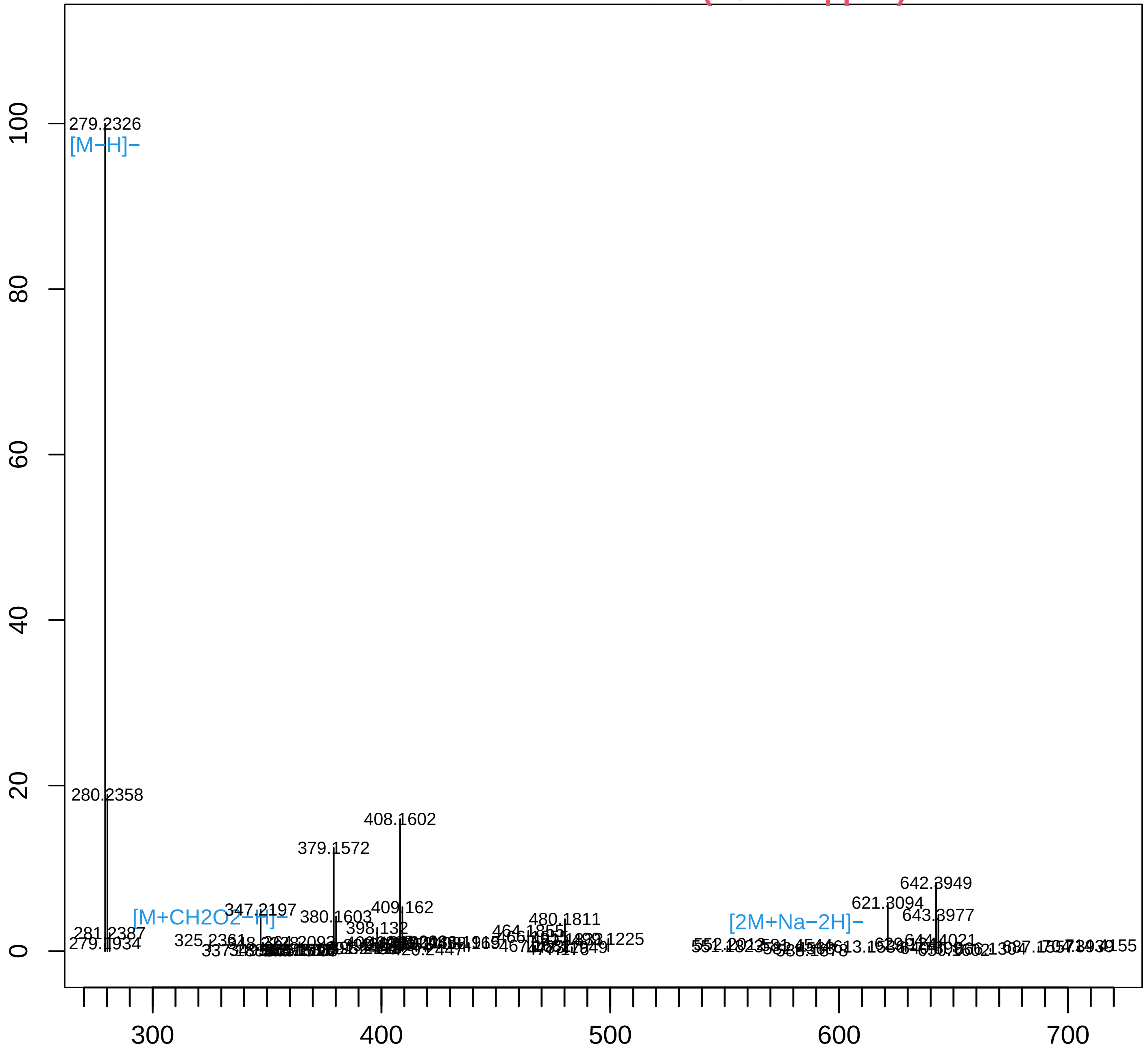

35 : M.findmain = 280.2399 (+/− 3.1 ppm)

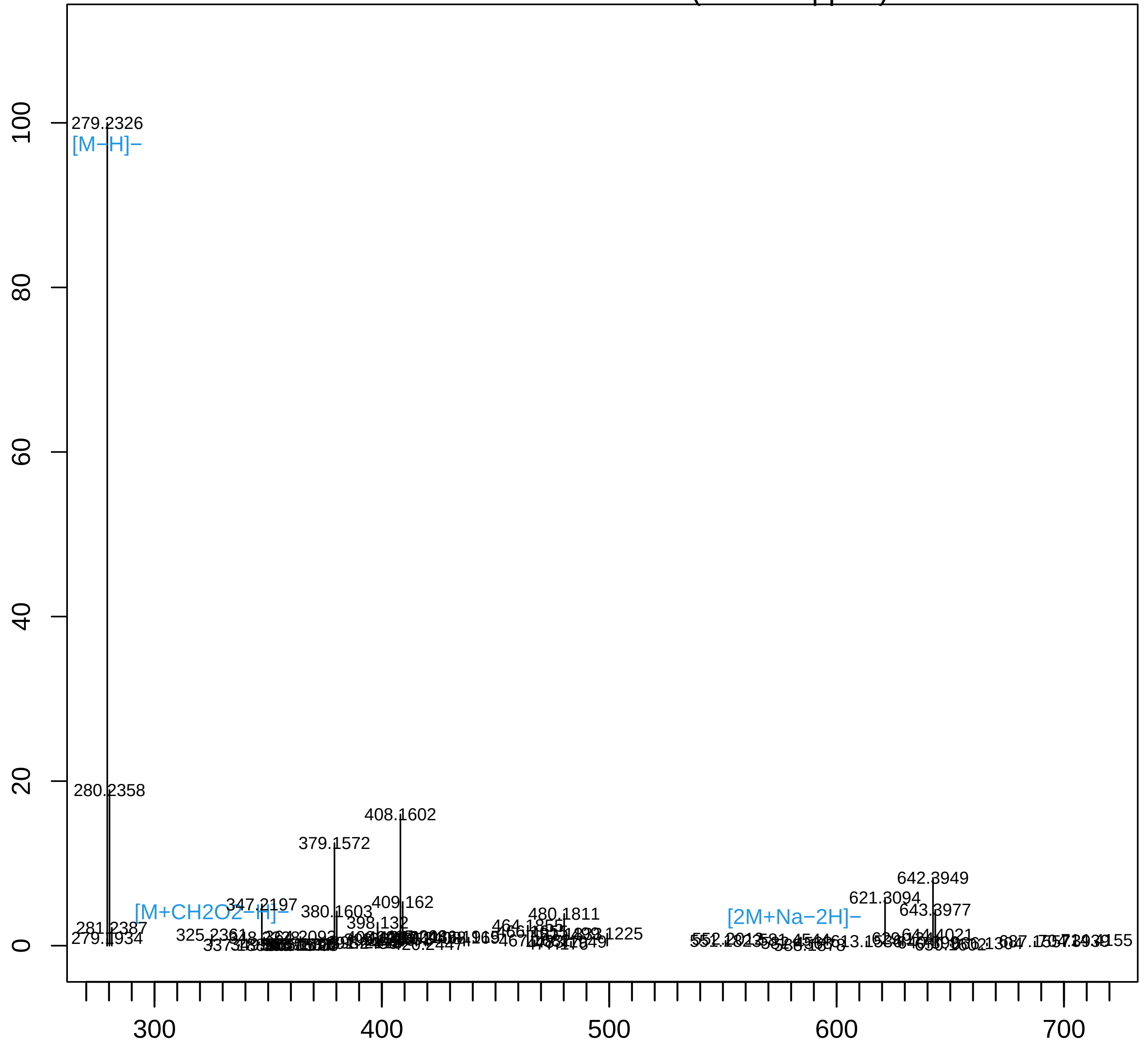

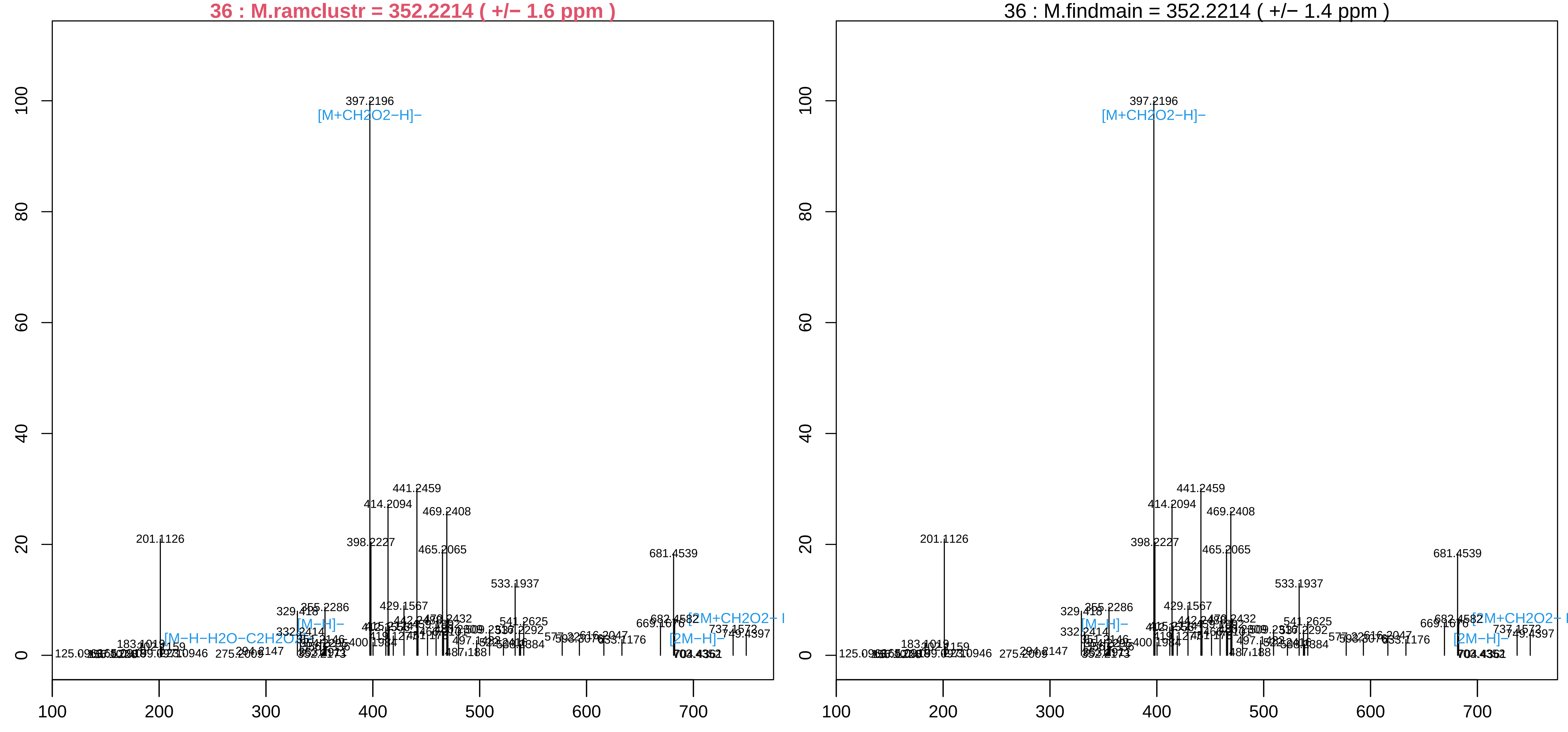

36 : M.ramclustr = 352.2214 (+/− 1.6 ppm)
397.2196
[M+CH2O2−H]−
441.2459
414.2094
469.2408
201.1126
398.2227
465.2065
681.4539
533.1937
429.1567
329.1418
255.2286
[M−H]−
541.2625
[M−H−H2O−C2H2O2]−
[2M−H]−
[2M+CH2O2−
737.1572
749.4397
669.1676
682.4582
633.1176
275.2009
294.2147
125.0986
487.188
704.4351
0
20
40
60
80
100
100
200
300
400
500
600
700
36 : M.findmain = 352.2214 (+/− 1.4 ppm)
397.2196
[M+CH2O2−H]−
441.2459
414.2094
469.2408
201.1126
398.2227
465.2065
681.4539
533.1937
429.1567
329.1418
255.2286
[M−H]−
541.2625
[2M−H]−
[2M+CH2O2−
737.1572
749.4397
669.1676
682.4582
633.1176
275.2009
294.2147
125.0986
487.188
704.4351
0
20
40
60
80
100
100
200
300
400
500
600
700

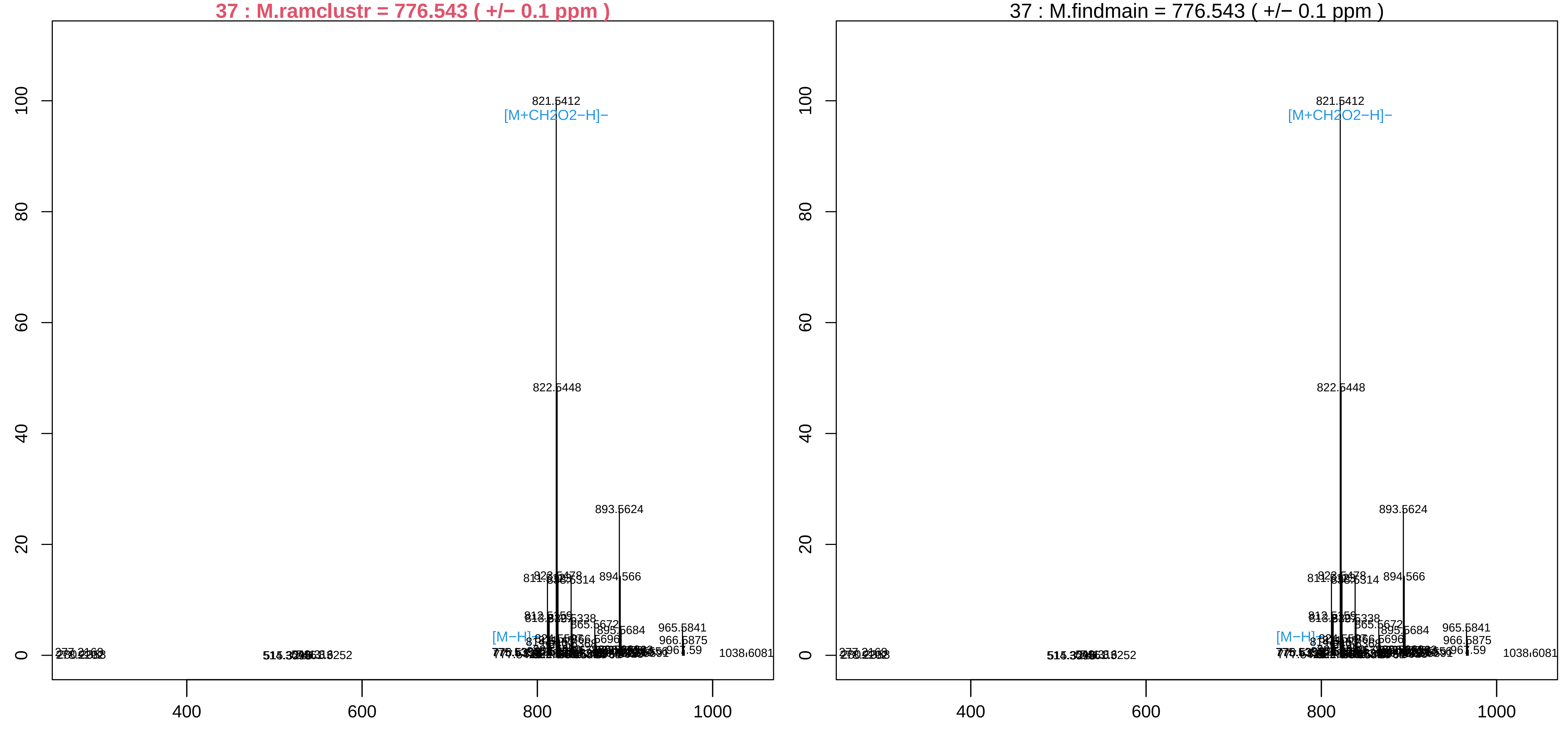
37 : M.ramclustr = 776.543 (+/− 0.1 ppm)
37 : M.findmain = 776.543 (+/− 0.1 ppm)
821.5412
[M+CH2O2−H]−
822.5448
893.5624
894.566
965.5841
966.5875
1038.6081
[M−H]−
0
20
40
60
80
100
400
600
800
1000

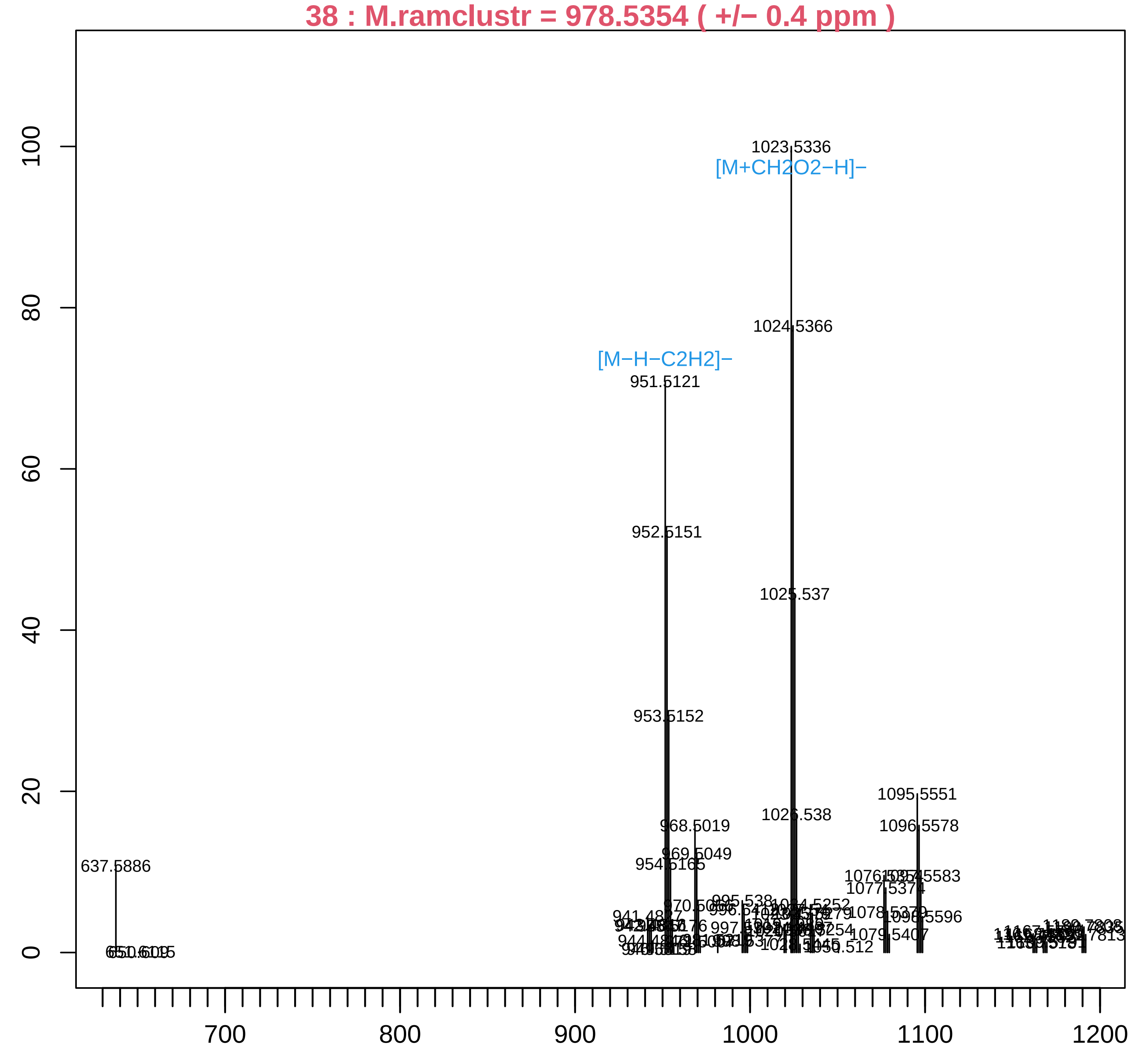
38 : M.ramclustr = 978.5354 (+/− 0.4 ppm)
1023.5336
[M+CH2O2−H]−
1024.5366
[M−H−C2H2]−
951.5121
952.5151
1025.537
953.5152
1026.538
1095.5551
1096.5578
968.5019
969.5049
954.5165
637.5886
1077.5374
1078.5379
1079.5407
995.538
1024.5252
0
20
40
60
80
100
700
800
900
1000
1100
1200

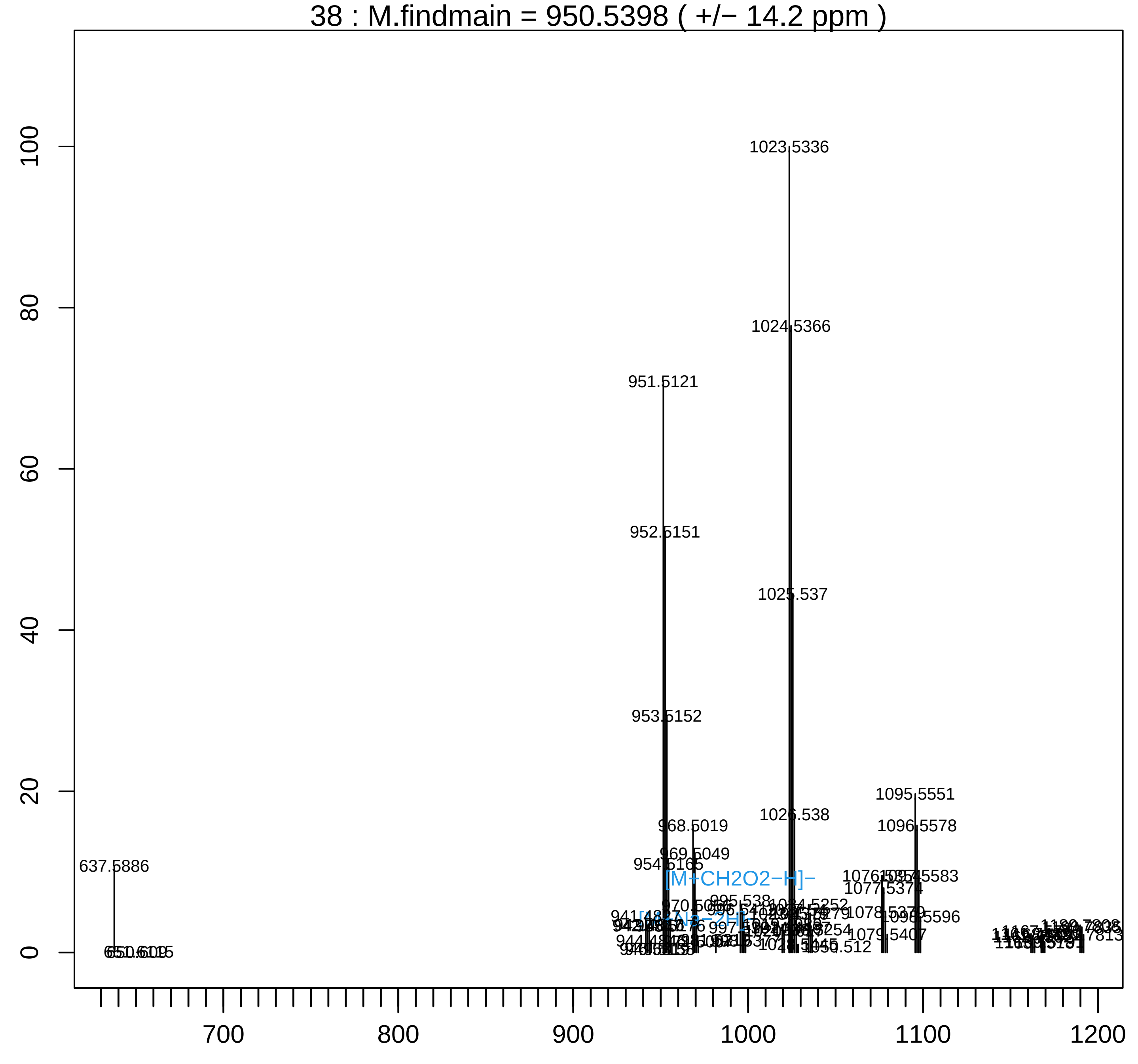
38 : M.findmain = 950.5398 (+/− 14.2 ppm)
1023.5336
1024.5366
951.5121
952.5151
1025.537
953.5152
1026.538
1095.5551
1096.5578
968.5019
969.5049
954.5165
637.5886
[M+CH2O2−H]−
1077.5374
1078.5379
1079.5407
995.538
1024.5252
0
20
40
60
80
100
700
800
900
1000
1100
1200

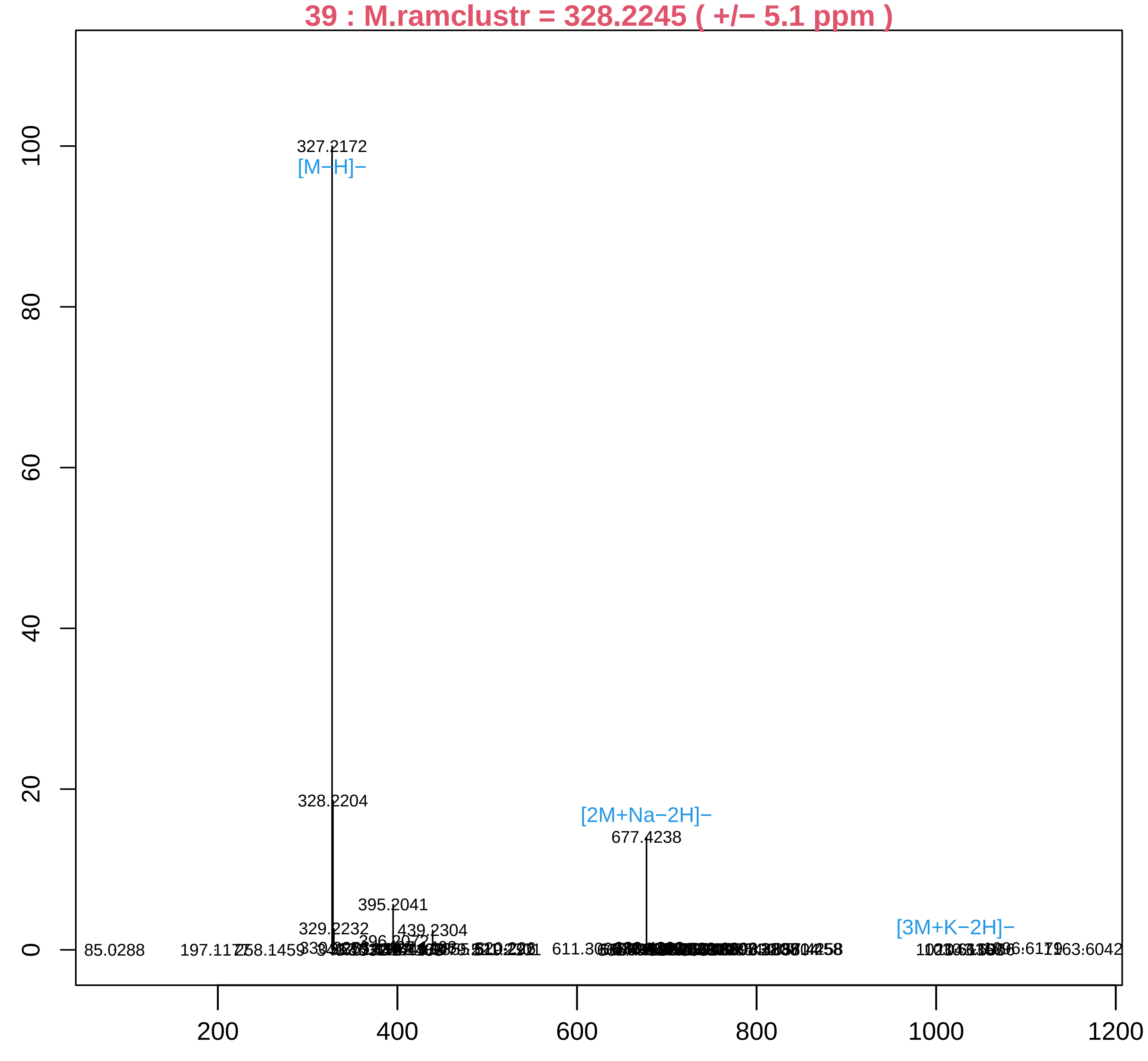
39 : M.ramclustr = 328.2245 (+/− 5.1 ppm)
327.2172
[M−H]−
328.2204
[2M+Na−2H]−
677.4238
395.2041
329.2232
439.2304
[3M+K−2H]−
85.0288
1163.6042
0
20
40
60
80
100
200
400
600
800
1000
1200

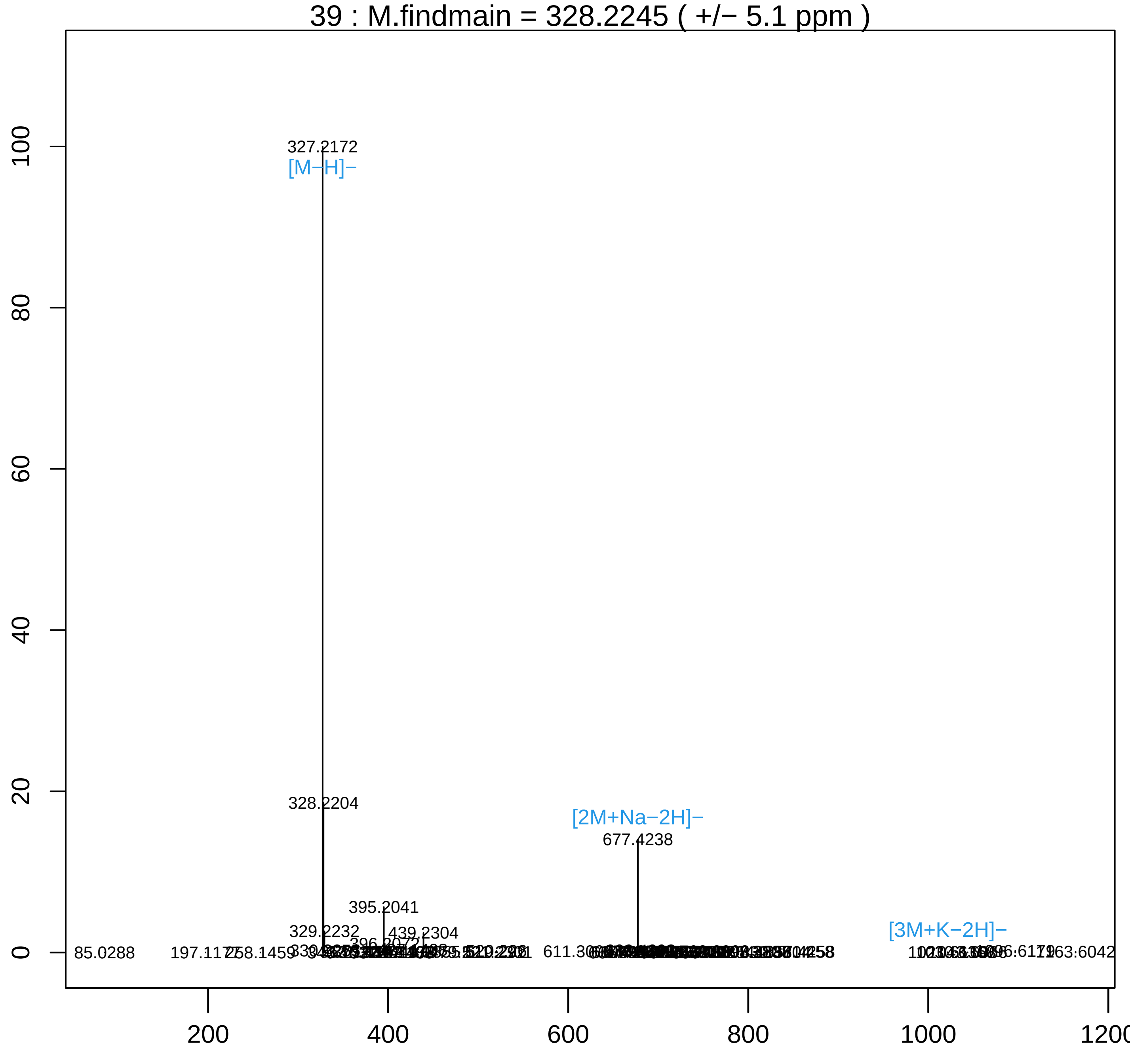
39 : M.findmain = 328.2245 (+/− 5.1 ppm)
327.2172
[M−H]−
328.2204
[2M+Na−2H]−
677.4238
395.2041
329.2232
439.2304
[3M+K−2H]−
85.0288
1163.6042
0
20
40
60
80
100
200
400
600
800
1000
1200

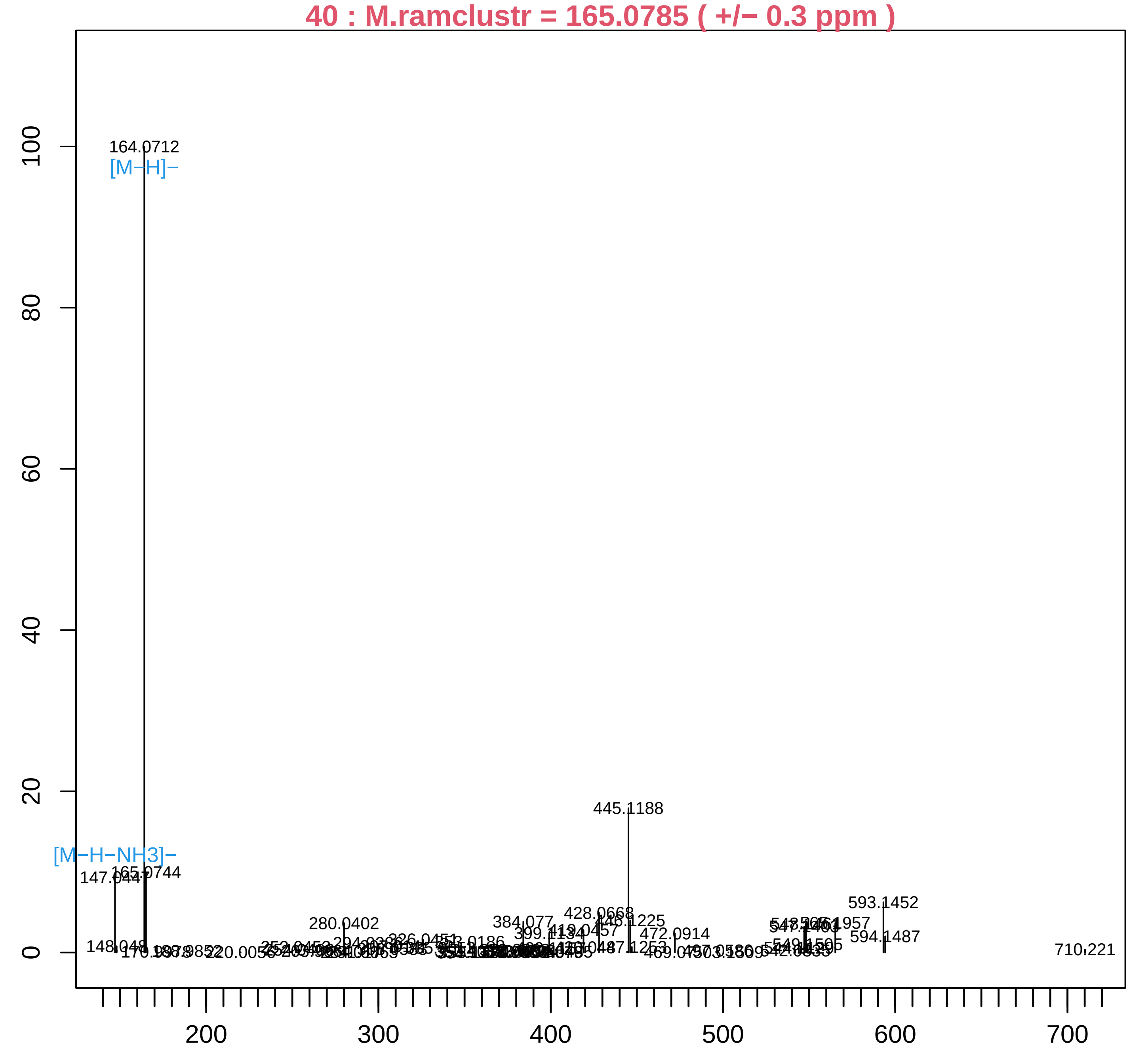
40 : M.ramclustr = 165.0785 (+/− 0.3 ppm)
164.0712
[M−H]−
[M−H−NH3]−
147.044
165.0744
445.1188
593.1452
280.0402
384.077
428.0668
472.0914
594.1487
710.221
0
20
40
60
80
100
200
300
400
500
600
700

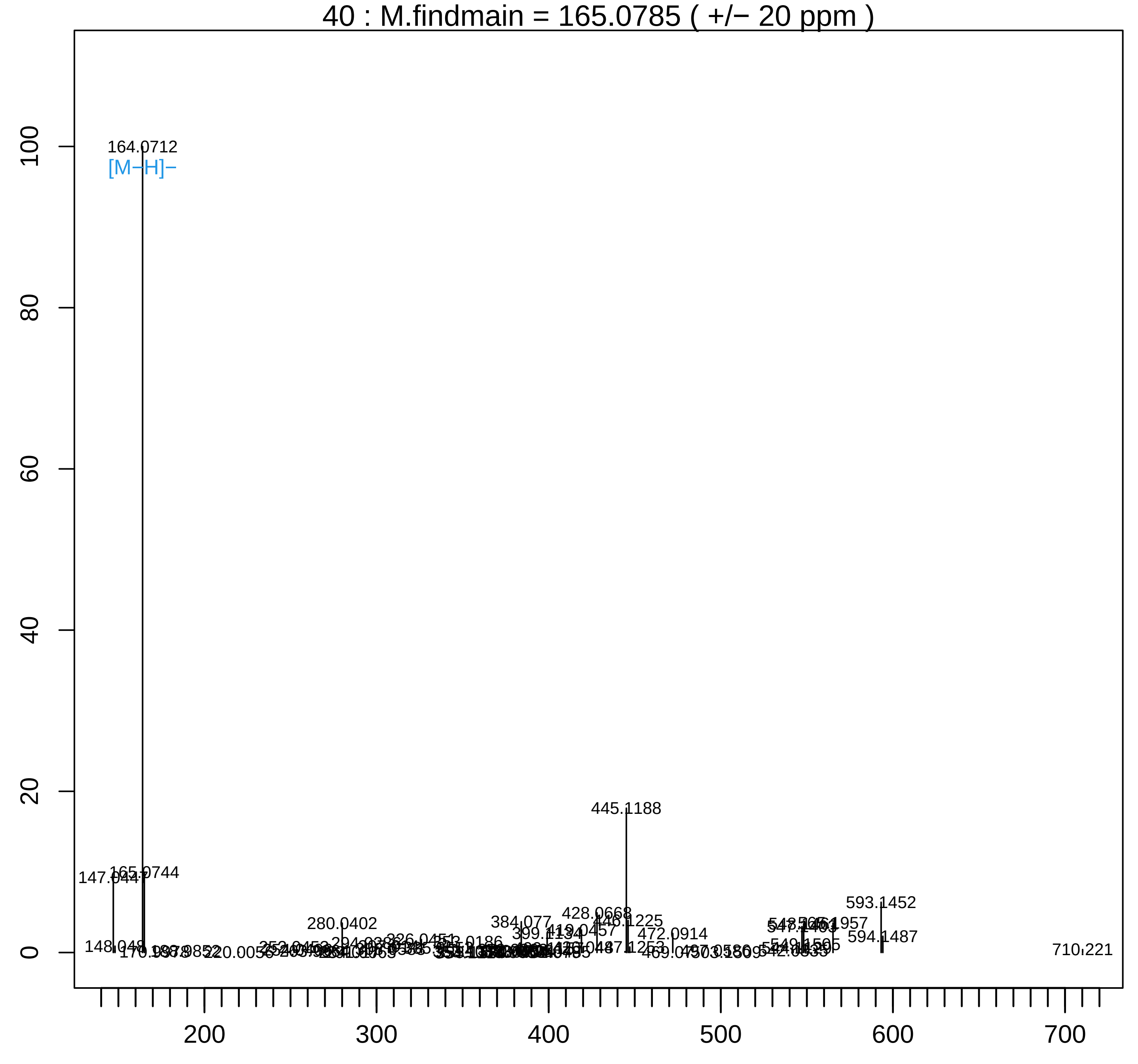
40 : M.findmain = 165.0785 (+/− 20 ppm)
164.0712
[M−H]−
147.044
165.0744
445.1188
593.1452
280.0402
384.077
428.0668
472.0914
594.1487
710.221
0
20
40
60
80
100
200
300
400
500
600
700

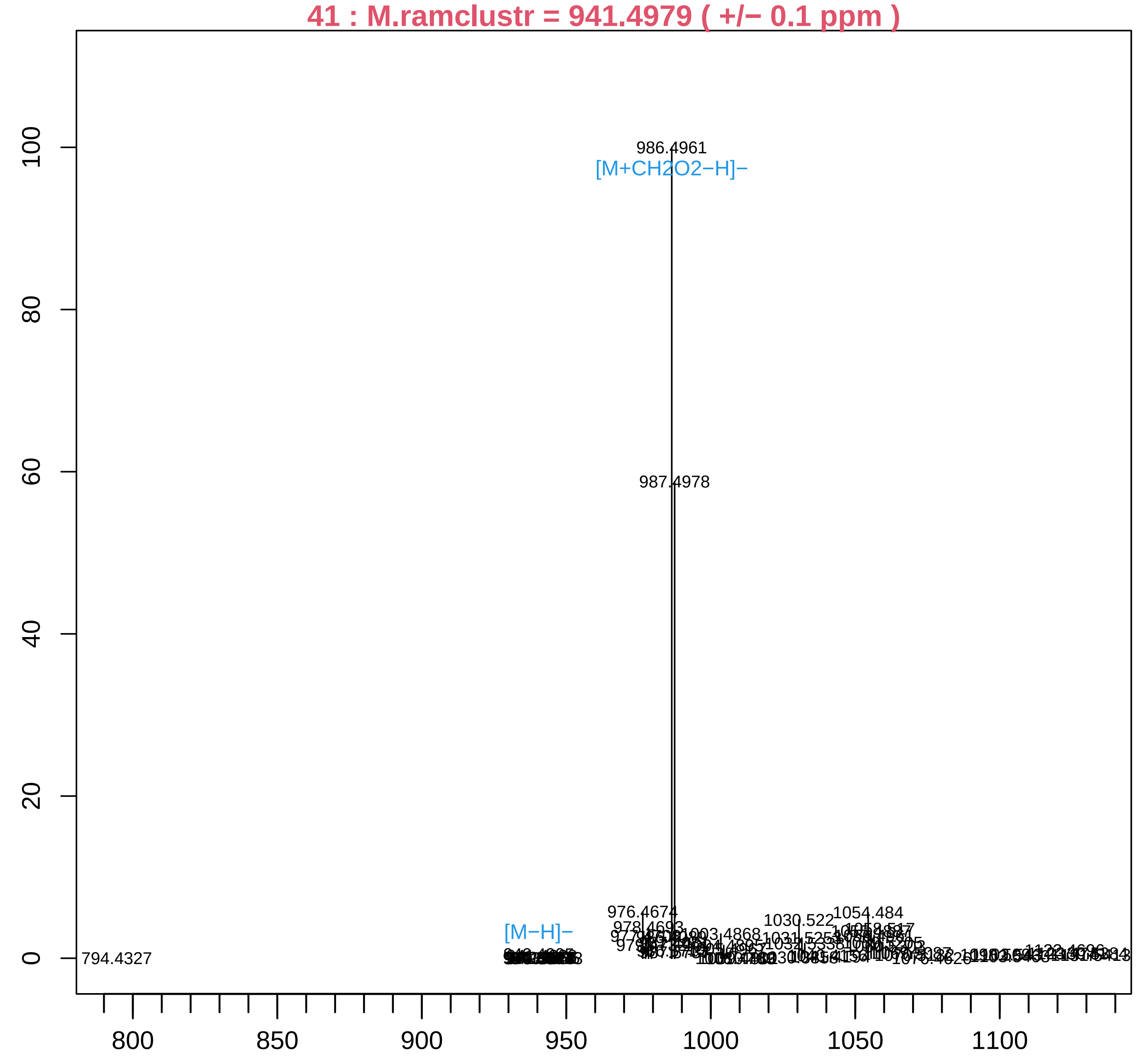
41 : M.ramclustr = 941.4979 (+/− 0.1 ppm)
986.4961
[M+CH2O2−H]−
987.4978
976.4674
1030.522
1054.484
[M−H]−
794.4327

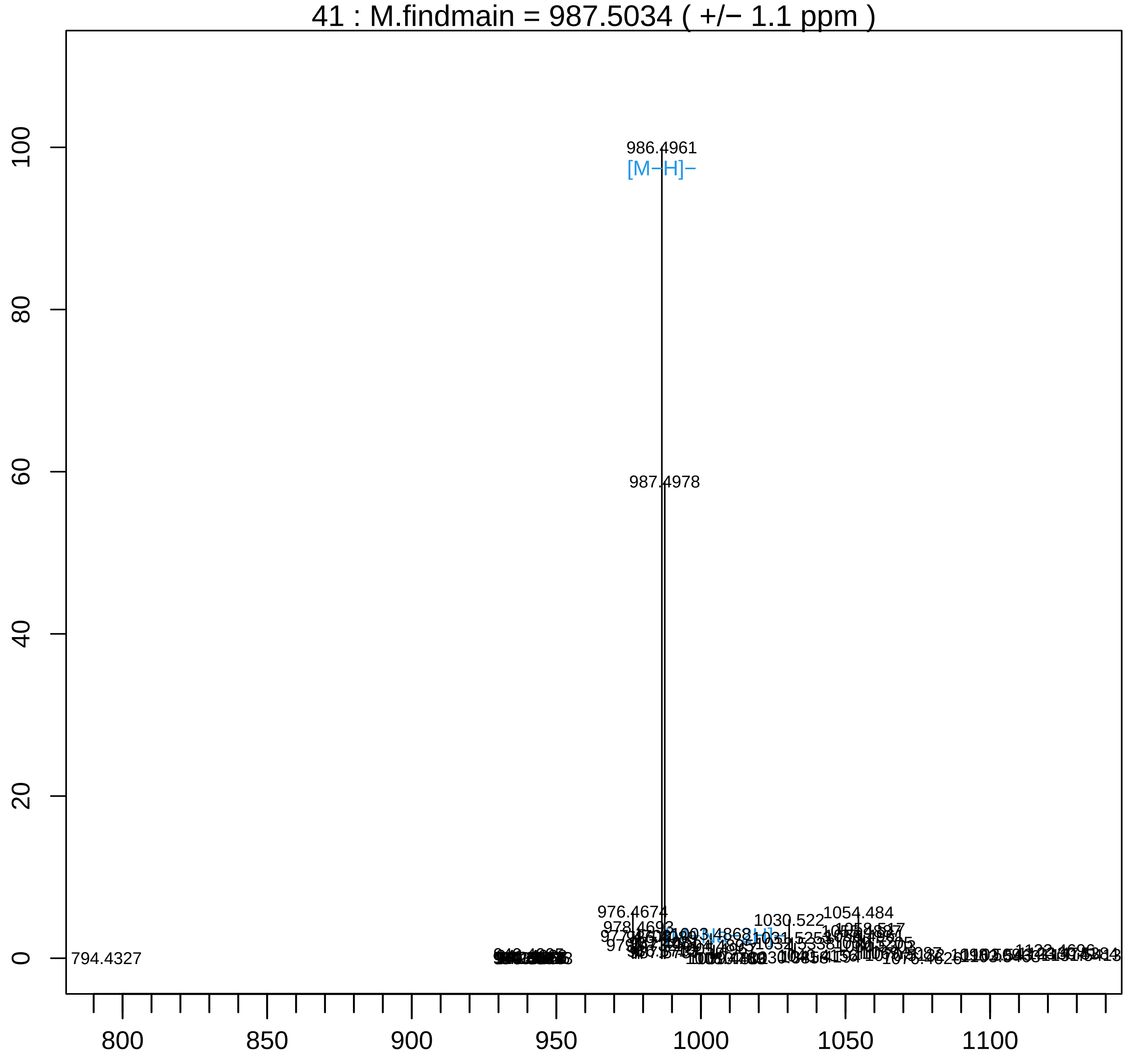
41 : M.findmain = 987.5034 (+/− 1.1 ppm)
986.4961
[M−H]−
987.4978
976.4674
1030.522
1054.484
794.4327

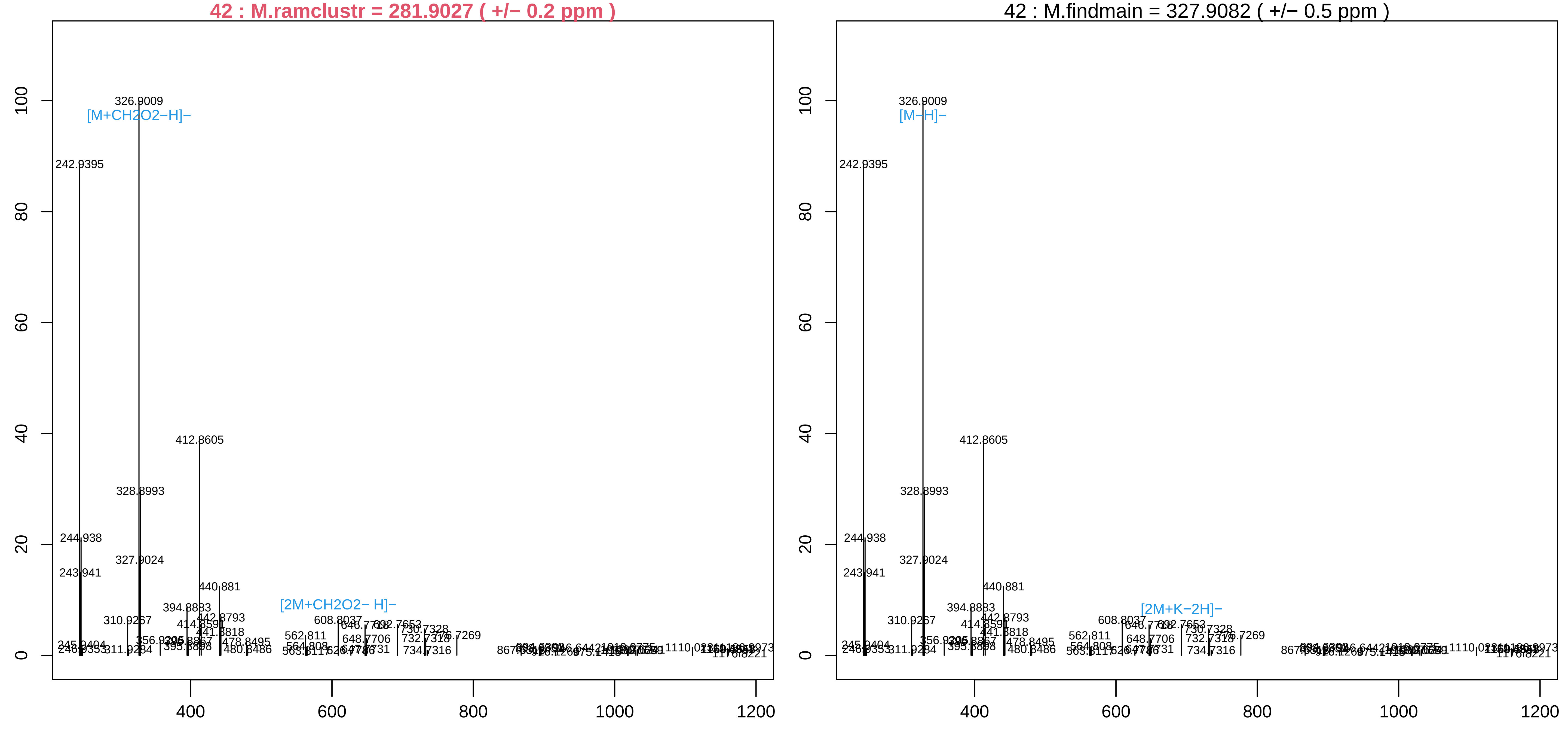
42 : M.ramclustr = 281.9027 (+/− 0.2 ppm)
[M+CH2O2−H]−
[2M+CH2O2− H]−
326.9009
242.9395
412.8605
328.8993
244.938
327.9024
243.941
440.881
394.8833
310.9267
442.8793
414.8591
441.8818
356.9205
478.8495
480.8486
395.8898
311.9284
245.9404
562.811
564.808
608.8037
648.7706
730.7328
732.7318
734.7316
776.7269
692.7653
1110.0
42 : M.findmain = 327.9082 (+/− 0.5 ppm)
[M−H]−
[2M+K−2H]−
0
20
40
60
80
100
400
600
800
1000
1200

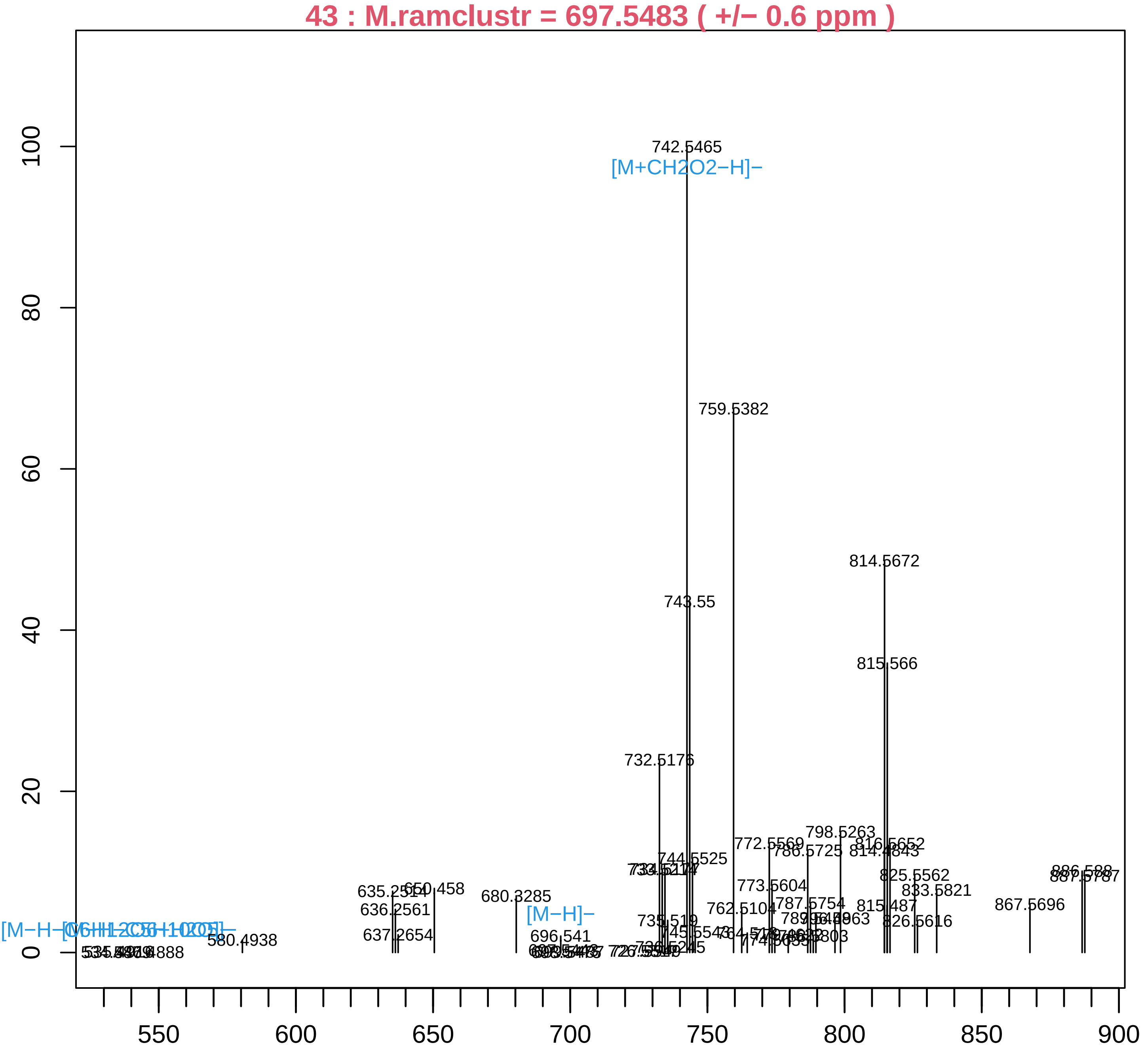

43 : M.ramclustr = 697.5483 (+/− 0.6 ppm)
742.5465
[M+CH2O2−H]−
759.5382
814.5672
743.55
815.566
732.5176
[M−H]−

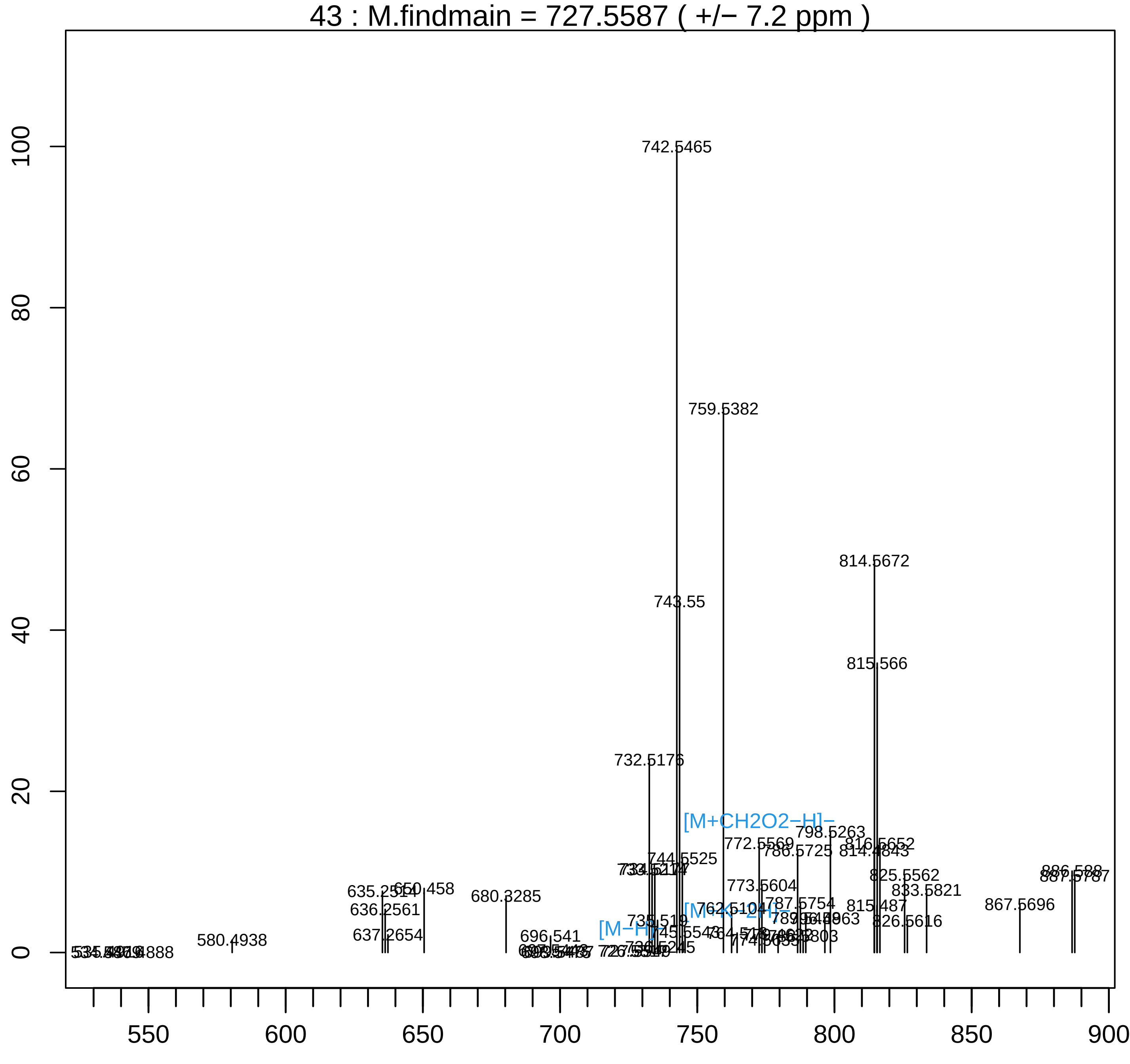

43 : M.findmain = 727.5587 (+/− 7.2 ppm)
742.5465
759.5382
814.5672
743.55
815.566
732.5176
[M+CH2O2−H]−

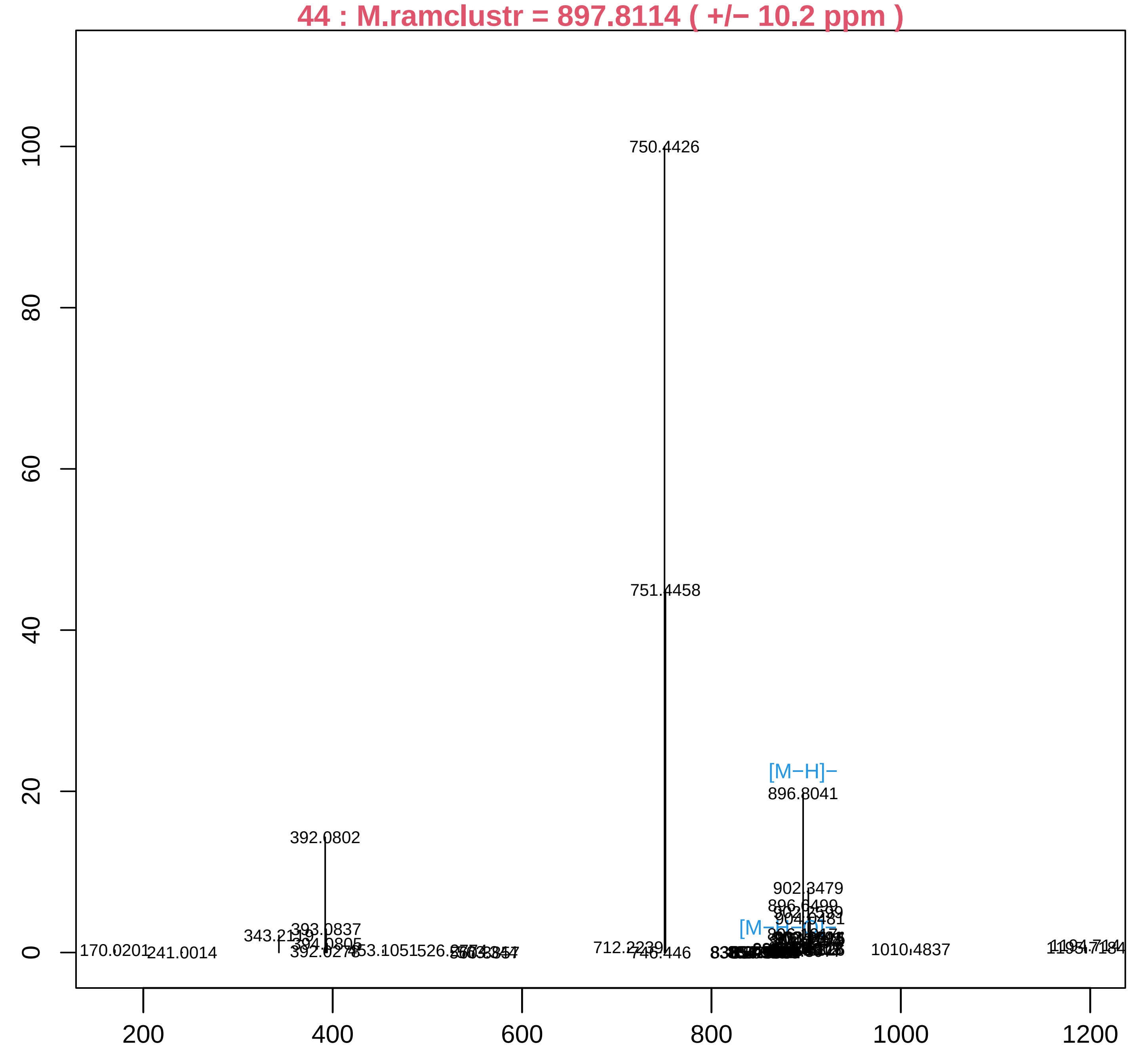
44 : M.ramclustr = 897.8114 (+/− 10.2 ppm)
750.4426
751.4458
[M−H]−
896.8041
392.0802
902.3479
393.0837
343.2119
712.2239
746.446
1010.4837
170.0201
241.0014
0
20
40
60
80
100
200
400
600
800
1000
1200

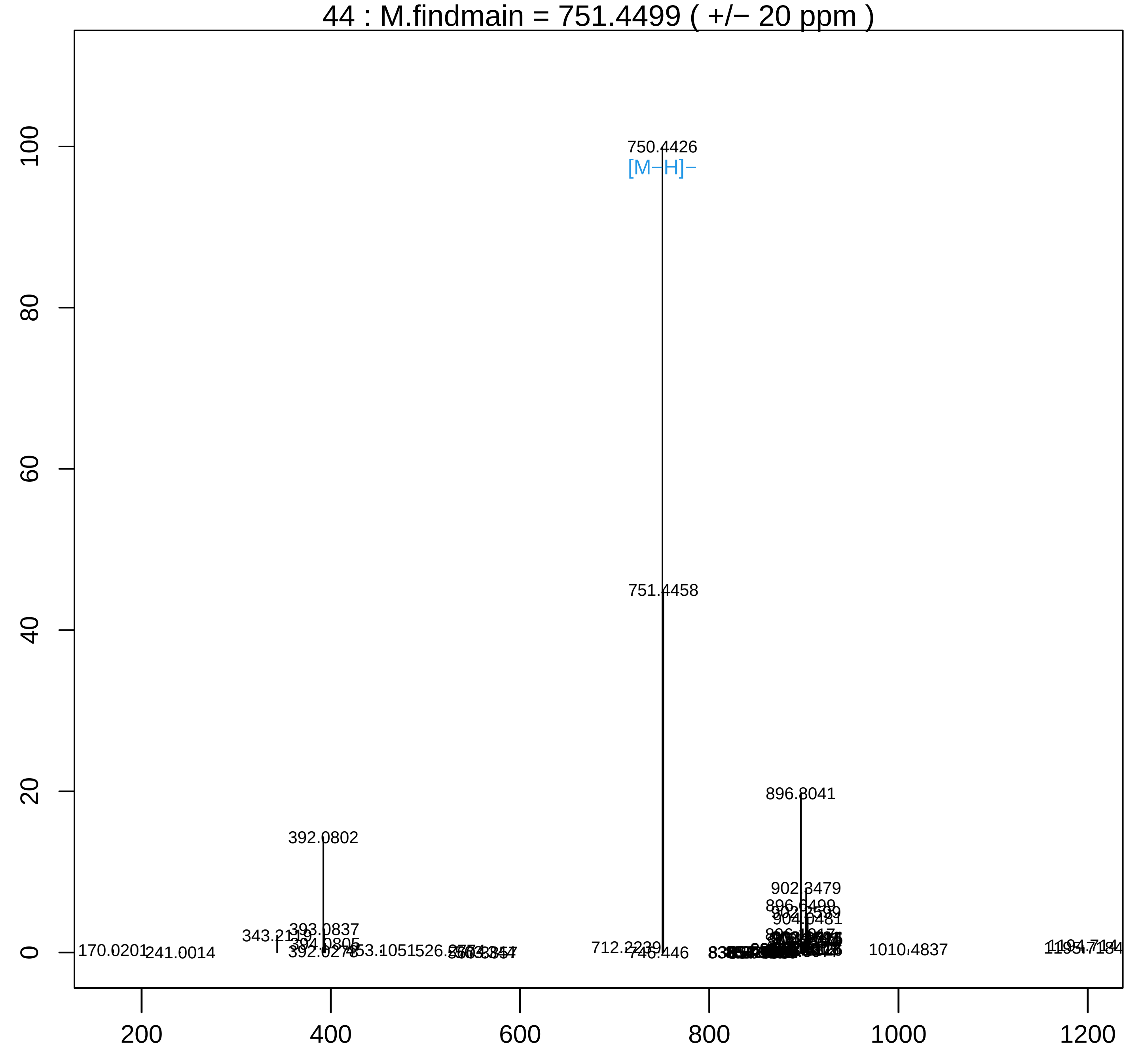
44 : M.findmain = 751.4499 (+/− 20 ppm)
750.4426
[M−H]−
751.4458
896.8041
392.0802
902.3479
393.0837
343.2119
712.2239
746.446
1010.4837
170.0201
241.0014
0
20
40
60
80
100
200
400
600
800
1000
1200

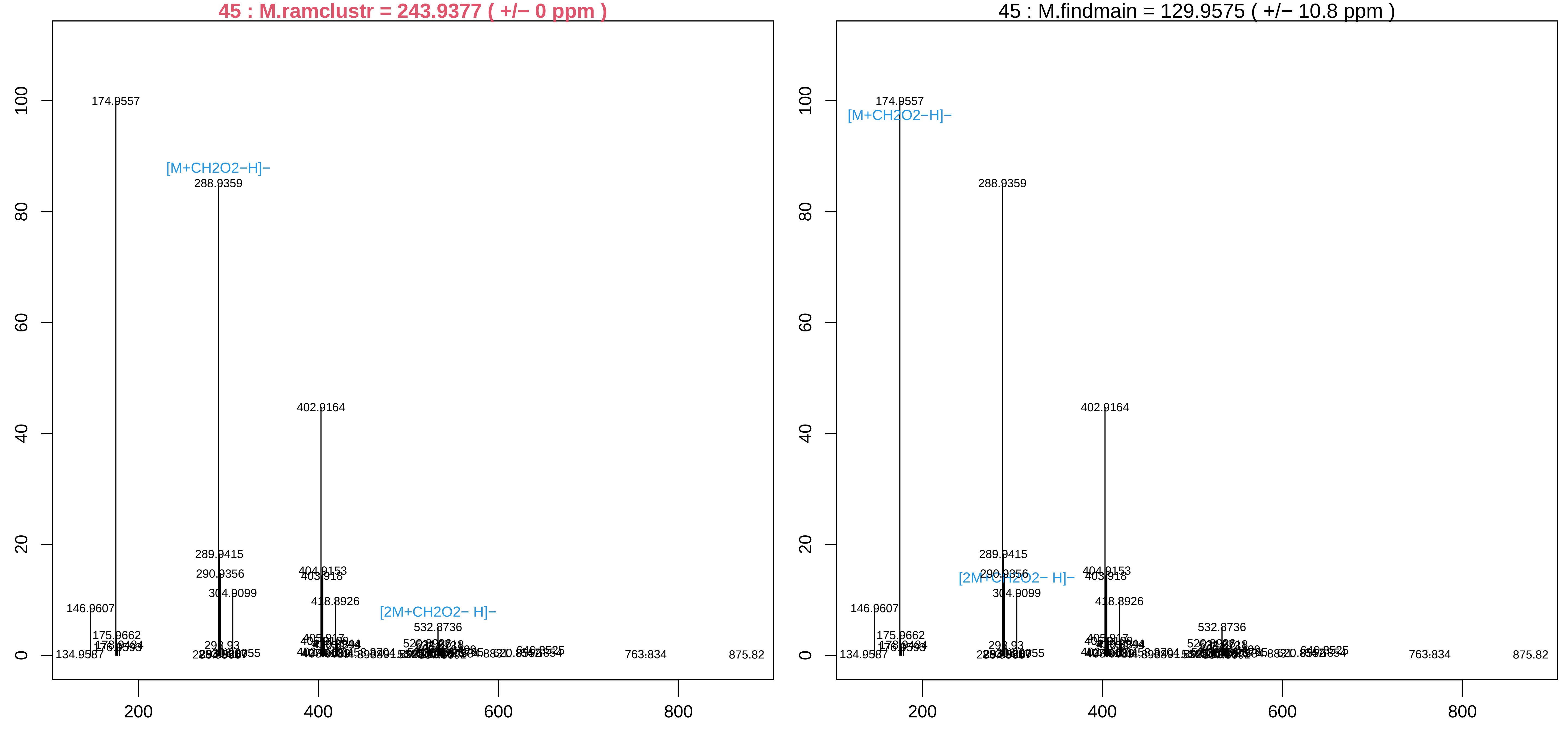
45 : M.ramclustr = 243.9377 (+/− 0 ppm)
45 : M.findmain = 129.9575 (+/− 10.8 ppm)
[M+CH2O2−H]−
[2M+CH2O2− H]−
174.9557
288.9359
402.9164
289.9415
290.9356
304.9099
404.9153
403.918
418.8926
532.8736
146.9607
175.9662
134.9587
763.834
875.82
646.8525
0
20
40
60
80
100
200
400
600
800

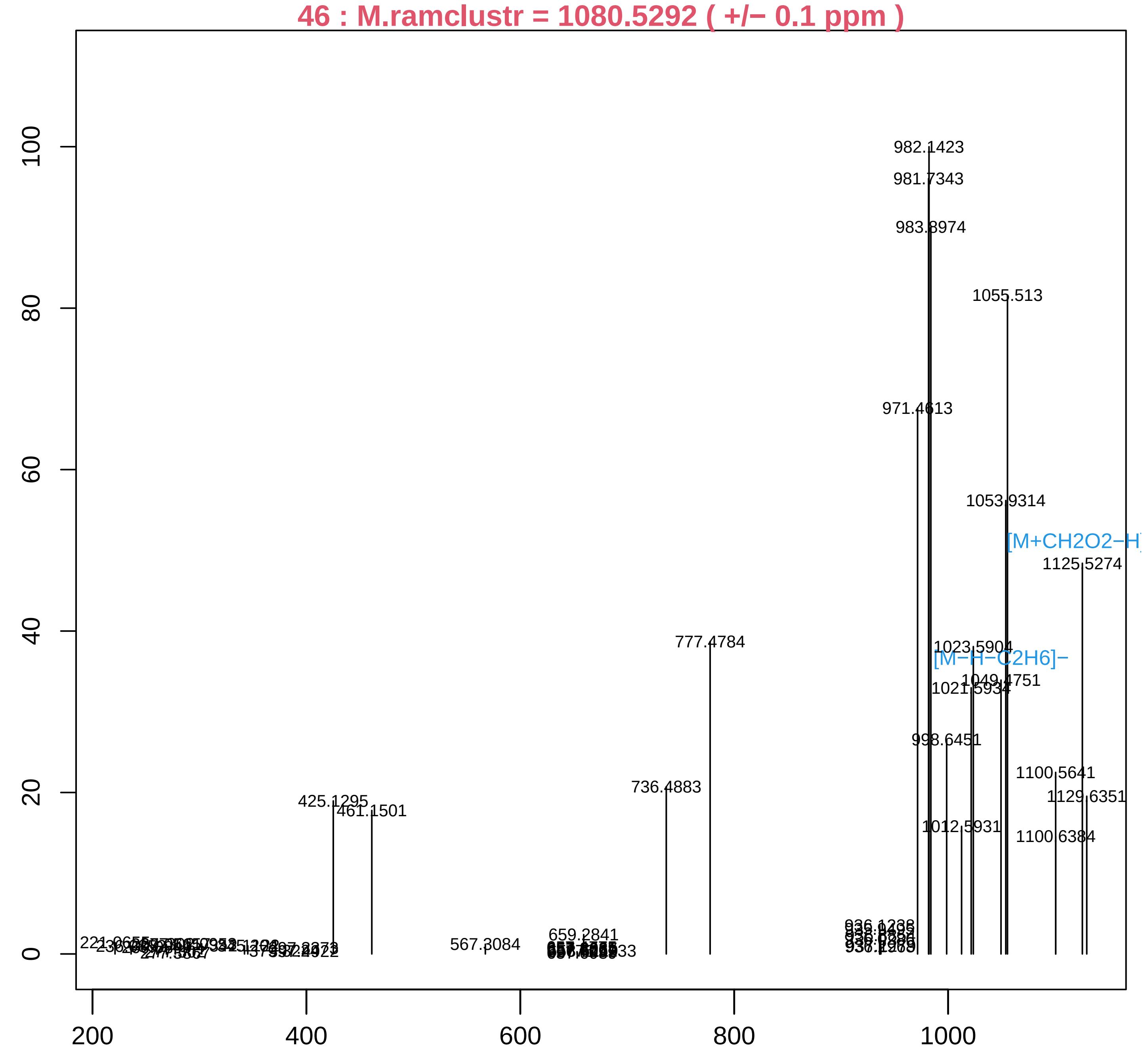

46 : M.ramclustr = 1080.5292 (+/− 0.1 ppm)
982.1423
981.7343
983.8974
1055.513
971.4613
1053.9314
[M+CH2O2−H]
1125.5274
777.4784
1023.5904
[M−H−C2H6]−
1049.4751
1021.5934
998.6451
1100.5641
736.4883
1129.6351
425.1295
461.1501
1012.5931
1100.6384
659.2841
567.3084
0
20
40
60
80
100
200
400
600
800
1000

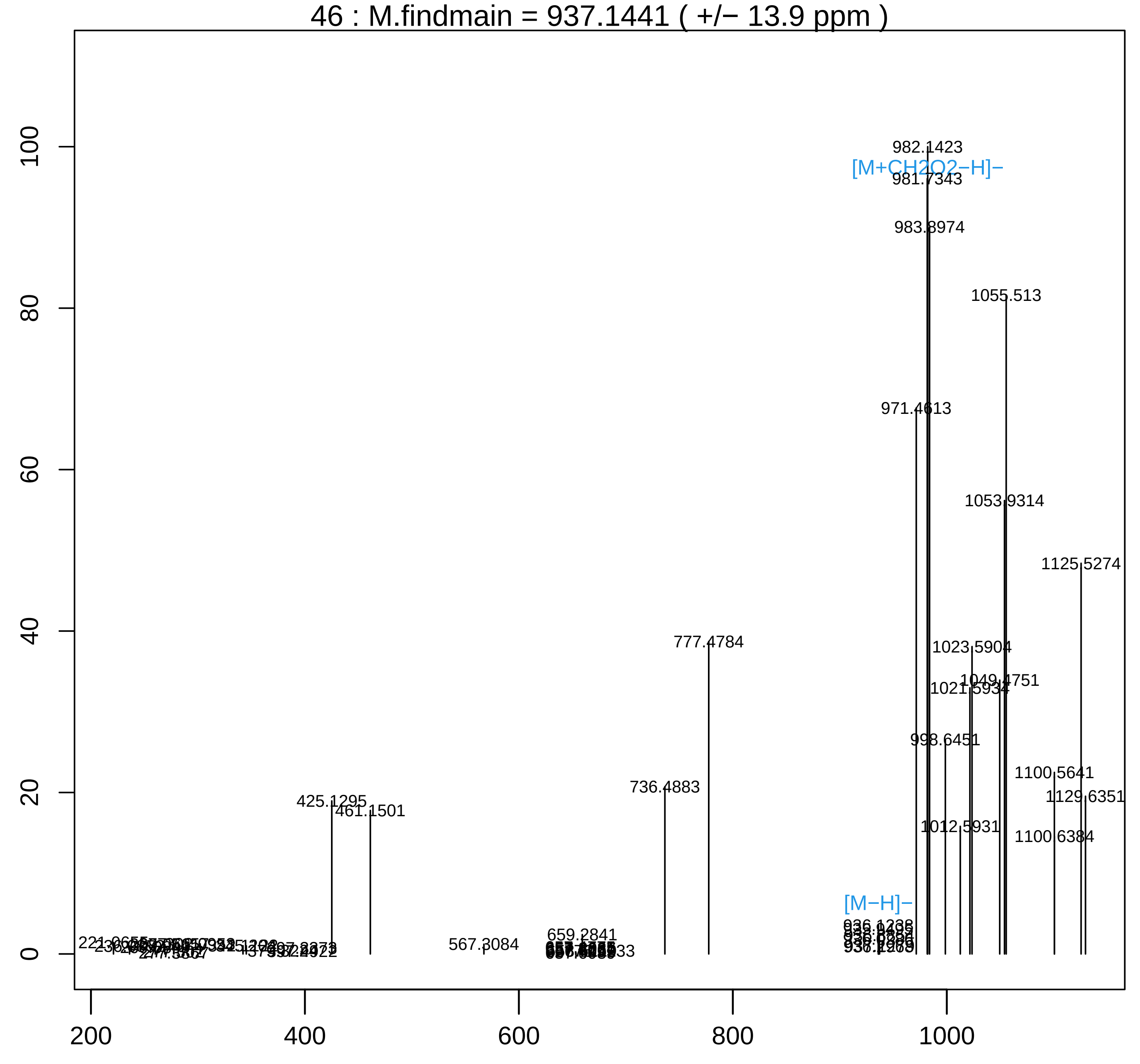

46 : M.findmain = 937.1441 (+/− 13.9 ppm)
982.1423
[M+CH2O2−H]−
981.7343
983.8974
1055.513
971.4613
1053.9314
1125.5274
777.4784
1023.5904
1049.4751
1021.5934
998.6451
1100.5641
736.4883
1129.6351
425.1295
461.1501
1012.5931
1100.6384
[M−H]−
659.2841
567.3084
0
20
40
60
80
100
200
400
600
800
1000

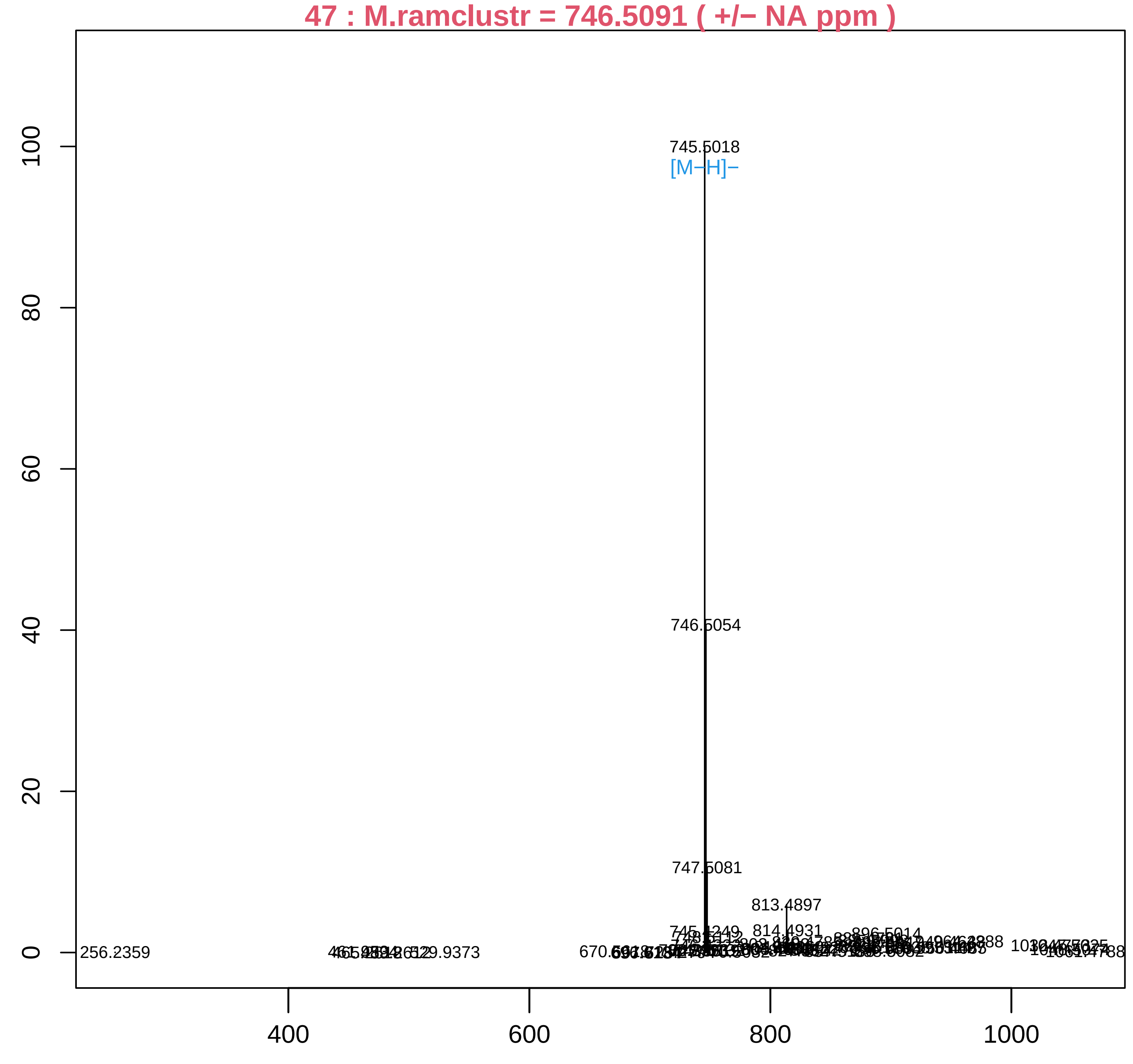
47 : M.ramclustr = 746.5091 (+/− NA ppm)
745.5018
[M−H]−
746.5054
747.5081
813.4897
814.4931
896.5014
256.2359
529.9373
0
20
40
60
80
100
400
600
800
1000

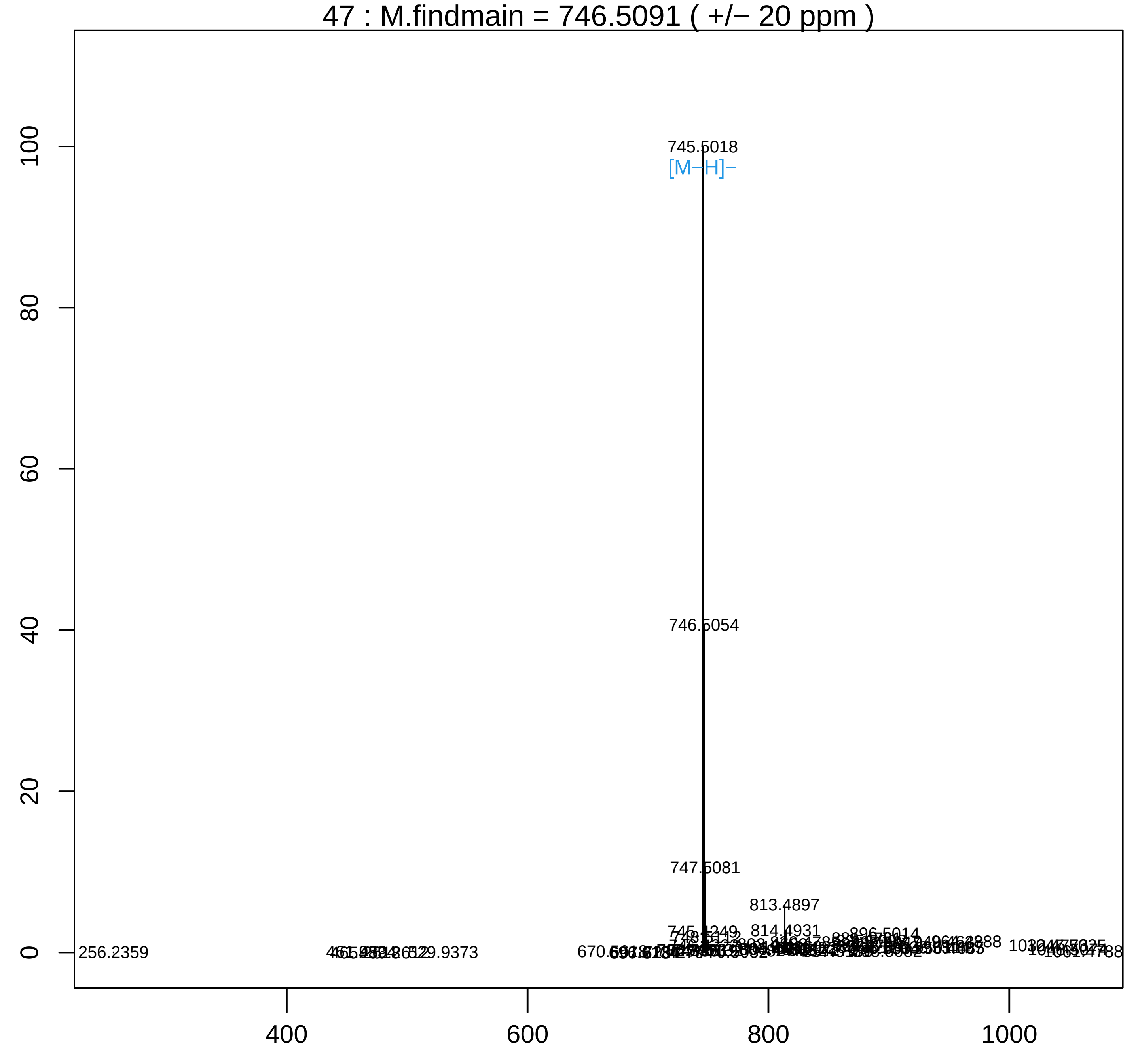
47 : M.findmain = 746.5091 (+/− 20 ppm)
745.5018
[M−H]−
746.5054
747.5081
813.4897
814.4931
896.5014
256.2359
529.9373
0
20
40
60
80
100
400
600
800
1000

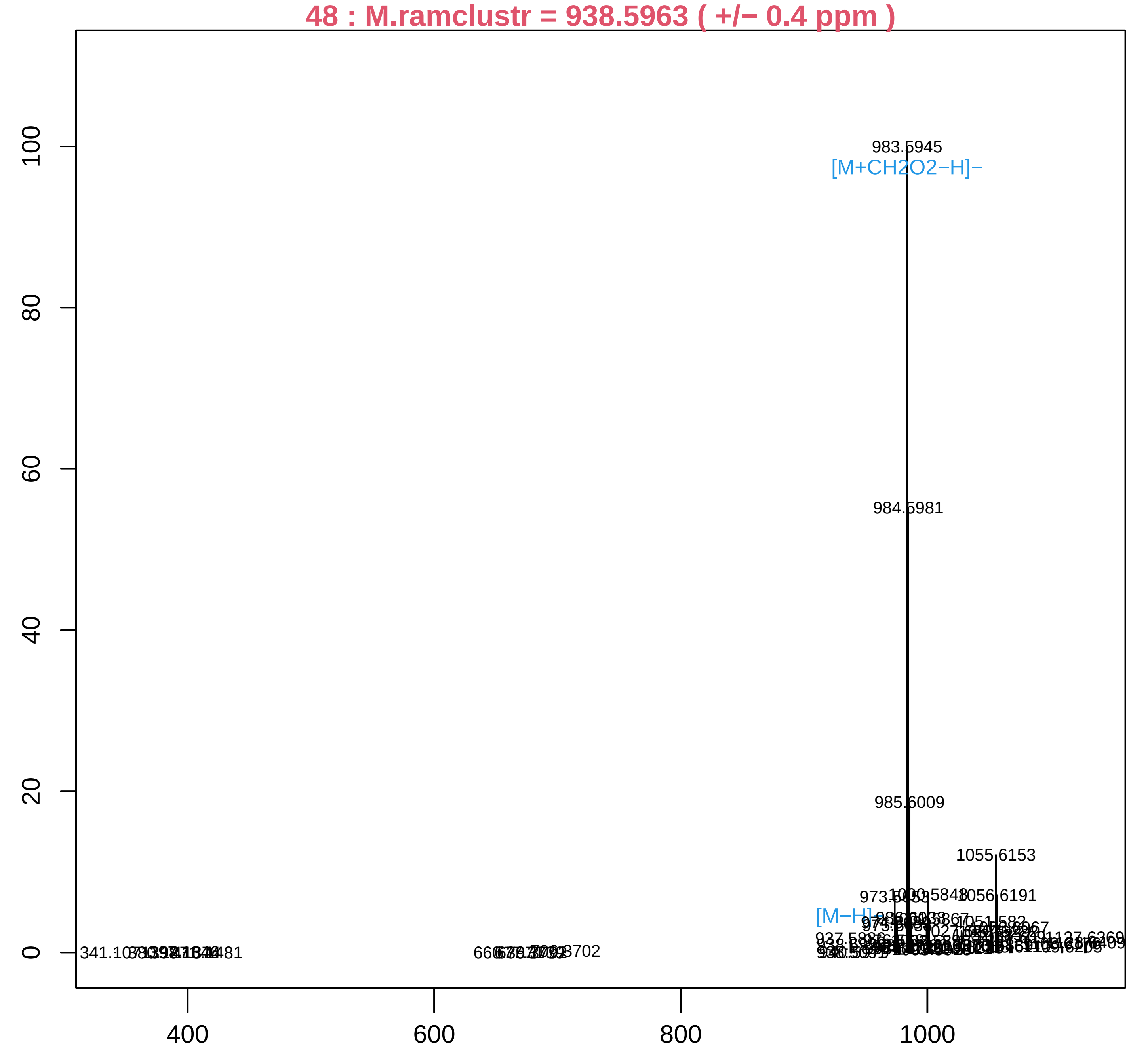
48 : M.ramclustr = 938.5963 (+/− 0.4 ppm)
983.5945
[M+CH2O2−H]−
984.5981
985.6009
1055.6153
1056.6191
[M−H]−
0
20
40
60
80
100
400
600
800
1000

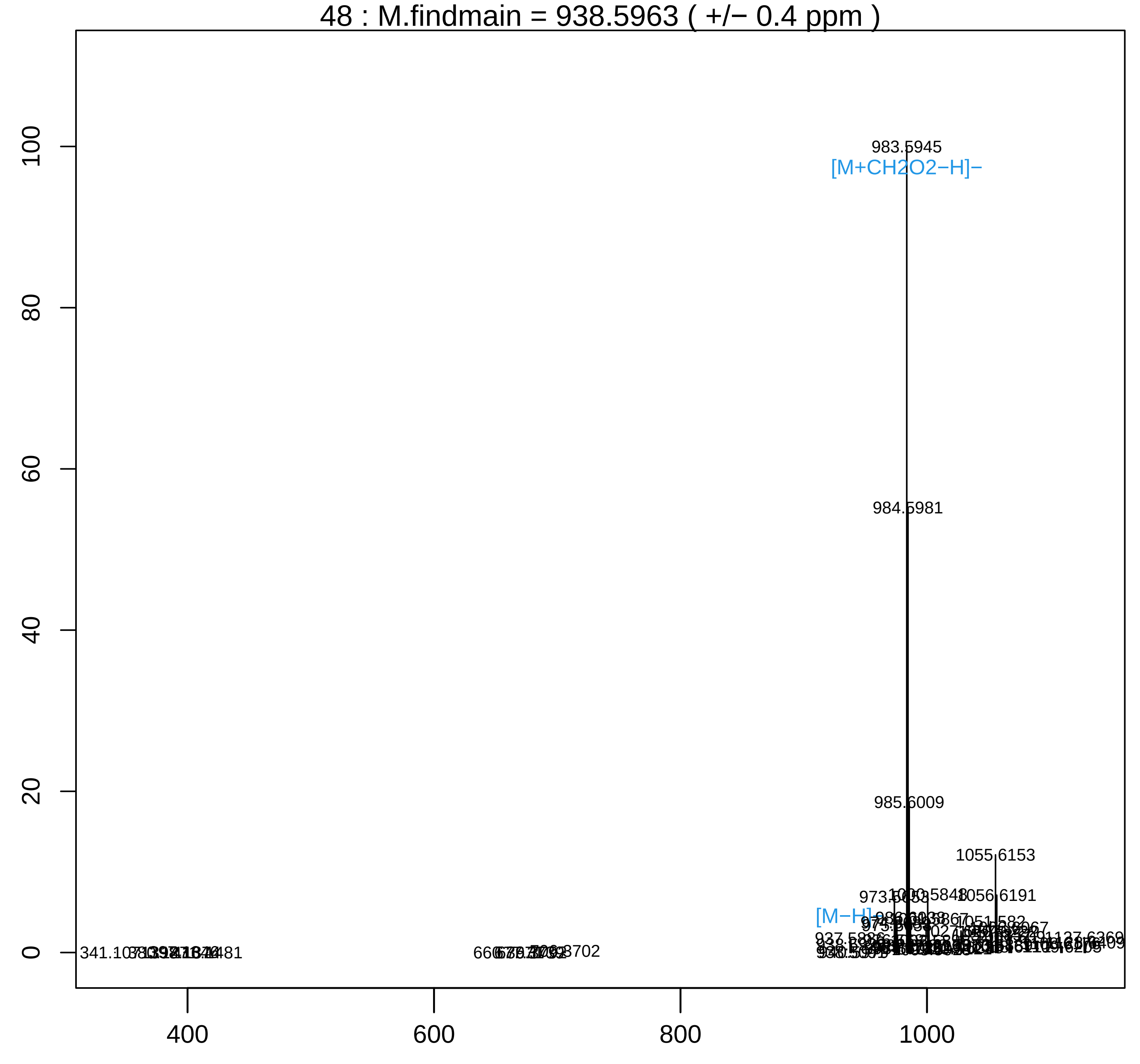
48 : M.findmain = 938.5963 (+/− 0.4 ppm)
983.5945
[M+CH2O2−H]−
984.5981
985.6009
1055.6153
1056.6191
[M−H]−
0
20
40
60
80
100
400
600
800
1000

49 : M.ramclustr = 300.0839 (+/− 0.3 ppm)

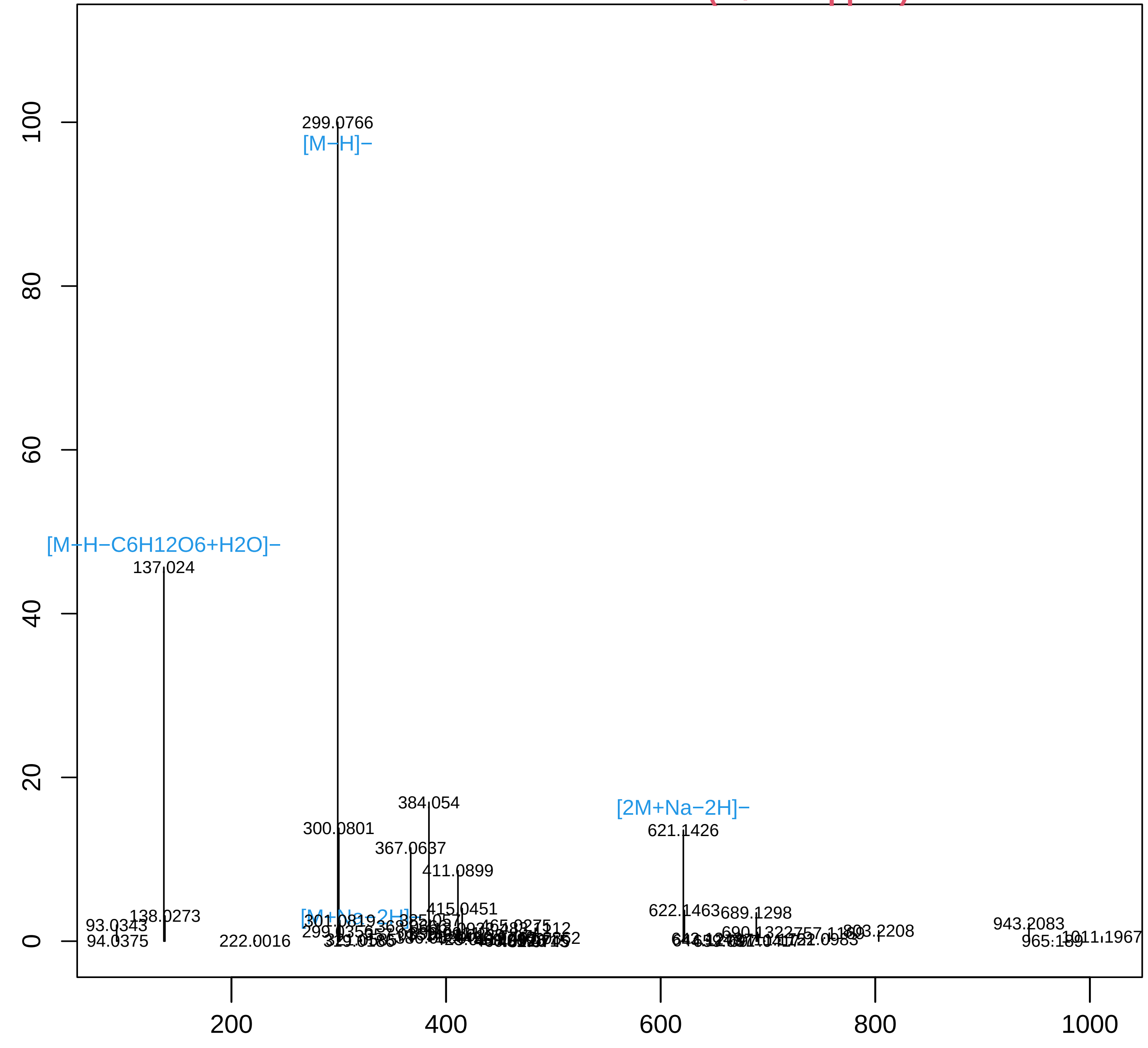

49 : M.findmain = 322.0655 (+/− 0.9 ppm)

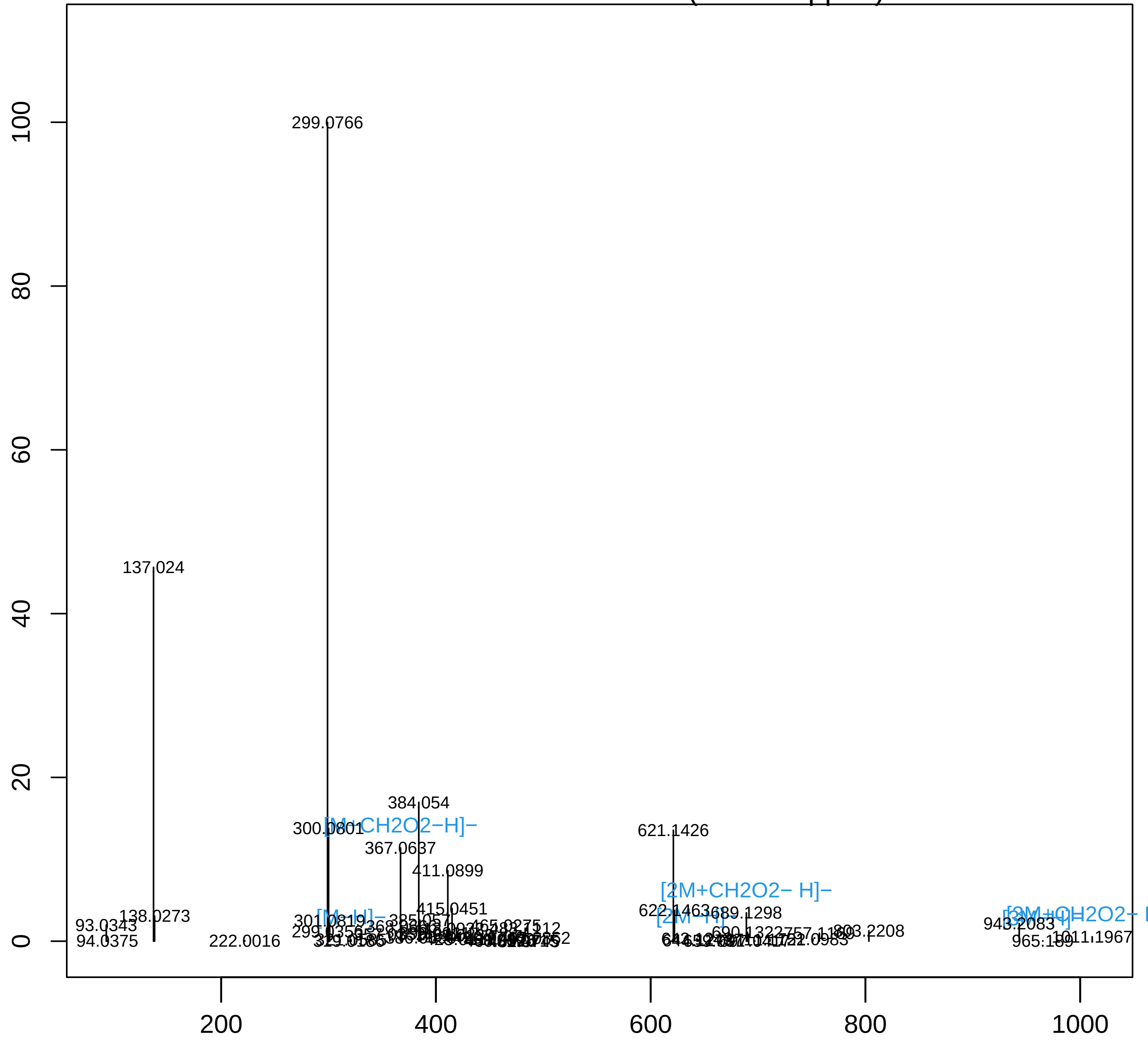

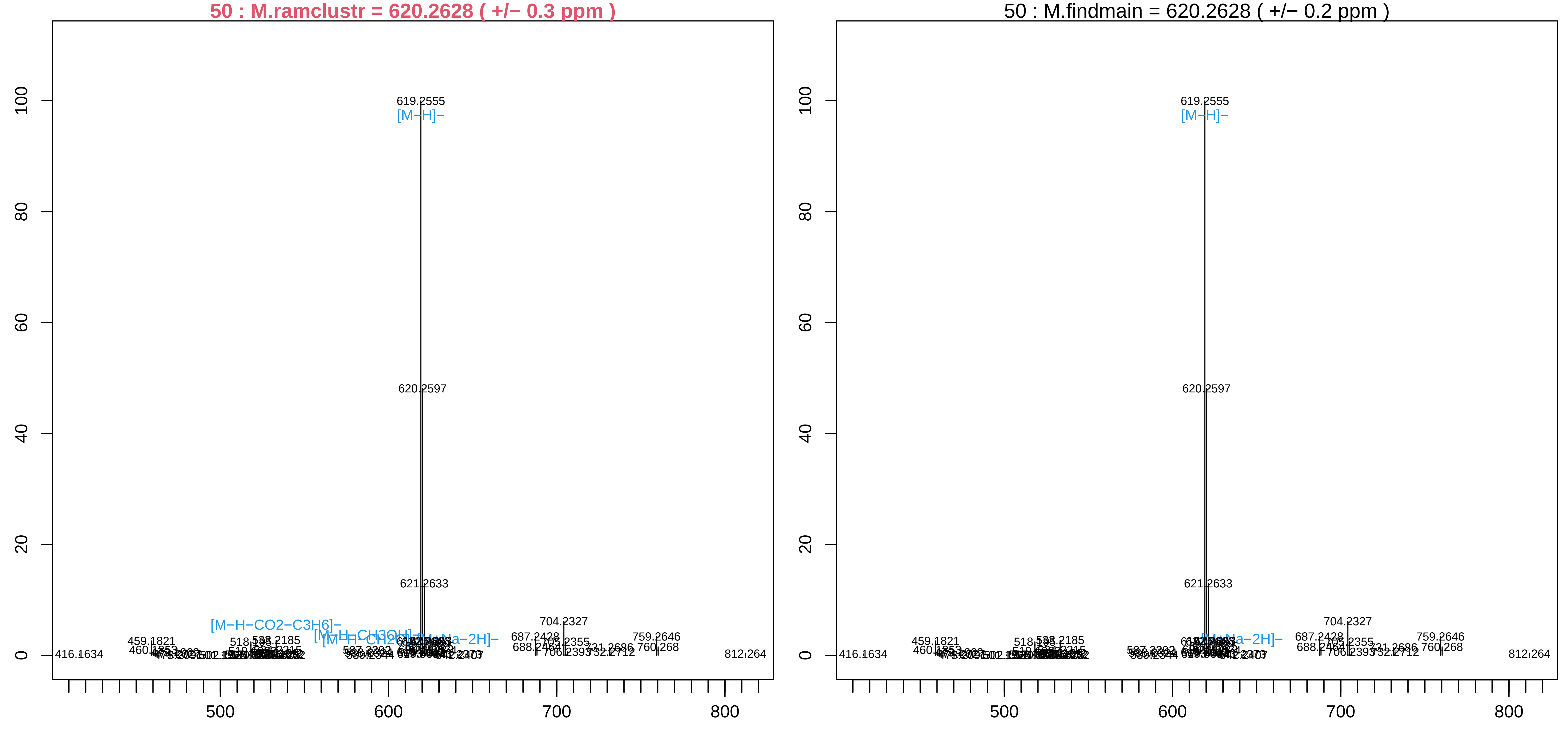
50 : M.ramclustr = 620.2628 (+/− 0.3 ppm)
50 : M.findmain = 620.2628 (+/− 0.2 ppm)
619.2555
[M−H]−
620.2597
621.2633
704.2327
[M−H−CO2−C3H6]−
687.2428
759.2646
760.268
459.1821
416.1634
812.264
0
20
40
60
80
100
500
600
700
800

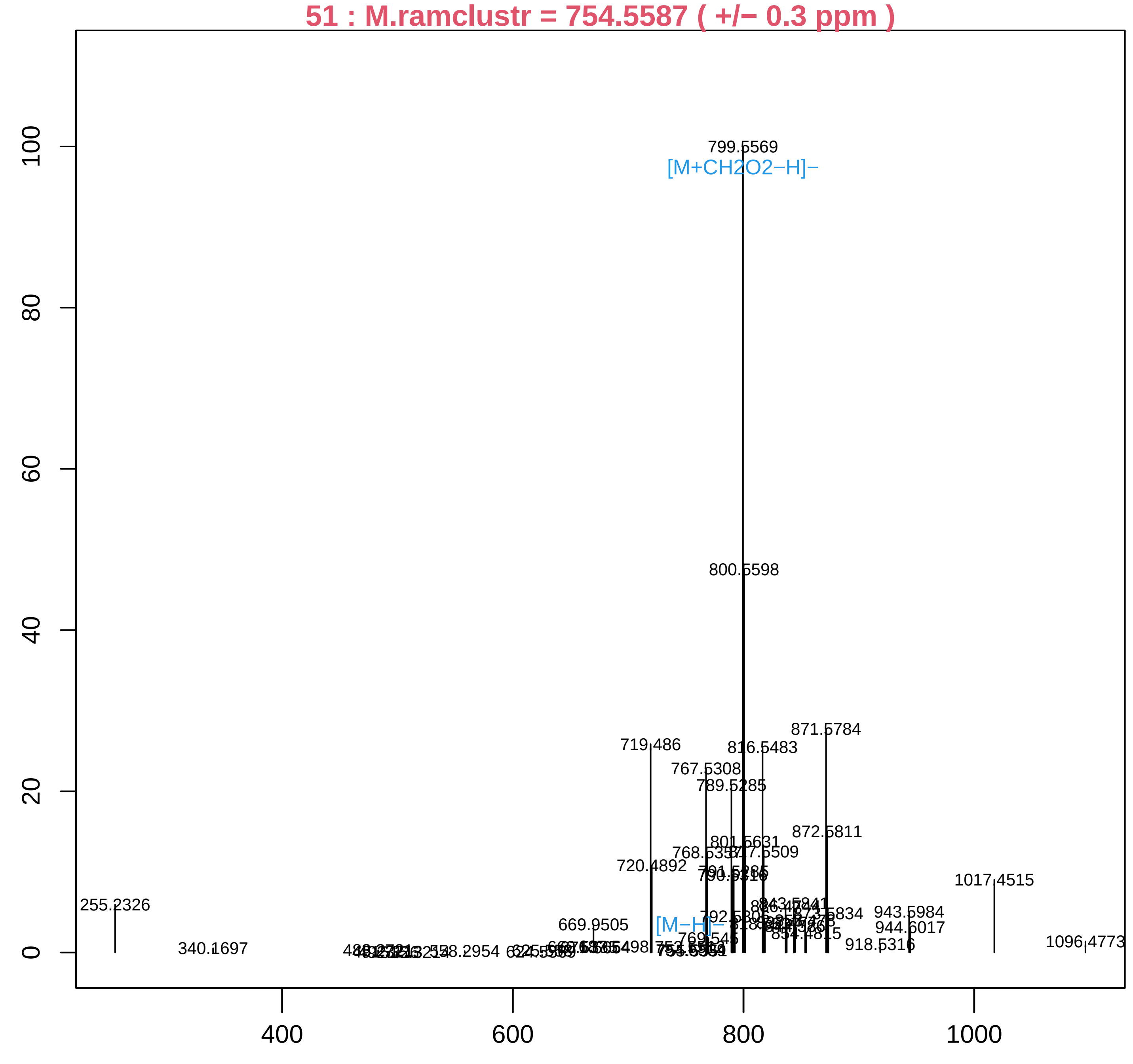
51 : M.ramclustr = 754.5587 (+/− 0.3 ppm)
799.5569
[M+CH2O2−H]−
800.5598
871.5784
719.486
816.5483
767.5308
789.5285
872.5811
801.5631
768.5337
817.5509
720.4892
1017.4515
255.2326
943.5984
944.6017
669.9505
[M−H]−
918.5316
1096.4773
340.1697
518.2954
0
20
40
60
80
100
400
600
800
1000

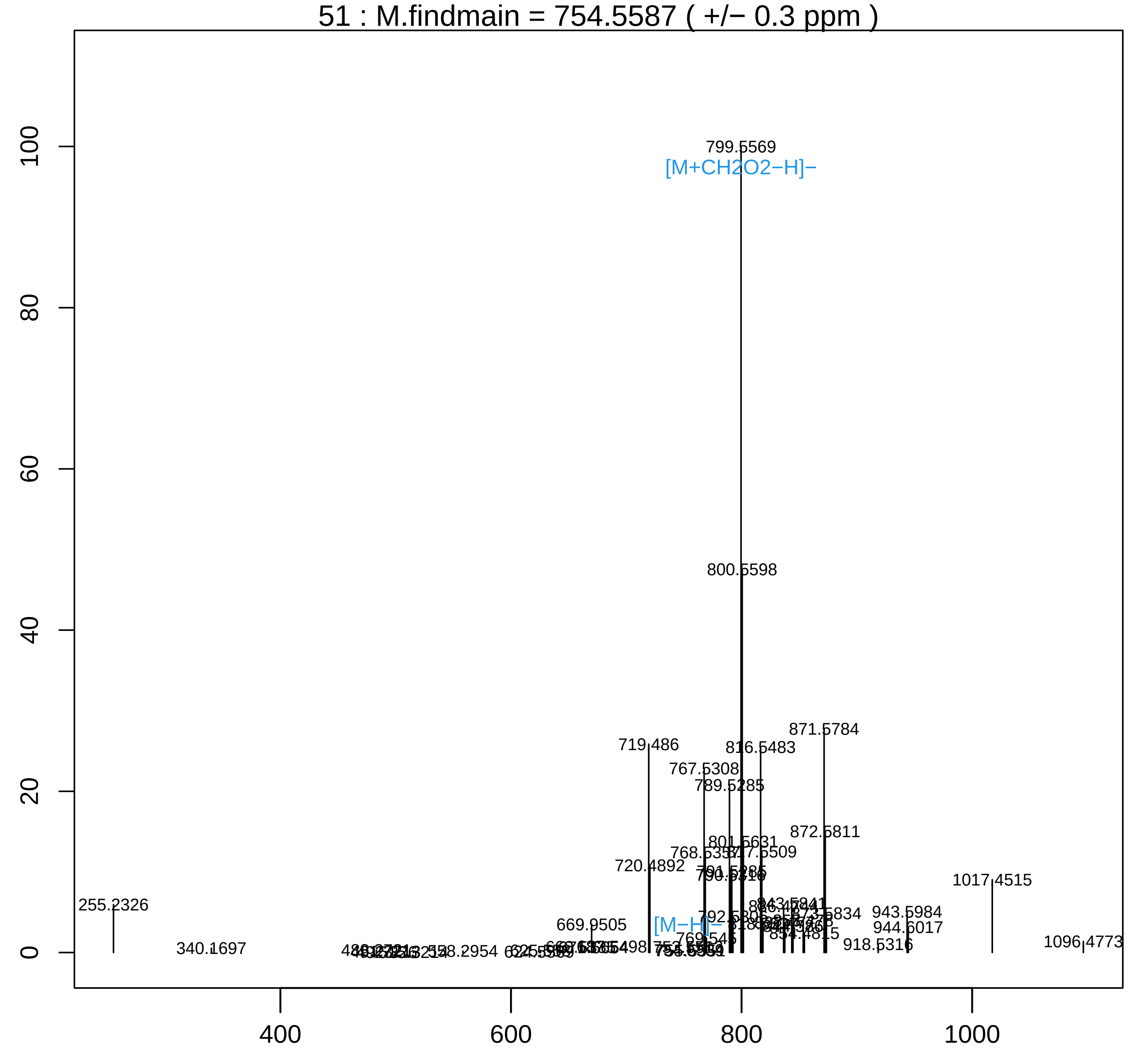
51 : M.findmain = 754.5587 (+/− 0.3 ppm)
799.5569
[M+CH2O2−H]−
800.5598
871.5784
719.486
816.5483
767.5308
789.5285
872.5811
801.5631
768.5337
817.5509
720.4892
1017.4515
255.2326
943.5984
944.6017
669.9505
[M−H]−
918.5316
1096.4773
340.1697
518.2954
0
20
40
60
80
100
400
600
800
1000

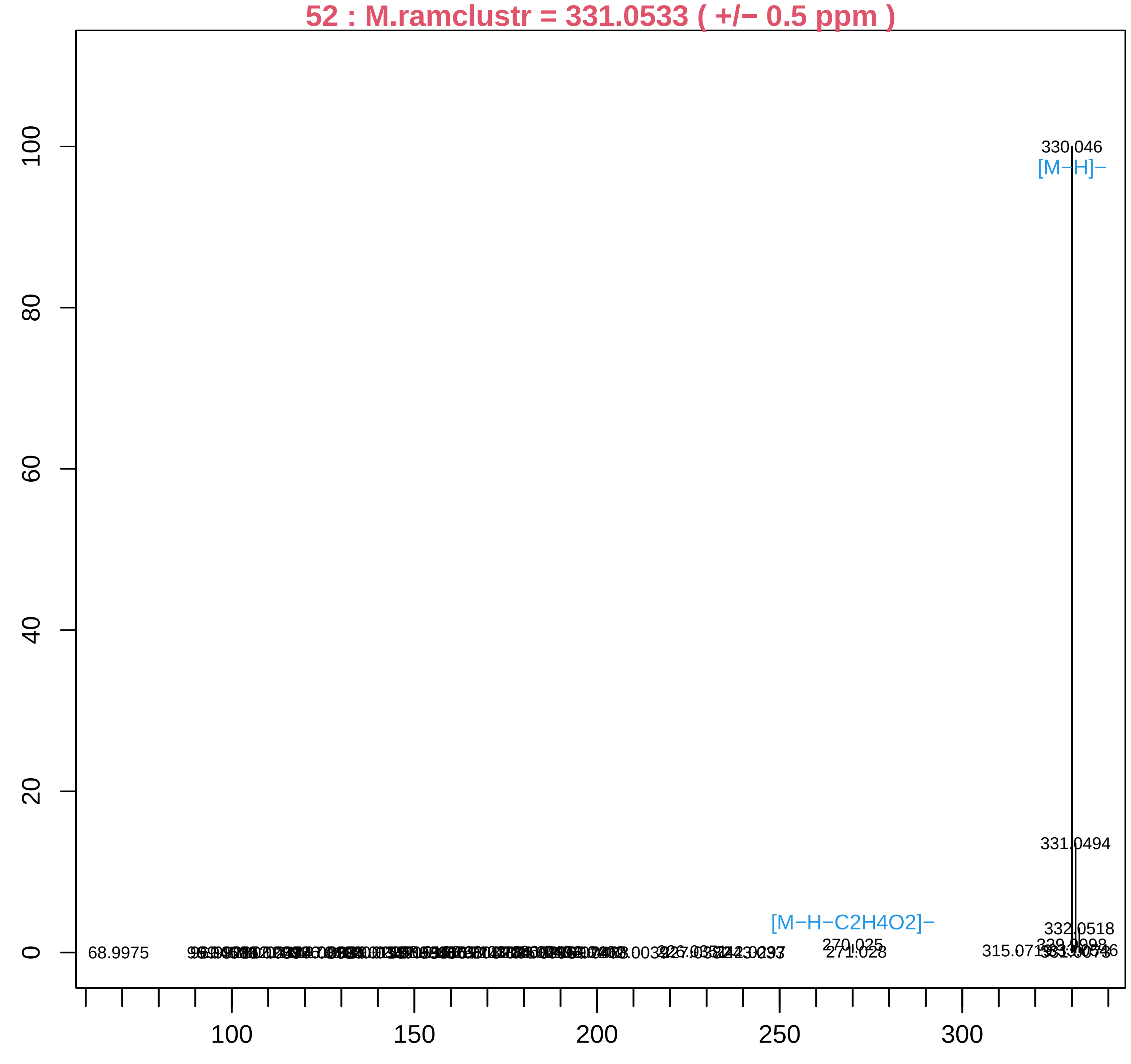
52 : M.ramclustr = 331.0533 (+/− 0.5 ppm)
330.046
[M−H]−
331.0494
332.0518
[M−H−C2H4O2]−
270.025
271.028
68.9975
315.071
0
20
40
60
80
100
100
150
200
250
300

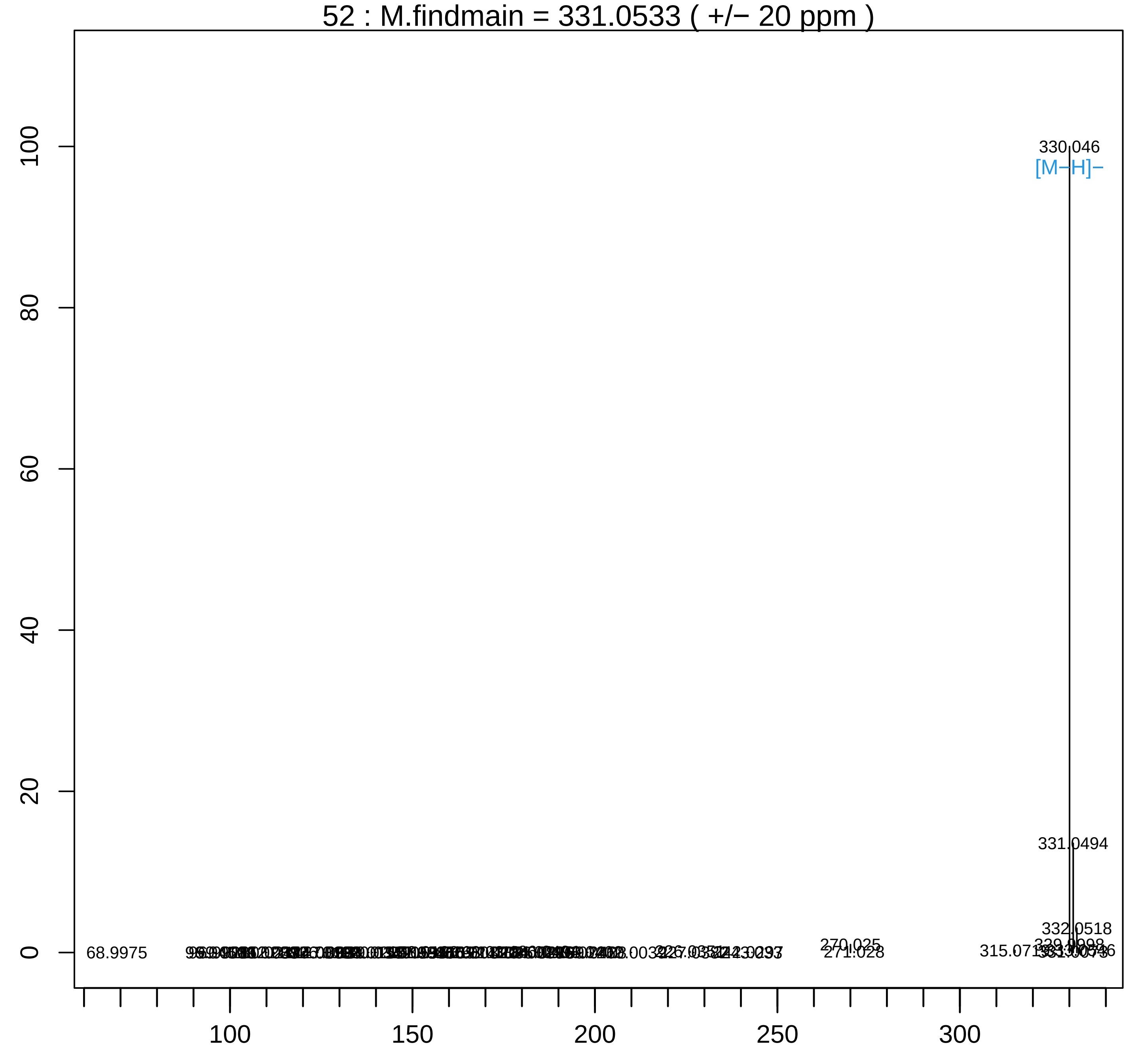
52 : M.findmain = 331.0533 (+/− 20 ppm)
330.046
[M−H]−
331.0494
332.0518
270.025
271.028
68.9975
315.071
0
20
40
60
80
100
100
150
200
250
300

53 : M.ramclustr = 368.1126 (+/− 1 ppm)

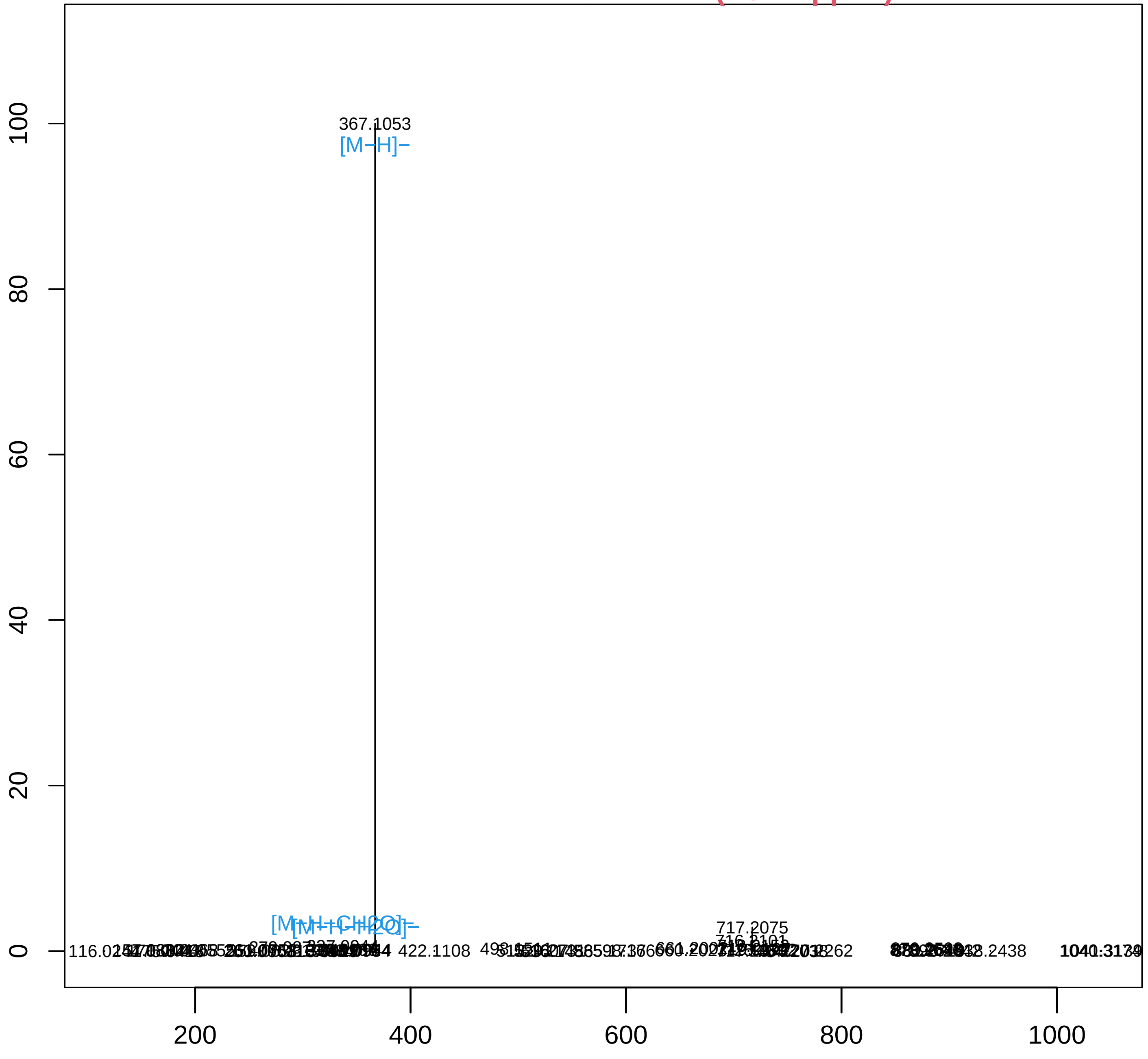

53 : M.findmain = 368.1126 (+/− 20 ppm)

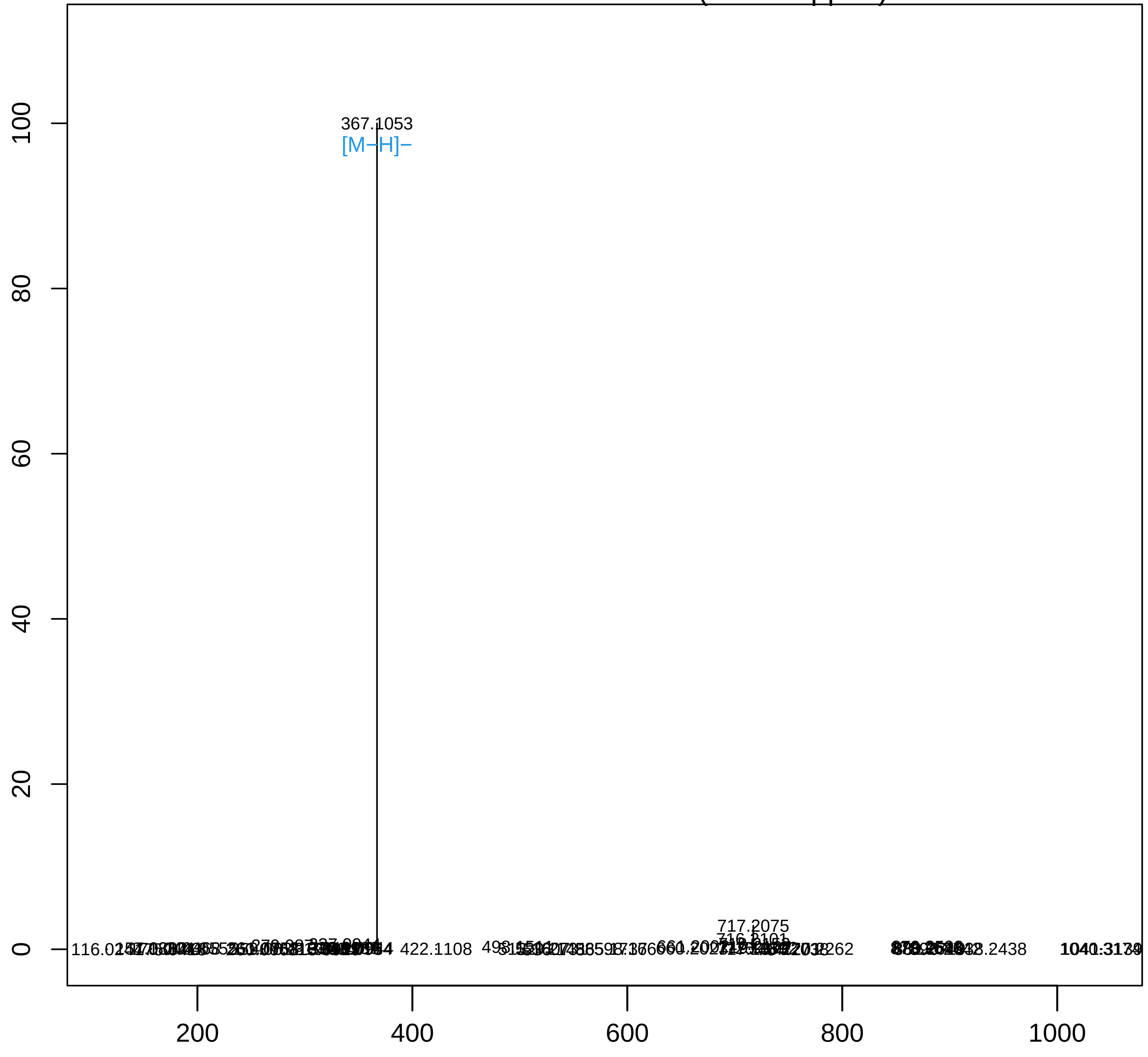

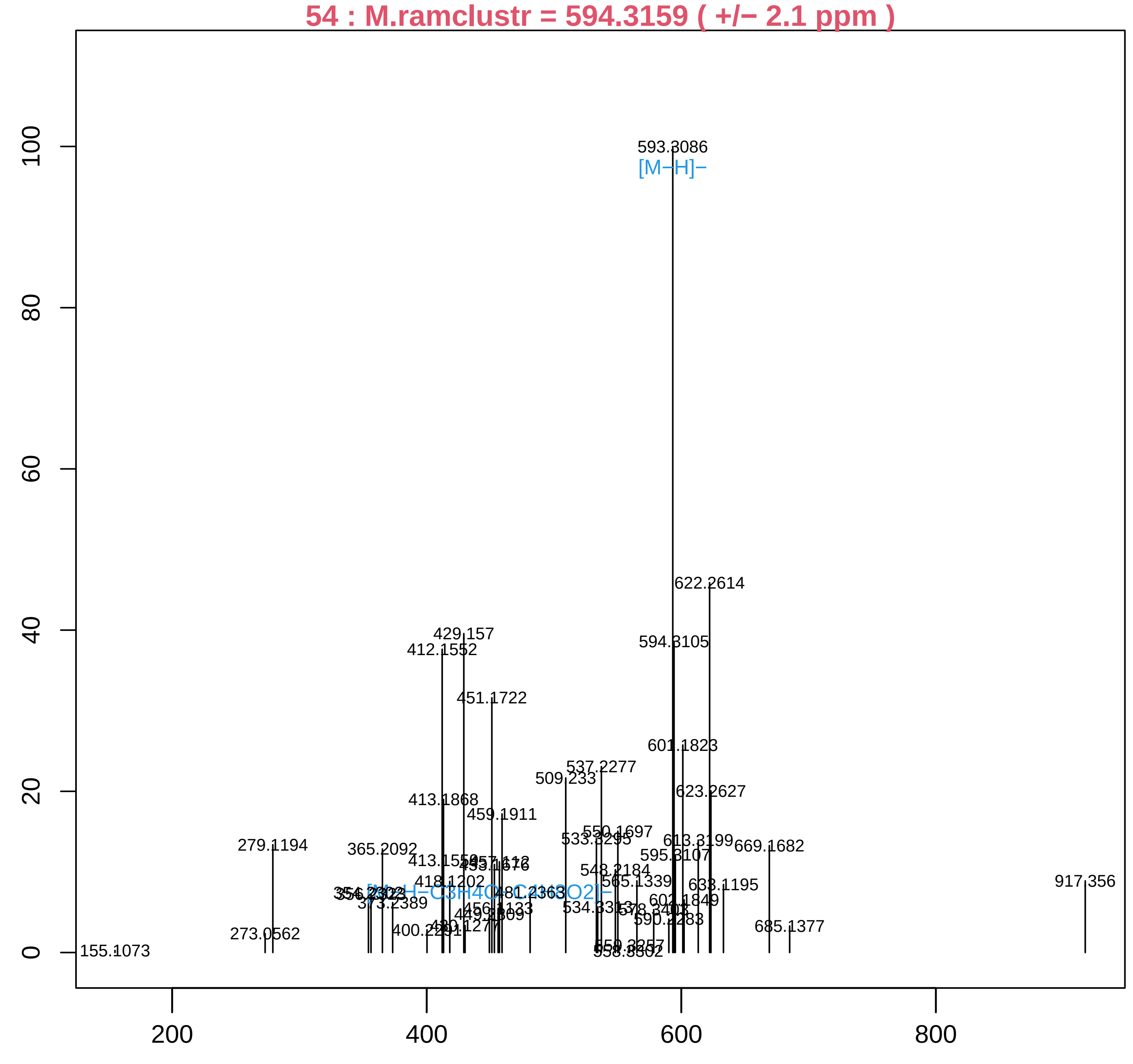

54 : M.ramclustr = 594.3159 (+/− 2.1 ppm)
593.3086
[M−H]−
622.2614
429.157
412.1552
594.3105
451.1722
601.1823
537.2277
509.233
623.2627
413.1868
459.1911
550.1697
533.3295
613.3199
279.1194
365.2092
669.1682
595.3107
548.2184
565.1339
418.1202
633.1195
917.356
373.2389
601.1849
590.2283
685.1377
400.2291
273.0562
155.1073
558.8802
0
20
40
60
80
100
200
400
600
800

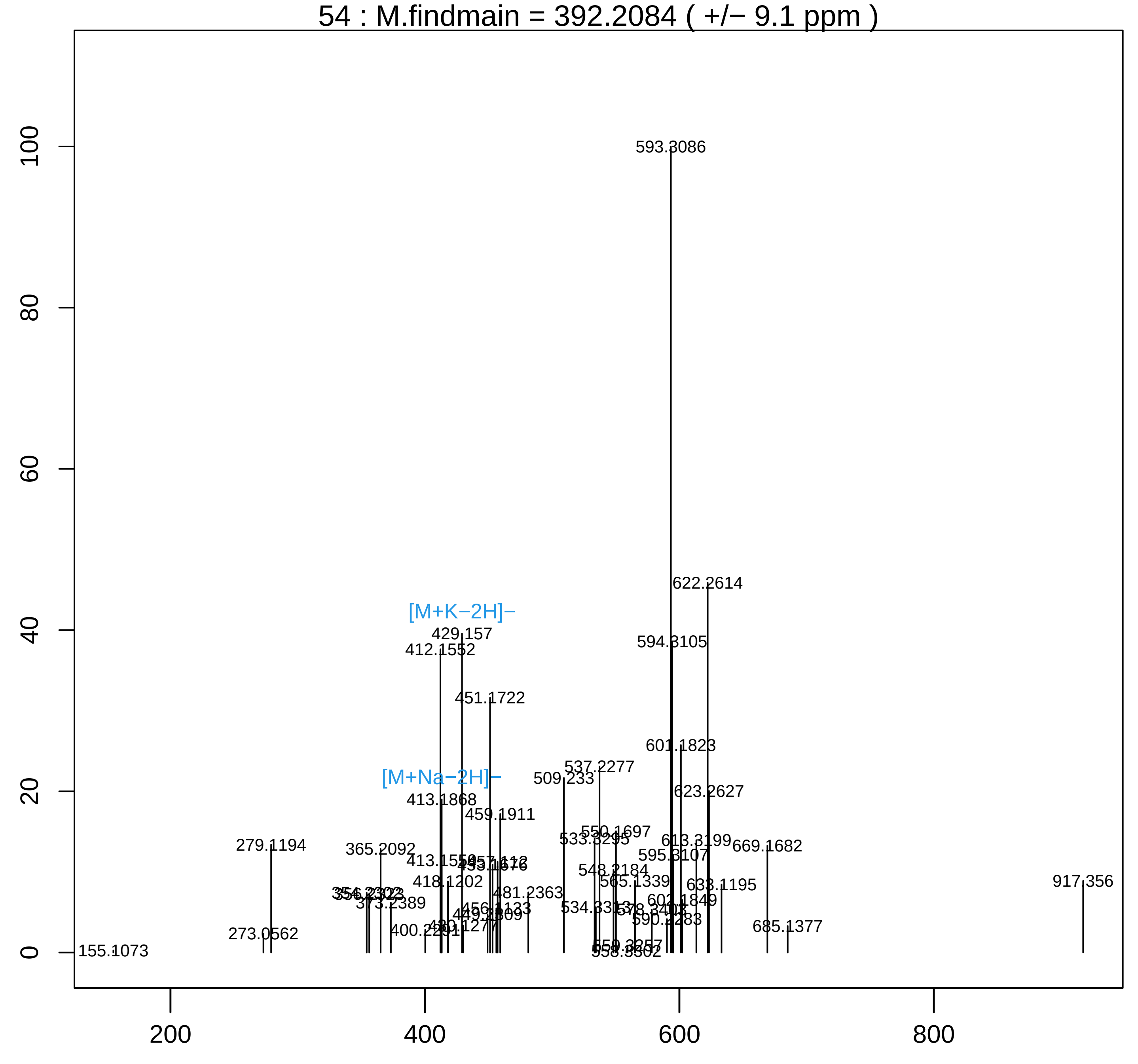

54 : M.findmain = 392.2084 (+/− 9.1 ppm)
593.3086
[M+K−2H]−
622.2614
429.157
412.1552
594.3105
451.1722
601.1823
[M+Na−2H]−
537.2277
509.233
623.2627
413.1868
459.1911
550.1697
533.3295
613.3199
279.1194
365.2092
669.1682
595.3107
548.2184
565.1339
418.1202
481.2363
633.1195
917.356
373.2389
601.1849
590.2283
685.1377
400.2291
273.0562
155.1073
558.8802
0
20
40
60
80
100
200
400
600
800

55 : M.ramclustr = 883.4923 (+/− 0.1 ppm)

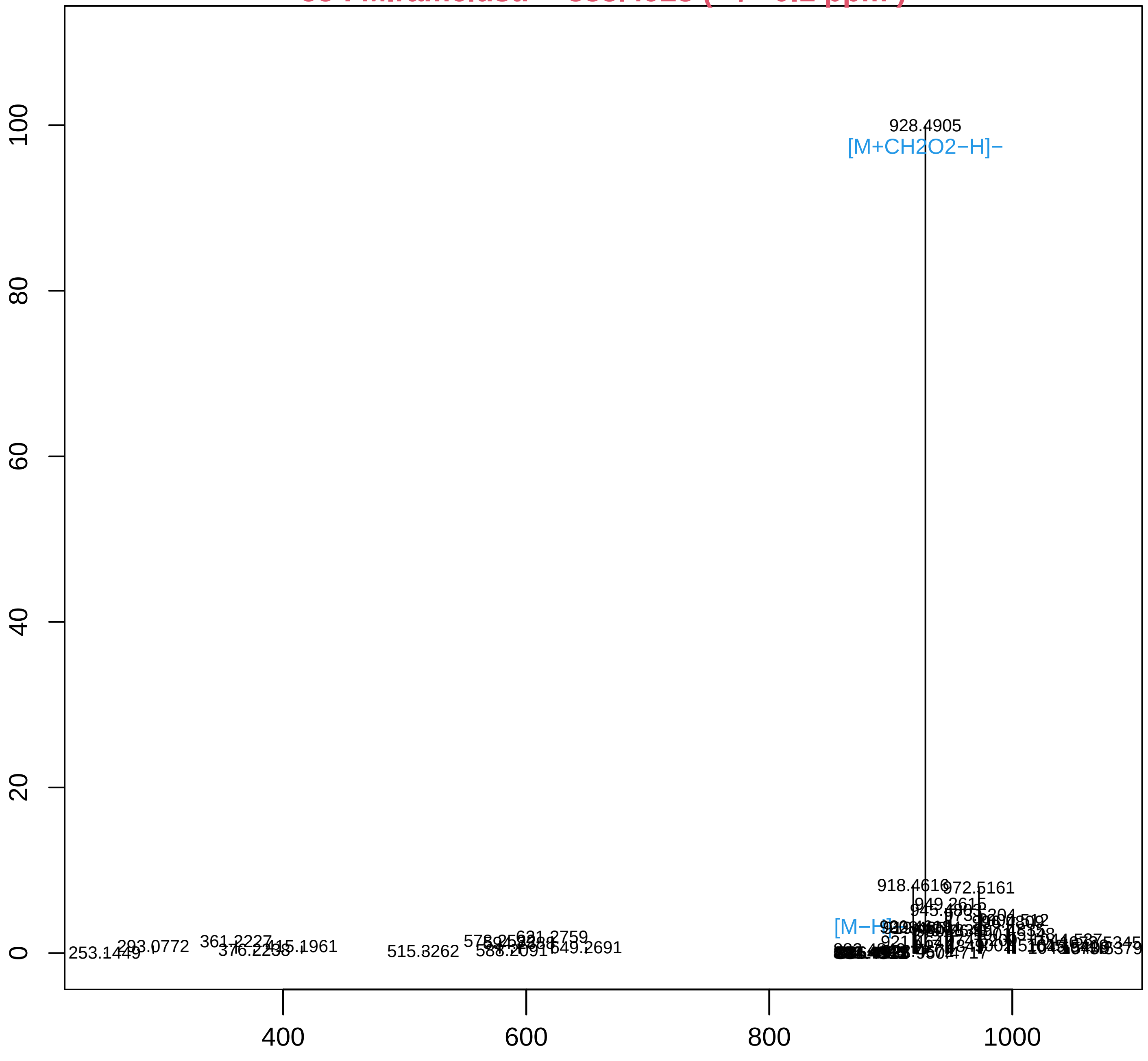

55 : M.findmain = 929.4978 (+/− 0.8 ppm)

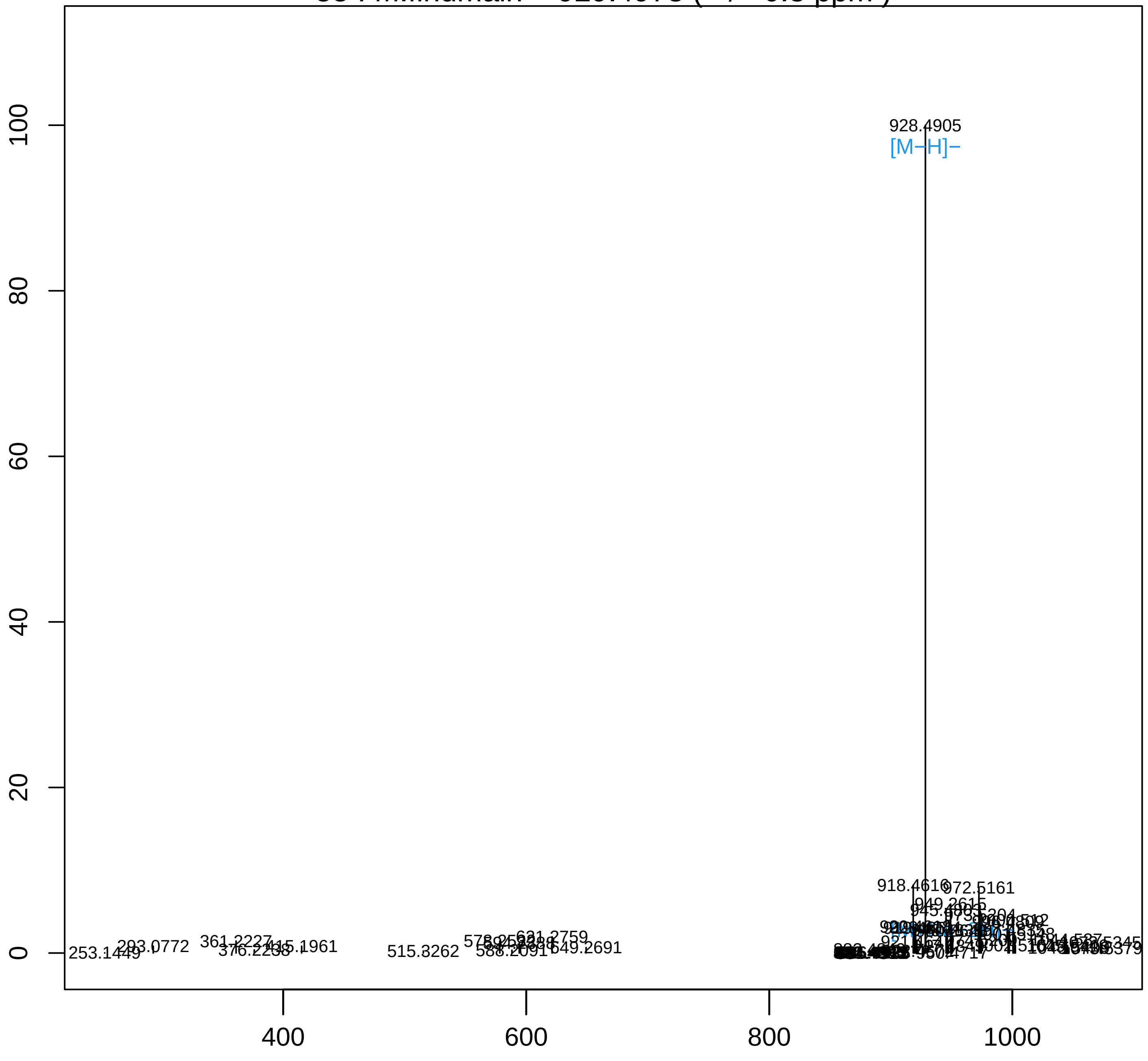

56 : M.ramclustr = 416.1311 (+/− 0.4 ppm)

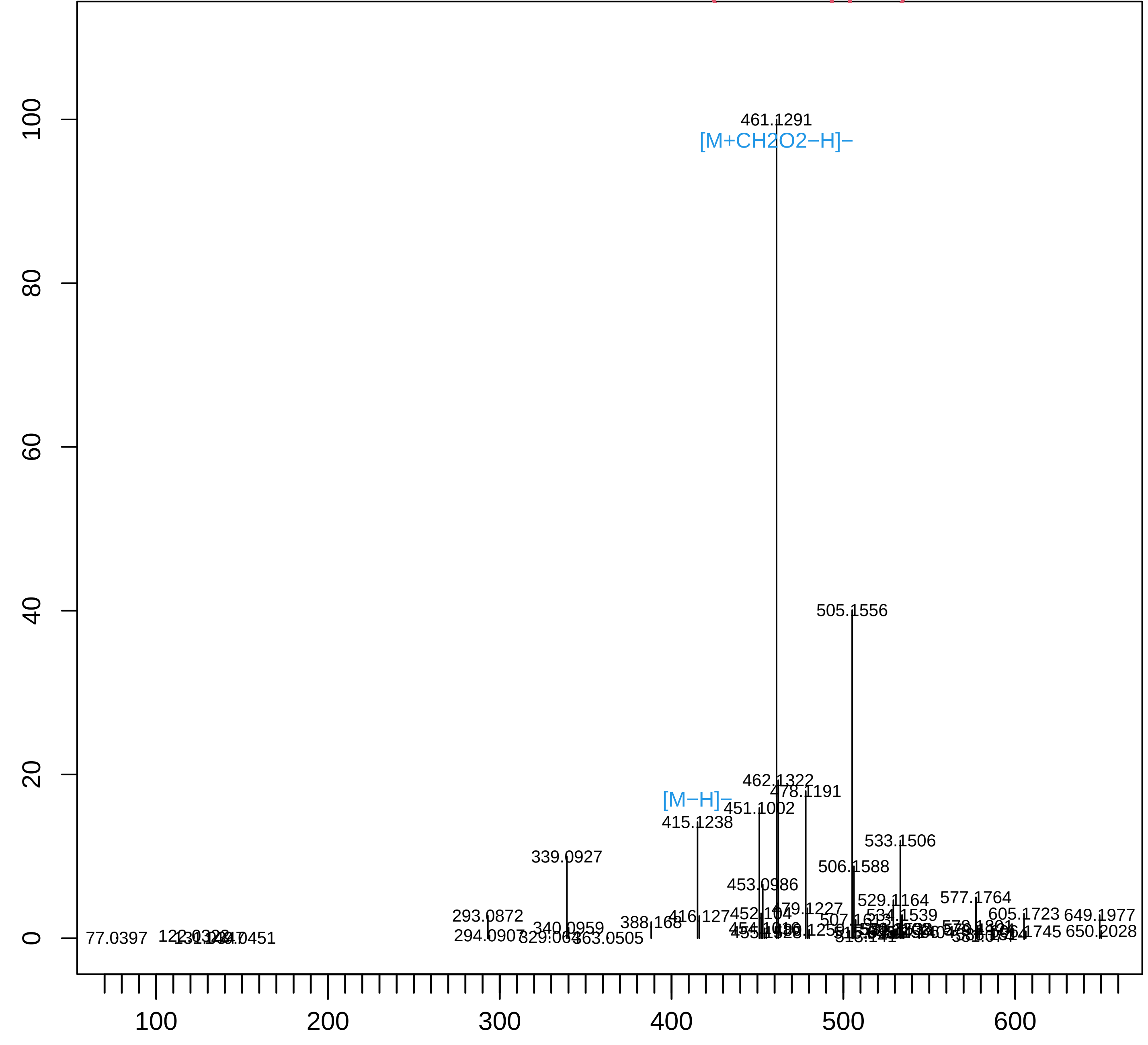

56 : M.findmain = 416.1309 (+/− 0.4 ppm)

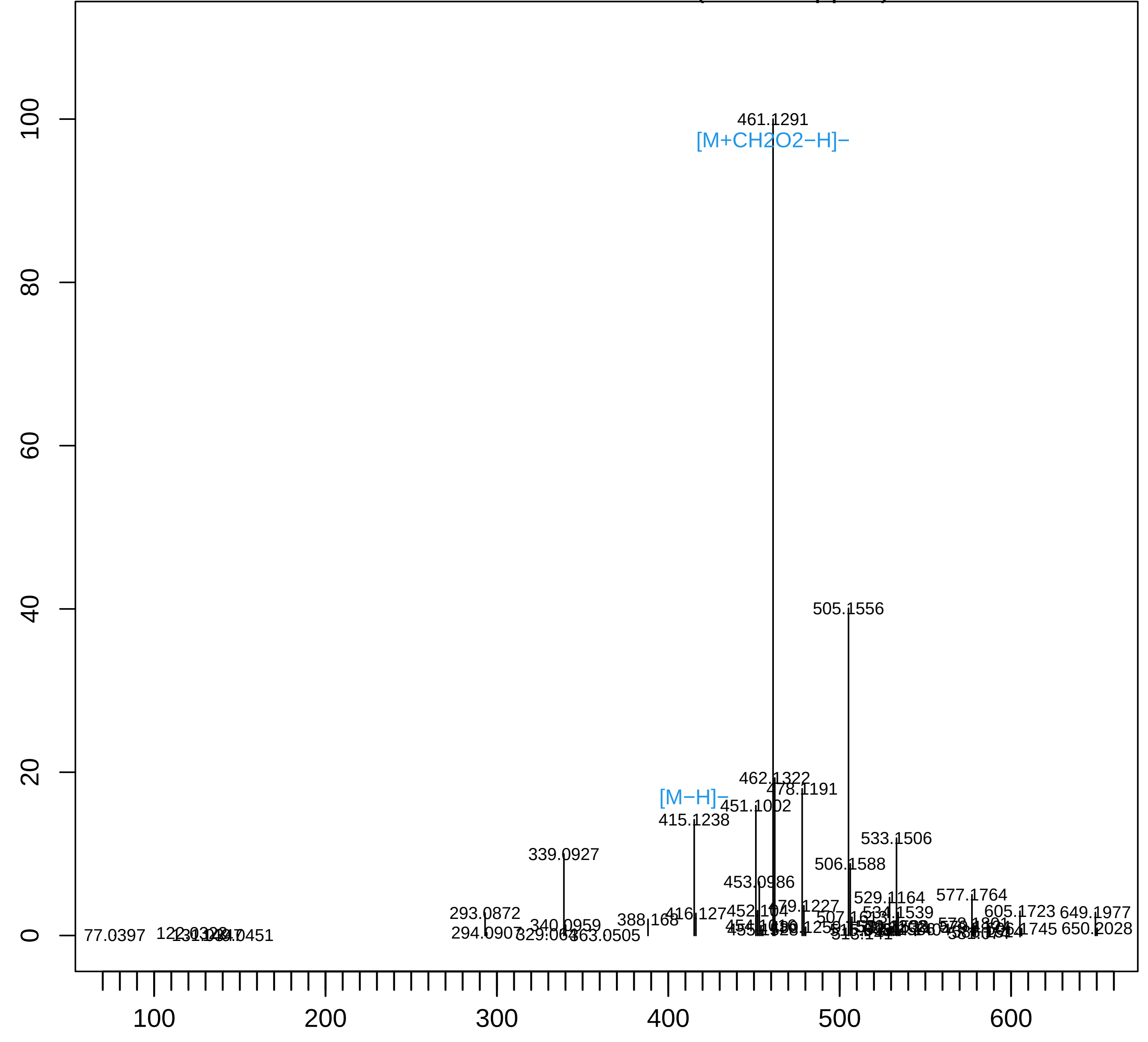

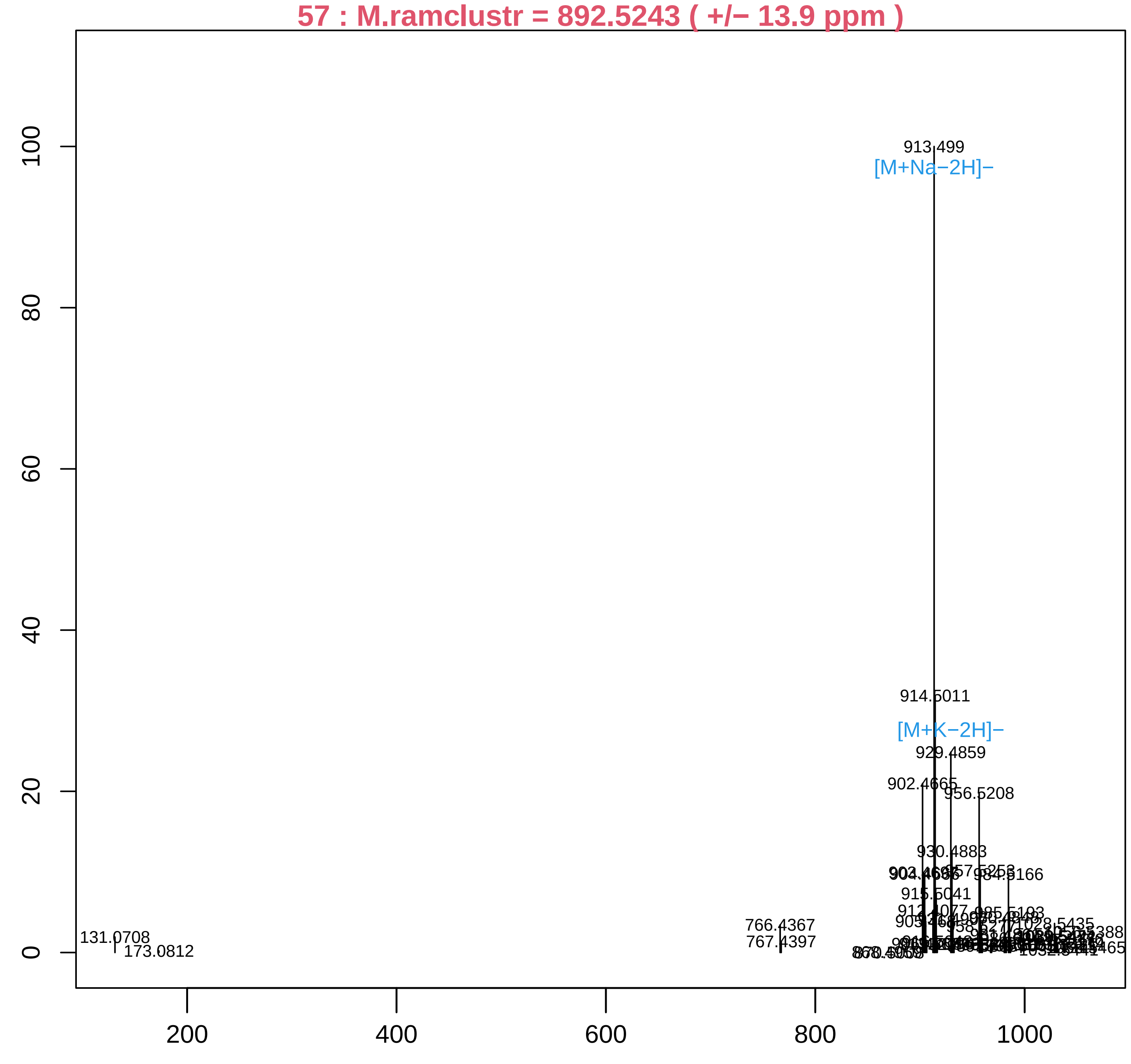
57 : M.ramclustr = 892.5243 (+/− 13.9 ppm)
913.499
[M+Na−2H]−
914.5011
[M+K−2H]−
929.4859
902.4665
956.5208
930.4883
984.5166
915.5041
766.4367
767.4397
131.0708
173.0812
0
20
40
60
80
100
200
400
600
800
1000

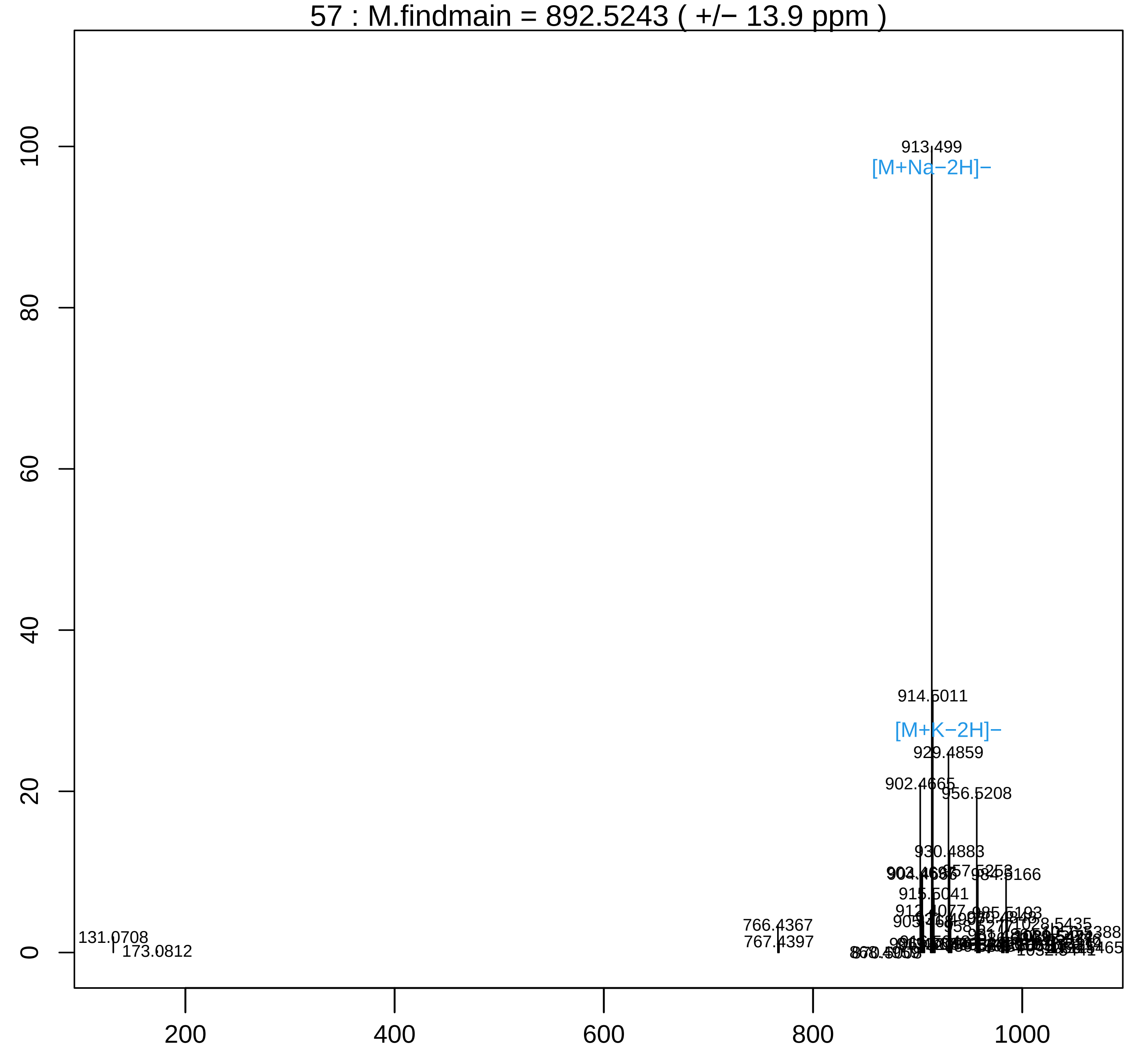
57 : M.findmain = 892.5243 (+/− 13.9 ppm)
913.499
[M+Na−2H]−
914.5011
[M+K−2H]−
929.4859
902.4665
956.5208
930.4883
984.5166
915.5041
766.4367
767.4397
131.0708
173.0812
0
20
40
60
80
100
200
400
600
800
1000

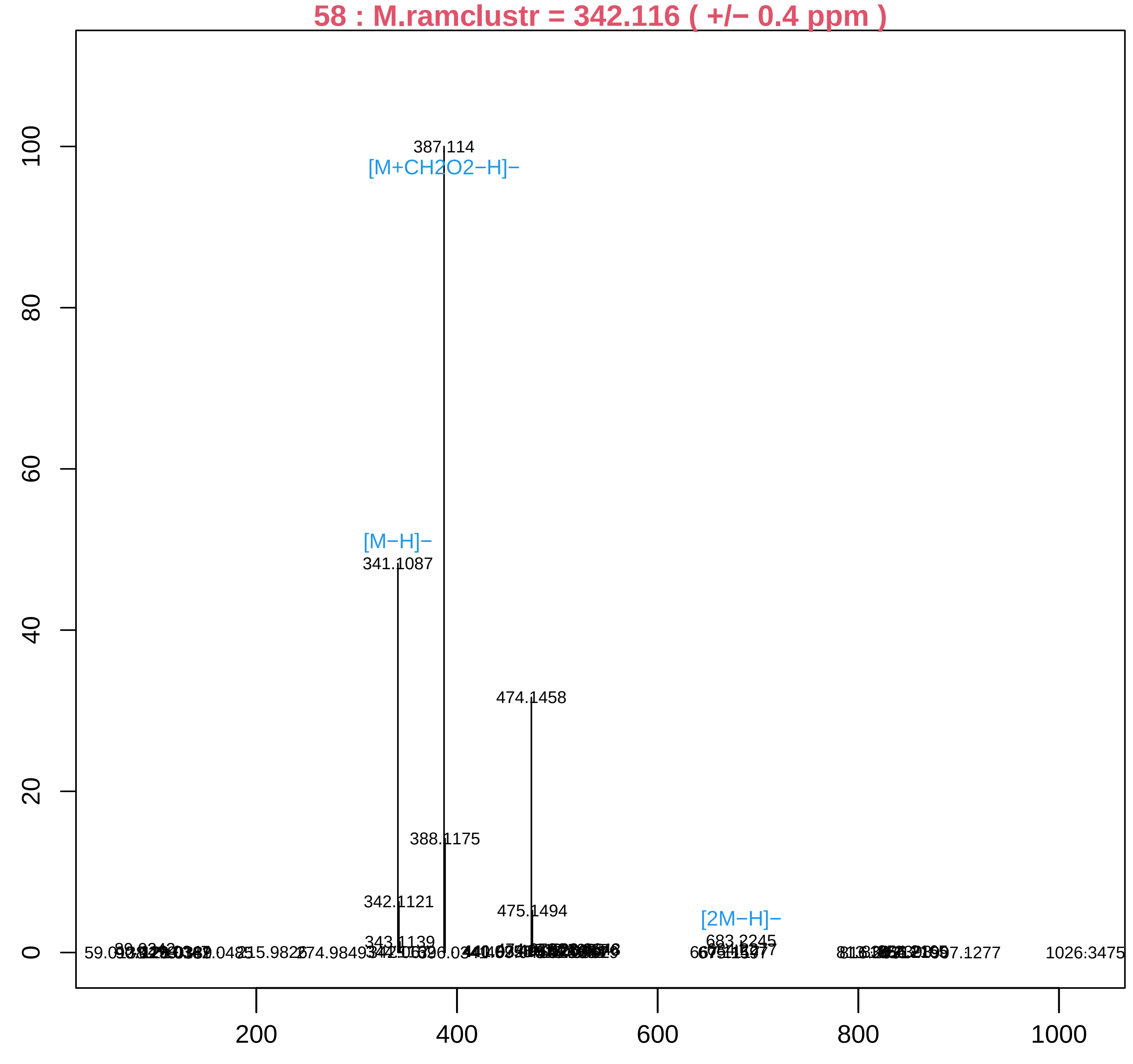
58 : M.ramclustr = 342.116 (+/− 0.4 ppm)
387.114
[M+CH2O2−H]−
[M−H]−
341.1087
474.1458
388.1175
342.1121
475.1494
[2M−H]−
683.2245
343.1139
1026.3475
0
20
40
60
80
100
200
400
600
800
1000

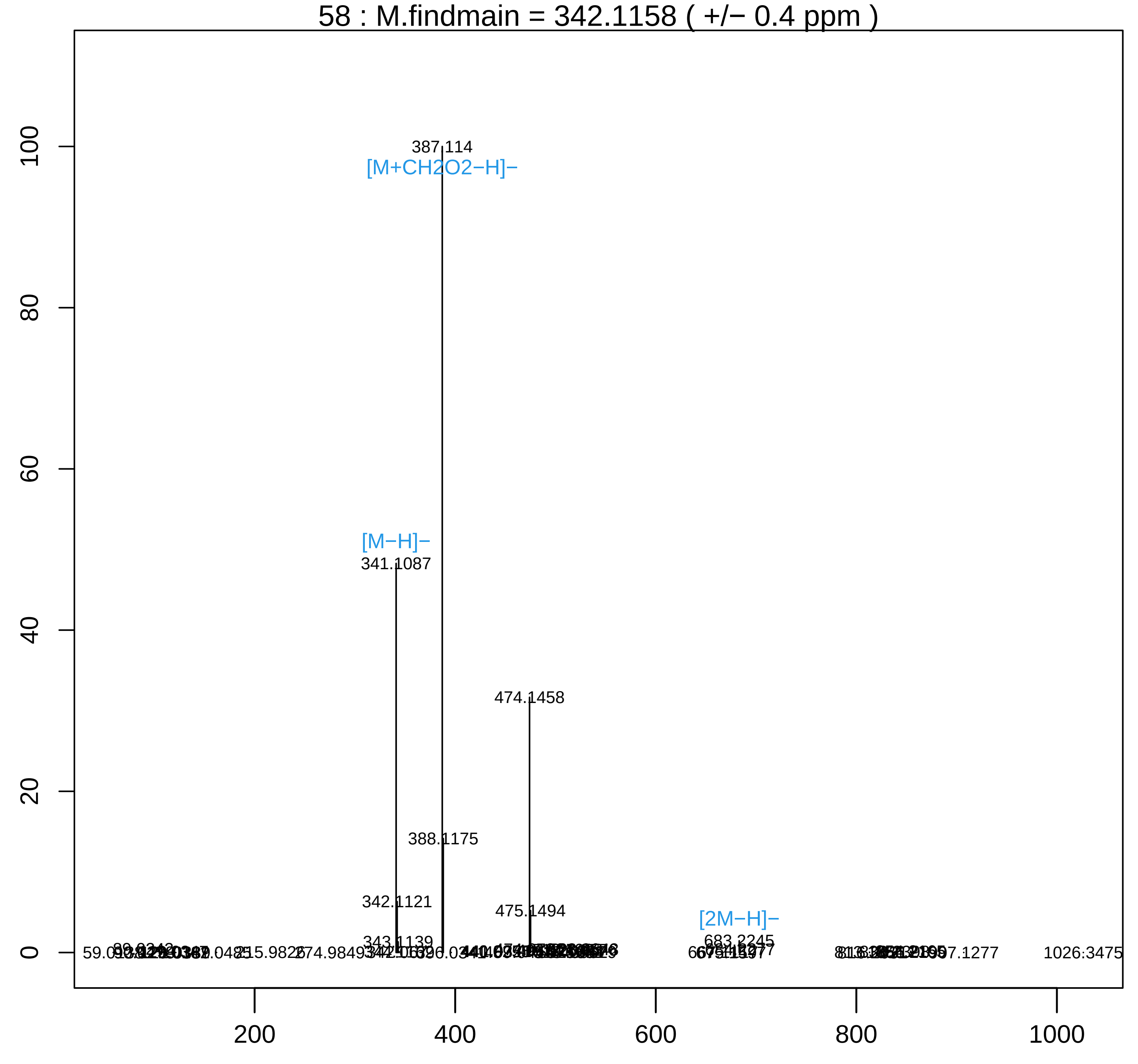
58 : M.findmain = 342.1158 (+/− 0.4 ppm)
387.114
[M+CH2O2−H]−
[M−H]−
341.1087
474.1458
388.1175
342.1121
475.1494
[2M−H]−
683.2245
343.1139
1026.3475
0
20
40
60
80
100
200
400
600
800
1000

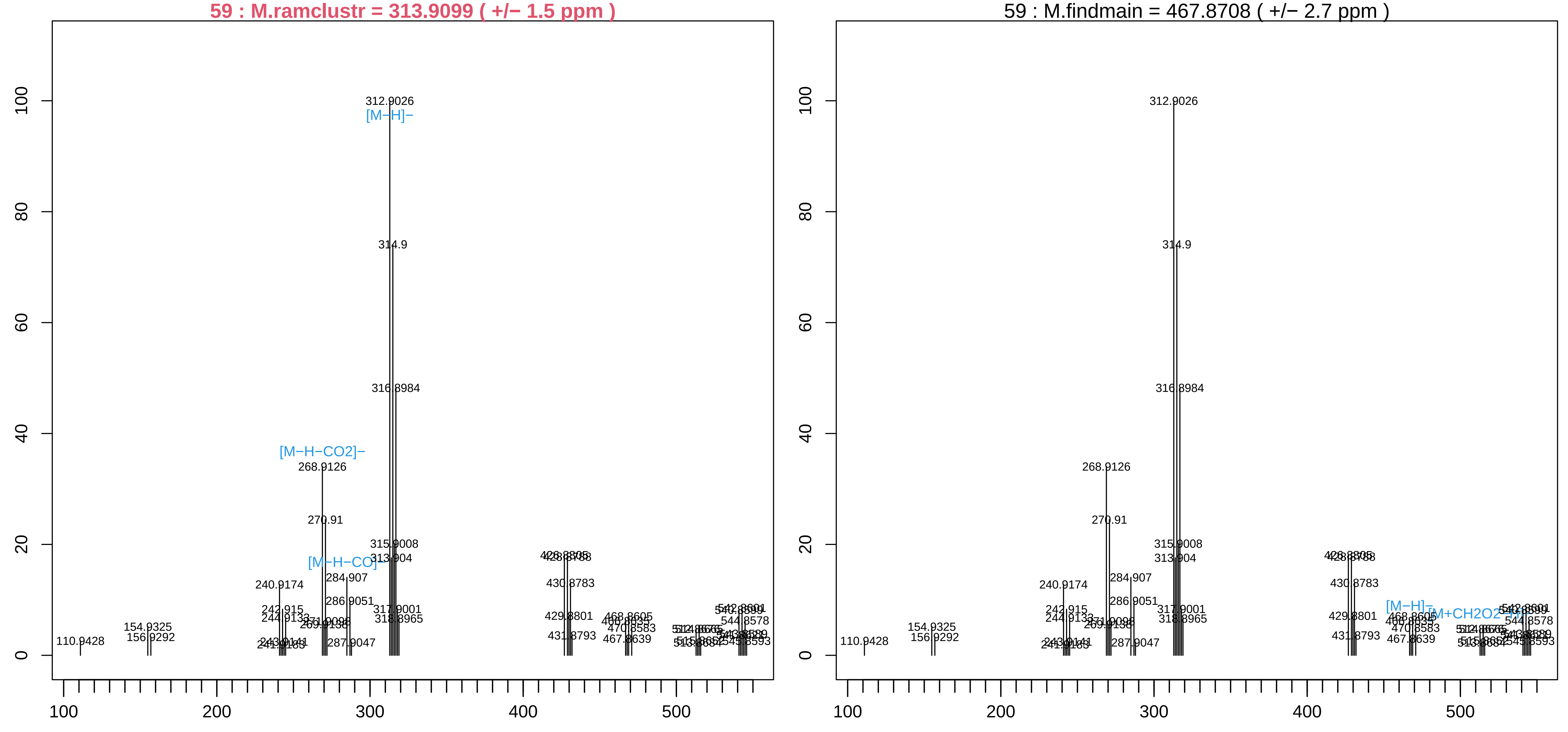
59 : M.ramclustr = 313.9099 (+/− 1.5 ppm)
59 : M.findmain = 467.8708 (+/− 2.7 ppm)
312.9026
[M−H]−
314.9
316.8984
[M−H−CO2]−
268.9126
270.91
315.9008
313.904
[M−H−CO]
284.907
240.9174
286.9051
242.915
244.9133
317.9001
318.8965
154.9325
156.9292
110.9428
287.9047
428.8805
430.8783
429.8801
431.8793
468.8605
470.8583
467.8639
542.8601
544.8578
[M−H]−
[M+CH2O2−H]−

60 : M.ramclustr = 392.0394 (+/− 1 ppm)

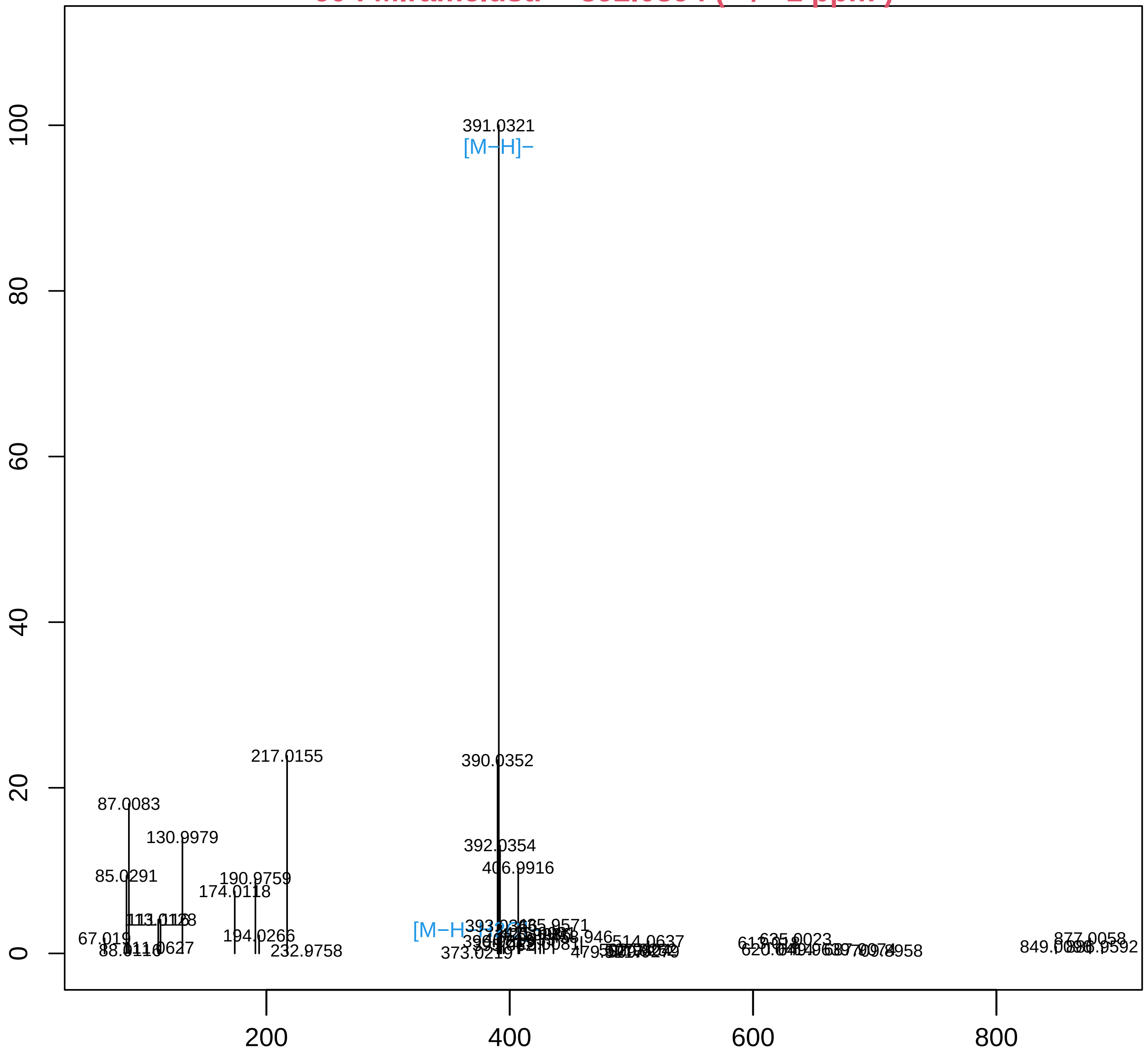

60 : M.findmain = 392.0394 (+/− 20 ppm)

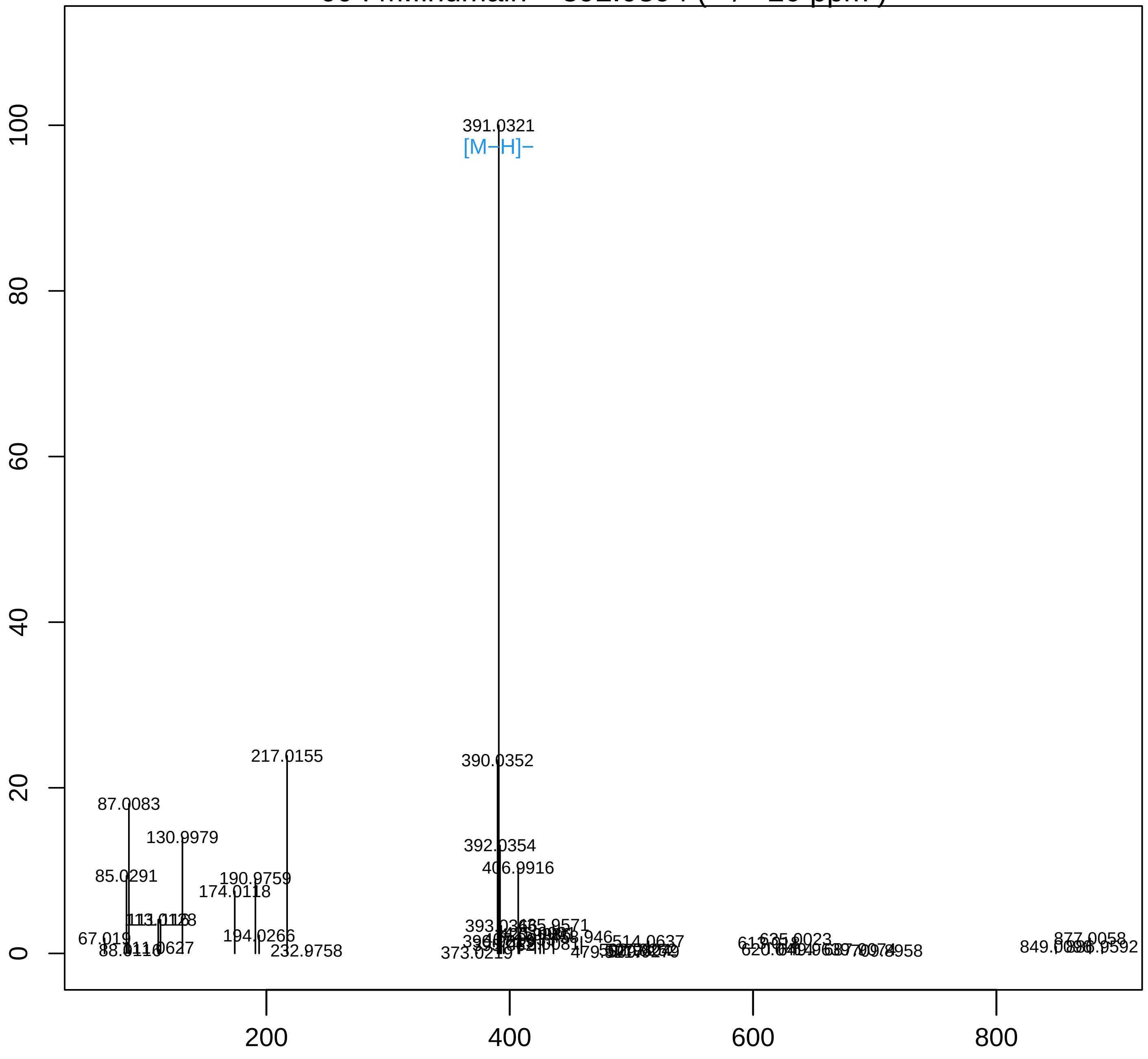

61 : M.ramclustr = 708.4724 (+/− 0.7 ppm)

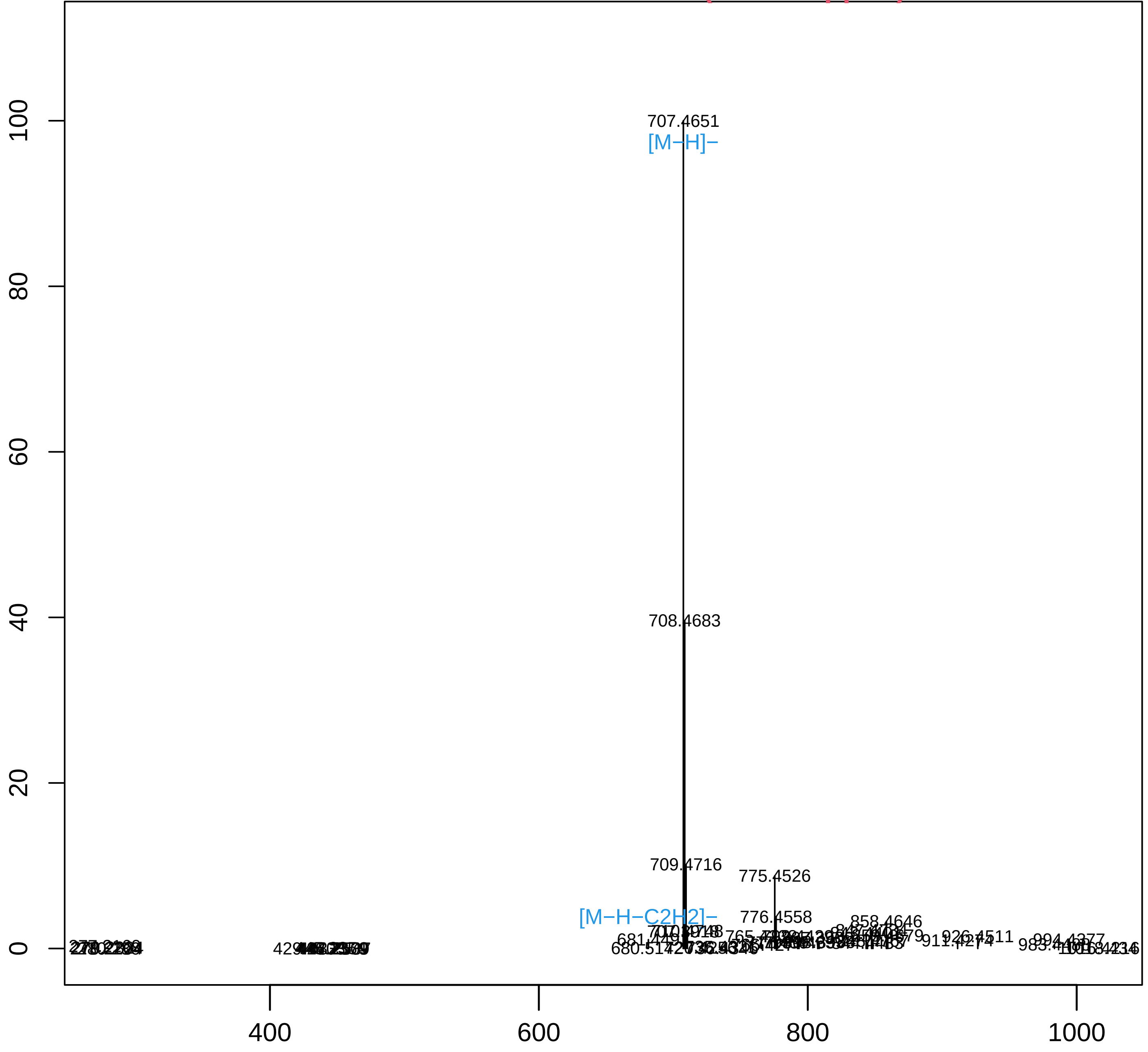

61 : M.findmain = 708.4724 (+/− 20 ppm)

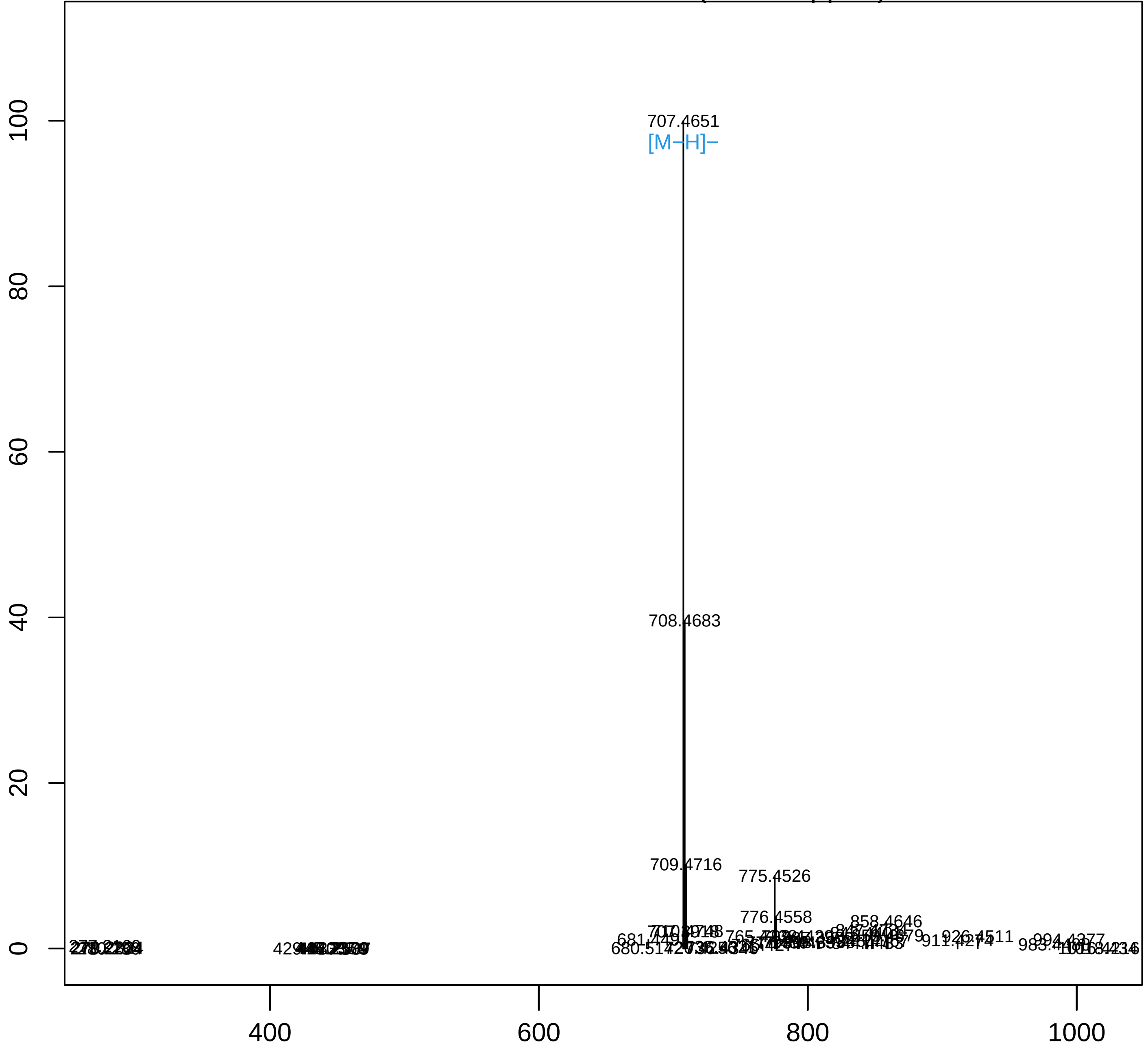

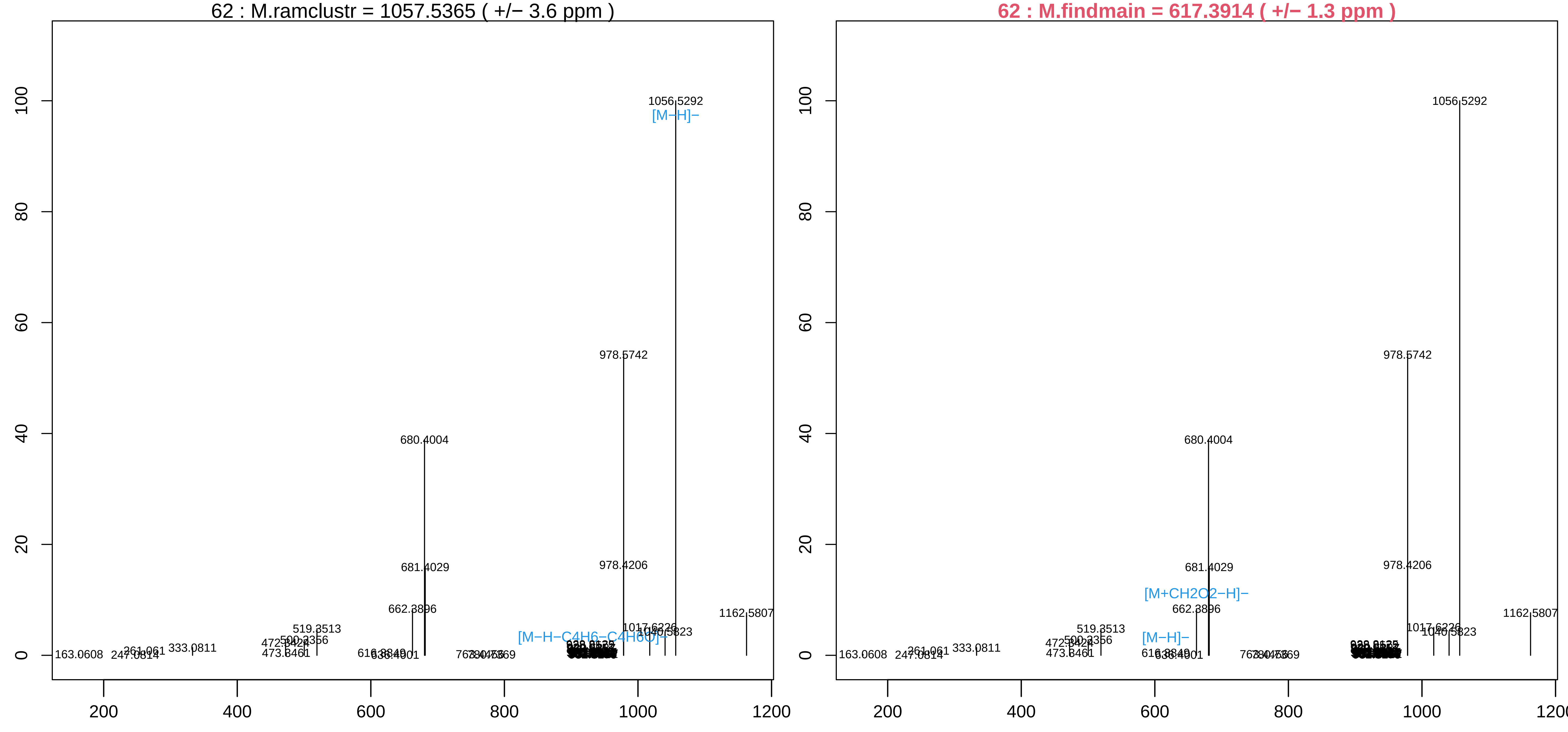
62 : M.ramclustr = 1057.5365 (+/− 3.6 ppm)
62 : M.findmain = 617.3914 (+/− 1.3 ppm)
1056.5292
[M−H]−
978.5742
680.4004
681.4029
978.4206
662.3896
1162.5807
1017.6226
1040.5823
519.3513
500.2356
473.3461
333.0811
261.061
247.0814
163.0608
636.4001
[M+CH2O2−H]−
[M−H]−
0
20
40
60
80
100
200
400
600
800
1000
1200

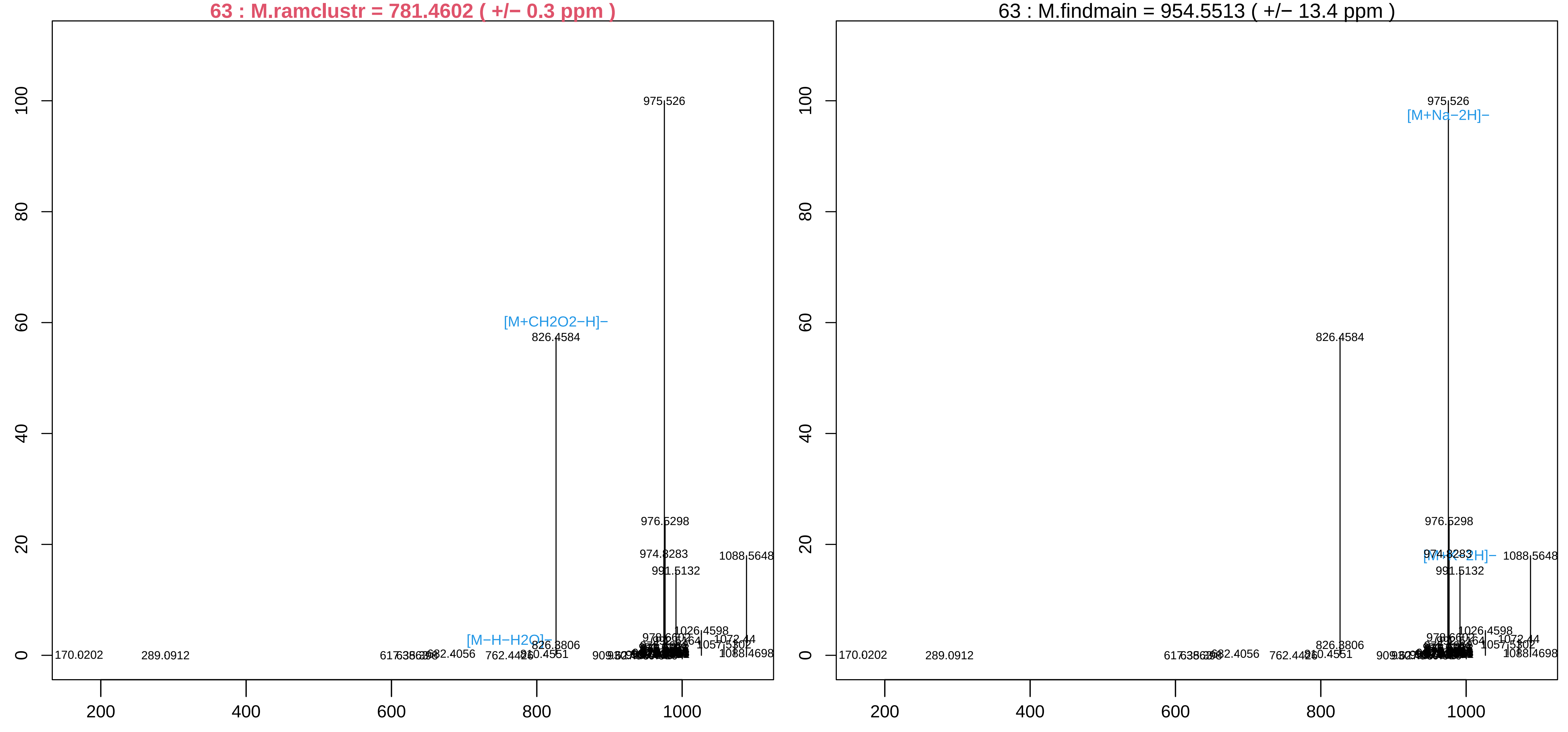
63 : M.ramclustr = 781.4602 (+/− 0.3 ppm)
975.526
[M+CH2O2−H]−
826.4584
976.5298
974.8283
991.5132
1088.5648
1026.4598
1072.44
1057.5302
1088.4698
[M−H−H2O]−
826.3806
170.0202
289.0912
682.4056
762.4426
910.4551
63 : M.findmain = 954.5513 (+/− 13.4 ppm)
[M+Na−2H]−
[M+K−2H]−
0
20
40
60
80
100
200
400
600
800
1000

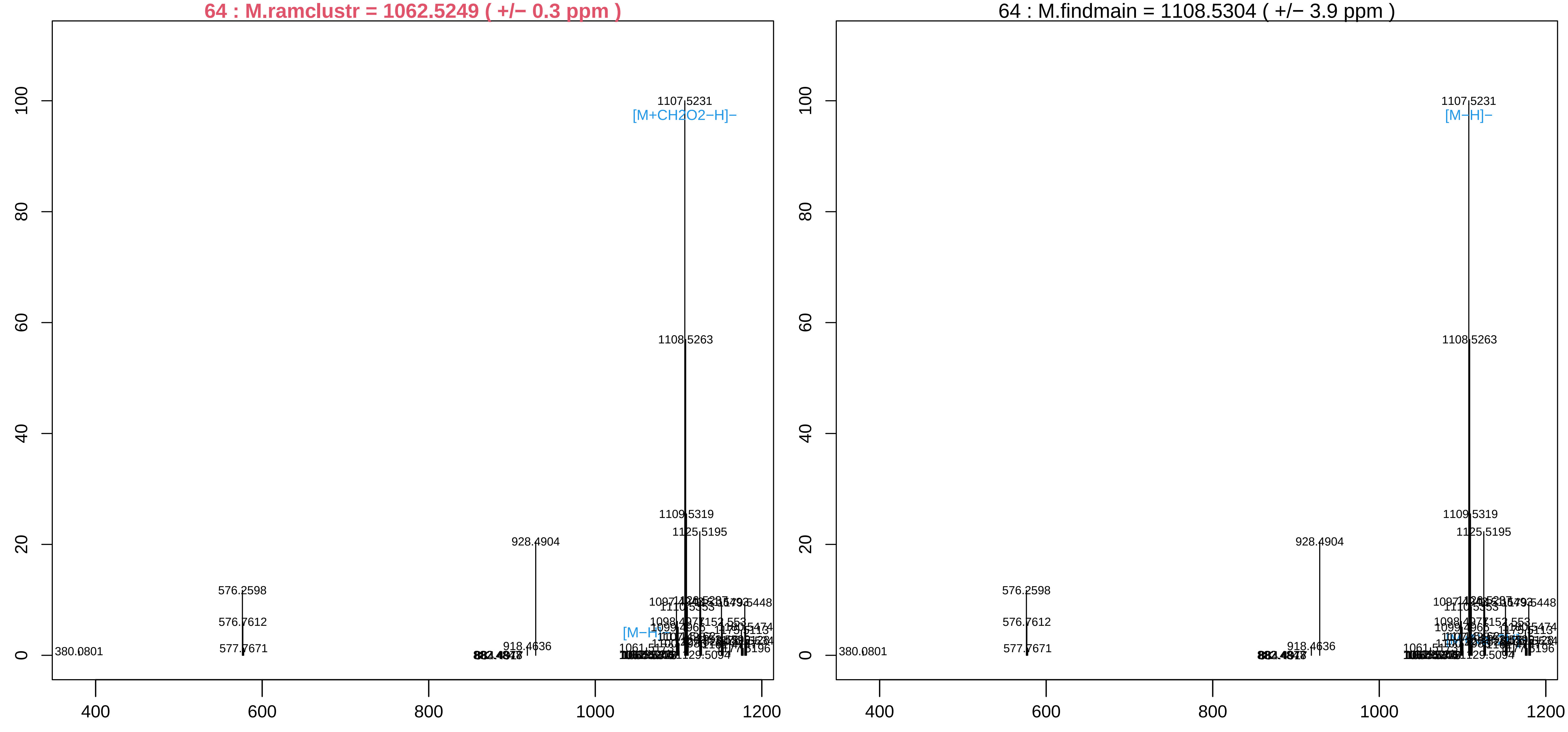
64 : M.ramclustr = 1062.5249 (+/− 0.3 ppm)
64 : M.findmain = 1108.5304 (+/− 3.9 ppm)
1107.5231
[M+CH2O2−H]−
[M−H]−
1108.5263
1109.5319
1125.5195
928.4904
576.2598
576.7612
577.7671
918.4636
380.0801
1110.5353
1152.553
1129.5094
0
20
40
60
80
100
400
600
800
1000
1200

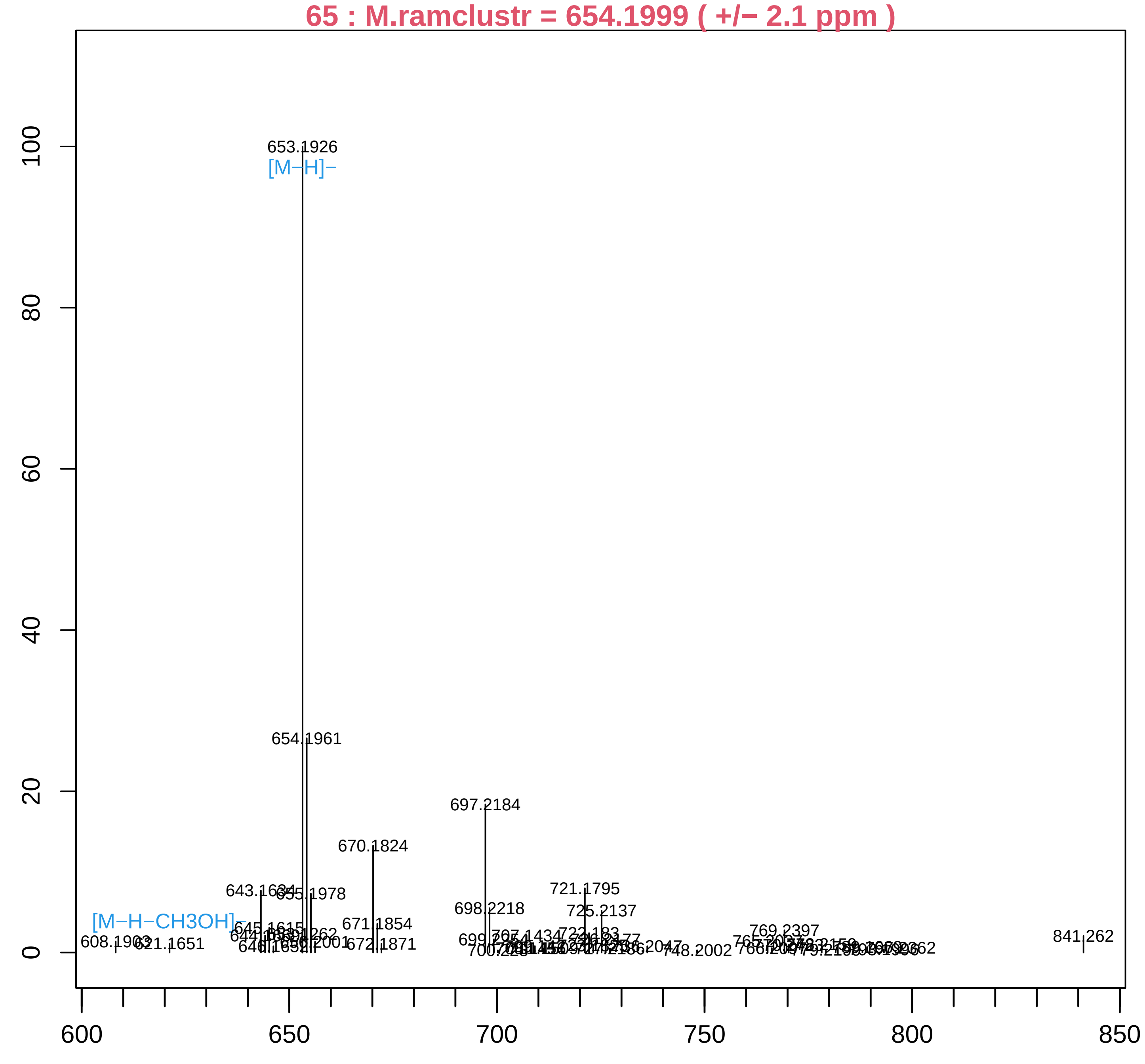
65 : M.ramclustr = 654.1999 (+/− 2.1 ppm)
653.1926
[M−H]−
654.1961
697.2184
670.1824
721.1795
725.2137
698.2218
643.1634
655.1978
671.1854
672.1871
[M−H−CH3OH]−
608.1903
621.1651
769.2397
748.2002
841.262
0
20
40
60
80
100
600
650
700
750
800
850

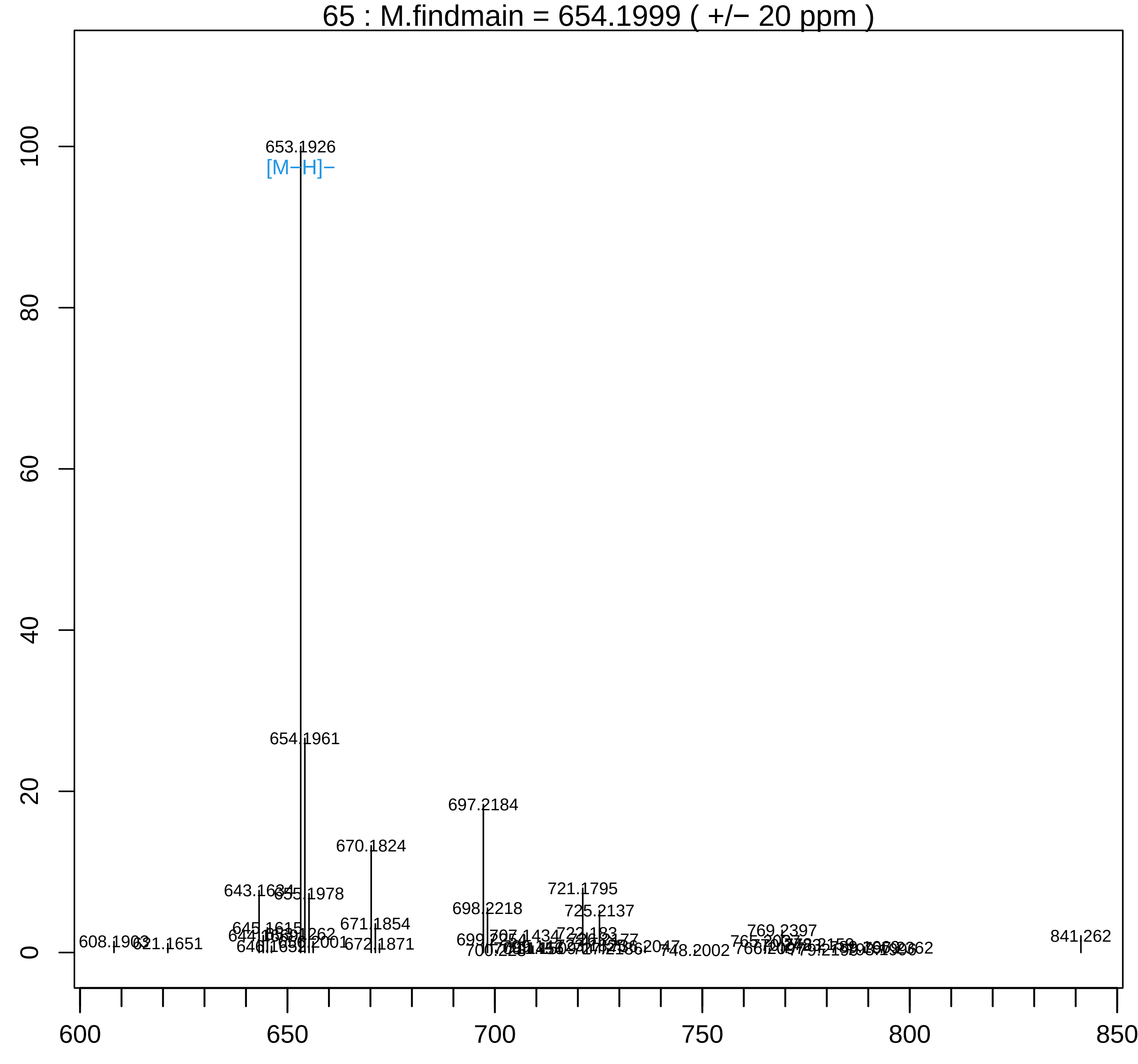
65 : M.findmain = 654.1999 (+/− 20 ppm)
653.1926
[M−H]−
654.1961
697.2184
670.1824
721.1795
725.2137
698.2218
643.1634
655.1978
671.1854
672.1871
608.1903
621.1651
769.2397
748.2002
841.262
0
20
40
60
80
100
600
650
700
750
800
850

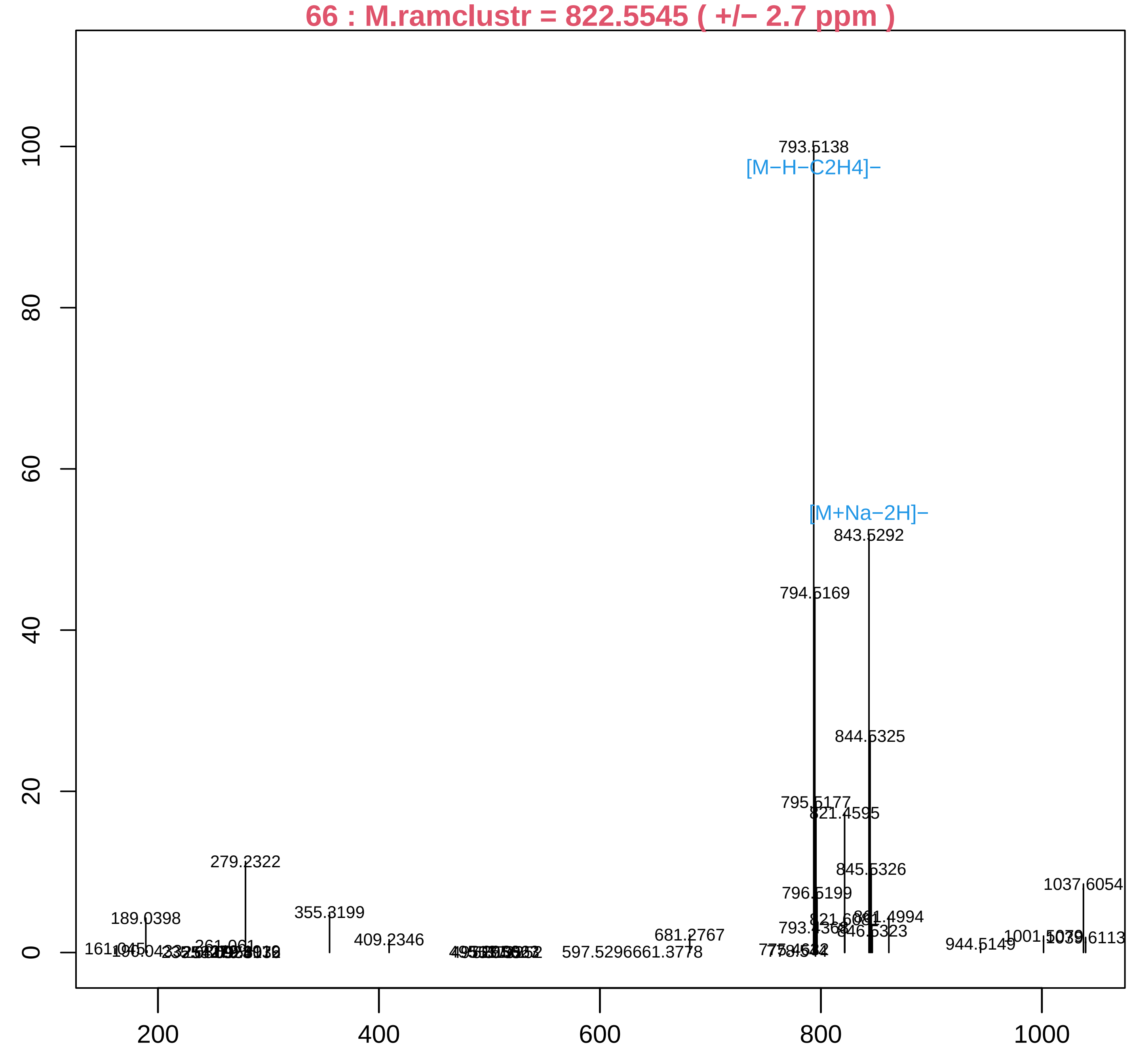
66 : M.ramclustr = 822.5545 (+/− 2.7 ppm)
793.5138
[M−H−C2H4]−
[M+Na−2H]−
843.5292
794.5169
844.5325
795.5177
821.4595
279.2322
845.5326
796.5199
1037.6054
189.0398
355.3199
409.2346
681.2767
597.5296
661.3778
944.5149

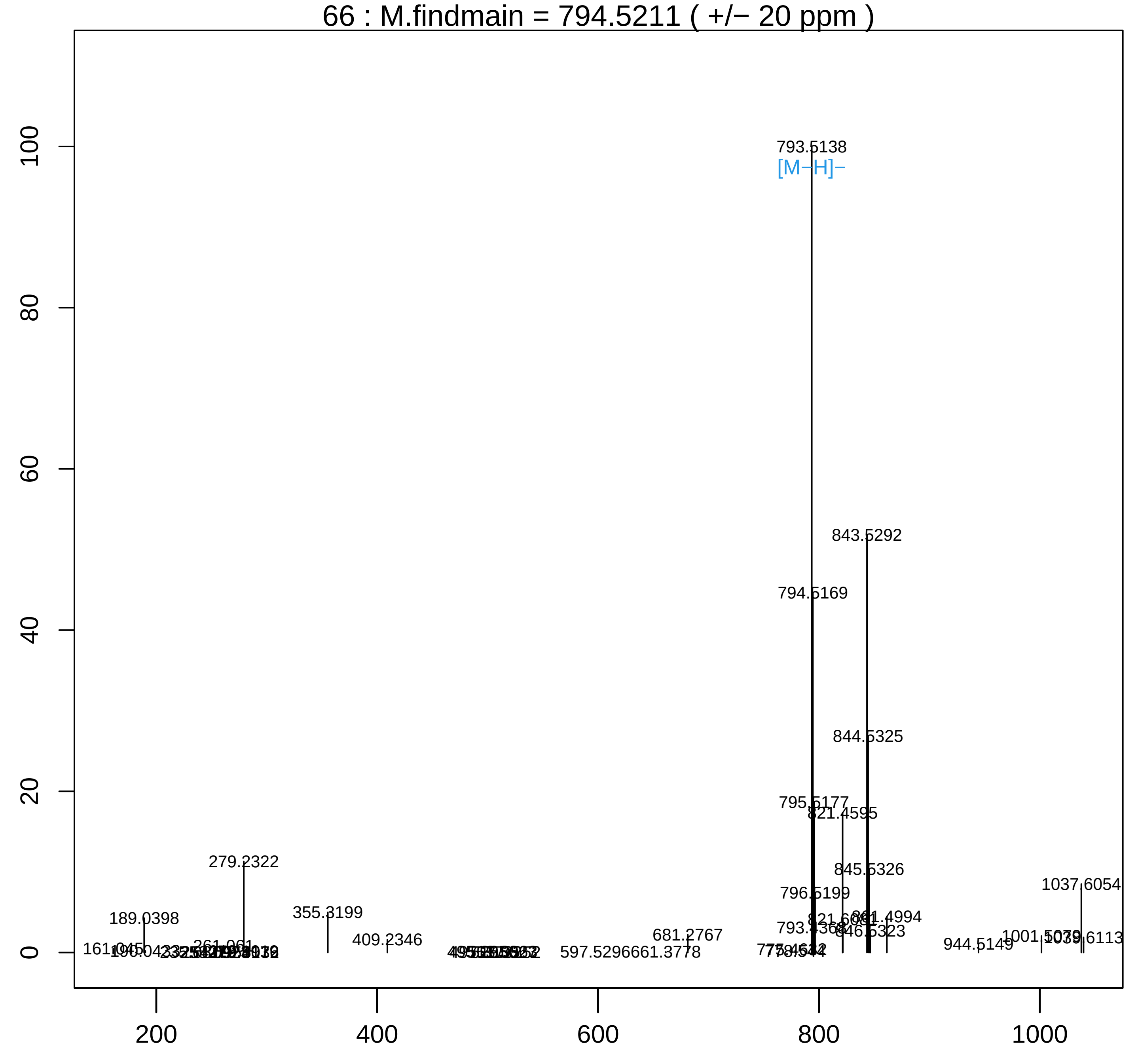
66 : M.findmain = 794.5211 (+/− 20 ppm)
793.5138
[M−H]−
843.5292
794.5169
844.5325
795.5177
821.4595
279.2322
845.5326
796.5199
1037.6054
189.0398
355.3199
409.2346
681.2767
597.5296
661.3778
944.5149

67 : M.ramclustr = 804.5646 (+/− 0.3 ppm)

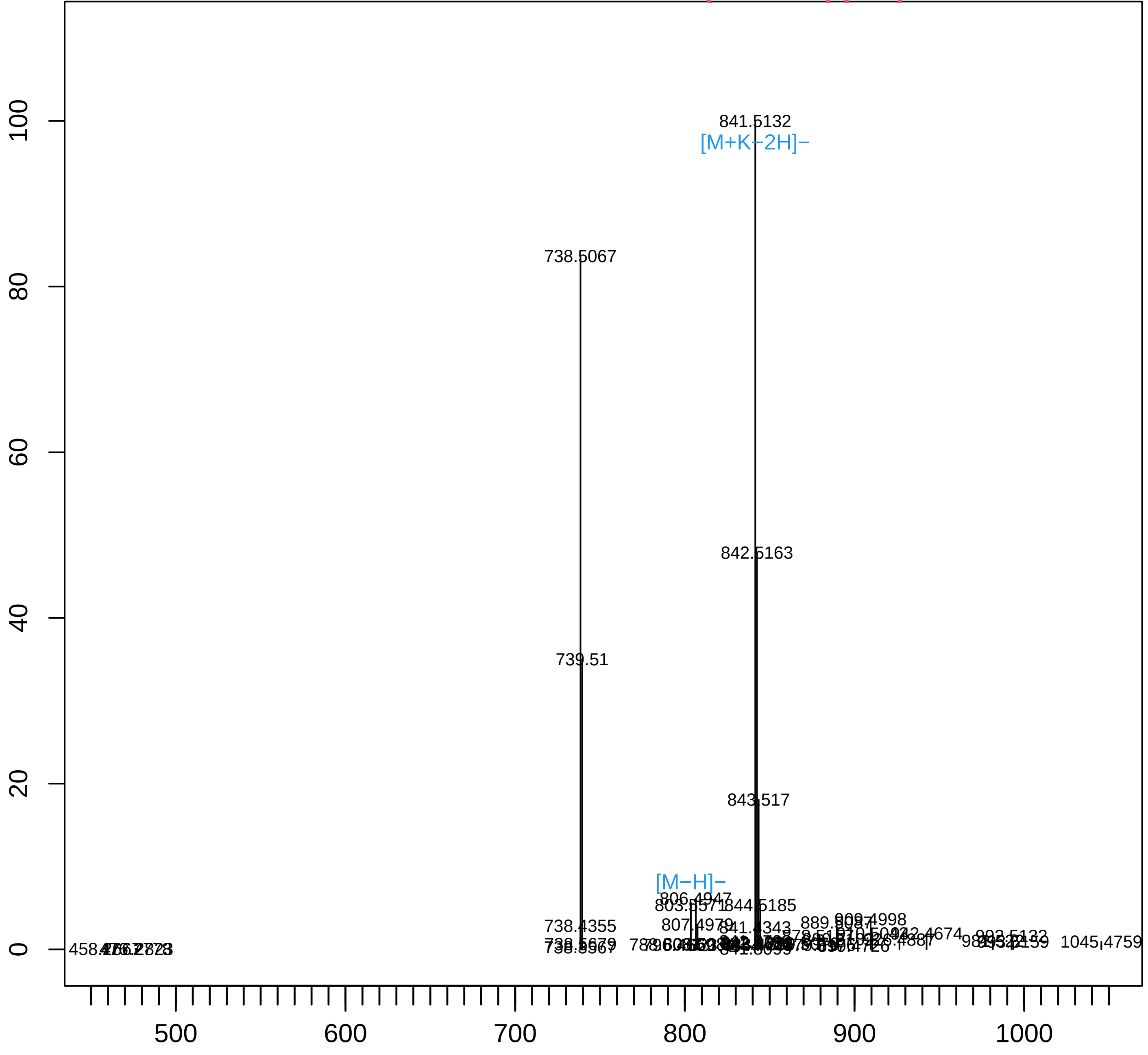

67 : M.findmain = 804.5646 (+/− 0.3 ppm)

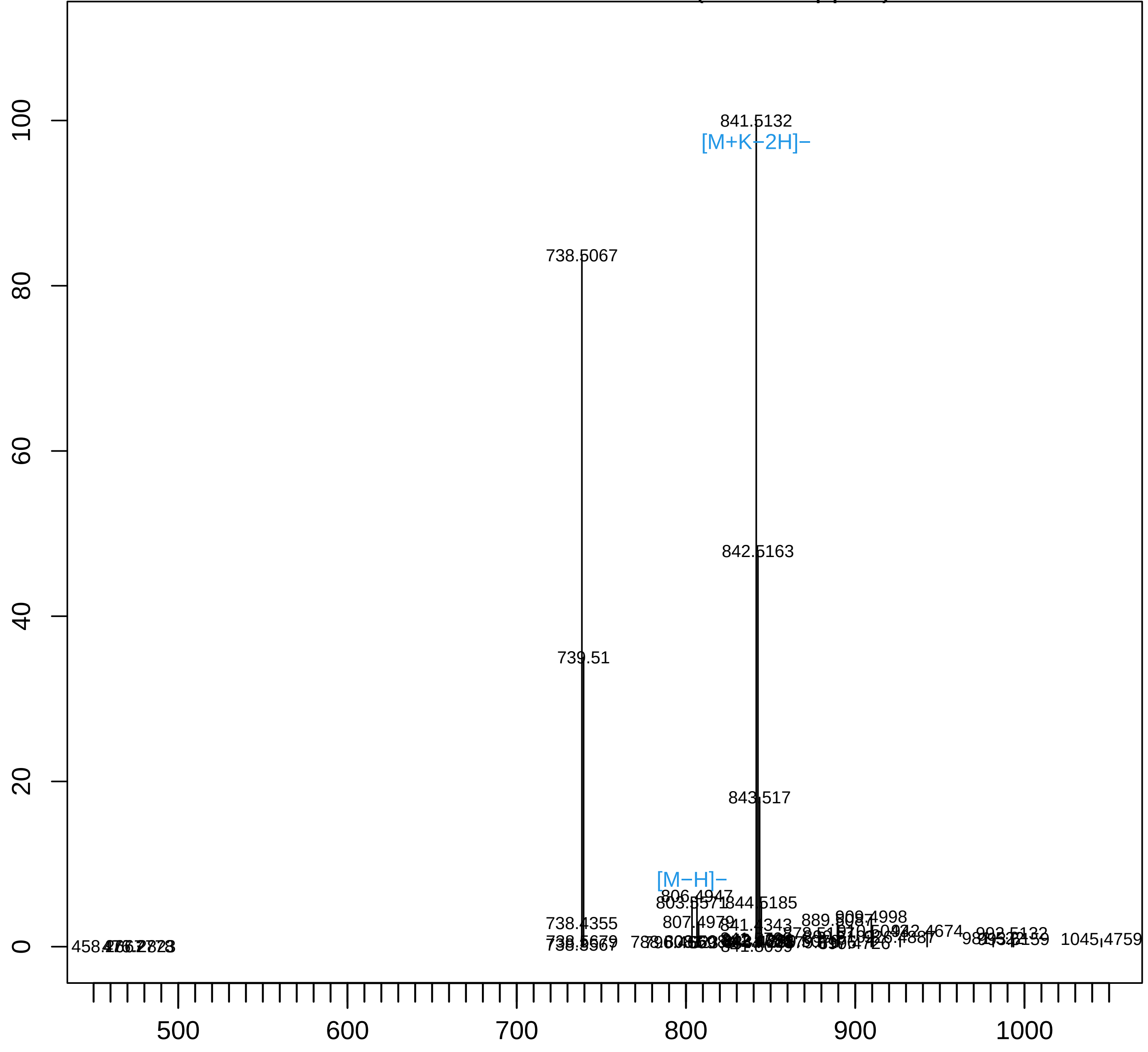

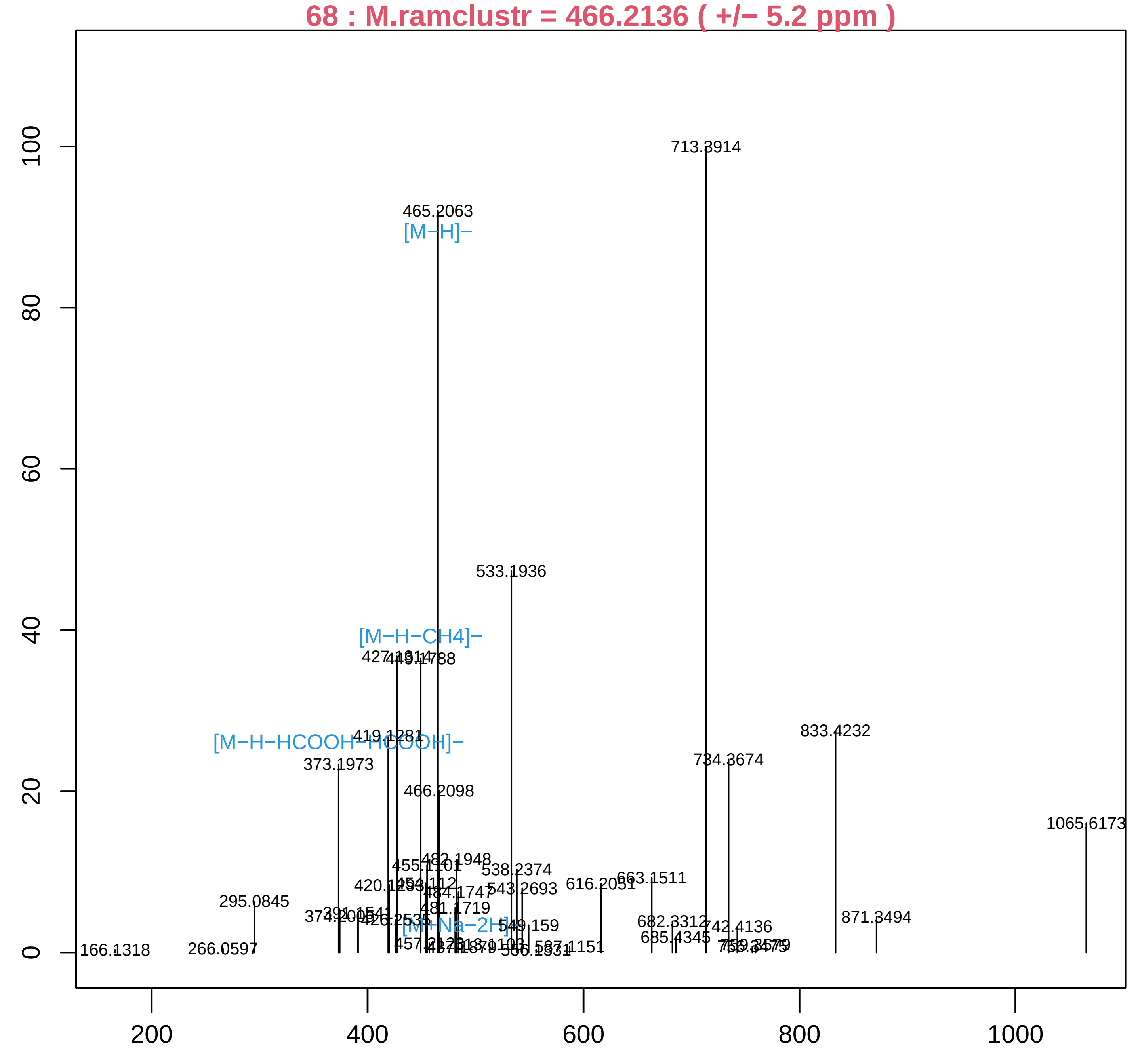
68 : M.ramclustr = 466.2136 (+/− 5.2 ppm)
713.3914
465.2063
[M−H]−
533.1936
[M−H−CH4]−
[M−H−HCOOH−HCOOH]−
419.1281
373.1973
833.4232
734.3674
466.2098
1065.6173
295.0845
616.2051
663.1511
871.3494
742.4136
682.3312
635.4345
166.1318
266.0597
0
20
40
60
80
100
200
400
600
800
1000

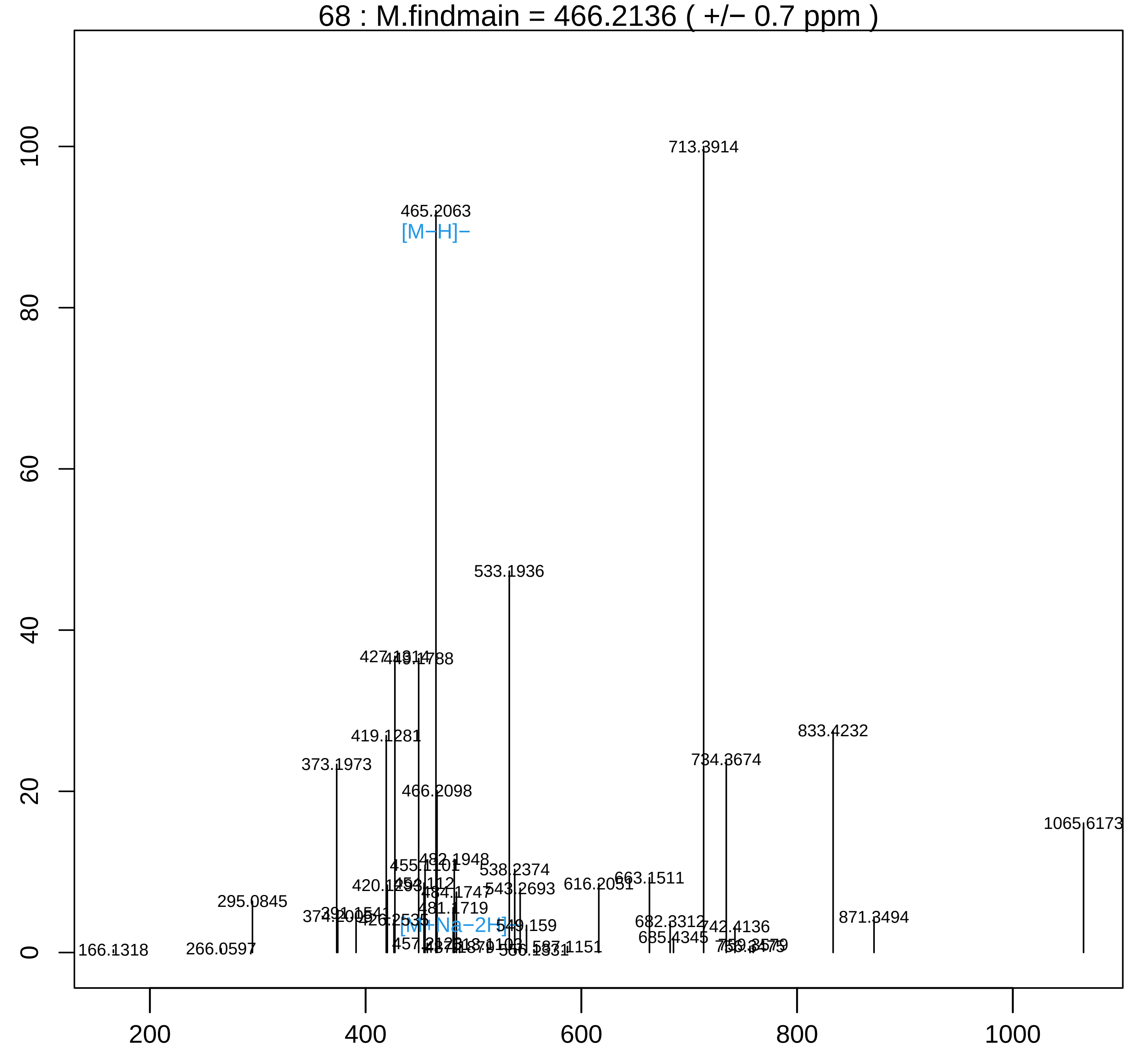
68 : M.findmain = 466.2136 (+/− 0.7 ppm)
713.3914
465.2063
[M−H]−
533.1936
419.1281
373.1973
833.4232
734.3674
466.2098
1065.6173
295.0845
616.2051
663.1511
871.3494
742.4136
682.3312
635.4345
166.1318
266.0597
0
20
40
60
80
100
200
400
600
800
1000

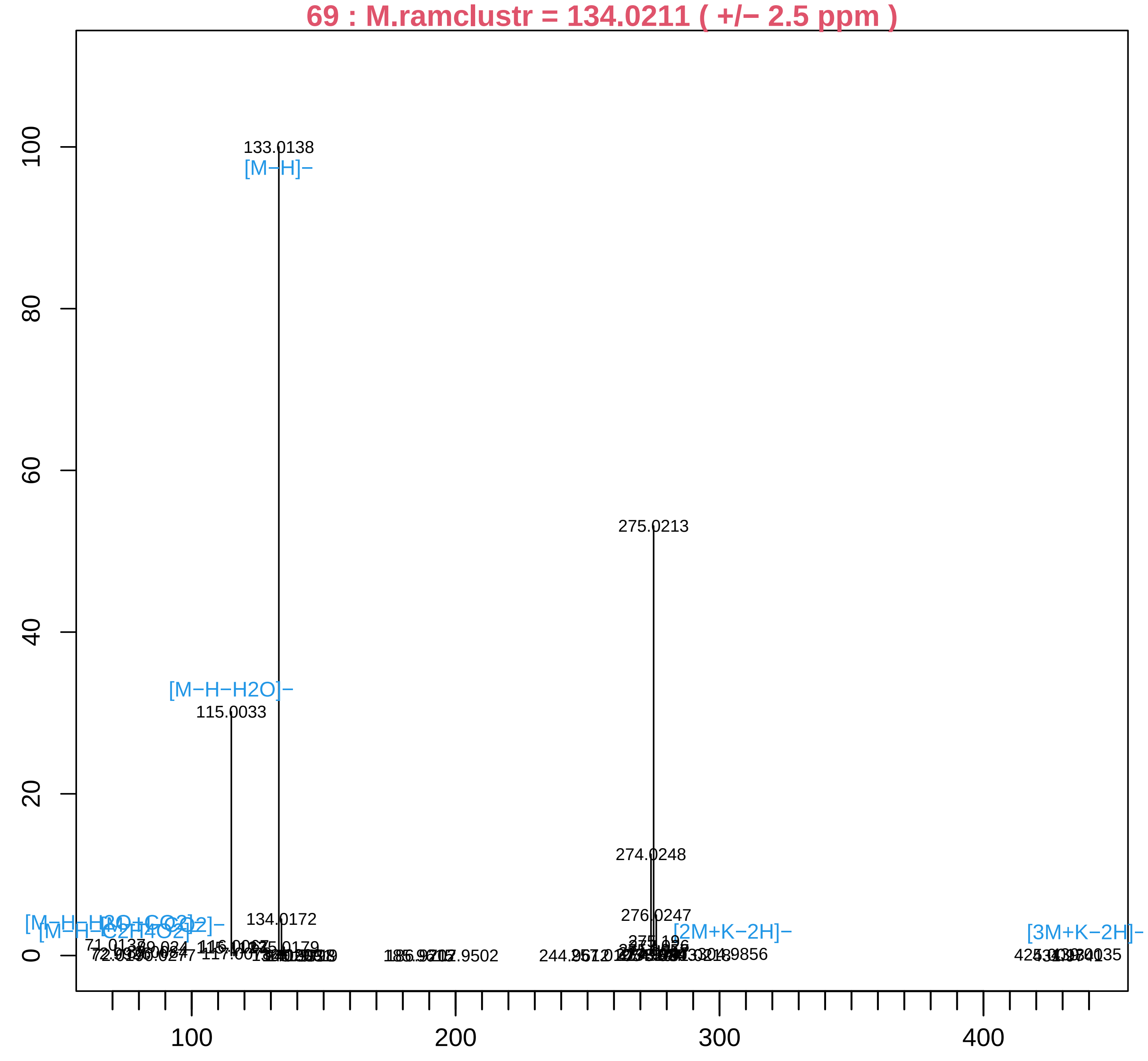
69 : M.ramclustr = 134.0211 (+/− 2.5 ppm)
133.0138
[M−H]−
275.0213
[M−H−H2O]−
115.0033
274.0248
134.0172
276.0247
[2M+K−2H]−
[3M+K−2H]−
0
20
40
60
80
100
100
200
300
400
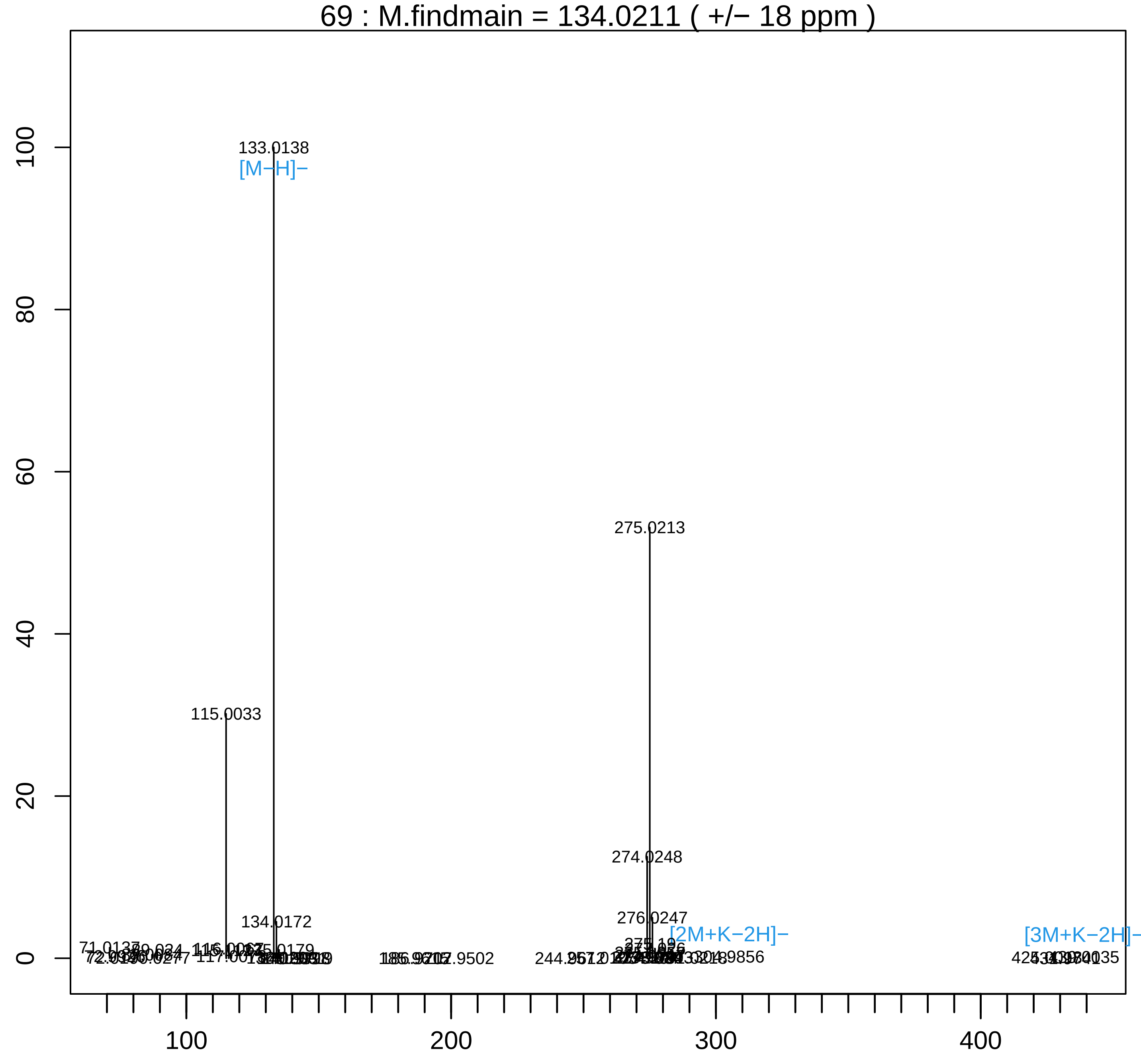
69 : M.findmain = 134.0211 (+/− 18 ppm)
133.0138
[M−H]−
275.0213
115.0033
274.0248
134.0172
276.0247
[2M+K−2H]−
[3M+K−2H]−
0
20
40
60
80
100
100
200
300
400

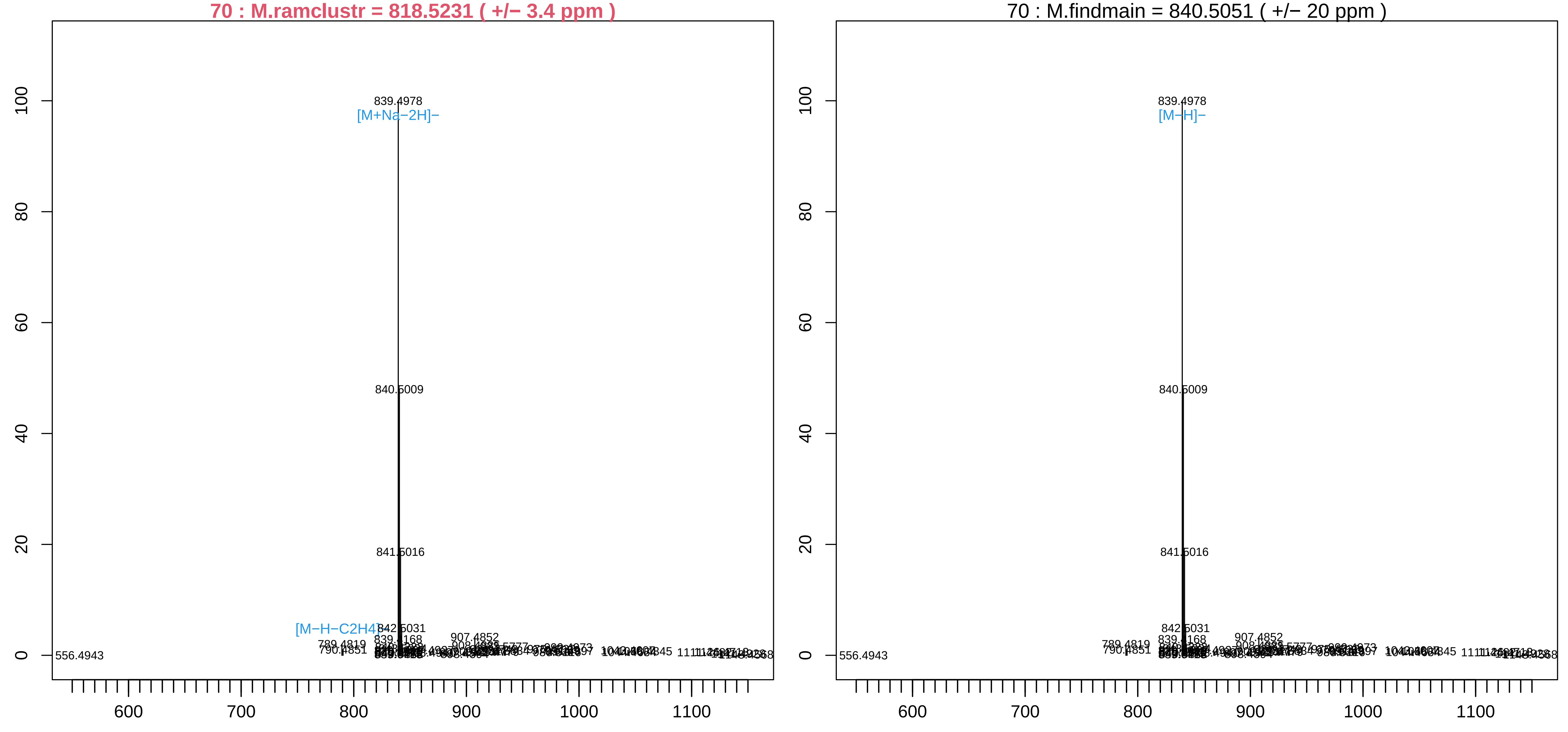
70 : M.ramclustr = 818.5231 (+/− 3.4 ppm)
839.4978
[M+Na−2H]−
840.5009
841.5016
[M−H−C2H4]
842.5031
907.4852
789.4819
790.4851
556.4943
0
20
40
60
80
100
600
700
800
900
1000
1100
70 : M.findmain = 840.5051 (+/− 20 ppm)
839.4978
[M−H]−
840.5009
841.5016
842.5031
907.4852
789.4819
790.4851
556.4943
0
20
40
60
80
100
600
700
800
900
1000
1100

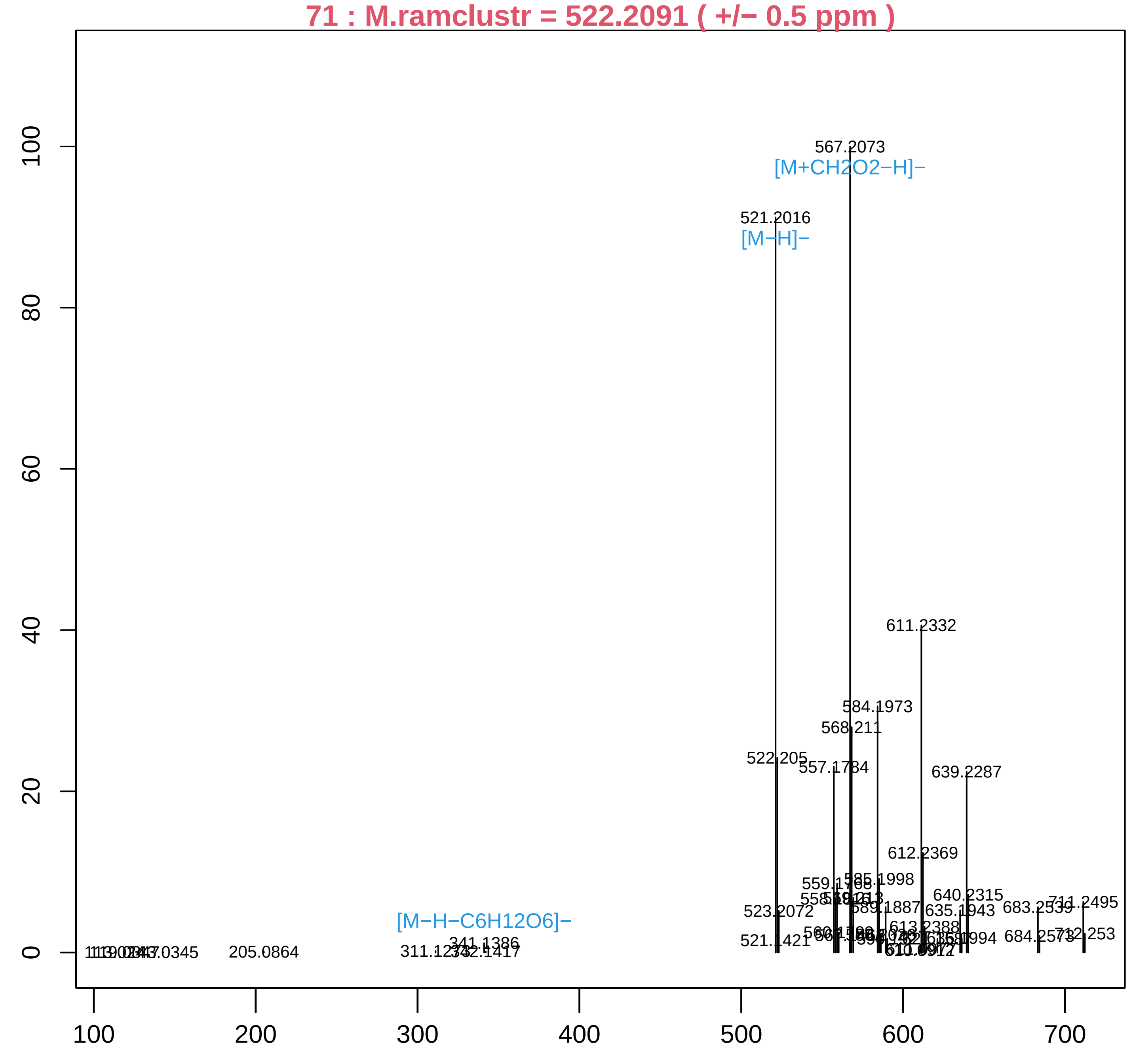
71 : M.ramclustr = 522.2091 (+/− 0.5 ppm)
567.2073
[M+CH2O2−H]−
521.2016
[M−H]−
611.2332
584.1973
568.211
522.205
557.1784
639.2287
612.2369
585.1998
640.2315
683.2539
711.2495
523.2072
589.1887
635.1943
613.2388
[M−H−C6H12O6]−
341.1386
521.1421
684.2573
712.253
205.0864
311.1237
332.1417
0
20
40
60
80
100
100
200
300
400
500
600
700
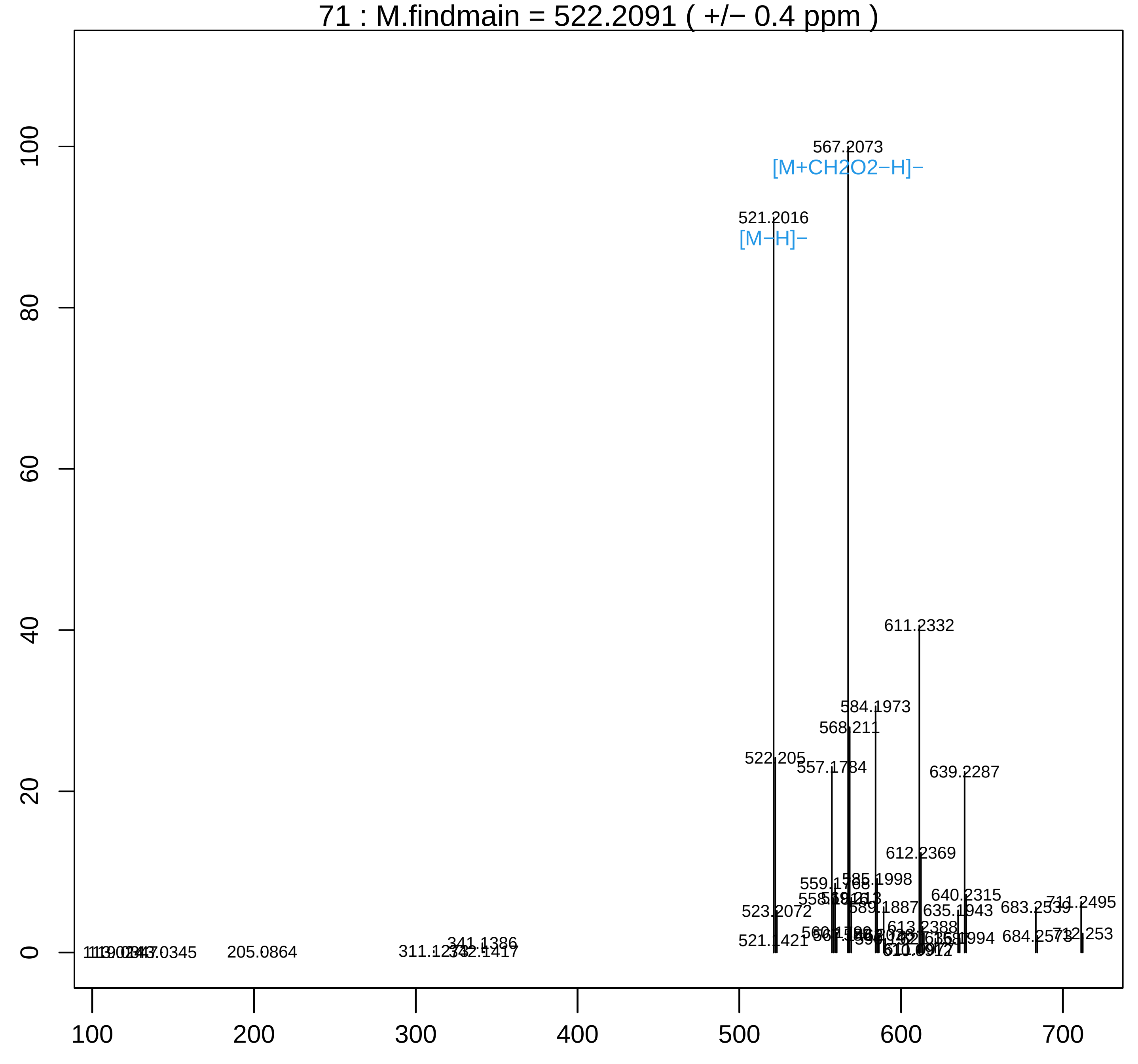
71 : M.findmain = 522.2091 (+/− 0.4 ppm)
567.2073
[M+CH2O2−H]−
521.2016
[M−H]−
611.2332
584.1973
568.211
522.205
557.1784
639.2287
612.2369
585.1998
640.2315
683.2539
711.2495
523.2072
589.1887
635.1943
613.2388
341.1386
521.1421
684.2573
712.253
205.0864
311.1237
332.1417
0
20
40
60
80
100
100
200
300
400
500
600
700

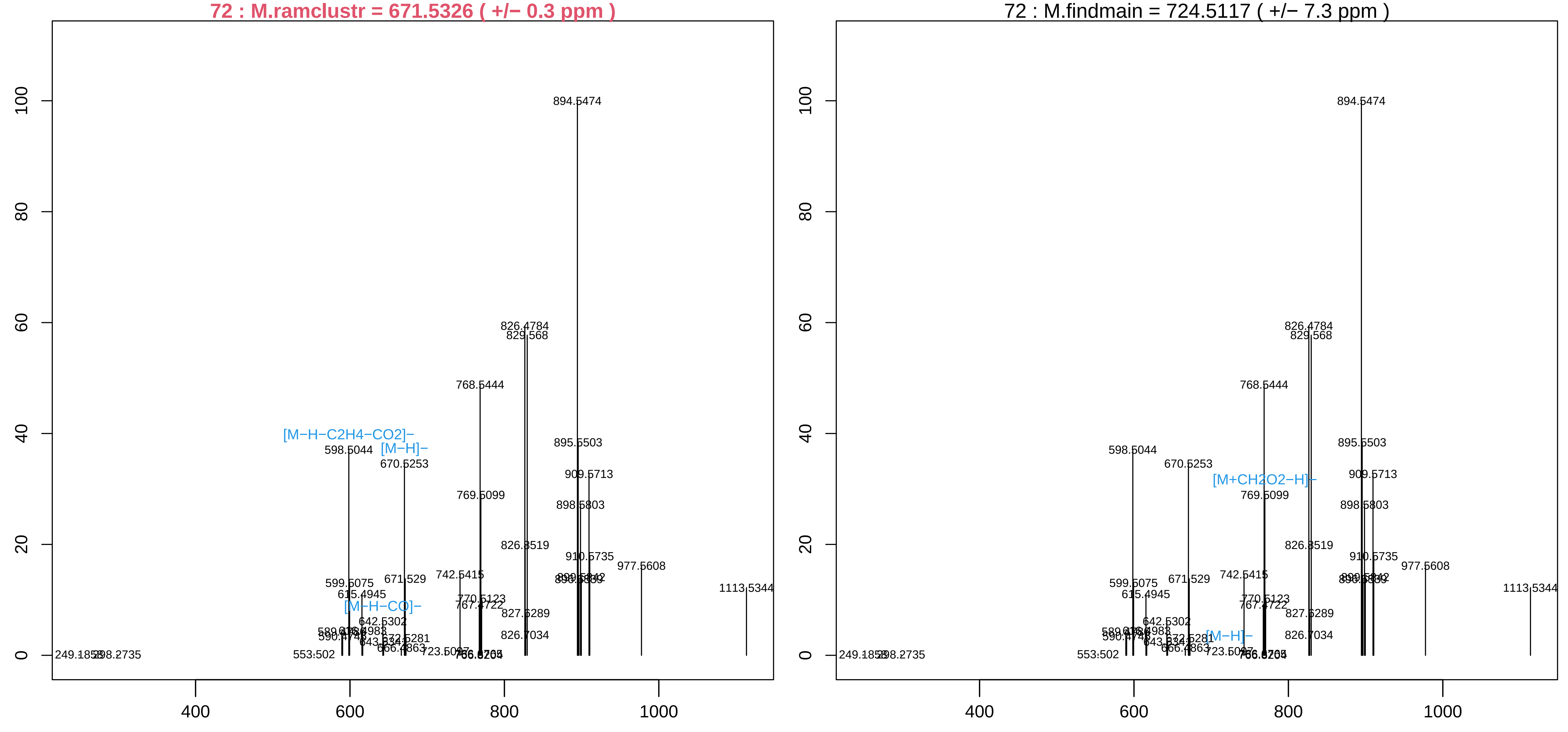
72 : M.ramclustr = 671.5326 (+/− 0.3 ppm)
72 : M.findmain = 724.5117 (+/− 7.3 ppm)
[M−H−C2H4−CO2]−
[M−H]−
[M−H−CO]−
[M+CH2O2−H]−
894.5474
826.4784
829.568
768.5444
598.5044
670.5253
895.5503
909.5713
769.5099
898.5803
826.3519
910.5735
977.5608
742.5415
671.529
599.5075
615.4945
1113.5344
770.5123
767.4722
827.6289
642.5302
826.7034
553.502
249.1858
298.2735
0
20
40
60
80
100
400
600
800
1000

73 : M.ramclustr = 452.02 (+/− 5.6 ppm)

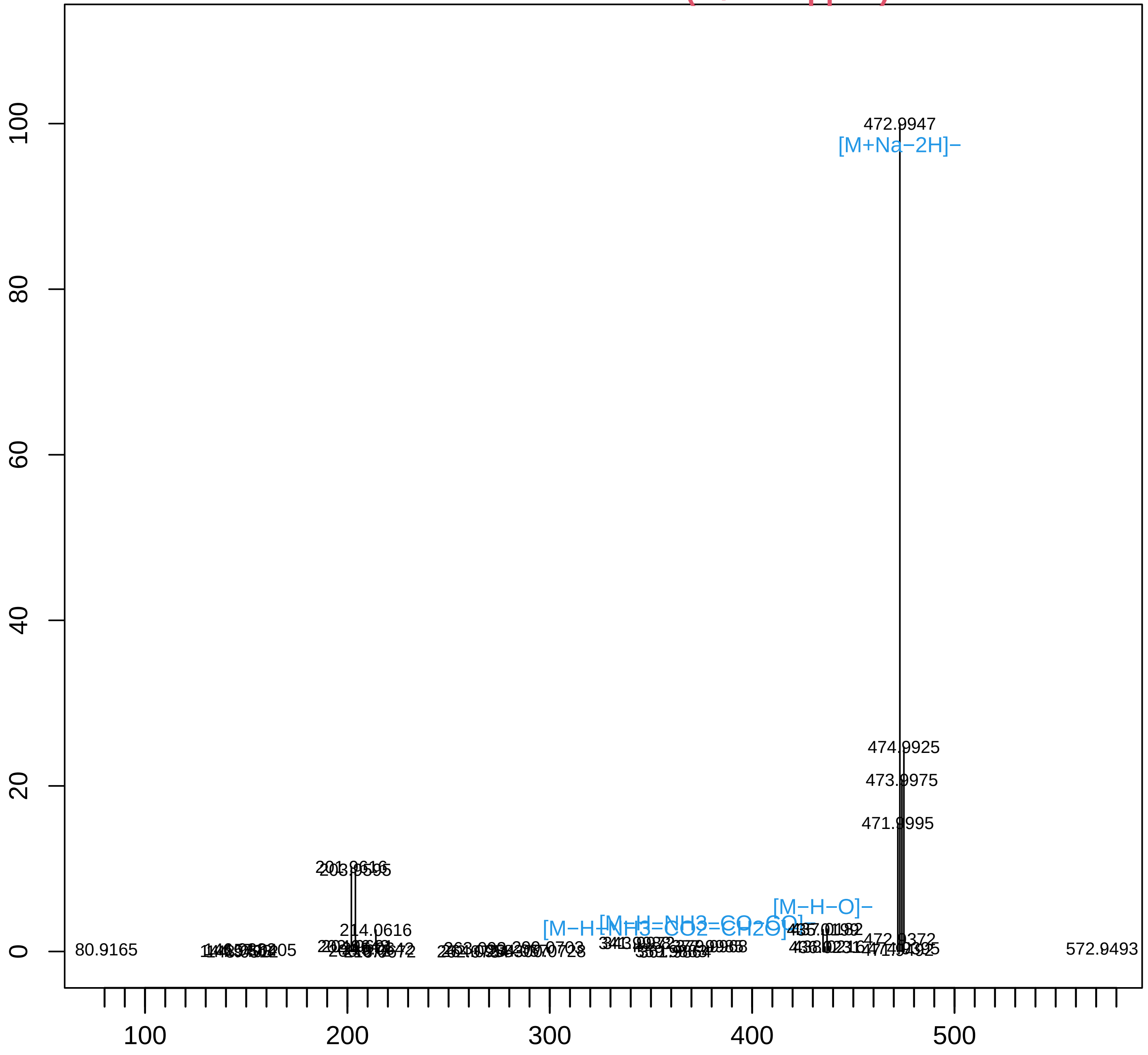

73 : M.findmain = 474.002 (+/− 20 ppm)

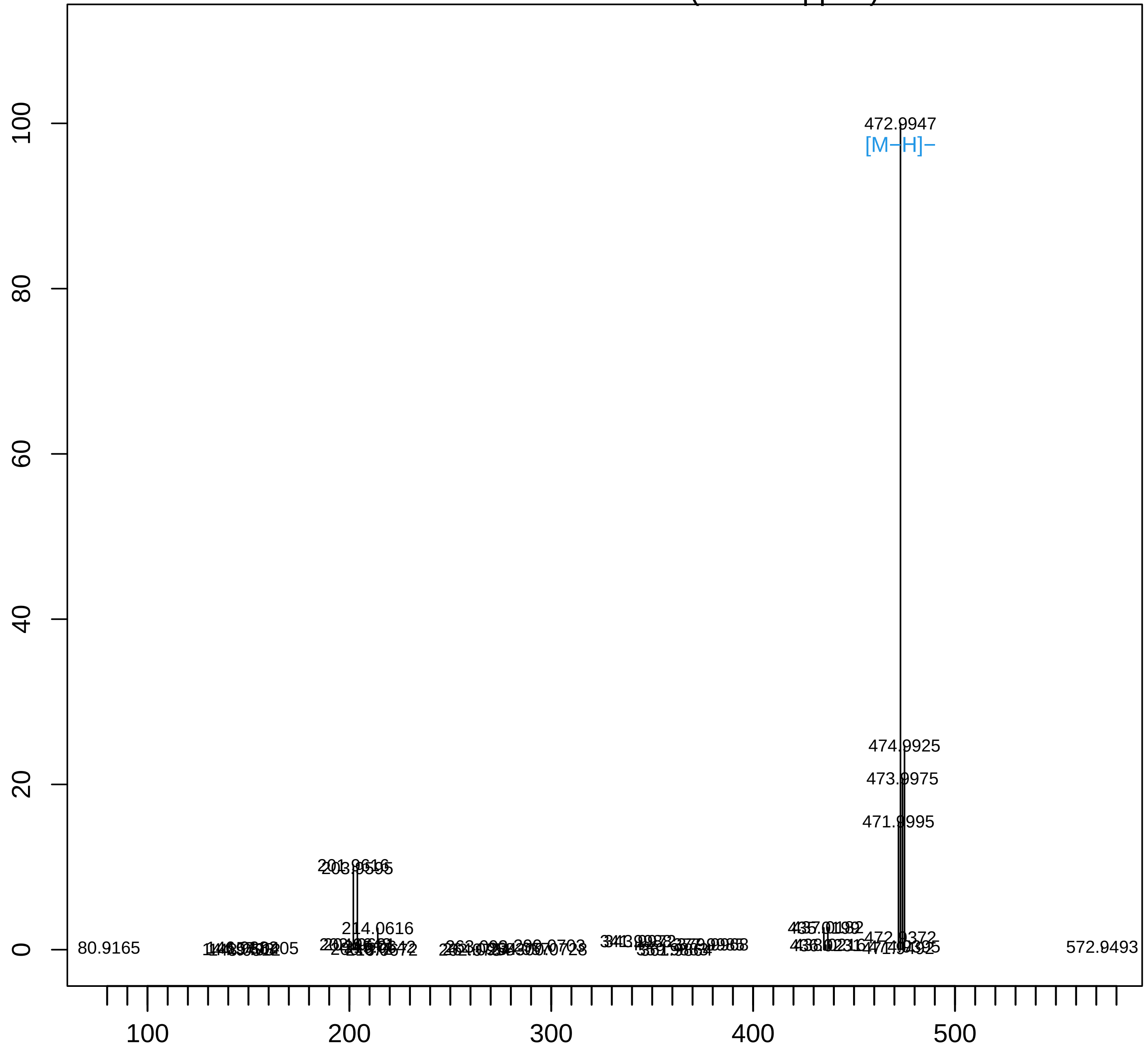

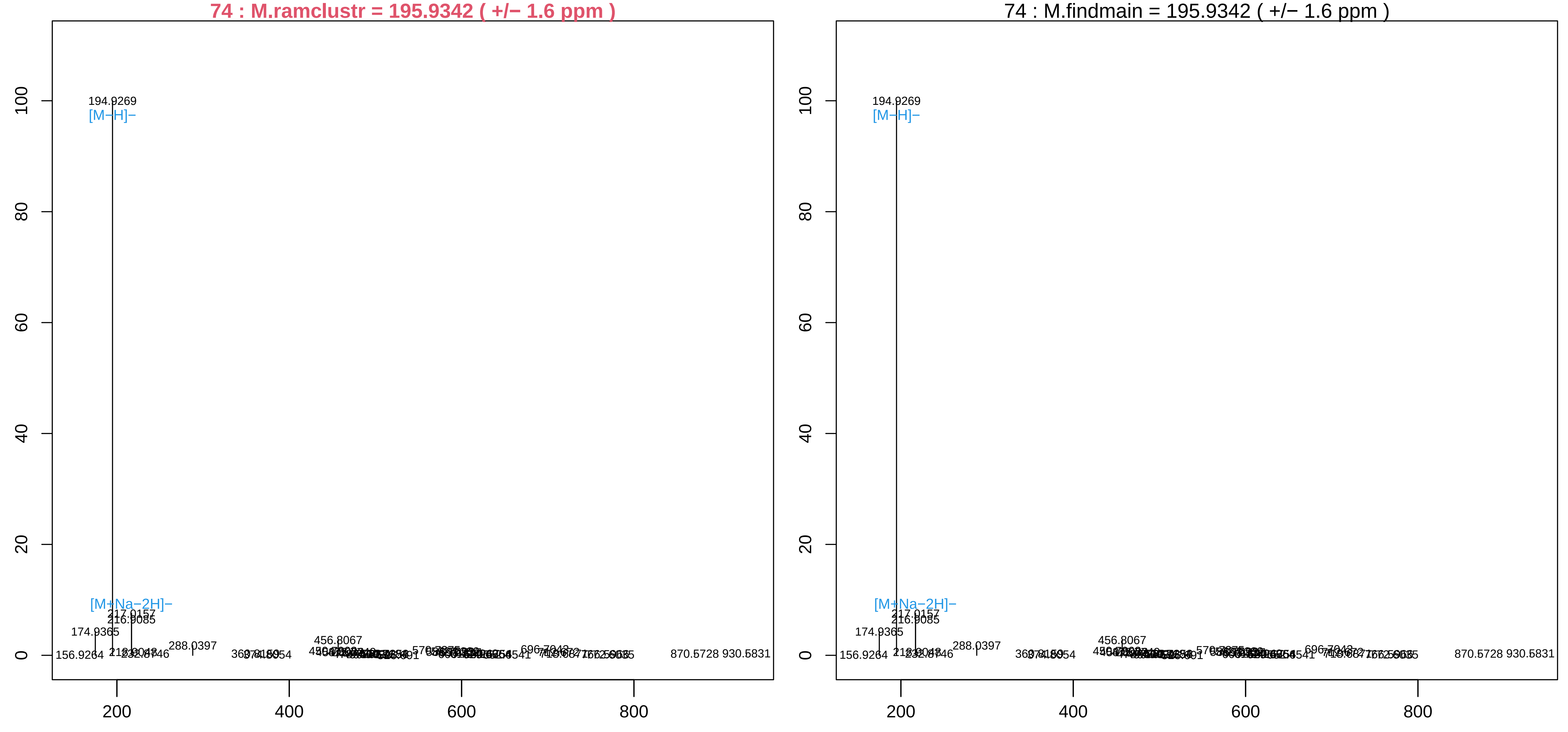

74 : M.ramclustr = 195.9342 (+/− 1.6 ppm)
74 : M.findmain = 195.9342 (+/− 1.6 ppm)
194.9269
[M−H]−
[M+Na−2H]−
217.0157
216.9085
174.9365
156.9264
288.0397
456.8067
696.7042
870.5728
930.5831
0
20
40
60
80
100
200
400
600
800

75 : M.ramclustr = 818.5208 (+/− NA ppm)

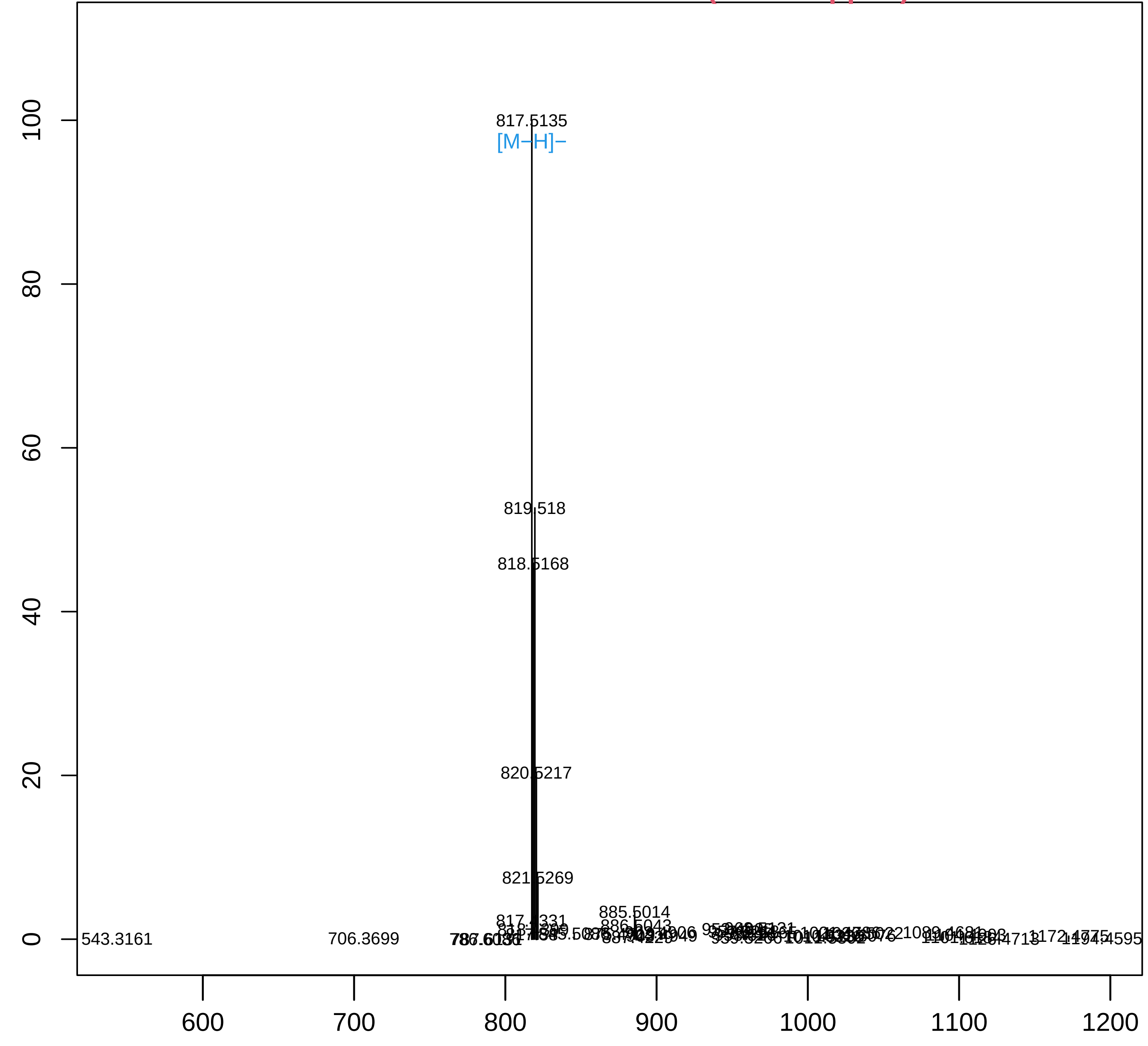

75 : M.findmain = 818.5208 (+/− 20 ppm)

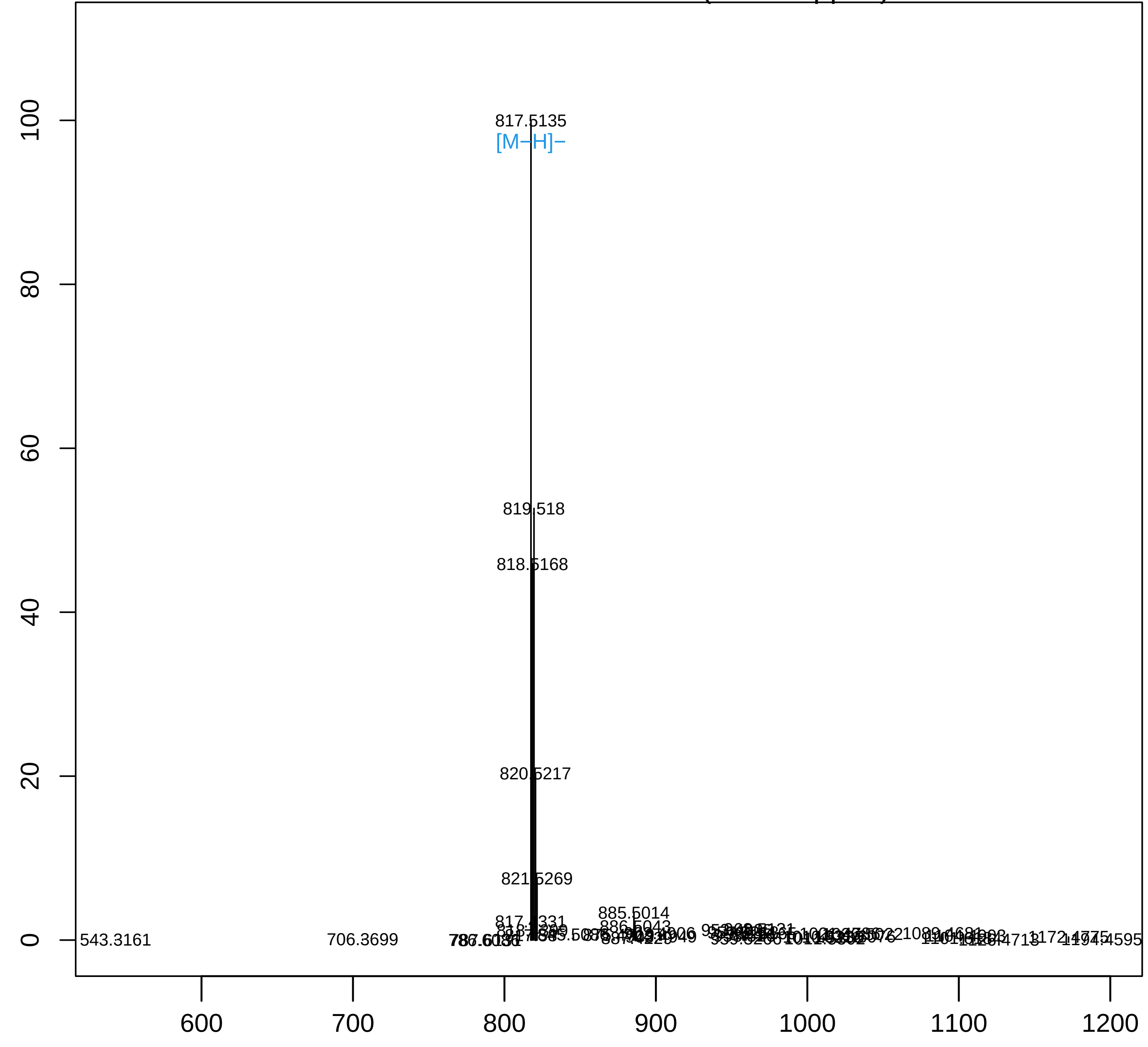

76 : M.ramclustr = 967.5501 (+/− 1.7 ppm)

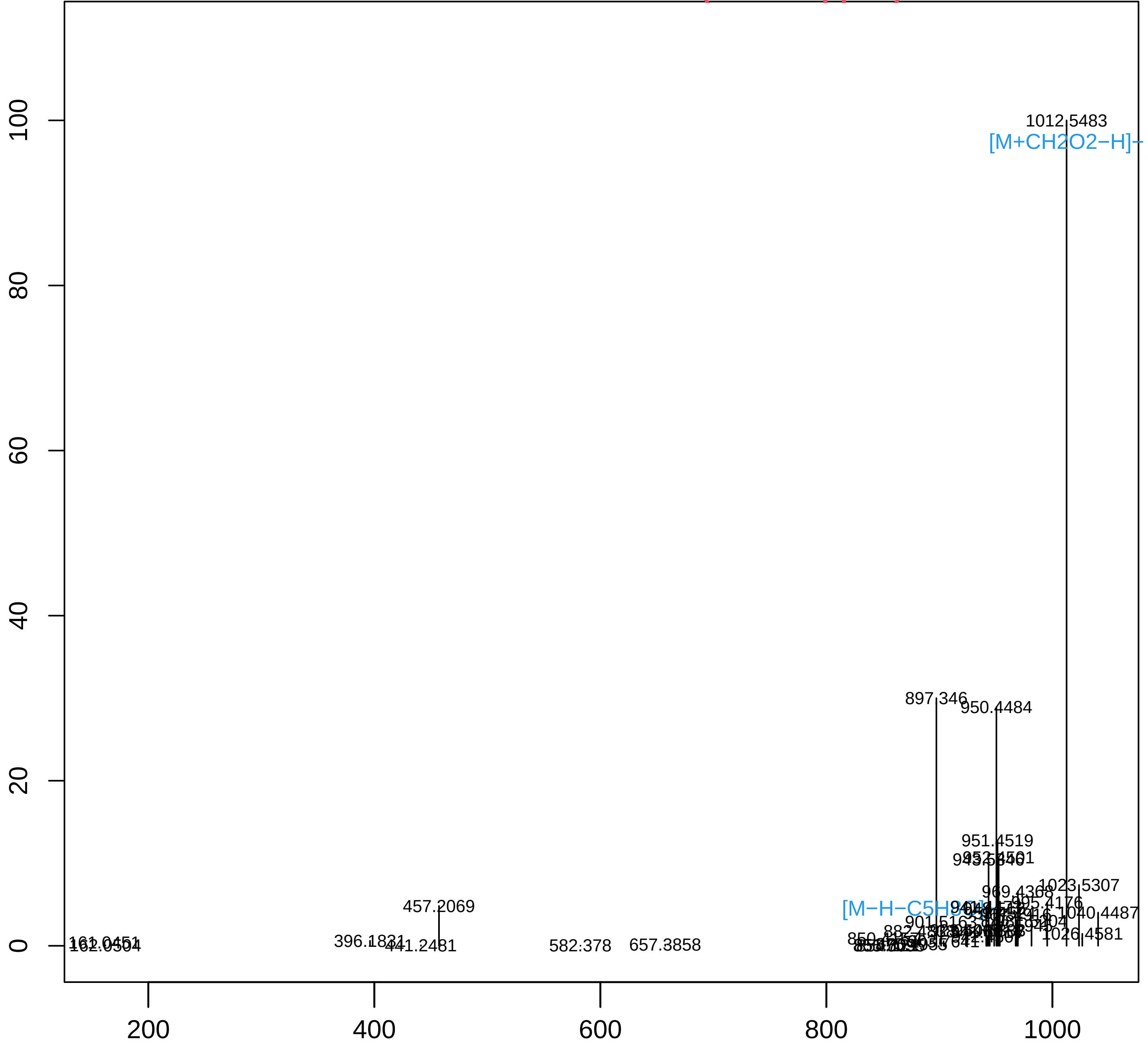

76 : M.findmain = 902.5236 (+/− 2.7 ppm)

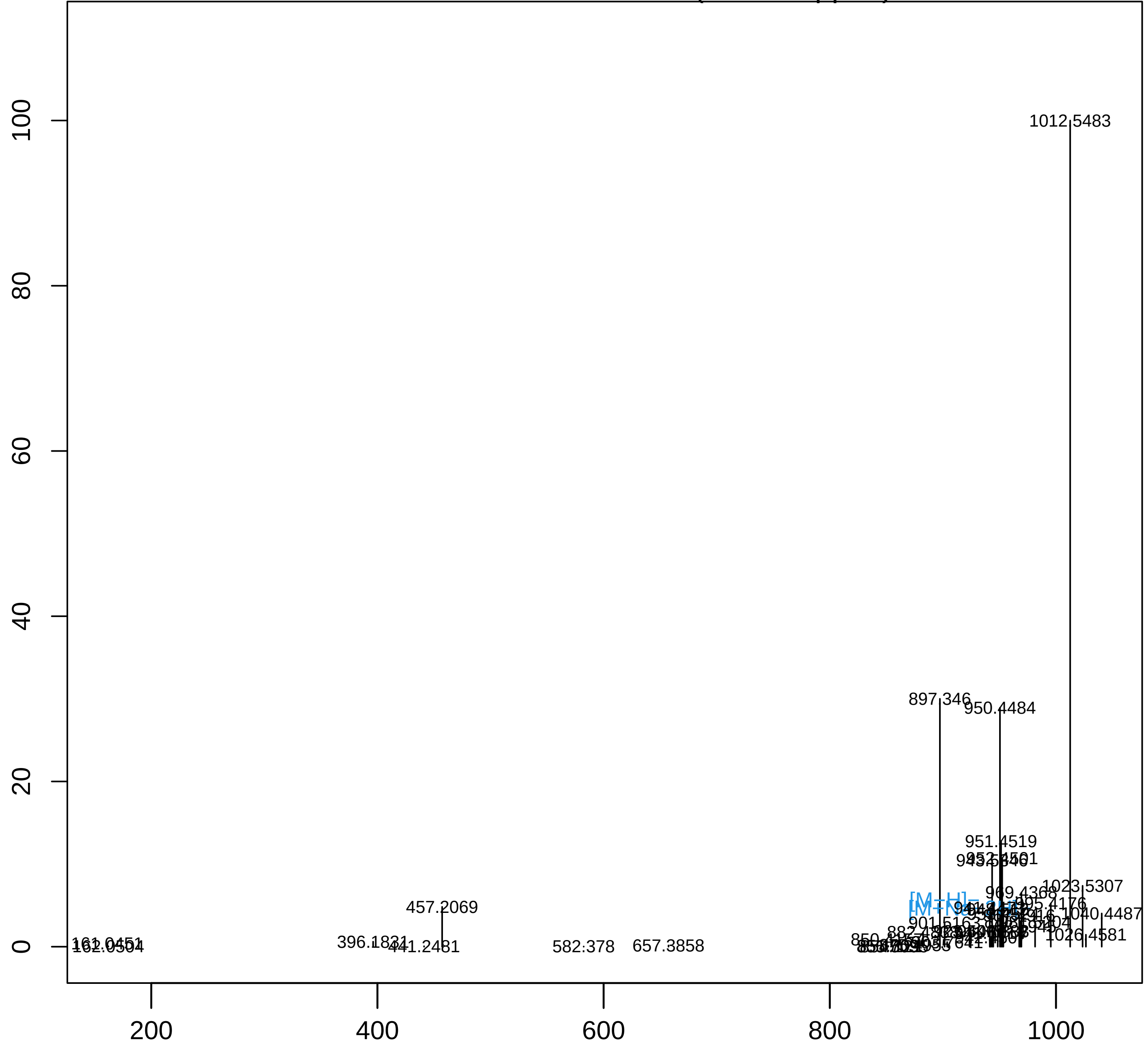

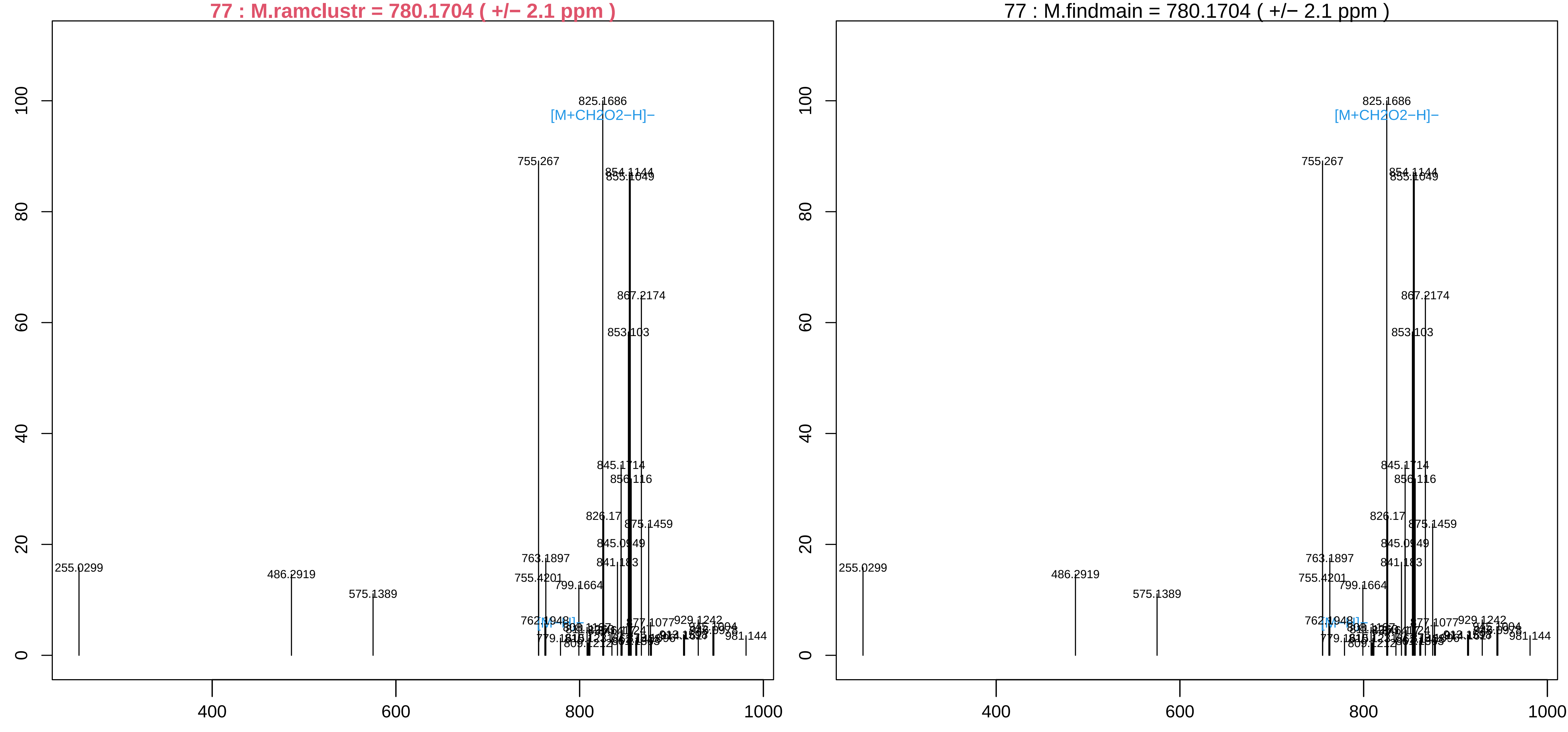
77 : M.ramclustr = 780.1704 (+/− 2.1 ppm)
77 : M.findmain = 780.1704 (+/− 2.1 ppm)
825.1686
[M+CH2O2−H]−
755.267
854.1144
855.1049
867.2174
853.103
845.1714
856.116
826.17
875.1459
845.0949
841.183
763.1897
255.0299
486.2919
575.1389
755.4201
799.1664
762.1948
929.1242
877.1077
981.144
0
20
40
60
80
100
400
600
800
1000

78 : M.ramclustr = 676.366 (+/− 0.3 ppm)

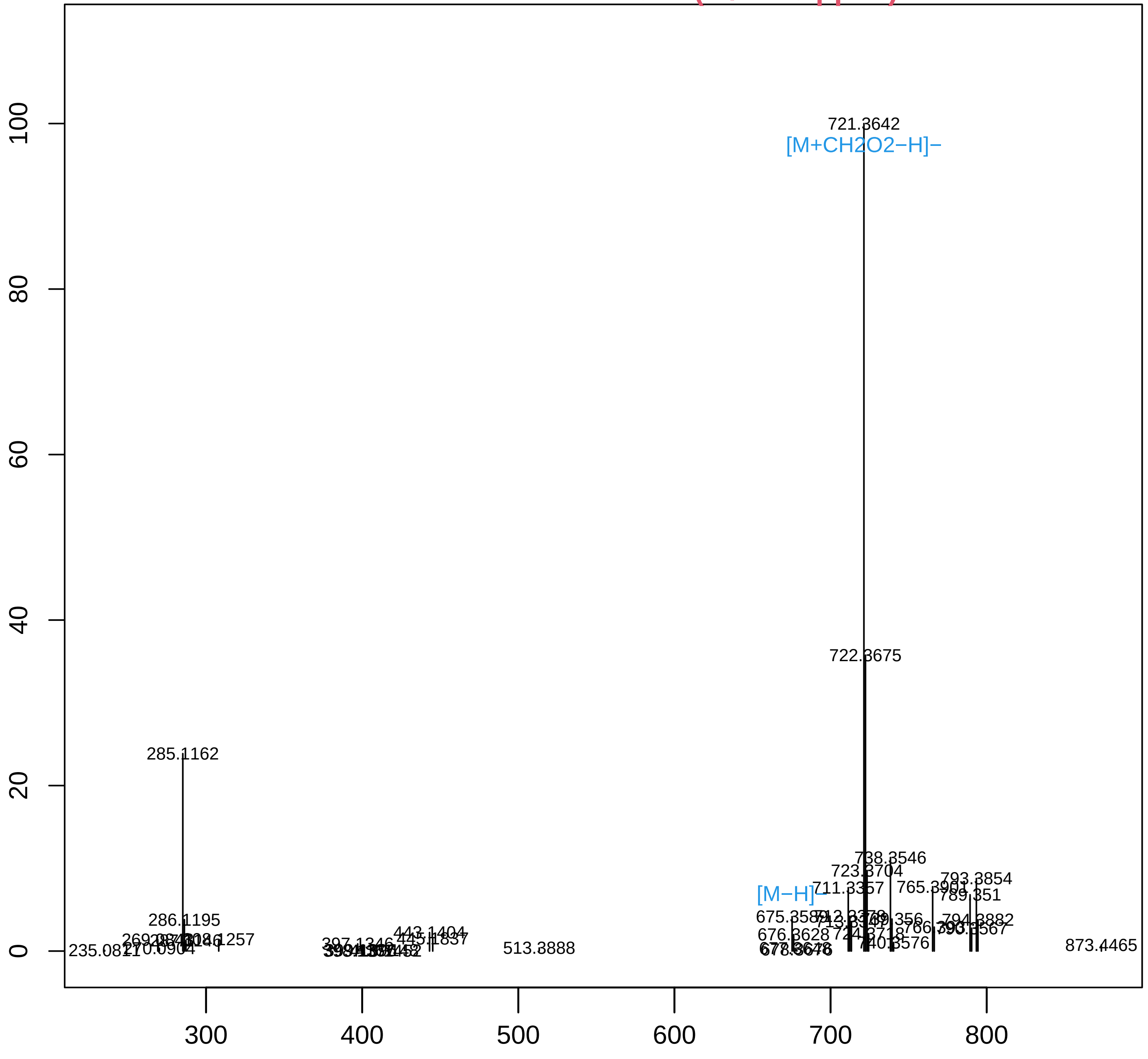

78 : M.findmain = 676.366 (+/− 0.3 ppm)

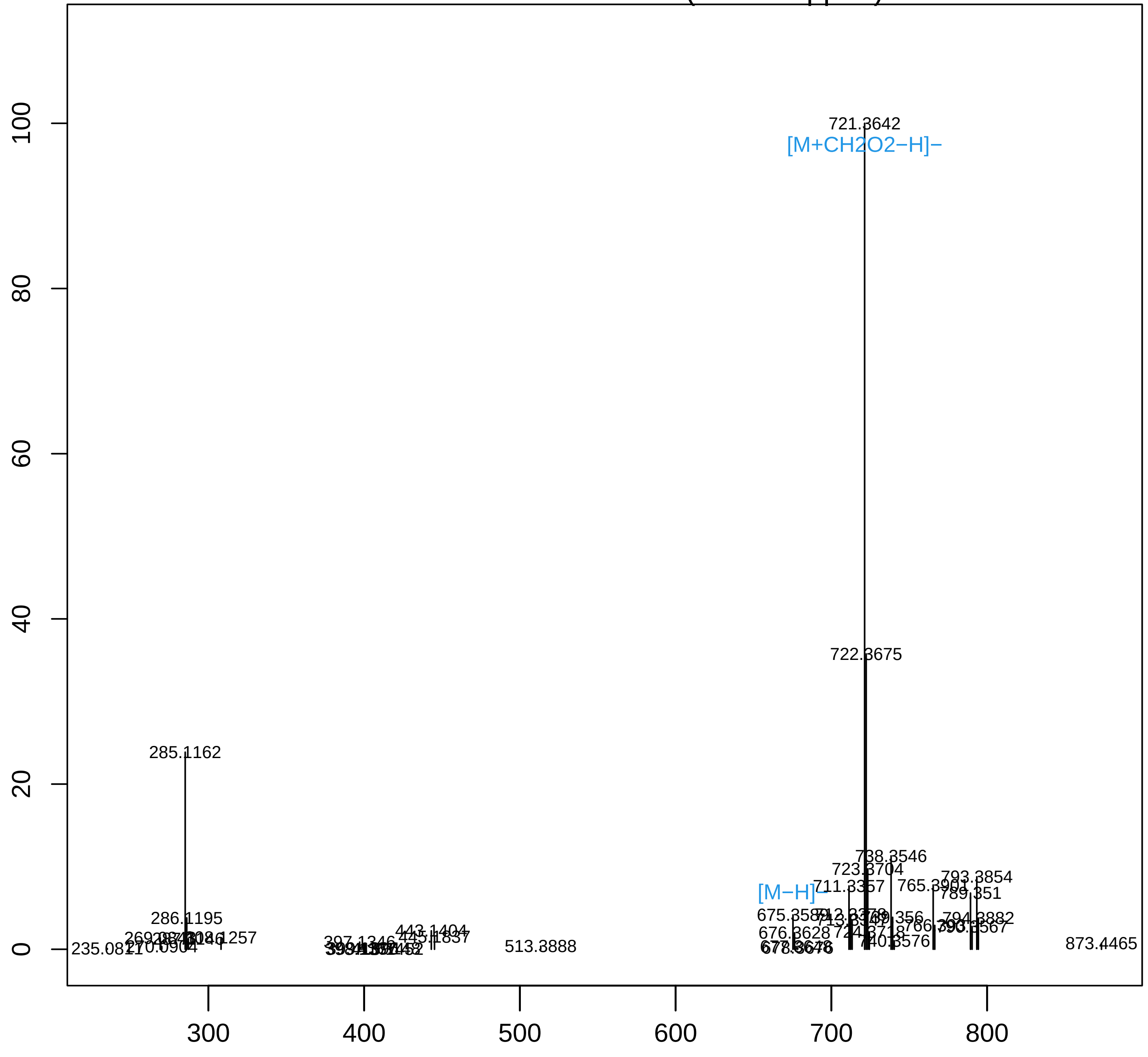

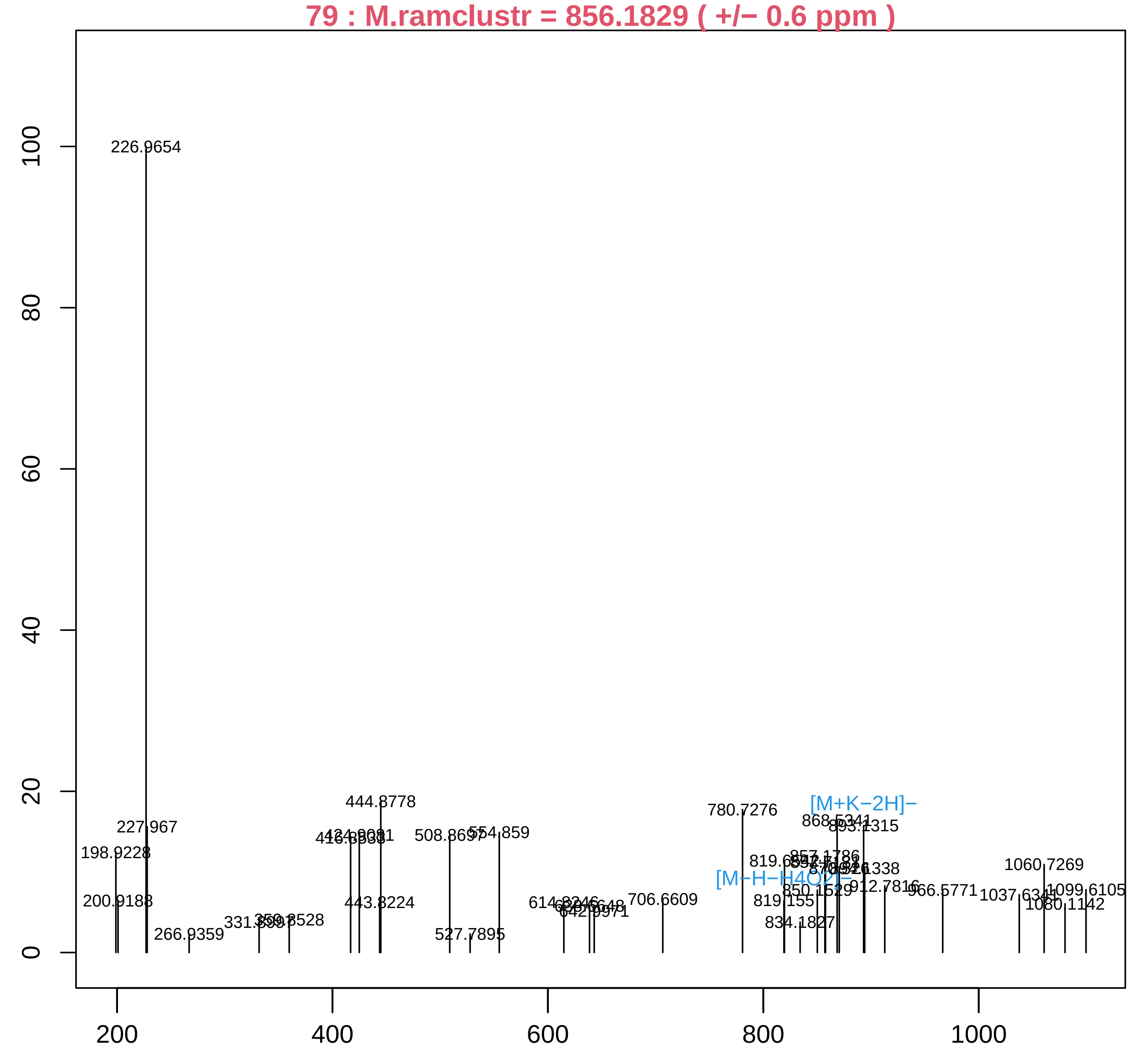
79 : M.ramclustr = 856.1829 (+/− 0.6 ppm)
226.9654
227.967
198.9228
200.9188
266.9359
359.8528
444.8778
443.8224
508.8697
554.859
527.7895
614.8246
706.6609
780.7276
868.5341
893.1315
857.1786
819.155
850.1529
834.1827
912.7816
966.5771
1060.7269
1099.6105
1080.1142
[M+K−2H]−
0
20
40
60
80
100
200
400
600
800
1000

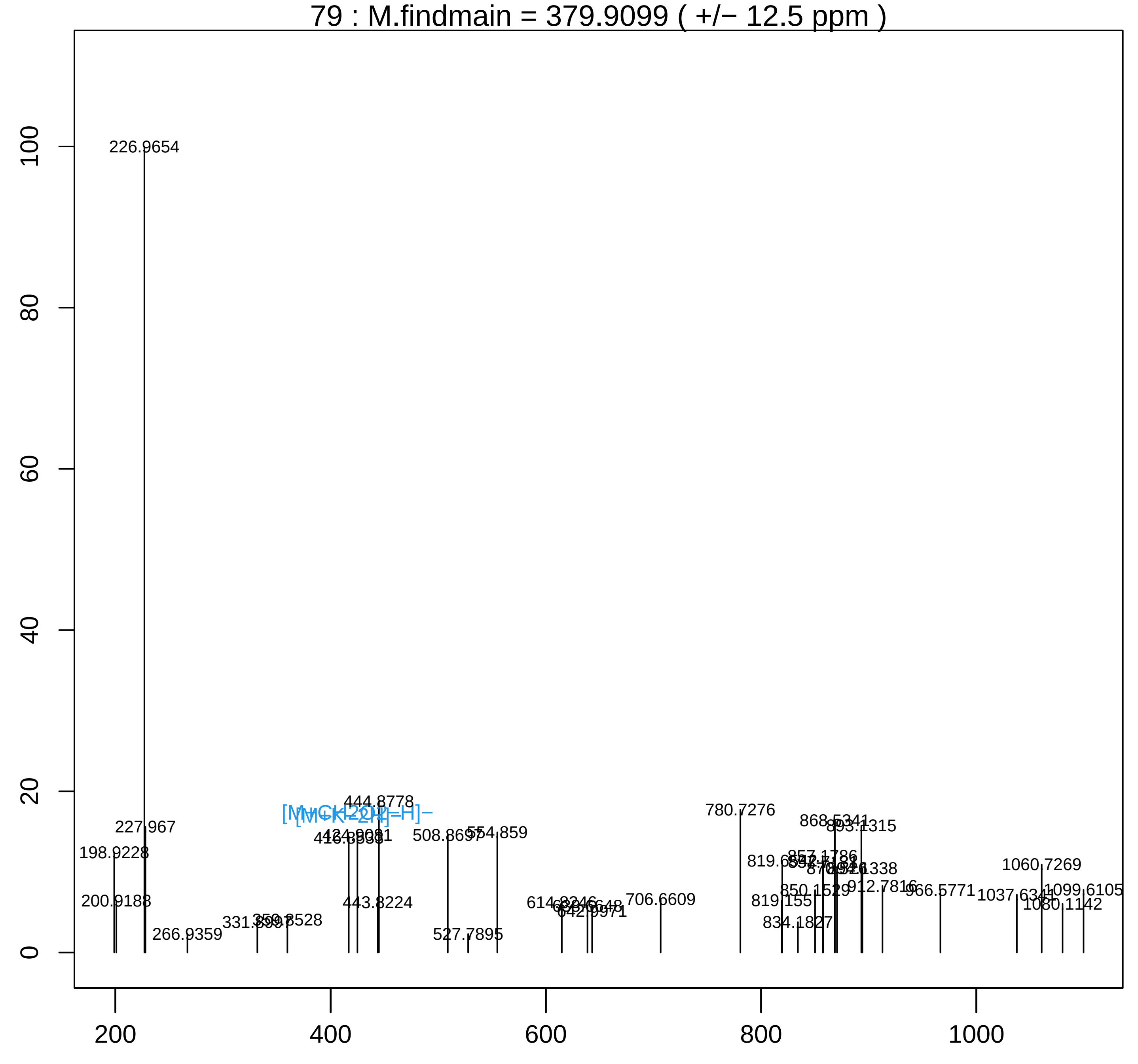
79 : M.findmain = 379.9099 (+/− 12.5 ppm)
226.9654
227.967
198.9228
200.9188
266.9359
359.8528
444.8778
443.8224
508.8697
554.859
527.7895
614.8246
706.6609
780.7276
868.5341
893.1315
857.1786
819.155
850.1529
834.1827
912.7816
966.5771
1060.7269
1099.6105
1080.1142
0
20
40
60
80
100
200
400
600
800
1000

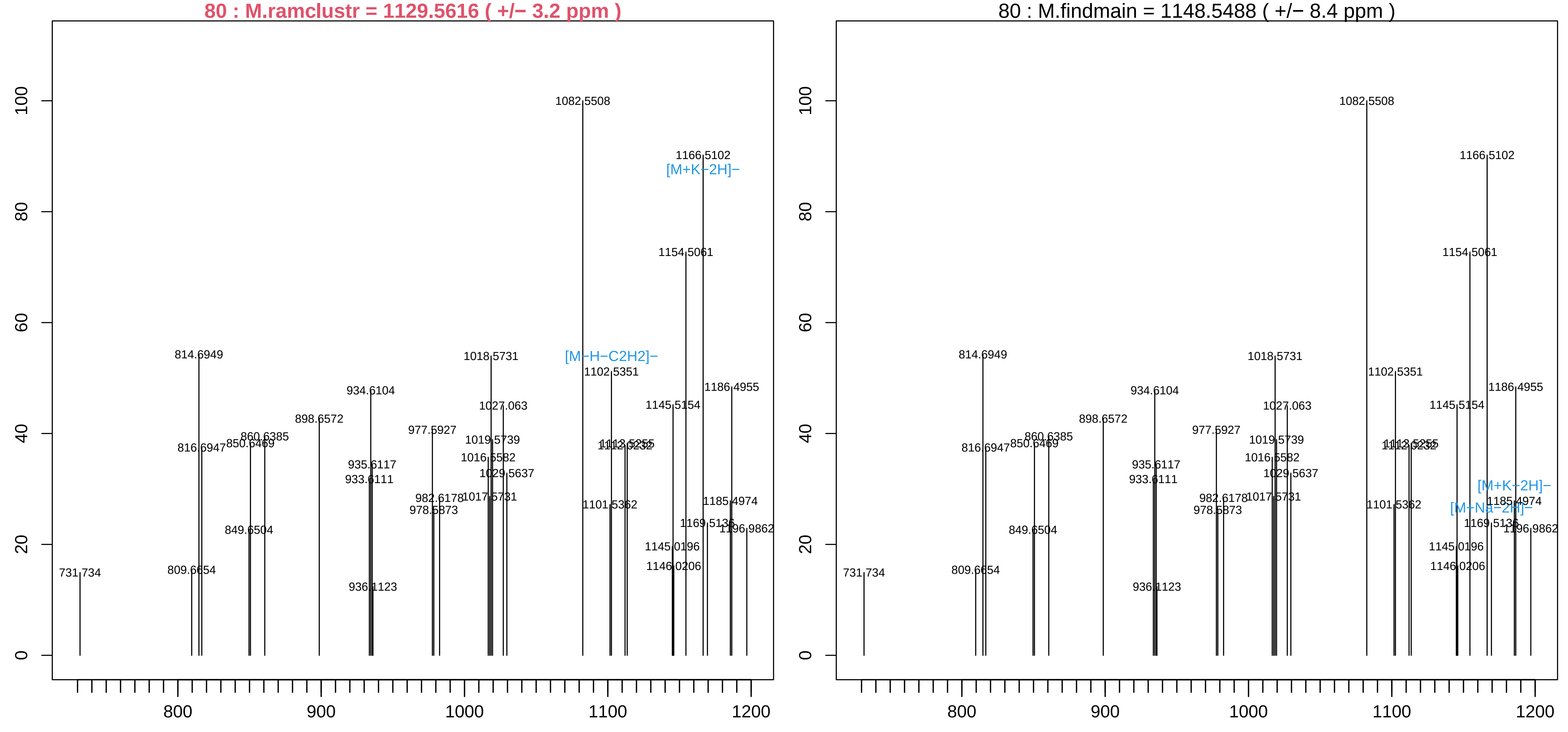

80 : M.ramclustr = 1129.5616 (+/− 3.2 ppm)
80 : M.findmain = 1148.5488 (+/− 8.4 ppm)
[M+K−2H]−
[M−H−C2H2]−
[M+Na−2H]−
1082.5508
1166.5102
1154.5061
814.6949
1018.5731
1102.5351
1186.4955
934.6104
1027.063
1145.5154
898.6572
977.5927
860.6385
850.6469
816.6947
1019.5739
1113.5255
1112.0232
1016.5582
935.6117
1029.5637
933.6111
982.6178
978.5873
1017.5731
1101.5362
1185.4974
1169.5136
1196.9862
849.6504
1145.0196
1146.0206
731.734
809.6654
936.1123
0
20
40
60
80
100
800
900
1000
1100
1200

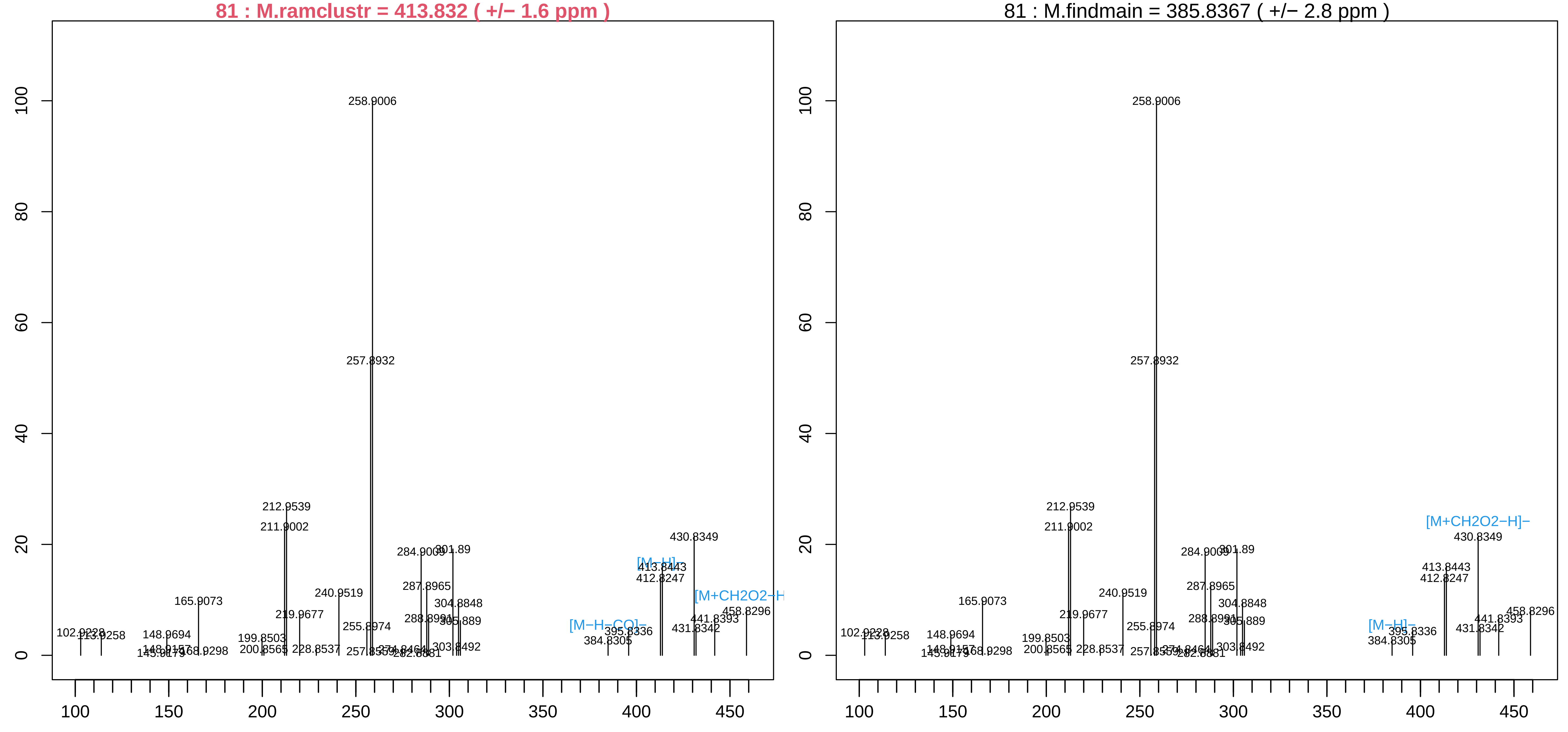

81 : M.ramclustr = 413.832 (+/− 1.6 ppm)
81 : M.findmain = 385.8367 (+/− 2.8 ppm)
258.9006
257.8932
212.9539
211.9002
165.9073
240.9519
219.9677
284.9009
301.89
287.8965
304.8848
288.8991
305.889
255.8974
199.8503
200.8565
228.8537
148.9694
102.9229
113.9258
303.8492
430.8349
413.8443
412.8247
441.8393
431.8342
458.8296
395.8336
384.8305
[M−H]−
[M+CH2O2−H]−
[M−H−CO]−
0
20
40
60
80
100
150
200
250
300
350
400
450

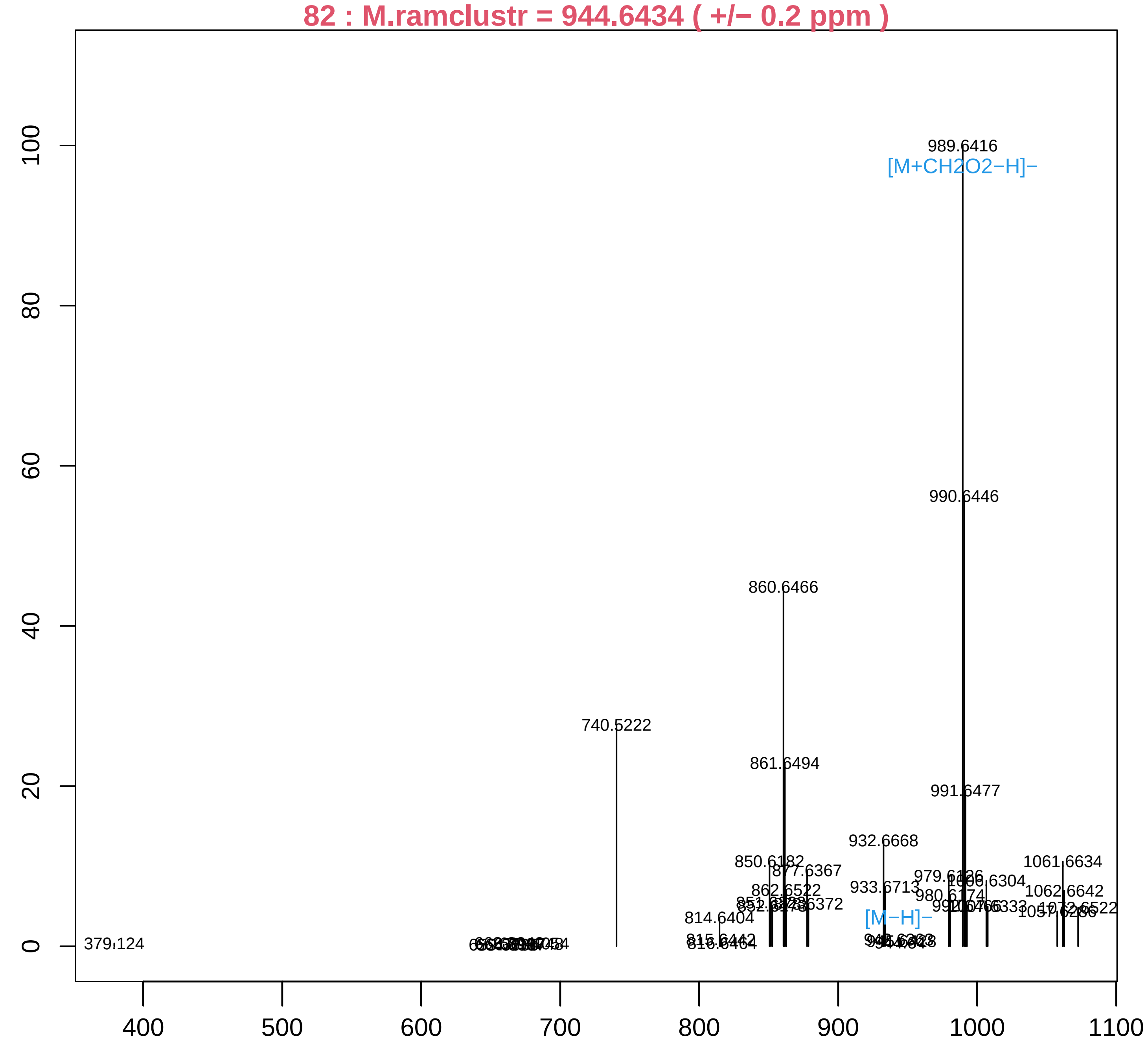
82 : M.ramclustr = 944.6434 (+/− 0.2 ppm)
989.6416
[M+CH2O2−H]−
990.6446
860.6466
740.5222
861.6494
991.6477
932.6668
850.6182
877.6367
1061.6634
933.6713
979.6126
1062.6642
[M−H]−
814.6404
379.124
0
20
40
60
80
100
400
500
600
700
800
900
1000
1100

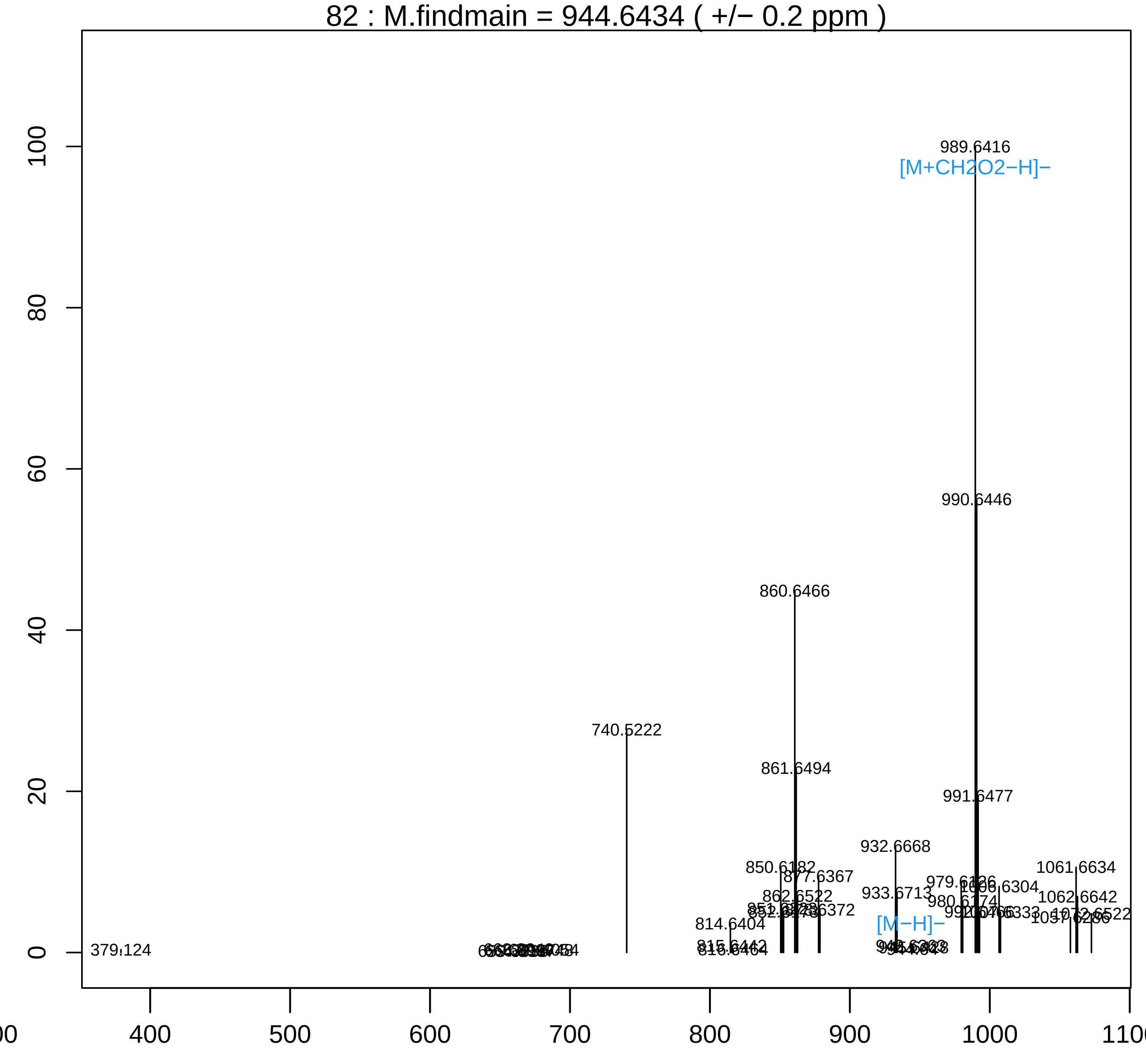
82 : M.findmain = 944.6434 (+/− 0.2 ppm)
989.6416
[M+CH2O2−H]−
990.6446
860.6466
740.5222
861.6494
991.6477
932.6668
850.6182
877.6367
1061.6634
933.6713
979.6126
1062.6642
[M−H]−
814.6404
379.124
0
20
40
60
80
100
400
500
600
700
800
900
1000

83 : M.ramclustr = 916.6122 (+/− 0.2 ppm) 83 : M.findmain = 916.6122 (+/− 0.2 ppm)

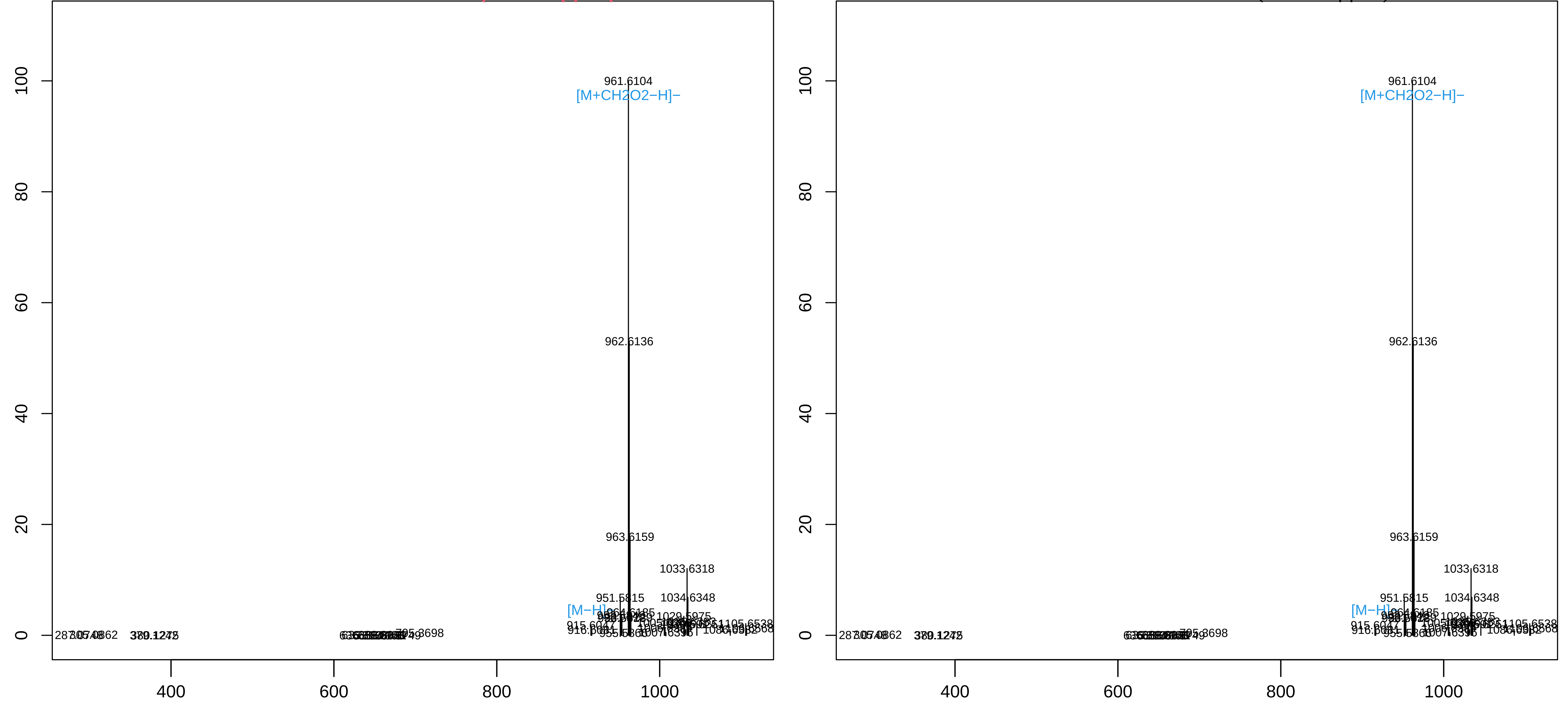

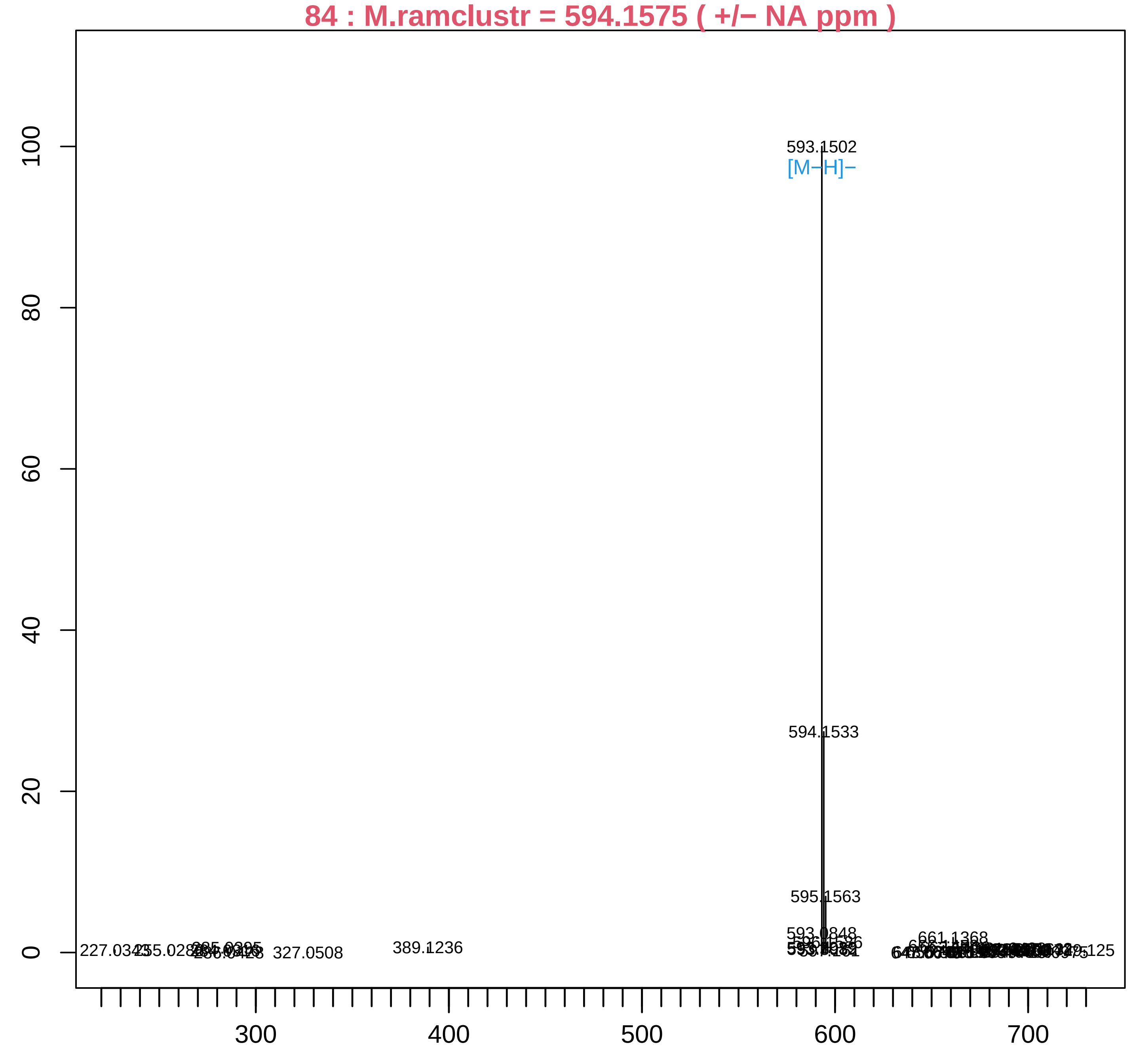
84 : M.ramclustr = 594.1575 (+/− NA ppm)
593.1502
[M−H]−
594.1533
595.1563
593.0848
661.1368
227.0343
327.0508
389.1236
0
20
40
60
80
100
300
400
500
600
700

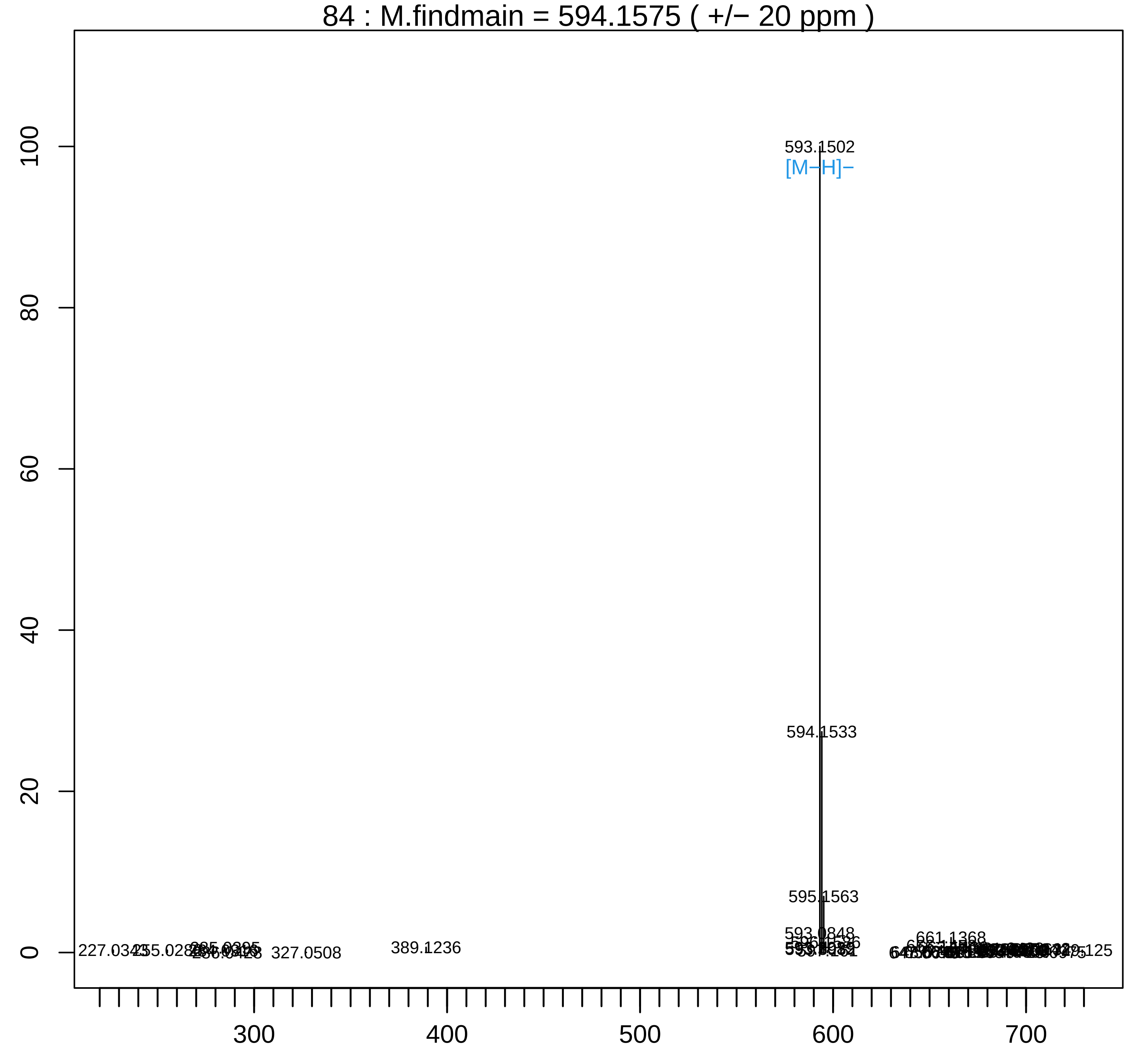
84 : M.findmain = 594.1575 (+/− 20 ppm)
593.1502
[M−H]−
594.1533
595.1563
593.0848
661.1368
227.0343
327.0508
389.1236
0
20
40
60
80
100
300
400
500
600
700

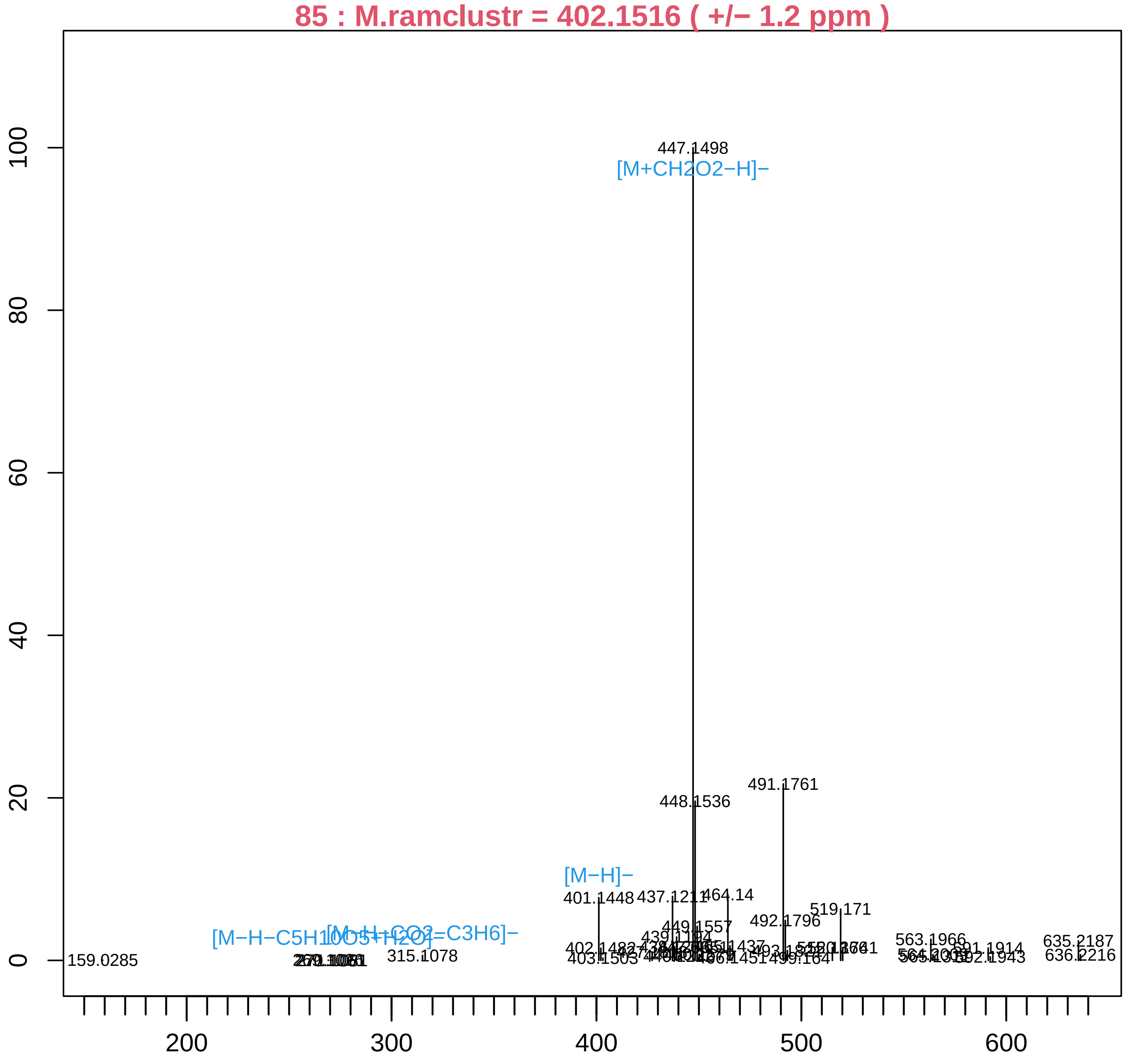
85 : M.ramclustr = 402.1516 (+/− 1.2 ppm)
447.1498
[M+CH2O2−H]−
448.1536
491.1761
[M−H]−
401.1448
437.1211
464.14
449.1557
492.1796
519.171
563.1966
635.2187
636.2216
159.0285
315.1078
403.1503
592.1943
0
20
40
60
80
100
200
300
400
500
600
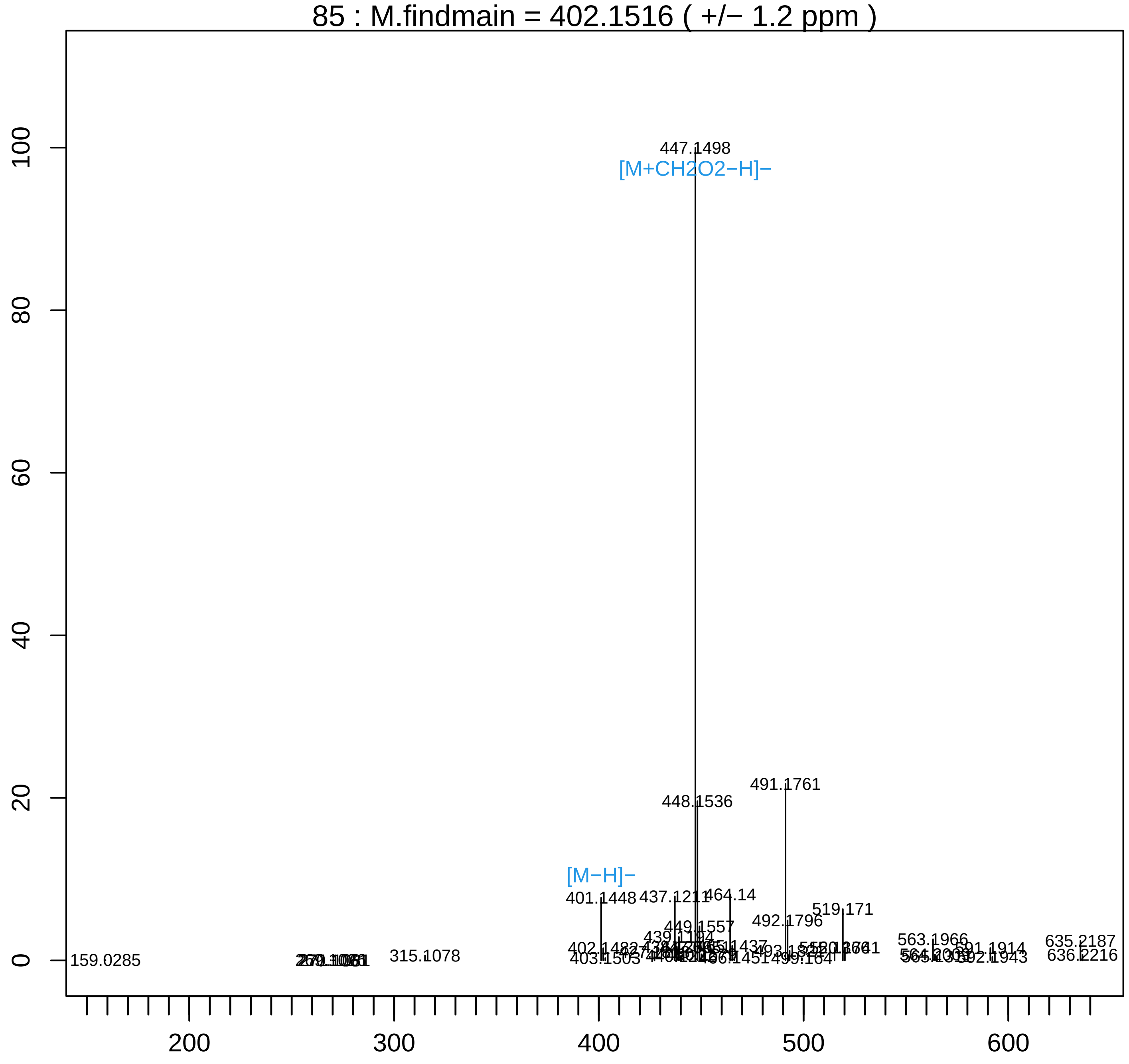
85 : M.findmain = 402.1516 (+/− 1.2 ppm)
447.1498
[M+CH2O2−H]−
448.1536
491.1761
[M−H]−
401.1448
437.1211
464.14
449.1557
492.1796
519.171
563.1966
635.2187
636.2216
159.0285
315.1078
403.1503
592.1943
0
20
40
60
80
100
200
300
400
500
600

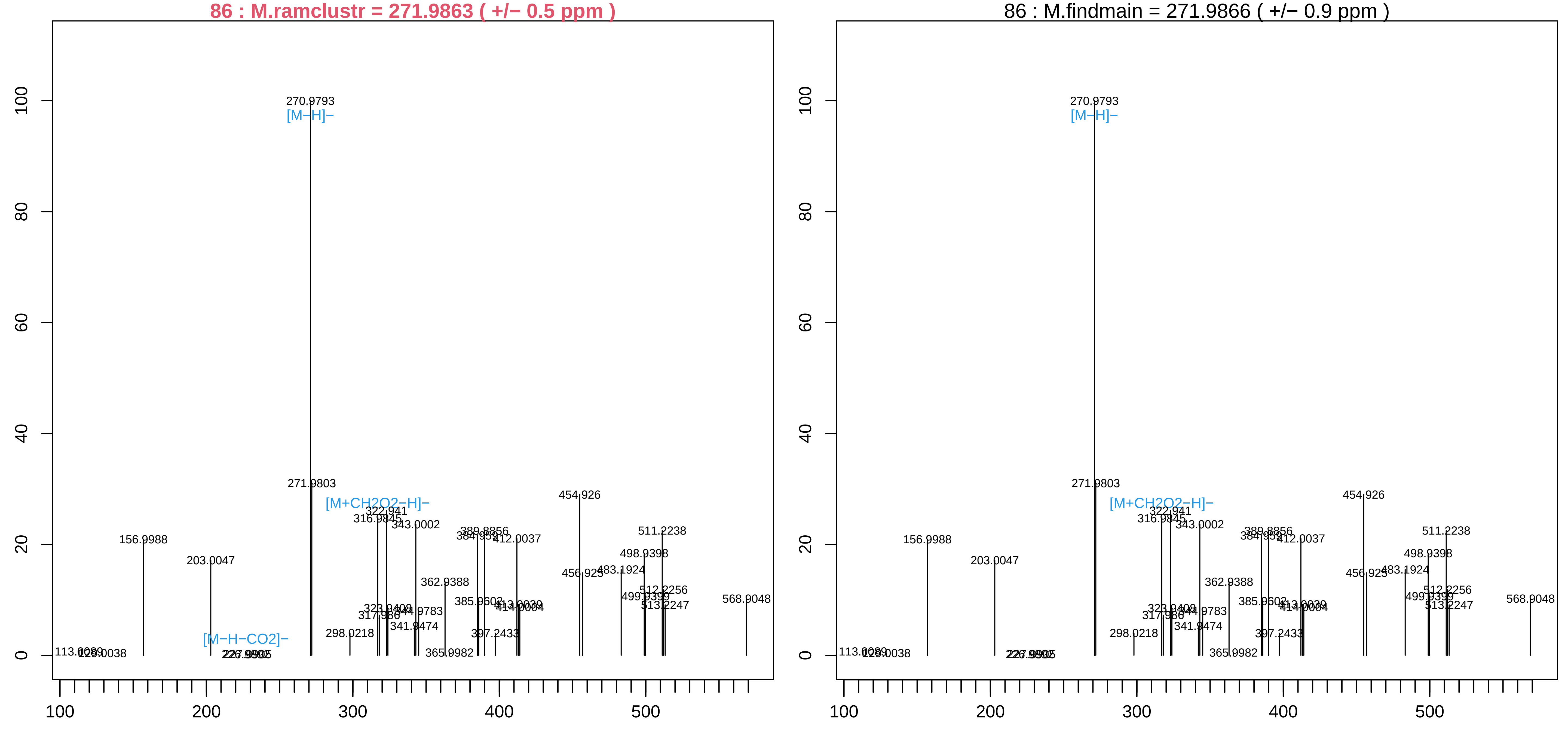
86 : M.ramclustr = 271.9863 (+/− 0.5 ppm)
86 : M.findmain = 271.9866 (+/− 0.9 ppm)
270.9793
[M−H]−
271.9803
[M+CH2O2−H]−
[M−H−CO2]−
156.9988
203.0047
298.0218
316.9845
322.941
343.0002
341.9474
344.9783
362.9388
365.9982
385.9602
389.8856
397.2433
412.0037
413.0039
414.0004
454.926
456.925
483.1924
498.9398
499.9399
511.2238
512.2256
513.2247
568.9048
100
200
300
400
500
0
20
40
60
80
100

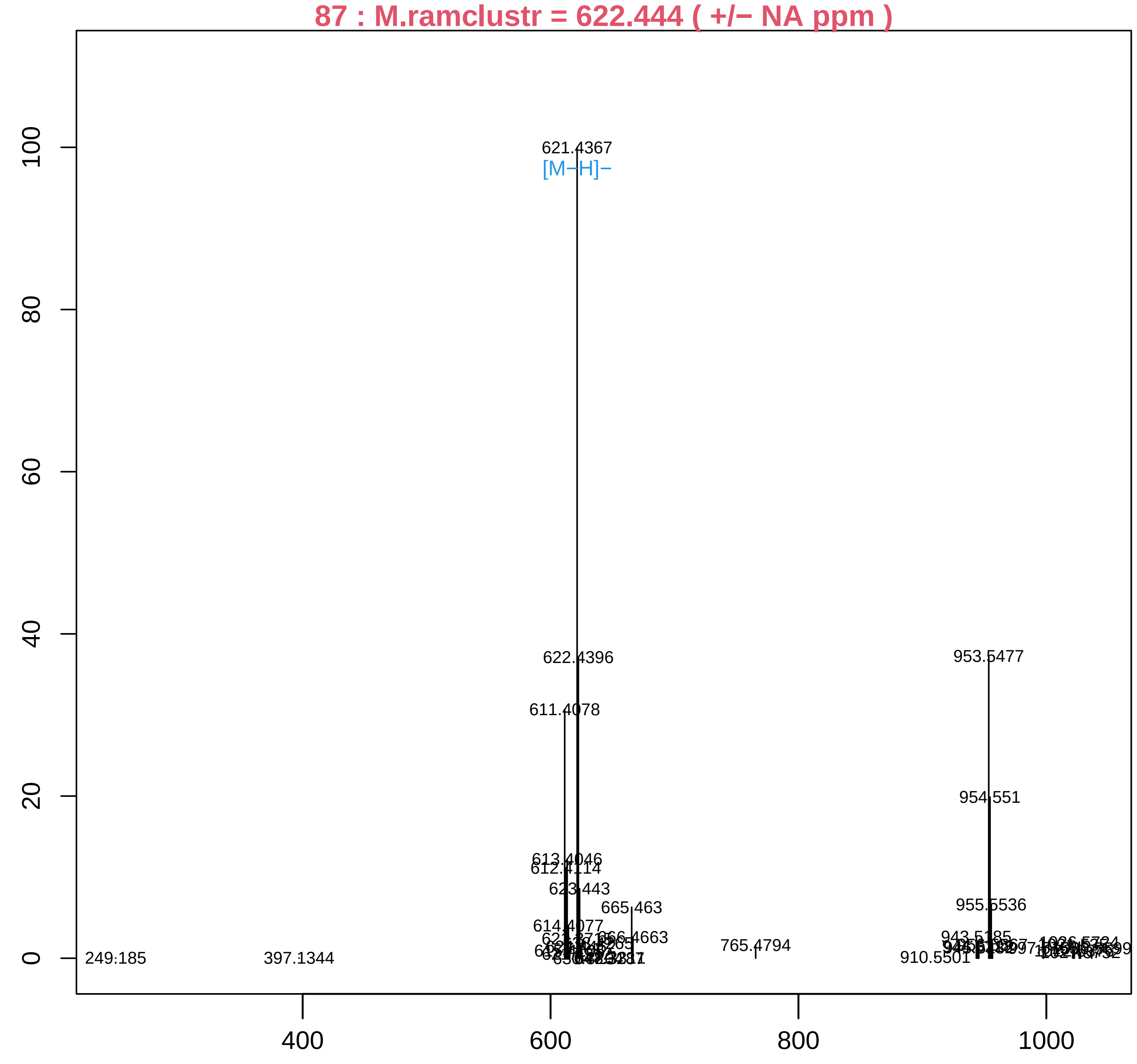

87 : M.ramclustr = 622.444 (+/− NA ppm)
621.4367
[M−H]−
622.4396
611.4078
613.4046
612.4114
623.443
665.463
614.4077
666.4663
765.4794
953.5477
954.551
955.5536
943.5185
910.5501
249.185
397.1344
0
20
40
60
80
100
400
600
800
1000

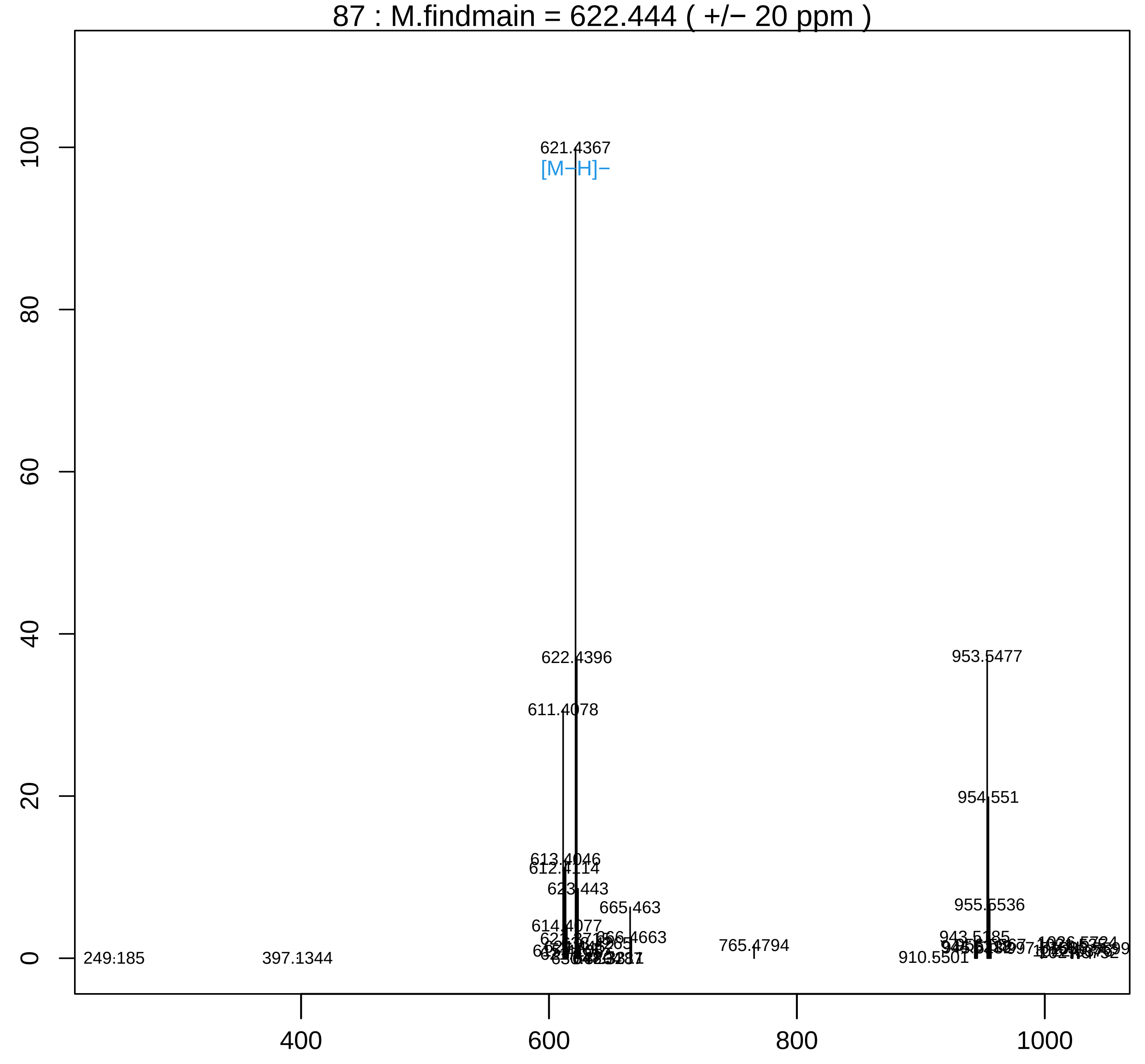

87 : M.findmain = 622.444 (+/− 20 ppm)
621.4367
[M−H]−
622.4396
611.4078
613.4046
612.4114
623.443
665.463
614.4077
666.4663
765.4794
953.5477
954.551
955.5536
943.5185
910.5501
249.185
397.1344
0
20
40
60
80
100
400
600
800
1000

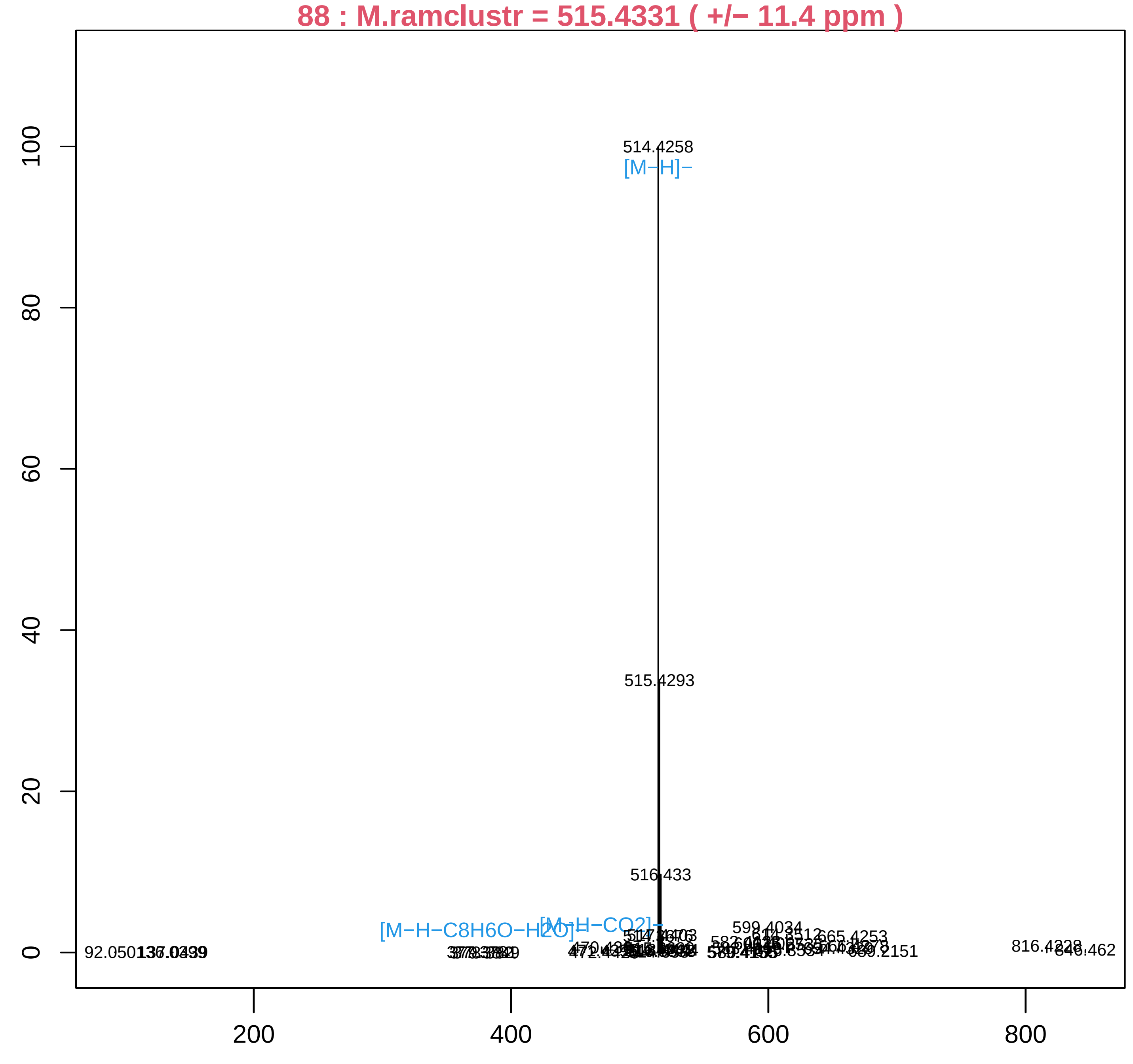
88 : M.ramclustr = 515.4331 (+/− 11.4 ppm)
514.4258
[M−H]−
515.4293
516.433
[M−H−C8H6O−H2O]
599.4034
665.4253
689.2151
816.4228
846.462
92.0501
0
20
40
60
80
100
200
400
600
800
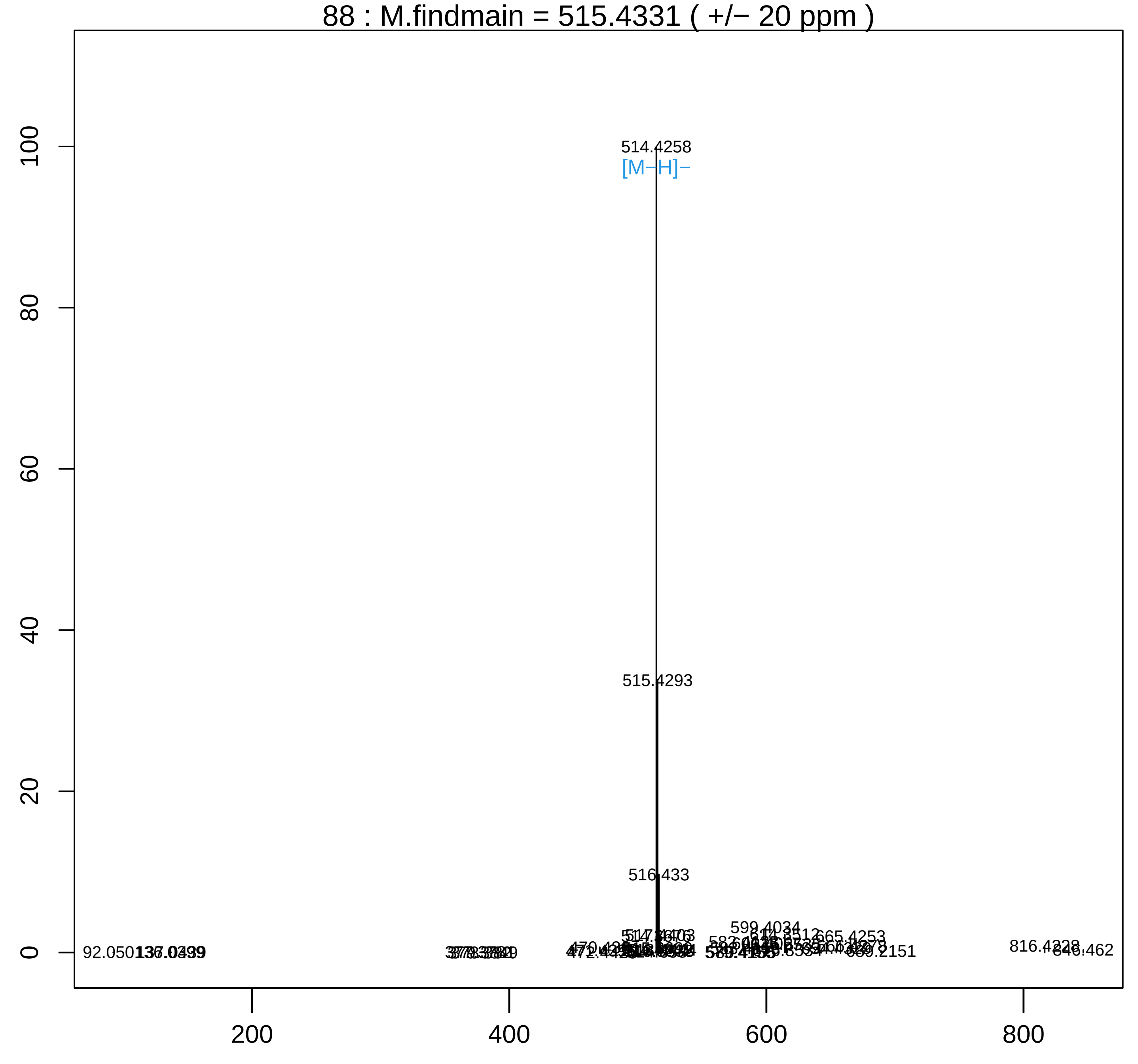
88 : M.findmain = 515.4331 (+/− 20 ppm)
514.4258
[M−H]−
515.4293
516.433
599.4034
665.4253
689.2151
816.4228
846.462
92.0501
0
20
40
60
80
100
200
400
600
800

89 : M.ramclustr = 865.4814 (+/− 0.3 ppm)

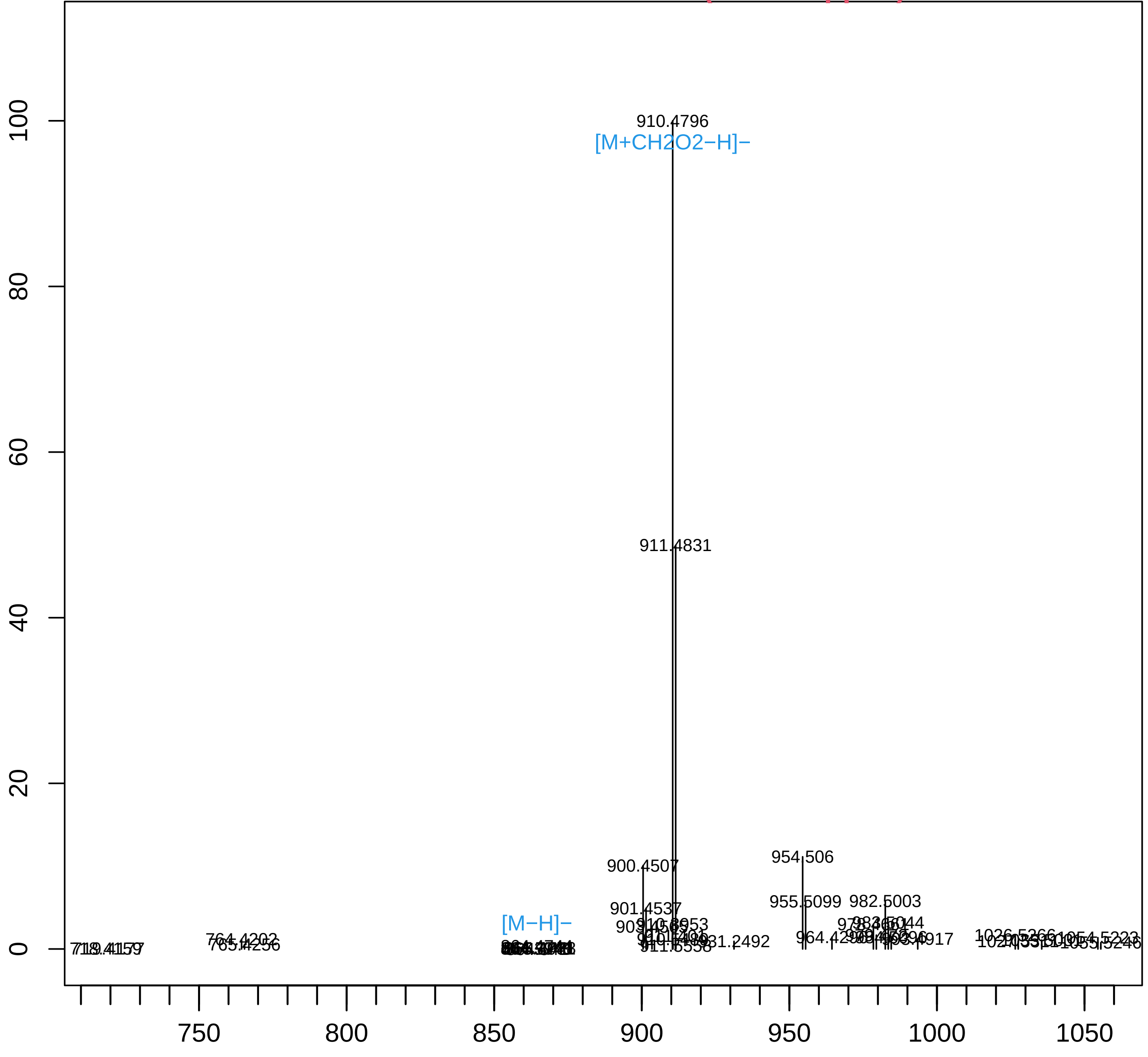

89 : M.findmain = 865.4814 (+/− 0.3 ppm)

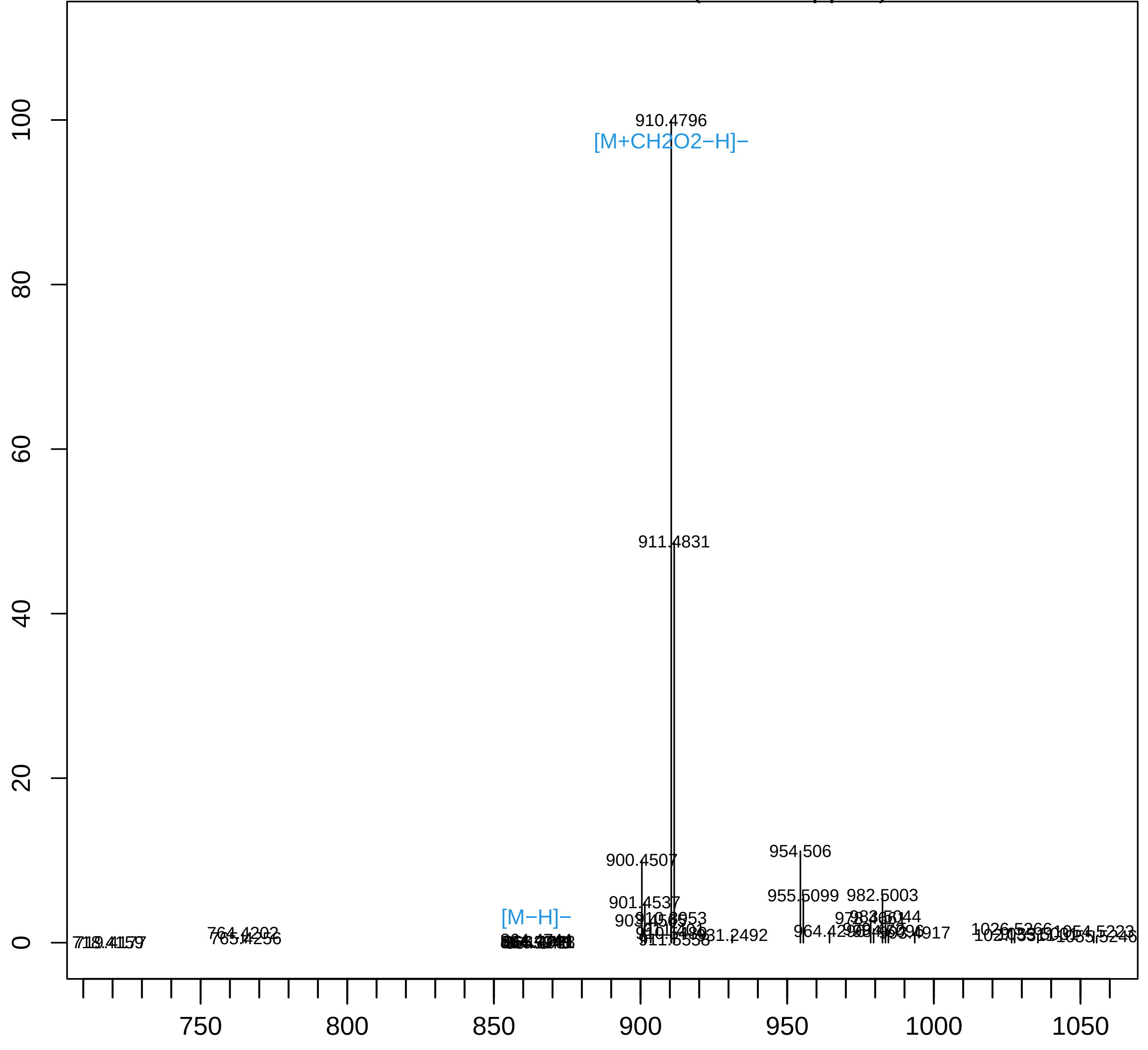

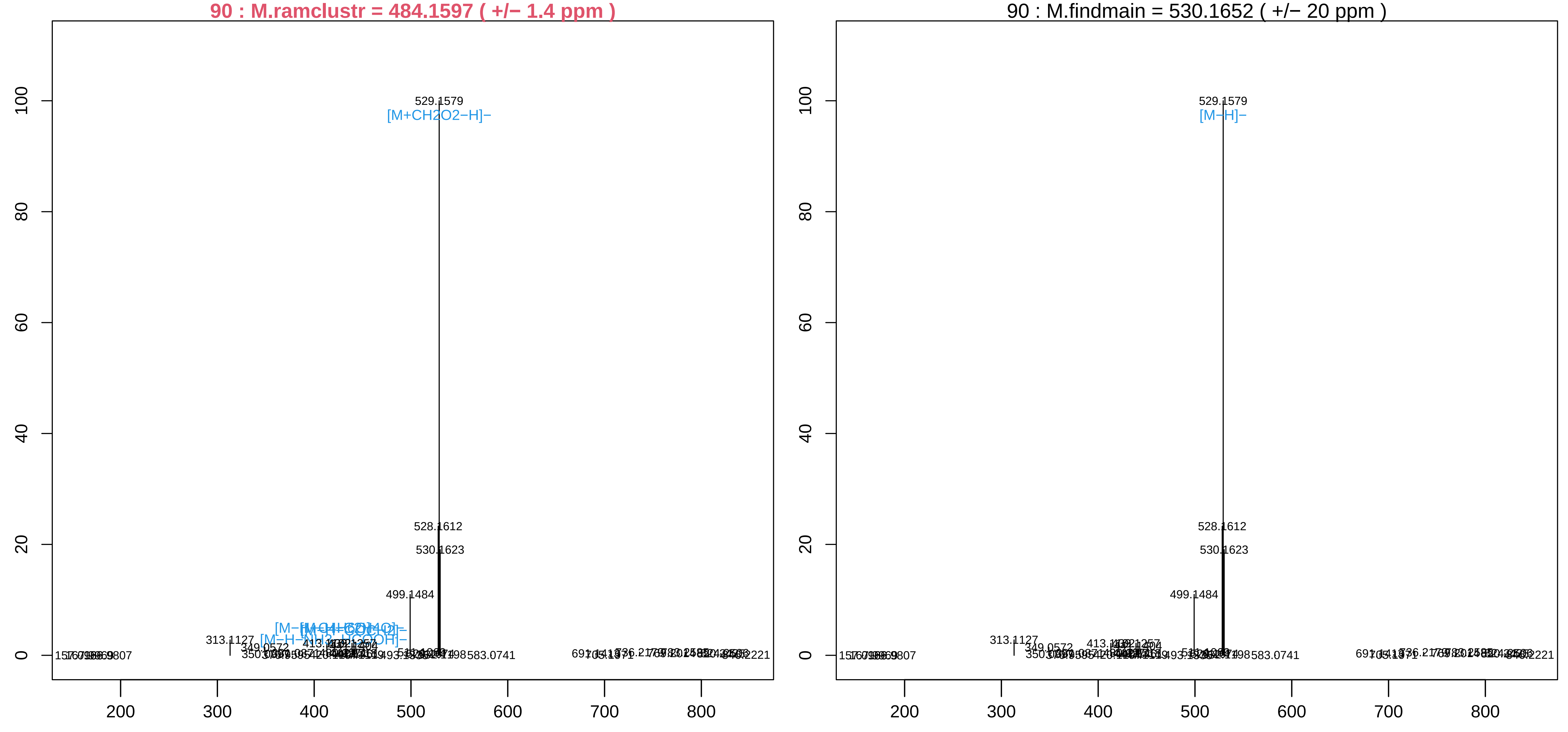

90 : M.ramclustr = 484.1597 (+/− 1.4 ppm)
529.1579
[M+CH2O2−H]−
528.1612
530.1623
499.1484
313.1127
349.0572
583.0741
90 : M.findmain = 530.1652 (+/− 20 ppm)
529.1579
[M−H]−
528.1612
530.1623
499.1484
313.1127
349.0572
583.0741

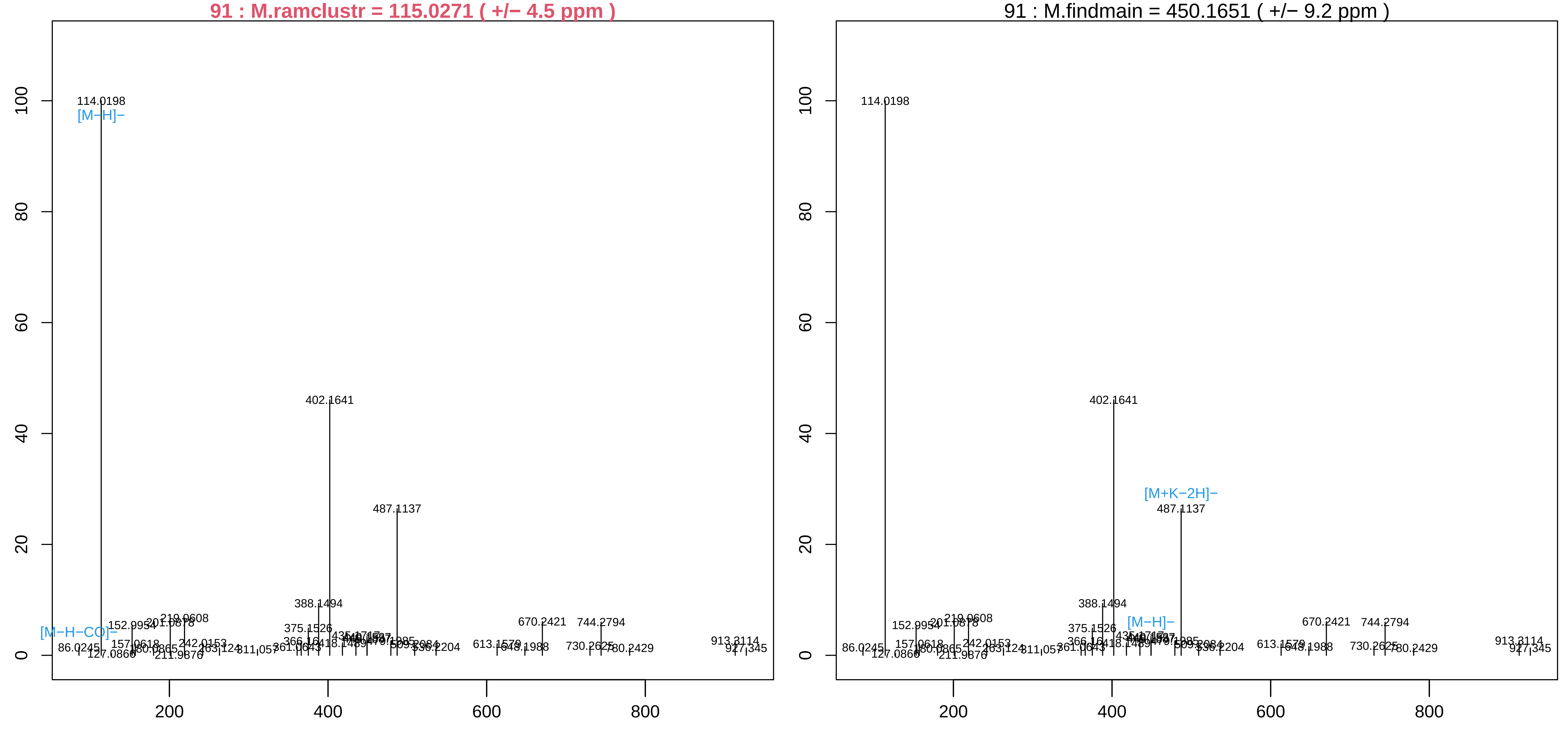

91 : M.ramclustr = 115.0271 (+/− 4.5 ppm)
91 : M.findmain = 450.1651 (+/− 9.2 ppm)
114.0198
[M−H]−
402.1641
487.1137
[M+K−2H]−
388.1494
[M−H−CO]−
670.2421
744.2794
219.0608
152.9954
375.1526
913.3114

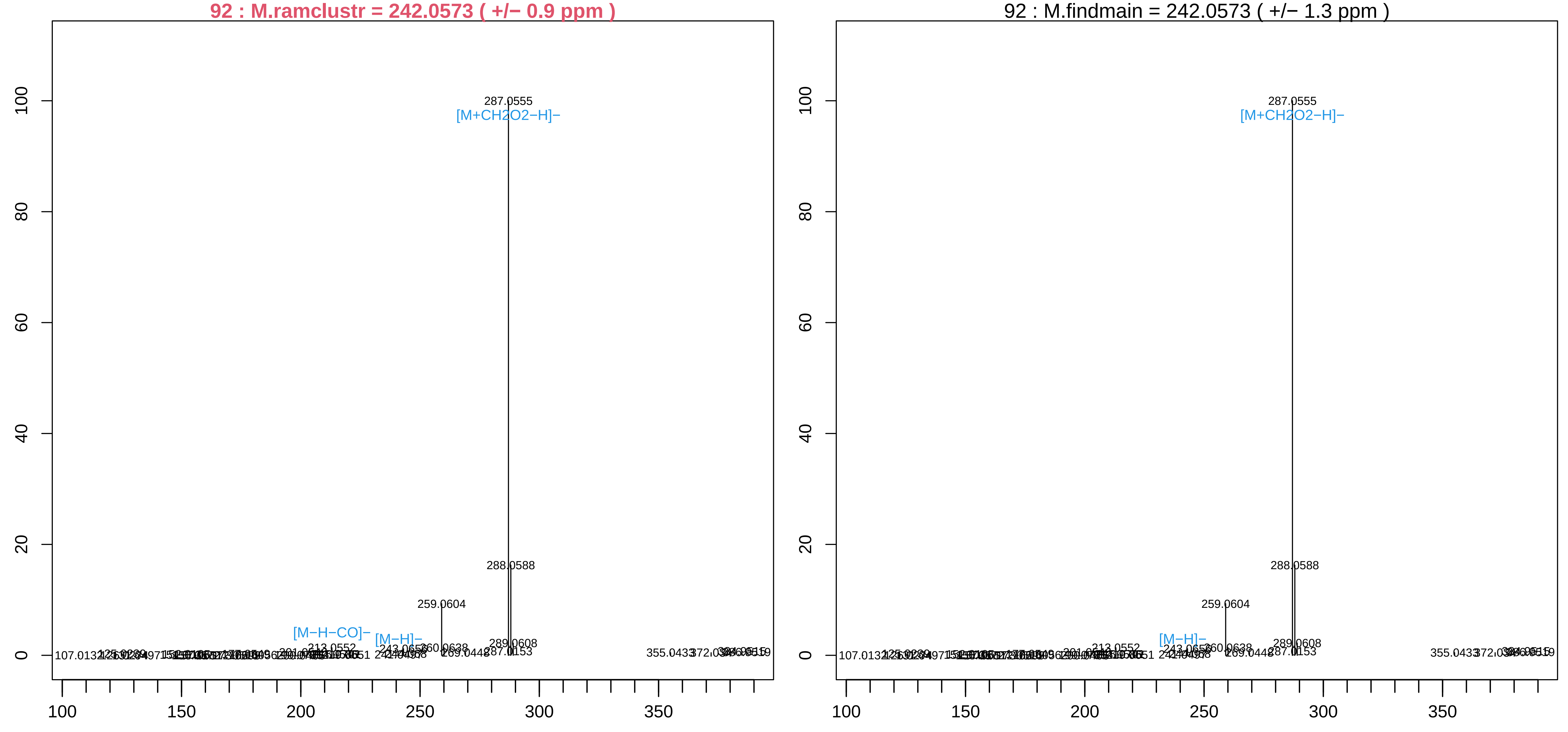
92 : M.ramclustr = 242.0573 (+/− 0.9 ppm)
287.0555
[M+CH2O2−H]−
288.0588
259.0604
[M−H−CO]−
[M−H]−
213.0552
260.0638
289.0608
287.0153
269.0448
355.0433
107.0132
92 : M.findmain = 242.0573 (+/− 1.3 ppm)
287.0555
[M+CH2O2−H]−
288.0588
259.0604
[M−H]−
213.0552
260.0638
289.0608
287.0153
269.0448
355.0433
107.0132
0
20
40
60
80
100
150
200
250
300
350

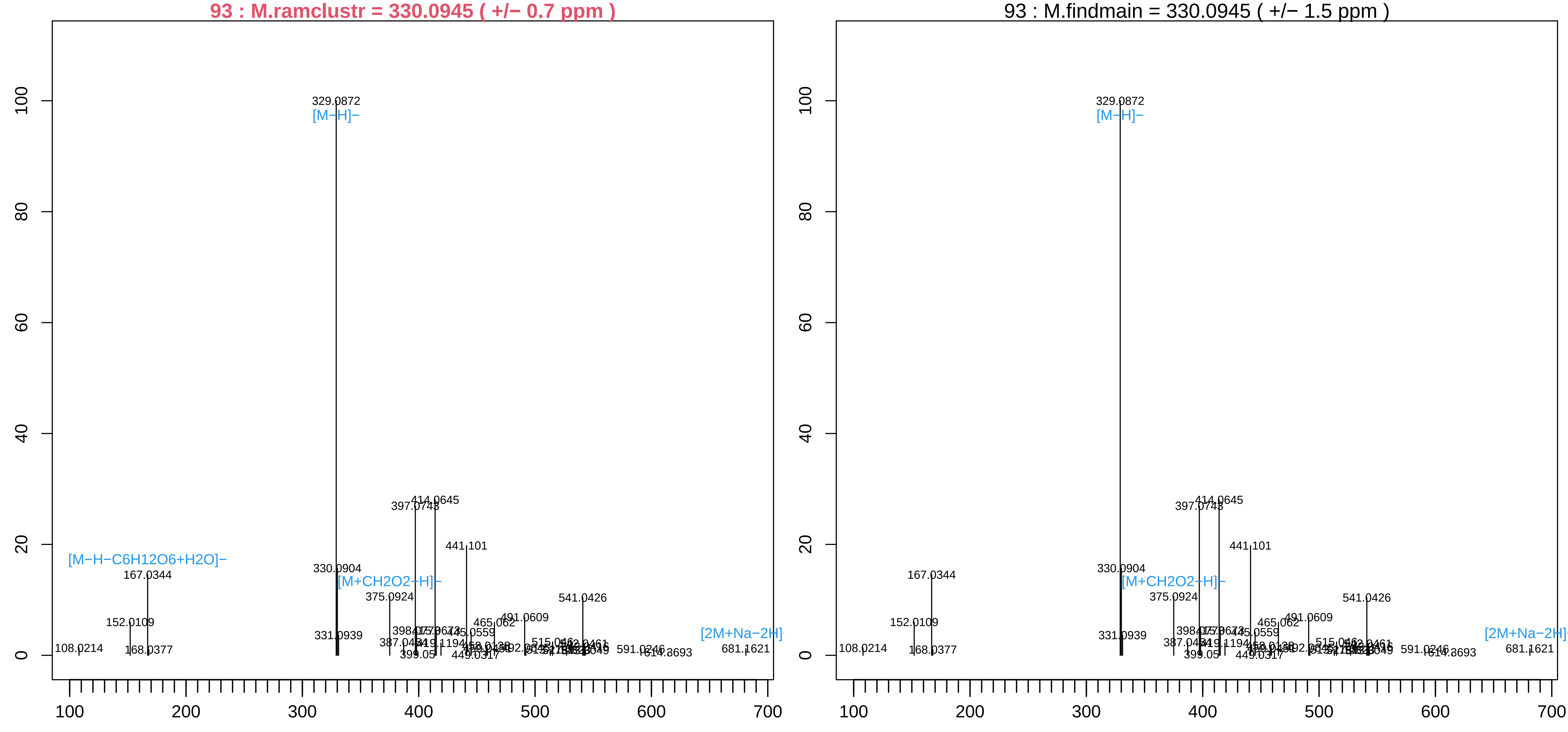
93 : M.ramclustr = 330.0945 (+/− 0.7 ppm)
329.0872
[M−H]−
[M−H−C6H12O6+H2O]−
167.0344
152.0109
108.0214
168.0377
330.0904
[M+CH2O2−H]−
331.0939
375.0924
397.0743
414.0645
441.101
465.062
491.0609
541.0426
591.0246
614.3693
681.1621
[2M+Na−2H]−
93 : M.findmain = 330.0945 (+/− 1.5 ppm)
329.0872
[M−H]−
167.0344
152.0109
108.0214
168.0377
330.0904
[M+CH2O2−H]−
331.0939
375.0924
397.0743
414.0645
441.101
465.062
491.0609
541.0426
591.0246
614.3693
681.1621
[2M+Na−2H]
0
20
40
60
80
100
200
300
400
500
600
700

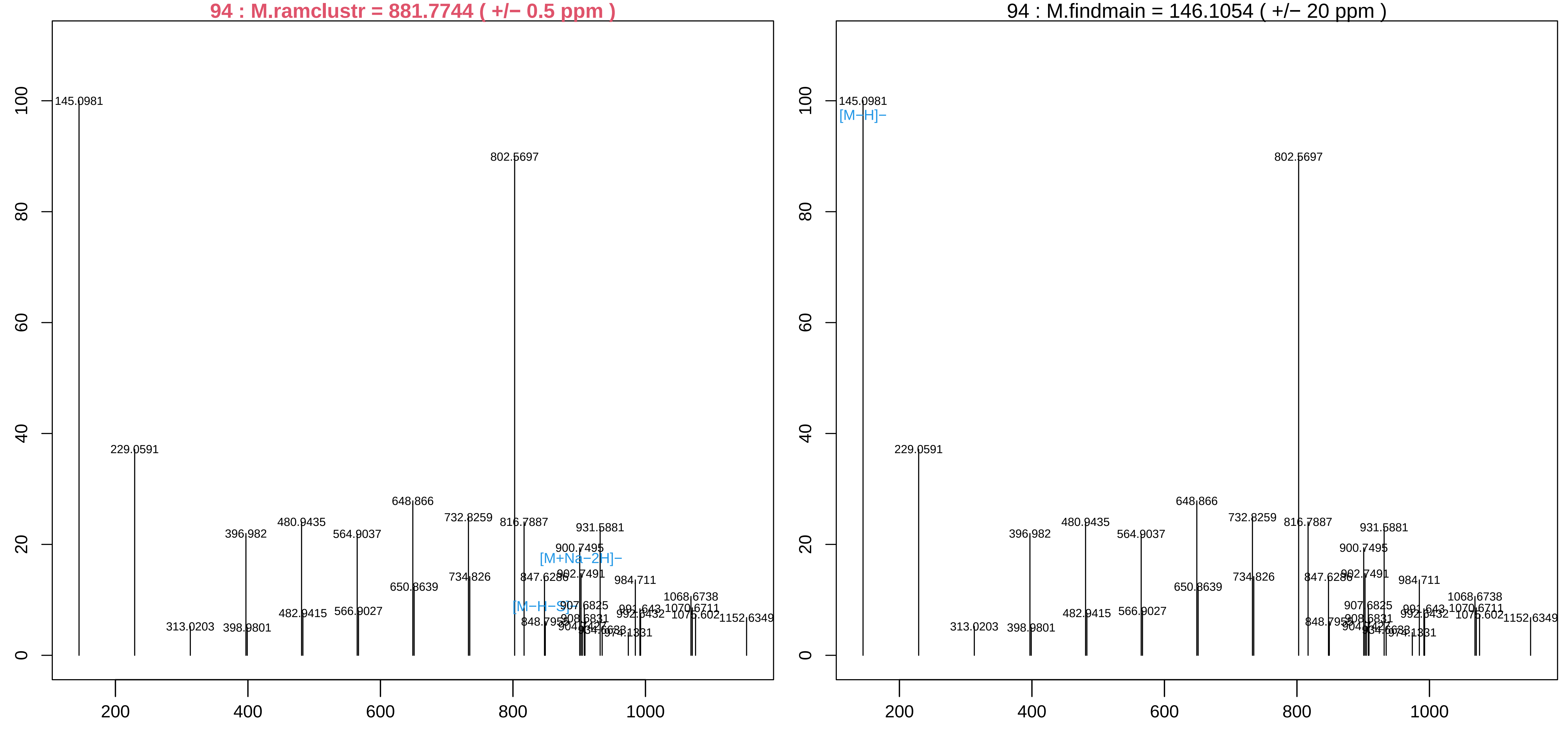
94 : M.ramclustr = 881.7744 (+/− 0.5 ppm)
145.0981
802.5697
229.0591
648.866
732.8259
816.7887
931.5881
480.9435
396.982
564.9037
900.7495
[M+Na−2H]−
902.7491
847.6286
734.826
984.711
650.8639
1068.6738
907.6825
1070.6711
992.6432
1075.602
1152.6349
482.9415
566.9027
313.0203
398.9801
908.6831
974.1331
94 : M.findmain = 146.1054 (+/− 20 ppm)
[M−H]−
0
20
40
60
80
100
200
400
600
800
1000

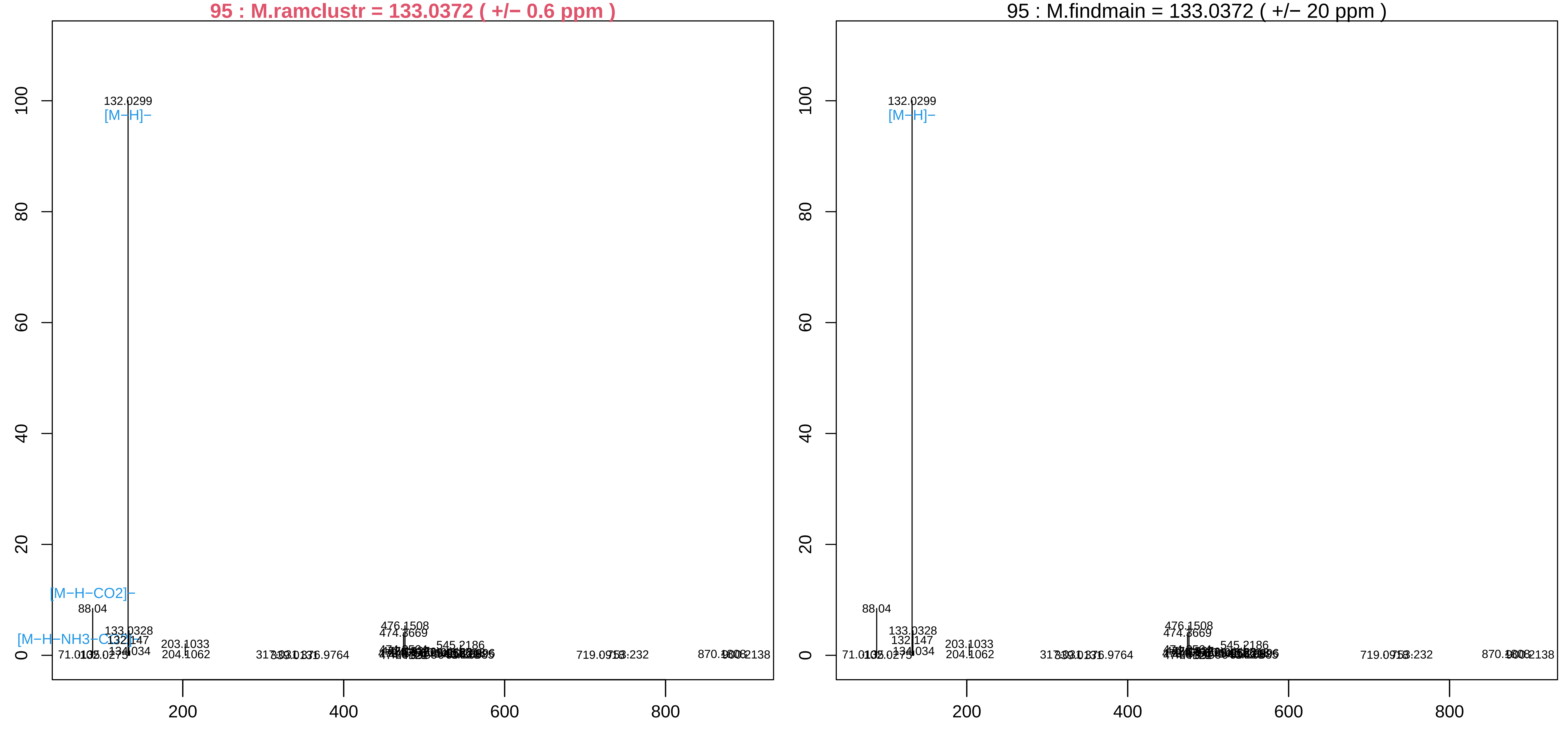

95 : M.ramclustr = 133.0372 (+/− 0.6 ppm)
132.0299
[M−H]−
[M−H−CO2]−
88.04
133.0328
[M−H−NH3−CO2]−
132.147
134.034
71.0105
203.1033
204.1062
476.1508
474.3669
545.2186
719.0953
870.9808
95 : M.findmain = 133.0372 (+/− 20 ppm)
132.0299
[M−H]−
88.04
133.0328
132.147
134.034
203.1033
204.1062
476.1508
474.3669
545.2186
719.0953
870.9808

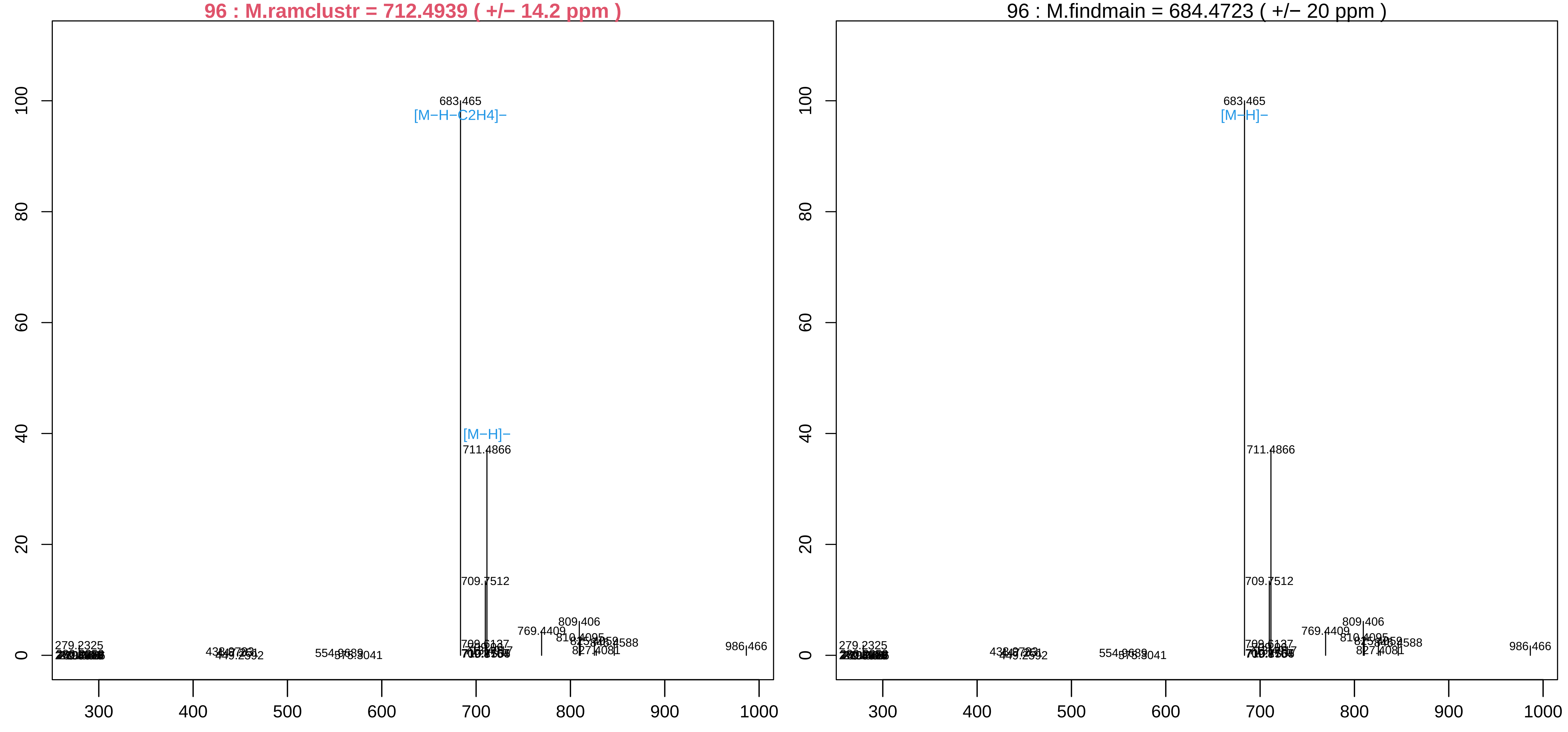

96 : M.ramclustr = 712.4939 (+/− 14.2 ppm)
683.465
[M−H−C2H4]−
[M−H]−
711.4866
709.7512
809.406
769.4409
279.2325
986.466
445.2592
575.3041
96 : M.findmain = 684.4723 (+/− 20 ppm)
683.465
[M−H]−
711.4866
709.7512
809.406
769.4409
279.2325
986.466
445.2592
575.3041

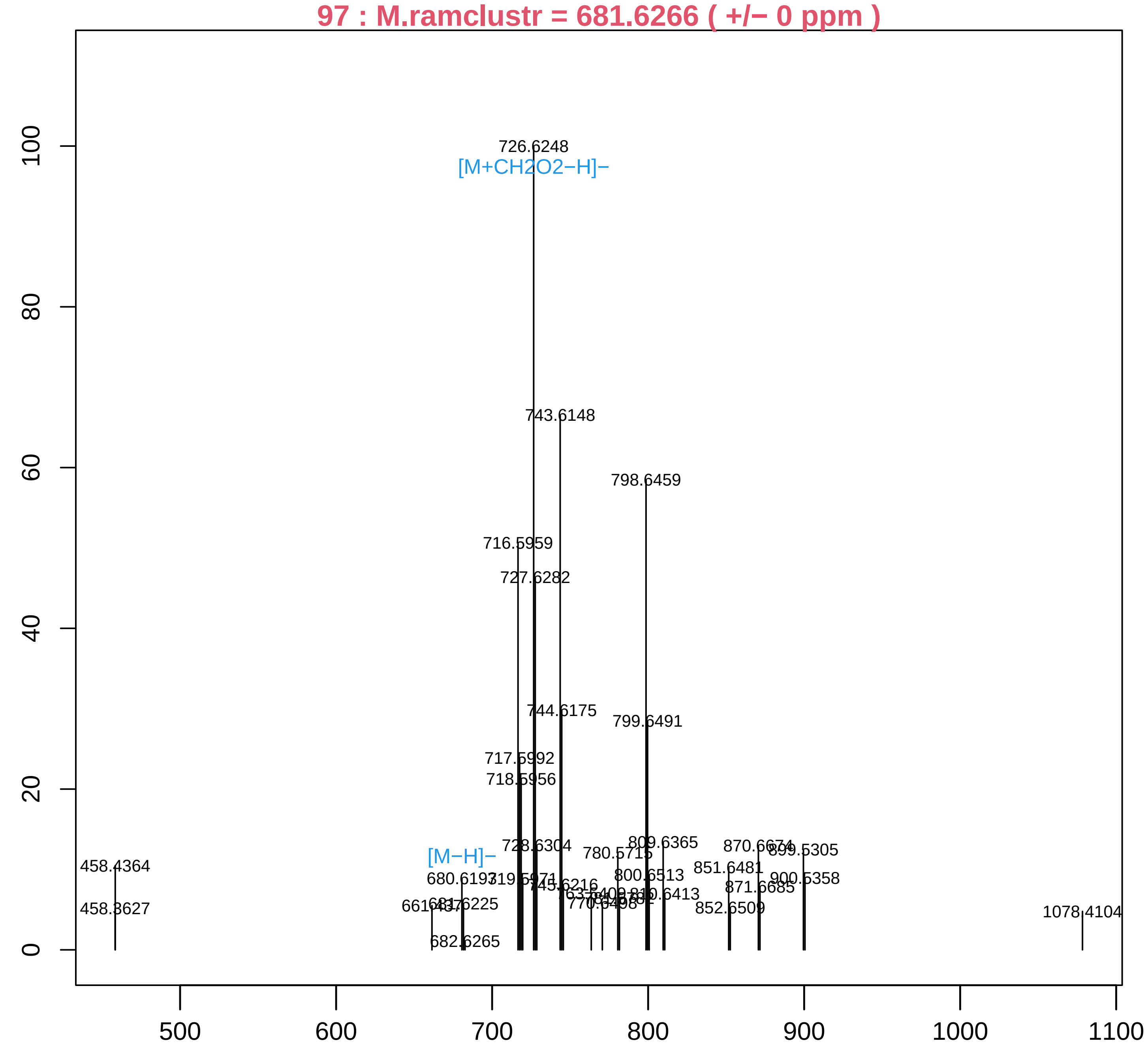
97 : M.ramclustr = 681.6266 (+/− 0 ppm)
726.6248
[M+CH2O2−H]−
743.6148
798.6459
716.5959
727.6282
744.6175
799.6491
717.5992
718.5956
728.6304
809.6365
870.6674
899.5305
780.5715
[M−H]−
458.4364
851.6481
680.6193
800.6513
900.5358
871.6685
458.3627
852.6509
1078.4104
682.6265
0
20
40
60
80
100
500
600
700
800
900
1000
1100
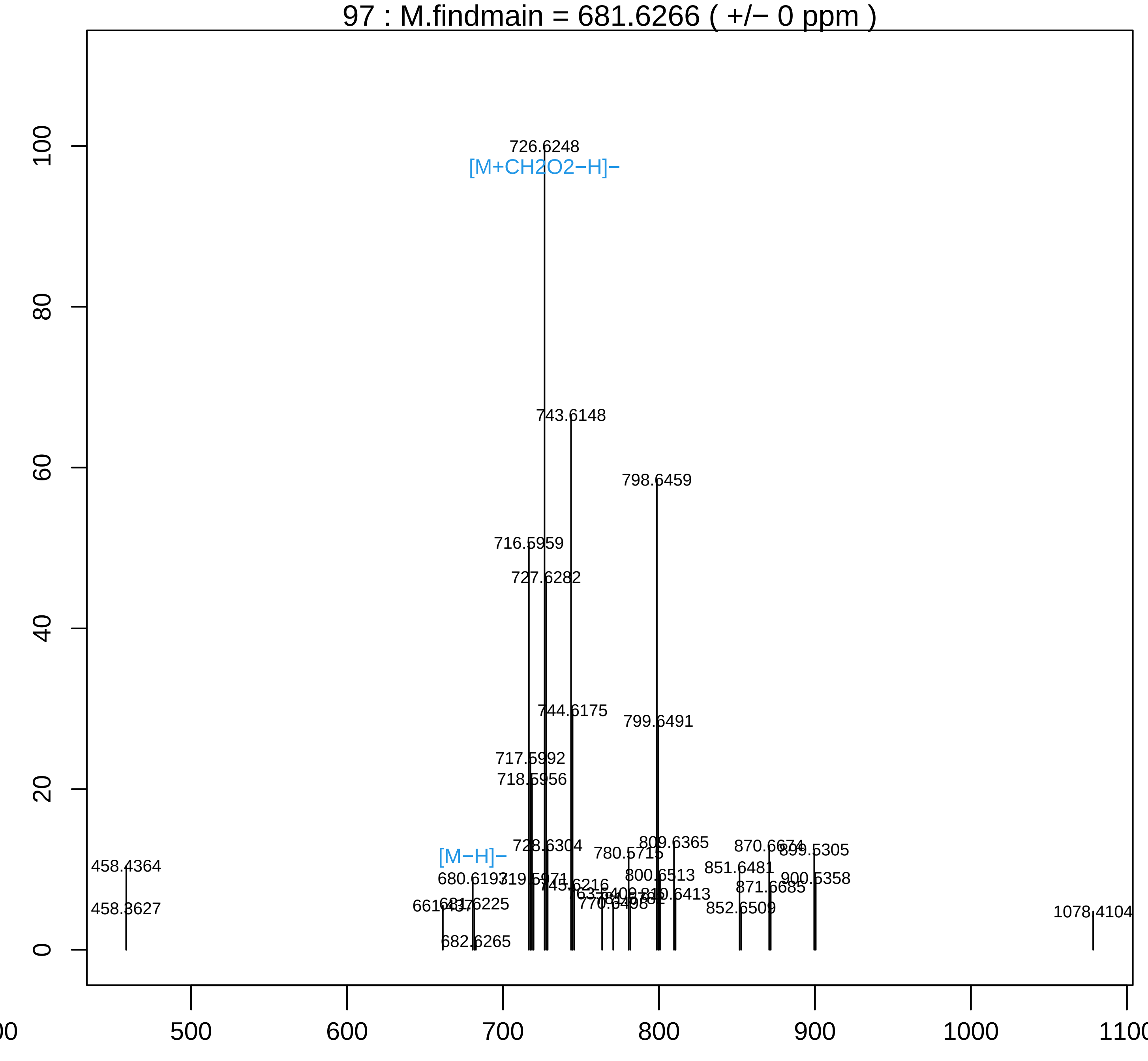
97 : M.findmain = 681.6266 (+/− 0 ppm)
726.6248
[M+CH2O2−H]−
743.6148
798.6459
716.5959
727.6282
744.6175
799.6491
717.5992
718.5956
728.6304
809.6365
870.6674
899.5305
780.5715
[M−H]−
458.4364
851.6481
680.6193
800.6513
900.5358
871.6685
458.3627
852.6509
1078.4104
682.6265
0
20
40
60
80
100
500
600
700
800
900
1000
1100

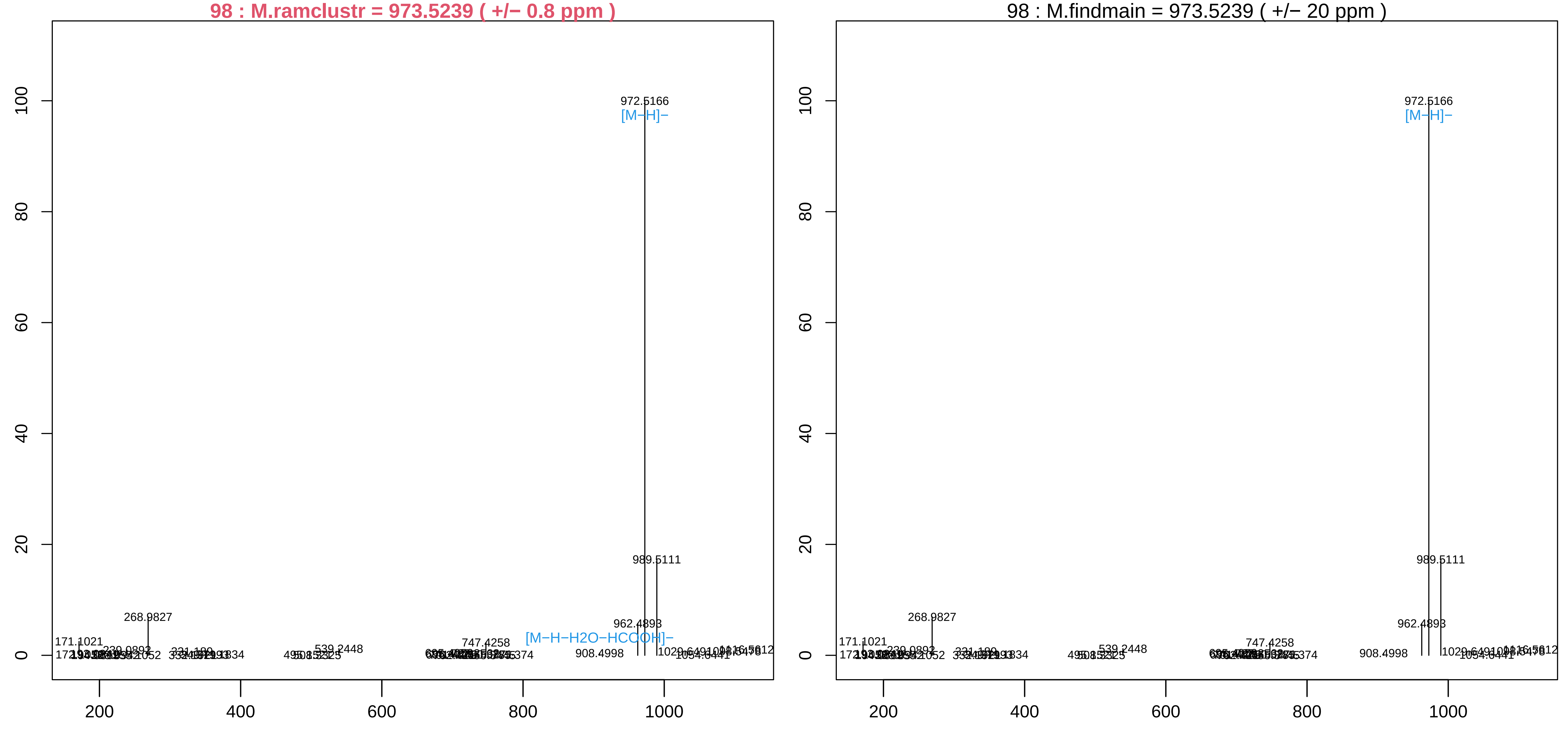

98 : M.ramclustr = 973.5239 (+/− 0.8 ppm)
98 : M.findmain = 973.5239 (+/− 20 ppm)
972.5166
[M−H]−
989.5111
962.4893
[M−H−H2O−HCOOH]−
268.9827
171.1021
747.4258
539.2448
908.4998
1054.6441

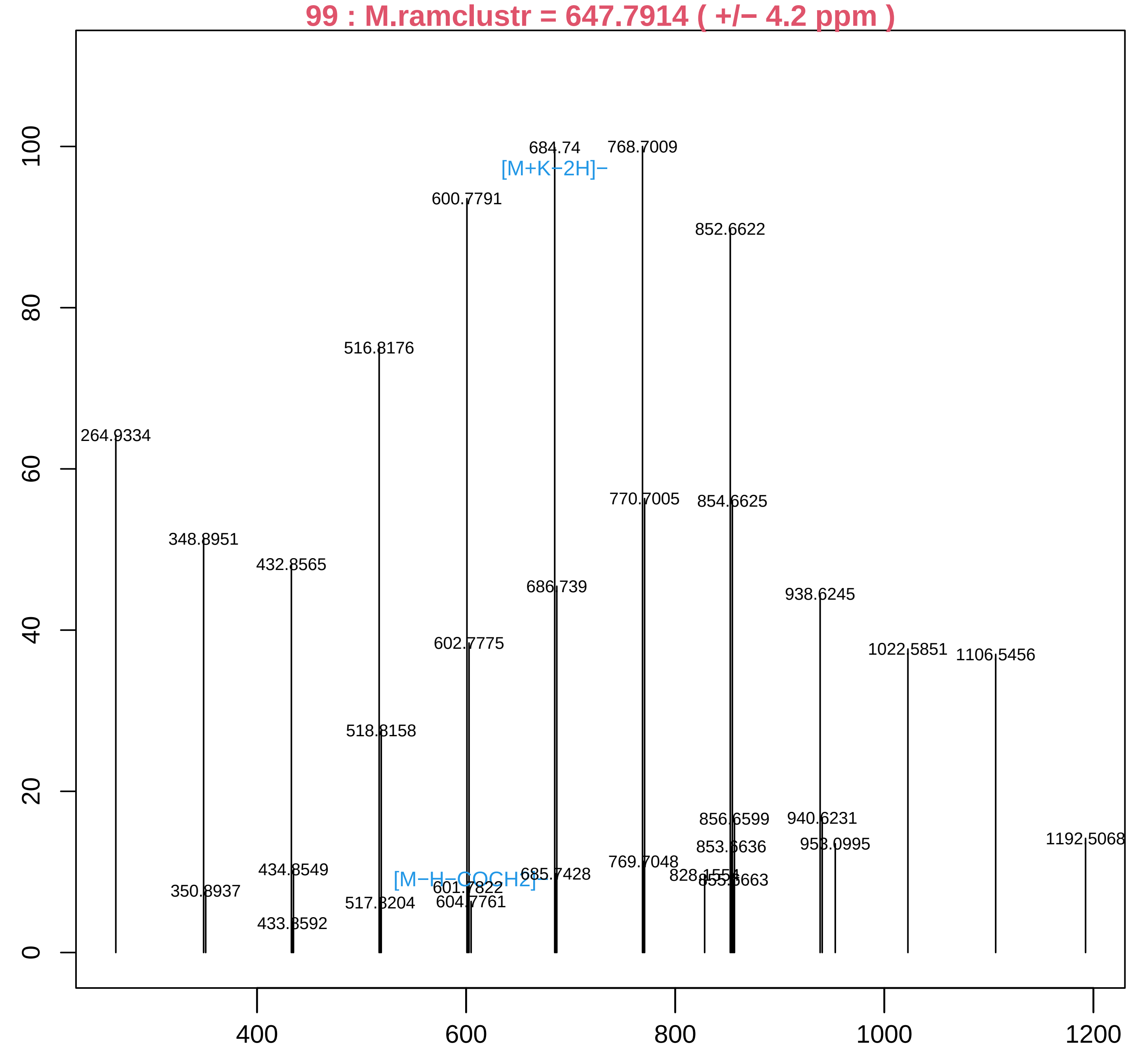
99 : M.ramclustr = 647.7914 (+/− 4.2 ppm)
[M+K−2H]−
[M−H−COCH2]−
684.74
768.7009
600.7791
852.6622
516.8176
264.9334
770.7005
854.6625
348.8951
432.8565
686.739
938.6245
602.7775
1022.5851
1106.5456
518.8158
856.6599
940.6231
953.0995
853.5636
1192.5068
769.7048
434.8549
685.7428
828.1554
855.5663
601.7822
350.8937
517.8204
604.7761
433.3592
0
20
40
60
80
100
400
600
800
1000
1200

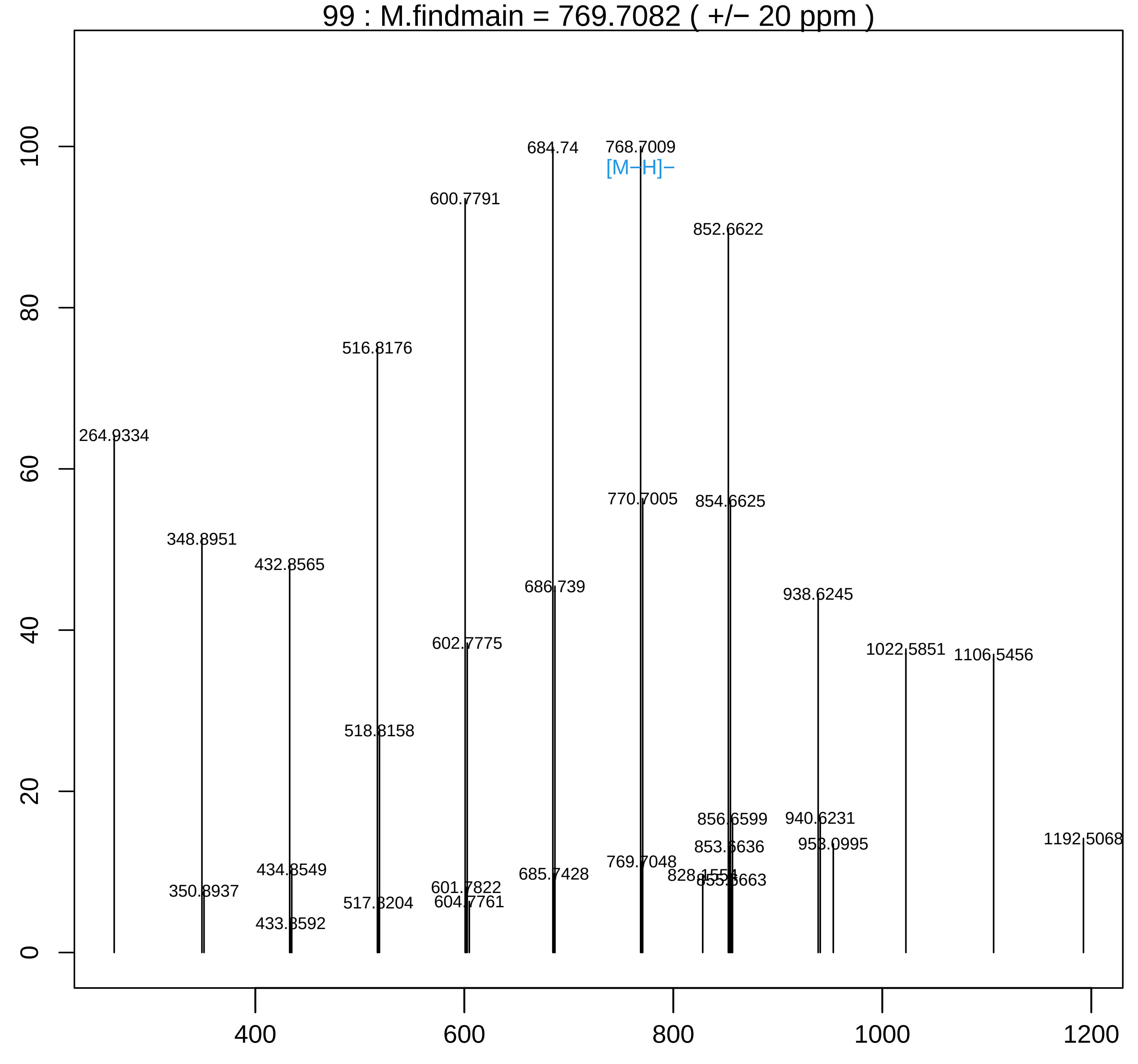
99 : M.findmain = 769.7082 (+/− 20 ppm)
[M−H]−
684.74
768.7009
600.7791
852.6622
516.8176
264.9334
770.7005
854.6625
348.8951
432.8565
686.739
938.6245
602.7775
1022.5851
1106.5456
518.8158
856.6599
940.6231
953.0995
853.5636
1192.5068
769.7048
434.8549
685.7428
828.1554
855.5663
601.7822
350.8937
517.8204
604.7761
433.3592
0
20
40
60
80
100
400
600
800
1000
1200

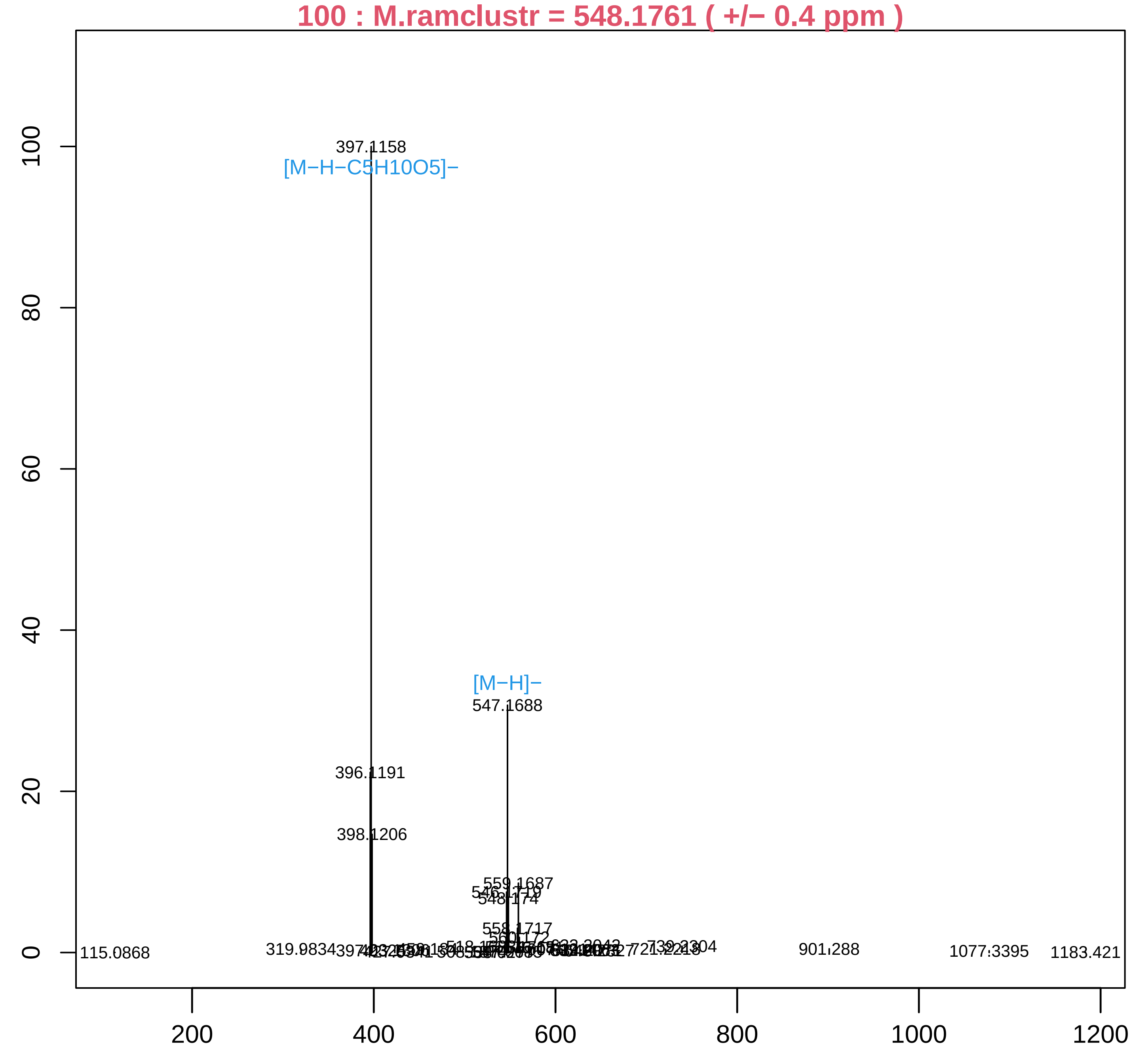

100 : M.ramclustr = 548.1761 (+/− 0.4 ppm)
397.1158
[M−H−C5H10O5]−
[M−H]−
547.1688
396.1191
398.1206
559.1687
548.174
558.1717
115.0868
319.9834
901.288
1077.3395
1183.421
0
20
40
60
80
100
200
400
600
800
1000
1200

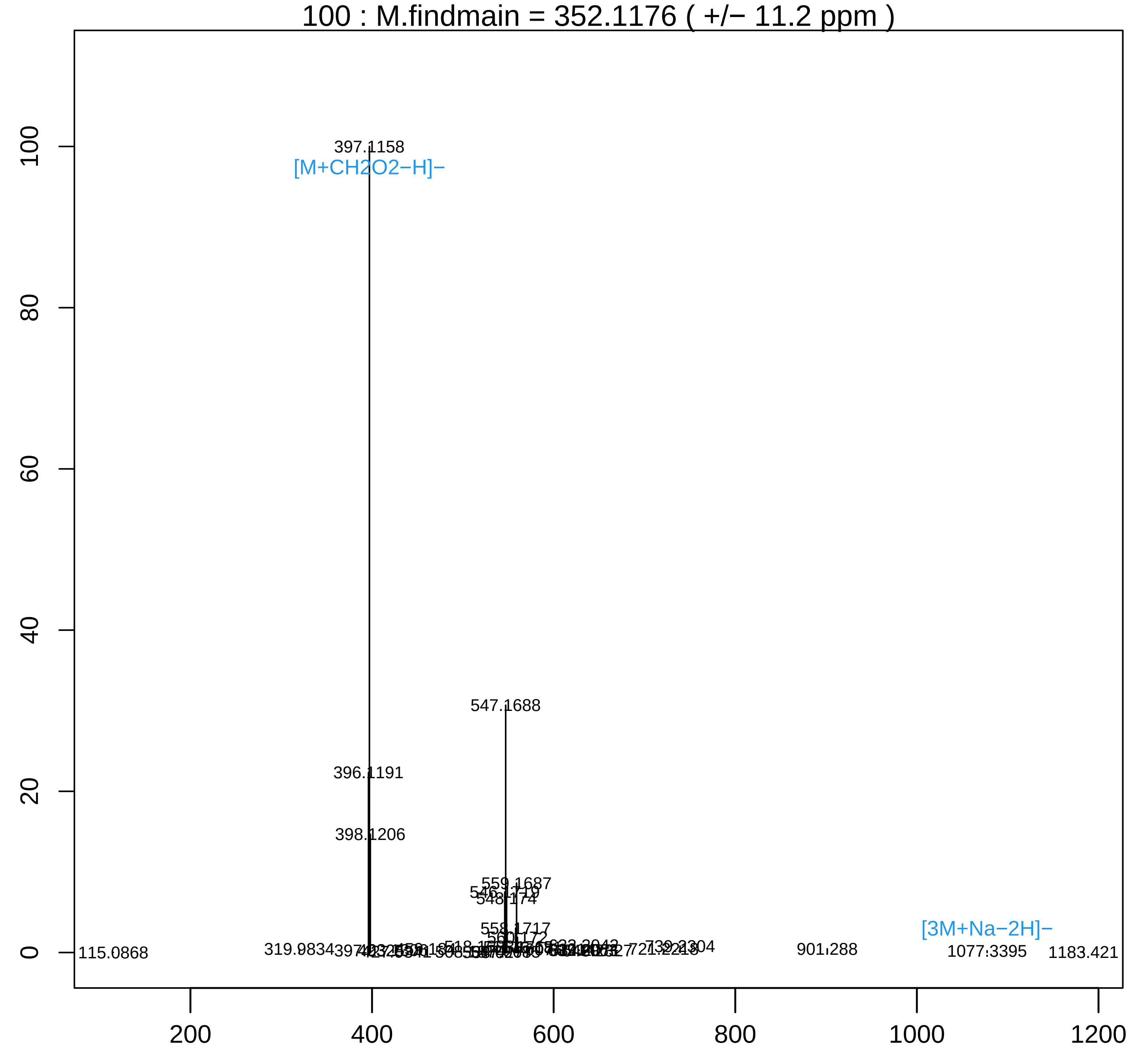

100 : M.findmain = 352.1176 (+/− 11.2 ppm)
397.1158
[M+CH2O2−H]−
547.1688
396.1191
398.1206
559.1687
548.174
558.1717
[3M+Na−2H]−
115.0868
319.9834
901.288
1077.3395
1183.421
0
20
40
60
80
100
200
400
600
800
1000
1200

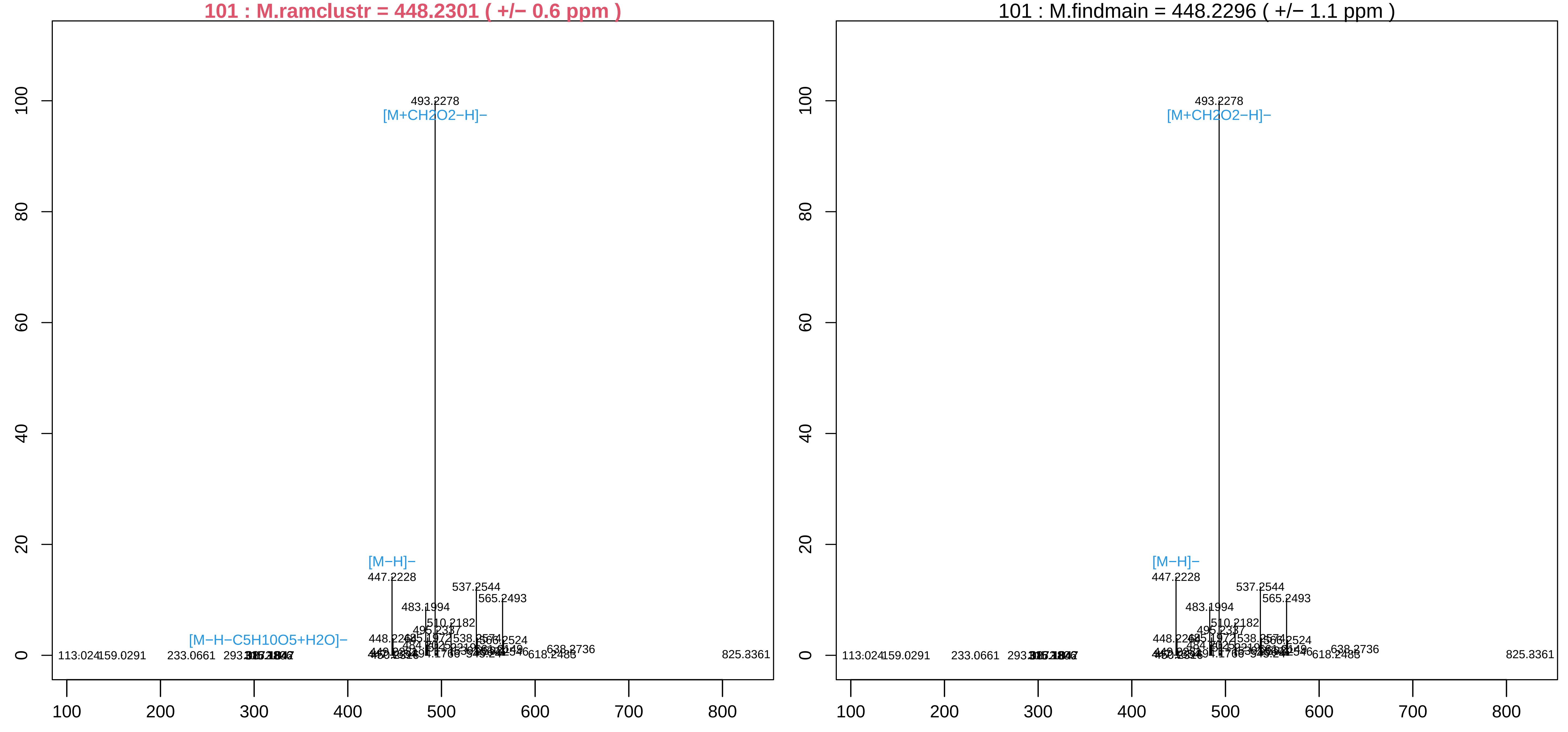

101 : M.ramclustr = 448.2301 (+/− 0.6 ppm)
101 : M.findmain = 448.2296 (+/− 1.1 ppm)
493.2278
[M+CH2O2−H]−
[M−H]−
447.2228
537.2544
565.2493
483.1994
510.2182
[M−H−C5H10O5+H2O]−
448.2248
638.2736
618.2488
825.3361
113.024
159.0291
233.0661
0
20
40
60
80
100
100
200
300
400
500
600
700
800

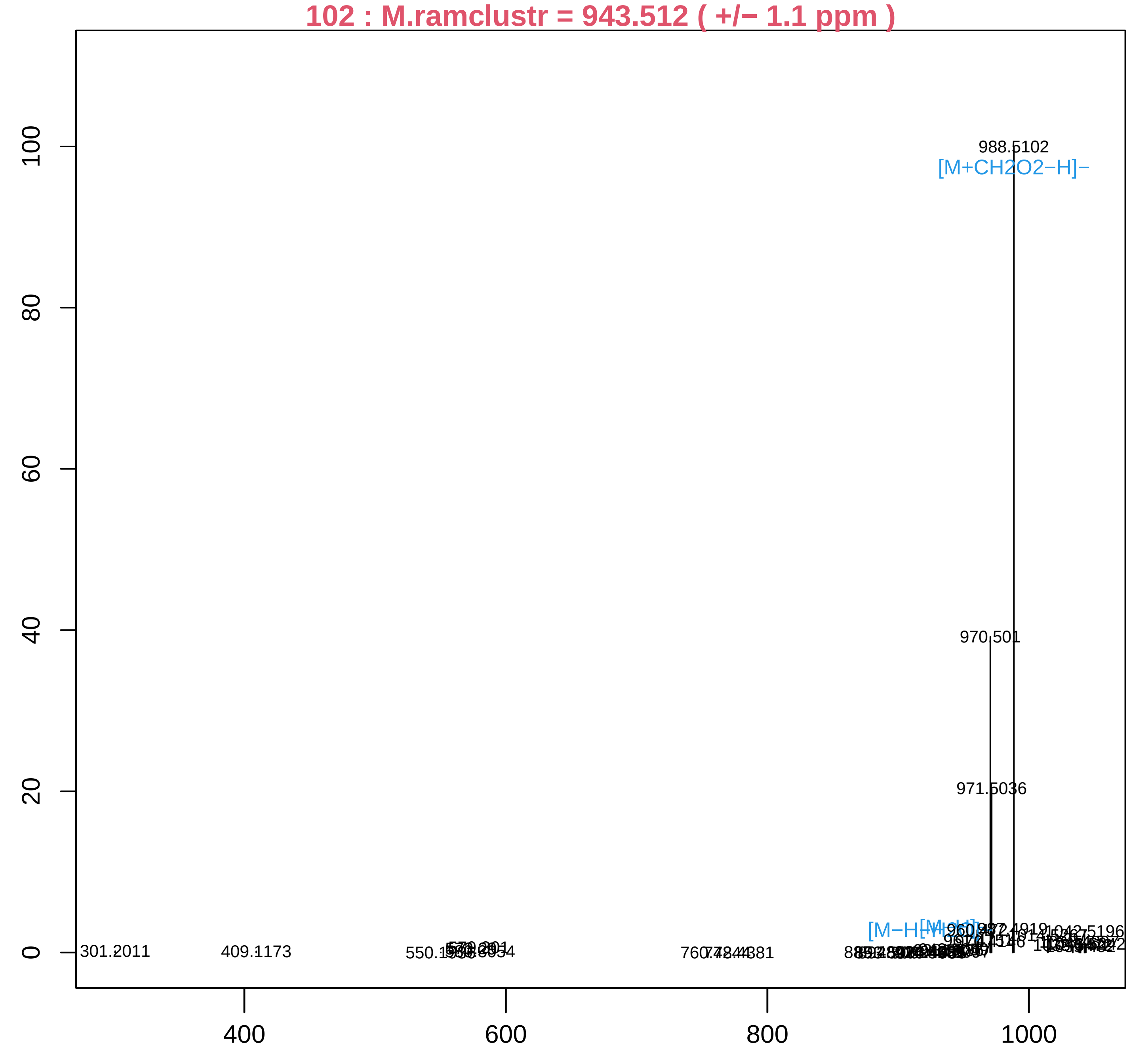

102 : M.ramclustr = 943.512 (+/− 1.1 ppm)
988.5102
[M+CH2O2−H]−
970.501
971.6036
301.2011
409.1173
760.7244
1042.5196
0
20
40
60
80
100
400
600
800
1000

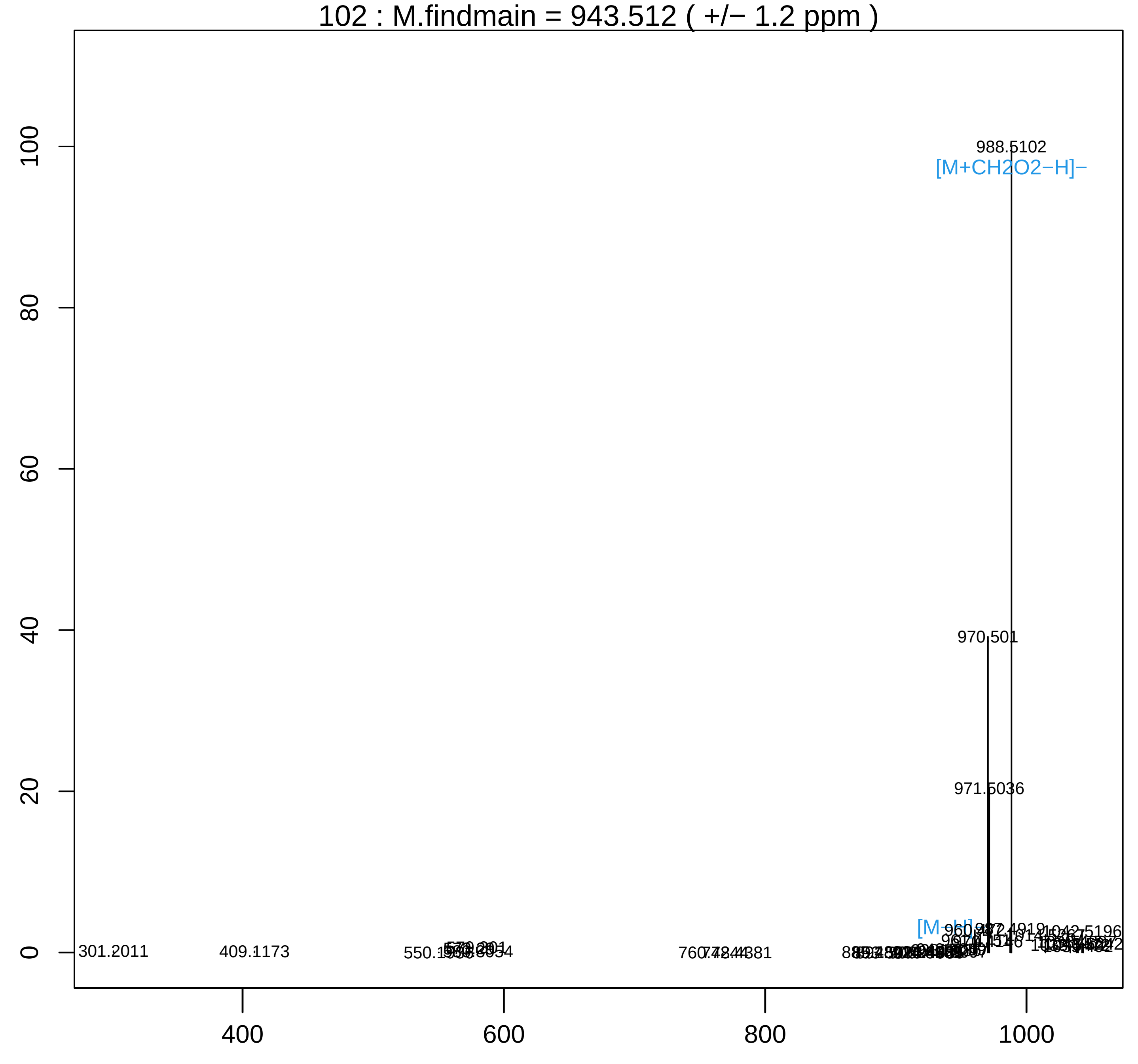

102 : M.findmain = 943.512 (+/− 1.2 ppm)
988.5102
[M+CH2O2−H]−
970.501
971.6036
301.2011
409.1173
1042.5196
0
20
40
60
80
100
400
600
800
1000

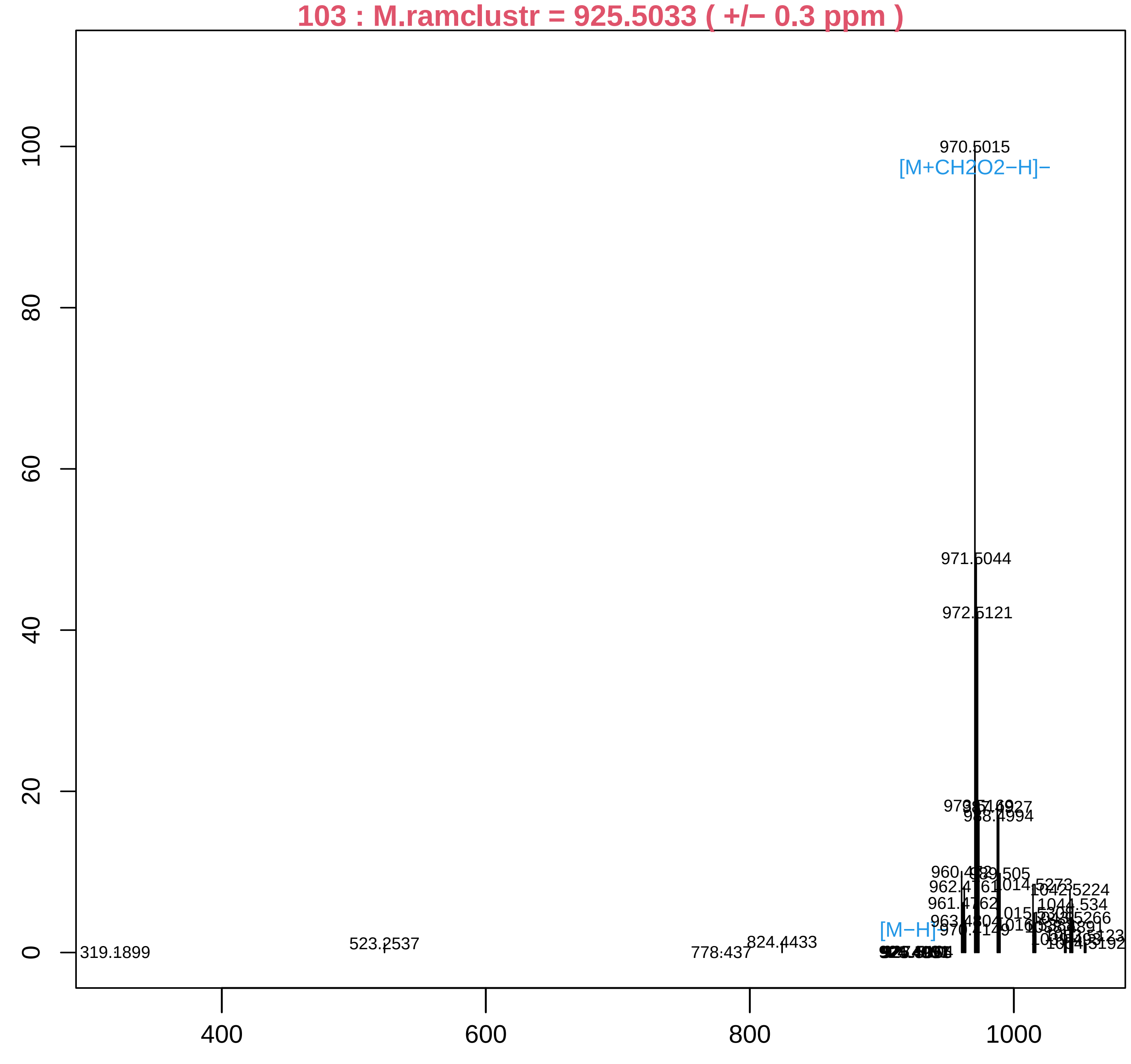

103 : M.ramclustr = 925.5033 (+/− 0.3 ppm)
970.5015
[M+CH2O2−H]−
971.5044
972.5121
[M−H]−
319.1899
523.2537
778.437
824.4433
0
20
40
60
80
100
400
600
800
1000

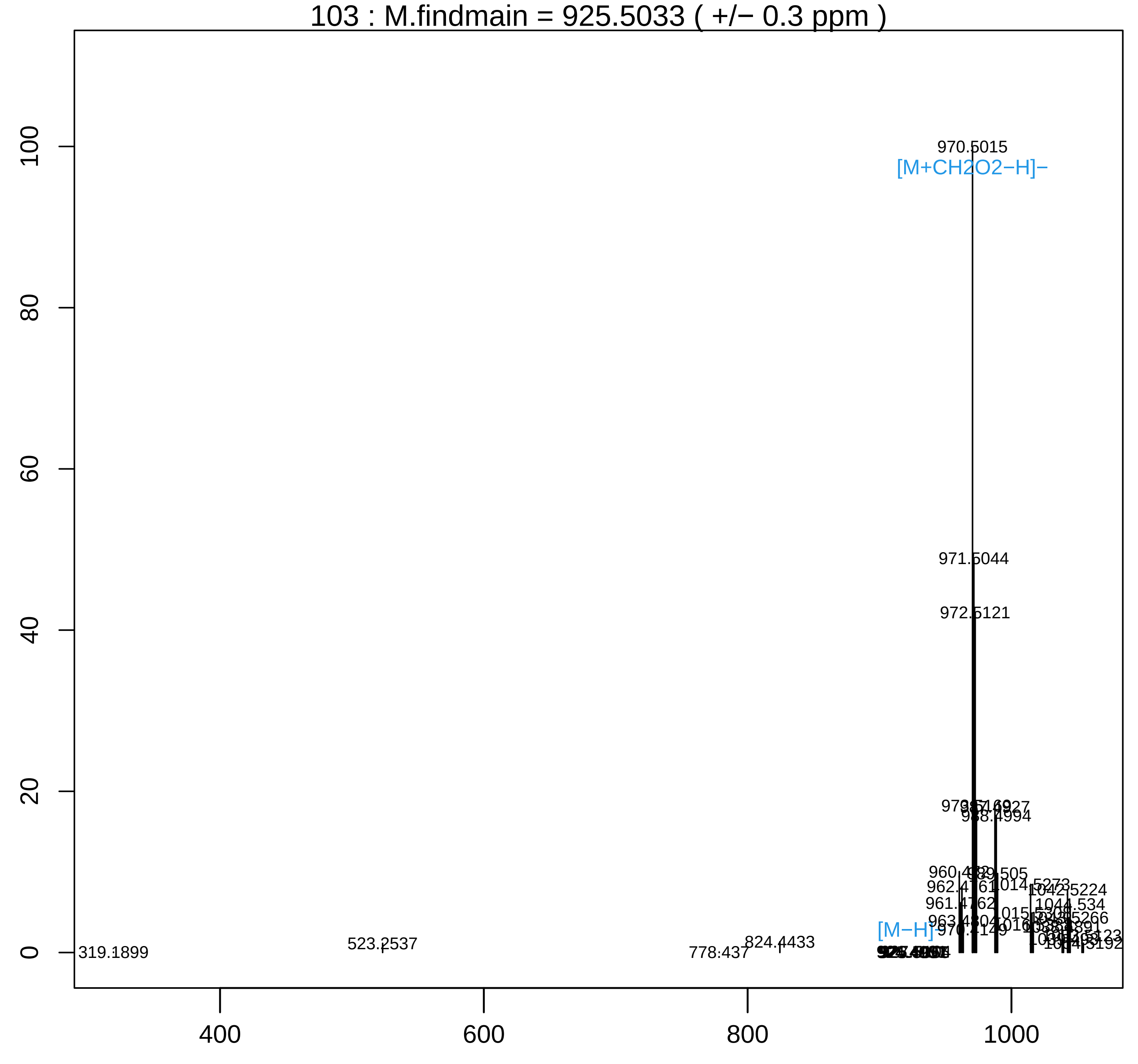

103 : M.findmain = 925.5033 (+/− 0.3 ppm)
970.5015
[M+CH2O2−H]−
971.5044
972.5121
[M−H]−
319.1899
523.2537
778.437
824.4433
0
20
40
60
80
100
400
600
800
1000

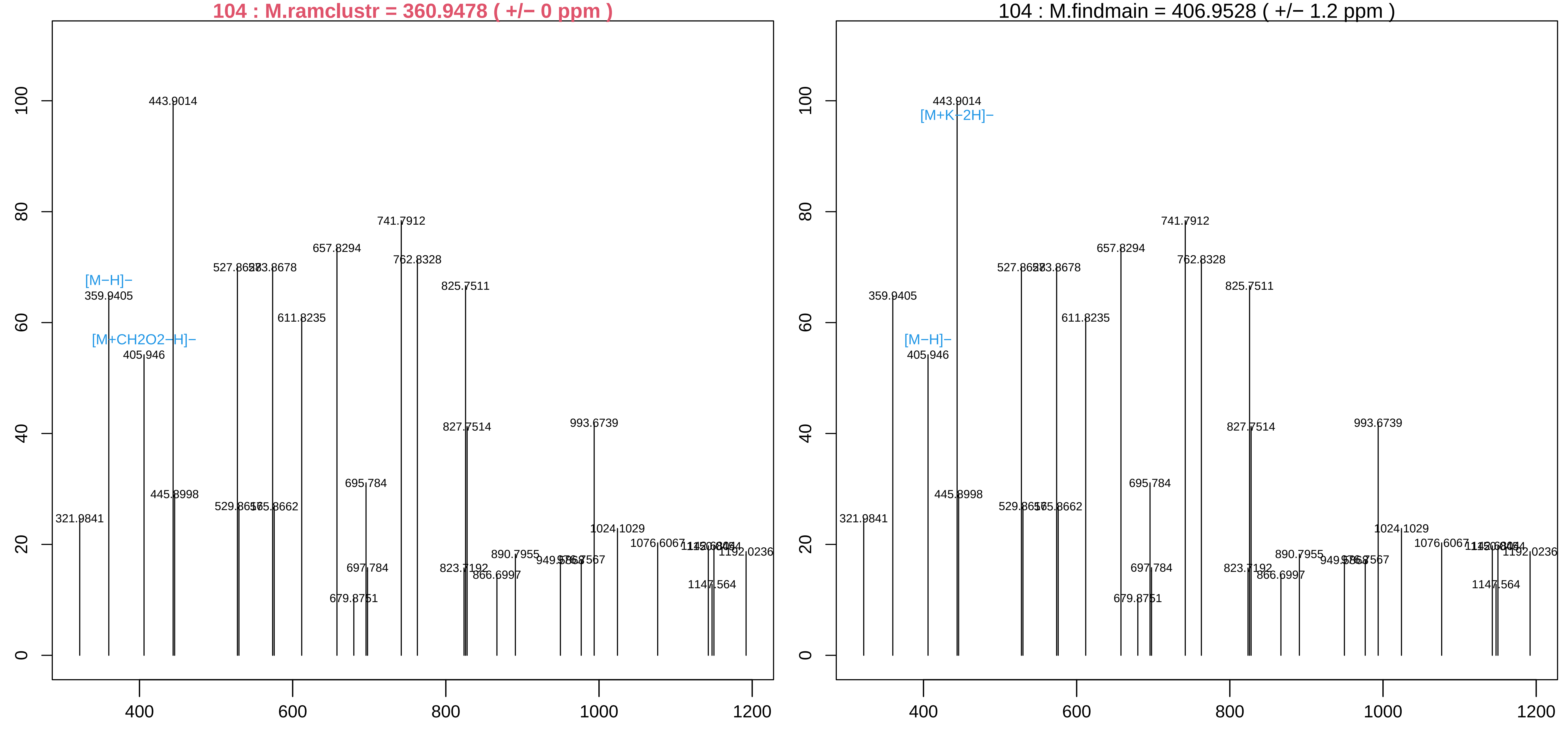
104 : M.ramclustr = 360.9478 (+/− 0 ppm)
104 : M.findmain = 406.9528 (+/− 1.2 ppm)
[M−H]−
[M+CH2O2−H]−
[M+K−2H]−
321.9841
359.9405
405.946
443.9014
445.8998
527.8658
529.8656
573.8678
575.8662
611.8235
657.8294
679.8751
695.784
697.784
741.7912
762.8328
823.7192
825.7511
827.7514
866.6997
890.7955
949.5868
976.7567
993.6739
1024.1029
1076.6067
1147.564
1192.0236
0
20
40
60
80
100
400
600
800
1000
1200

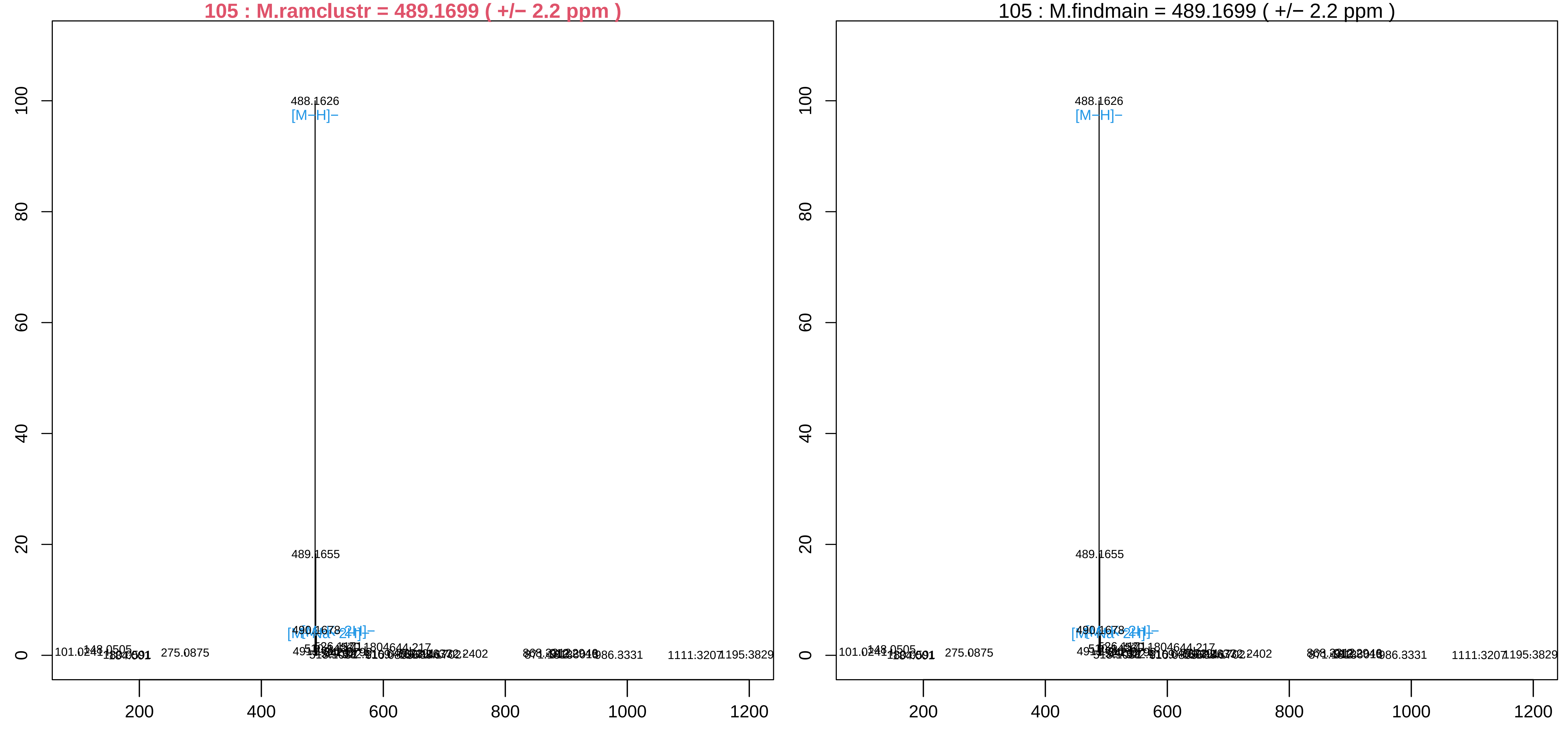
105 : M.ramclustr = 489.1699 (+/− 2.2 ppm)
105 : M.findmain = 489.1699 (+/− 2.2 ppm)
488.1626
[M−H]−
489.1655
490.1678
101.0241
148.0505
275.0875
986.3331
1111.3207
1195.3829
0
20
40
60
80
100
200
400
600
800
1000
1200

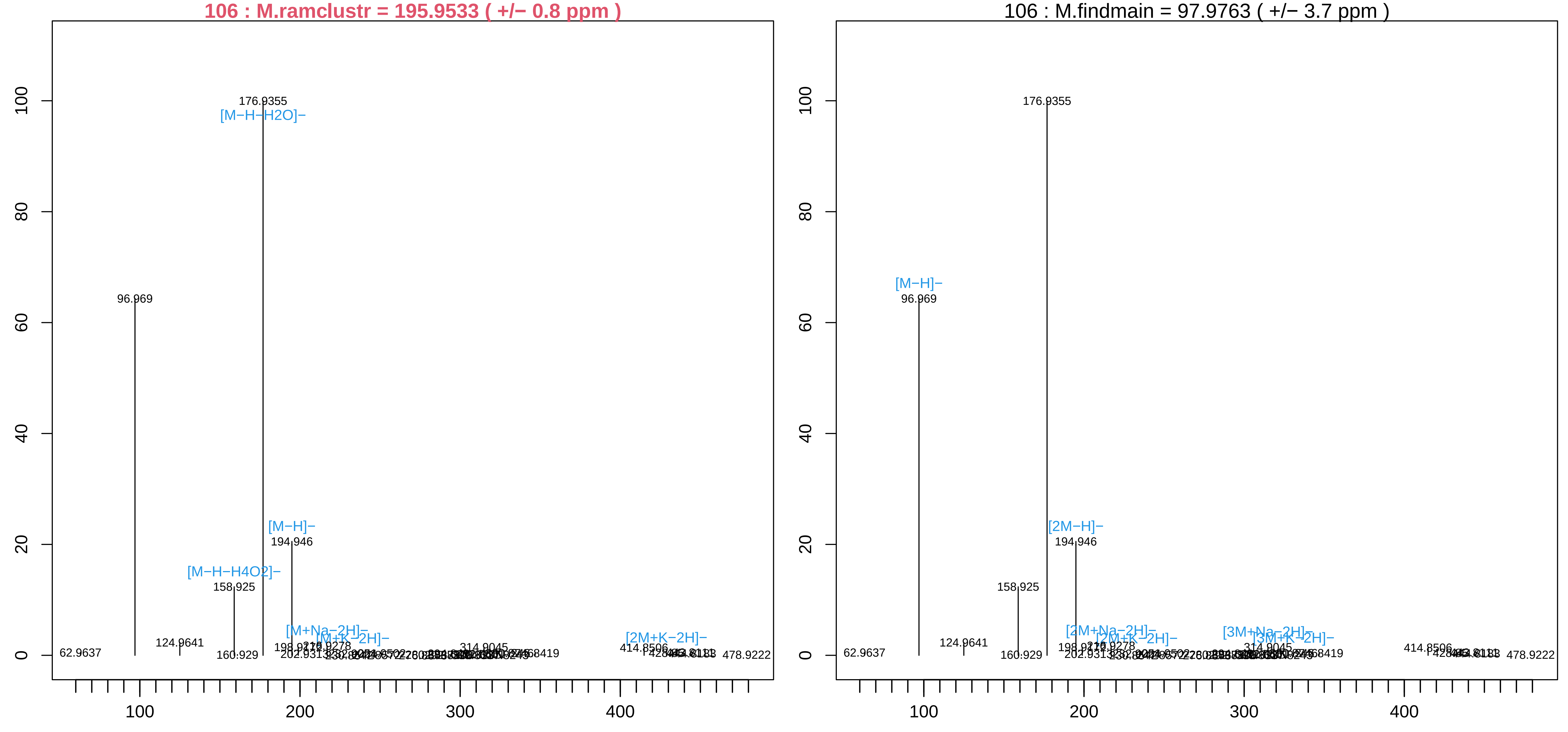
106 : M.ramclustr = 195.9533 (+/− 0.8 ppm)
176.9355
[M−H−H2O]−
96.969
[M−H]−
194.946
[M−H−H4O2]−
158.925
[M+Na−2H]−
[M+K−2H]−
124.9641
62.9637
160.929
314.9045
[2M+K−2H]−
414.8506
478.9222
106 : M.findmain = 97.9763 (+/− 3.7 ppm)
176.9355
[M−H]−
96.969
[2M−H]−
194.946
158.925
[2M+Na−2H]−
[2M+K−2H]−
[3M+Na−2H]−
[3M+K−2H]−
124.9641
62.9637
160.929
314.9045
414.8506
478.9222
0
20
40
60
80
100
200
300
400

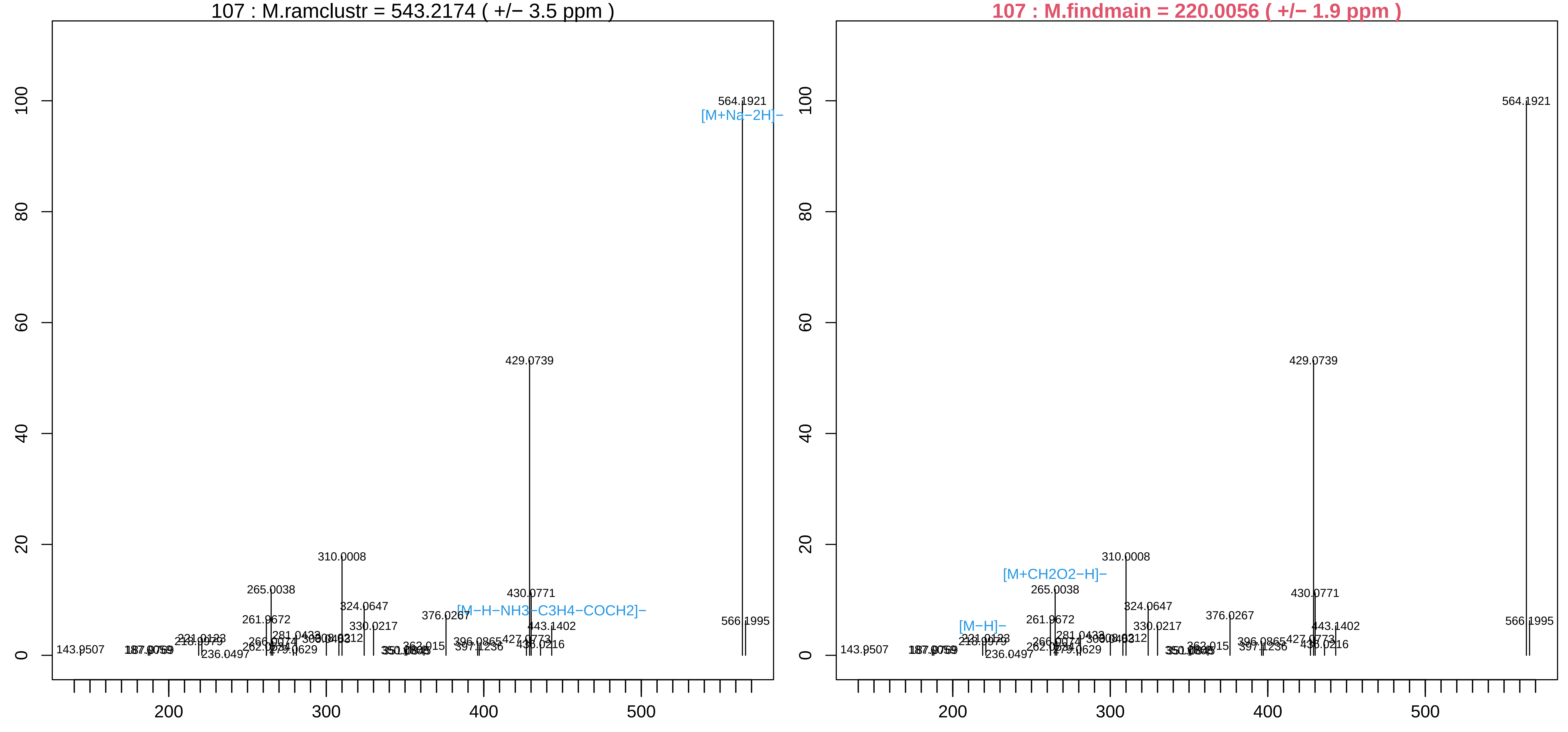
107 : M.ramclustr = 543.2174 (+/− 3.5 ppm)
564.1921
[M+Na−2H]−
429.0739
310.0008
265.0038
430.0771
324.0647
[M−H−NH3−C3H4−COCH2]−
376.0267
261.9672
330.0217
443.1402
566.1995
143.9507
236.0497
397.1236
107 : M.findmain = 220.0056 (+/− 1.9 ppm)
[M+CH2O2−H]−
[M−H]−

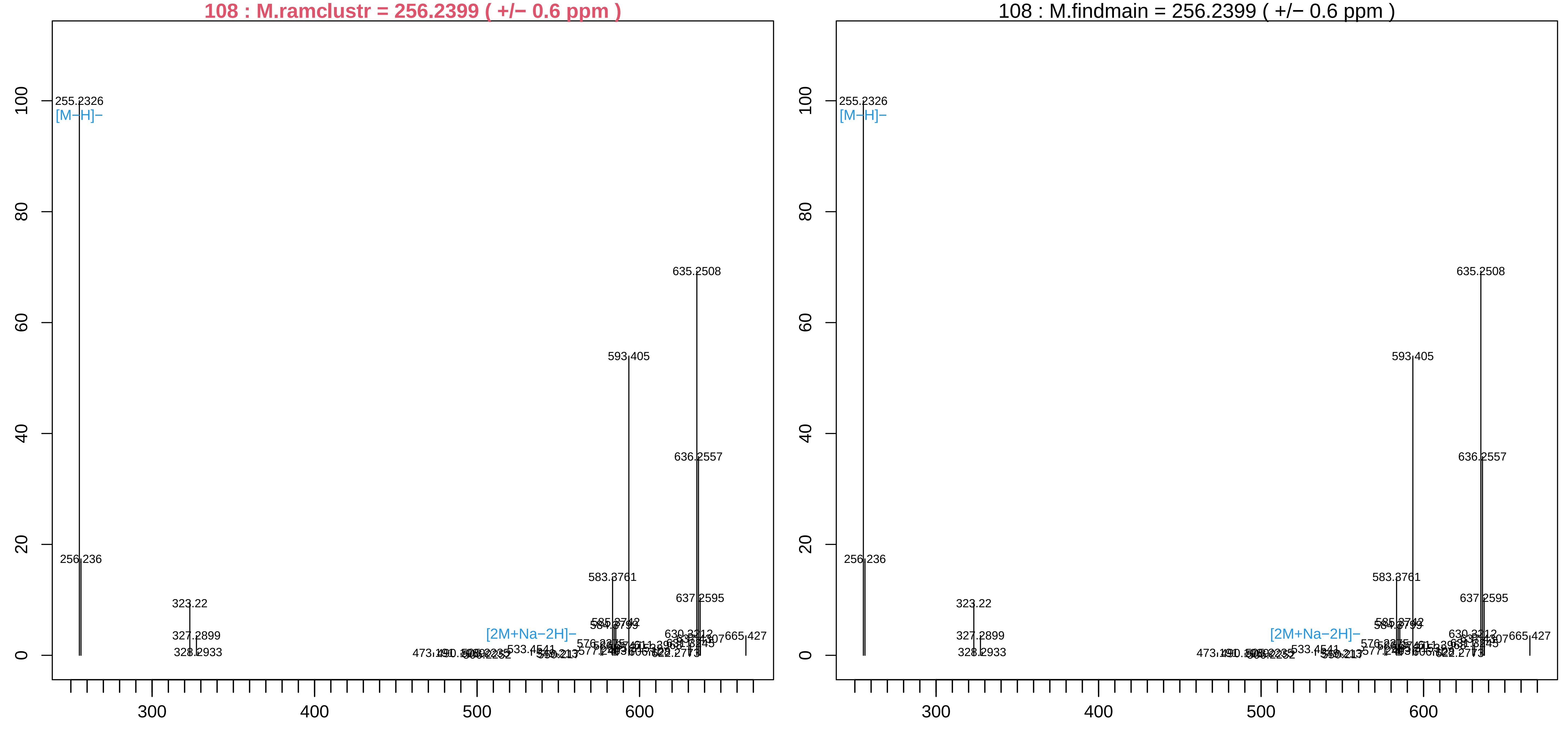

108 : M.ramclustr = 256.2399 (+/− 0.6 ppm)
108 : M.findmain = 256.2399 (+/− 0.6 ppm)
255.2326
[M−H]−
256.236
323.22
327.2899
328.2933
533.4541
583.3761
593.405
635.2508
636.2557
637.2595
665.427
[2M+Na−2H]−
0
20
40
60
80
100
300
400
500
600

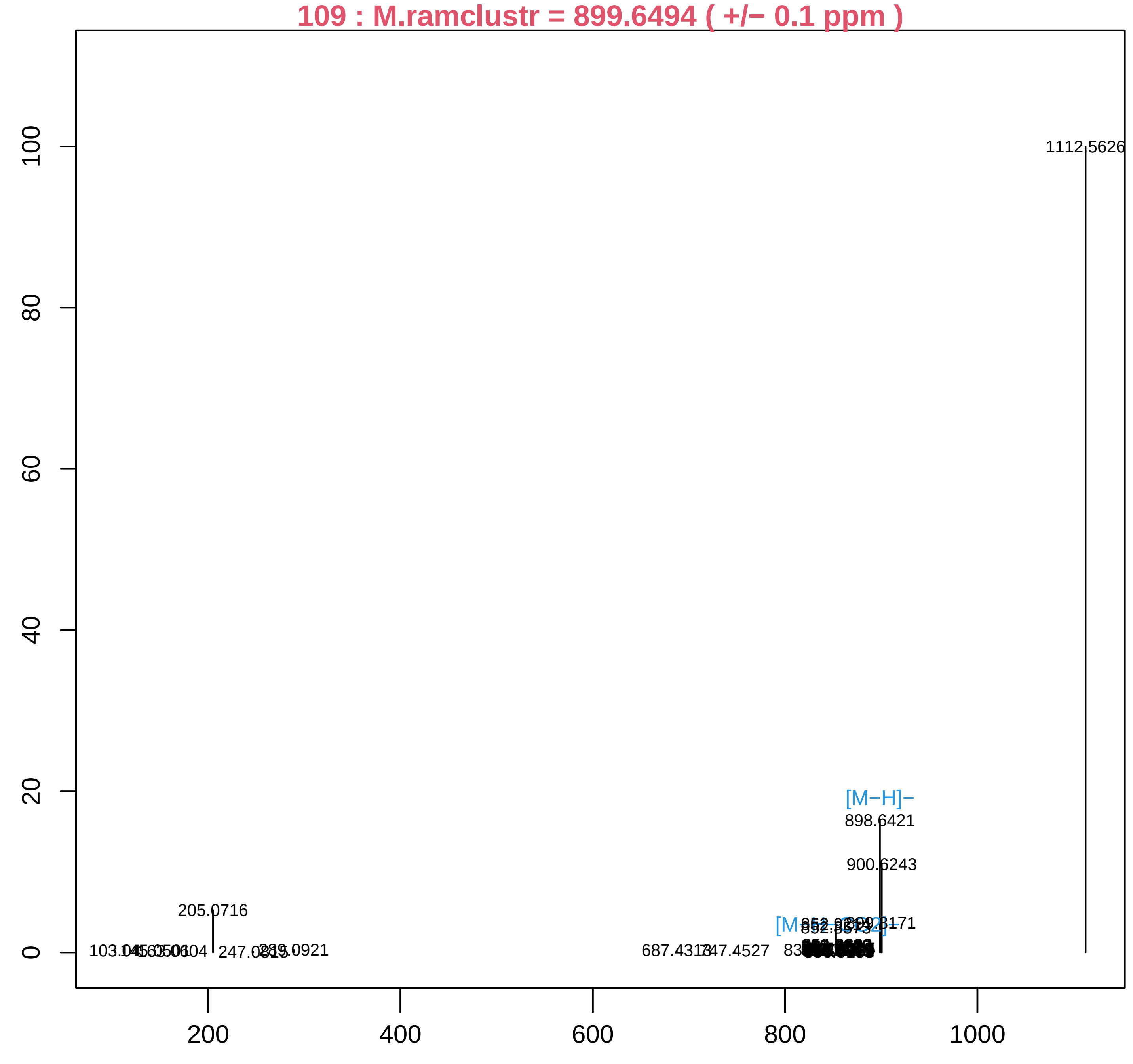
109 : M.ramclustr = 899.6494 (+/− 0.1 ppm)
1112.5626
[M−H]−
898.6421
900.6243
205.0716
687.4313
747.4527
0
20
40
60
80
100
200
400
600
800
1000

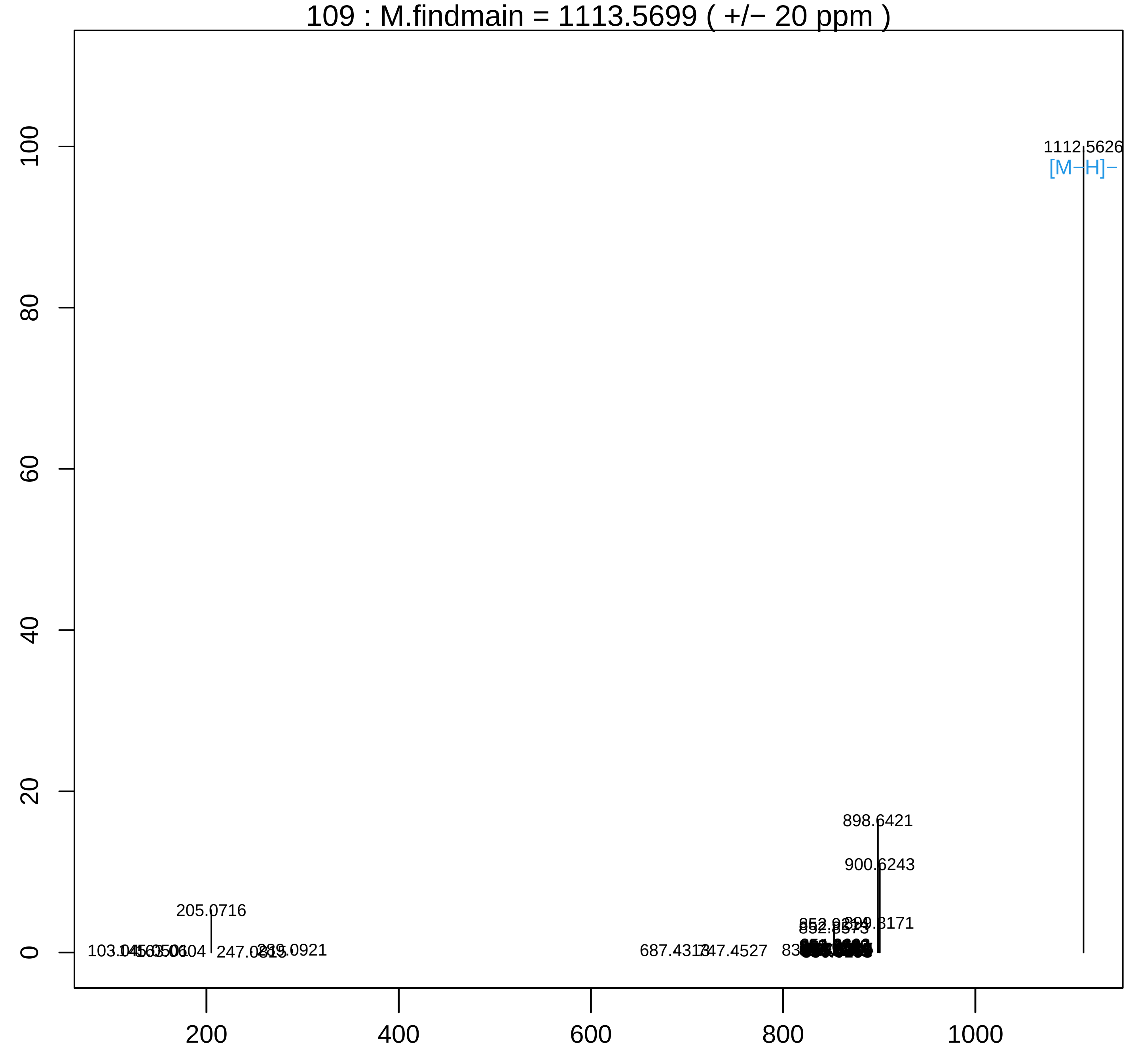
109 : M.findmain = 1113.5699 (+/− 20 ppm)
1112.5626
[M−H]−
898.6421
900.6243
205.0716
687.4313
747.4527
0
20
40
60
80
100
200
400
600
800
1000

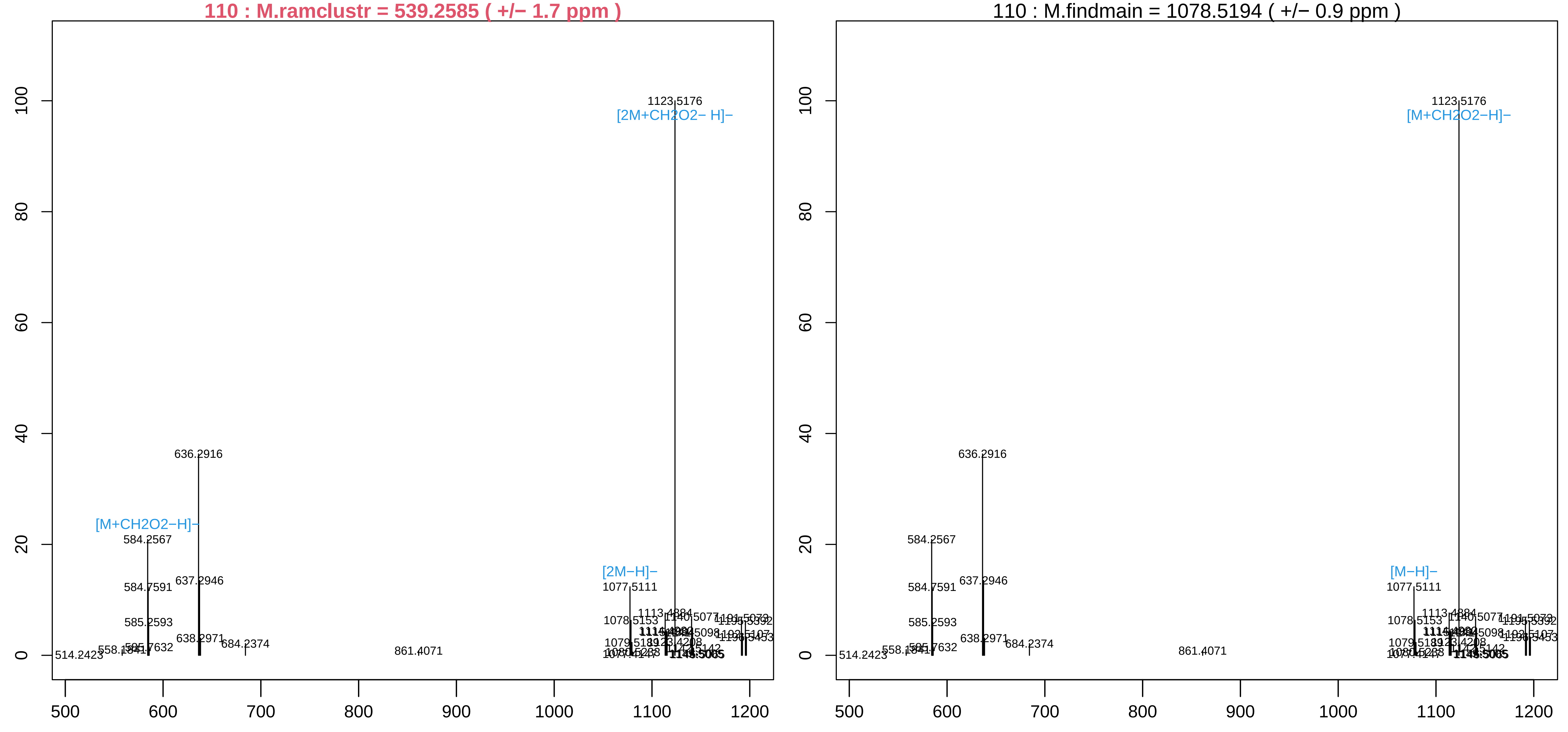

110 : M.ramclustr = 539.2585 (+/− 1.7 ppm)
1123.5176
[2M+CH2O2− H]−
636.2916
[M+CH2O2−H]−
584.2567
[2M−H]−
1077.5111
584.7591
637.2946
585.2593
638.2971
684.2374
514.2423
861.4071
110 : M.findmain = 1078.5194 (+/− 0.9 ppm)
[M+CH2O2−H]−
[M−H]−

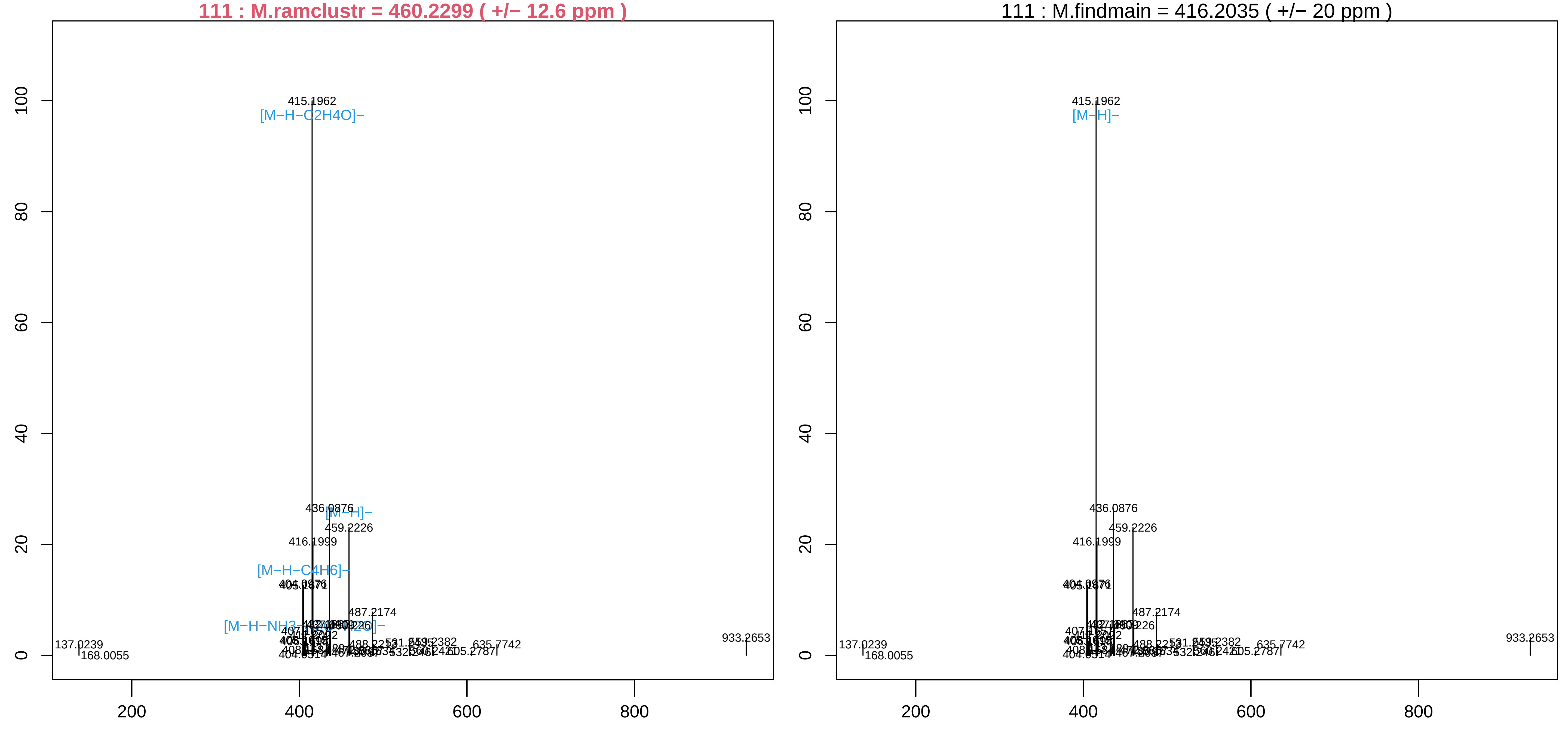
111 : M.ramclustr = 460.2299 (+/− 12.6 ppm)
415.1962
[M−H−C2H4O]−
436.0876
[M−H]−
459.2226
416.1999
[M−H−C4H6]−
487.2174
137.0239
168.0055
933.2653
635.7742
111 : M.findmain = 416.2035 (+/− 20 ppm)
415.1962
[M−H]−
436.0876
459.2226
416.1999
487.2174
137.0239
168.0055
933.2653
635.7742
0
20
40
60
80
100
200
400
600
800

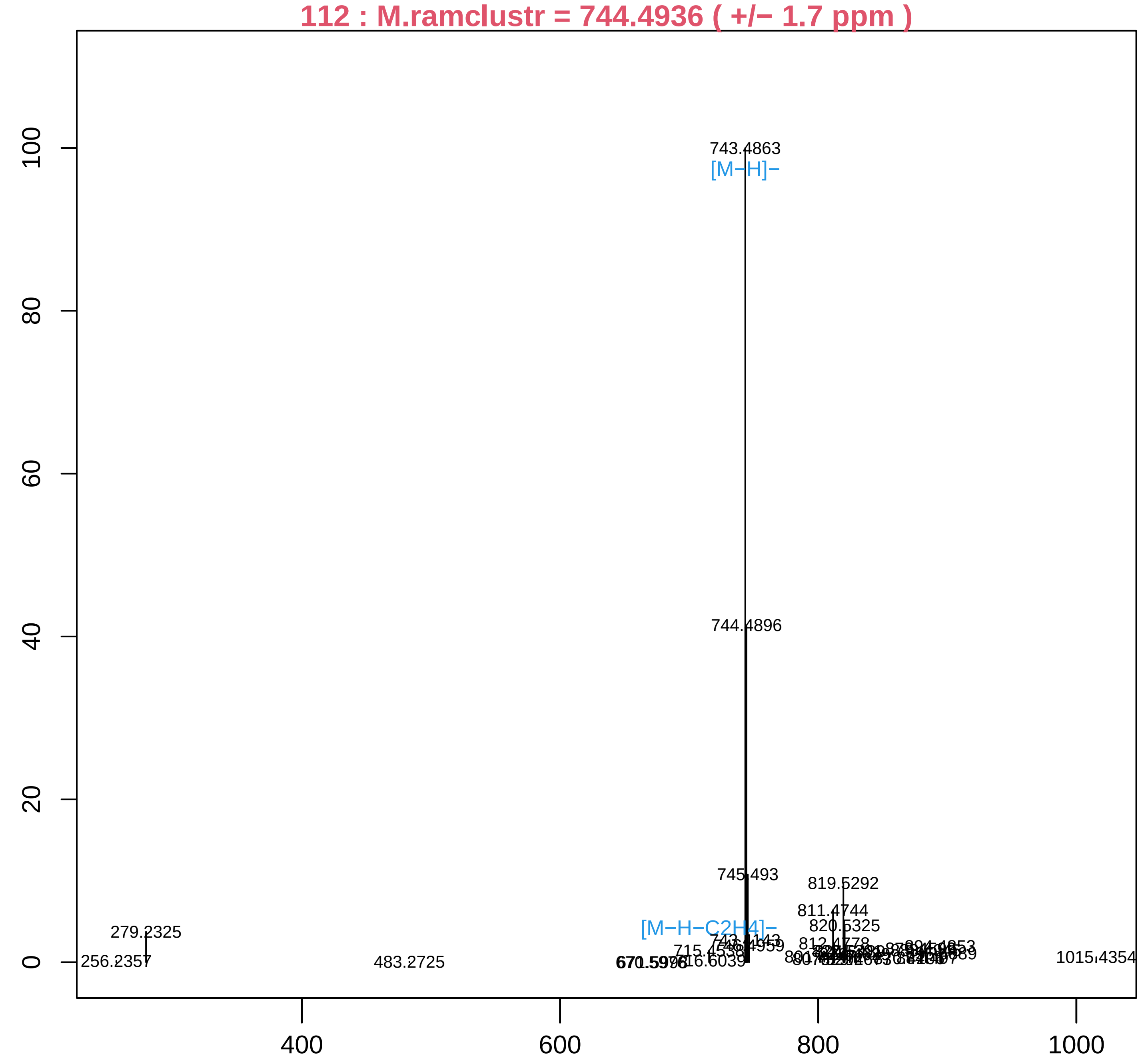
112 : M.ramclustr = 744.4936 (+/− 1.7 ppm)
743.4863
[M−H]−
744.4896
745.493
819.5292
811.4744
820.5325
[M−H−C2H4]−
279.2325
256.2357
483.2725
1015.4354
0
20
40
60
80
100
400
600
800
1000

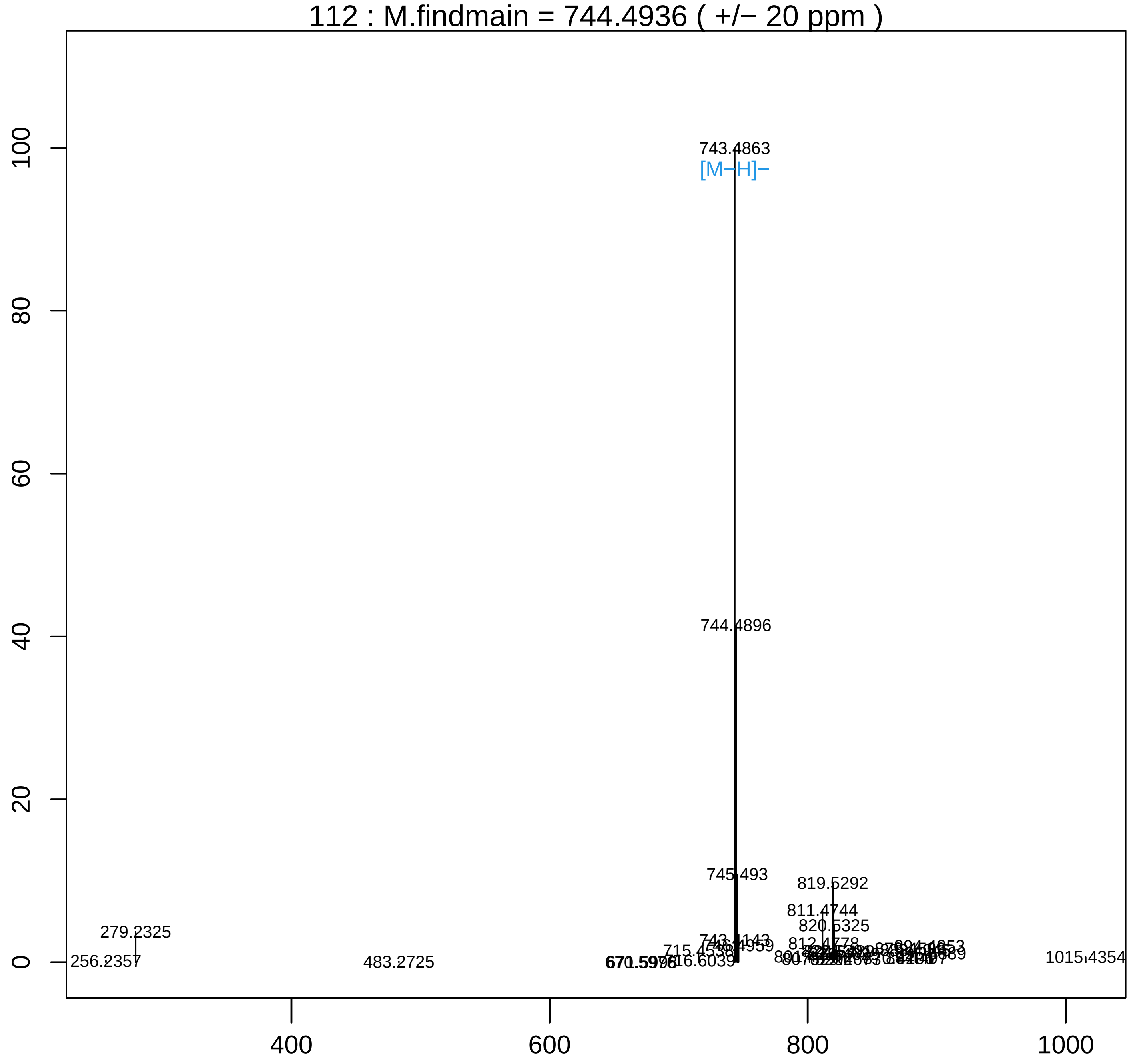
112 : M.findmain = 744.4936 (+/− 20 ppm)
743.4863
[M−H]−
744.4896
745.493
819.5292
811.4744
820.5325
279.2325
256.2357
483.2725
1015.4354
0
20
40
60
80
100
400
600
800
1000

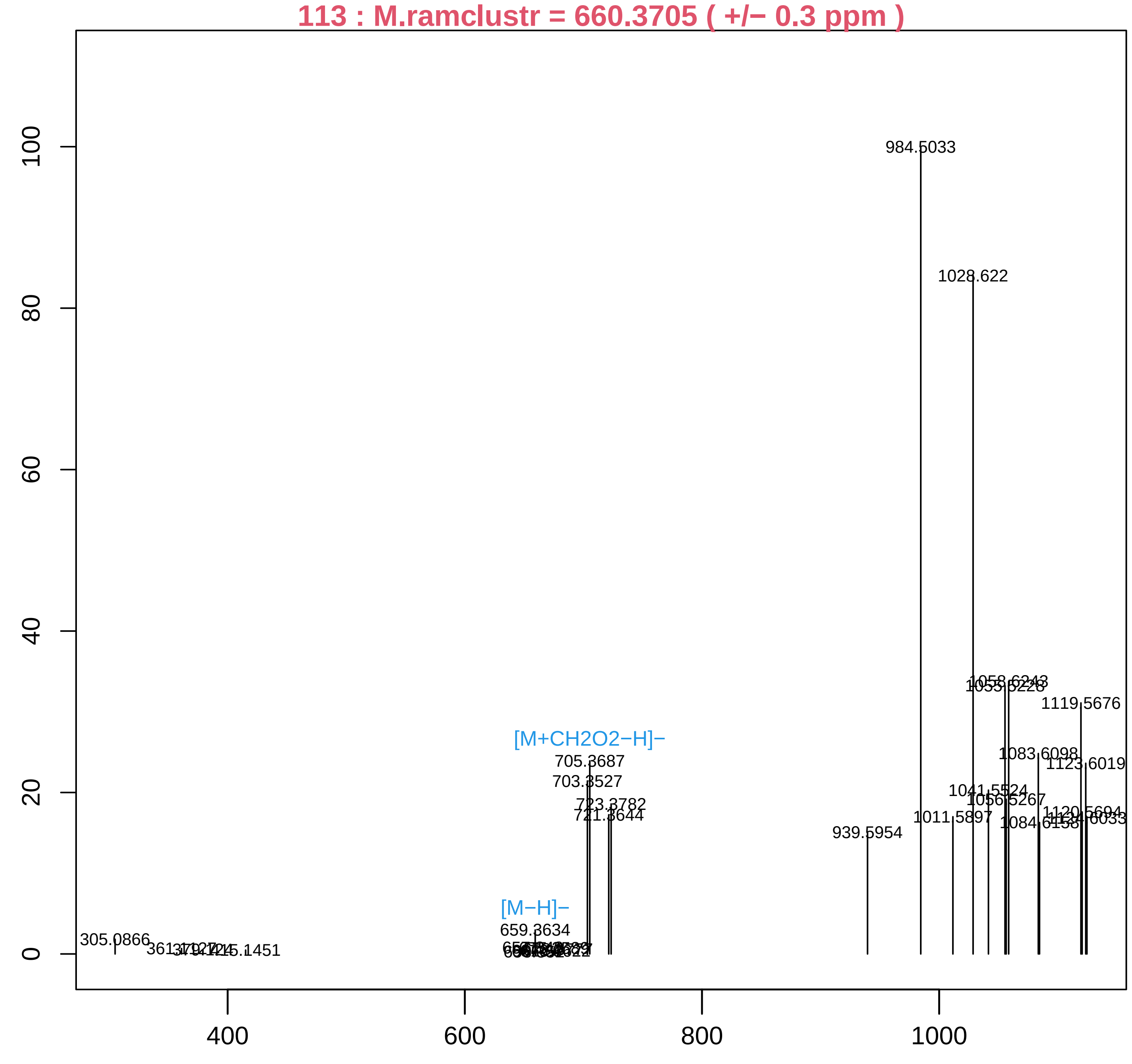
113 : M.ramclustr = 660.3705 (+/− 0.3 ppm)
984.5033
1028.622
1058.6243
1055.5226
1119.5676
[M+CH2O2−H]−
705.3687
1083.6098
1123.6019
703.3527
1041.5524
1056.5267
723.3782
721.3644
1120.5694
1011.5897
1084.6158
939.5954
[M−H]−
659.3634
305.0866
415.1451
0
20
40
60
80
100
400
600
800
1000

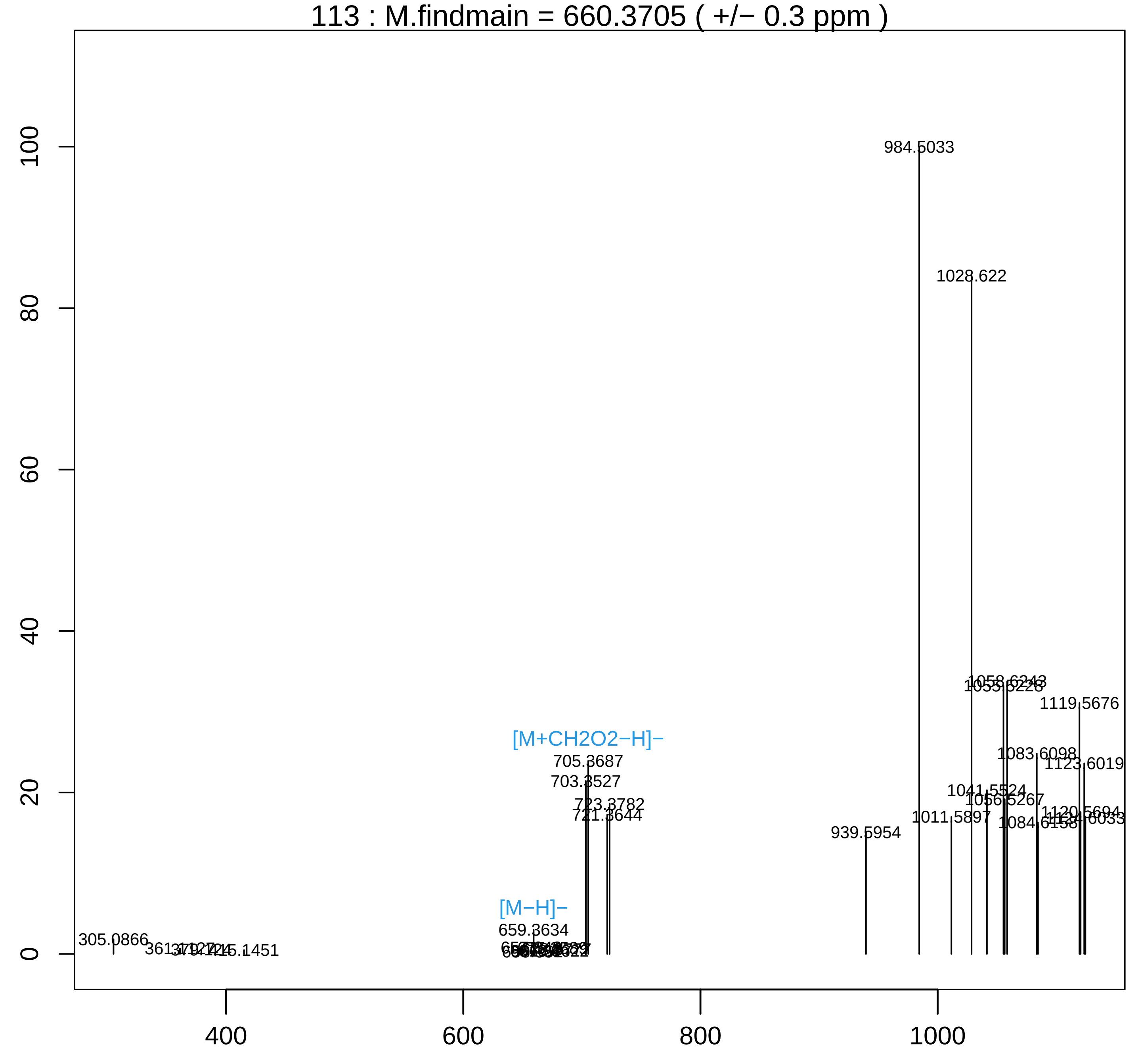
113 : M.findmain = 660.3705 (+/− 0.3 ppm)
984.5033
1028.622
1058.6243
1055.5226
1119.5676
[M+CH2O2−H]−
705.3687
1083.6098
1123.6019
703.3527
1041.5524
1056.5267
723.3782
721.3644
1120.5694
1011.5897
1084.6158
939.5954
[M−H]−
659.3634
305.0866
415.1451
0
20
40
60
80
100
400
600
800
1000

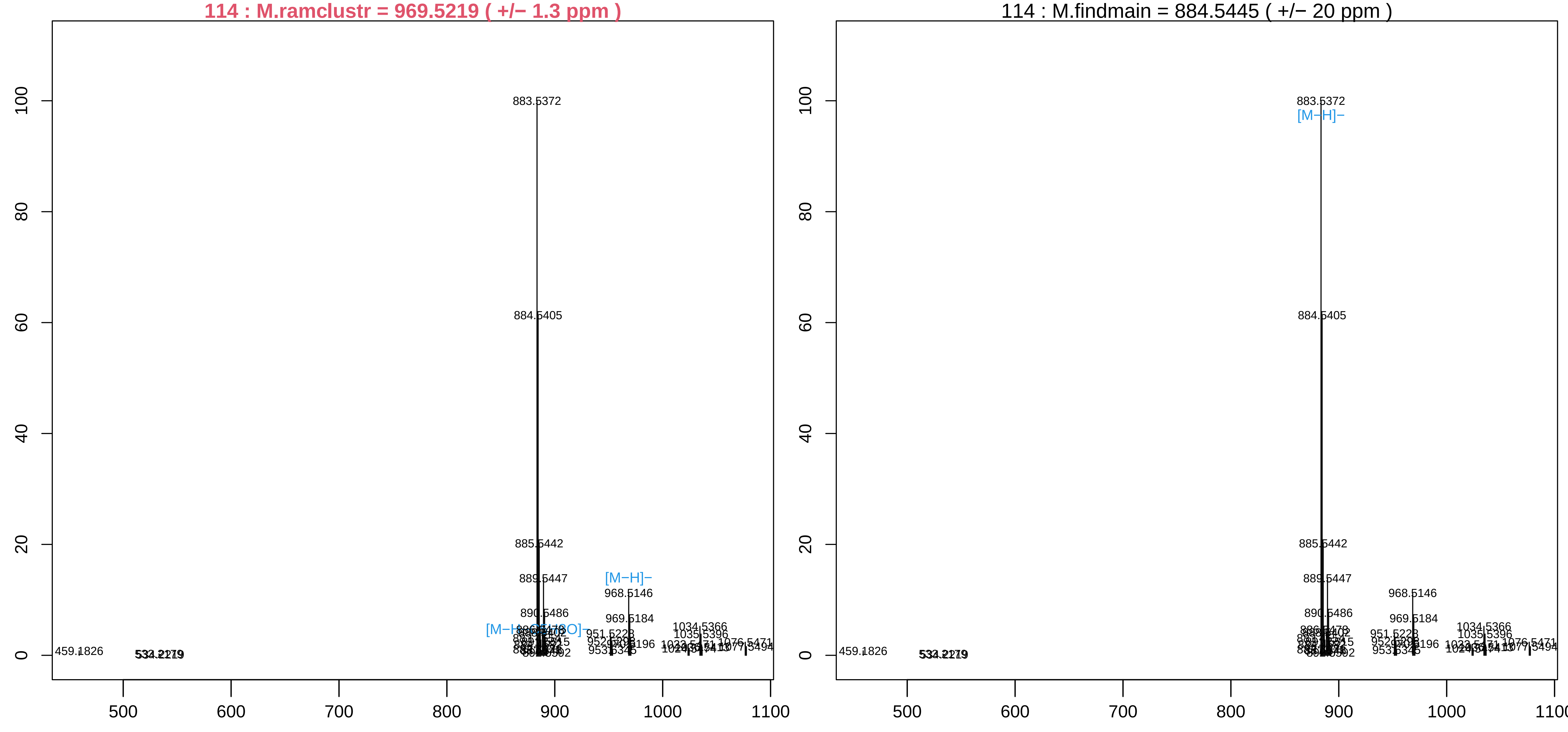
114 : M.ramclustr = 969.5219 (+/− 1.3 ppm)
883.5372
884.5405
885.5442
889.5447
[M−H]−
968.5146
890.5486
969.5184
1034.5366
1035.5396
951.5223
1076.5471
459.1826
533.2179
114 : M.findmain = 884.5445 (+/− 20 ppm)
[M−H]−
0
20
40
60
80
100
500
600
700
800
900
1000
1100

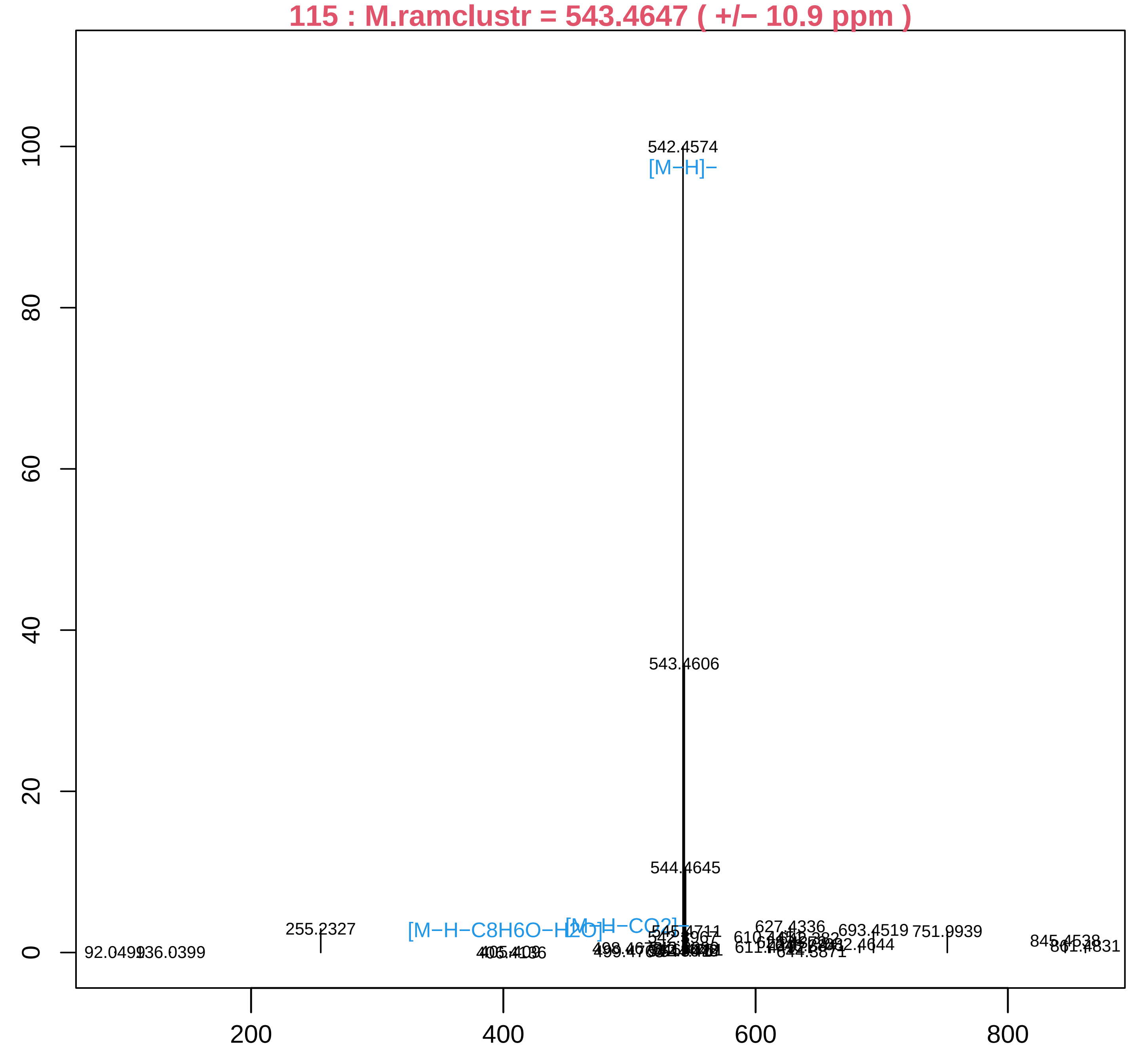
115 : M.ramclustr = 543.4647 (+/− 10.9 ppm)
542.4574
[M−H]−
543.4606
544.4645
255.2327
[M−H−C8H6O−H2O]−
[M−H−CO2]−
627.4336
693.4519
751.9939
845.4538
861.4831
92.0499
136.0399
405.4136
644.3871

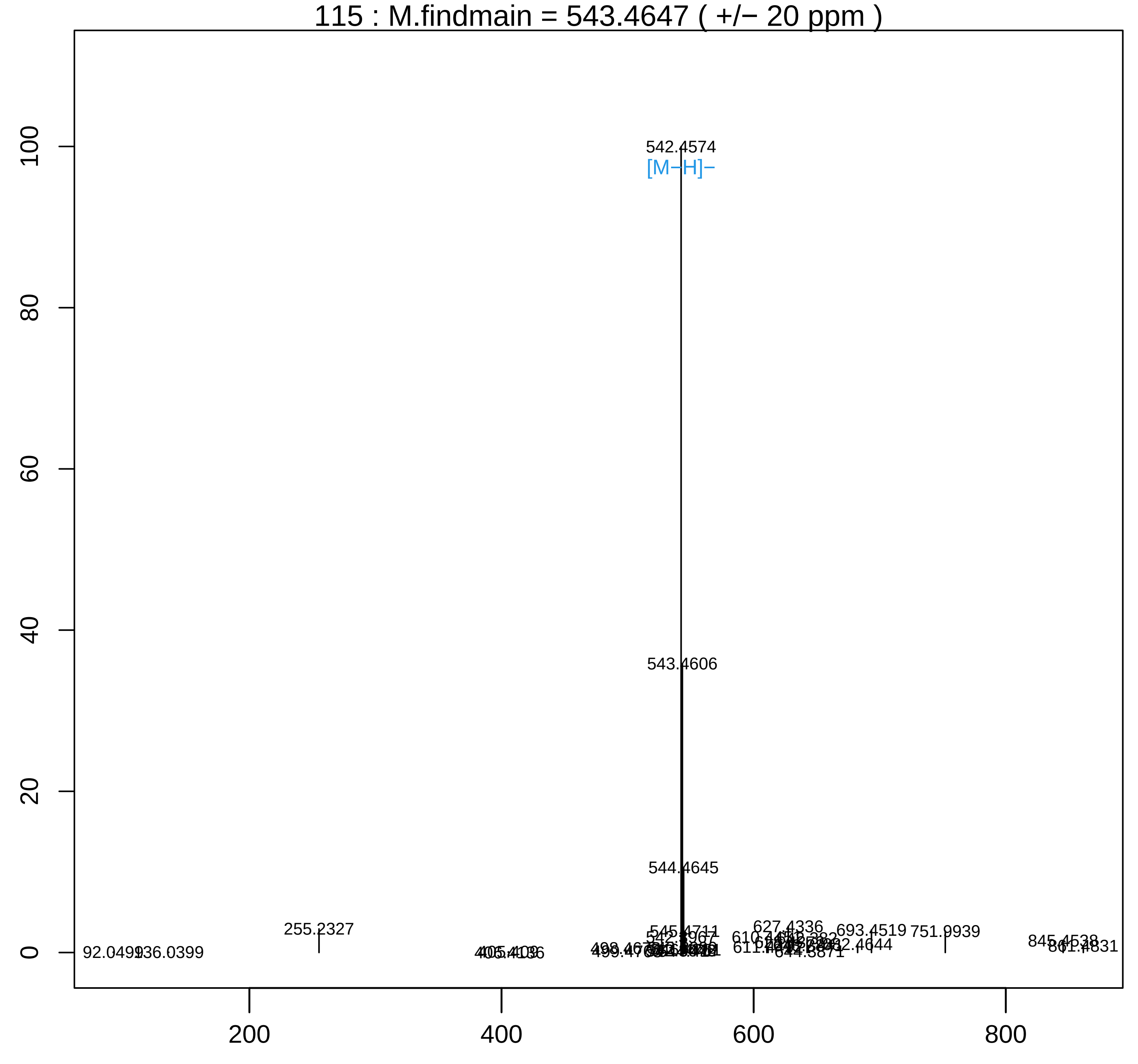
115 : M.findmain = 543.4647 (+/− 20 ppm)
542.4574
[M−H]−
543.4606
544.4645
255.2327
627.4336
693.4519
751.9939
845.4538
861.4831
92.0499
136.0399
405.4136
644.3871

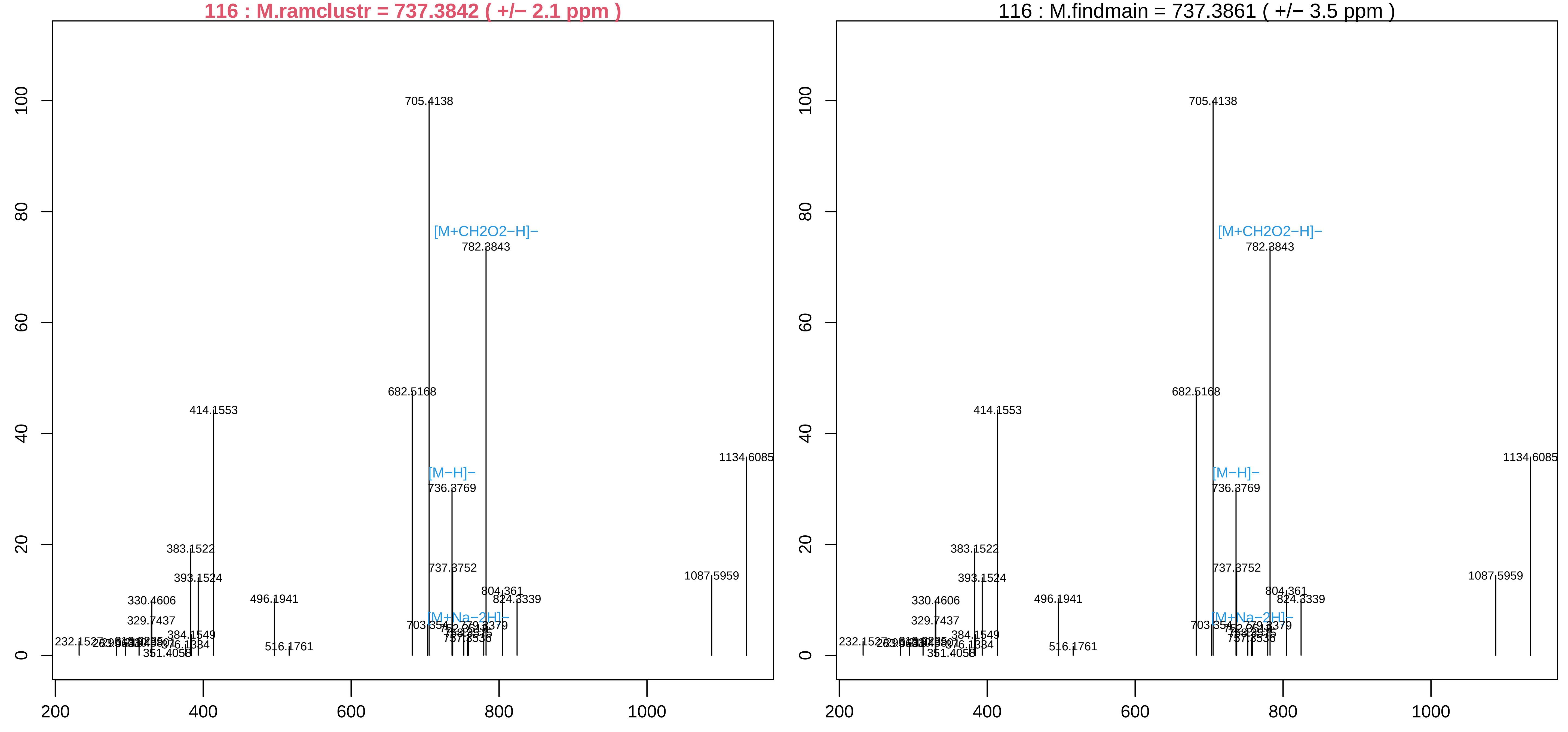
116 : M.ramclustr = 737.3842 (+/− 2.1 ppm)
116 : M.findmain = 737.3861 (+/− 3.5 ppm)
705.4138
[M+CH2O2−H]−
782.3843
682.5168
414.1553
1134.6085
[M−H]−
736.3769
383.1522
737.3752
393.1524
1087.5959
330.4606
496.1941
804.361
824.3339
329.7437
[M+Na−2H]−
384.1549
232.1527
516.1761
351.4058
0
20
40
60
80
100
200
400
600
800
1000

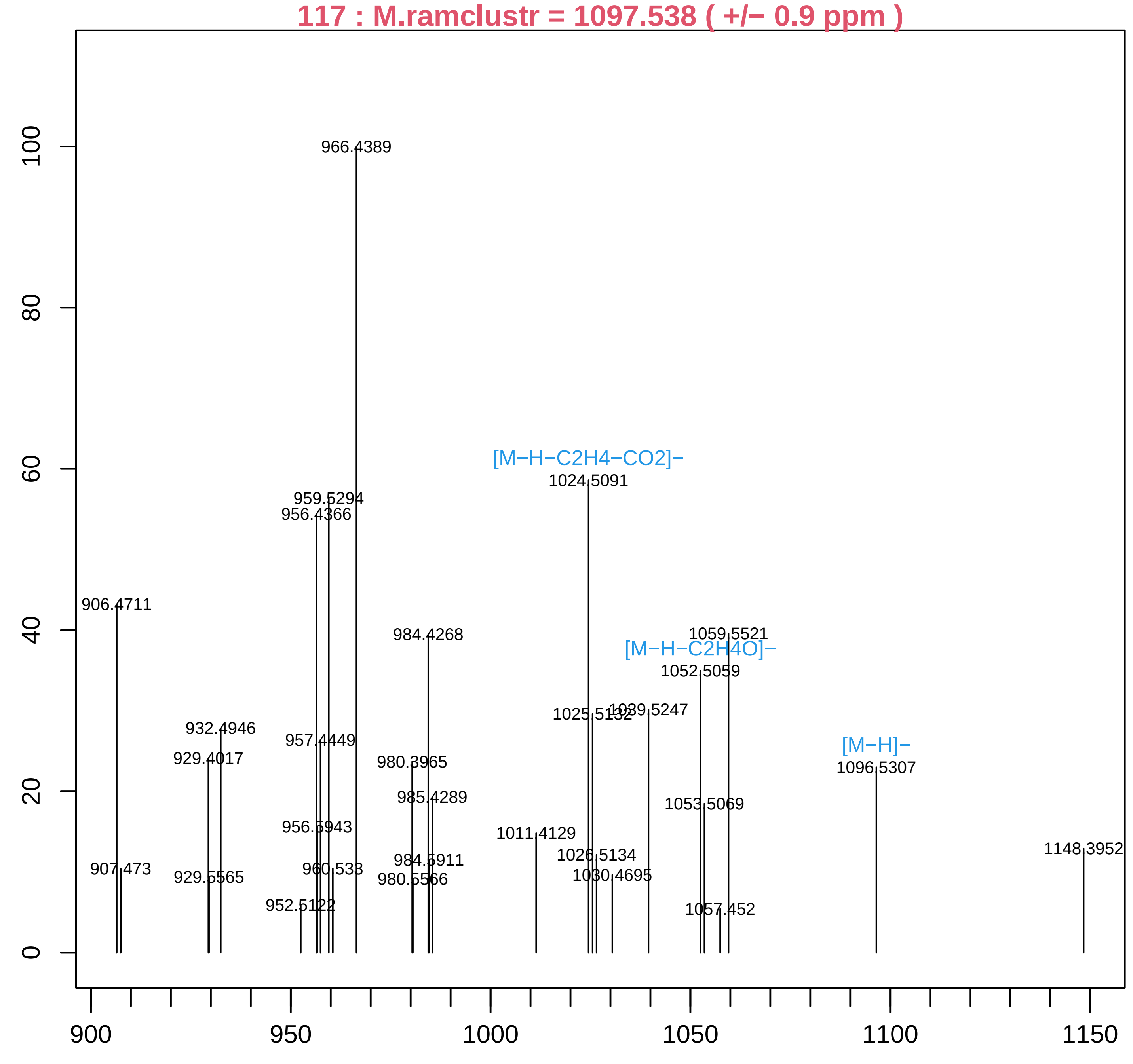

117 : M.ramclustr = 1097.538 (+/− 0.9 ppm)
[M−H−C2H4−CO2]−
[M−H−C2H4O]−
[M−H]−
966.4389
1024.5091
959.5294
956.4366
906.4711
984.4268
1059.5521
1052.5059
1025.5132
1039.5247
932.4946
957.4449
929.4017
980.3965
1096.5307
985.4289
1053.5069
956.5943
1011.4129
1026.5134
907.473
960.533
984.5911
929.5565
980.5566
1030.4695
1148.3952
952.5122
1057.452

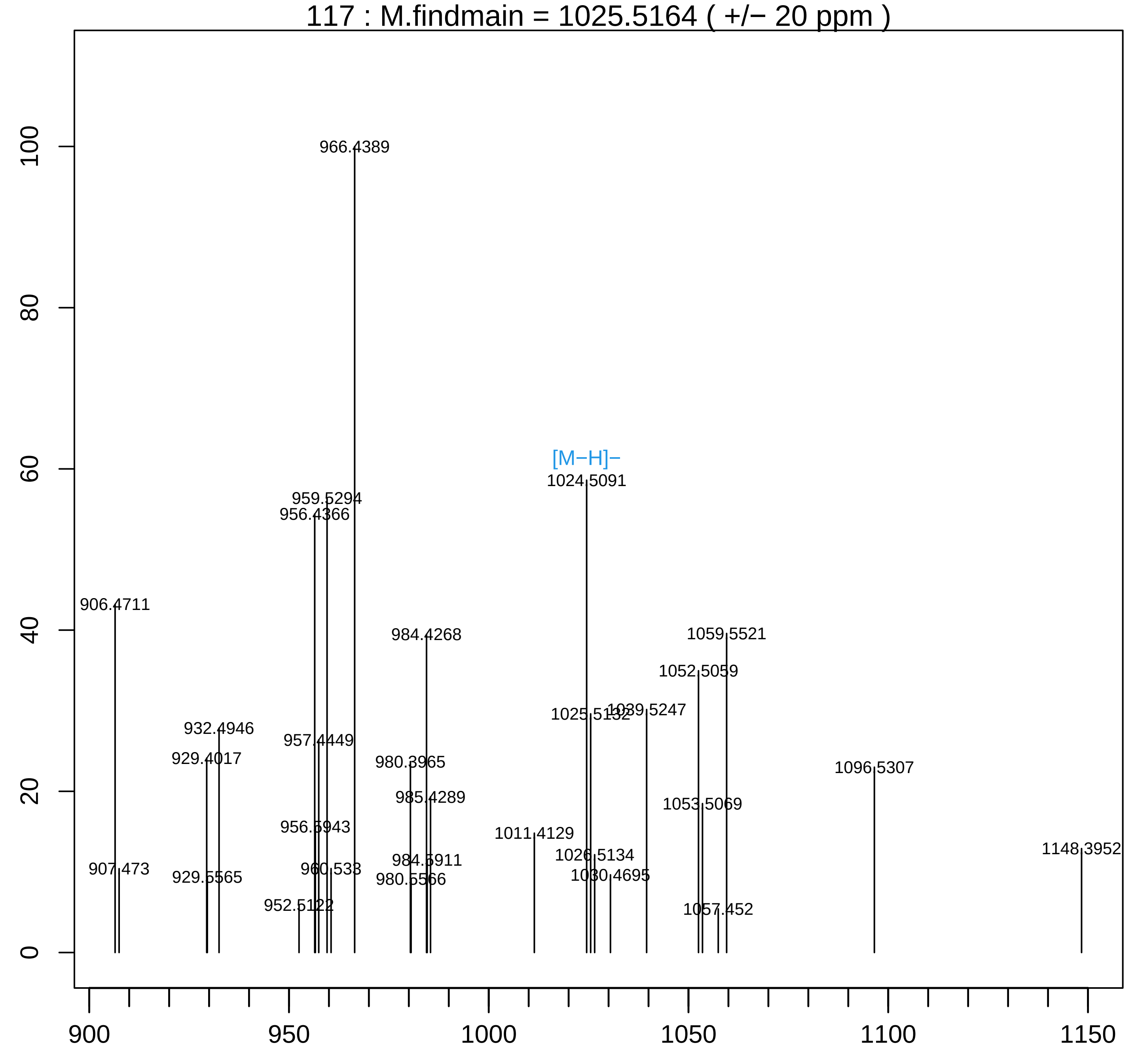

117 : M.findmain = 1025.5164 (+/− 20 ppm)
[M−H]−
966.4389
1024.5091
959.5294
956.4366
906.4711
984.4268
1059.5521
1052.5059
1025.5132
1039.5247
932.4946
957.4449
929.4017
980.3965
1096.5307
985.4289
1053.5069
956.5943
1011.4129
1026.5134
907.473
960.533
984.5911
929.5565
980.5566
1030.4695
1148.3952
952.5122
1057.452

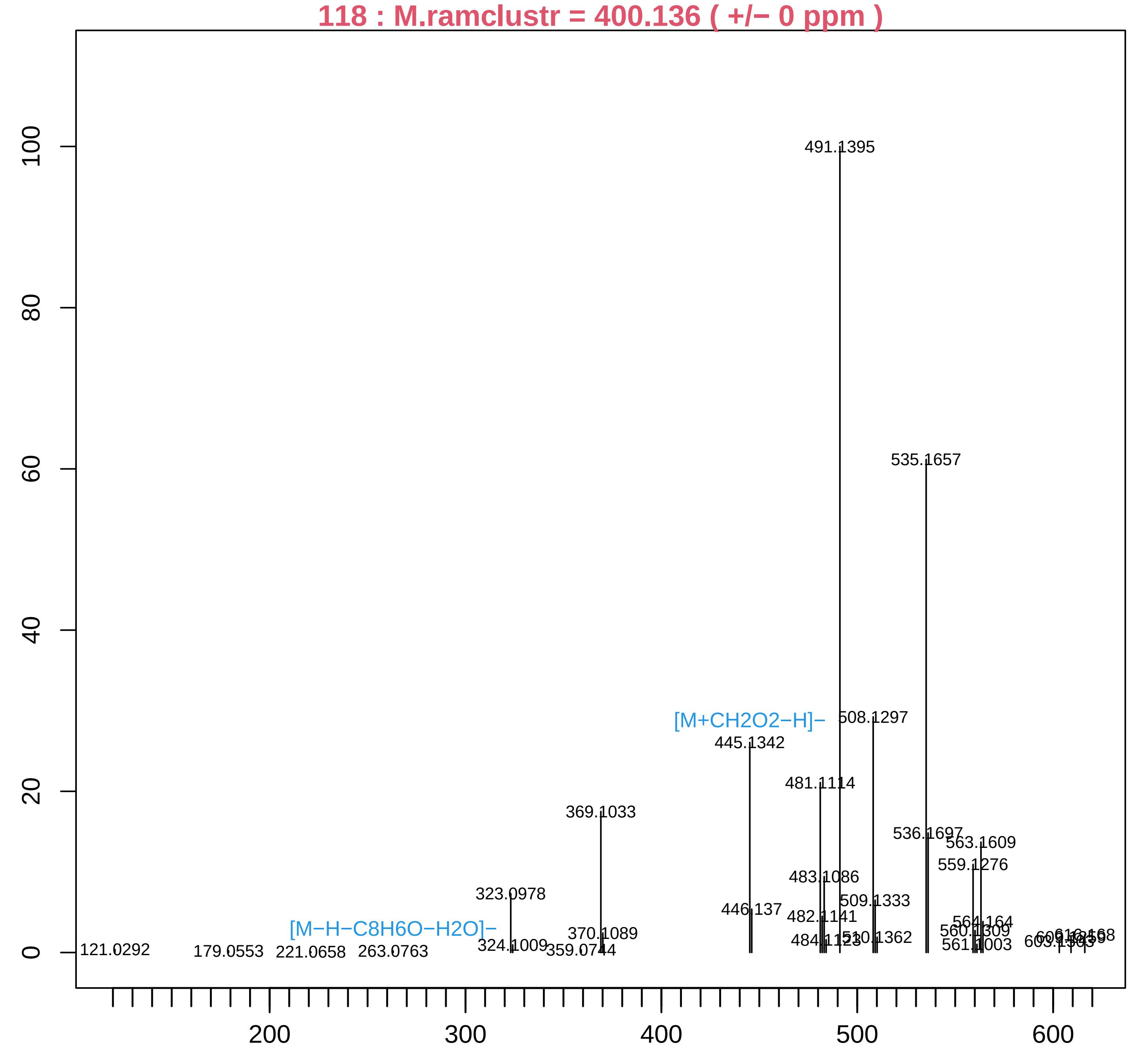
118 : M.ramclustr = 400.136 (+/− 0 ppm)
491.1395
535.1657
[M+CH2O2−H]−
445.1342
508.1297
481.1114
369.1033
536.1697
563.1609
559.1276
483.1086
323.0978
509.1333
446.137
482.1141
564.164
[M−H−C8H6O−H2O]−
370.1089
560.1309
510.1362
121.0292
179.0553
221.0658
263.0763
324.1009
359.0744
561.1003
0
20
40
60
80
100
200
300
400
500
600

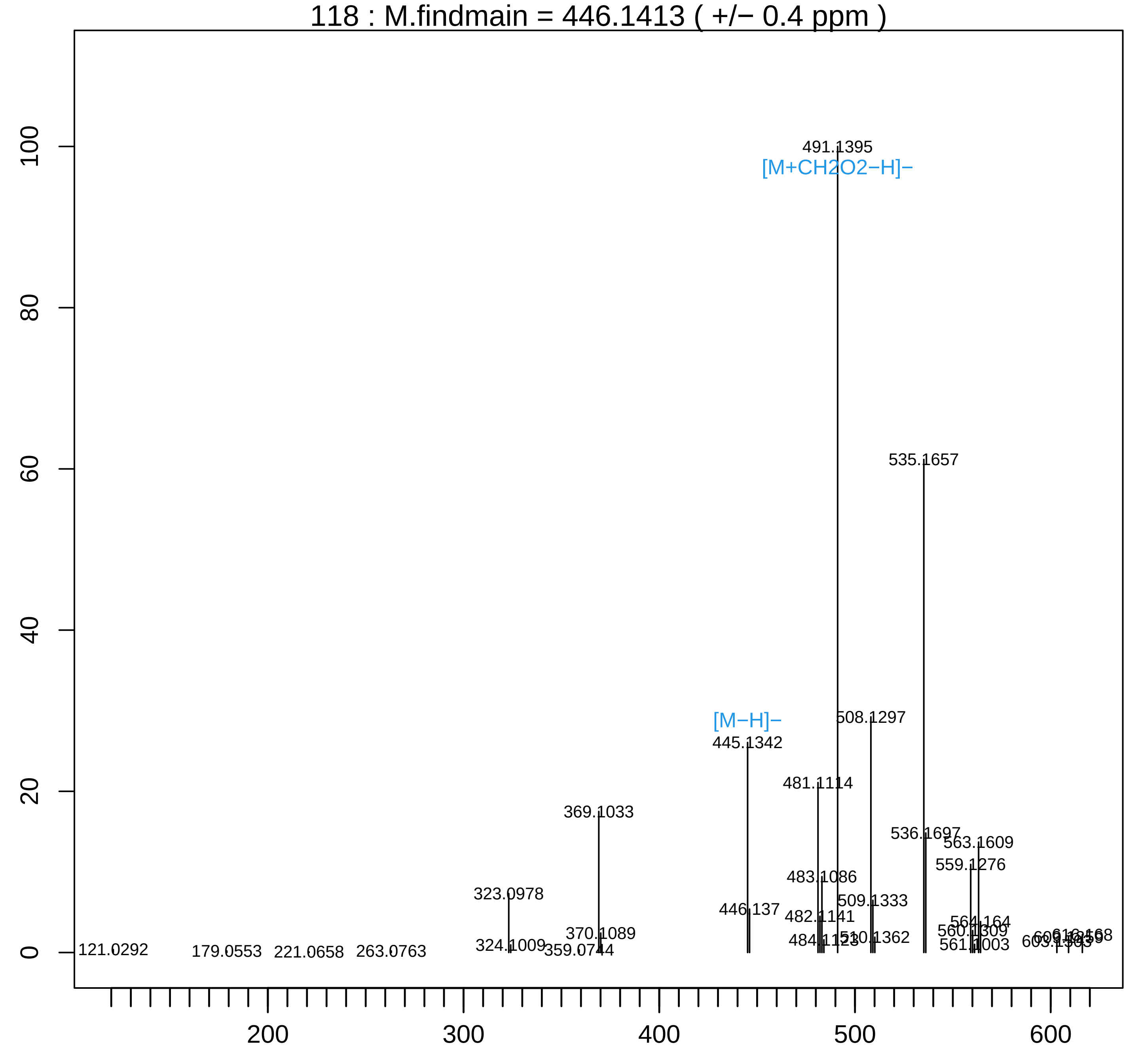
118 : M.findmain = 446.1413 (+/− 0.4 ppm)
491.1395
[M+CH2O2−H]−
535.1657
[M−H]−
445.1342
508.1297
481.1114
369.1033
536.1697
563.1609
559.1276
483.1086
323.0978
509.1333
446.137
482.1141
564.164
370.1089
560.1309
510.1362
121.0292
179.0553
221.0658
263.0763
324.1009
359.0744
561.1003
0
20
40
60
80
100
200
300
400
500
600

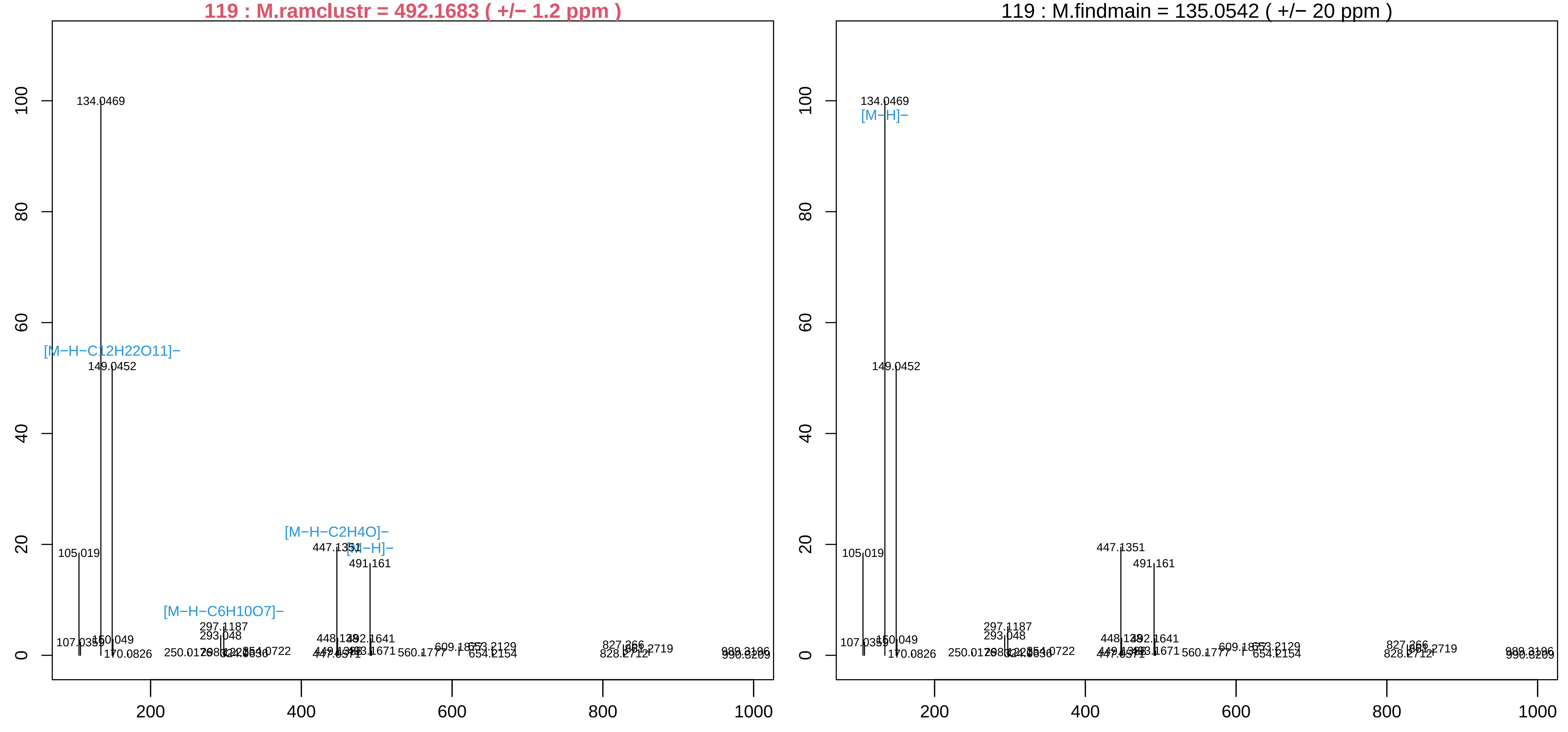

119 : M.ramclustr = 492.1683 (+/− 1.2 ppm)
[M−H−C12H22O11]−
[M−H−C2H4O]−
[M−H]−
[M−H−C6H10O7]−
134.0469
149.0452
105.019
447.1351
491.161
297.1187
293.048
107.0359
150.049
170.0826
560.1777
654.2154
828.2712
119 : M.findmain = 135.0542 (+/− 20 ppm)
[M−H]−

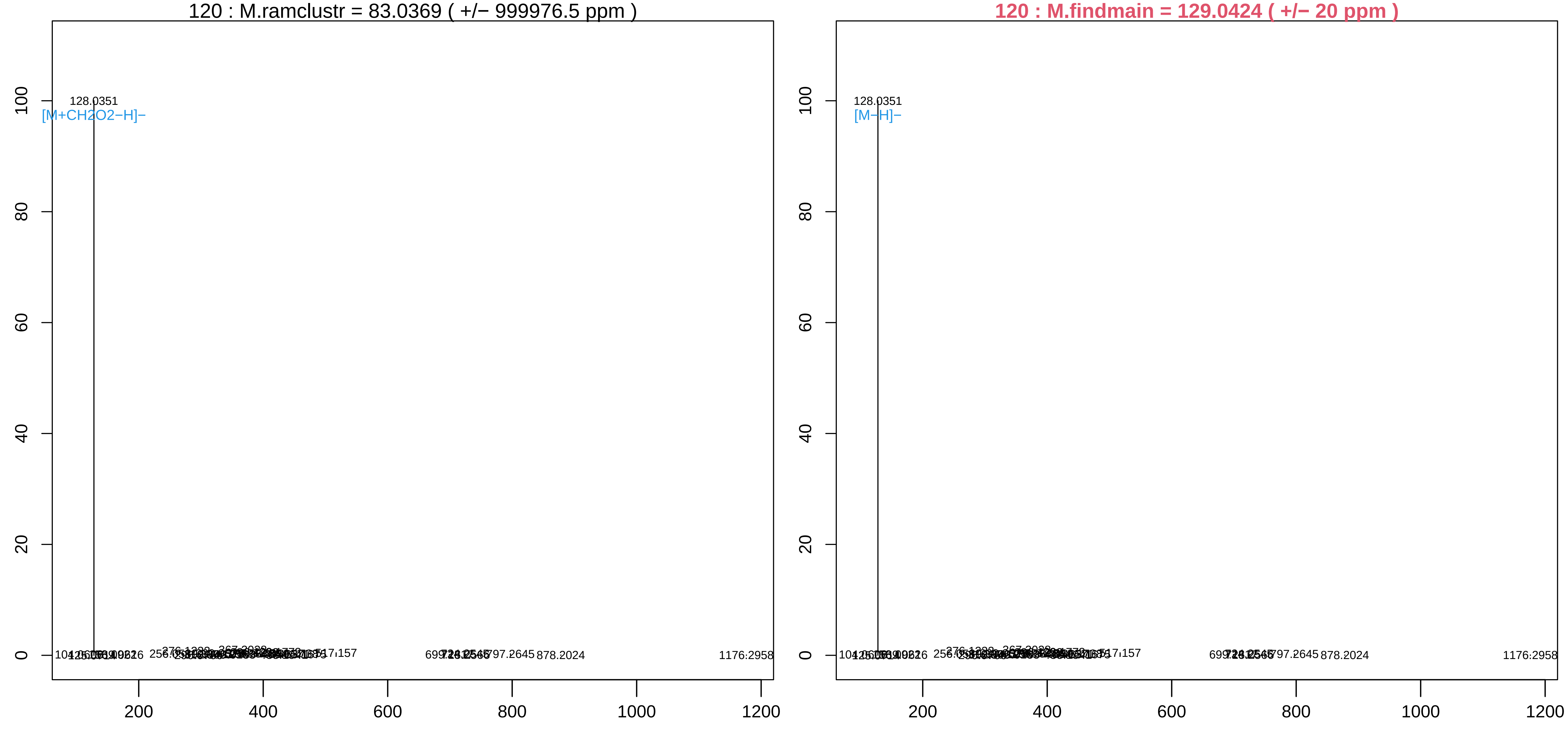
120 : M.ramclustr = 83.0369 (+/− 999976.5 ppm)
128.0351
[M+CH2O2−H]−
120 : M.findmain = 129.0424 (+/− 20 ppm)
128.0351
[M−H]−
797.2645
878.2024
1176.2958

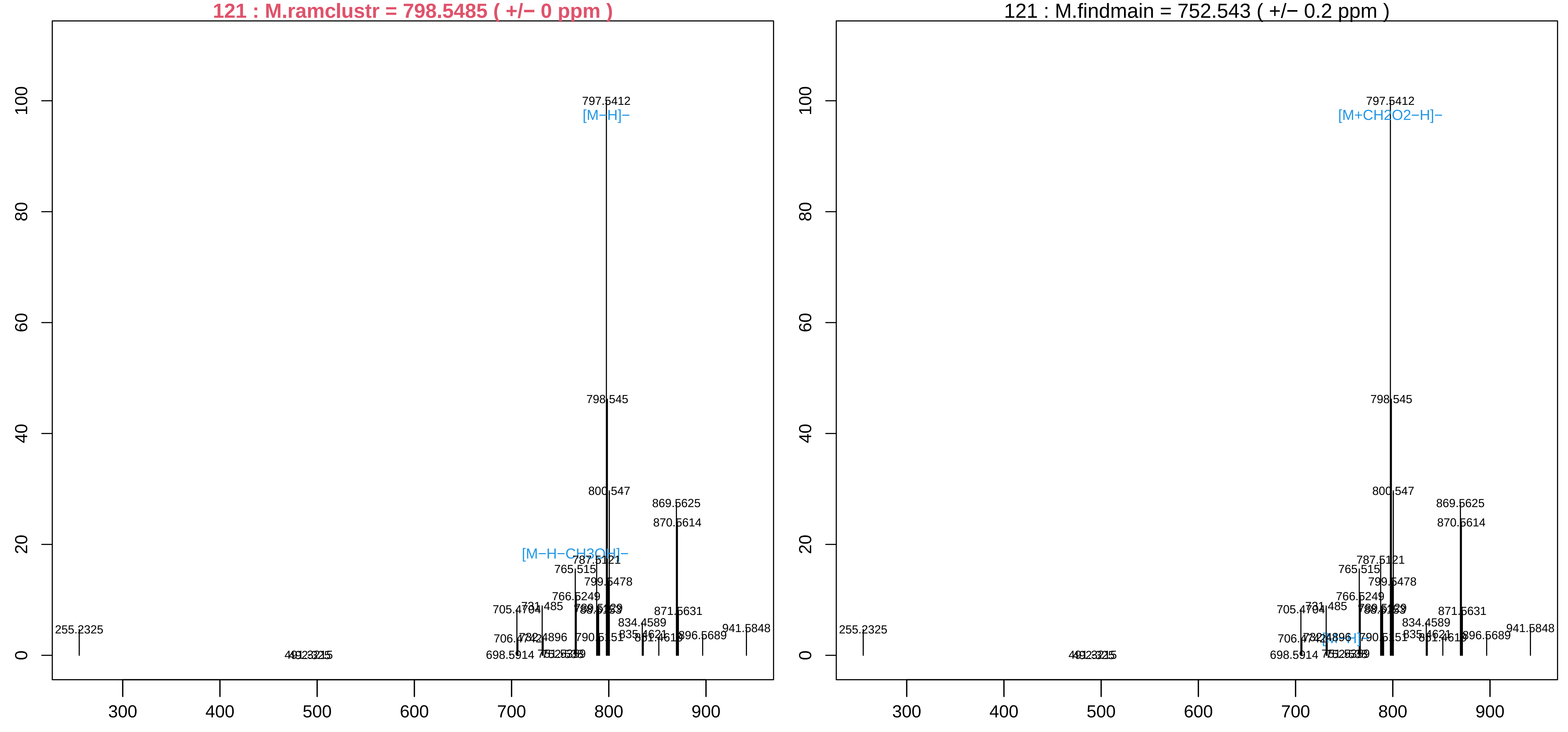
121 : M.ramclustr = 798.5485 (+/− 0 ppm)
121 : M.findmain = 752.543 (+/− 0.2 ppm)
797.5412
[M−H]−
[M+CH2O2−H]−
798.545
800.547
869.5625
870.5614
[M−H−CH3OH]−
787.5121
765.515
799.5478
766.5249
705.4704
731.485
871.5631
834.4589
255.2325
941.5848
896.5689
698.5914
0
20
40
60
80
100
300
400
500
600
700
800
900

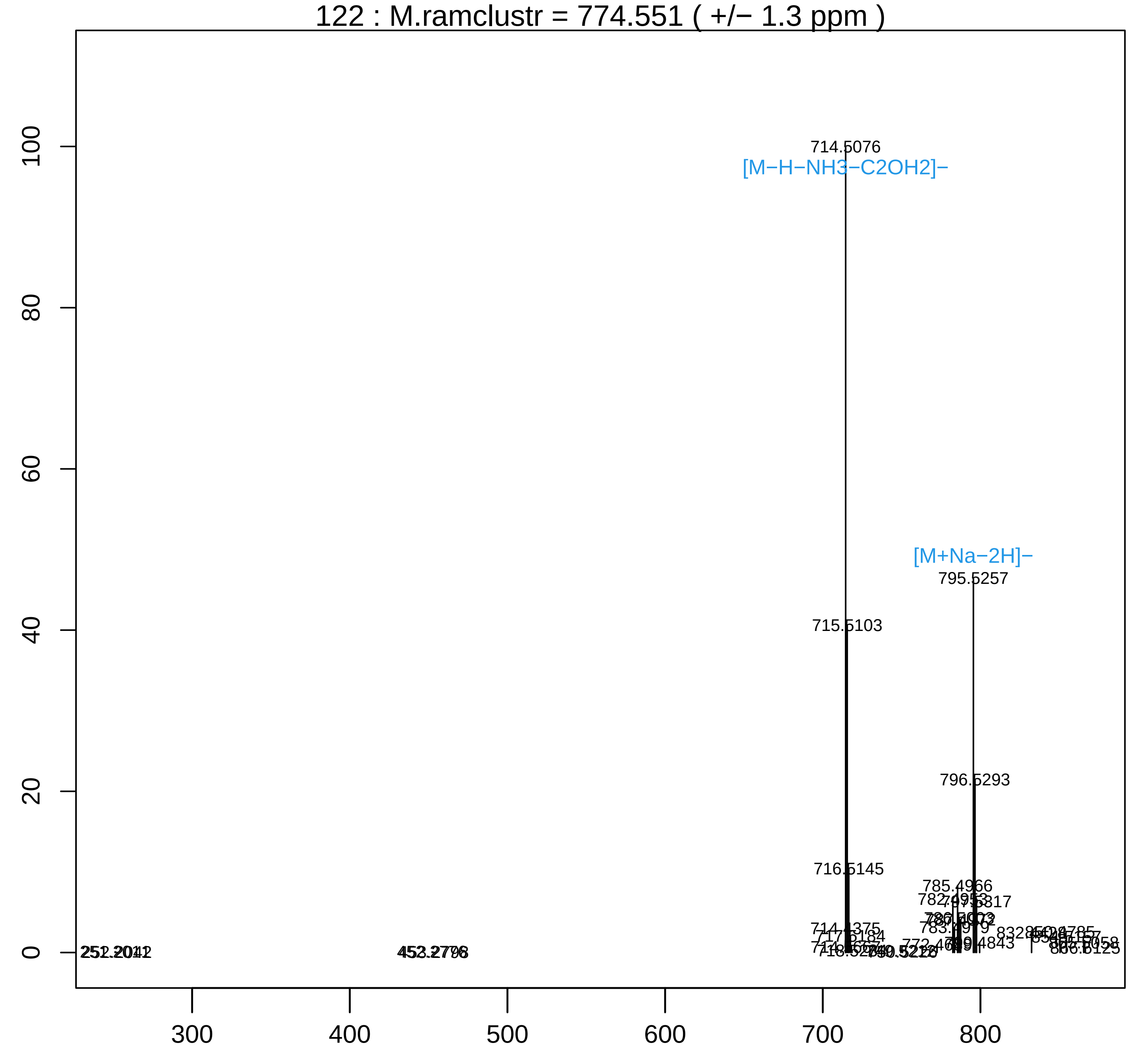
122 : M.ramclustr = 774.551 (+/− 1.3 ppm)
714.5076
[M−H−NH3−C2OH2]−
715.5103
716.5145
795.5257
[M+Na−2H]−
796.5293
785.4966
714.4375
251.2041
453.2798
0
20
40
60
80
100
300
400
500
600
700
800
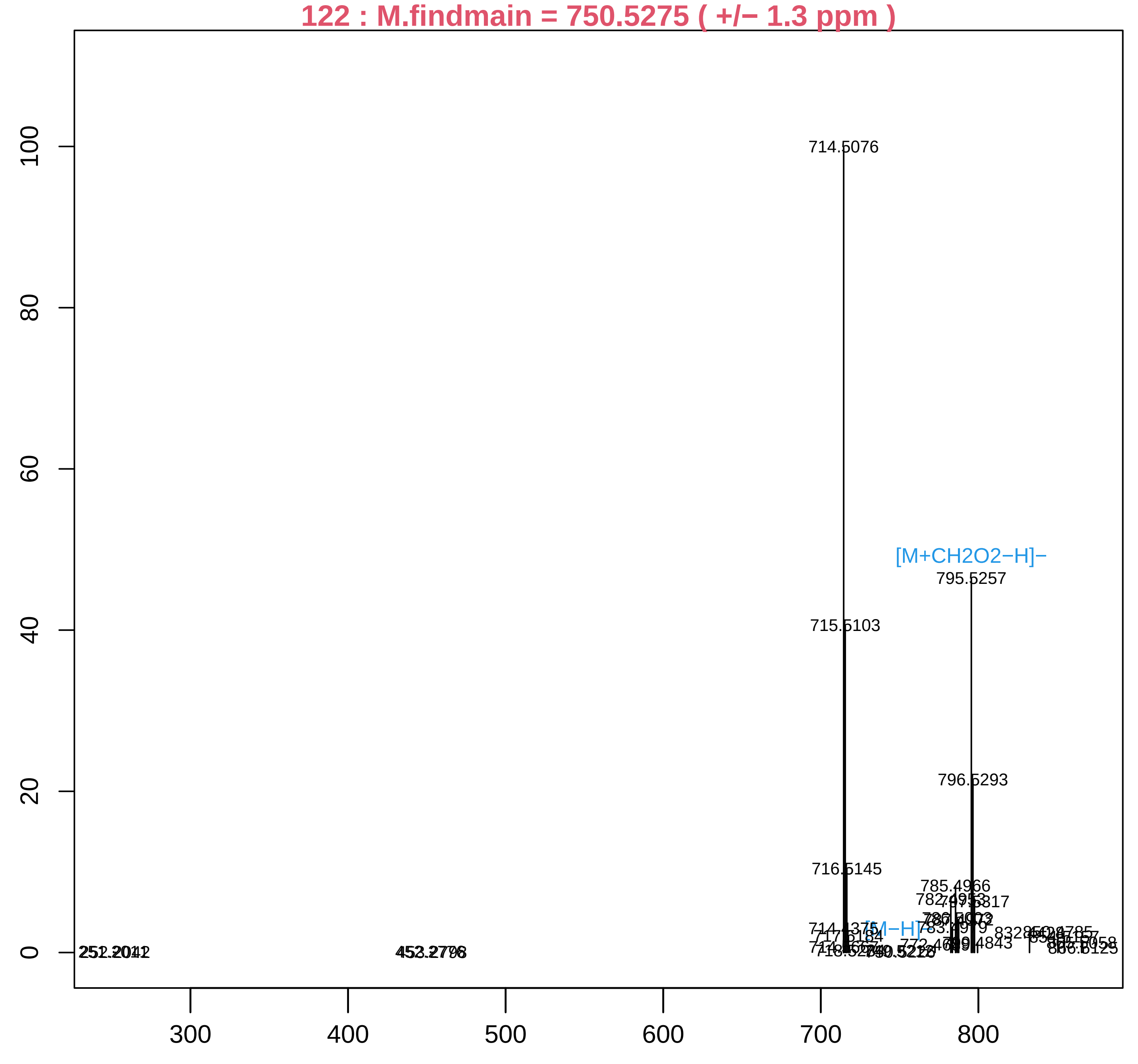
122 : M.findmain = 750.5275 (+/− 1.3 ppm)
714.5076
715.5103
716.5145
795.5257
[M+CH2O2−H]−
796.5293
785.4966
714.4375
251.2041
453.2798
0
20
40
60
80
100
300
400
500
600
700
800

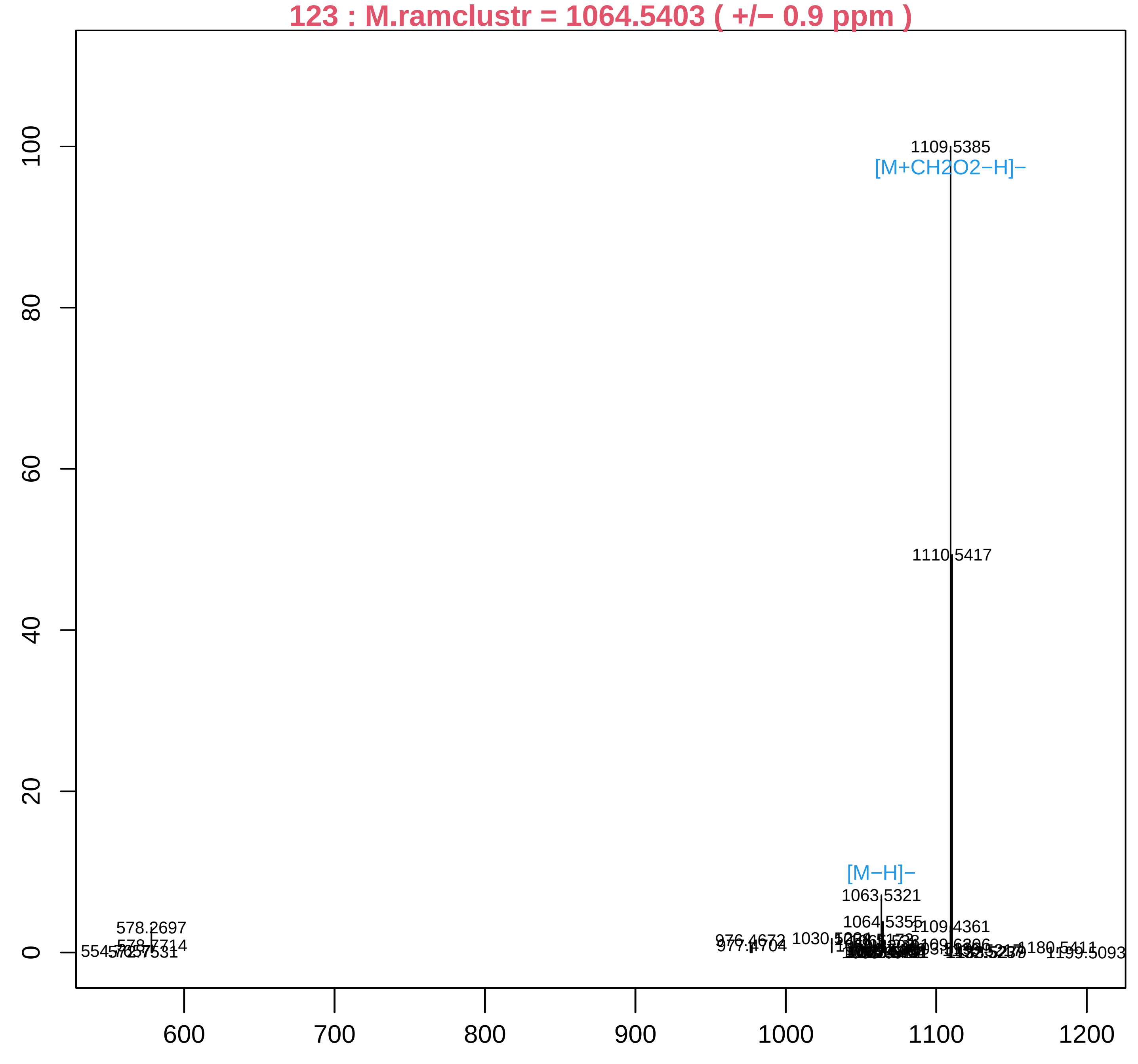

123 : M.ramclustr = 1064.5403 (+/− 0.9 ppm)
1109.5385
[M+CH2O2−H]−
1110.5417
[M−H]−
1063.5321
1064.5355
1109.4361
578.2697
1180.5411
1199.5093
976.4672
977.4704
0
20
40
60
80
100
600
700
800
900
1000
1100
1200

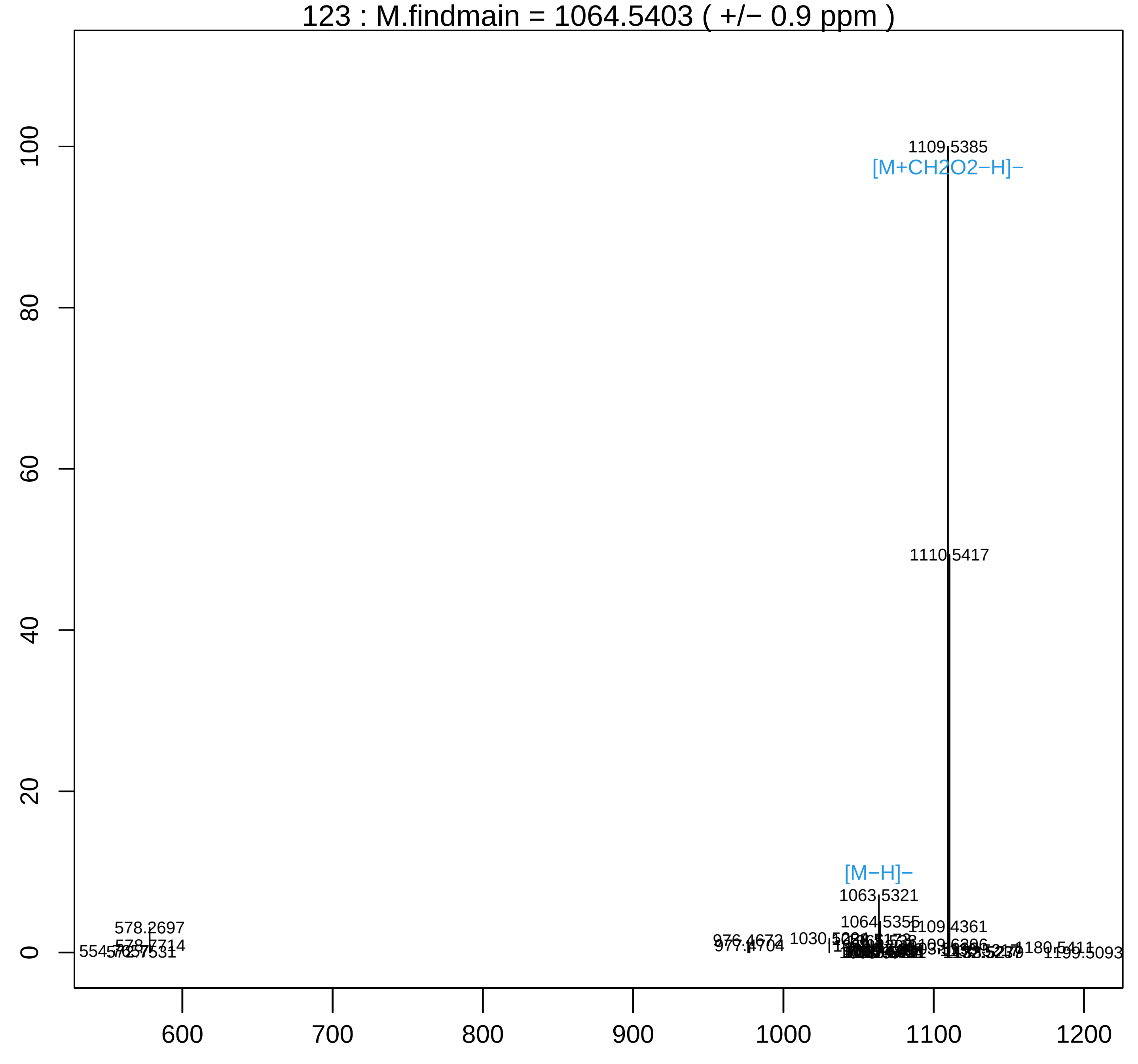

123 : M.findmain = 1064.5403 (+/− 0.9 ppm)
1109.5385
[M+CH2O2−H]−
1110.5417
[M−H]−
1063.5321
1064.5355
1109.4361
578.2697
1180.5411
1199.5093
976.4672
977.4704
0
20
40
60
80
100
600
700
800
900
1000
1100
1200

124 : M.ramclustr = 607.0805 (+/− 1.3 ppm)

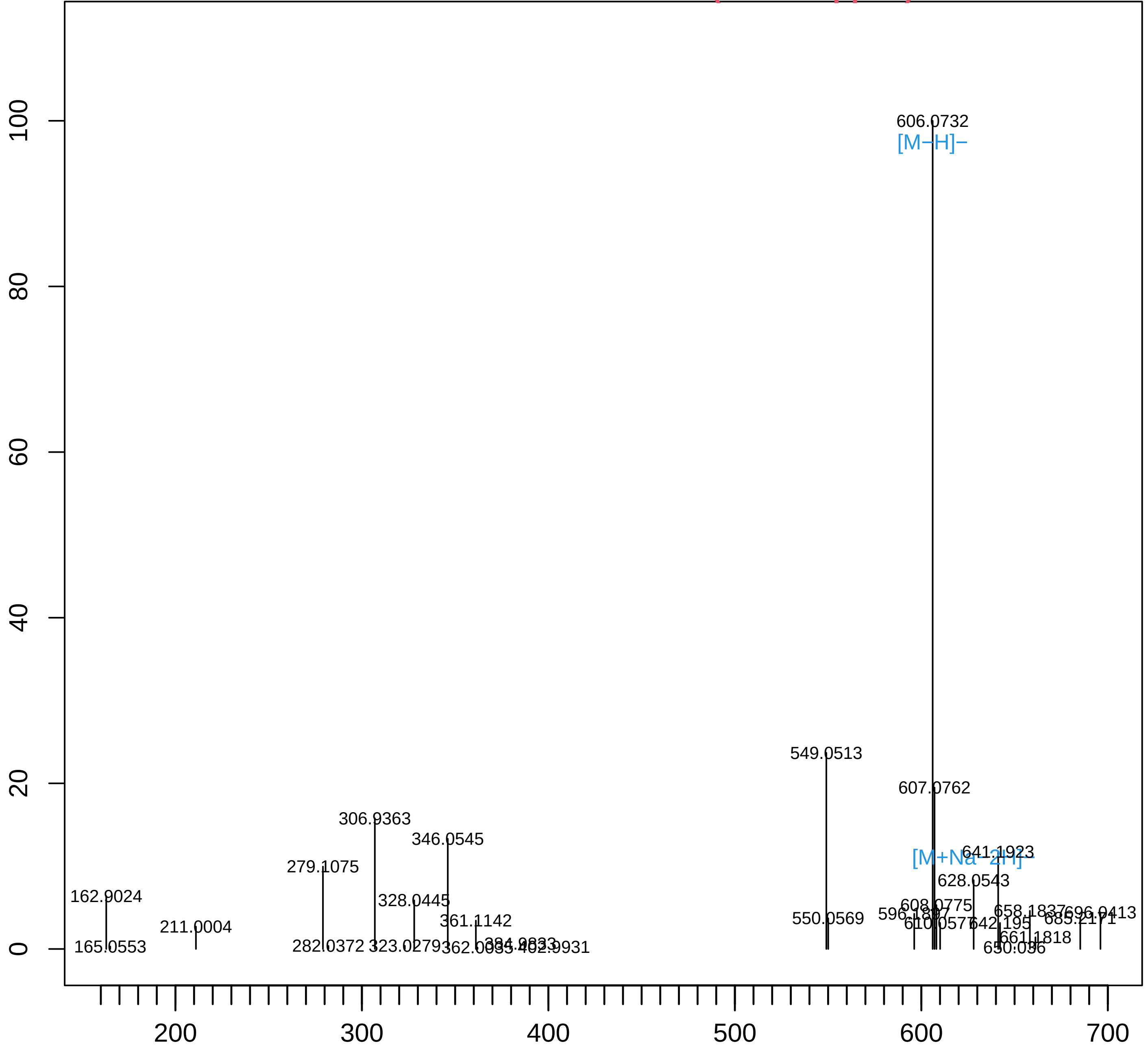

124 : M.findmain = 607.0805 (+/− 1.3 ppm)

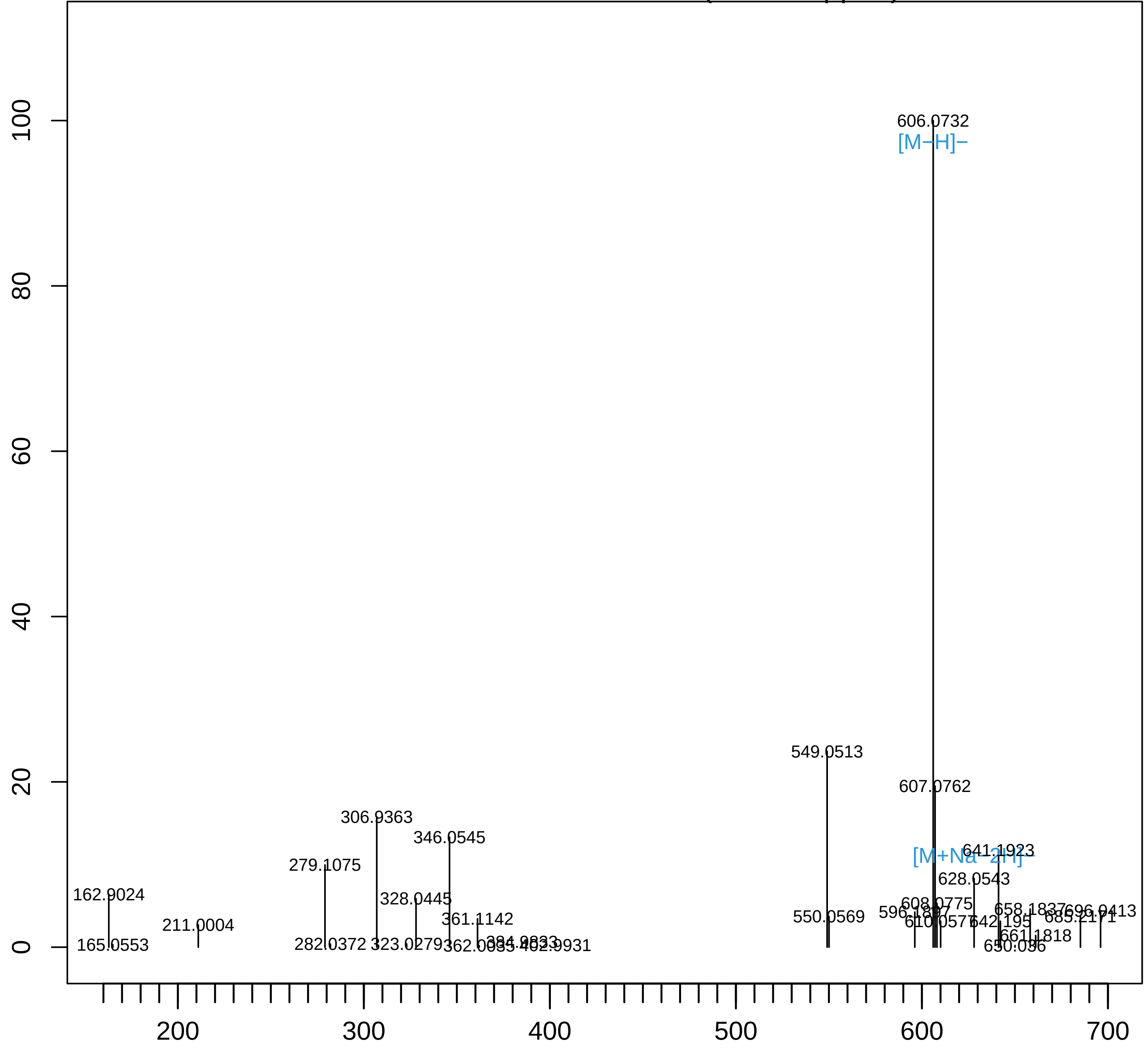

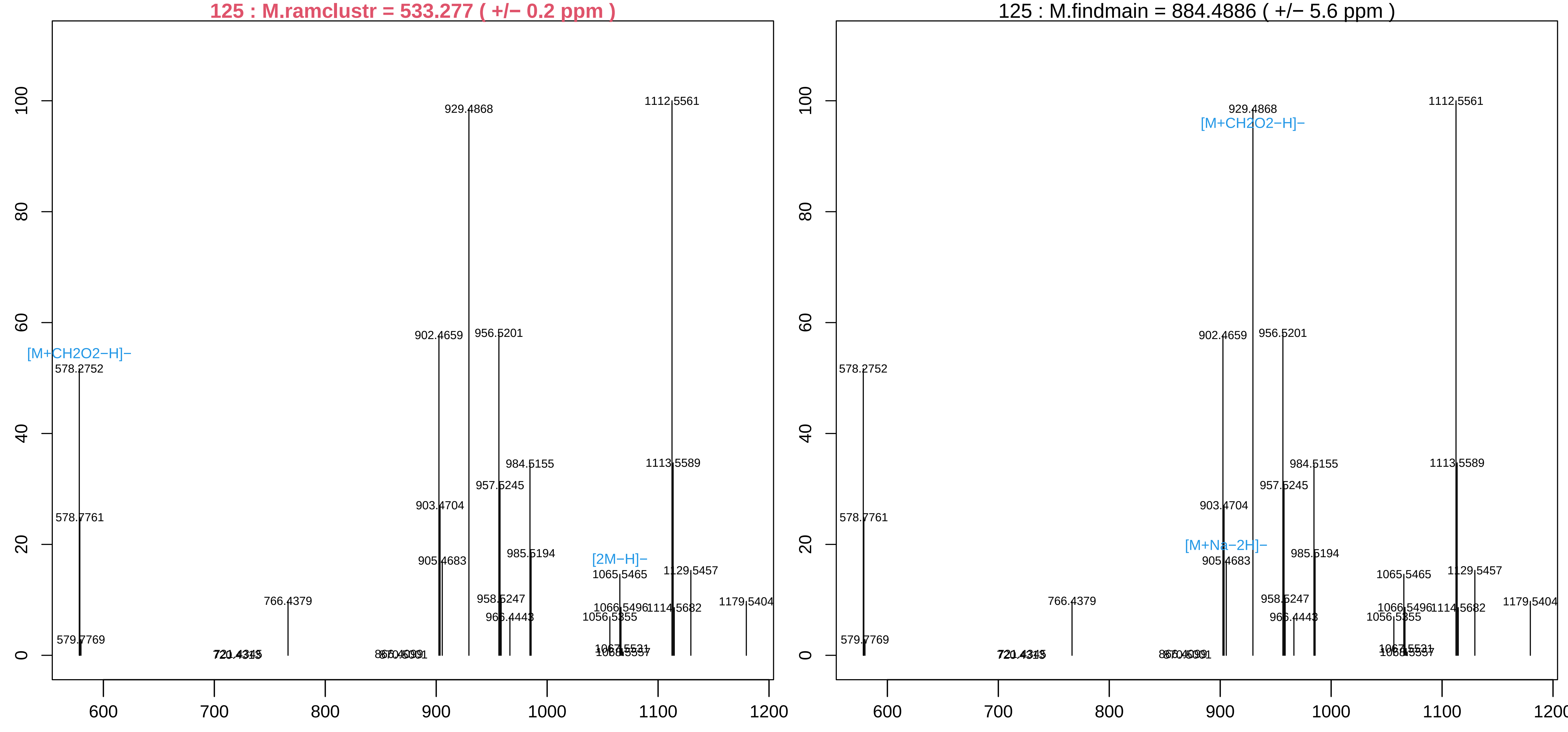
125 : M.ramclustr = 533.277 (+/− 0.2 ppm)
[M+CH2O2−H]−
578.2752
578.7761
579.7769
766.4379
902.4659
903.4704
905.4683
929.4868
956.5201
957.5245
958.5247
966.4443
984.5155
985.5194
[2M−H]−
1065.5465
1066.5496
1056.5355
1112.5561
1113.5589
1114.5682
1129.5457
1179.5404
125 : M.findmain = 884.4886 (+/− 5.6 ppm)
[M+CH2O2−H]−
[M+Na−2H]−
0
20
40
60
80
100
600
700
800
900
1000
1100
1200

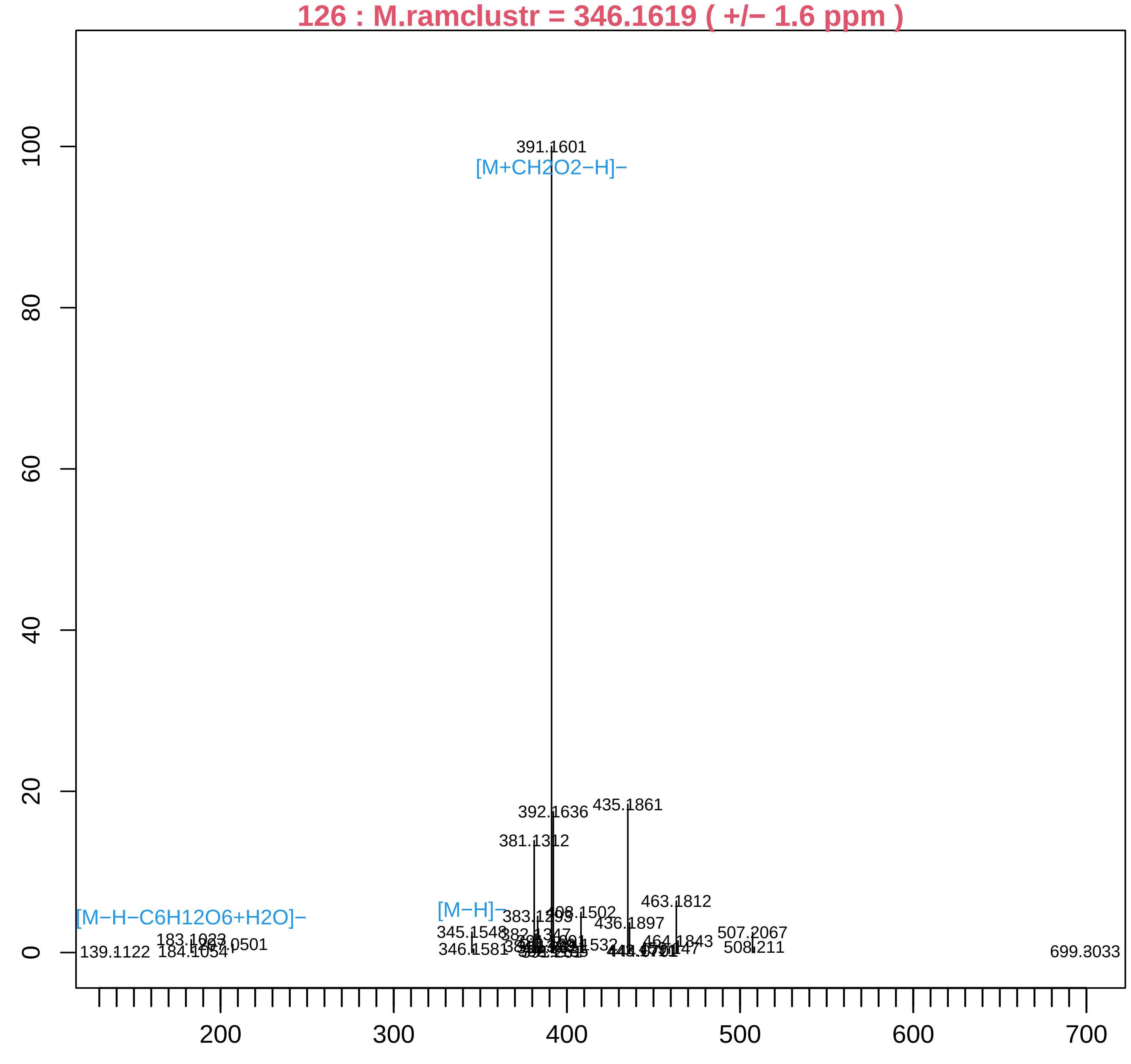
126 : M.ramclustr = 346.1619 (+/− 1.6 ppm)
391.1601
[M+CH2O2−H]−
392.1636
435.1861
381.1312
463.1812
[M−H]−
383.1299
408.1502
436.1897
[M−H−C6H12O6+H2O]−
345.1548
507.2067
183.1023
464.1843
346.1581
508.1211
139.1122
184.1054
699.3033
0
20
40
60
80
100
200
300
400
500
600
700

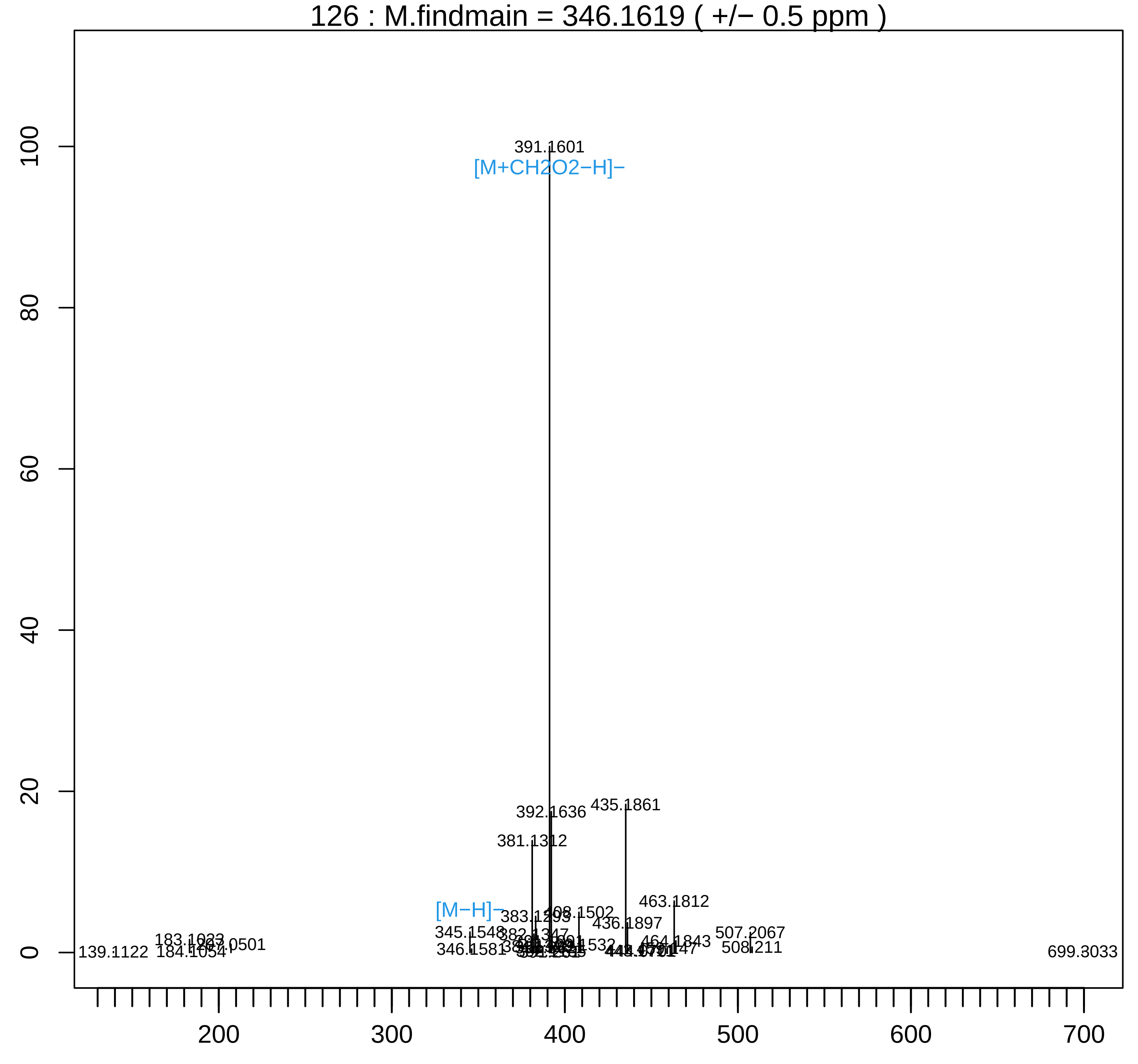
126 : M.findmain = 346.1619 (+/− 0.5 ppm)
391.1601
[M+CH2O2−H]−
392.1636
435.1861
381.1312
463.1812
[M−H]−
383.1299
408.1502
436.1897
345.1548
507.2067
183.1023
464.1843
346.1581
508.1211
139.1122
184.1054
699.3033
0
20
40
60
80
100
200
300
400
500
600
700

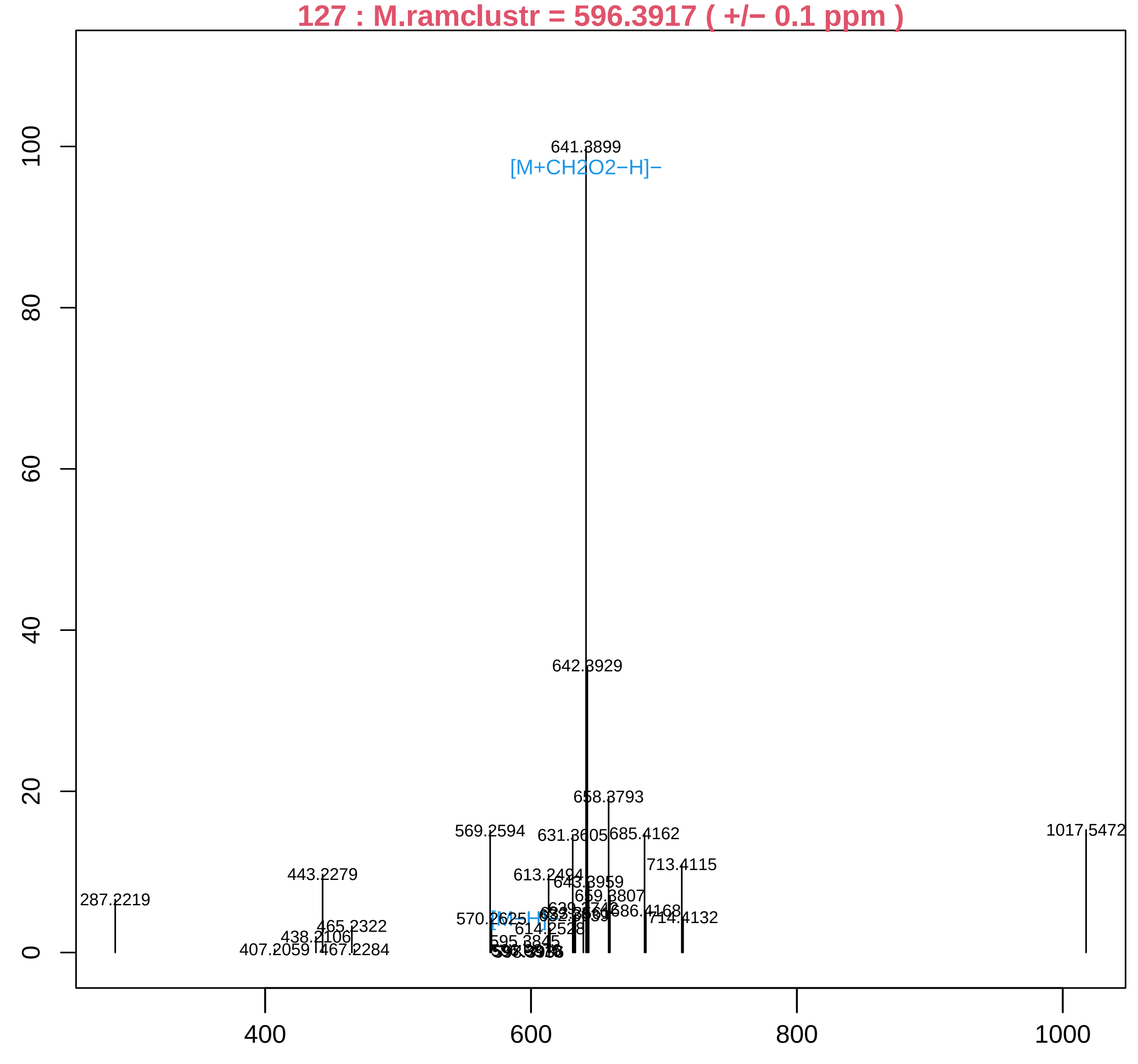
127 : M.ramclustr = 596.3917 (+/− 0.1 ppm)
641.3899
[M+CH2O2−H]−
642.3929
658.3793
569.2594
631.3605
685.4162
1017.5472
713.4115
443.2279
613.2494
643.3959
659.3807
287.2219
686.4168
714.4132
570.2625
465.2322
614.2528
438.2106
595.3845
407.2059
467.2284
0
20
40
60
80
100
400
600
800
1000
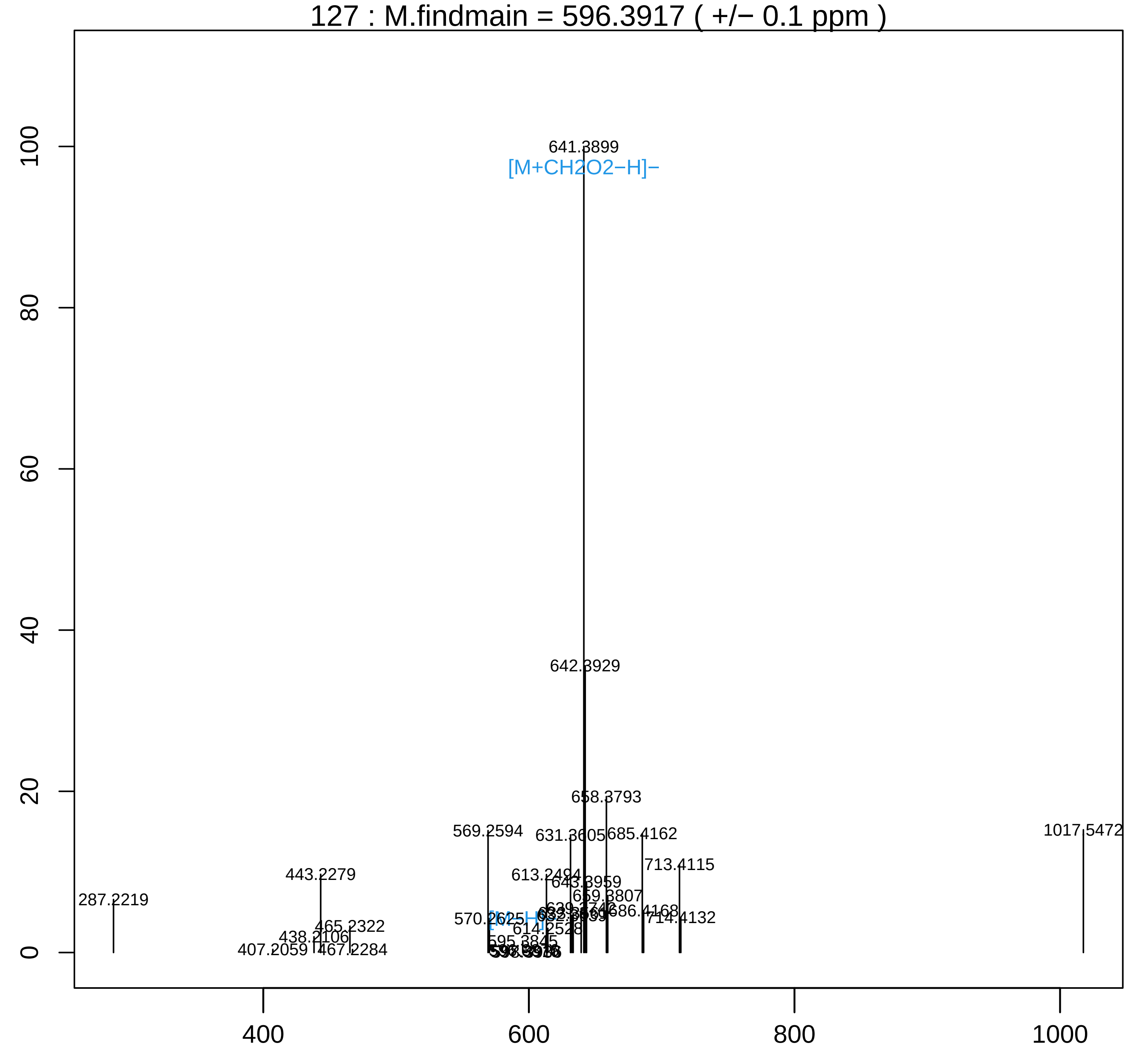
127 : M.findmain = 596.3917 (+/− 0.1 ppm)
641.3899
[M+CH2O2−H]−
642.3929
658.3793
569.2594
631.3605
685.4162
1017.5472
713.4115
443.2279
613.2494
643.3959
659.3807
287.2219
686.4168
714.4132
570.2625
465.2322
614.2528
438.2106
595.3845
407.2059
467.2284
0
20
40
60
80
100
400
600
800
1000

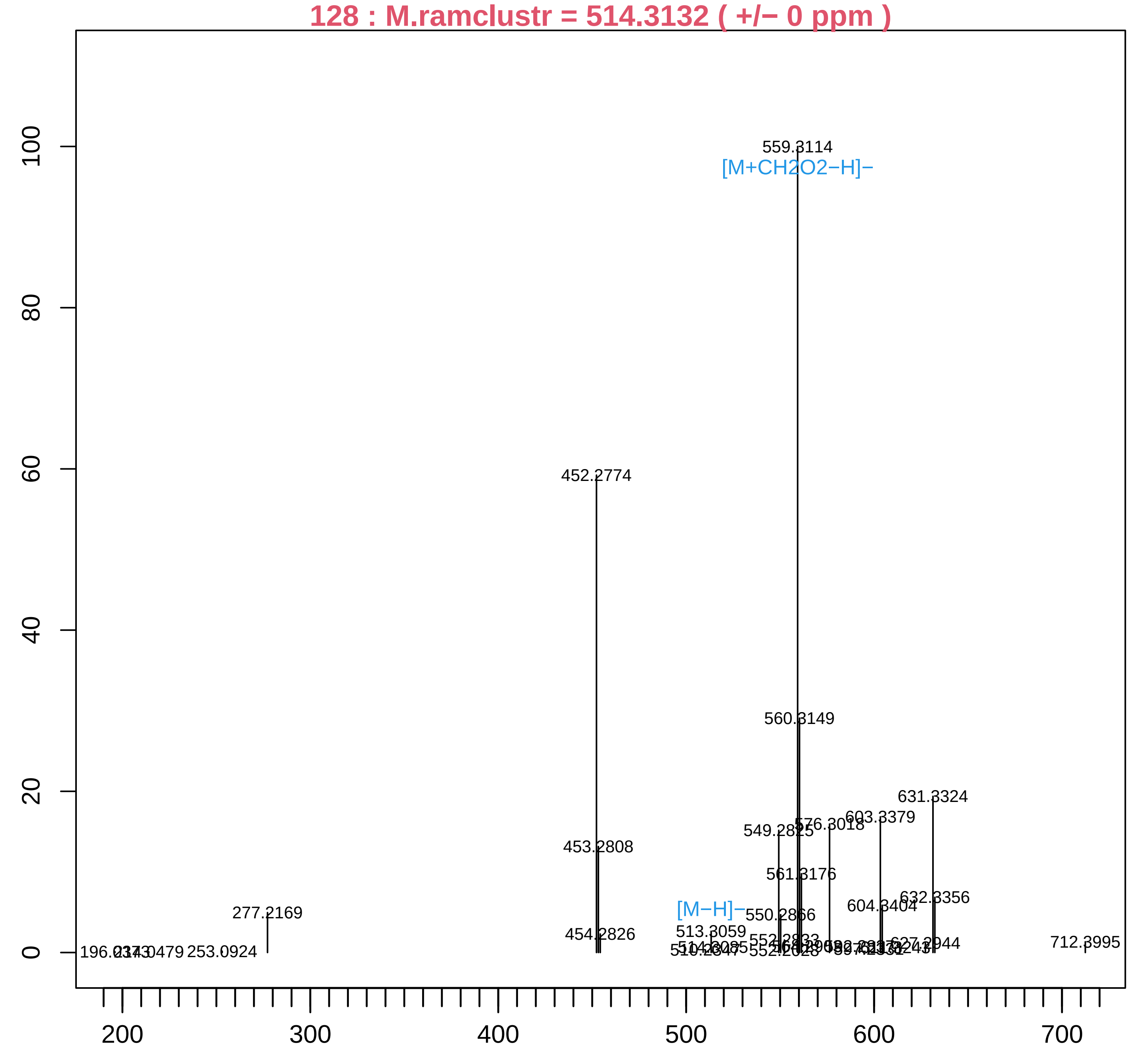
128 : M.ramclustr = 514.3132 (+/− 0 ppm)
559.3114
[M+CH2O2−H]−
452.2774
560.3149
631.3324
603.3379
576.3018
549.2825
453.2808
561.3176
632.3356
604.3404
[M−H]−
550.2866
277.2169
454.2826
513.3059
627.2944
712.3995
253.0924
0
20
40
60
80
100
200
300
400
500
600
700

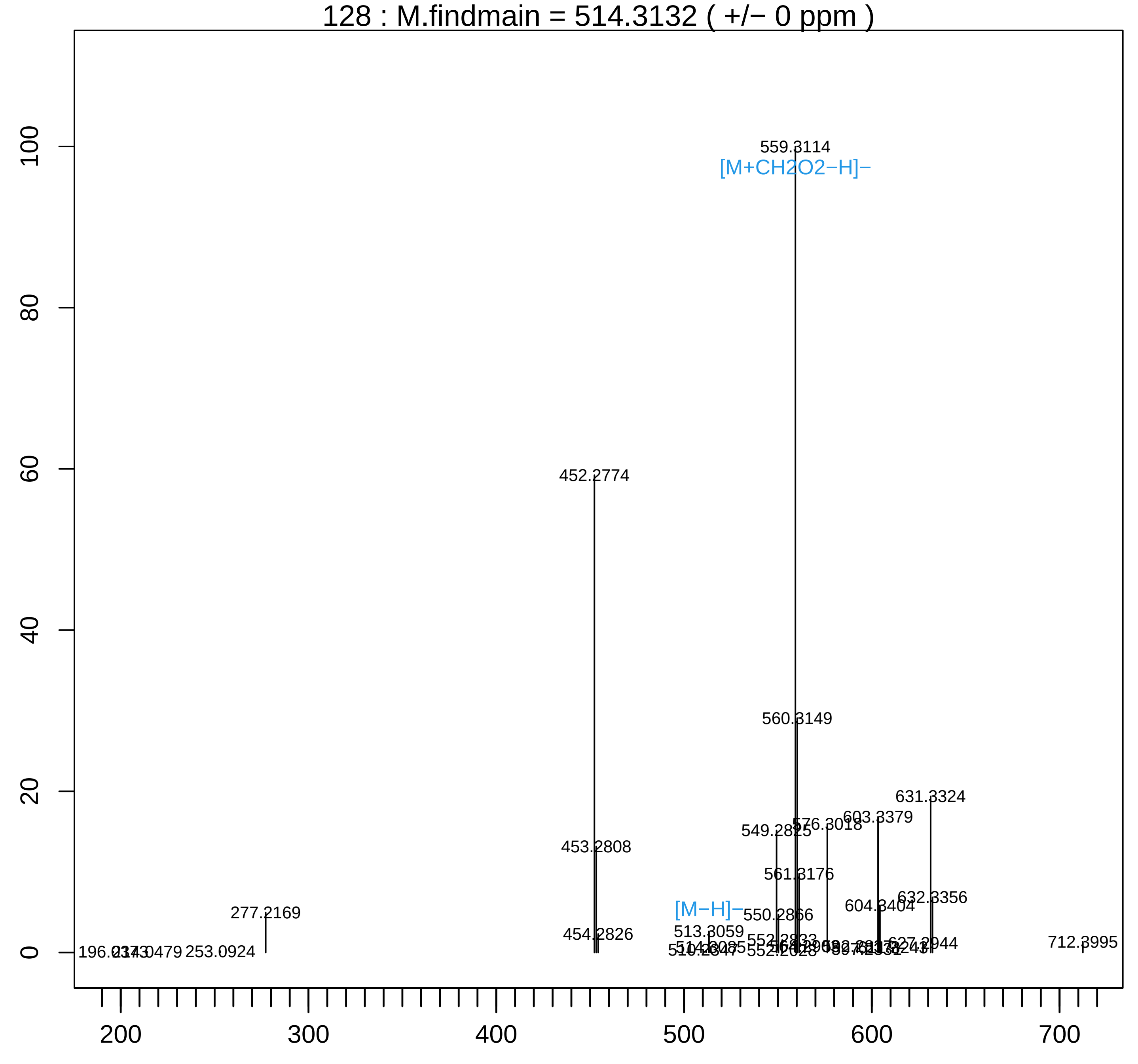
128 : M.findmain = 514.3132 (+/− 0 ppm)
559.3114
[M+CH2O2−H]−
452.2774
560.3149
631.3324
603.3379
576.3018
549.2825
453.2808
561.3176
632.3356
604.3404
[M−H]−
550.2866
277.2169
454.2826
513.3059
627.2944
712.3995
253.0924
0
20
40
60
80
100
200
300
400
500
600
700

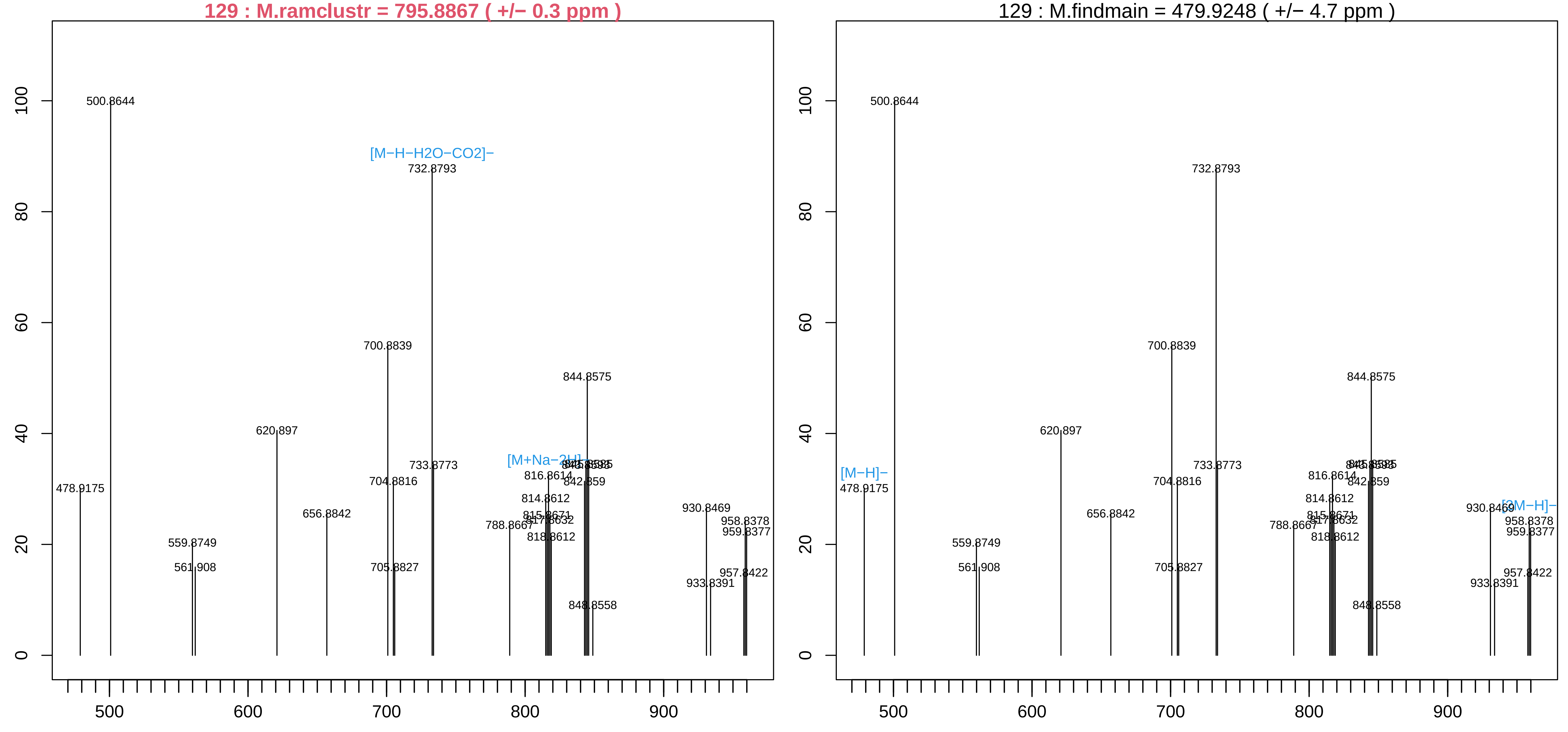
129 : M.ramclustr = 795.8867 (+/− 0.3 ppm)
[M−H−H2O−CO2]−
[M+Na−2H]−
500.8644
732.8793
700.8839
844.8575
620.897
733.8773
816.8614
704.8816
842.859
478.9175
814.8612
815.8671
817.8632
818.8612
788.8667
930.8469
958.8378
959.8377
656.8842
559.8749
561.908
705.8827
957.8422
933.8391
848.8558
129 : M.findmain = 479.9248 (+/− 4.7 ppm)
[M−H]−
[2M−H]−
0
20
40
60
80
100
500
600
700
800
900

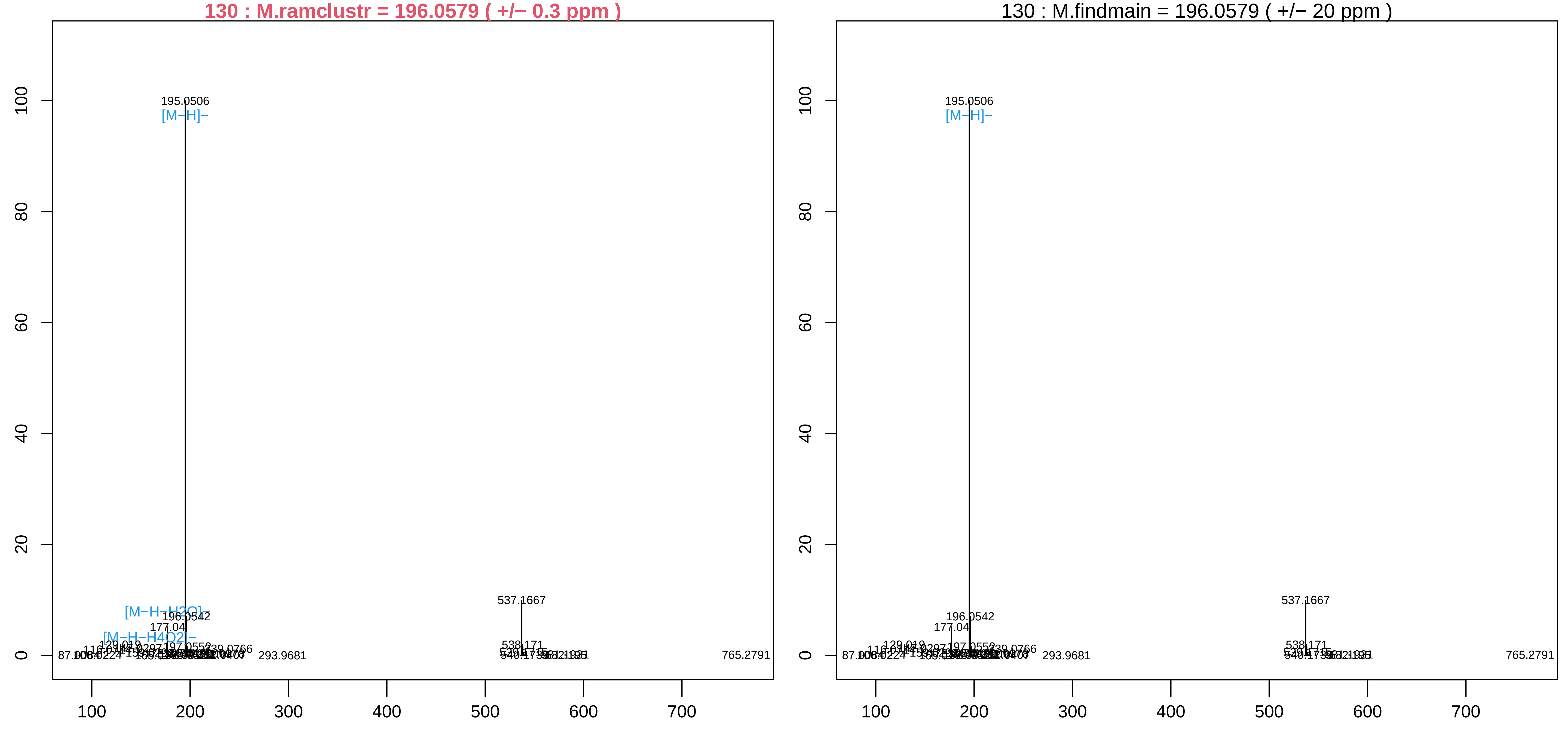

130 : M.ramclustr = 196.0579 (+/− 0.3 ppm)
195.0506
[M−H]−
[M−H−H2O]−
196.0542
177.04
129.019
537.1667
538.171
293.9681
765.2791
130 : M.findmain = 196.0579 (+/− 20 ppm)
195.0506
[M−H]−
196.0542
177.04
129.019
537.1667
538.171
293.9681
765.2791
0
20
40
60
80
100
200
300
400
500
600
700

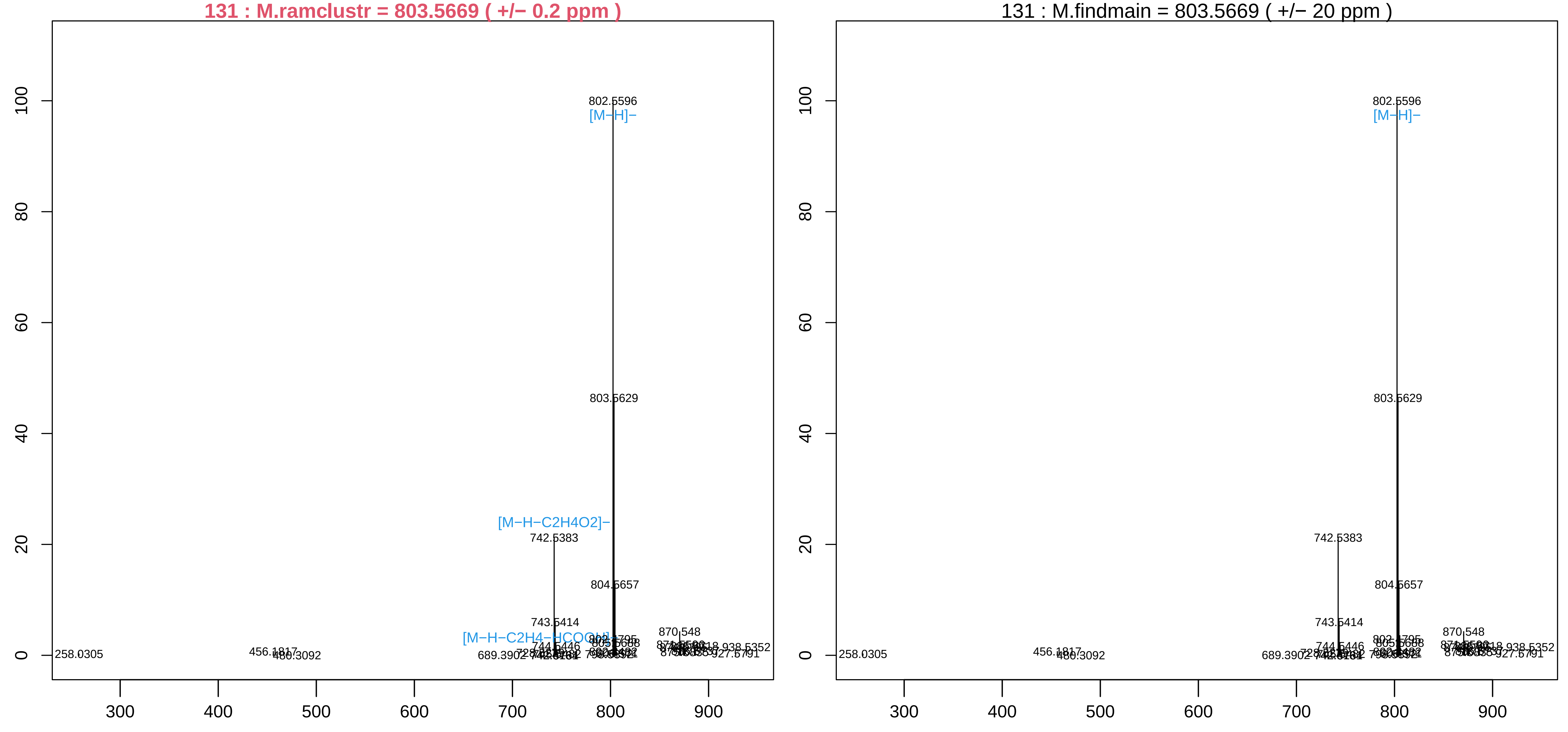
131 : M.ramclustr = 803.5669 (+/− 0.2 ppm)
802.5596
[M−H]−
803.5629
[M−H−C2H4O2]−
742.5383
804.5657
743.5414
[M−H−C2H4−HCOOH]−
870.548
938.5352
927.5791
258.0305
456.1817
480.3092
689.3902
131 : M.findmain = 803.5669 (+/− 20 ppm)
0
20
40
60
80
100
300
400
500
600
700
800
900

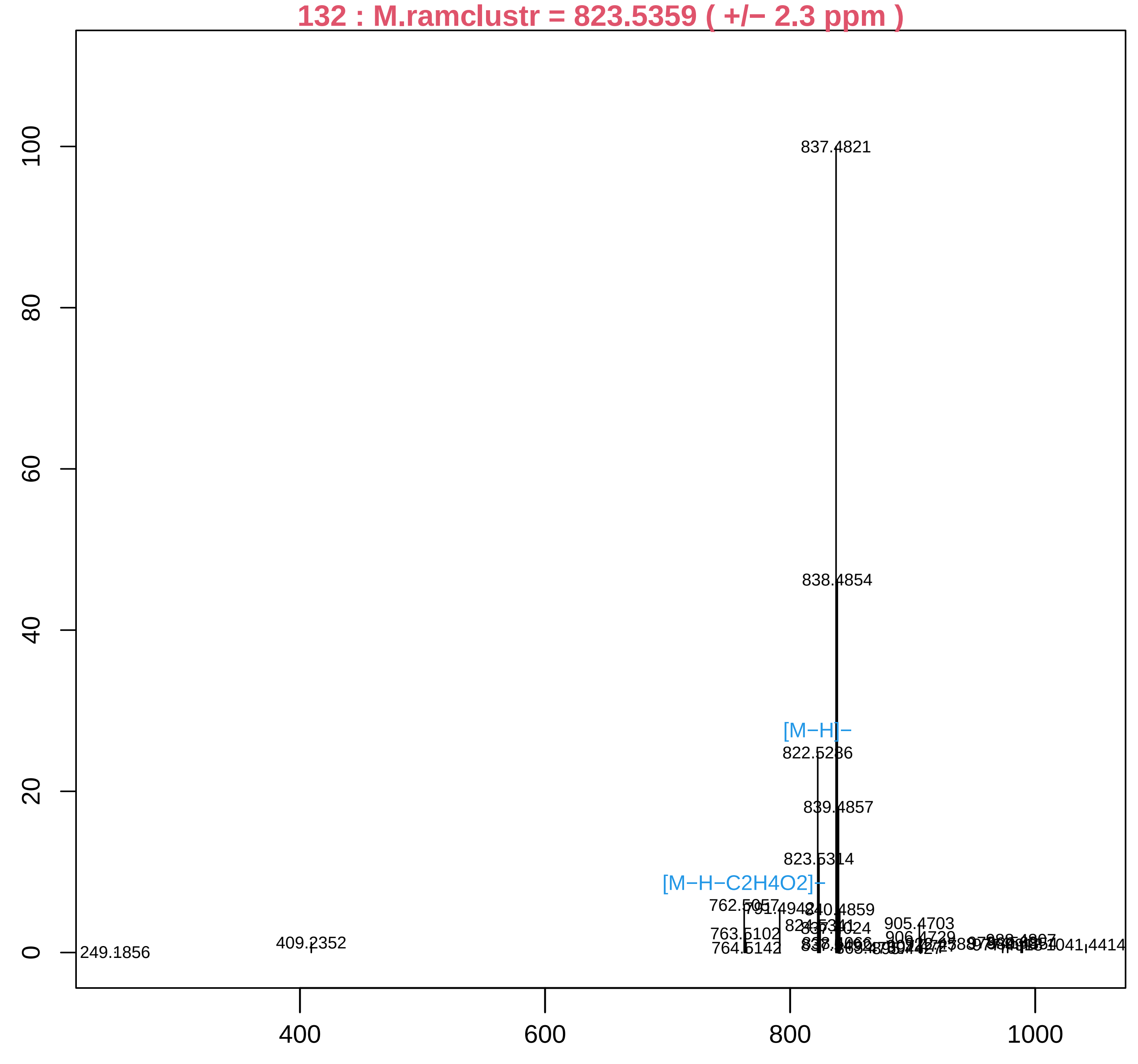
132 : M.ramclustr = 823.5359 (+/− 2.3 ppm)
837.4821
838.4854
[M−H]−
822.5236
839.4857
823.5314
[M−H−C2H4O2]−
762.5057
840.4859
905.4703
906.4729
763.5102
764.5142
409.2352
249.1856
0
20
40
60
80
100
400
600
800
1000

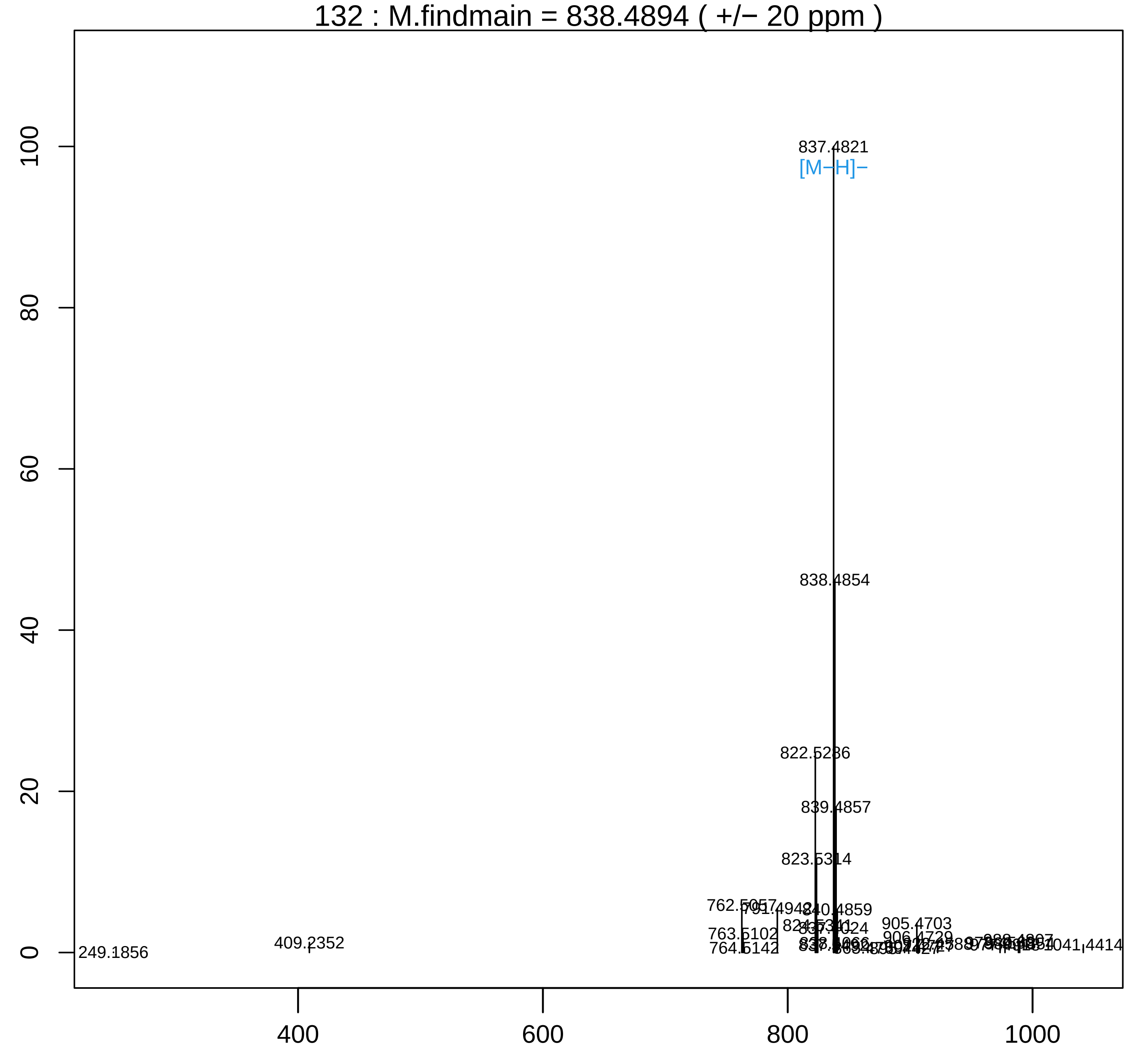
132 : M.findmain = 838.4894 (+/− 20 ppm)
837.4821
[M−H]−
838.4854
822.5236
839.4857
823.5314
762.5057
840.4859
905.4703
906.4729
763.5102
764.5142
409.2352
249.1856
0
20
40
60
80
100
400
600
800
1000

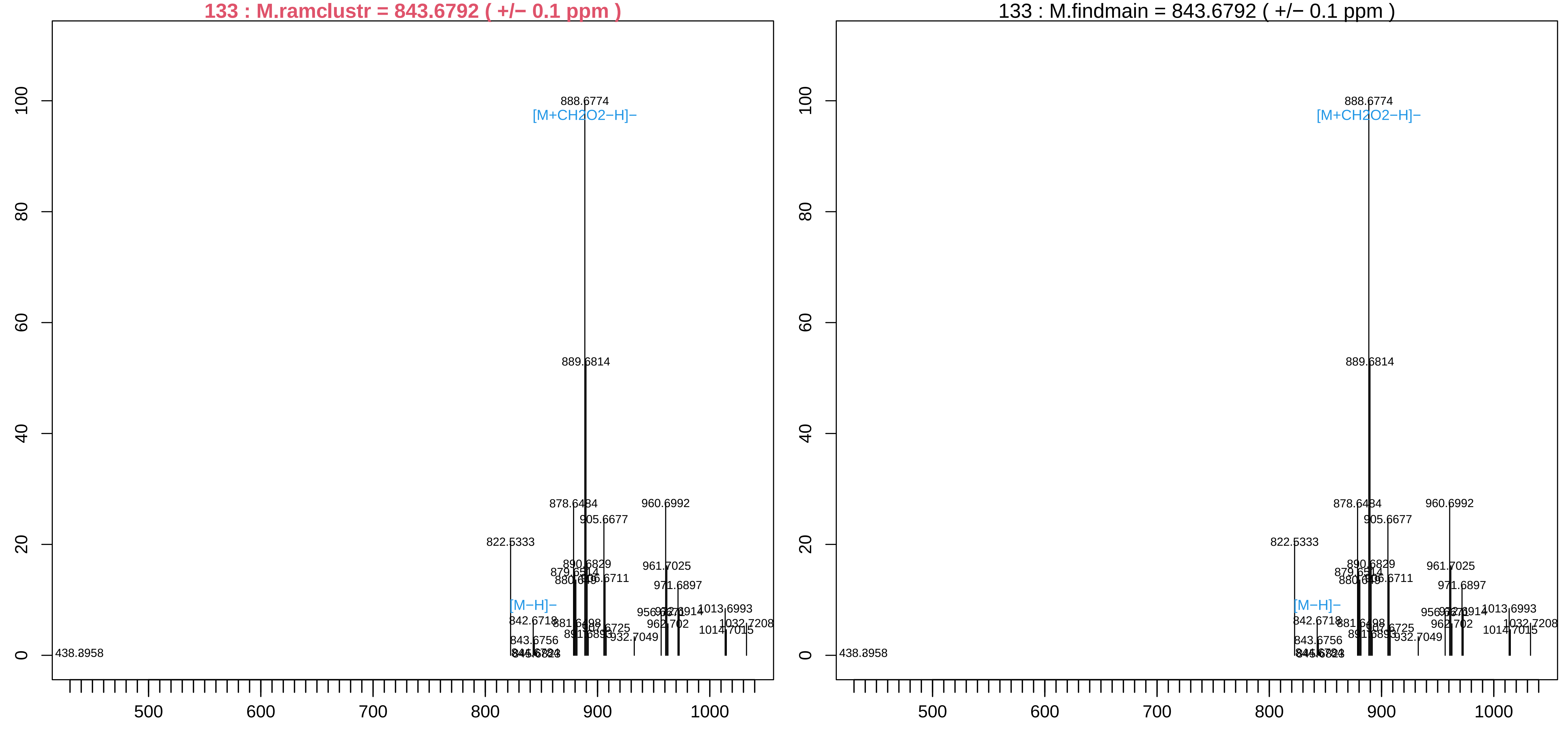
133 : M.ramclustr = 843.6792 (+/− 0.1 ppm)
133 : M.findmain = 843.6792 (+/− 0.1 ppm)
888.6774
[M+CH2O2−H]−
889.6814
878.6484
960.6992
905.6677
822.5333
890.6829
961.7025
971.6897
[M−H]−
842.6718
1013.6993
1032.7208
1014.7015
932.7049
843.6756
438.3958
0
20
40
60
80
100
500
600
700
800
900
1000

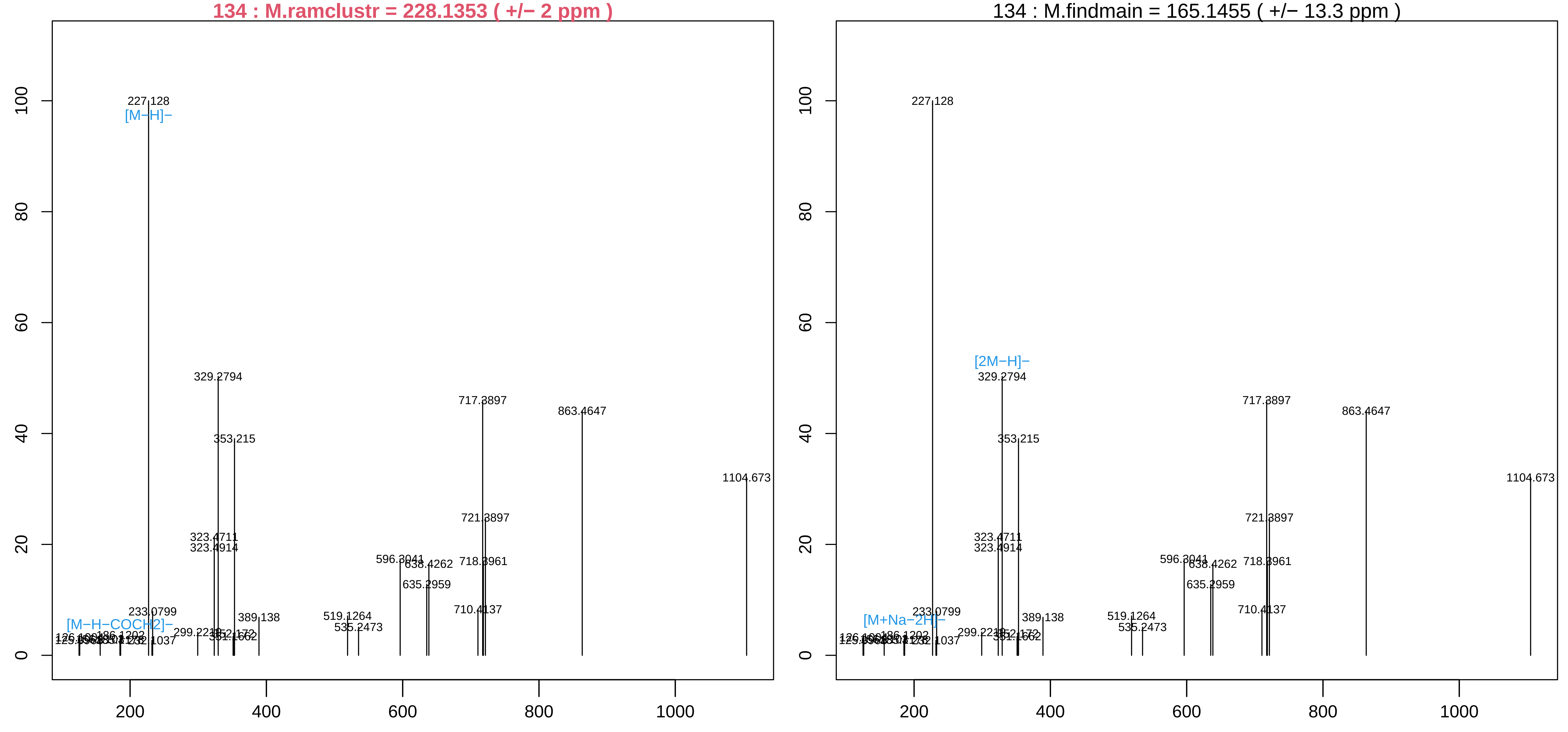
134 : M.ramclustr = 228.1353 (+/− 2 ppm)
227.128
[M−H]−
329.2794
353.215
717.3897
863.4647
1104.673
721.3897
323.4711
323.4914
596.3041
638.4262
718.3961
635.2959
710.4137
233.0799
389.138
519.1264
535.2473
[M−H−COCH2]−
299.2219
134 : M.findmain = 165.1455 (+/− 13.3 ppm)
[2M−H]−
[M+Na−2H]−
0
20
40
60
80
100
200
400
600
800
1000

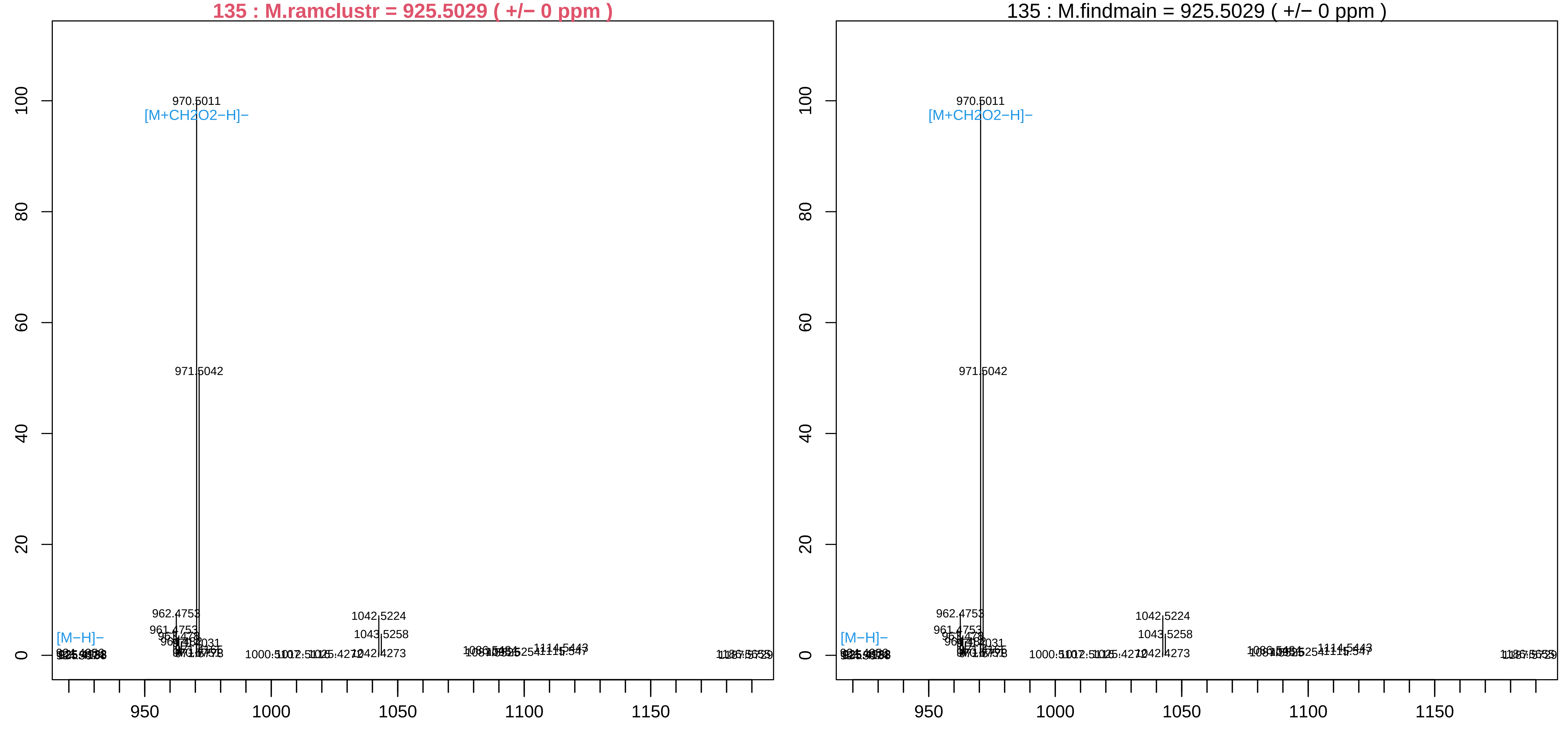

135 : M.ramclustr = 925.5029 (+/− 0 ppm)
970.5011
[M+CH2O2−H]−
971.5042
962.4753
1042.5224
1043.5258
[M−H]−
135 : M.findmain = 925.5029 (+/− 0 ppm)
970.5011
[M+CH2O2−H]−
971.5042
962.4753
1042.5224
1043.5258
[M−H]−

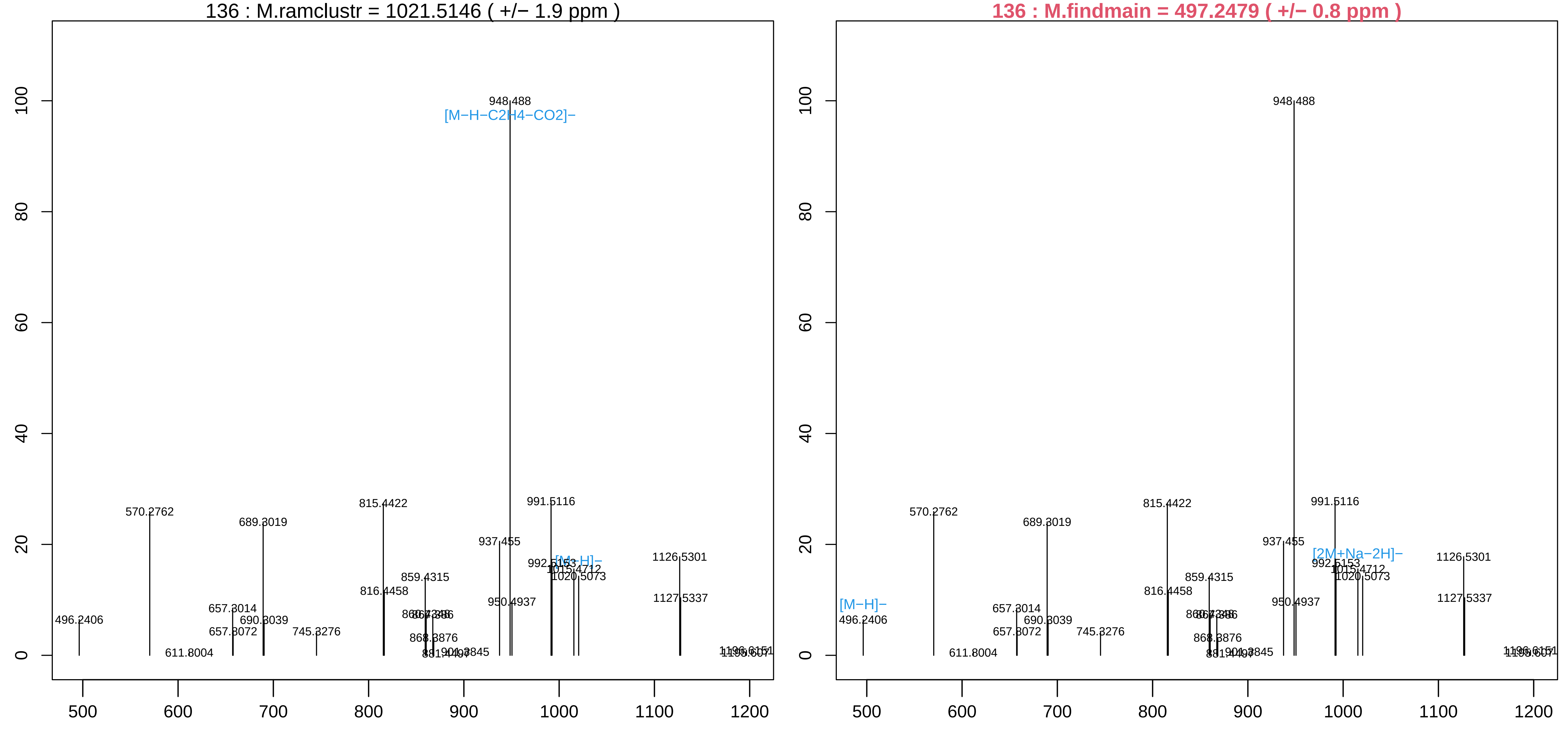
136 : M.ramclustr = 1021.5146 (+/− 1.9 ppm)
136 : M.findmain = 497.2479 (+/− 0.8 ppm)
948.488
[M−H−C2H4−CO2]−
[M−H]−
[2M+Na−2H]−
991.5116
815.4422
570.2762
689.3019
937.455
1126.5301
992.5153
1015.4712
1020.5073
859.4315
816.4458
1127.5337
950.4937
657.3014
690.3039
496.2406
657.3072
745.3276
868.3876
611.8004
901.3845
1196.6151
1195.607
0
20
40
60
80
100
500
600
700
800
900
1000
1100
1200

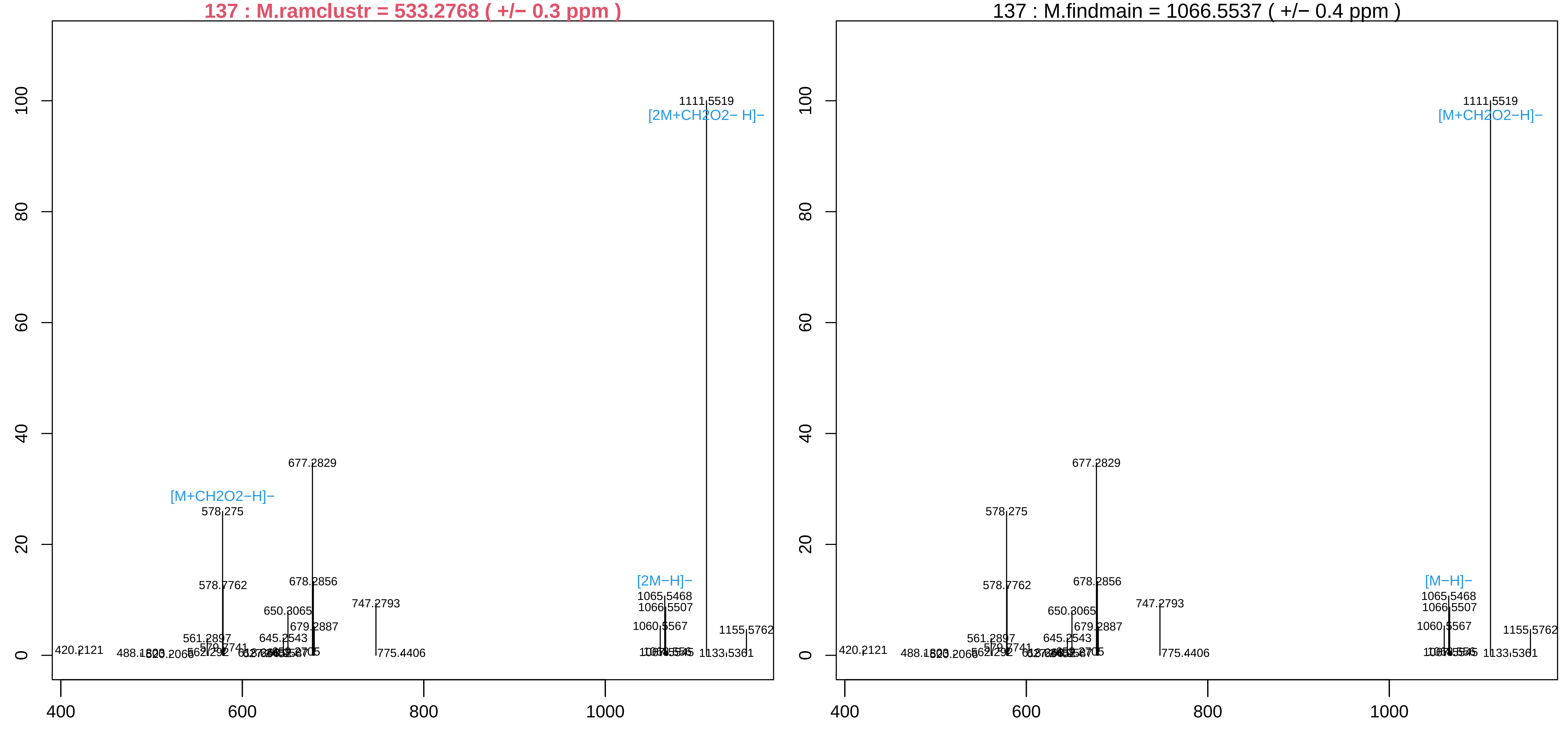
137 : M.ramclustr = 533.2768 (+/− 0.3 ppm)
137 : M.findmain = 1066.5537 (+/− 0.4 ppm)
1111.5519
[2M+CH2O2− H]−
[M+CH2O2−H]−
[2M−H]−
[M−H]−
677.2829
578.275
578.7762
678.2856
747.2793
650.3065
679.2887
561.2897
645.2543
579.7741
420.2121
775.4406
1065.5468
1066.5507
1060.5567
1155.5762
1133.5361
0
20
40
60
80
100
400
600
800
1000

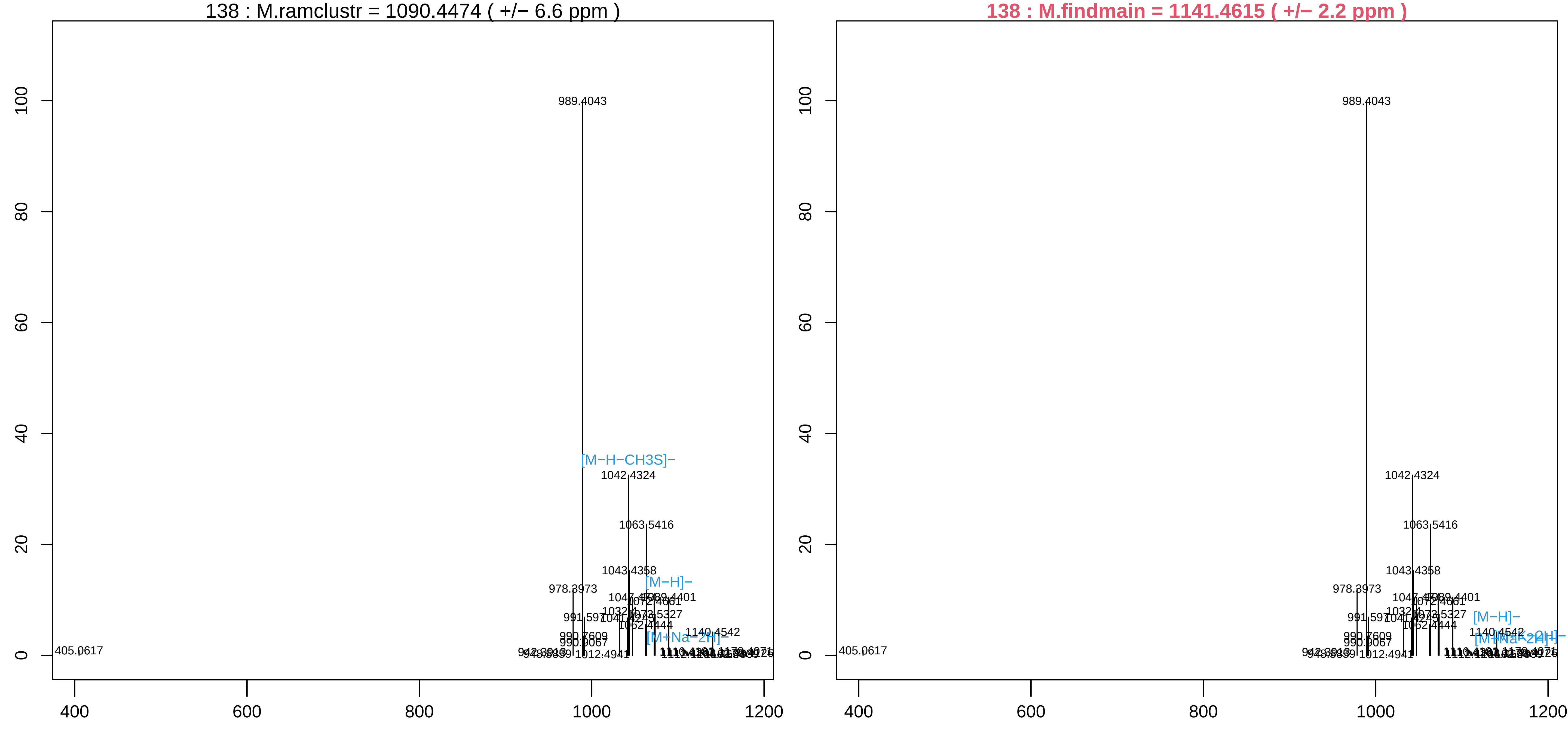
138 : M.ramclustr = 1090.4474 (+/− 6.6 ppm)
138 : M.findmain = 1141.4615 (+/− 2.2 ppm)
989.4043
[M−H−CH3S]−
1042.4324
1063.5416
1043.4358
[M−H]−
978.3973
1140.4542
[M+Na−2H]−
405.0617
990.7609
990.9067
1012.4941
0
20
40
60
80
100
400
600
800
1000
1200

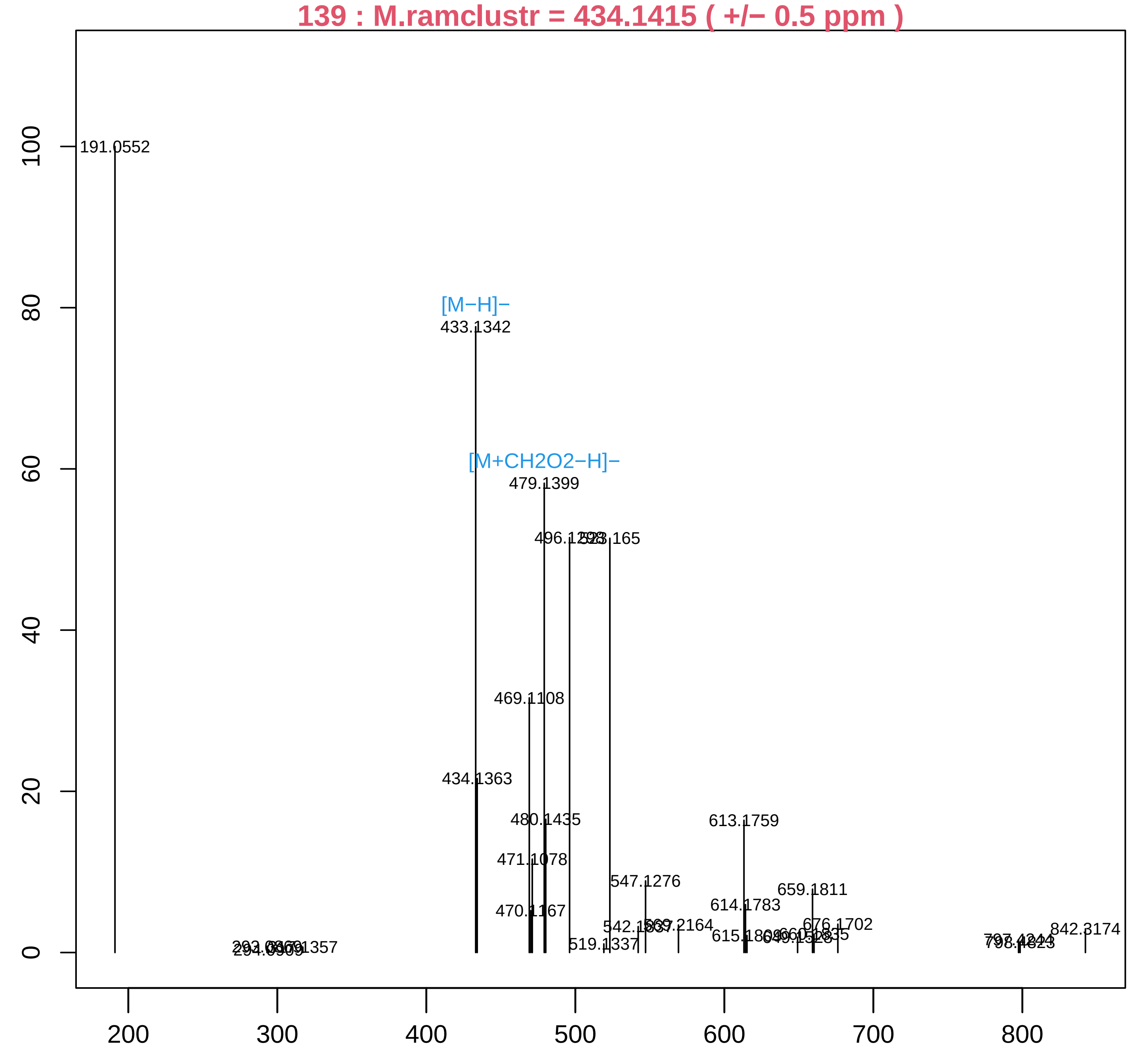
139 : M.ramclustr = 434.1415 (+/− 0.5 ppm)
[M−H]−
[M+CH2O2−H]−
191.0552
433.1342
479.1399
469.1108
434.1363
480.1435
471.1078
470.1167
613.1759
614.1783
659.1811
547.1276
676.1702
842.3174
519.1337
0
20
40
60
80
100
200
300
400
500
600
700
800

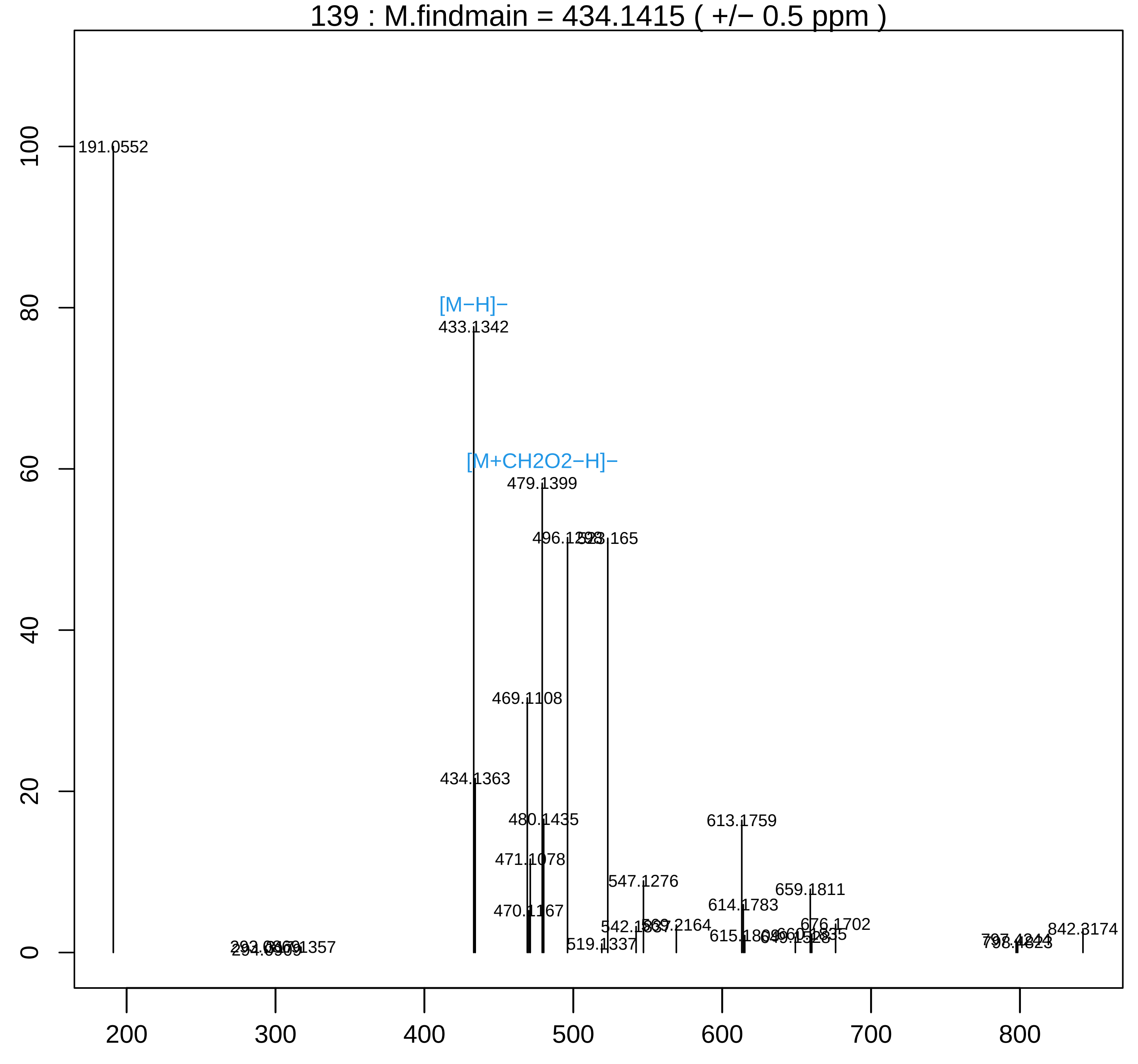
139 : M.findmain = 434.1415 (+/− 0.5 ppm)
[M−H]−
[M+CH2O2−H]−
191.0552
433.1342
479.1399
469.1108
434.1363
480.1435
471.1078
470.1167
613.1759
614.1783
659.1811
547.1276
676.1702
842.3174
519.1337
0
20
40
60
80
100
200
300
400
500
600
700
800

140 : M.ramclustr = 867.4972 (+/− 0.7 ppm)

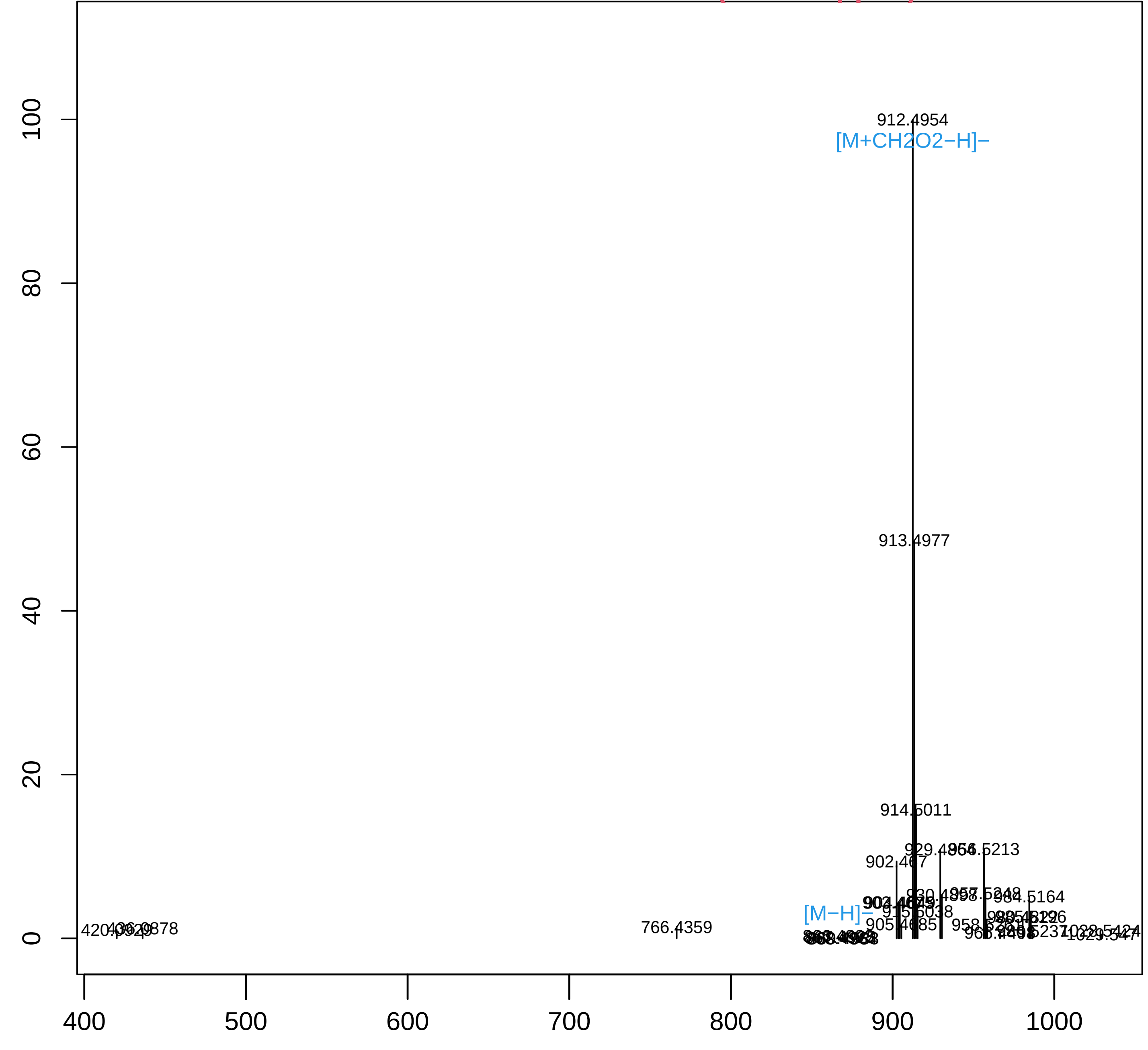

140 : M.findmain = 867.4972 (+/− 0.7 ppm)

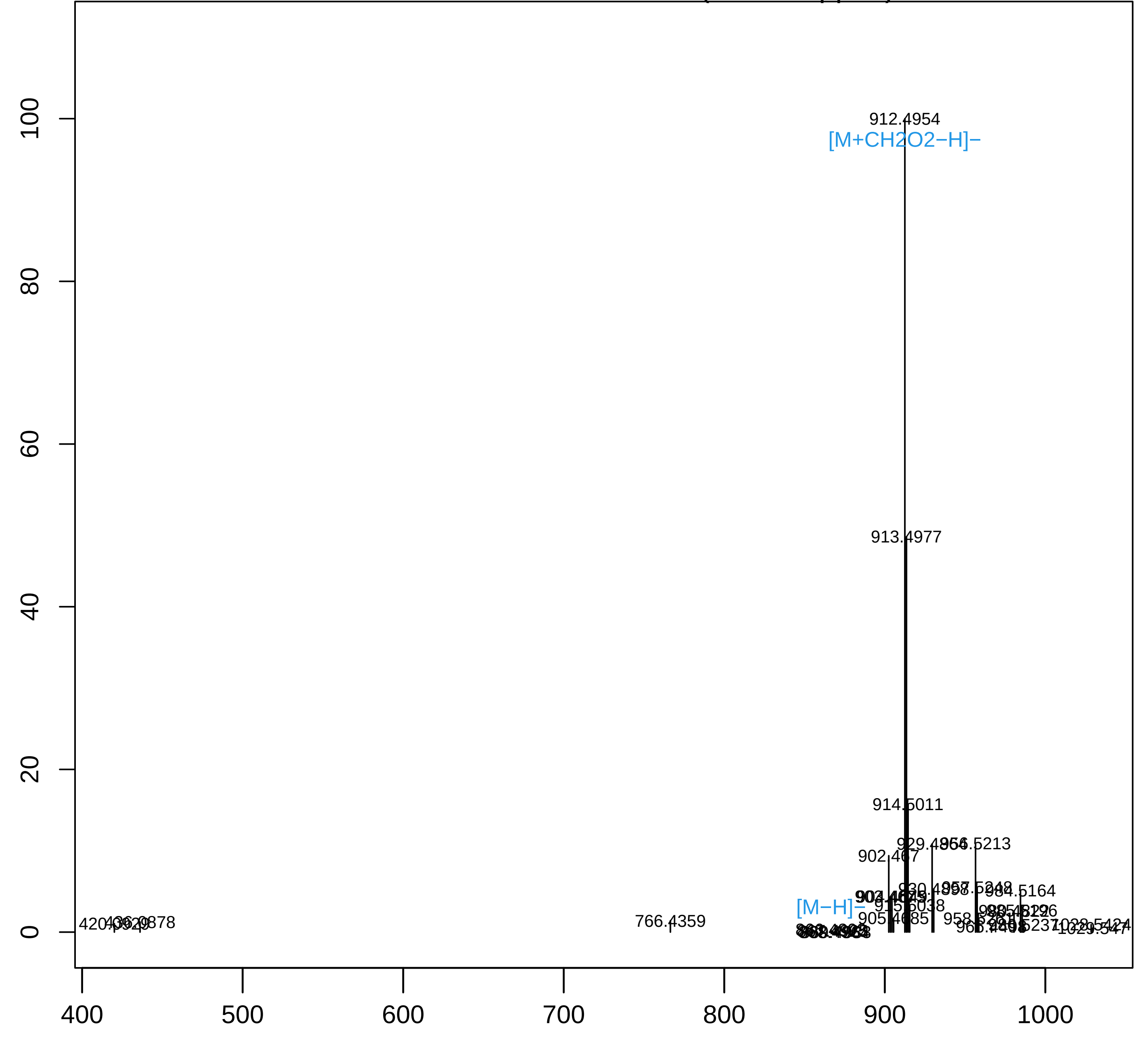

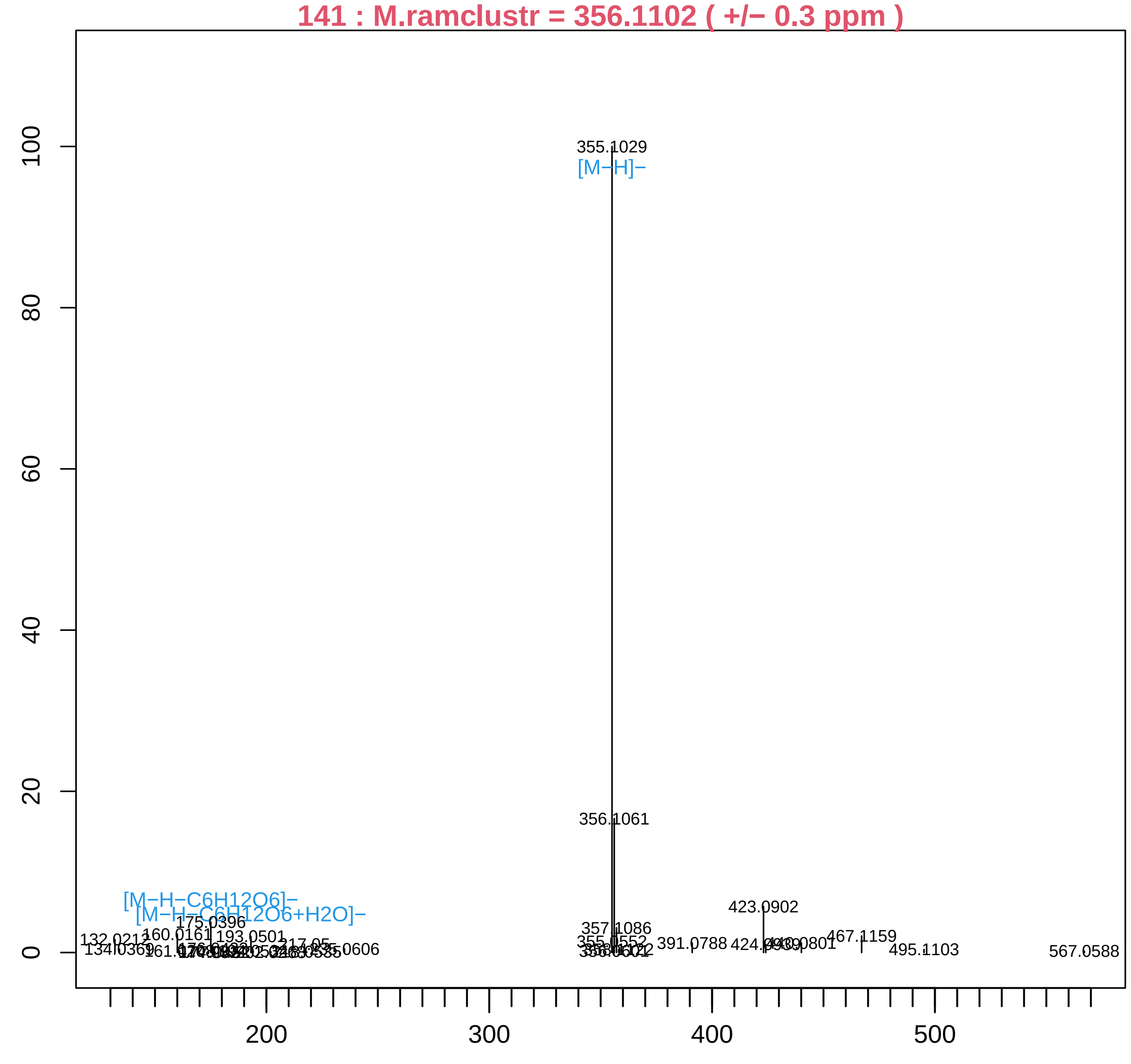
141 : M.ramclustr = 356.1102 (+/− 0.3 ppm)
355.1029
[M−H]−
356.1061
[M−H−C6H12O6]−
[M−H−C6H12O6+H2O]−
175.0396
160.0161
193.0501
132.0212
357.1086
355.0552
391.0788
423.0902
467.1159
495.1103
567.0588
0
20
40
60
80
100
200
300
400
500

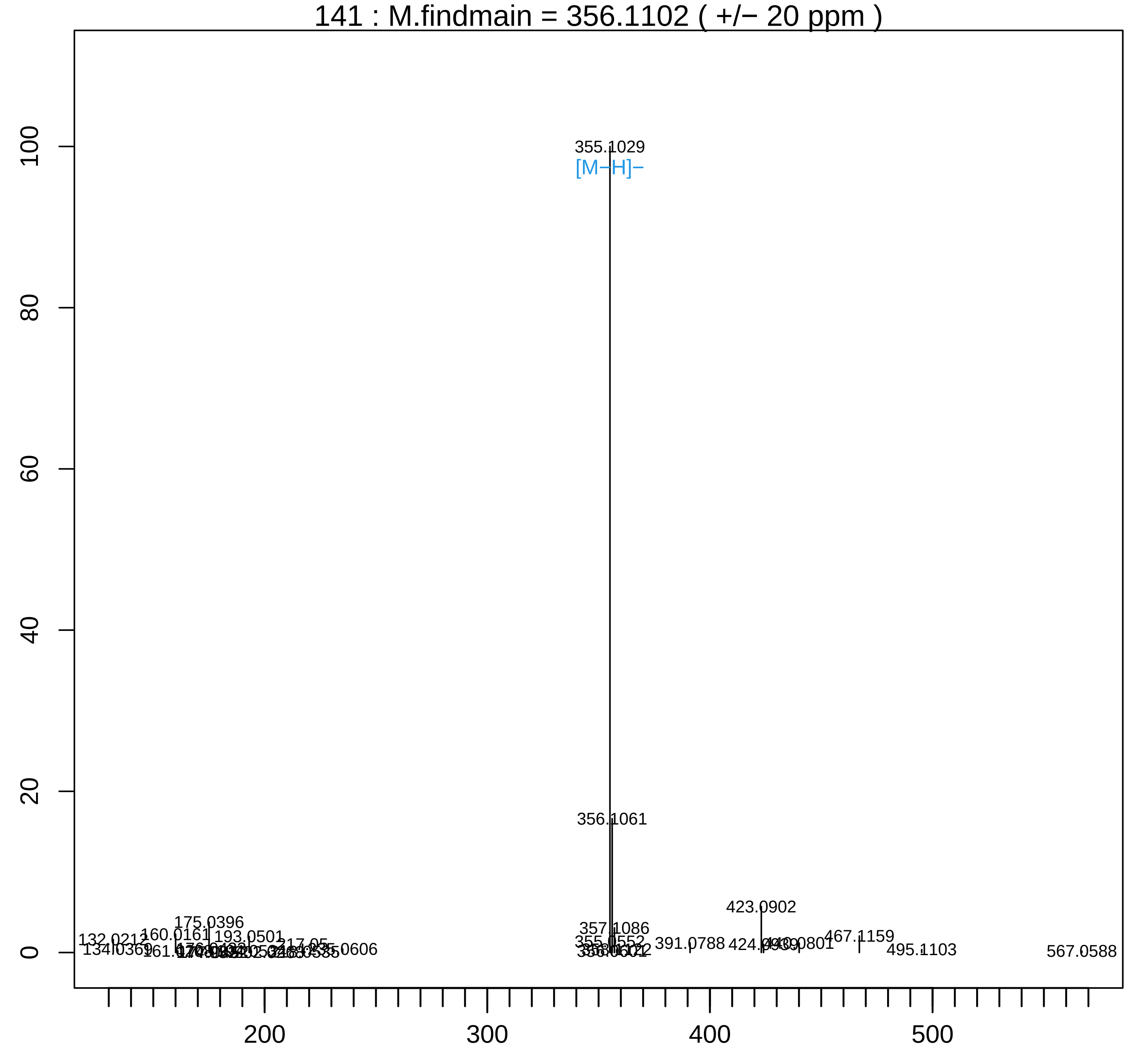
141 : M.findmain = 356.1102 (+/− 20 ppm)
355.1029
[M−H]−
356.1061
175.0396
160.0161
193.0501
132.0212
357.1086
355.0552
391.0788
423.0902
467.1159
495.1103
567.0588
0
20
40
60
80
100
200
300
400
500

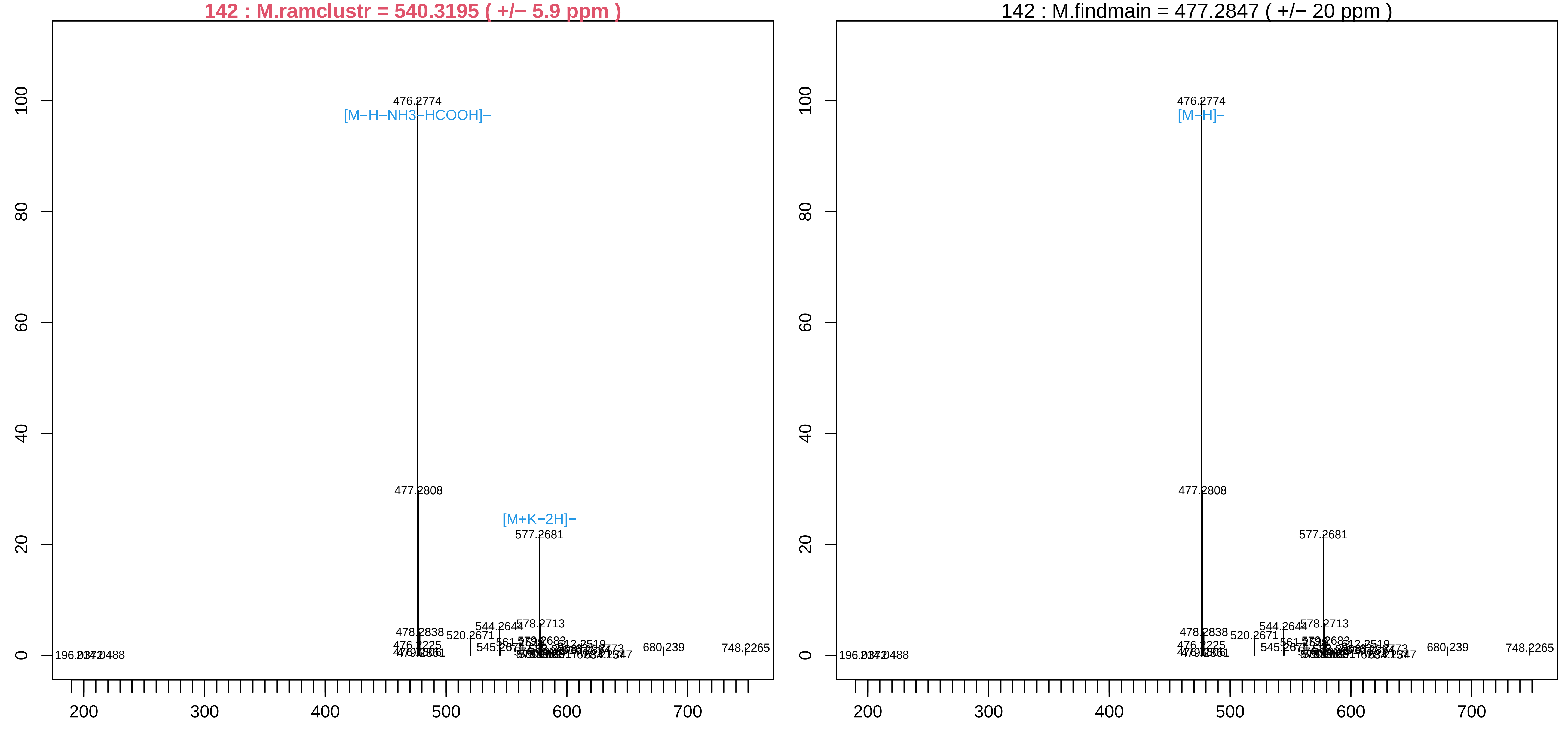
142 : M.ramclustr = 540.3195 (+/− 5.9 ppm)
476.2774
[M−H−NH3−HCOOH]−
477.2808
[M+K−2H]−
577.2681
478.2838
476.2225
520.2671
544.2644
578.2713
680.239
748.2265
0
20
40
60
80
100
200
300
400
500
600
700
142 : M.findmain = 477.2847 (+/− 20 ppm)
476.2774
[M−H]−
477.2808
577.2681
478.2838
476.2225
520.2671
544.2644
578.2713
680.239
748.2265
0
20
40
60
80
100
200
300
400
500
600
700

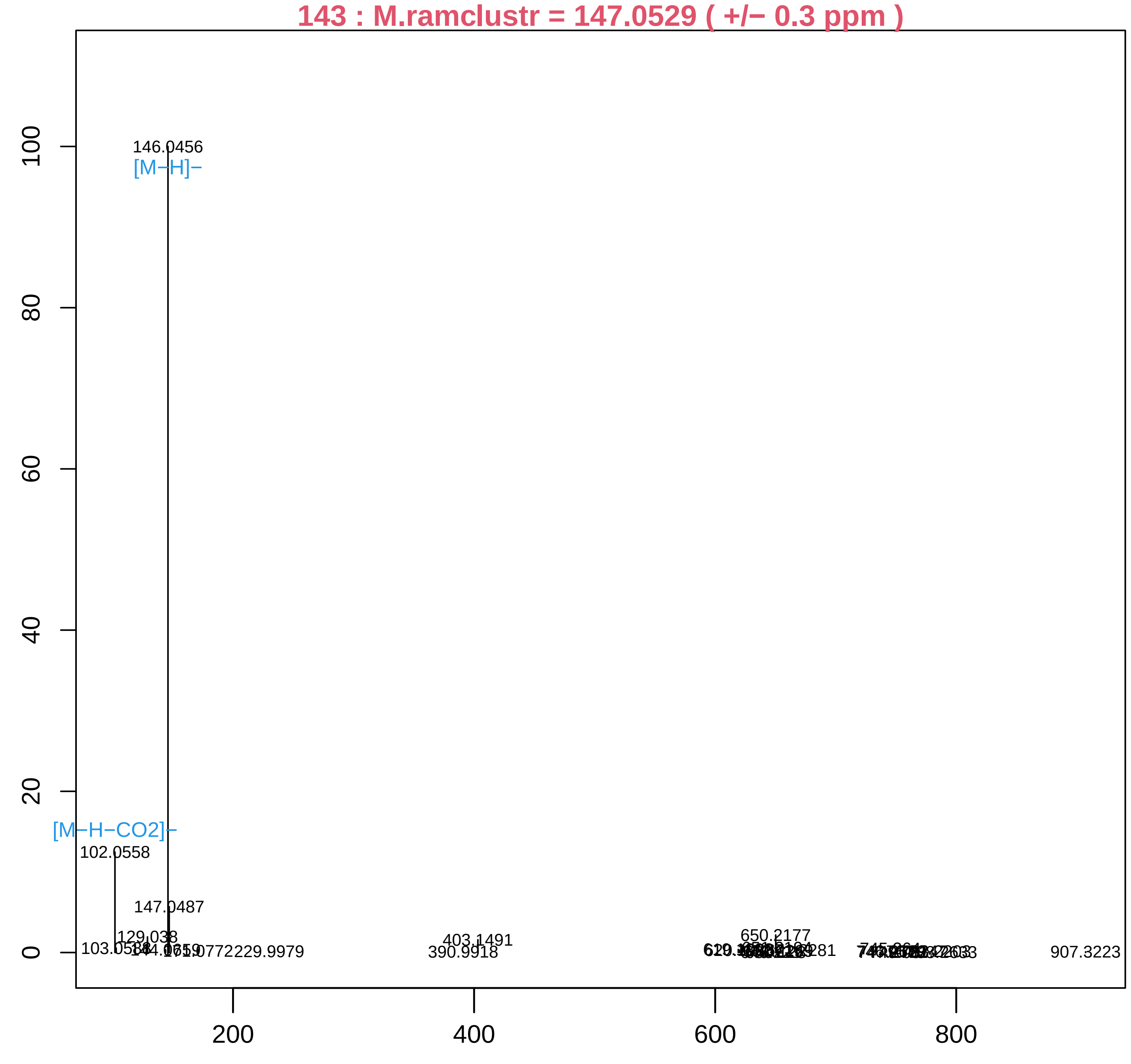
143 : M.ramclustr = 147.0529 (+/− 0.3 ppm)
146.0456
[M−H]−
[M−H−CO2]−
102.0558
147.0487
129.038
403.1491
390.9918
650.2177
907.3223
0
20
40
60
80
100
200
400
600
800

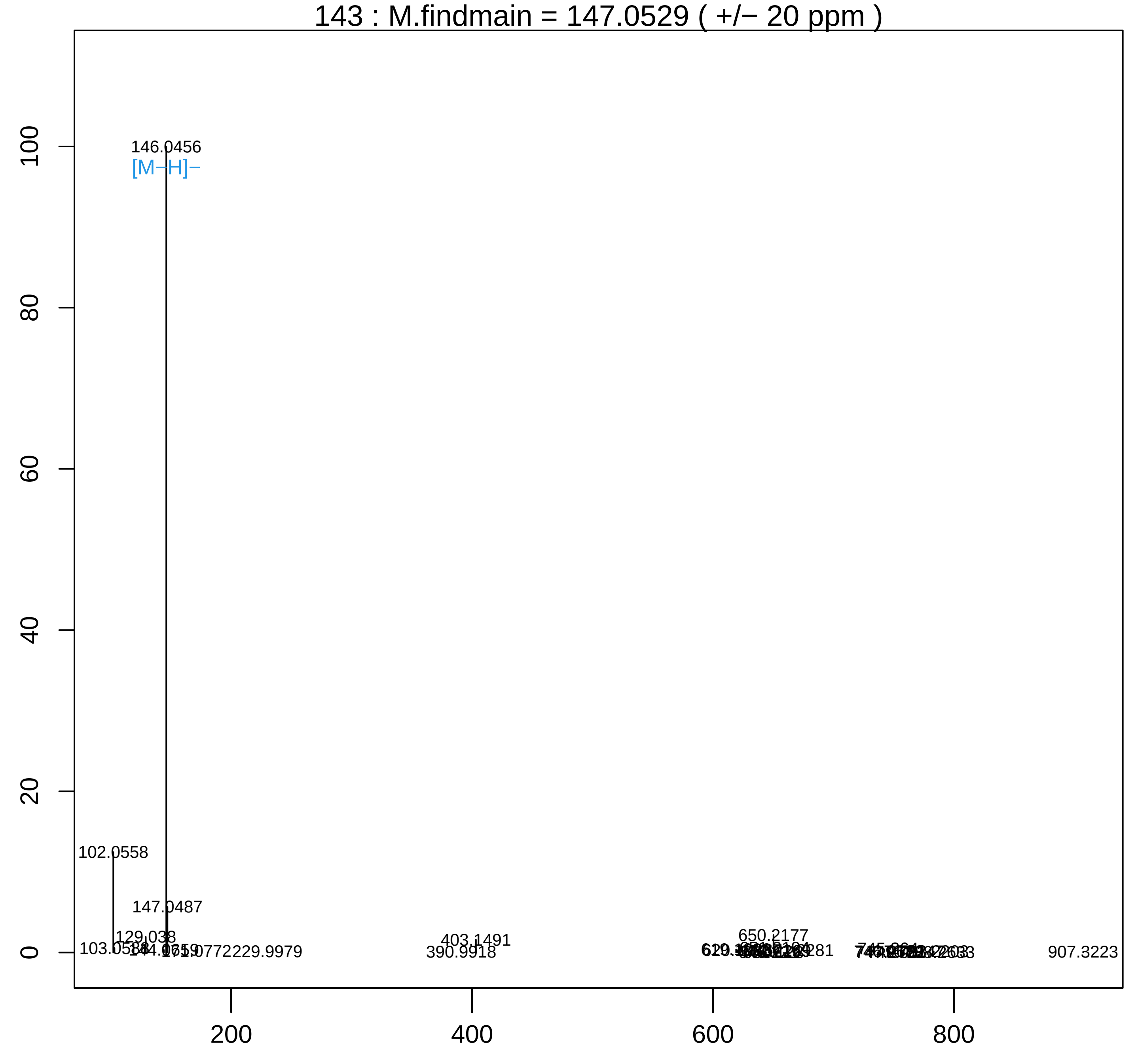
143 : M.findmain = 147.0529 (+/− 20 ppm)
146.0456
[M−H]−
102.0558
147.0487
129.038
403.1491
390.9918
650.2177
907.3223
0
20
40
60
80
100
200
400
600
800

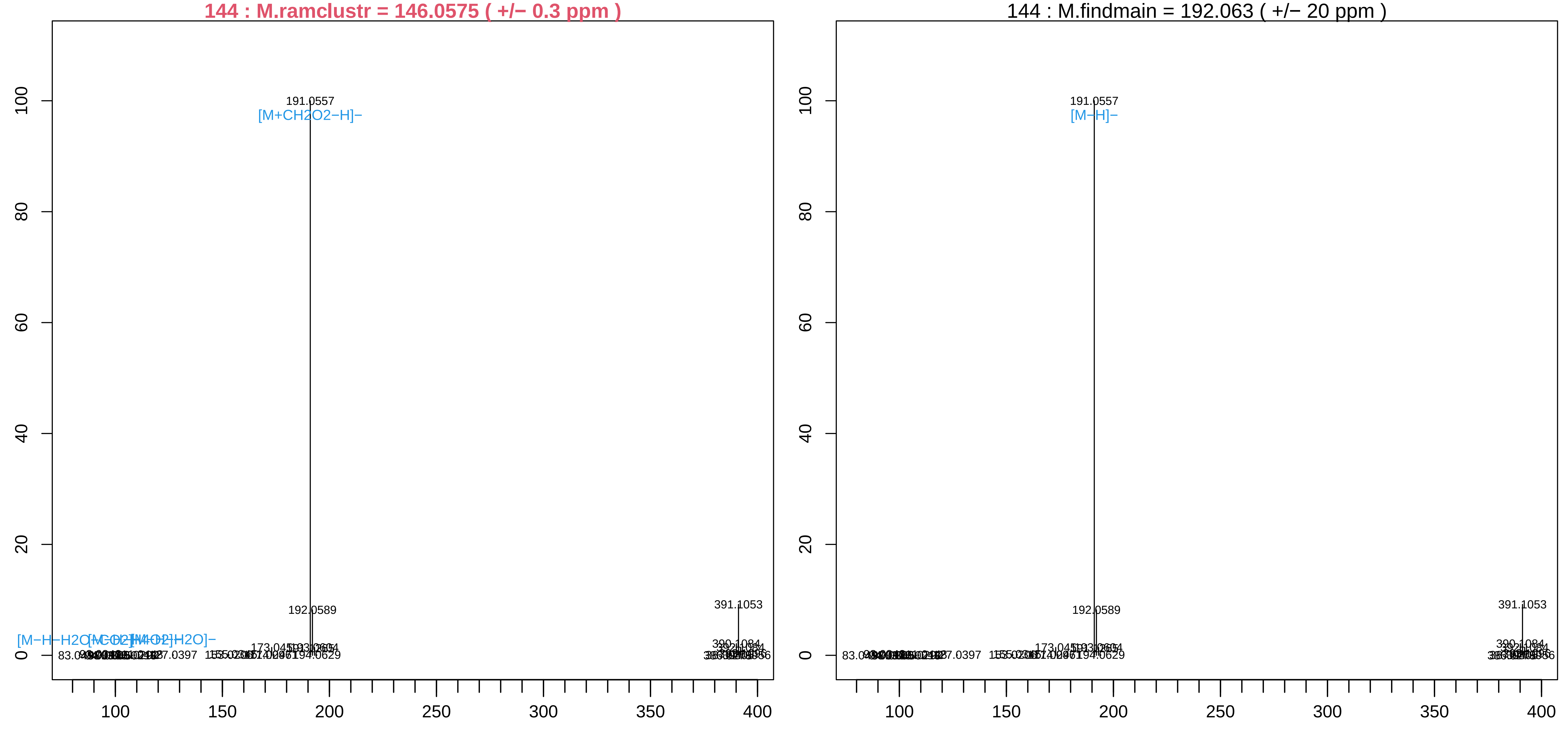
144 : M.ramclustr = 146.0575 (+/− 0.3 ppm)
191.0557
[M+CH2O2−H]−
192.0589
391.1053
390.1084
173.0451
194.0629
83.0
137.0397
144 : M.findmain = 192.063 (+/− 20 ppm)
191.0557
[M−H]−
192.0589
391.1053
390.1084
173.0451
194.0629
83.0
137.0397
0
20
40
60
80
100
150
200
250
300
350
400

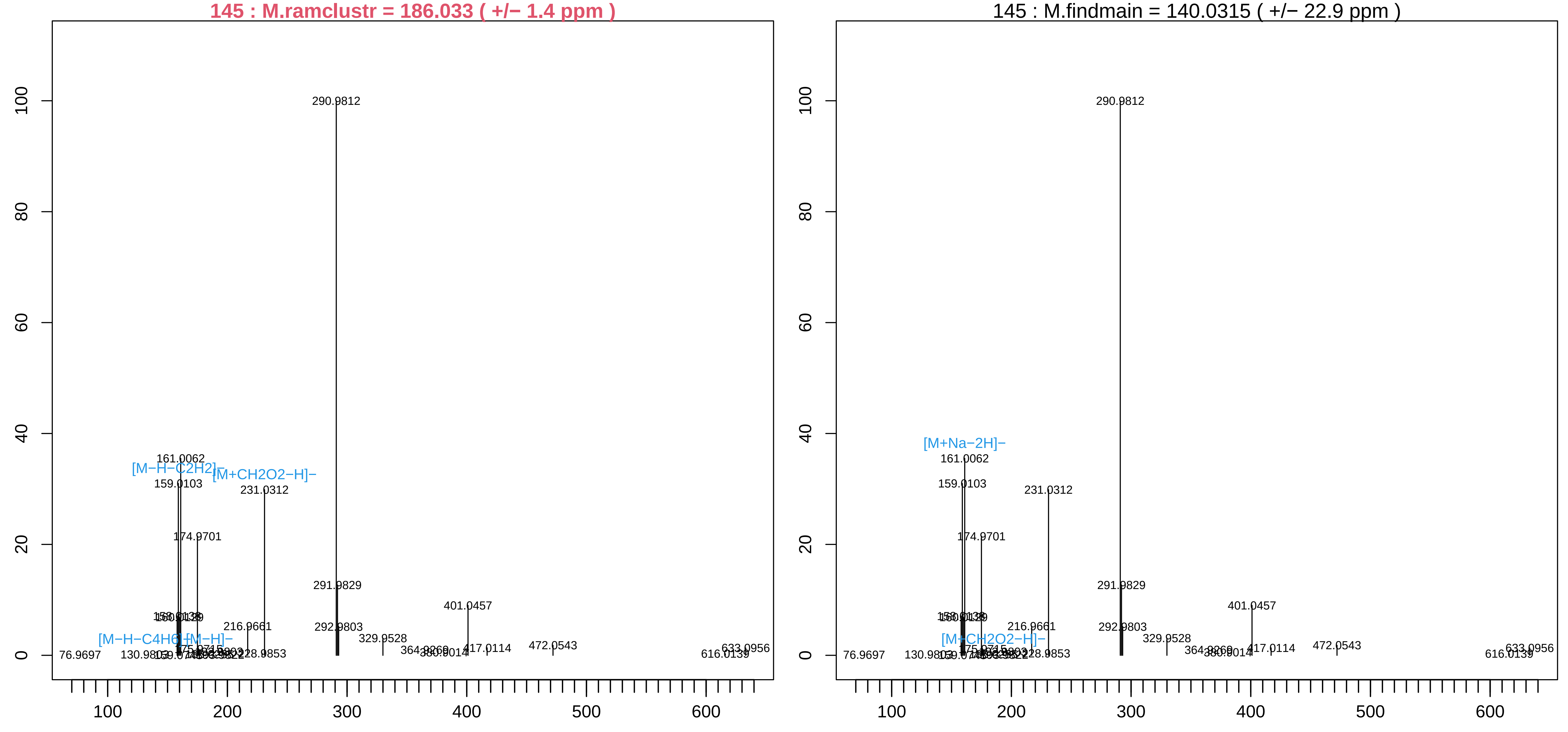

145 : M.ramclustr = 186.033 (+/− 1.4 ppm)
290.9812
161.0062
[M−H−C2H2]−
[M+CH2O2−H]−
159.0103
231.0312
174.9701
291.9829
401.0457
216.9661
292.9803
329.9528
[M−H−C4H6]−
[M−H]−
472.0543
417.0114
633.0956
616.0139
76.9697
228.9853
100
200
300
400
500
600
145 : M.findmain = 140.0315 (+/− 22.9 ppm)
[M+Na−2H]−
[M+CH2O2−H]−

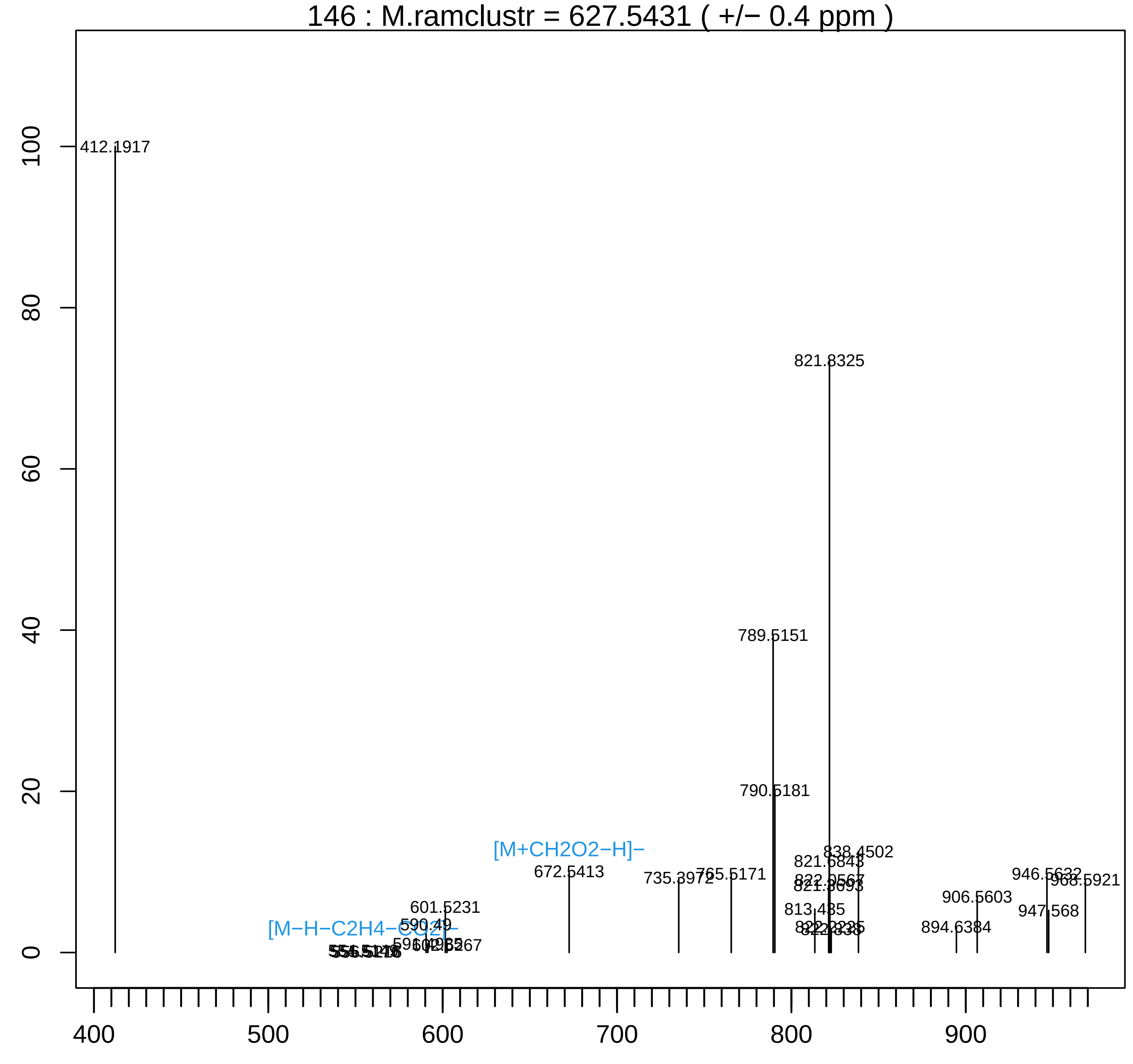

146 : M.ramclustr = 627.5431 (+/− 0.4 ppm)
412.1917
821.8325
789.5151
790.5181
[M+CH2O2−H]−
672.5413
735.3972
765.5171
838.4502
821.6843
822.0567
821.8693
813.435
906.5603
946.5632
968.5921
947.568
894.6384
601.5231
590.49
[M−H−C2H4−CO2]−
556.5218
0
20
40
60
80
100
400
500
600
700
800
900

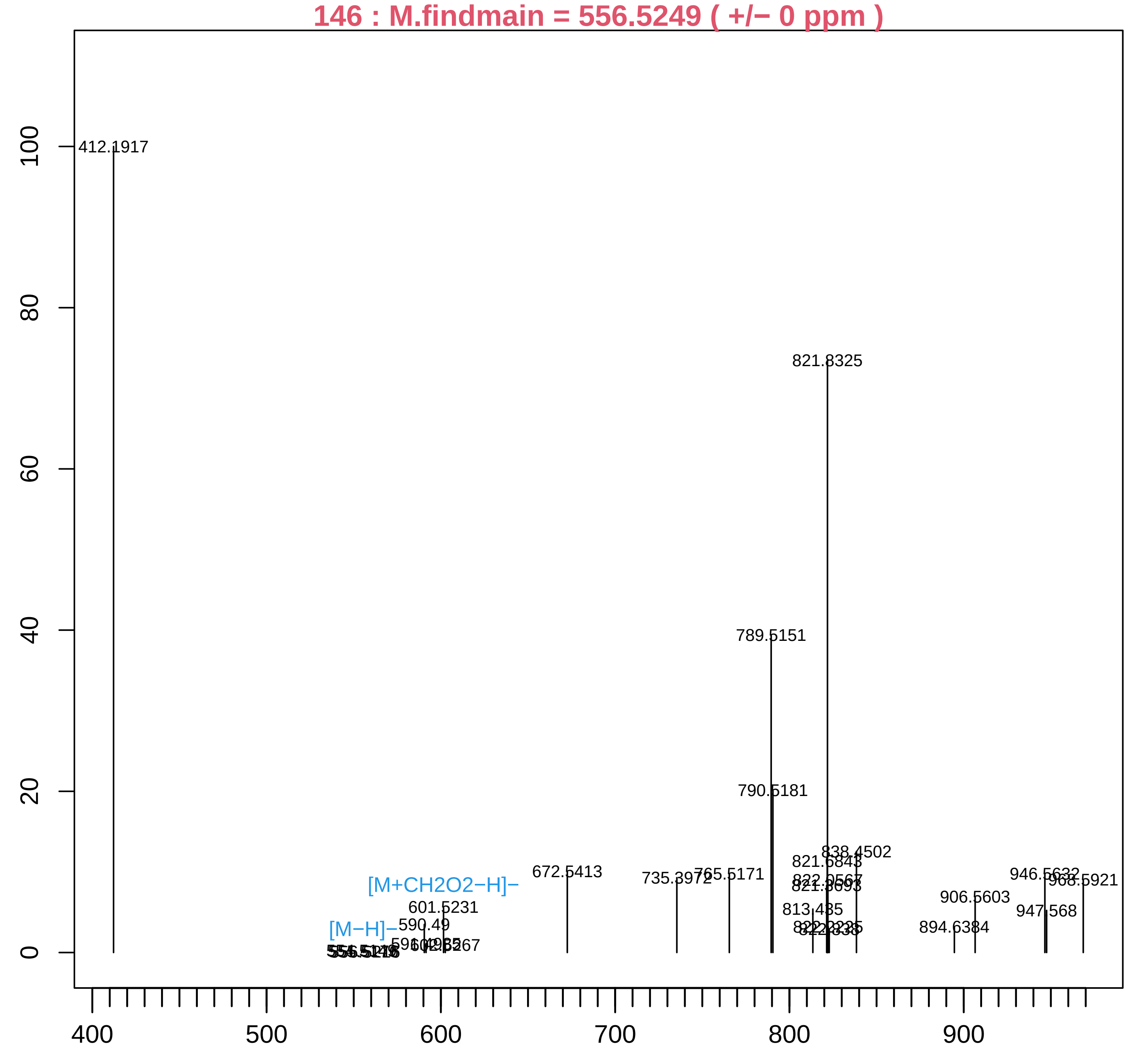

146 : M.findmain = 556.5249 (+/− 0 ppm)
412.1917
821.8325
789.5151
790.5181
[M+CH2O2−H]−
672.5413
735.3972
765.5171
838.4502
821.6843
822.0567
821.8693
813.435
906.5603
946.5632
968.5921
947.568
894.6384
601.5231
590.49
[M−H]−
556.5218
0
20
40
60
80
100
400
500
600
700
800
900

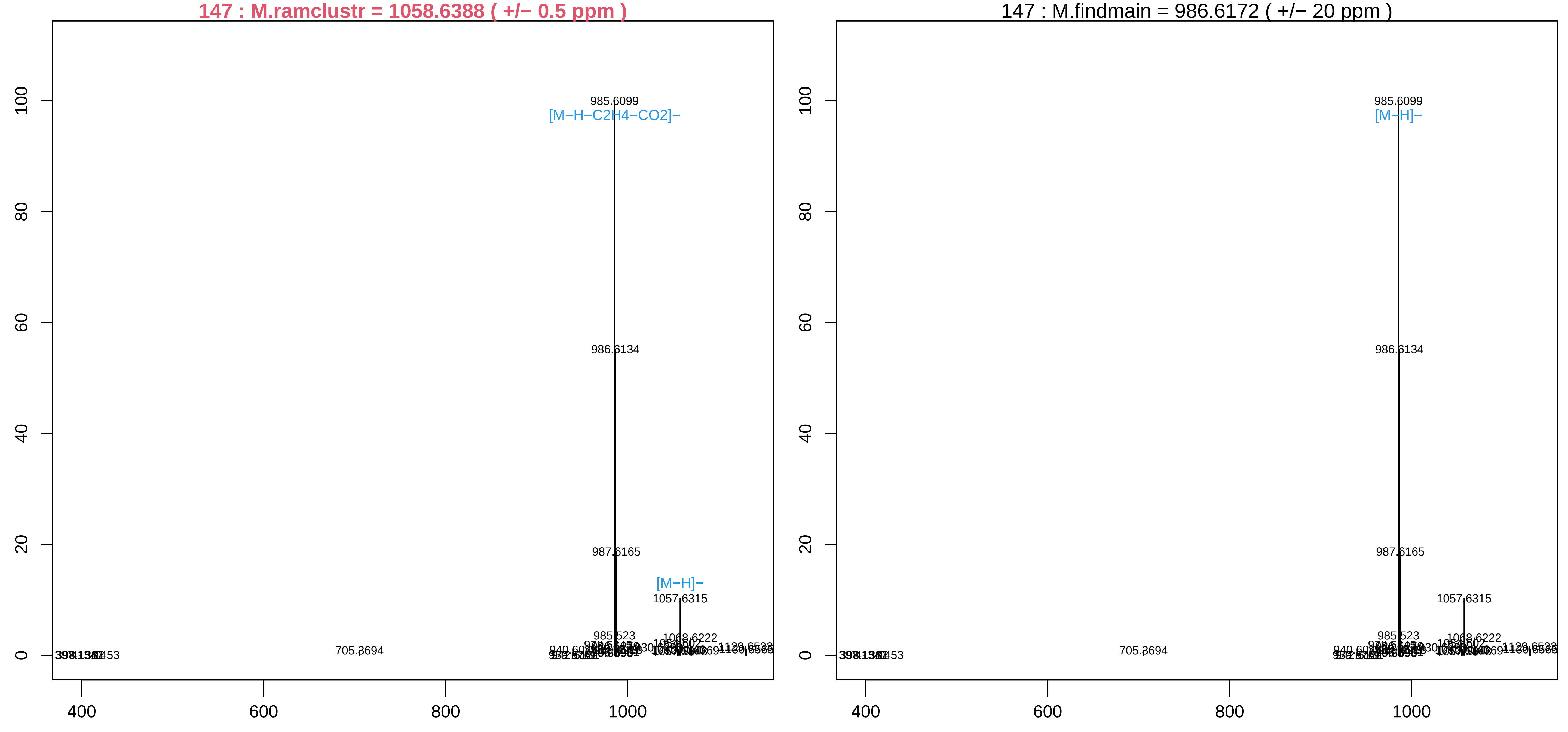

147 : M.ramclustr = 1058.6388 (+/− 0.5 ppm)
985.6099
[M−H−C2H4−CO2]−
986.6134
987.6165
[M−H]−
1057.6315
1068.6222
705.3694
147 : M.findmain = 986.6172 (+/− 20 ppm)
985.6099
[M−H]−
986.6134
987.6165
1057.6315
1068.6222
705.3694
0
20
40
60
80
100
400
600
800
1000

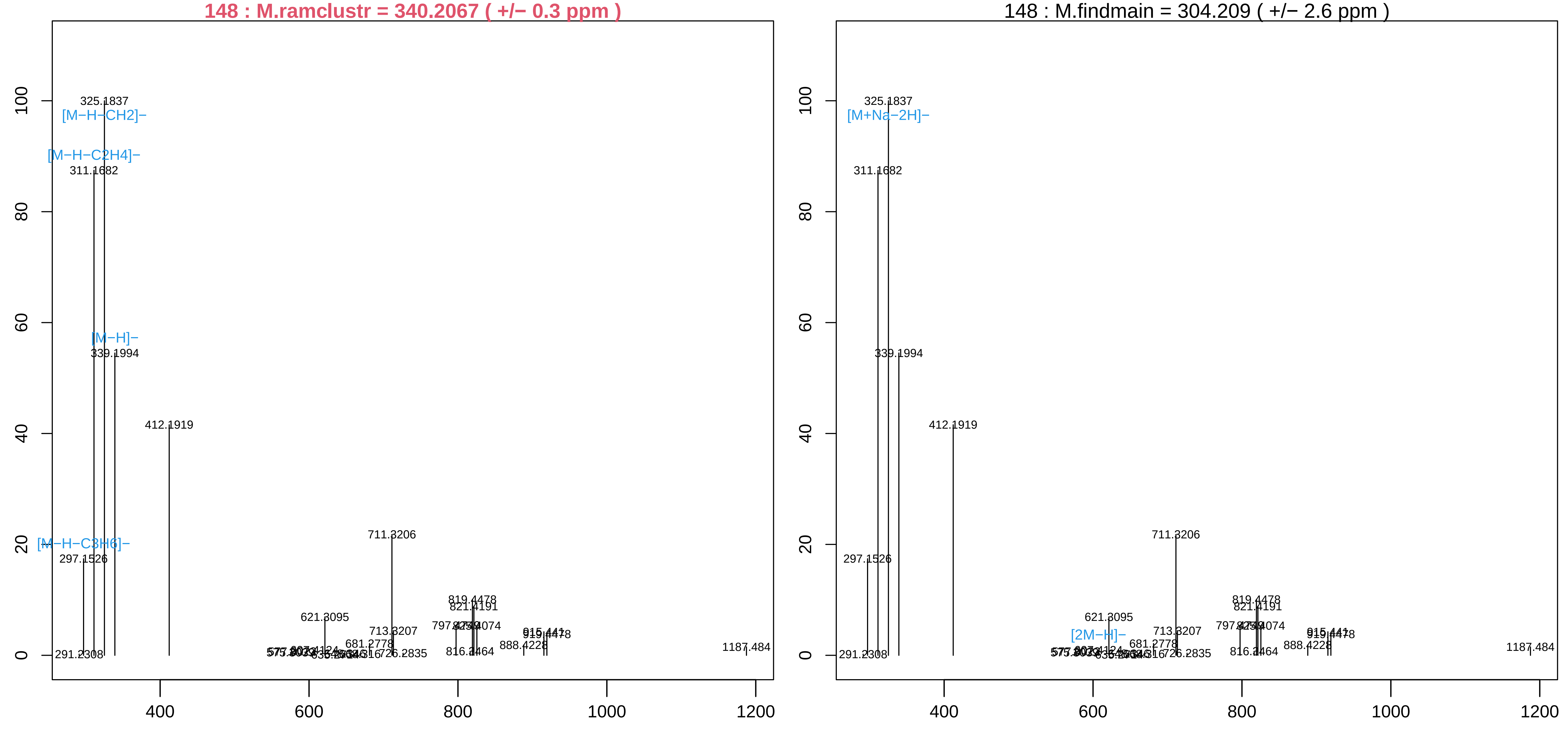
148 : M.ramclustr = 340.2067 (+/− 0.3 ppm)
325.1837
[M−H−CH2]−
[M−H−C2H4]−
311.1682
[M−H]−
339.1994
412.1919
[M−H−C3H6]−
297.1526
291.2308
711.3206
621.3095
713.3207
681.2778
726.2835
819.4478
821.4191
816.4464
888.4228
915.441
919.4478
1187.484
148 : M.findmain = 304.209 (+/− 2.6 ppm)
[M+Na−2H]−
[2M−H]−
0
20
40
60
80
100
400
600
800
1000
1200

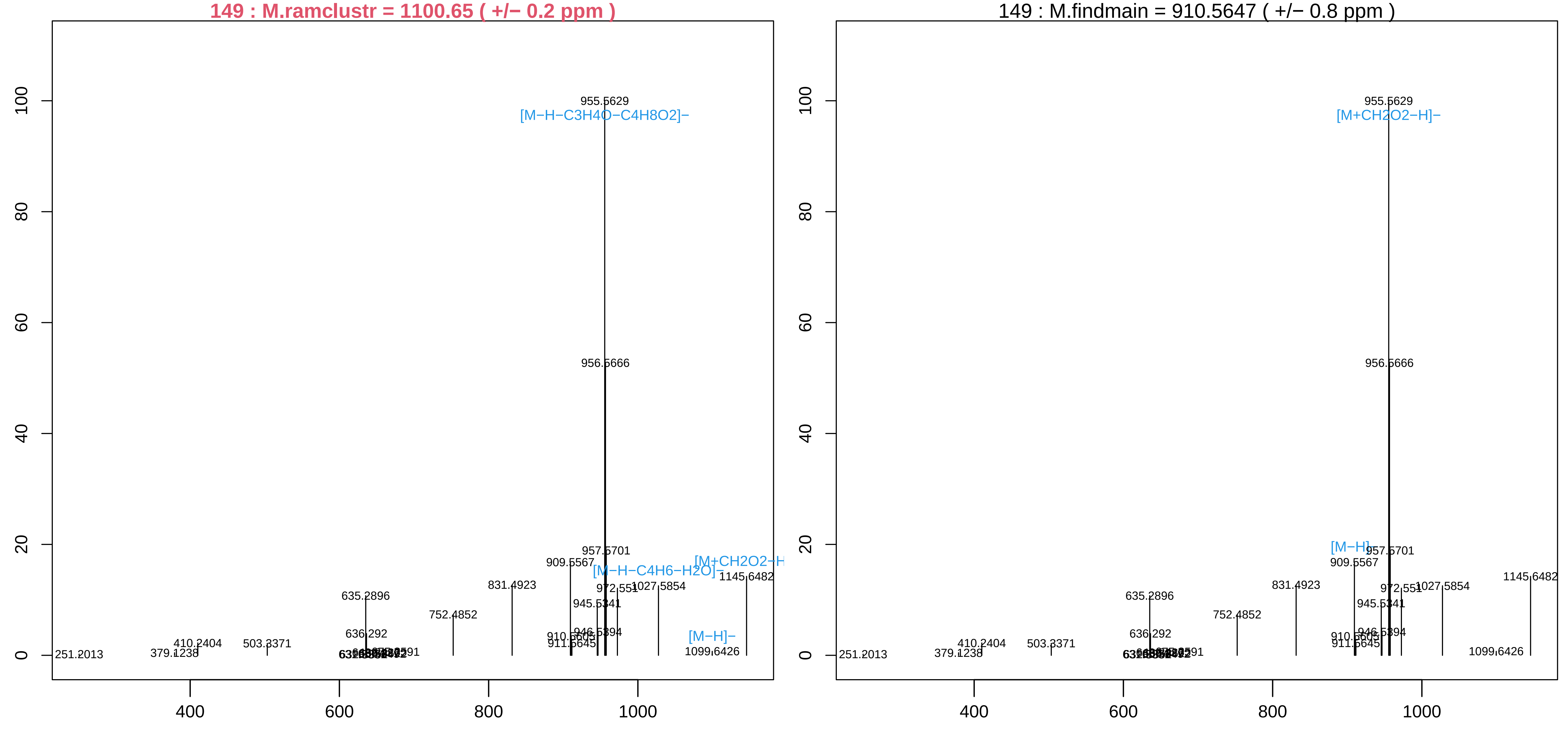

149 : M.ramclustr = 1100.65 (+/− 0.2 ppm)
149 : M.findmain = 910.5647 (+/− 0.8 ppm)
955.5629
[M−H−C3H4O−C4H8O2]−
[M+CH2O2−H]−
956.5666
957.5701
909.5567
[M−H−C4H6−H2O]−
[M−H]−
1145.6482
831.4923
972.551
1027.5854
635.2896
945.5341
752.4852
636.292
946.5394
910.5605
911.5645
410.2404
503.3371
379.1238
251.2013
1099.6426

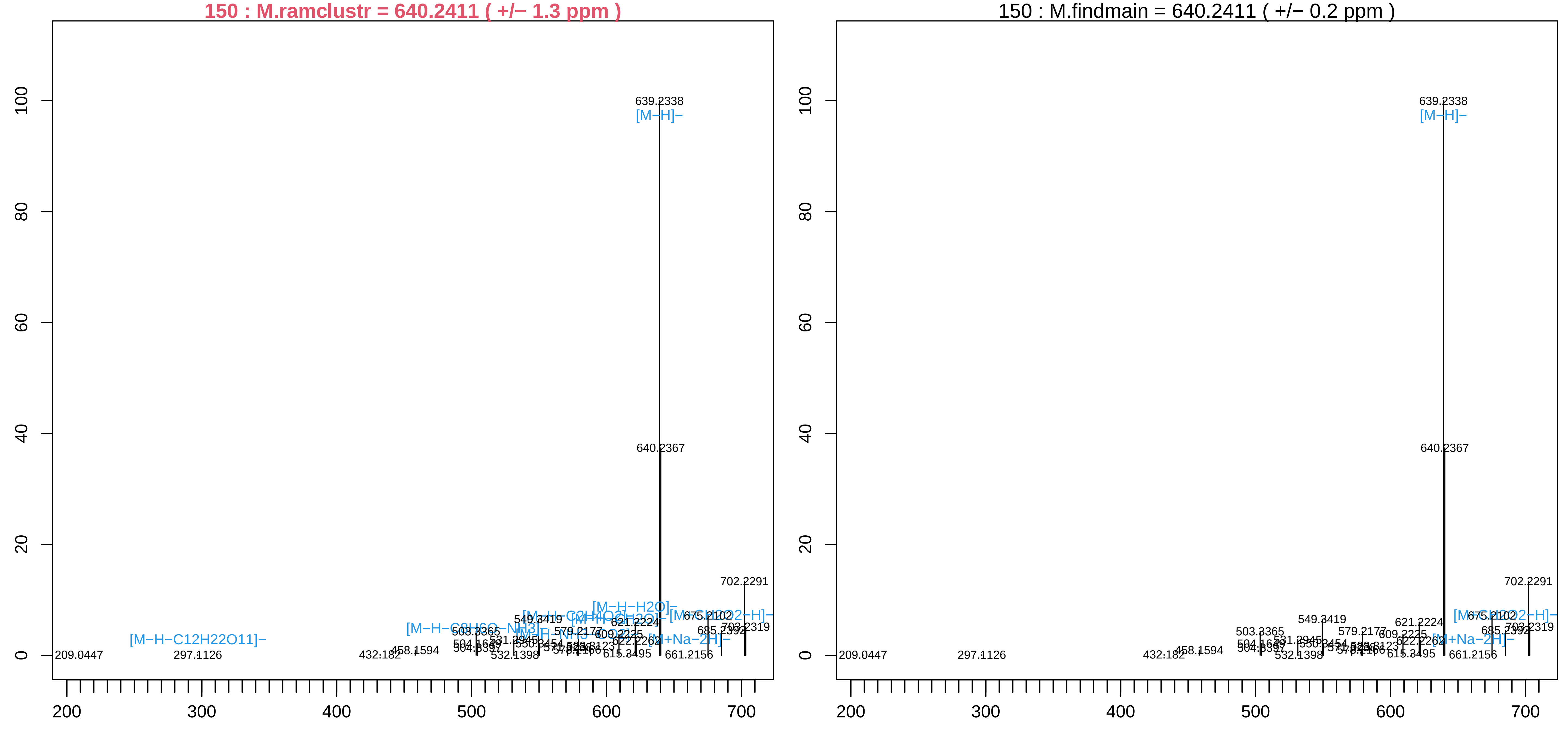
150 : M.ramclustr = 640.2411 (+/− 1.3 ppm)
150 : M.findmain = 640.2411 (+/− 0.2 ppm)
639.2338
[M−H]−
640.2367
702.2291
[M−H−H2O]−
[M−H−C12H22O11]−
[M+Na−2H]−
675.2102
685.2392
703.2319
621.2224
549.3419
503.3365
579.2177
609.2225
209.0447
297.1126
432.182
458.1594
532.1398
615.3495
661.2156
0
20
40
60
80
100
200
300
400
500
600
700

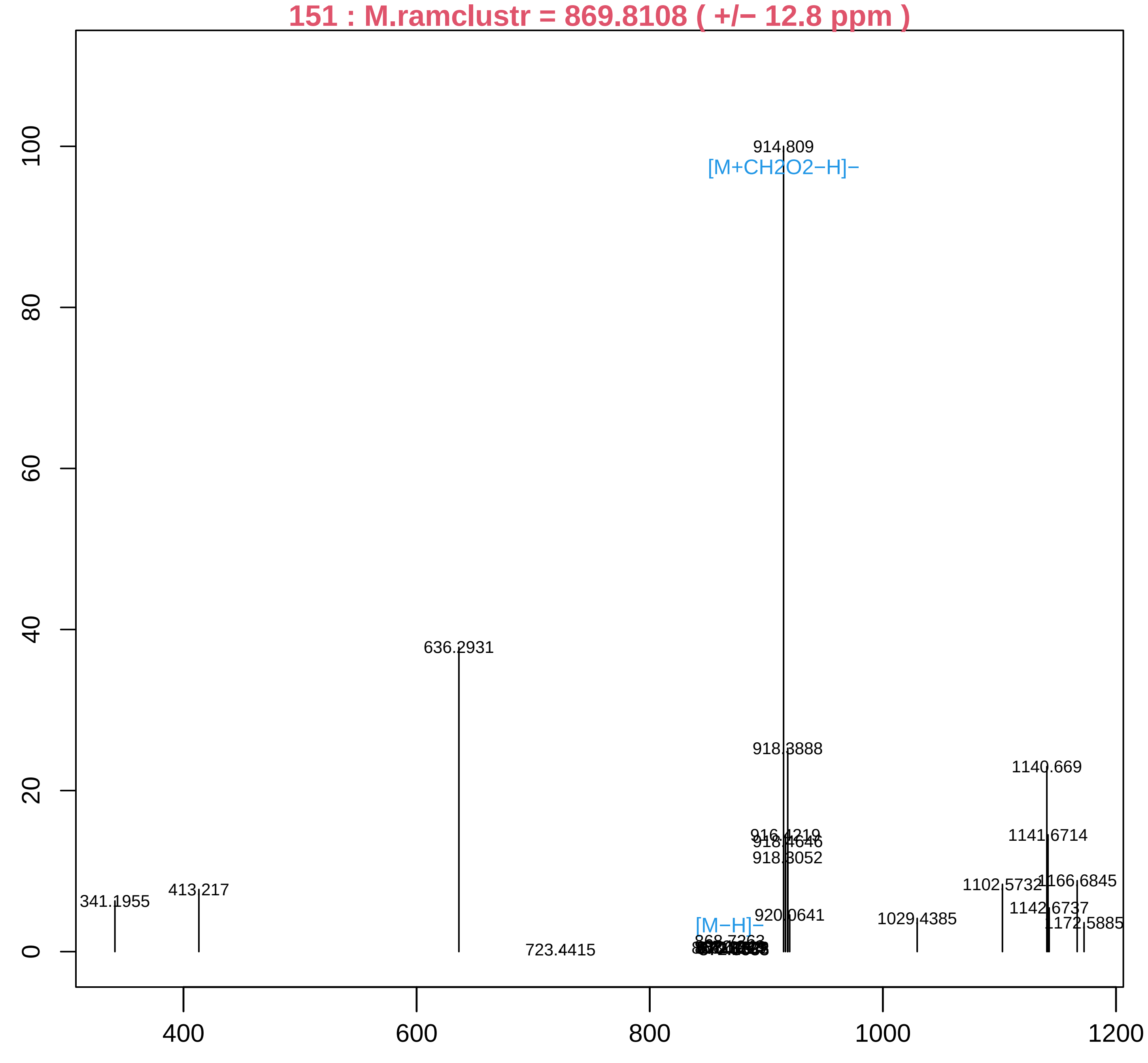

151 : M.ramclustr = 869.8108 (+/− 12.8 ppm)
914.809
[M+CH2O2−H]−
636.2931
918.3888
1140.669
916.4219
918.4646
918.3052
1141.6714
1102.5732
1166.6845
341.1955
413.217
1142.6737
920.0641
1029.4385
1172.5885
[M−H]−
868.7263
723.4415
0 20 40 60 80 100
400 600 800 1000 1200

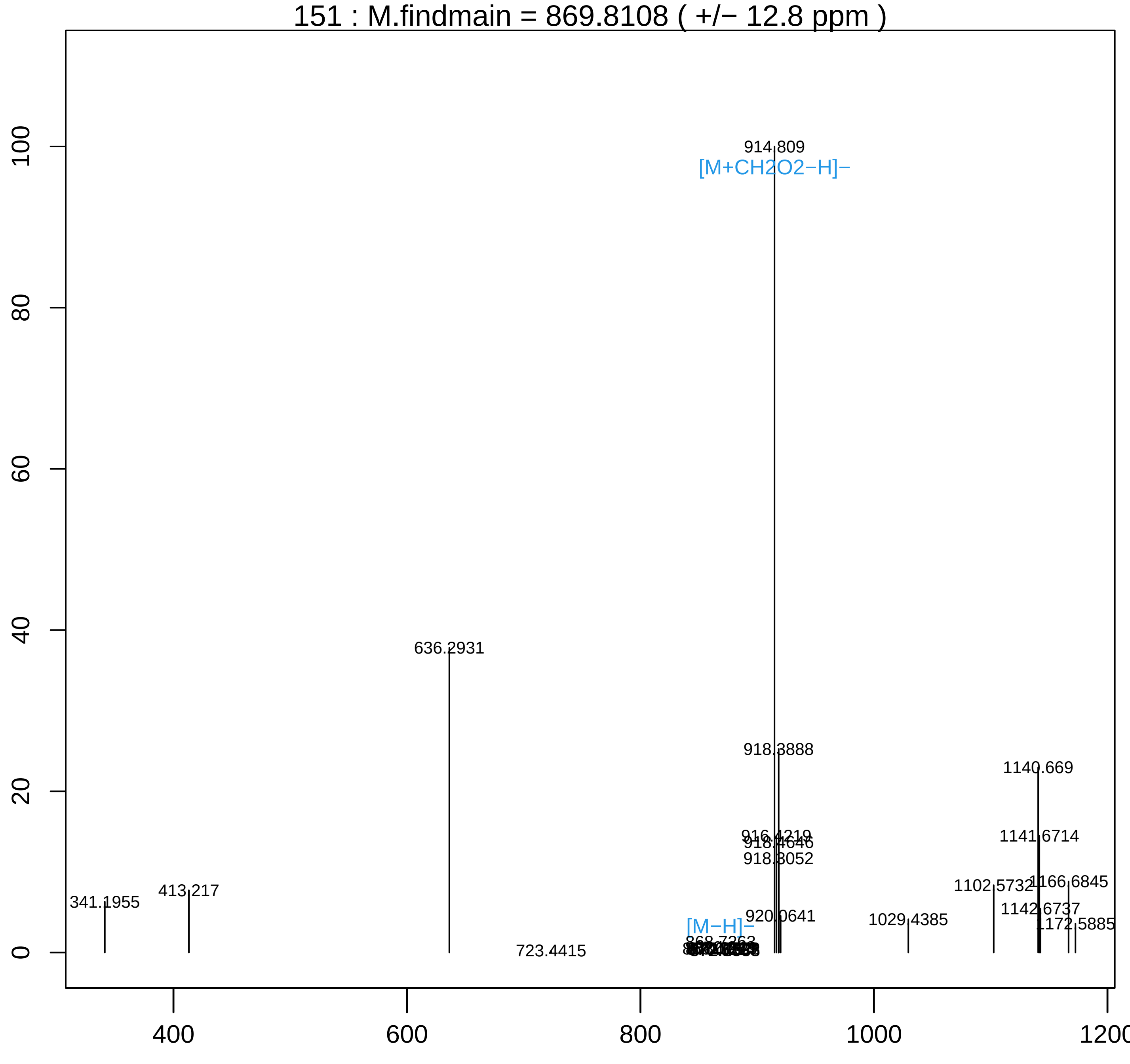

151 : M.findmain = 869.8108 (+/− 12.8 ppm)
914.809
[M+CH2O2−H]−
636.2931
918.3888
1140.669
916.4219
918.4646
918.3052
1141.6714
1102.5732
1166.6845
341.1955
413.217
1142.6737
920.0641
1029.4385
1172.5885
[M−H]−
868.7263
723.4415
0 20 40 60 80 100
400 600 800 1000 1200

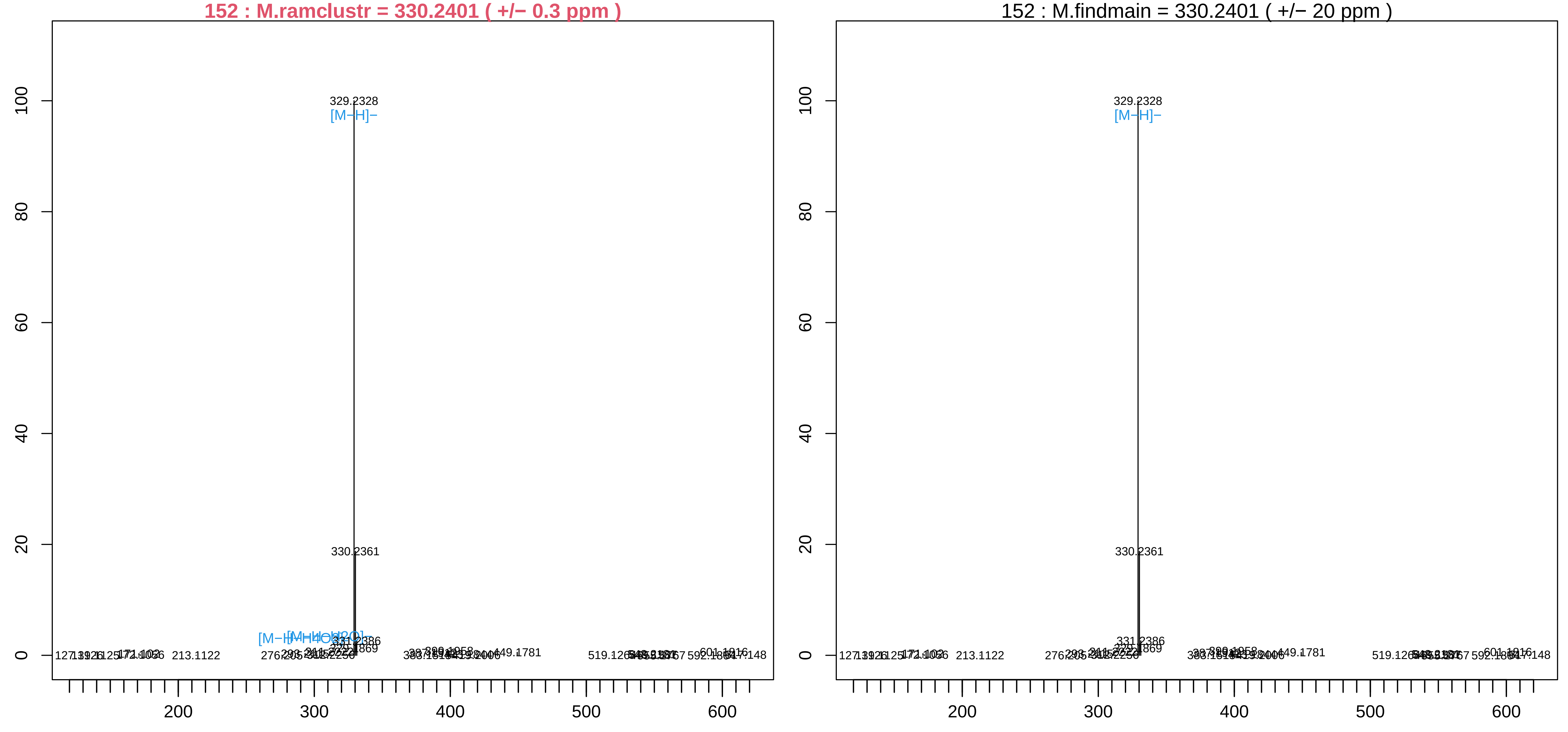

152 : M.ramclustr = 330.2401 (+/− 0.3 ppm)
152 : M.findmain = 330.2401 (+/− 20 ppm)
329.2328
[M−H]−
330.2361
331.2386
213.1122
449.1781

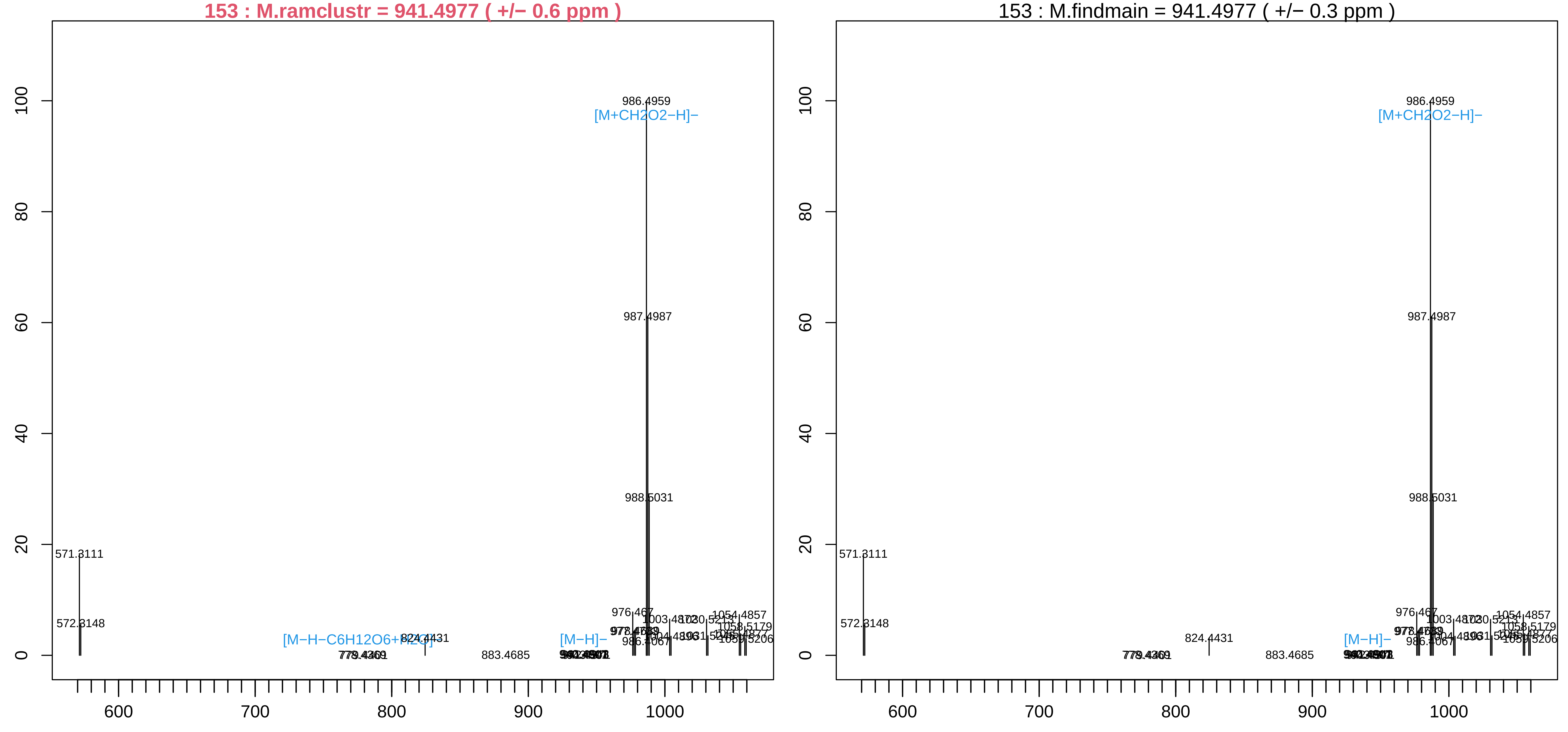

153 : M.ramclustr = 941.4977 (+/− 0.6 ppm)
153 : M.findmain = 941.4977 (+/− 0.3 ppm)
986.4959
[M+CH2O2−H]−
987.4987
988.5031
571.3111
572.3148
[M−H−C6H12O6+H2O]−
824.4431
883.4685
[M−H]−
976.467
1054.4857
1058.5179
986.4067
0
20
40
60
80
100
600
700
800
900
1000

154 : M.ramclustr = 514.3134 (+/− 0.3 ppm)

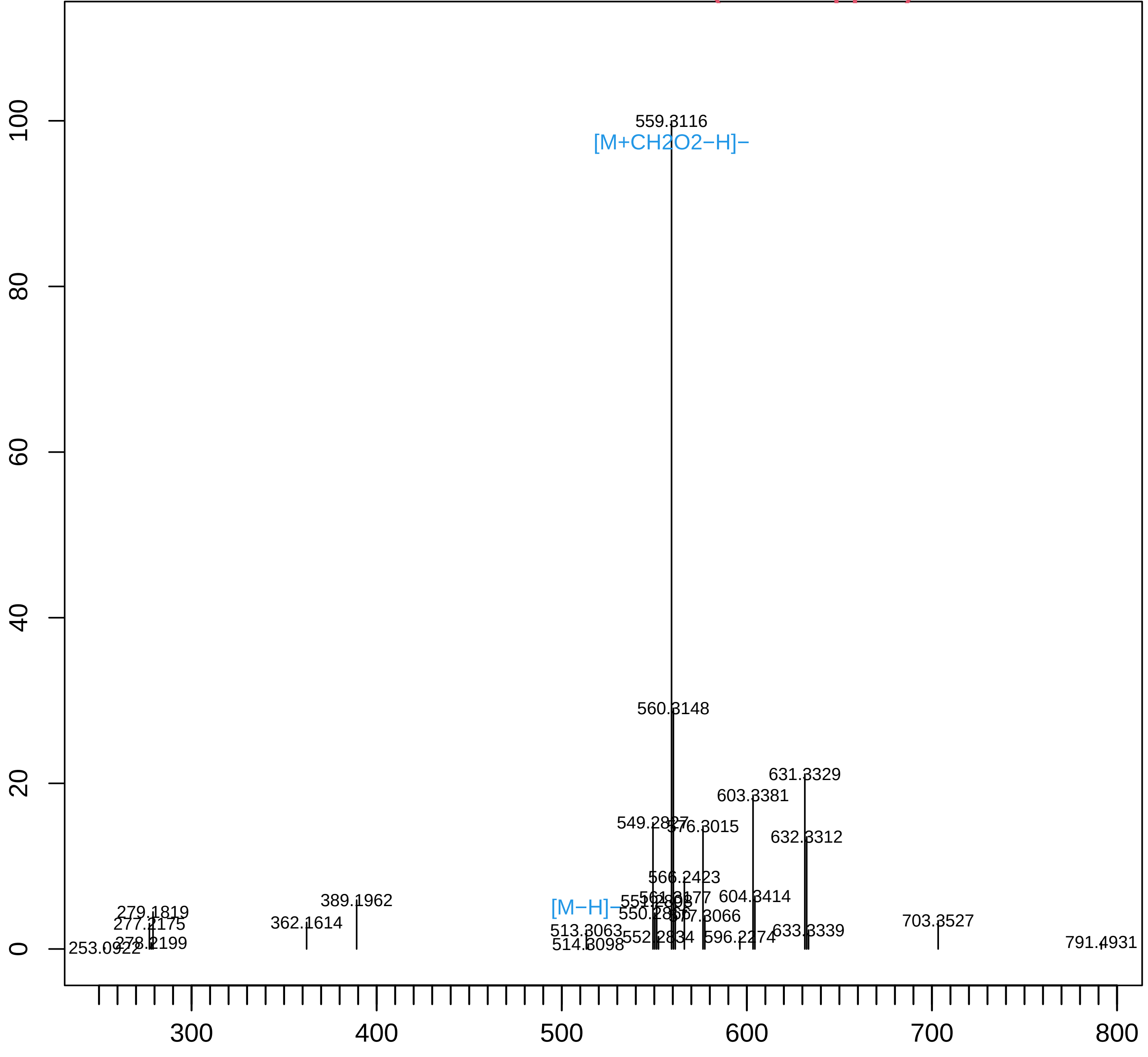

154 : M.findmain = 514.3134 (+/− 0.3 ppm)

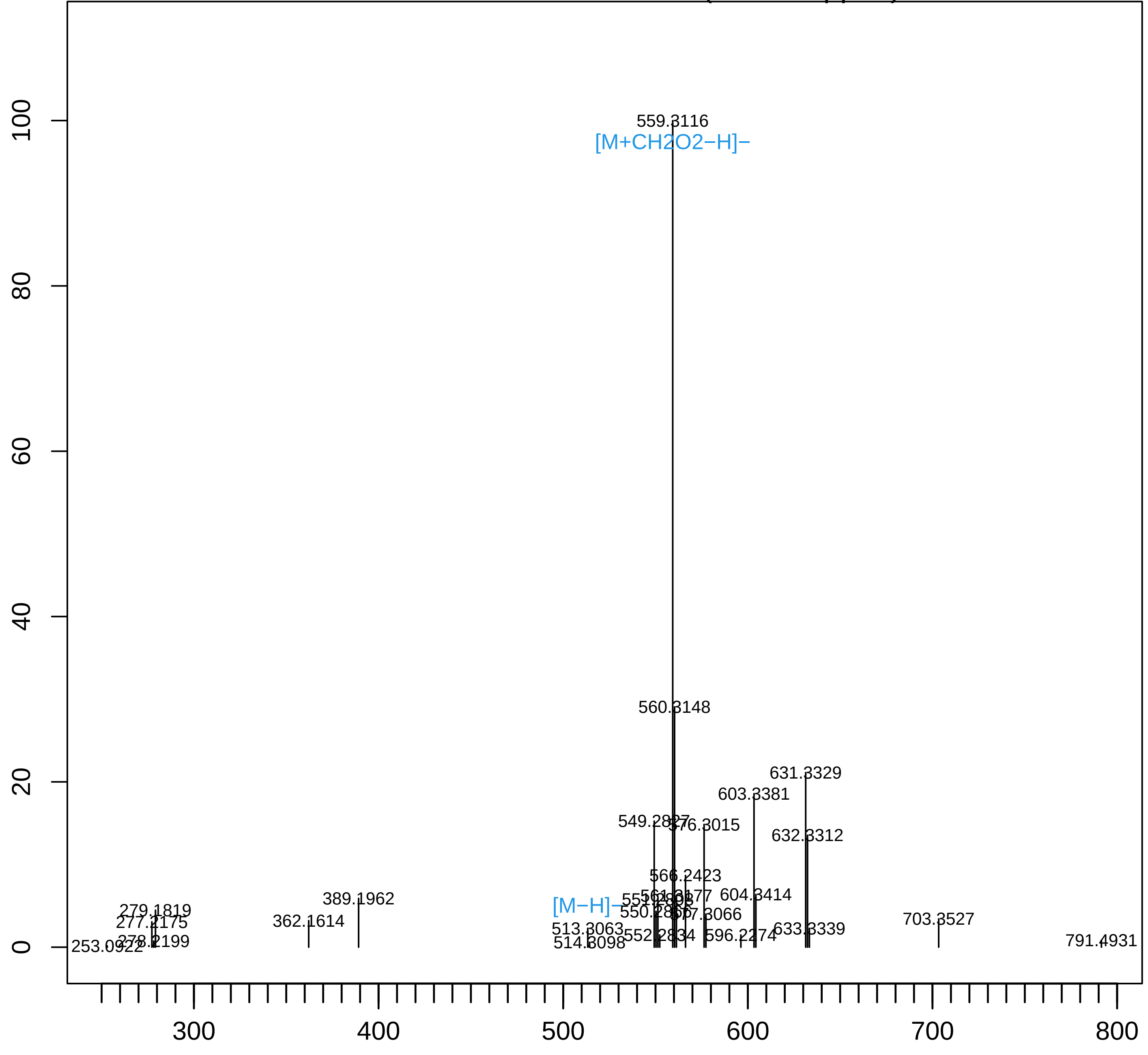

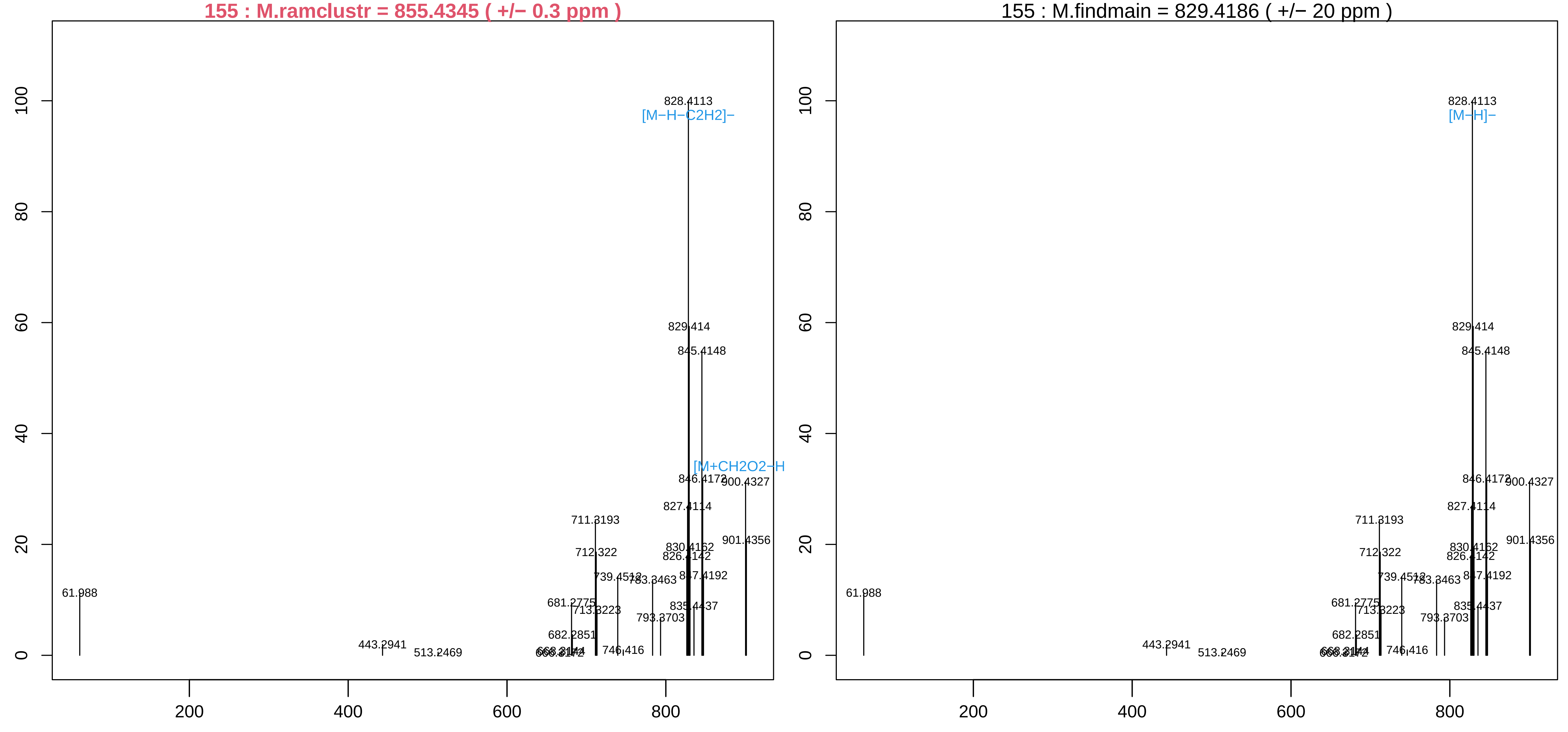
155 : M.ramclustr = 855.4345 (+/− 0.3 ppm)
155 : M.findmain = 829.4186 (+/− 20 ppm)
828.4113
[M−H−C2H2]−
[M−H]−
829.414
845.4148
[M+CH2O2−H
846.4172
900.4327
827.4114
711.3193
901.4356
830.4162
712.322
826.4142
739.4512
783.3463
847.4192
61.988
681.2775
713.3223
835.4437
793.3703
682.2851
443.2941
513.2469
746.416
0
20
40
60
80
100
200
400
600
800

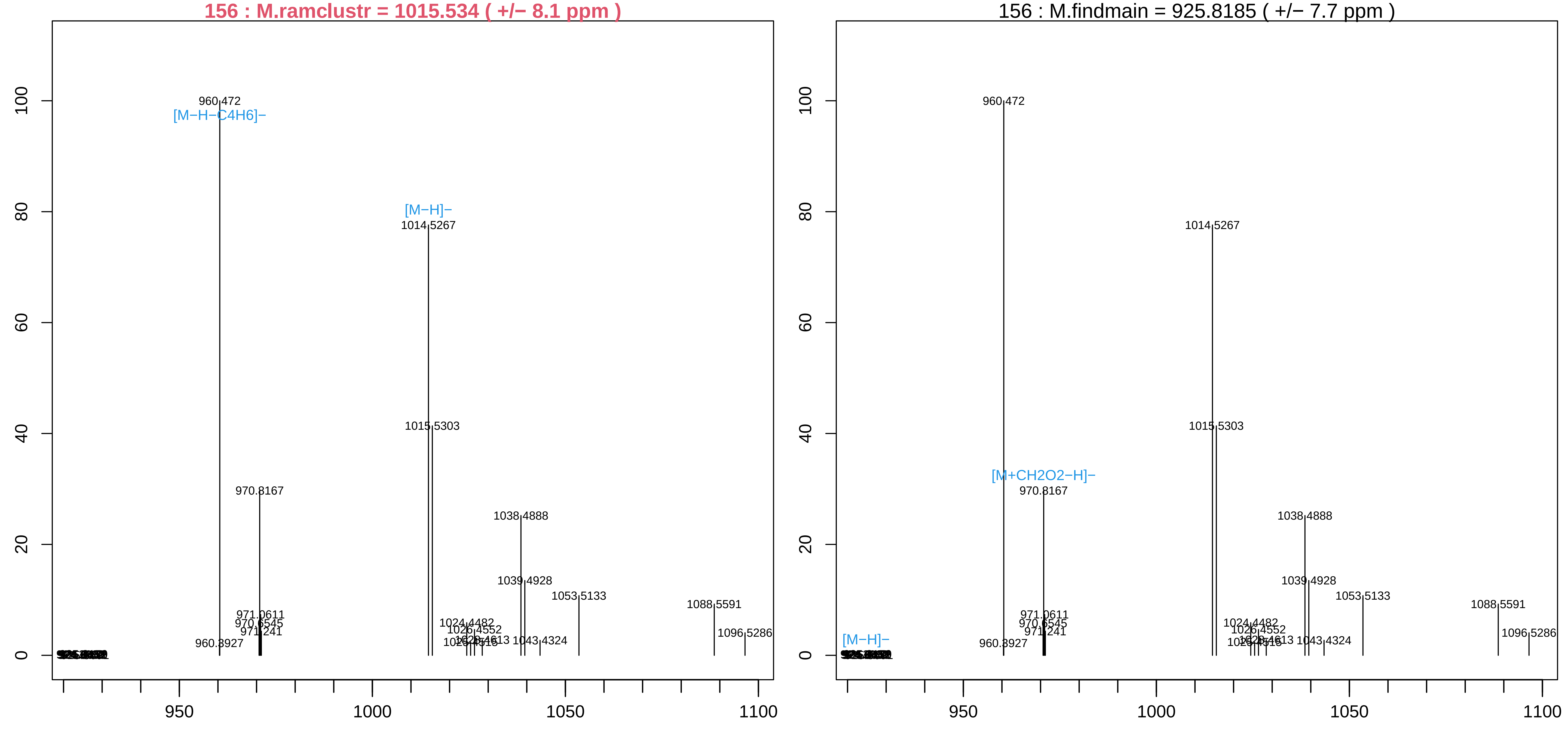

156 : M.ramclustr = 1015.534 (+/− 8.1 ppm)
[M−H−C4H6]−
960.472
[M−H]−
1014.5267
1015.5303
970.8167
1038.4888
1039.4928
1053.5133
1088.5591
1096.5286
971.0611
970.6545
971.241
960.3927
1024.4482
1026.4552
1043.4324
0
20
40
60
80
100
950
1000
1050
1100
156 : M.findmain = 925.8185 (+/− 7.7 ppm)
960.472
1014.5267
1015.5303
[M+CH2O2−H]−
970.8167
1038.4888
1039.4928
1053.5133
1088.5591
1096.5286
971.0611
970.6545
971.241
960.3927
1024.4482
1026.4552
1043.4324
[M−H]−

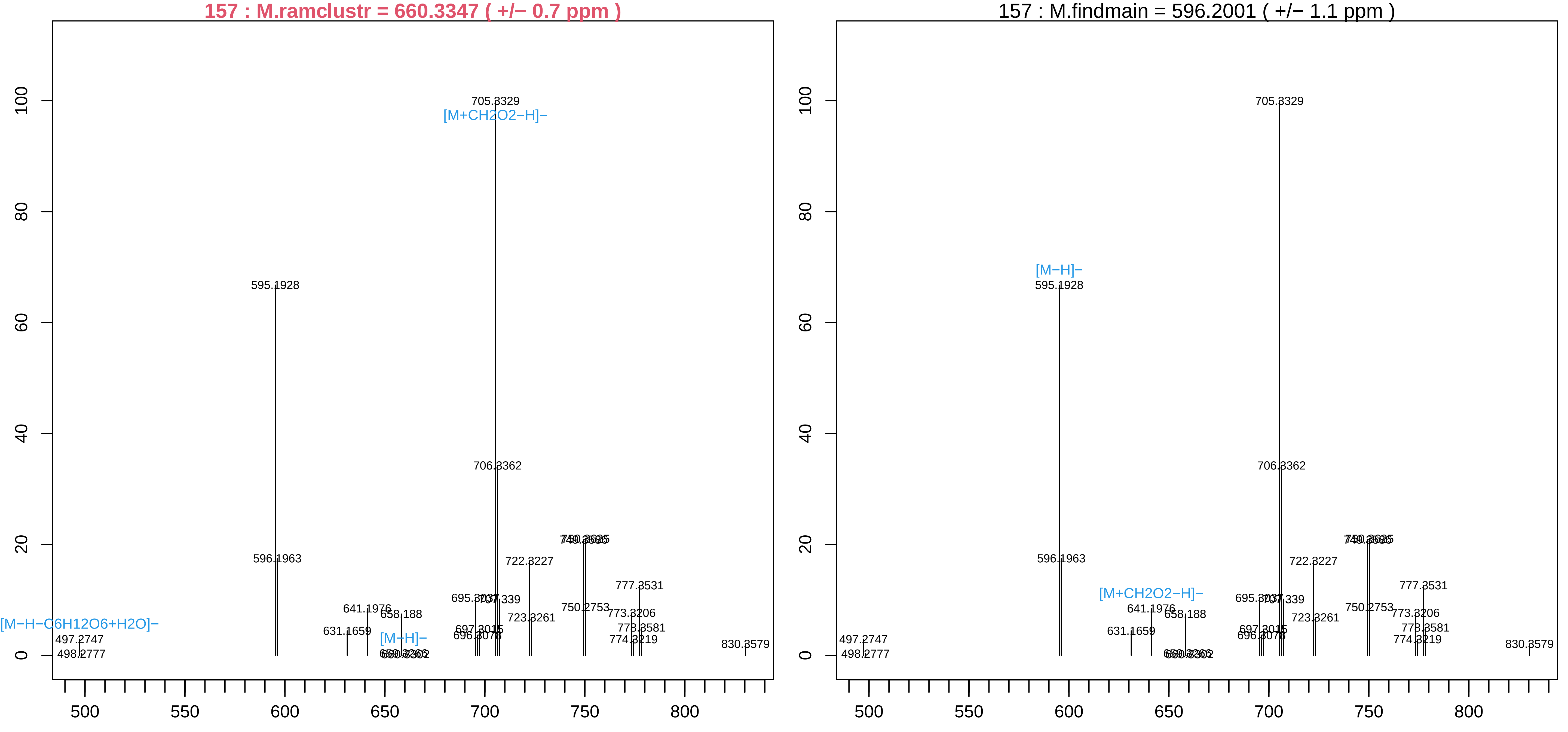
157 : M.ramclustr = 660.3347 (+/− 0.7 ppm)
157 : M.findmain = 596.2001 (+/− 1.1 ppm)
705.3329
[M+CH2O2−H]−
595.1928
[M−H]−
706.3362
596.1963
722.3227
777.3531
695.3037
707.339
641.1976
658.188
750.2753
773.3206
631.1659
723.3261
697.3015
696.3078
778.3581
774.3219
[M−H−C6H12O6+H2O]−
497.2747
498.2777
830.3579
0
20
40
60
80
100
500
550
600
650
700
750
800

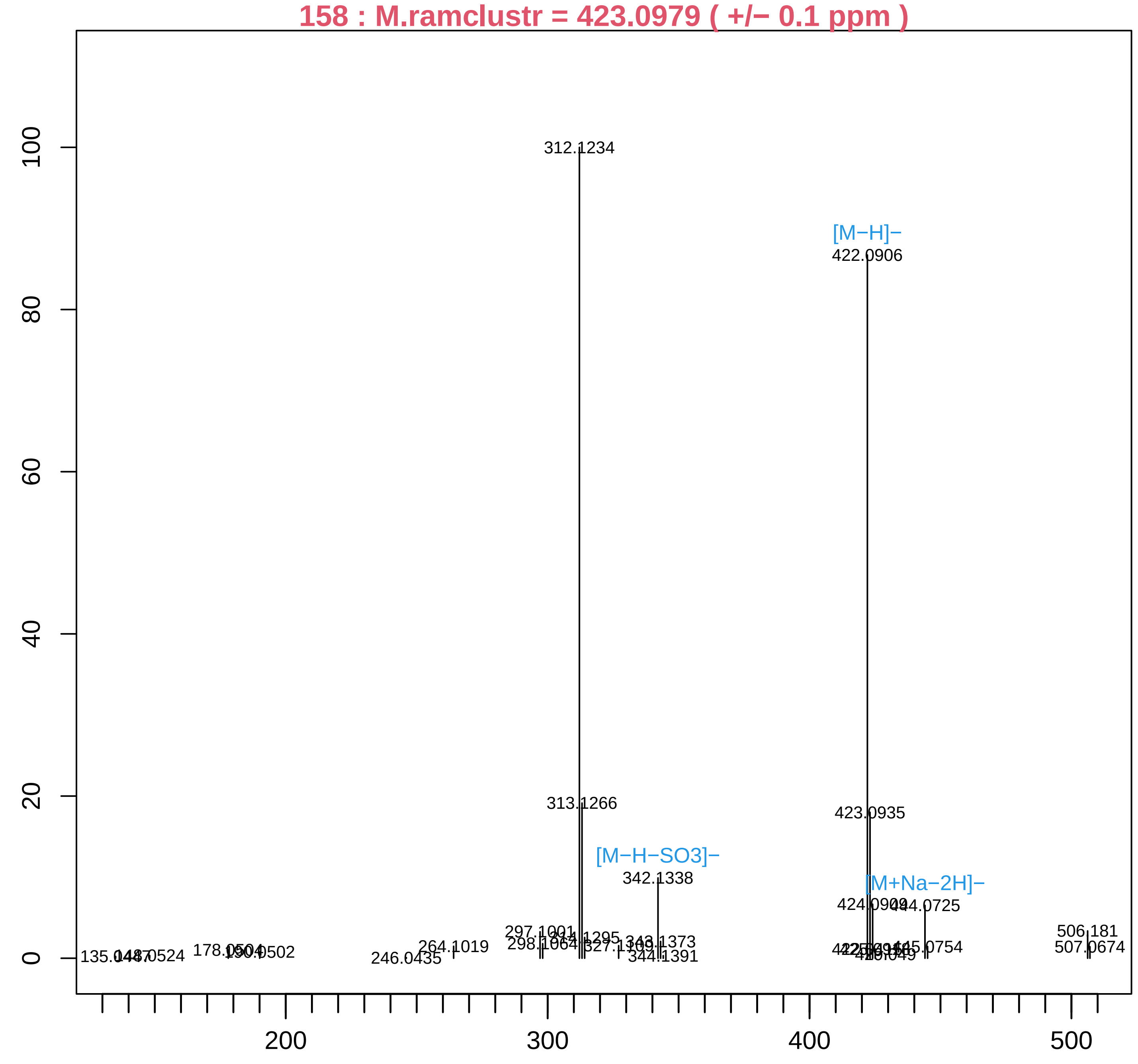
158 : M.ramclustr = 423.0979 (+/− 0.1 ppm)
312.1234
[M−H]−
422.0906
313.1266
423.0935
[M−H−SO3]−
342.1338
[M+Na−2H]−
444.0725
424.0909
297.1001
298.1064
343.1373
344.1391
264.1019
246.0435
178.0504
190.0502
135.0487
445.0754
506.181
507.0674
0
20
40
60
80
100
200
300
400
500

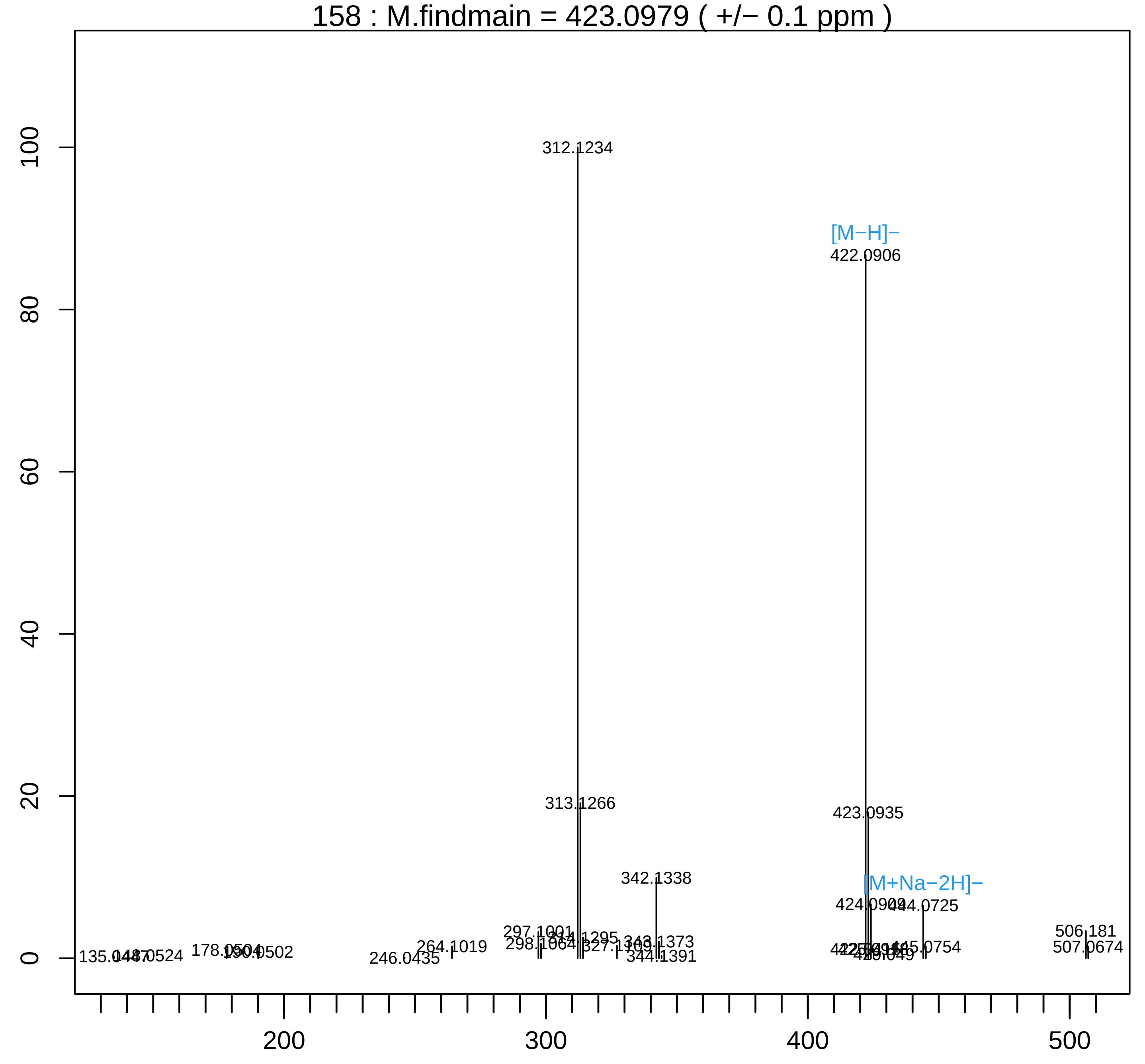
158 : M.findmain = 423.0979 (+/− 0.1 ppm)
312.1234
[M−H]−
422.0906
313.1266
423.0935
342.1338
[M+Na−2H]−
444.0725
424.0909
297.1001
298.1064
343.1373
344.1391
264.1019
246.0435
178.0504
190.0502
135.0487
445.0754
506.181
507.0674
0
20
40
60
80
100
200
300
400
500

159 : M.ramclustr = 1132.5282 (+/− 6.4 ppm)

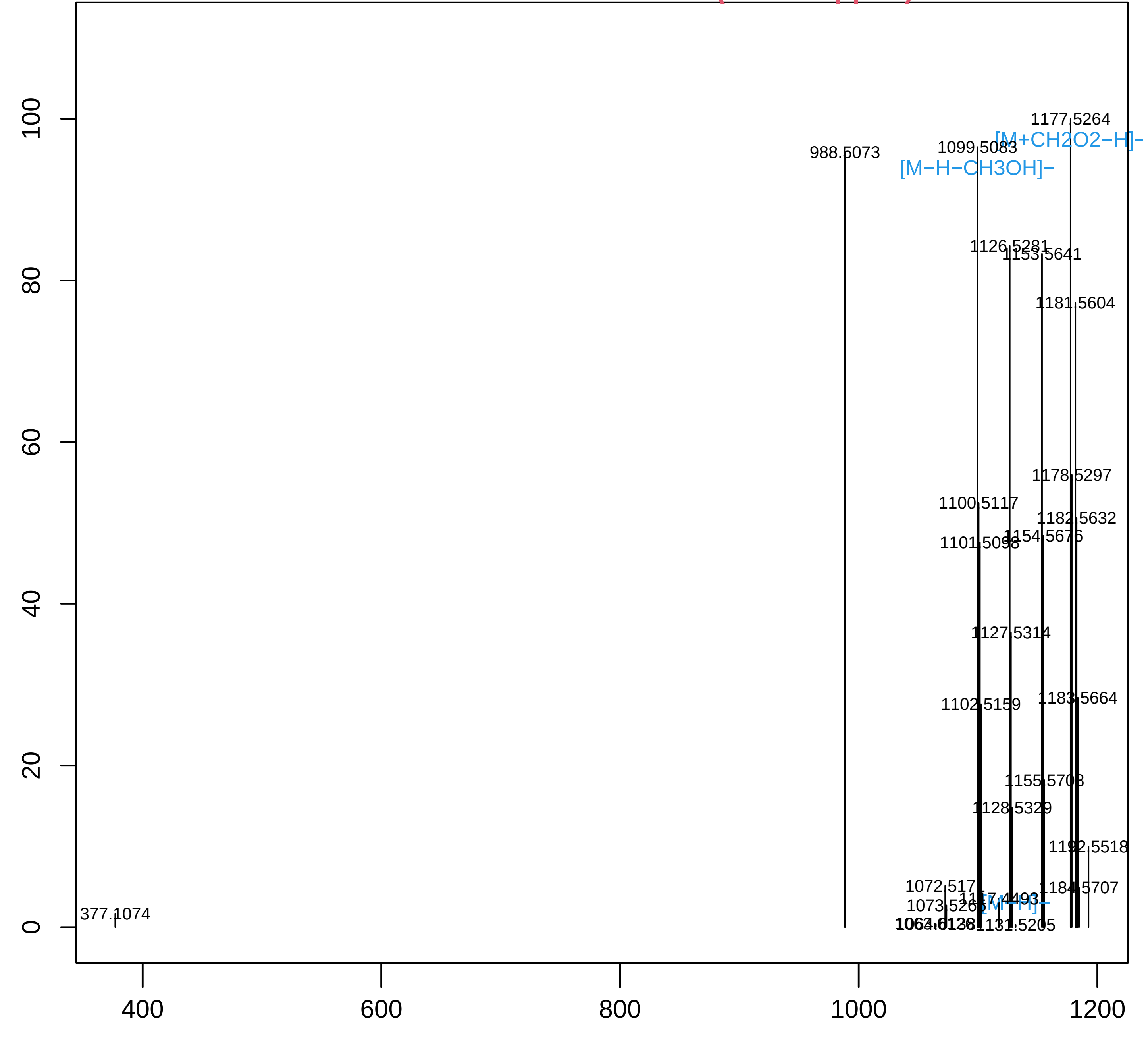

159 : M.findmain = 1132.5282 (+/− 0.4 ppm)

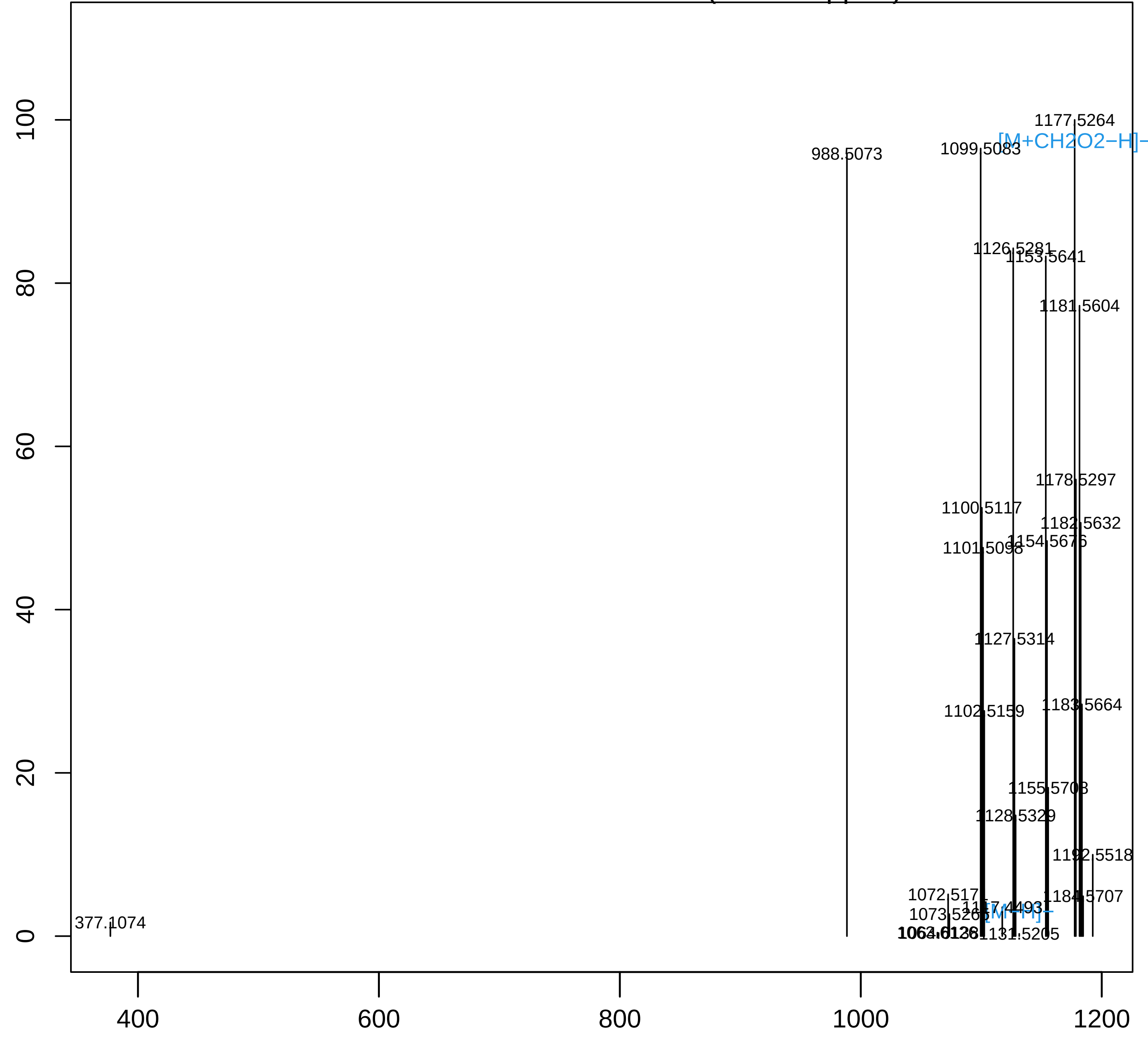

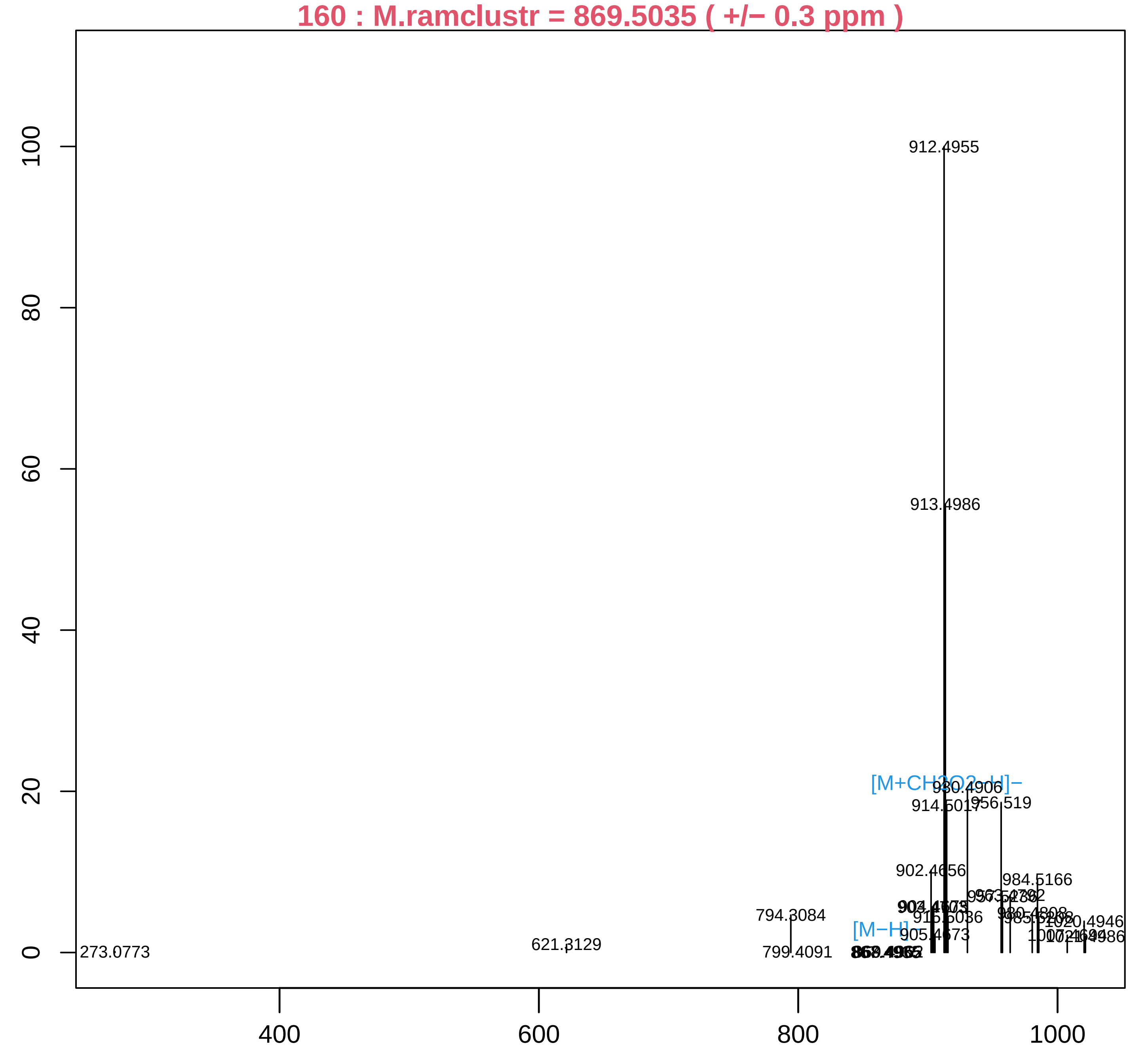
160 : M.ramclustr = 869.5035 (+/− 0.3 ppm)
912.4955
913.4986
[M+CH2O2-H]−
930.4906
914.5017
956.519
902.4656
984.5166
794.3084
[M−H]−
905.4673
621.3129
273.0773
799.4091
1020.4946
0
20
40
60
80
100
400
600
800
1000

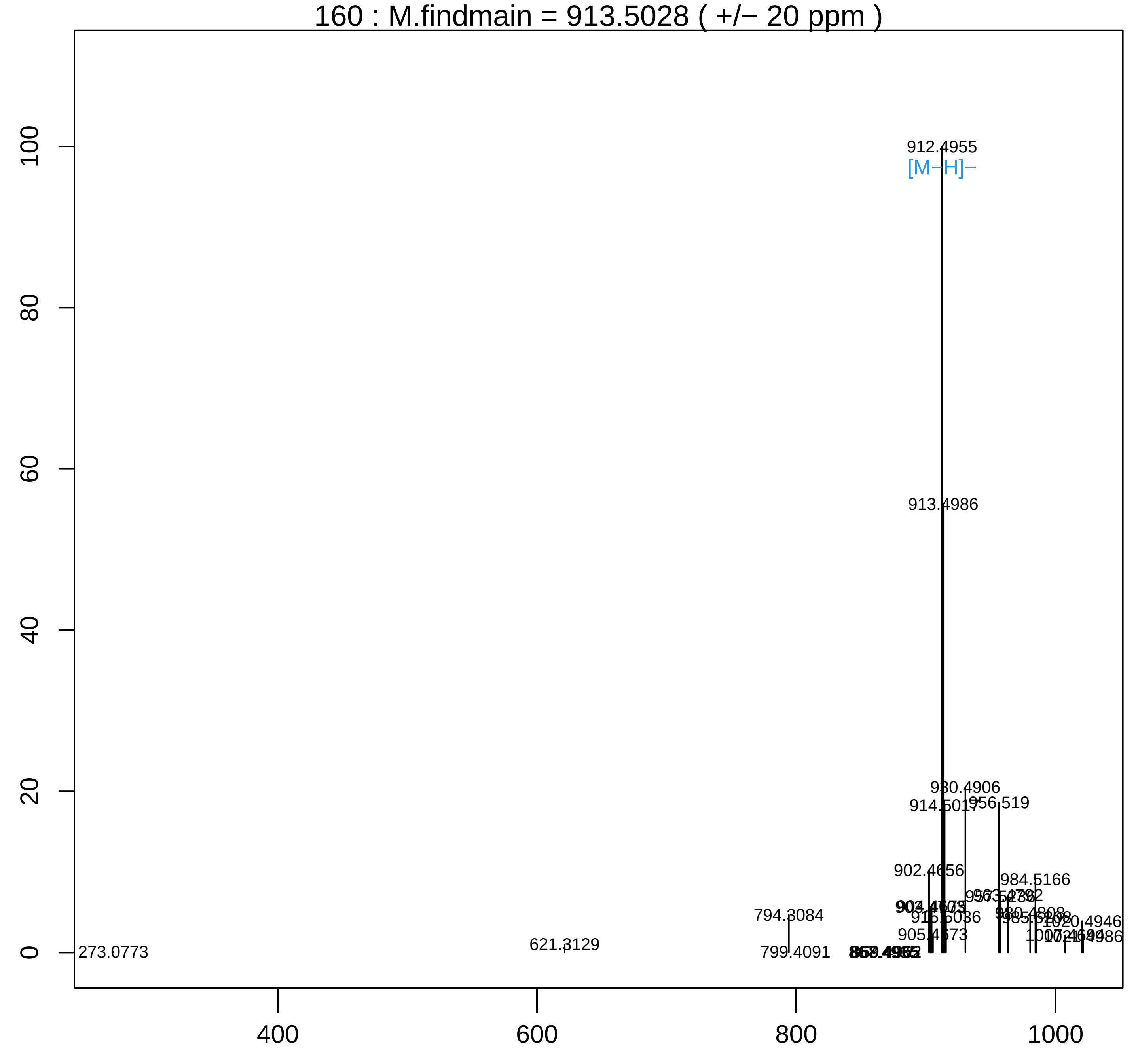
160 : M.findmain = 913.5028 (+/− 20 ppm)
912.4955
[M−H]−
913.4986
930.4906
914.5017
956.519
902.4656
984.5166
794.3084
905.4673
621.3129
273.0773
799.4091
1020.4946
0
20
40
60
80
100
400
600
800
1000

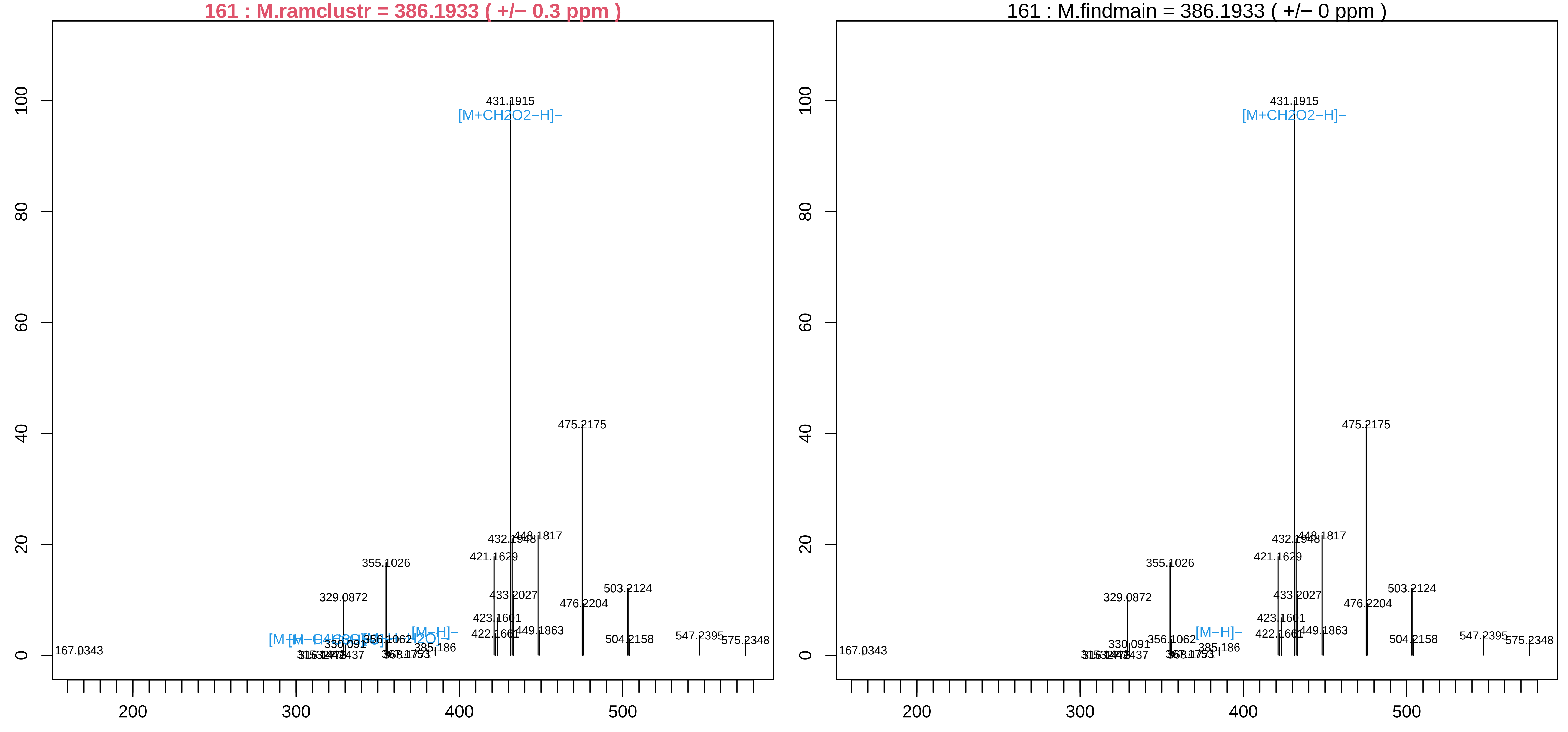

161 : M.ramclustr = 386.1933 (+/− 0.3 ppm)
161 : M.findmain = 386.1933 (+/− 0 ppm)
431.1915
[M+CH2O2−H]−
475.2175
448.1817
432.1948
421.1629
355.1026
433.2027
503.2124
329.0872
476.2204
423.1601
[M−H]−
449.1863
422.1661
356.1062
504.2158
547.2395
575.2348
385.186
167.0343
330.091
0
20
40
60
80
100
200
300
400
500

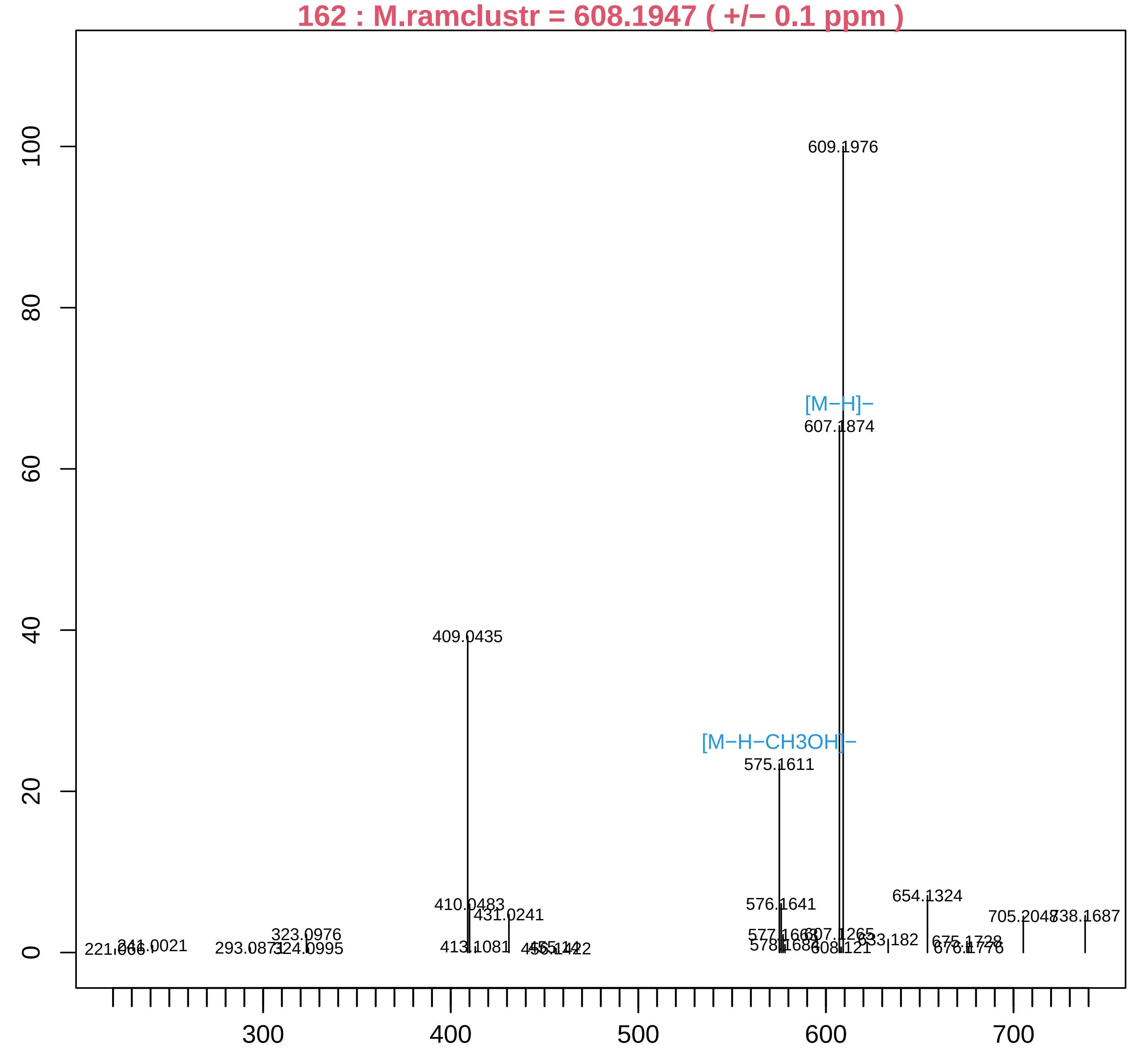
162 : M.ramclustr = 608.1947 (+/− 0.1 ppm)
[M−H]−
[M−H−CH3OH]−
609.1976
607.1874
409.0435
575.1611
410.0483
431.0241
576.1641
654.1324
705.2048
738.1687
633.182
323.0976
241.0021
293.0873
324.0995
221.066
413.1081
455.1422

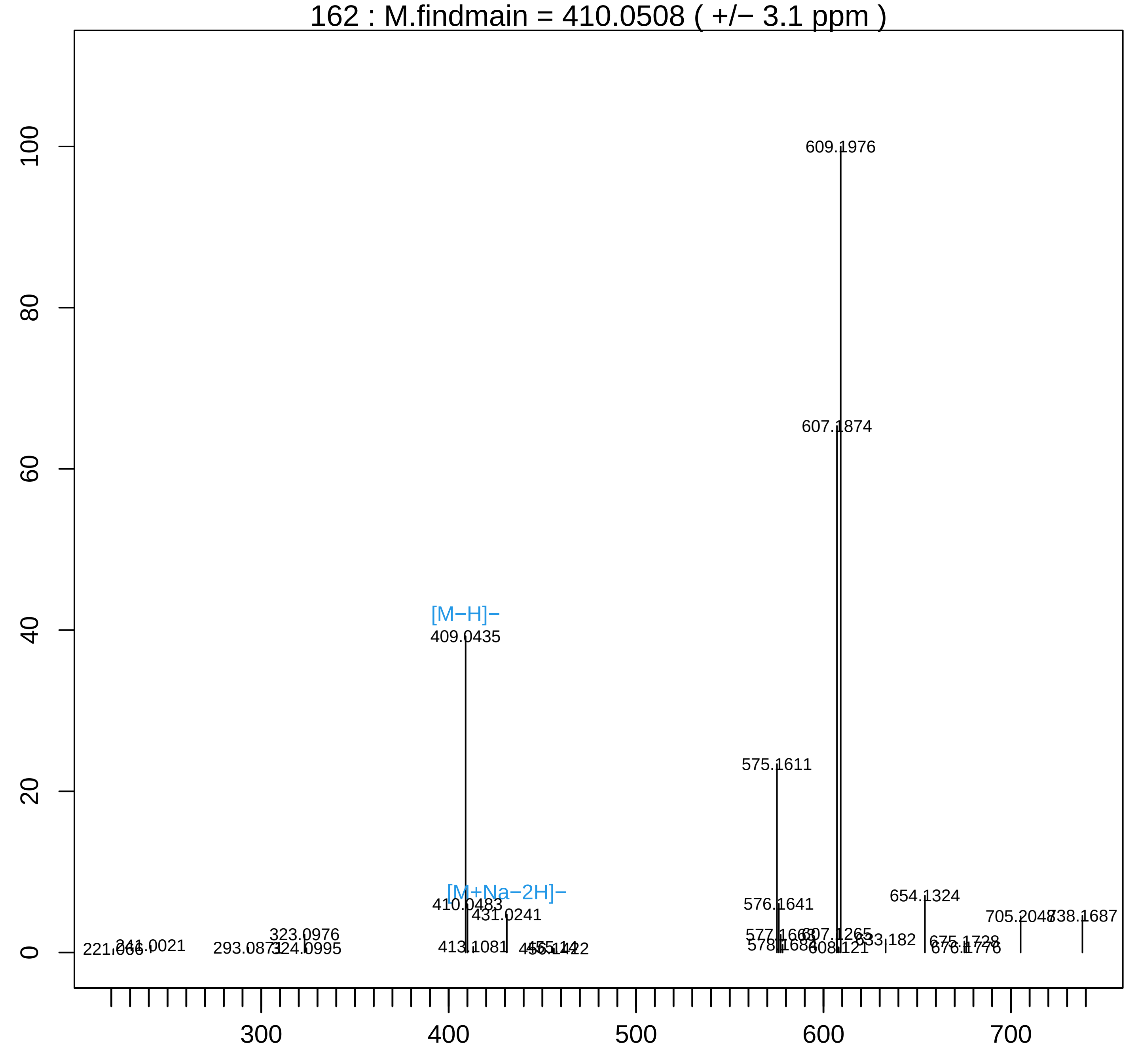
162 : M.findmain = 410.0508 (+/− 3.1 ppm)
[M−H]−
[M+Na−2H]−
609.1976
607.1874
409.0435
575.1611
410.0483
431.0241
576.1641
654.1324
705.2048
738.1687
633.182
323.0976
241.0021
293.0873
324.0995
221.066
413.1081
455.1422

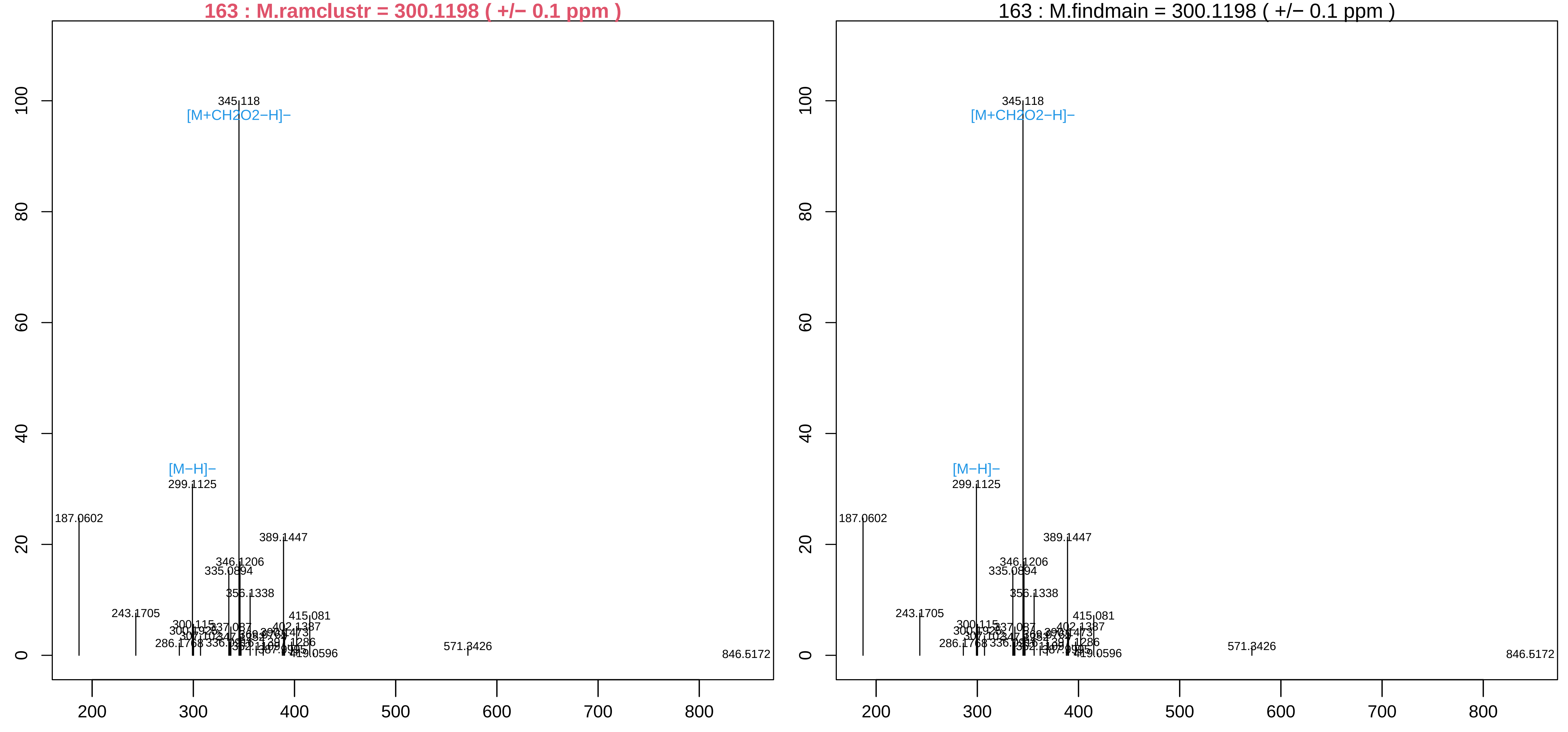
163 : M.ramclustr = 300.1198 (+/− 0.1 ppm)
163 : M.findmain = 300.1198 (+/− 0.1 ppm)
345.118
[M+CH2O2−H]−
[M−H]−
299.1125
187.0602
389.1447
346.1206
335.0894
356.1338
243.1705
415.081
402.1387
286.1768
571.3426
846.5172
419.0596

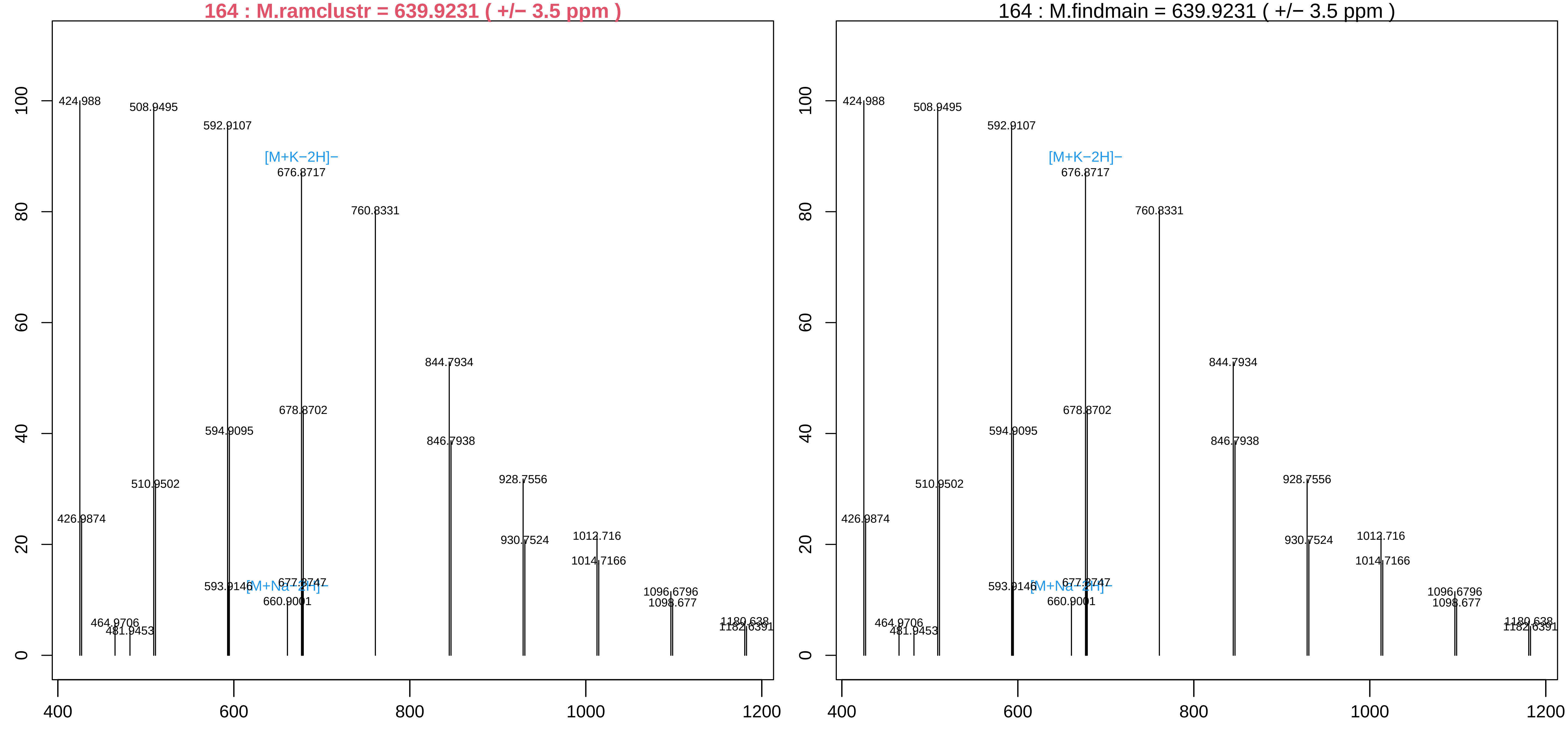
164 : M.ramclustr = 639.9231 (+/− 3.5 ppm)
164 : M.findmain = 639.9231 (+/− 3.5 ppm)
[M+K−2H]−
[M+Na−2H]−
424.988
426.9874
464.9706
481.9453
508.9495
510.9502
592.9107
593.9146
594.9095
660.9001
676.8717
677.8747
678.8702
760.8331
844.7934
846.7938
928.7556
930.7524
1012.716
1014.7166
1096.6796
1098.677
1180.638
1182.6391
0
20
40
60
80
100
400
600
800
1000
1200

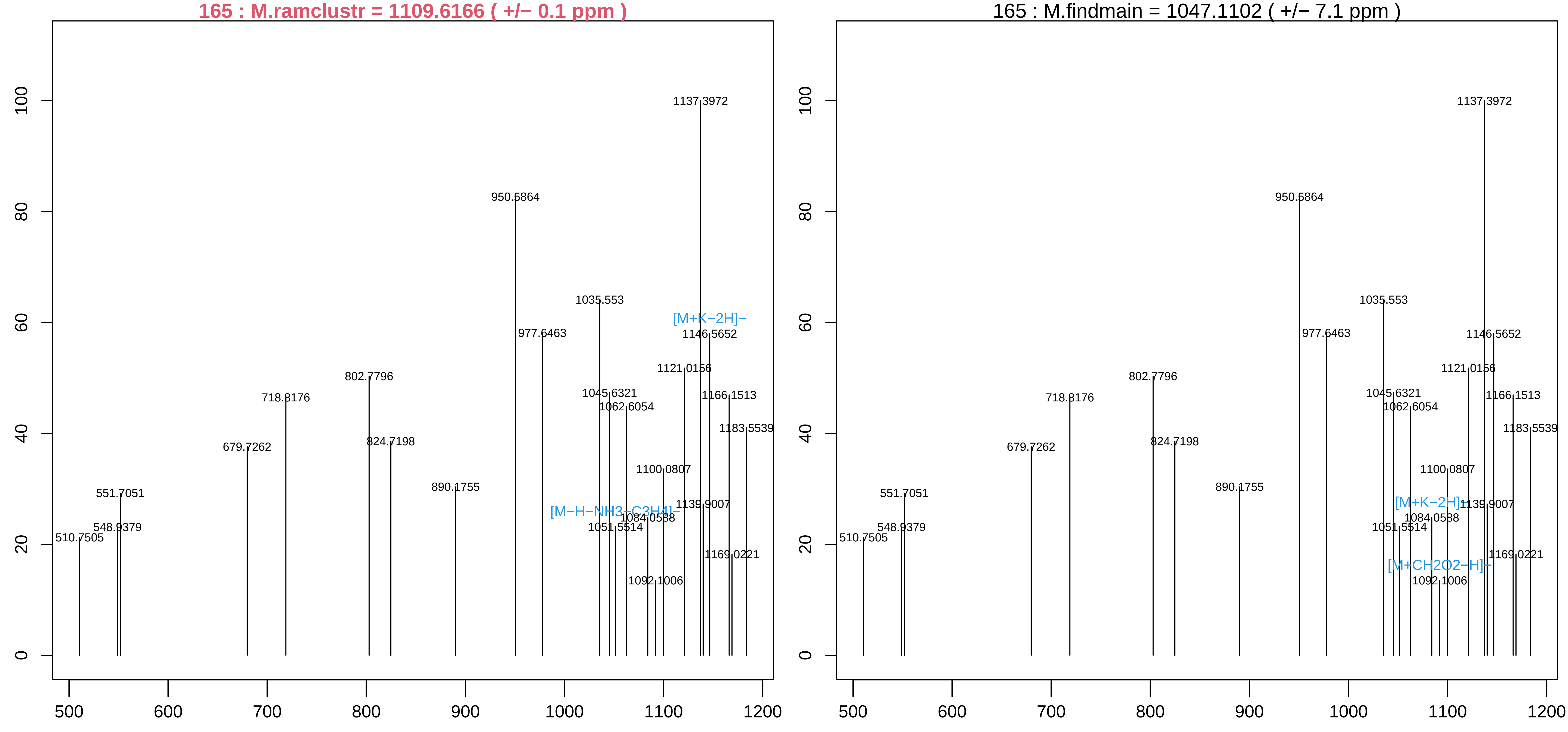
165 : M.ramclustr = 1109.6166 (+/− 0.1 ppm)
165 : M.findmain = 1047.1102 (+/− 7.1 ppm)
[M+K−2H]−
[M−H−NH3−C3H4]−
[M+CH2O2−H]−
1137.3972
950.5864
1035.553
977.6463
1146.5652
1121.0156
802.7796
718.8176
1045.6321
1062.6054
1166.1513
1183.5539
679.7262
824.7198
1100.0807
890.1755
551.7051
1139.9007
1084.0588
1051.5514
548.9379
510.7505
1169.0221
1092.1006
0
20
40
60
80
100
500
600
700
800
900
1000
1100
1200

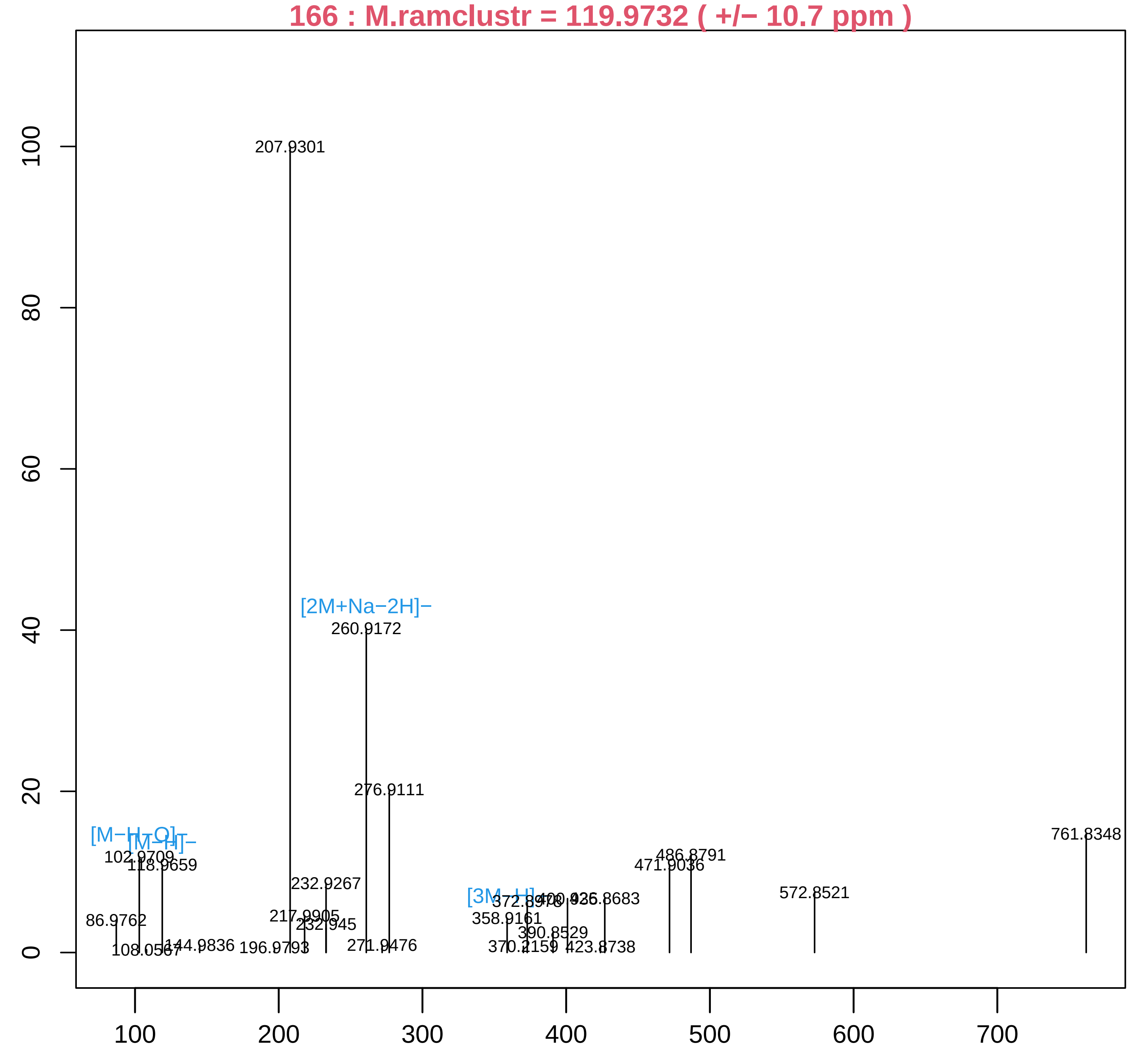
166 : M.ramclustr = 119.9732 (+/− 10.7 ppm)
[2M+Na−2H]−
[M−H]−
[3M−H]−
207.9301
260.9172
276.9111
761.8348
486.8791
471.9036
572.8521
232.9267
102.9709
118.9659
86.9762
217.9905
232.945
358.9161
390.8529
423.8738
370.2159
271.9476
196.9793
144.9836
108.0567
0
20
40
60
80
100
200
300
400
500
600
700

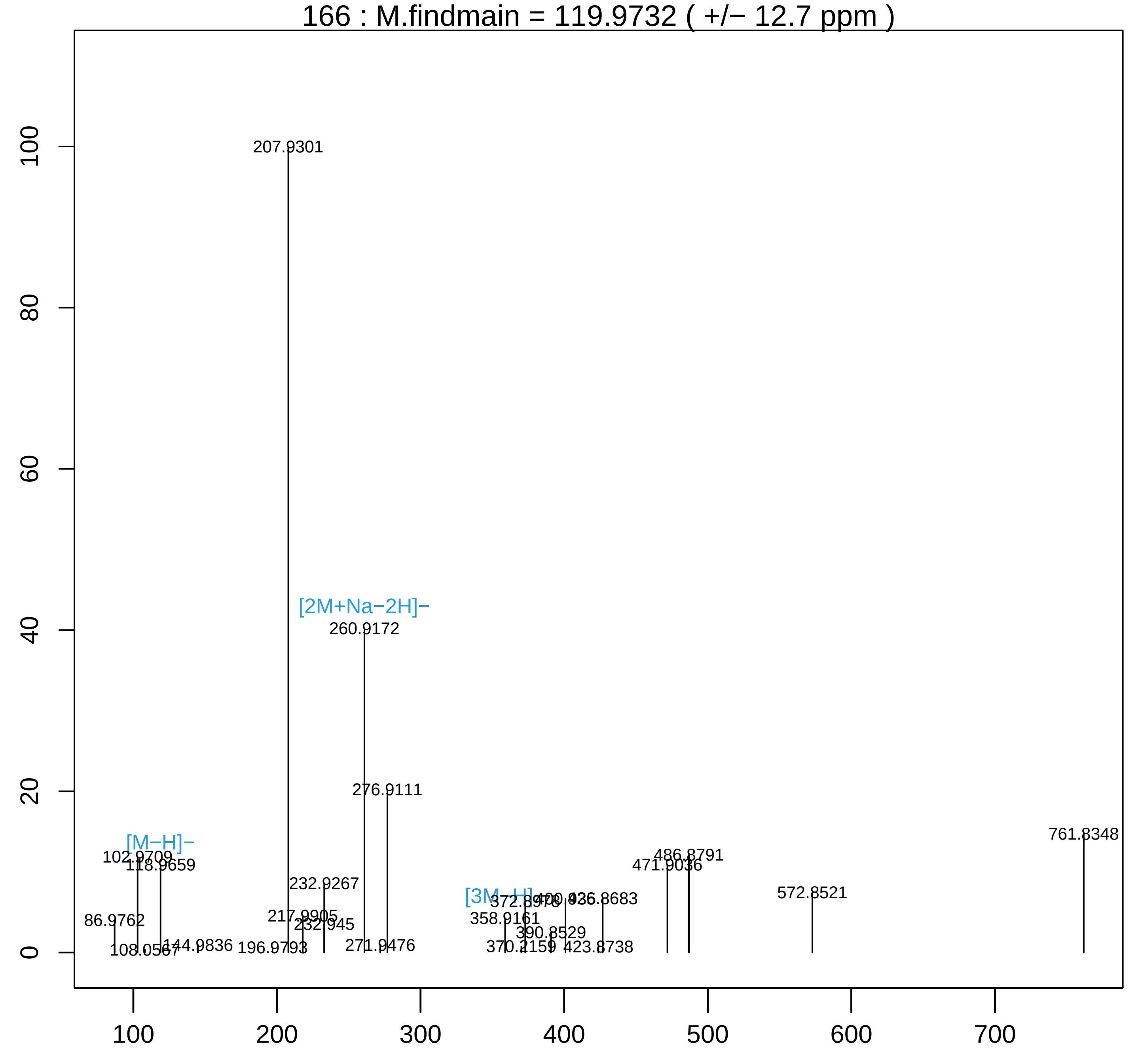
166 : M.findmain = 119.9732 (+/− 12.7 ppm)
[2M+Na−2H]−
[M−H]−
[3M−H]−
207.9301
260.9172
276.9111
761.8348
486.8791
471.9036
572.8521
232.9267
102.9709
118.9659
86.9762
217.9905
232.945
358.9161
390.8529
423.8738
370.2159
271.9476
196.9793
144.9836
108.0567
0
20
40
60
80
100
200
300
400
500
600
700

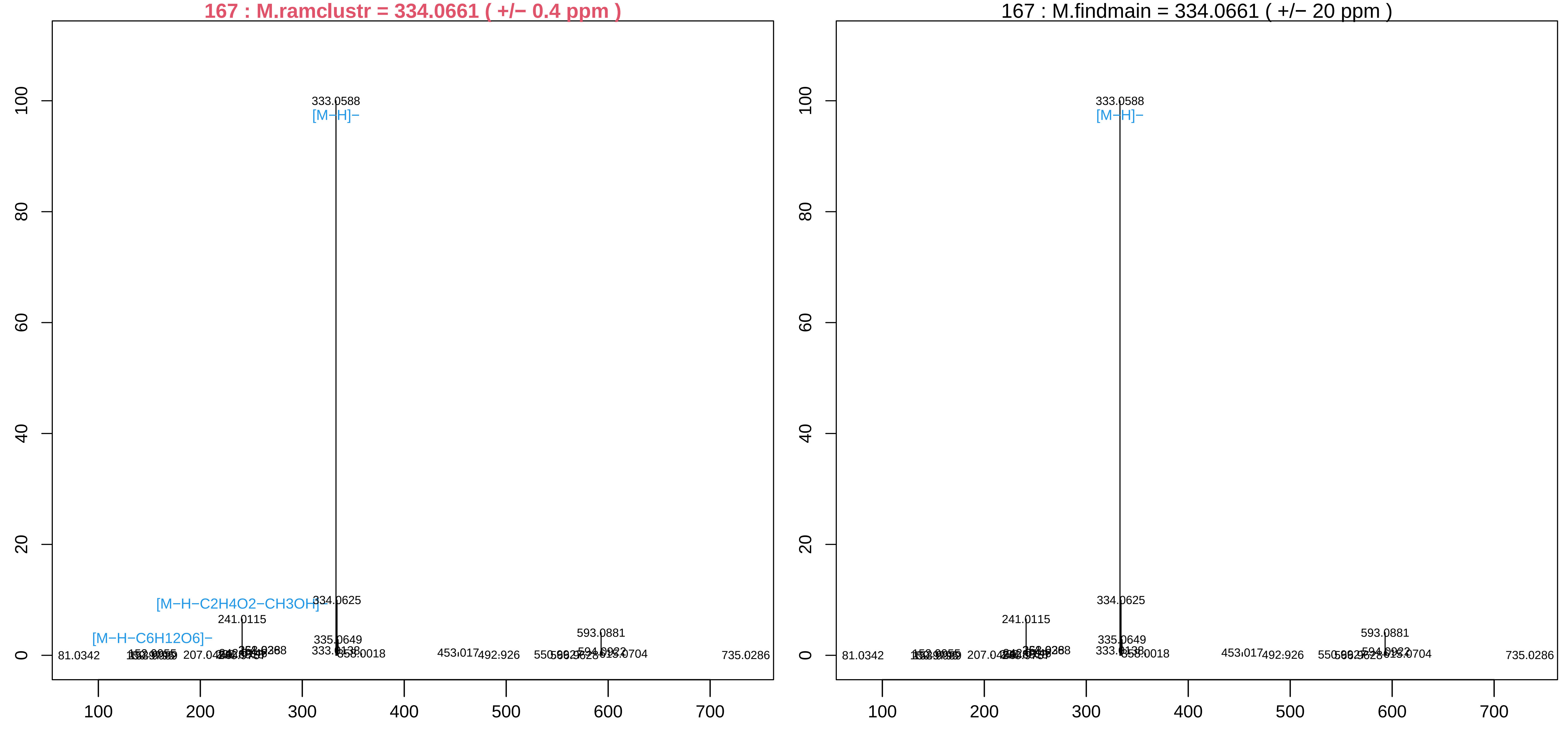
167 : M.ramclustr = 334.0661 (+/− 0.4 ppm)
333.0588
[M−H]−
[M−H−C2H4O2−CH3OH]−
334.0625
241.0115
[M−H−C6H12O6]−
335.0649
593.0881
81.0342
453.017
492.926
735.0286
167 : M.findmain = 334.0661 (+/− 20 ppm)
333.0588
[M−H]−
334.0625
241.0115
335.0649
593.0881
81.0342
453.017
492.926
735.0286
0
20
40
60
80
100
100
200
300
400
500
600
700

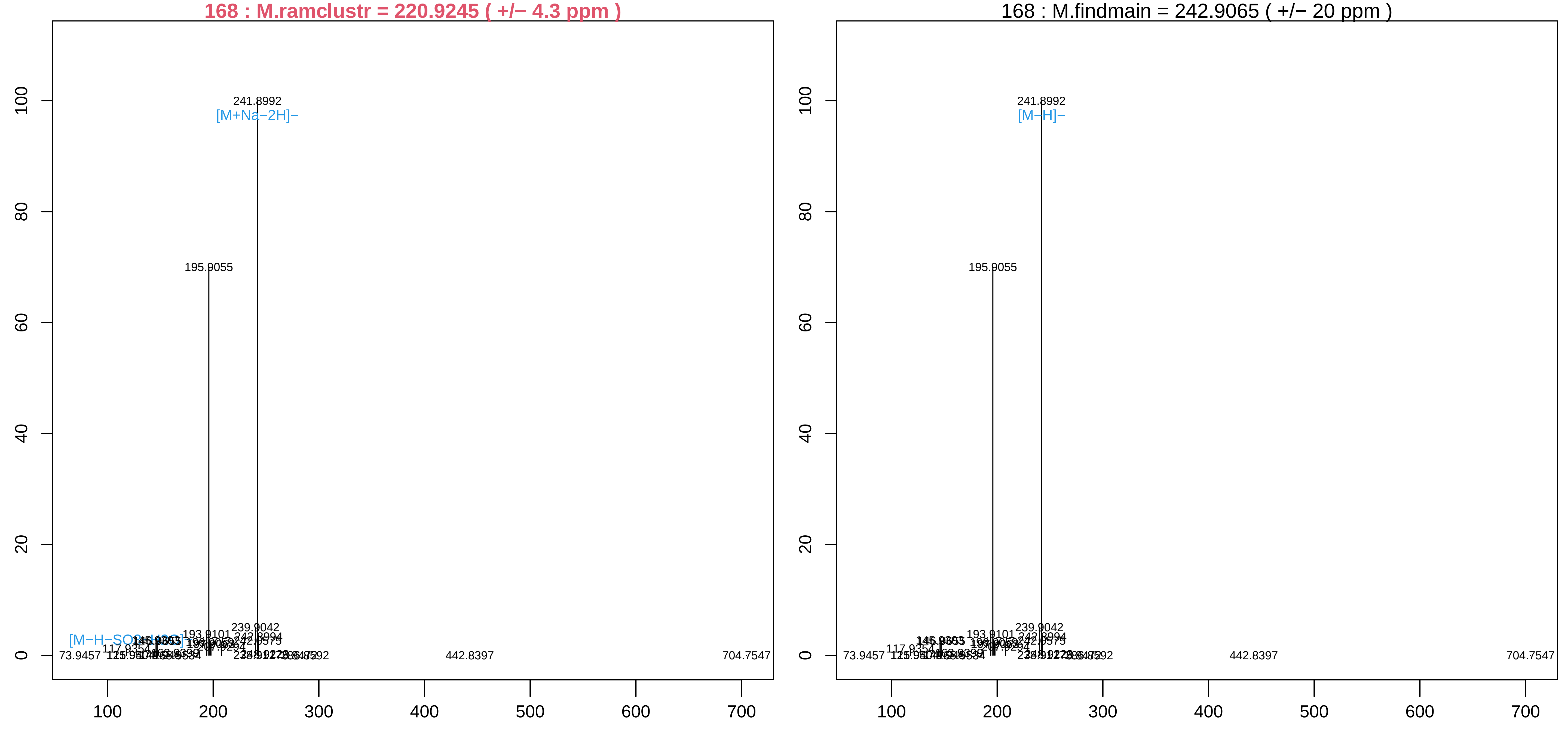
168 : M.ramclustr = 220.9245 (+/− 4.3 ppm)
241.8992
[M+Na−2H]−
195.9055
239.9042
73.9457
442.8397
704.7547
168 : M.findmain = 242.9065 (+/− 20 ppm)
241.8992
[M−H]−
195.9055
239.9042
73.9457
442.8397
704.7547

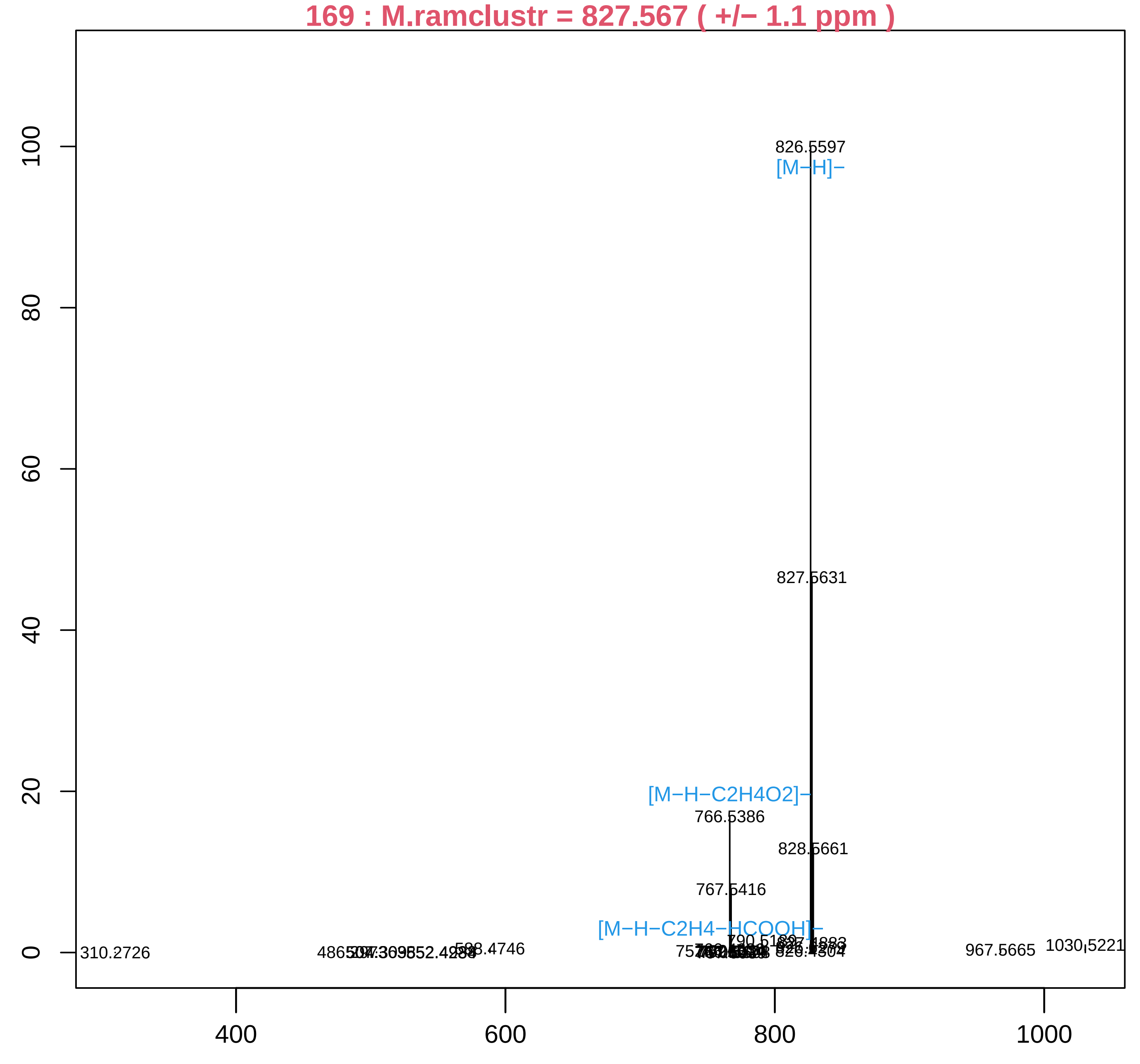

169 : M.ramclustr = 827.567 (+/− 1.1 ppm)
826.5597
[M−H]−
827.5631
[M−H−C2H4O2]−
766.5386
828.5661
767.5416
[M−H−C2H4−HCOOH]−
310.2726
598.4746
967.5665
1030.5221

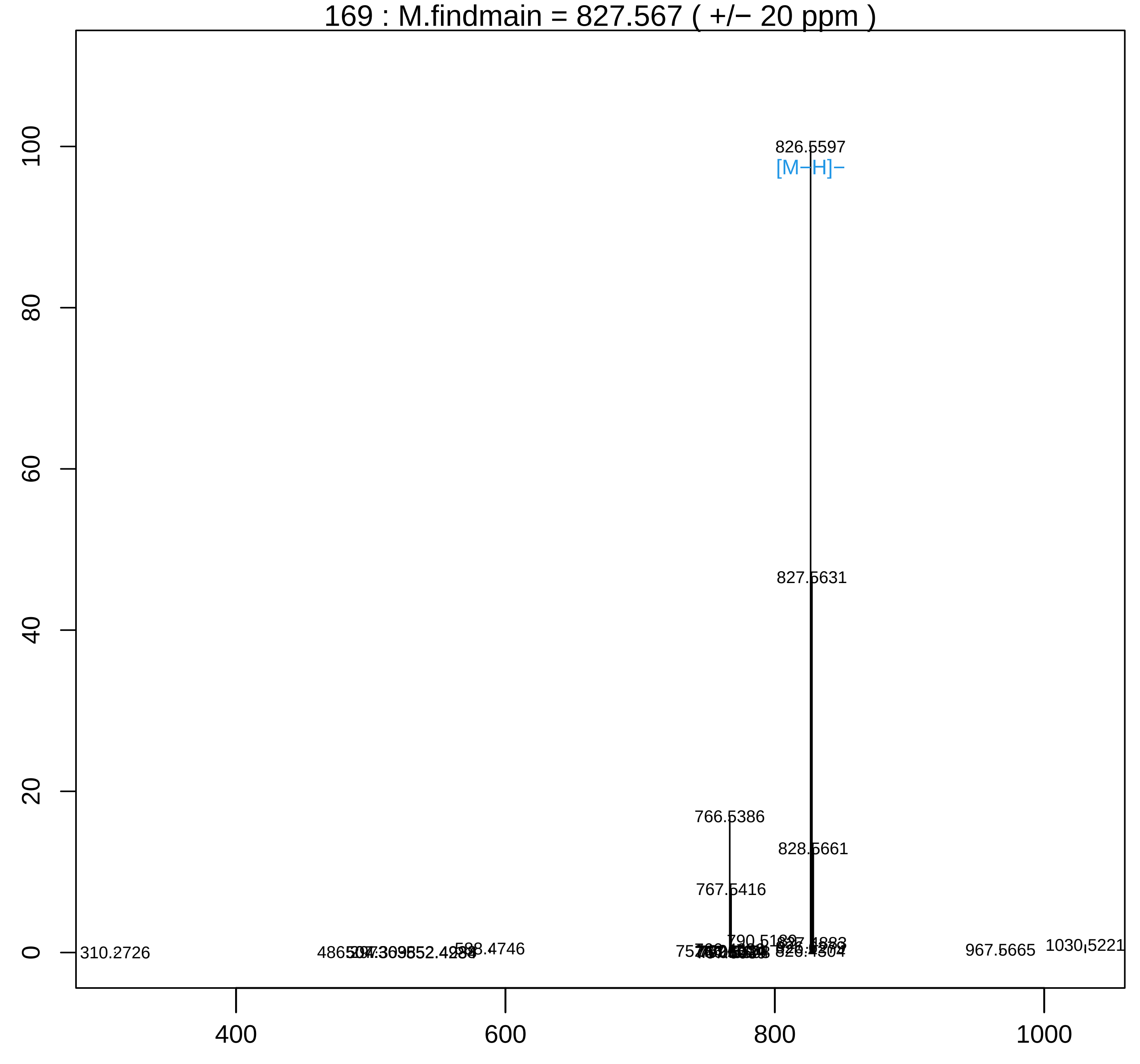

169 : M.findmain = 827.567 (+/− 20 ppm)
826.5597
[M−H]−
827.5631
766.5386
828.5661
767.5416
310.2726
598.4746
967.5665
1030.5221

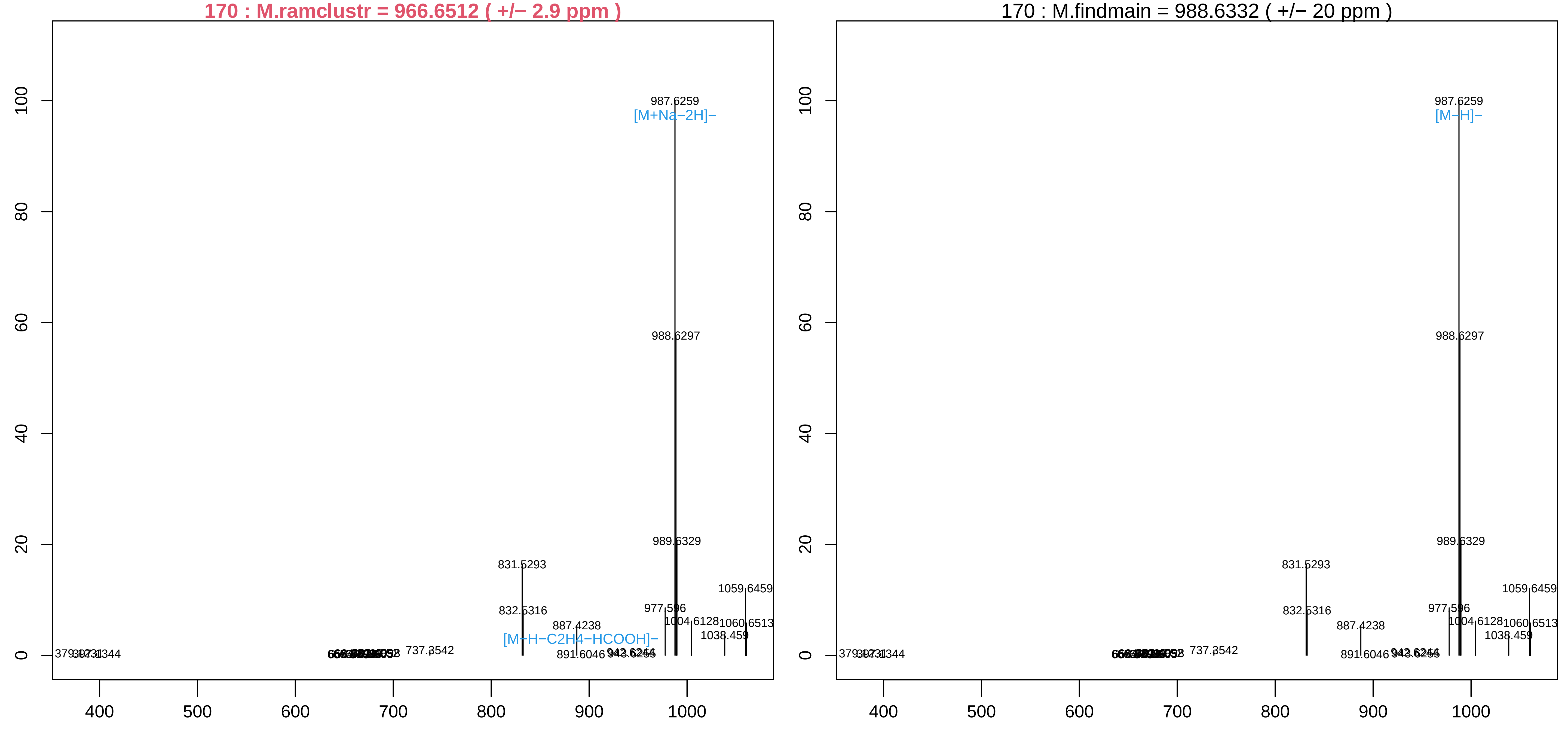

170 : M.ramclustr = 966.6512 (+/− 2.9 ppm)
987.6259
[M+Na−2H]−
988.6297
989.6329
831.5293
832.5316
887.4238
977.596
1004.6128
1059.6459
1060.6513
1038.459
[M−H−C2H4−HCOOH]−
737.3542
891.6046
942.6244
943.6255
170 : M.findmain = 988.6332 (+/− 20 ppm)
987.6259
[M−H]−
988.6297
989.6329
831.5293
832.5316
887.4238
977.596
1004.6128
1059.6459
1060.6513
1038.459
737.3542
891.6046
942.6244
943.6255

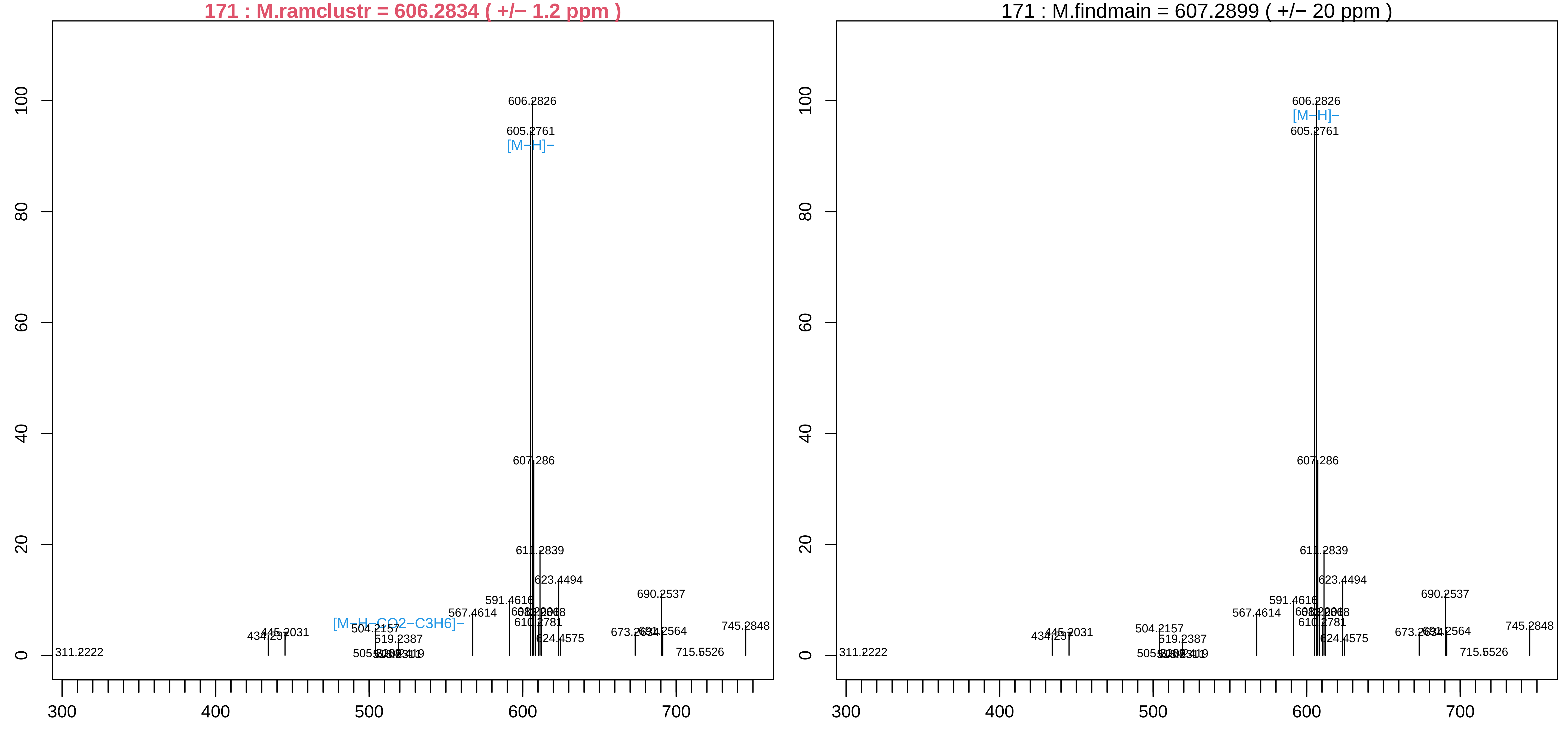
171 : M.ramclustr = 606.2834 (+/− 1.2 ppm)
606.2826
605.2761
[M−H]−
607.286
611.2839
623.4494
690.2537
591.4616
567.4614
[M−H−CO2−C3H6]−
504.2157
610.2781
519.2387
445.2031
434.237
673.2634
691.2564
745.2848
624.4575
715.5526
311.2222
171 : M.findmain = 607.2899 (+/− 20 ppm)
606.2826
[M−H]−
605.2761
607.286
611.2839
623.4494
690.2537
591.4616
567.4614
504.2157
610.2781
519.2387
445.2031
434.237
673.2634
691.2564
745.2848
624.4575
715.5526
311.2222
0
20
40
60
80
100
300
400
500
600
700

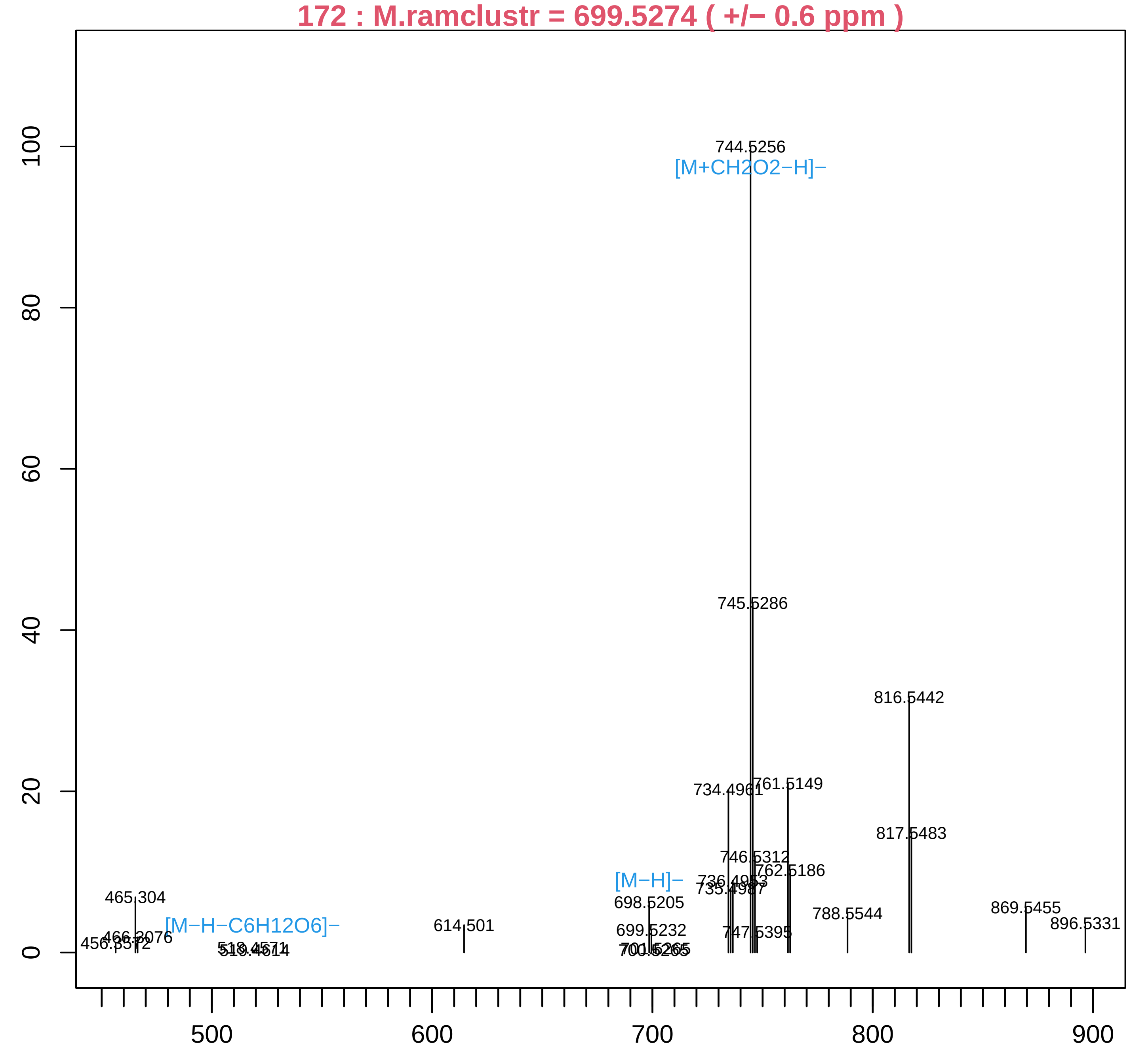
172 : M.ramclustr = 699.5274 (+/− 0.6 ppm)
744.5256
[M+CH2O2−H]−
745.5286
816.5442
734.4961
761.5149
817.5483
746.5312
762.5186
[M−H]−
736.4983
735.4987
465.304
698.5205
788.5544
869.5455
[M−H−C6H12O6]−
614.501
699.5232
896.5331
747.5395
466.3076
456.3572
518.4571
519.4614
701.5265
700.5265
0
20
40
60
80
100
500
600
700
800
900

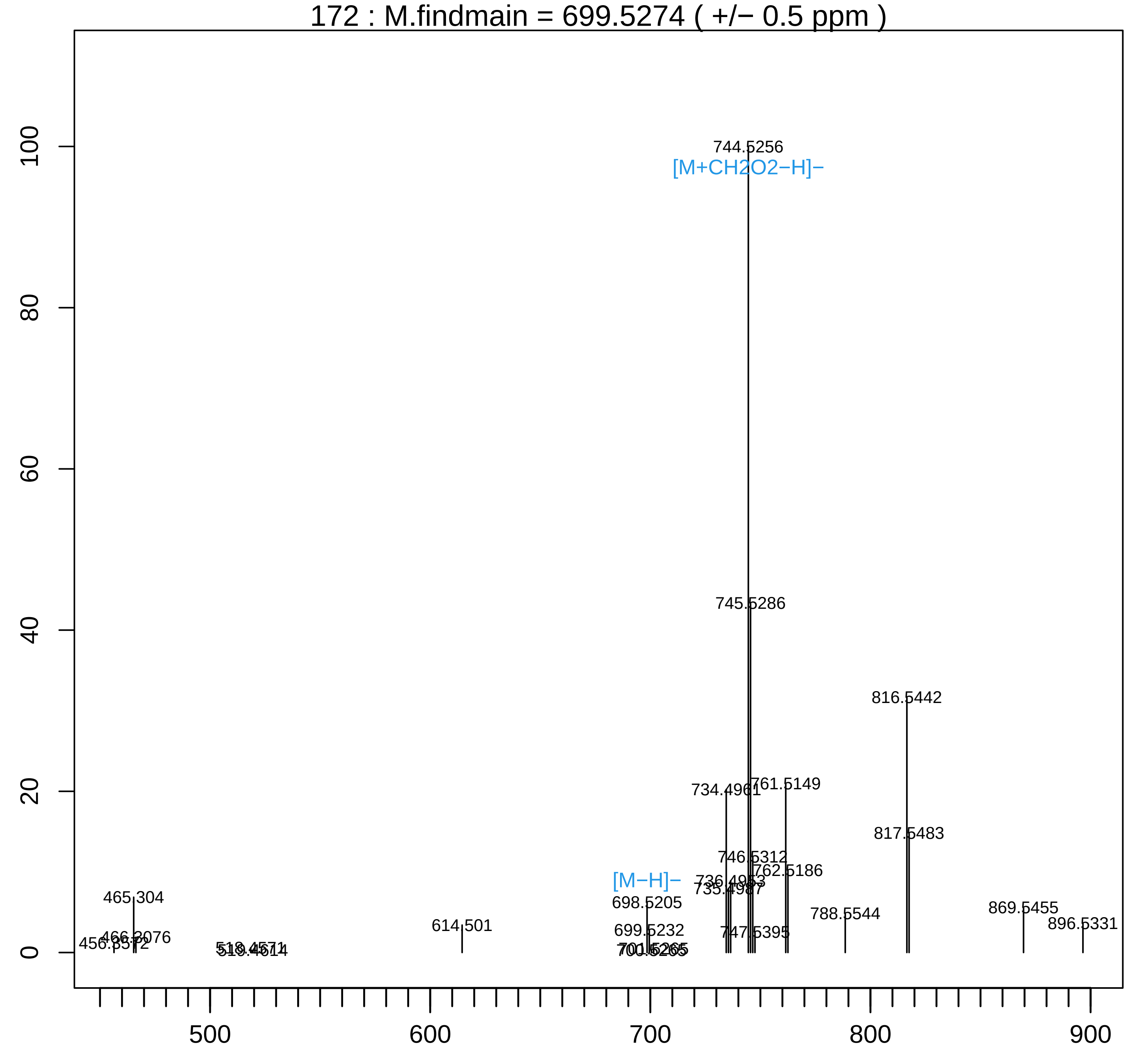
172 : M.findmain = 699.5274 (+/− 0.5 ppm)
744.5256
[M+CH2O2−H]−
745.5286
816.5442
734.4961
761.5149
817.5483
746.5312
762.5186
[M−H]−
736.4983
735.4987
465.304
698.5205
788.5544
869.5455
614.501
699.5232
896.5331
747.5395
466.3076
456.3572
518.4571
519.4614
701.5265
700.5265
0
20
40
60
80
100
500
600
700
800
900

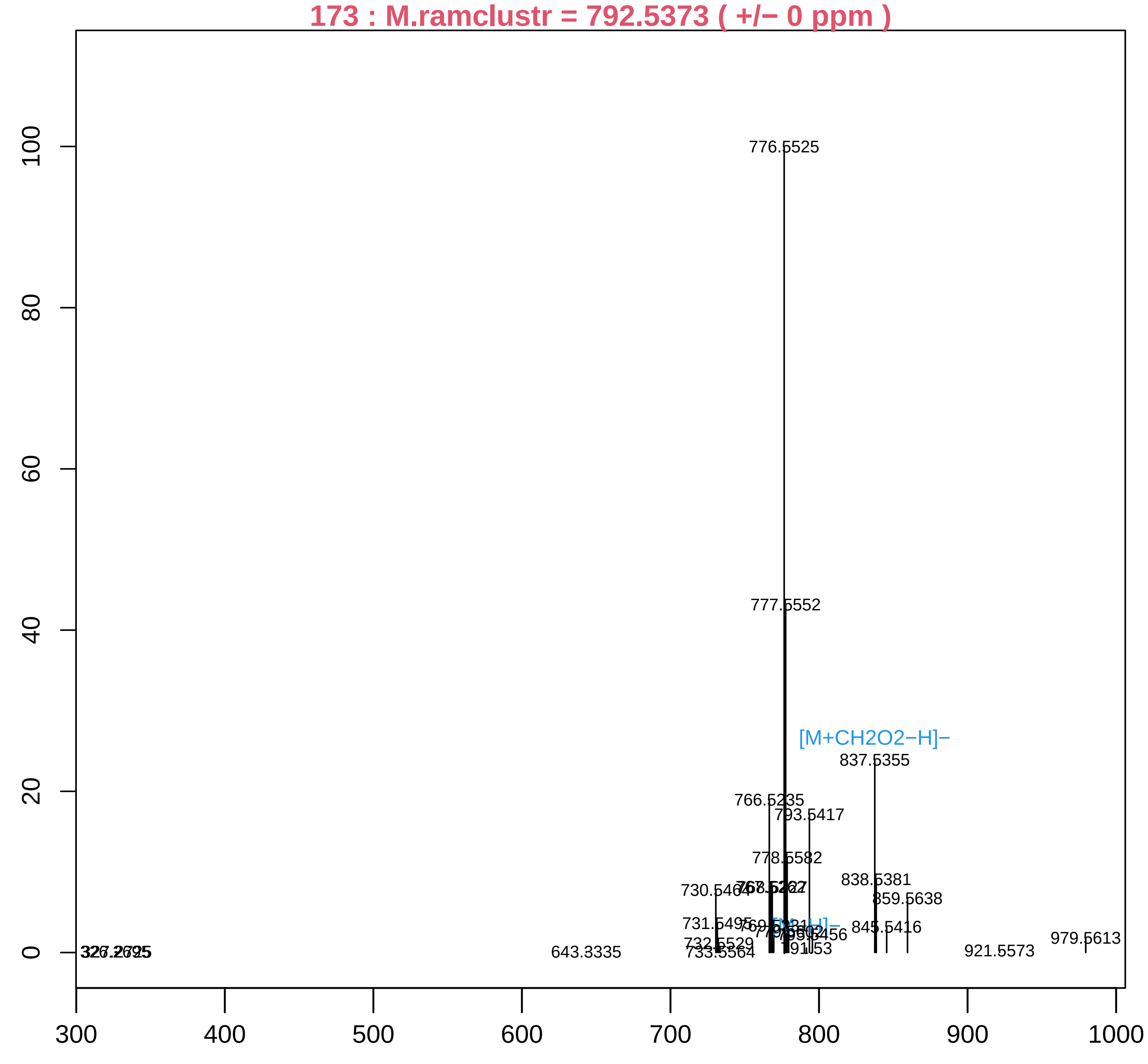
173 : M.ramclustr = 792.5373 (+/− 0 ppm)
776.5525
777.5552
[M+CH2O2−H]−
837.5355
766.5235
793.5417
778.5582
838.5381
730.5464
859.5638
731.5495
845.5416
979.5613
732.5529
733.5564
643.3335
921.5573
0
20
40
60
80
100
300
400
500
600
700
800
900
1000

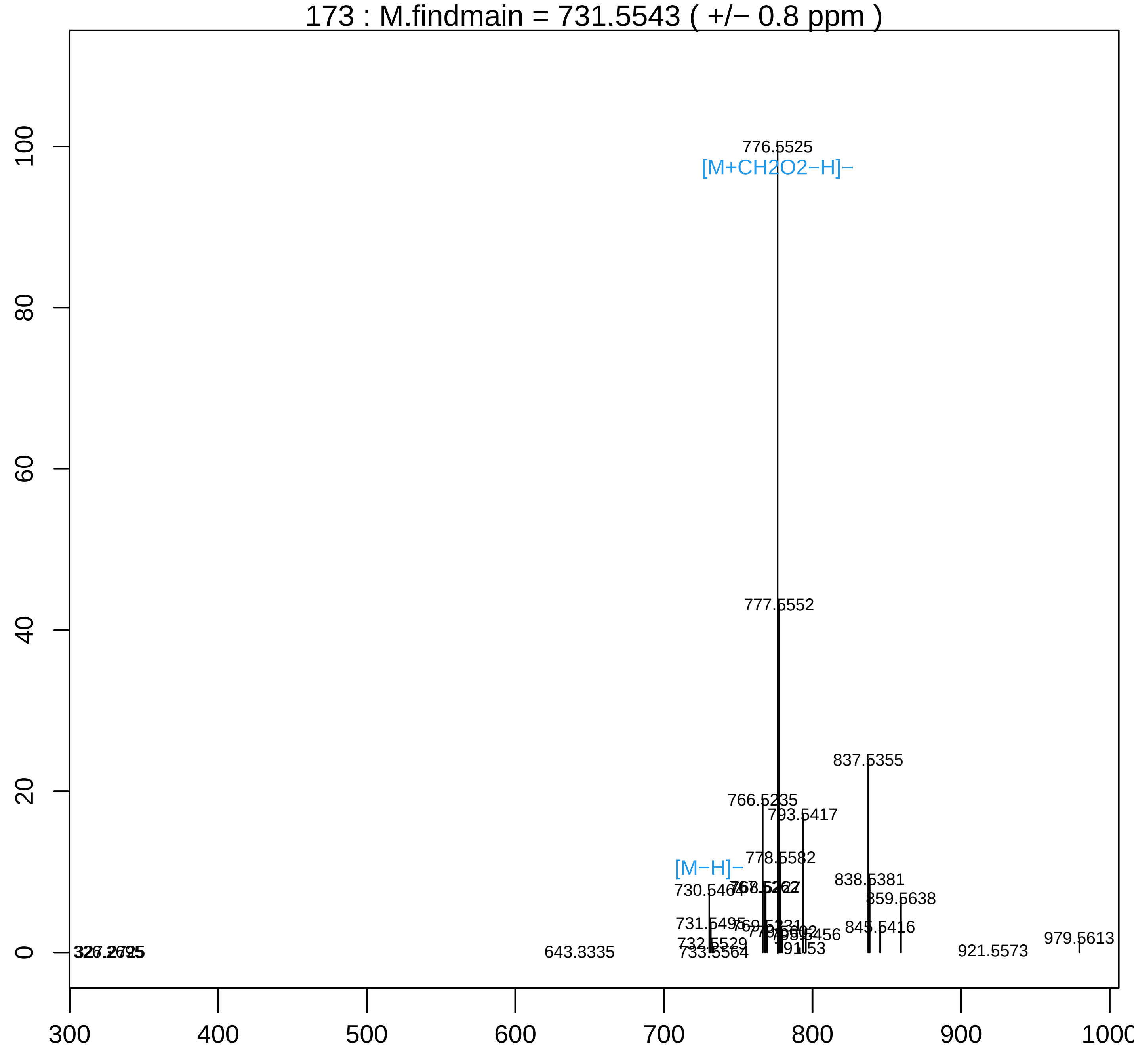
173 : M.findmain = 731.5543 (+/− 0.8 ppm)
776.5525
[M+CH2O2−H]−
777.5552
837.5355
766.5235
793.5417
[M−H]−
778.5582
838.5381
730.5464
859.5638
731.5495
845.5416
979.5613
732.5529
733.5564
643.3335
921.5573
0
20
40
60
80
100
300
400
500
600
700
800
900
1000

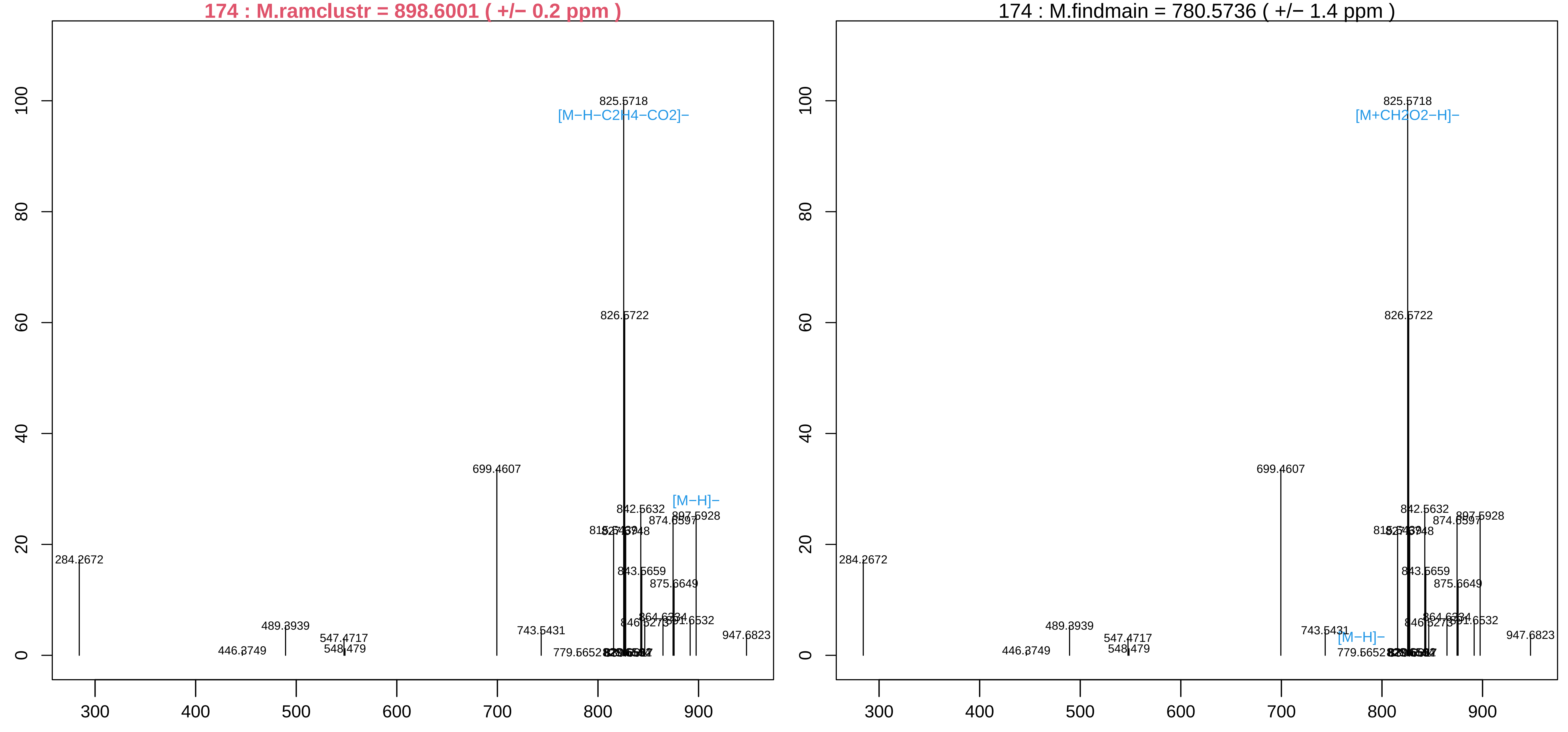
174 : M.ramclustr = 898.6001 (+/− 0.2 ppm)
174 : M.findmain = 780.5736 (+/− 1.4 ppm)
825.5718
[M−H−C2H4−CO2]−
[M+CH2O2−H]−
826.5722
699.4607
842.5632
[M−H]−
897.5928
874.5597
284.2672
843.5659
875.6649
489.3939
547.4717
548.479
446.3749
743.5431
947.6823
779.6652

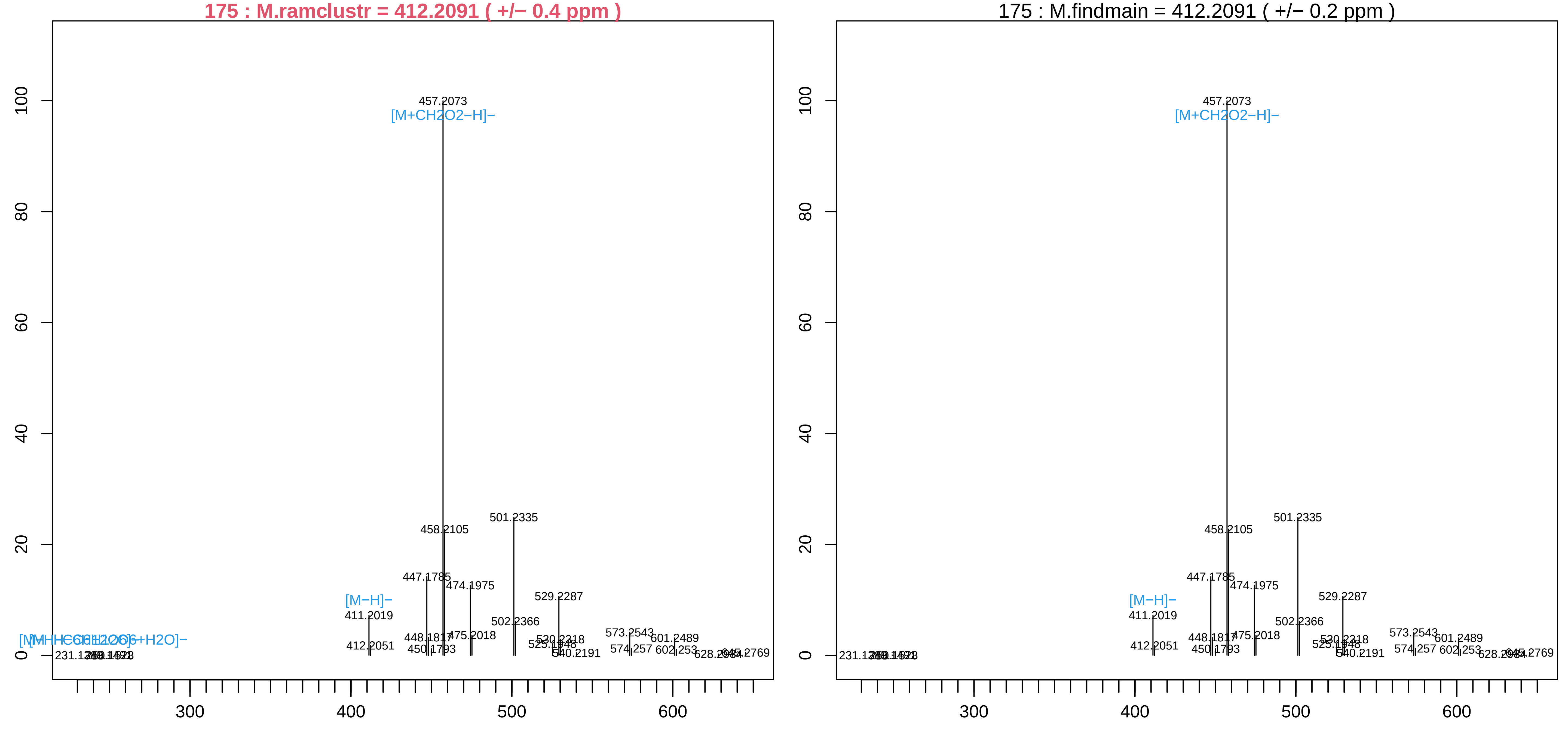
175 : M.ramclustr = 412.2091 (+/− 0.4 ppm)
457.2073
[M+CH2O2−H]−
501.2335
458.2105
447.1785
474.1975
529.2287
[M−H]−
411.2019
502.2366
448.1817
475.2018
530.2318
573.2543
601.2489
412.2051
450.1793
574.257
602.253
540.2191
645.2769
0
20
40
60
80
100
300
400
500
600
175 : M.findmain = 412.2091 (+/− 0.2 ppm)
457.2073
[M+CH2O2−H]−
501.2335
458.2105
447.1785
474.1975
529.2287
[M−H]−
411.2019
502.2366
448.1817
475.2018
530.2318
573.2543
601.2489
412.2051
450.1793
574.257
602.253
540.2191
645.2769
0
20
40
60
80
100
300
400
500
600

176 : M.ramclustr = 569.2511 (+/− 1.4 ppm)

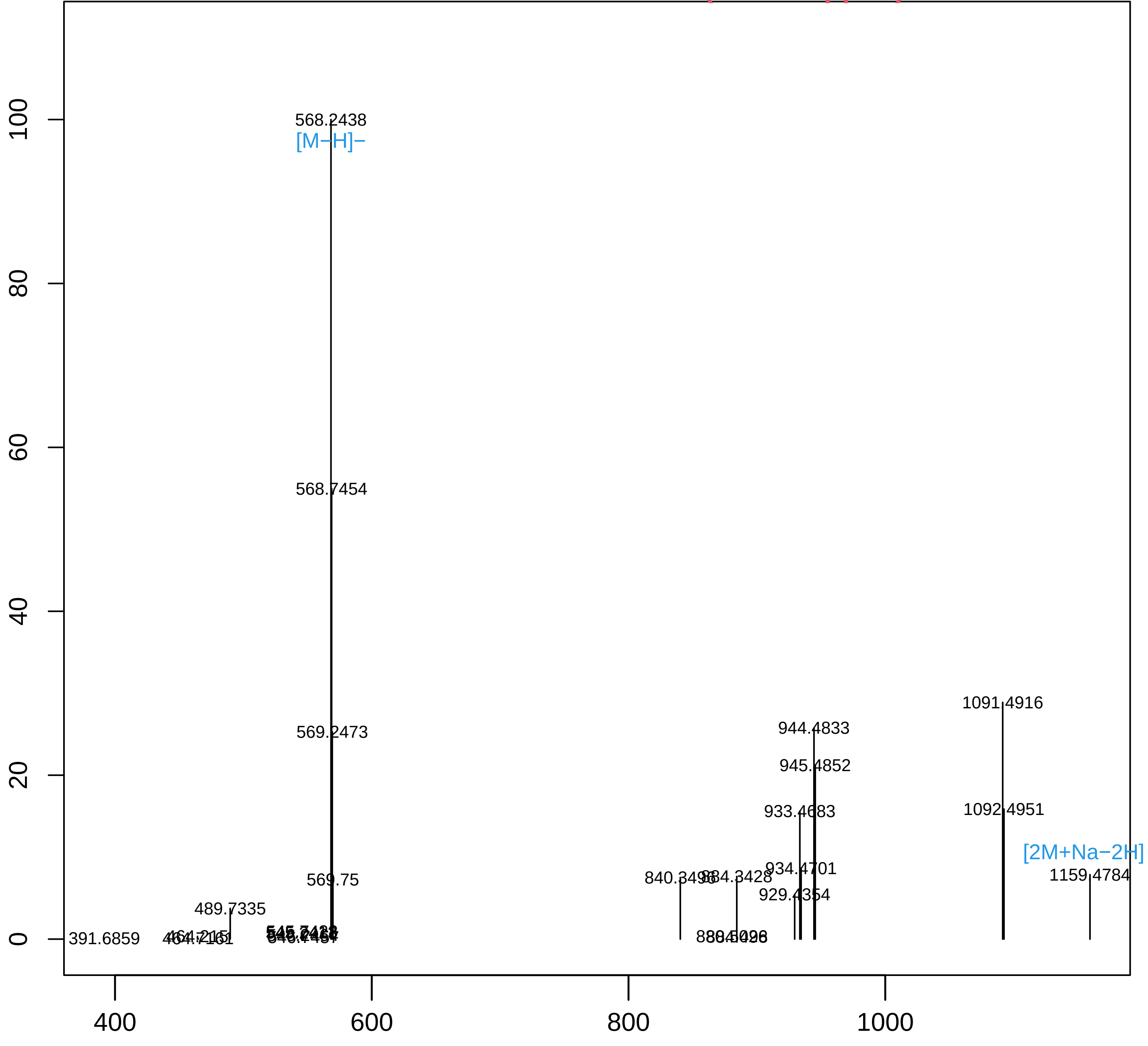

176 : M.findmain = 569.2511 (+/− 1.4 ppm)

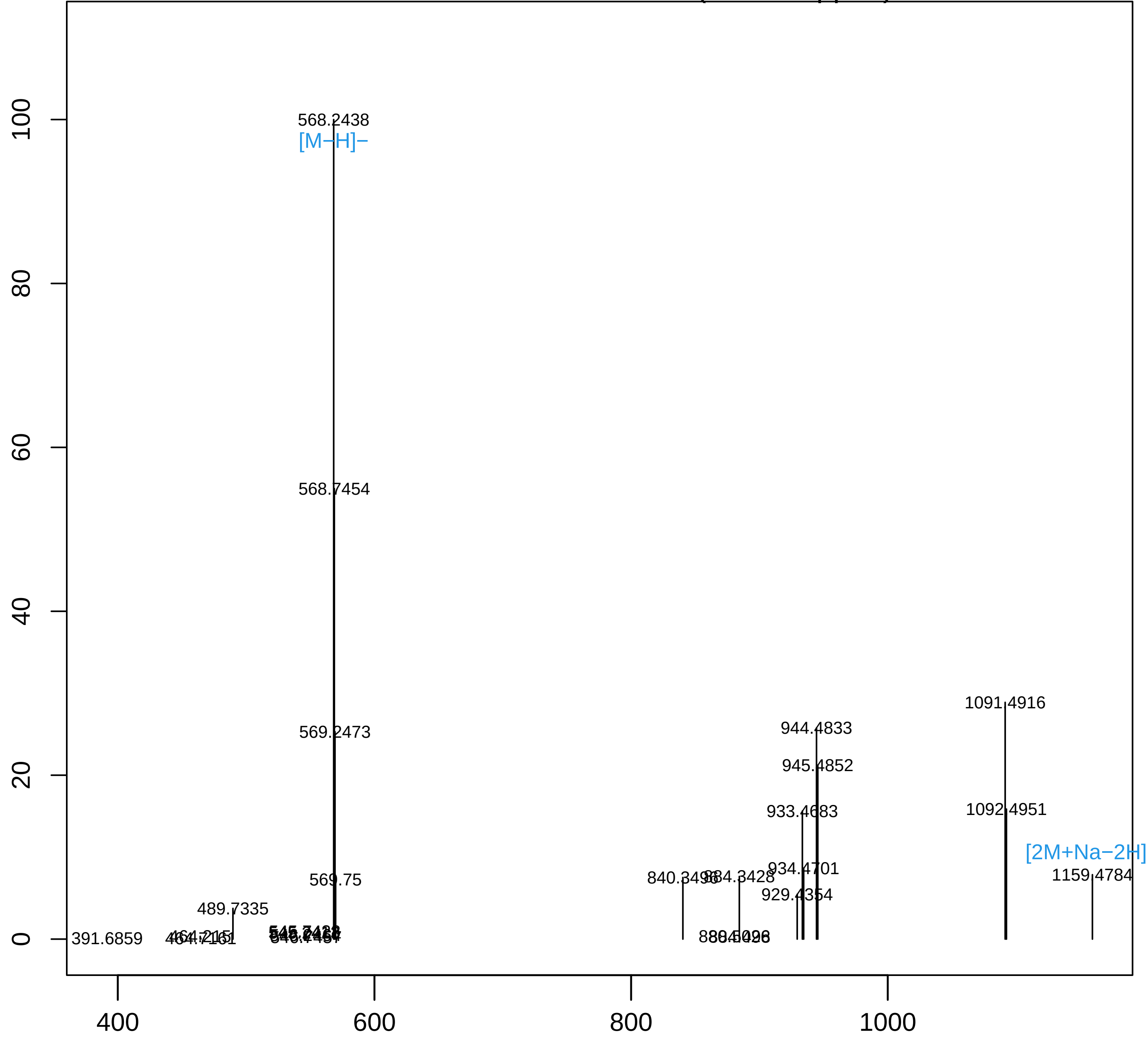

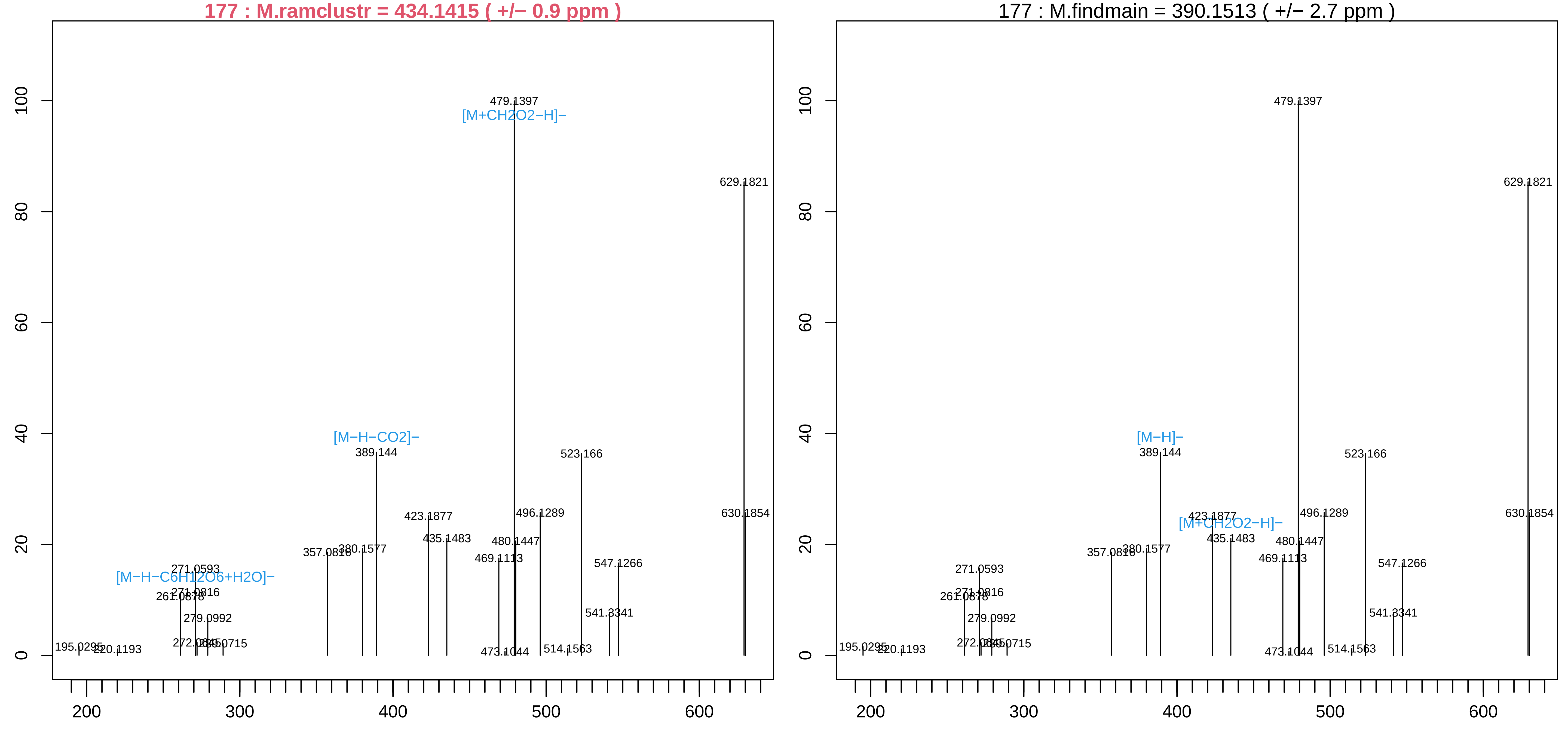
177 : M.ramclustr = 434.1415 (+/− 0.9 ppm)
177 : M.findmain = 390.1513 (+/− 2.7 ppm)
479.1397
[M+CH2O2−H]−
629.1821
[M−H−CO2]−
389.144
523.166
[M−H]−
423.1877
496.1289
630.1854
435.1483
480.1447
469.1113
357.0816
380.1577
547.1266
271.0593
[M−H−C6H12O6+H2O]−
271.0816
261.0878
279.0992
541.3341
195.0295
220.1193
473.1044
514.1563
0
20
40
60
80
100
200
300
400
500
600

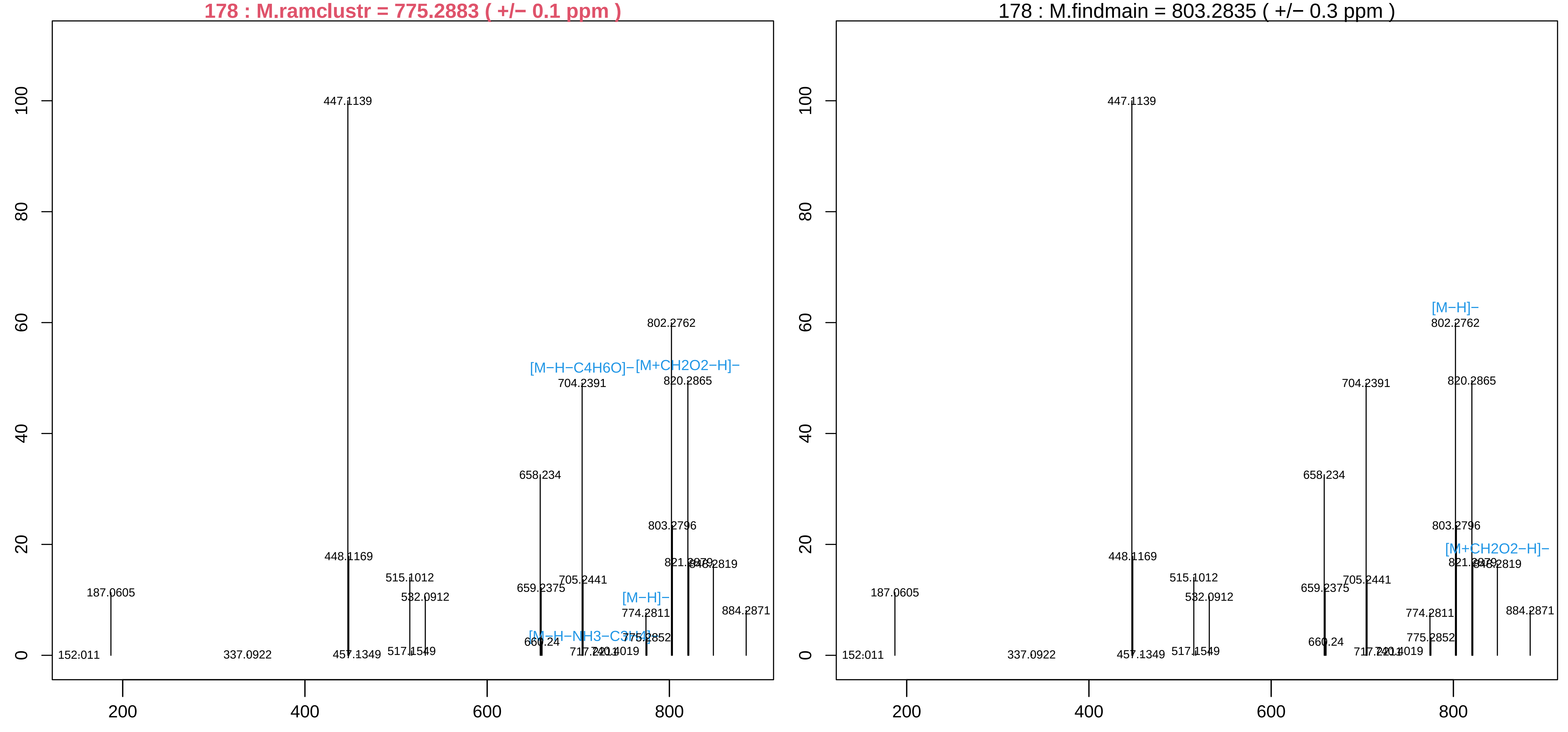
178 : M.ramclustr = 775.2883 (+/− 0.1 ppm)
178 : M.findmain = 803.2835 (+/− 0.3 ppm)
447.1139
802.2762
[M−H−C4H6O]−
[M+CH2O2−H]−
704.2391
820.2865
658.234
803.2796
448.1169
821.2979
848.2819
515.1012
187.0605
532.0912
659.2375
705.2441
[M−H]−
774.2811
884.2871
[M−H−NH3−C3H4]
775.2852
660.24
152.011
337.0922
457.1349
517.1549
717.2401
720.4019
0
20
40
60
80
100
200
400
600
800

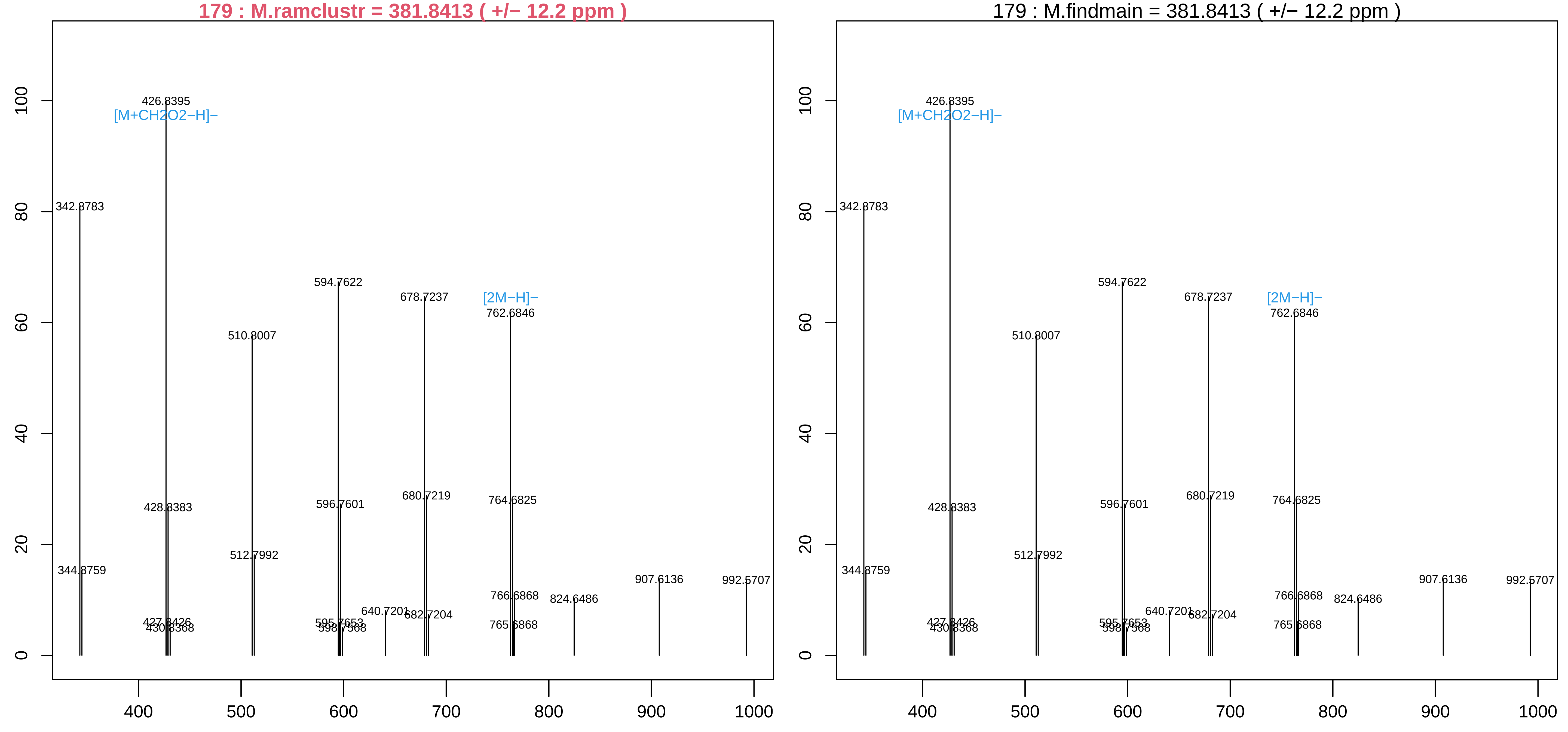
179 : M.ramclustr = 381.8413 (+/− 12.2 ppm)
426.8395
[M+CH2O2−H]−
342.8783
594.7622
678.7237
[2M−H]−
762.6846
510.8007
428.8383
596.7601
680.7219
764.6825
512.7992
344.8759
907.6136
992.5707
766.6868
824.6486
640.7201
682.7204
427.8426
430.8368
595.7653
598.7568
765.6868
179 : M.findmain = 381.8413 (+/− 12.2 ppm)
426.8395
[M+CH2O2−H]−
342.8783
594.7622
678.7237
[2M−H]−
762.6846
510.8007
428.8383
596.7601
680.7219
764.6825
512.7992
344.8759
907.6136
992.5707
766.6868
824.6486
640.7201
682.7204
427.8426
430.8368
595.7653
598.7568
765.6868

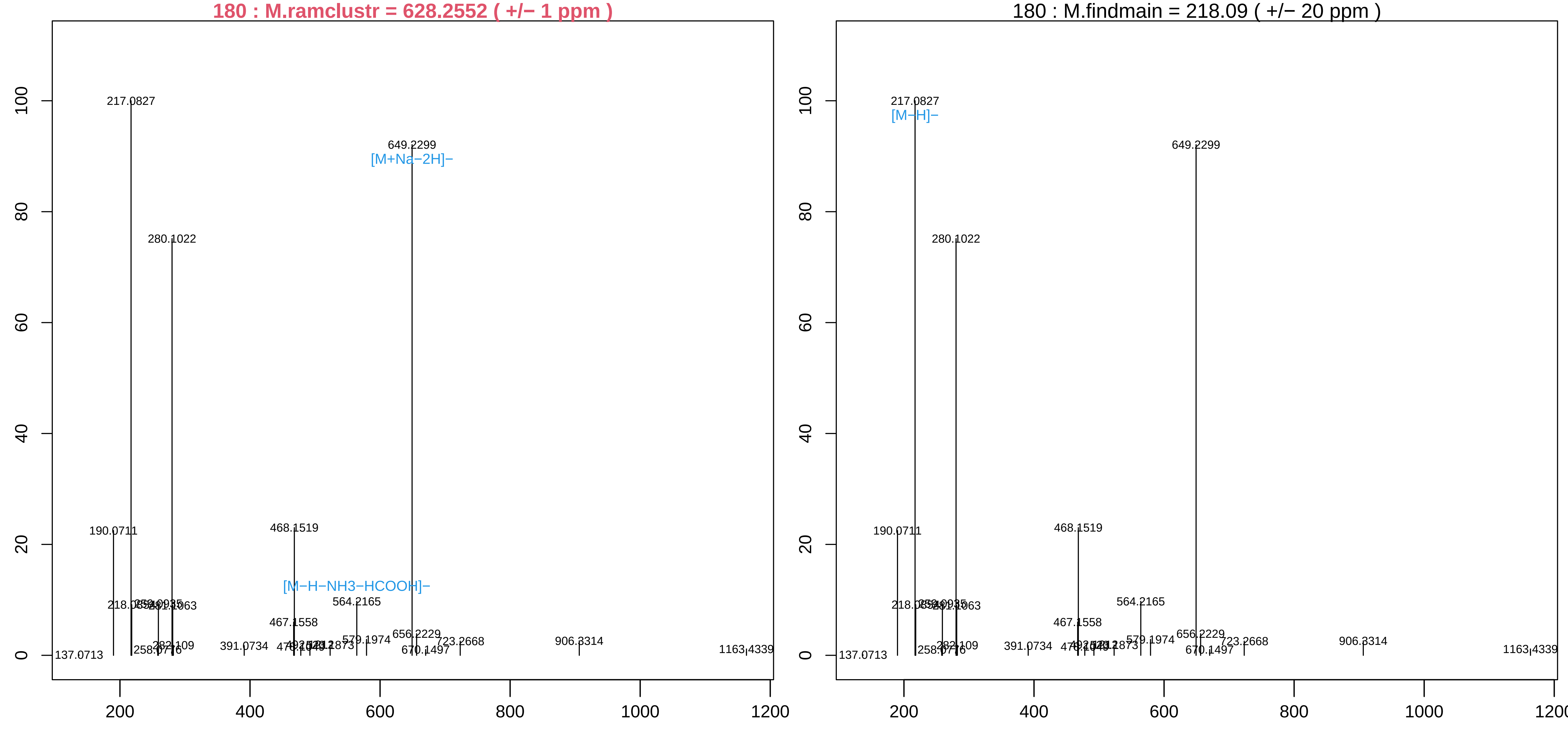
180 : M.ramclustr = 628.2552 (+/− 1 ppm)
180 : M.findmain = 218.09 (+/− 20 ppm)
217.0827
[M−H]−
649.2299
[M+Na−2H]−
280.1022
190.0711
468.1519
[M−H−NH3−HCOOH]−
564.2165
467.1558
579.1974
656.2229
723.2668
670.1497
906.3314
1163.4339
391.0734
282.109
258.0716
137.0713
281.1063
218.0854
0
20
40
60
80
100
200
400
600
800
1000
1200

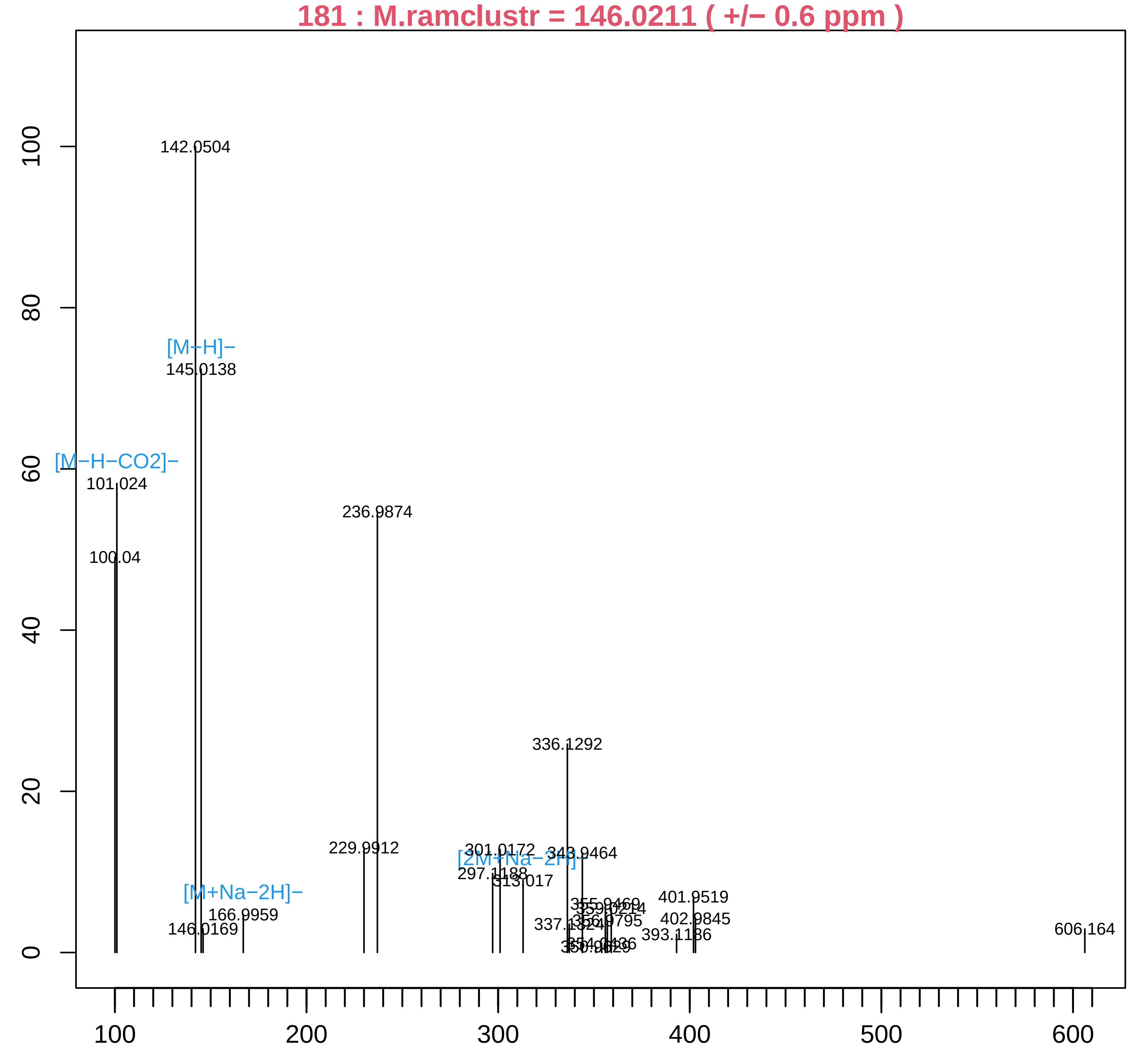
181 : M.ramclustr = 146.0211 (+/− 0.6 ppm)
142.0504
[M−H]−
145.0138
[M−H−CO2]−
101.024
100.04
236.9874
336.1292
229.9912
301.0172
[2M+Na−2H]−
343.9464
297.1188
313.017
[M+Na−2H]−
166.9959
146.0169
401.9519
402.9845
393.1136
606.164
0
20
40
60
80
100
200
300
400
500
600

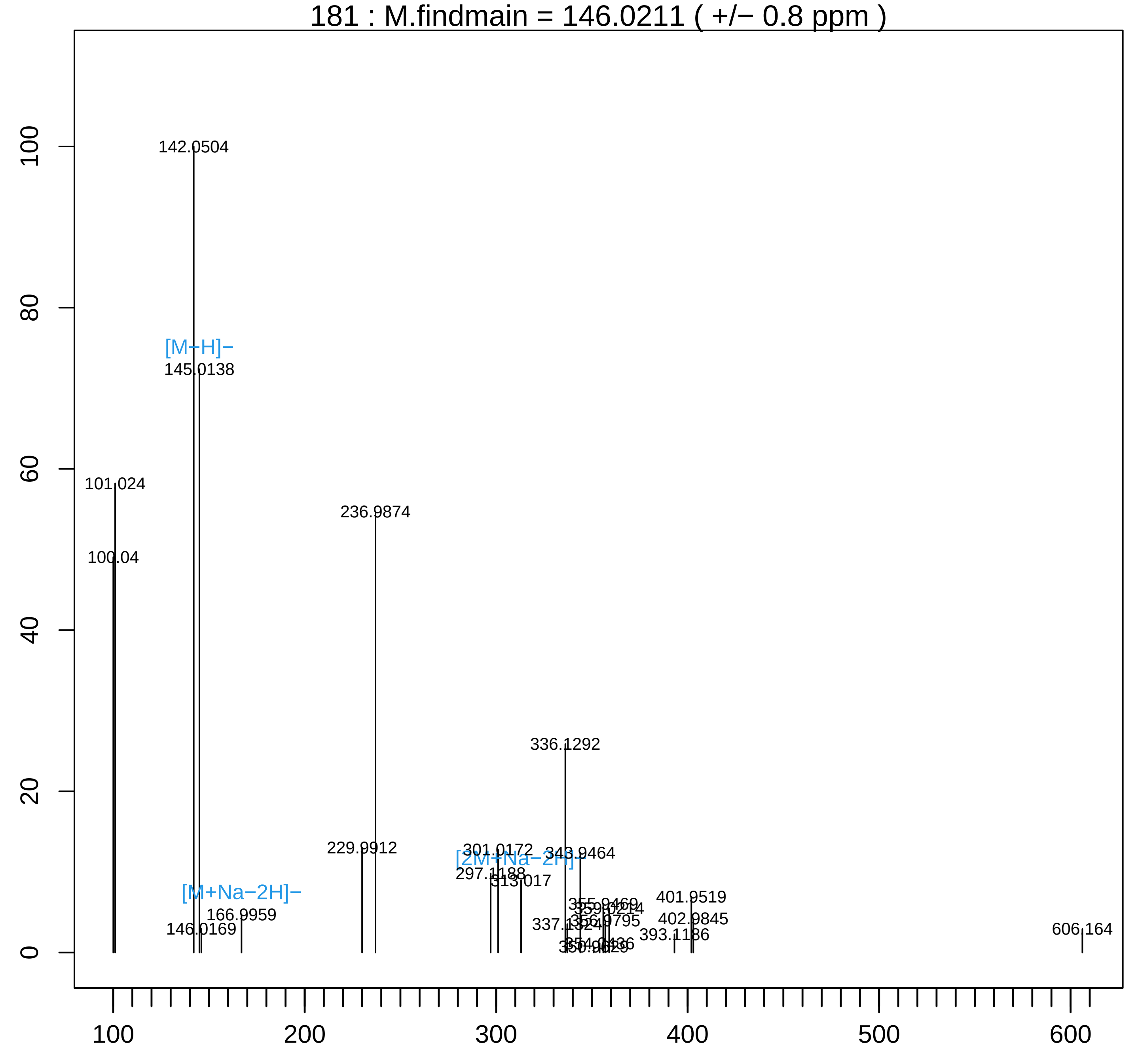
181 : M.findmain = 146.0211 (+/− 0.8 ppm)
142.0504
[M−H]−
145.0138
101.024
100.04
236.9874
336.1292
229.9912
301.0172
[2M+Na−2H]−
343.9464
297.1188
313.017
[M+Na−2H]−
166.9959
146.0169
401.9519
402.9845
393.1136
606.164
0
20
40
60
80
100
200
300
400
500
600

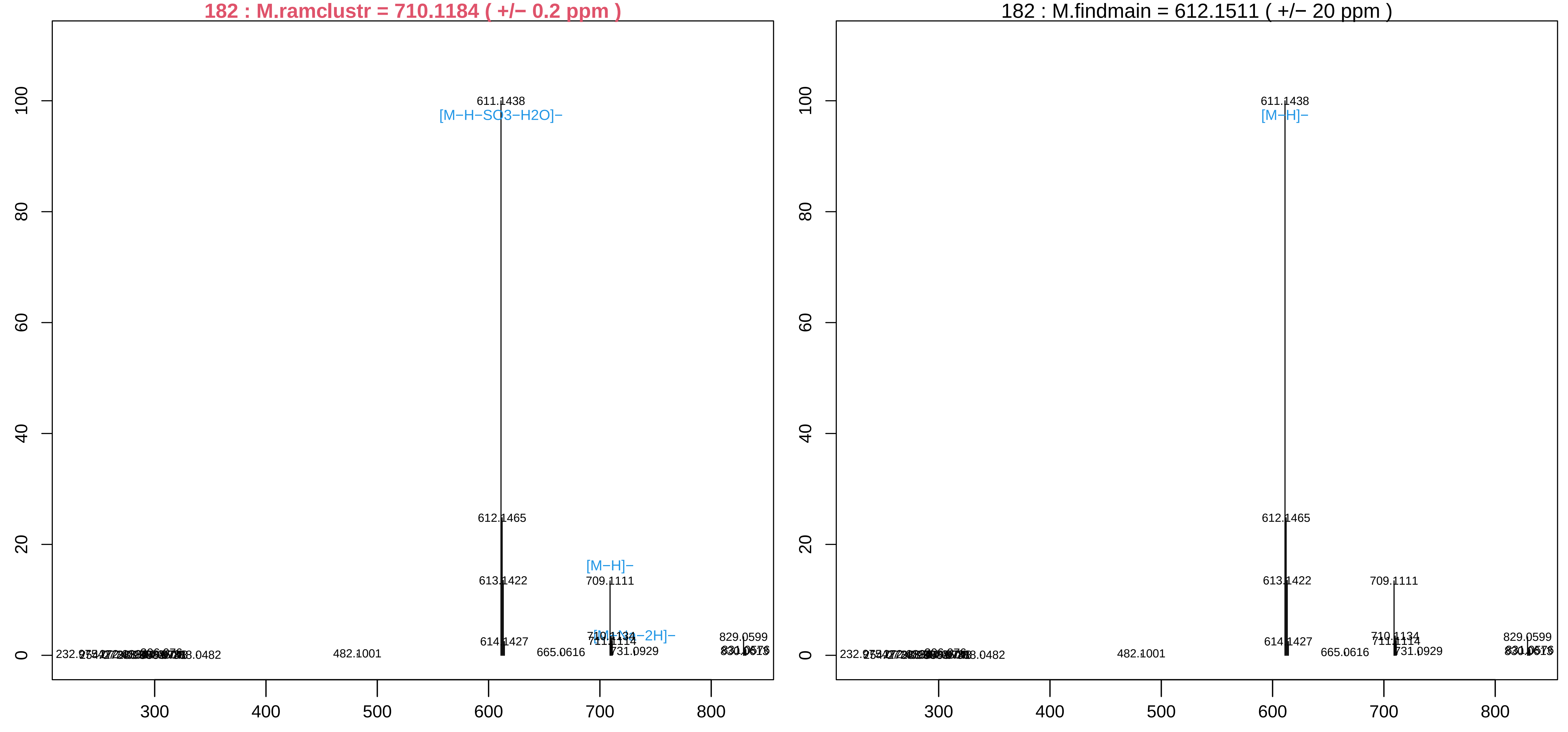
182 : M.ramclustr = 710.1184 (+/− 0.2 ppm)
611.1438
[M−H−SO3−H2O]−
612.1465
613.1422
614.1427
[M−H]−
709.1111
731.0929
665.0616
482.1001
829.0599
182 : M.findmain = 612.1511 (+/− 20 ppm)
611.1438
[M−H]−
612.1465
613.1422
614.1427
709.1111
710.1134
711.1114
731.0929
665.0616
482.1001
829.0599
0
20
40
60
80
100
300
400
500
600
700
800

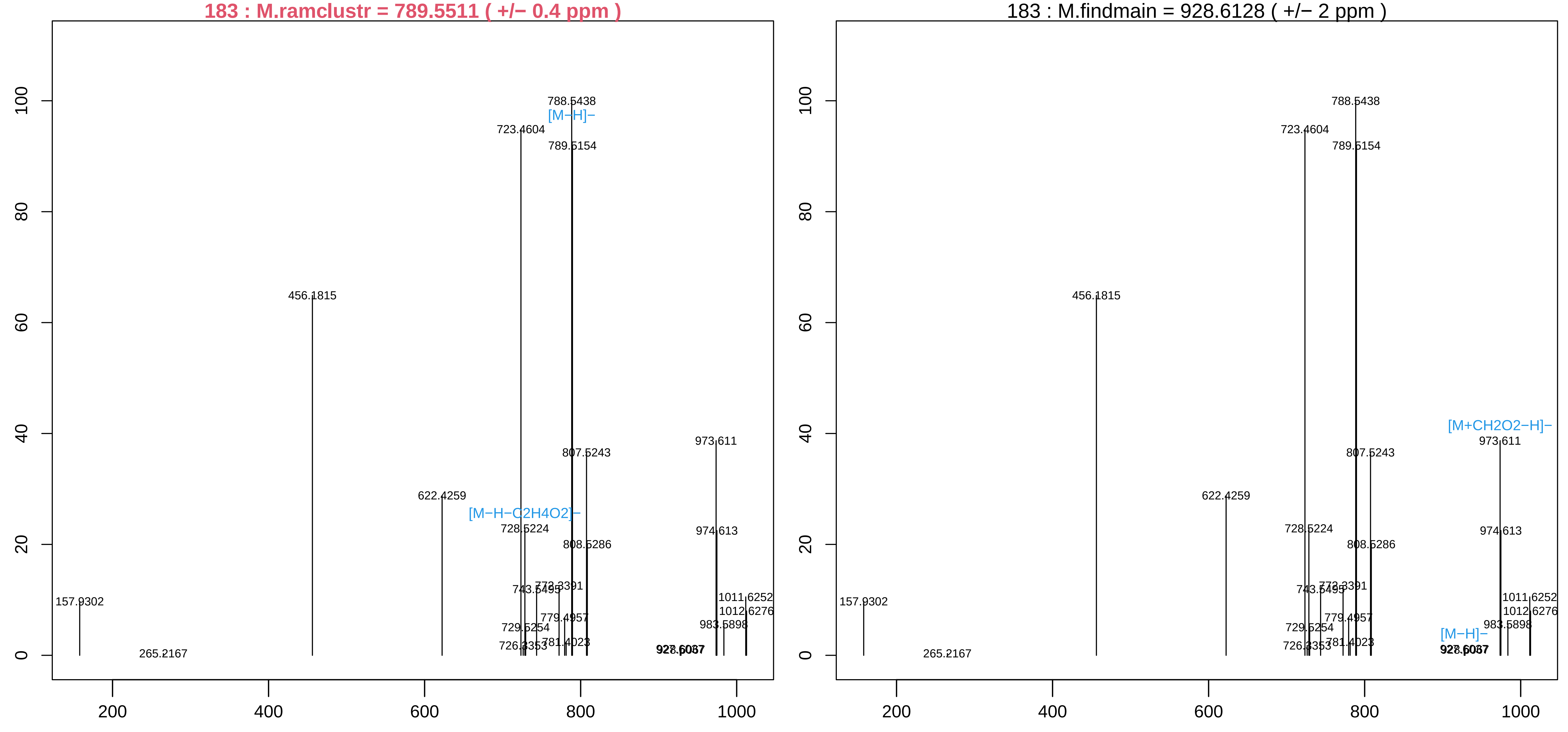
183 : M.ramclustr = 789.5511 (+/− 0.4 ppm)
788.5438
[M−H]−
723.4604
789.5154
456.1815
973.611
807.5243
622.4259
[M−H−C2H4O2]−
728.5224
974.613
808.5286
772.3391
743.5495
1011.6252
157.9302
1012.6276
779.4957
983.5898
729.5254
781.4023
726.3353
928.6087
265.2167
183 : M.findmain = 928.6128 (+/− 2 ppm)
[M+CH2O2−H]−
[M−H]−

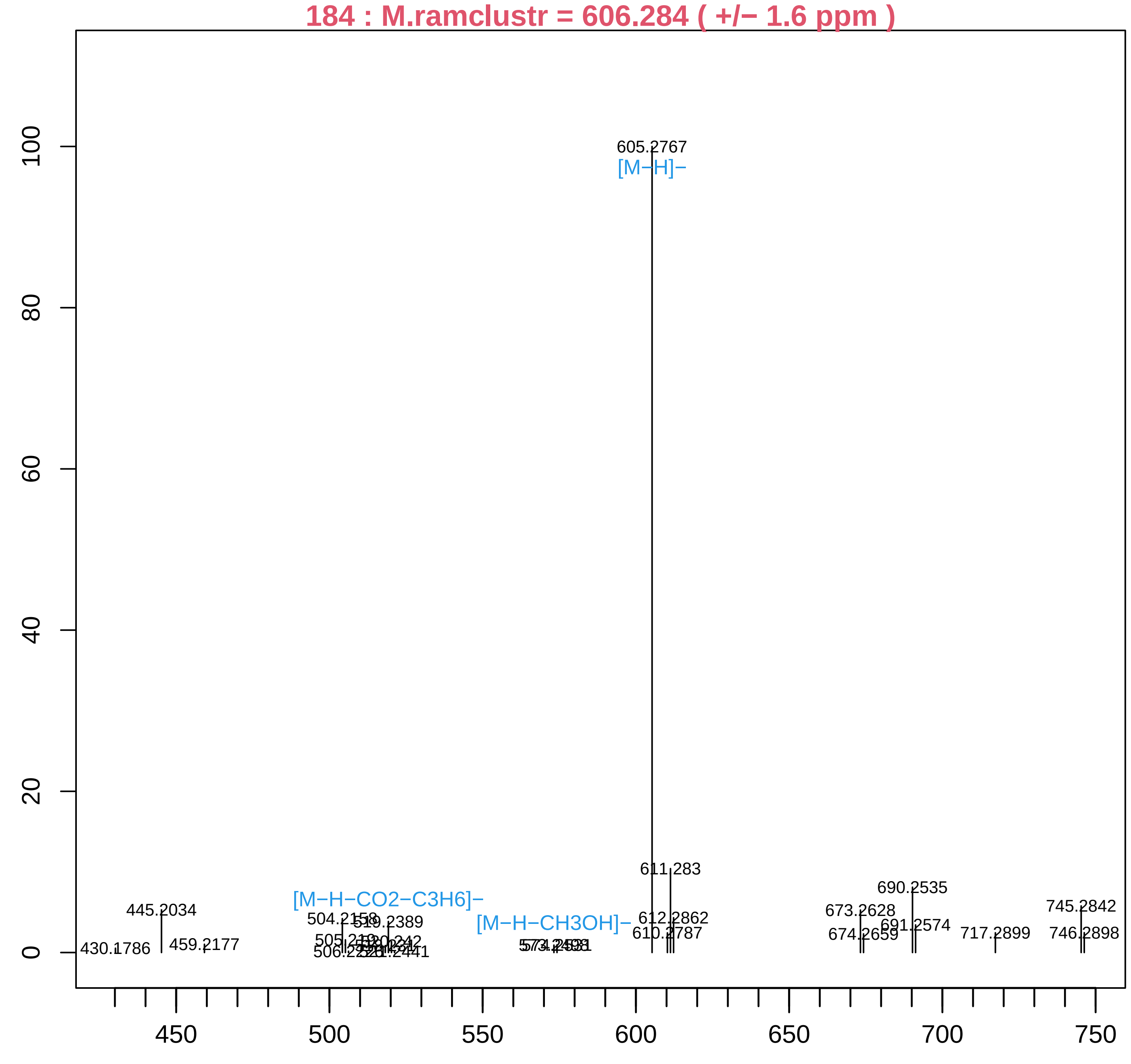
184 : M.ramclustr = 606.284 (+/− 1.6 ppm)
605.2767
[M−H]−
611.283
612.2862
610.2787
445.2034
430.1786
459.2177
504.2159
519.2389
[M−H−CO2−C3H6]−
[M−H−CH3OH]−
673.2628
674.2659
690.2535
691.2574
717.2899
745.2842
746.2898
0
20
40
60
80
100
450
500
550
600
650
700
750

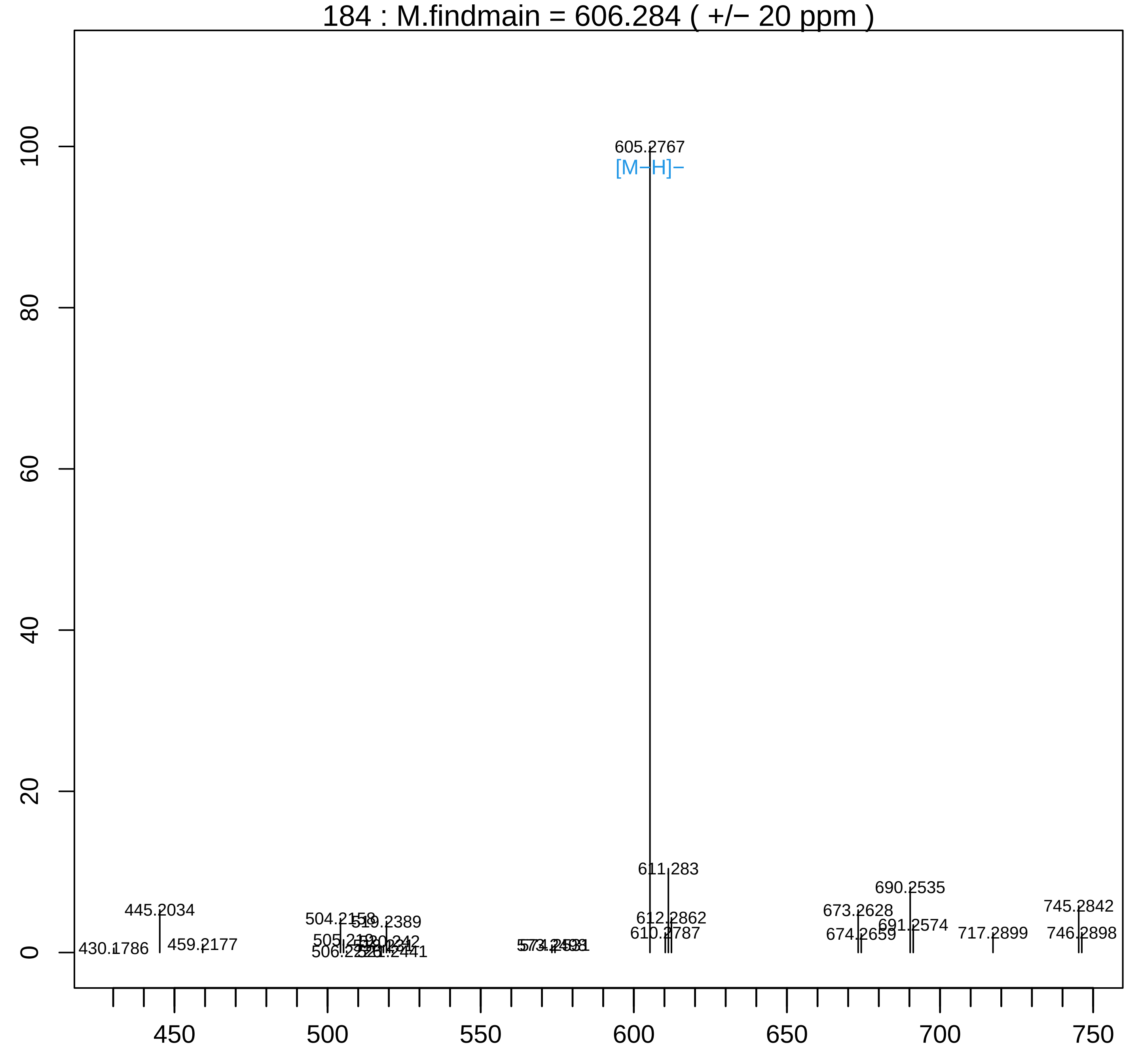
184 : M.findmain = 606.284 (+/− 20 ppm)
605.2767
[M−H]−
611.283
612.2862
610.2787
445.2034
430.1786
459.2177
504.2159
519.2389
673.2628
674.2659
690.2535
691.2574
717.2899
745.2842
746.2898
0
20
40
60
80
100
450
500
550
600
650
700
750

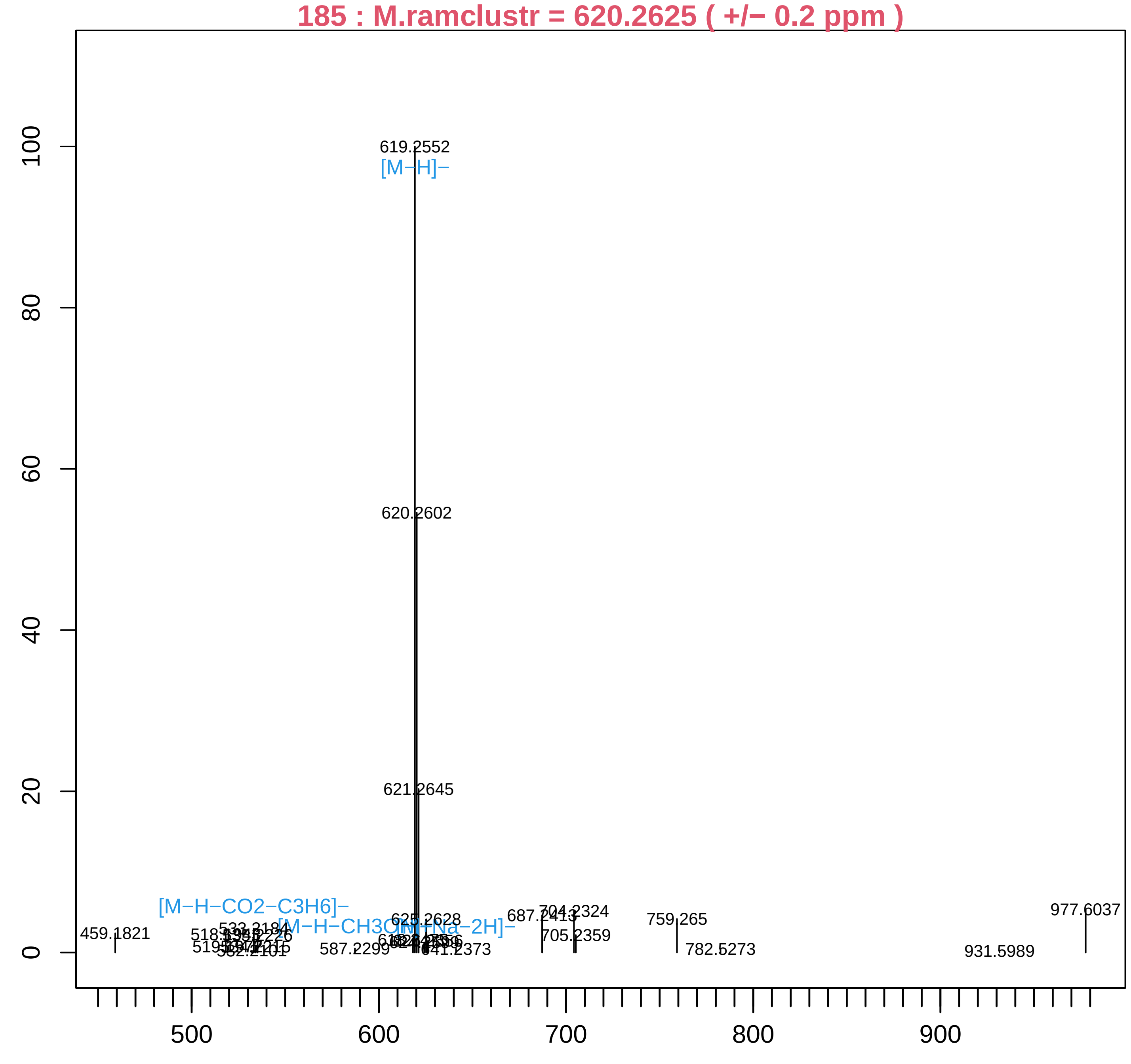
185 : M.ramclustr = 620.2625 (+/− 0.2 ppm)
619.2552
[M−H]−
620.2602
621.2645
[M−H−CO2−C3H6]−
[M−H−CH3OH]−
[M+Na−2H]−
459.1821
533.2184
587.2299
625.2628
641.2373
687.2413
704.2324
705.2359
759.265
782.5273
931.5989
977.6037
0
20
40
60
80
100
500
600
700
800
900

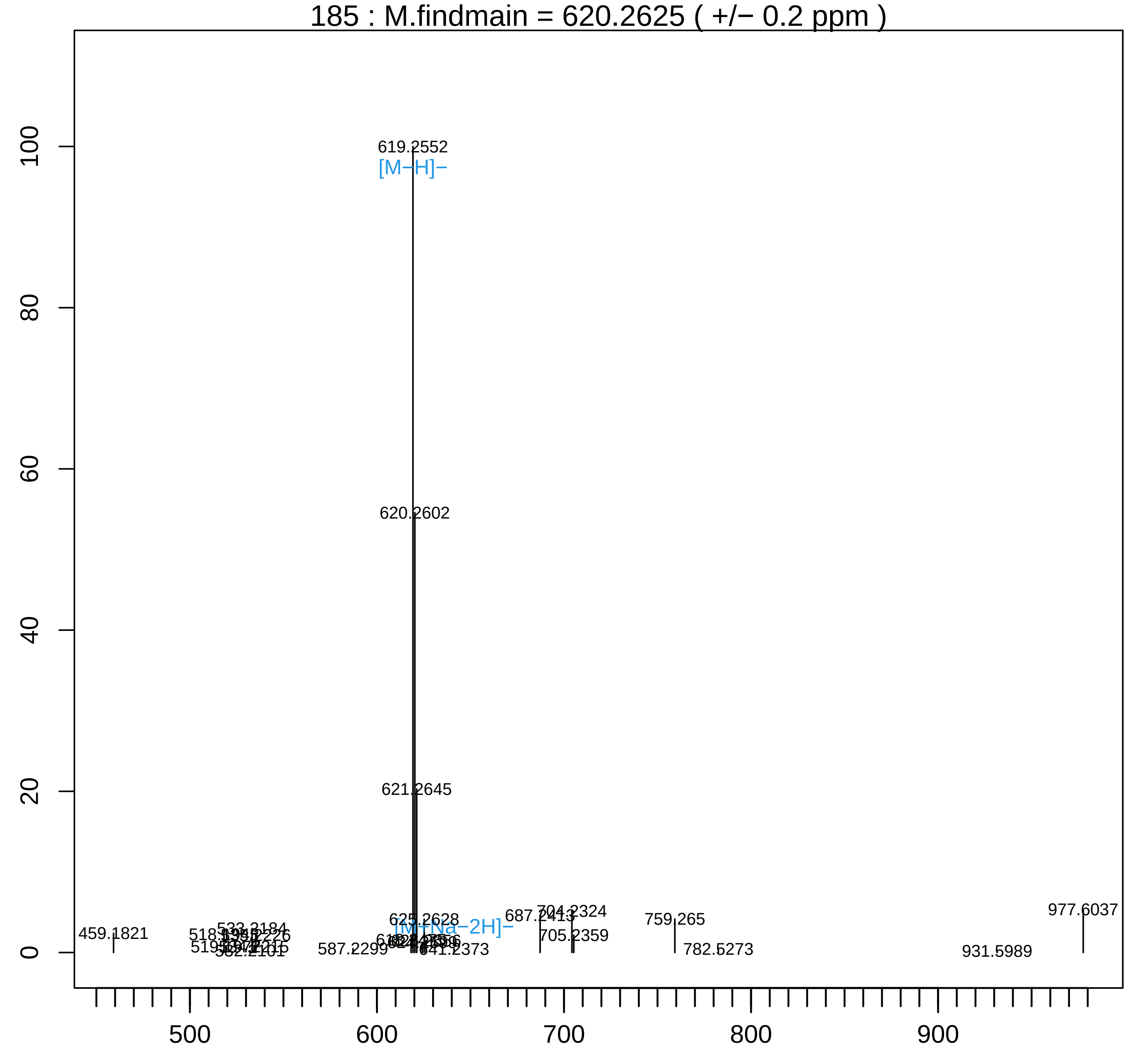
185 : M.findmain = 620.2625 (+/− 0.2 ppm)
619.2552
[M−H]−
620.2602
621.2645
[M+Na−2H]−
459.1821
533.2184
587.2299
625.2628
641.2373
687.2413
704.2324
705.2359
759.265
782.5273
931.5989
977.6037
0
20
40
60
80
100
500
600
700
800
900

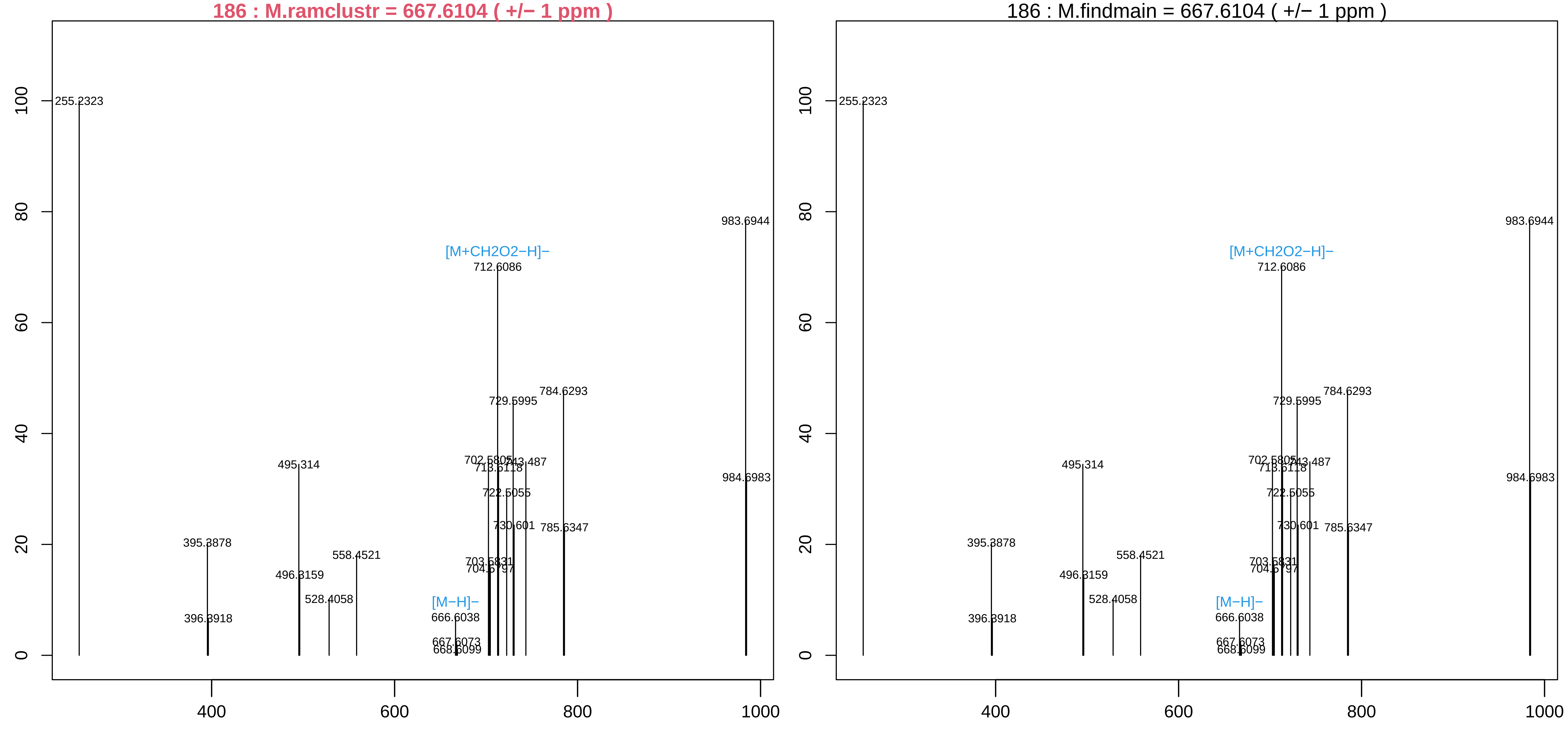
186 : M.ramclustr = 667.6104 (+/− 1 ppm)
186 : M.findmain = 667.6104 (+/− 1 ppm)
0
20
40
60
80
100
400
600
800
1000
255.2323
395.3878
396.3918
495.314
496.3159
528.4058
558.4521
[M−H]−
666.6038
667.6073
668.6099
702.5805
703.5831
704.5797
[M+CH2O2−H]−
712.6086
713.6118
722.5055
729.5995
730.601
743.487
784.6293
785.6347
983.6944
984.6983

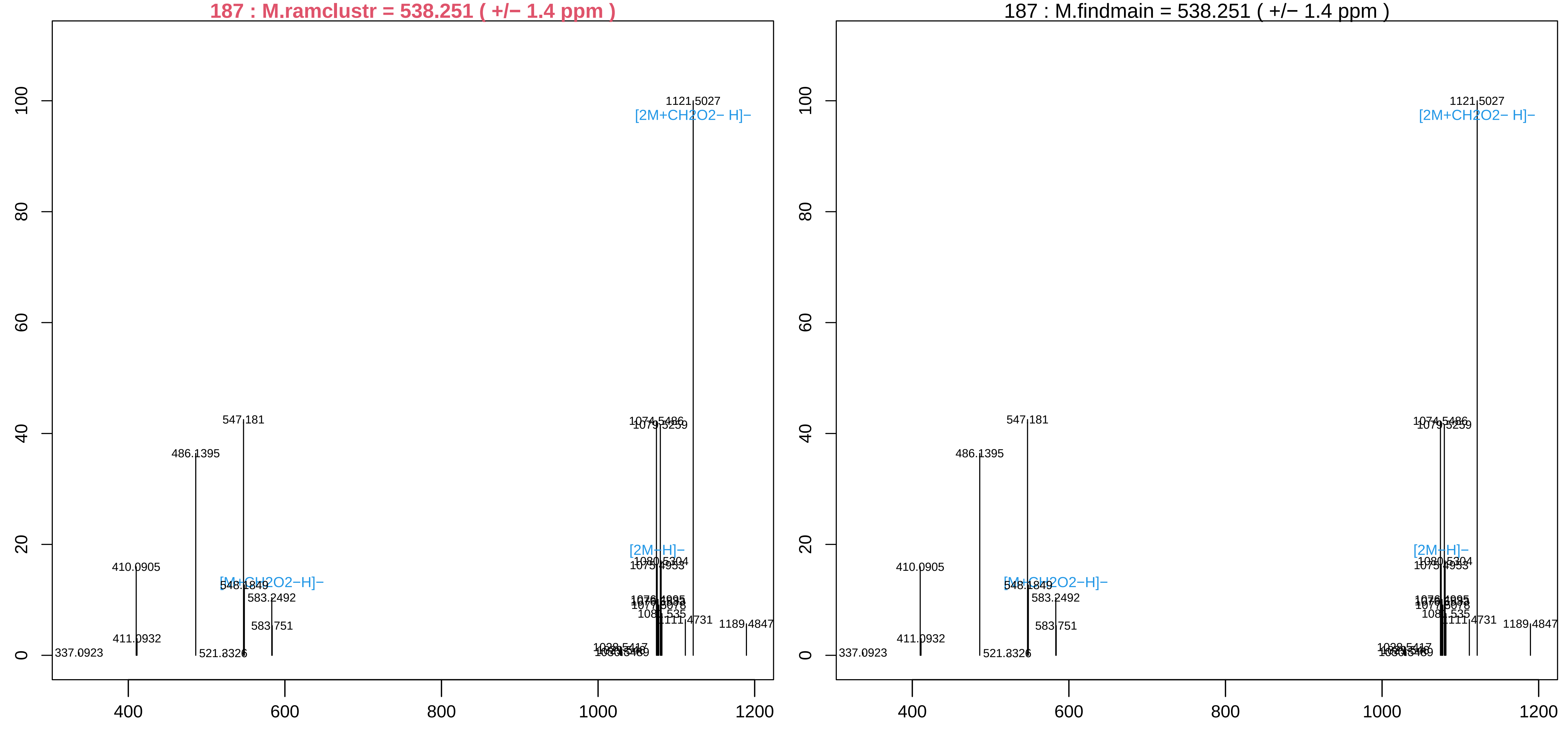
187 : M.ramclustr = 538.251 (+/− 1.4 ppm)
187 : M.findmain = 538.251 (+/− 1.4 ppm)
1121.5027
[2M+CH2O2− H]−
547.181
486.1395
1074.5486
1079.5259
[2M−H]−
1080.5304
1075.4953
410.0905
[M+CH2O2−H]−
548.1849
583.2492
583.751
1111.4731
1189.4847
411.0932
337.0923
521.3326
0
20
40
60
80
100
400
600
800
1000
1200

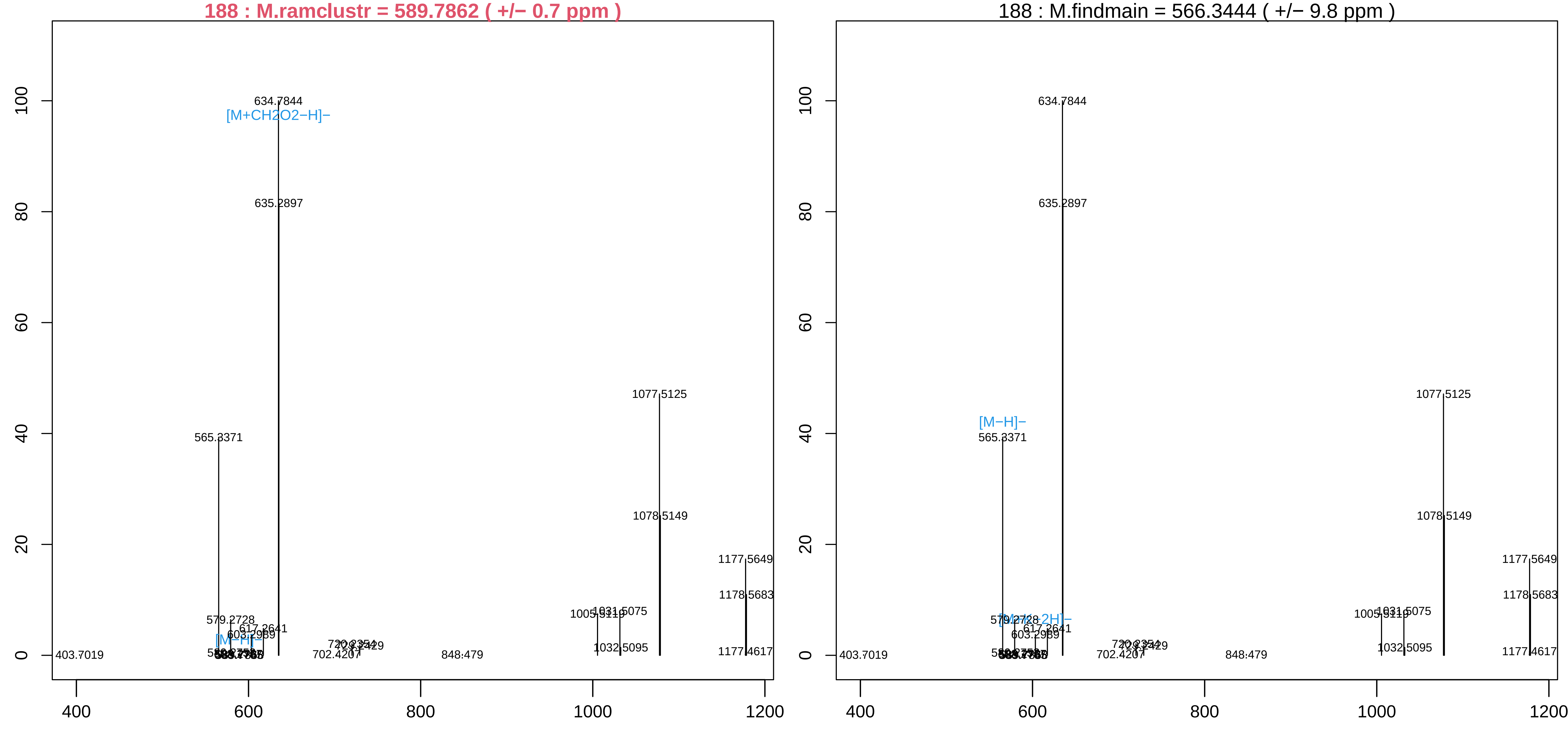
188 : M.ramclustr = 589.7862 (+/− 0.7 ppm)
634.7844
[M+CH2O2−H]−
635.2897
565.3371
1077.5125
1078.5149
1177.5649
1178.5683
579.2728
617.2641
603.2959
[M−H]−
403.7019
702.4207
848.479
1032.5095
1177.4617
188 : M.findmain = 566.3444 (+/− 9.8 ppm)
634.7844
635.2897
[M−H]−
565.3371
1077.5125
1078.5149
1177.5649
1178.5683
617.2641
603.2959
403.7019
702.4207
848.479
1032.5095
1177.4617
0
20
40
60
80
100
400
600
800
1000
1200

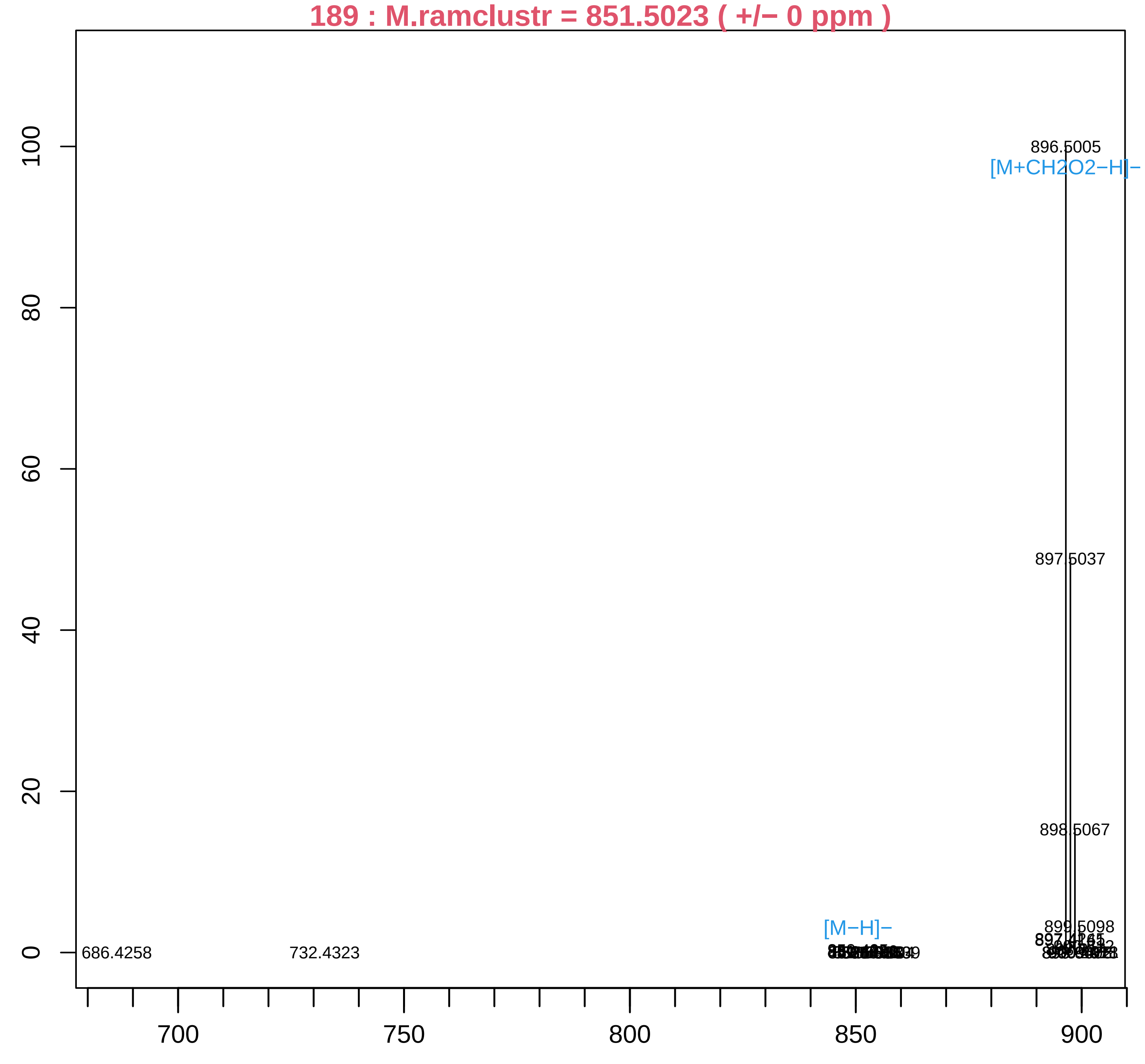

189 : M.ramclustr = 851.5023 (+/− 0 ppm)
896.5005
[M+CH2O2−H]−
897.5037
898.5067
899.5098
[M−H]−
686.4258
732.4323
0
20
40
60
80
100
700
750
800
850
900

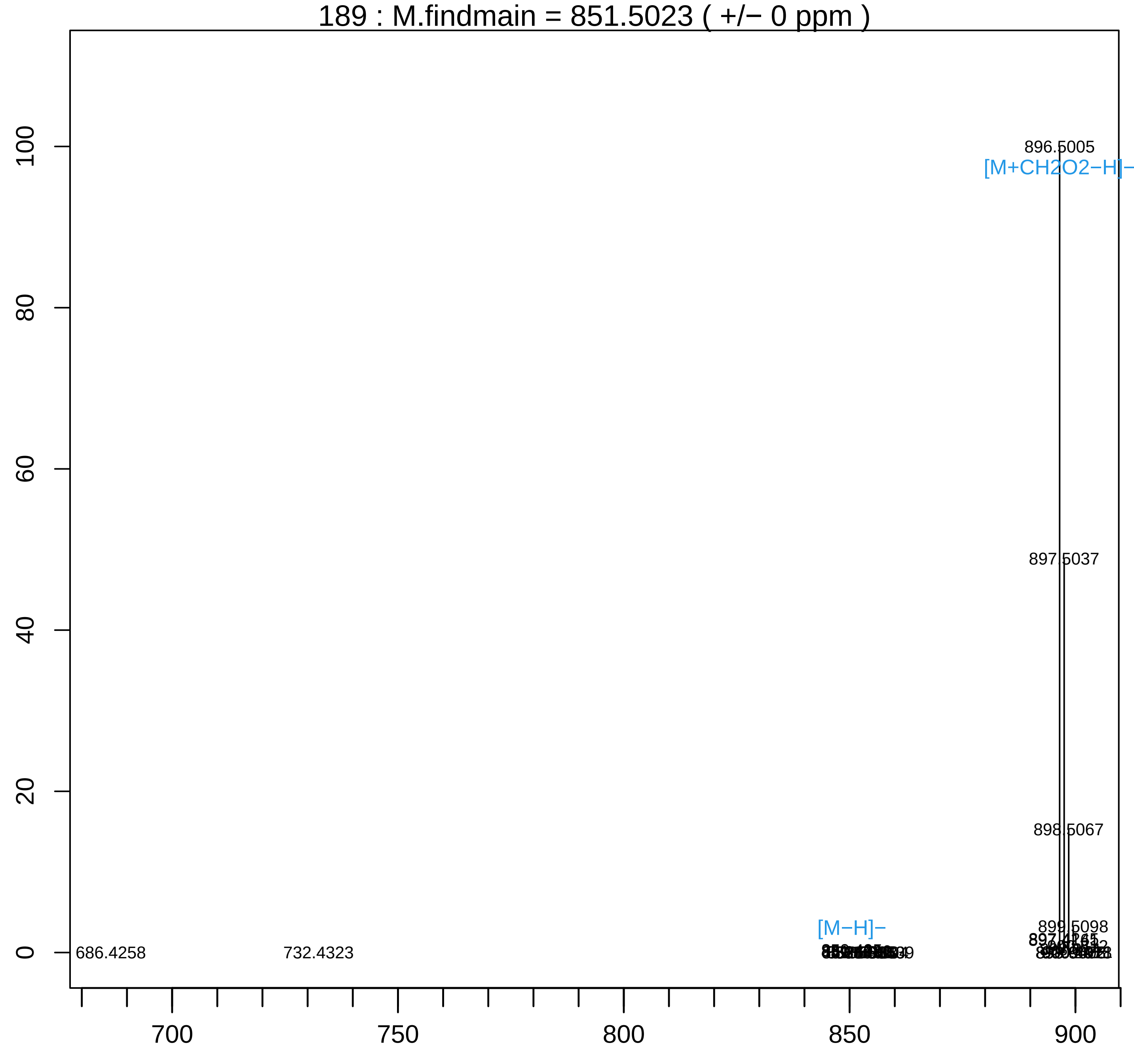

189 : M.findmain = 851.5023 (+/− 0 ppm)
896.5005
[M+CH2O2−H]−
897.5037
898.5067
899.5098
[M−H]−
686.4258
732.4323
0
20
40
60
80
100
700
750
800
850
900

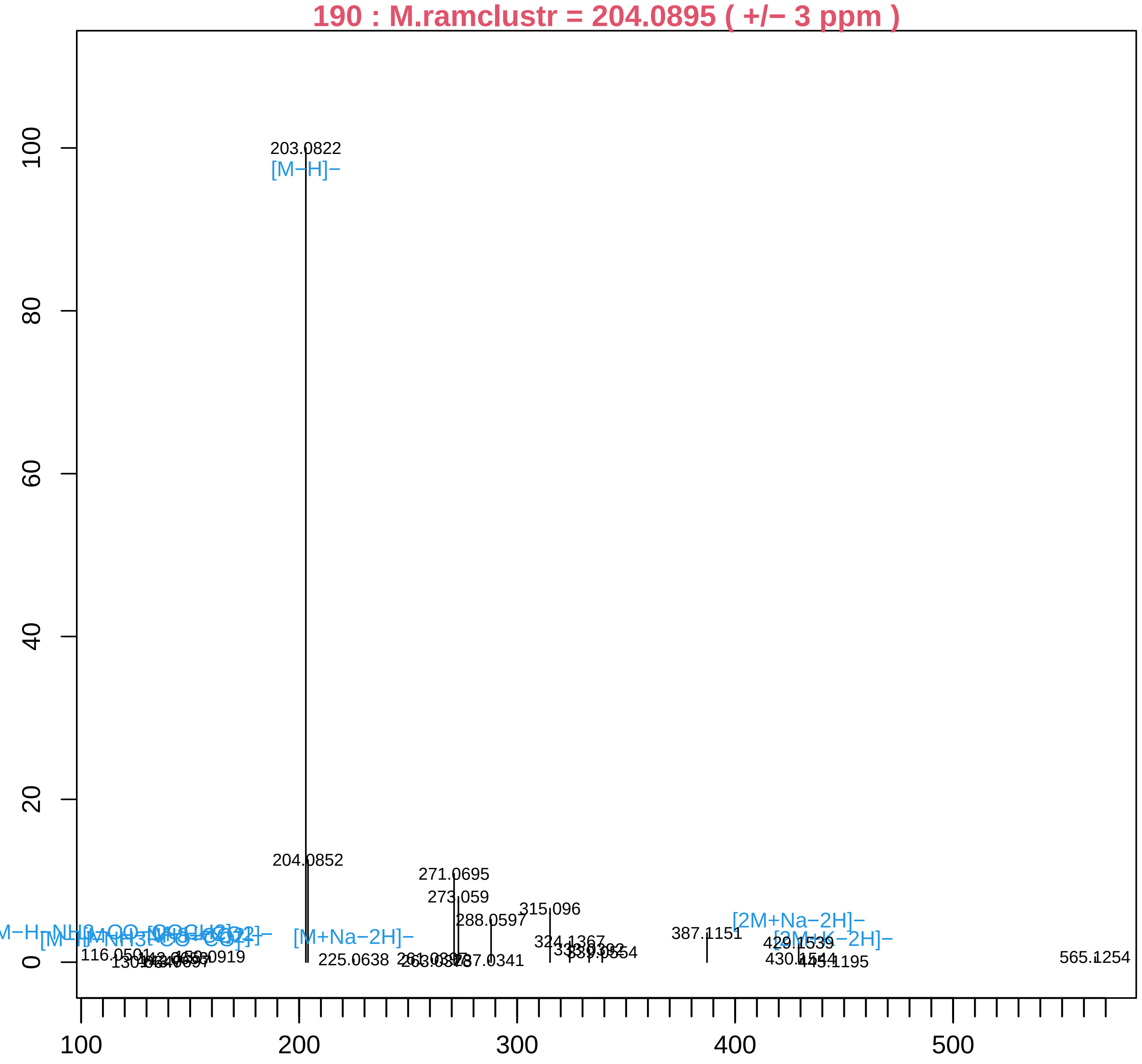
190 : M.ramclustr = 204.0895 (+/− 3 ppm)
203.0822
[M−H]−
204.0852
271.0695
273.059
288.0597
315.096
324.1367
387.1151
[2M+Na−2H]−
[2M+K−2H]−
429.1539
430.1544
445.1195
565.1254
[M+Na−2H]−
225.0638
116.0501
0
20
40
60
80
100
100
200
300
400
500

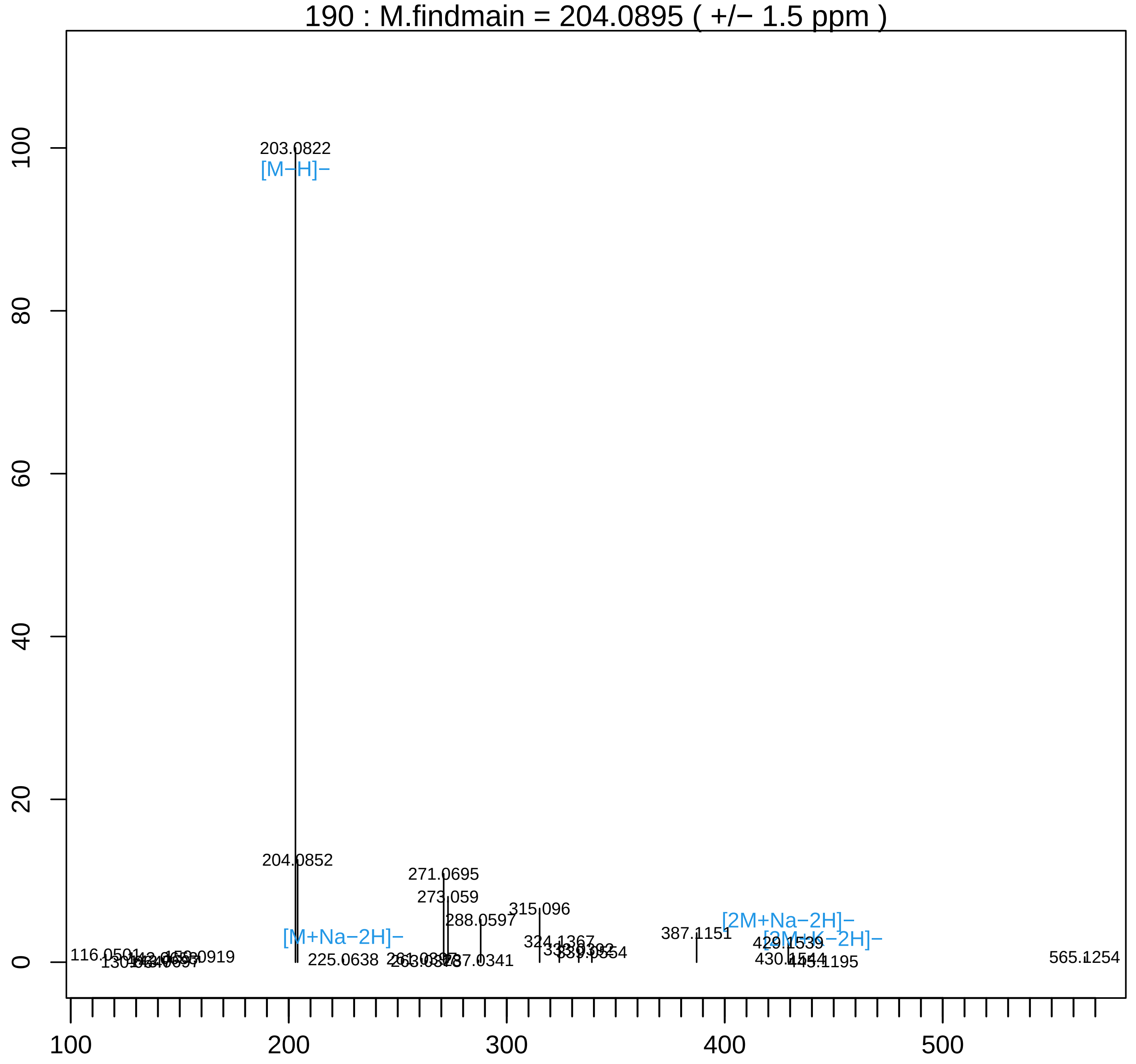
190 : M.findmain = 204.0895 (+/− 1.5 ppm)
203.0822
[M−H]−
204.0852
271.0695
273.059
288.0597
315.096
324.1367
387.1151
[2M+Na−2H]−
[2M+K−2H]−
429.1539
430.1544
445.1195
565.1254
[M+Na−2H]−
225.0638
116.0501
0
20
40
60
80
100
100
200
300
400
500

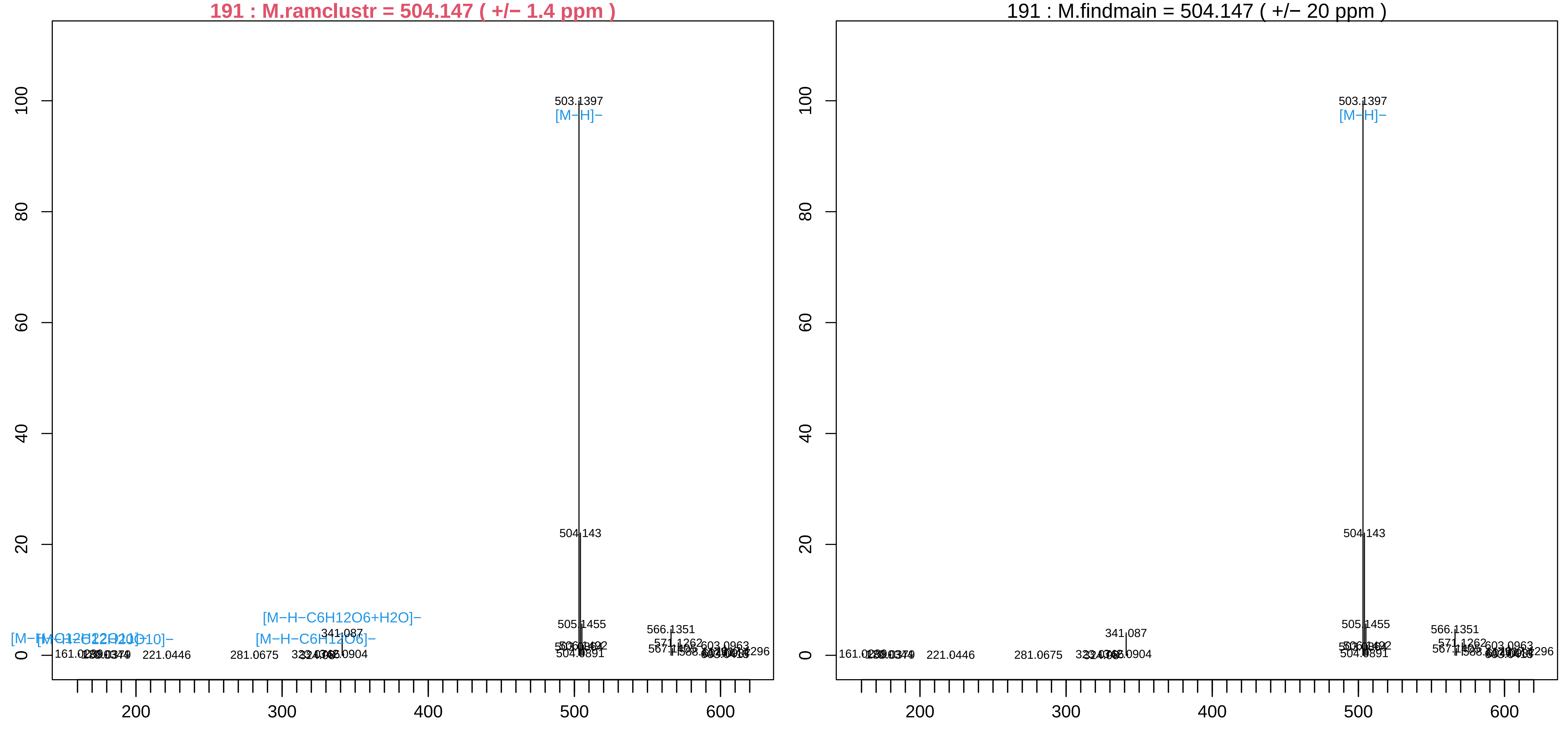
191 : M.ramclustr = 504.147 (+/− 1.4 ppm)
503.1397
[M−H]−
504.143
505.1455
566.1351
571.1262
603.0963
341.087
[M−H−C6H12O6+H2O]−
[M−H−C6H12O6]−
161.0
221.0446
281.0675
504.0891
191 : M.findmain = 504.147 (+/− 20 ppm)
0
20
40
60
80
100
200
300
400
500
600

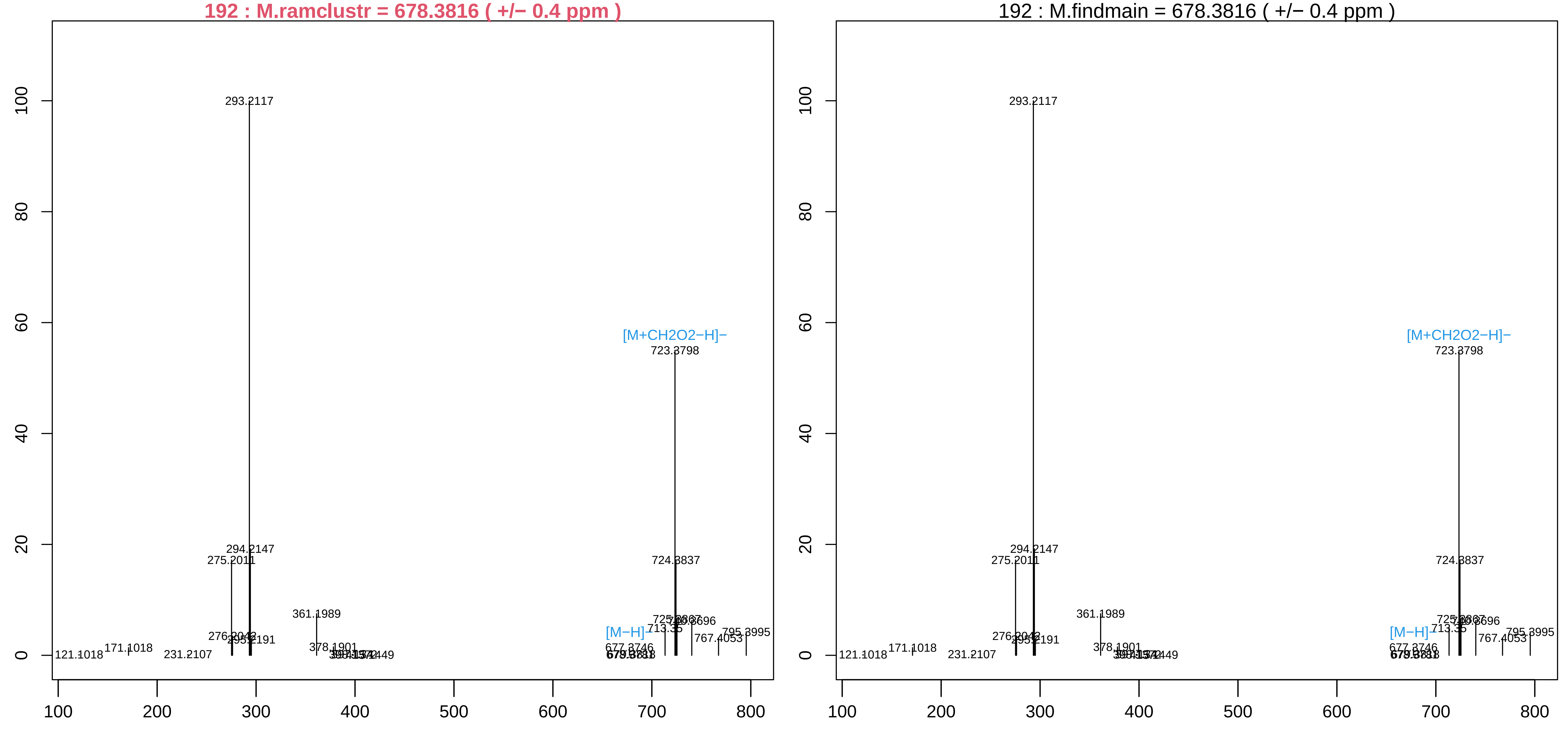

192 : M.ramclustr = 678.3816 (+/− 0.4 ppm)
192 : M.findmain = 678.3816 (+/− 0.4 ppm)
293.2117
[M+CH2O2−H]−
723.3798
294.2147
275.2011
724.3837
361.1989
[M−H]−
713.35
740.3696
795.3995
767.4053
677.3746
276.2042
295.2191
378.1901
121.1018
171.1018
231.2107
0
20
40
60
80
100
200
300
400
500
600
700
800

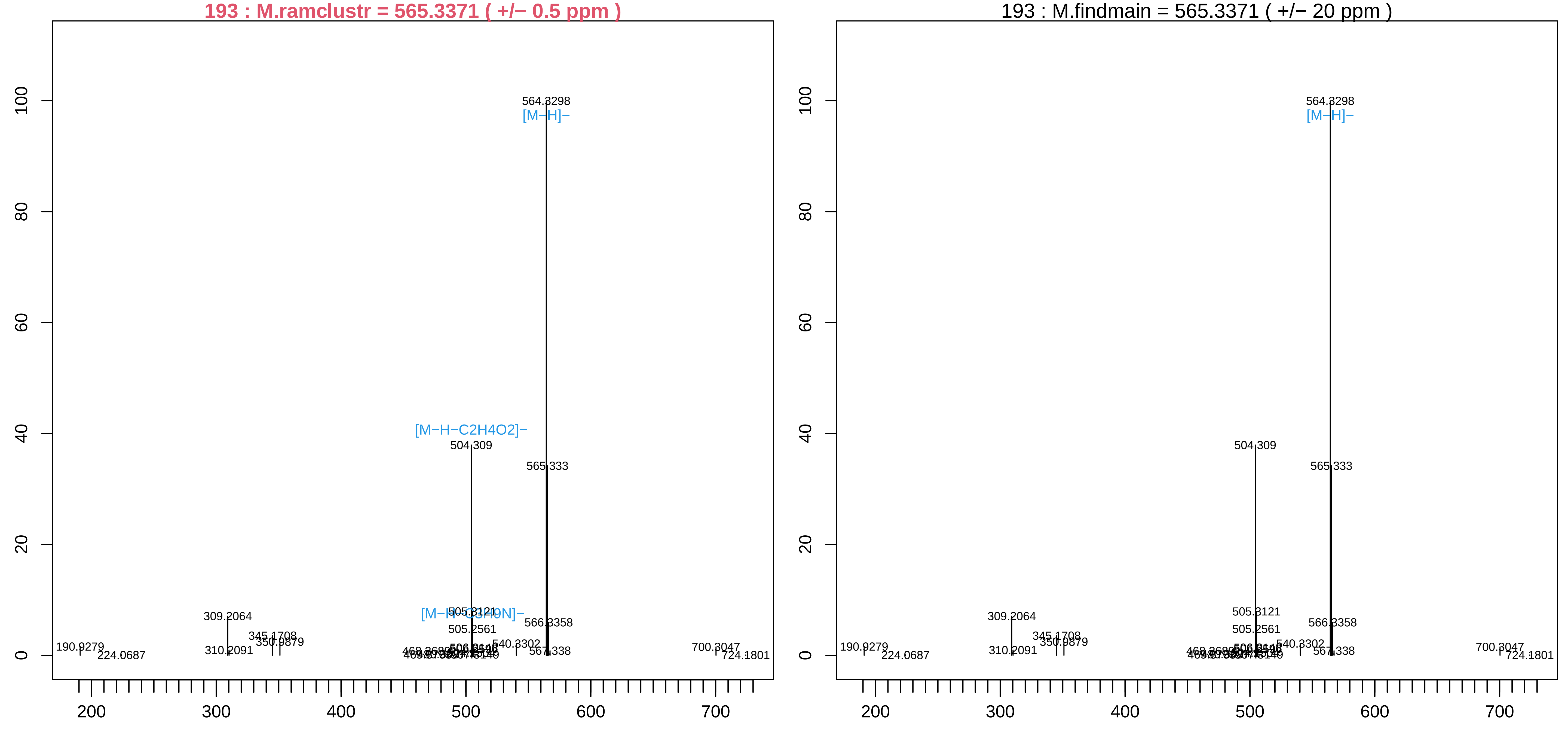
193 : M.ramclustr = 565.3371 (+/− 0.5 ppm)
564.3298
[M−H]−
[M−H−C2H4O2]−
504.309
565.333
309.2064
505.3121
505.2561
566.3358
345.1708
350.9879
540.3302
190.9279
224.0687
310.2091
567.338
700.3047
724.1801
193 : M.findmain = 565.3371 (+/− 20 ppm)
0
20
40
60
80
100
200
300
400
500
600
700

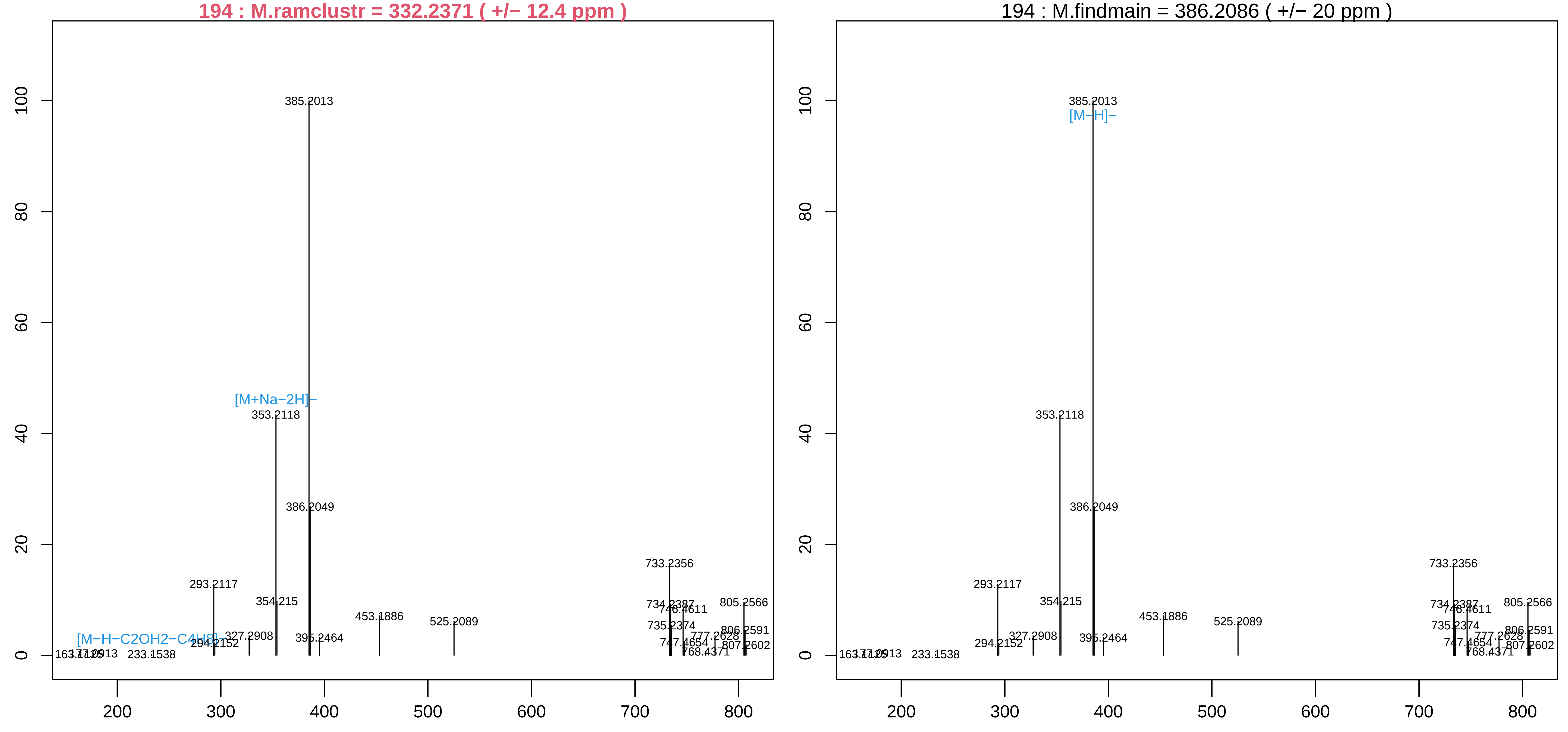

194 : M.ramclustr = 332.2371 (+/− 12.4 ppm)
194 : M.findmain = 386.2086 (+/− 20 ppm)
385.2013
[M−H]−
[M+Na−2H]−
353.2118
386.2049
733.2356
293.2117
354.215
805.2566
734.2387
746.4611
453.1886
525.2089
735.2374
806.2591
777.2628
327.2908
395.2464
[M−H−C2OH2−C4H8]−
294.2152
747.4654
807.2602
768.4371
163.1125
177.0913
233.1538
0
20
40
60
80
100
200
300
400
500
600
700
800

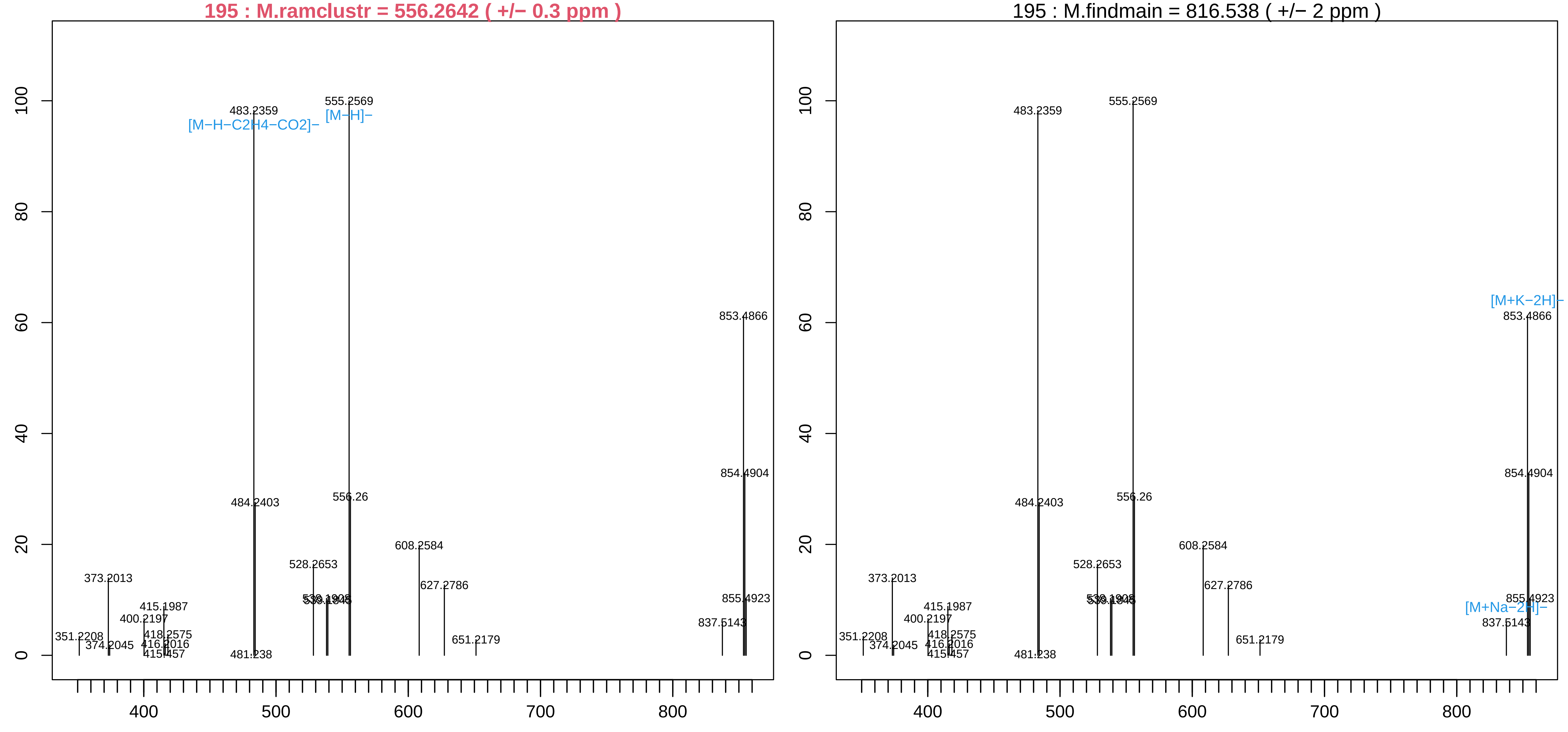
195 : M.ramclustr = 556.2642 (+/− 0.3 ppm)
195 : M.findmain = 816.538 (+/− 2 ppm)
[M−H−C2H4−CO2]−
[M−H]−
[M+K−2H]−
[M+Na−2H]−
483.2359
555.2569
853.4866
854.4904
484.2403
556.26
608.2584
528.2653
373.2013
627.2786
855.4923
415.1987
400.2197
418.2575
416.2016
415.1457
351.2208
374.2045
481.238
837.5143
651.2179
0
20
40
60
80
100
400
500
600
700
800

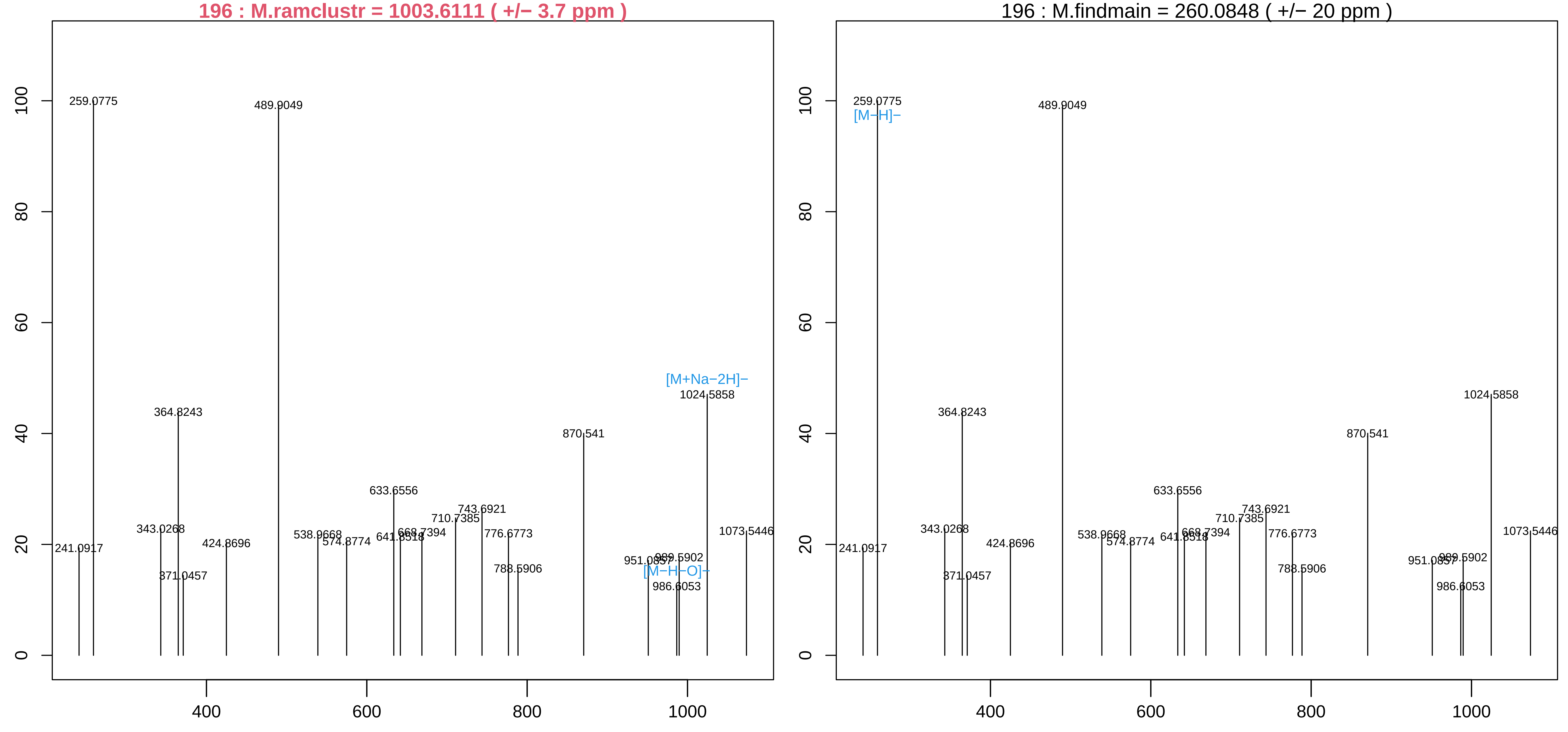

196 : M.ramclustr = 1003.6111 (+/− 3.7 ppm)
259.0775
489.9049
364.8243
870.541
1024.5858
[M+Na−2H]−
633.6556
743.6921
710.7385
343.0268
538.9668
574.8774
641.8518
668.7394
776.6773
424.8696
241.0917
1073.5446
951.0857
989.5902
[M−H−O]−
871.0457
788.5906
986.6053
196 : M.findmain = 260.0848 (+/− 20 ppm)
[M−H]−

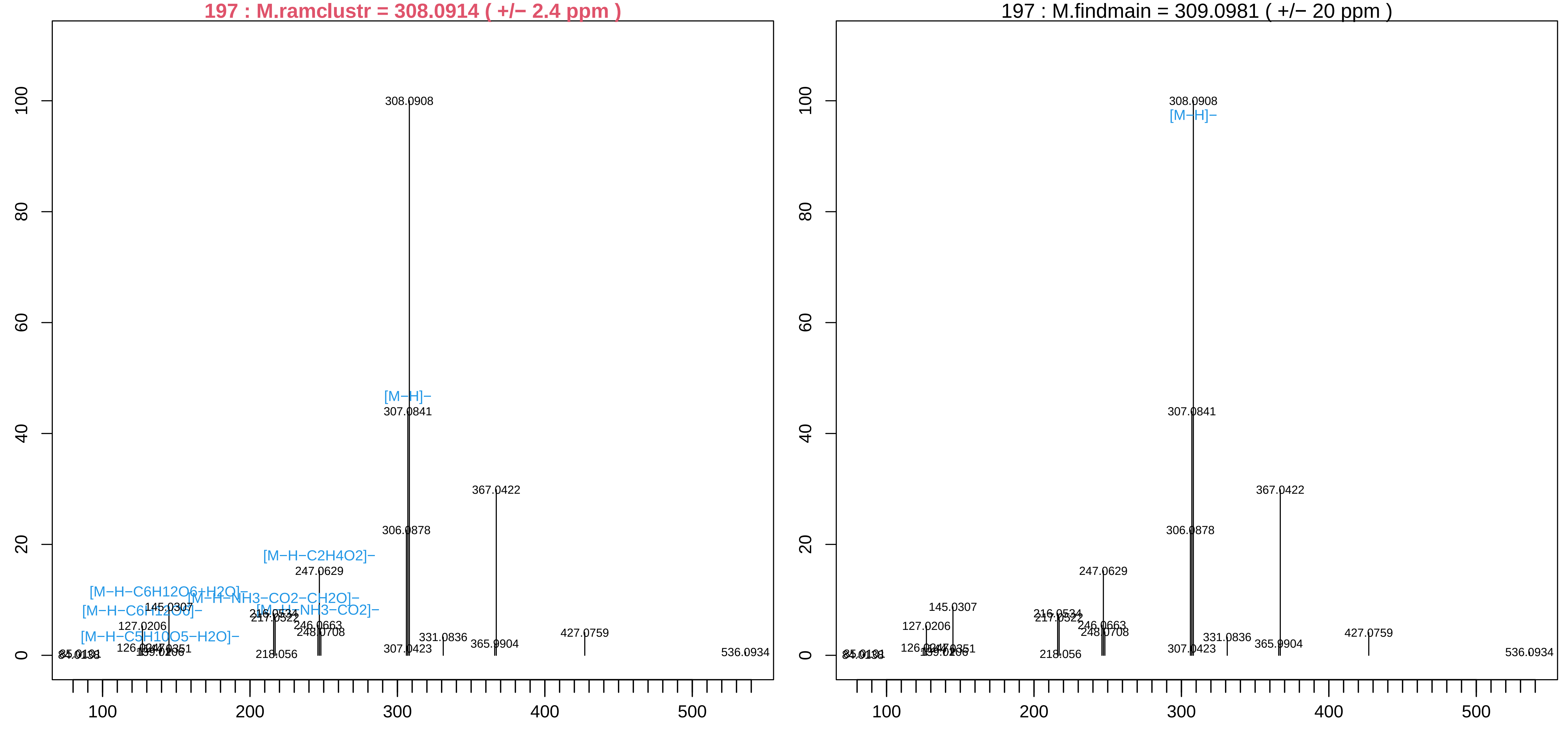
197 : M.ramclustr = 308.0914 (+/− 2.4 ppm)
308.0908
[M−H]−
307.0841
367.0422
306.0878
[M−H−C2H4O2]−
247.0629
[M−H−C6H12O6+H2O]−
[M−H−NH3−CO2−CH2O]−
[M−H−C6H12O6]−
145.0307
[M−H−NH3−CO2]−
127.0206
246.0663
248.0708
[M−H−C5H10O5−H2O]−
331.0836
427.0759
365.9904
307.0423
218.056
536.0934
197 : M.findmain = 309.0981 (+/− 20 ppm)
308.0908
[M−H]−
307.0841
367.0422
306.0878
247.0629
145.0307
216.0534
217.0522
127.0206
246.0663
248.0708
331.0836
427.0759
365.9904
307.0423
218.056
536.0934
0
20
40
60
80
100
200
300
400
500

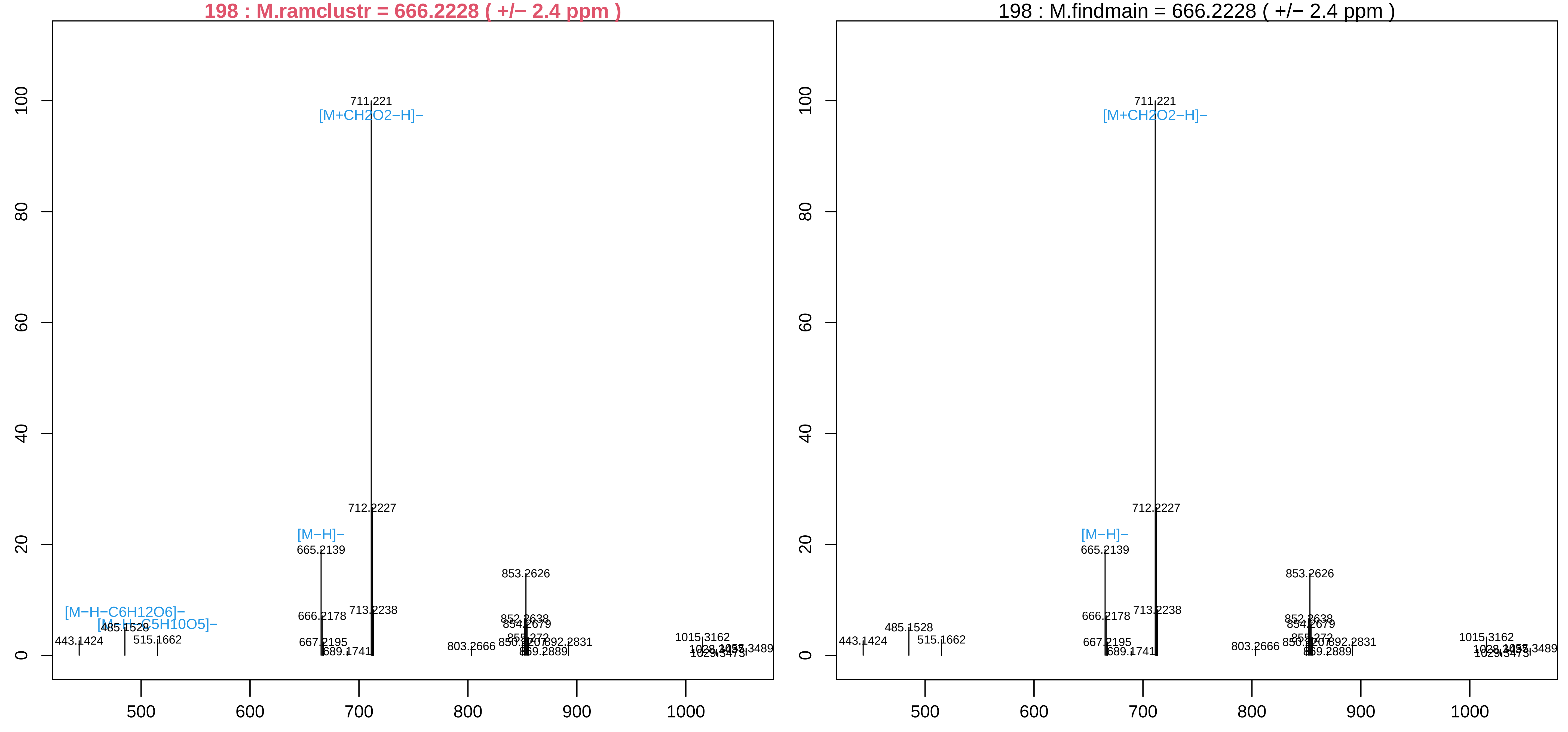
198 : M.ramclustr = 666.2228 (+/− 2.4 ppm)
198 : M.findmain = 666.2228 (+/− 2.4 ppm)
711.221
[M+CH2O2−H]−
712.2227
[M−H]−
665.2139
853.2626
713.2238
666.2178
[M−H−C6H12O6]−
[M−H−C5H10O5]−
485.1528
443.1424
515.1662
667.2195
689.1741
803.2666
852.2638
854.2679
855.272
850.2207
892.2831
859.2889
1015.3162
1055.3489
0
20
40
60
80
100
500
600
700
800
900
1000

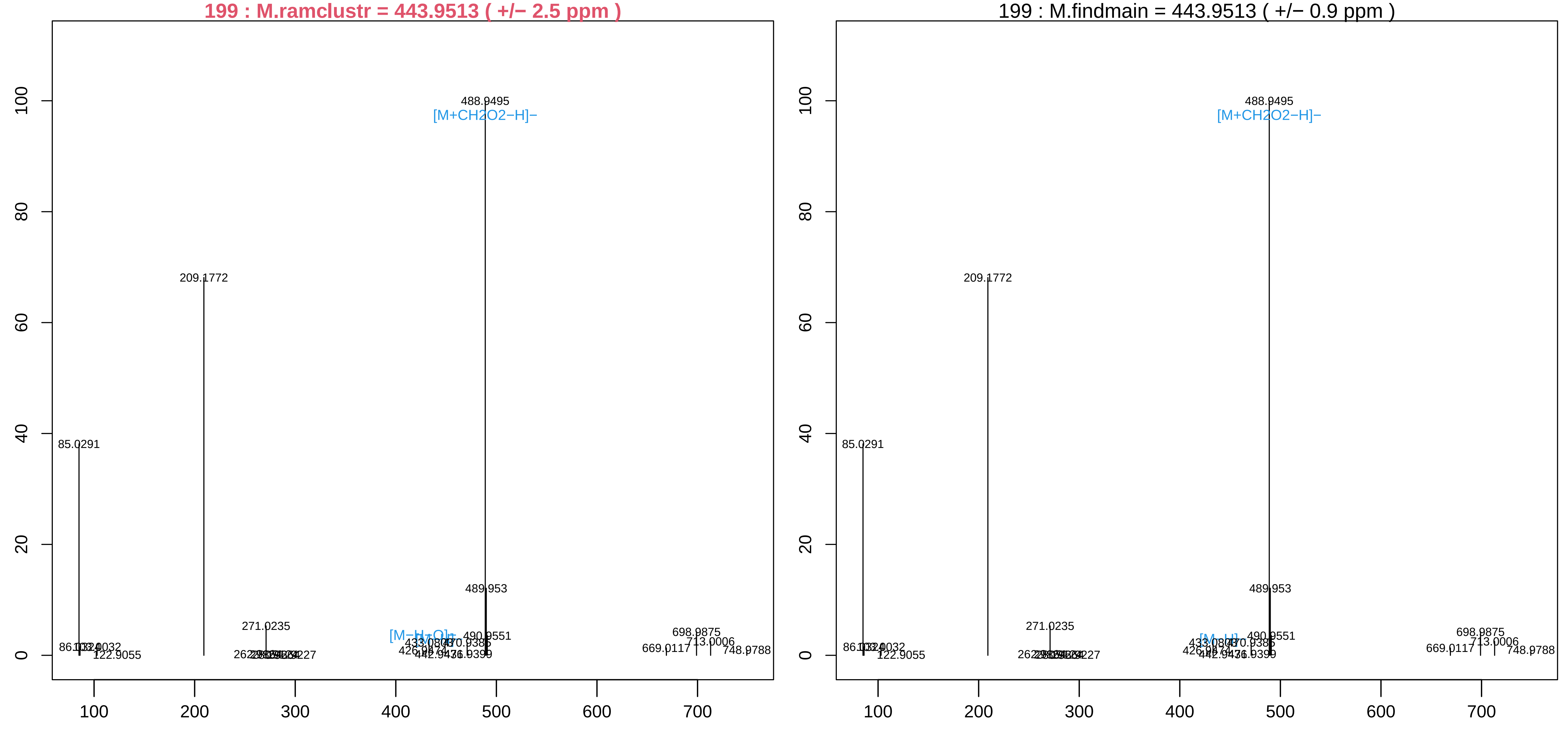
199 : M.ramclustr = 443.9513 (+/− 2.5 ppm)
488.9495
[M+CH2O2−H]−
209.1772
85.0291
271.0235
489.953
490.9551
698.9875
713.0006
669.0117
748.9788
122.9055
0
20
40
60
80
100
100
200
300
400
500
600
700
199 : M.findmain = 443.9513 (+/− 0.9 ppm)
488.9495
[M+CH2O2−H]−
209.1772
85.0291
271.0235
489.953
490.9551
698.9875
713.0006
669.0117
748.9788
122.9055
0
20
40
60
80
100
100
200
300
400
500
600
700

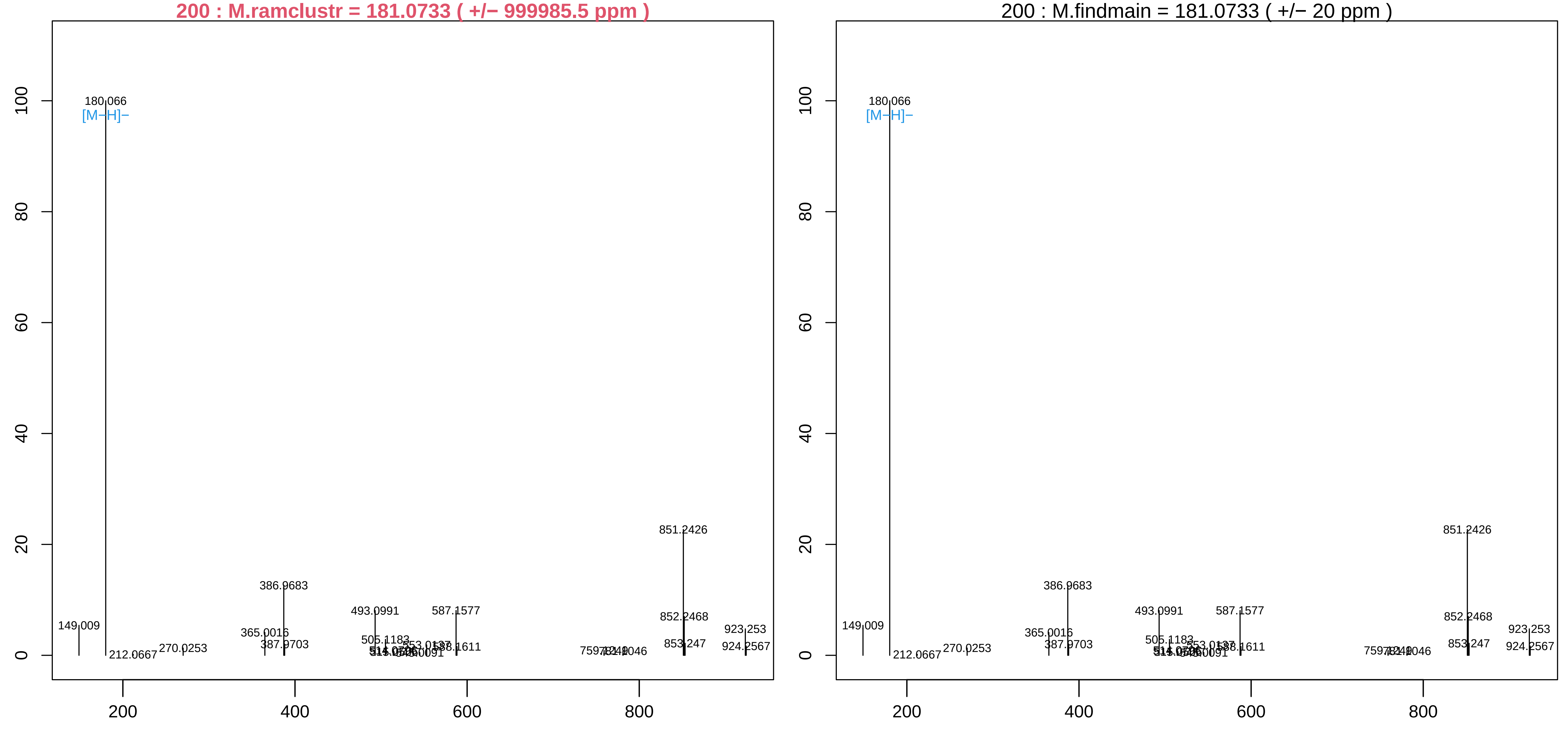
200 : M.ramclustr = 181.0733 (+/− 999985.5 ppm)
180.066
[M−H]−
149.009
212.0667
270.0253
365.0016
386.9683
387.9703
493.0991
505.1183
587.1577
588.1611
851.2426
852.2468
853.247
923.253
924.2567
200 : M.findmain = 181.0733 (+/− 20 ppm)
180.066
[M−H]−
149.009
212.0667
270.0253
365.0016
386.9683
387.9703
493.0991
505.1183
587.1577
588.1611
851.2426
852.2468
853.247
923.253
924.2567
0
20
40
60
80
100
200
400
600
800

201 : M.ramclustr = 174.0158 (+/− 2.2 ppm)

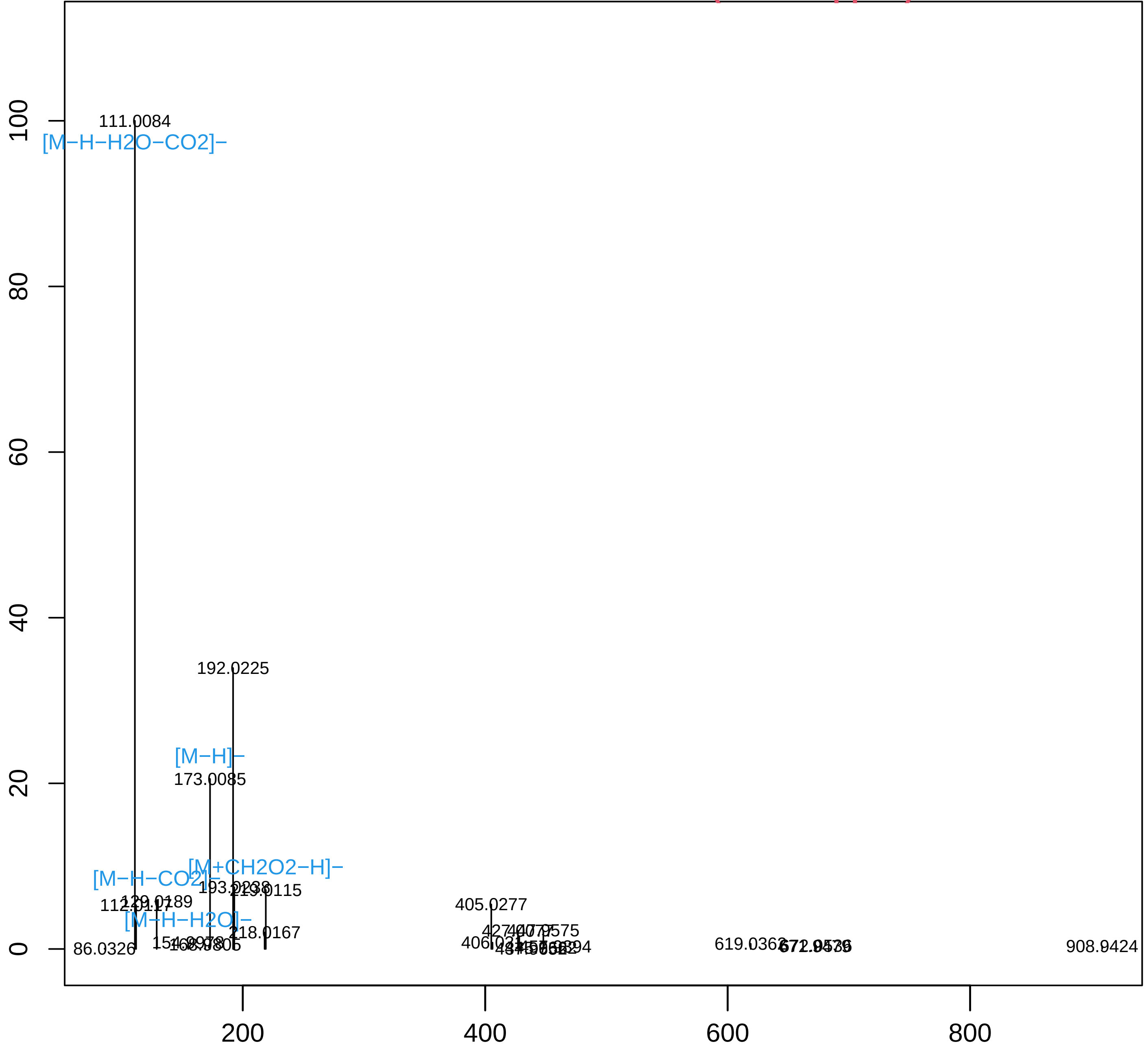

201 : M.findmain = 66.0102 (+/− 28.5 ppm)

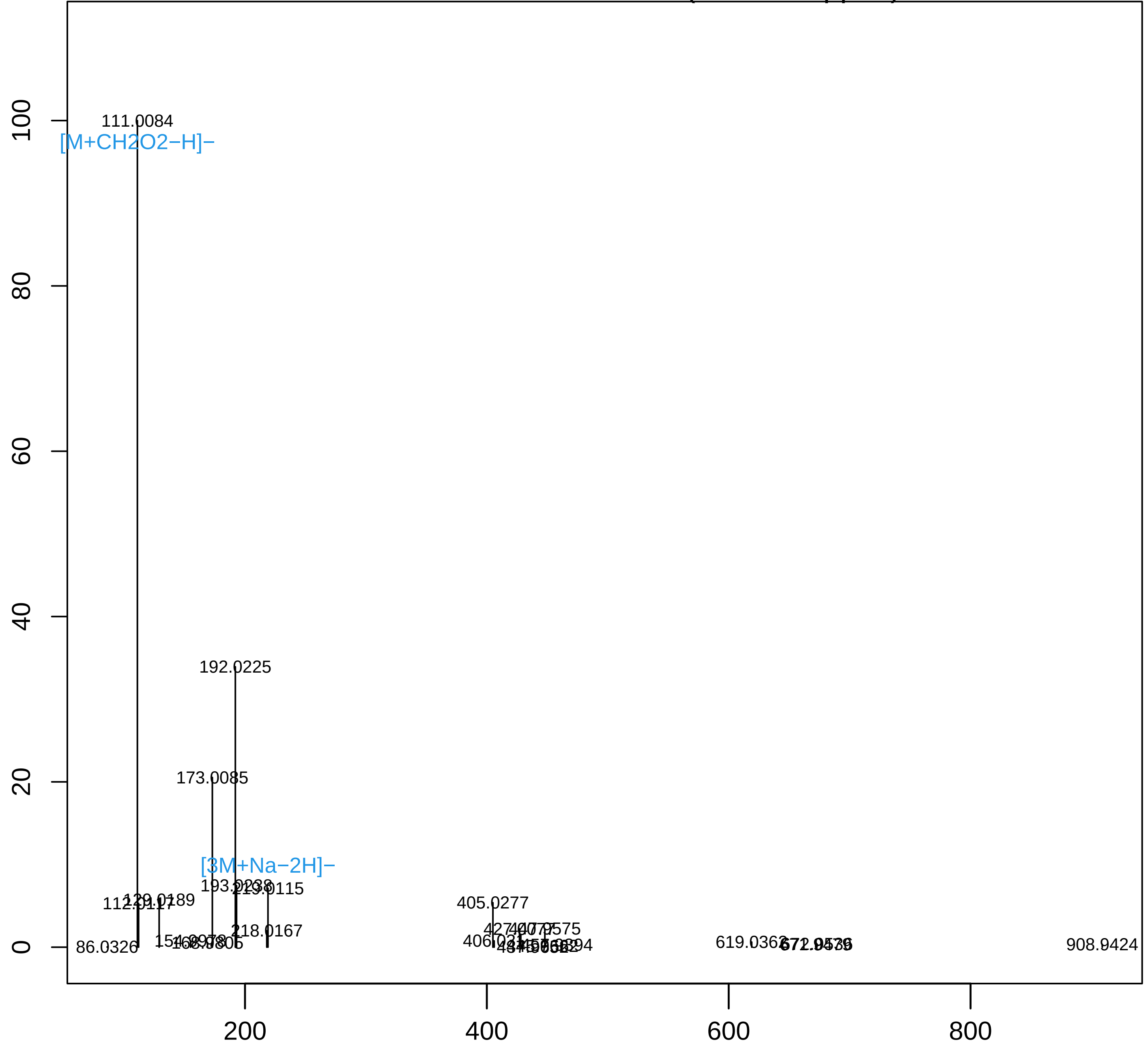

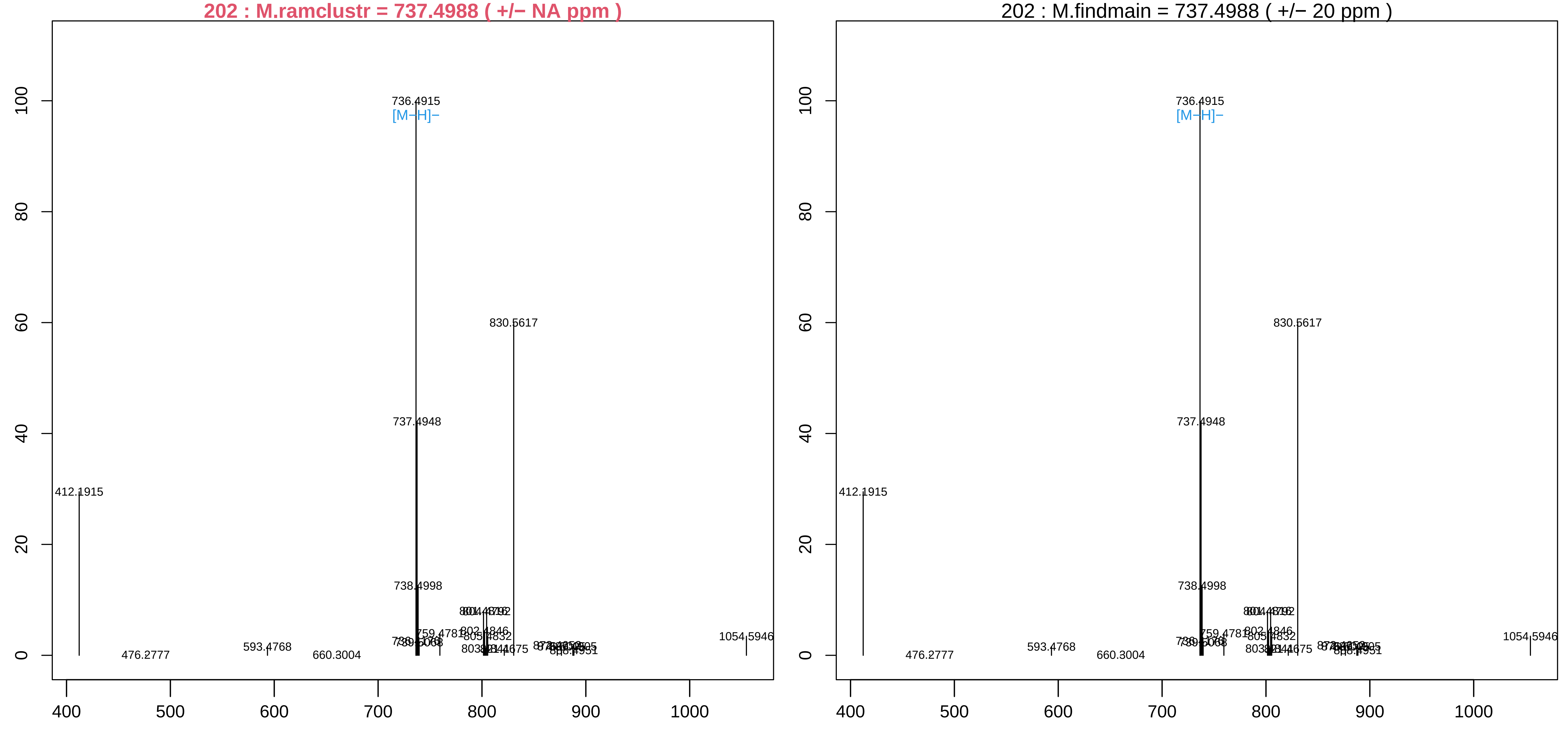
202 : M.ramclustr = 737.4988 (+/− NA ppm)
202 : M.findmain = 737.4988 (+/− 20 ppm)
736.4915
[M−H]−
830.5617
737.4948
412.1915
738.4998
759.4781
802.4846
805.4832
1054.5946
593.4768
476.2777
660.3004
0
20
40
60
80
100
400
500
600
700
800
900
1000

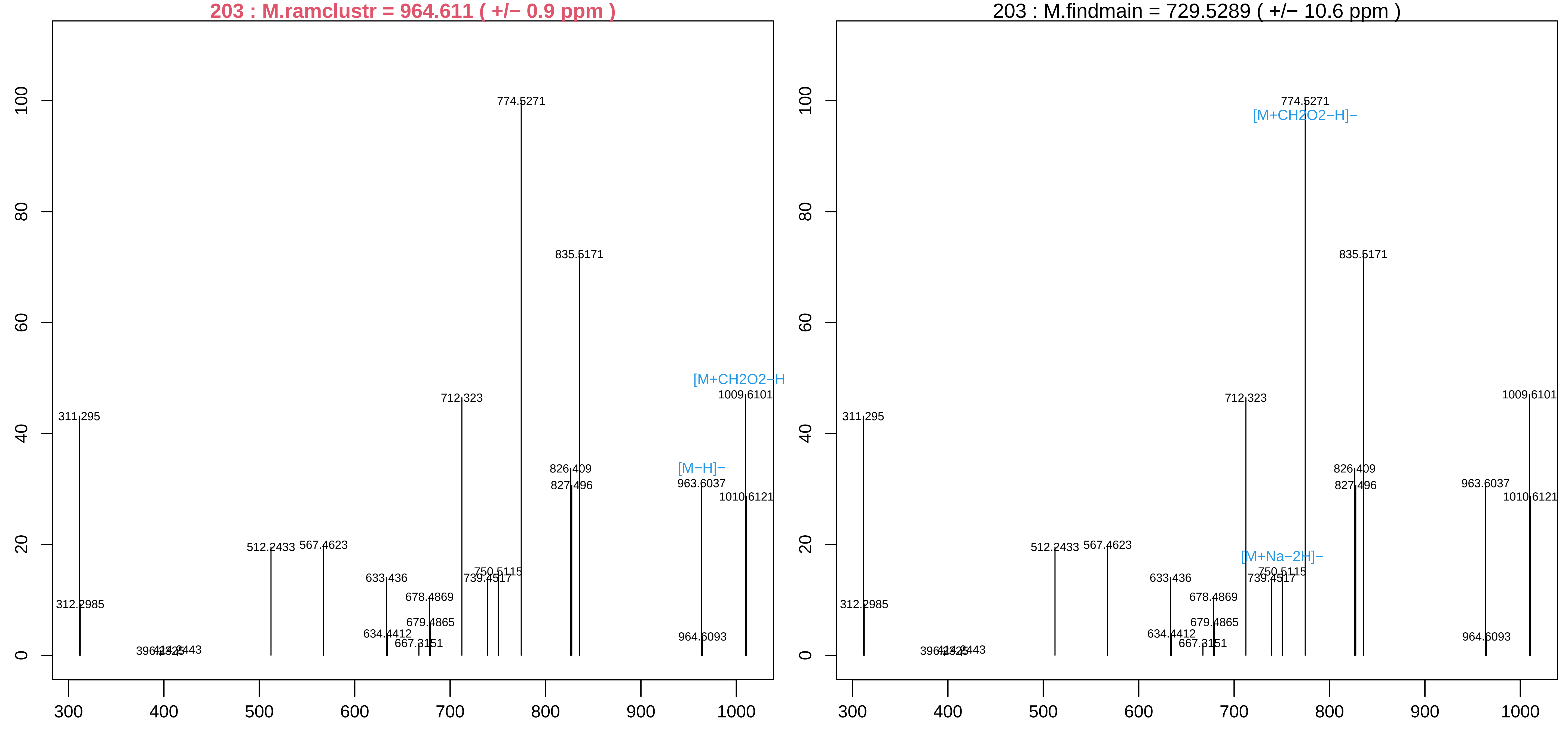

203 : M.ramclustr = 964.611 (+/− 0.9 ppm)
774.5271
835.5171
712.323
311.295
[M+CH2O2−H
1009.6101
[M−H]−
963.6037
826.409
827.496
1010.6121
512.2433
567.4623
633.436
750.5115
739.4517
678.4869
679.4865
634.4412
667.3151
312.2985
964.6093
396.4325
412.2443
203 : M.findmain = 729.5289 (+/− 10.6 ppm)
[M+CH2O2−H]−
[M+Na−2H]−
0
20
40
60
80
100
300
400
500
600
700
800
900
1000

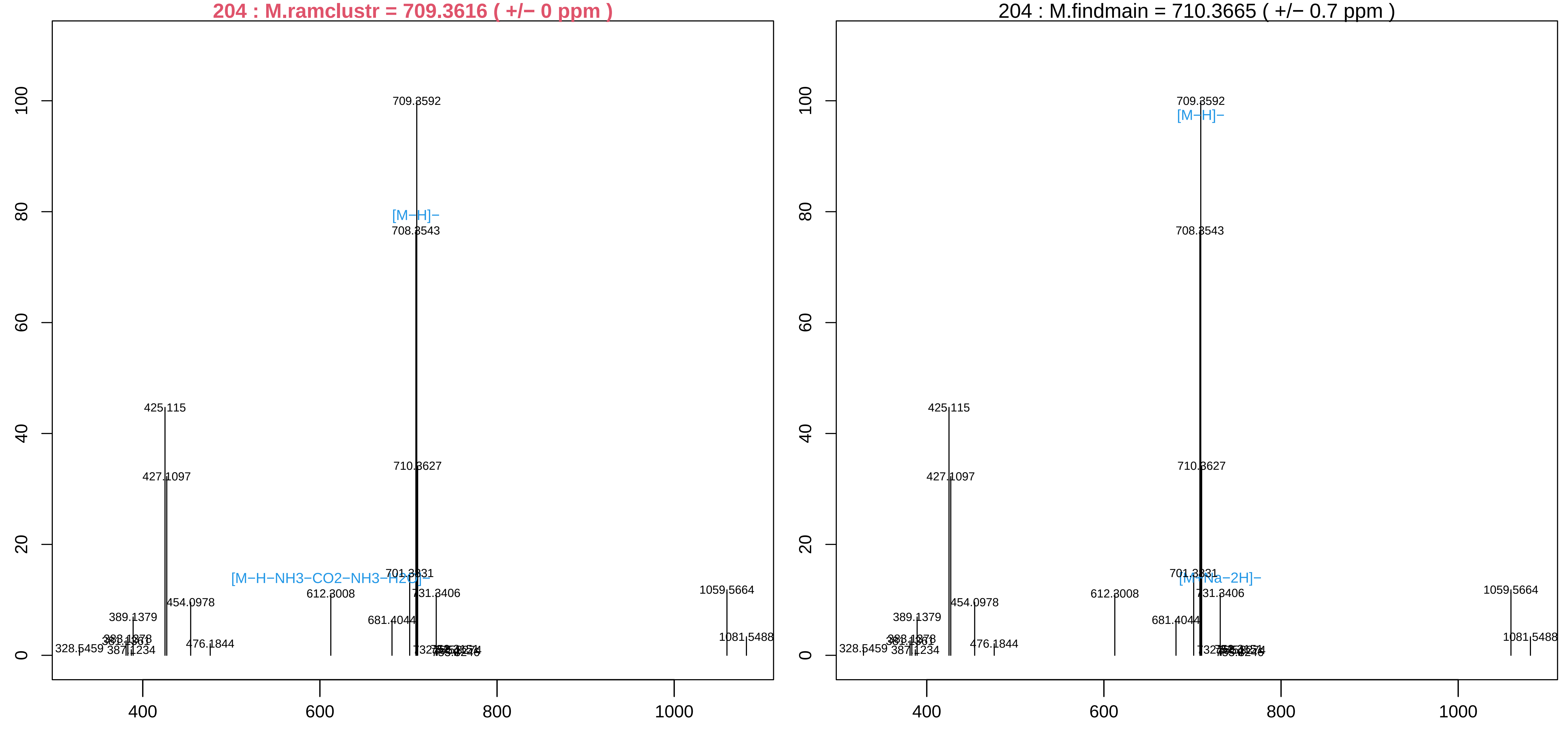
204 : M.ramclustr = 709.3616 (+/− 0 ppm)
709.3592
[M−H]−
708.3543
425.115
427.1097
710.3627
[M−H−NH3−CO2−NH3−H2O]−
701.3831
612.3008
731.3406
454.0978
389.1379
681.4044
476.1844
328.5459
387.1234
1059.5664
1081.5488
204 : M.findmain = 710.3665 (+/− 0.7 ppm)
709.3592
[M−H]−
708.3543
425.115
427.1097
710.3627
701.3831
612.3008
731.3406
454.0978
389.1379
681.4044
476.1844
328.5459
387.1234
1059.5664
1081.5488
0
20
40
60
80
100
400
600
800
1000

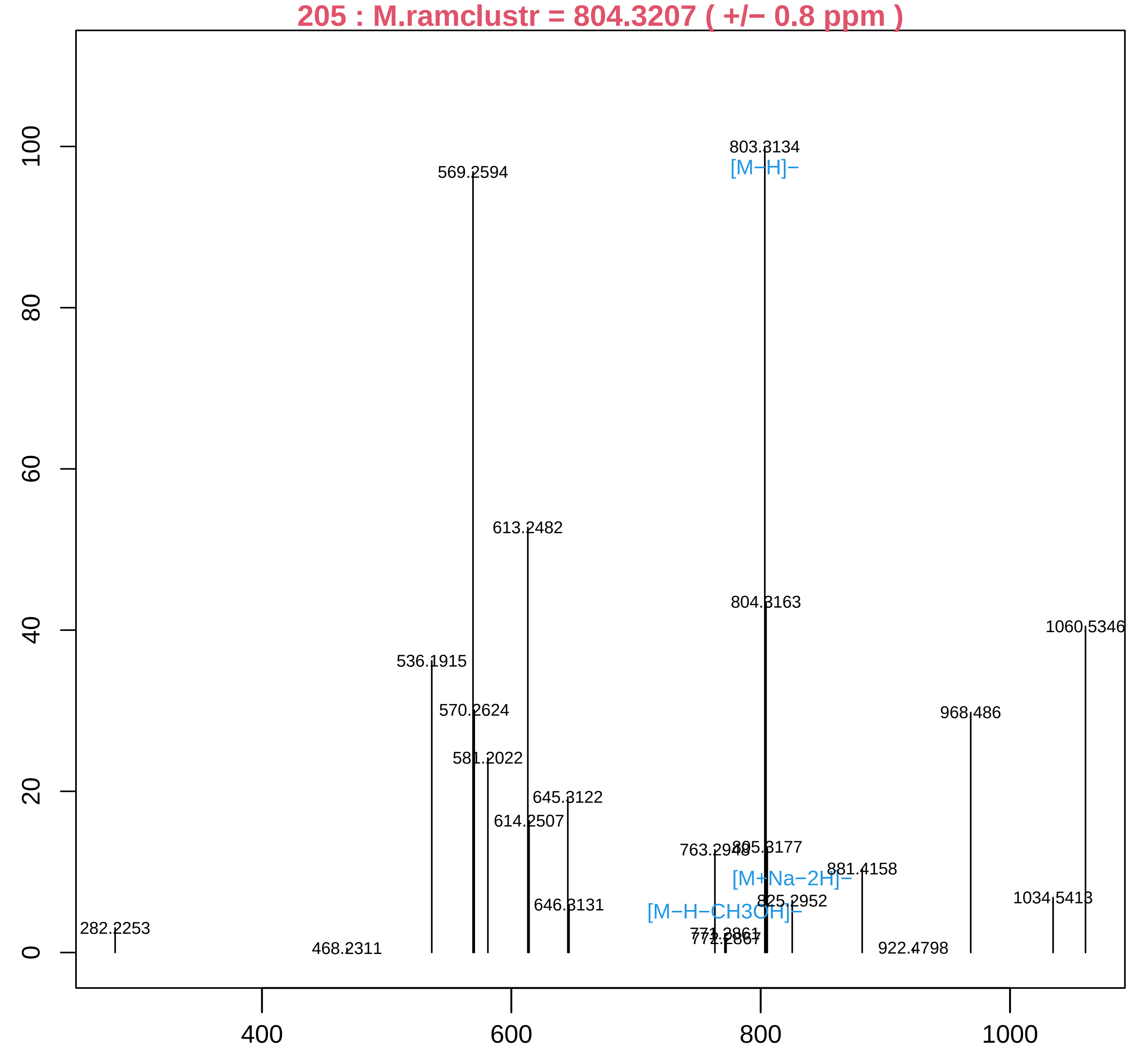
205 : M.ramclustr = 804.3207 (+/− 0.8 ppm)
803.3134
[M−H]−
569.2594
613.2482
804.3163
1060.5346
536.1915
570.2624
968.486
581.2022
645.3122
614.2507
763.2948
805.3177
881.4158
[M+Na−2H]−
1034.5413
825.2952
646.3131
[M−H−CH3OH]−
282.2253
771.2861
772.2867
468.2311
922.4798
0
20
40
60
80
100
400
600
800
1000

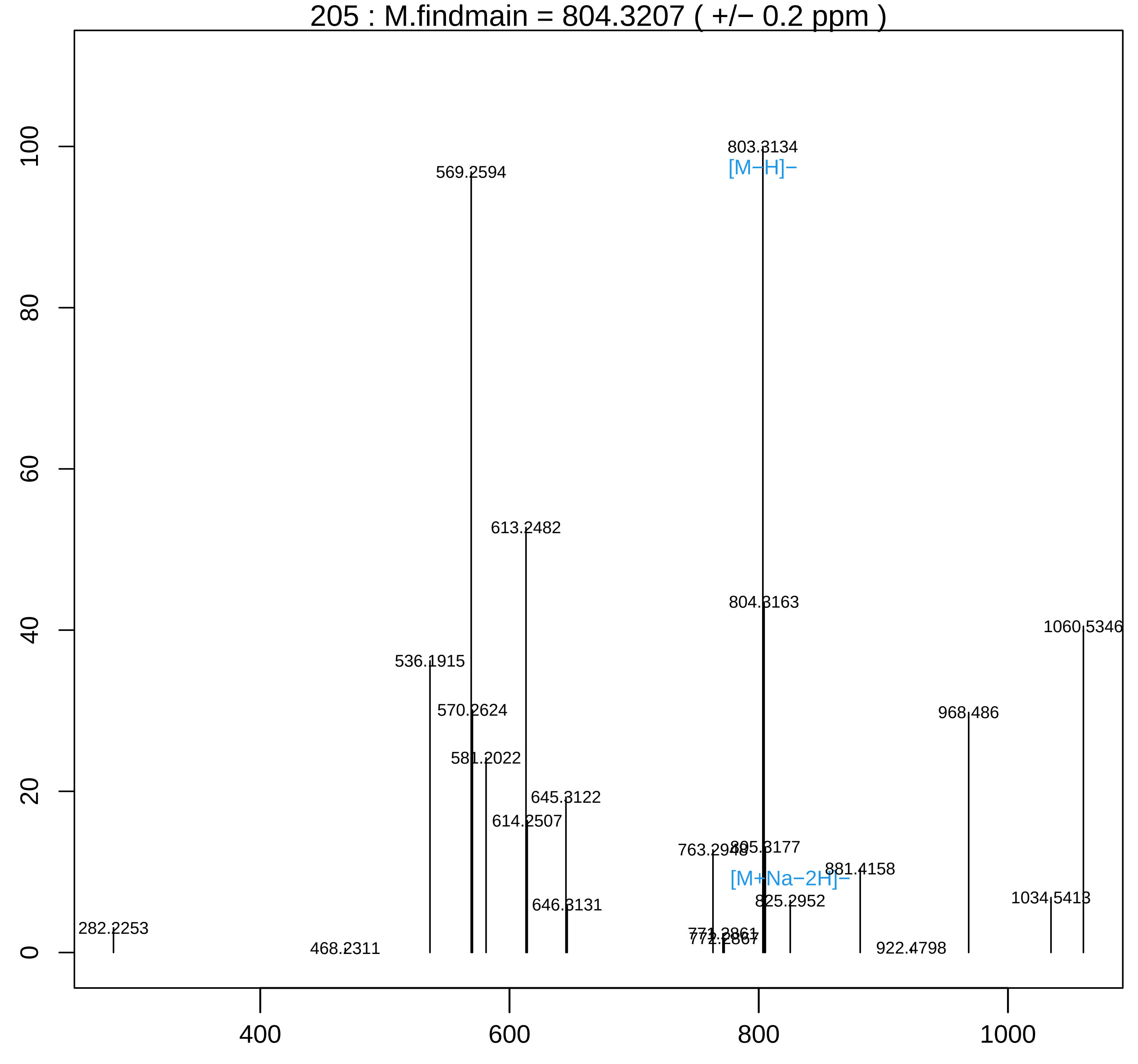
205 : M.findmain = 804.3207 (+/− 0.2 ppm)
803.3134
[M−H]−
569.2594
613.2482
804.3163
1060.5346
536.1915
570.2624
968.486
581.2022
645.3122
614.2507
763.2948
805.3177
881.4158
[M+Na−2H]−
1034.5413
825.2952
646.3131
282.2253
771.2861
772.2867
468.2311
922.4798
0
20
40
60
80
100
400
600
800
1000

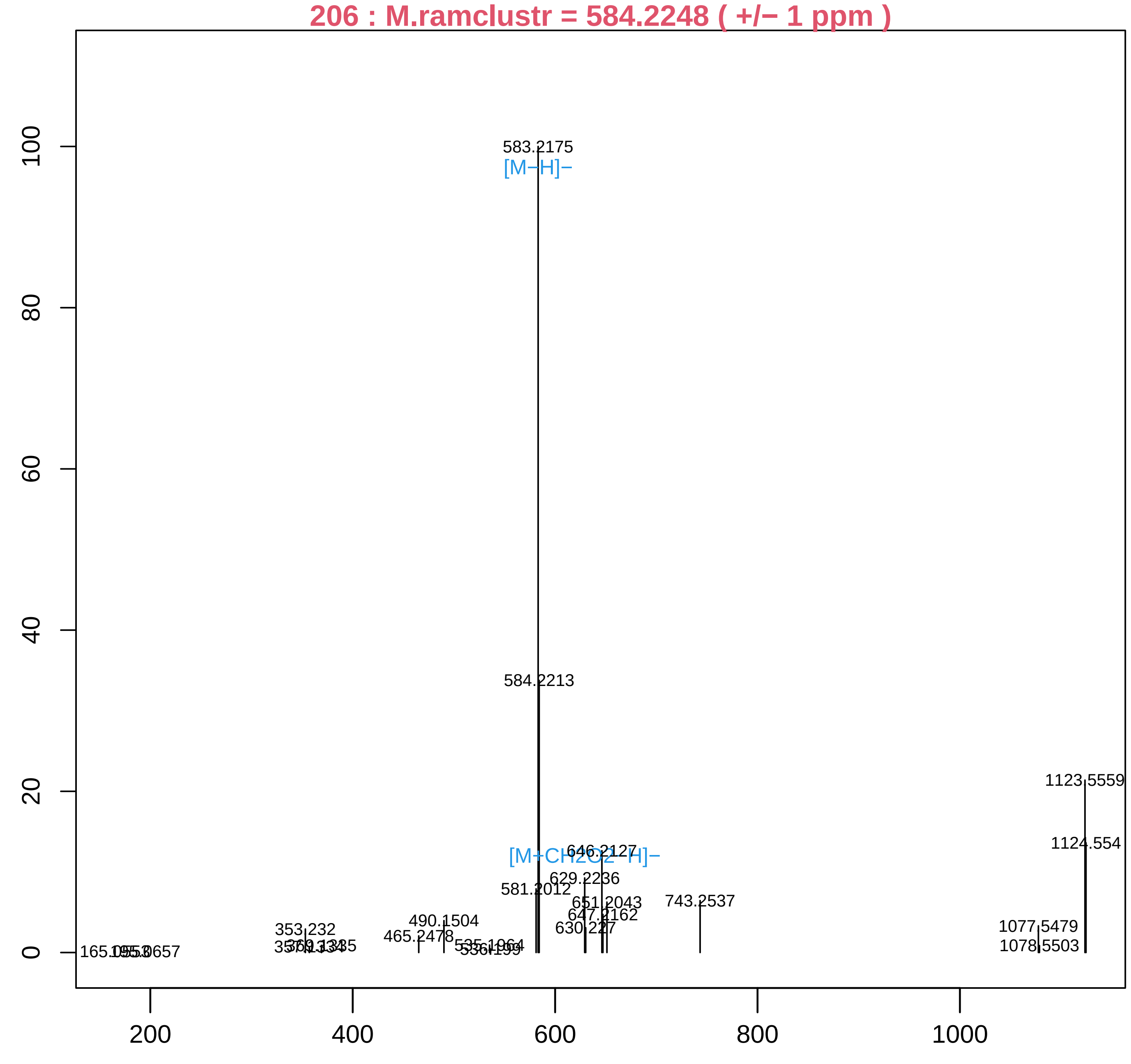
206 : M.ramclustr = 584.2248 (+/− 1 ppm)
583.2175
[M−H]−
584.2213
1123.5559
1124.554
[M+CH2O2−H]−
646.2127
629.2236
581.2012
743.2537
651.2043
647.2162
630.227
490.1504
465.2478
353.232
1077.5479
1078.5503
0
20
40
60
80
100
200
400
600
800
1000

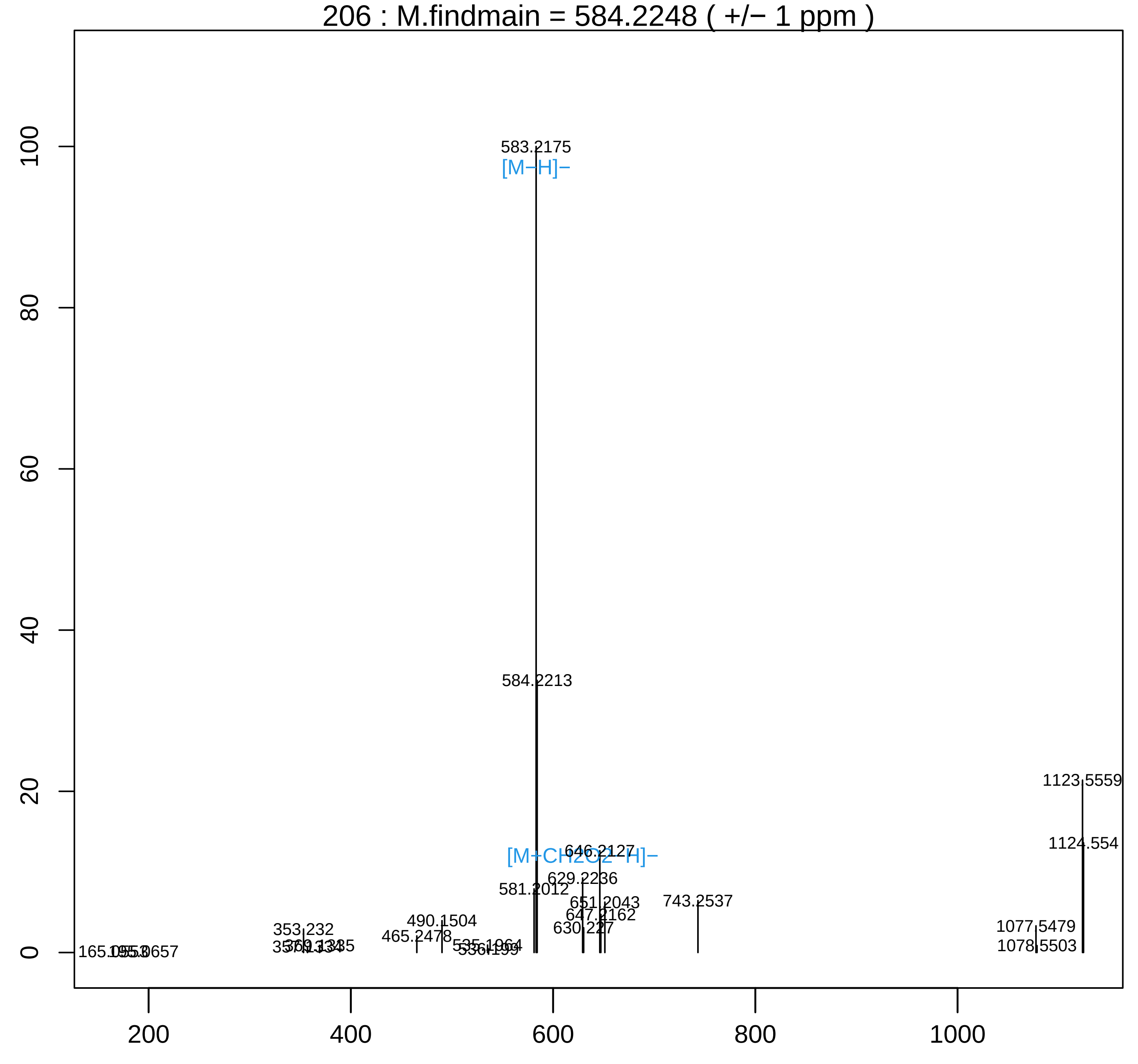
206 : M.findmain = 584.2248 (+/− 1 ppm)
583.2175
[M−H]−
584.2213
1123.5559
1124.554
[M+CH2O2−H]−
646.2127
629.2236
581.2012
743.2537
651.2043
647.2162
630.227
490.1504
465.2478
353.232
1077.5479
1078.5503
0
20
40
60
80
100
200
400
600
800
1000

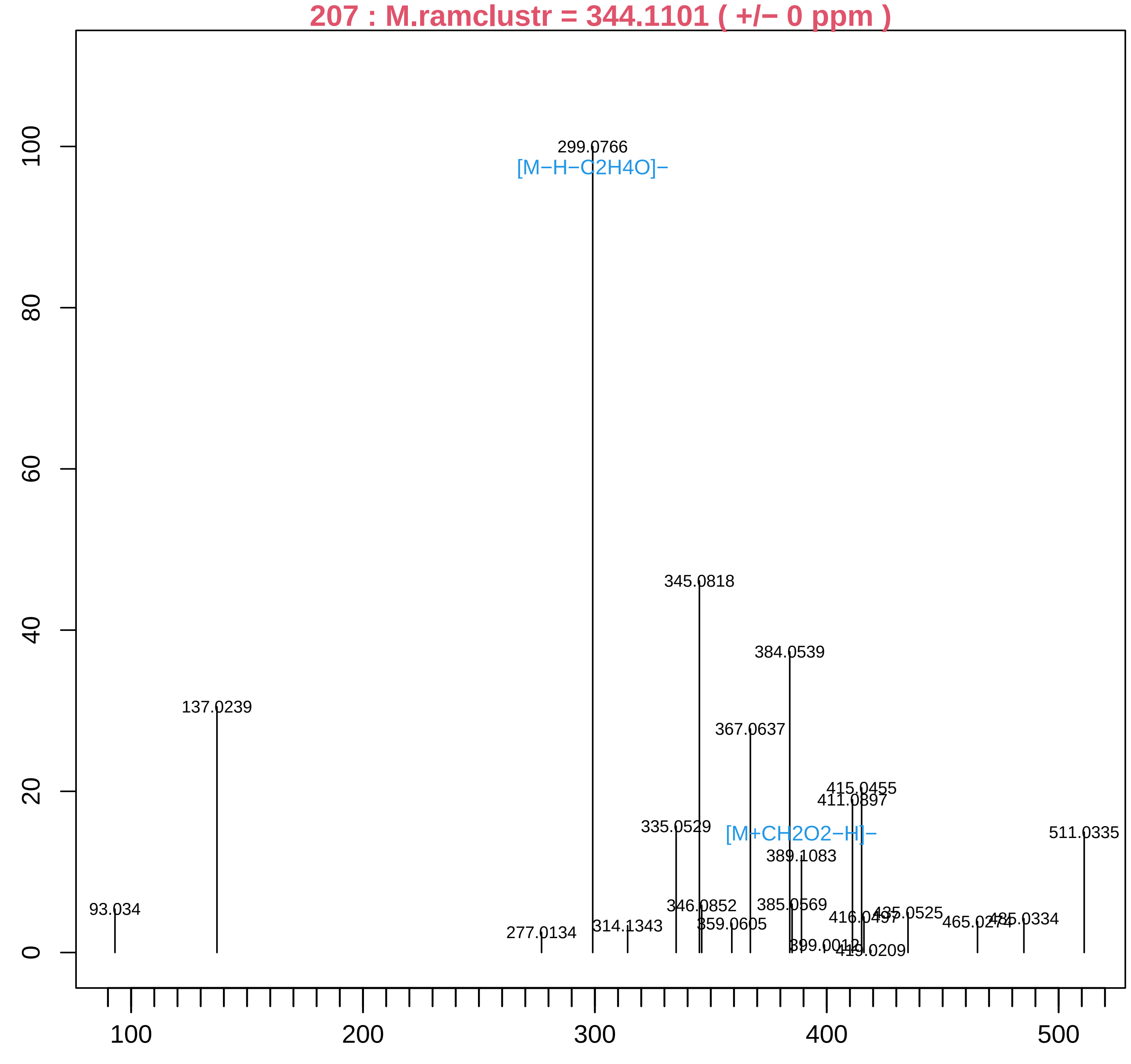
207 : M.ramclustr = 344.1101 (+/− 0 ppm)
299.0766
[M−H−C2H4O]−
345.0818
384.0539
137.0239
367.0637
415.0455
411.0897
335.0529
[M+CH2O2−H]−
511.0335
389.1083
93.034
346.0852
385.0569
416.0497
435.0525
277.0134
314.1343
359.0605
465.0274
485.0334
399.0012
419.0209
0
20
40
60
80
100
200
300
400
500

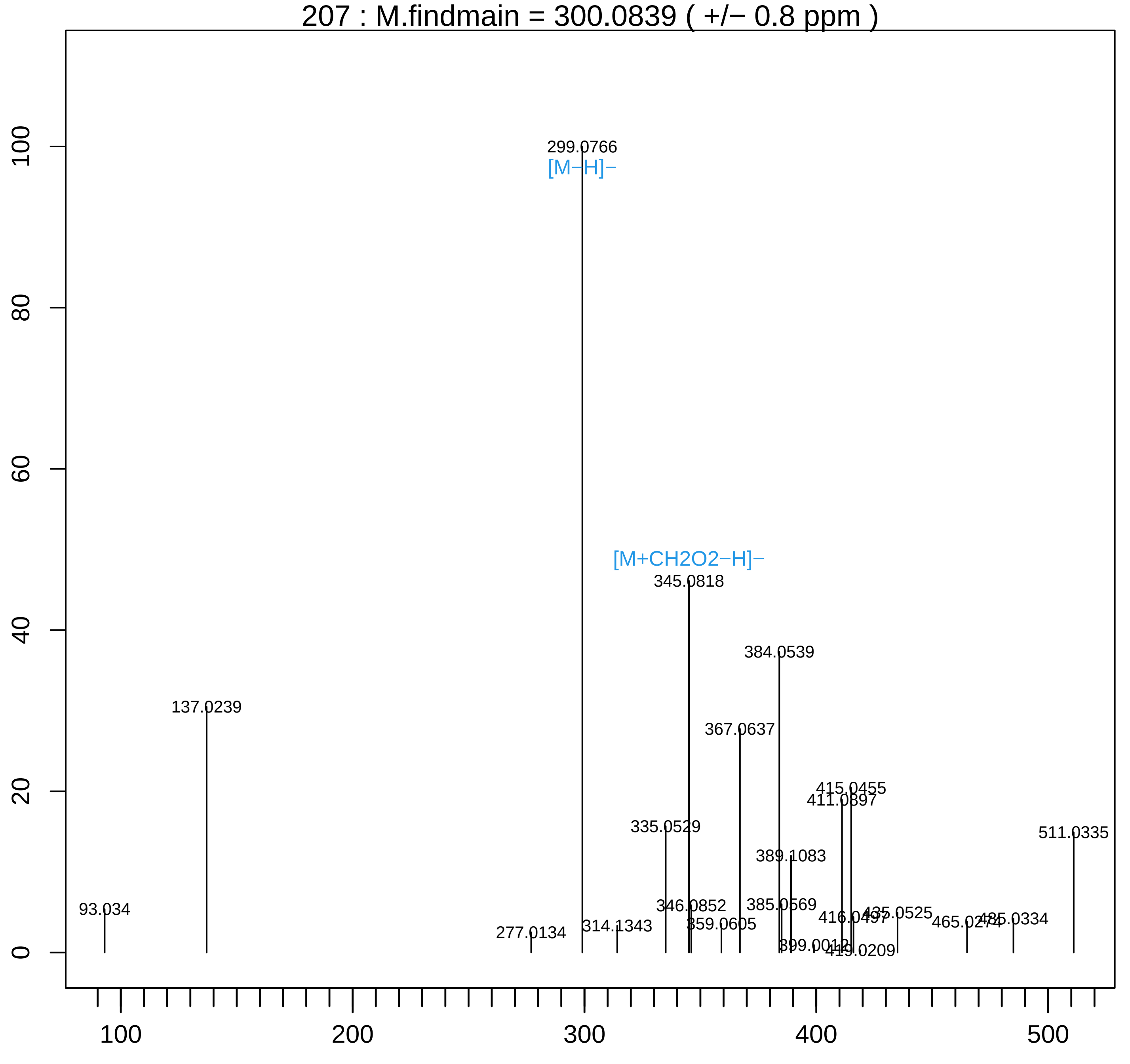
207 : M.findmain = 300.0839 (+/− 0.8 ppm)
299.0766
[M−H]−
[M+CH2O2−H]−
345.0818
384.0539
137.0239
367.0637
415.0455
411.0897
335.0529
511.0335
389.1083
93.034
346.0852
385.0569
416.0497
435.0525
277.0134
314.1343
359.0605
465.0274
485.0334
399.0012
419.0209
0
20
40
60
80
100
200
300
400
500

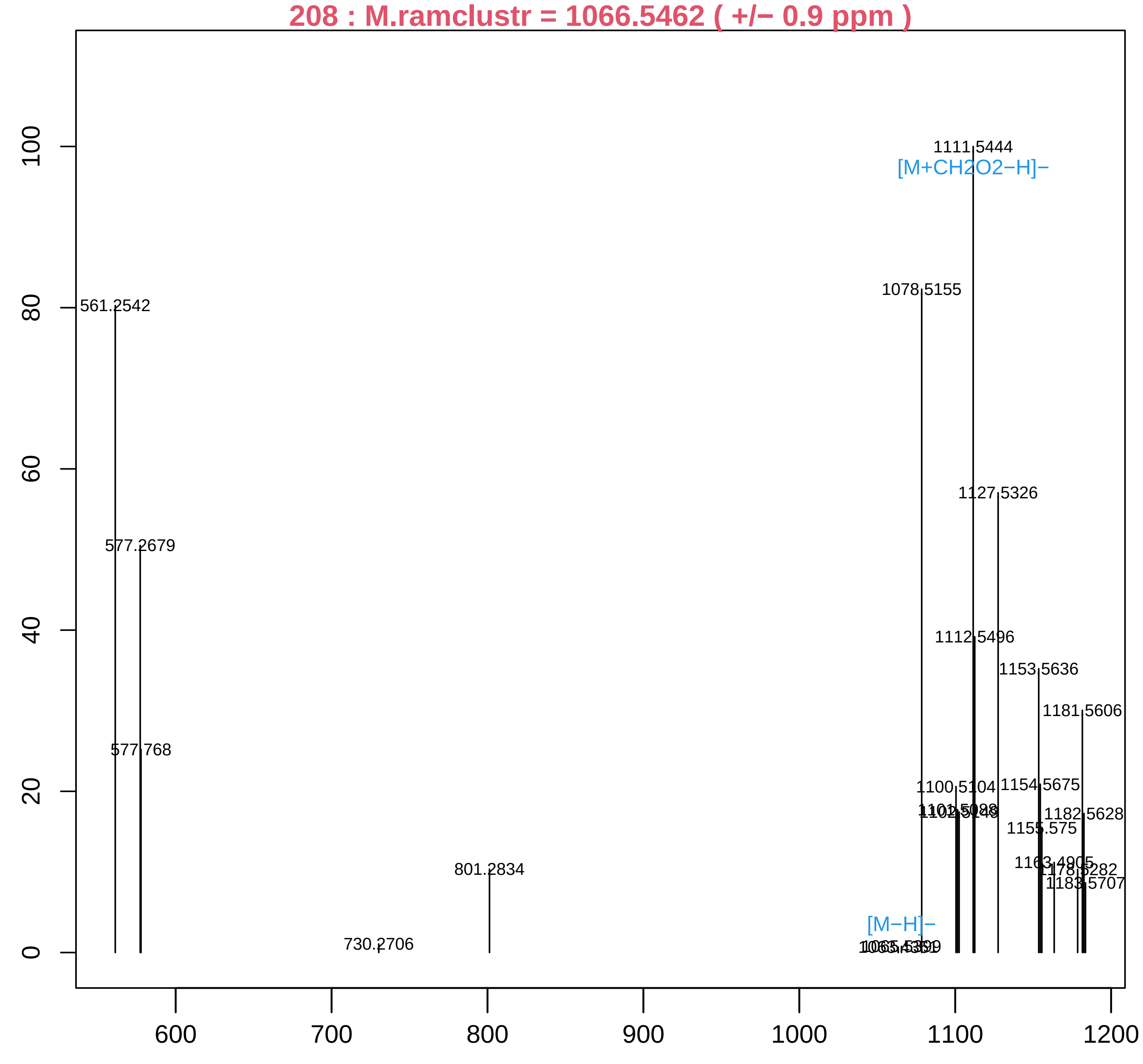
208 : M.ramclustr = 1066.5462 (+/− 0.9 ppm)
0
20
40
60
80
100
600
700
800
900
1000
1100
1200
561.2542
577.2679
577.768
730.2706
801.2834
[M−H]−
1078.5155
1100.5104
1111.5444
[M+CH2O2−H]−
1112.5496
1127.5326
1153.5636
1154.5675
1155.575
1163.4905
1181.5606
1182.5628
1183.5707

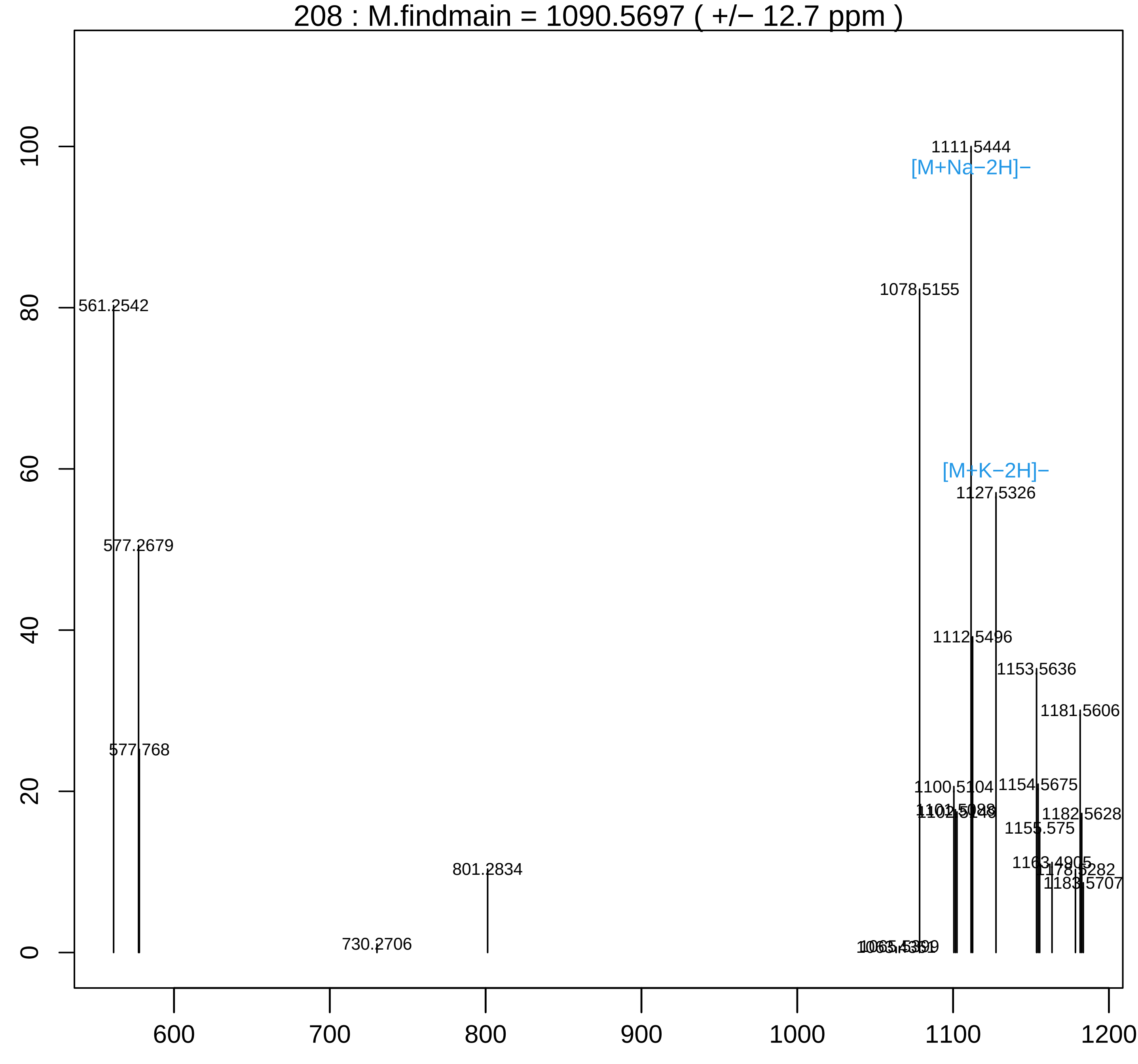
208 : M.findmain = 1090.5697 (+/− 12.7 ppm)
0
20
40
60
80
100
600
700
800
900
1000
1100
1200
561.2542
577.2679
577.768
730.2706
801.2834
1078.5155
1100.5104
1111.5444
[M+Na−2H]−
1112.5496
[M+K−2H]−
1127.5326
1153.5636
1154.5675
1155.575
1163.4905
1181.5606
1182.5628
1183.5707

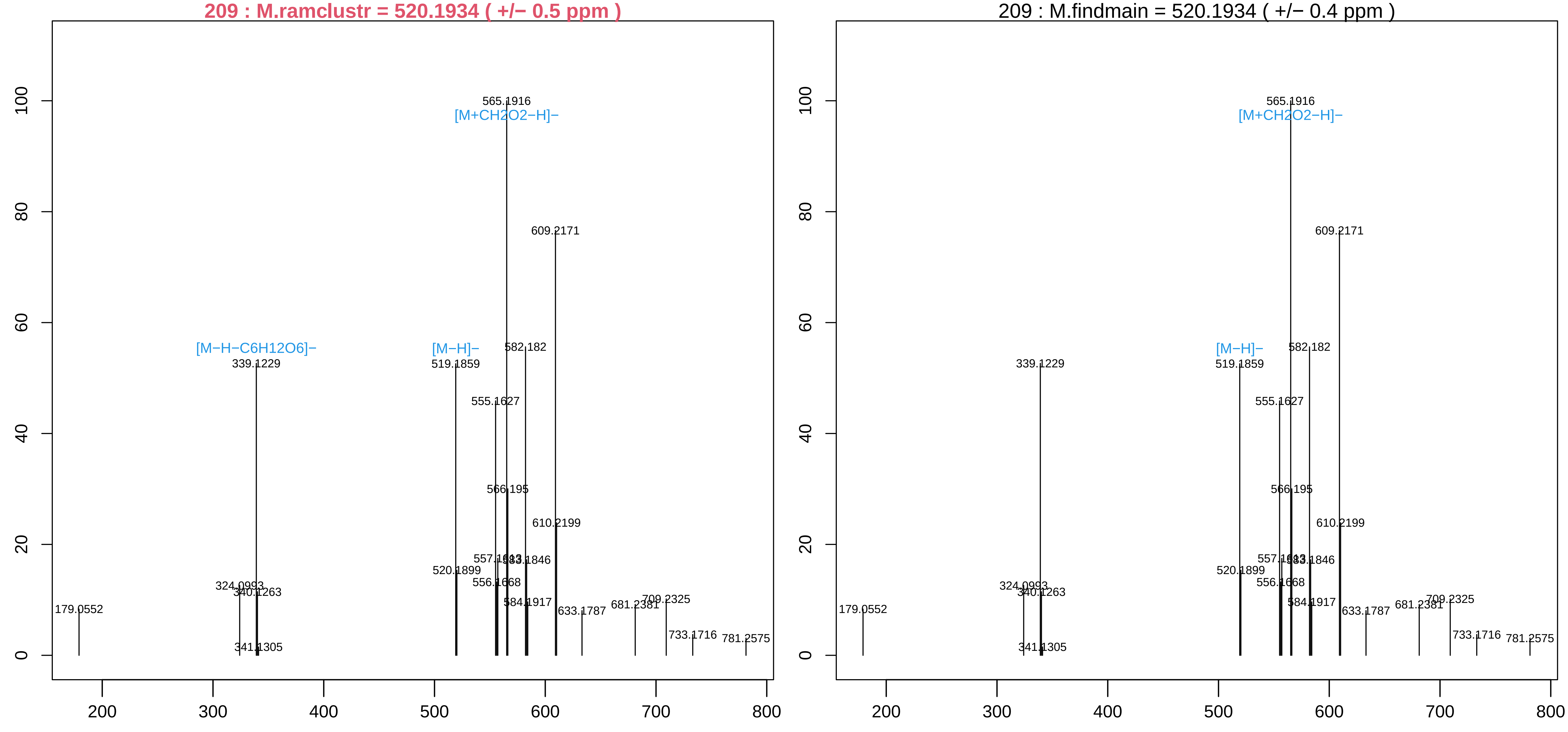

209 : M.ramclustr = 520.1934 (+/− 0.5 ppm)
209 : M.findmain = 520.1934 (+/− 0.4 ppm)
565.1916
[M+CH2O2−H]−
609.2171
[M−H−C6H12O6]−
339.1229
[M−H]−
519.1859
582.182
555.1627
566.195
610.2199
557.1613
583.1846
520.1899
556.1668
324.0993
340.1263
179.0552
584.1917
633.1787
681.2381
709.2325
733.1716
781.2575
341.1305

210 : M.ramclustr = 885.5073 (+/− 0.1 ppm)

210 : M.findmain = 885.5073 (+/− 0.1 ppm)

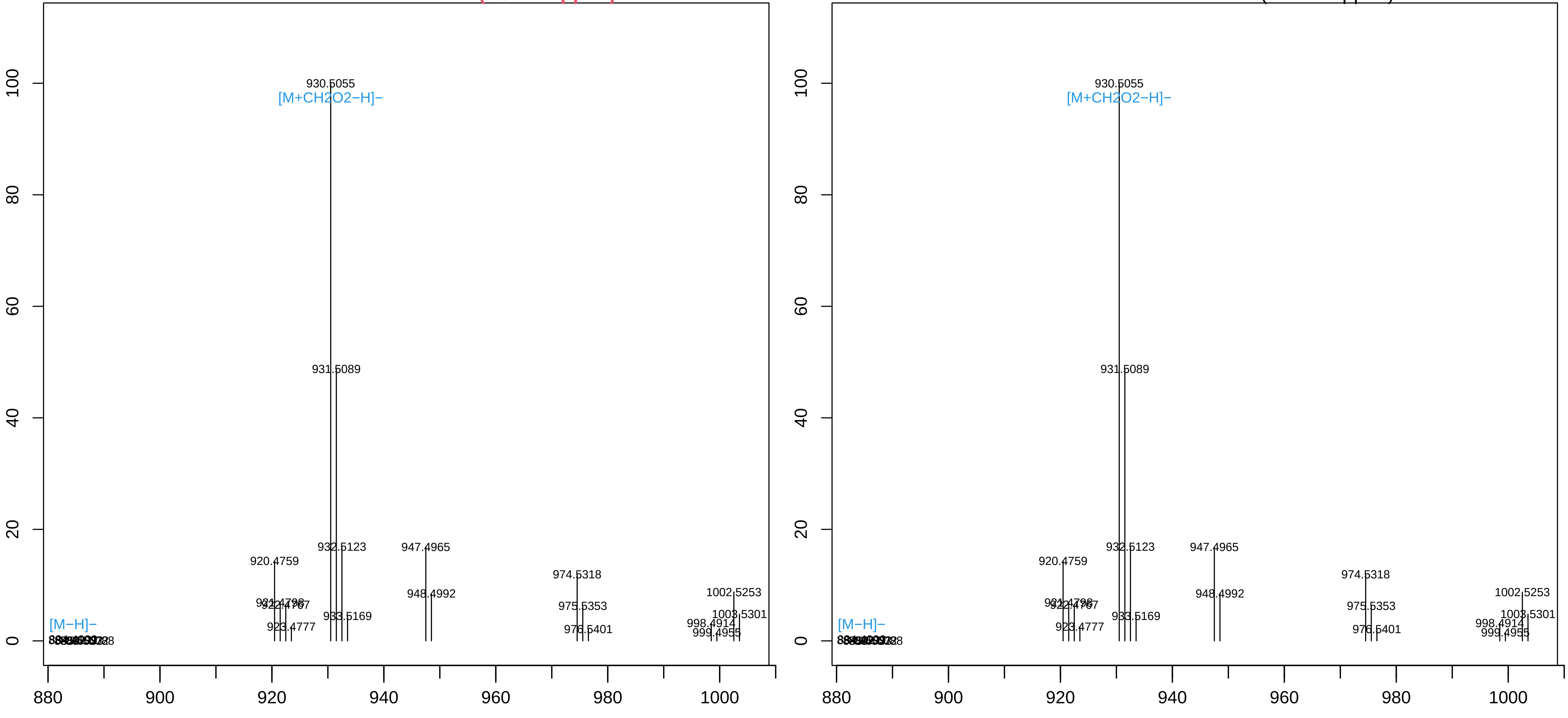

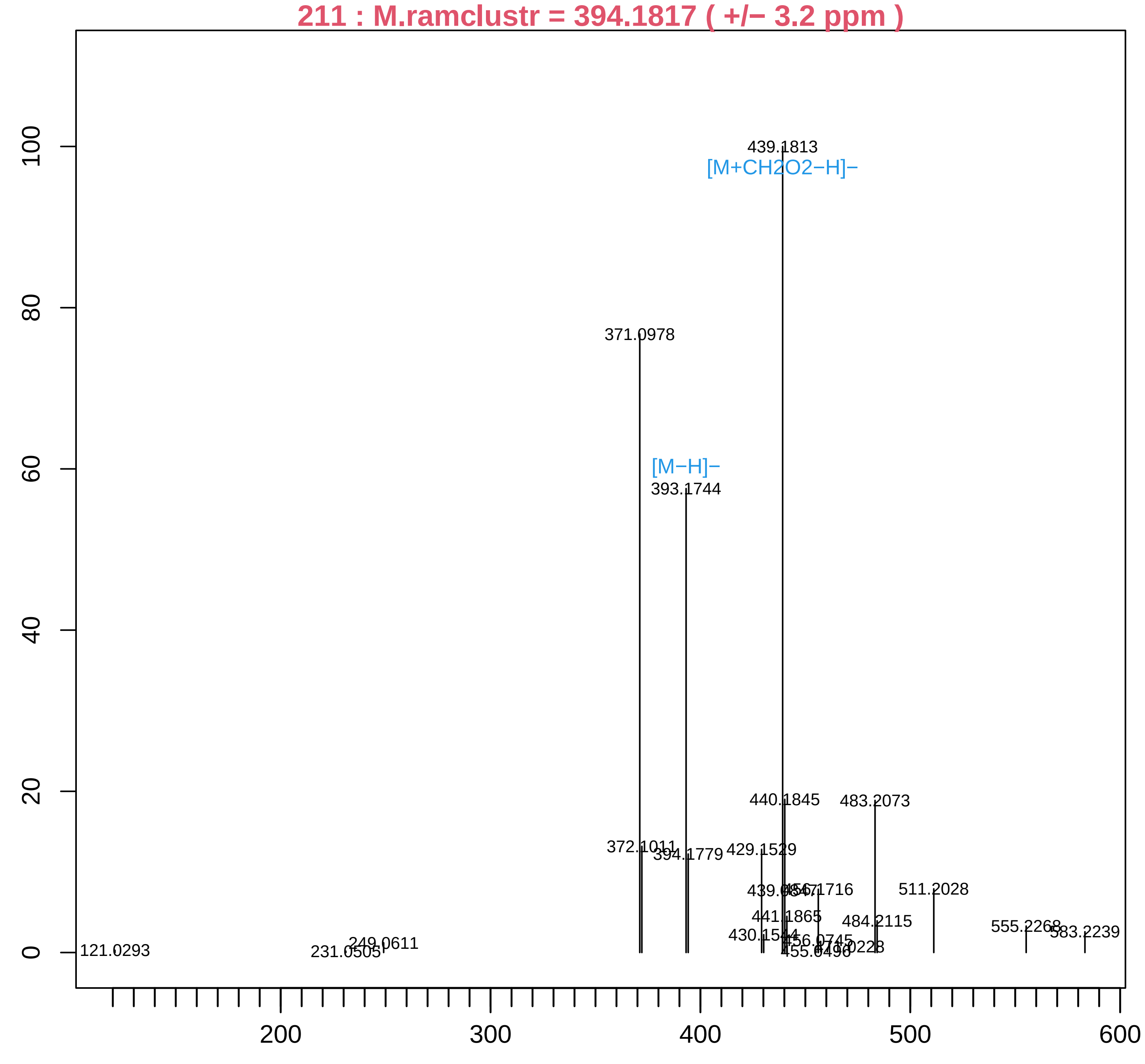
211 : M.ramclustr = 394.1817 (+/− 3.2 ppm)
439.1813
[M+CH2O2−H]−
371.0978
[M−H]−
393.1744
440.1845
483.2073
372.1011
394.1779
429.1529
511.2028
441.1865
484.2115
555.2268
583.2239
121.0293
249.0611
231.0505
0
20
40
60
80
100
200
300
400
500
600

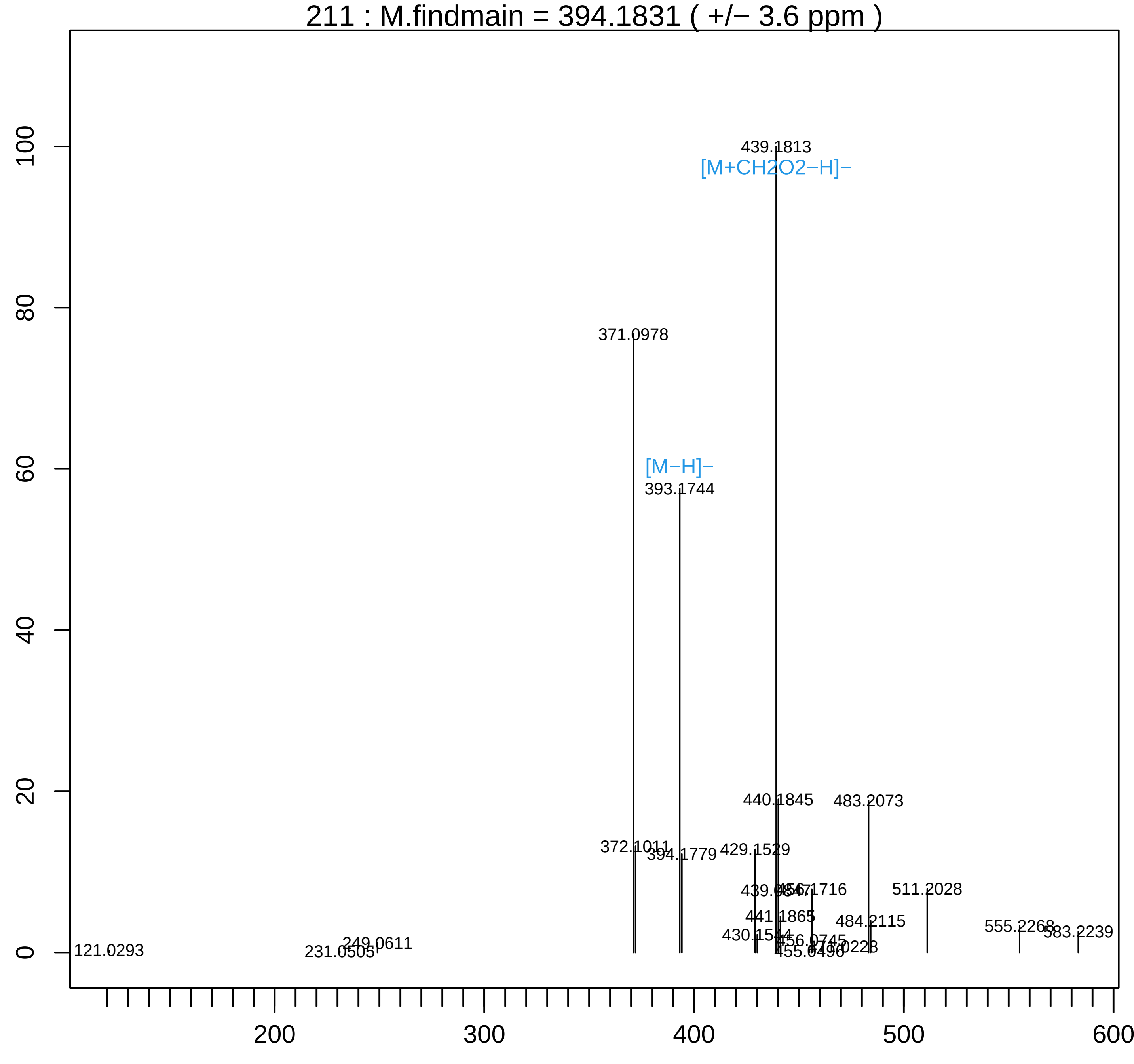
211 : M.findmain = 394.1831 (+/− 3.6 ppm)
439.1813
[M+CH2O2−H]−
371.0978
[M−H]−
393.1744
440.1845
483.2073
372.1011
394.1779
429.1529
511.2028
441.1865
484.2115
555.2268
583.2239
121.0293
249.0611
231.0505
0
20
40
60
80
100
200
300
400
500
600

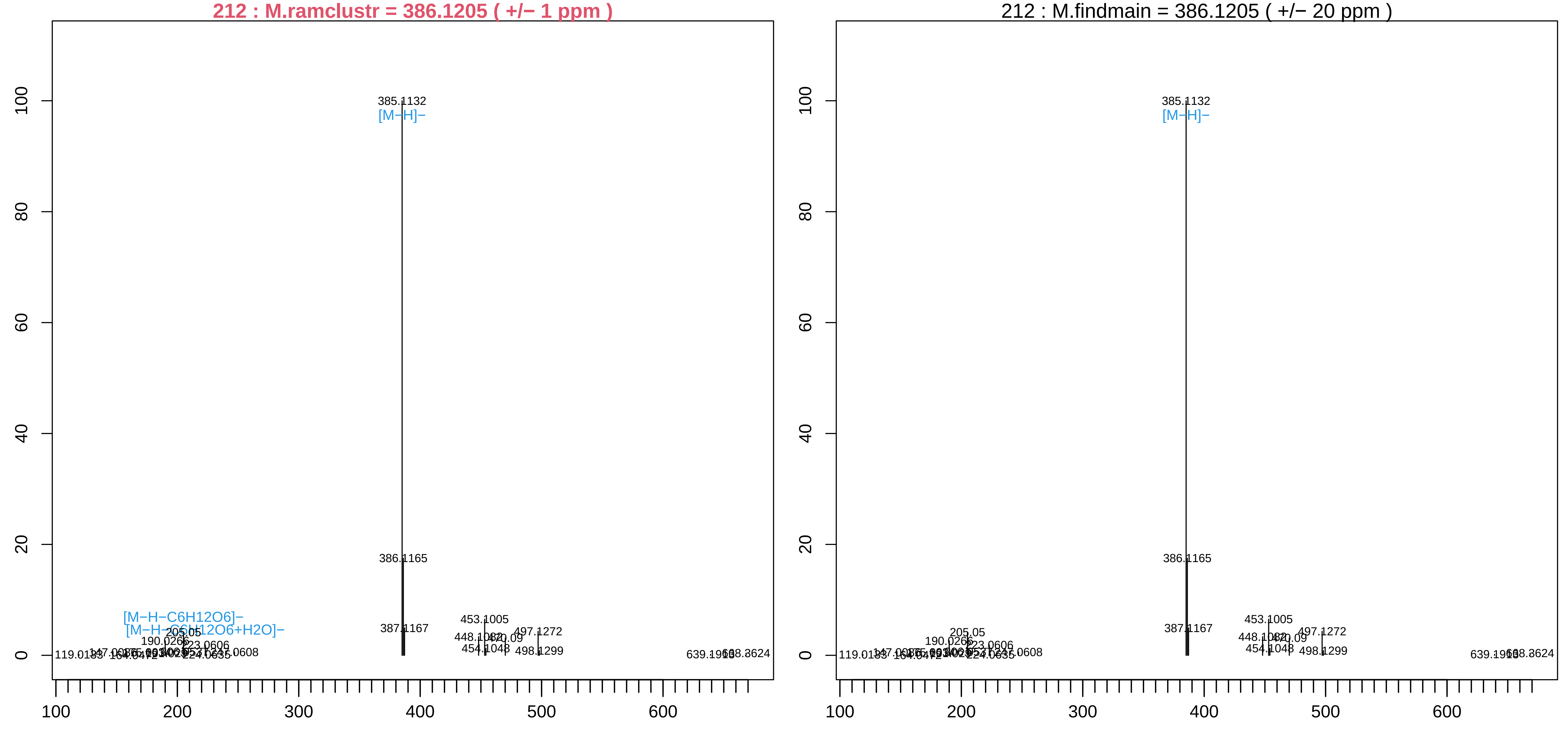
212 : M.ramclustr = 386.1205 (+/− 1 ppm)
212 : M.findmain = 386.1205 (+/− 20 ppm)
385.1132
[M−H]−
386.1165
387.1167
453.1005
497.1272
448.1062
454.1048
498.1299
205.05
190.0266
223.0606
119.0133
164.0472
224.0635
639.1968
658.3624
[M−H−C6H12O6]−
[M−H−C6H12O6+H2O]−
0
20
40
60
80
100
200
300
400
500
600

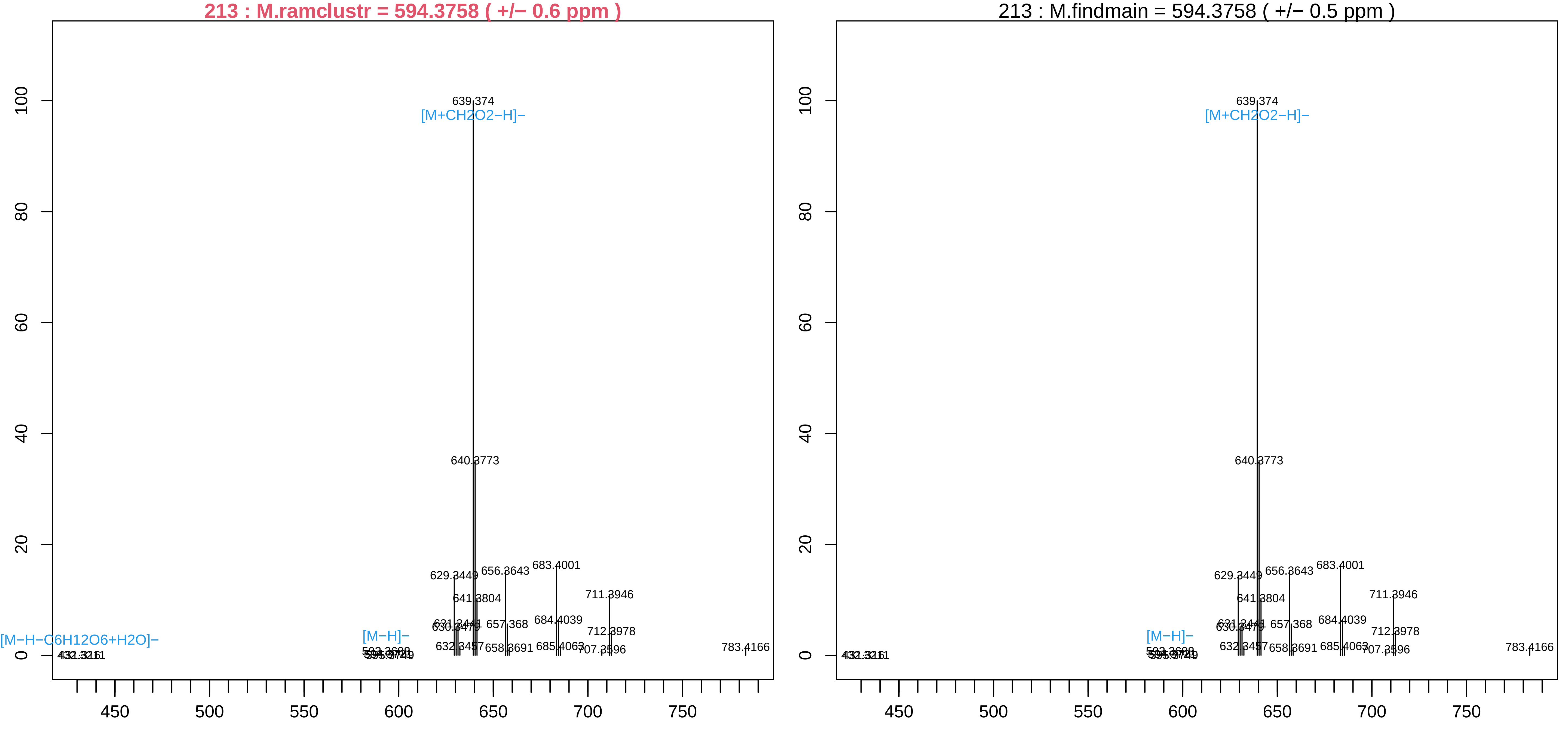

213 : M.ramclustr = 594.3758 (+/− 0.6 ppm)
213 : M.findmain = 594.3758 (+/− 0.5 ppm)
639.374
[M+CH2O2−H]−
640.3773
629.3449
656.3643
683.4001
641.3804
711.3946
631.3441
630.3476
657.368
684.4039
712.3978
632.3457
658.3691
685.4063
707.3596
783.4166
[M−H]−
[M−H−C6H12O6+H2O]−
431.3161
0
20
40
60
80
100
450
500
550
600
650
700
750

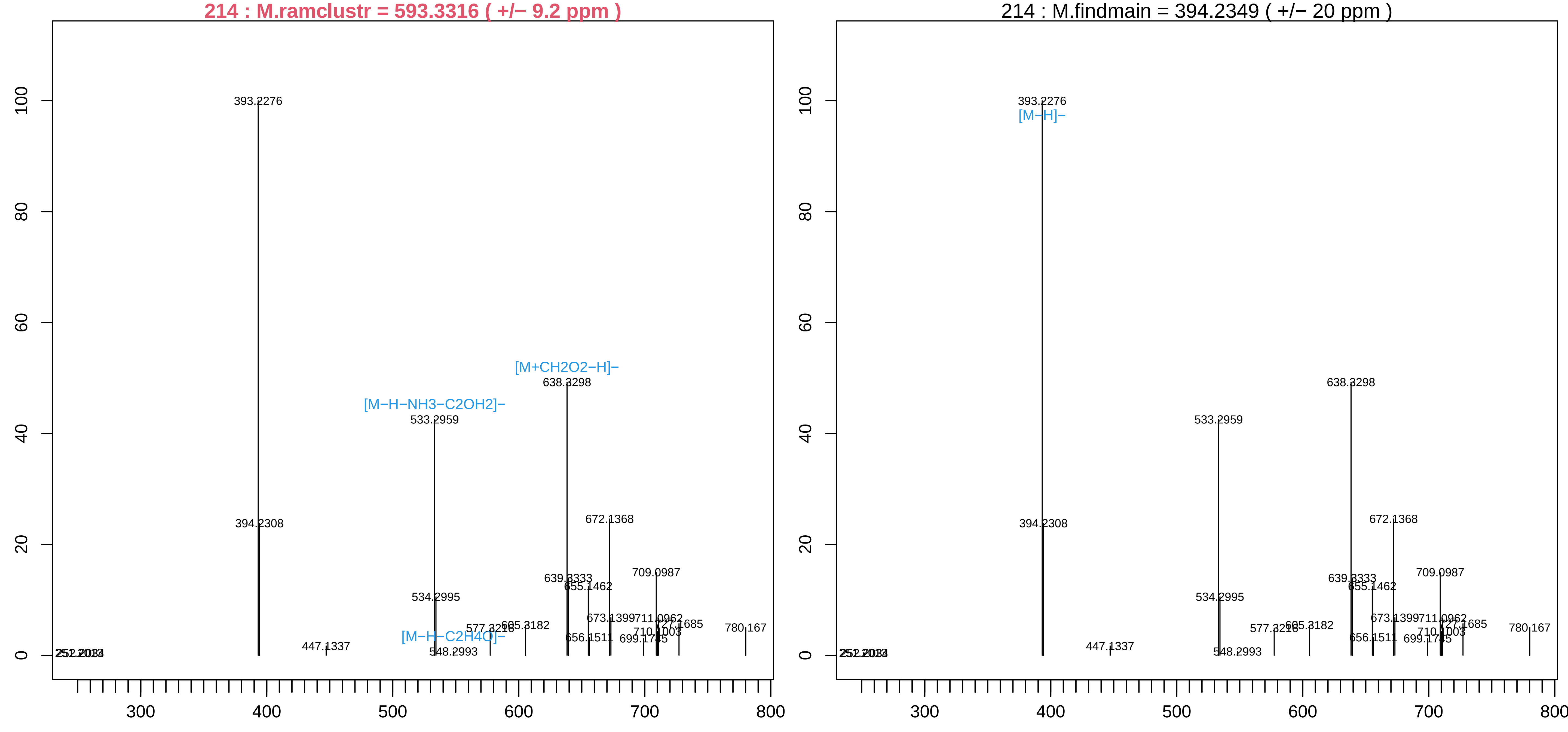

214 : M.ramclustr = 593.3316 (+/− 9.2 ppm)
393.2276
394.2308
[M−H−NH3−C2OH2]−
533.2959
534.2995
[M−H−C2H4O]−
548.2993
[M+CH2O2−H]−
638.3298
639.3333
655.1462
656.1511
672.1368
673.1399
577.3216
605.3182
447.1337
699.1745
709.0987
710.1003
711.0962
727.1685
780.167
214 : M.findmain = 394.2349 (+/− 20 ppm)
[M−H]−
0
20
40
60
80
100
300
400
500
600
700
800

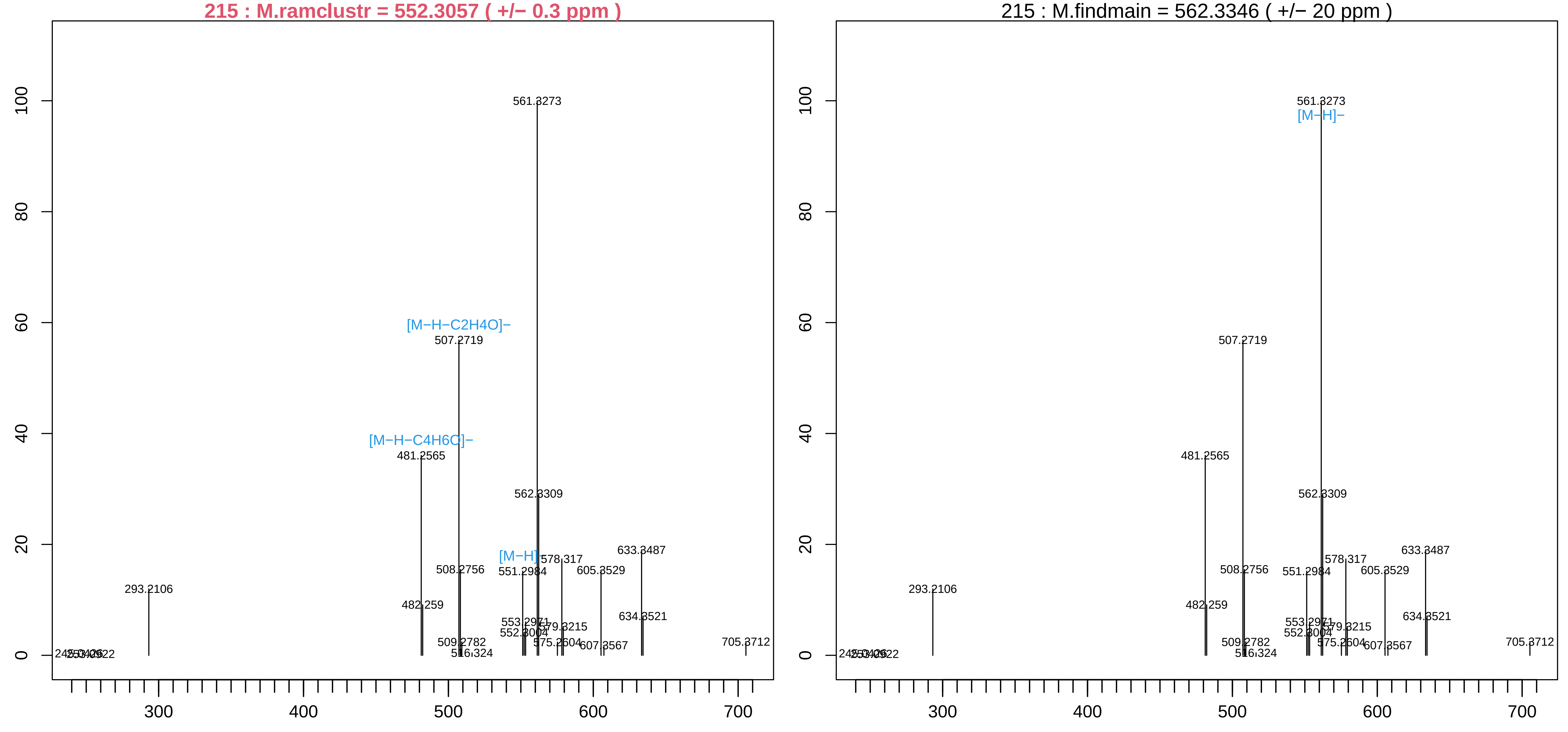

215 : M.ramclustr = 552.3057 (+/− 0.3 ppm)
561.3273
[M−H−C2H4O]−
507.2719
[M−H−C4H6O]−
481.2565
562.3309
[M−H]−
578.317
508.2756
551.2984
605.3529
633.3487
293.2106
482.259
553.2971
552.3004
579.3215
634.3521
509.2782
575.2604
607.3567
516.324
705.3712
215 : M.findmain = 562.3346 (+/− 20 ppm)
561.3273
[M−H]−
507.2719
481.2565
562.3309
578.317
508.2756
551.2984
605.3529
633.3487
293.2106
482.259
553.2971
552.3004
579.3215
634.3521
509.2782
575.2604
607.3567
516.324
705.3712
0
20
40
60
80
100
300
400
500
600
700

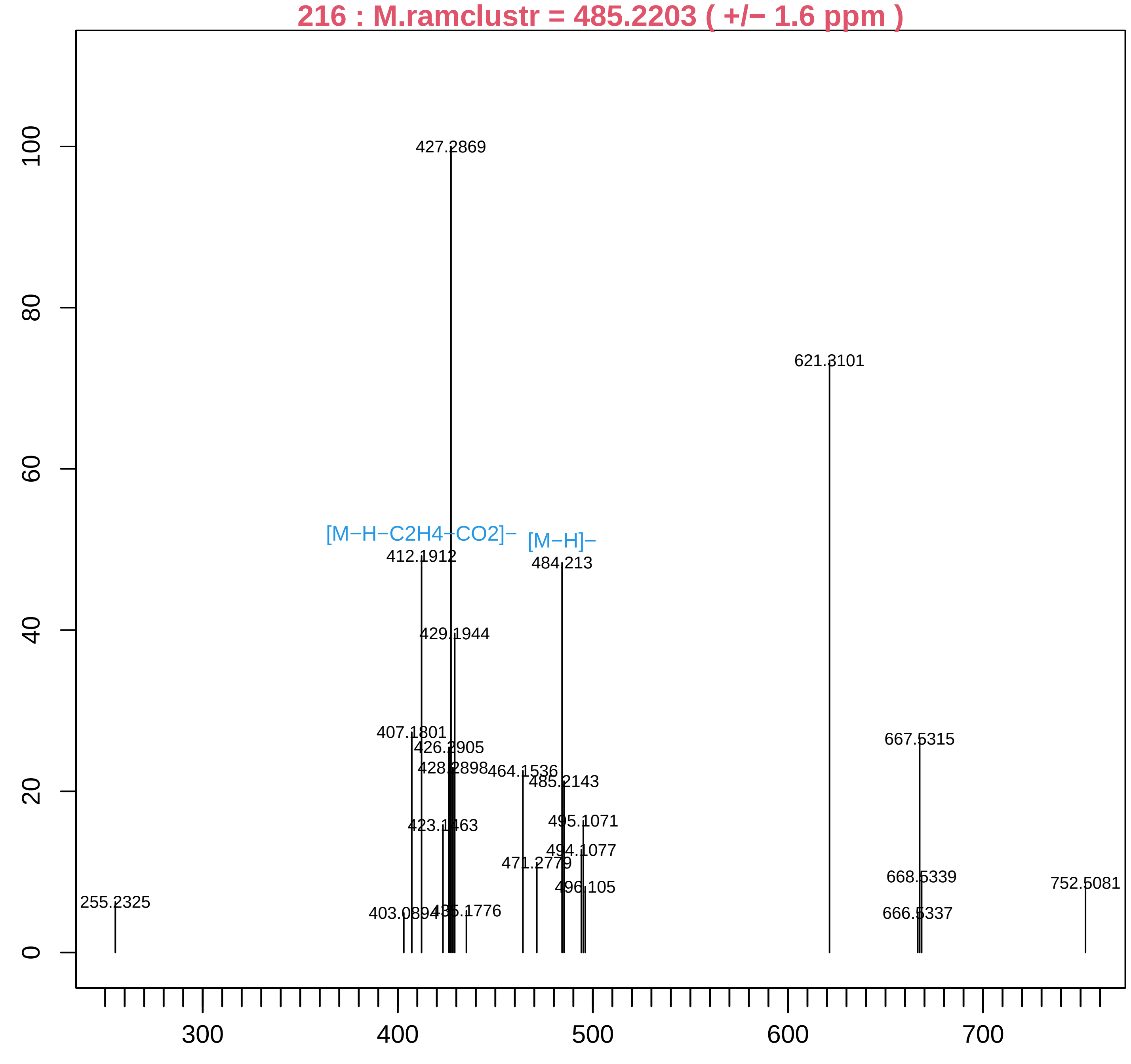
216 : M.ramclustr = 485.2203 (+/− 1.6 ppm)
[M−H−C2H4−CO2]−
[M−H]−
427.2869
621.3101
412.1912
484.213
429.1944
407.1801
426.2905
428.2898
464.1536
485.2143
667.5315
423.1463
495.1071
494.1077
471.2779
668.5339
752.5081
496.105
255.2325
403.0894
435.1776
666.5337

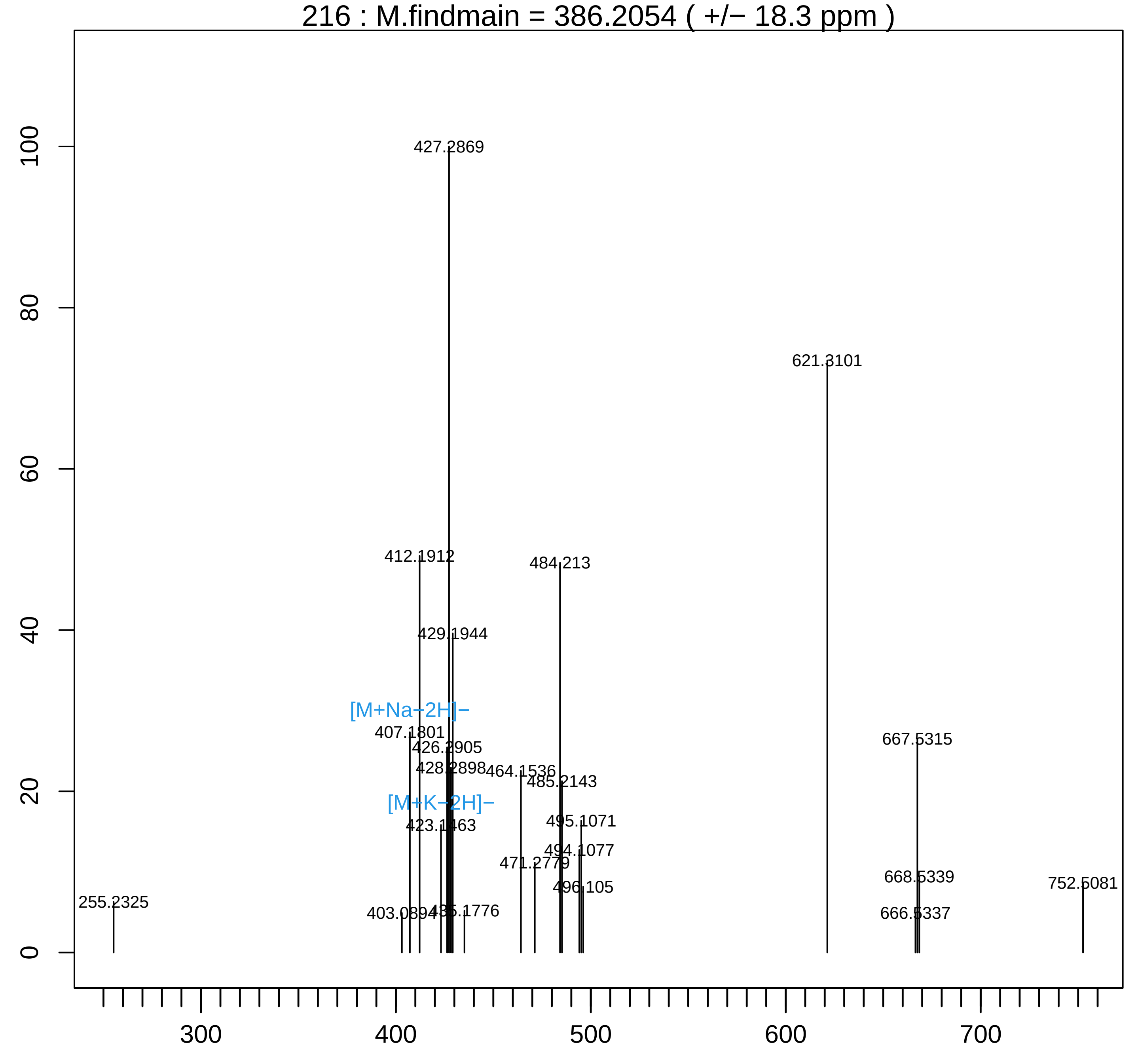
216 : M.findmain = 386.2054 (+/− 18.3 ppm)
[M+Na−2H]−
[M+K−2H]−
427.2869
621.3101
412.1912
484.213
429.1944
407.1801
426.2905
428.2898
464.1536
485.2143
667.5315
423.1463
495.1071
494.1077
471.2779
668.5339
752.5081
496.105
255.2325
403.0894
435.1776
666.5337

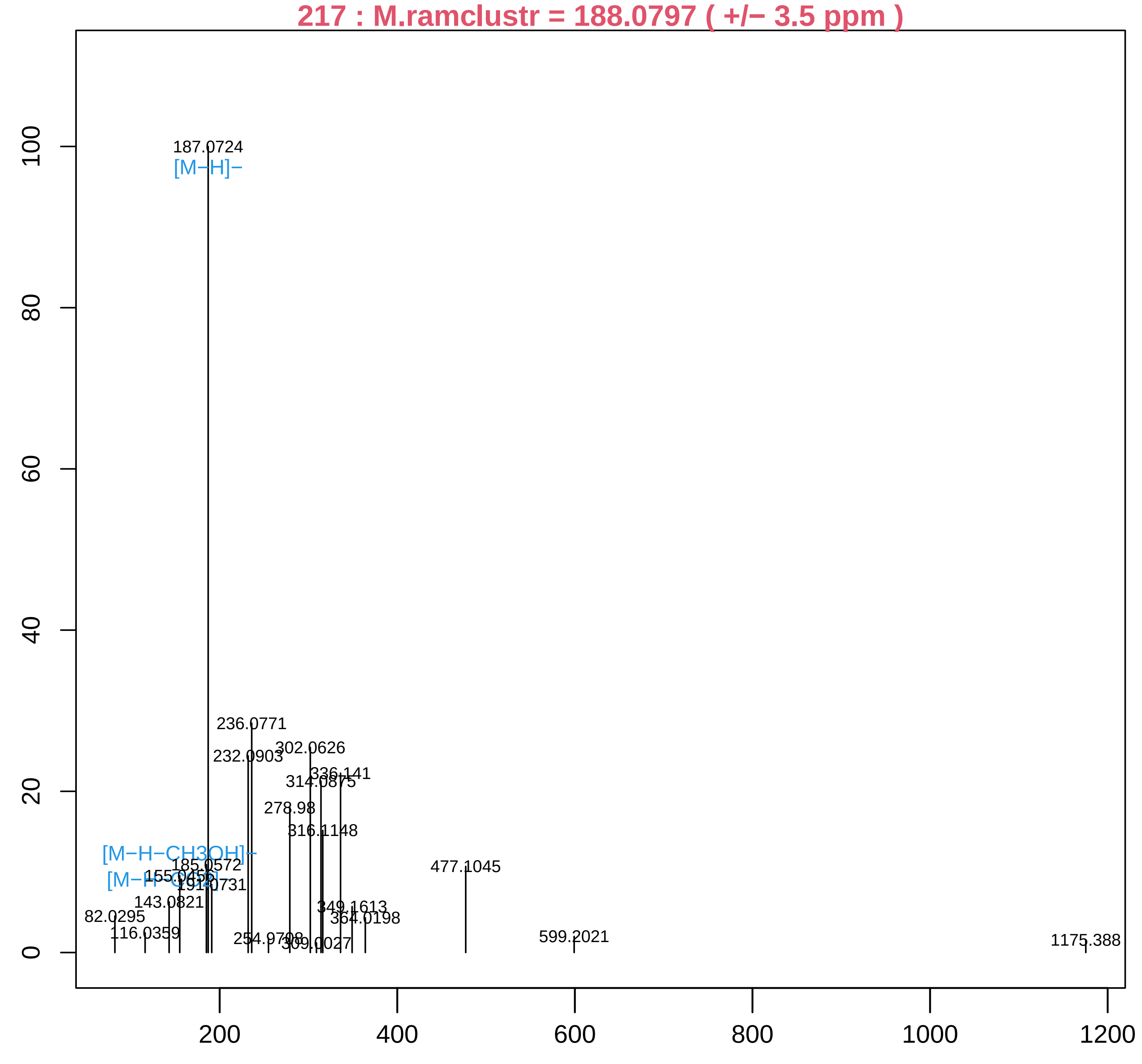
217 : M.ramclustr = 188.0797 (+/− 3.5 ppm)
187.0724
[M−H]−
[M−H−CH3OH]−
236.0771
232.0903
302.0626
336.141
314.0875
278.98
316.1148
185.0572
155.0456
191.0731
143.0821
82.0295
116.0359
254.9708
309.0027
349.1613
364.0198
477.1045
599.2021
1175.388
0
20
40
60
80
100
200
400
600
800
1000
1200

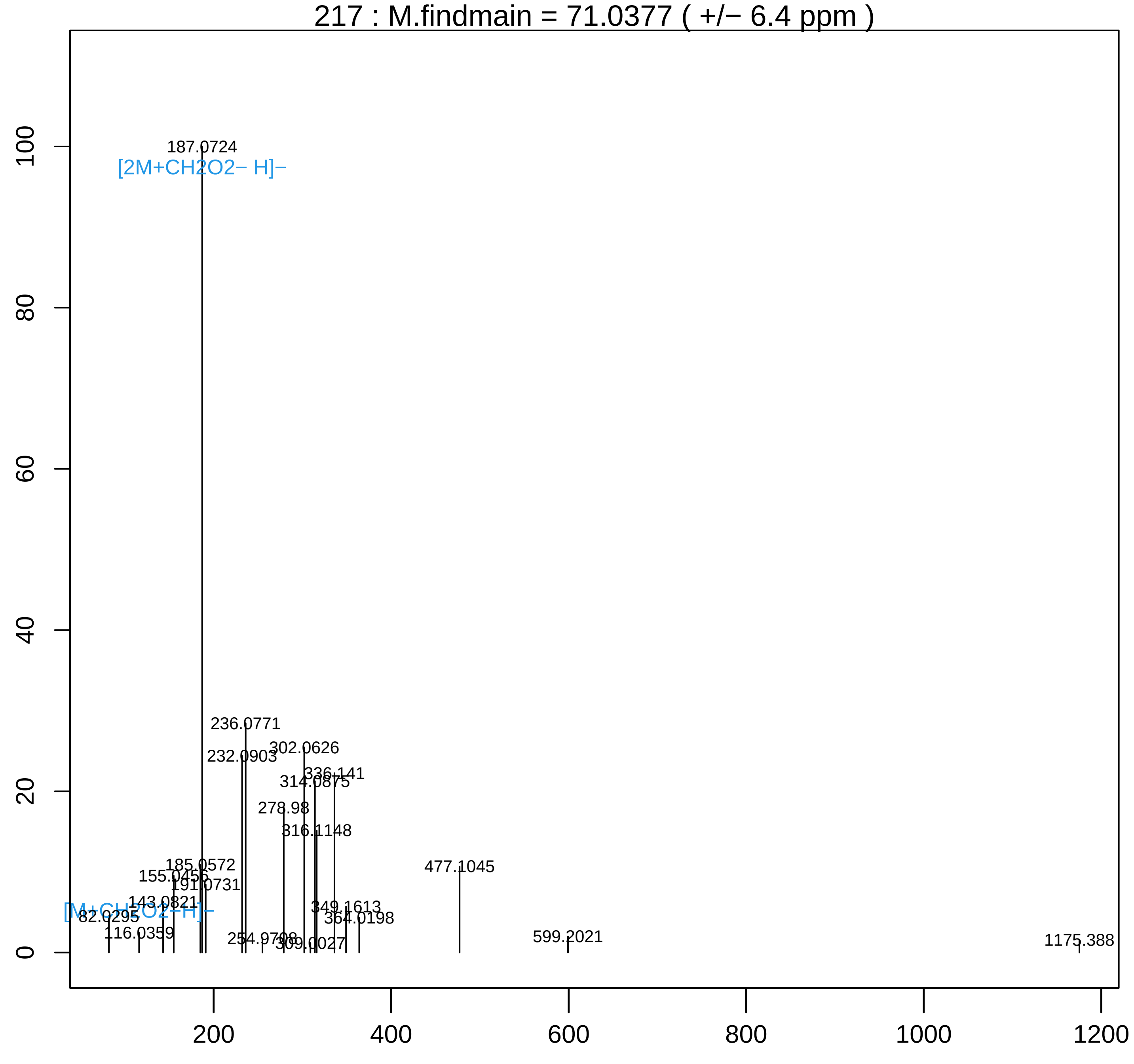
217 : M.findmain = 71.0377 (+/− 6.4 ppm)
187.0724
[2M+CH2O2− H]−
236.0771
232.0903
302.0626
336.141
314.0875
278.98
316.1148
185.0572
155.0456
191.0731
143.0821
82.0295
116.0359
254.9708
309.0027
349.1613
364.0198
477.1045
599.2021
1175.388
0
20
40
60
80
100
200
400
600
800
1000
1200

218 : M.ramclustr = 458.144 (+/− 0.1 ppm)

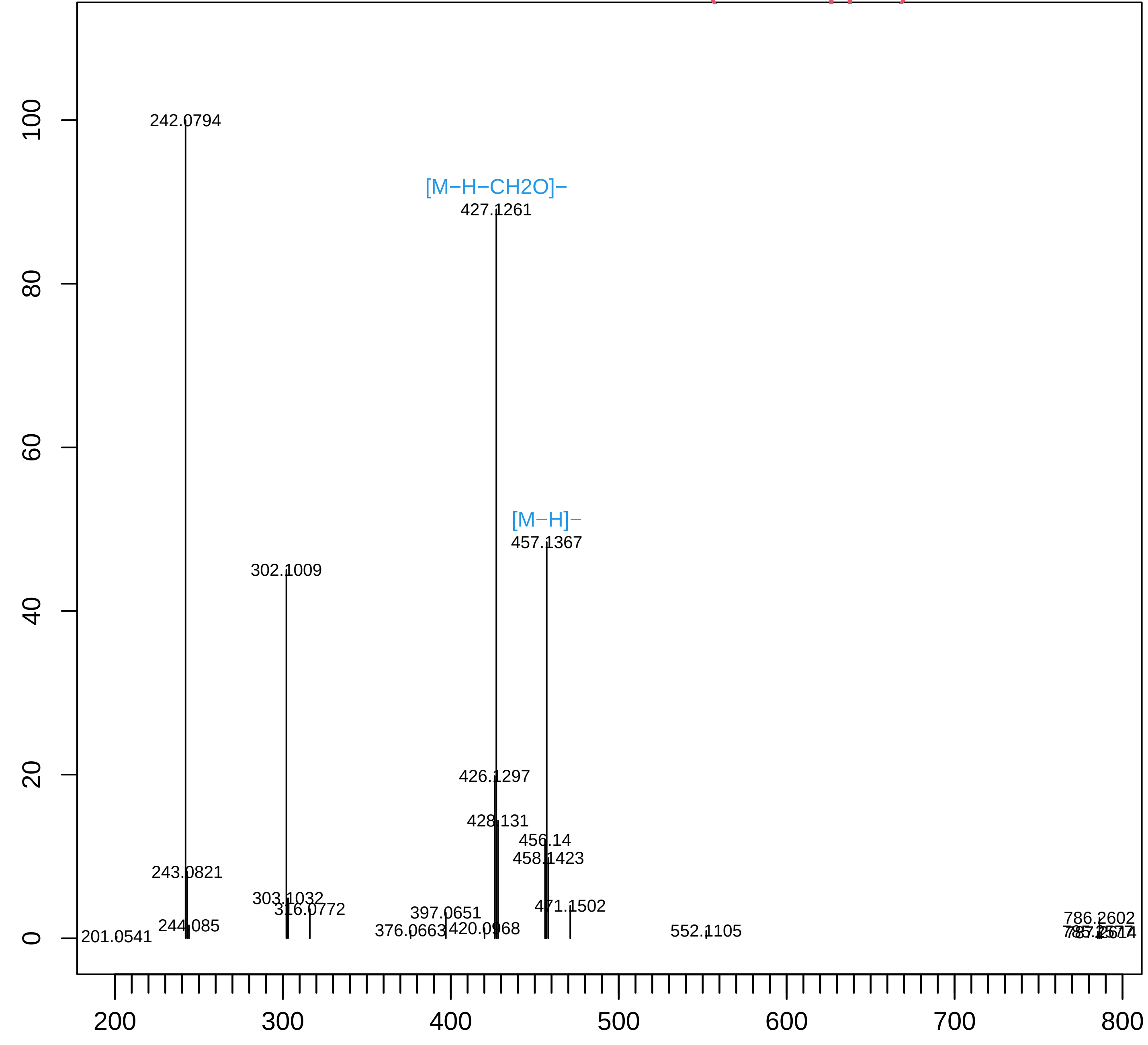

218 : M.findmain = 383.1482 (+/− 12.3 ppm)

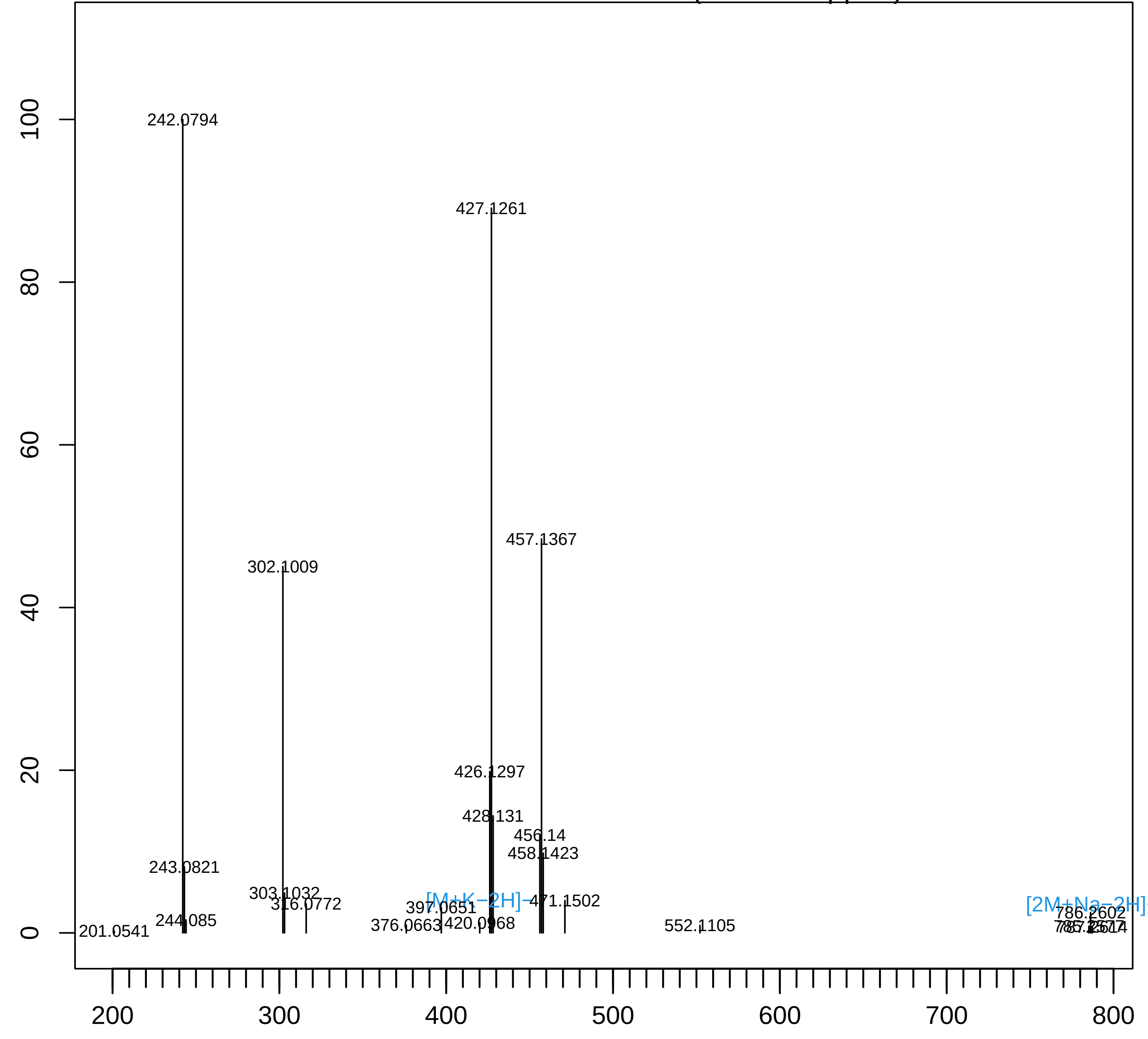

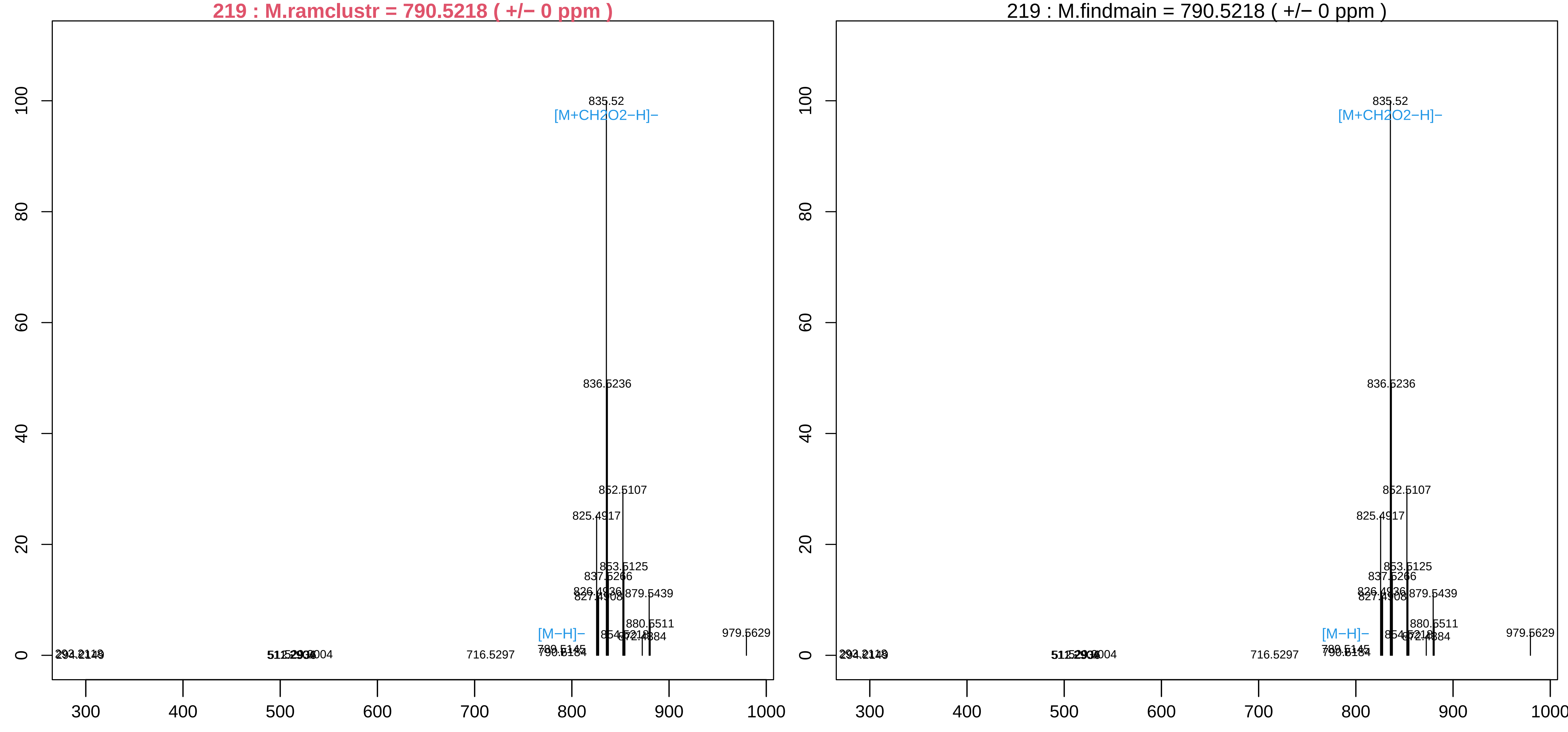
219 : M.ramclustr = 790.5218 (+/− 0 ppm)
219 : M.findmain = 790.5218 (+/− 0 ppm)
835.52
[M+CH2O2−H]−
836.5236
852.5107
825.4917
853.5125
837.5266
879.5439
880.5511
979.5629
[M−H]−
716.5297
0
20
40
60
80
100
300
400
500
600
700
800
900
1000

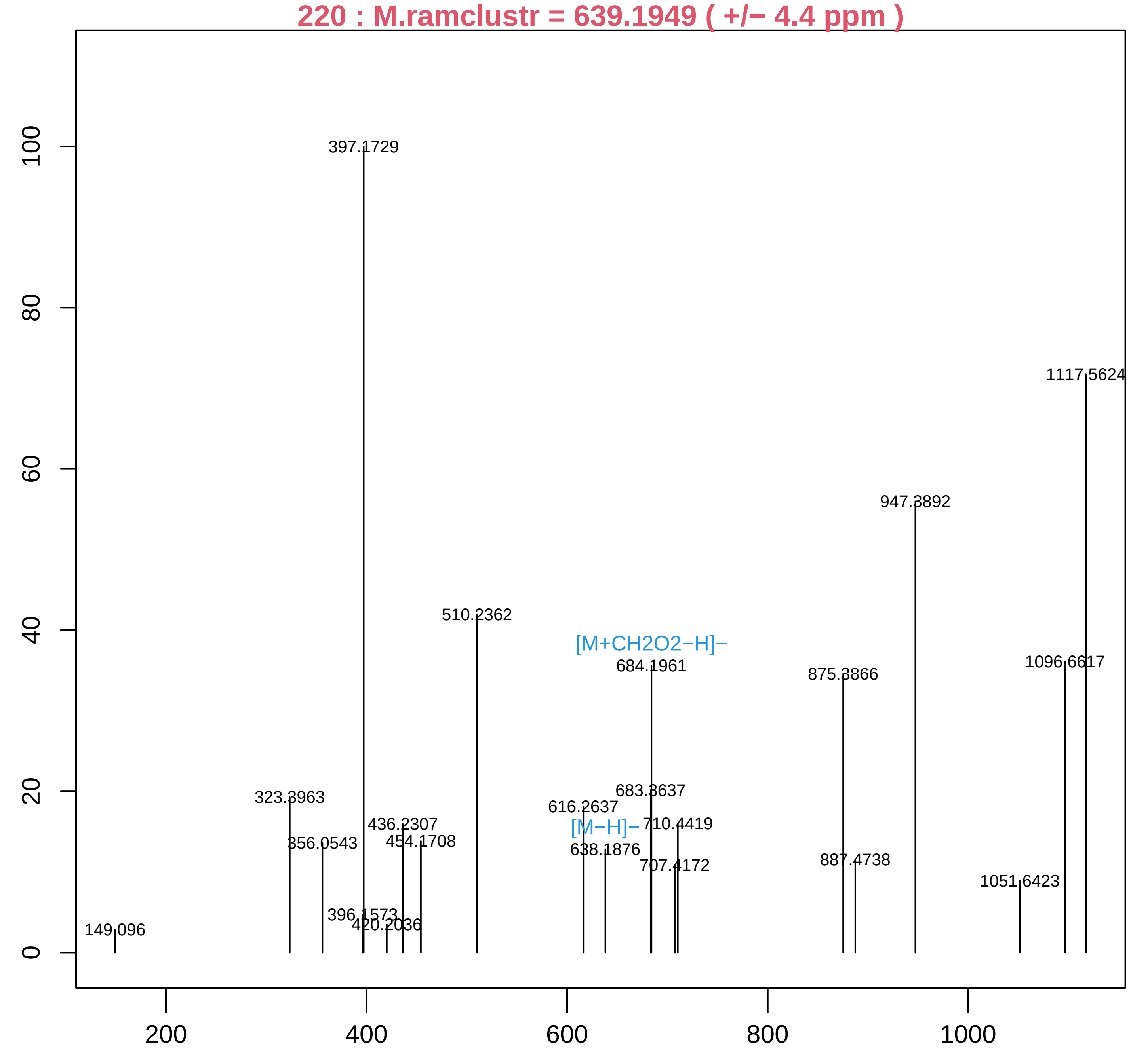

220 : M.ramclustr = 639.1949 (+/− 4.4 ppm)
397.1729
1117.5624
947.3892
510.2362
[M+CH2O2−H]−
684.1961
875.3866
1096.6617
323.3963
683.3637
616.2637
436.2307
710.4419
[M−H]−
356.0543
454.1708
638.1876
707.4172
887.4738
1051.6423
396.1573
420.2036
149.096

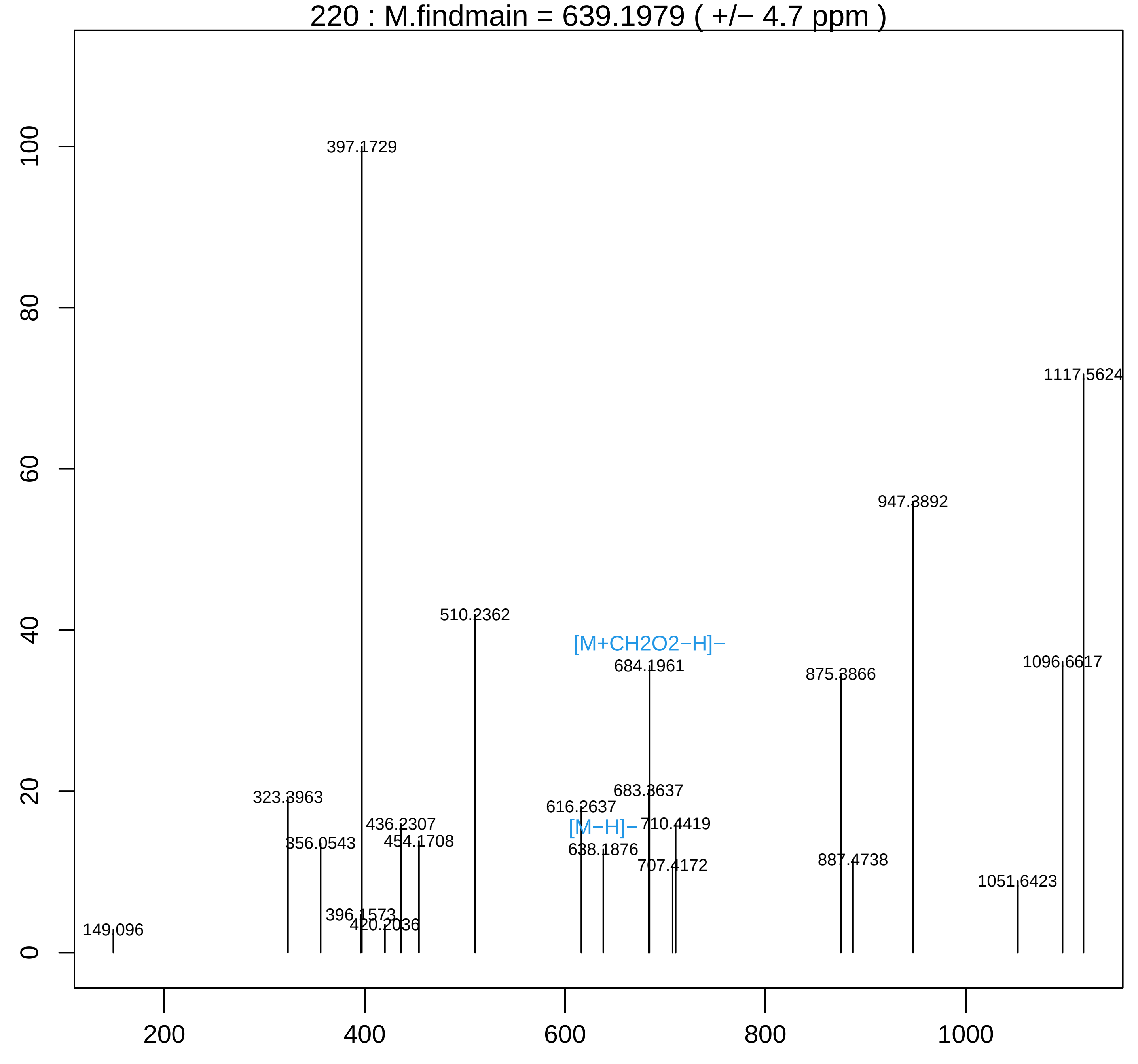

220 : M.findmain = 639.1979 (+/− 4.7 ppm)
397.1729
1117.5624
947.3892
510.2362
[M+CH2O2−H]−
684.1961
875.3866
1096.6617
323.3963
683.3637
616.2637
436.2307
710.4419
[M−H]−
356.0543
454.1708
638.1876
707.4172
887.4738
1051.6423
396.1573
420.2036
149.096

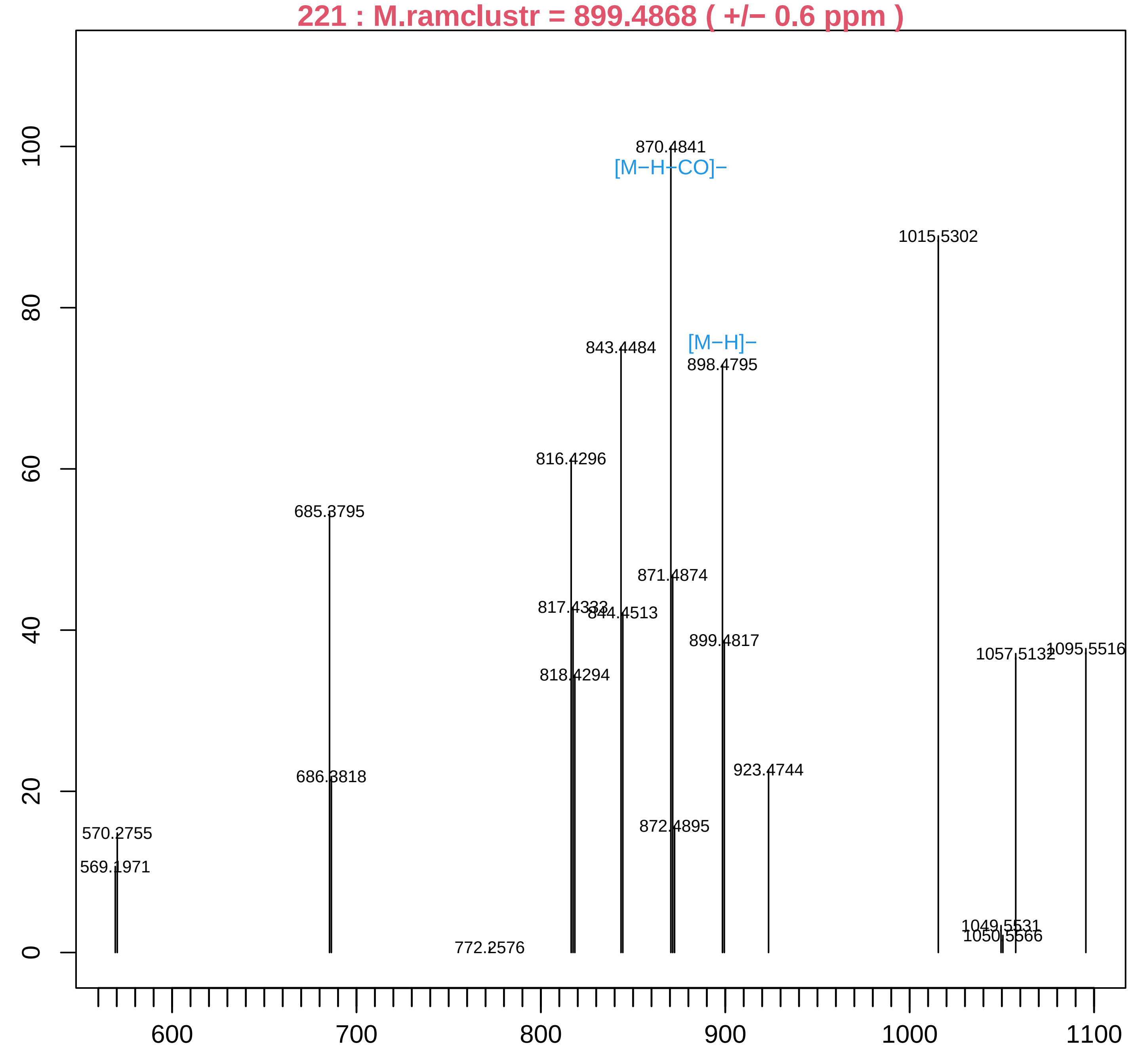
221 : M.ramclustr = 899.4868 (+/− 0.6 ppm)
870.4841
[M−H−CO]−
1015.5302
843.4484
[M−H]−
898.4795
816.4296
685.3795
871.4874
817.4332
844.4513
899.4817
1057.5132
1095.5516
818.4294
686.3818
923.4744
872.4895
570.2755
569.1971
1049.5531
1050.5566
772.2576
0
20
40
60
80
100
600
700
800
900
1000
1100

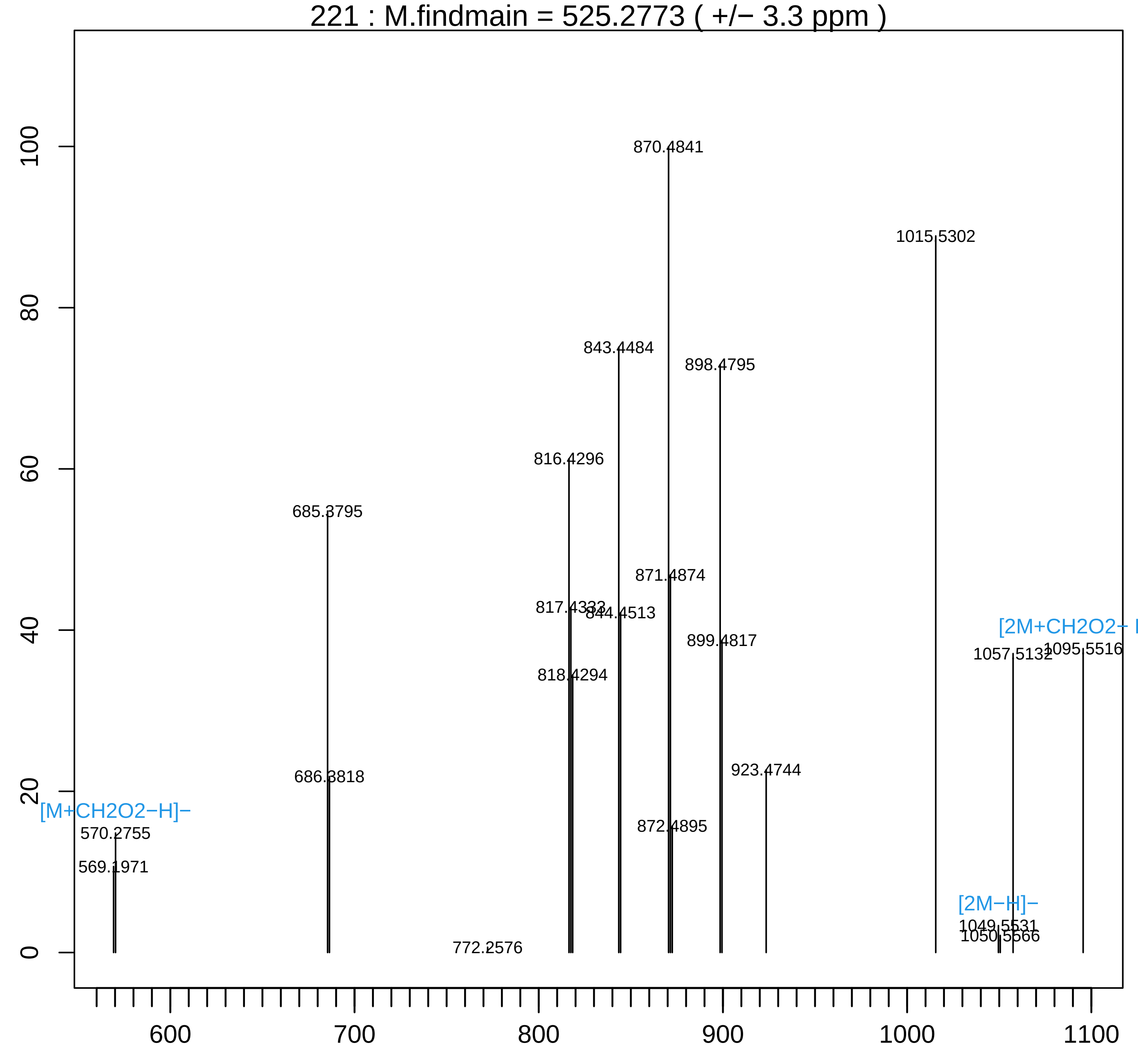
221 : M.findmain = 525.2773 (+/− 3.3 ppm)
870.4841
1015.5302
843.4484
898.4795
816.4296
685.3795
871.4874
817.4332
844.4513
[2M+CH2O2−
899.4817
1057.5132
1095.5516
818.4294
686.3818
923.4744
[M+CH2O2−H]−
872.4895
570.2755
569.1971
[2M−H]−
1049.5531
1050.5566
772.2576
0
20
40
60
80
100
600
700
800
900
1000
1100

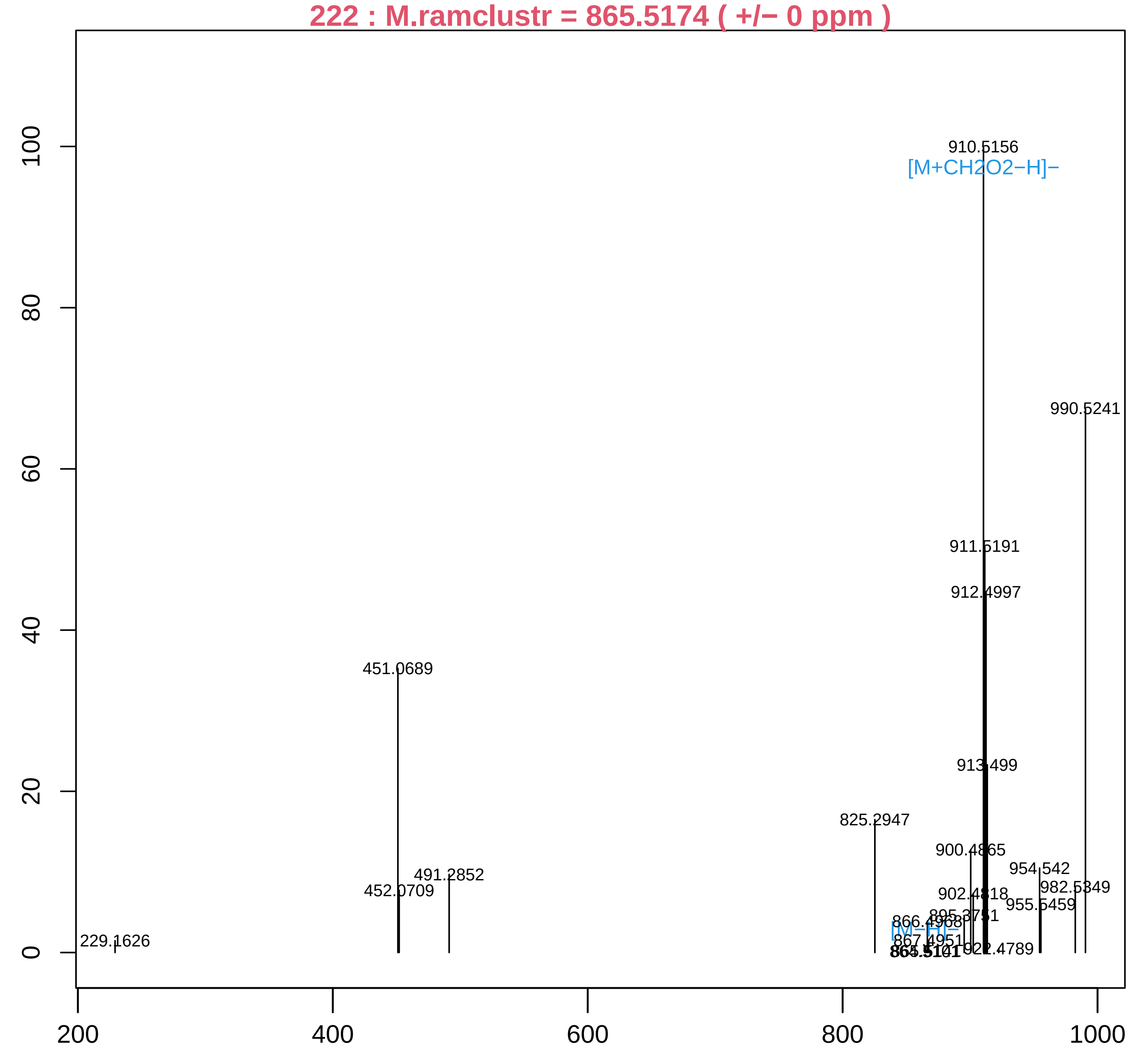

222 : M.ramclustr = 865.5174 (+/− 0 ppm)
910.5156
[M+CH2O2−H]−
990.5241
911.5191
912.4997
451.0689
913.499
825.2947
900.4865
954.542
491.2852
982.5349
452.0709
902.4818
955.5459
895.3751
866.4968
[M−H]−
867.4951
229.1626
865.5161
922.4789
0
20
40
60
80
100
200
400
600
800
1000

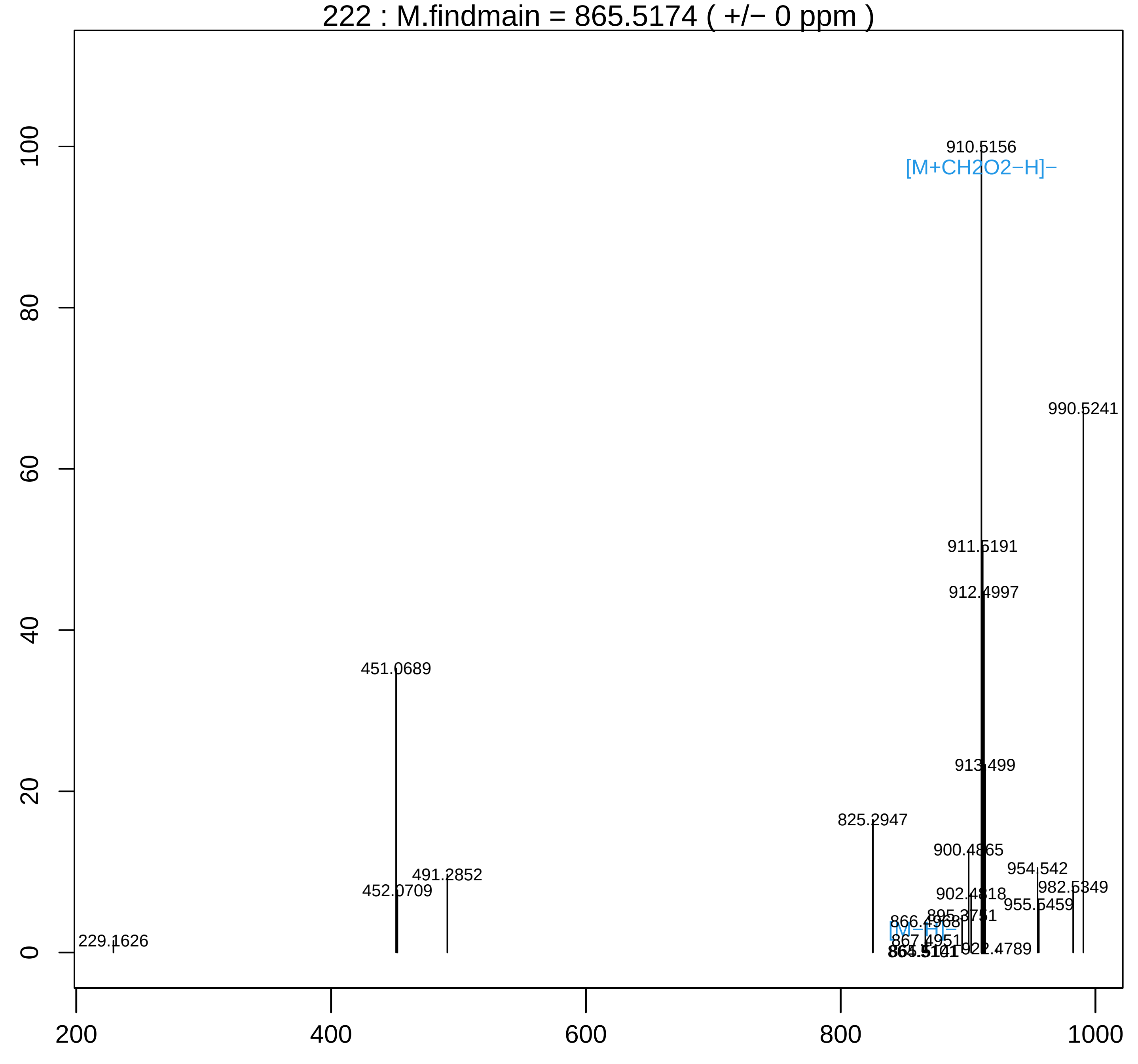

222 : M.findmain = 865.5174 (+/− 0 ppm)
910.5156
[M+CH2O2−H]−
990.5241
911.5191
912.4997
451.0689
913.499
825.2947
900.4865
954.542
491.2852
982.5349
452.0709
902.4818
955.5459
895.3751
866.4968
[M−H]−
867.4951
229.1626
865.5161
922.4789
0
20
40
60
80
100
200
400
600
800
1000

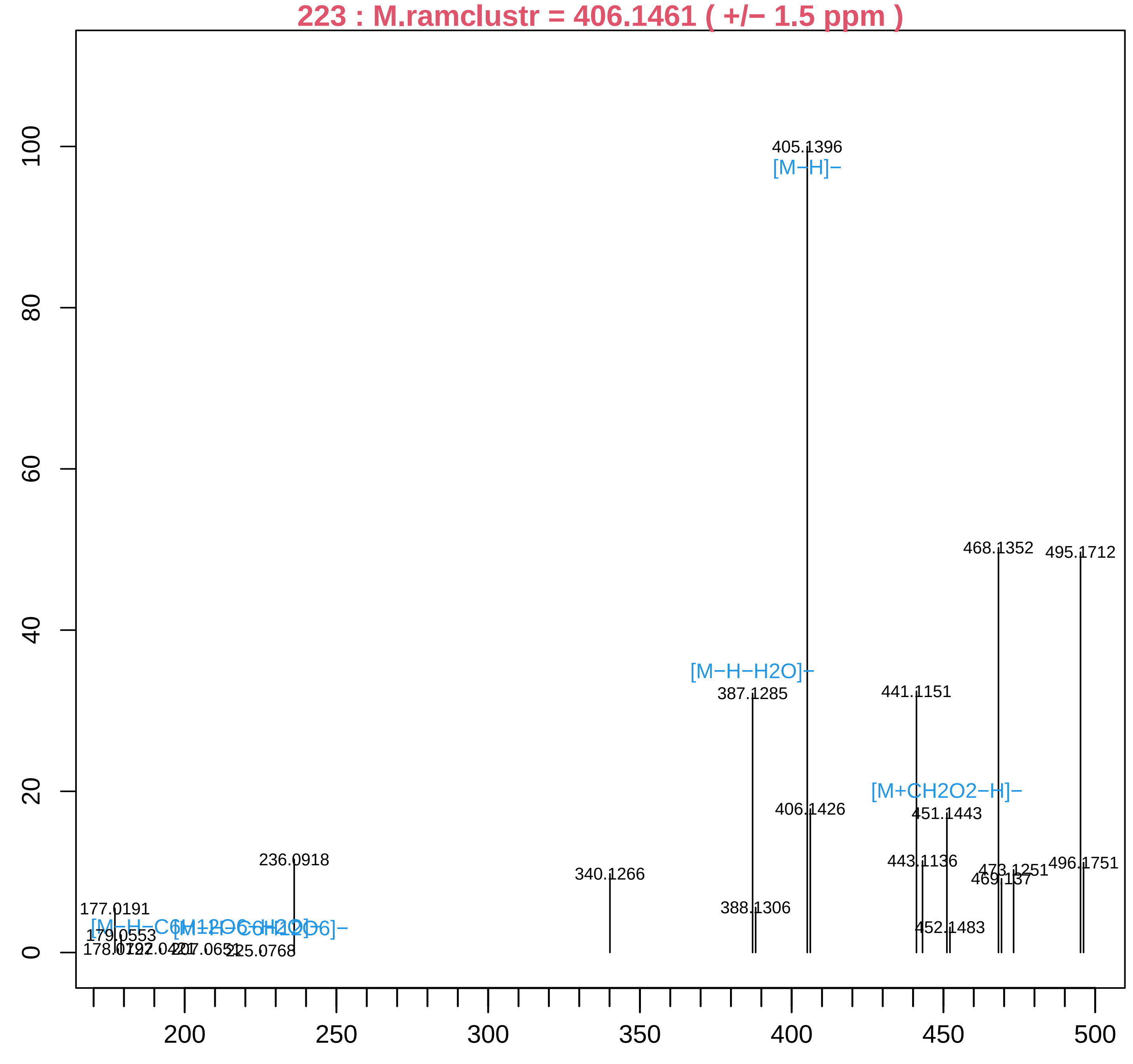
223 : M.ramclustr = 406.1461 (+/− 1.5 ppm)
405.1396
[M−H]−
468.1352
495.1712
[M−H−H2O]−
387.1285
441.1151
[M+CH2O2−H]−
406.1426
451.1443
236.0918
443.1136
473.1251
469.137
496.1751
340.1266
388.1306
177.0191
179.0553
452.1483
225.0768
0
20
40
60
80
100
200
250
300
350
400
450
500
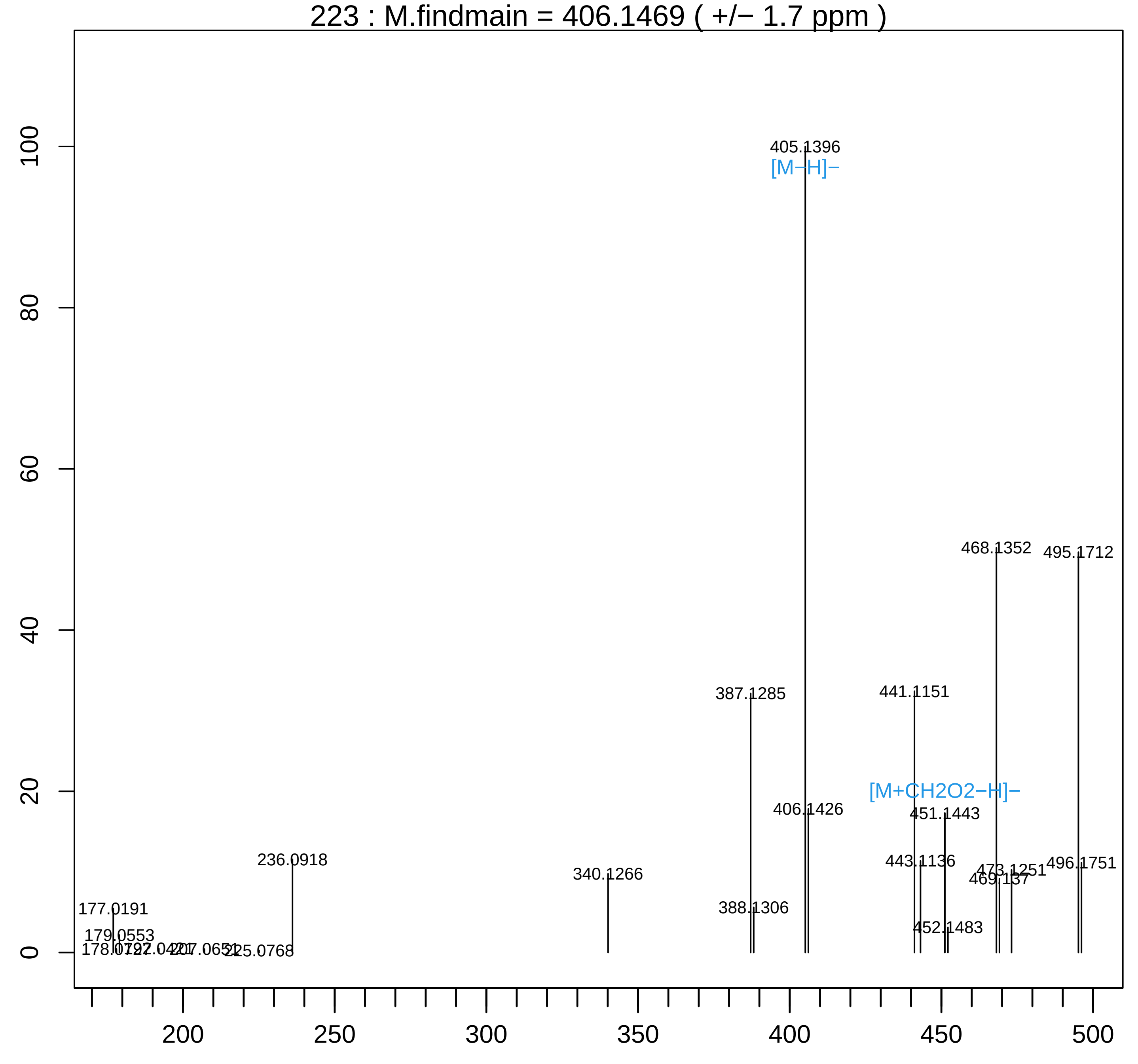
223 : M.findmain = 406.1469 (+/− 1.7 ppm)
405.1396
[M−H]−
468.1352
495.1712
387.1285
441.1151
[M+CH2O2−H]−
406.1426
451.1443
236.0918
443.1136
473.1251
469.137
496.1751
340.1266
388.1306
177.0191
179.0553
452.1483
225.0768
0
20
40
60
80
100
200
250
300
350
400
450
500

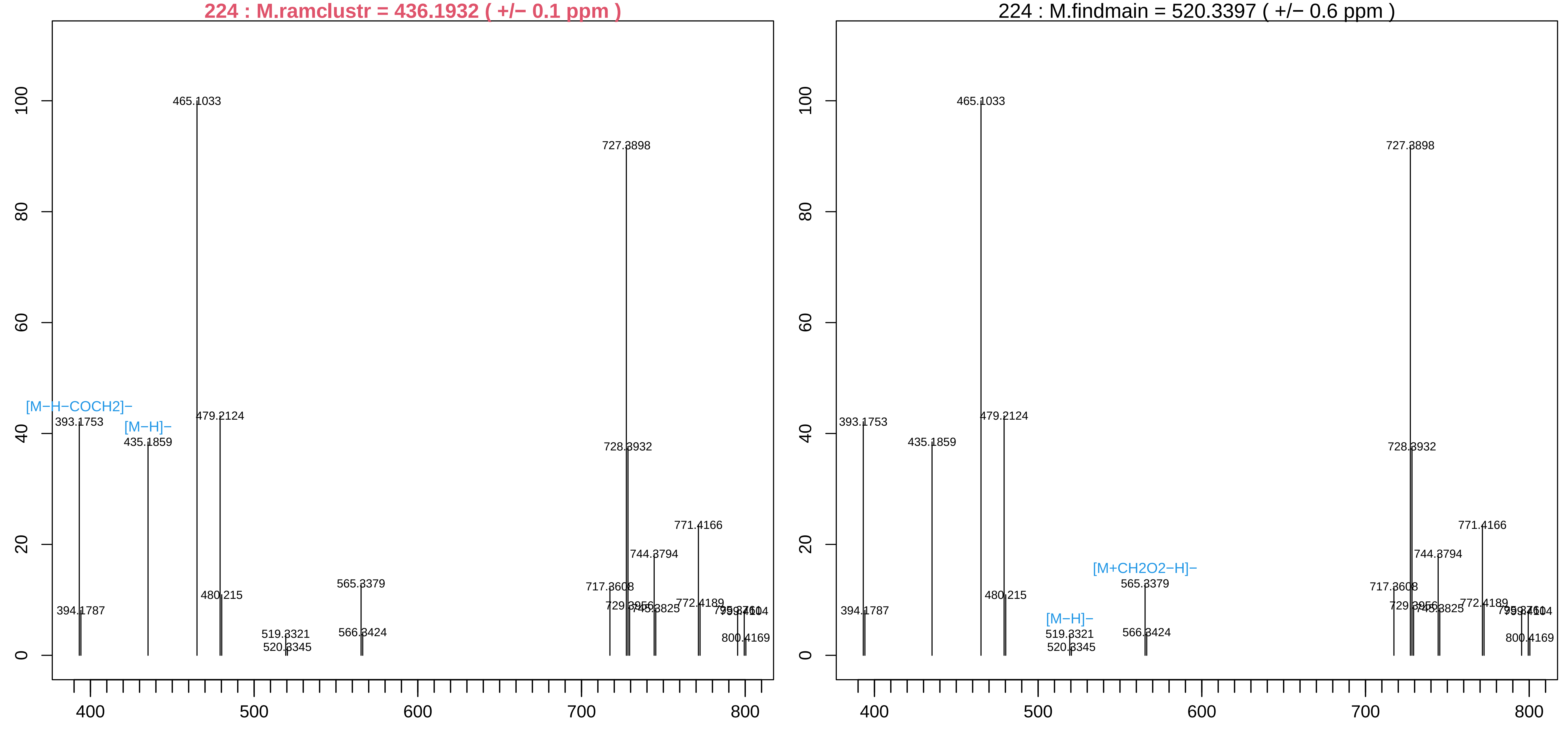
224 : M.ramclustr = 436.1932 (+/− 0.1 ppm)
[M−H−COCH2]−
[M−H]−
393.1753
394.1787
435.1859
465.1033
479.2124
480.215
519.3321
520.3345
565.3379
566.3424
717.3608
727.3898
728.3932
729.3956
744.3794
771.4166
772.4189
800.4169
224 : M.findmain = 520.3397 (+/− 0.6 ppm)
[M−H]−
[M+CH2O2−H]−
393.1753
394.1787
435.1859
465.1033
479.2124
480.215
519.3321
520.3345
565.3379
566.3424
717.3608
727.3898
728.3932
729.3956
744.3794
771.4166
772.4189
800.4169

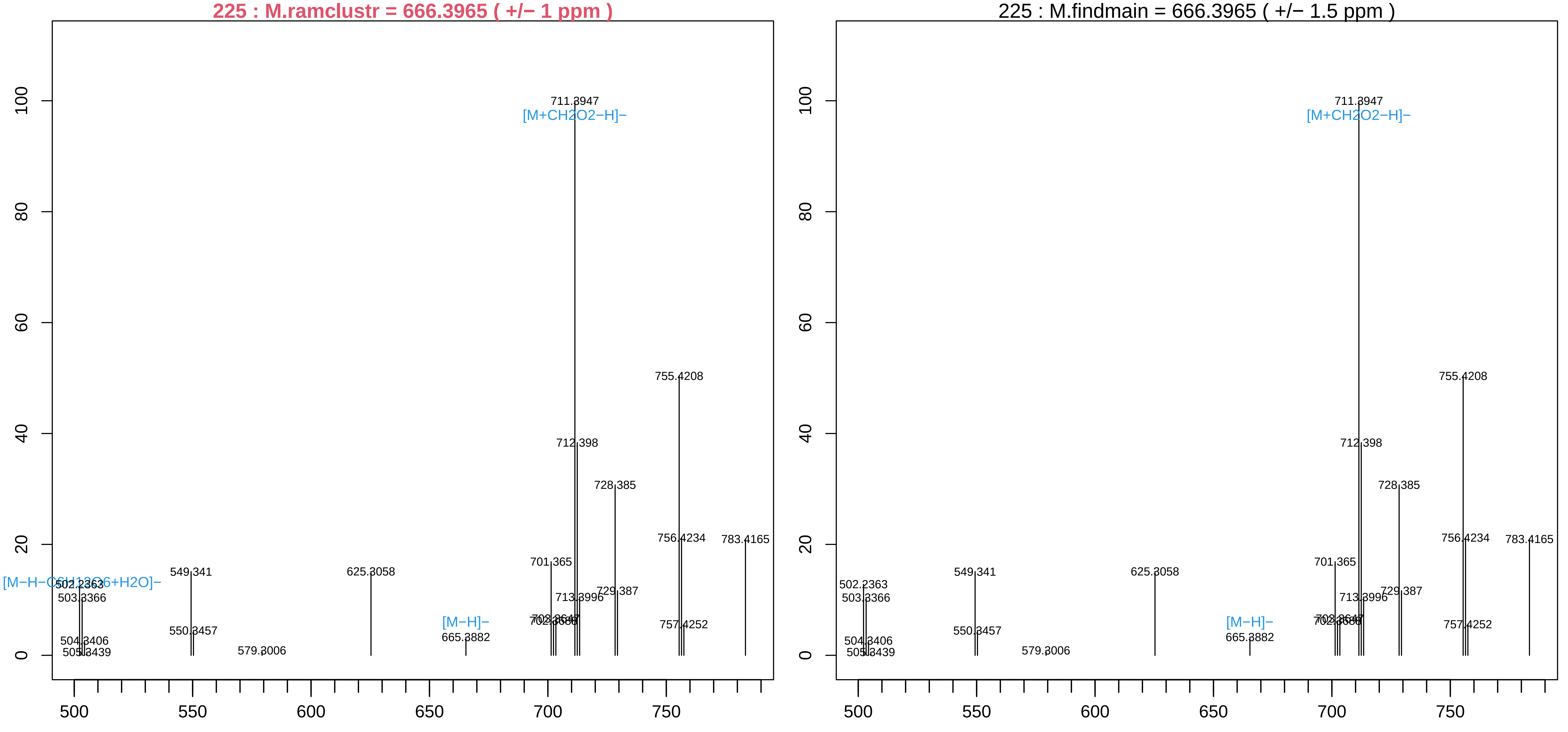
225 : M.ramclustr = 666.3965 (+/− 1 ppm)
[M+CH2O2−H]−
[M−H]−
[M−H−C6H12O6+H2O]−
711.3947
755.4208
712.398
728.385
756.4234
783.4165
701.365
549.341
625.3058
502.2363
503.3366
713.3996
729.387
702.3647
703.3686
550.3457
757.4252
665.3882
504.3406
505.3439
579.3006
225 : M.findmain = 666.3965 (+/− 1.5 ppm)
[M+CH2O2−H]−
[M−H]−
0
20
40
60
80
100
500
550
600
650
700
750

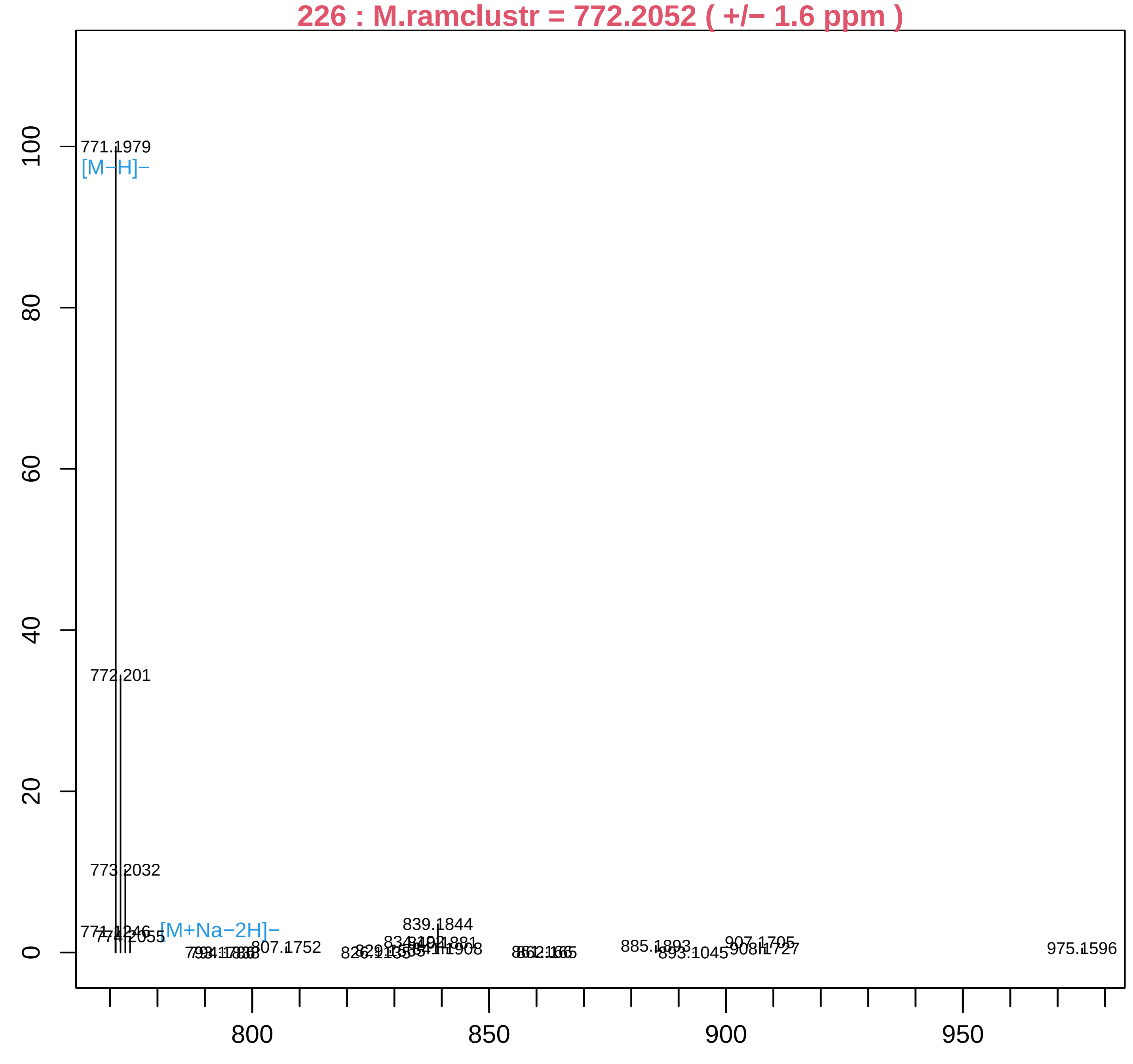

226 : M.ramclustr = 772.2052 (+/− 1.6 ppm)
771.1979
[M−H]−
772.201
773.2032
[M+Na−2H]−
839.1844
807.1752
885.1893
893.1045
907.1705
975.1596
0
20
40
60
80
100
800
850
900
950

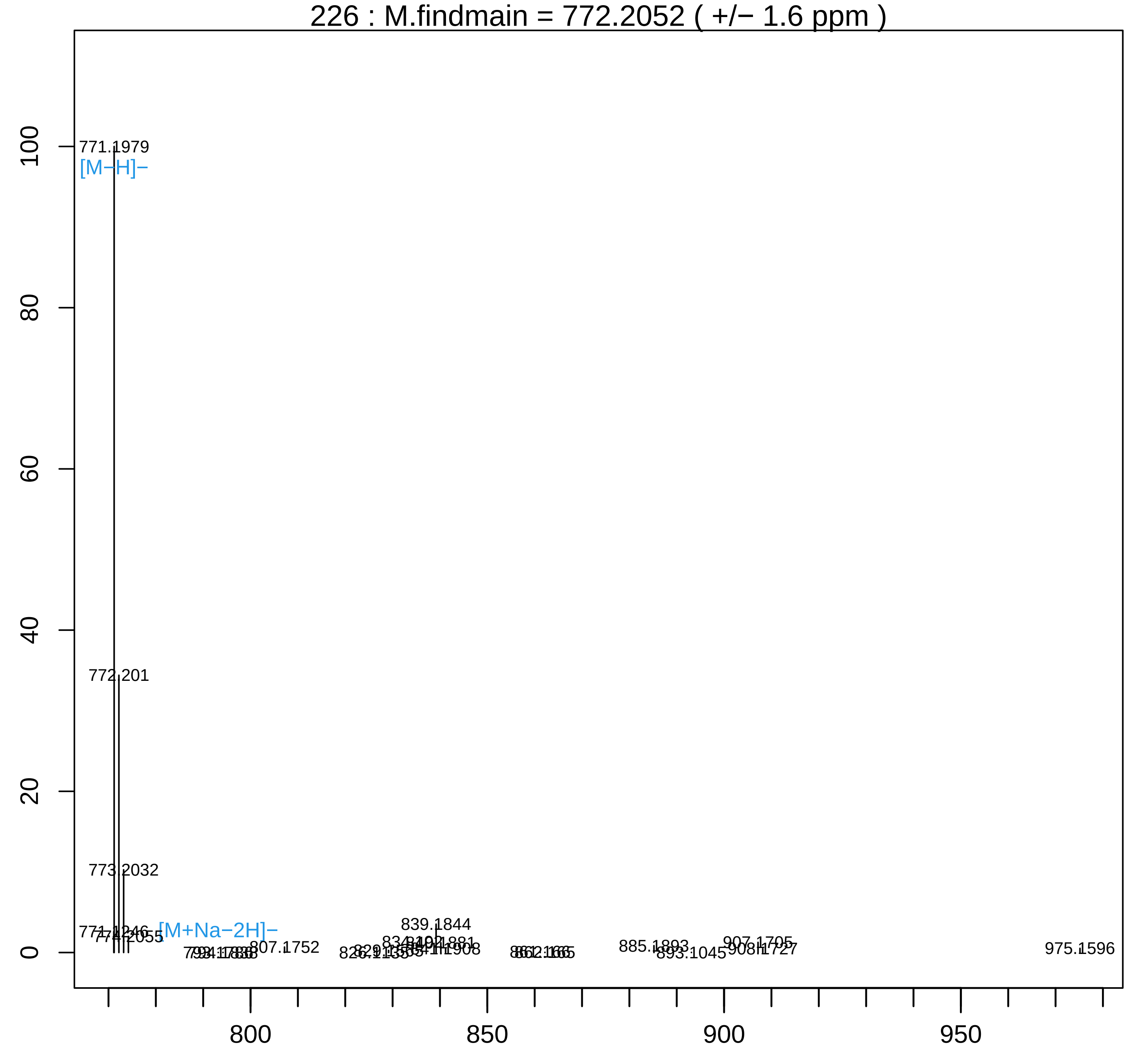

226 : M.findmain = 772.2052 (+/− 1.6 ppm)
771.1979
[M−H]−
772.201
773.2032
[M+Na−2H]−
839.1844
807.1752
885.1893
893.1045
907.1705
975.1596
0
20
40
60
80
100
800
850
900
950

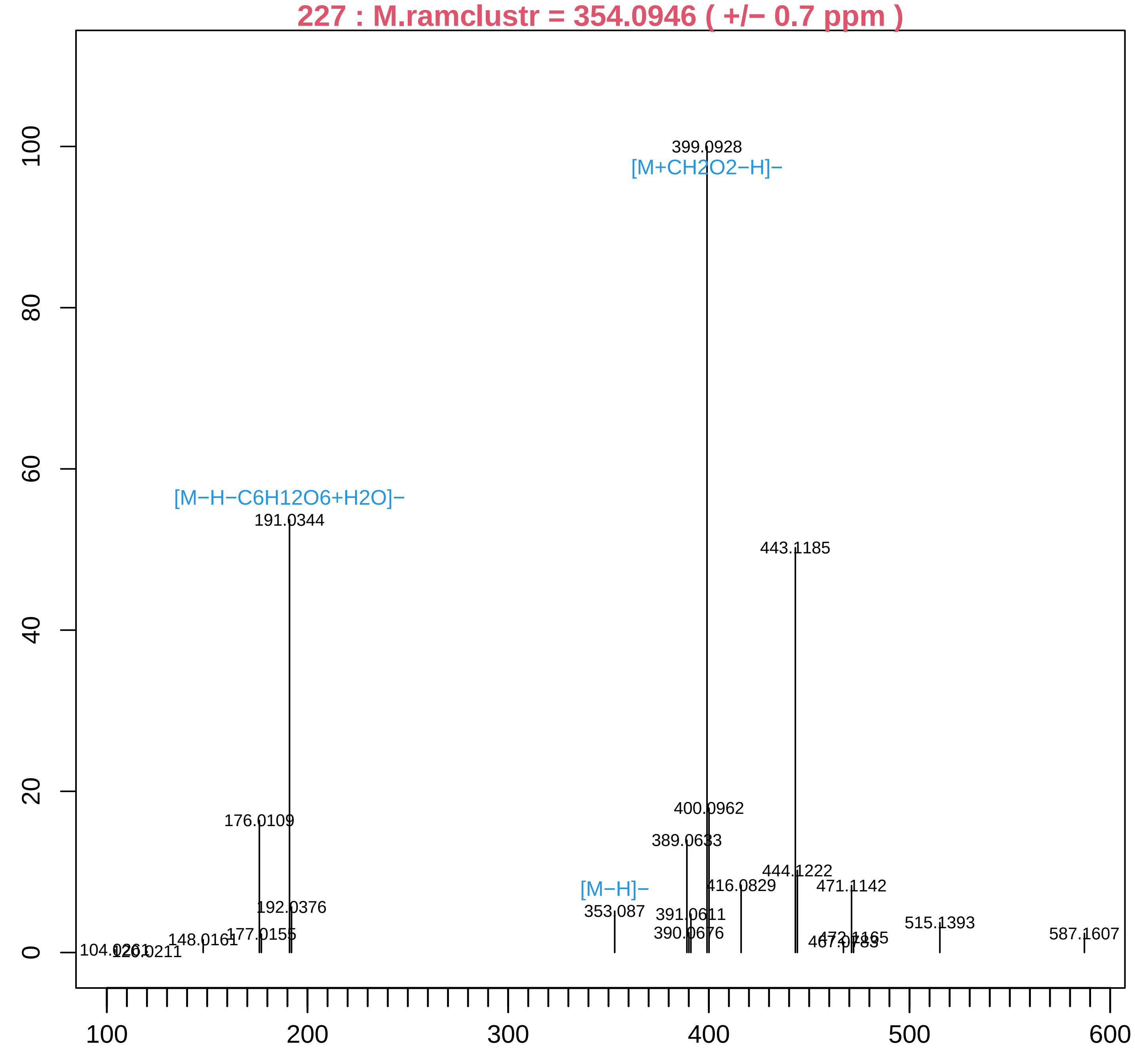
227 : M.ramclustr = 354.0946 (+/− 0.7 ppm)
399.0928
[M+CH2O2−H]−
[M−H−C6H12O6+H2O]−
191.0344
443.1185
176.0109
400.0962
389.0633
444.1222
416.0829
471.1142
192.0376
[M−H]−
353.087
391.0611
390.0676
515.1393
587.1607
148.0161
177.0155
104.0261
120.0211
472.1165
467.0783
0
20
40
60
80
100
100
200
300
400
500
600

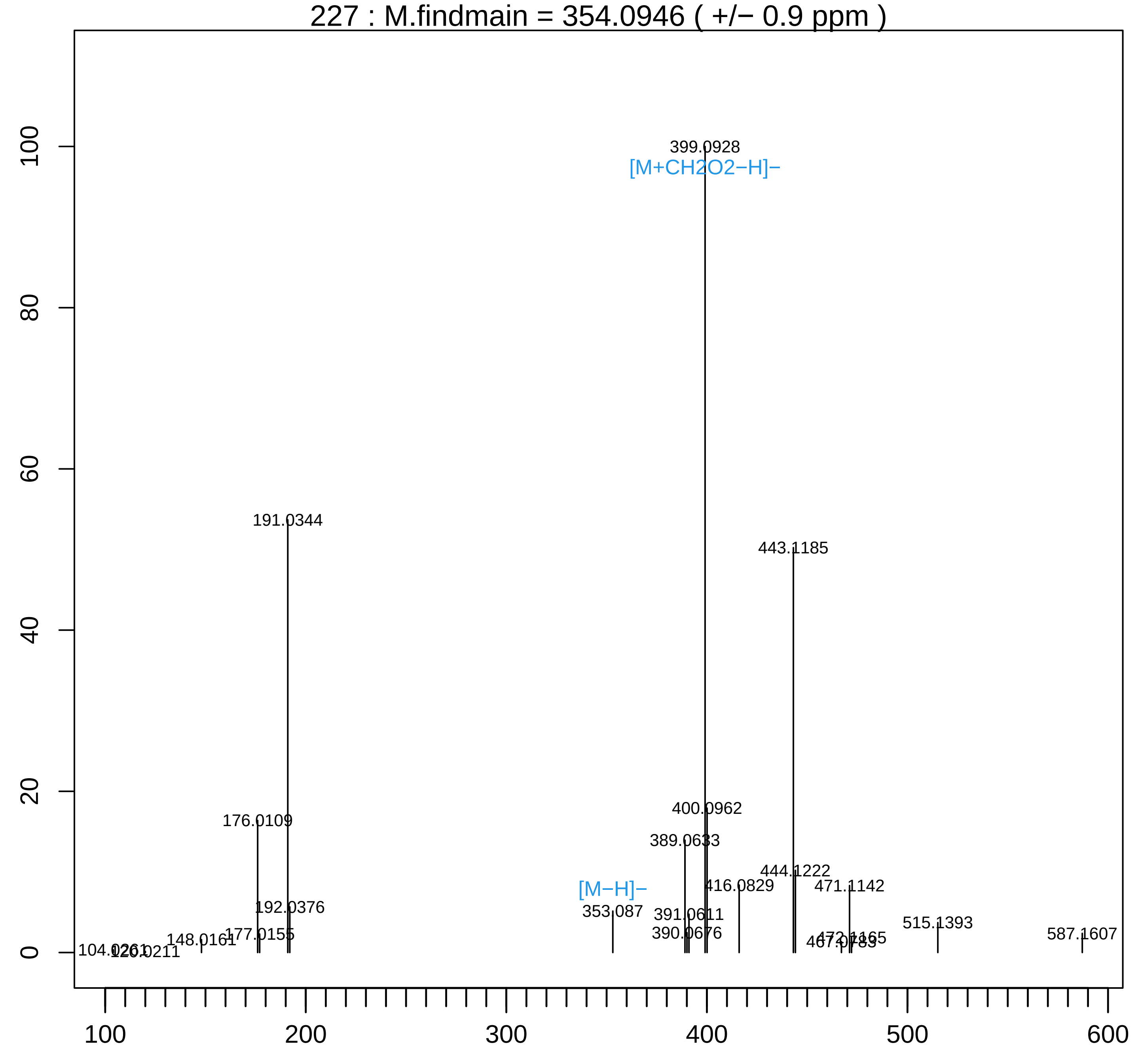
227 : M.findmain = 354.0946 (+/− 0.9 ppm)
399.0928
[M+CH2O2−H]−
191.0344
443.1185
176.0109
400.0962
389.0633
444.1222
416.0829
471.1142
192.0376
[M−H]−
353.087
391.0611
390.0676
515.1393
587.1607
148.0161
177.0155
104.0261
120.0211
472.1165
467.0783
0
20
40
60
80
100
100
200
300
400
500
600

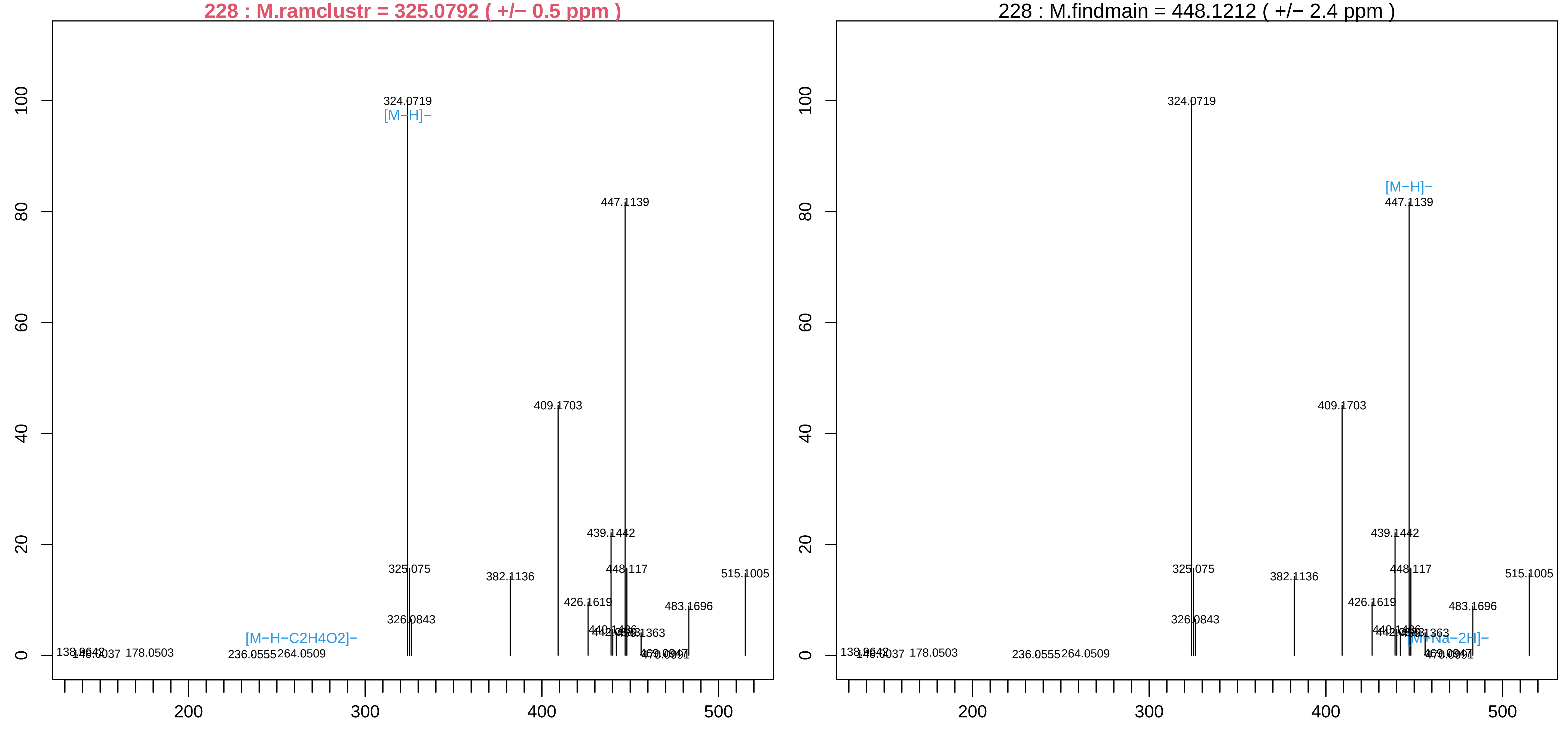

228 : M.ramclustr = 325.0792 (+/− 0.5 ppm)
324.0719
[M−H]−
447.1139
409.1703
439.1442
325.075
382.1136
448.117
515.1005
426.1619
483.1696
326.0843
[M−H−C2H4O2]−
138.9642
178.0503
236.0555
264.0509
228 : M.findmain = 448.1212 (+/− 2.4 ppm)
324.0719
[M−H]−
447.1139
409.1703
439.1442
325.075
382.1136
448.117
515.1005
426.1619
483.1696
326.0843
138.9642
178.0503
236.0555
264.0509

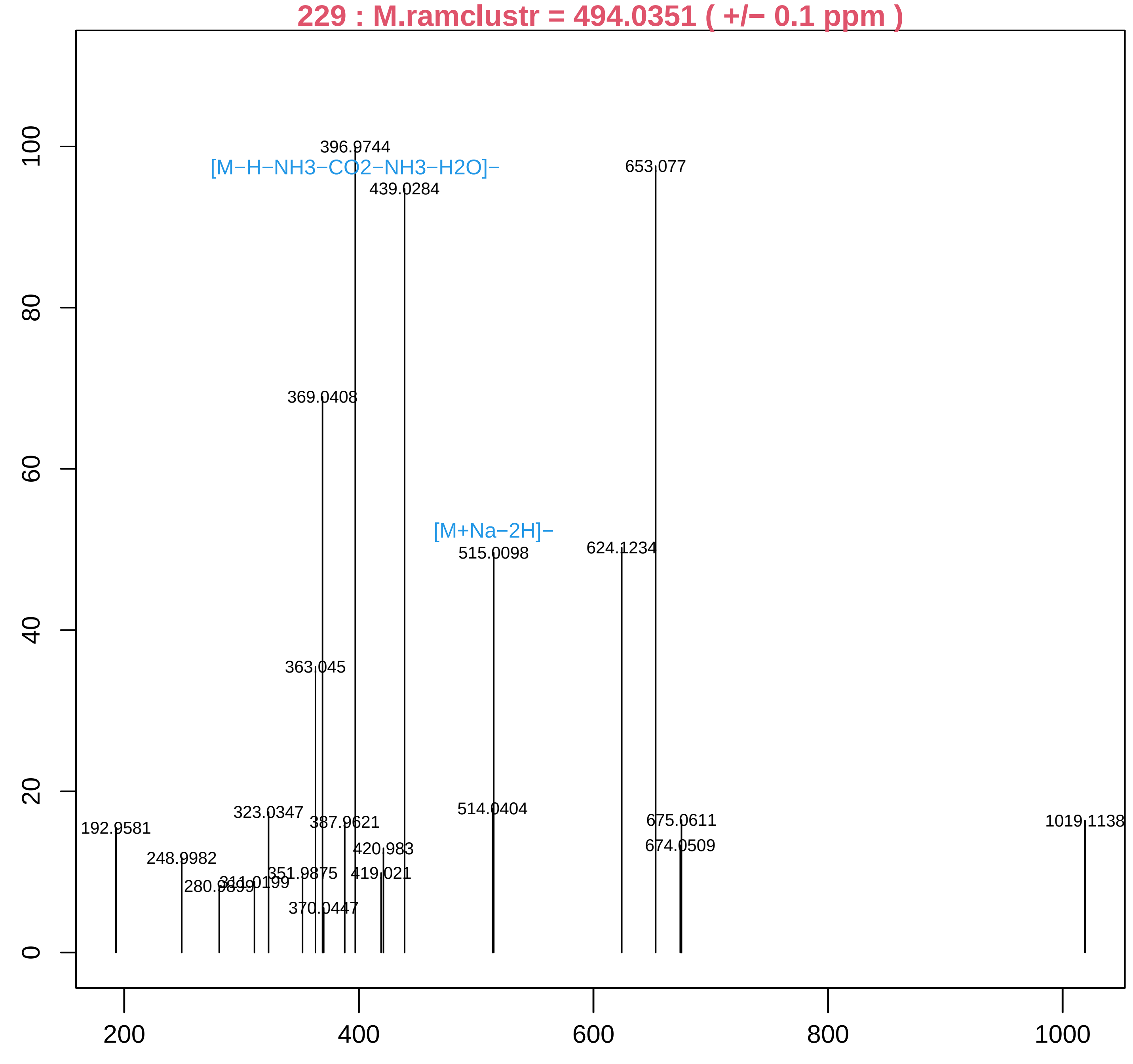
229 : M.ramclustr = 494.0351 (+/− 0.1 ppm)
[M−H−NH3−CO2−NH3−H2O]−
[M+Na−2H]−
396.9744
653.077
439.0284
369.0408
515.0098
624.1234
363.045
192.9581
248.9982
280.9899
311.0199
323.0347
351.9875
370.0447
387.9621
420.983
419.021
514.0404
675.0611
674.0509
1019.1138
0
20
40
60
80
100
200
400
600
800
1000

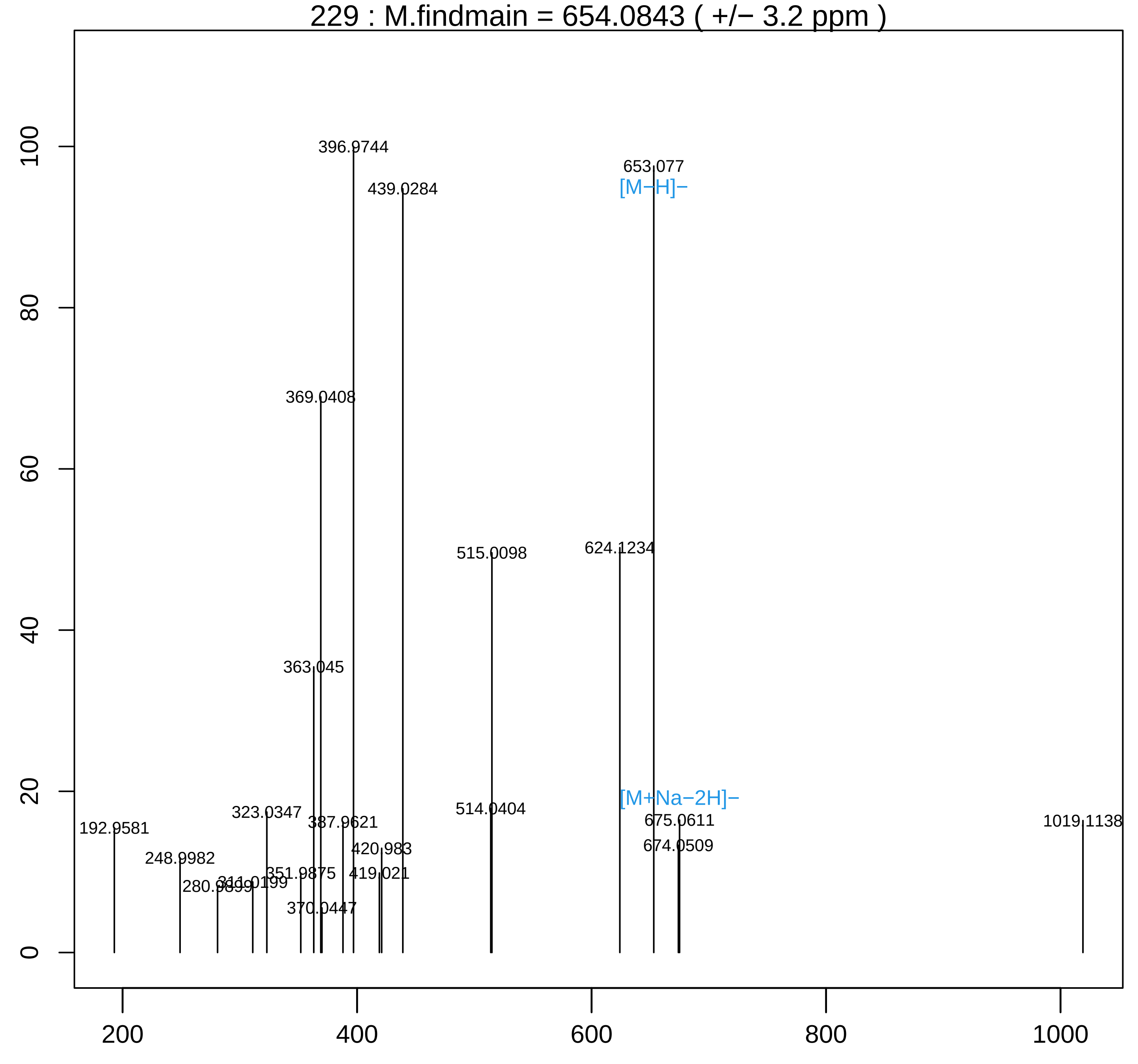
229 : M.findmain = 654.0843 (+/− 3.2 ppm)
[M−H]−
[M+Na−2H]−
396.9744
653.077
439.0284
369.0408
515.0098
624.1234
363.045
192.9581
248.9982
280.9899
311.0199
323.0347
351.9875
370.0447
387.9621
420.983
419.021
514.0404
675.0611
674.0509
1019.1138
0
20
40
60
80
100
200
400
600
800
1000

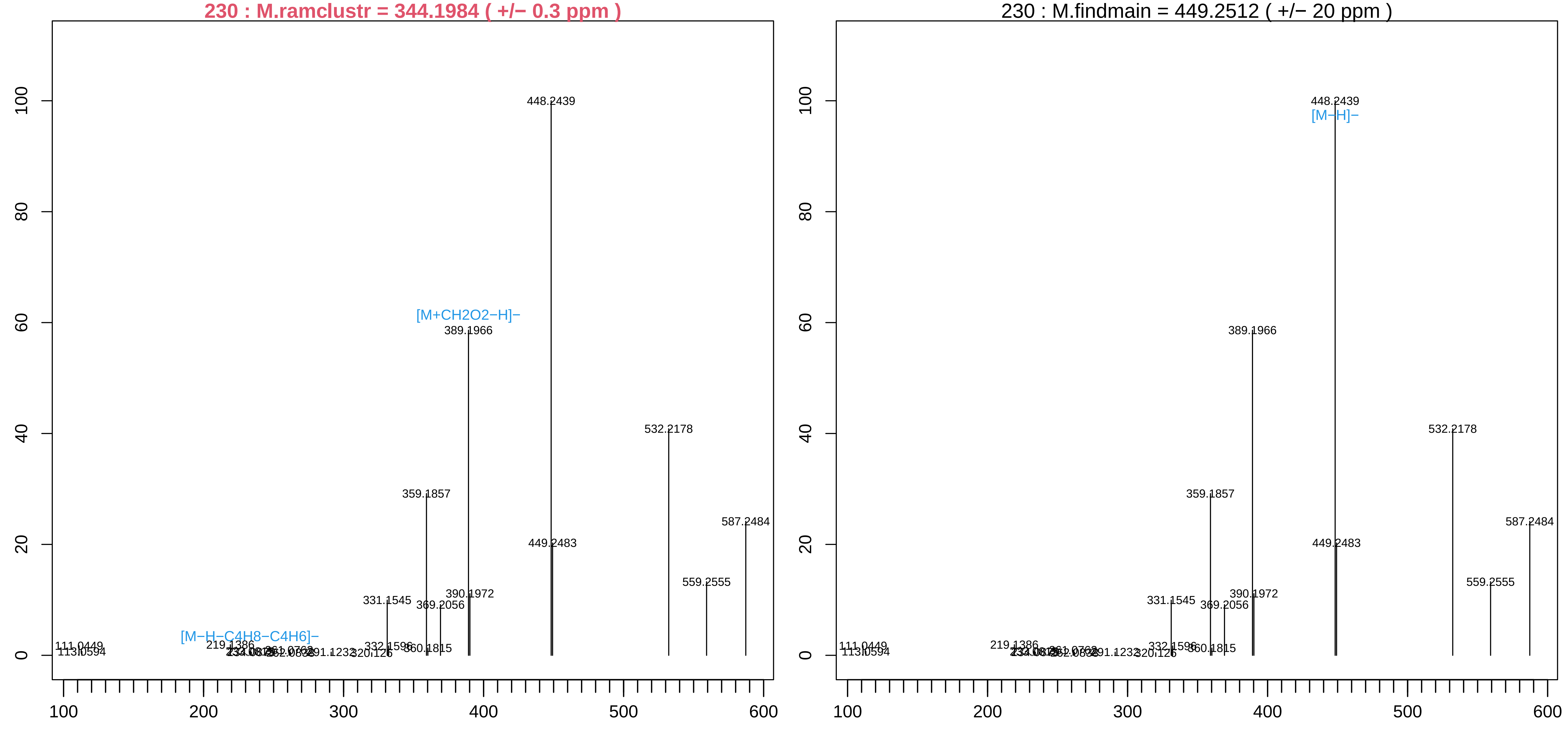
230 : M.ramclustr = 344.1984 (+/− 0.3 ppm)
[M+CH2O2−H]−
[M−H−C4H8−C4H6]−
448.2439
389.1966
359.1857
532.2178
587.2484
449.2483
559.2555
390.1972
331.1545
369.2056
332.1596
360.1815
219.1386
261.0762
291.1232
320.126
111.0449
113.0594
230 : M.findmain = 449.2512 (+/− 20 ppm)
[M−H]−

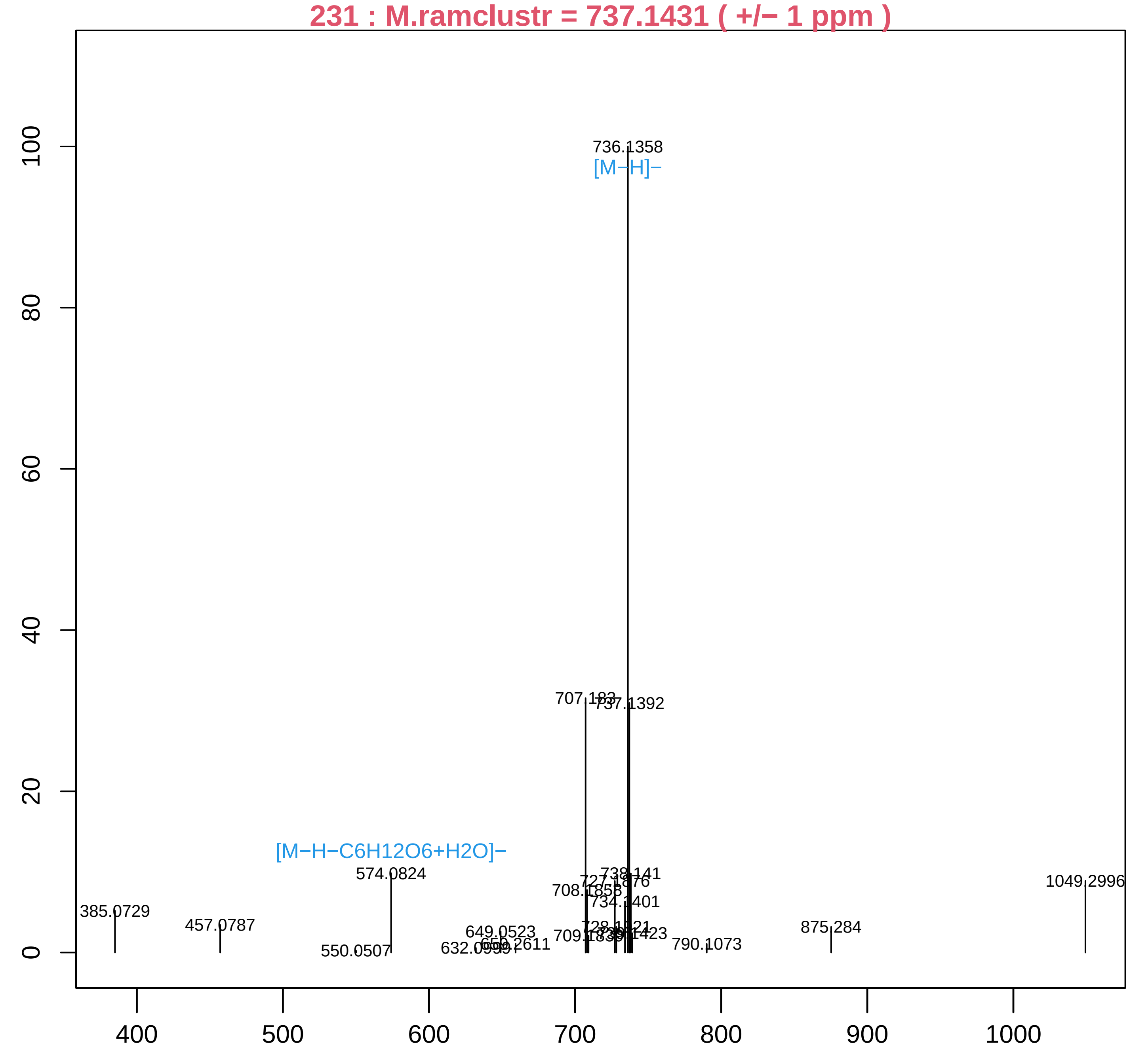
231 : M.ramclustr = 737.1431 (+/− 1 ppm)
736.1358
[M−H]−
707.183
737.1392
[M−H−C6H12O6+H2O]−
574.0824
738.141
708.1855
734.1401
385.0729
457.0787
550.0507
649.0523
632.0599
659.2611
709.1839
790.1073
875.284
1049.2996
0
20
40
60
80
100
400
500
600
700
800
900
1000

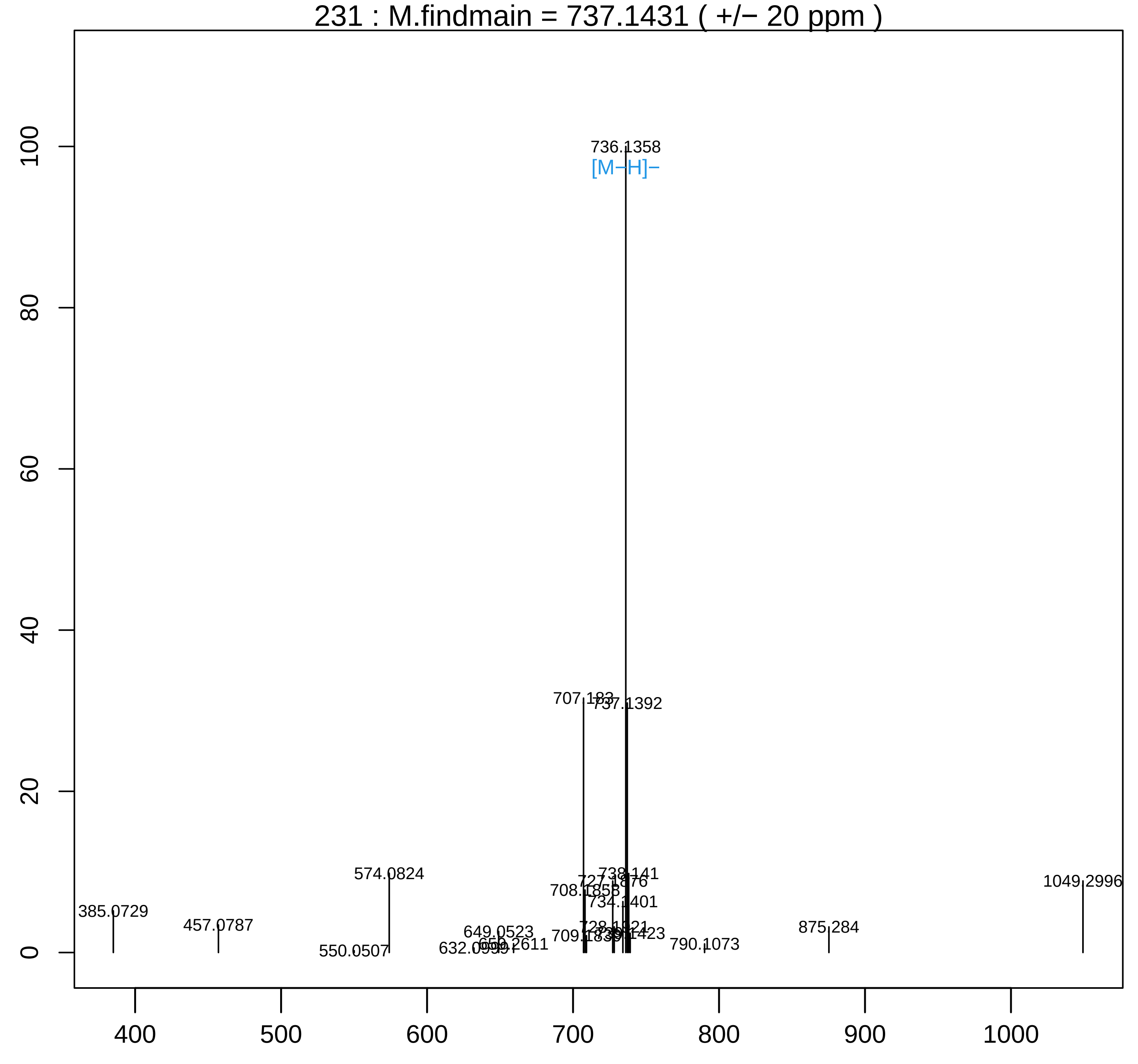
231 : M.findmain = 737.1431 (+/− 20 ppm)
736.1358
[M−H]−
707.183
737.1392
574.0824
738.141
708.1855
734.1401
385.0729
457.0787
550.0507
649.0523
632.0599
659.2611
709.1839
790.1073
875.284
1049.2996
0
20
40
60
80
100
400
500
600
700
800
900
1000

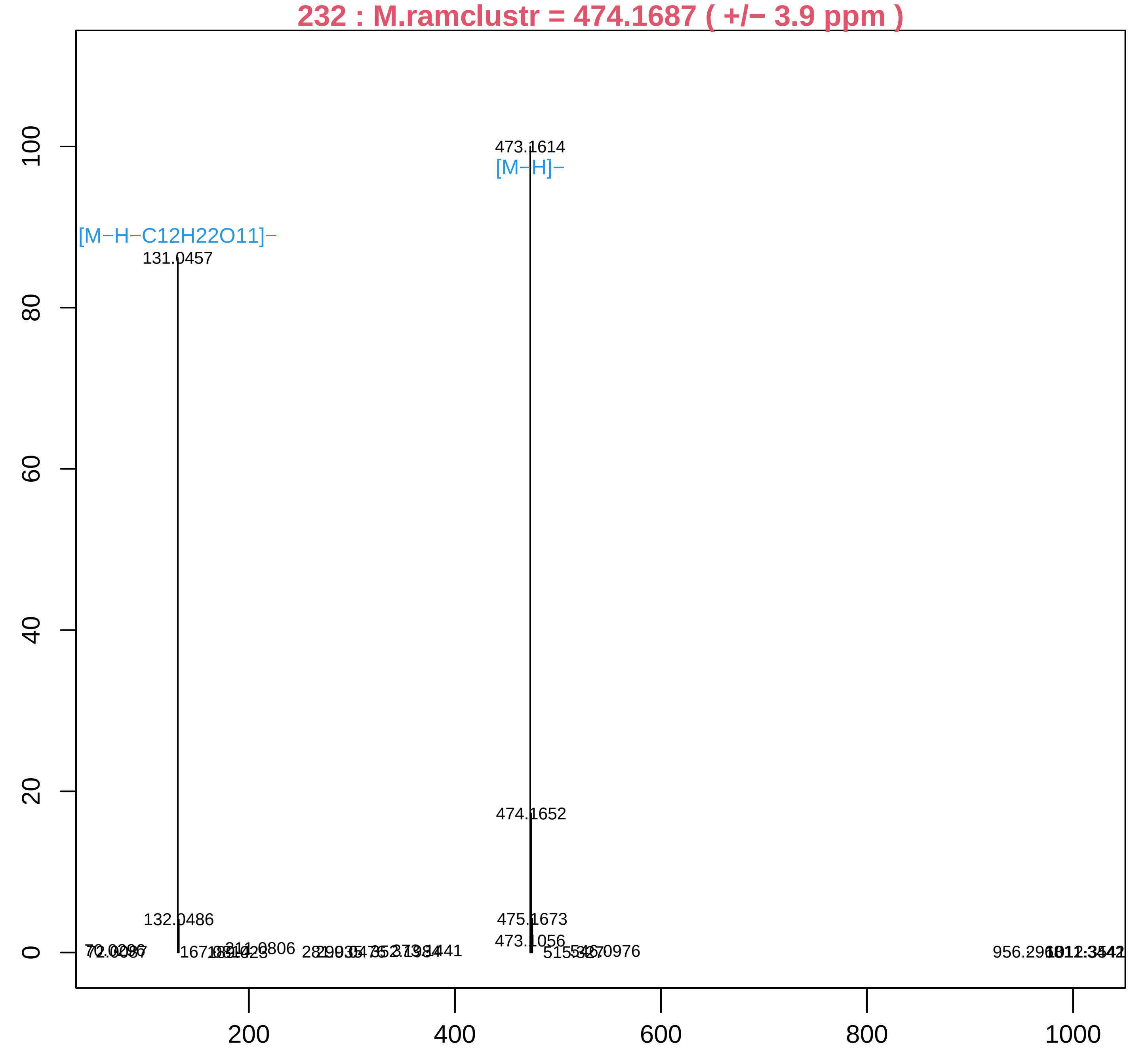
232 : M.ramclustr = 474.1687 (+/− 3.9 ppm)
473.1614
[M−H]−
[M−H−C12H22O11]−
131.0457
474.1652
132.0486
475.1673
473.1056
0
20
40
60
80
100
200
400
600
800
1000

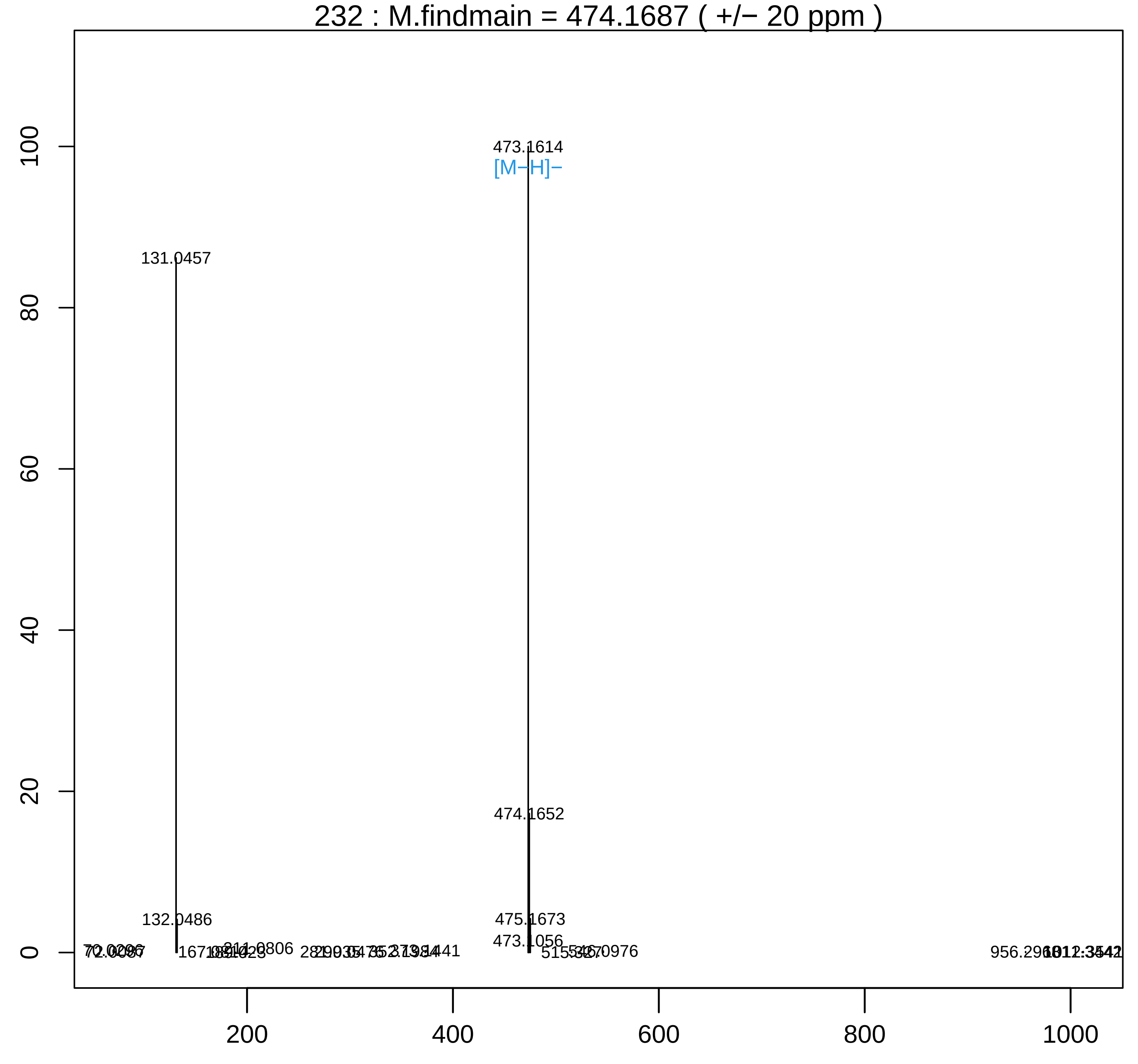
232 : M.findmain = 474.1687 (+/− 20 ppm)
473.1614
[M−H]−
131.0457
474.1652
132.0486
475.1673
473.1056
0
20
40
60
80
100
200
400
600
800
1000

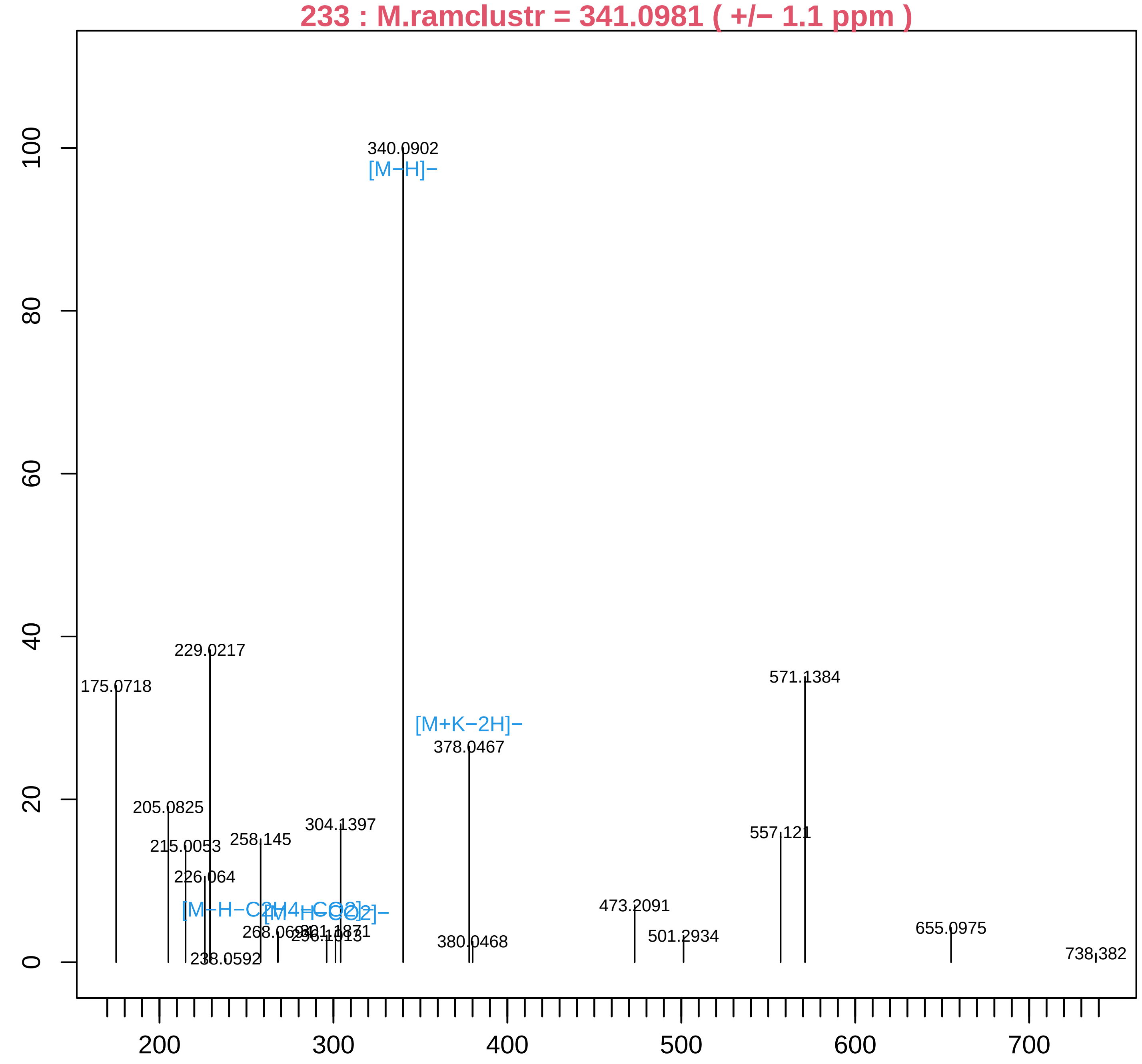

233 : M.ramclustr = 341.0981 (+/− 1.1 ppm)
340.0902
[M−H]−
229.0217
175.0718
571.1384
[M+K−2H]−
378.0467
205.0825
304.1397
258.145
215.0053
557.121
226.064
473.2091
655.0975
501.2934
380.0468
238.0592
738.382
0
20
40
60
80
100
200
300
400
500
600
700

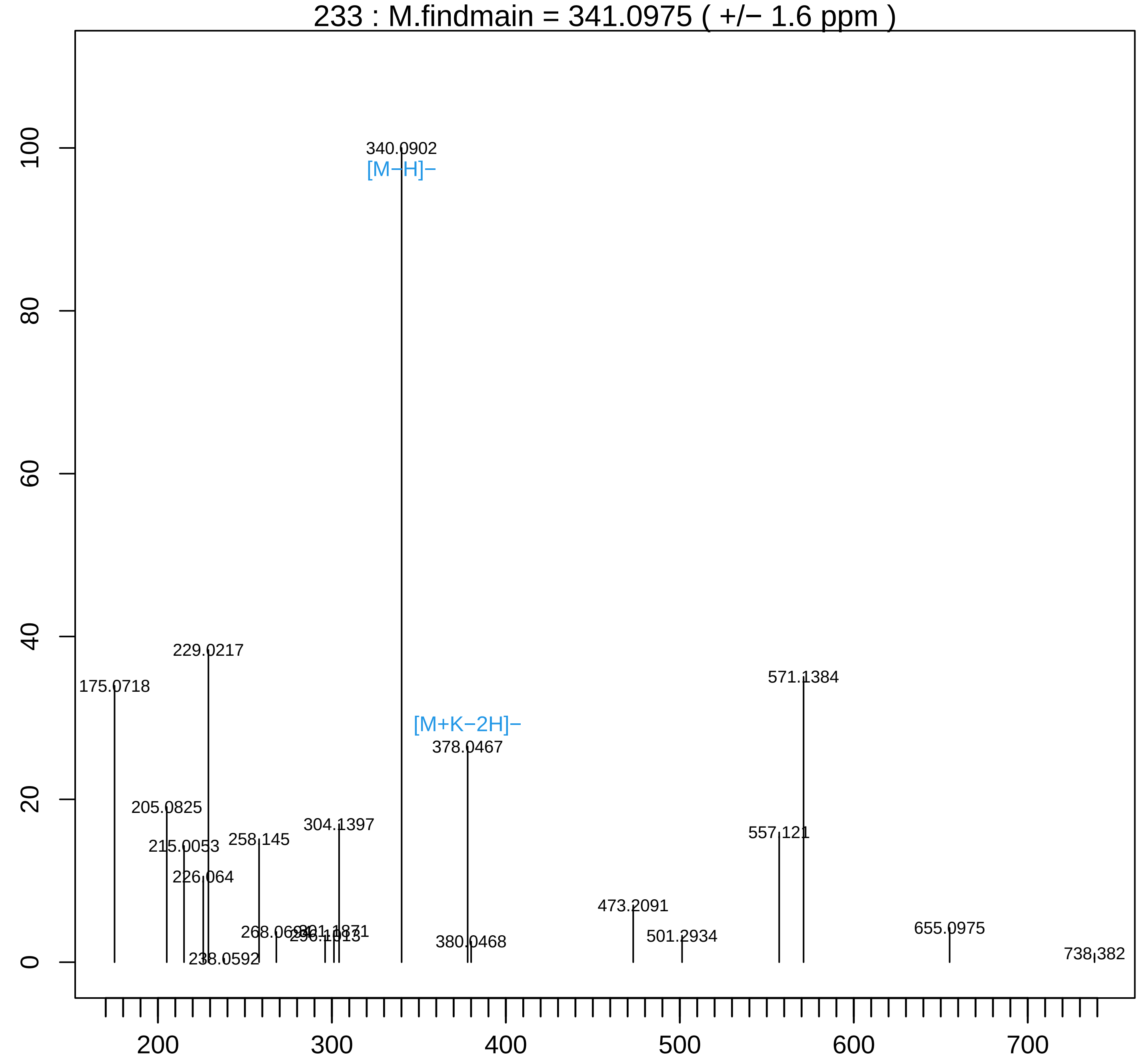

233 : M.findmain = 341.0975 (+/− 1.6 ppm)
340.0902
[M−H]−
229.0217
175.0718
571.1384
[M+K−2H]−
378.0467
205.0825
304.1397
258.145
215.0053
557.121
226.064
473.2091
655.0975
501.2934
380.0468
238.0592
738.382
0
20
40
60
80
100
200
300
400
500
600
700

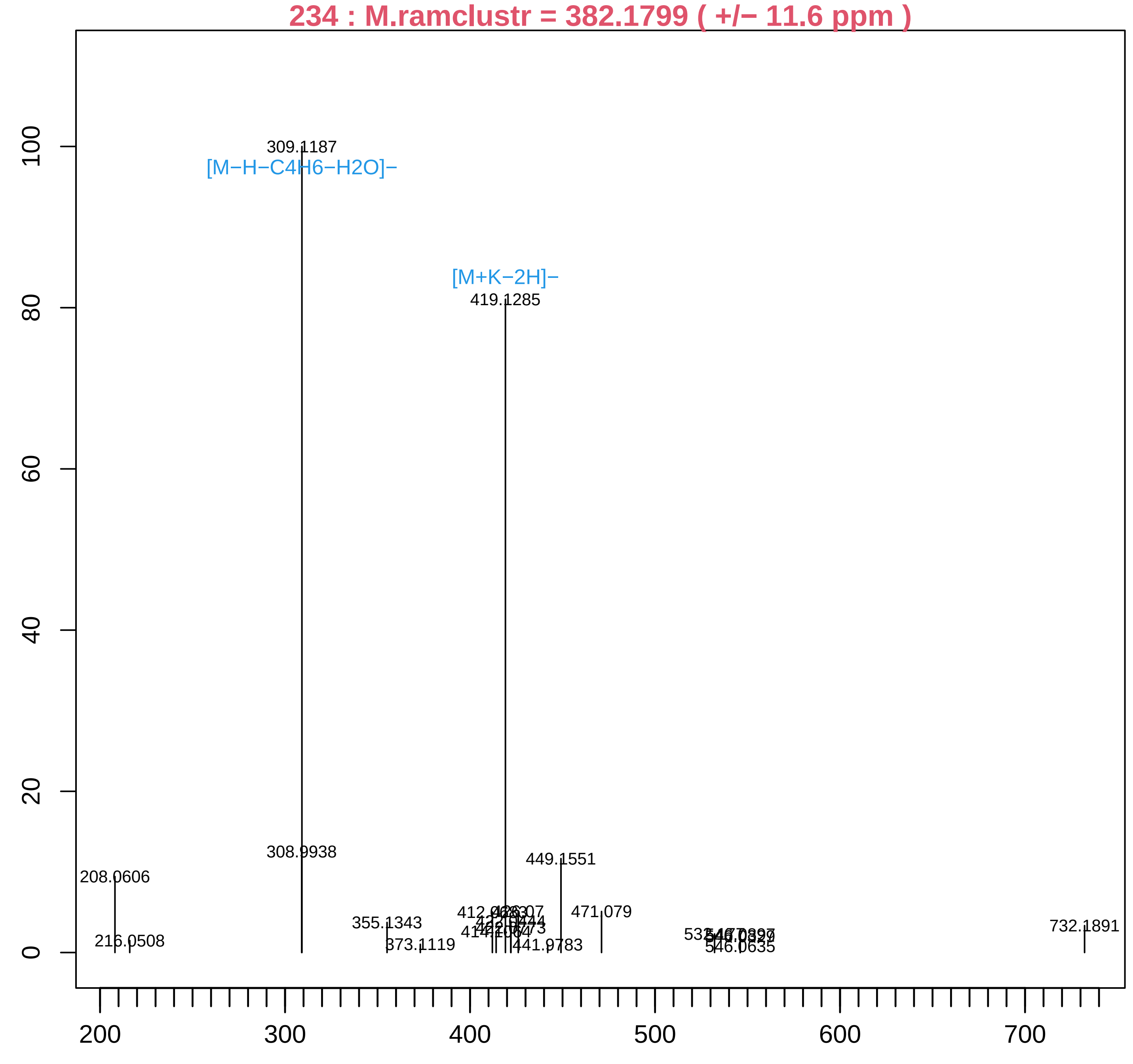
234 : M.ramclustr = 382.1799 (+/− 11.6 ppm)
309.1187
[M−H−C4H6−H2O]−
[M+K−2H]−
419.1285
449.1551
471.079
308.9938
208.0606
216.0508
355.1343
373.1119
441.9783
732.1891
546.0635
0
20
40
60
80
100
200
300
400
500
600
700

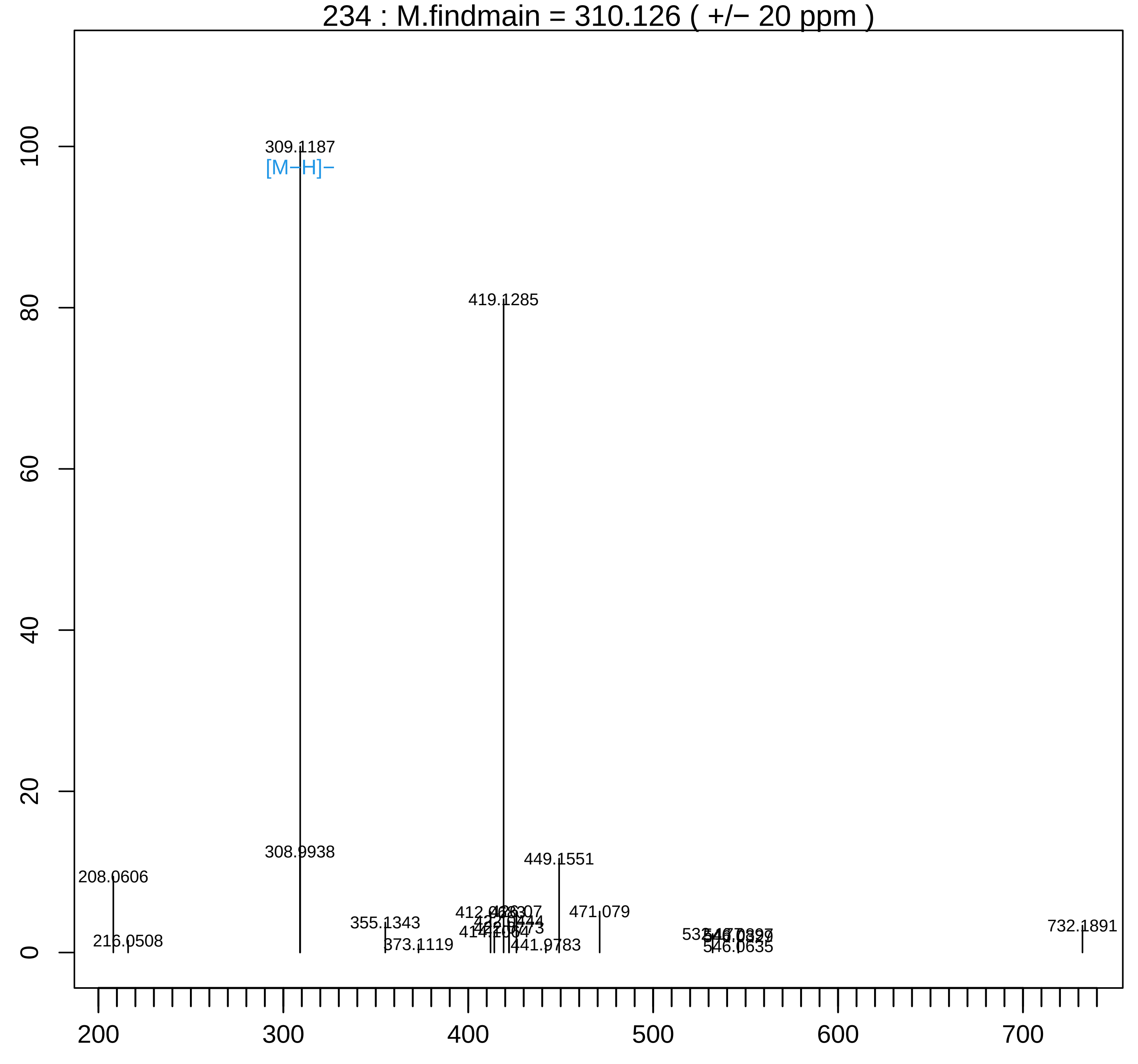
234 : M.findmain = 310.126 (+/− 20 ppm)
309.1187
[M−H]−
419.1285
449.1551
471.079
308.9938
208.0606
216.0508
355.1343
373.1119
441.9783
732.1891
546.0635
0
20
40
60
80
100
200
300
400
500
600
700

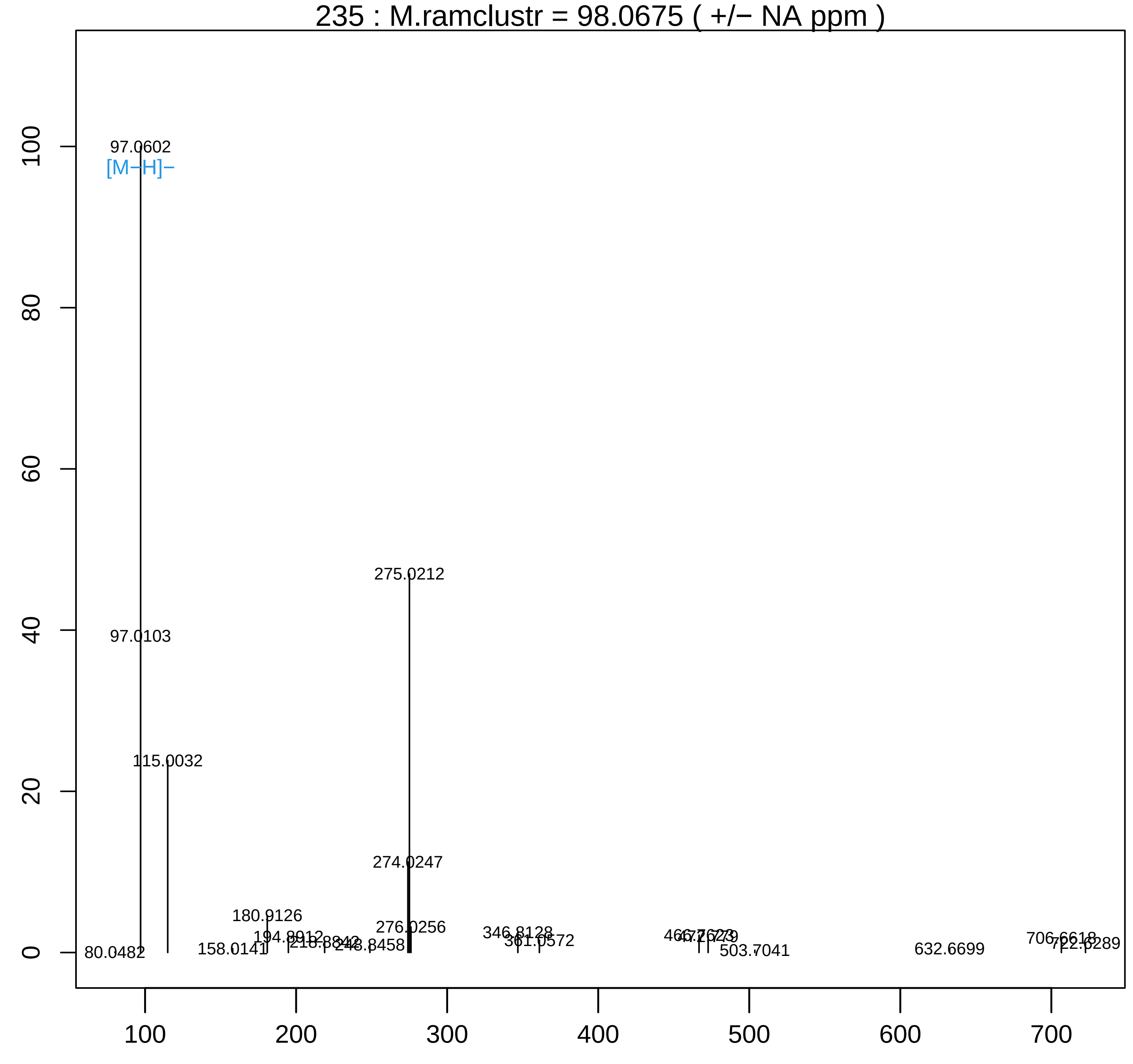

235 : M.ramclustr = 98.0675 (+/− NA ppm)
97.0602
[M−H]−
97.0103
115.0032
275.0212
274.0247
276.0256
180.9126
194.8912
216.8842
248.8458
158.0141
80.0482
346.8128
361.0572
466.7629
503.7041
632.6699
706.6618
722.6289
0
20
40
60
80
100
100
200
300
400
500
600
700

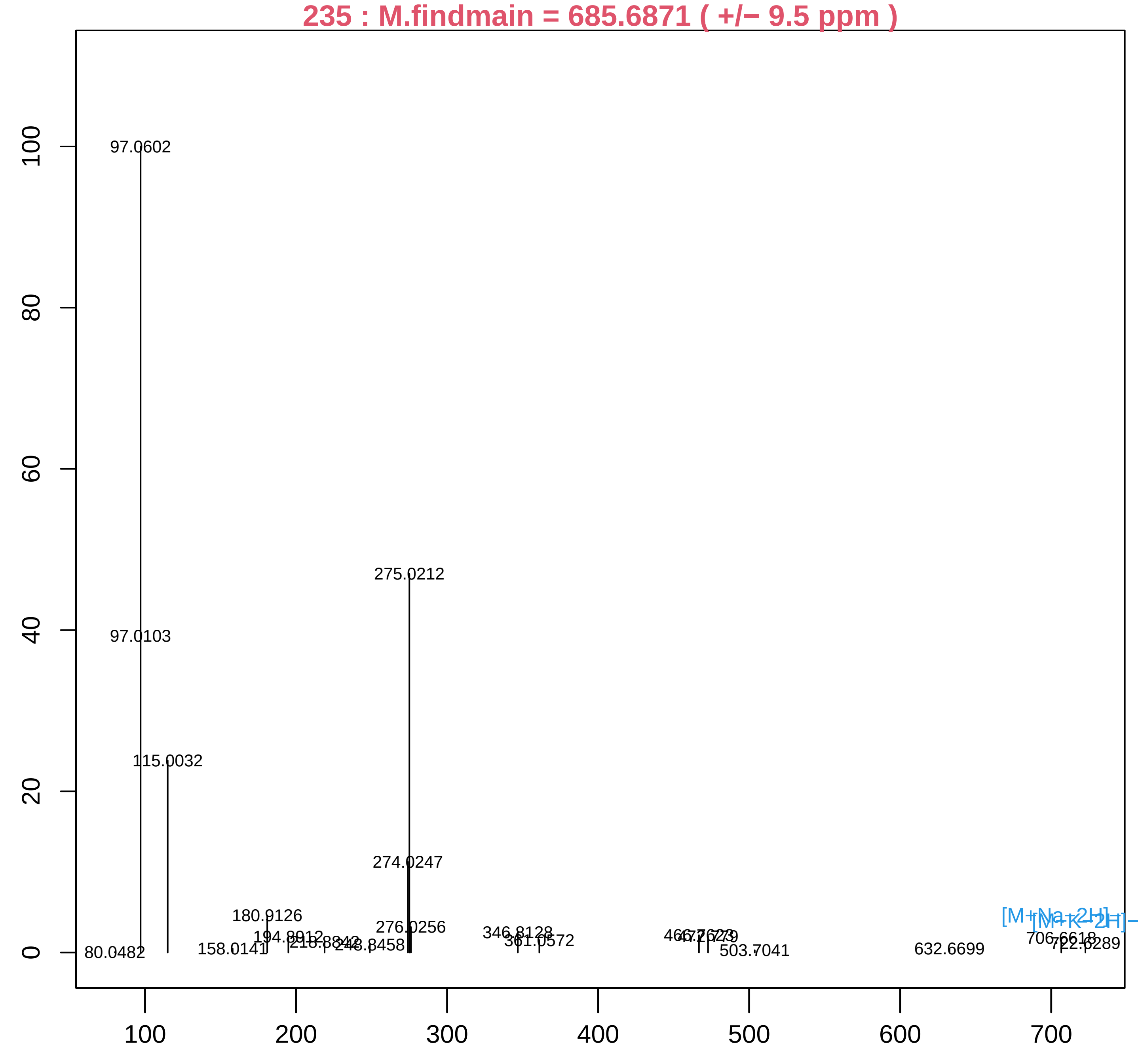

235 : M.findmain = 685.6871 (+/− 9.5 ppm)
97.0602
97.0103
115.0032
275.0212
274.0247
276.0256
180.9126
194.8912
216.8842
248.8458
158.0141
80.0482
346.8128
361.0572
466.7629
503.7041
632.6699
706.6618
722.6289
[M+Na−2H]−
[M+K−2H]−
0
20
40
60
80
100
100
200
300
400
500
600
700

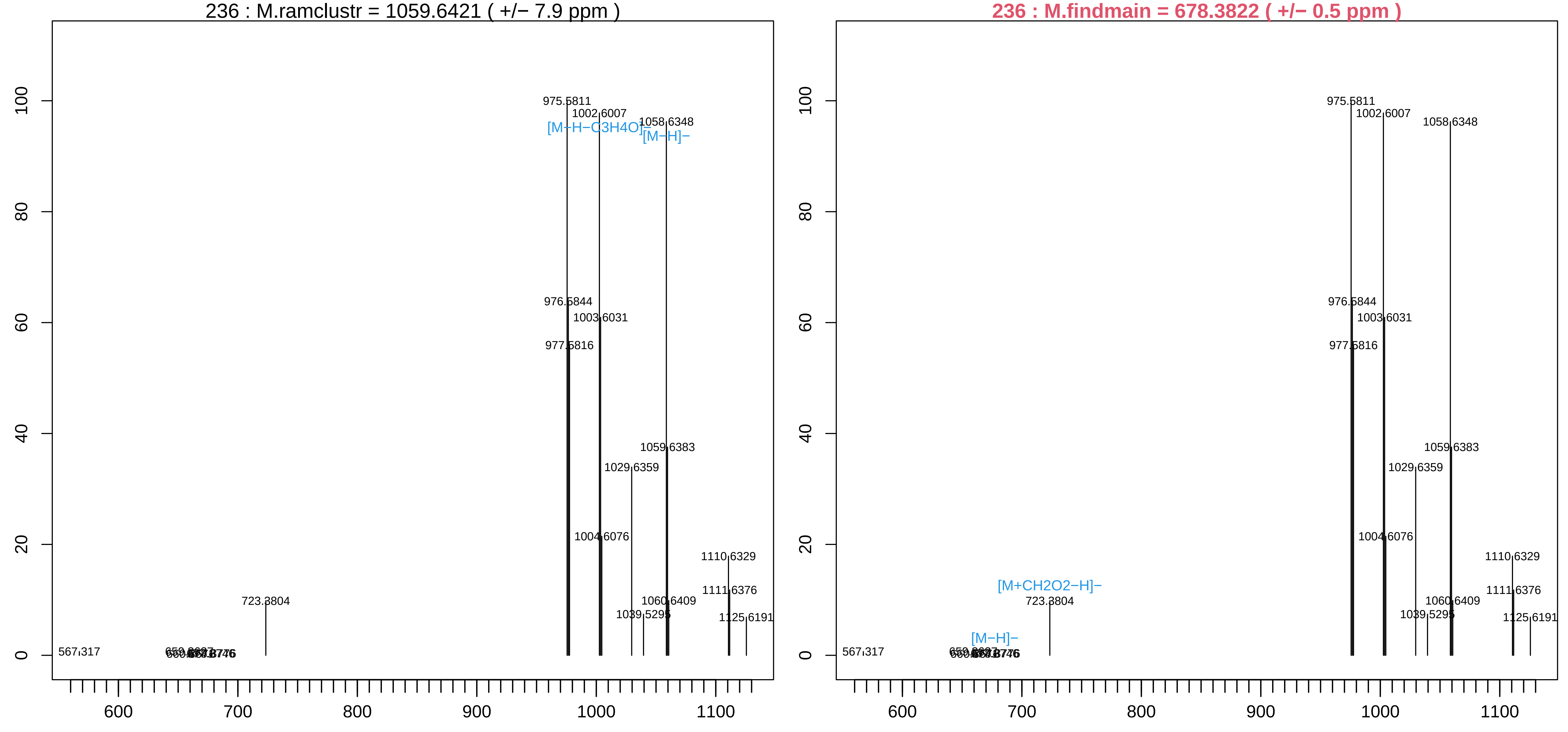

236 : M.ramclustr = 1059.6421 (+/− 7.9 ppm)
236 : M.findmain = 678.3822 (+/− 0.5 ppm)
975.5811
1002.6007
1058.6348
[M−H−C3H4O]−
[M−H]−
976.5844
1003.6031
977.5816
1059.6383
1029.6359
1004.6076
1110.6329
1111.6376
723.3804
1060.6409
1039.5295
1125.6191
567.317
[M+CH2O2−H]−
0
20
40
60
80
100
600
700
800
900
1000
1100

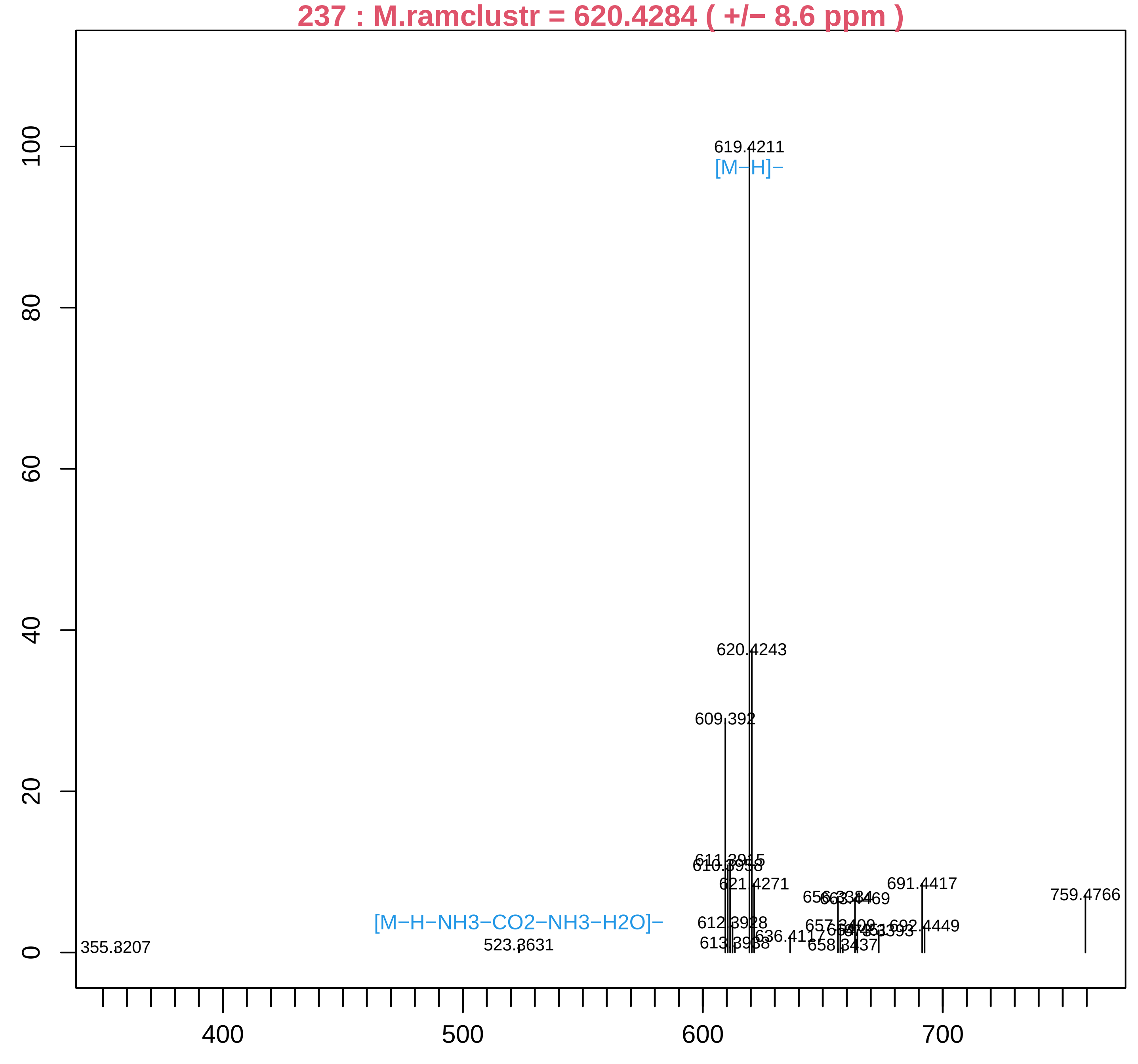
237 : M.ramclustr = 620.4284 (+/− 8.6 ppm)
619.4211
[M−H]−
620.4243
609.392
691.4417
759.4766
612.3928
[M−H−NH3−CO2−NH3−H2O]−
523.3631
355.3207
692.4449
658.3437
636.4117
0
20
40
60
80
100
400
500
600
700

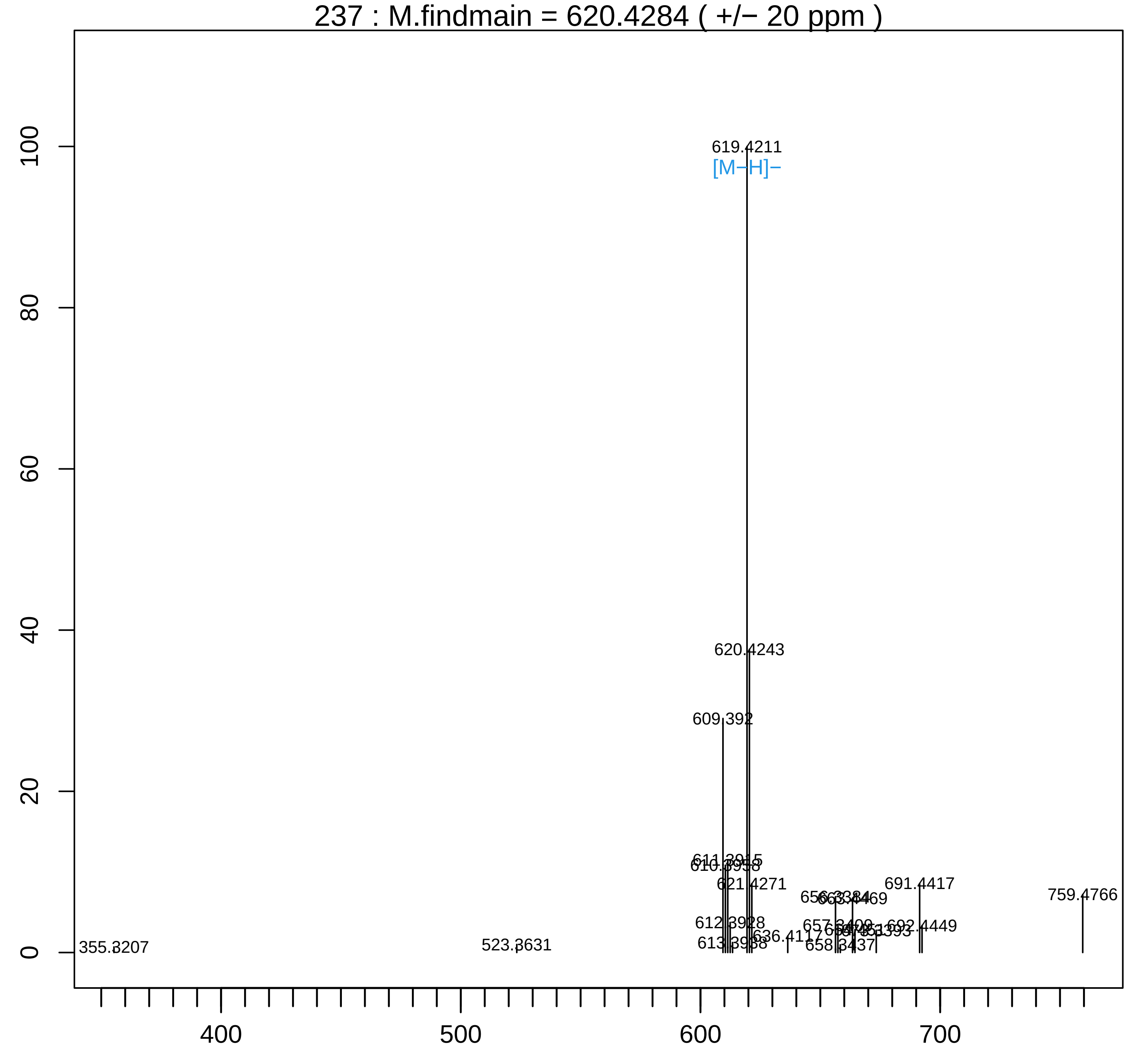
237 : M.findmain = 620.4284 (+/− 20 ppm)
619.4211
[M−H]−
620.4243
609.392
691.4417
759.4766
612.3928
523.3631
355.3207
692.4449
658.3437
636.4117
0
20
40
60
80
100
400
500
600
700

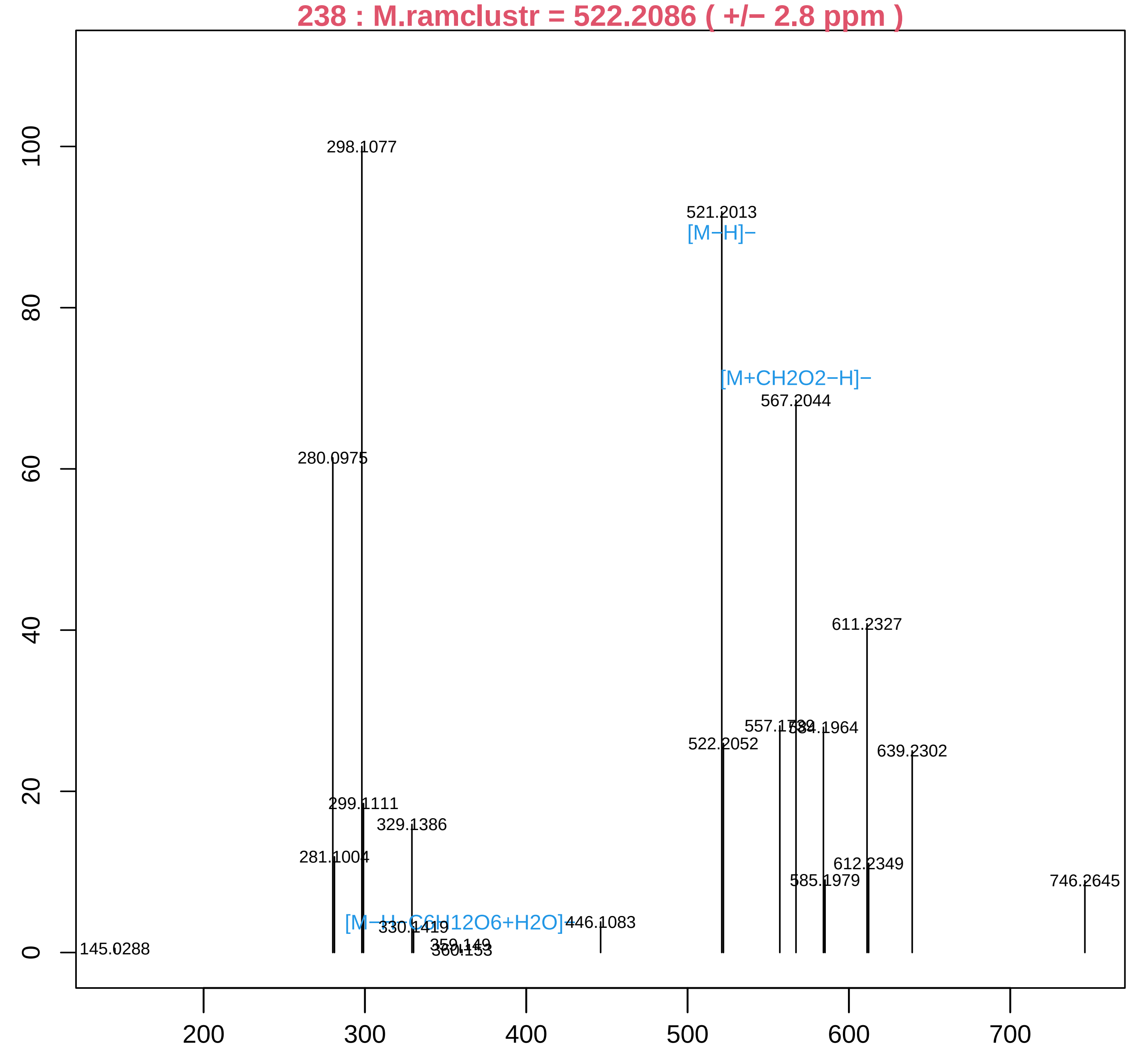

238 : M.ramclustr = 522.2086 (+/− 2.8 ppm)
298.1077
521.2013
[M−H]−
[M+CH2O2−H]−
567.2044
280.0975
611.2327
557.1789
584.1964
522.2052
639.2302
299.1111
329.1386
281.1004
612.2349
585.1979
746.2645
[M−H−C6H12O6+H2O]−
446.1083
330.1419
359.149
360.153
145.0288
0
20
40
60
80
100
200
300
400
500
600
700

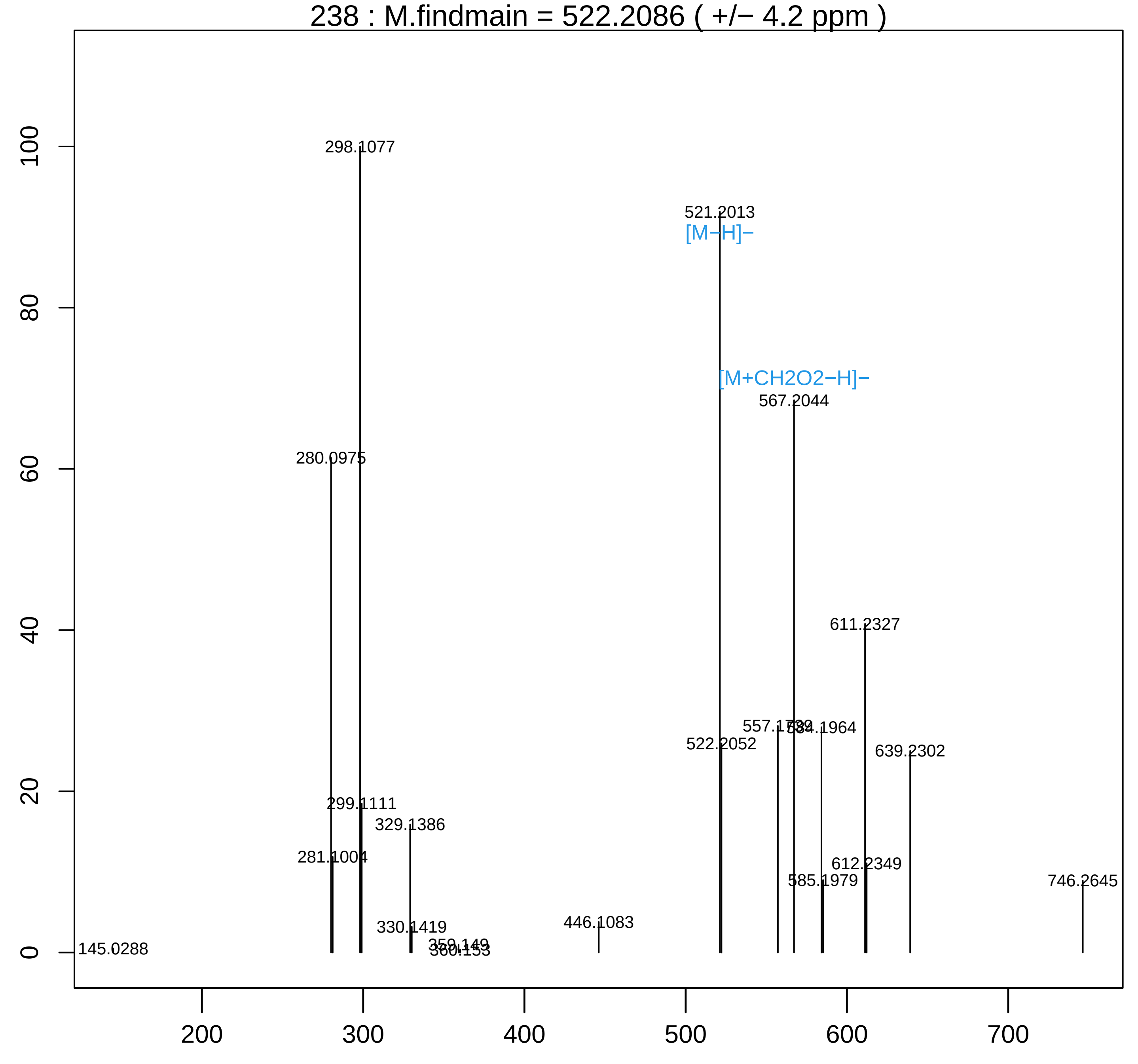

238 : M.findmain = 522.2086 (+/− 4.2 ppm)
298.1077
521.2013
[M−H]−
[M+CH2O2−H]−
567.2044
280.0975
611.2327
557.1789
584.1964
522.2052
639.2302
299.1111
329.1386
281.1004
612.2349
585.1979
746.2645
446.1083
330.1419
359.149
360.153
145.0288
0
20
40
60
80
100
200
300
400
500
600
700

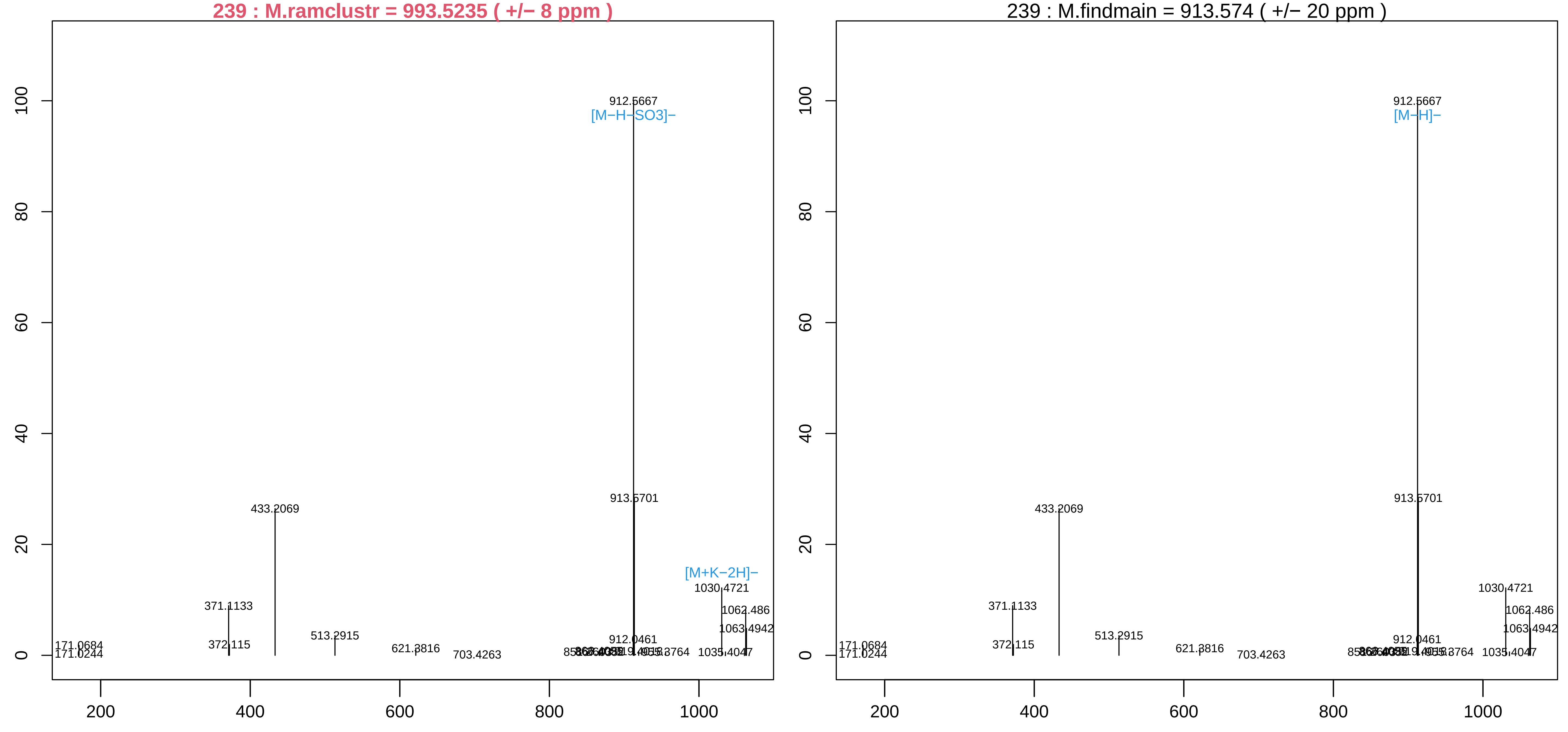

239 : M.ramclustr = 993.5235 (+/− 8 ppm)
912.5667
[M−H−SO3]−
913.5701
433.2069
[M+K−2H]−
1030.4721
1062.486
1063.4942
371.1133
372.115
513.2915
621.3816
703.4263
912.0461
1035.4047
171.0684
171.0244
239 : M.findmain = 913.574 (+/− 20 ppm)
912.5667
[M−H]−
913.5701
433.2069
1030.4721
1062.486
1063.4942
371.1133
372.115
513.2915
621.3816
703.4263
912.0461
1035.4047
171.0684
171.0244

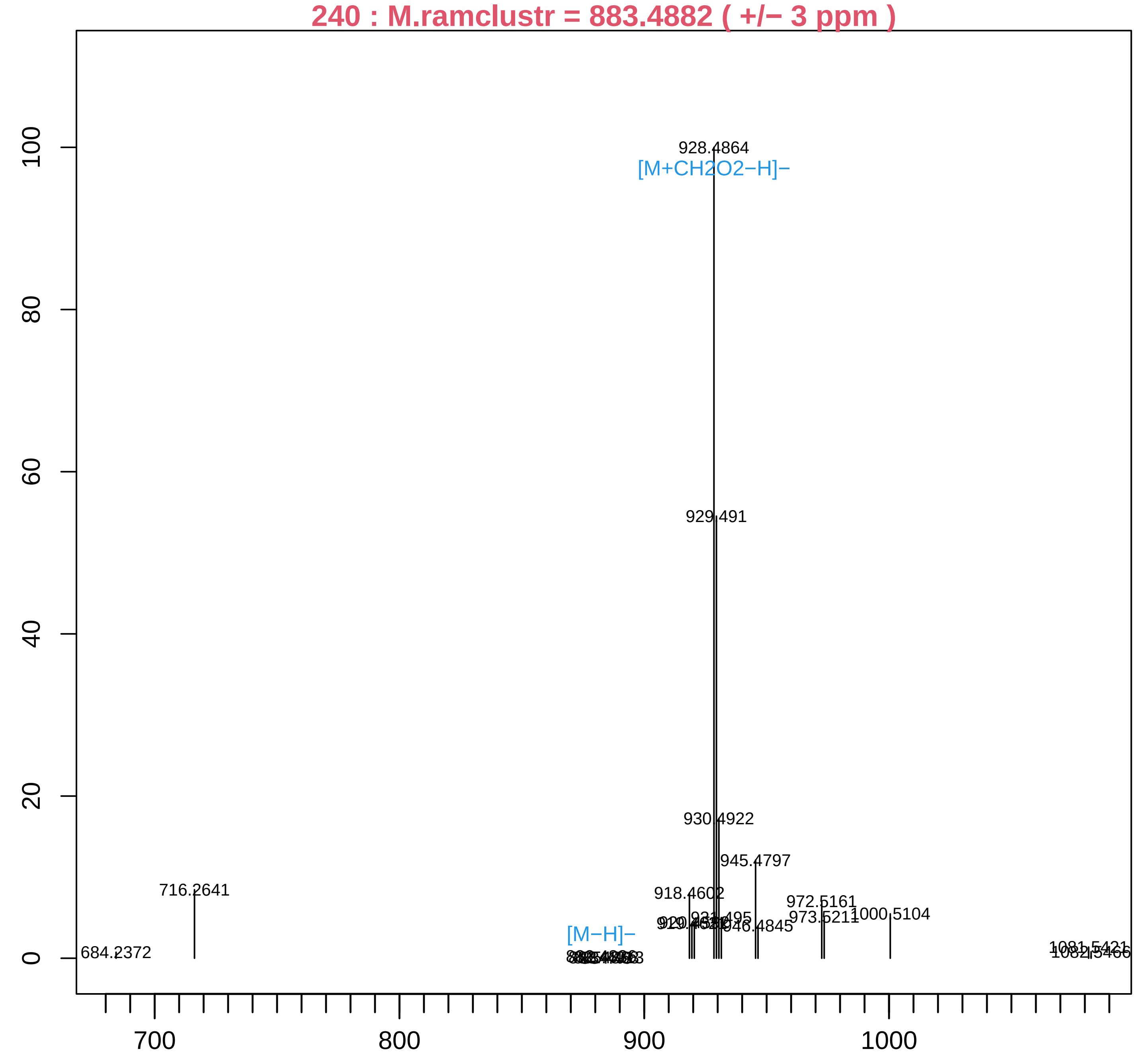
240 : M.ramclustr = 883.4882 (+/− 3 ppm)
928.4864
[M+CH2O2−H]−
929.491
930.4922
945.4797
918.4602
972.5161
973.5211
1000.5104
716.2641
684.2372
[M−H]−
1081.5421
1082.5466
0
20
40
60
80
100
700
800
900
1000

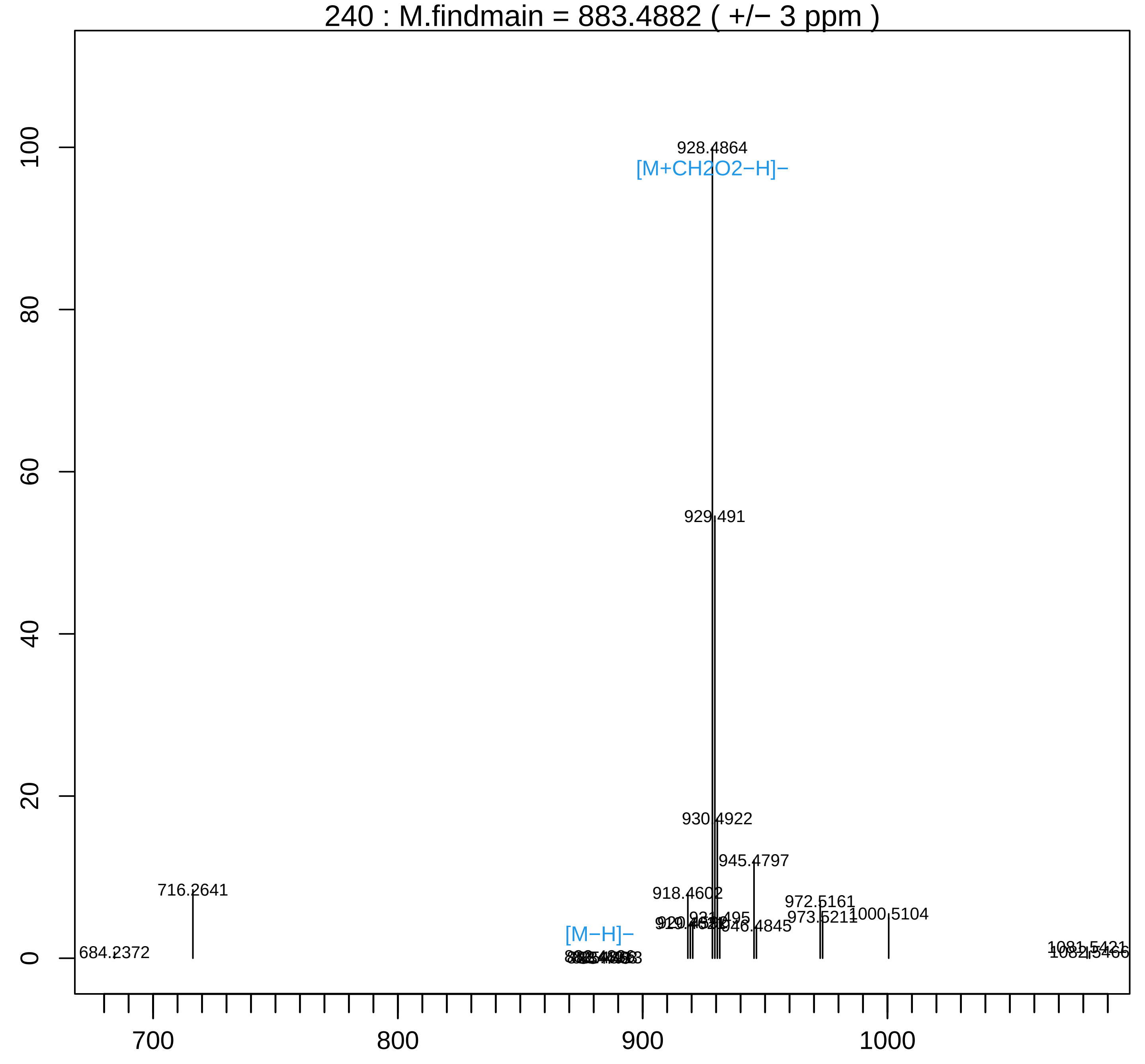
240 : M.findmain = 883.4882 (+/− 3 ppm)
928.4864
[M+CH2O2−H]−
929.491
930.4922
945.4797
918.4602
972.5161
973.5211
1000.5104
716.2641
684.2372
[M−H]−
1081.5421
1082.5466
0
20
40
60
80
100
700
800
900
1000

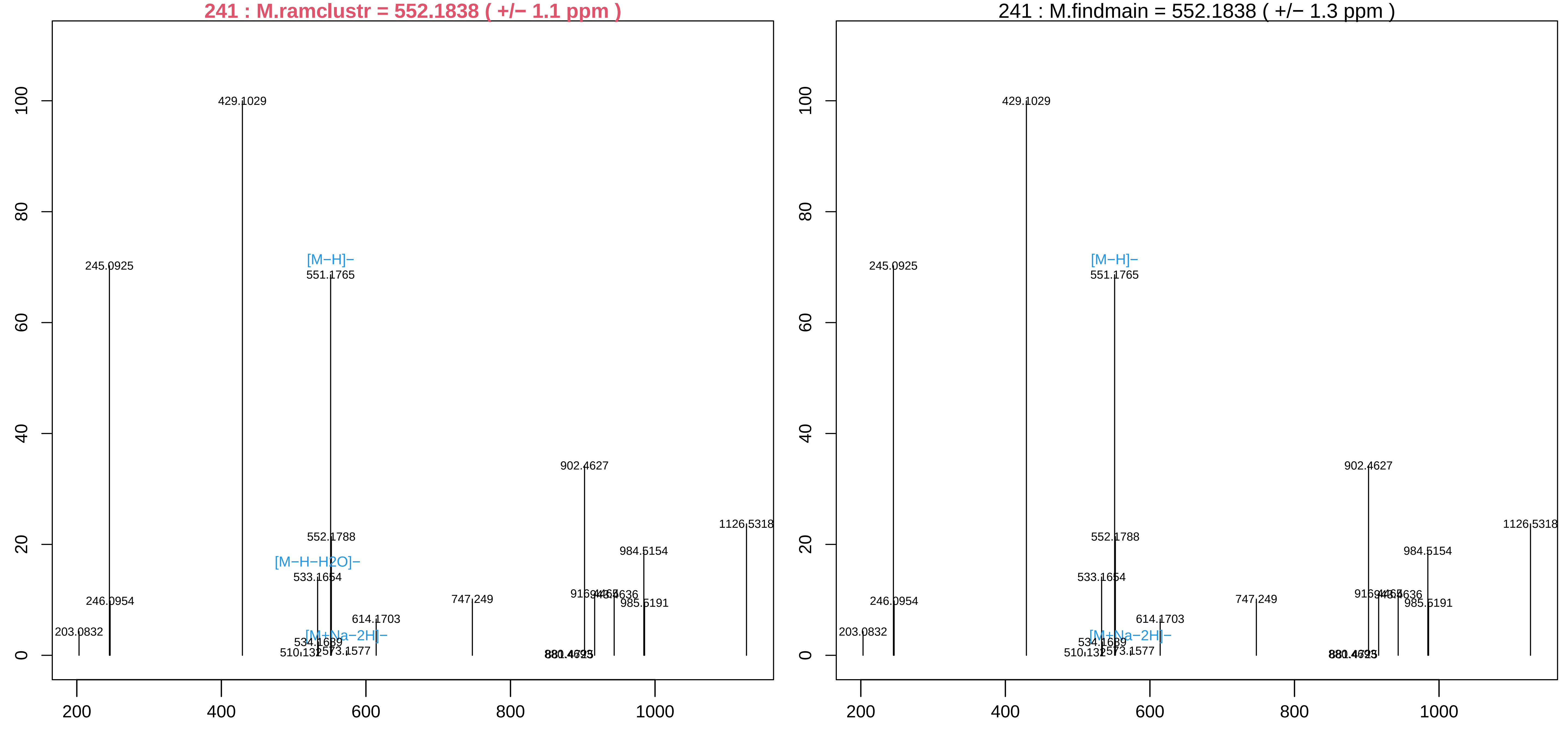

241 : M.ramclustr = 552.1838 (+/− 1.1 ppm)
429.1029
245.0925
[M−H]−
551.1765
552.1788
[M−H−H2O]−
533.1654
246.0954
203.0832
[M+Na−2H]−
534.1689
510.132
573.1577
614.1703
747.249
902.4627
984.5154
985.5191
1126.5318
880.4695
241 : M.findmain = 552.1838 (+/− 1.3 ppm)
0
20
40
60
80
100
200
400
600
800
1000

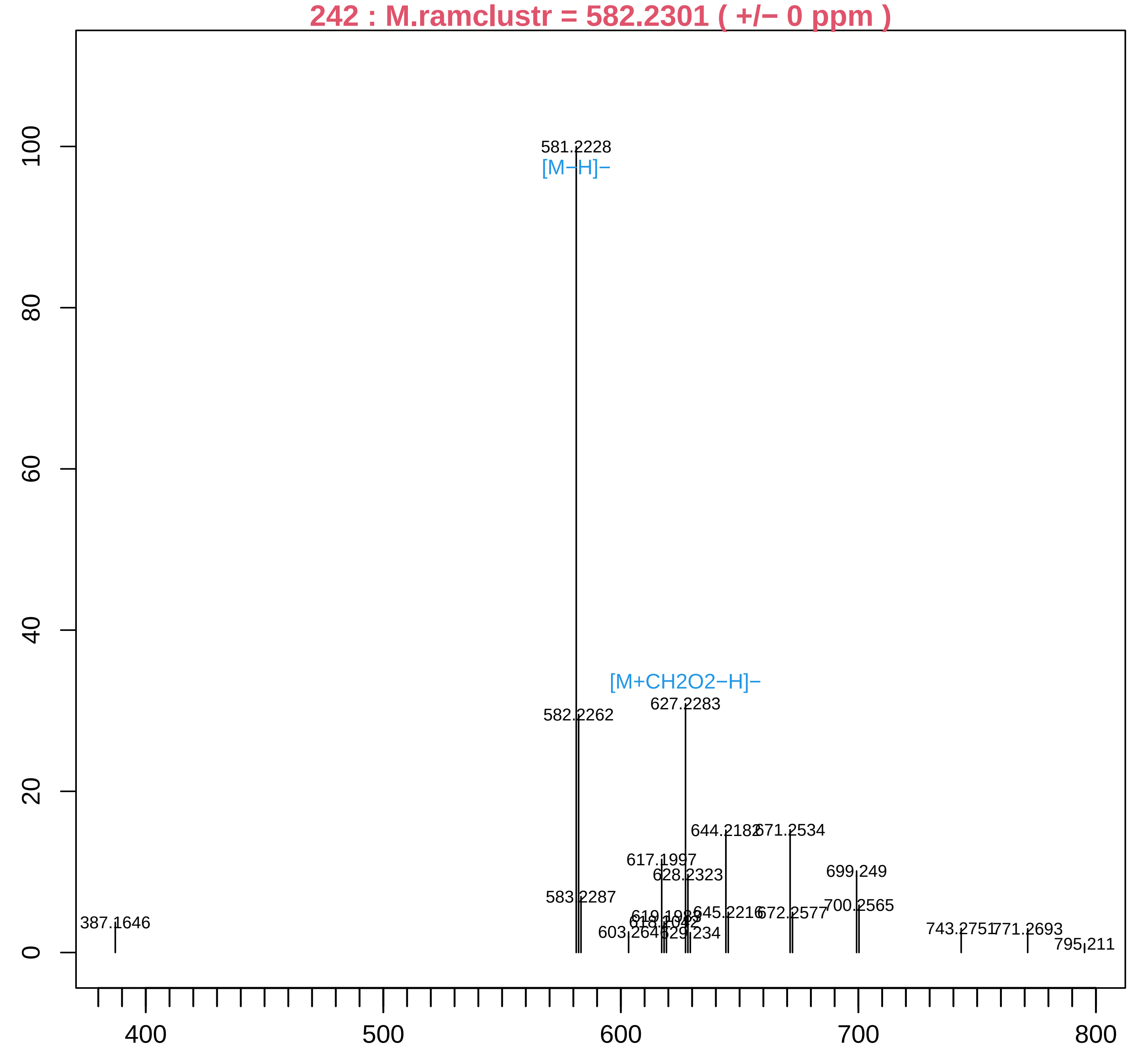
242 : M.ramclustr = 582.2301 (+/− 0 ppm)
581.2228
[M−H]−
[M+CH2O2−H]−
627.2283
582.2262
644.2183
671.2534
617.1997
628.2323
699.249
583.2287
645.2216
672.2577
700.2565
387.1646
603.2645
629.234
743.2751
771.2693
795.211
0
20
40
60
80
100
400
500
600
700
800

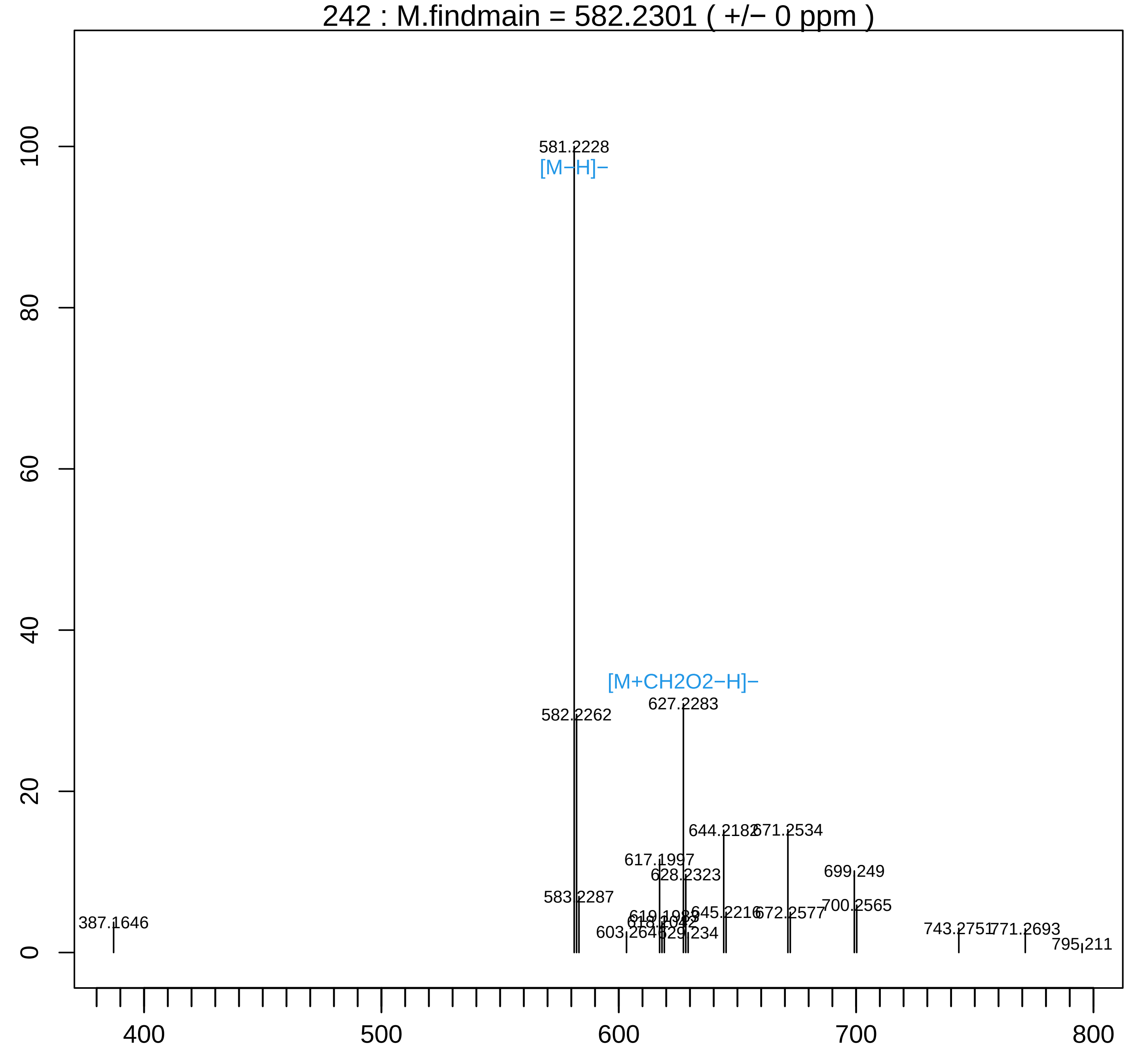
242 : M.findmain = 582.2301 (+/− 0 ppm)
581.2228
[M−H]−
[M+CH2O2−H]−
627.2283
582.2262
644.2183
671.2534
617.1997
628.2323
699.249
583.2287
645.2216
672.2577
700.2565
387.1646
603.2645
629.234
743.2751
771.2693
795.211
0
20
40
60
80
100
400
500
600
700
800

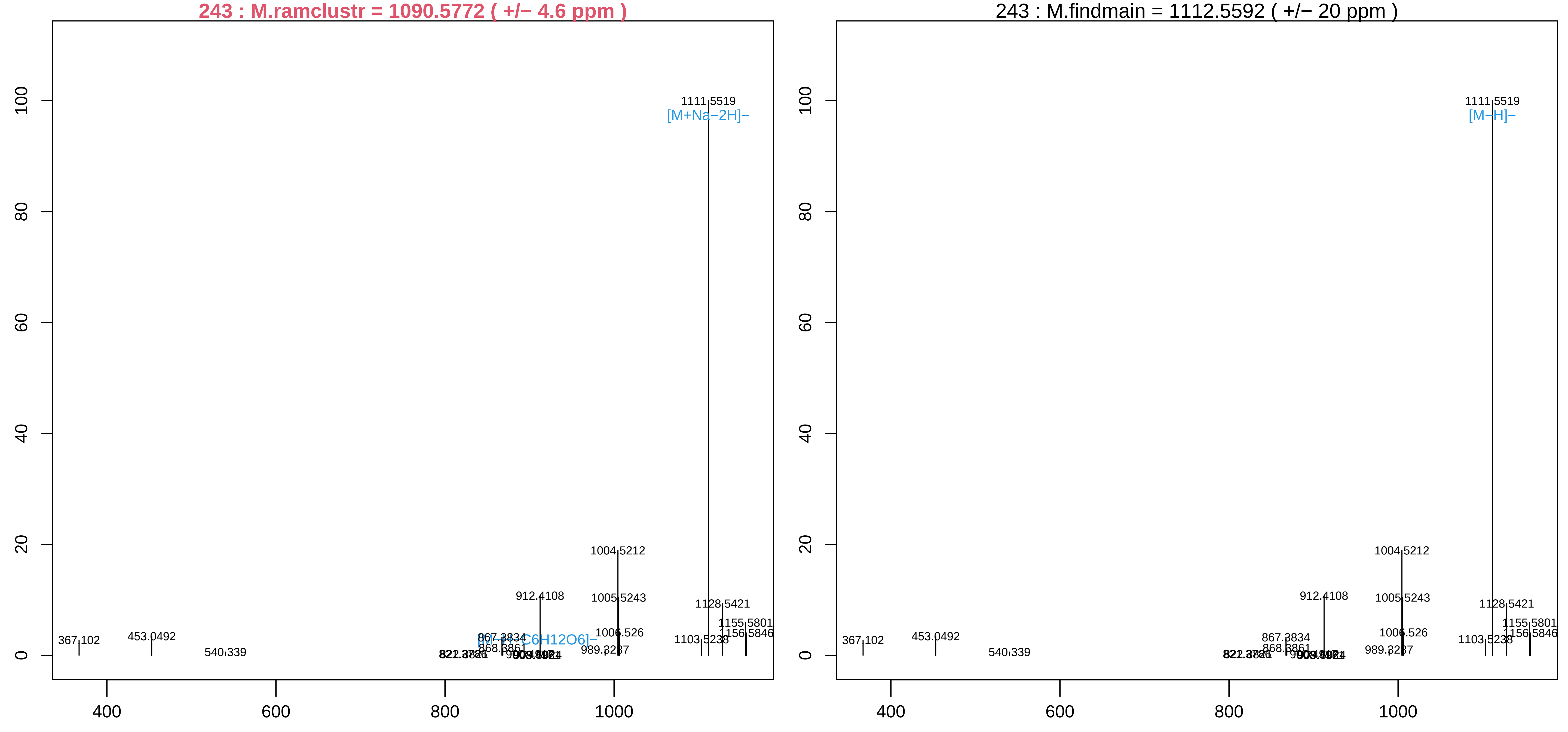
243 : M.ramclustr = 1090.5772 (+/− 4.6 ppm)
243 : M.findmain = 1112.5592 (+/− 20 ppm)
1111.5519
[M+Na−2H]−
[M−H]−
1004.5212
912.4108
1005.5243
1128.5421
1155.5801
1156.5846
1006.526
1103.5238
989.3237
867.3834
868.3861
367.102
453.0492
540.339
0
20
40
60
80
100
400
600
800
1000

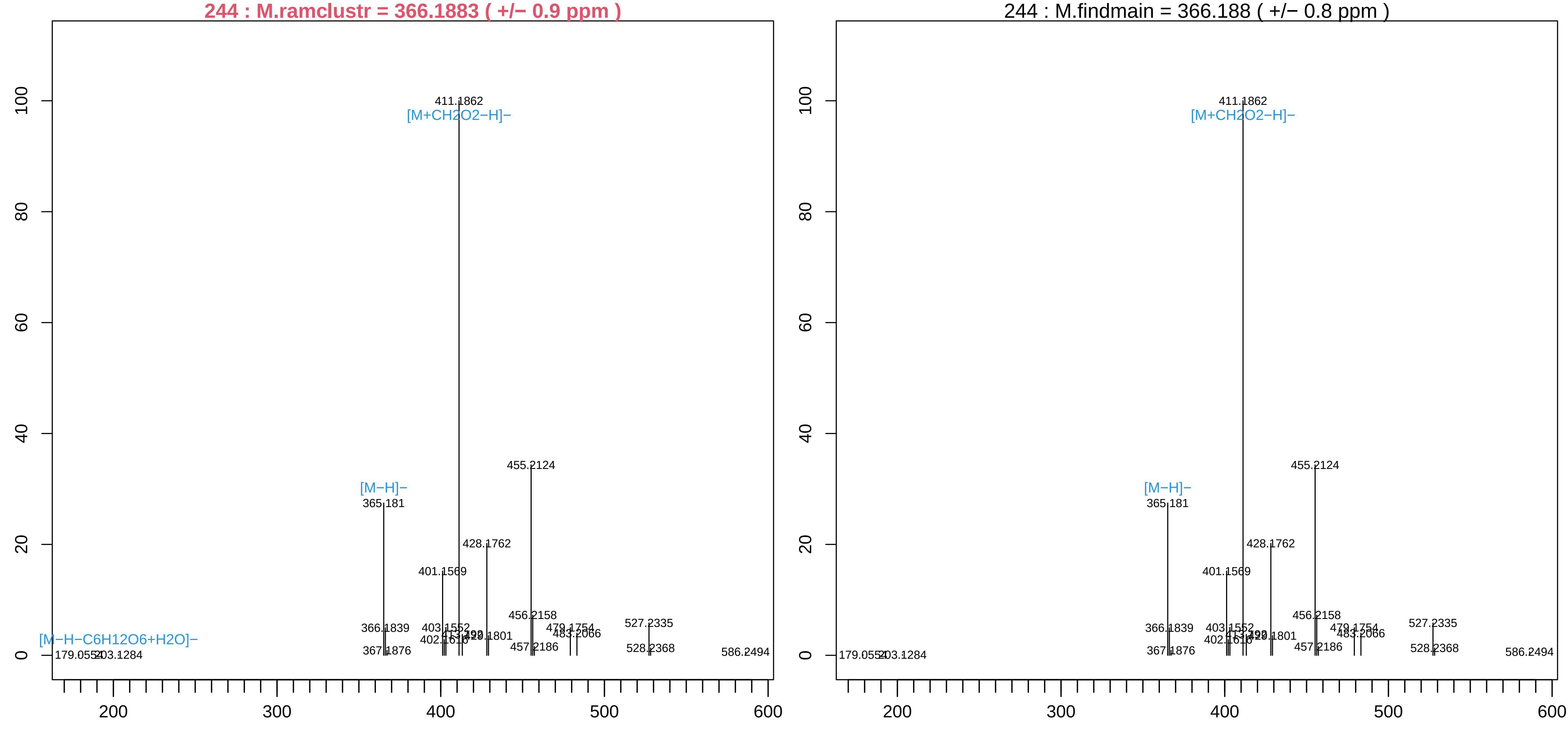
244 : M.ramclustr = 366.1883 (+/− 0.9 ppm)
244 : M.findmain = 366.188 (+/− 0.8 ppm)
411.1862
[M+CH2O2−H]−
455.2124
[M−H]−
365.181
428.1762
401.1569
456.2158
366.1839
403.1552
479.1754
483.2066
527.2335
402.1616
429.1801
457.2186
528.2368
367.1876
586.2494
[M−H−C6H12O6+H2O]−
179.0552
203.1284
0
20
40
60
80
100
200
300
400
500
600

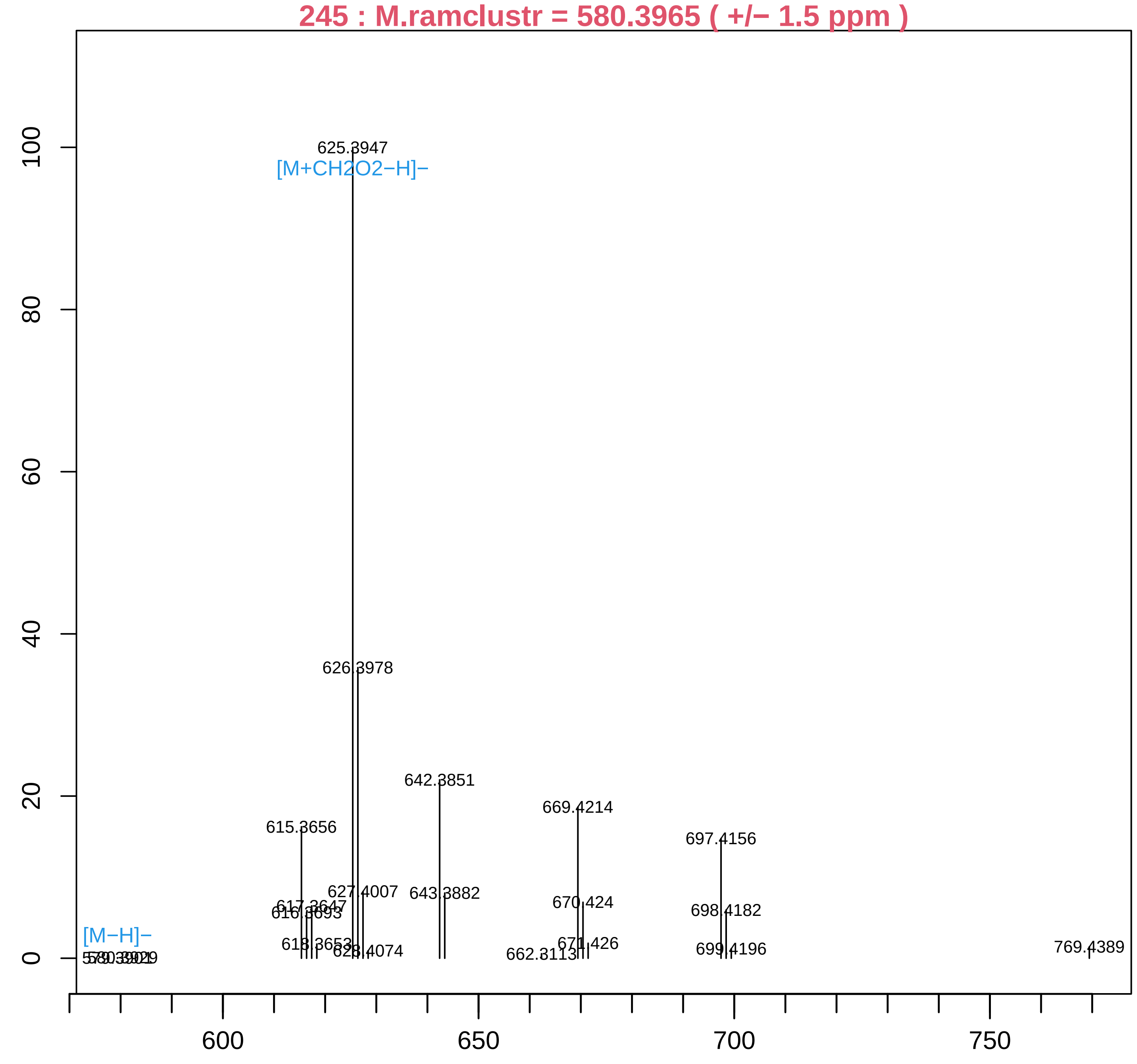
245 : M.ramclustr = 580.3965 (+/− 1.5 ppm)
625.3947
[M+CH2O2−H]−
626.3978
642.3851
669.4214
615.3656
697.4156
627.4007
643.3882
617.3647
616.3693
670.424
698.4182
[M−H]−
618.3653
628.4074
671.426
699.4196
662.8113
769.4389
0
20
40
60
80
100
600
650
700
750

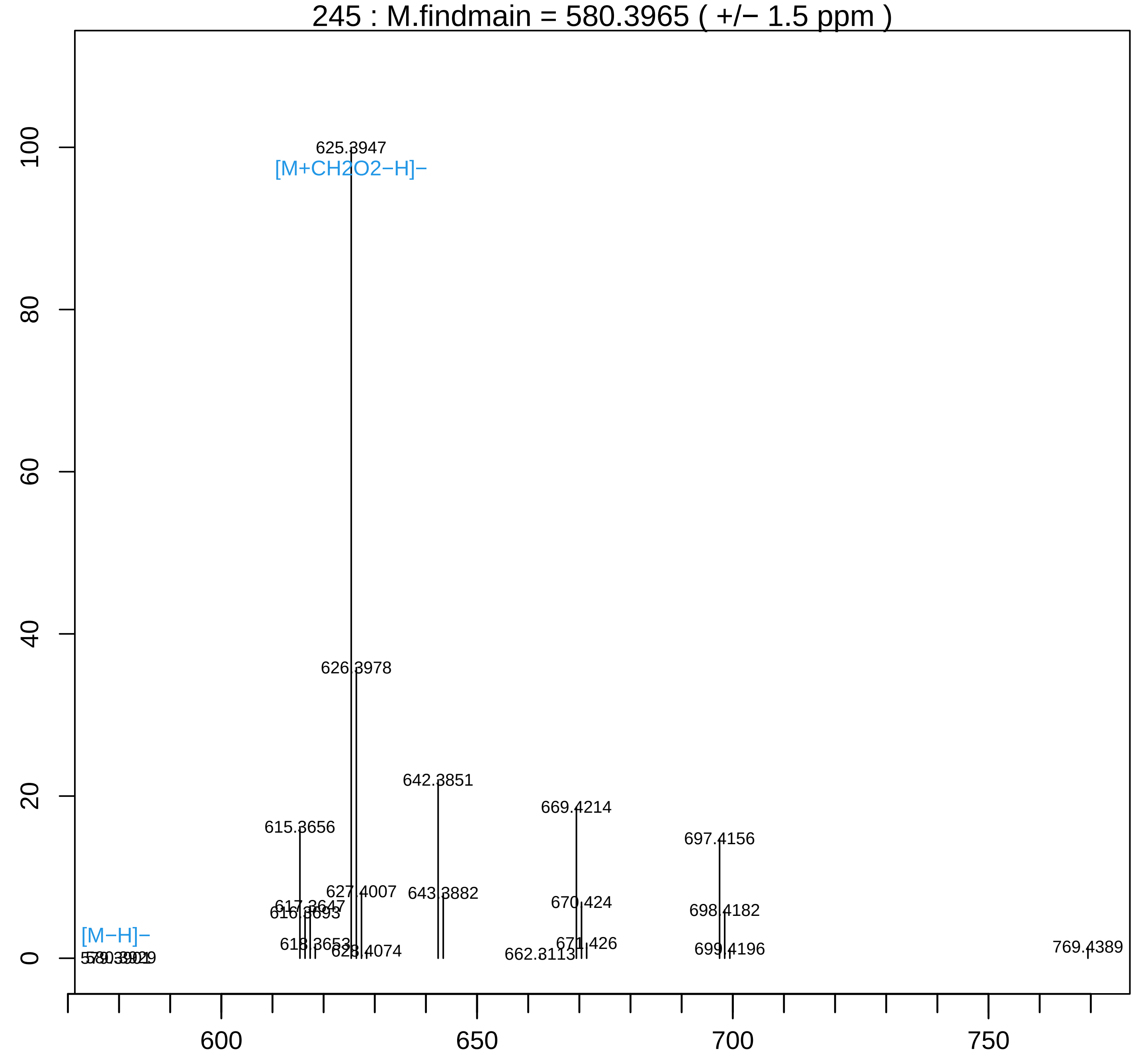
245 : M.findmain = 580.3965 (+/− 1.5 ppm)
625.3947
[M+CH2O2−H]−
626.3978
642.3851
669.4214
615.3656
697.4156
627.4007
643.3882
617.3647
616.3693
670.424
698.4182
[M−H]−
618.3653
628.4074
671.426
699.4196
662.8113
769.4389
0
20
40
60
80
100
600
650
700
750

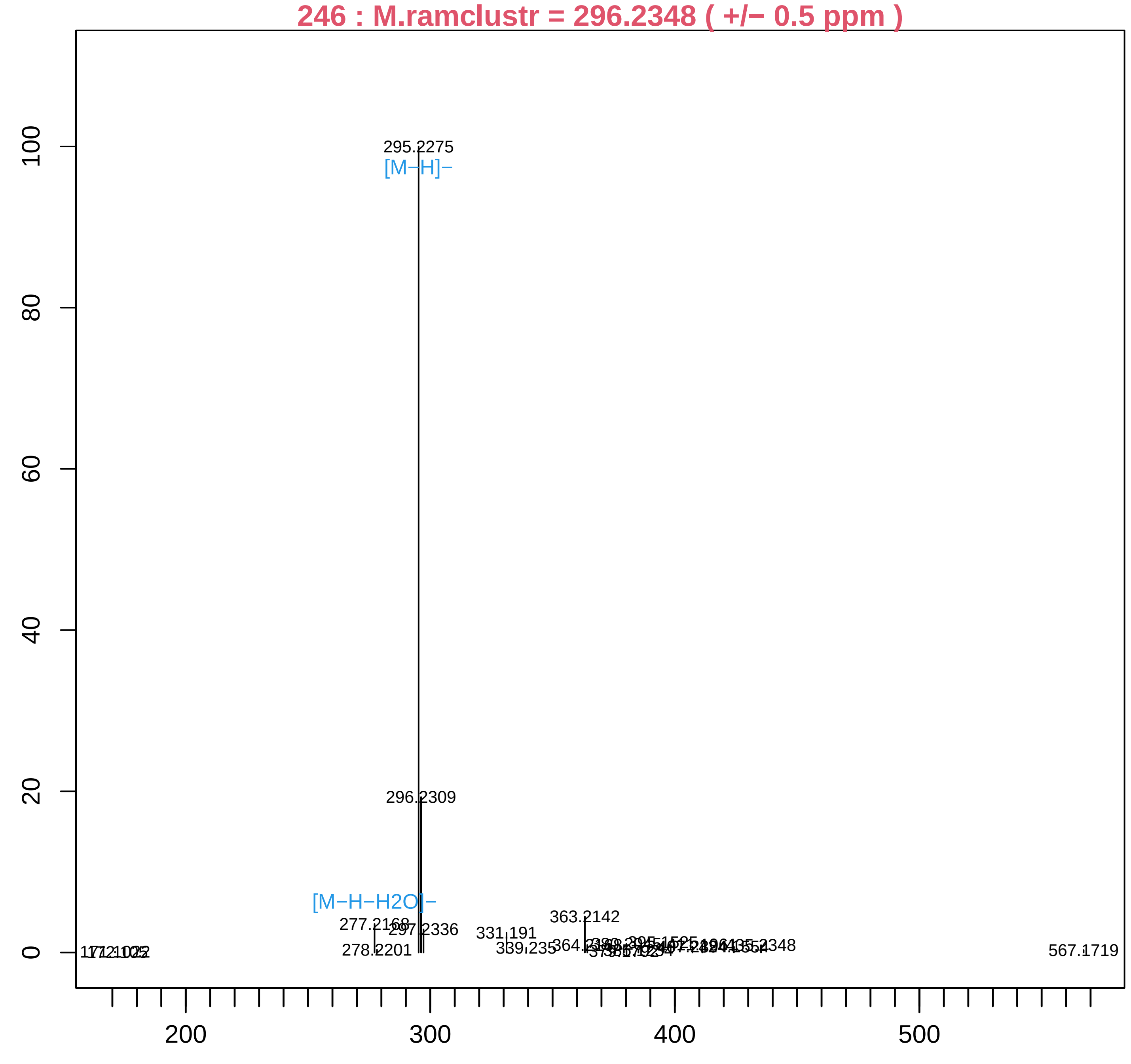
246 : M.ramclustr = 296.2348 (+/− 0.5 ppm)
295.2275
[M−H]−
296.2309
[M−H−H2O]−
277.2168
297.2336
278.2201
331.191
339.235
363.2142
567.1719
0
20
40
60
80
100
200
300
400
500

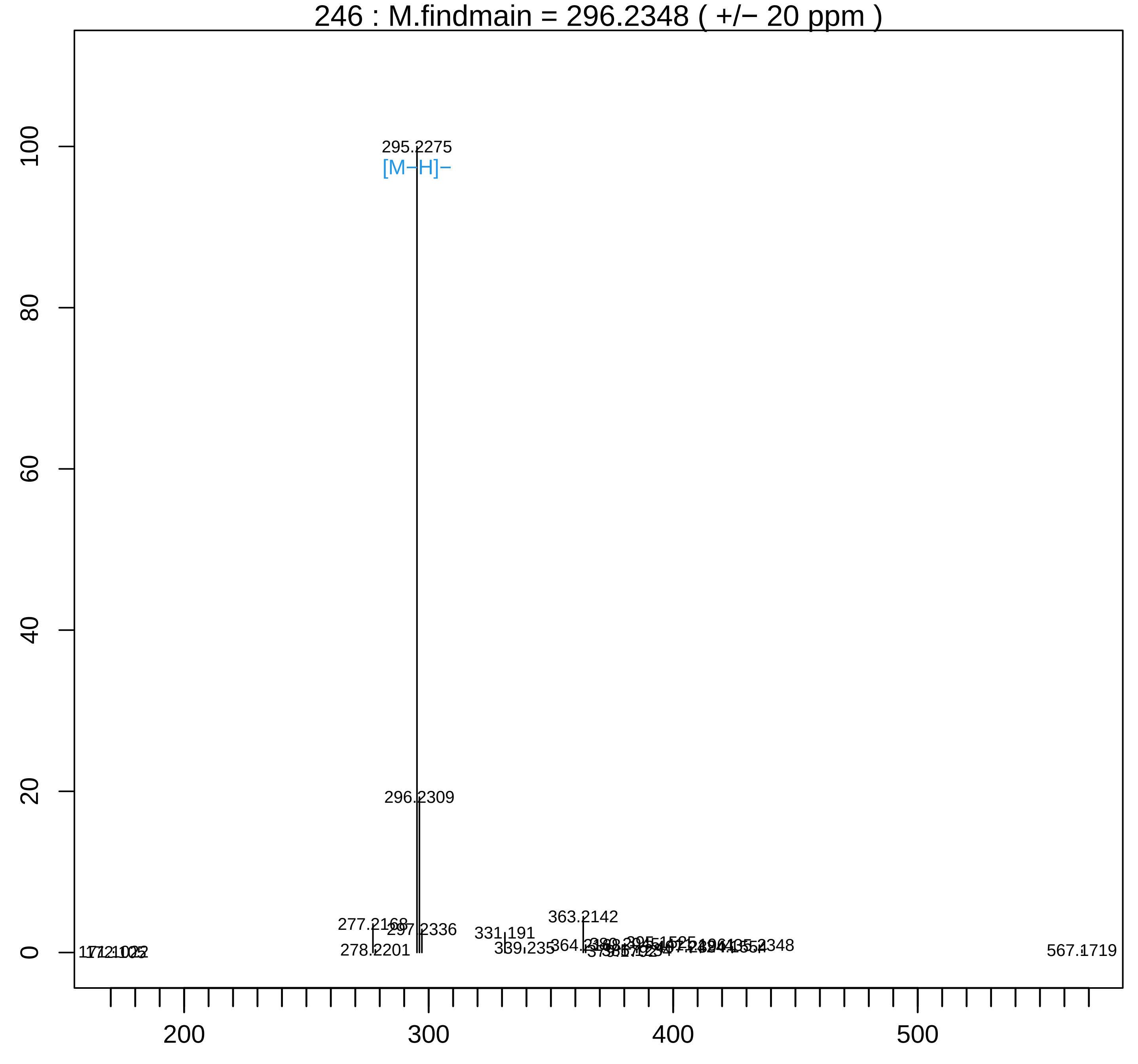
246 : M.findmain = 296.2348 (+/− 20 ppm)
295.2275
[M−H]−
296.2309
277.2168
297.2336
278.2201
331.191
339.235
363.2142
567.1719
0
20
40
60
80
100
200
300
400
500

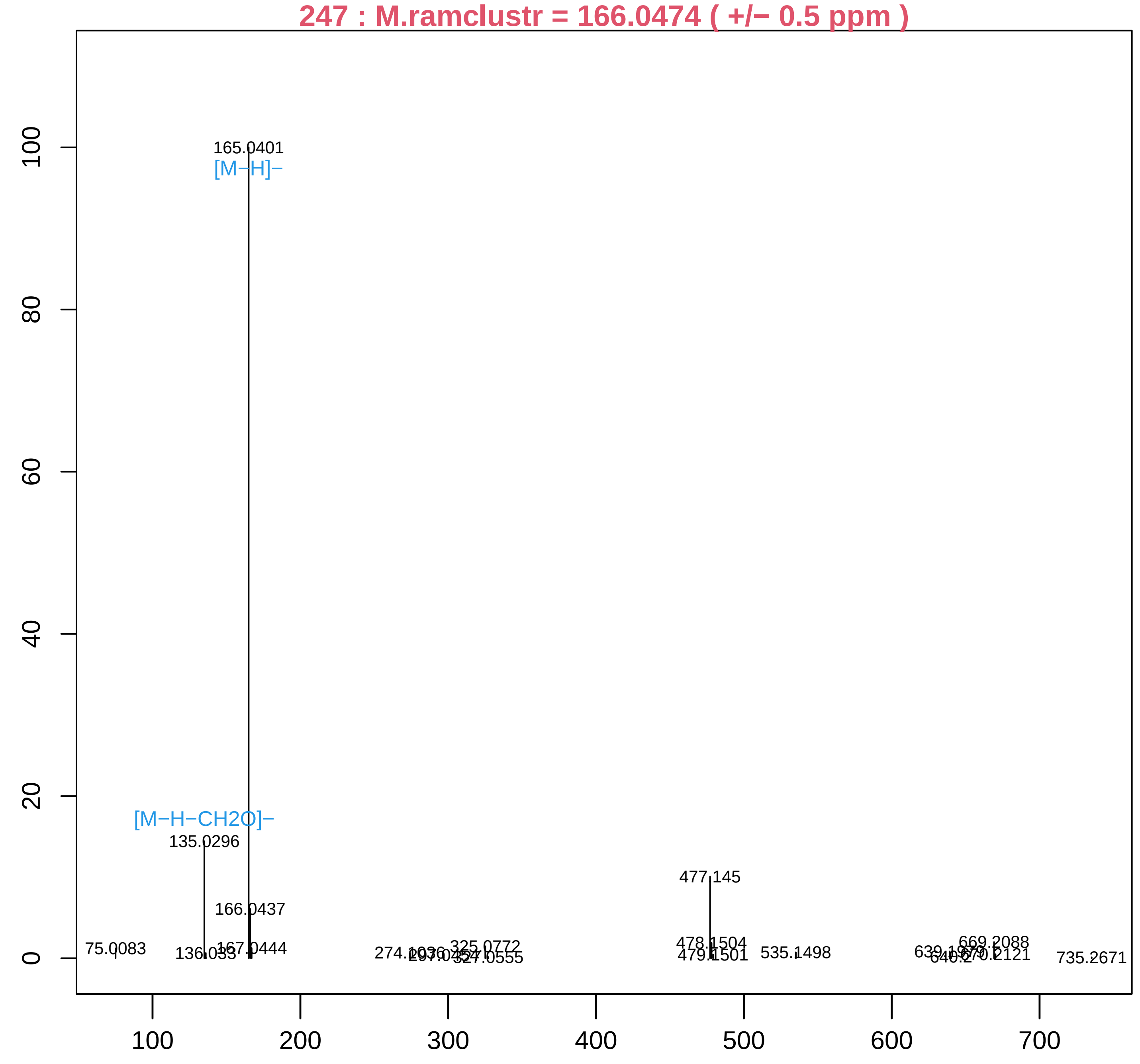
247 : M.ramclustr = 166.0474 (+/− 0.5 ppm)
165.0401
[M−H]−
[M−H−CH2O]−
135.0296
166.0437
477.145
75.0083
136.033
167.0444
325.0772
327.0555
478.1504
479.1501
535.1498
669.2088
735.2671
0
20
40
60
80
100
100
200
300
400
500
600
700

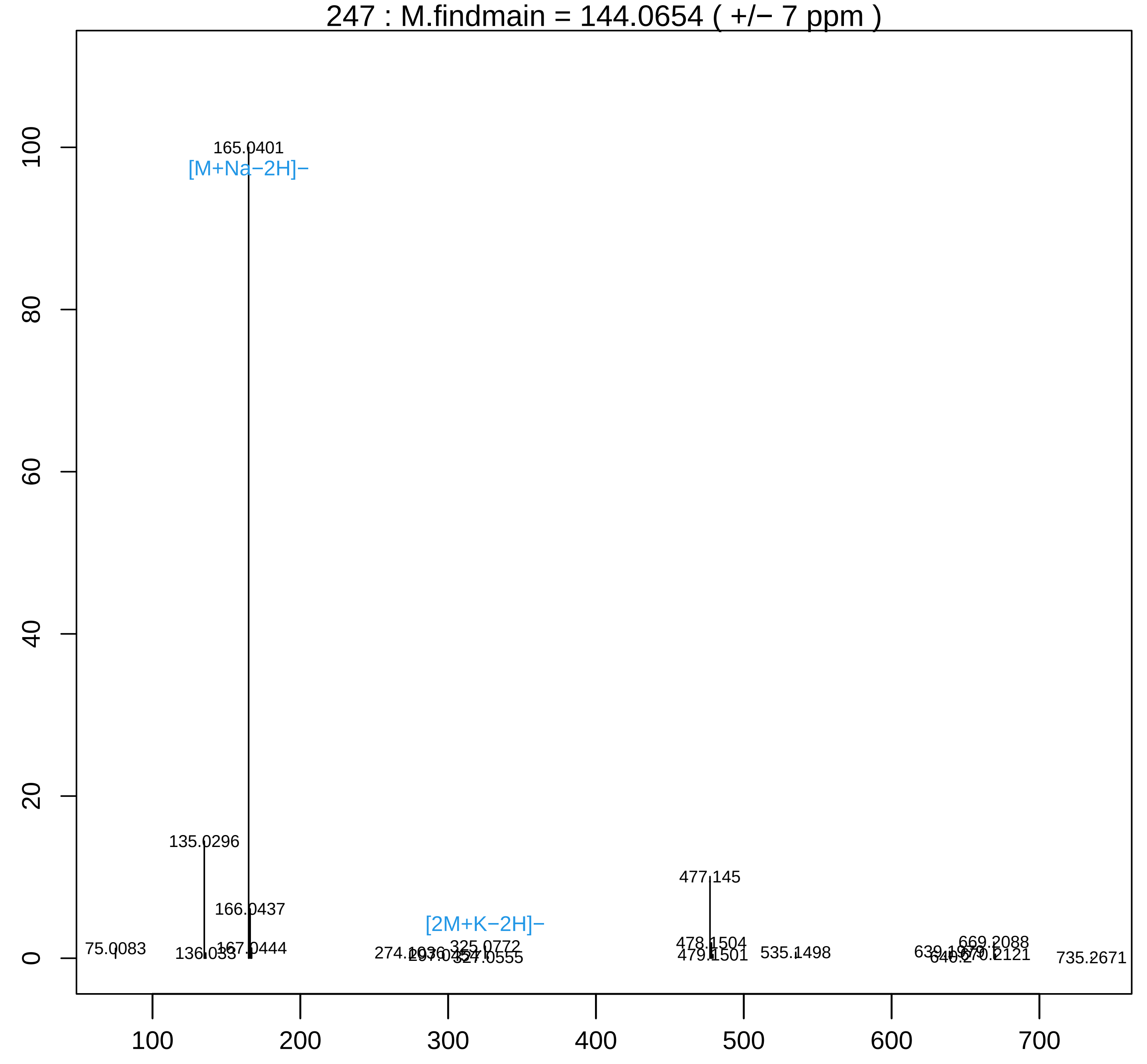
247 : M.findmain = 144.0654 (+/− 7 ppm)
165.0401
[M+Na−2H]−
135.0296
166.0437
[2M+K−2H]−
477.145
75.0083
136.033
167.0444
325.0772
327.0555
478.1504
479.1501
535.1498
669.2088
735.2671
0
20
40
60
80
100
100
200
300
400
500
600
700

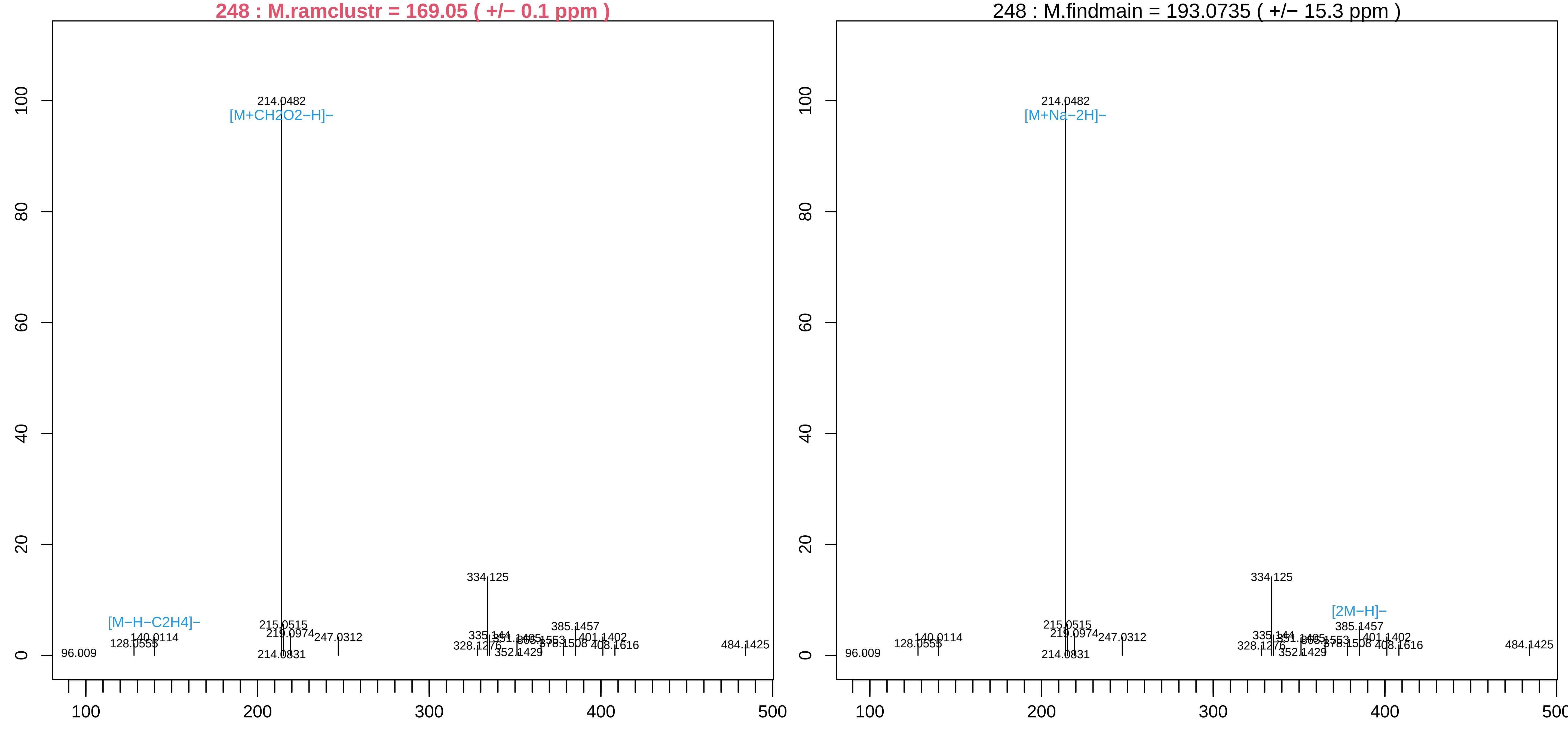
248 : M.ramclustr = 169.05 (+/− 0.1 ppm)
248 : M.findmain = 193.0735 (+/− 15.3 ppm)
214.0482
[M+CH2O2−H]−
[M+Na−2H]−
[M−H−C2H4]−
[2M−H]−
334.125
385.1457
215.0515
219.0974
247.0312
140.0114
128.0555
96.009
214.0831
328.1276
352.1429
378.1508
401.1402
408.1616
484.1425
0
20
40
60
80
100
200
300
400
500

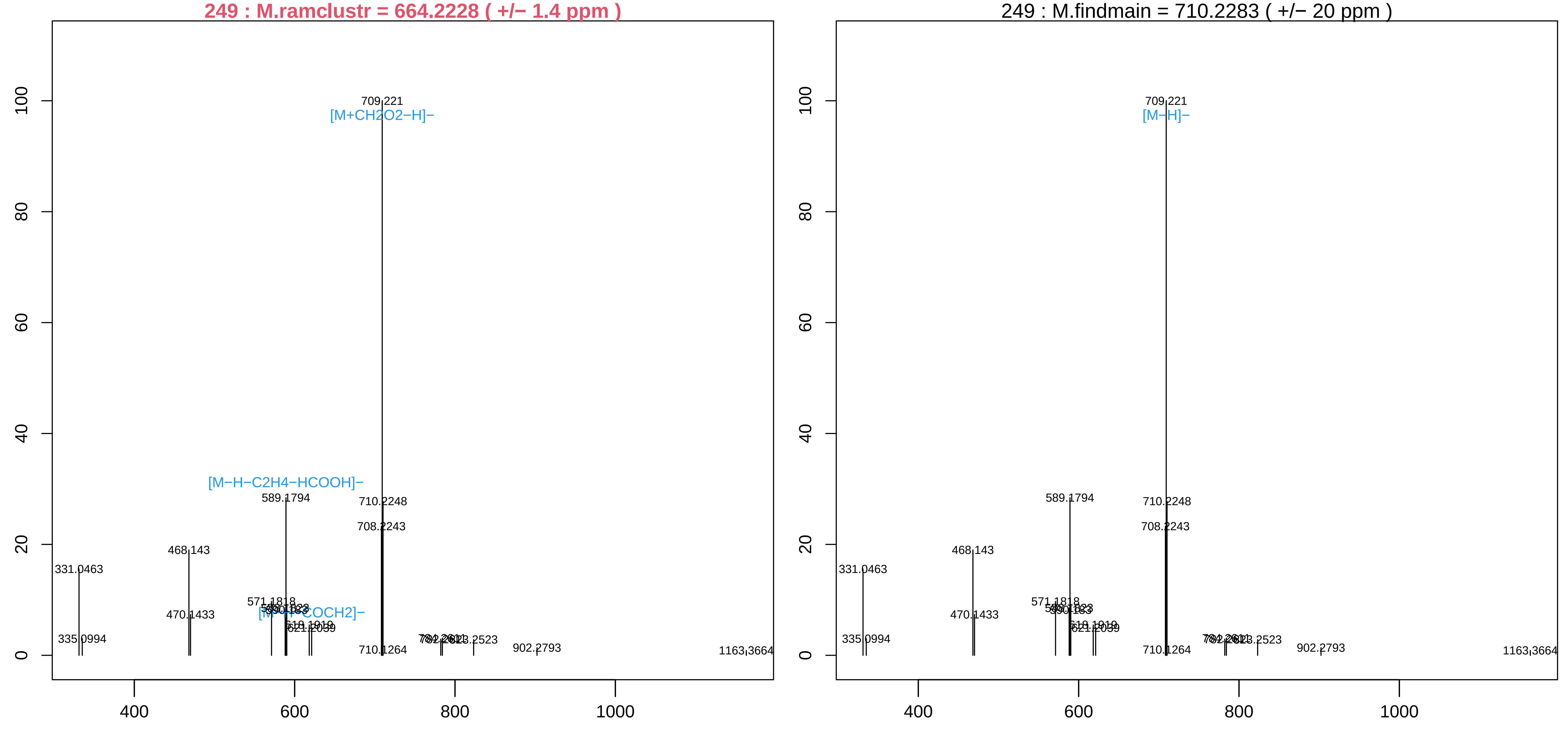
249 : M.ramclustr = 664.2228 (+/− 1.4 ppm)
709.221
[M+CH2O2−H]−
[M−H−C2H4−HCOOH]−
589.1794
710.2248
708.2243
468.143
331.0463
571.1818
470.1433
619.1919
621.2059
335.0994
710.1264
823.2523
902.2793
1163.3664
249 : M.findmain = 710.2283 (+/− 20 ppm)
709.221
[M−H]−
589.1794
710.2248
708.2243
468.143
331.0463
571.1818
470.1433
619.1919
621.2059
335.0994
710.1264
823.2523
902.2793
1163.3664
0
20
40
60
80
100
400
600
800
1000

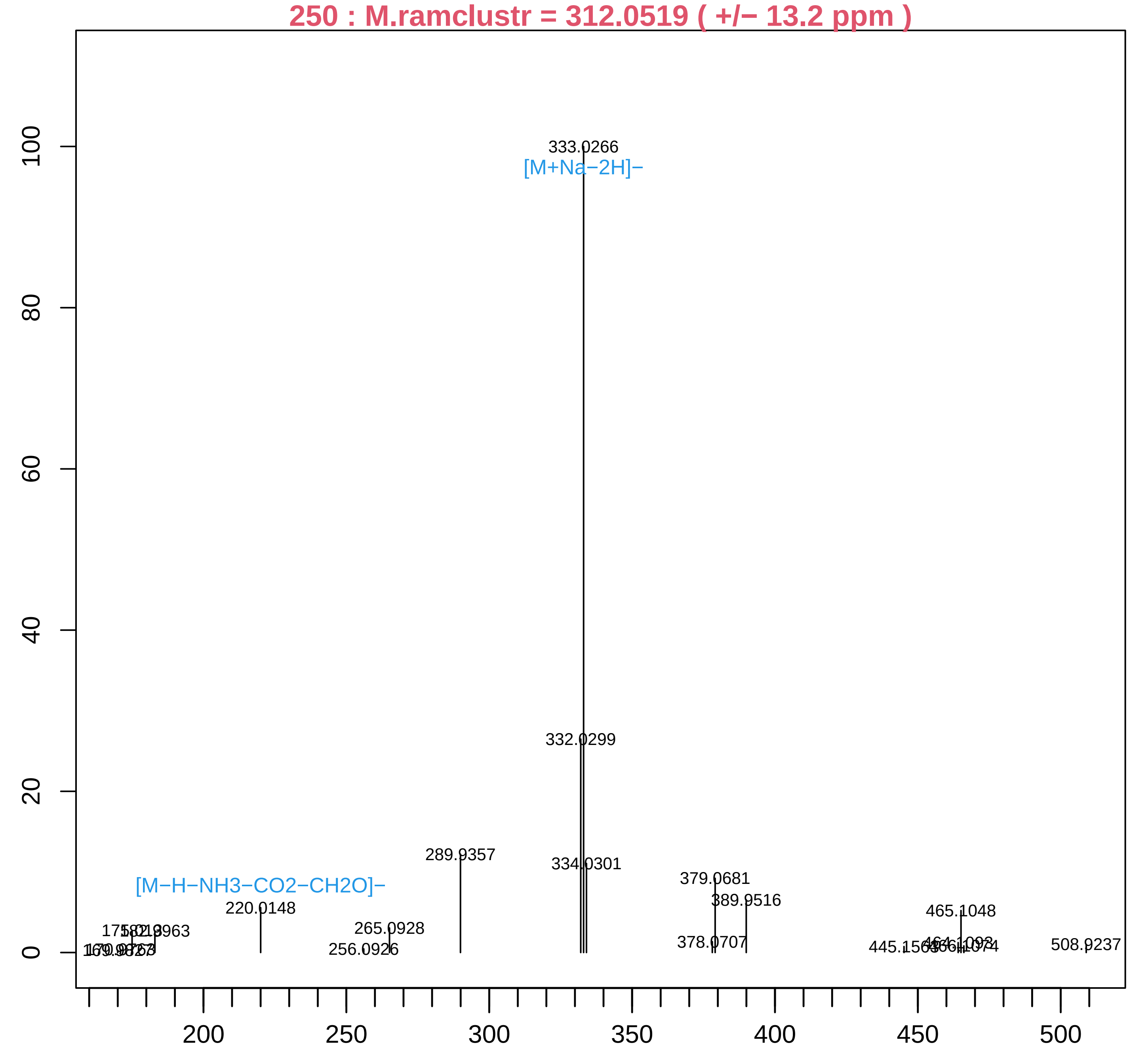
250 : M.ramclustr = 312.0519 (+/− 13.2 ppm)
333.0266
[M+Na−2H]−
332.0299
289.9357
334.0301
379.0681
389.9516
378.0707
[M−H−NH3−CO2−CH2O]−
220.0148
265.0928
256.0926
465.1048
508.9237
0
20
40
60
80
100
200
250
300
350
400
450
500

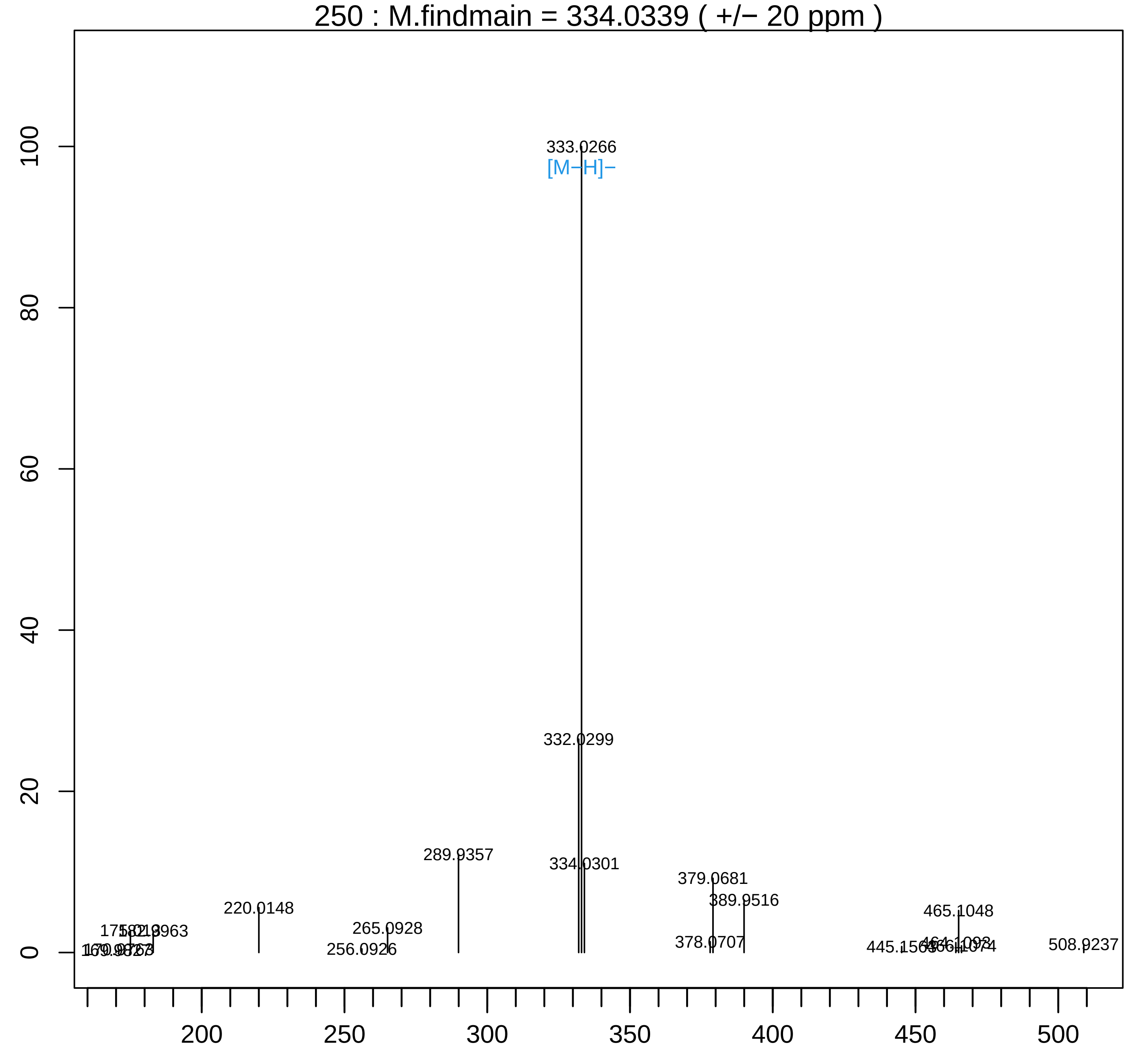
250 : M.findmain = 334.0339 (+/− 20 ppm)
333.0266
[M−H]−
332.0299
289.9357
334.0301
379.0681
389.9516
378.0707
220.0148
265.0928
256.0926
465.1048
508.9237
0
20
40
60
80
100
200
250
300
350
400
450
500

251 : M.ramclustr = 538.1889 (+/− 0 ppm)

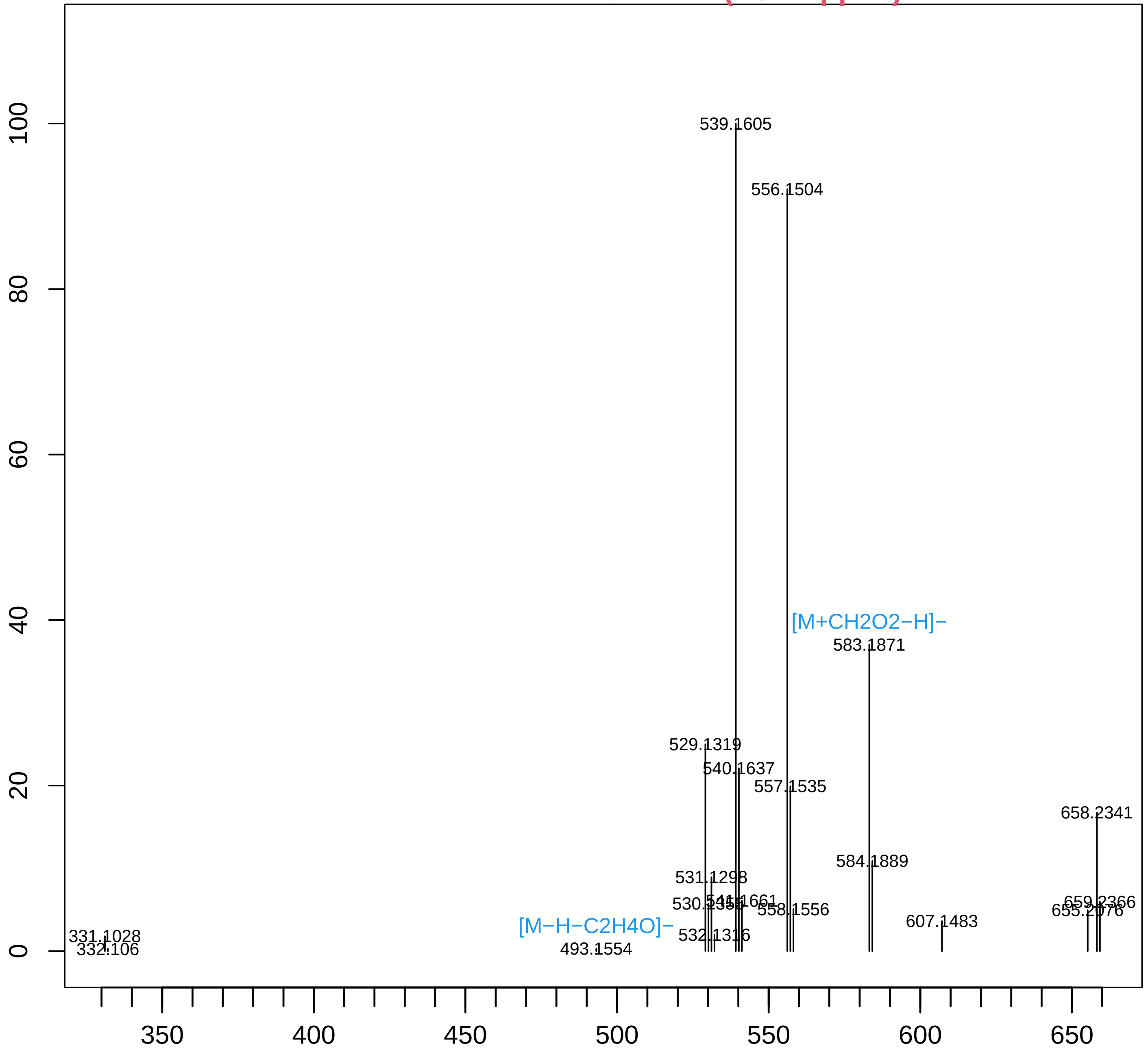

251 : M.findmain = 494.1623 (+/− 0.8 ppm)

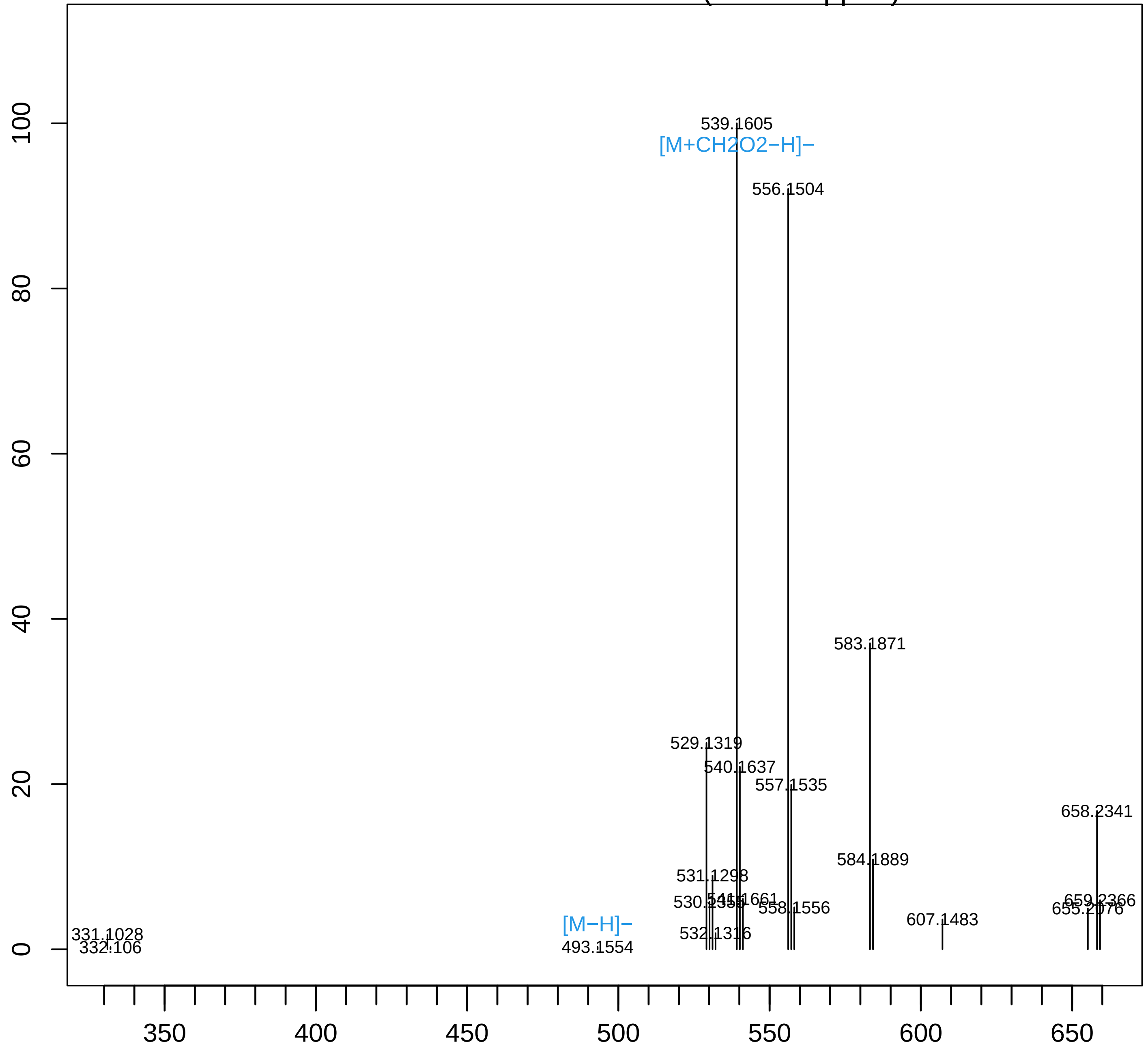

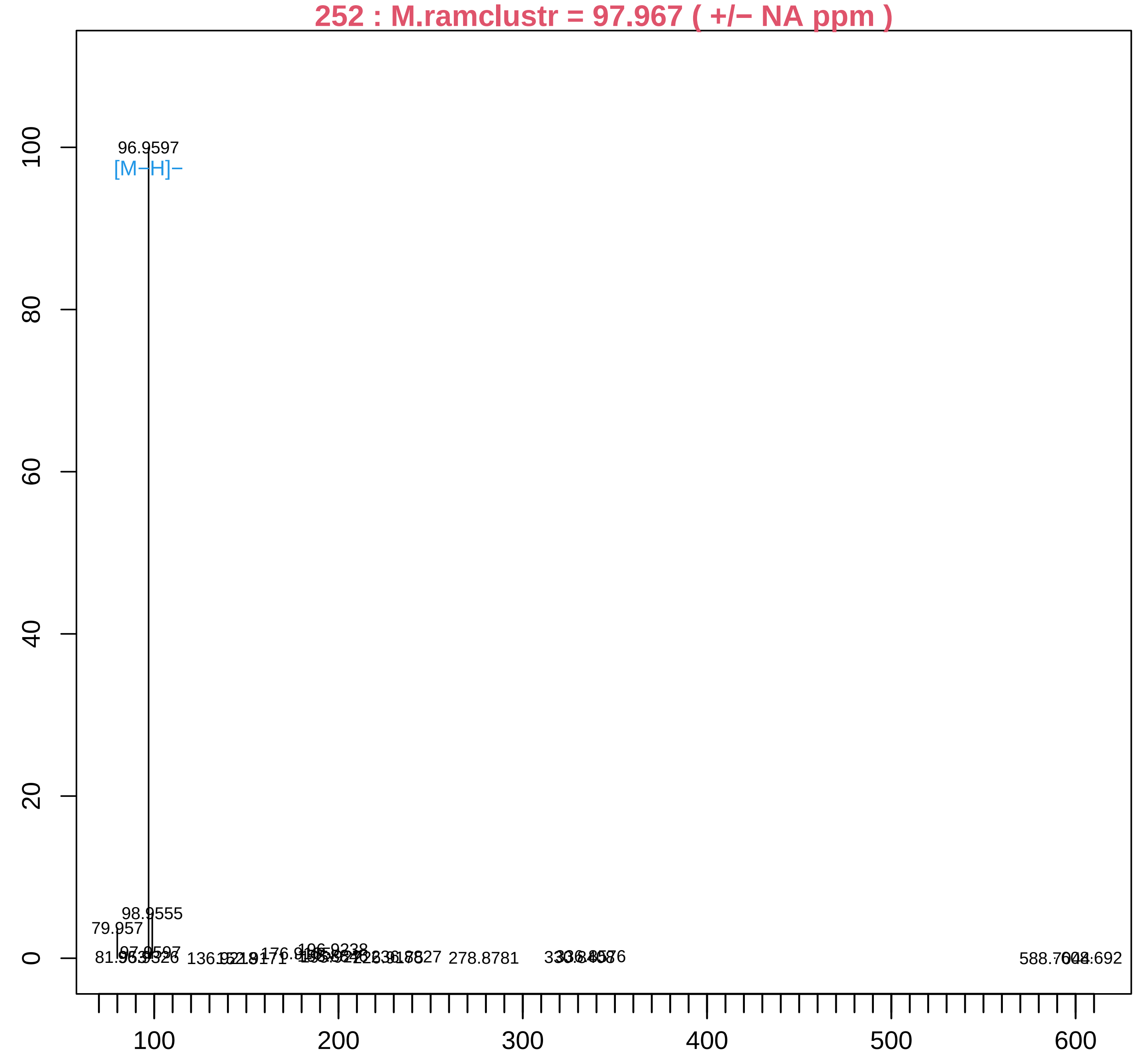
252 : M.ramclustr = 97.967 (+/− NA ppm)
96.9597
[M−H]−
98.9555
79.957
278.8781
100
200
300
400
500
600
0
20
40
60
80
100

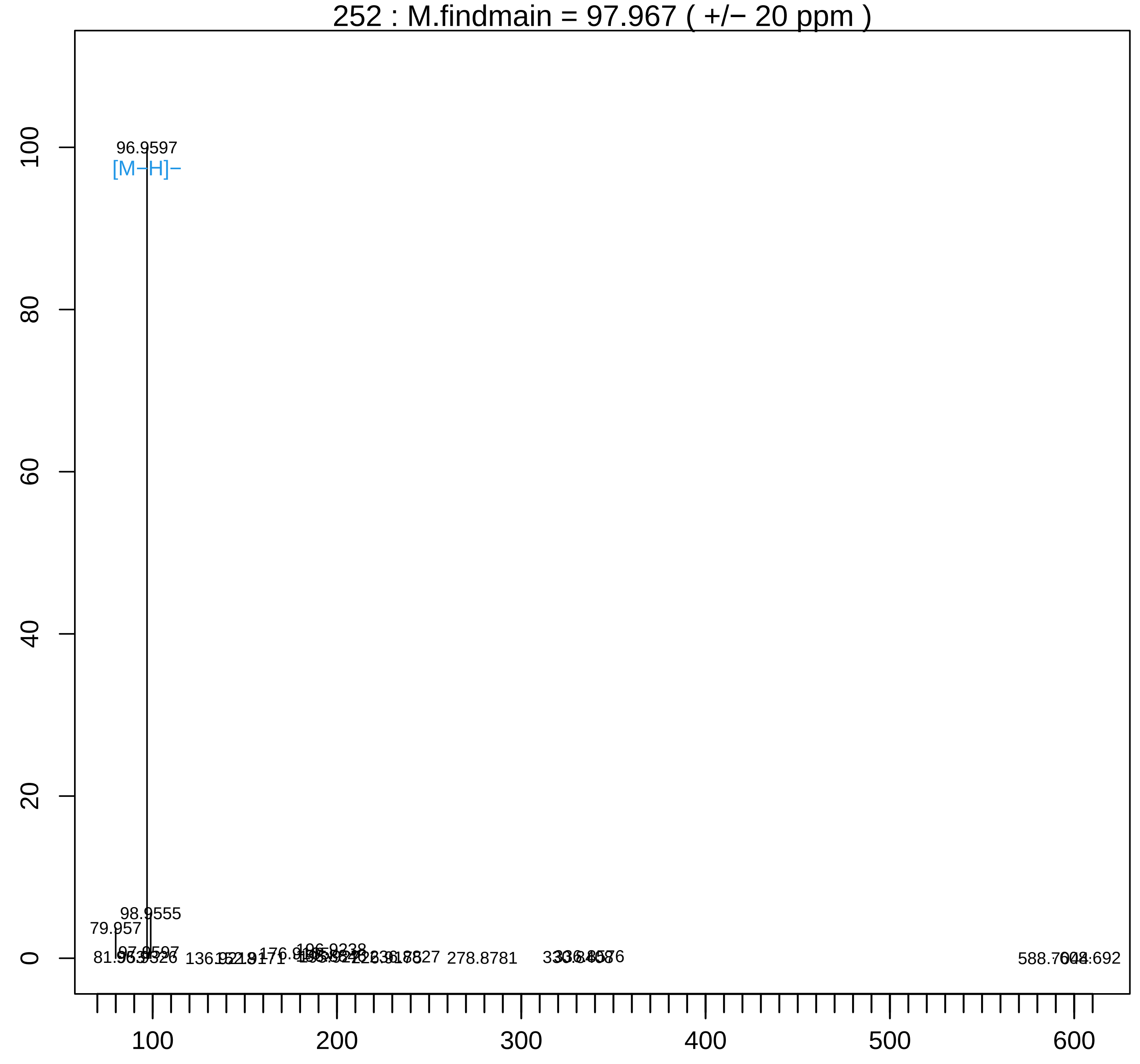
252 : M.findmain = 97.967 (+/− 20 ppm)
96.9597
[M−H]−
98.9555
79.957
278.8781
100
200
300
400
500
600
0
20
40
60
80
100

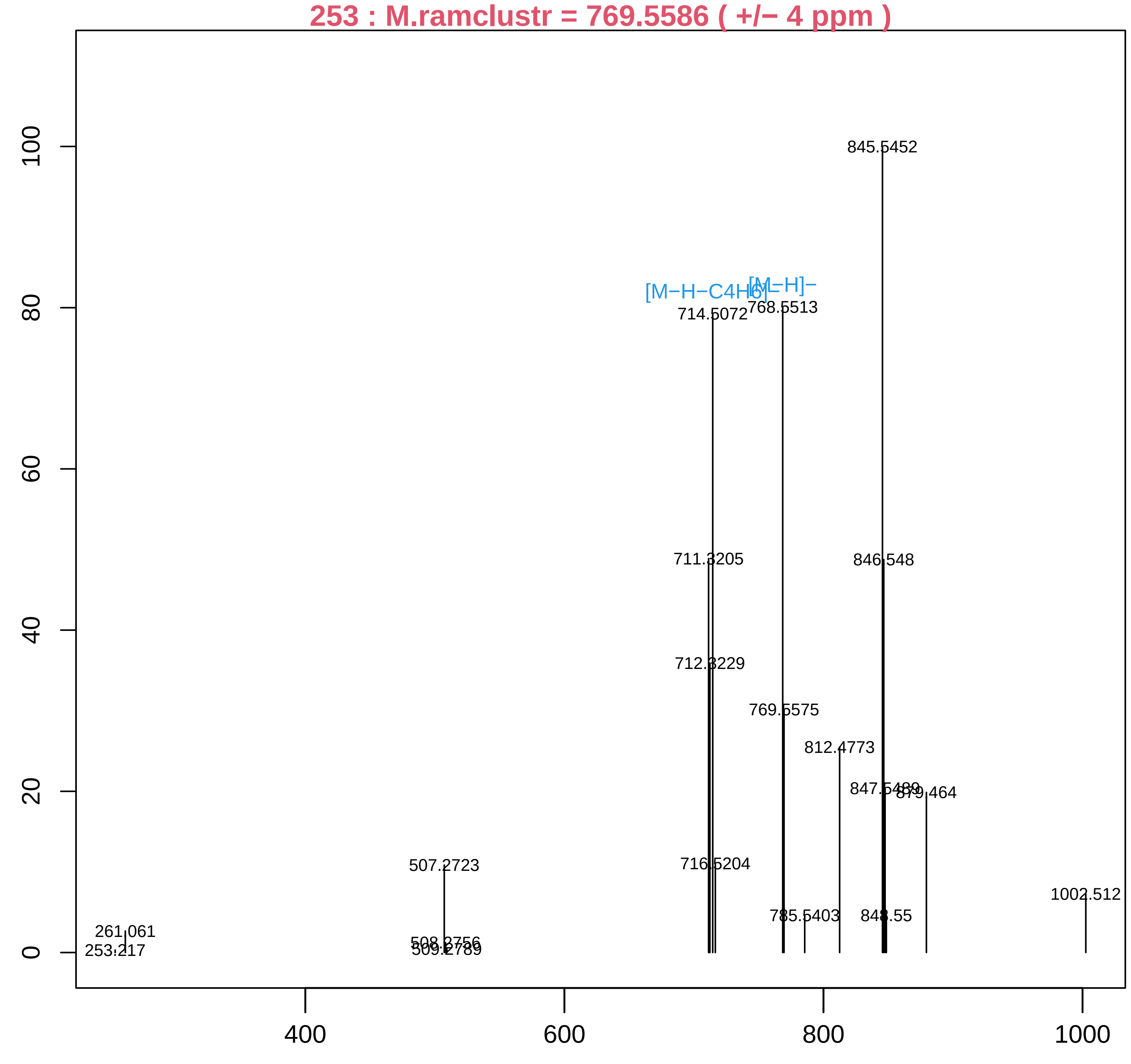

253 : M.ramclustr = 769.5586 (+/− 4 ppm)
[M−H−C4H6]−
[M−H]−
845.5452
714.5072
768.5513
711.3205
846.548
712.3229
769.5575
812.4773
847.5489
879.464
507.2723
716.6204
1002.512
785.5403
848.55
261.061
253.217
508.2756
509.2789
0
20
40
60
80
100
400
600
800
1000

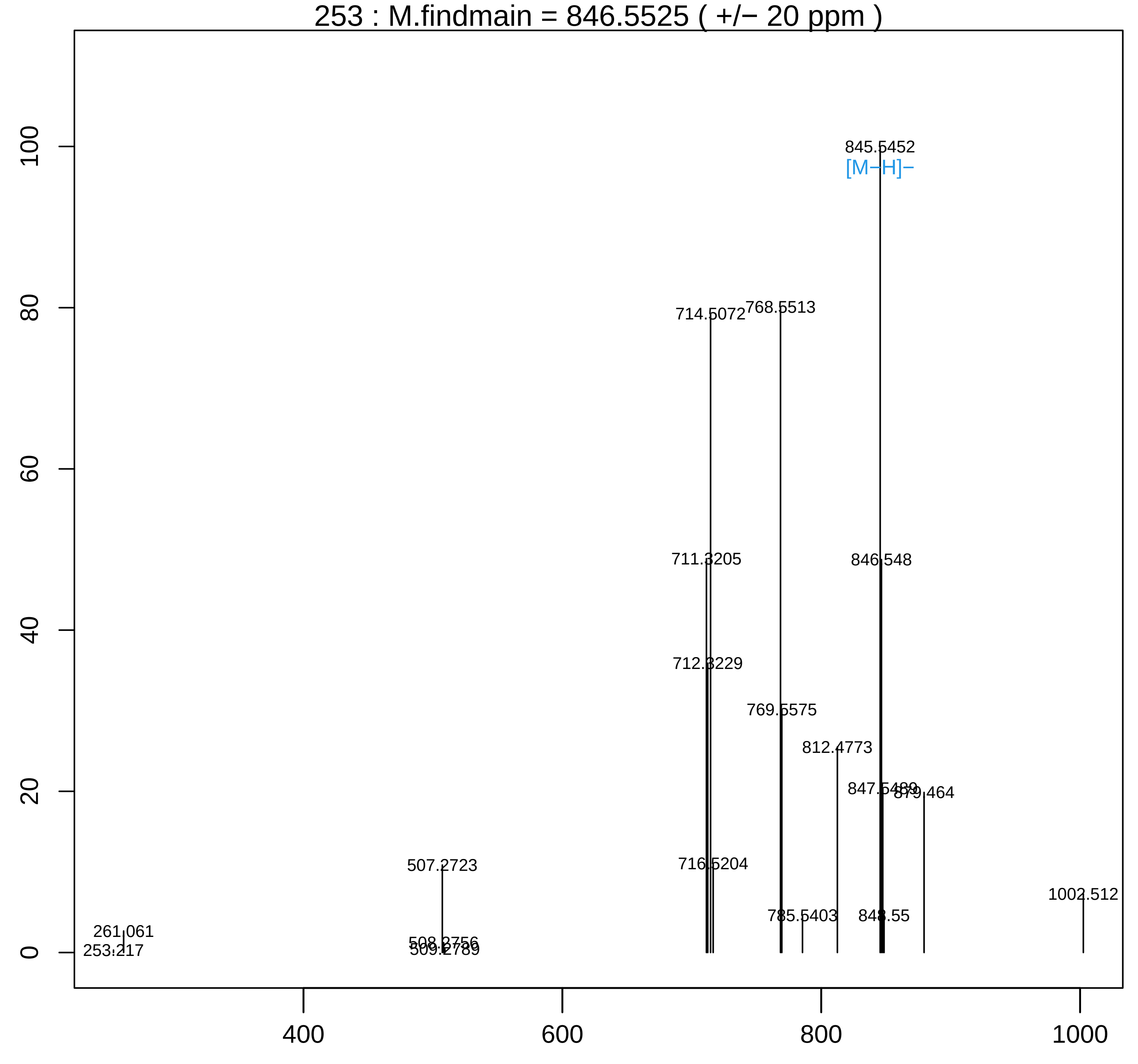

253 : M.findmain = 846.5525 (+/− 20 ppm)
845.5452
[M−H]−
714.5072
768.5513
711.3205
846.548
712.3229
769.5575
812.4773
847.5489
879.464
507.2723
716.6204
1002.512
785.5403
848.55
261.061
253.217
508.2756
509.2789
0
20
40
60
80
100
400
600
800
1000

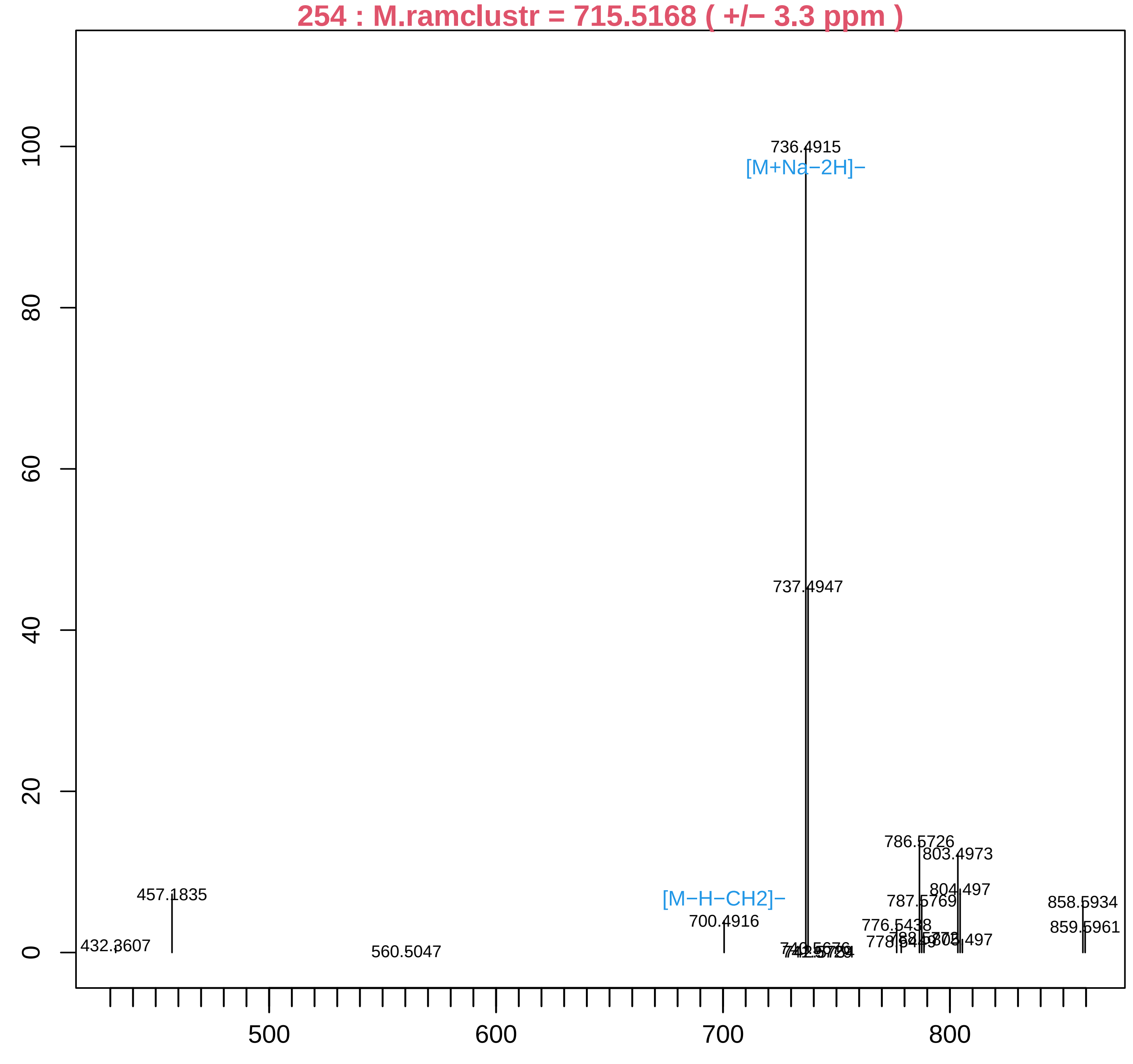
254 : M.ramclustr = 715.5168 (+/− 3.3 ppm)
736.4915
[M+Na−2H]−
737.4947
786.5726
803.4973
804.497
787.5769
[M−H−CH2]−
700.4916
776.5438
457.1835
432.3607
560.5047
858.5934
859.5961
0
20
40
60
80
100
500
600
700
800

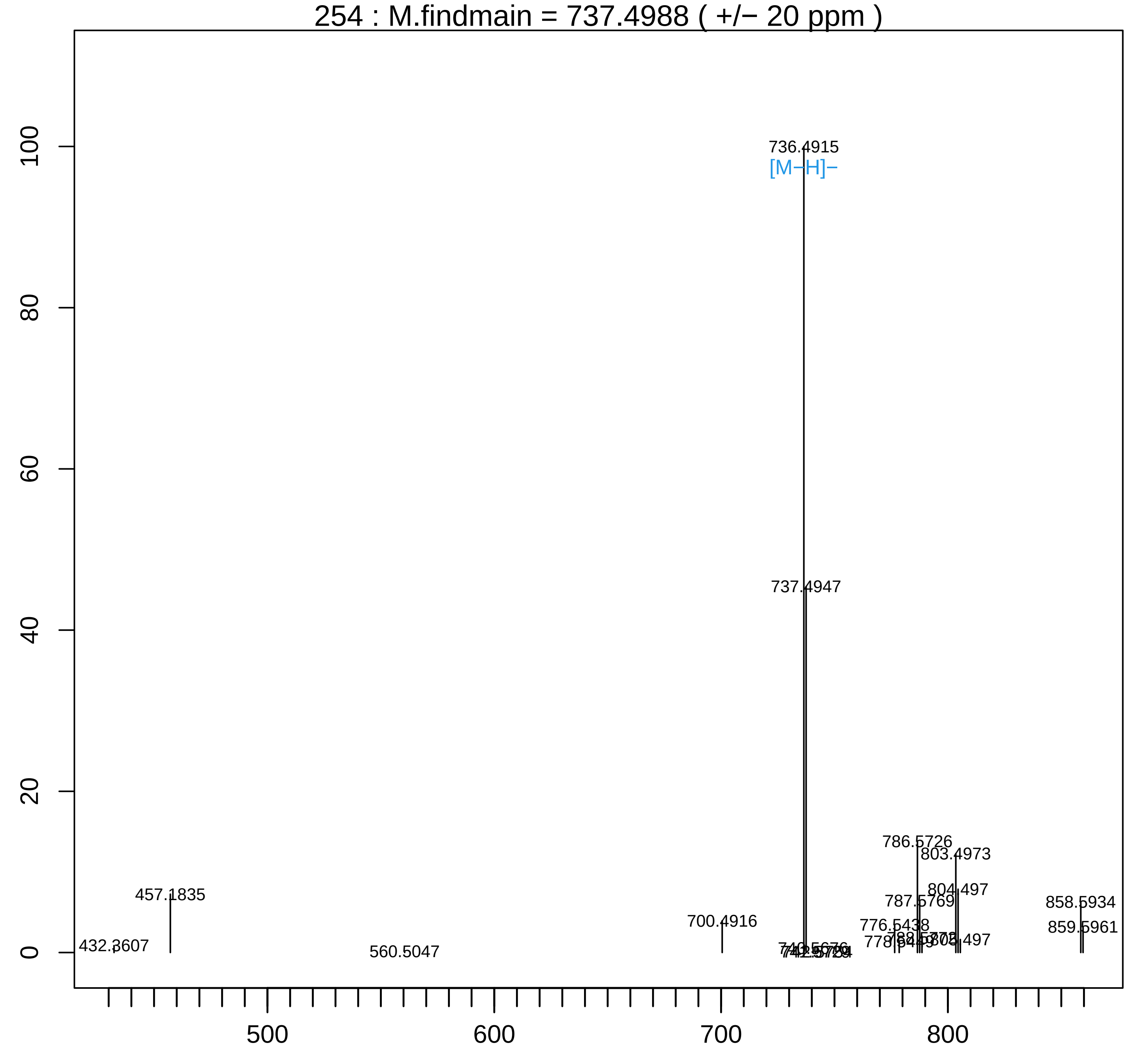
254 : M.findmain = 737.4988 (+/− 20 ppm)
736.4915
[M−H]−
737.4947
786.5726
803.4973
804.497
787.5769
700.4916
776.5438
457.1835
432.3607
560.5047
858.5934
859.5961
0
20
40
60
80
100
500
600
700
800

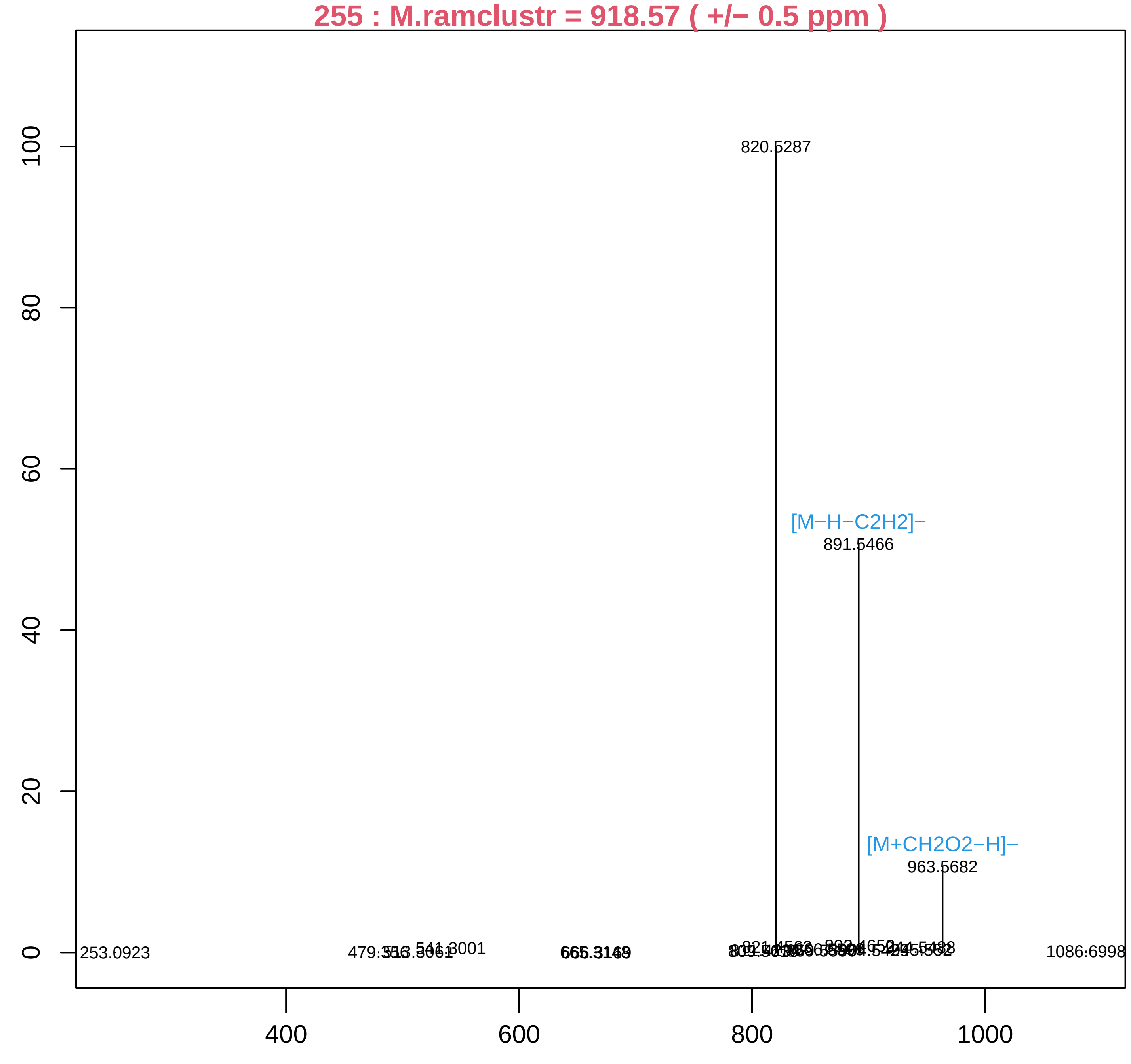
255 : M.ramclustr = 918.57 (+/− 0.5 ppm)
820.5287
[M−H−C2H2]−
891.5466
[M+CH2O2−H]−
963.5682
253.0923
541.3001
665.3149
1086.6998

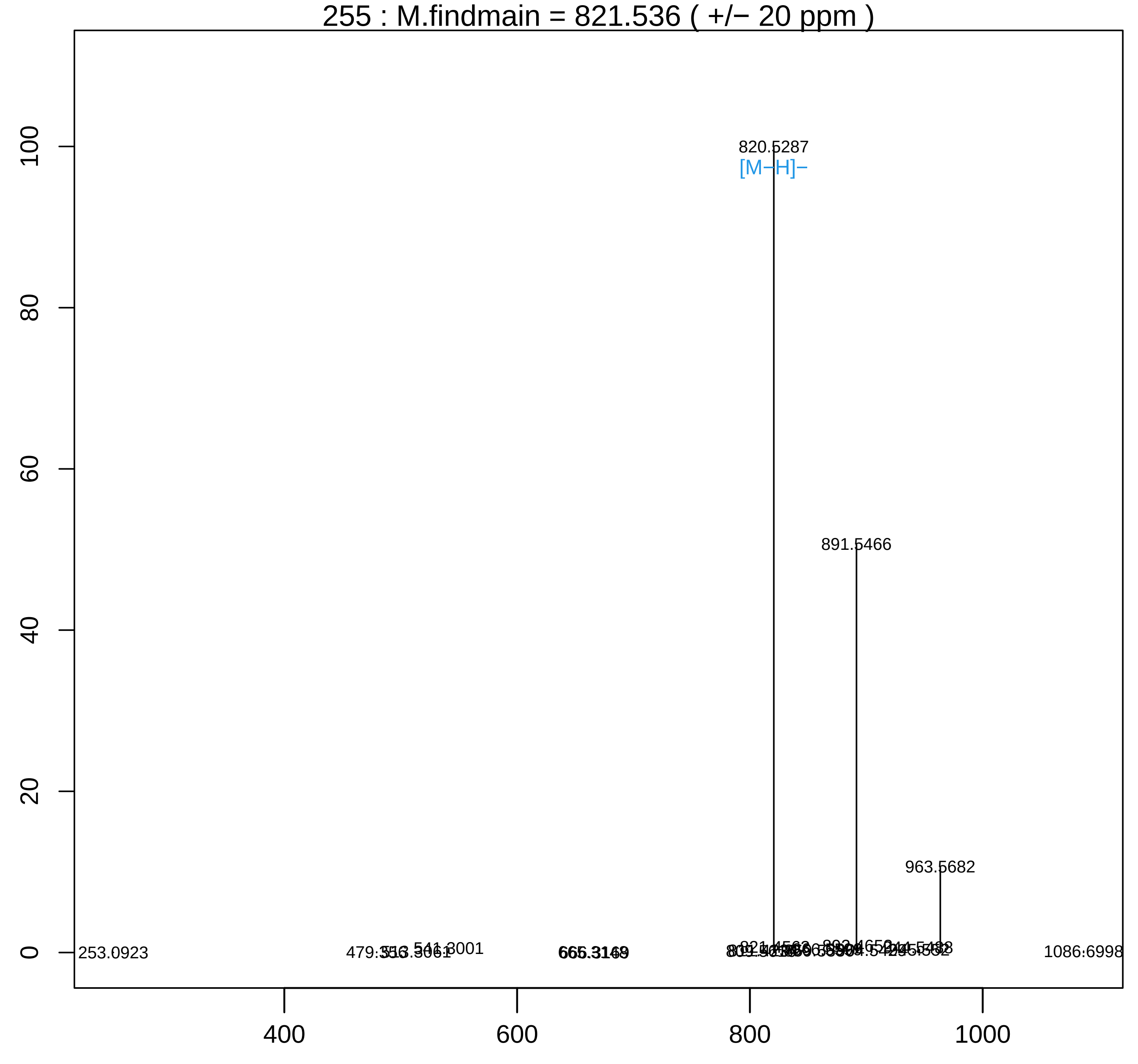
255 : M.findmain = 821.536 (+/− 20 ppm)
820.5287
[M−H]−
891.5466
963.5682
253.0923
541.3001
665.3149
1086.6998

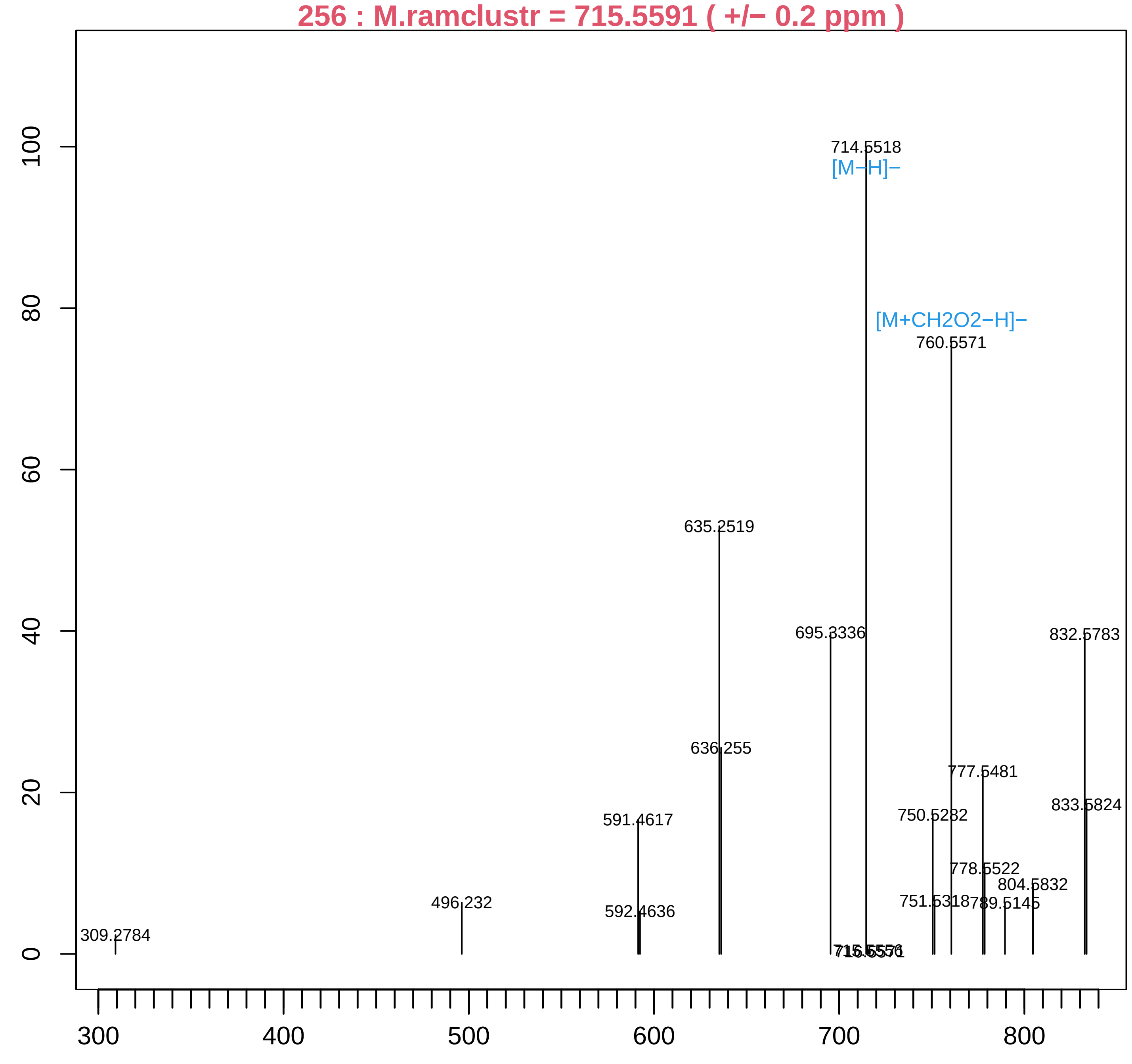
256 : M.ramclustr = 715.5591 (+/− 0.2 ppm)
714.5518
[M−H]−
[M+CH2O2−H]−
760.5571
635.2519
695.3336
832.5783
636.255
777.5481
591.4617
750.5282
833.5824
778.5522
804.5832
751.5318
789.5145
496.232
592.4636
309.2784
0
20
40
60
80
100
300
400
500
600
700
800

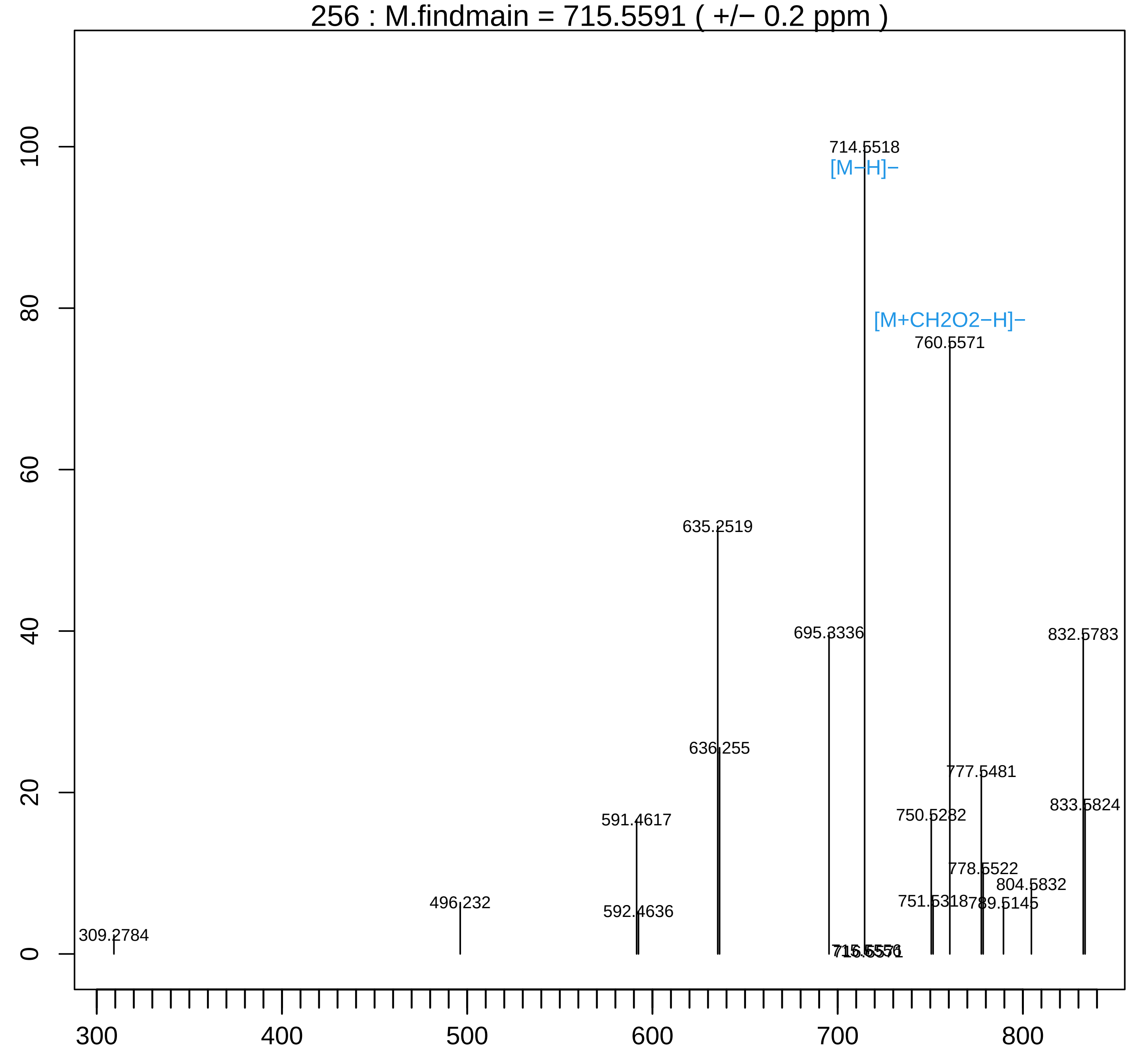
256 : M.findmain = 715.5591 (+/− 0.2 ppm)
714.5518
[M−H]−
[M+CH2O2−H]−
760.5571
635.2519
695.3336
832.5783
636.255
777.5481
591.4617
750.5282
833.5824
778.5522
804.5832
751.5318
789.5145
496.232
592.4636
309.2784
0
20
40
60
80
100
300
400
500
600
700
800

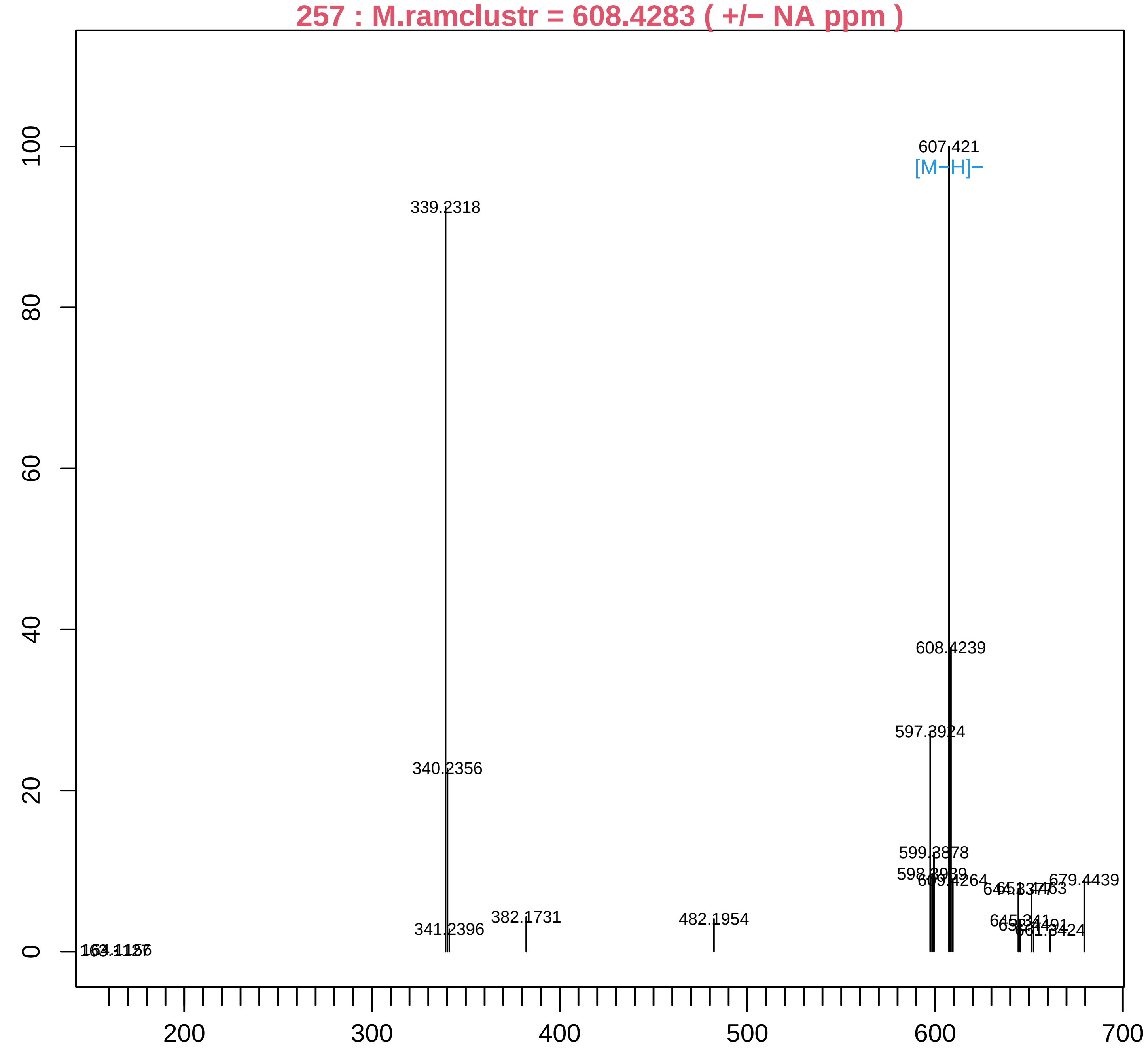
257 : M.ramclustr = 608.4283 (+/− NA ppm)
607.421
[M−H]−
339.2318
608.4239
597.3924
340.2356
599.3878
679.4439
382.1731
482.1954
341.2396
0
20
40
60
80
100
200
300
400
500
600
700

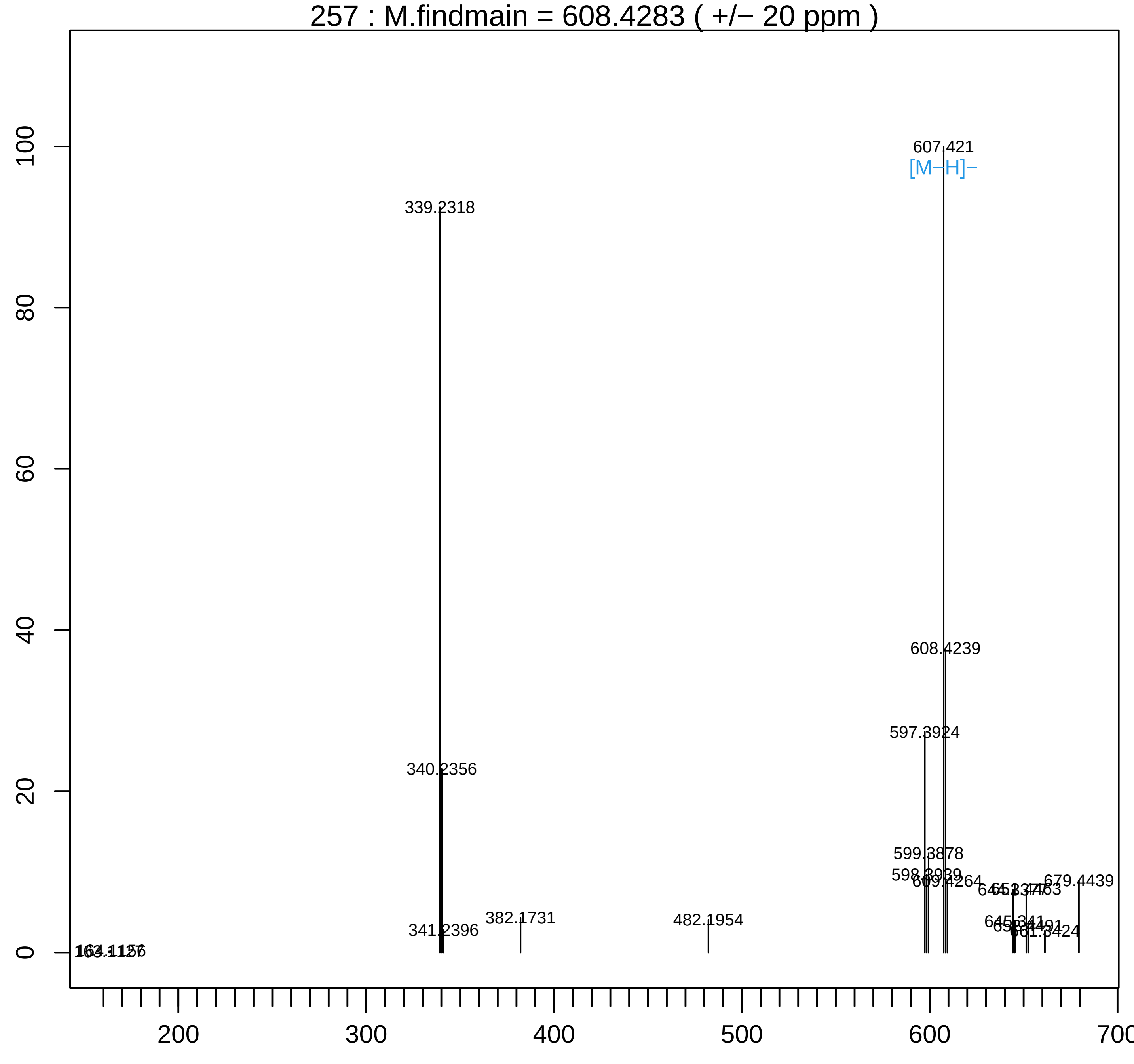
257 : M.findmain = 608.4283 (+/− 20 ppm)
607.421
[M−H]−
339.2318
608.4239
597.3924
340.2356
599.3878
679.4439
382.1731
482.1954
341.2396
0
20
40
60
80
100
200
300
400
500
600
700

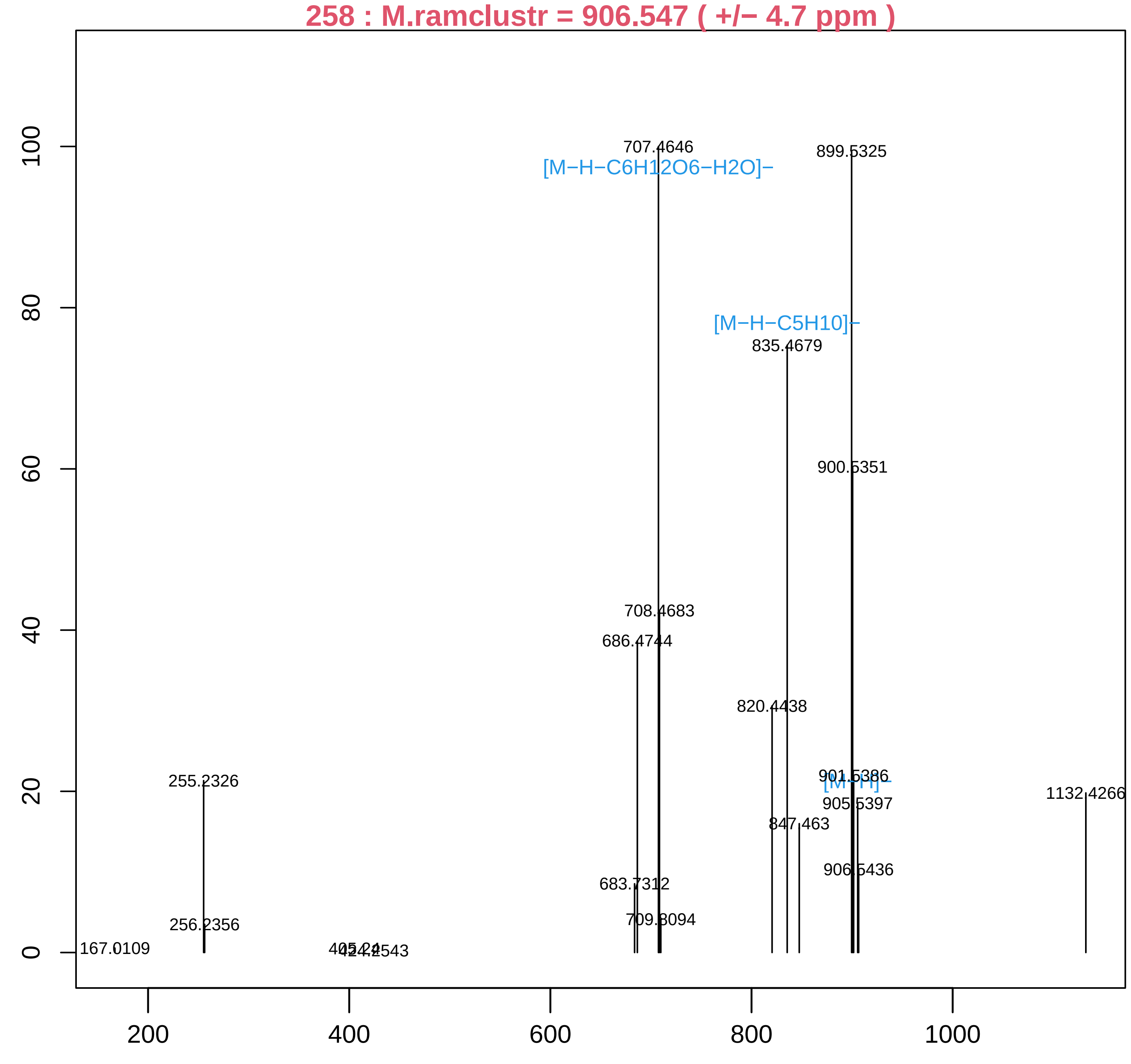
258 : M.ramclustr = 906.547 (+/− 4.7 ppm)
707.4646
[M−H−C6H12O6−H2O]−
899.5325
[M−H−C5H10]−
835.4679
900.5351
708.4683
686.4744
820.4438
901.5386
[M−H]−
905.5397
847.463
1132.4266
255.2326
906.5436
683.7312
709.8094
256.2356
167.0109
405.24
424.2543
0
20
40
60
80
100
200
400
600
800
1000

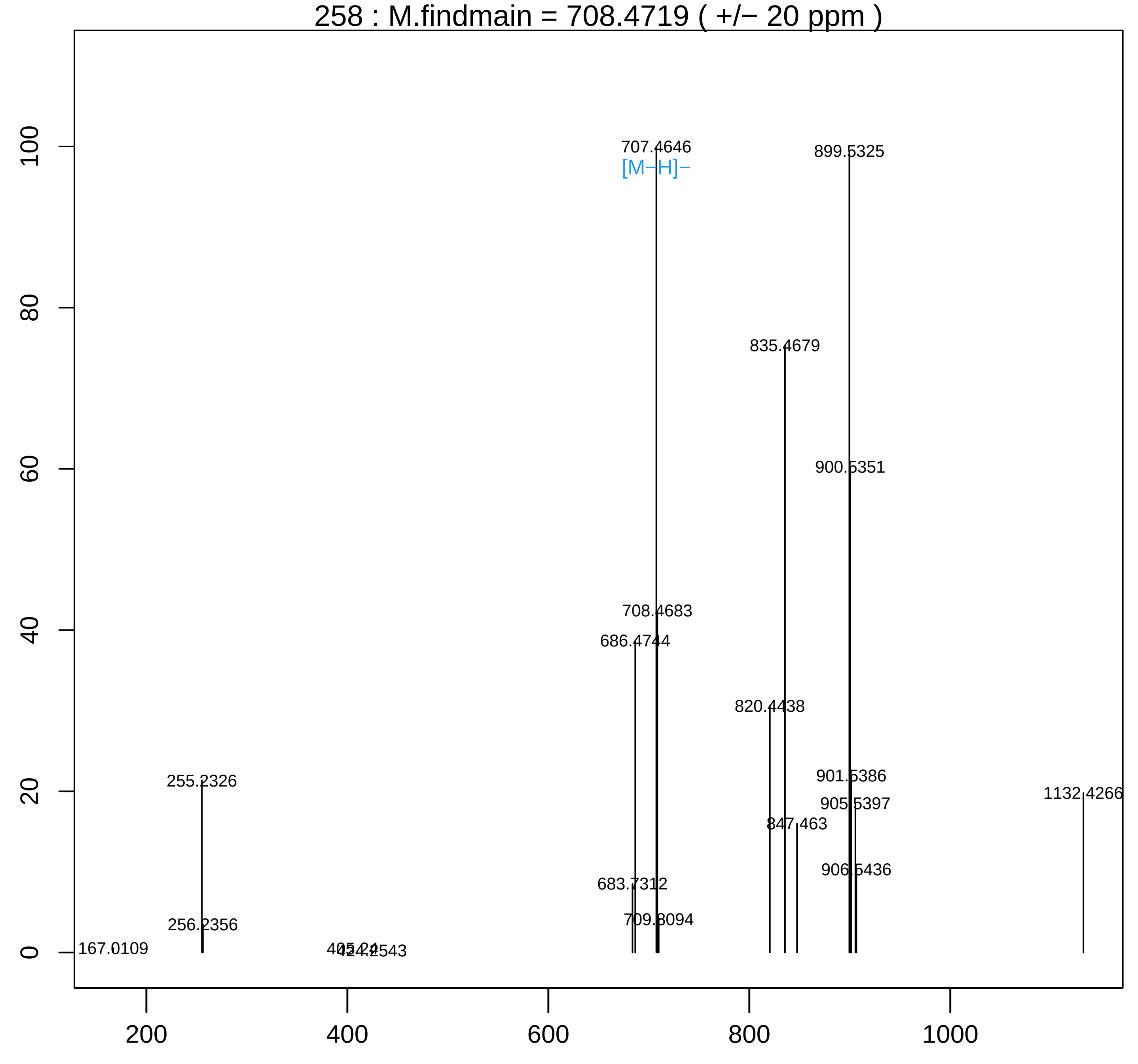
258 : M.findmain = 708.4719 (+/− 20 ppm)
707.4646
[M−H]−
899.5325
835.4679
900.5351
708.4683
686.4744
820.4438
901.5386
905.5397
847.463
1132.4266
255.2326
906.5436
683.7312
709.8094
256.2356
167.0109
405.24
424.2543
0
20
40
60
80
100
200
400
600
800
1000

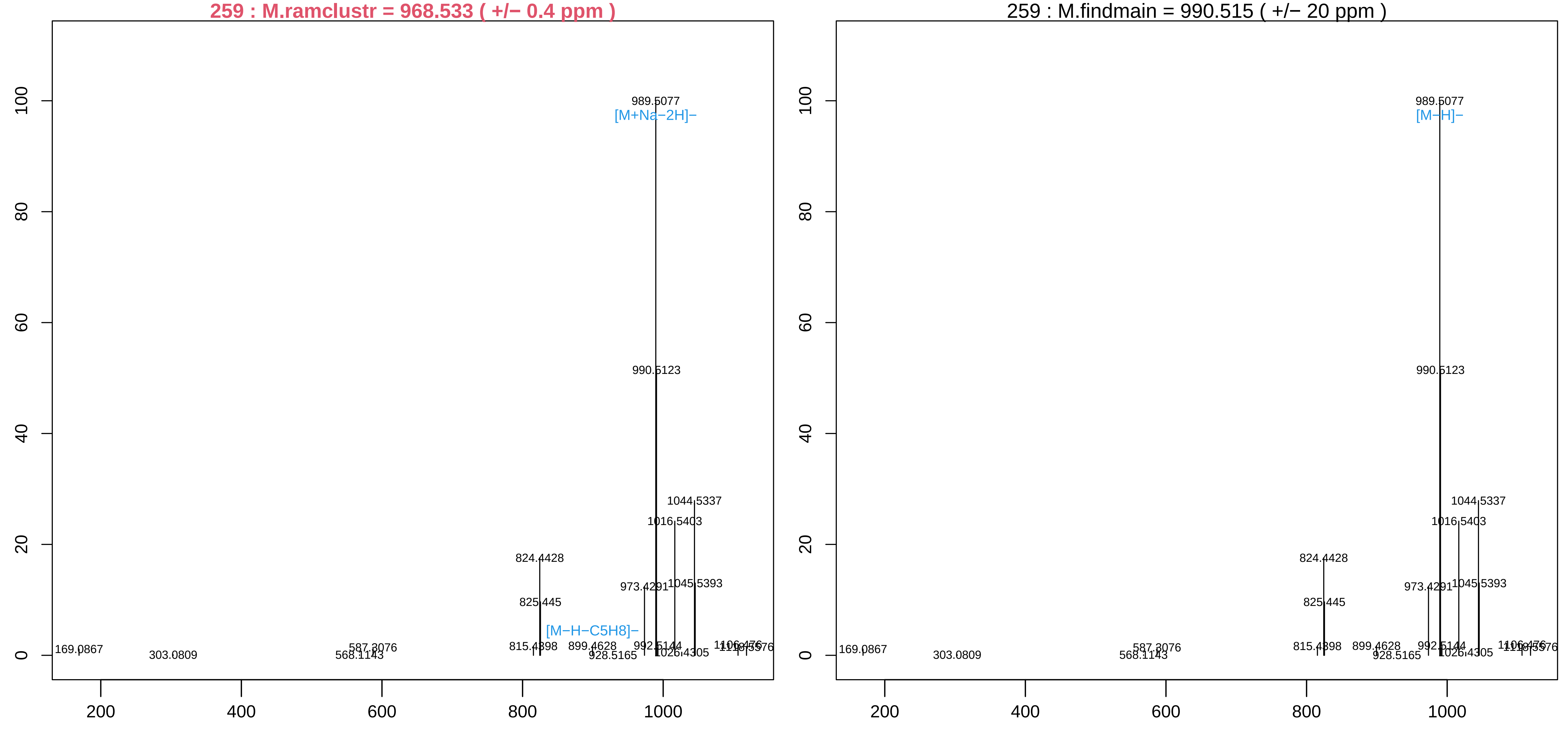
259 : M.ramclustr = 968.533 (+/− 0.4 ppm)
989.5077
[M+Na−2H]−
990.5123
1044.5337
1016.5403
824.4428
973.4291
1045.5393
825.445
[M−H−C5H8]−
169.0867
303.0809
587.3076
568.1143
815.4398
899.4628
928.5165
992.5144
1026.4305
259 : M.findmain = 990.515 (+/− 20 ppm)
[M−H]−
0
20
40
60
80
100
200
400
600
800
1000

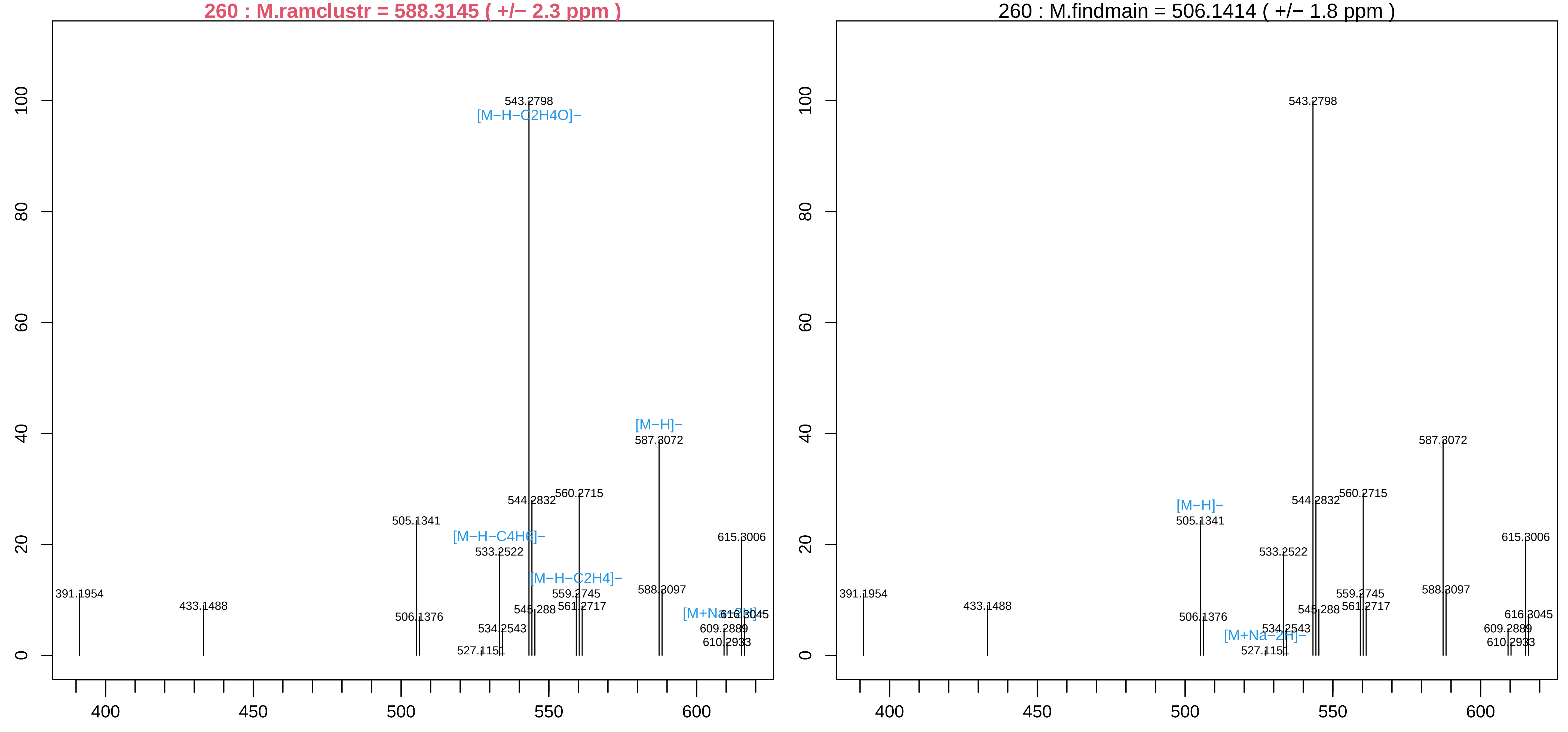

260 : M.ramclustr = 588.3145 (+/− 2.3 ppm)
543.2798
[M−H−C2H4O]−
[M−H]−
587.3072
560.2715
544.2832
505.1341
[M−H−C4H6]−
533.2522
615.3006
[M−H−C2H4]−
559.2745
588.3097
391.1954
433.1488
545.288
561.2717
[M+Na−2H]−
616.3045
506.1376
534.2543
609.2889
610.2933
527.1151
260 : M.findmain = 506.1414 (+/− 1.8 ppm)
543.2798
587.3072
[M−H]−
505.1341
560.2715
544.2832
533.2522
615.3006
559.2745
588.3097
391.1954
433.1488
545.288
561.2717
616.3045
506.1376
534.2543
[M+Na−2H]−
609.2889
610.2933
527.1151
0
20
40
60
80
100
400
450
500
550
600

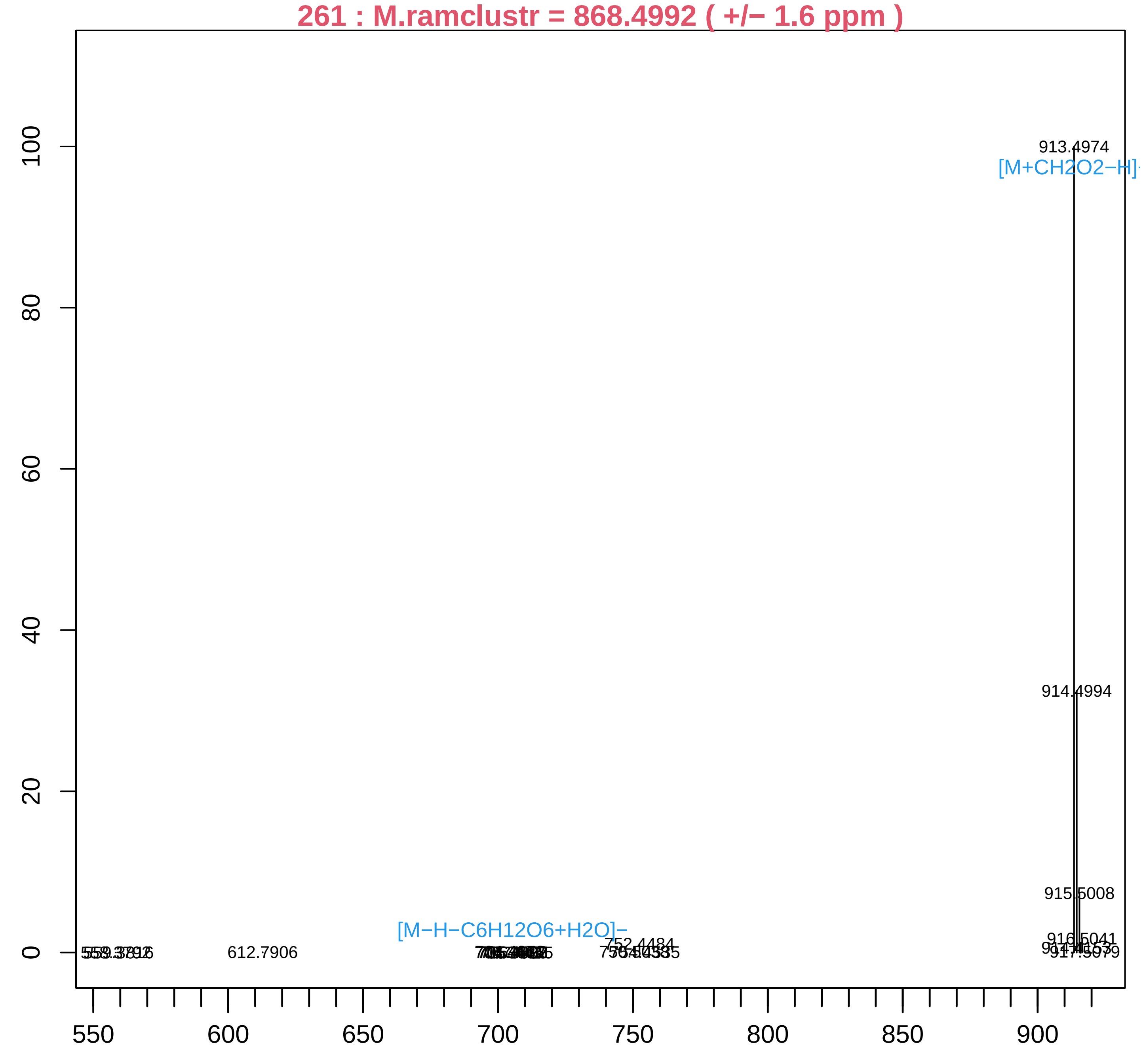

261 : M.ramclustr = 868.4992 (+/− 1.6 ppm)
913.4974
[M+CH2O2−H]−
914.4994
915.5008
916.5041
[M−H−C6H12O6+H2O]−
752.4484
612.7906

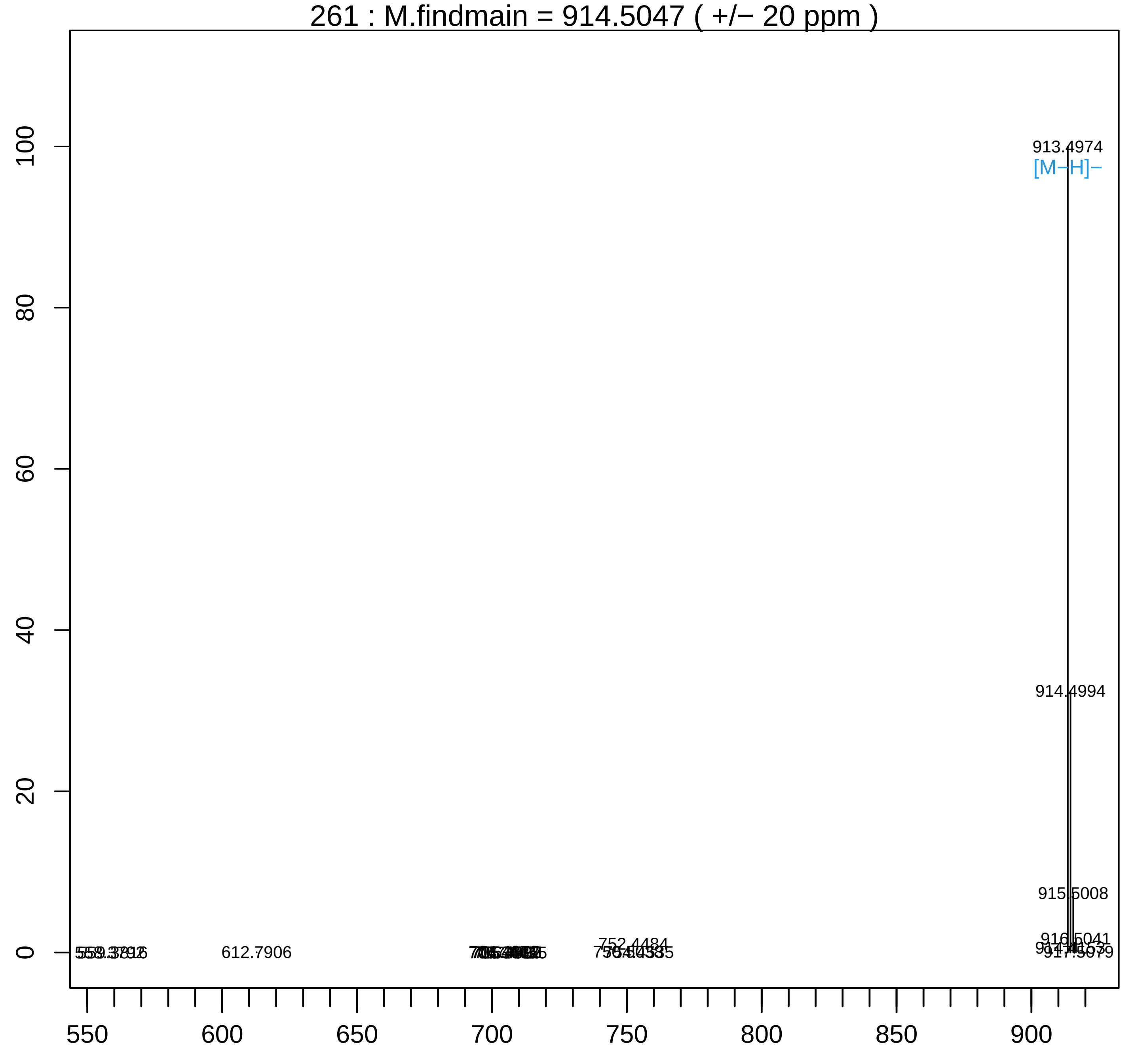

261 : M.findmain = 914.5047 (+/− 20 ppm)
913.4974
[M−H]−
914.4994
915.5008
916.5041
752.4484
612.7906

262 : M.ramclustr = 1046.5301 (+/− 0.1 ppm)

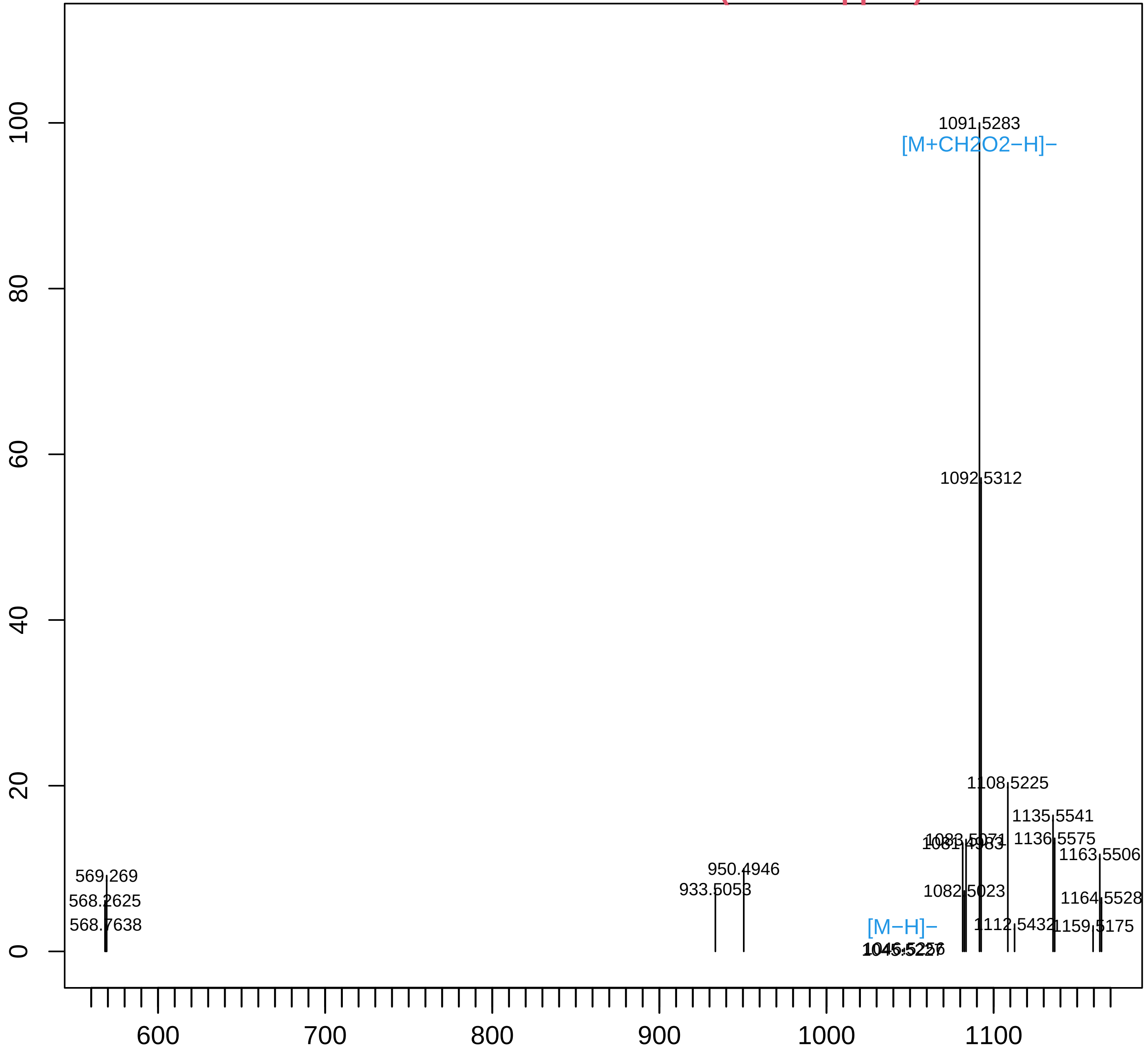

262 : M.findmain = 1046.5301 (+/− 0.1 ppm)

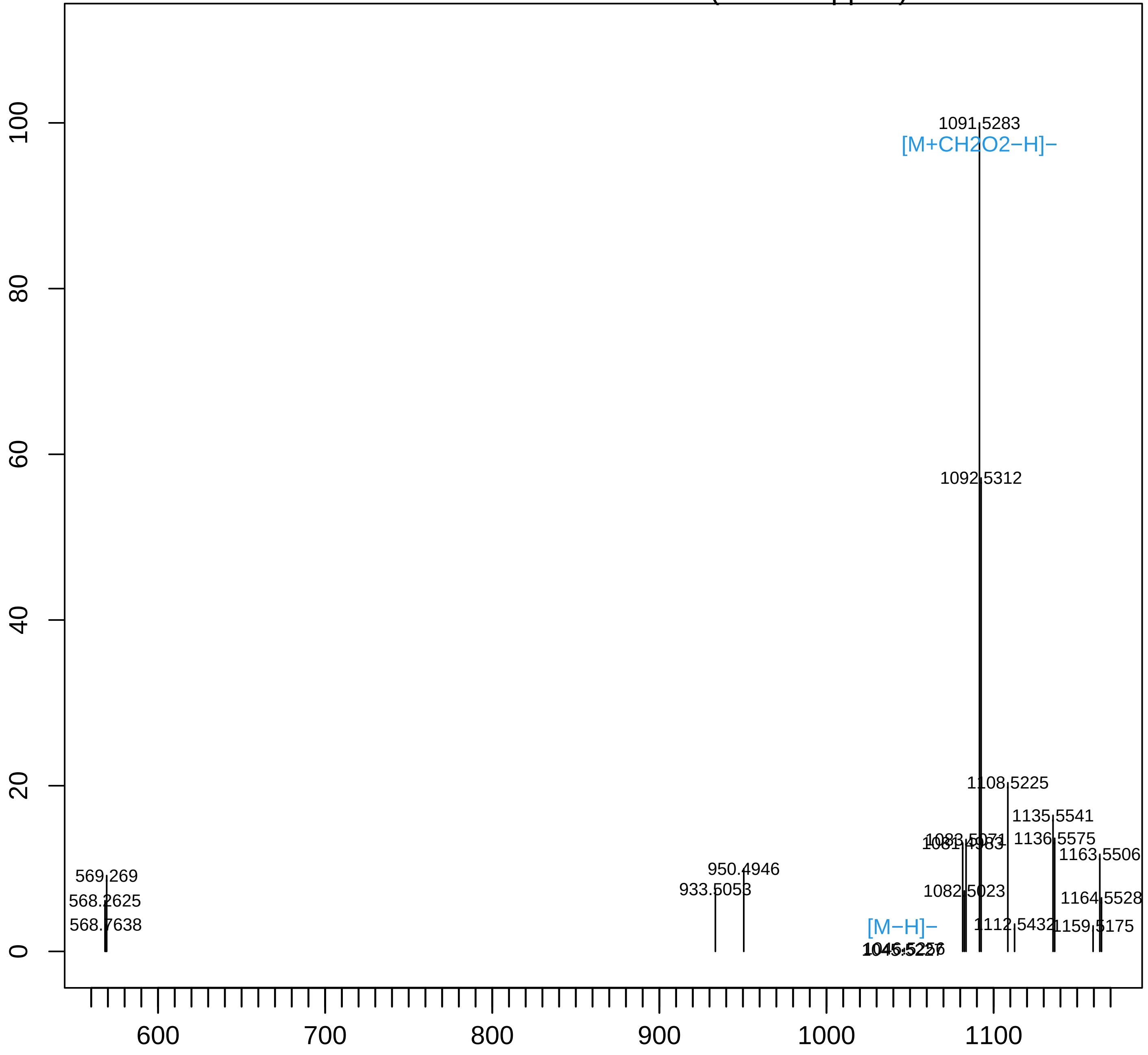

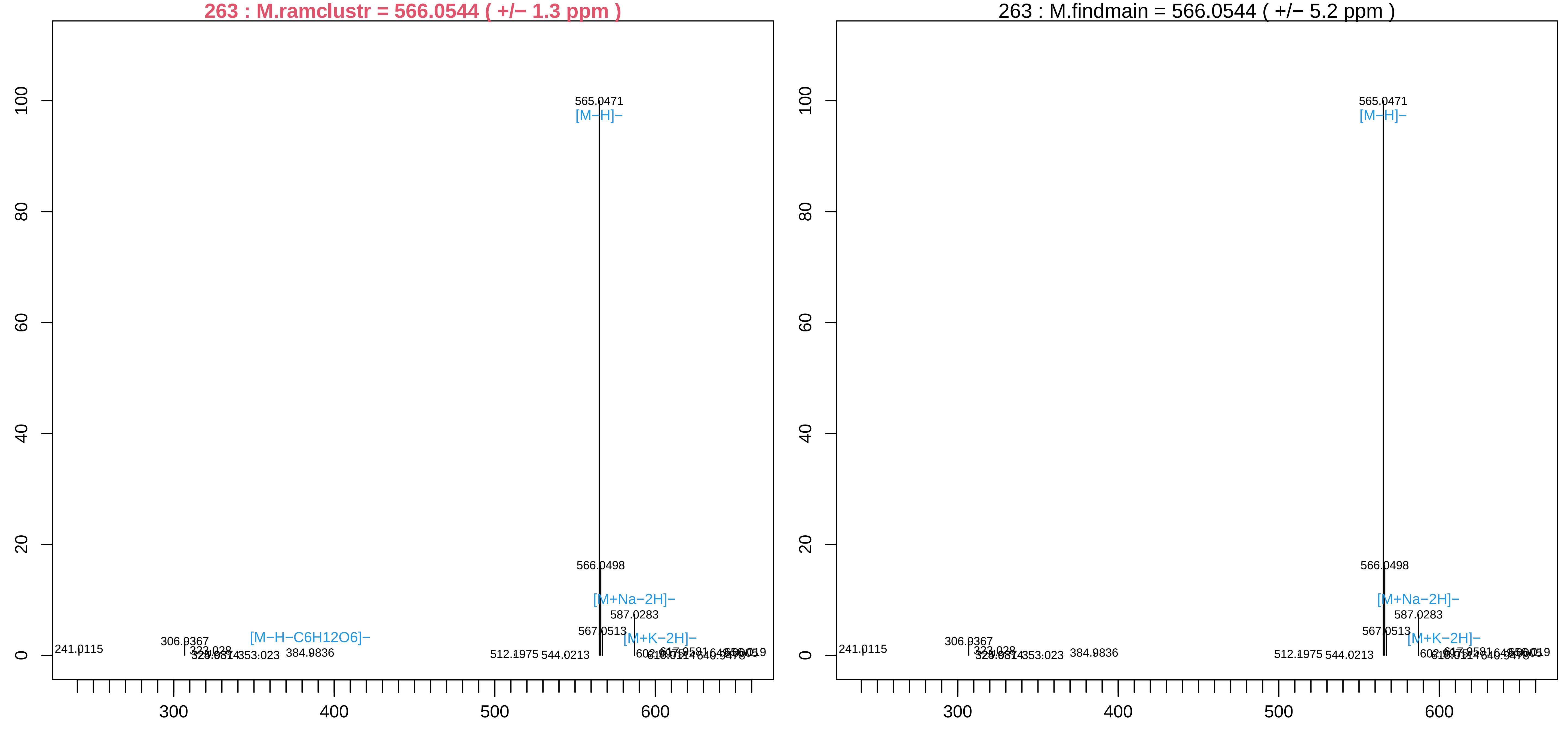
263 : M.ramclustr = 566.0544 (+/− 1.3 ppm)
565.0471
[M−H]−
566.0498
[M+Na−2H]−
587.0283
567.0513
[M+K−2H]−
[M−H−C6H12O6]−
241.0115
306.9367
353.023
384.9836
512.1975
544.0213
263 : M.findmain = 566.0544 (+/− 5.2 ppm)
0
20
40
60
80
100
300
400
500
600

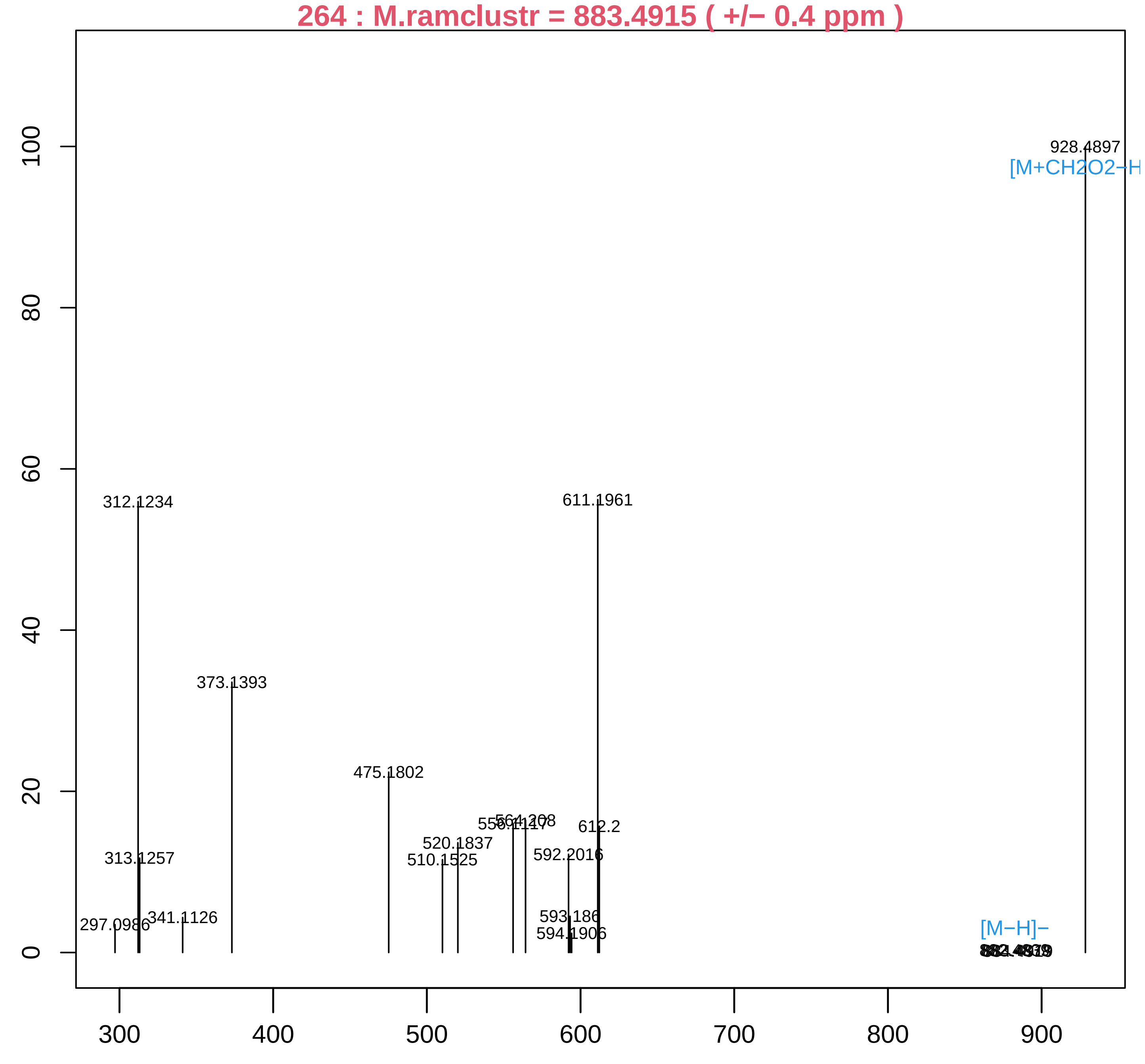
264 : M.ramclustr = 883.4915 (+/− 0.4 ppm)
928.4897
[M+CH2O2−H
312.1234
611.1961
373.1393
475.1802
564.208
520.1837
510.1525
612.2
592.2016
313.1257
297.0986
341.1126
593.186
594.1906
[M−H]−
0
20
40
60
80
100
300
400
500
600
700
800
900

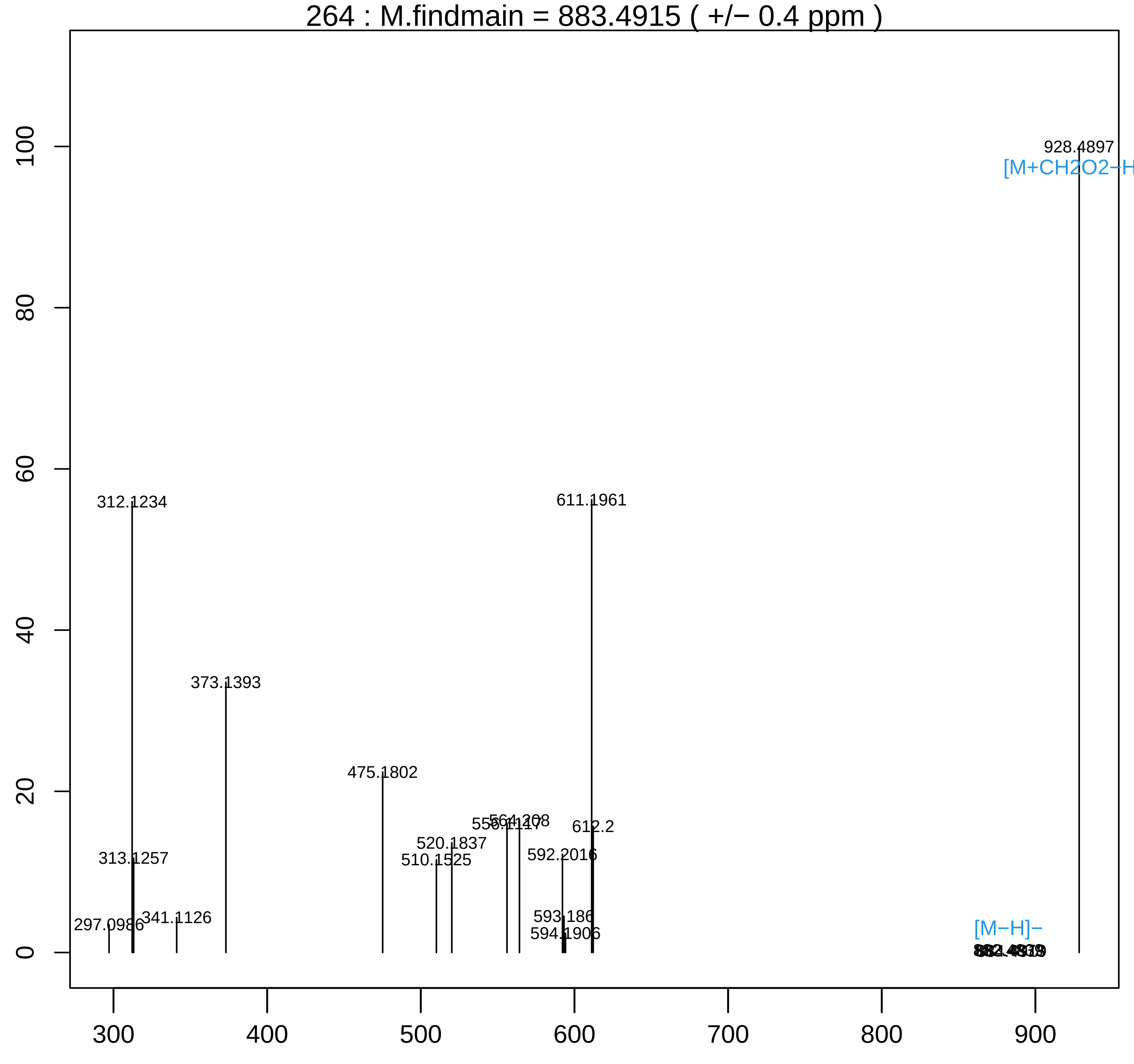
264 : M.findmain = 883.4915 (+/− 0.4 ppm)
928.4897
[M+CH2O2−H
312.1234
611.1961
373.1393
475.1802
564.208
520.1837
510.1525
612.2
592.2016
313.1257
297.0986
341.1126
593.186
594.1906
[M−H]−
0
20
40
60
80
100
300
400
500
600
700
800
900

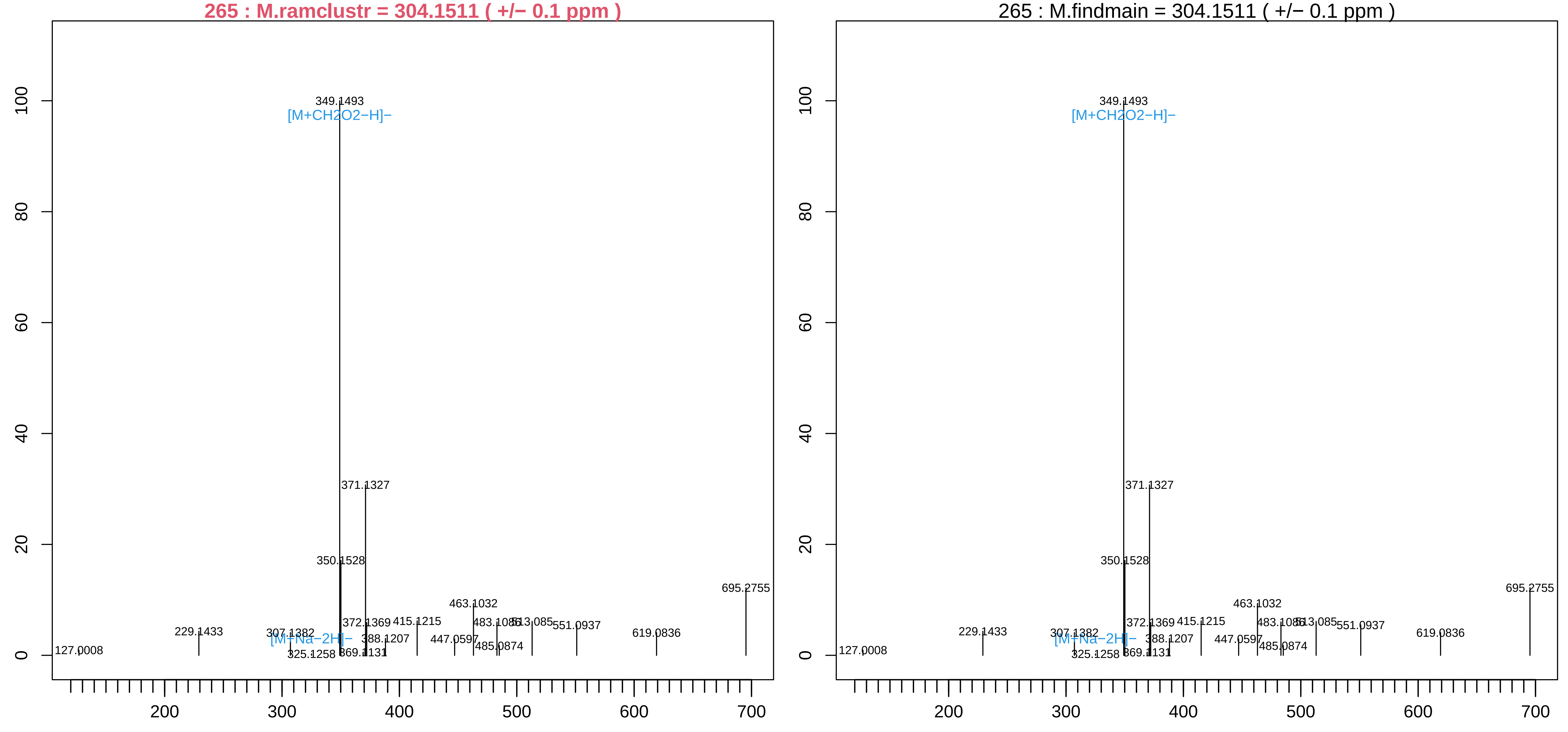
265 : M.ramclustr = 304.1511 (+/− 0.1 ppm)
265 : M.findmain = 304.1511 (+/− 0.1 ppm)
349.1493
[M+CH2O2−H]−
371.1327
350.1528
695.2755
463.1032
372.1369
415.1215
483.1086
513.085
551.0937
229.1433
307.1382
[M+Na−2H]−
388.1207
447.0597
619.0836
485.0874
127.0008
325.1258
369.1131
0
20
40
60
80
100
200
300
400
500
600
700

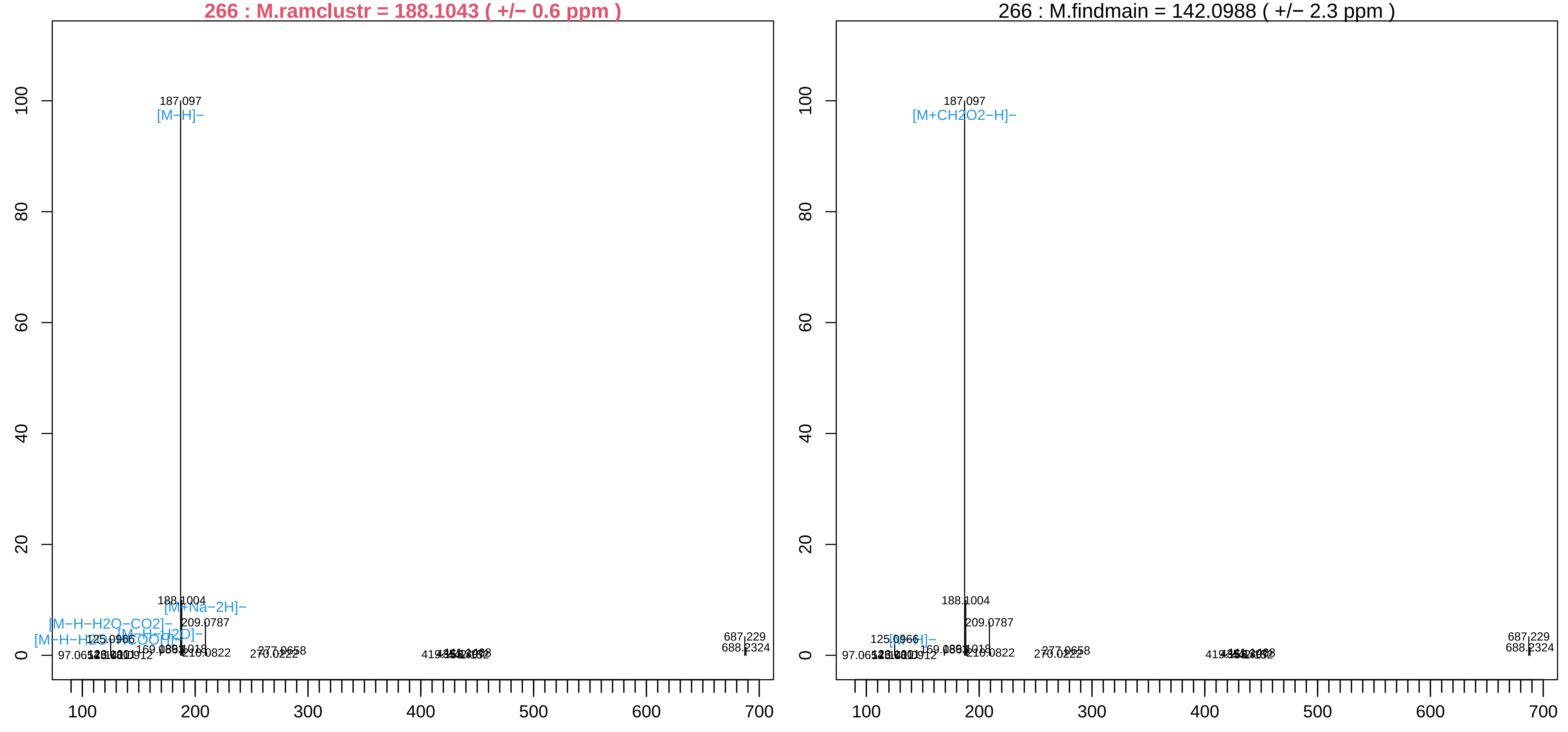

266 : M.ramclustr = 188.1043 (+/− 0.6 ppm)
187.097
[M−H]−
188.1004
[M+Na−2H]−
209.0787
[M−H−H2O−CO2]−
[M−H−H2O]−
125.0966
169.0861
210.0822
270.0222
277.0658
687.229
688.2324
97.0651
266 : M.findmain = 142.0988 (+/− 2.3 ppm)
[M+CH2O2−H]−
0
20
40
60
80
100
200
300
400
500
600
700

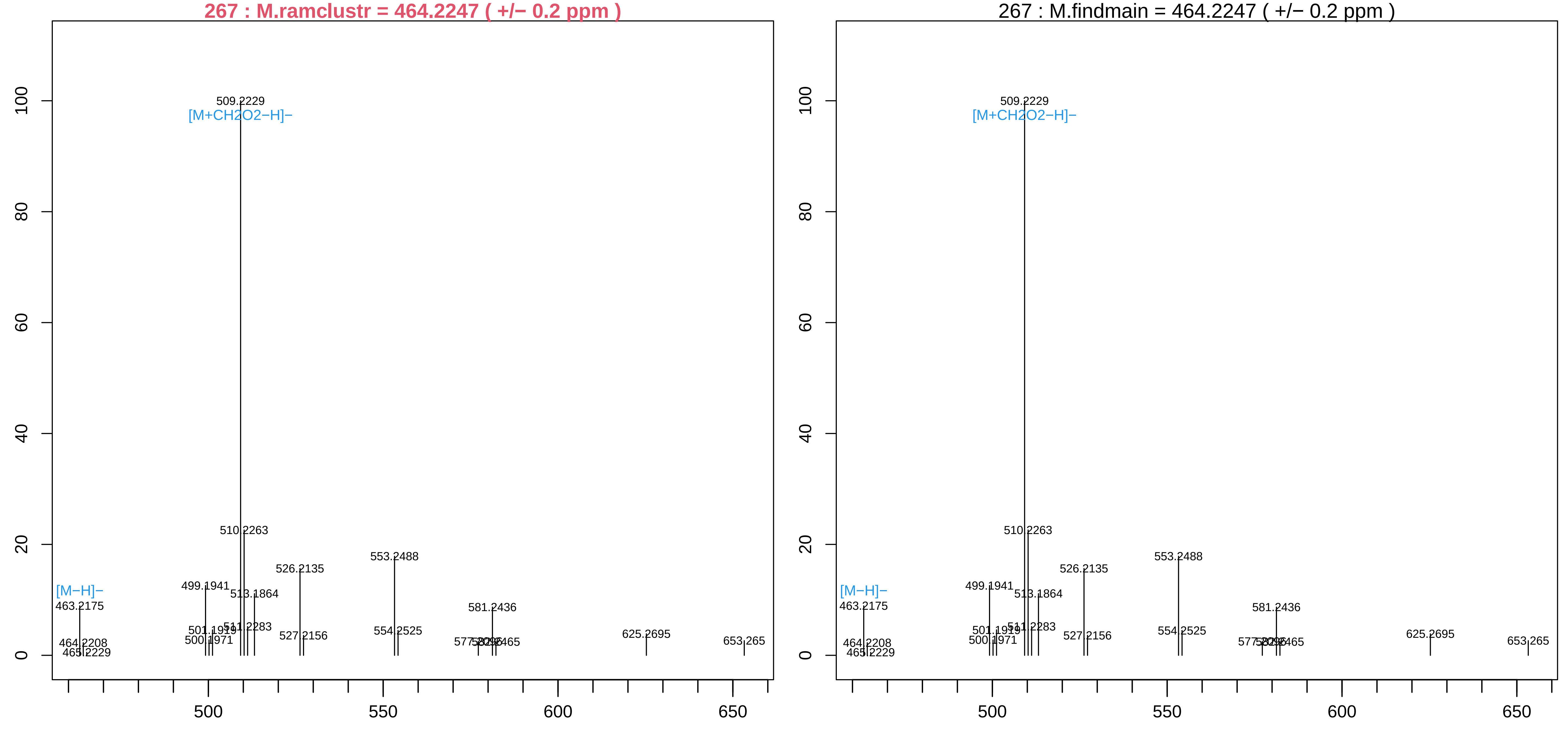
267 : M.ramclustr = 464.2247 (+/− 0.2 ppm)
509.2229
[M+CH2O2−H]−
510.2263
553.2488
526.2135
499.1941
513.1864
[M−H]−
463.2175
581.2436
501.1919
511.2283
500.1971
527.2156
554.2525
464.2208
465.2229
577.2296
582.2465
625.2695
653.265
0
20
40
60
80
100
500
550
600
650
267 : M.findmain = 464.2247 (+/− 0.2 ppm)

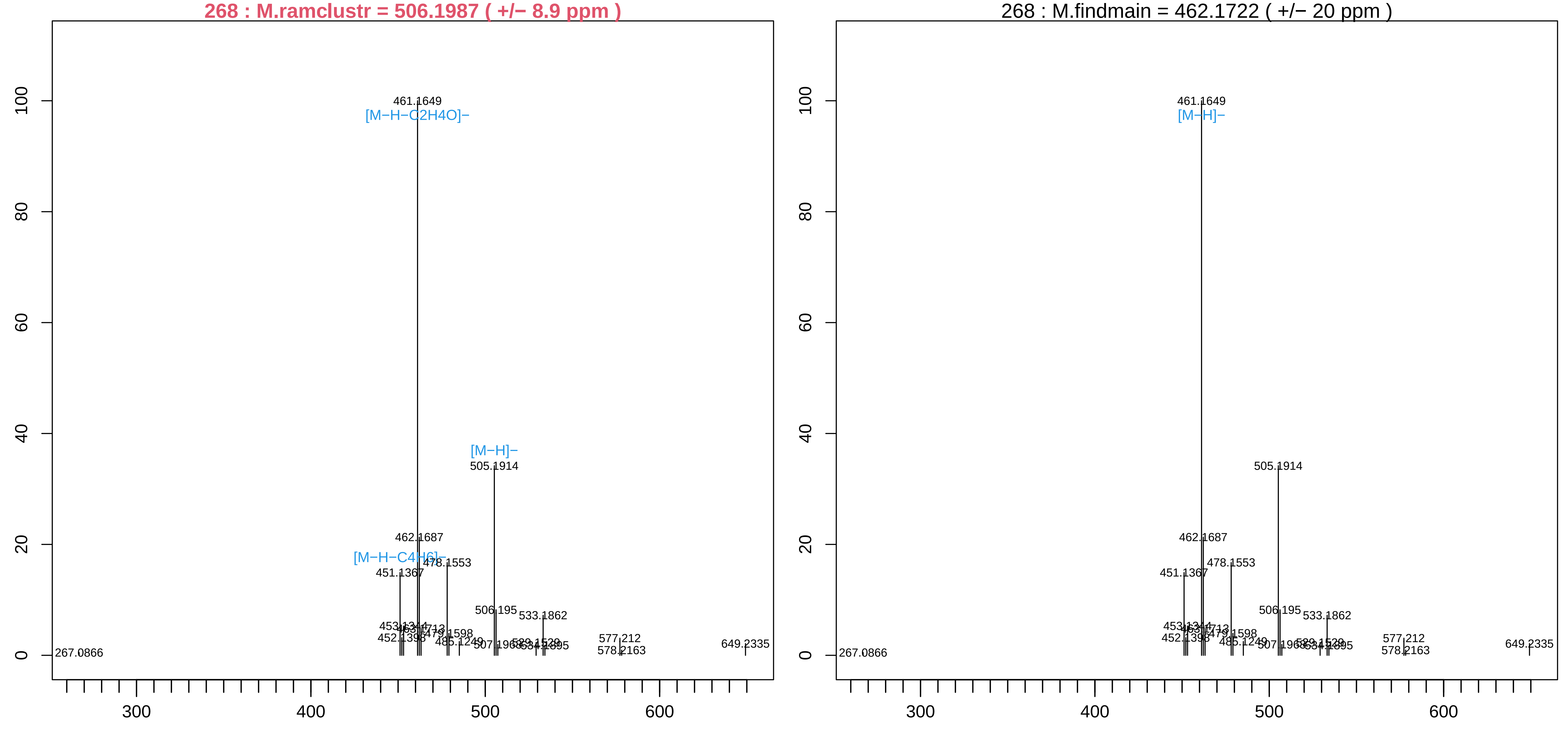

268 : M.ramclustr = 506.1987 (+/− 8.9 ppm)
461.1649
[M−H−C2H4O]−
[M−H]−
505.1914
462.1687
[M−H−C4H6]−
478.1553
451.1367
506.195
533.1862
453.1344
463.1713
479.1598
452.1395
485.1249
507.1968
529.1529
534.1895
577.212
578.2163
649.2335
267.0866
268 : M.findmain = 462.1722 (+/− 20 ppm)
461.1649
[M−H]−
505.1914
462.1687
478.1553
451.1367
506.195
533.1862
0
20
40
60
80
100
300
400
500
600

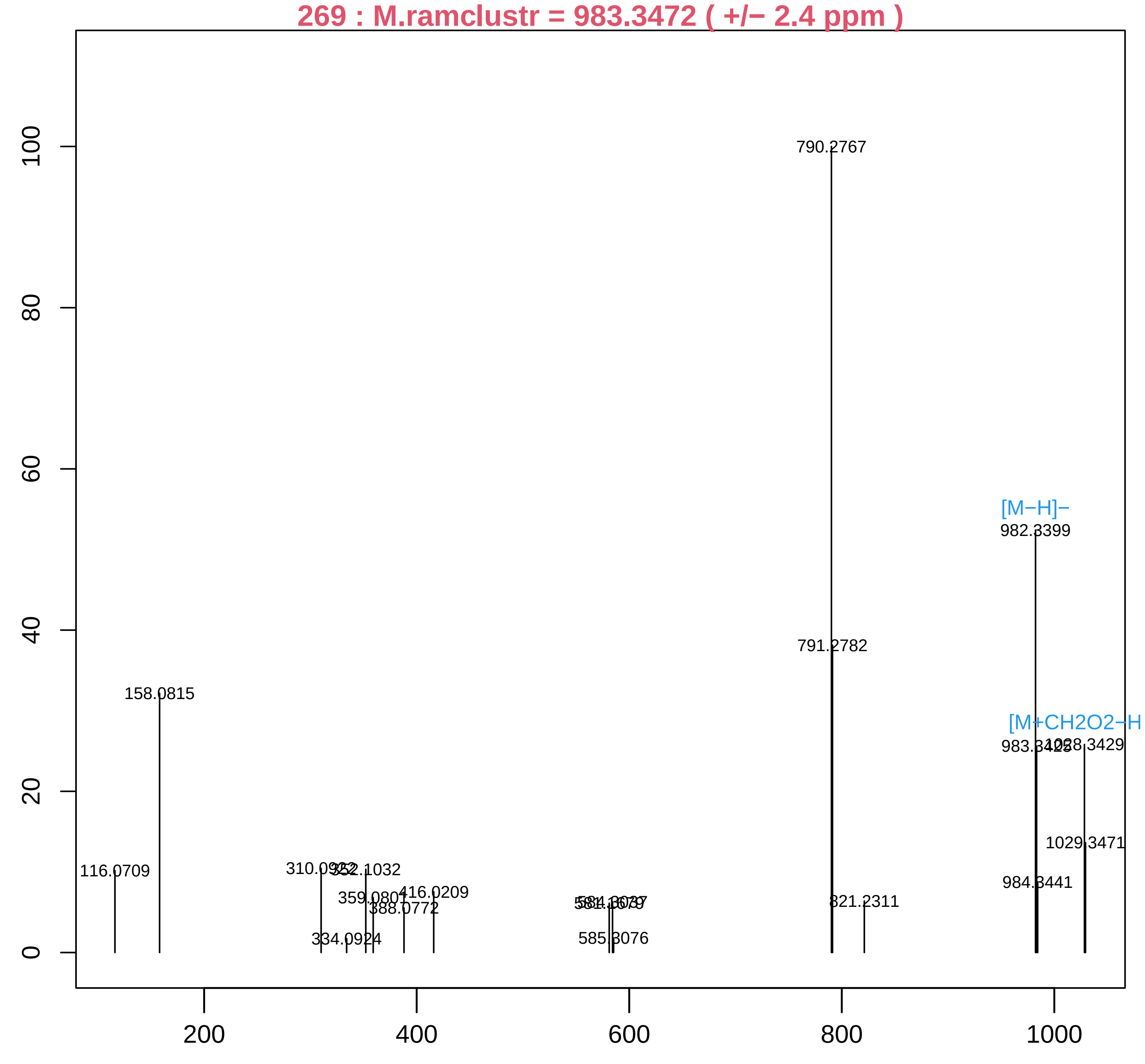
269 : M.ramclustr = 983.3472 (+/− 2.4 ppm)
790.2767
791.2782
158.0815
116.0709
310.0922
352.1032
359.0801
388.0772
416.0209
334.0924
585.3076
821.2311
[M−H]−
982.3399
[M+CH2O2−H
983.3428
1028.3429
1029.3471
984.3441
0
20
40
60
80
100
200
400
600
800
1000

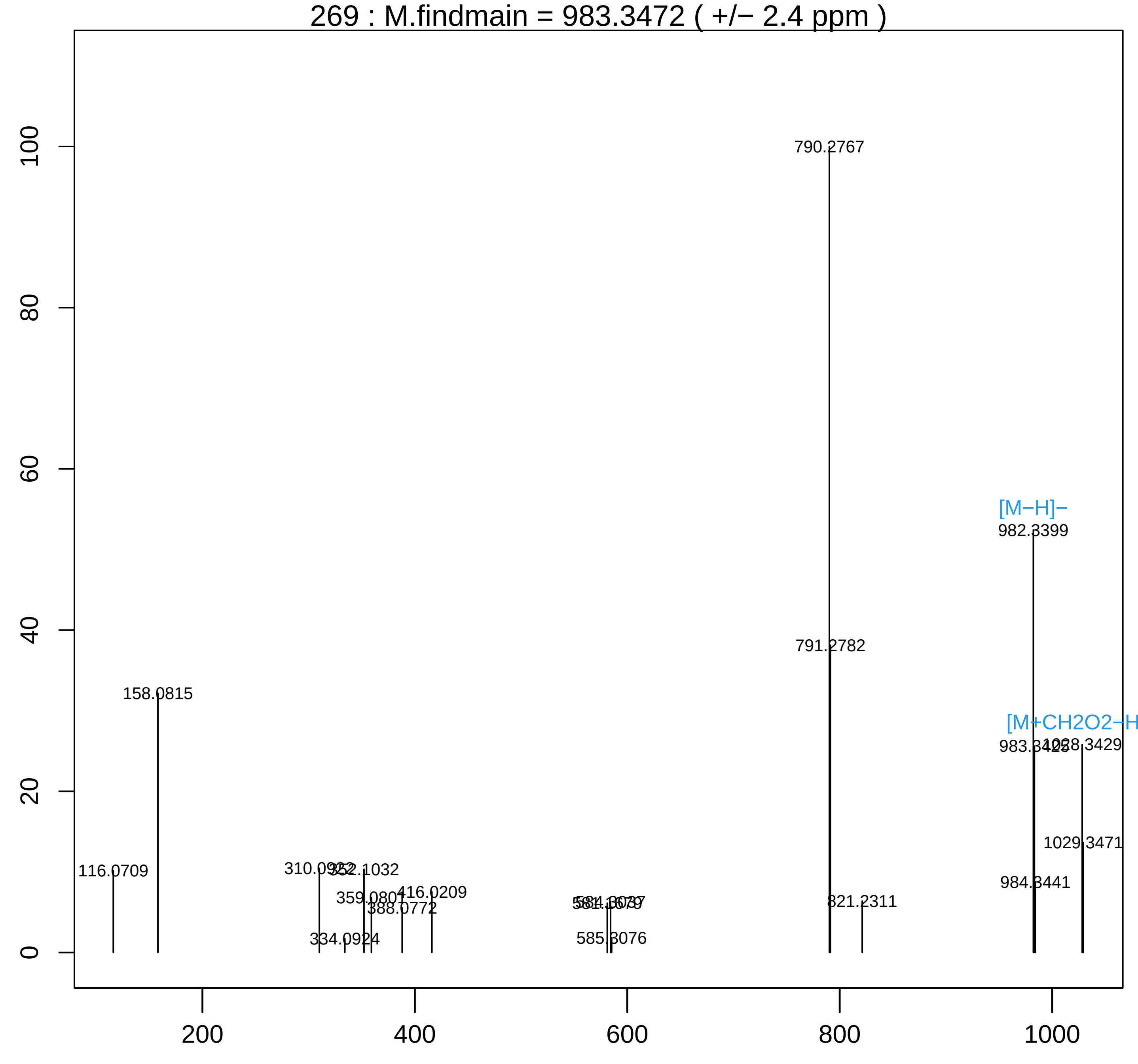
269 : M.findmain = 983.3472 (+/− 2.4 ppm)
790.2767
791.2782
158.0815
116.0709
310.0922
352.1032
359.0801
388.0772
416.0209
334.0924
585.3076
821.2311
[M−H]−
982.3399
[M+CH2O2−H
983.3428
1028.3429
1029.3471
984.3441
0
20
40
60
80
100
200
400
600
800
1000

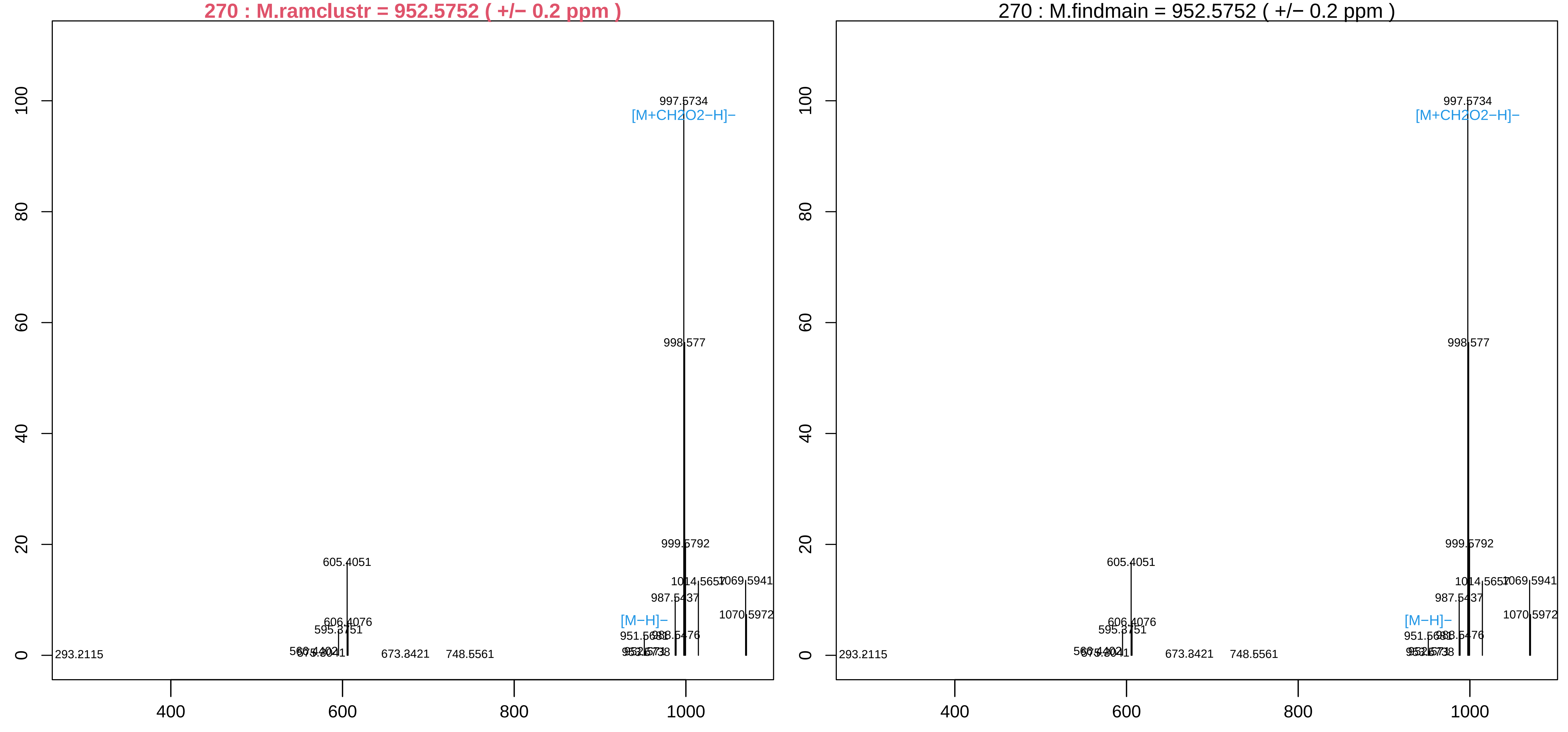

270 : M.ramclustr = 952.5752 (+/− 0.2 ppm)
997.5734
[M+CH2O2−H]−
998.577
999.5792
605.4051
1014.5651
1069.5941
987.5437
1070.5972
[M−H]−
606.4076
595.3751
951.5688
988.5476
293.2115
673.3421
748.5561
0
20
40
60
80
100
400
600
800
1000
270 : M.findmain = 952.5752 (+/− 0.2 ppm)
997.5734
[M+CH2O2−H]−
998.577
999.5792
605.4051
1014.5651
1069.5941
987.5437
1070.5972
[M−H]−
606.4076
595.3751
951.5688
988.5476
293.2115
673.3421
748.5561
0
20
40
60
80
100
400
600
800
1000

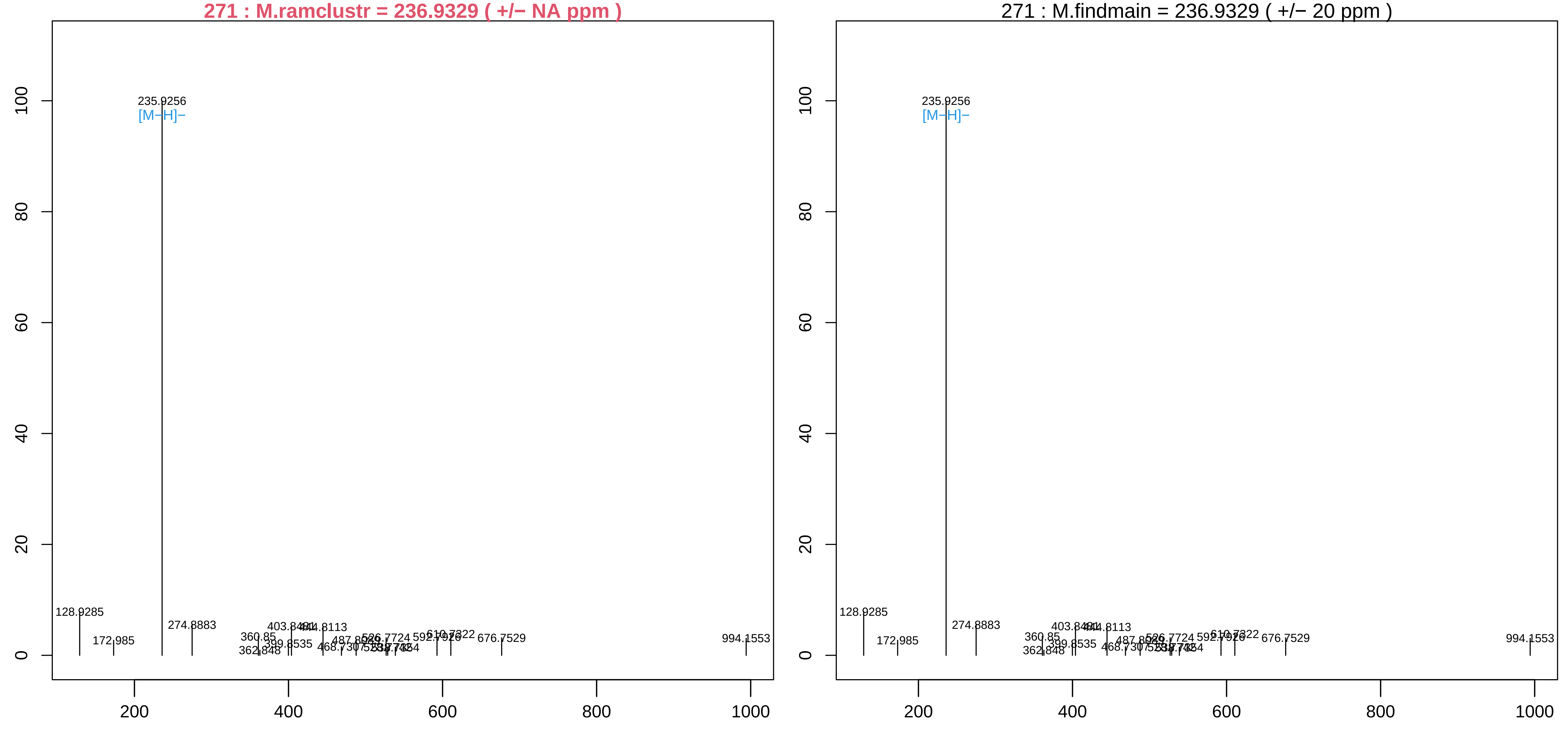
271 : M.ramclustr = 236.9329 (+/− NA ppm)
271 : M.findmain = 236.9329 (+/− 20 ppm)
235.9256
[M−H]−
128.9285
172.985
274.8883
360.85
362.848
399.8535
403.8481
444.8113
468.7307
487.8529
526.7724
538.7454
592.7926
610.7322
676.7529
994.1553

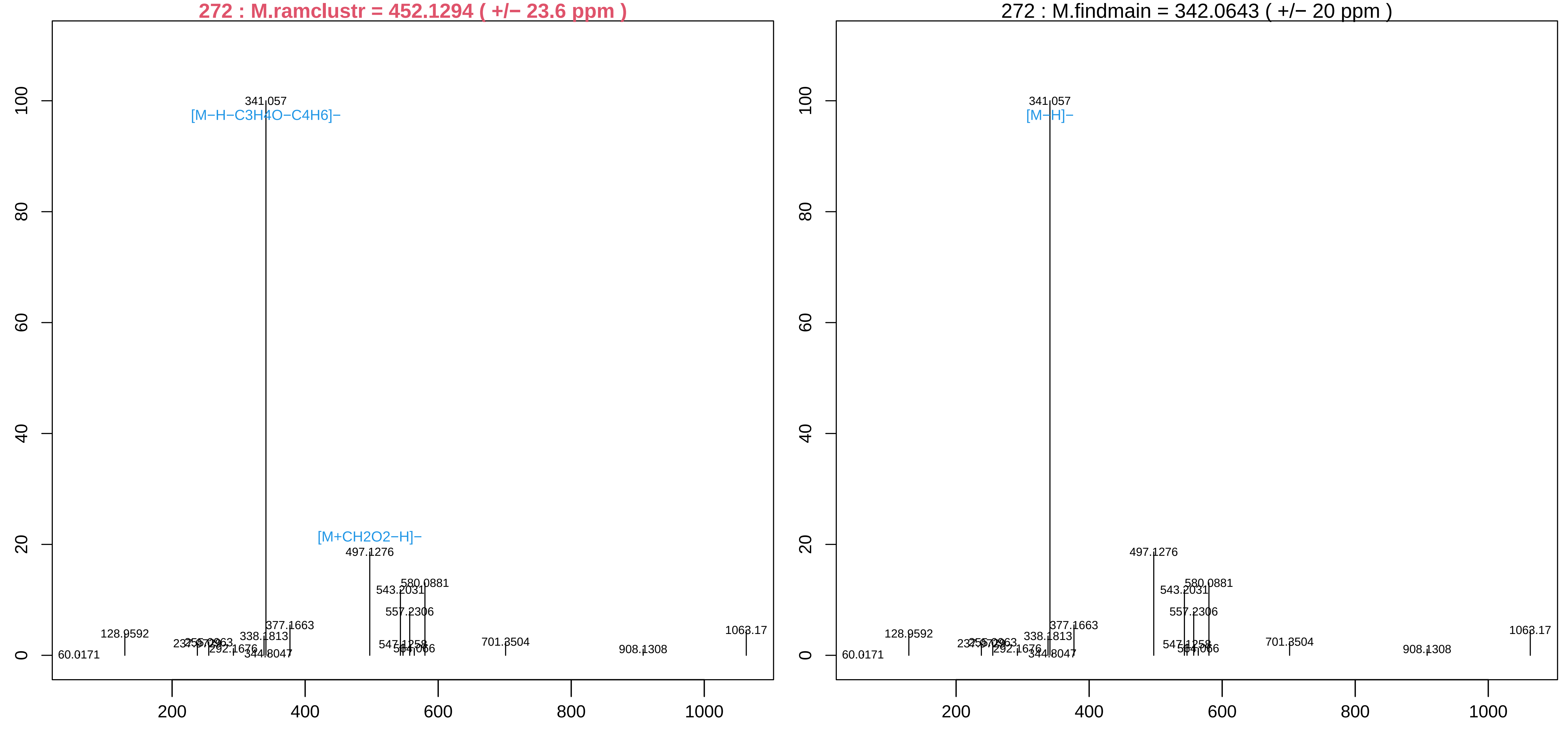
272 : M.ramclustr = 452.1294 (+/− 23.6 ppm)
341.057
[M−H−C3H4O−C4H6]−
[M+CH2O2−H]−
497.1276
580.0881
543.2031
557.2306
377.1663
338.1813
128.9592
547.1258
564.066
701.3504
1063.17
908.1308
292.1676
344.8047
60.0171
272 : M.findmain = 342.0643 (+/− 20 ppm)
341.057
[M−H]−
497.1276
580.0881
543.2031
557.2306
377.1663
338.1813
128.9592
547.1258
564.066
701.3504
1063.17
908.1308
292.1676
344.8047
60.0171

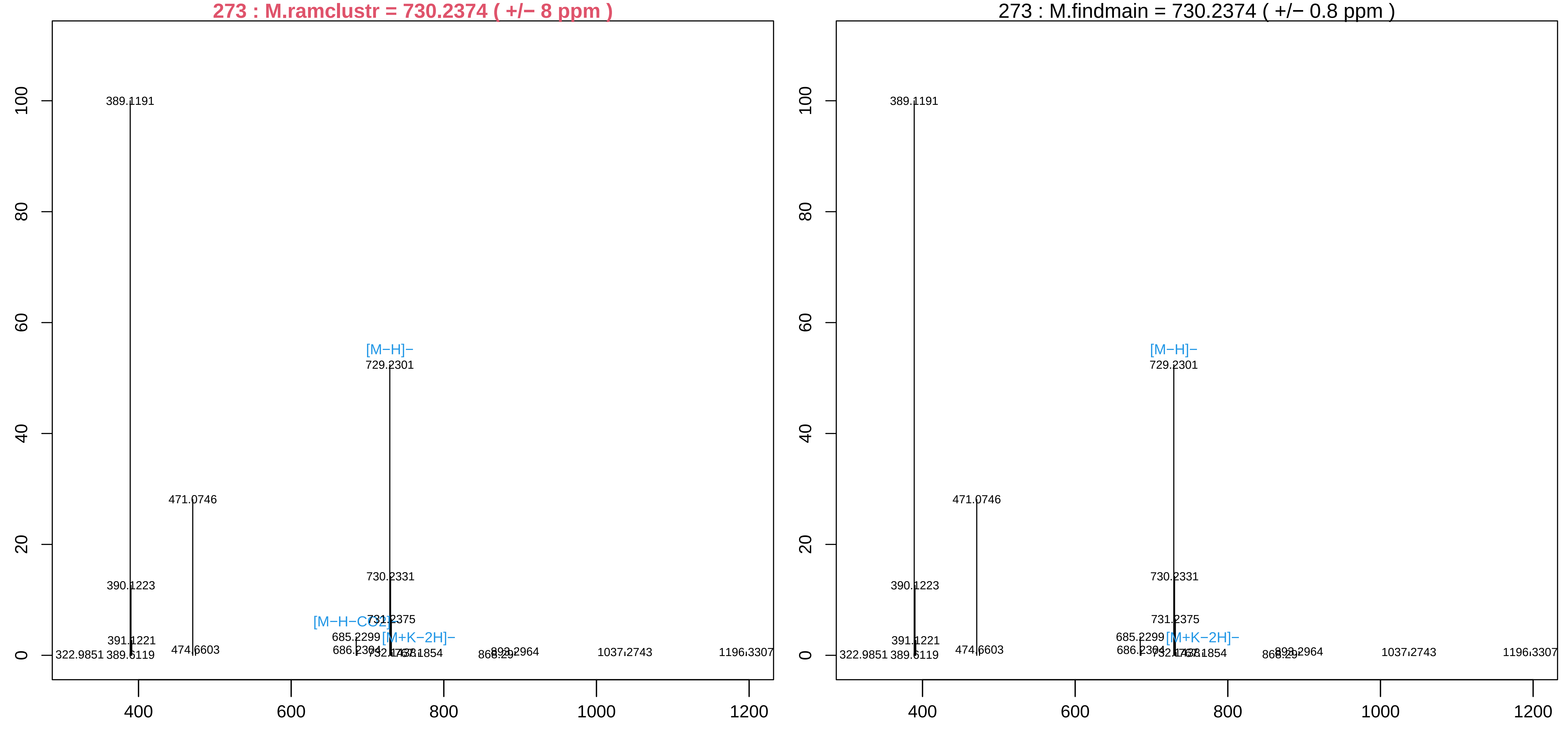
273 : M.ramclustr = 730.2374 (+/− 8 ppm)
273 : M.findmain = 730.2374 (+/− 0.8 ppm)
389.1191
[M−H]−
729.2301
471.0746
730.2331
390.1223
731.2375
[M−H−CO2]−
685.2299
[M+K−2H]−
391.1221
686.2304
474.6603
322.9851
389.5119
893.2964
1037.2743
1196.3307
0
20
40
60
80
100
400
600
800
1000
1200

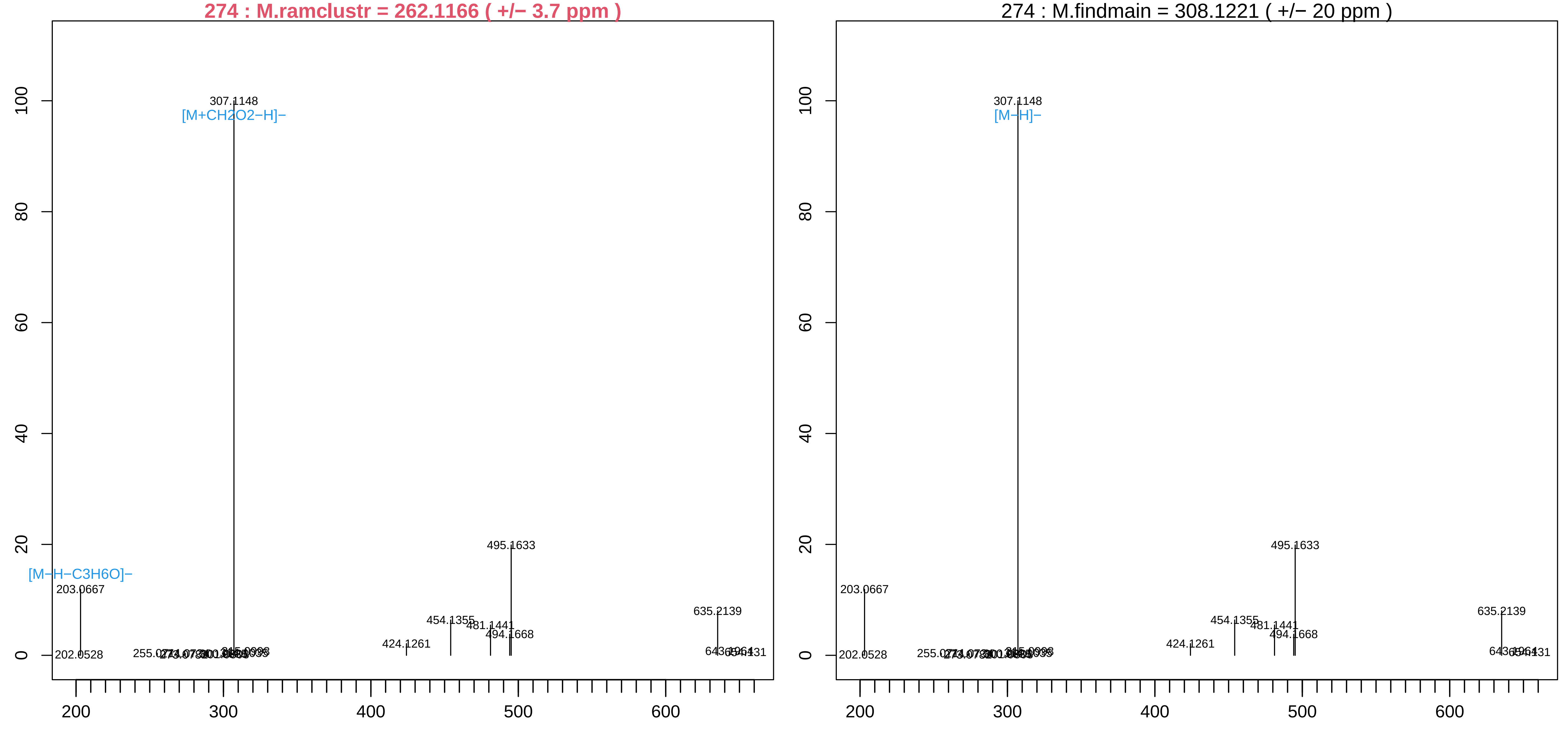

274 : M.ramclustr = 262.1166 (+/− 3.7 ppm)
307.1148
[M+CH2O2−H]−
[M−H−C3H6O]−
203.0667
202.0528
255.0
424.1261
454.1355
481.1441
494.1668
495.1633
635.2139
274 : M.findmain = 308.1221 (+/− 20 ppm)
307.1148
[M−H]−
203.0667
202.0528
424.1261
454.1355
481.1441
494.1668
495.1633
635.2139
0
20
40
60
80
100
200
300
400
500
600

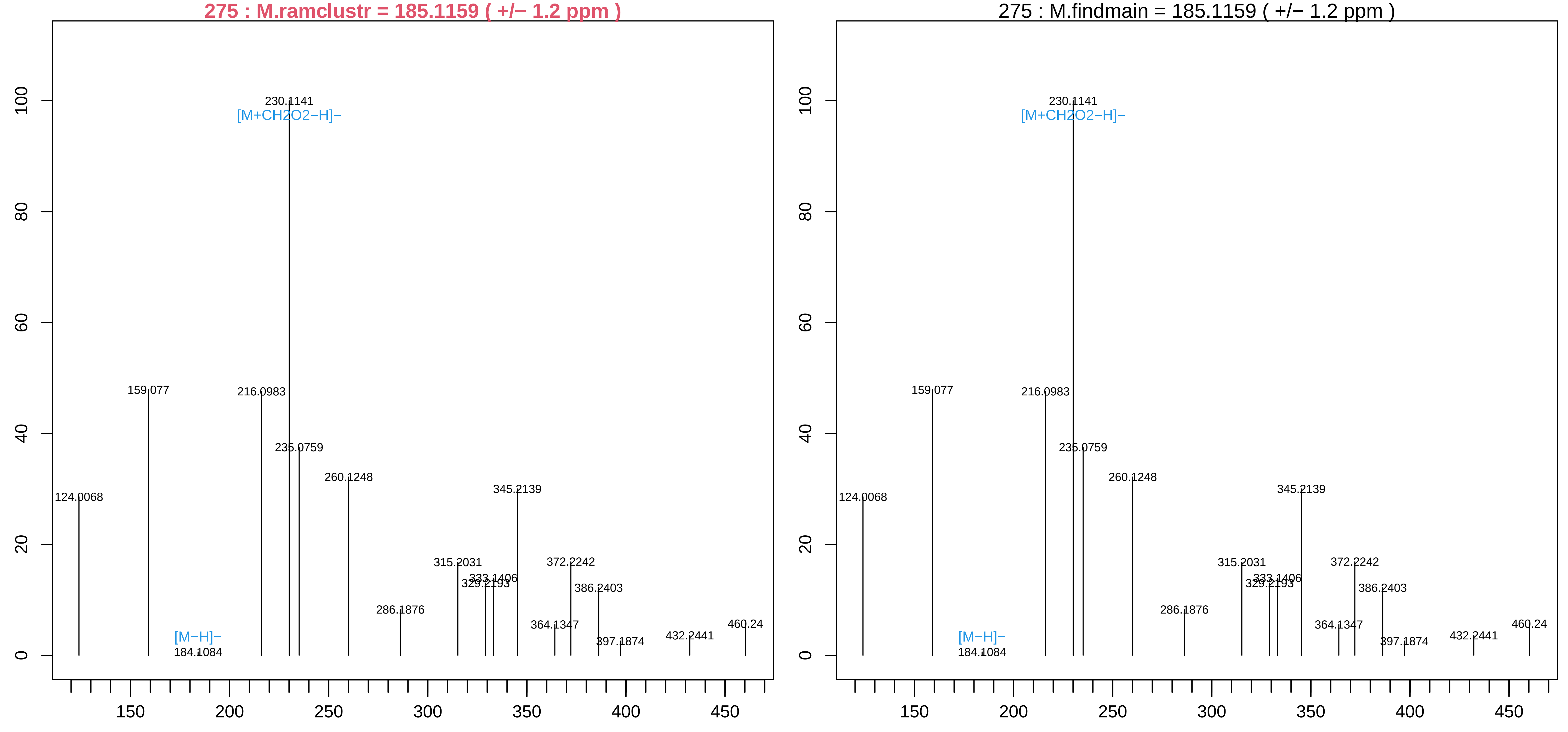
275 : M.ramclustr = 185.1159 (+/− 1.2 ppm)
275 : M.findmain = 185.1159 (+/− 1.2 ppm)
230.1141
[M+CH2O2−H]−
159.077
216.0983
235.0759
260.1248
124.0068
345.2139
315.2031
333.1406
329.2193
372.2242
386.2403
286.1876
364.1347
460.24
432.2441
397.1874
[M−H]−
184.1084
0
20
40
60
80
100
150
200
250
300
350
400
450

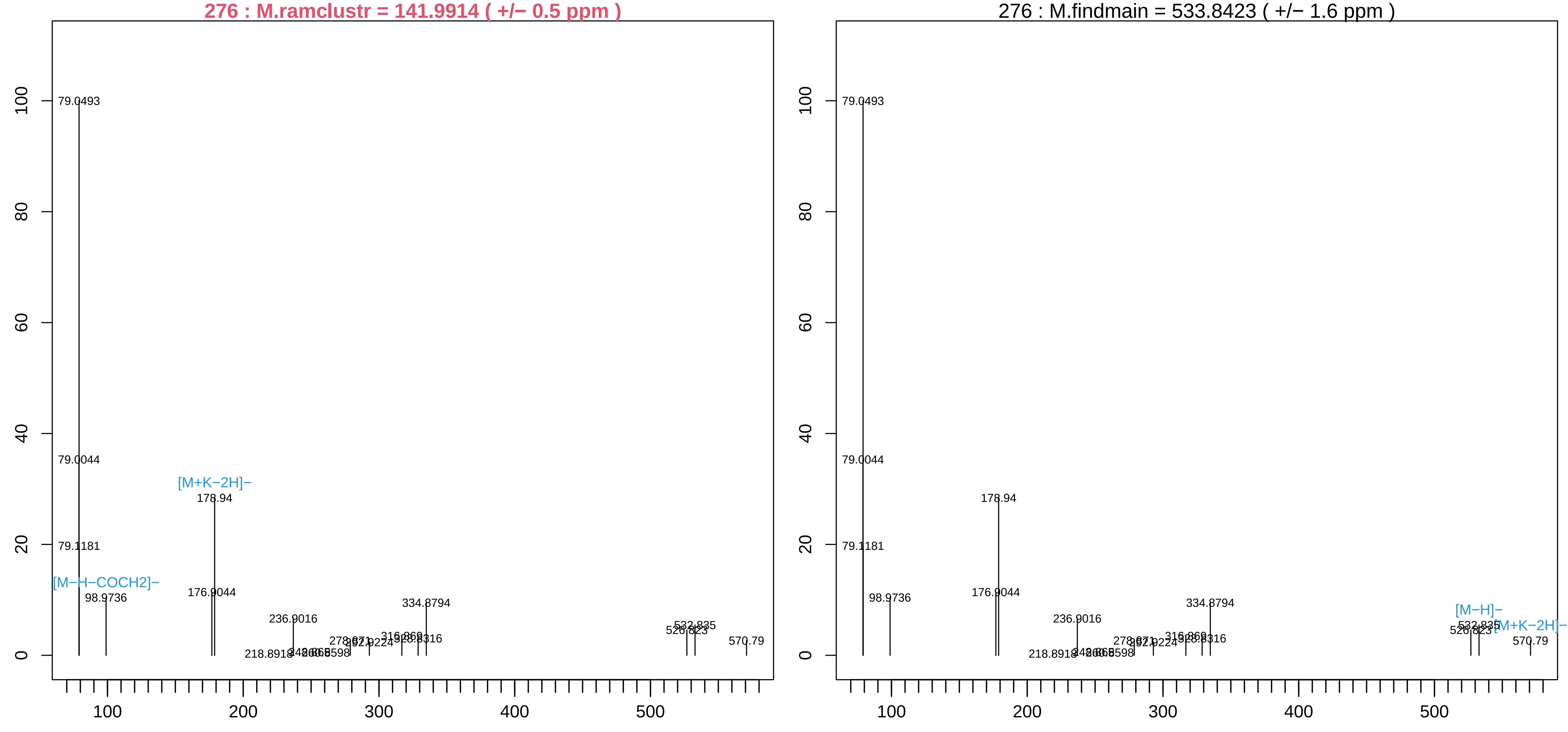

276 : M.ramclustr = 141.9914 (+/− 0.5 ppm)
276 : M.findmain = 533.8423 (+/− 1.6 ppm)
79.0493
79.0044
79.1181
98.9736
[M−H−COCH2]−
[M+K−2H]−
178.94
176.9044
236.9016
218.8918
334.8794
570.79
[M−H]−
0
20
40
60
80
100
200
300
400
500

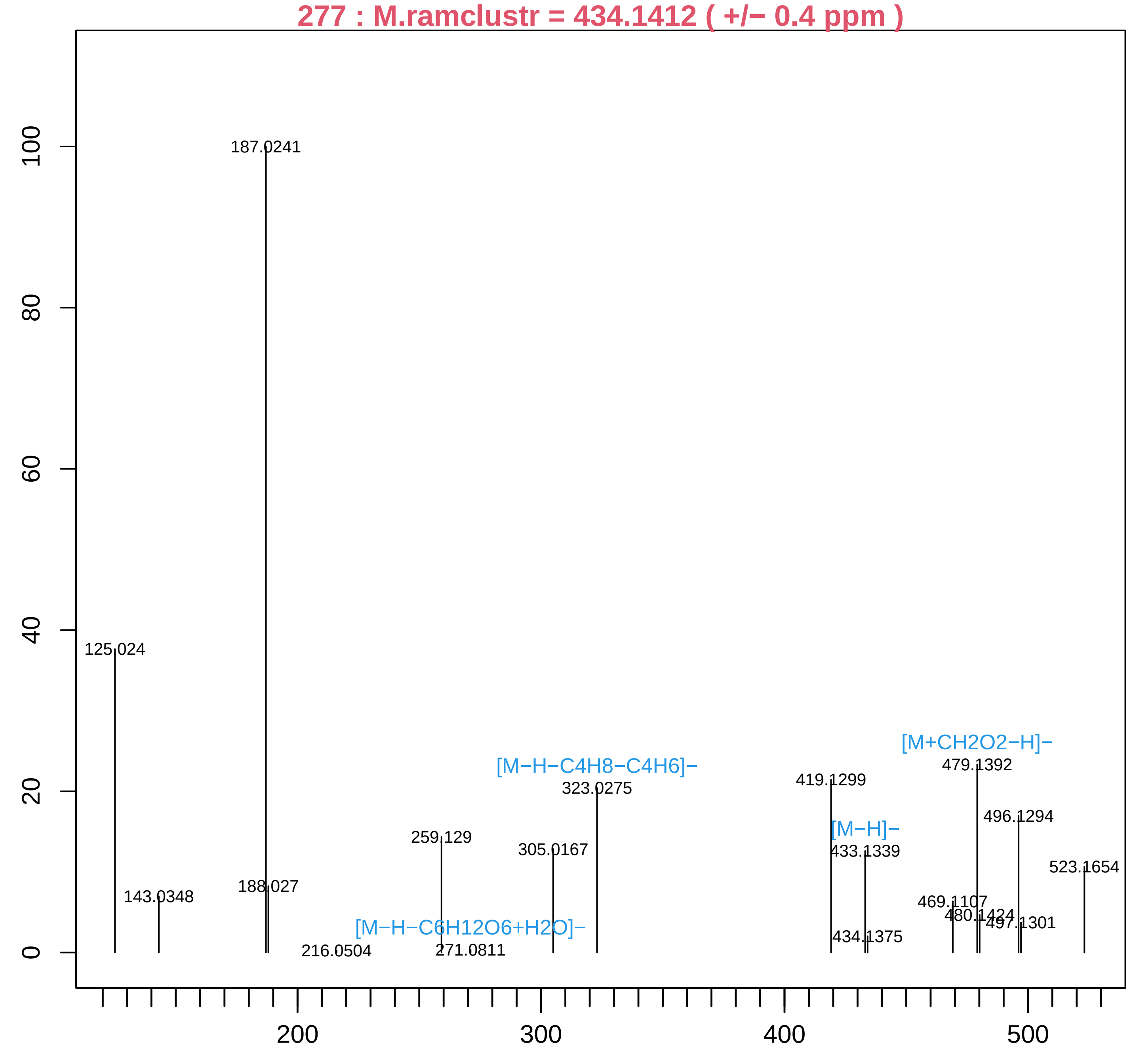

277 : M.ramclustr = 434.1412 (+/− 0.4 ppm)
187.0241
125.024
143.0348
188.027
216.0504
259.129
271.0811
305.0167
323.0275
[M−H−C4H8−C4H6]−
[M−H−C6H12O6+H2O]−
419.1299
[M−H]−
433.1339
434.1375
469.1107
480.1424
479.1392
[M+CH2O2−H]−
496.1294
497.1301
523.1654
0
20
40
60
80
100
200
300
400
500

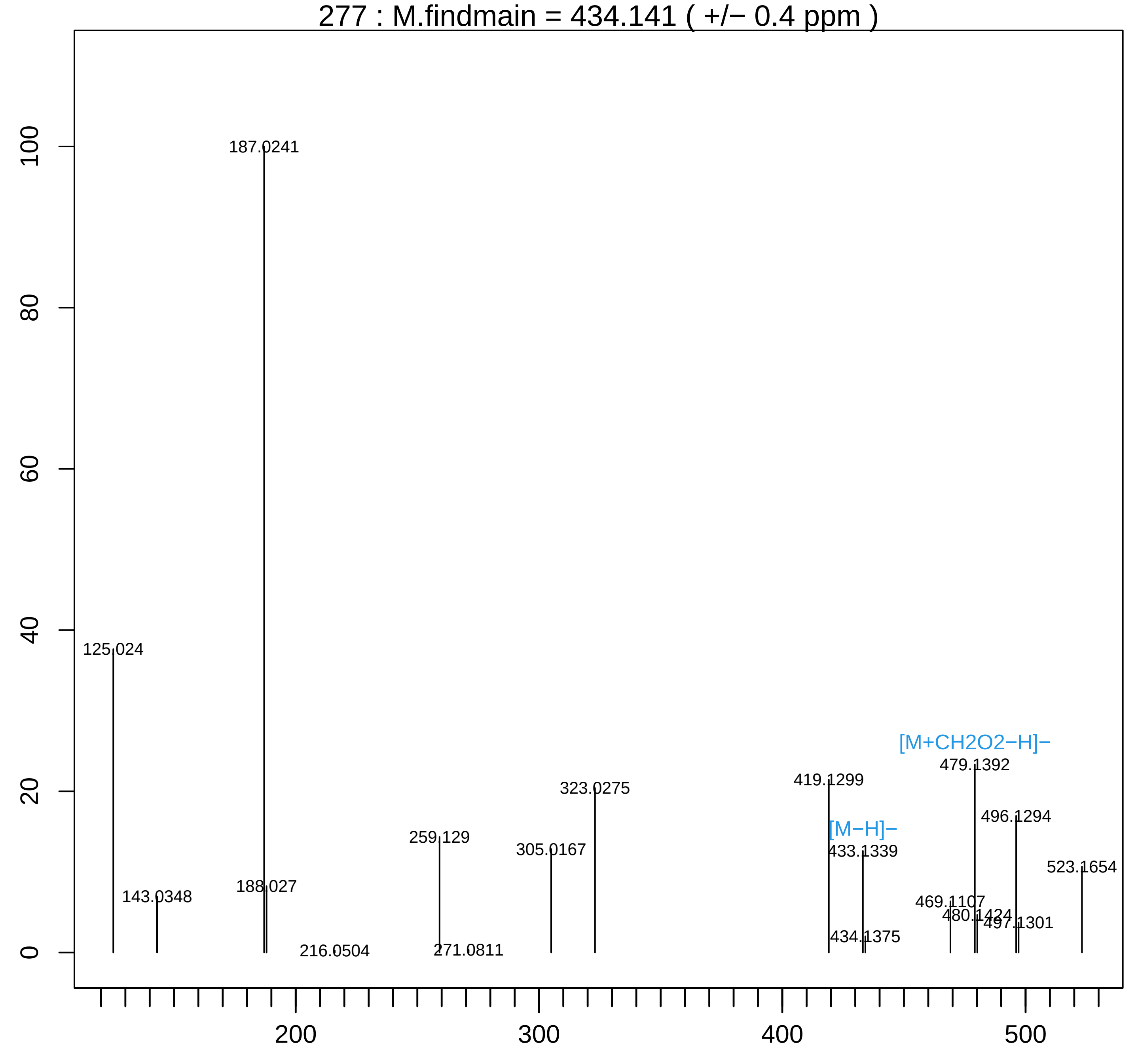

277 : M.findmain = 434.141 (+/− 0.4 ppm)
187.0241
125.024
143.0348
188.027
216.0504
259.129
271.0811
305.0167
323.0275
419.1299
[M−H]−
433.1339
434.1375
469.1107
480.1424
479.1392
[M+CH2O2−H]−
496.1294
497.1301
523.1654
0
20
40
60
80
100
200
300
400
500

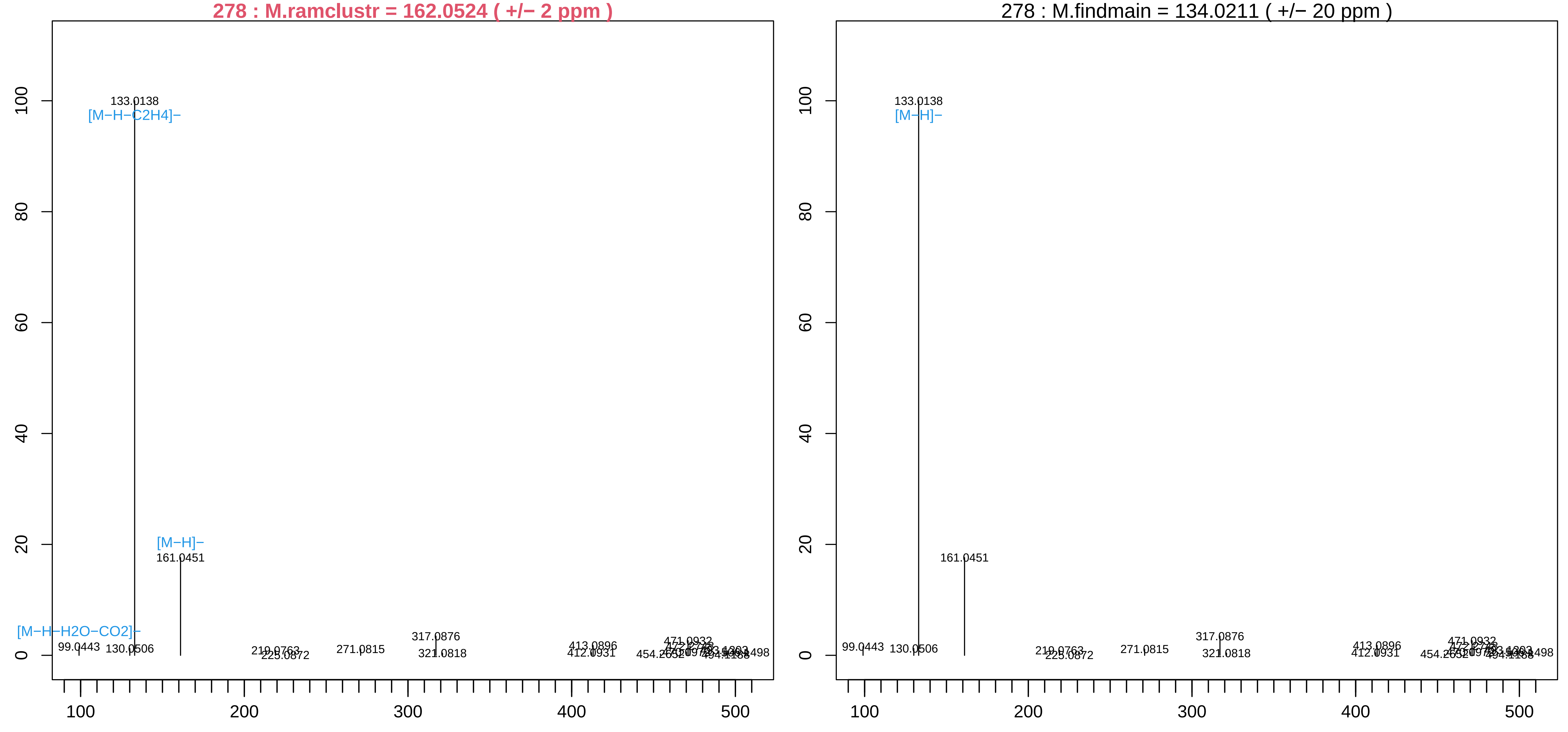

278 : M.ramclustr = 162.0524 (+/− 2 ppm)
133.0138
[M−H−C2H4]−
[M−H]−
161.0451
[M−H−H2O−CO2]−
99.0443
130.0506
219.0763
225.0872
271.0815
317.0876
321.0818
413.0896
412.0931
454.2652
471.0932
278 : M.findmain = 134.0211 (+/− 20 ppm)
133.0138
[M−H]−
161.0451

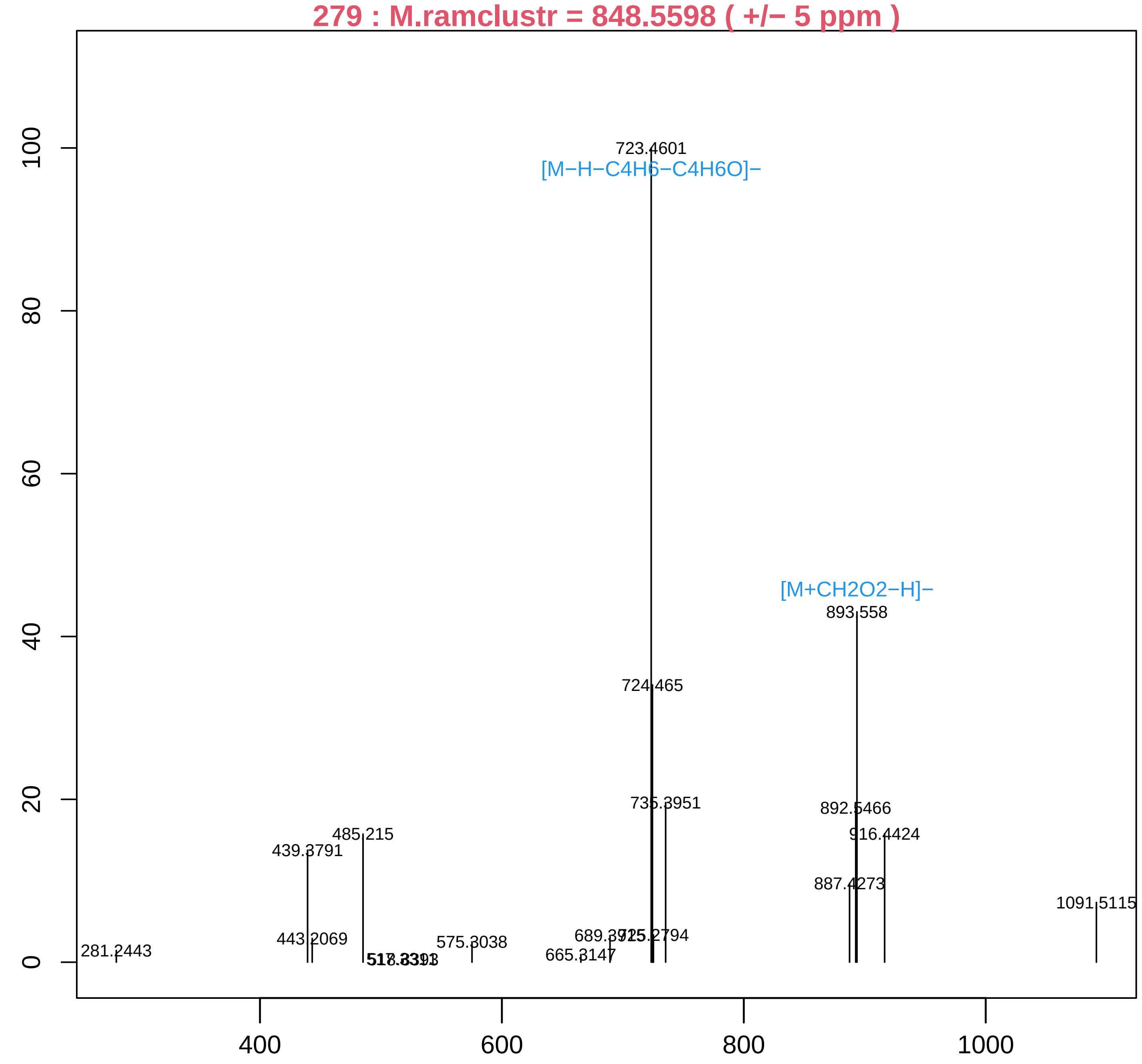
279 : M.ramclustr = 848.5598 (+/− 5 ppm)
[M−H−C4H6−C4H6O]−
723.4601
[M+CH2O2−H]−
893.558
724.465
735.3951
892.5466
916.4424
887.4273
1091.5115
485.215
439.3791
443.2069
575.3038
689.3975
715.2794
665.3147
281.2443
0
20
40
60
80
100
400
600
800
1000

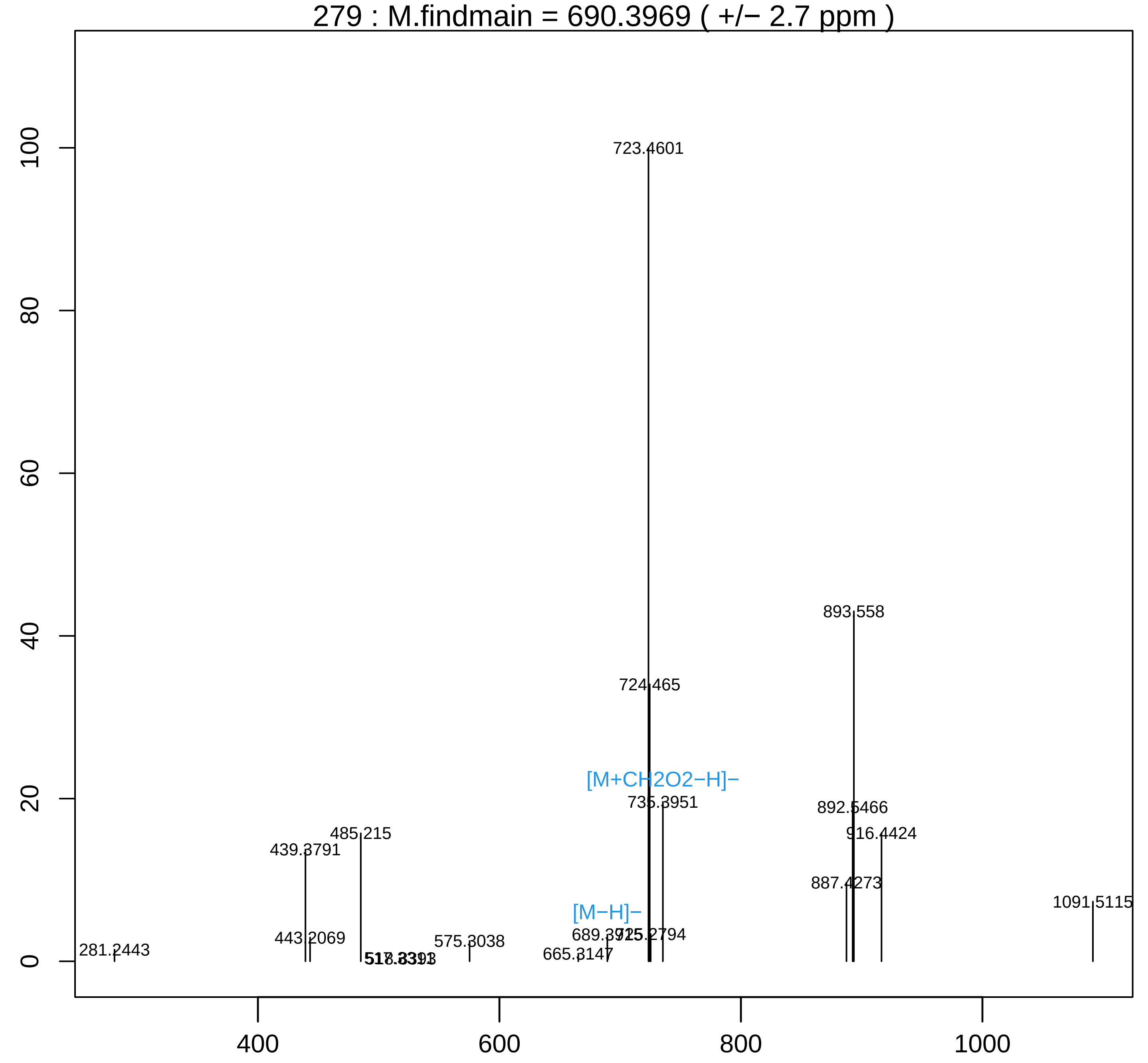
279 : M.findmain = 690.3969 (+/− 2.7 ppm)
723.4601
893.558
724.465
[M+CH2O2−H]−
735.3951
892.5466
916.4424
887.4273
1091.5115
[M−H]−
485.215
439.3791
443.2069
575.3038
689.3975
715.2794
665.3147
281.2443
0
20
40
60
80
100
400
600
800
1000

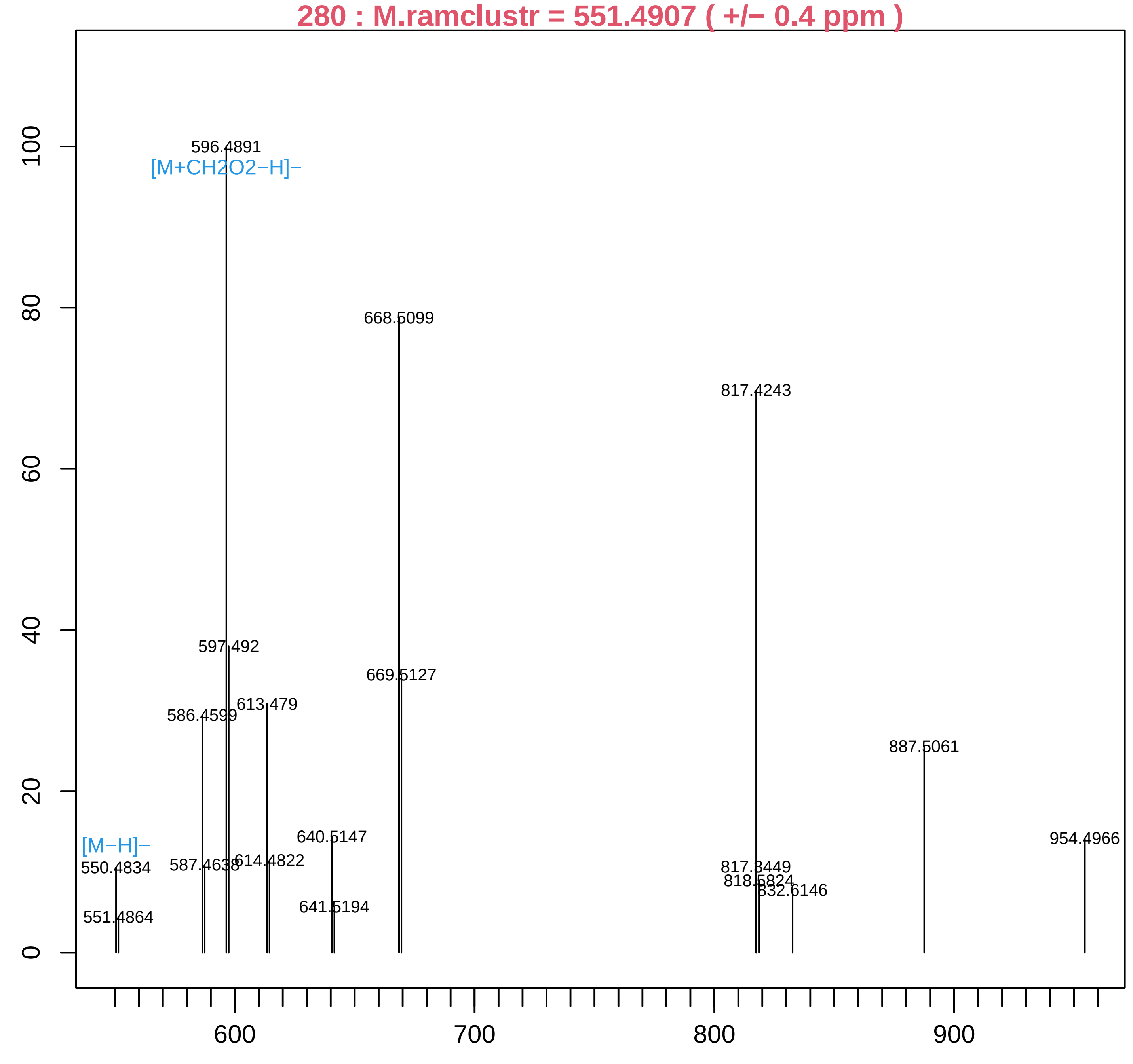

280 : M.ramclustr = 551.4907 (+/− 0.4 ppm)
596.4891
[M+CH2O2−H]−
668.5099
817.4243
597.492
669.5127
586.4599
613.479
887.5061
640.5147
954.4966
[M−H]−
550.4834
587.4638
614.4822
817.3449
818.5824
832.6146
641.5194
551.4864
0
20
40
60
80
100
600
700
800
900

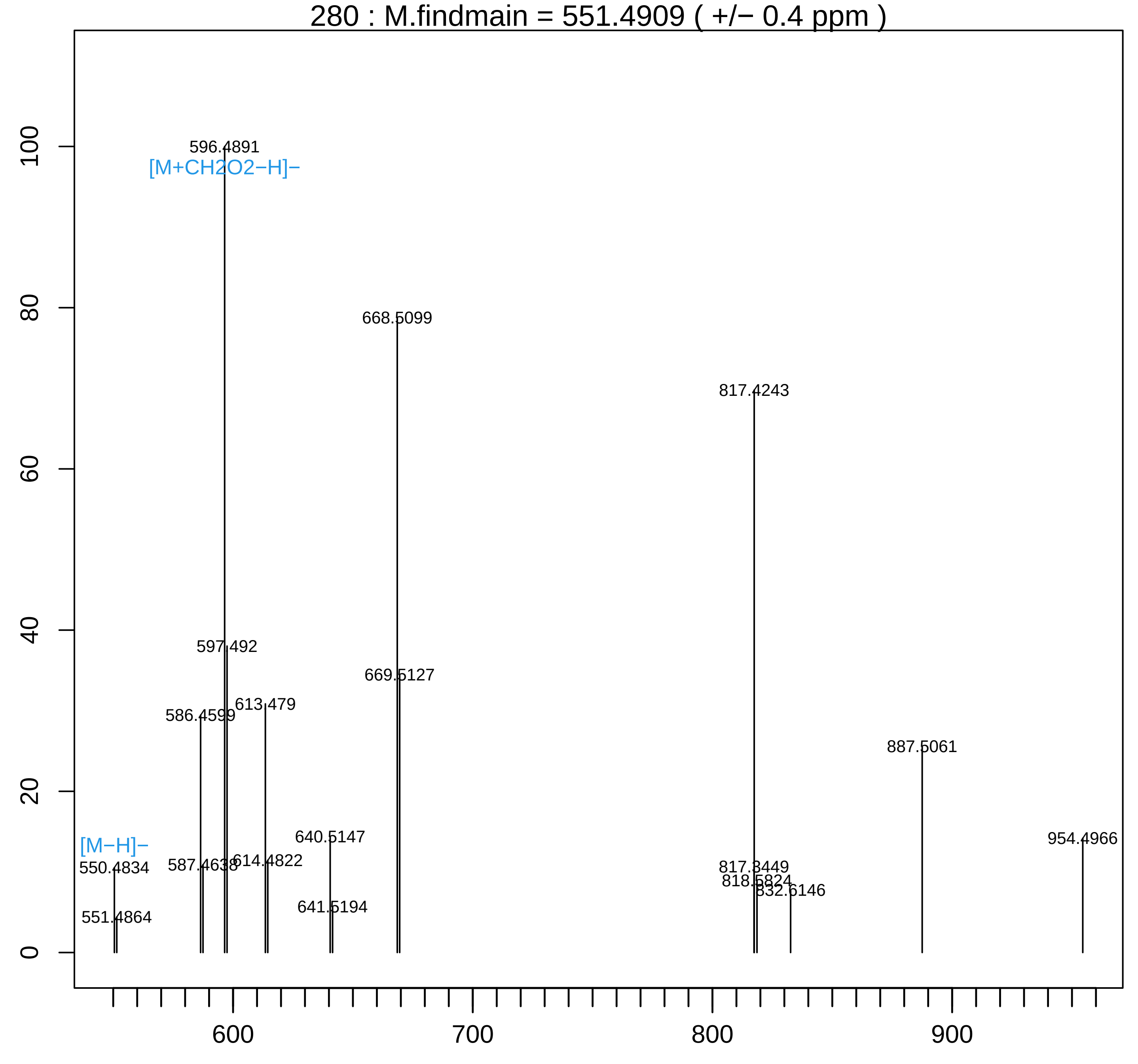

280 : M.findmain = 551.4909 (+/− 0.4 ppm)
596.4891
[M+CH2O2−H]−
668.5099
817.4243
597.492
669.5127
586.4599
613.479
887.5061
640.5147
954.4966
[M−H]−
550.4834
587.4638
614.4822
817.3449
818.5824
832.6146
641.5194
551.4864
0
20
40
60
80
100
600
700
800
900

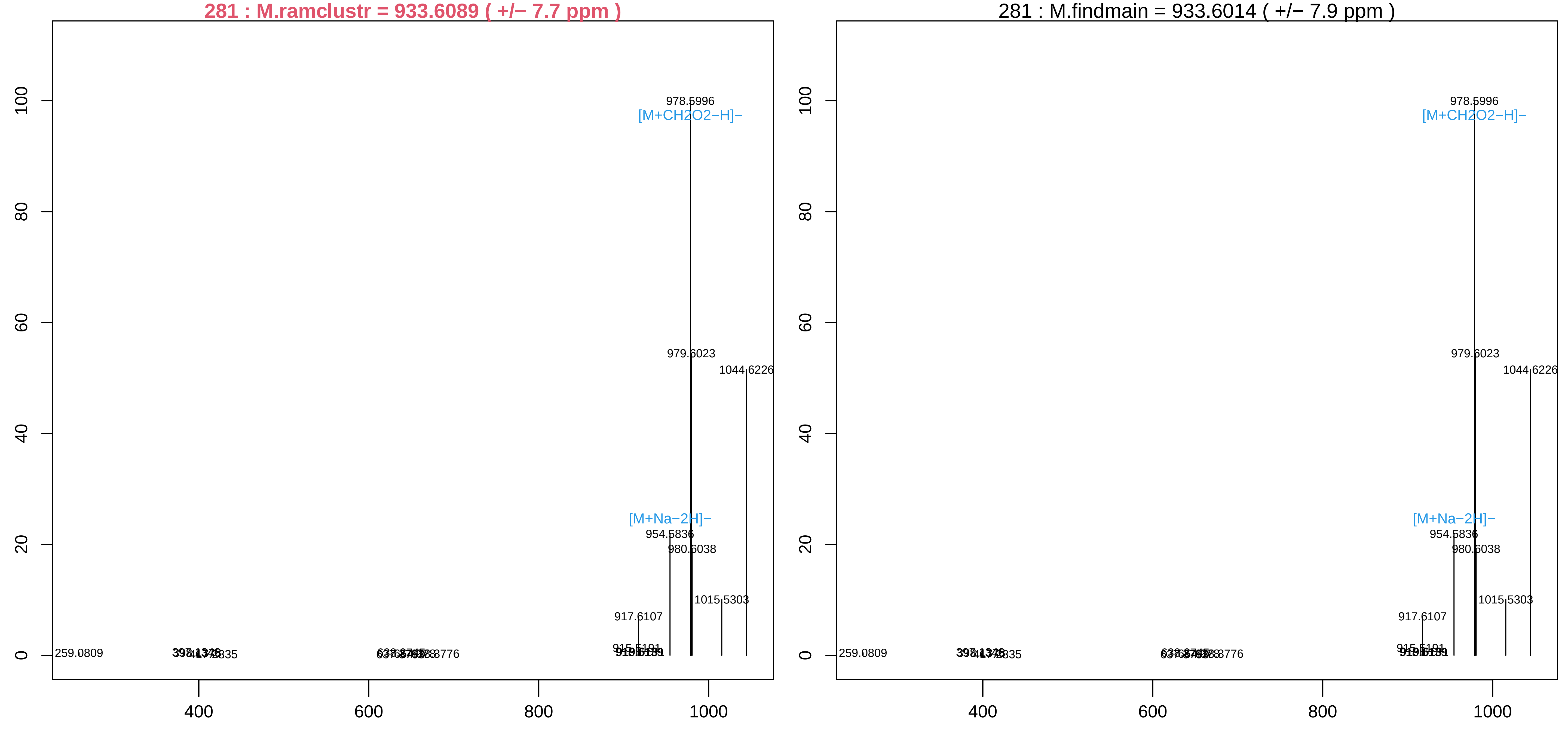
281 : M.ramclustr = 933.6089 (+/− 7.7 ppm)
978.5996
[M+CH2O2−H]−
979.6023
1044.6226
[M+Na−2H]−
954.5836
980.6038
1015.5303
917.6107
915.5101
259.0809
0
20
40
60
80
100
400
600
800
1000
281 : M.findmain = 933.6014 (+/− 7.9 ppm)
978.5996
[M+CH2O2−H]−
979.6023
1044.6226
[M+Na−2H]−
954.5836
980.6038
1015.5303
917.6107
915.5101
259.0809
0
20
40
60
80
100
400
600
800
1000

282 : M.ramclustr = 1038.5212 (+/− 5.6 ppm)

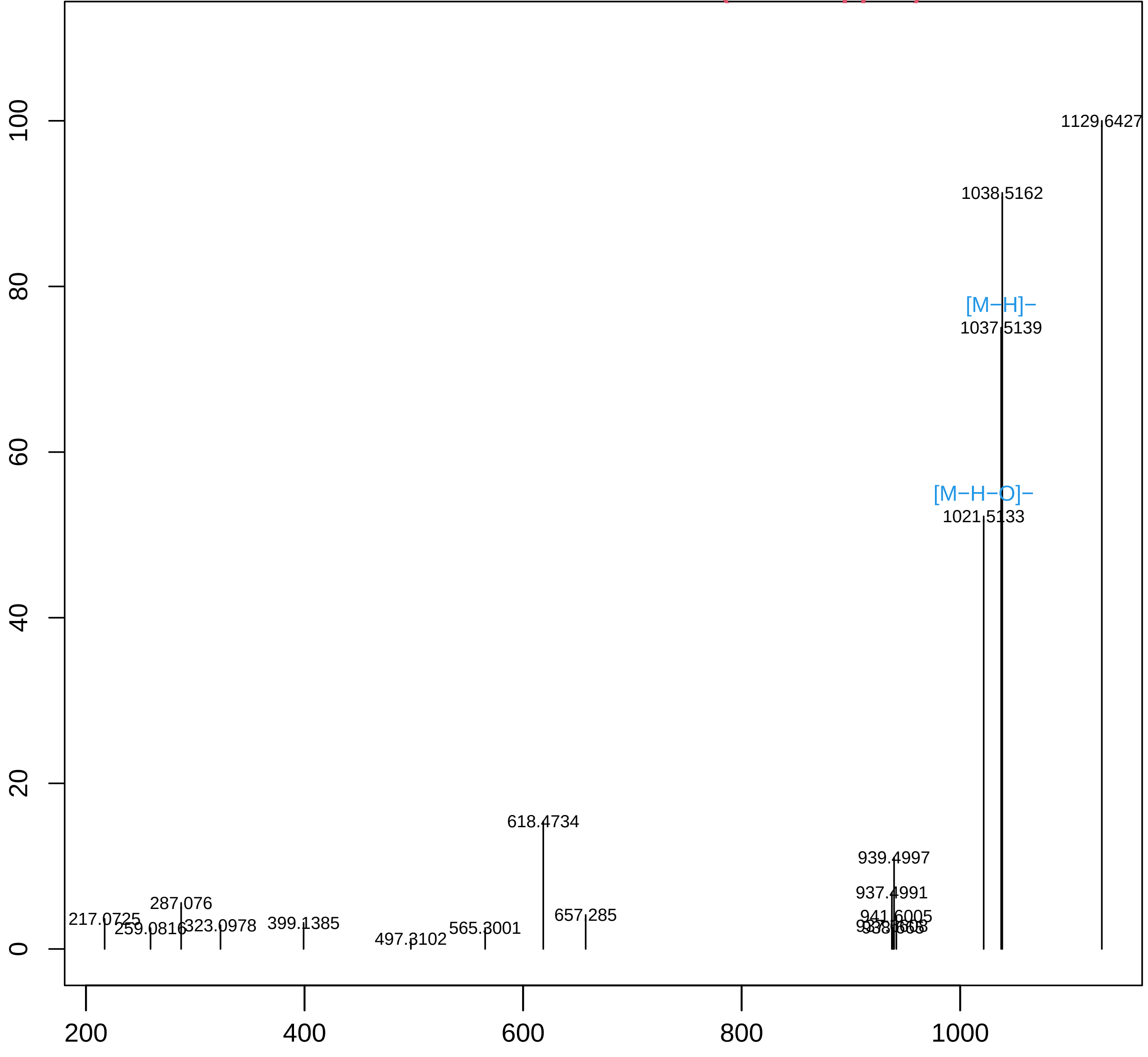

282 : M.findmain = 1039.5235 (+/− 20 ppm)

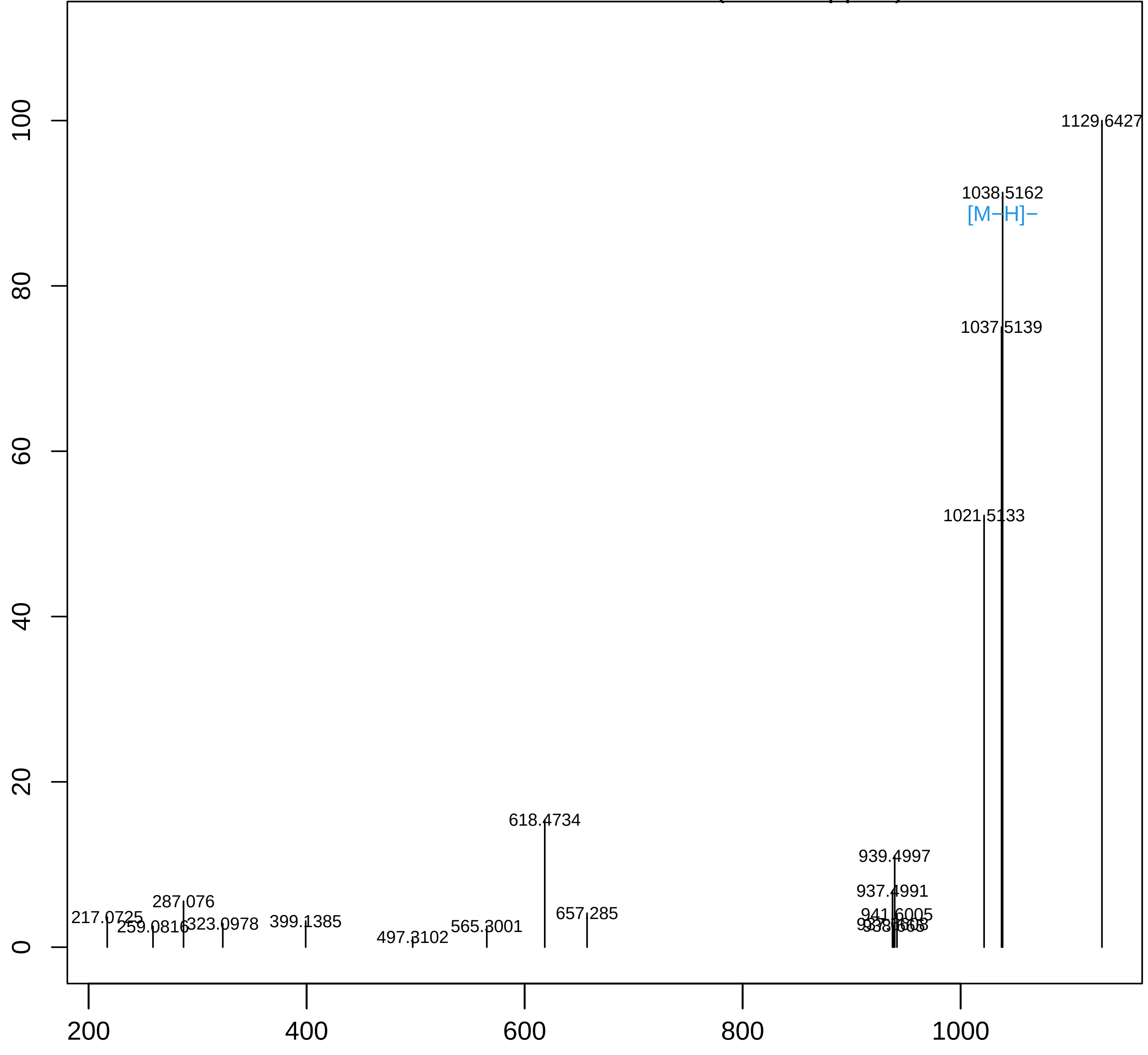

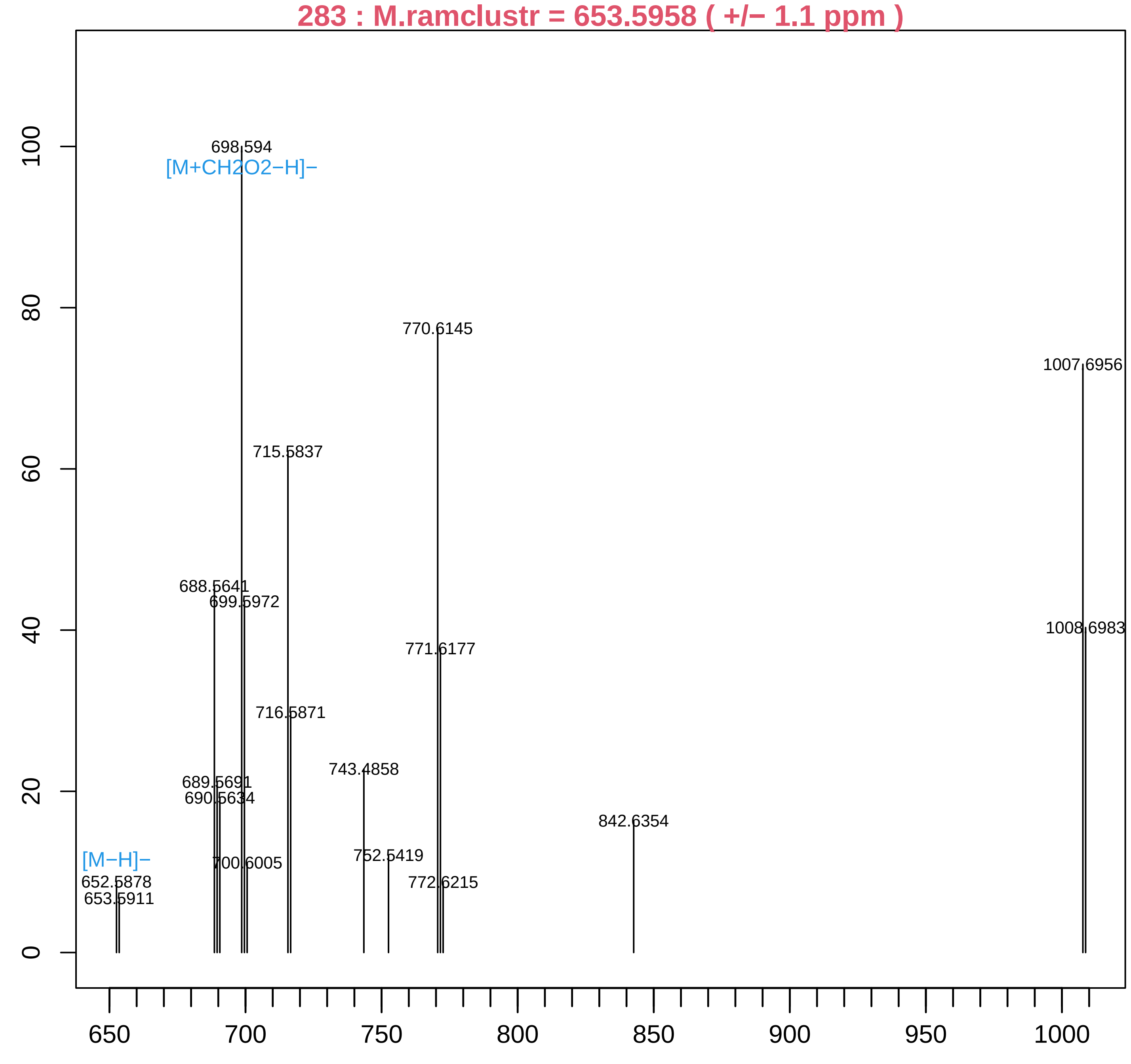

283 : M.ramclustr = 653.5958 (+/− 1.1 ppm)
698.594
[M+CH2O2−H]−
770.6145
1007.6956
715.5837
688.5641
699.5972
1008.6983
771.6177
716.5871
743.4858
689.5691
690.6634
842.6354
[M−H]−
700.6005
752.5419
652.5878
772.6215
653.5911
0
20
40
60
80
100
650
700
750
800
850
900
950
1000

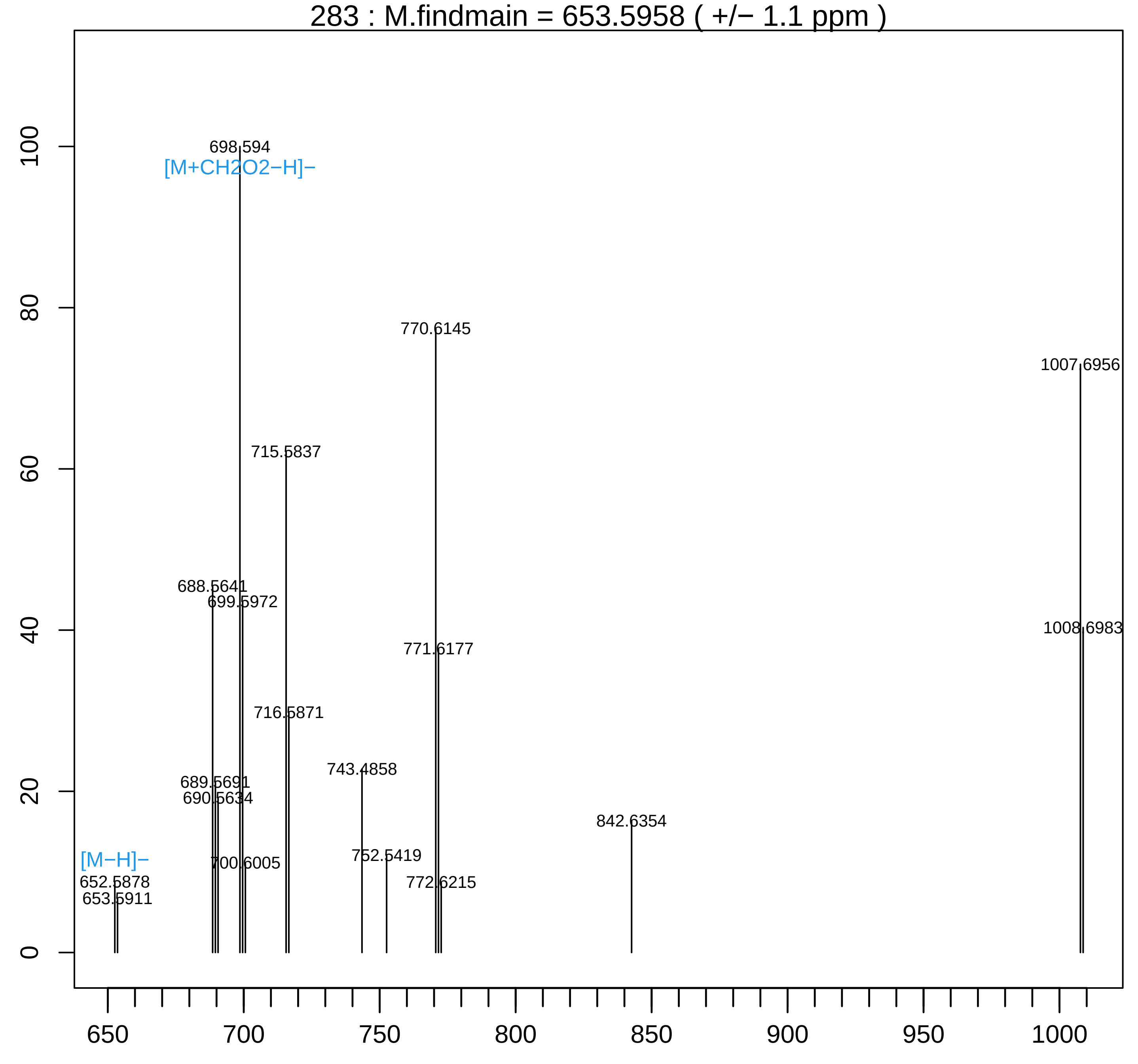

283 : M.findmain = 653.5958 (+/− 1.1 ppm)
698.594
[M+CH2O2−H]−
770.6145
1007.6956
715.5837
688.5641
699.5972
1008.6983
771.6177
716.5871
743.4858
689.5691
690.6634
842.6354
[M−H]−
700.6005
752.5419
652.5878
772.6215
653.5911
0
20
40
60
80
100
650
700
750
800
850
900
950
1000

284 : M.ramclustr = 150.9989 (+/− 0.9 ppm)

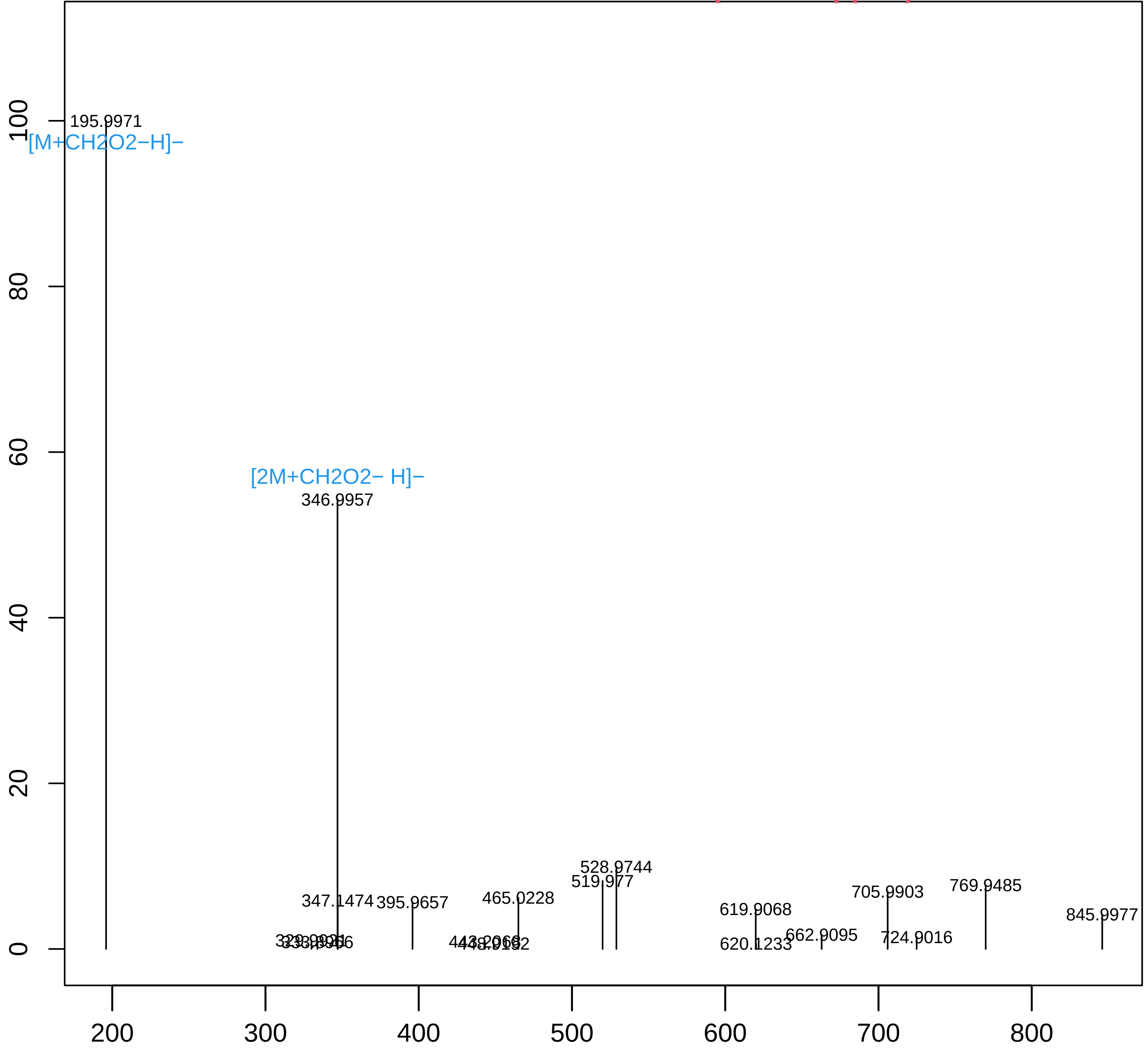

284 : M.findmain = 150.9989 (+/− 0.9 ppm)

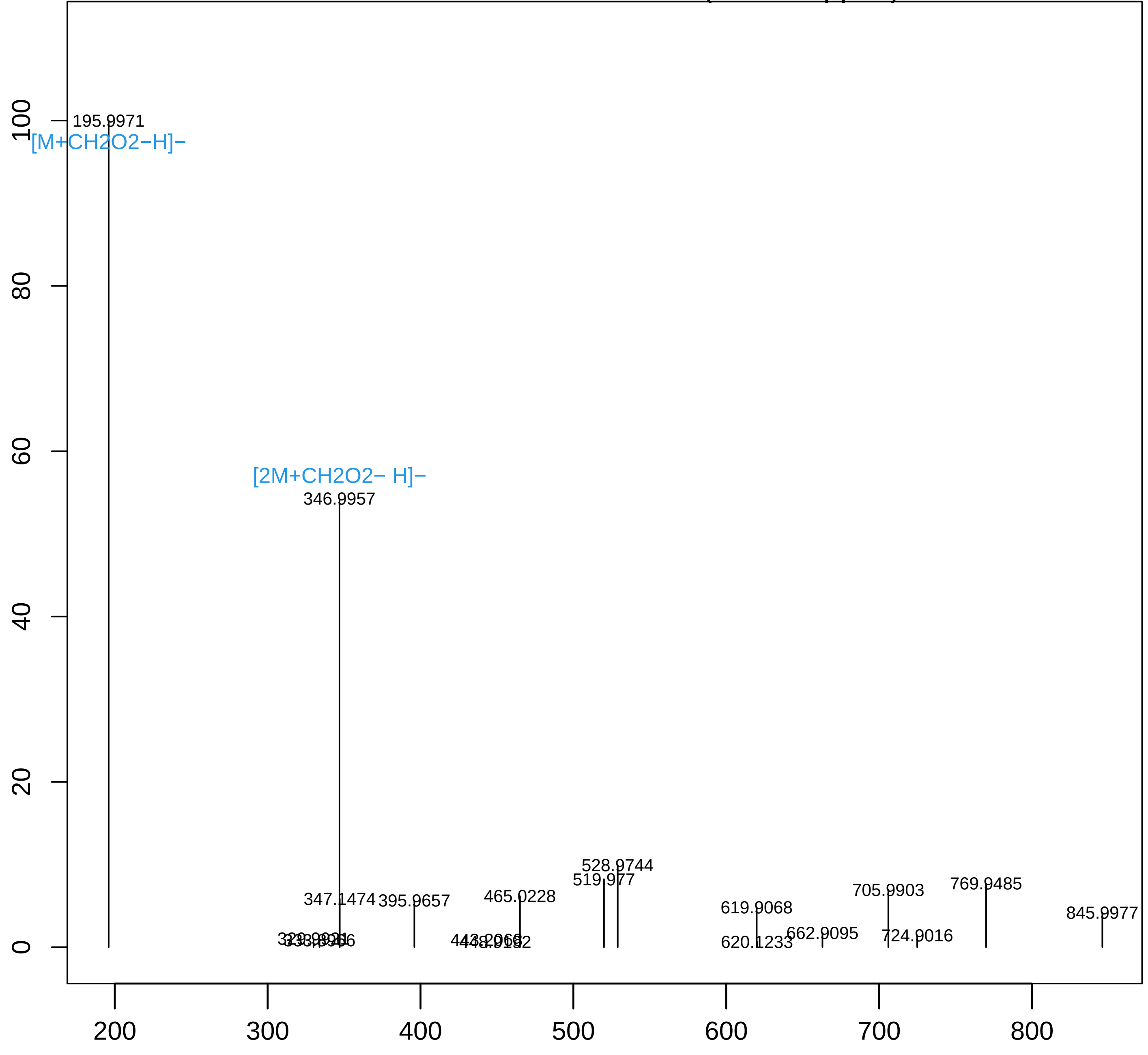

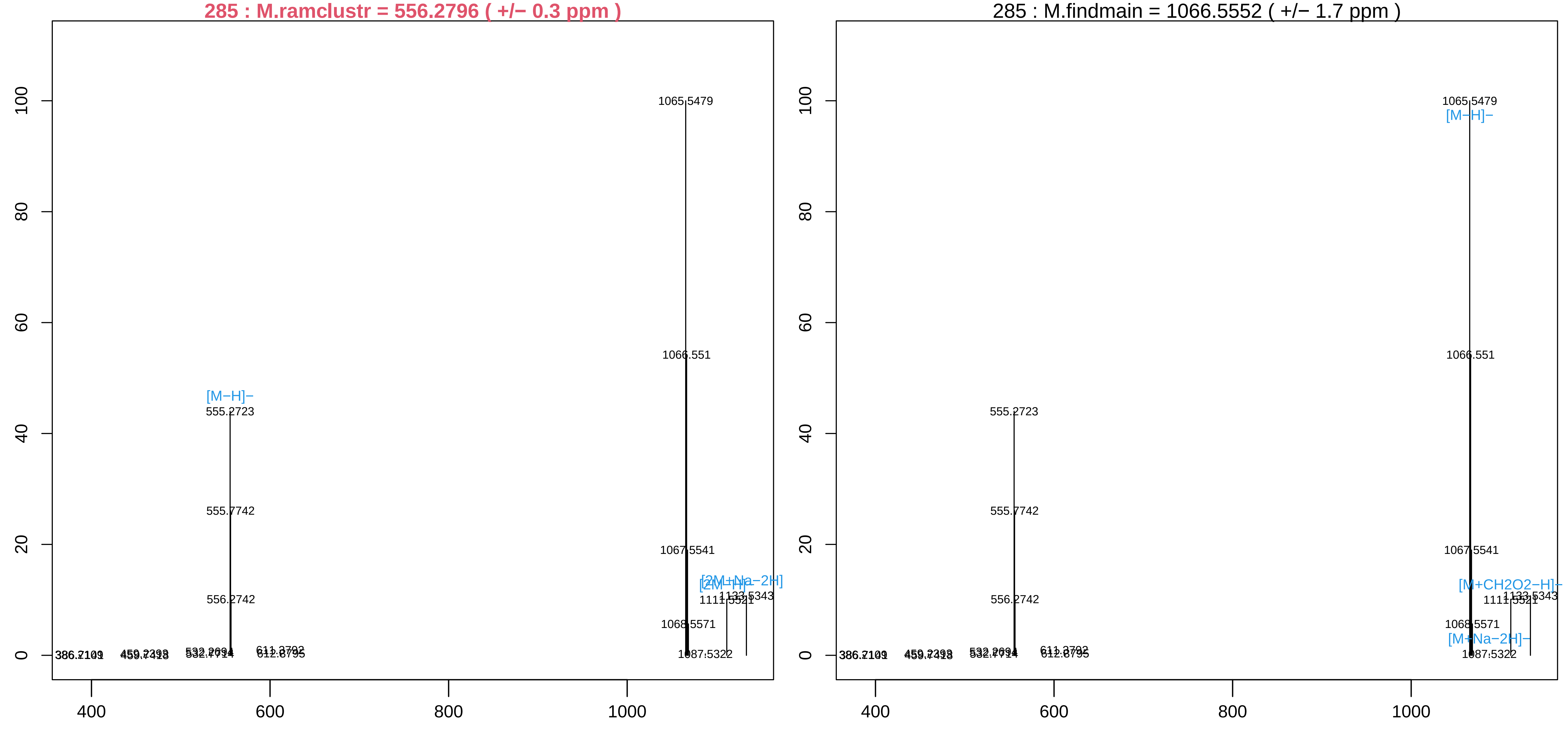
285 : M.ramclustr = 556.2796 (+/− 0.3 ppm)
1065.5479
1066.551
[M−H]−
555.2723
555.7742
1067.5541
[2M+Na−2H]
556.2742
1133.5343
1111.5521
1068.5571
1087.5322
386.2101
459.2293
532.2694
532.7714
611.2792
612.6795
285 : M.findmain = 1066.5552 (+/− 1.7 ppm)
[M−H]−
[M+CH2O2−H]−
[M+Na−2H]−
0
20
40
60
80
100
400
600
800
1000

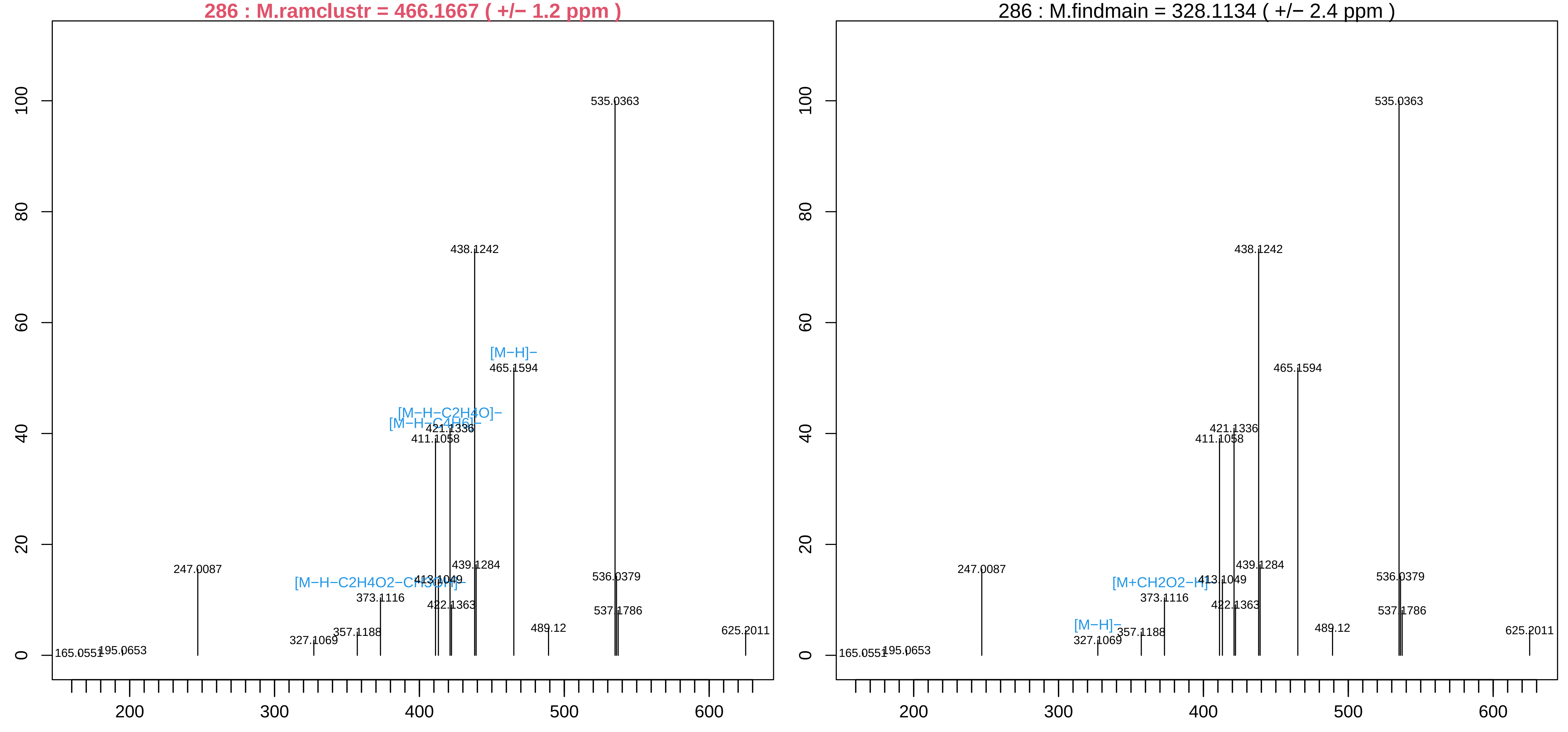

286 : M.ramclustr = 466.1667 (+/− 1.2 ppm)
286 : M.findmain = 328.1134 (+/− 2.4 ppm)
535.0363
438.1242
[M−H]−
465.1594
[M−H−C2H4O]−
[M−H−C4H6]−
421.1336
411.1058
439.1284
413.1049
[M−H−C2H4O2−CH3OH]−
[M+CH2O2−H]−
[M−H]−
536.0379
537.1786
373.1116
422.1363
489.12
625.2011
357.1188
327.1069
247.0087
165.0551
195.0653
0
20
40
60
80
100
200
300
400
500
600

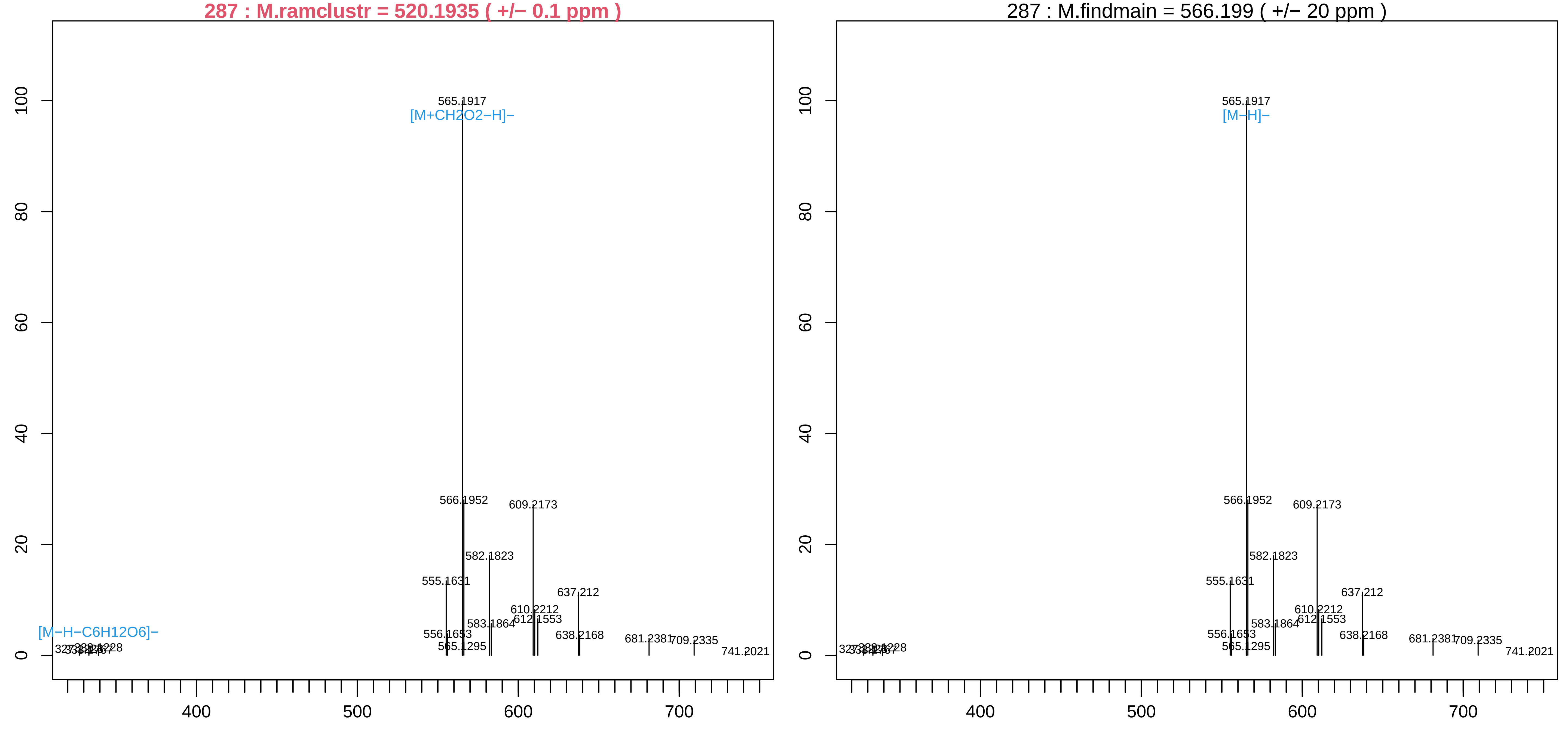
287 : M.ramclustr = 520.1935 (+/− 0.1 ppm)
565.1917
[M+CH2O2−H]−
566.1952
609.2173
582.1823
555.1631
637.212
610.2212
612.1553
583.1864
556.1653
638.2168
565.1295
681.2381
709.2335
741.2021
[M−H−C6H12O6]−
287 : M.findmain = 566.199 (+/− 20 ppm)
[M−H]−
0
20
40
60
80
100
400
500
600
700

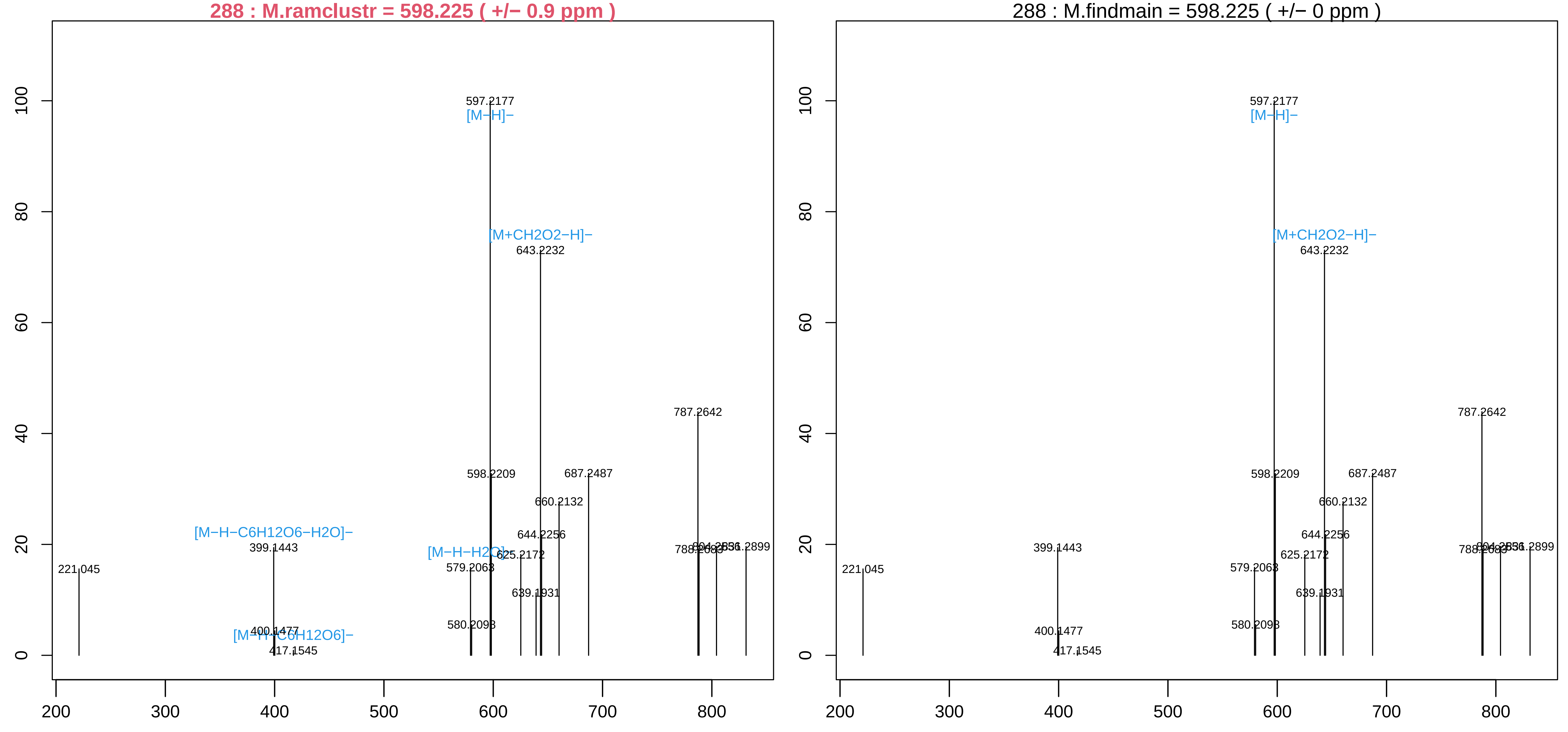

288 : M.ramclustr = 598.225 (+/− 0.9 ppm)
288 : M.findmain = 598.225 (+/− 0 ppm)
597.2177
[M−H]−
[M+CH2O2−H]−
643.2232
787.2642
598.2209
687.2487
660.2132
644.2256
[M−H−C6H12O6−H2O]−
399.1443
[M−H−H2O]−
625.2172
579.2063
221.045
639.1931
580.2093
400.1477
[M−H−C6H12O6]−
417.1545
788.2683
804.2856
831.2899
0
20
40
60
80
100
200
300
400
500
600
700
800

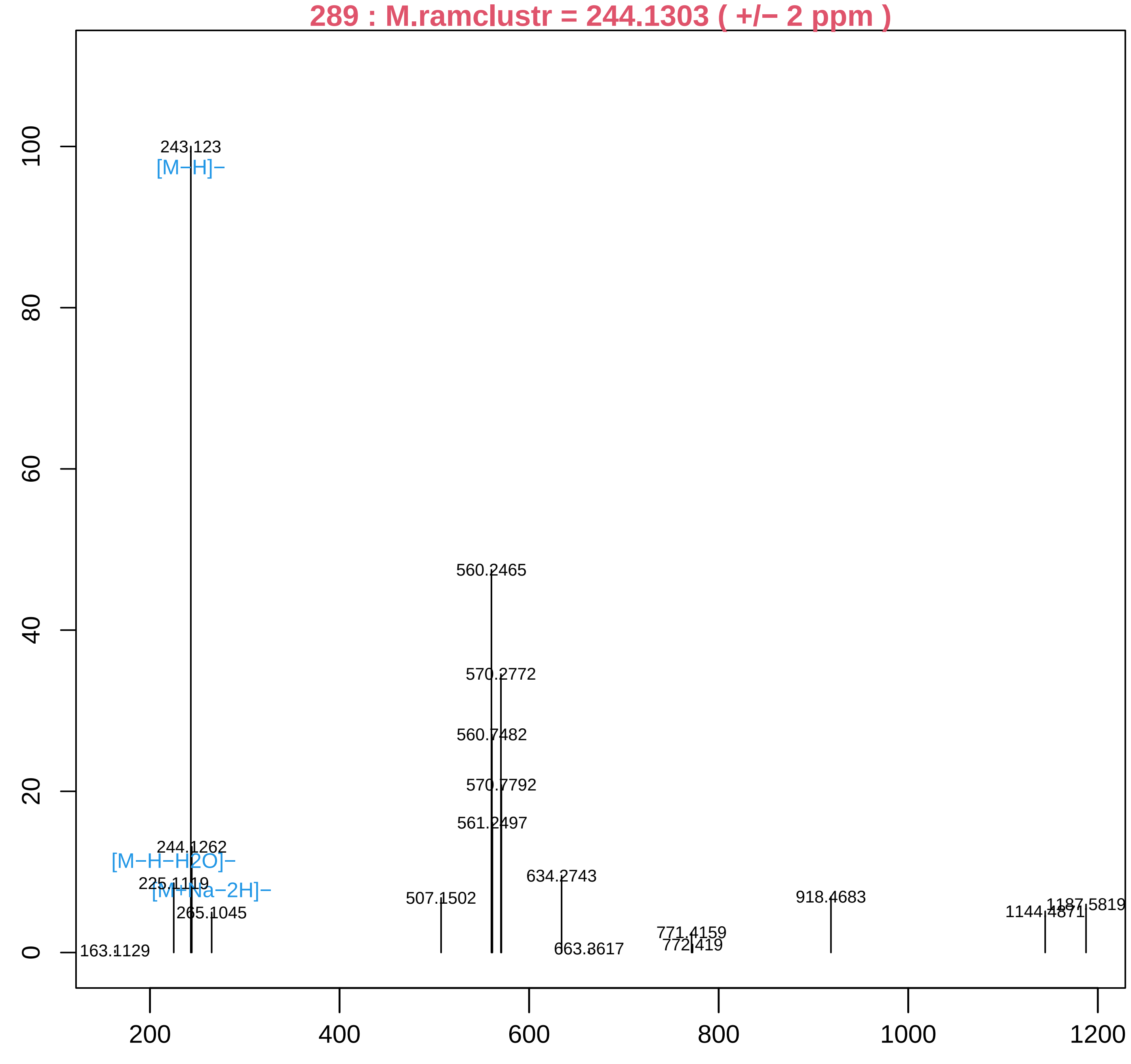
289 : M.ramclustr = 244.1303 (+/− 2 ppm)
243.123
[M−H]−
560.2465
570.2772
560.7482
570.7792
561.2497
244.1262
[M−H−H2O]−
225.1119
[M+Na−2H]−
265.1045
634.2743
507.1502
918.4683
1187.5819
1144.4871
771.4159
772.419
663.3617
163.1129
0
20
40
60
80
100
200
400
600
800
1000
1200

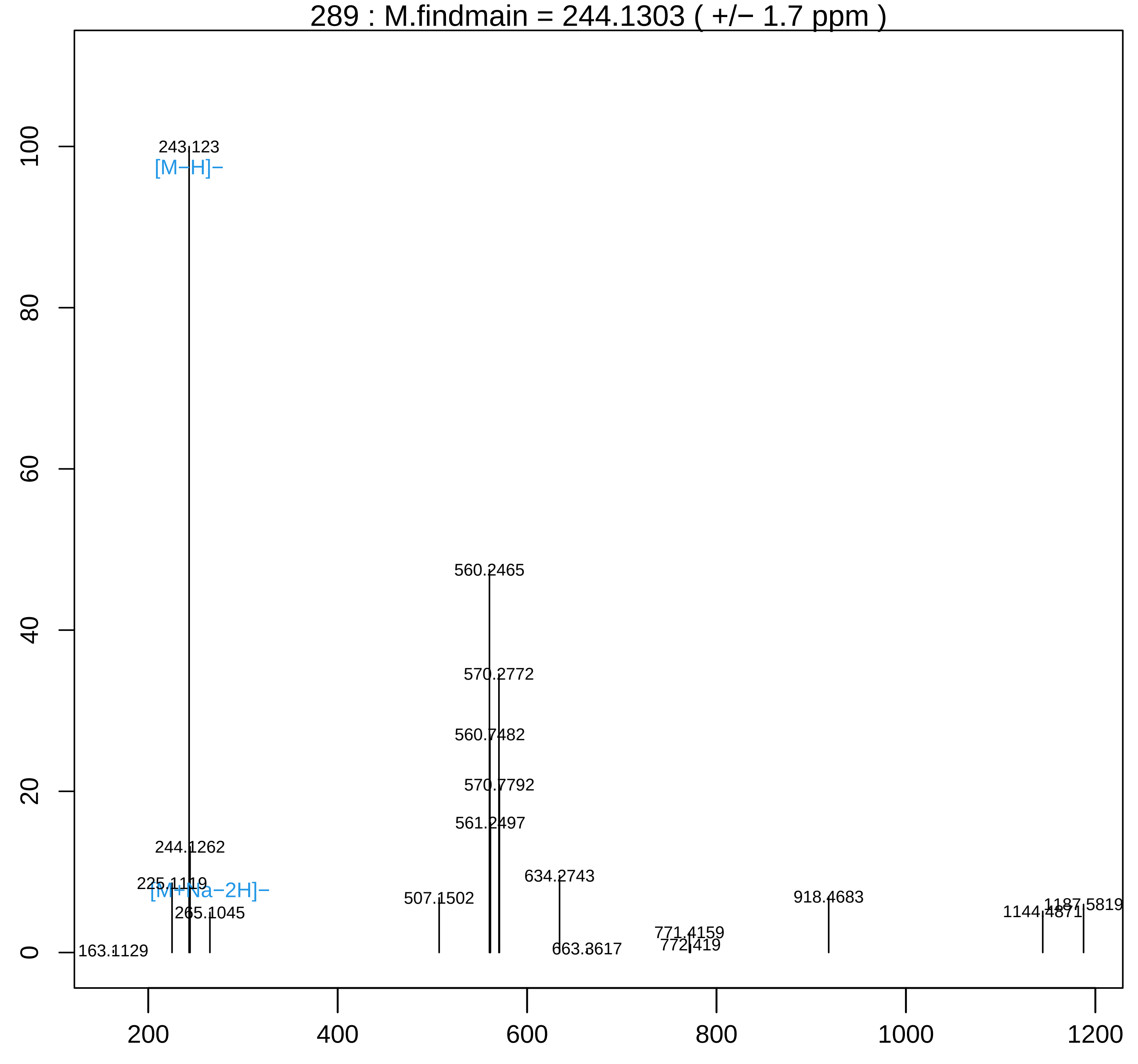
289 : M.findmain = 244.1303 (+/− 1.7 ppm)
243.123
[M−H]−
560.2465
570.2772
560.7482
570.7792
561.2497
244.1262
225.1119
[M+Na−2H]−
265.1045
634.2743
507.1502
918.4683
1187.5819
1144.4871
771.4159
772.419
663.3617
163.1129
0
20
40
60
80
100
200
400
600
800
1000
1200

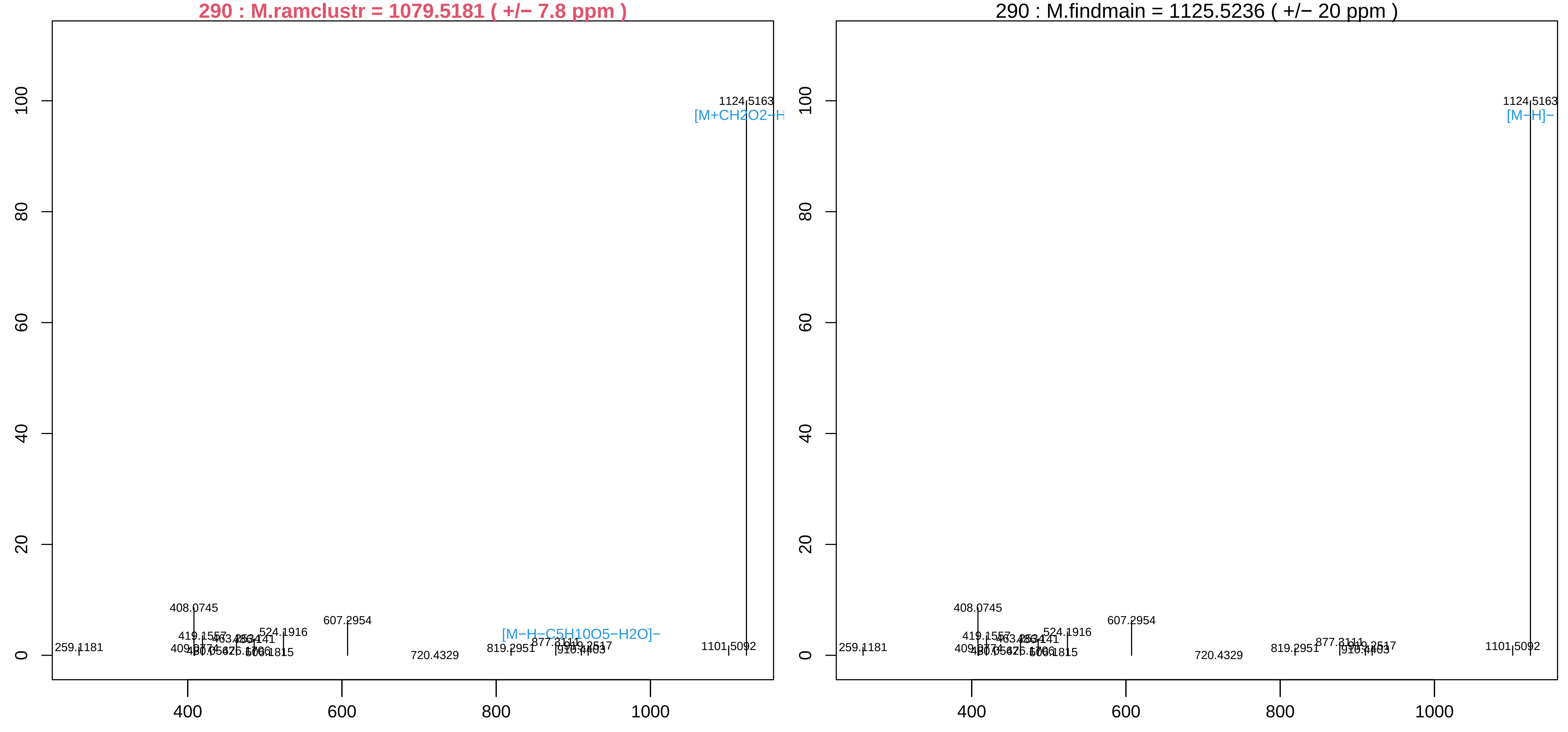

290 : M.ramclustr = 1079.5181 (+/− 7.8 ppm)
1124.5163
[M+CH2O2−H
408.0745
419.1557
463.2564
524.1916
607.2954
720.4329
819.2951
877.3111
919.2517
910.4403
1101.5092
259.1181
[M−H−C5H10O5−H2O]−
290 : M.findmain = 1125.5236 (+/− 20 ppm)
[M−H]−
0
20
40
60
80
100
400
600
800
1000

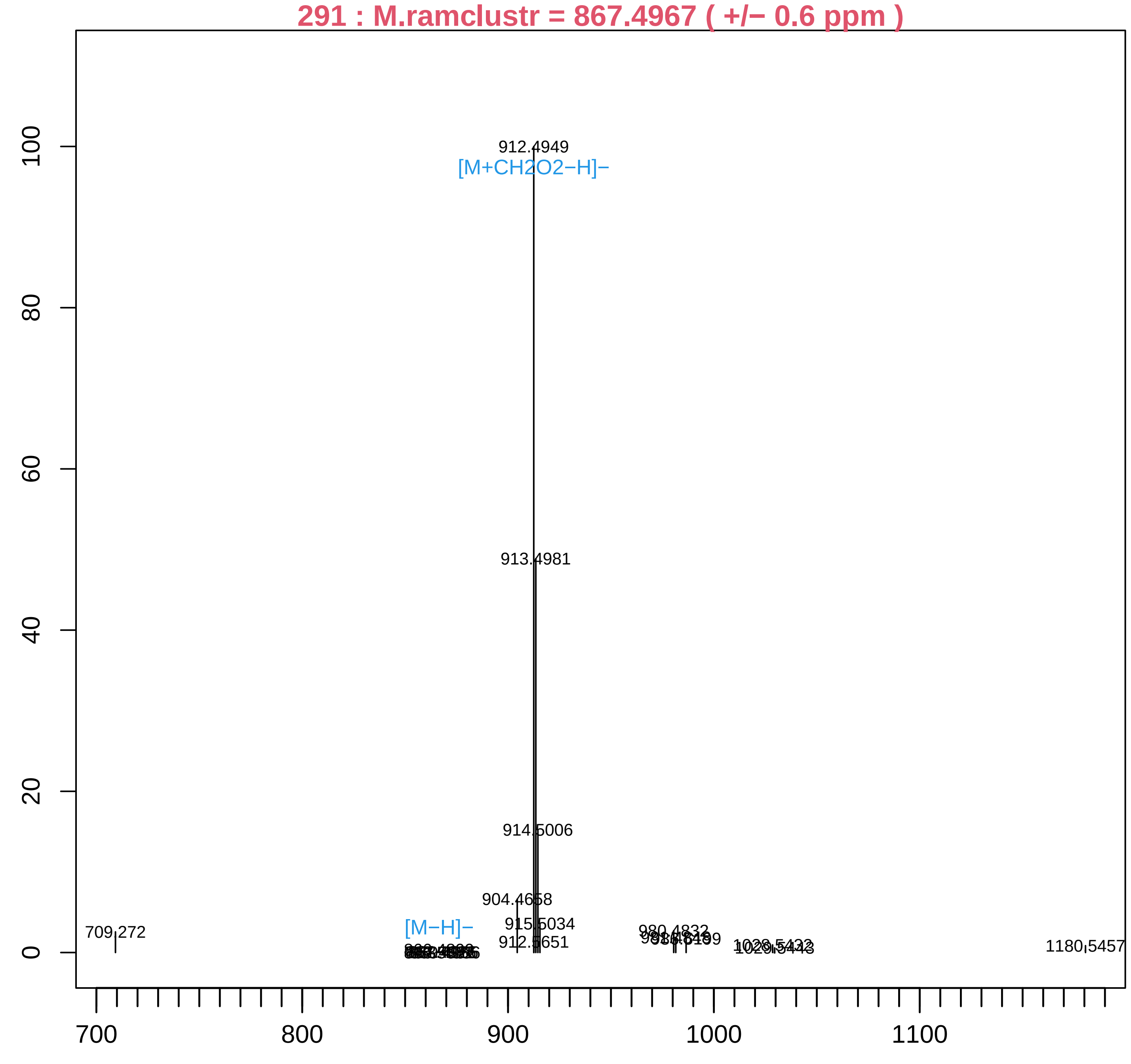
291 : M.ramclustr = 867.4967 (+/− 0.6 ppm)
912.4949
[M+CH2O2−H]−
913.4981
914.5006
904.4658
915.5034
912.7651
[M−H]−
709.272
980.4832
1029.5422
1180.5457
0
20
40
60
80
100
700
800
900
1000
1100
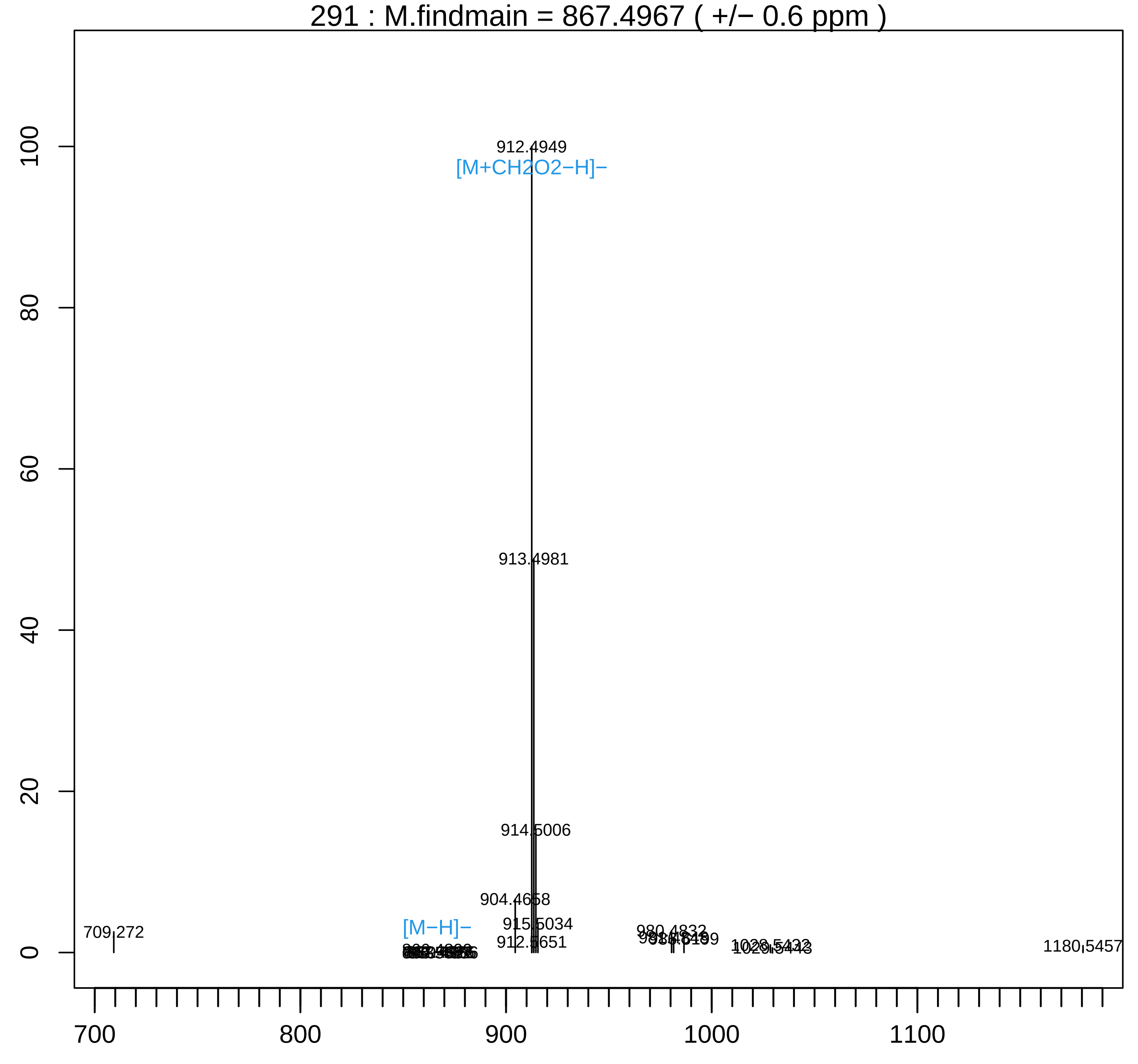
291 : M.findmain = 867.4967 (+/− 0.6 ppm)
912.4949
[M+CH2O2−H]−
913.4981
914.5006
904.4658
915.5034
912.7651
[M−H]−
709.272
980.4832
1029.5422
1180.5457
0
20
40
60
80
100
700
800
900
1000
1100

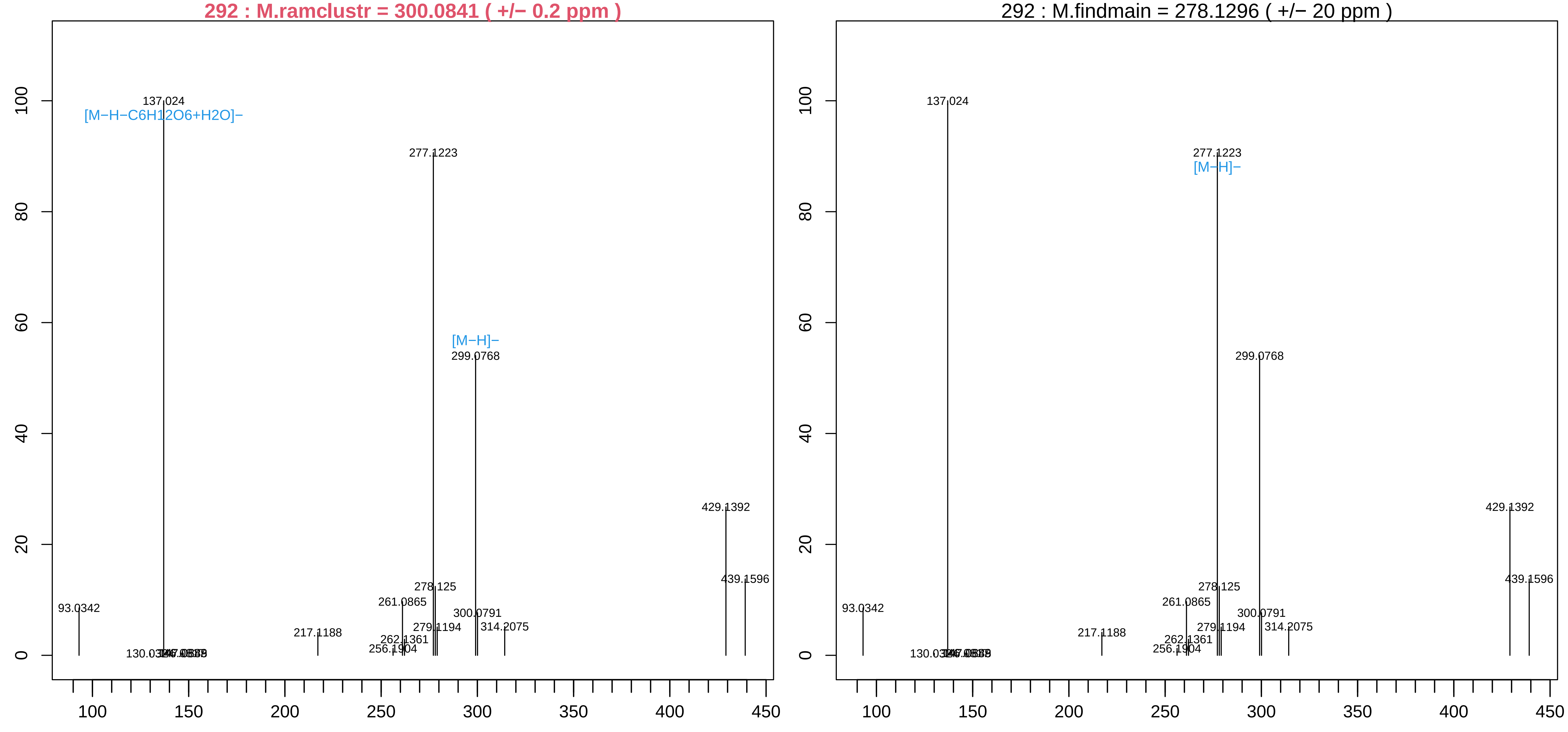
292 : M.ramclustr = 300.0841 (+/− 0.2 ppm)
[M−H−C6H12O6+H2O]−
137.024
277.1223
[M−H]−
299.0768
429.1392
439.1596
93.0342
278.125
261.0865
300.0791
314.2075
279.1194
217.1188
262.1361
256.1904
292 : M.findmain = 278.1296 (+/− 20 ppm)
[M−H]−

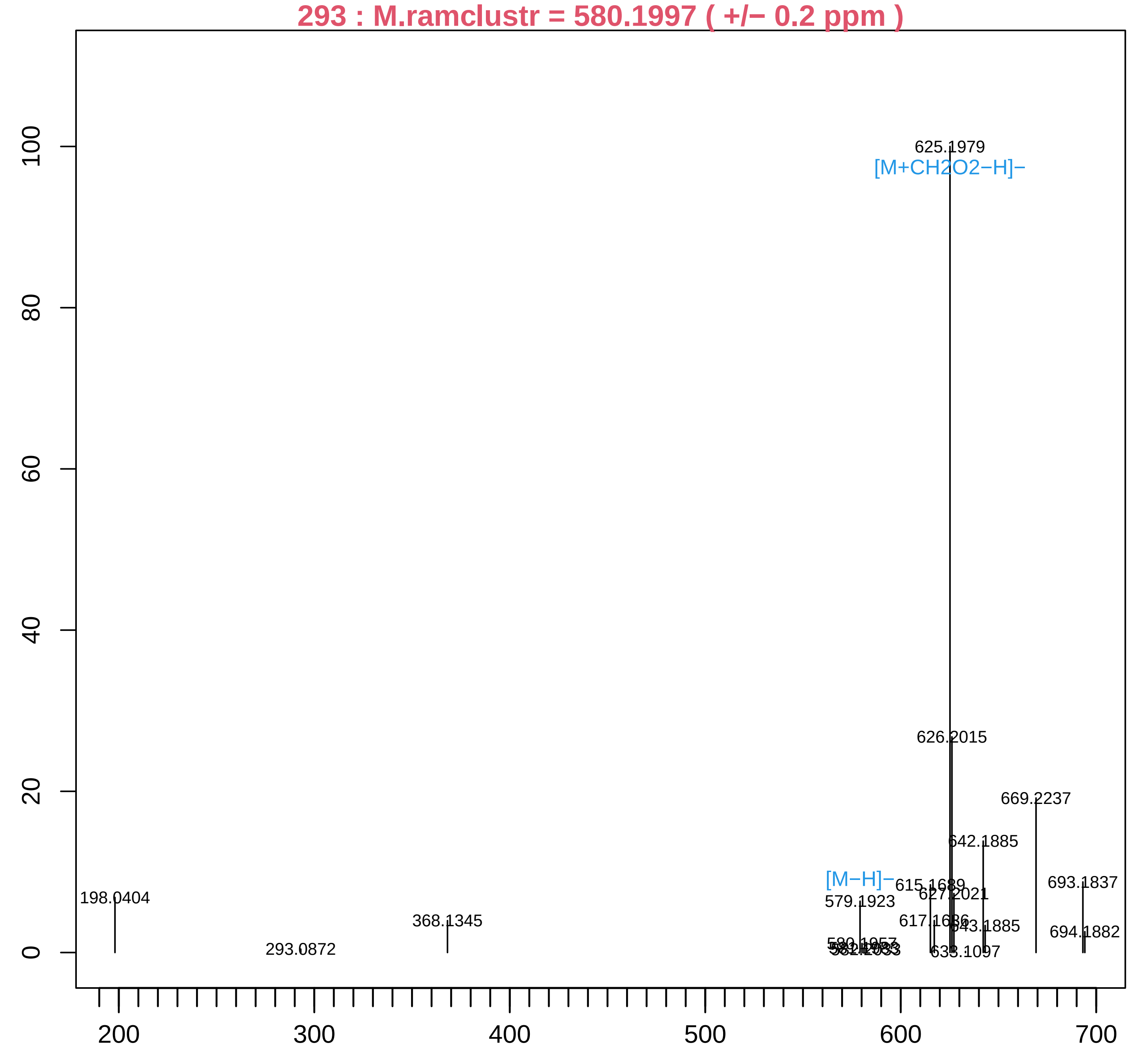
293 : M.ramclustr = 580.1997 (+/− 0.2 ppm)
625.1979
[M+CH2O2−H]−
626.2015
669.2237
642.1885
[M−H]−
615.1689
627.2021
693.1837
198.0404
579.1923
368.1345
617.1686
643.1885
694.1882
293.0872
633.1097
0
20
40
60
80
100
200
300
400
500
600
700

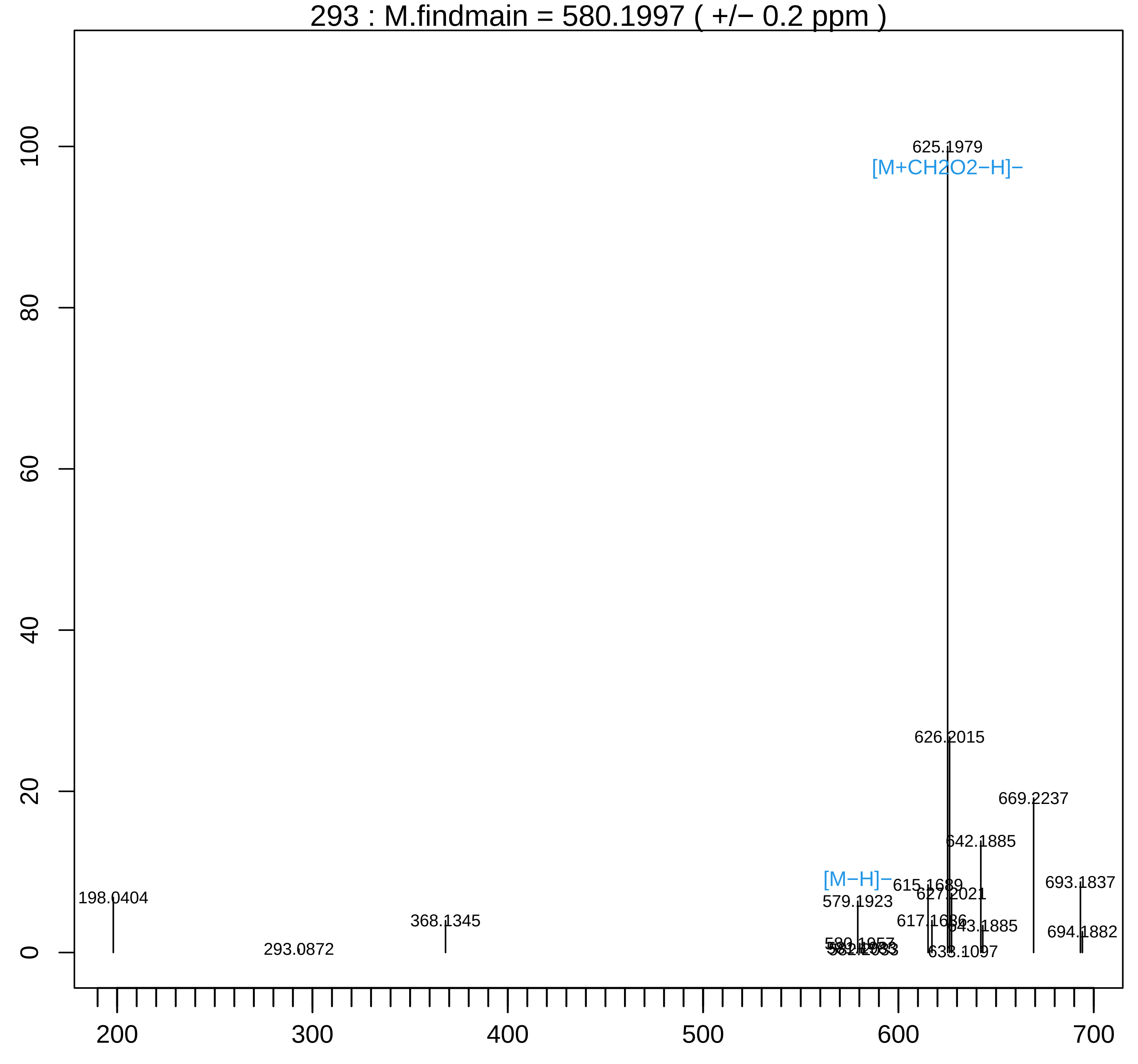
293 : M.findmain = 580.1997 (+/− 0.2 ppm)
625.1979
[M+CH2O2−H]−
626.2015
669.2237
642.1885
[M−H]−
615.1689
627.2021
693.1837
198.0404
579.1923
368.1345
617.1686
643.1885
694.1882
293.0872
633.1097
0
20
40
60
80
100
200
300
400
500
600
700

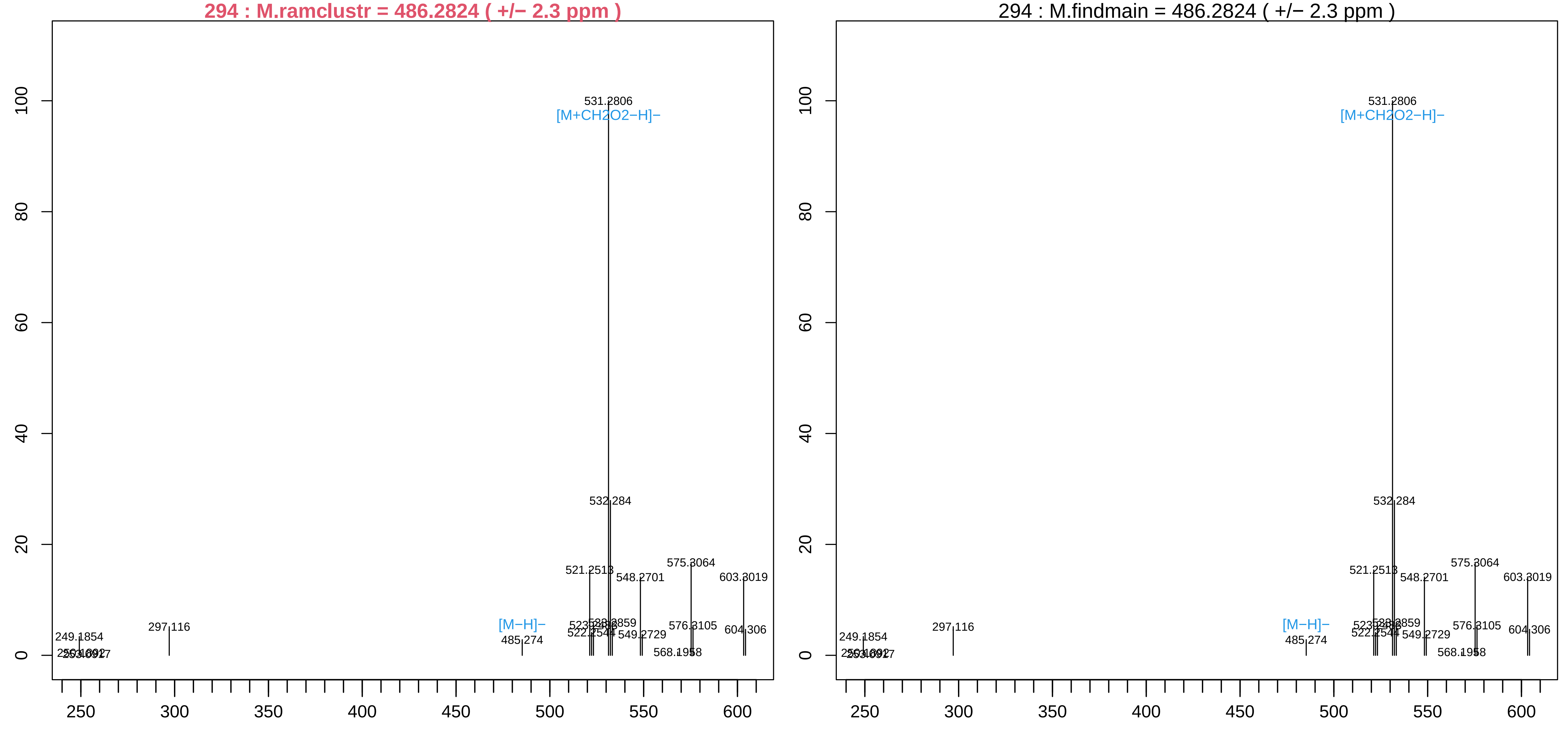

294 : M.ramclustr = 486.2824 (+/− 2.3 ppm)
294 : M.findmain = 486.2824 (+/− 2.3 ppm)
531.2806
[M+CH2O2−H]−
532.284
521.2513
548.2701
575.3064
603.3019
297.116
249.1854
[M−H]−
485.274
576.3105
604.306
549.2729
568.1958
522.2544
0
20
40
60
80
100
250
300
350
400
450
500
550
600

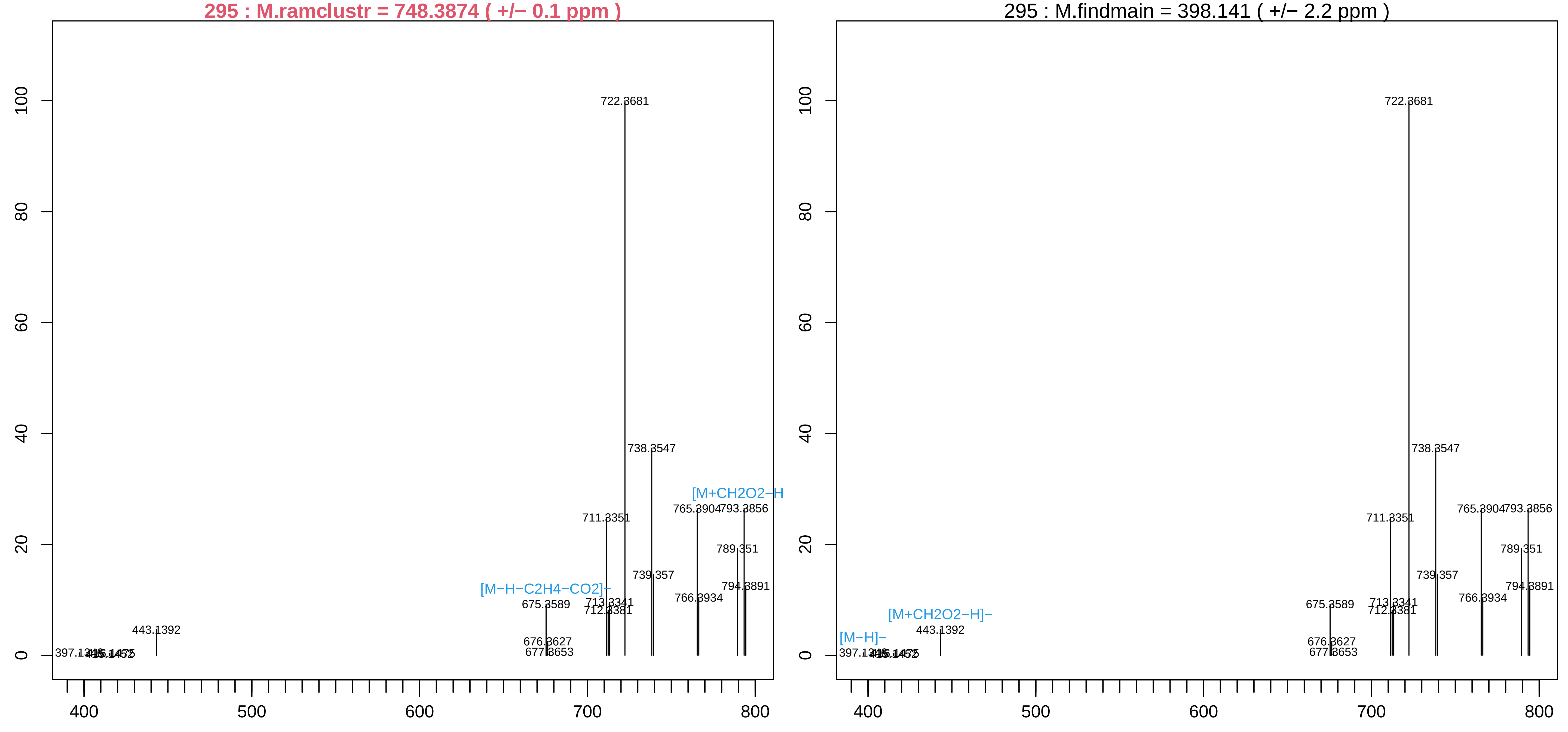

295 : M.ramclustr = 748.3874 (+/− 0.1 ppm)
295 : M.findmain = 398.141 (+/− 2.2 ppm)
722.3681
738.3547
[M+CH2O2−H
765.3904
793.3856
711.3351
789.351
739.357
794.3891
[M−H−C2H4−CO2]−
766.3934
713.3341
675.3589
712.3381
443.1392
676.3627
677.3653
[M+CH2O2−H]−
[M−H]−
397.1346
0
20
40
60
80
100
400
500
600
700
800

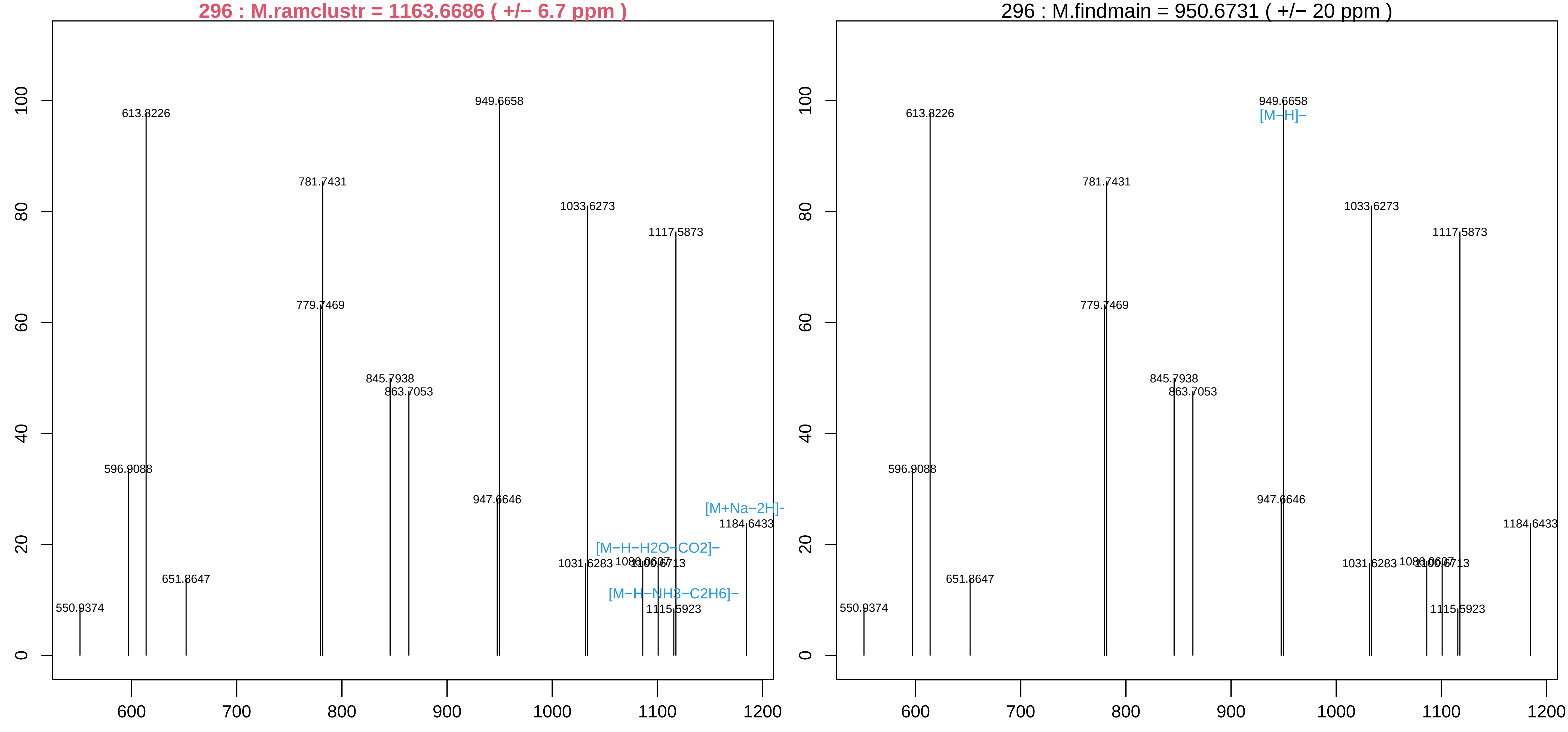
296 : M.ramclustr = 1163.6686 (+/− 6.7 ppm)
613.8226
949.6658
781.7431
1033.6273
1117.5873
779.7469
845.7938
863.7053
596.9088
947.6646
[M+Na−2H]-
1184.6433
[M−H−H2O−CO2]−
1031.6283
1100.6713
651.8647
[M−H−NH3−C2H6]−
1115.5923
550.9374
296 : M.findmain = 950.6731 (+/− 20 ppm)
[M−H]−
0
20
40
60
80
100
600
700
800
900
1000
1100
1200

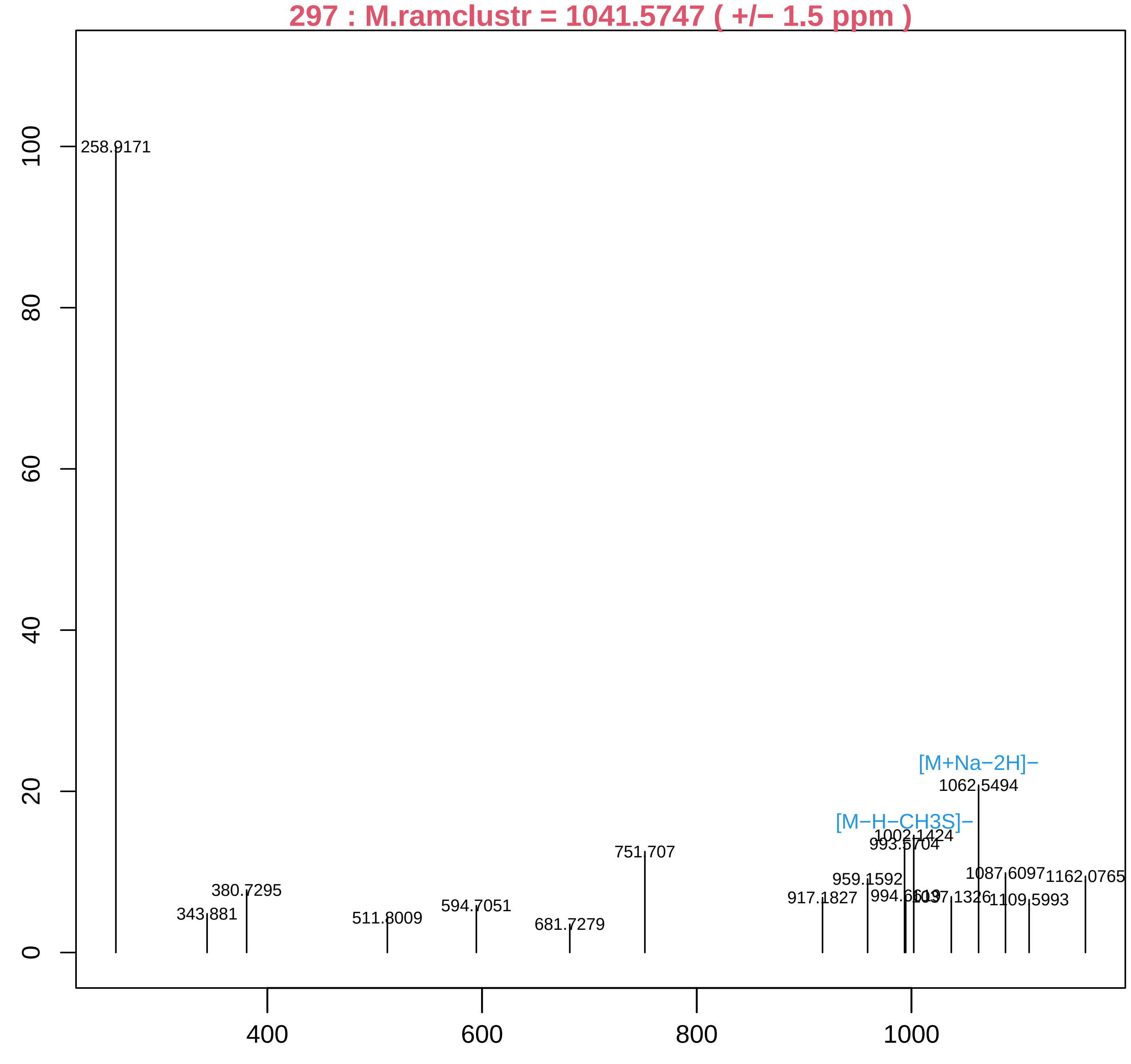
297 : M.ramclustr = 1041.5747 (+/− 1.5 ppm)
258.9171
343.881
380.7295
511.8009
594.7051
681.7279
751.707
917.1827
959.1592
993.5704
1002.1424
[M−H−CH3S]−
1062.5494
[M+Na−2H]−
1087.6097
1109.5993
1162.0765
0
20
40
60
80
100
400
600
800
1000

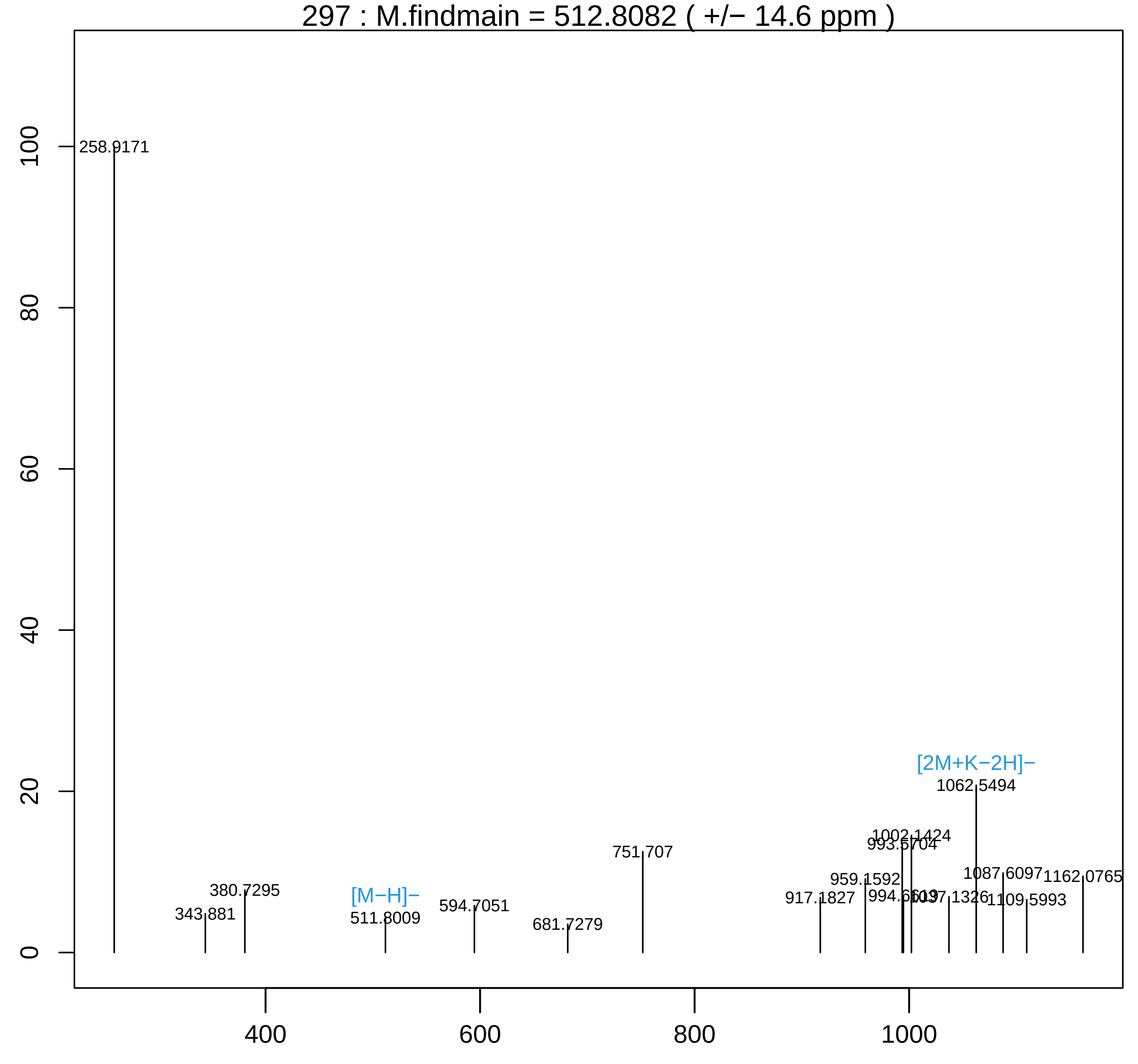
297 : M.findmain = 512.8082 (+/− 14.6 ppm)
258.9171
343.881
380.7295
[M−H]−
511.8009
594.7051
681.7279
751.707
917.1827
959.1592
993.5704
1002.1424
1062.5494
[2M+K−2H]−
1087.6097
1109.5993
1162.0765
0
20
40
60
80
100
400
600
800
1000

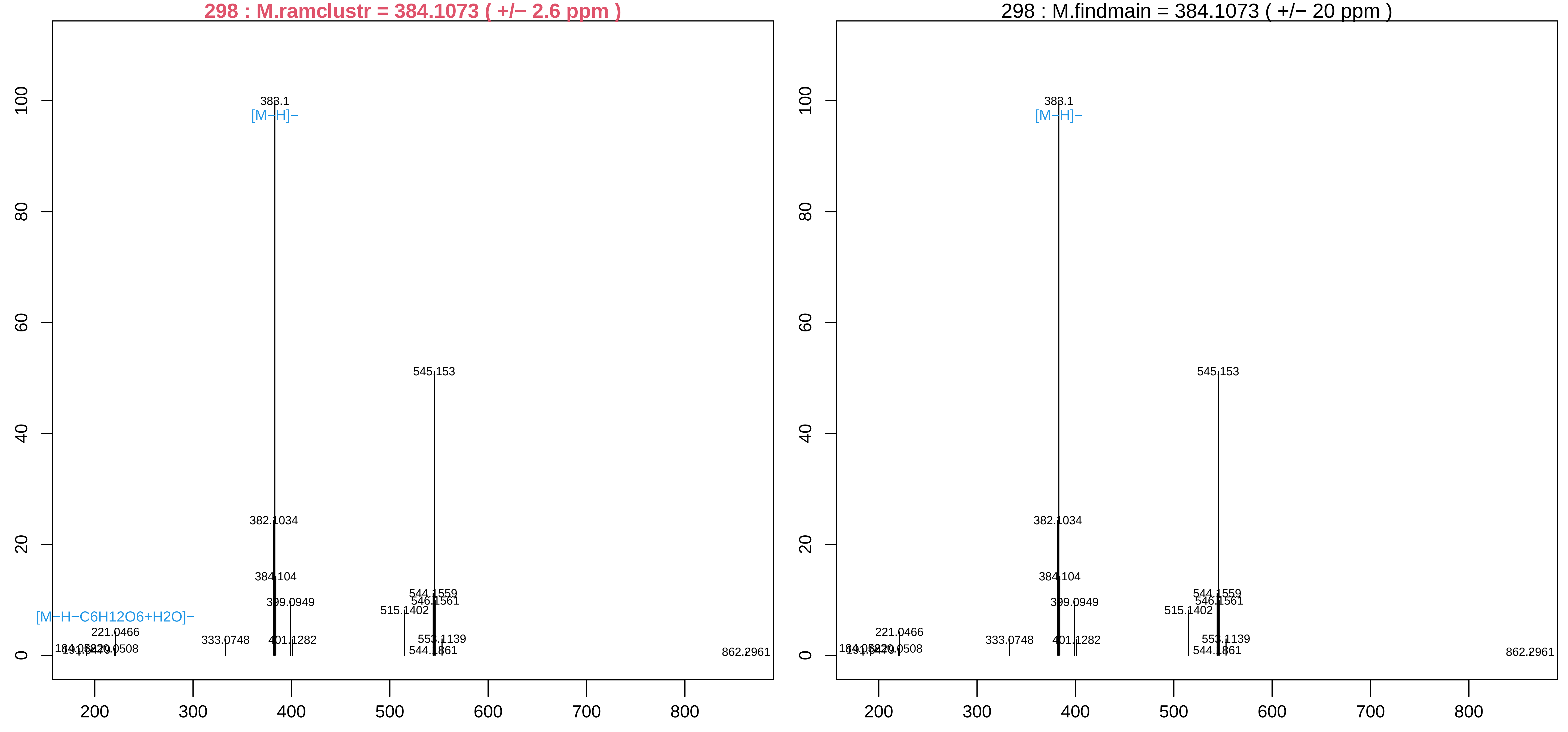

298 : M.ramclustr = 384.1073 (+/− 2.6 ppm)
298 : M.findmain = 384.1073 (+/− 20 ppm)
383.1
[M−H]−
545.153
382.1034
384.104
544.1559
546.1561
399.0949
515.1402
[M−H−C6H12O6+H2O]−
221.0466
333.0748
401.1282
553.1139
544.1861
862.2961
0
20
40
60
80
100
200
300
400
500
600
700
800

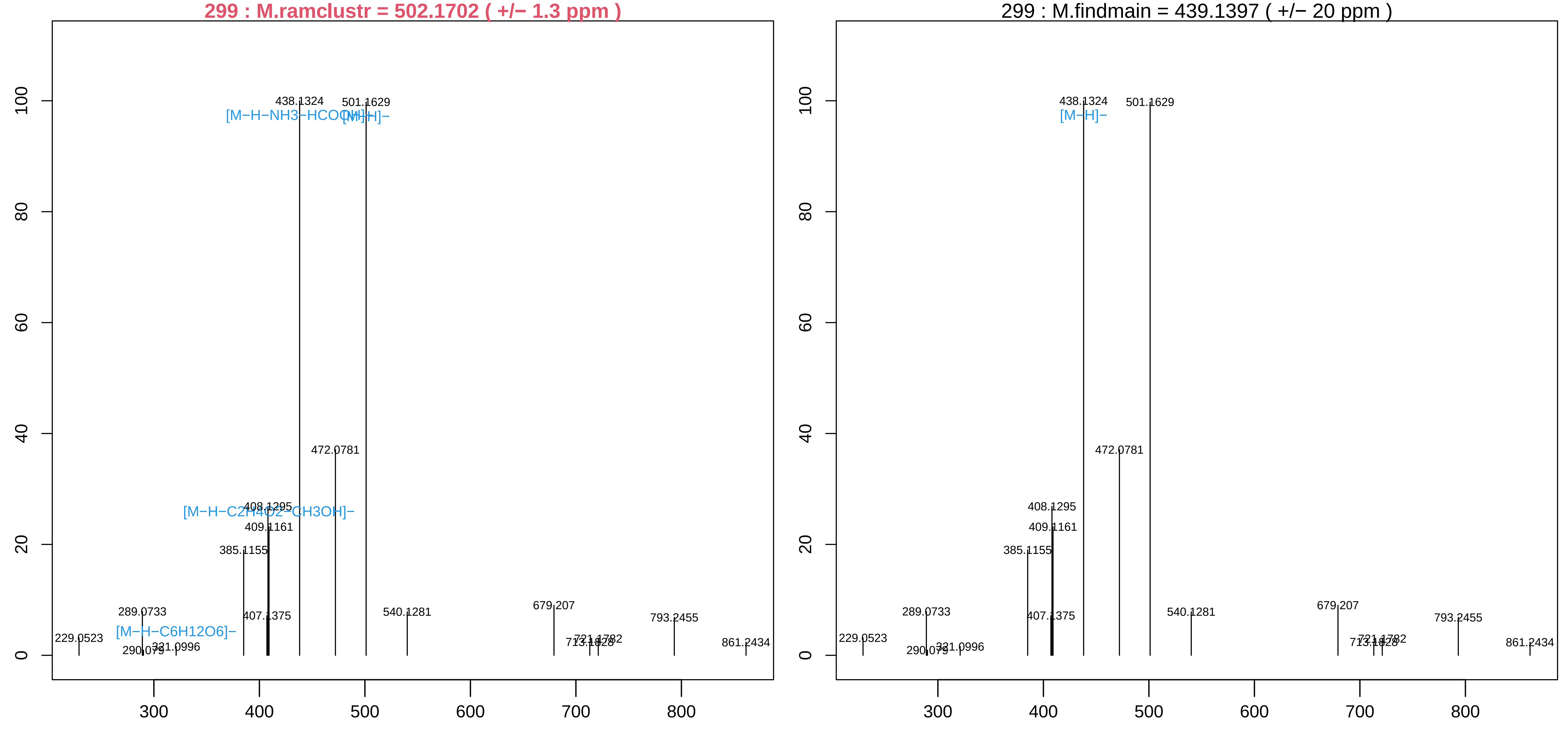

299 : M.ramclustr = 502.1702 (+/− 1.3 ppm)
299 : M.findmain = 439.1397 (+/− 20 ppm)
438.1324
501.1629
[M−H−NH3−HCOOH]−
[M−H]−
472.0781
408.1295
[M−H−C2H4O2−CH3OH]−
409.1161
385.1155
289.0733
407.1375
540.1281
679.207
793.2455
[M−H−C6H12O6]−
229.0523
721.1782
713.1828
861.2434
290.079
321.0996
0
20
40
60
80
100
300
400
500
600
700
800

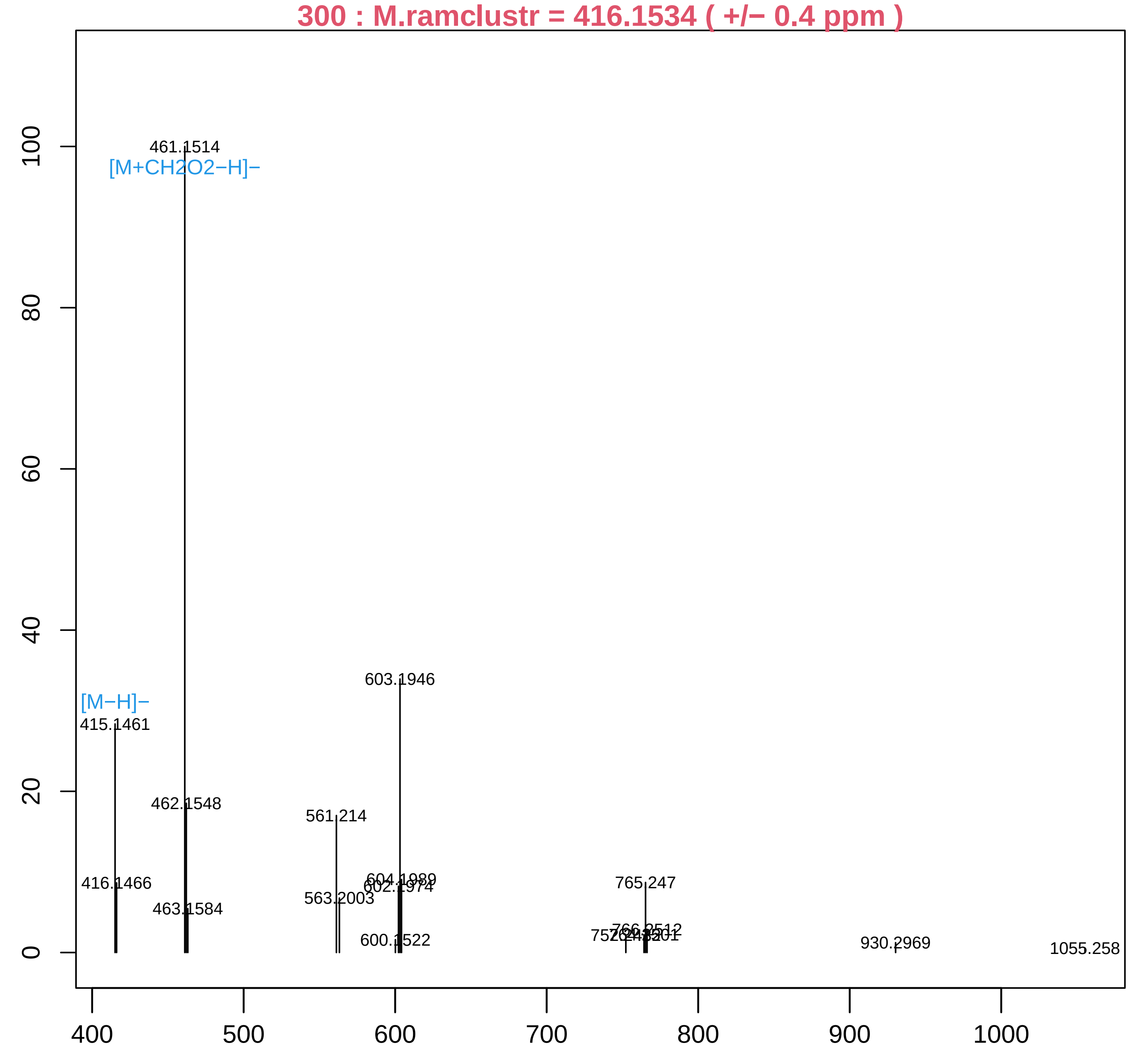
300 : M.ramclustr = 416.1534 (+/− 0.4 ppm)
461.1514
[M+CH2O2−H]−
[M−H]−
415.1461
603.1946
462.1548
561.214
416.1466
604.1989
602.1974
765.247
563.2003
463.1584
600.1522
766.2512
930.2969
1055.258

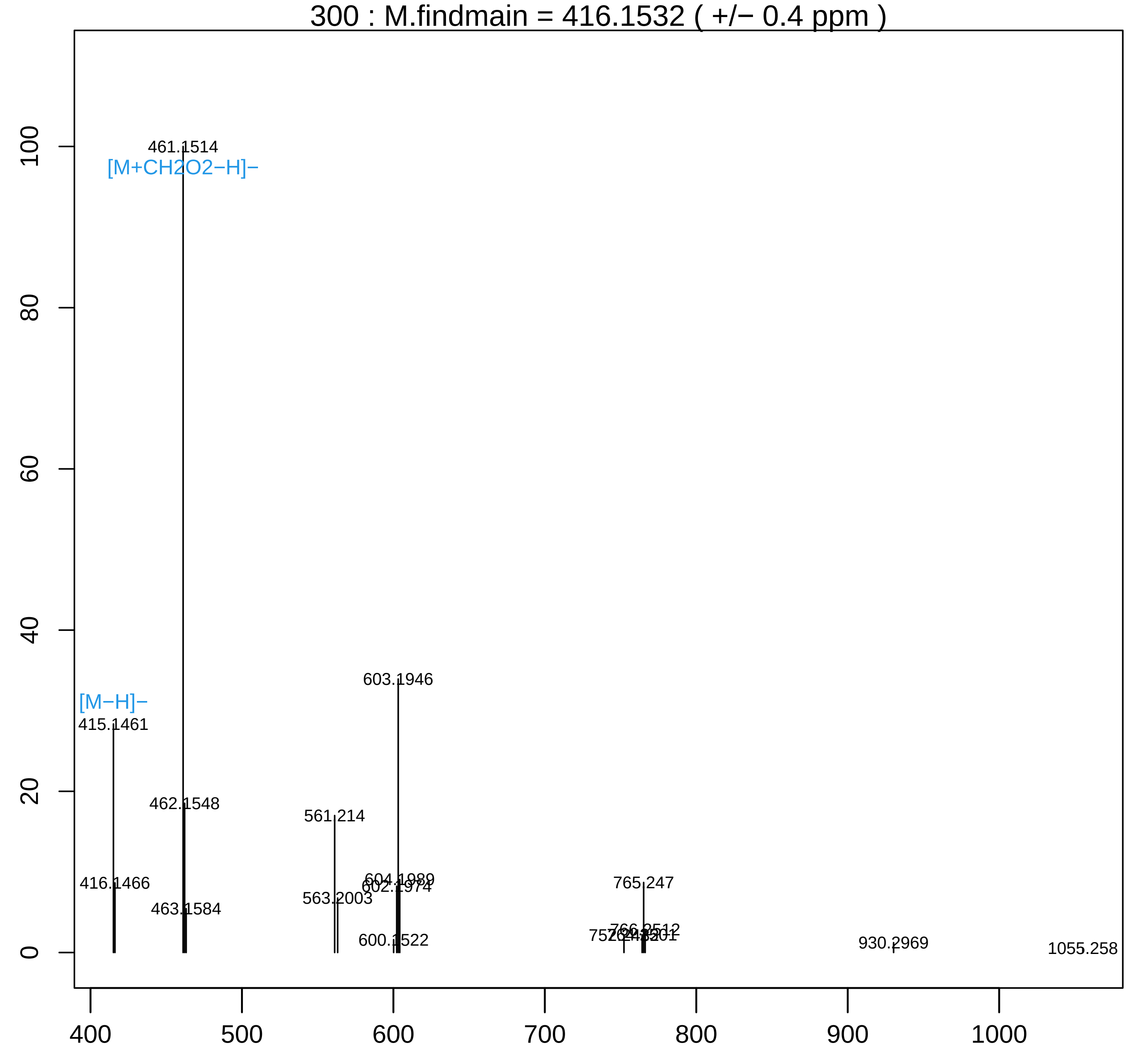
300 : M.findmain = 416.1532 (+/− 0.4 ppm)
461.1514
[M+CH2O2−H]−
[M−H]−
415.1461
603.1946
462.1548
561.214
416.1466
604.1989
602.1974
765.247
563.2003
463.1584
600.1522
766.2512
930.2969
1055.258

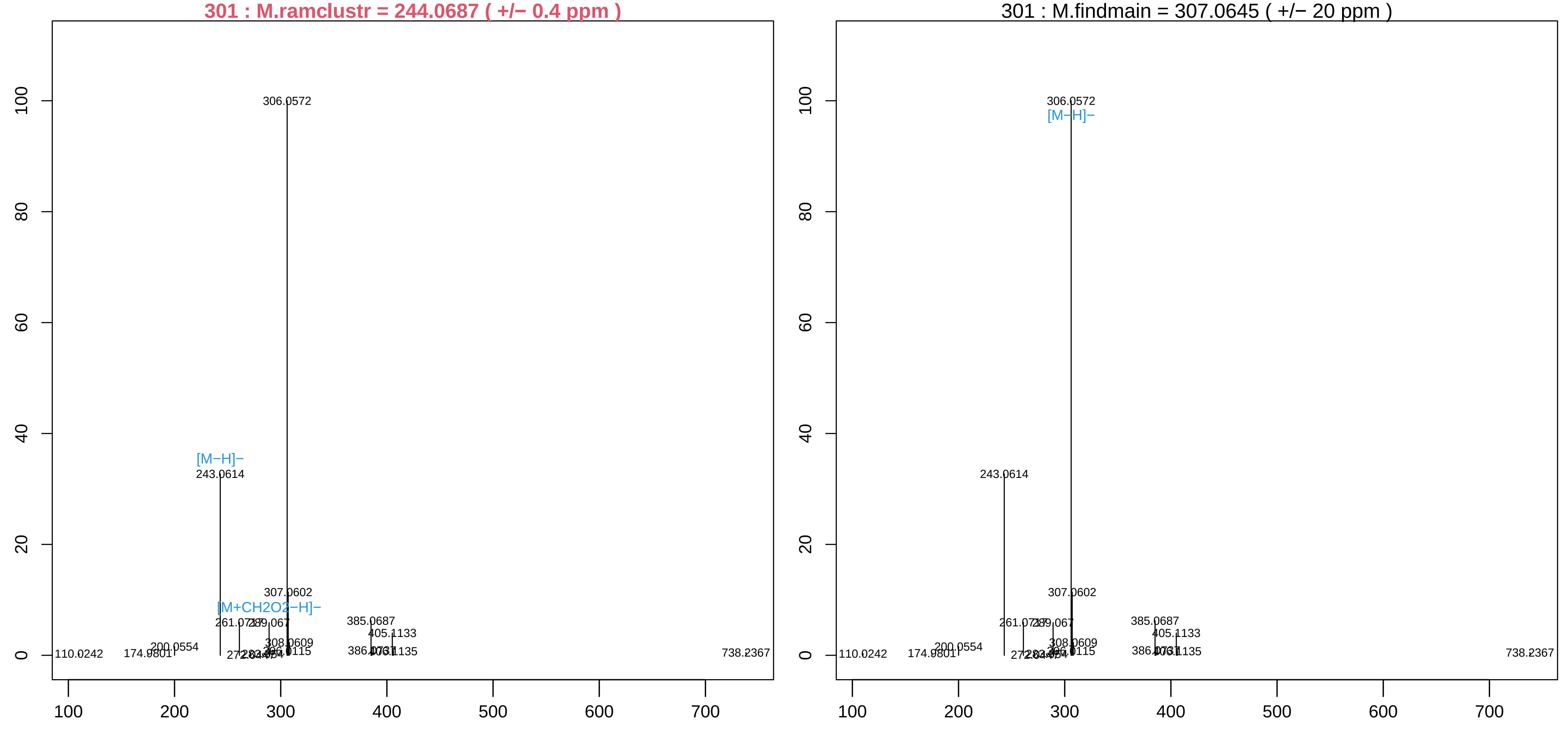
301 : M.ramclustr = 244.0687 (+/− 0.4 ppm)
306.0572
[M−H]−
243.0614
307.0602
[M+CH2O2−H]−
385.0687
405.1133
308.0609
200.0554
110.0242
174.9801
386.0731
738.2367
301 : M.findmain = 307.0645 (+/− 20 ppm)
306.0572
[M−H]−
243.0614
307.0602
385.0687
405.1133
308.0609
200.0554
110.0242
174.9801
738.2367
0
20
40
60
80
100
100
200
300
400
500
600
700

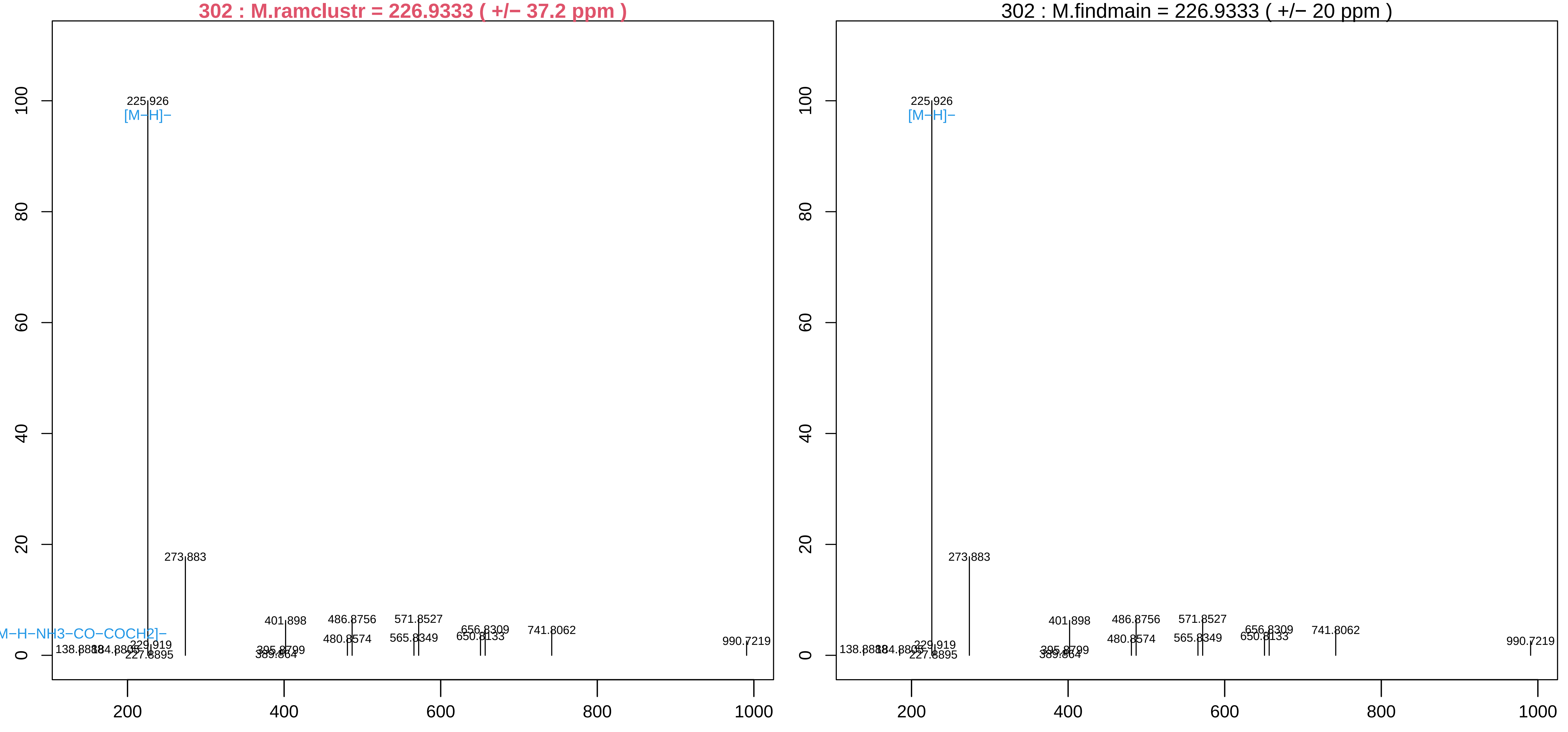
302 : M.ramclustr = 226.9333 (+/− 37.2 ppm)
302 : M.findmain = 226.9333 (+/− 20 ppm)
225.926
[M−H]−
273.883
401.898
486.8756
571.8527
656.8309
741.8062
990.7219
480.8574
565.8349
650.8133
395.8799
389.864
229.919
227.8895
138.8818
184.8805
[M−H−NH3−CO−COCH2]−
0
20
40
60
80
100
200
400
600
800
1000

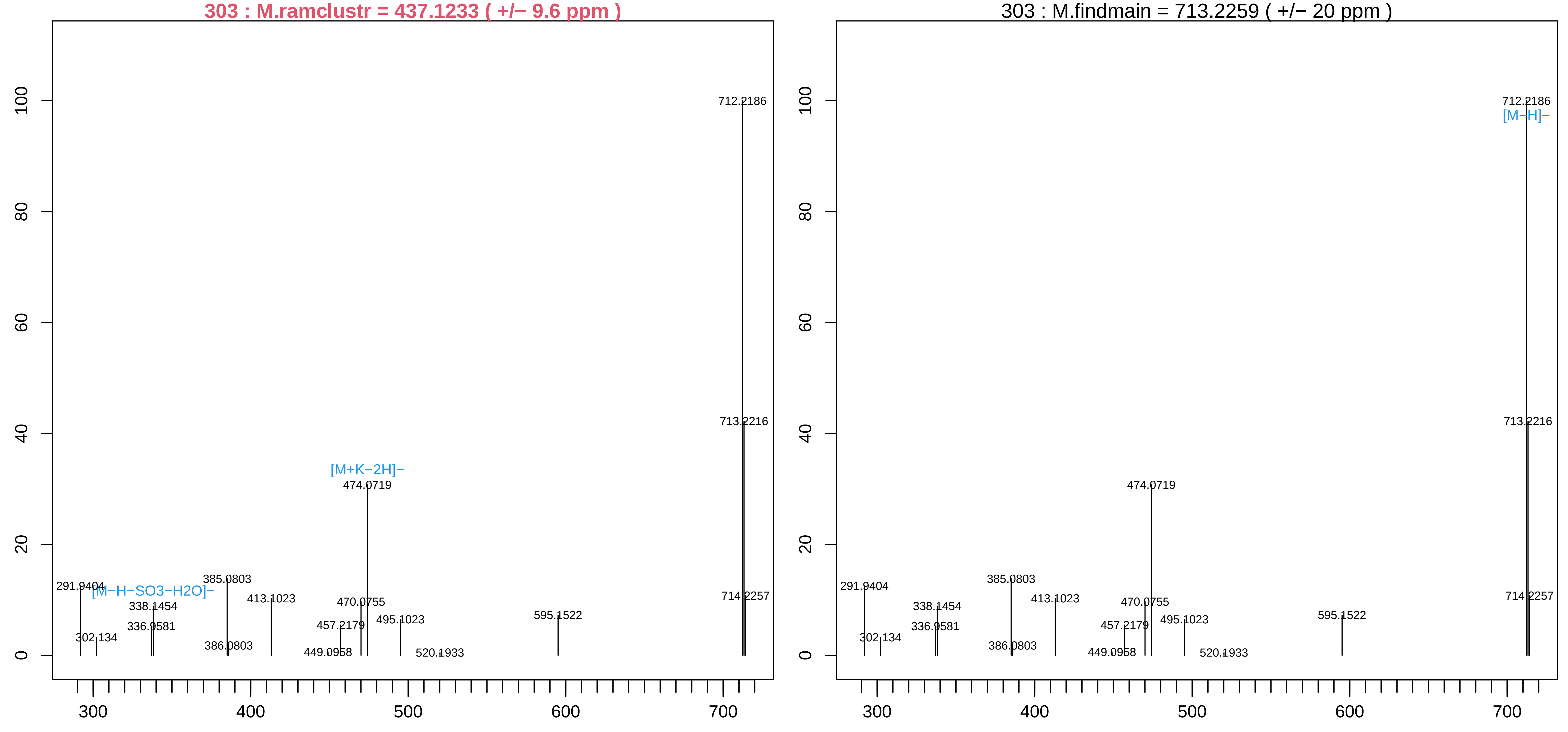
303 : M.ramclustr = 437.1233 (+/− 9.6 ppm)
712.2186
713.2216
714.2257
[M+K−2H]−
474.0719
291.9404
[M−H−SO3−H2O]−
338.1454
336.9581
302.134
385.0803
386.0803
413.1023
470.0755
457.2179
449.0958
495.1023
520.1933
595.1522
303 : M.findmain = 713.2259 (+/− 20 ppm)
712.2186
[M−H]−
713.2216
714.2257
474.0719
291.9404
338.1454
336.9581
302.134
385.0803
386.0803
413.1023
470.0755
457.2179
449.0958
495.1023
520.1933
595.1522
0
20
40
60
80
100
300
400
500
600
700

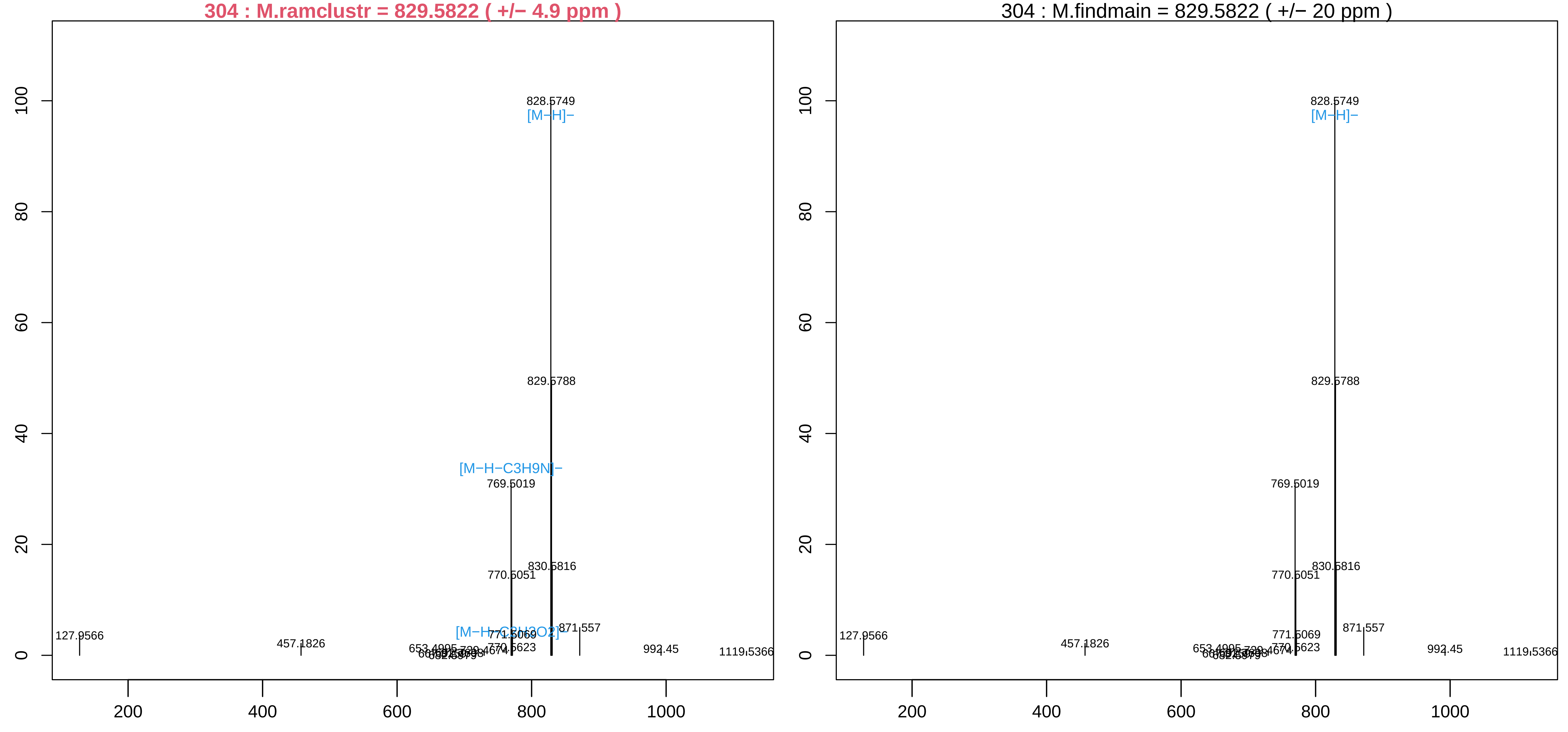

304 : M.ramclustr = 829.5822 (+/− 4.9 ppm)
828.5749
[M−H]−
829.5788
[M−H−C3H9N]−
769.5019
830.5816
770.5051
871.557
[M−H−C3H3O2]−
771.5069
770.5623
127.9566
457.1826
653.4995
992.45
1119.5366
304 : M.findmain = 829.5822 (+/− 20 ppm)
828.5749
[M−H]−
829.5788
769.5019
830.5816
770.5051
871.557
771.5069
770.5623
127.9566
457.1826
653.4995
992.45
1119.5366

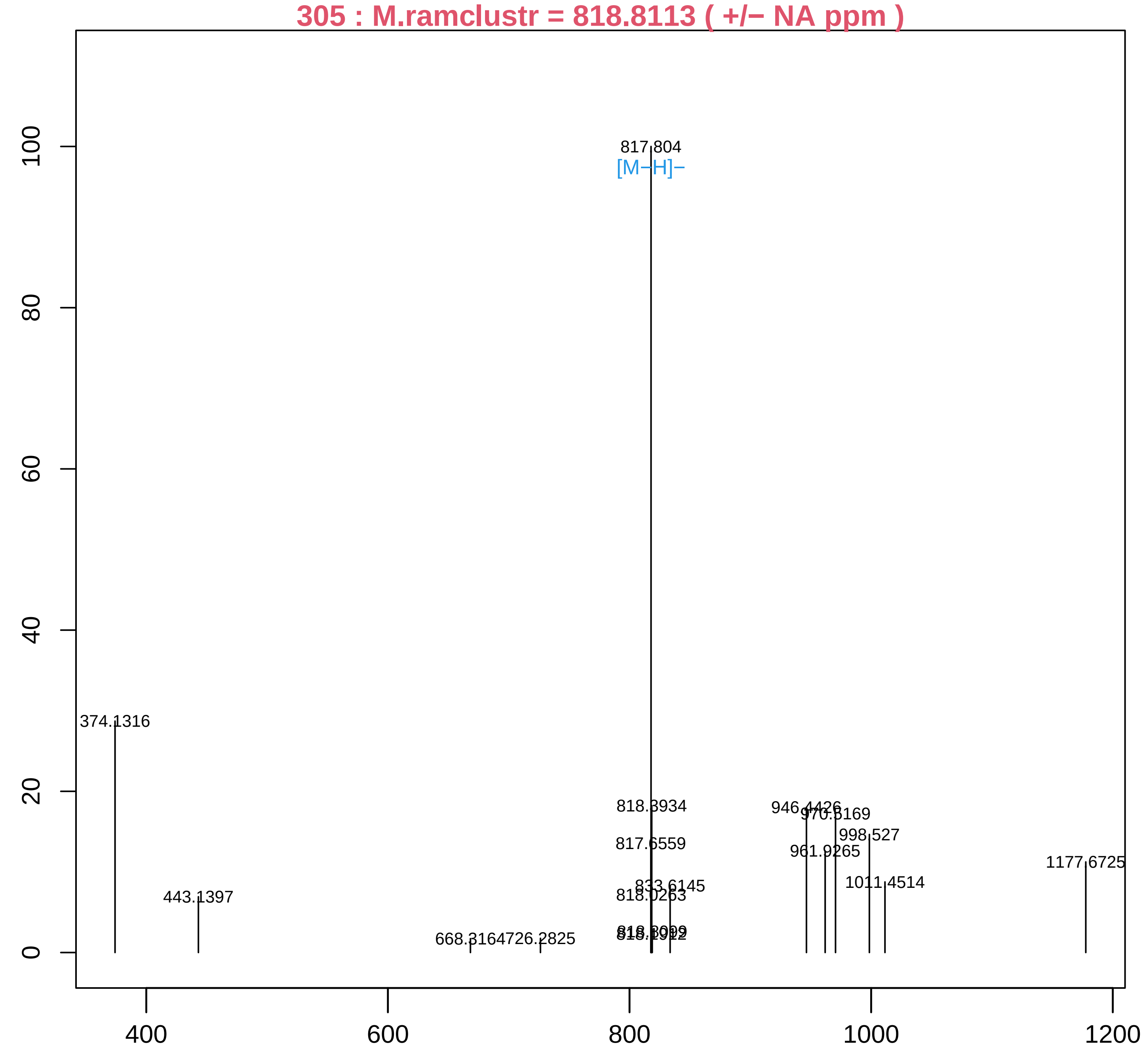

305 : M.ramclustr = 818.8113 (+/− NA ppm)
817.804
[M−H]−
374.1316
443.1397
668.3164
726.2825
818.3934
817.6559
818.0263
833.6145
946.4426
970.5169
998.527
961.9265
1011.4514
1177.6725
0
20
40
60
80
100
400
600
800
1000
1200

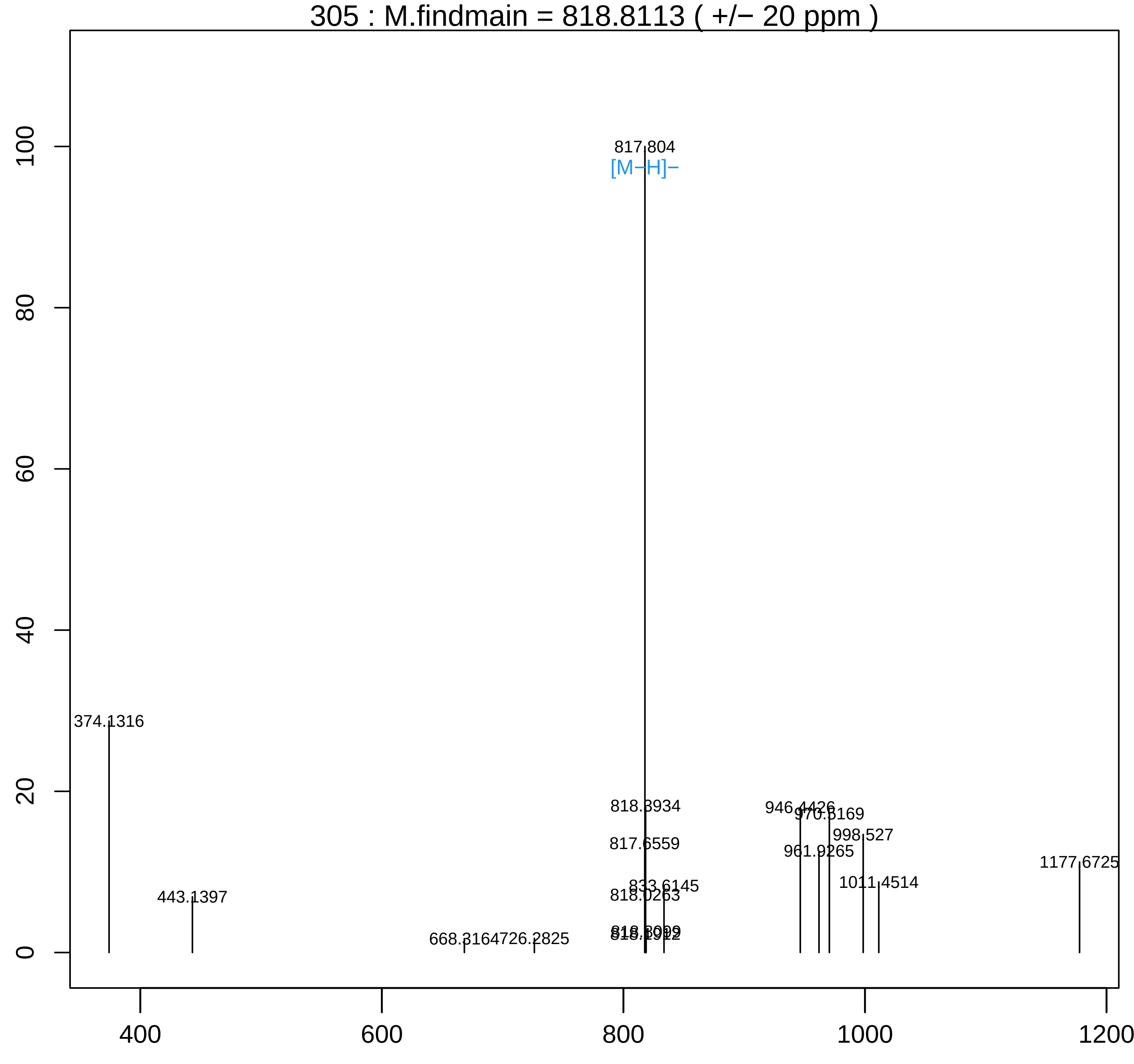

305 : M.findmain = 818.8113 (+/− 20 ppm)
817.804
[M−H]−
374.1316
443.1397
668.3164
726.2825
818.3934
817.6559
818.0263
833.6145
946.4426
970.5169
998.527
961.9265
1011.4514
1177.6725
0
20
40
60
80
100
400
600
800
1000
1200

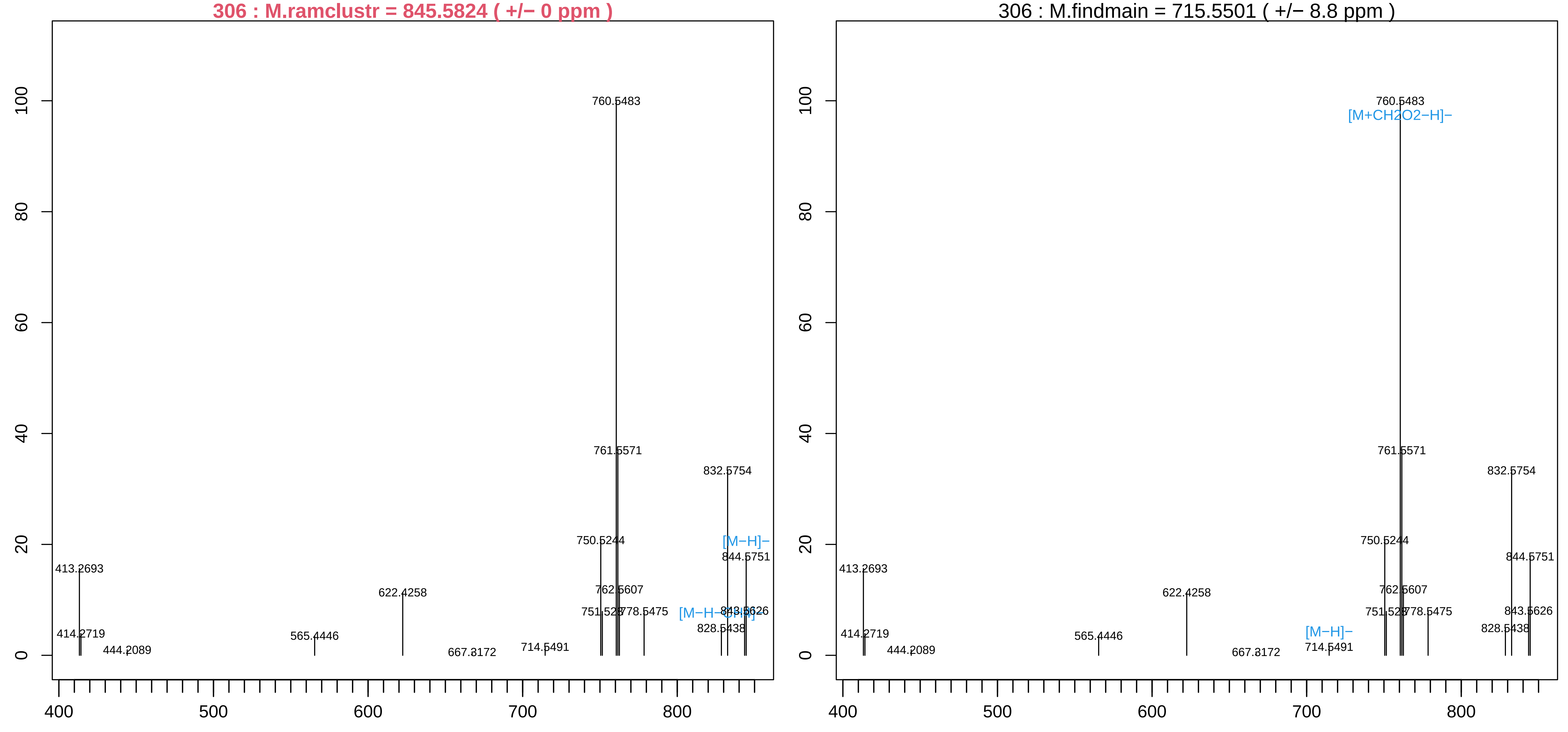

306 : M.ramclustr = 845.5824 (+/− 0 ppm)
760.5483
761.5571
832.5754
750.5244
[M−H]−
844.5751
413.2693
622.4258
762.5607
751.528
778.5475
[M−H−CH3]−
843.5626
828.5438
414.2719
565.4446
444.2089
714.5491
667.8172
306 : M.findmain = 715.5501 (+/− 8.8 ppm)
[M+CH2O2−H]−
[M−H]−
0
20
40
60
80
100
400
500
600
700
800

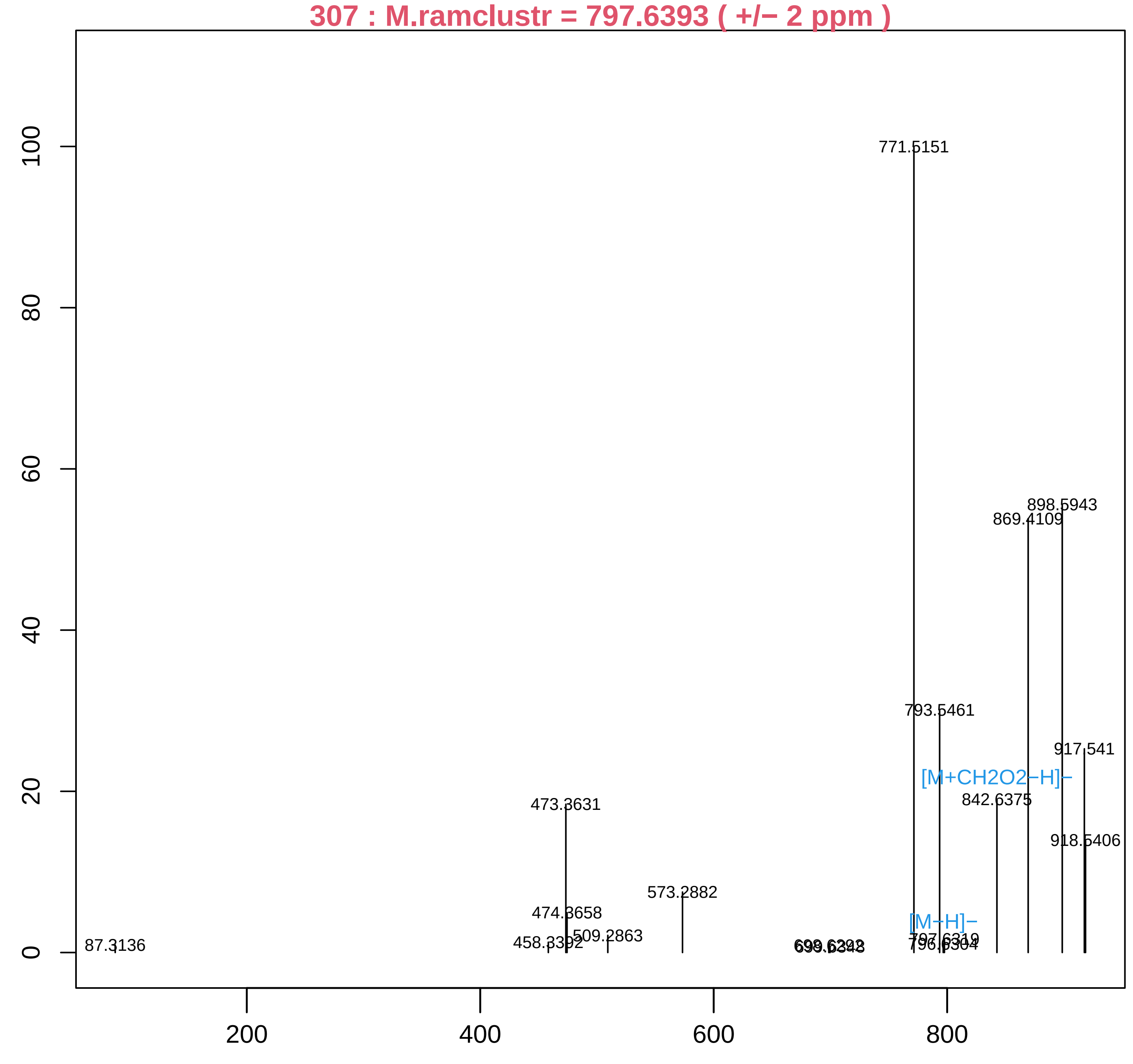
307 : M.ramclustr = 797.6393 (+/− 2 ppm)
771.5151
898.5943
869.4109
793.5461
917.541
[M+CH2O2-H]−
842.6375
473.3631
918.5406
573.2882
474.3658
[M-H]−
509.2863
458.3392
87.3136
797.6319
796.6304
0
20
40
60
80
100
200
400
600
800

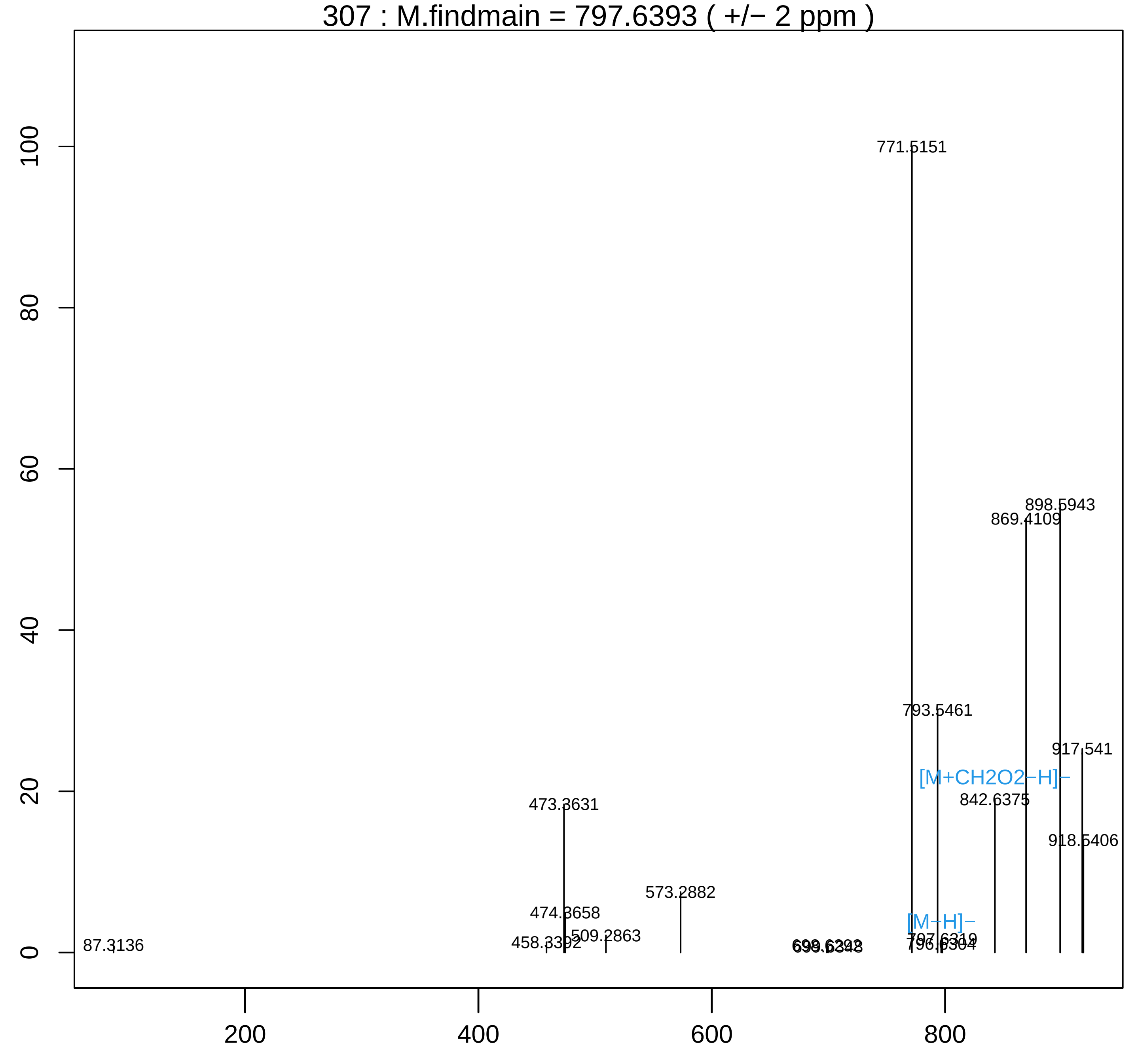
307 : M.findmain = 797.6393 (+/− 2 ppm)
771.5151
898.5943
869.4109
793.5461
917.541
[M+CH2O2-H]−
842.6375
473.3631
918.5406
573.2882
474.3658
[M-H]−
509.2863
458.3392
87.3136
797.6319
796.6304
0
20
40
60
80
100
200
400
600
800

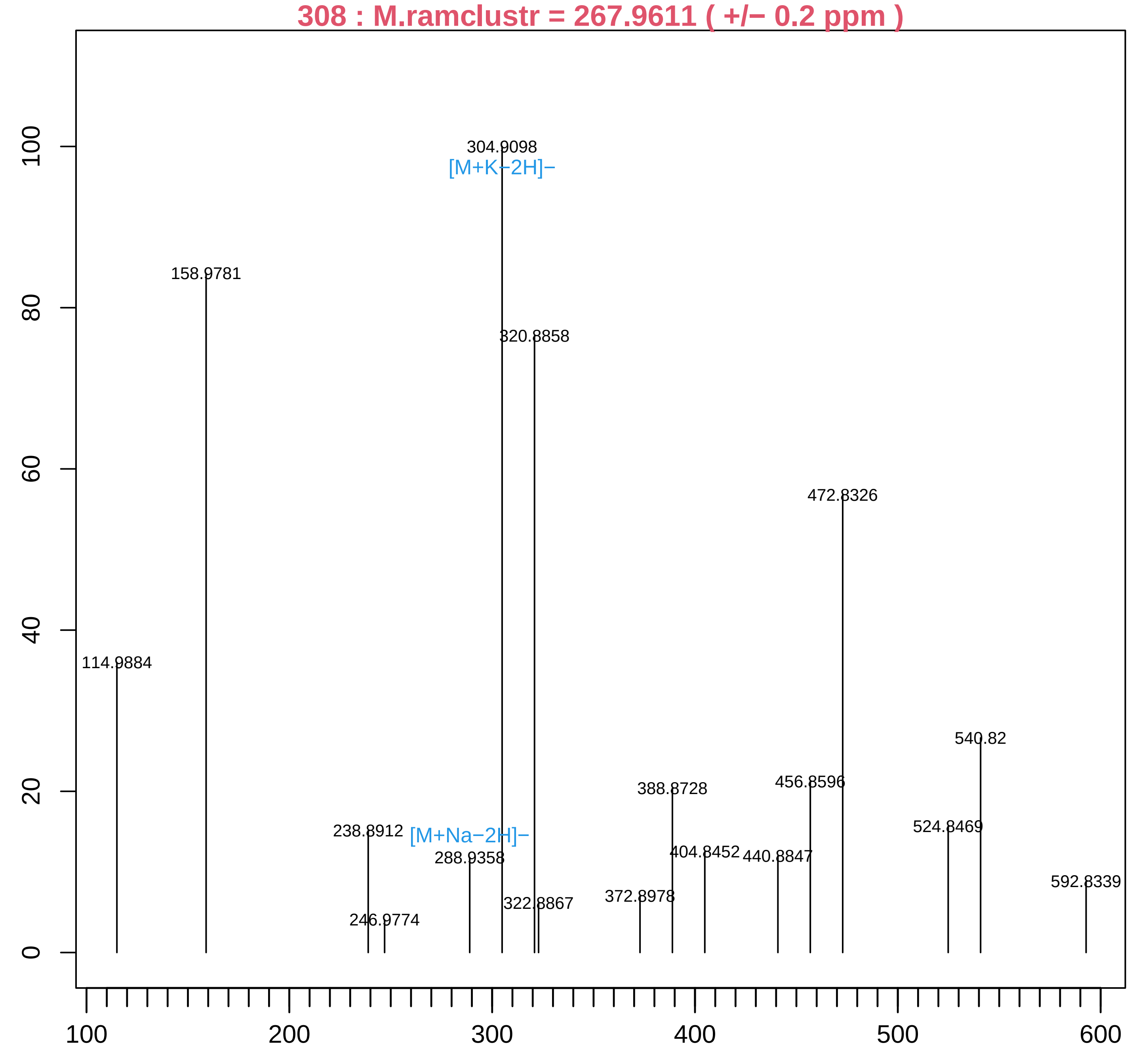
308 : M.ramclustr = 267.9611 (+/− 0.2 ppm)
304.9098
[M+K−2H]−
158.9781
320.8858
472.8326
114.9884
540.82
388.8728
456.8596
238.8912
[M+Na−2H]−
524.8469
288.9358
404.8452
440.8847
592.8339
322.8867
372.8978
246.9774

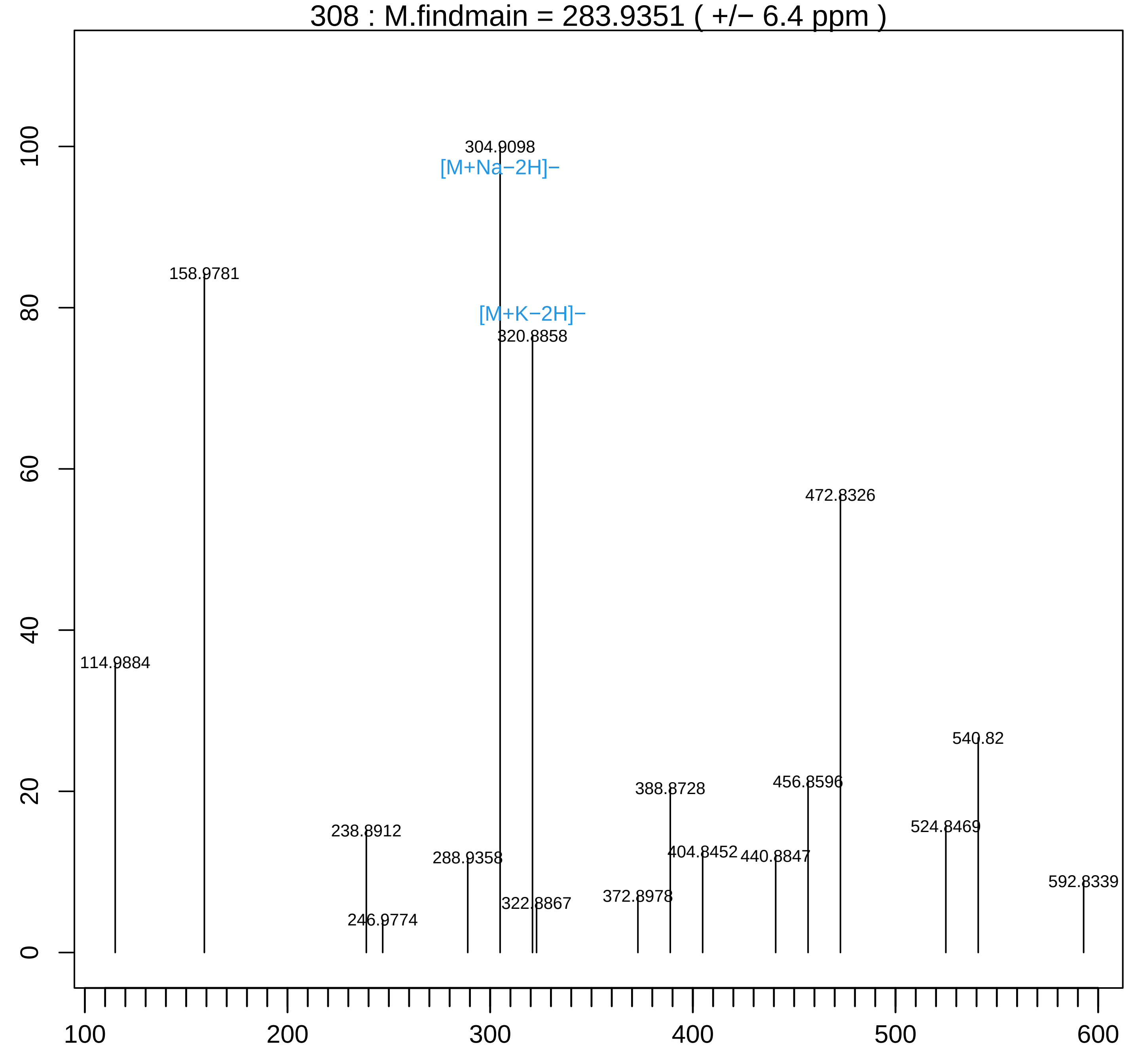
308 : M.findmain = 283.9351 (+/− 6.4 ppm)
304.9098
[M+Na−2H]−
158.9781
[M+K−2H]−
320.8858
472.8326
114.9884
540.82
388.8728
456.8596
238.8912
524.8469
288.9358
404.8452
440.8847
592.8339
322.8867
372.8978
246.9774

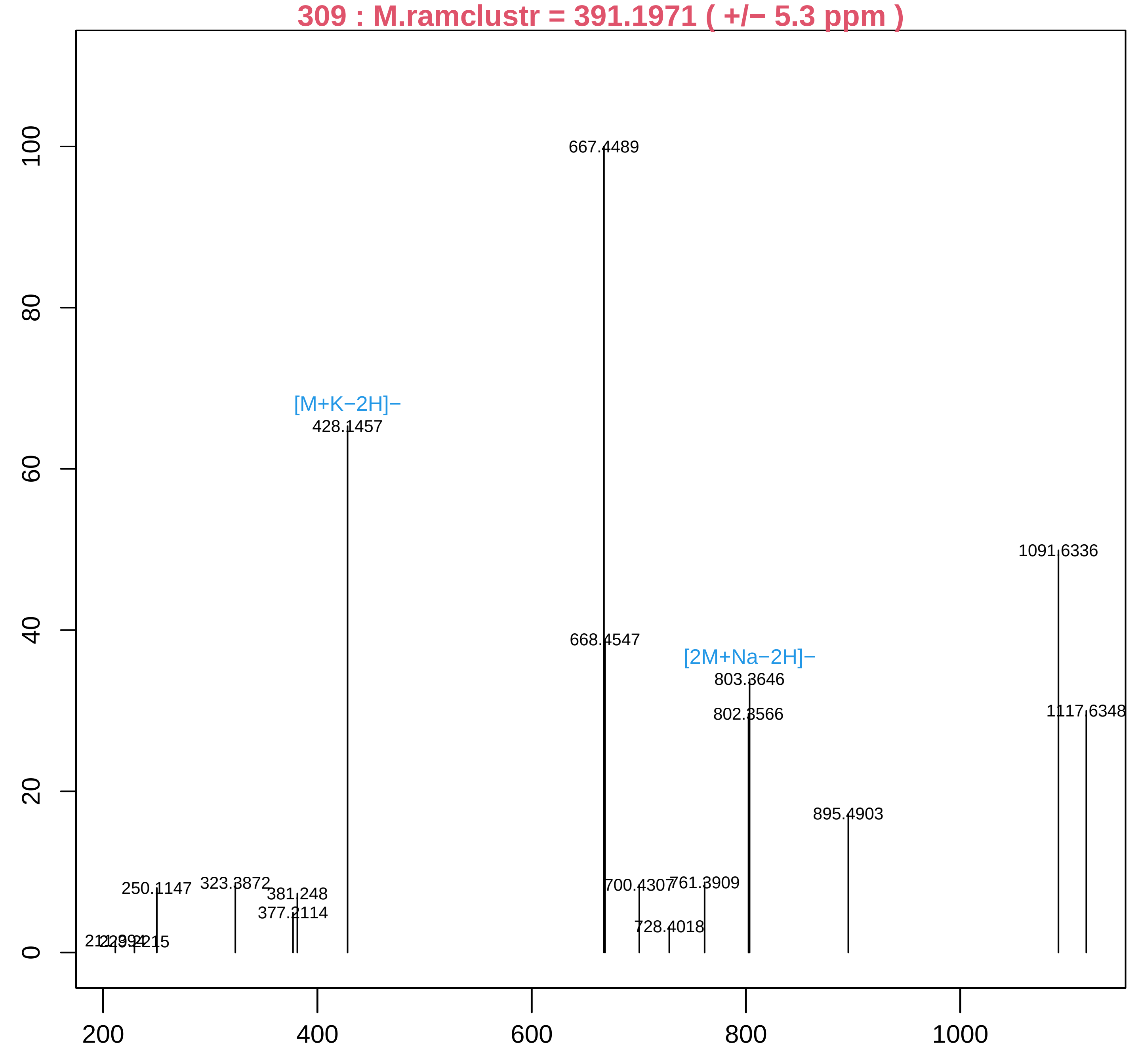
309 : M.ramclustr = 391.1971 (+/− 5.3 ppm)
667.4489
[M+K−2H]−
428.1457
1091.6336
668.4547
[2M+Na−2H]−
803.3646
802.3566
1117.6348
895.4903
250.1147
323.3872
381.248
377.2114
700.4307
761.3909
728.4018
0
20
40
60
80
100
200
400
600
800
1000

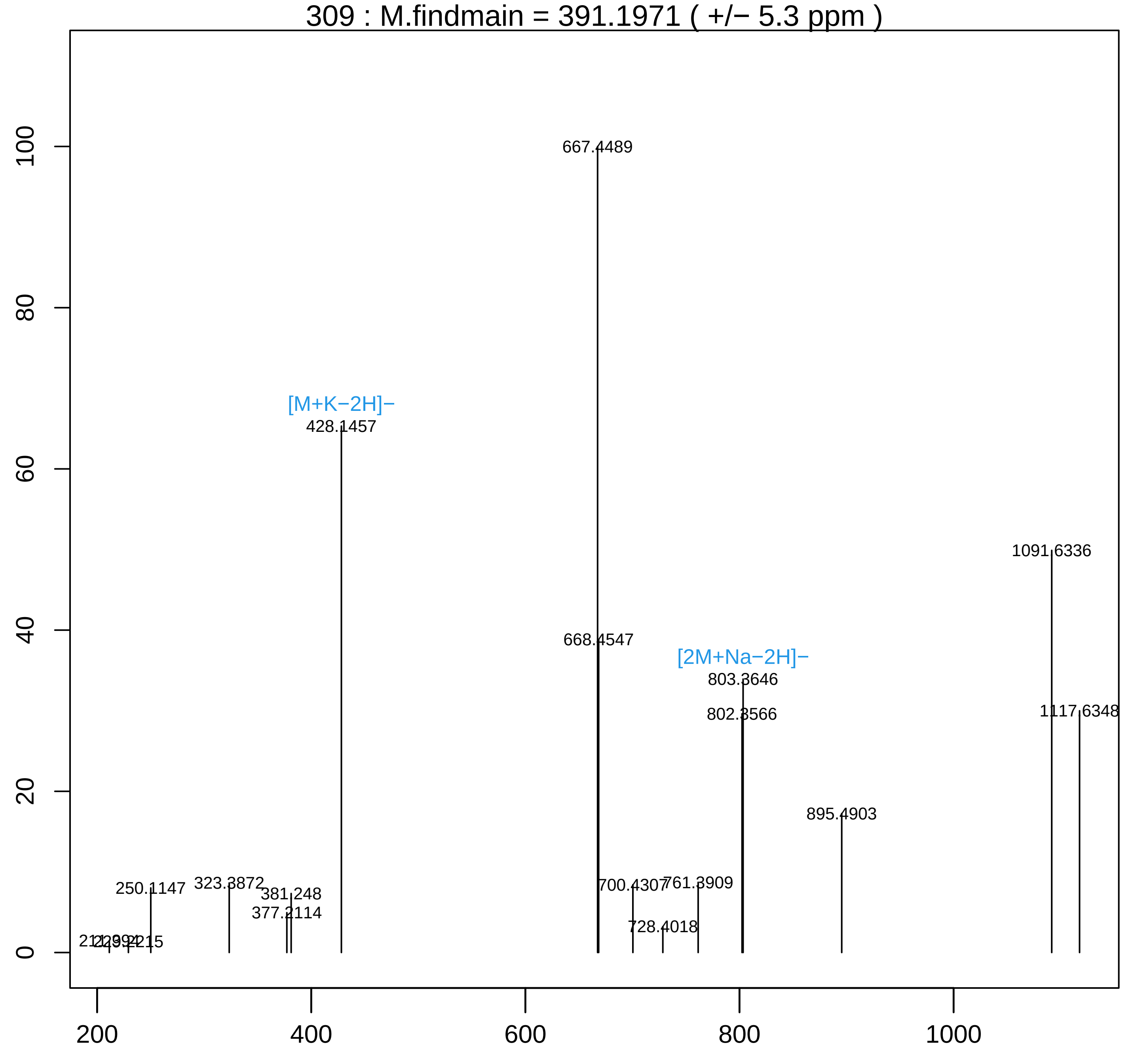
309 : M.findmain = 391.1971 (+/− 5.3 ppm)
667.4489
[M+K−2H]−
428.1457
1091.6336
668.4547
[2M+Na−2H]−
803.3646
802.3566
1117.6348
895.4903
250.1147
323.3872
381.248
377.2114
700.4307
761.3909
728.4018
0
20
40
60
80
100
200
400
600
800
1000

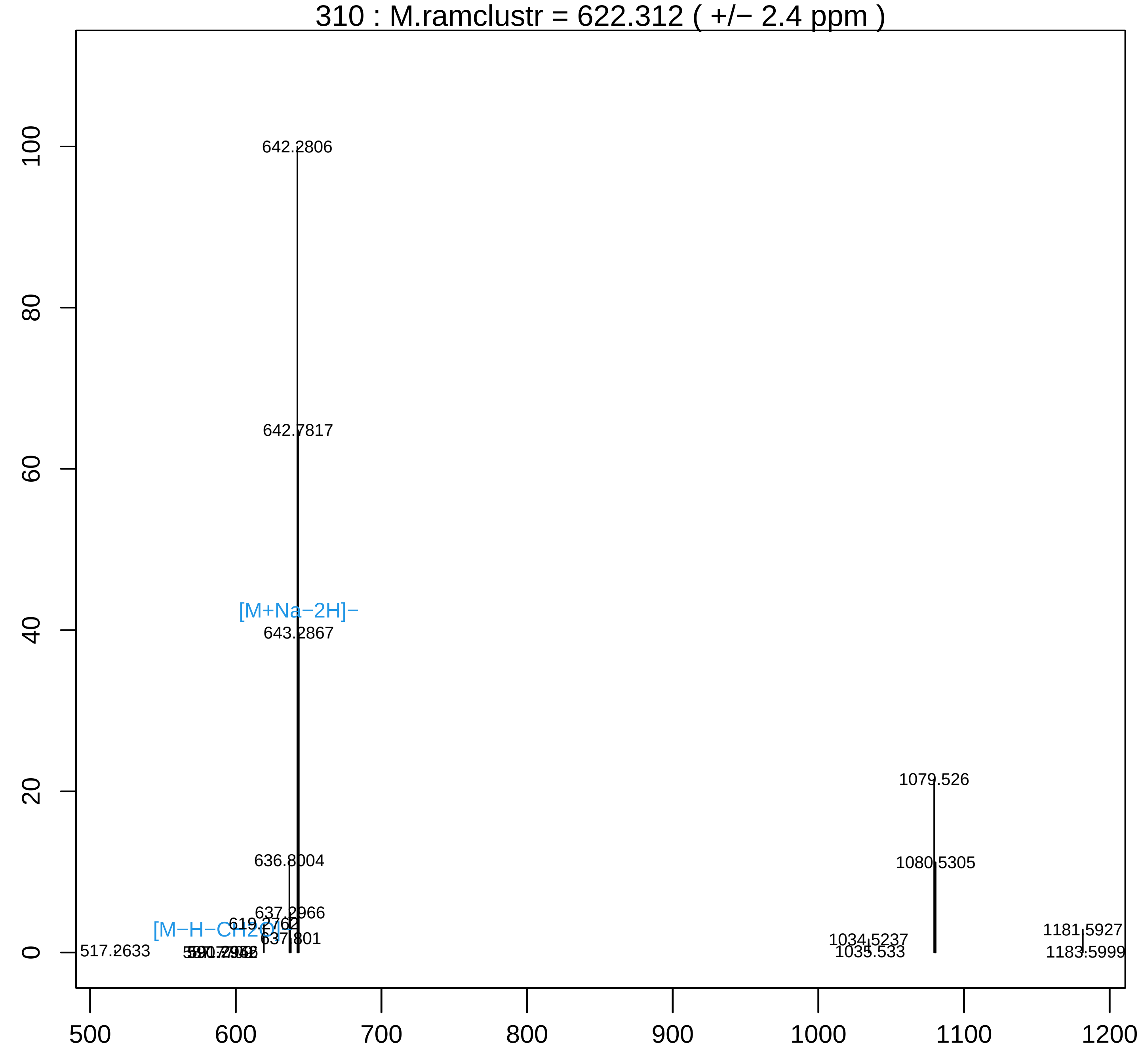
310 : M.ramclustr = 622.312 (+/− 2.4 ppm)
642.2806
642.7817
[M+Na−2H]−
643.2867
1079.526
636.8004
1080.5305
637.2966
[M−H−CH2O]−
619.2762
637.801
1034.5237
1035.533
1181.5927
1183.5999
517.2633
0
20
40
60
80
100
500
600
700
800
900
1000
1100
1200

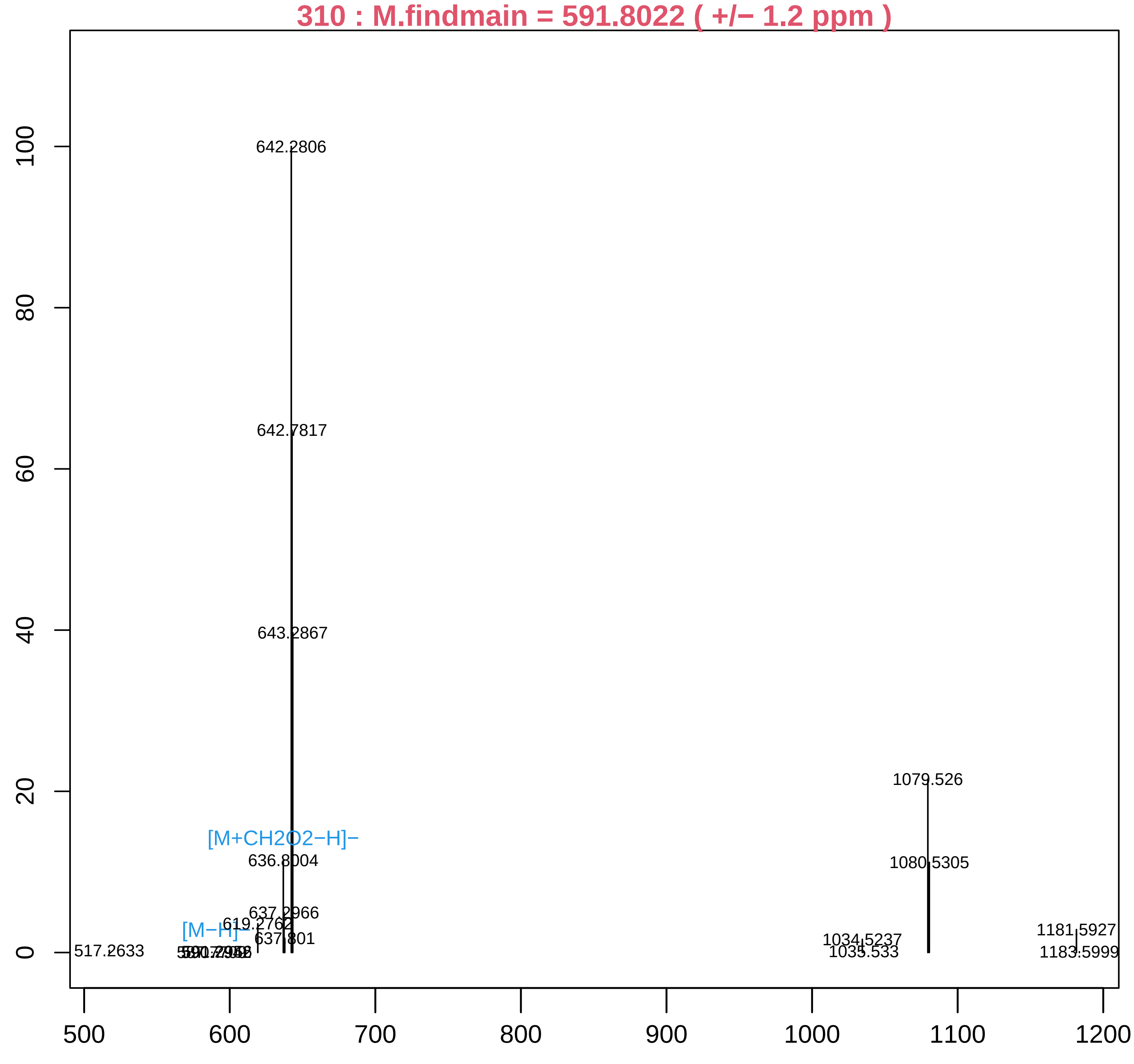
310 : M.findmain = 591.8022 (+/− 1.2 ppm)
642.2806
642.7817
643.2867
1079.526
[M+CH2O2−H]−
636.8004
1080.5305
637.2966
[M−H]−
619.2762
637.801
1034.5237
1035.533
1181.5927
1183.5999
517.2633
0
20
40
60
80
100
500
600
700
800
900
1000
1100
1200

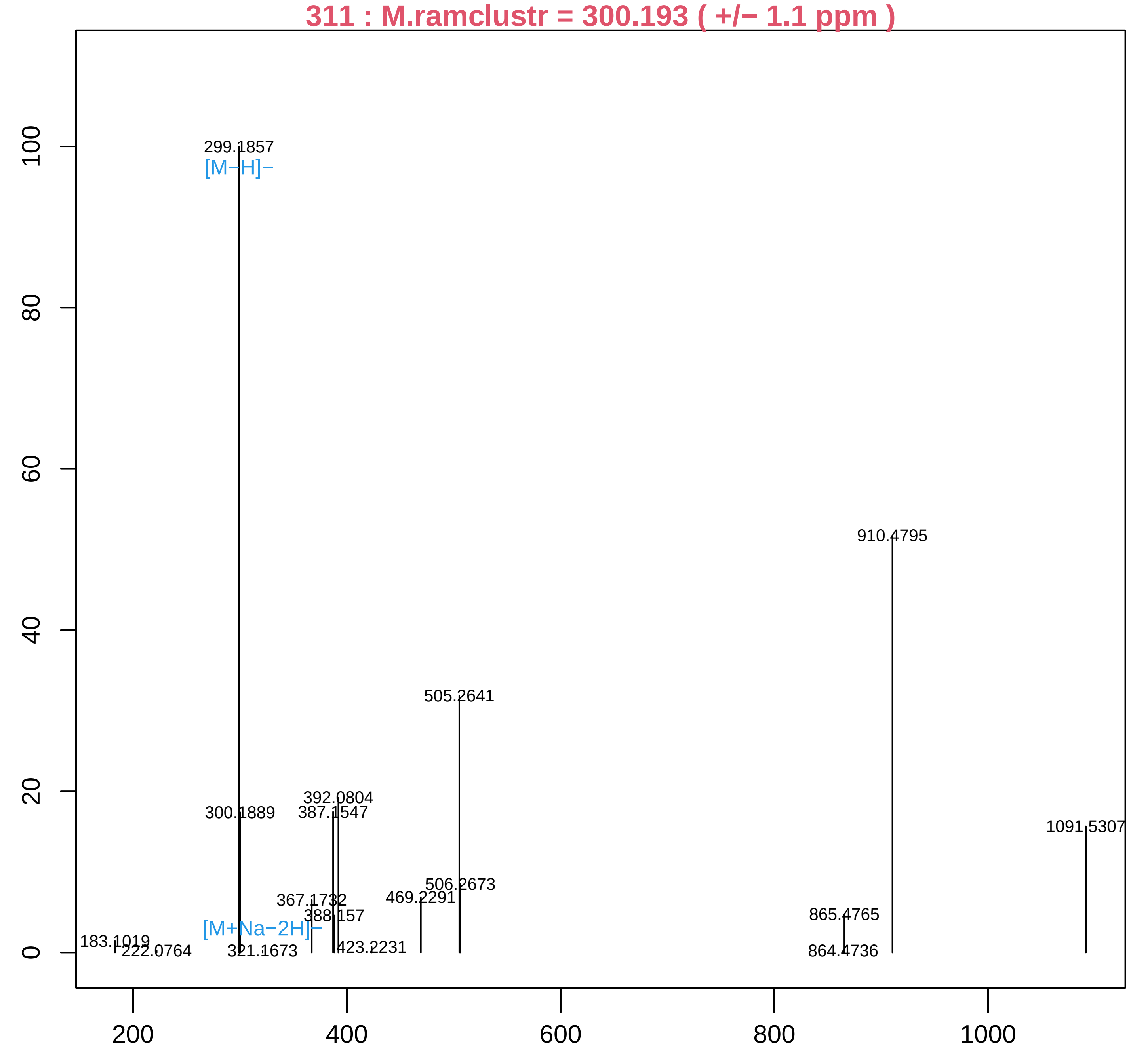
311 : M.ramclustr = 300.193 (+/− 1.1 ppm)
299.1857
[M−H]−
910.4795
505.2641
392.0804
300.1889
387.1547
1091.5307
506.2673
367.1732
469.2291
865.4765
388.157
183.1019
[M+Na−2H]−
222.0764
321.1673
423.2231
864.4736
0
20
40
60
80
100
200
400
600
800
1000

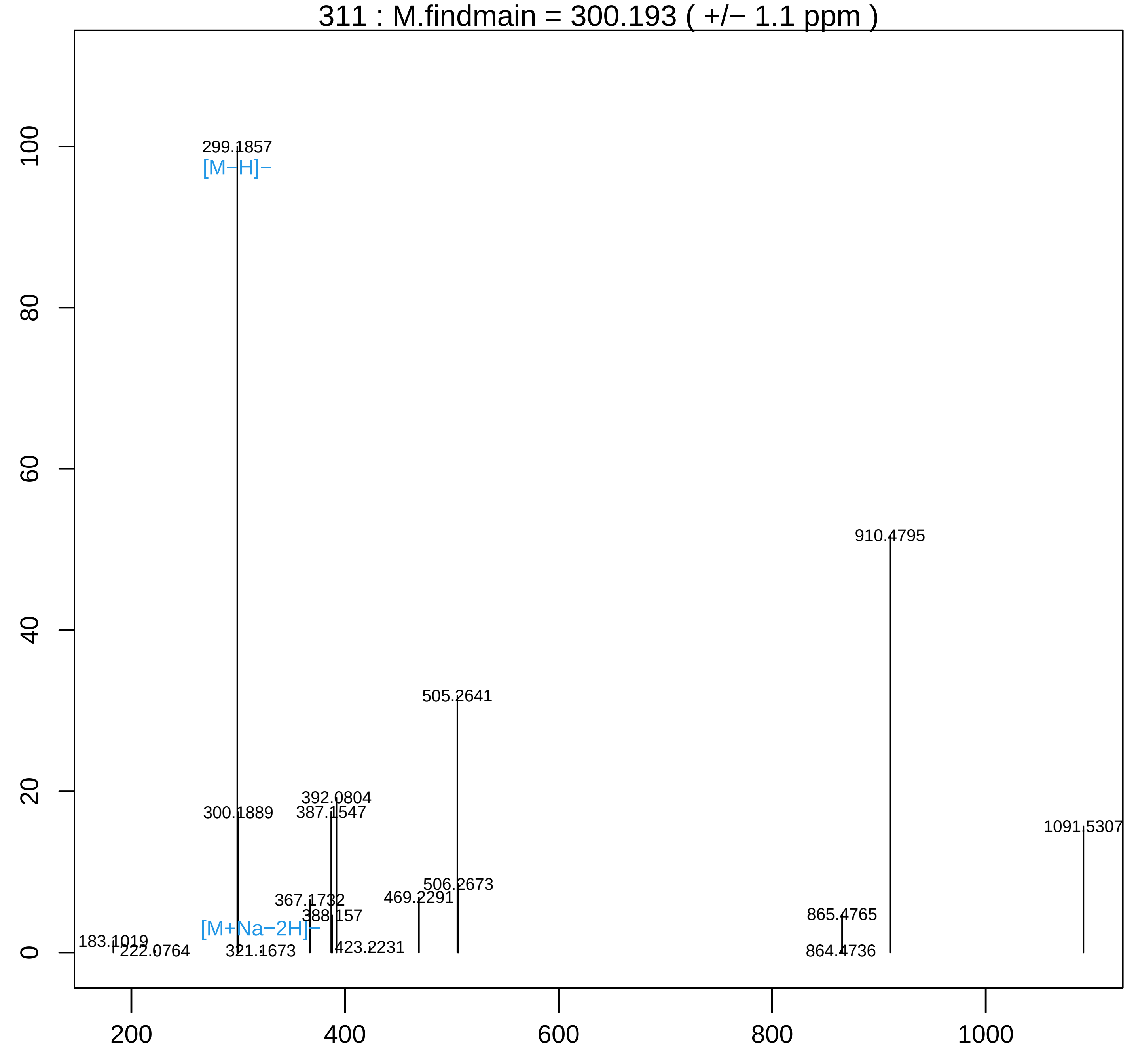
311 : M.findmain = 300.193 (+/− 1.1 ppm)
299.1857
[M−H]−
910.4795
505.2641
392.0804
300.1889
387.1547
1091.5307
506.2673
367.1732
469.2291
865.4765
388.157
183.1019
[M+Na−2H]−
222.0764
321.1673
423.2231
864.4736
0
20
40
60
80
100
200
400
600
800
1000

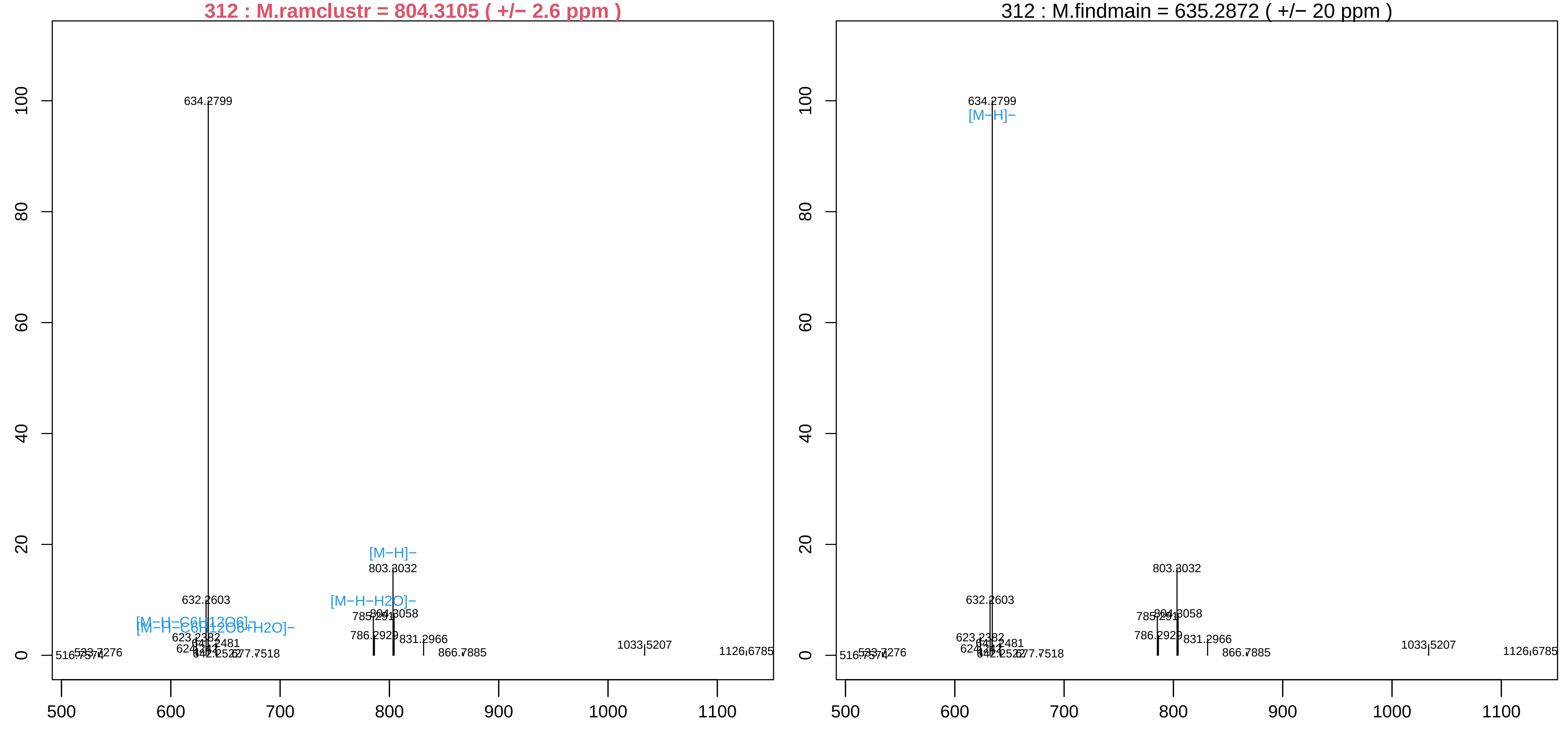
312 : M.ramclustr = 804.3105 (+/− 2.6 ppm)
634.2799
632.2603
623.2382
641.2481
642.2522
677.7518
516.7374
533.7276
[M−H]−
803.3032
[M−H−H2O]−
785.2918
804.3058
786.2929
831.2966
866.7885
1033.5207
1126.6785
312 : M.findmain = 635.2872 (+/− 20 ppm)
634.2799
[M−H]−
803.3032
632.2603
0
20
40
60
80
100
500
600
700
800
900
1000
1100

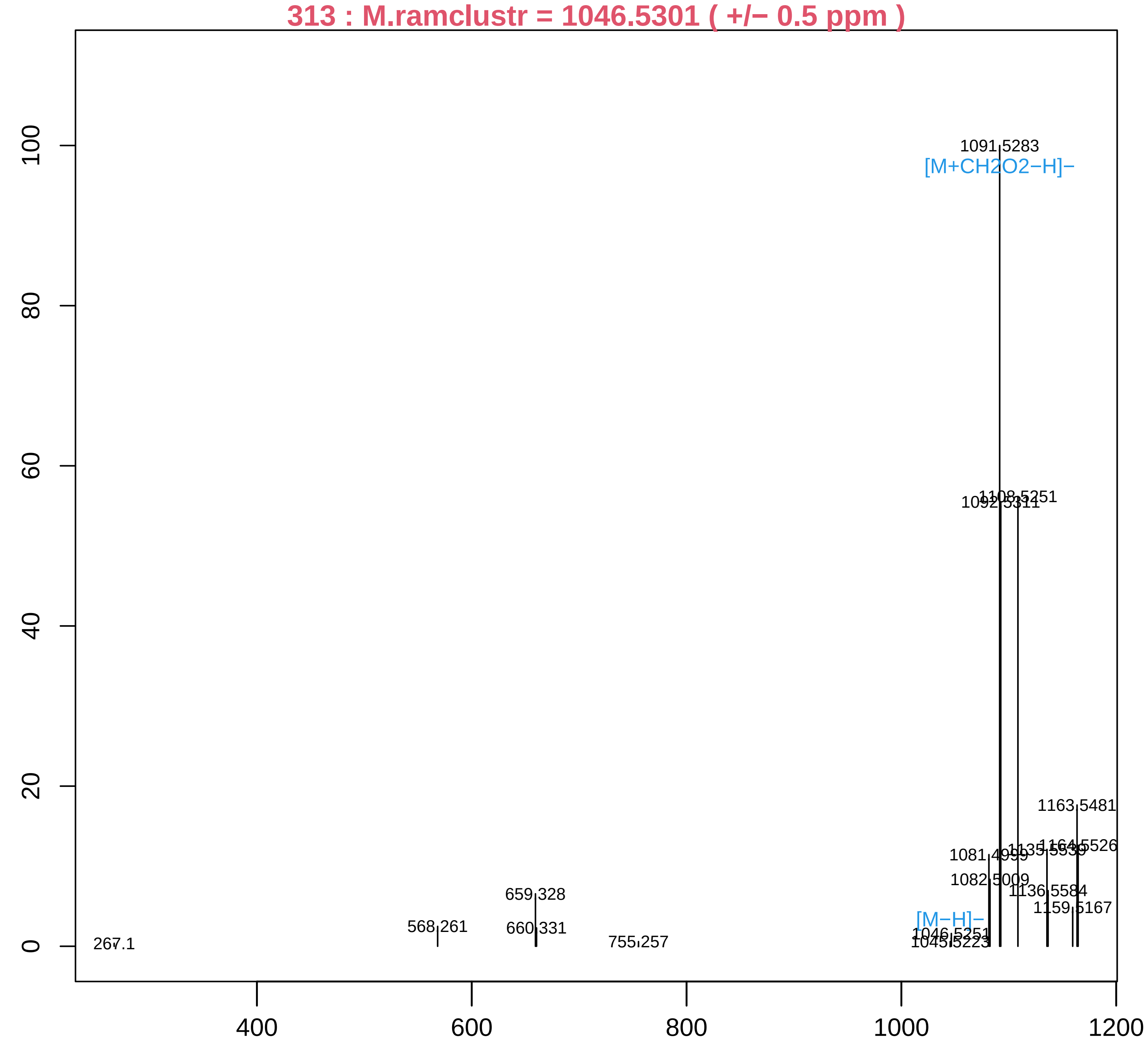

313 : M.ramclustr = 1046.5301 (+/− 0.5 ppm)
1091.5283
[M+CH2O2−H]−
1108.5251
1092.5311
1163.5481
1164.5526
1135.5539
1081.4959
1082.5009
1136.5584
1159.5167
659.328
[M−H]−
568.261
660.331
1046.5251
1045.5223
755.257
267.1
0
20
40
60
80
100
400
600
800
1000
1200

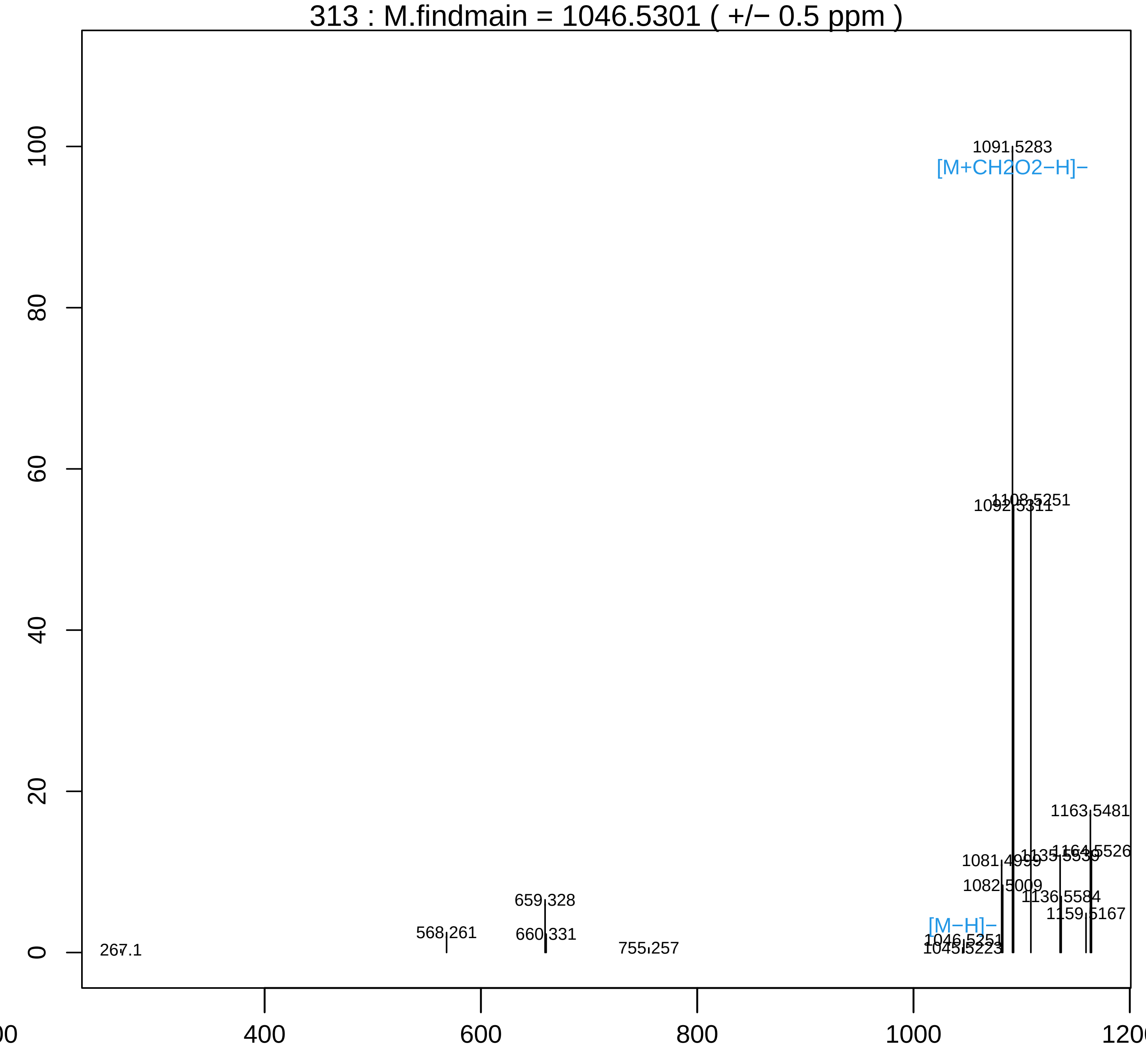

313 : M.findmain = 1046.5301 (+/− 0.5 ppm)
1091.5283
[M+CH2O2−H]−
1108.5251
1092.5311
1163.5481
1164.5526
1135.5539
1081.4959
1082.5009
1136.5584
1159.5167
659.328
[M−H]−
568.261
660.331
1046.5251
1045.5223
755.257
267.1
0
20
40
60
80
100
400
600
800
1000

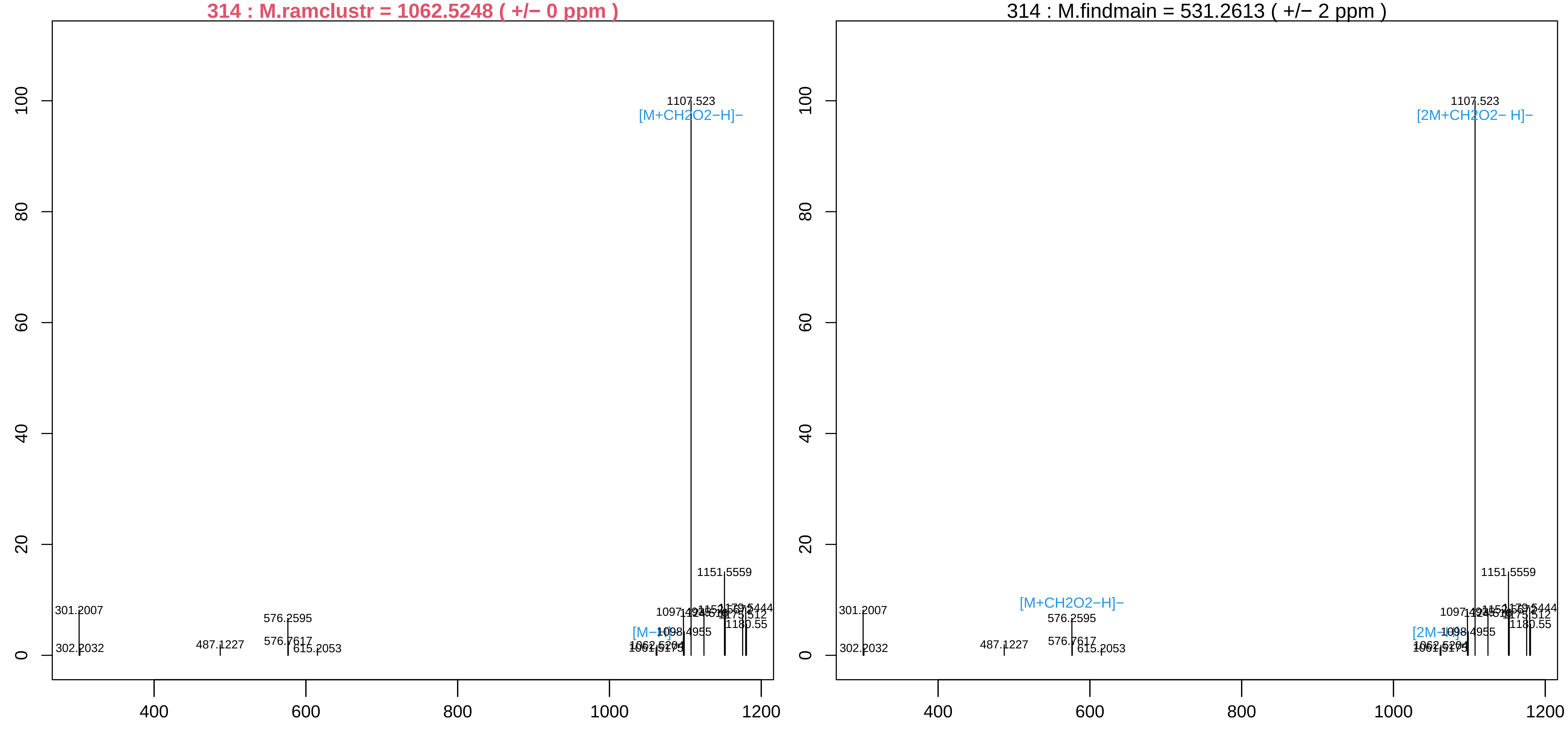
314 : M.ramclustr = 1062.5248 (+/− 0 ppm)
1107.523
[M+CH2O2−H]−
1151.5559
301.2007
576.2595
487.1227
576.7617
615.2053
302.2032
[M−H]−
1098.4955
1180.55
314 : M.findmain = 531.2613 (+/− 2 ppm)
[2M+CH2O2− H]−
[M+CH2O2−H]−
[2M−H]−

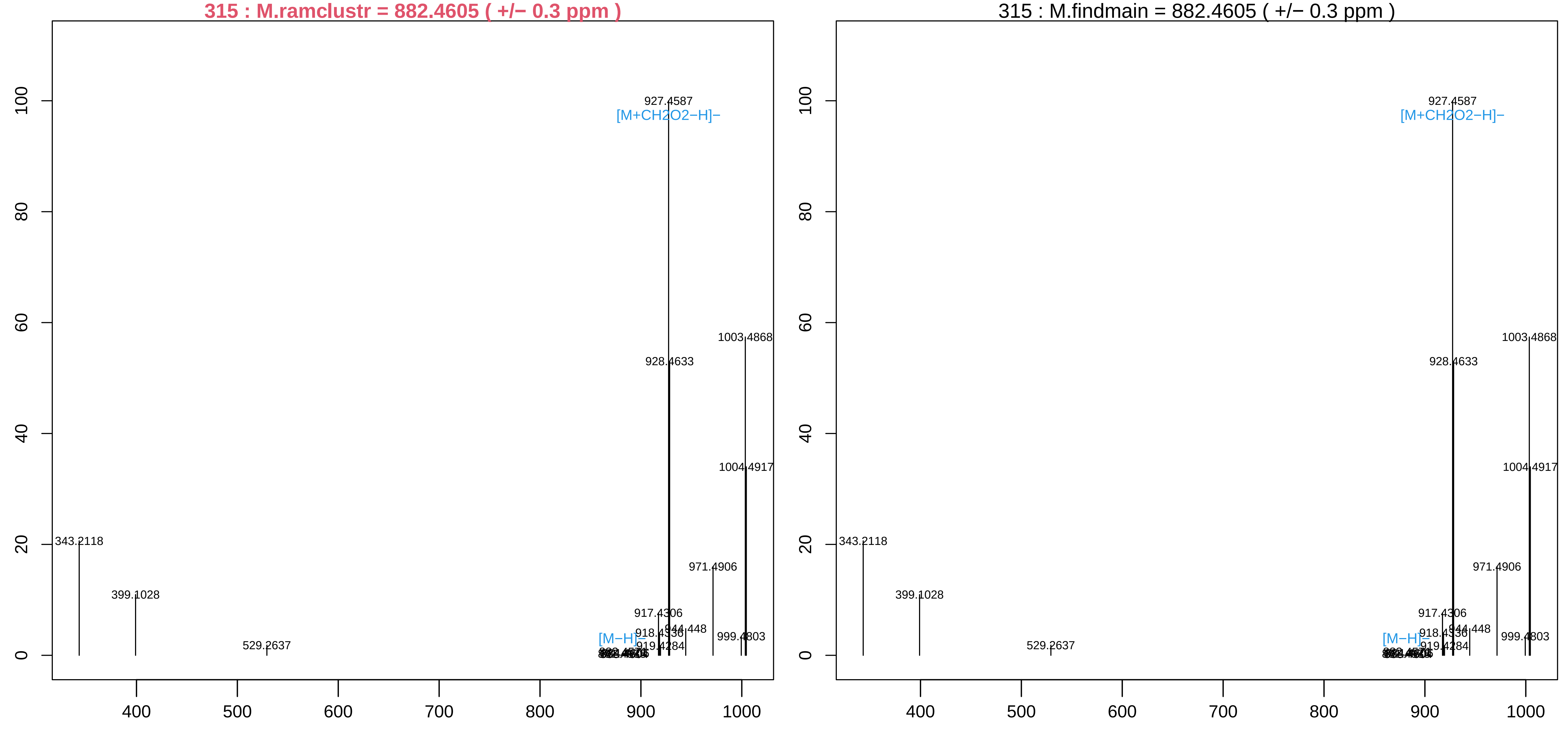

315 : M.ramclustr = 882.4605 (+/− 0.3 ppm)
927.4587
[M+CH2O2−H]−
1003.4868
928.4633
1004.4917
343.2118
399.1028
971.4906
917.4306
918.4336
944.448
999.4803
[M−H]−
919.4284
529.2637
0
20
40
60
80
100
400
500
600
700
800
900
1000
315 : M.findmain = 882.4605 (+/− 0.3 ppm)

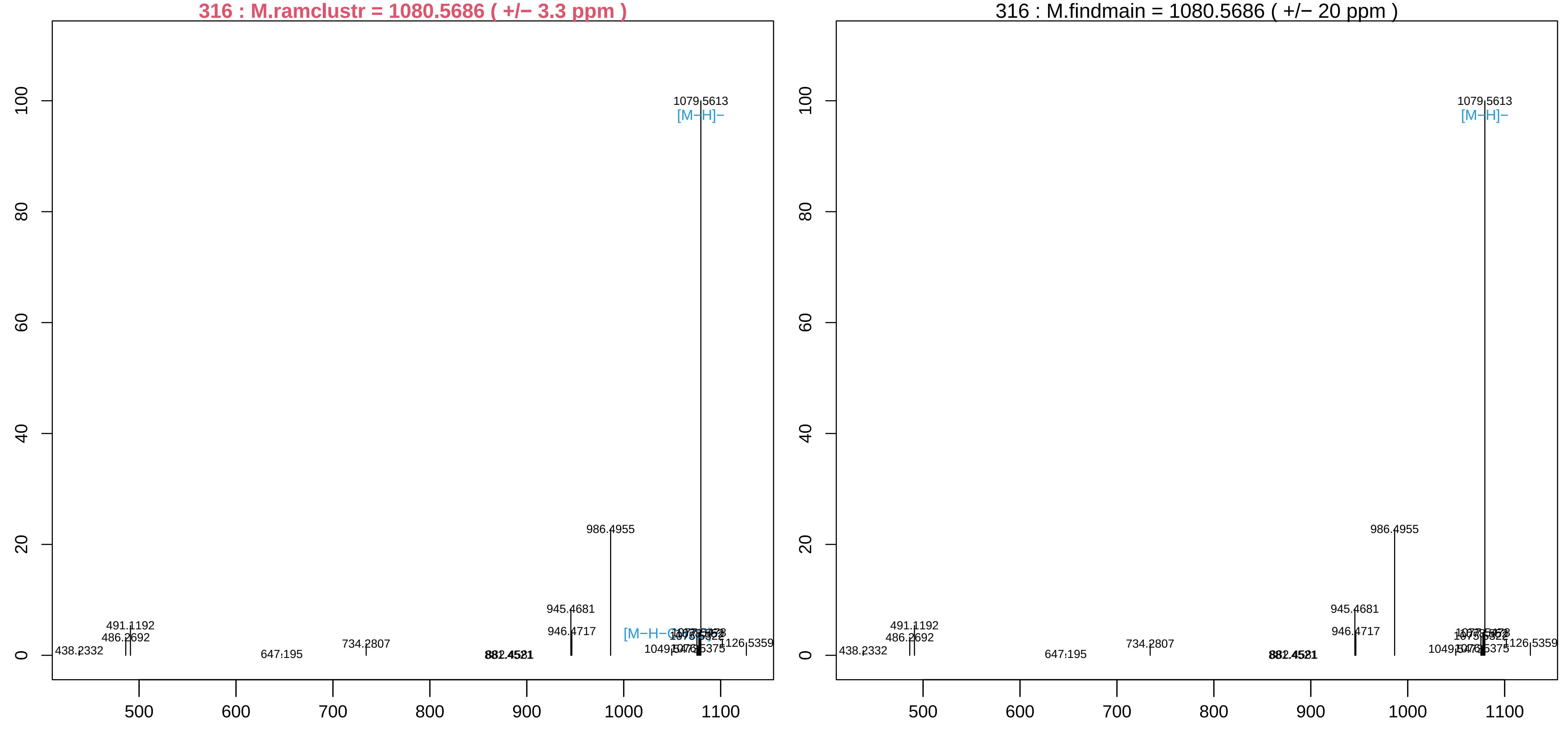
316 : M.ramclustr = 1080.5686 (+/− 3.3 ppm)
316 : M.findmain = 1080.5686 (+/− 20 ppm)
1079.5613
[M−H]−
986.4955
945.4681
946.4717
491.1192
486.2692
438.2332
647.195
734.2807
882.4521
1126.5359
0
20
40
60
80
100
500
600
700
800
900
1000
1100

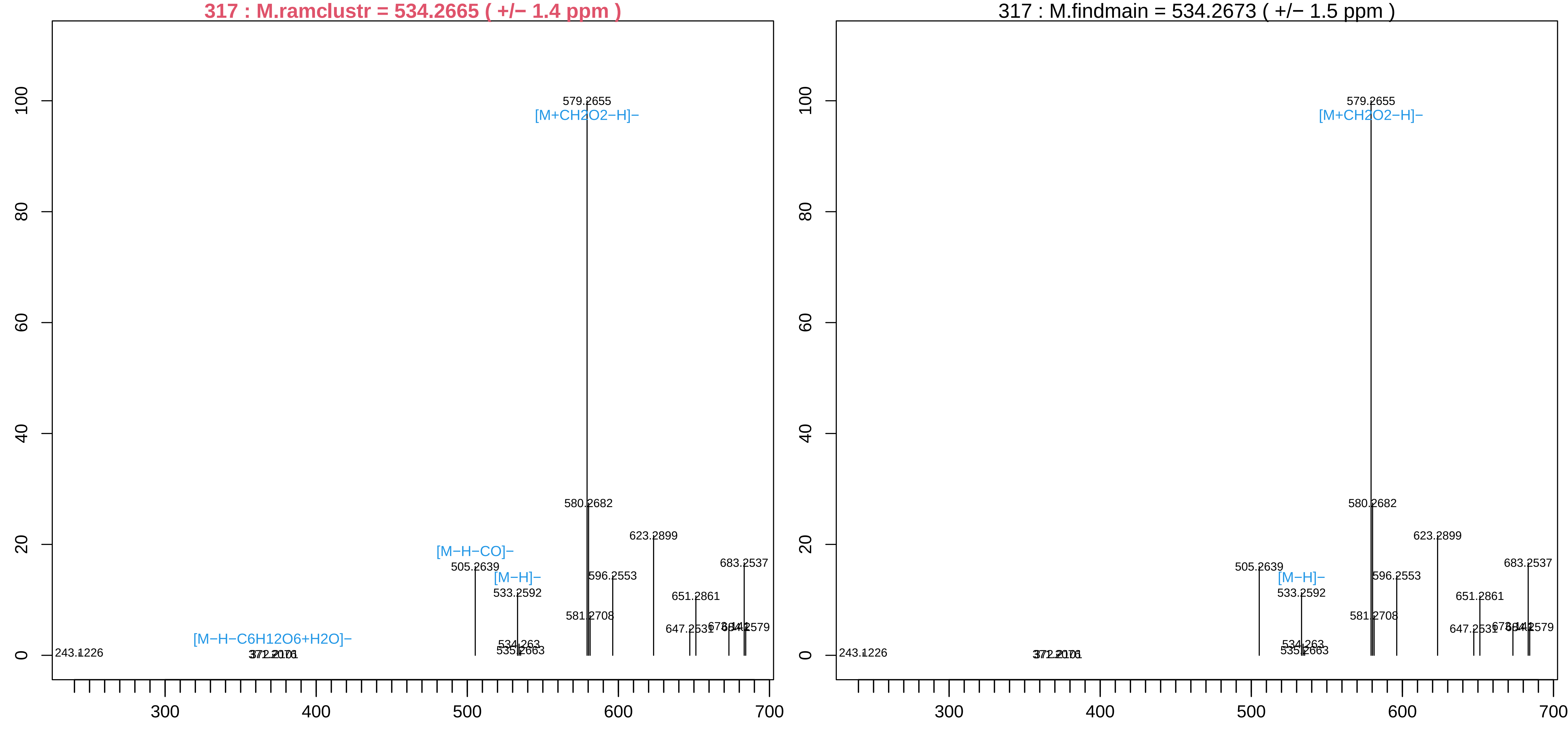
317 : M.ramclustr = 534.2665 (+/− 1.4 ppm)
579.2655
[M+CH2O2−H]−
580.2682
623.2899
[M−H−CO]−
505.2639
[M−H]−
533.2592
596.2553
683.2537
651.2861
581.2708
647.2531
534.263
535.2663
[M−H−C6H12O6+H2O]−
243.1226
317 : M.findmain = 534.2673 (+/− 1.5 ppm)
579.2655
[M+CH2O2−H]−
580.2682
623.2899
505.2639
[M−H]−
533.2592
596.2553
683.2537
651.2861
581.2708
647.2531
534.263
535.2663
243.1226
0
20
40
60
80
100
300
400
500
600
700

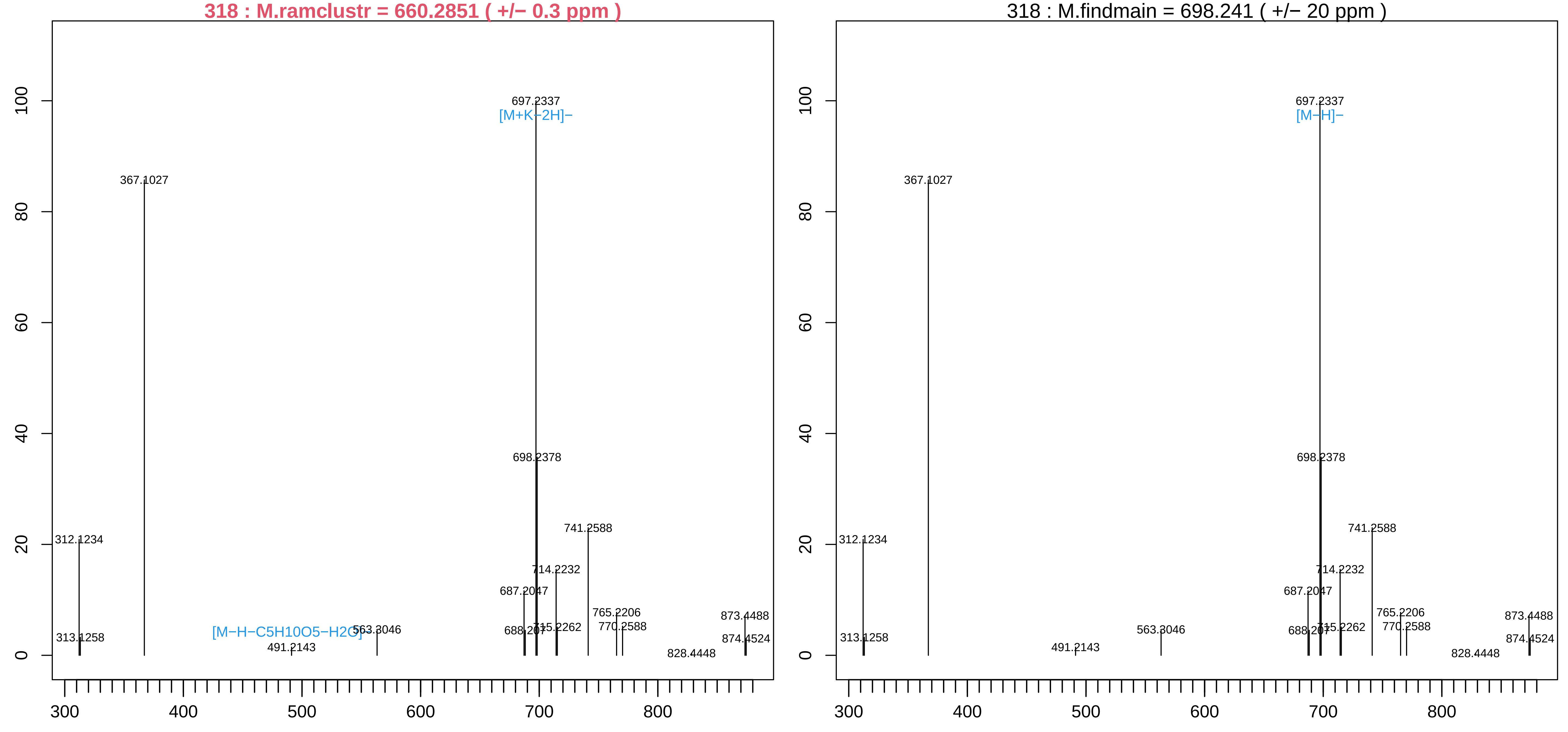

318 : M.ramclustr = 660.2851 (+/− 0.3 ppm)
697.2337
[M+K−2H]−
367.1027
698.2378
741.2588
312.1234
714.2232
687.2047
765.2206
873.4488
313.1258
[M−H−C5H10O5−H2O]−
563.3046
688.207
715.2262
770.2588
874.4524
491.2143
828.4448
318 : M.findmain = 698.241 (+/− 20 ppm)
[M−H]−

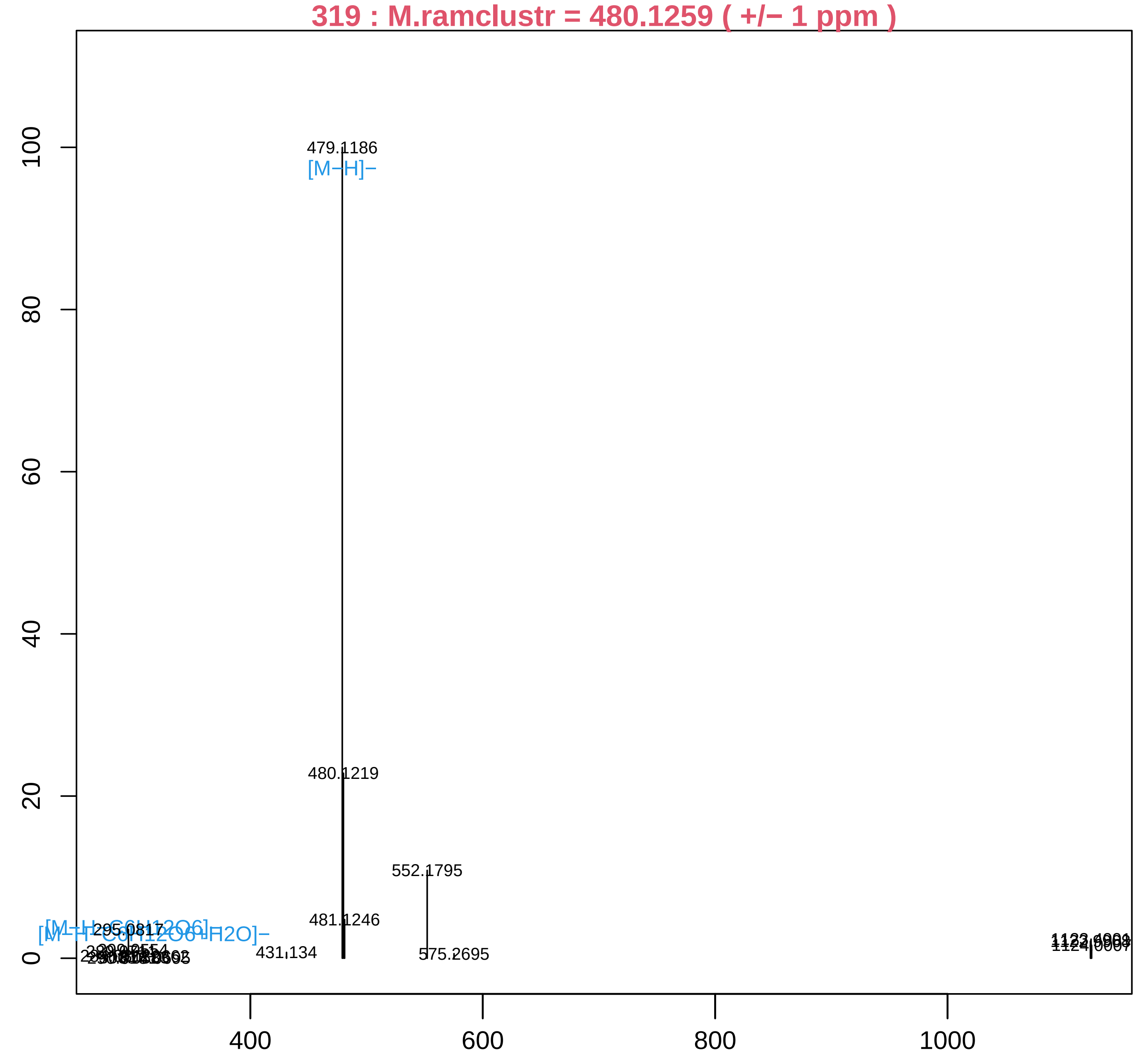
319 : M.ramclustr = 480.1259 (+/− 1 ppm)
479.1186
[M−H]−
480.1219
552.1795
481.1246
295.0817
431.134
575.2695
0
20
40
60
80
100
400
600
800
1000

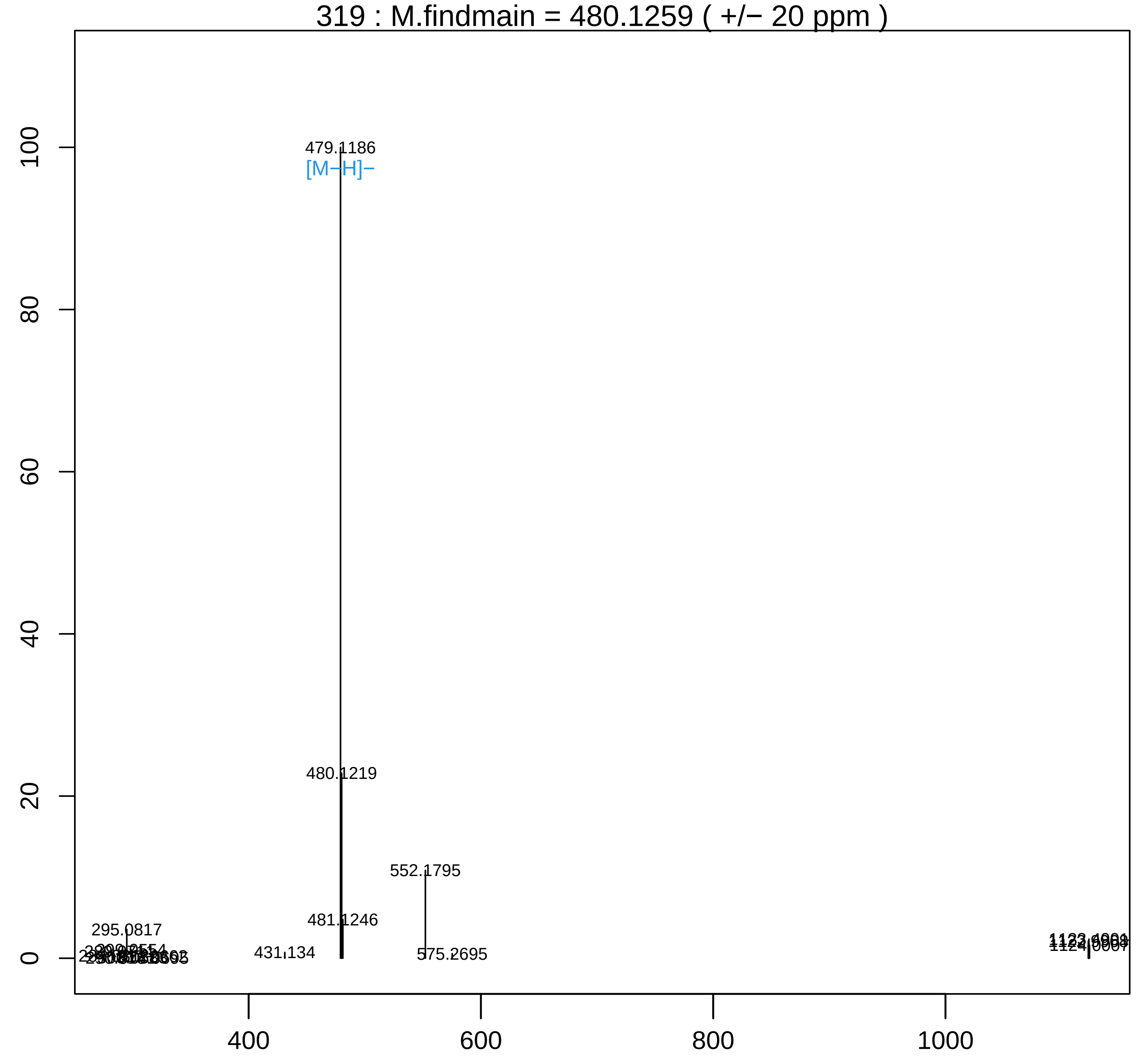
319 : M.findmain = 480.1259 (+/− 20 ppm)
479.1186
[M−H]−
480.1219
552.1795
481.1246
295.0817
431.134
575.2695
0
20
40
60
80
100
400
600
800
1000

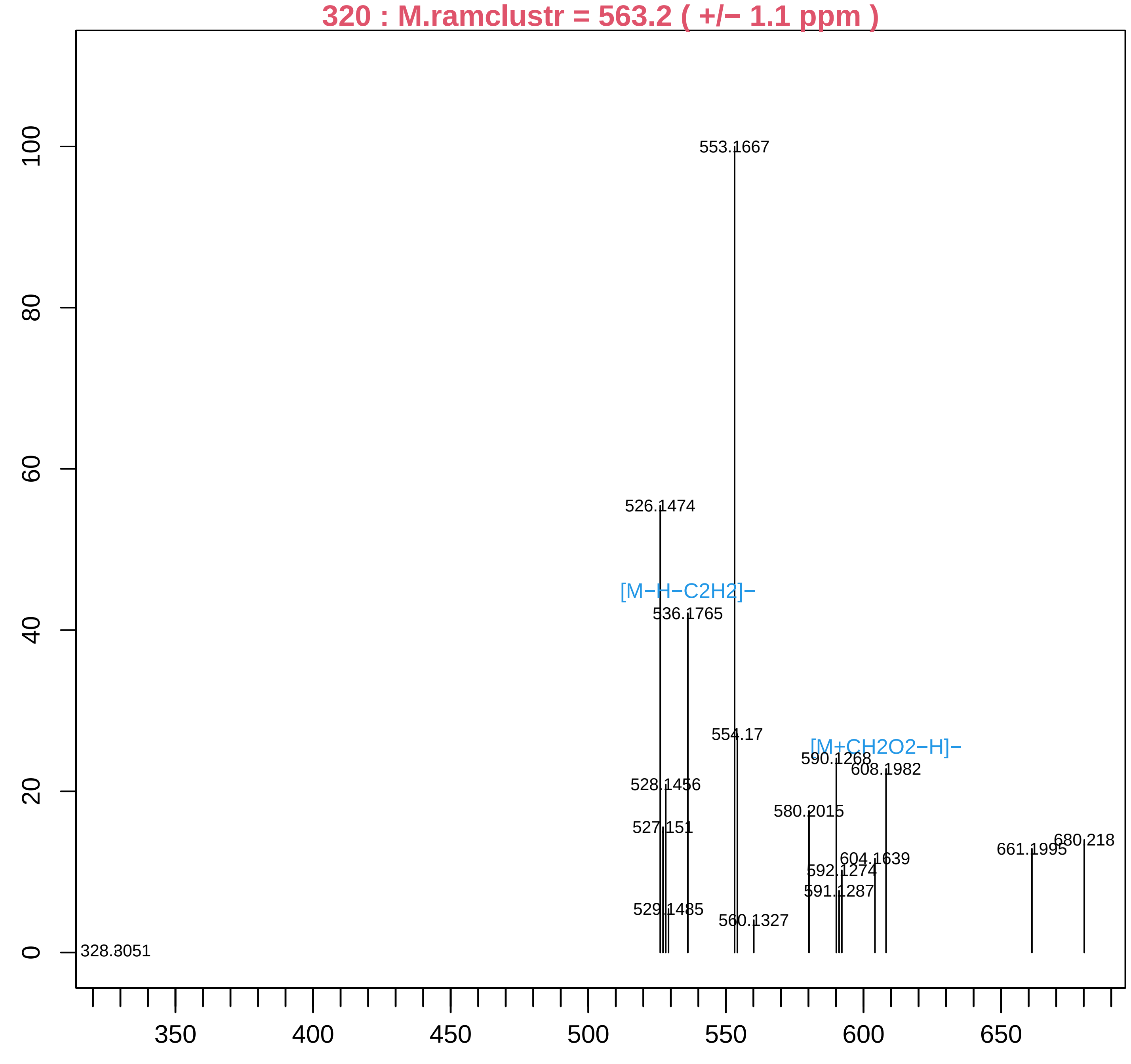
320 : M.ramclustr = 563.2 (+/− 1.1 ppm)
553.1667
526.1474
[M−H−C2H2]−
536.1765
554.17
[M+CH2O2−H]−
590.1268
608.1982
528.1456
580.2015
527.151
680.218
661.1995
604.1639
592.1274
591.1287
529.1485
560.1327
328.3051
0
20
40
60
80
100
350
400
450
500
550
600
650

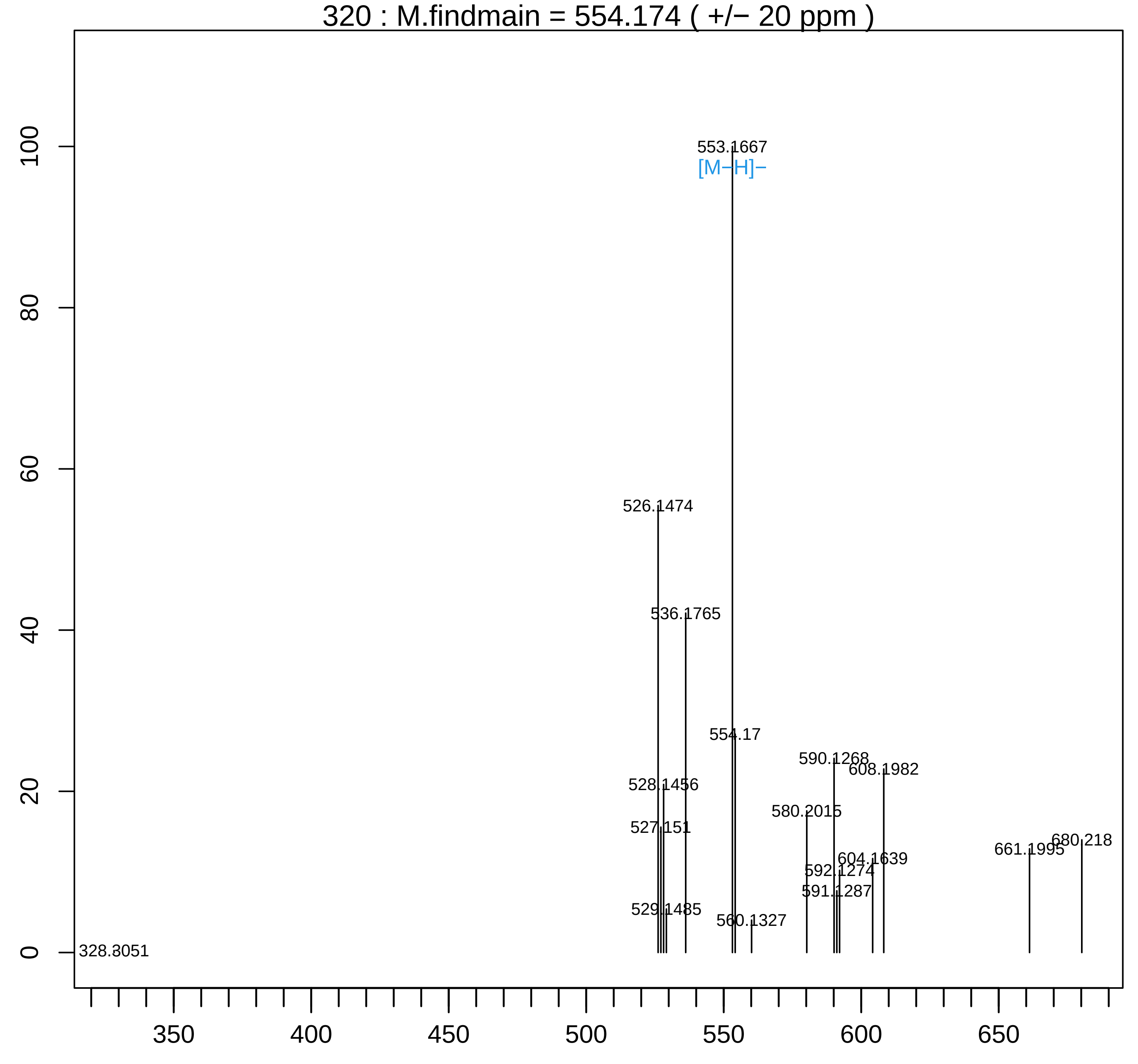
320 : M.findmain = 554.174 (+/− 20 ppm)
553.1667
[M−H]−
526.1474
536.1765
554.17
590.1268
608.1982
528.1456
580.2015
527.151
680.218
661.1995
604.1639
592.1274
591.1287
529.1485
560.1327
328.3051
0
20
40
60
80
100
350
400
450
500
550
600
650

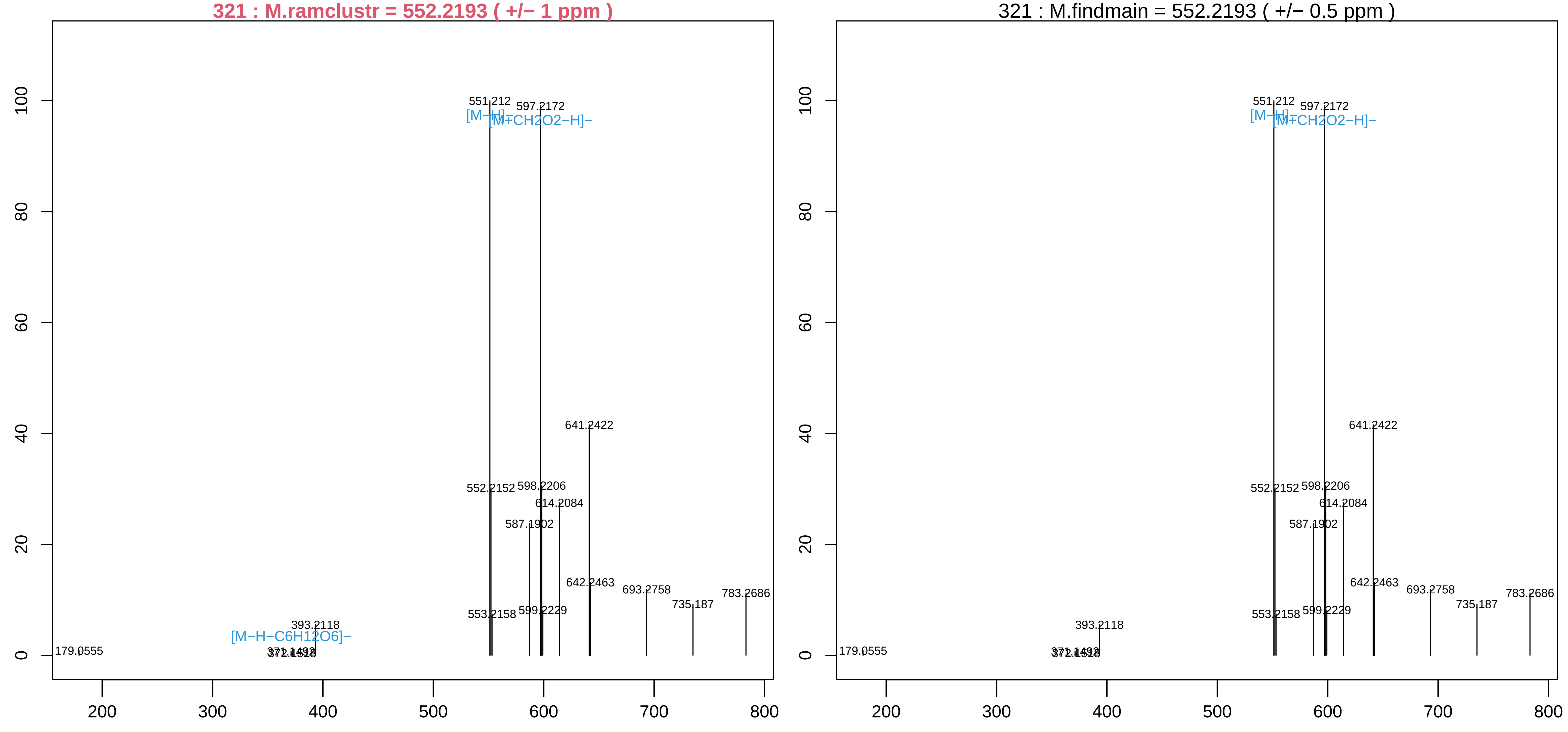
321 : M.ramclustr = 552.2193 (+/− 1 ppm)
321 : M.findmain = 552.2193 (+/− 0.5 ppm)
551.212
[M−H]−
597.2172
[M+CH2O2−H]−
641.2422
552.2152
598.2206
614.2084
587.1902
642.2463
693.2758
735.187
783.2686
553.2158
599.2229
393.2118
[M−H−C6H12O6]−
371.1492
372.1518
179.0555
0
20
40
60
80
100
200
300
400
500
600
700
800

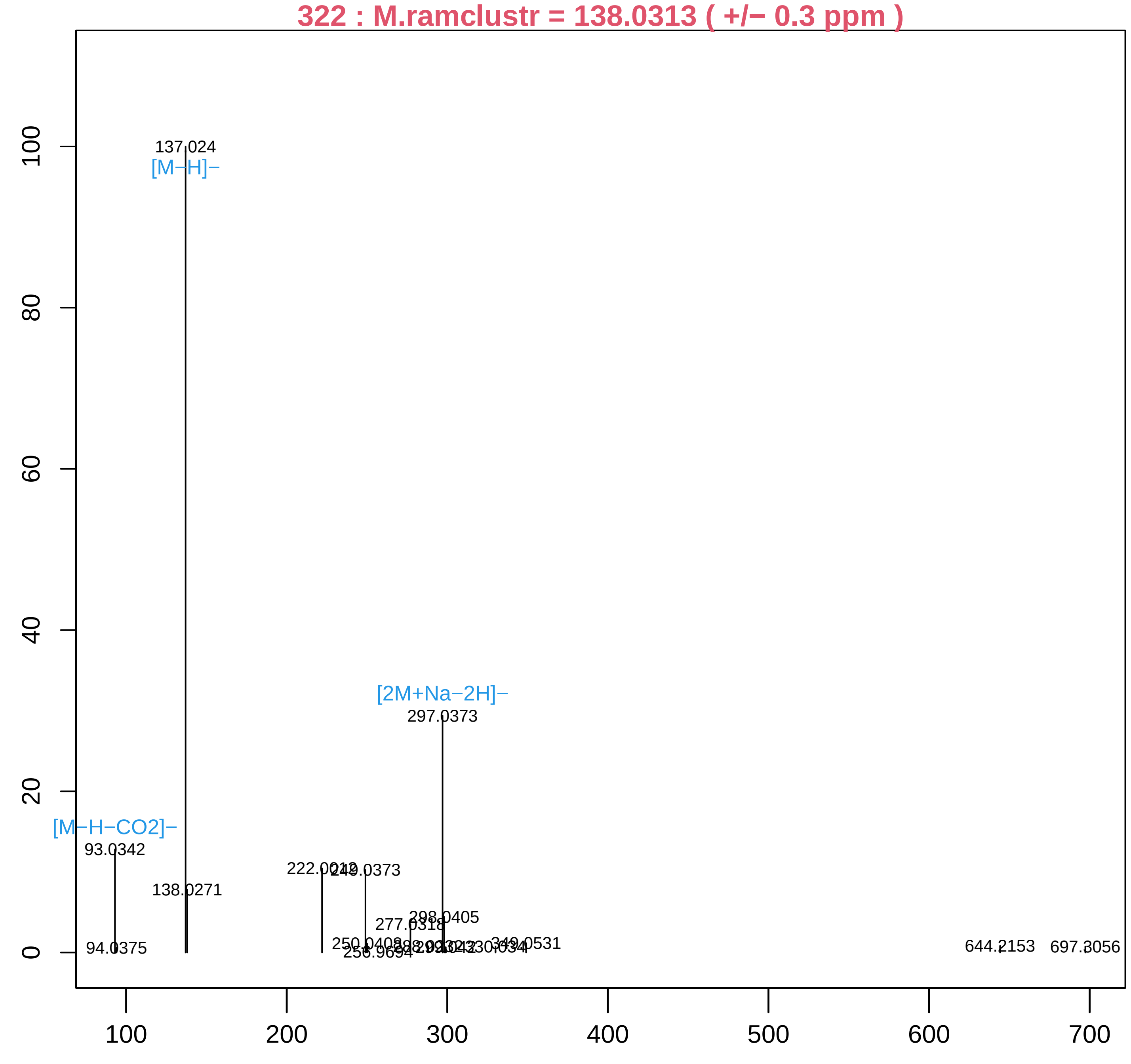
322 : M.ramclustr = 138.0313 (+/− 0.3 ppm)
137.024
[M−H]−
[2M+Na−2H]−
297.0373
[M−H−CO2]−
93.0342
138.0271
222.0012
249.0373
298.0405
277.0318
349.0531
94.0375
644.2153
697.3056
0
20
40
60
80
100
100
200
300
400
500
600
700

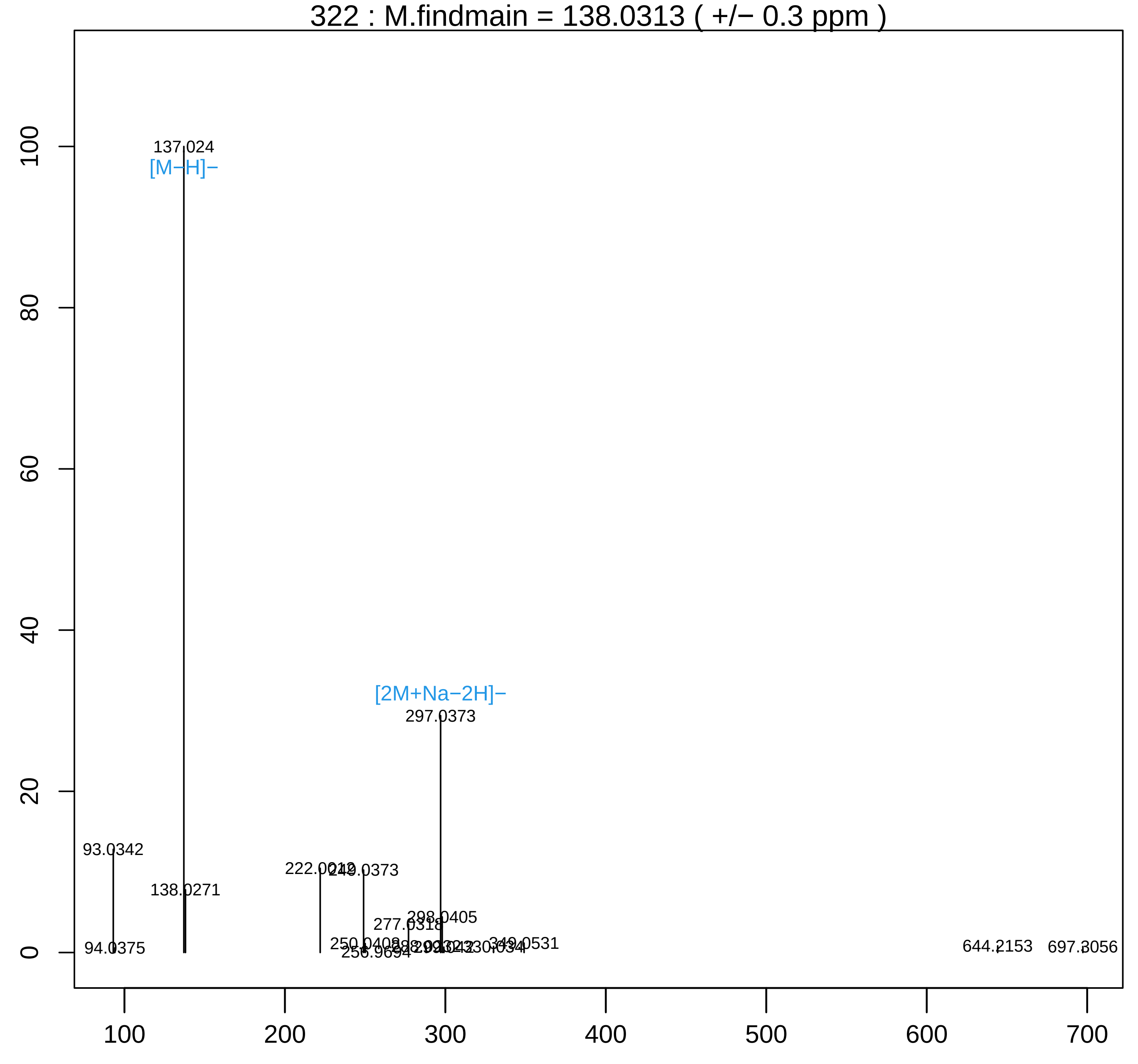
322 : M.findmain = 138.0313 (+/− 0.3 ppm)
137.024
[M−H]−
[2M+Na−2H]−
297.0373
93.0342
138.0271
222.0012
249.0373
298.0405
277.0318
349.0531
94.0375
644.2153
697.3056
0
20
40
60
80
100
100
200
300
400
500
600
700

323 : M.ramclustr = 388.1725 (+/− 0.9 ppm)

323 : M.findmain = 388.1725 (+/− 0.3 ppm)

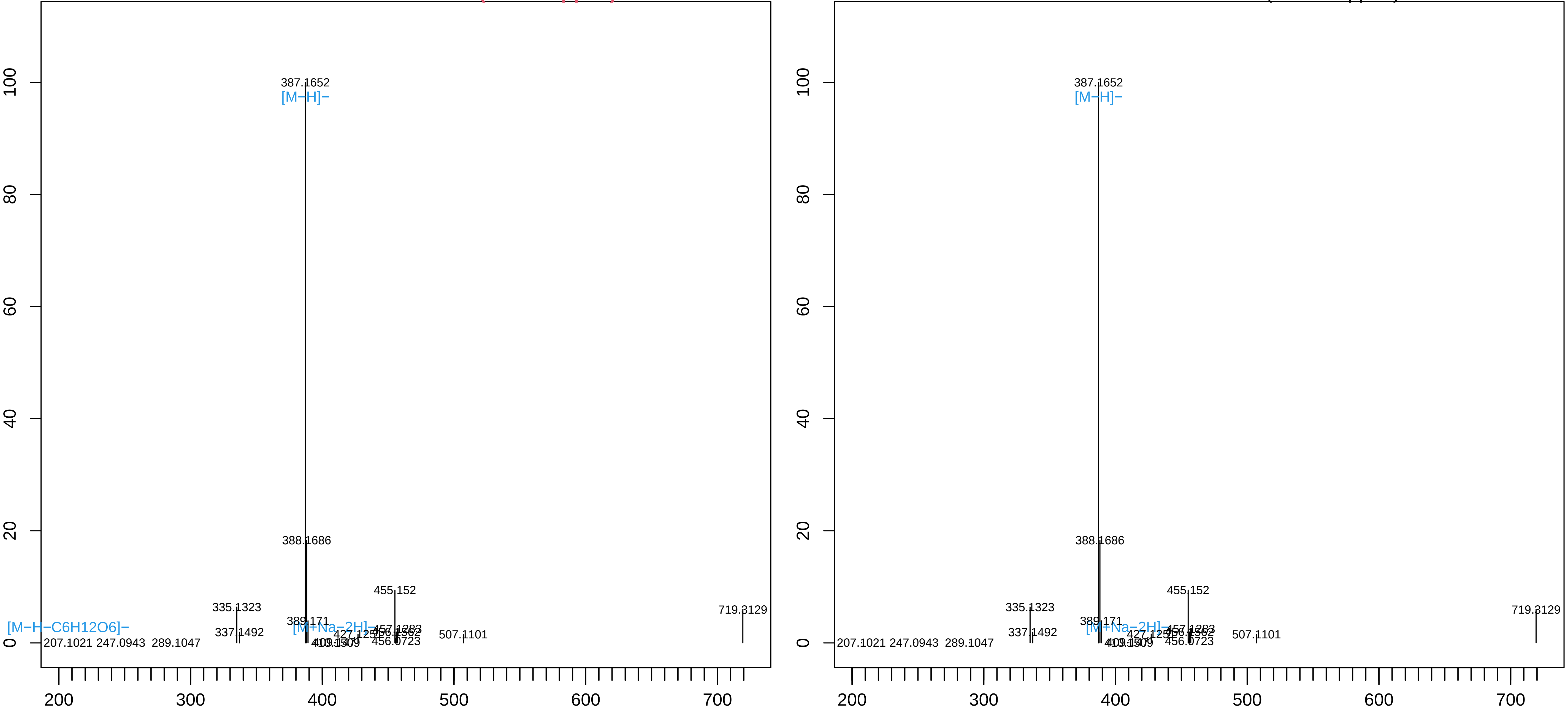

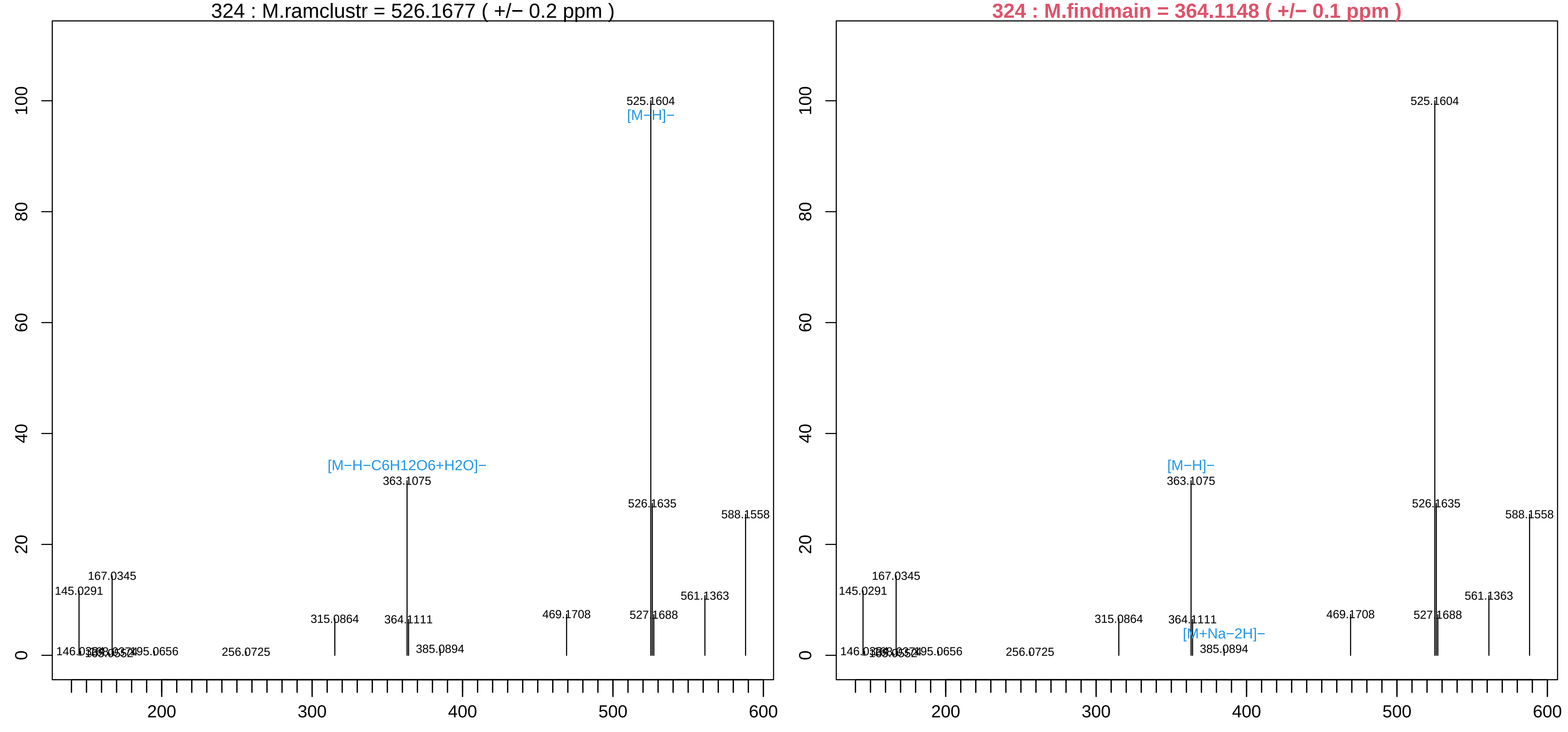

324 : M.ramclustr = 526.1677 (+/− 0.2 ppm)
525.1604
[M−H]−
[M−H−C6H12O6+H2O]−
363.1075
526.1635
588.1558
167.0345
145.0291
561.1363
315.0864
364.1111
469.1708
527.1688
385.0894
256.0725
195.0656
324 : M.findmain = 364.1148 (+/− 0.1 ppm)
[M−H]−
[M+Na−2H]−
0
20
40
60
80
100
200
300
400
500
600

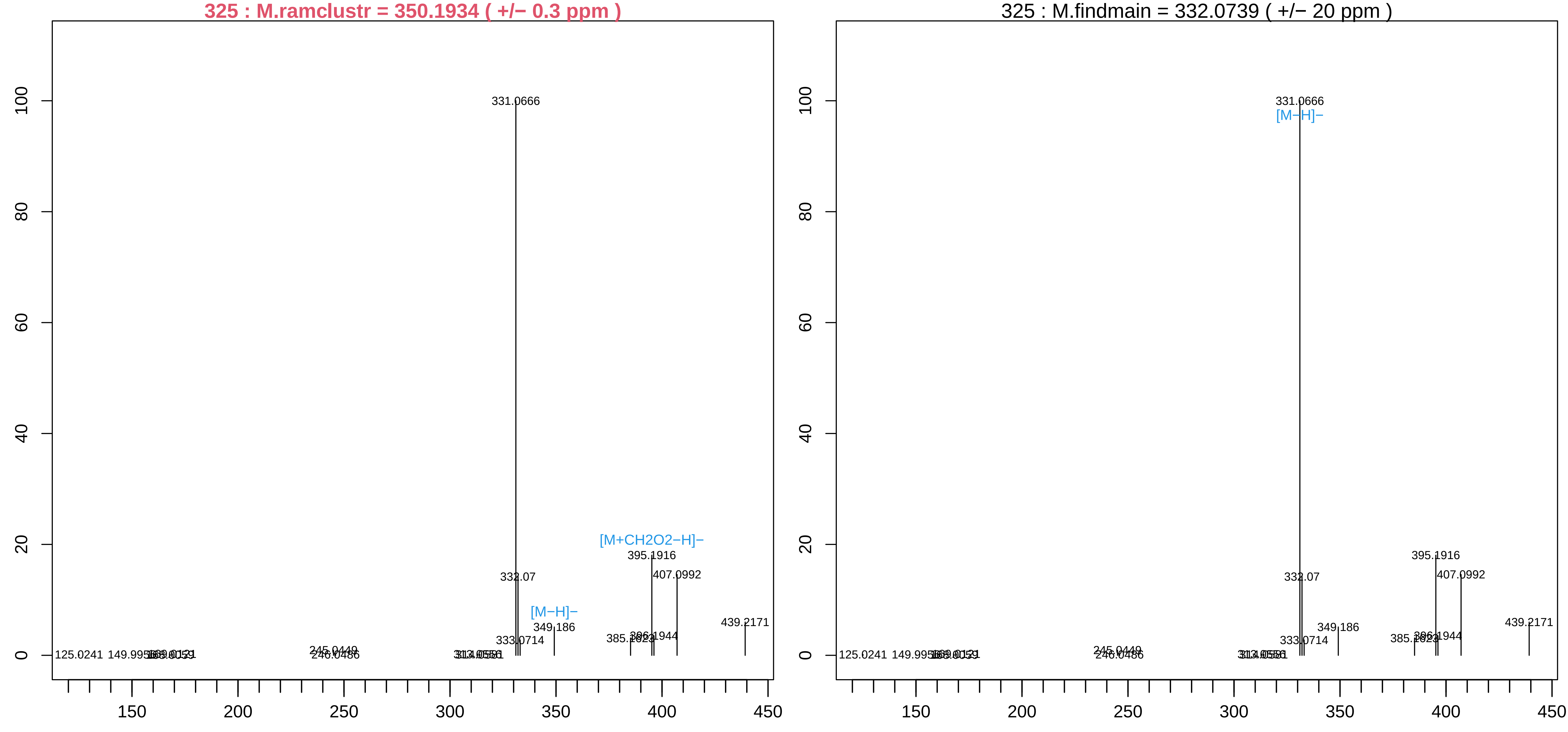
325 : M.ramclustr = 350.1934 (+/− 0.3 ppm)
331.0666
[M+CH2O2−H]−
395.1916
407.0992
332.07
[M−H]−
349.186
439.2171
333.0714
385.1623
125.0241
245.0449
246.0486
325 : M.findmain = 332.0739 (+/− 20 ppm)
331.0666
[M−H]−
395.1916
407.0992
332.07
349.186
439.2171
333.0714
385.1623
125.0241
245.0449
246.0486
0
20
40
60
80
100
150
200
250
300
350
400
450

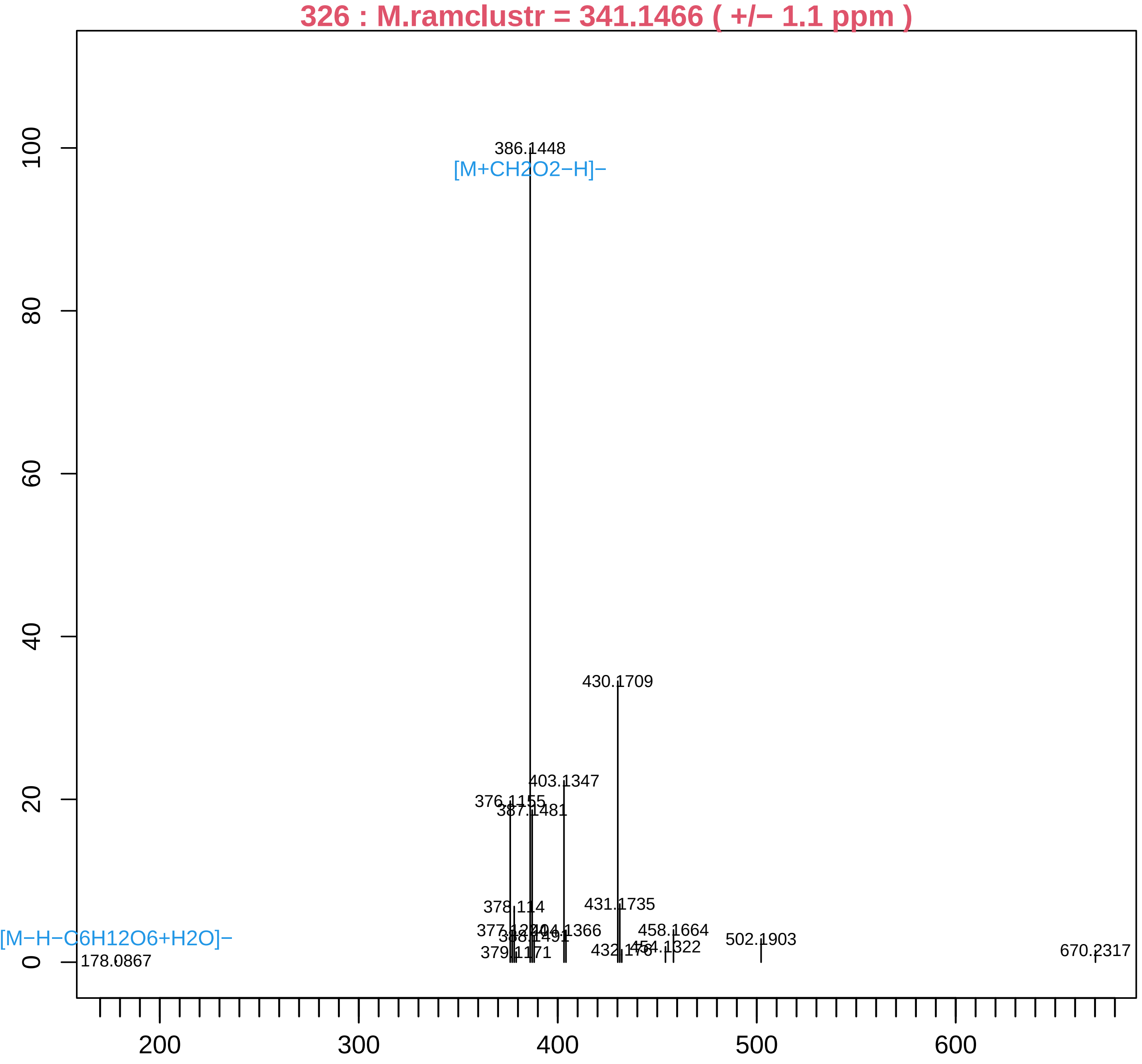
326 : M.ramclustr = 341.1466 (+/− 1.1 ppm)
386.1448
[M+CH2O2−H]−
430.1709
403.1347
376.1155
387.1481
378.114
431.1735
377.1204
404.1366
388.1491
458.1664
502.1903
379.1171
432.176
454.1322
670.2317
[M−H−C6H12O6+H2O]−
178.0867
0
20
40
60
80
100
200
300
400
500
600
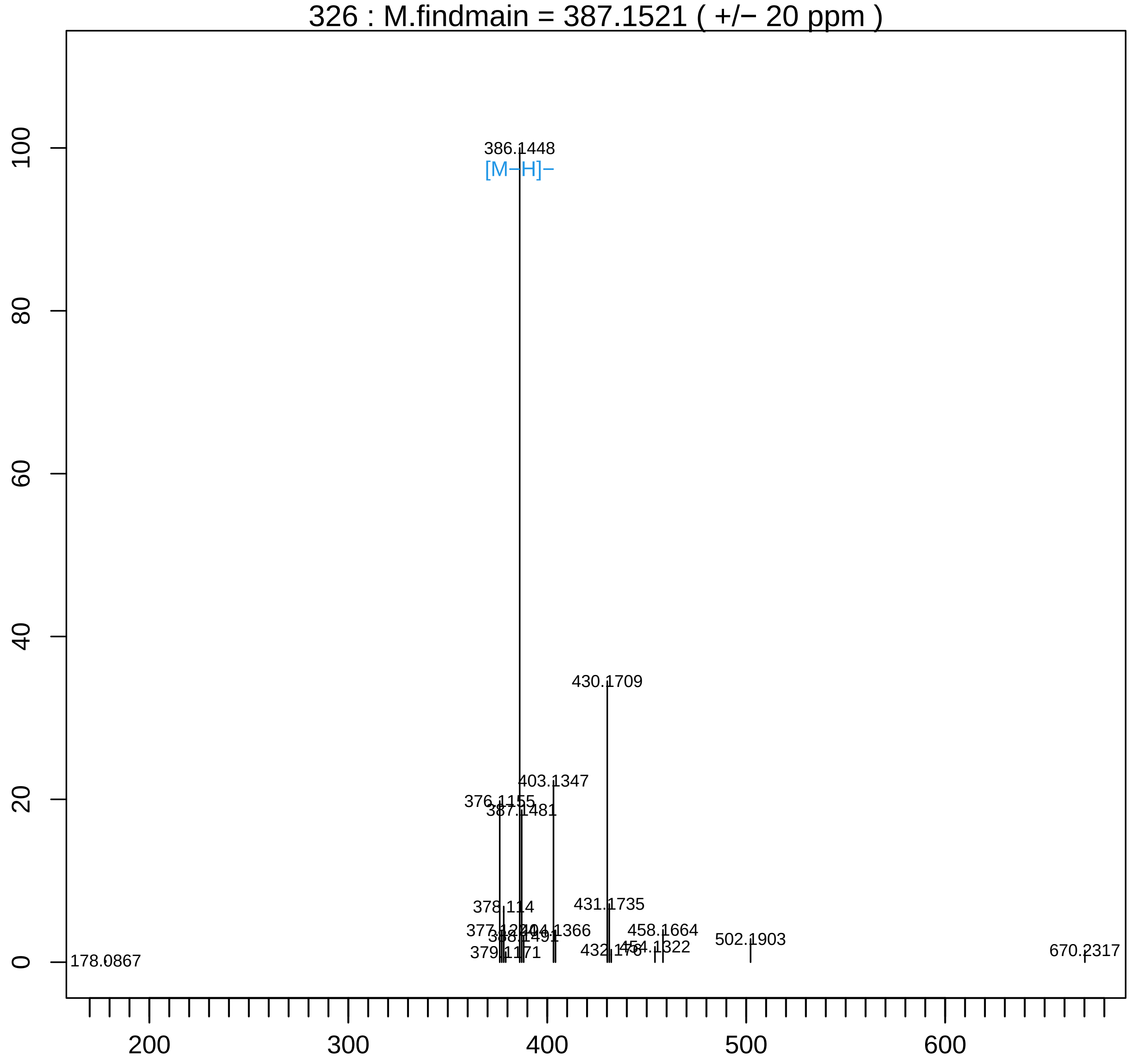
326 : M.findmain = 387.1521 (+/− 20 ppm)
386.1448
[M−H]−
430.1709
403.1347
376.1155
387.1481
378.114
431.1735
377.1204
404.1366
388.1491
458.1664
502.1903
379.1171
432.176
454.1322
670.2317
178.0867
0
20
40
60
80
100
200
300
400
500
600

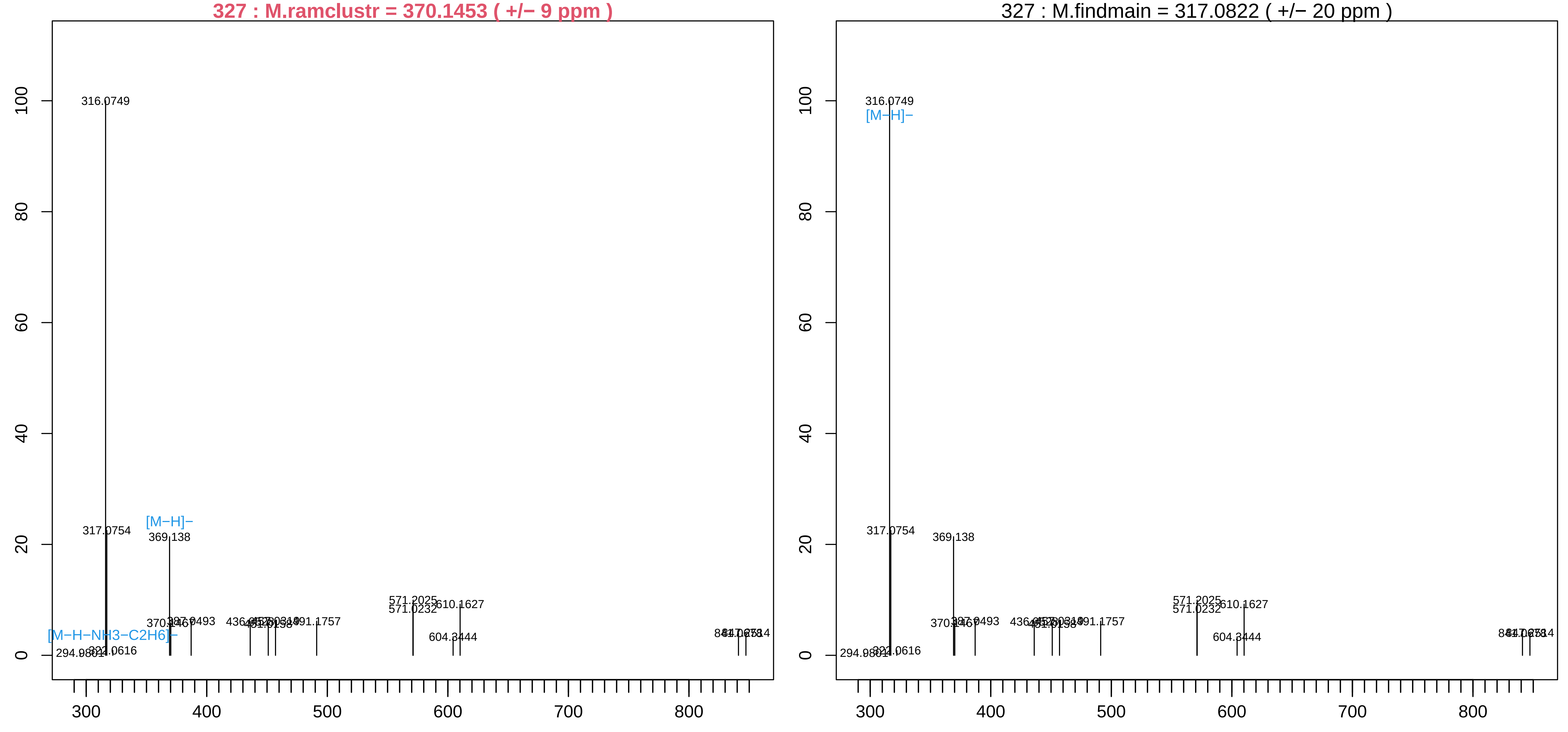
327 : M.ramclustr = 370.1453 (+/− 9 ppm)
316.0749
317.0754
[M−H]−
369.138
370.1467
387.0493
436.0457
451.0158
491.1757
571.2025
571.0232
610.1627
604.3444
[M−H−NH3−C2H6]−
294.9801
322.0616
327 : M.findmain = 317.0822 (+/− 20 ppm)
316.0749
[M−H]−
317.0754
369.138
370.1467
387.0493
436.0457
451.0158
491.1757
571.2025
571.0232
610.1627
604.3444
294.9801
322.0616
0
20
40
60
80
100
300
400
500
600
700
800

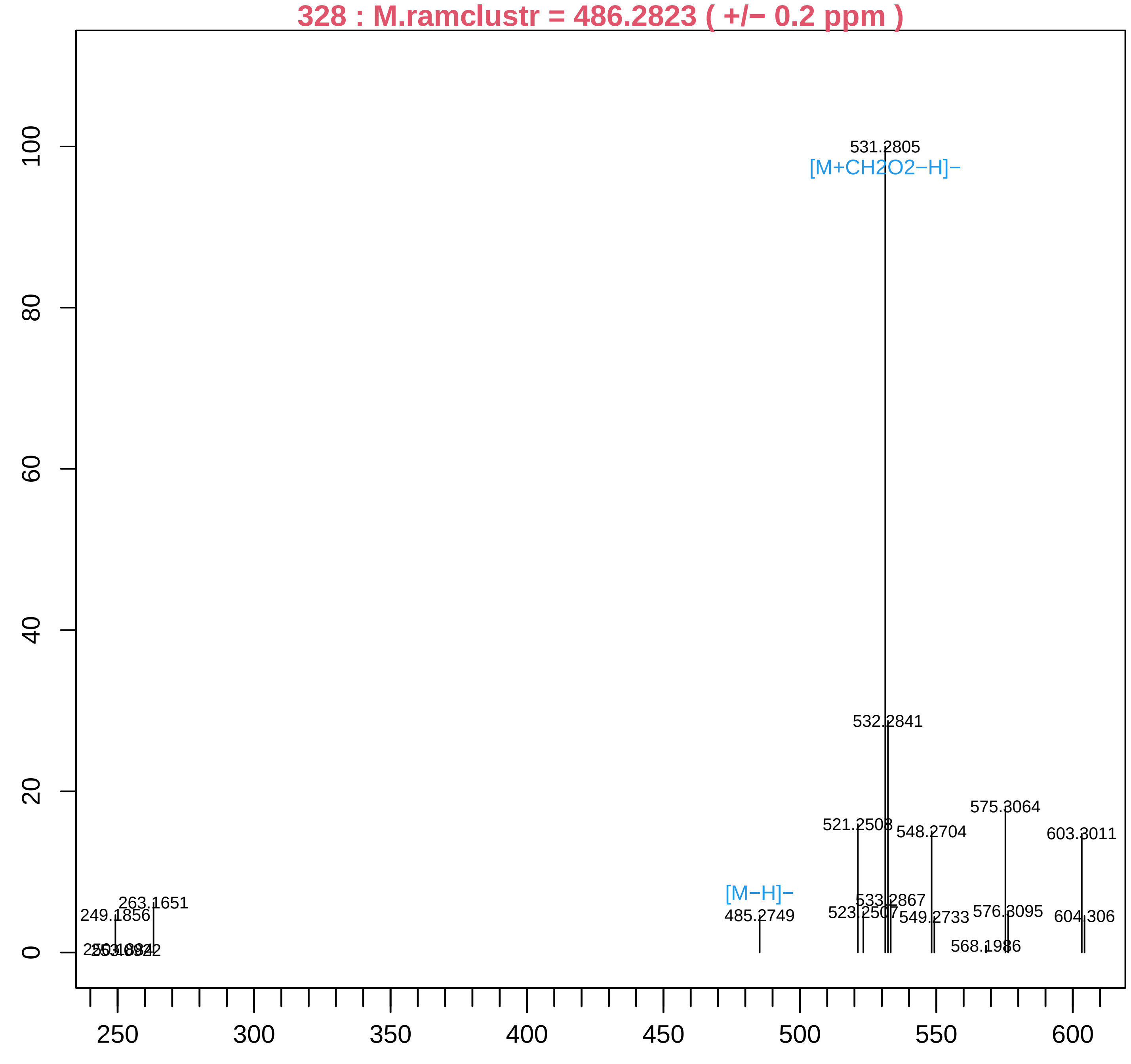
328 : M.ramclustr = 486.2823 (+/− 0.2 ppm)
531.2805
[M+CH2O2−H]−
532.2841
521.2503
548.2704
575.3064
603.3011
[M−H]−
485.2749
533.2867
523.2507
549.2733
576.3095
604.306
568.1986
263.1651
249.1856
0
20
40
60
80
100
250
300
350
400
450
500
550
600

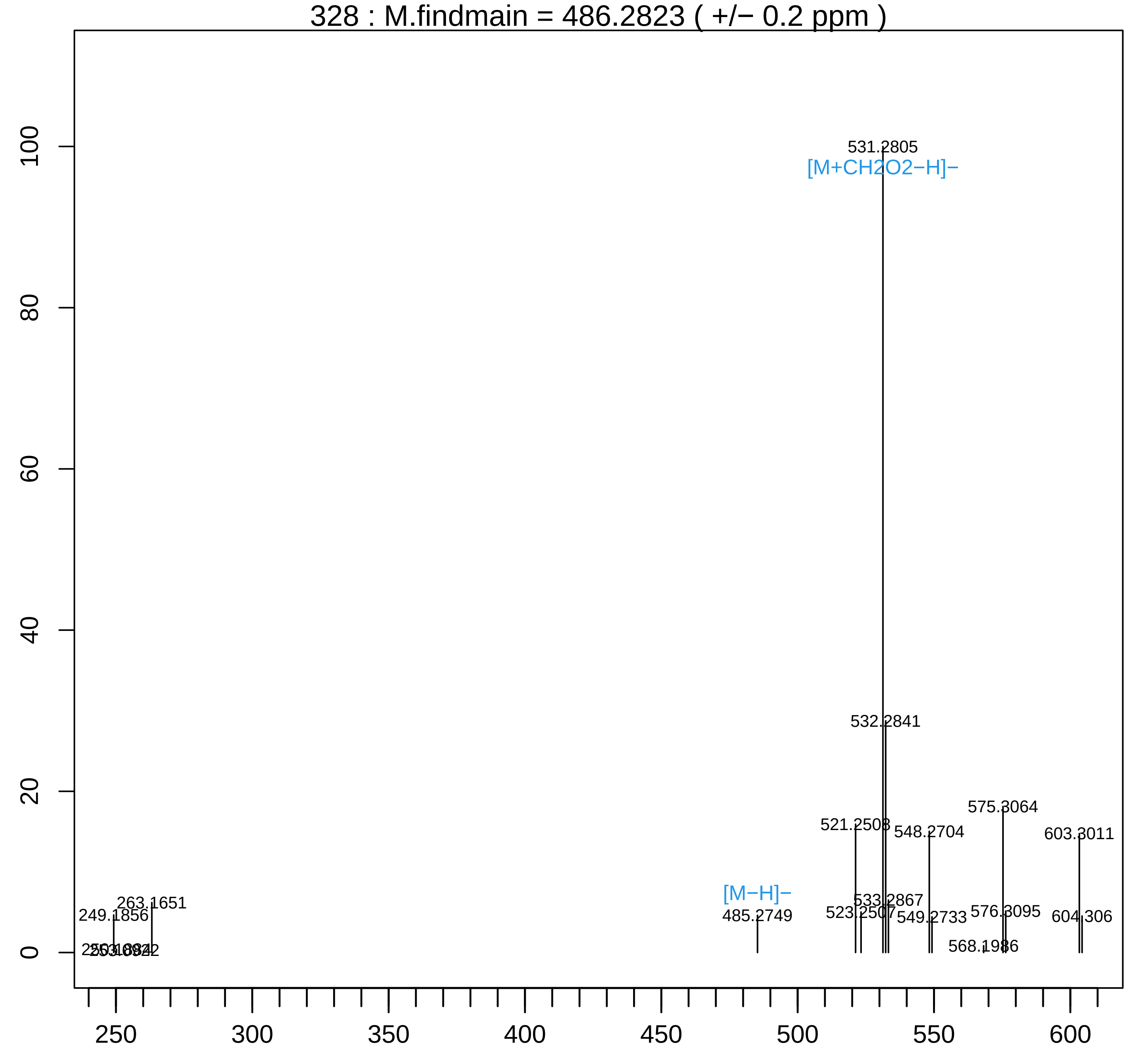
328 : M.findmain = 486.2823 (+/− 0.2 ppm)
531.2805
[M+CH2O2−H]−
532.2841
521.2503
548.2704
575.3064
603.3011
[M−H]−
485.2749
533.2867
523.2507
549.2733
576.3095
604.306
568.1986
263.1651
249.1856
0
20
40
60
80
100
250
300
350
400
450
500
550
600

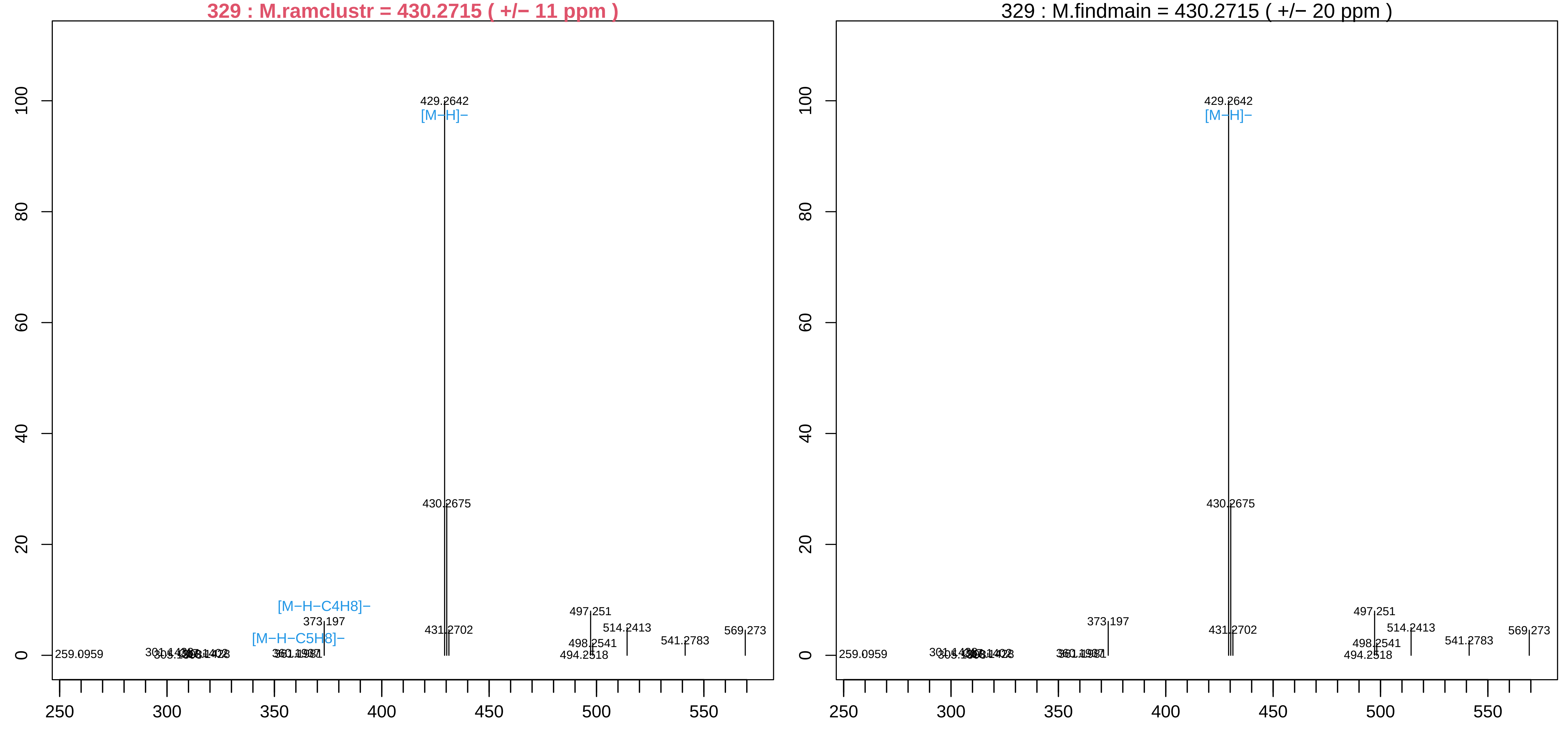
329 : M.ramclustr = 430.2715 (+/− 11 ppm)
329 : M.findmain = 430.2715 (+/− 20 ppm)
429.2642
[M−H]−
430.2675
431.2702
[M−H−C4H8]−
373.197
[M−H−C5H8]−
497.251
514.2413
498.2541
494.2518
541.2783
569.273
259.0959
361.1987
0
20
40
60
80
100
250
300
350
400
450
500
550

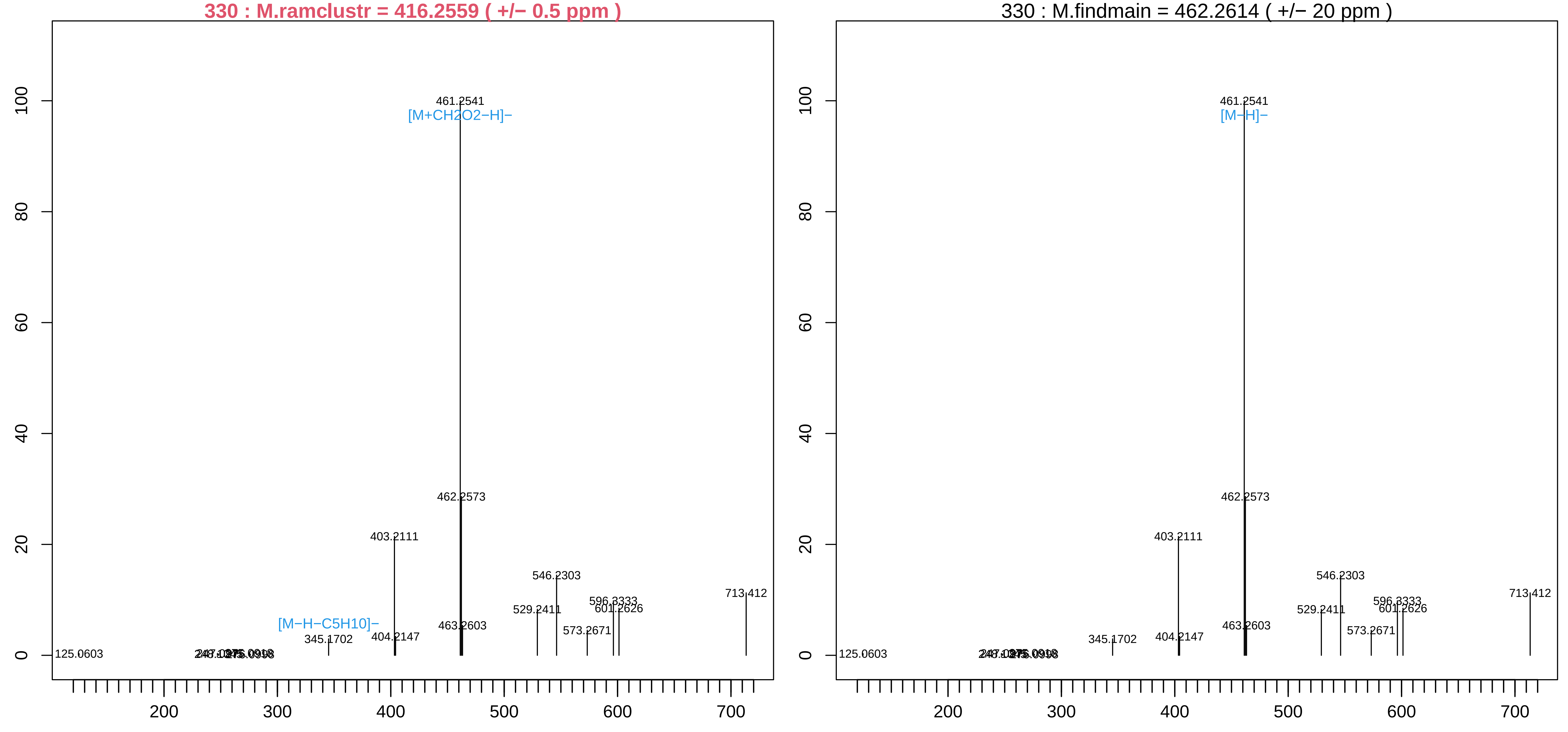
330 : M.ramclustr = 416.2559 (+/− 0.5 ppm)
461.2541
[M+CH2O2−H]−
462.2573
403.2111
546.2303
713.412
596.3333
601.2626
529.2411
[M−H−C5H10]−
463.2603
573.2671
345.1702
404.2147
125.0603
0
20
40
60
80
100
200
300
400
500
600
700
330 : M.findmain = 462.2614 (+/− 20 ppm)
461.2541
[M−H]−
462.2573
403.2111
546.2303
713.412
596.3333
601.2626
529.2411
463.2603
573.2671
345.1702
404.2147
125.0603

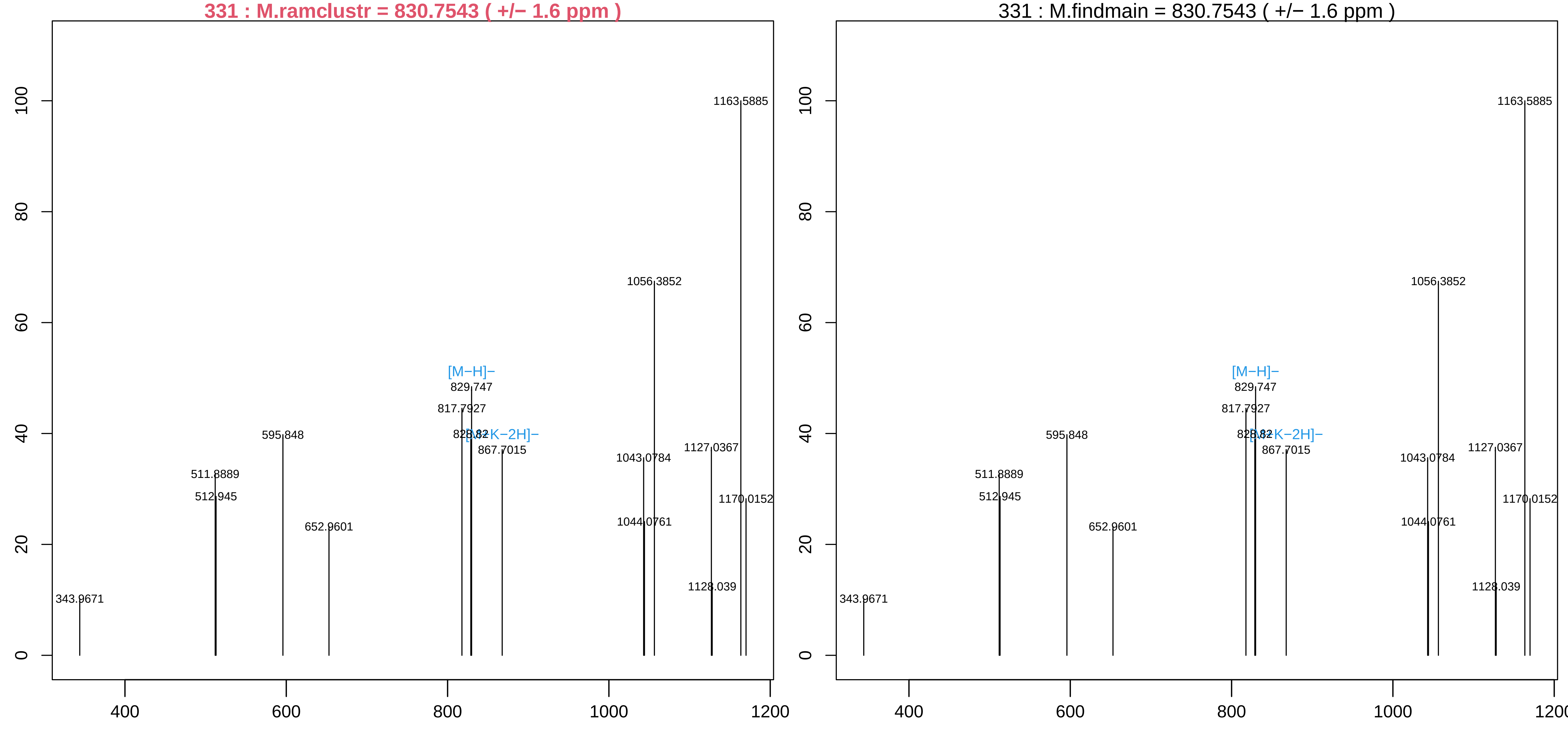

331 : M.ramclustr = 830.7543 (+/− 1.6 ppm)
331 : M.findmain = 830.7543 (+/− 1.6 ppm)
0
20
40
60
80
100
400
600
800
1000
1200
1163.5885
1056.3852
[M−H]−
829.747
817.7927
828.82
[M+K−2H]−
867.7015
595.848
511.8889
512.945
652.9601
343.9671
1043.0784
1044.0761
1127.0367
1128.039
1170.0152

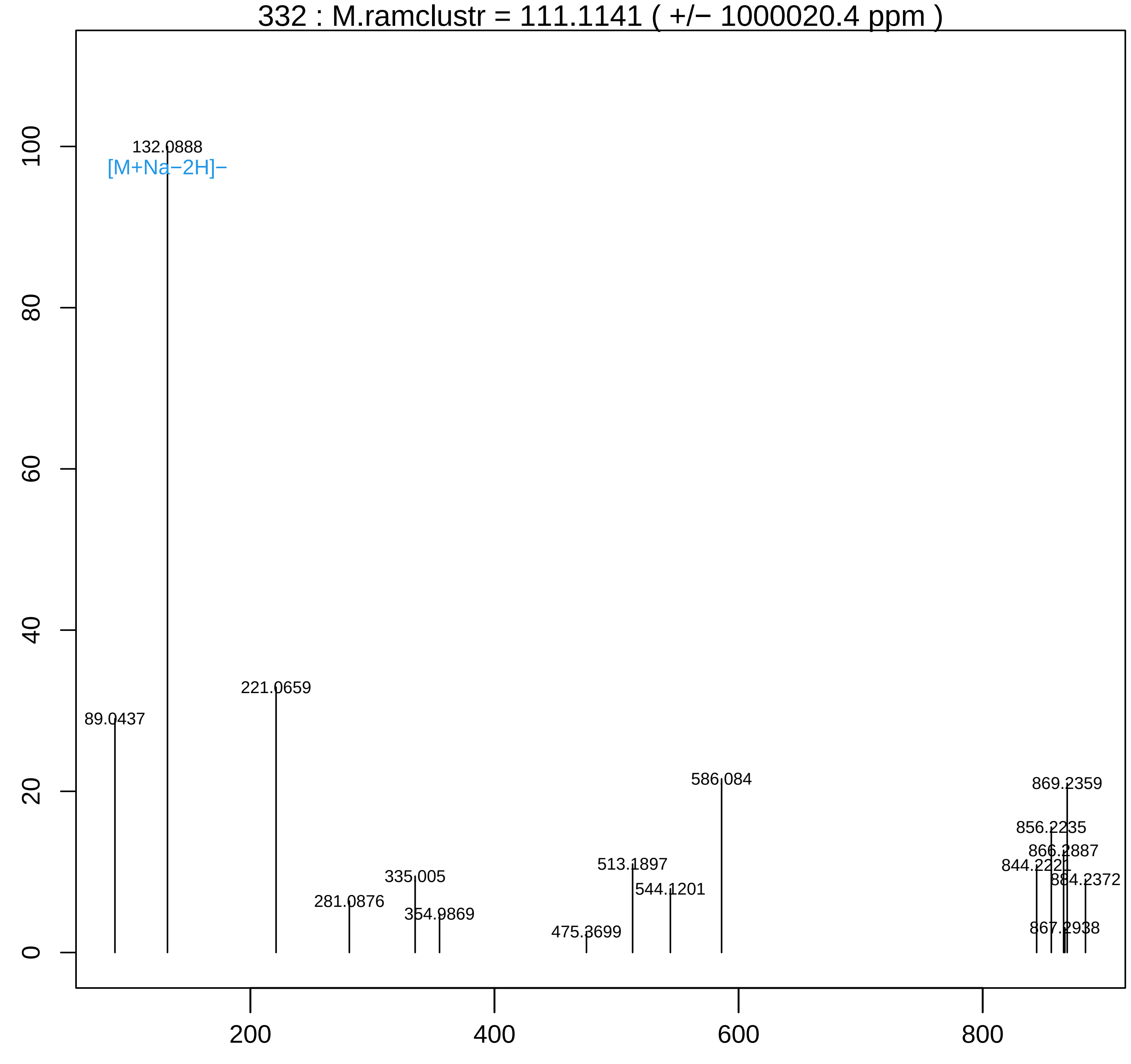
332 : M.ramclustr = 111.1141 (+/− 1000020.4 ppm)
132.0888
[M+Na−2H]−
89.0437
221.0659
281.0876
335.005
354.9869
475.3699
513.1897
544.1201
586.084
844.2221
856.2235
866.2887
867.2938
869.2359
884.2372
0
20
40
60
80
100
200
400
600
800
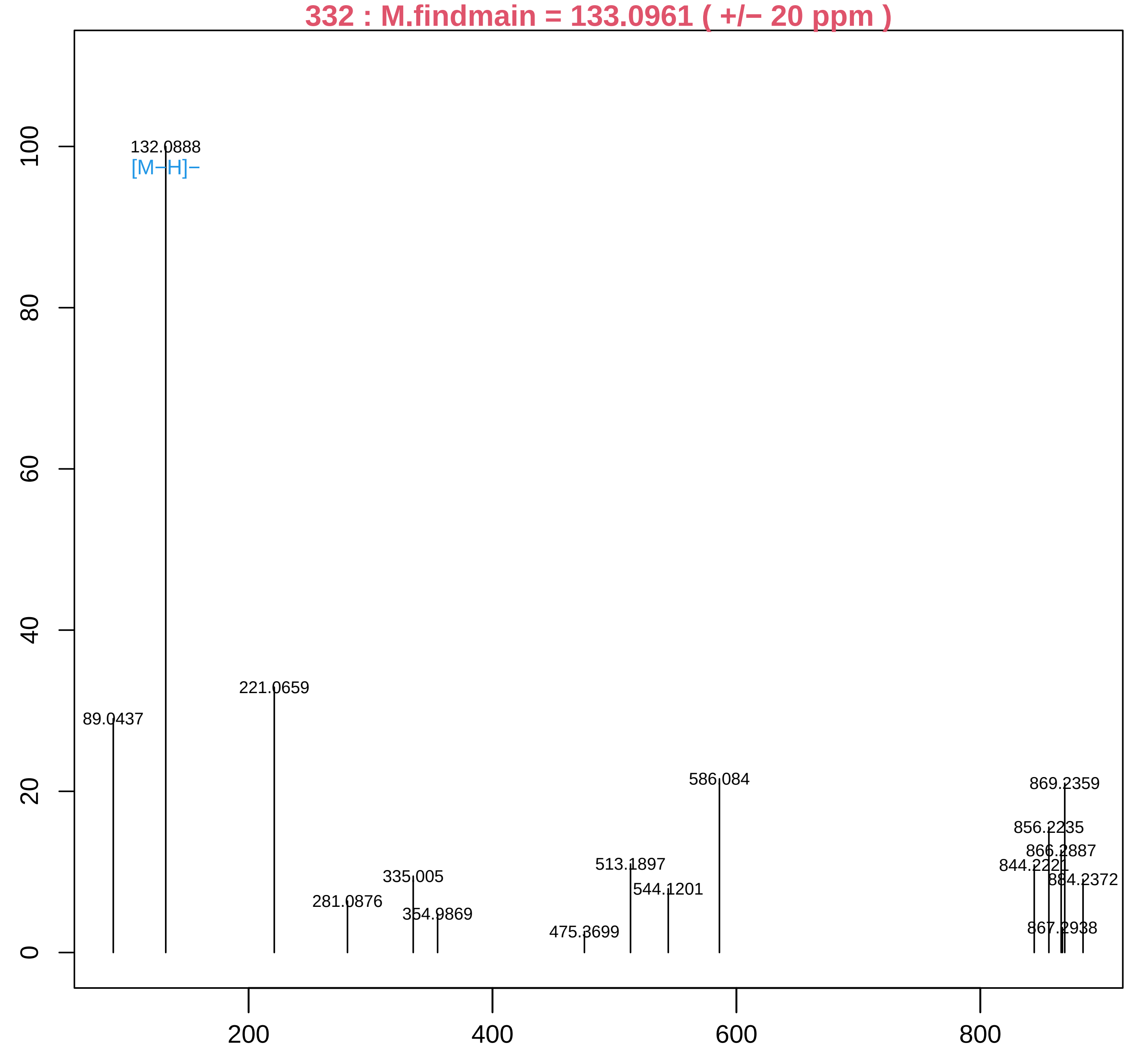
332 : M.findmain = 133.0961 (+/− 20 ppm)
132.0888
[M−H]−
89.0437
221.0659
281.0876
335.005
354.9869
475.3699
513.1897
544.1201
586.084
844.2221
856.2235
866.2887
867.2938
869.2359
884.2372
0
20
40
60
80
100
200
400
600
800

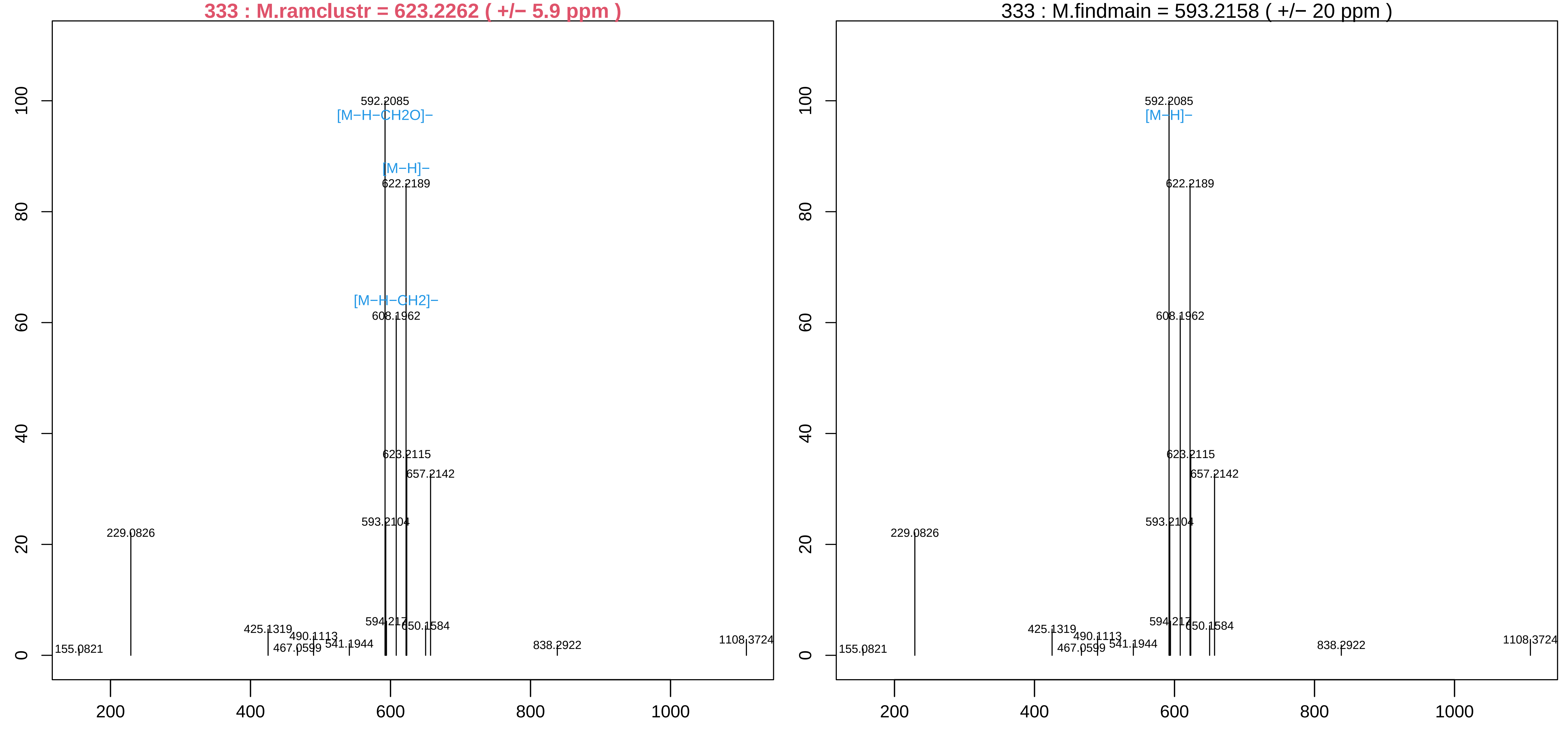

333 : M.ramclustr = 623.2262 (+/− 5.9 ppm)
592.2085
[M−H−CH2O]−
[M−H]−
622.2189
[M−H−CH2]−
608.1962
623.2115
657.2142
229.0826
593.2104
594.217
650.1584
425.1319
490.1113
467.0599
541.1944
155.0821
838.2922
1108.3724
333 : M.findmain = 593.2158 (+/− 20 ppm)
592.2085
[M−H]−
622.2189
608.1962
623.2115
657.2142
229.0826
593.2104
594.217
650.1584
425.1319
490.1113
467.0599
541.1944
155.0821
838.2922
1108.3724
0
20
40
60
80
100
200
400
600
800
1000

334 : M.ramclustr = 182.0782 (+/− 0.4 ppm)

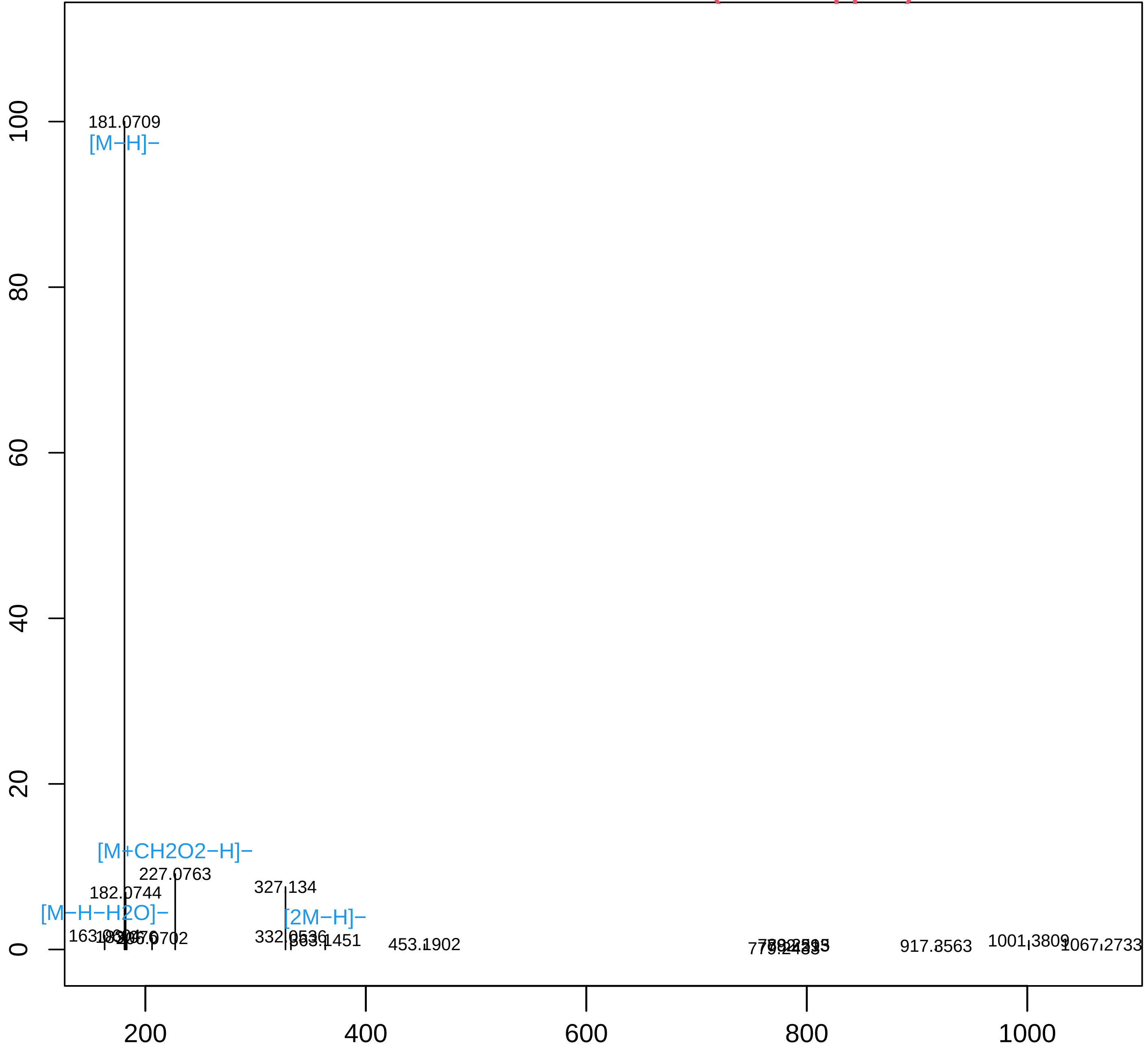

334 : M.findmain = 182.0782 (+/− 5.7 ppm)

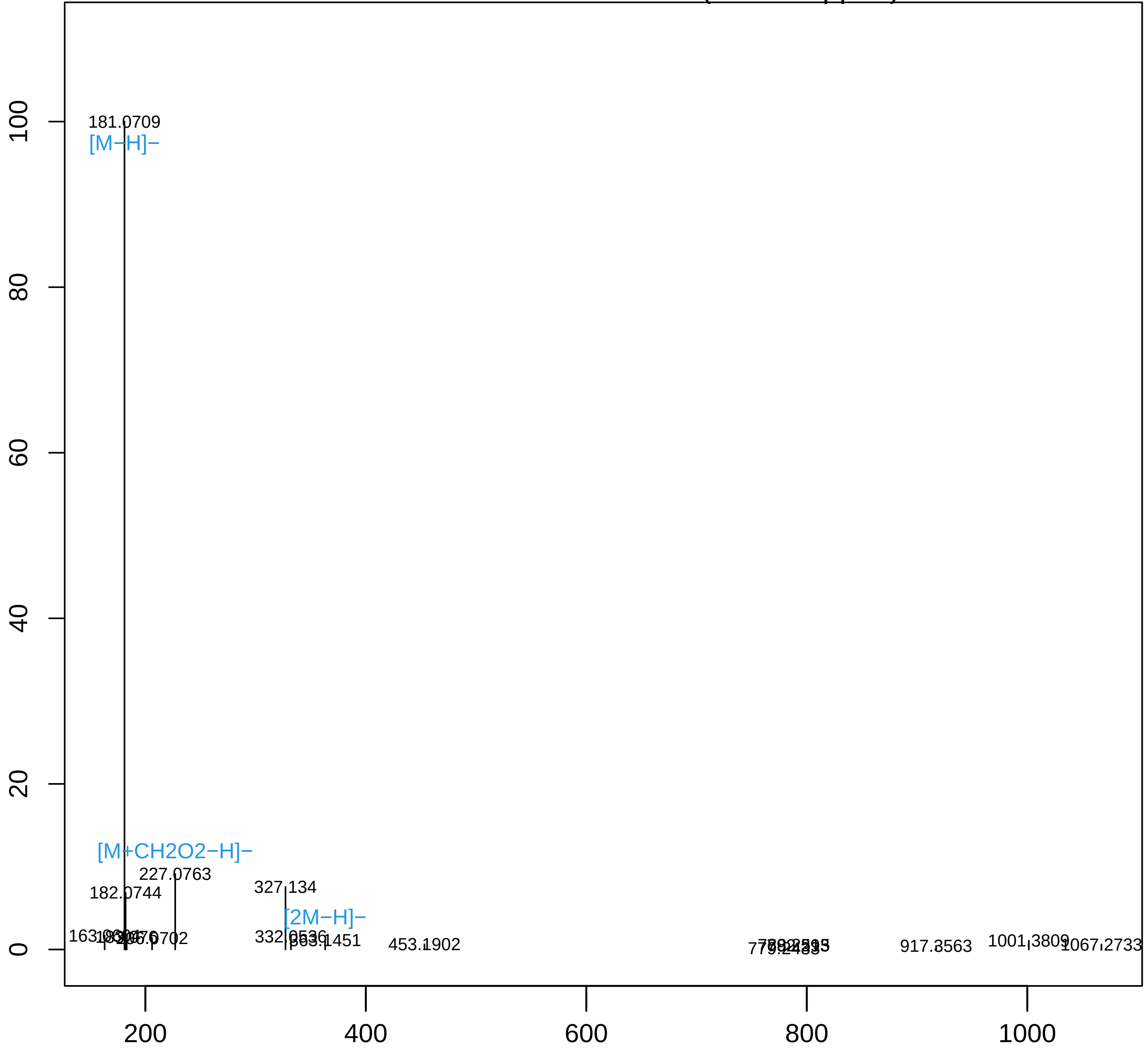

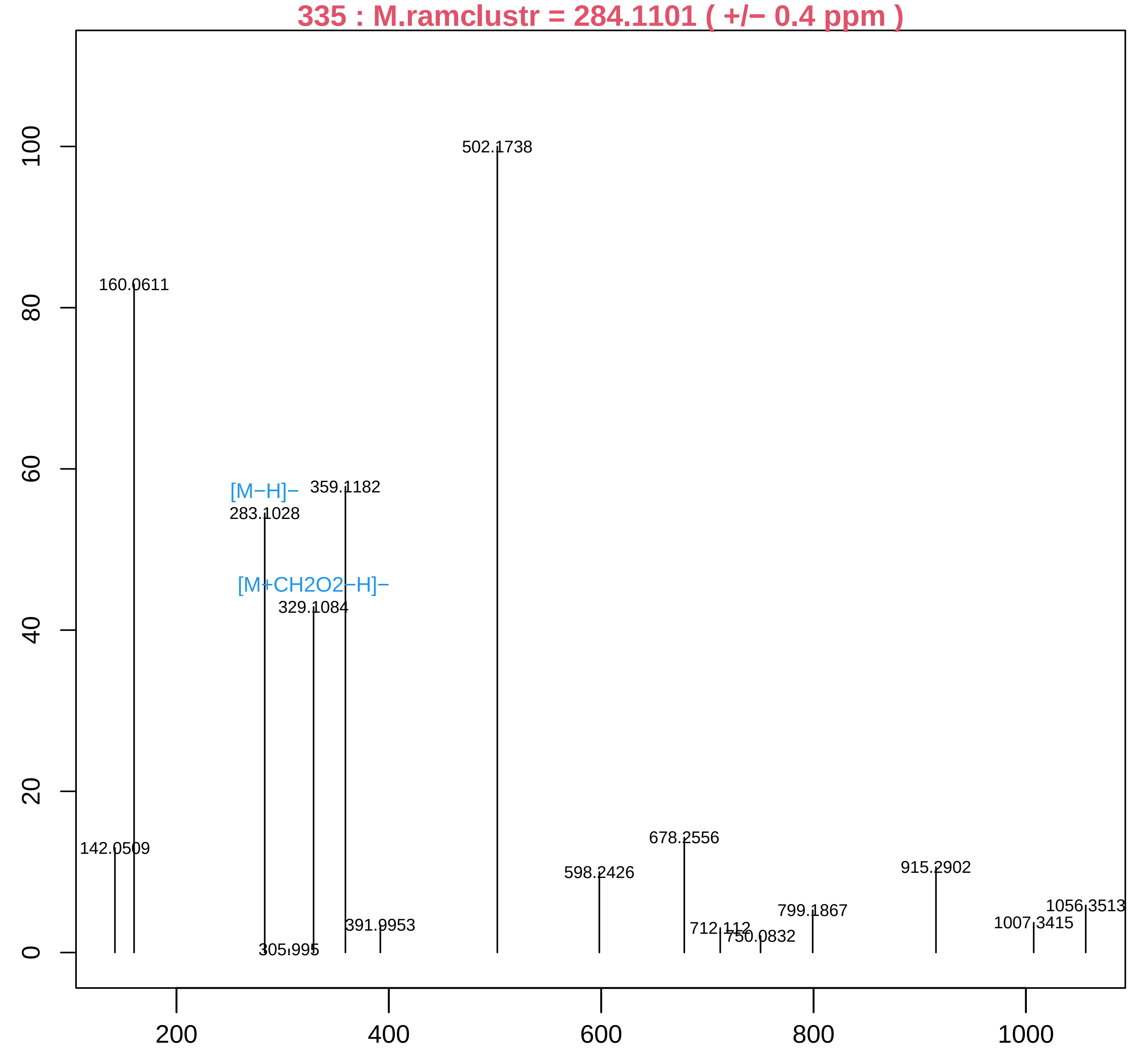
335 : M.ramclustr = 284.1101 (+/− 0.4 ppm)
0
20
40
60
80
100
200
400
600
800
1000
502.1738
160.0611
[M−H]−
283.1028
359.1182
[M+CH2O2−H]−
329.1084
142.0509
305.995
391.9953
598.2426
678.2556
712.112
750.0832
799.1867
915.2902
1007.3415
1056.3513

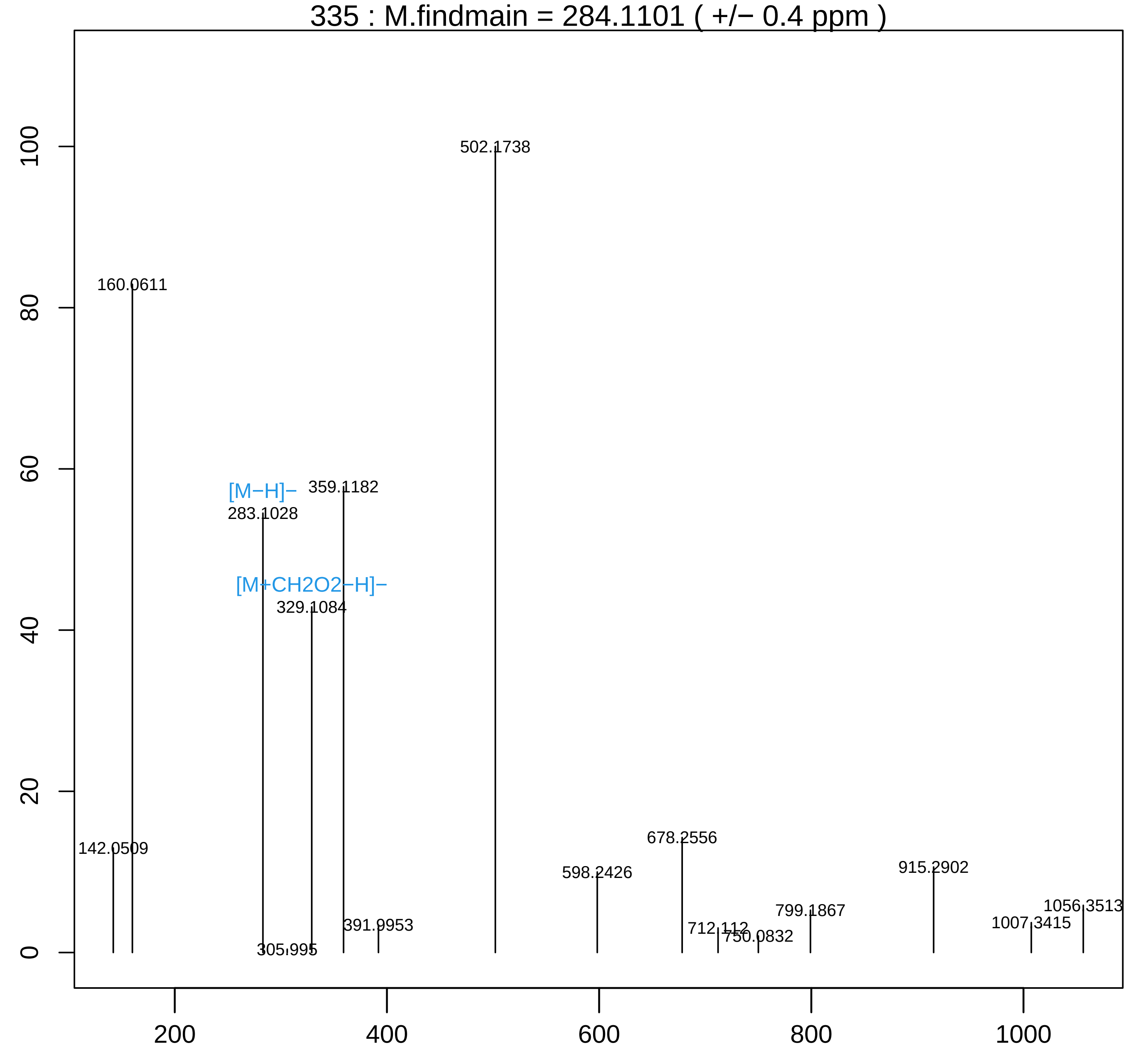
335 : M.findmain = 284.1101 (+/− 0.4 ppm)
0
20
40
60
80
100
200
400
600
800
1000
502.1738
160.0611
[M−H]−
283.1028
359.1182
[M+CH2O2−H]−
329.1084
142.0509
305.995
391.9953
598.2426
678.2556
712.112
750.0832
799.1867
915.2902
1007.3415
1056.3513

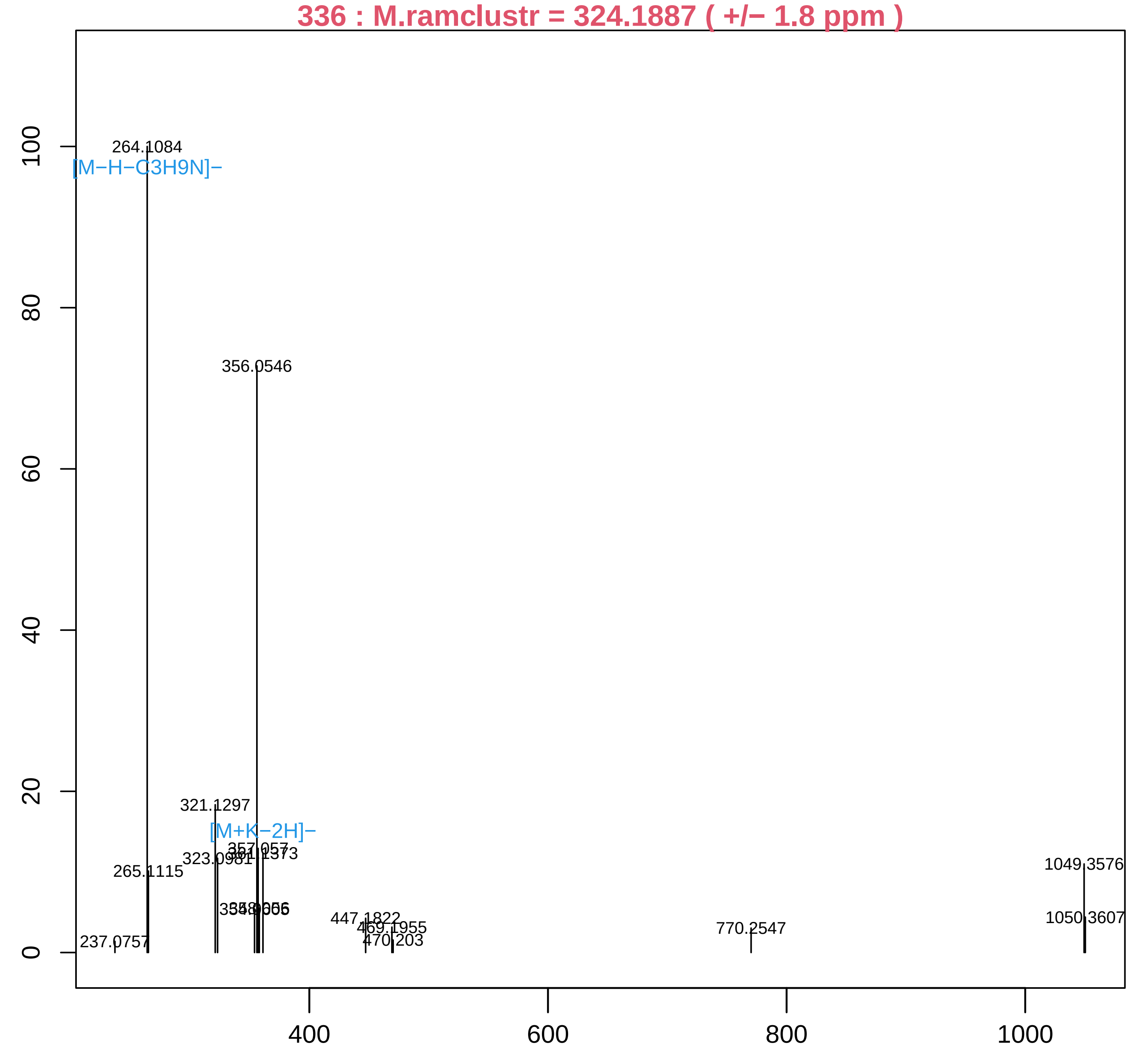

336 : M.ramclustr = 324.1887 (+/− 1.8 ppm)
264.1084
[M−H−C3H9N]−
356.0546
321.1297
[M+K−2H]−
357.057
323.0981
361.1373
265.1115
1049.3576
1050.3607
447.1822
469.1955
470.203
770.2547
237.0757
0
20
40
60
80
100
400
600
800
1000

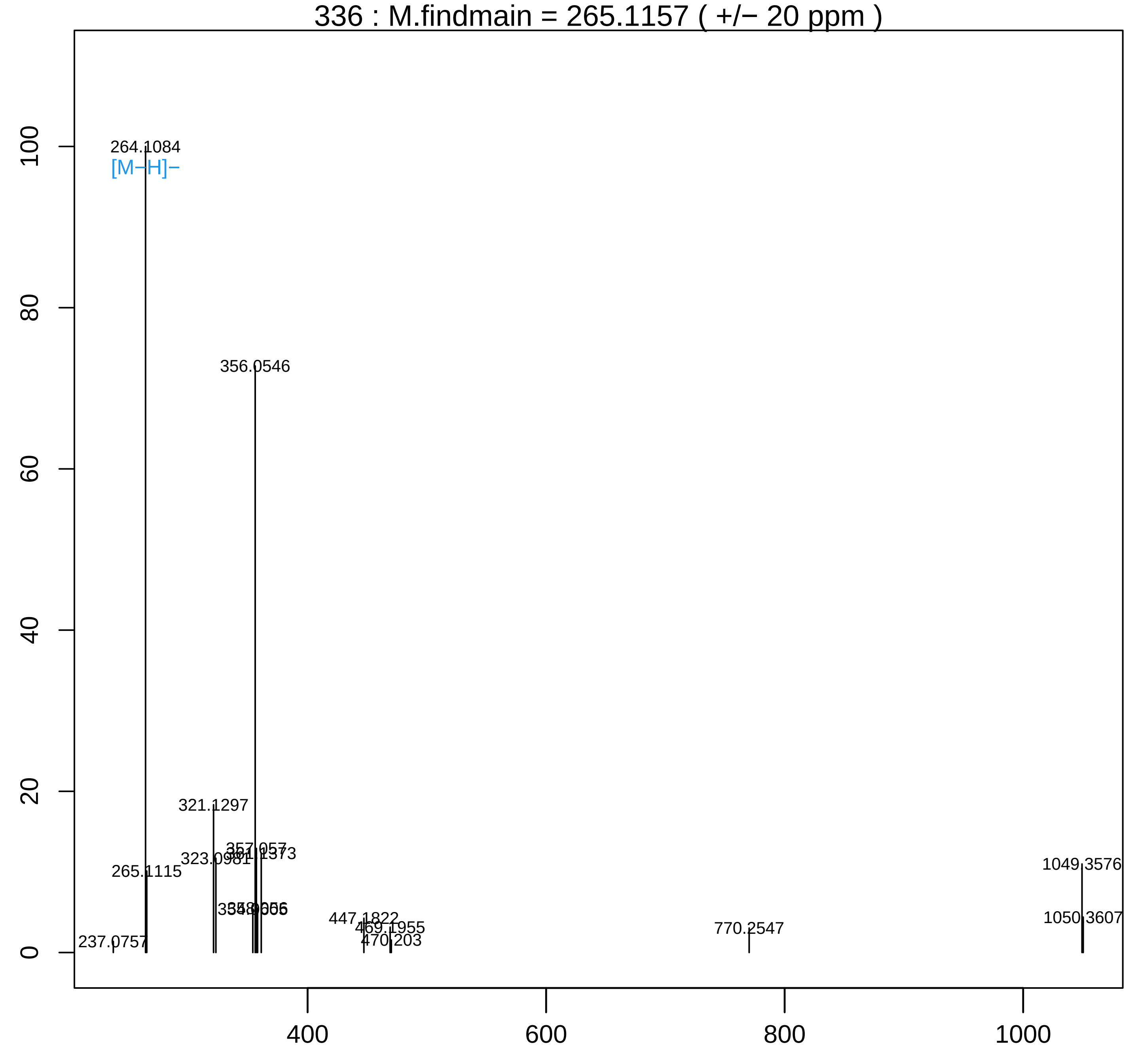

336 : M.findmain = 265.1157 (+/− 20 ppm)
264.1084
[M−H]−
356.0546
321.1297
357.057
323.0981
361.1373
265.1115
1049.3576
1050.3607
447.1822
469.1955
470.203
770.2547
237.0757
0
20
40
60
80
100
400
600
800
1000

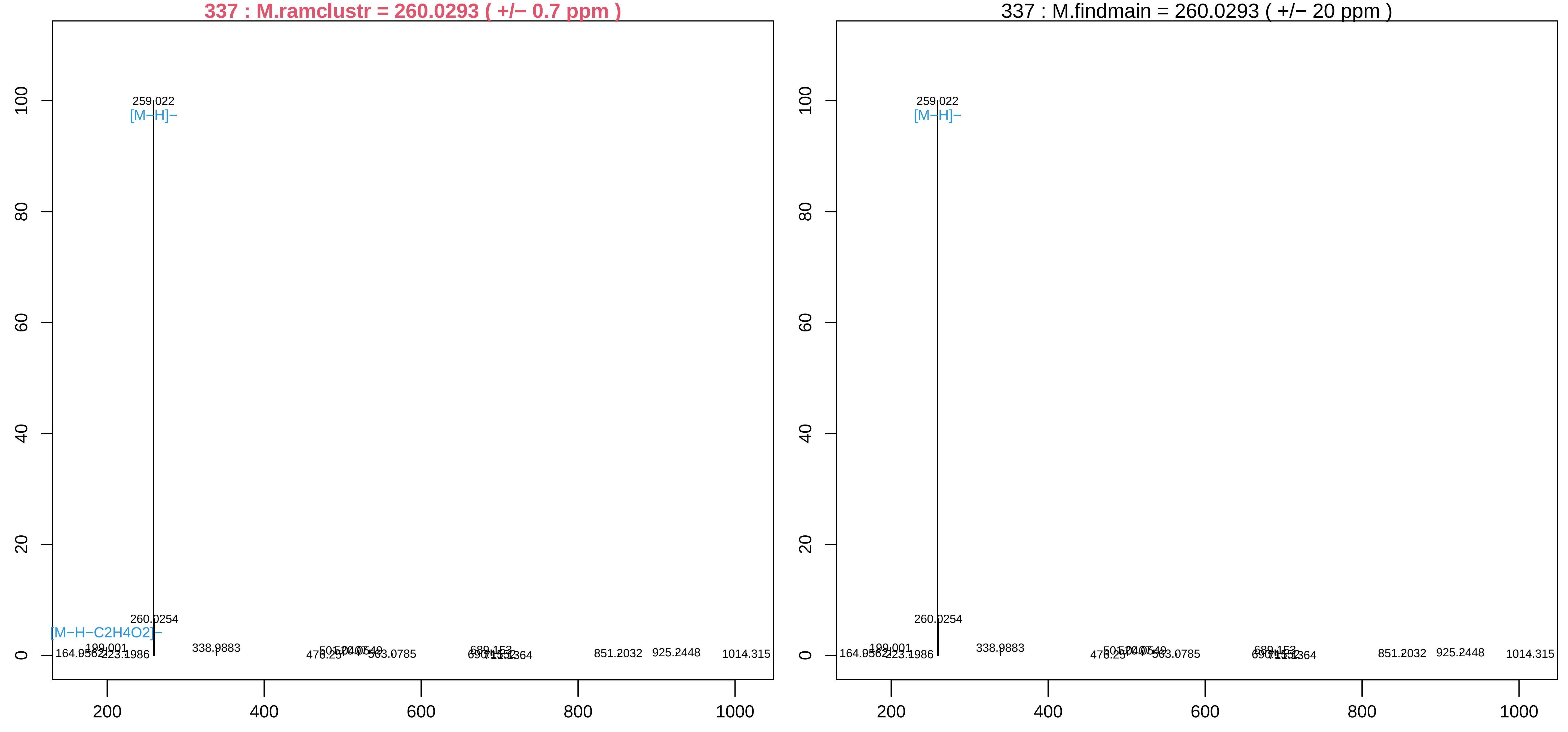

337 : M.ramclustr = 260.0293 (+/− 0.7 ppm)
259.022
[M−H]−
260.0254
[M−H−C2H4O2]−
199.001
164.9562
223.1986
338.9883
476.25
563.0785
689.153
851.2032
925.2448
1014.315
337 : M.findmain = 260.0293 (+/− 20 ppm)
259.022
[M−H]−
260.0254
199.001
164.9562
223.1986
338.9883
476.25
563.0785
689.153
851.2032
925.2448
1014.315

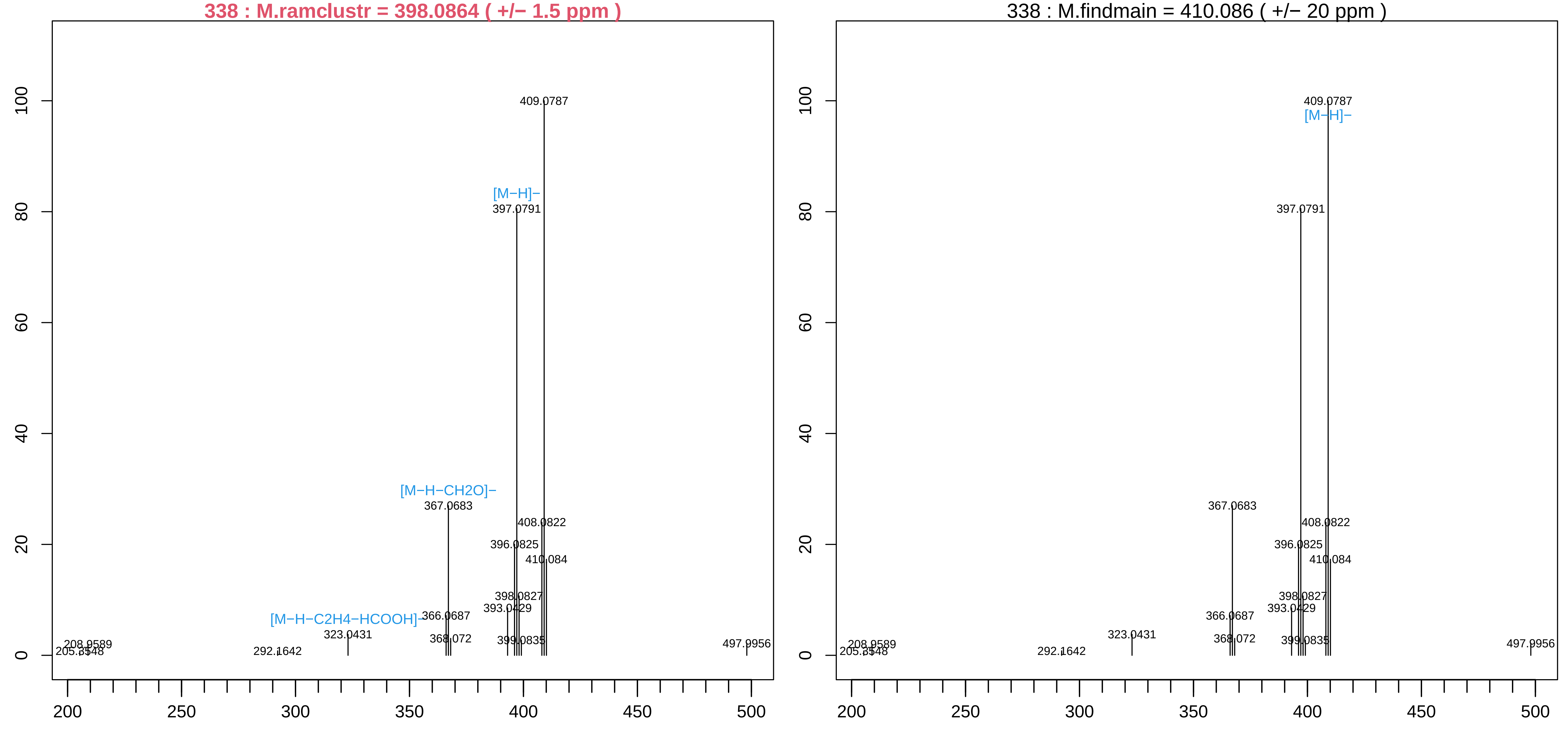
338 : M.ramclustr = 398.0864 (+/− 1.5 ppm)
409.0787
[M−H]−
397.0791
[M−H−CH2O]−
367.0683
408.0822
396.0825
410.084
398.0827
393.0429
[M−H−C2H4−HCOOH]−
366.0687
323.0431
368.072
399.0835
208.9589
205.3548
292.1642
497.9956
338 : M.findmain = 410.086 (+/− 20 ppm)
409.0787
[M−H]−
397.0791
367.0683
408.0822
396.0825
410.084
398.0827
393.0429
366.0687
323.0431
368.072
399.0835
208.9589
205.3548
292.1642
497.9956

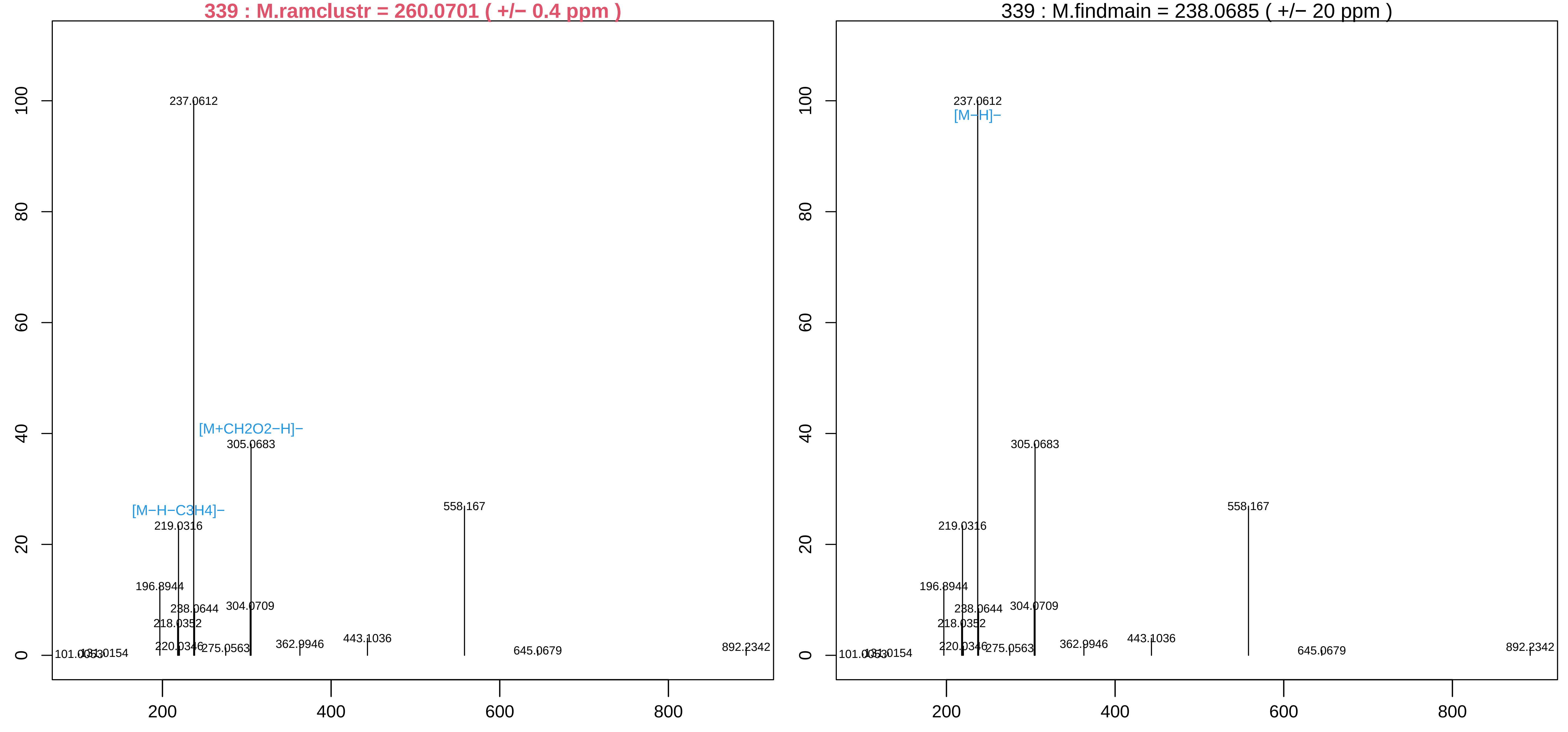

339 : M.ramclustr = 260.0701 (+/− 0.4 ppm)
237.0612
[M+CH2O2−H]−
305.0683
[M−H−C3H4]−
219.0316
558.167
196.8944
238.0644
304.0709
218.0352
220.0346
275.0563
362.9946
443.1036
645.0679
892.2342
101.0053
103.0154
339 : M.findmain = 238.0685 (+/− 20 ppm)
[M−H]−
0
20
40
60
80
100
200
400
600
800

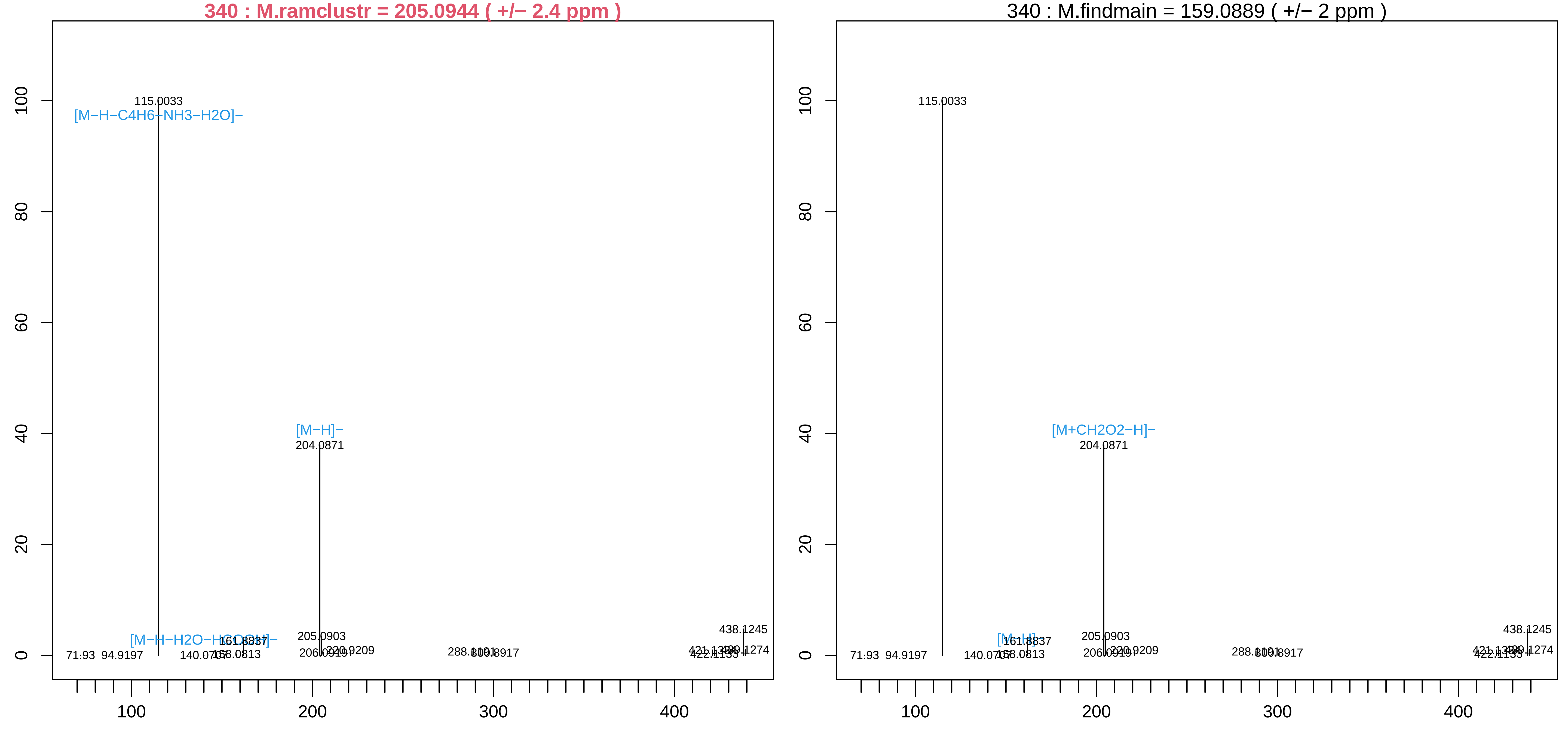

340 : M.ramclustr = 205.0944 (+/− 2.4 ppm)
115.0033
[M−H−C4H6−NH3−H2O]−
[M−H]−
204.0871
205.0903
220.9209
206.0919
288.1091
300.8917
438.1245
421.1389
439.1274
422.1133
71.93
94.9197
140.0715
158.0813
161.8837
[M−H−H2O−HCOOH]−
340 : M.findmain = 159.0889 (+/− 2 ppm)
[M+CH2O2−H]−
[M−H]−
0
20
40
60
80
100
200
300
400

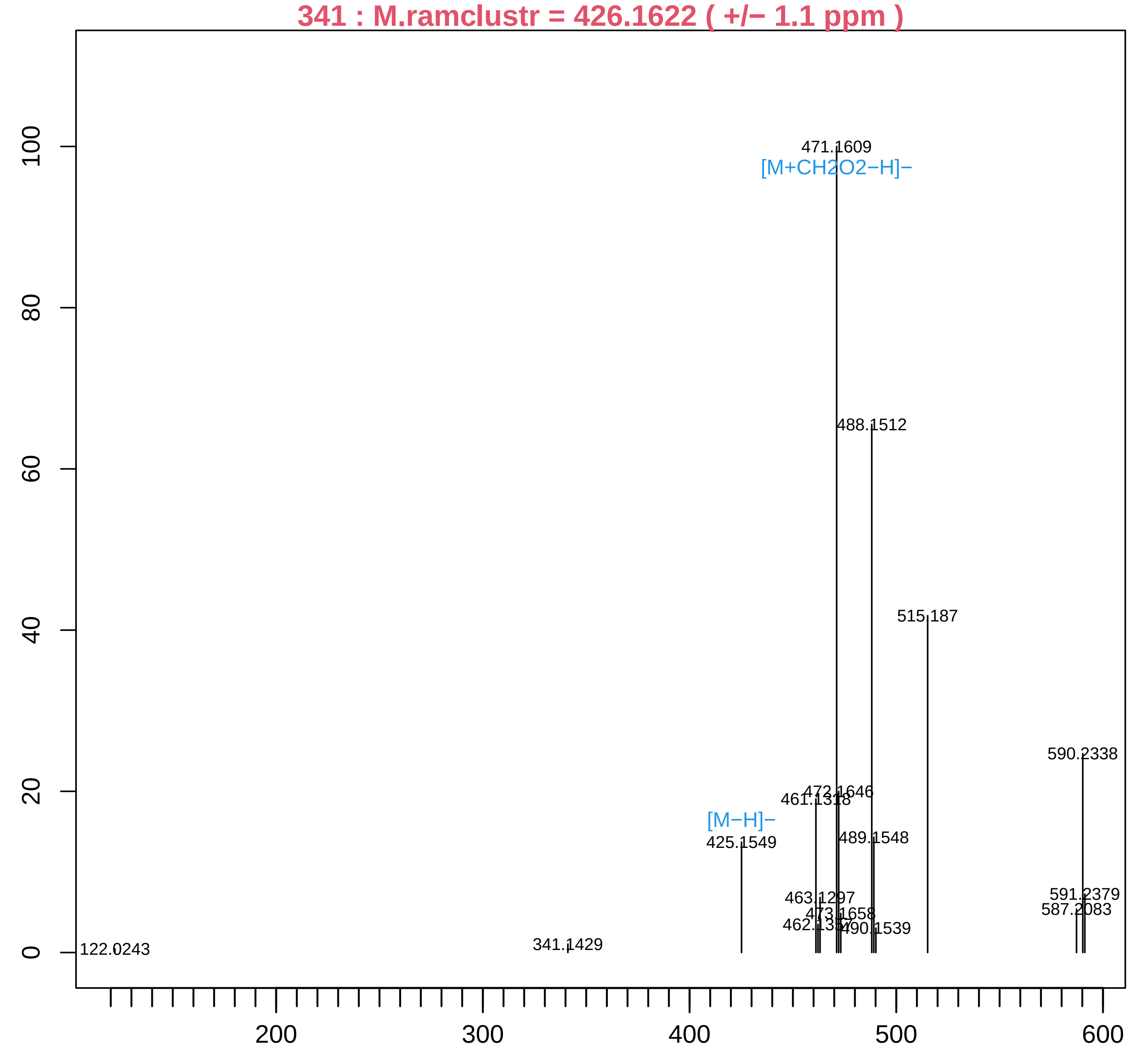
341 : M.ramclustr = 426.1622 (+/− 1.1 ppm)
471.1609
[M+CH2O2−H]−
488.1512
515.187
590.2338
472.1646
461.1318
[M−H]−
425.1549
489.1548
463.1297
473.1658
462.1357
490.1539
591.2379
587.2083
341.1429
122.0243

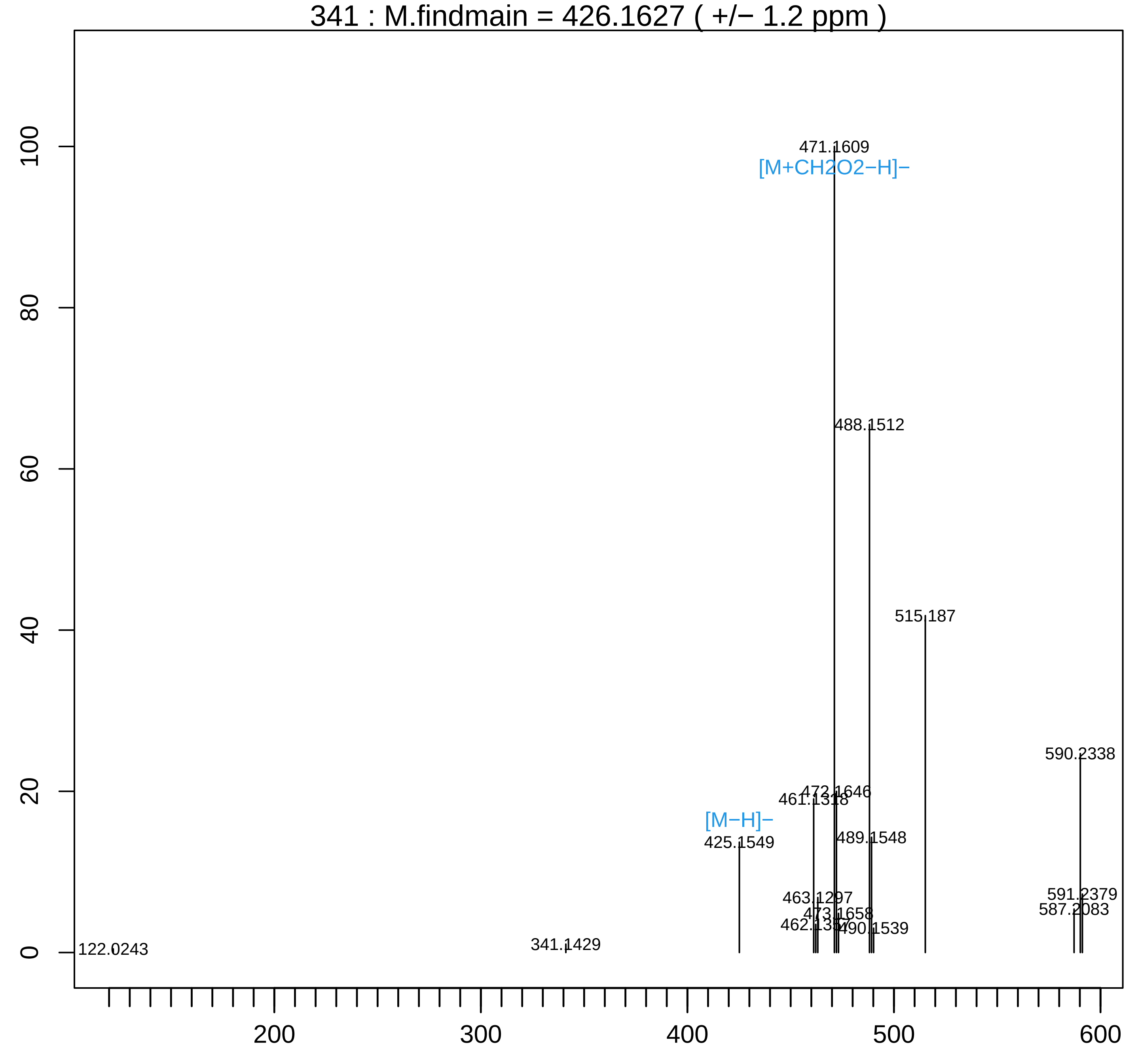
341 : M.findmain = 426.1627 (+/− 1.2 ppm)
471.1609
[M+CH2O2−H]−
488.1512
515.187
590.2338
472.1646
461.1318
[M−H]−
425.1549
489.1548
463.1297
473.1658
462.1357
490.1539
591.2379
587.2083
341.1429
122.0243

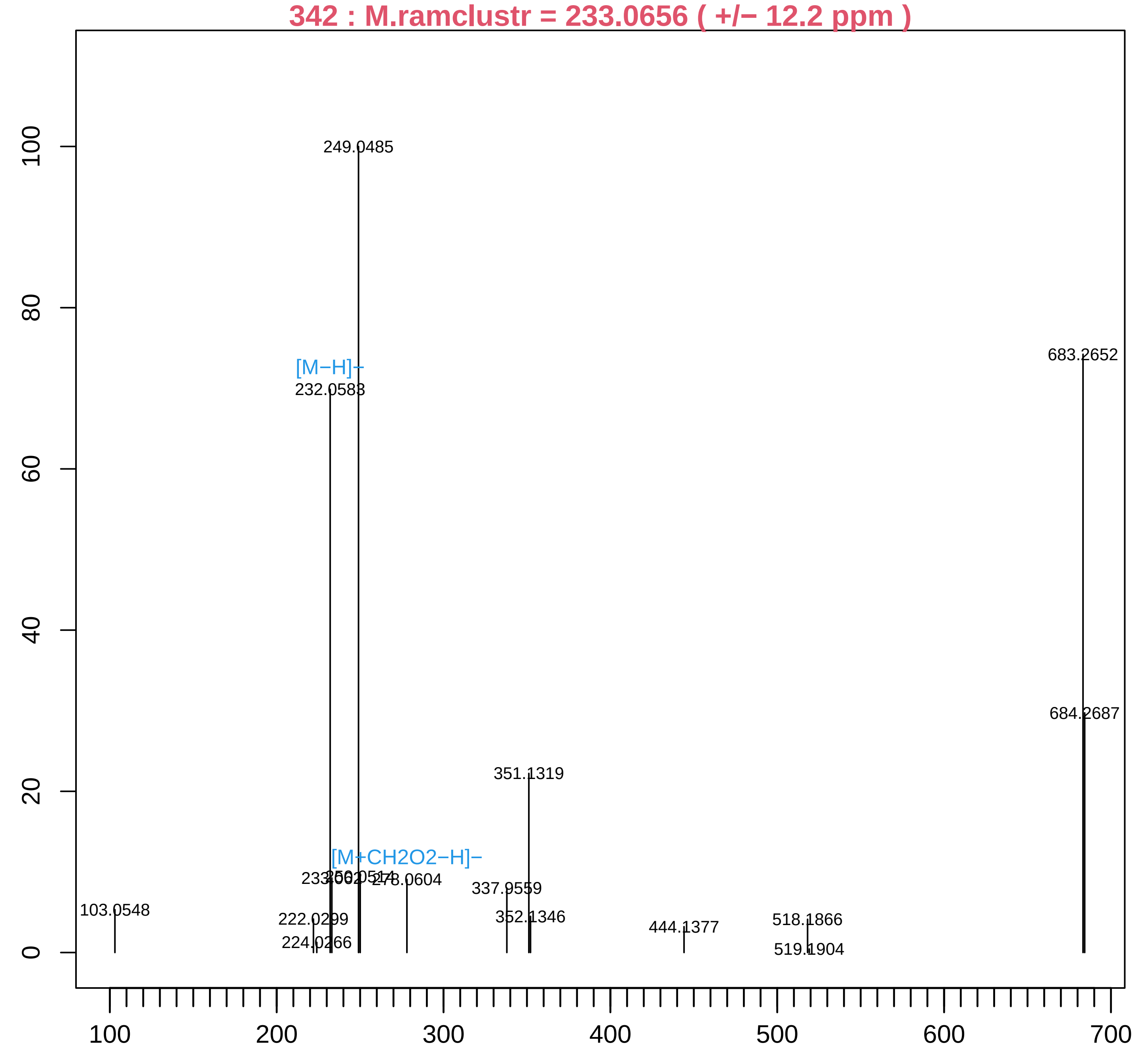
342 : M.ramclustr = 233.0656 (+/− 12.2 ppm)
249.0485
[M−H]−
232.0588
683.2652
684.2687
351.1319
[M+CH2O2−H]−
278.0604
337.9559
103.0548
222.0299
224.0266
352.1346
444.1377
518.1866
519.1904
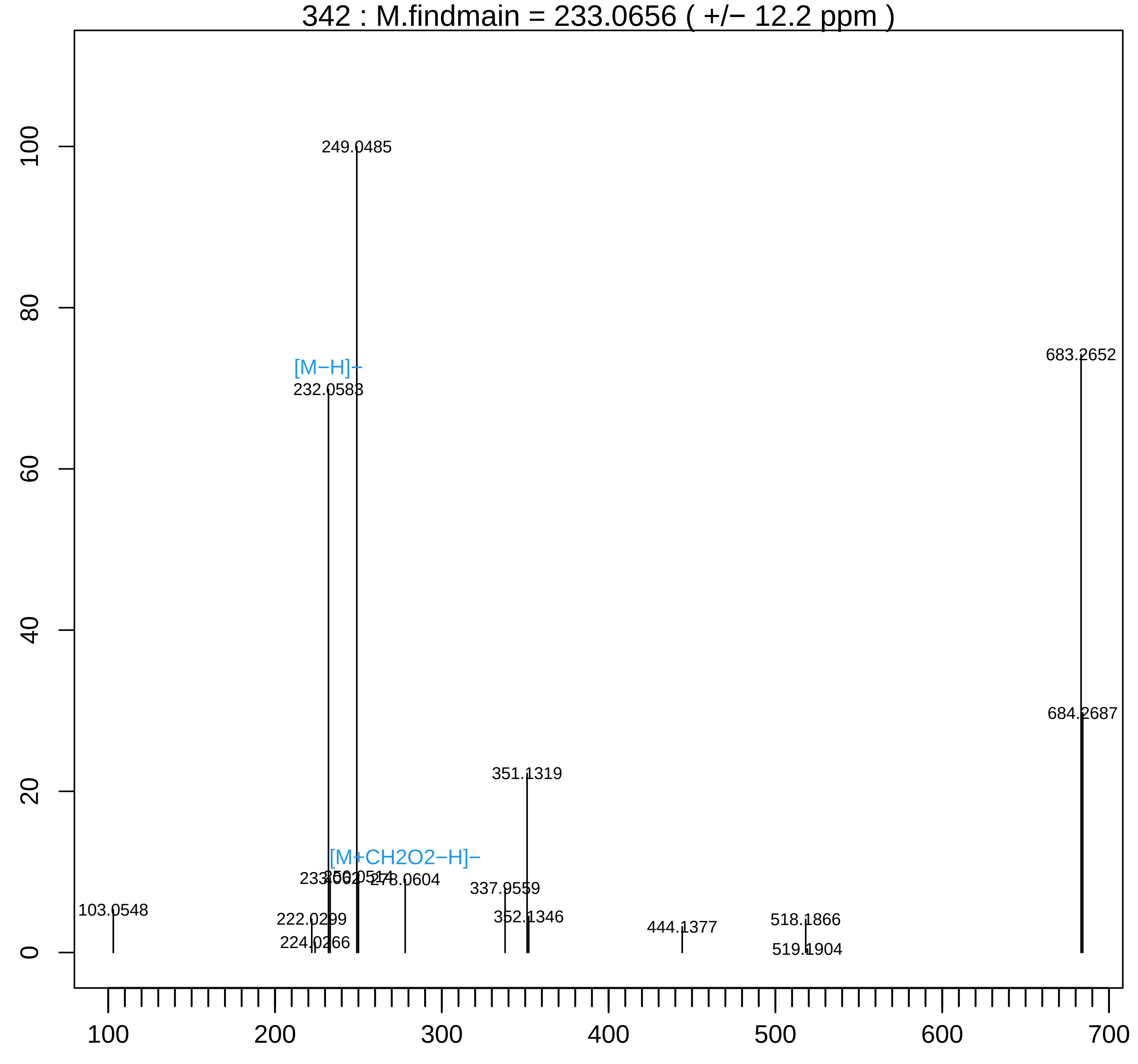
342 : M.findmain = 233.0656 (+/− 12.2 ppm)
249.0485
[M−H]−
232.0588
683.2652
684.2687
351.1319
[M+CH2O2−H]−
278.0604
337.9559
103.0548
222.0299
224.0266
352.1346
444.1377
518.1866
519.1904

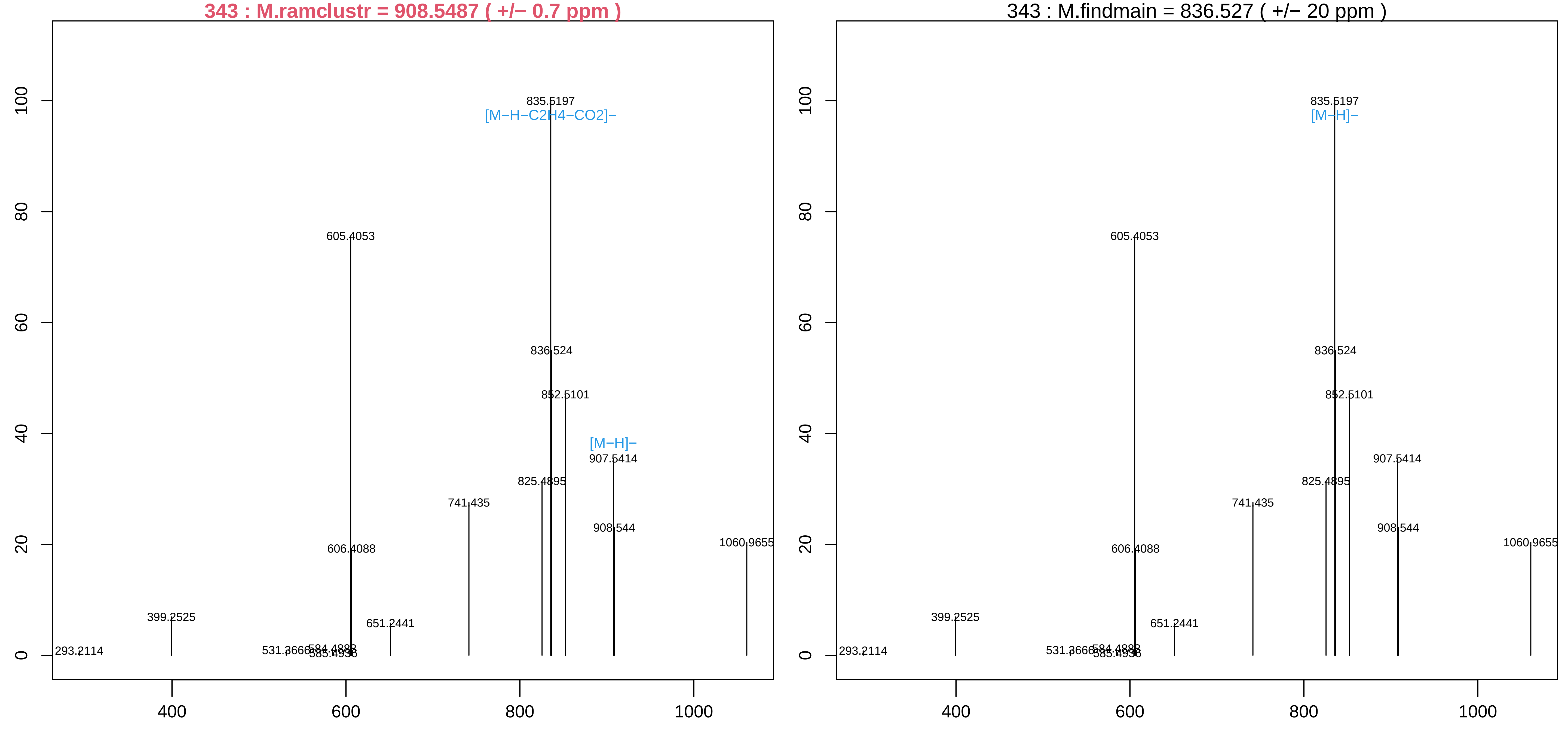
343 : M.ramclustr = 908.5487 (+/− 0.7 ppm)
835.5197
[M−H−C2H4−CO2]−
605.4053
836.524
852.5101
[M−H]−
907.5414
825.4895
741.435
908.544
606.4088
1060.9655
399.2525
651.2441
293.2114
531.3666
584.4888
585.4936
343 : M.findmain = 836.527 (+/− 20 ppm)
835.5197
[M−H]−
605.4053
836.524
852.5101
907.5414
825.4895
741.435
908.544
606.4088
1060.9655
399.2525
651.2441
293.2114
531.3666
584.4888
585.4936
0
20
40
60
80
100
400
600
800
1000

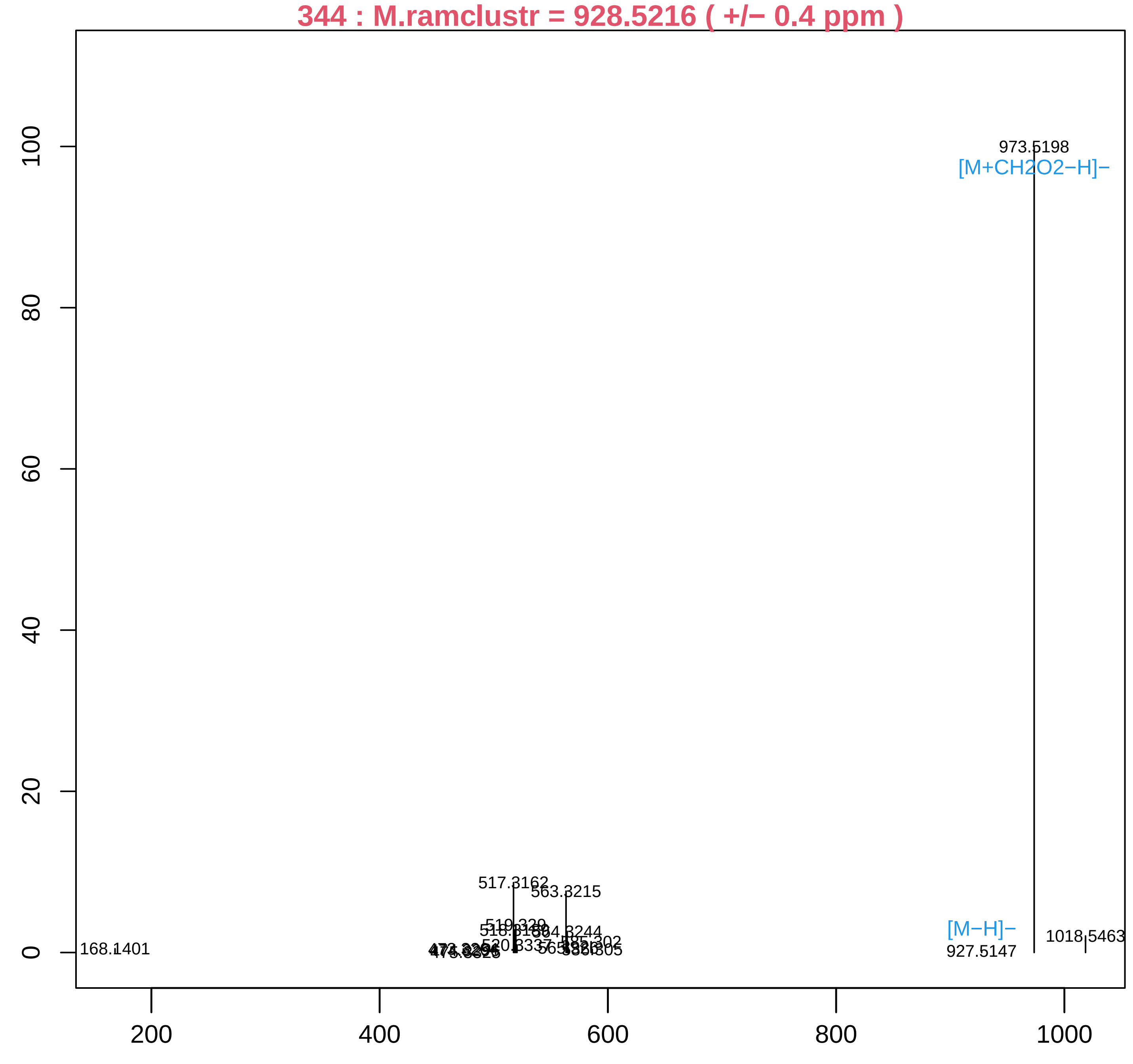
344 : M.ramclustr = 928.5216 (+/− 0.4 ppm)
973.5198
[M+CH2O2−H]−
517.3162
563.3215
[M−H]−
927.5147
1018.5463
168.1401

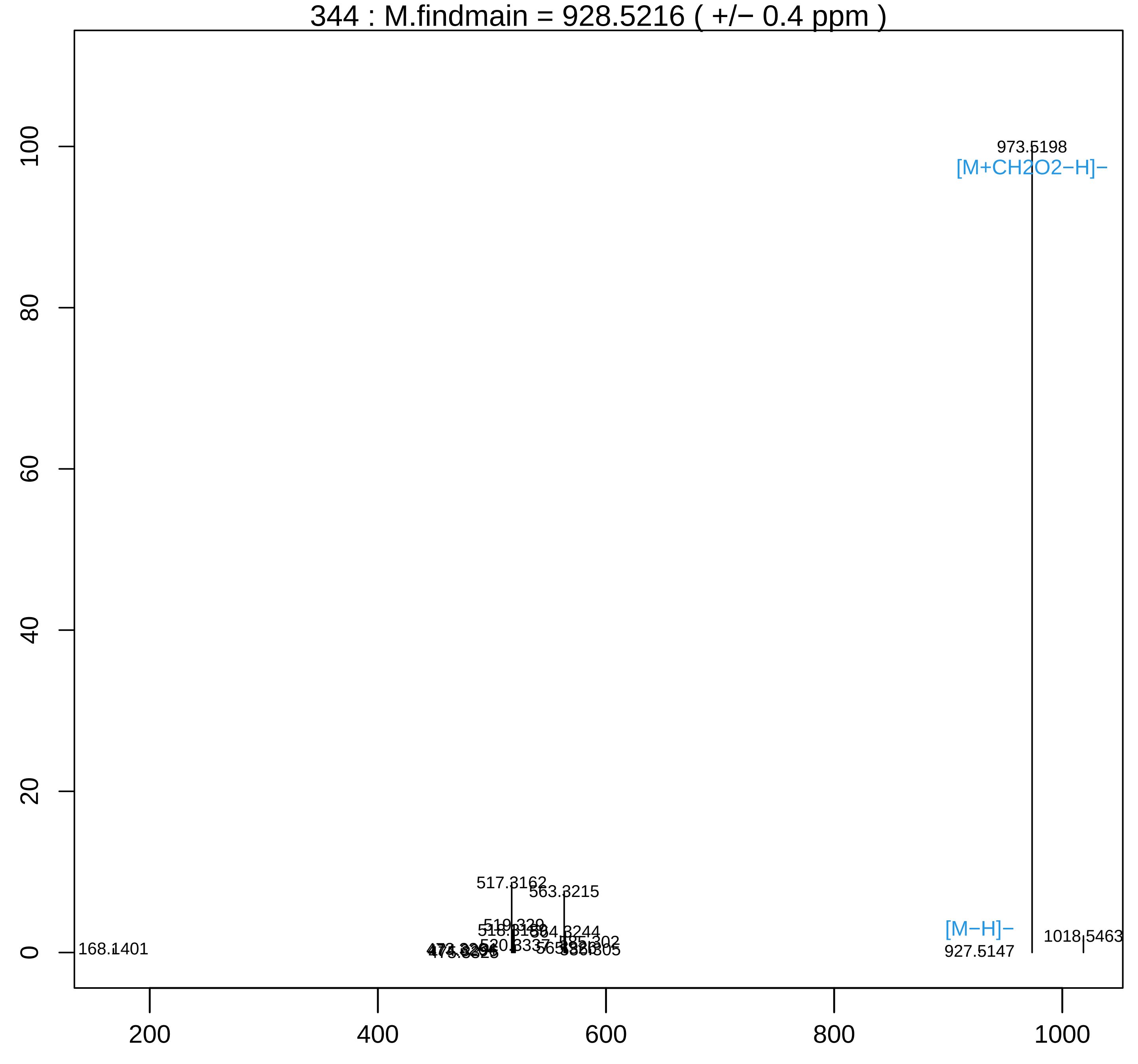
344 : M.findmain = 928.5216 (+/− 0.4 ppm)
973.5198
[M+CH2O2−H]−
517.3162
563.3215
[M−H]−
927.5147
1018.5463
168.1401

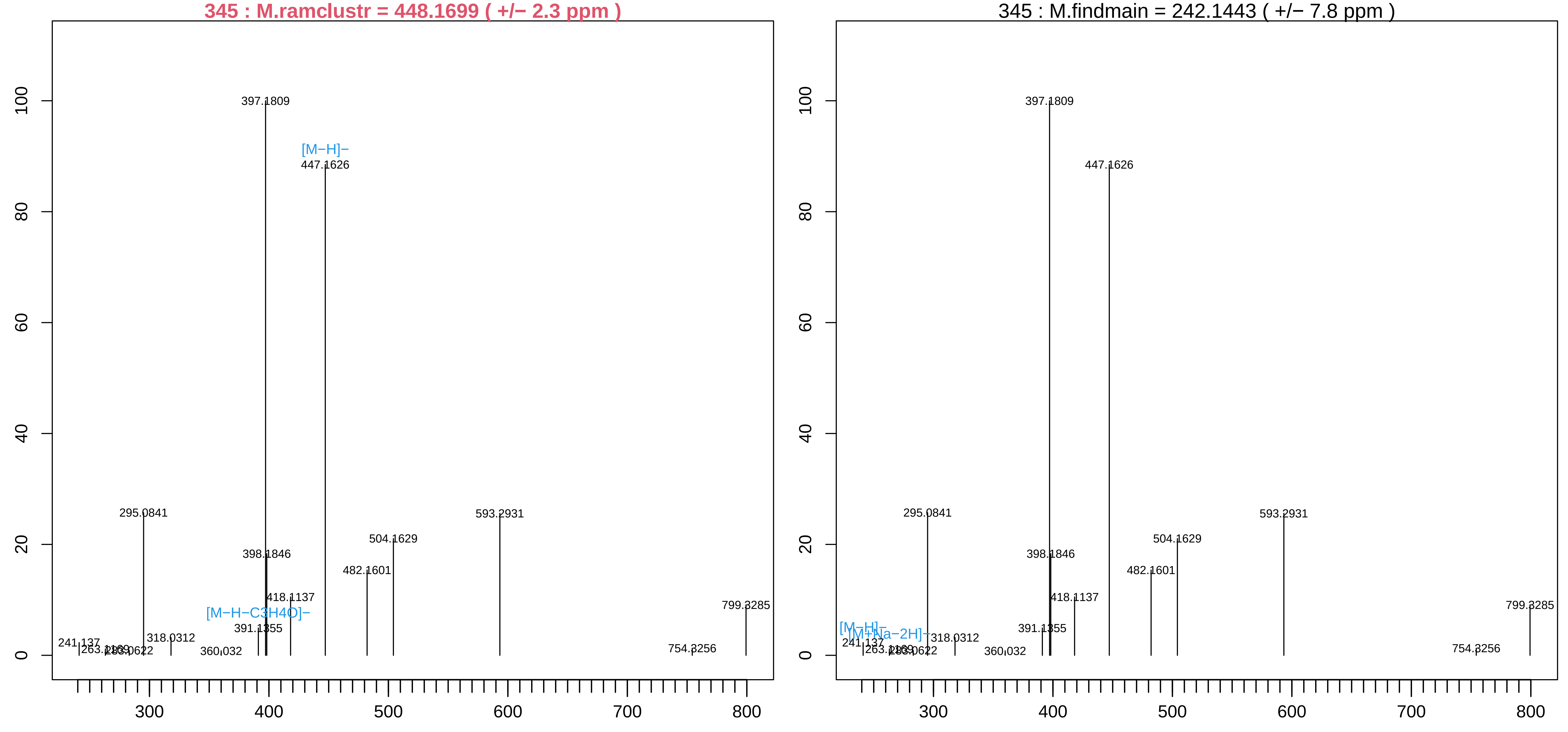
345 : M.ramclustr = 448.1699 (+/− 2.3 ppm)
397.1809
[M−H]−
447.1626
295.0841
593.2931
504.1629
398.1846
482.1601
418.1137
[M−H−C3H4O]−
391.1355
799.3285
241.137
318.0312
360.032
754.3256
345 : M.findmain = 242.1443 (+/− 7.8 ppm)
[M−H]−
[M+Na−2H]−
300
400
500
600
700
800
0
20
40
60
80
100

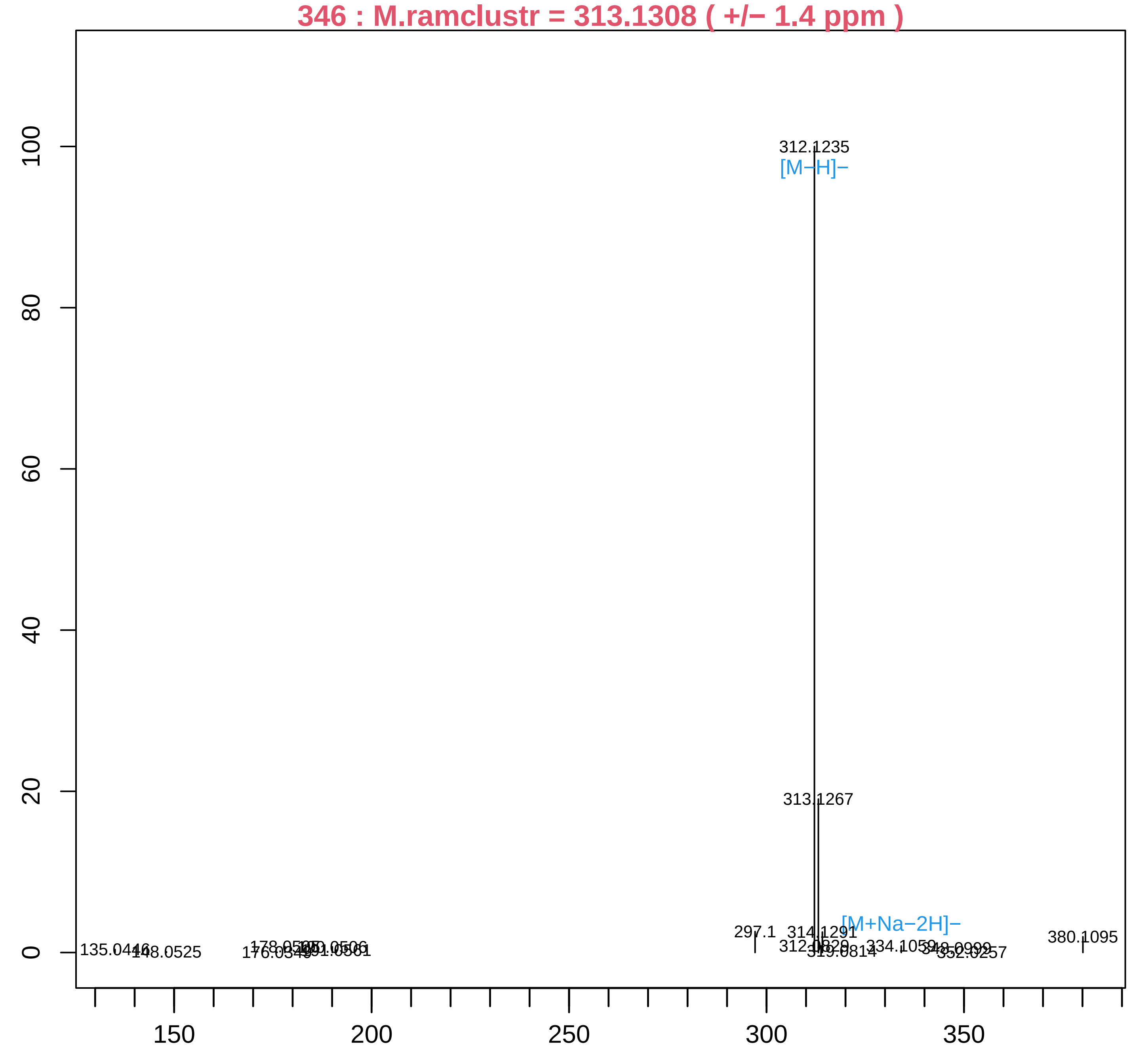
346 : M.ramclustr = 313.1308 (+/− 1.4 ppm)
312.1235
[M−H]−
313.1267
[M+Na−2H]−
297.1
314.1291
380.1095
135.0446
148.0525
176.0343
334.1059
332.0257
0
20
40
60
80
100
150
200
250
300
350

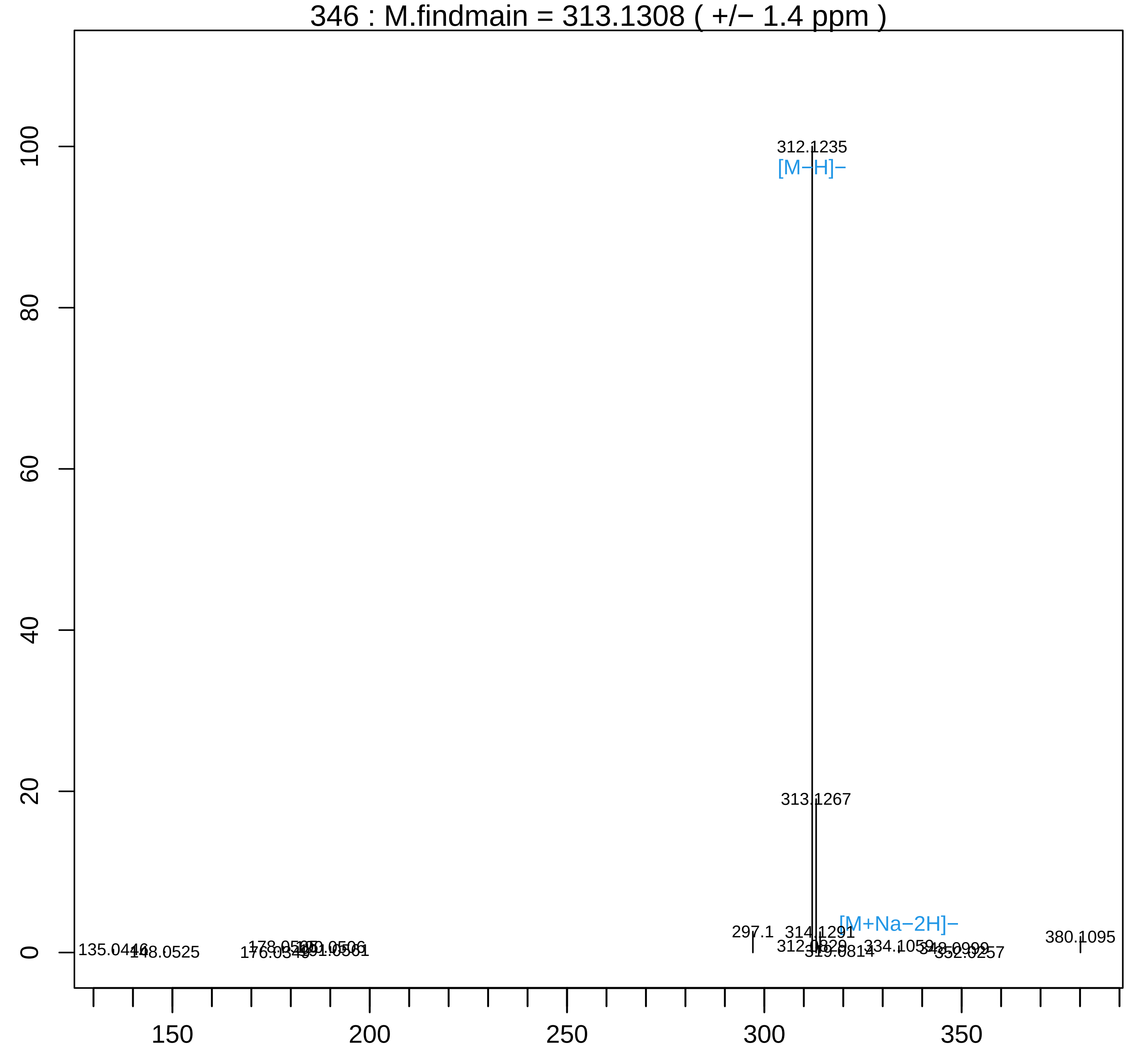
346 : M.findmain = 313.1308 (+/− 1.4 ppm)
312.1235
[M−H]−
313.1267
[M+Na−2H]−
297.1
314.1291
380.1095
135.0446
148.0525
176.0343
334.1059
332.0257
0
20
40
60
80
100
150
200
250
300
350

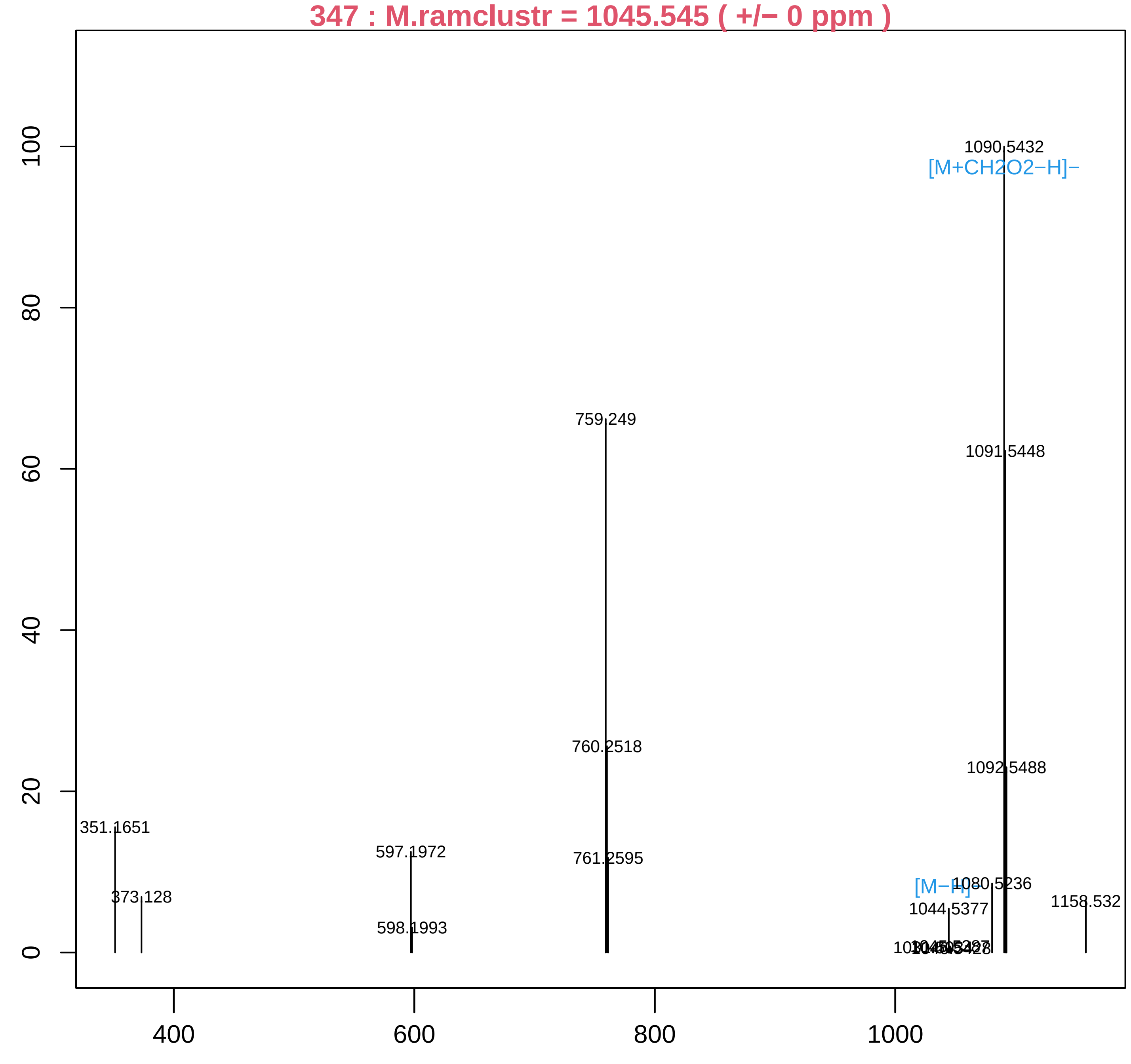

347 : M.ramclustr = 1045.545 (+/− 0 ppm)
1090.5432
[M+CH2O2−H]−
759.249
1091.5448
760.2518
1092.5488
351.1651
597.1972
761.2595
[M−H]−
1080.5236
373.128
1044.5377
1158.532
598.1993
0
20
40
60
80
100
400
600
800
1000

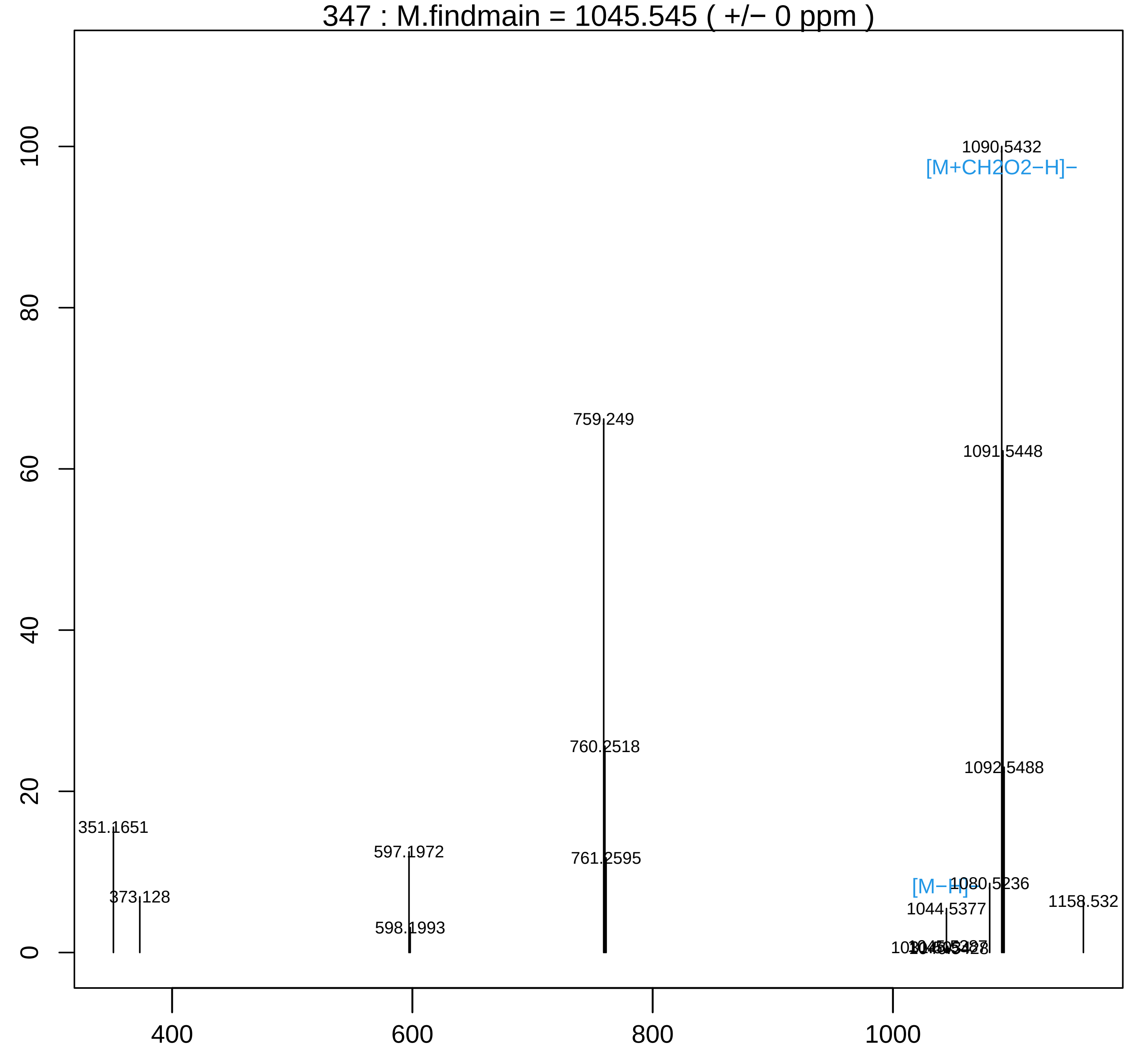

347 : M.findmain = 1045.545 (+/− 0 ppm)
1090.5432
[M+CH2O2−H]−
759.249
1091.5448
760.2518
1092.5488
351.1651
597.1972
761.2595
[M−H]−
1080.5236
373.128
1044.5377
1158.532
598.1993
0
20
40
60
80
100
400
600
800
1000

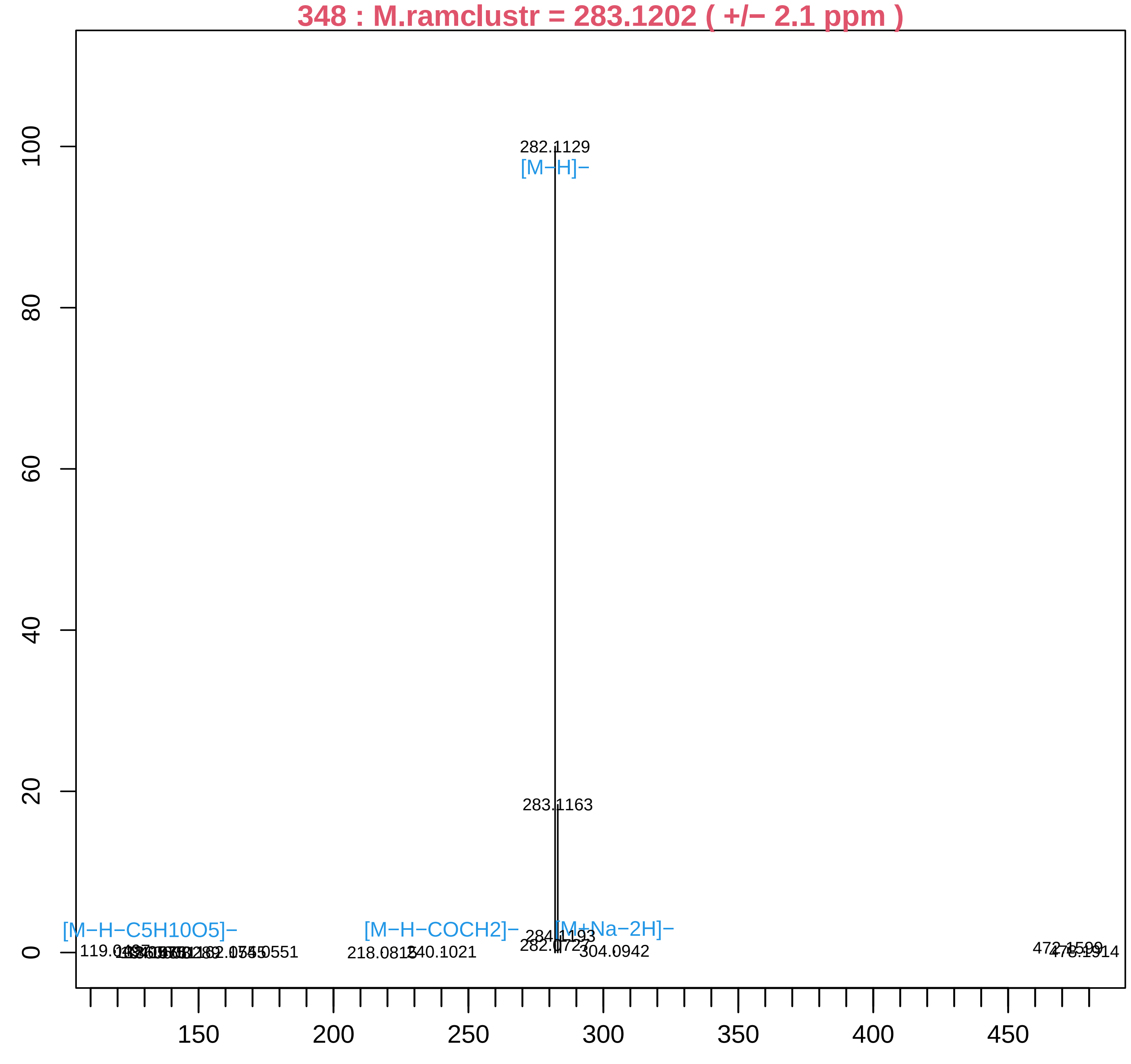

348 : M.ramclustr = 283.1202 (+/− 2.1 ppm)
282.1129
[M−H]−
283.1163
[M−H−C5H10O5]−
[M−H−COCH2]−
[M+Na−2H]−
284.1193
282.0727
304.0942
218.0812
240.1021
472.1599
478.1914
0
20
40
60
80
100
150
200
250
300
350
400
450

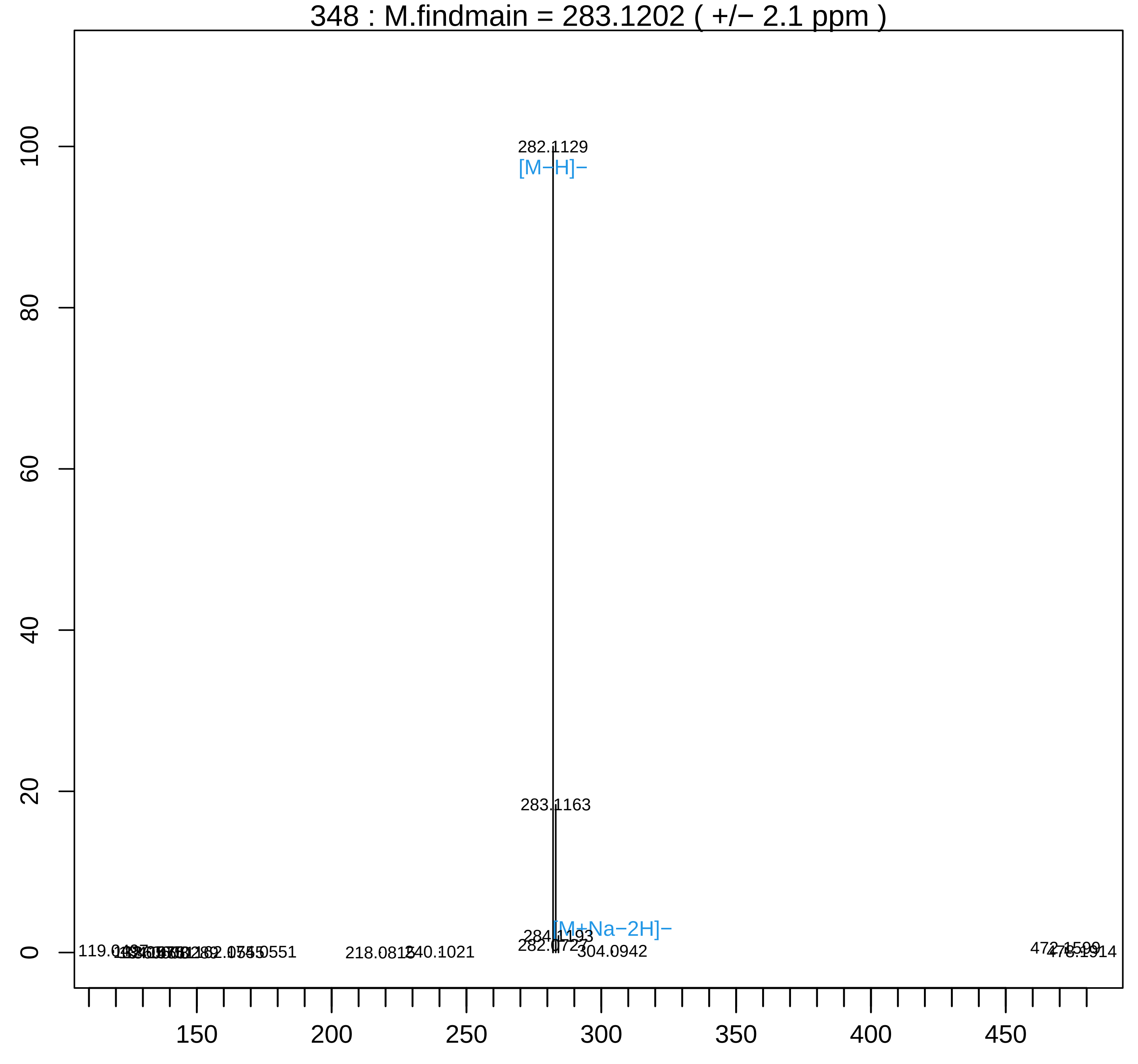

348 : M.findmain = 283.1202 (+/− 2.1 ppm)
282.1129
[M−H]−
283.1163
[M+Na−2H]−
284.1193
282.0727
304.0942
218.0812
240.1021
472.1599
478.1914
0
20
40
60
80
100
150
200
250
300
350
400
450

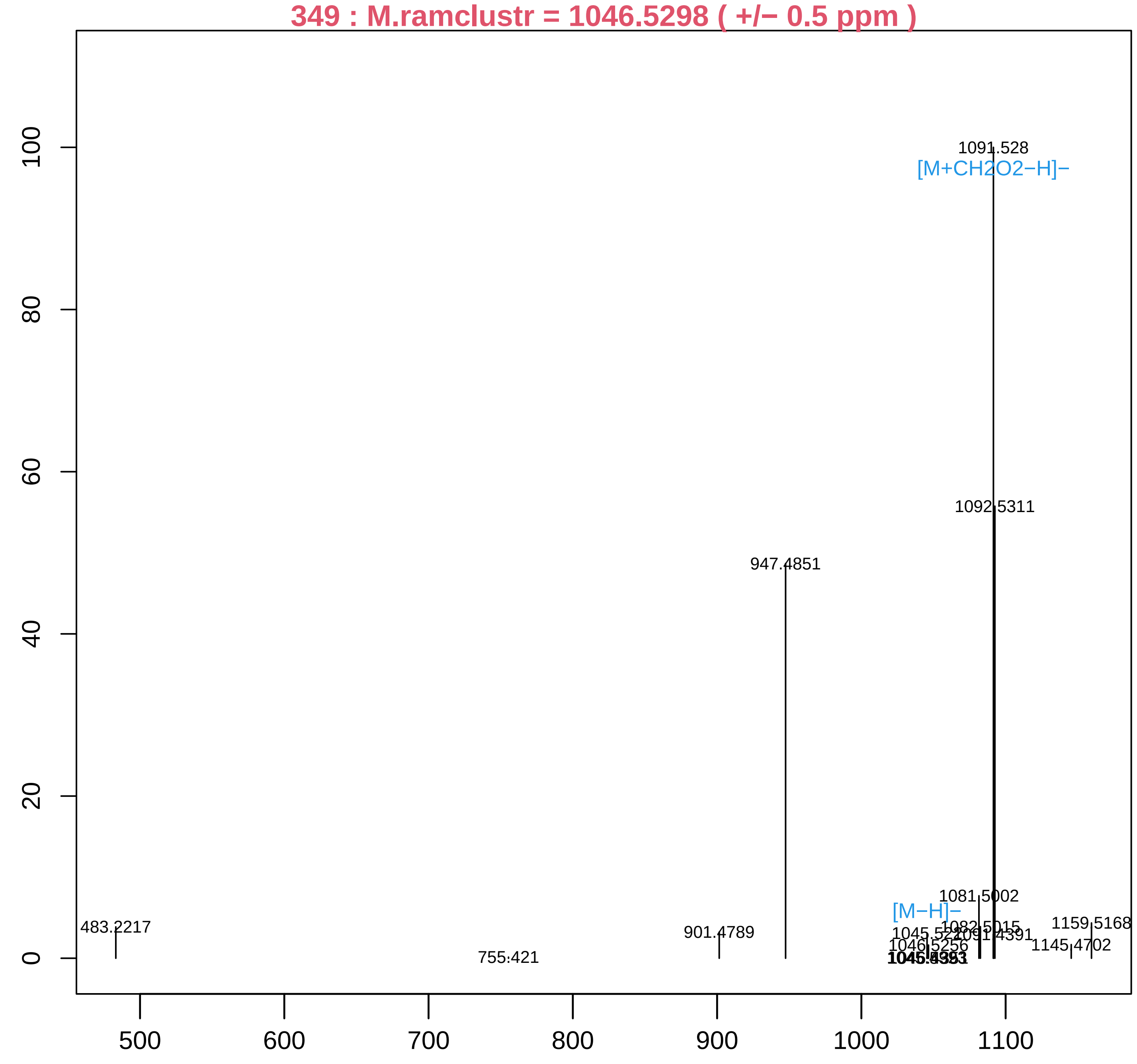
349 : M.ramclustr = 1046.5298 (+/− 0.5 ppm)
1091.528
[M+CH2O2−H]−
1092.5311
947.4851
1081.5002
[M−H]−
483.2217
901.4789
1082.5015
1159.5168
1145.4702
755.421
0
20
40
60
80
100
500
600
700
800
900
1000
1100

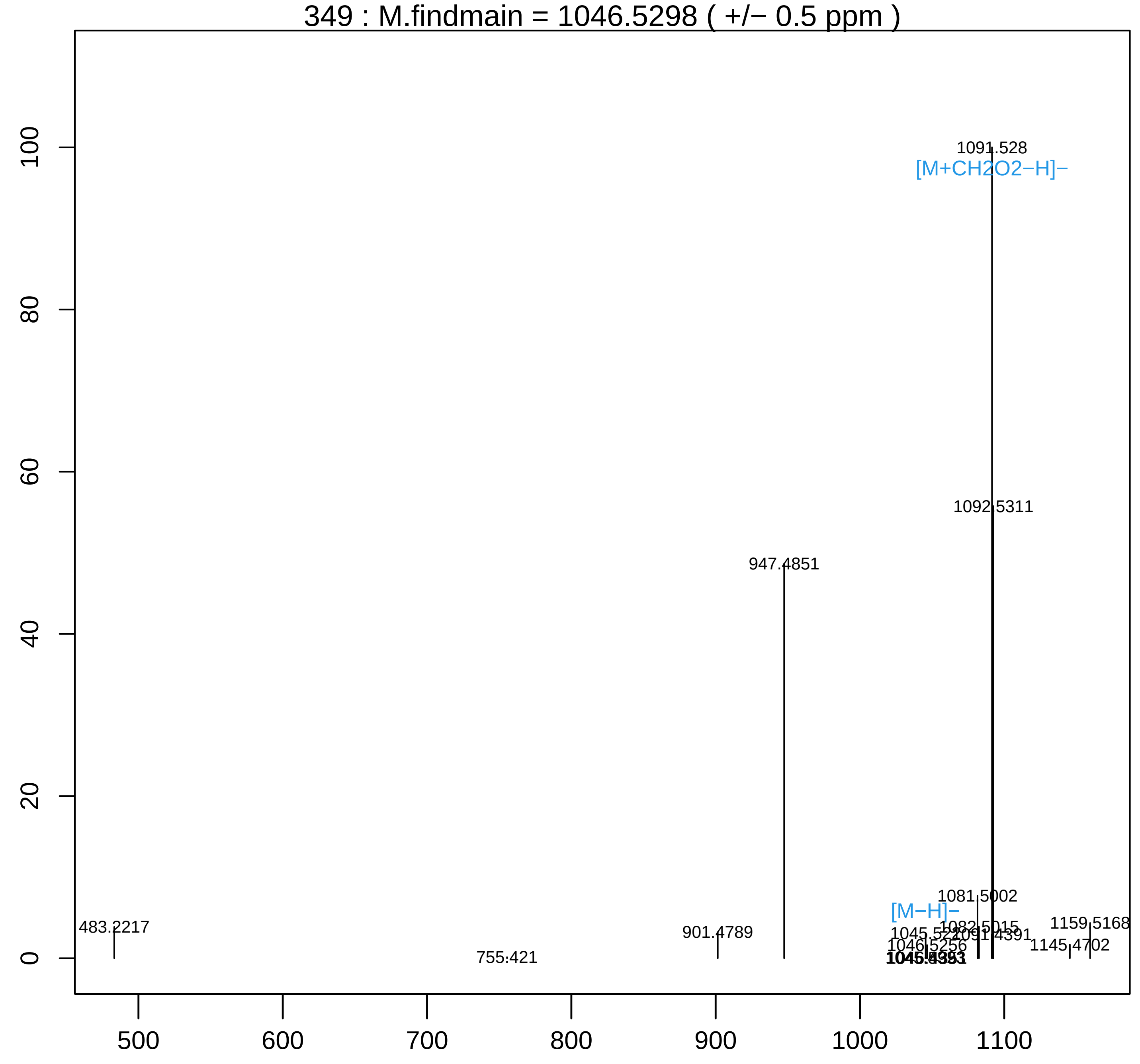
349 : M.findmain = 1046.5298 (+/− 0.5 ppm)
1091.528
[M+CH2O2−H]−
1092.5311
947.4851
1081.5002
[M−H]−
483.2217
901.4789
1082.5015
1159.5168
1145.4702
755.421
0
20
40
60
80
100
500
600
700
800
900
1000
1100

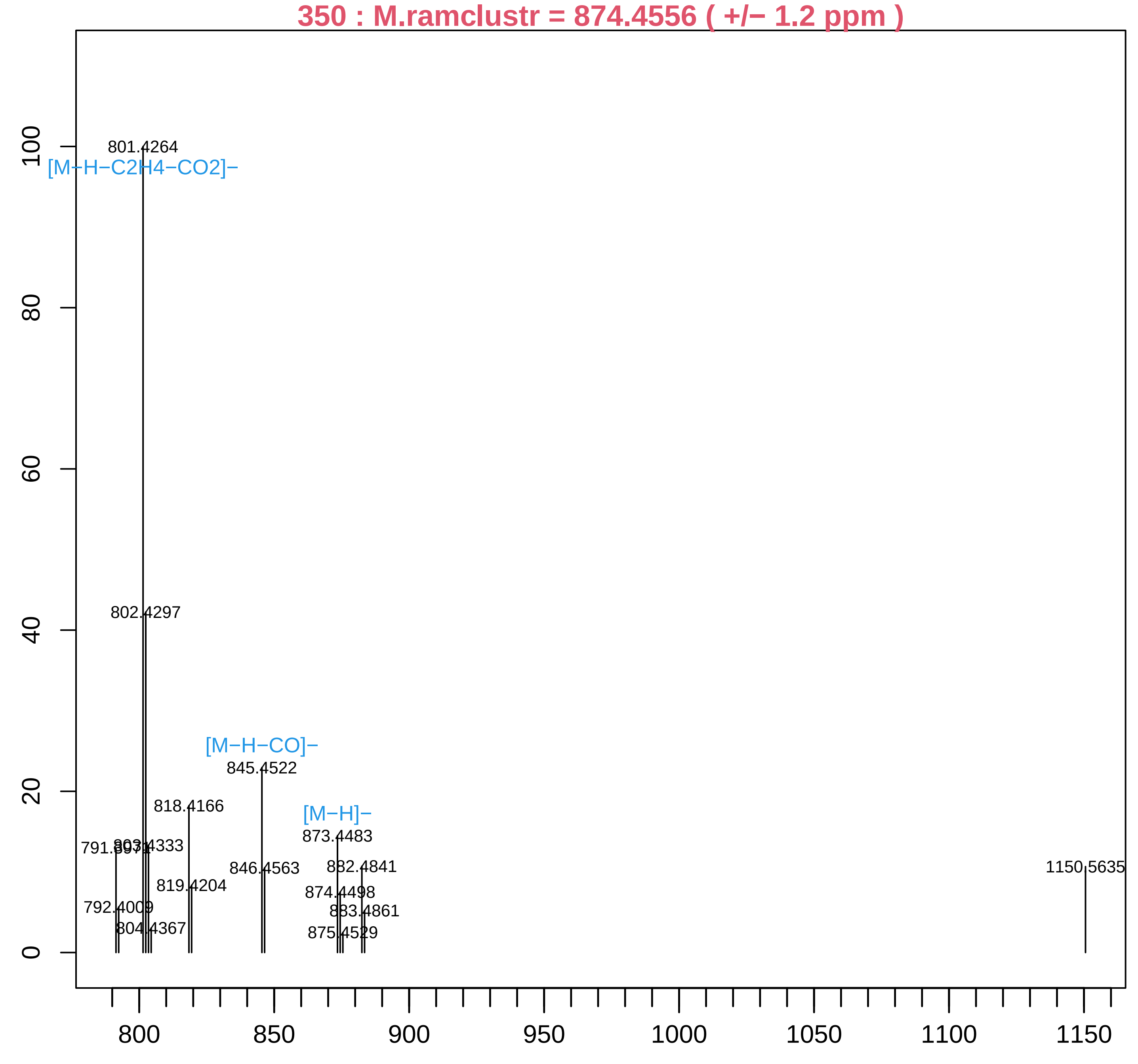
350 : M.ramclustr = 874.4556 (+/− 1.2 ppm)
801.4264
[M−H−C2H4−CO2]−
802.4297
[M−H−CO]−
845.4522
818.4166
[M−H]−
873.4483
791.3971
803.4333
846.4563
882.4841
819.4204
874.4498
792.4009
883.4861
804.4367
875.4529
1150.5635
0
20
40
60
80
100
800
850
900
950
1000
1050
1100
1150

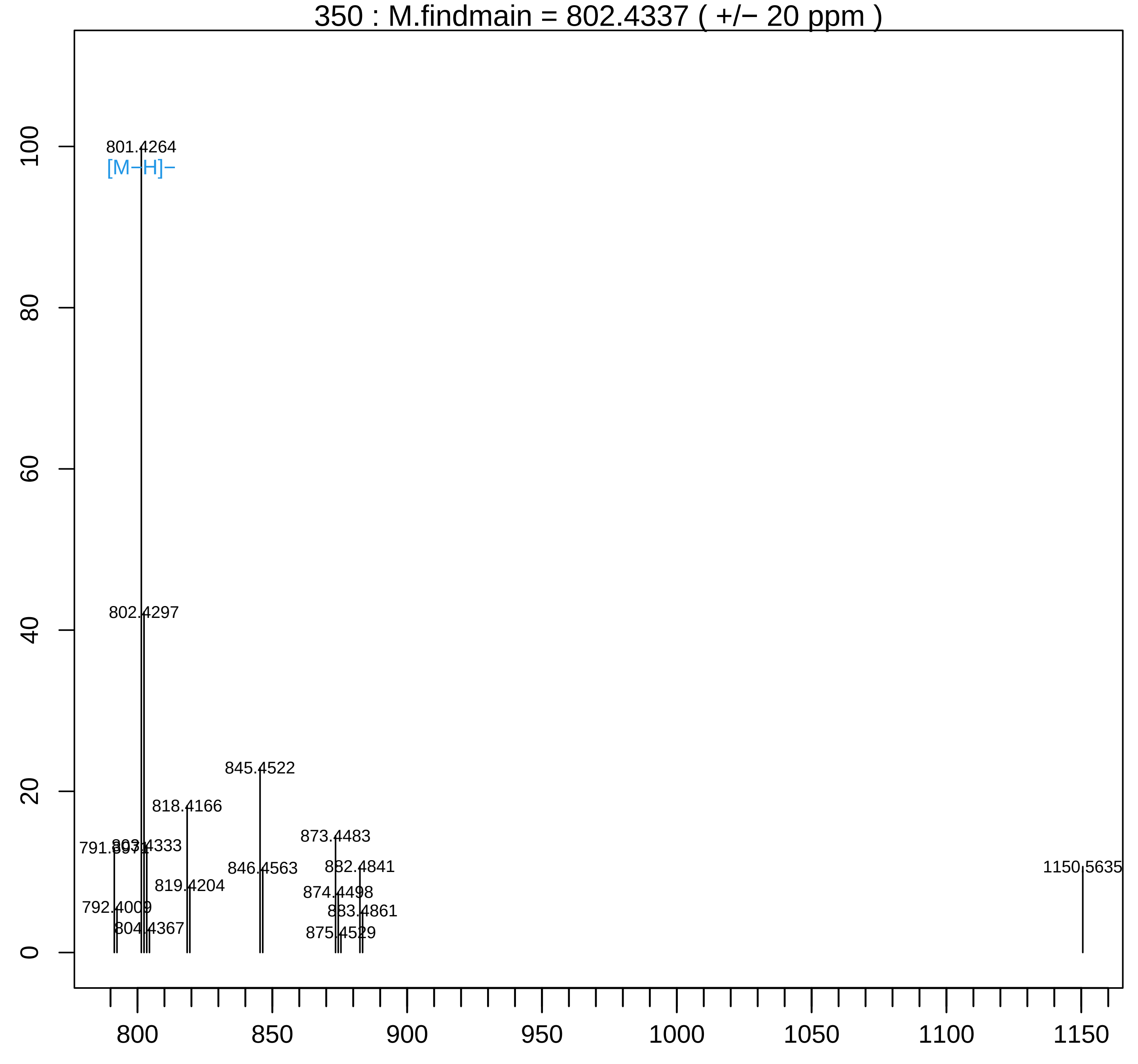
350 : M.findmain = 802.4337 (+/− 20 ppm)
801.4264
[M−H]−
802.4297
845.4522
818.4166
873.4483
791.3971
803.4333
846.4563
882.4841
819.4204
874.4498
792.4009
883.4861
804.4367
875.4529
1150.5635
0
20
40
60
80
100
800
850
900
950
1000
1050
1100
1150

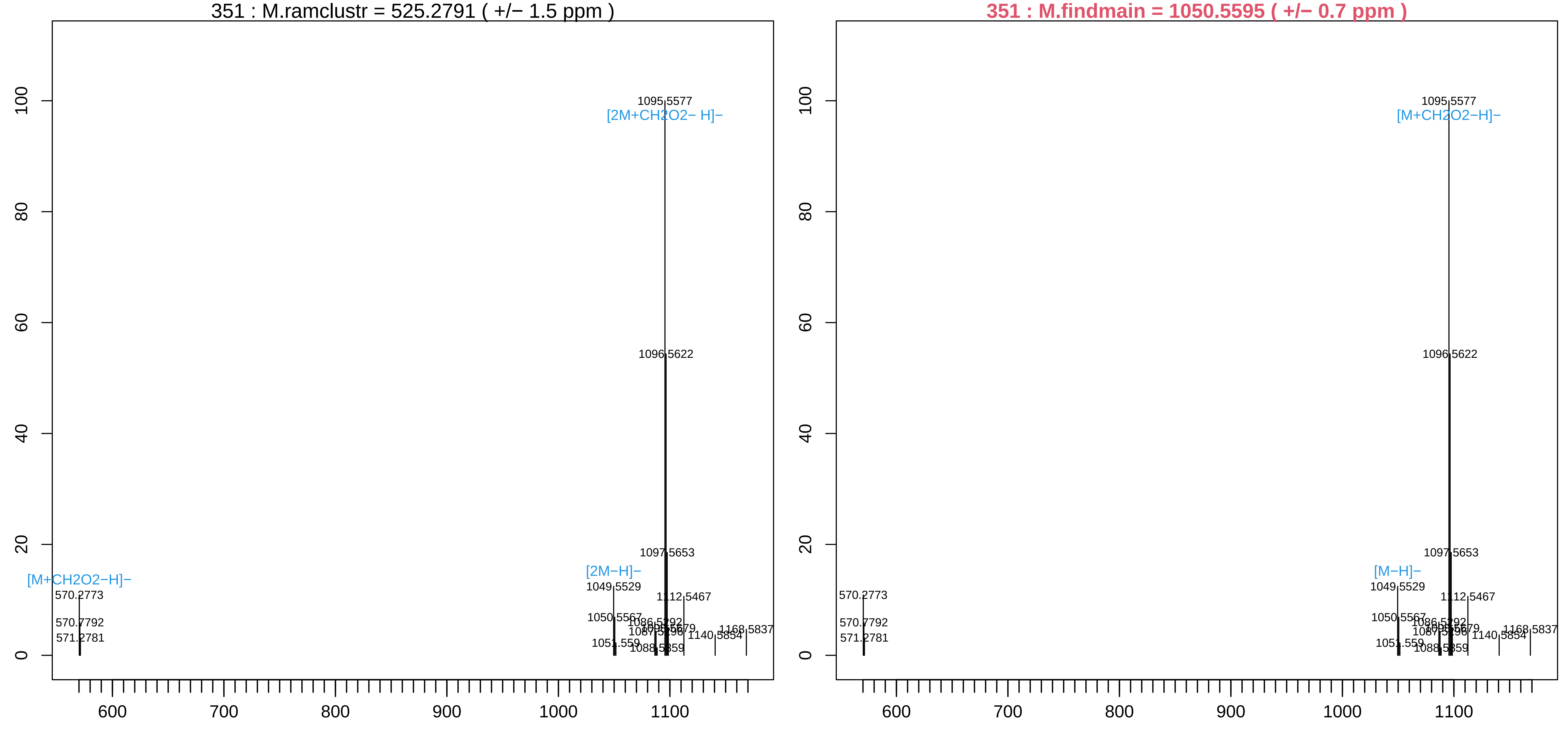
351 : M.ramclustr = 525.2791 (+/− 1.5 ppm)
[2M+CH2O2− H]−
[M+CH2O2−H]−
[2M−H]−
1095.5577
1096.5622
1097.5653
1112.5467
1049.5529
1050.5567
1051.559
1086.5292
1087.5196
1088.5359
1140.5854
1168.5837
570.2773
570.7792
571.2781
351 : M.findmain = 1050.5595 (+/− 0.7 ppm)
[M+CH2O2−H]−
[M−H]−
0
20
40
60
80
100
600
700
800
900
1000
1100

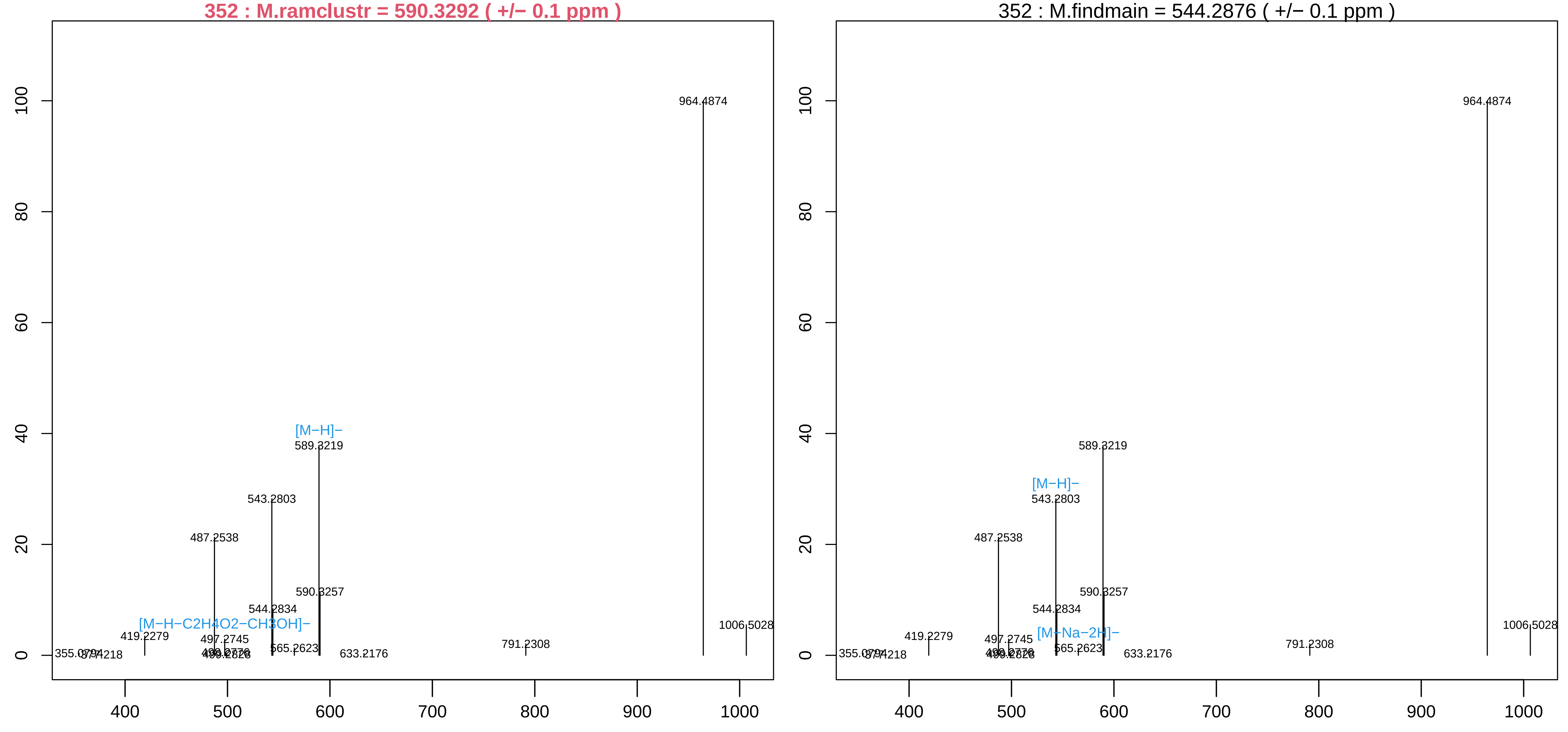
352 : M.ramclustr = 590.3292 (+/− 0.1 ppm)
964.4874
[M−H]−
589.3219
543.2803
487.2538
590.3257
544.2834
[M−H−C2H4O2−CH3OH]−
1006.5028
419.2279
497.2745
565.2623
791.2308
633.2176
352 : M.findmain = 544.2876 (+/− 0.1 ppm)
964.4874
589.3219
[M−H]−
543.2803
487.2538
590.3257
544.2834
[M+Na−2H]−
1006.5028
419.2279
497.2745
565.2623
791.2308
633.2176
0
20
40
60
80
100
400
500
600
700
800
900
1000

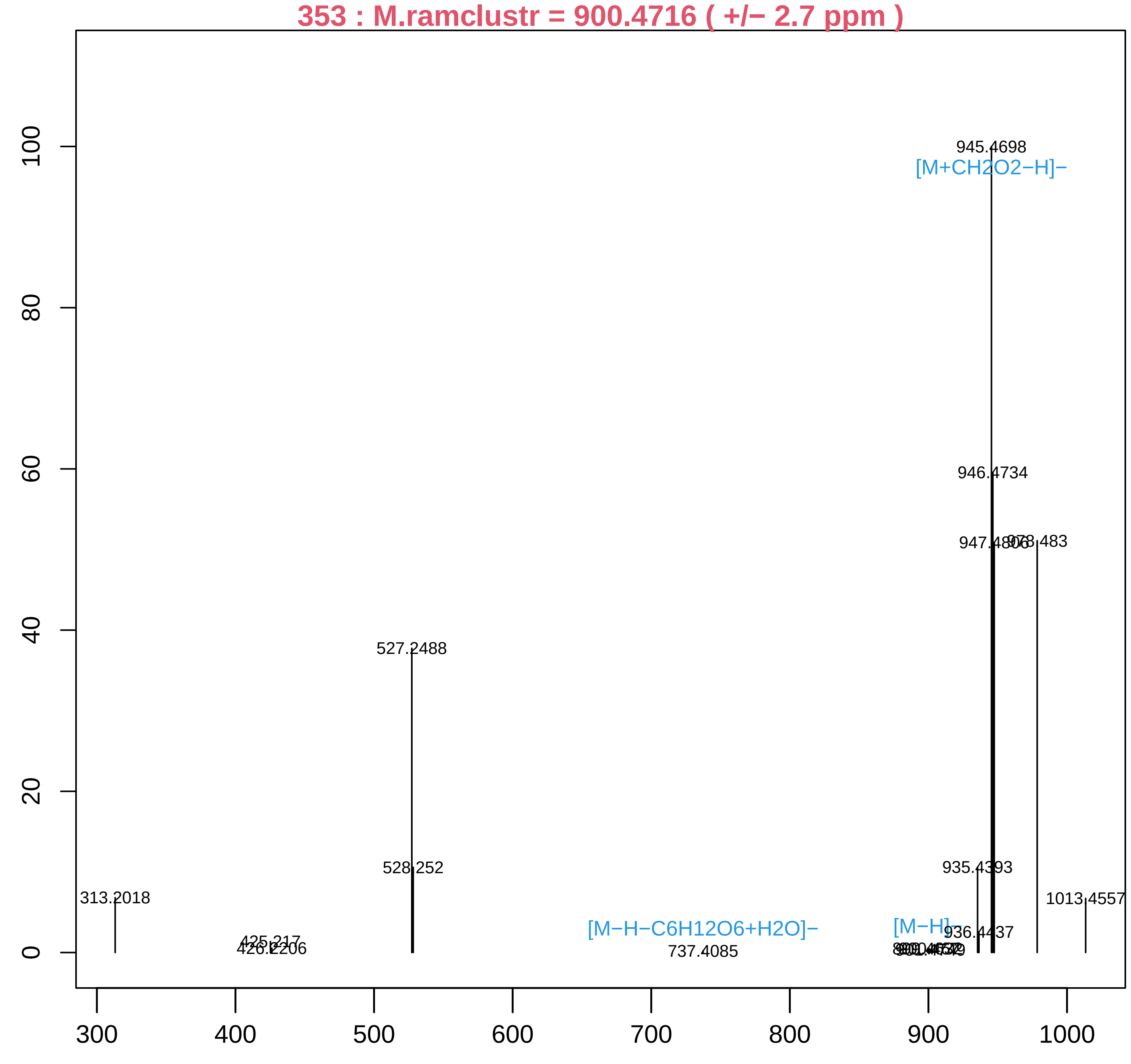
353 : M.ramclustr = 900.4716 (+/− 2.7 ppm)
945.4698
[M+CH2O2−H]−
946.4734
947.4806
978.483
527.2488
528.252
935.4393
313.2018
1013.4557
425.217
426.2206
[M−H−C6H12O6+H2O]−
737.4085
[M−H]−
936.4437
0
20
40
60
80
100
300
400
500
600
700
800
900
1000

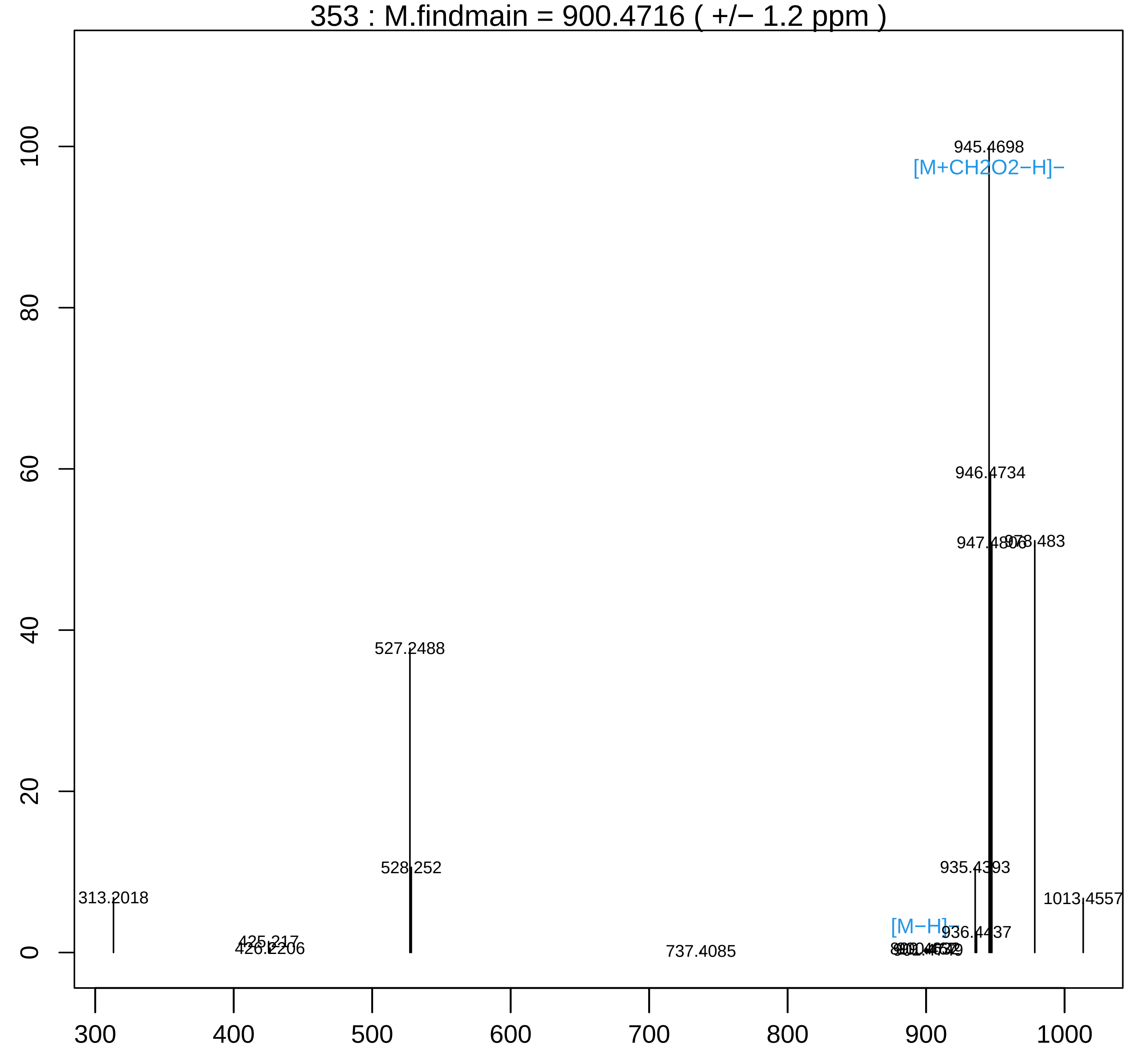
353 : M.findmain = 900.4716 (+/− 1.2 ppm)
945.4698
[M+CH2O2−H]−
946.4734
947.4806
978.483
527.2488
528.252
935.4393
313.2018
1013.4557
425.217
426.2206
737.4085
[M−H]−
936.4437
0
20
40
60
80
100
300
400
500
600
700
800
900
1000

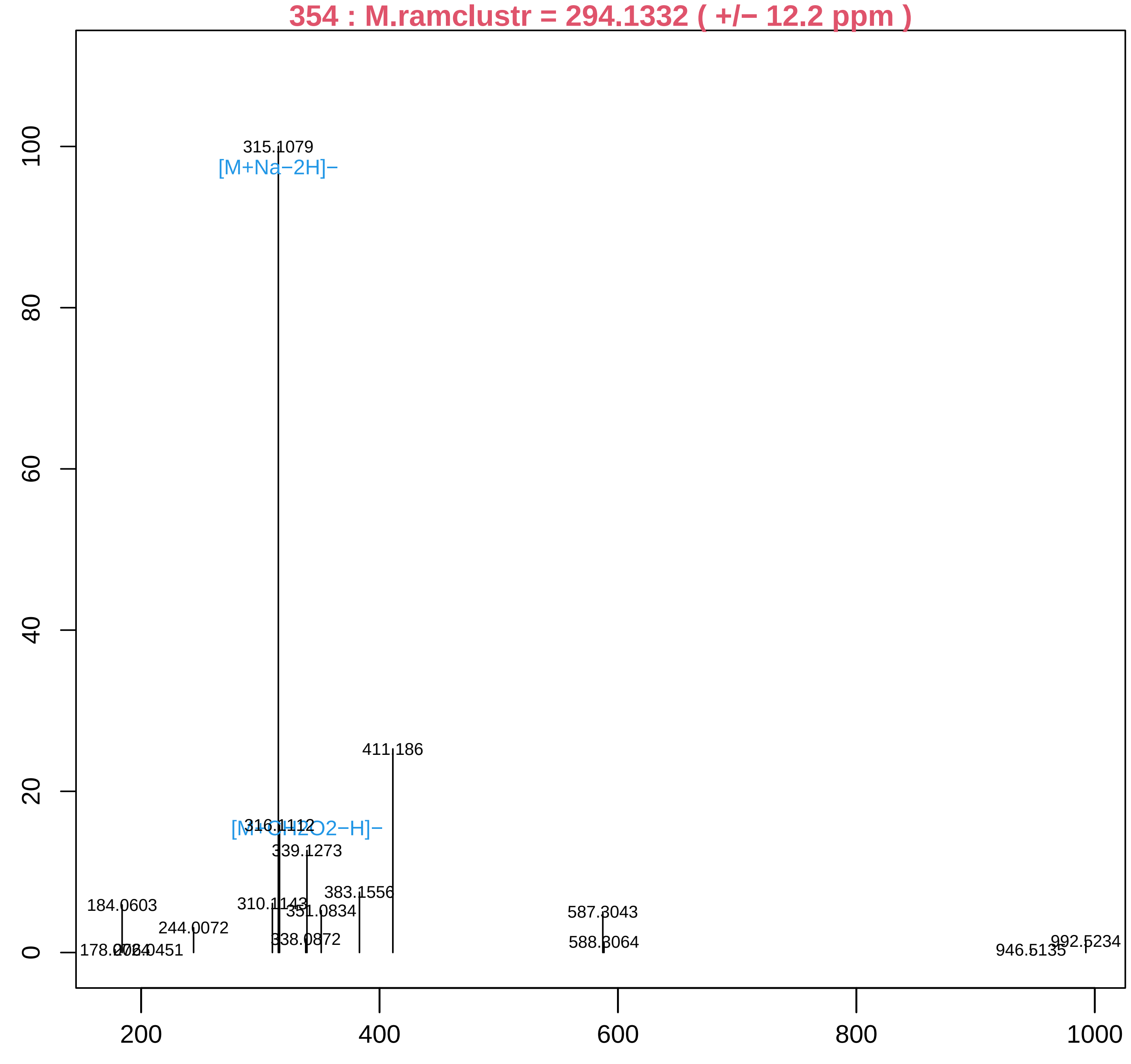
354 : M.ramclustr = 294.1332 (+/− 12.2 ppm)
315.1079
[M+Na−2H]−
411.186
316.1112
[M+CH2O2−H]−
339.1273
383.1556
184.0603
310.1143
351.0834
587.3043
244.0072
338.0872
588.3064
992.5234
178.0724
204.0451
946.5135
0
20
40
60
80
100
200
400
600
800
1000

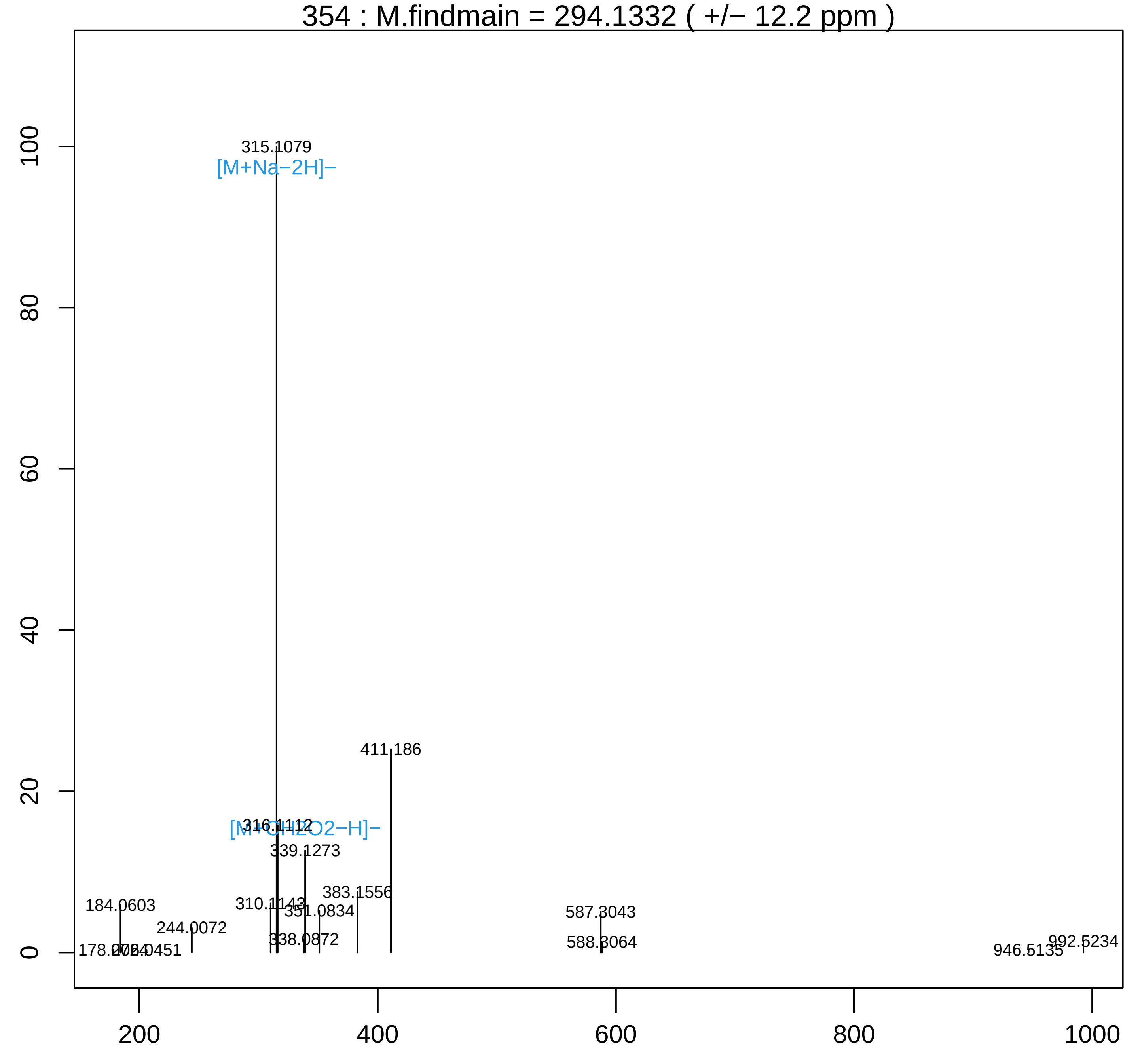
354 : M.findmain = 294.1332 (+/− 12.2 ppm)
315.1079
[M+Na−2H]−
411.186
316.1112
[M+CH2O2−H]−
339.1273
383.1556
184.0603
310.1143
351.0834
587.3043
244.0072
338.0872
588.3064
992.5234
178.0724
204.0451
946.5135
0
20
40
60
80
100
200
400
600
800
1000

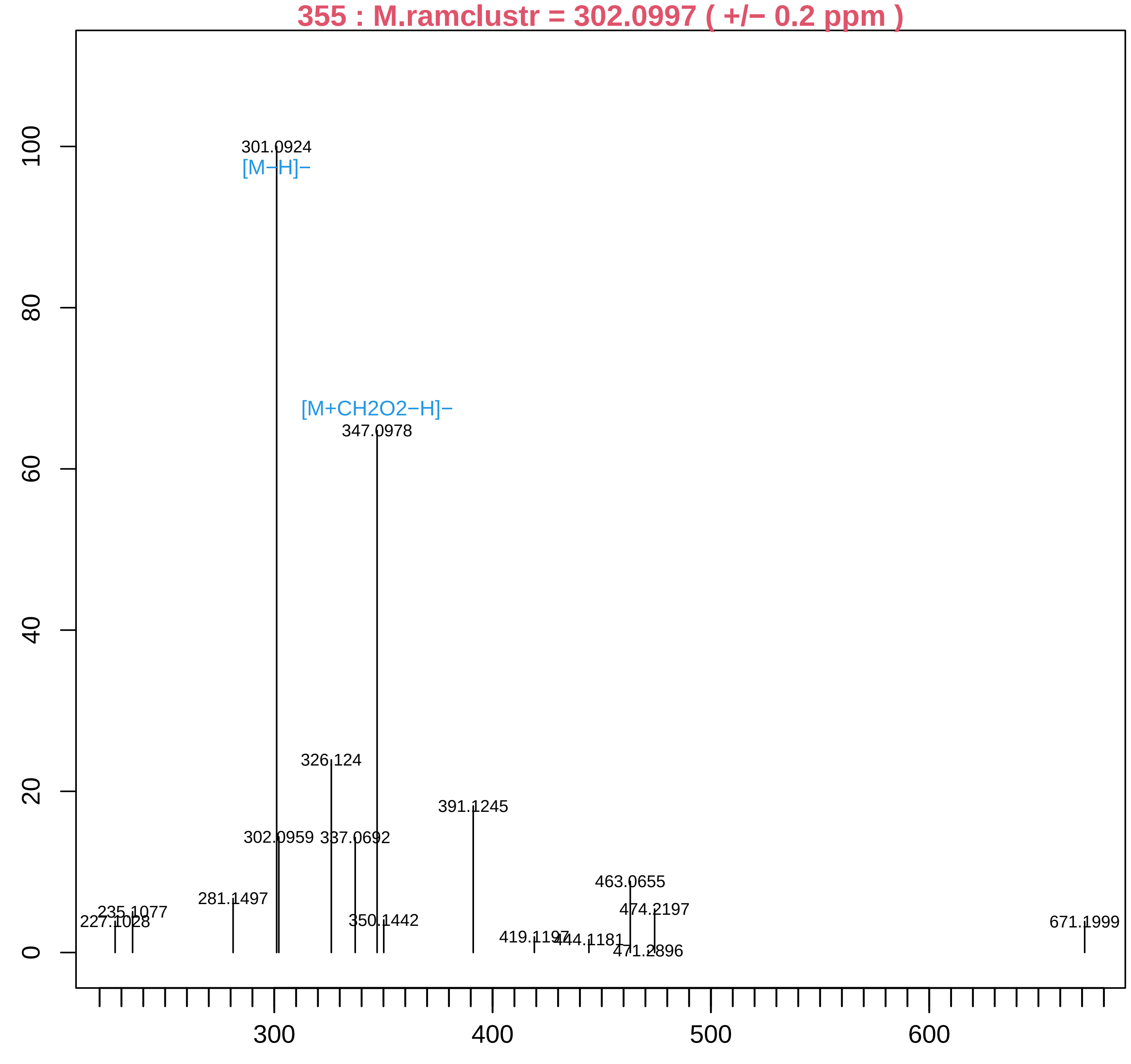
355 : M.ramclustr = 302.0997 (+/− 0.2 ppm)
301.0924
[M−H]−
[M+CH2O2−H]−
347.0978
326.124
391.1245
302.0959
337.0692
463.0655
474.2197
281.1497
235.1077
227.1028
350.1442
419.1197
444.1181
471.2896
671.1999
0
20
40
60
80
100
300
400
500
600

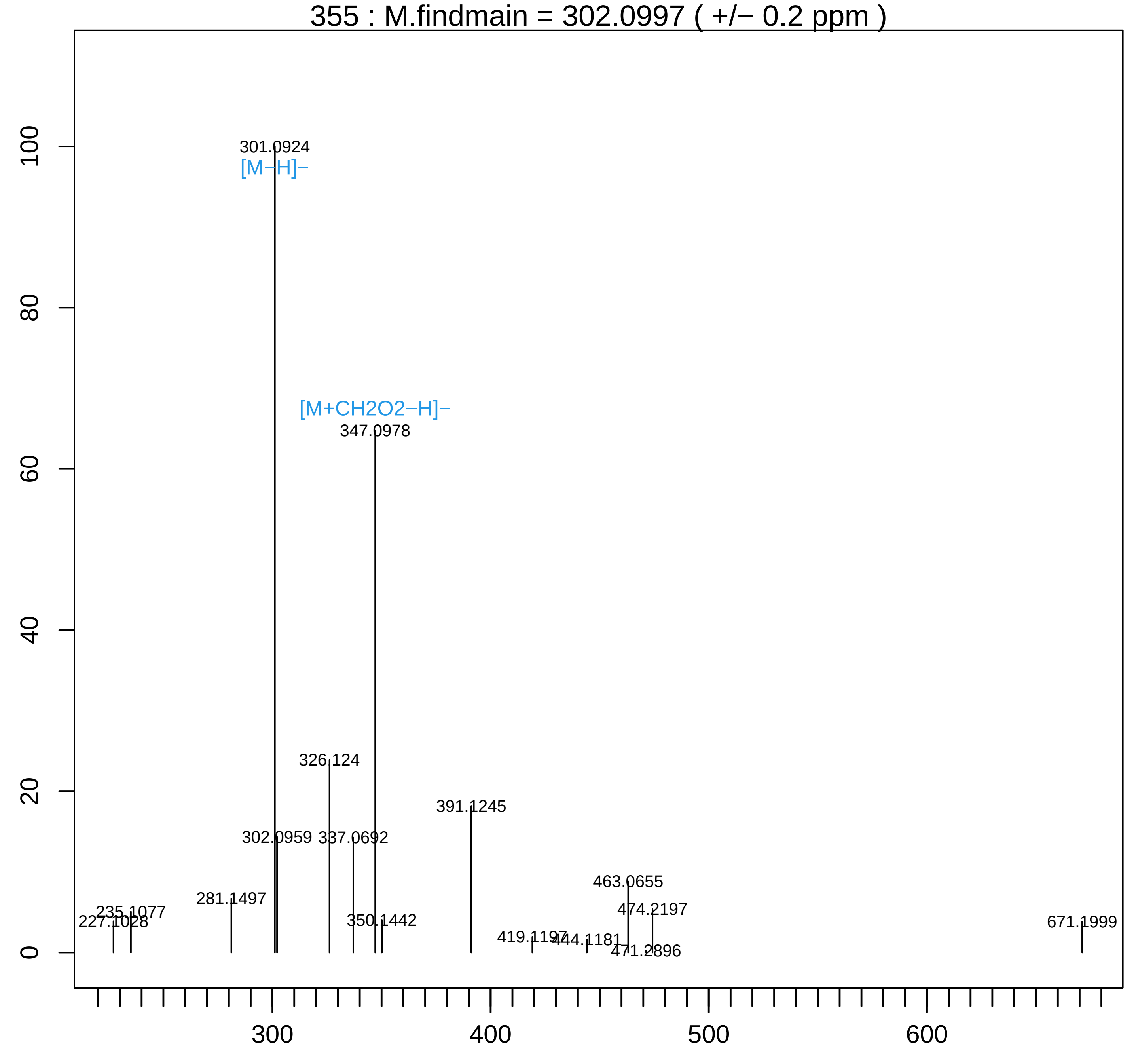
355 : M.findmain = 302.0997 (+/− 0.2 ppm)
301.0924
[M−H]−
[M+CH2O2−H]−
347.0978
326.124
391.1245
302.0959
337.0692
463.0655
474.2197
281.1497
235.1077
227.1028
350.1442
419.1197
444.1181
471.2896
671.1999
0
20
40
60
80
100
300
400
500
600

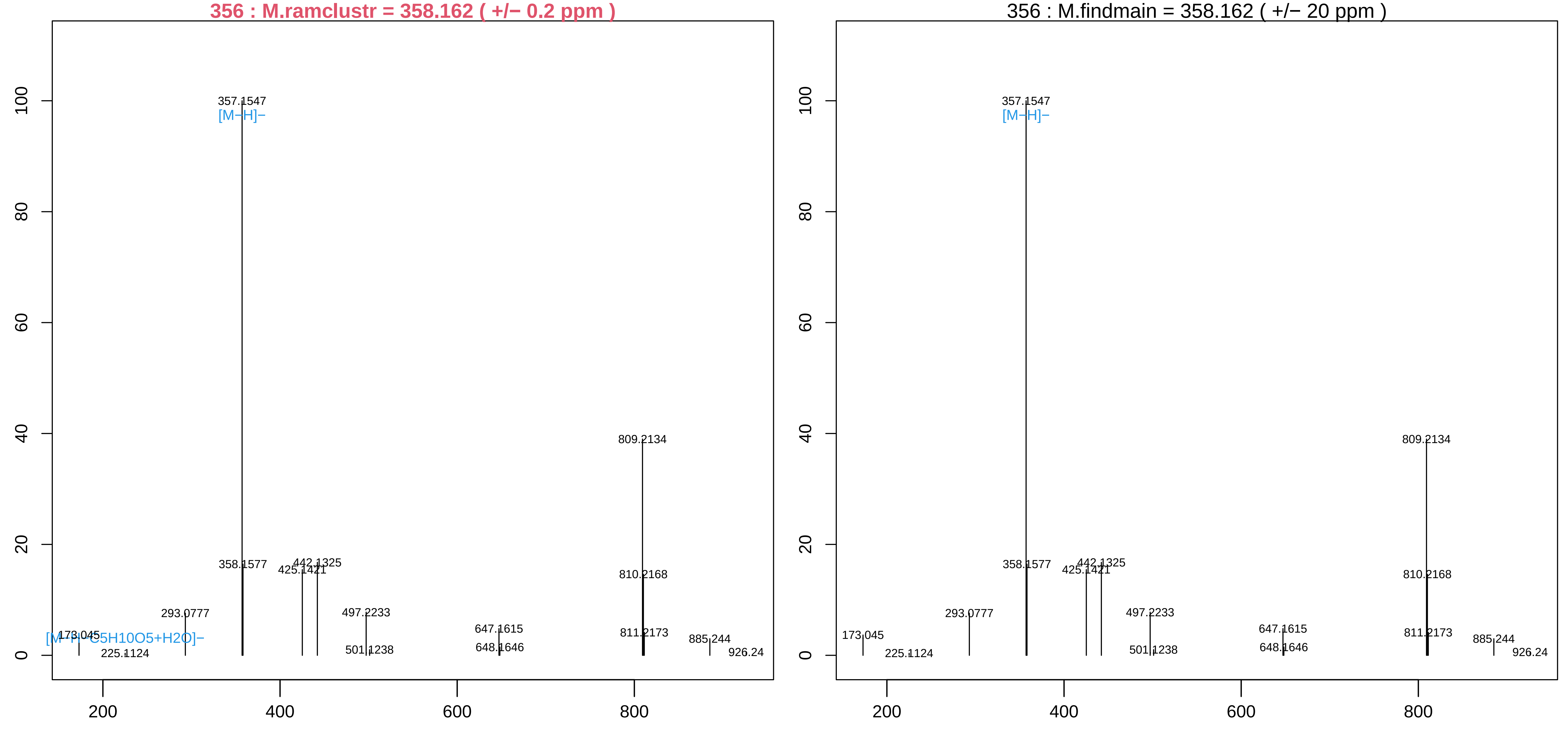
356 : M.ramclustr = 358.162 (+/− 0.2 ppm)
357.1547
[M−H]−
358.1577
425.1421
442.1325
293.0777
497.2233
501.1238
647.1615
648.1646
809.2134
810.2168
811.2173
885.244
926.24
173.045
[M−H−C5H10O5+H2O]−
225.1124
356 : M.findmain = 358.162 (+/− 20 ppm)
357.1547
[M−H]−
358.1577
425.1421
442.1325
293.0777
497.2233
501.1238
647.1615
648.1646
809.2134
810.2168
811.2173
885.244
926.24
173.045
225.1124
0
20
40
60
80
100
200
400
600
800

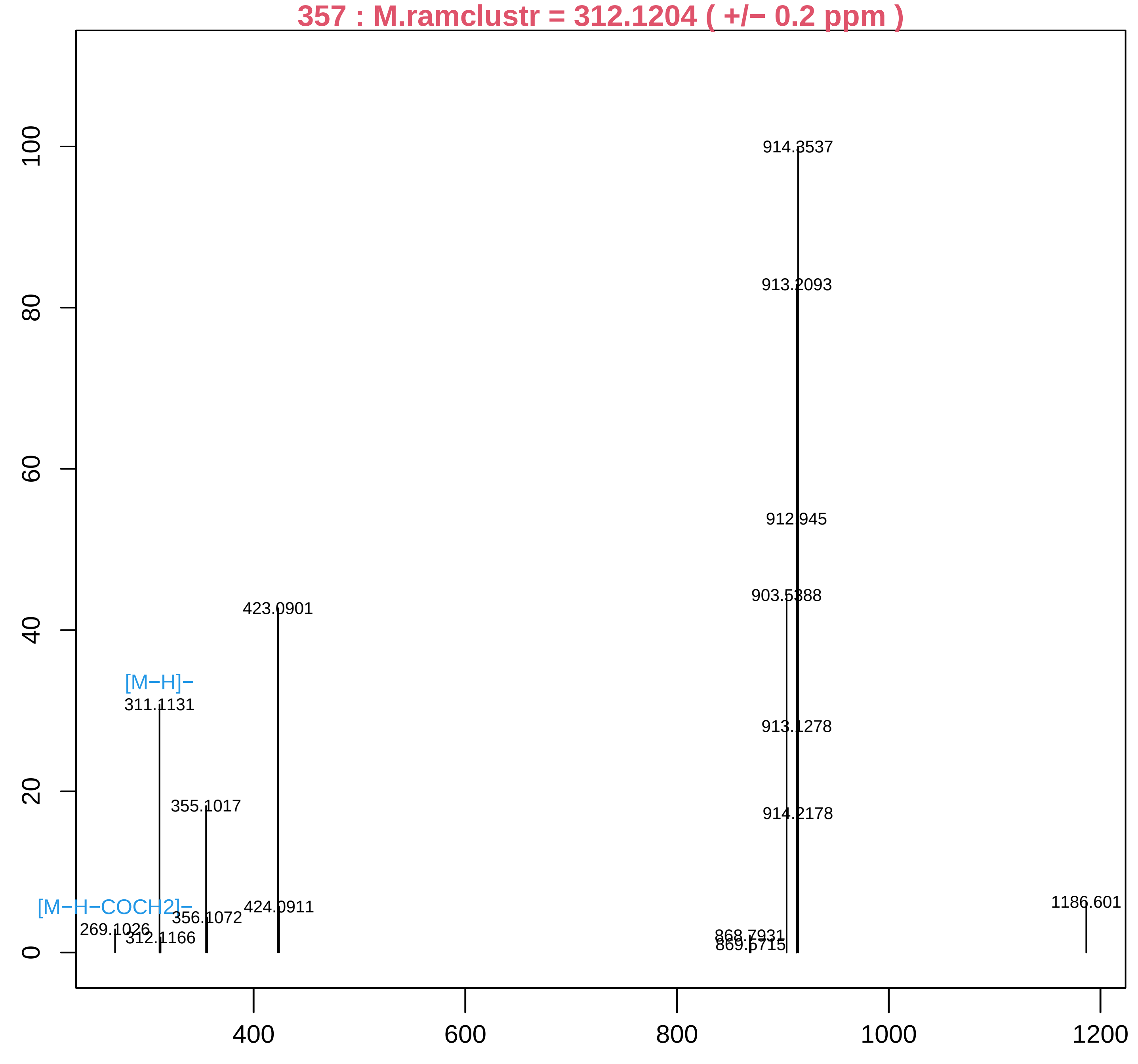
357 : M.ramclustr = 312.1204 (+/− 0.2 ppm)
914.3537
913.2093
912.945
903.5388
423.0901
[M−H]−
311.1131
913.1278
355.1017
914.2178
[M−H−COCH2]−
424.0911
356.1072
269.1026
312.1166
868.7931
869.5715
1186.601
0
20
40
60
80
100
400
600
800
1000
1200

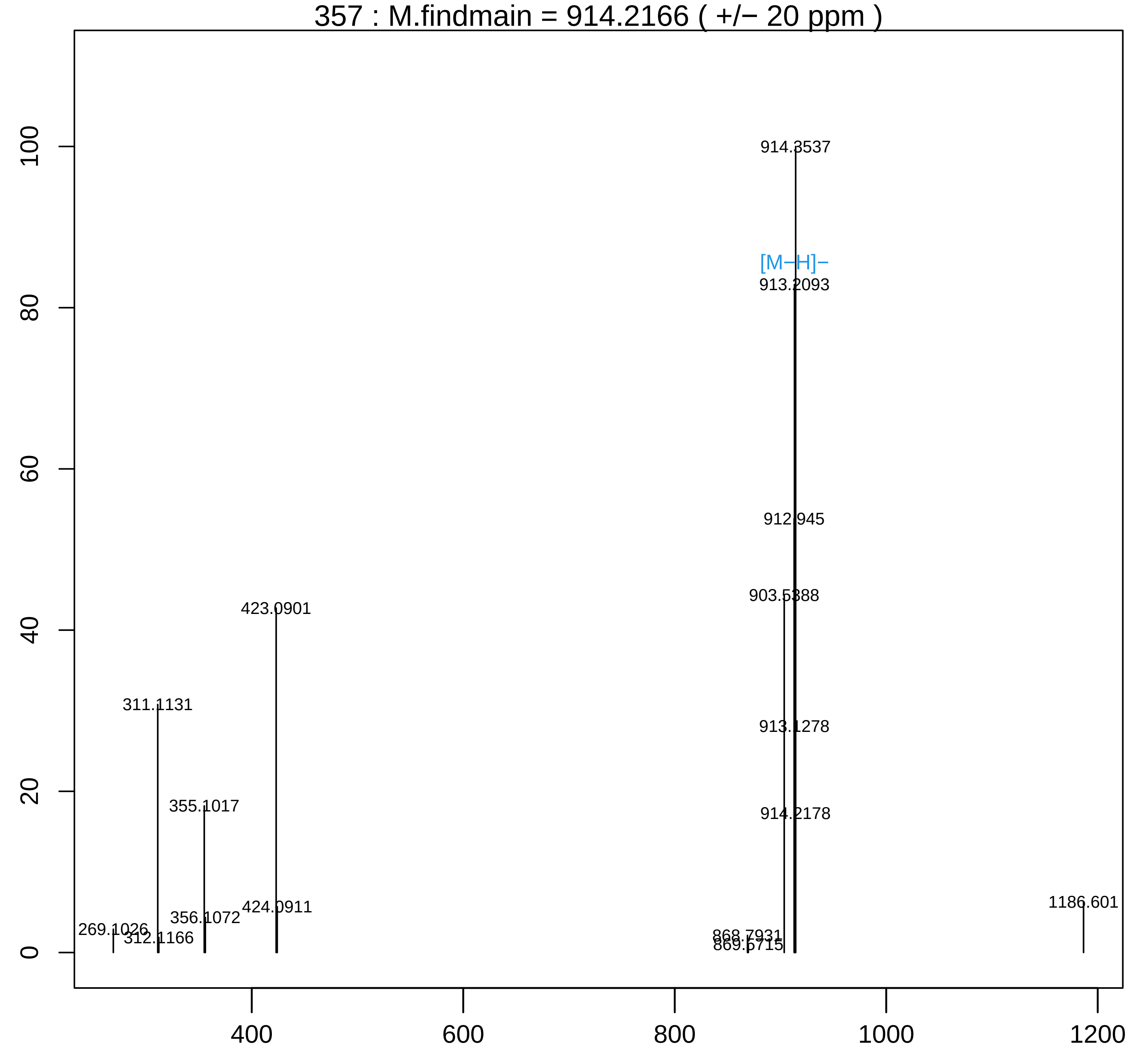
357 : M.findmain = 914.2166 (+/− 20 ppm)
914.3537
[M−H]−
913.2093
912.945
903.5388
423.0901
311.1131
913.1278
355.1017
914.2178
424.0911
356.1072
269.1026
312.1166
868.7931
869.5715
1186.601
0
20
40
60
80
100
400
600
800
1000
1200

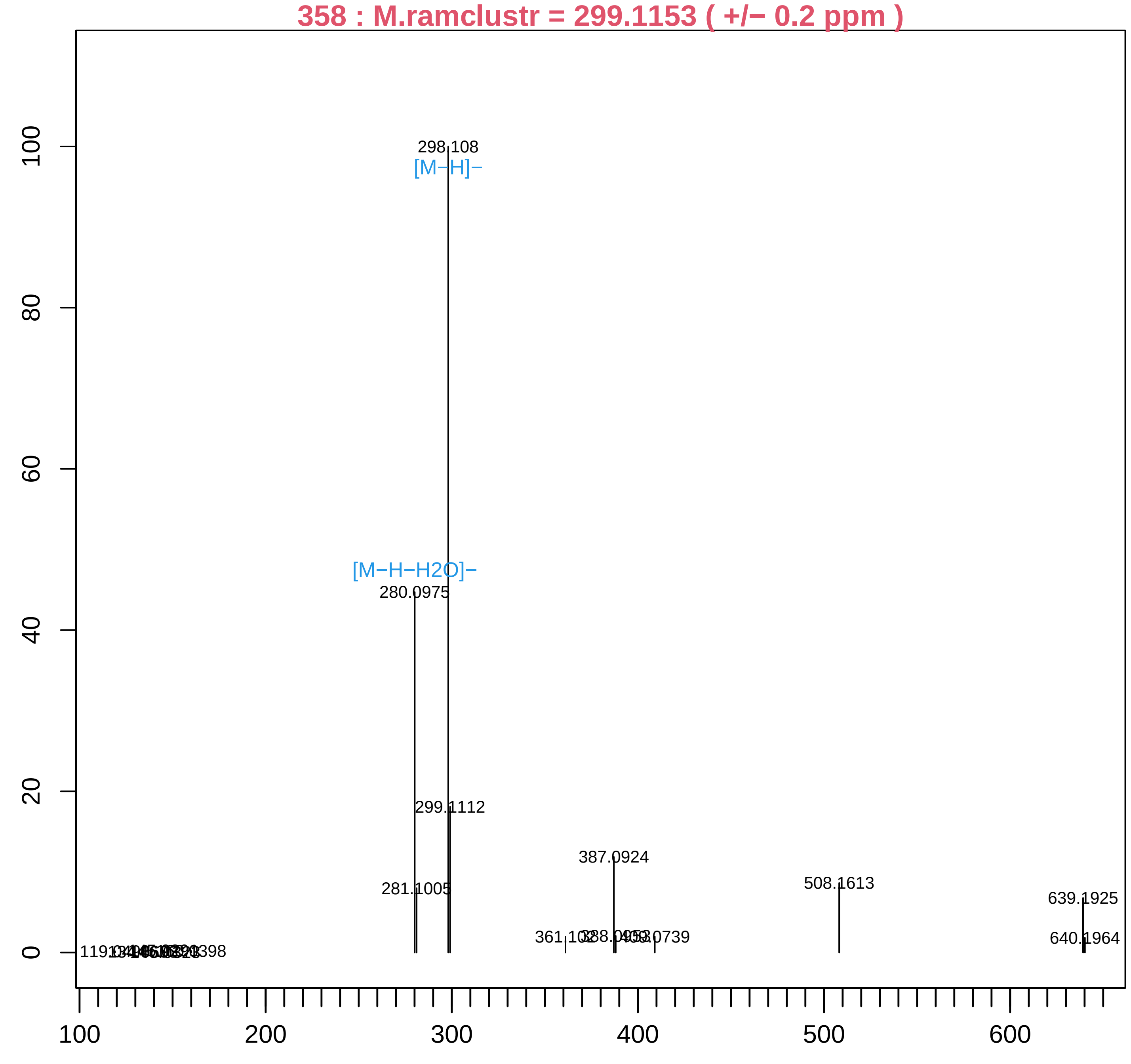

358 : M.ramclustr = 299.1153 (+/− 0.2 ppm)
298.108
[M−H]−
[M−H−H2O]−
280.0975
299.1112
281.1005
387.0924
508.1613
639.1925
640.1964
361.1032
409.0739

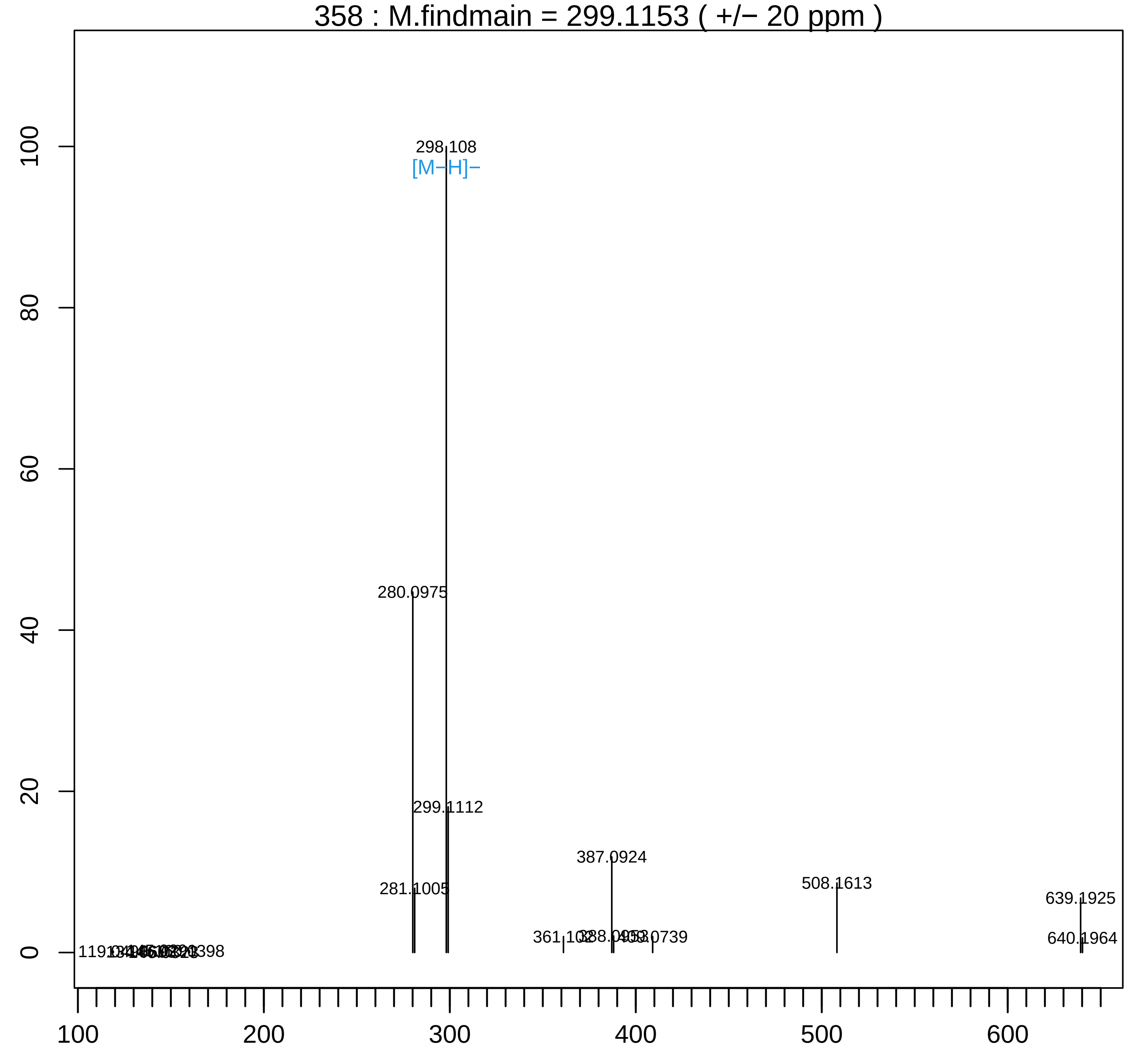

358 : M.findmain = 299.1153 (+/− 20 ppm)
298.108
[M−H]−
280.0975
299.1112
281.1005
387.0924
508.1613
639.1925
640.1964
361.1032
409.0739

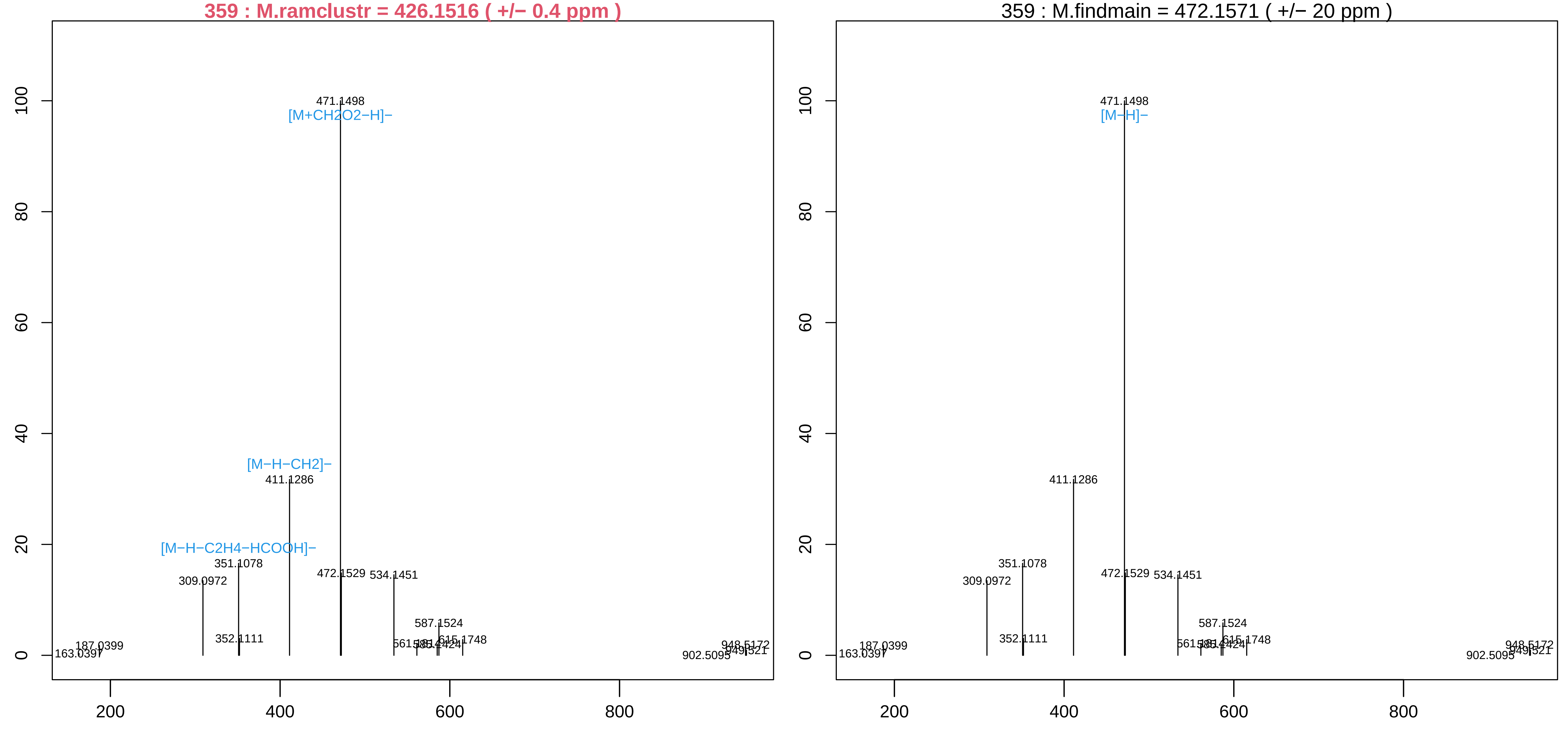

359 : M.ramclustr = 426.1516 (+/− 0.4 ppm)
471.1498
[M+CH2O2−H]−
[M−H−CH2]−
411.1286
[M−H−C2H4−HCOOH]−
351.1078
309.0972
472.1529
534.1451
587.1524
352.1111
615.1748
187.0399
163.0397
948.5172
949.521
902.5095
359 : M.findmain = 472.1571 (+/− 20 ppm)
471.1498
[M−H]−
411.1286
351.1078
309.0972
472.1529
534.1451
587.1524
352.1111
615.1748
187.0399
163.0397
948.5172
949.521
902.5095
0
20
40
60
80
100
200
400
600
800

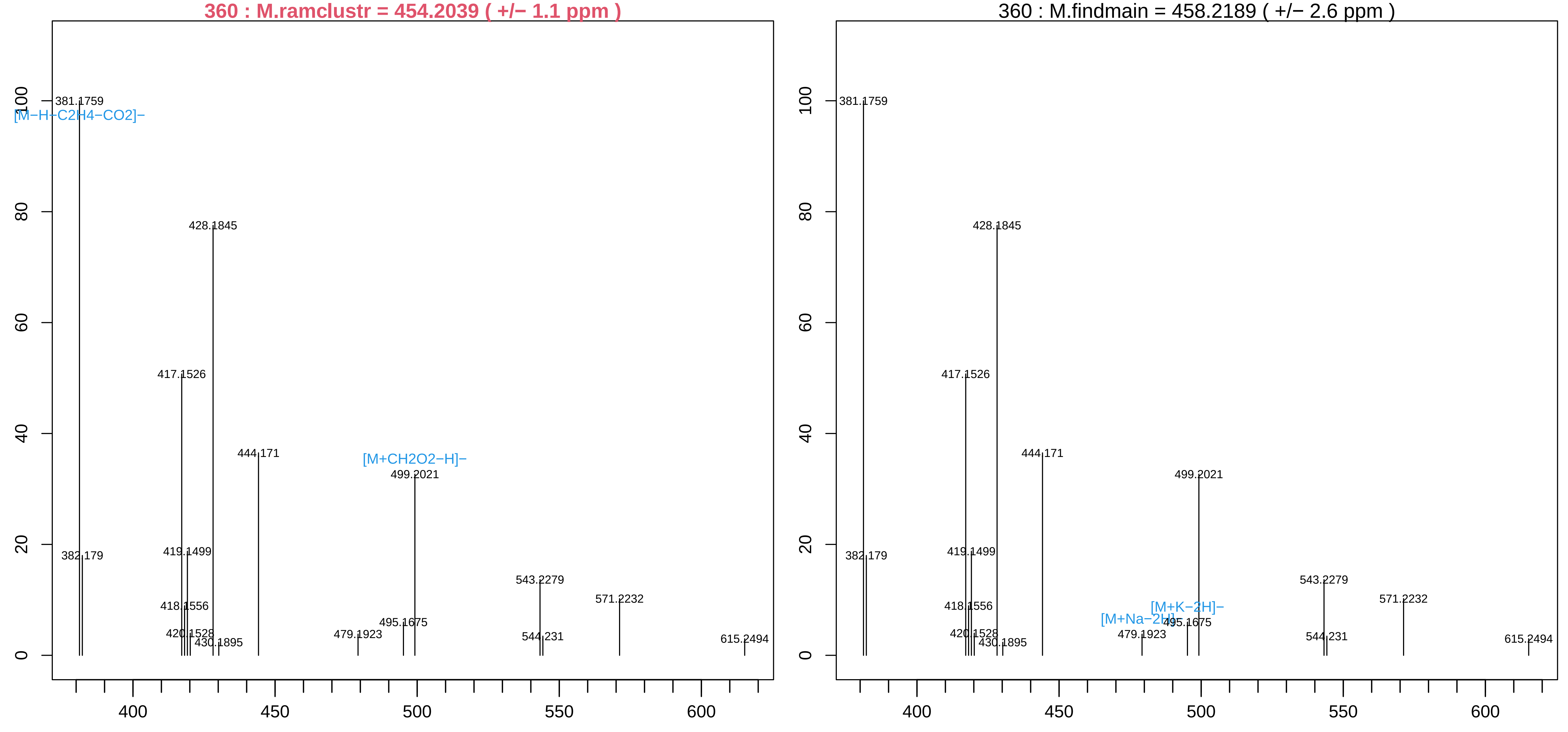
360 : M.ramclustr = 454.2039 (+/− 1.1 ppm)
381.1759
[M−H−C2H4−CO2]−
428.1845
417.1526
444.171
[M+CH2O2−H]−
499.2021
382.179
419.1499
418.1556
420.1528
430.1895
479.1923
495.1675
543.2279
544.231
571.2232
615.2494
360 : M.findmain = 458.2189 (+/− 2.6 ppm)
[M+K−2H]−
[M+Na−2H]−
0
20
40
60
80
100
400
450
500
550
600

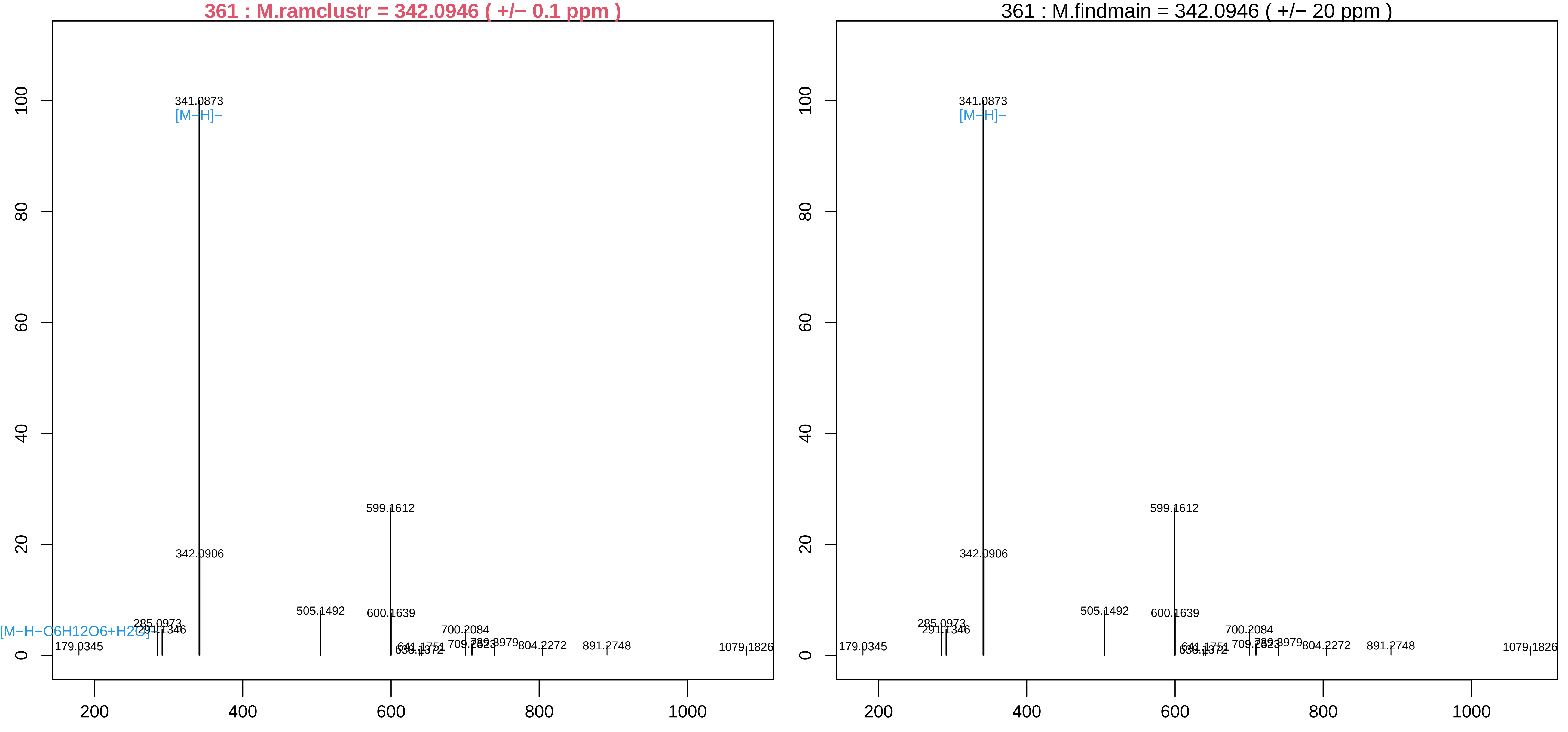

361 : M.ramclustr = 342.0946 (+/− 0.1 ppm)
341.0873
[M−H]−
342.0906
599.1612
600.1639
505.1492
285.0973
291.1346
[M−H−C6H12O6+H2O]−
179.0345
700.2084
709.2523
739.3979
641.1751
636.1372
804.2272
891.2748
1079.1826
361 : M.findmain = 342.0946 (+/− 20 ppm)

362 : M.ramclustr = 868.4816 (+/− 0.6 ppm)

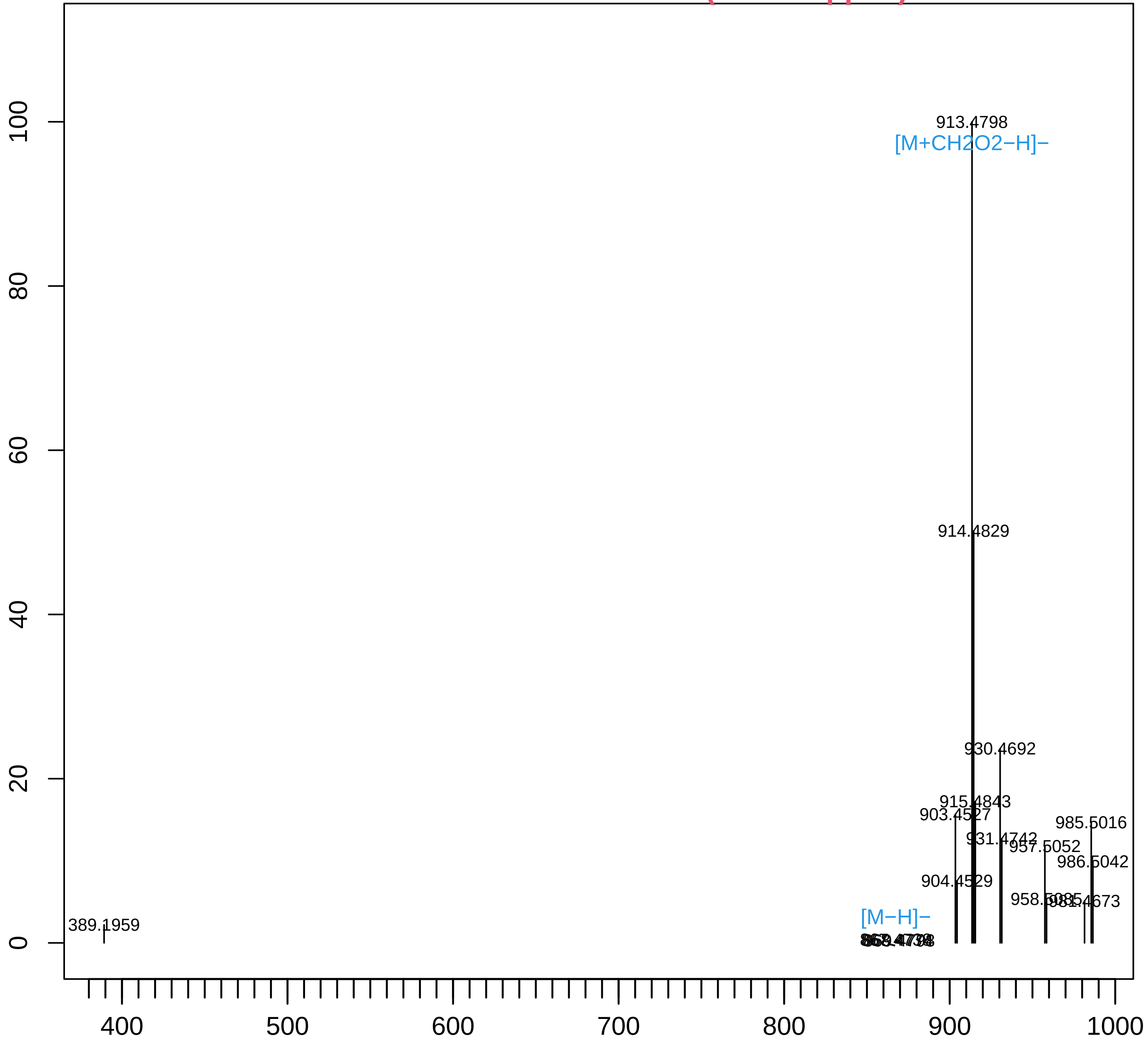

362 : M.findmain = 868.4816 (+/− 0.6 ppm)

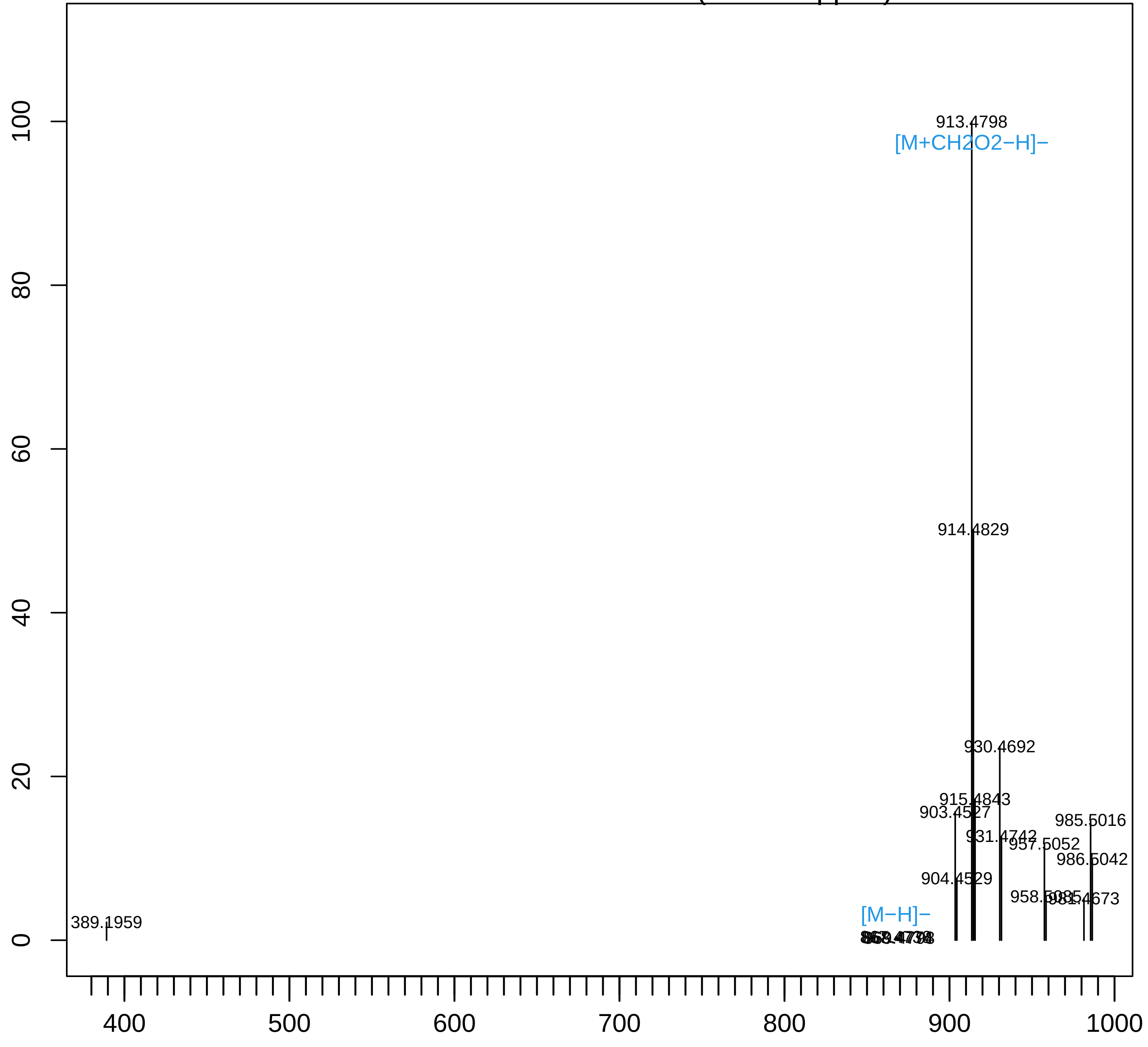

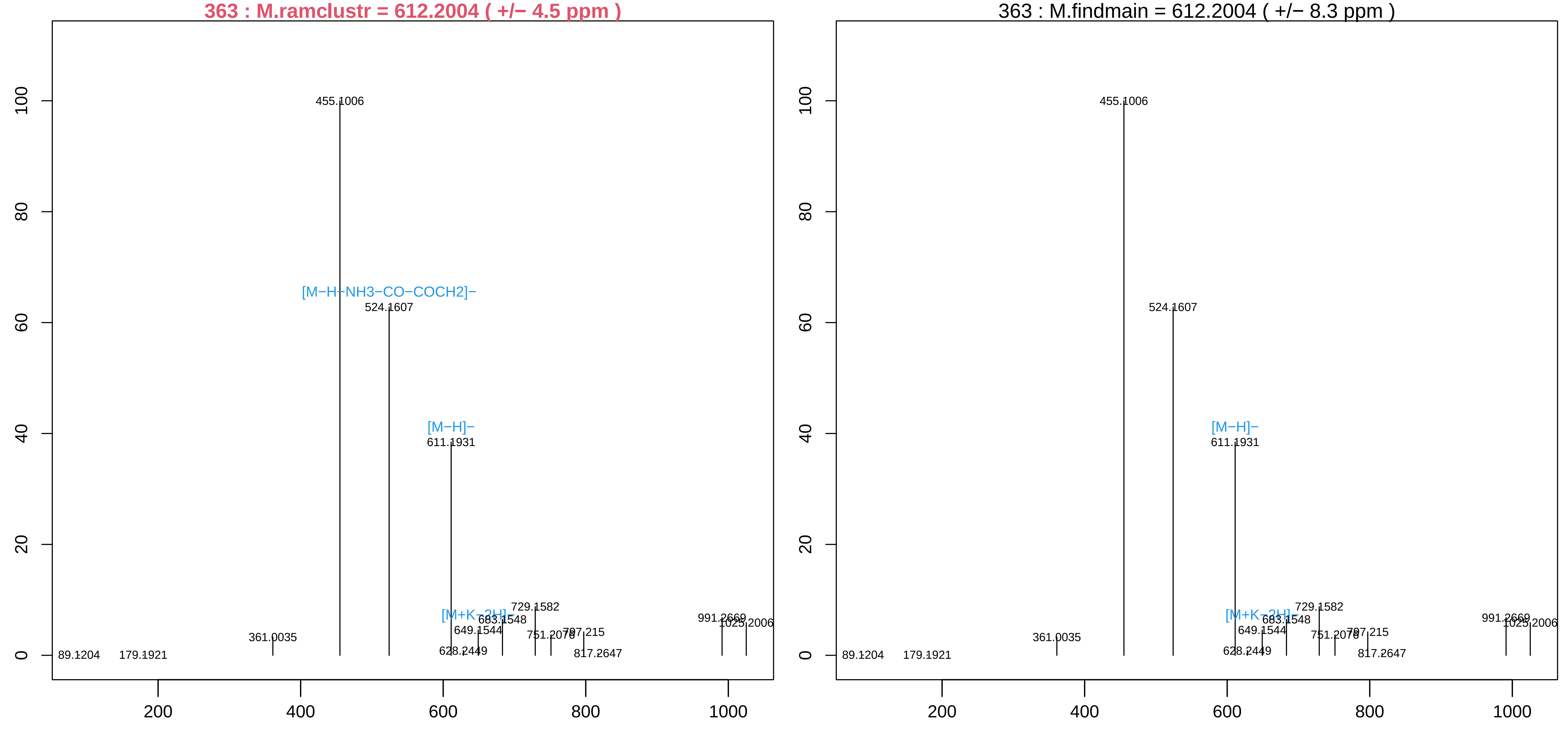
363 : M.ramclustr = 612.2004 (+/− 4.5 ppm)
455.1006
[M−H−NH3−CO−COCH2]−
524.1607
[M−H]−
611.1931
[M+K−2H]−
729.1582
683.1548
649.1544
751.2078
797.215
628.2449
817.2647
991.2669
1025.2006
361.0035
89.1204
179.1921
363 : M.findmain = 612.2004 (+/− 8.3 ppm)
455.1006
524.1607
[M−H]−
611.1931
[M+K−2H]−
729.1582
683.1548
649.1544
751.2078
797.215
628.2449
817.2647
991.2669
1025.2006
361.0035
89.1204
179.1921
0
20
40
60
80
100
200
400
600
800
1000

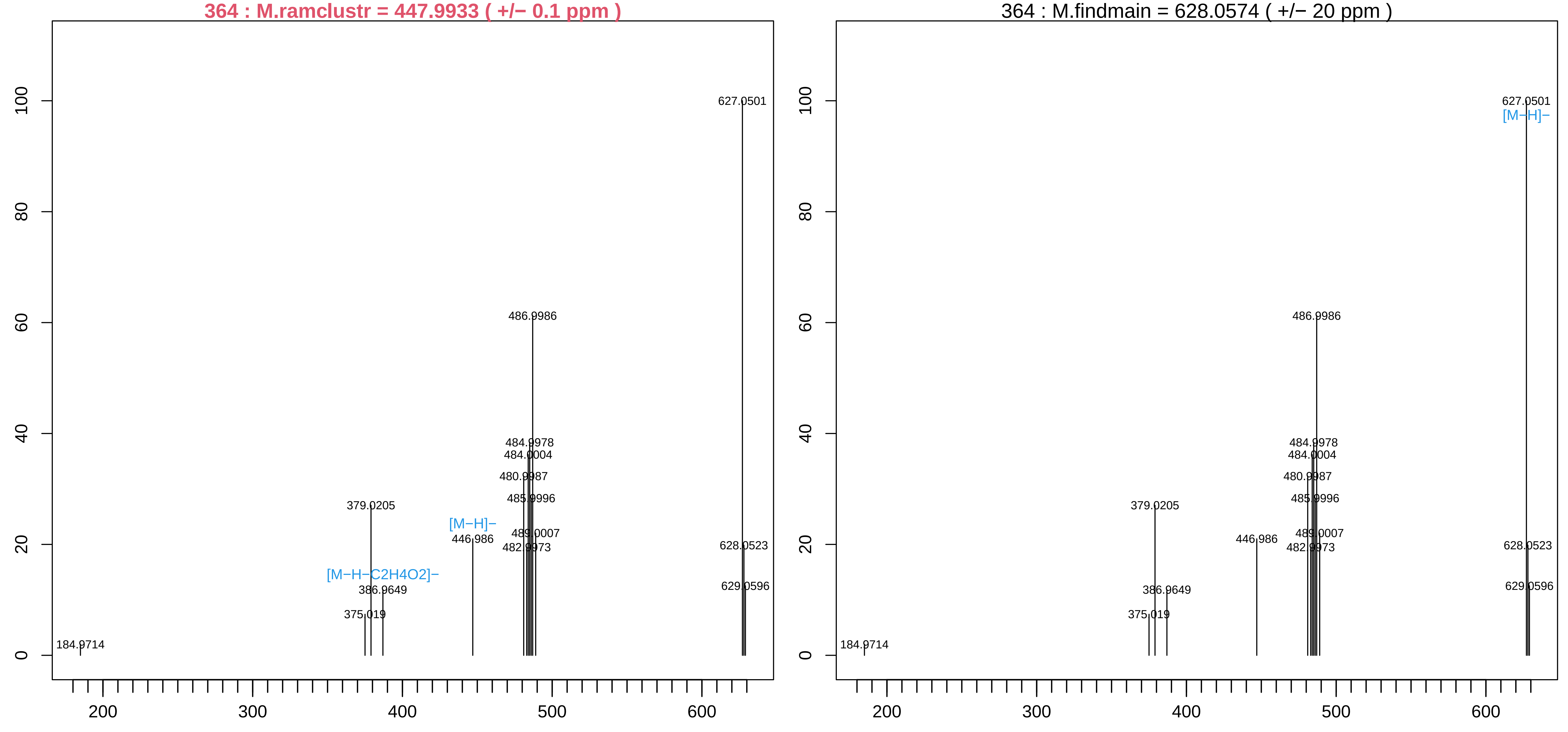

364 : M.ramclustr = 447.9933 (+/− 0.1 ppm)
627.0501
486.9986
484.9978
484.0004
480.9987
485.9996
489.0007
482.9973
379.0205
[M−H]−
446.986
628.0523
629.0596
[M−H−C2H4O2]−
386.9649
375.019
184.9714
364 : M.findmain = 628.0574 (+/− 20 ppm)
627.0501
[M−H]−
486.9986
484.9978
484.0004
480.9987
485.9996
489.0007
482.9973
379.0205
446.986
628.0523
629.0596
386.9649
375.019
184.9714
0
20
40
60
80
100
200
300
400
500
600

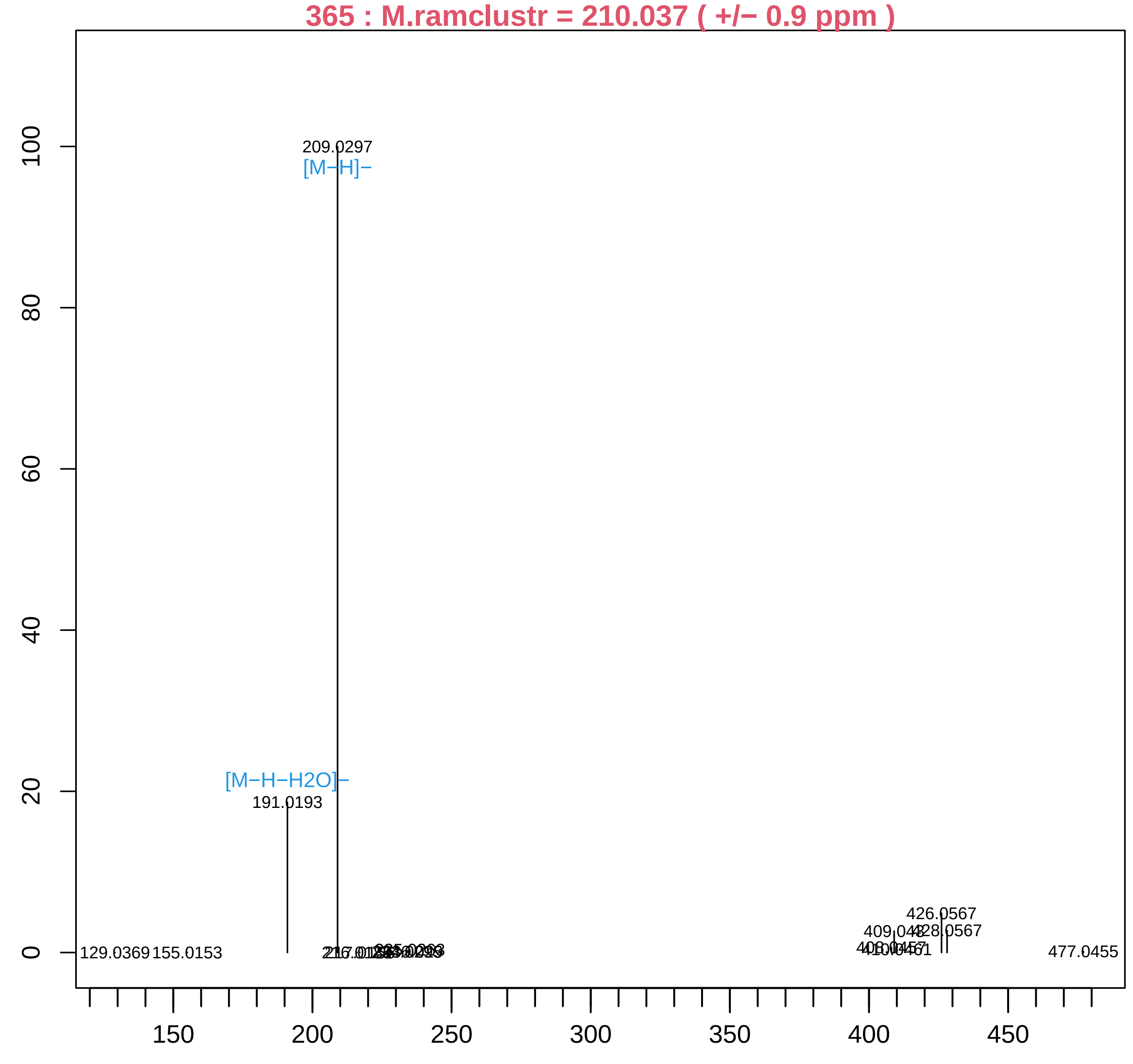
365 : M.ramclustr = 210.037 (+/− 0.9 ppm)
209.0297
[M−H]−
[M−H−H2O]−
191.0193
426.0567
428.0567
129.0369
155.0153
477.0455
0
20
40
60
80
100
150
200
250
300
350
400
450

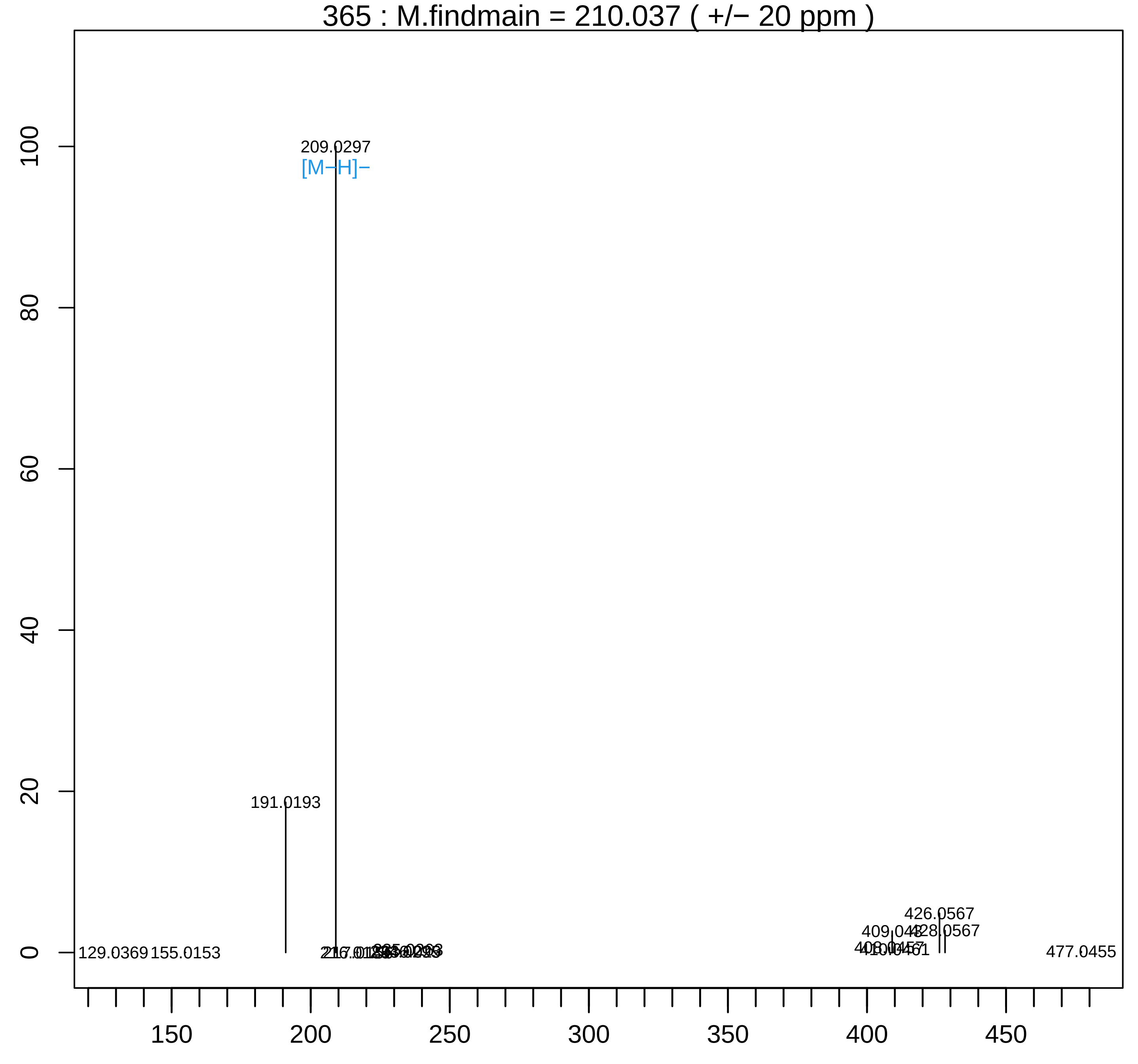
365 : M.findmain = 210.037 (+/− 20 ppm)
209.0297
[M−H]−
191.0193
426.0567
428.0567
129.0369
155.0153
477.0455
0
20
40
60
80
100
150
200
250
300
350
400
450

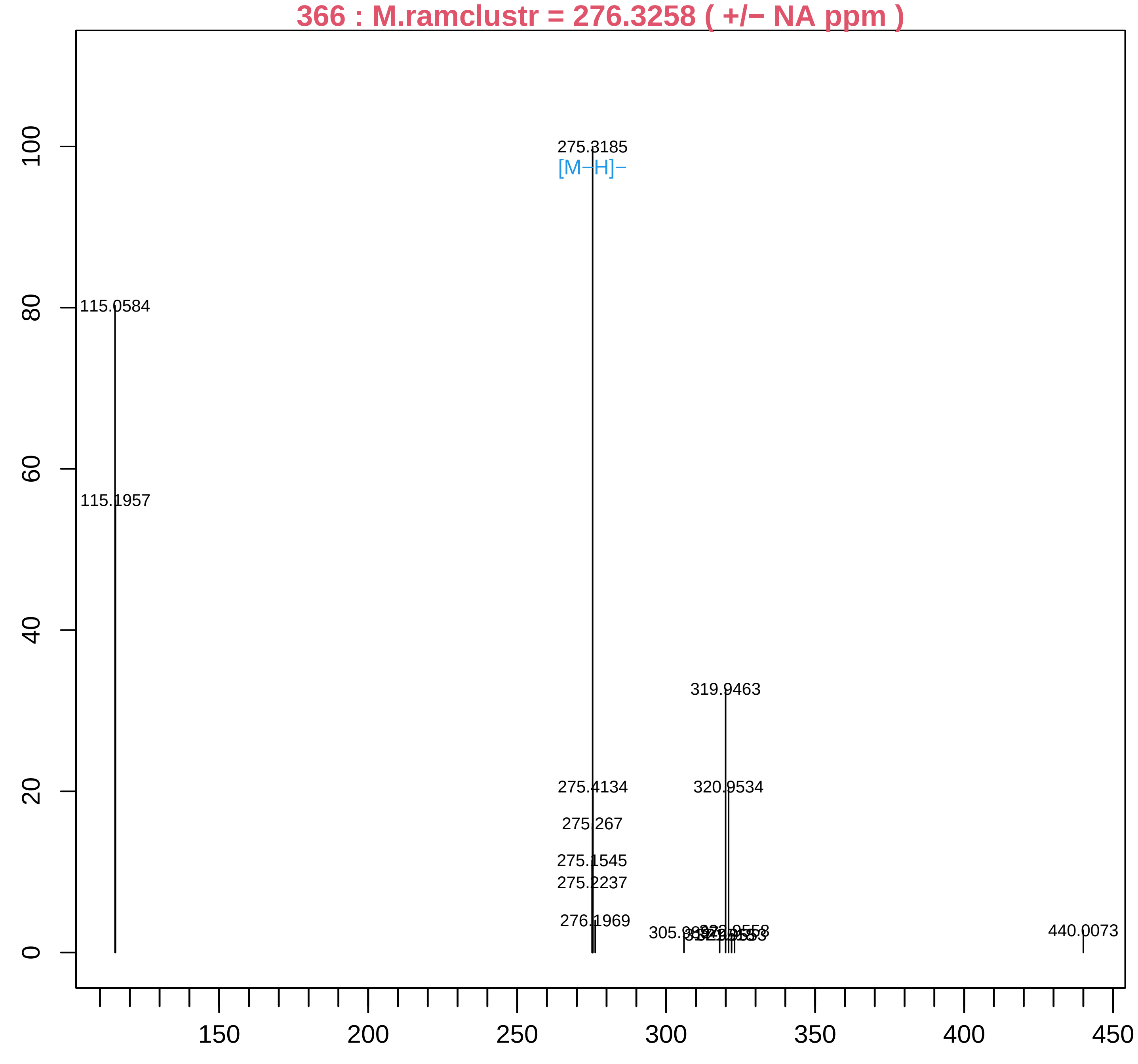
366 : M.ramclustr = 276.3258 (+/− NA ppm)
275.3185
[M−H]−
115.0584
115.1957
319.9463
275.4134
320.9534
275.267
275.1545
275.2237
276.1969
440.0073
0
20
40
60
80
100
150
200
250
300
350
400
450

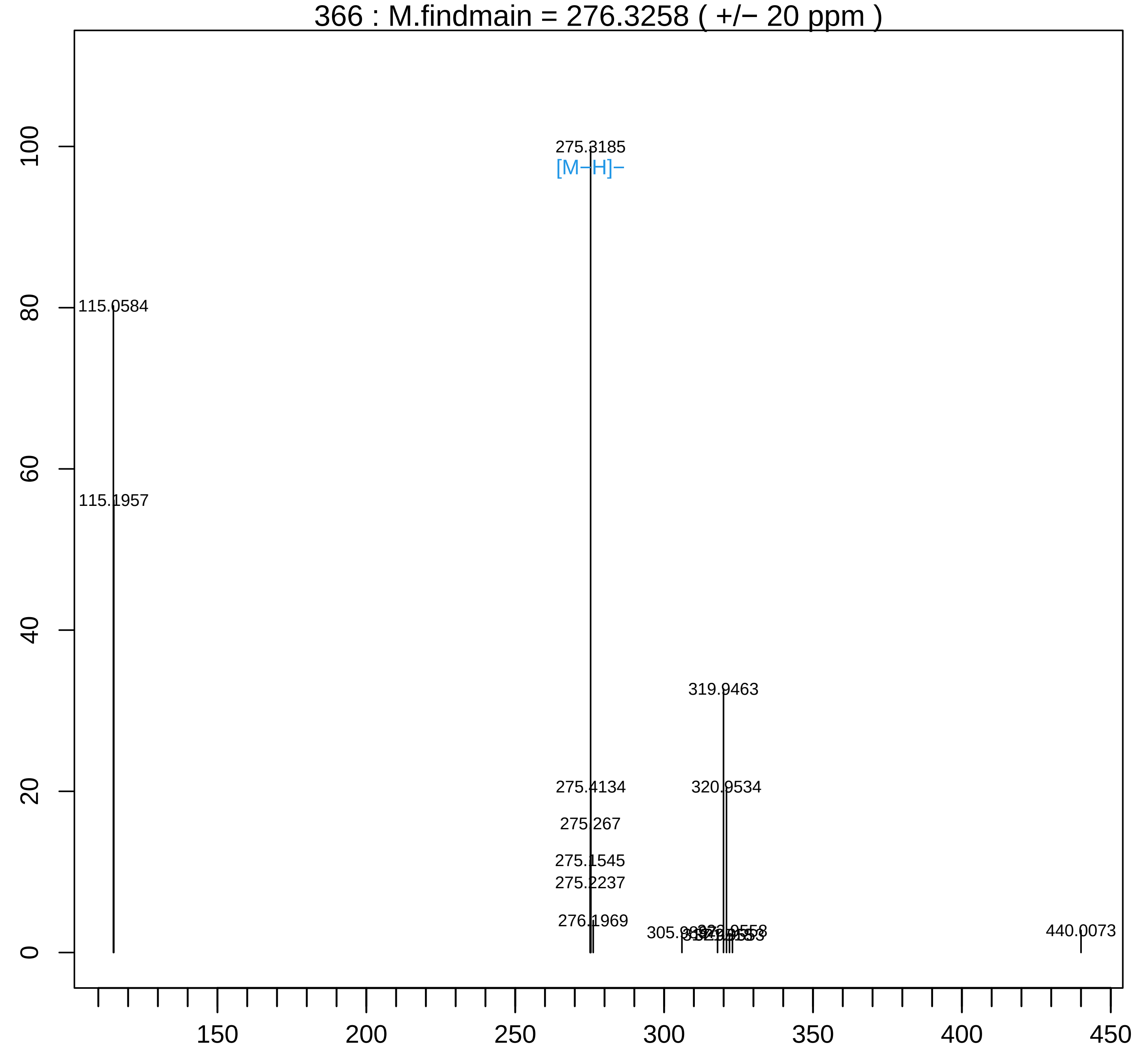
366 : M.findmain = 276.3258 (+/− 20 ppm)
275.3185
[M−H]−
115.0584
115.1957
319.9463
275.4134
320.9534
275.267
275.1545
275.2237
276.1969
440.0073
0
20
40
60
80
100
150
200
250
300
350
400
450

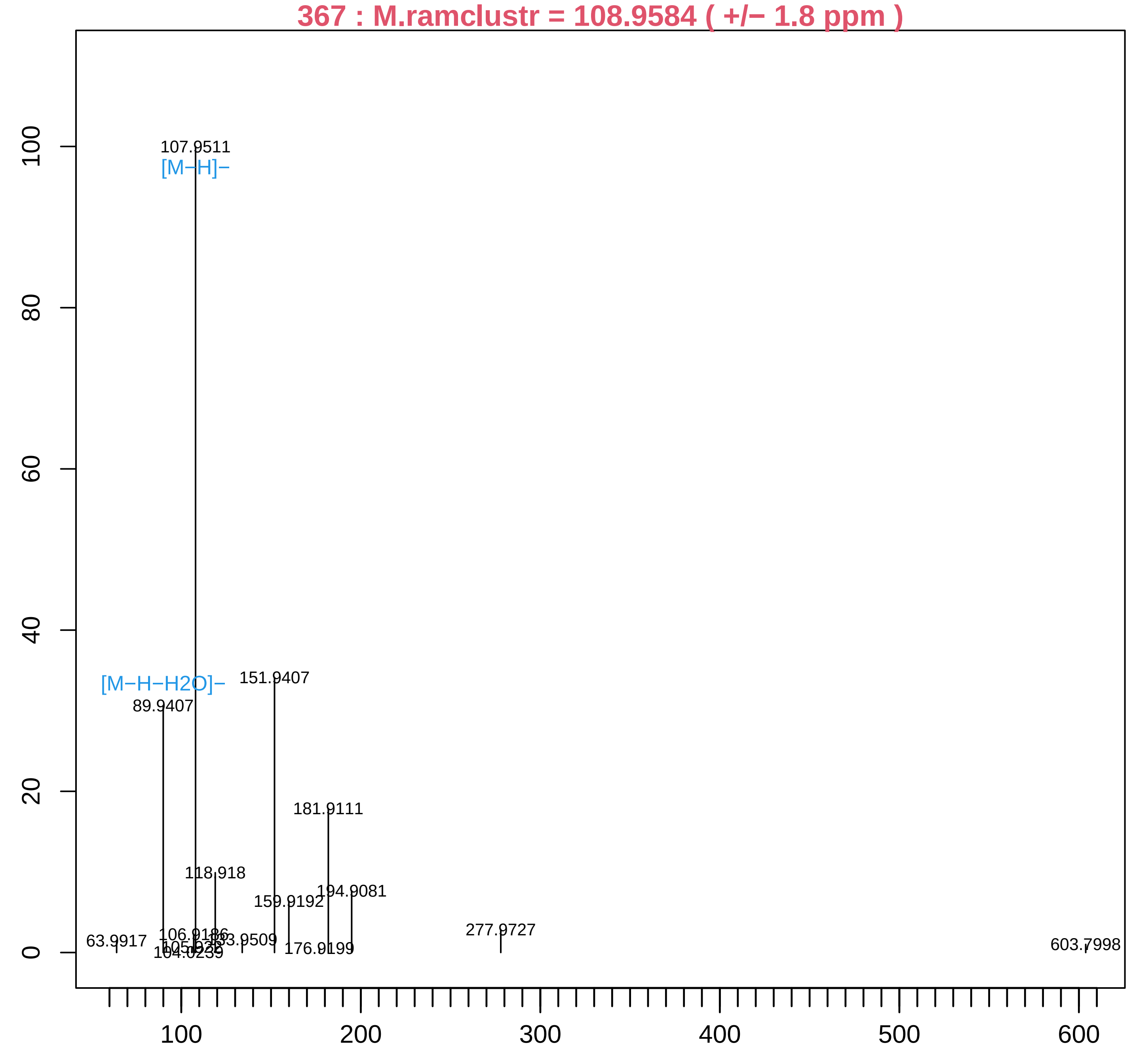
367 : M.ramclustr = 108.9584 (+/− 1.8 ppm)
107.9511
[M−H]−
[M−H−H2O]−
89.9407
151.9407
181.9111
118.918
159.9192
194.9081
63.9917
106.9186
105.9238
104.0239
133.9509
176.9199
277.9727
603.7998
0
20
40
60
80
100
100
200
300
400
500
600

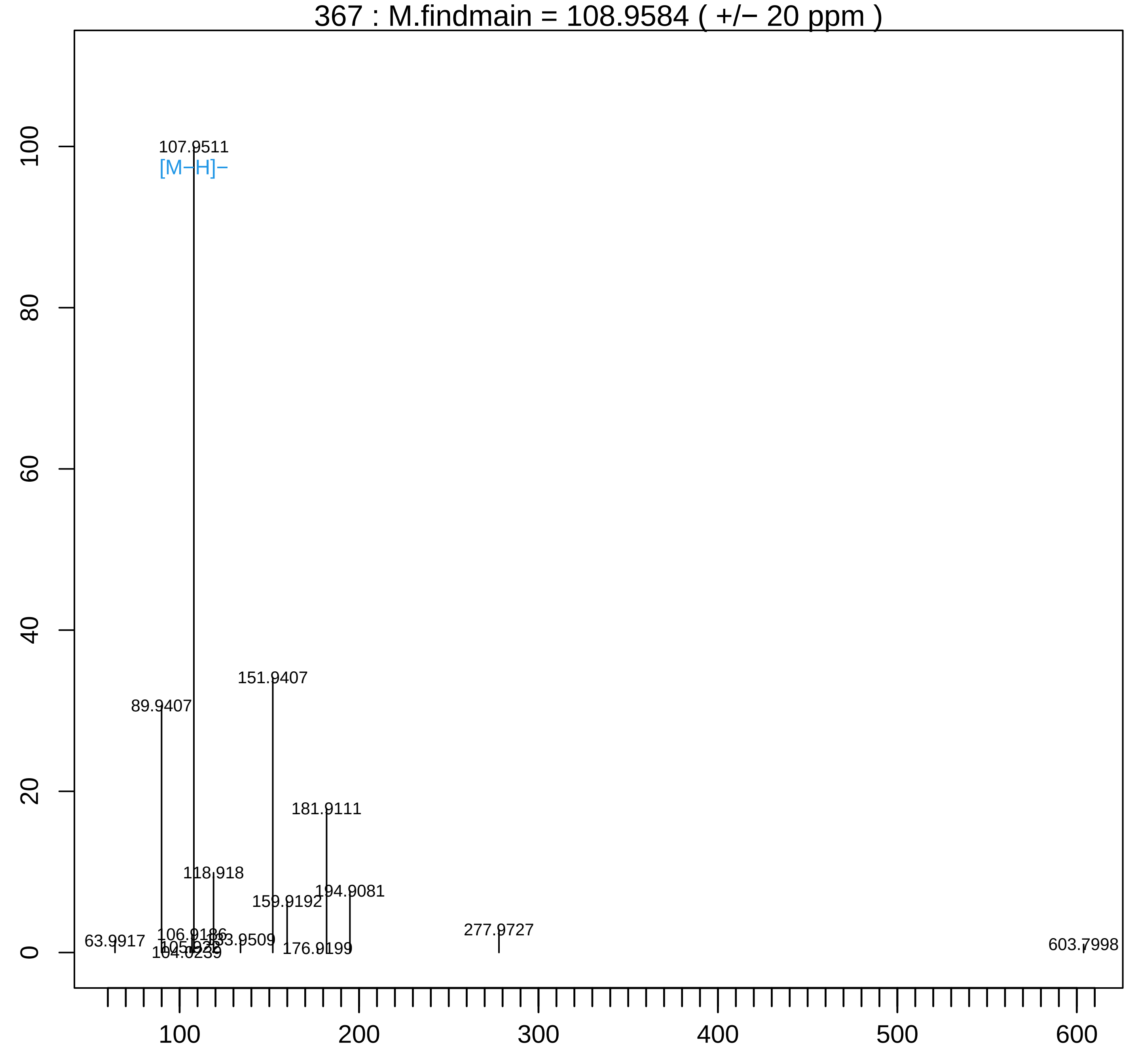
367 : M.findmain = 108.9584 (+/− 20 ppm)
107.9511
[M−H]−
89.9407
151.9407
181.9111
118.918
159.9192
194.9081
63.9917
106.9186
105.9238
104.0239
133.9509
176.9199
277.9727
603.7998
0
20
40
60
80
100
100
200
300
400
500
600

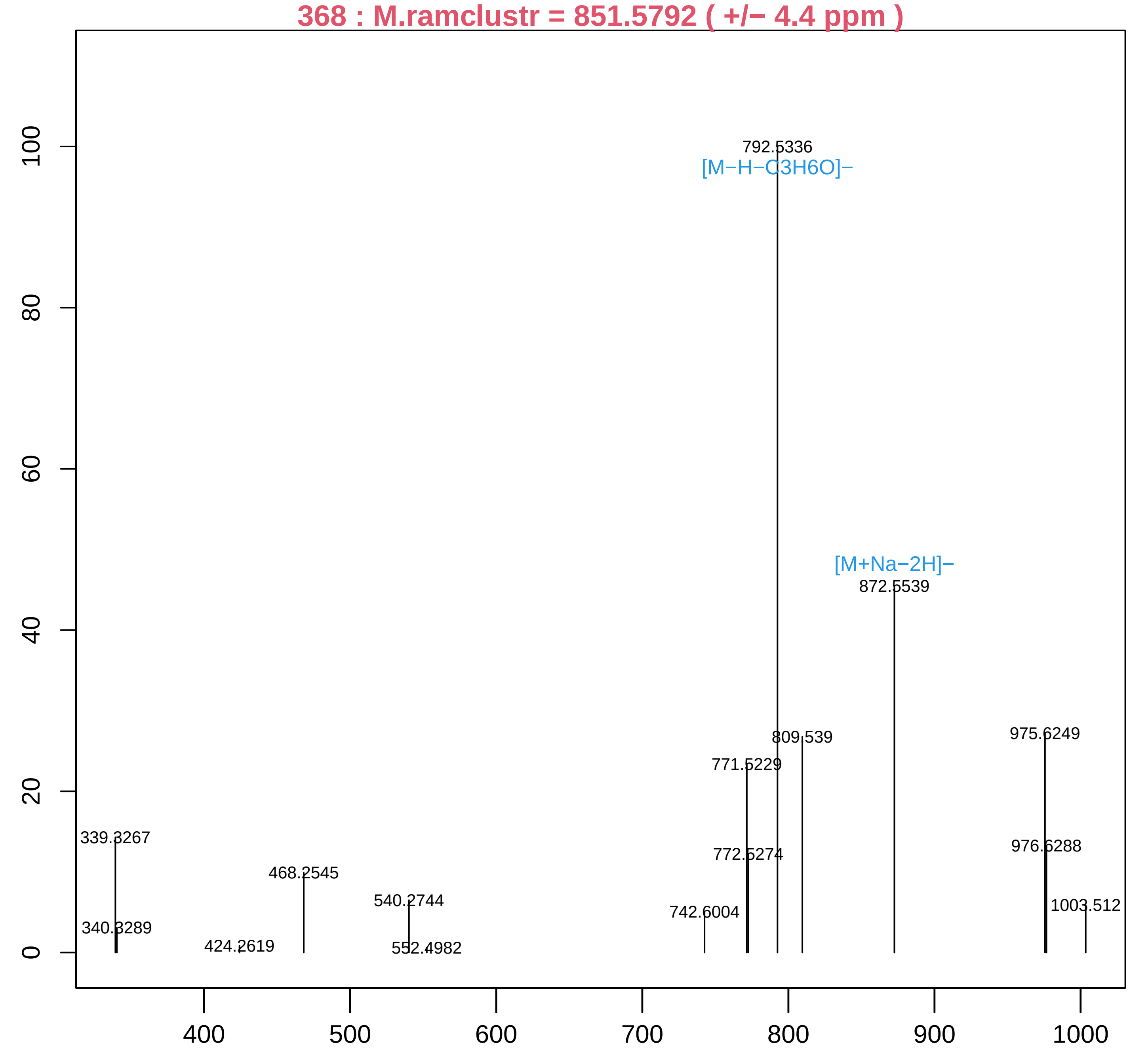

368 : M.ramclustr = 851.5792 (+/− 4.4 ppm)
792.5336
[M−H−C3H6O]−
[M+Na−2H]−
872.5539
809.539
771.5229
975.6249
976.6288
772.5274
339.3267
468.2545
540.2744
742.6004
1003.512
340.3289
424.2619
552.4982
0
20
40
60
80
100
400
500
600
700
800
900
1000

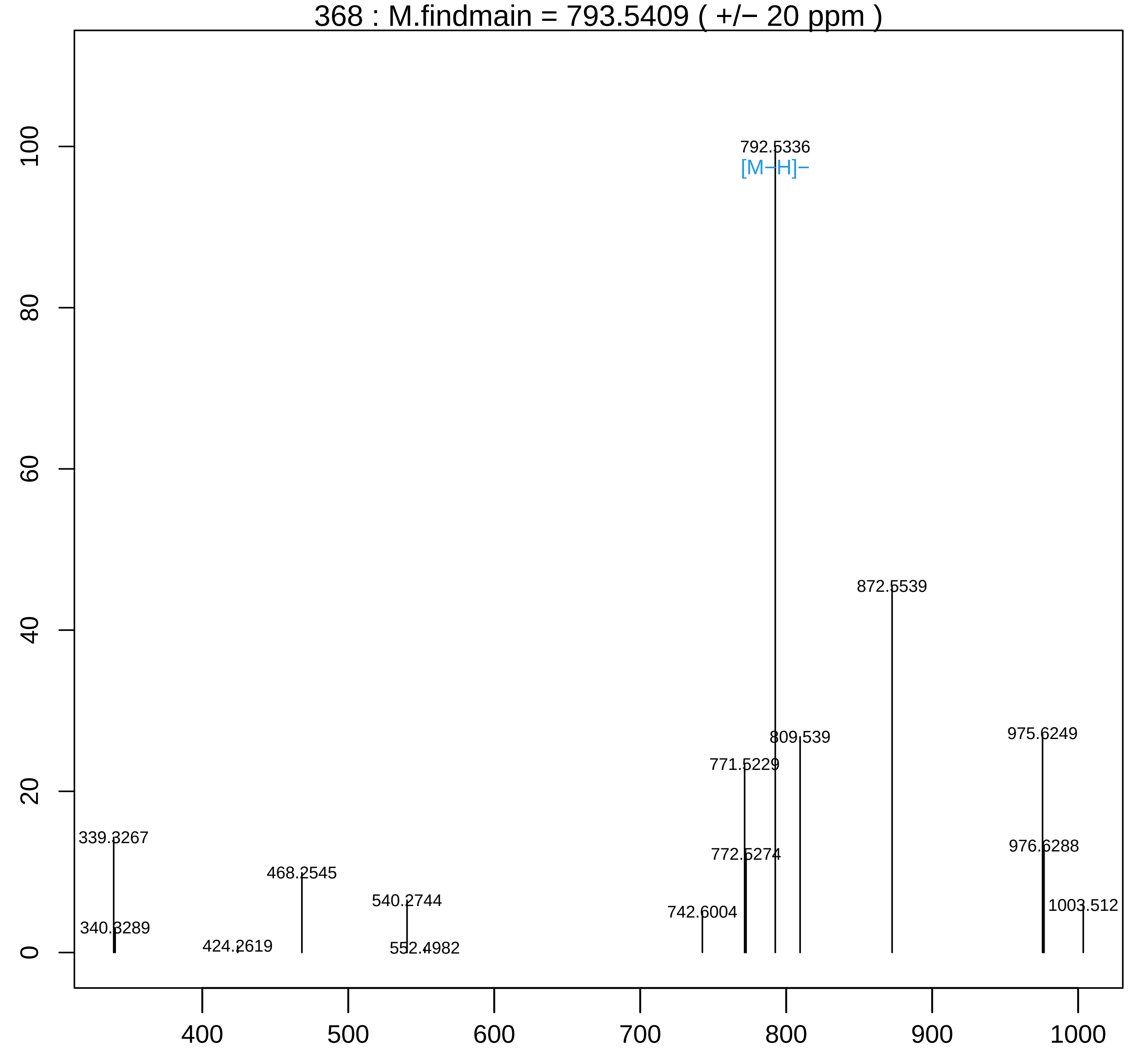

368 : M.findmain = 793.5409 (+/− 20 ppm)
792.5336
[M−H]−
872.5539
809.539
771.5229
975.6249
976.6288
772.5274
339.3267
468.2545
540.2744
742.6004
1003.512
340.3289
424.2619
552.4982
0
20
40
60
80
100
400
500
600
700
800
900
1000

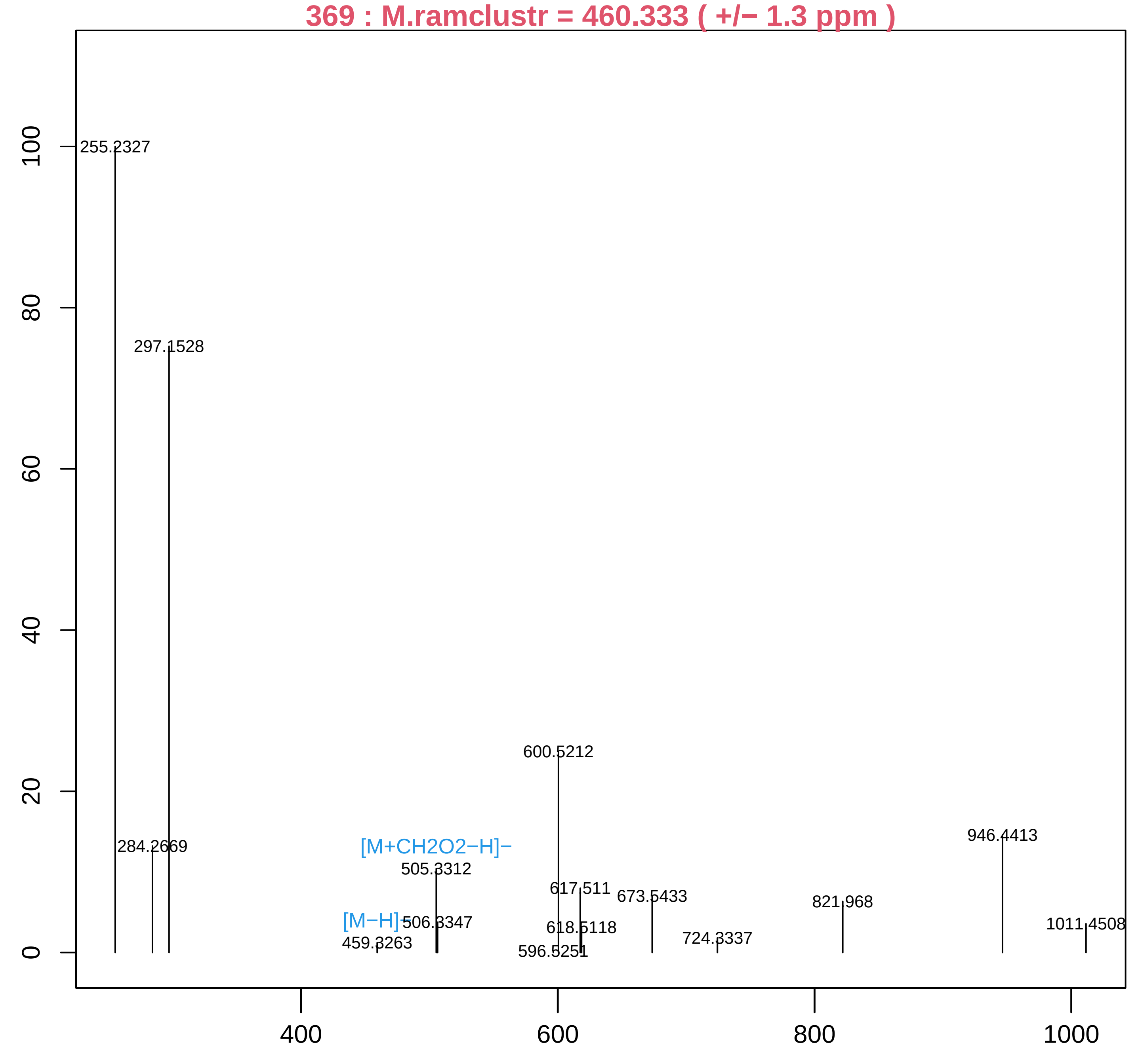
369 : M.ramclustr = 460.333 (+/− 1.3 ppm)
255.2327
297.1528
284.2669
[M+CH2O2−H]−
505.3312
[M−H]−
506.3347
459.3263
600.5212
617.511
618.5118
596.5251
673.5433
724.3337
821.968
946.4413
1011.4508
0
20
40
60
80
100
400
600
800
1000

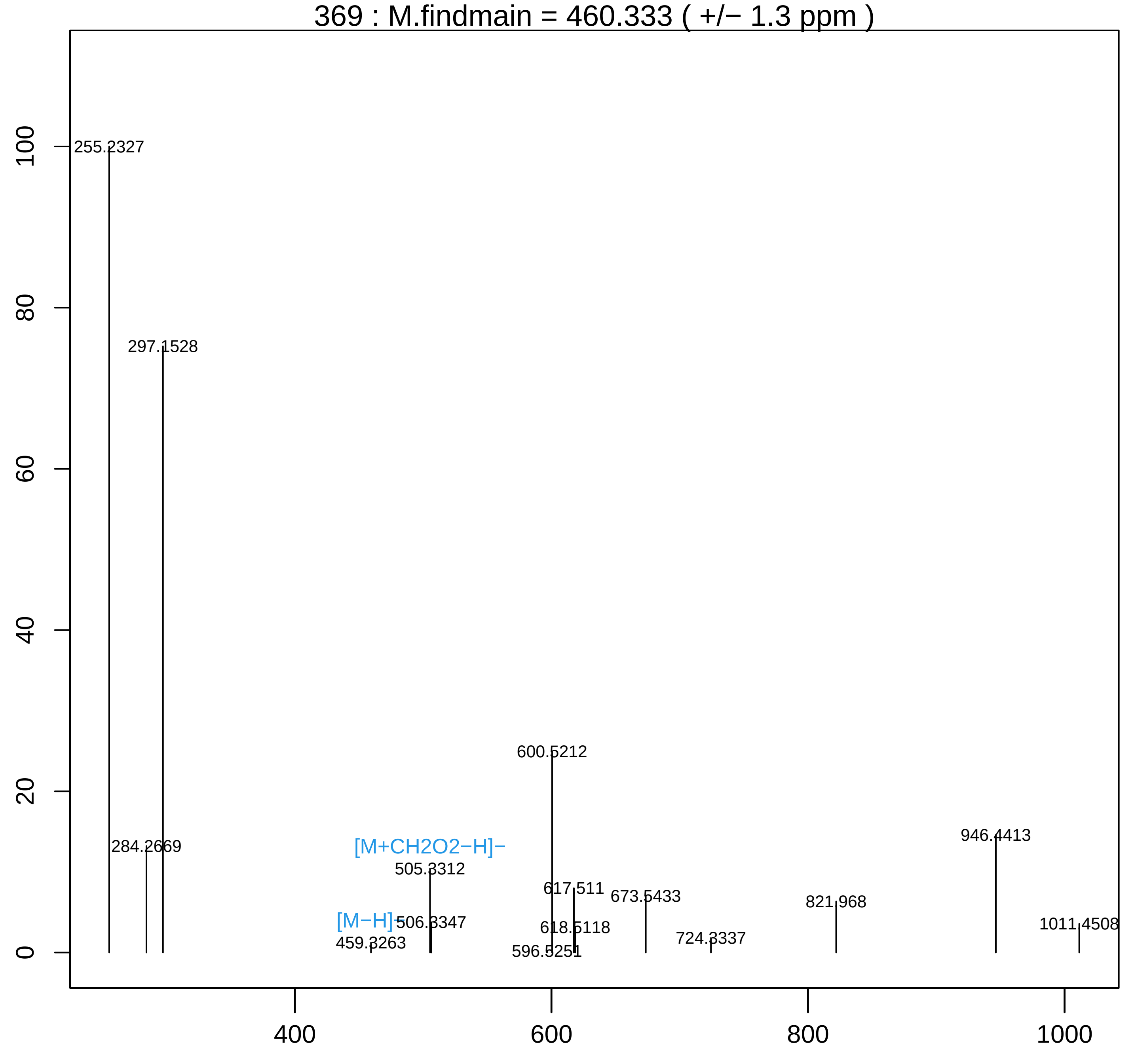
369 : M.findmain = 460.333 (+/− 1.3 ppm)
255.2327
297.1528
284.2669
[M+CH2O2−H]−
505.3312
[M−H]−
506.3347
459.3263
600.5212
617.511
618.5118
596.5251
673.5433
724.3337
821.968
946.4413
1011.4508
0
20
40
60
80
100
400
600
800
1000

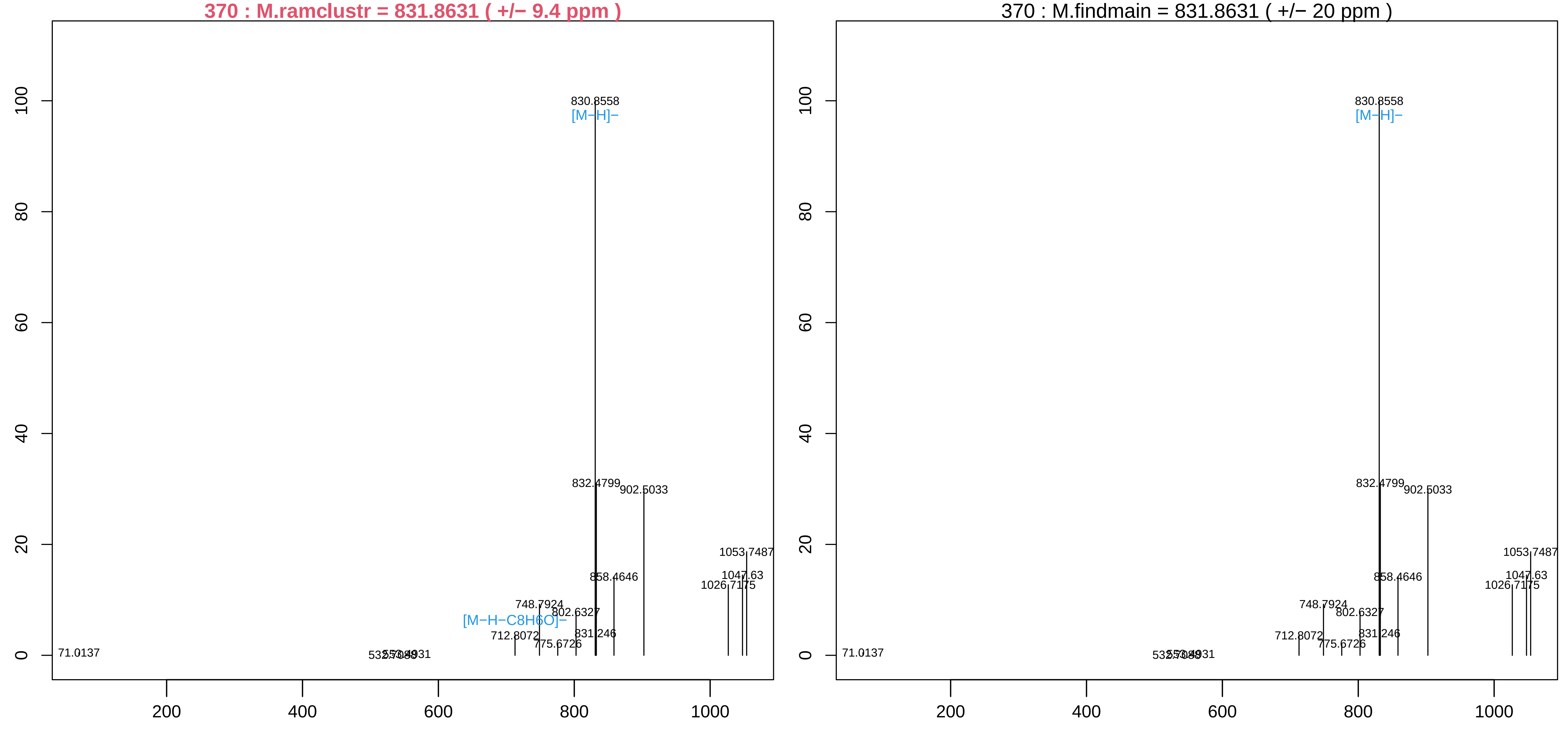

370 : M.ramclustr = 831.8631 (+/− 9.4 ppm)
830.8558
[M−H]−
832.4799
902.5033
1053.7487
1047.63
858.4646
1026.7175
748.7924
802.6327
[M−H−C8H6O]−
712.8072
831.246
775.6726
71.0137
0
20
40
60
80
100
200
400
600
800
1000
370 : M.findmain = 831.8631 (+/− 20 ppm)
830.8558
[M−H]−
832.4799
902.5033
1053.7487
1047.63
858.4646
1026.7175
748.7924
802.6327
712.8072
831.246
775.6726
71.0137
0
20
40
60
80
100
200
400
600
800
1000

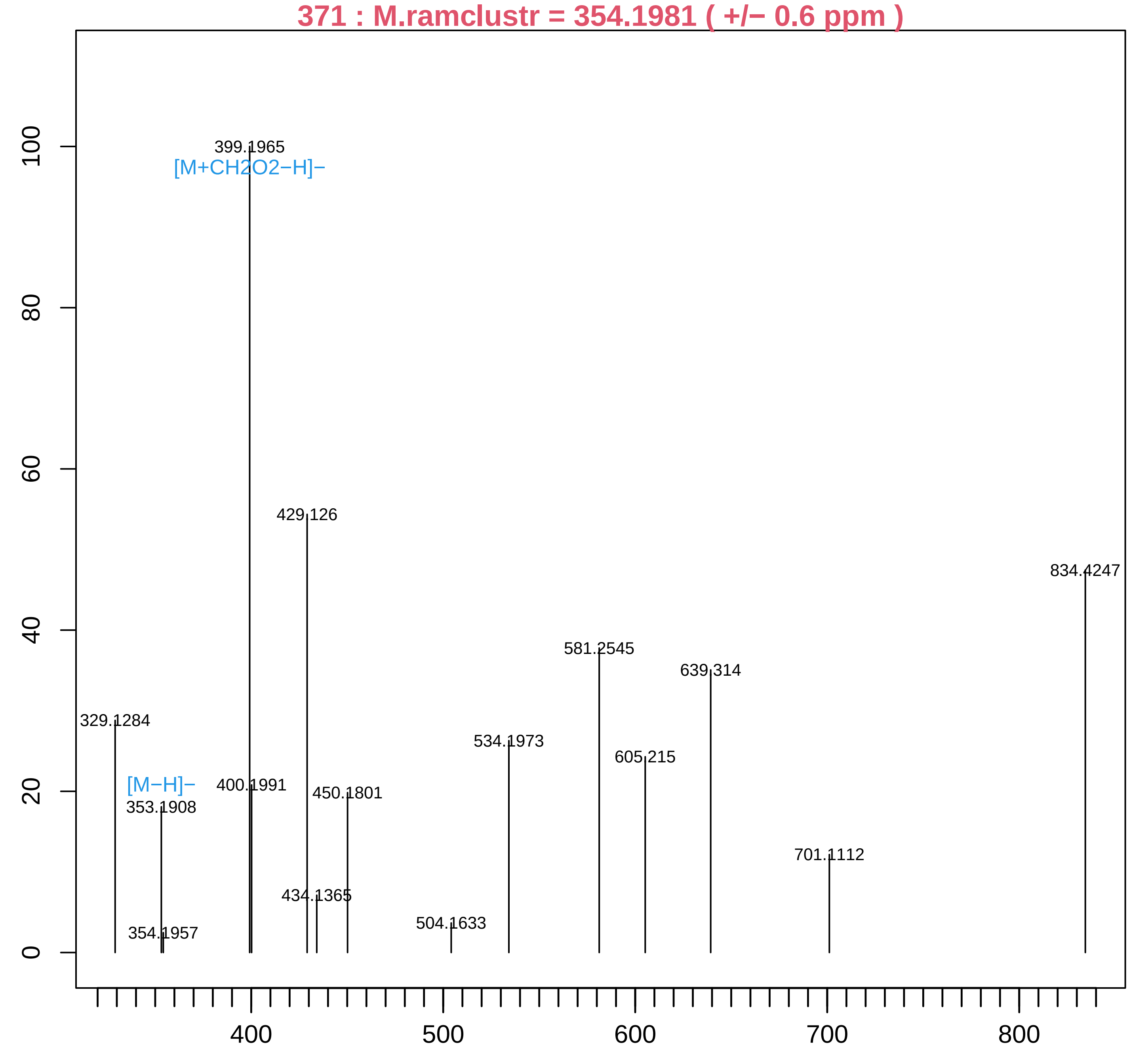
371 : M.ramclustr = 354.1981 (+/− 0.6 ppm)
399.1965
[M+CH2O2−H]−
429.126
834.4247
581.2545
639.314
329.1284
534.1973
605.215
[M−H]−
400.1991
353.1908
450.1801
701.1112
434.1365
504.1633
354.1957
0
20
40
60
80
100
400
500
600
700
800

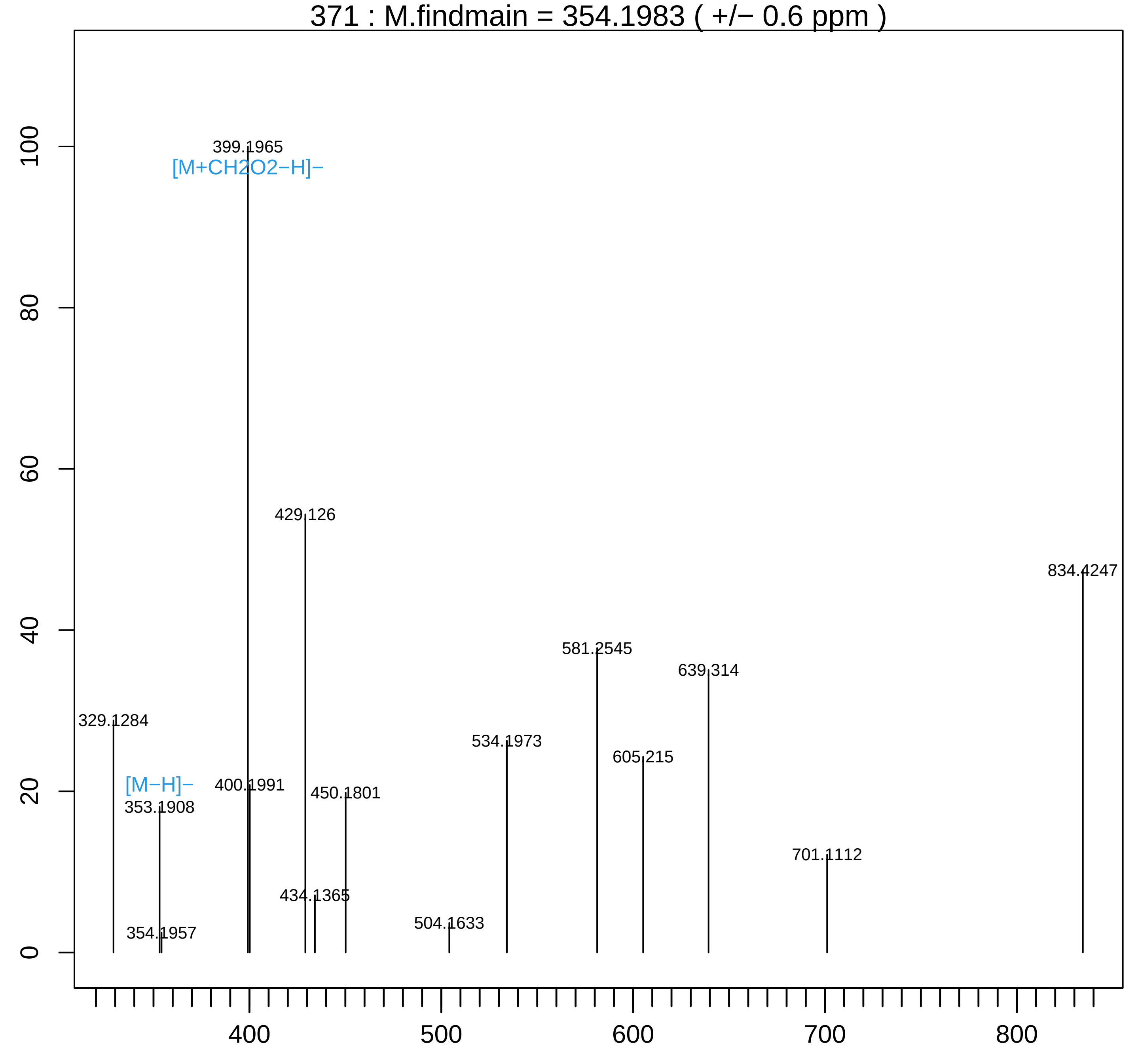
371 : M.findmain = 354.1983 (+/− 0.6 ppm)
399.1965
[M+CH2O2−H]−
429.126
834.4247
581.2545
639.314
329.1284
534.1973
605.215
[M−H]−
400.1991
353.1908
450.1801
701.1112
434.1365
504.1633
354.1957
0
20
40
60
80
100
400
500
600
700
800

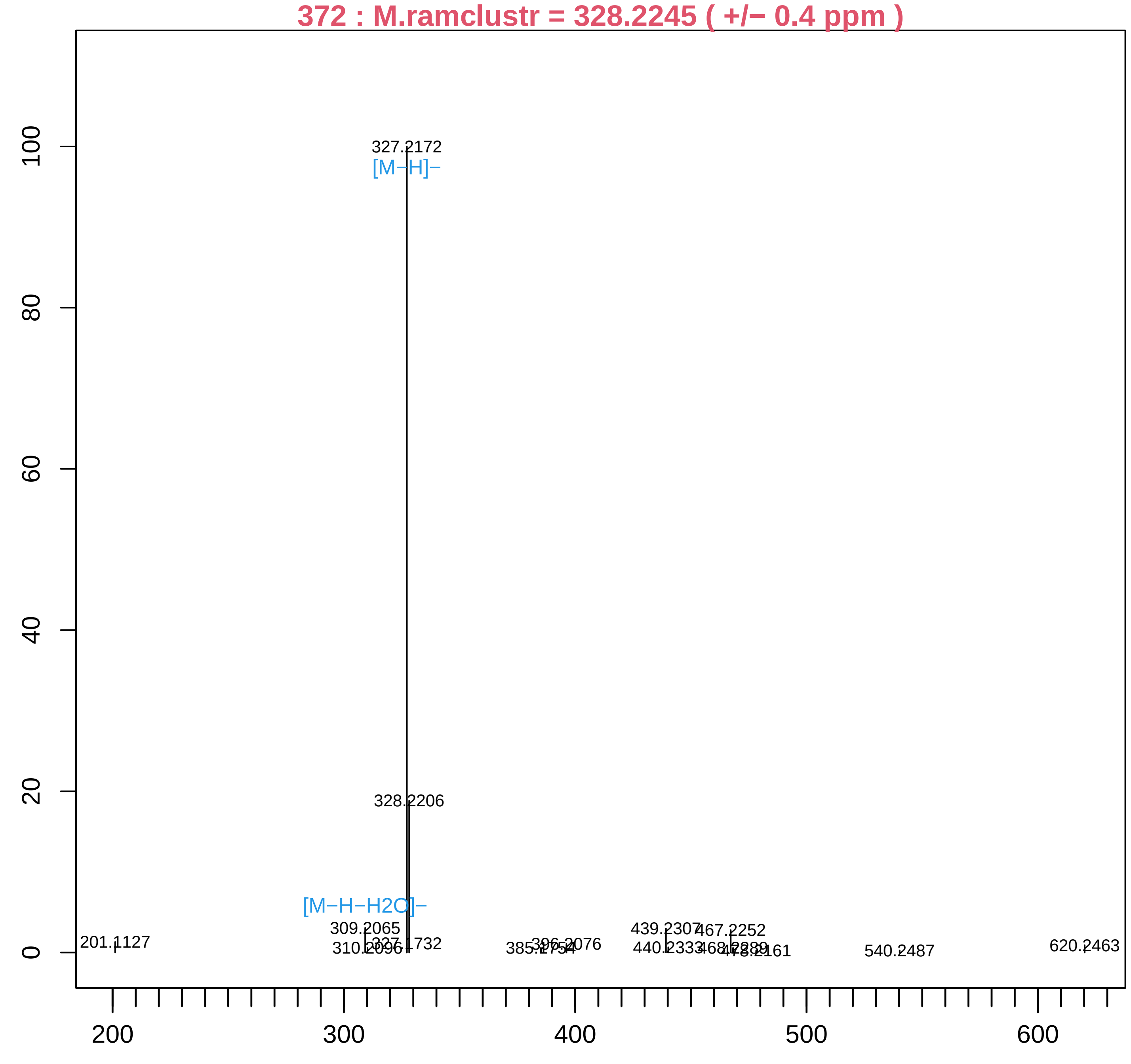
372 : M.ramclustr = 328.2245 (+/− 0.4 ppm)
327.2172
[M−H]−
328.2206
[M−H−H2O]−
309.2065
310.2096
327.1732
201.1127
385.1754
396.2076
439.2307
440.2333
467.2252
468.2289
478.2161
540.2487
620.2463
0
20
40
60
80
100
200
300
400
500
600

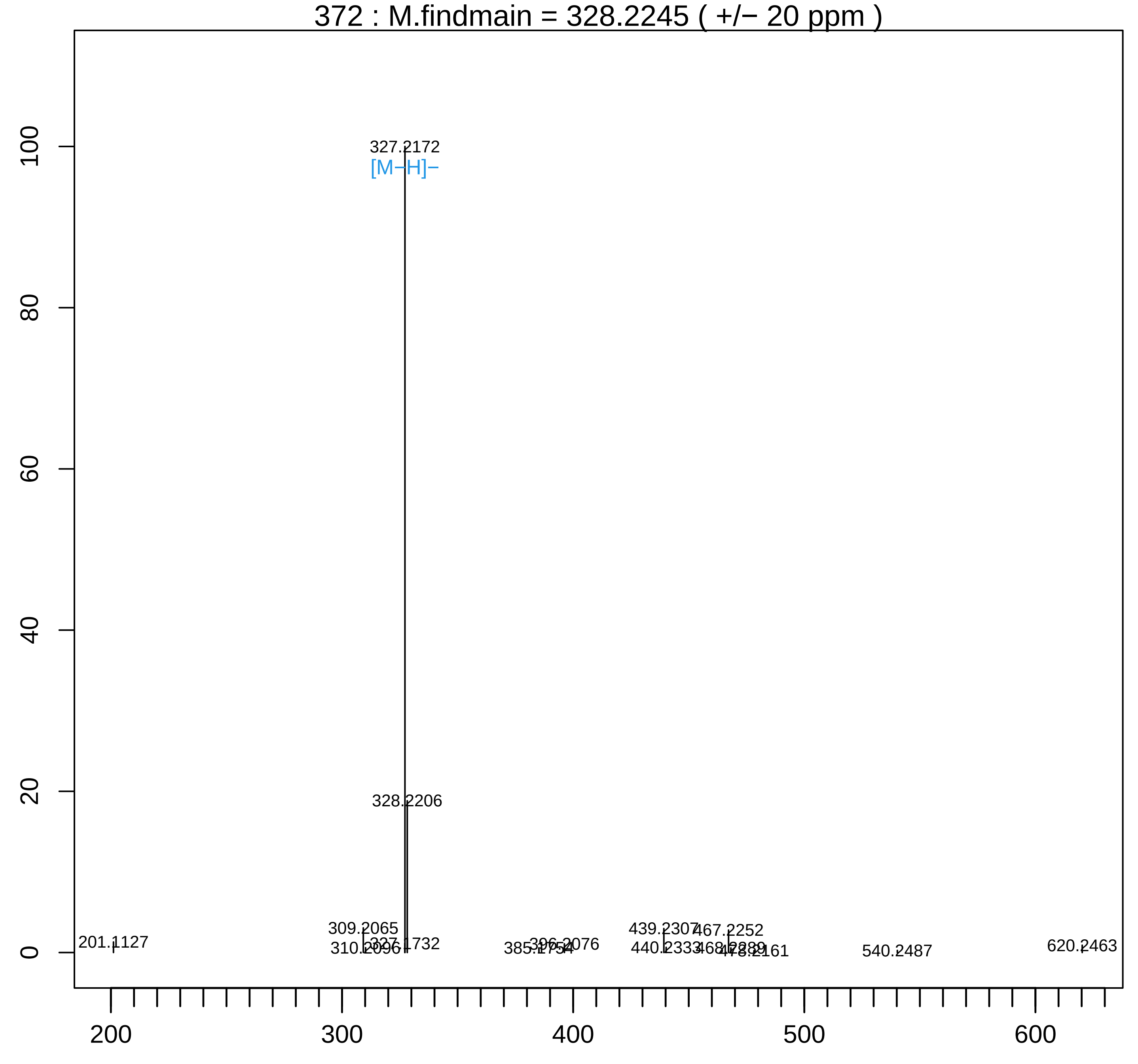
372 : M.findmain = 328.2245 (+/− 20 ppm)
327.2172
[M−H]−
328.2206
309.2065
310.2096
327.1732
201.1127
385.1754
396.2076
439.2307
440.2333
467.2252
468.2289
478.2161
540.2487
620.2463
0
20
40
60
80
100
200
300
400
500
600

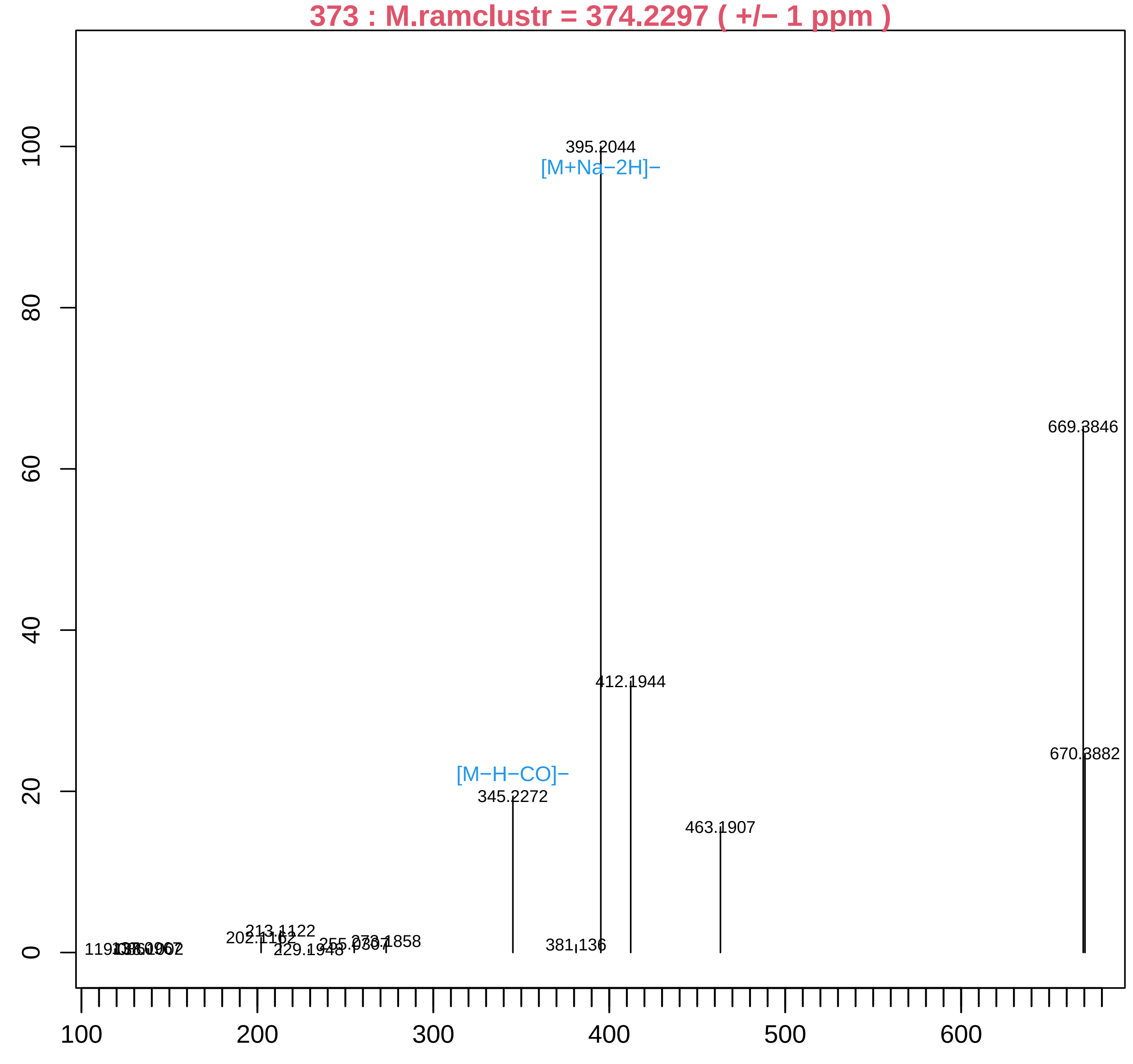

373 : M.ramclustr = 374.2297 (+/− 1 ppm)
395.2044
[M+Na−2H]−
669.3846
412.1944
670.3882
[M−H−CO]−
345.2272
463.1907
213.1122
202.1162
229.1948
273.1858
381.136

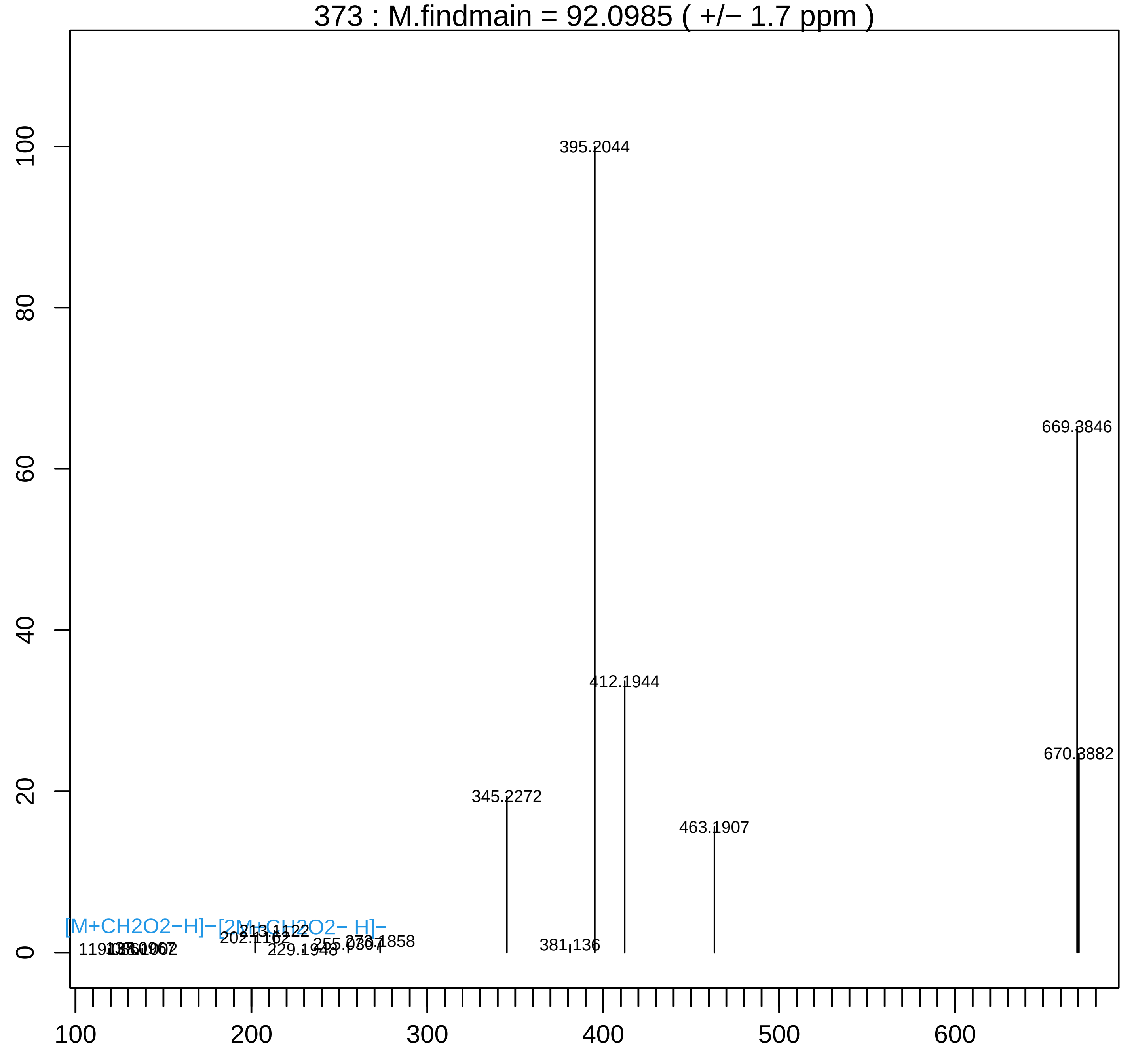

373 : M.findmain = 92.0985 (+/− 1.7 ppm)
395.2044
669.3846
412.1944
670.3882
345.2272
463.1907
381.136
273.1858
229.1948

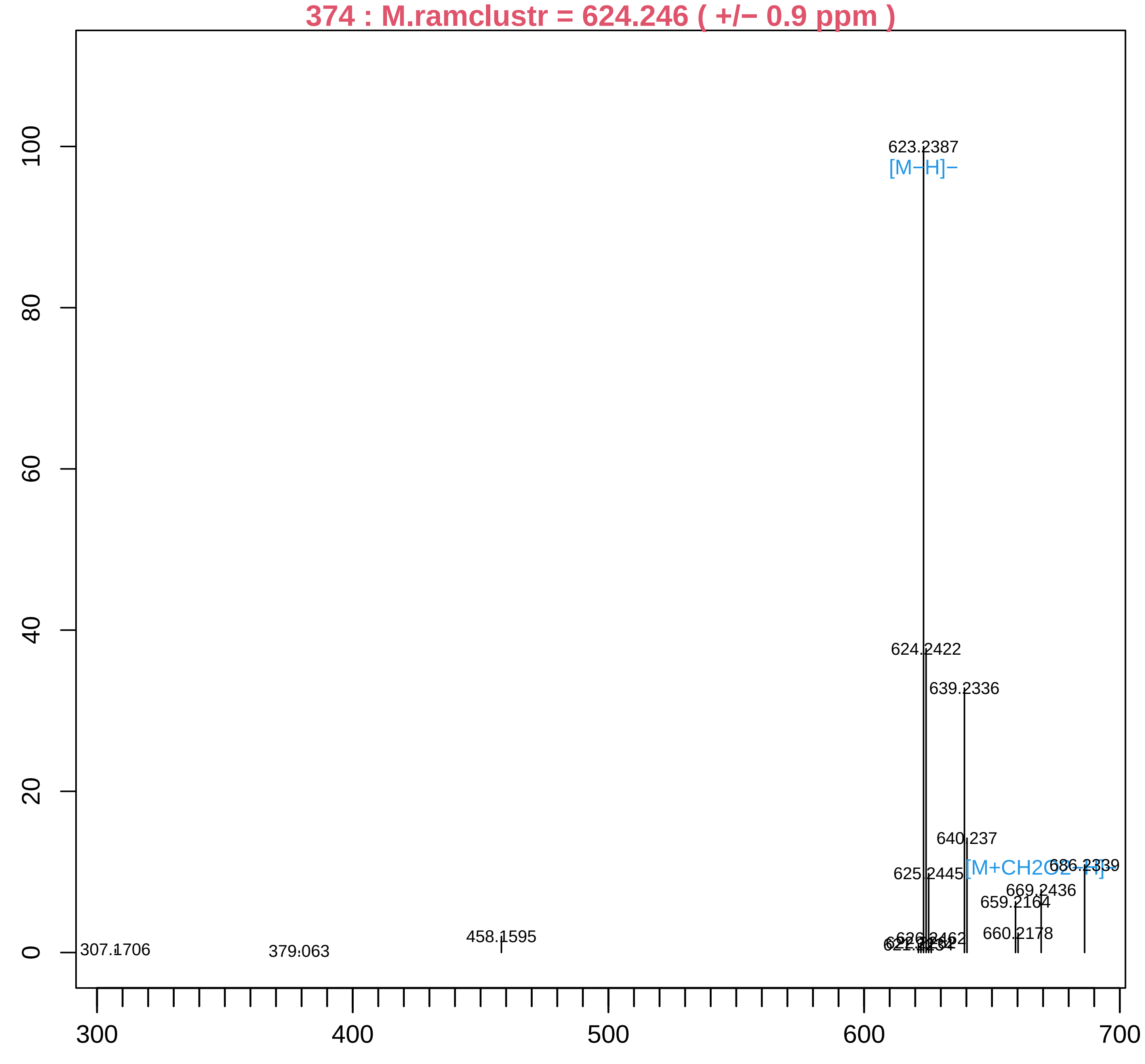
374 : M.ramclustr = 624.246 (+/− 0.9 ppm)
623.2387
[M−H]−
624.2422
639.2336
640.237
625.2445
[M+CH2O2−H]−
686.2339
669.2436
659.2164
660.2178
626.2462
458.1595
307.1706
379.063
0
20
40
60
80
100
300
400
500
600
700

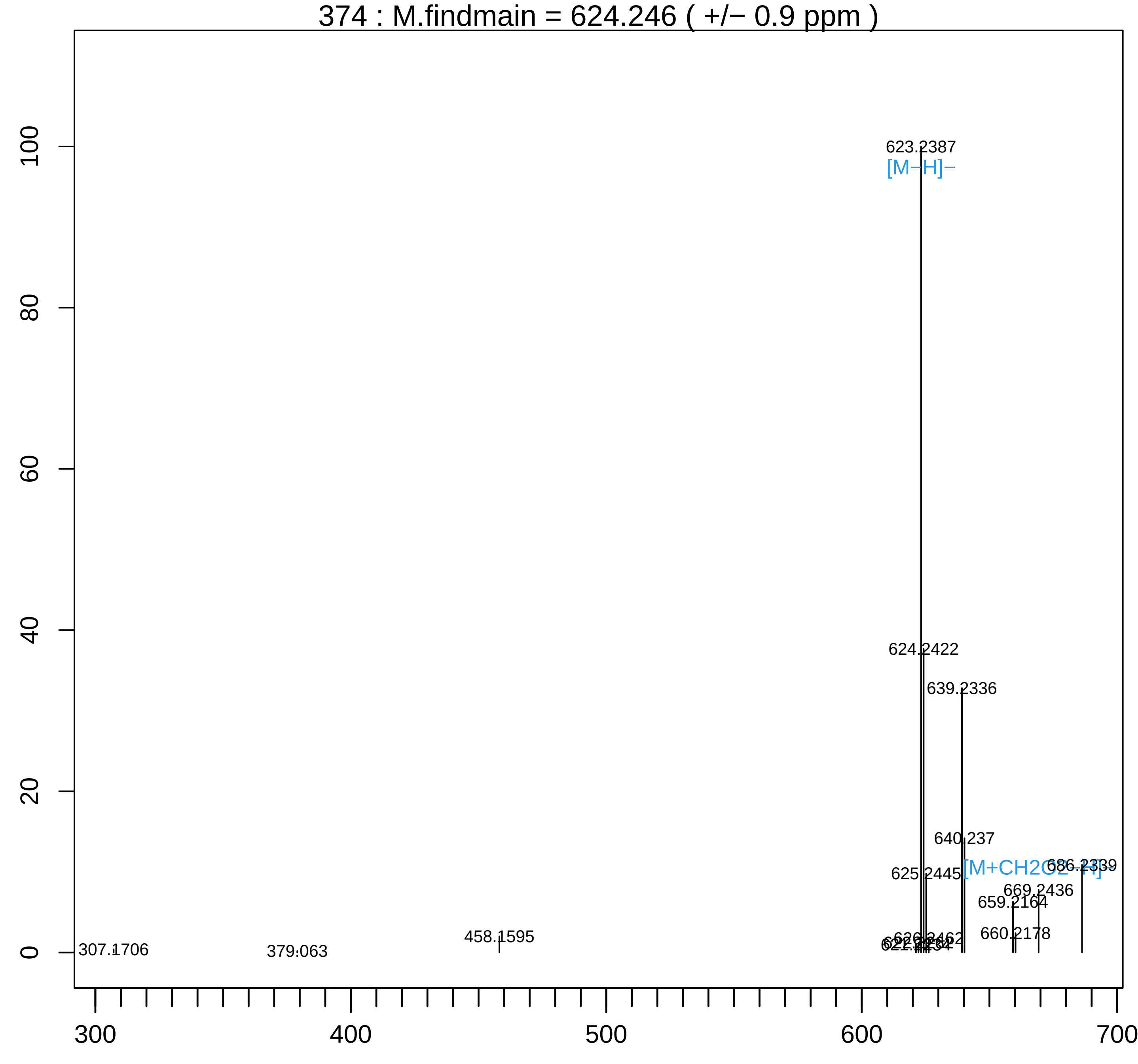
374 : M.findmain = 624.246 (+/− 0.9 ppm)
623.2387
[M−H]−
624.2422
639.2336
640.237
625.2445
[M+CH2O2−H]−
686.2339
669.2436
659.2164
660.2178
626.2462
458.1595
307.1706
379.063
0
20
40
60
80
100
300
400
500
600
700

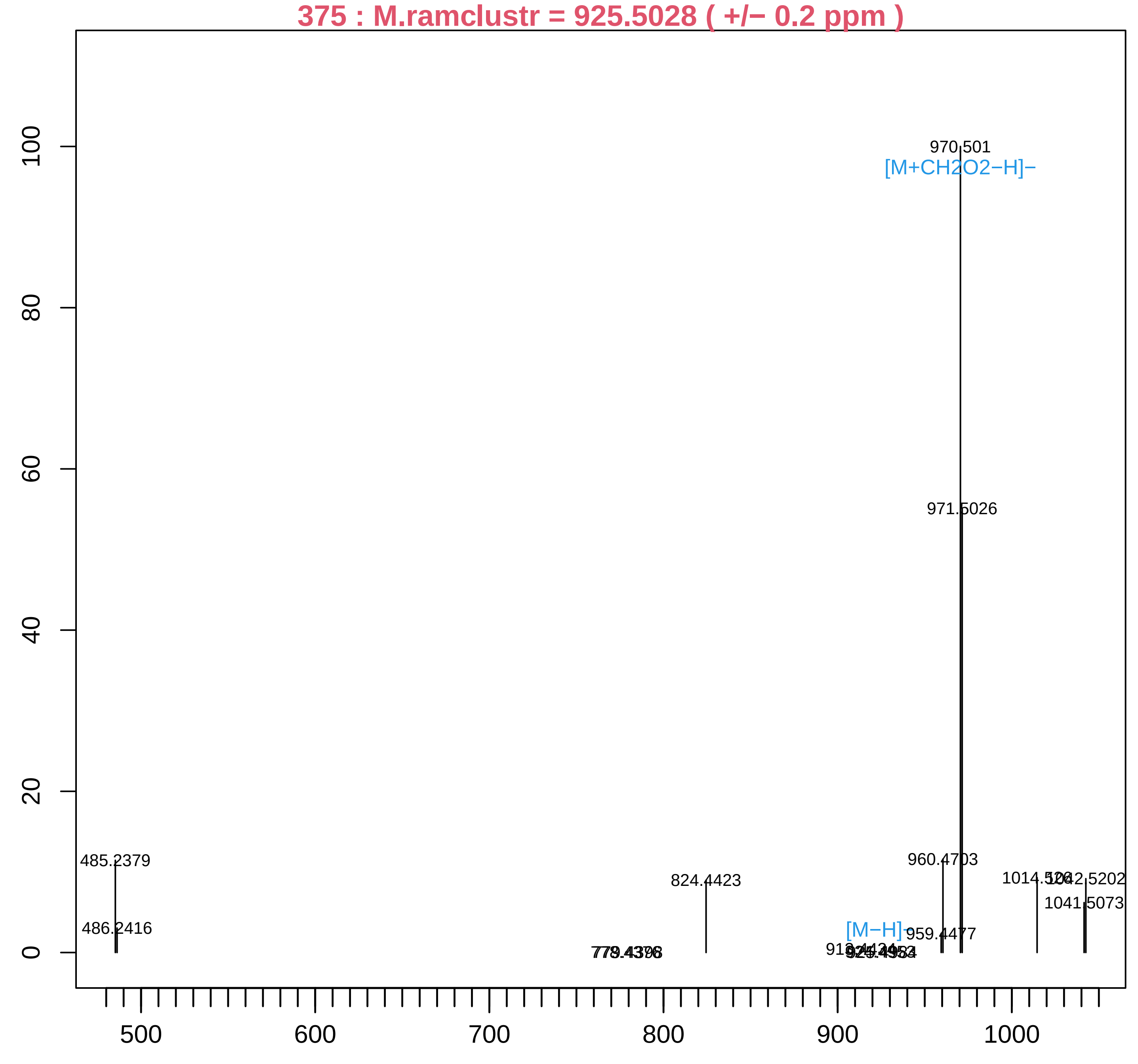
375 : M.ramclustr = 925.5028 (+/− 0.2 ppm)
970.501
[M+CH2O2−H]−
971.5026
485.2379
486.2416
824.4423
960.4703
959.4477
[M−H]−
1014.5262
1042.5202
1041.5073
0
20
40
60
80
100
500
600
700
800
900
1000

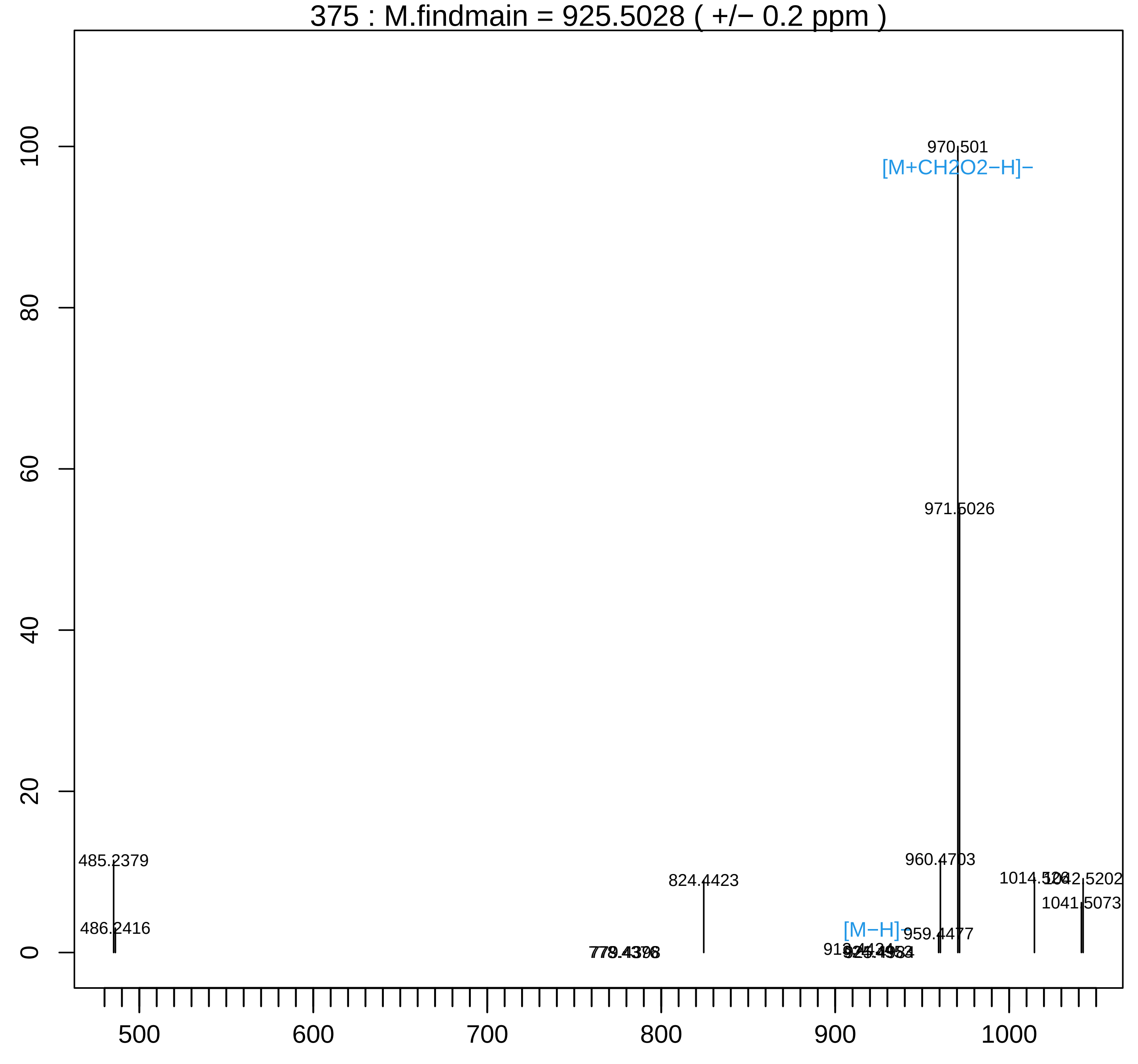
375 : M.findmain = 925.5028 (+/− 0.2 ppm)
970.501
[M+CH2O2−H]−
971.5026
485.2379
486.2416
824.4423
960.4703
959.4477
[M−H]−
1014.5262
1042.5202
1041.5073
0
20
40
60
80
100
500
600
700
800
900
1000

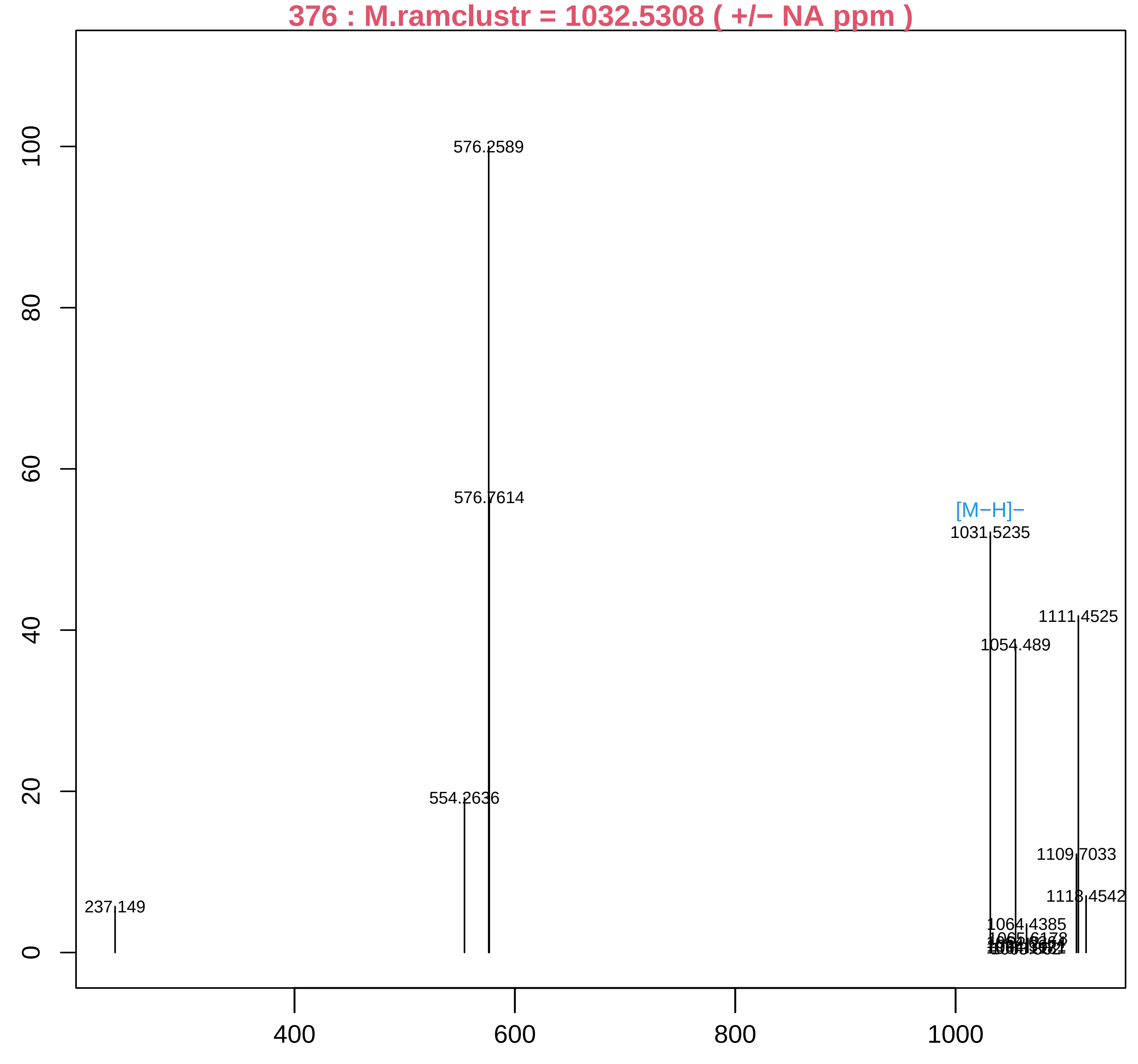
376 : M.ramclustr = 1032.5308 (+/− NA ppm)
576.2589
576.7614
554.2636
237.149
[M−H]−
1031.5235
1111.4525
1054.489
1109.7033
1118.4542
1064.4385
1065.6178
0
20
40
60
80
100
400
600
800
1000

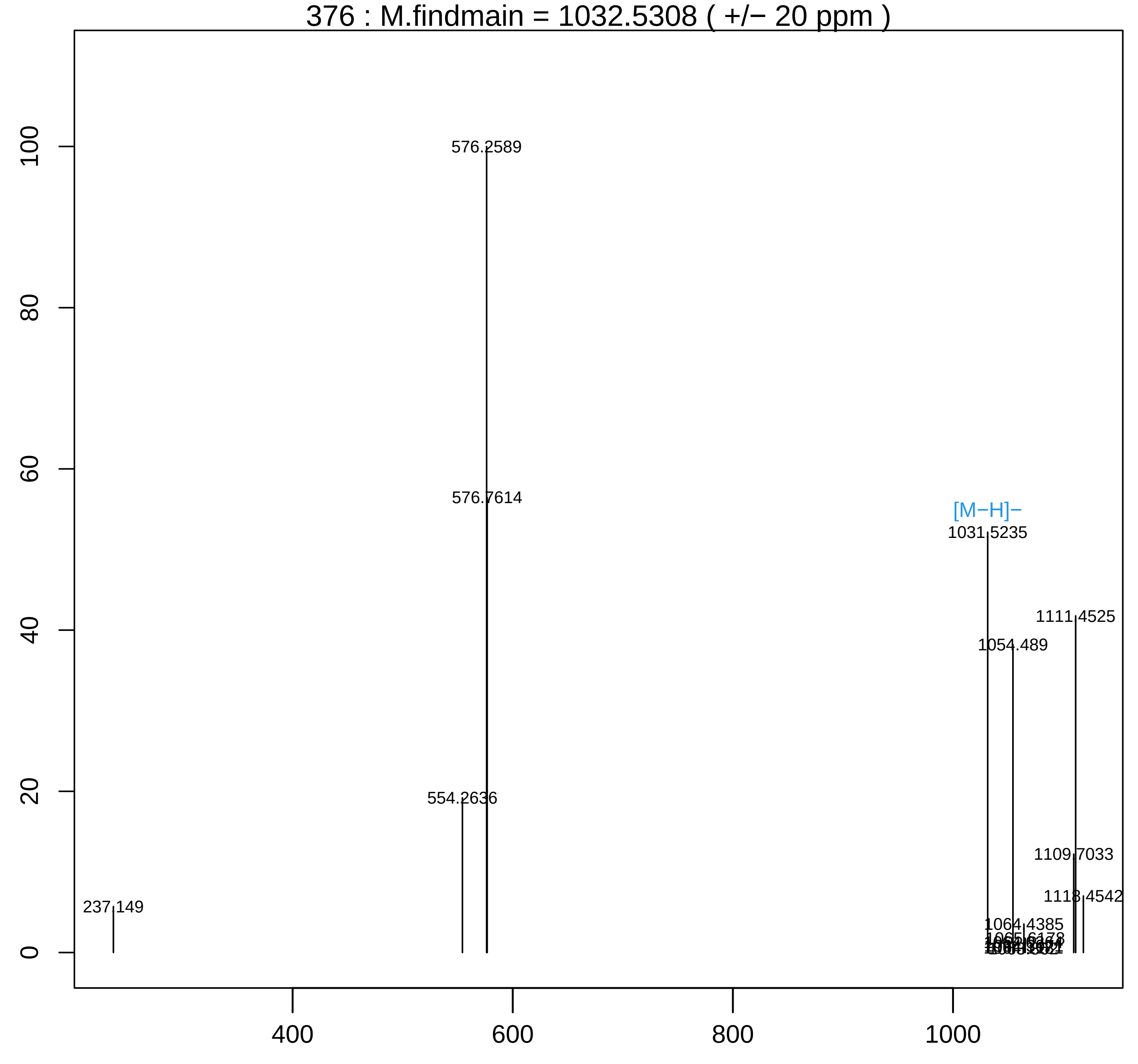
376 : M.findmain = 1032.5308 (+/− 20 ppm)
576.2589
576.7614
554.2636
237.149
[M−H]−
1031.5235
1111.4525
1054.489
1109.7033
1118.4542
1064.4385
1065.6178
0
20
40
60
80
100
400
600
800
1000

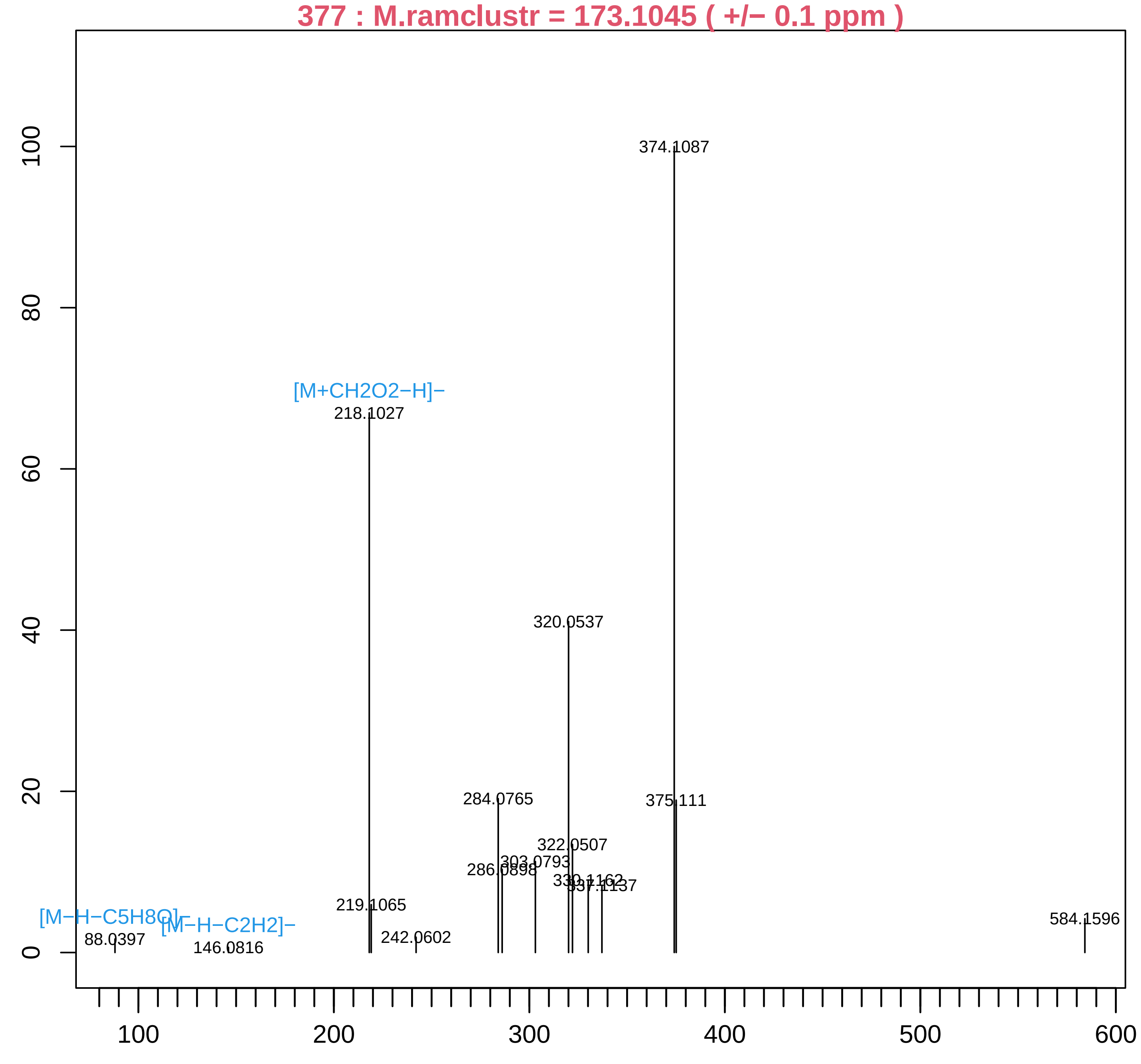
377 : M.ramclustr = 173.1045 (+/− 0.1 ppm)
[M+CH2O2−H]−
218.1027
374.1087
320.0537
284.0765
375.111
322.0507
303.0793
286.0898
330.1162
337.1137
219.1065
[M−H−C5H8O]−
[M−H−C2H2]−
88.0397
146.0816
242.0602
584.1596
0
20
40
60
80
100
100
200
300
400
500
600

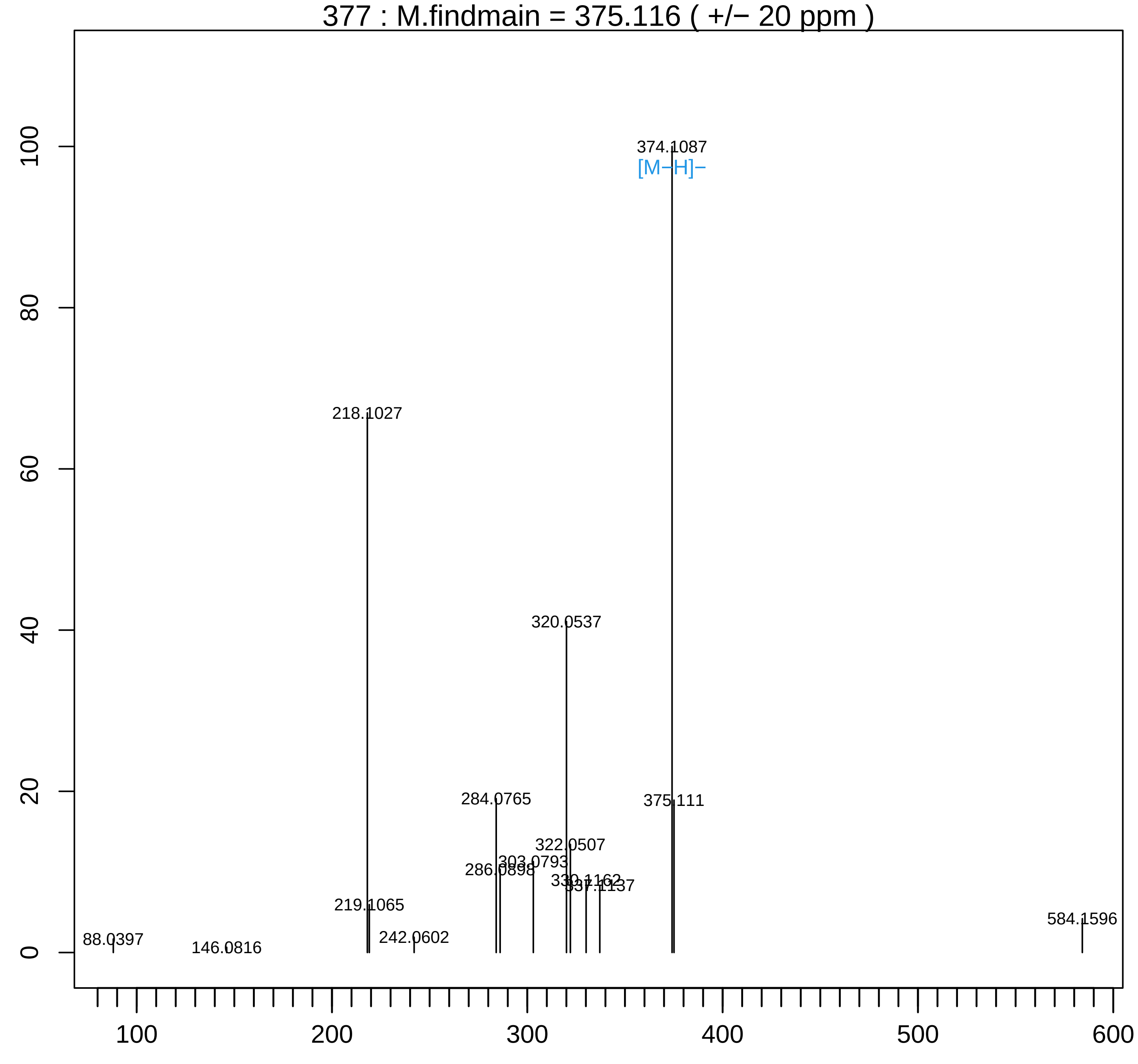
377 : M.findmain = 375.116 (+/− 20 ppm)
374.1087
[M−H]−
218.1027
320.0537
284.0765
375.111
322.0507
303.0793
286.0898
330.1162
337.1137
219.1065
88.0397
146.0816
242.0602
584.1596
0
20
40
60
80
100
100
200
300
400
500
600

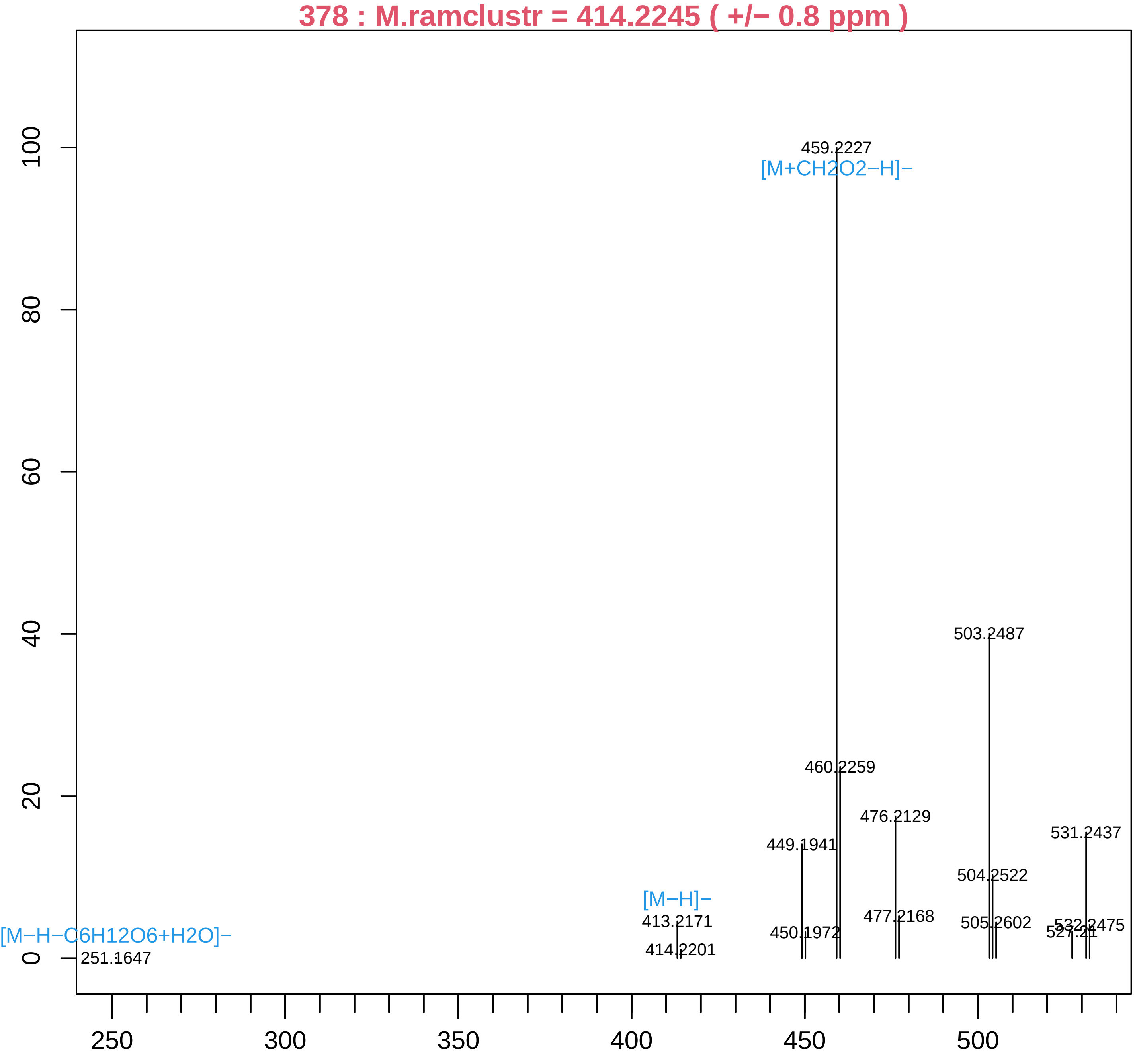

378 : M.ramclustr = 414.2245 (+/− 0.8 ppm)
459.2227
[M+CH2O2−H]−
503.2487
460.2259
476.2129
449.1941
531.2437
504.2522
[M−H]−
413.2171
477.2168
505.2602
532.2475
527.21
450.1972
[M−H−C6H12O6+H2O]−
414.2201
251.1647
0
20
40
60
80
100
250
300
350
400
450
500

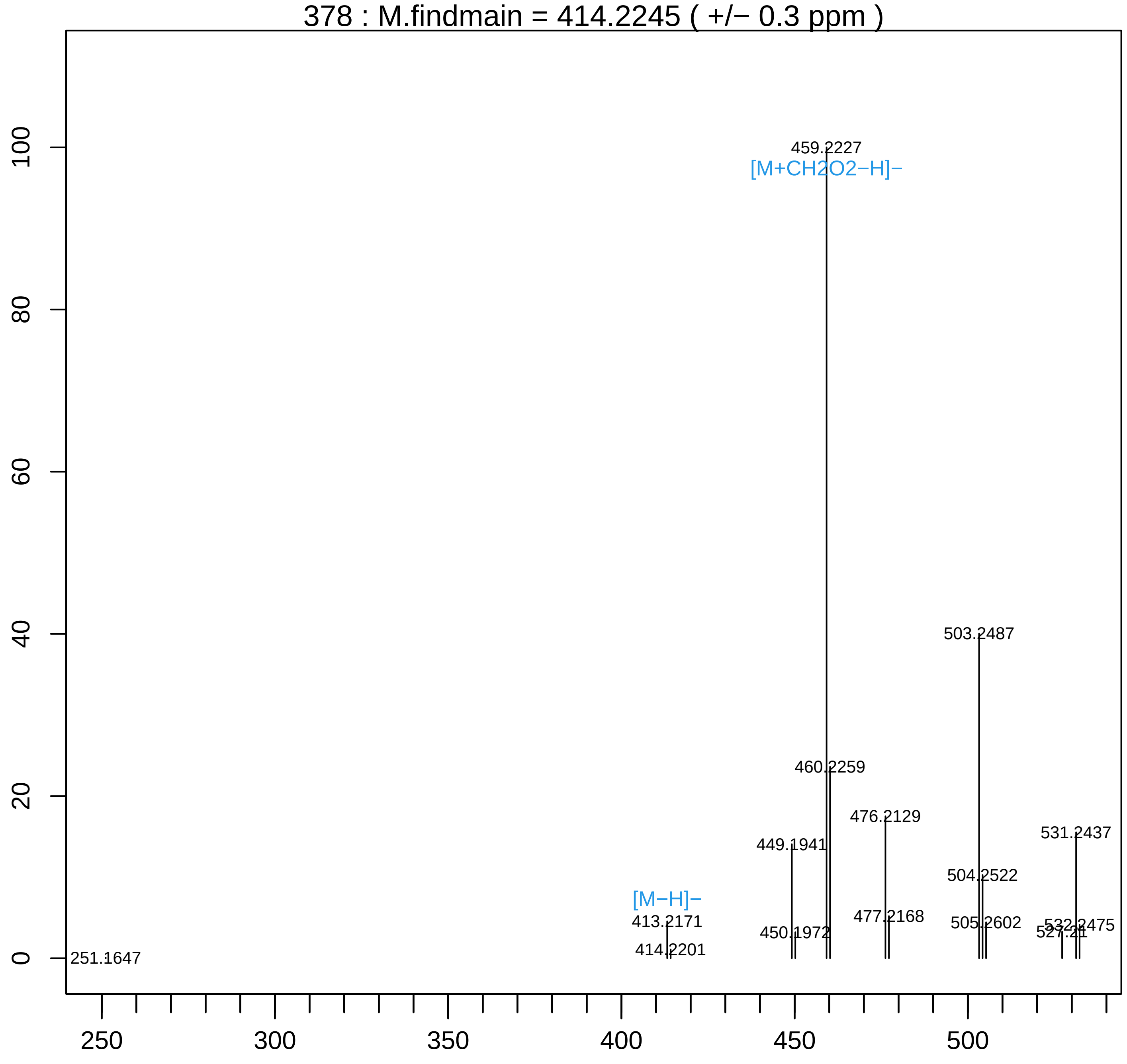

378 : M.findmain = 414.2245 (+/− 0.3 ppm)
459.2227
[M+CH2O2−H]−
503.2487
460.2259
476.2129
449.1941
531.2437
504.2522
[M−H]−
413.2171
477.2168
505.2602
532.2475
527.21
450.1972
414.2201
251.1647
0
20
40
60
80
100
250
300
350
400
450
500

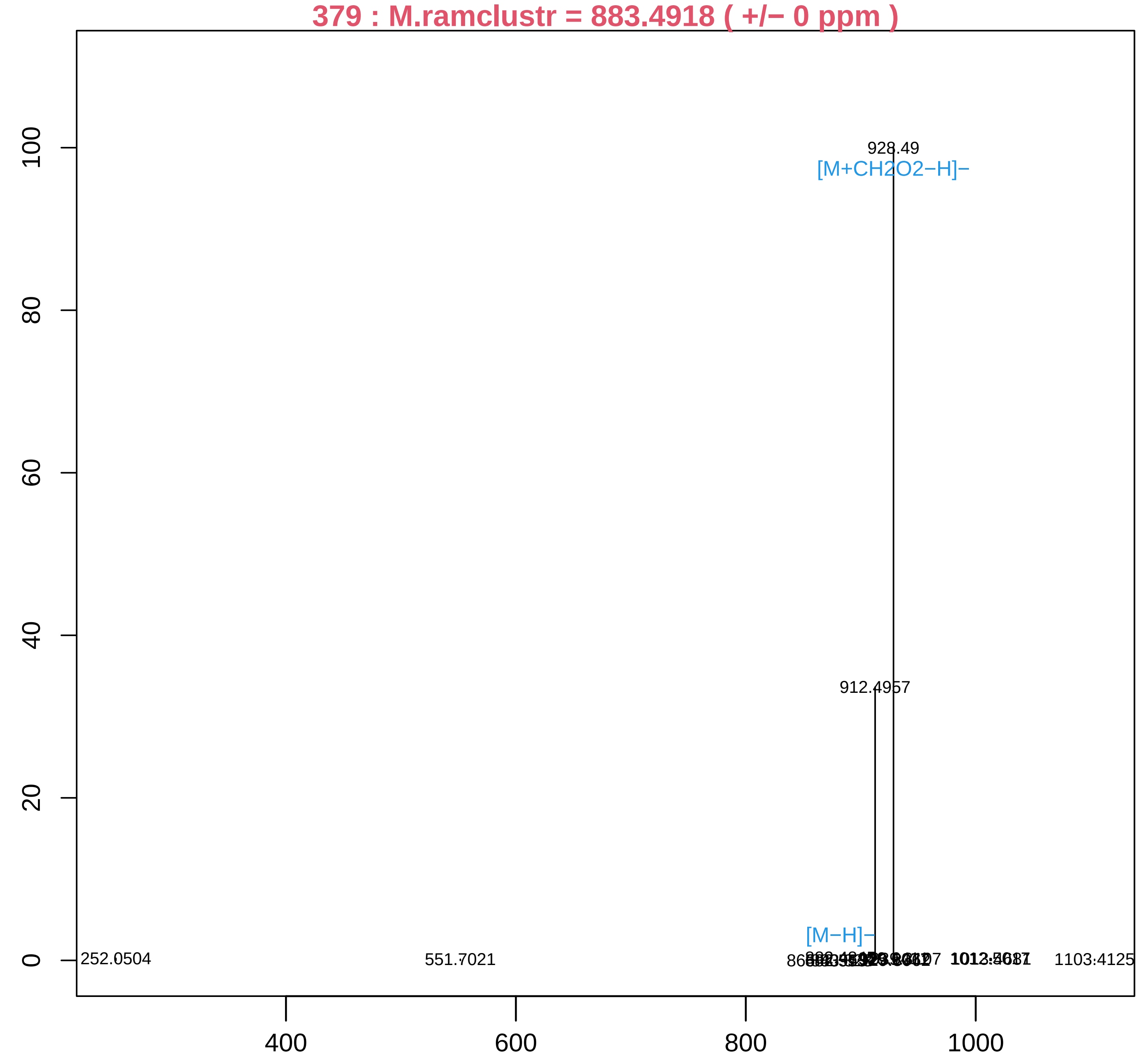
379 : M.ramclustr = 883.4918 (+/− 0 ppm)
928.49
[M+CH2O2−H]−
912.4957
[M−H]−
252.0504
551.7021
1103.4125
0
20
40
60
80
100
400
600
800
1000

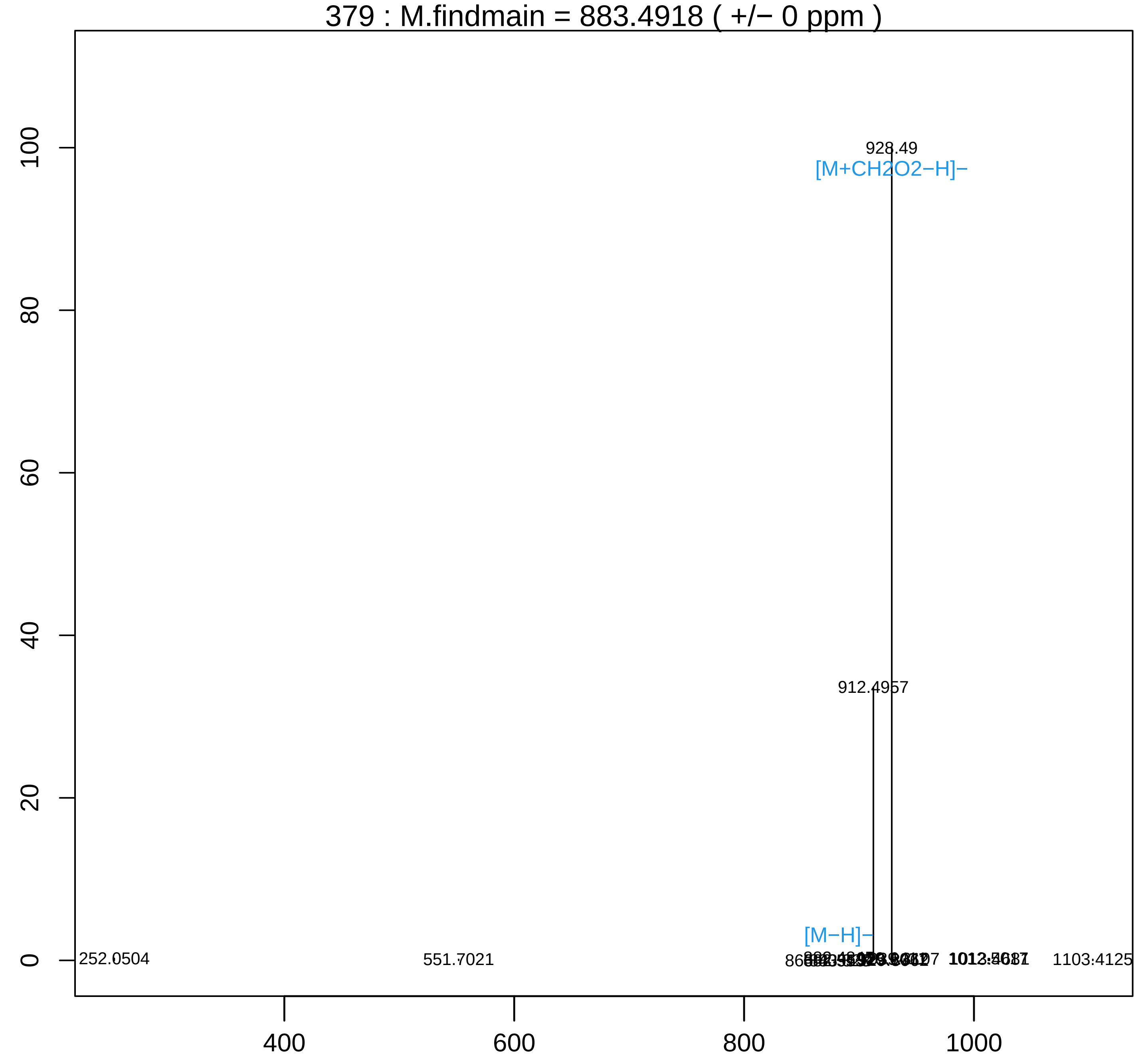
379 : M.findmain = 883.4918 (+/− 0 ppm)
928.49
[M+CH2O2−H]−
912.4957
[M−H]−
252.0504
551.7021
1103.4125
0
20
40
60
80
100
400
600
800
1000

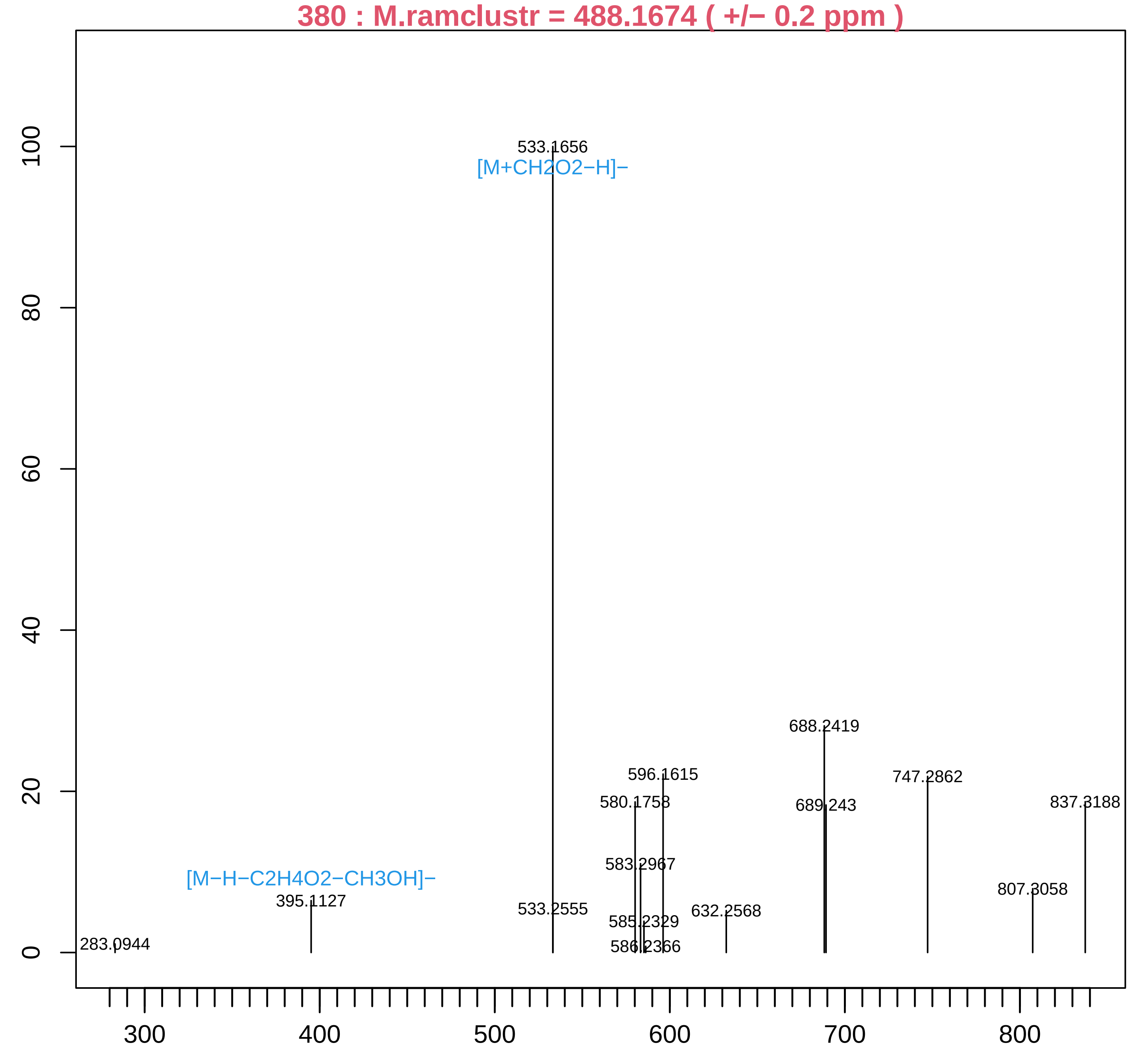

380 : M.ramclustr = 488.1674 (+/− 0.2 ppm)
533.1656
[M+CH2O2−H]−
596.1615
580.1758
583.2967
585.2329
586.2366
533.2555
632.2568
688.2419
689.243
747.2862
807.3058
837.3188
395.1127
[M−H−C2H4O2−CH3OH]−
283.0944
0
20
40
60
80
100
300
400
500
600
700
800

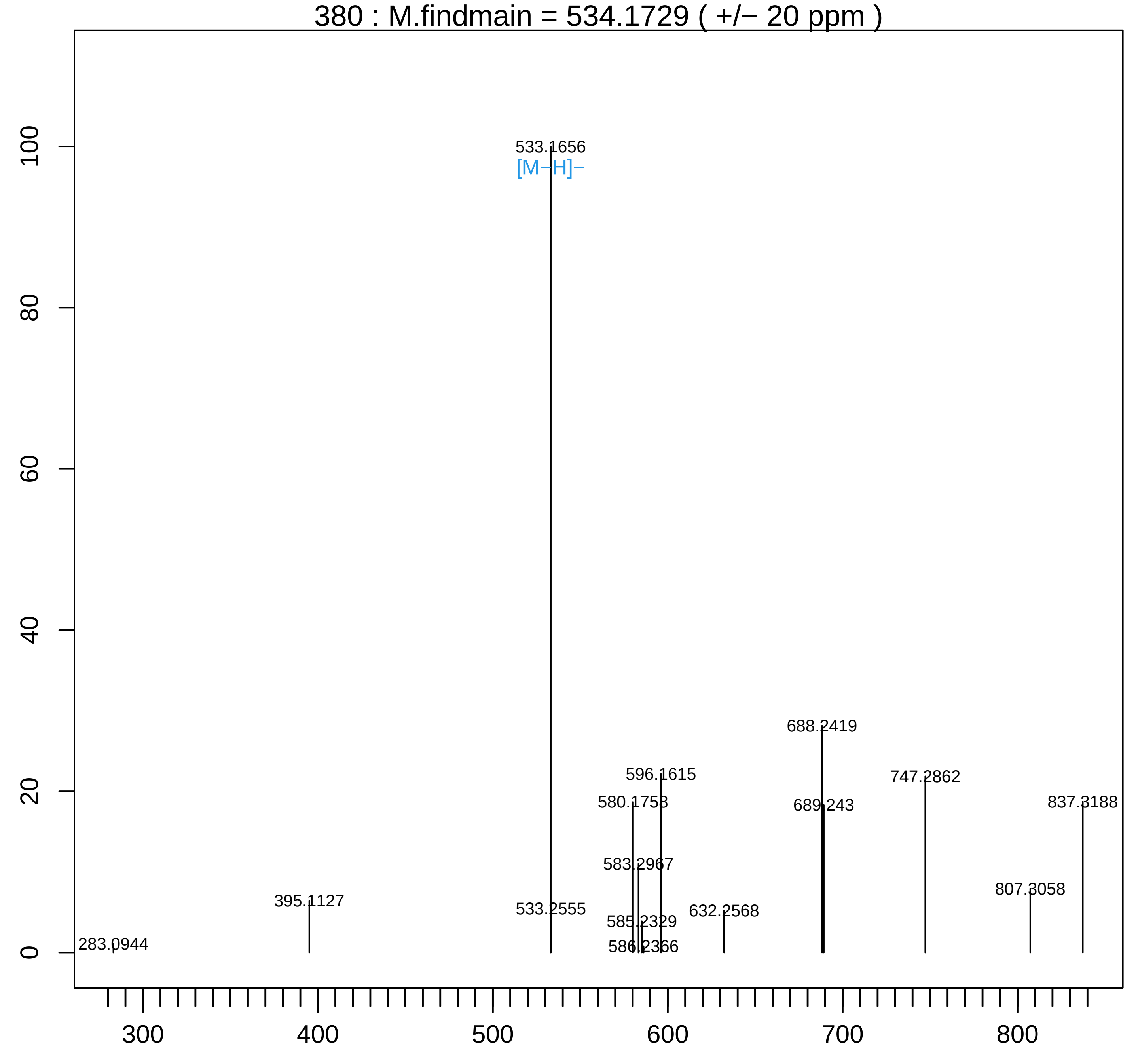

380 : M.findmain = 534.1729 (+/− 20 ppm)
533.1656
[M−H]−
596.1615
580.1758
583.2967
585.2329
586.2366
533.2555
632.2568
688.2419
689.243
747.2862
807.3058
837.3188
395.1127
283.0944
0
20
40
60
80
100
300
400
500
600
700
800

381 : M.ramclustr = 799.3511 (+/− 2.4 ppm)

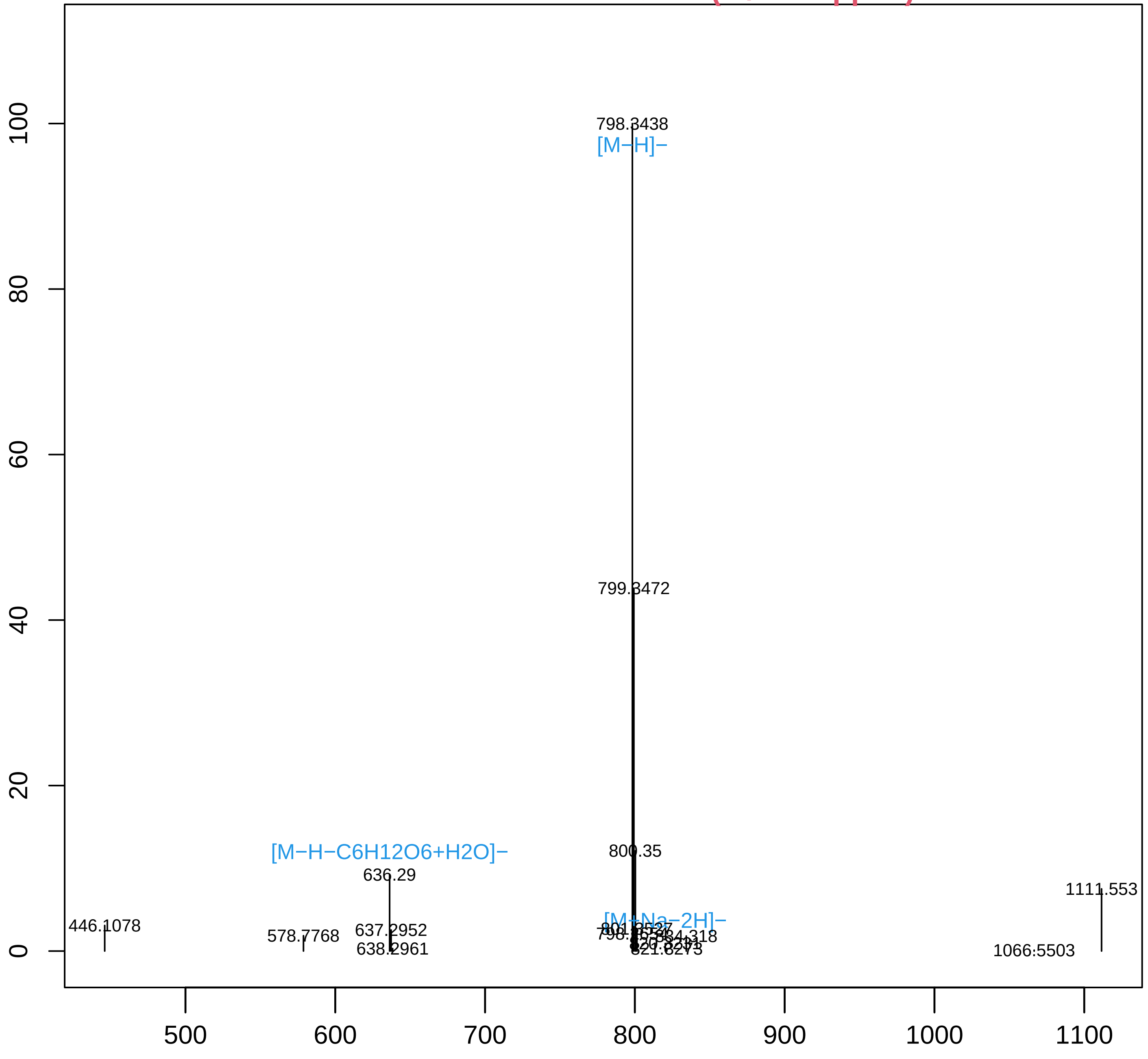

381 : M.findmain = 799.3511 (+/− 3.2 ppm)

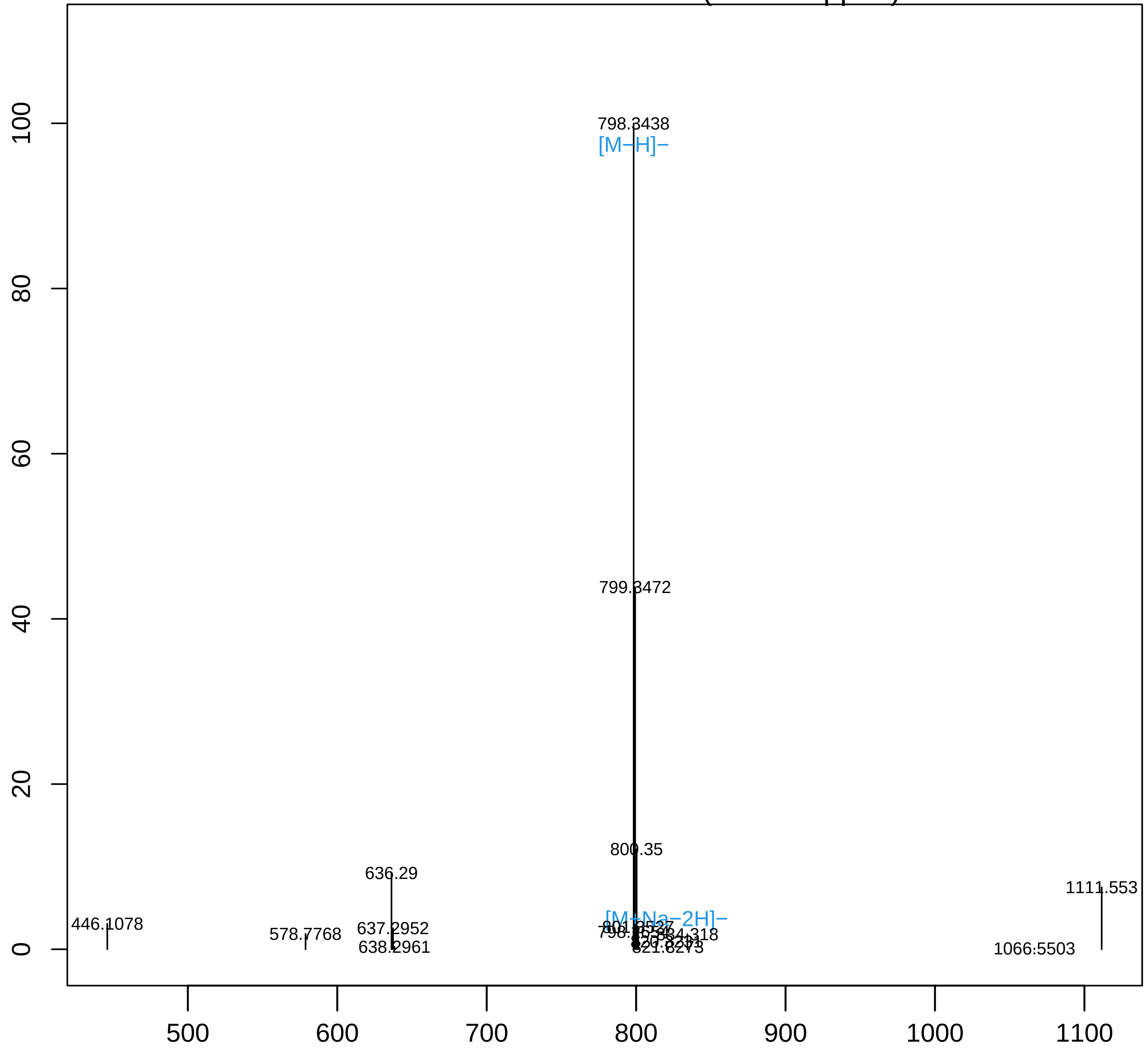

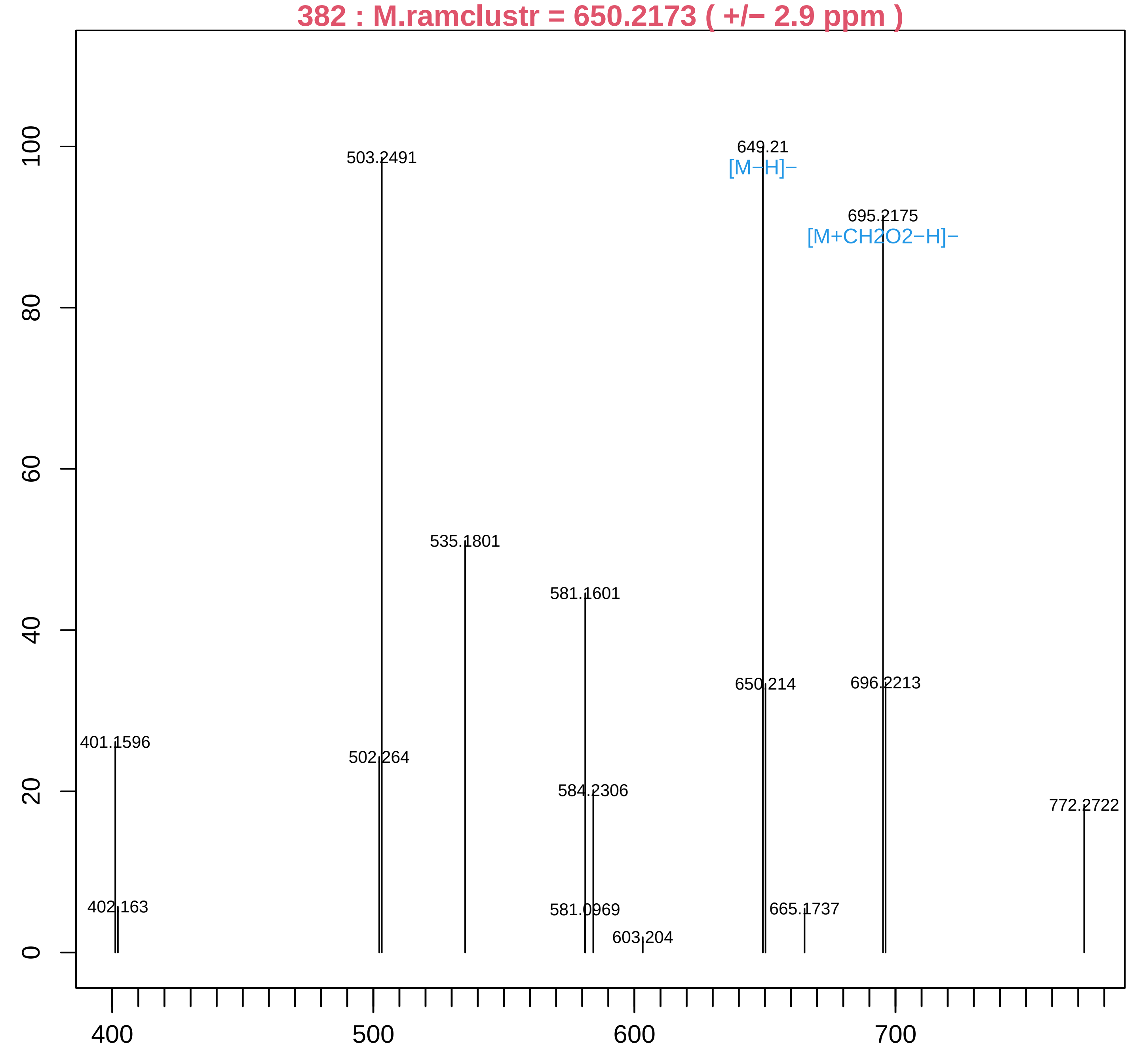
382 : M.ramclustr = 650.2173 (+/− 2.9 ppm)
503.2491
649.21
[M−H]−
695.2175
[M+CH2O2−H]−
535.1801
581.1601
650.214
696.2213
401.1596
502.264
584.2306
772.2722
402.163
581.0969
665.1737
603.204
0
20
40
60
80
100
400
500
600
700

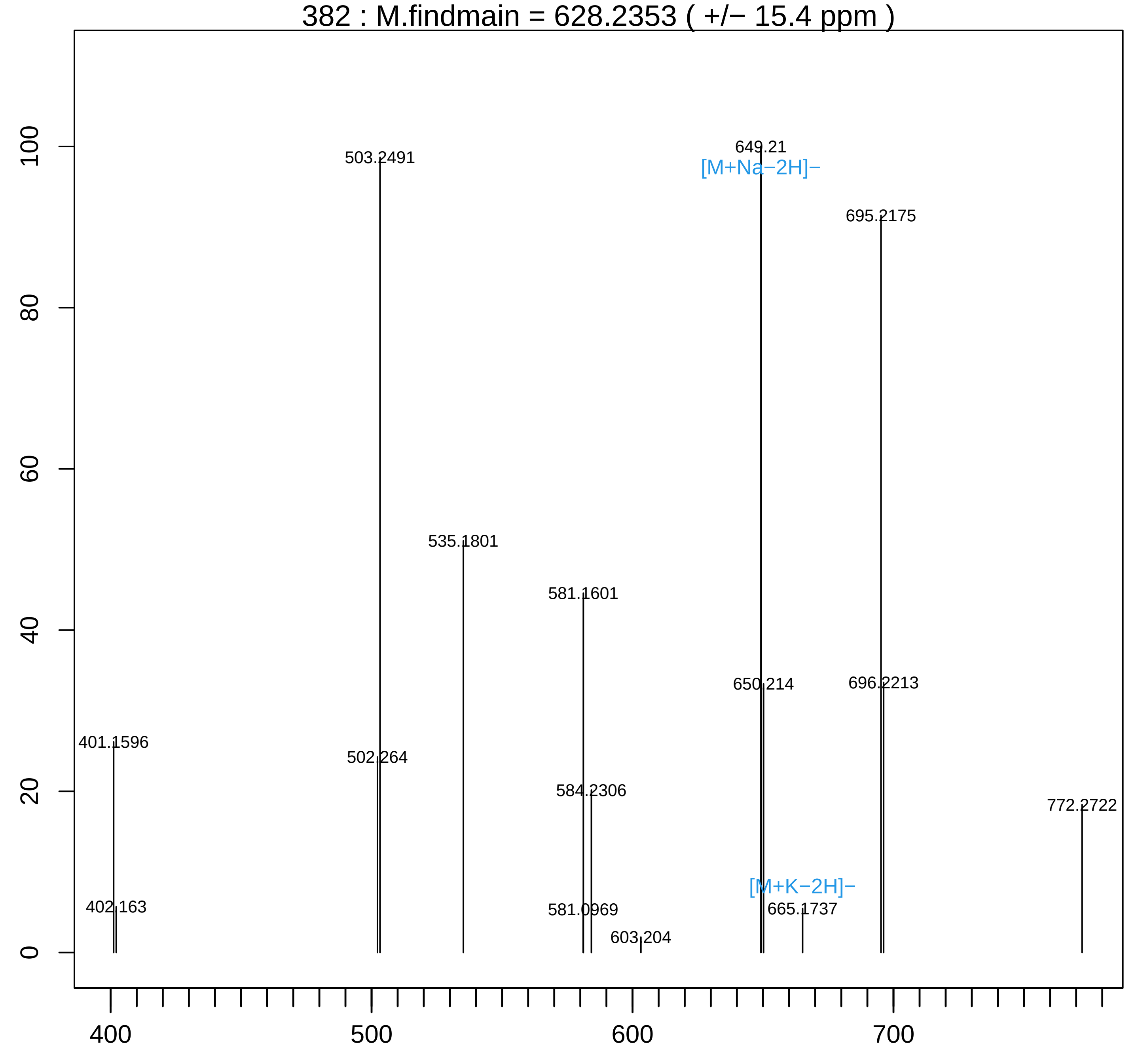
382 : M.findmain = 628.2353 (+/− 15.4 ppm)
503.2491
649.21
[M+Na−2H]−
695.2175
535.1801
581.1601
650.214
696.2213
401.1596
502.264
584.2306
772.2722
[M+K−2H]−
402.163
581.0969
665.1737
603.204
0
20
40
60
80
100
400
500
600
700

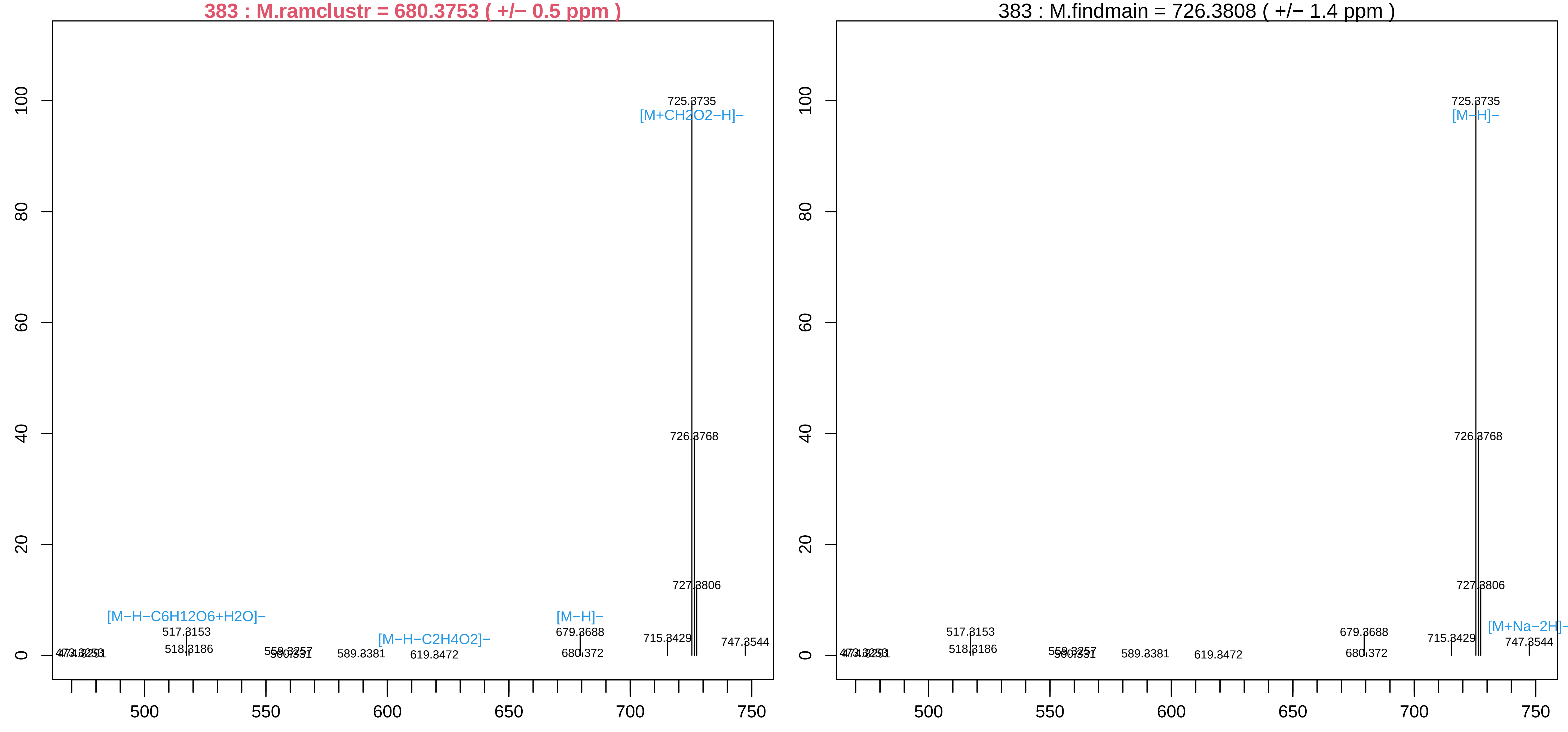
383 : M.ramclustr = 680.3753 (+/− 0.5 ppm)
725.3735
[M+CH2O2−H]−
726.3768
727.3806
[M−H−C6H12O6+H2O]−
517.3153
518.3186
[M−H−C2H4O2]−
589.3381
619.3472
[M−H]−
679.3688
680.372
715.3429
747.3544
383 : M.findmain = 726.3808 (+/− 1.4 ppm)
725.3735
[M−H]−
726.3768
727.3806
[M+Na−2H]−
517.3153
518.3186
589.3381
619.3472
679.3688
680.372
715.3429
747.3544
0
20
40
60
80
100
500
550
600
650
700
750

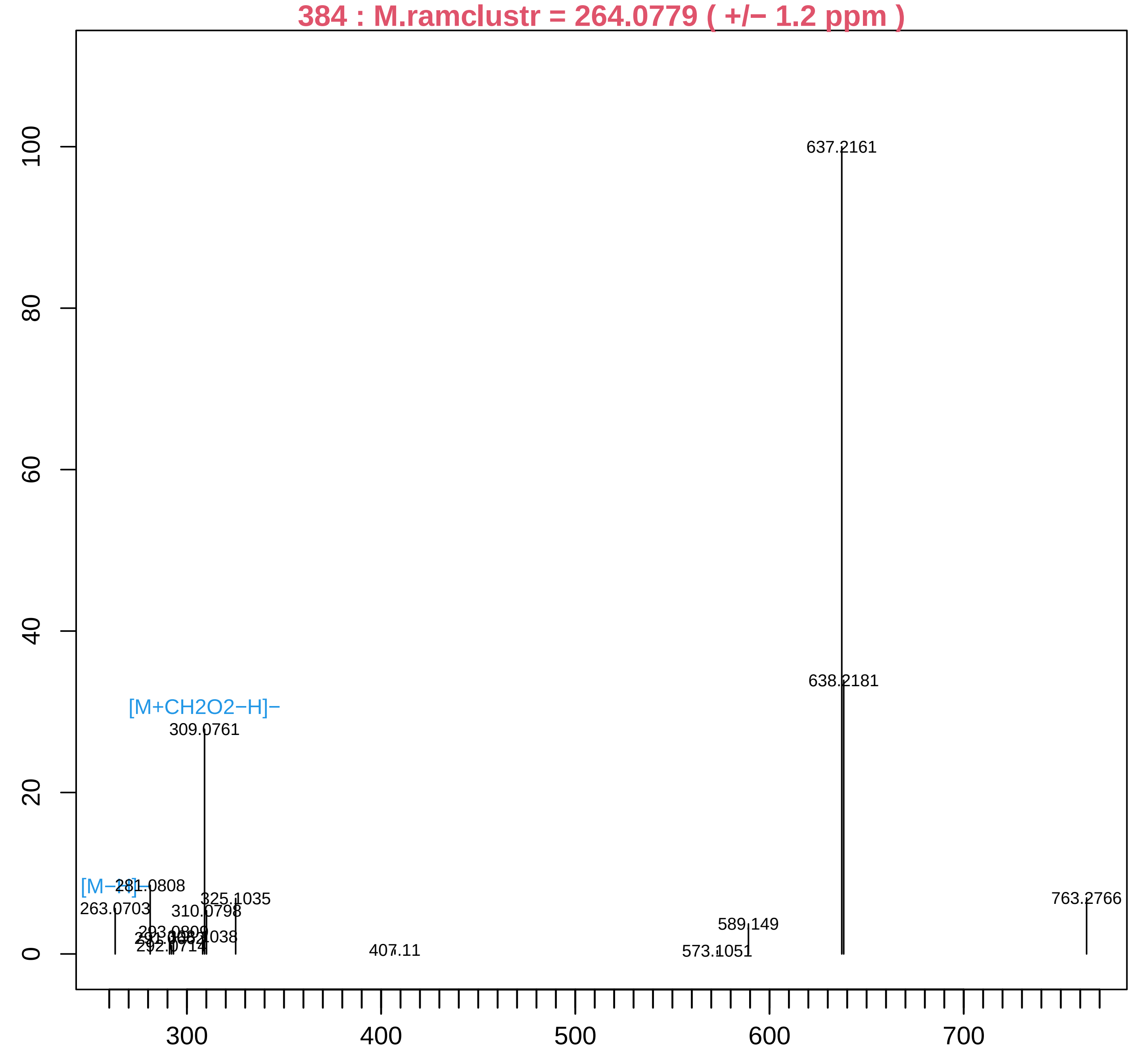

384 : M.ramclustr = 264.0779 (+/− 1.2 ppm)
637.2161
638.2181
[M+CH2O2−H]−
309.0761
[M−H]−
281.0808
263.0703
325.1035
310.0798
763.2766
589.149
407.11
573.1051
0
20
40
60
80
100
300
400
500
600
700

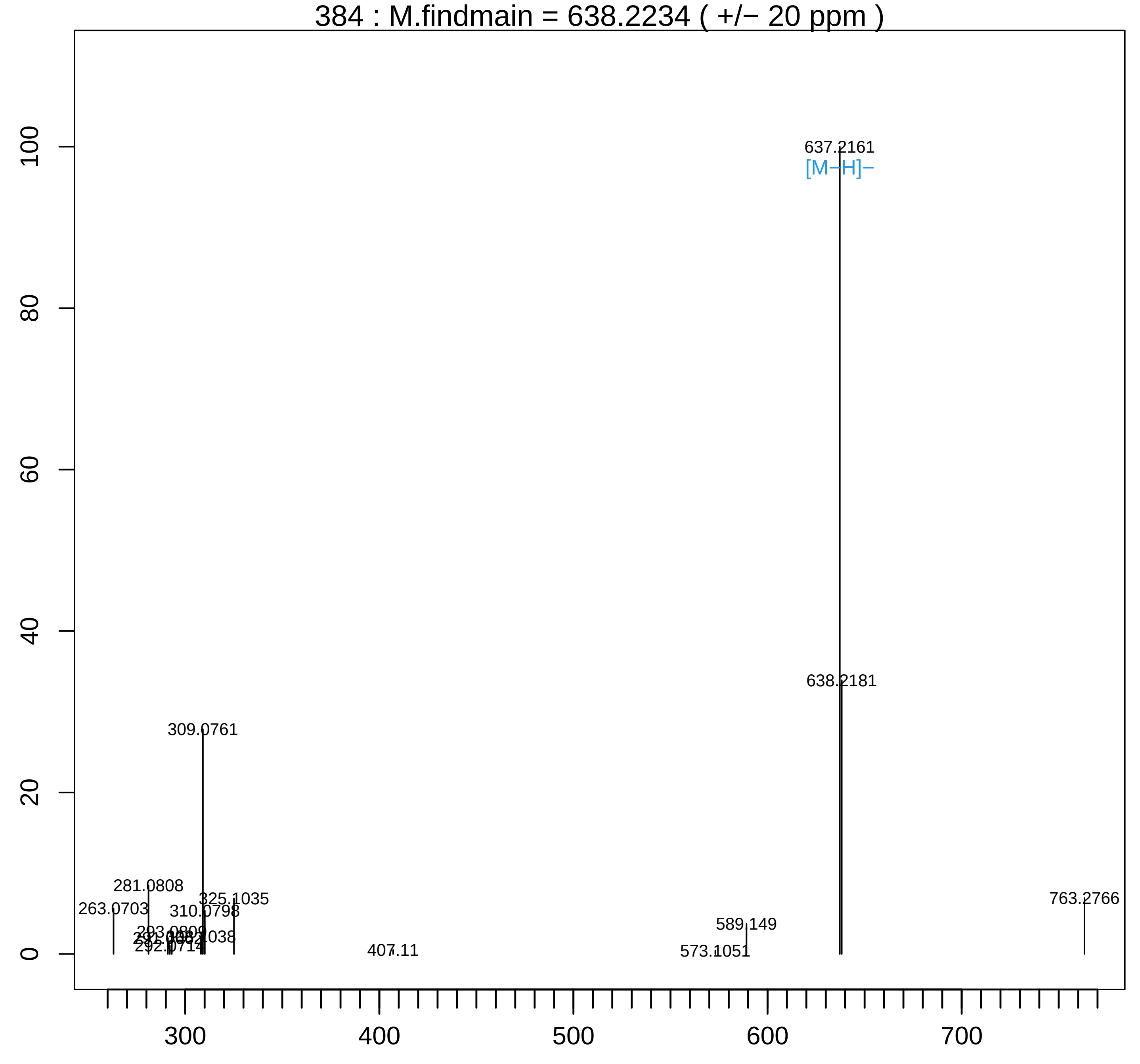

384 : M.findmain = 638.2234 (+/− 20 ppm)
637.2161
[M−H]−
638.2181
309.0761
281.0808
263.0703
325.1035
310.0798
763.2766
589.149
407.11
573.1051
0
20
40
60
80
100
300
400
500
600
700

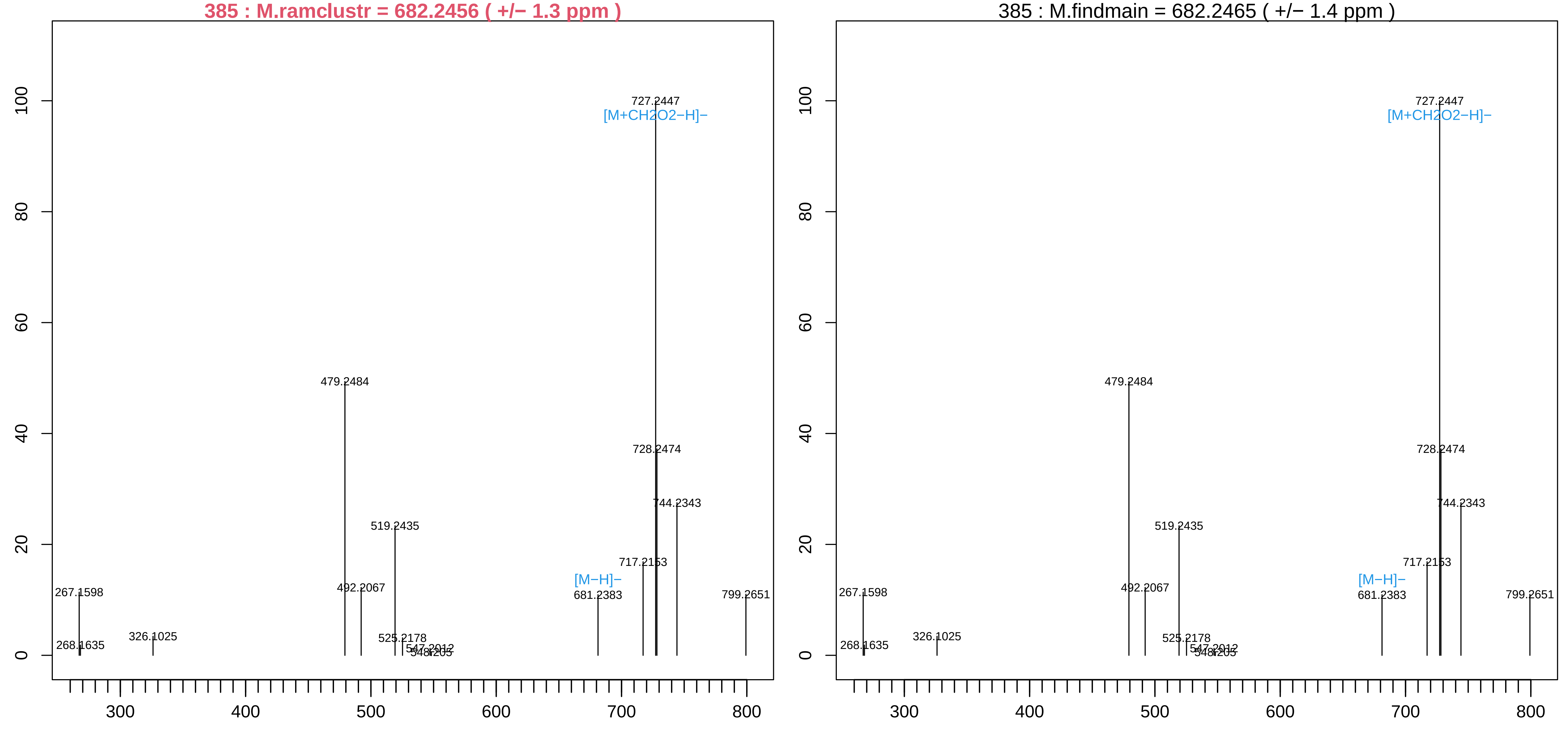
385 : M.ramclustr = 682.2456 (+/− 1.3 ppm)
385 : M.findmain = 682.2465 (+/− 1.4 ppm)
727.2447
[M+CH2O2−H]−
479.2484
728.2474
744.2343
519.2435
717.2153
[M−H]−
681.2383
267.1598
492.2067
799.2651
326.1025
268.1635
525.2178
547.2012
548.205
0
20
40
60
80
100
300
400
500
600
700
800

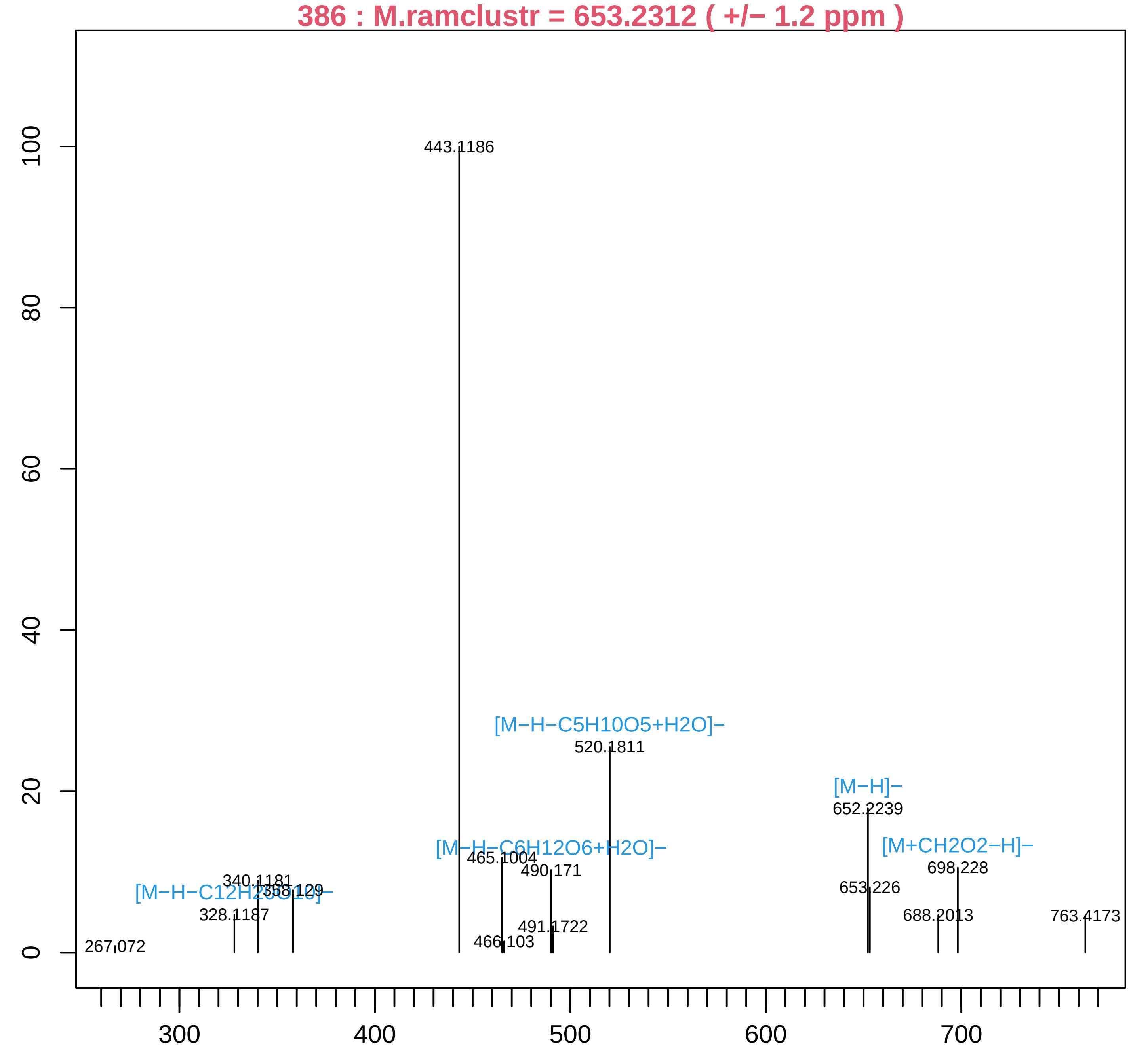
386 : M.ramclustr = 653.2312 (+/− 1.2 ppm)
443.1186
[M−H−C5H10O5+H2O]−
520.1811
[M−H]−
652.2239
[M+CH2O2−H]−
698.228
653.226
688.2013
763.4173
[M−H−C6H12O6+H2O]−
465.1004
490.171
491.1722
466.103
340.1181
358.129
328.1187
267.072
0
20
40
60
80
100
300
400
500
600
700

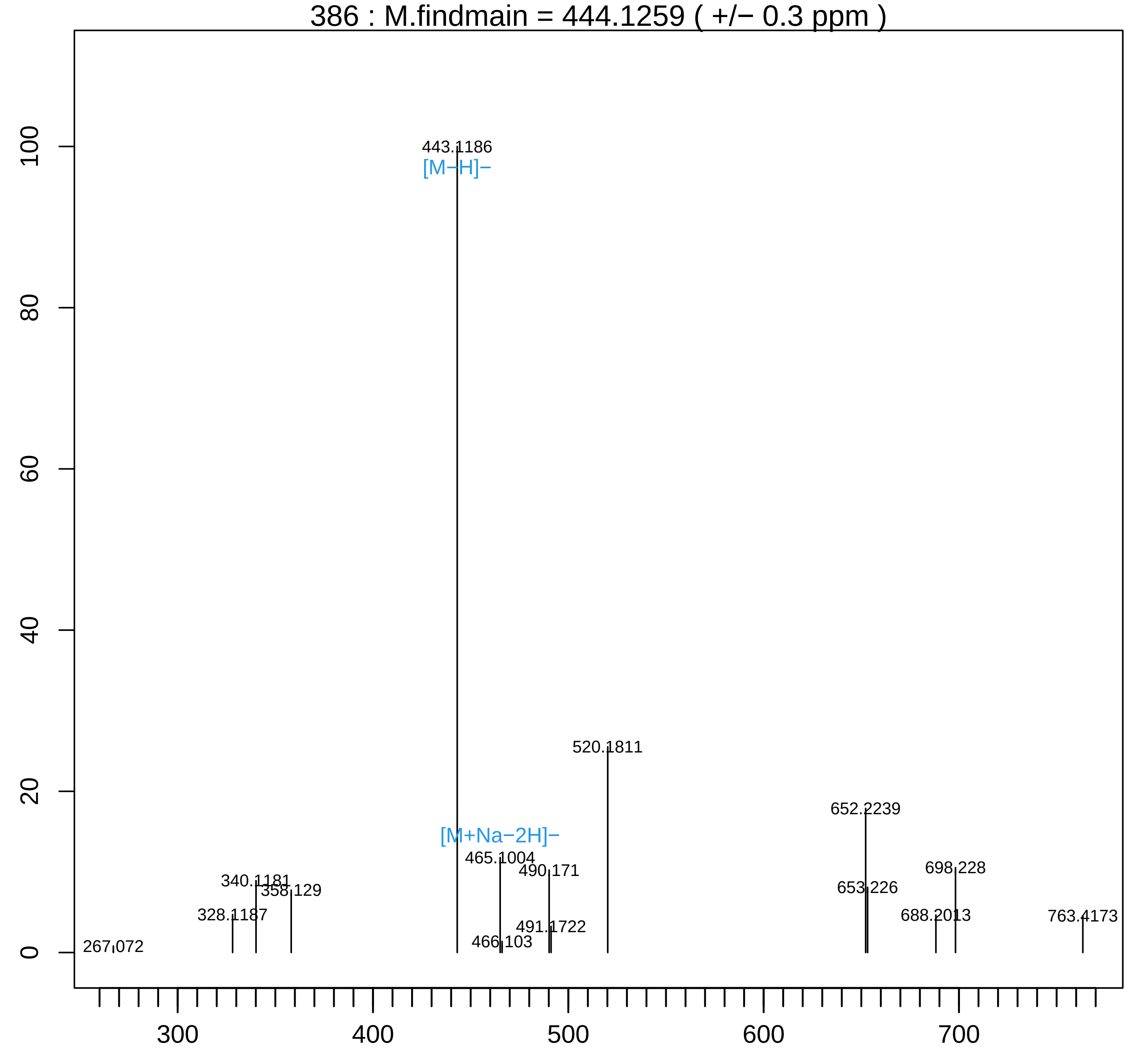
386 : M.findmain = 444.1259 (+/− 0.3 ppm)
443.1186
[M−H]−
520.1811
652.2239
[M+Na−2H]−
465.1004
490.171
491.1722
466.103
698.228
653.226
688.2013
763.4173
340.1181
358.129
328.1187
267.072
0
20
40
60
80
100
300
400
500
600
700

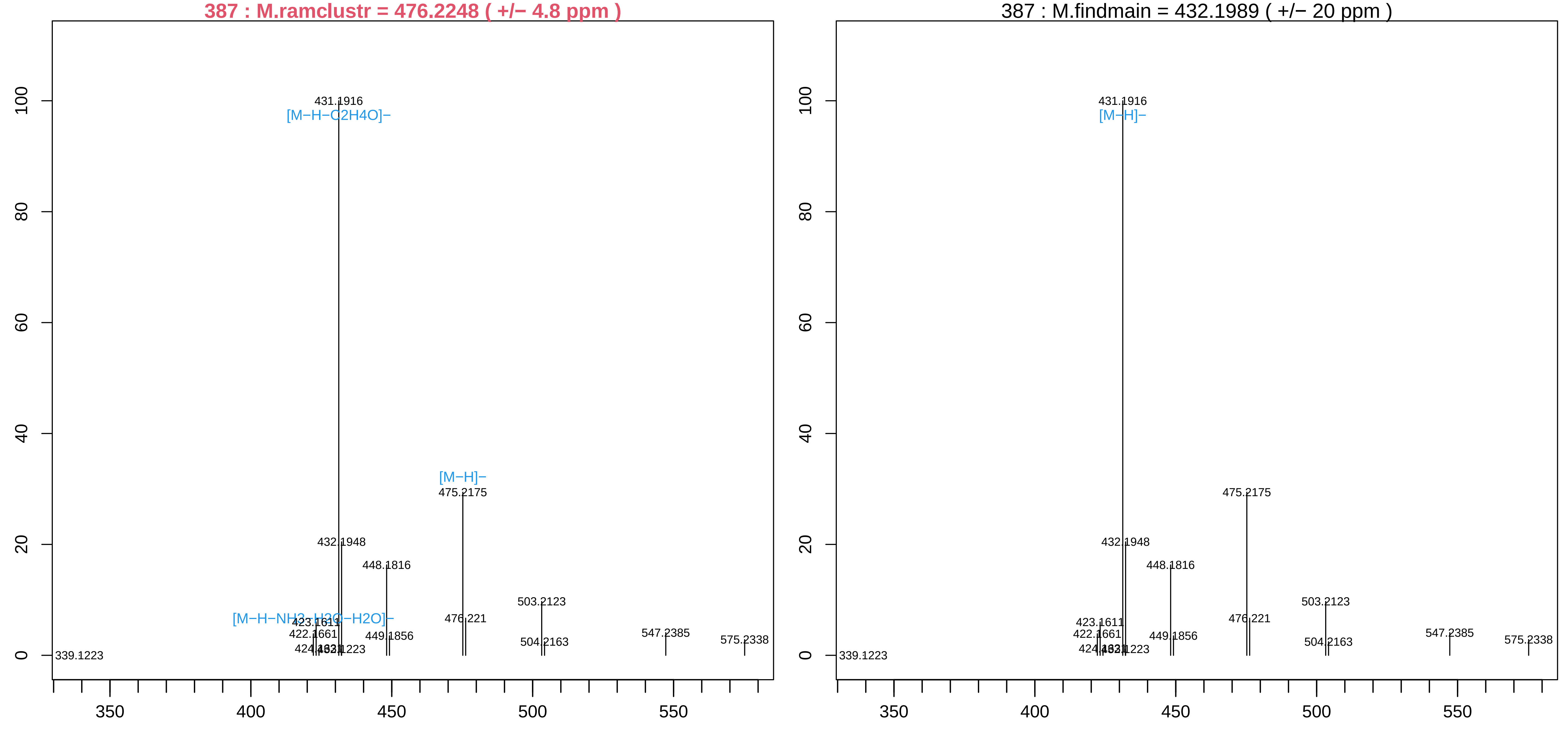
387 : M.ramclustr = 476.2248 (+/− 4.8 ppm)
431.1916
[M−H−C2H4O]−
[M−H]−
475.2175
432.1948
448.1816
503.2123
476.221
423.1611
422.1661
449.1856
504.2163
547.2385
575.2338
339.1223
387 : M.findmain = 432.1989 (+/− 20 ppm)
431.1916
[M−H]−
475.2175
432.1948
448.1816
503.2123
476.221
423.1611
422.1661
449.1856
504.2163
547.2385
575.2338
339.1223
0
20
40
60
80
100
350
400
450
500
550

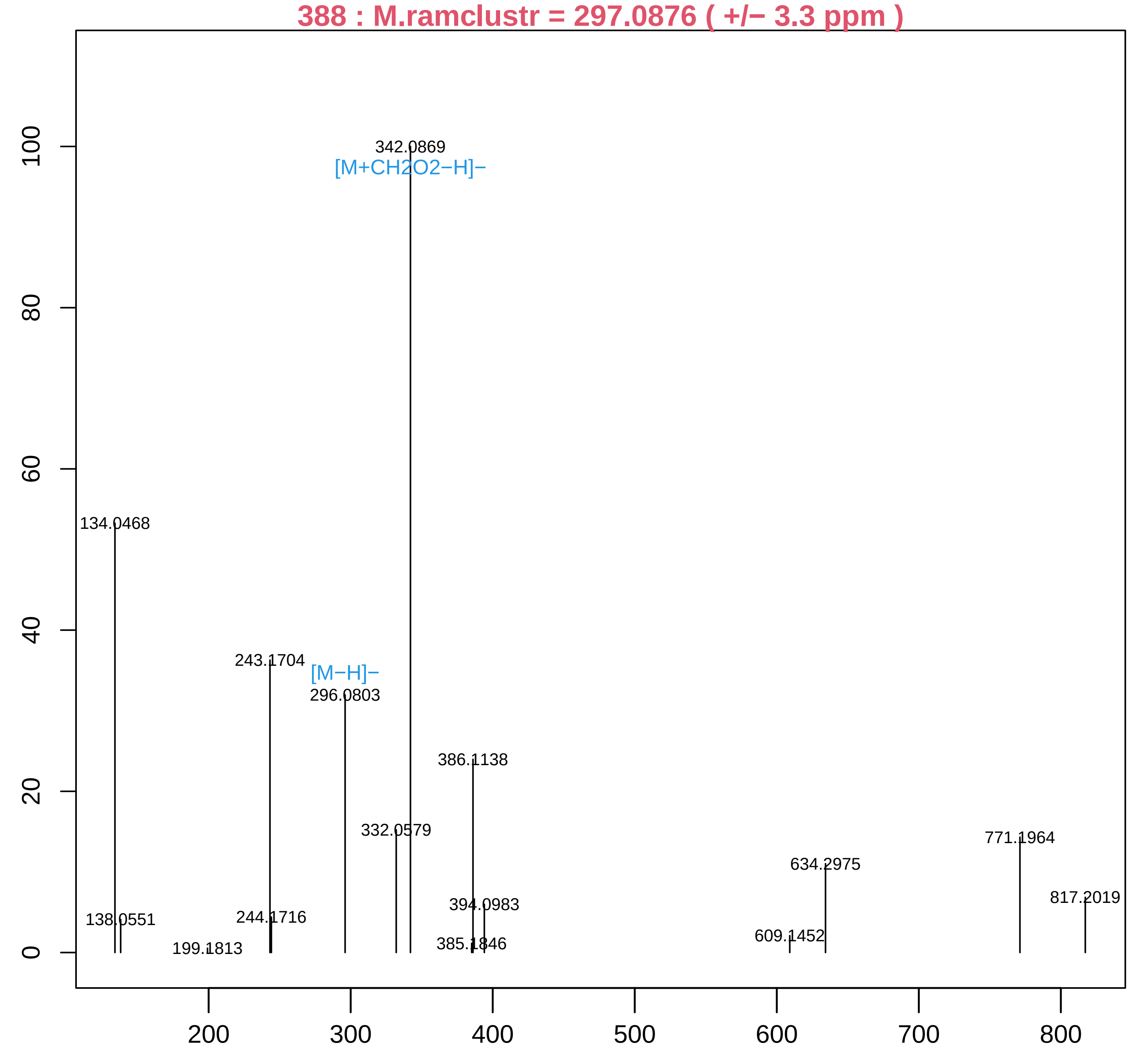

388 : M.ramclustr = 297.0876 (+/− 3.3 ppm)
342.0869
[M+CH2O2−H]−
134.0468
243.1704
[M−H]−
296.0803
386.1138
332.0579
771.1964
634.2975
817.2019
394.0983
244.1716
138.0551
609.1452
385.1846
199.1813

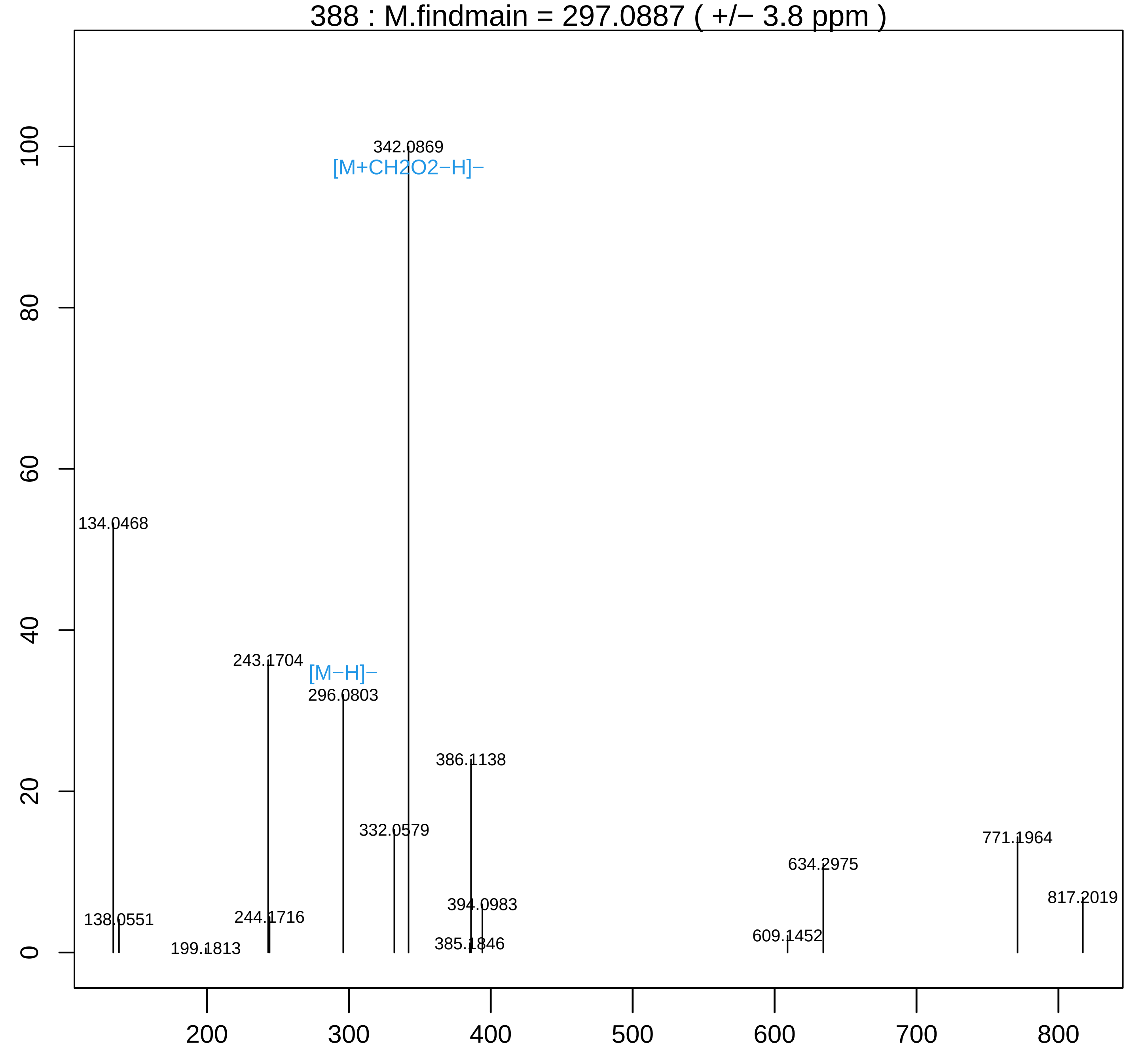

388 : M.findmain = 297.0887 (+/− 3.8 ppm)
342.0869
[M+CH2O2−H]−
134.0468
243.1704
[M−H]−
296.0803
386.1138
332.0579
771.1964
634.2975
817.2019
394.0983
244.1716
138.0551
609.1452
385.1846
199.1813

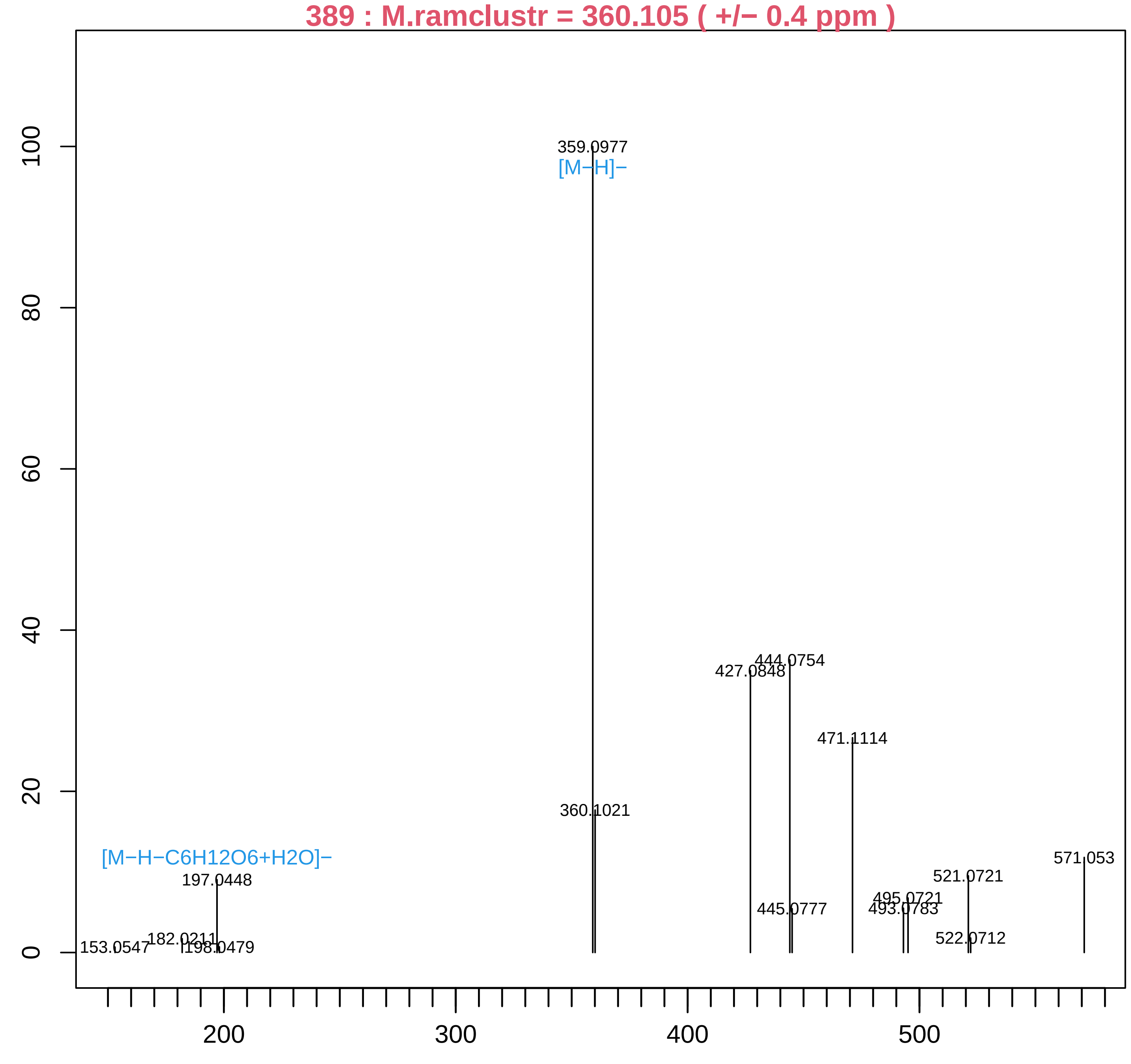
389 : M.ramclustr = 360.105 (+/− 0.4 ppm)
359.0977
[M−H]−
360.1021
427.0848
444.0754
445.0777
471.1114
495.0721
493.0783
521.0721
522.0712
571.053
[M−H−C6H12O6+H2O]−
197.0448
182.0211
153.0547
198.0479
0
20
40
60
80
100
200
300
400
500

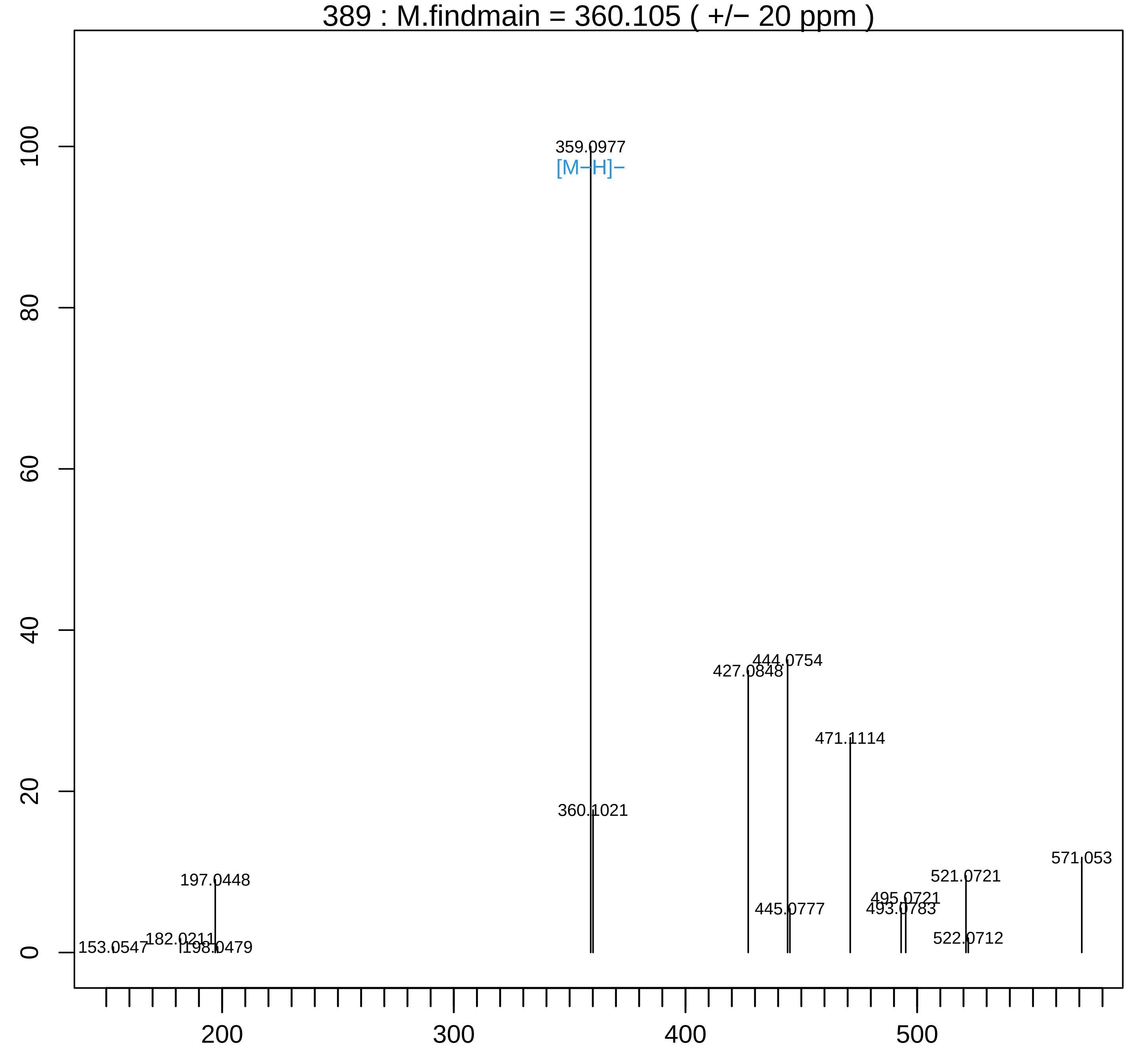
389 : M.findmain = 360.105 (+/− 20 ppm)
359.0977
[M−H]−
360.1021
427.0848
444.0754
445.0777
471.1114
495.0721
493.0783
521.0721
522.0712
571.053
197.0448
182.0211
153.0547
198.0479
0
20
40
60
80
100
200
300
400
500

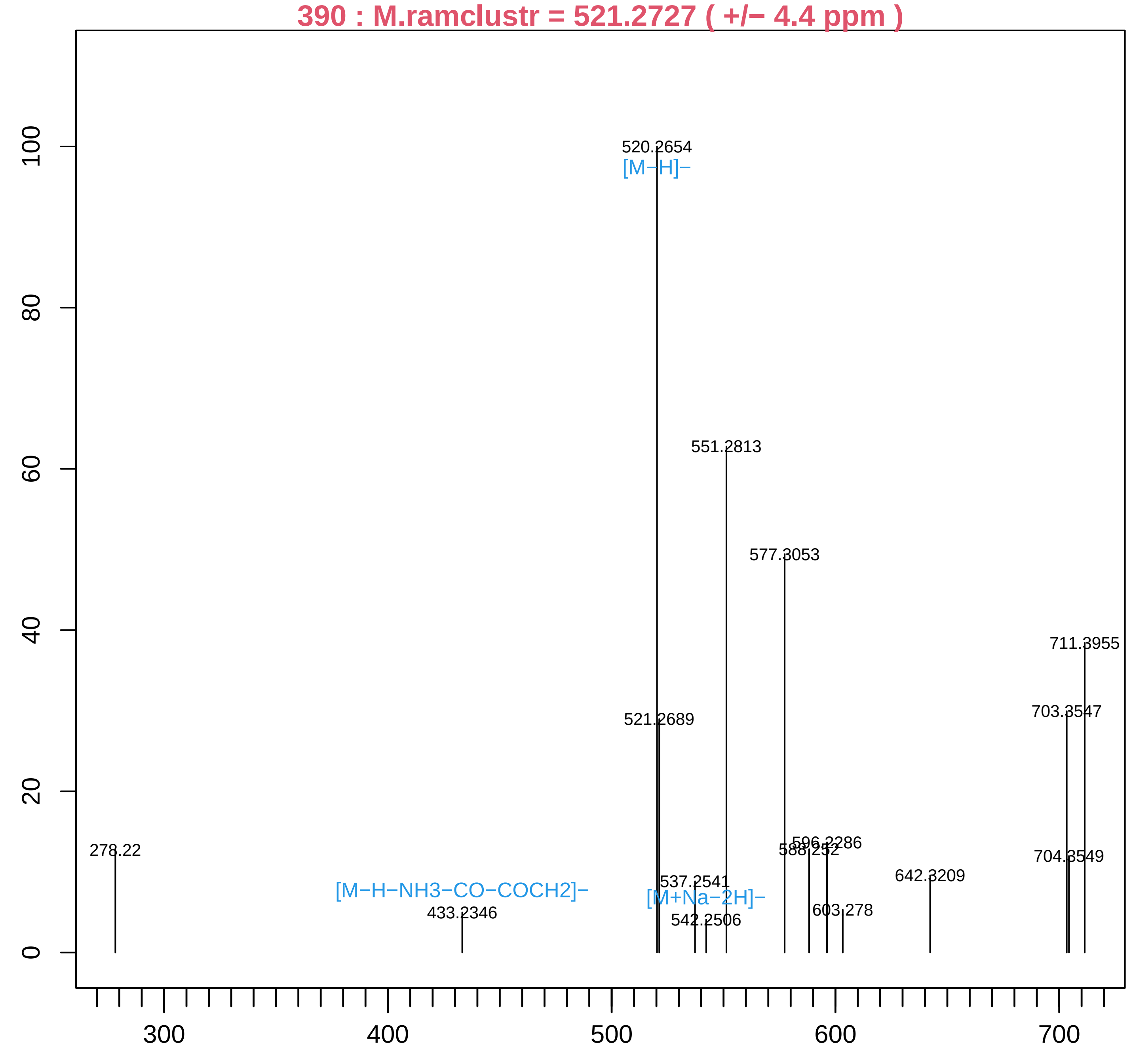
390 : M.ramclustr = 521.2727 (+/− 4.4 ppm)
520.2654
[M−H]−
551.2813
577.3053
711.3955
521.2689
703.3547
278.22
596.2286
588.252
704.3549
642.3209
537.2541
[M−H−NH3−CO−COCH2]−
[M+Na−2H]−
433.2346
603.278
542.2506
0
20
40
60
80
100
300
400
500
600
700

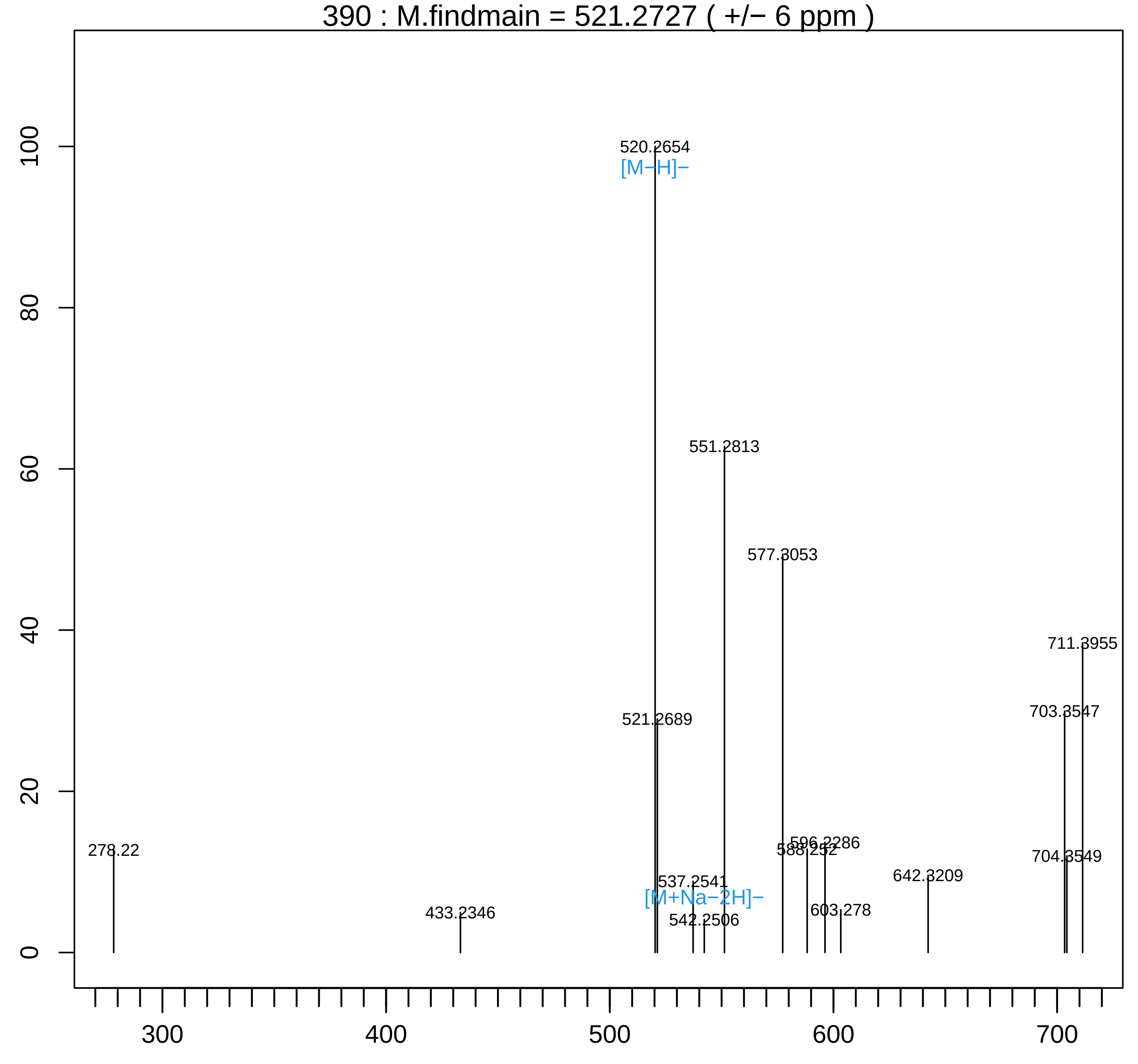
390 : M.findmain = 521.2727 (+/− 6 ppm)
520.2654
[M−H]−
551.2813
577.3053
711.3955
521.2689
703.3547
278.22
596.2286
588.252
704.3549
642.3209
537.2541
[M+Na−2H]−
433.2346
603.278
542.2506
0
20
40
60
80
100
300
400
500
600
700

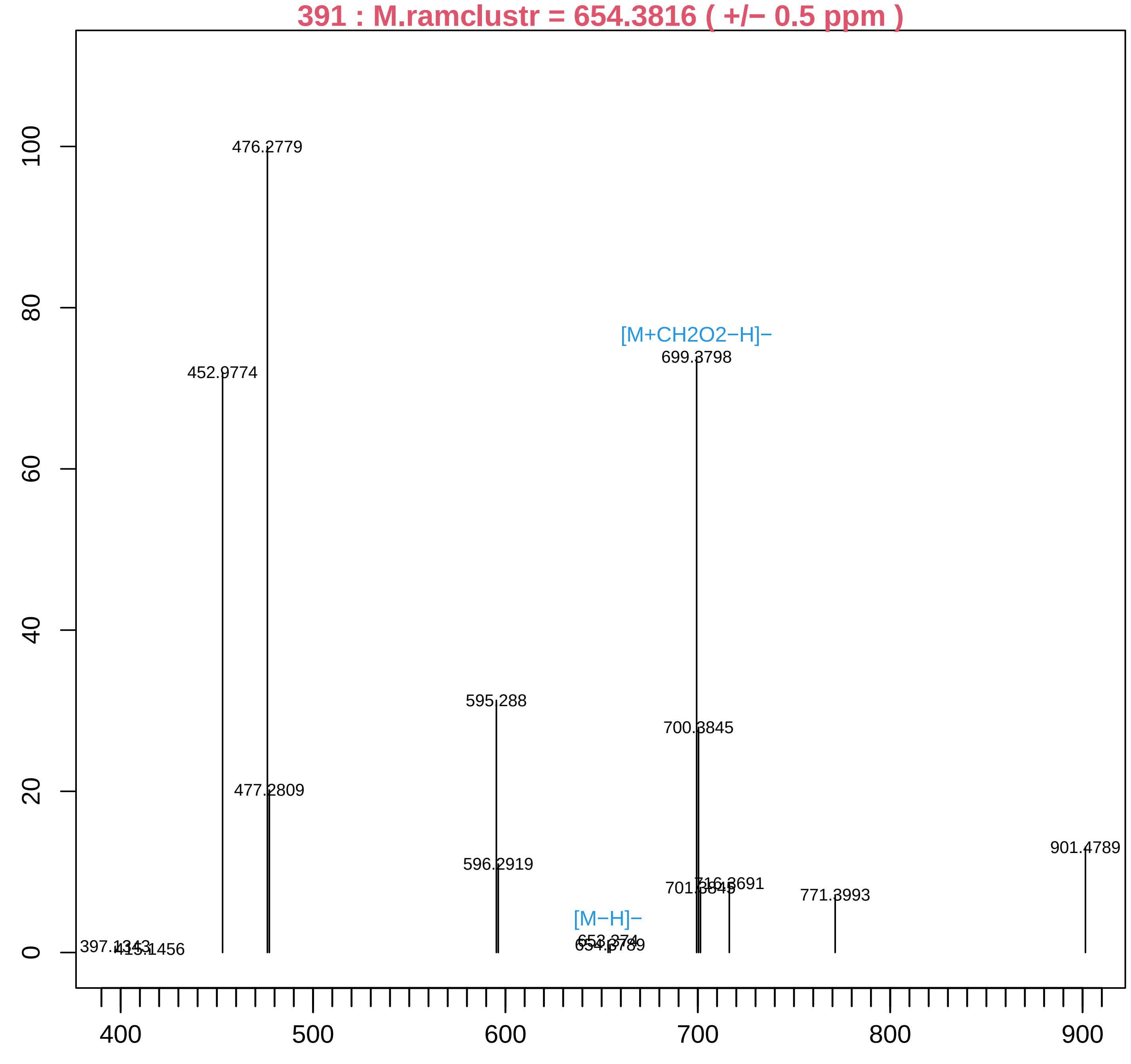
391 : M.ramclustr = 654.3816 (+/− 0.5 ppm)
[M+CH2O2−H]−
[M−H]−
476.2779
452.9774
699.3798
595.288
700.3845
477.2809
596.2919
901.4789
701.3848
716.3691
771.3993
653.374
654.3789
397.1343
415.1456
0
20
40
60
80
100
400
500
600
700
800
900

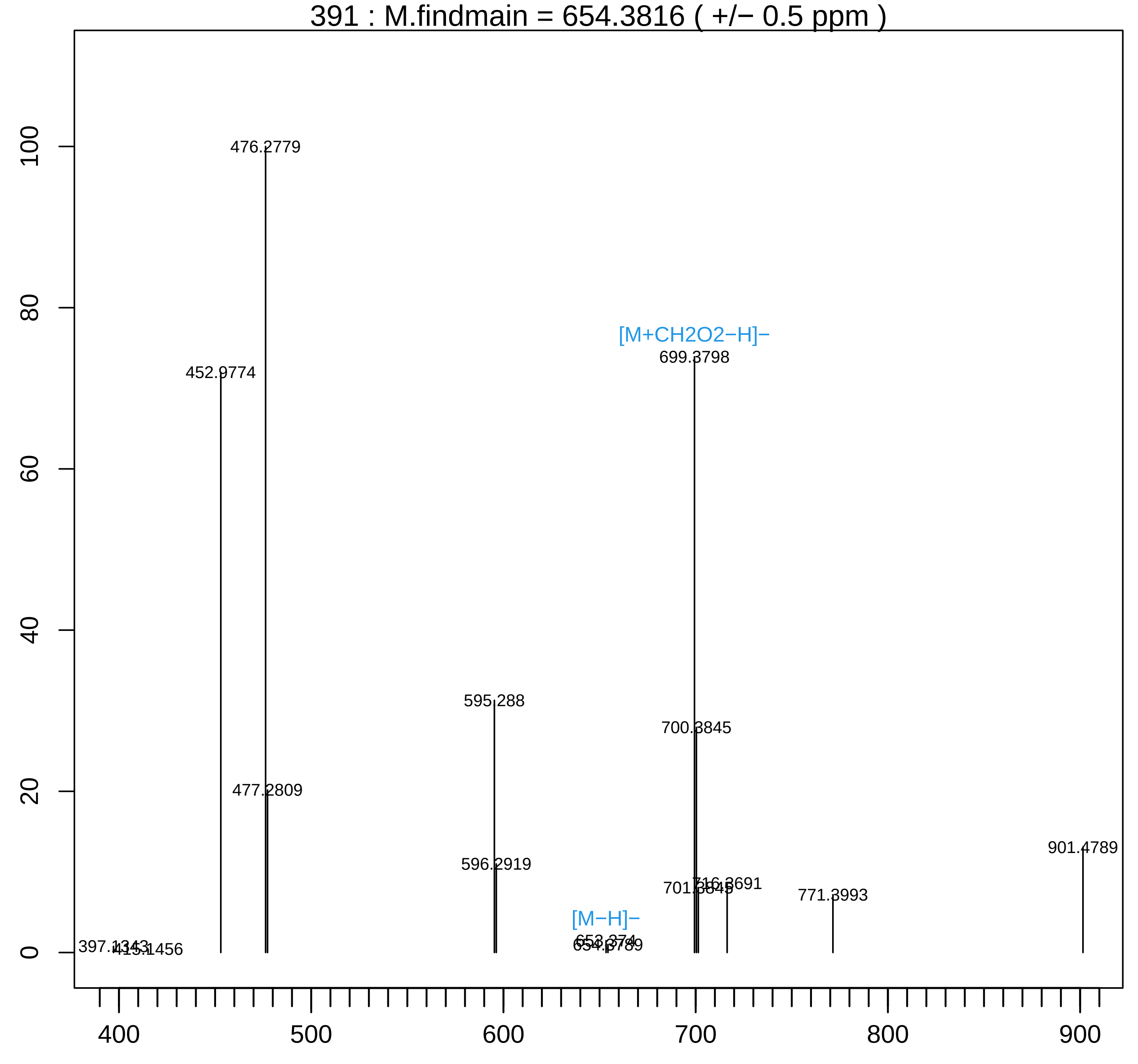
391 : M.findmain = 654.3816 (+/− 0.5 ppm)
[M+CH2O2−H]−
[M−H]−
476.2779
452.9774
699.3798
595.288
700.3845
477.2809
596.2919
901.4789
701.3848
716.3691
771.3993
653.374
654.3789
397.1343
415.1456
0
20
40
60
80
100
400
500
600
700
800
900

392 : M.ramclustr = 266.1547 (+/− NA ppm)

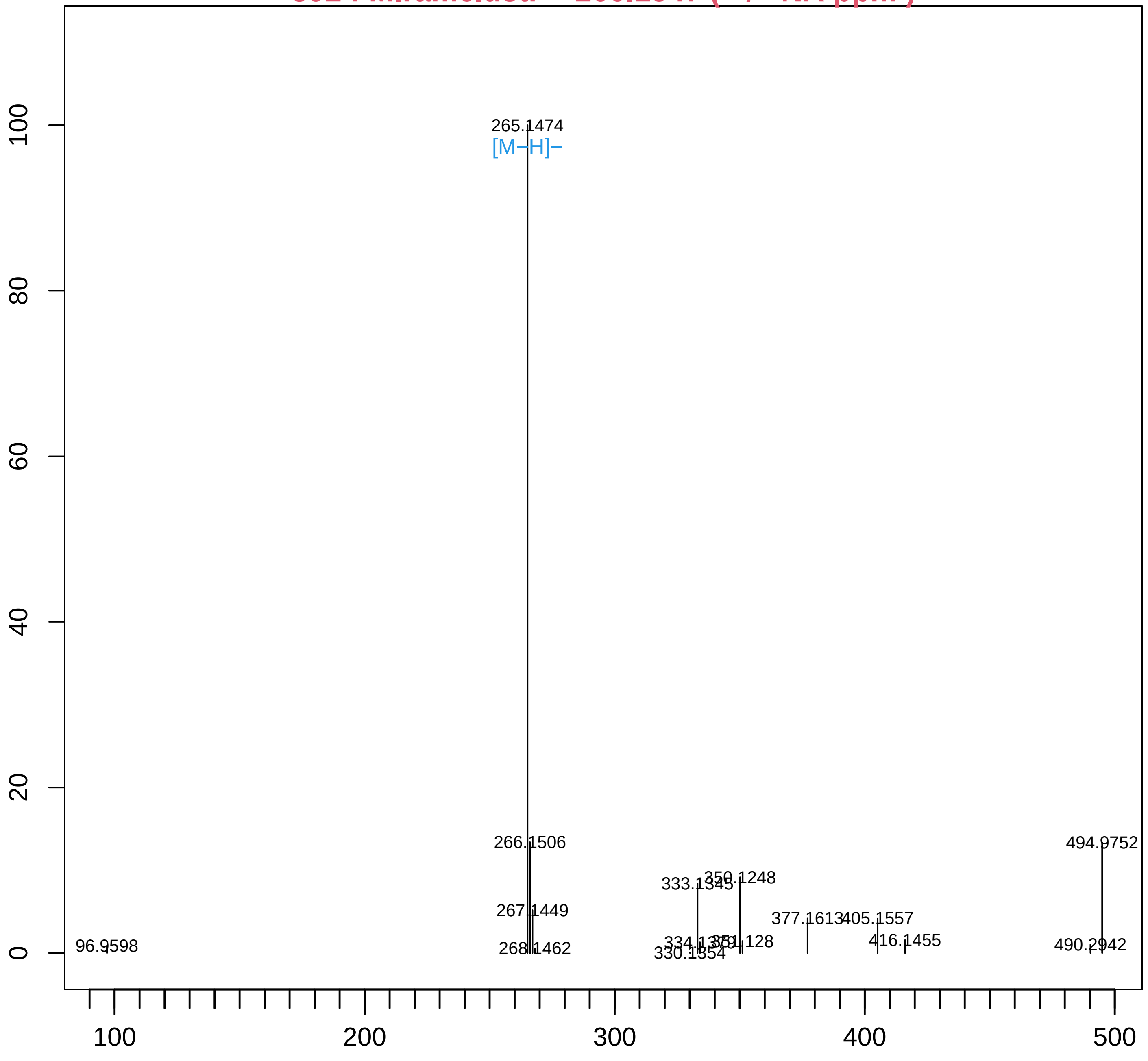

392 : M.findmain = 266.1547 (+/− 20 ppm)

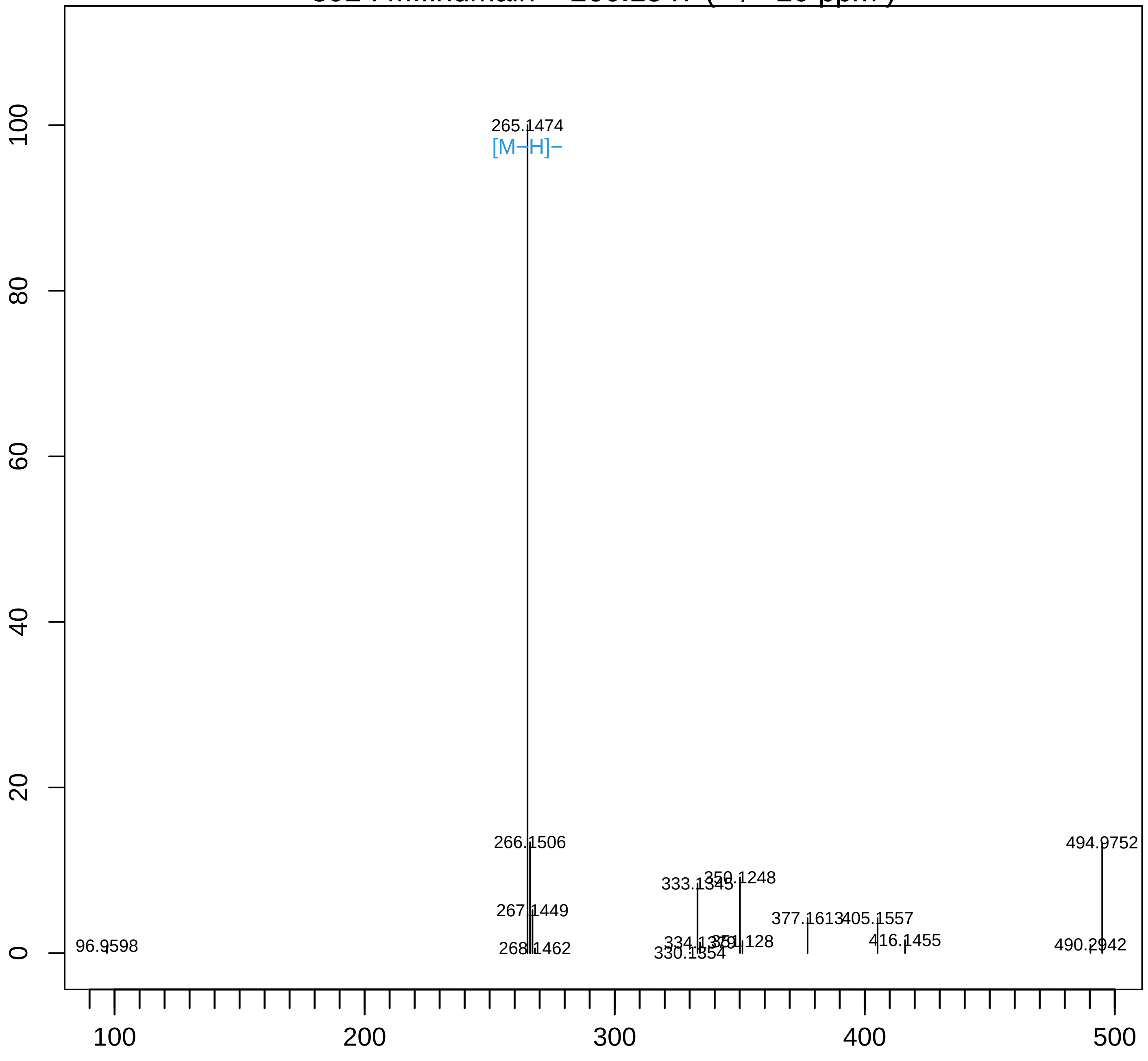

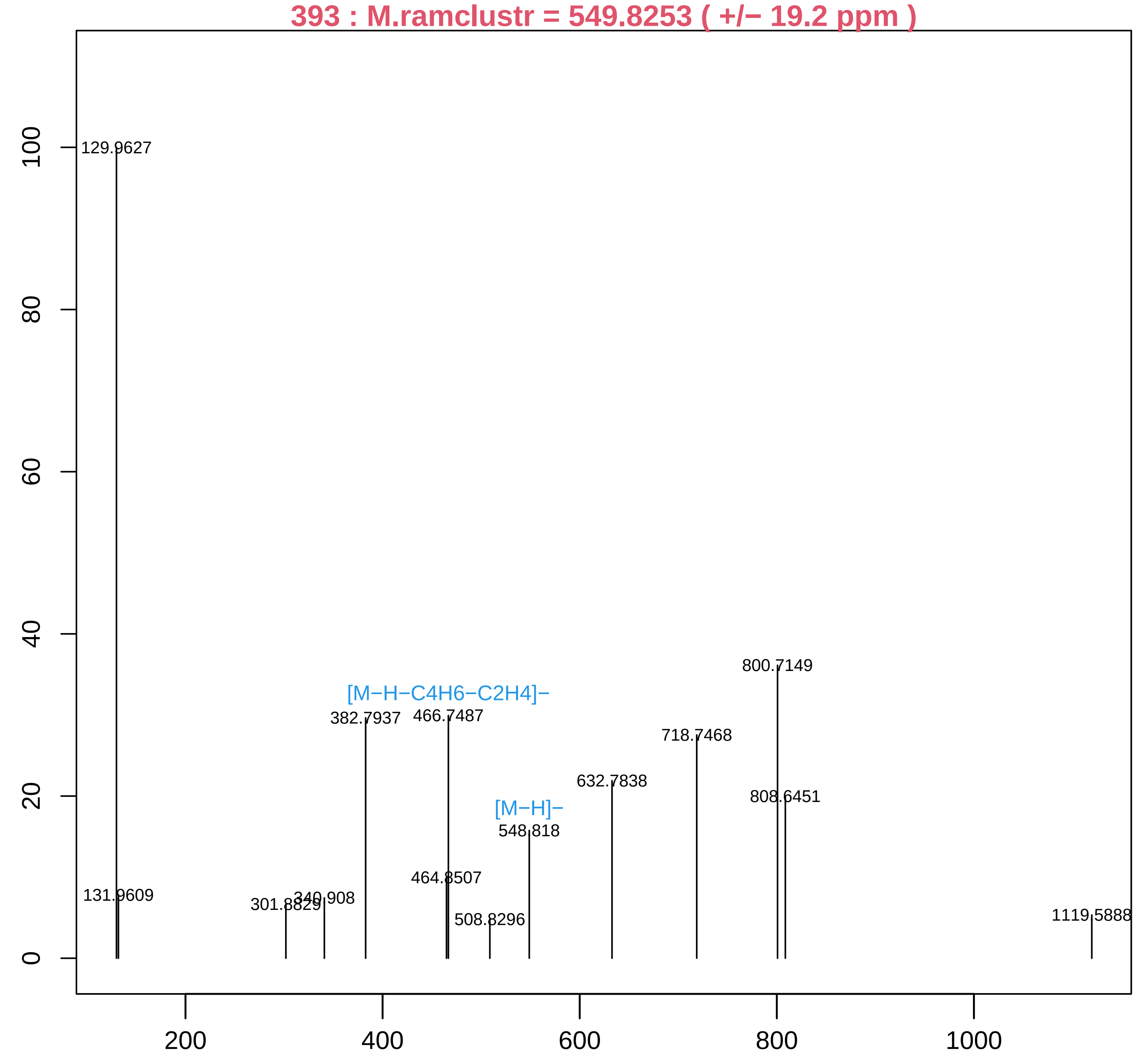
393 : M.ramclustr = 549.8253 (+/− 19.2 ppm)
129.9627
131.9609
301.8829
340.908
382.7937
[M−H−C4H6−C2H4]−
466.7487
464.8507
508.8296
[M−H]−
548.818
632.7838
718.7468
800.7149
808.6451
1119.5888
0
20
40
60
80
100
200
400
600
800
1000

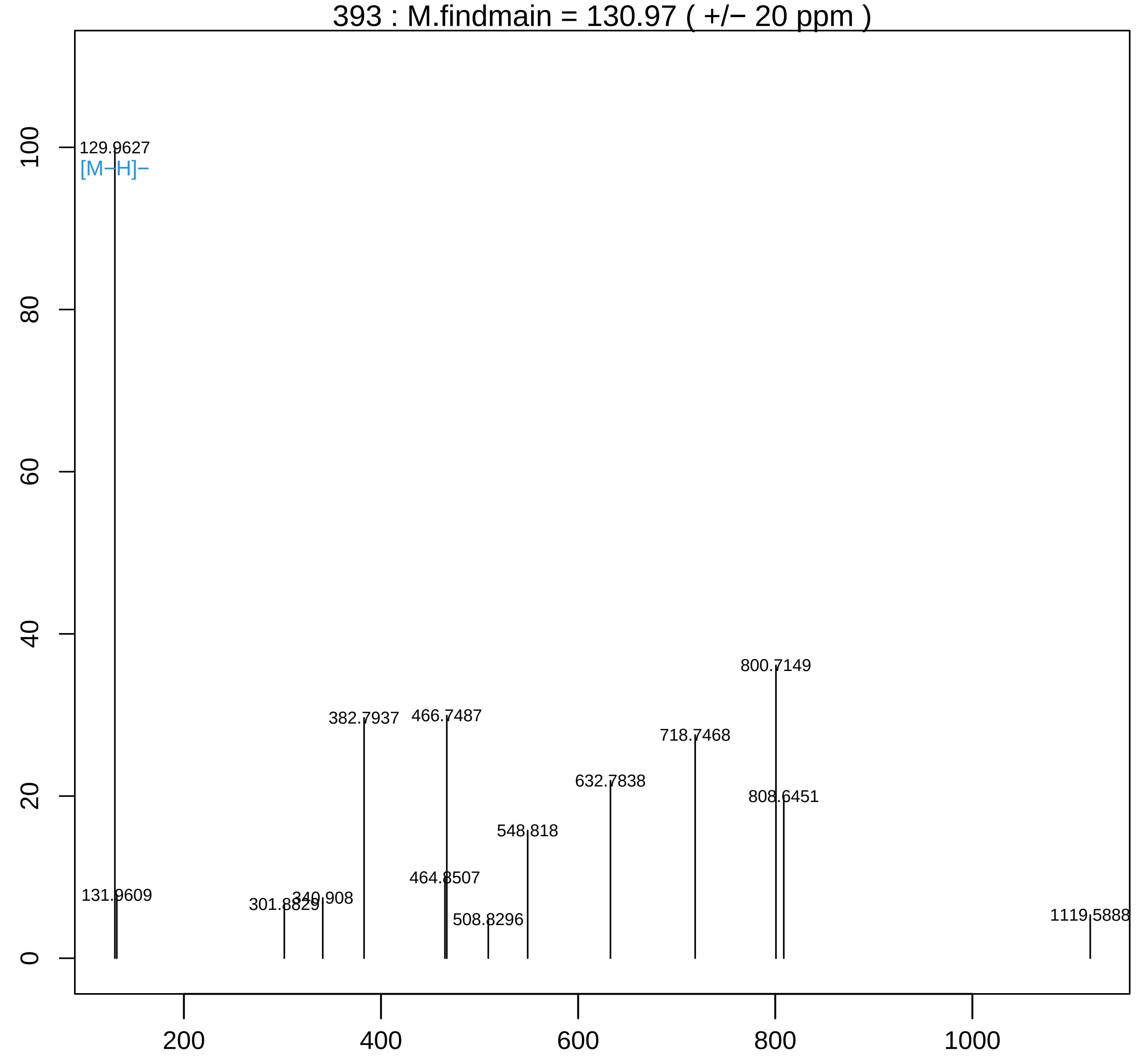
393 : M.findmain = 130.97 (+/− 20 ppm)
129.9627
[M−H]−
131.9609
301.8829
340.908
382.7937
466.7487
464.8507
508.8296
548.818
632.7838
718.7468
800.7149
808.6451
1119.5888
0
20
40
60
80
100
200
400
600
800
1000

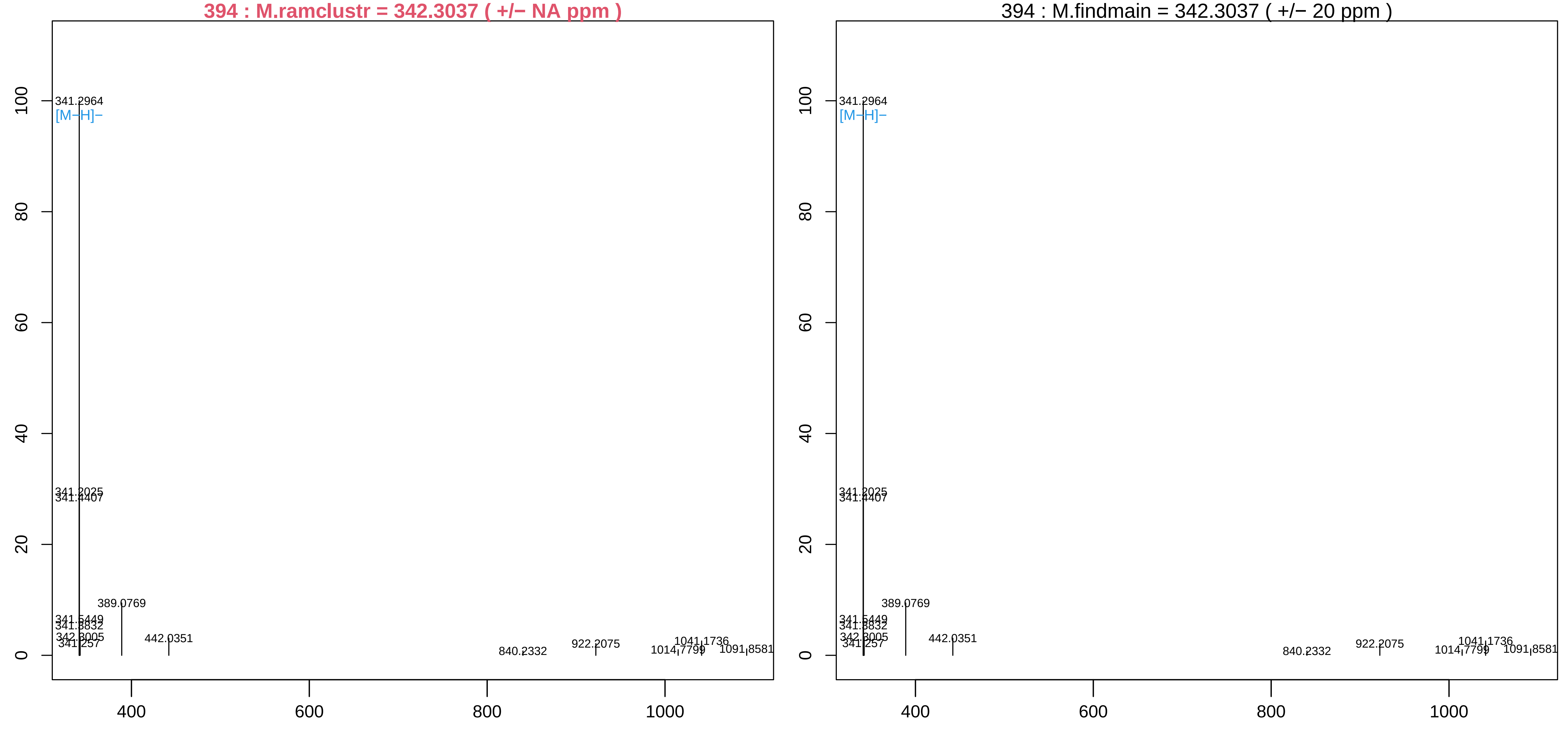
394 : M.ramclustr = 342.3037 (+/− NA ppm)
394 : M.findmain = 342.3037 (+/− 20 ppm)
341.2964
[M−H]−
341.2025
341.4407
389.0769
341.5449
341.3832
342.3005
341.257
442.0351
840.2332
922.2075
1014.7799
1041.1736
1091.8581
0
20
40
60
80
100
400
600
800
1000

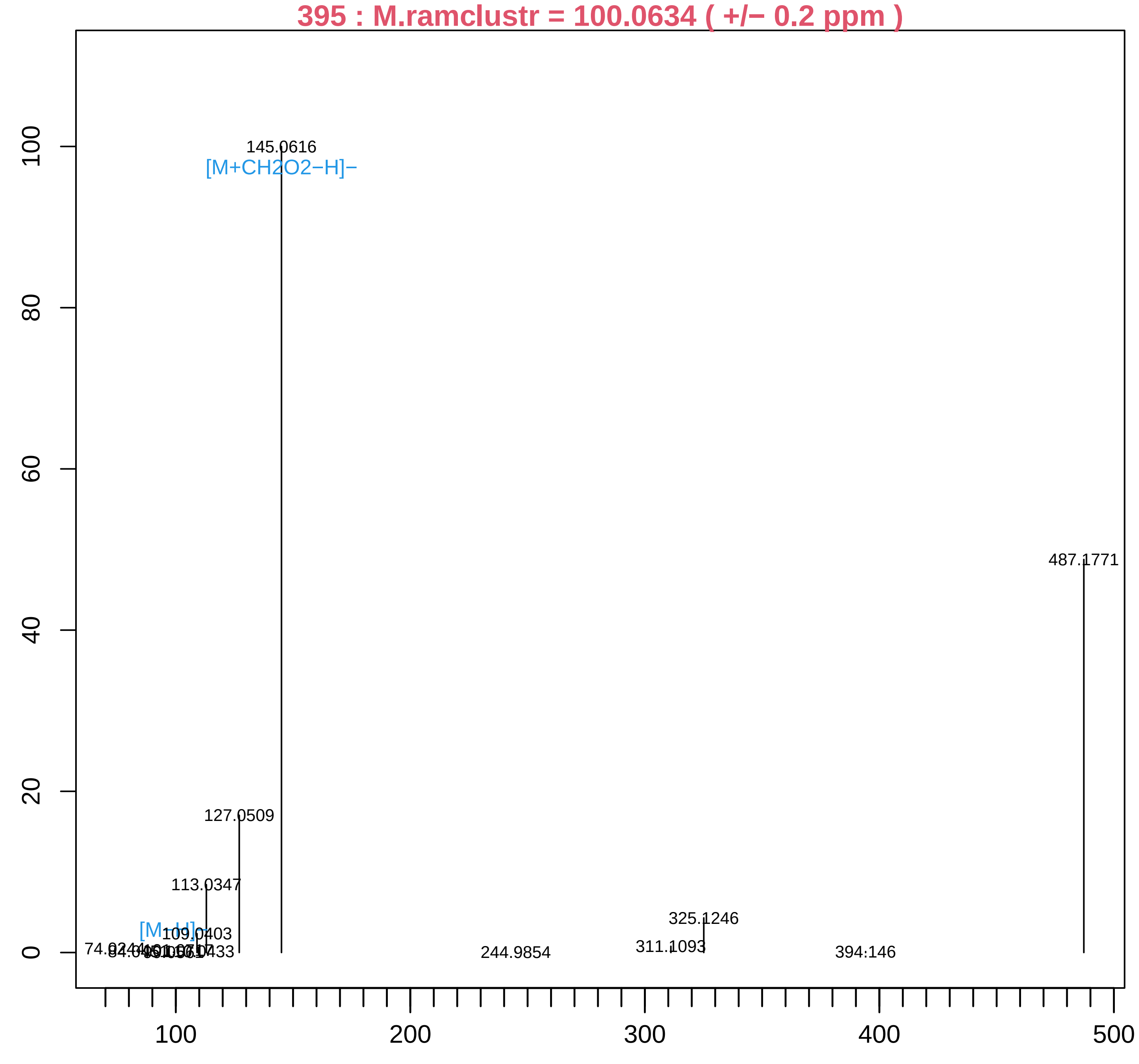
395 : M.ramclustr = 100.0634 (+/− 0.2 ppm)
145.0616
[M+CH2O2−H]−
487.1771
127.0509
113.0347
109.0403
325.1246
311.1093
244.9854
394:146
0
20
40
60
80
100
100
200
300
400
500

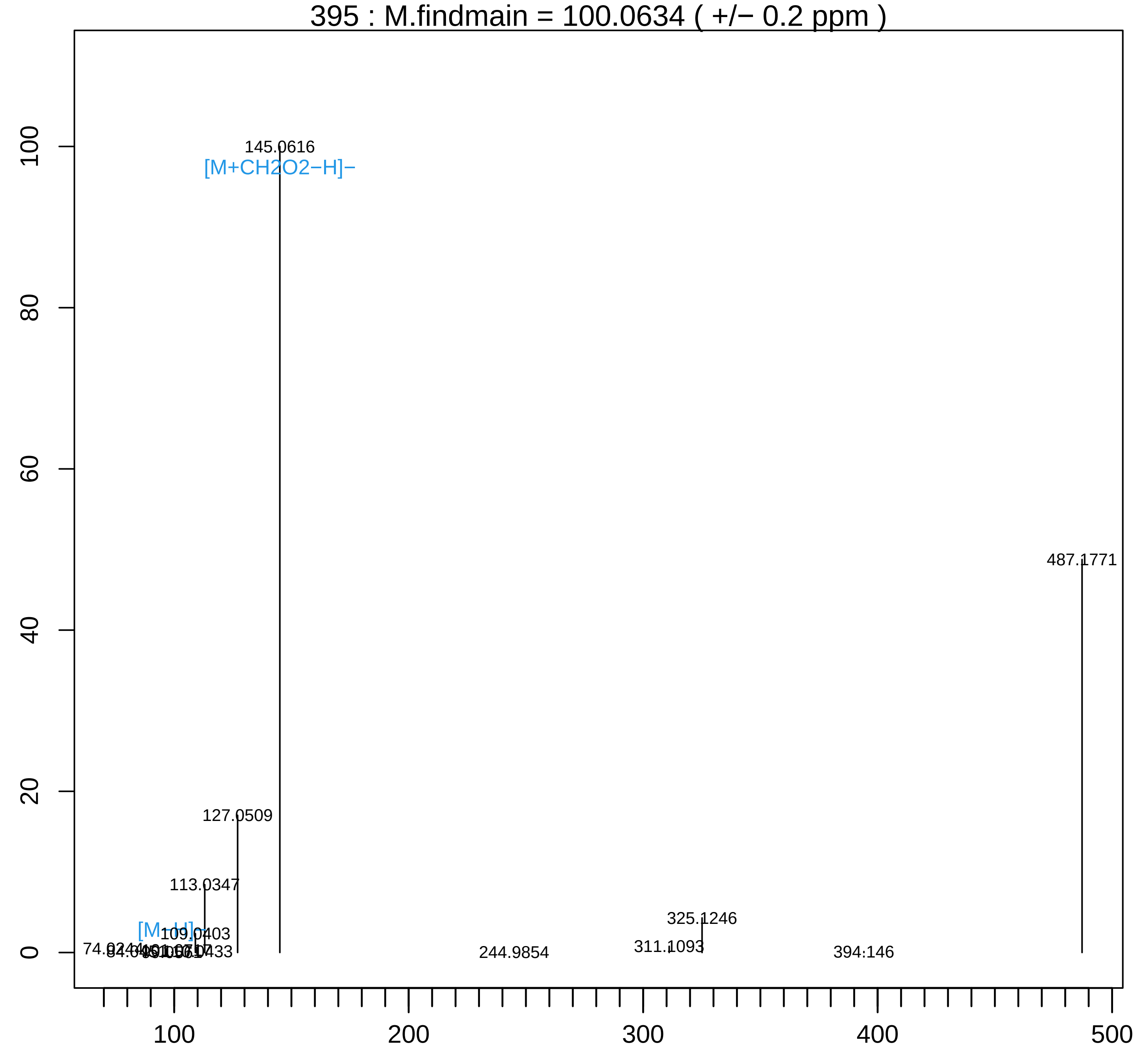
395 : M.findmain = 100.0634 (+/− 0.2 ppm)
145.0616
[M+CH2O2−H]−
487.1771
127.0509
113.0347
109.0403
325.1246
311.1093
244.9854
394:146
0
20
40
60
80
100
100
200
300
400
500

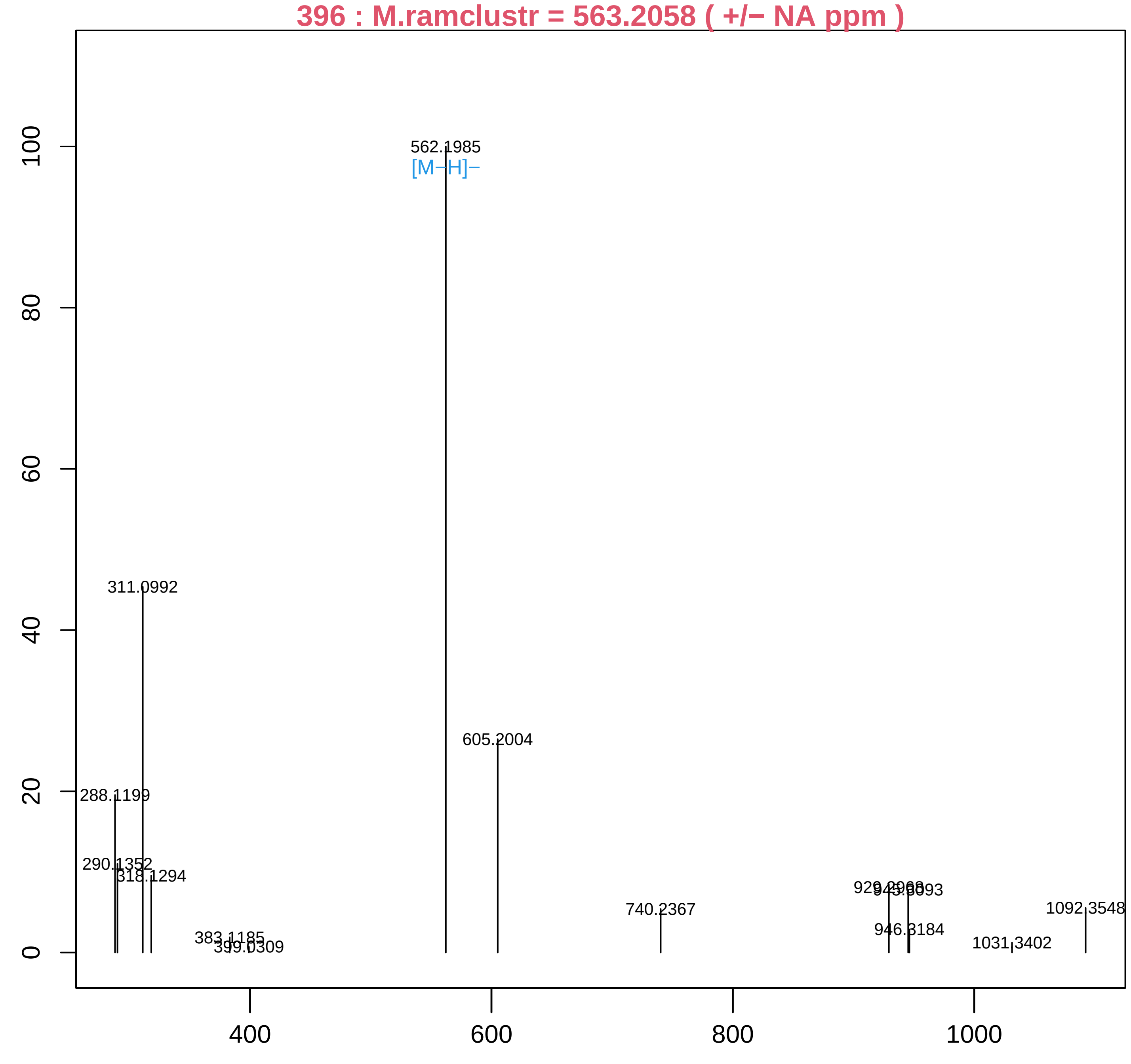
396 : M.ramclustr = 563.2058 (+/− NA ppm)
562.1985
[M−H]−
311.0992
605.2004
288.1199
290.1352
318.1294
383.1185
399.0309
740.2367
929.2068
943.3093
946.3184
1031.3402
1092.3548
0
20
40
60
80
100
400
600
800
1000

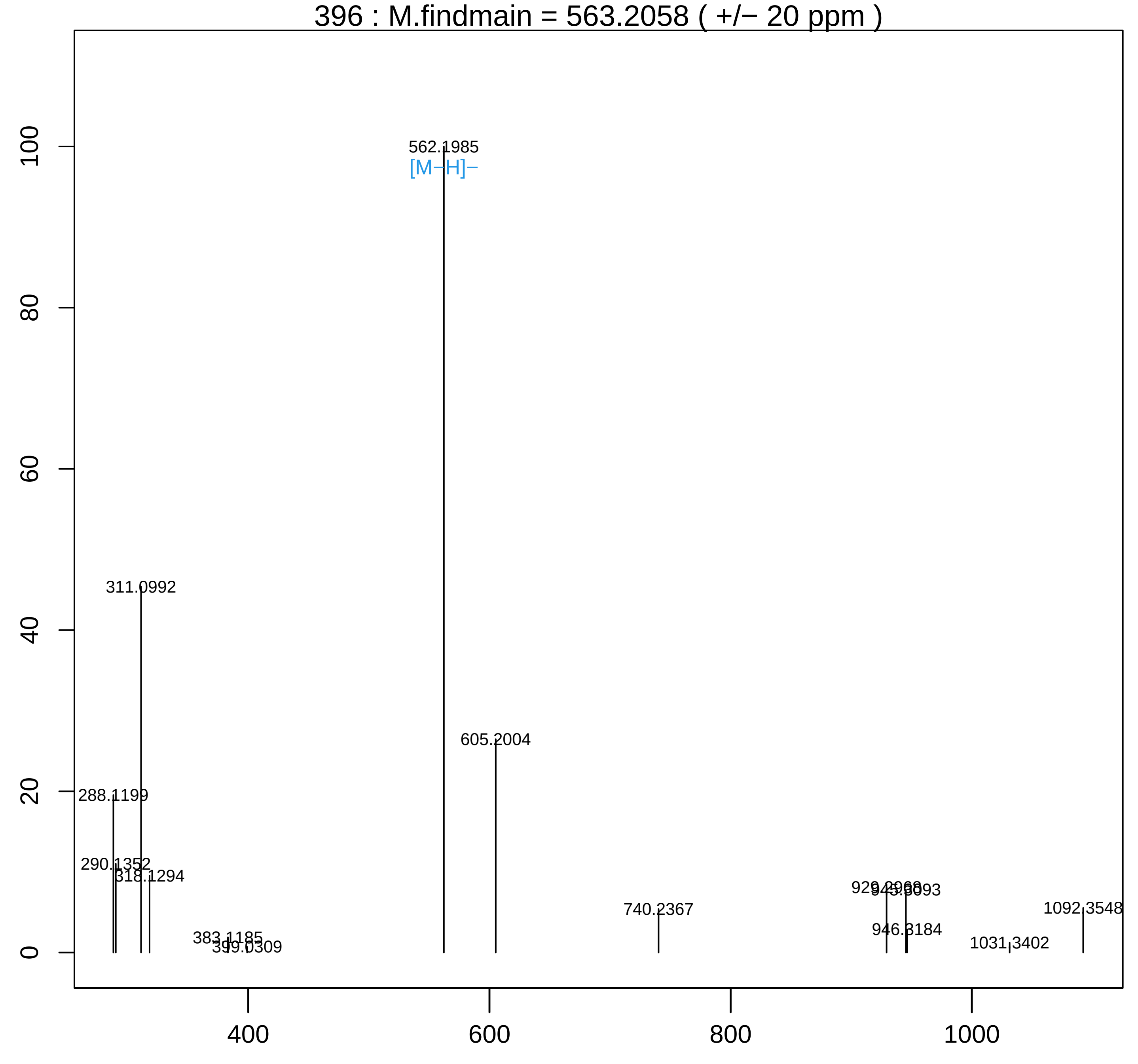
396 : M.findmain = 563.2058 (+/− 20 ppm)
562.1985
[M−H]−
311.0992
605.2004
288.1199
290.1352
318.1294
383.1185
399.0309
740.2367
929.2068
943.3093
946.3184
1031.3402
1092.3548
0
20
40
60
80
100
400
600
800
1000

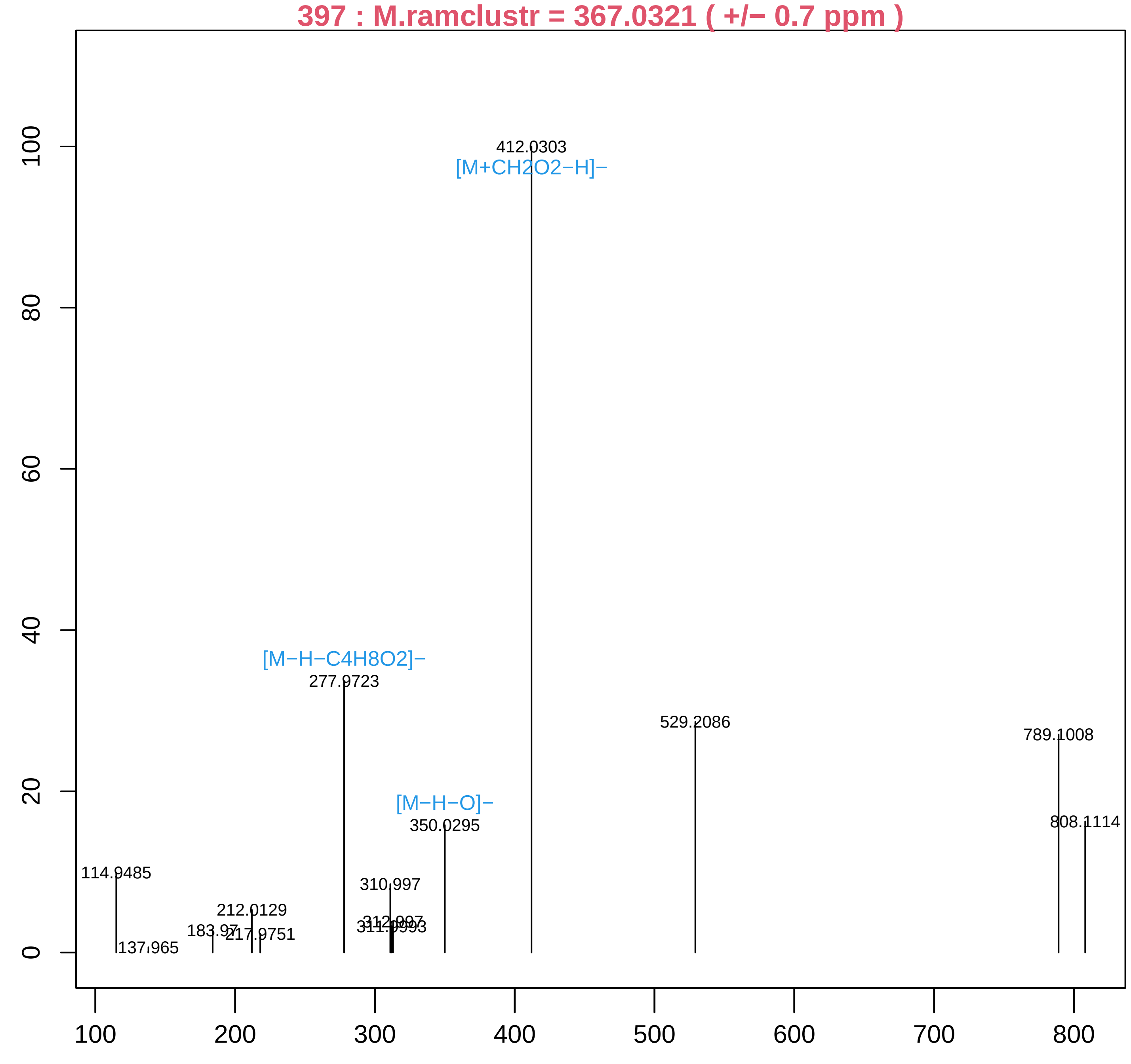

397 : M.ramclustr = 367.0321 (+/− 0.7 ppm)
412.0303
[M+CH2O2−H]−
[M−H−C4H8O2]−
277.9723
529.2086
789.1008
[M−H−O]−
350.0295
808.1114
114.9485
310.997
212.0129
183.97
217.9751
312.997
311.0993
137.965

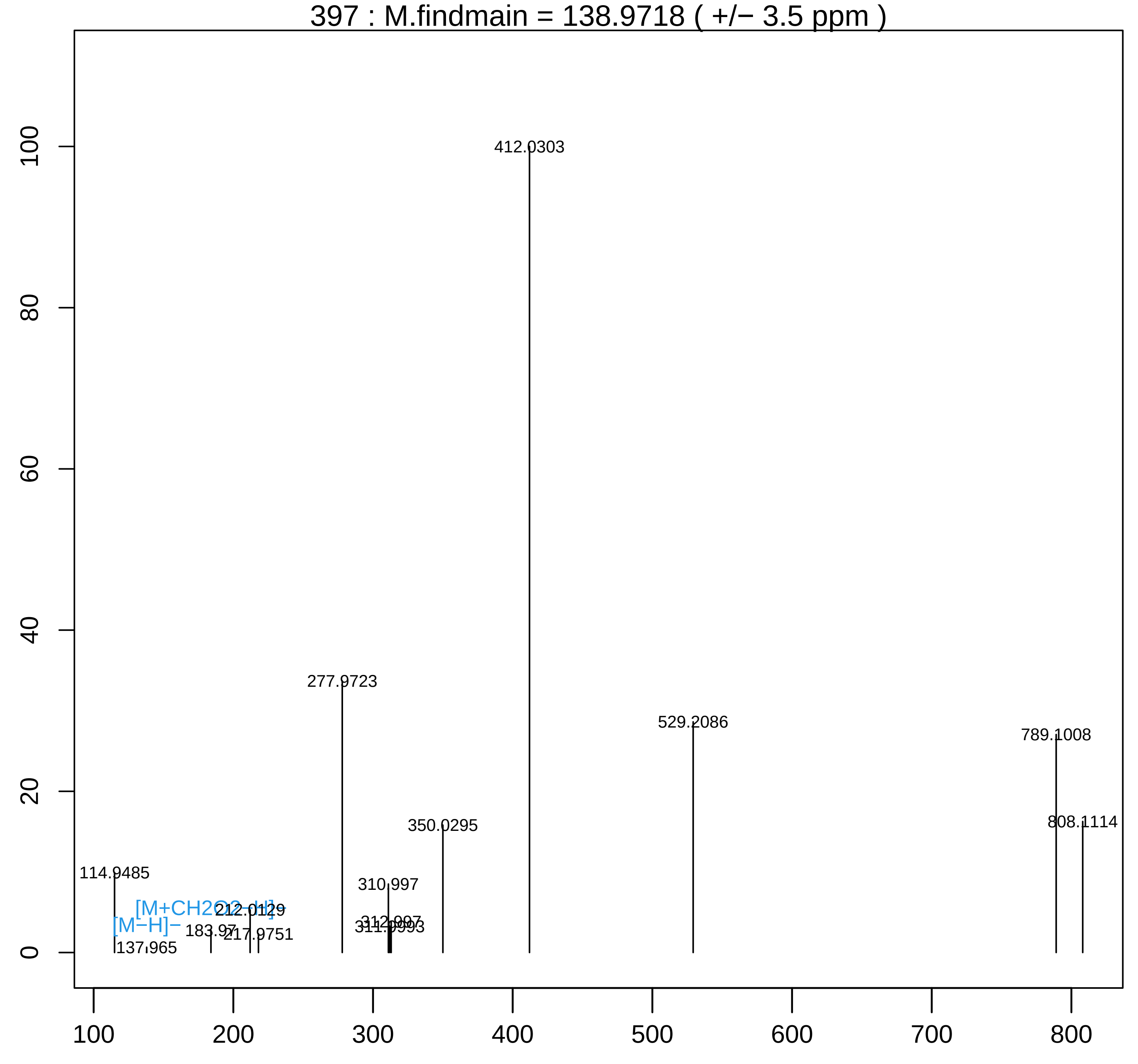

397 : M.findmain = 138.9718 (+/− 3.5 ppm)
412.0303
277.9723
529.2086
789.1008
350.0295
808.1114
114.9485
310.997
[M+CH2O2−H]−
212.0129
[M−H]−
183.97
217.9751
312.997
311.0993
137.965

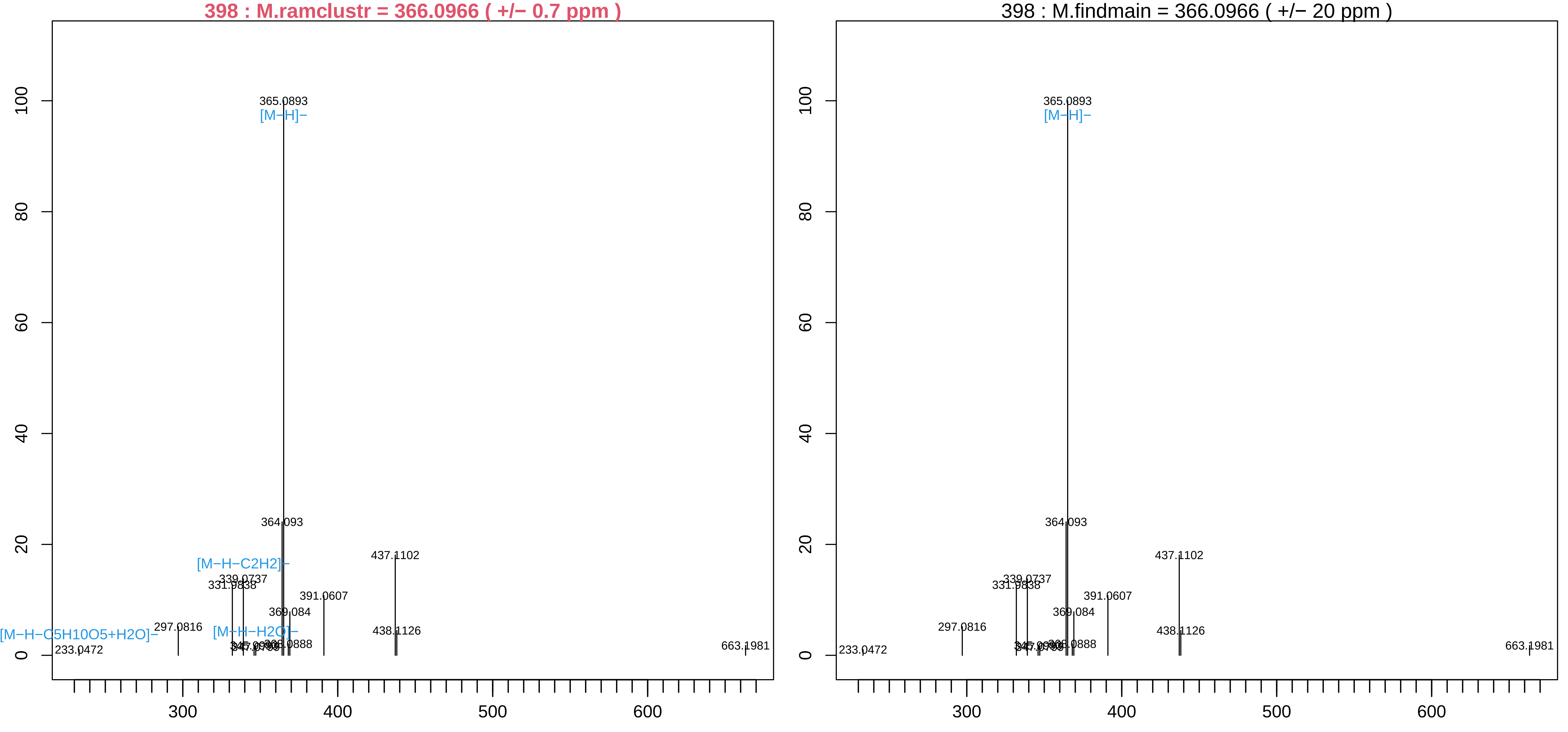

398 : M.ramclustr = 366.0966 (+/− 0.7 ppm)
365.0893
[M−H]−
364.093
[M−H−C2H2]−
339.0737
331.9838
391.0607
369.084
437.1102
438.1126
297.0816
[M−H−C5H10O5+H2O]−
[M−H−H2O]−
233.0472
663.1981
0
20
40
60
80
100
300
400
500
600
398 : M.findmain = 366.0966 (+/− 20 ppm)
365.0893
[M−H]−
364.093
339.0737
331.9838
391.0607
369.084
437.1102
438.1126
297.0816
233.0472
663.1981
0
20
40
60
80
100
300
400
500
600

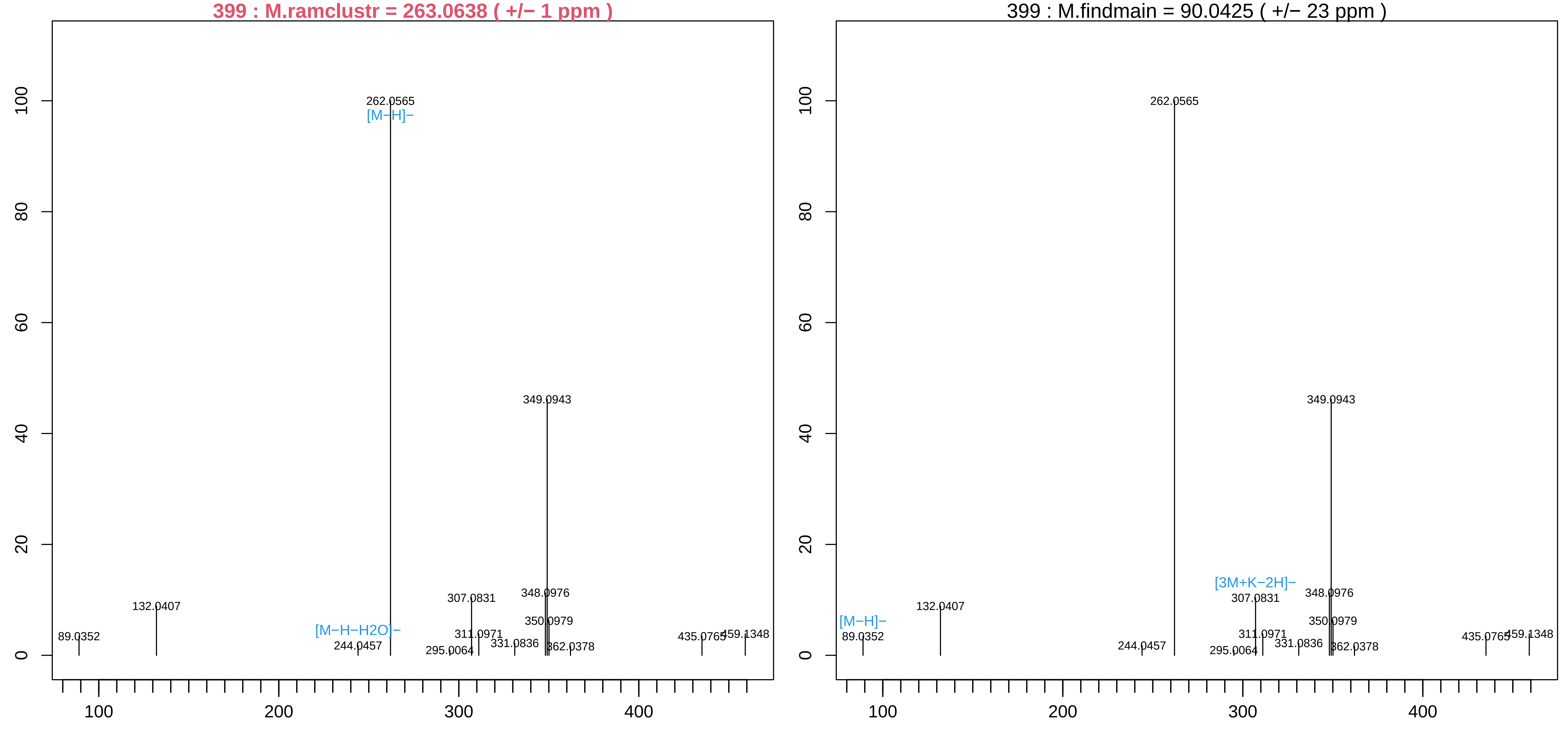

399 : M.ramclustr = 263.0638 (+/− 1 ppm)
262.0565
[M−H]−
349.0943
307.0831
348.0976
132.0407
350.0979
[M−H−H2O]−
311.0971
89.0352
331.0836
244.0457
362.0378
295.0064
435.0765
459.1348
399 : M.findmain = 90.0425 (+/− 23 ppm)
[3M+K−2H]−
[M−H]−

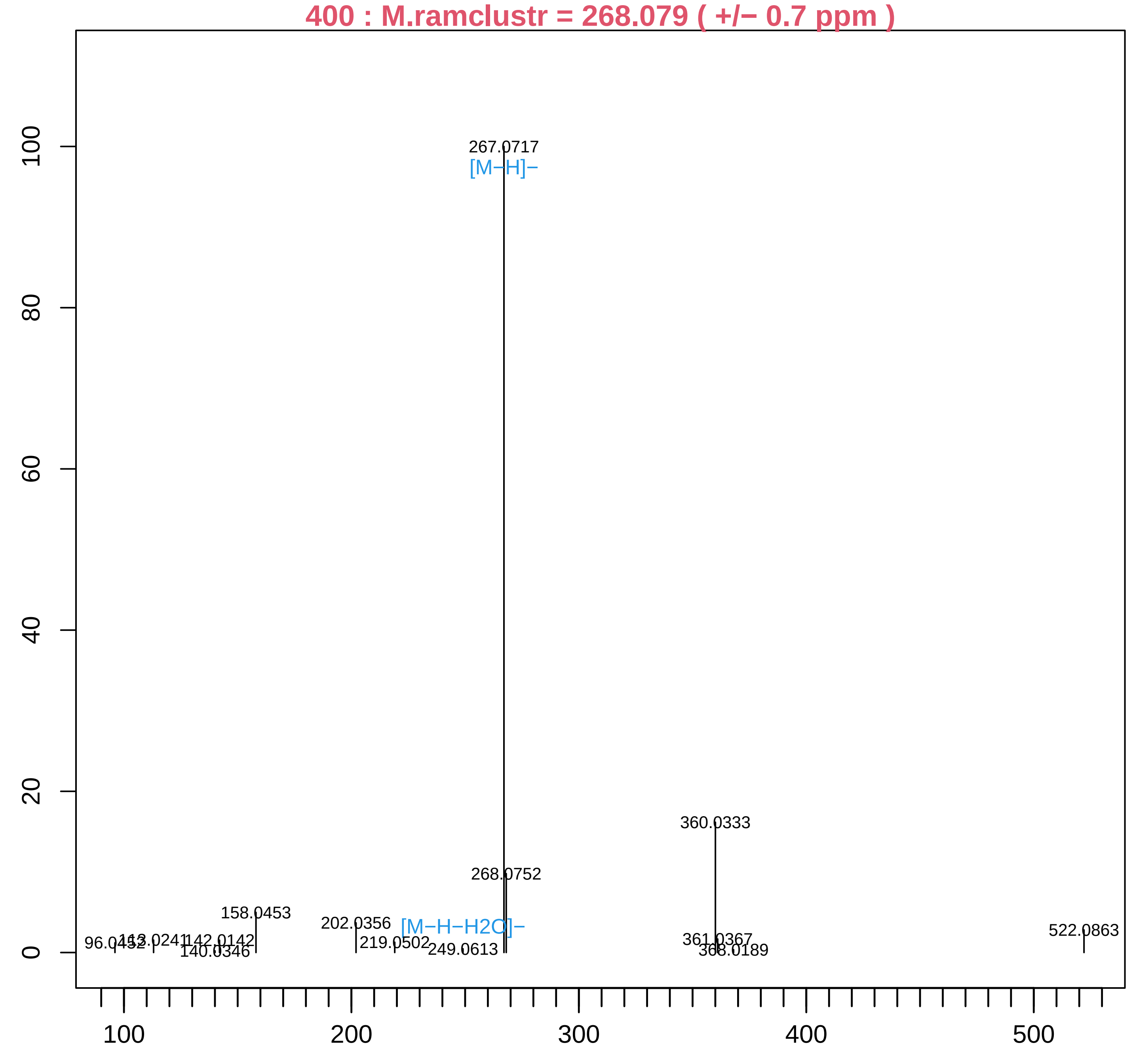
400 : M.ramclustr = 268.079 (+/− 0.7 ppm)
267.0717
[M−H]−
360.0333
268.0752
158.0453
202.0356
[M−H−H2O]−
96.0432
113.0241
142.0142
140.0346
219.0502
249.0613
361.0367
368.0189
522.0863
0
20
40
60
80
100
100
200
300
400
500
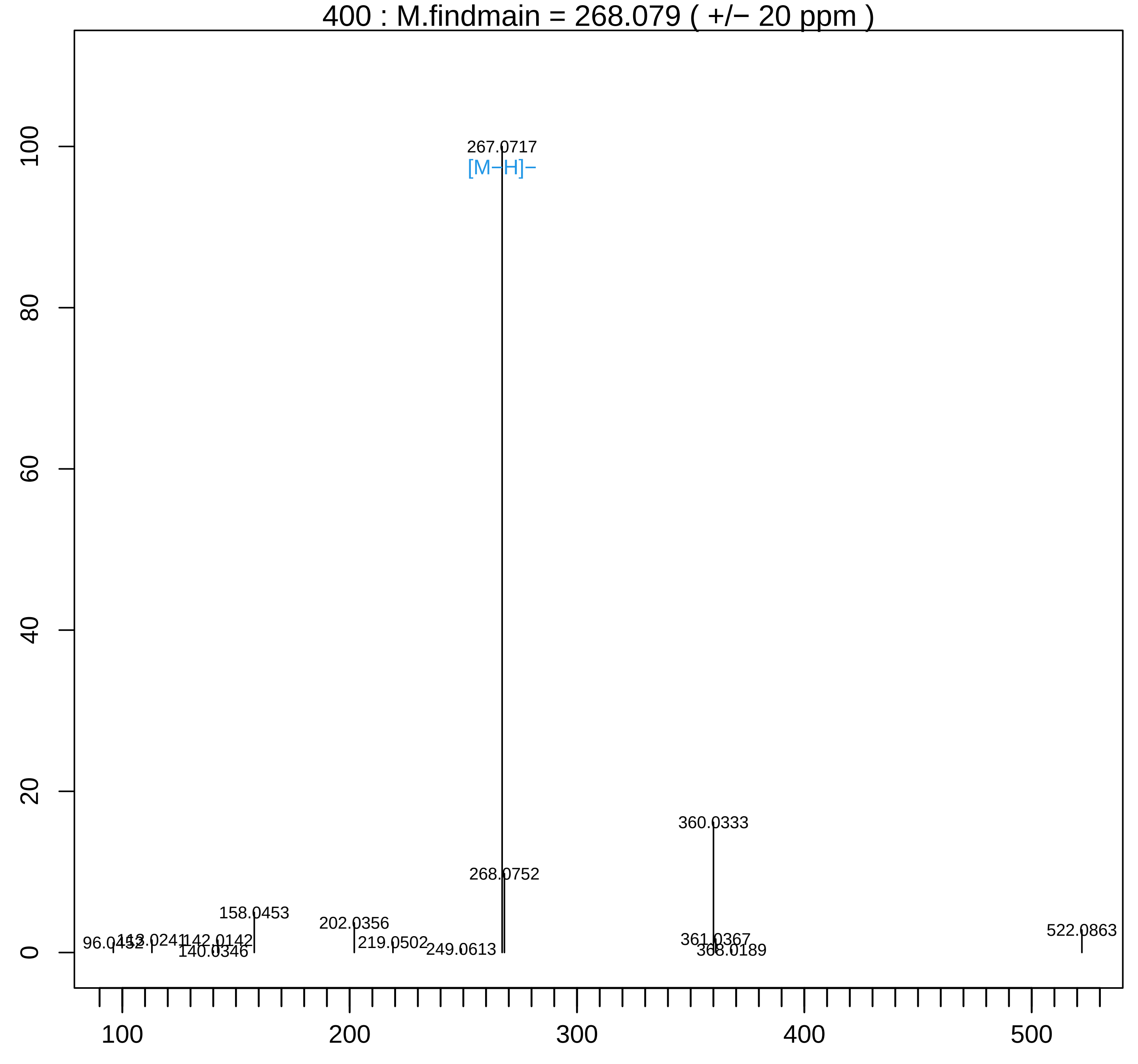
400 : M.findmain = 268.079 (+/− 20 ppm)
267.0717
[M−H]−
360.0333
268.0752
158.0453
202.0356
96.0432
113.0241
142.0142
140.0346
219.0502
249.0613
361.0367
368.0189
522.0863
0
20
40
60
80
100
100
200
300
400
500

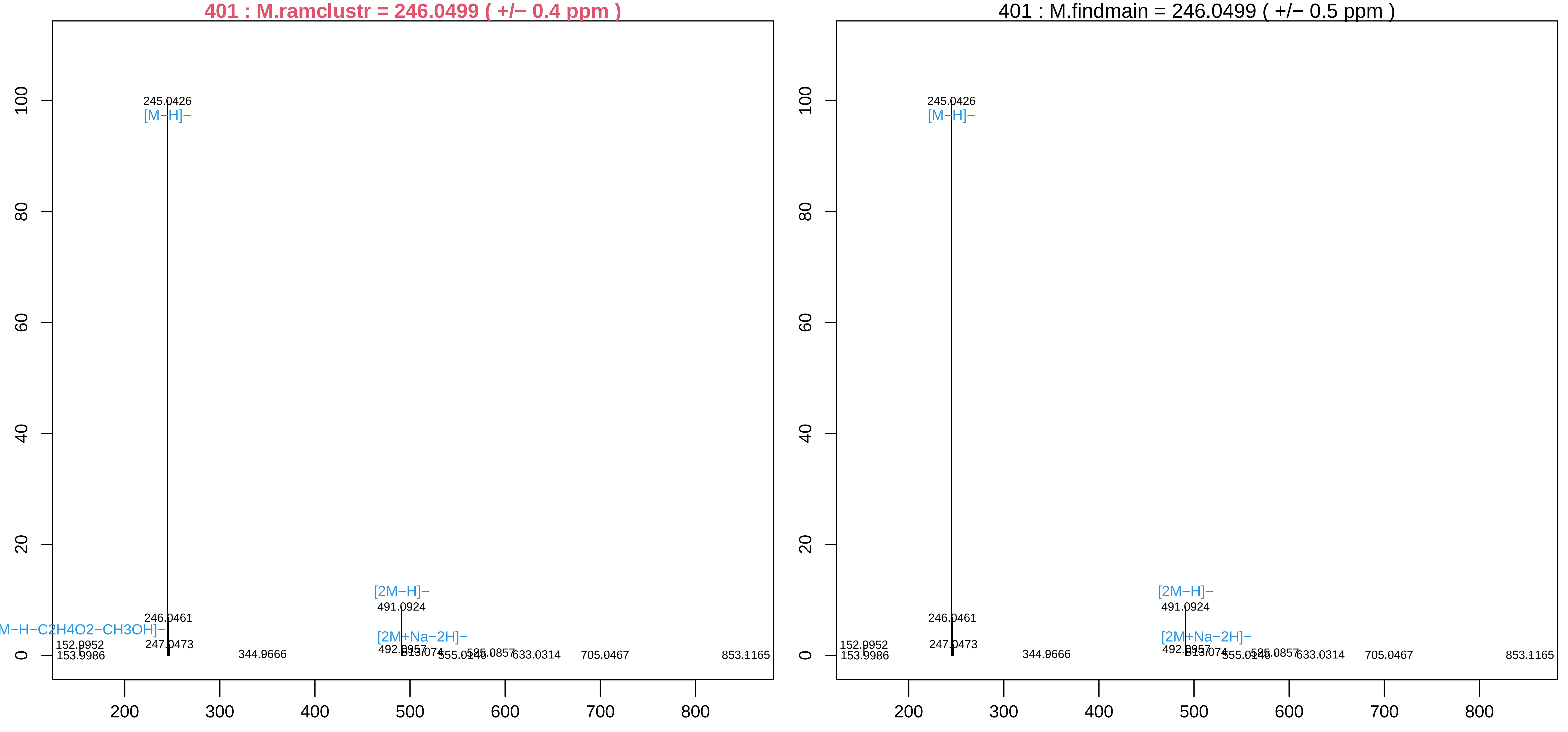

401 : M.ramclustr = 246.0499 (+/− 0.4 ppm)
245.0426
[M−H]−
[2M−H]−
491.0924
246.0461
[M−H−C2H4O2−CH3OH]−
152.9952
247.0473
153.9986
344.9666
[2M+Na−2H]−
492.0957
513.074
555.0146
585.0857
633.0314
705.0467
853.1165
401 : M.findmain = 246.0499 (+/− 0.5 ppm)
245.0426
[M−H]−
[2M−H]−
491.0924
246.0461
152.9952
247.0473
153.9986
344.9666
[2M+Na−2H]−
492.0957
513.074
555.0146
585.0857
633.0314
705.0467
853.1165

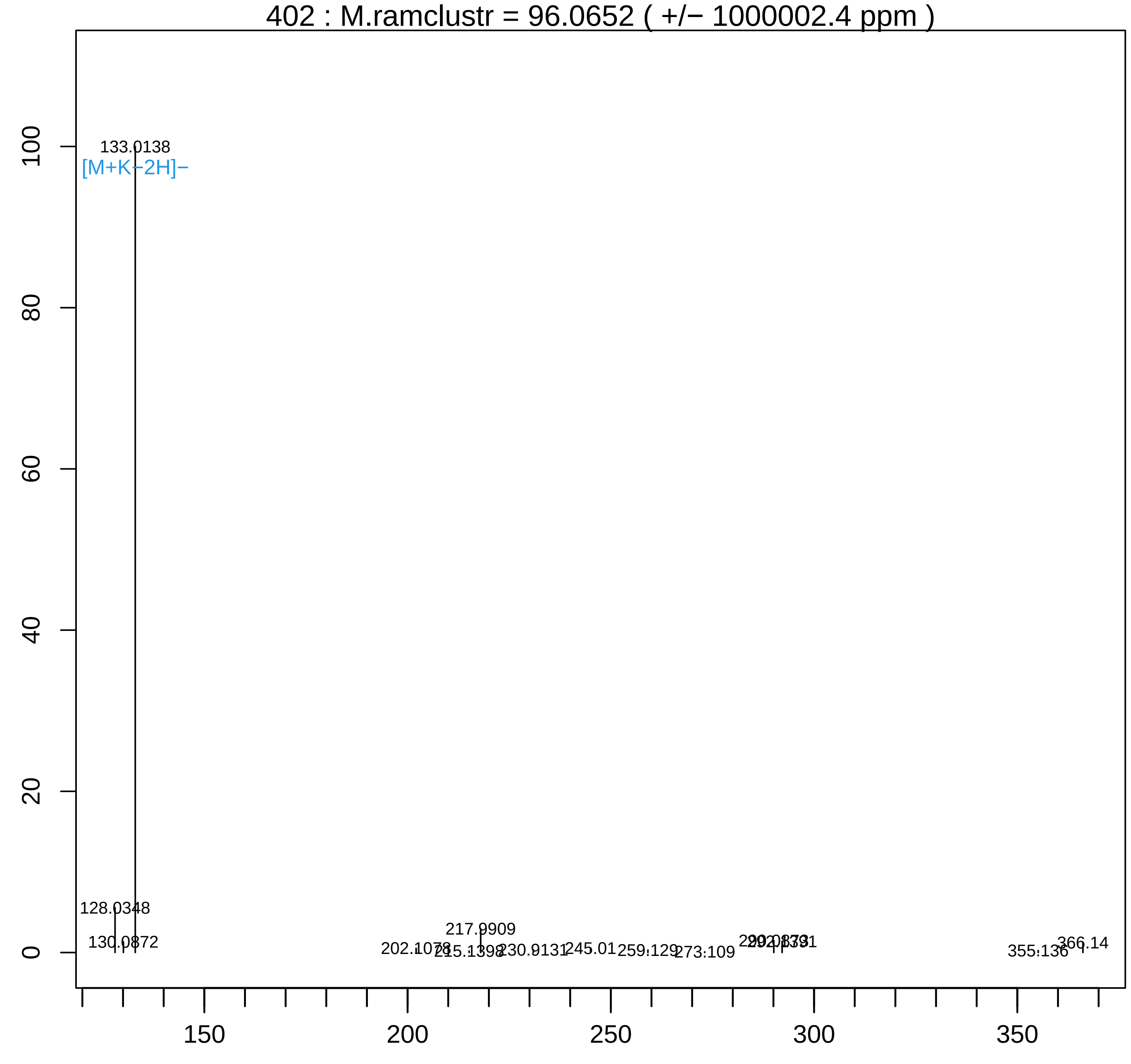

402 : M.ramclustr = 96.0652 (+/− 1000002.4 ppm)
133.0138
[M+K-2H]−
128.0348
130.0872
202.1078
215.1398
217.9909
230.9131
245.01
259.129
273.109
355.136
366.14

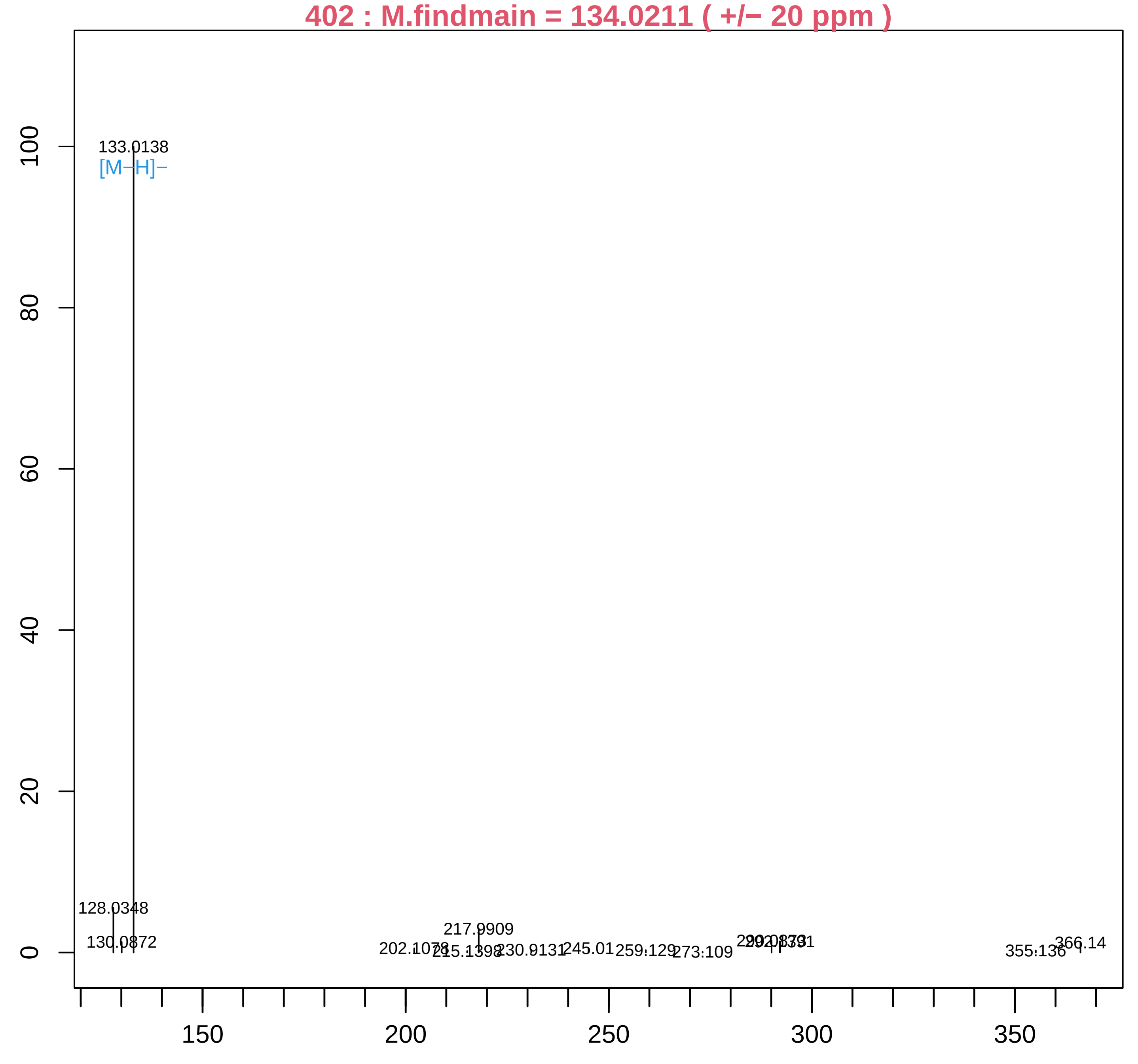

402 : M.findmain = 134.0211 (+/− 20 ppm)
133.0138
[M-H]−
128.0348
130.0872
202.1078
215.1398
217.9909
230.9131
245.01
259.129
273.109
355.136
366.14

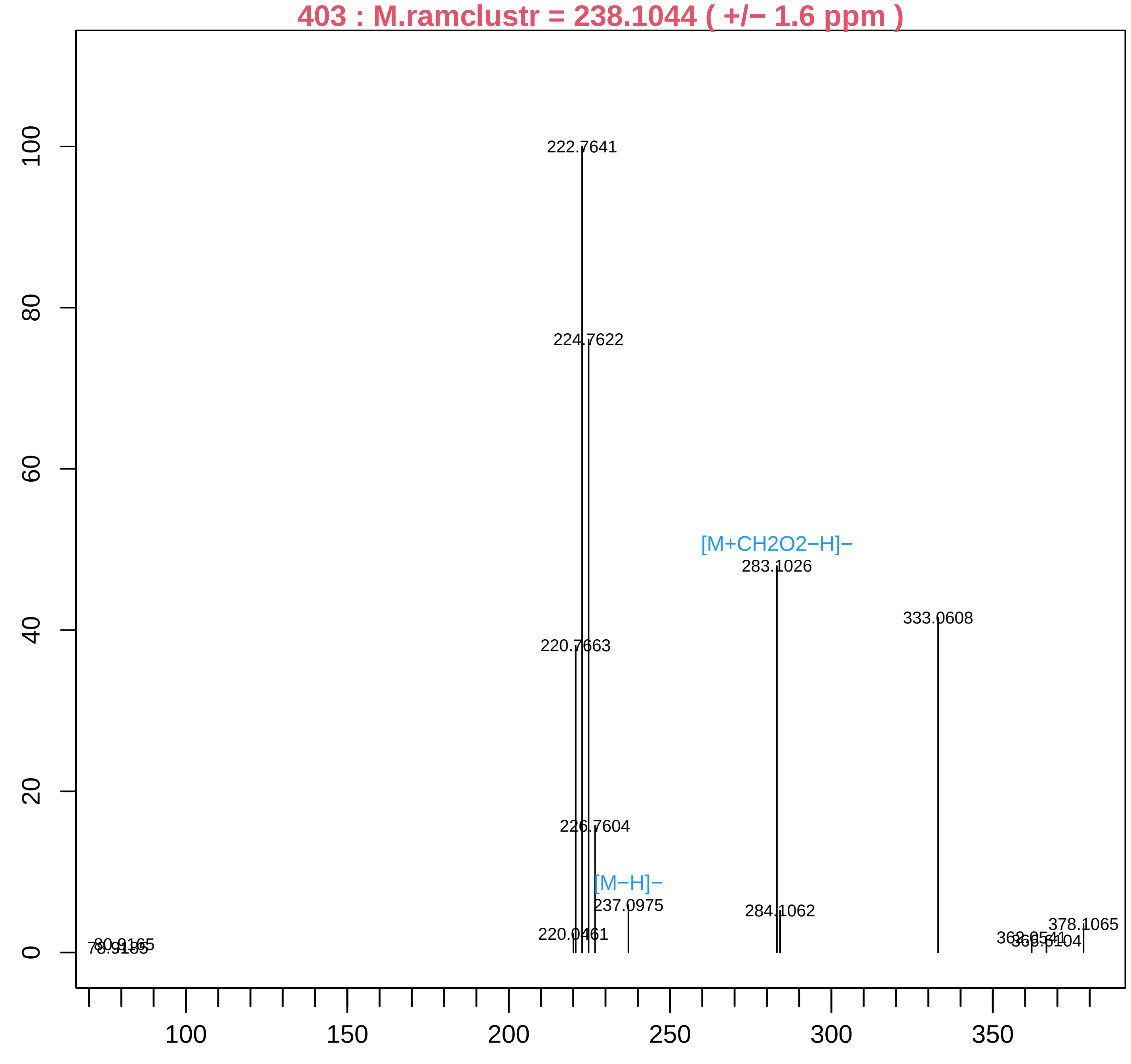
403 : M.ramclustr = 238.1044 (+/− 1.6 ppm)
222.7641
224.7622
220.7663
226.7604
[M−H]−
237.0975
220.0461
[M+CH2O2−H]−
283.1026
284.1062
333.0608
378.1065
80.9165
78.9185
0
20
40
60
80
100
100
150
200
250
300
350
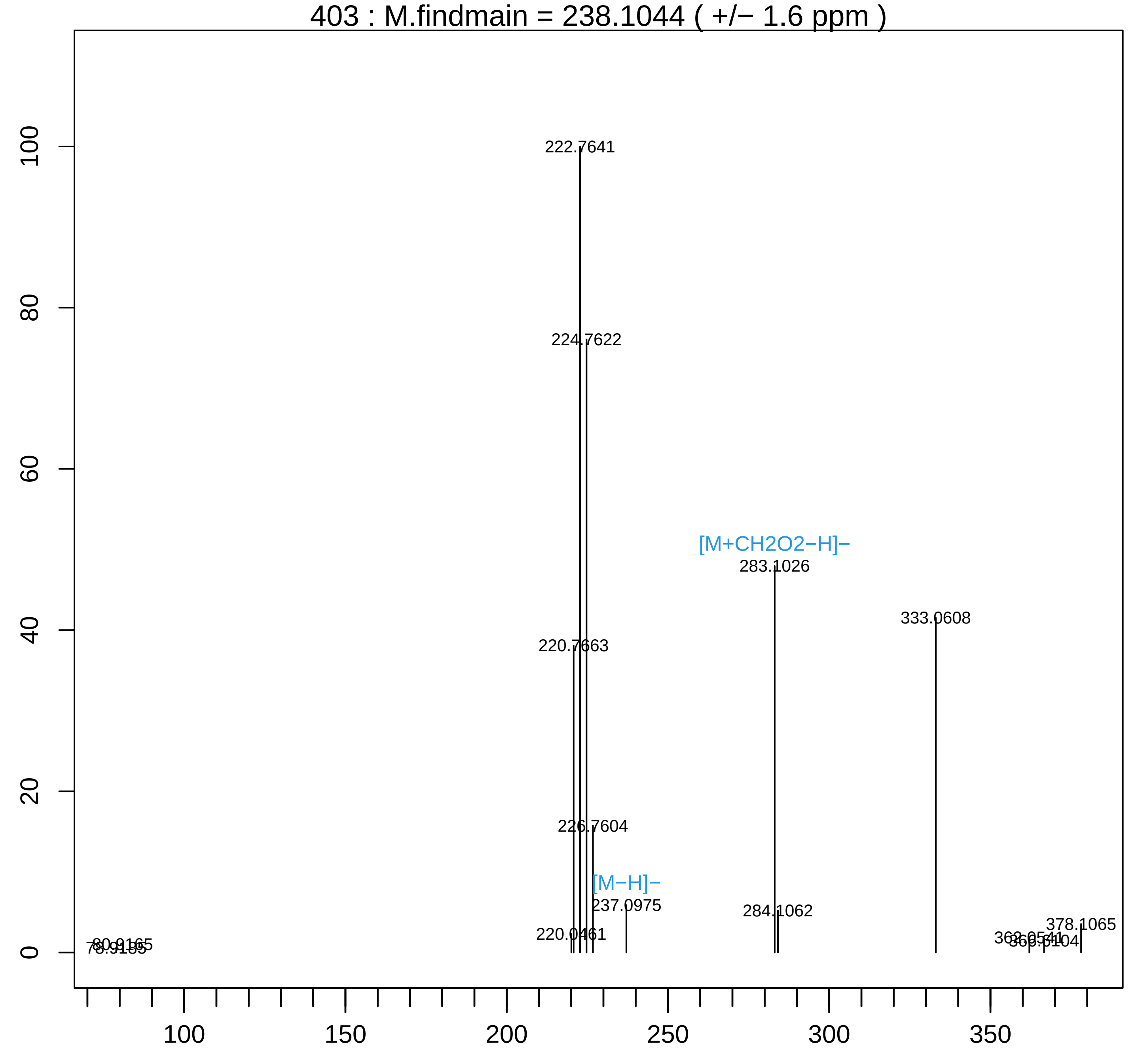
403 : M.findmain = 238.1044 (+/− 1.6 ppm)
222.7641
224.7622
220.7663
226.7604
[M−H]−
237.0975
220.0461
[M+CH2O2−H]−
283.1026
284.1062
333.0608
378.1065
80.9165
78.9185
0
20
40
60
80
100
100
150
200
250
300
350

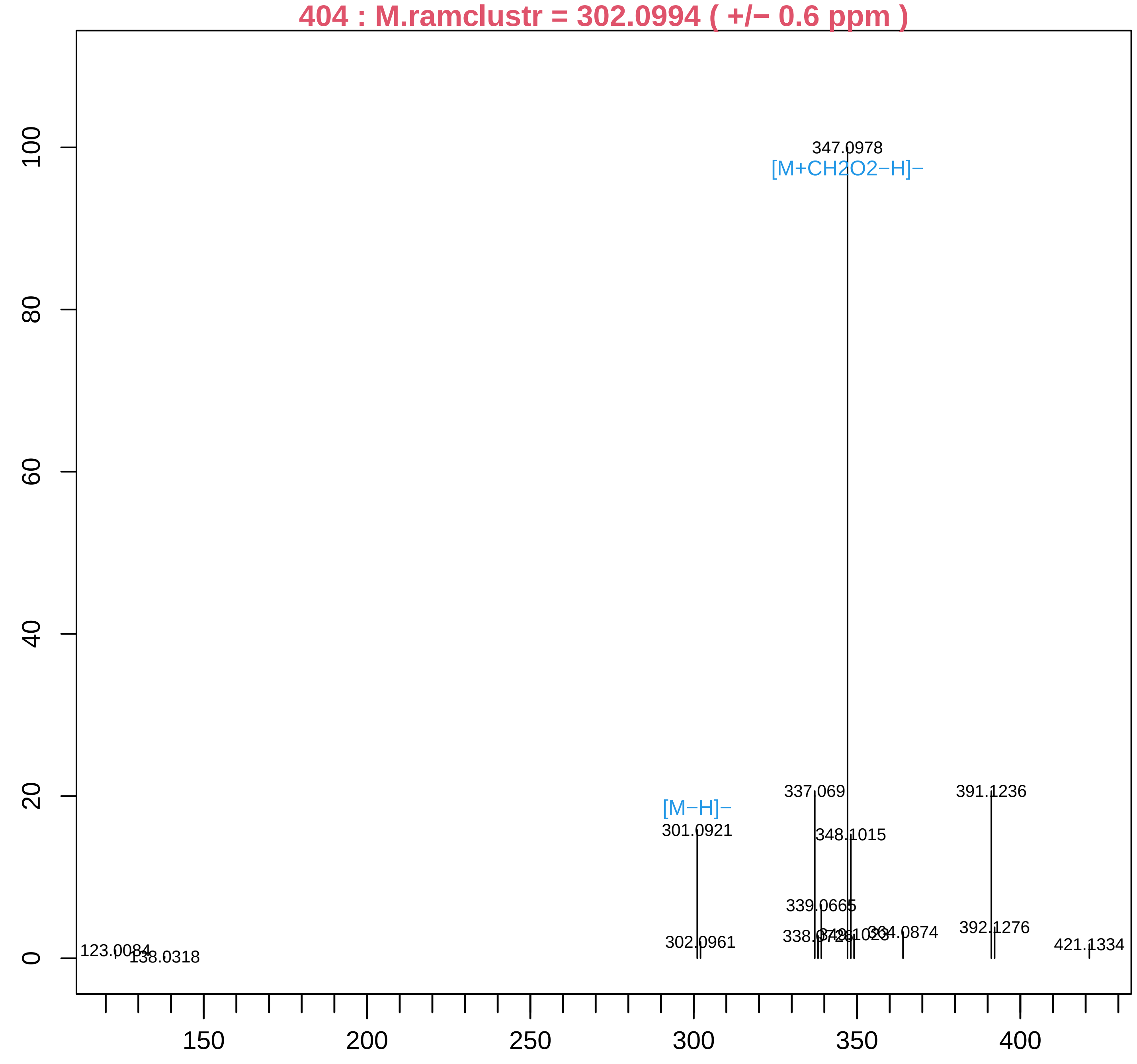
404 : M.ramclustr = 302.0994 (+/− 0.6 ppm)
347.0978
[M+CH2O2−H]−
[M−H]−
301.0921
337.069
391.1236
348.1015
339.0665
302.0961
338.0726
349.1023
364.0874
392.1276
421.1334
123.0084
138.0318
0
20
40
60
80
100
150
200
250
300
350
400

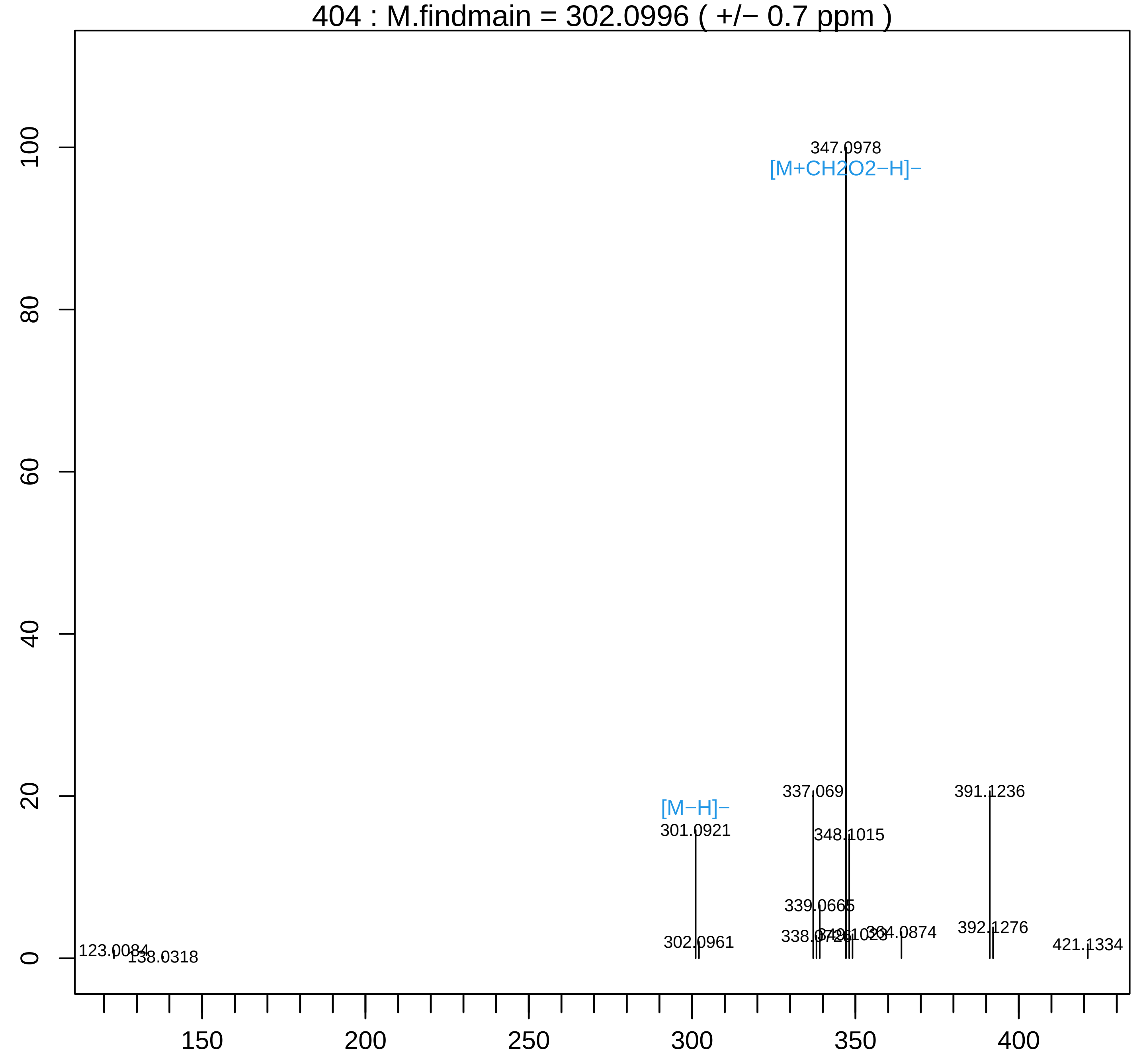
404 : M.findmain = 302.0996 (+/− 0.7 ppm)
347.0978
[M+CH2O2−H]−
[M−H]−
301.0921
337.069
391.1236
348.1015
339.0665
302.0961
338.0726
349.1023
364.0874
392.1276
421.1334
123.0084
138.0318
0
20
40
60
80
100
150
200
250
300
350
400

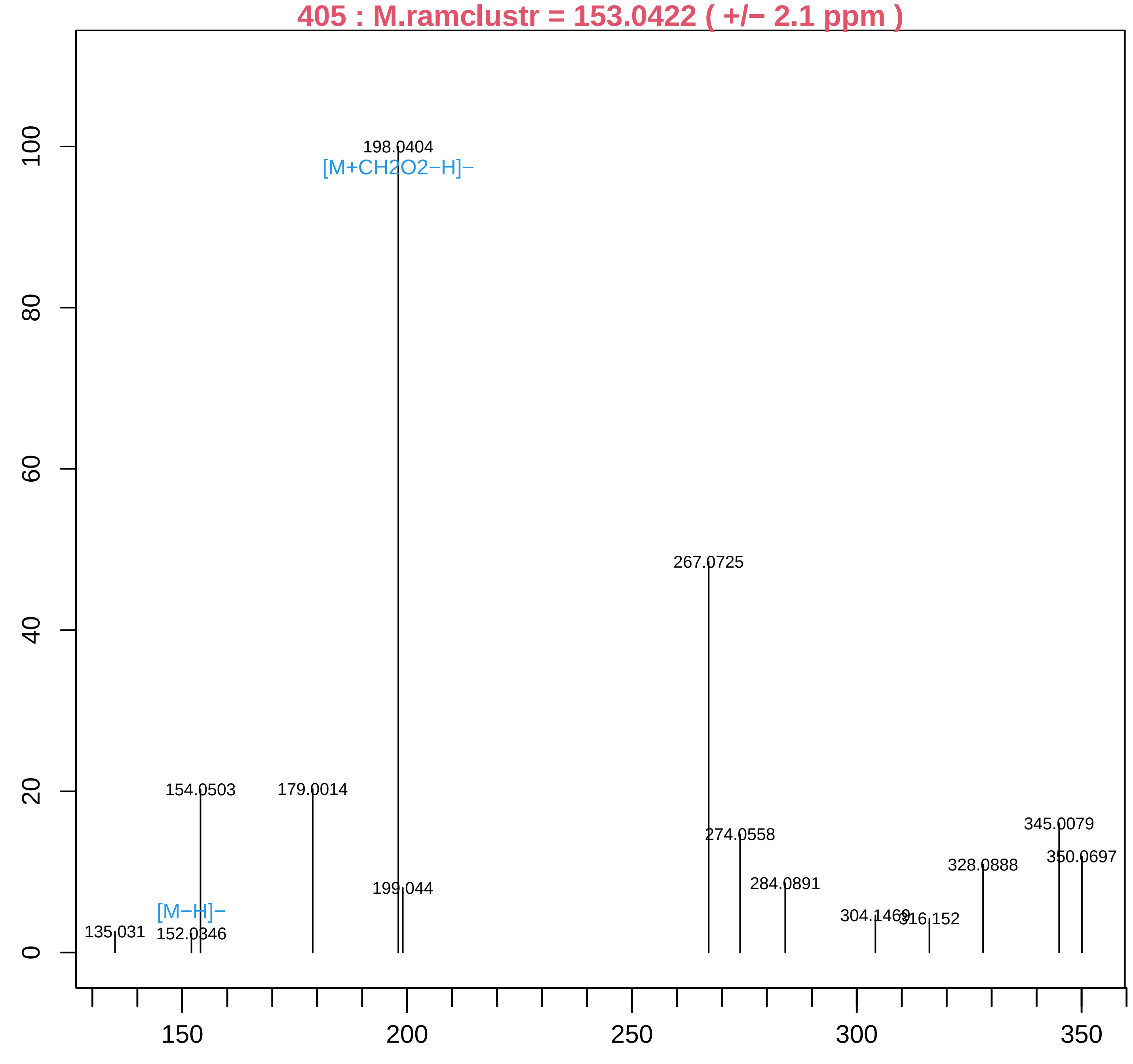
405 : M.ramclustr = 153.0422 (+/− 2.1 ppm)
198.0404
[M+CH2O2−H]−
267.0725
154.0503
179.0014
274.0558
345.0079
350.0697
328.0888
199.044
284.0891
[M−H]−
304.1469
316.152
135.031
152.0346
0
20
40
60
80
100
150
200
250
300
350

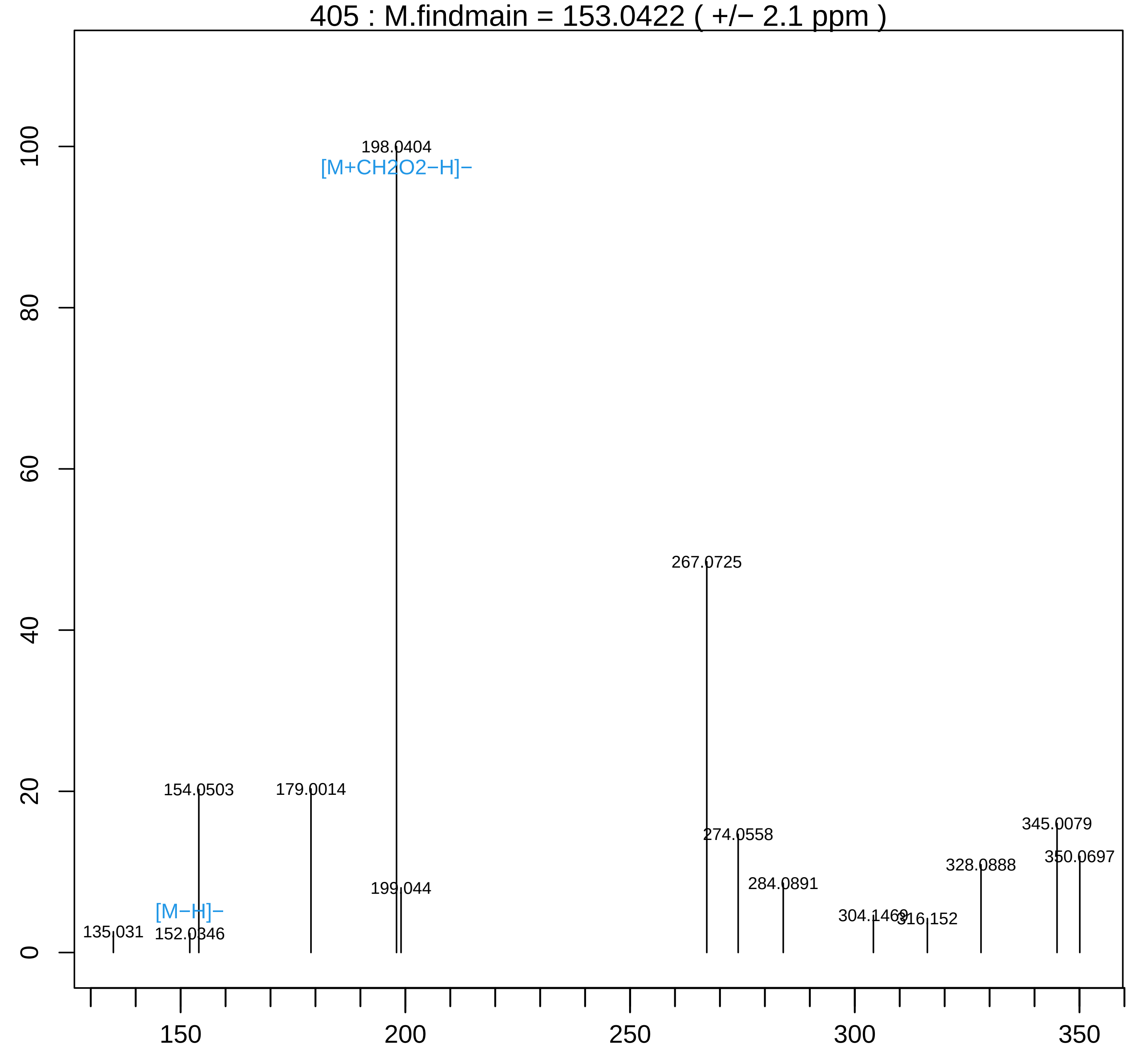
405 : M.findmain = 153.0422 (+/− 2.1 ppm)
198.0404
[M+CH2O2−H]−
267.0725
154.0503
179.0014
274.0558
345.0079
350.0697
328.0888
199.044
284.0891
[M−H]−
304.1469
316.152
135.031
152.0346
0
20
40
60
80
100
150
200
250
300
350

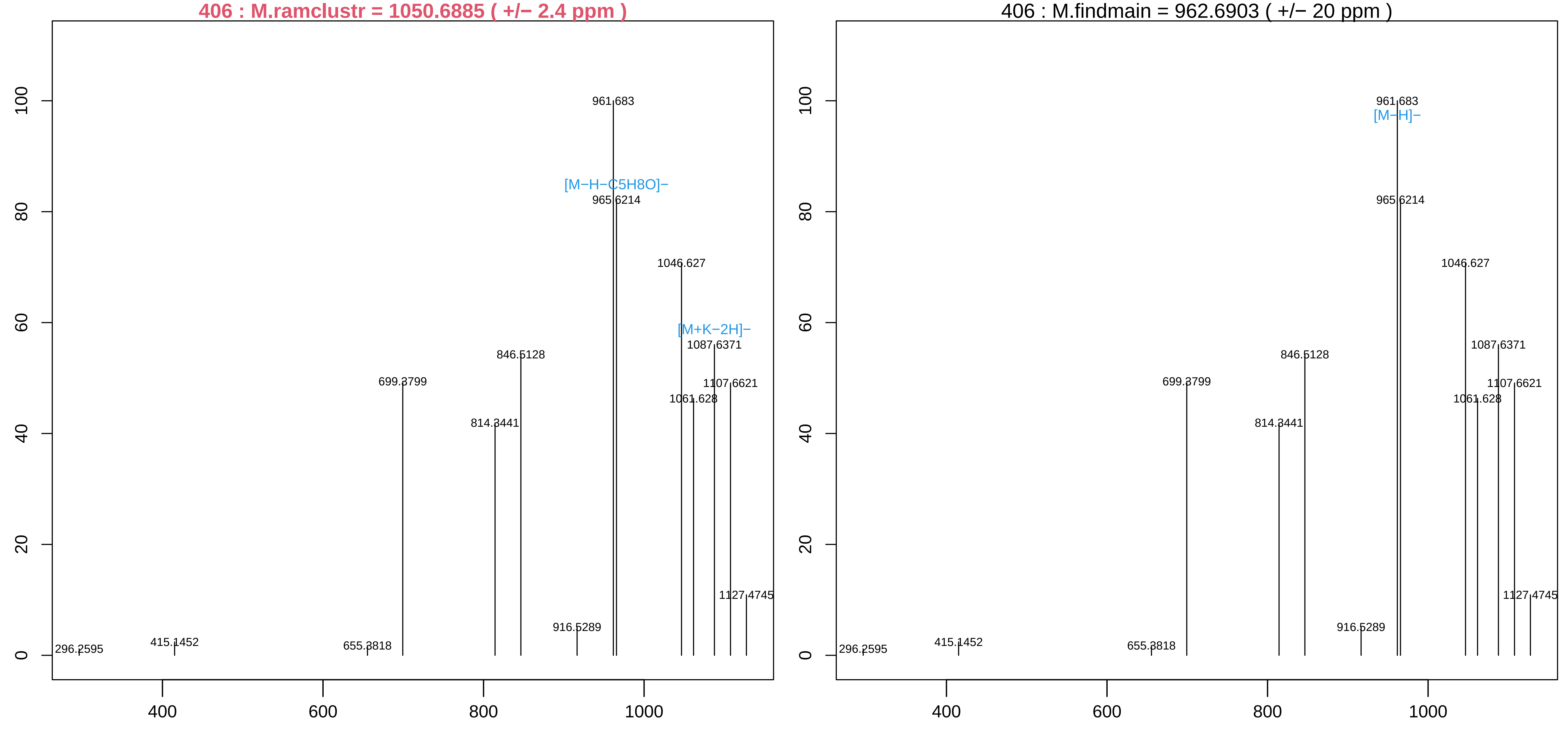

406 : M.ramclustr = 1050.6885 (+/− 2.4 ppm)
406 : M.findmain = 962.6903 (+/− 20 ppm)
961.683
[M−H−C5H8O]−
965.6214
1046.627
[M+K−2H]−
1087.6371
846.5128
699.3799
1107.6621
1061.628
814.3441
1127.4745
916.5289
415.1452
655.3818
296.2595
[M−H]−
0
20
40
60
80
100
400
600
800
1000

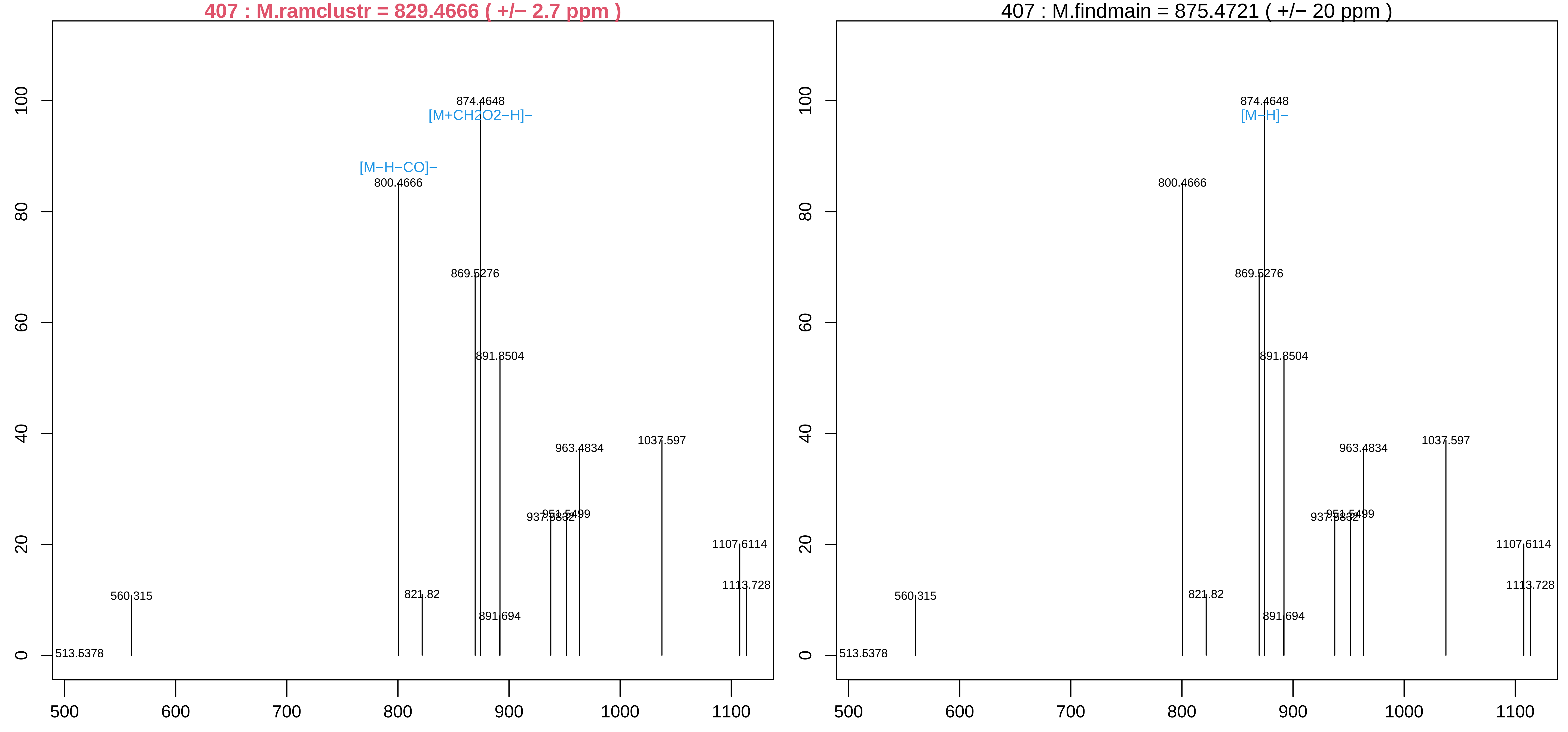

407 : M.ramclustr = 829.4666 (+/− 2.7 ppm)
874.4648
[M+CH2O2−H]−
[M−H−CO]−
800.4666
869.5276
891.8504
963.4834
1037.597
937.5832
951.5499
1107.6114
1113.728
560.315
821.82
891.694
513.5378
407 : M.findmain = 875.4721 (+/− 20 ppm)
874.4648
[M−H]−
800.4666
869.5276
891.8504
963.4834
1037.597
937.5832
951.5499
1107.6114
1113.728
560.315
821.82
891.694
513.5378
0
20
40
60
80
100
500
600
700
800
900
1000
1100

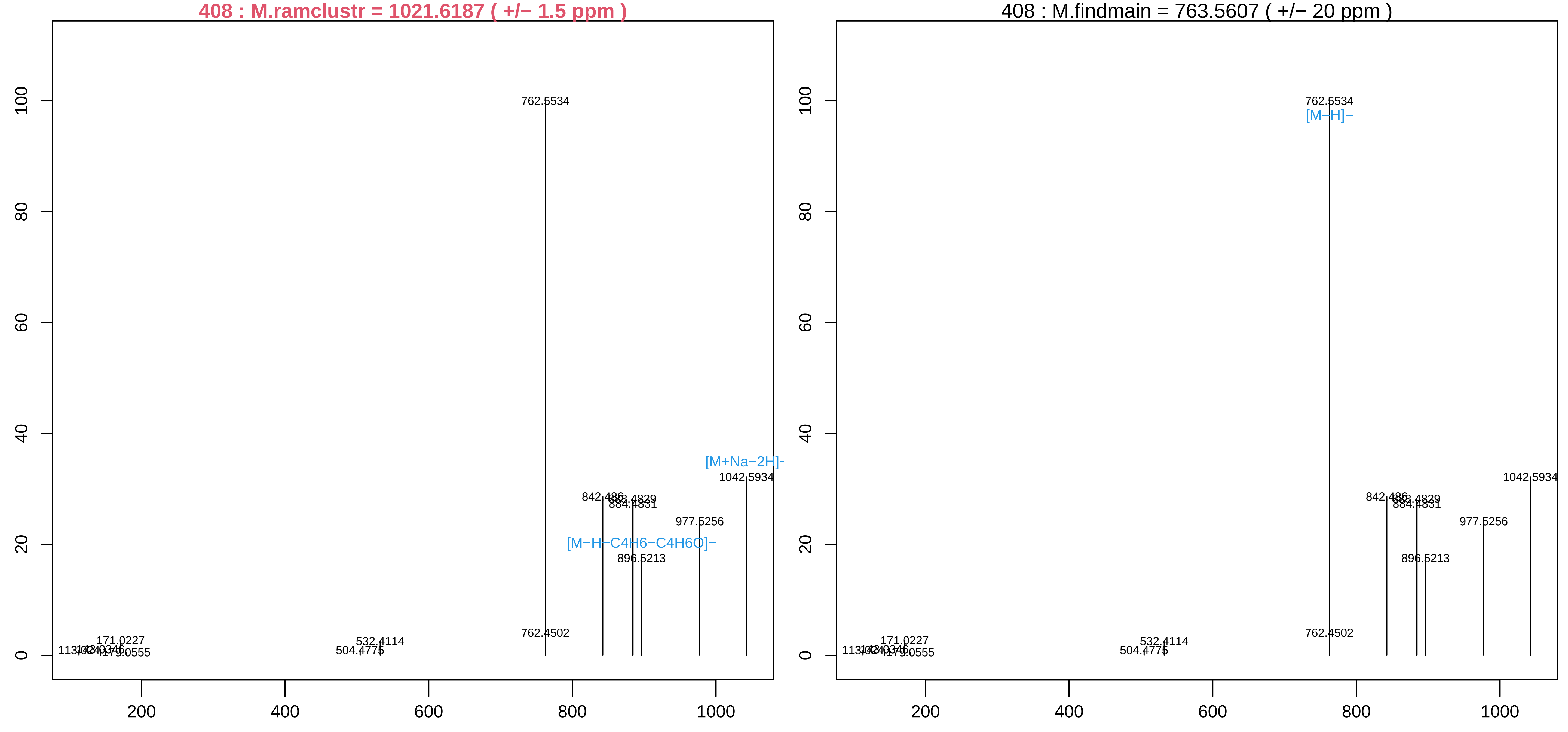

408 : M.ramclustr = 1021.6187 (+/− 1.5 ppm)
762.5534
[M+Na−2H]-
1042.5934
842.486
868.4829
884.4831
977.5256
[M−H−C4H6−C4H6O]−
896.5213
762.4502
171.0227
532.4114
504.4775
179.0555
408 : M.findmain = 763.5607 (+/− 20 ppm)
762.5534
[M−H]−
1042.5934
842.486
868.4829
884.4831
977.5256
896.5213
762.4502
171.0227
532.4114
504.4775
179.0555

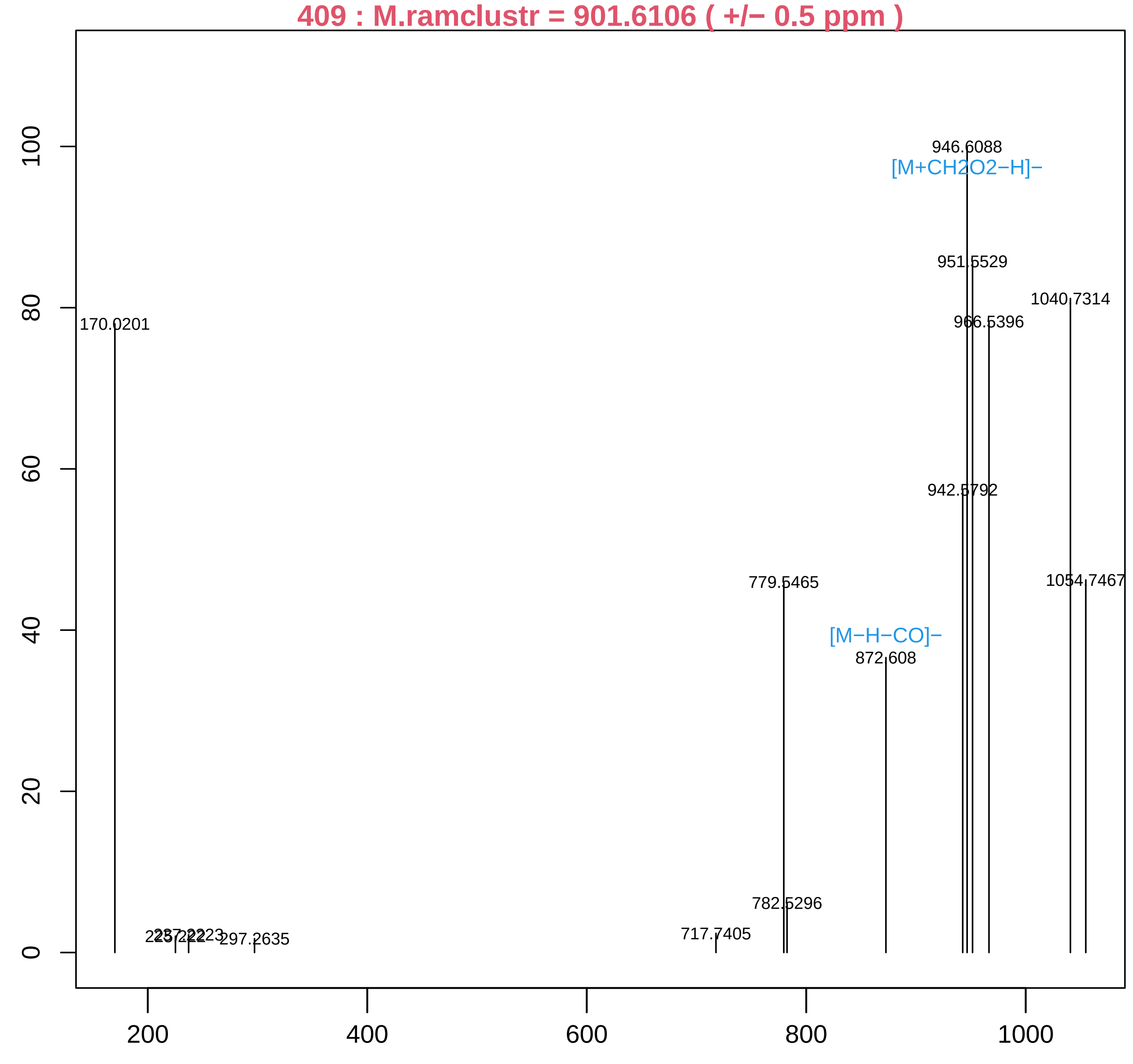
409 : M.ramclustr = 901.6106 (+/− 0.5 ppm)
946.6088
[M+CH2O2−H]−
951.5529
1040.7314
170.0201
966.5396
942.5792
779.5465
1054.7467
[M−H−CO]−
872.608
782.5296
717.7405
297.2635

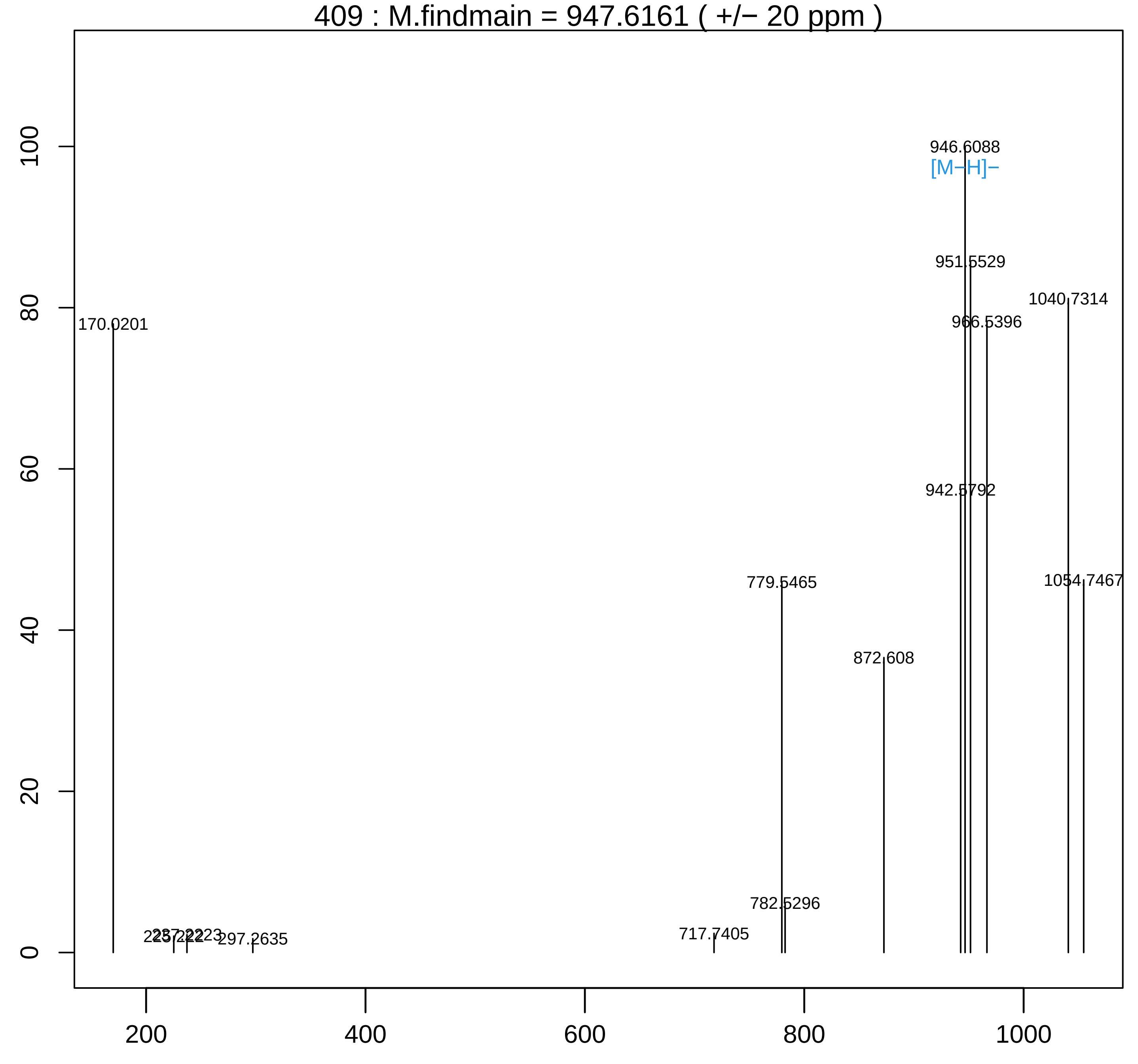
409 : M.findmain = 947.6161 (+/− 20 ppm)
946.6088
[M−H]−
951.5529
1040.7314
170.0201
966.5396
942.5792
779.5465
1054.7467
872.608
782.5296
717.7405
297.2635

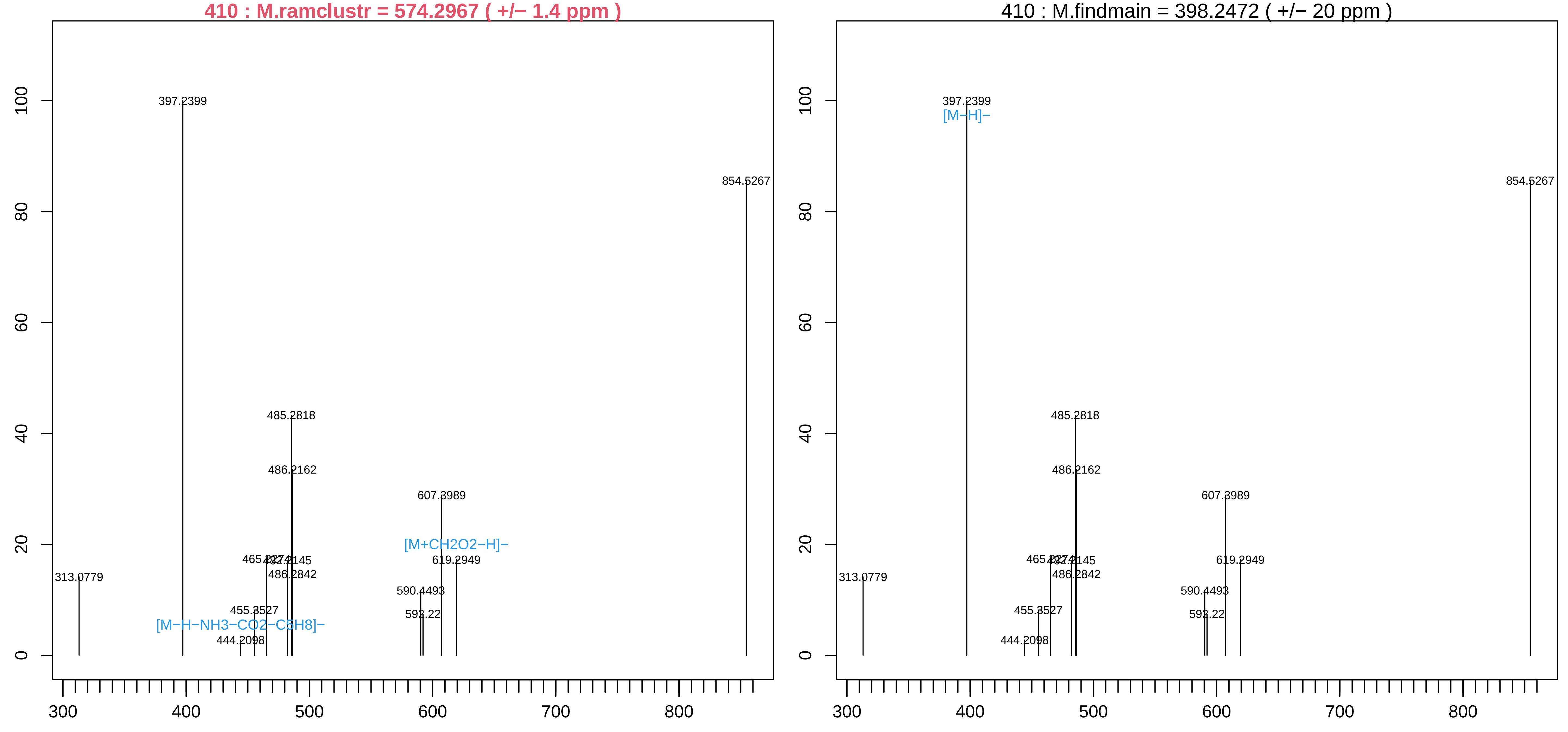

410 : M.ramclustr = 574.2967 (+/− 1.4 ppm)
397.2399
854.5267
485.2818
486.2162
607.3989
[M+CH2O2−H]−
465.2274
482.2145
619.2949
486.2842
313.0779
590.4493
455.3527
592.22
[M−H−NH3−CO2−C5H8]−
444.2098
0
20
40
60
80
100
300
400
500
600
700
800
410 : M.findmain = 398.2472 (+/− 20 ppm)
397.2399
[M−H]−
854.5267
485.2818
486.2162
607.3989
465.2274
482.2145
619.2949
486.2842
313.0779
590.4493
455.3527
592.22
444.2098
0
20
40
60
80
100
300
400
500
600
700
800

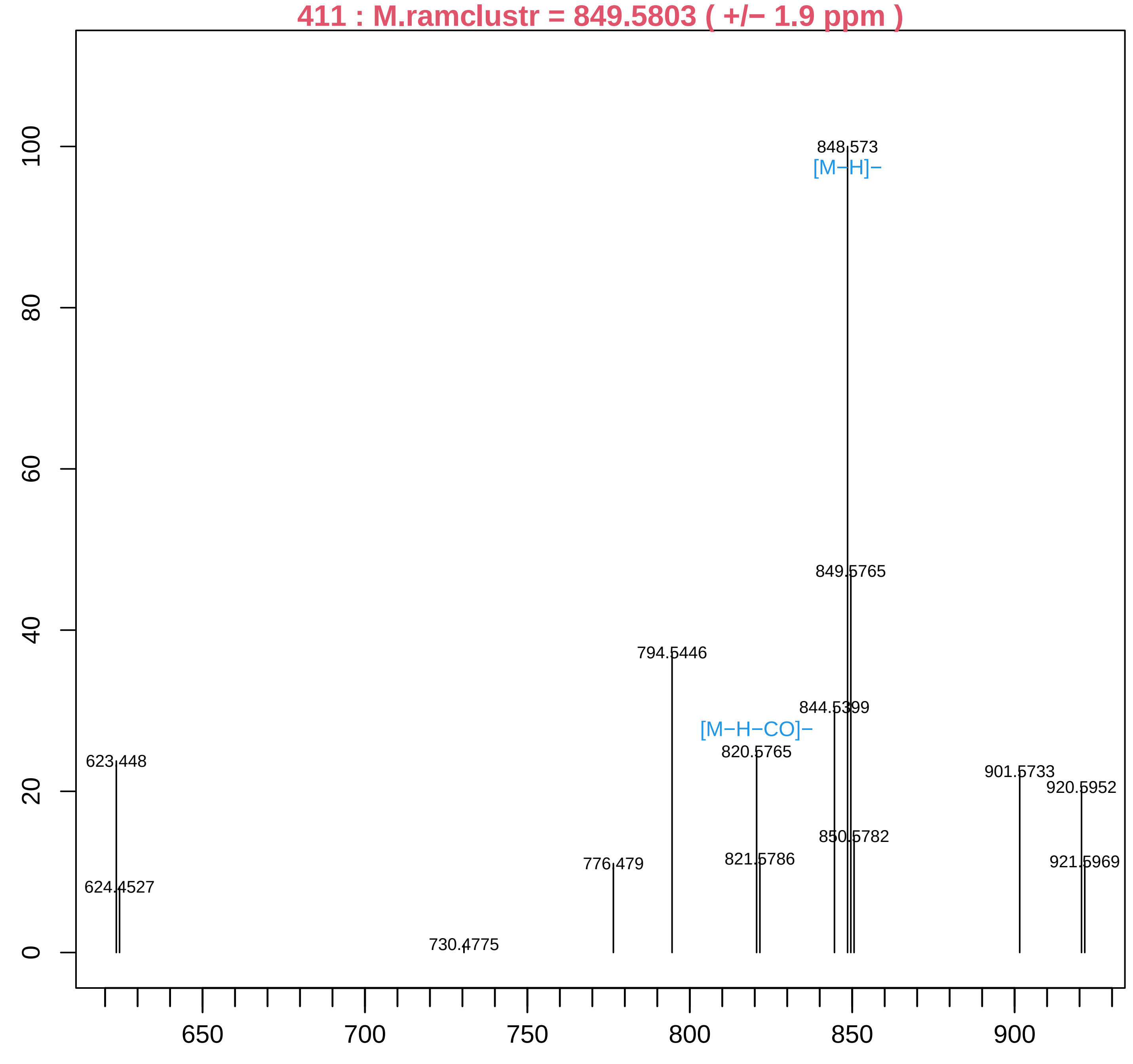

411 : M.ramclustr = 849.5803 (+/− 1.9 ppm)
848.573
[M−H]−
849.5765
794.5446
844.5399
[M−H−CO]−
820.5765
623.448
901.5733
920.5952
850.5782
821.5786
776.479
921.5969
624.4527
730.4775
0
20
40
60
80
100
650
700
750
800
850
900

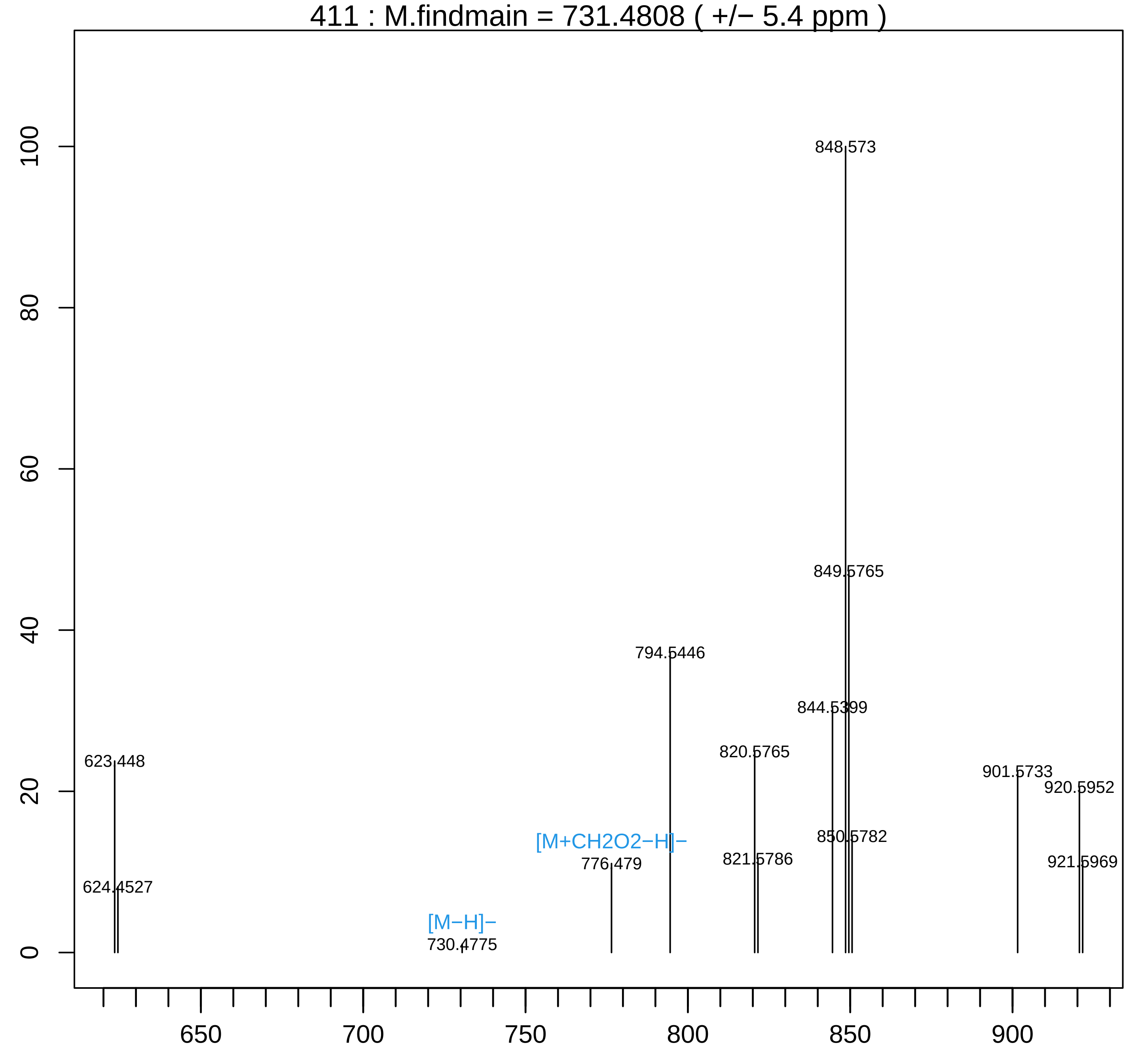

411 : M.findmain = 731.4808 (+/− 5.4 ppm)
848.573
849.5765
794.5446
844.5399
820.5765
623.448
901.5733
920.5952
850.5782
[M+CH2O2−H]−
776.479
821.5786
921.5969
624.4527
[M−H]−
730.4775
0
20
40
60
80
100
650
700
750
800
850
900

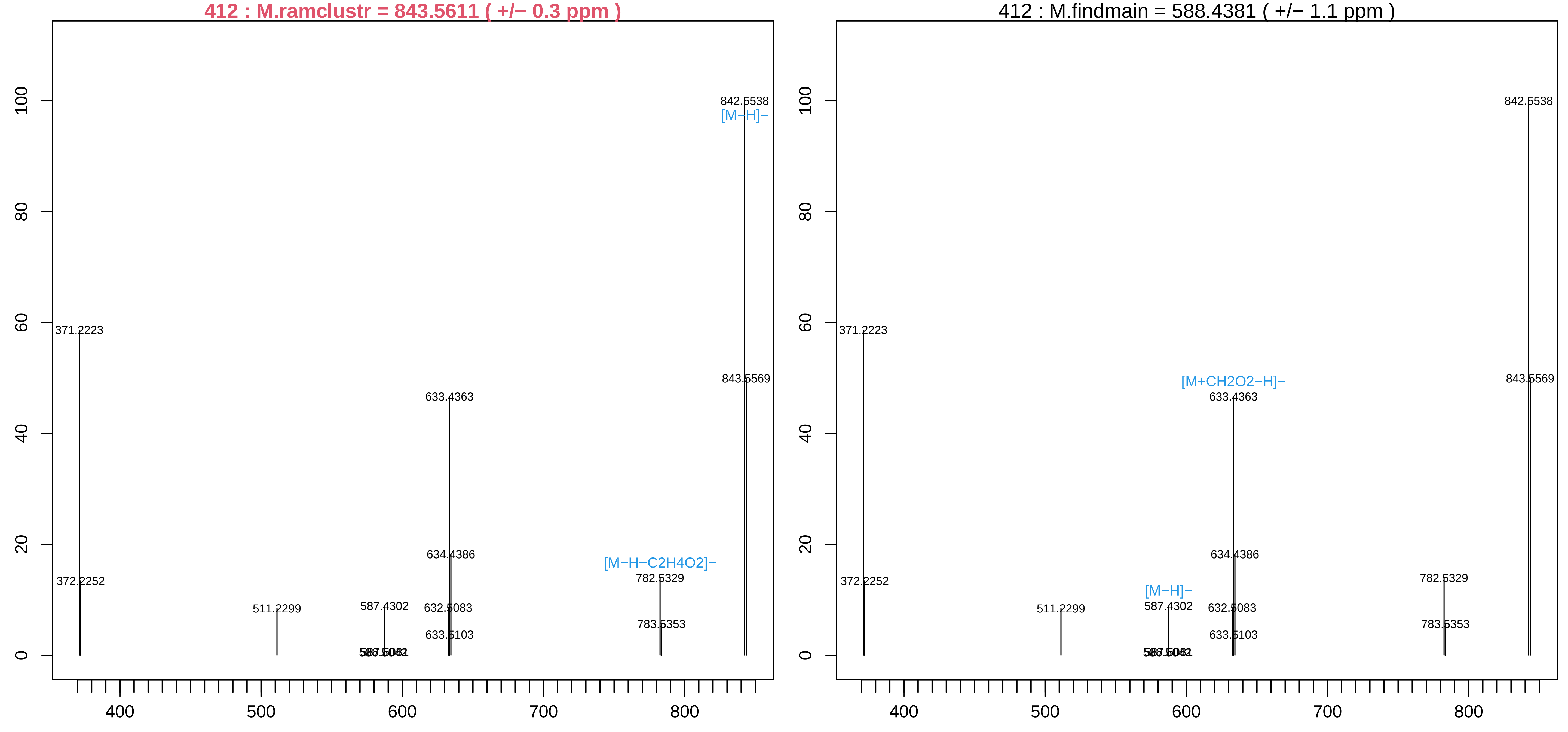

412 : M.ramclustr = 843.5611 (+/− 0.3 ppm)
842.5538
[M−H]−
371.2223
843.5569
633.4363
634.4386
[M−H−C2H4O2]−
782.5329
372.2252
511.2299
587.4302
632.5083
783.5353
633.5103
412 : M.findmain = 588.4381 (+/− 1.1 ppm)
842.5538
371.2223
843.5569
[M+CH2O2−H]−
633.4363
634.4386
782.5329
372.2252
[M−H]−
511.2299
587.4302
632.5083
783.5353
633.5103
0
20
40
60
80
100
400
500
600
700
800

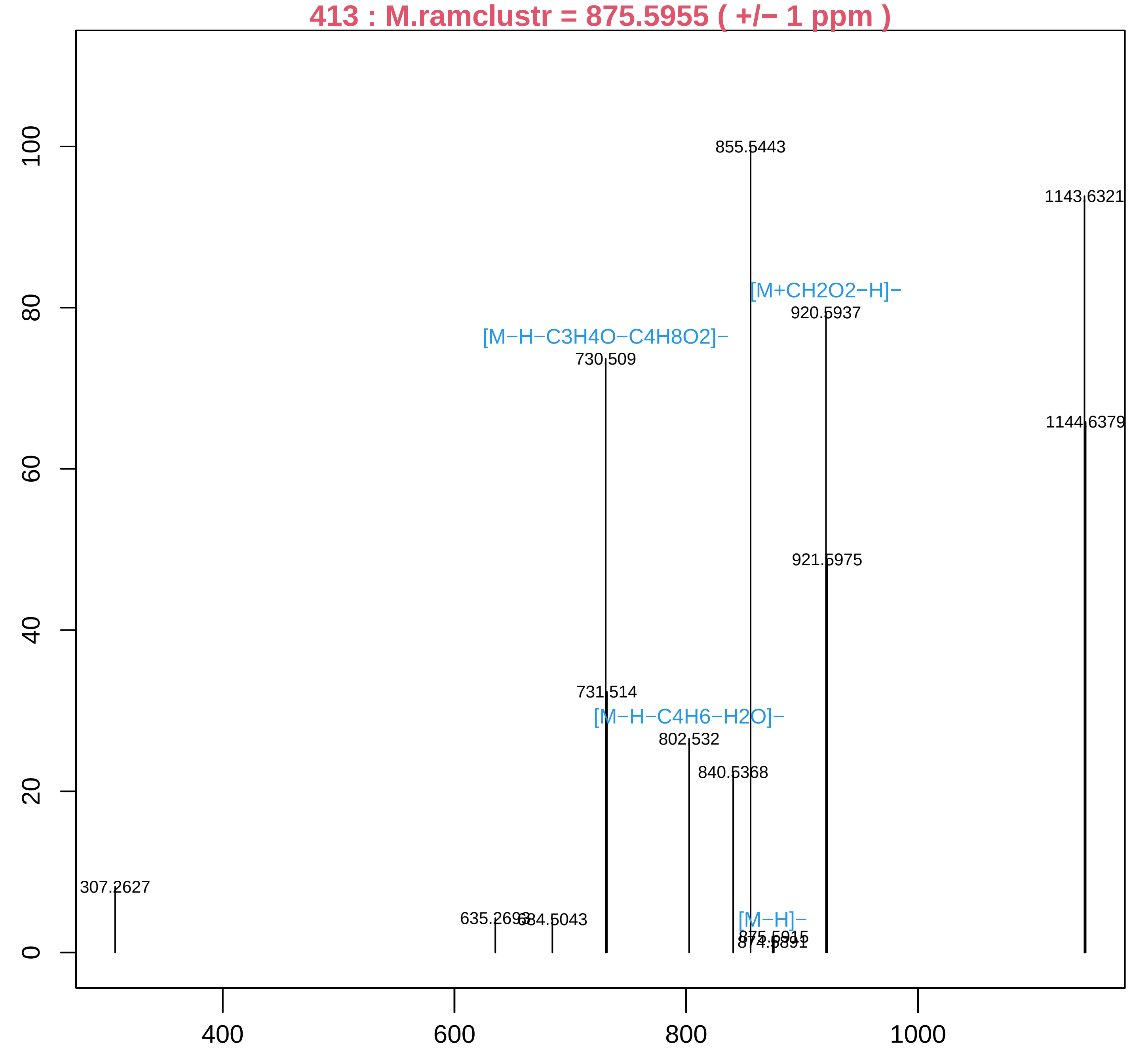

413 : M.ramclustr = 875.5955 (+/− 1 ppm)
855.5443
1143.6321
[M+CH2O2−H]−
920.5937
[M−H−C3H4O−C4H8O2]−
730.509
1144.6379
921.5975
731.514
[M−H−C4H6−H2O]−
802.532
840.5368
307.2627
635.2693
684.5043
[M−H]−
875.5915
874.5891
0
20
40
60
80
100
400
600
800
1000

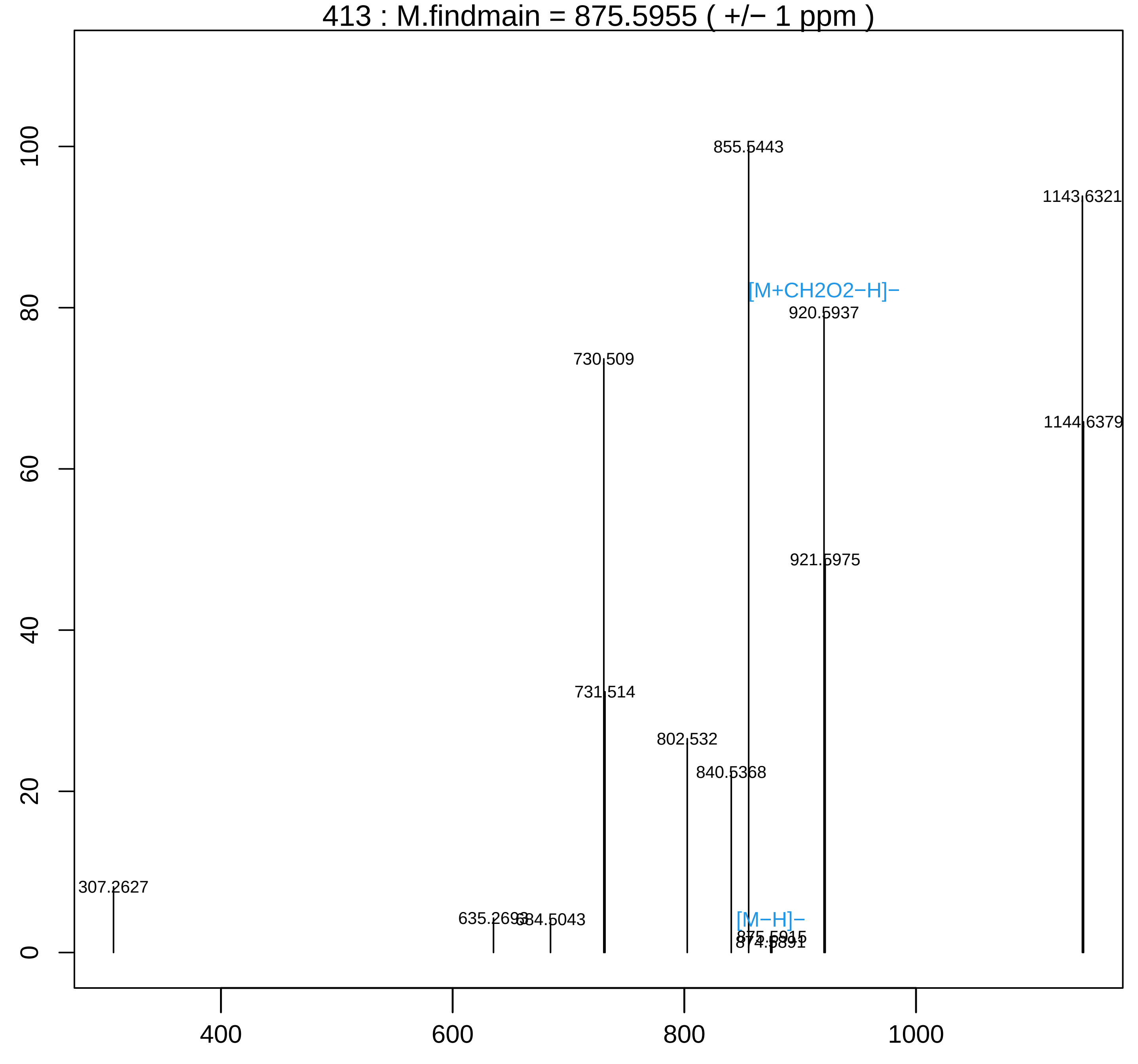

413 : M.findmain = 875.5955 (+/− 1 ppm)
855.5443
1143.6321
[M+CH2O2−H]−
920.5937
730.509
1144.6379
921.5975
731.514
802.532
840.5368
307.2627
635.2693
684.5043
[M−H]−
875.5915
874.5891
0
20
40
60
80
100
400
600
800
1000

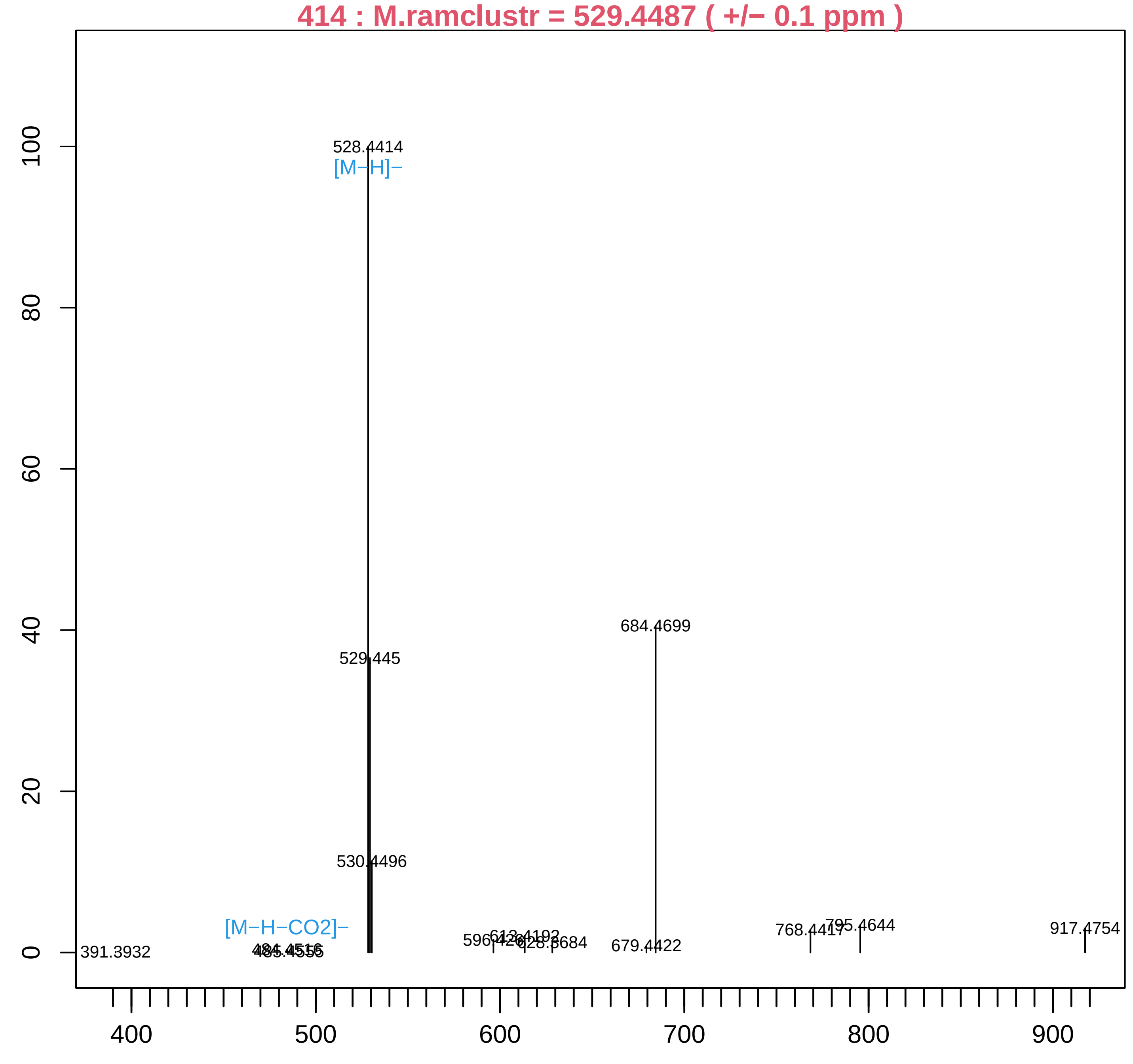
414 : M.ramclustr = 529.4487 (+/− 0.1 ppm)
528.4414
[M−H]−
684.4699
529.445
530.4496
[M−H−CO2]−
484.4516
485.4555
391.3932
596.4201
612.4192
628.3684
679.4422
768.4417
795.4644
917.4754
0
20
40
60
80
100
400
500
600
700
800
900

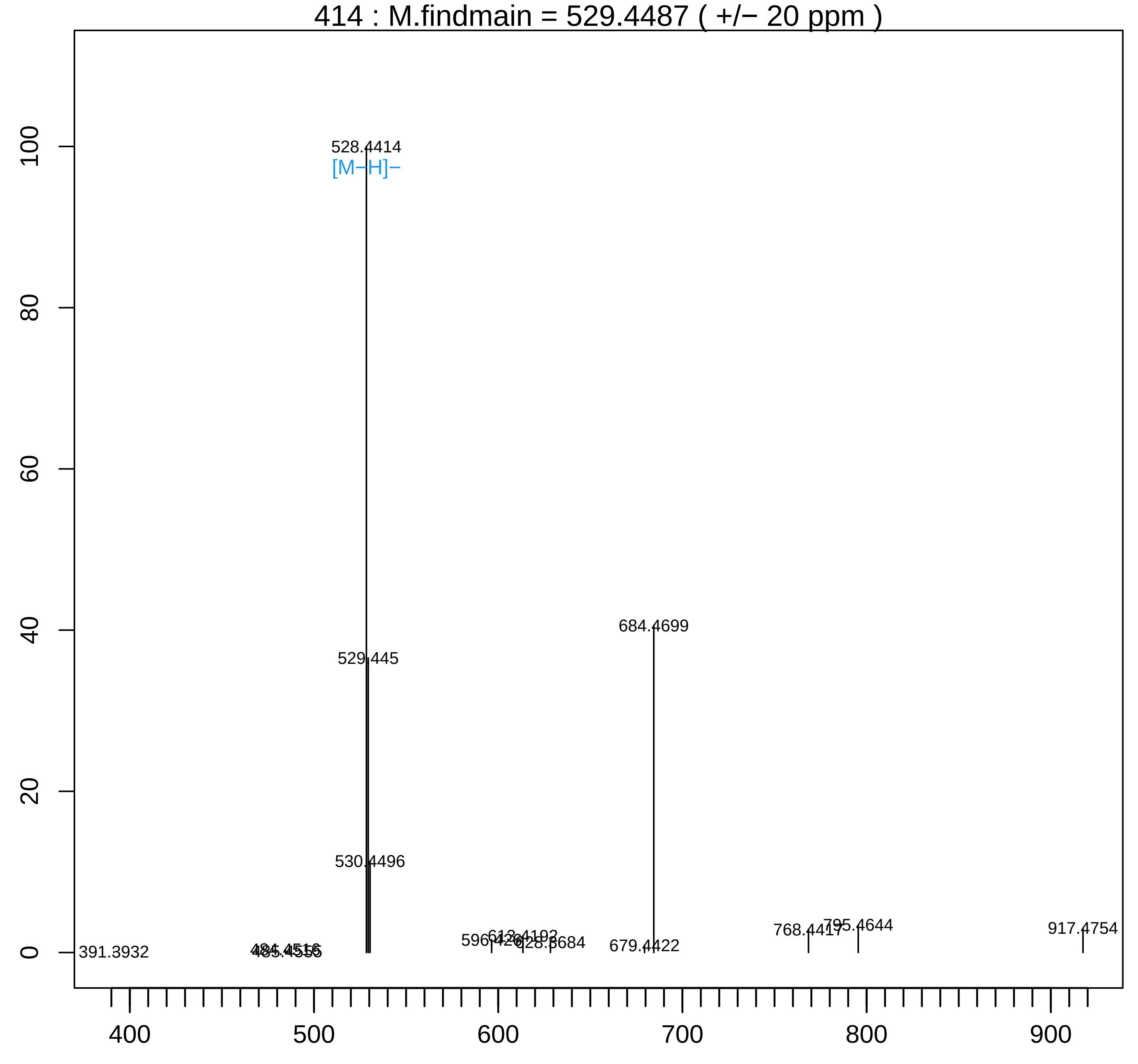
414 : M.findmain = 529.4487 (+/− 20 ppm)
528.4414
[M−H]−
684.4699
529.445
530.4496
484.4516
485.4555
391.3932
596.4201
612.4192
628.3684
679.4422
768.4417
795.4644
917.4754
0
20
40
60
80
100
400
500
600
700
800
900

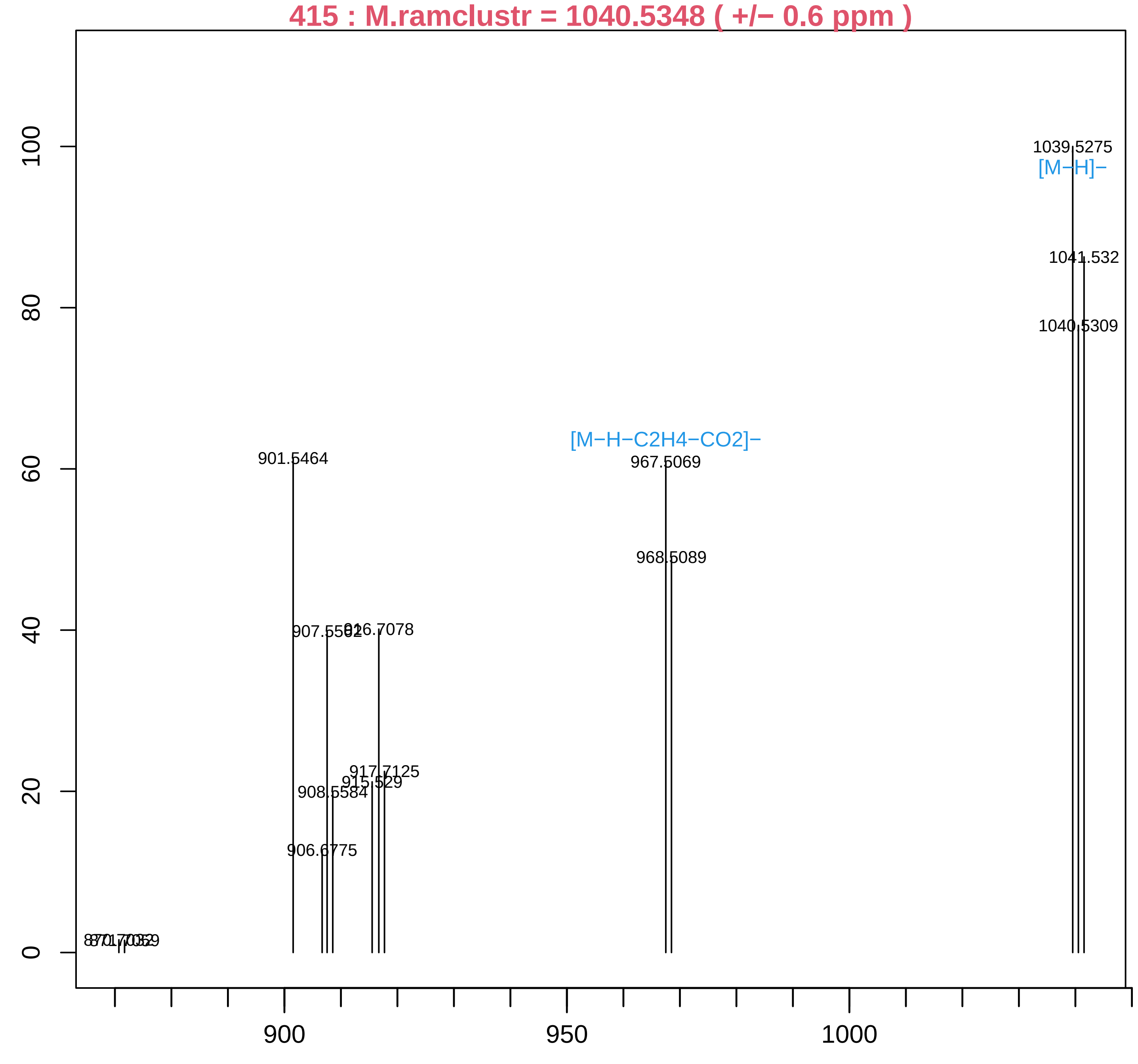

415 : M.ramclustr = 1040.5348 (+/− 0.6 ppm)
1039.5275
[M−H]−
1041.532
1040.5309
[M−H−C2H4−CO2]−
967.5069
901.5464
968.5089
907.5502
916.7078
917.7125
915.529
908.5584
906.6775
0
20
40
60
80
100
900
950
1000

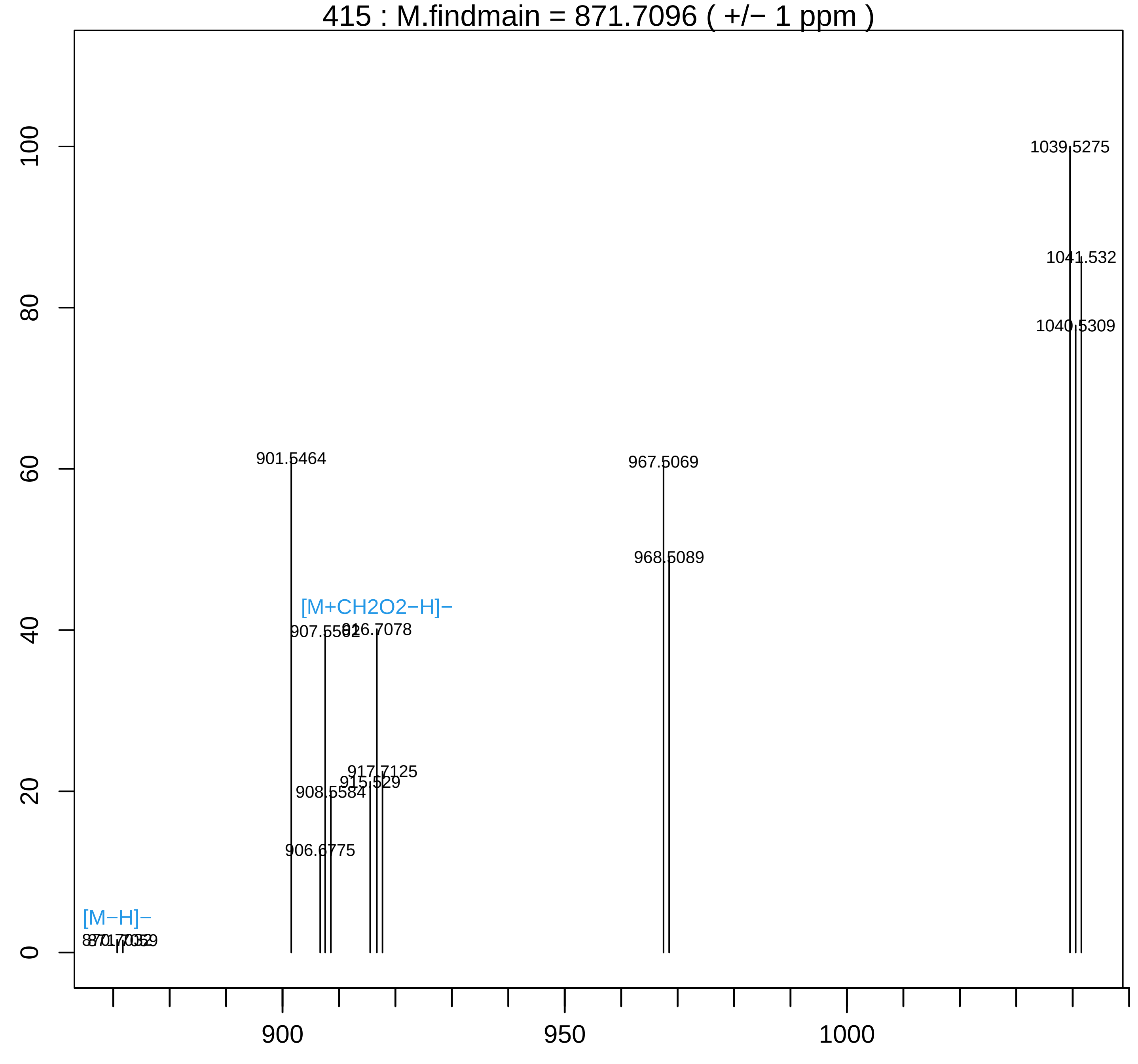

415 : M.findmain = 871.7096 (+/− 1 ppm)
1039.5275
1041.532
1040.5309
901.5464
967.5069
968.5089
[M+CH2O2−H]−
907.5502
916.7078
917.7125
915.529
908.5584
906.6775
[M−H]−
0
20
40
60
80
100
900
950
1000

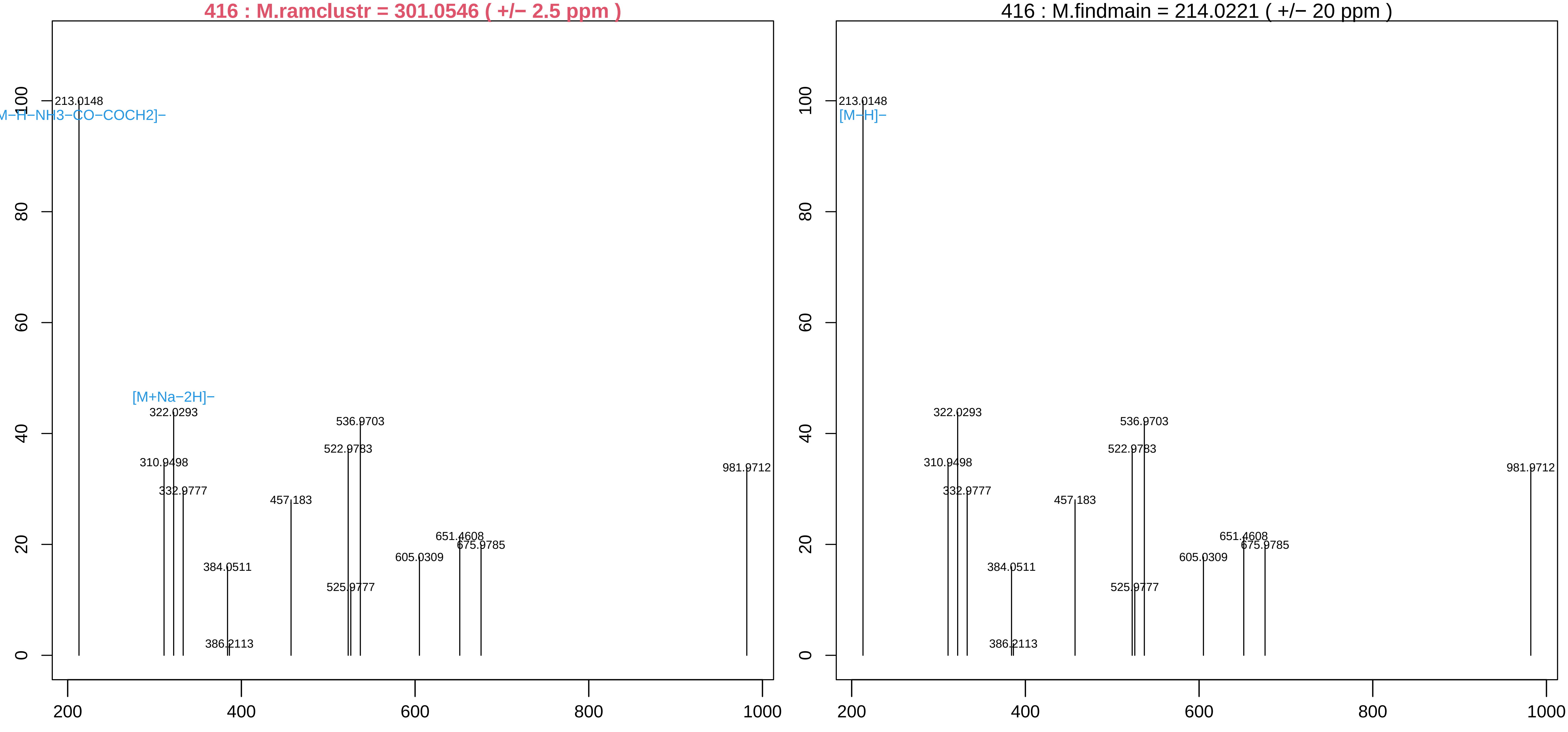
416 : M.ramclustr = 301.0546 (+/− 2.5 ppm)
213.0148
[M−H−NH3−CO−COCH2]−
[M+Na−2H]−
322.0293
310.9498
332.9777
384.0511
386.2113
457.183
522.9783
536.9703
525.9777
605.0309
651.4608
675.9785
981.9712
416 : M.findmain = 214.0221 (+/− 20 ppm)
213.0148
[M−H]−
322.0293
310.9498
332.9777
384.0511
386.2113
457.183
522.9783
536.9703
525.9777
605.0309
651.4608
675.9785
981.9712
0
20
40
60
80
100
200
400
600
800
1000

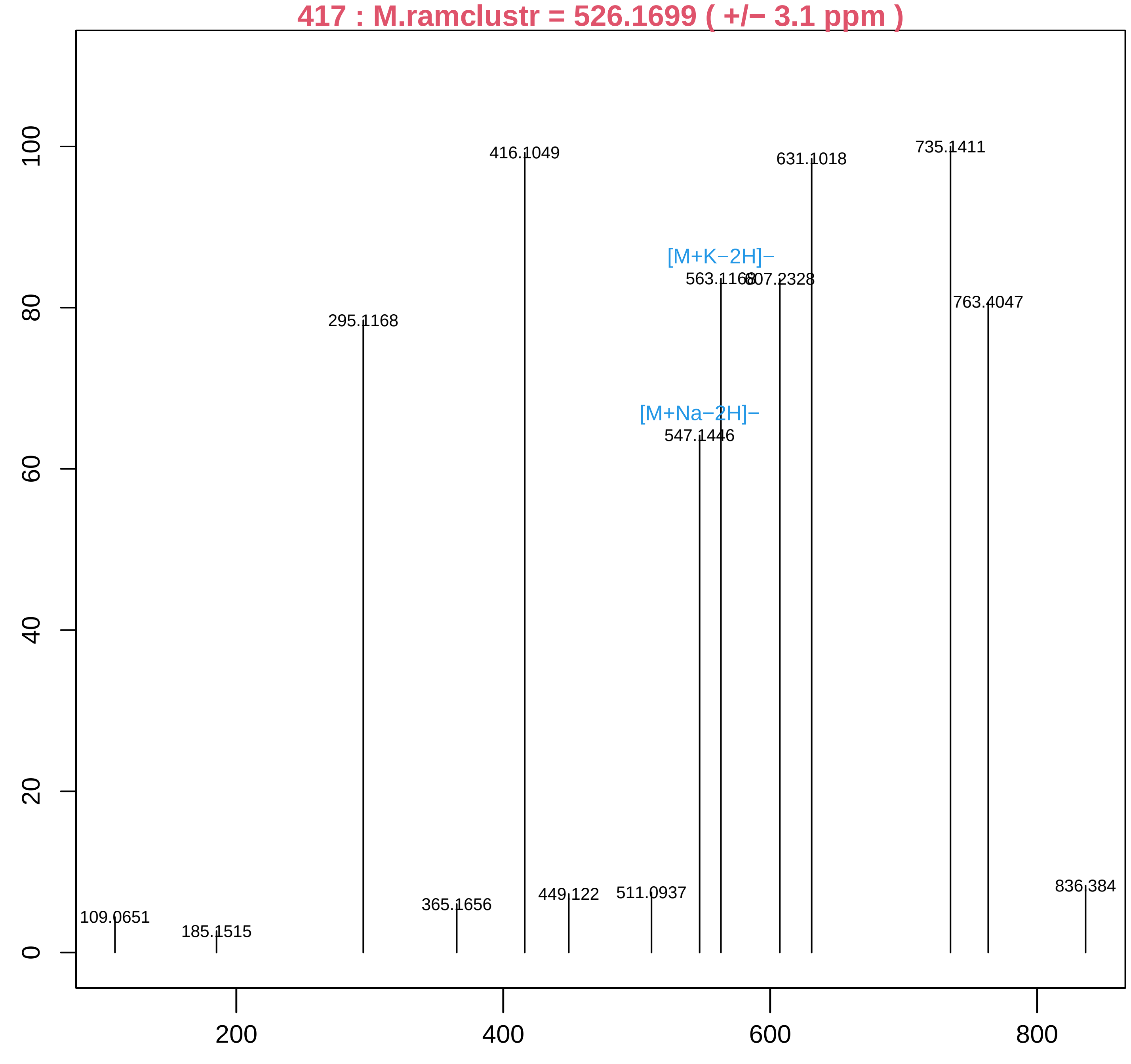
417 : M.ramclustr = 526.1699 (+/− 3.1 ppm)
0
20
40
60
80
100
200
400
600
800
109.0651
185.1515
295.1168
365.1656
416.1049
449.122
511.0937
[M+Na−2H]−
547.1446
[M+K−2H]−
563.1168
607.2328
631.1018
735.1411
763.4047
836.384

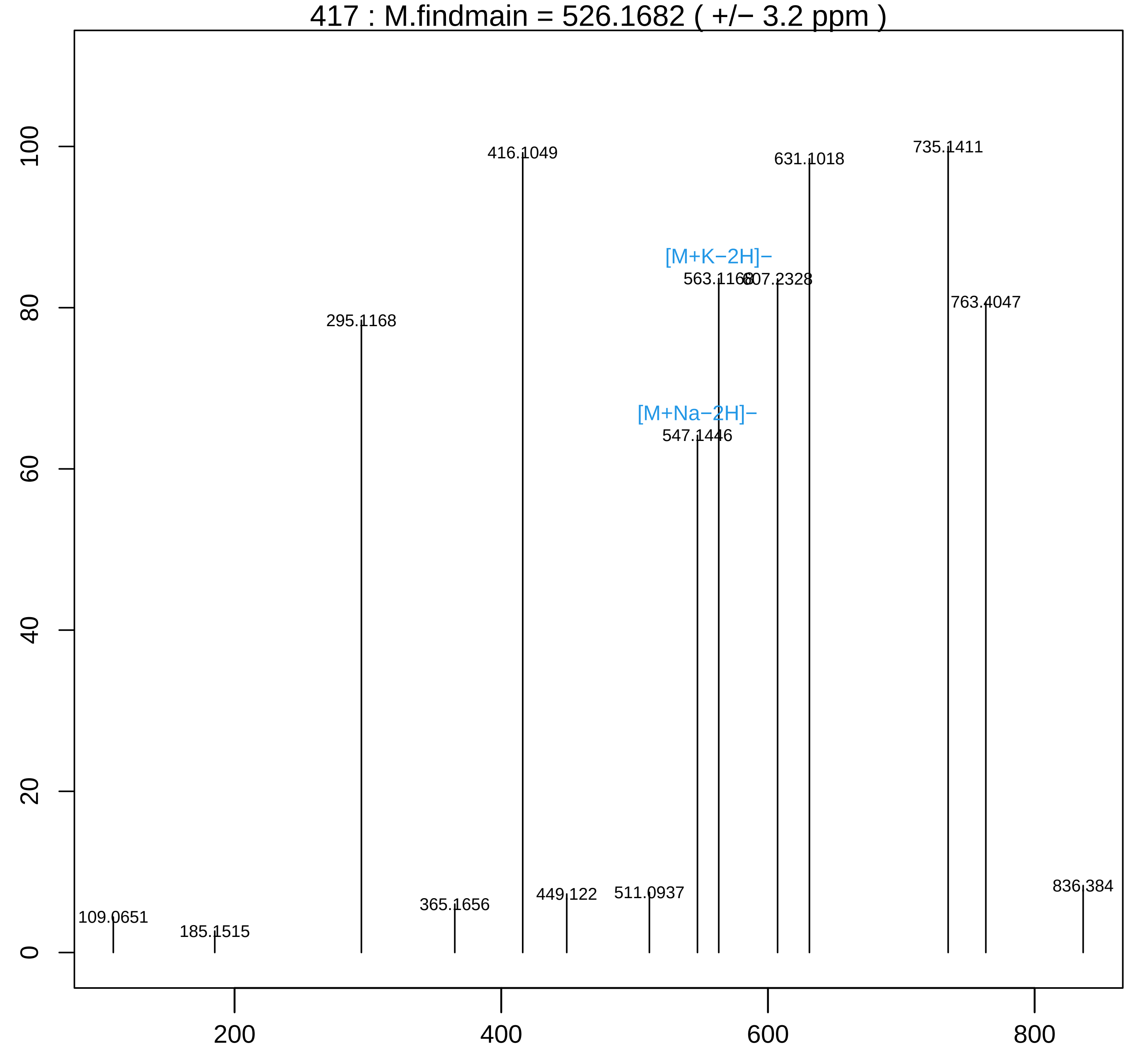
417 : M.findmain = 526.1682 (+/− 3.2 ppm)
0
20
40
60
80
100
200
400
600
800
109.0651
185.1515
295.1168
365.1656
416.1049
449.122
511.0937
[M+Na−2H]−
547.1446
[M+K−2H]−
563.1168
607.2328
631.1018
735.1411
763.4047
836.384

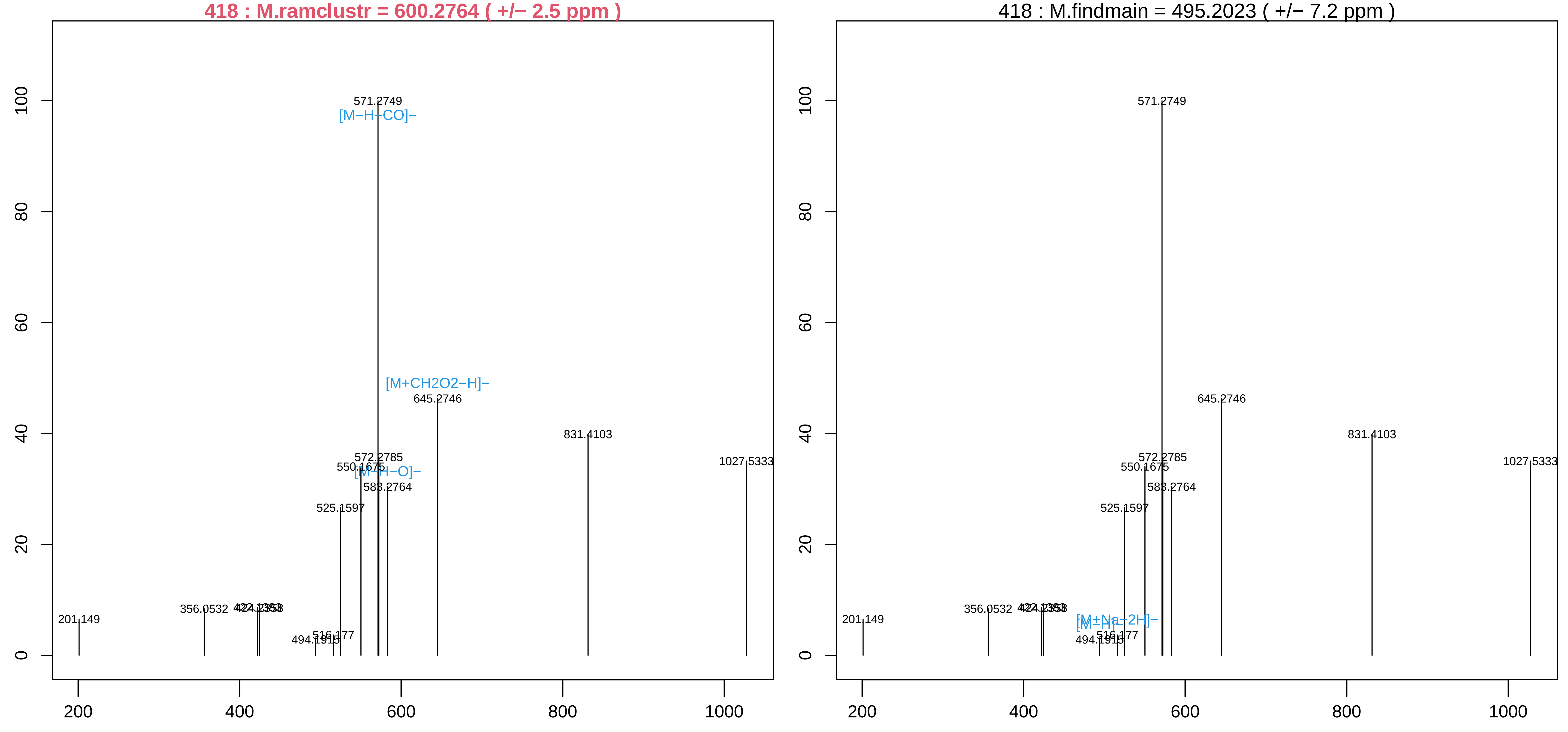

418 : M.ramclustr = 600.2764 (+/− 2.5 ppm)
571.2749
[M−H−CO]−
[M+CH2O2−H]−
645.2746
831.4103
1027.5333
572.2785
550.1675
[M−H−O]−
583.2764
525.1597
201.149
356.0532
494.1915
516.177
418 : M.findmain = 495.2023 (+/− 7.2 ppm)
[M+Na−2H]−
[M−H]−
0
20
40
60
80
100
200
400
600
800
1000

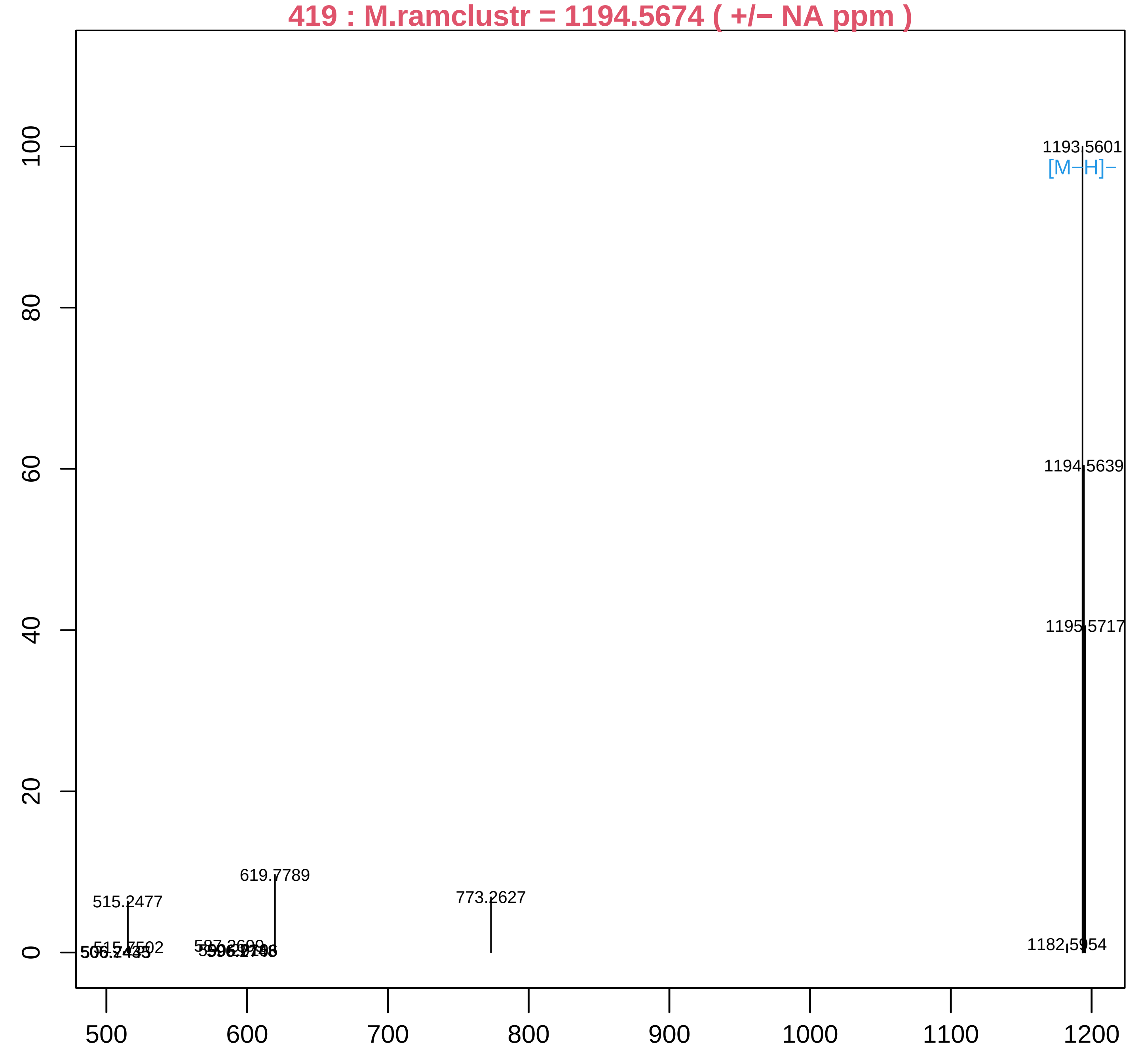

419 : M.ramclustr = 1194.5674 (+/− NA ppm)
1193.5601
[M−H]−
1194.5639
1195.5717
1182.5954
619.7789
515.2477
773.2627
0
20
40
60
80
100
500
600
700
800
900
1000
1100
1200

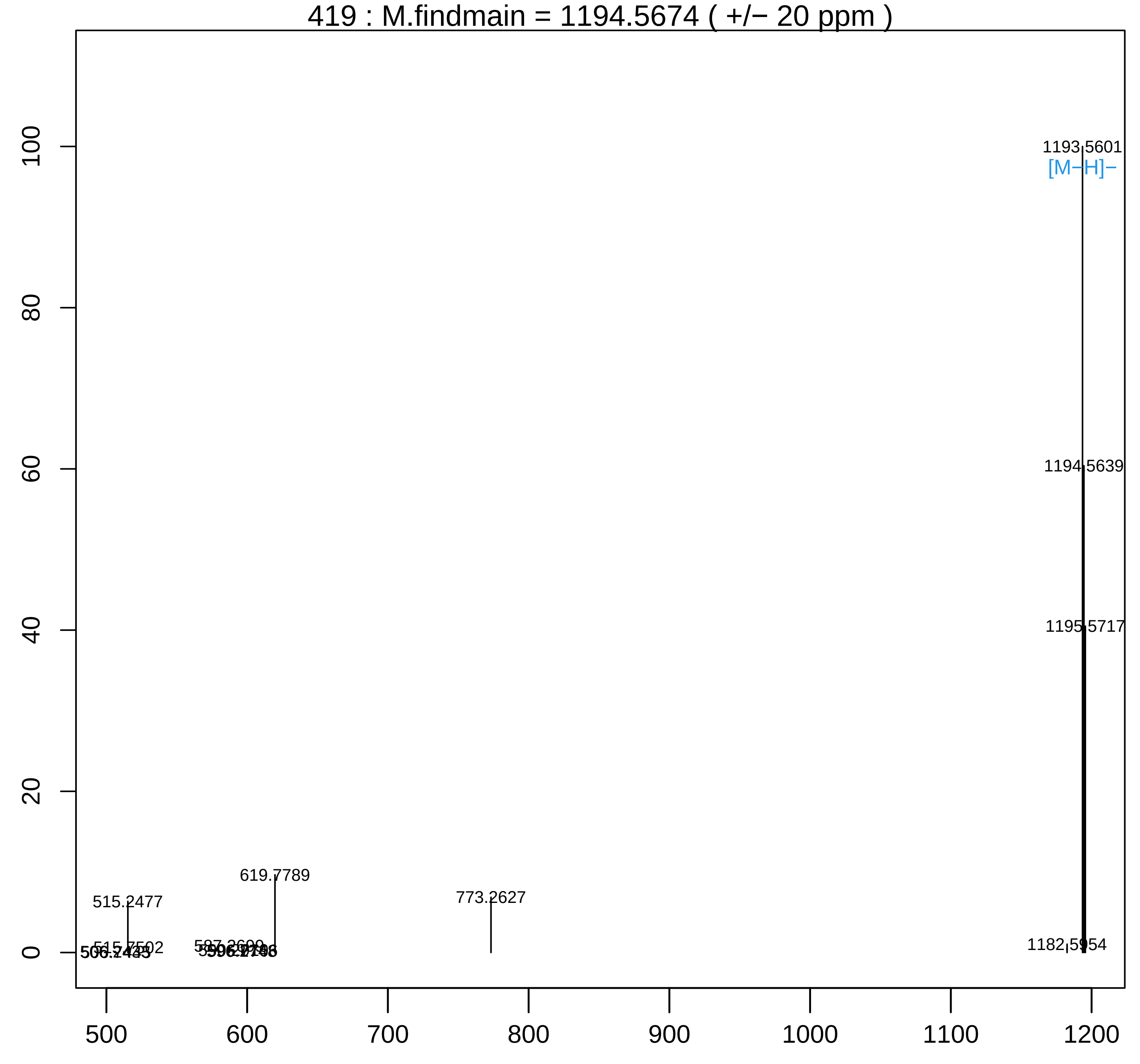

419 : M.findmain = 1194.5674 (+/− 20 ppm)
1193.5601
[M−H]−
1194.5639
1195.5717
1182.5954
619.7789
515.2477
773.2627
0
20
40
60
80
100
500
600
700
800
900
1000
1100
1200

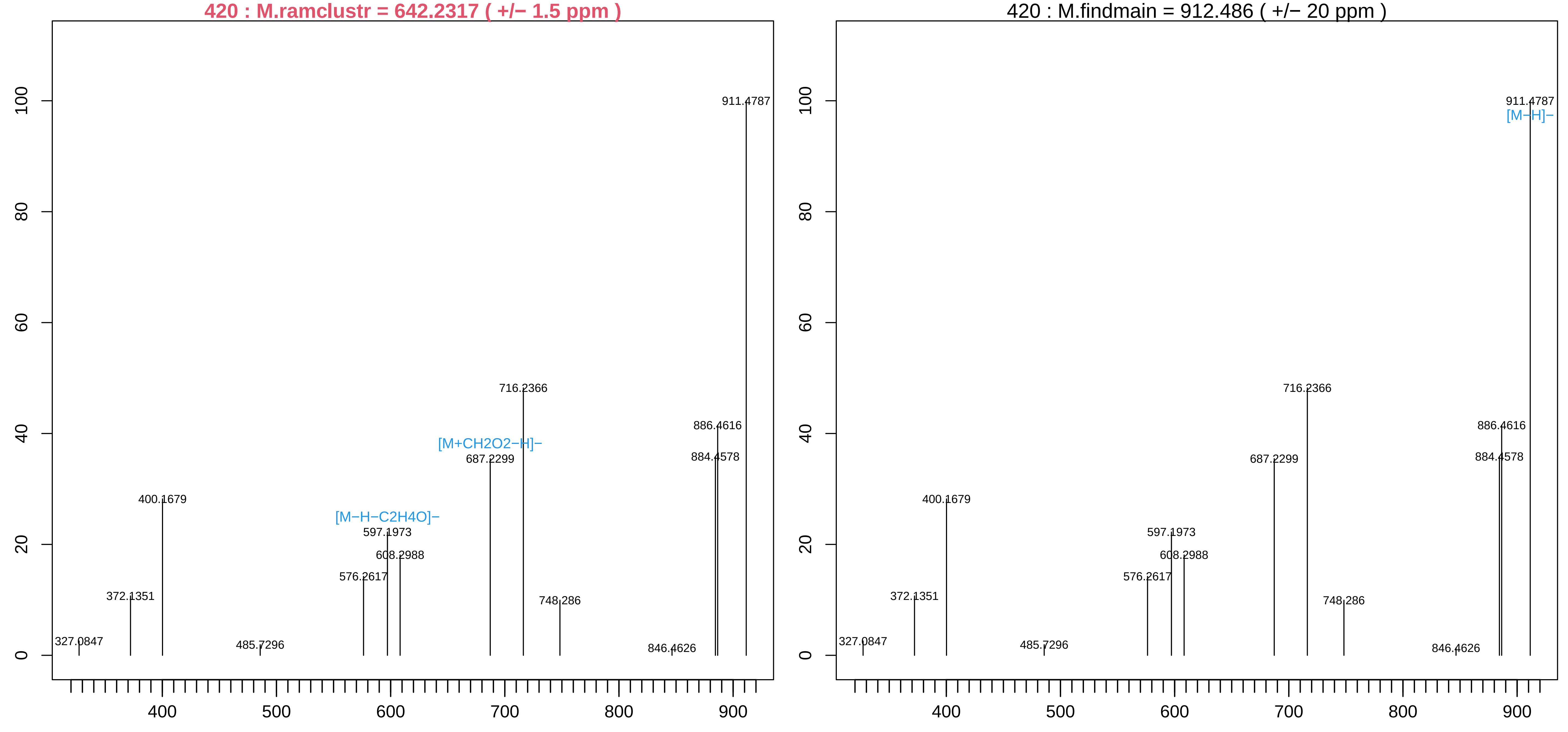
420 : M.ramclustr = 642.2317 (+/− 1.5 ppm)
420 : M.findmain = 912.486 (+/− 20 ppm)
911.4787
[M−H]−
716.2366
886.4616
884.4578
[M+CH2O2−H]−
687.2299
400.1679
[M−H−C2H4O]−
597.1973
608.2988
576.2617
372.1351
748.286
327.0847
485.7296
846.4626
0
20
40
60
80
100
400
500
600
700
800
900

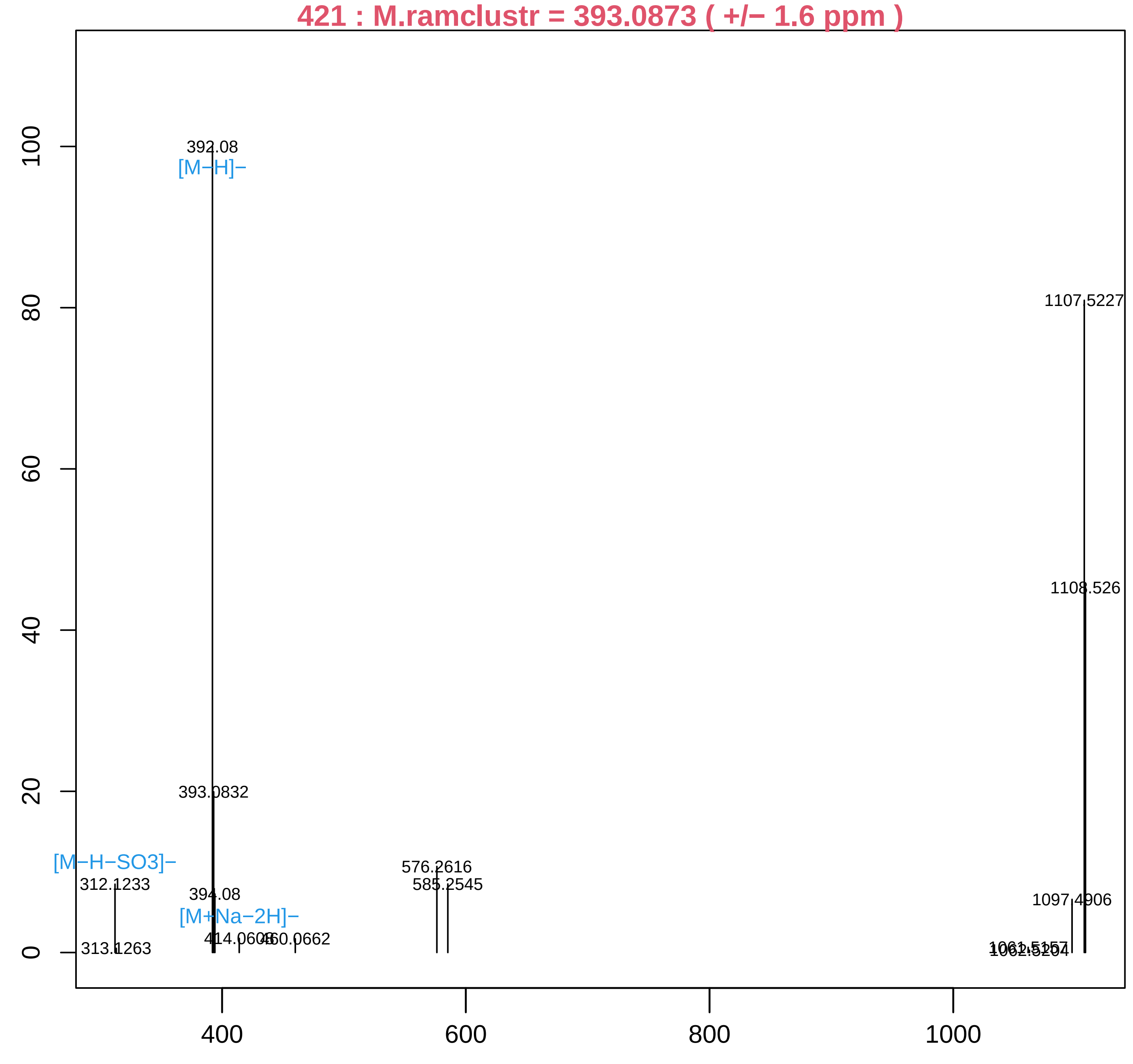
421 : M.ramclustr = 393.0873 (+/− 1.6 ppm)
392.08
[M−H]−
393.0832
[M−H−SO3]−
312.1233
313.1263
394.08
[M+Na−2H]−
414.0609
460.0662
576.2616
585.2545
1107.5227
1108.526
1097.4906
1061.5157
1062.5204
0
20
40
60
80
100
400
600
800
1000
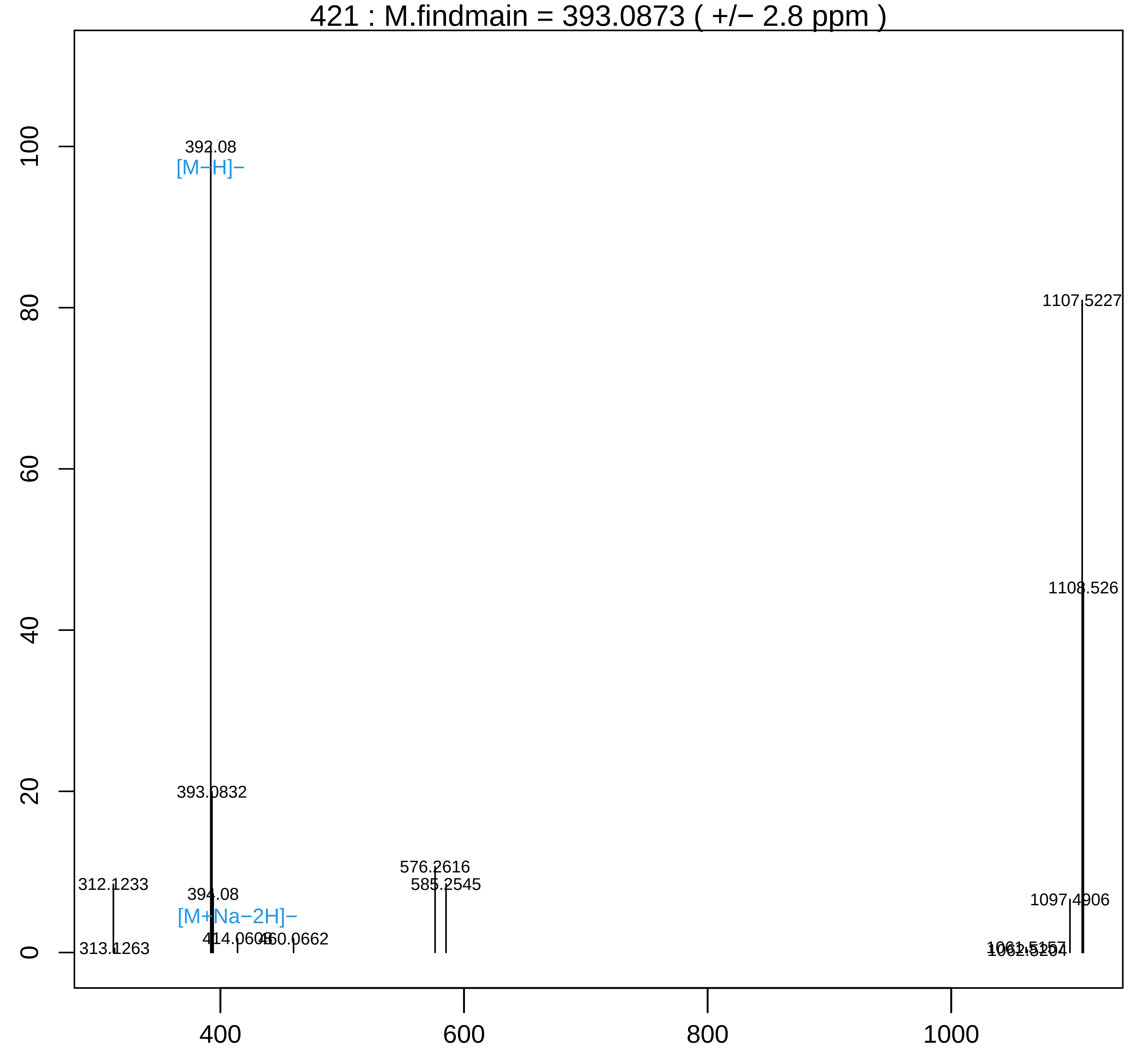
421 : M.findmain = 393.0873 (+/− 2.8 ppm)
392.08
[M−H]−
393.0832
312.1233
313.1263
394.08
[M+Na−2H]−
414.0609
460.0662
576.2616
585.2545
1107.5227
1108.526
1097.4906
1061.5157
1062.5204
0
20
40
60
80
100
400
600
800
1000

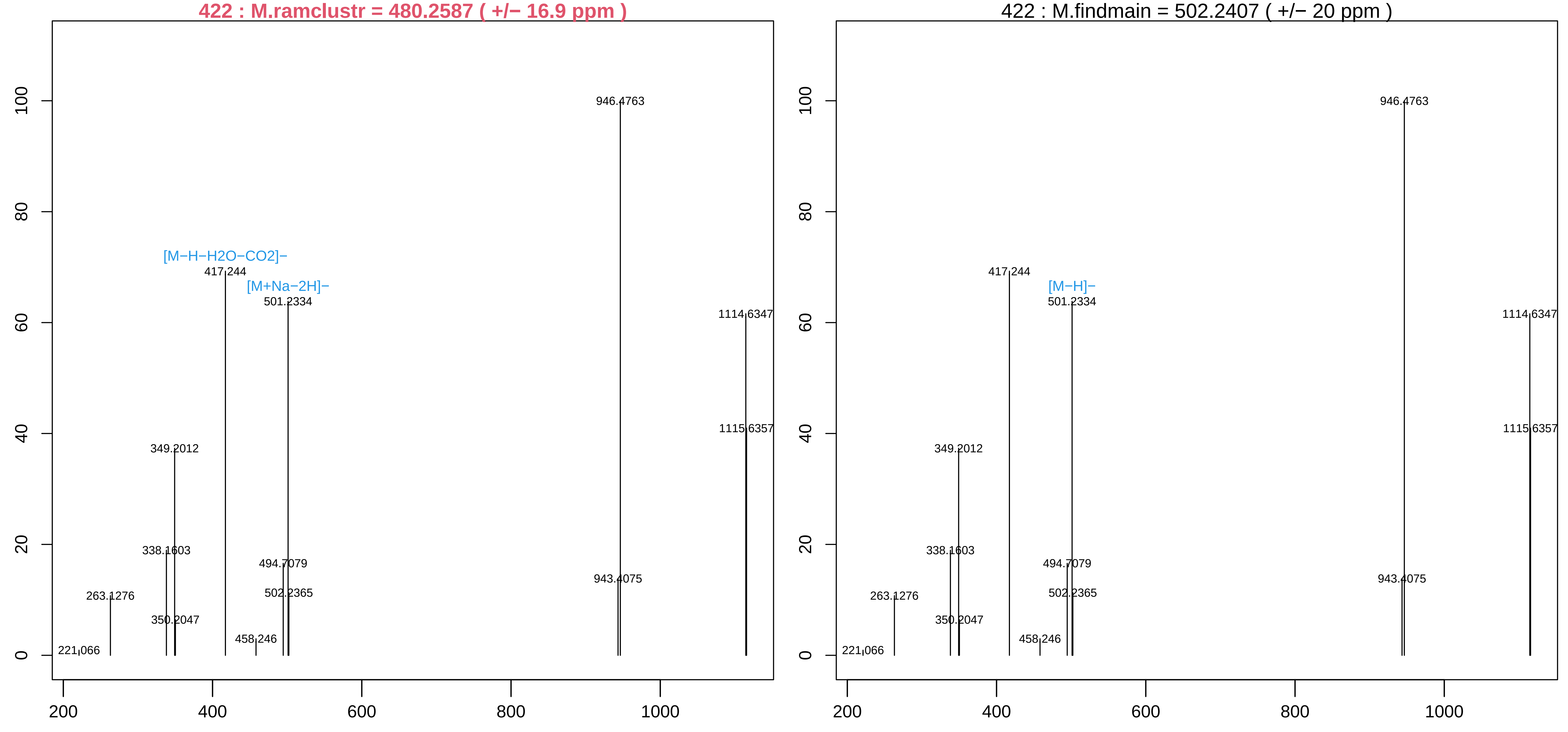

422 : M.ramclustr = 480.2587 (+/− 16.9 ppm)
[M−H−H2O−CO2]−
[M+Na−2H]−
946.4763
417.244
501.2334
1114.6347
1115.6357
349.2012
338.1603
494.7079
943.4075
502.2365
263.1276
350.2047
458.246
221.066
422 : M.findmain = 502.2407 (+/− 20 ppm)
[M−H]−

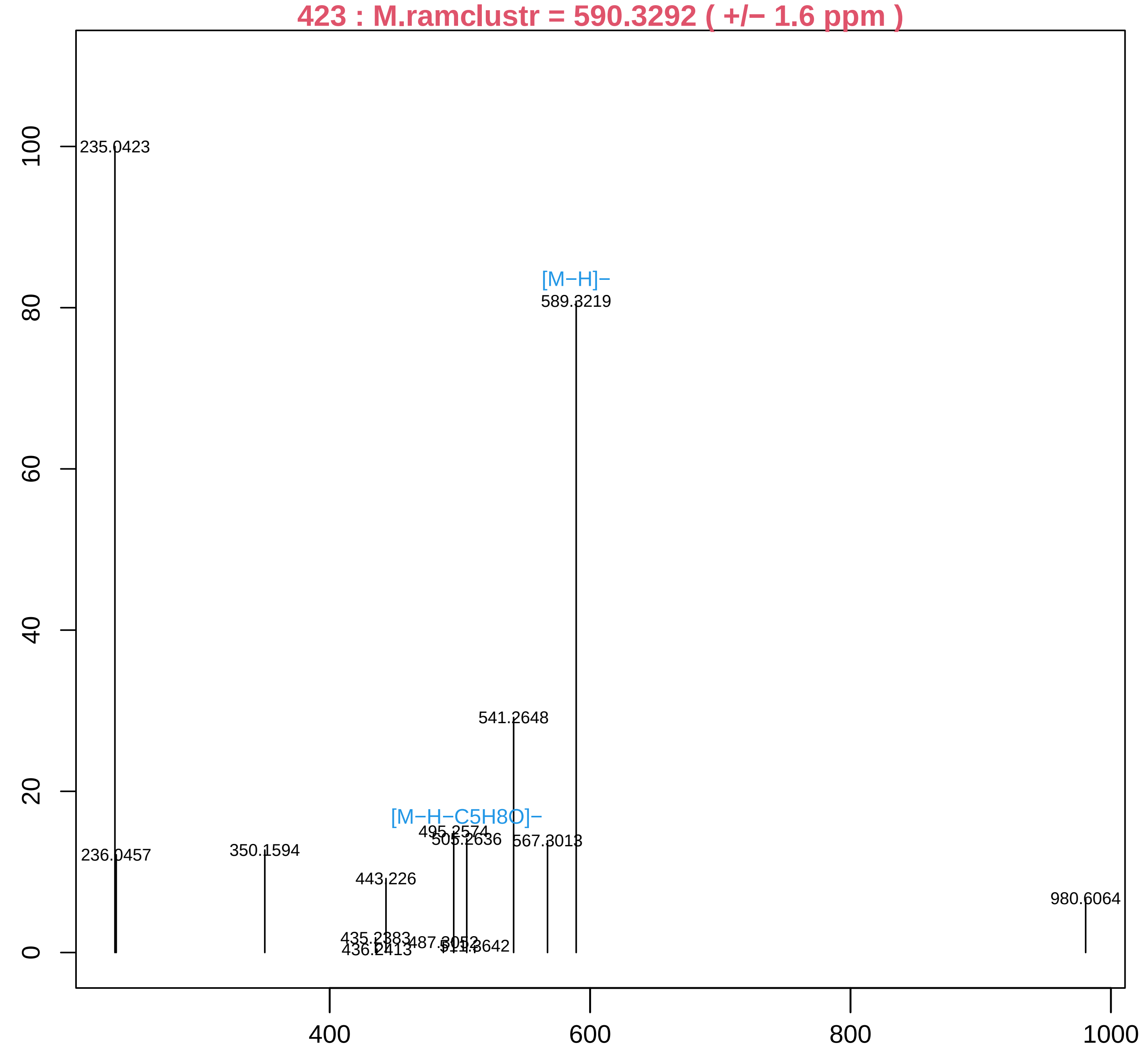
423 : M.ramclustr = 590.3292 (+/− 1.6 ppm)
[M−H]−
589.3219
235.0423
541.2648
[M−H−C5H8O]−
495.2574
505.2636
567.3013
236.0457
350.1594
443.226
435.2383
436.2413
487.3052
511.3642
980.6064
0
20
40
60
80
100
400
600
800
1000

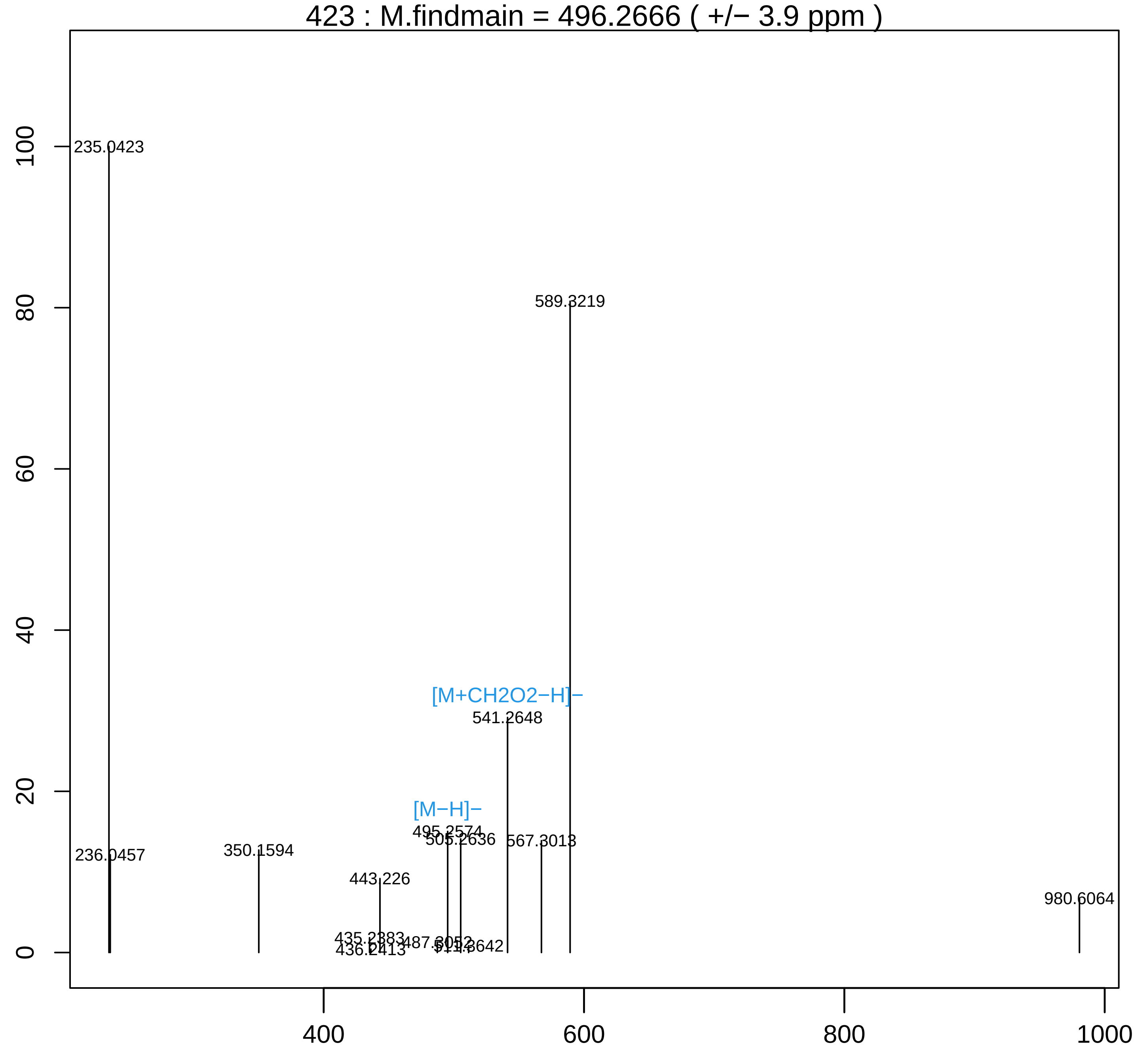
423 : M.findmain = 496.2666 (+/− 3.9 ppm)
235.0423
589.3219
[M+CH2O2−H]−
541.2648
[M−H]−
495.2574
505.2636
567.3013
236.0457
350.1594
443.226
435.2383
436.2413
487.3052
511.3642
980.6064
0
20
40
60
80
100
400
600
800
1000

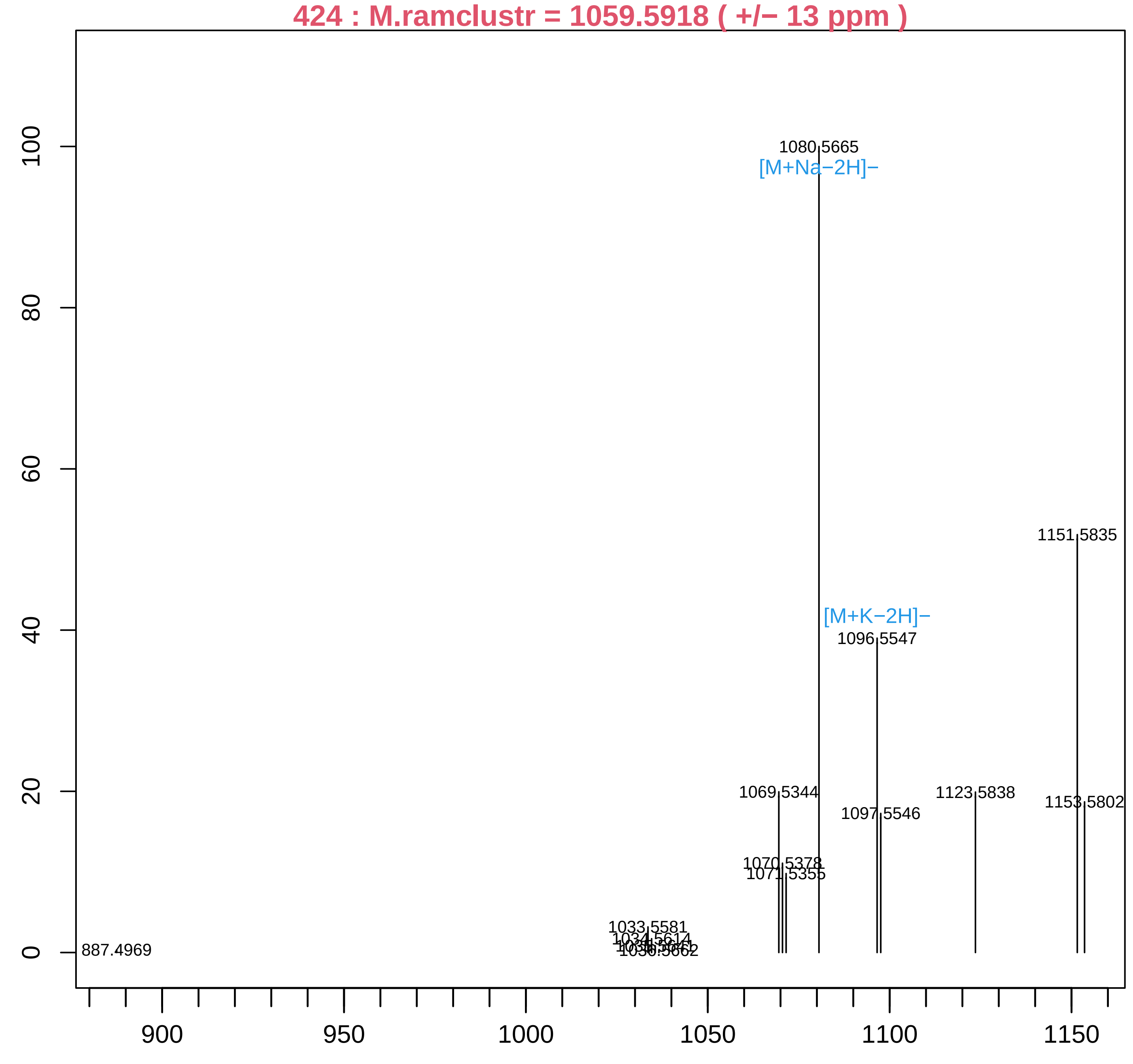
424 : M.ramclustr = 1059.5918 (+/− 13 ppm)
1080.5665
[M+Na−2H]−
1151.5835
[M+K−2H]−
1096.5547
1069.5344
1123.5838
1097.5546
1153.5802
1070.5378
1071.5355
1033.5581
1034.5614
1036.5662
887.4969
0
20
40
60
80
100
900
950
1000
1050
1100
1150

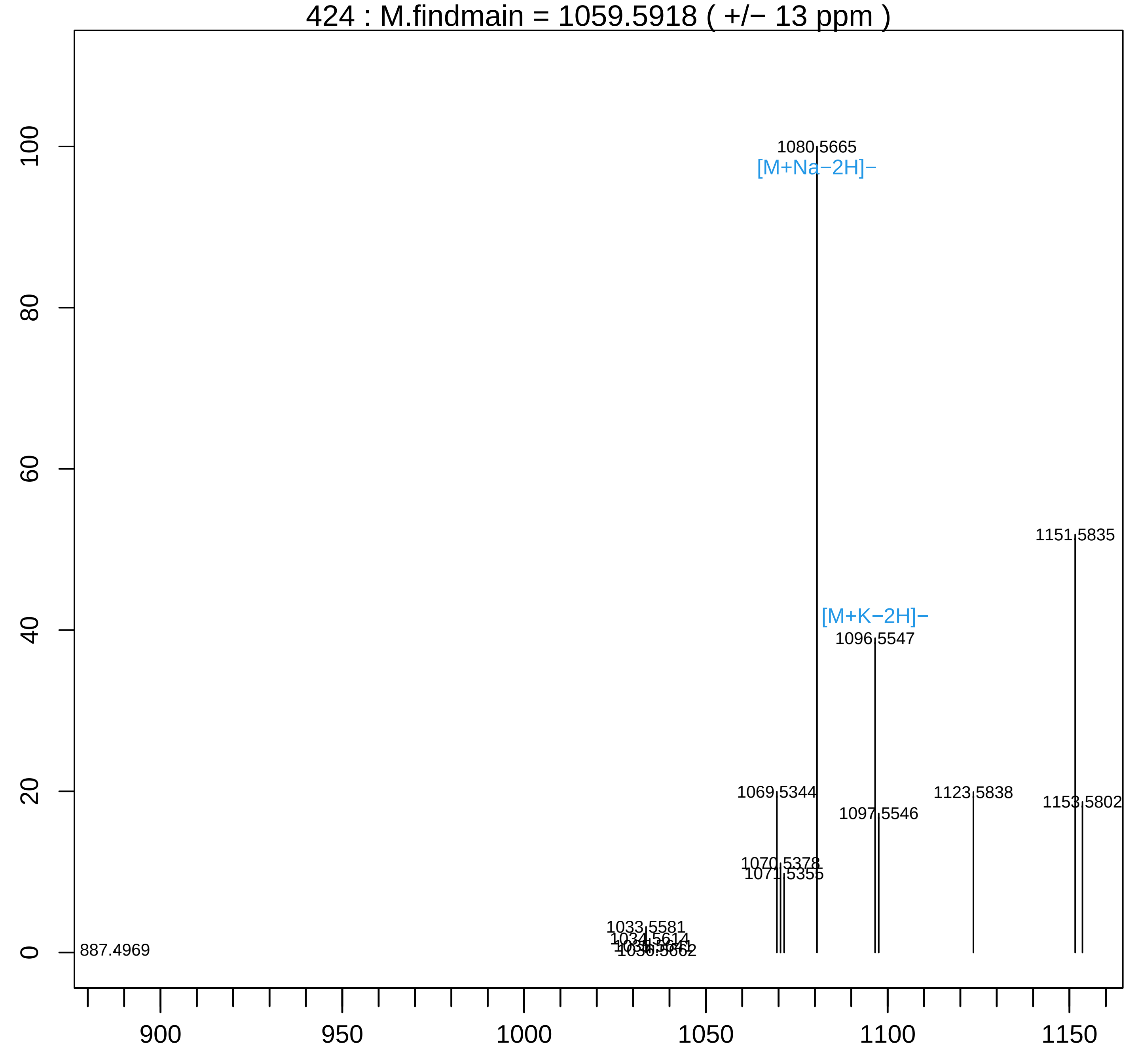
424 : M.findmain = 1059.5918 (+/− 13 ppm)
1080.5665
[M+Na−2H]−
1151.5835
[M+K−2H]−
1096.5547
1069.5344
1123.5838
1097.5546
1153.5802
1070.5378
1071.5355
1033.5581
1034.5614
1036.5662
887.4969
0
20
40
60
80
100
900
950
1000
1050
1100
1150

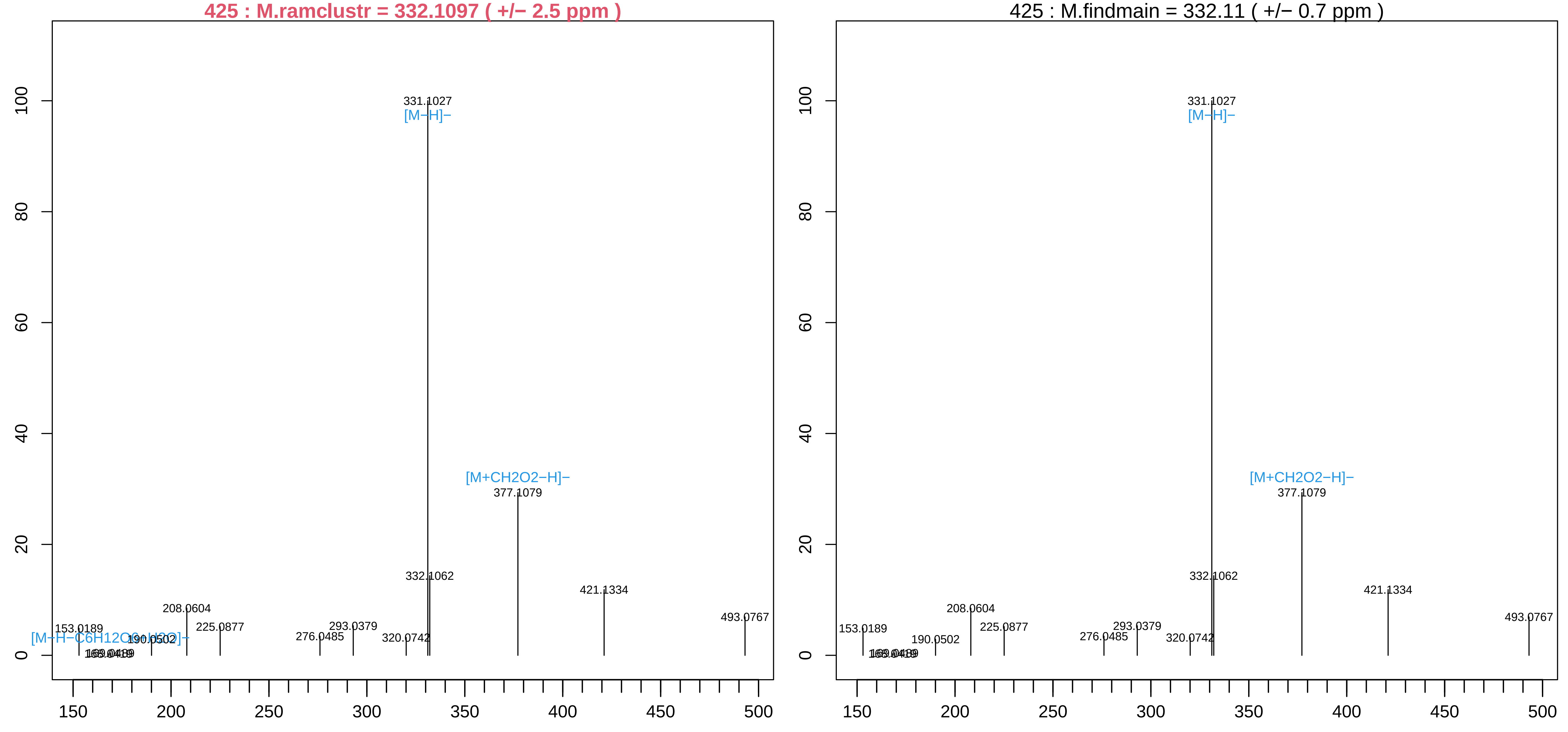

425 : M.ramclustr = 332.1097 (+/− 2.5 ppm)
425 : M.findmain = 332.11 (+/− 0.7 ppm)
331.1027
[M−H]−
[M+CH2O2−H]−
377.1079
332.1062
421.1334
208.0604
493.0767
153.0189
225.0877
293.0379
276.0485
320.0742
190.0502
[M−H−C6H12O6+H2O]−

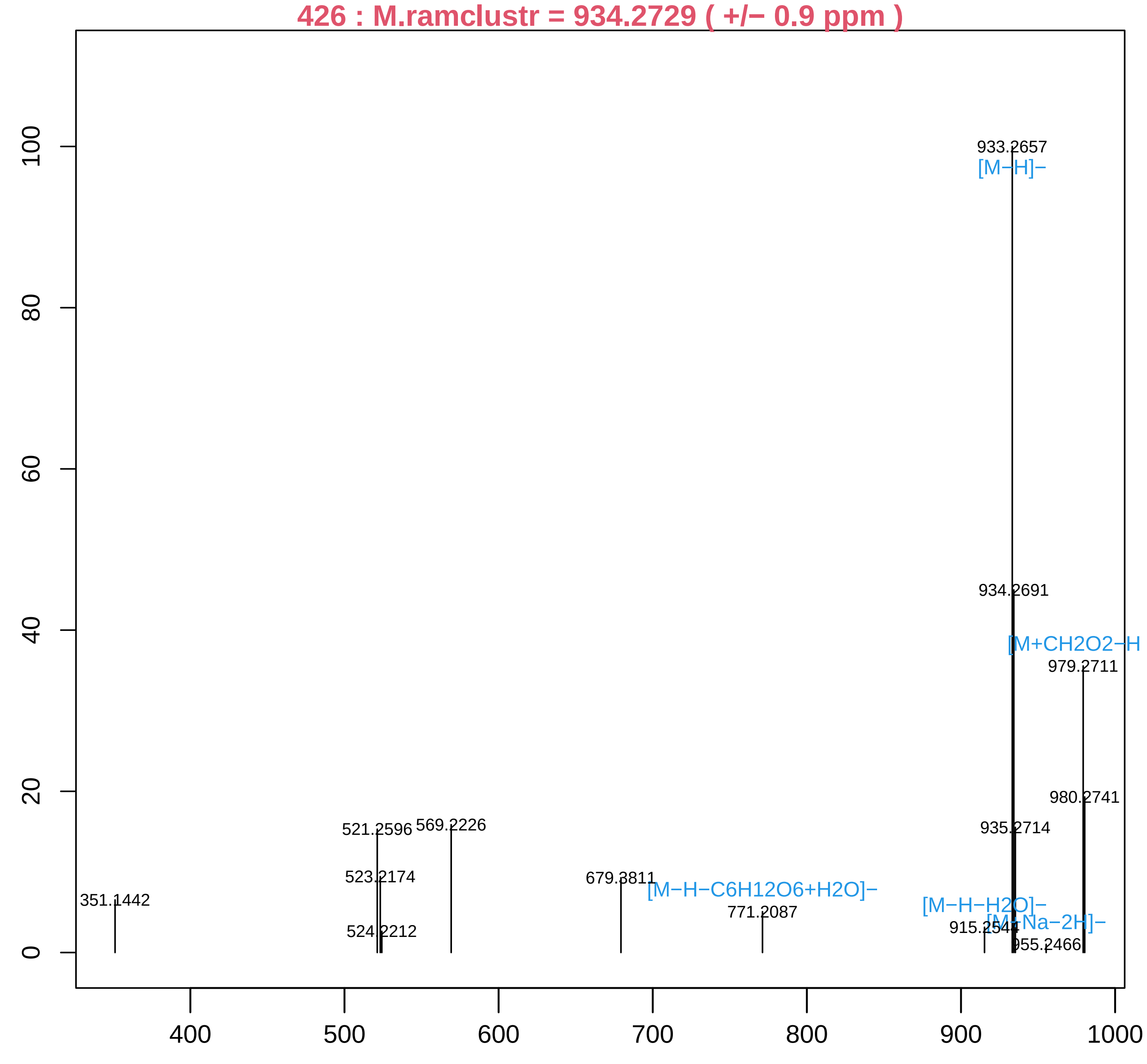

426 : M.ramclustr = 934.2729 (+/− 0.9 ppm)
933.2657
[M−H]−
934.2691
[M+CH2O2−H
979.2711
980.2741
935.2714
521.2596
569.2226
523.2174
524.2212
351.1442
679.3811
[M−H−C6H12O6+H2O]−
771.2087
[M−H−H2O]−
915.2544
[M+Na−2H]−
955.2466
0
20
40
60
80
100
400
500
600
700
800
900
1000

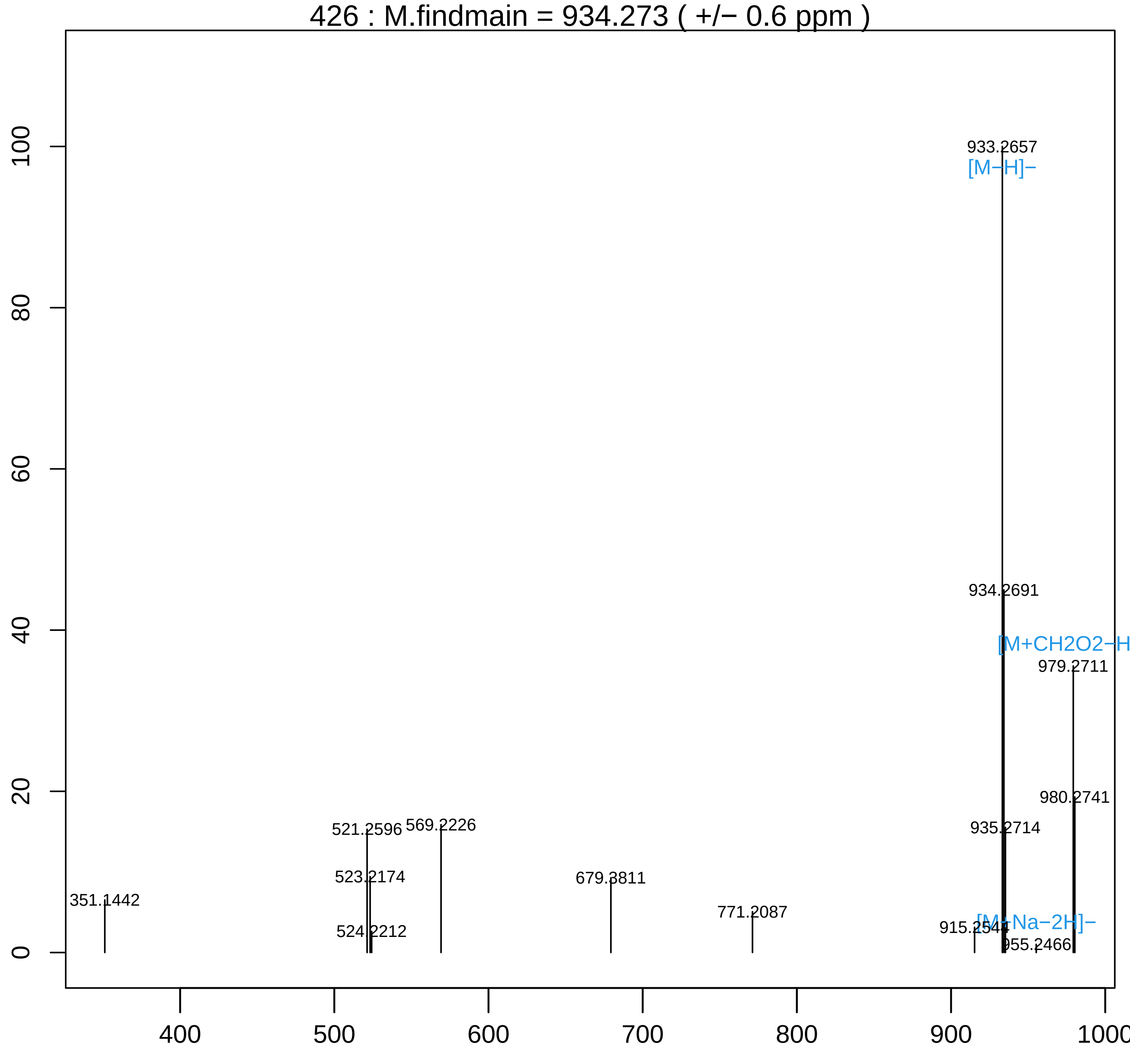

426 : M.findmain = 934.273 (+/− 0.6 ppm)
933.2657
[M−H]−
934.2691
[M+CH2O2−H
979.2711
980.2741
935.2714
521.2596
569.2226
523.2174
524.2212
351.1442
679.3811
771.2087
915.2544
[M+Na−2H]−
955.2466
0
20
40
60
80
100
400
500
600
700
800
900
1000

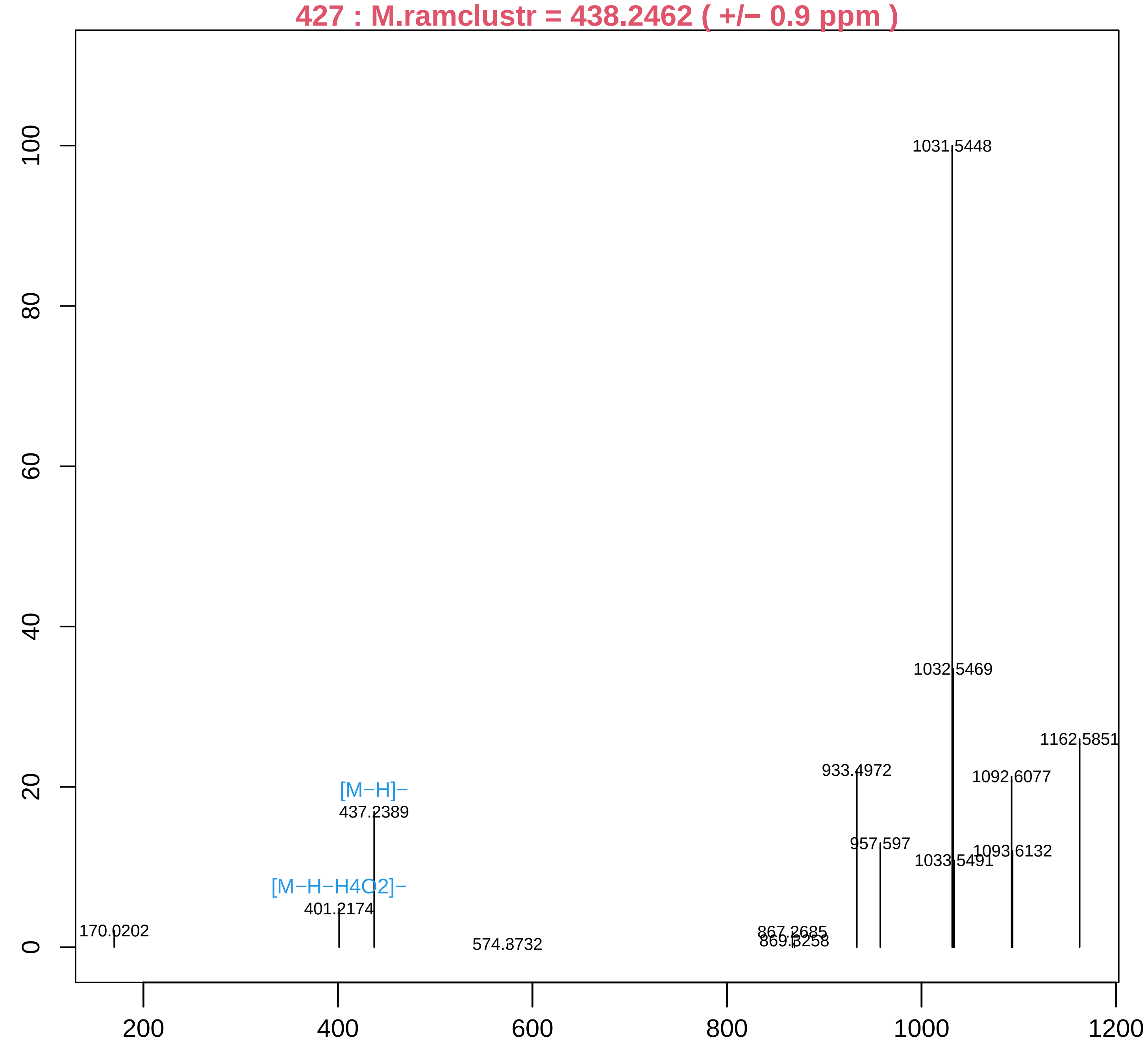

427 : M.ramclustr = 438.2462 (+/− 0.9 ppm)
1031.5448
1032.5469
1162.5851
933.4972
1092.6077
[M−H]−
437.2389
957.597
1033.5491
1093.6132
[M−H−H4O2]−
401.2174
170.0202
574.8732
867.2685
869.6258
0
20
40
60
80
100
200
400
600
800
1000
1200

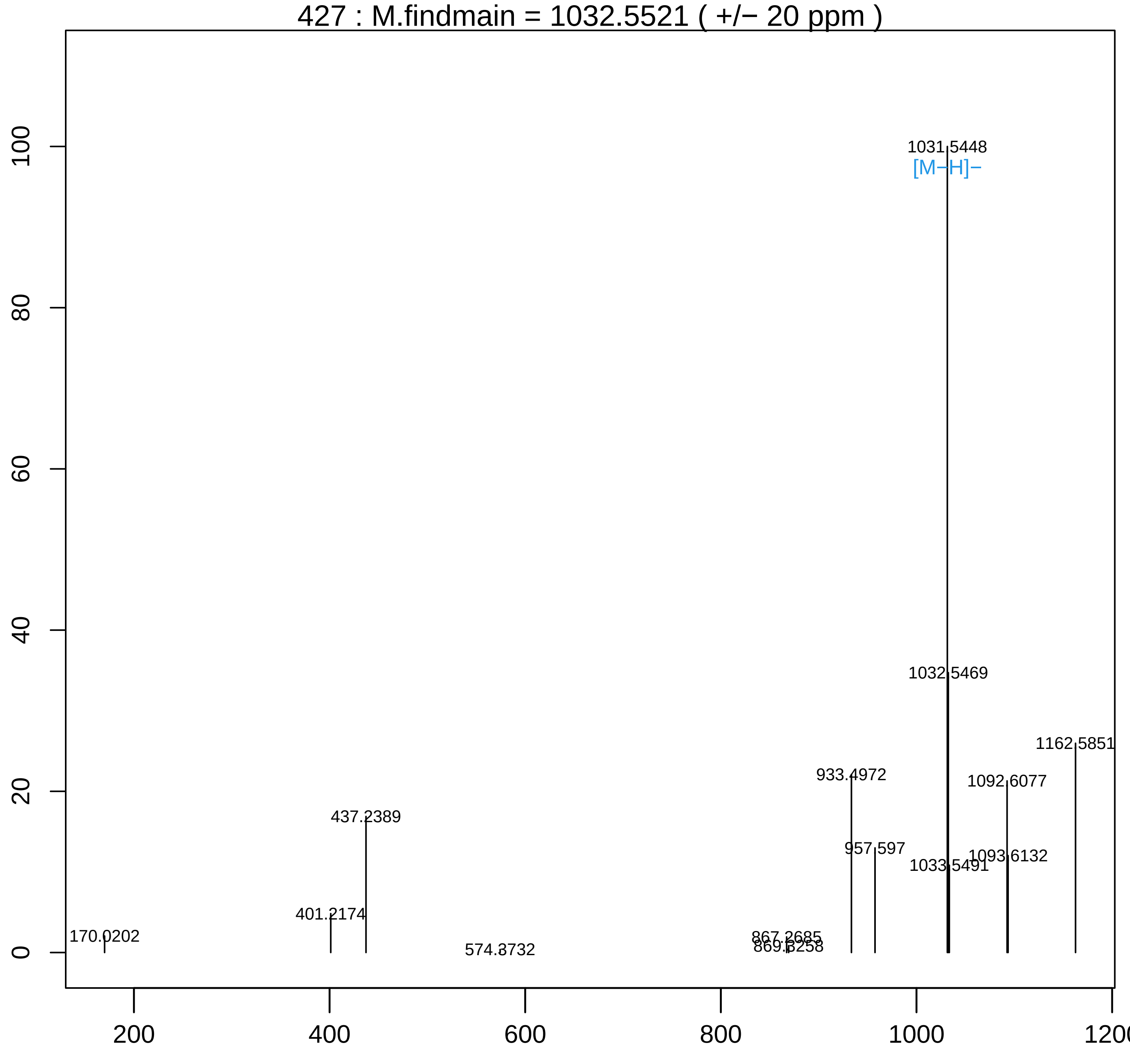

427 : M.findmain = 1032.5521 (+/− 20 ppm)
1031.5448
[M−H]−
1032.5469
1162.5851
933.4972
1092.6077
437.2389
957.597
1033.5491
1093.6132
401.2174
170.0202
574.8732
867.2685
869.6258
0
20
40
60
80
100
200
400
600
800
1000
1200

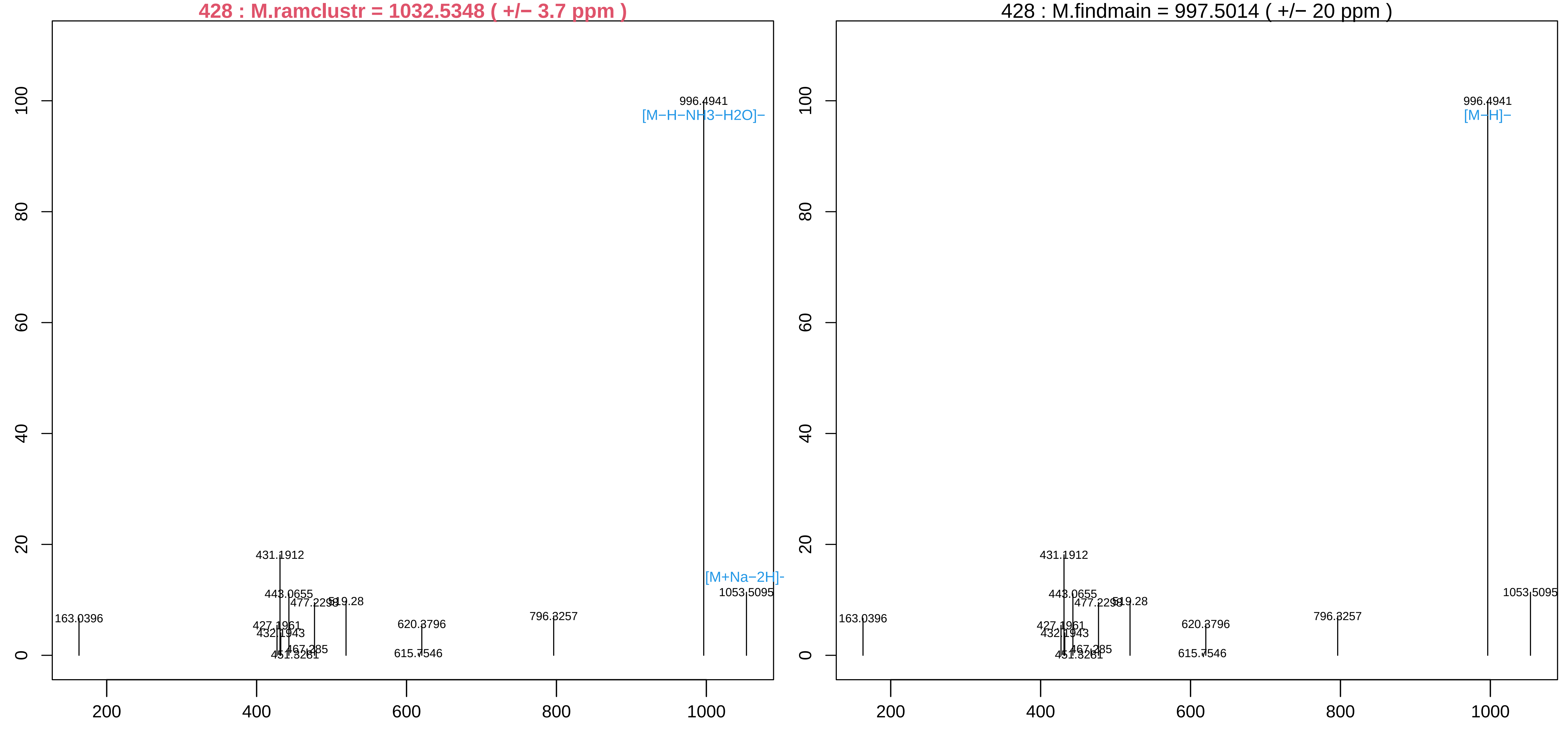
428 : M.ramclustr = 1032.5348 (+/− 3.7 ppm)
996.4941
[M−H−NH3−H2O]−
431.1912
[M+Na−2H]-
1053.5095
443.0655
477.2298
519.28
163.0396
427.1961
432.1943
467.285
451.3261
620.3796
615.7546
796.3257
428 : M.findmain = 997.5014 (+/− 20 ppm)
996.4941
[M−H]−
431.1912
443.0655
477.2298
519.28
1053.5095
163.0396
427.1961
432.1943
467.285
451.3261
620.3796
615.7546
796.3257
0
20
40
60
80
100
200
400
600
800
1000

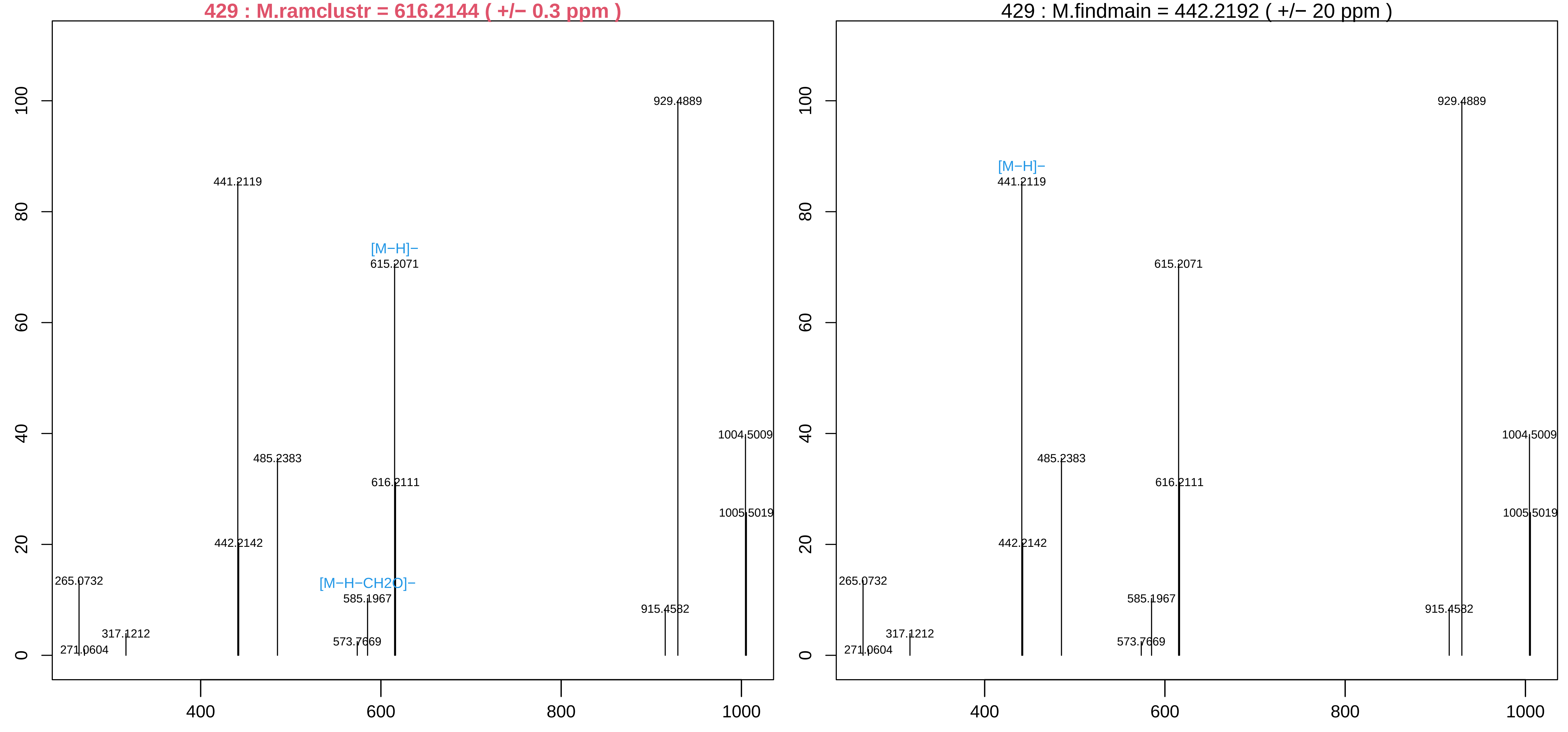

429 : M.ramclustr = 616.2144 (+/− 0.3 ppm)
929.4889
441.2119
[M−H]−
615.2071
485.2383
616.2111
1004.5009
1005.5019
442.2142
265.0732
[M−H−CH2O]−
585.1967
915.4582
317.1212
573.7669
271.0604
429 : M.findmain = 442.2192 (+/− 20 ppm)
[M−H]−
441.2119
615.2071
485.2383
616.2111
1004.5009
1005.5019
442.2142
265.0732
585.1967
915.4582
317.1212
573.7669
271.0604
0
20
40
60
80
100
400
600
800
1000

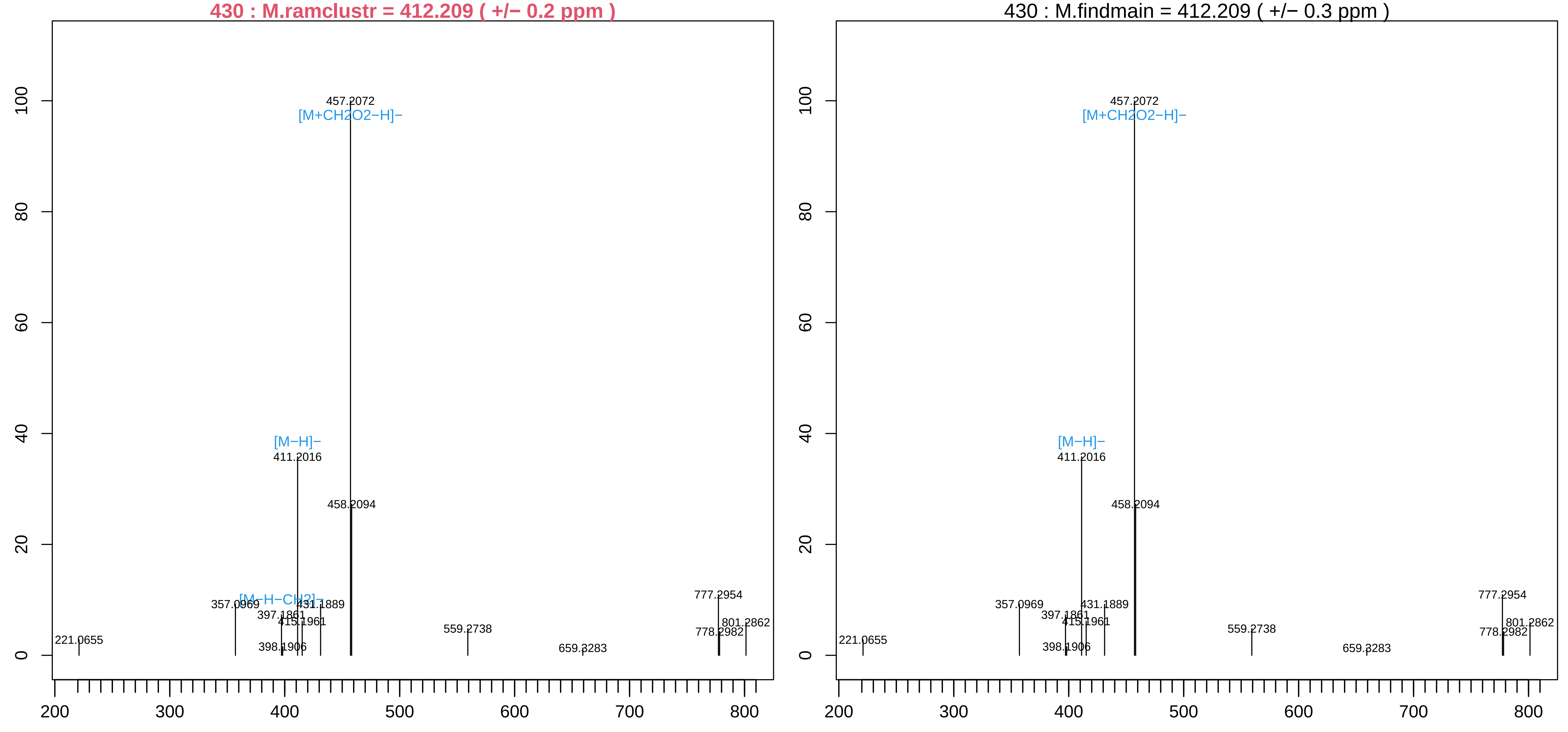
430 : M.ramclustr = 412.209 (+/− 0.2 ppm)
430 : M.findmain = 412.209 (+/− 0.3 ppm)
457.2072
[M+CH2O2−H]−
[M−H]−
411.2016
458.2094
[M−H−CH2]−
357.0969
397.1861
431.1889
415.1961
398.1906
221.0655
559.2738
659.3283
777.2954
778.2982
801.2862
0
20
40
60
80
100
200
300
400
500
600
700
800

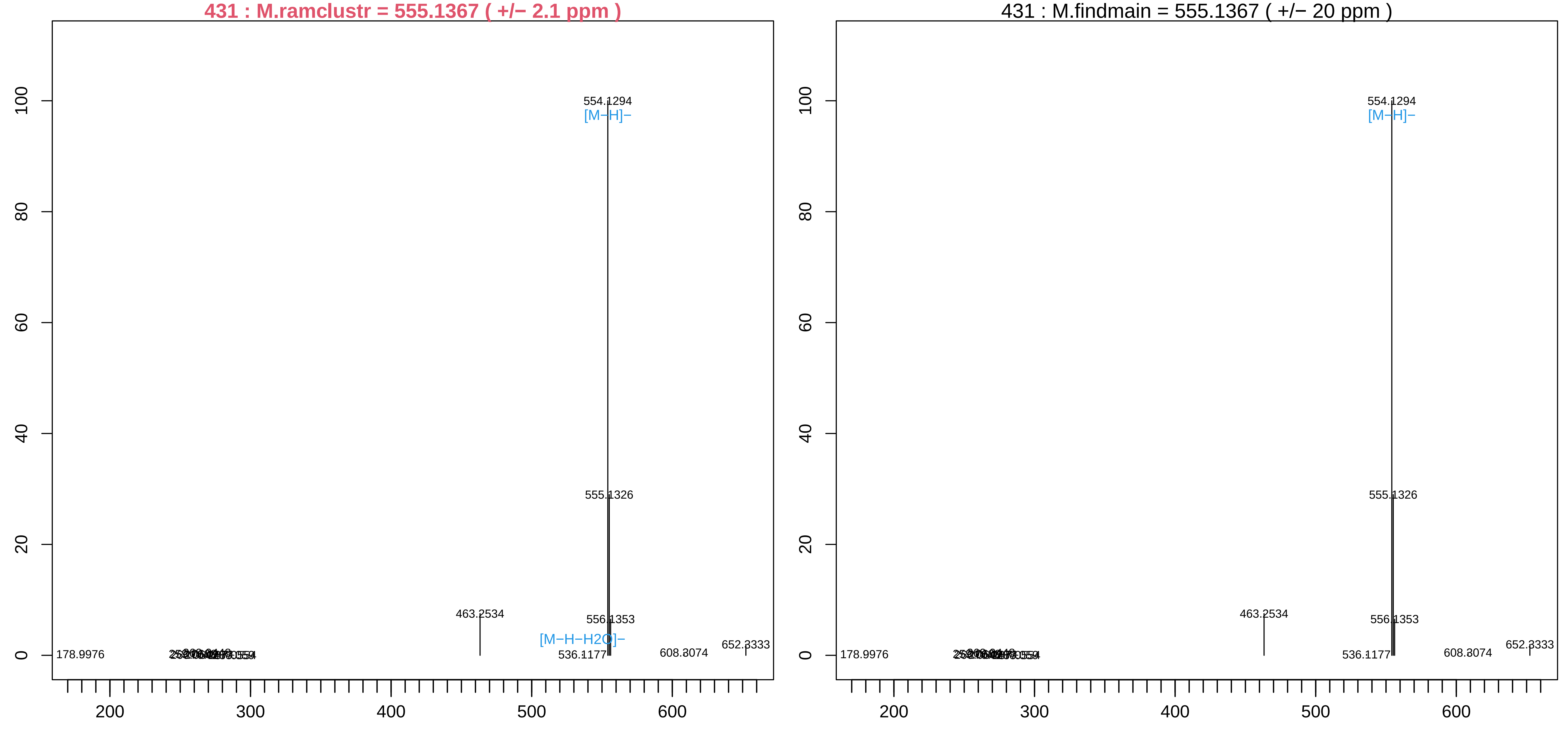
431 : M.ramclustr = 555.1367 (+/− 2.1 ppm)
554.1294
[M−H]−
555.1326
463.2534
556.1353
[M−H−H2O]−
652.3333
178.9976
536.1177
608.8074
431 : M.findmain = 555.1367 (+/− 20 ppm)
554.1294
[M−H]−
555.1326
463.2534
556.1353
652.3333
178.9976
536.1177
608.8074
0
20
40
60
80
100
200
300
400
500
600

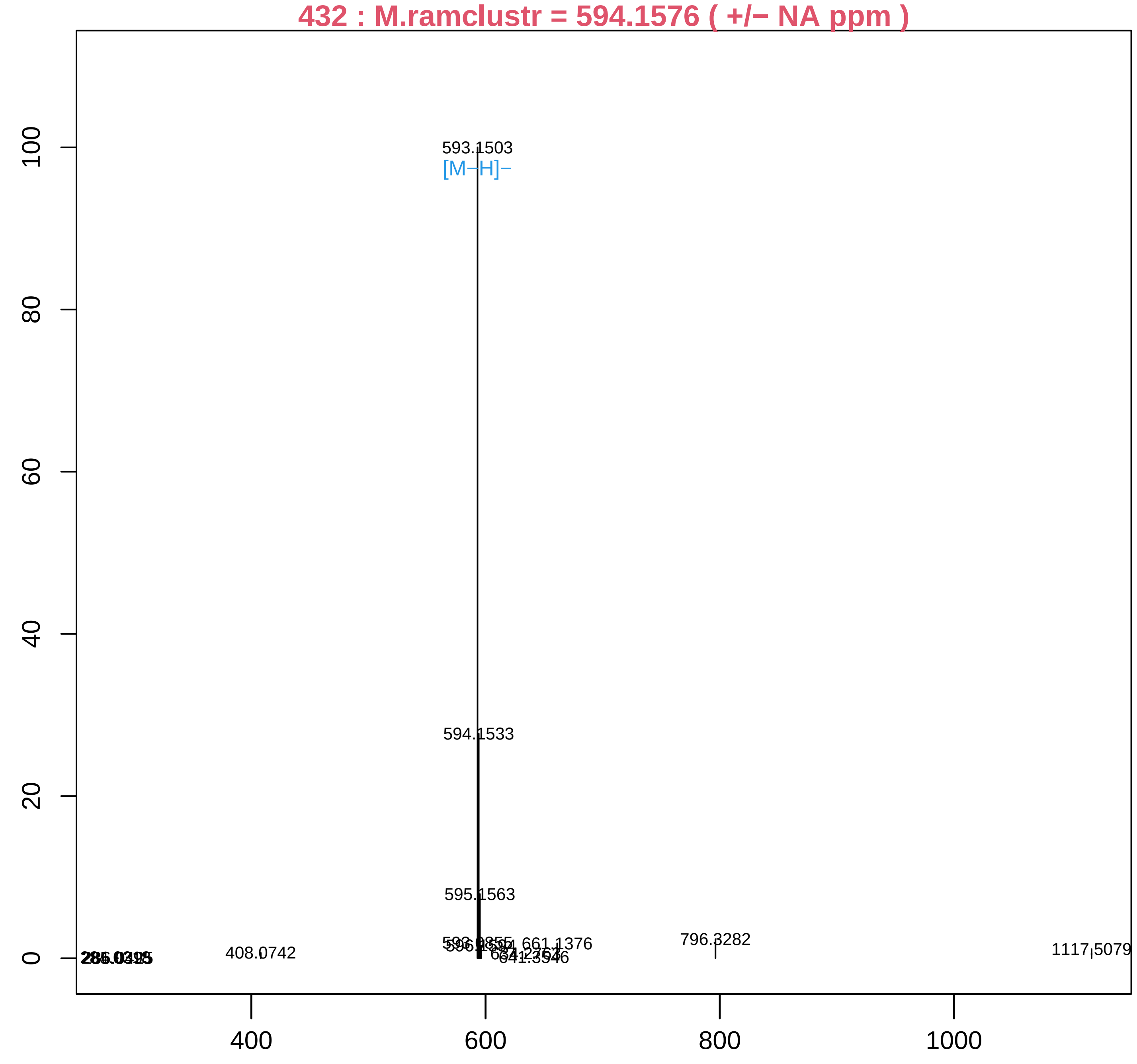
432 : M.ramclustr = 594.1576 (+/− NA ppm)
593.1503
[M−H]−
594.1533
595.1563
661.1376
796.3282
408.0742
1117.5079
641.3546
0
20
40
60
80
100
400
600
800
1000

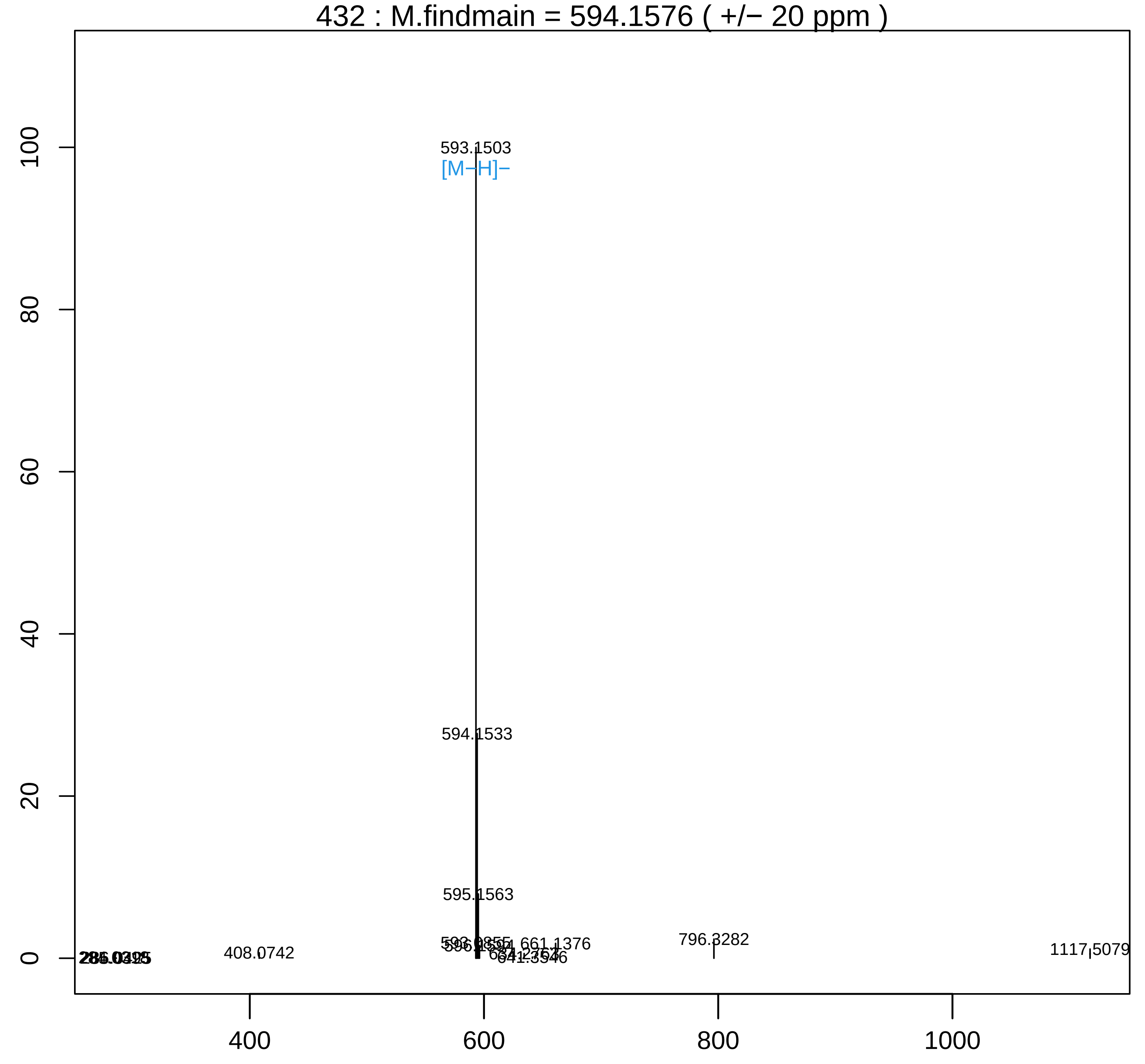
432 : M.findmain = 594.1576 (+/− 20 ppm)
593.1503
[M−H]−
594.1533
595.1563
661.1376
796.3282
408.0742
1117.5079
641.3546
0
20
40
60
80
100
400
600
800
1000

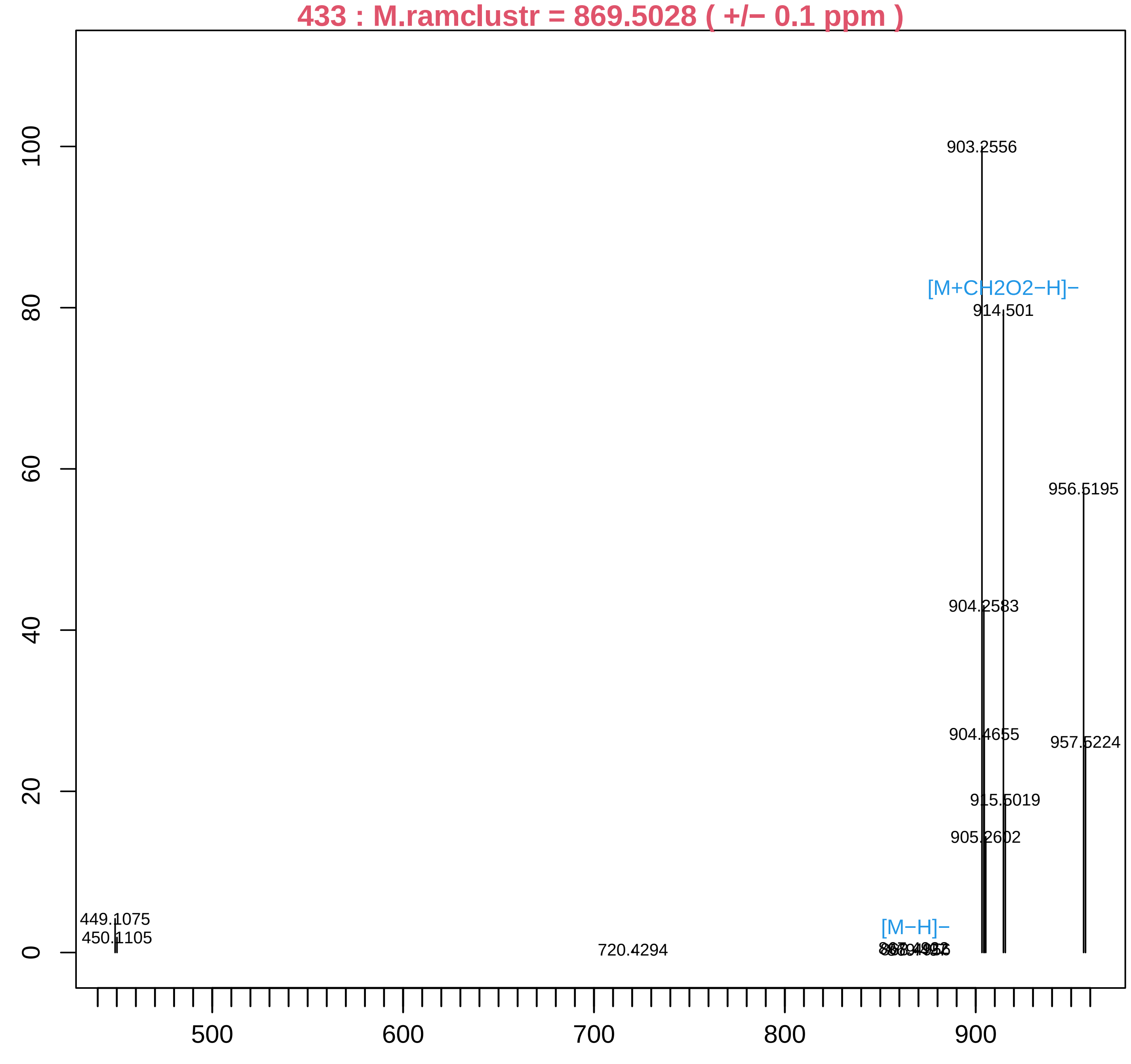
433 : M.ramclustr = 869.5028 (+/− 0.1 ppm)
903.2556
[M+CH2O2−H]−
914.501
956.5195
904.2583
904.4655
957.5224
915.5019
905.2602
449.1075
450.1105
720.4294
[M−H]−
0
20
40
60
80
100
500
600
700
800
900

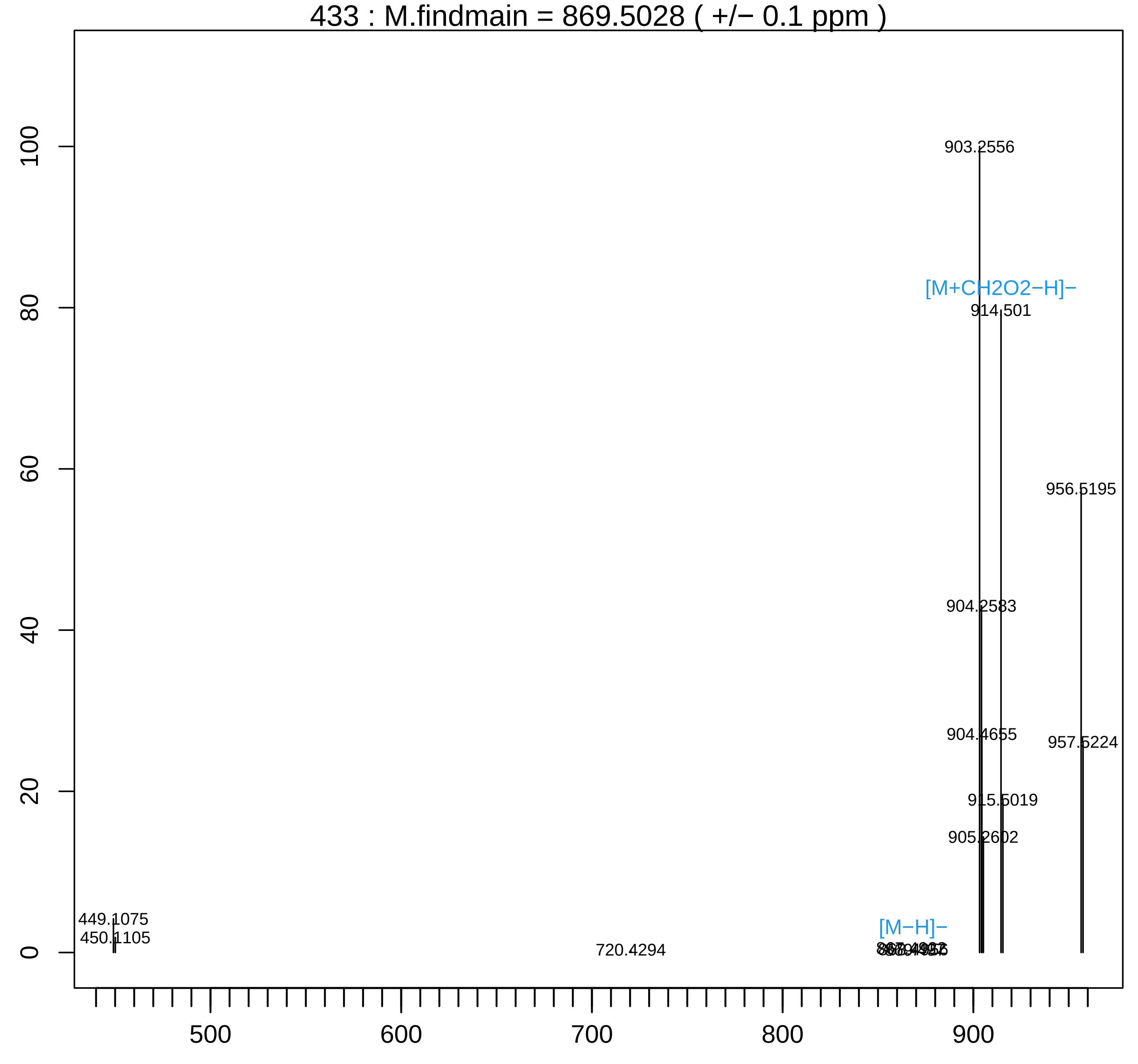
433 : M.findmain = 869.5028 (+/− 0.1 ppm)
903.2556
[M+CH2O2−H]−
914.501
956.5195
904.2583
904.4655
957.5224
915.5019
905.2602
449.1075
450.1105
720.4294
[M−H]−
0
20
40
60
80
100
500
600
700
800
900

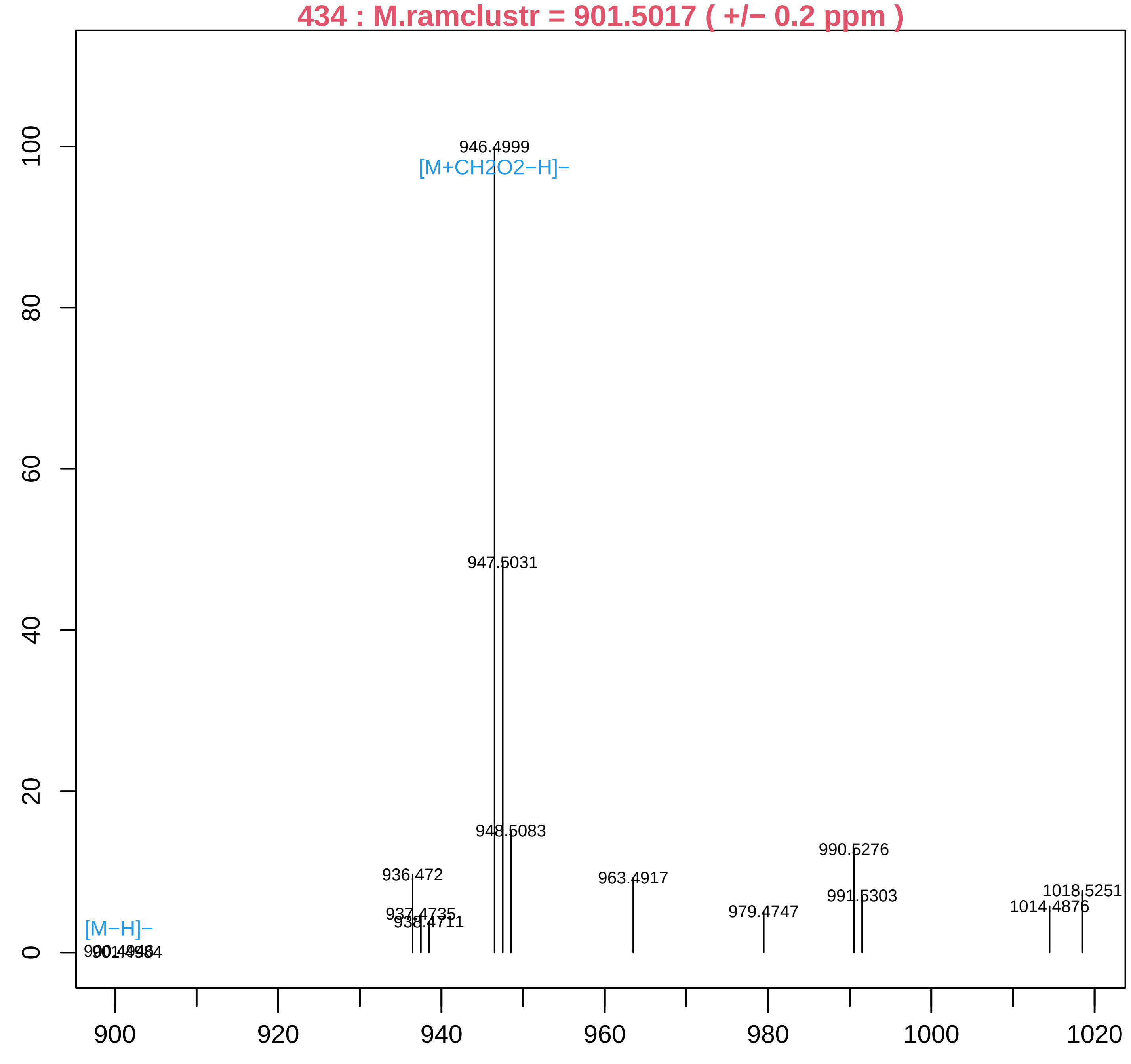
434 : M.ramclustr = 901.5017 (+/− 0.2 ppm)
946.4999
[M+CH2O2−H]−
947.5031
948.5083
936.472
937.4735
938.4711
963.4917
979.4747
990.5276
991.5303
1014.4876
1018.5251
[M−H]−
0
20
40
60
80
100
900
920
940
960
980
1000
1020

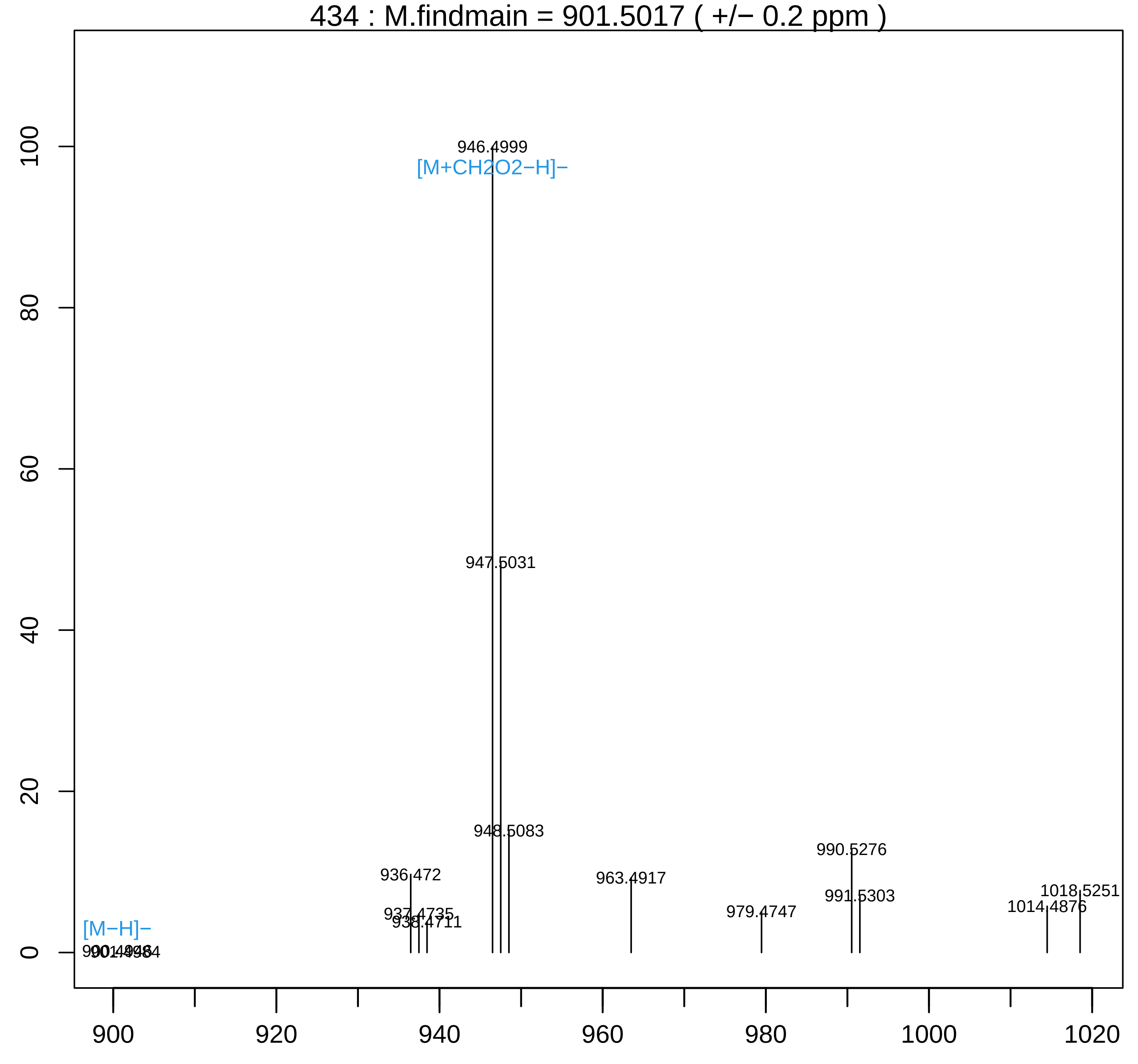
434 : M.findmain = 901.5017 (+/− 0.2 ppm)
946.4999
[M+CH2O2−H]−
947.5031
948.5083
936.472
937.4735
938.4711
963.4917
979.4747
990.5276
991.5303
1014.4876
1018.5251
[M−H]−
0
20
40
60
80
100
900
920
940
960
980
1000
1020

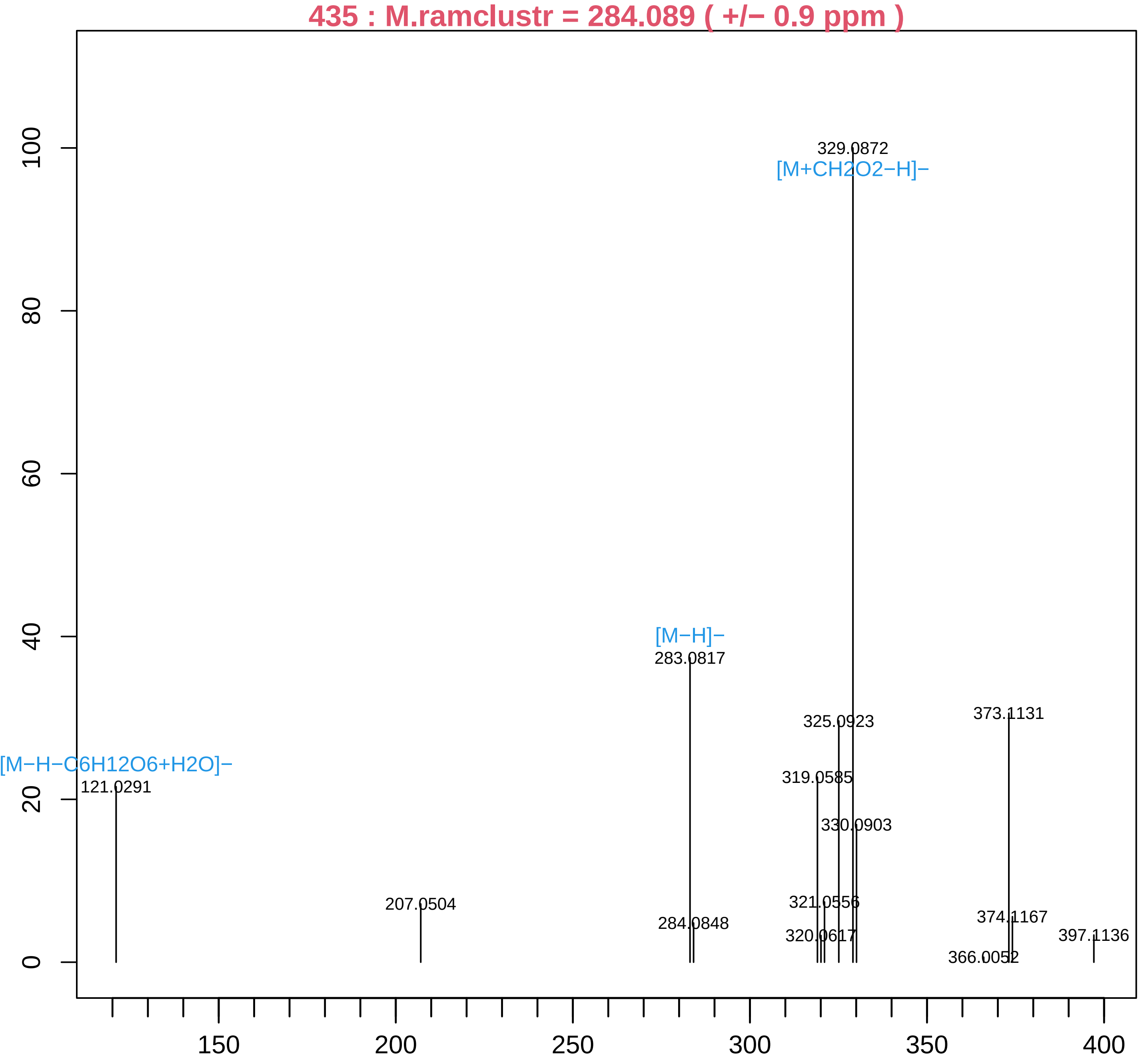
435 : M.ramclustr = 284.089 (+/− 0.9 ppm)
329.0872
[M+CH2O2−H]−
[M−H]−
283.0817
325.0923
373.1131
[M−H−C6H12O6+H2O]−
121.0291
319.0585
330.0903
207.0504
321.0556
284.0848
374.1167
320.0617
397.1136
366.0052
0
20
40
60
80
100
150
200
250
300
350
400

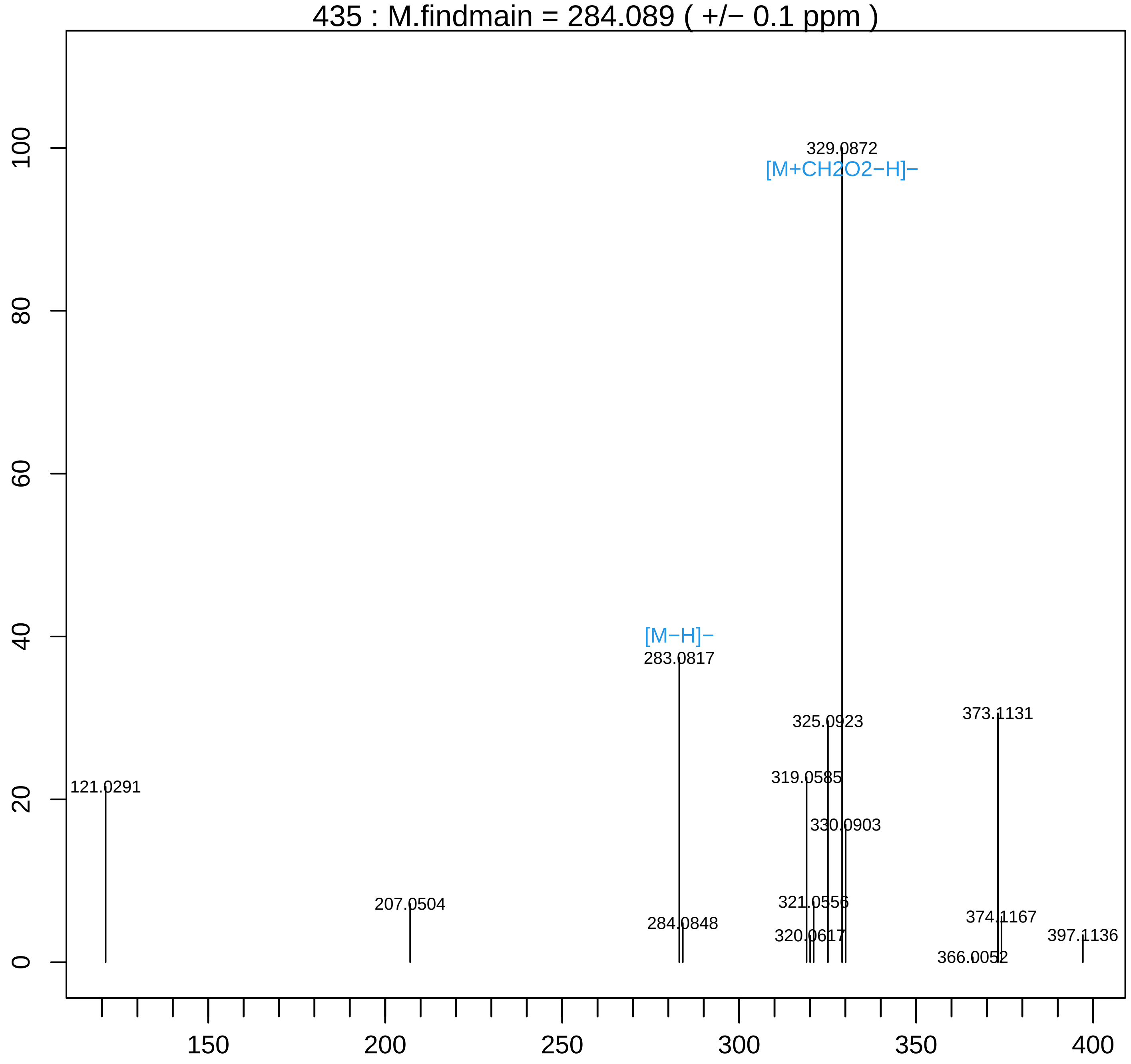
435 : M.findmain = 284.089 (+/− 0.1 ppm)
329.0872
[M+CH2O2−H]−
[M−H]−
283.0817
325.0923
373.1131
121.0291
319.0585
330.0903
207.0504
321.0556
284.0848
374.1167
320.0617
397.1136
366.0052
0
20
40
60
80
100
150
200
250
300
350
400

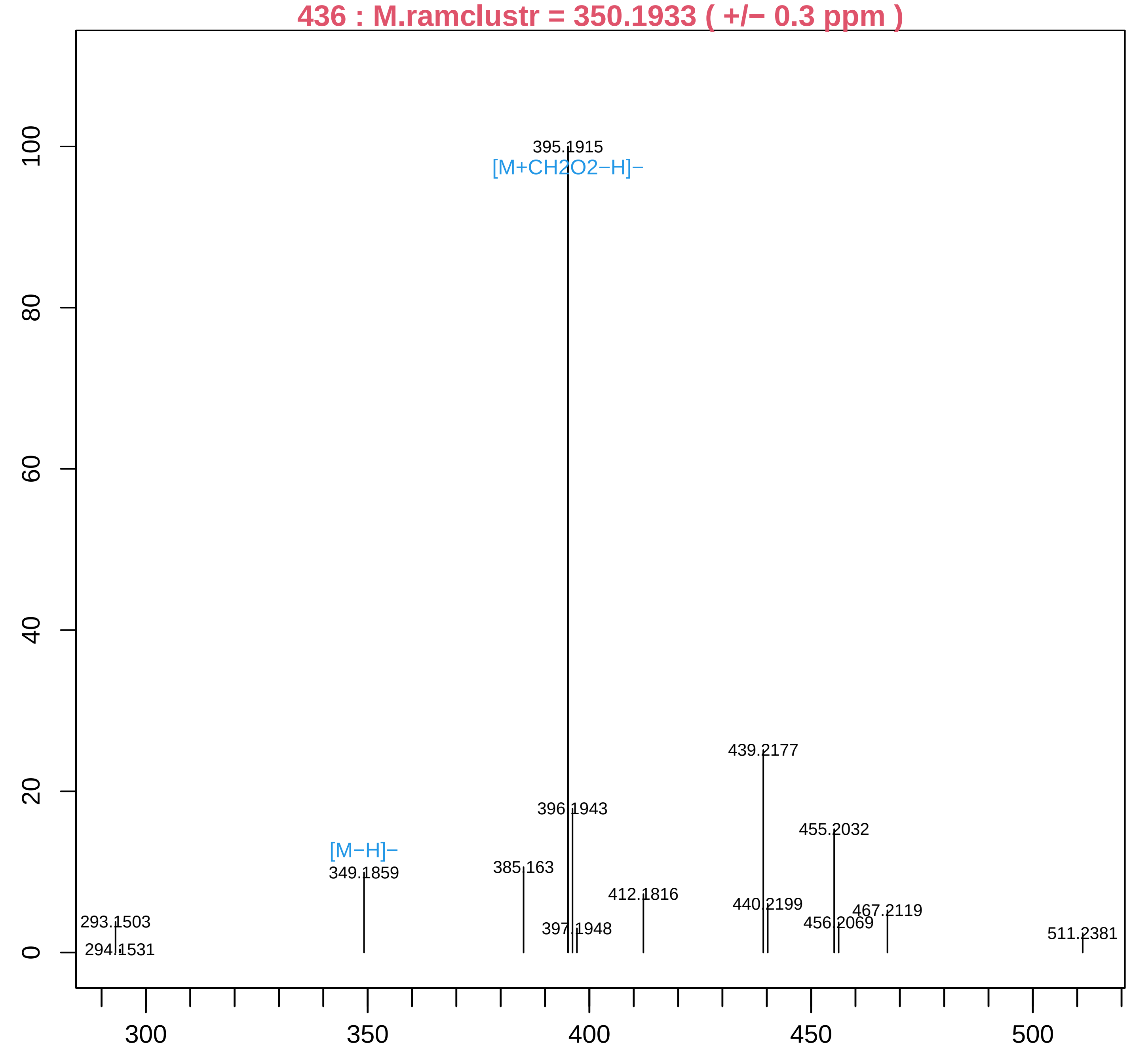

436 : M.ramclustr = 350.1933 (+/− 0.3 ppm)
395.1915
[M+CH2O2−H]−
439.2177
396.1943
455.2032
[M−H]−
349.1859
385.163
412.1816
440.2199
467.2119
456.2069
397.1948
293.1503
294.1531
511.2381
0
20
40
60
80
100
300
350
400
450
500

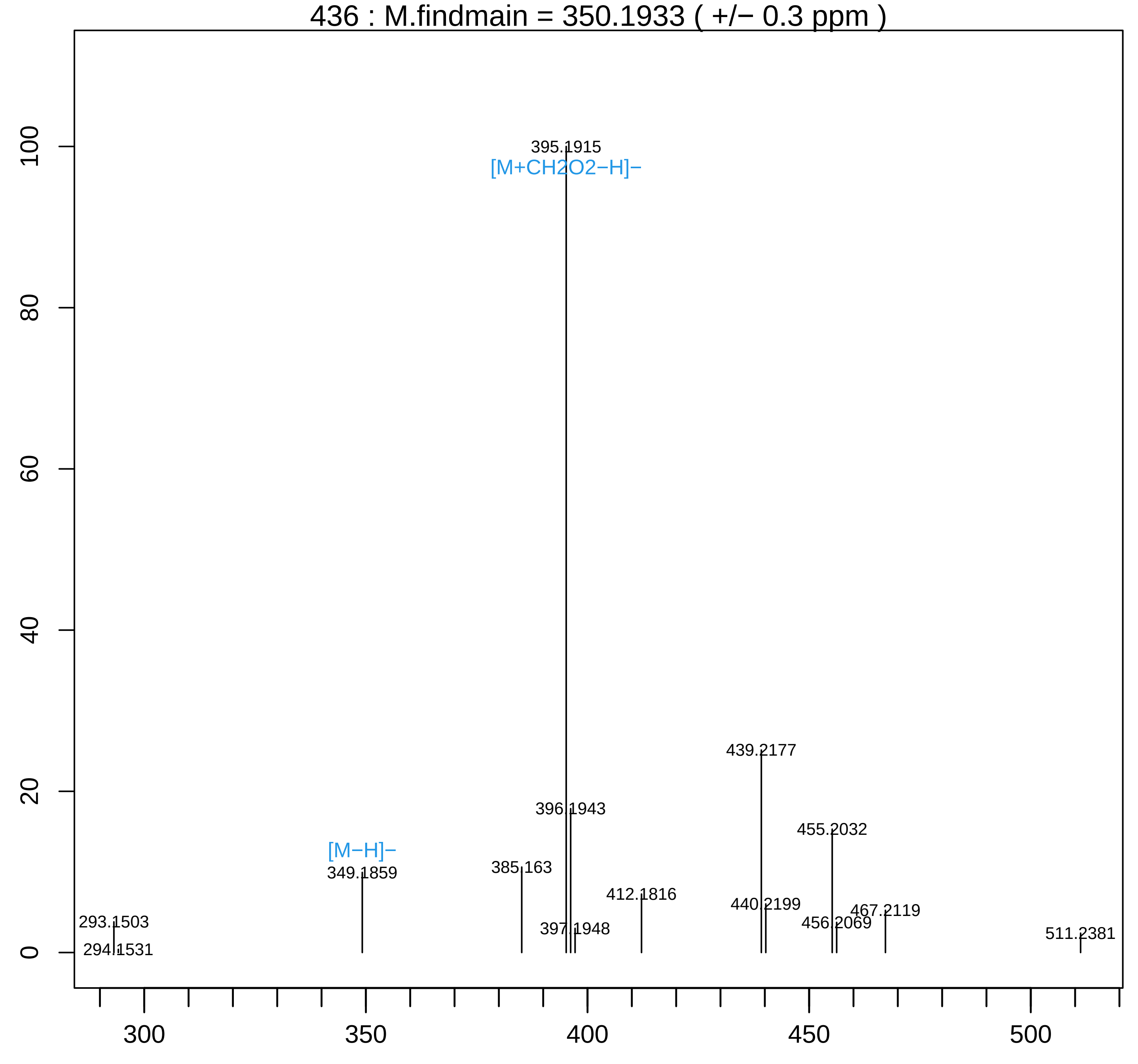

436 : M.findmain = 350.1933 (+/− 0.3 ppm)
395.1915
[M+CH2O2−H]−
439.2177
396.1943
455.2032
[M−H]−
349.1859
385.163
412.1816
440.2199
467.2119
456.2069
397.1948
293.1503
294.1531
511.2381
0
20
40
60
80
100
300
350
400
450
500

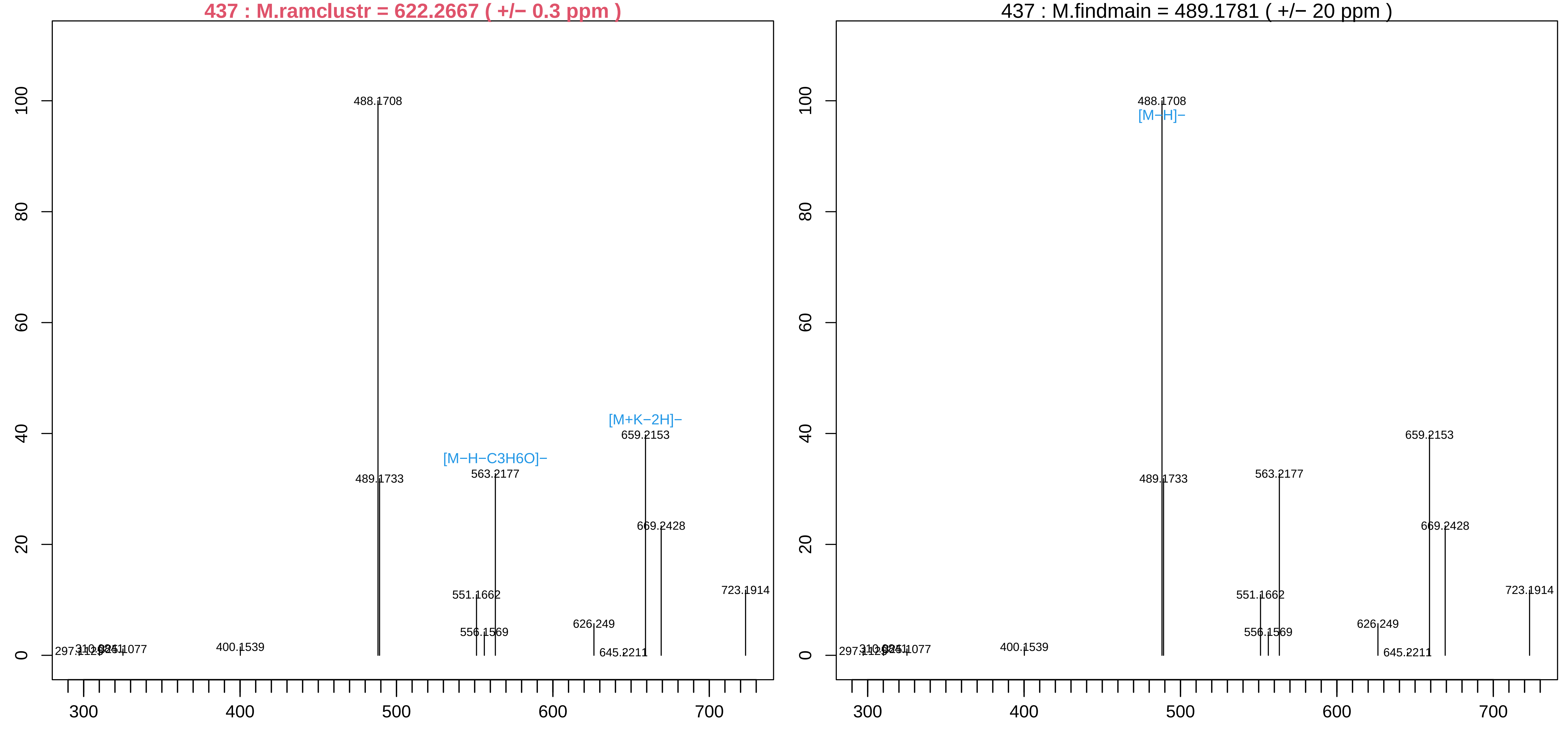
437 : M.ramclustr = 622.2667 (+/− 0.3 ppm)
488.1708
[M−H−C3H6O]−
563.2177
[M+K−2H]−
659.2153
489.1733
669.2428
551.1662
556.1569
626.249
645.2211
723.1914
400.1539
297.1125
310.0925
324.1077
437 : M.findmain = 489.1781 (+/− 20 ppm)
488.1708
[M−H]−
489.1733
563.2177
659.2153
669.2428
551.1662
556.1569
626.249
645.2211
723.1914
400.1539
0
20
40
60
80
100
300
400
500
600
700

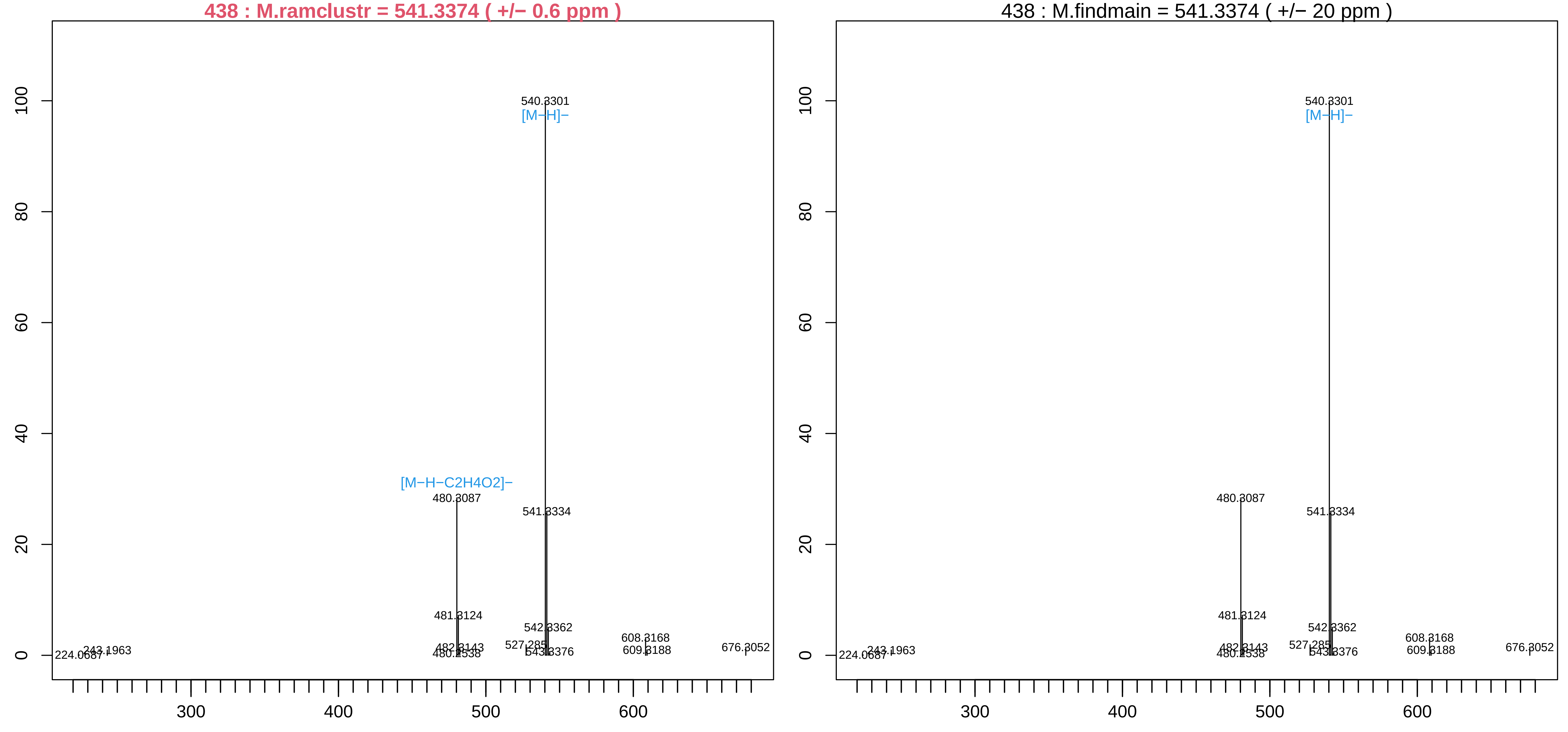
438 : M.ramclustr = 541.3374 (+/− 0.6 ppm)
540.3301
[M−H]−
[M−H−C2H4O2]−
480.3087
541.3334
481.3124
542.3362
527.285
482.3143
480.2538
543.3376
608.3168
609.3188
676.3052
224.0687
243.1963
438 : M.findmain = 541.3374 (+/− 20 ppm)
0
20
40
60
80
100
300
400
500
600

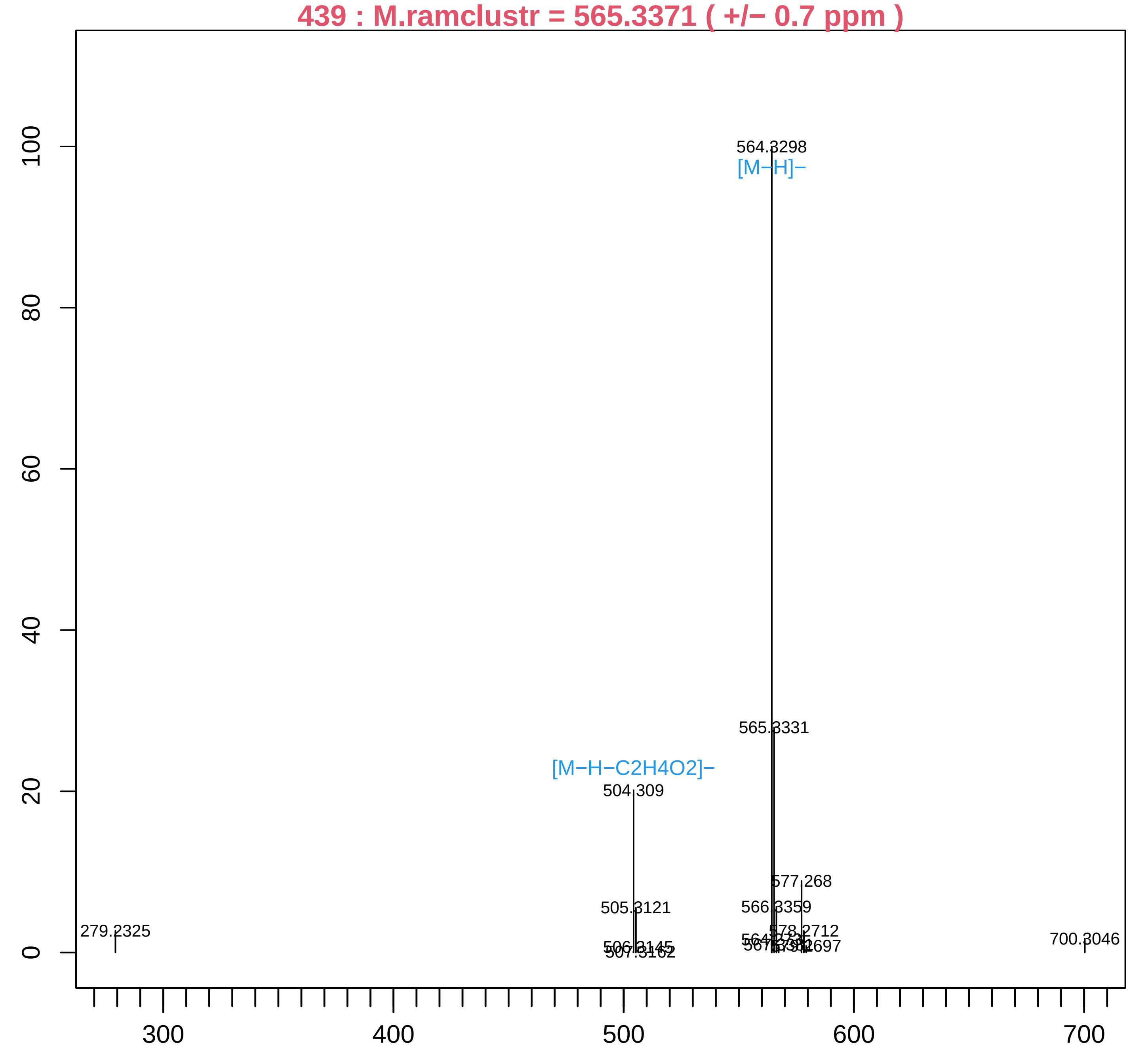
439 : M.ramclustr = 565.3371 (+/− 0.7 ppm)
564.3298
[M−H]−
565.3331
[M−H−C2H4O2]−
504.309
577.268
505.3121
566.3359
279.2325
578.2712
700.3046
506.3145
507.3162
0
20
40
60
80
100
300
400
500
600
700

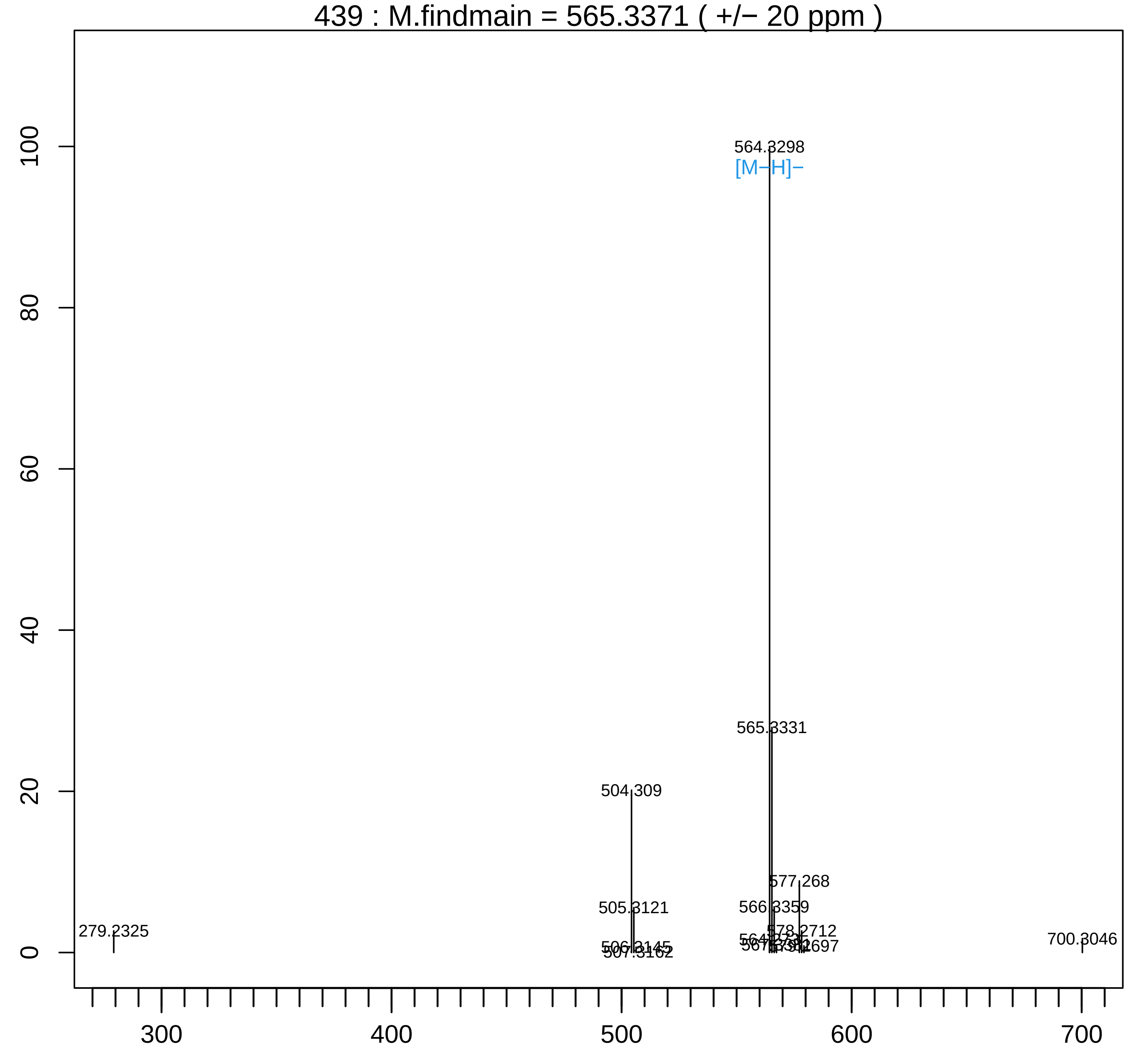
439 : M.findmain = 565.3371 (+/− 20 ppm)
564.3298
[M−H]−
565.3331
504.309
577.268
505.3121
566.3359
279.2325
578.2712
700.3046
506.3145
507.3162
0
20
40
60
80
100
300
400
500
600
700

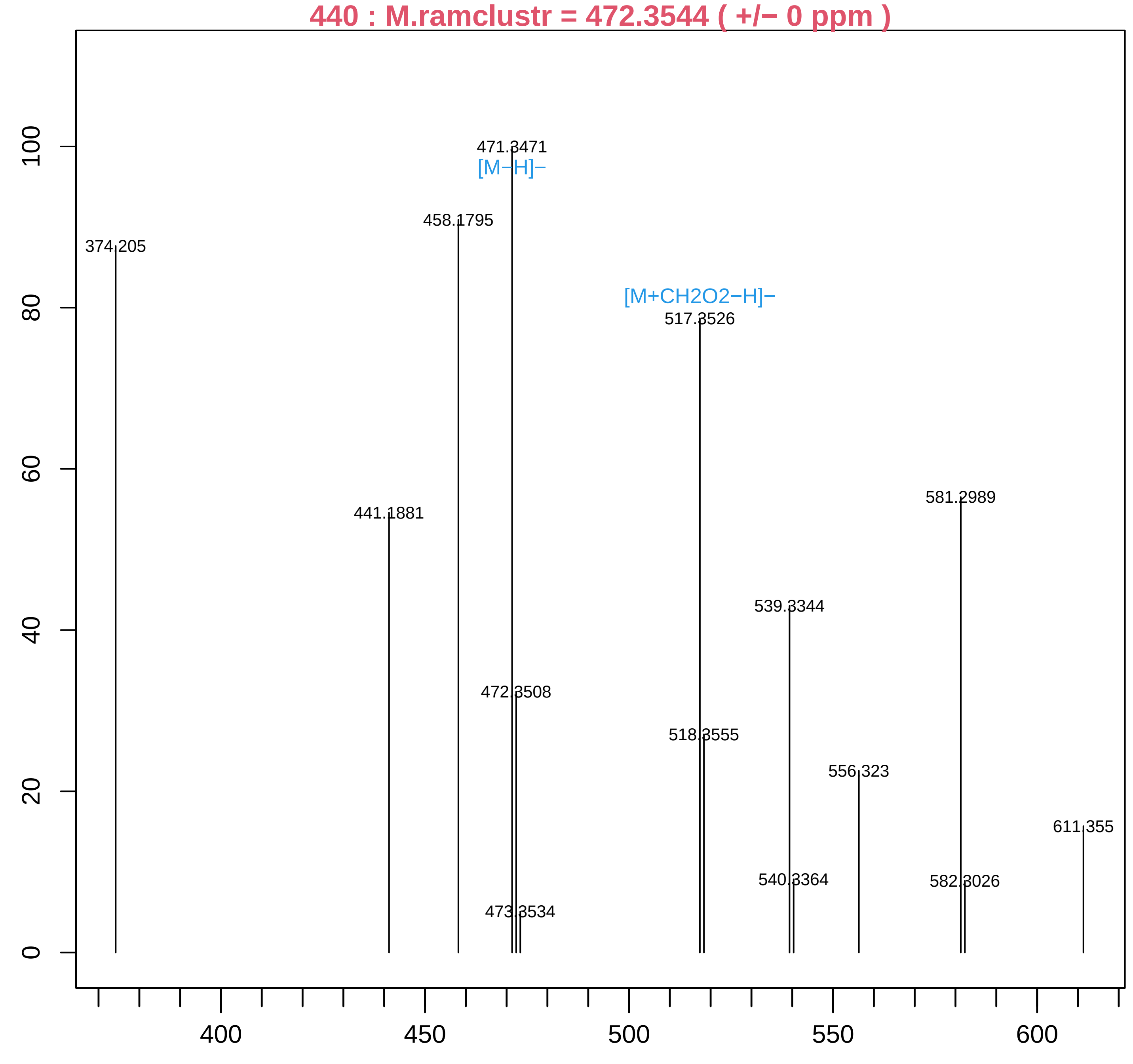
440 : M.ramclustr = 472.3544 (+/− 0 ppm)
471.3471
[M−H]−
458.1795
374.205
[M+CH2O2−H]−
517.3526
441.1881
581.2989
539.3344
472.3508
518.3555
556.323
611.355
540.3364
582.3026
473.3534
0
20
40
60
80
100
400
450
500
550
600

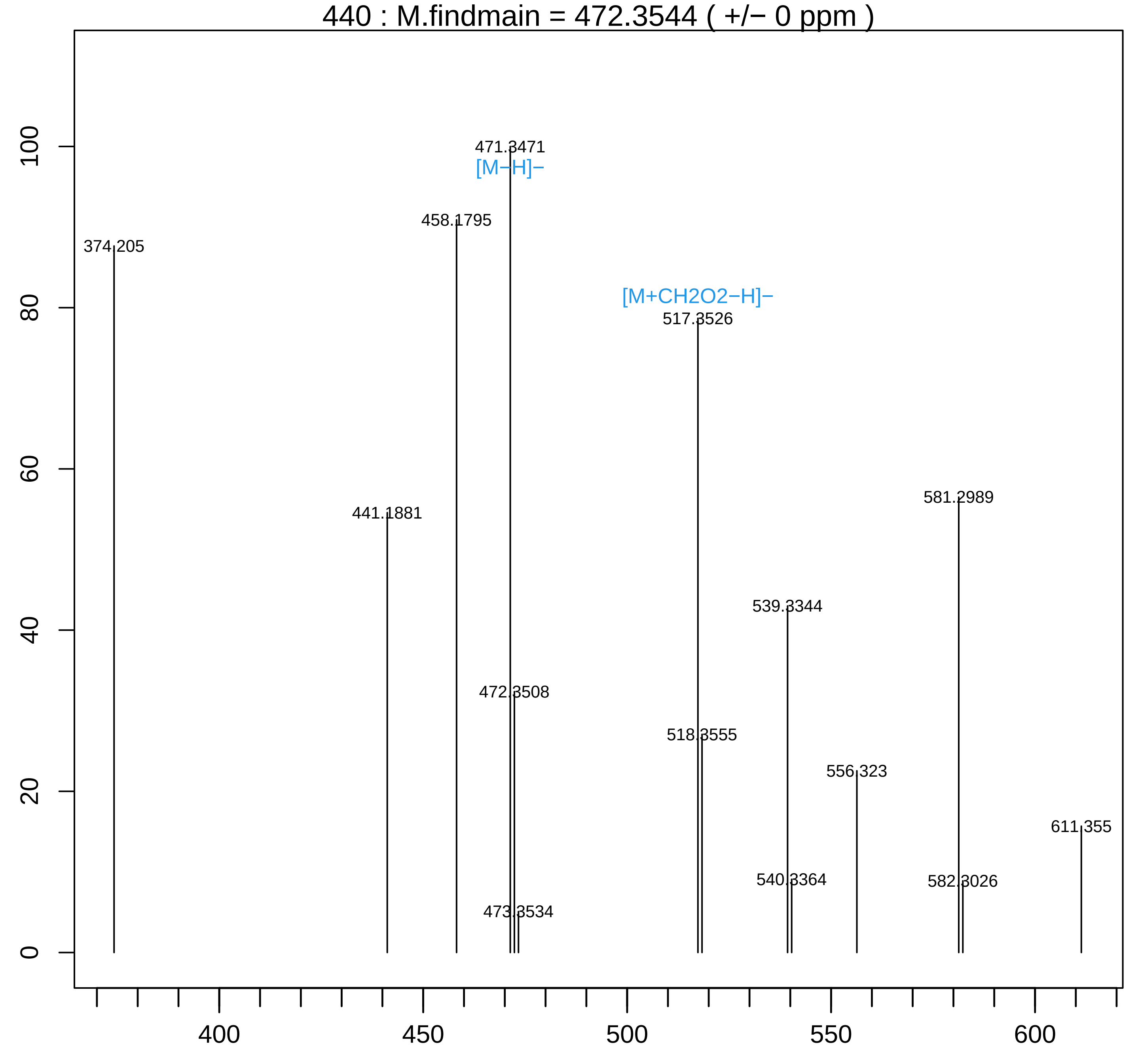
440 : M.findmain = 472.3544 (+/− 0 ppm)
471.3471
[M−H]−
458.1795
374.205
[M+CH2O2−H]−
517.3526
441.1881
581.2989
539.3344
472.3508
518.3555
556.323
611.355
540.3364
582.3026
473.3534
0
20
40
60
80
100
400
450
500
550
600

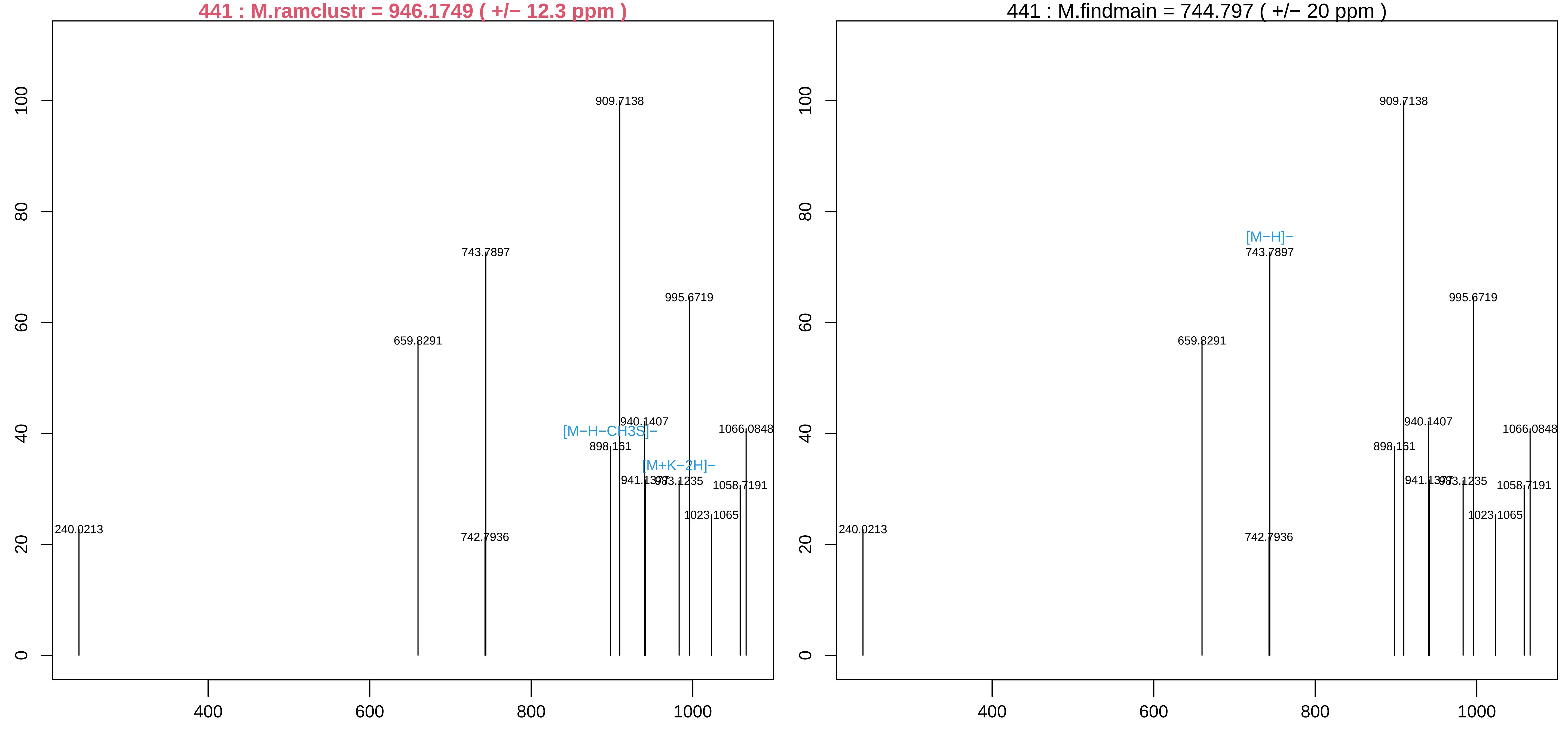
441 : M.ramclustr = 946.1749 (+/− 12.3 ppm)
441 : M.findmain = 744.797 (+/− 20 ppm)
0
20
40
60
80
100
400
600
800
1000
909.7138
743.7897
995.6719
659.8291
940.1407
1066.0848
898.161
941.1377
983.1235
1058.7191
1023.1065
240.0213
742.7936
[M−H−CH3S]−
[M+K−2H]−
[M−H]−

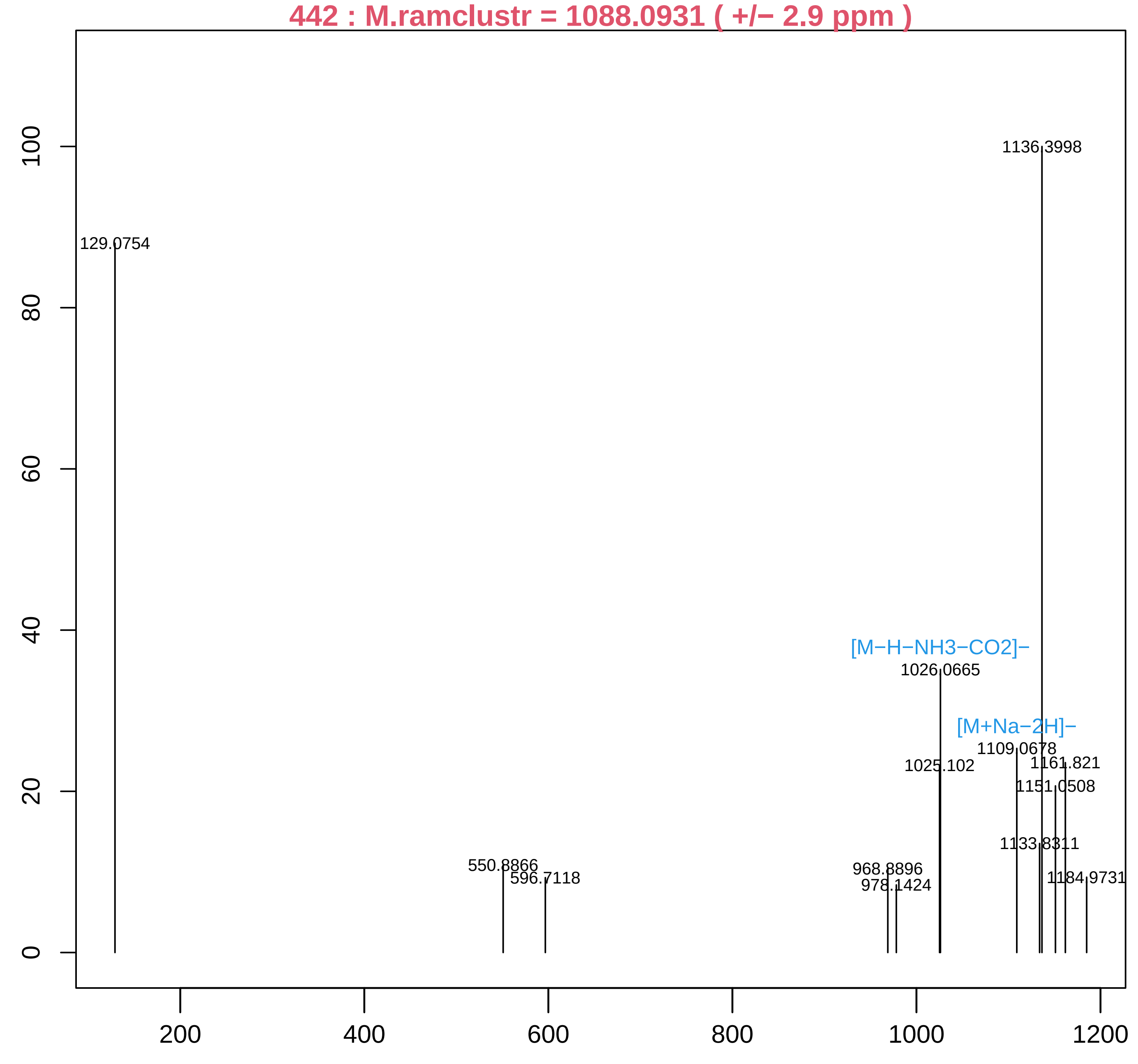

442 : M.ramclustr = 1088.0931 (+/− 2.9 ppm)
1136.3998
129.0754
[M−H−NH3−CO2]−
1026.0665
[M+Na−2H]−
1109.0678
1161.821
1025.102
1151.0508
1133.8311
550.8866
596.7118
968.8896
978.1424
1184.9731
0
20
40
60
80
100
200
400
600
800
1000
1200

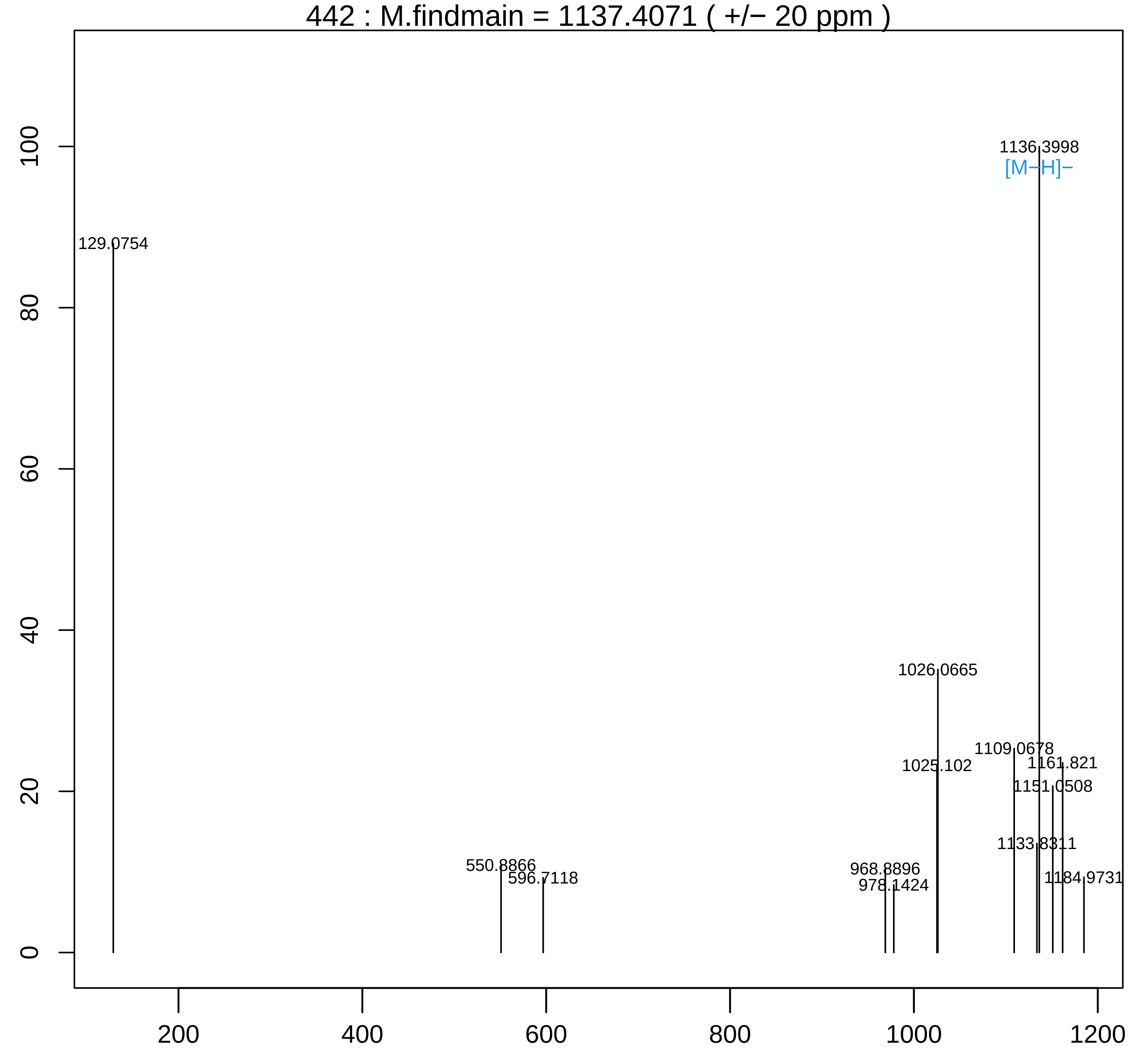

442 : M.findmain = 1137.4071 (+/− 20 ppm)
1136.3998
[M−H]−
129.0754
1026.0665
1109.0678
1161.821
1025.102
1151.0508
1133.8311
550.8866
596.7118
968.8896
978.1424
1184.9731
0
20
40
60
80
100
200
400
600
800
1000
1200

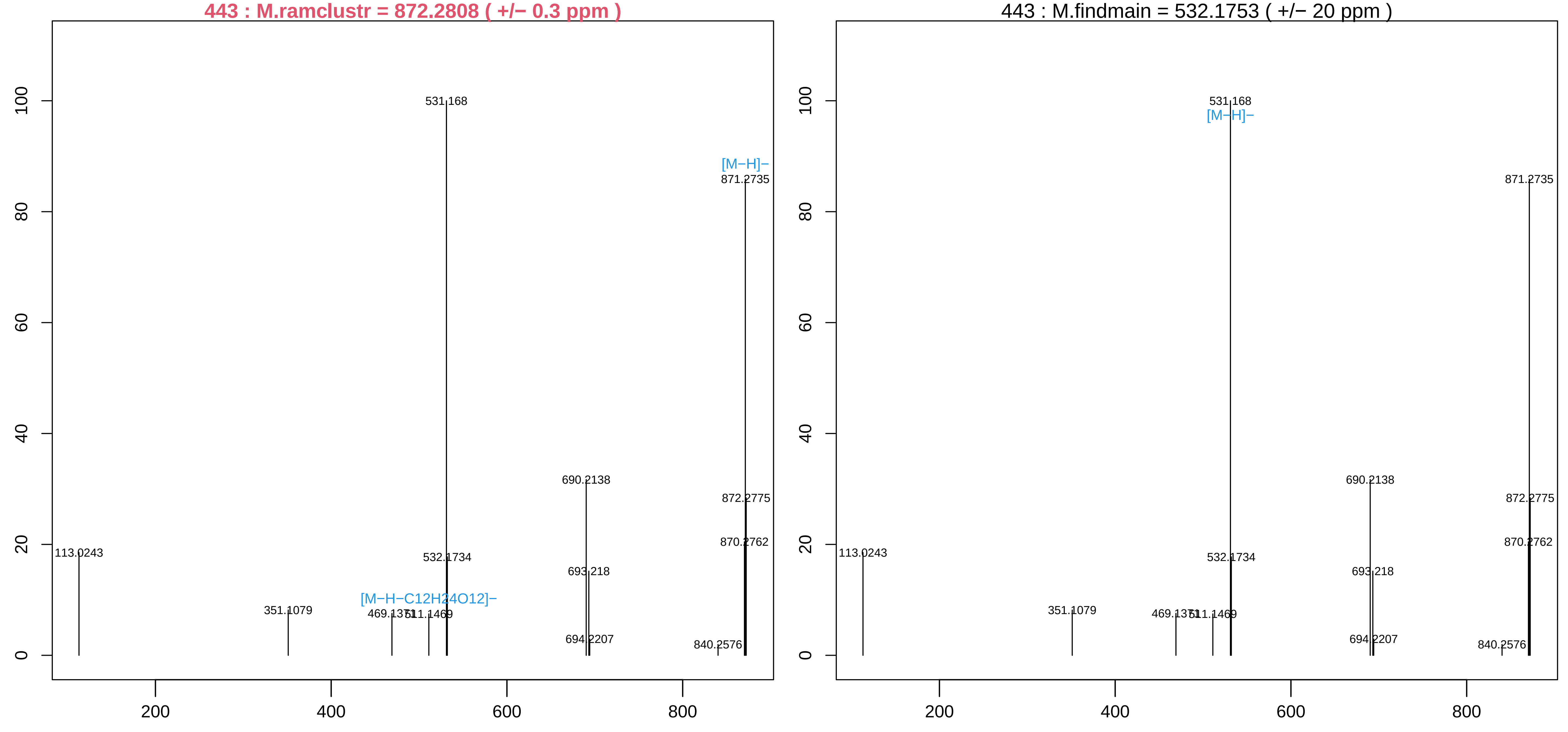

443 : M.ramclustr = 872.2808 (+/− 0.3 ppm)
531.168
[M−H]−
871.2735
690.2138
872.2775
870.2762
113.0243
532.1734
693.218
[M−H−C12H24O12]−
351.1079
469.1371
511.1469
694.2207
840.2576
443 : M.findmain = 532.1753 (+/− 20 ppm)
531.168
[M−H]−
871.2735
690.2138
872.2775
870.2762
113.0243
532.1734
693.218
351.1079
469.1371
511.1469
694.2207
840.2576
0
20
40
60
80
100
200
400
600
800

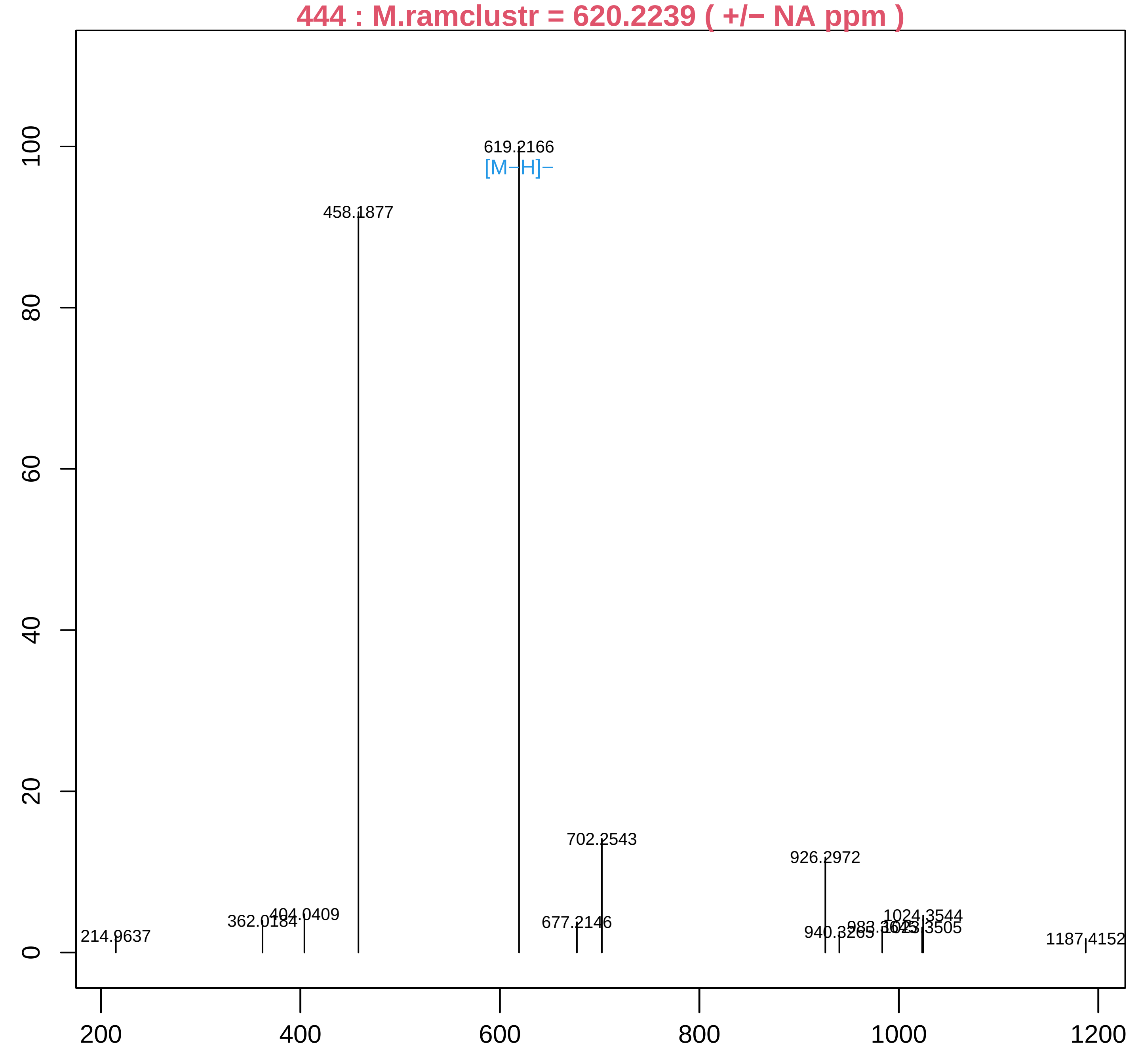
444 : M.ramclustr = 620.2239 (+/− NA ppm)
619.2166
[M−H]−
458.1877
702.2543
926.2972
214.9637
362.0184
404.0409
677.2146
940.3265
983.3625
1024.3544
1025.3505
1187.4152
0
20
40
60
80
100
200
400
600
800
1000
1200

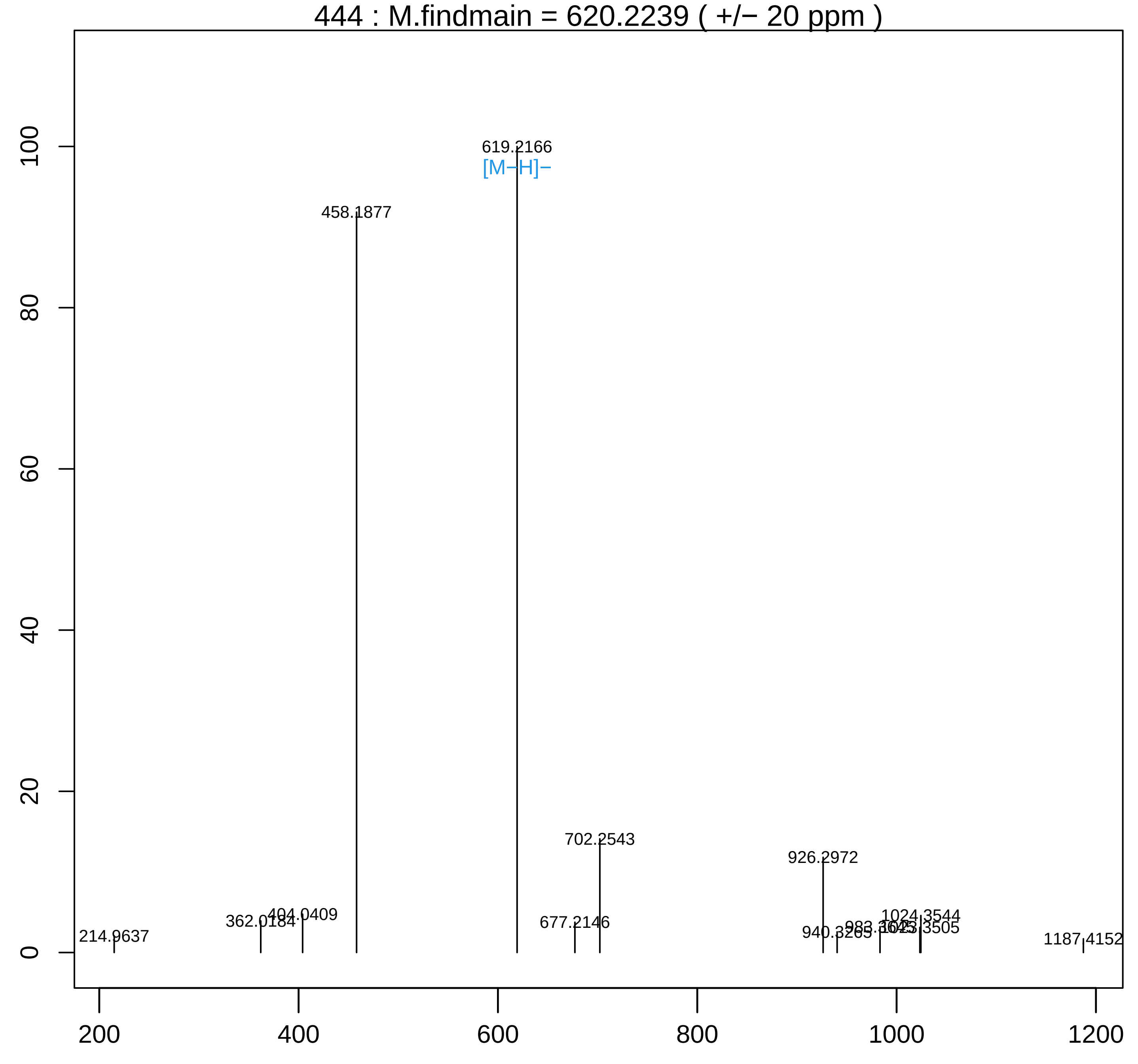
444 : M.findmain = 620.2239 (+/− 20 ppm)
619.2166
[M−H]−
458.1877
702.2543
926.2972
214.9637
362.0184
404.0409
677.2146
940.3265
983.3625
1024.3544
1025.3505
1187.4152
0
20
40
60
80
100
200
400
600
800
1000
1200

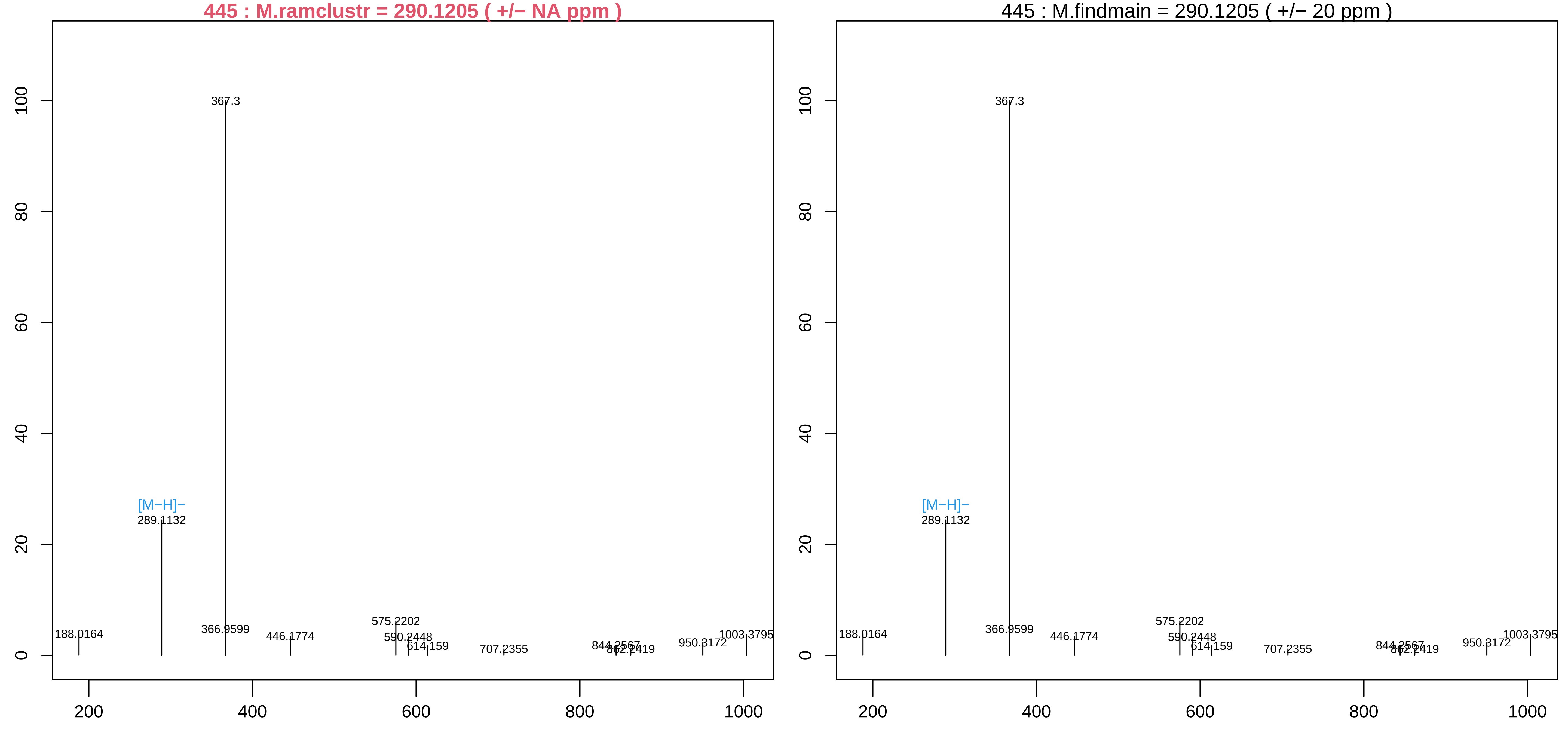
445 : M.ramclustr = 290.1205 (+/− NA ppm)
445 : M.findmain = 290.1205 (+/− 20 ppm)
367.3
[M−H]−
289.1132
188.0164
366.9599
446.1774
575.2202
590.2448
614.159
707.2355
844.2567
862.2419
950.3172
1003.3795
0
20
40
60
80
100
200
400
600
800
1000

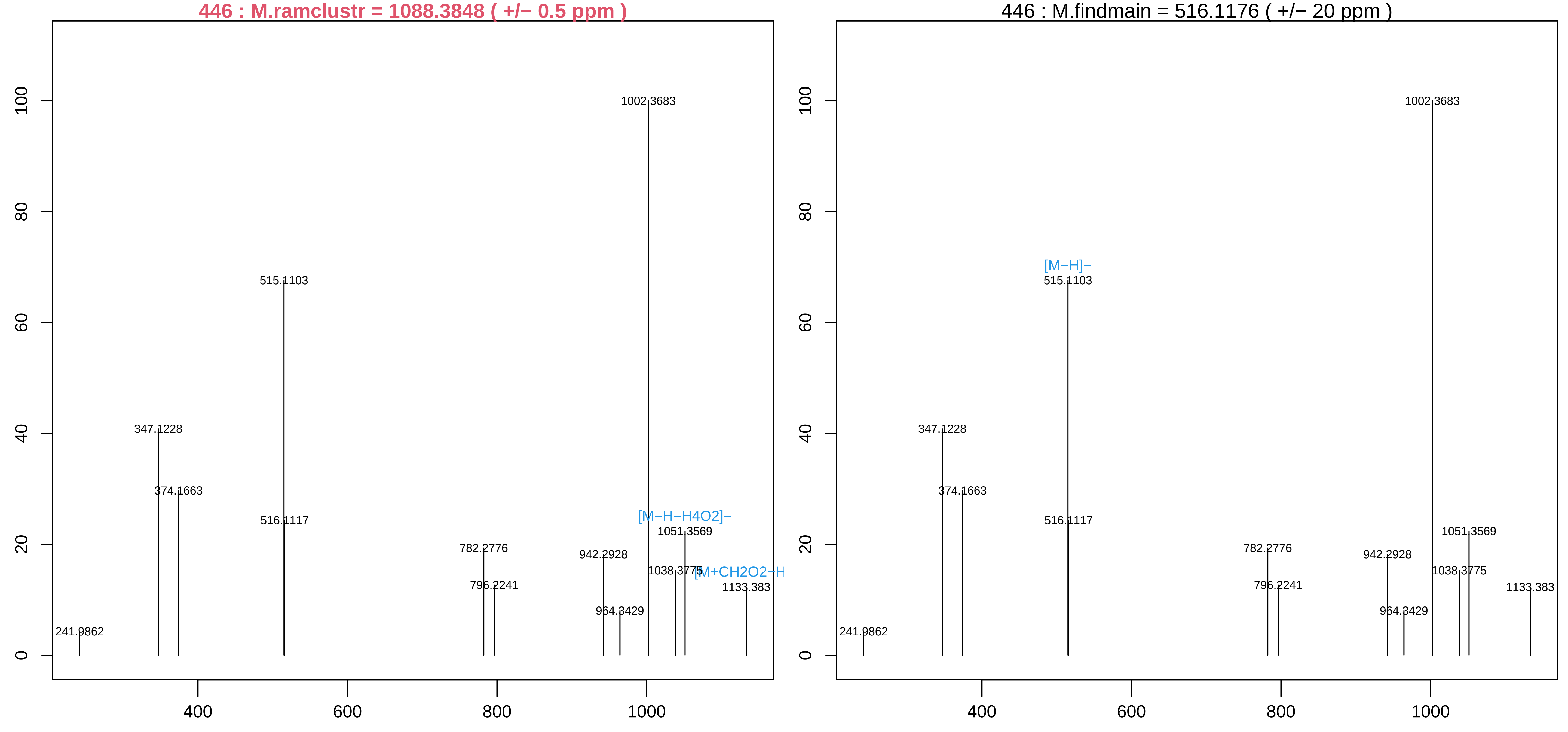
446 : M.ramclustr = 1088.3848 (+/− 0.5 ppm)
446 : M.findmain = 516.1176 (+/− 20 ppm)
0
20
40
60
80
100
400
600
800
1000
241.9862
347.1228
374.1663
515.1103
516.1117
782.2776
796.2241
942.2928
964.3429
1002.3683
1038.3775
1051.3569
1133.383
[M−H−H4O2]−
[M+CH2O2−H
[M−H]−

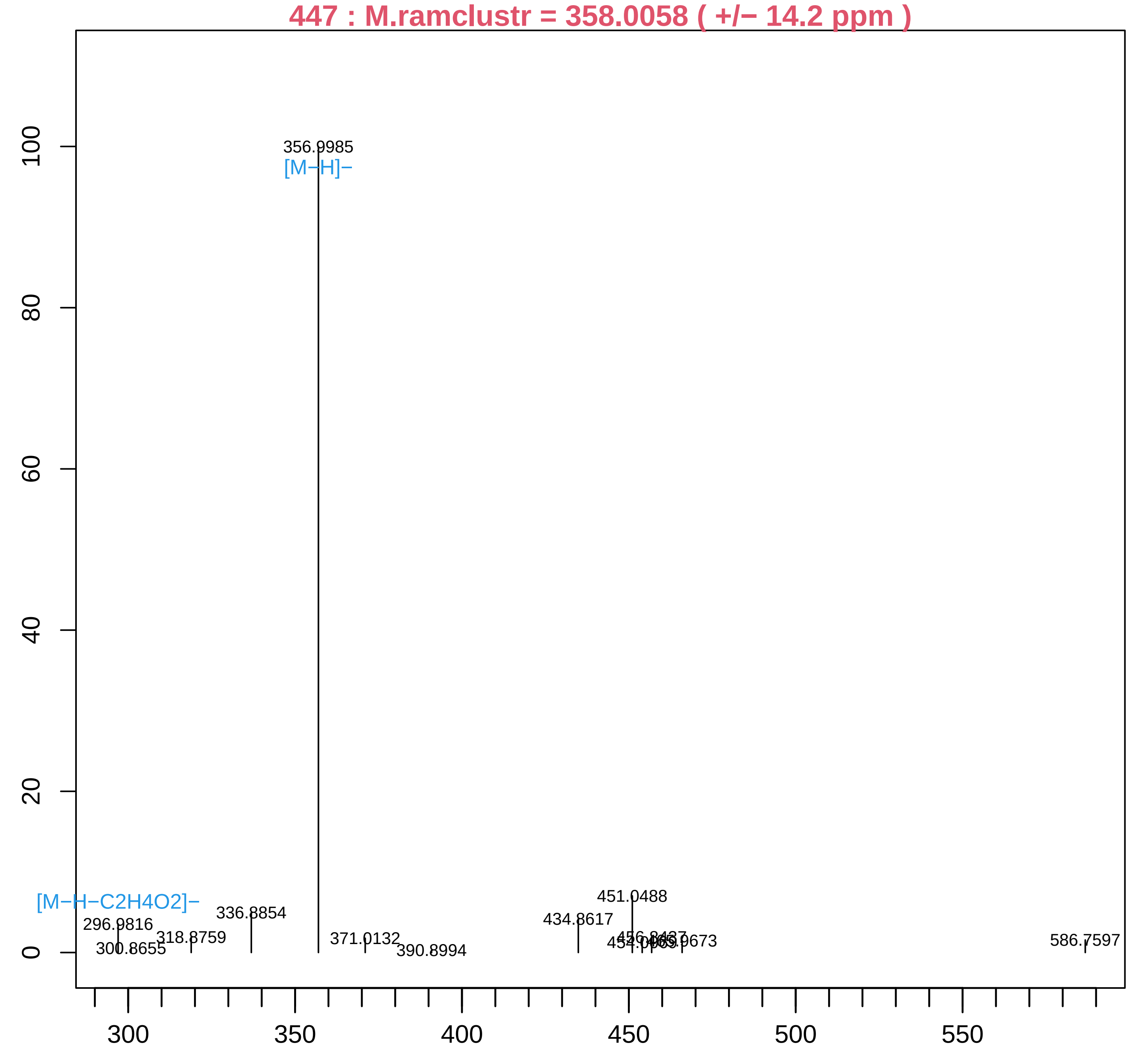

447 : M.ramclustr = 358.0058 (+/− 14.2 ppm)
356.9985
[M−H]−
[M−H−C2H4O2]−
296.9816
300.8655
318.8759
336.8854
371.0132
390.8994
434.8617
451.0488
456.8437
454.9965
465.0673
586.7597

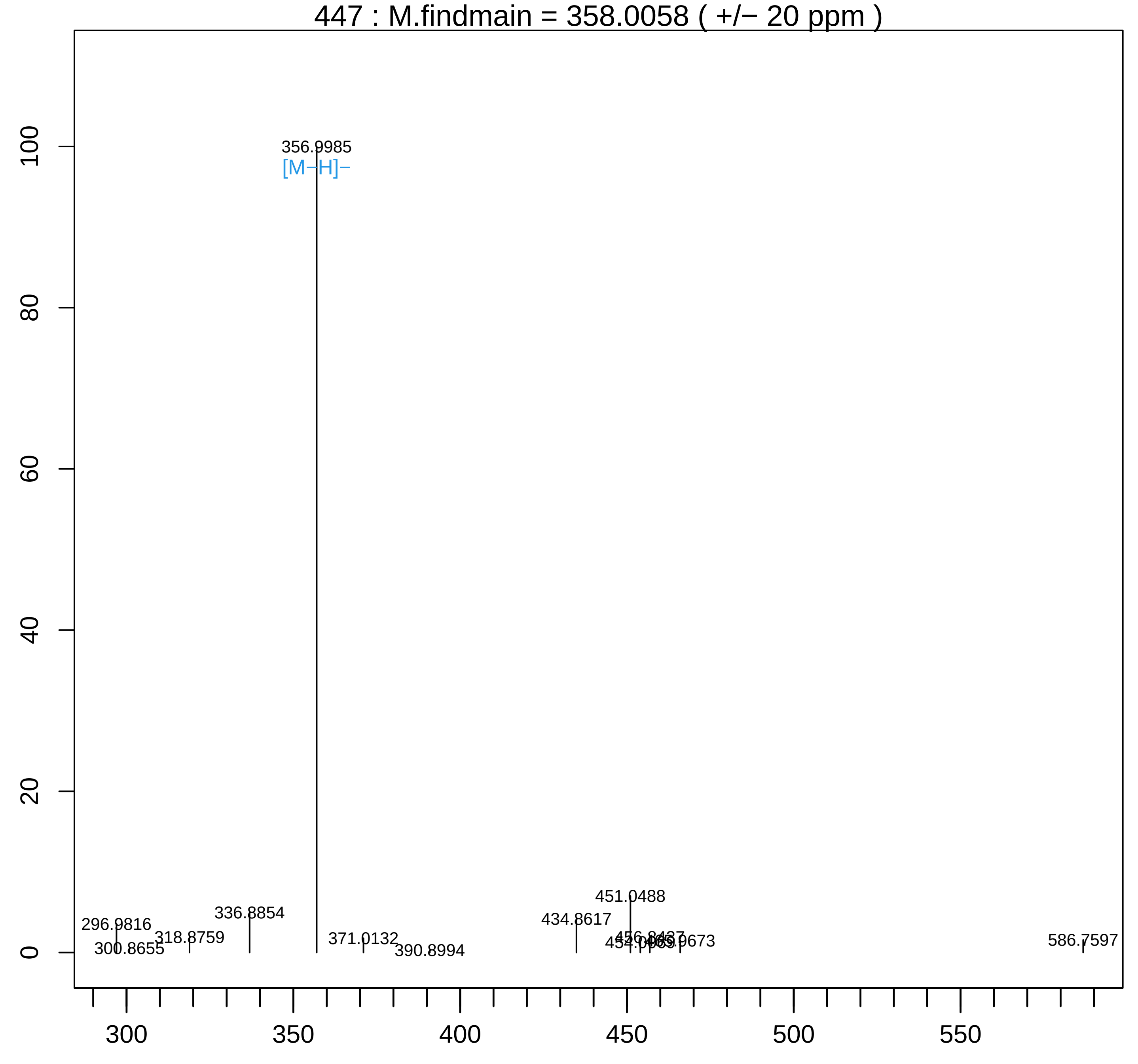

447 : M.findmain = 358.0058 (+/− 20 ppm)
356.9985
[M−H]−
296.9816
300.8655
318.8759
336.8854
371.0132
390.8994
434.8617
451.0488
456.8437
454.9965
465.0673
586.7597

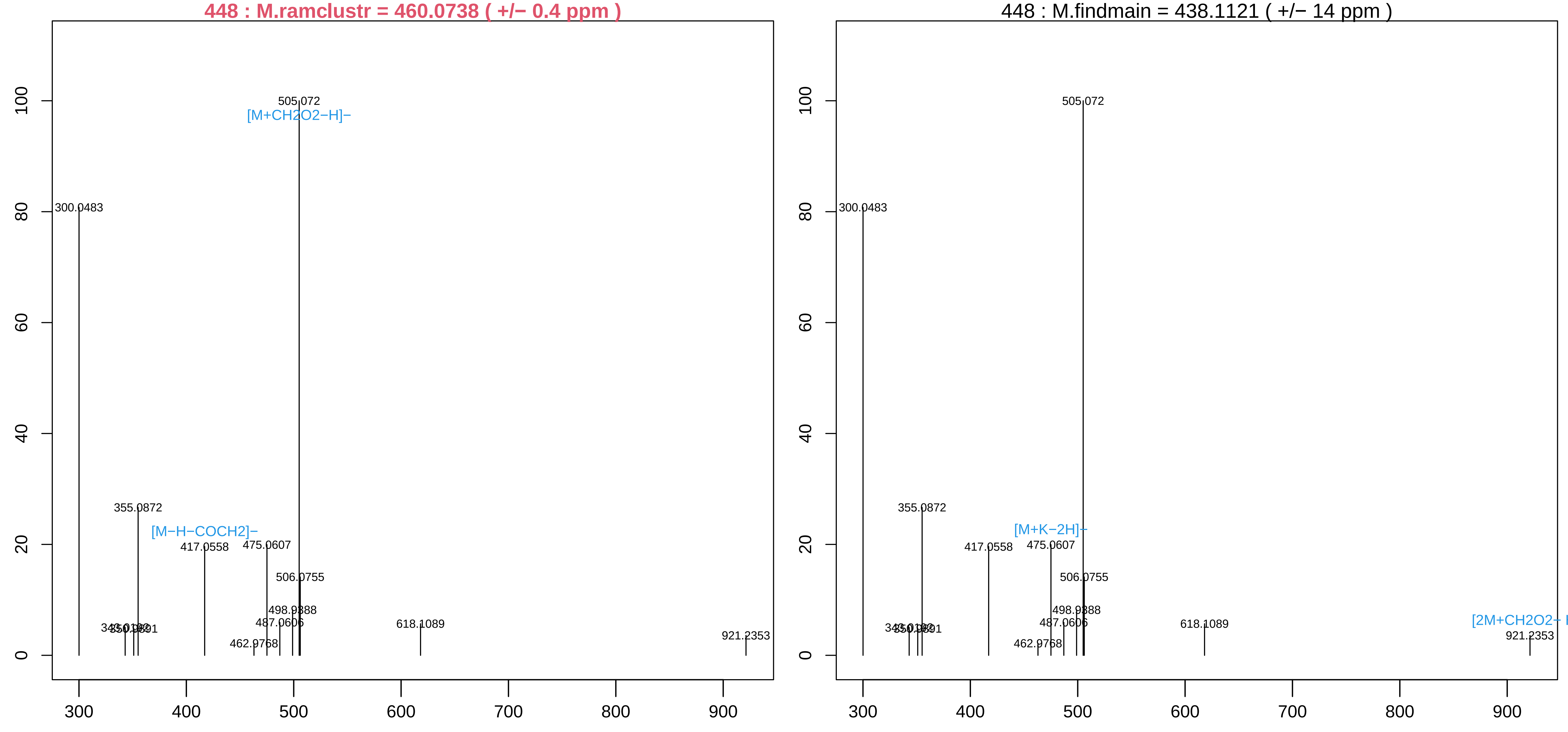
448 : M.ramclustr = 460.0738 (+/− 0.4 ppm)
505.072
[M+CH2O2−H]−
300.0483
355.0872
[M−H−COCH2]−
417.0558
475.0607
506.0755
498.9388
487.0606
462.9768
618.1089
921.2353
448 : M.findmain = 438.1121 (+/− 14 ppm)
505.072
300.0483
355.0872
[M+K−2H]−
417.0558
475.0607
506.0755
498.9388
487.0606
462.9768
618.1089
921.2353

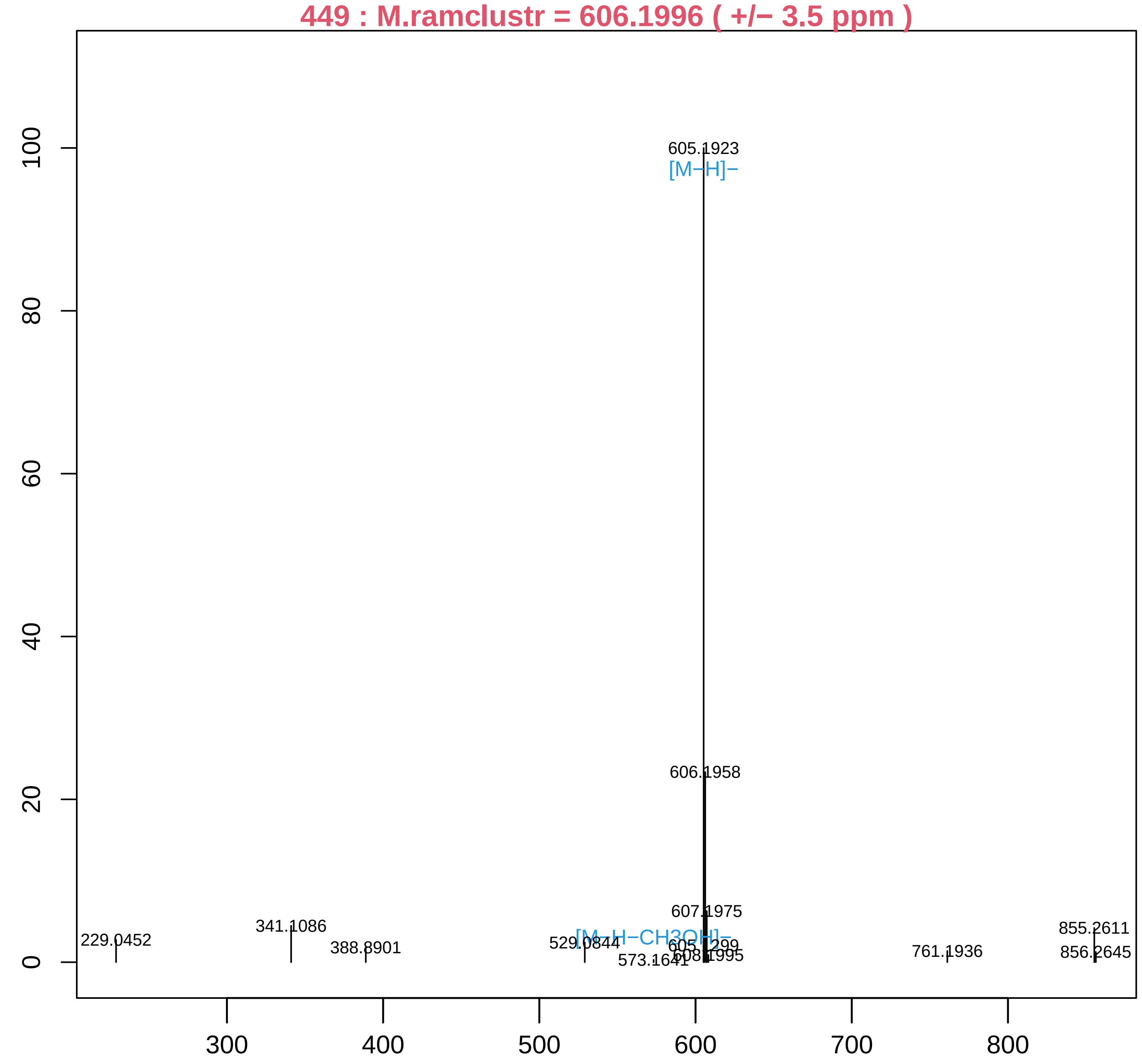
449 : M.ramclustr = 606.1996 (+/− 3.5 ppm)
605.1923
[M−H]−
606.1958
607.1975
229.0452
341.1086
388.8901
529.0844
[M−H−CH3OH]−
573.1641
605.1299
608.1995
761.1936
855.2611
856.2645
0
20
40
60
80
100
300
400
500
600
700
800
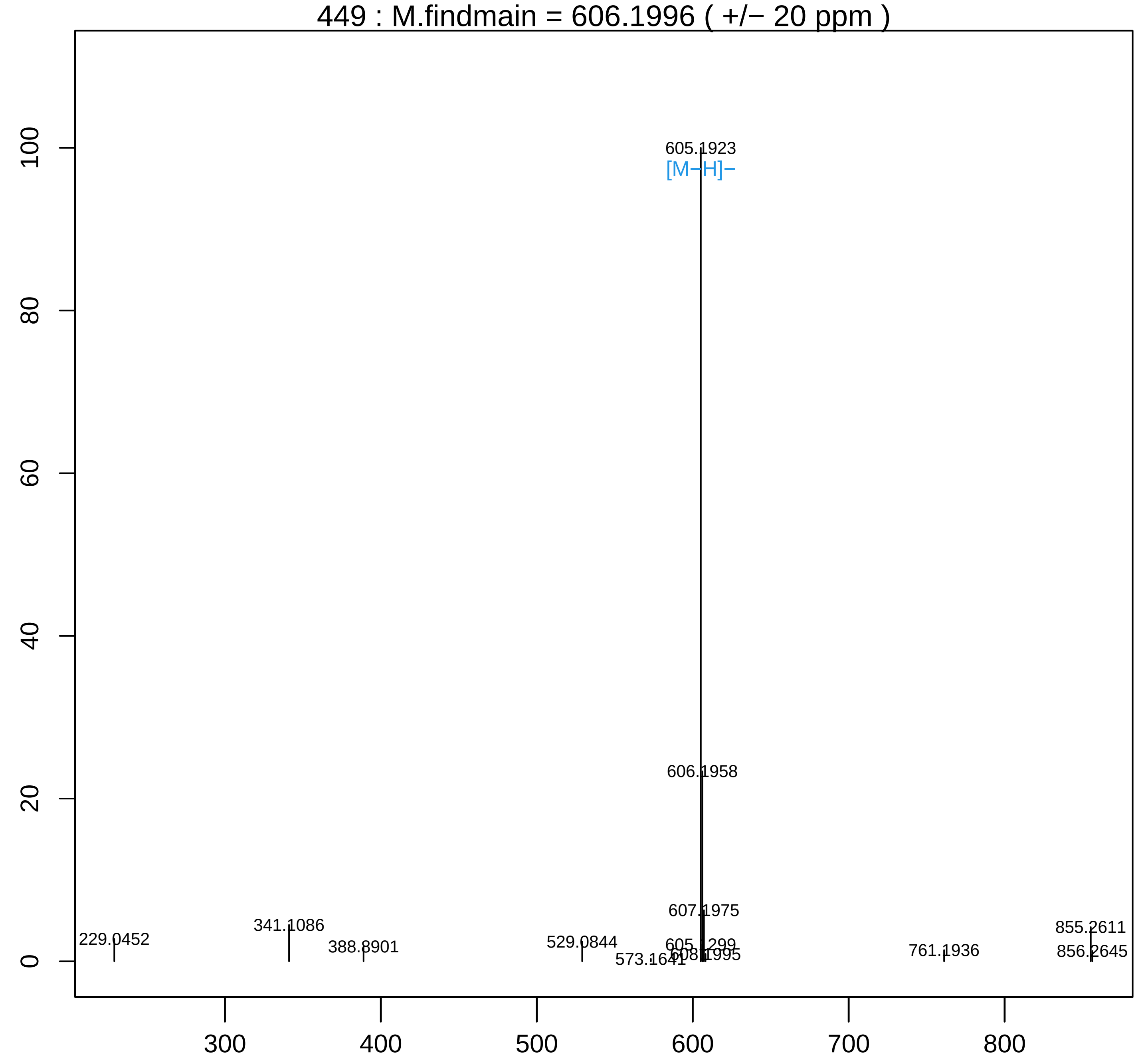
449 : M.findmain = 606.1996 (+/− 20 ppm)
605.1923
[M−H]−
606.1958
607.1975
229.0452
341.1086
388.8901
529.0844
573.1641
605.1299
608.1995
761.1936
855.2611
856.2645
0
20
40
60
80
100
300
400
500
600
700
800

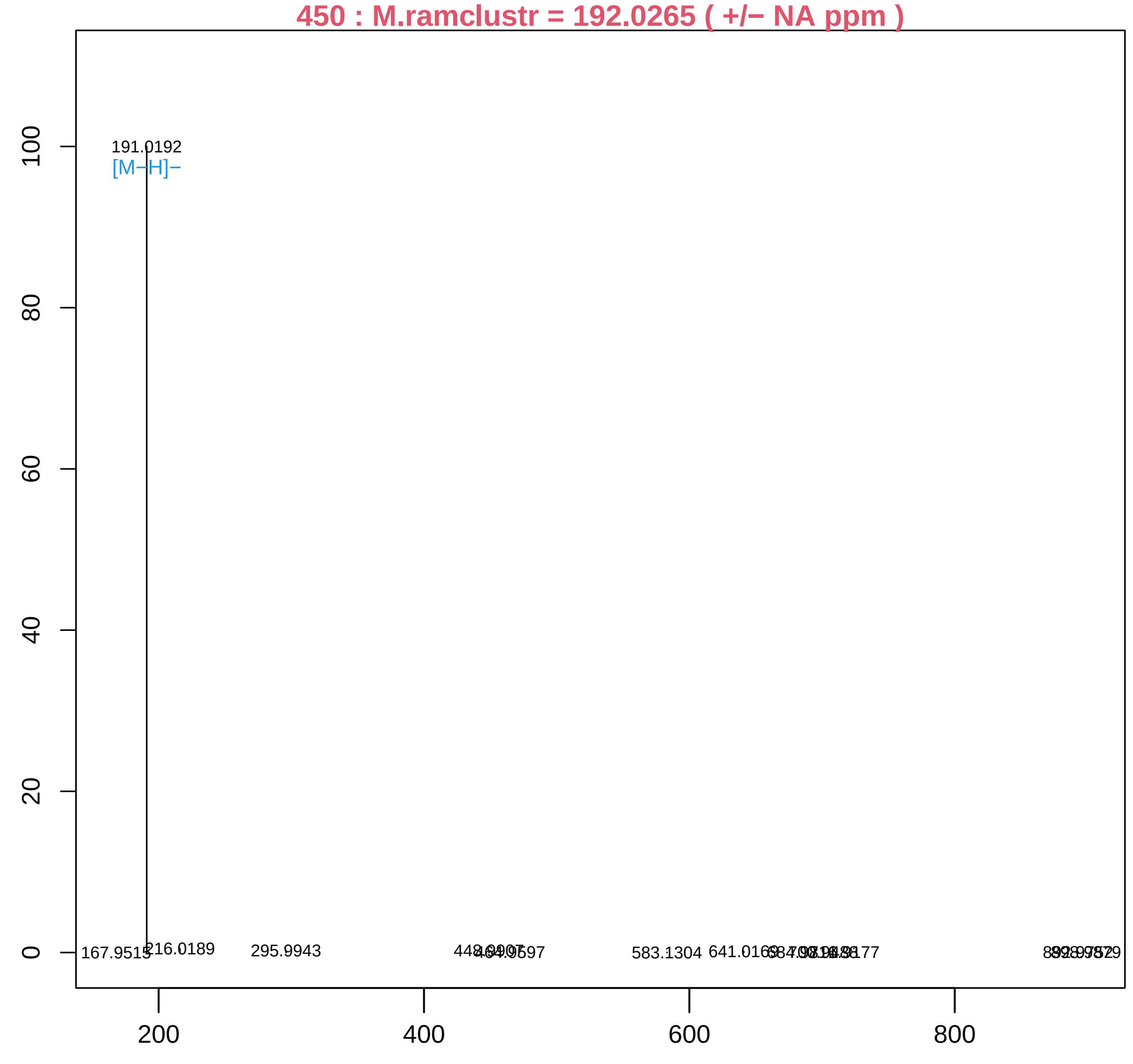
450 : M.ramclustr = 192.0265 (+/− NA ppm)
191.0192
[M−H]−
167.9515
216.0189
295.9943
583.1304
0
20
40
60
80
100
200
400
600
800

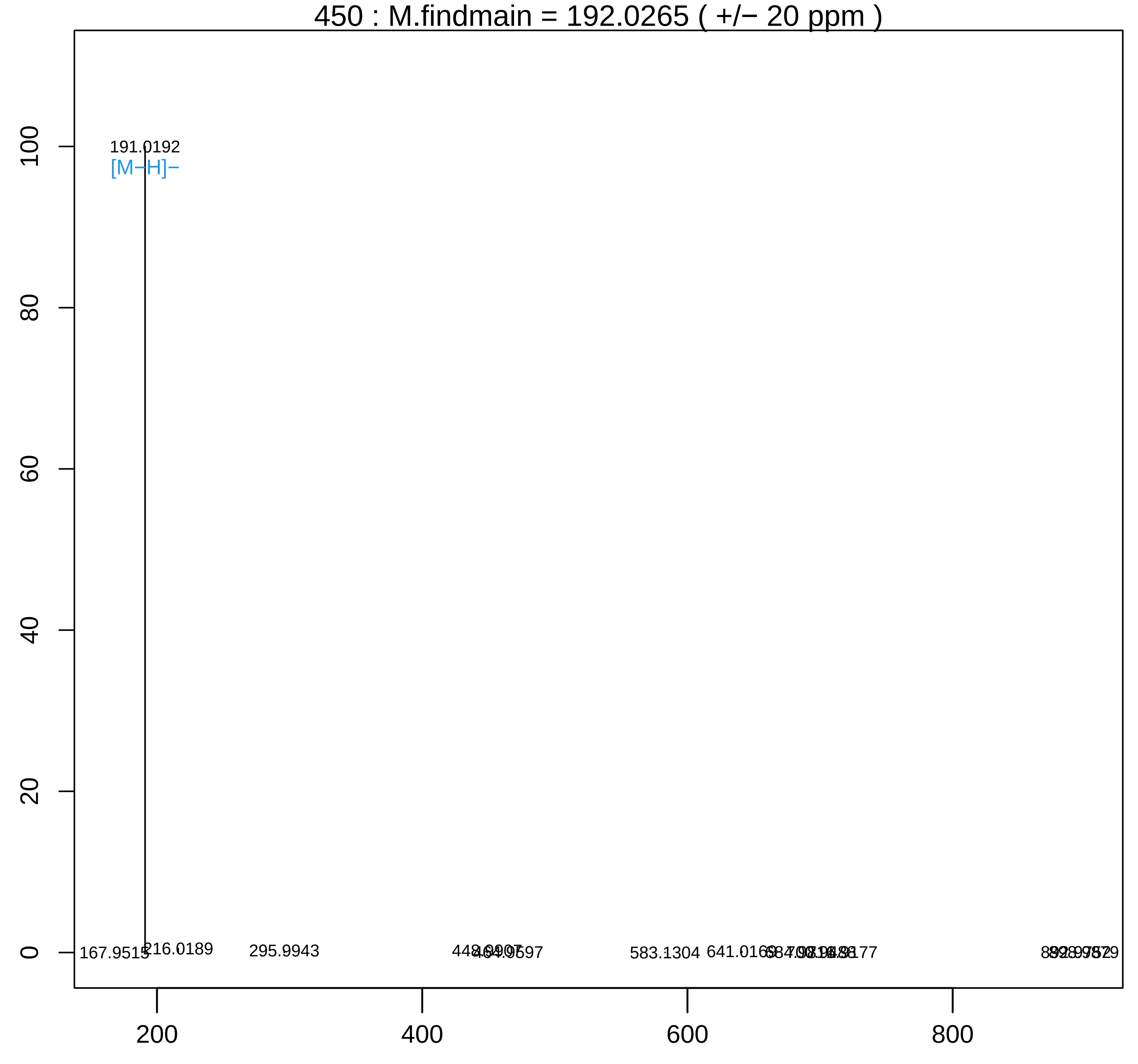
450 : M.findmain = 192.0265 (+/− 20 ppm)
191.0192
[M−H]−
167.9515
216.0189
295.9943
583.1304
0
20
40
60
80
100
200
400
600
800

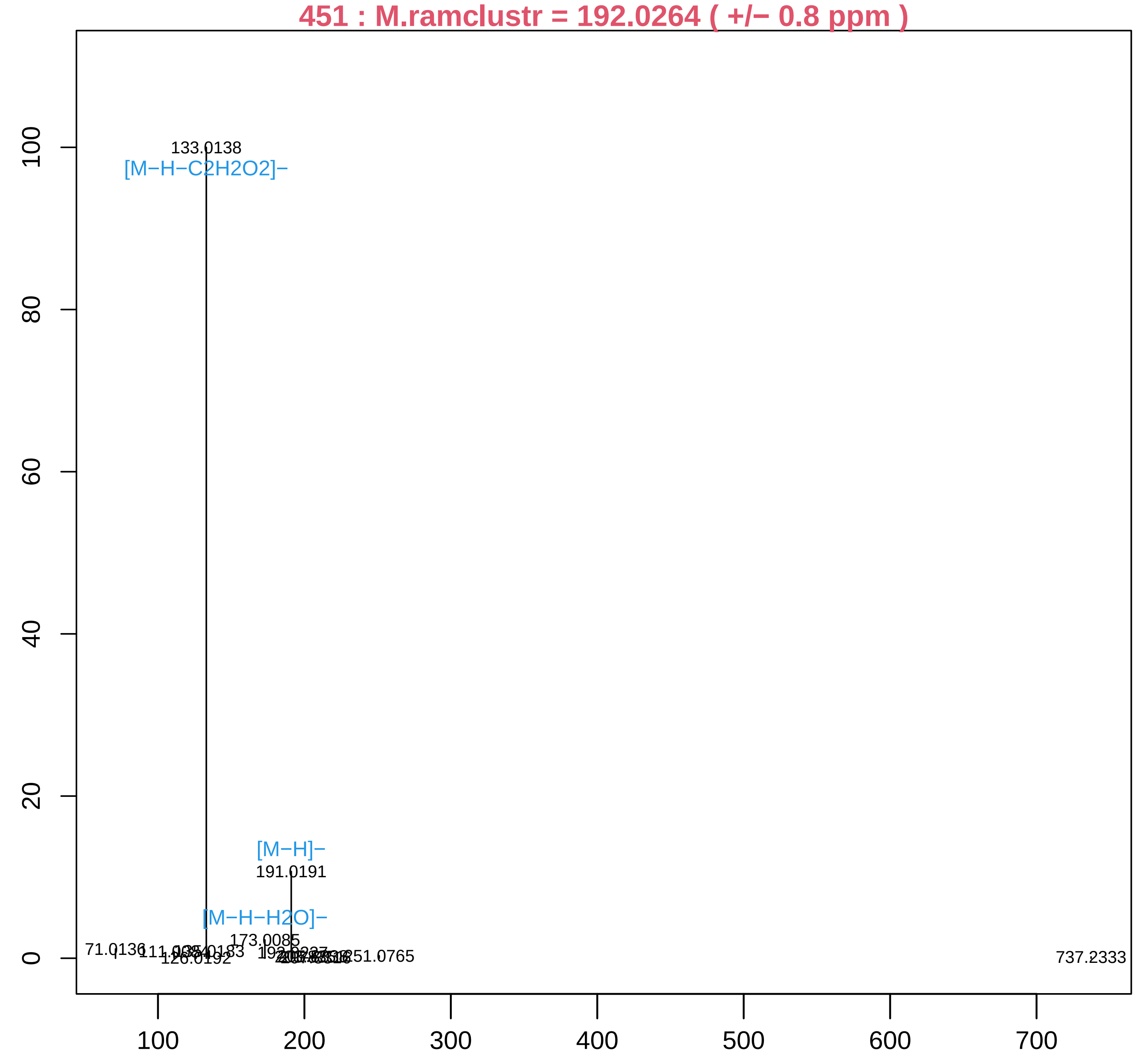
451 : M.ramclustr = 192.0264 (+/− 0.8 ppm)
133.0138
[M−H−C2H2O2]−
[M−H]−
191.0191
[M−H−H2O]−
173.0085
71.0136
126.0192
737.2333
0
20
40
60
80
100
100
200
300
400
500
600
700

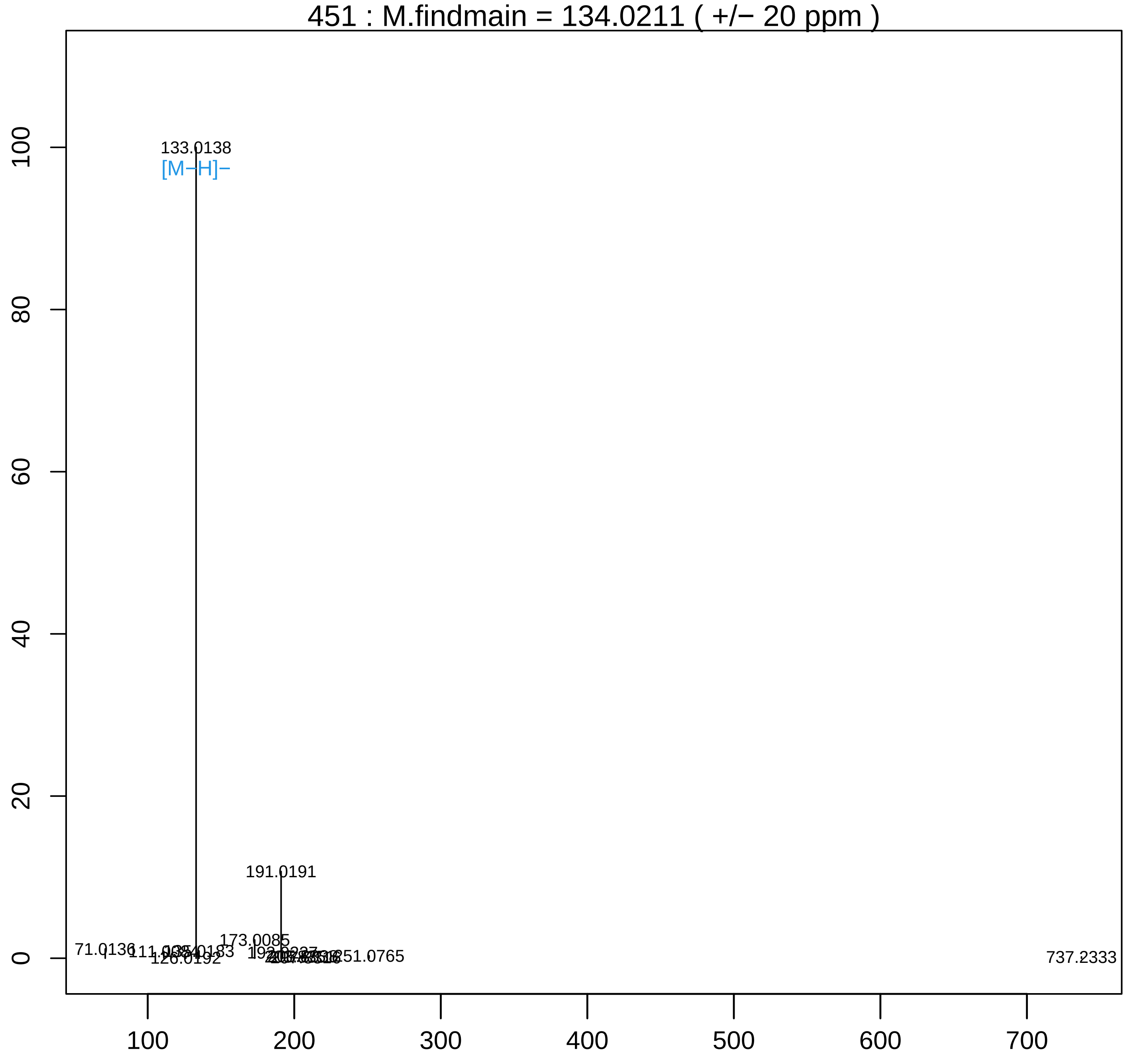
451 : M.findmain = 134.0211 (+/− 20 ppm)
133.0138
[M−H]−
191.0191
173.0085
71.0136
126.0192
737.2333
0
20
40
60
80
100
100
200
300
400
500
600
700

452 : M.ramclustr = 918.6277 (+/− 0.1 ppm)

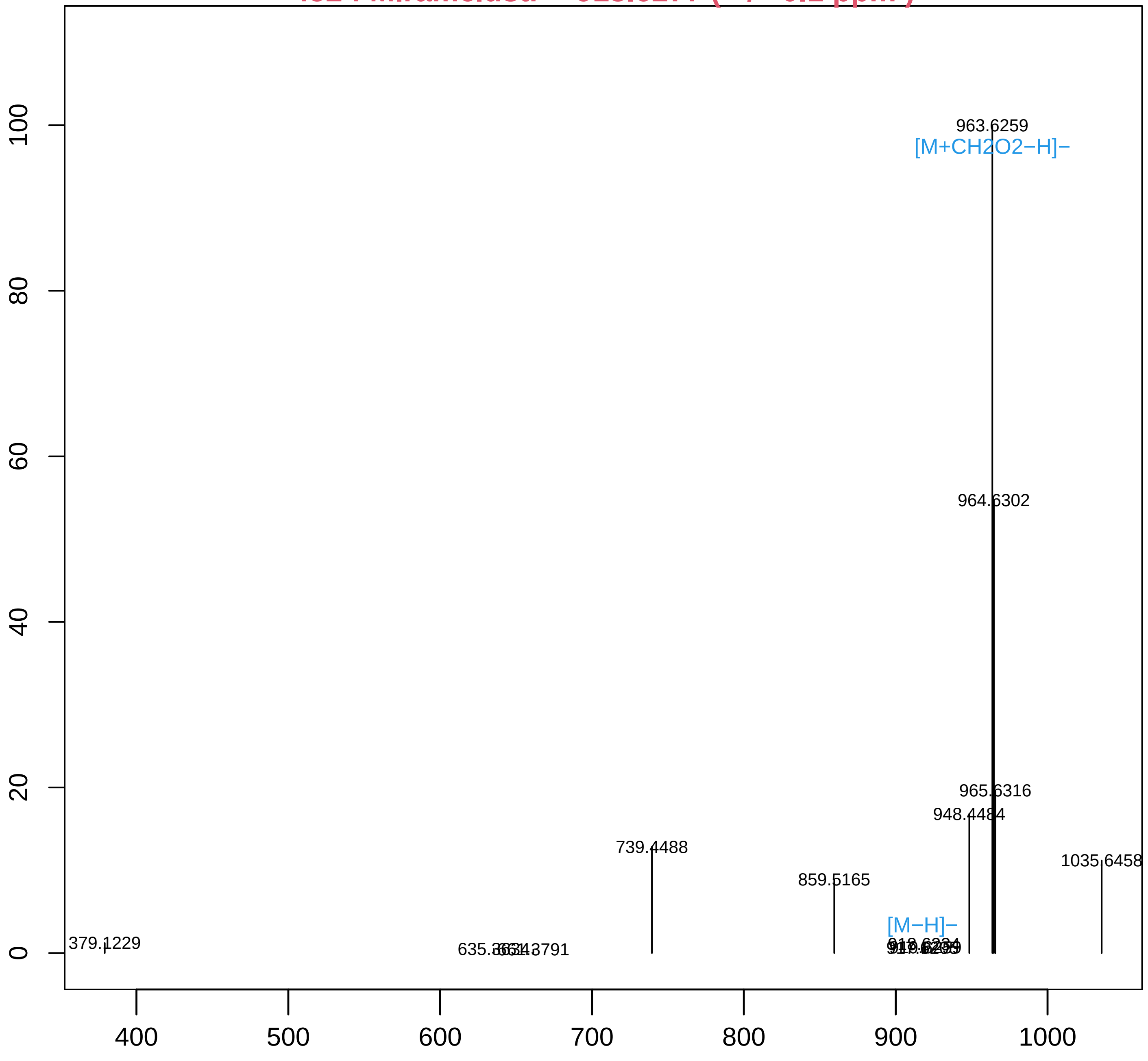

452 : M.findmain = 918.6277 (+/− 0.1 ppm)

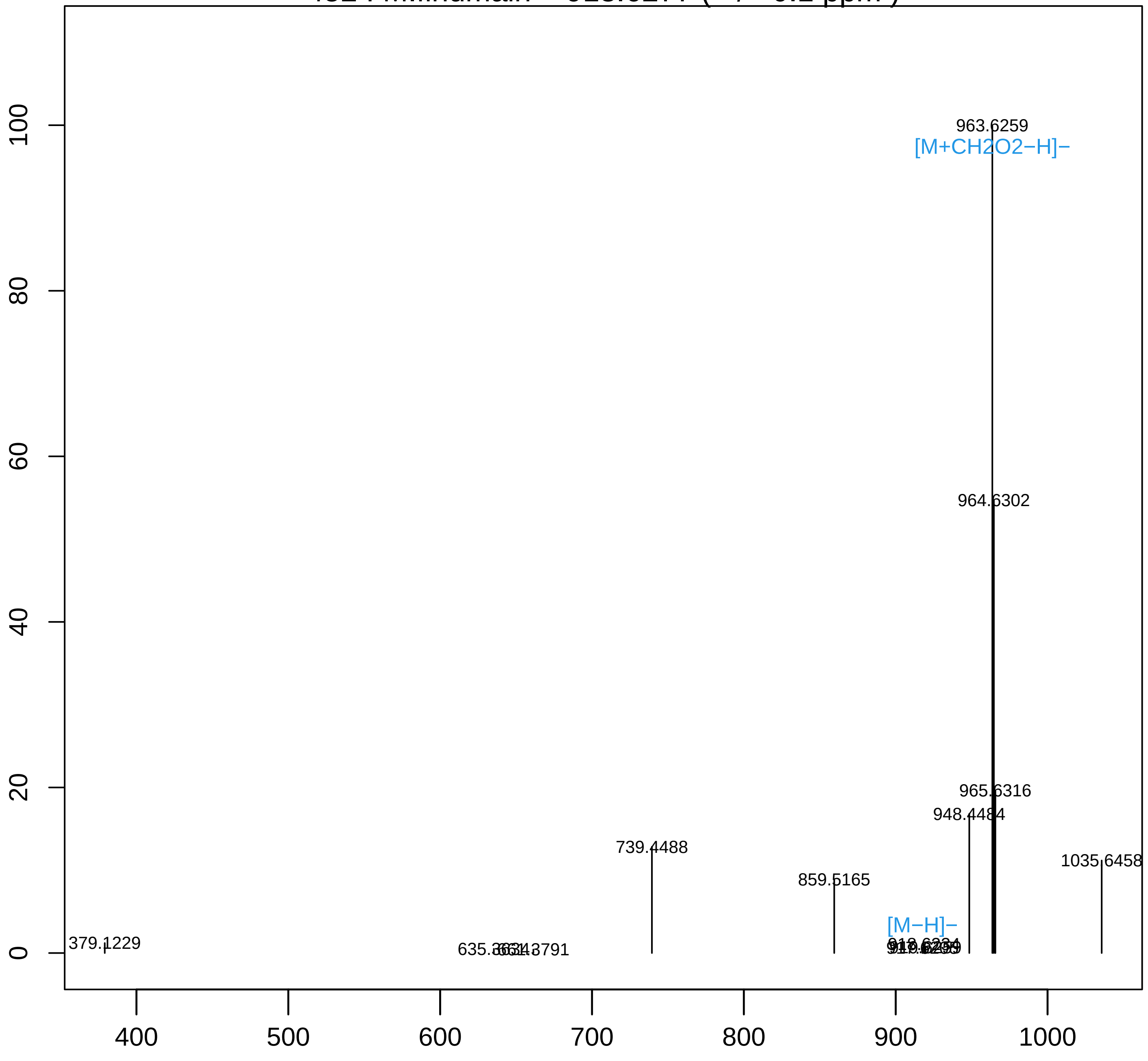

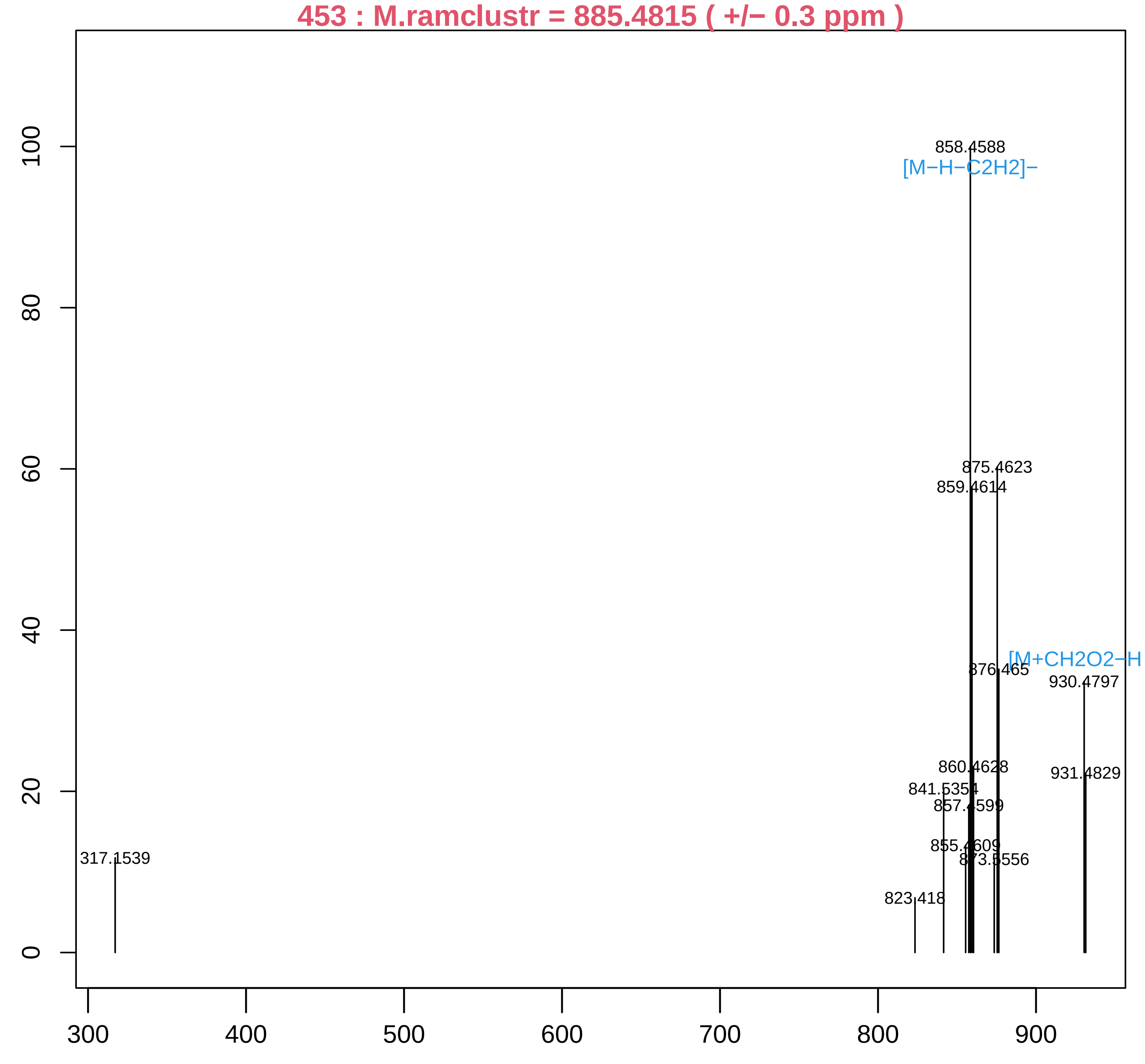

453 : M.ramclustr = 885.4815 (+/− 0.3 ppm)
858.4588
[M−H−C2H2]−
875.4623
859.4614
[M+CH2O2−H
876.465
930.4797
860.4628
931.4829
841.5354
857.4599
855.4609
873.5556
317.1539
823.418
0
20
40
60
80
100
300
400
500
600
700
800
900

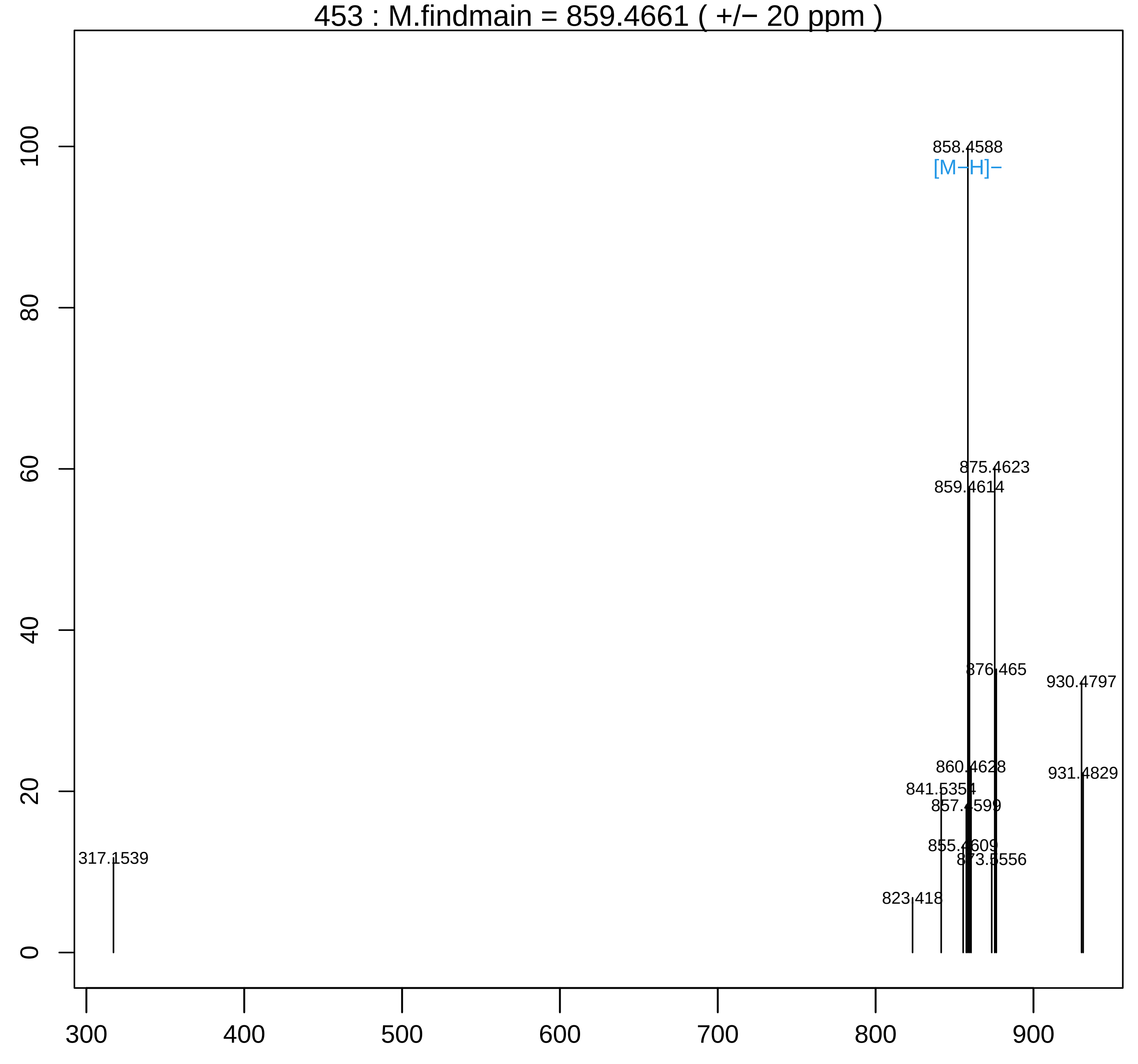

453 : M.findmain = 859.4661 (+/− 20 ppm)
858.4588
[M−H]−
875.4623
859.4614
876.465
930.4797
860.4628
931.4829
841.5354
857.4599
855.4609
873.5556
317.1539
823.418
0
20
40
60
80
100
300
400
500
600
700
800
900

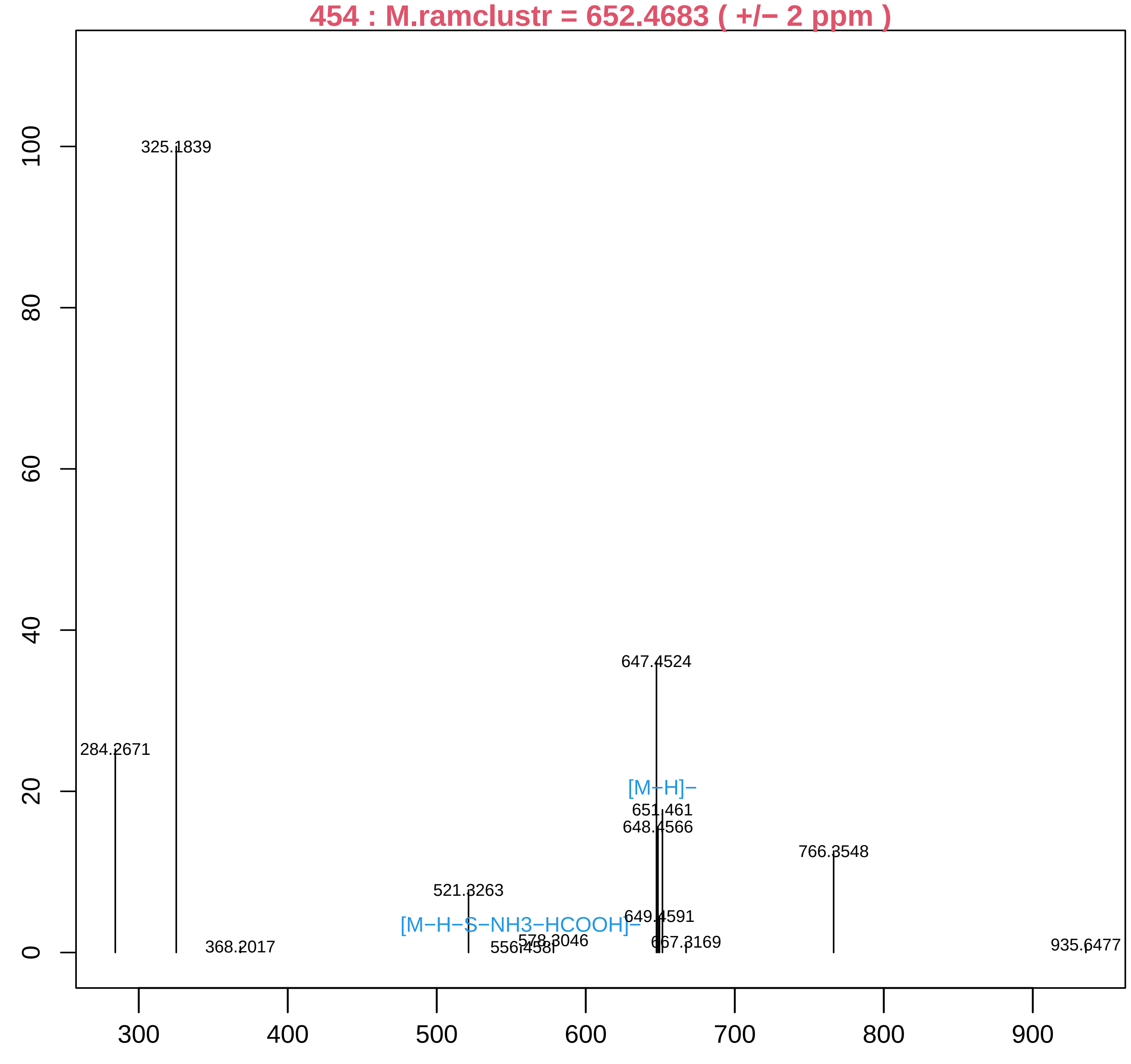
454 : M.ramclustr = 652.4683 (+/− 2 ppm)
325.1839
647.4524
284.2671
[M−H]−
651.461
648.4566
766.3548
521.3263
[M−H−S−NH3−HCOOH]−
649.4591
578.3046
556.458
667.3169
368.2017
935.6477
0
20
40
60
80
100
300
400
500
600
700
800
900
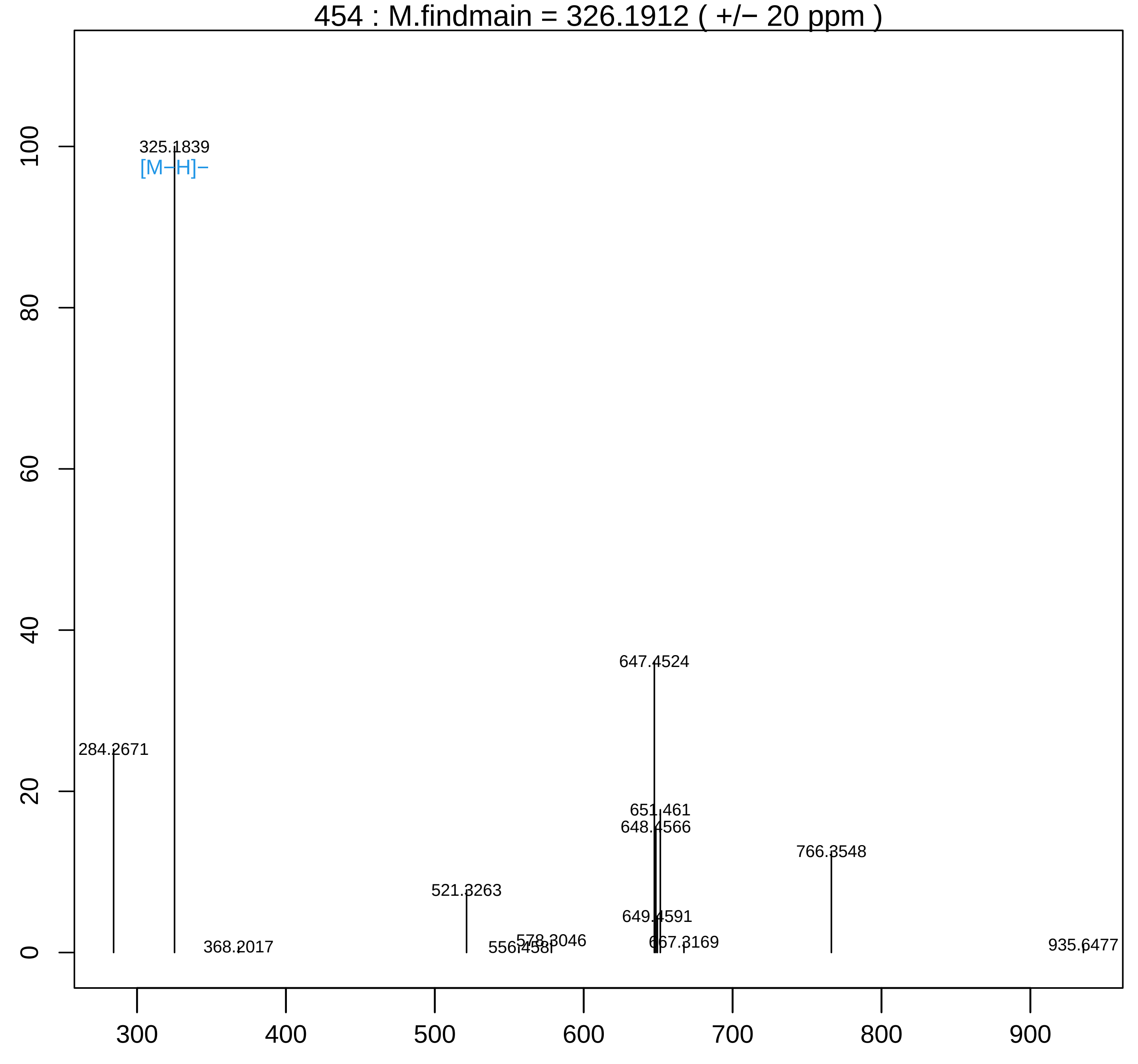
454 : M.findmain = 326.1912 (+/− 20 ppm)
325.1839
[M−H]−
647.4524
284.2671
651.461
648.4566
766.3548
521.3263
649.4591
578.3046
556.458
667.3169
368.2017
935.6477
0
20
40
60
80
100
300
400
500
600
700
800
900

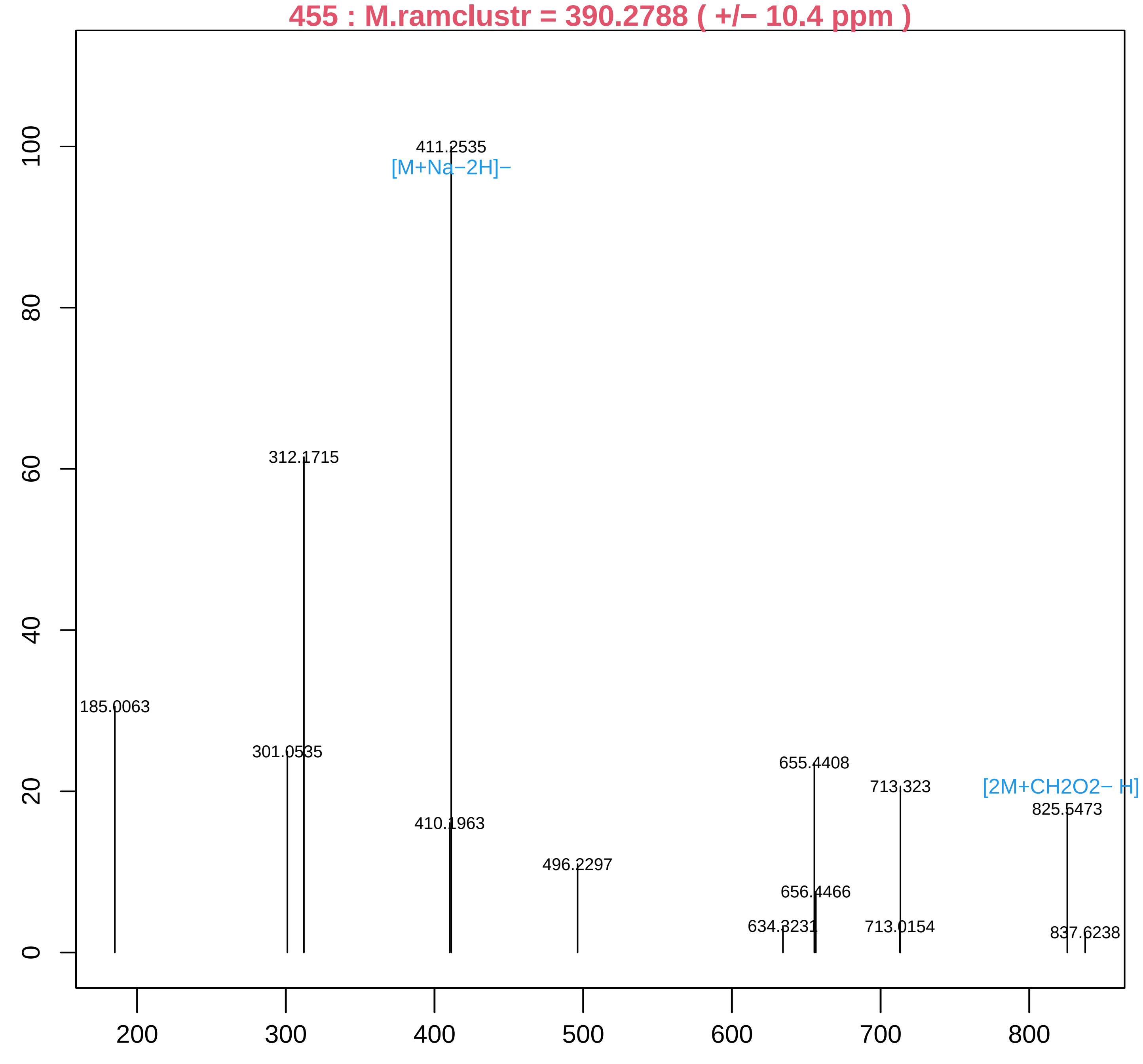
455 : M.ramclustr = 390.2788 (+/− 10.4 ppm)
411.2535
[M+Na−2H]−
312.1715
185.0063
301.0535
655.4408
713.323
[2M+CH2O2− H]
825.5473
410.1963
496.2297
656.4466
634.3231
713.0154
837.6238
0
20
40
60
80
100
200
300
400
500
600
700
800

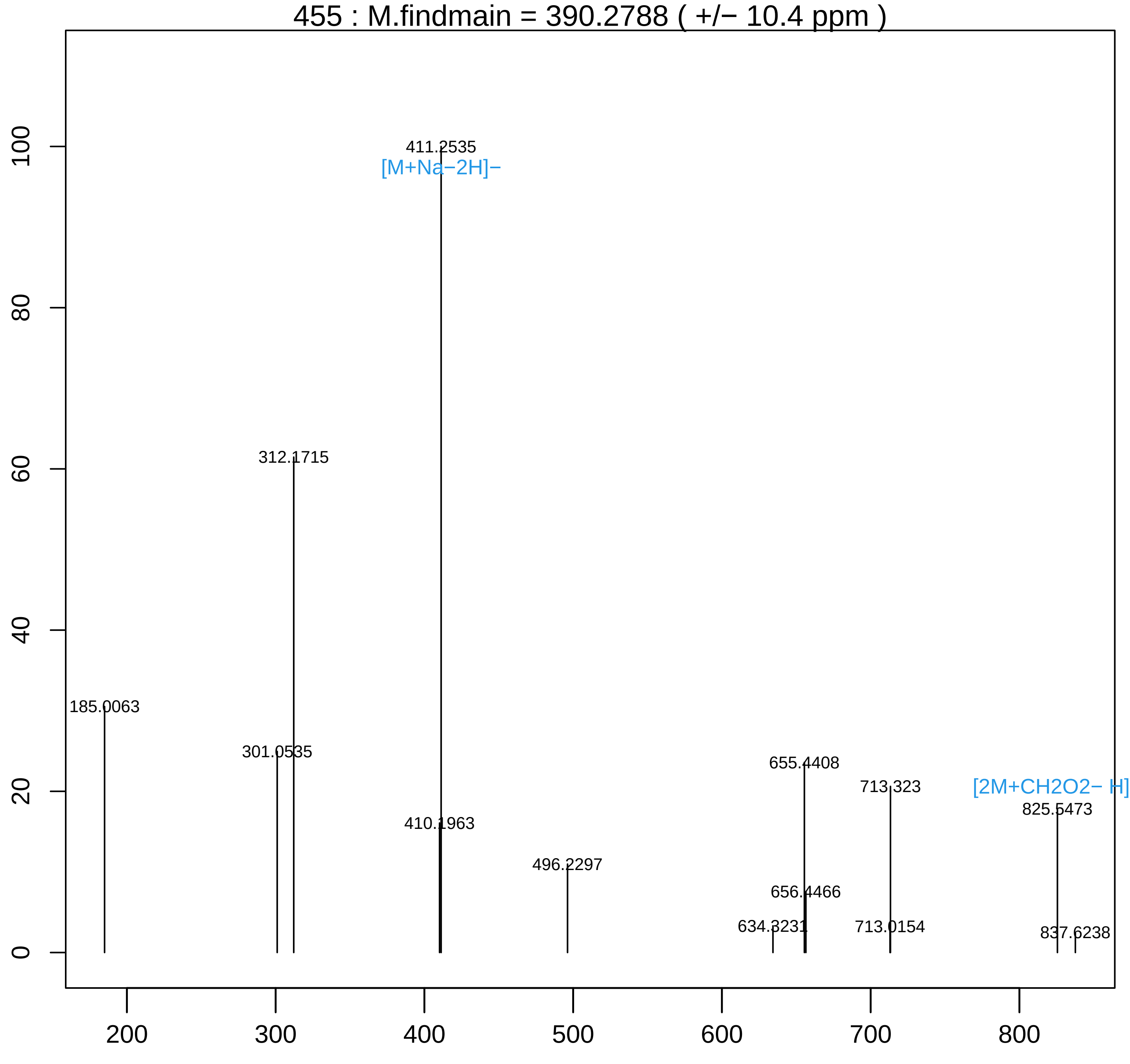
455 : M.findmain = 390.2788 (+/− 10.4 ppm)
411.2535
[M+Na−2H]−
312.1715
185.0063
301.0535
655.4408
713.323
[2M+CH2O2− H]
825.5473
410.1963
496.2297
656.4466
634.3231
713.0154
837.6238
0
20
40
60
80
100
200
300
400
500
600
700
800

456 : M.ramclustr = 852.5224 (+/− 0.3 ppm)

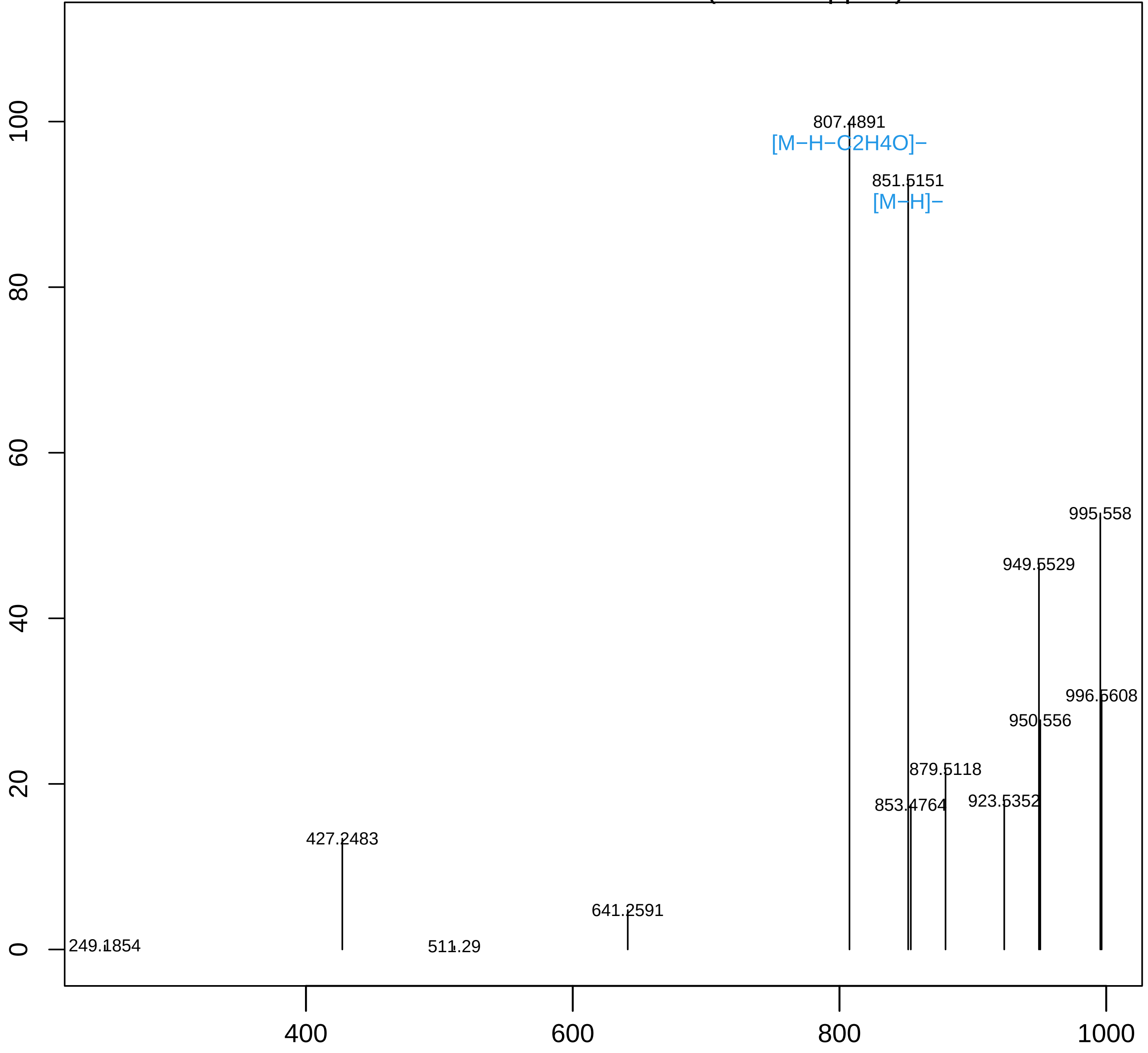

456 : M.findmain = 950.5598 (+/− 0.4 ppm)

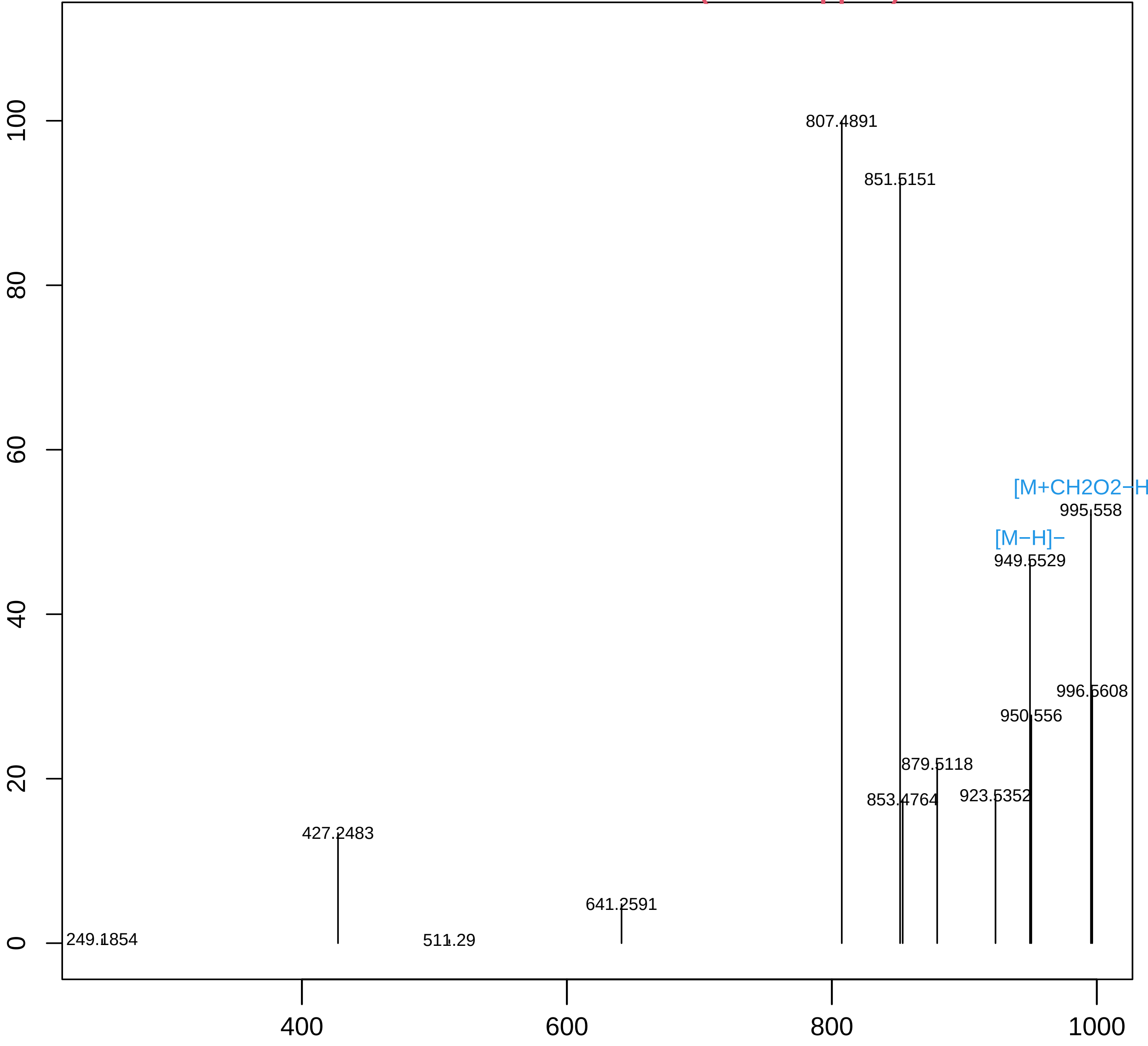

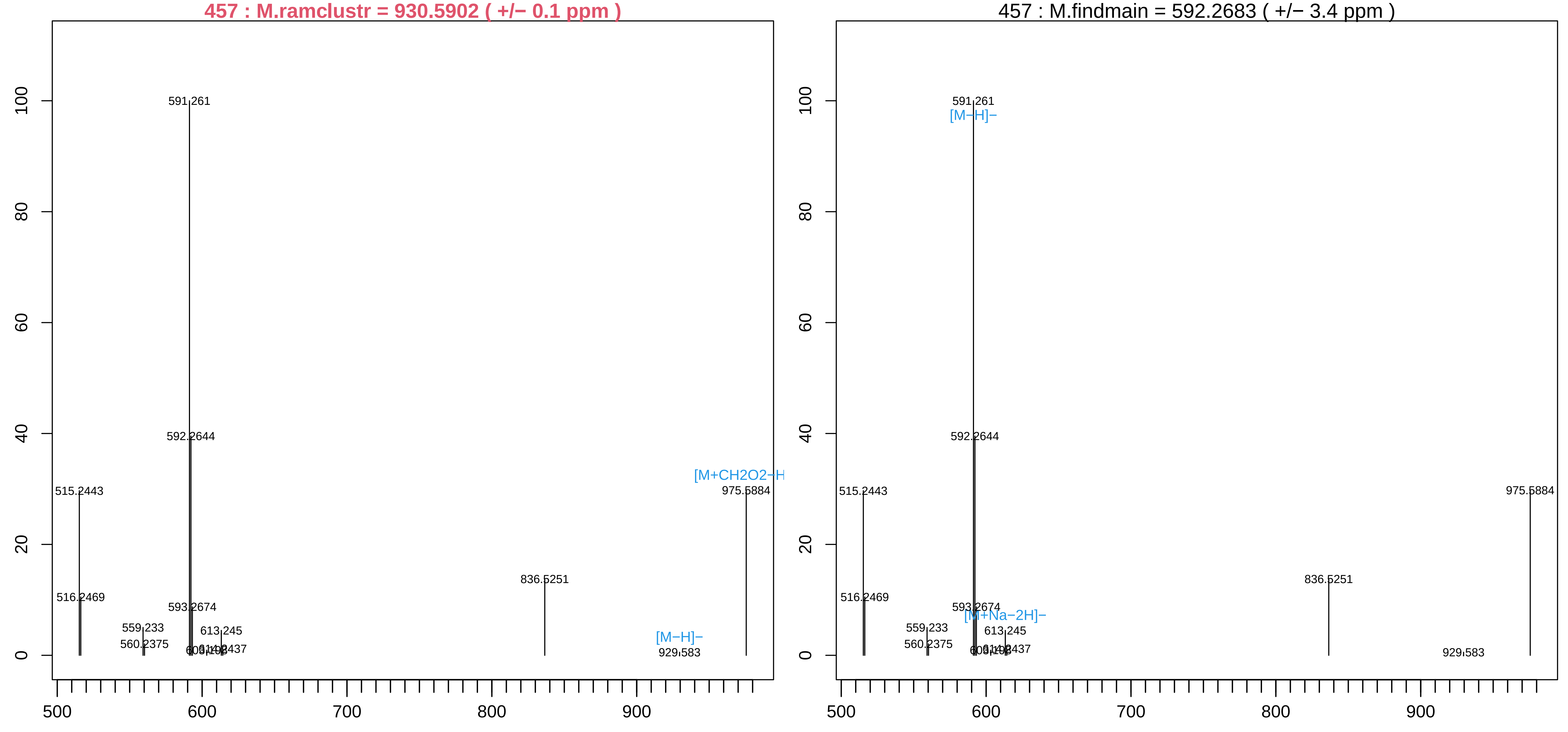

457 : M.ramclustr = 930.5902 (+/− 0.1 ppm)
591.261
592.2644
515.2443
516.2469
559.233
560.2375
593.2674
613.245
614.2437
836.5251
[M+CH2O2−H
975.5884
[M−H]−
929.583
457 : M.findmain = 592.2683 (+/− 3.4 ppm)
591.261
[M−H]−
592.2644
515.2443
516.2469
559.233
560.2375
593.2674
[M+Na−2H]−
613.245
614.2437
836.5251
929.583
975.5884
0
20
40
60
80
100
500
600
700
800
900

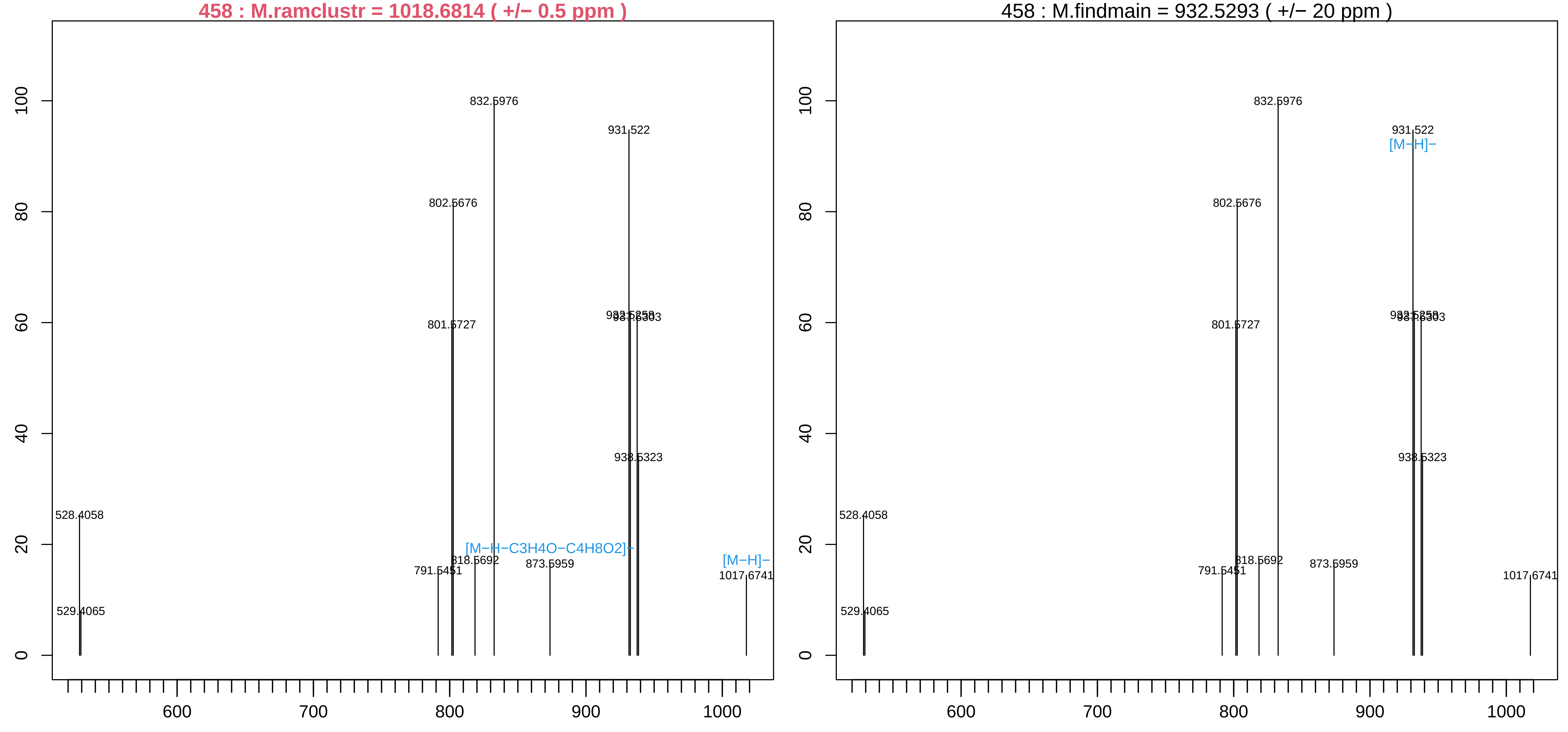
458 : M.ramclustr = 1018.6814 (+/− 0.5 ppm)
458 : M.findmain = 932.5293 (+/− 20 ppm)
832.5976
931.522
802.5676
801.5727
938.5323
528.4058
529.4065
791.5451
818.5692
873.5959
1017.6741
[M−H−C3H4O−C4H8O2]−
[M−H]−

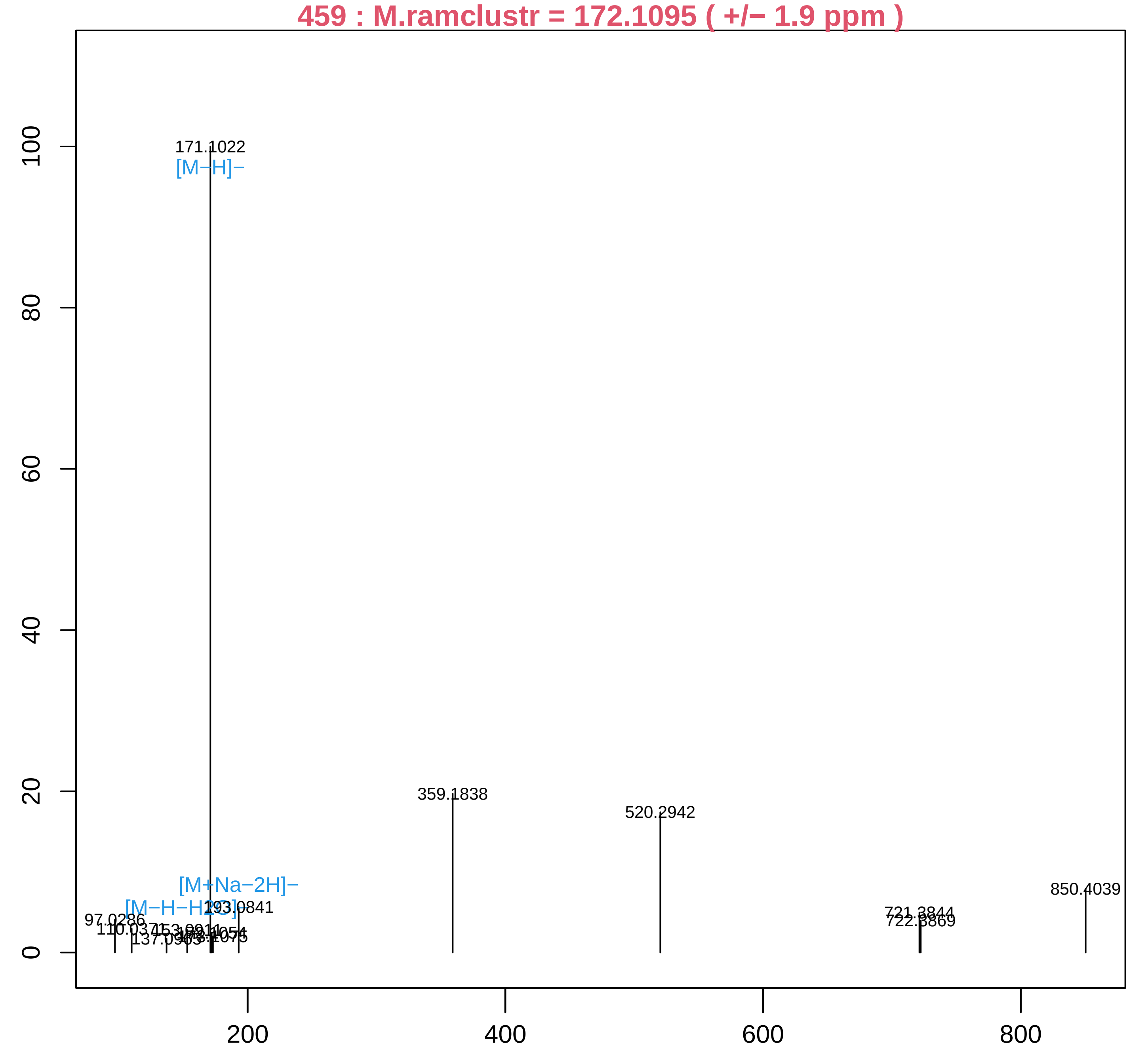
459 : M.ramclustr = 172.1095 (+/− 1.9 ppm)
171.1022
[M−H]−
359.1838
520.2942
850.4039
721.3844
722.3869
[M+Na−2H]−
193.0841
[M−H−H2O]−
97.0286
110.0371
153.0911
137.0903
0
20
40
60
80
100
200
400
600
800

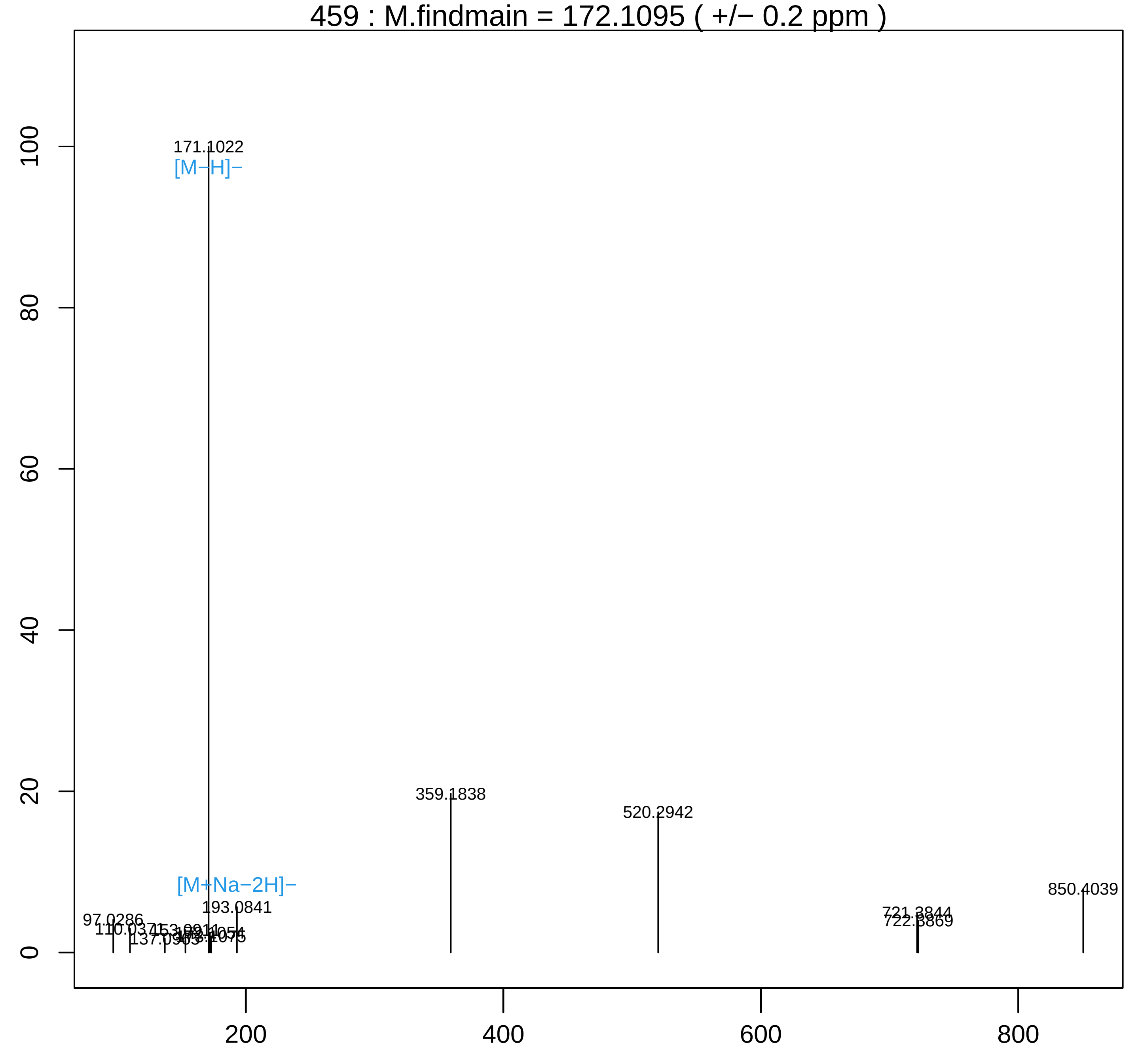
459 : M.findmain = 172.1095 (+/− 0.2 ppm)
171.1022
[M−H]−
359.1838
520.2942
850.4039
721.3844
722.3869
[M+Na−2H]−
193.0841
97.0286
110.0371
153.0911
137.0903
0
20
40
60
80
100
200
400
600
800

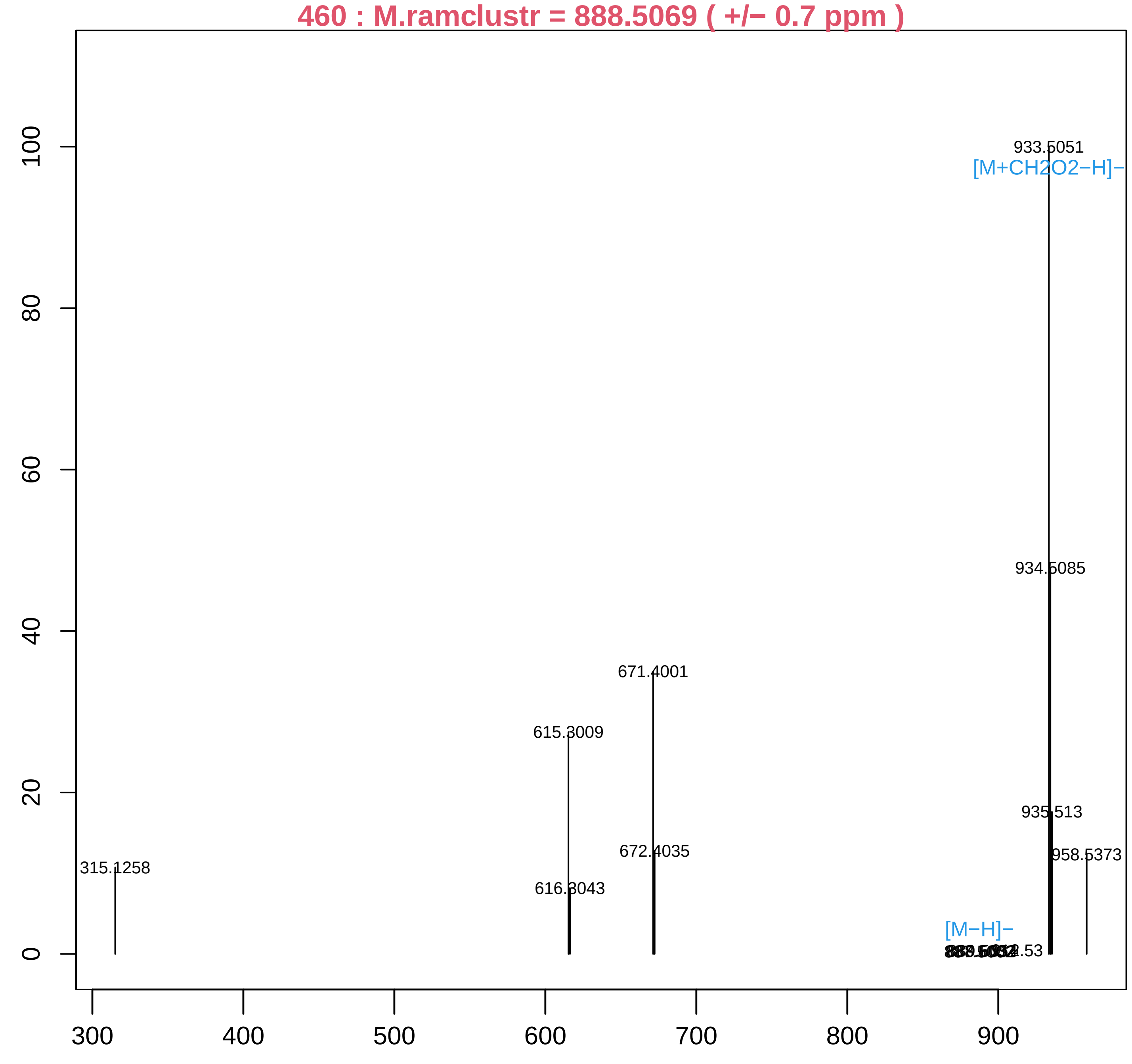

460 : M.ramclustr = 888.5069 (+/− 0.7 ppm)
933.5051
[M+CH2O2−H]−
934.5085
671.4001
615.3009
935.513
672.4035
958.5373
315.1258
616.3043
[M−H]−
0
20
40
60
80
100
300
400
500
600
700
800
900

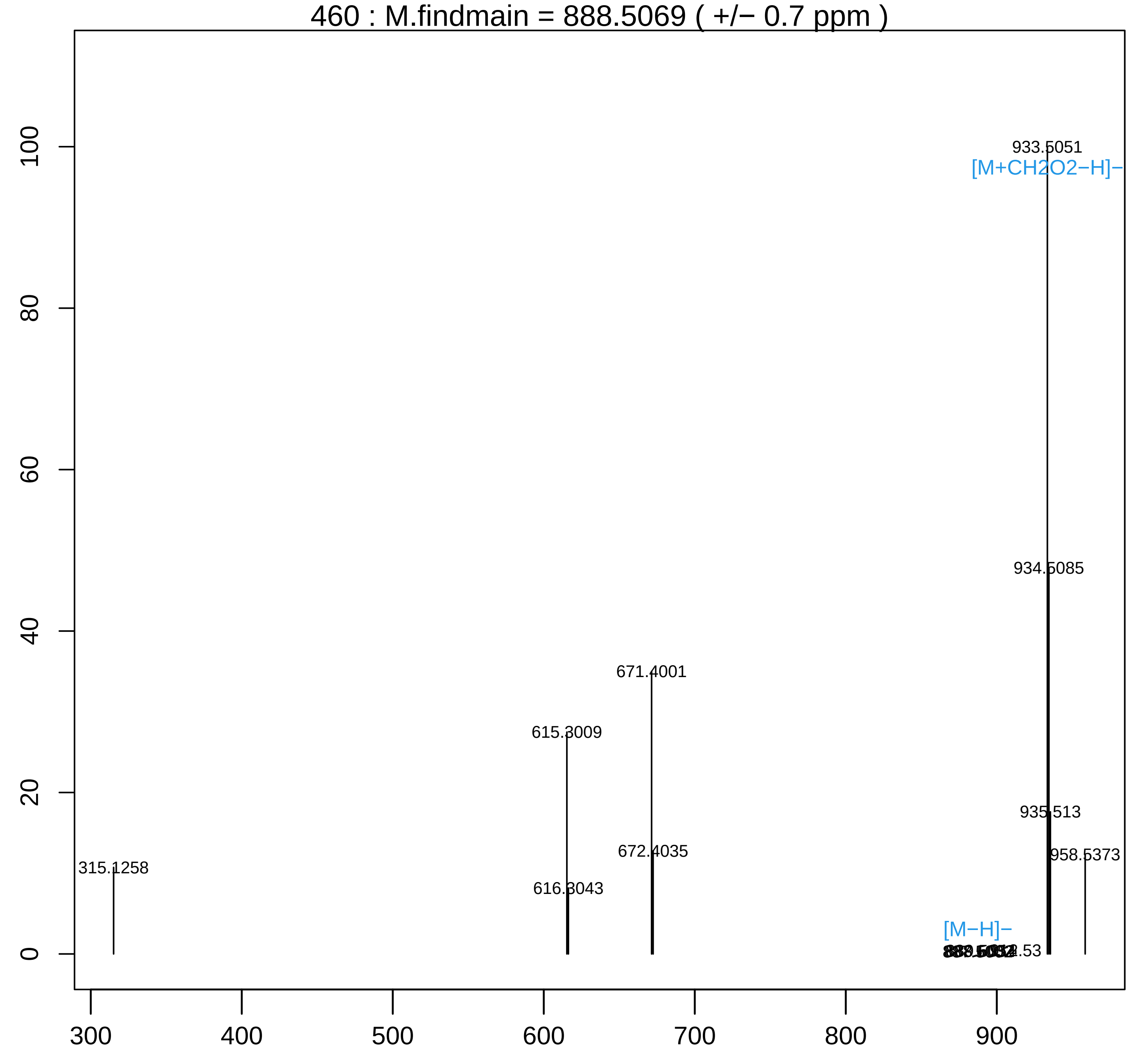

460 : M.findmain = 888.5069 (+/− 0.7 ppm)
933.5051
[M+CH2O2−H]−
934.5085
671.4001
615.3009
935.513
672.4035
958.5373
315.1258
616.3043
[M−H]−
0
20
40
60
80
100
300
400
500
600
700
800
900

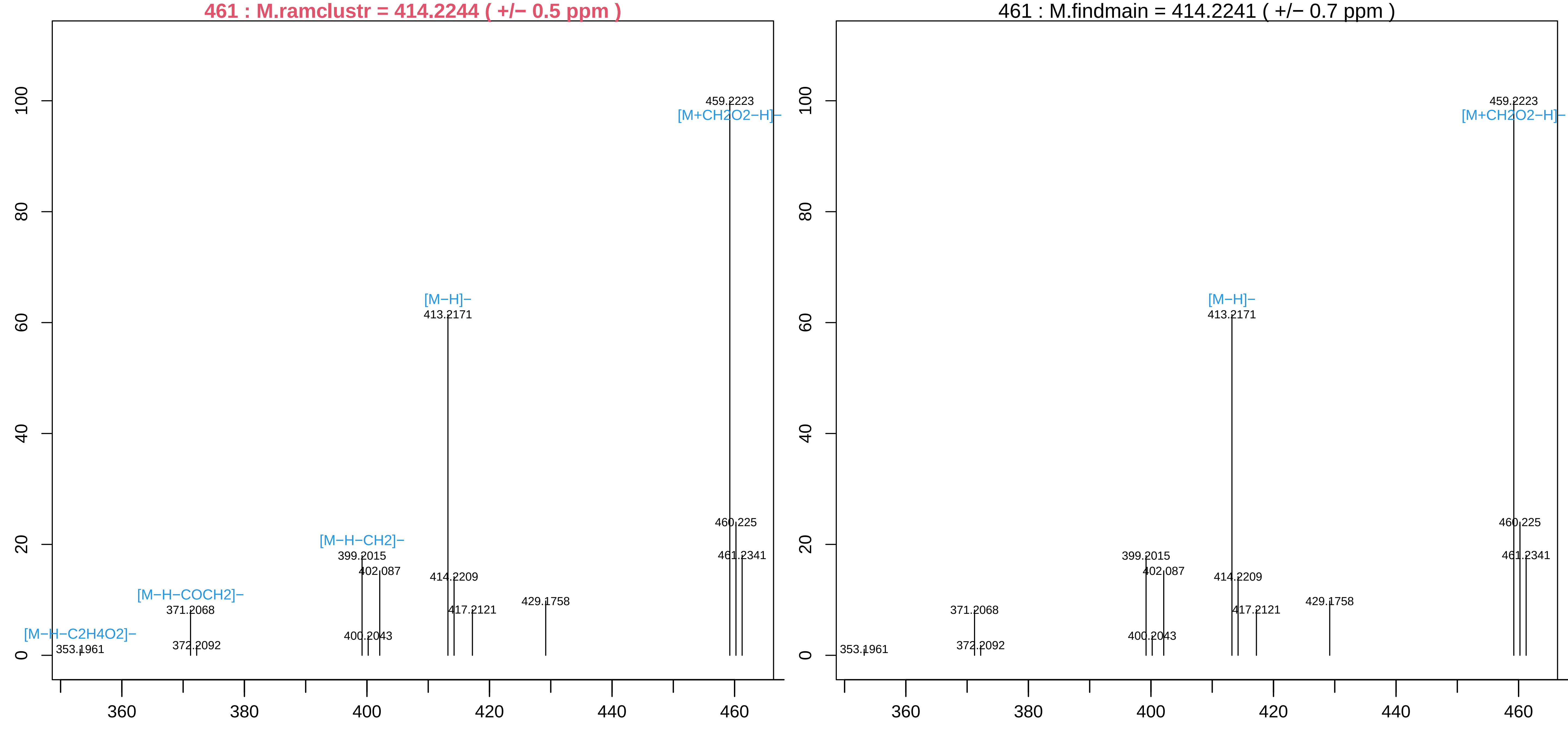
461 : M.ramclustr = 414.2244 (+/− 0.5 ppm)
459.2223
[M+CH2O2−H]−
[M−H]−
413.2171
460.225
461.2341
[M−H−CH2]−
399.2015
402.087
414.2209
[M−H−COCH2]−
371.2068
417.2121
429.1758
[M−H−C2H4O2]−
353.1961
372.2092
400.2043
461 : M.findmain = 414.2241 (+/− 0.7 ppm)
459.2223
[M+CH2O2−H]−
[M−H]−
413.2171
460.225
461.2341
399.2015
402.087
414.2209
371.2068
417.2121
429.1758
353.1961
372.2092
400.2043
0
20
40
60
80
100
360
380
400
420
440
460

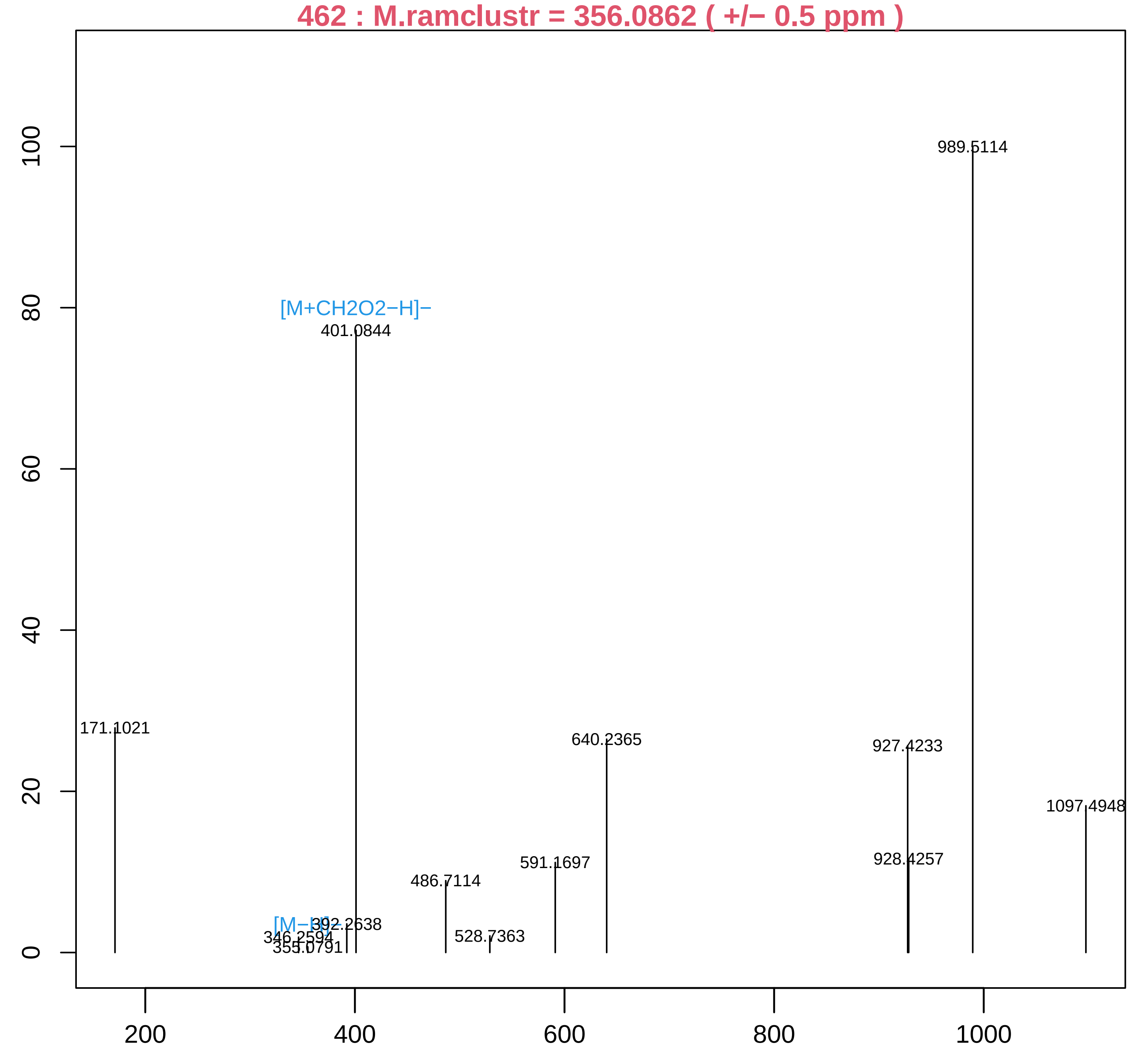
462 : M.ramclustr = 356.0862 (+/− 0.5 ppm)
[M+CH2O2−H]−
401.0844
989.5114
171.1021
640.2365
927.4233
1097.4948
928.4257
591.1697
486.7114
[M−H]−
392.2638
346.2594
355.0791
528.7363
0
20
40
60
80
100
200
400
600
800
1000

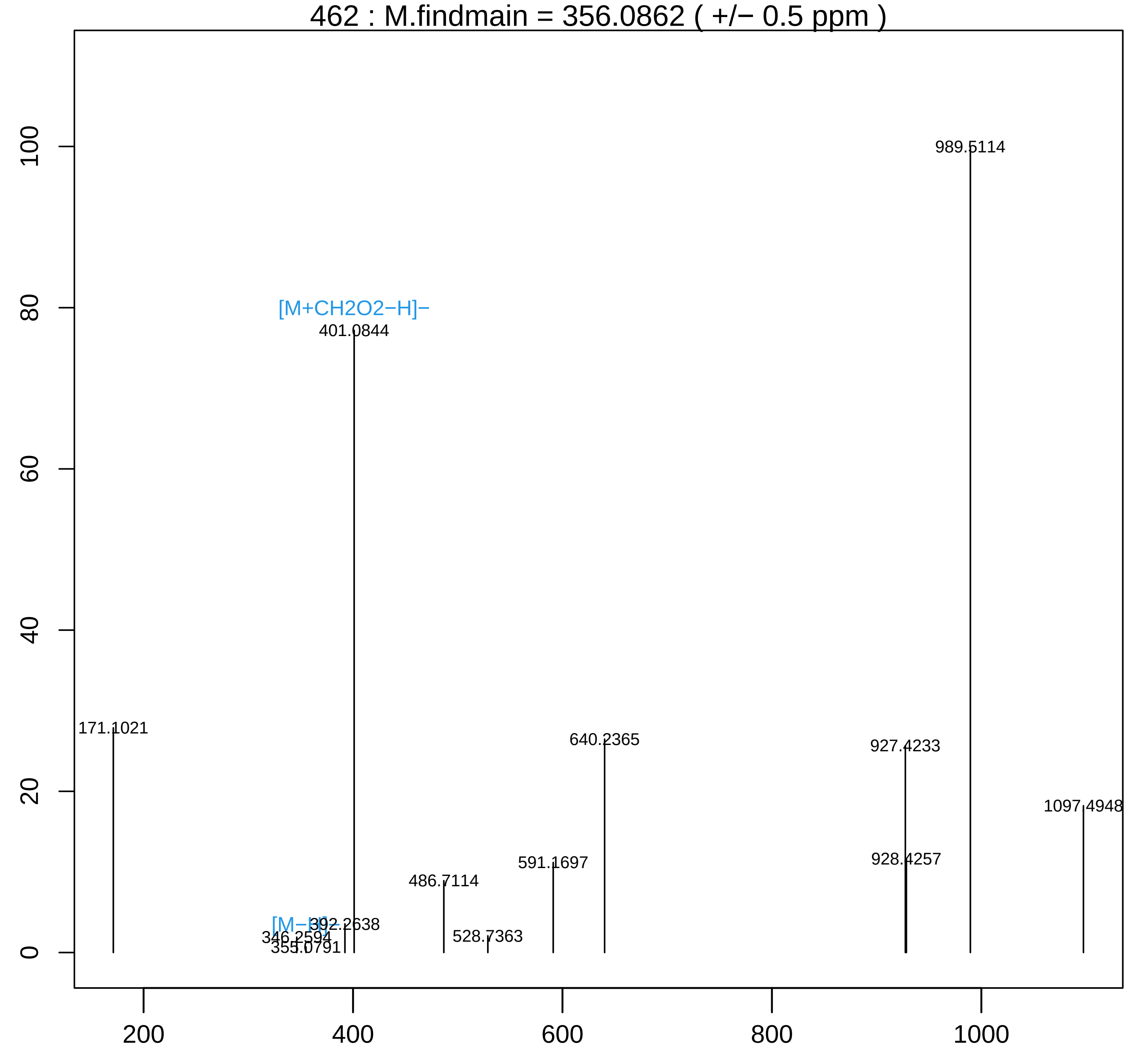
462 : M.findmain = 356.0862 (+/− 0.5 ppm)
[M+CH2O2−H]−
401.0844
989.5114
171.1021
640.2365
927.4233
1097.4948
928.4257
591.1697
486.7114
[M−H]−
392.2638
346.2594
355.0791
528.7363
0
20
40
60
80
100
200
400
600
800
1000

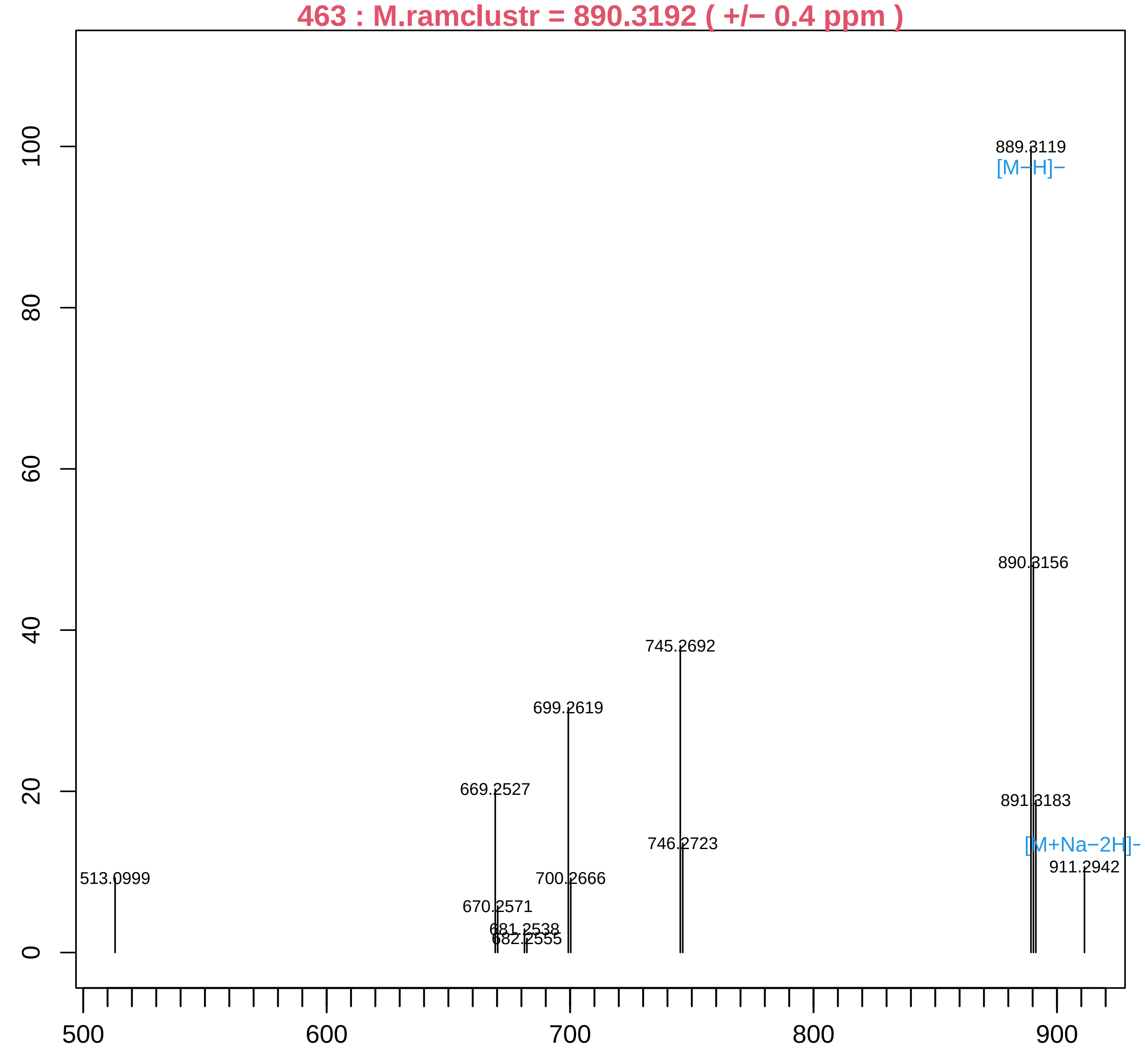
463 : M.ramclustr = 890.3192 (+/− 0.4 ppm)
889.3119
[M−H]−
890.3156
745.2692
699.2619
669.2527
891.3183
746.2723
[M+Na−2H]-
911.2942
513.0999
700.2666
670.2571
681.2538
682.2555
0
20
40
60
80
100
500
600
700
800
900

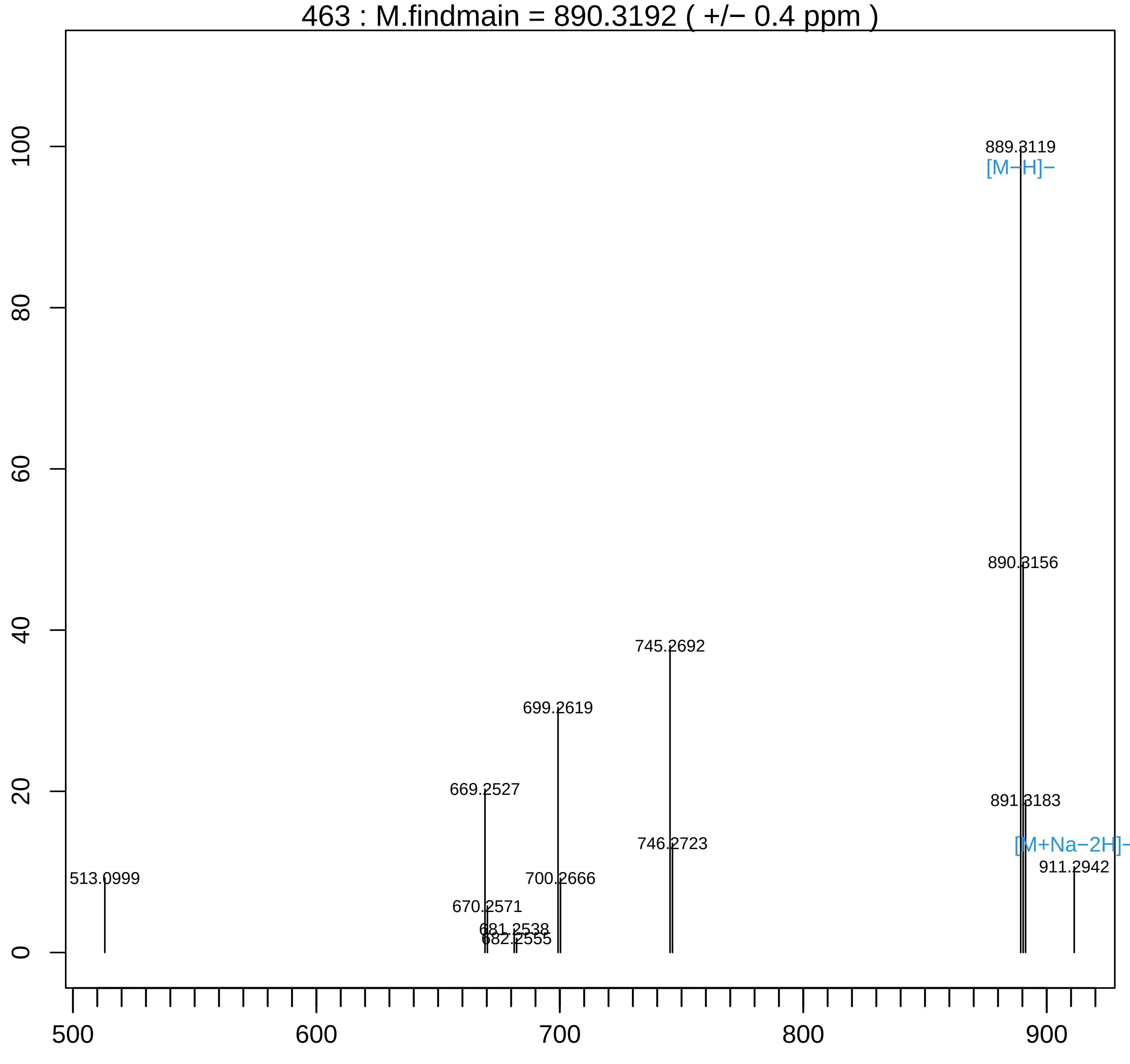
463 : M.findmain = 890.3192 (+/− 0.4 ppm)
889.3119
[M−H]−
890.3156
745.2692
699.2619
669.2527
891.3183
746.2723
[M+Na−2H]-
911.2942
513.0999
700.2666
670.2571
681.2538
682.2555
0
20
40
60
80
100
500
600
700
800
900

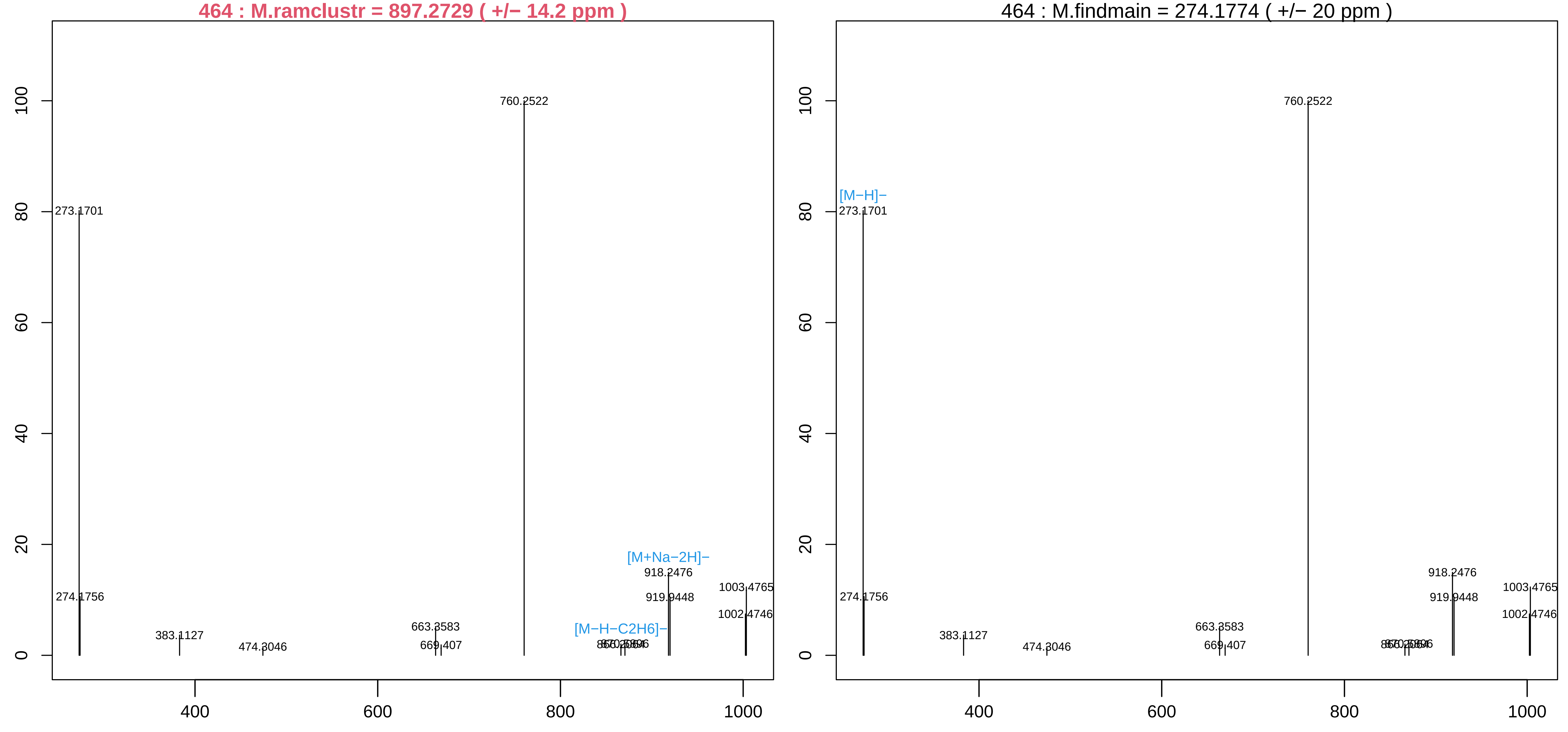

464 : M.ramclustr = 897.2729 (+/− 14.2 ppm)
273.1701
274.1756
383.1127
474.3046
663.3583
669.407
760.2522
[M−H−C2H6]−
[M+Na−2H]−
918.2476
919.9448
1003.4765
1002.4746
464 : M.findmain = 274.1774 (+/− 20 ppm)
[M−H]−
273.1701
274.1756
383.1127
474.3046
663.3583
669.407
760.2522
918.2476
919.9448
1003.4765
1002.4746
0
20
40
60
80
100
400
600
800
1000

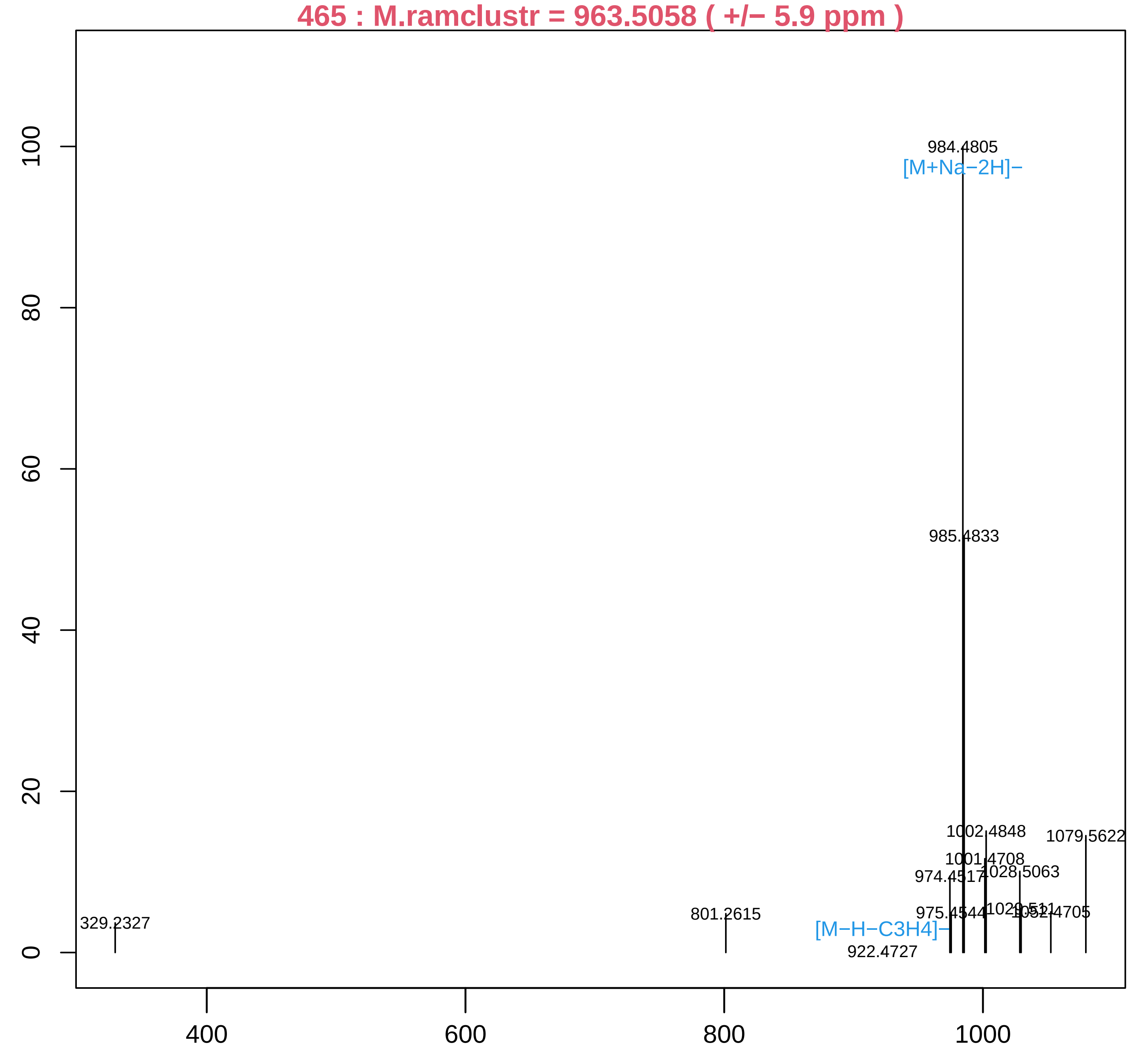
465 : M.ramclustr = 963.5058 (+/− 5.9 ppm)
984.4805
[M+Na−2H]−
985.4833
1002.4848
1079.5622
1001.4708
974.4517
1028.5063
975.4544
1029.511
1052.4705
801.2615
329.2327
[M−H−C3H4]−
922.4727
0
20
40
60
80
100
400
600
800
1000

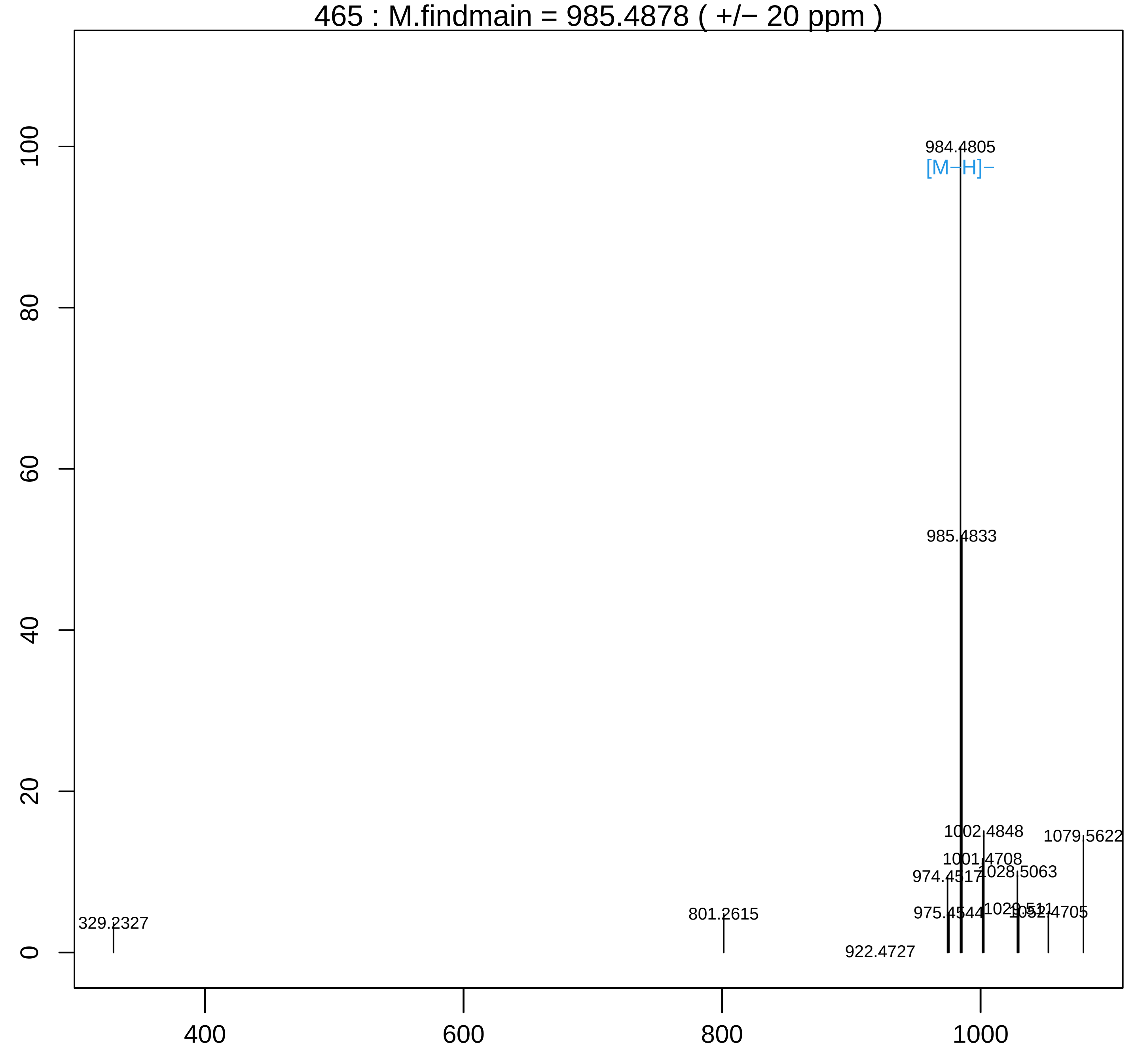
465 : M.findmain = 985.4878 (+/− 20 ppm)
984.4805
[M−H]−
985.4833
1002.4848
1079.5622
1001.4708
974.4517
1028.5063
975.4544
1029.511
1052.4705
801.2615
329.2327
922.4727
0
20
40
60
80
100
400
600
800
1000

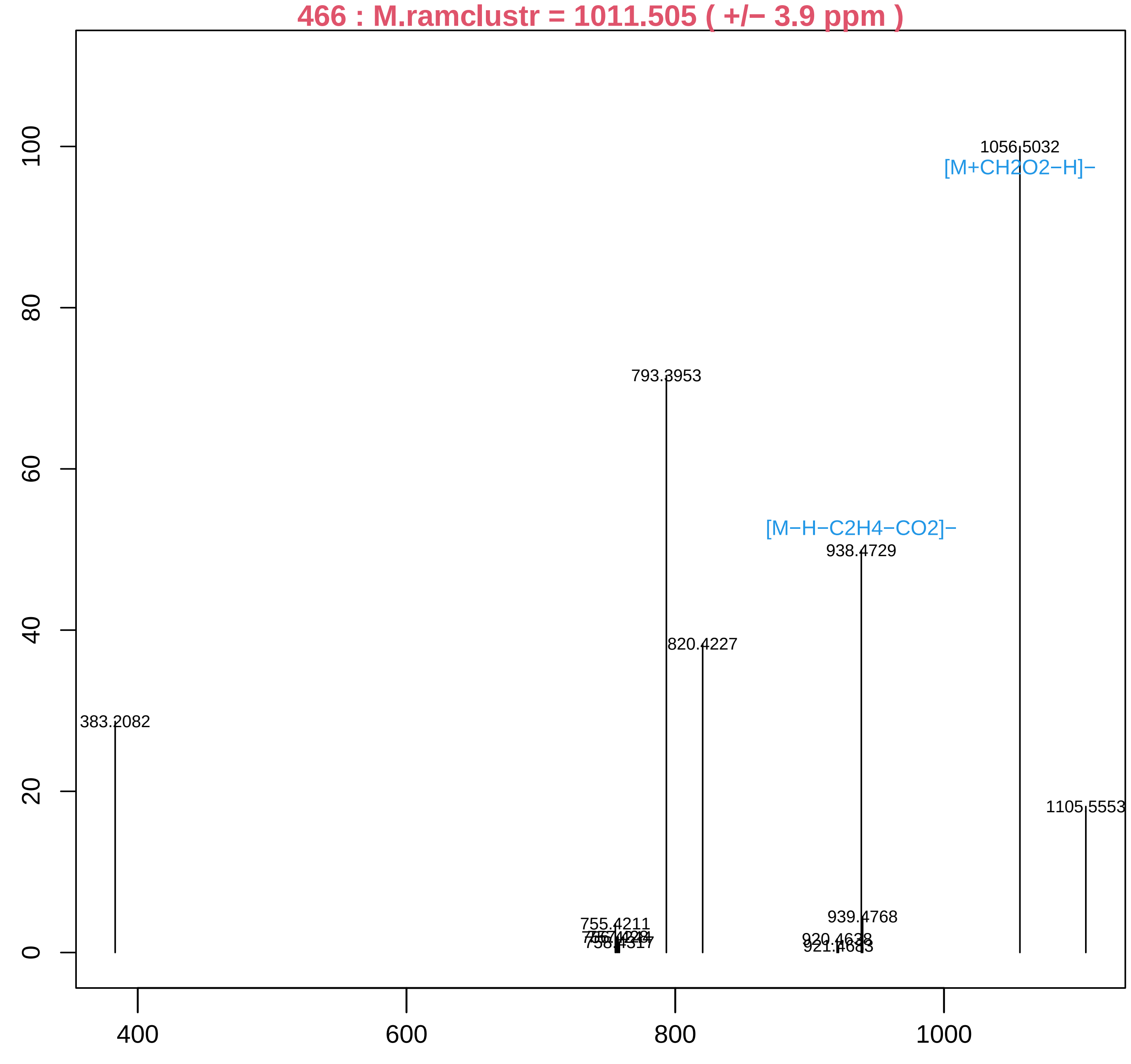
466 : M.ramclustr = 1011.505 (+/− 3.9 ppm)
1056.5032
[M+CH2O2−H]−
793.3953
[M−H−C2H4−CO2]−
938.4729
820.4227
383.2082
1105.5553
755.4211
939.4768
920.4628
921.4683
758.4317

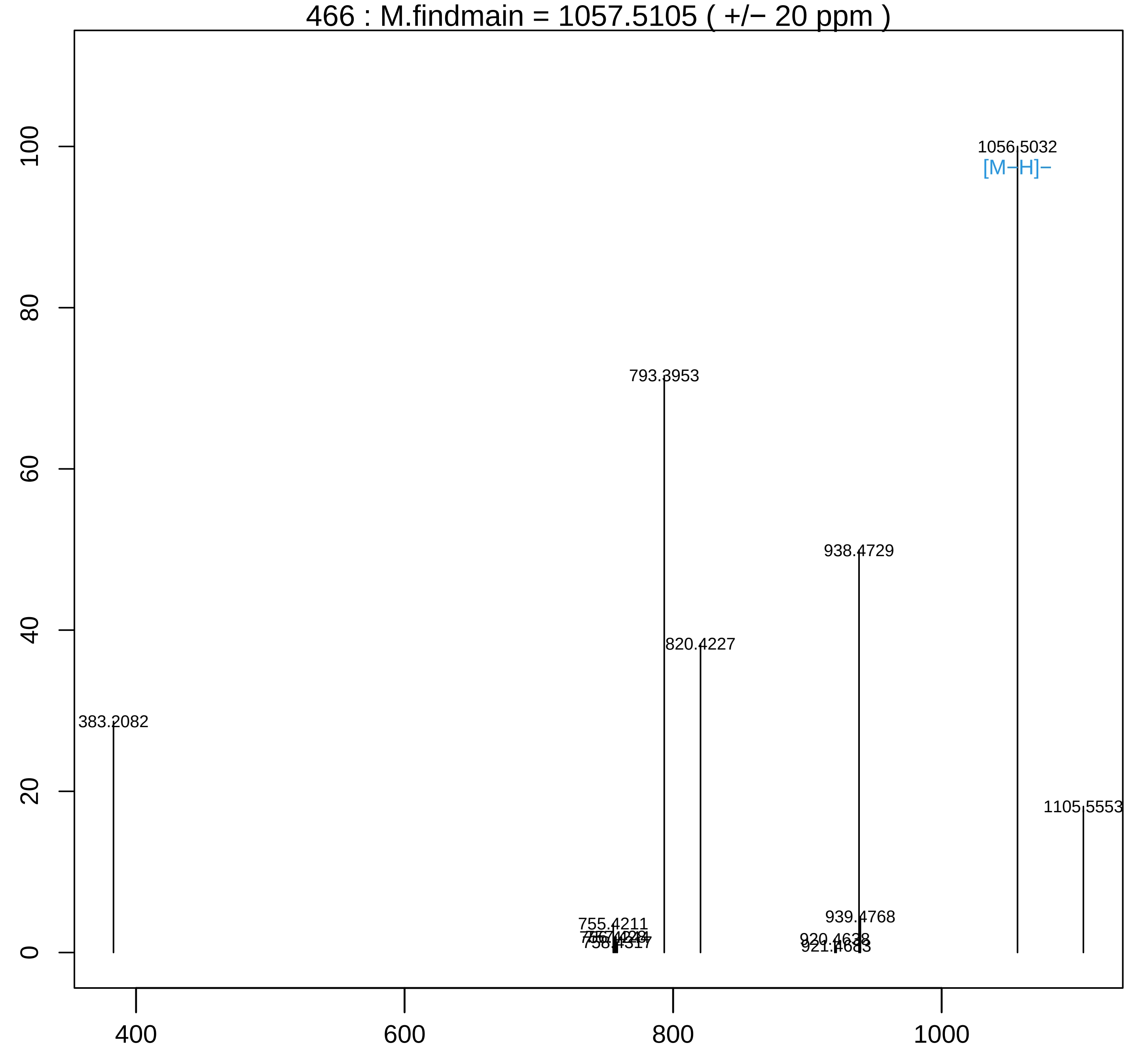
466 : M.findmain = 1057.5105 (+/− 20 ppm)
1056.5032
[M−H]−
793.3953
938.4729
820.4227
383.2082
1105.5553
755.4211
939.4768
920.4628
921.4683
758.4317

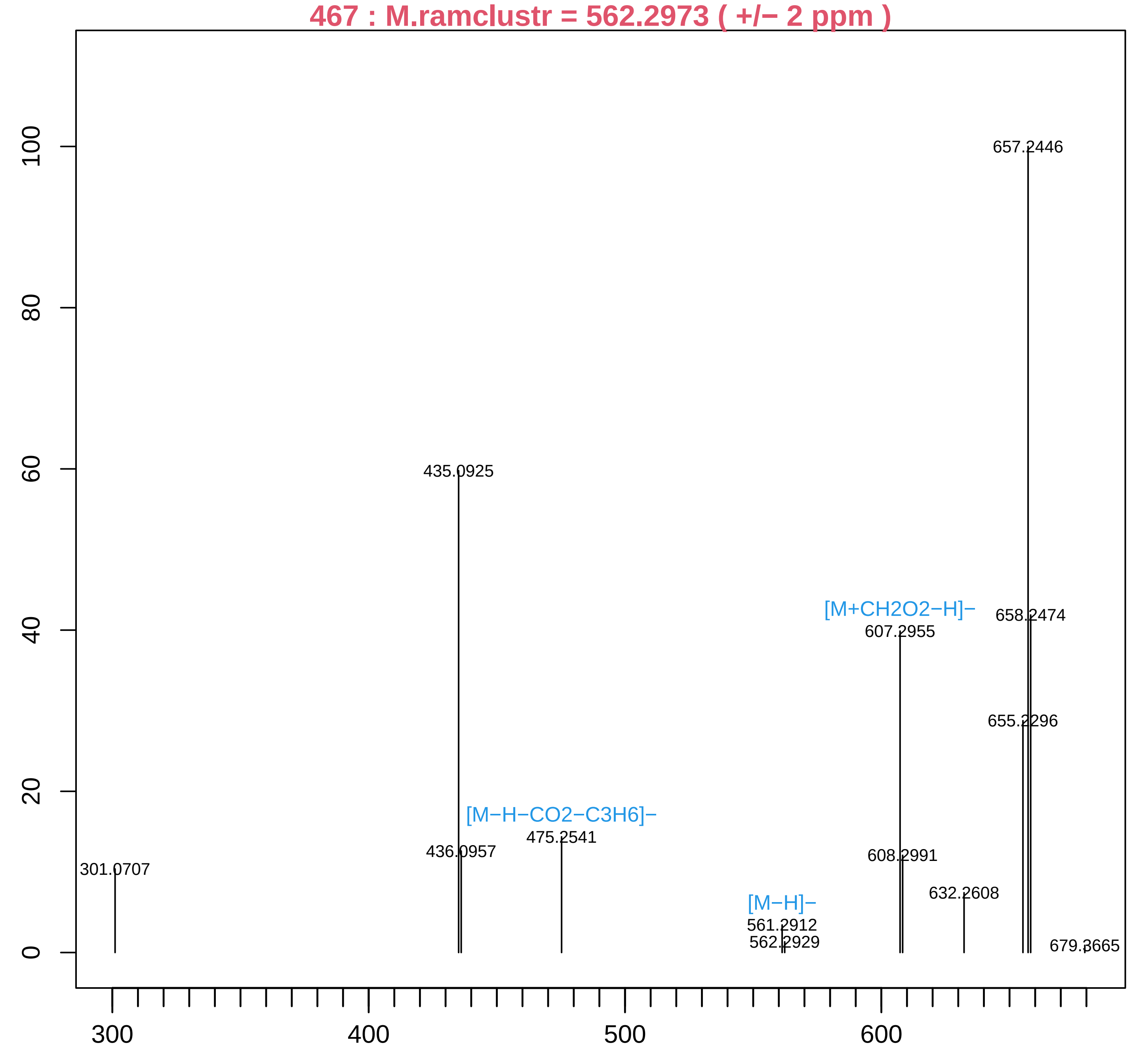

467 : M.ramclustr = 562.2973 (+/− 2 ppm)
657.2446
435.0925
[M+CH2O2−H]−
607.2955
658.2474
655.2296
[M−H−CO2−C3H6]−
475.2541
436.0957
608.2991
301.0707
632.2608
[M−H]−
561.2912
562.2929
679.3665
0
20
40
60
80
100
300
400
500
600

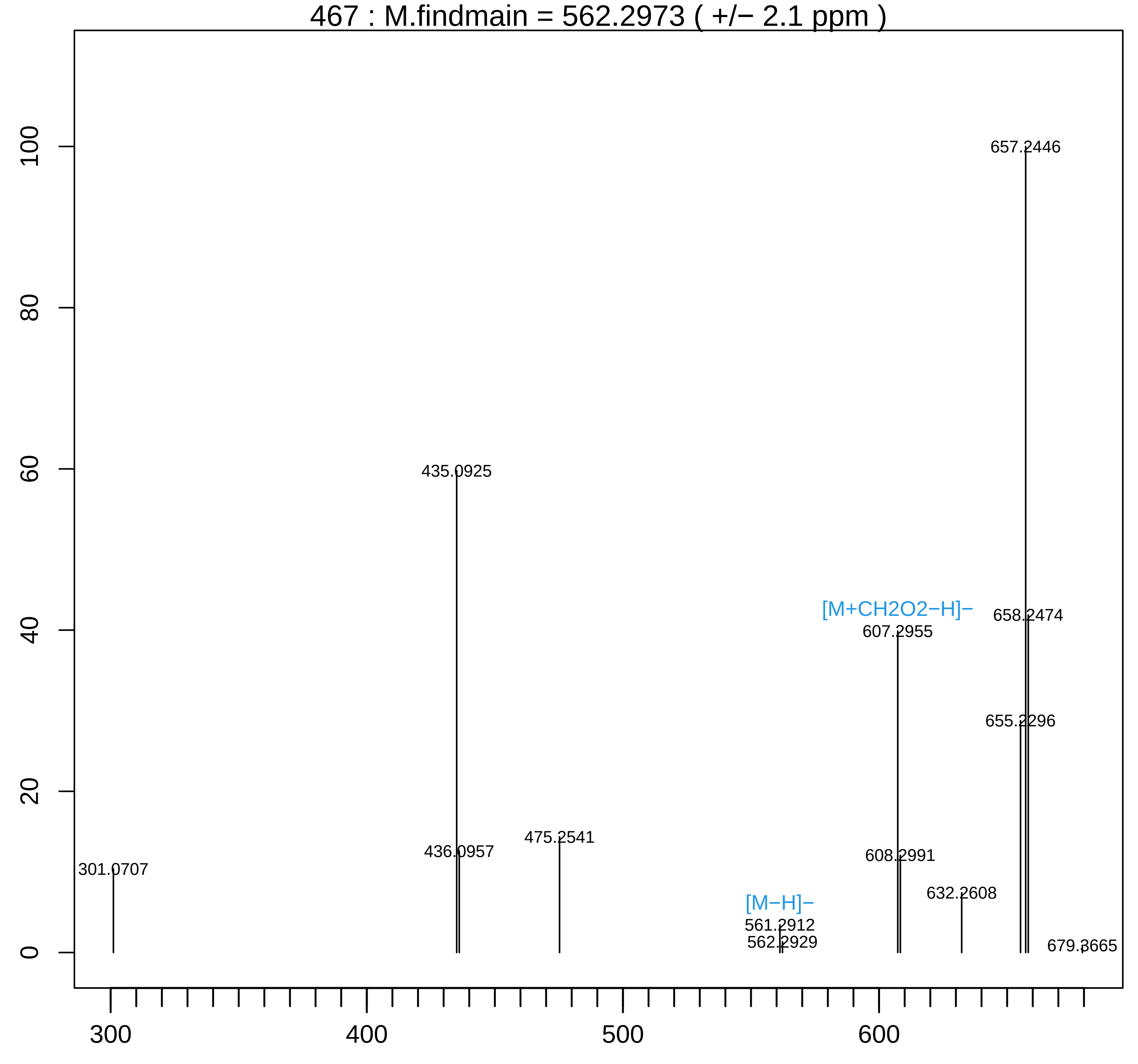

467 : M.findmain = 562.2973 (+/− 2.1 ppm)
657.2446
435.0925
[M+CH2O2−H]−
607.2955
658.2474
655.2296
475.2541
436.0957
608.2991
301.0707
632.2608
[M−H]−
561.2912
562.2929
679.3665
0
20
40
60
80
100
300
400
500
600

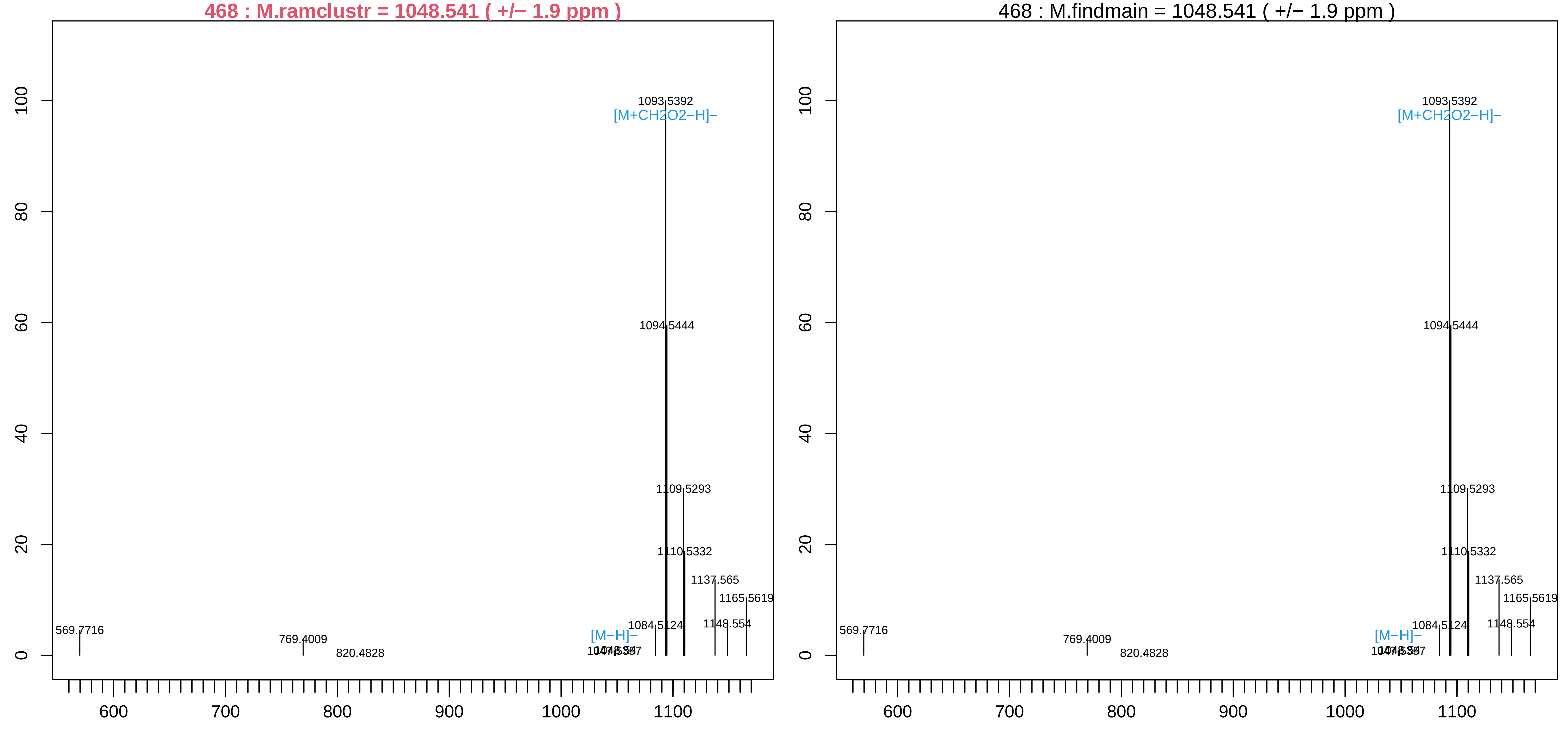

468 : M.ramclustr = 1048.541 (+/− 1.9 ppm)
468 : M.findmain = 1048.541 (+/− 1.9 ppm)
1093.5392
[M+CH2O2−H]−
1094.5444
1109.5293
1110.5332
1137.565
1165.5619
1148.554
1084.5124
[M−H]−
569.7716
769.4009
820.4828
0
20
40
60
80
100
600
700
800
900
1000
1100

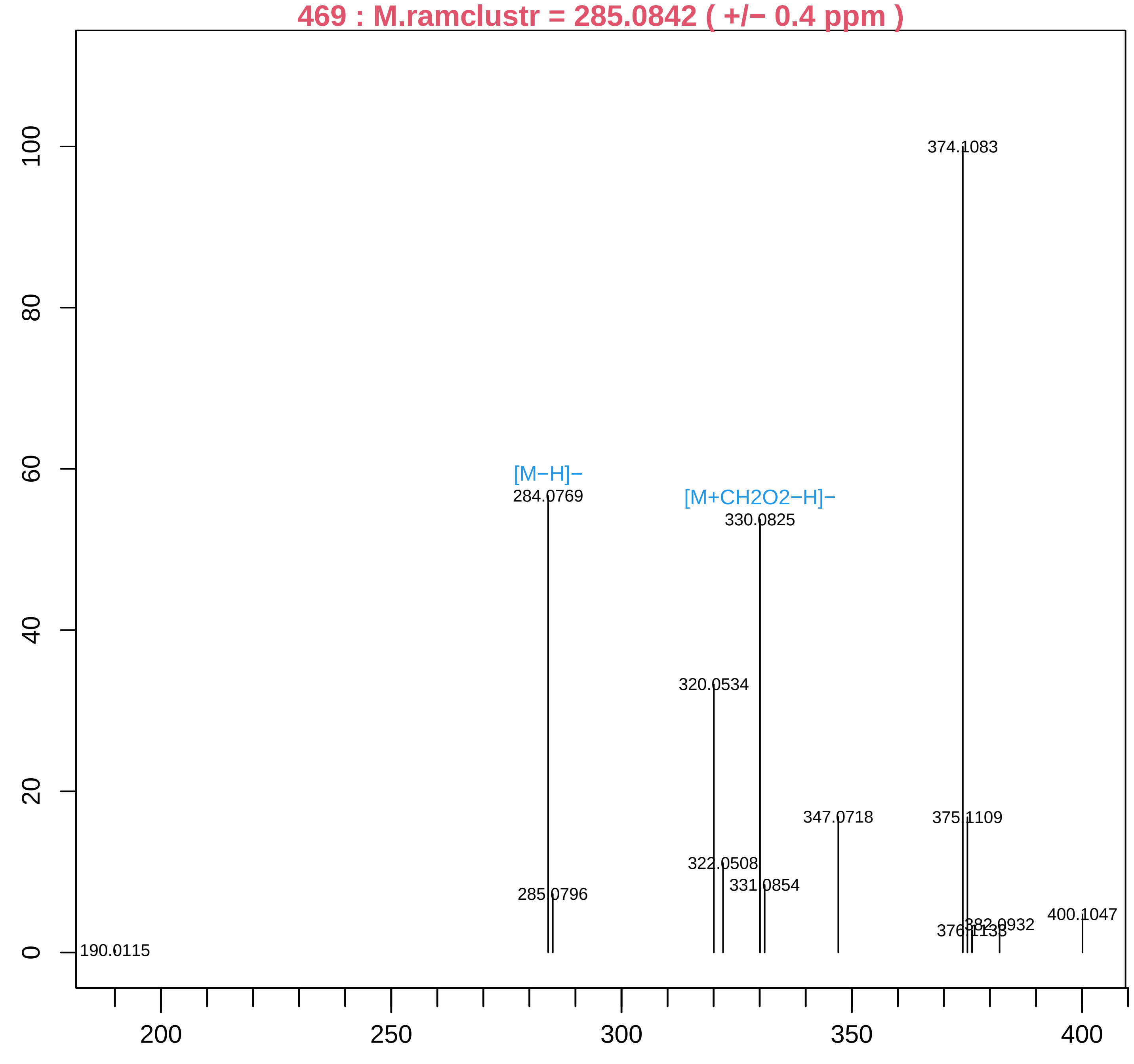
469 : M.ramclustr = 285.0842 (+/− 0.4 ppm)
[M−H]−
284.0769
[M+CH2O2−H]−
330.0825
374.1083
320.0534
347.0718
375.1109
322.0508
331.0854
285.0796
400.1047
382.0932
376.1133
190.0115
0
20
40
60
80
100
200
250
300
350
400

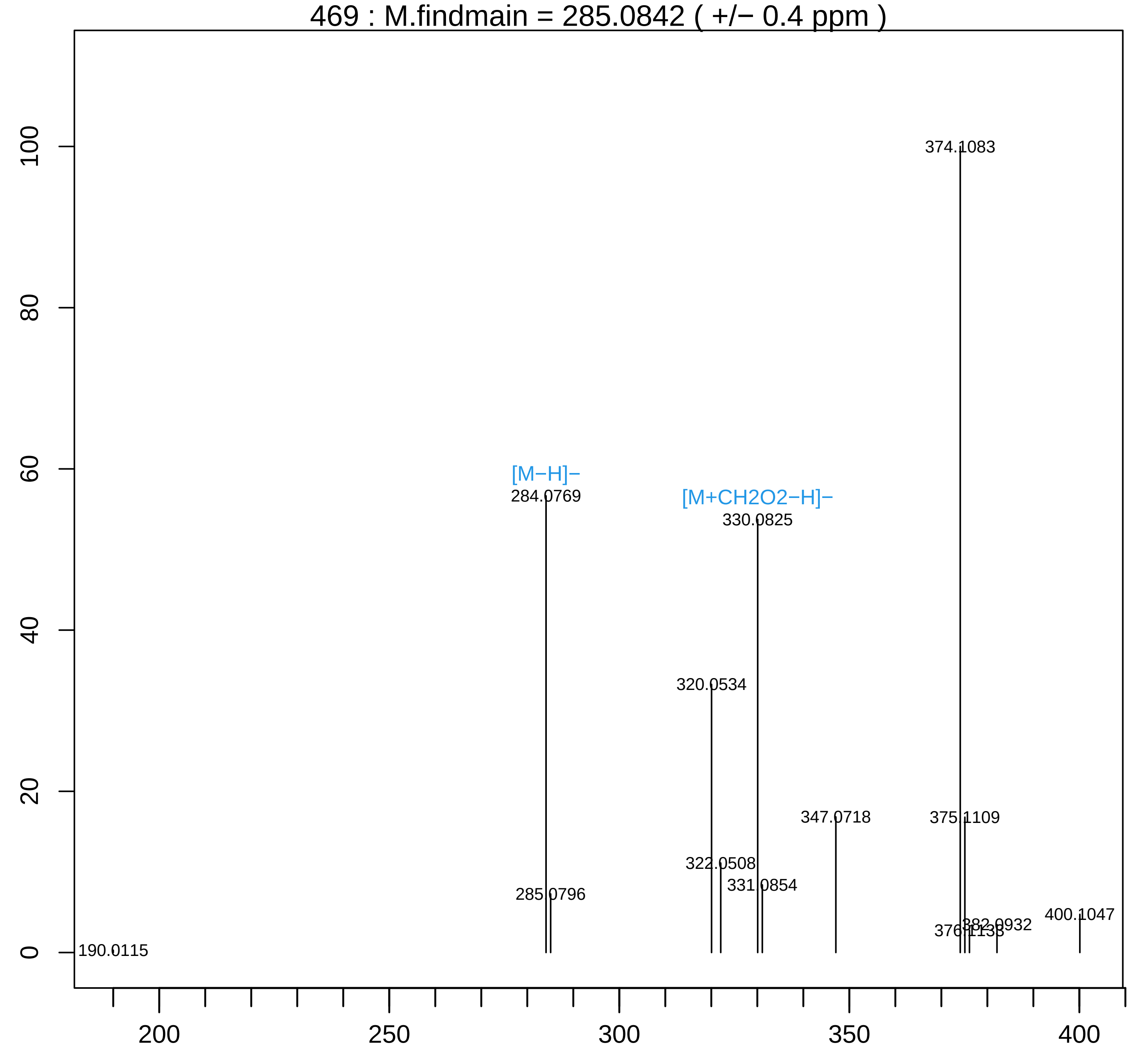
469 : M.findmain = 285.0842 (+/− 0.4 ppm)
[M−H]−
284.0769
[M+CH2O2−H]−
330.0825
374.1083
320.0534
347.0718
375.1109
322.0508
331.0854
285.0796
400.1047
382.0932
376.1133
190.0115
0
20
40
60
80
100
200
250
300
350
400

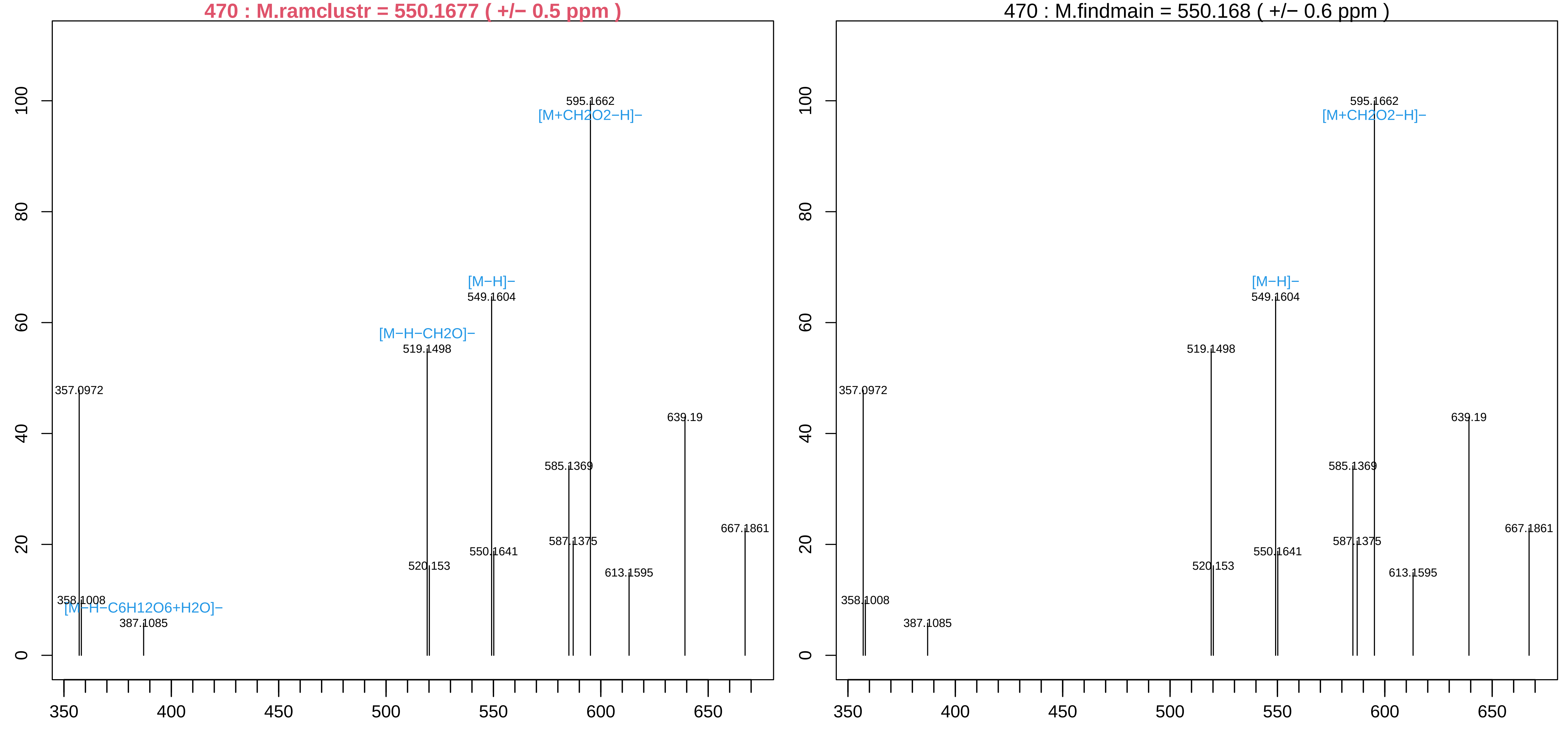
470 : M.ramclustr = 550.1677 (+/− 0.5 ppm)
595.1662
[M+CH2O2−H]−
[M−H]−
549.1604
[M−H−CH2O]−
519.1498
357.0972
639.19
585.1369
667.1861
587.1375
550.1641
520.153
613.1595
358.1008
[M−H−C6H12O6+H2O]−
387.1085
470 : M.findmain = 550.168 (+/− 0.6 ppm)
595.1662
[M+CH2O2−H]−
[M−H]−
549.1604
519.1498
357.0972
639.19
585.1369
667.1861
587.1375
550.1641
520.153
613.1595
358.1008
387.1085
0
20
40
60
80
100
350
400
450
500
550
600
650

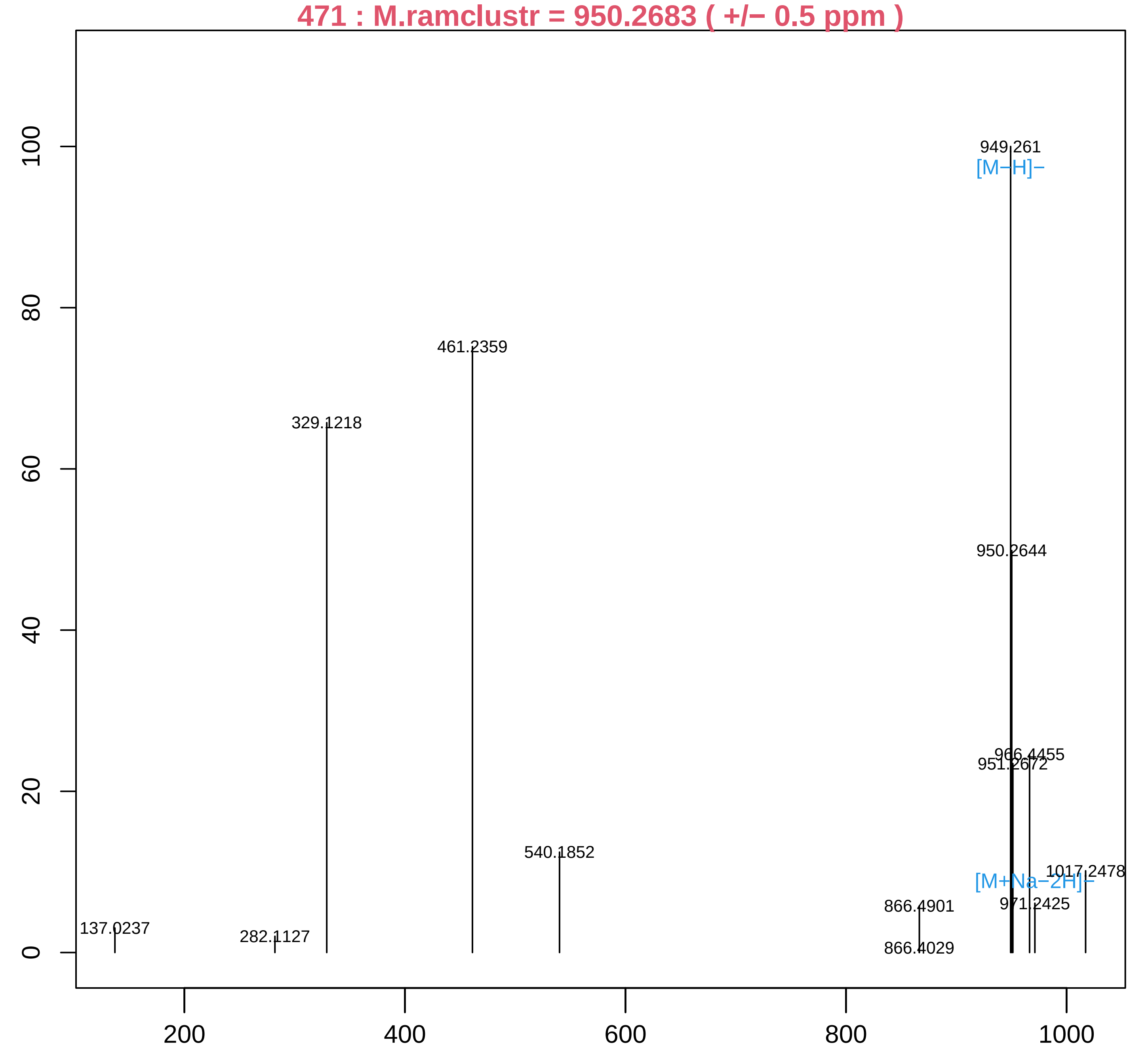

471 : M.ramclustr = 950.2683 (+/− 0.5 ppm)
949.261
[M−H]−
461.2359
329.1218
950.2644
966.4455
951.2672
540.1852
1017.2478
[M+Na−2H]−
866.4901
971.2425
137.0237
282.1127
866.4029
0
20
40
60
80
100
200
400
600
800
1000

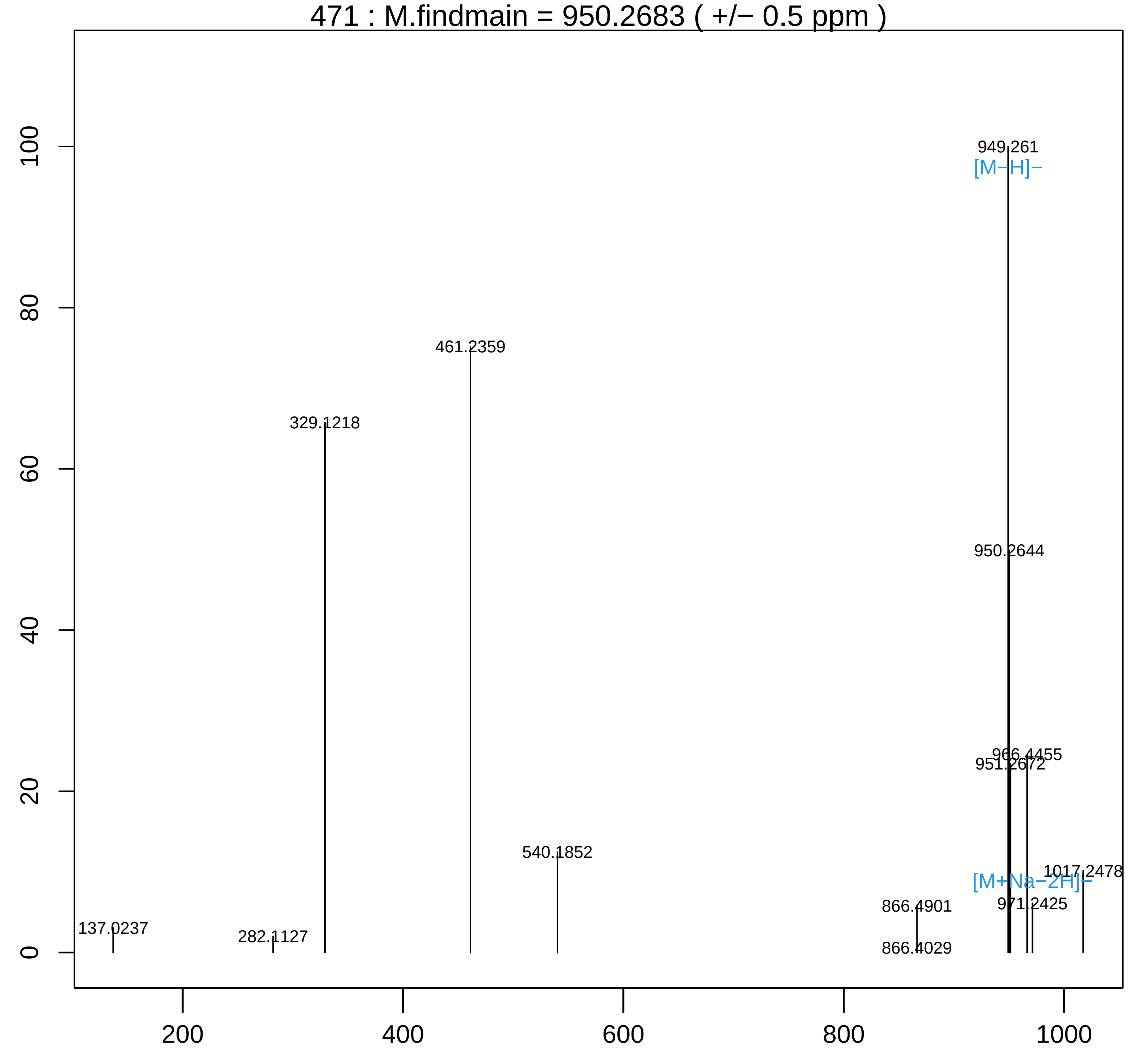

471 : M.findmain = 950.2683 (+/− 0.5 ppm)
949.261
[M−H]−
461.2359
329.1218
950.2644
966.4455
951.2672
540.1852
1017.2478
[M+Na−2H]−
866.4901
971.2425
137.0237
282.1127
866.4029
0
20
40
60
80
100
200
400
600
800
1000

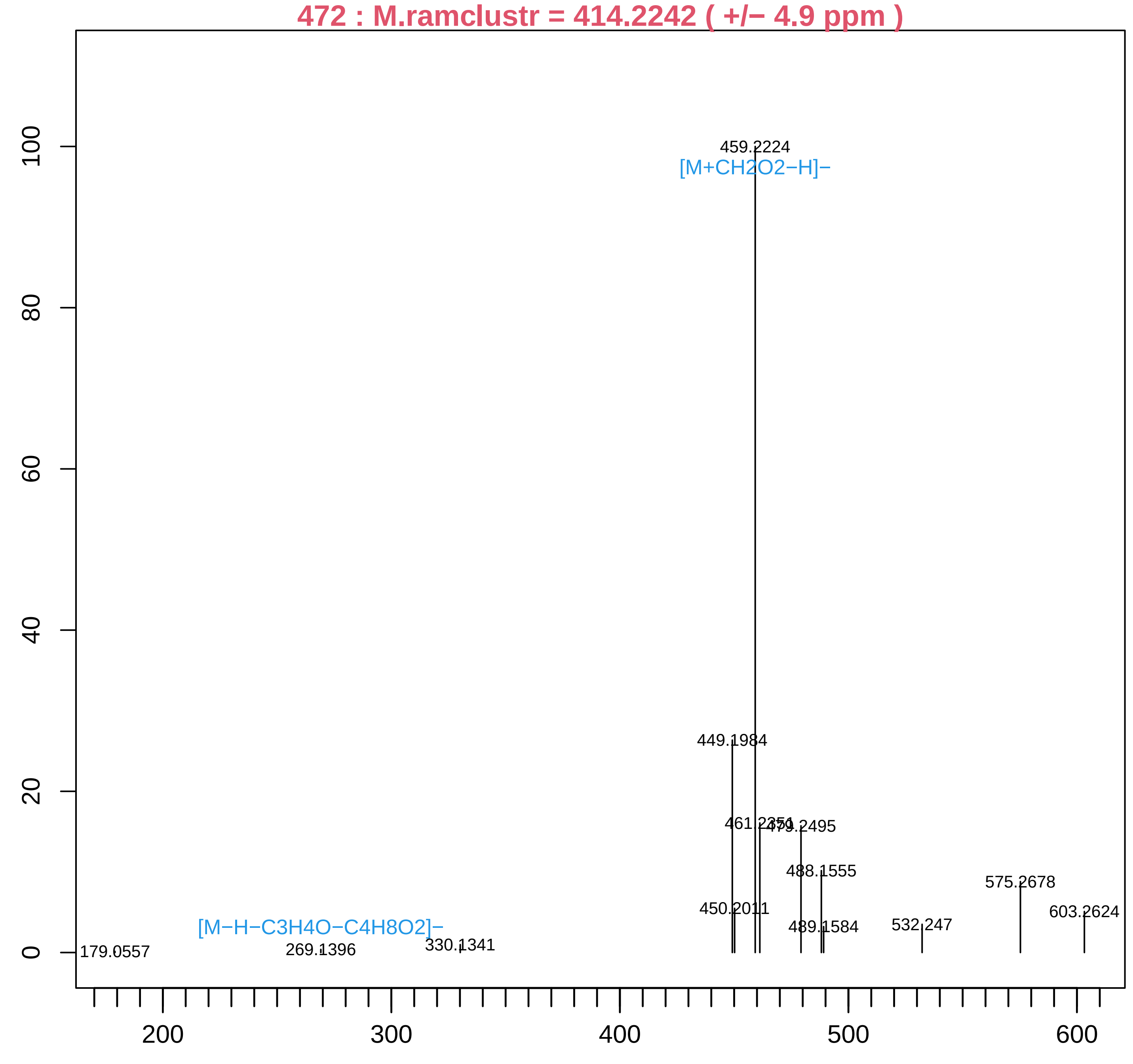

472 : M.ramclustr = 414.2242 (+/− 4.9 ppm)
459.2224
[M+CH2O2−H]−
449.1984
461.2351
479.2495
488.1555
450.2011
489.1584
532.247
575.2678
603.2624
[M−H−C3H4O−C4H8O2]−
179.0557
269.1396
330.1341
0
20
40
60
80
100
200
300
400
500
600

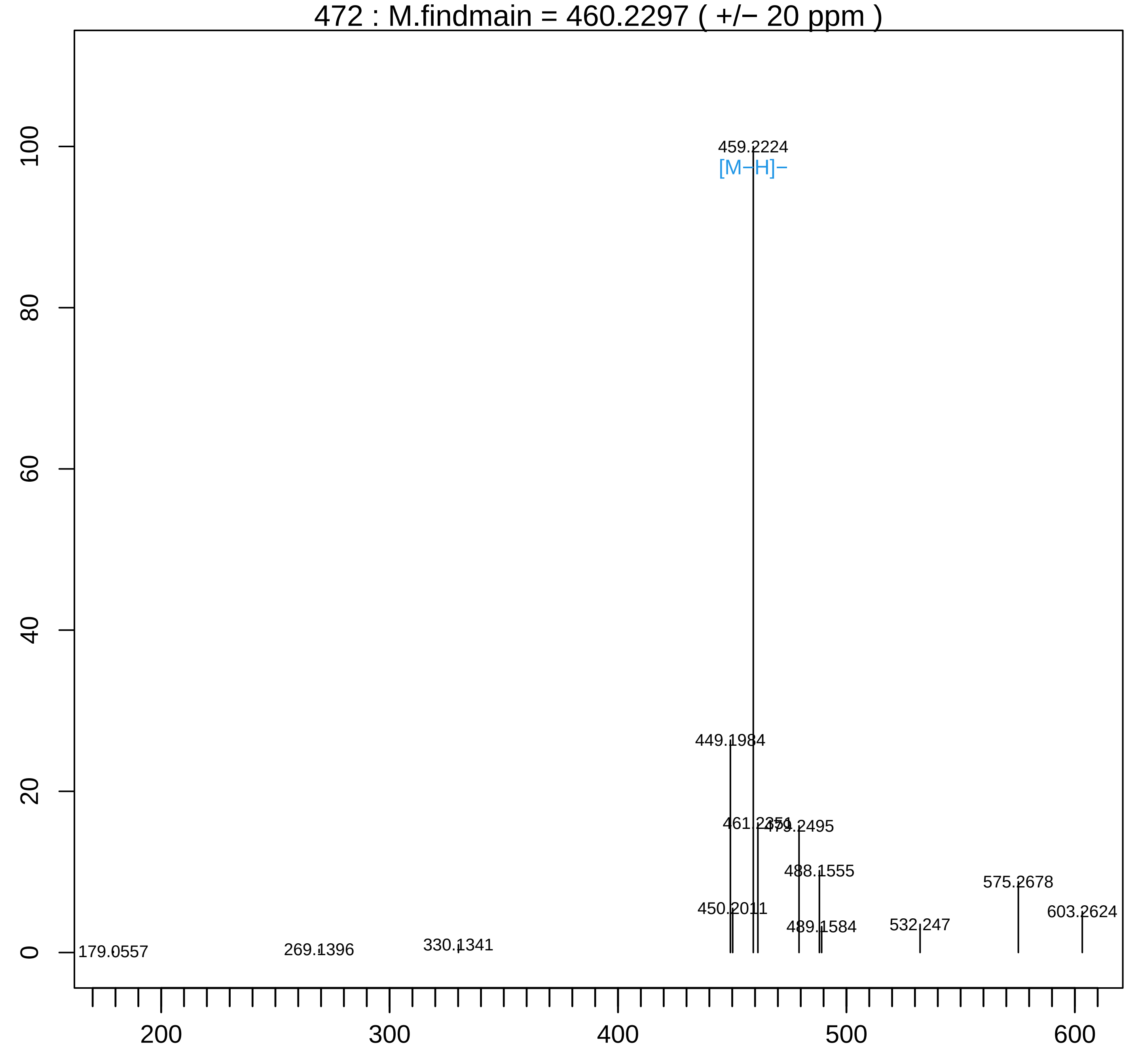

472 : M.findmain = 460.2297 (+/− 20 ppm)
459.2224
[M−H]−
449.1984
461.2351
479.2495
488.1555
450.2011
489.1584
532.247
575.2678
603.2624
179.0557
269.1396
330.1341
0
20
40
60
80
100
200
300
400
500
600

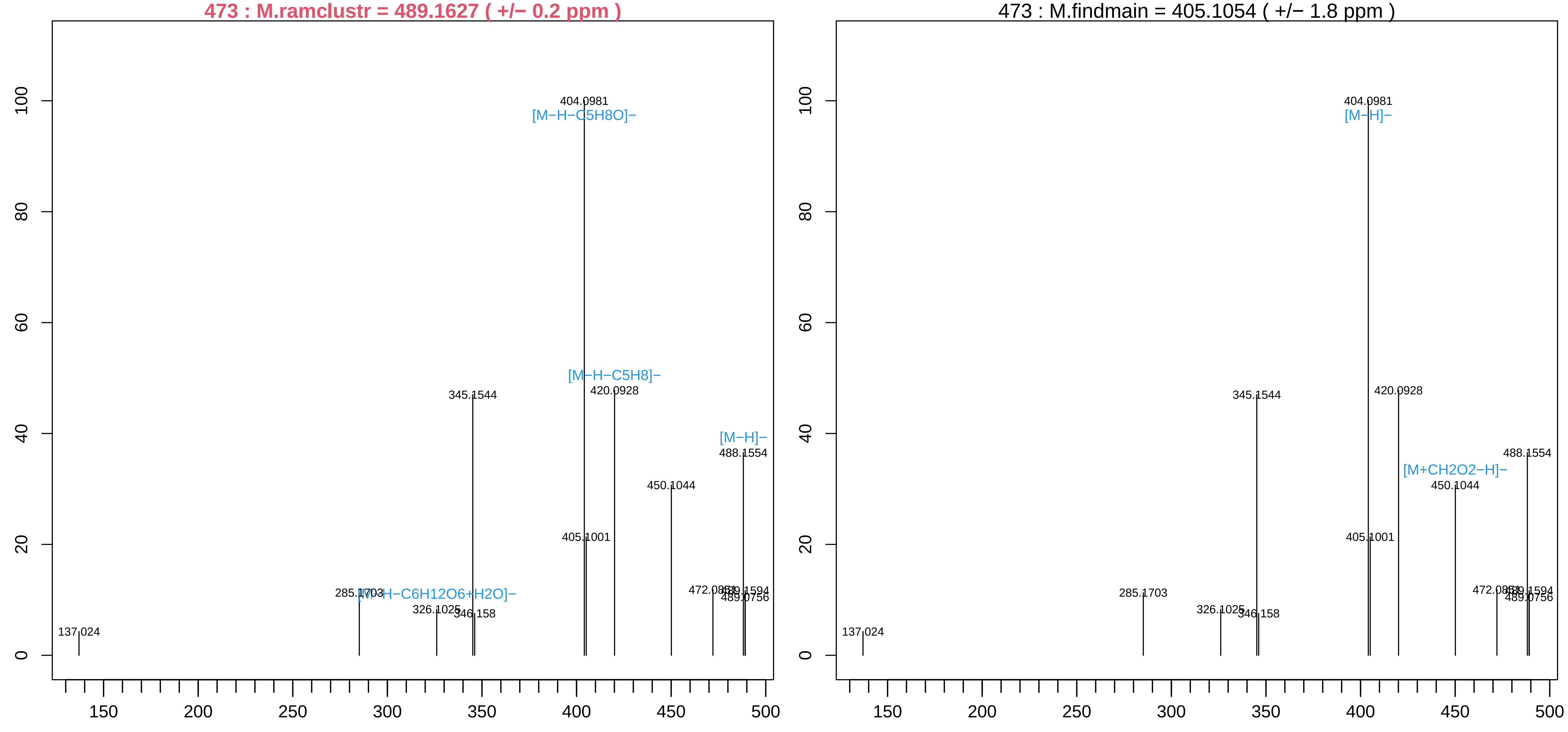
473 : M.ramclustr = 489.1627 (+/− 0.2 ppm)
404.0981
[M−H−C5H8O]−
[M−H−C5H8]−
420.0928
345.1544
[M−H]−
488.1554
450.1044
405.1001
285.1703
[M−H−C6H12O6+H2O]−
326.1025
346.158
472.0851
489.1594
489.0756
137.024
473 : M.findmain = 405.1054 (+/− 1.8 ppm)
404.0981
[M−H]−
420.0928
345.1544
488.1554
[M+CH2O2−H]−
450.1044
405.1001
285.1703
326.1025
346.158
472.0851
489.1594
489.0756
137.024

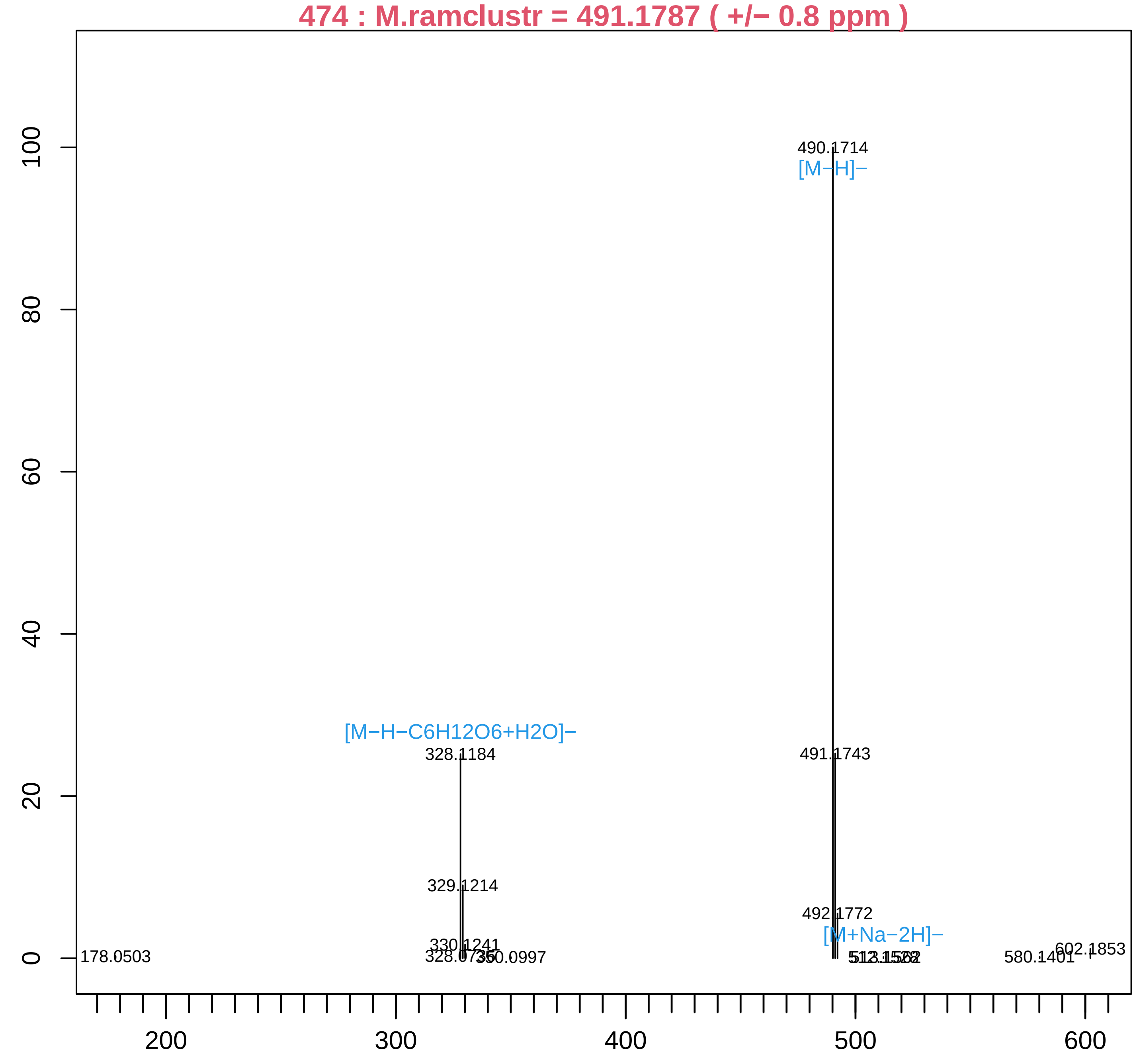

474 : M.ramclustr = 491.1787 (+/− 0.8 ppm)
490.1714
[M−H]−
[M−H−C6H12O6+H2O]−
328.1184
491.1743
329.1214
492.1772
[M+Na−2H]−
330.1241
328.0735
350.0997
178.0503
512.1582
580.1401
602.1853
0
20
40
60
80
100
200
300
400
500
600

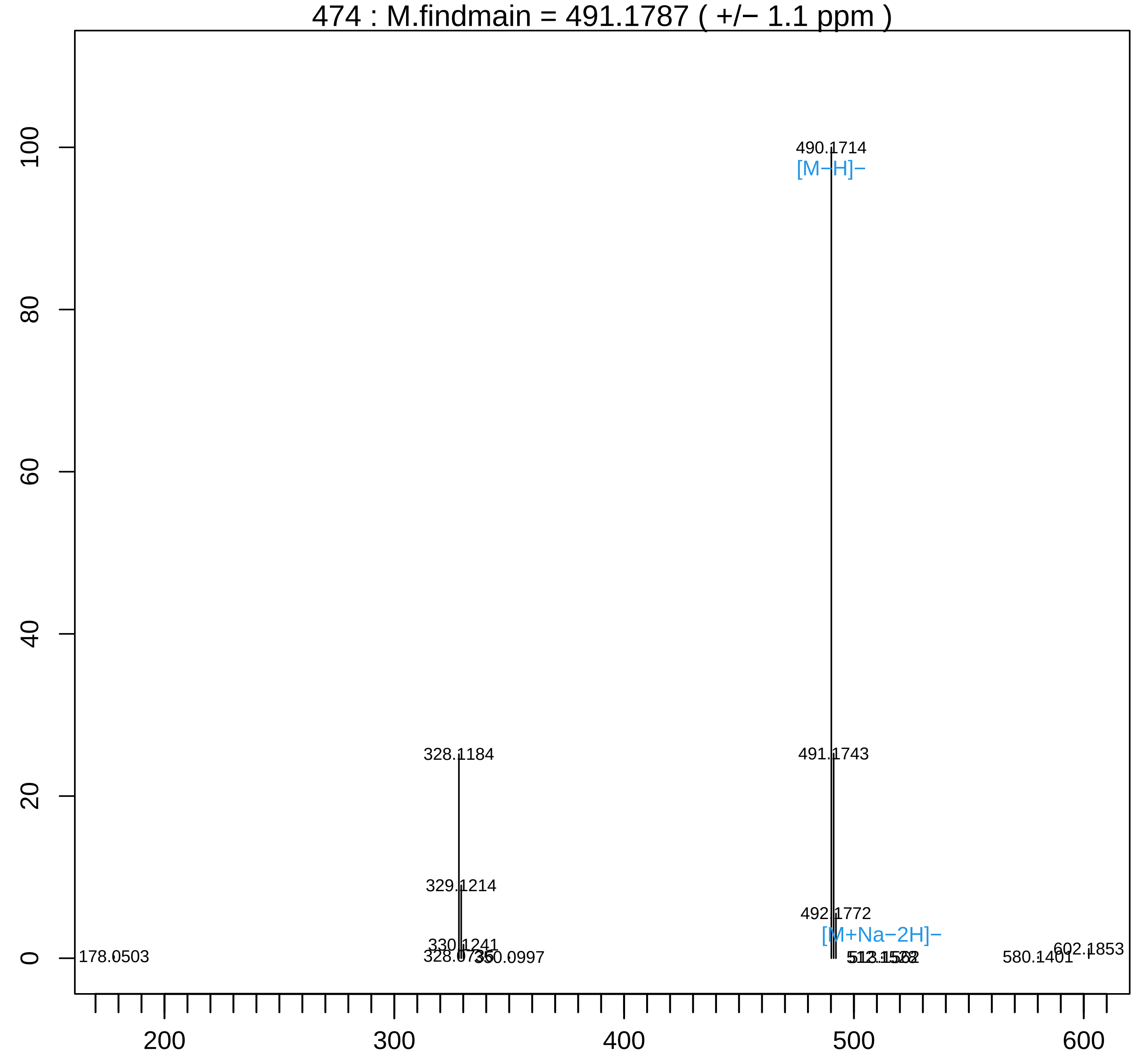

474 : M.findmain = 491.1787 (+/− 1.1 ppm)
490.1714
[M−H]−
328.1184
491.1743
329.1214
492.1772
[M+Na−2H]−
330.1241
328.0735
350.0997
178.0503
512.1582
580.1401
602.1853
0
20
40
60
80
100
200
300
400
500
600

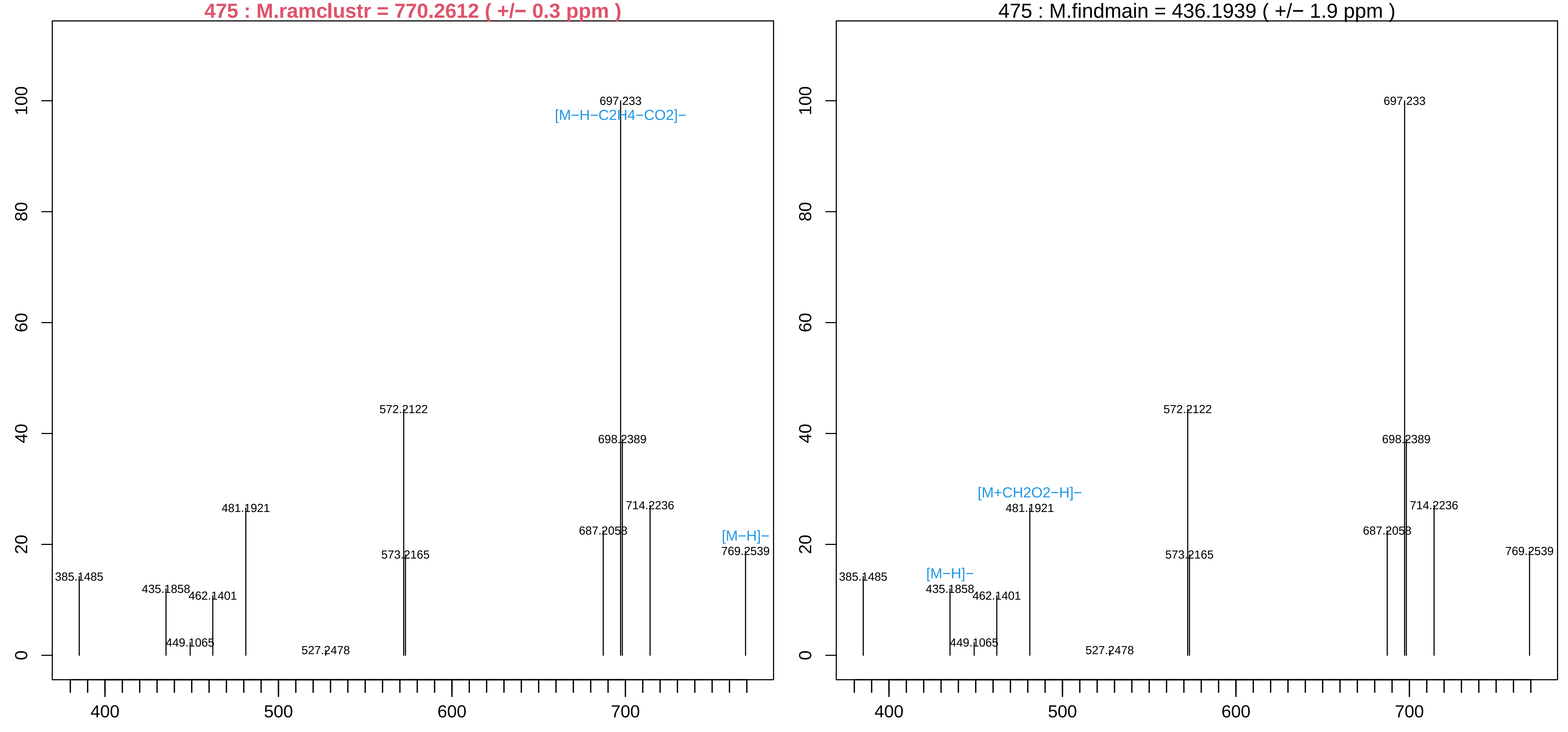
475 : M.ramclustr = 770.2612 (+/− 0.3 ppm)
475 : M.findmain = 436.1939 (+/− 1.9 ppm)
697.233
[M−H−C2H4−CO2]−
572.2122
698.2389
714.2236
481.1921
687.2058
[M−H]−
769.2539
573.2165
385.1485
435.1858
462.1401
449.1065
527.2478
[M+CH2O2−H]−
0
20
40
60
80
100
400
500
600
700

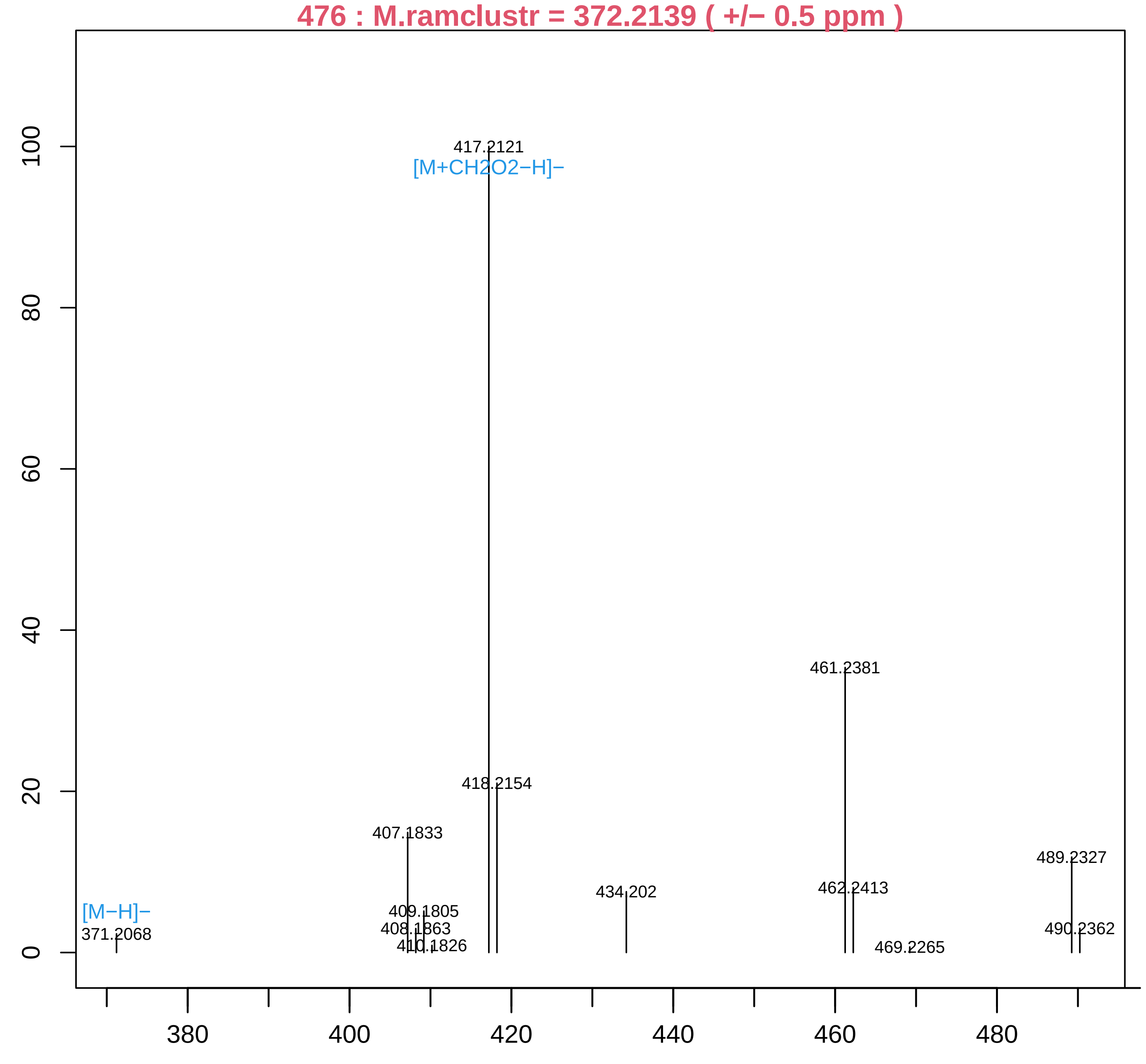
476 : M.ramclustr = 372.2139 (+/− 0.5 ppm)
417.2121
[M+CH2O2−H]−
461.2381
418.2154
407.1833
489.2327
434.202
462.2413
[M−H]−
409.1805
408.1863
490.2362
371.2068
410.1826
469.2265
0
20
40
60
80
100
380
400
420
440
460
480

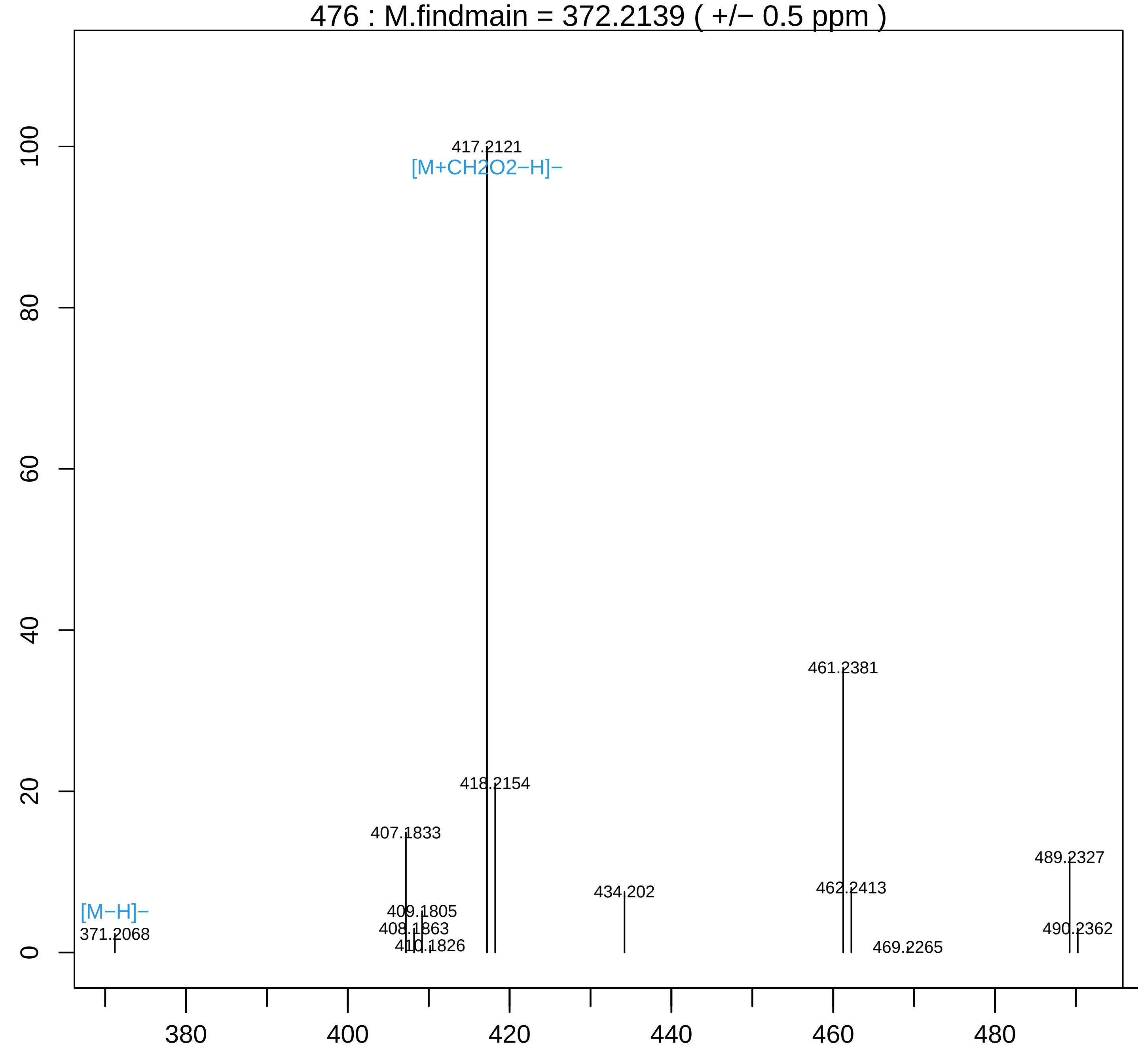
476 : M.findmain = 372.2139 (+/− 0.5 ppm)
417.2121
[M+CH2O2−H]−
461.2381
418.2154
407.1833
489.2327
434.202
462.2413
[M−H]−
409.1805
408.1863
490.2362
371.2068
410.1826
469.2265
0
20
40
60
80
100
380
400
420
440
460
480

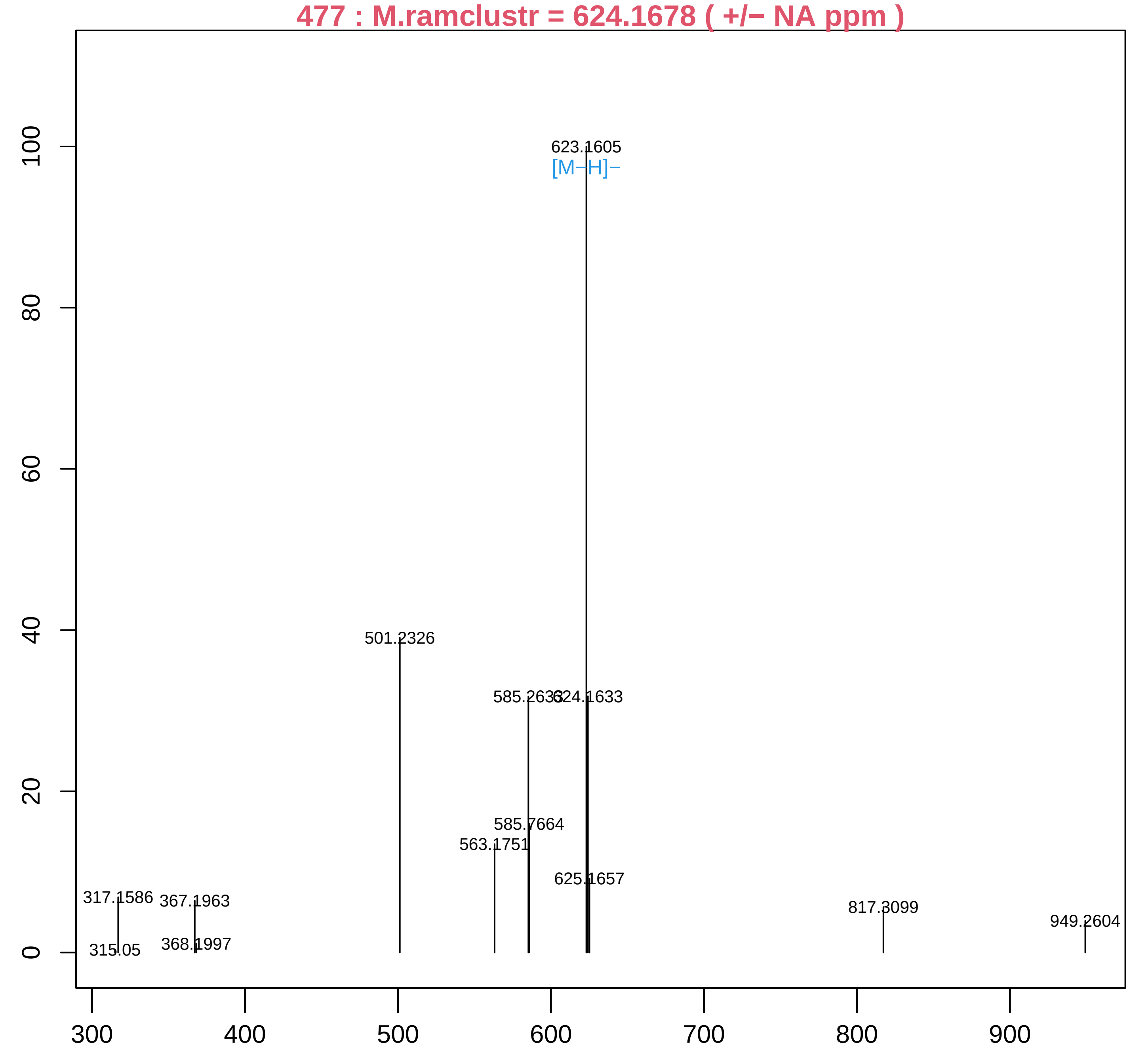
477 : M.ramclustr = 624.1678 (+/− NA ppm)
623.1605
[M−H]−
501.2326
585.2636
624.1633
585.7664
563.1751
625.1657
317.1586
367.1963
817.3099
949.2604
315.05
368.1997
0
20
40
60
80
100
300
400
500
600
700
800
900

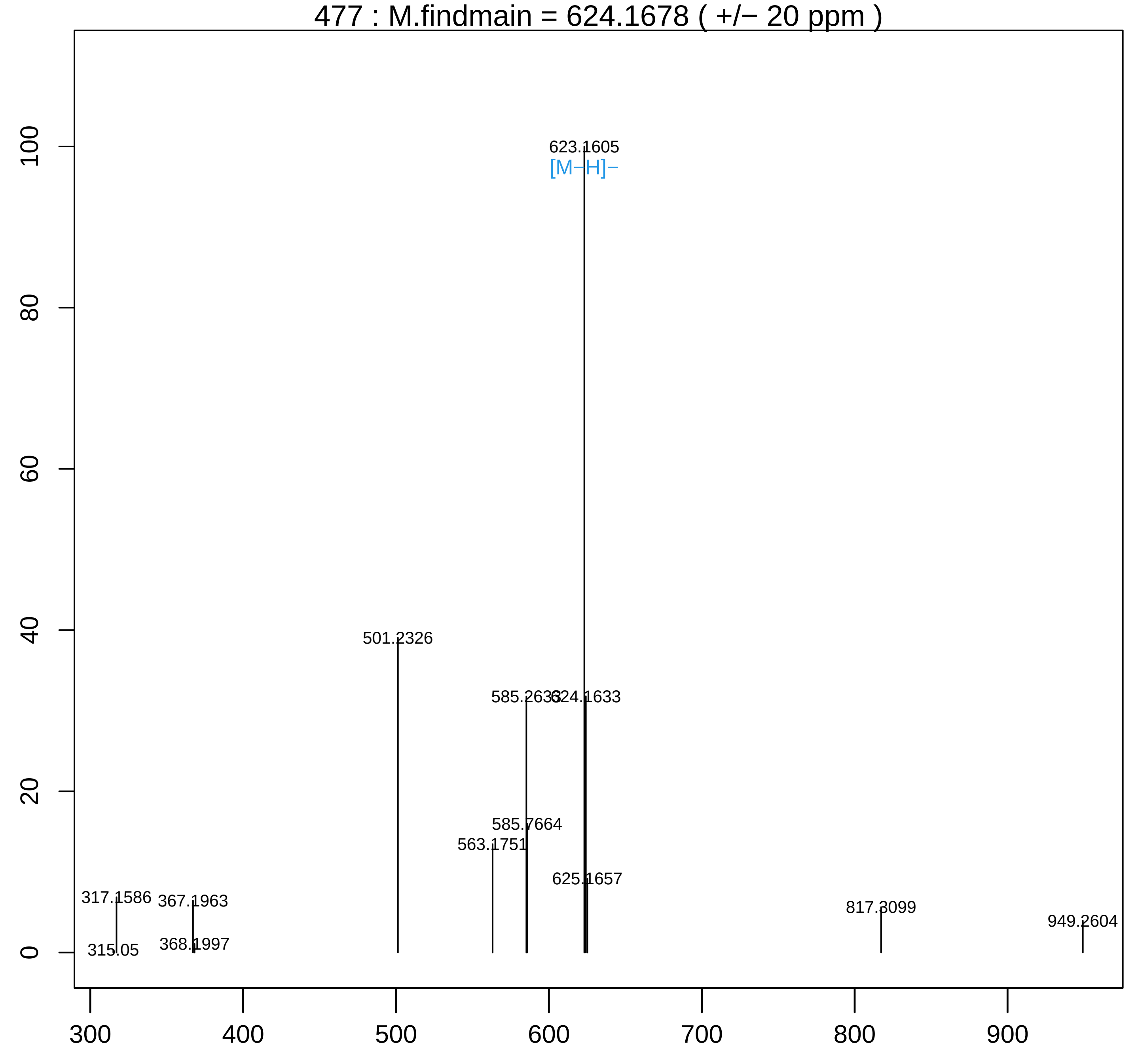
477 : M.findmain = 624.1678 (+/− 20 ppm)
623.1605
[M−H]−
501.2326
585.2636
624.1633
585.7664
563.1751
625.1657
317.1586
367.1963
817.3099
949.2604
315.05
368.1997
0
20
40
60
80
100
300
400
500
600
700
800
900

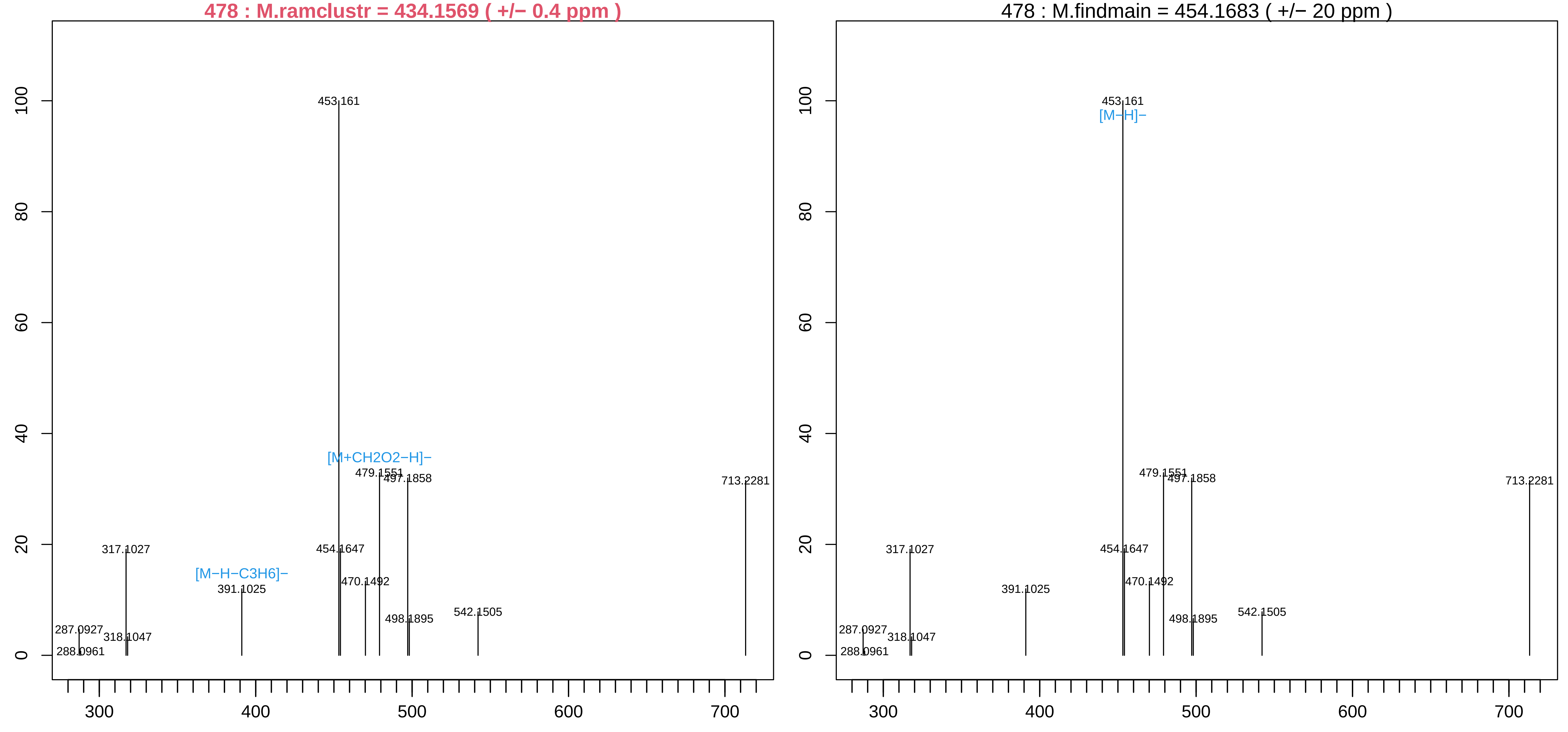
478 : M.ramclustr = 434.1569 (+/− 0.4 ppm)
453.161
[M+CH2O2−H]−
479.1551
497.1858
713.2281
317.1027
454.1647
[M−H−C3H6]−
391.1025
470.1492
542.1505
498.1895
287.0927
318.1047
288.0961
0
20
40
60
80
100
300
400
500
600
700
478 : M.findmain = 454.1683 (+/− 20 ppm)
453.161
[M−H]−
479.1551
497.1858
713.2281
317.1027
454.1647
391.1025
470.1492
542.1505
498.1895
287.0927
318.1047
288.0961
0
20
40
60
80
100
300
400
500
600
700

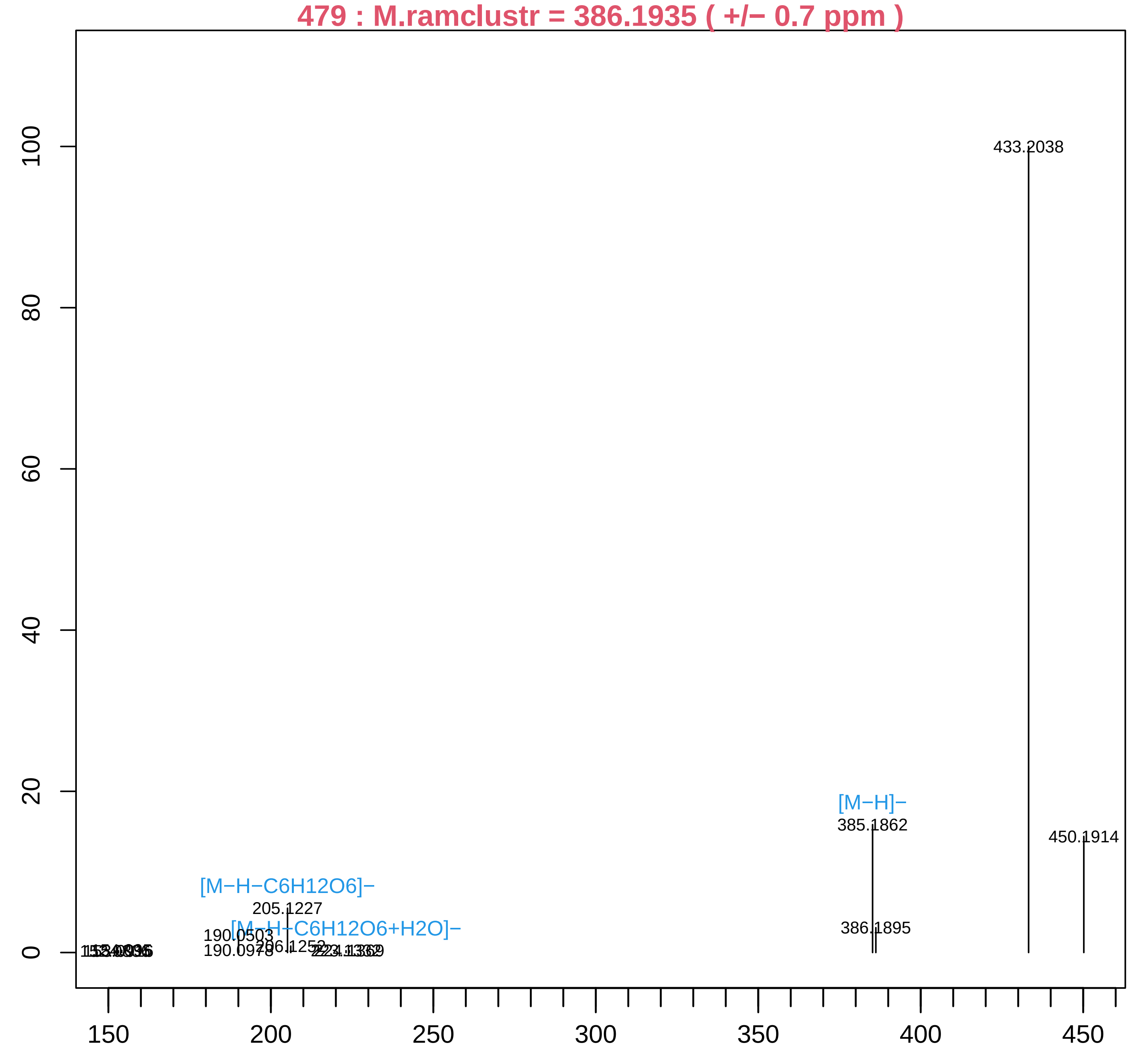
479 : M.ramclustr = 386.1935 (+/− 0.7 ppm)
433.2038
[M−H]−
385.1862
450.1914
386.1895
[M−H−C6H12O6]−
205.1227
190.0503
[M−H−C6H12O6+H2O]−
190.0978
206.1252
0
20
40
60
80
100
150
200
250
300
350
400
450

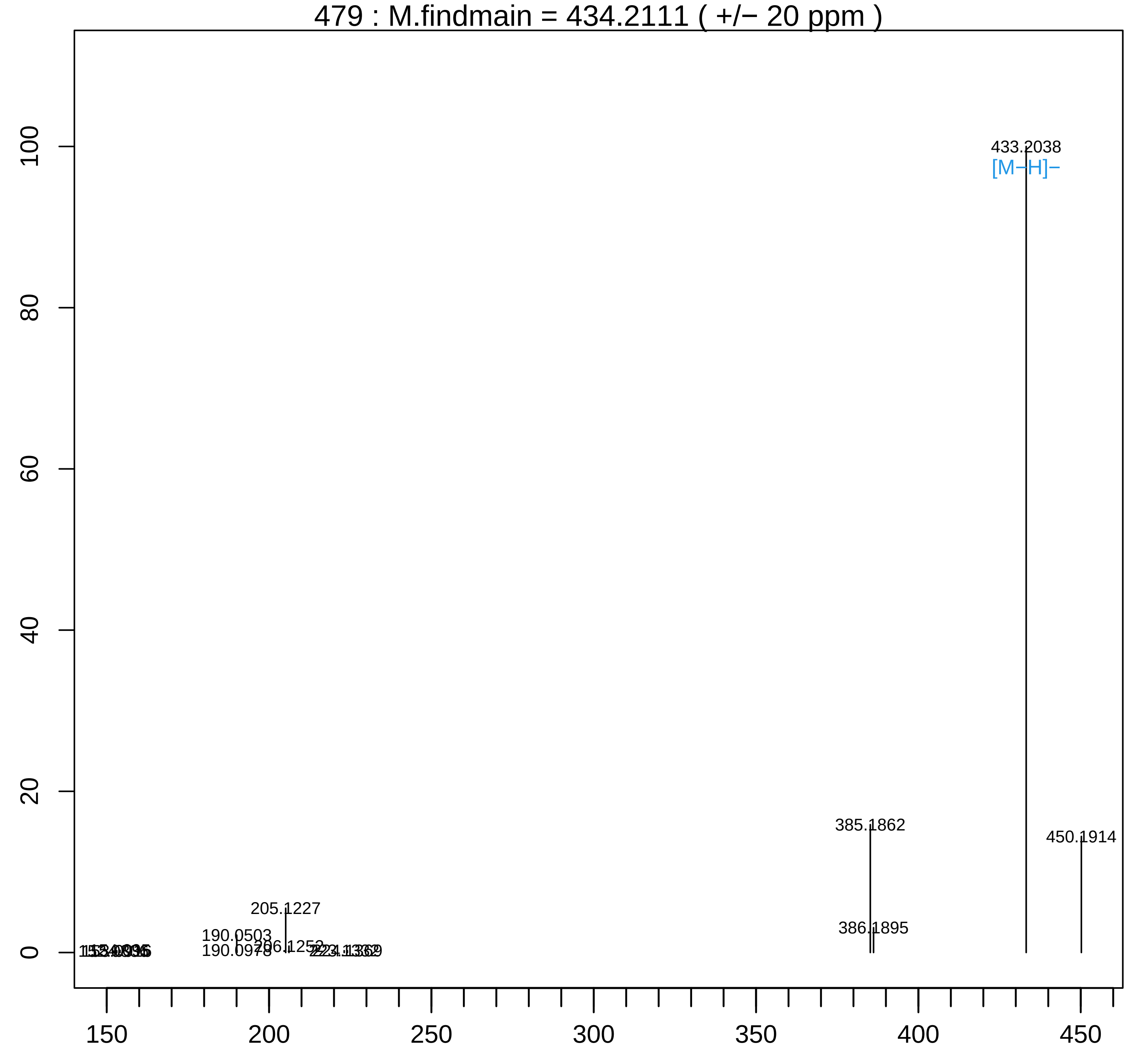
479 : M.findmain = 434.2111 (+/− 20 ppm)
433.2038
[M−H]−
385.1862
450.1914
386.1895
205.1227
190.0503
190.0978
206.1252
0
20
40
60
80
100
150
200
250
300
350
400
450

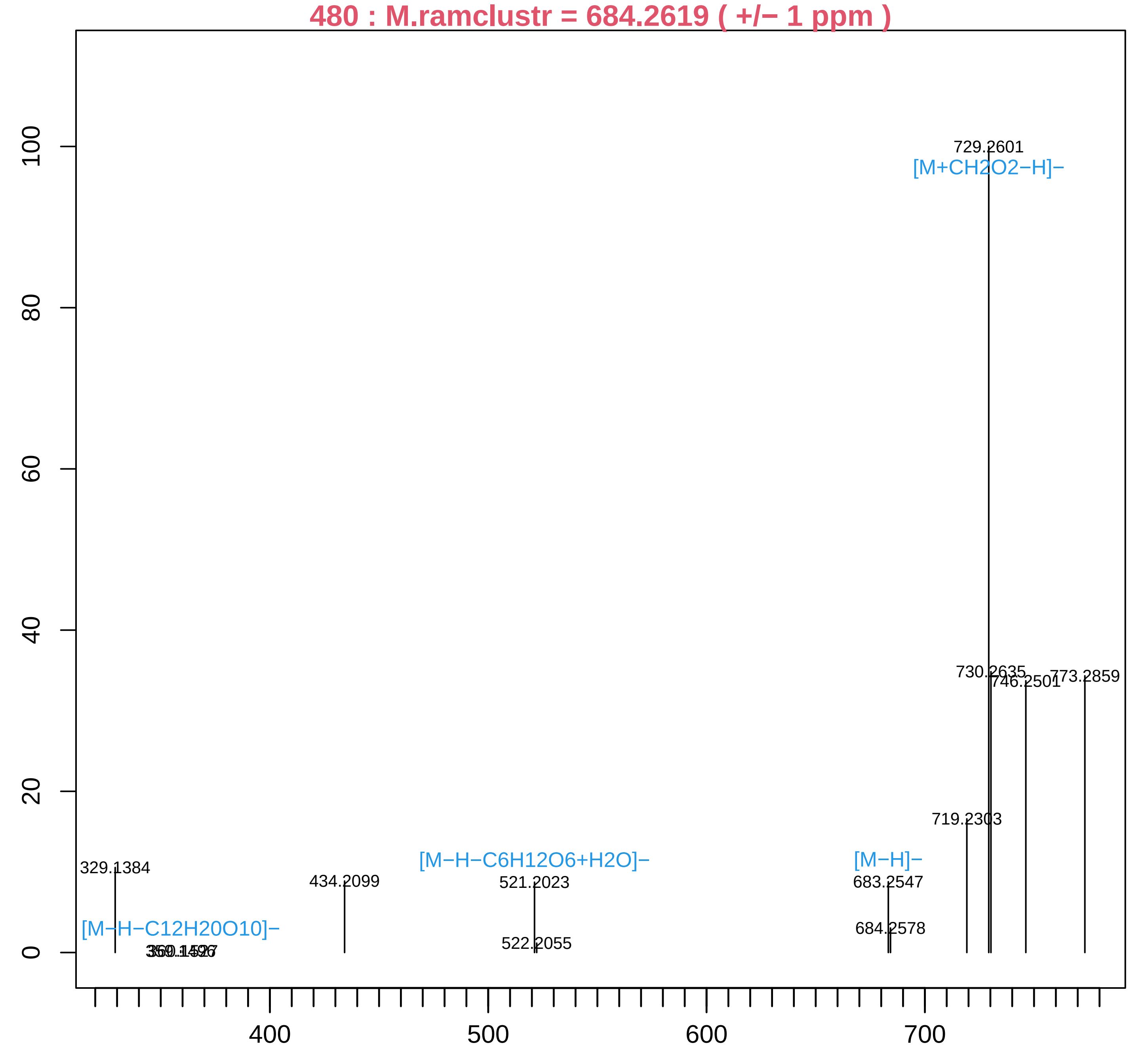
480 : M.ramclustr = 684.2619 (+/− 1 ppm)
729.2601
[M+CH2O2−H]−
730.2635
746.2501
773.2859
719.2303
329.1384
434.2099
[M−H−C6H12O6+H2O]−
521.2023
[M−H]−
683.2547
684.2578
[M−H−C12H20O10]−
522.2055
0
20
40
60
80
100
400
500
600
700
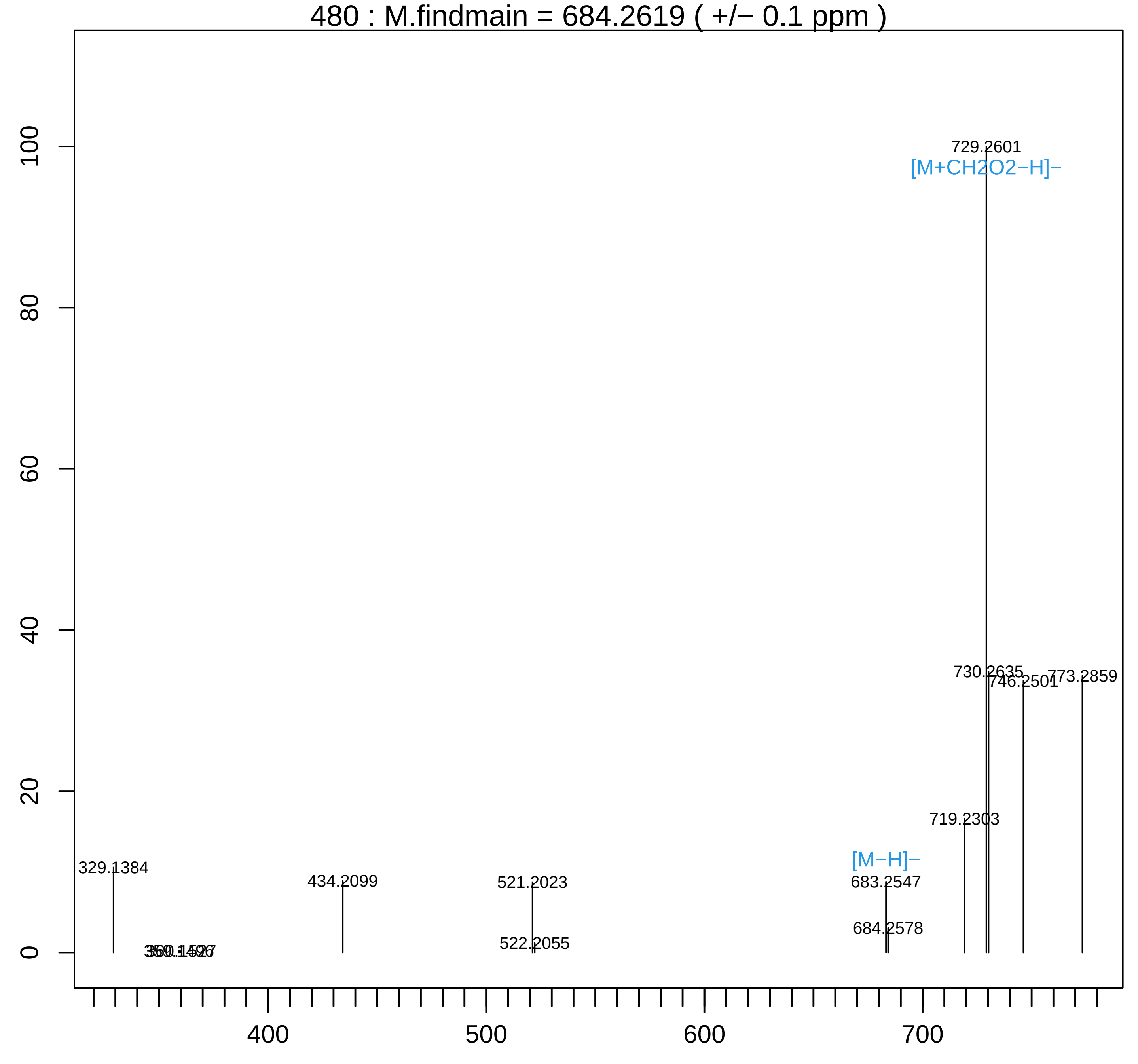
480 : M.findmain = 684.2619 (+/− 0.1 ppm)
729.2601
[M+CH2O2−H]−
730.2635
746.2501
773.2859
719.2303
329.1384
434.2099
521.2023
[M−H]−
683.2547
684.2578
522.2055
0
20
40
60
80
100
400
500
600
700

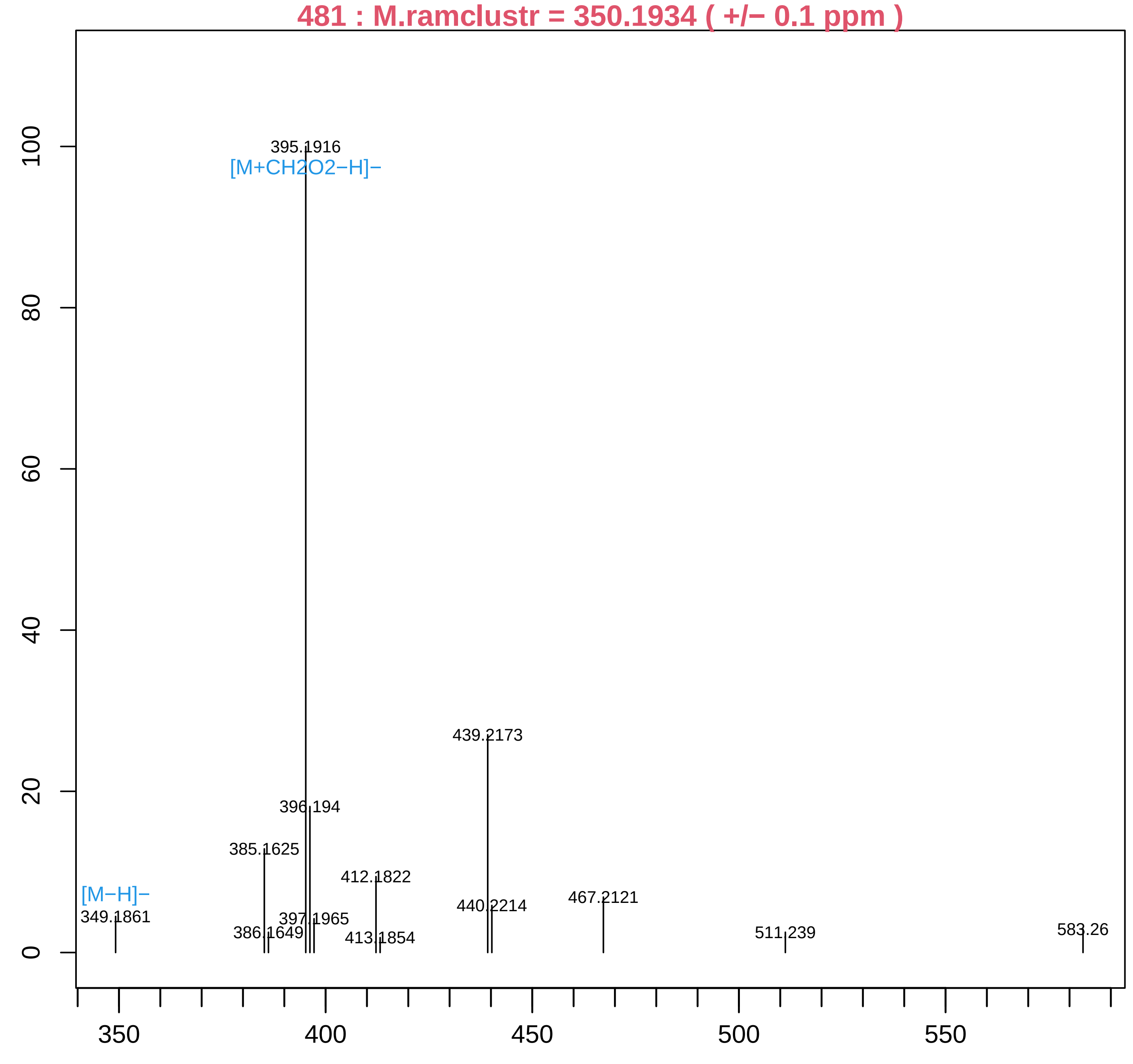
481 : M.ramclustr = 350.1934 (+/− 0.1 ppm)
395.1916
[M+CH2O2−H]−
439.2173
396.194
385.1625
412.1822
[M−H]−
349.1861
397.1965
440.2214
467.2121
386.1649
413.1854
511.239
583.26
0
20
40
60
80
100
350
400
450
500
550

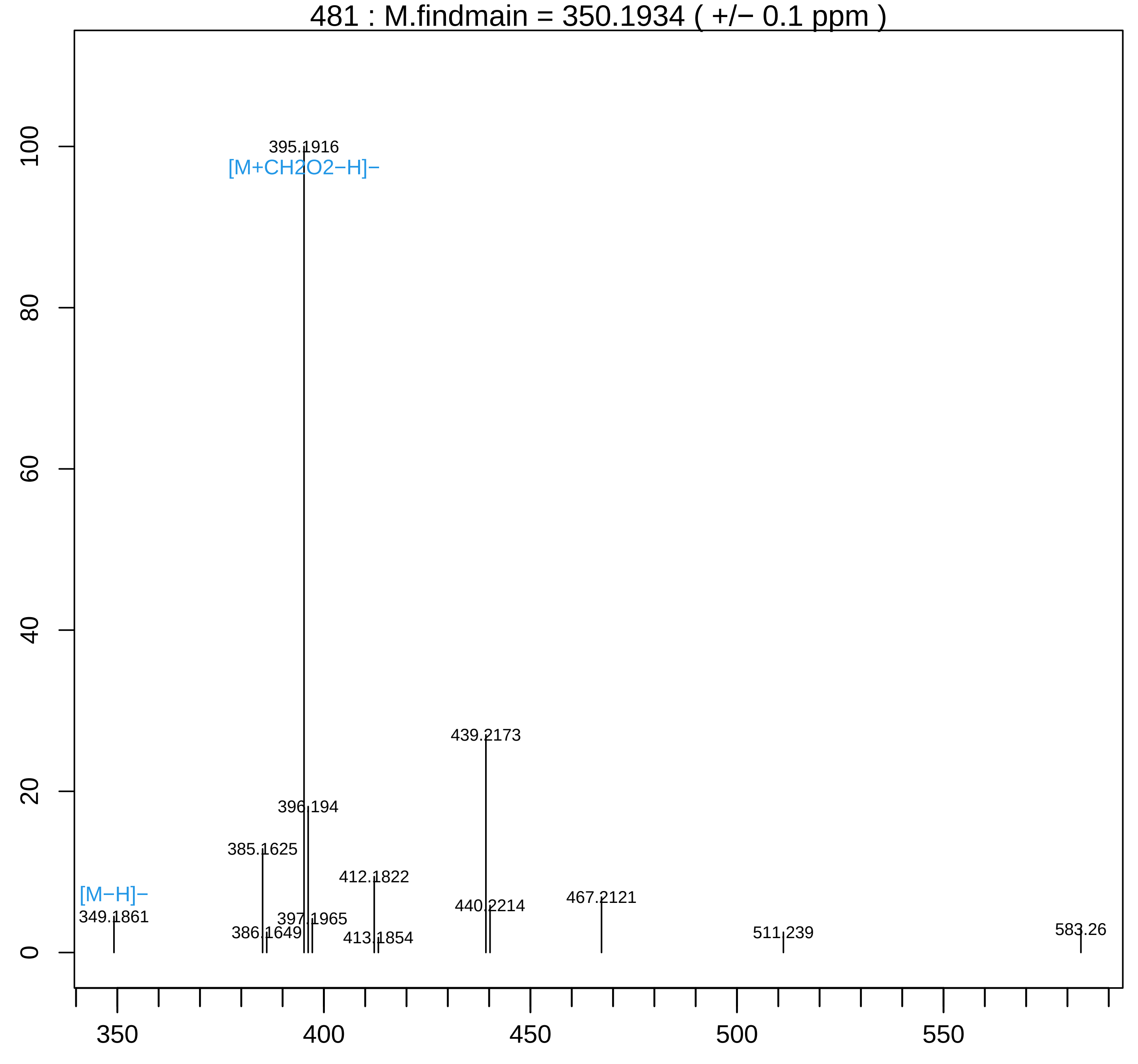
481 : M.findmain = 350.1934 (+/− 0.1 ppm)
395.1916
[M+CH2O2−H]−
439.2173
396.194
385.1625
412.1822
[M−H]−
349.1861
397.1965
440.2214
467.2121
386.1649
413.1854
511.239
583.26
0
20
40
60
80
100
350
400
450
500
550

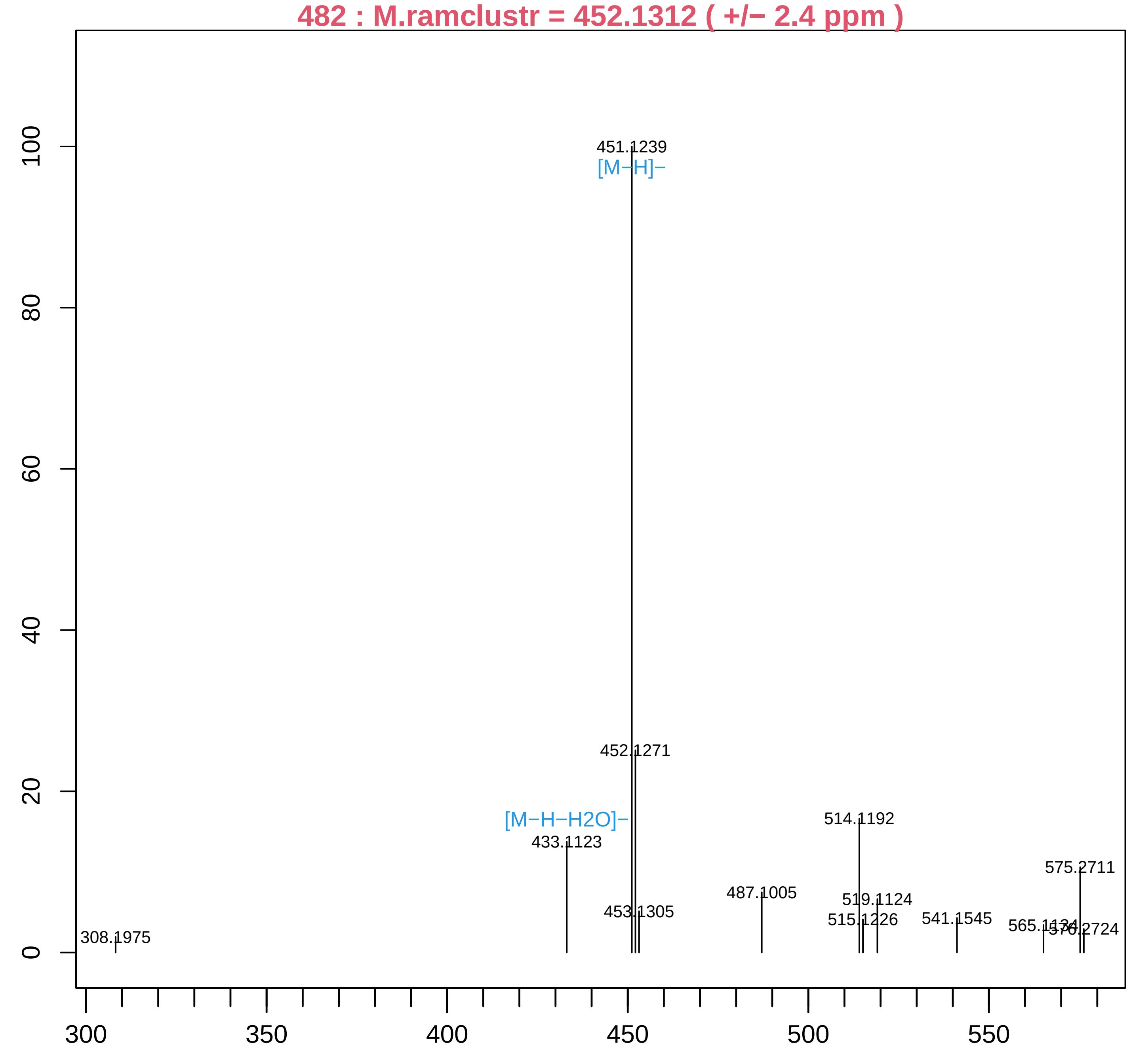
482 : M.ramclustr = 452.1312 (+/− 2.4 ppm)
451.1239
[M−H]−
452.1271
[M−H−H2O]−
433.1123
514.1192
575.2711
487.1005
519.1124
453.1305
515.1226
541.1545
565.1434
576.2724
308.1975
0
20
40
60
80
100
300
350
400
450
500
550

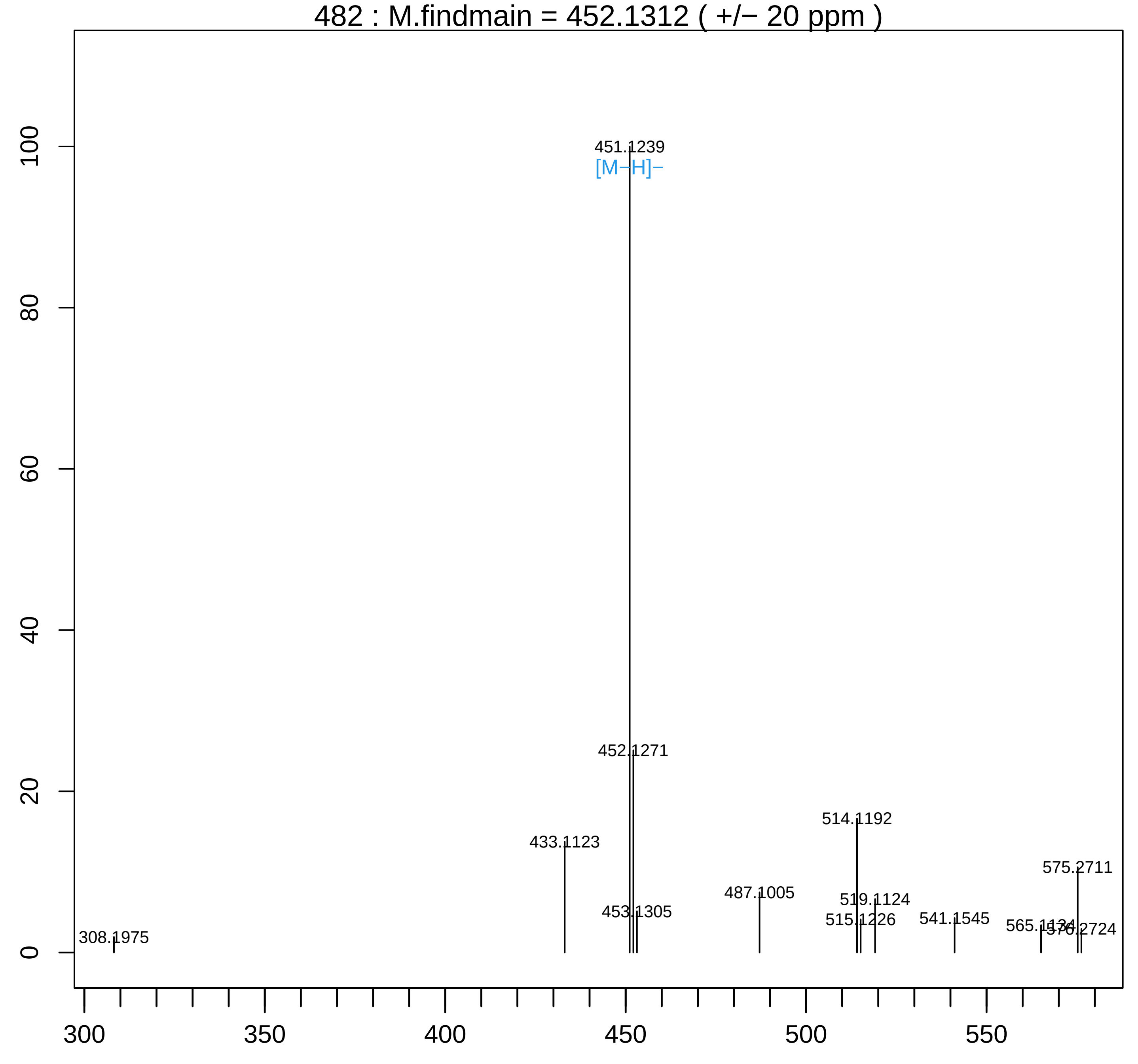
482 : M.findmain = 452.1312 (+/− 20 ppm)
451.1239
[M−H]−
452.1271
433.1123
514.1192
575.2711
487.1005
519.1124
453.1305
515.1226
541.1545
565.1434
576.2724
308.1975
0
20
40
60
80
100
300
350
400
450
500
550

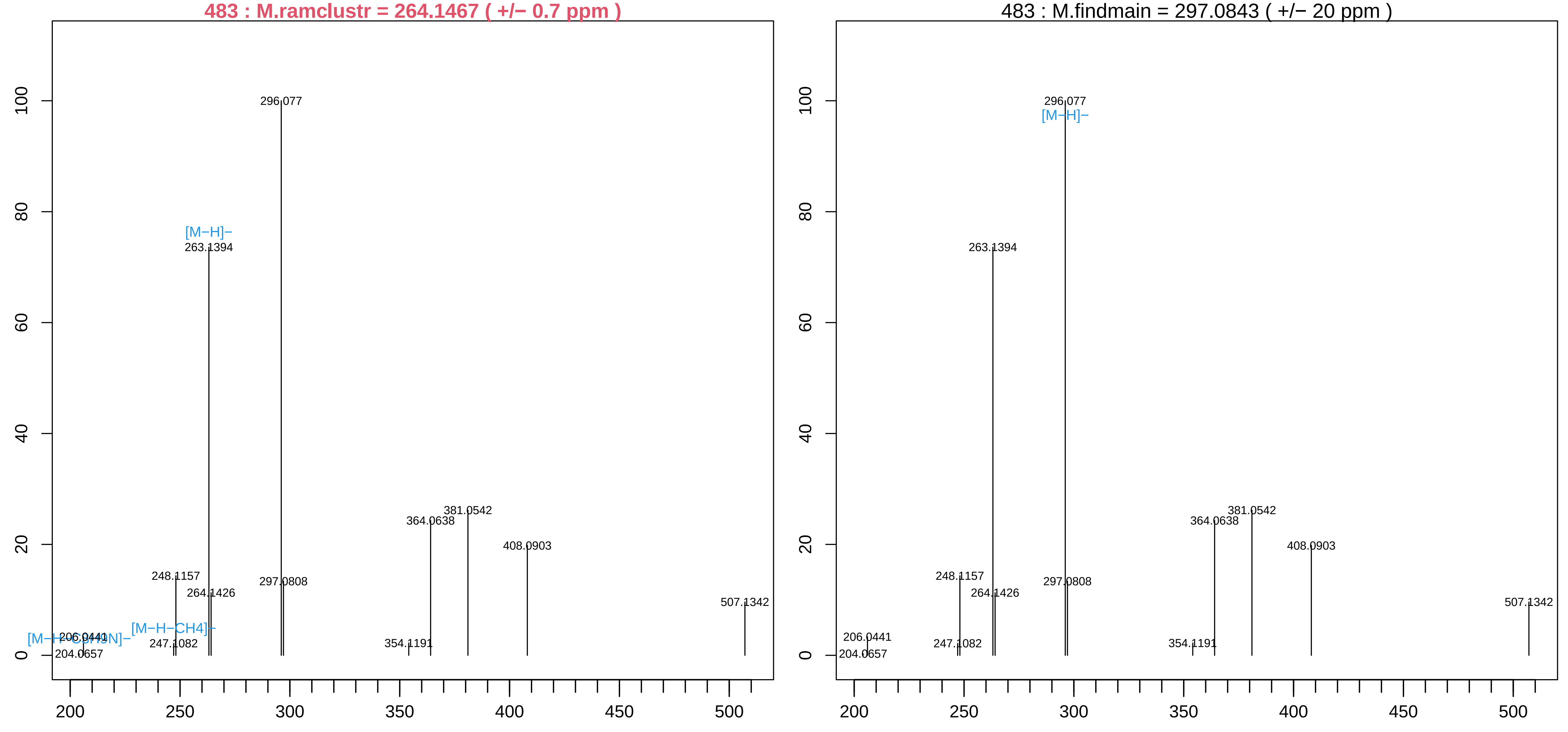
483 : M.ramclustr = 264.1467 (+/− 0.7 ppm)
[M−H]−
263.1394
296.077
248.1157
264.1426
297.0808
[M−H−CH4]−
206.0441
247.1082
204.0657
354.1191
364.0638
381.0542
408.0903
507.1342
483 : M.findmain = 297.0843 (+/− 20 ppm)
296.077
[M−H]−
263.1394
248.1157
264.1426
297.0808
206.0441
247.1082
204.0657
354.1191
364.0638
381.0542
408.0903
507.1342
0
20
40
60
80
100
200
250
300
350
400
450
500

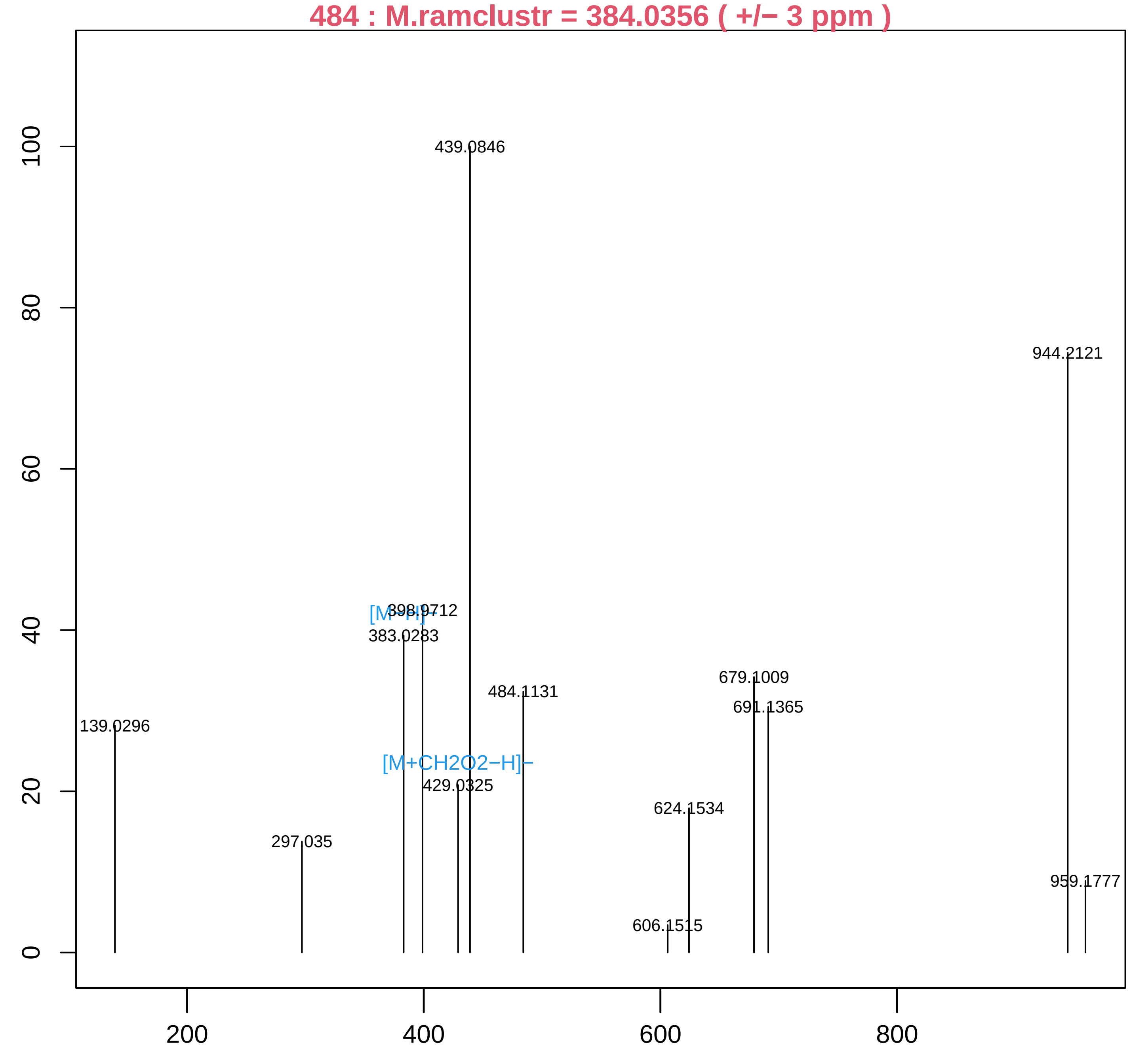

484 : M.ramclustr = 384.0356 (+/− 3 ppm)
0
20
40
60
80
100
200
400
600
800
439.0846
944.2121
[M−H]−
398.9712
383.0283
484.1131
679.1009
691.1365
139.0296
[M+CH2O2−H]−
429.0325
624.1534
297.035
959.1777
606.1515

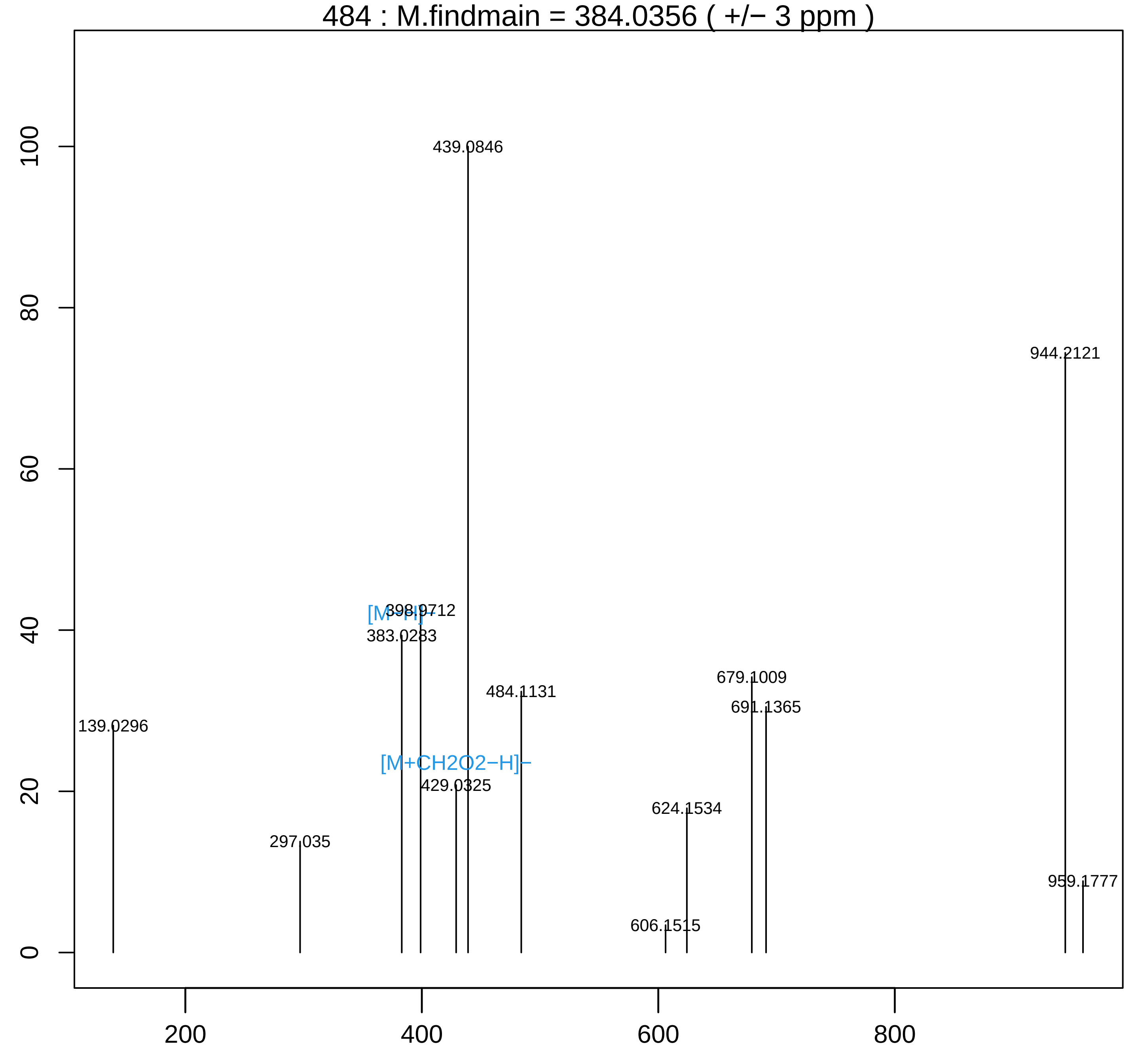

484 : M.findmain = 384.0356 (+/− 3 ppm)
0
20
40
60
80
100
200
400
600
800
439.0846
944.2121
[M−H]−
398.9712
383.0283
484.1131
679.1009
691.1365
139.0296
[M+CH2O2−H]−
429.0325
624.1534
297.035
959.1777
606.1515

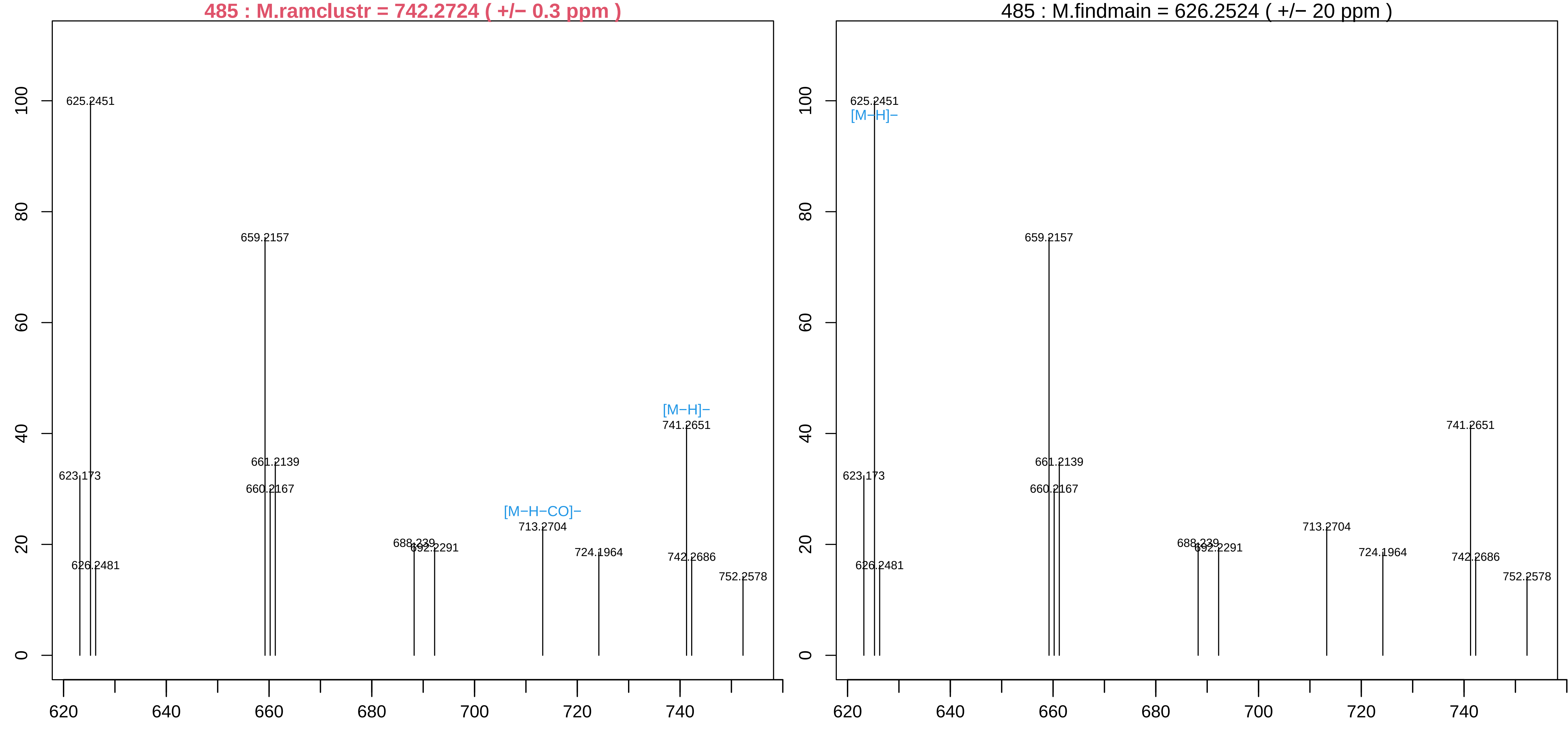
485 : M.ramclustr = 742.2724 (+/− 0.3 ppm)
625.2451
659.2157
[M−H]−
741.2651
661.2139
623.173
660.2167
[M−H−CO]−
713.2704
688.239
692.2291
724.1964
742.2686
626.2481
752.2578
485 : M.findmain = 626.2524 (+/− 20 ppm)
625.2451
[M−H]−
659.2157
741.2651
661.2139
623.173
660.2167
713.2704
688.239
692.2291
724.1964
742.2686
626.2481
752.2578

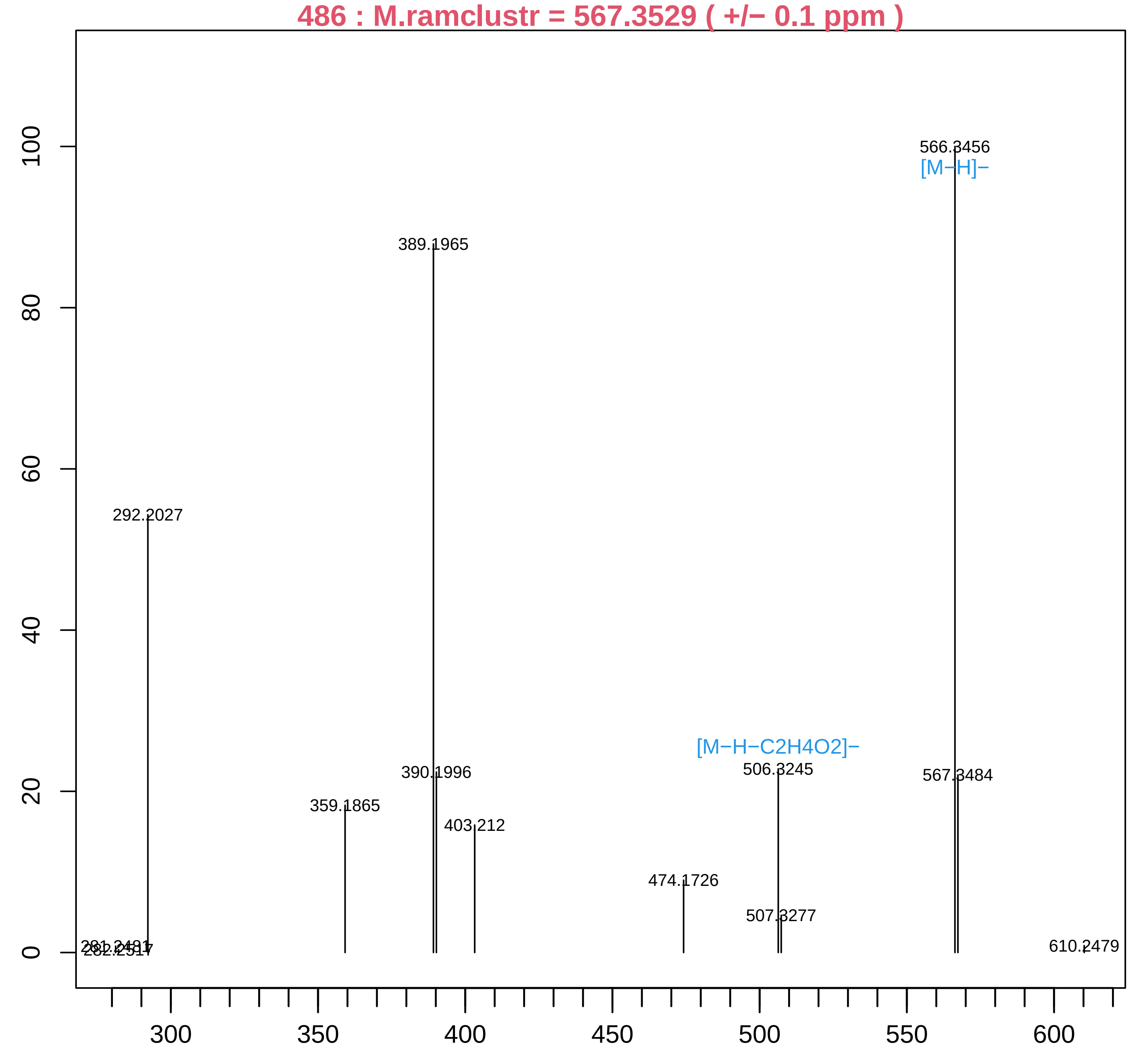
486 : M.ramclustr = 567.3529 (+/− 0.1 ppm)
566.3456
[M−H]−
389.1965
292.2027
[M−H−C2H4O2]−
506.3245
390.1996
567.3484
359.1865
403.212
474.1726
507.3277
281.2491
282.2517
610.2479
0
20
40
60
80
100
300
350
400
450
500
550
600

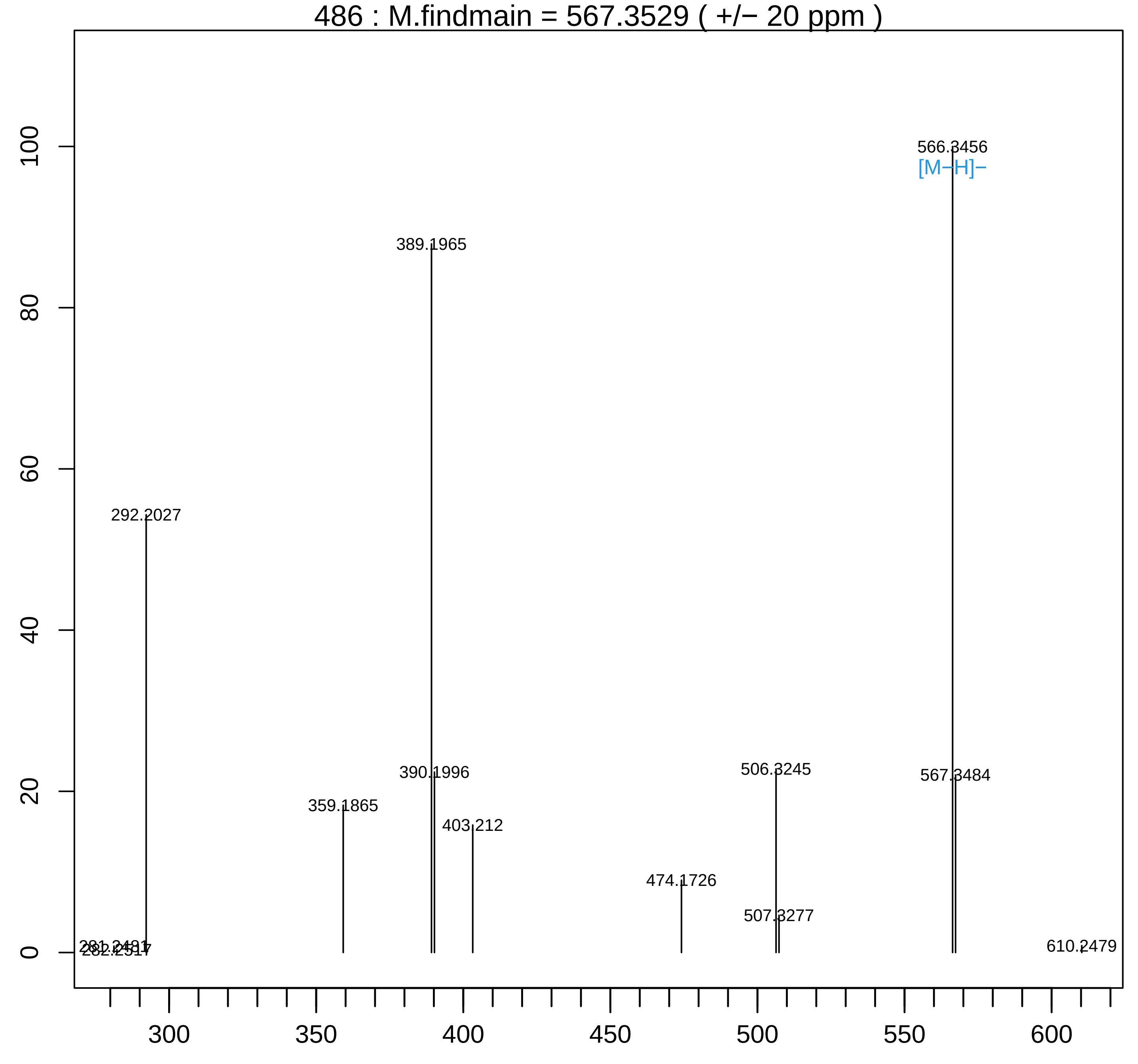
486 : M.findmain = 567.3529 (+/− 20 ppm)
566.3456
[M−H]−
389.1965
292.2027
506.3245
390.1996
567.3484
359.1865
403.212
474.1726
507.3277
281.2491
282.2517
610.2479
0
20
40
60
80
100
300
350
400
450
500
550
600

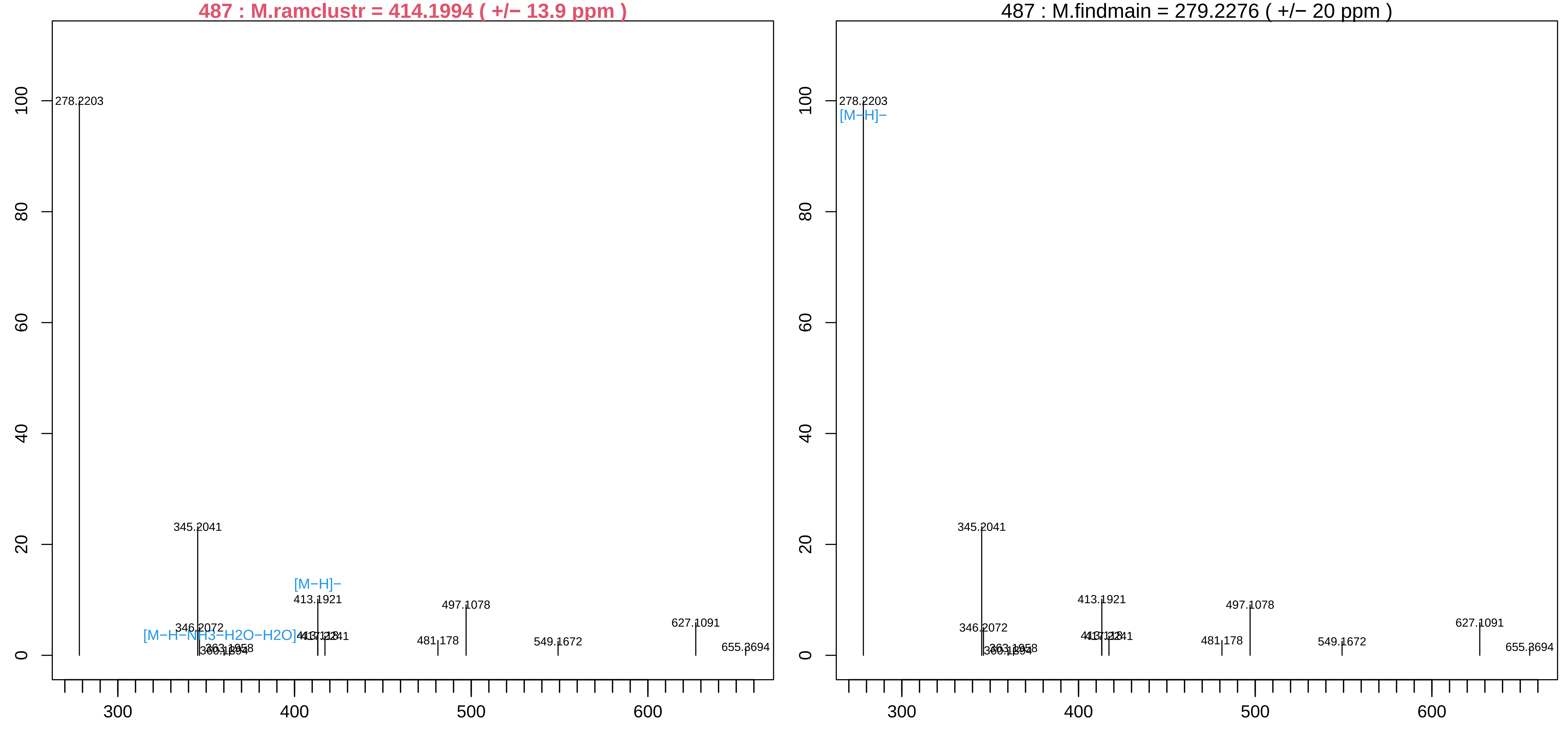
487 : M.ramclustr = 414.1994 (+/− 13.9 ppm)
278.2203
345.2041
[M−H]−
413.1921
497.1078
627.1091
655.3694
346.2072
[M−H−NH3−H2O−H2O]−
481.178
549.1672
487 : M.findmain = 279.2276 (+/− 20 ppm)
278.2203
[M−H]−
345.2041
413.1921
497.1078
627.1091
346.2072
481.178
549.1672
655.3694
0
20
40
60
80
100
300
400
500
600

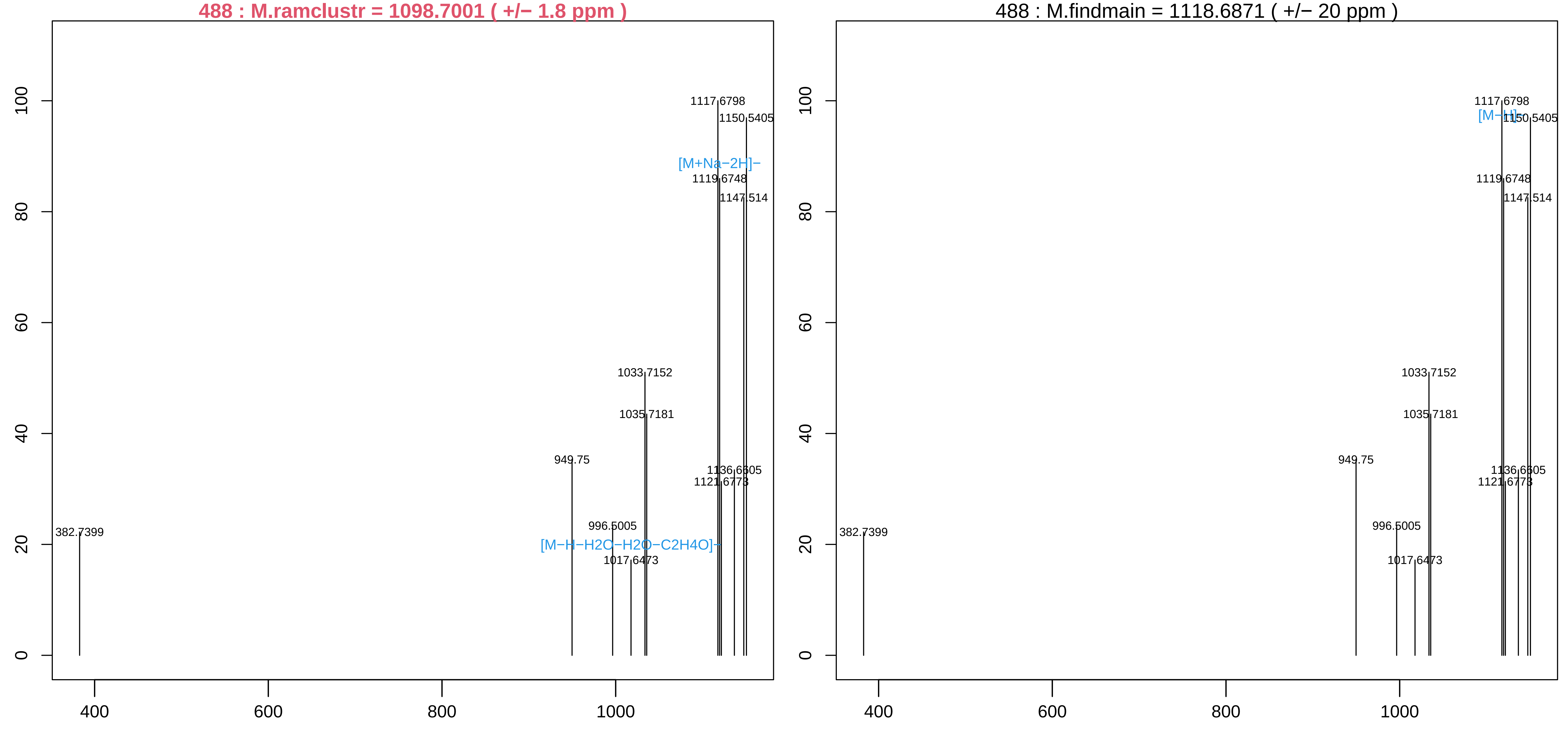

488 : M.ramclustr = 1098.7001 (+/− 1.8 ppm)
1117.6798
1150.5405
[M+Na−2H]−
1119.6748
1147.514
1033.7152
1035.7181
949.75
1136.6605
1121.6773
996.5005
[M−H−H2O−H2O−C2H4O]−
1017.6473
382.7399
488 : M.findmain = 1118.6871 (+/− 20 ppm)
1117.6798
[M−H]−
1150.5405
1119.6748
1147.514
1033.7152
1035.7181
949.75
1136.6605
1121.6773
996.5005
1017.6473
382.7399
0
20
40
60
80
100
400
600
800
1000

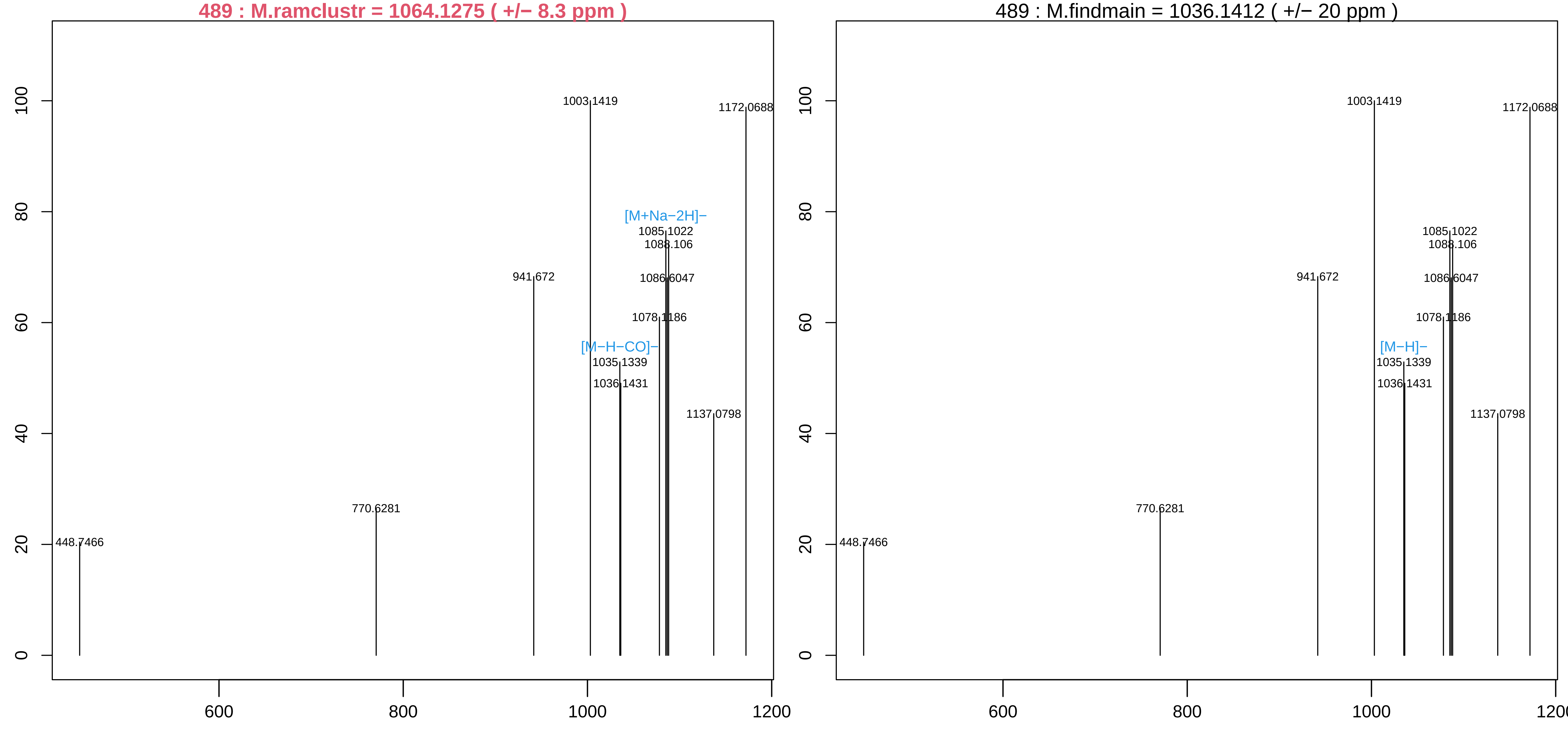

489 : M.ramclustr = 1064.1275 (+/− 8.3 ppm)
489 : M.findmain = 1036.1412 (+/− 20 ppm)
0
20
40
60
80
100
600
800
1000
1200
448.7466
770.6281
941.672
1003.1419
[M−H−CO]−
1035.1339
1036.1431
1078.1186
[M+Na−2H]−
1085.1022
1088.106
1086.6047
1137.0798
1172.0688
[M−H]−

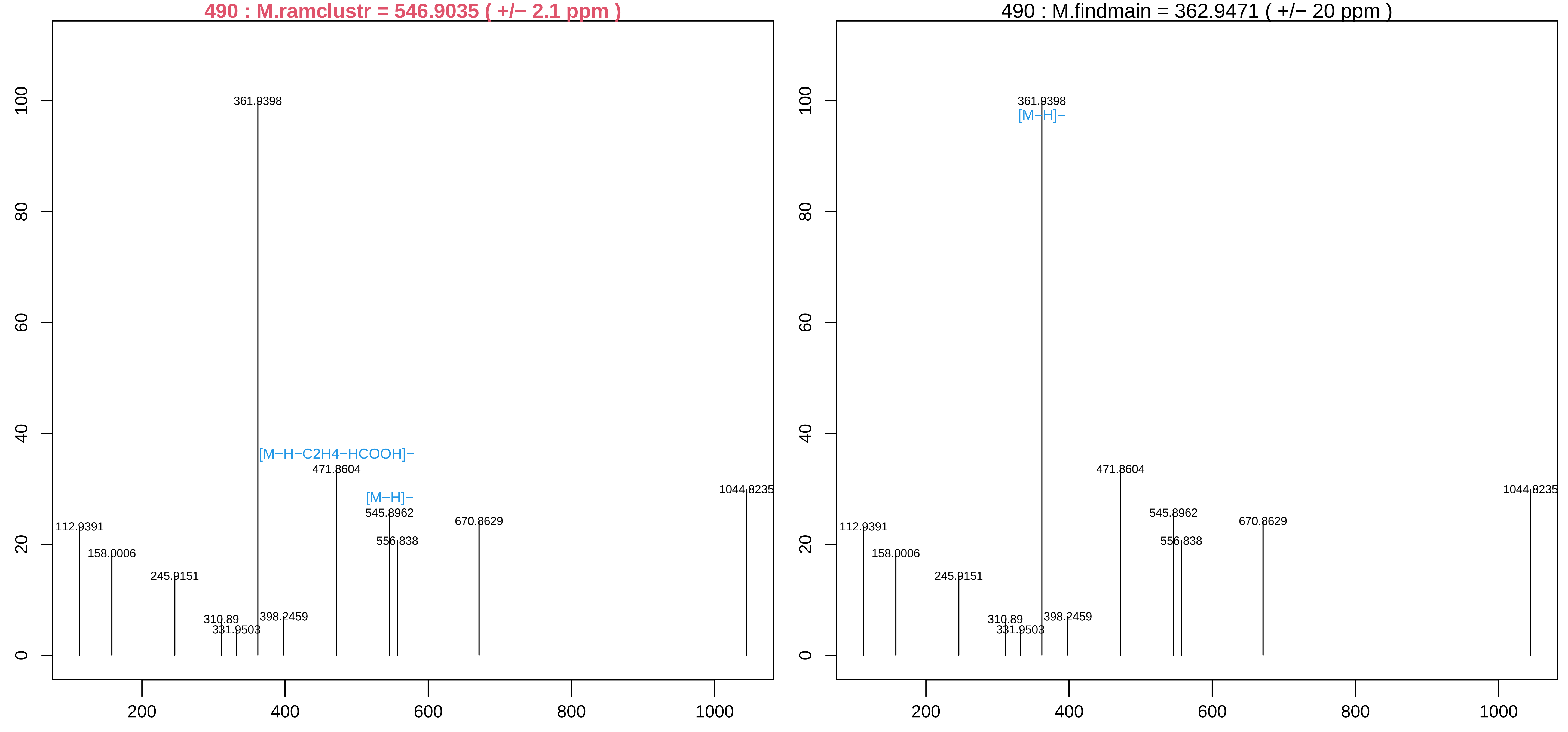

490 : M.ramclustr = 546.9035 (+/− 2.1 ppm)
361.9398
[M−H−C2H4−HCOOH]−
471.8604
[M−H]−
545.8962
556.838
670.8629
1044.8235
112.9391
158.0006
245.9151
310.89
331.9503
398.2459
0
20
40
60
80
100
200
400
600
800
1000
490 : M.findmain = 362.9471 (+/− 20 ppm)
361.9398
[M−H]−
471.8604
545.8962
556.838
670.8629
1044.8235
112.9391
158.0006
245.9151
310.89
331.9503
398.2459

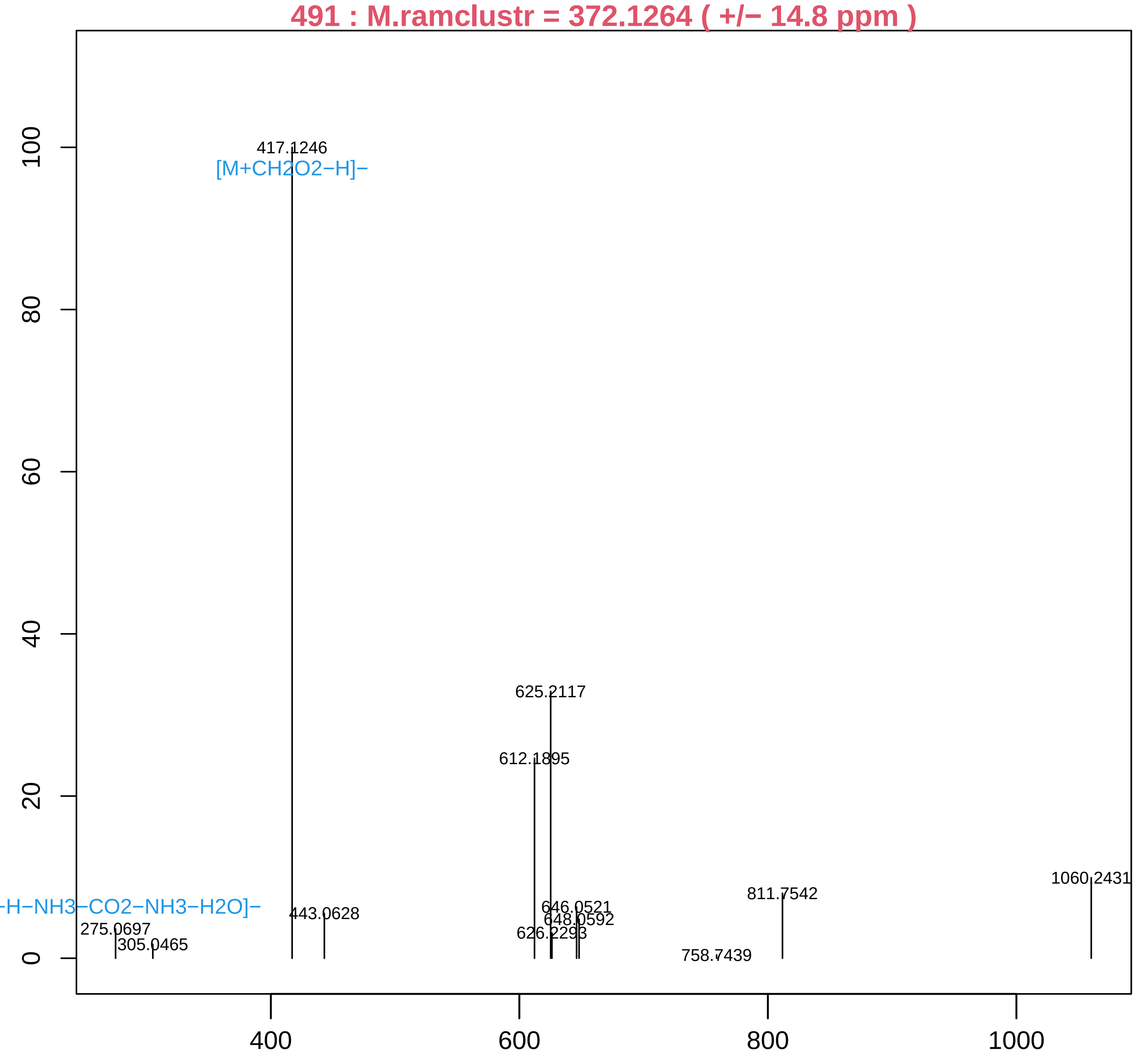
491 : M.ramclustr = 372.1264 (+/− 14.8 ppm)
417.1246
[M+CH2O2−H]−
-H−NH3−CO2−NH3−H2O]−
275.0697
305.0465
443.0628
612.1895
625.2117
626.2293
646.0521
648.0592
758.7439
811.7542
1060.2431
0
20
40
60
80
100
400
600
800
1000

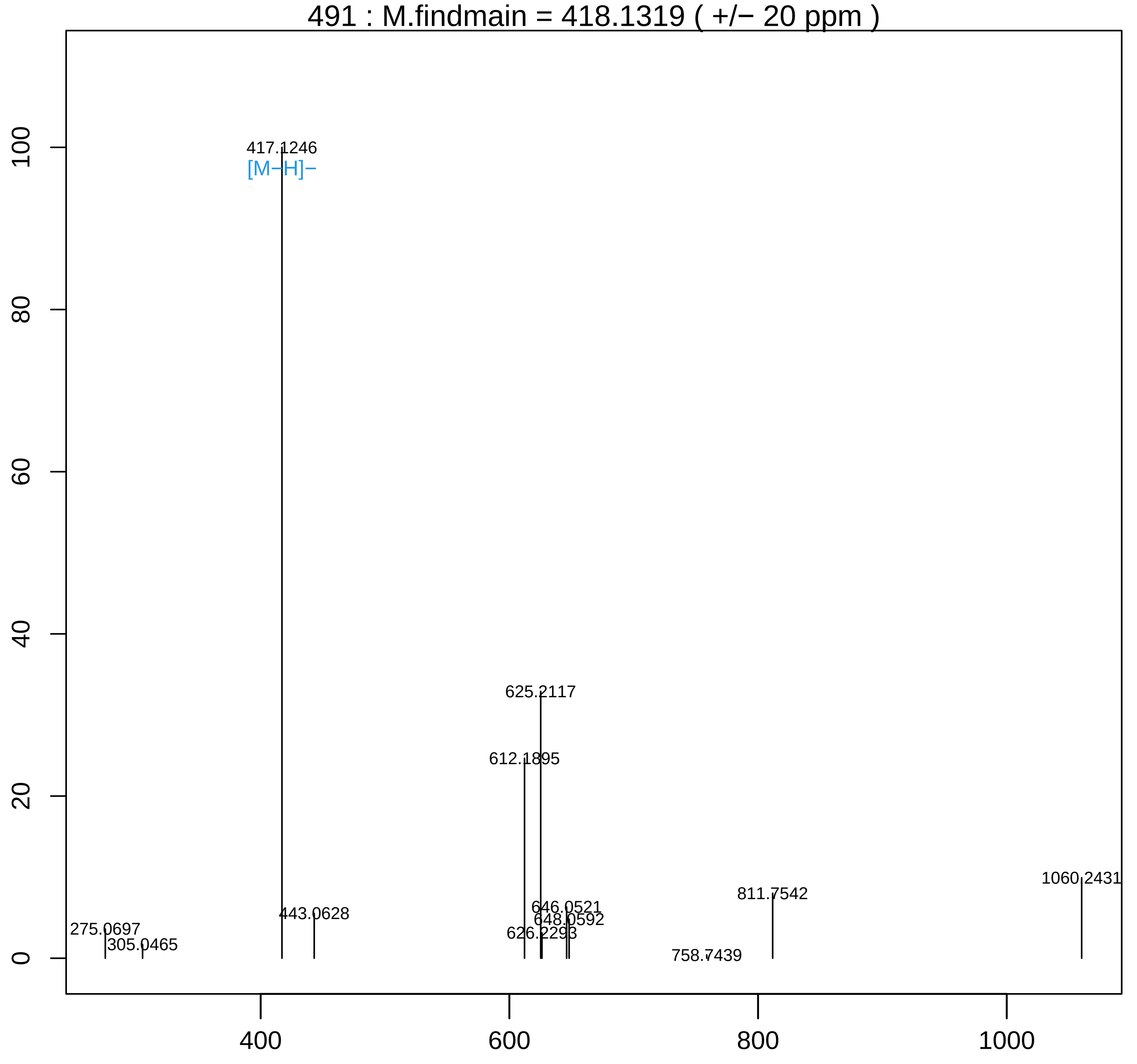
491 : M.findmain = 418.1319 (+/− 20 ppm)
417.1246
[M−H]−
275.0697
305.0465
443.0628
612.1895
625.2117
626.2293
646.0521
648.0592
758.7439
811.7542
1060.2431
0
20
40
60
80
100
400
600
800
1000

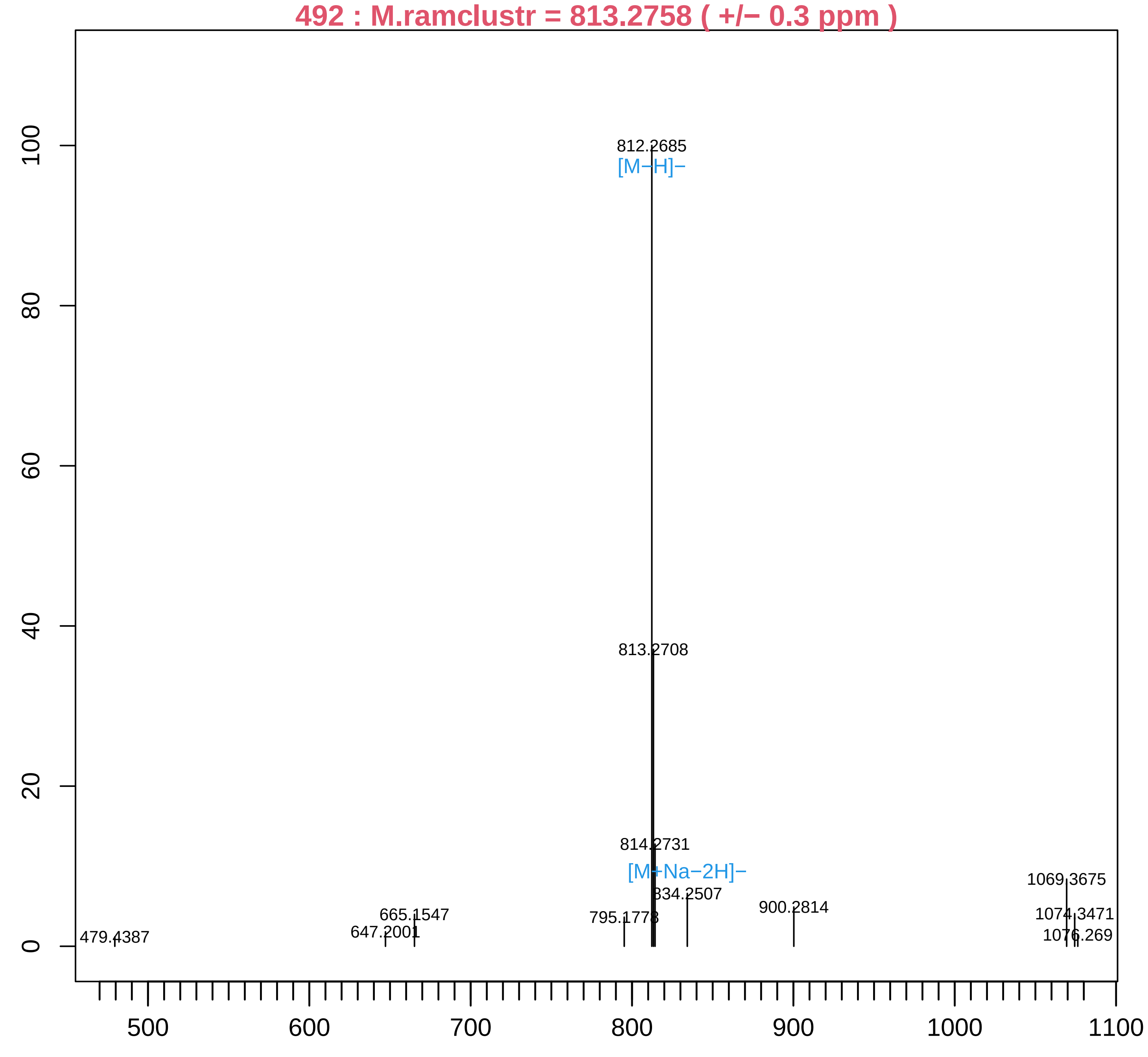

492 : M.ramclustr = 813.2758 (+/− 0.3 ppm)
812.2685
[M−H]−
813.2708
814.2731
[M+Na−2H]−
834.2507
900.2814
795.1773
665.1547
647.2001
479.4387
1069.3675
1074.3471
1076.269
0
20
40
60
80
100
500
600
700
800
900
1000
1100

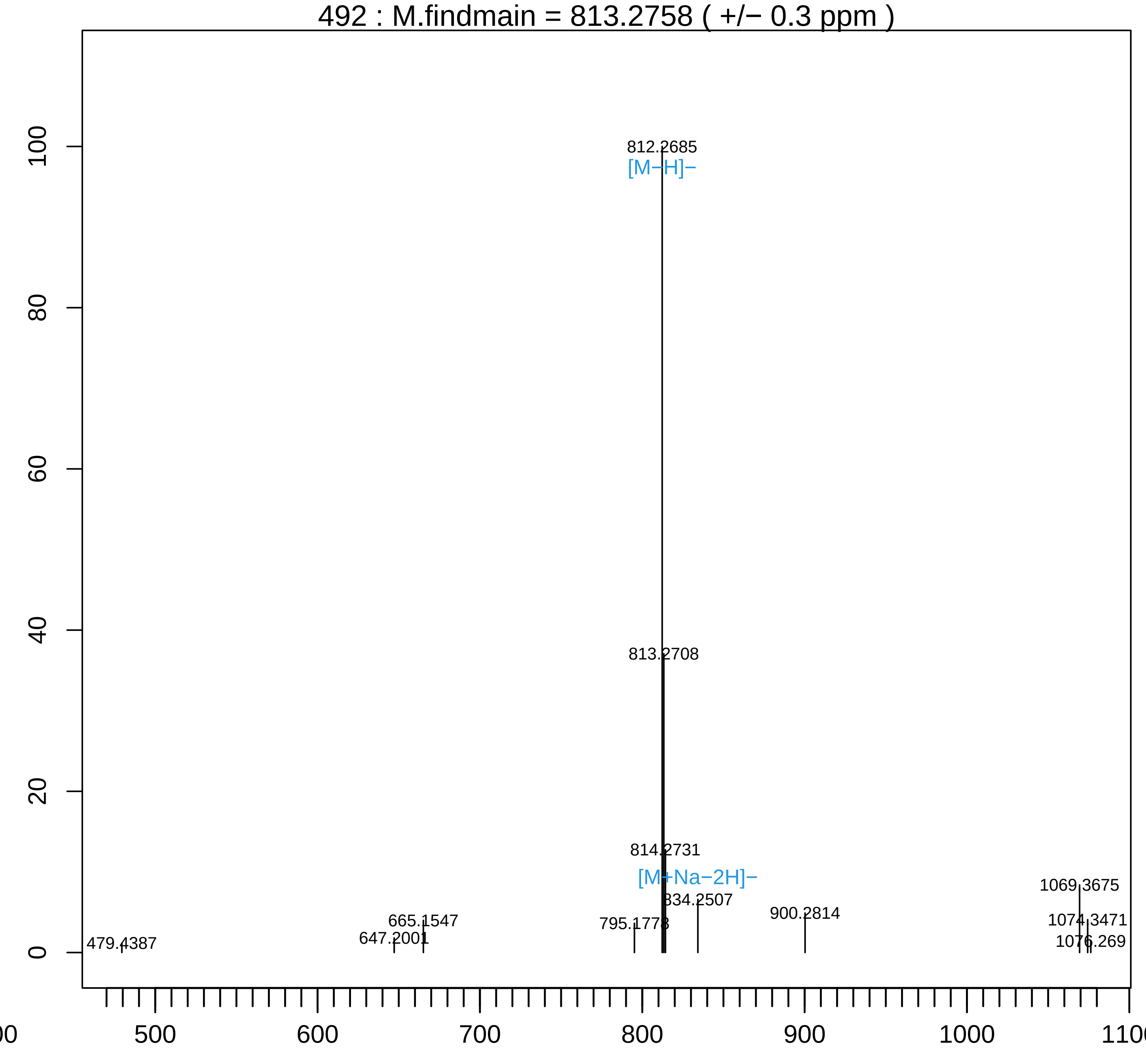

492 : M.findmain = 813.2758 (+/− 0.3 ppm)
812.2685
[M−H]−
813.2708
814.2731
[M+Na−2H]−
834.2507
900.2814
795.1773
665.1547
647.2001
479.4387
1069.3675
1074.3471
1076.269
0
20
40
60
80
100
500
600
700
800
900
1000
1100

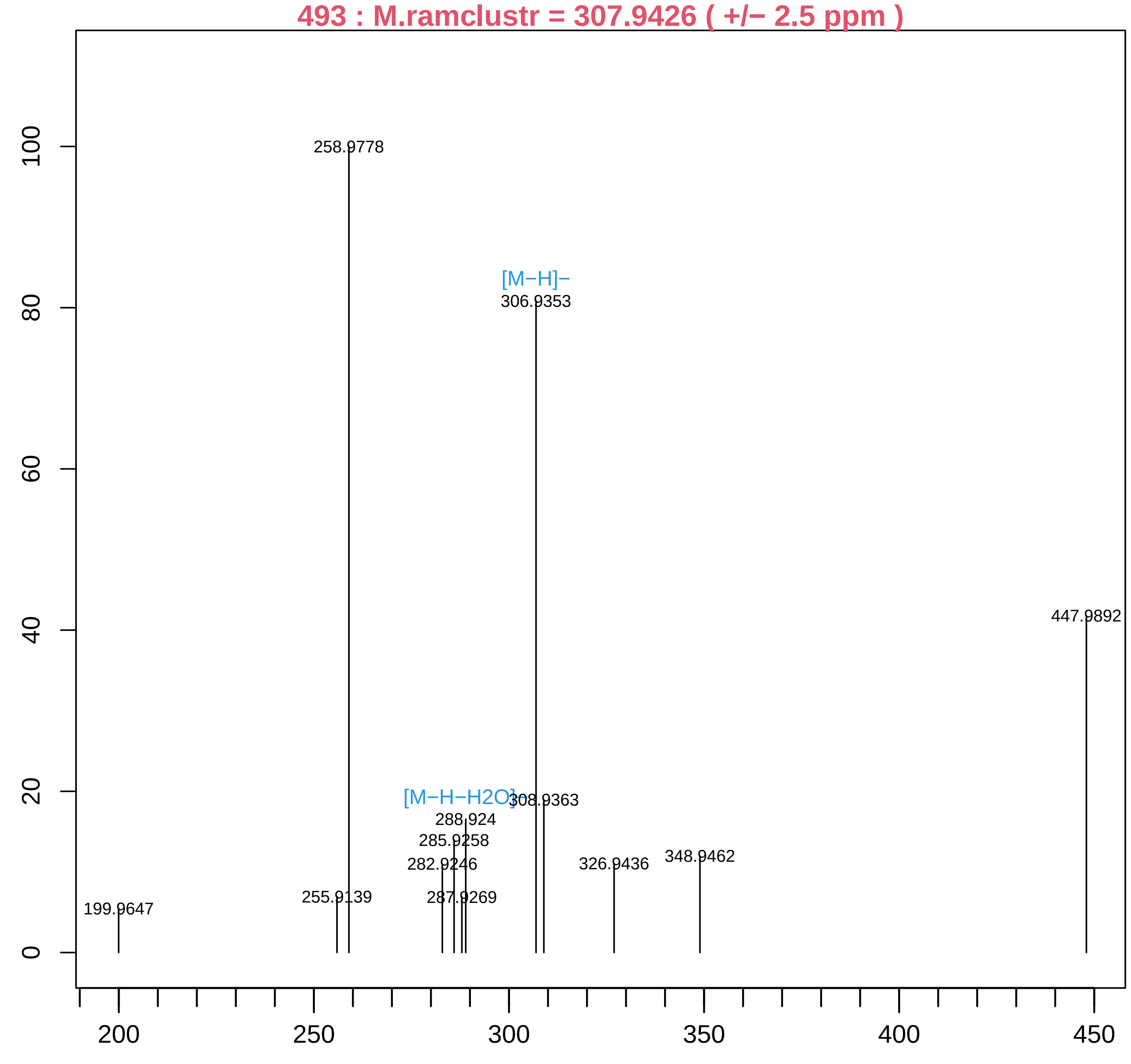

493 : M.ramclustr = 307.9426 (+/− 2.5 ppm)
258.9778
[M−H]−
306.9353
447.9892
[M−H−H2O]
308.9363
288.924
285.9258
282.9246
326.9436
348.9462
199.9647
255.9139
287.9269
0
20
40
60
80
100
200
250
300
350
400
450

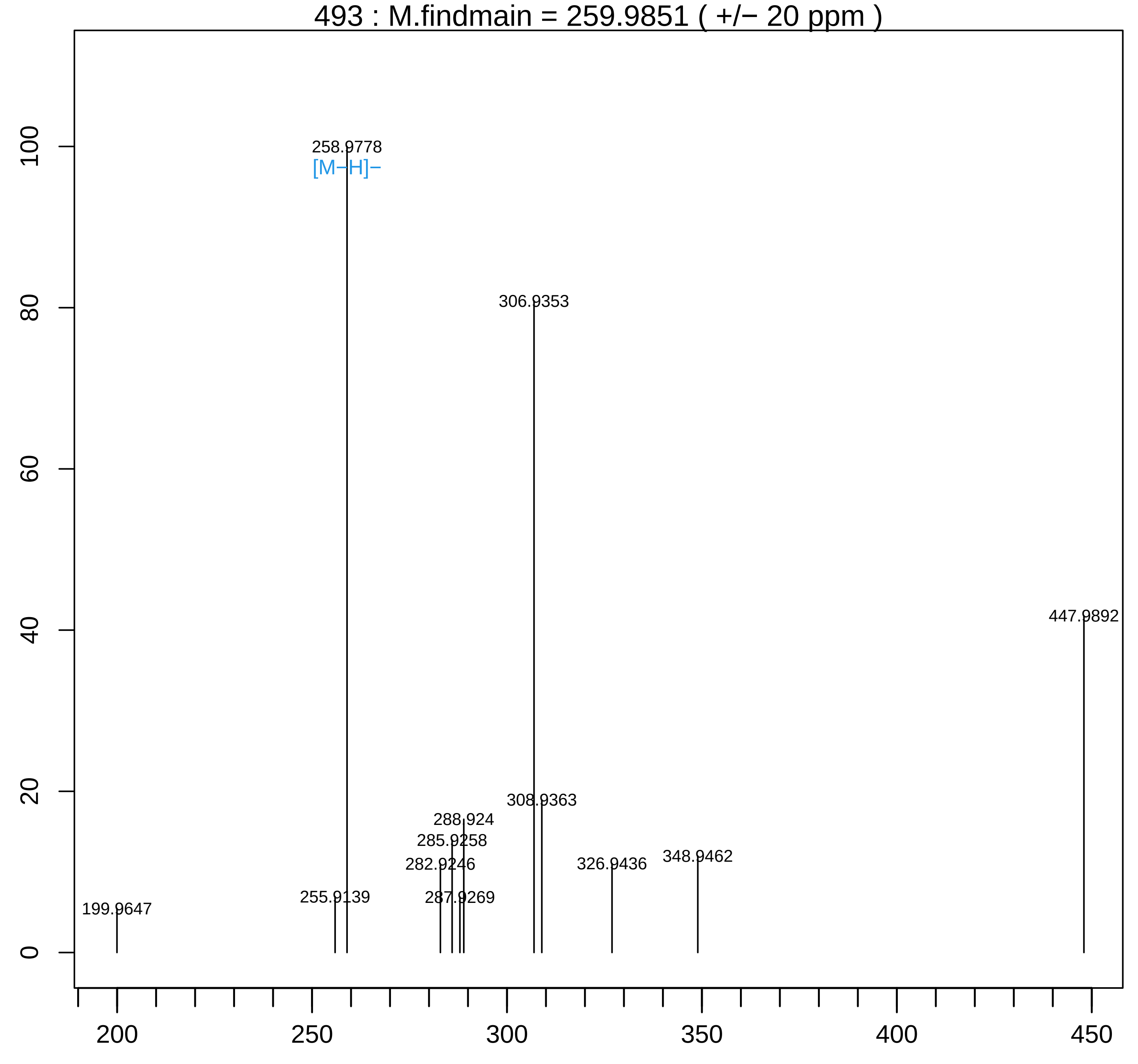

493 : M.findmain = 259.9851 (+/− 20 ppm)
258.9778
[M−H]−
306.9353
447.9892
308.9363
288.924
285.9258
282.9246
326.9436
348.9462
199.9647
255.9139
287.9269
0
20
40
60
80
100
200
250
300
350
400
450

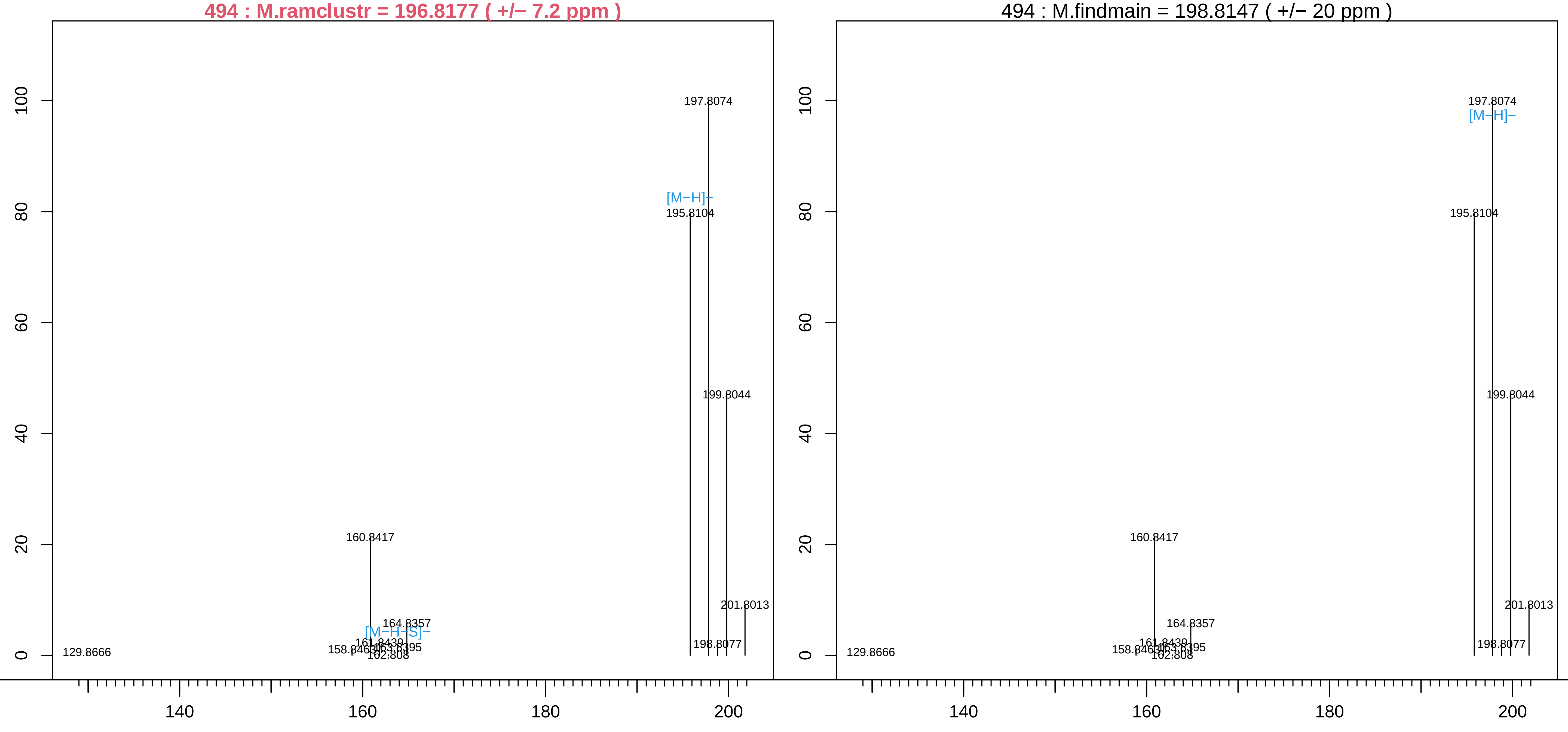
494 : M.ramclustr = 196.8177 (+/− 7.2 ppm)
197.8074
[M−H]−
195.8104
199.8044
160.8417
201.8013
164.8357
[M−H−S]−
161.8439
158.8463
163.8395
162.808
198.8077
129.8666
494 : M.findmain = 198.8147 (+/− 20 ppm)
197.8074
[M−H]−
195.8104
199.8044
160.8417
201.8013
164.8357
161.8439
158.8463
163.8395
162.808
198.8077
129.8666
0
20
40
60
80
100
140
160
180
200

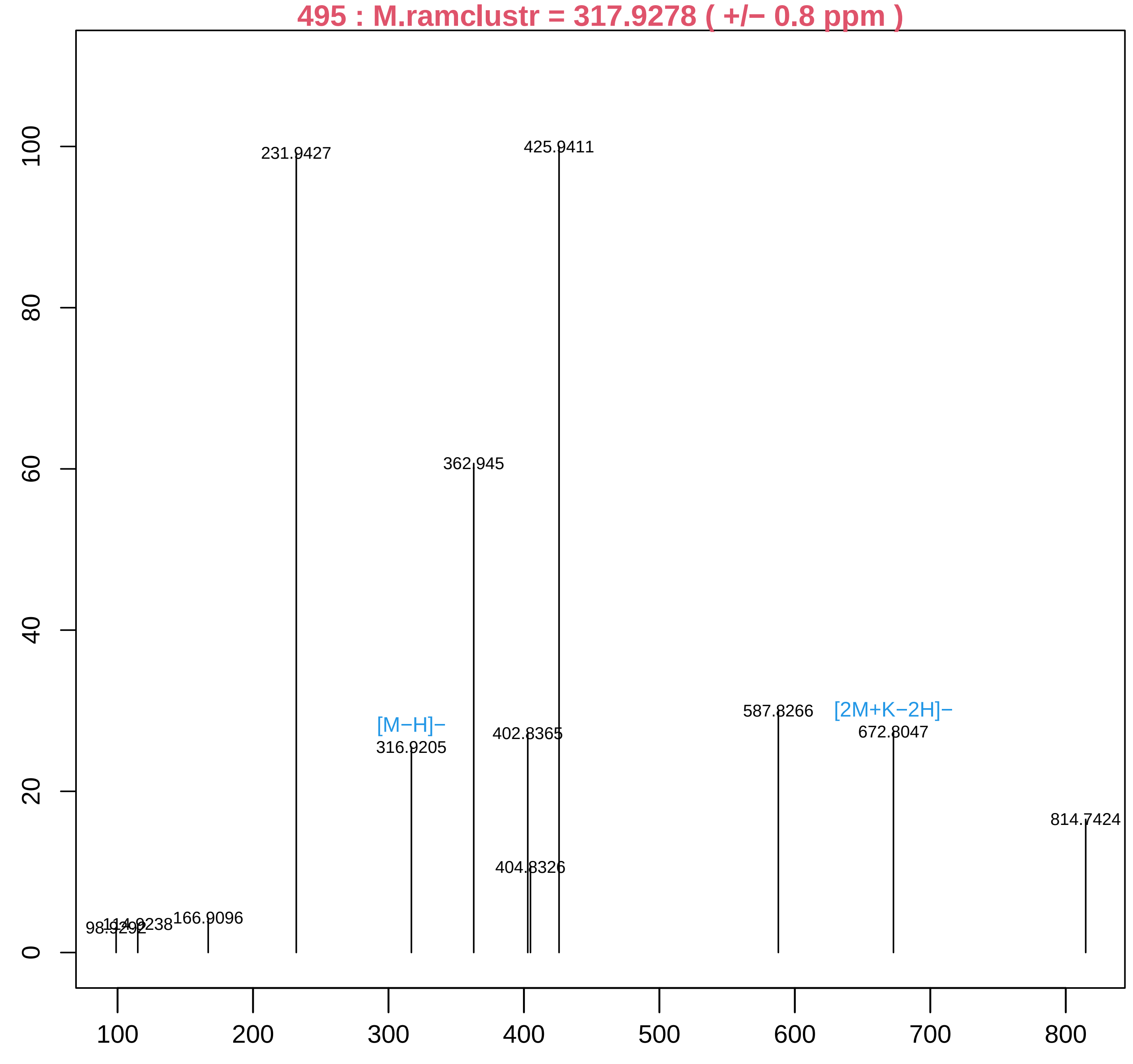
495 : M.ramclustr = 317.9278 (+/− 0.8 ppm)
231.9427
425.9411
362.945
[M−H]−
316.9205
402.8365
404.8326
587.8266
[2M+K−2H]−
672.8047
814.7424
98.9292
114.9238
166.9096
0
20
40
60
80
100
100
200
300
400
500
600
700
800

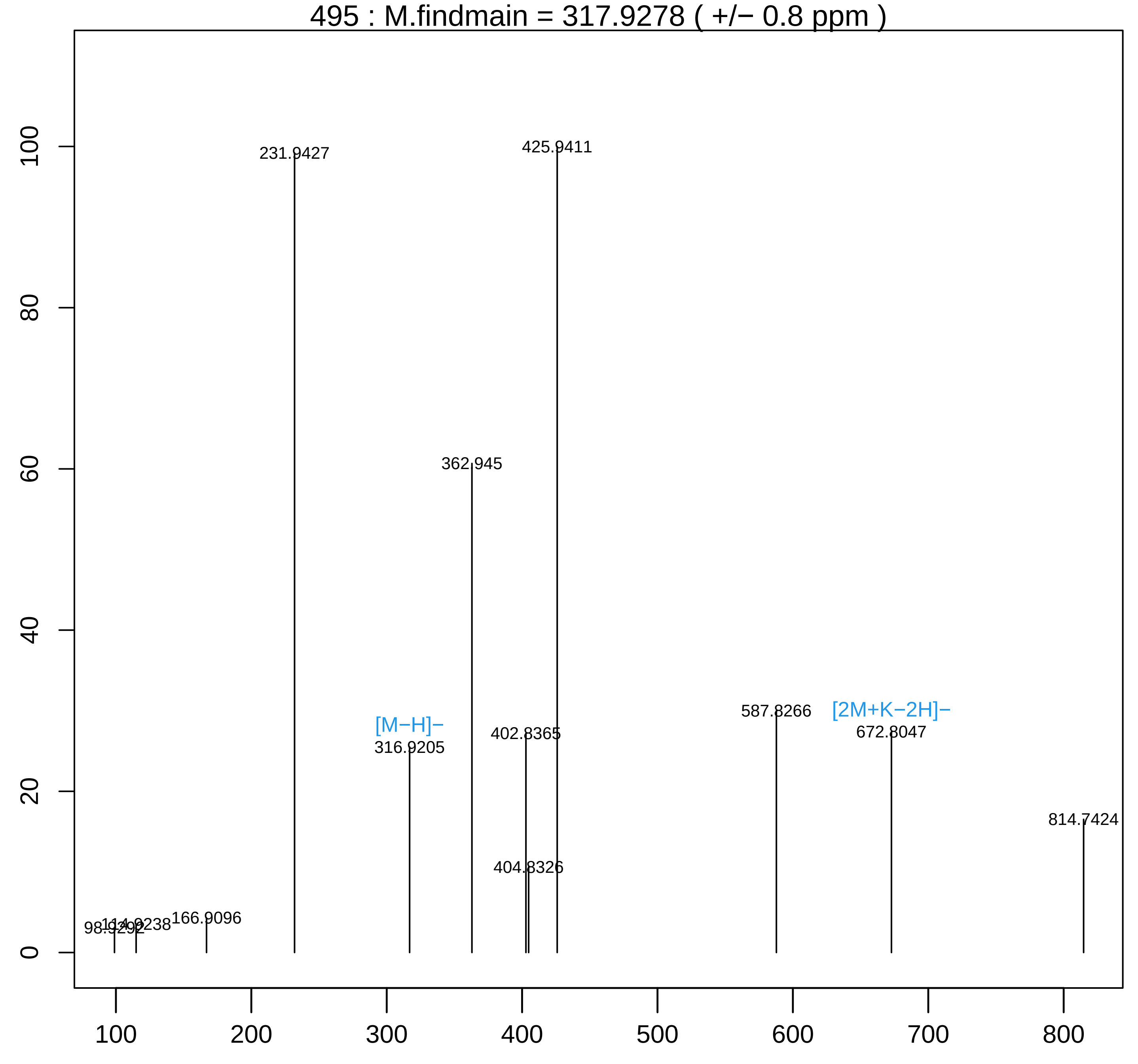
495 : M.findmain = 317.9278 (+/− 0.8 ppm)
231.9427
425.9411
362.945
[M−H]−
316.9205
402.8365
404.8326
587.8266
[2M+K−2H]−
672.8047
814.7424
98.9292
114.9238
166.9096
0
20
40
60
80
100
100
200
300
400
500
600
700
800

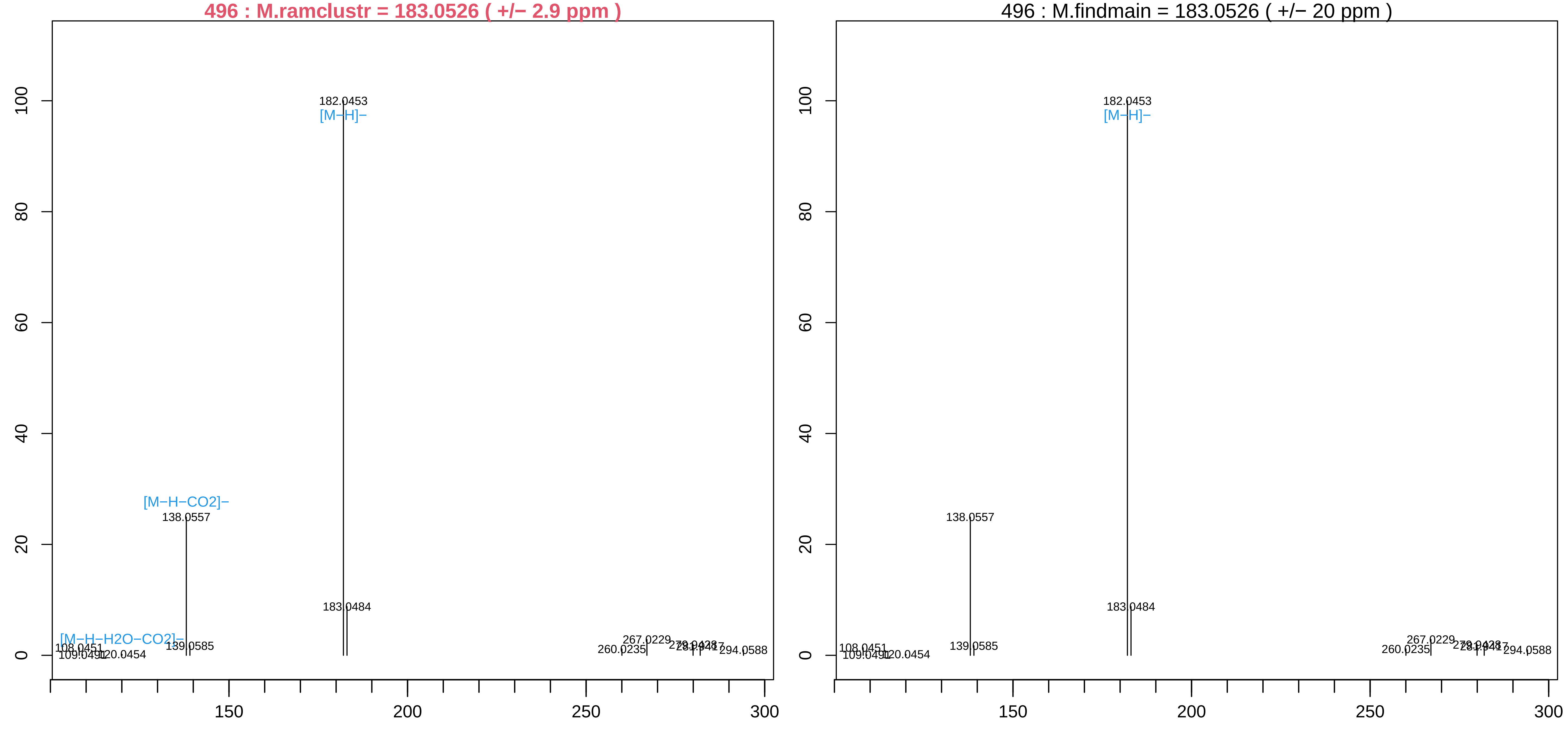

496 : M.ramclustr = 183.0526 (+/− 2.9 ppm)
496 : M.findmain = 183.0526 (+/− 20 ppm)
182.0453
[M−H]−
[M−H−CO2]−
138.0557
183.0484
[M−H−H2O−CO2]−
108.0451
109.0491
120.0454
139.0585
260.0235
267.0229
294.0588
0
20
40
60
80
100
150
200
250
300

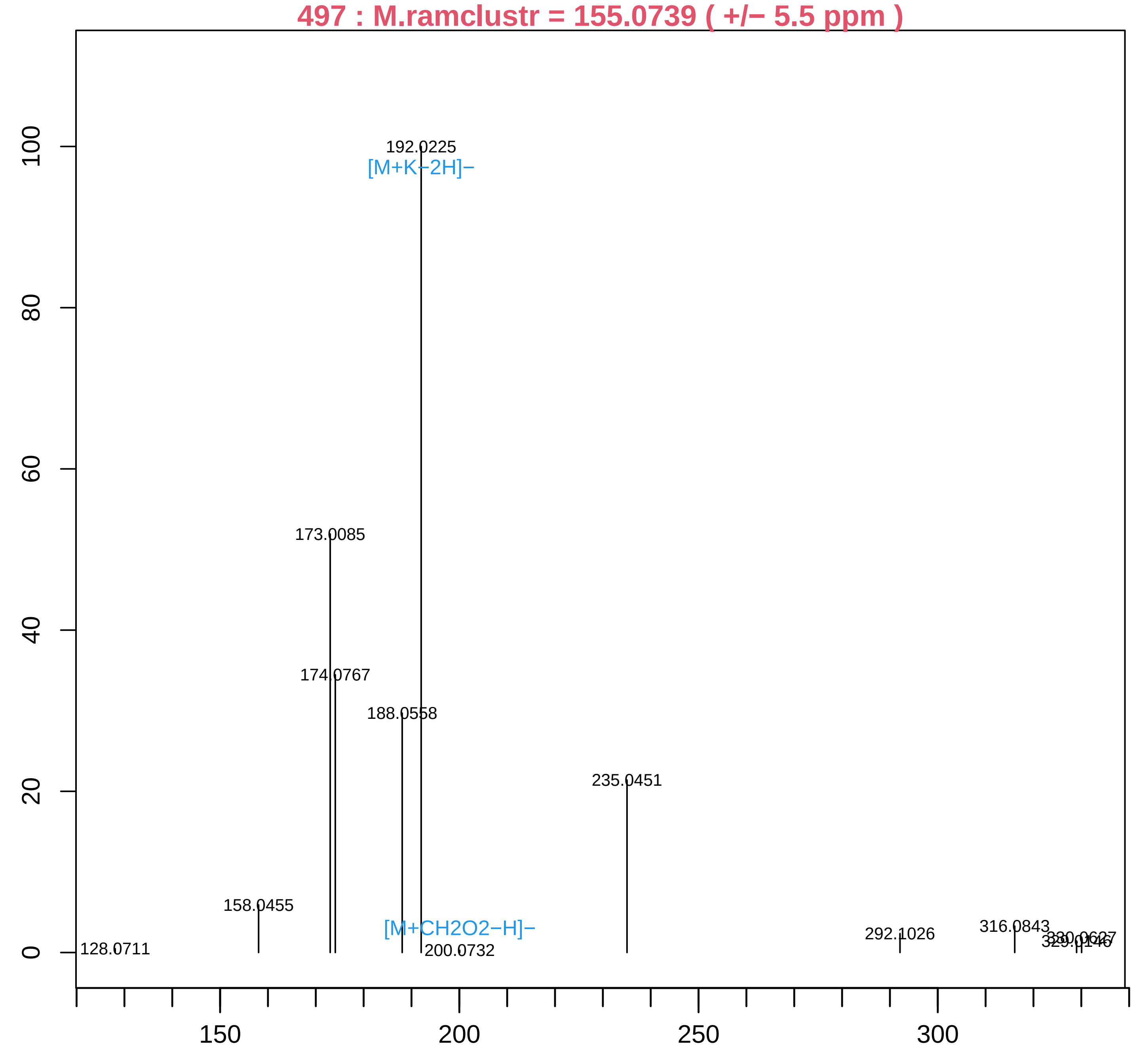
497 : M.ramclustr = 155.0739 (+/− 5.5 ppm)
192.0225
[M+K−2H]−
173.0085
174.0767
188.0558
235.0451
158.0455
[M+CH2O2−H]−
128.0711
200.0732
292.1026
316.0843
330.0627
329.0146
0
20
40
60
80
100
150
200
250
300

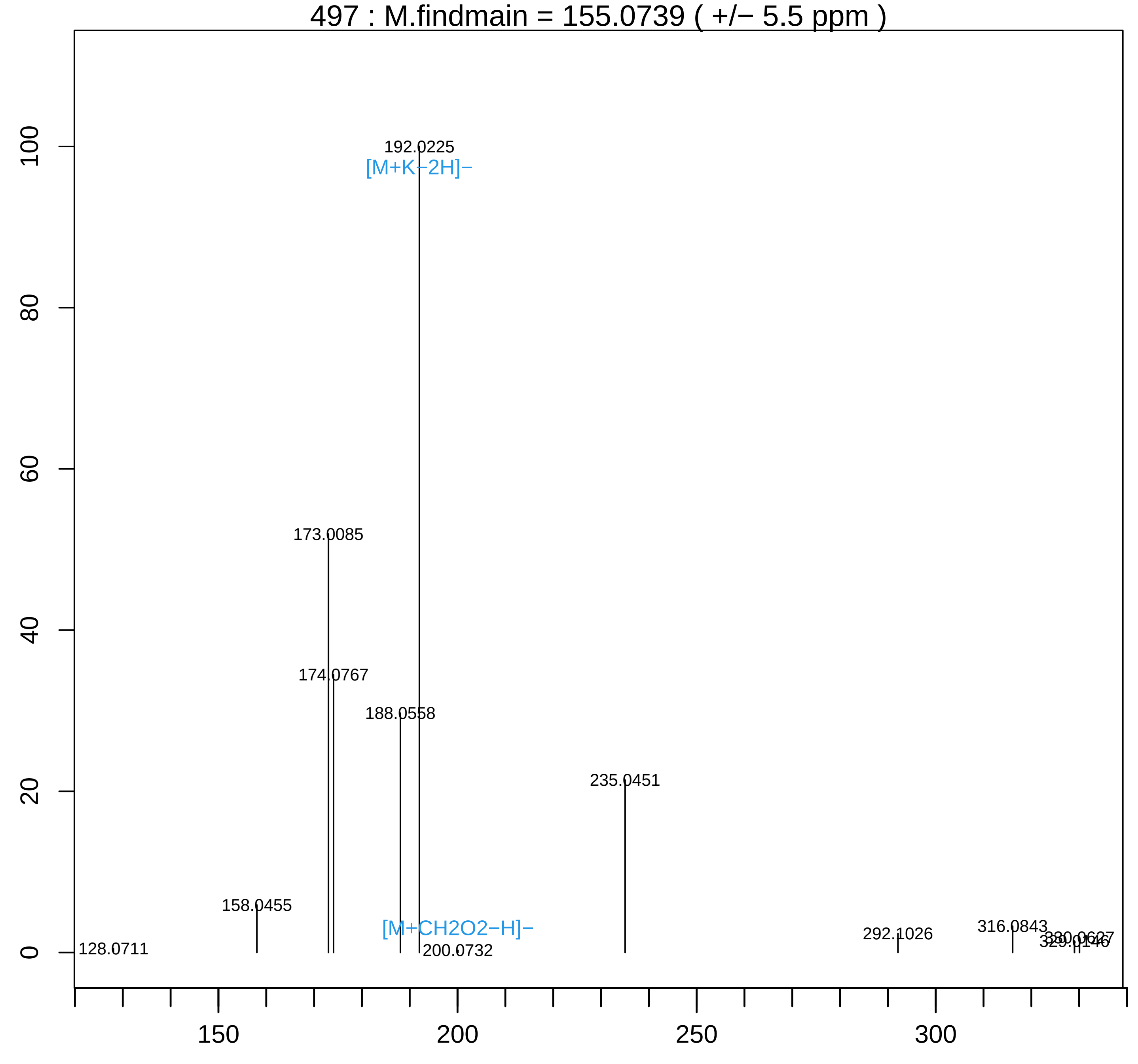
497 : M.findmain = 155.0739 (+/− 5.5 ppm)
192.0225
[M+K−2H]−
173.0085
174.0767
188.0558
235.0451
158.0455
[M+CH2O2−H]−
128.0711
200.0732
292.1026
316.0843
330.0627
329.0146
0
20
40
60
80
100
150
200
250
300

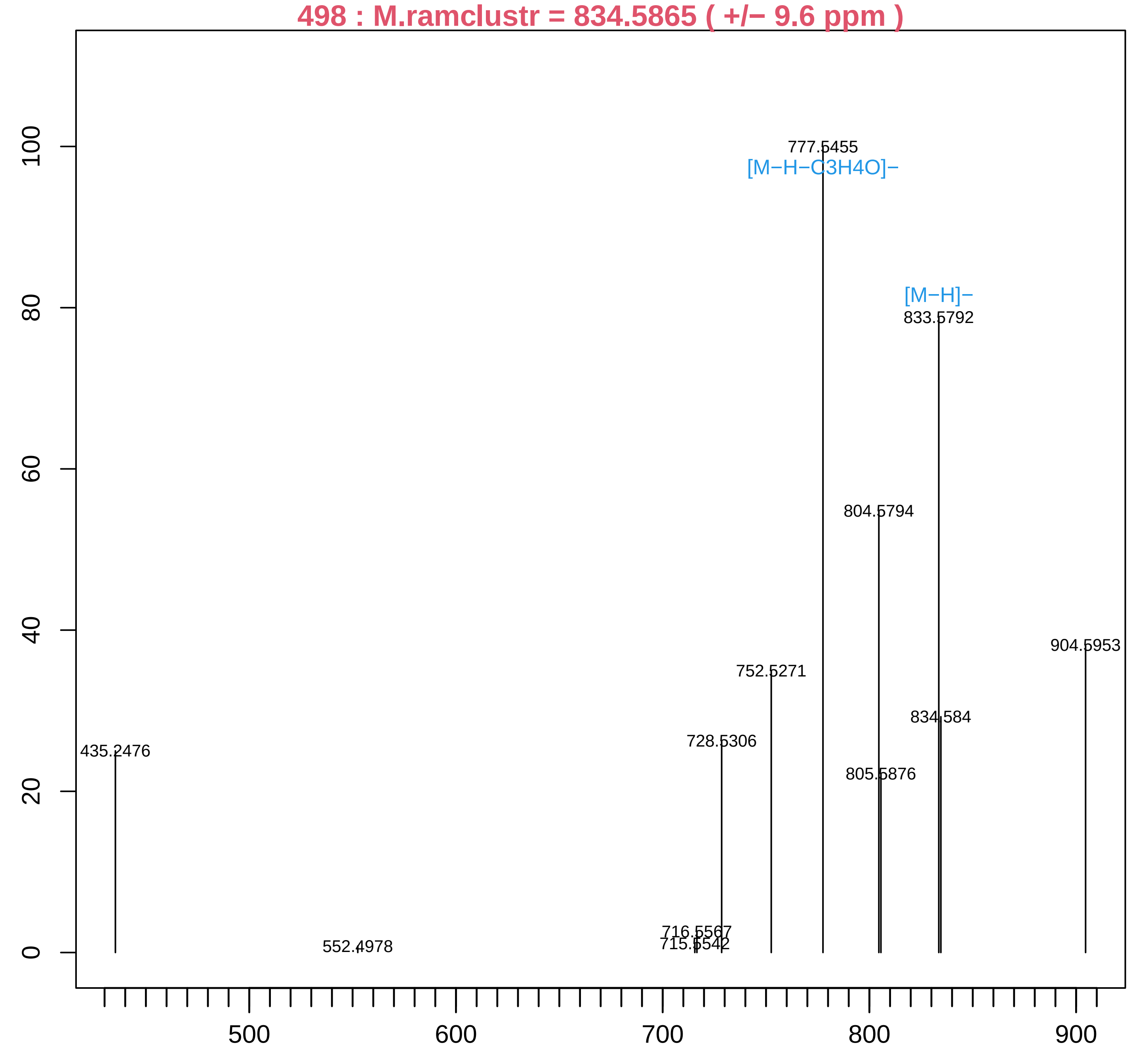

498 : M.ramclustr = 834.5865 (+/− 9.6 ppm)
777.5455
[M−H−C3H4O]−
[M−H]−
833.5792
804.5794
752.5271
904.5953
834.584
728.5306
435.2476
805.5876
716.5567
715.5542
552.4978
0
20
40
60
80
100
500
600
700
800
900

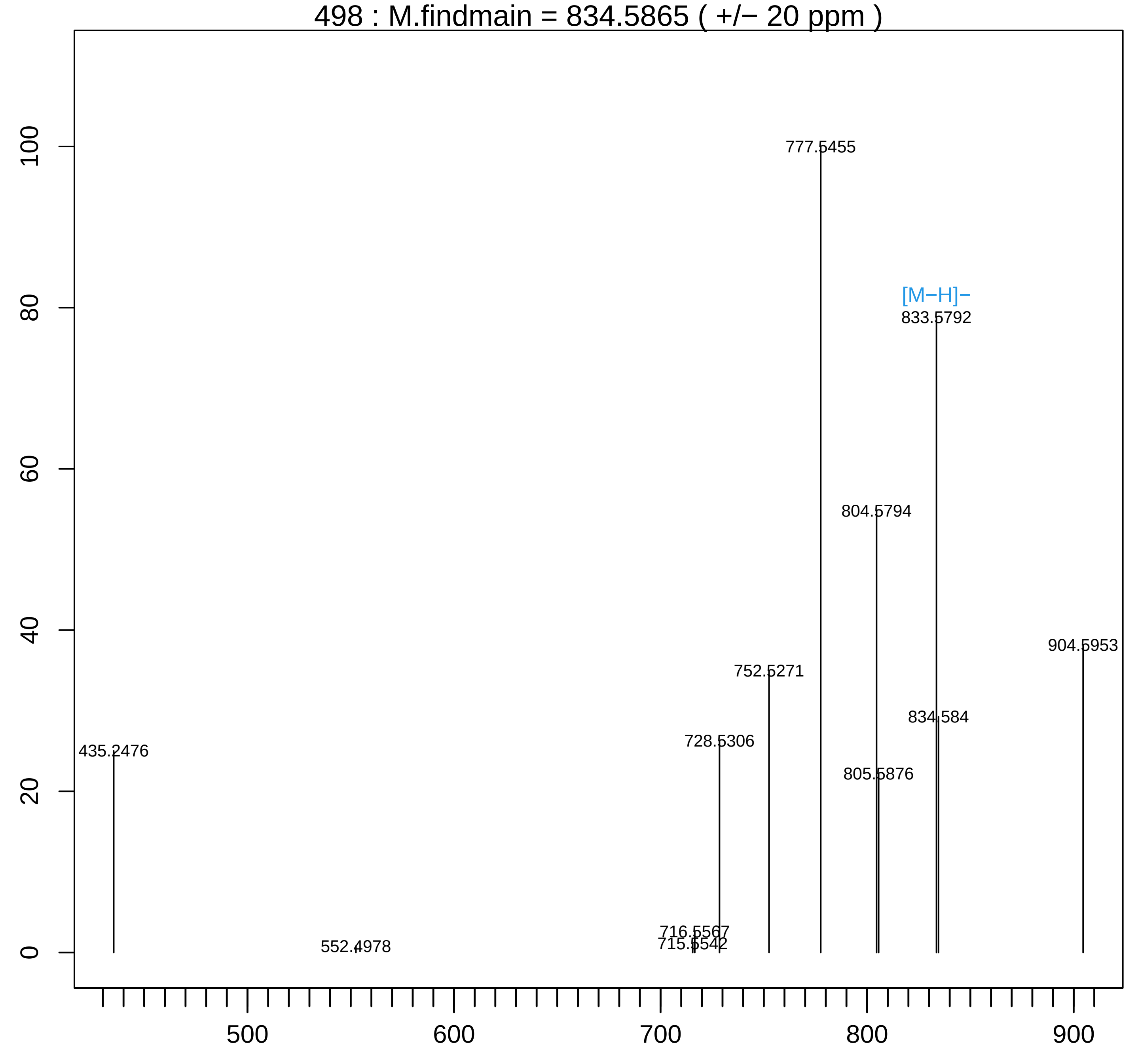

498 : M.findmain = 834.5865 (+/− 20 ppm)
777.5455
[M−H]−
833.5792
804.5794
752.5271
904.5953
834.584
728.5306
435.2476
805.5876
716.5567
715.5542
552.4978
0
20
40
60
80
100
500
600
700
800
900

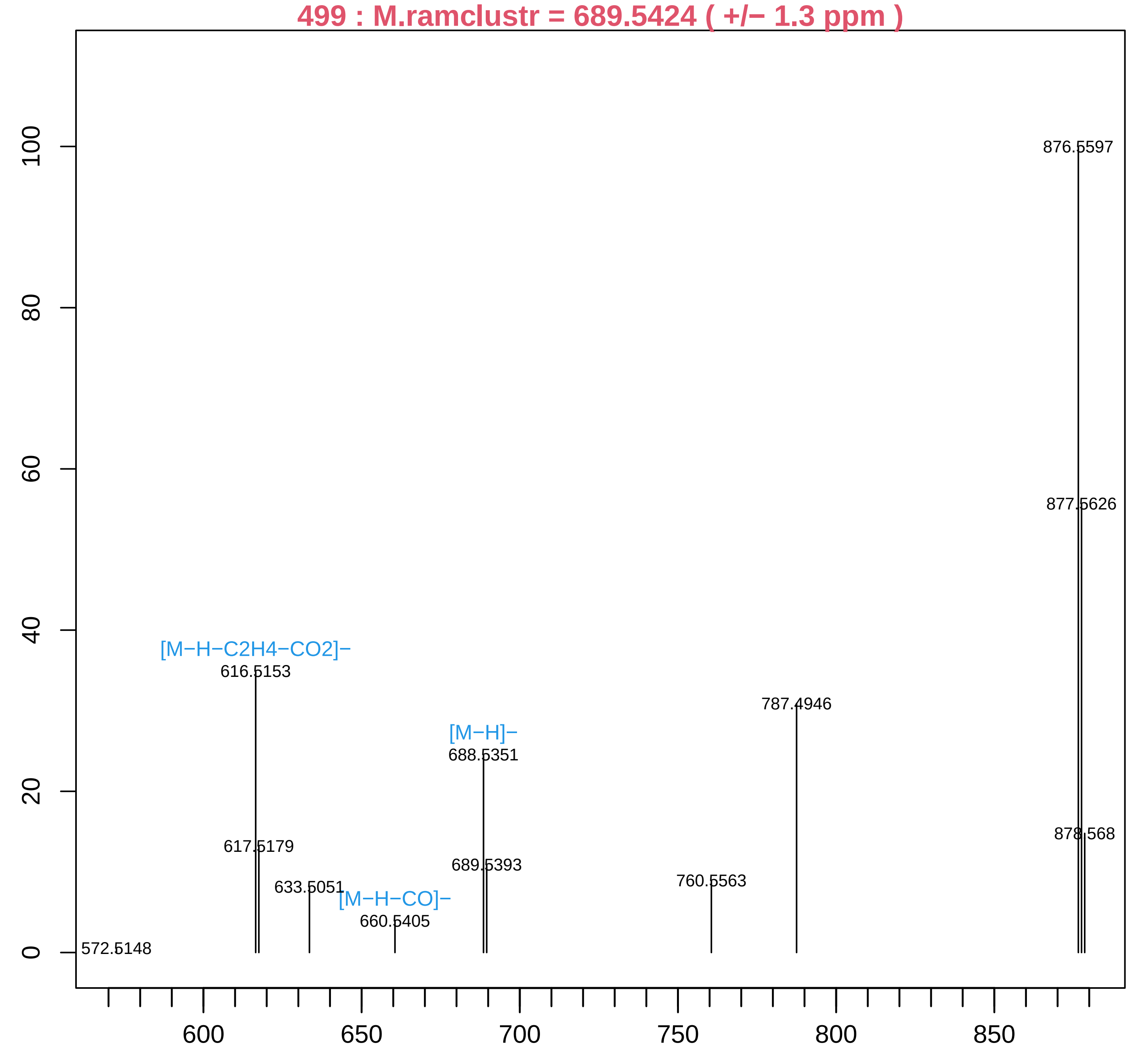
499 : M.ramclustr = 689.5424 (+/− 1.3 ppm)
876.5597
877.5626
878.568
[M−H−C2H4−CO2]−
616.5153
617.5179
633.5051
[M−H−CO]−
660.5405
[M−H]−
688.5351
689.5393
760.5563
787.4946
572.5148
0
20
40
60
80
100
600
650
700
750
800
850

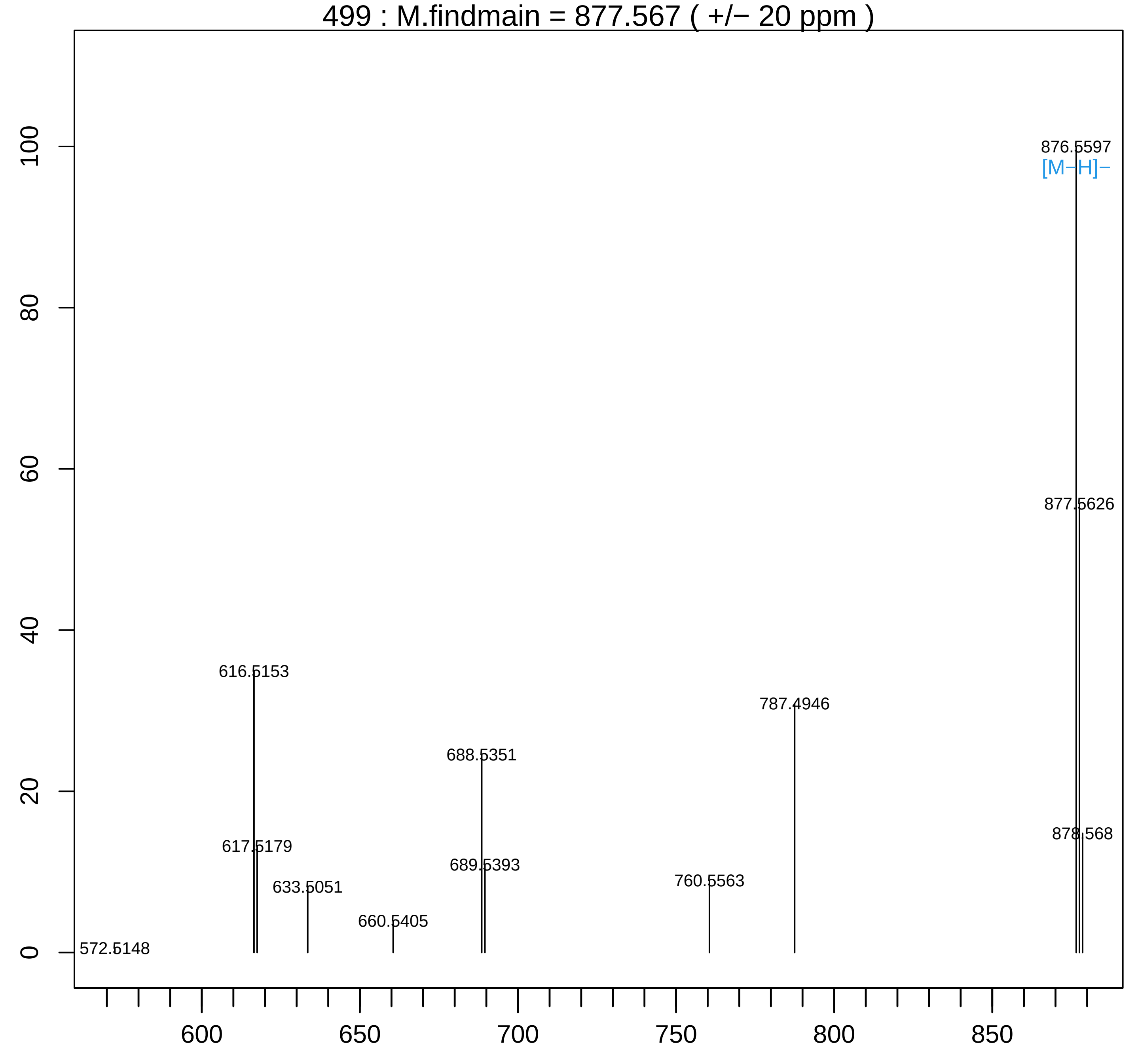
499 : M.findmain = 877.567 (+/− 20 ppm)
876.5597
[M−H]−
877.5626
878.568
616.5153
617.5179
633.5051
660.5405
688.5351
689.5393
760.5563
787.4946
572.5148
0
20
40
60
80
100
600
650
700
750
800
850

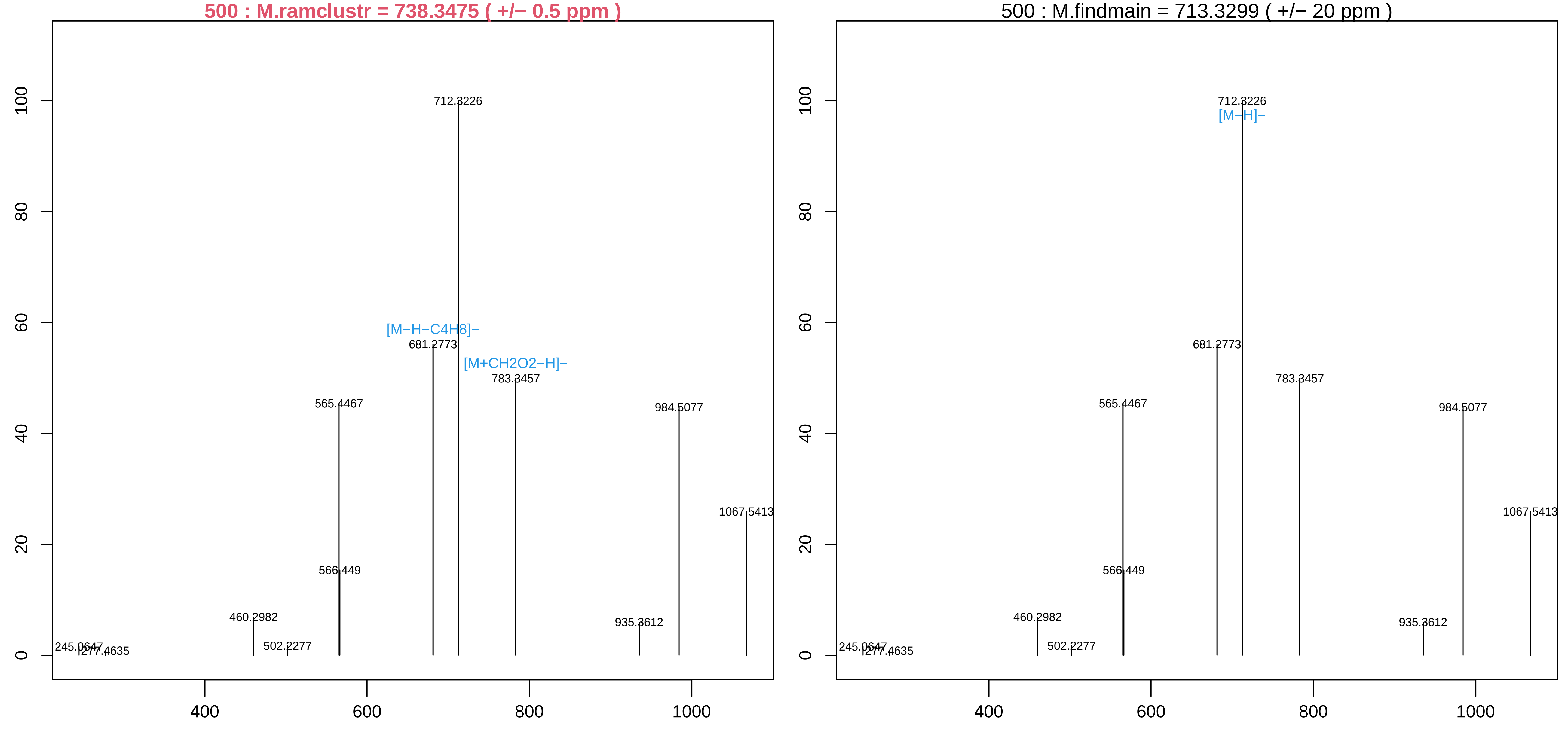

500 : M.ramclustr = 738.3475 (+/− 0.5 ppm)
500 : M.findmain = 713.3299 (+/− 20 ppm)
712.3226
[M−H]−
[M−H−C4H8]−
681.2773
[M+CH2O2−H]−
783.3457
565.4467
984.5077
1067.5413
566.449
460.2982
935.3612
502.2277
245.0647
277.4635

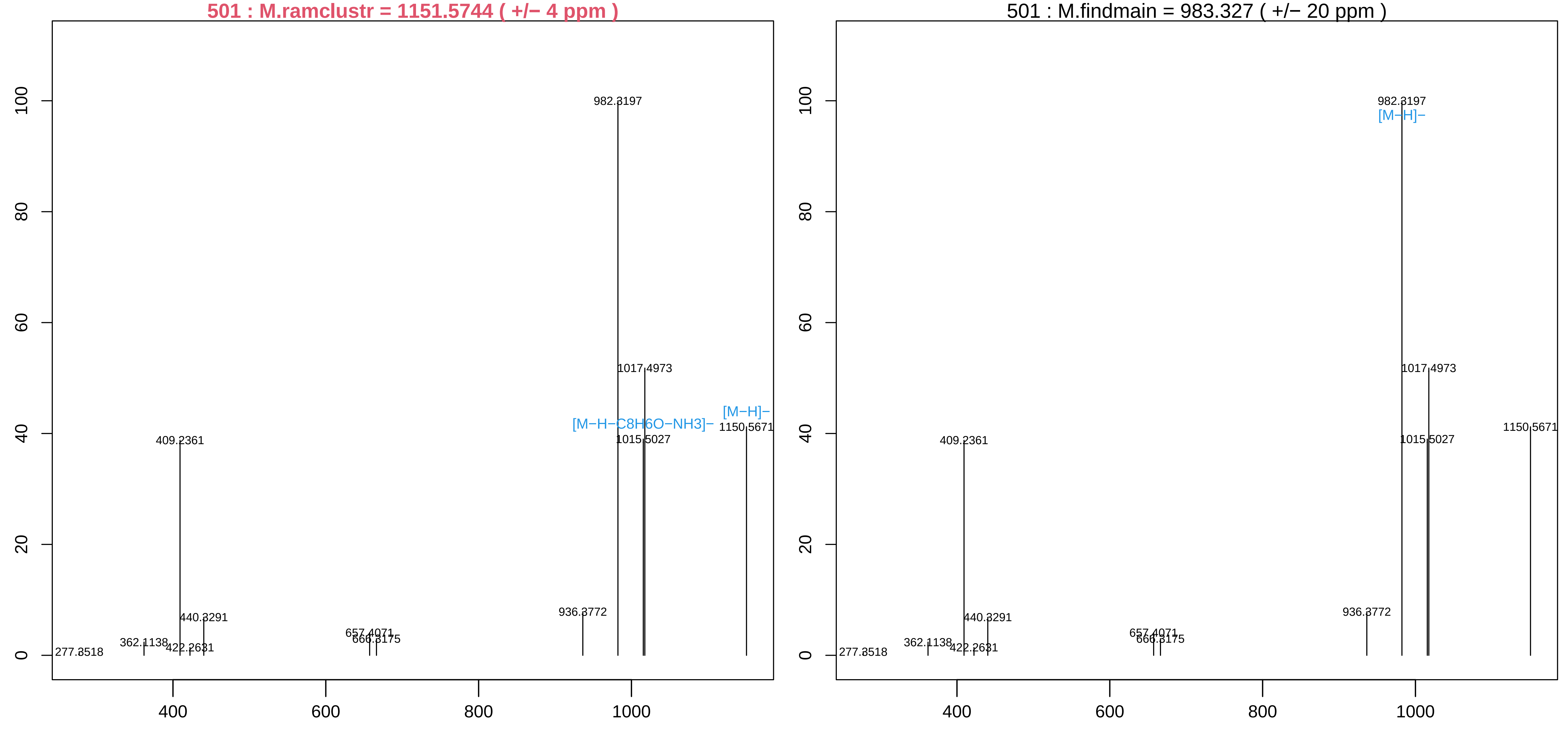

501 : M.ramclustr = 1151.5744 (+/− 4 ppm)
982.3197
1017.4973
[M−H]−
[M−H−C8H6O−NH3]−
1150.5671
1015.5027
409.2361
440.3291
936.3772
657.4071
666.3175
362.1138
422.2631
277.8518
501 : M.findmain = 983.327 (+/− 20 ppm)
982.3197
[M−H]−
1017.4973
1150.5671
1015.5027
409.2361
440.3291
936.3772
657.4071
666.3175
362.1138
422.2631
277.8518

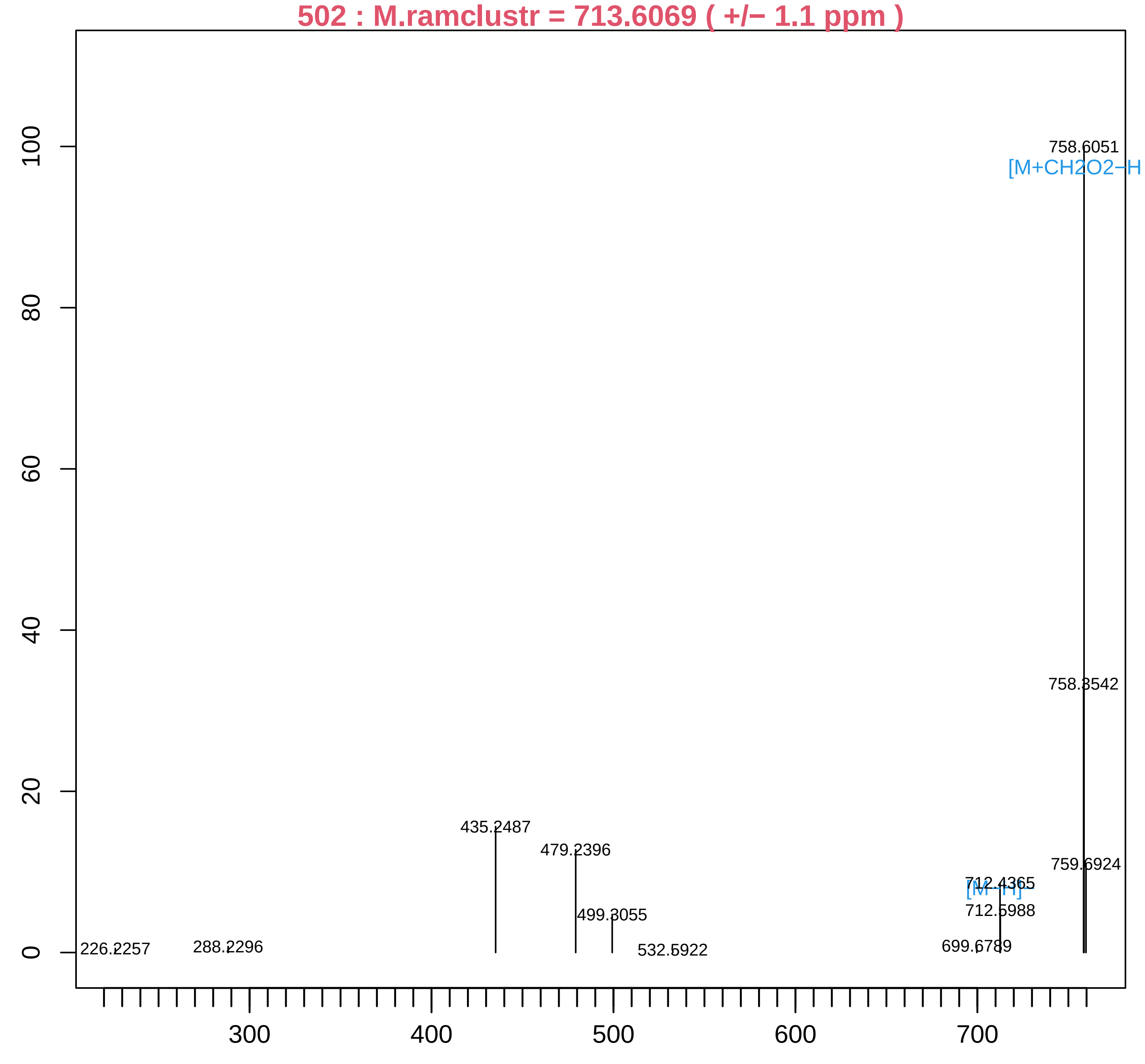

502 : M.ramclustr = 713.6069 (+/− 1.1 ppm)
758.6051
[M+CH2O2−H
758.3542
759.6924
712.4365
[M−H]
712.5988
699.6789
435.2487
479.2396
499.3055
532.5922
226.2257
288.2296
0
20
40
60
80
100
300
400
500
600
700

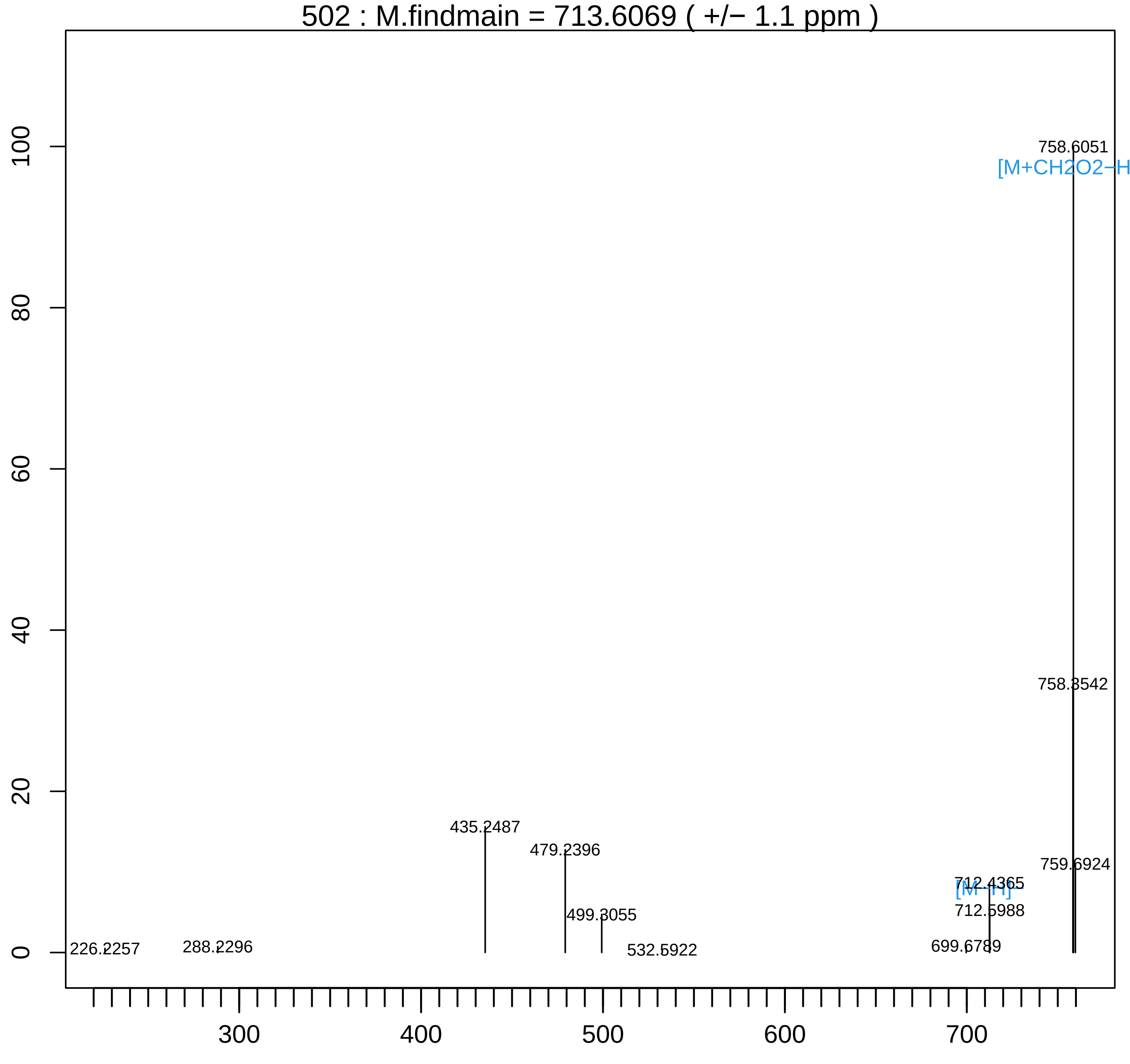

502 : M.findmain = 713.6069 (+/− 1.1 ppm)
758.6051
[M+CH2O2−H
758.3542
759.6924
712.4365
[M−H]
712.5988
699.6789
435.2487
479.2396
499.3055
532.5922
226.2257
288.2296
0
20
40
60
80
100
300
400
500
600
700

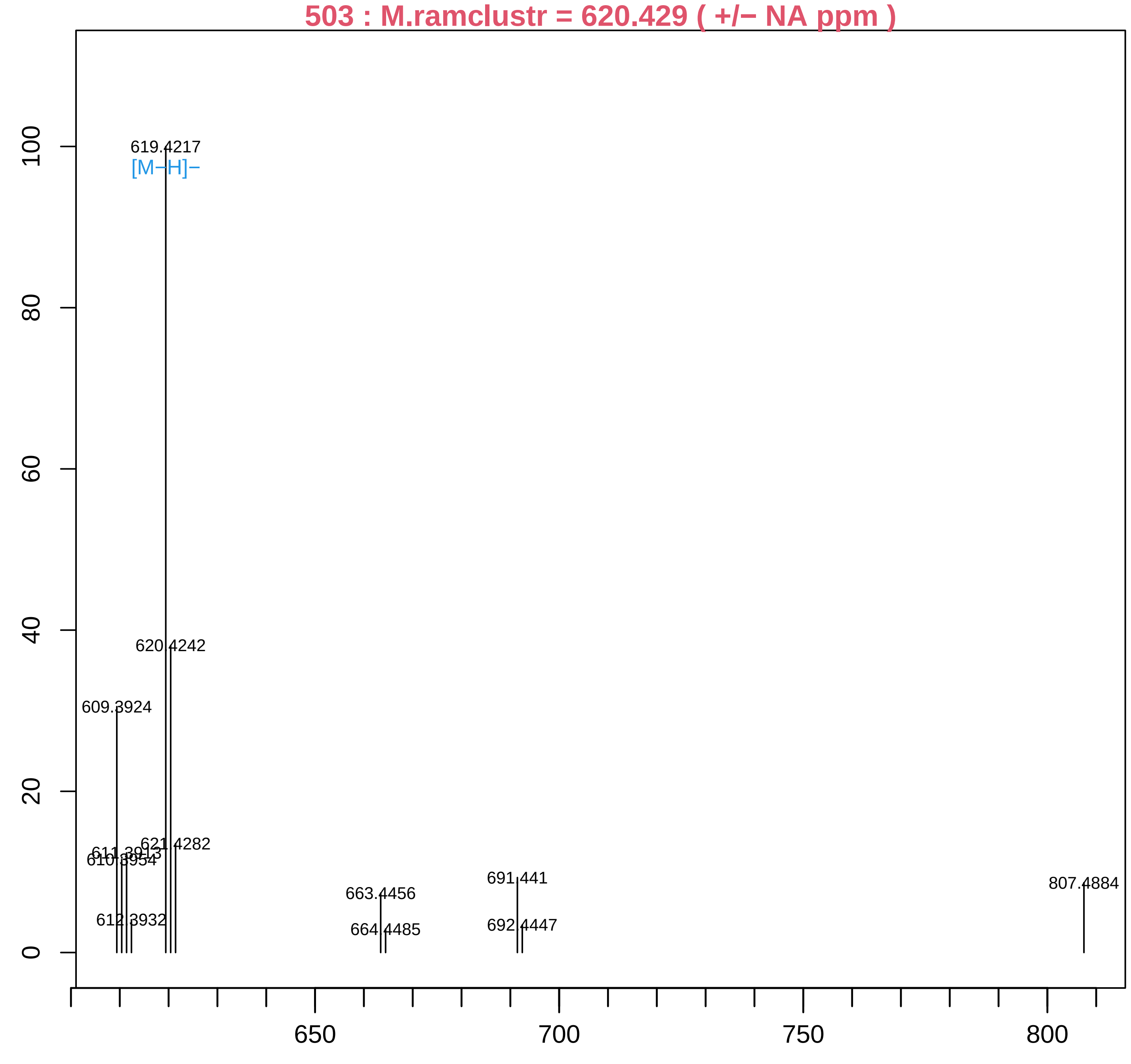
503 : M.ramclustr = 620.429 (+/− NA ppm)
619.4217
[M−H]−
620.4242
609.3924
621.4282
611.3913
610.3954
612.3932
663.4456
664.4485
691.441
692.4447
807.4884
0
20
40
60
80
100
650
700
750
800

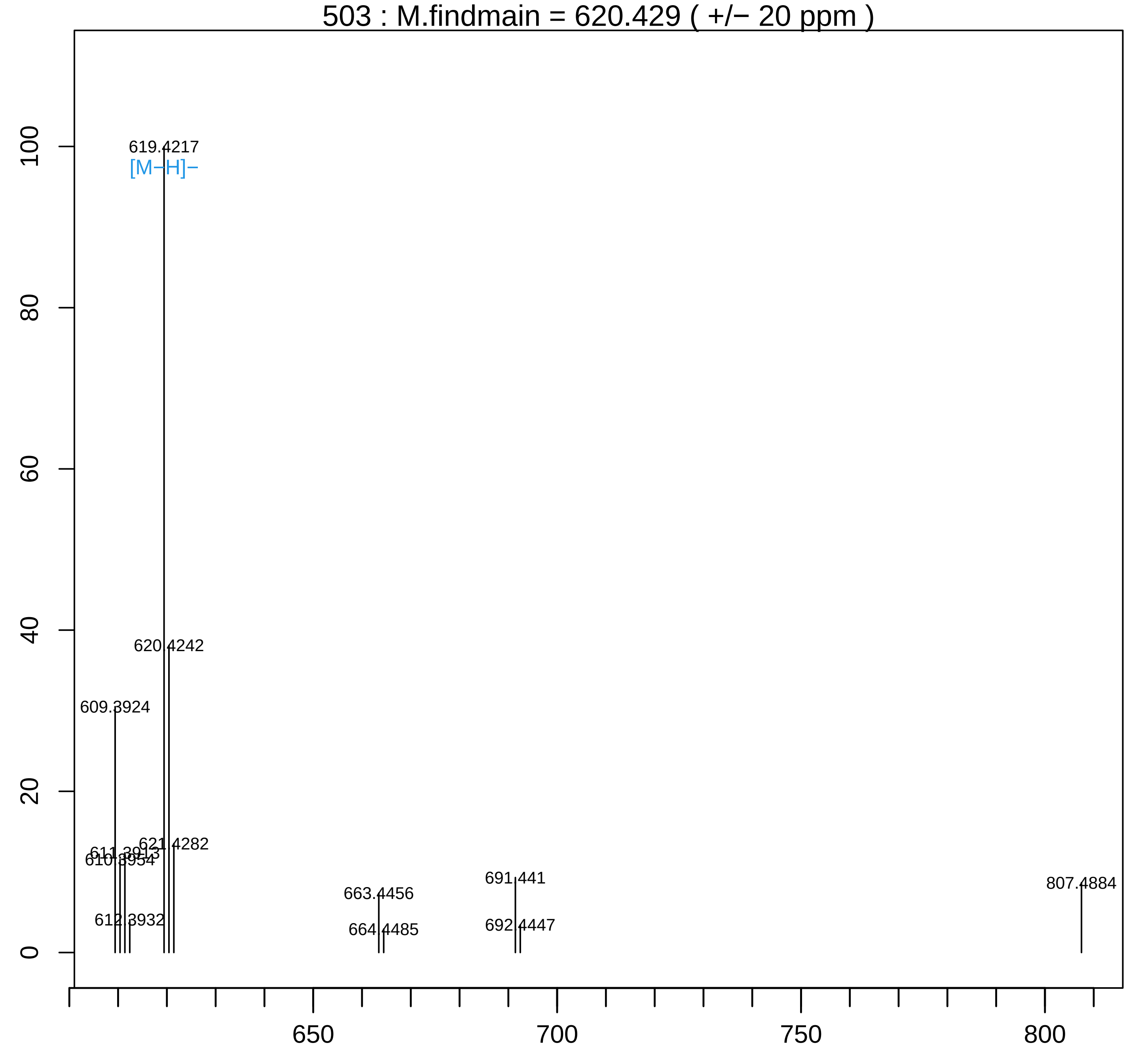
503 : M.findmain = 620.429 (+/− 20 ppm)
619.4217
[M−H]−
620.4242
609.3924
621.4282
611.3913
610.3954
612.3932
663.4456
664.4485
691.441
692.4447
807.4884
0
20
40
60
80
100
650
700
750
800

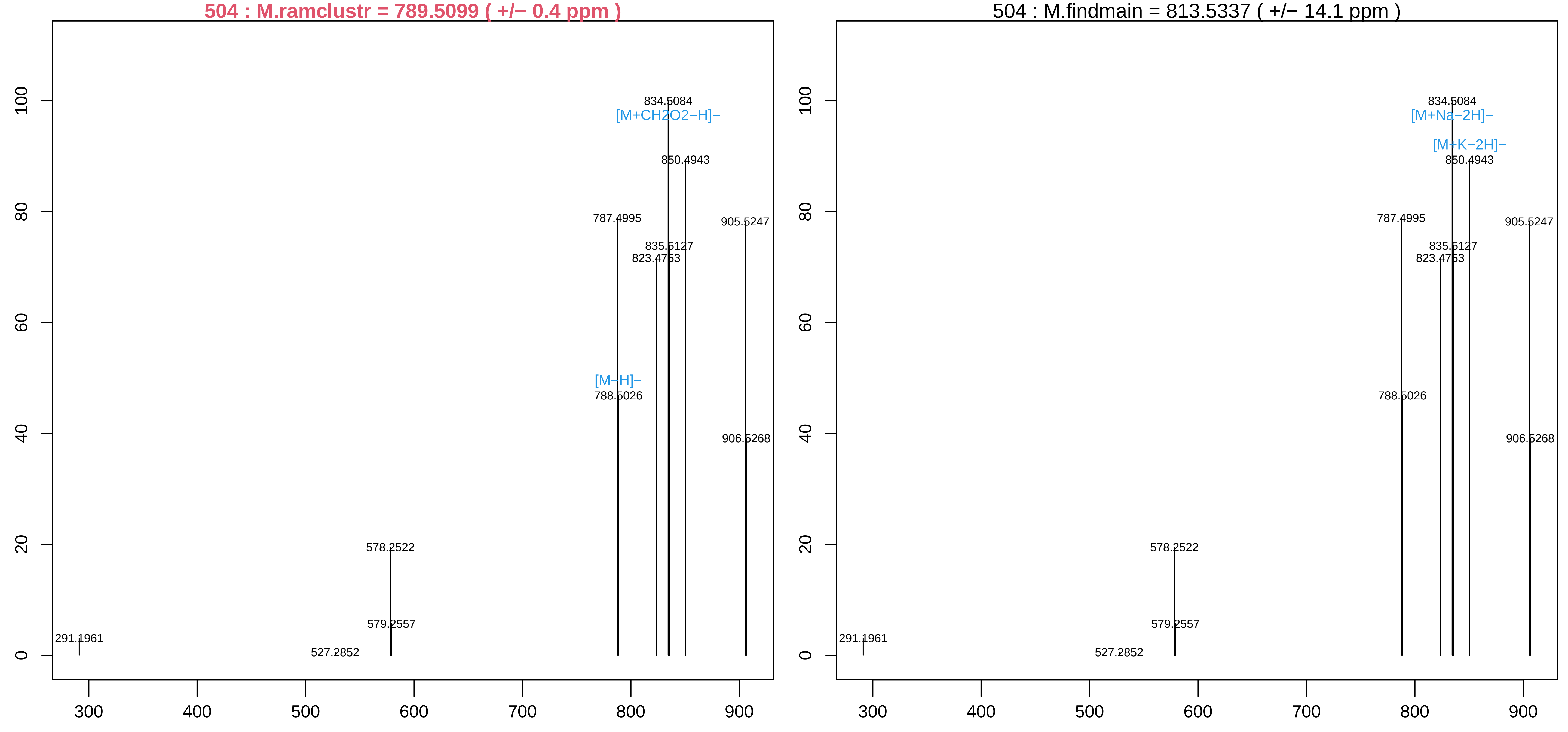

504 : M.ramclustr = 789.5099 (+/− 0.4 ppm)
834.5084
[M+CH2O2−H]−
850.4943
787.4995
905.5247
835.5127
823.4753
[M−H]−
788.5026
906.5268
578.2522
579.2557
291.1961
527.2852
504 : M.findmain = 813.5337 (+/− 14.1 ppm)
834.5084
[M+Na−2H]−
[M+K−2H]−
850.4943
787.4995
905.5247
835.5127
823.4753
788.5026
906.5268
578.2522
579.2557
291.1961
527.2852
0
20
40
60
80
100
300
400
500
600
700
800
900

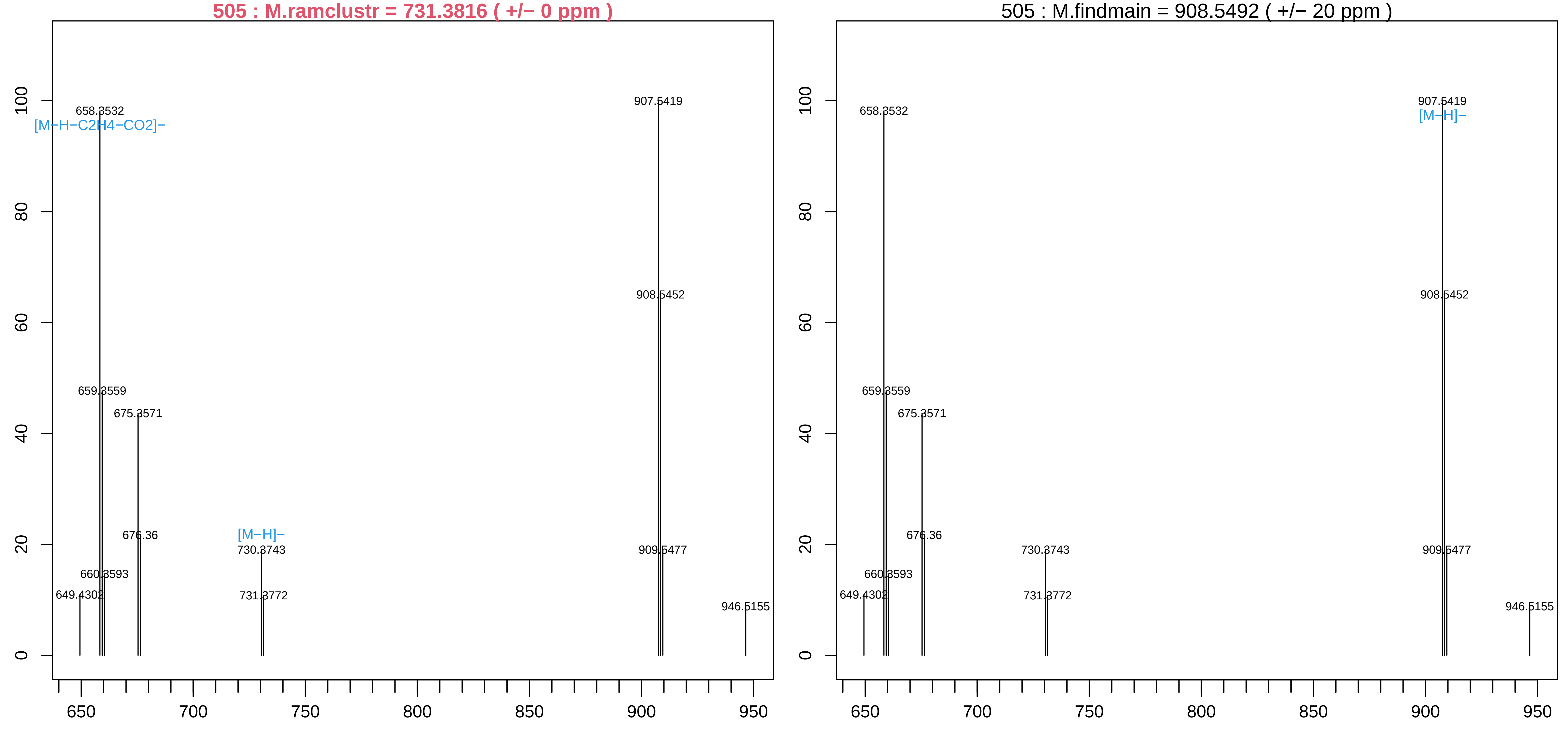
505 : M.ramclustr = 731.3816 (+/− 0 ppm)
658.3532
[M−H−C2H4−CO2]−
907.5419
908.5452
659.3559
675.3571
676.36
[M−H]−
730.3743
909.5477
660.3593
649.4302
731.3772
946.5155
505 : M.findmain = 908.5492 (+/− 20 ppm)
658.3532
907.5419
[M−H]−
908.5452
659.3559
675.3571
676.36
730.3743
909.5477
660.3593
649.4302
731.3772
946.5155
0
20
40
60
80
100
650
700
750
800
850
900
950

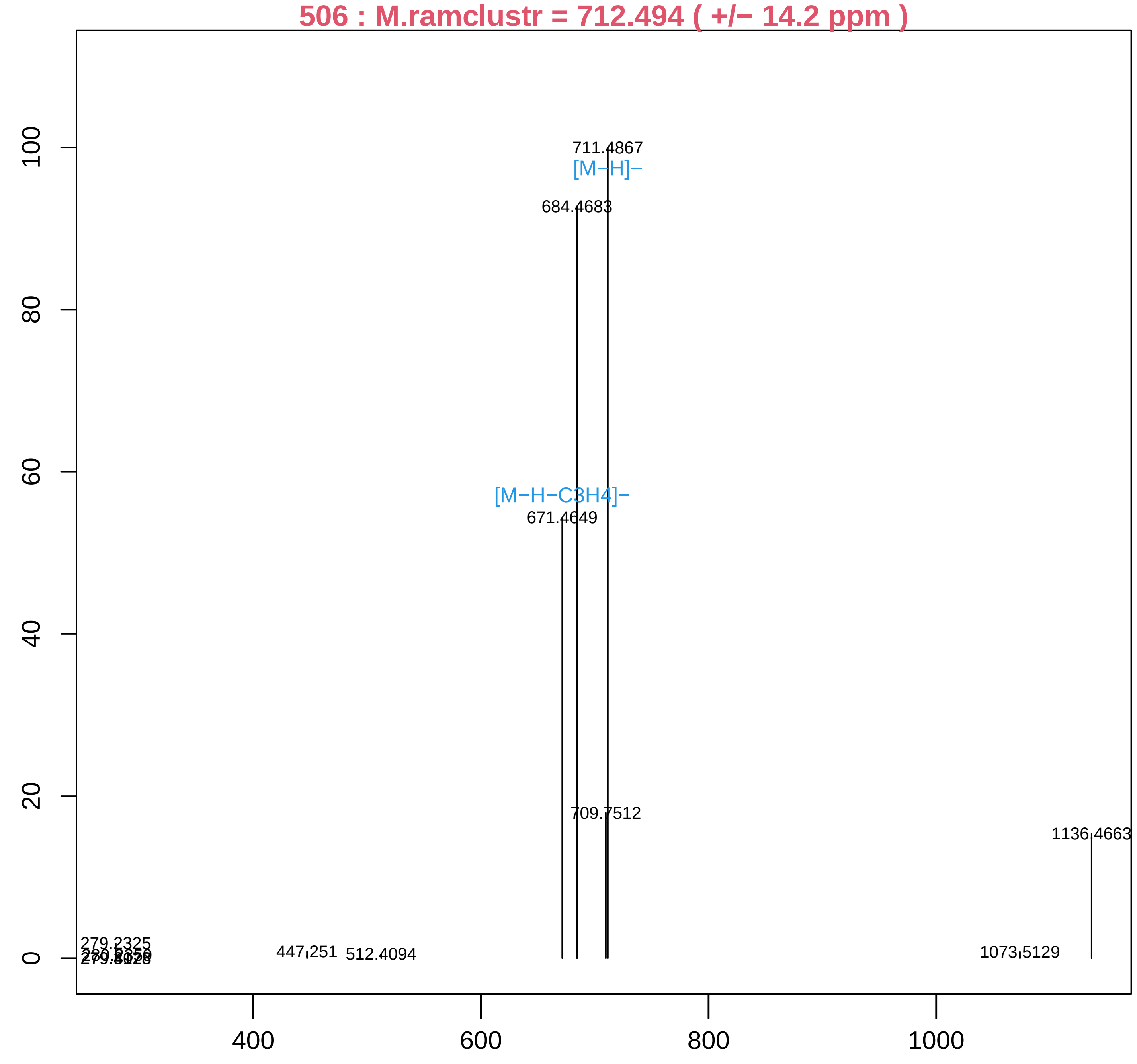
506 : M.ramclustr = 712.494 (+/− 14.2 ppm)
711.4867
[M−H]−
684.4683
[M−H−C3H4]−
671.4649
709.7512
1136.4663
279.2325
447.251
512.4094
1073.5129
0
20
40
60
80
100
400
600
800
1000

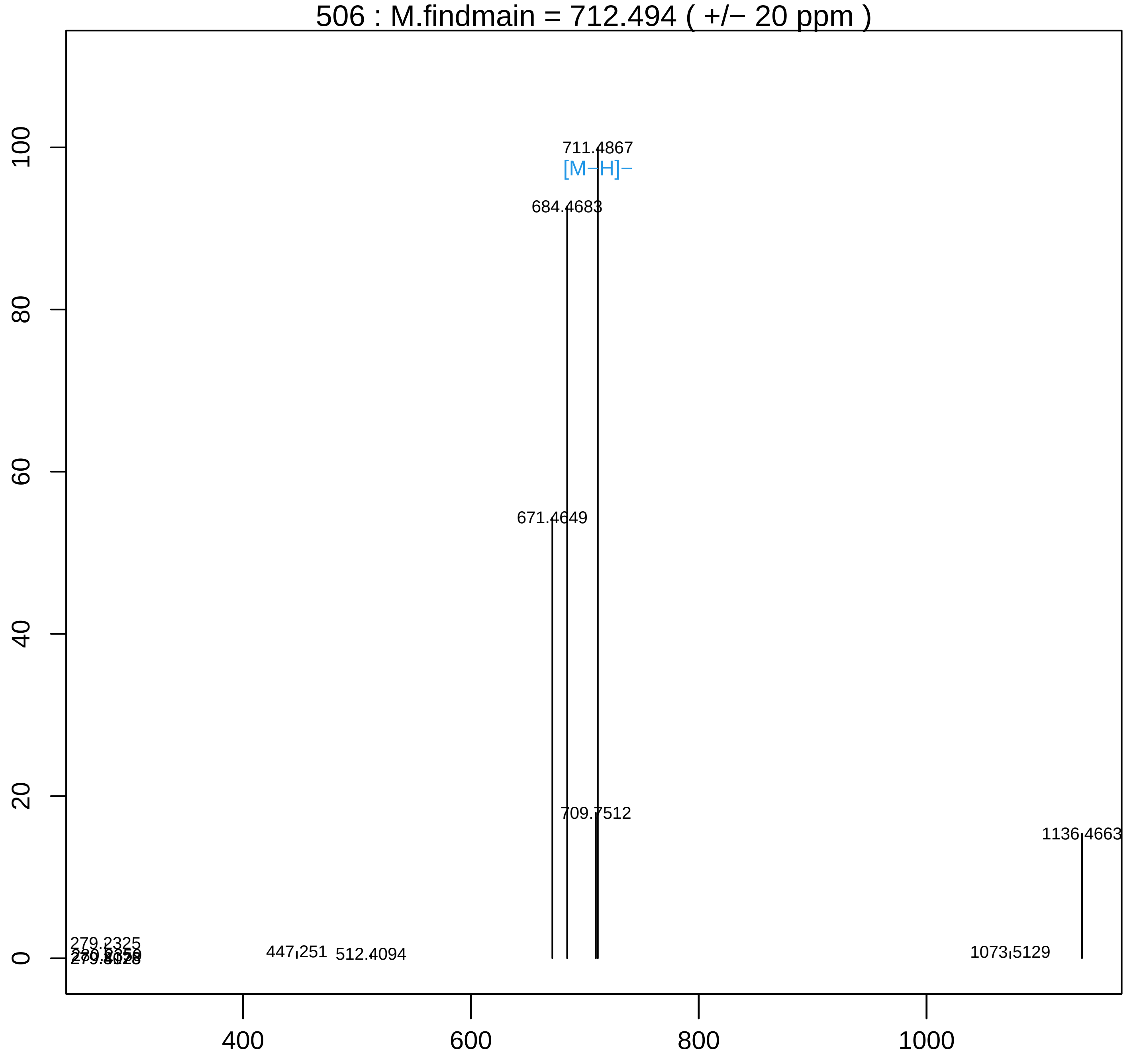
506 : M.findmain = 712.494 (+/− 20 ppm)
711.4867
[M−H]−
684.4683
671.4649
709.7512
1136.4663
279.2325
447.251
512.4094
1073.5129
0
20
40
60
80
100
400
600
800
1000

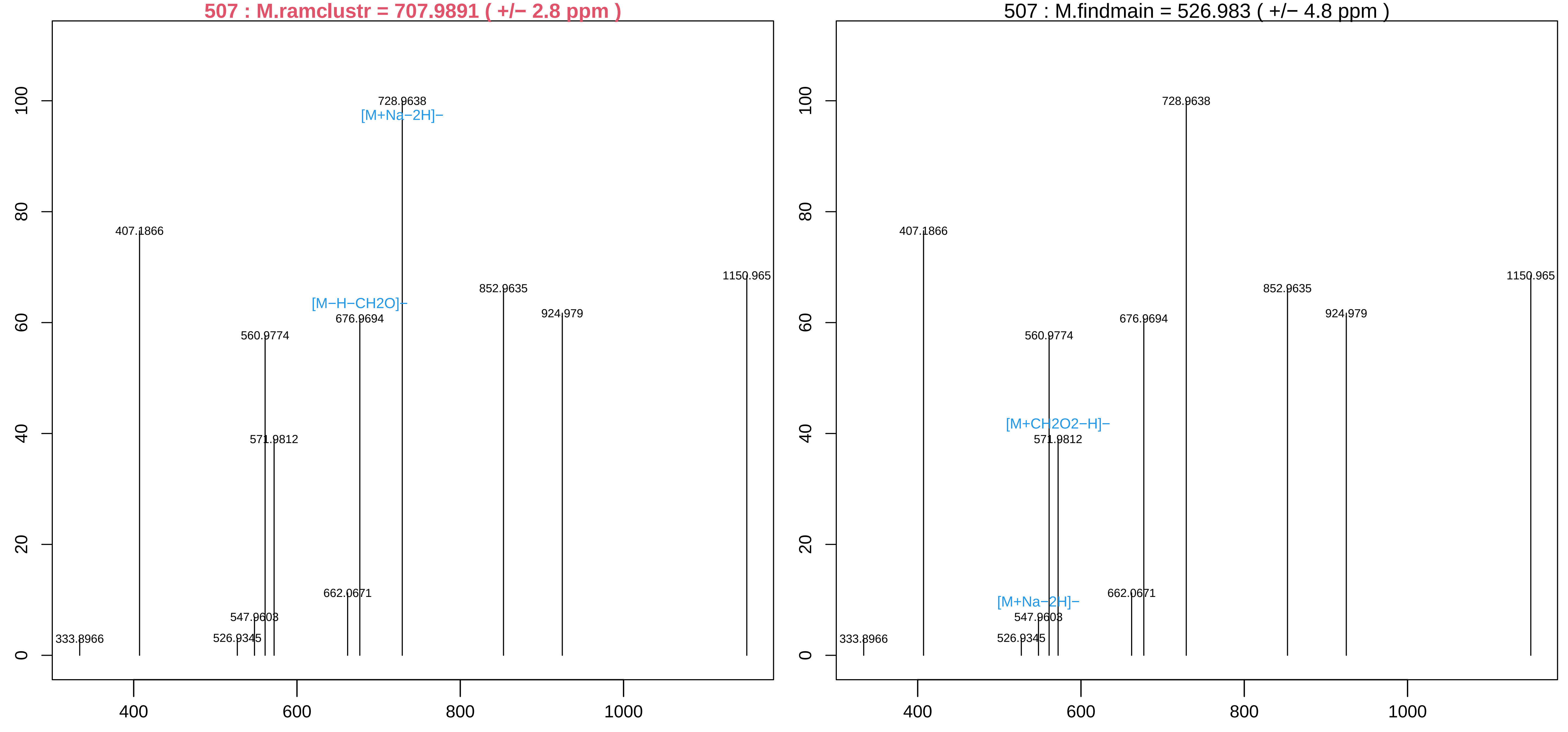
507 : M.ramclustr = 707.9891 (+/− 2.8 ppm)
728.9638
[M+Na−2H]−
407.1866
[M−H−CH2O]−
676.9694
852.9635
924.979
1150.965
560.9774
571.9812
662.0671
547.9603
526.9345
333.8966
507 : M.findmain = 526.983 (+/− 4.8 ppm)
[M+CH2O2−H]−
[M+Na−2H]−
0
20
40
60
80
100
400
600
800
1000

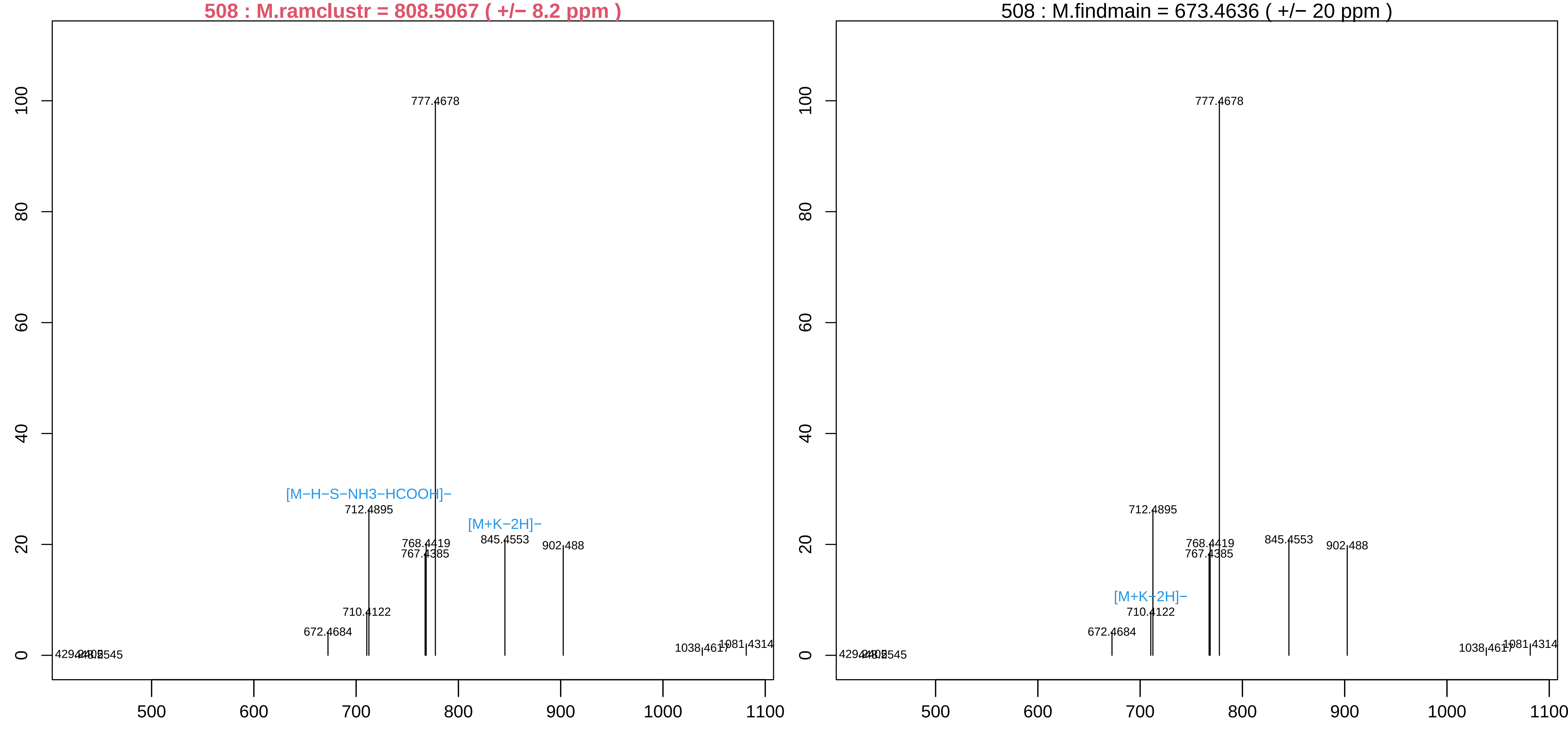
508 : M.ramclustr = 808.5067 (+/− 8.2 ppm)
777.4678
[M−H−S−NH3−HCOOH]−
712.4895
[M+K−2H]−
845.4553
768.4419
767.4385
902.488
710.4122
672.4684
1038.4617
1081.4314
0
20
40
60
80
100
500
600
700
800
900
1000
1100
508 : M.findmain = 673.4636 (+/− 20 ppm)
777.4678
712.4895
768.4419
767.4385
845.4553
902.488
[M+K−2H]−
710.4122
672.4684
1038.4617
1081.4314

509 : M.ramclustr = 665.6303 (+/− 0.7 ppm)

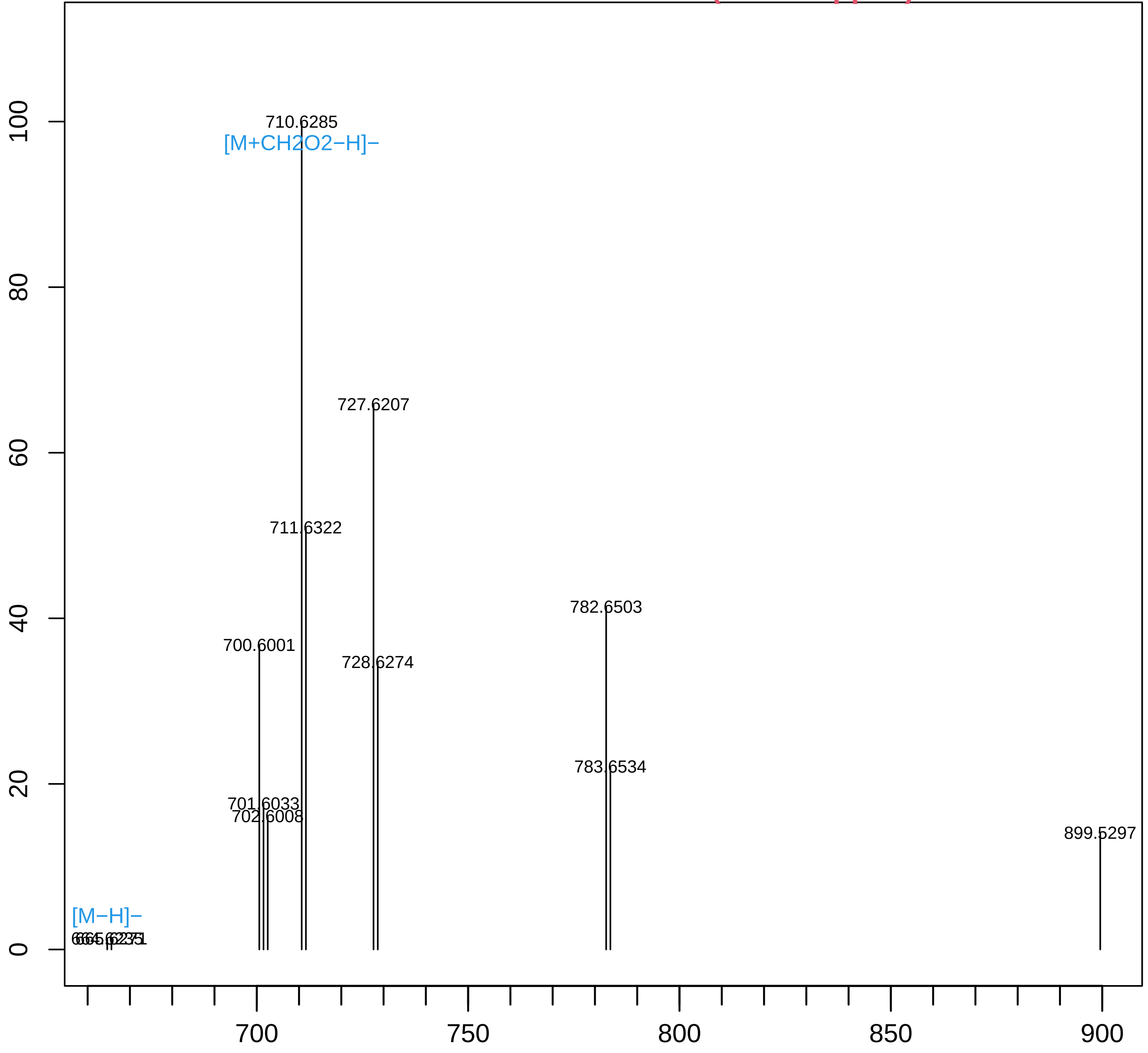

509 : M.findmain = 665.6303 (+/− 0.7 ppm)

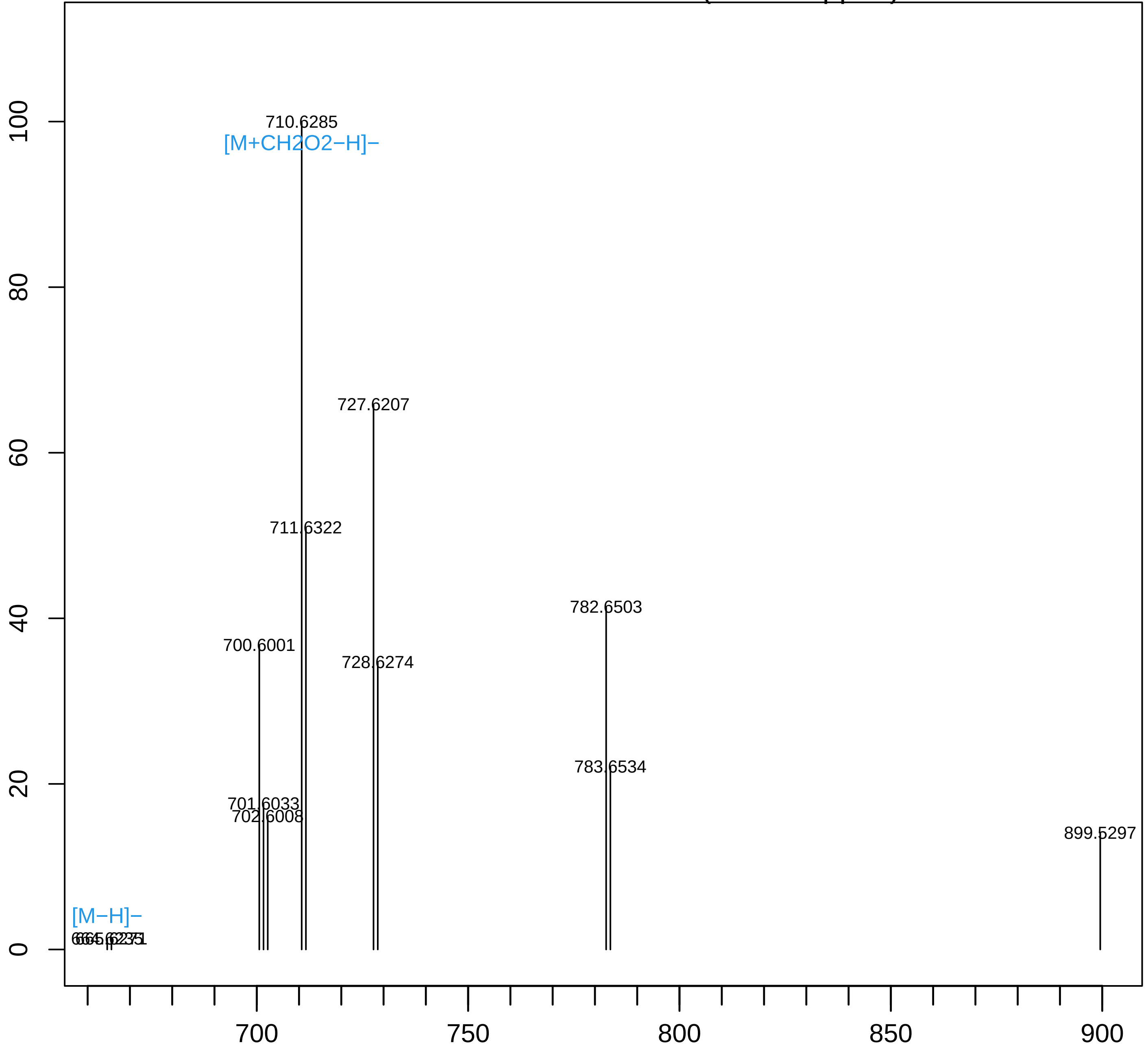

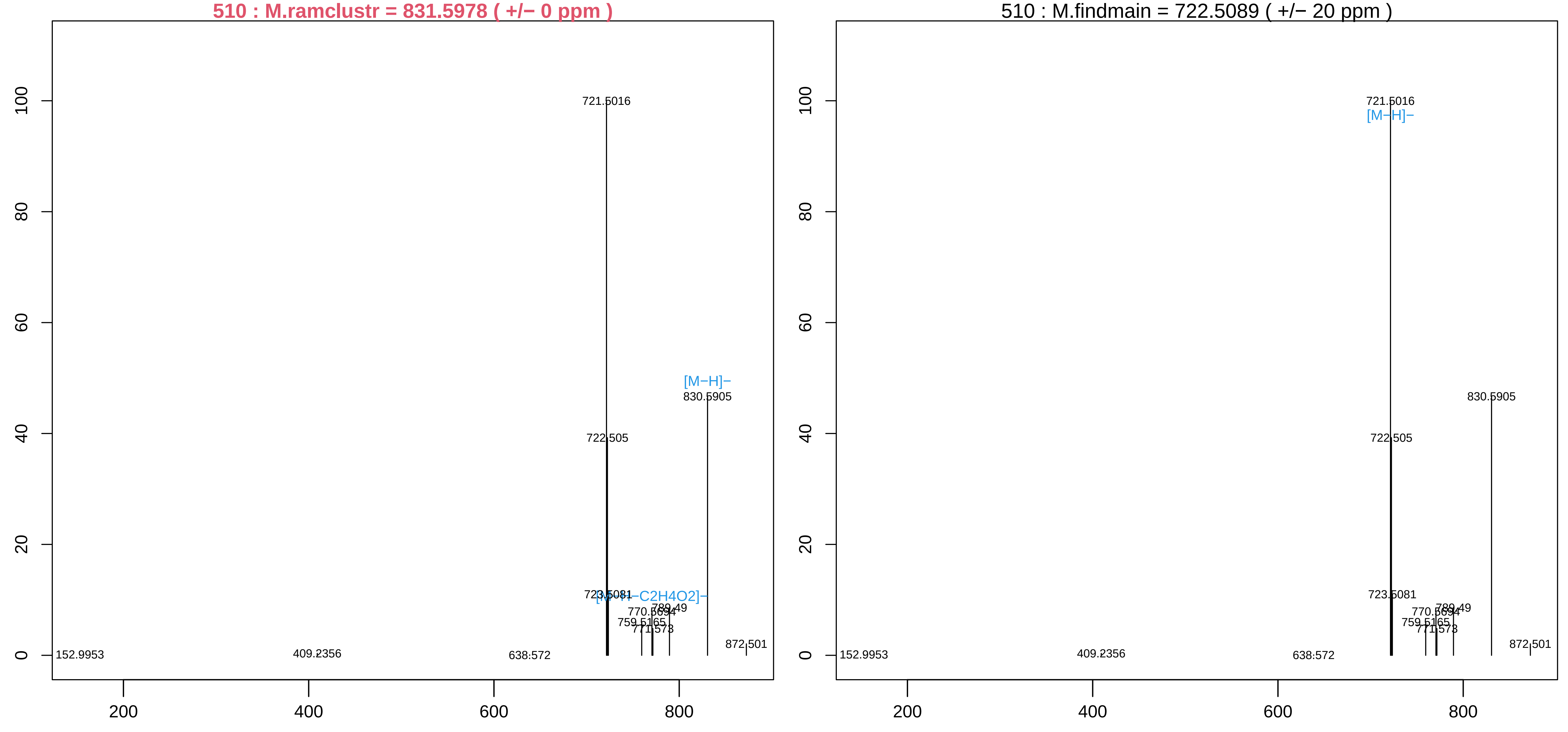
510 : M.ramclustr = 831.5978 (+/− 0 ppm)
721.5016
[M−H]−
830.5905
722.505
723.5081
[M−H−C2H4O2]−
770.5694
789.49
759.5165
771.573
872.501
152.9953
409.2356
638.572
510 : M.findmain = 722.5089 (+/− 20 ppm)
721.5016
[M−H]−
830.5905
722.505
723.5081
770.5694
789.49
759.5165
771.573
872.501
152.9953
409.2356
638.572
0
20
40
60
80
100
200
400
600
800

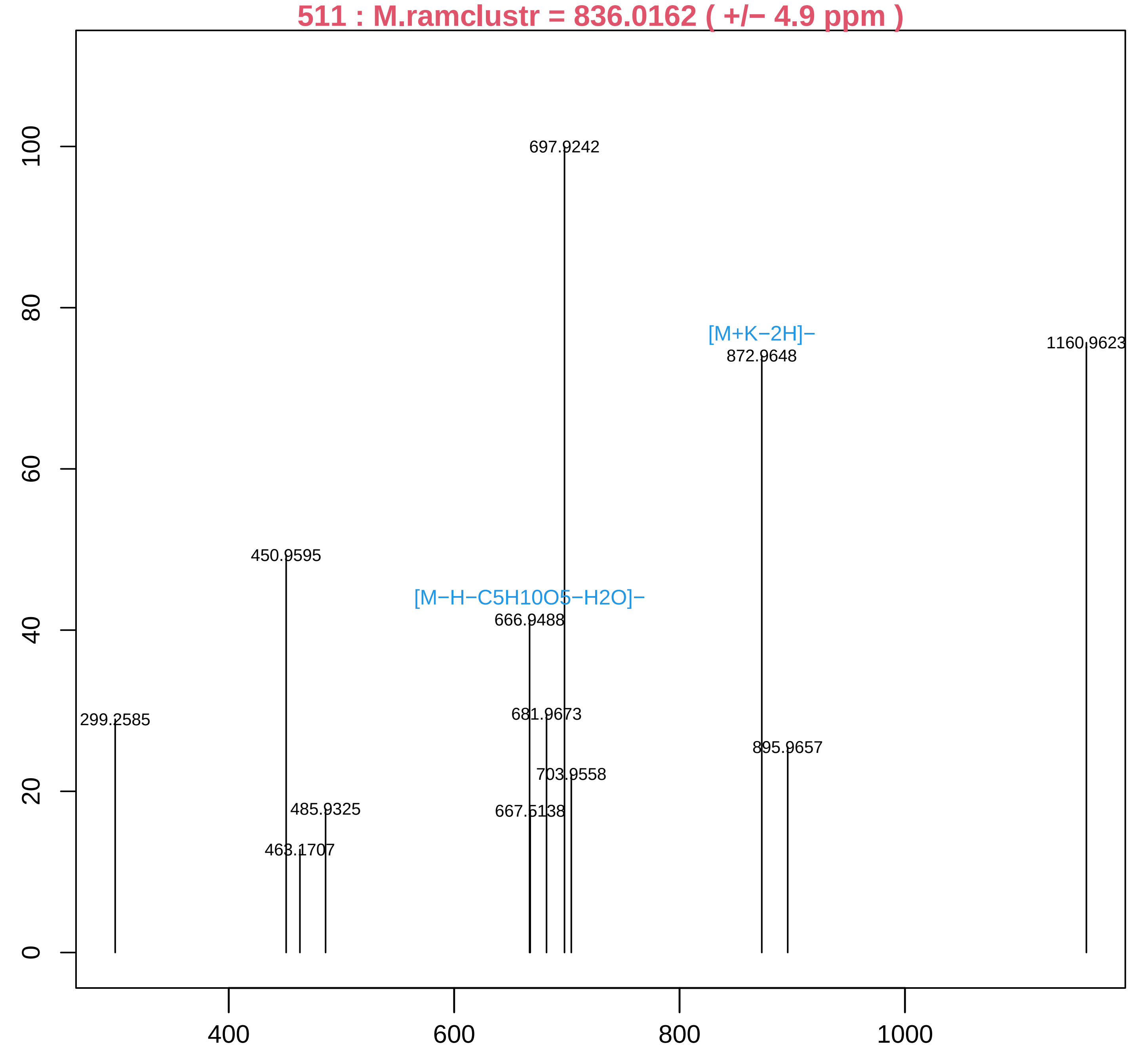
511 : M.ramclustr = 836.0162 (+/− 4.9 ppm)
697.9242
[M+K−2H]−
872.9648
1160.9623
450.9595
[M−H−C5H10O5−H2O]−
666.9488
299.2585
681.9673
895.9657
703.9558
485.9325
667.5138
463.1707
0
20
40
60
80
100
400
600
800
1000

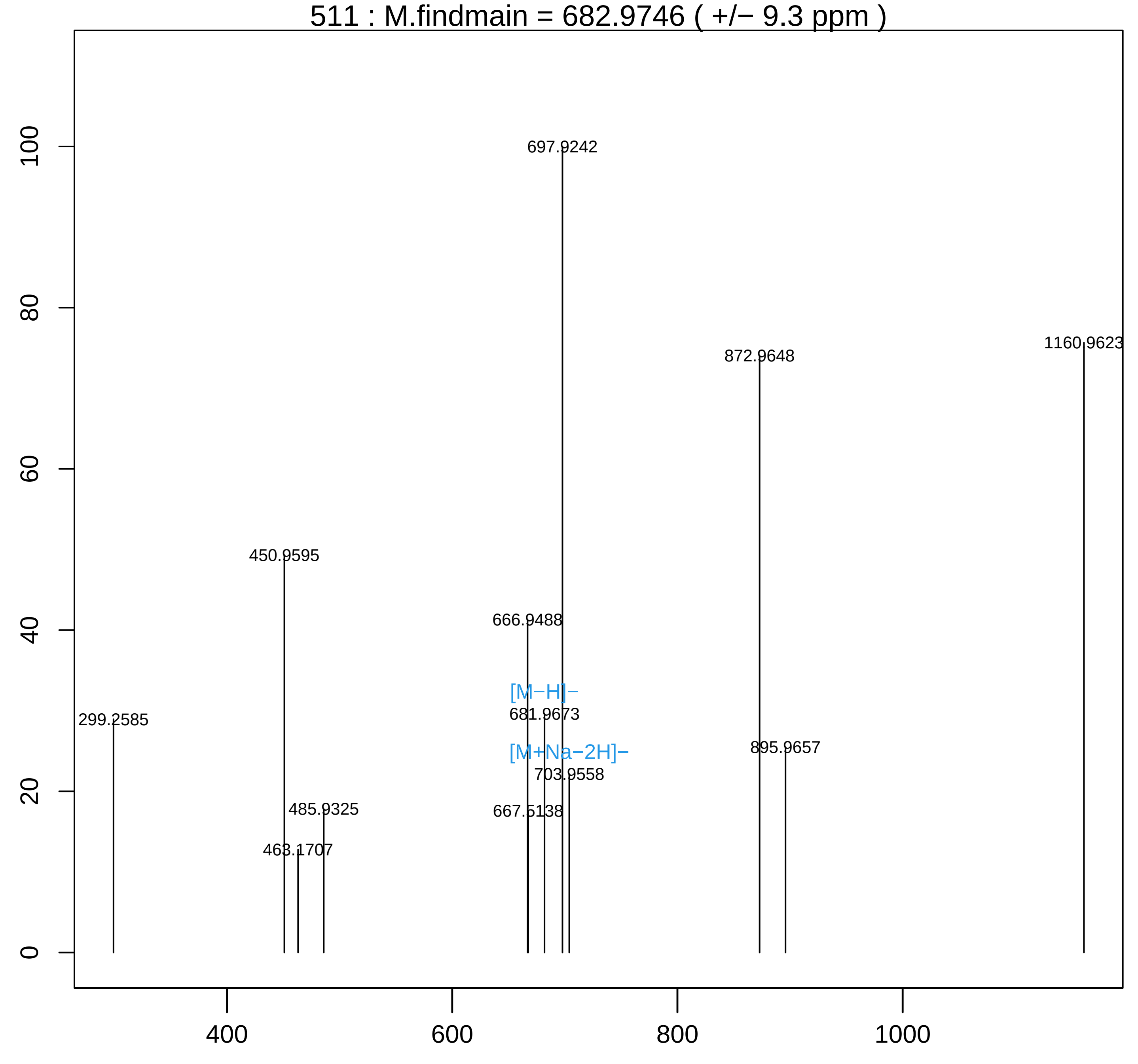
511 : M.findmain = 682.9746 (+/− 9.3 ppm)
697.9242
872.9648
1160.9623
450.9595
666.9488
[M−H]−
681.9673
299.2585
[M+Na−2H]−
895.9657
703.9558
485.9325
667.5138
463.1707
0
20
40
60
80
100
400
600
800
1000

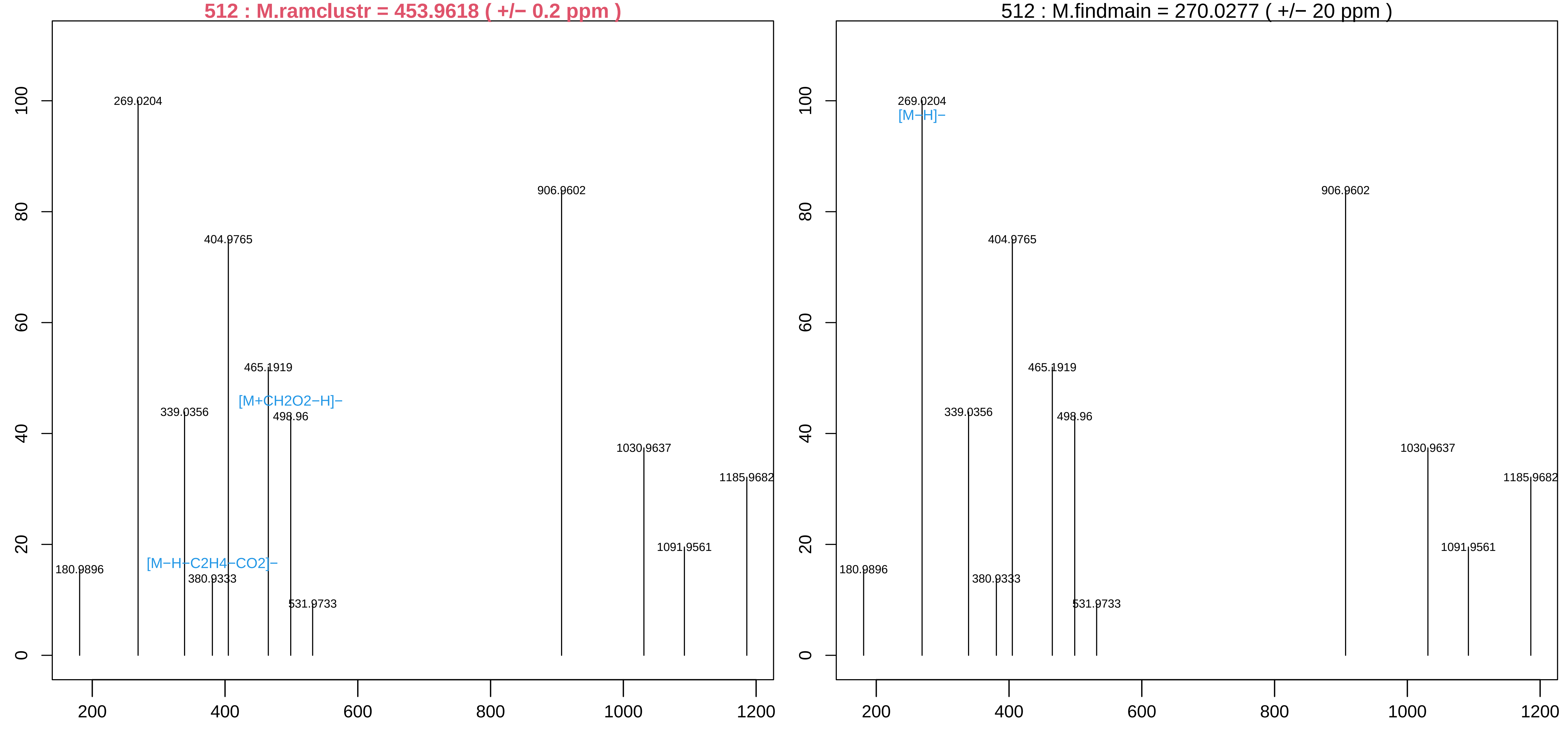
512 : M.ramclustr = 453.9618 (+/− 0.2 ppm)
269.0204
906.9602
404.9765
465.1919
[M+CH2O2−H]−
339.0356
498.96
1030.9637
1185.9682
1091.9561
180.9896
[M−H−C2H4−CO2]−
380.9333
531.9733
0
20
40
60
80
100
200
400
600
800
1000
1200
512 : M.findmain = 270.0277 (+/− 20 ppm)
269.0204
[M−H]−
906.9602
404.9765
465.1919
339.0356
498.96
1030.9637
1185.9682
1091.9561
180.9896
380.9333
531.9733
0
20
40
60
80
100
200
400
600
800
1000
1200

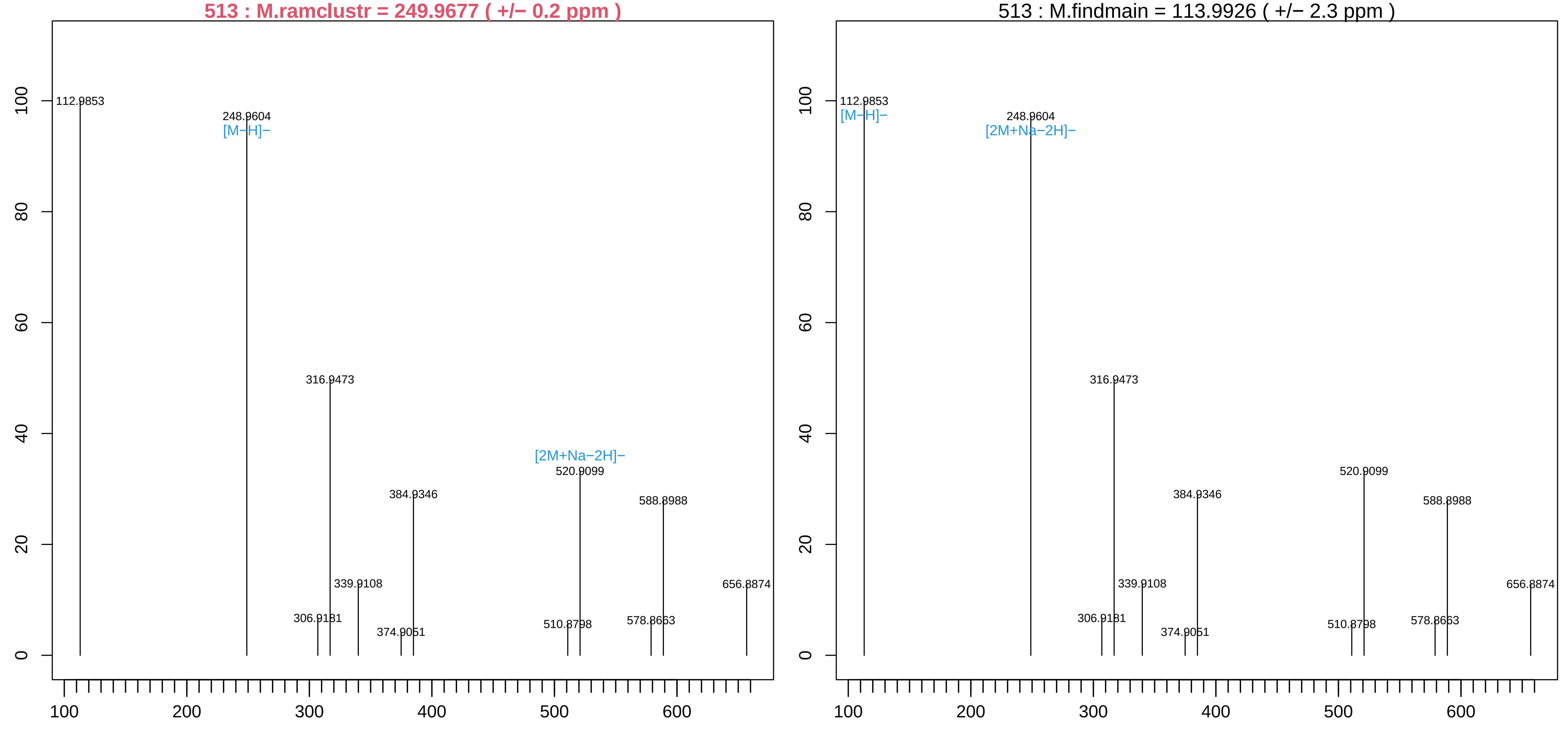

513 : M.ramclustr = 249.9677 (+/− 0.2 ppm)
112.9853
248.9604
[M−H]−
316.9473
306.9181
339.9108
384.9346
374.9051
[2M+Na−2H]−
520.9099
510.8798
588.8988
578.8663
656.8874
513 : M.findmain = 113.9926 (+/− 2.3 ppm)
112.9853
[M−H]−
248.9604
[2M+Na−2H]−
316.9473
306.9181
339.9108
384.9346
374.9051
520.9099
510.8798
588.8988
578.8663
656.8874

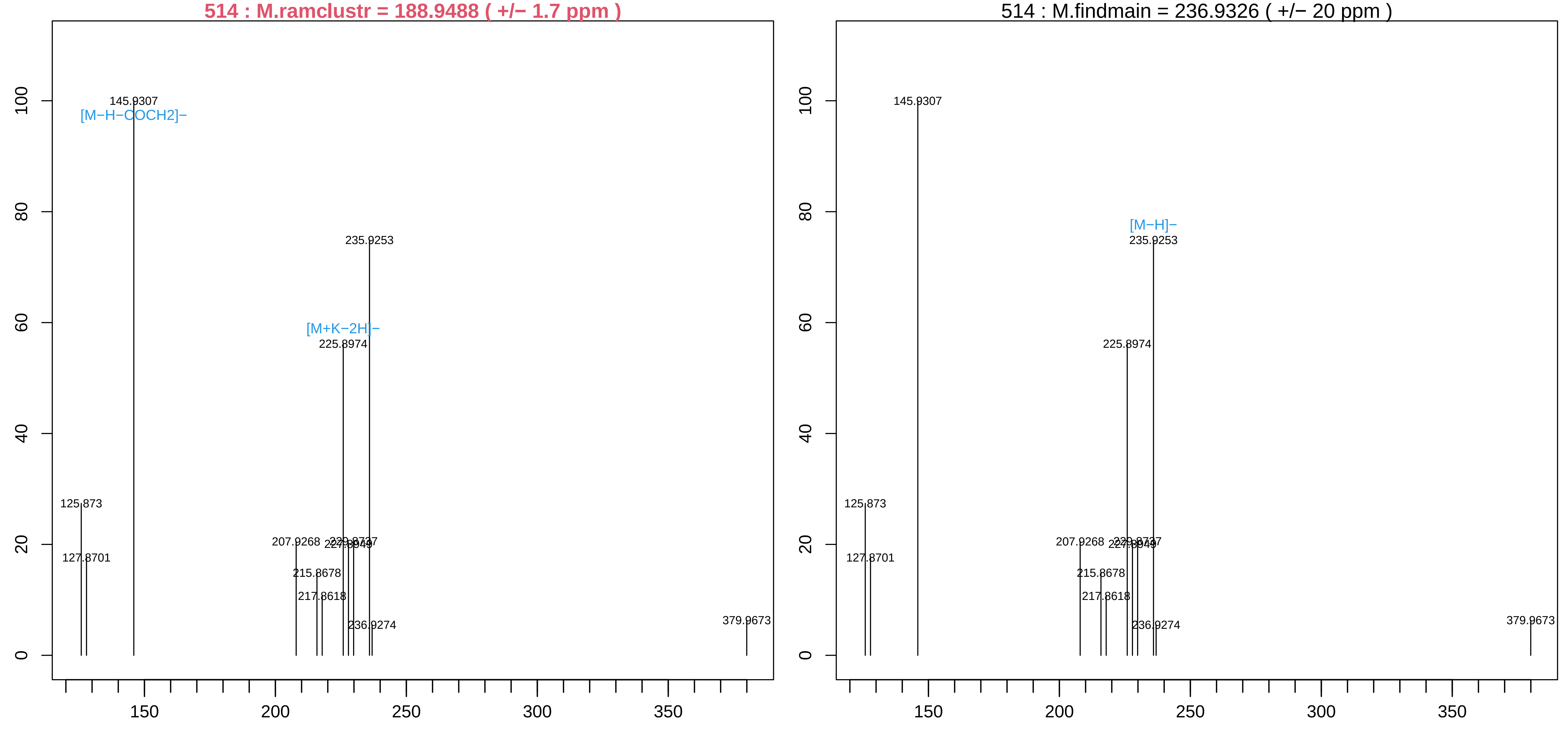
514 : M.ramclustr = 188.9488 (+/− 1.7 ppm)
[M−H−COCH2]−
145.9307
[M+K−2H]−
225.8974
235.9253
125.873
127.8701
207.9268
229.8737
227.8949
215.8678
217.8618
236.9274
379.9673
514 : M.findmain = 236.9326 (+/− 20 ppm)
145.9307
[M−H]−
235.9253
225.8974
125.873
127.8701
207.9268
229.8737
227.8949
215.8678
217.8618
236.9274
379.9673
0
20
40
60
80
100
150
200
250
300
350

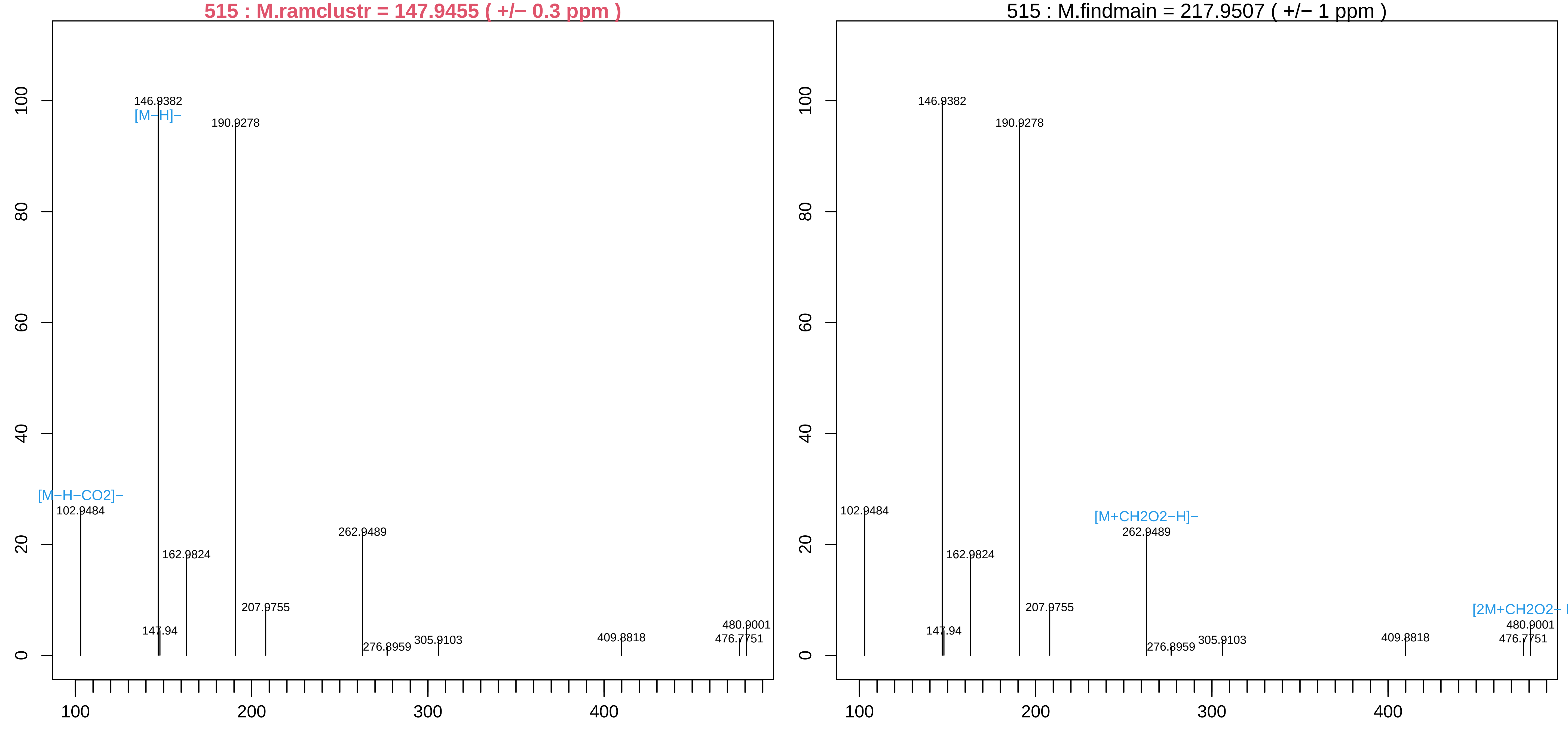

515 : M.ramclustr = 147.9455 (+/− 0.3 ppm)
146.9382
[M−H]−
190.9278
[M−H−CO2]−
102.9484
262.9489
162.9824
207.9755
147.94
276.8959
305.9103
409.8818
480.9001
476.7751
515 : M.findmain = 217.9507 (+/− 1 ppm)
[M+CH2O2−H]−
[2M+CH2O2−
0
20
40
60
80
100
200
300
400

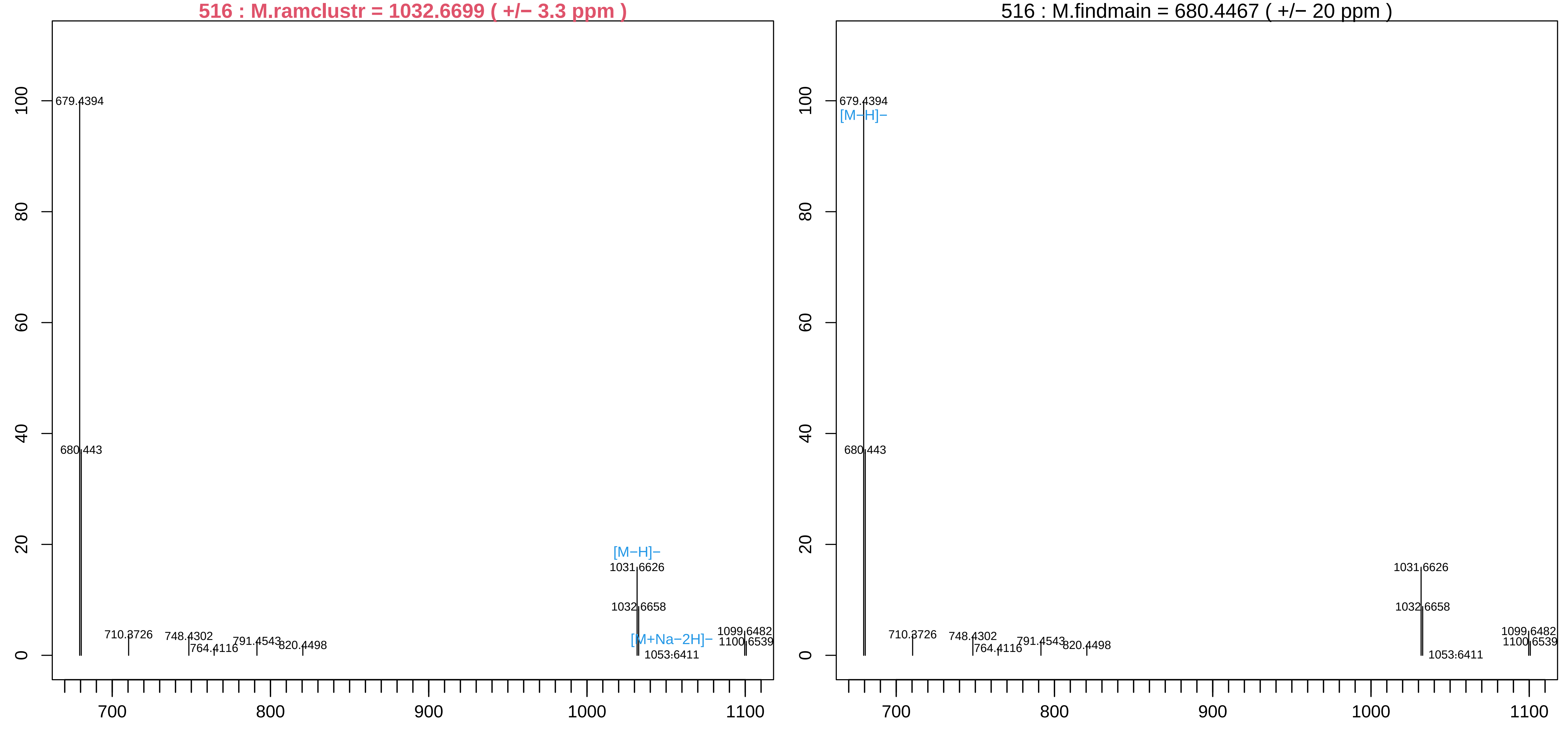

516 : M.ramclustr = 1032.6699 (+/− 3.3 ppm)
679.4394
680.443
710.3726
748.4302
764.4116
791.4543
820.4498
[M−H]−
1031.6626
1032.6658
[M+Na−2H]−
1053.6411
1099.6482
1100.6539
516 : M.findmain = 680.4467 (+/− 20 ppm)
679.4394
[M−H]−
680.443
710.3726
748.4302
764.4116
791.4543
820.4498
1031.6626
1032.6658
1053.6411
1099.6482
1100.6539

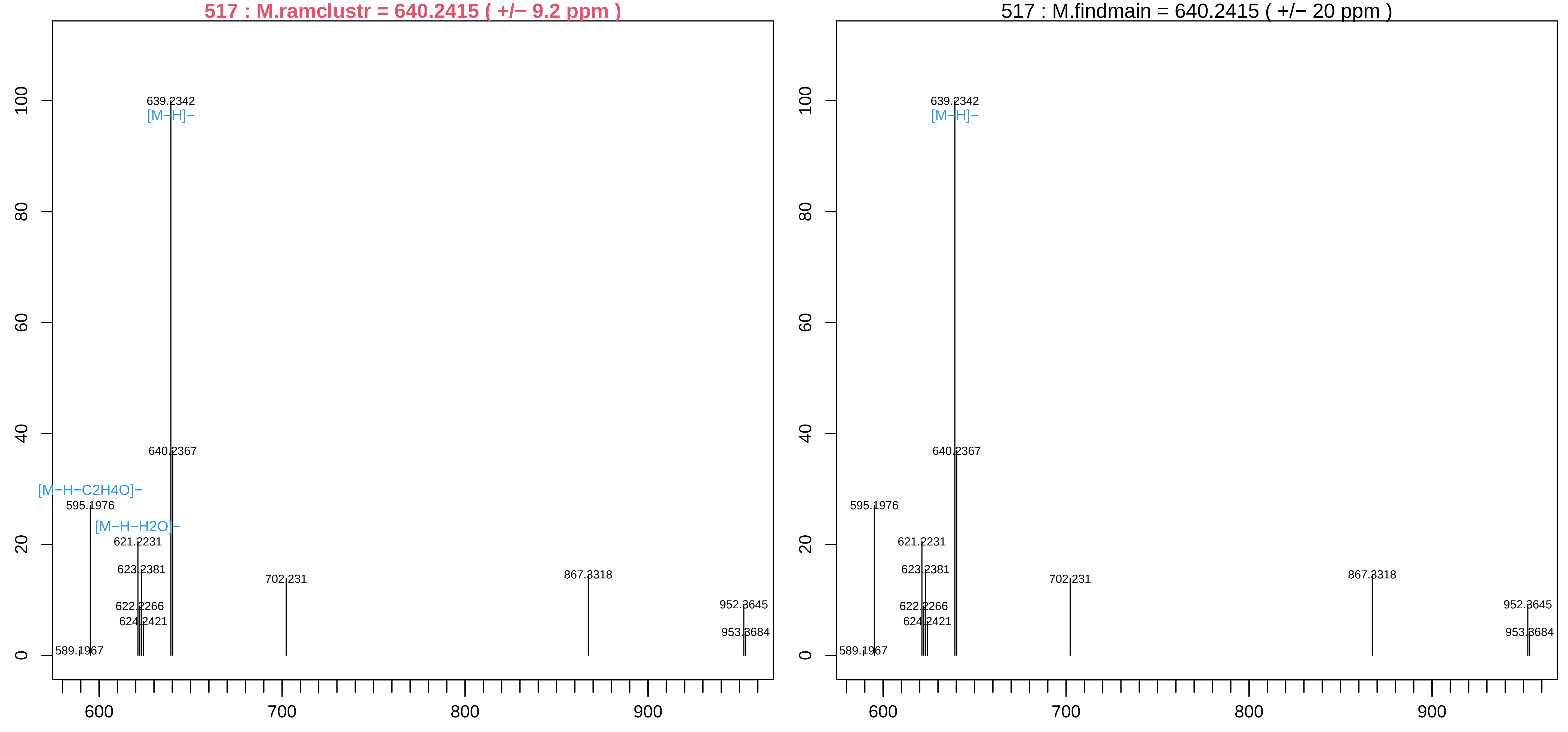
517 : M.ramclustr = 640.2415 (+/− 9.2 ppm)
639.2342
[M−H]−
640.2367
[M−H−C2H4O]−
595.1976
[M−H−H2O]−
621.2231
623.2381
702.231
867.3318
622.2266
624.2421
952.3645
953.3684
589.1967
517 : M.findmain = 640.2415 (+/− 20 ppm)
0
20
40
60
80
100
600
700
800
900

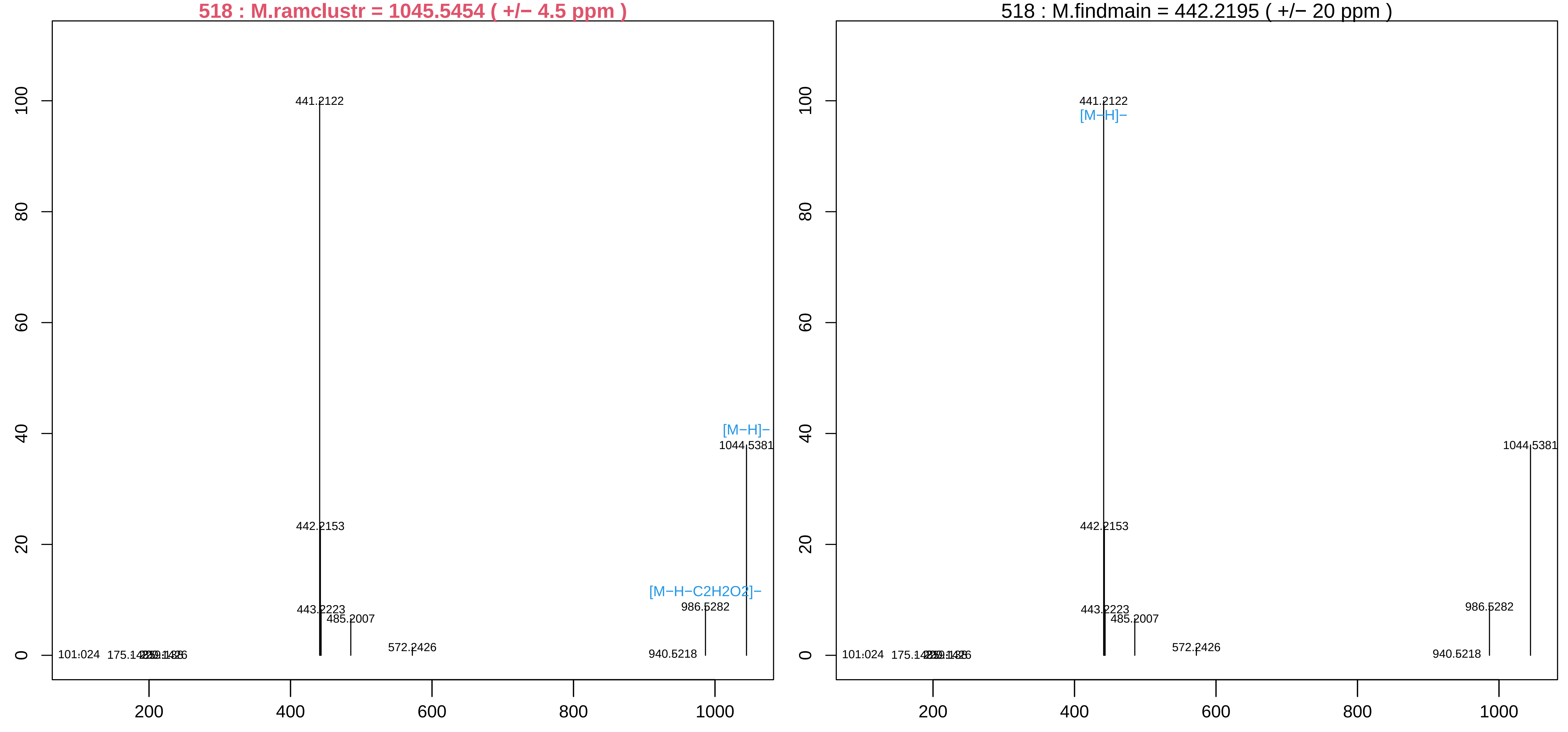

518 : M.ramclustr = 1045.5454 (+/− 4.5 ppm)
441.2122
442.2153
443.2223
485.2007
572.2426
940.5218
986.5282
[M−H−C2H2O2]−
1044.5381
[M−H]−
101.024
0
20
40
60
80
100
200
400
600
800
1000
518 : M.findmain = 442.2195 (+/− 20 ppm)
441.2122
[M−H]−
442.2153
443.2223
485.2007
572.2426
940.5218
986.5282
1044.5381
101.024

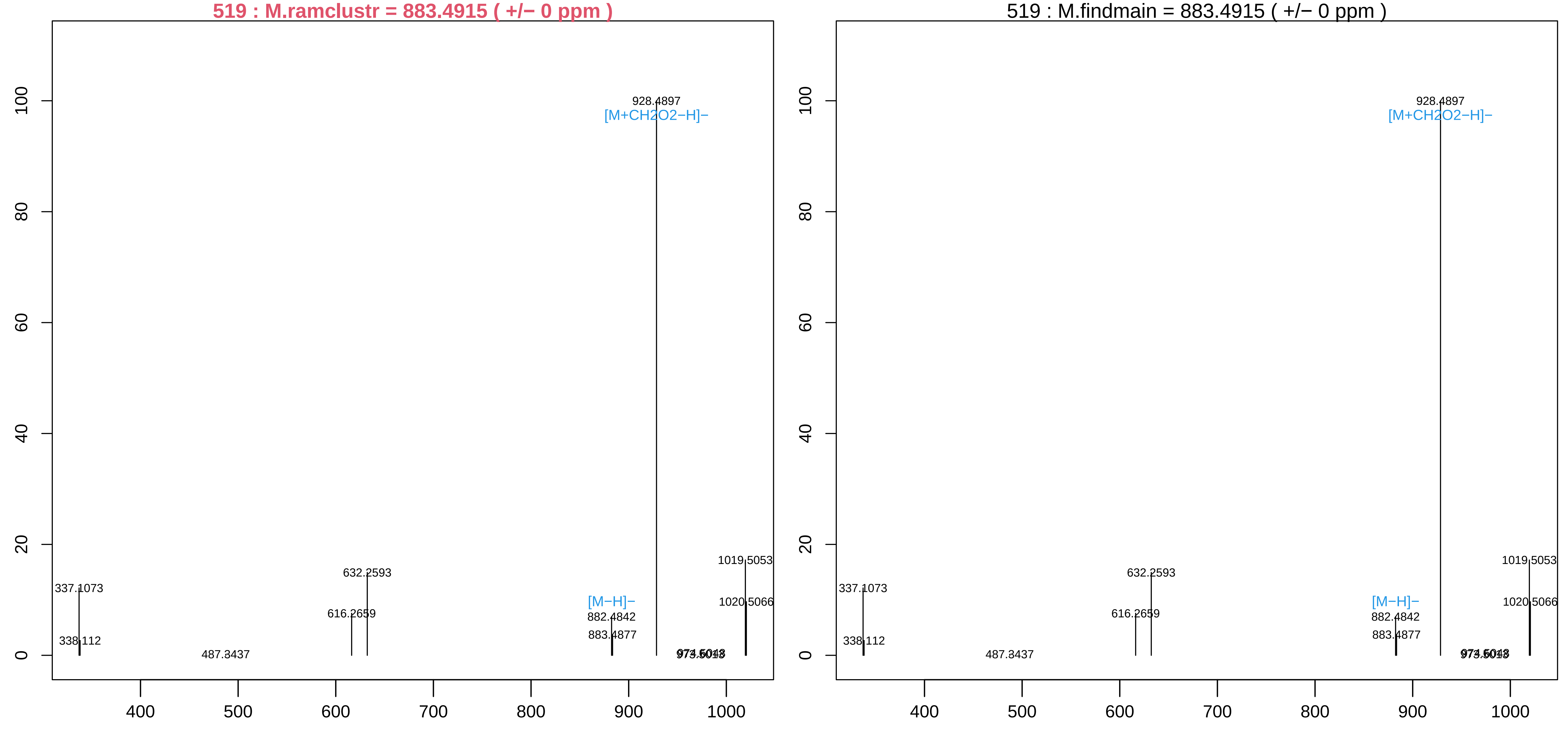

519 : M.ramclustr = 883.4915 (+/− 0 ppm)
519 : M.findmain = 883.4915 (+/− 0 ppm)
928.4897
[M+CH2O2−H]−
1019.5053
1020.5066
632.2593
337.1073
616.2659
[M−H]−
882.4842
883.4877
338.112
487.3437
974.5043
973.5013
0
20
40
60
80
100
400
500
600
700
800
900
1000

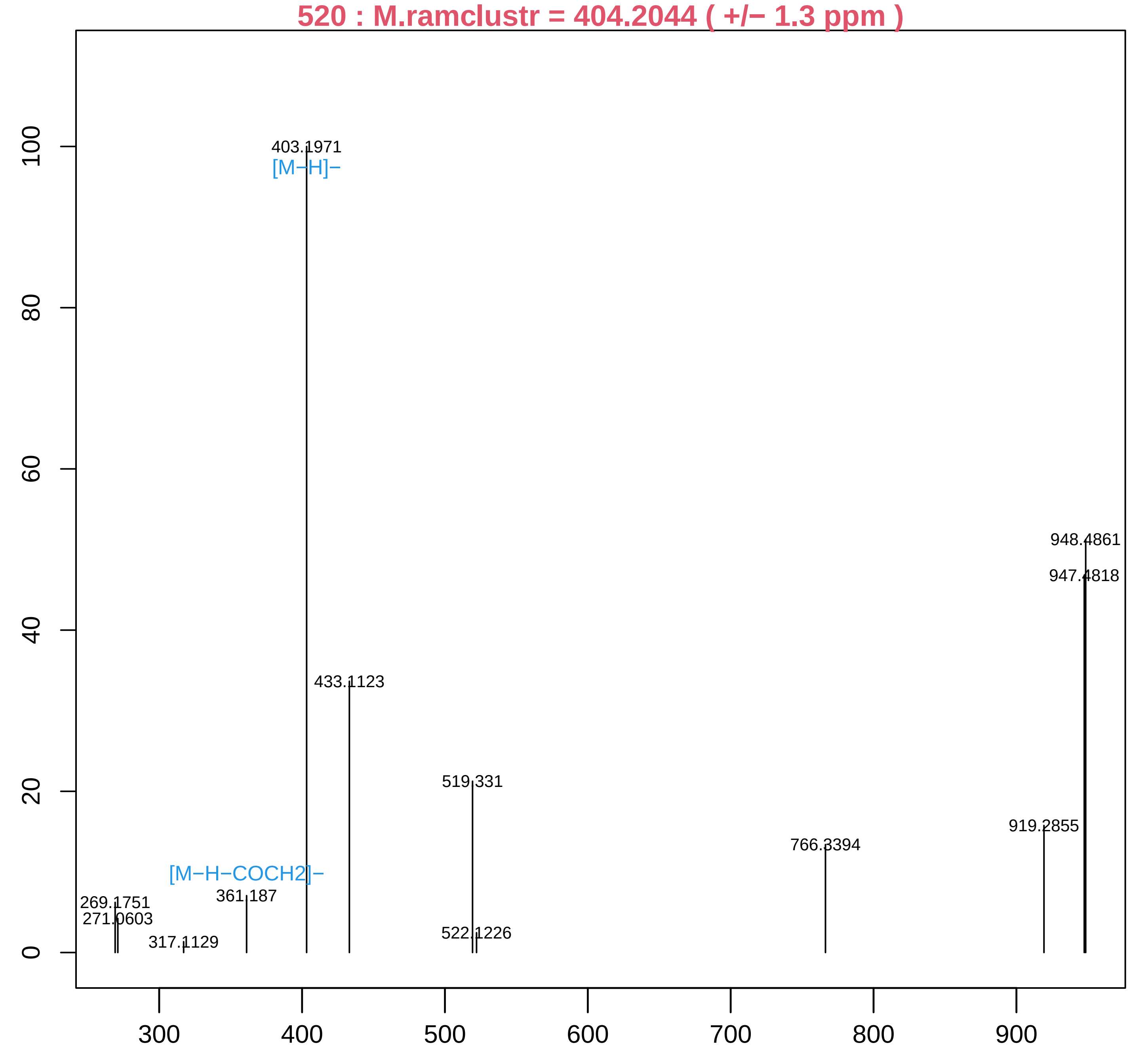
520 : M.ramclustr = 404.2044 (+/− 1.3 ppm)
403.1971
[M−H]−
433.1123
519.331
522.1226
766.3394
919.2855
948.4861
947.4818
[M−H−COCH2]−
361.187
269.1751
271.0603
317.1129

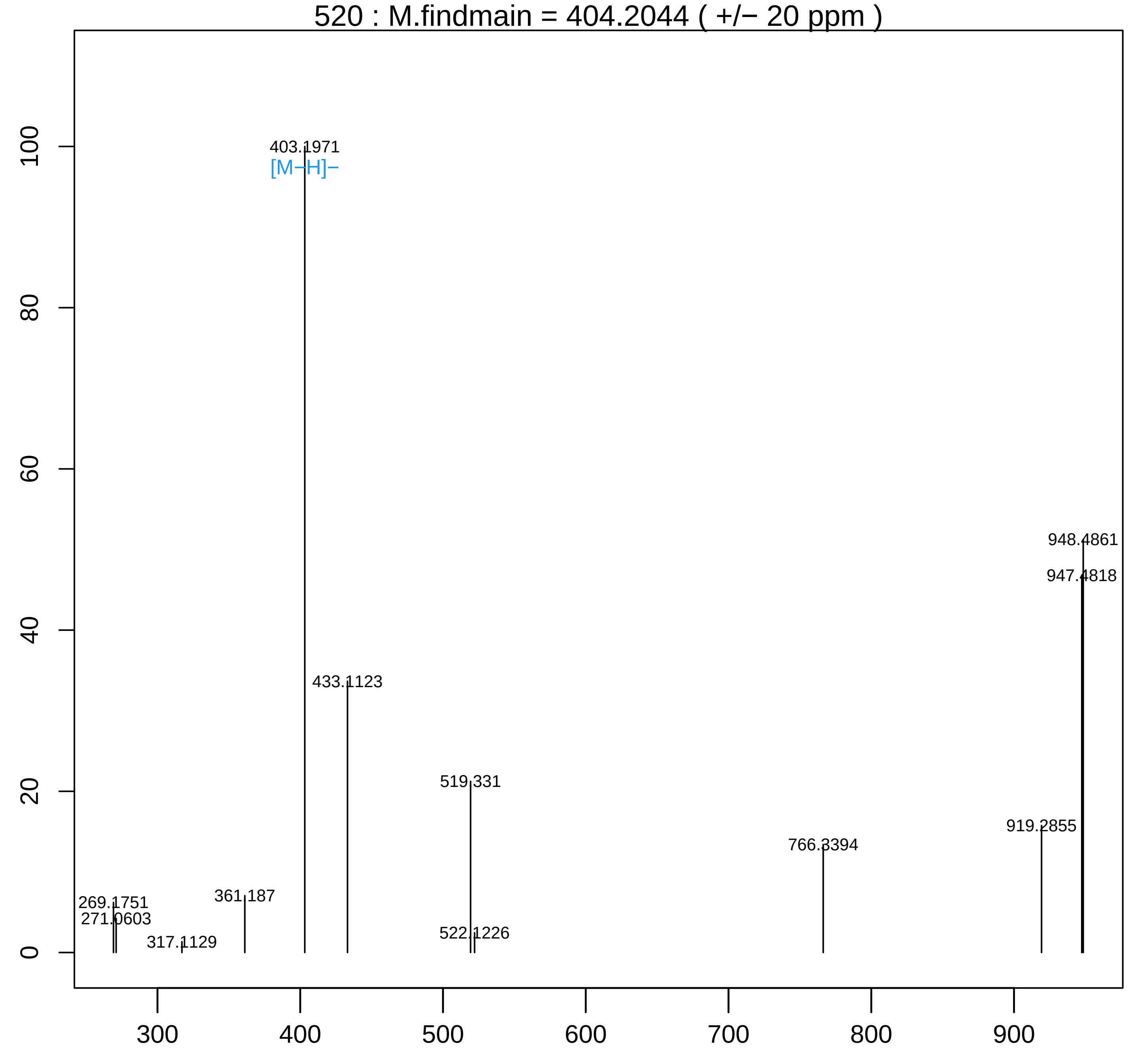
520 : M.findmain = 404.2044 (+/− 20 ppm)
403.1971
[M−H]−
433.1123
519.331
522.1226
766.3394
919.2855
948.4861
947.4818
361.187
269.1751
271.0603
317.1129

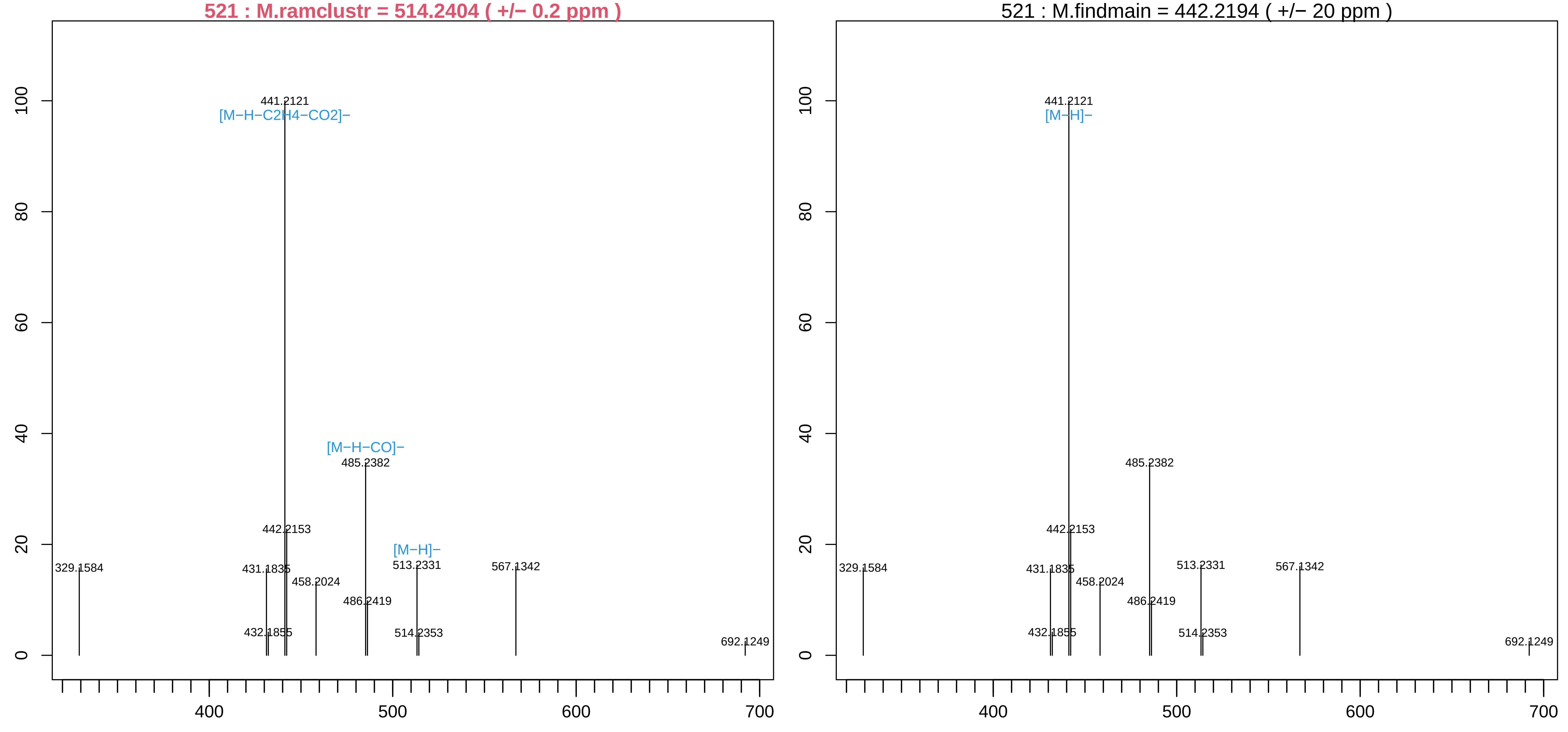
521 : M.ramclustr = 514.2404 (+/− 0.2 ppm)
441.2121
[M−H−C2H4−CO2]−
[M−H−CO]−
485.2382
442.2153
[M−H]−
329.1584
431.1835
458.2024
513.2331
567.1342
486.2419
432.1855
514.2353
692.1249
521 : M.findmain = 442.2194 (+/− 20 ppm)
441.2121
[M−H]−
485.2382
442.2153
329.1584
431.1835
458.2024
513.2331
567.1342
486.2419
432.1855
514.2353
692.1249
0
20
40
60
80
100
400
500
600
700

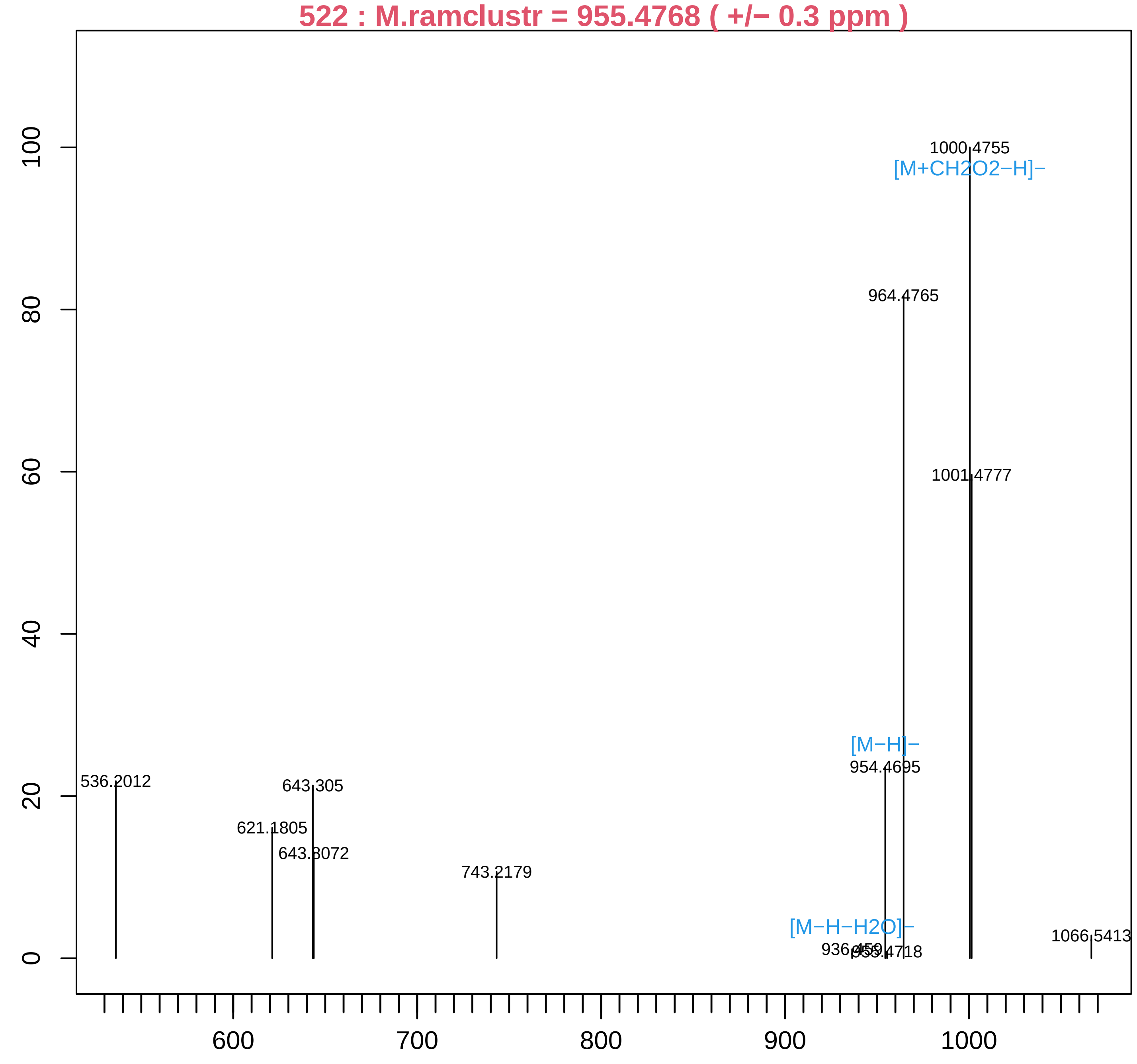
522 : M.ramclustr = 955.4768 (+/− 0.3 ppm)
1000.4755
[M+CH2O2−H]−
964.4765
1001.4777
[M−H]−
954.4695
536.2012
643.305
621.1805
643.3072
743.2179
[M−H−H2O]−
936.459
955.4718
1066.5413
0
20
40
60
80
100
600
700
800
900
1000
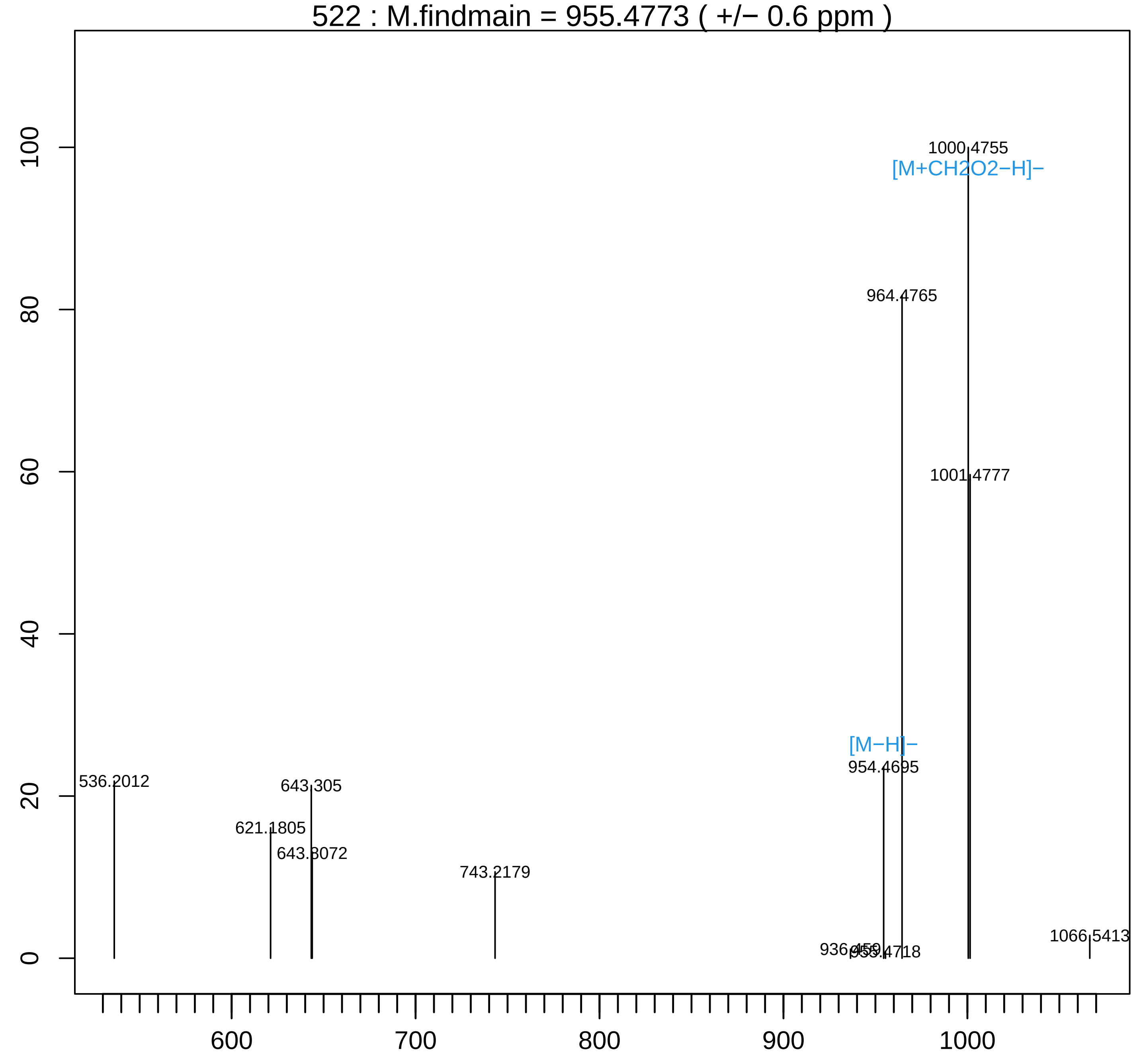
522 : M.findmain = 955.4773 (+/− 0.6 ppm)
1000.4755
[M+CH2O2−H]−
964.4765
1001.4777
[M−H]−
954.4695
536.2012
643.305
621.1805
643.3072
743.2179
936.459
955.4718
1066.5413
0
20
40
60
80
100
600
700
800
900
1000

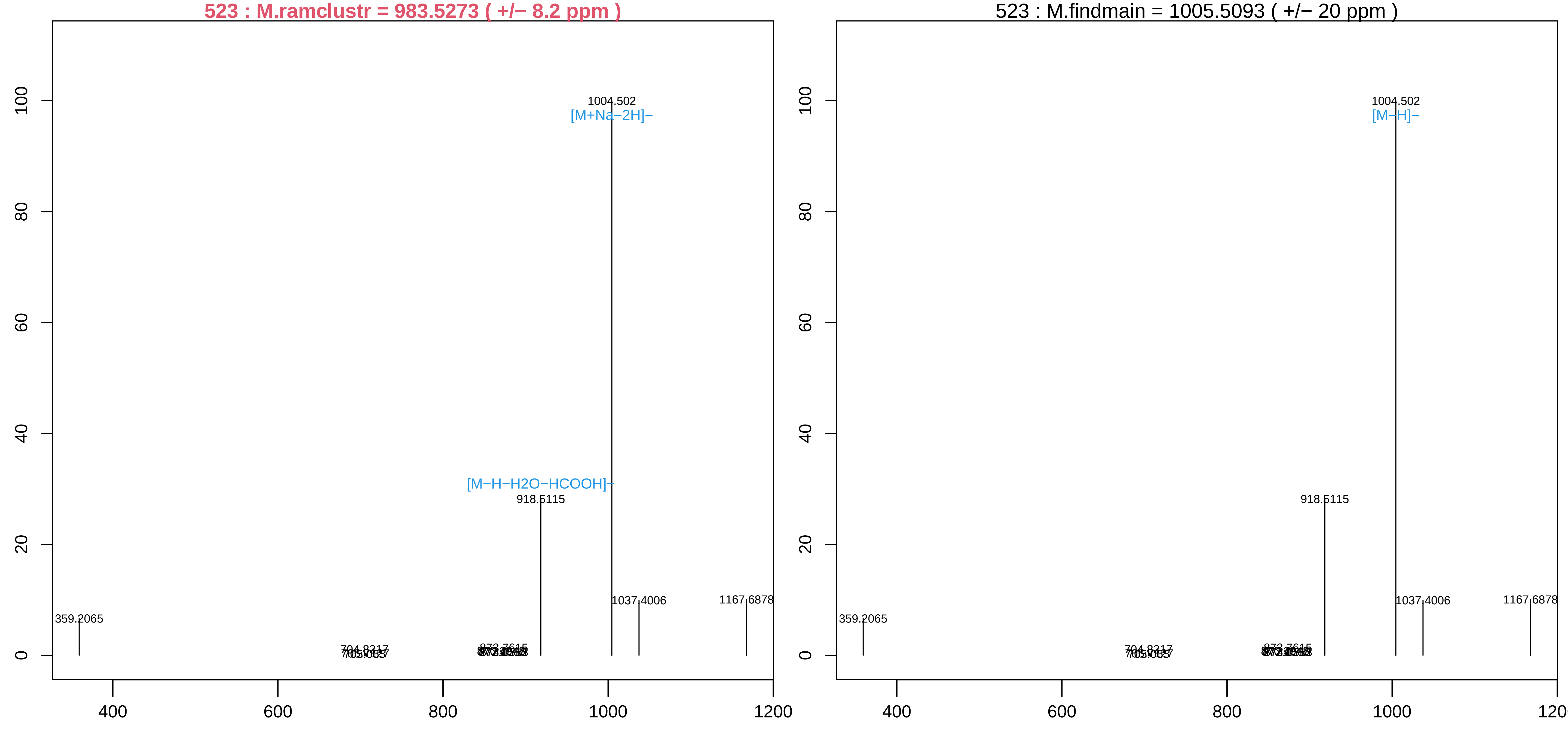
523 : M.ramclustr = 983.5273 (+/− 8.2 ppm)
1004.502
[M+Na−2H]−
[M−H−H2O−HCOOH]−
918.5115
1037.4006
1167.6878
359.2065
704.8317
872.7615
0
20
40
60
80
100
400
600
800
1000
1200
523 : M.findmain = 1005.5093 (+/− 20 ppm)
1004.502
[M−H]−
918.5115
1037.4006
1167.6878
359.2065
704.8317
872.7615

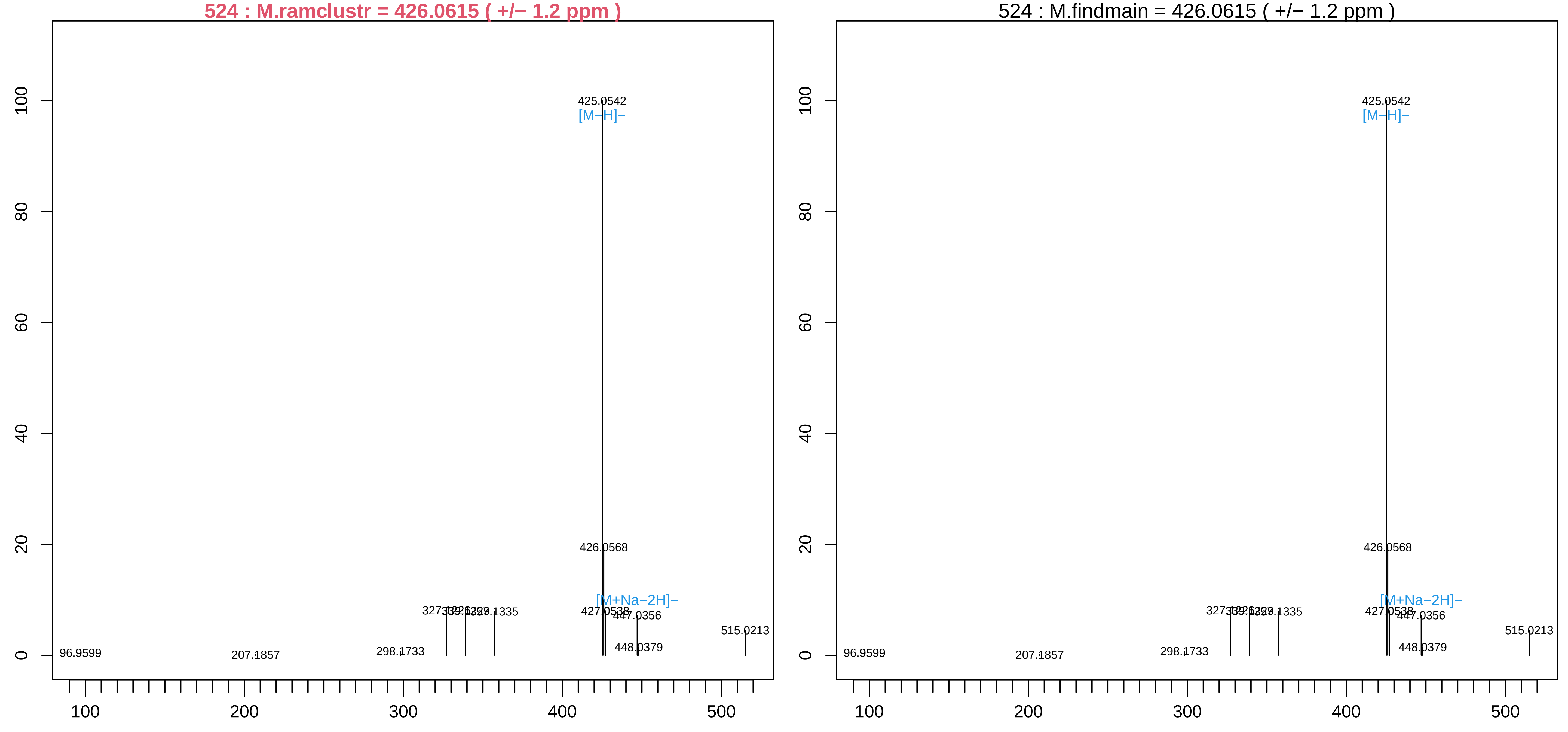

524 : M.ramclustr = 426.0615 (+/− 1.2 ppm)
425.0542
[M−H]−
426.0568
[M+Na−2H]−
427.0538
447.0356
448.0379
515.0213
96.9599
207.1857
298.1733
357.1335
0
20
40
60
80
100
100
200
300
400
500
524 : M.findmain = 426.0615 (+/− 1.2 ppm)
425.0542
[M−H]−
426.0568
[M+Na−2H]−
427.0538
447.0356
448.0379
515.0213
96.9599
207.1857
298.1733
357.1335
0
20
40
60
80
100
100
200
300
400
500

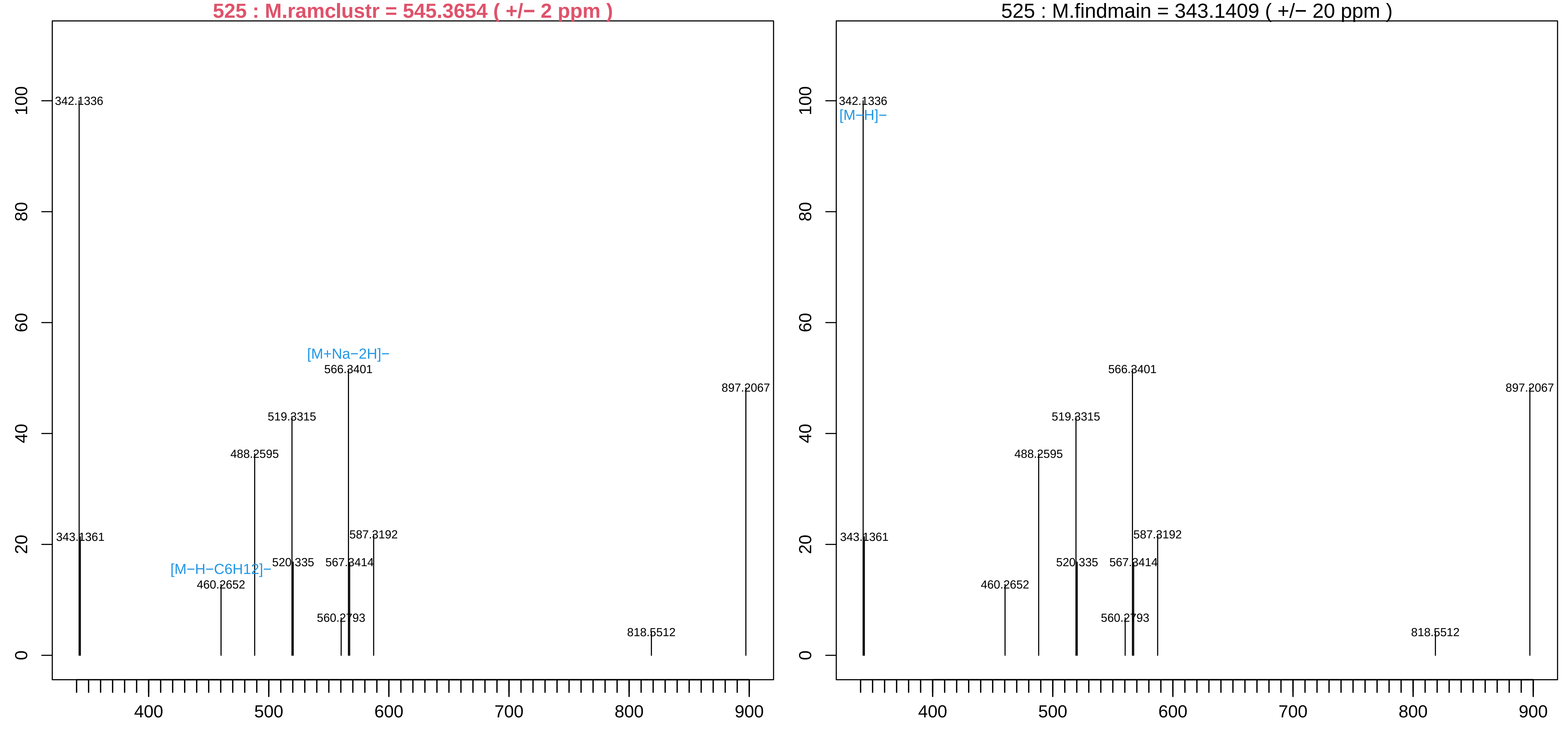

525 : M.ramclustr = 545.3654 (+/− 2 ppm)
342.1336
343.1361
[M−H−C6H12]−
460.2652
488.2595
519.3315
520.335
[M+Na−2H]−
566.3401
567.3414
560.2793
587.3192
818.5512
897.2067
525 : M.findmain = 343.1409 (+/− 20 ppm)
342.1336
[M−H]−
343.1361
460.2652
488.2595
519.3315
520.335
566.3401
567.3414
560.2793
587.3192
818.5512
897.2067

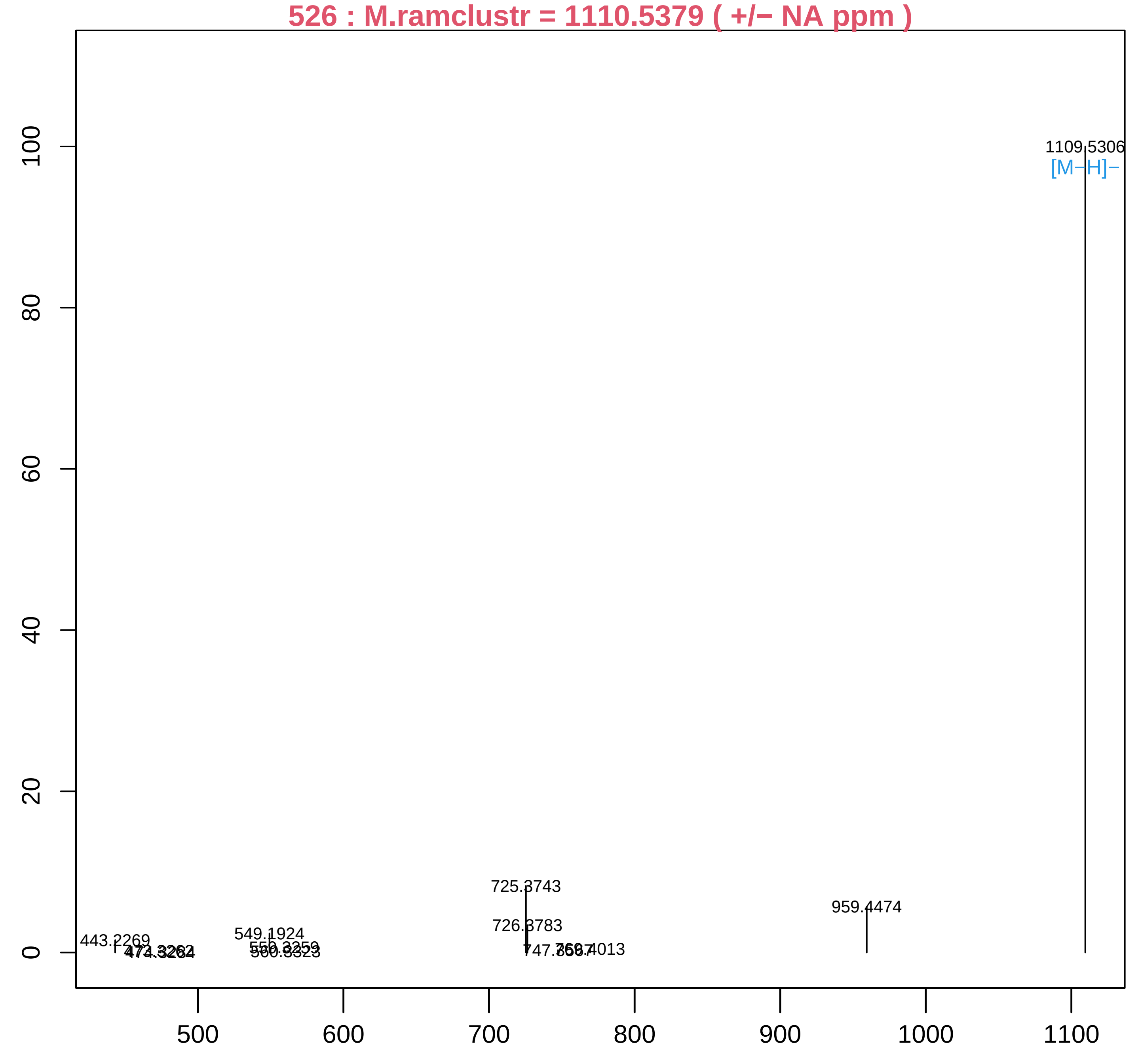
526 : M.ramclustr = 1110.5379 (+/− NA ppm)
1109.5306
[M−H]−
725.3743
726.3783
959.4474
443.2269
549.1924
0
20
40
60
80
100
500
600
700
800
900
1000
1100

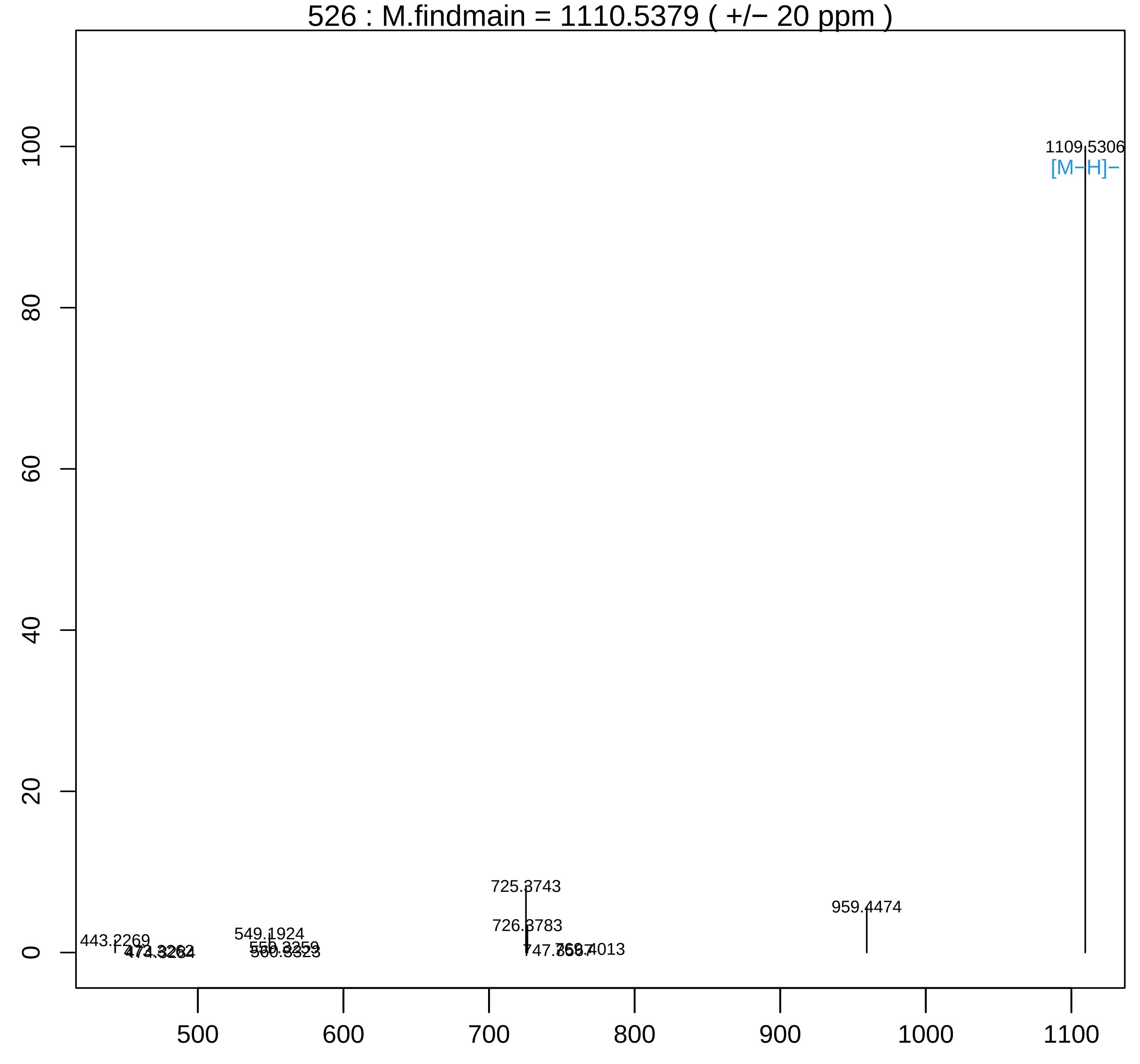
526 : M.findmain = 1110.5379 (+/− 20 ppm)
1109.5306
[M−H]−
725.3743
726.3783
959.4474
443.2269
549.1924
0
20
40
60
80
100
500
600
700
800
900
1000
1100

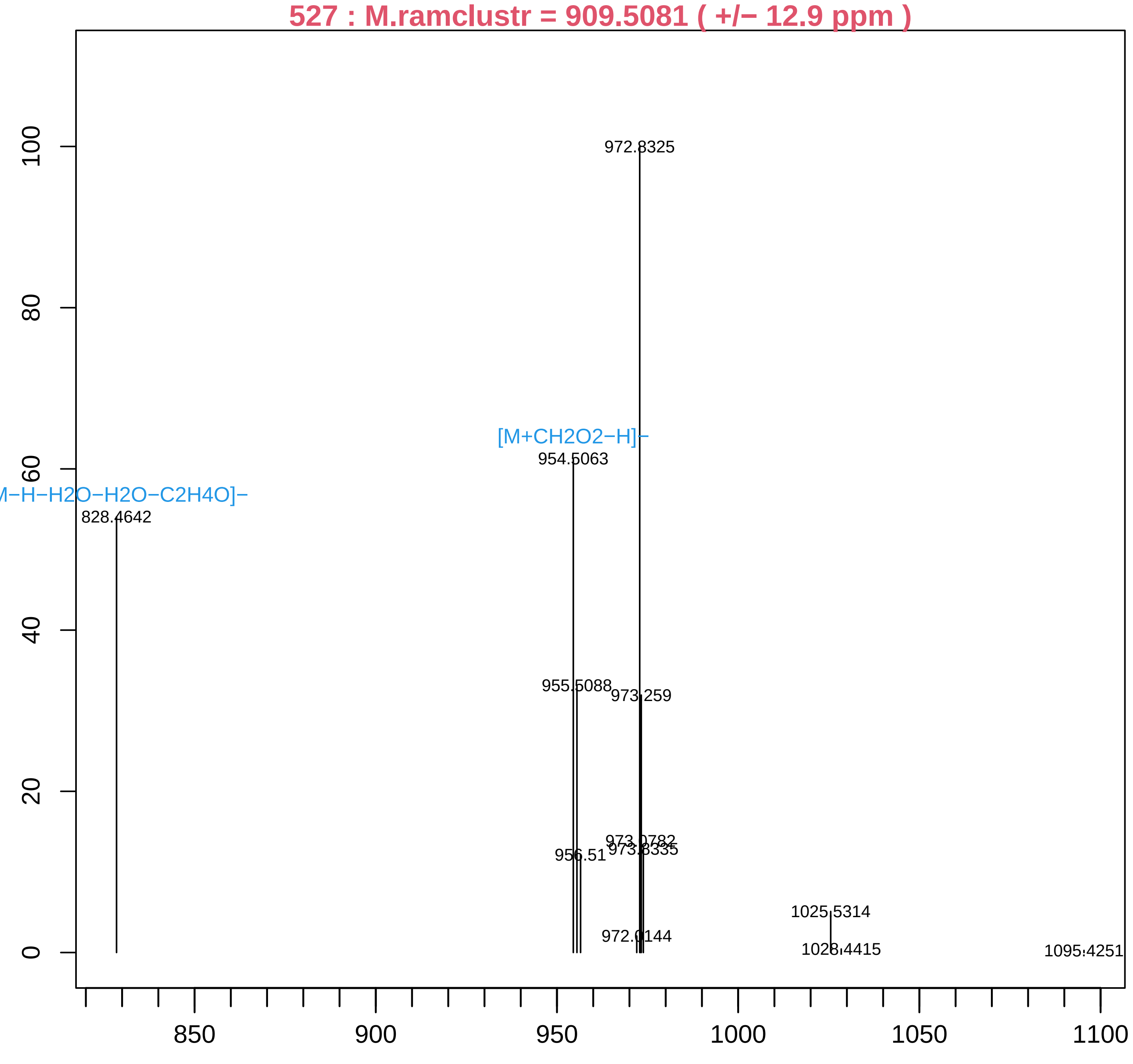

527 : M.ramclustr = 909.5081 (+/− 12.9 ppm)
972.8325
[M+CH2O2−H]−
954.5063
M−H−H2O−H2O−C2H4O]−
828.4642
955.5088
973.259
973.0782
973.3335
956.51
1025.5314
972.0144
1028.4415
1095.4251
0
20
40
60
80
100
850
900
950
1000
1050
1100

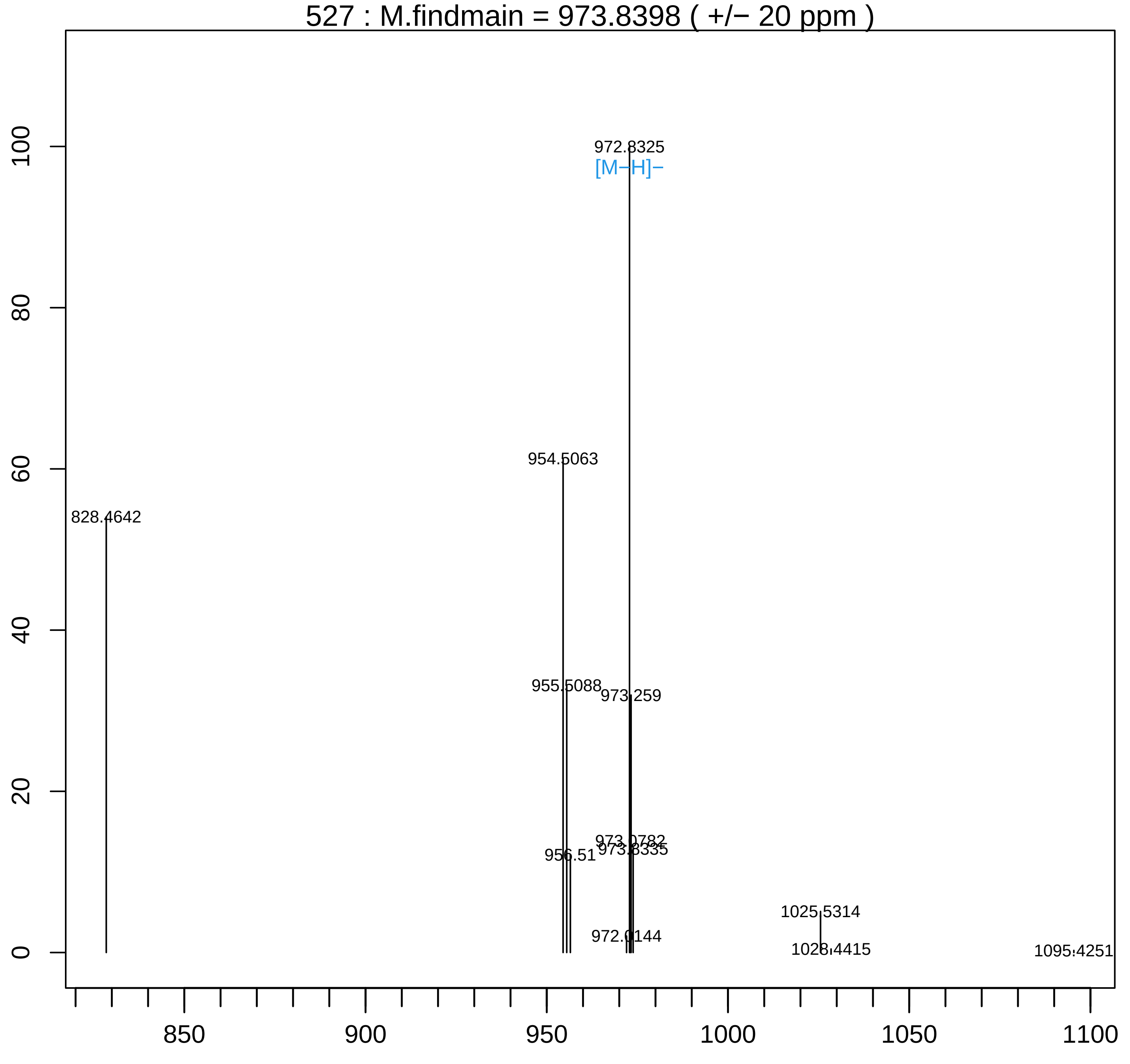

527 : M.findmain = 973.8398 (+/− 20 ppm)
972.8325
[M−H]−
954.5063
828.4642
955.5088
973.259
973.0782
973.3335
956.51
1025.5314
972.0144
1028.4415
1095.4251
0
20
40
60
80
100
850
900
950
1000
1050
1100

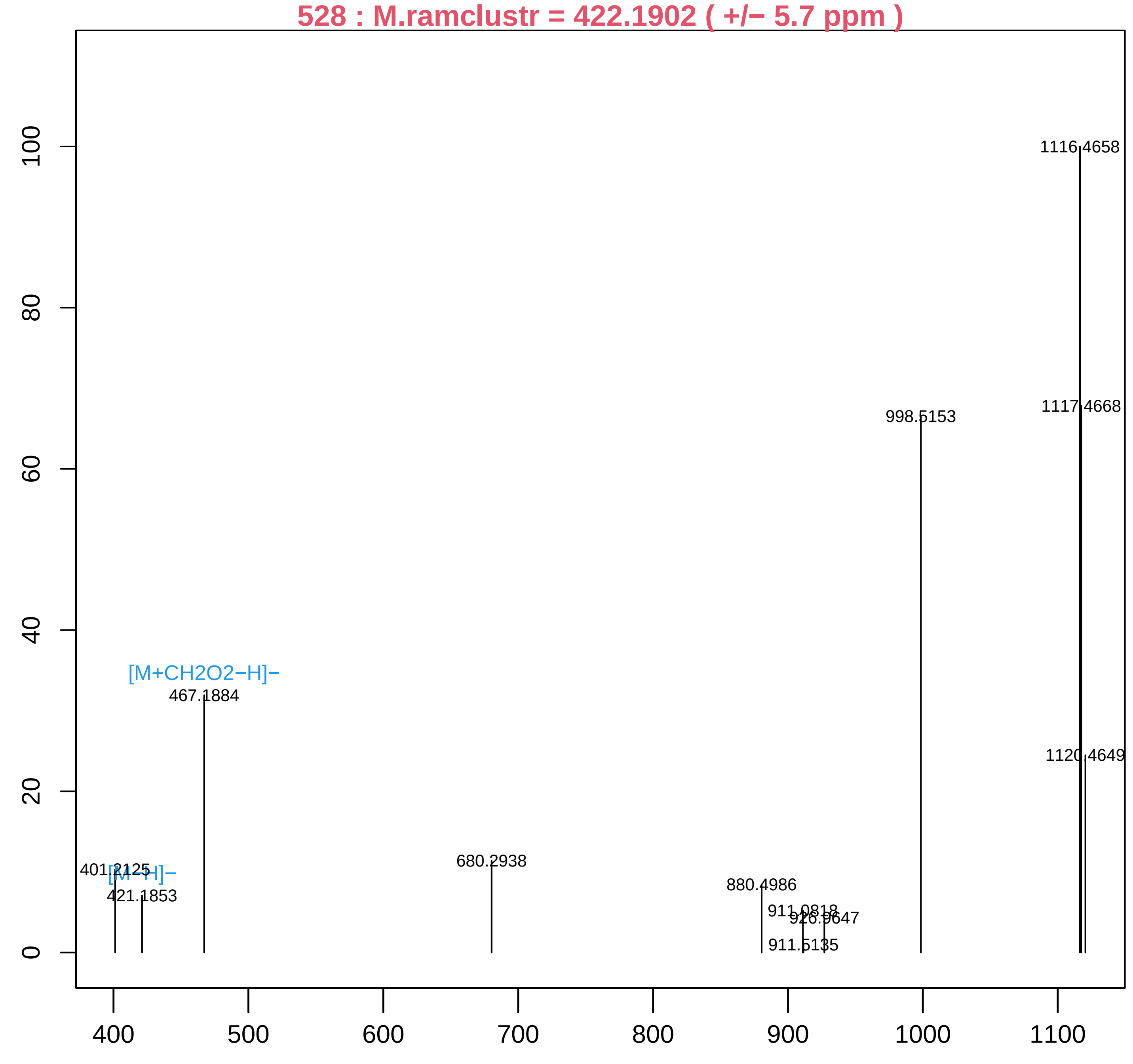
528 : M.ramclustr = 422.1902 (+/− 5.7 ppm)
1116.4658
1117.4668
998.5153
1120.4649
[M+CH2O2−H]−
467.1884
680.2938
401.2125
[M−H]−
421.1853
880.4986
911.0818
926.9647
911.5135
0
20
40
60
80
100
400
500
600
700
800
900
1000
1100

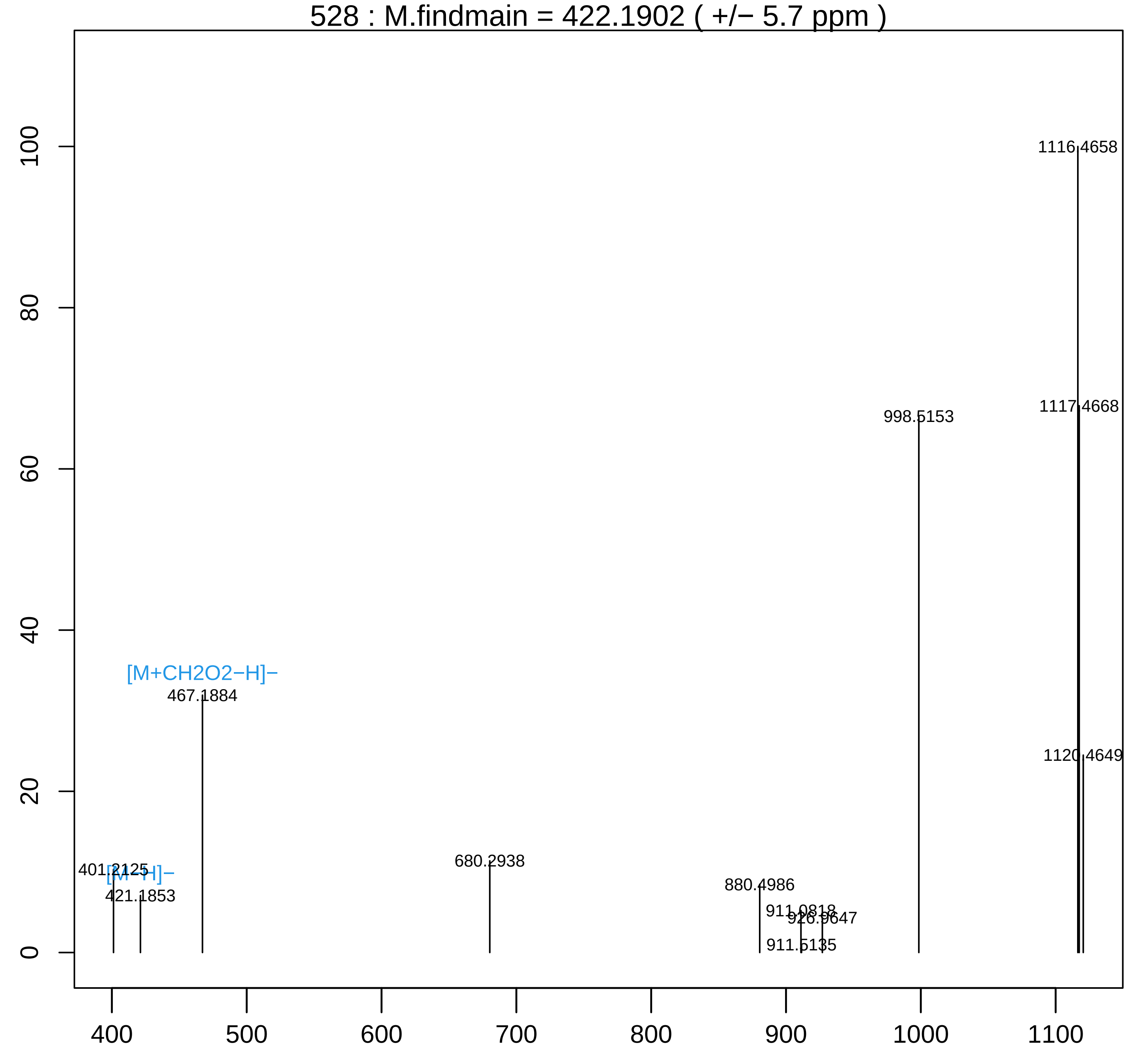
528 : M.findmain = 422.1902 (+/− 5.7 ppm)
1116.4658
1117.4668
998.5153
1120.4649
[M+CH2O2−H]−
467.1884
680.2938
401.2125
[M−H]−
421.1853
880.4986
911.0818
926.9647
911.5135
0
20
40
60
80
100
400
500
600
700
800
900
1000
1100

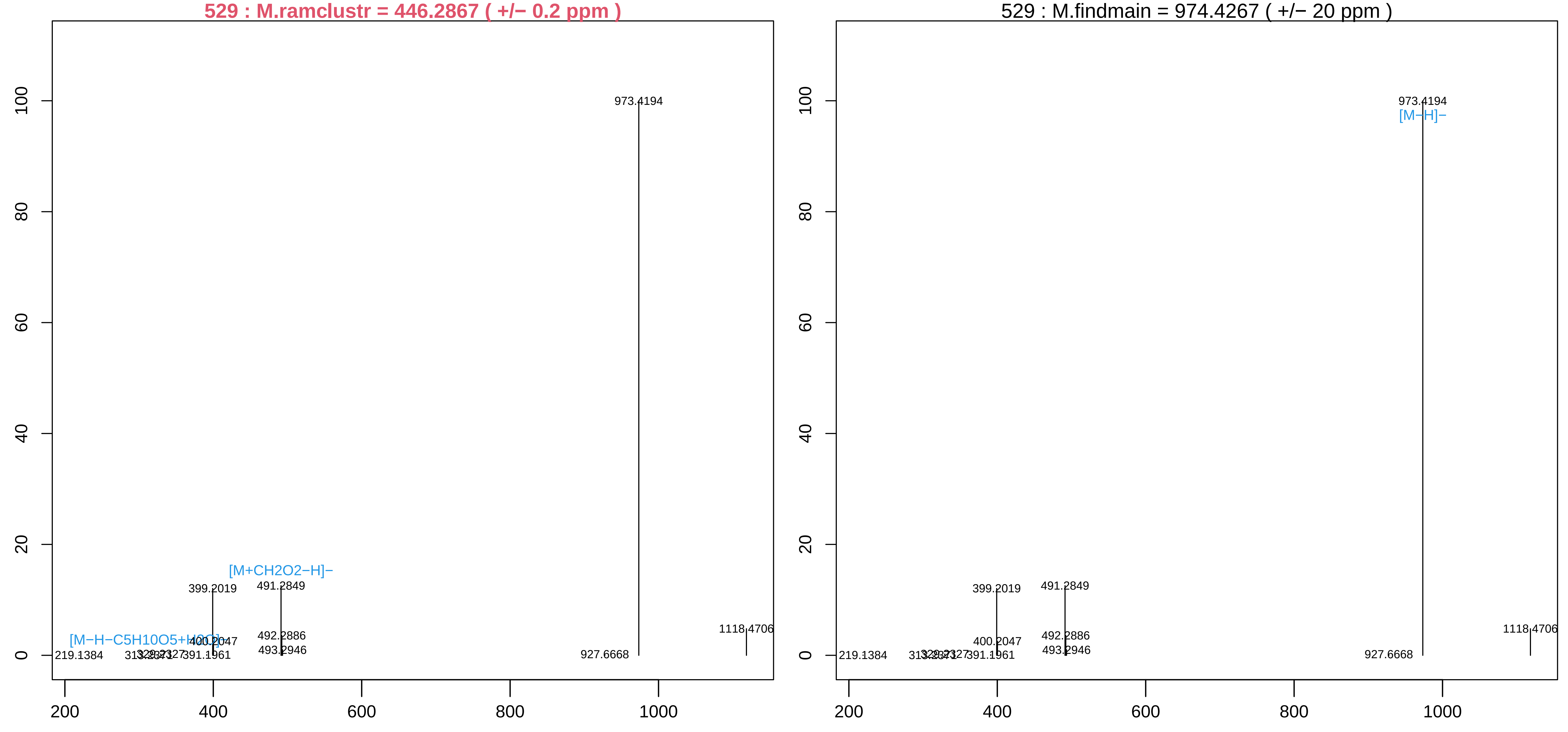

529 : M.ramclustr = 446.2867 (+/− 0.2 ppm)
973.4194
[M+CH2O2−H]−
399.2019
491.2849
[M−H−C5H10O5+H2O]−
400.2047
492.2886
493.2946
219.1384
391.1961
927.6668
1118.4706
529 : M.findmain = 974.4267 (+/− 20 ppm)
973.4194
[M−H]−
399.2019
491.2849
400.2047
492.2886
493.2946
219.1384
391.1961
927.6668
1118.4706

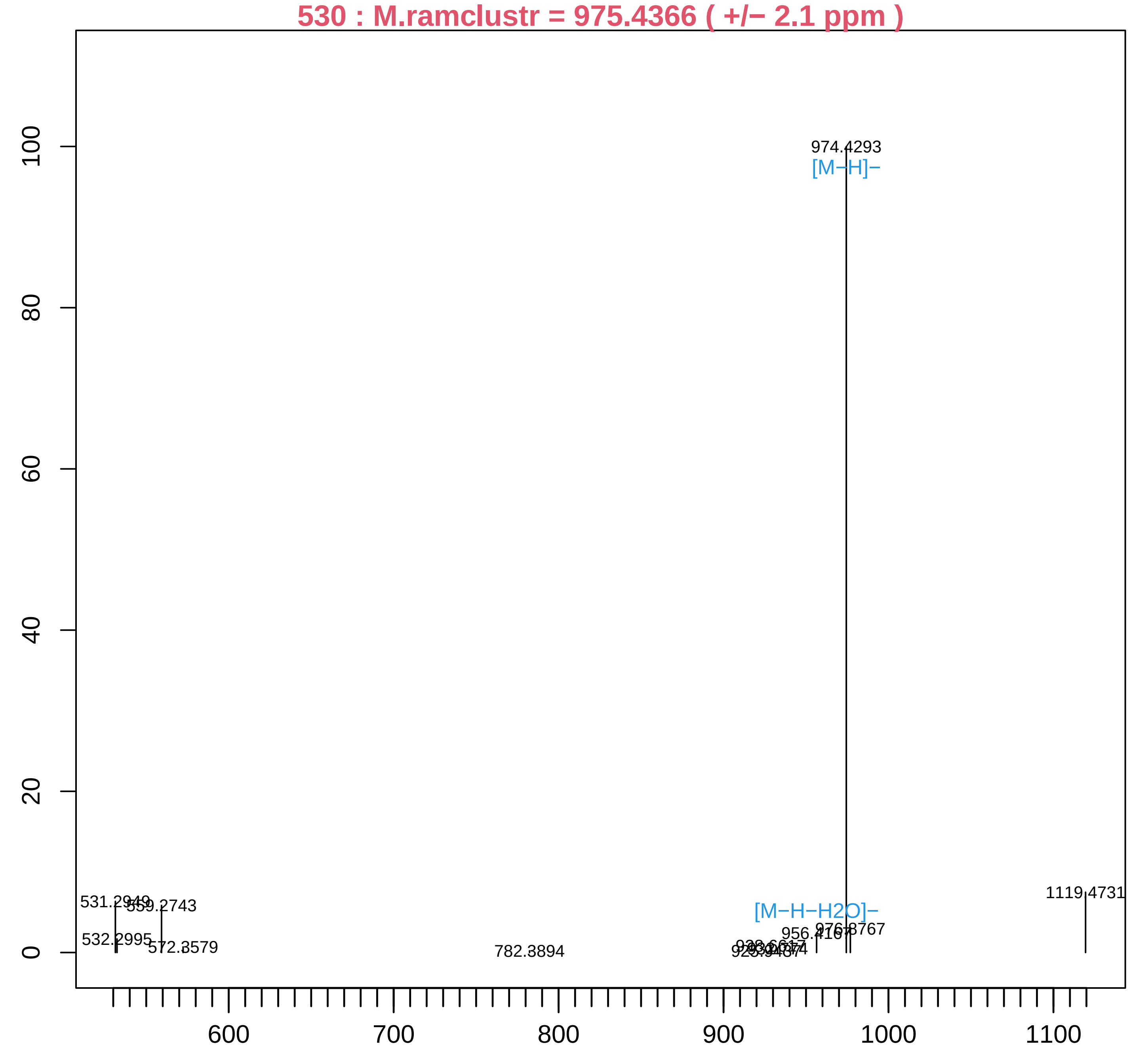
530 : M.ramclustr = 975.4366 (+/− 2.1 ppm)
974.4293
[M−H]−
[M−H−H2O]−
531.2949
559.2743
532.2995
572.8579
782.8894
956.4167
976.8767
1119.4731
0
20
40
60
80
100
600
700
800
900
1000
1100

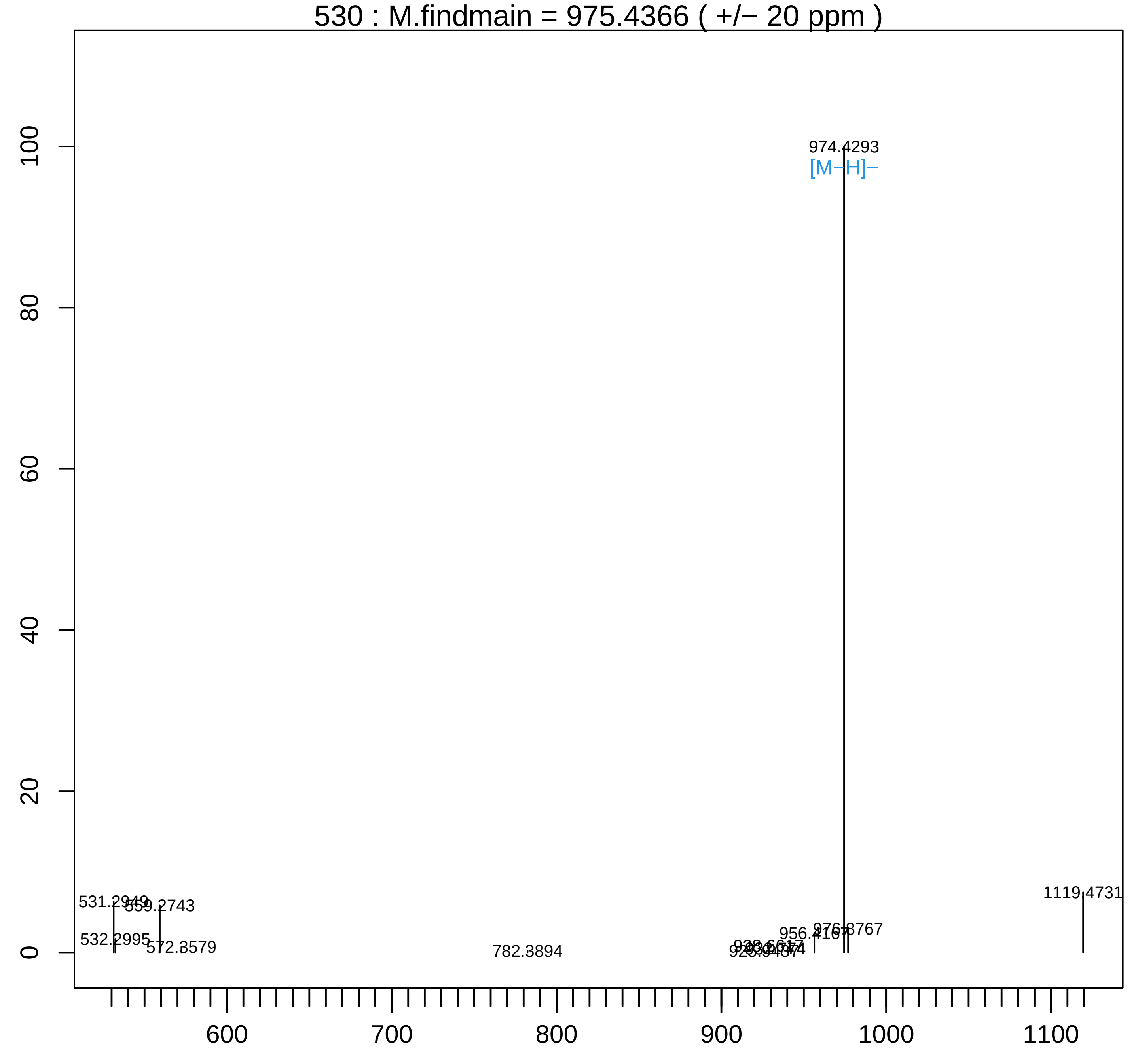
530 : M.findmain = 975.4366 (+/− 20 ppm)
974.4293
[M−H]−
531.2949
559.2743
532.2995
572.8579
782.8894
956.4167
976.8767
1119.4731
0
20
40
60
80
100
600
700
800
900
1000
1100

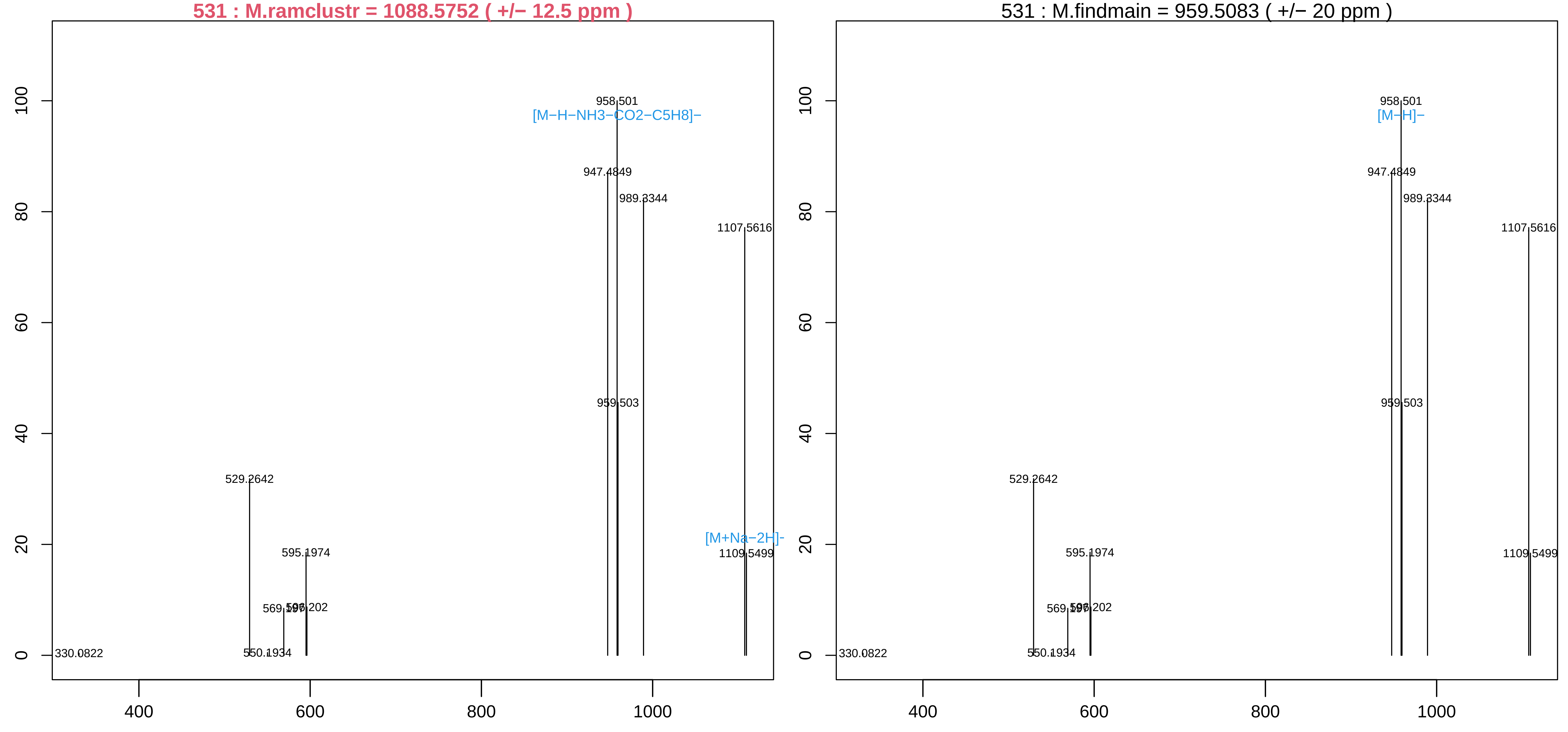
531 : M.ramclustr = 1088.5752 (+/− 12.5 ppm)
958.501
[M−H−NH3−CO2−C5H8]−
947.4849
989.3344
1107.5616
959.503
529.2642
[M+Na−2H]-
595.1974
1109.5499
569.197
598.202
330.0822
550.1934
531 : M.findmain = 959.5083 (+/− 20 ppm)
958.501
[M−H]−
947.4849
989.3344
1107.5616
959.503
529.2642
595.1974
1109.5499
569.197
598.202
330.0822
550.1934
0
20
40
60
80
100
400
600
800
1000

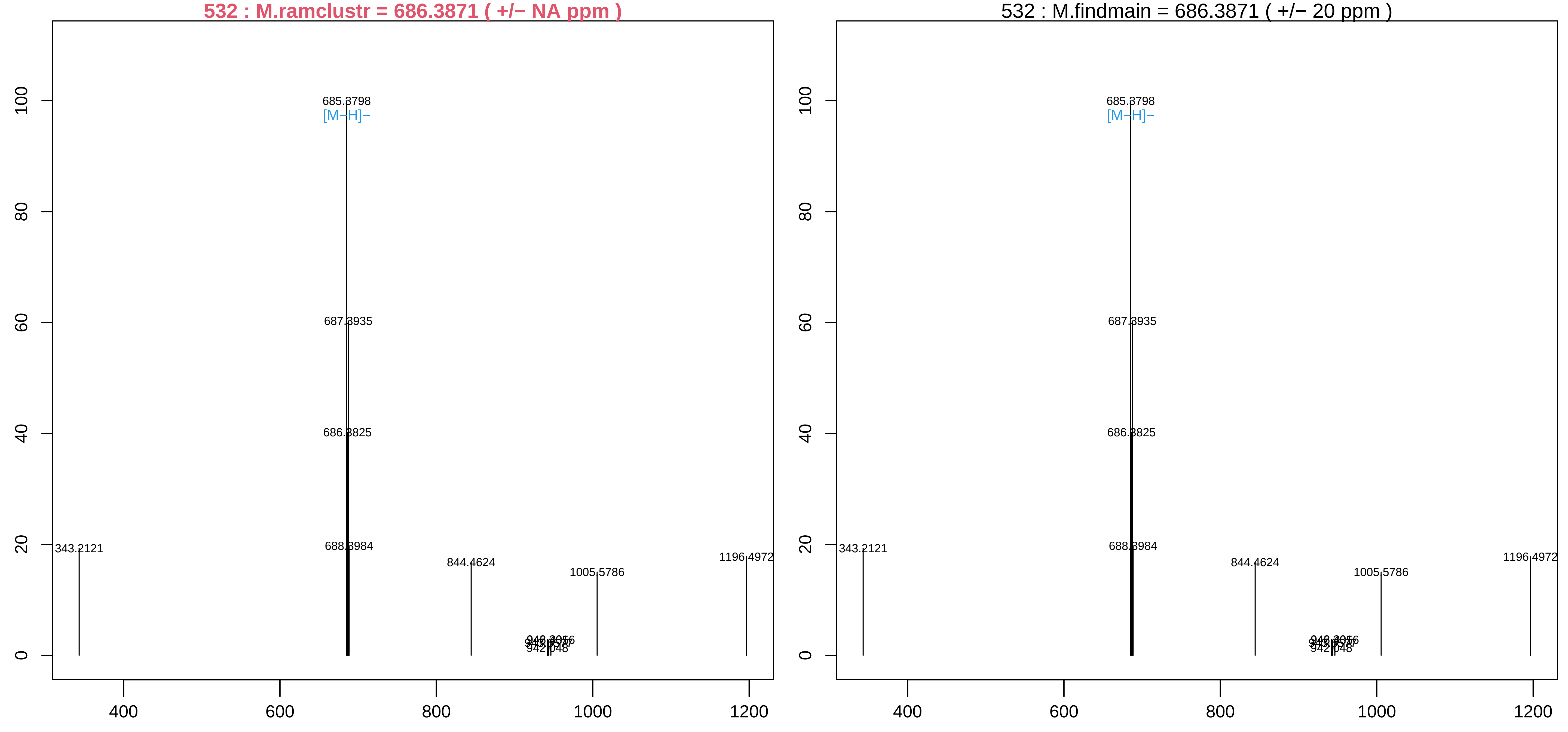
532 : M.ramclustr = 686.3871 (+/− NA ppm)
532 : M.findmain = 686.3871 (+/− 20 ppm)
685.3798
[M−H]−
687.3935
686.3825
688.3984
343.2121
844.4624
1005.5786
1196.4972
942.3048
0
20
40
60
80
100
400
600
800
1000
1200

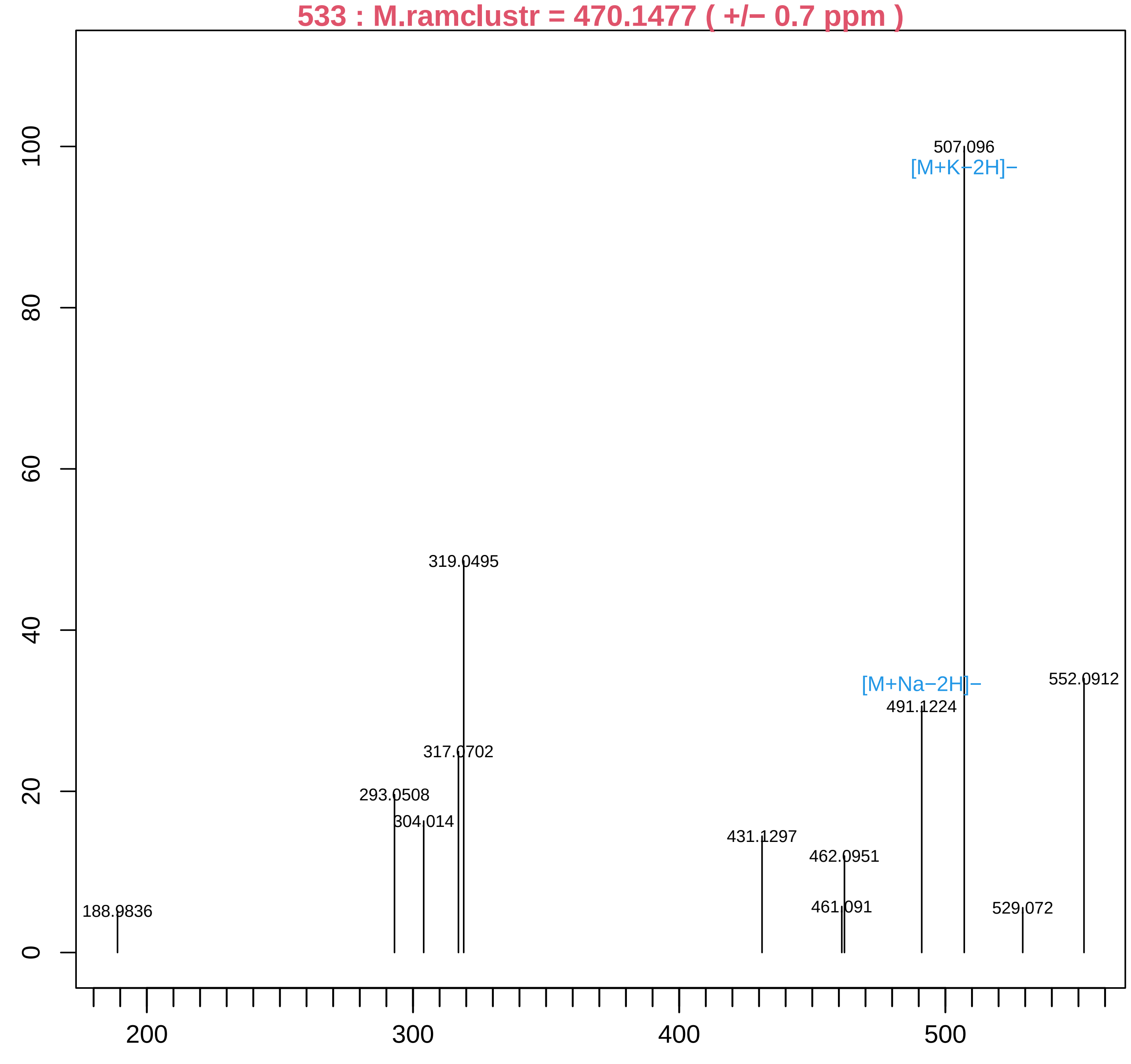

533 : M.ramclustr = 470.1477 (+/− 0.7 ppm)
507.096
[M+K−2H]−
319.0495
[M+Na−2H]−
552.0912
491.1224
317.0702
293.0508
304.014
431.1297
462.0951
461.091
529.072
188.9836
0
20
40
60
80
100
200
300
400
500

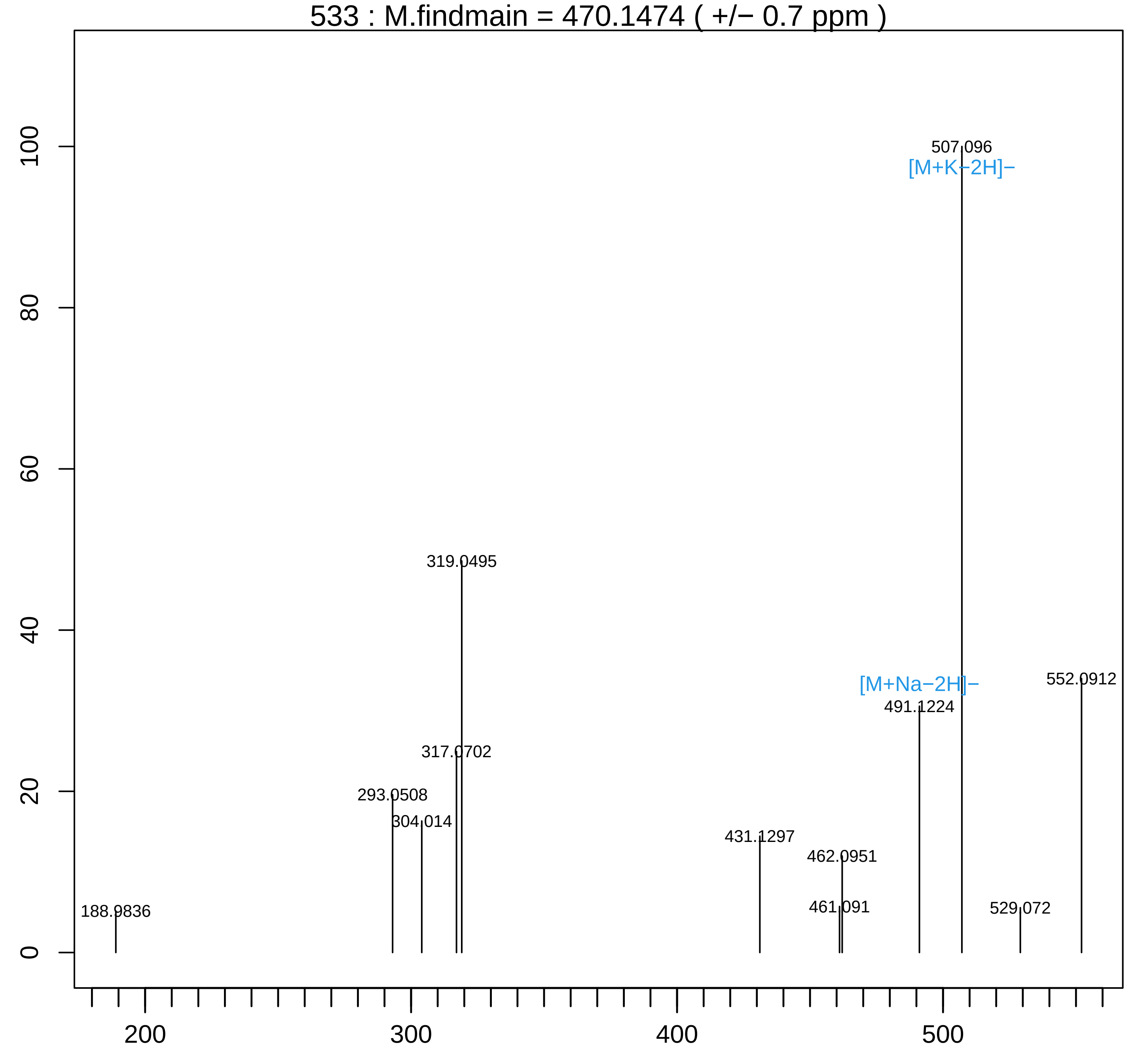

533 : M.findmain = 470.1474 (+/− 0.7 ppm)
507.096
[M+K−2H]−
319.0495
[M+Na−2H]−
552.0912
491.1224
317.0702
293.0508
304.014
431.1297
462.0951
461.091
529.072
188.9836
0
20
40
60
80
100
200
300
400
500

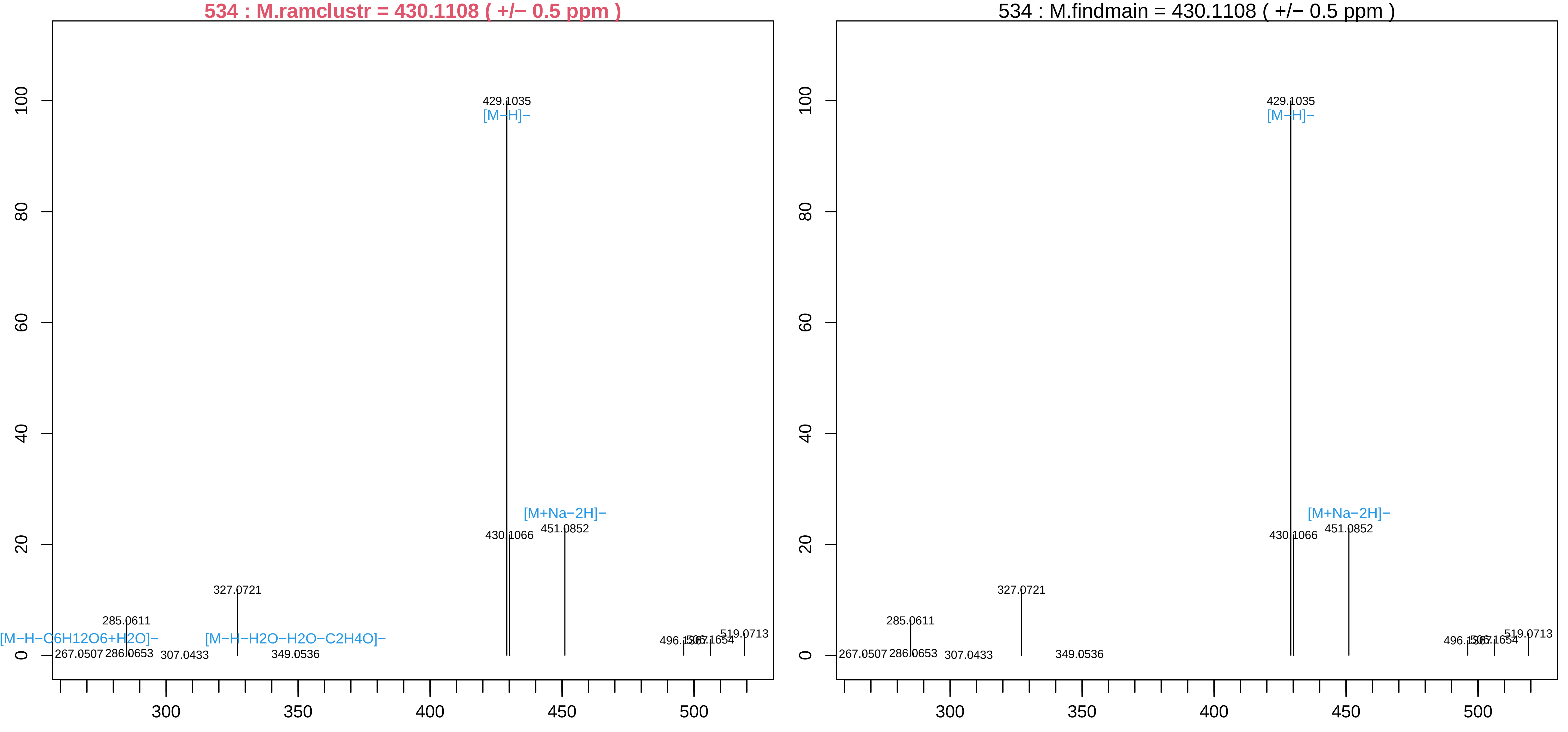

534 : M.ramclustr = 430.1108 (+/− 0.5 ppm)
429.1035
[M−H]−
[M+Na−2H]−
430.1066
451.0852
327.0721
285.0611
[M−H−C6H12O6+H2O]−
[M−H−H2O−H2O−C2H4O]−
267.0507
286.0653
307.0433
349.0536
496.1306
506.1654
519.0713
534 : M.findmain = 430.1108 (+/− 0.5 ppm)
429.1035
[M−H]−
[M+Na−2H]−
430.1066
451.0852
327.0721
285.0611
267.0507
286.0653
307.0433
349.0536
496.1306
506.1654
519.0713

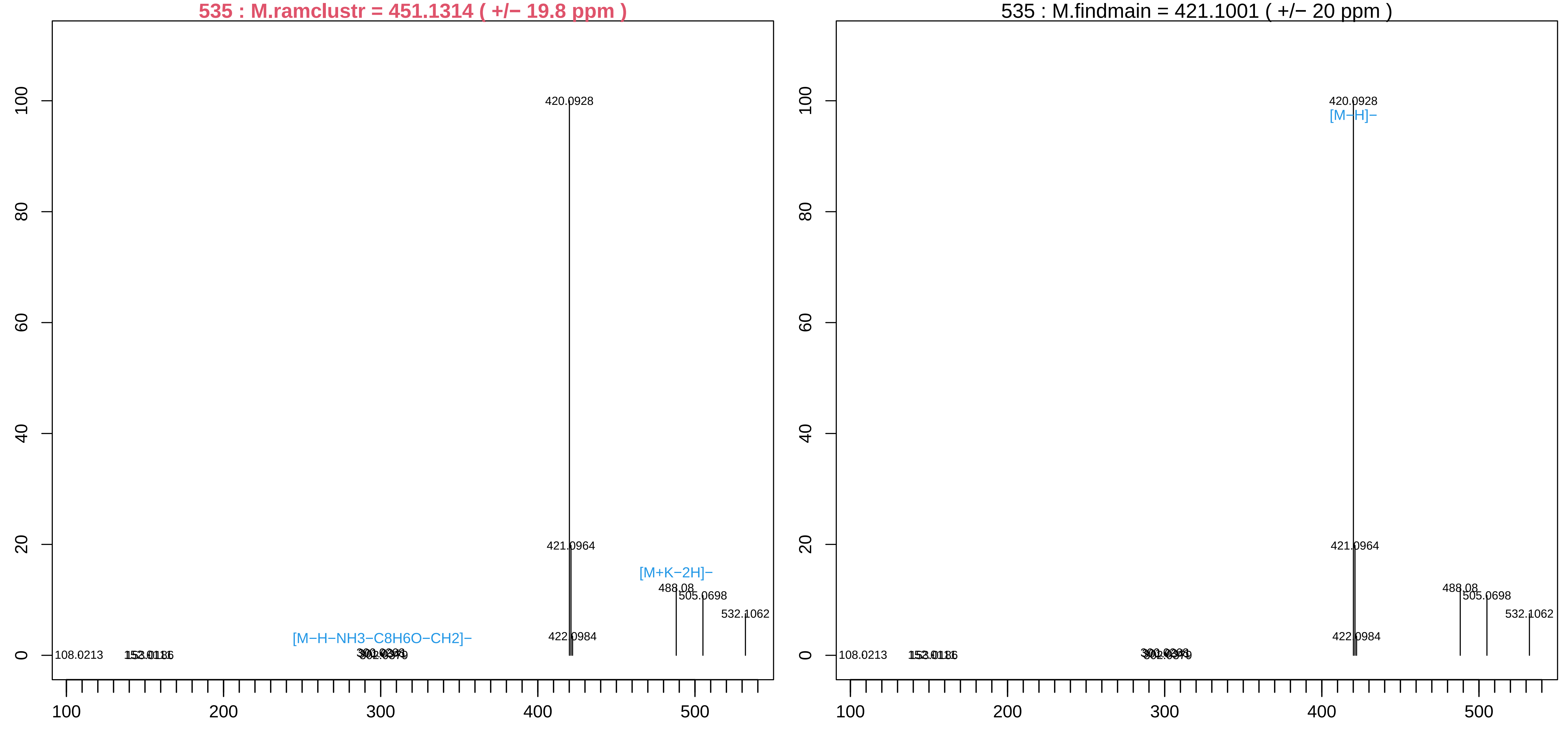

535 : M.ramclustr = 451.1314 (+/− 19.8 ppm)
420.0928
421.0964
422.0984
[M+K−2H]−
488.08
505.0698
532.1062
[M−H−NH3−C8H6O−CH2]−
108.0213
0
20
40
60
80
100
200
300
400
500
535 : M.findmain = 421.1001 (+/− 20 ppm)
420.0928
[M−H]−
421.0964
422.0984
488.08
505.0698
532.1062
108.0213

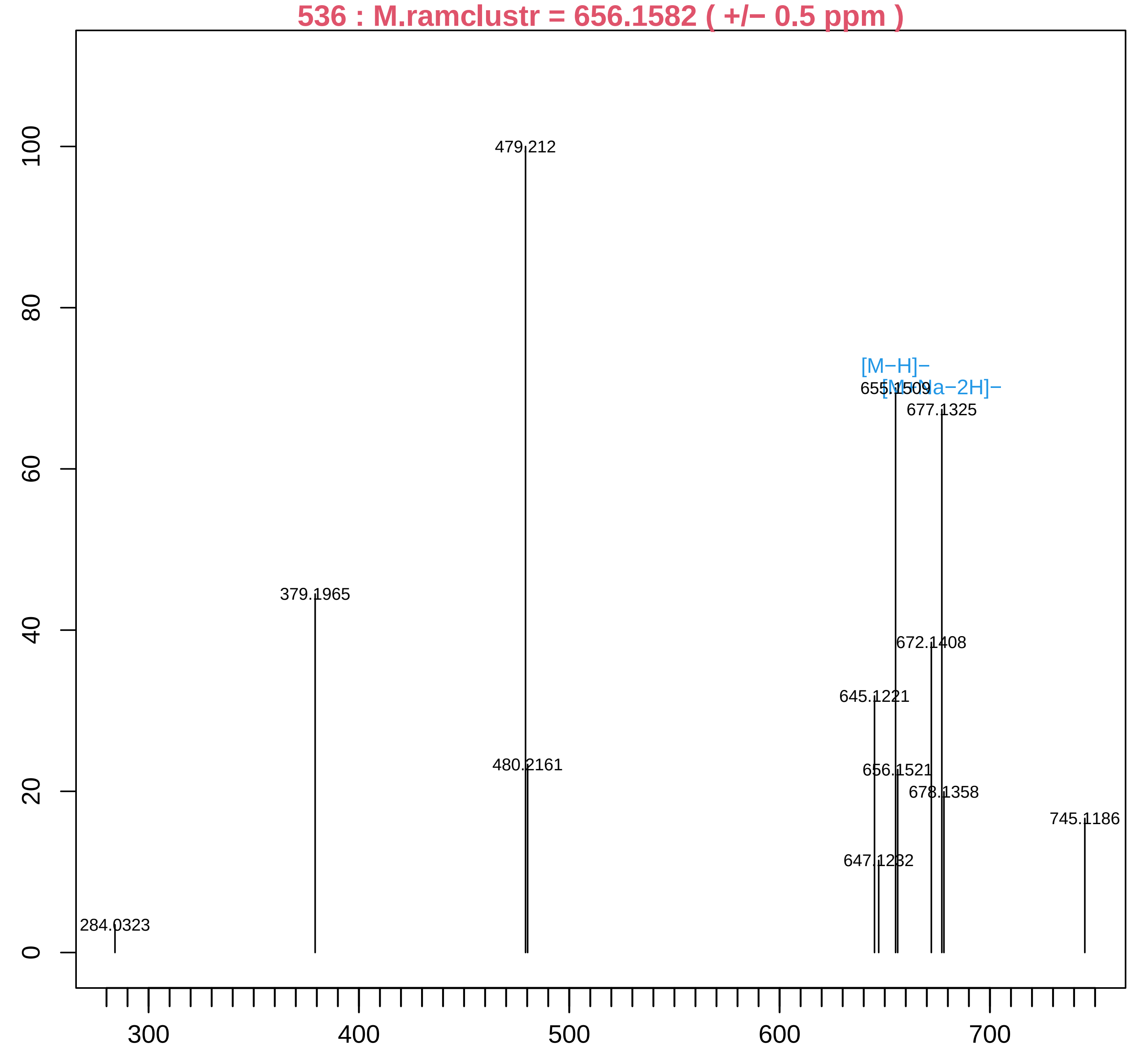
536 : M.ramclustr = 656.1582 (+/− 0.5 ppm)
479.212
379.1965
[M−H]−
655.1509
[M+Na−2H]−
677.1325
672.1408
645.1221
480.2161
656.1521
678.1358
745.1186
647.1232
284.0323
0
20
40
60
80
100
300
400
500
600
700

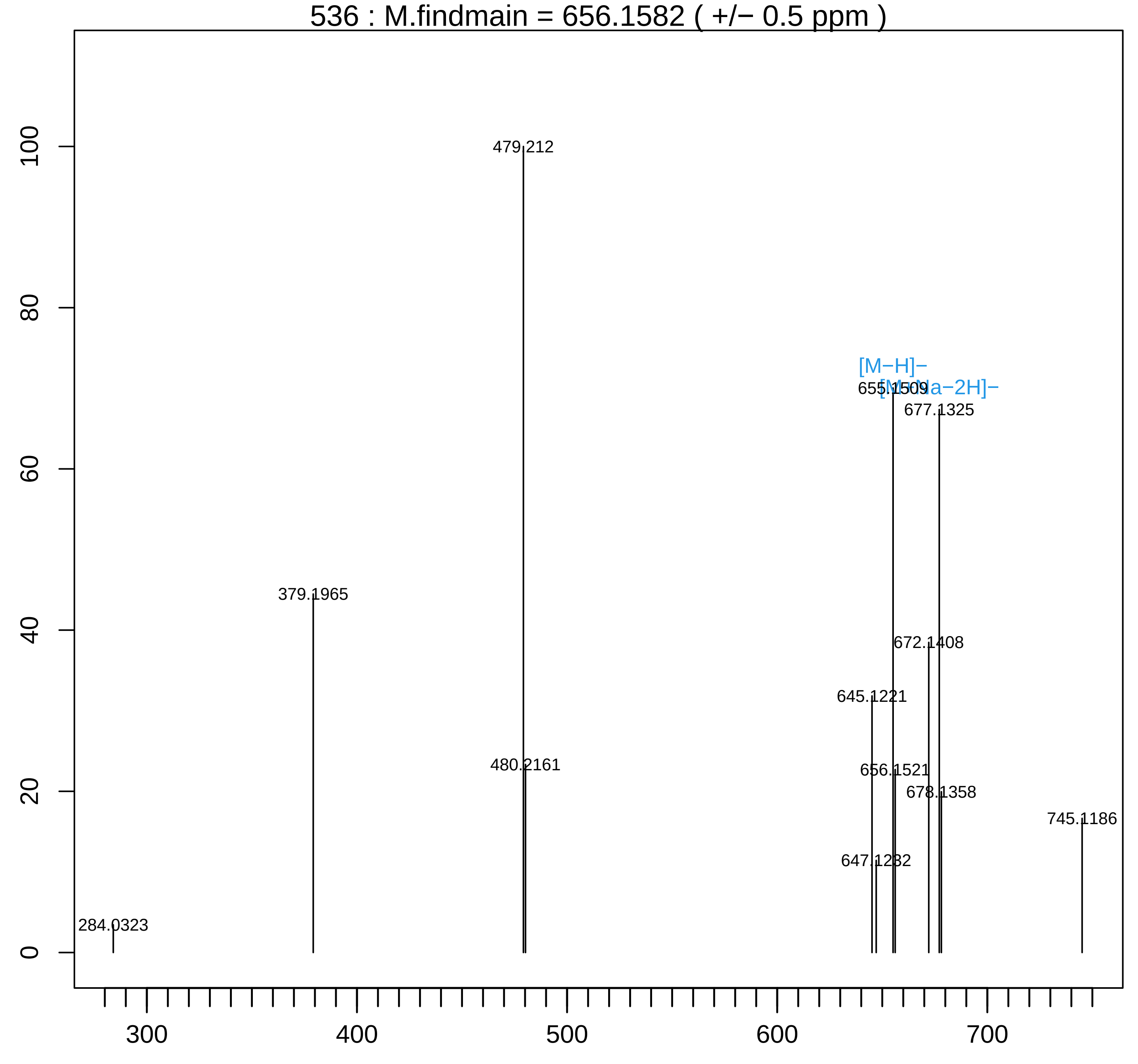
536 : M.findmain = 656.1582 (+/− 0.5 ppm)
479.212
379.1965
[M−H]−
655.1509
[M+Na−2H]−
677.1325
672.1408
645.1221
480.2161
656.1521
678.1358
745.1186
647.1232
284.0323
0
20
40
60
80
100
300
400
500
600
700

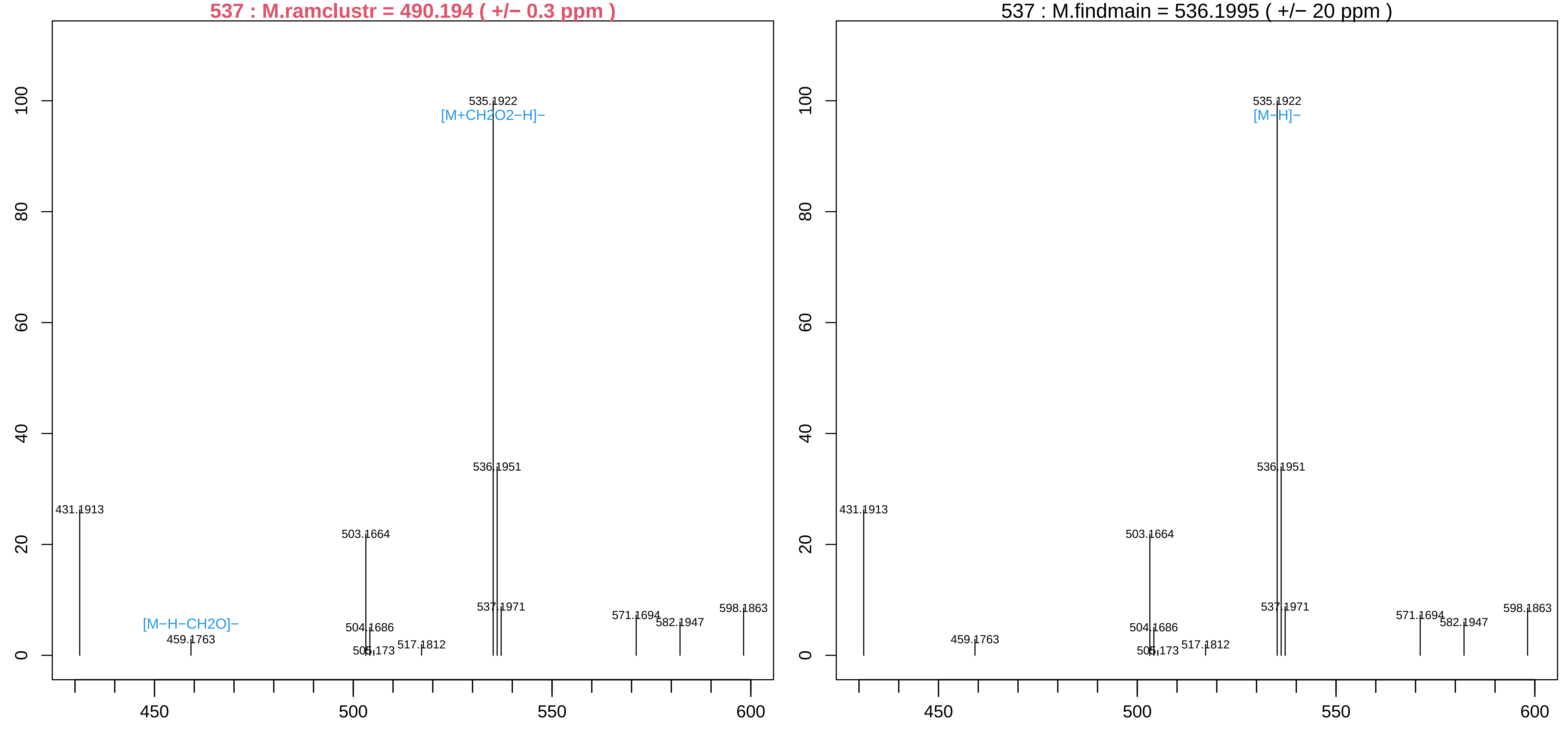
537 : M.ramclustr = 490.194 (+/− 0.3 ppm)
535.1922
[M+CH2O2−H]−
536.1951
431.1913
503.1664
537.1971
571.1694
582.1947
598.1863
[M−H−CH2O]−
504.1686
459.1763
517.1812
505.173
537 : M.findmain = 536.1995 (+/− 20 ppm)
535.1922
[M−H]−
536.1951
431.1913
503.1664
537.1971
571.1694
582.1947
598.1863
504.1686
459.1763
517.1812
505.173
0
20
40
60
80
100
450
500
550
600

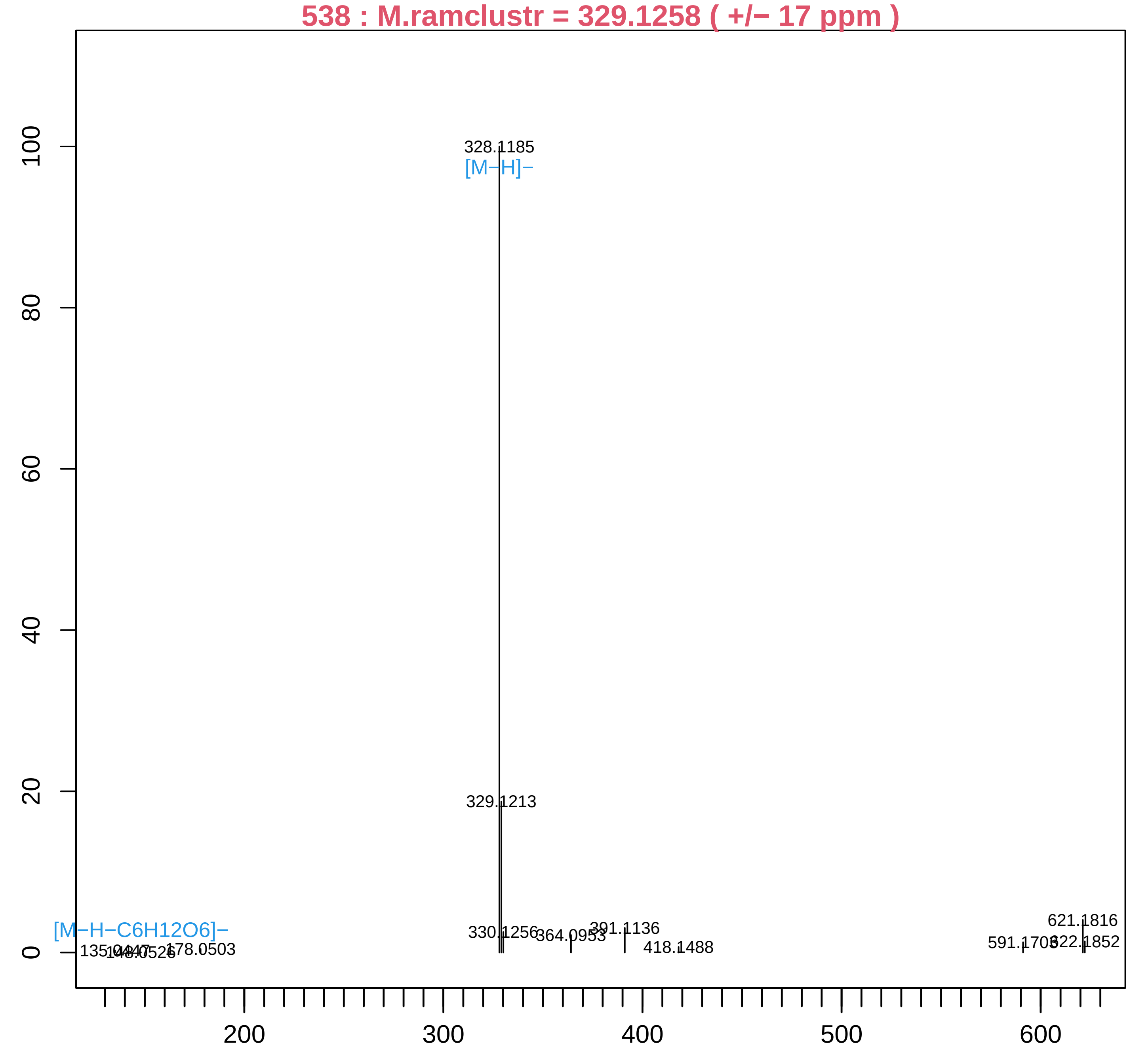
538 : M.ramclustr = 329.1258 (+/− 17 ppm)
328.1185
[M−H]−
329.1213
330.1256
364.0953
391.1136
418.1488
591.1708
621.1816
622.1852
[M−H−C6H12O6]−
135.0447
148.0526
178.0503
0
20
40
60
80
100
200
300
400
500
600

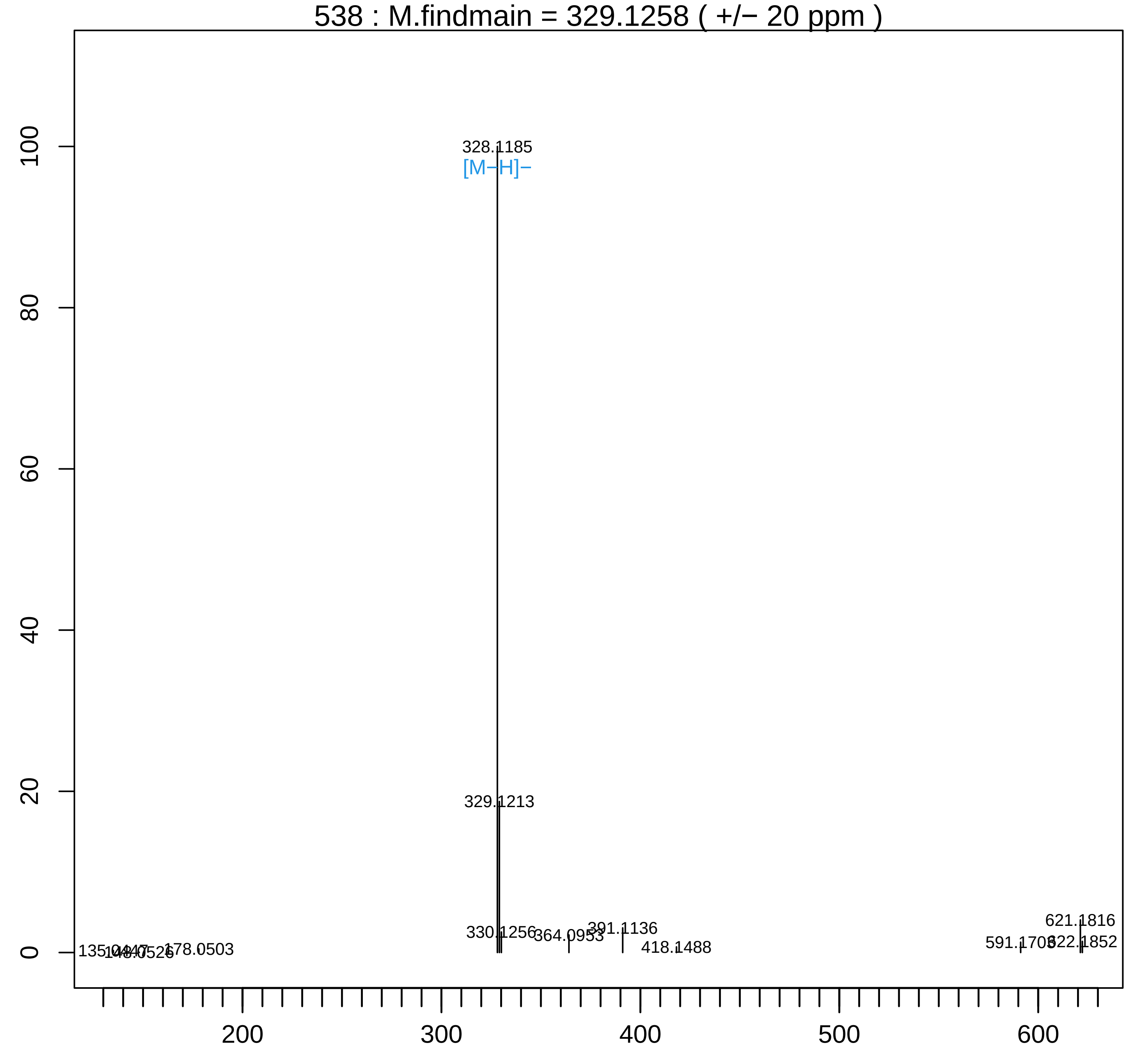
538 : M.findmain = 329.1258 (+/− 20 ppm)
328.1185
[M−H]−
329.1213
330.1256
364.0953
391.1136
418.1488
591.1708
621.1816
622.1852
135.0447
148.0526
178.0503
0
20
40
60
80
100
200
300
400
500
600

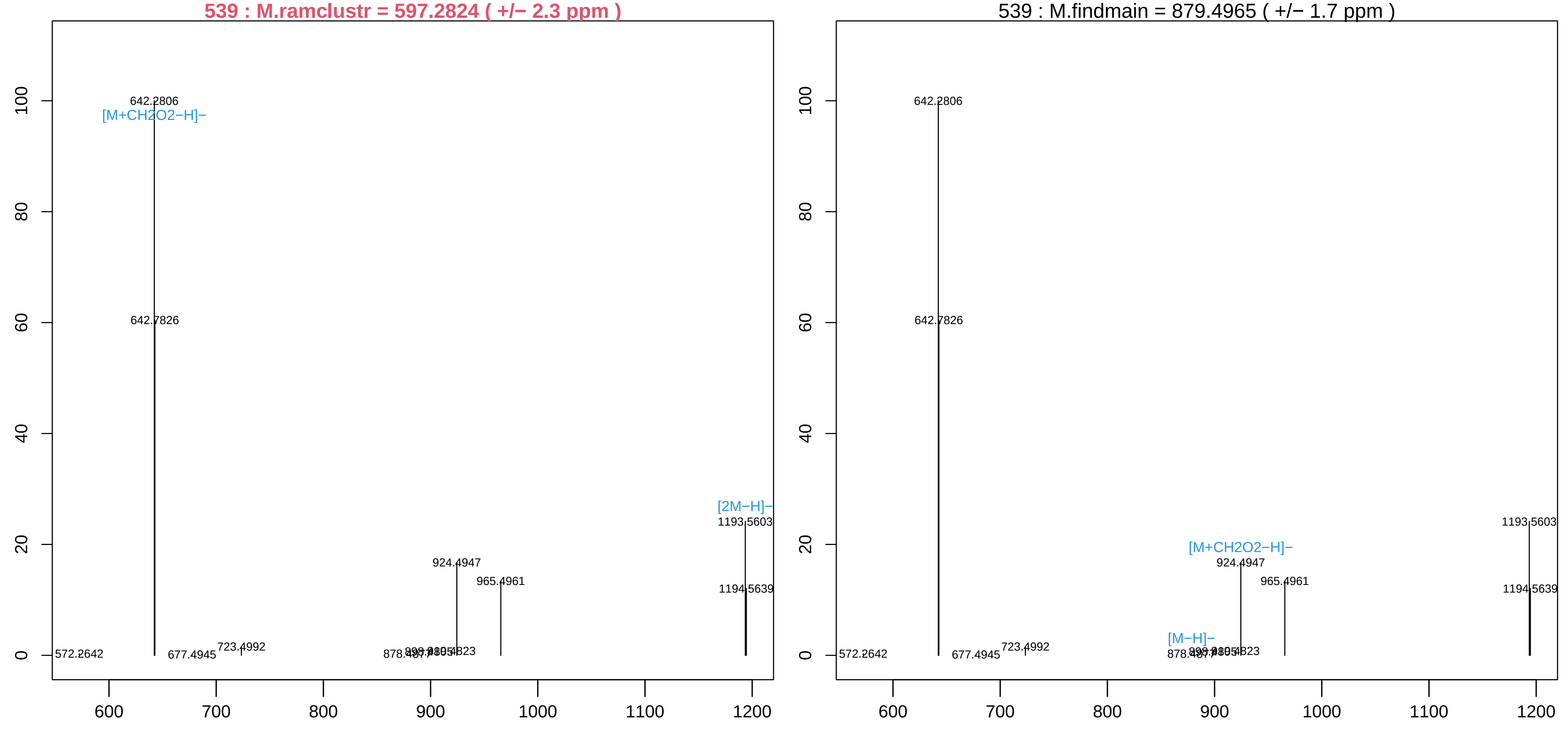

539 : M.ramclustr = 597.2824 (+/− 2.3 ppm)
642.2806
[M+CH2O2−H]−
642.7826
[2M−H]−
1193.5603
924.4947
965.4961
1194.5639
572.2642
677.4945
723.4992
878.4877
0
20
40
60
80
100
600
700
800
900
1000
1100
1200
539 : M.findmain = 879.4965 (+/− 1.7 ppm)
642.2806
642.7826
1193.5603
[M+CH2O2−H]−
924.4947
965.4961
1194.5639
[M−H]−
572.2642
677.4945
723.4992

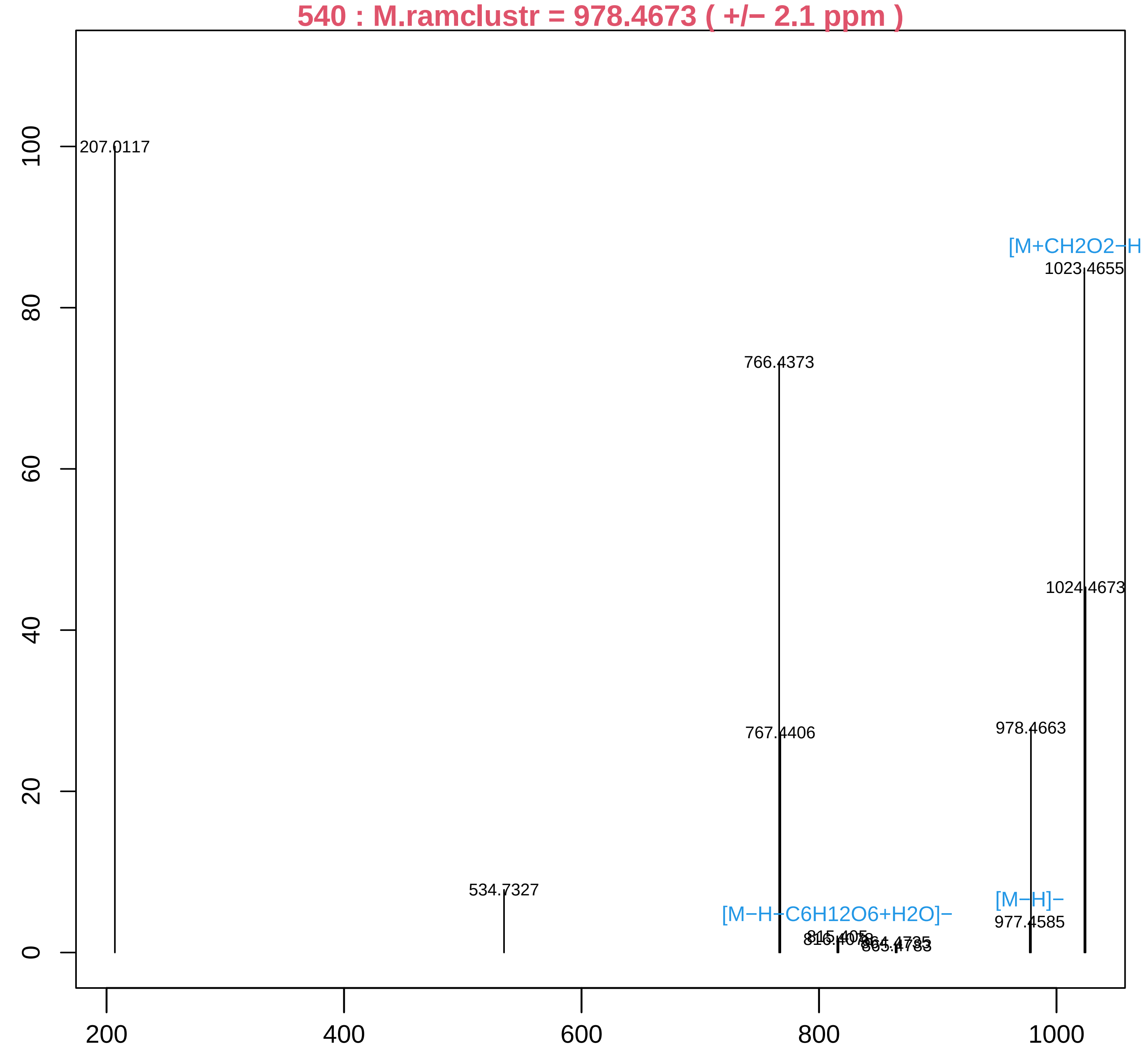
540 : M.ramclustr = 978.4673 (+/− 2.1 ppm)
207.0117
[M+CH2O2−H
1023.4655
766.4373
1024.4673
767.4406
978.4663
534.7327
[M−H−C6H12O6+H2O]−
[M−H]−
977.4585
0
20
40
60
80
100
200
400
600
800
1000

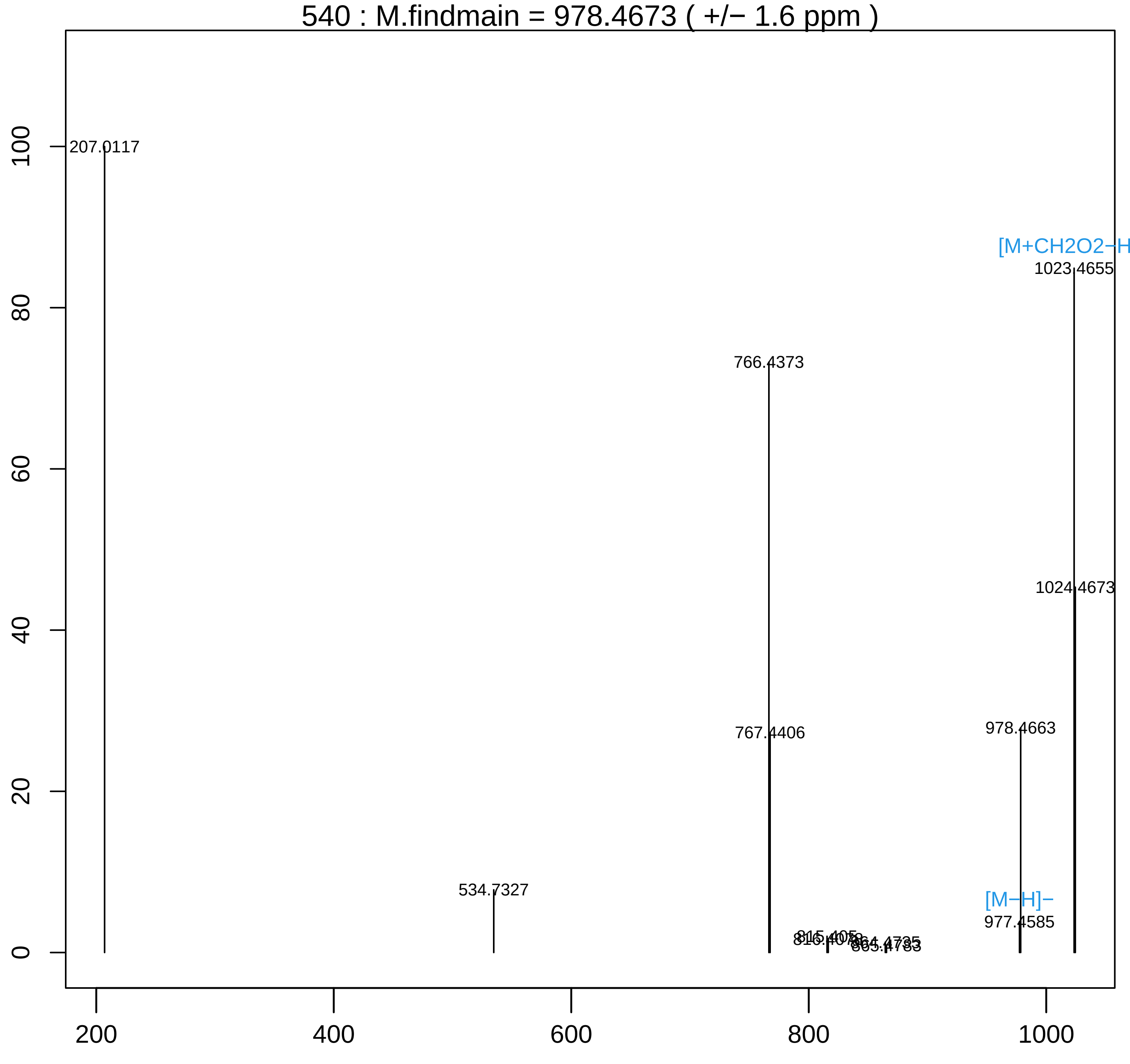
540 : M.findmain = 978.4673 (+/− 1.6 ppm)
207.0117
[M+CH2O2−H
1023.4655
766.4373
1024.4673
767.4406
978.4663
534.7327
[M−H]−
977.4585
0
20
40
60
80
100
200
400
600
800
1000

541 : M.ramclustr = 881.5123 (+/− 0.8 ppm)

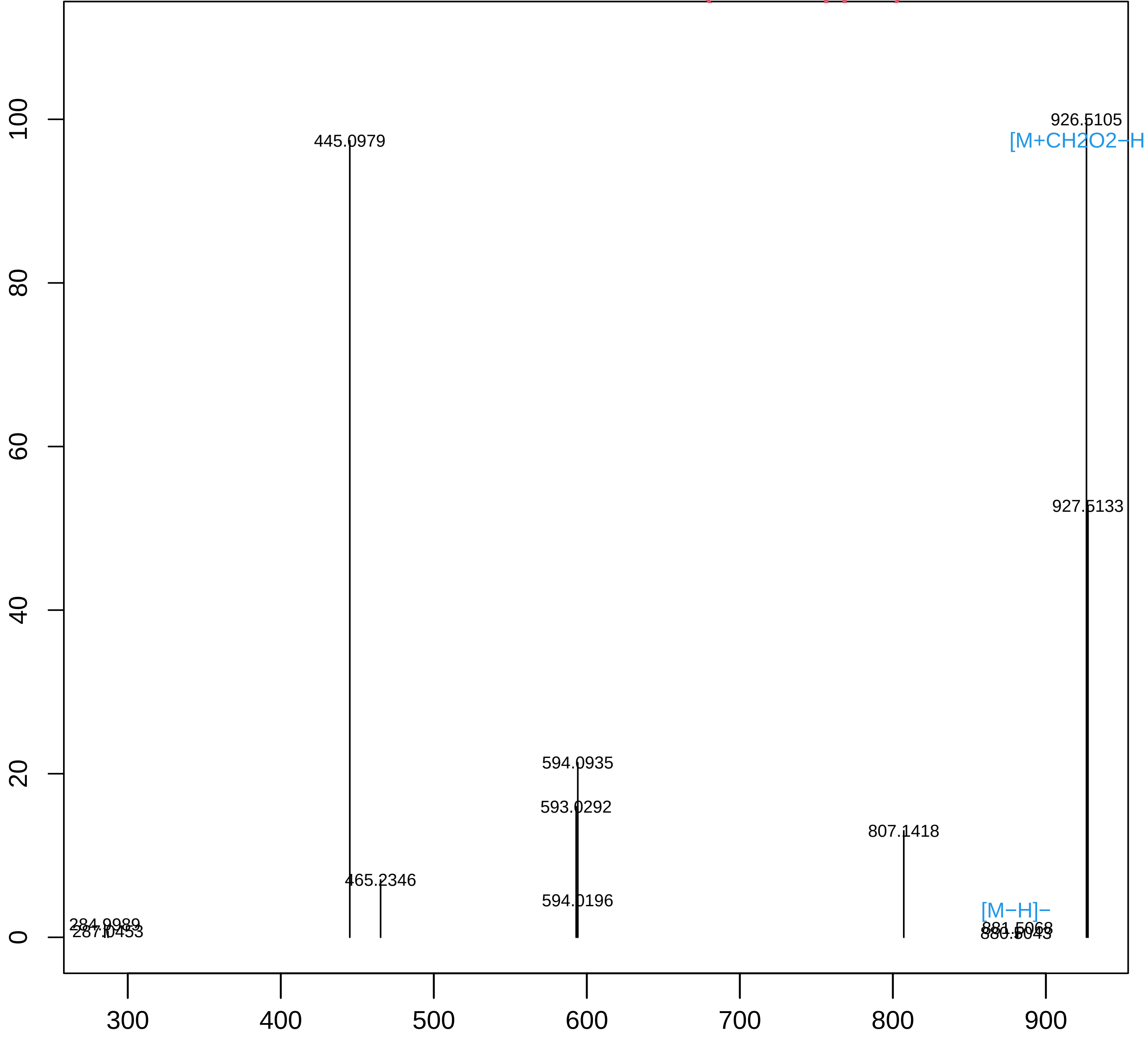

541 : M.findmain = 881.5123 (+/− 0.8 ppm)

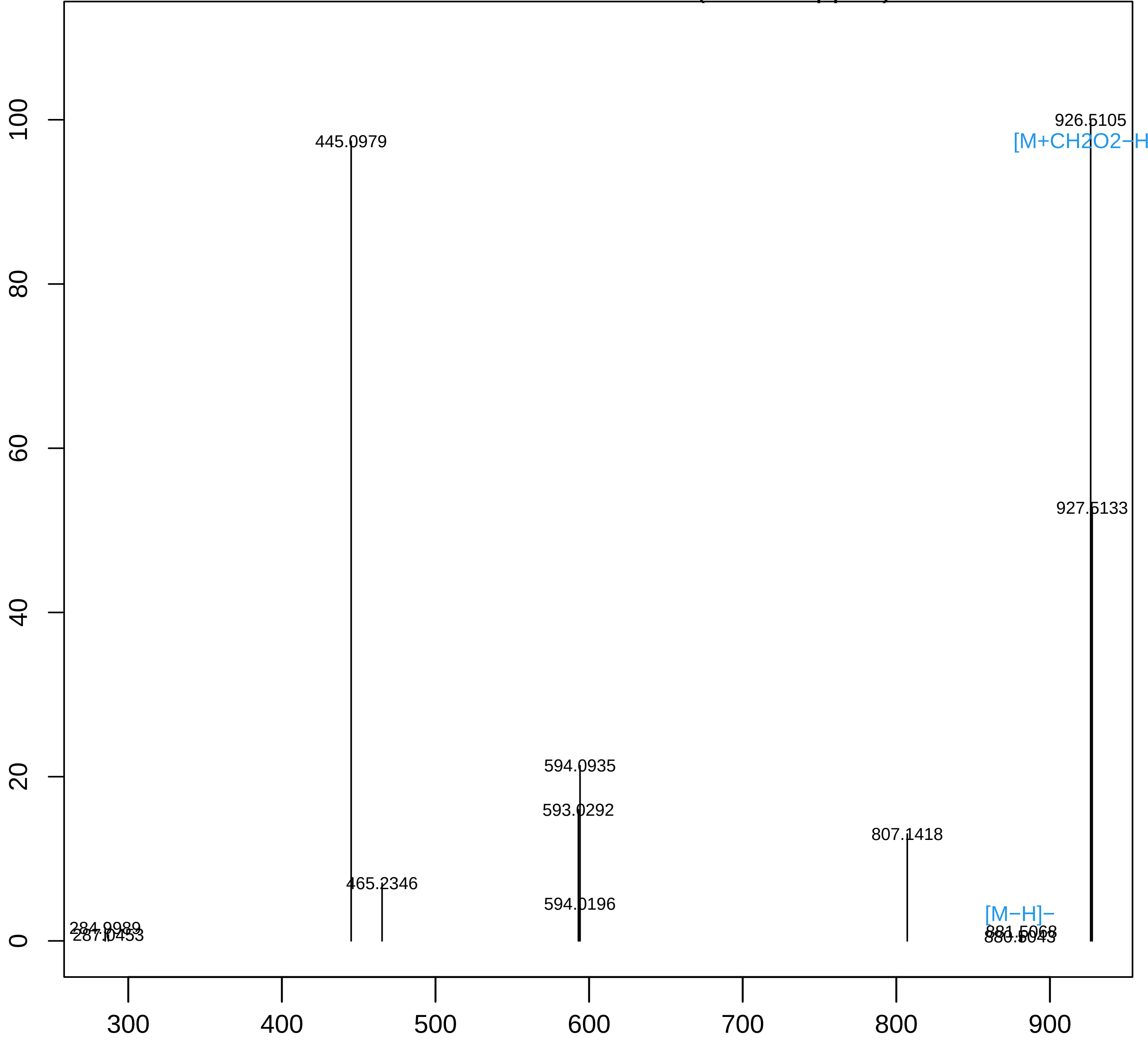

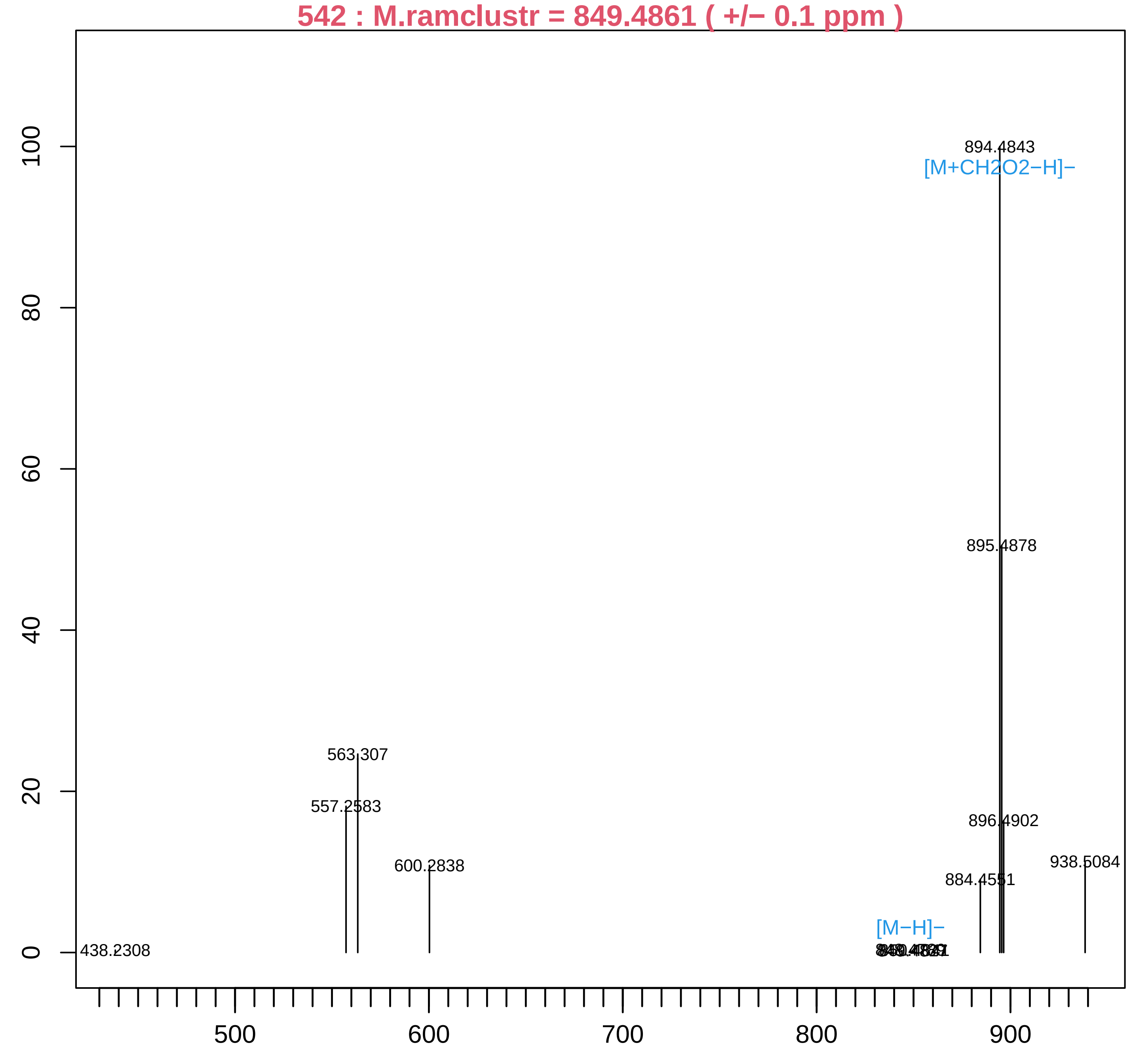
542 : M.ramclustr = 849.4861 (+/− 0.1 ppm)
894.4843
[M+CH2O2−H]−
895.4878
563.307
557.2583
896.4902
600.2838
938.5084
884.4551
[M−H]−
438.2308
0
20
40
60
80
100
500
600
700
800
900

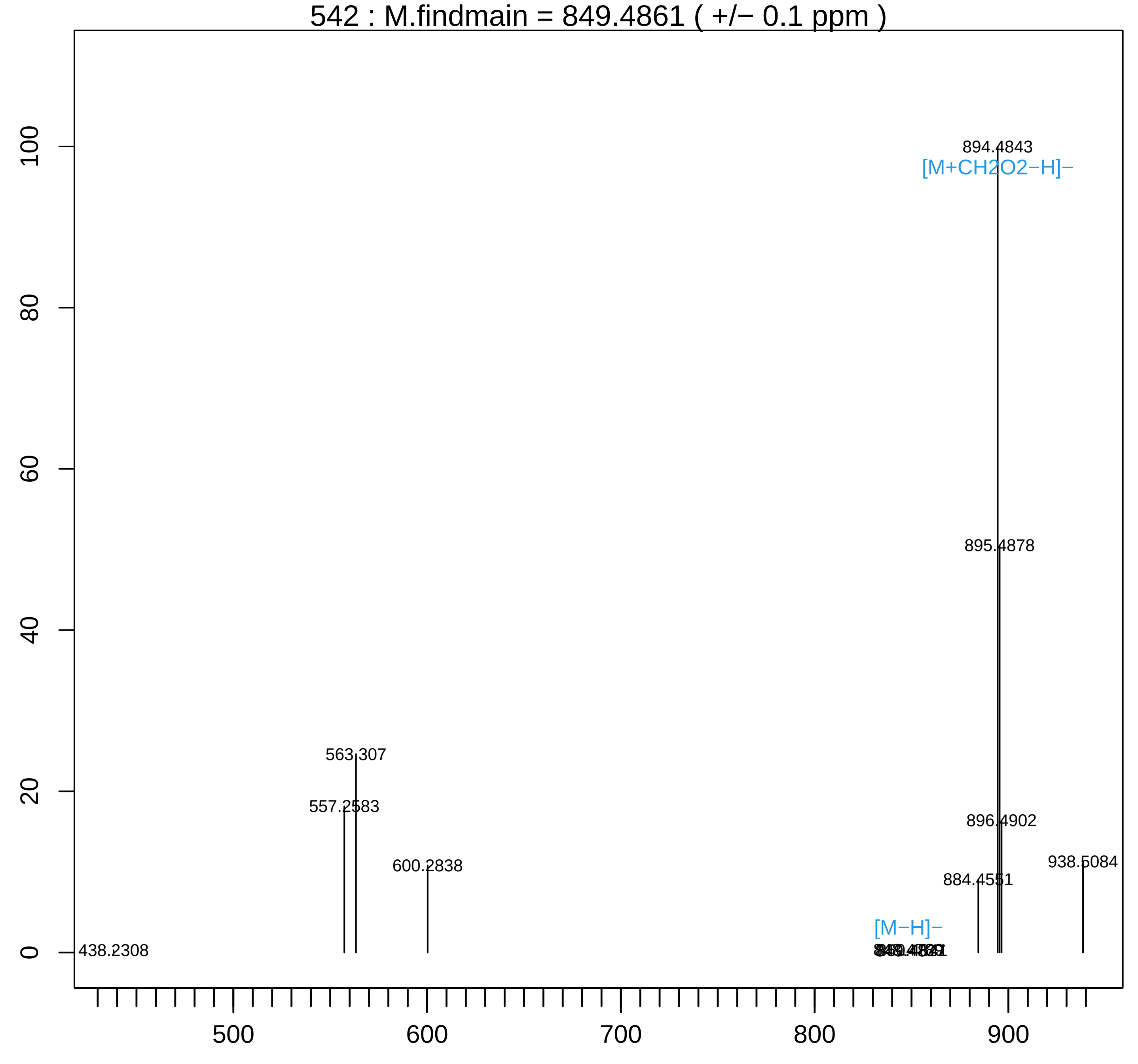
542 : M.findmain = 849.4861 (+/− 0.1 ppm)
894.4843
[M+CH2O2−H]−
895.4878
563.307
557.2583
896.4902
600.2838
938.5084
884.4551
[M−H]−
438.2308
0
20
40
60
80
100
500
600
700
800
900

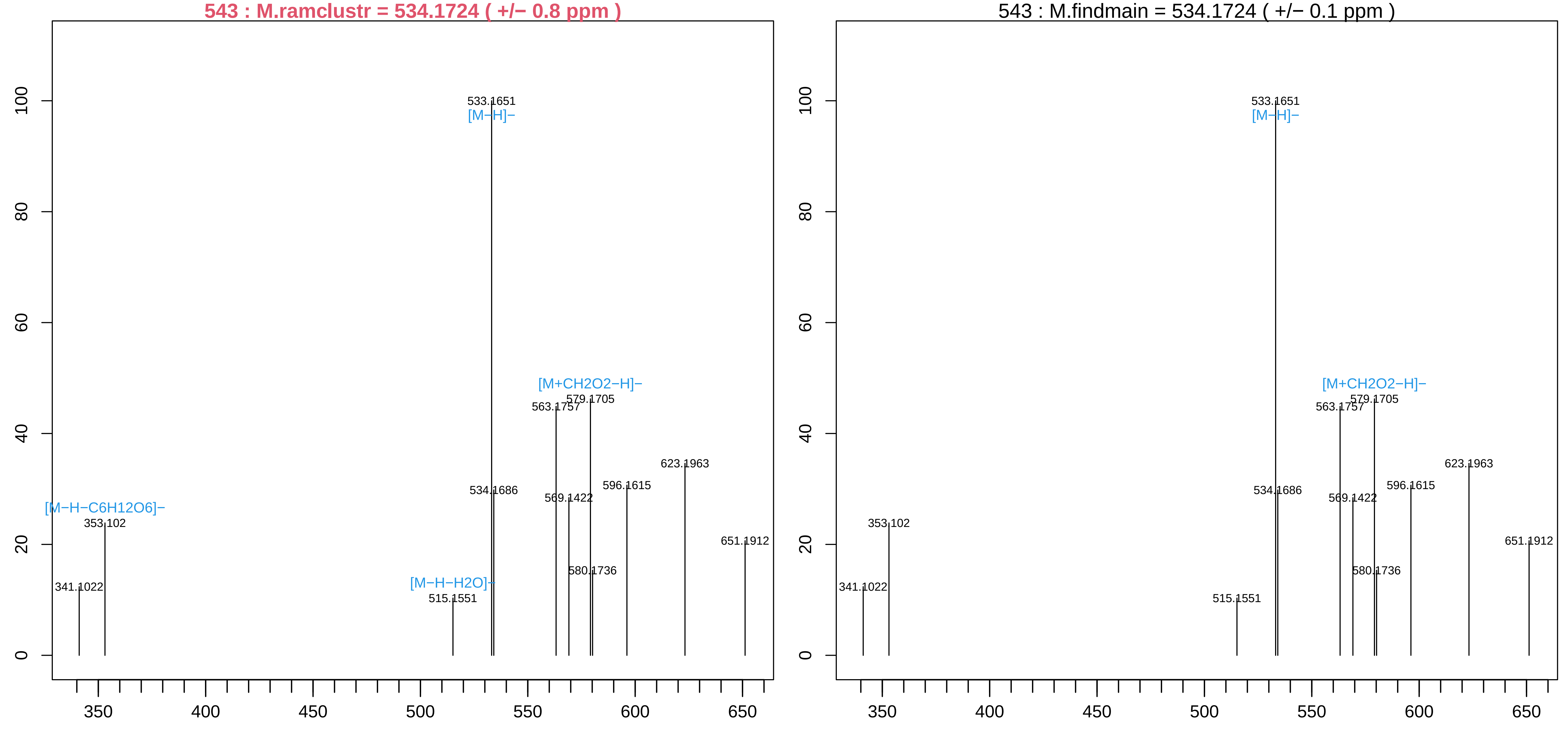
543 : M.ramclustr = 534.1724 (+/− 0.8 ppm)
543 : M.findmain = 534.1724 (+/− 0.1 ppm)
533.1651
[M−H]−
[M+CH2O2−H]−
563.1757
579.1705
623.1963
534.1686
596.1615
569.1422
[M−H−C6H12O6]−
353.102
651.1912
580.1736
[M−H−H2O]−
341.1022
515.1551
0
20
40
60
80
100
350
400
450
500
550
600
650

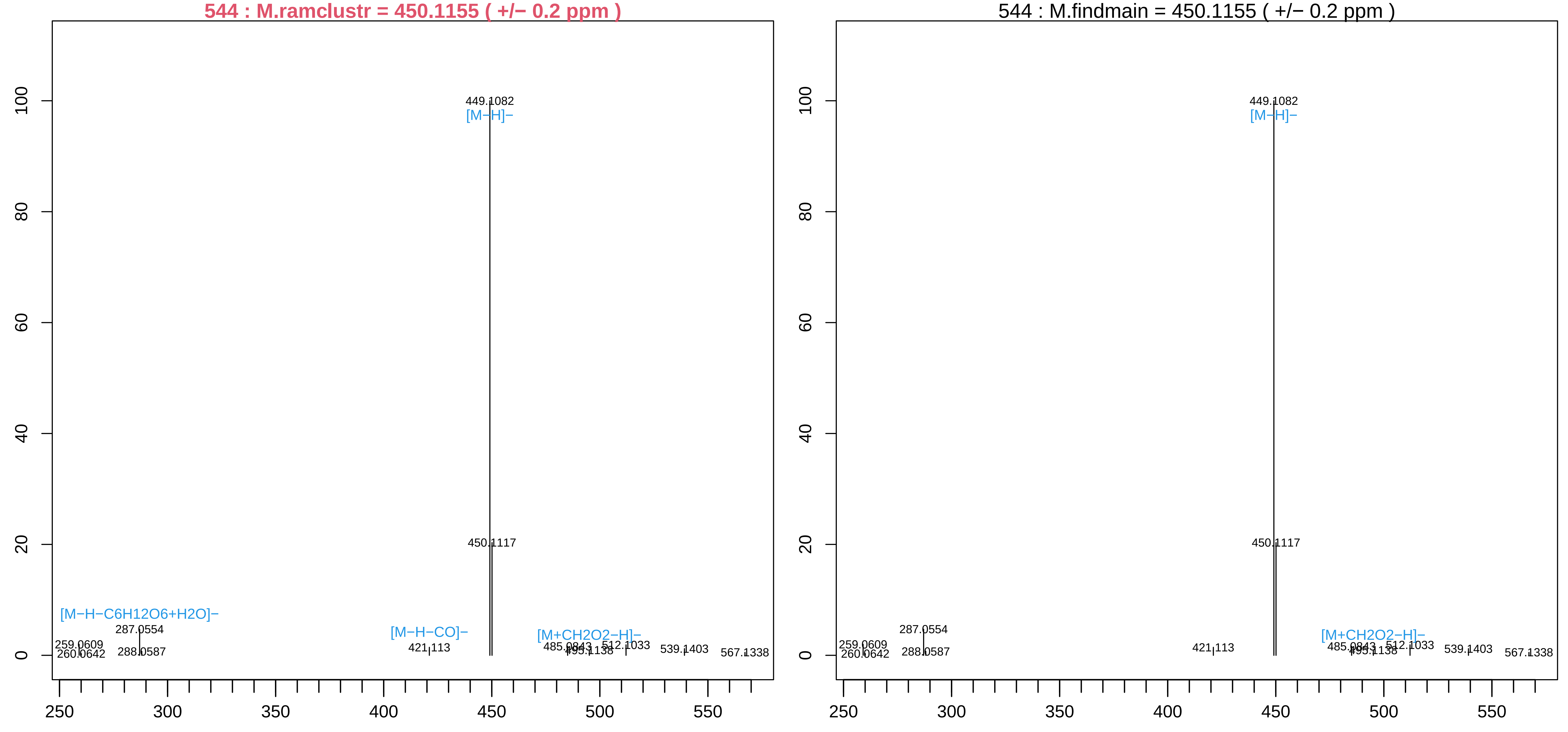
544 : M.ramclustr = 450.1155 (+/− 0.2 ppm)
544 : M.findmain = 450.1155 (+/− 0.2 ppm)
449.1082
[M−H]−
450.1117
[M−H−C6H12O6+H2O]−
287.0554
259.0609
260.0642
288.0587
[M−H−CO]−
421.113
[M+CH2O2−H]−
485.0843
495.1138
512.1033
539.1403
567.1338
0
20
40
60
80
100
250
300
350
400
450
500
550

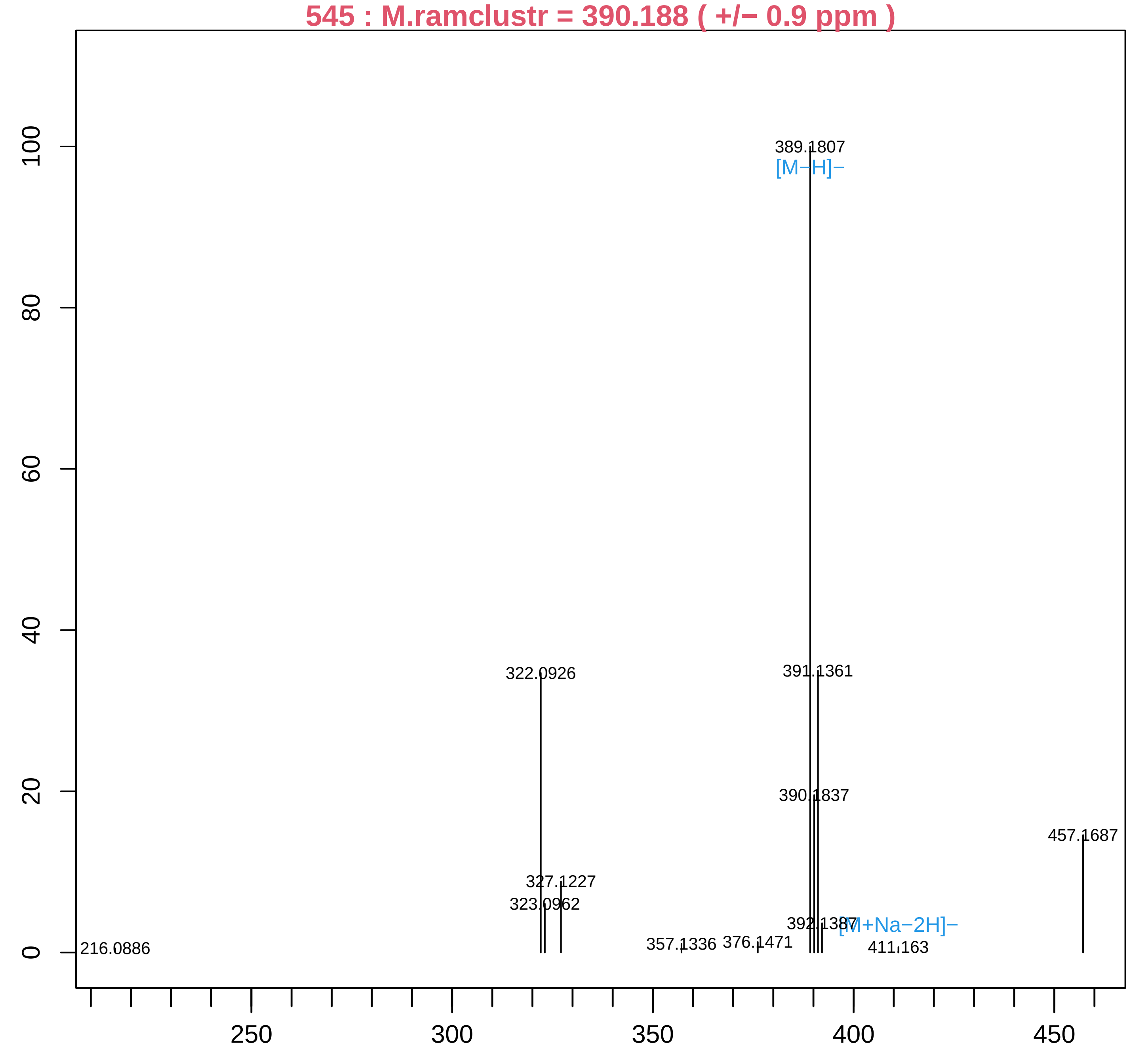
545 : M.ramclustr = 390.188 (+/− 0.9 ppm)
389.1807
[M−H]−
322.0926
391.1361
390.1837
457.1687
327.1227
323.0962
392.1387
[M+Na−2H]−
216.0886
357.1336
376.1471
411.163
0
20
40
60
80
100
250
300
350
400
450

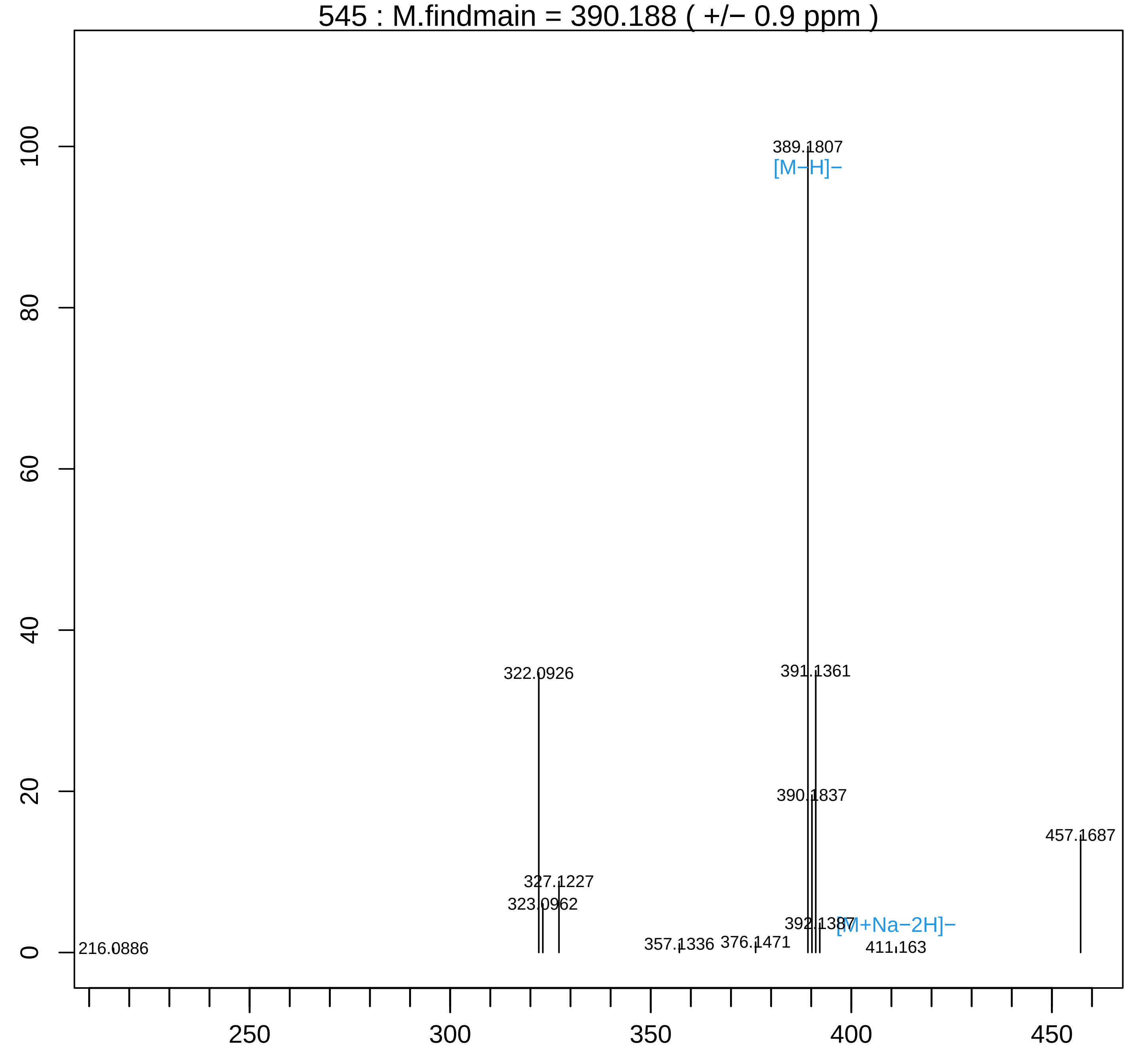
545 : M.findmain = 390.188 (+/− 0.9 ppm)
389.1807
[M−H]−
322.0926
391.1361
390.1837
457.1687
327.1227
323.0962
392.1387
[M+Na−2H]−
216.0886
357.1336
376.1471
411.163
0
20
40
60
80
100
250
300
350
400
450

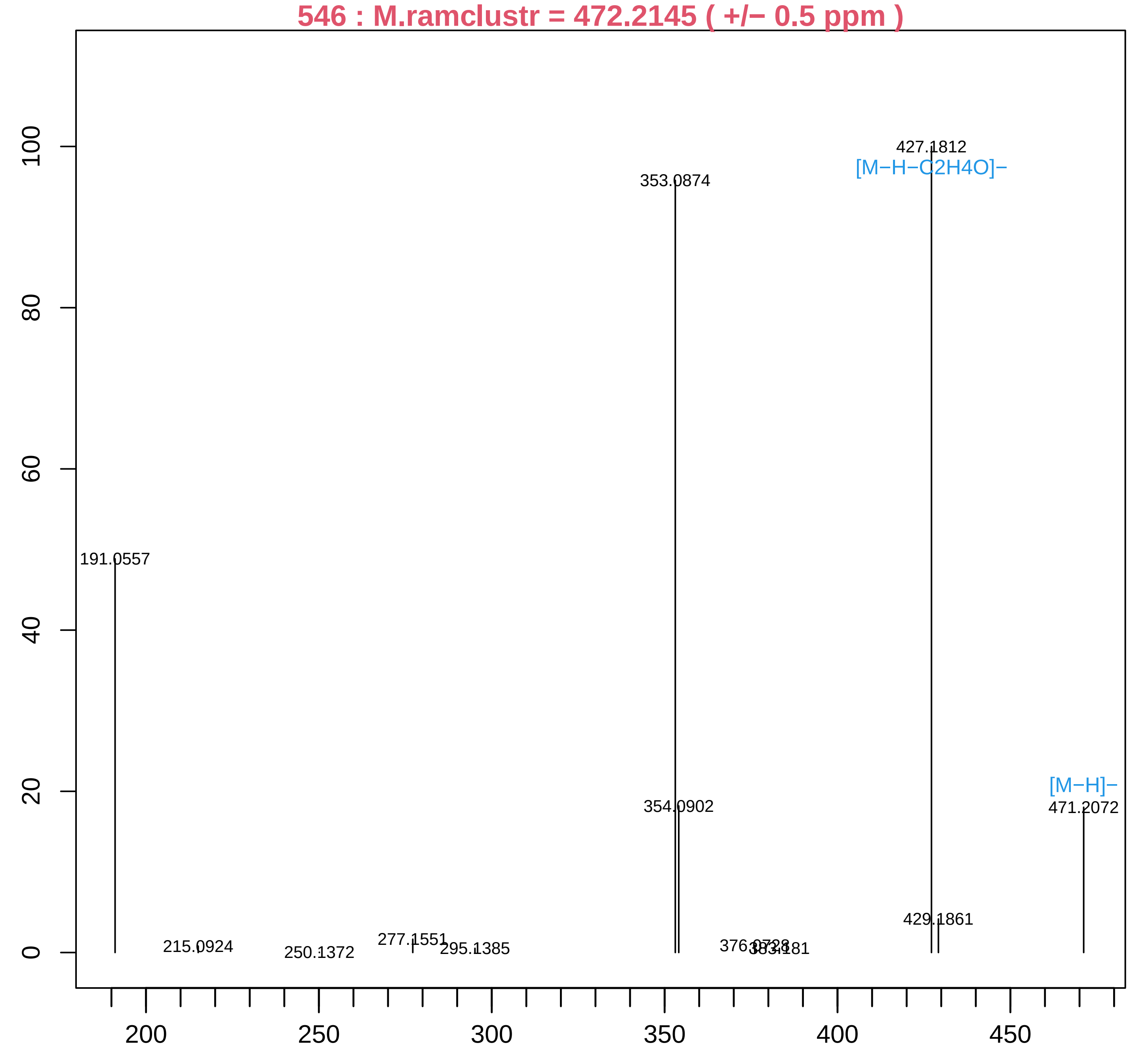
546 : M.ramclustr = 472.2145 (+/− 0.5 ppm)
[M−H−C2H4O]−
[M−H]−
427.1812
353.0874
191.0557
354.0902
471.2072
429.1861
215.0924
250.1372
277.1551
295.1385
376.0728
383.181
0
20
40
60
80
100
200
250
300
350
400
450

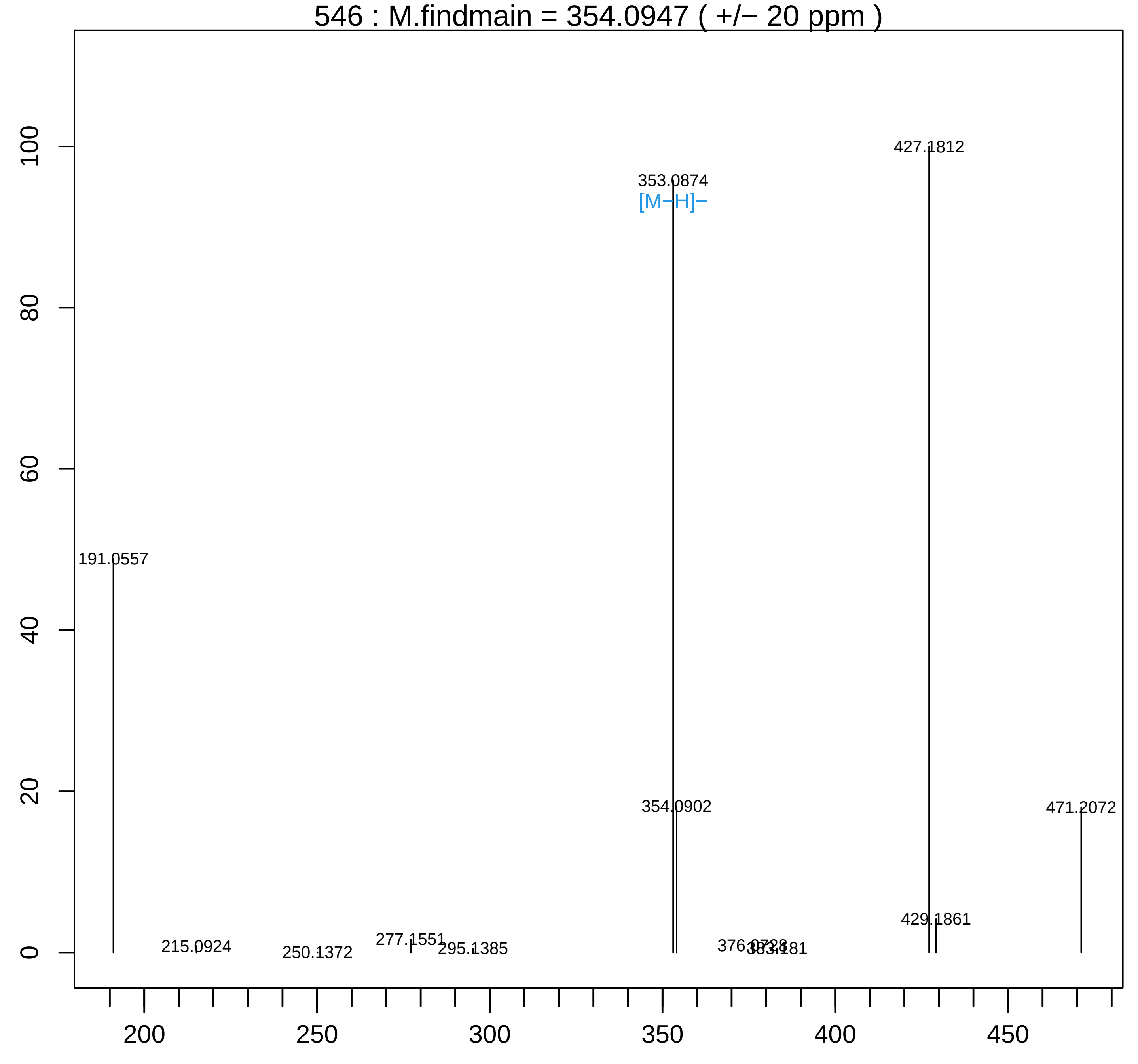
546 : M.findmain = 354.0947 (+/− 20 ppm)
[M−H]−
427.1812
353.0874
191.0557
354.0902
471.2072
429.1861
215.0924
250.1372
277.1551
295.1385
376.0728
383.181
0
20
40
60
80
100
200
250
300
350
400
450

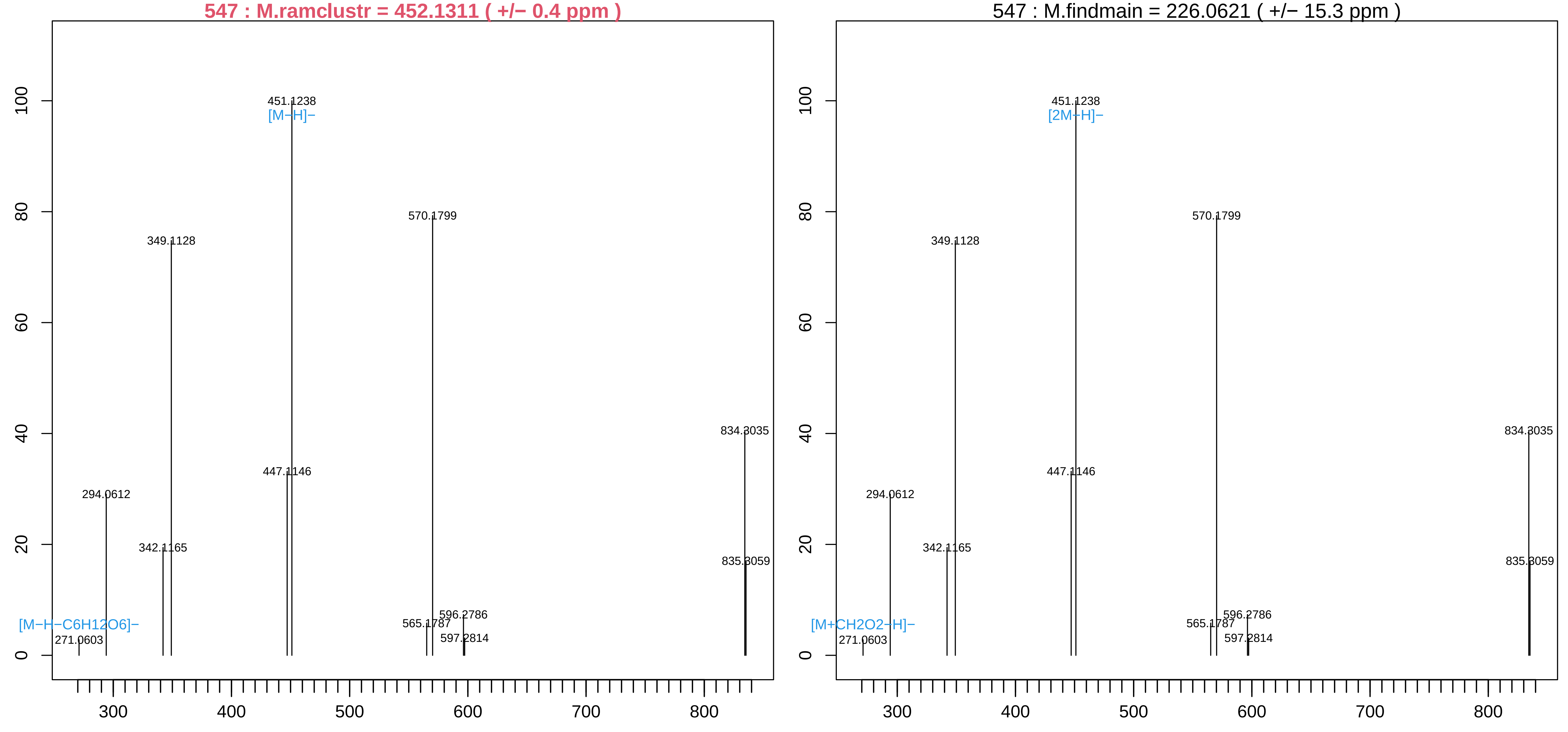
547 : M.ramclustr = 452.1311 (+/− 0.4 ppm)
451.1238
[M−H]−
570.1799
349.1128
834.3035
447.1146
294.0612
342.1165
835.3059
596.2786
[M−H−C6H12O6]−
565.1787
271.0603
597.2814
547 : M.findmain = 226.0621 (+/− 15.3 ppm)
451.1238
[2M−H]−
570.1799
349.1128
834.3035
447.1146
294.0612
342.1165
835.3059
596.2786
[M+CH2O2−H]−
565.1787
271.0603
597.2814
0
20
40
60
80
100
300
400
500
600
700
800

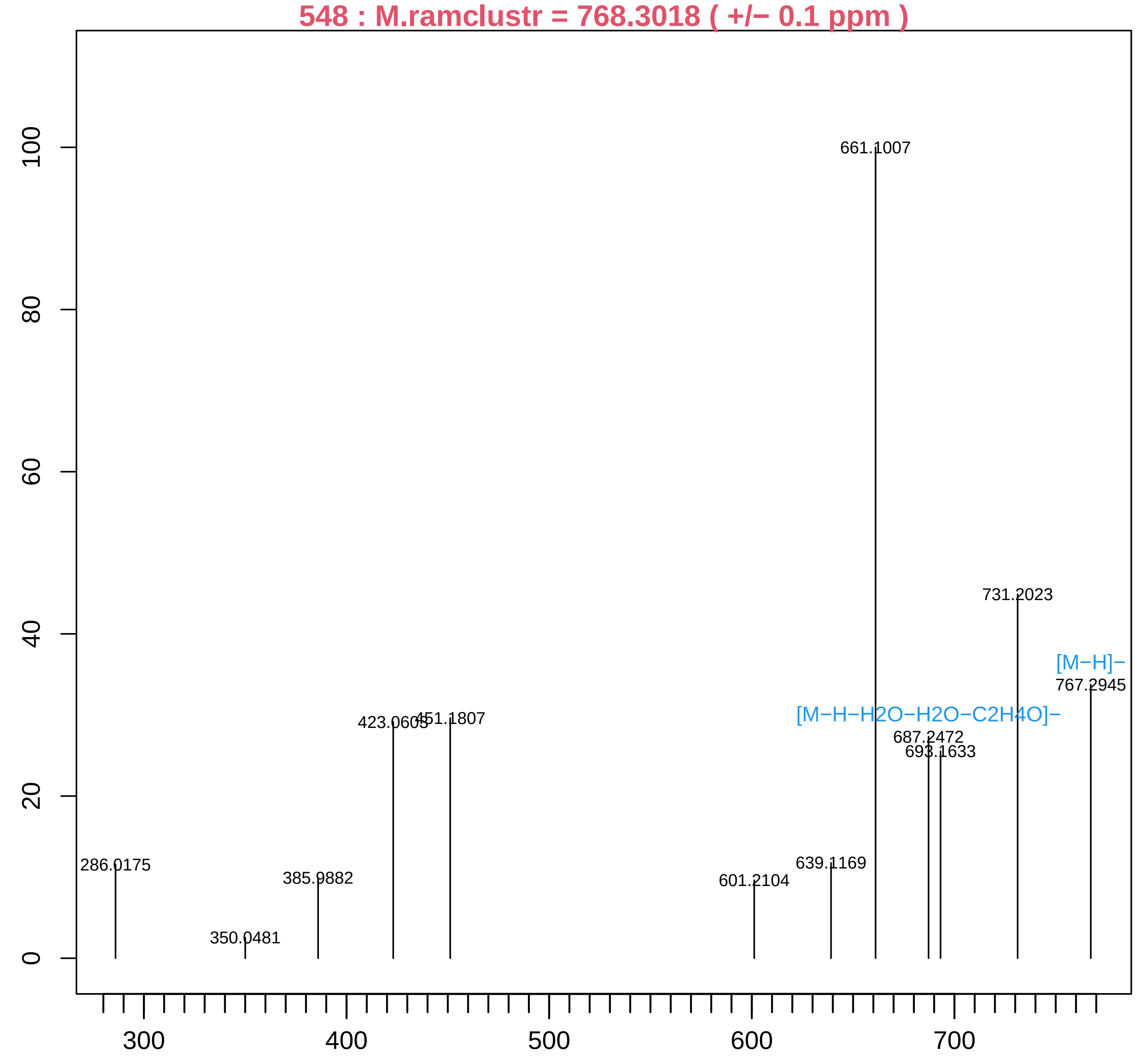
548 : M.ramclustr = 768.3018 (+/− 0.1 ppm)
661.1007
731.2023
[M−H]−
767.2945
[M−H−H2O−H2O−C2H4O]−
687.2472
693.1633
423.0605
451.1807
286.0175
385.9882
350.0481
601.2104
639.1169
0
20
40
60
80
100
300
400
500
600
700

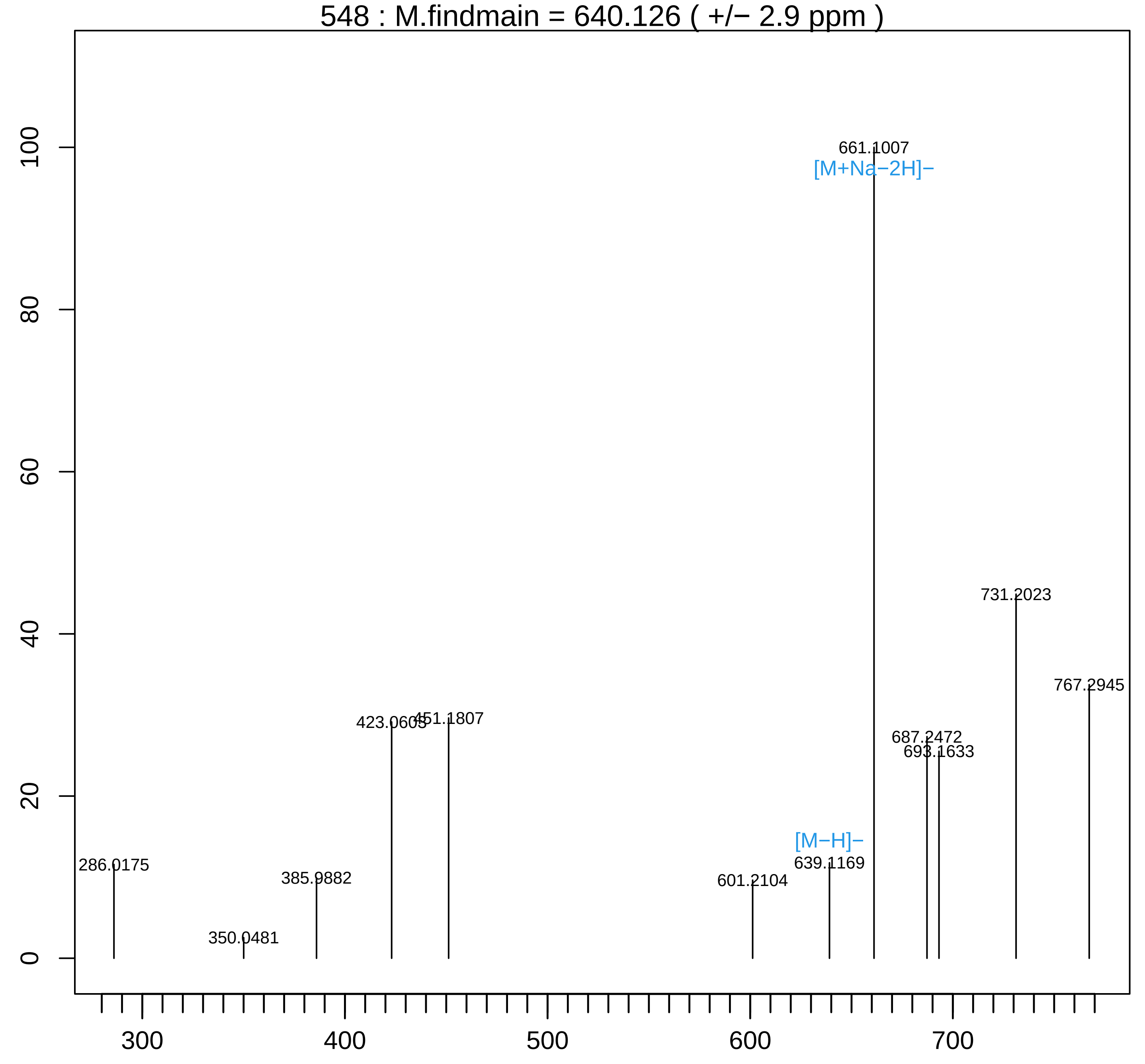
548 : M.findmain = 640.126 (+/− 2.9 ppm)
661.1007
[M+Na−2H]−
731.2023
767.2945
687.2472
693.1633
423.0605
451.1807
[M−H]−
639.1169
286.0175
385.9882
601.2104
350.0481
0
20
40
60
80
100
300
400
500
600
700

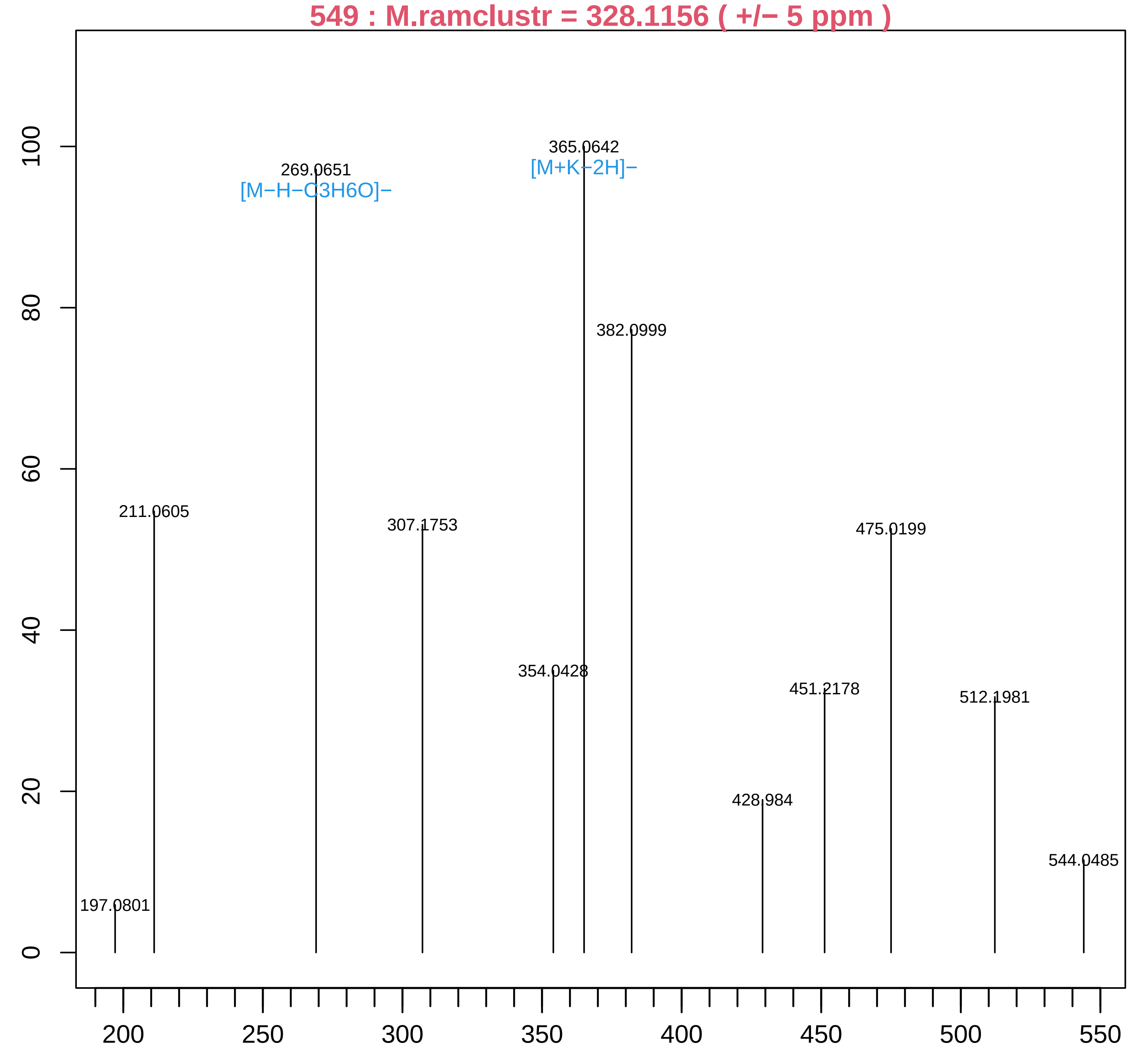
549 : M.ramclustr = 328.1156 (+/− 5 ppm)
197.0801
211.0605
269.0651
[M−H−C3H6O]−
307.1753
354.0428
365.0642
[M+K−2H]−
382.0999
428.984
451.2178
475.0199
512.1981
544.0485
0
20
40
60
80
100
200
250
300
350
400
450
500
550

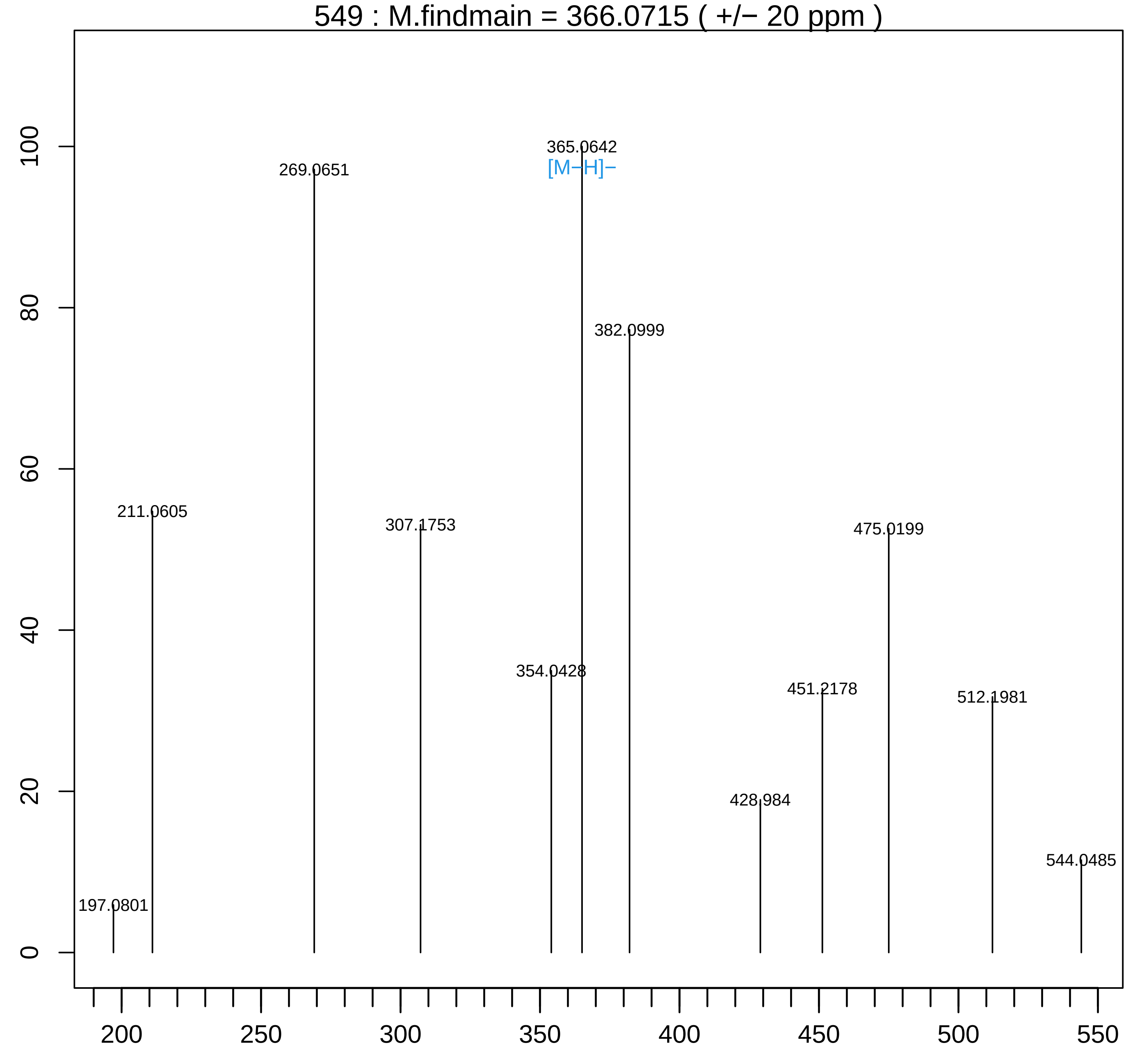
549 : M.findmain = 366.0715 (+/− 20 ppm)
197.0801
211.0605
269.0651
307.1753
354.0428
365.0642
[M−H]−
382.0999
428.984
451.2178
475.0199
512.1981
544.0485
0
20
40
60
80
100
200
250
300
350
400
450
500
550

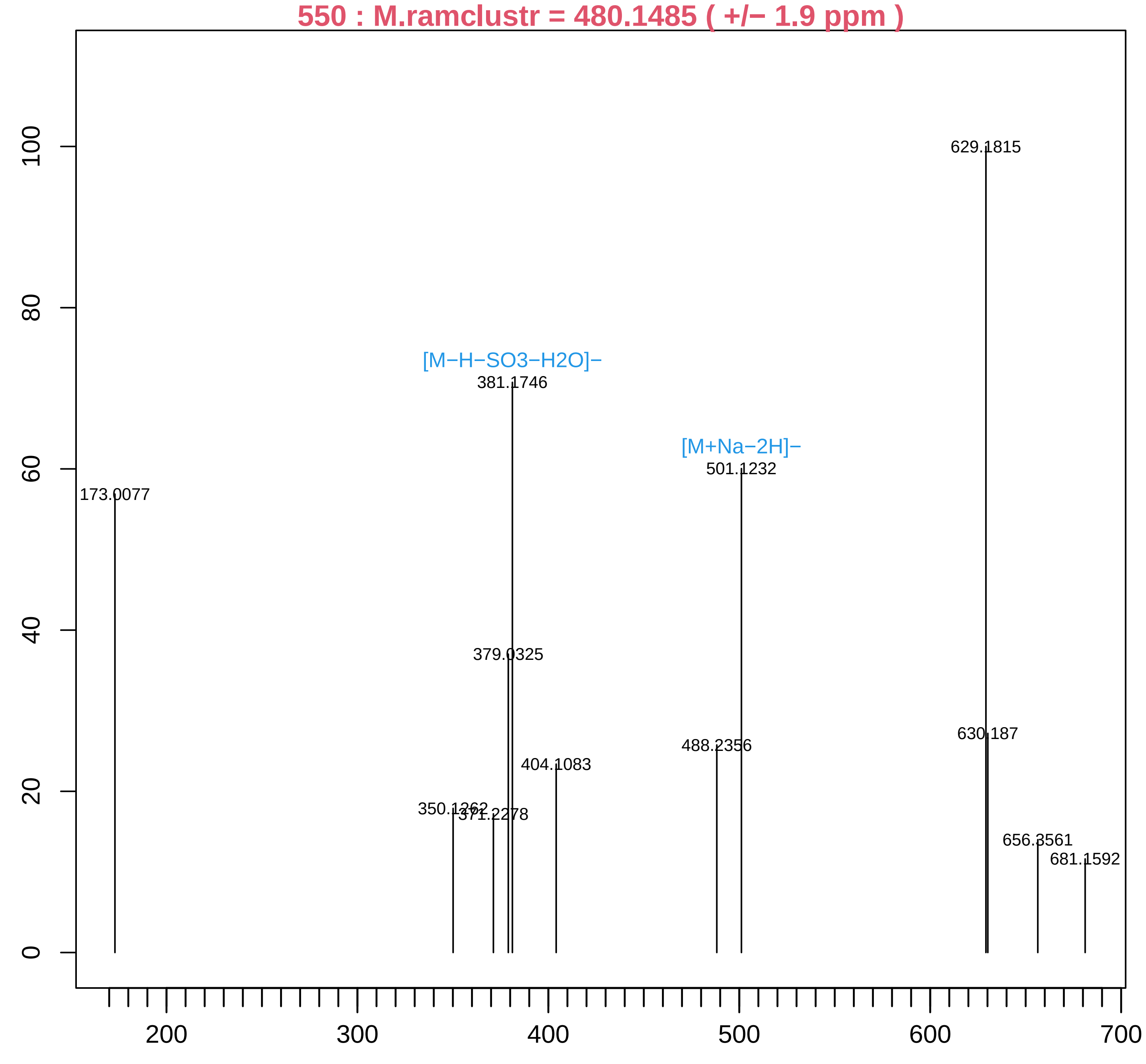
550 : M.ramclustr = 480.1485 (+/− 1.9 ppm)
[M−H−SO3−H2O]−
381.1746
[M+Na−2H]−
501.1232
629.1815
173.0077
379.0325
630.187
488.2356
404.1083
350.1262
371.2278
656.3561
681.1592
0
20
40
60
80
100
200
300
400
500
600
700

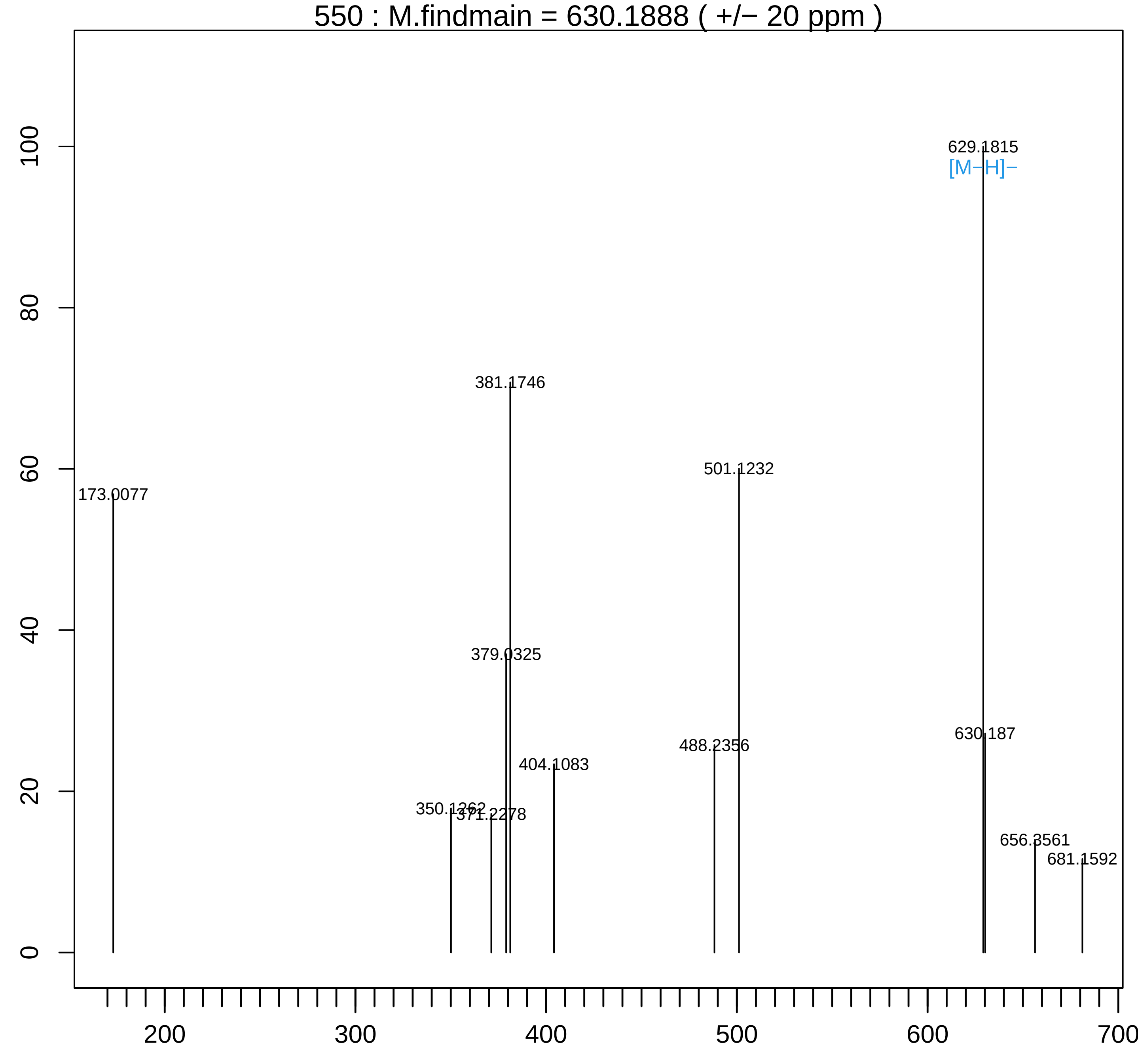
550 : M.findmain = 630.1888 (+/− 20 ppm)
629.1815
[M−H]−
381.1746
501.1232
173.0077
379.0325
630.187
488.2356
404.1083
350.1262
371.2278
656.3561
681.1592
0
20
40
60
80
100
200
300
400
500
600
700

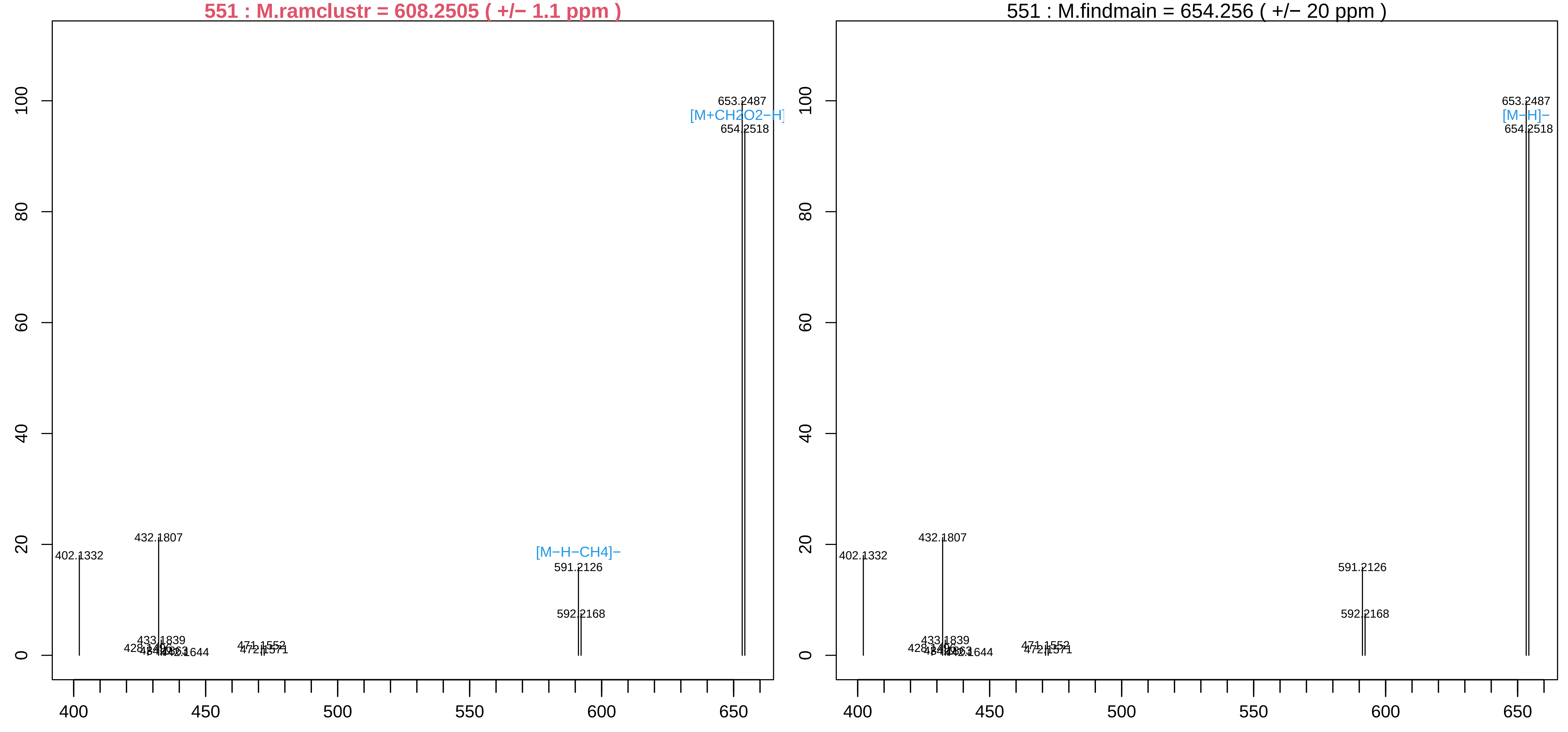
551 : M.ramclustr = 608.2505 (+/− 1.1 ppm)
653.2487
[M+CH2O2−H]
654.2518
[M−H−CH4]−
591.2126
592.2168
432.1807
402.1332
433.1839
471.1552
472.1571
442.1644
551 : M.findmain = 654.256 (+/− 20 ppm)
653.2487
[M−H]−
654.2518
591.2126
592.2168
432.1807
402.1332
433.1839
471.1552
472.1571
442.1644

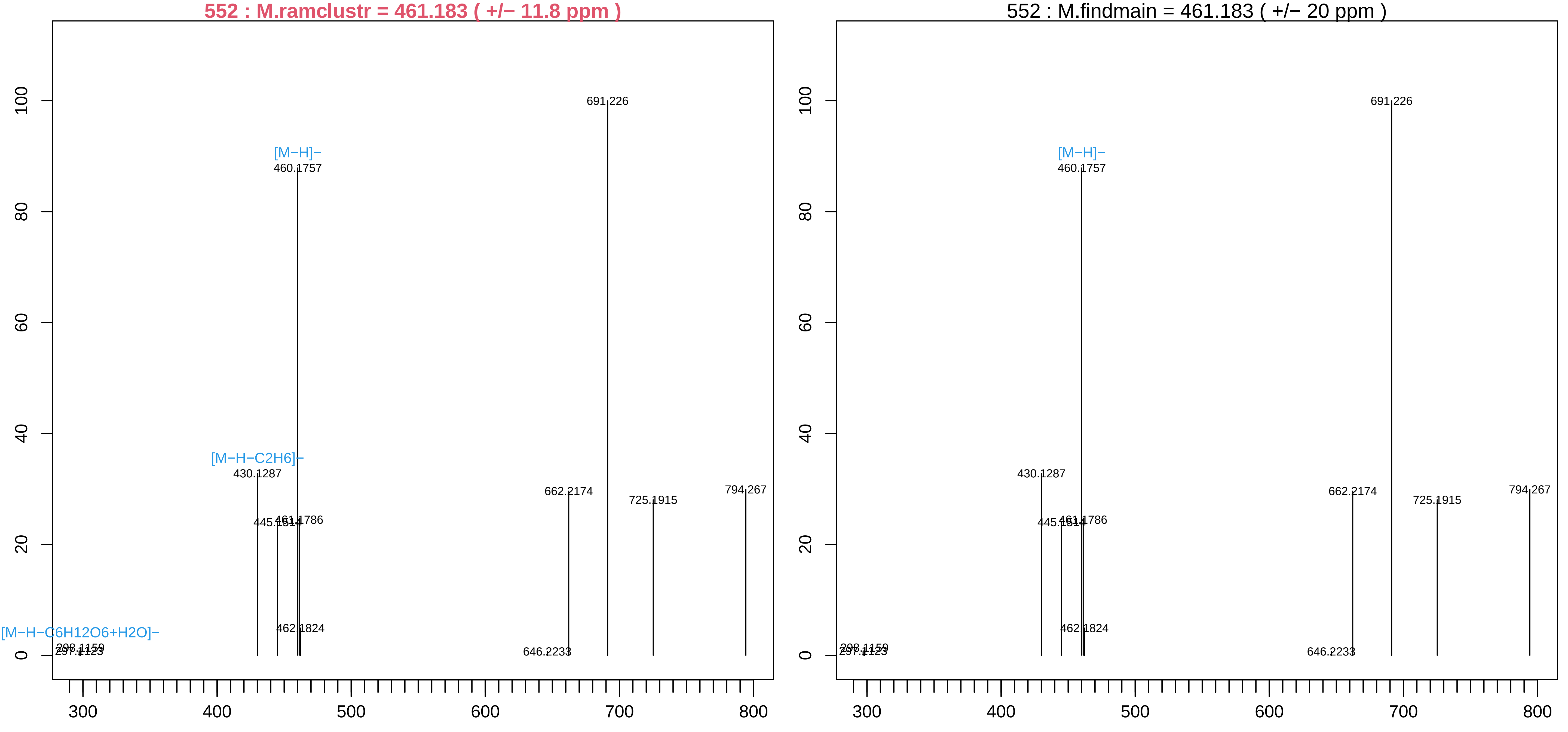
552 : M.ramclustr = 461.183 (+/− 11.8 ppm)
[M−H]−
460.1757
691.226
[M−H−C2H6]−
430.1287
445.1514
461.1786
462.1824
662.2174
646.2233
725.1915
794.267
[M−H−C6H12O6+H2O]−
298.1159
297.1123
552 : M.findmain = 461.183 (+/− 20 ppm)
[M−H]−
460.1757
691.226
430.1287
445.1514
461.1786
462.1824
662.2174
646.2233
725.1915
794.267
298.1159
297.1123
0
20
40
60
80
100
300
400
500
600
700
800

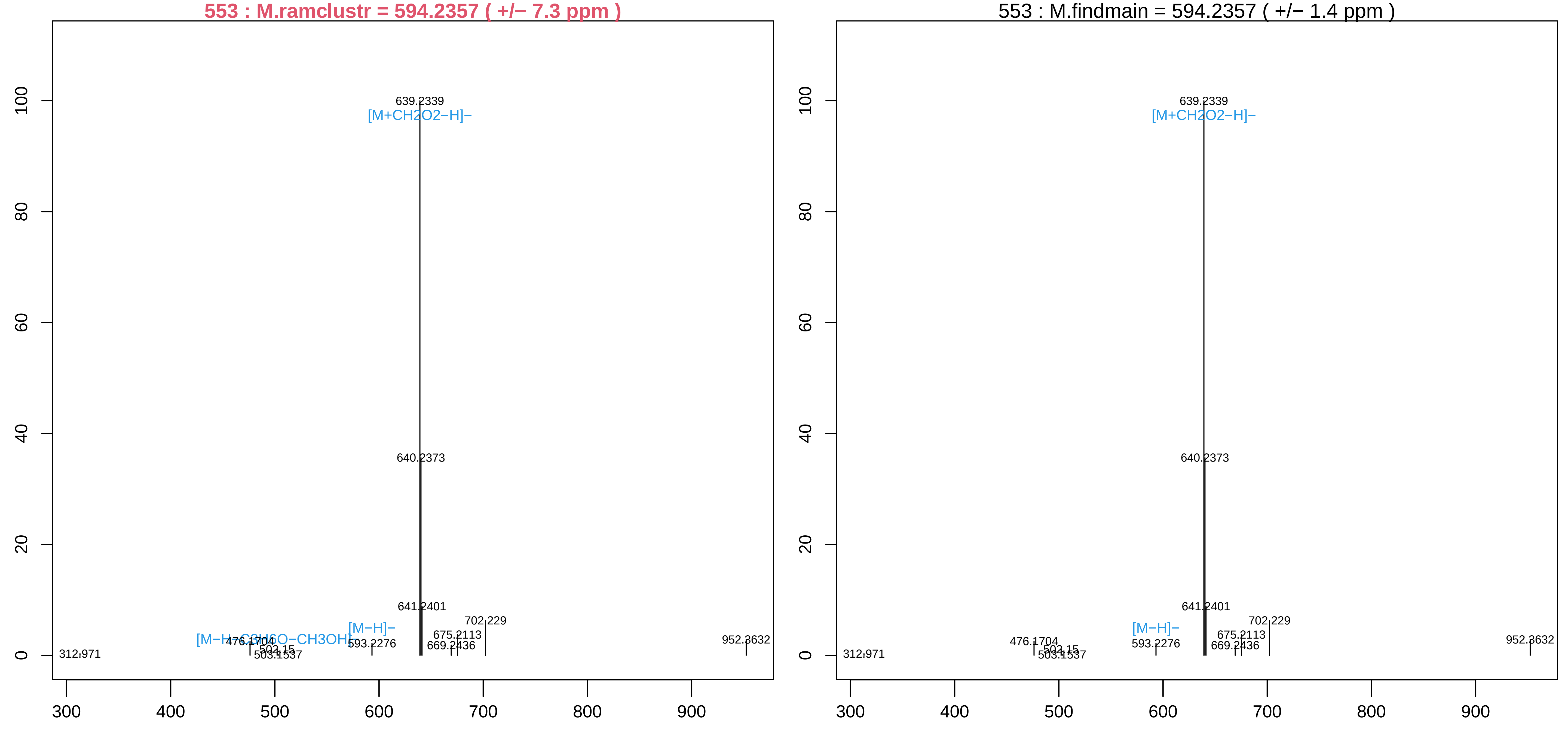
553 : M.ramclustr = 594.2357 (+/− 7.3 ppm)
639.2339
[M+CH2O2−H]−
640.2373
641.2401
702.229
[M−H]−
675.2113
[M−H−C3H6O−CH3OH]−
476.1704
502.15
593.2276
669.2436
952.3632
312.971
503.1537
0
20
40
60
80
100
300
400
500
600
700
800
900
553 : M.findmain = 594.2357 (+/− 1.4 ppm)
639.2339
[M+CH2O2−H]−
640.2373
641.2401
702.229
[M−H]−
675.2113
476.1704
502.15
593.2276
669.2436
952.3632
312.971
503.1537
0
20
40
60
80
100
300
400
500
600
700
800
900

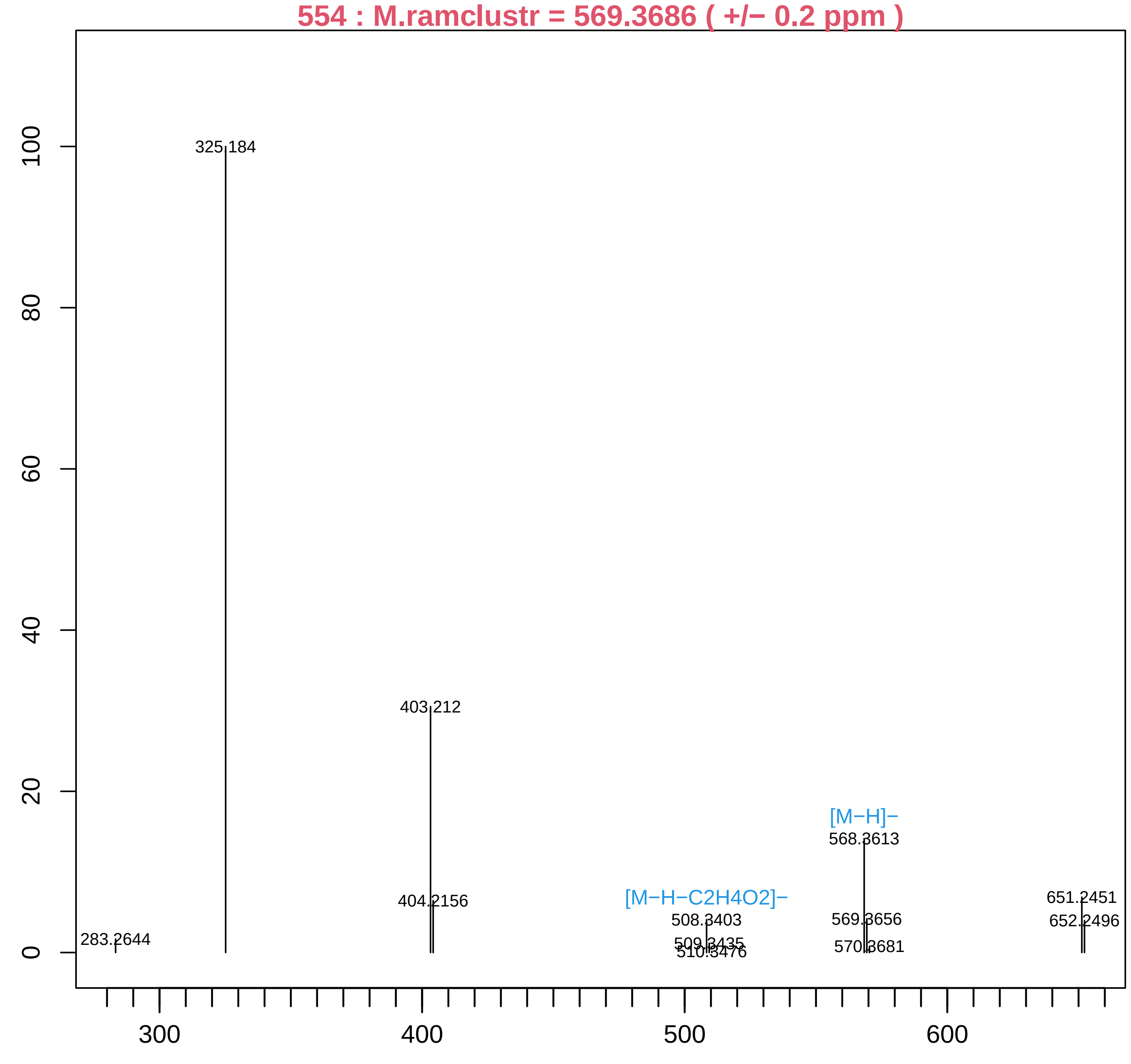
554 : M.ramclustr = 569.3686 (+/− 0.2 ppm)
325.184
403.212
404.2156
283.2644
[M−H−C2H4O2]−
508.3403
509.3435
510.3476
[M−H]−
568.3613
569.3656
570.3681
651.2451
652.2496
0
20
40
60
80
100
300
400
500
600

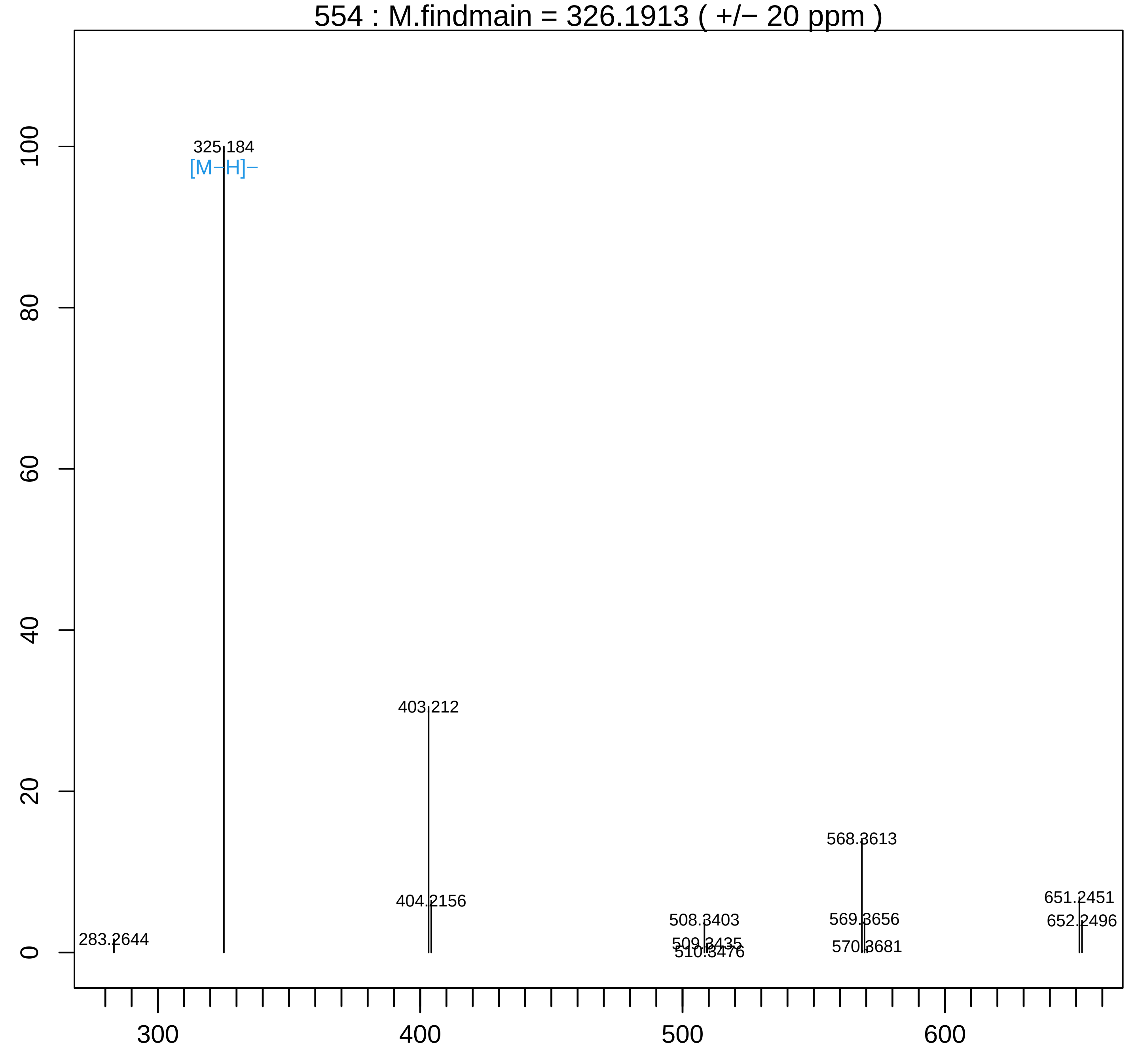
554 : M.findmain = 326.1913 (+/− 20 ppm)
325.184
[M−H]−
403.212
404.2156
283.2644
508.3403
509.3435
510.3476
568.3613
569.3656
570.3681
651.2451
652.2496
0
20
40
60
80
100
300
400
500
600

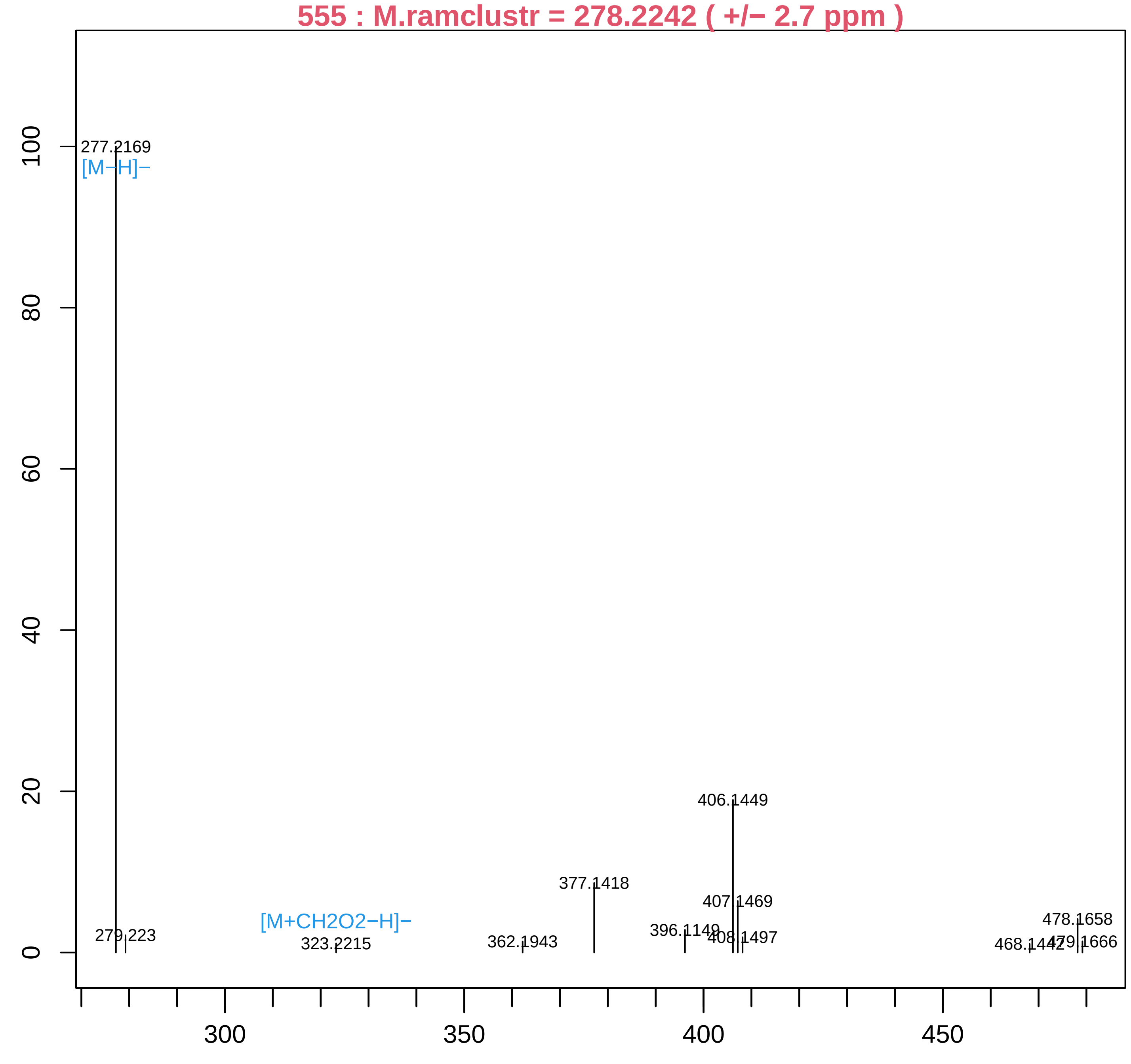
555 : M.ramclustr = 278.2242 (+/− 2.7 ppm)
277.2169
[M−H]−
279.223
[M+CH2O2−H]−
323.2215
362.1943
377.1418
396.1149
406.1449
407.1469
408.1497
468.1442
478.1658
479.1666
0
20
40
60
80
100
300
350
400
450

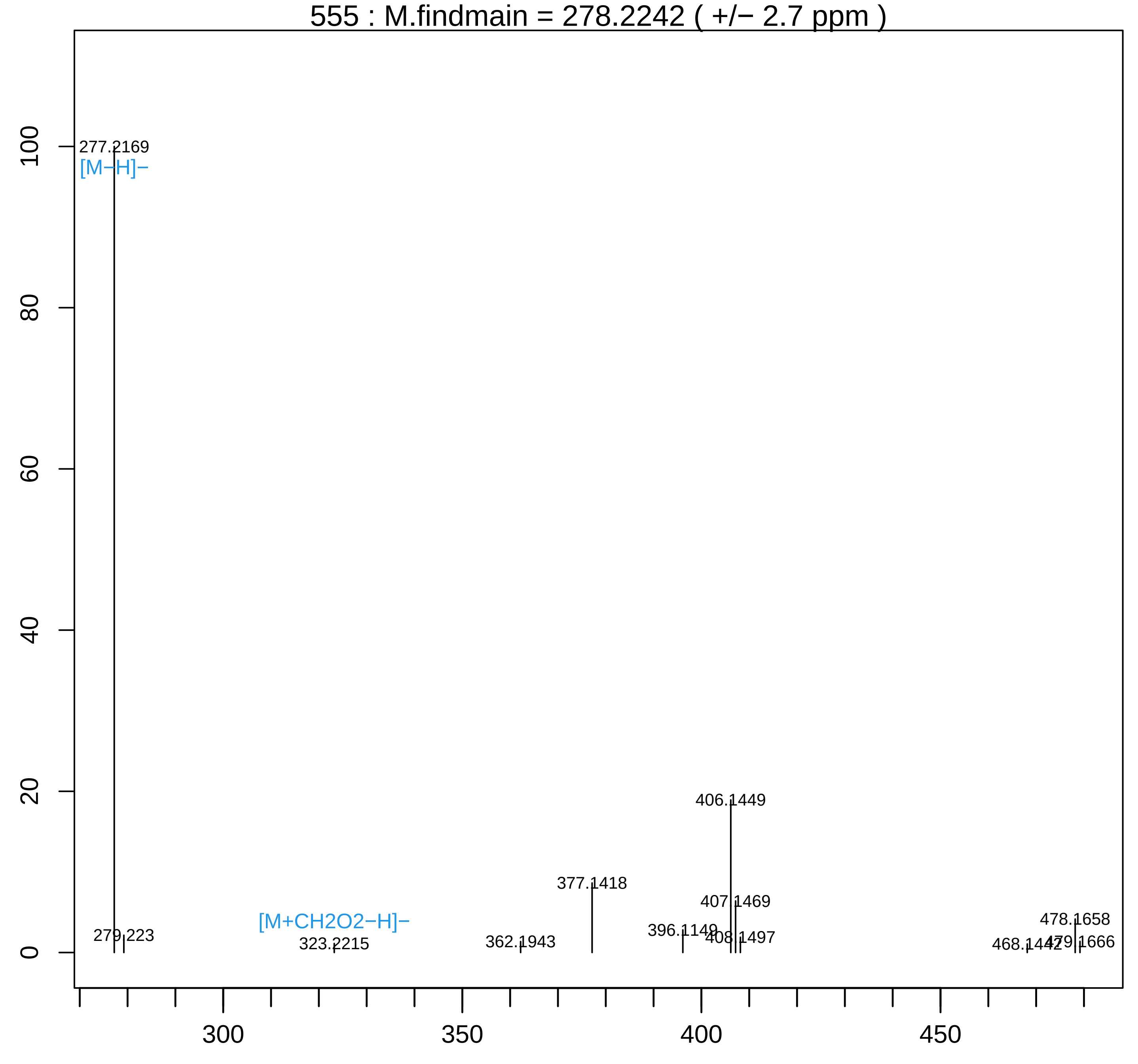
555 : M.findmain = 278.2242 (+/− 2.7 ppm)
277.2169
[M−H]−
279.223
[M+CH2O2−H]−
323.2215
362.1943
377.1418
396.1149
406.1449
407.1469
408.1497
468.1442
478.1658
479.1666
0
20
40
60
80
100
300
350
400
450

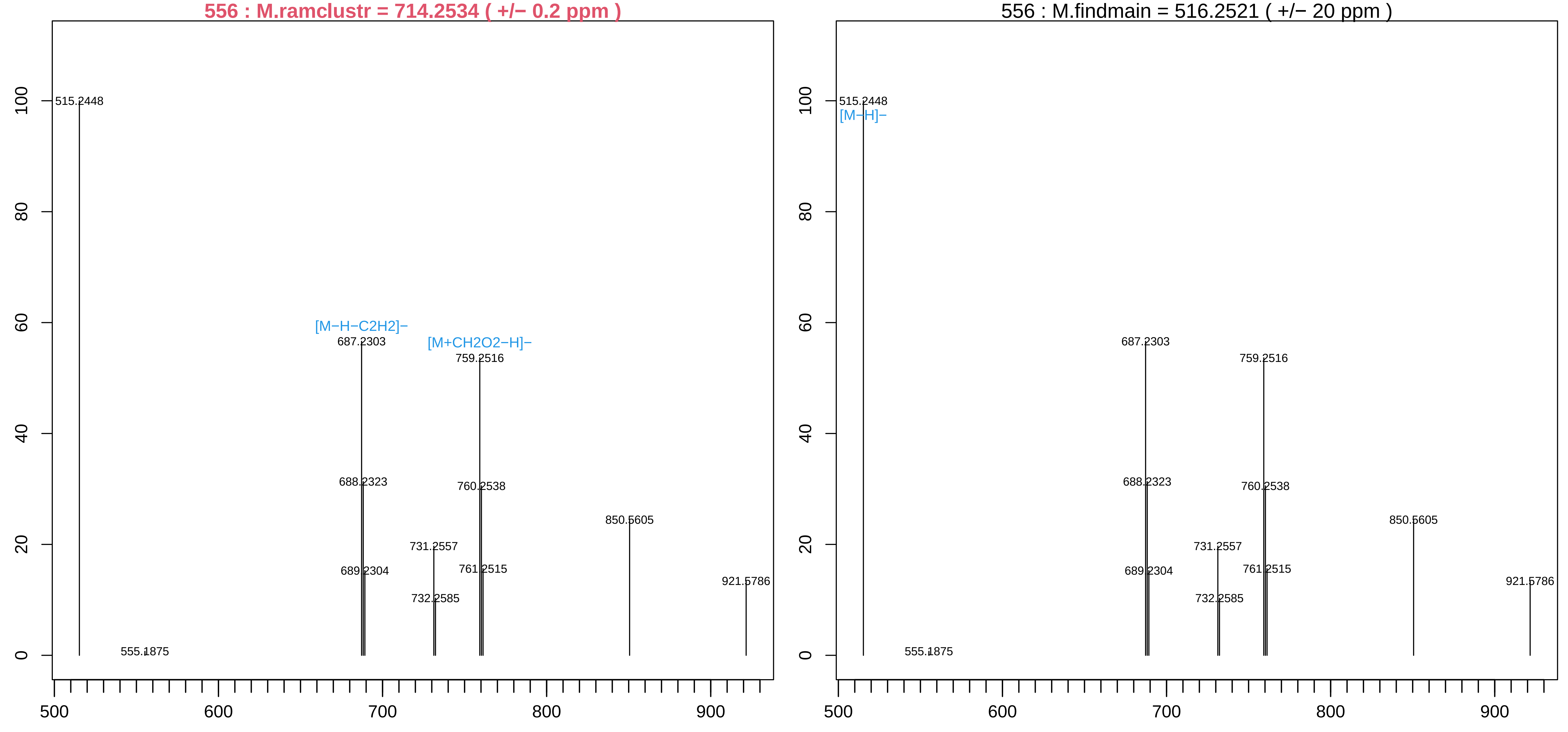

556 : M.ramclustr = 714.2534 (+/− 0.2 ppm)
515.2448
[M−H−C2H2]−
687.2303
[M+CH2O2−H]−
759.2516
688.2323
760.2538
850.5605
731.2557
689.2304
761.2515
921.5786
732.2585
555.1875
556 : M.findmain = 516.2521 (+/− 20 ppm)
515.2448
[M−H]−
687.2303
759.2516
688.2323
760.2538
850.5605
731.2557
689.2304
761.2515
921.5786
732.2585
555.1875
0
20
40
60
80
100
500
600
700
800
900

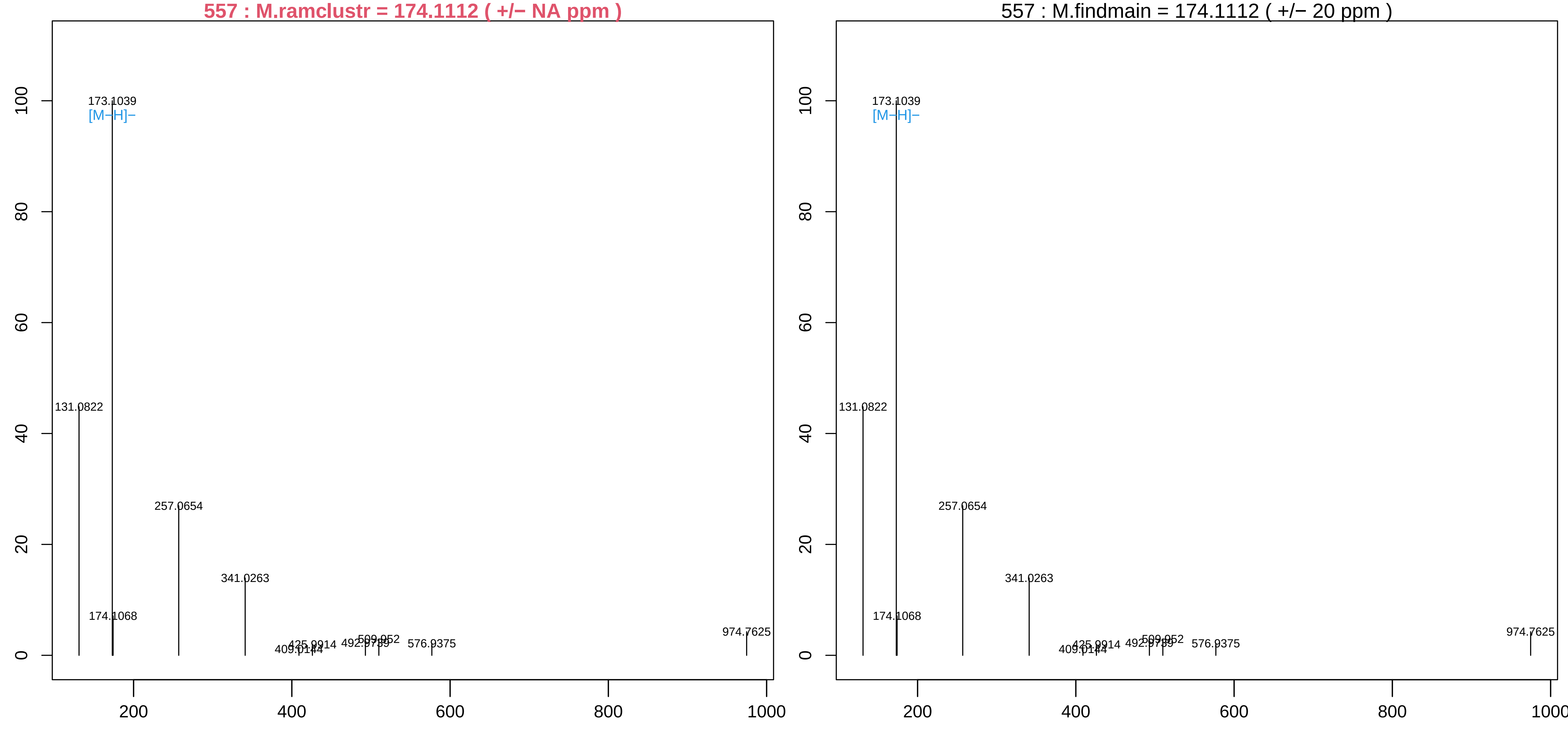
557 : M.ramclustr = 174.1112 (+/− NA ppm)
173.1039
[M−H]−
131.0822
257.0654
341.0263
174.1068
409.0144
425.9914
492.9739
509.952
576.9375
974.7625
0
20
40
60
80
100
200
400
600
800
1000
557 : M.findmain = 174.1112 (+/− 20 ppm)
173.1039
[M−H]−
131.0822
257.0654
341.0263
174.1068
409.0144
425.9914
492.9739
509.952
576.9375
974.7625
0
20
40
60
80
100
200
400
600
800
1000

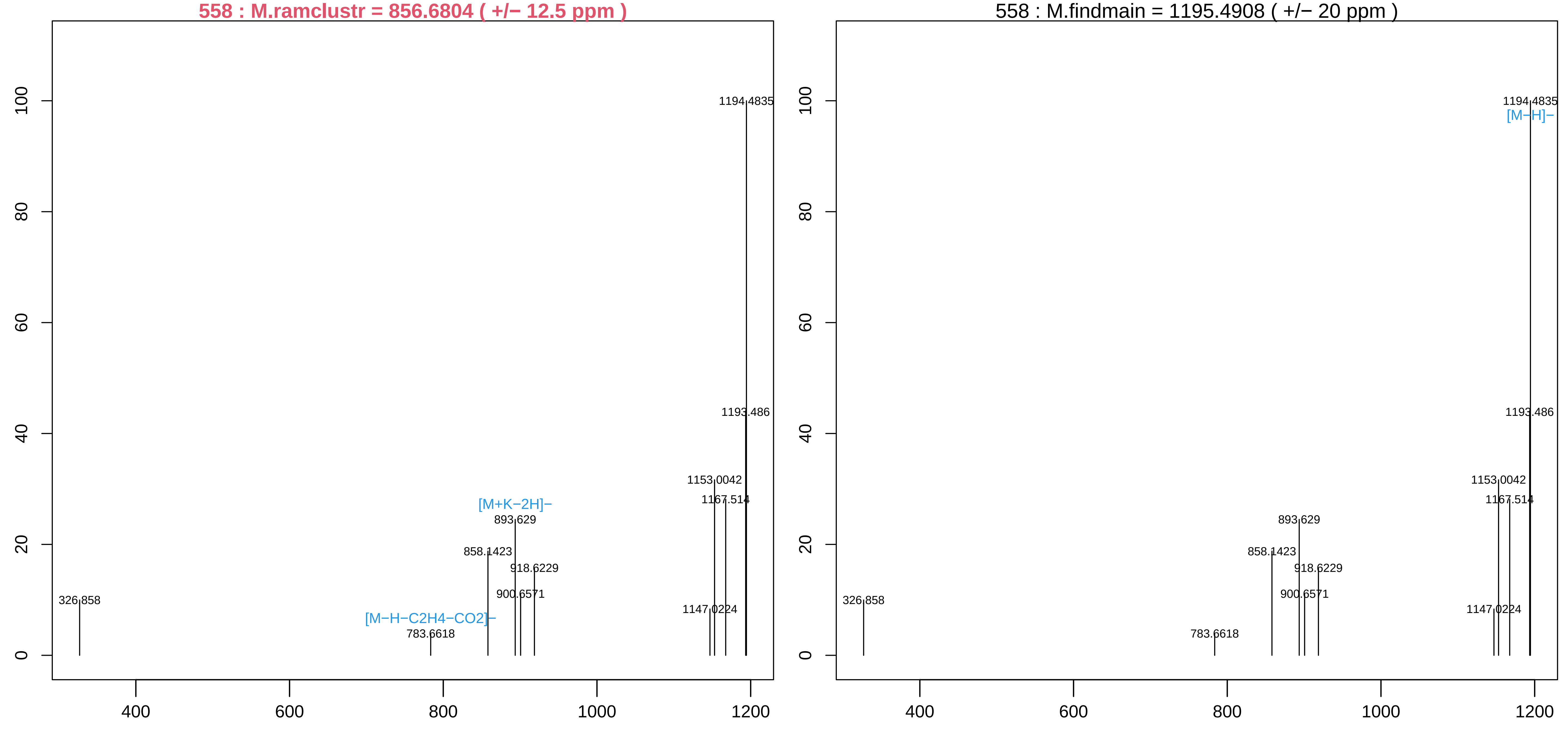

558 : M.ramclustr = 856.6804 (+/− 12.5 ppm)
558 : M.findmain = 1195.4908 (+/− 20 ppm)
1194.4835
[M−H]−
1193.486
1153.0042
1167.514
[M+K−2H]−
893.629
858.1423
918.6229
900.6571
1147.0224
326.858
[M−H−C2H4−CO2]−
783.6618
0
20
40
60
80
100
400
600
800
1000
1200

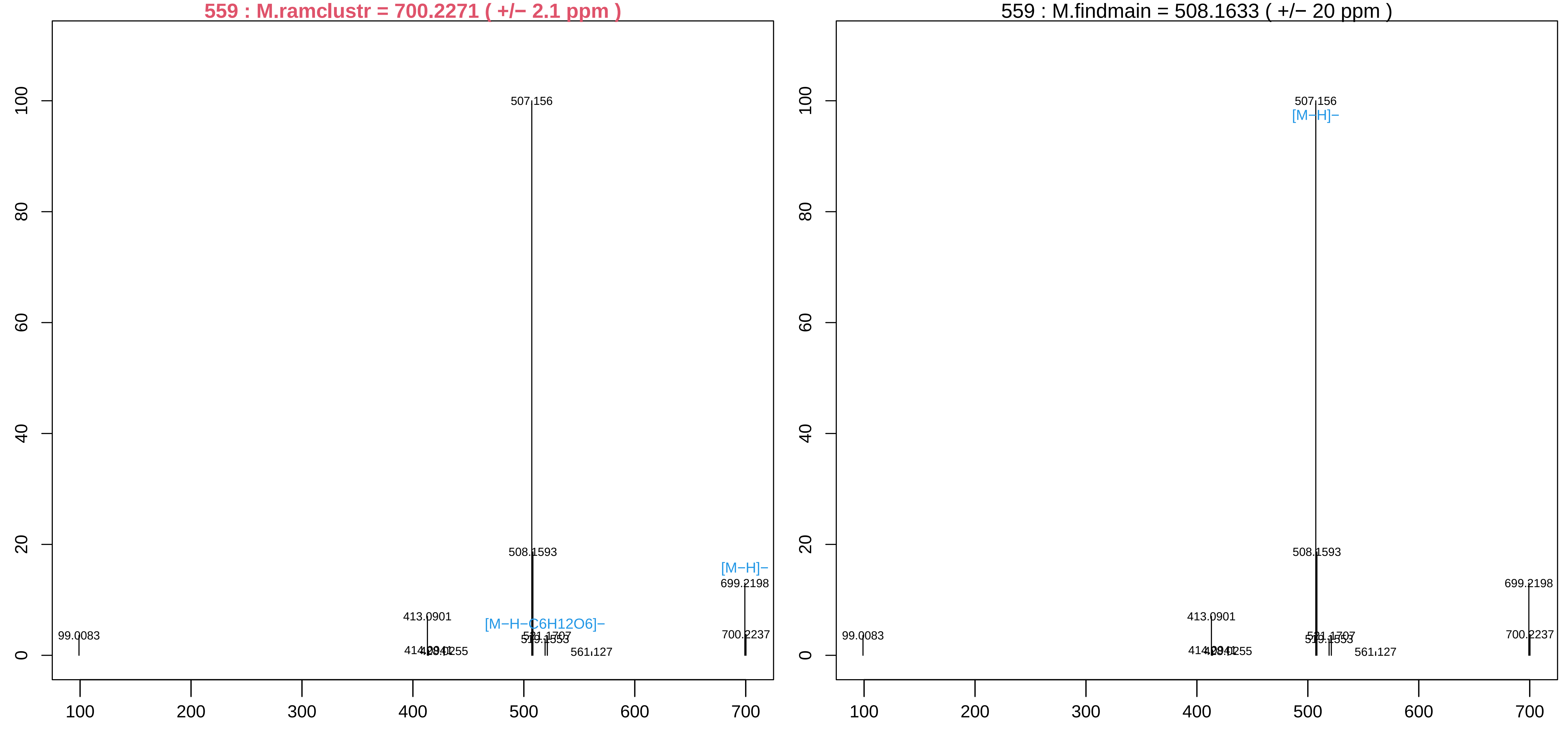

559 : M.ramclustr = 700.2271 (+/− 2.1 ppm)
507.156
508.1593
413.0901
99.0083
[M−H−C6H12O6]−
521.1707
519.1553
561.127
[M−H]−
699.2198
700.2237
559 : M.findmain = 508.1633 (+/− 20 ppm)
507.156
[M−H]−
508.1593
413.0901
99.0083
521.1707
519.1553
561.127
699.2198
700.2237

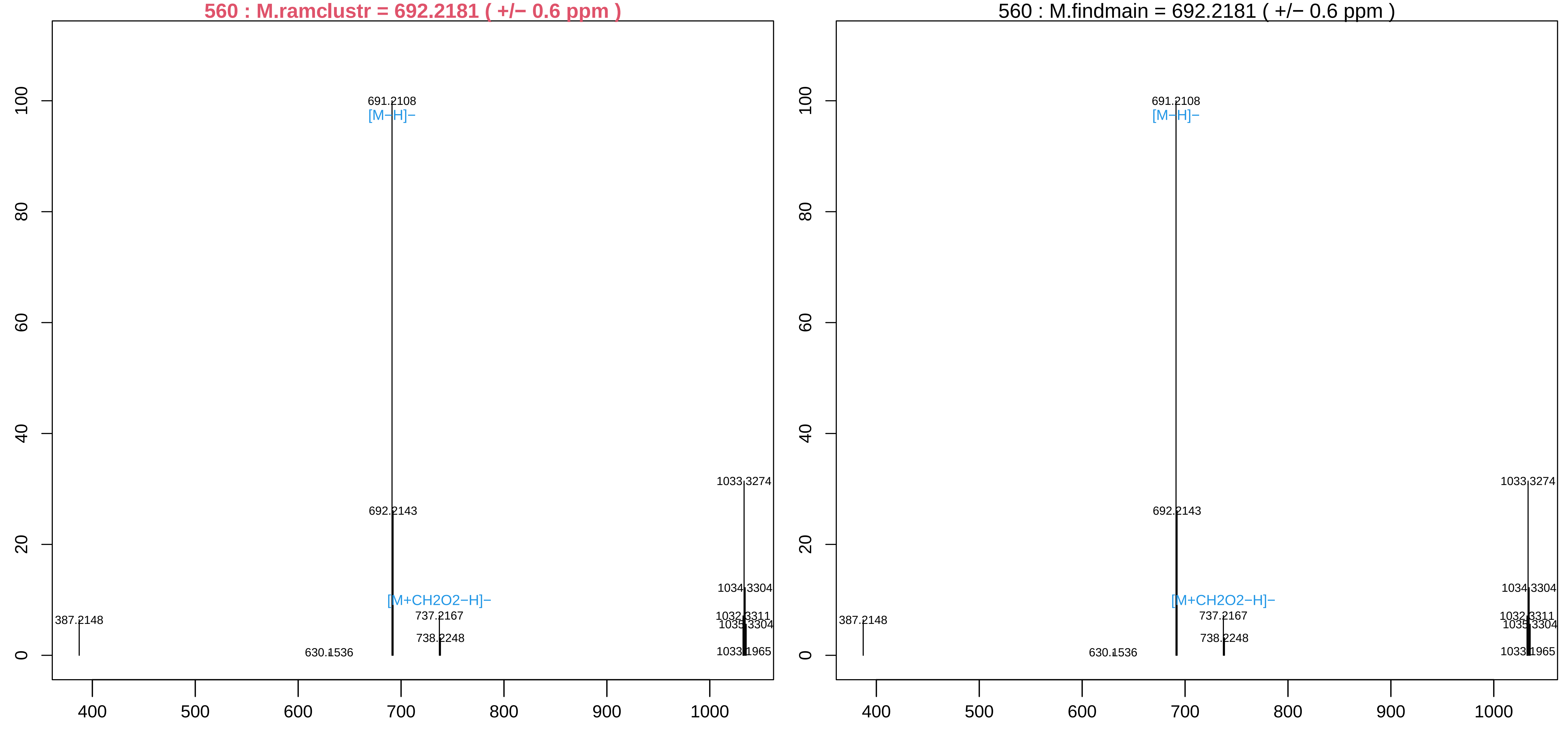
560 : M.ramclustr = 692.2181 (+/− 0.6 ppm)
560 : M.findmain = 692.2181 (+/− 0.6 ppm)
691.2108
[M−H]−
692.2143
[M+CH2O2−H]−
737.2167
738.2248
387.2148
630.1536
1033.3274
1034.3304
1032.3311
1035.3304
1033.1965
0
20
40
60
80
100
400
500
600
700
800
900
1000

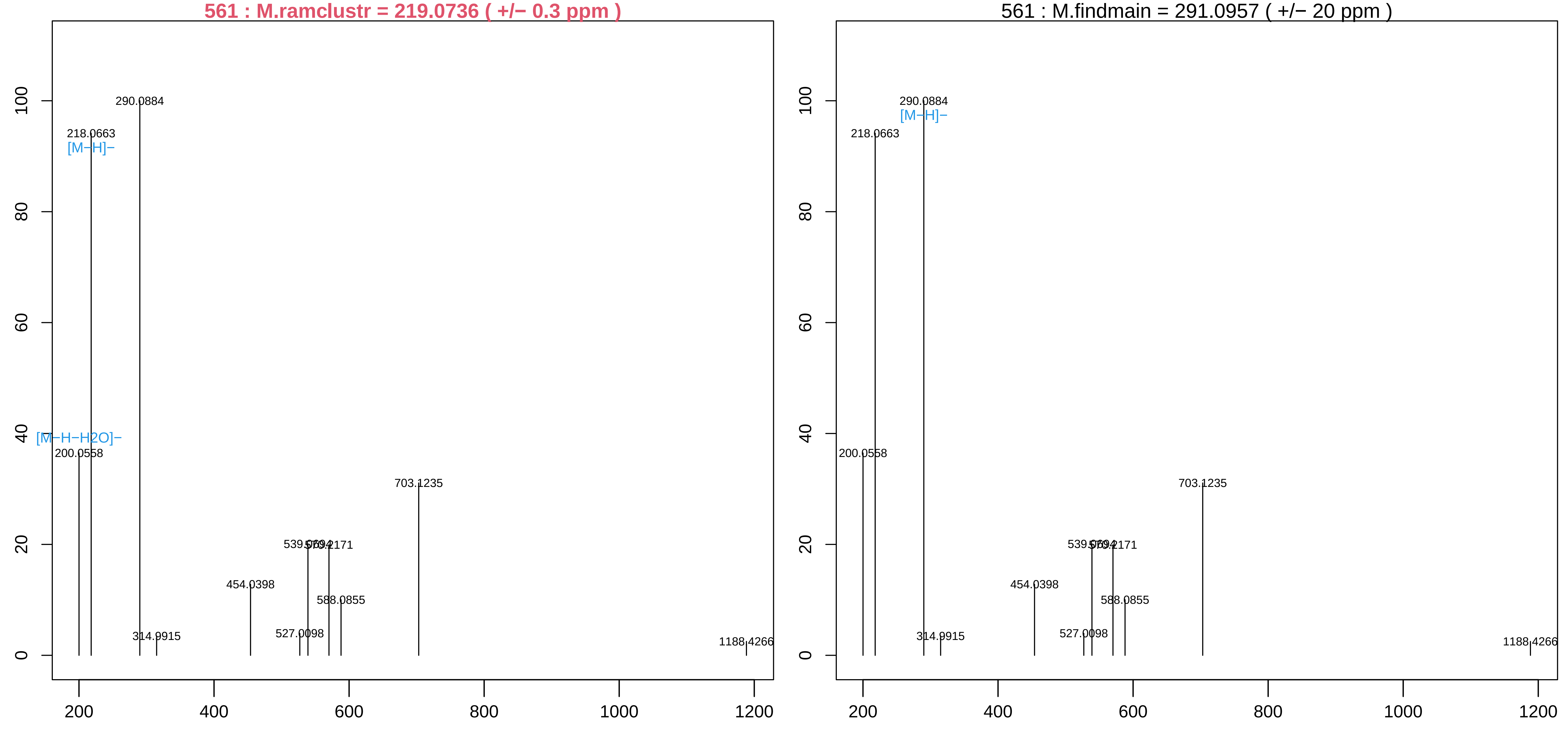

561 : M.ramclustr = 219.0736 (+/− 0.3 ppm)
290.0884
218.0663
[M−H]−
[M−H−H2O]−
200.0558
703.1235
539.0694
570.2171
454.0398
588.0855
527.0098
314.9915
1188.4266
0
20
40
60
80
100
200
400
600
800
1000
1200
561 : M.findmain = 291.0957 (+/− 20 ppm)
290.0884
[M−H]−
218.0663
200.0558
703.1235
539.0694
570.2171
454.0398
588.0855
527.0098
314.9915
1188.4266
0
20
40
60
80
100
200
400
600
800
1000
1200

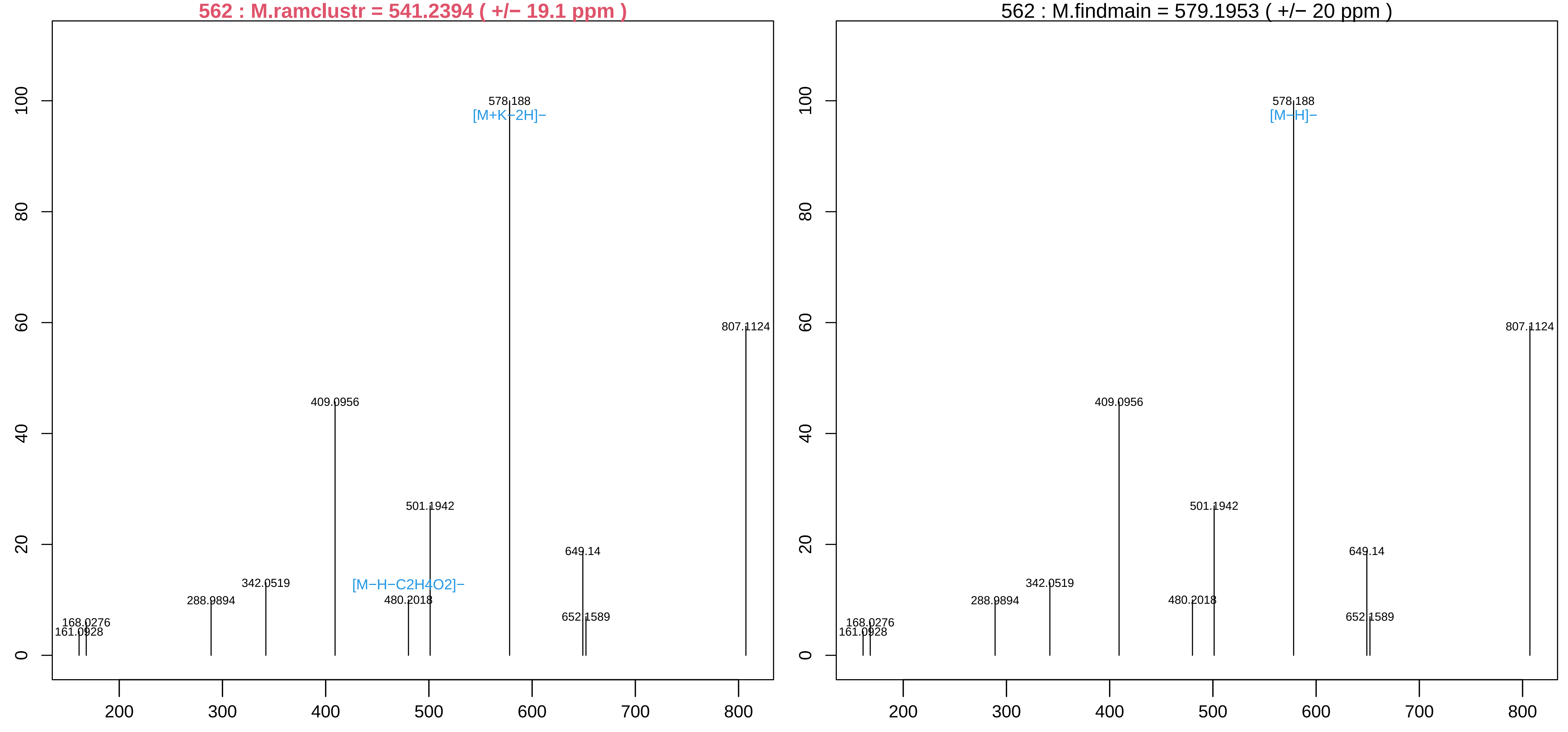
562 : M.ramclustr = 541.2394 (+/− 19.1 ppm)
578.188
[M+K−2H]−
807.1124
409.0956
501.1942
649.14
342.0519
[M−H−C2H4O2]−
288.9894
480.2018
652.1589
168.0276
161.0928
562 : M.findmain = 579.1953 (+/− 20 ppm)
578.188
[M−H]−
807.1124
409.0956
501.1942
649.14
342.0519
288.9894
480.2018
652.1589
168.0276
161.0928
0
20
40
60
80
100
200
300
400
500
600
700
800

563 : M.ramclustr = 294.1055 (+/− 3.4 ppm)

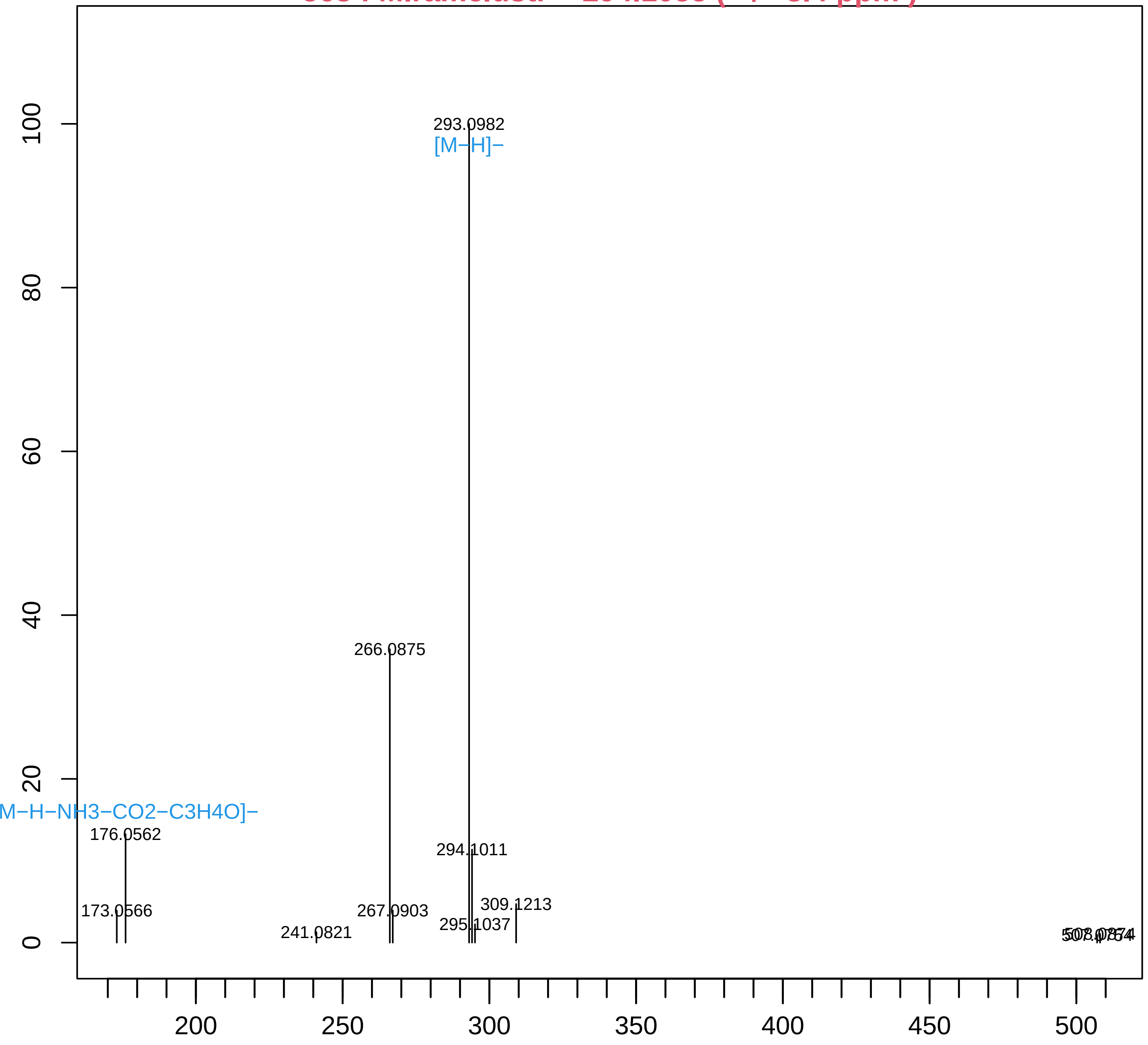

563 : M.findmain = 294.1055 (+/− 20 ppm)

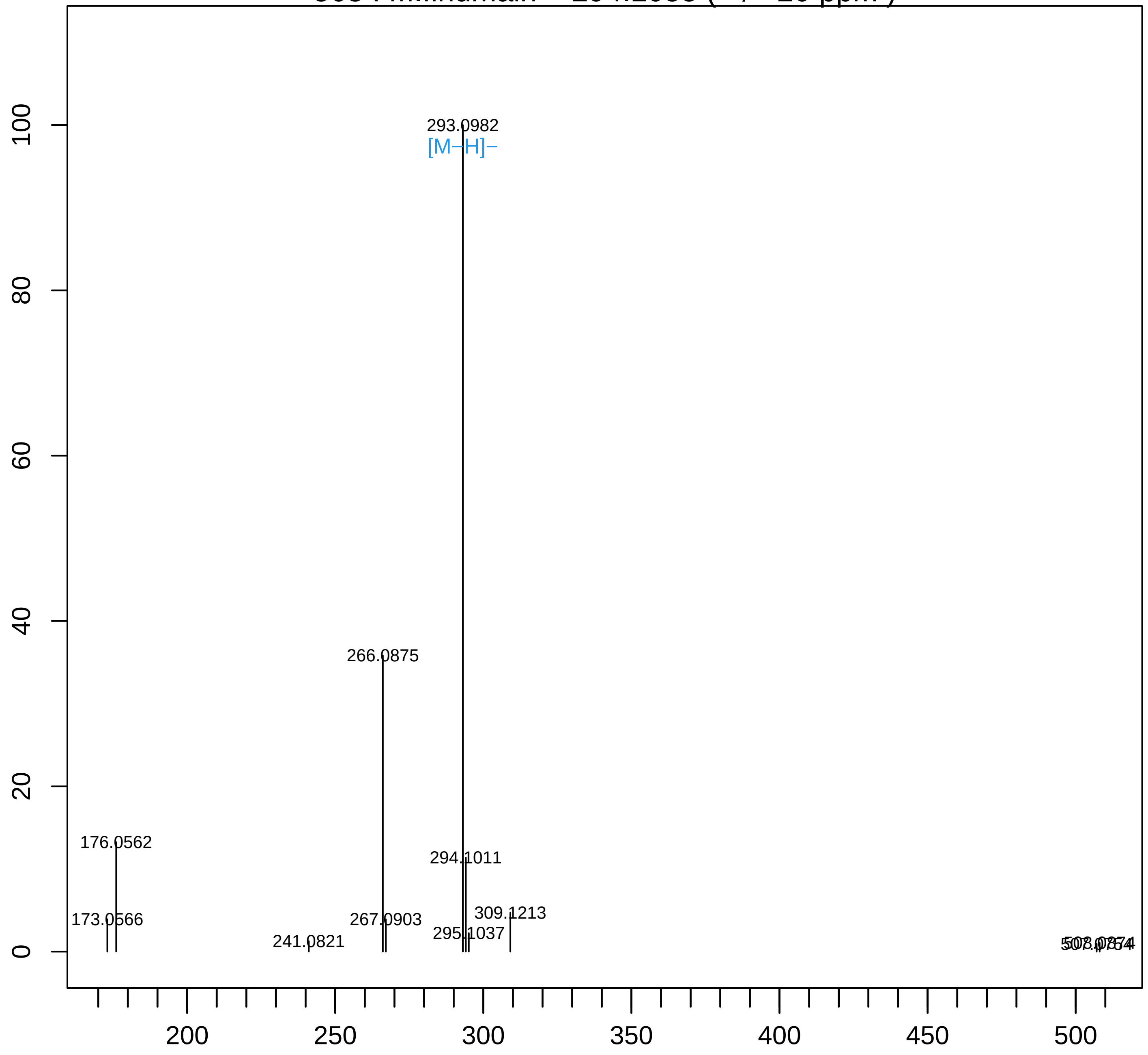

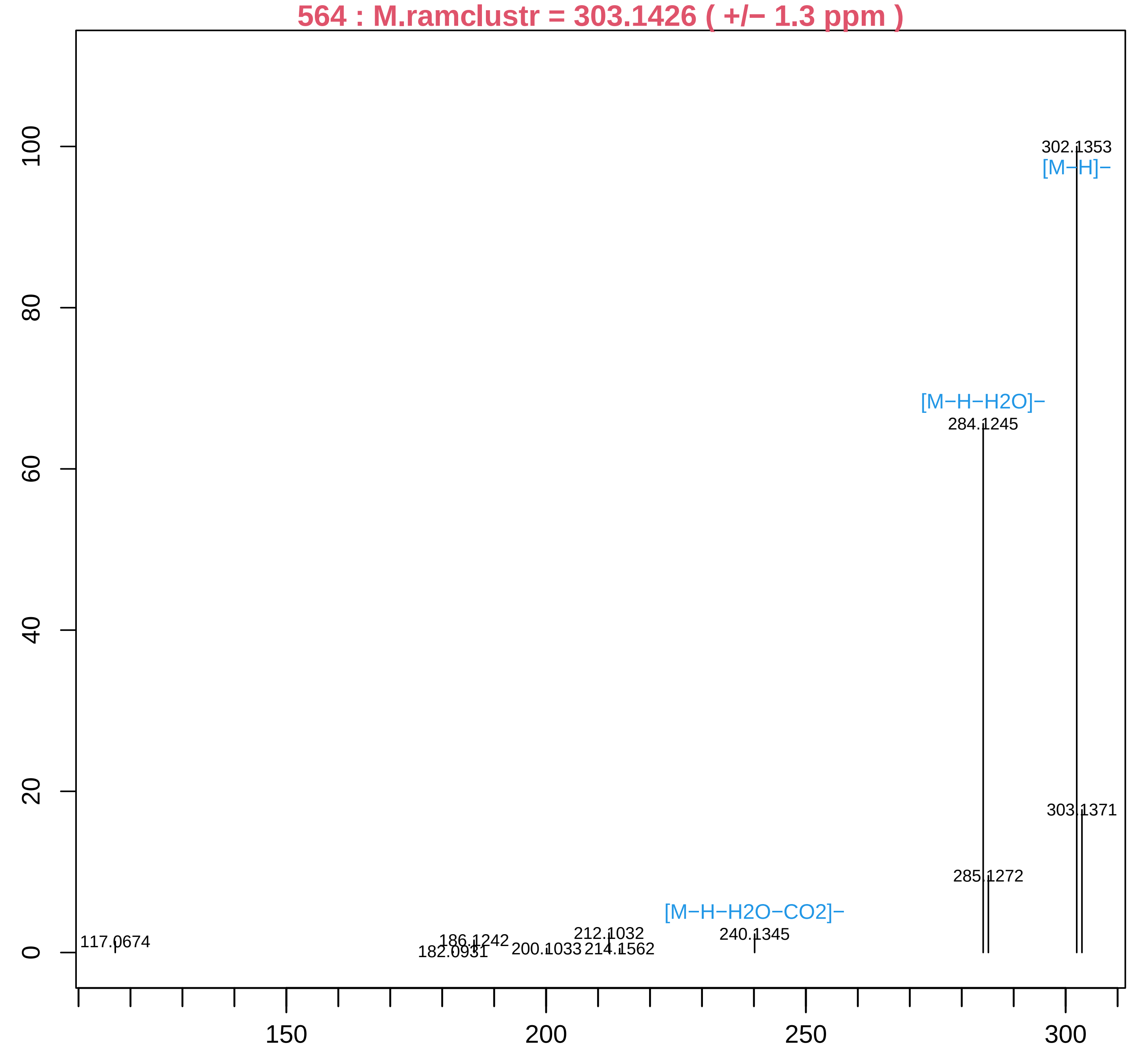
564 : M.ramclustr = 303.1426 (+/− 1.3 ppm)
302.1353
[M−H]−
[M−H−H2O]−
284.1245
303.1371
285.1272
[M−H−H2O−CO2]−
117.0674
186.1242
182.0931
200.1033
212.1032
214.1562
240.1345
0
20
40
60
80
100
150
200
250
300

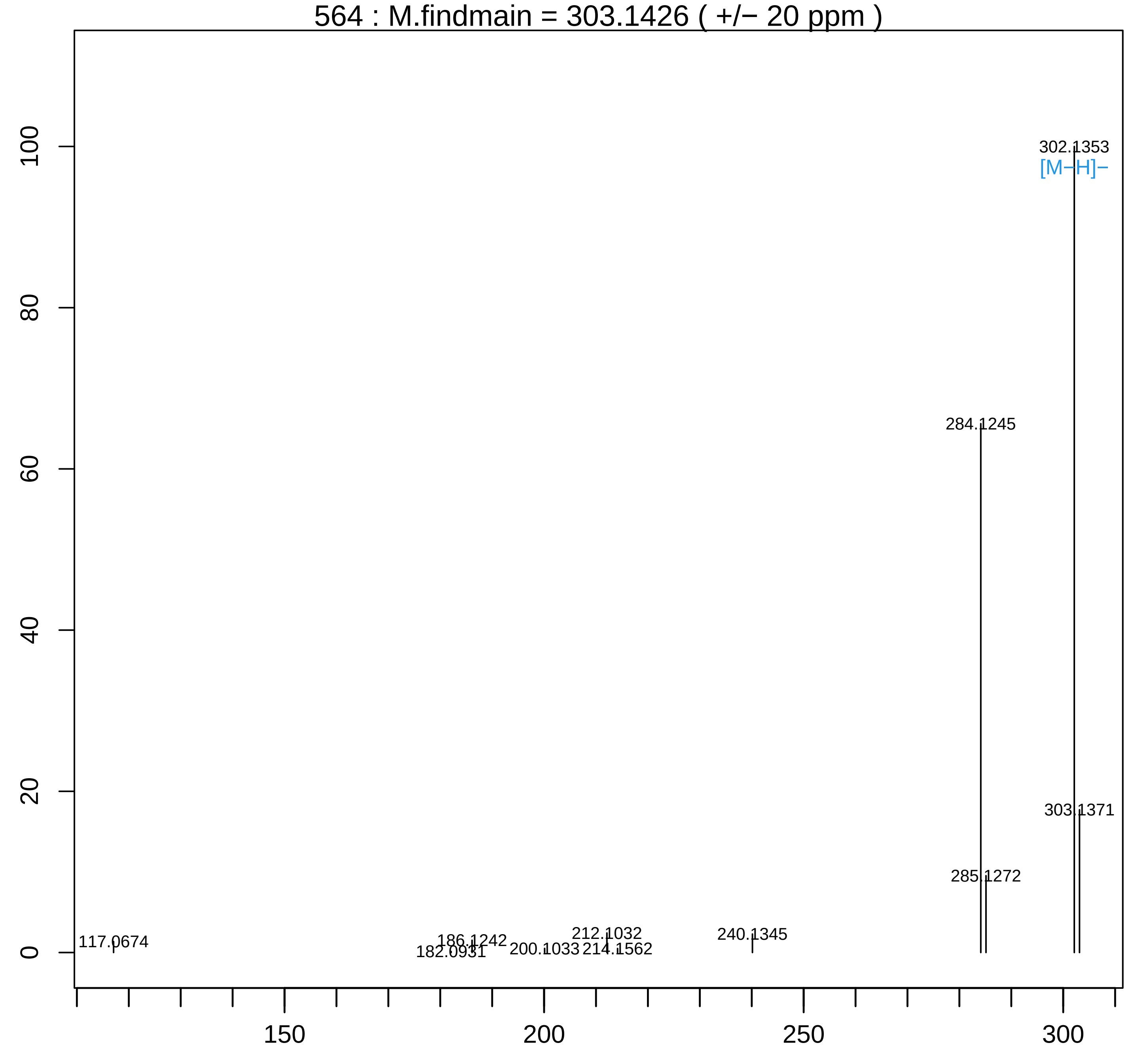
564 : M.findmain = 303.1426 (+/− 20 ppm)
302.1353
[M−H]−
284.1245
303.1371
285.1272
117.0674
186.1242
182.0931
200.1033
212.1032
214.1562
240.1345
0
20
40
60
80
100
150
200
250
300

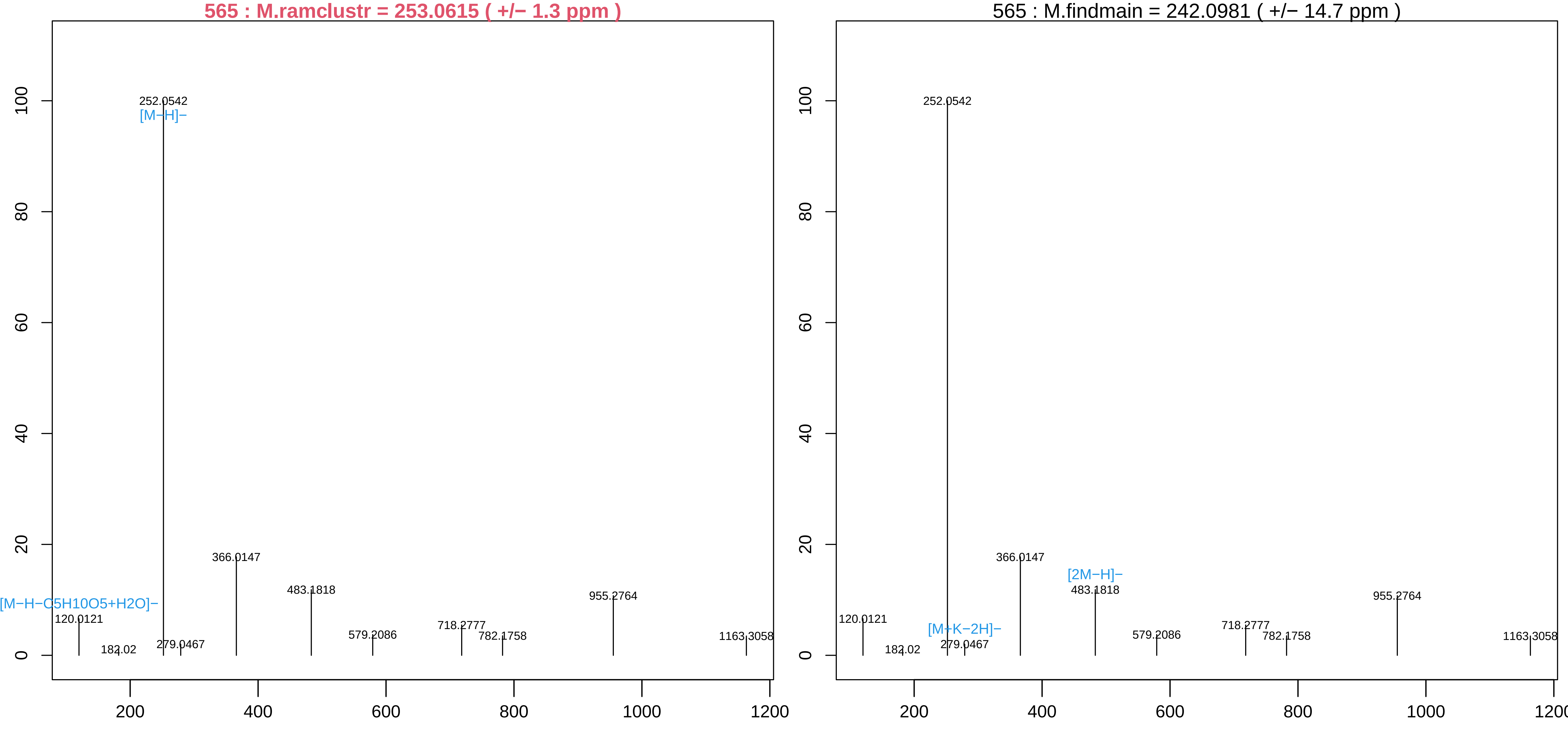

565 : M.ramclustr = 253.0615 (+/− 1.3 ppm)
252.0542
[M−H]−
366.0147
483.1818
[M−H−C5H10O5+H2O]−
120.0121
182.02
279.0467
579.2086
718.2777
782.1758
955.2764
1163.3058
565 : M.findmain = 242.0981 (+/− 14.7 ppm)
252.0542
366.0147
[2M−H]−
483.1818
120.0121
[M+K−2H]−
182.02
279.0467
579.2086
718.2777
782.1758
955.2764
1163.3058
0
20
40
60
80
100
200
400
600
800
1000
1200

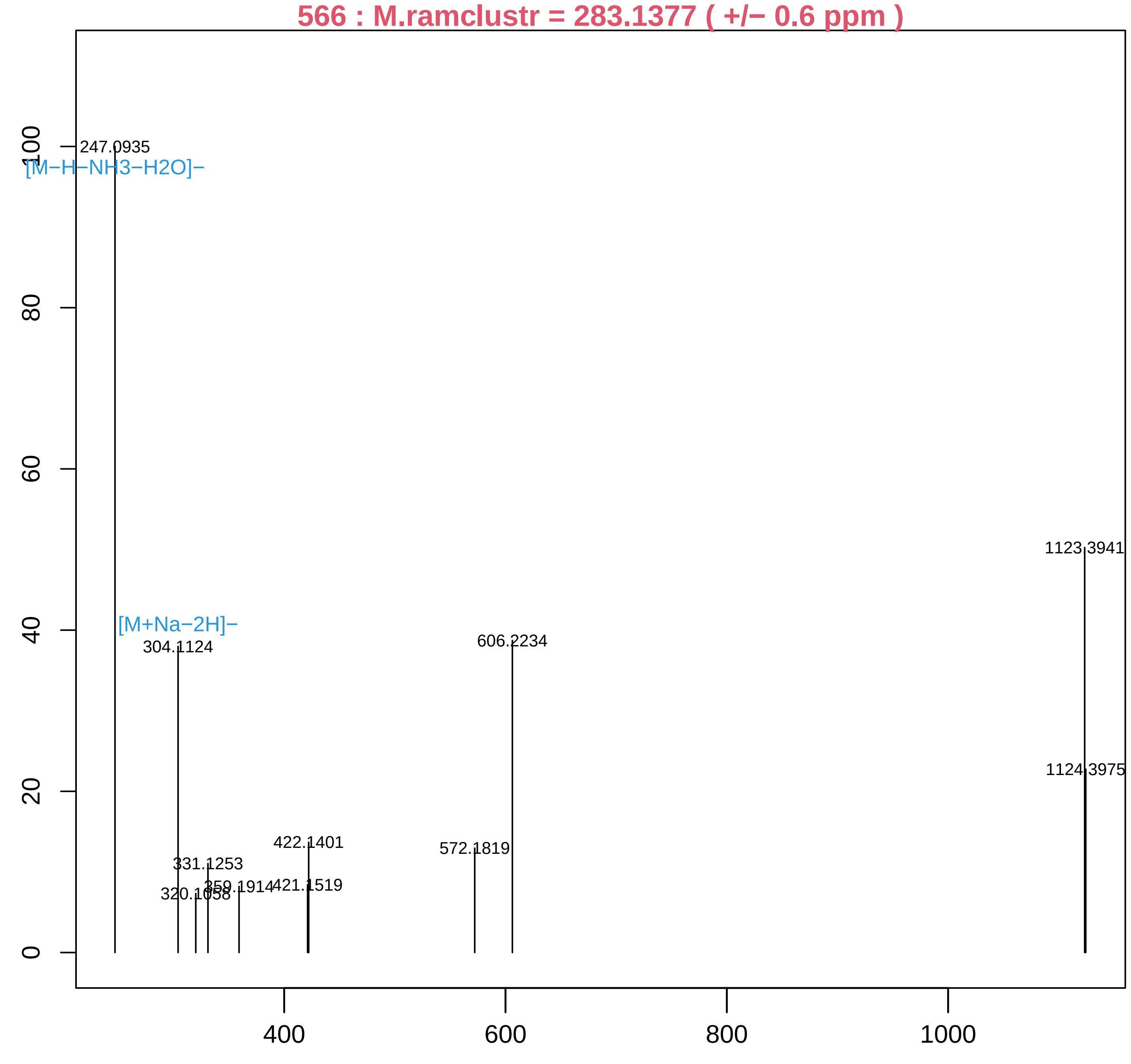

566 : M.ramclustr = 283.1377 (+/− 0.6 ppm)
247.0935
[M−H−NH3−H2O]−
[M+Na−2H]−
304.1124
606.2234
1123.3941
1124.3975
422.1401
331.1253
320.1058
359.1914
421.1519
572.1819
0
20
40
60
80
100
400
600
800
1000

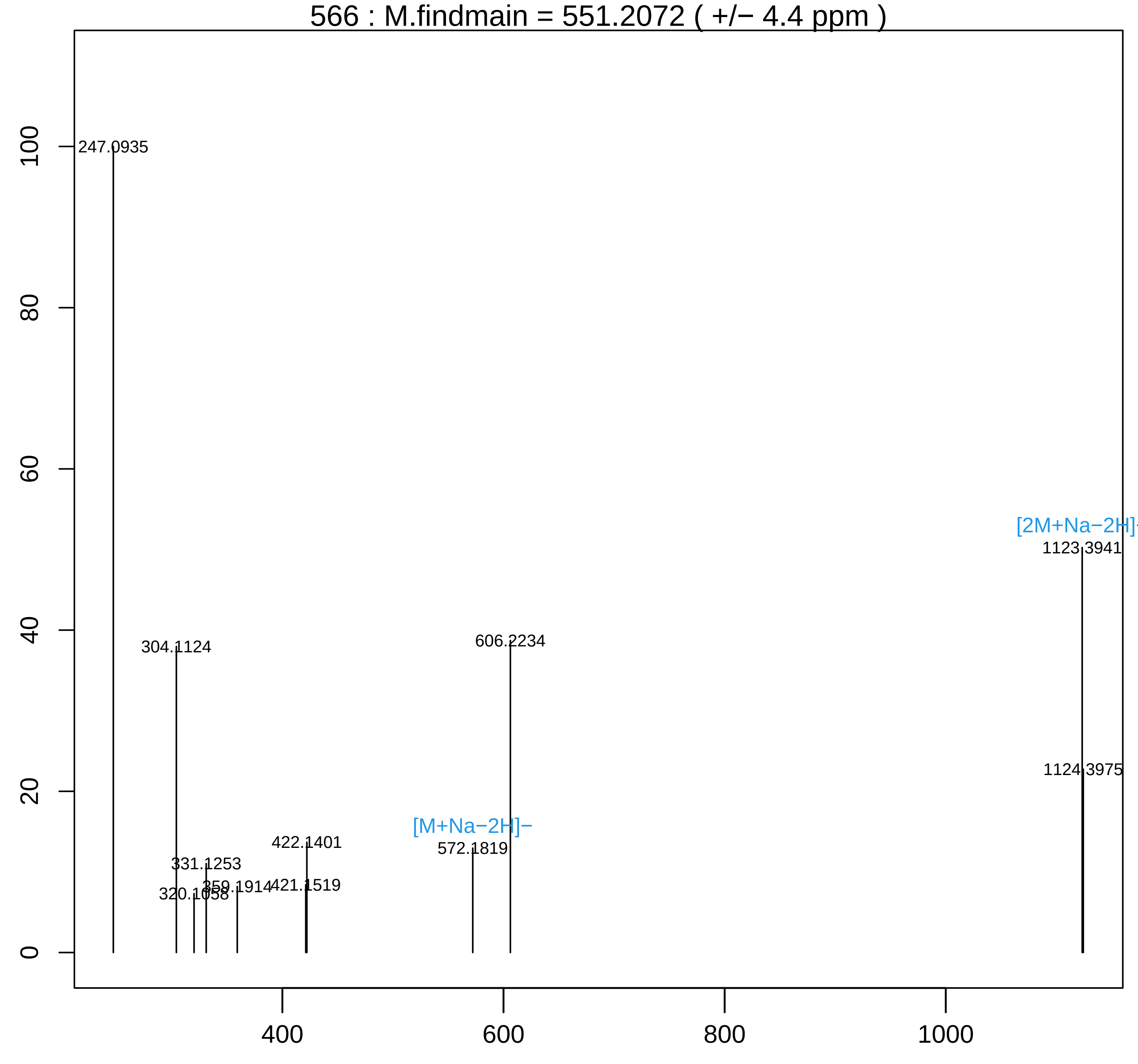

566 : M.findmain = 551.2072 (+/− 4.4 ppm)
247.0935
[2M+Na−2H]
1123.3941
304.1124
606.2234
1124.3975
[M+Na−2H]−
572.1819
422.1401
331.1253
320.1058
359.1914
421.1519
0
20
40
60
80
100
400
600
800
1000

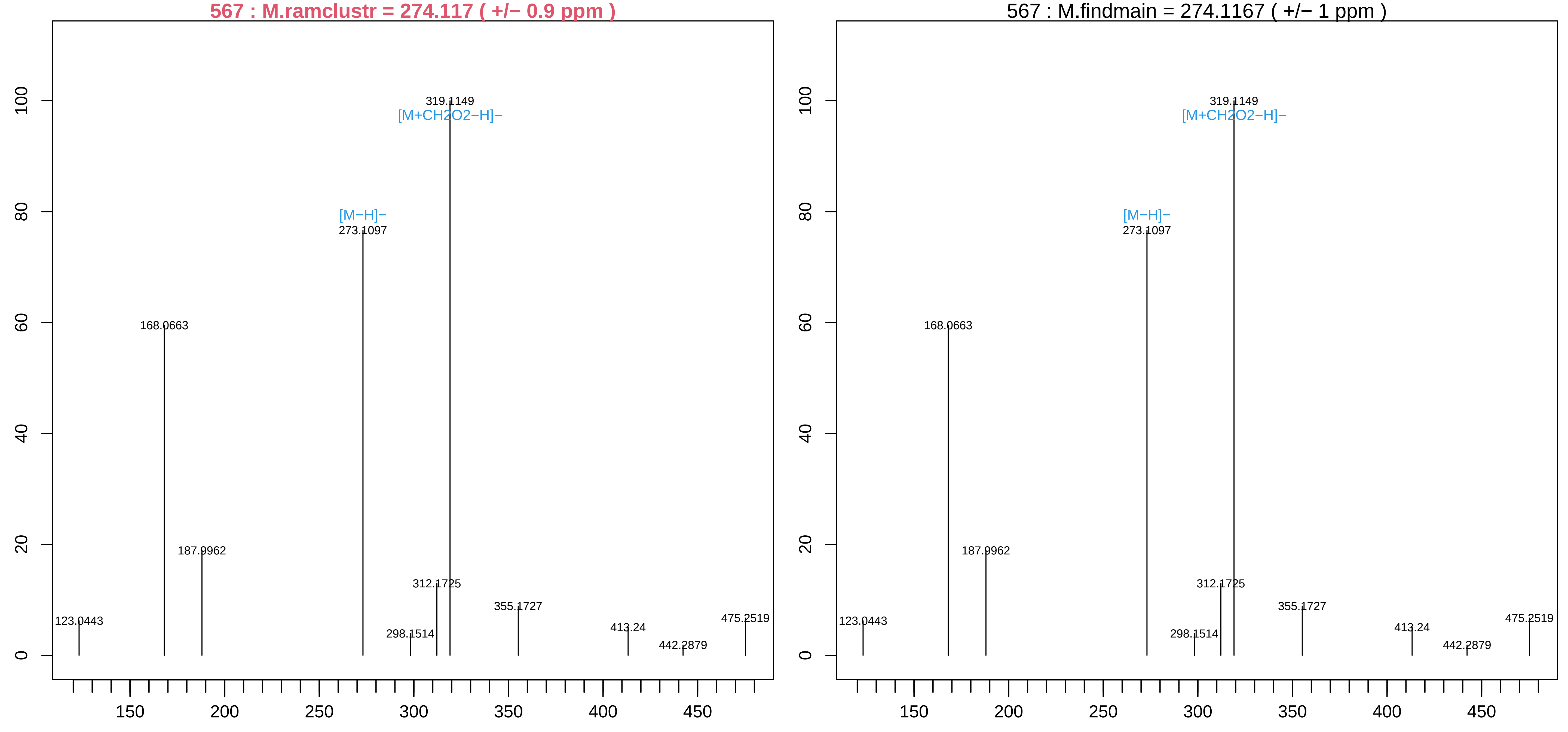
567 : M.ramclustr = 274.117 (+/− 0.9 ppm)
567 : M.findmain = 274.1167 (+/− 1 ppm)
319.1149
[M+CH2O2−H]−
[M−H]−
273.1097
168.0663
187.9962
312.1725
355.1727
123.0443
298.1514
413.24
442.2879
475.2519
0
20
40
60
80
100
150
200
250
300
350
400
450

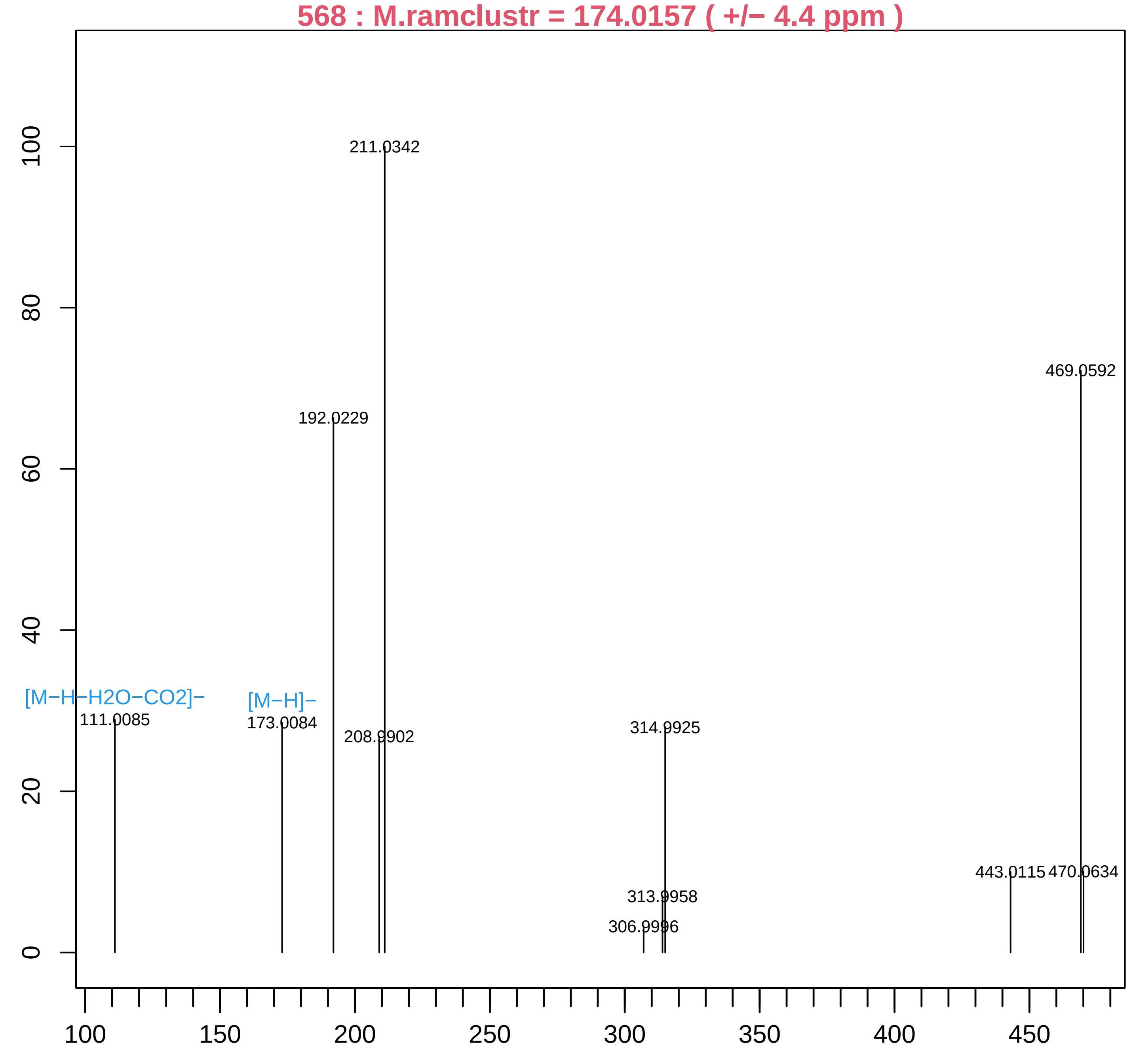
568 : M.ramclustr = 174.0157 (+/− 4.4 ppm)
[M−H−H2O−CO2]−
111.0085
[M−H]−
173.0084
192.0229
211.0342
208.9902
314.9925
313.9958
306.9996
443.0115
469.0592
470.0634
0
20
40
60
80
100
100
150
200
250
300
350
400
450

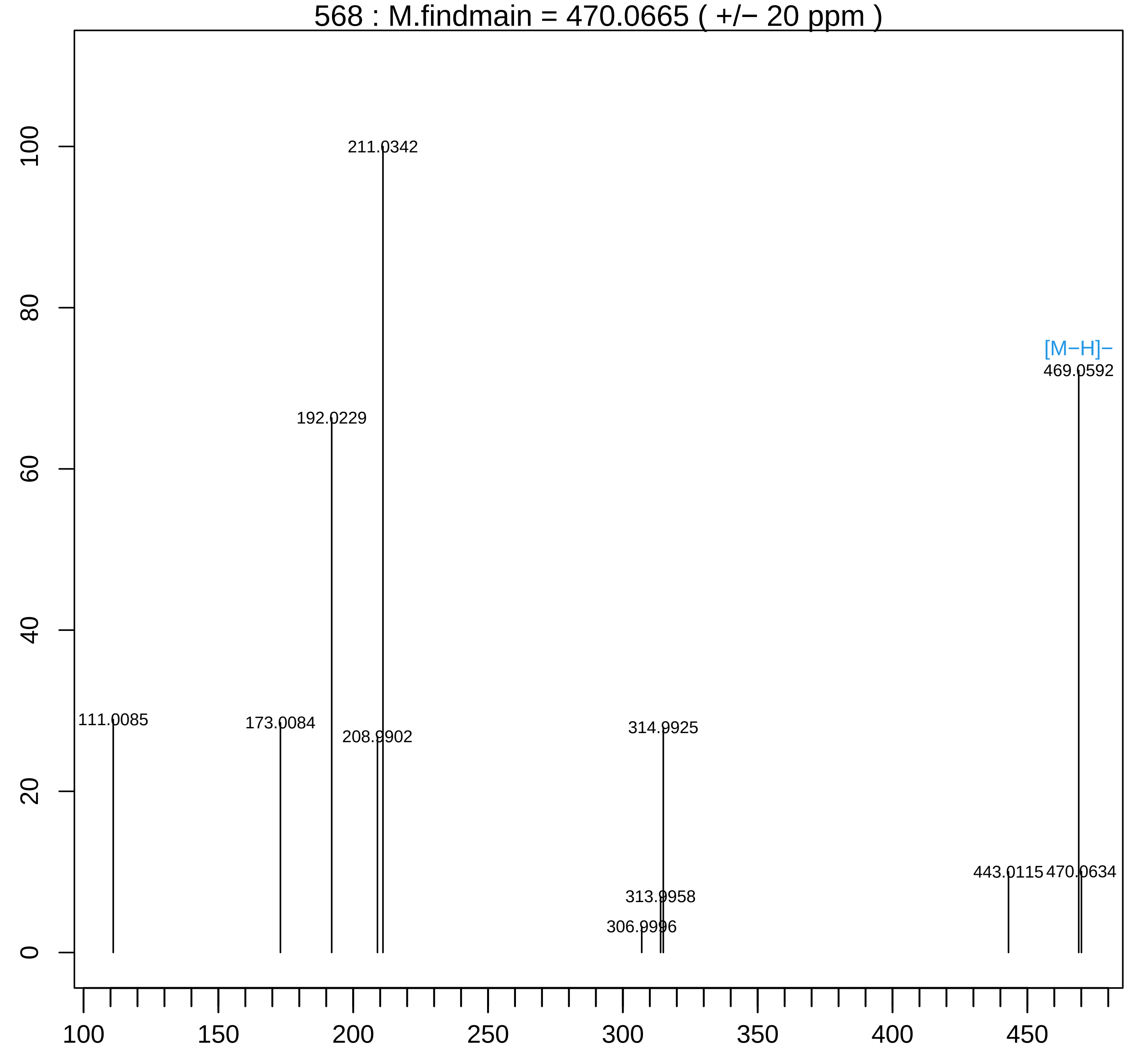
568 : M.findmain = 470.0665 (+/− 20 ppm)
111.0085
173.0084
192.0229
211.0342
208.9902
314.9925
313.9958
306.9996
443.0115
[M−H]−
469.0592
470.0634
0
20
40
60
80
100
100
150
200
250
300
350
400
450

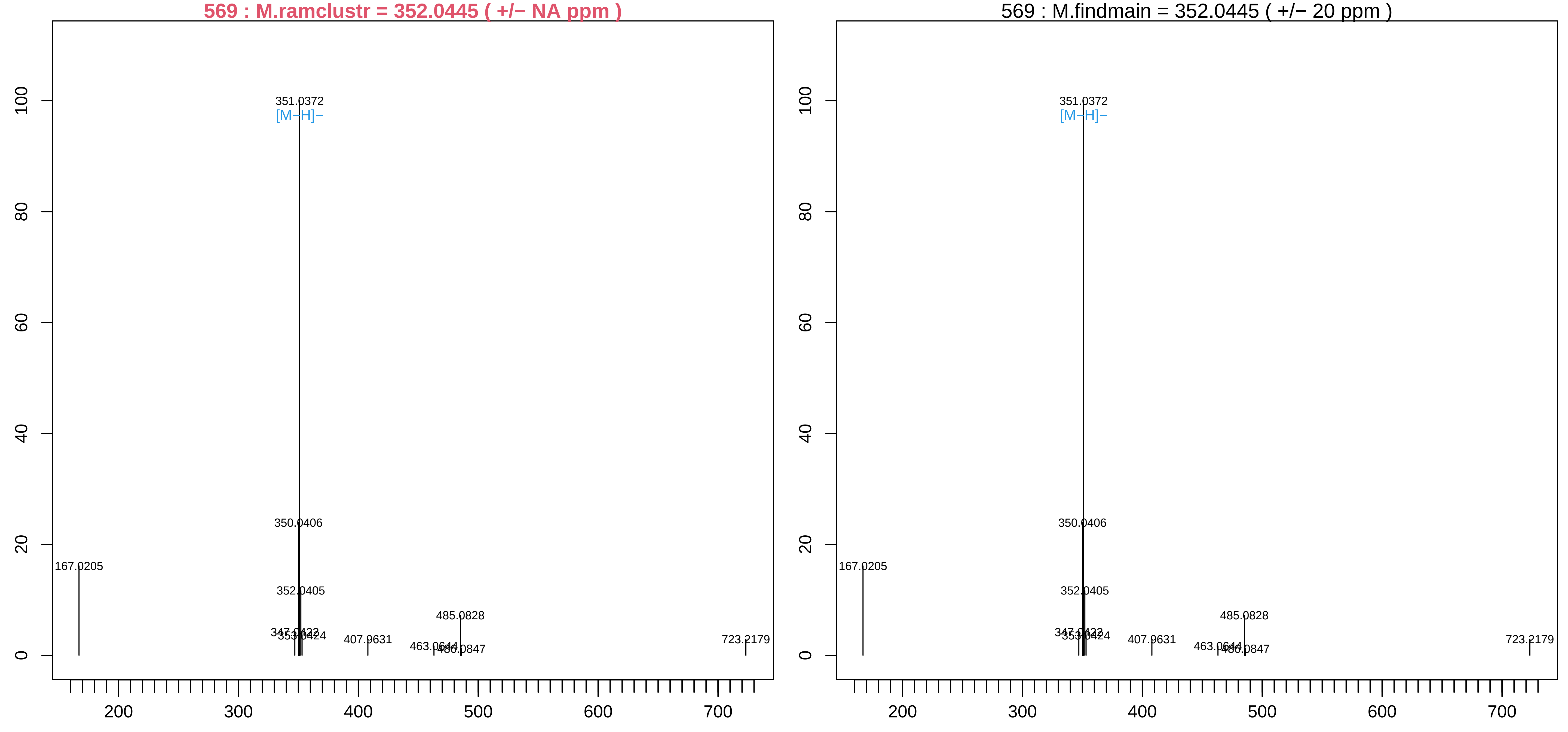
569 : M.ramclustr = 352.0445 (+/− NA ppm)
351.0372
[M−H]−
350.0406
167.0205
352.0405
485.0828
347.0422
353.0424
407.9631
463.0644
486.0847
723.2179
569 : M.findmain = 352.0445 (+/− 20 ppm)
351.0372
[M−H]−
350.0406
167.0205
352.0405
485.0828
347.0422
353.0424
407.9631
463.0644
486.0847
723.2179

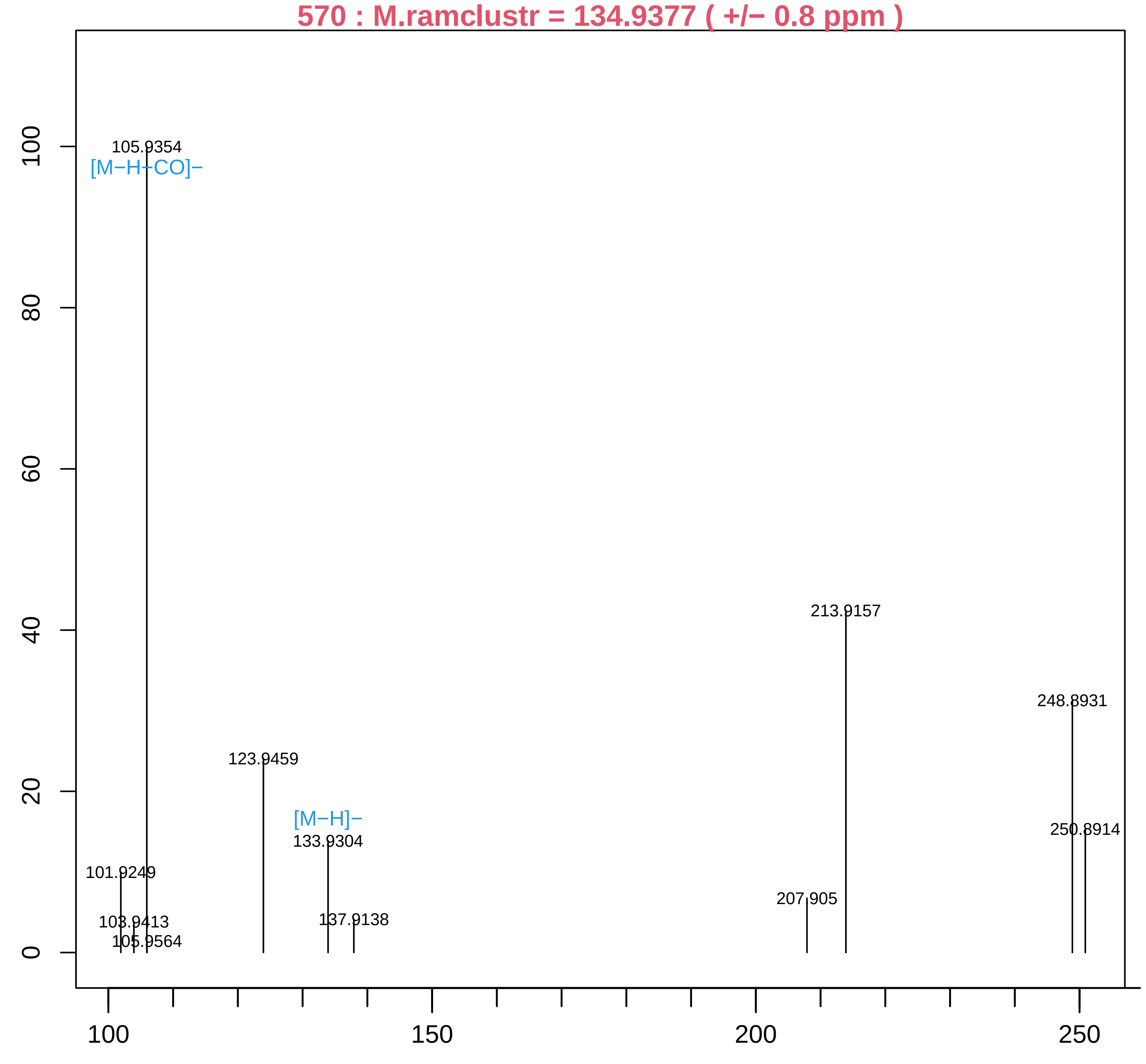
570 : M.ramclustr = 134.9377 (+/− 0.8 ppm)
105.9354
[M−H−CO]−
213.9157
248.8931
123.9459
[M−H]−
133.9304
250.8914
101.9249
207.905
103.9413
137.9138
105.9564
0
20
40
60
80
100
100
150
200
250

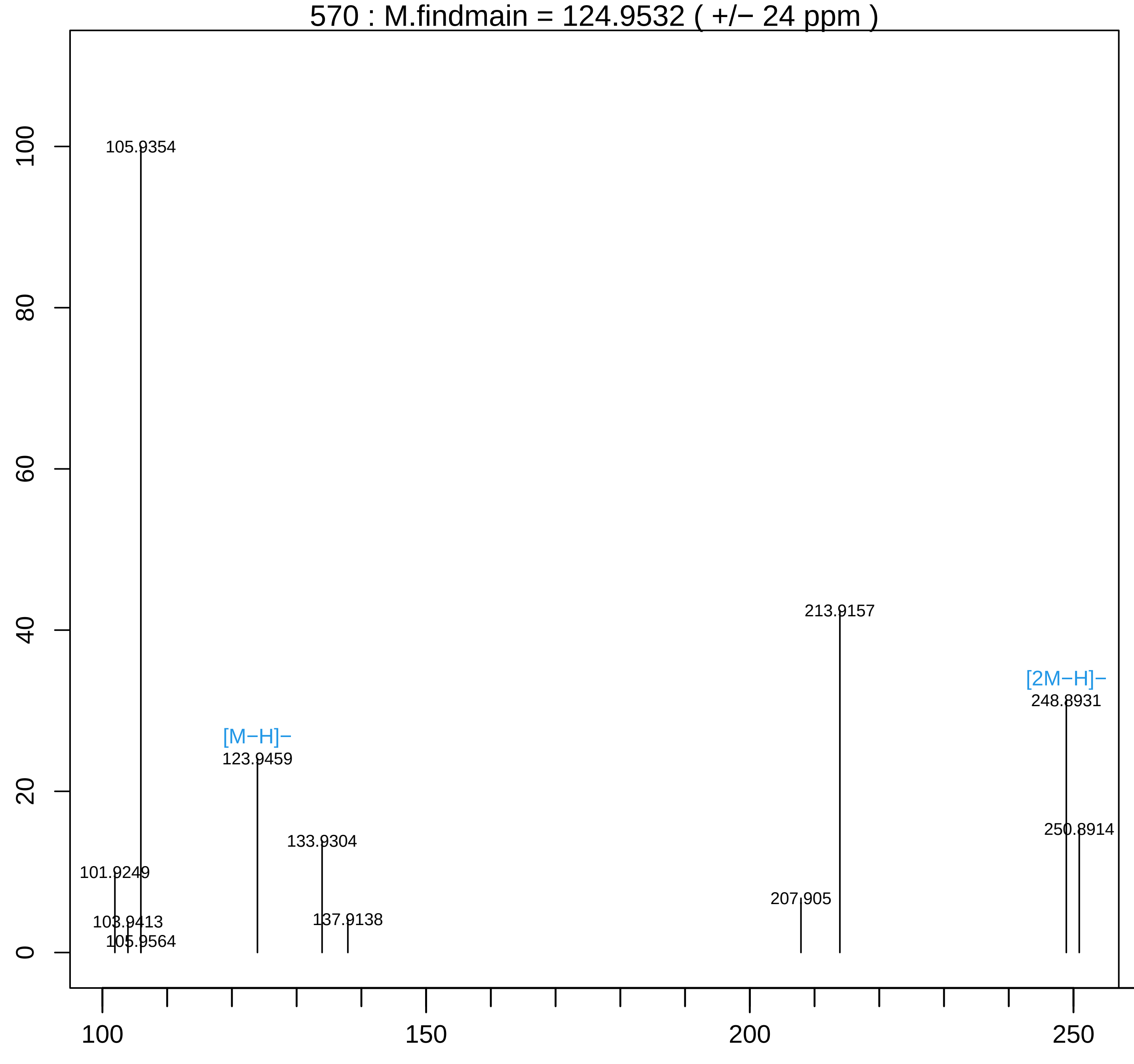
570 : M.findmain = 124.9532 (+/− 24 ppm)
105.9354
213.9157
[2M−H]−
248.8931
[M−H]−
123.9459
133.9304
250.8914
101.9249
207.905
103.9413
137.9138
105.9564
0
20
40
60
80
100
100
150
200
250

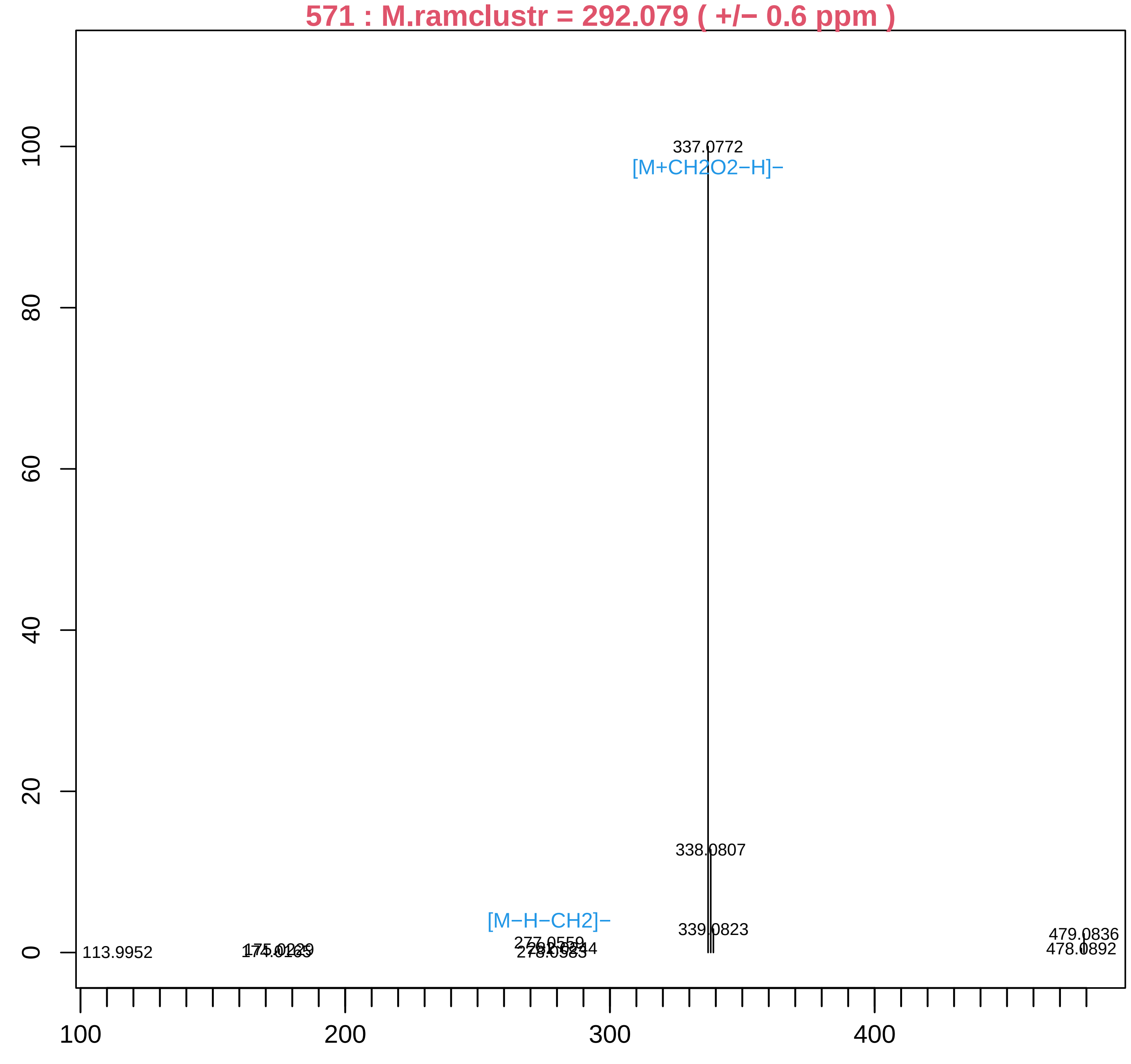

571 : M.ramclustr = 292.079 (+/− 0.6 ppm)
337.0772
[M+CH2O2−H]−
338.0807
339.0823
[M−H−CH2]−
113.9952
479.0836
478.0892
0
20
40
60
80
100
100
200
300
400

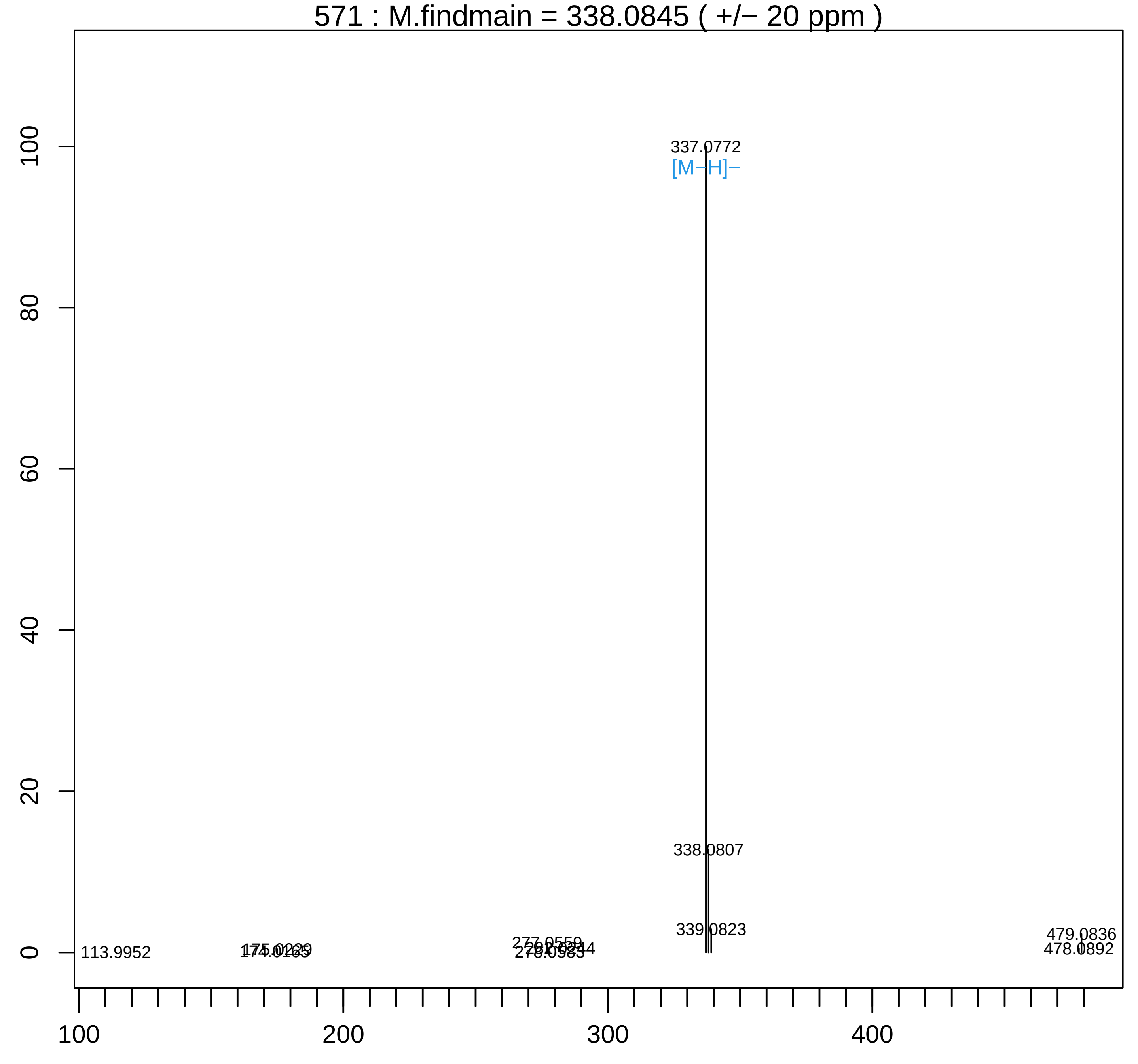

571 : M.findmain = 338.0845 (+/− 20 ppm)
337.0772
[M−H]−
338.0807
339.0823
113.9952
479.0836
478.0892
0
20
40
60
80
100
100
200
300
400

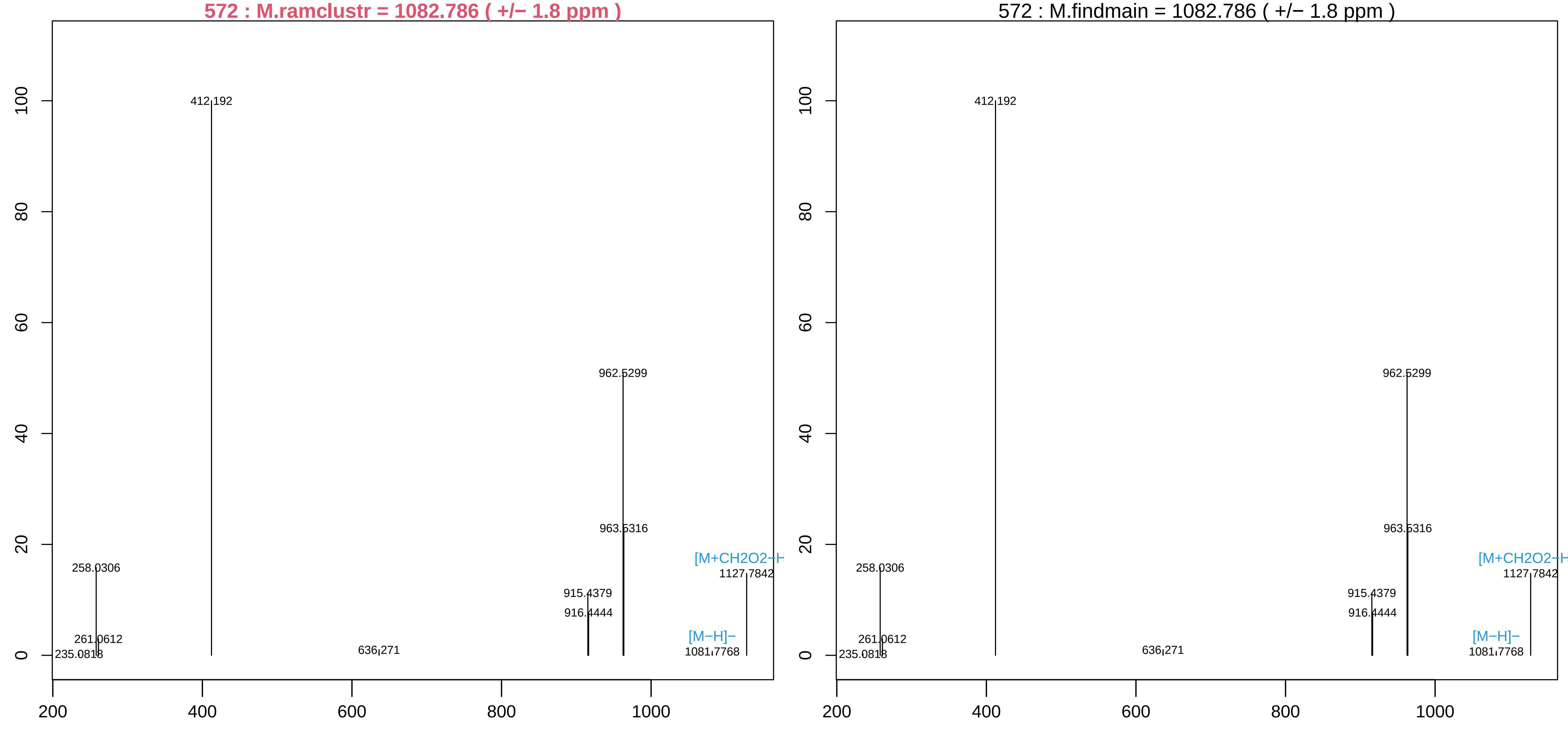

572 : M.ramclustr = 1082.786 (+/− 1.8 ppm)
572 : M.findmain = 1082.786 (+/− 1.8 ppm)
412.192
962.5299
963.5316
258.0306
915.4379
916.4444
261.0612
235.0818
636.271
[M+CH2O2−H
1127.7842
[M−H]−
1081.7768
0
20
40
60
80
100
200
400
600
800
1000

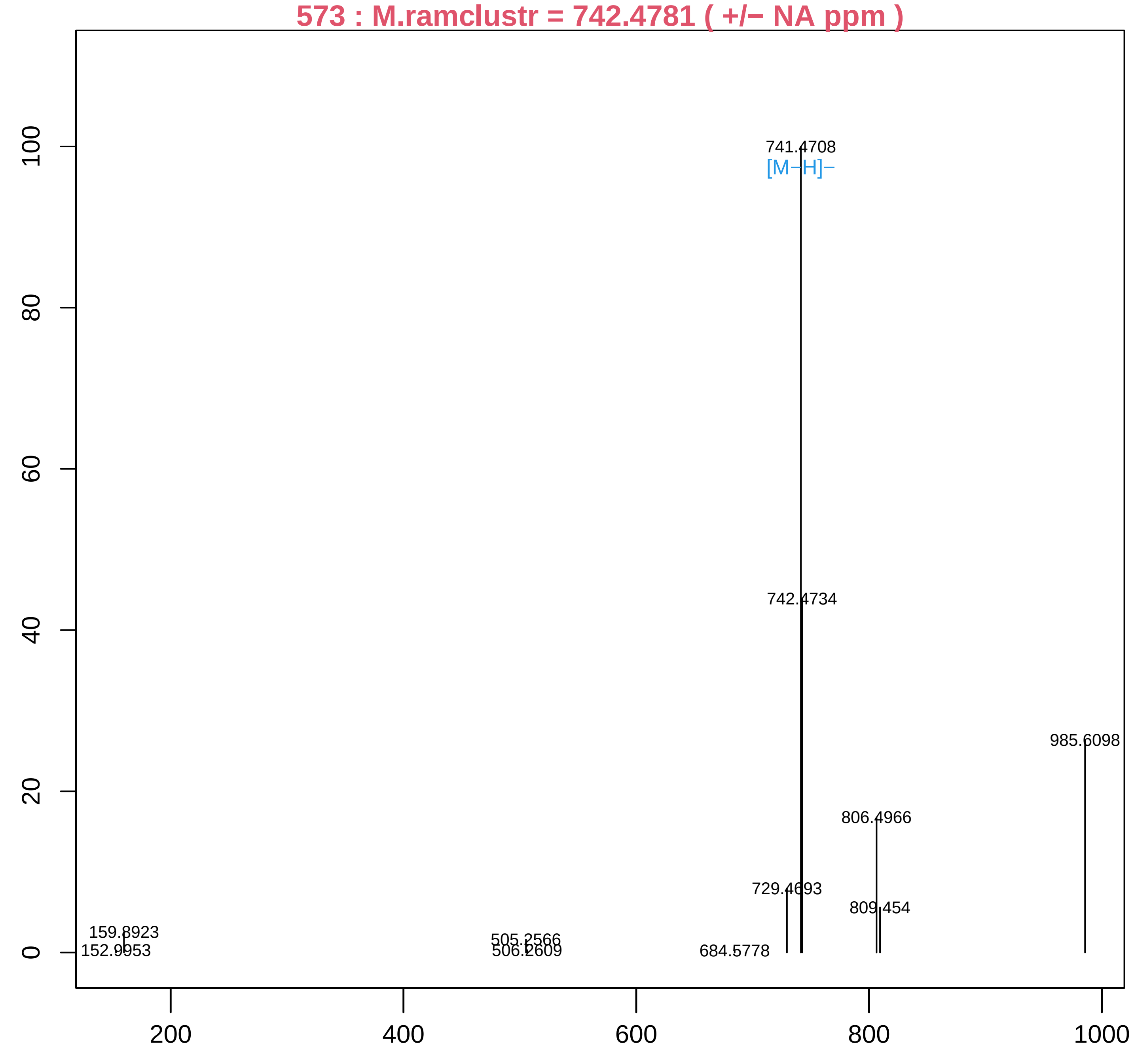
573 : M.ramclustr = 742.4781 (+/− NA ppm)
741.4708
[M−H]−
742.4734
985.6098
806.4966
729.4693
809.454
159.8923
152.9953
505.2566
506.2609
684.5778
0
20
40
60
80
100
200
400
600
800
1000

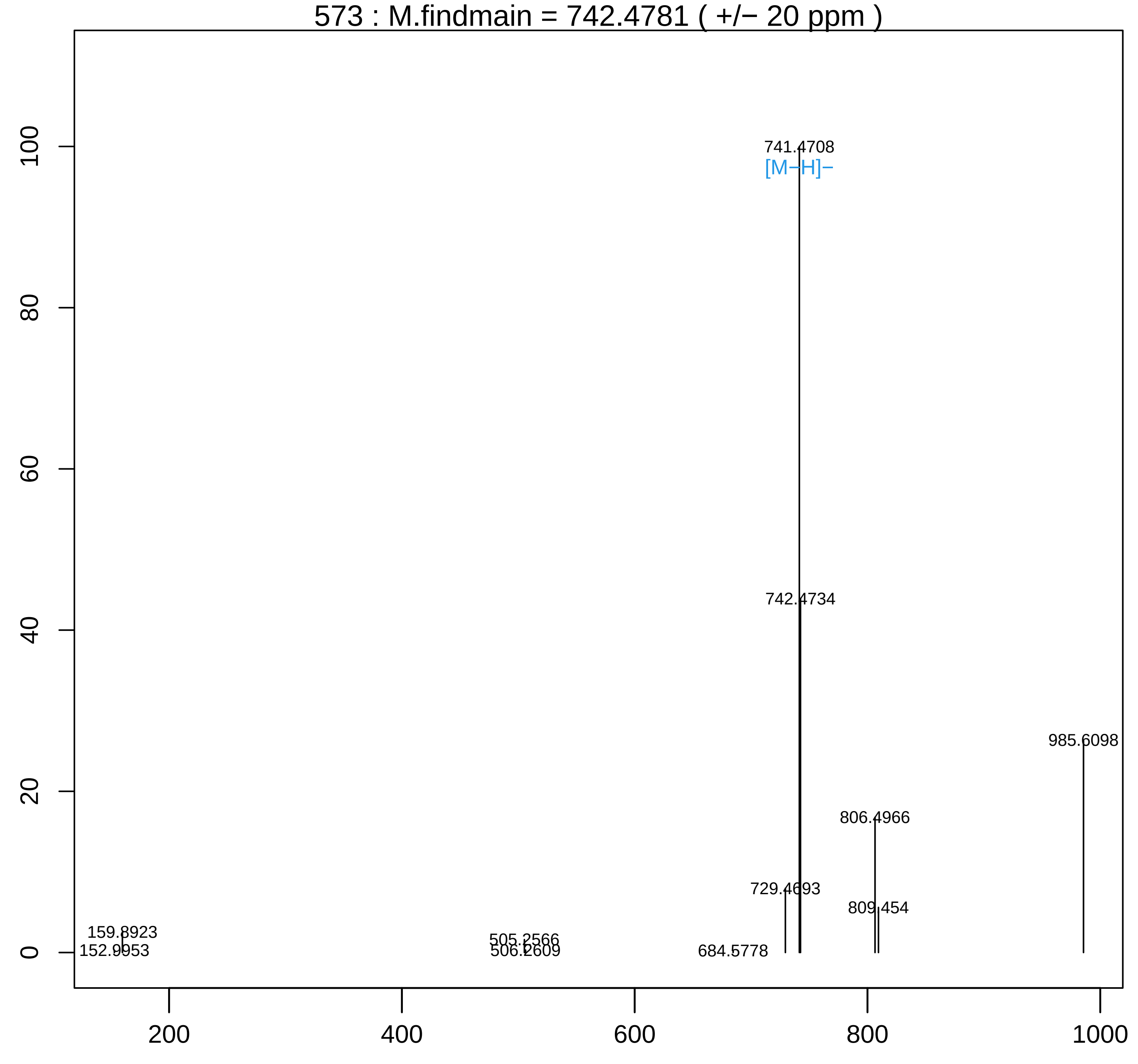
573 : M.findmain = 742.4781 (+/− 20 ppm)
741.4708
[M−H]−
742.4734
985.6098
806.4966
729.4693
809.454
159.8923
152.9953
505.2566
506.2609
684.5778
0
20
40
60
80
100
200
400
600
800
1000

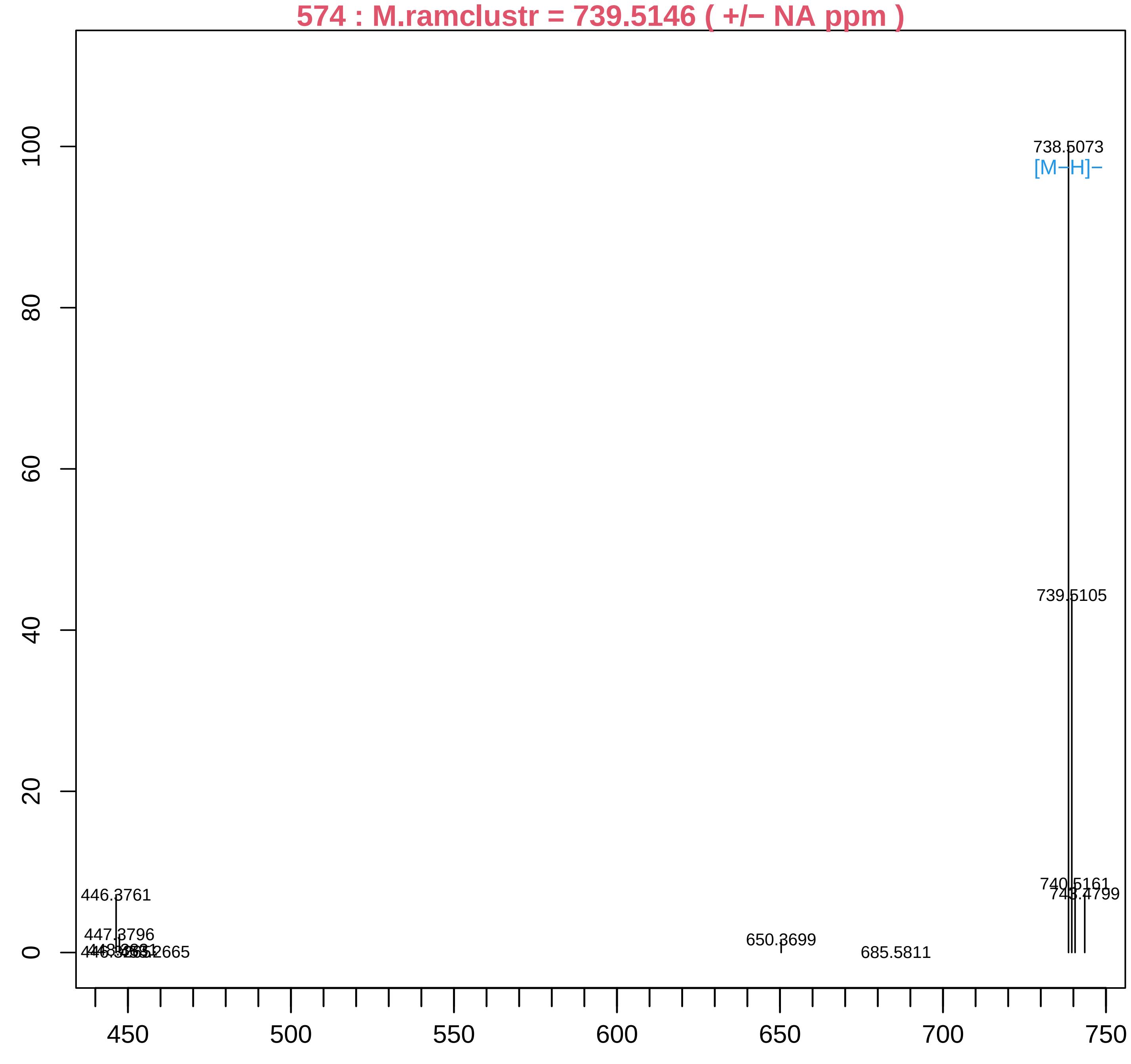
574 : M.ramclustr = 739.5146 (+/− NA ppm)
738.5073
[M−H]−
739.5105
740.5161
743.4799
446.3761
447.3796
650.3699
685.5811
0
20
40
60
80
100
450
500
550
600
650
700
750

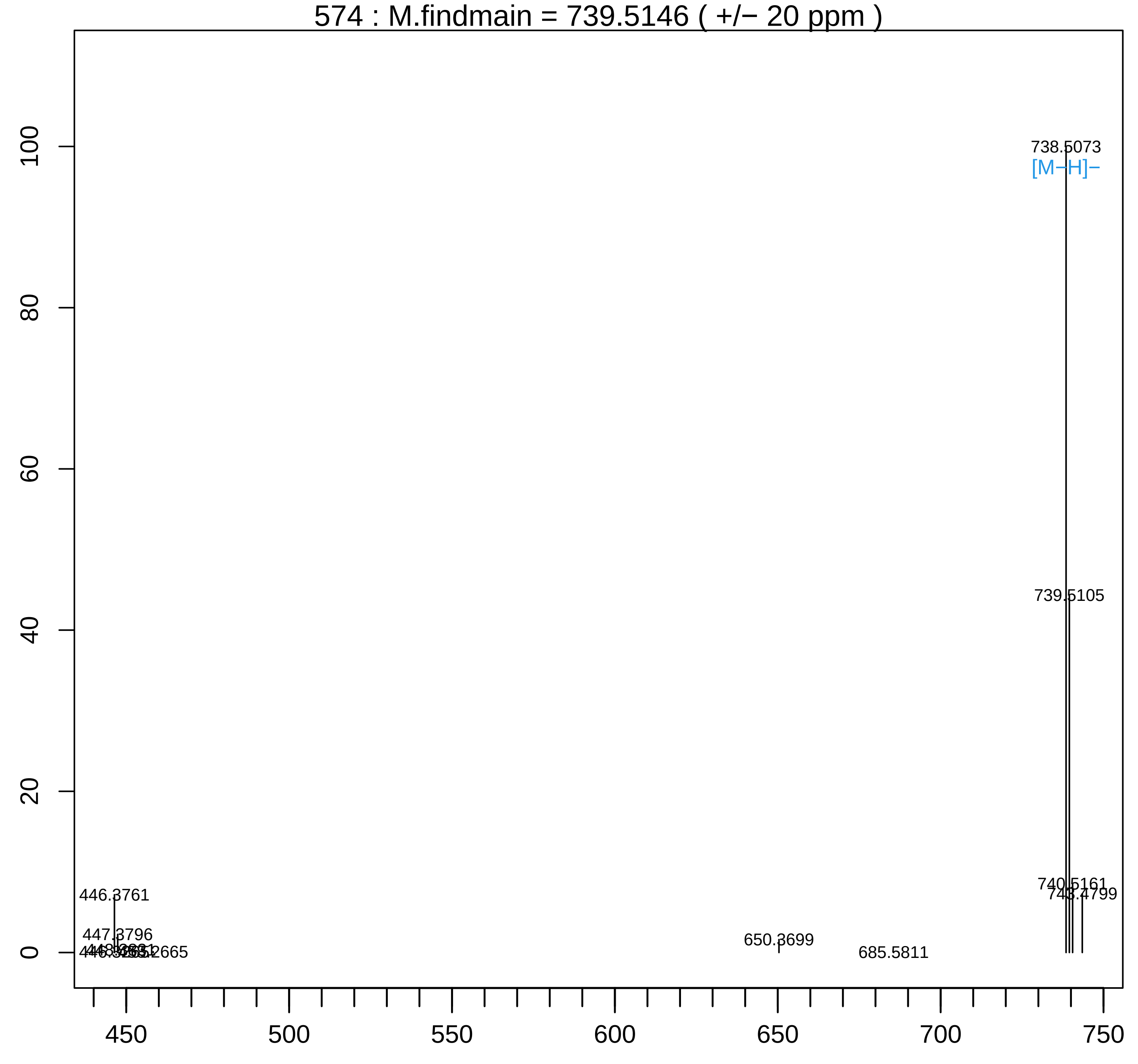
574 : M.findmain = 739.5146 (+/− 20 ppm)
738.5073
[M−H]−
739.5105
740.5161
743.4799
446.3761
447.3796
650.3699
685.5811
0
20
40
60
80
100
450
500
550
600
650
700
750

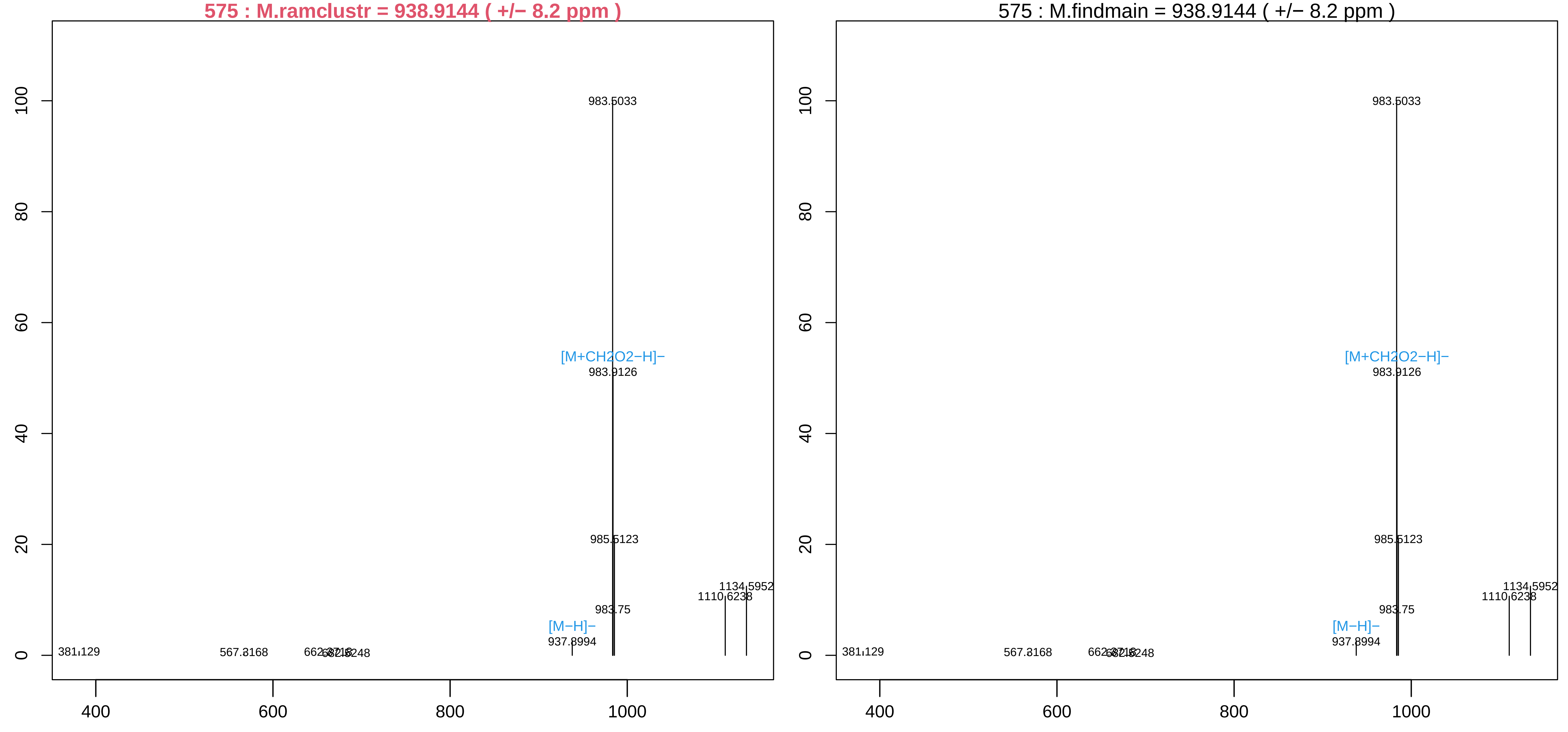
575 : M.ramclustr = 938.9144 (+/− 8.2 ppm)
983.5033
[M+CH2O2−H]−
983.9126
985.5123
1134.5952
1110.6238
983.75
[M−H]−
937.8994
381.129
567.8168
662.3718
682.8248
575 : M.findmain = 938.9144 (+/− 8.2 ppm)
983.5033
[M+CH2O2−H]−
983.9126
985.5123
1134.5952
1110.6238
983.75
[M−H]−
937.8994
381.129
567.8168
662.3718
682.8248

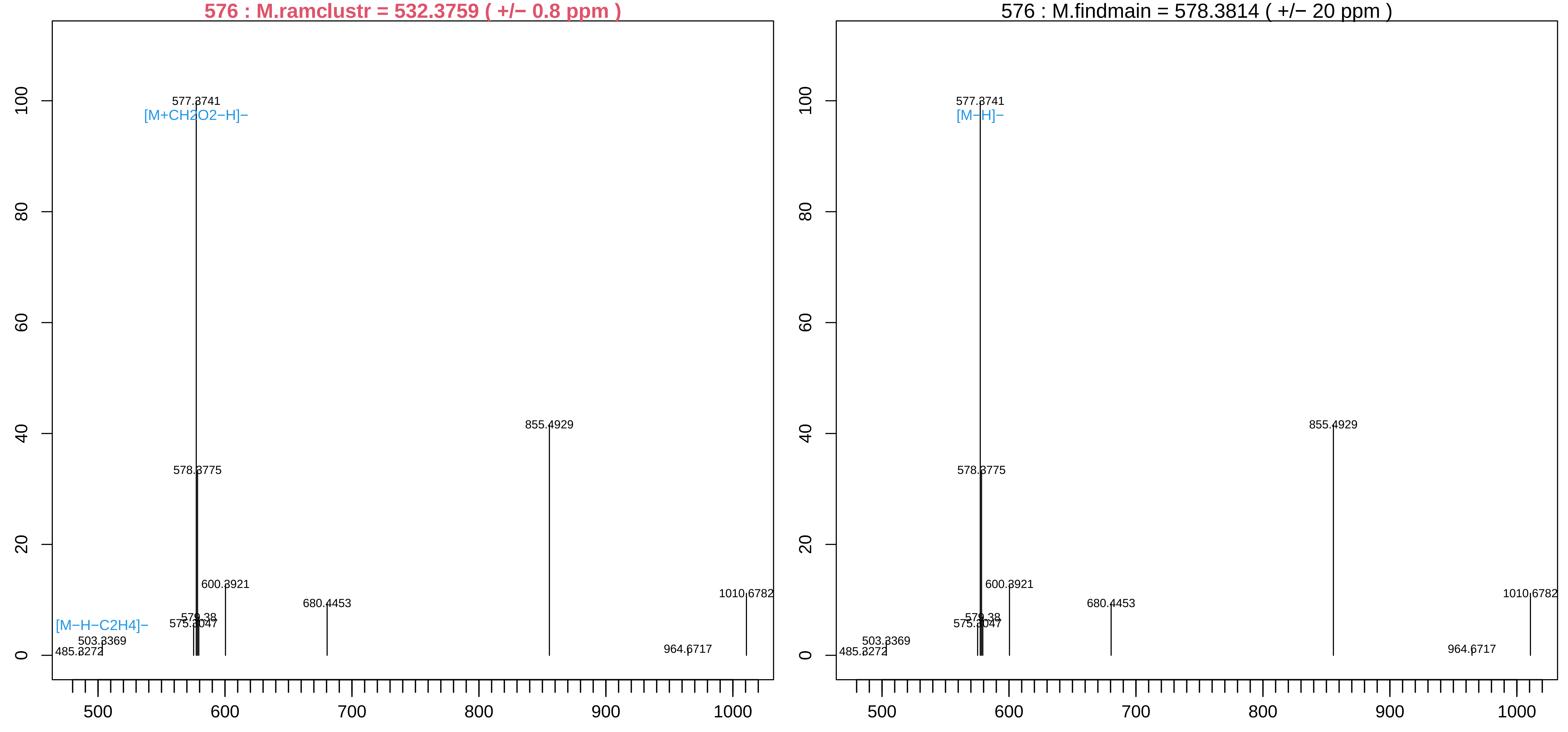
576 : M.ramclustr = 532.3759 (+/− 0.8 ppm)
577.3741
[M+CH2O2−H]−
855.4929
578.3775
600.3921
1010.6782
680.4453
579.38
[M−H−C2H4]−
575.3047
503.3369
964.6717
485.3272
576 : M.findmain = 578.3814 (+/− 20 ppm)
577.3741
[M−H]−
855.4929
578.3775
600.3921
1010.6782
680.4453
579.38
575.3047
503.3369
964.6717
485.3272
0
20
40
60
80
100
500
600
700
800
900
1000

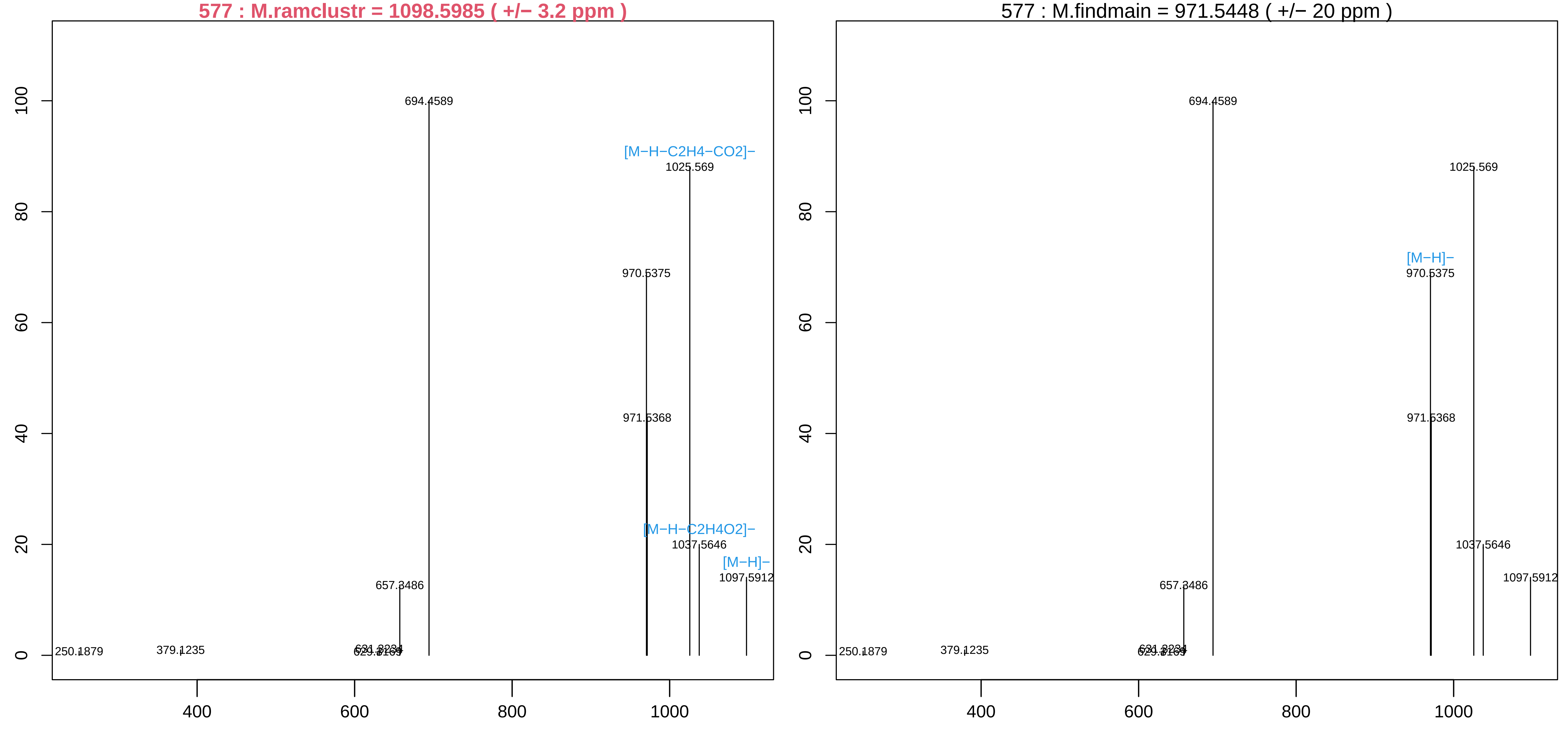

577 : M.ramclustr = 1098.5985 (+/− 3.2 ppm)
577 : M.findmain = 971.5448 (+/− 20 ppm)
[M−H−C2H4−CO2]−
[M−H−C2H4O2]−
[M−H]−
694.4589
1025.569
970.5375
971.5368
1037.5646
1097.5912
657.3486
250.1879
379.1235
0
20
40
60
80
100
400
600
800
1000

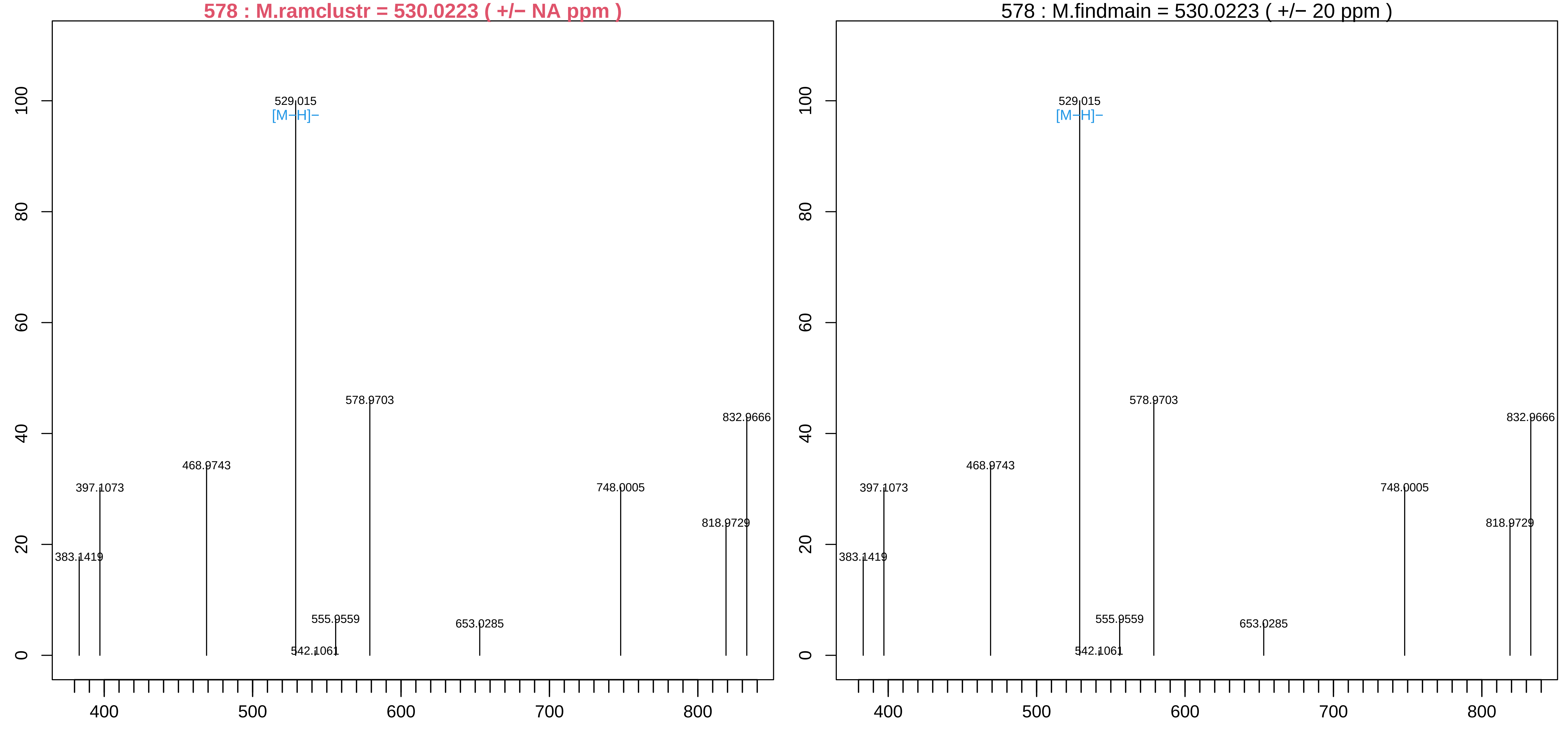

578 : M.ramclustr = 530.0223 (+/− NA ppm)
578 : M.findmain = 530.0223 (+/− 20 ppm)
529.015
[M−H]−
578.9703
832.9666
468.9743
397.1073
748.0005
818.9729
383.1419
555.9559
653.0285
542.1061
0
20
40
60
80
100
400
500
600
700
800

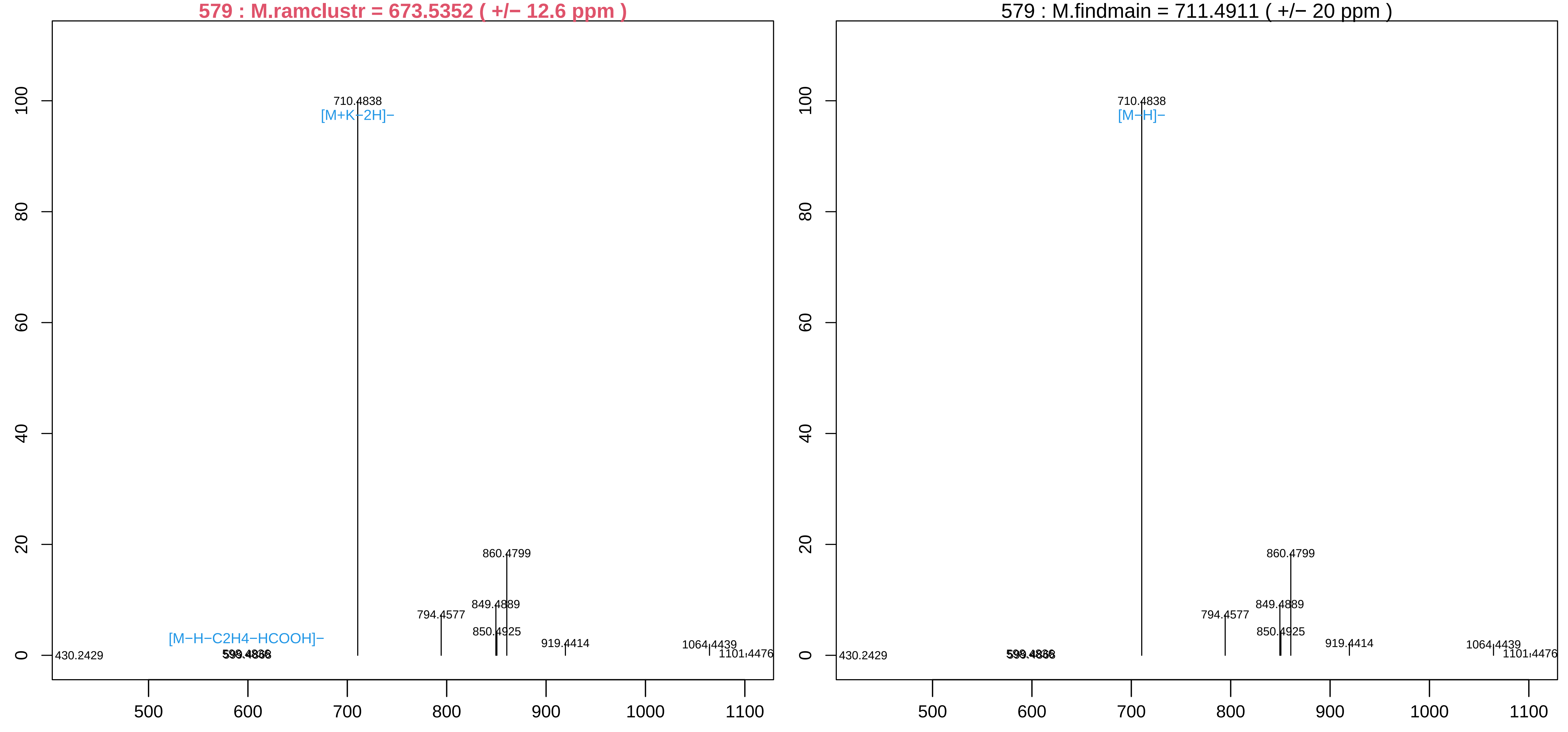

579 : M.ramclustr = 673.5352 (+/− 12.6 ppm)
710.4838
[M+K−2H]−
860.4799
849.4889
794.4577
850.4925
919.4414
1064.4439
1101.4476
430.2429
[M−H−C2H4−HCOOH]−
579 : M.findmain = 711.4911 (+/− 20 ppm)
[M−H]−

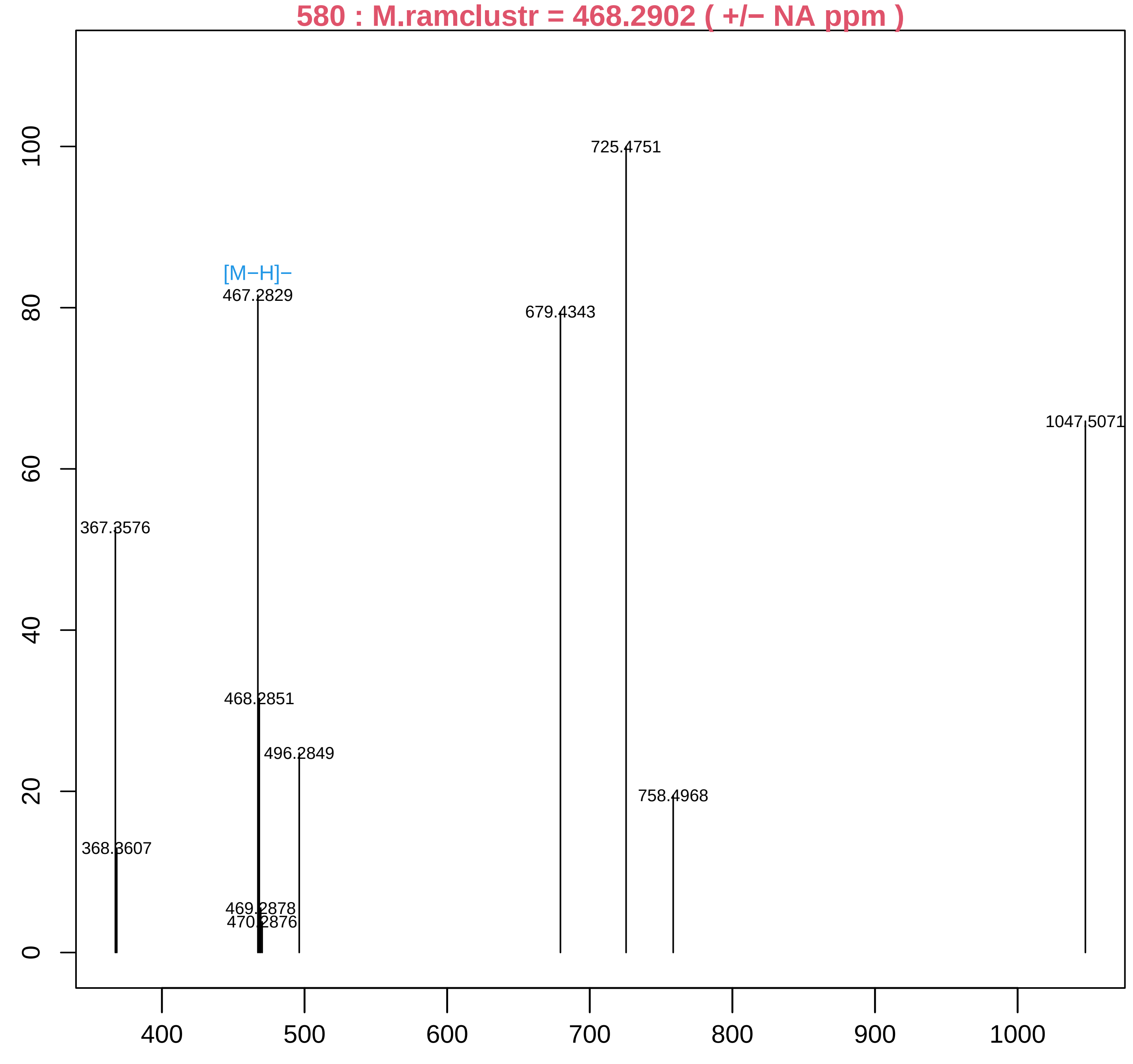

580 : M.ramclustr = 468.2902 (+/− NA ppm)
[M−H]−
467.2829
725.4751
679.4343
1047.5071
367.3576
468.2851
496.2849
758.4968
368.3607
469.2878
470.2876
0
20
40
60
80
100
400
500
600
700
800
900
1000

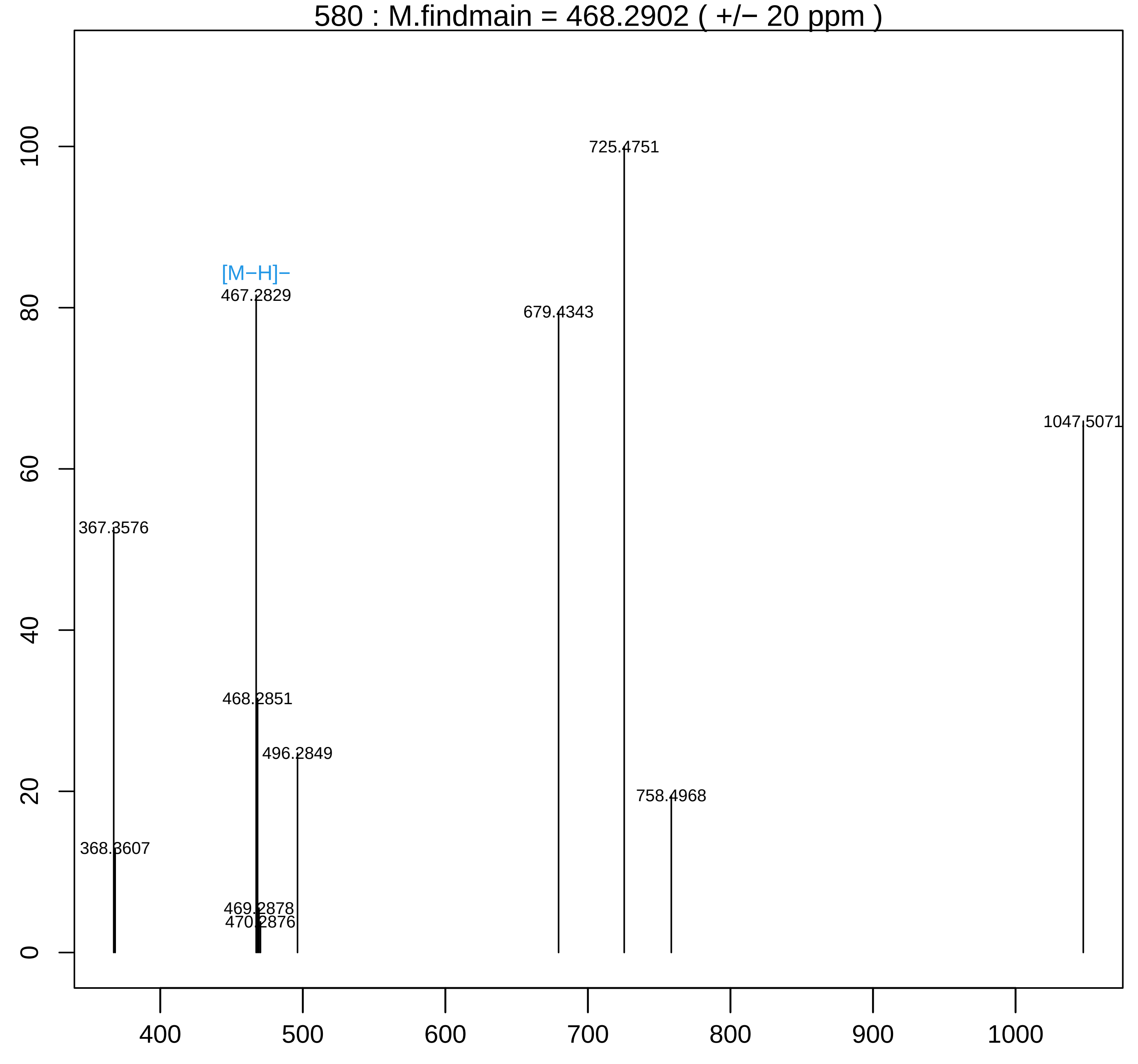

580 : M.findmain = 468.2902 (+/− 20 ppm)
[M−H]−
467.2829
725.4751
679.4343
1047.5071
367.3576
468.2851
496.2849
758.4968
368.3607
469.2878
470.2876
0
20
40
60
80
100
400
500
600
700
800
900
1000

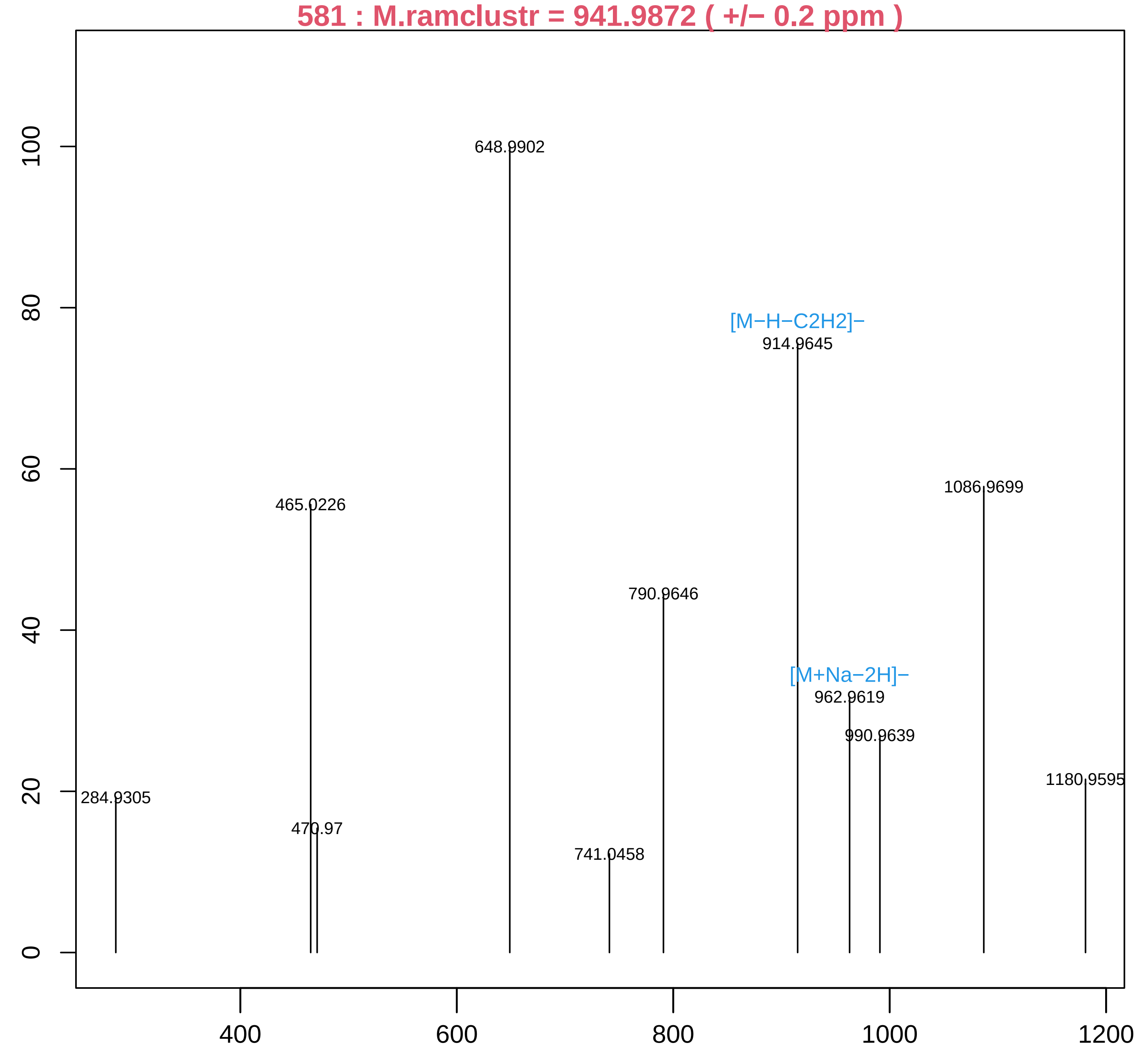
581 : M.ramclustr = 941.9872 (+/− 0.2 ppm)
[M−H−C2H2]−
[M+Na−2H]−
284.9305
465.0226
470.97
648.9902
741.0458
790.9646
914.9645
962.9619
990.9639
1086.9699
1180.9595
0
20
40
60
80
100
400
600
800
1000
1200

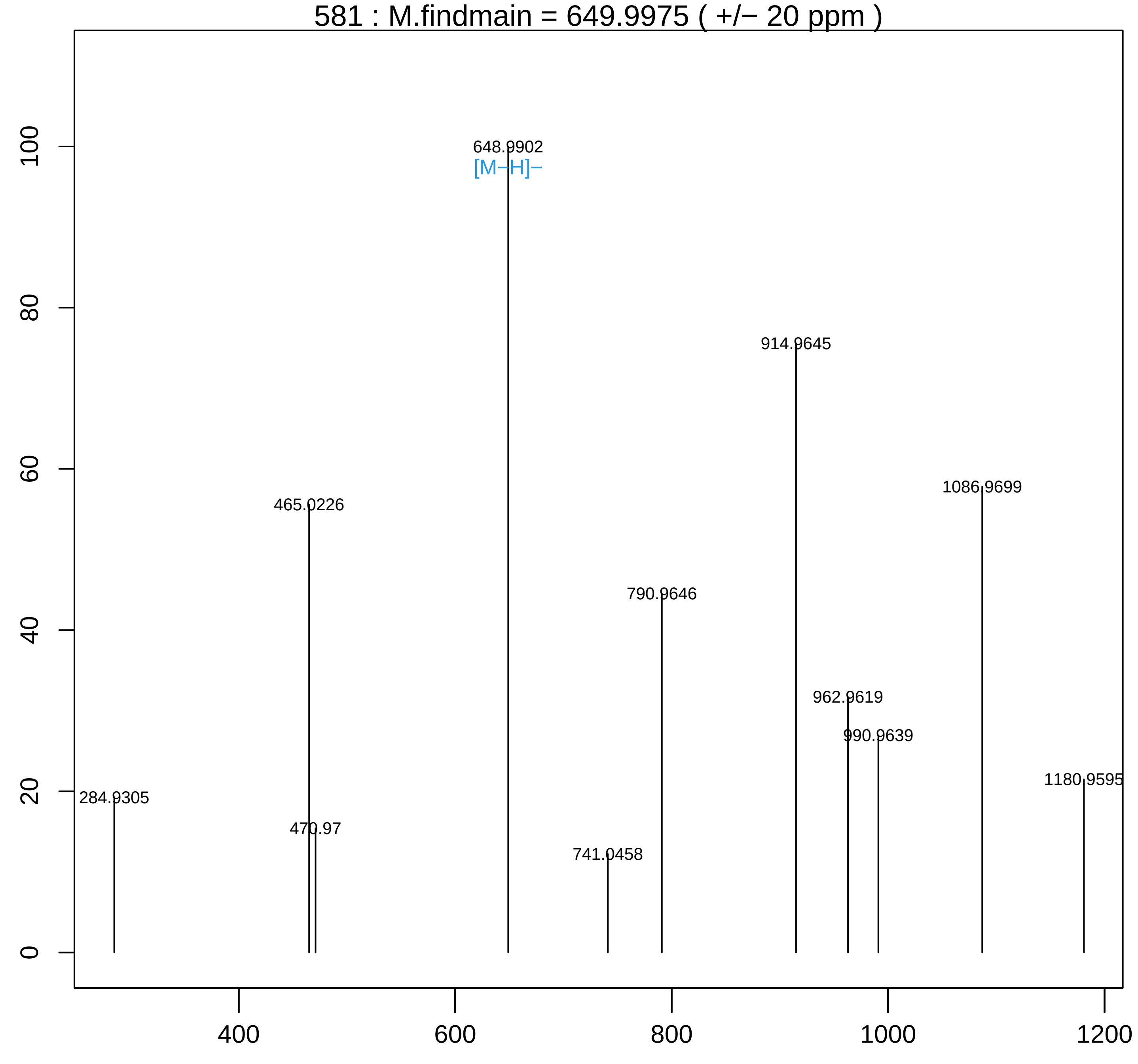
581 : M.findmain = 649.9975 (+/− 20 ppm)
[M−H]−
284.9305
465.0226
470.97
648.9902
741.0458
790.9646
914.9645
962.9619
990.9639
1086.9699
1180.9595
0
20
40
60
80
100
400
600
800
1000
1200

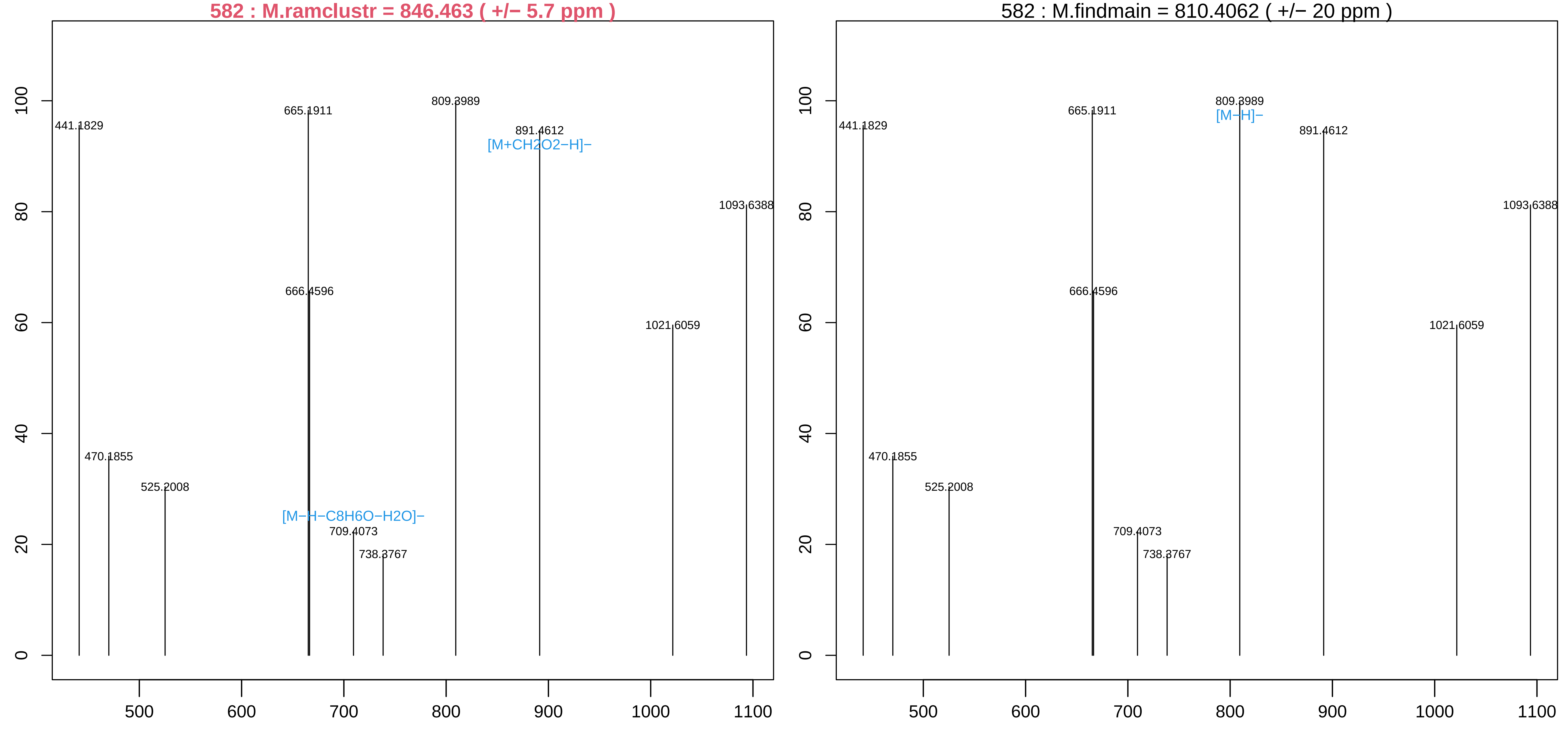

582 : M.ramclustr = 846.463 (+/− 5.7 ppm)
441.1829
470.1855
525.2008
665.1911
666.4596
[M−H−C8H6O−H2O]−
709.4073
738.3767
809.3989
891.4612
[M+CH2O2−H]−
1021.6059
1093.6388
582 : M.findmain = 810.4062 (+/− 20 ppm)
441.1829
470.1855
525.2008
665.1911
666.4596
709.4073
738.3767
809.3989
[M−H]−
891.4612
1021.6059
1093.6388
0
20
40
60
80
100
500
600
700
800
900
1000
1100

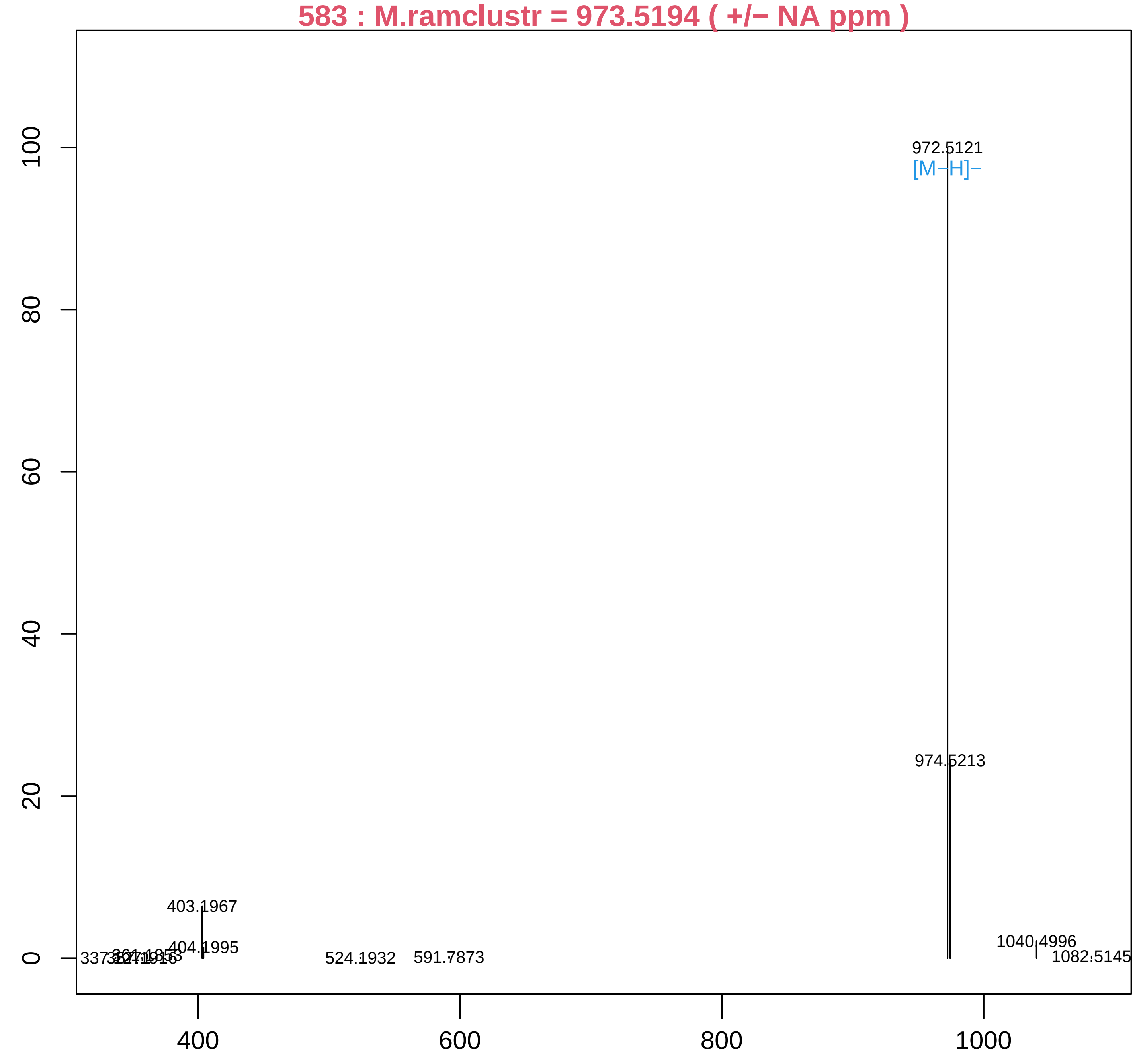
583 : M.ramclustr = 973.5194 (+/− NA ppm)
972.5121
[M−H]−
974.5213
403.1967
404.1995
524.1932
591.7873
1040.4996
1082.5145
0
20
40
60
80
100
400
600
800
1000

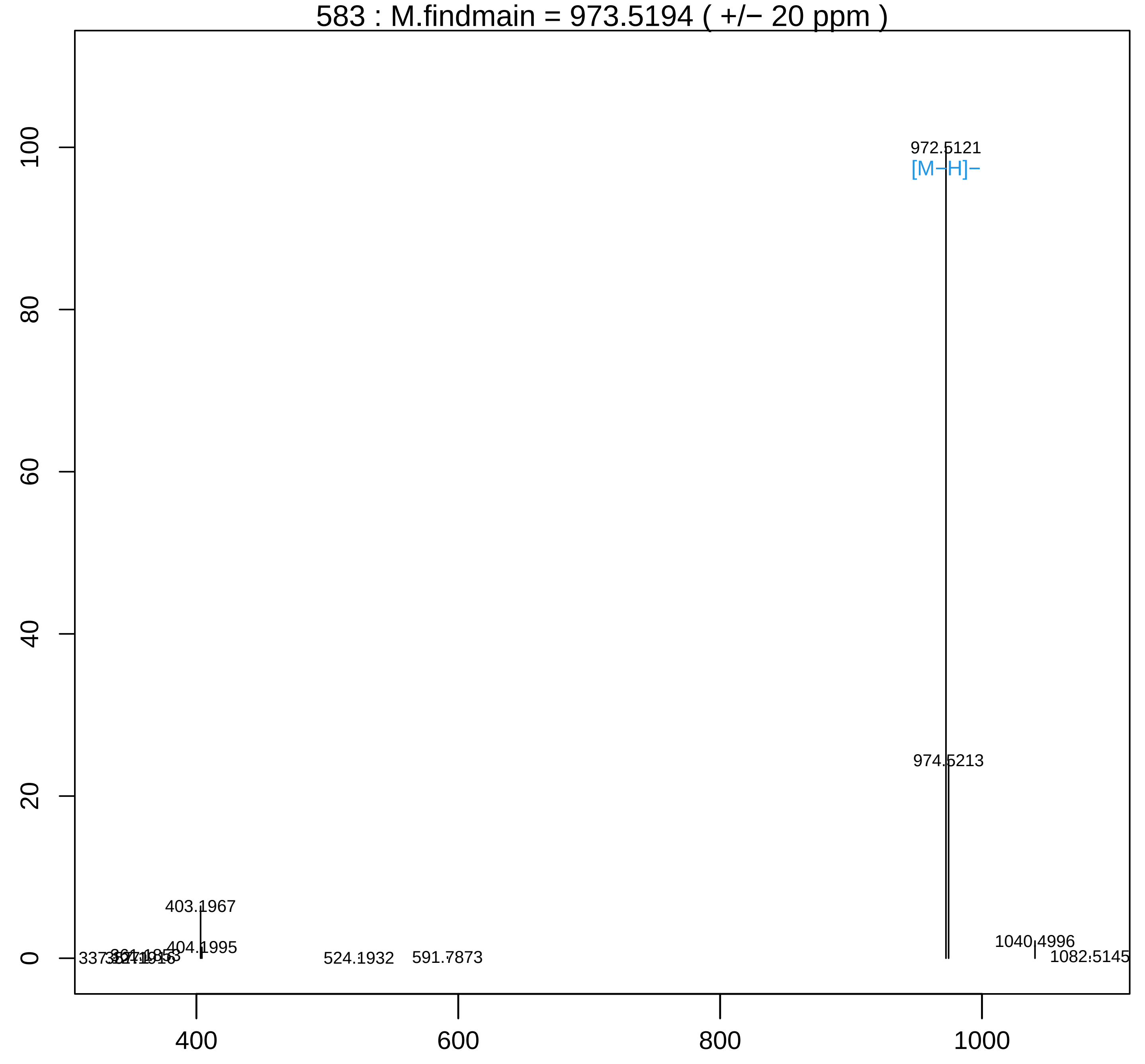
583 : M.findmain = 973.5194 (+/− 20 ppm)
972.5121
[M−H]−
974.5213
403.1967
404.1995
524.1932
591.7873
1040.4996
1082.5145
0
20
40
60
80
100
400
600
800
1000

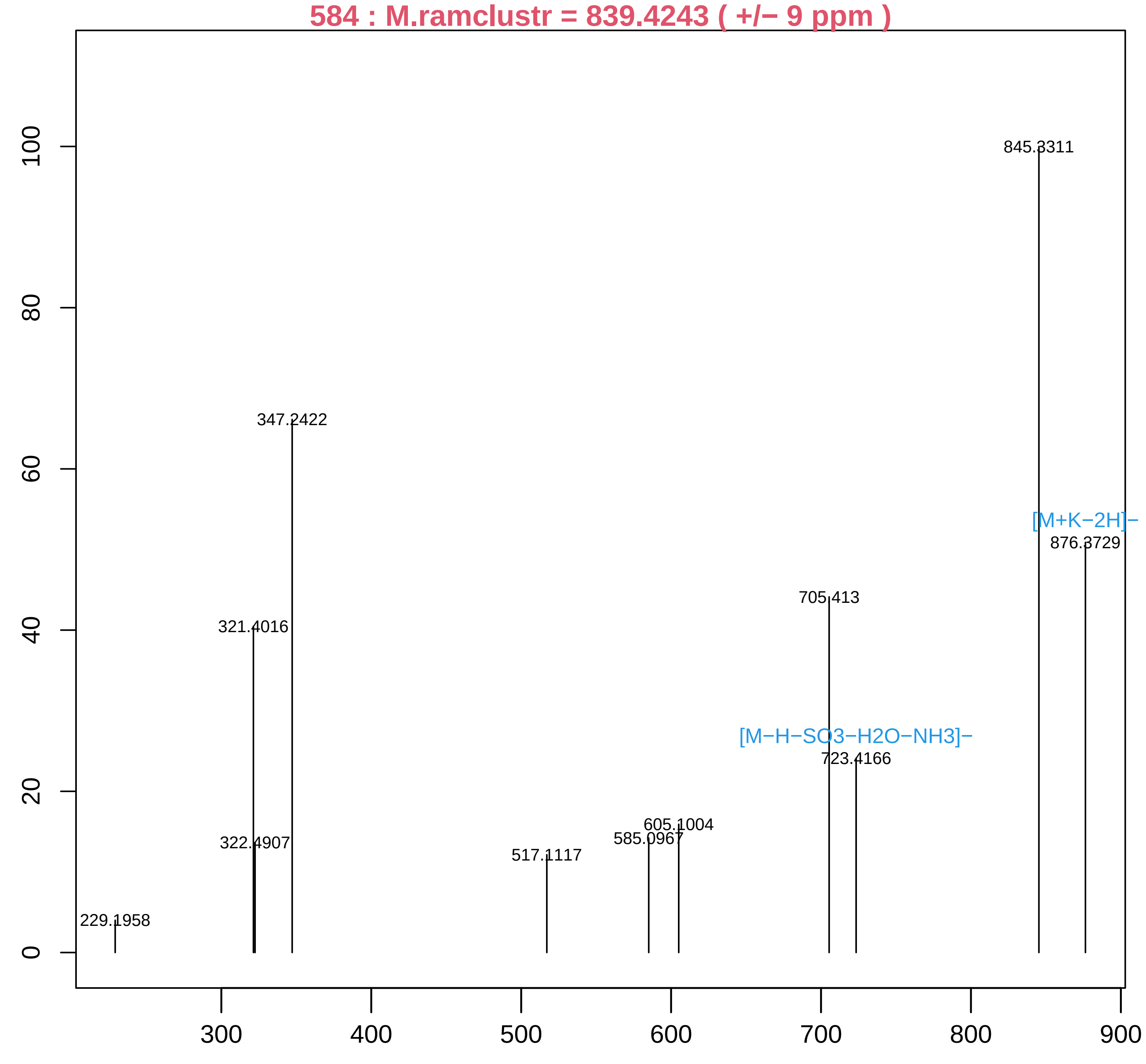
584 : M.ramclustr = 839.4243 (+/− 9 ppm)
845.3311
347.2422
[M+K−2H]−
876.3729
705.413
321.4016
[M−H−SO3−H2O−NH3]−
723.4166
605.1004
585.0967
322.4907
517.1117
229.1958
0
20
40
60
80
100
300
400
500
600
700
800
900
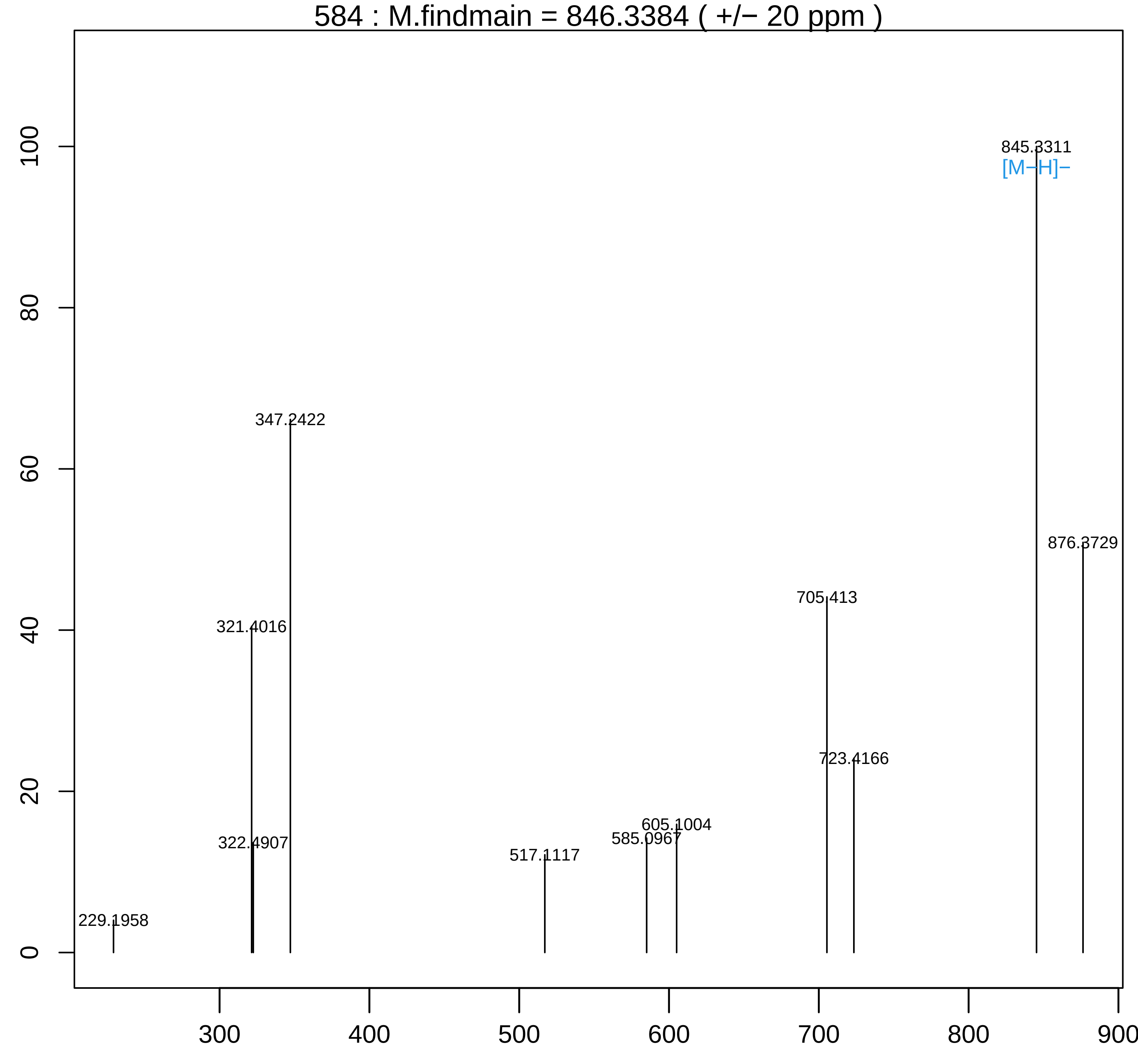
584 : M.findmain = 846.3384 (+/− 20 ppm)
845.3311
[M−H]−
347.2422
876.3729
705.413
321.4016
723.4166
605.1004
585.0967
322.4907
517.1117
229.1958
0
20
40
60
80
100
300
400
500
600
700
800
900

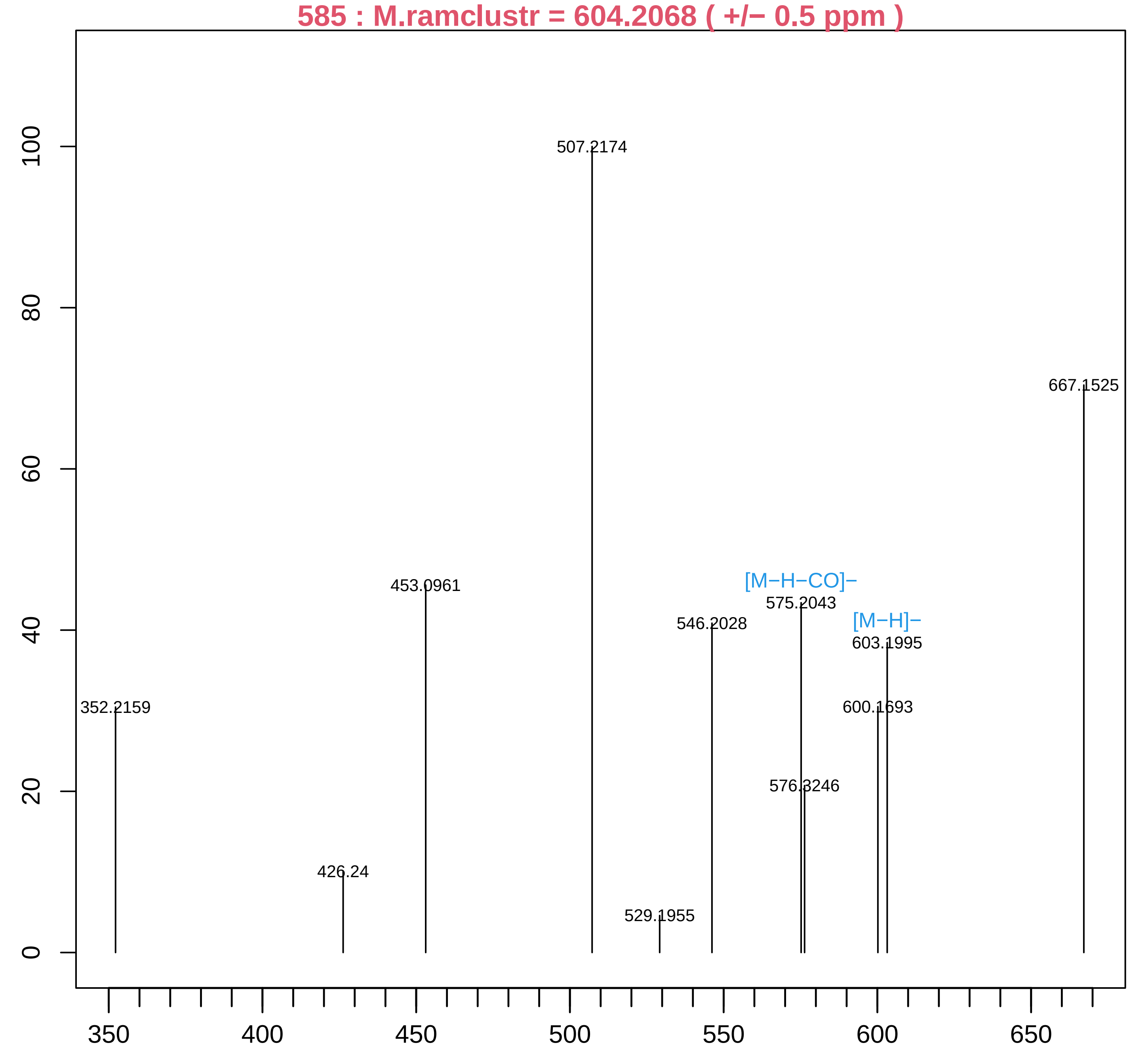
585 : M.ramclustr = 604.2068 (+/− 0.5 ppm)
507.2174
667.1525
453.0961
[M−H−CO]−
575.2043
546.2028
[M−H]−
603.1995
352.2159
600.1693
576.3246
426.24
529.1955
0
20
40
60
80
100
350
400
450
500
550
600
650

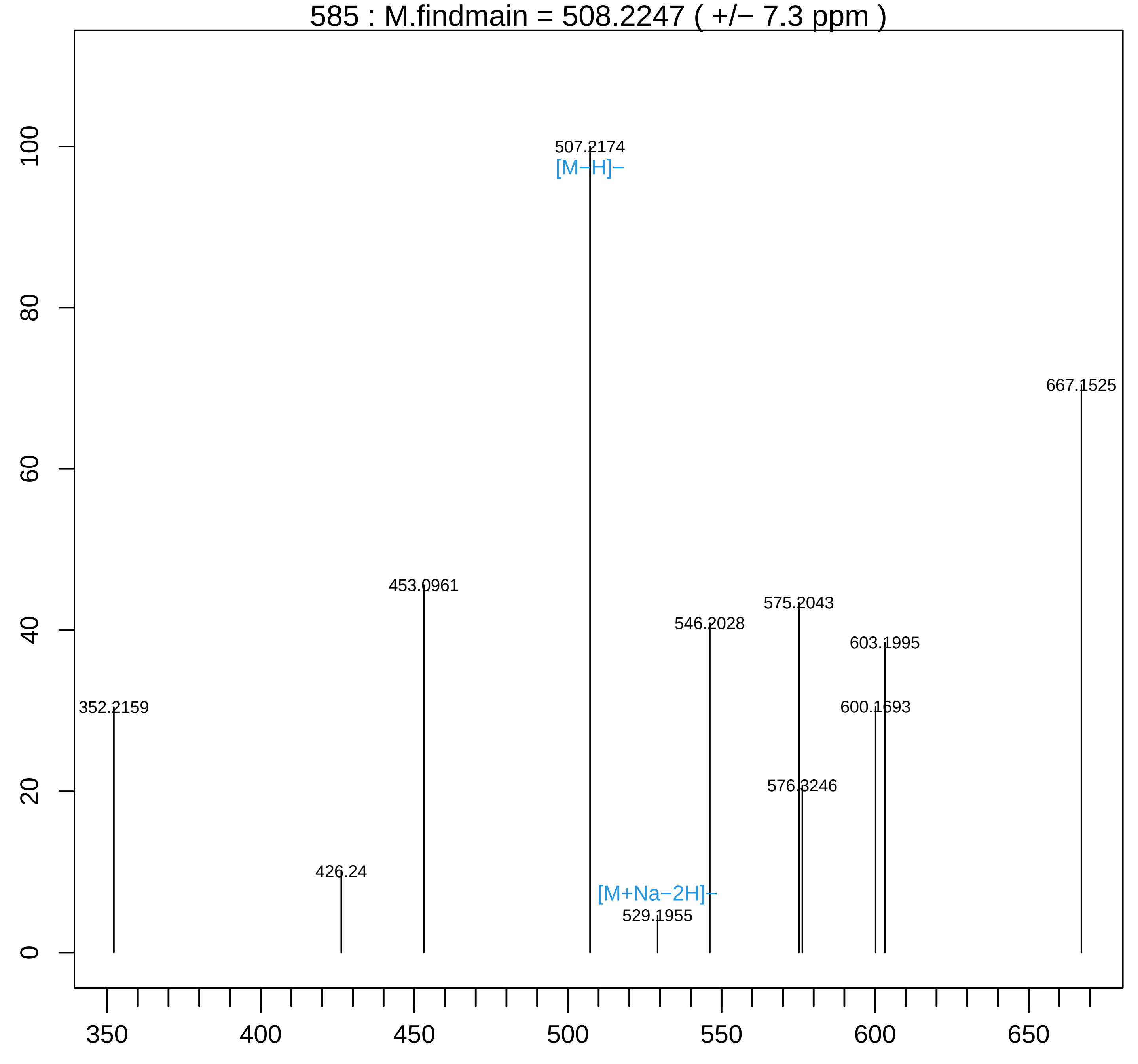
585 : M.findmain = 508.2247 (+/− 7.3 ppm)
507.2174
[M−H]−
667.1525
453.0961
575.2043
546.2028
603.1995
352.2159
600.1693
576.3246
426.24
[M+Na−2H]−
529.1955
0
20
40
60
80
100
350
400
450
500
550
600
650

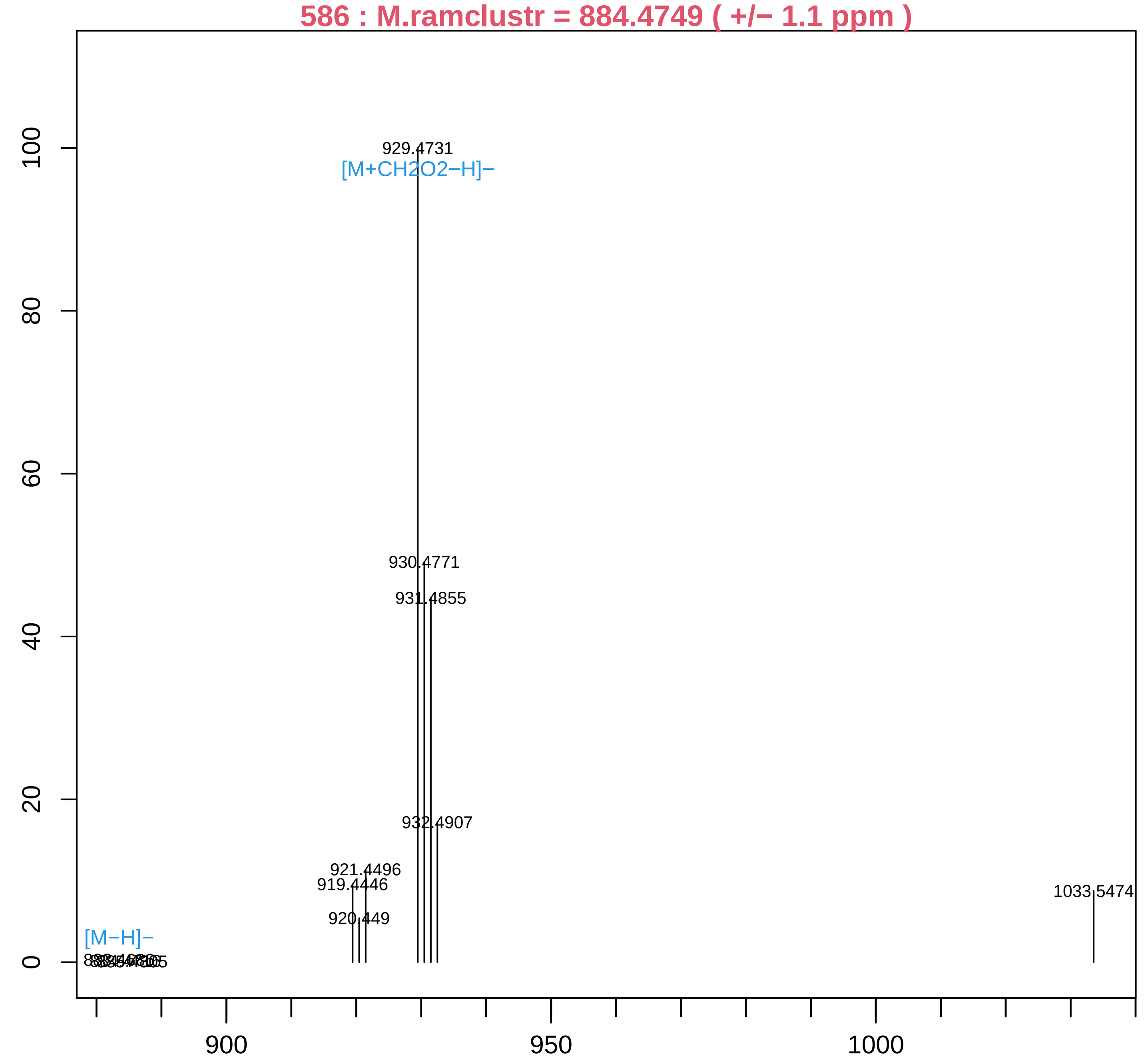
586 : M.ramclustr = 884.4749 (+/− 1.1 ppm)
929.4731
[M+CH2O2−H]−
930.4771
931.4855
932.4907
921.4496
919.4446
920.449
1033.5474
[M−H]−
0
20
40
60
80
100
900
950
1000

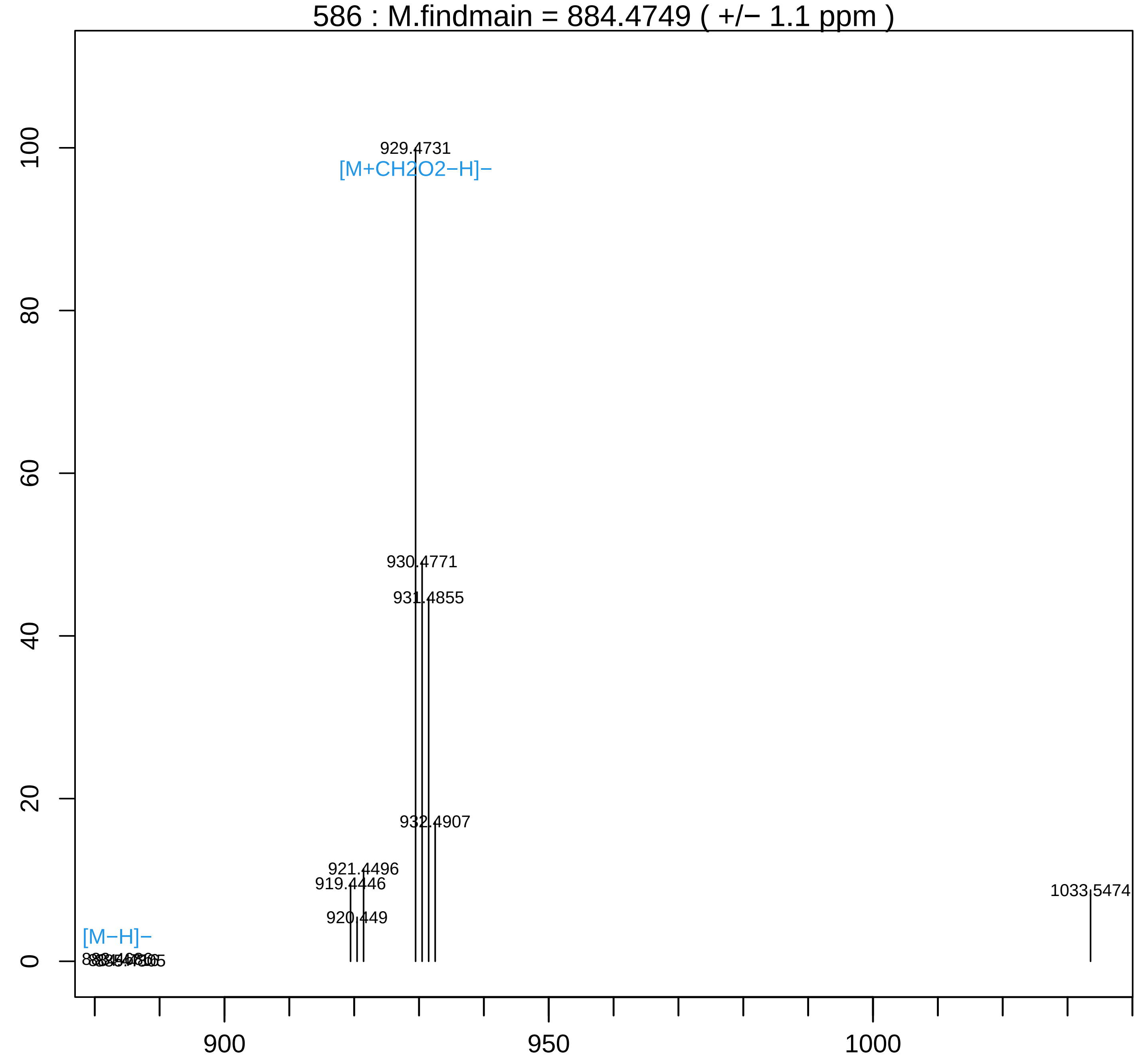
586 : M.findmain = 884.4749 (+/− 1.1 ppm)
929.4731
[M+CH2O2−H]−
930.4771
931.4855
932.4907
921.4496
919.4446
920.449
1033.5474
[M−H]−
0
20
40
60
80
100
900
950
1000

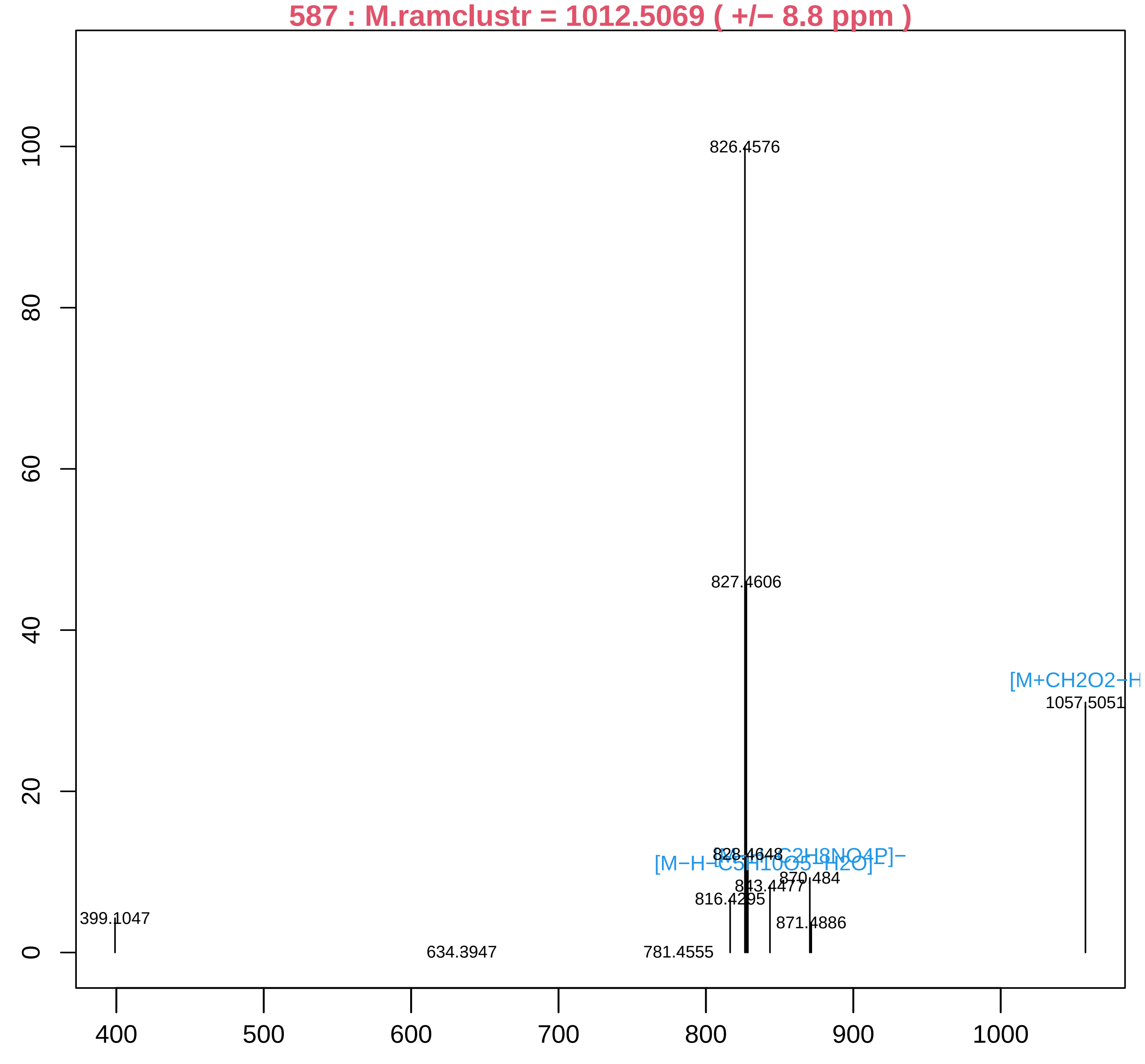
587 : M.ramclustr = 1012.5069 (+/− 8.8 ppm)
826.4576
827.4606
[M+CH2O2−H
1057.5051
828.4648
[M−H−C2H8NO4P]−
[M−H−C5H10O5−H2O]−
870.484
843.4477
816.4295
871.4886
399.1047
634.3947
781.4555
0
20
40
60
80
100
400
500
600
700
800
900
1000

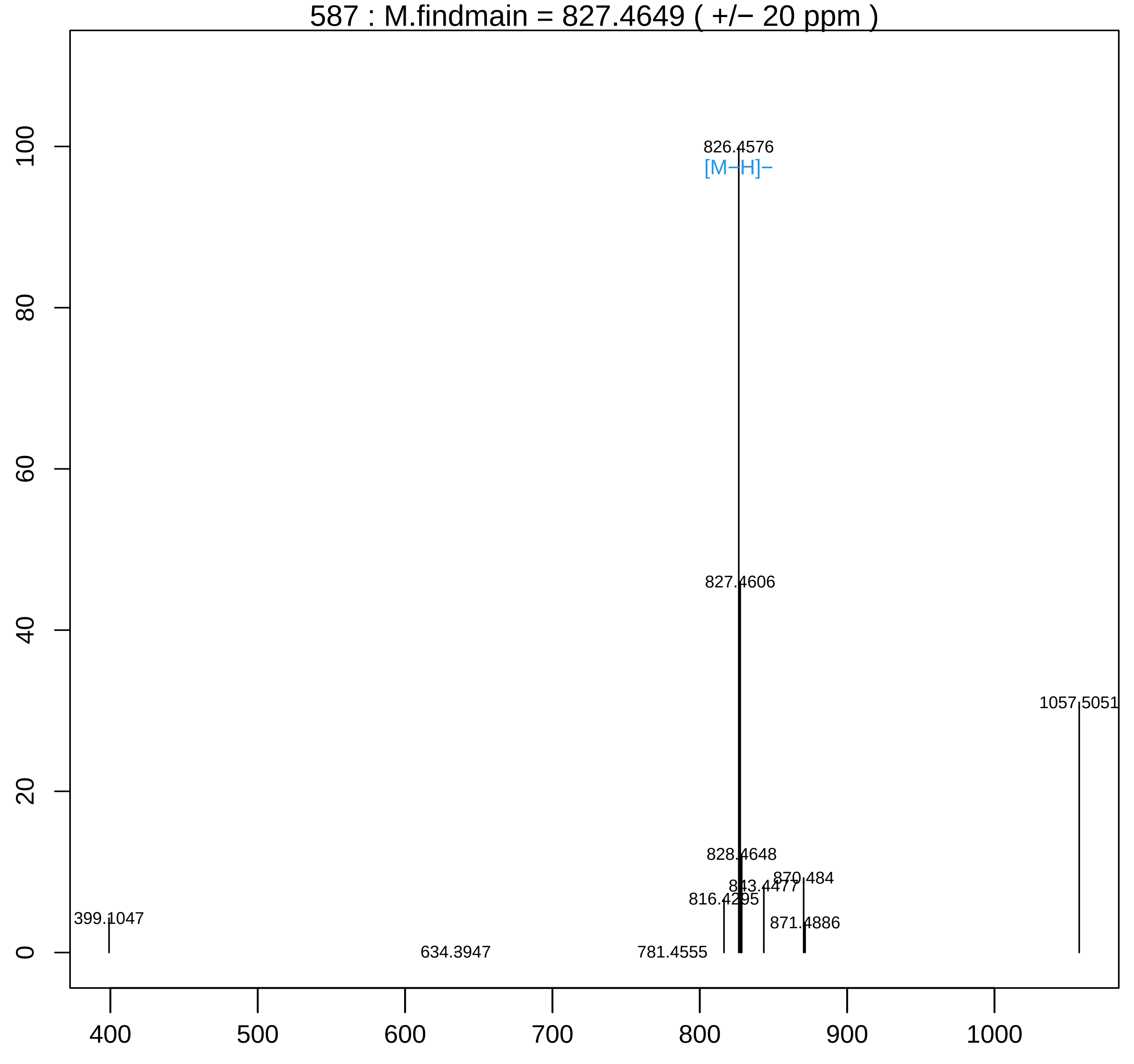
587 : M.findmain = 827.4649 (+/− 20 ppm)
826.4576
[M−H]−
827.4606
1057.5051
828.4648
870.484
843.4477
816.4295
871.4886
399.1047
634.3947
781.4555
0
20
40
60
80
100
400
500
600
700
800
900
1000

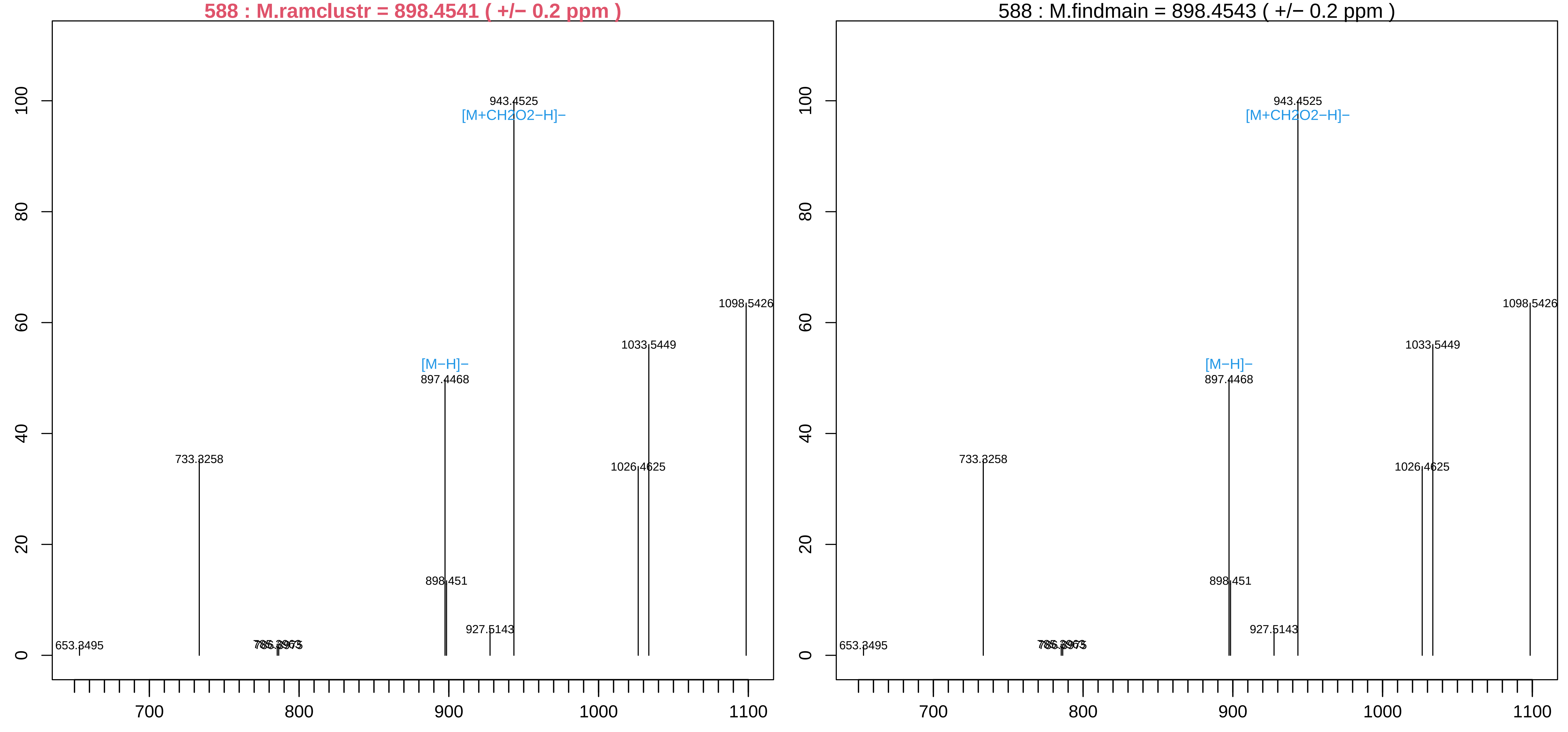

588 : M.ramclustr = 898.4541 (+/− 0.2 ppm)
943.4525
[M+CH2O2−H]−
1098.5426
1033.5449
[M−H]−
897.4468
733.3258
1026.4625
898.451
927.5143
653.3495
785.3967
0
20
40
60
80
100
700
800
900
1000
1100
588 : M.findmain = 898.4543 (+/− 0.2 ppm)
943.4525
[M+CH2O2−H]−
1098.5426
1033.5449
[M−H]−
897.4468
733.3258
1026.4625
898.451
927.5143
653.3495
785.3967
0
20
40
60
80
100
700
800
900
1000
1100

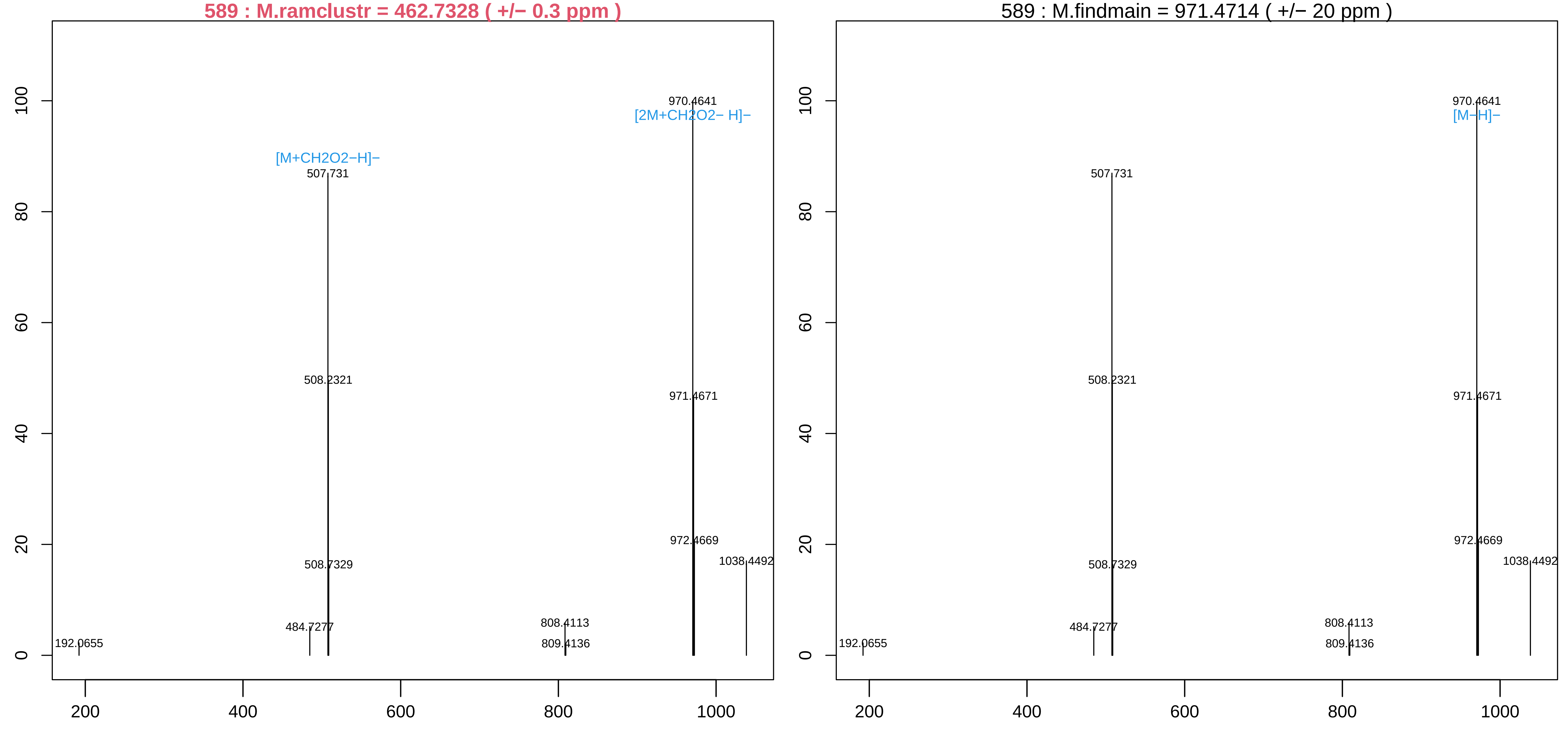

589 : M.ramclustr = 462.7328 (+/− 0.3 ppm)
[M+CH2O2−H]−
[2M+CH2O2− H]−
970.4641
507.731
508.2321
971.4671
972.4669
508.7329
1038.4492
808.4113
484.7277
809.4136
192.0655
589 : M.findmain = 971.4714 (+/− 20 ppm)
[M−H]−
0
20
40
60
80
100
200
400
600
800
1000

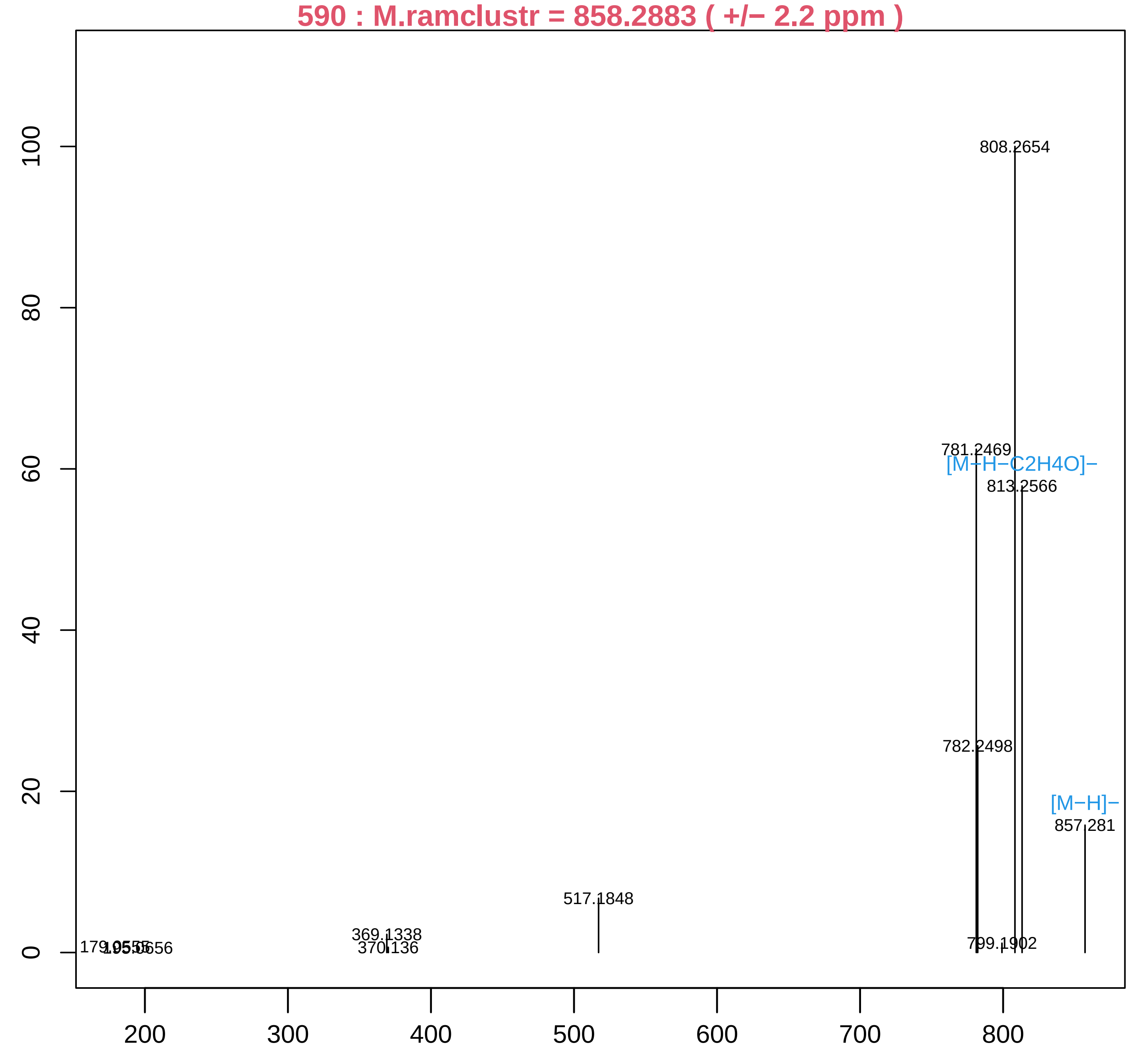

590 : M.ramclustr = 858.2883 (+/− 2.2 ppm)
808.2654
781.2469
[M−H−C2H4O]−
813.2566
782.2498
[M−H]−
857.281
517.1848
369.1338
370.1136
799.1902
0
20
40
60
80
100
200
300
400
500
600
700
800

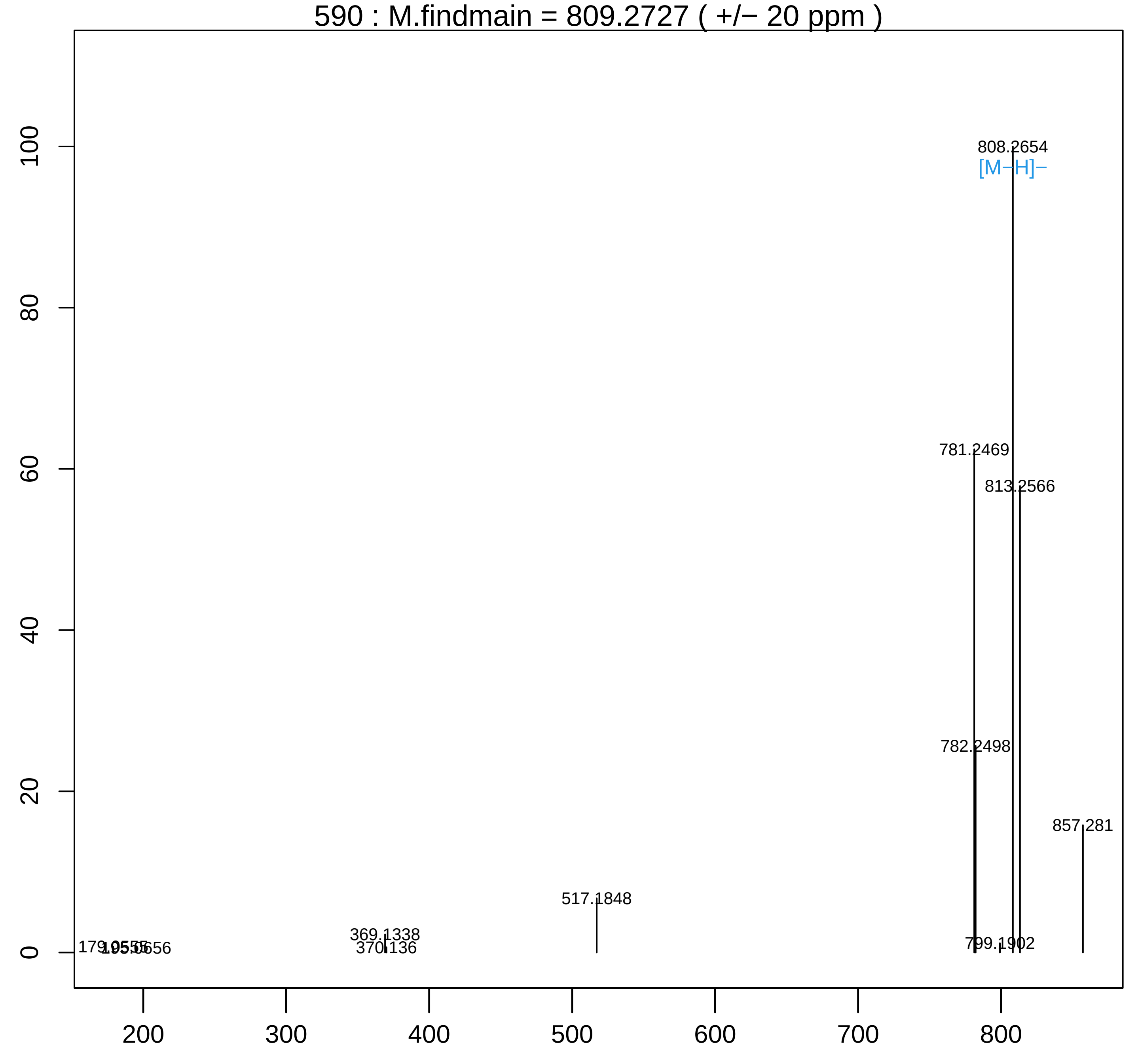

590 : M.findmain = 809.2727 (+/− 20 ppm)
808.2654
[M−H]−
781.2469
813.2566
782.2498
857.281
517.1848
369.1338
370.1136
799.1902
0
20
40
60
80
100
200
300
400
500
600
700
800

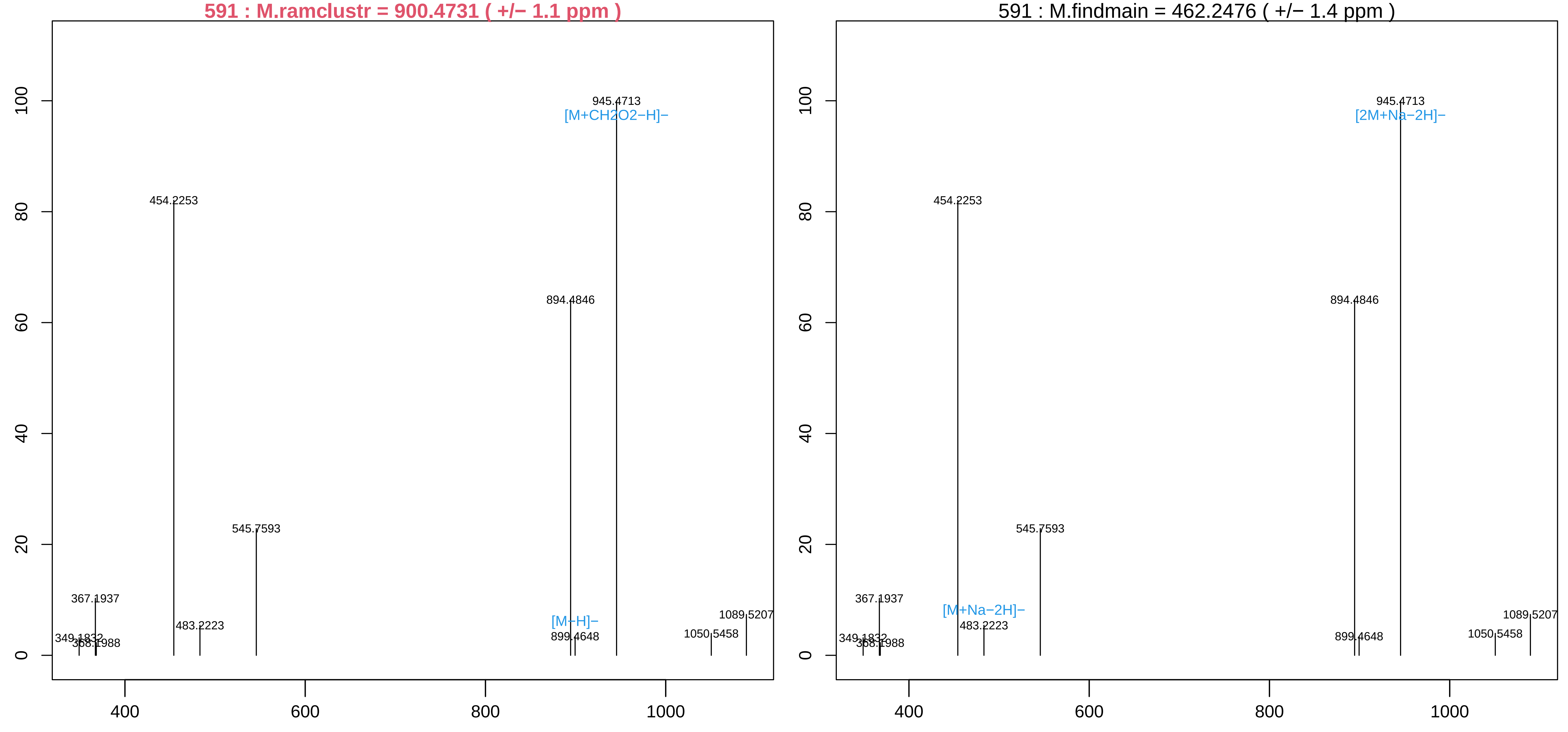

591 : M.ramclustr = 900.4731 (+/− 1.1 ppm)
945.4713
[M+CH2O2−H]−
454.2253
894.4846
545.7593
367.1937
483.2223
[M−H]−
899.4648
349.1832
368.1988
1050.5458
1089.5207
0
20
40
60
80
100
400
600
800
1000
591 : M.findmain = 462.2476 (+/− 1.4 ppm)
945.4713
[2M+Na−2H]−
454.2253
894.4846
545.7593
367.1937
[M+Na−2H]−
483.2223
899.4648
349.1832
368.1988
1050.5458
1089.5207
0
20
40
60
80
100
400
600
800
1000

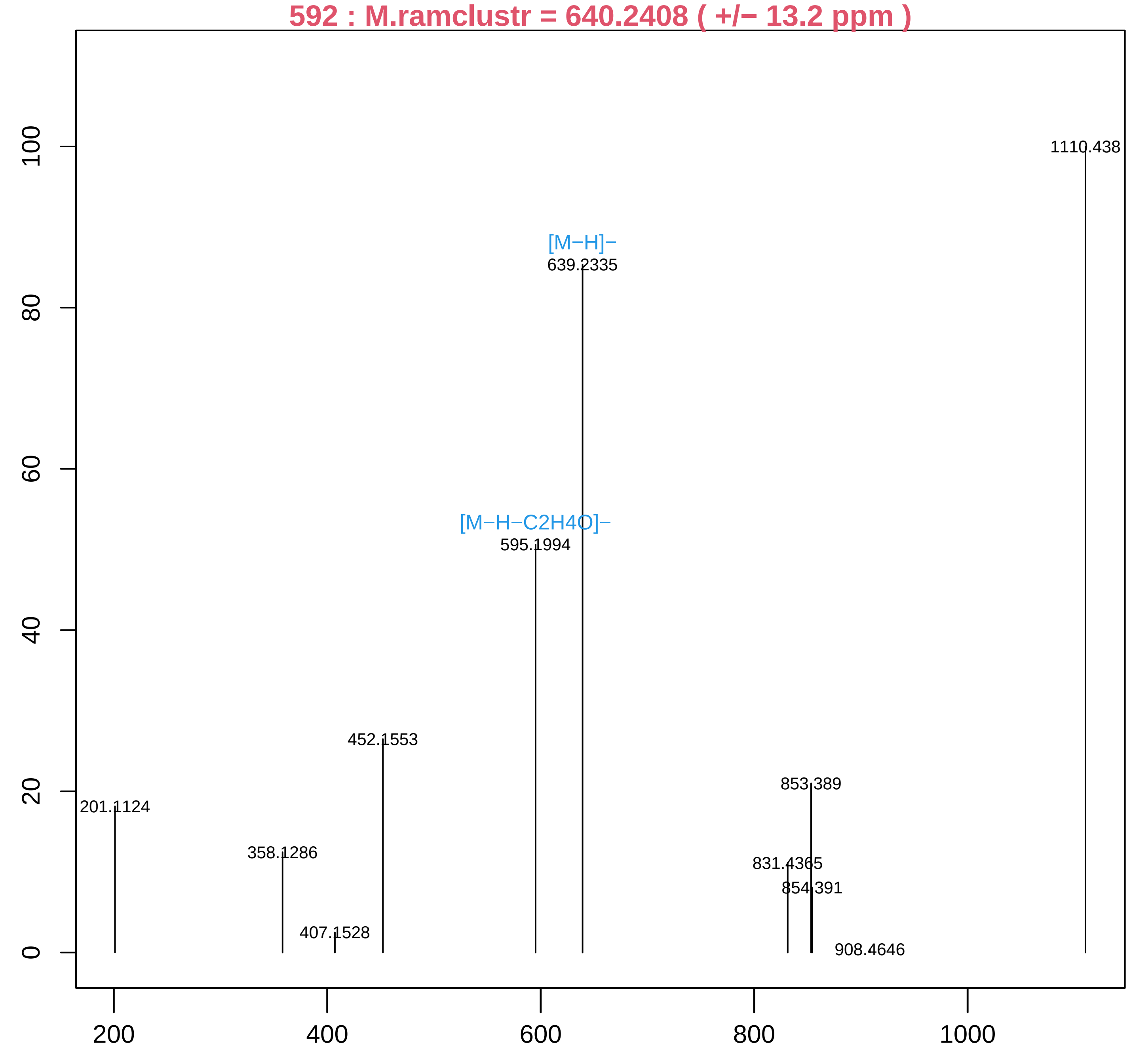
592 : M.ramclustr = 640.2408 (+/− 13.2 ppm)
[M−H]−
[M−H−C2H4O]−
1110.438
639.2335
595.1994
452.1553
201.1124
358.1286
407.1528
853.389
831.4365
854.391
908.4646
0
20
40
60
80
100
200
400
600
800
1000

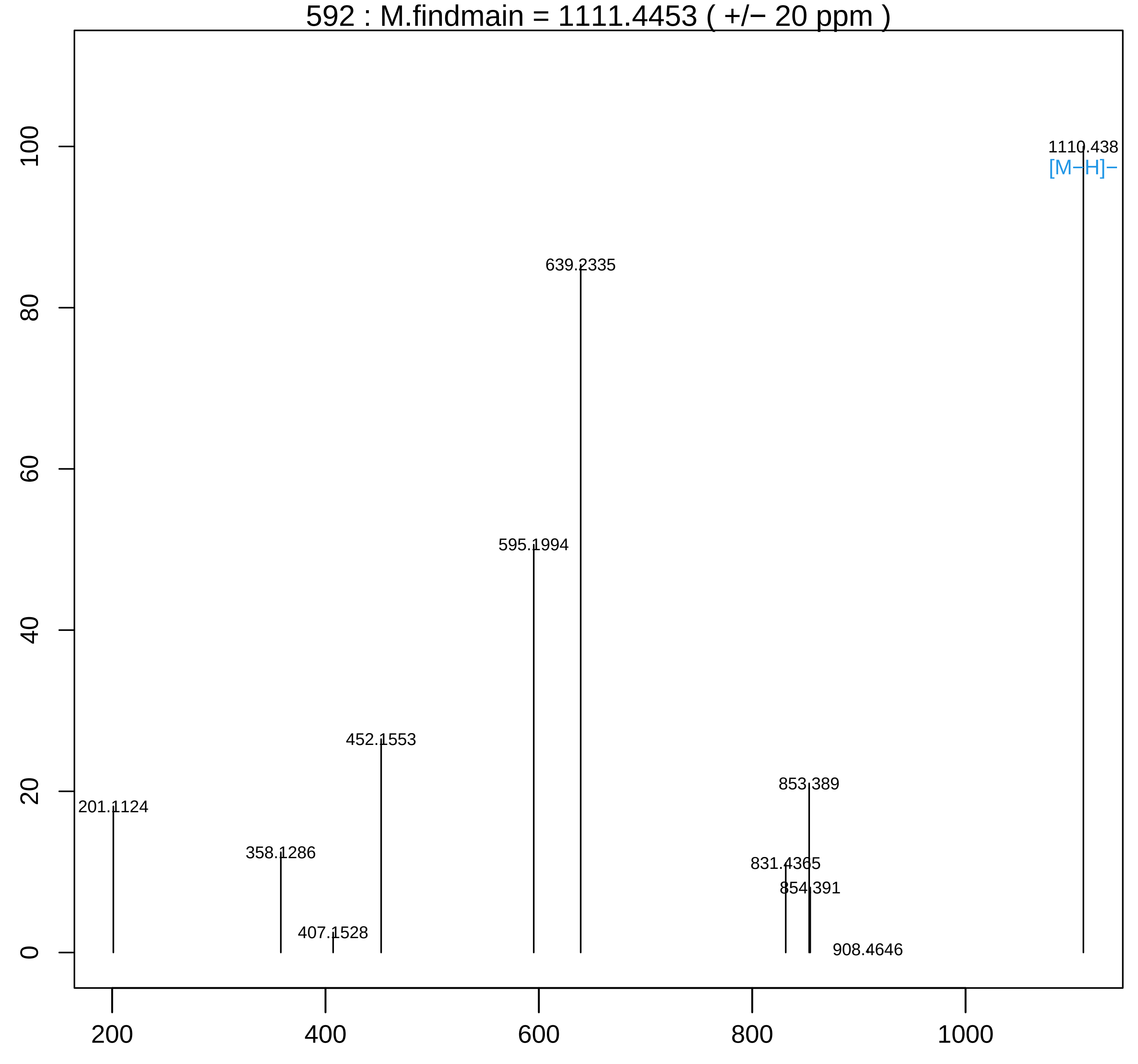
592 : M.findmain = 1111.4453 (+/− 20 ppm)
1110.438
[M−H]−
639.2335
595.1994
452.1553
201.1124
358.1286
407.1528
853.389
831.4365
854.391
908.4646
0
20
40
60
80
100
200
400
600
800
1000

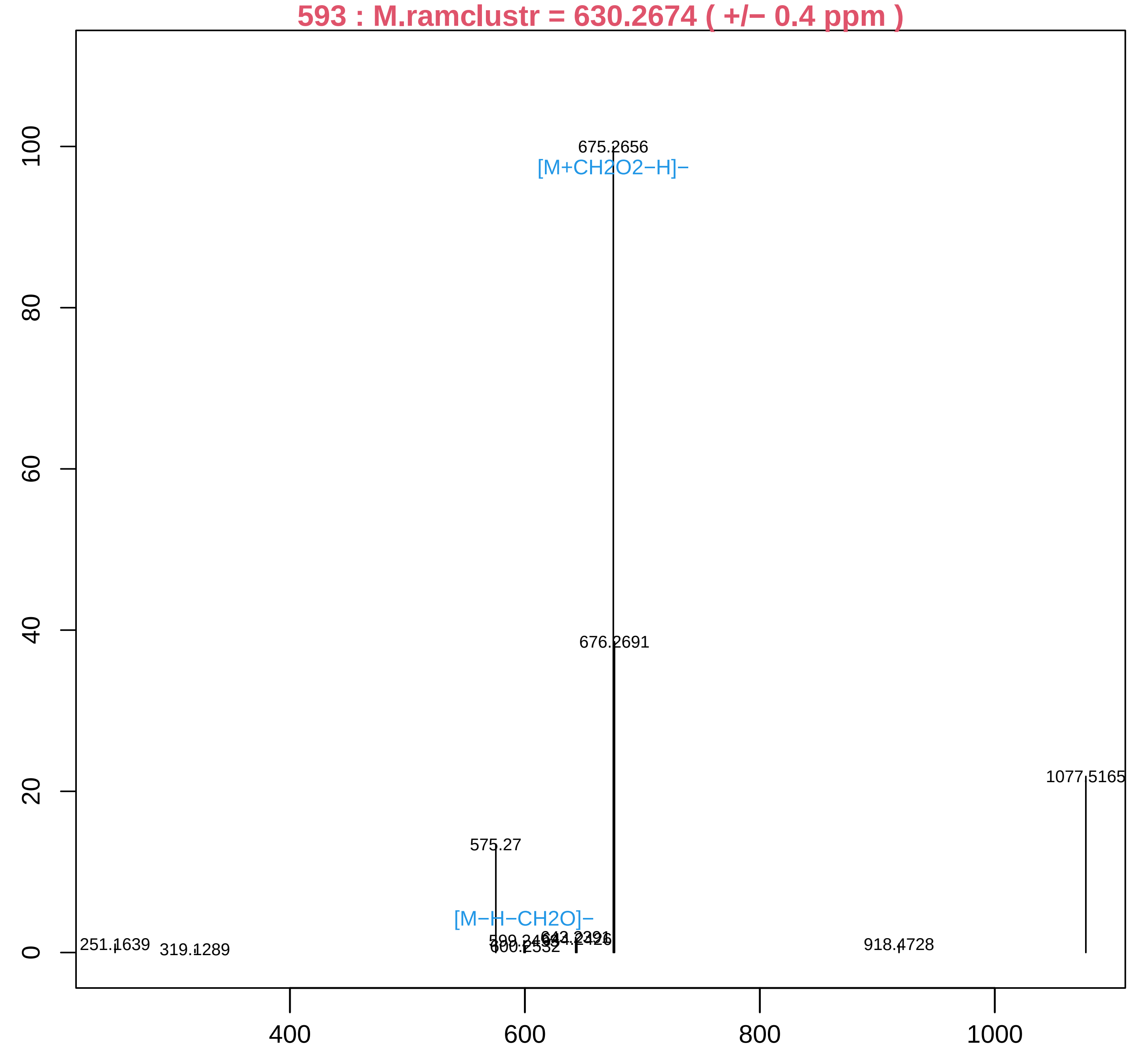
593 : M.ramclustr = 630.2674 (+/− 0.4 ppm)
675.2656
[M+CH2O2−H]−
676.2691
575.27
[M−H−CH2O]−
1077.5165
251.1639
319.1289
599.2464
600.2532
642.2291
644.2426
918.4728
0
20
40
60
80
100
400
600
800
1000

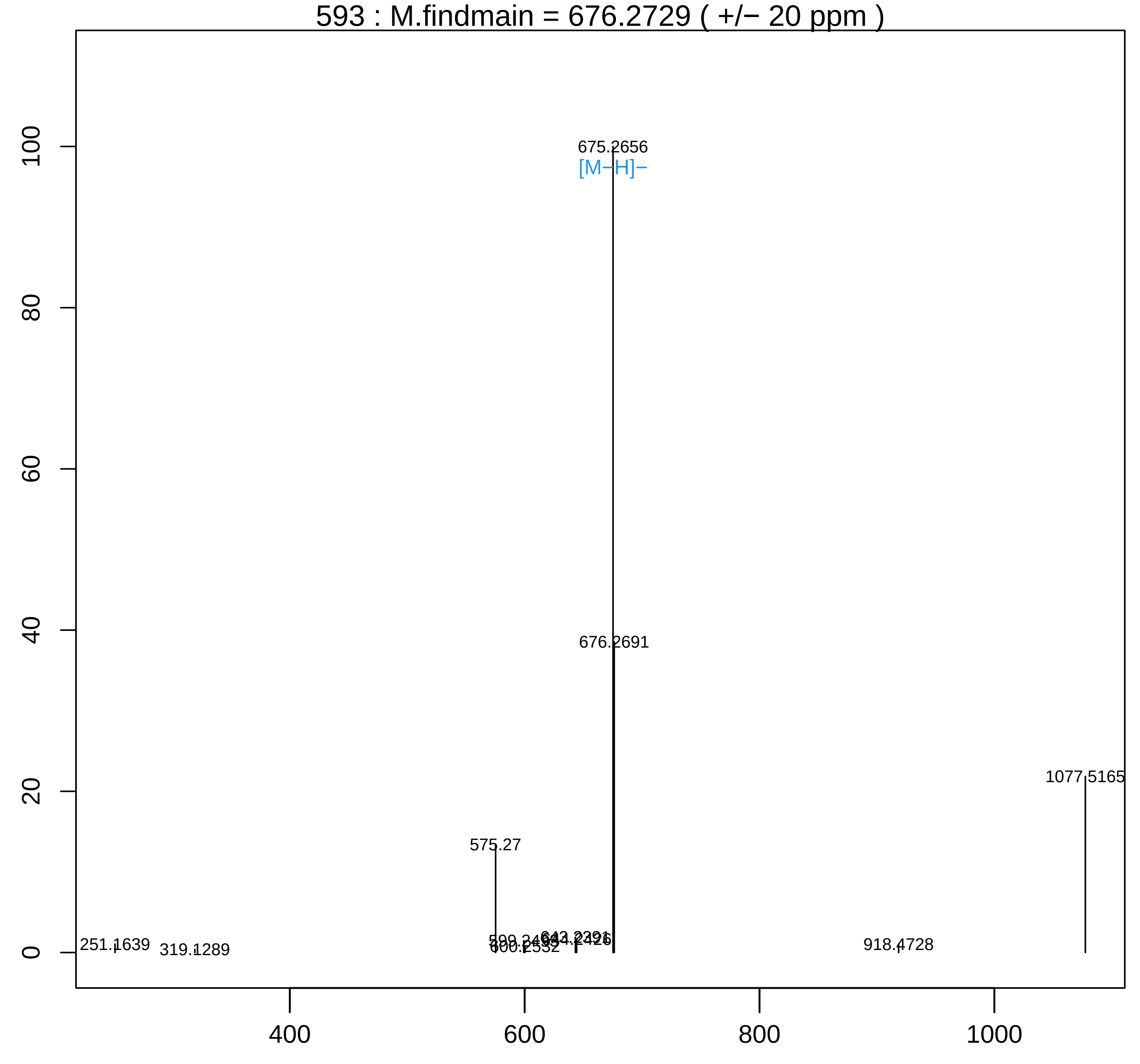
593 : M.findmain = 676.2729 (+/− 20 ppm)
675.2656
[M−H]−
676.2691
575.27
1077.5165
251.1639
319.1289
599.2464
600.2532
642.2291
644.2426
918.4728
0
20
40
60
80
100
400
600
800
1000

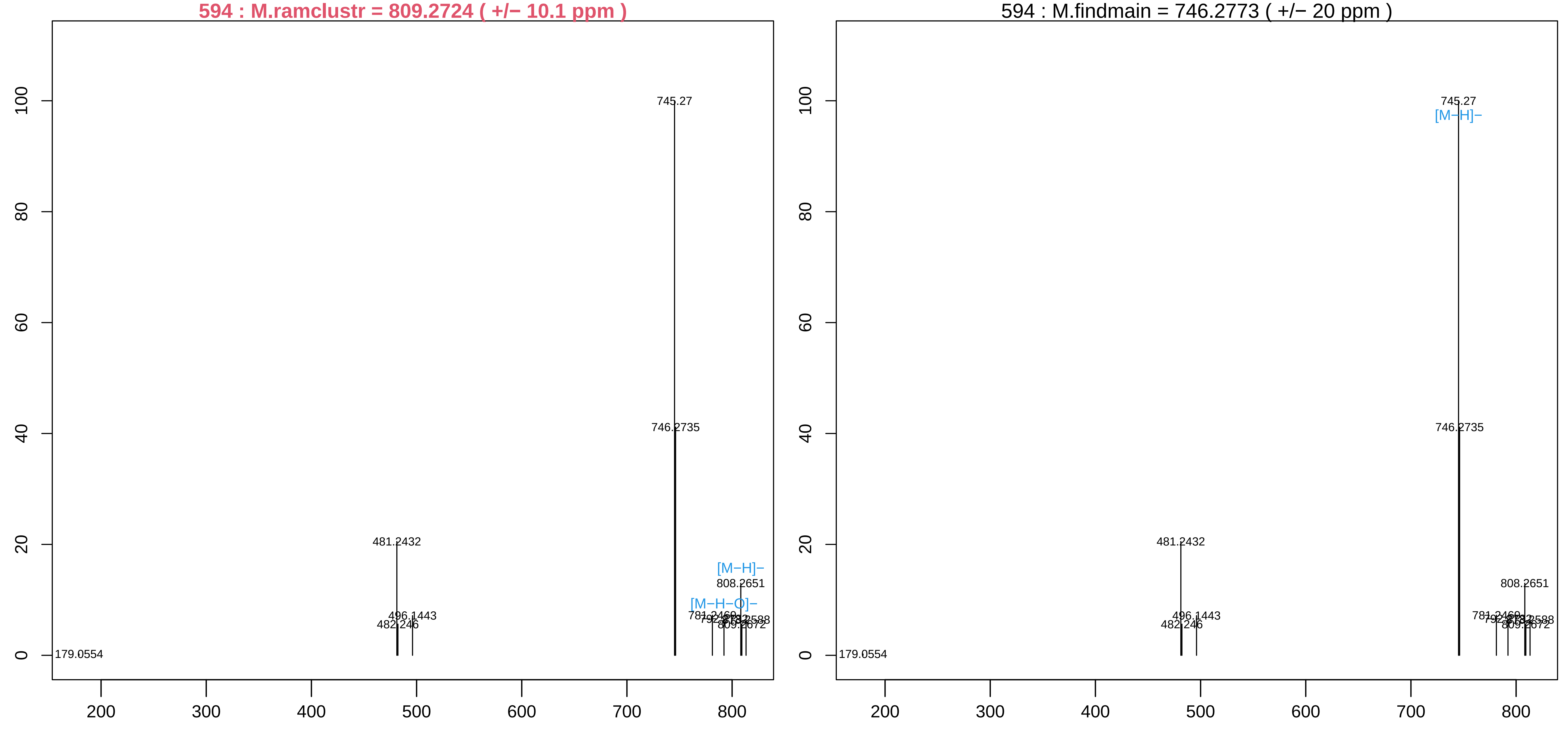
594 : M.ramclustr = 809.2724 (+/− 10.1 ppm)
745.27
746.2735
481.2432
496.1443
482.246
179.0554
[M−H]−
808.2651
[M−H−O]−
781.2460
809.2672
594 : M.findmain = 746.2773 (+/− 20 ppm)
745.27
[M−H]−
746.2735
481.2432
496.1443
482.246
179.0554
808.2651
781.2460
809.2672

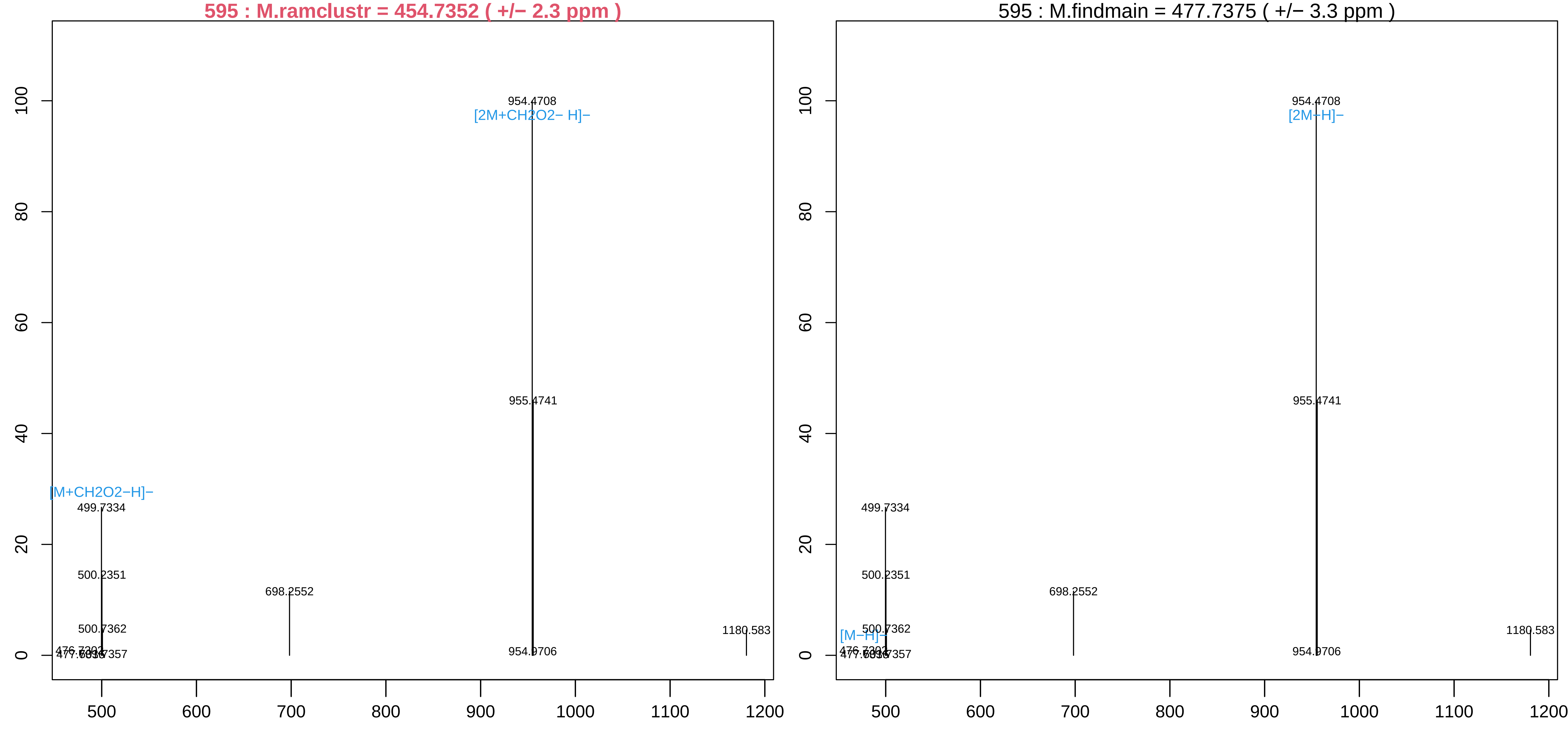
595 : M.ramclustr = 454.7352 (+/− 2.3 ppm)
954.4708
[2M+CH2O2− H]−
955.4741
[M+CH2O2−H]−
499.7334
500.2351
698.2552
500.7362
1180.583
954.9706
476.7302
477.7357
595 : M.findmain = 477.7375 (+/− 3.3 ppm)
954.4708
[2M−H]−
955.4741
499.7334
500.2351
698.2552
500.7362
[M−H]−
1180.583
954.9706
476.7302
477.7357

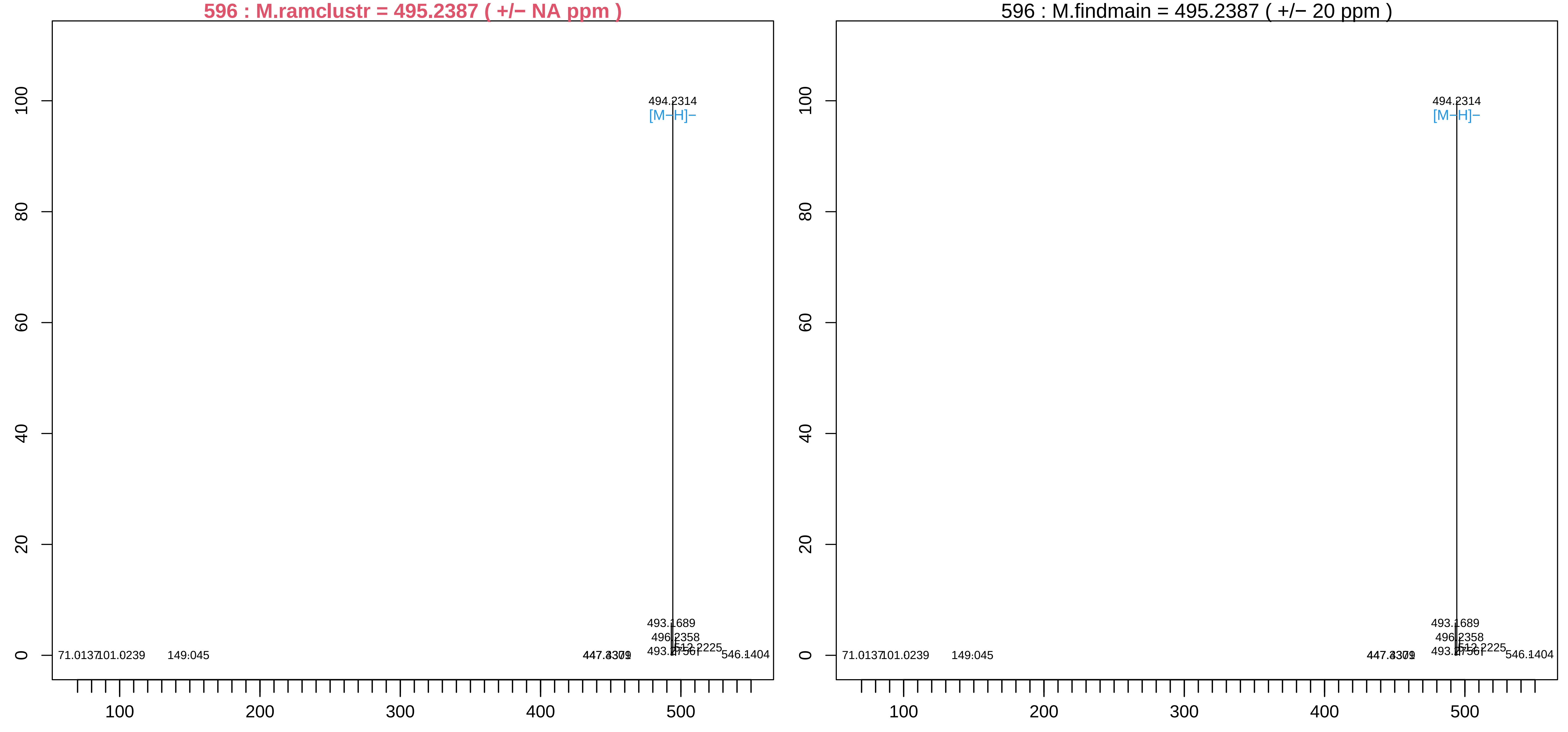

596 : M.ramclustr = 495.2387 (+/− NA ppm)
596 : M.findmain = 495.2387 (+/− 20 ppm)
494.2314
[M−H]−
493.1689
496.2358
512.2225
546.1404
71.0137
101.0239
149.045
0
20
40
60
80
100
200
300
400
500

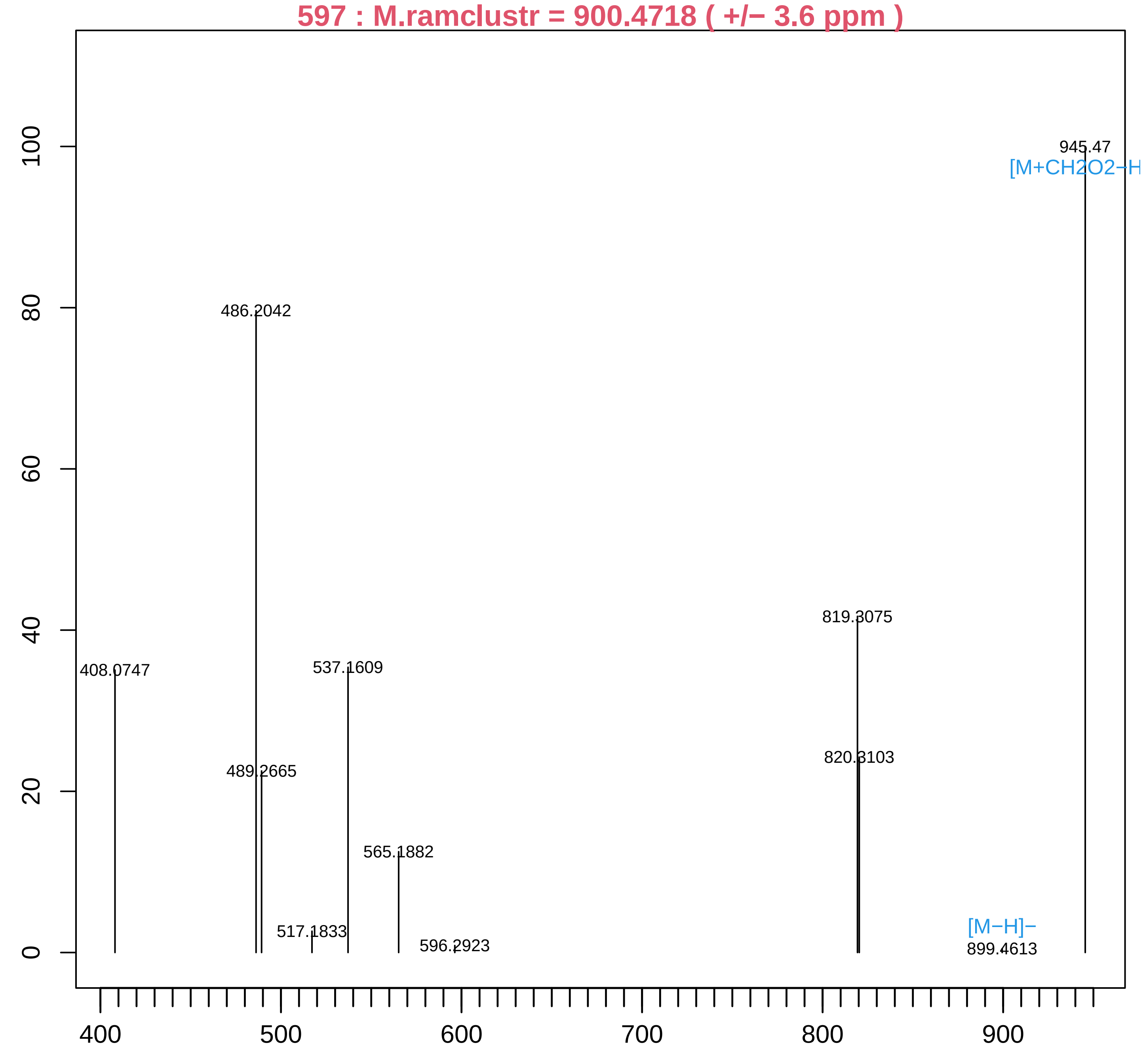
597 : M.ramclustr = 900.4718 (+/− 3.6 ppm)
945.47
[M+CH2O2−H
486.2042
819.3075
408.0747
537.1609
489.2665
820.3103
565.1882
517.1833
596.2923
[M−H]−
899.4613
0
20
40
60
80
100
400
500
600
700
800
900

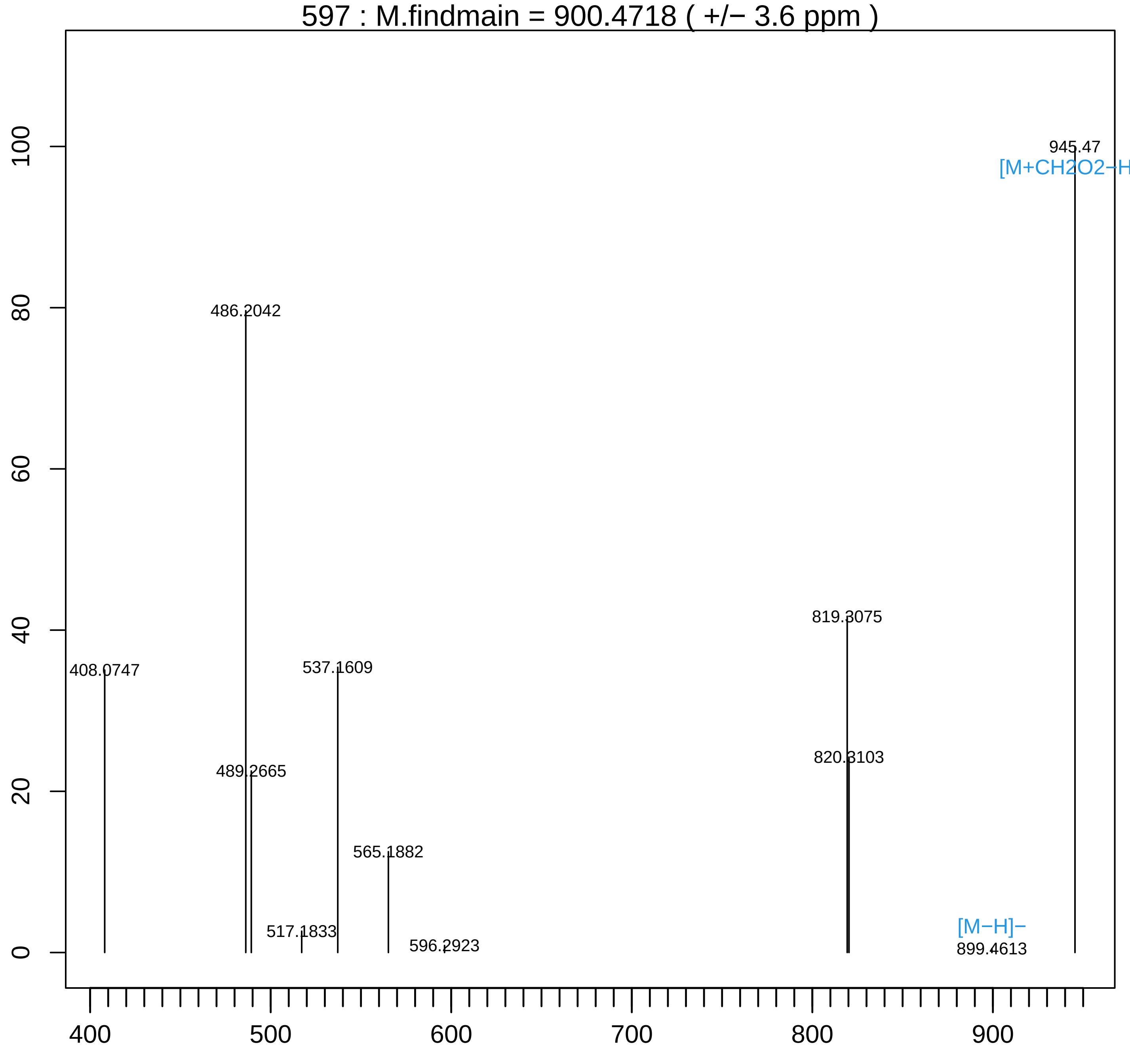
597 : M.findmain = 900.4718 (+/− 3.6 ppm)
945.47
[M+CH2O2−H
486.2042
819.3075
408.0747
537.1609
489.2665
820.3103
565.1882
517.1833
596.2923
[M−H]−
899.4613
0
20
40
60
80
100
400
500
600
700
800
900

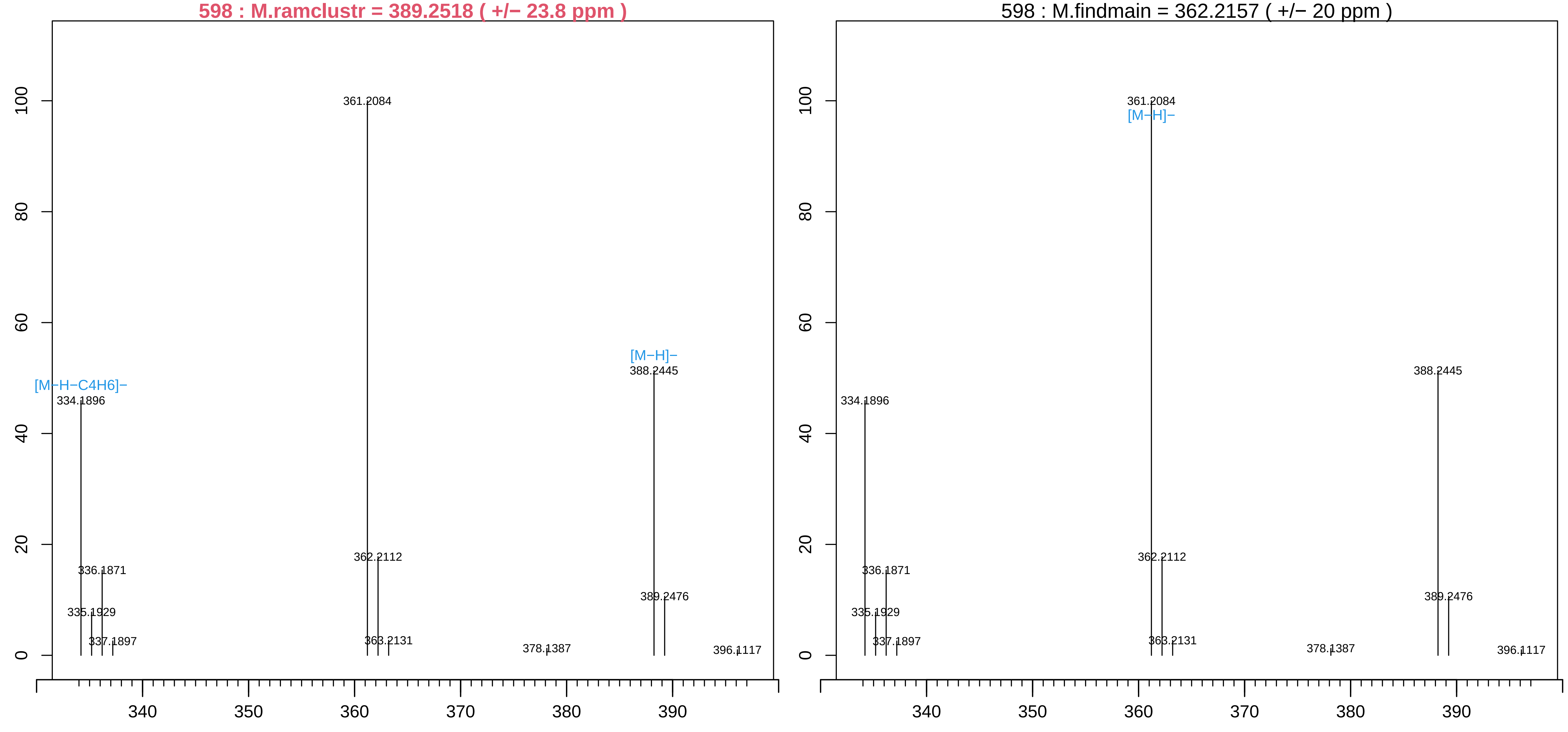
598 : M.ramclustr = 389.2518 (+/− 23.8 ppm)
361.2084
[M−H]−
388.2445
[M−H−C4H6]−
334.1896
362.2112
336.1871
389.2476
335.1929
337.1897
363.2131
378.1387
396.1117
598 : M.findmain = 362.2157 (+/− 20 ppm)
361.2084
[M−H]−
388.2445
334.1896
362.2112
336.1871
389.2476
335.1929
337.1897
363.2131
378.1387
396.1117

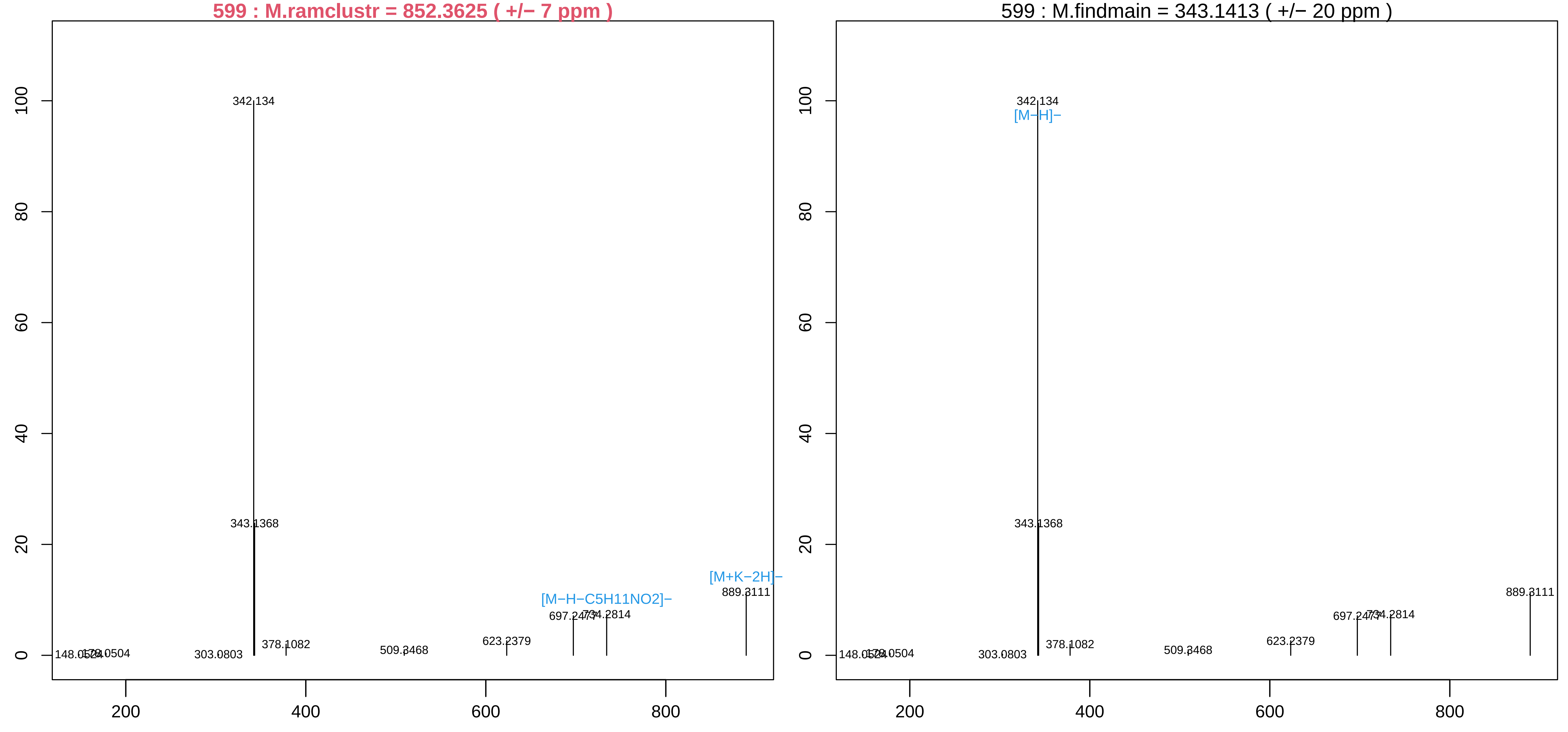
599 : M.ramclustr = 852.3625 (+/− 7 ppm)
342.134
343.1368
[M+K−2H]−
889.3111
[M−H−C5H11NO2]−
697.2473
734.2814
623.2379
378.1082
509.3468
148.0524
178.0504
303.0803
599 : M.findmain = 343.1413 (+/− 20 ppm)
342.134
[M−H]−
343.1368
889.3111
697.2473
734.2814
623.2379
378.1082
509.3468
148.0524
178.0504
303.0803
0
20
40
60
80
100
200
400
600
800

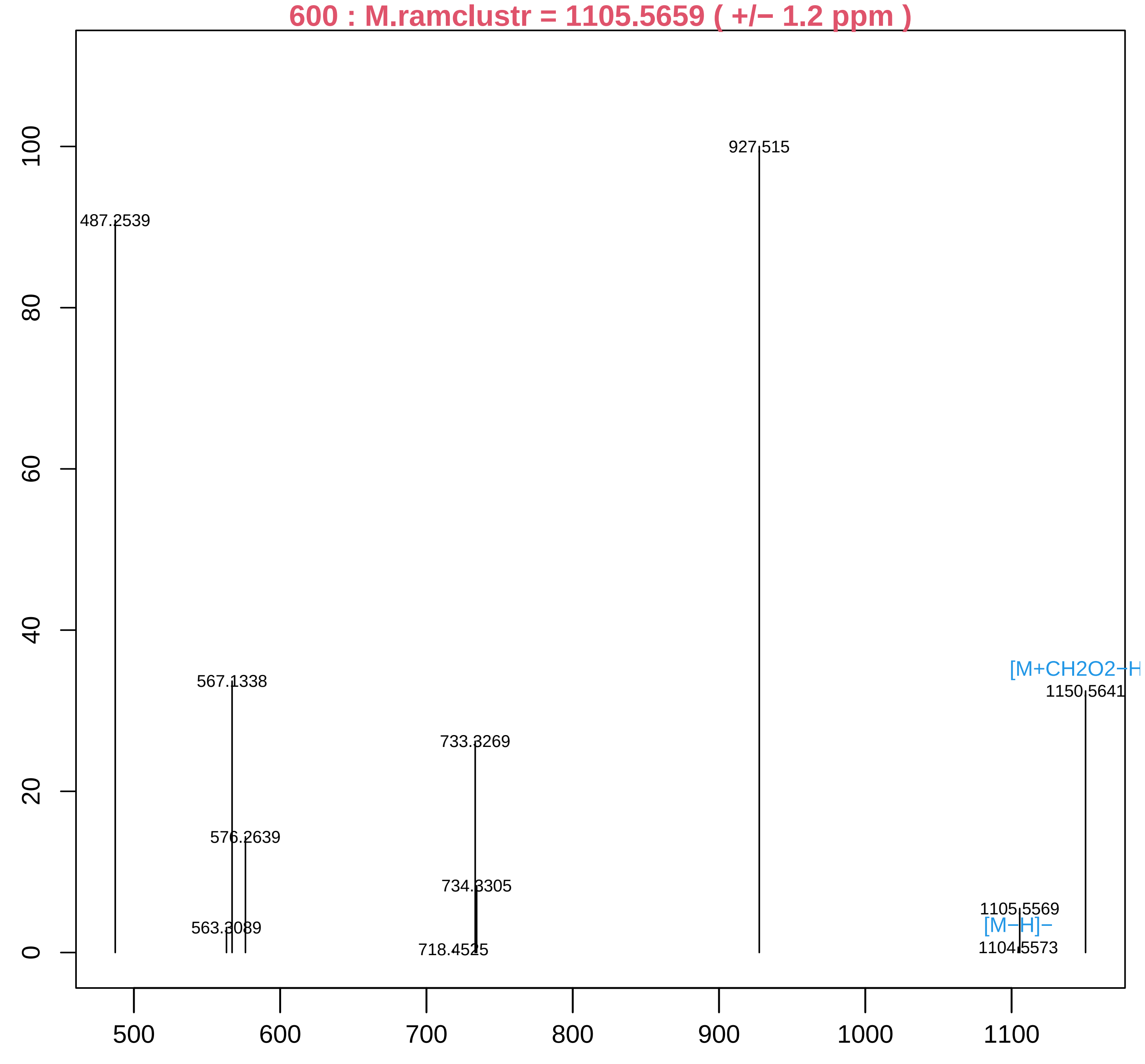
600 : M.ramclustr = 1105.5659 (+/− 1.2 ppm)
927.515
487.2539
567.1338
733.3269
576.2639
734.3305
563.3089
718.4525
1105.5569
[M−H]−
1104.5573
[M+CH2O2−H
1150.5641
0
20
40
60
80
100
500
600
700
800
900
1000
1100

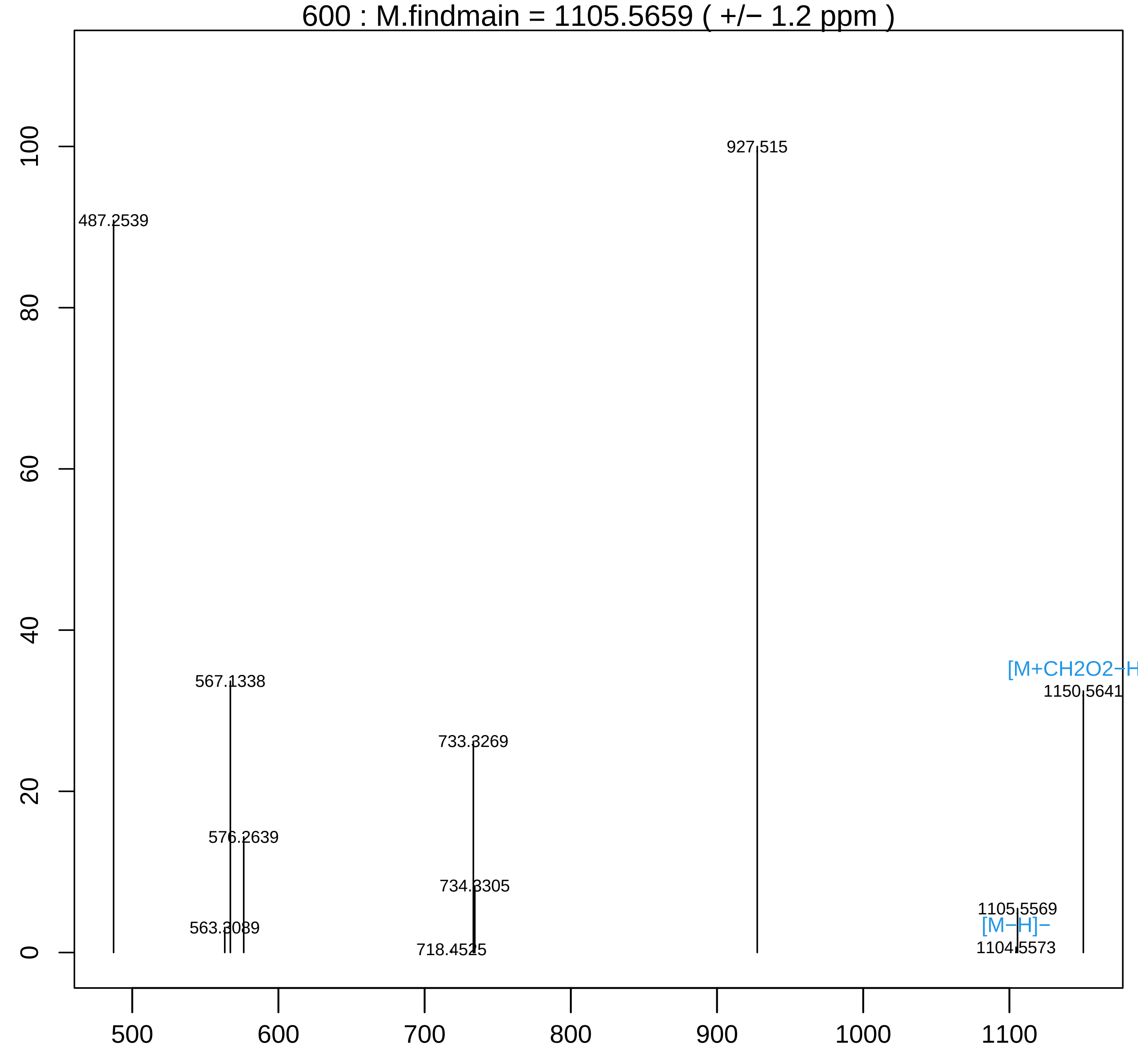
600 : M.findmain = 1105.5659 (+/− 1.2 ppm)
927.515
487.2539
567.1338
733.3269
576.2639
734.3305
563.3089
718.4525
1105.5569
[M−H]−
1104.5573
[M+CH2O2−H
1150.5641
0
20
40
60
80
100
500
600
700
800
900
1000
1100

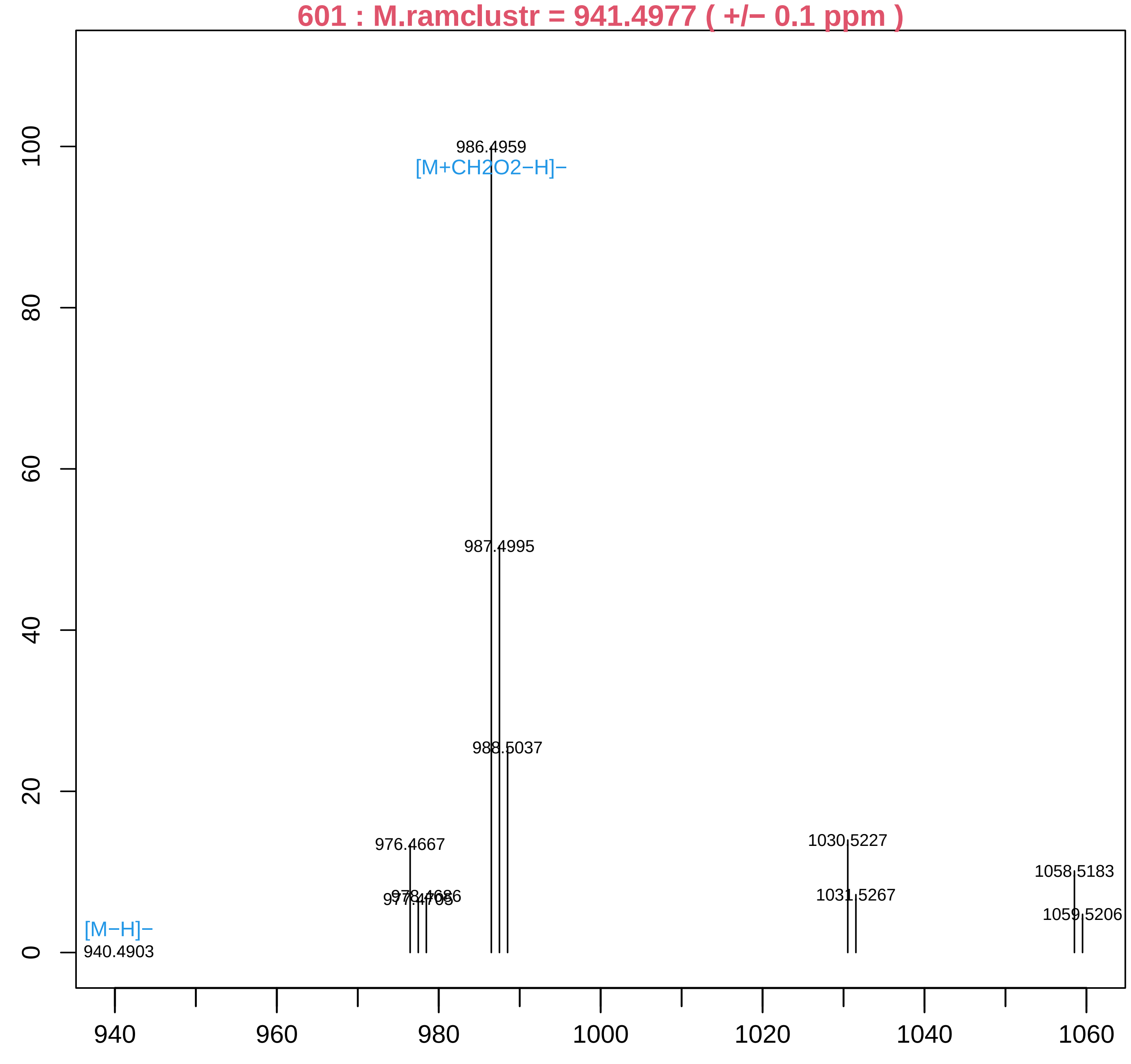
601 : M.ramclustr = 941.4977 (+/− 0.1 ppm)
986.4959
[M+CH2O2−H]−
987.4995
988.5037
976.4667
978.4686
977.4705
1030.5227
1031.5267
1058.5183
1059.5206
[M−H]−
940.4903
0
20
40
60
80
100
940
960
980
1000
1020
1040
1060

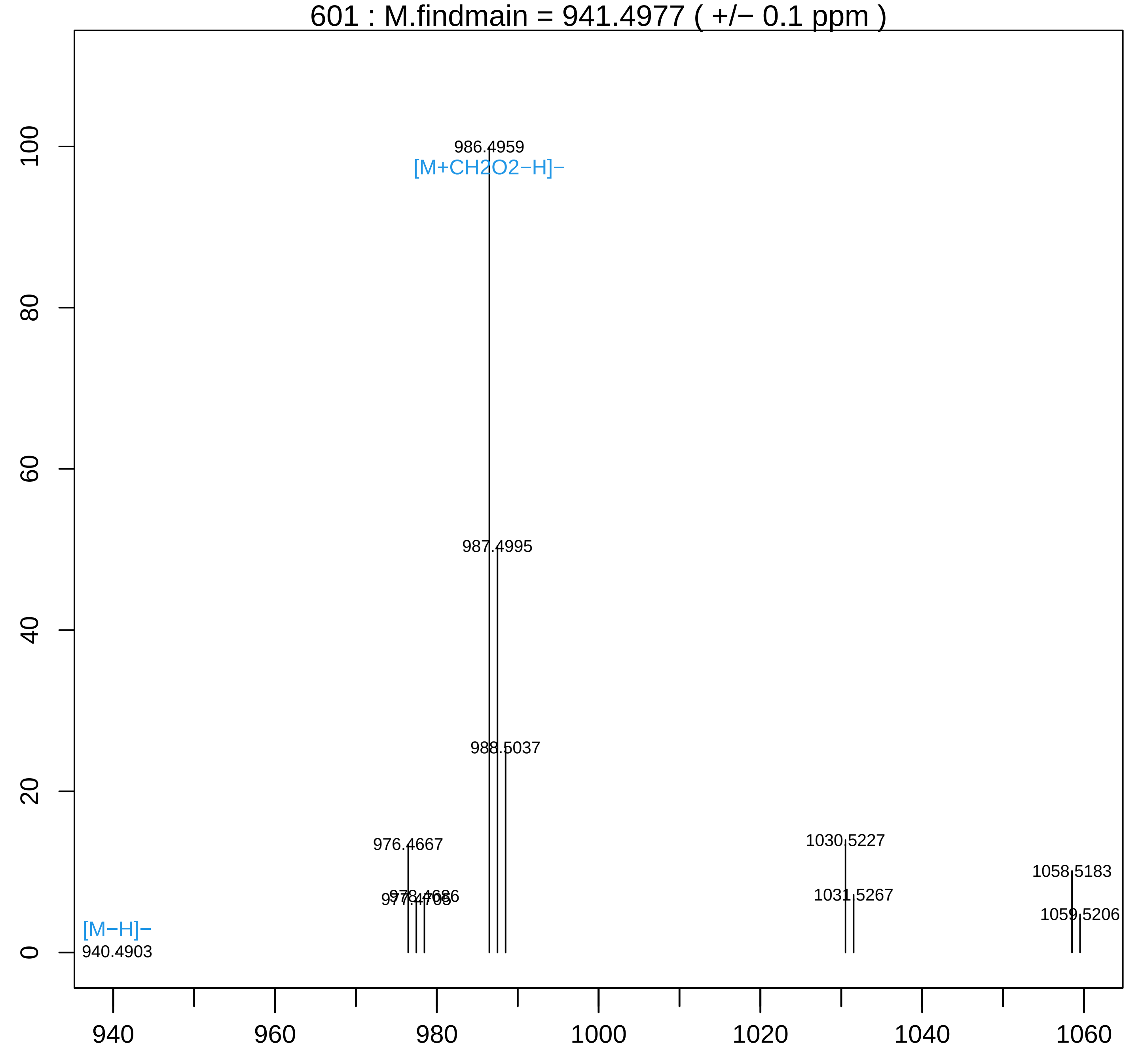
601 : M.findmain = 941.4977 (+/− 0.1 ppm)
986.4959
[M+CH2O2−H]−
987.4995
988.5037
976.4667
978.4686
977.4705
1030.5227
1031.5267
1058.5183
1059.5206
[M−H]−
940.4903
0
20
40
60
80
100
940
960
980
1000
1020
1040
1060

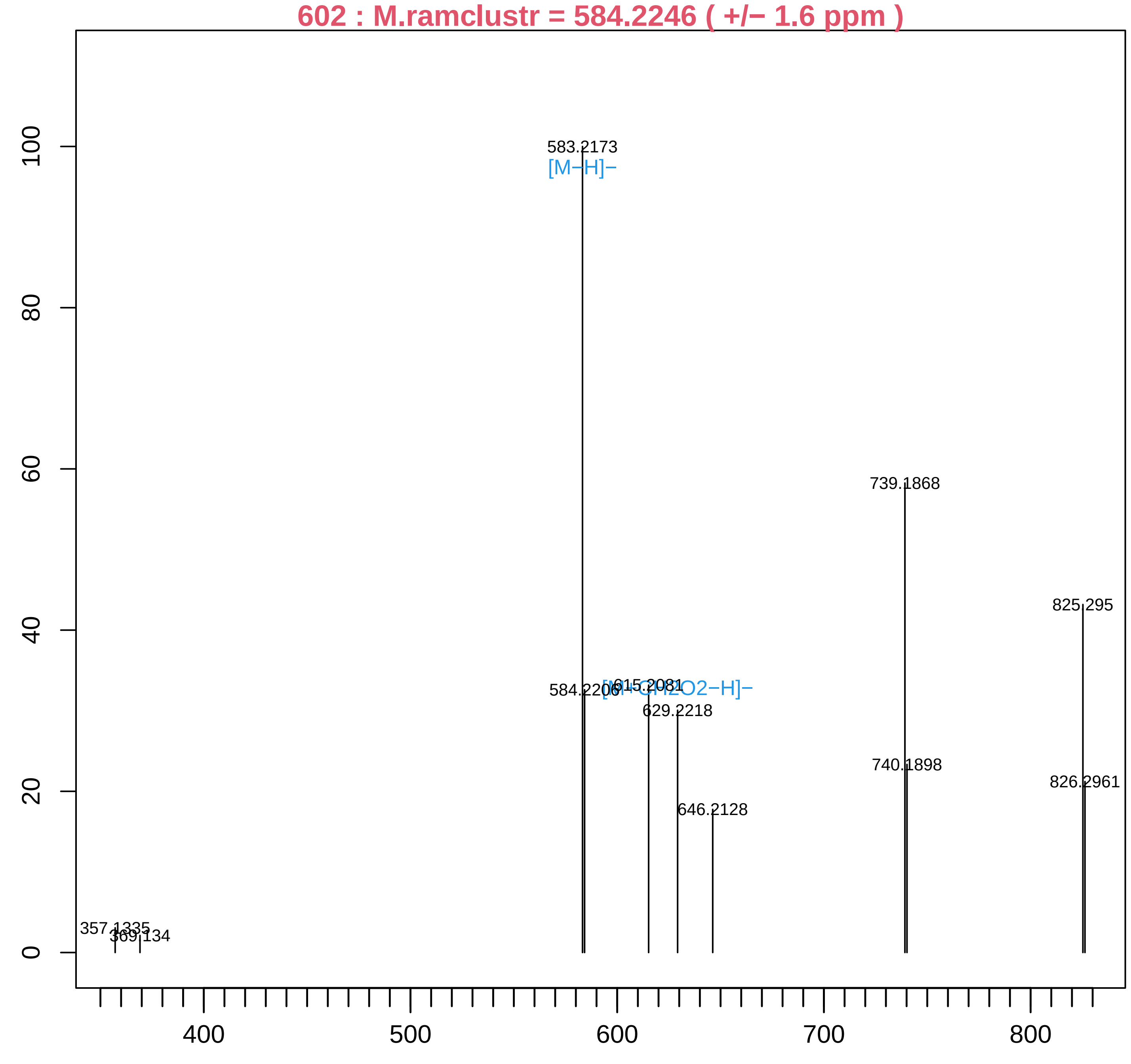
602 : M.ramclustr = 584.2246 (+/− 1.6 ppm)
583.2173
[M−H]−
739.1868
825.295
584.2206
[M+CH2O2−H]−
615.2081
629.2218
740.1898
826.2961
646.2128
357.1335
369.134
0
20
40
60
80
100
400
500
600
700
800

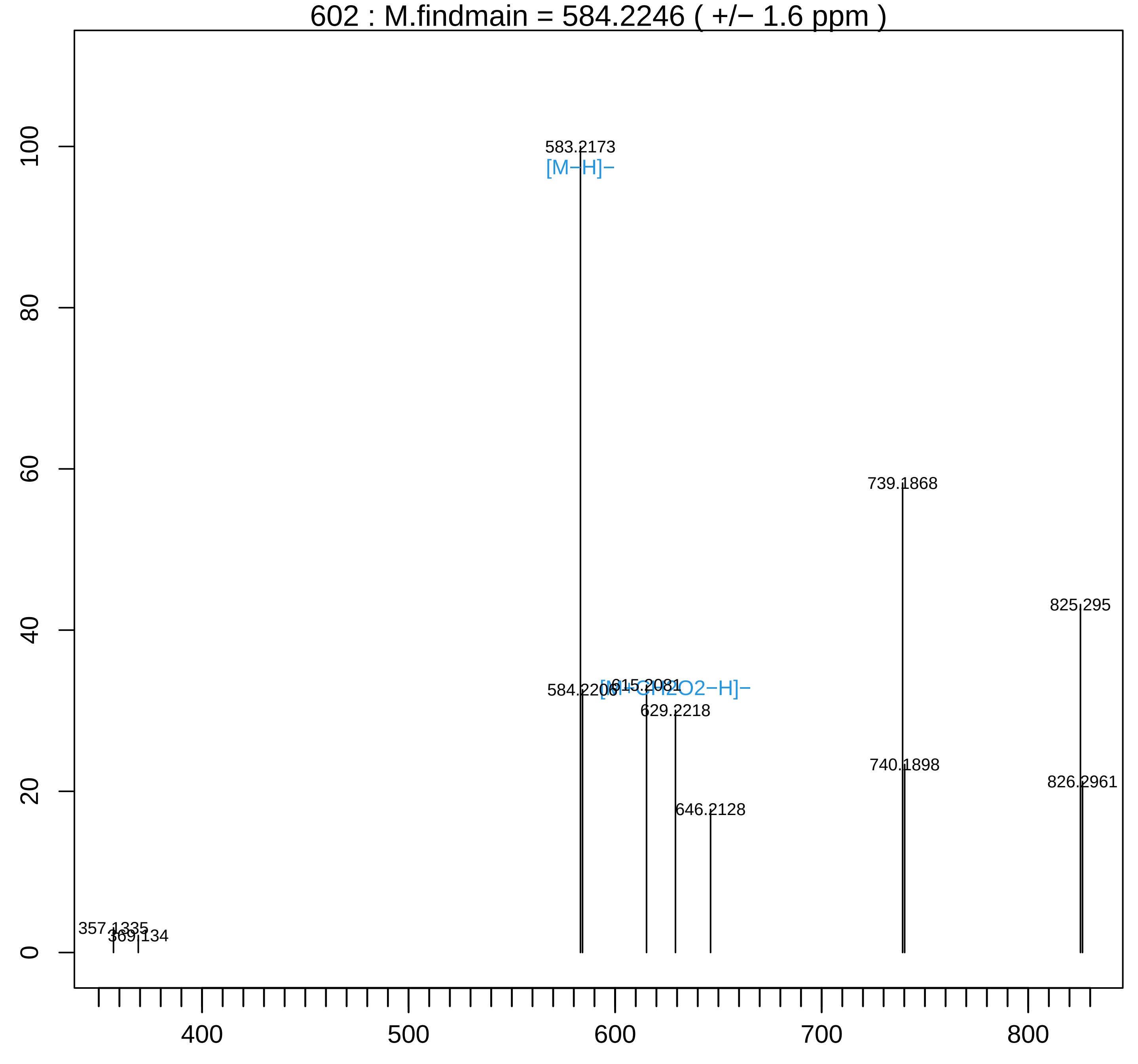
602 : M.findmain = 584.2246 (+/− 1.6 ppm)
583.2173
[M−H]−
739.1868
825.295
584.2206
[M+CH2O2−H]−
615.2081
629.2218
740.1898
826.2961
646.2128
357.1335
369.134
0
20
40
60
80
100
400
500
600
700
800

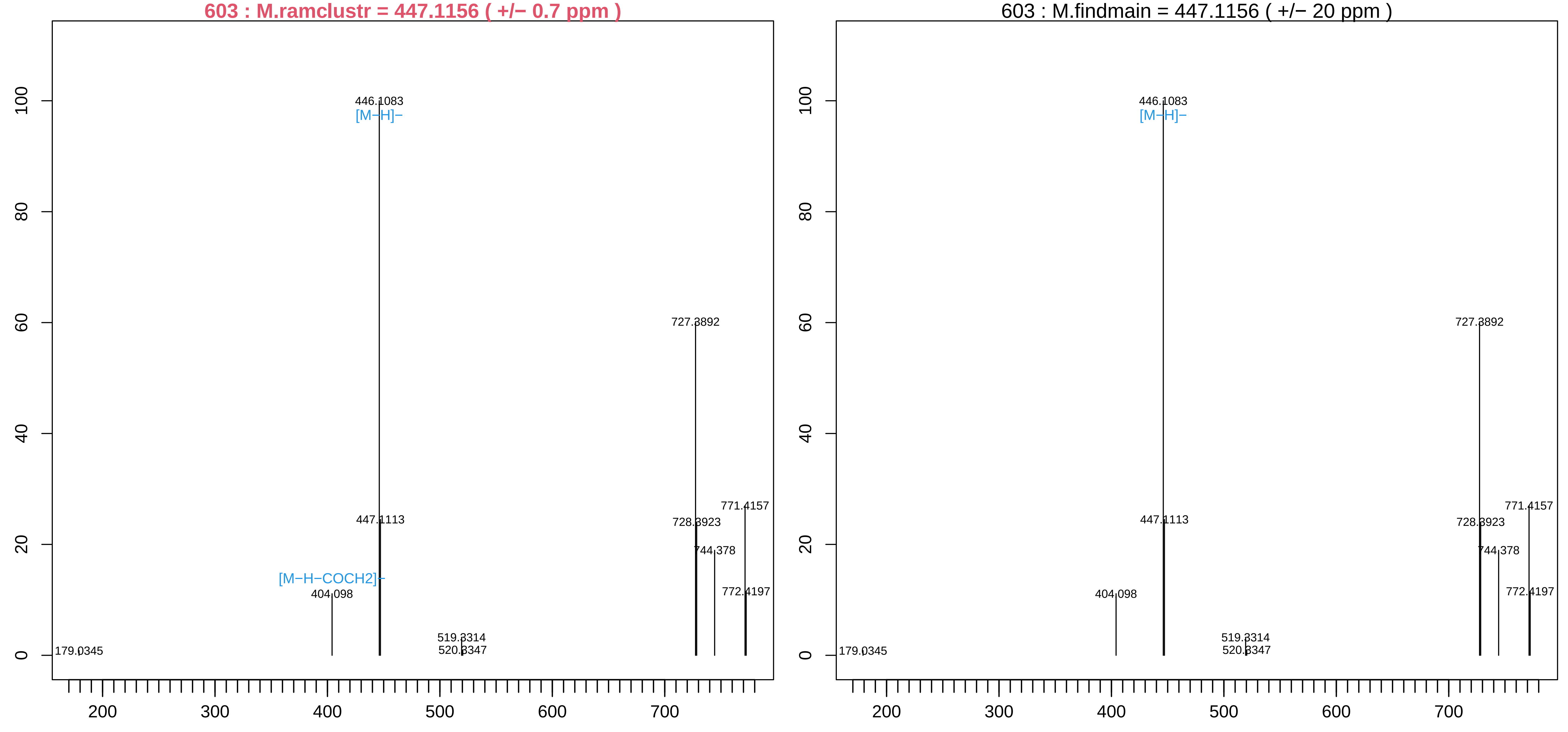
603 : M.ramclustr = 447.1156 (+/− 0.7 ppm)
446.1083
[M−H]−
727.3892
771.4157
447.1113
728.3923
744.378
[M−H−COCH2]−
404.098
772.4197
519.3314
520.3347
179.0345
603 : M.findmain = 447.1156 (+/− 20 ppm)
446.1083
[M−H]−
727.3892
771.4157
447.1113
728.3923
744.378
404.098
772.4197
519.3314
520.3347
179.0345
0
20
40
60
80
100
200
300
400
500
600
700

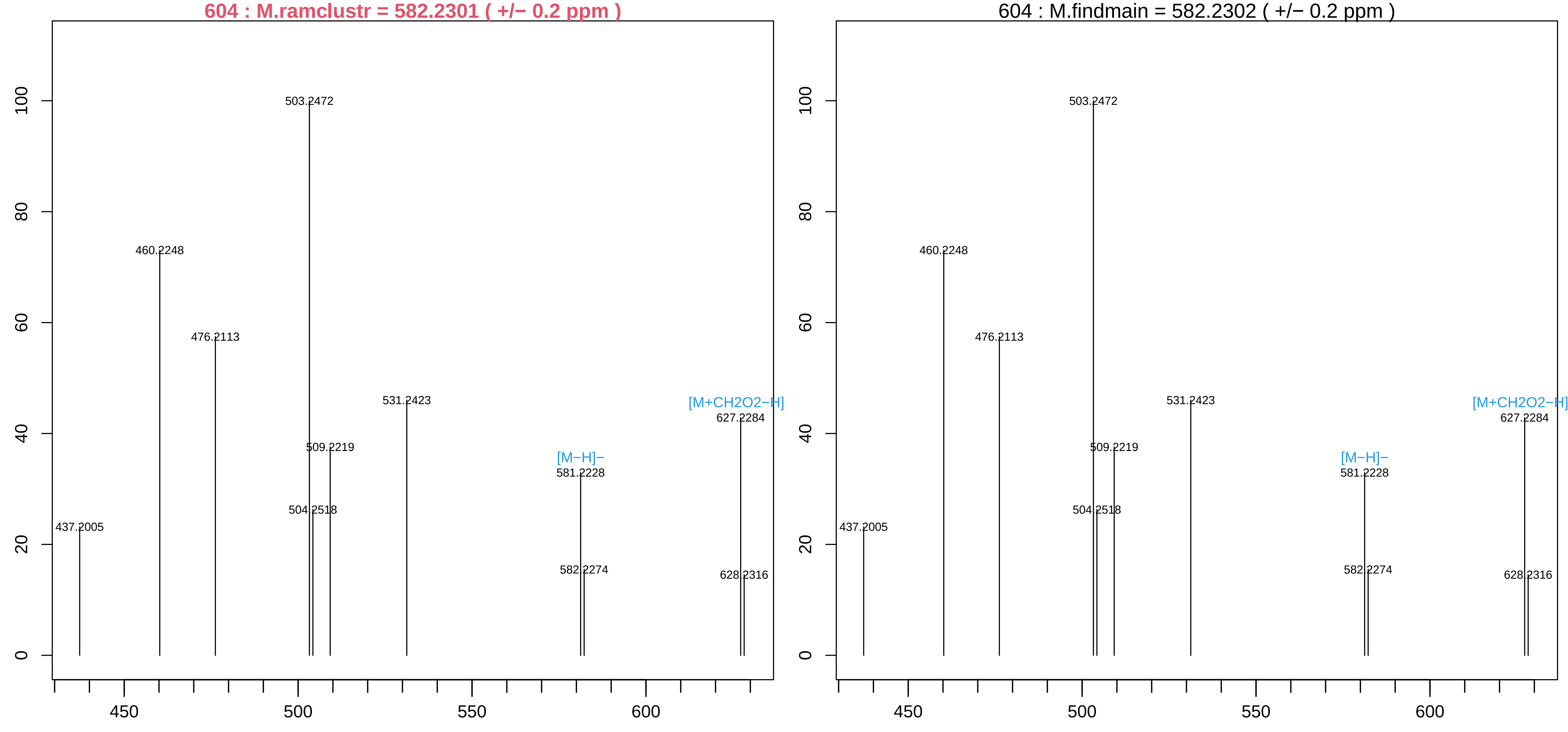

604 : M.ramclustr = 582.2301 (+/− 0.2 ppm)
503.2472
460.2248
476.2113
531.2423
[M+CH2O2−H]
627.2284
509.2219
[M−H]−
581.2228
504.2518
437.2005
582.2274
628.2316
604 : M.findmain = 582.2302 (+/− 0.2 ppm)
503.2472
460.2248
476.2113
531.2423
[M+CH2O2−H]
627.2284
509.2219
[M−H]−
581.2228
504.2518
437.2005
582.2274
628.2316

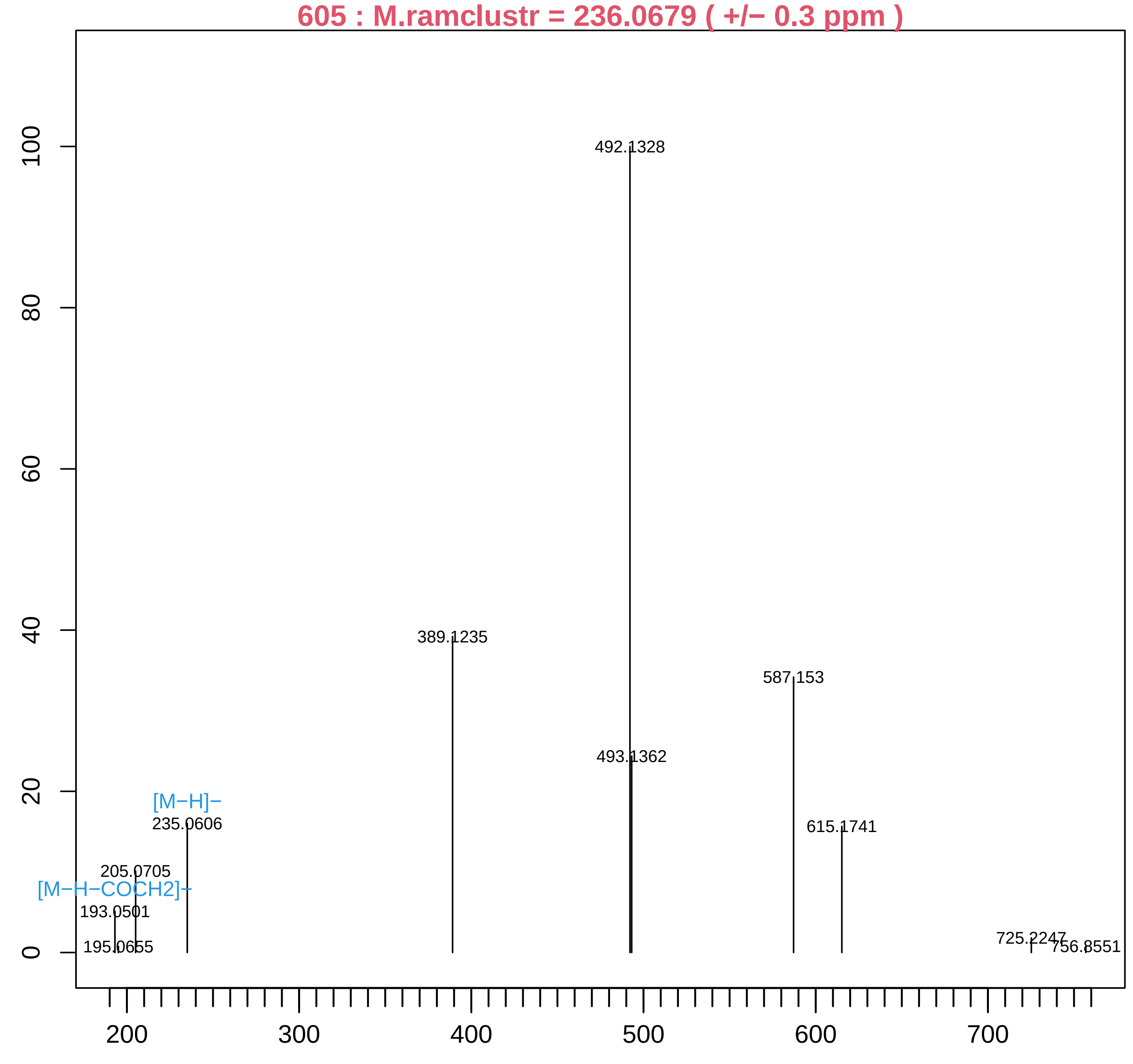
605 : M.ramclustr = 236.0679 (+/− 0.3 ppm)
492.1328
389.1235
587.153
493.1362
615.1741
[M−H]−
235.0606
205.0705
[M−H−COCH2]−
193.0501
195.0655
725.2247
756.8551
0
20
40
60
80
100
200
300
400
500
600
700

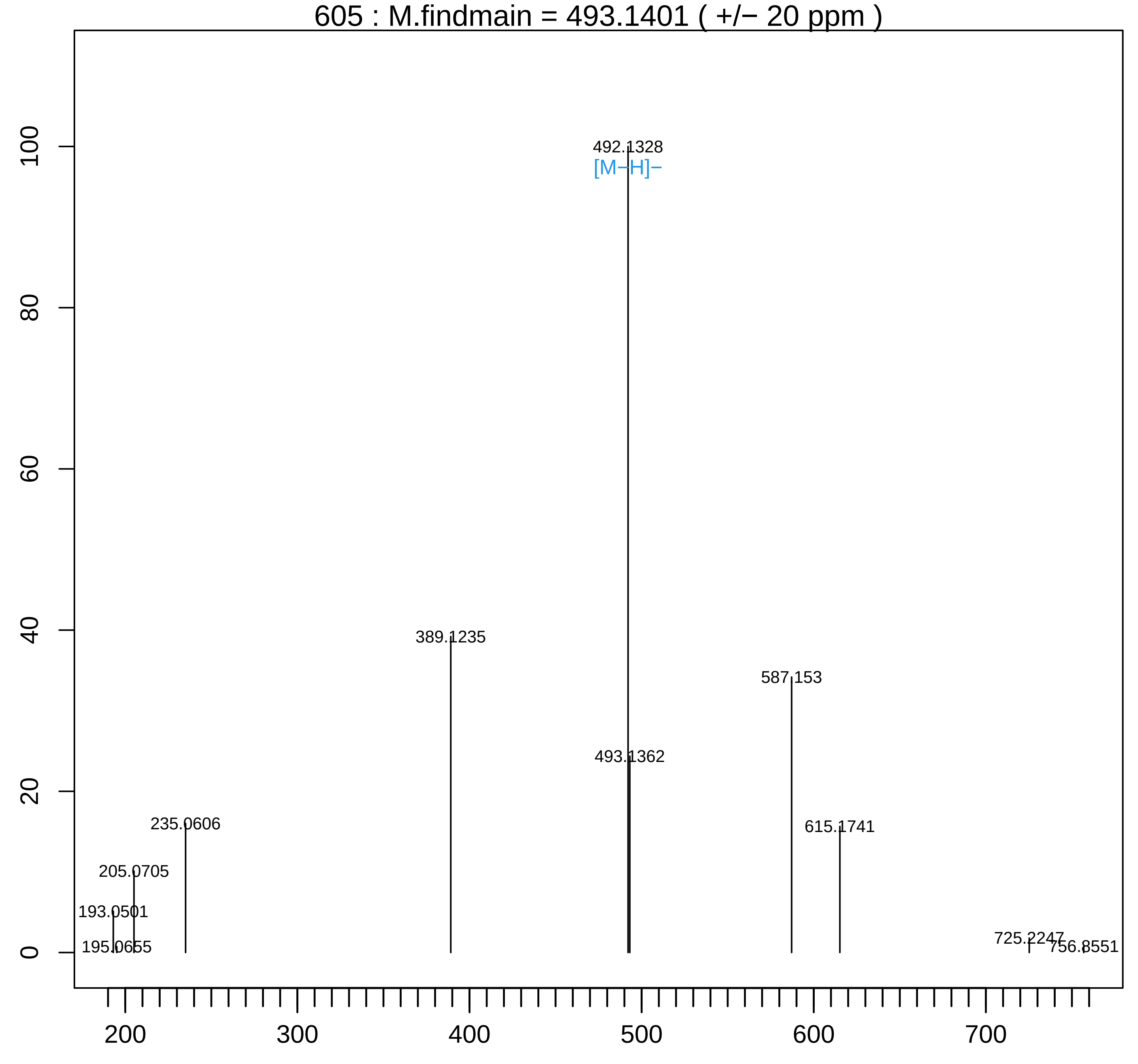
605 : M.findmain = 493.1401 (+/− 20 ppm)
492.1328
[M−H]−
389.1235
587.153
493.1362
235.0606
615.1741
205.0705
193.0501
195.0655
725.2247
756.8551
0
20
40
60
80
100
200
300
400
500
600
700

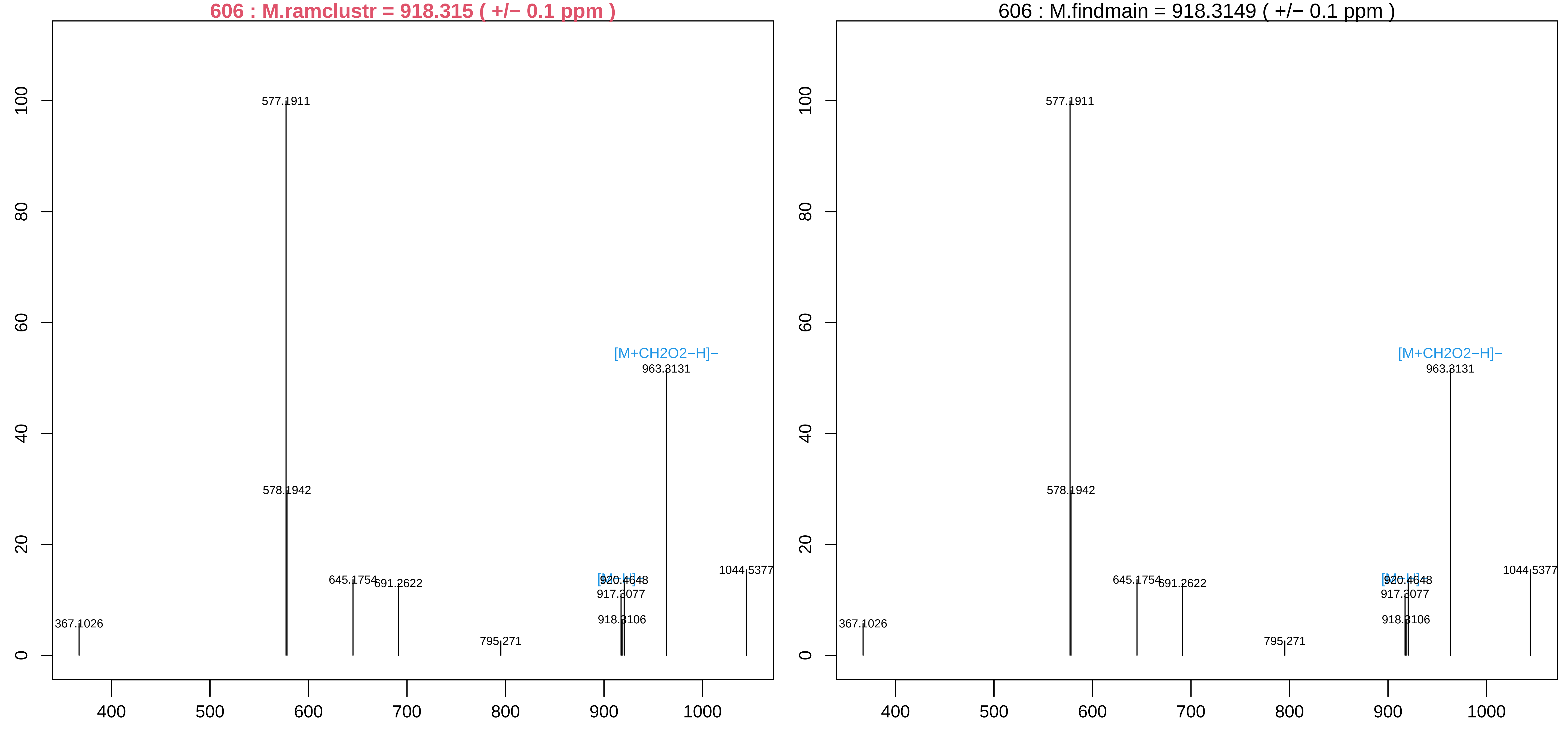
606 : M.ramclustr = 918.315 (+/− 0.1 ppm)
577.1911
578.1942
645.1754
691.2622
795.271
917.3077
918.3106
920.4648
[M+H]
[M+CH2O2−H]−
963.3131
1044.5377
367.1026
0
20
40
60
80
100
400
500
600
700
800
900
1000
606 : M.findmain = 918.3149 (+/− 0.1 ppm)
577.1911
578.1942
645.1754
691.2622
795.271
917.3077
918.3106
920.4648
[M+H]
[M+CH2O2−H]−
963.3131
1044.5377
367.1026
0
20
40
60
80
100
400
500
600
700
800
900
1000

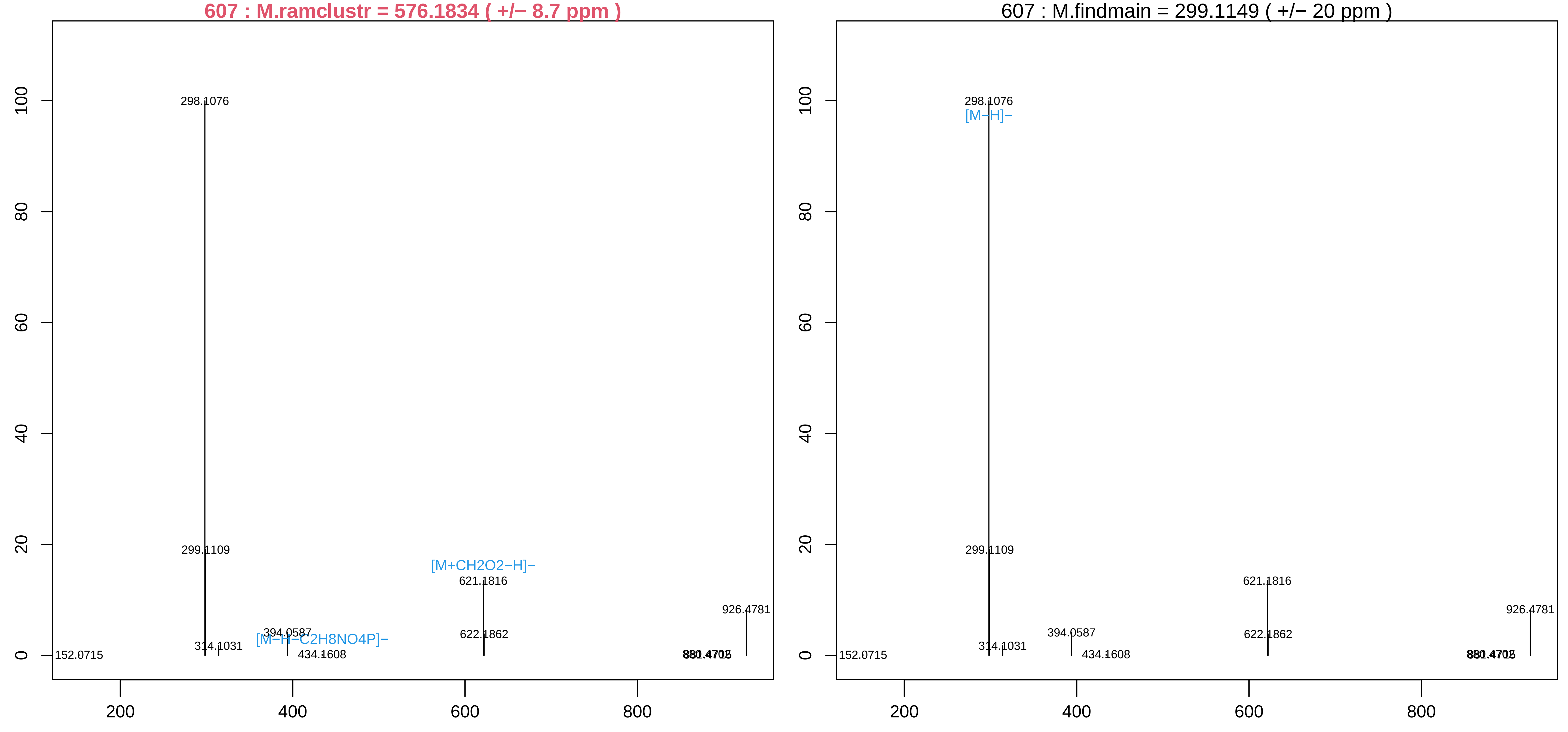

607 : M.ramclustr = 576.1834 (+/− 8.7 ppm)
298.1076
299.1109
[M+CH2O2−H]−
621.1816
926.4781
394.0587
[M−H−C2H8NO4P]−
314.1031
622.1862
152.0715
434.1608
880.4702
0
20
40
60
80
100
200
400
600
800
607 : M.findmain = 299.1149 (+/− 20 ppm)
298.1076
[M−H]−
299.1109
621.1816
926.4781
394.0587
622.1862
314.1031
152.0715
434.1608
880.4702
0
20
40
60
80
100
200
400
600
800

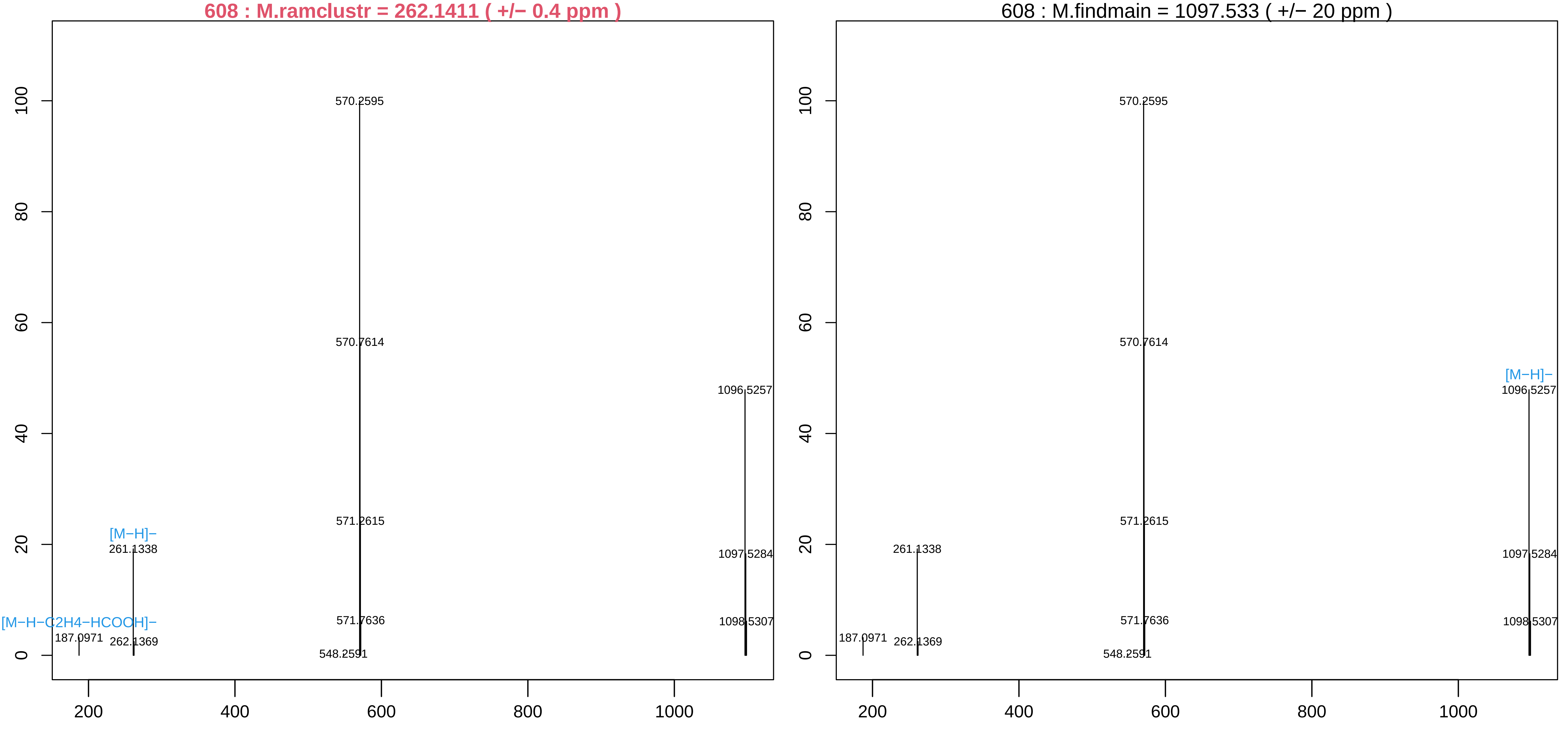
608 : M.ramclustr = 262.1411 (+/− 0.4 ppm)
570.2595
570.7614
1096.5257
571.2615
[M−H]−
261.1338
1097.5284
[M−H−C2H4−HCOOH]−
571.7636
1098.5307
187.0971
262.1369
548.2591
0
20
40
60
80
100
200
400
600
800
1000
608 : M.findmain = 1097.533 (+/− 20 ppm)
570.2595
570.7614
[M−H]−
1096.5257
571.2615
261.1338
1097.5284
571.7636
1098.5307
187.0971
262.1369
548.2591
0
20
40
60
80
100
200
400
600
800
1000

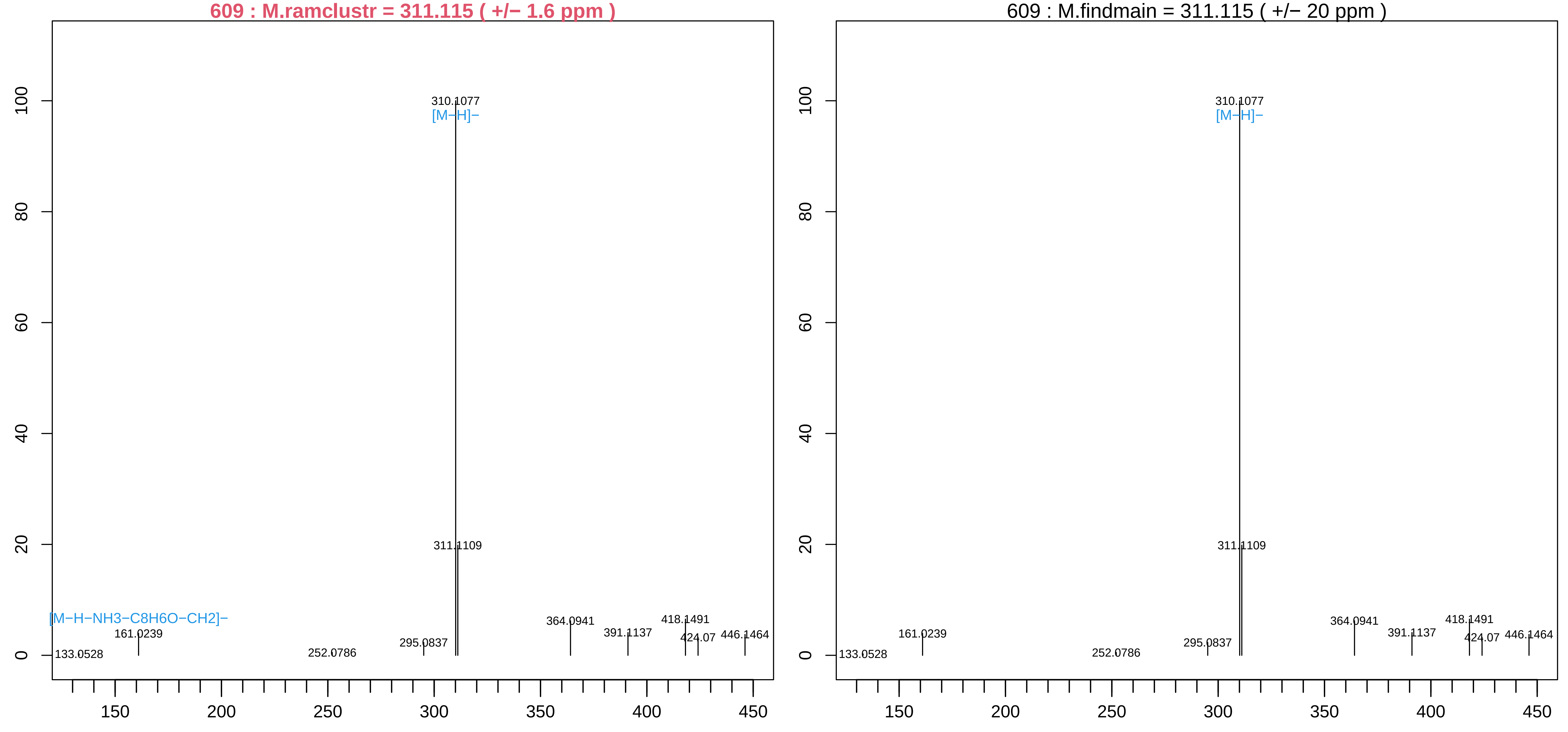

609 : M.ramclustr = 311.115 (+/− 1.6 ppm)
310.1077
[M−H]−
311.1109
[M−H−NH3−C8H6O−CH2]−
161.0239
133.0528
252.0786
295.0837
364.0941
391.1137
418.1491
424.07
446.1464
609 : M.findmain = 311.115 (+/− 20 ppm)
310.1077
[M−H]−
311.1109
161.0239
133.0528
252.0786
295.0837
364.0941
391.1137
418.1491
424.07
446.1464

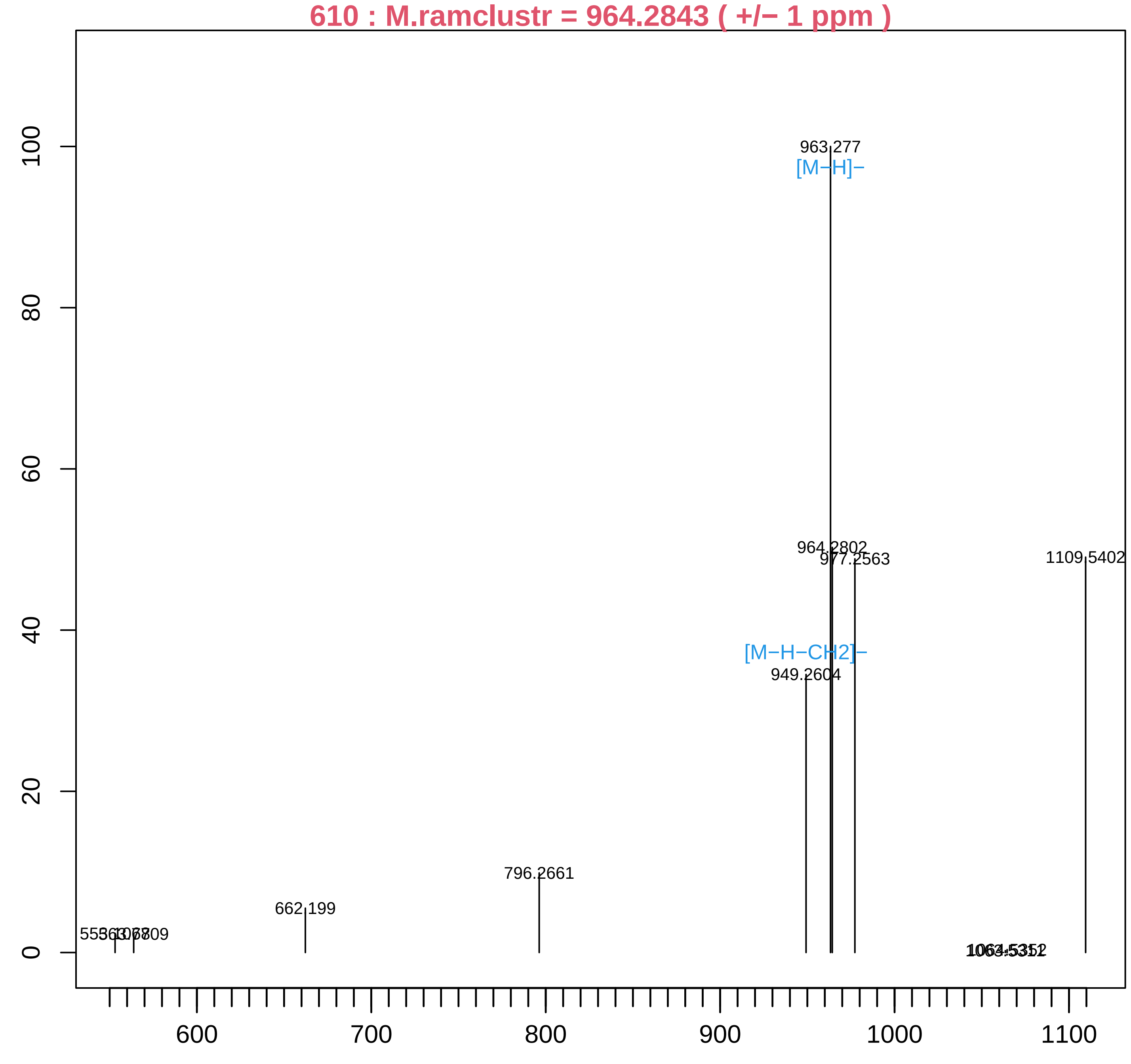
610 : M.ramclustr = 964.2843 (+/− 1 ppm)
963.277
[M−H]−
964.2802
977.2563
1109.5402
[M−H−CH2]−
949.2604
796.2661
662.199
0
20
40
60
80
100
600
700
800
900
1000
1100

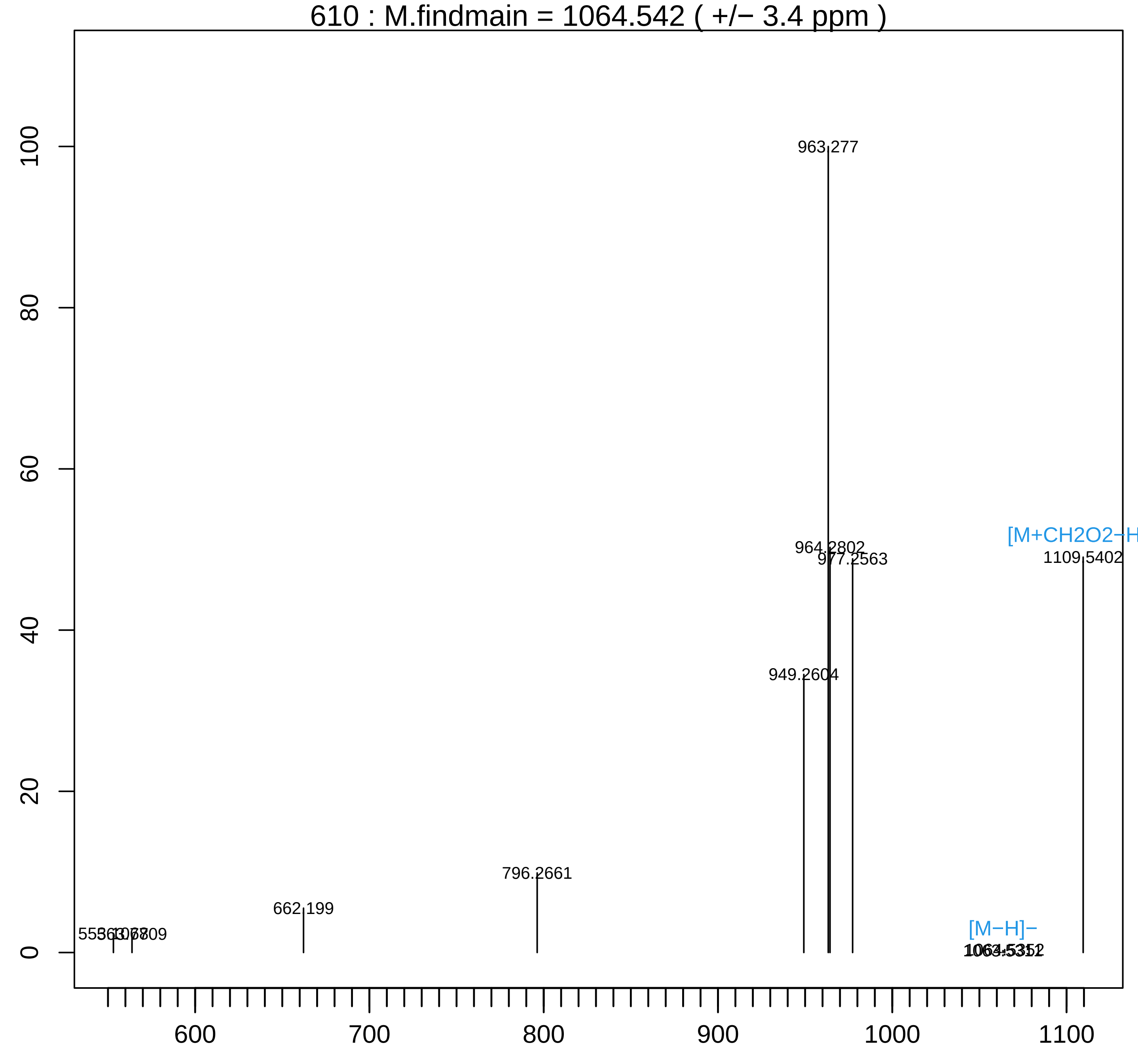
610 : M.findmain = 1064.542 (+/− 3.4 ppm)
963.277
964.2802
977.2563
[M+CH2O2−H
1109.5402
949.2604
796.2661
662.199
[M−H]−
0
20
40
60
80
100
600
700
800
900
1000
1100

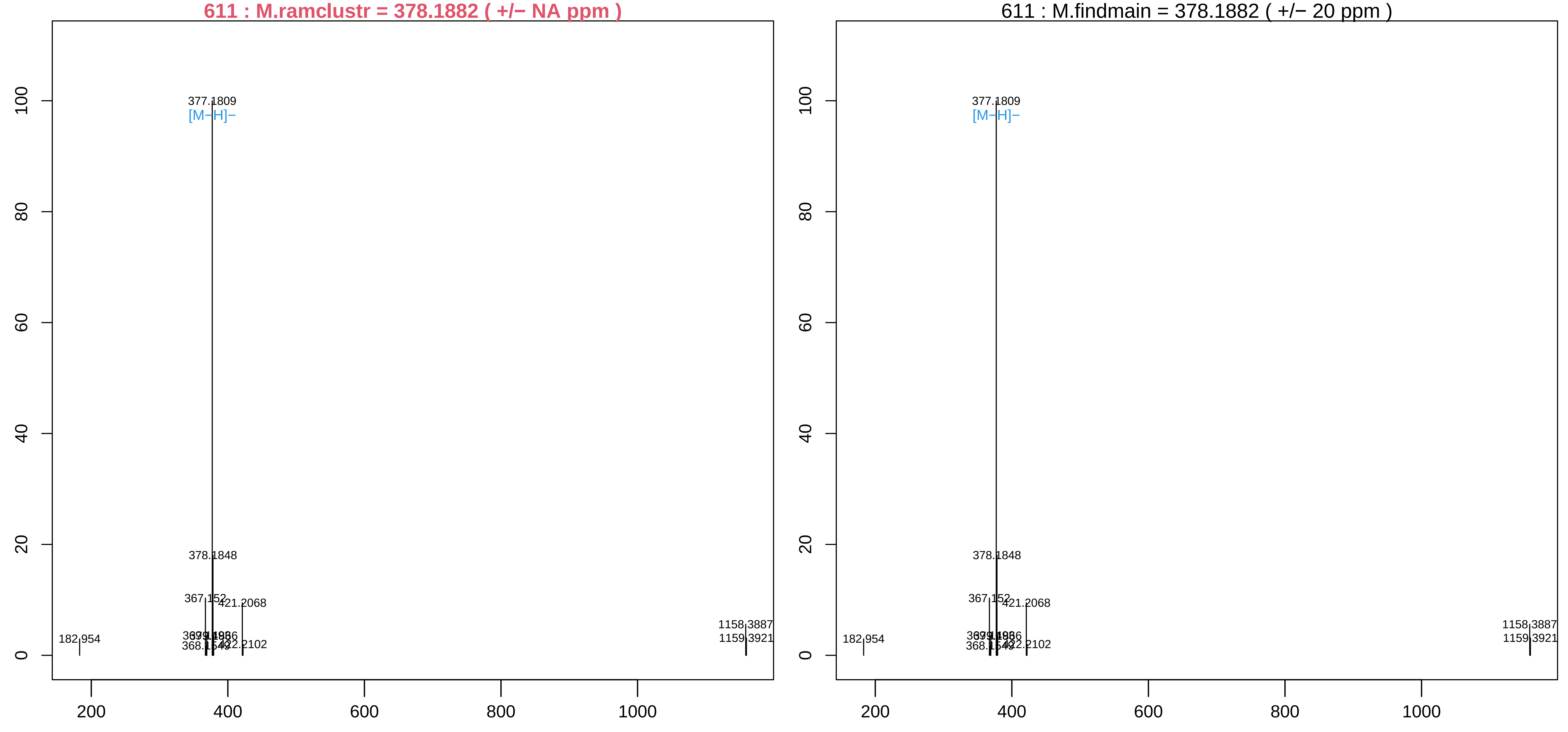

611 : M.ramclustr = 378.1882 (+/− NA ppm)
377.1809
[M−H]−
378.1848
367.152
421.2068
182.954
368.1549
422.2102
1158.3887
1159.3921
611 : M.findmain = 378.1882 (+/− 20 ppm)
377.1809
[M−H]−
378.1848
367.152
421.2068
182.954
368.1549
422.2102
1158.3887
1159.3921

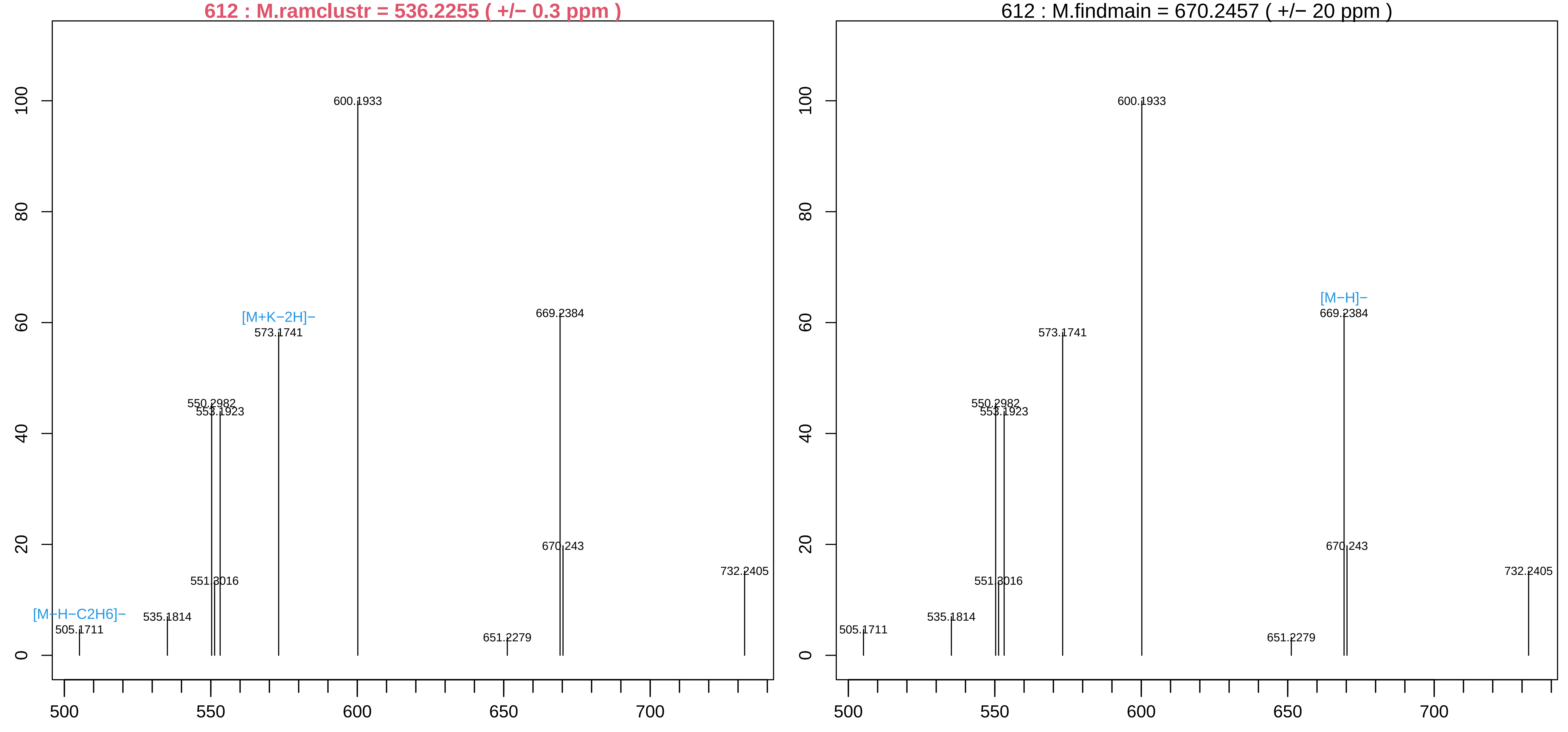

612 : M.ramclustr = 536.2255 (+/− 0.3 ppm)
[M−H−C2H6]−
505.1711
535.1814
550.2982
553.1923
551.3016
[M+K−2H]−
573.1741
600.1933
651.2279
669.2384
670.243
732.2405
612 : M.findmain = 670.2457 (+/− 20 ppm)
505.1711
535.1814
550.2982
553.1923
551.3016
573.1741
600.1933
651.2279
[M−H]−
669.2384
670.243
732.2405
0
20
40
60
80
100
500
550
600
650
700

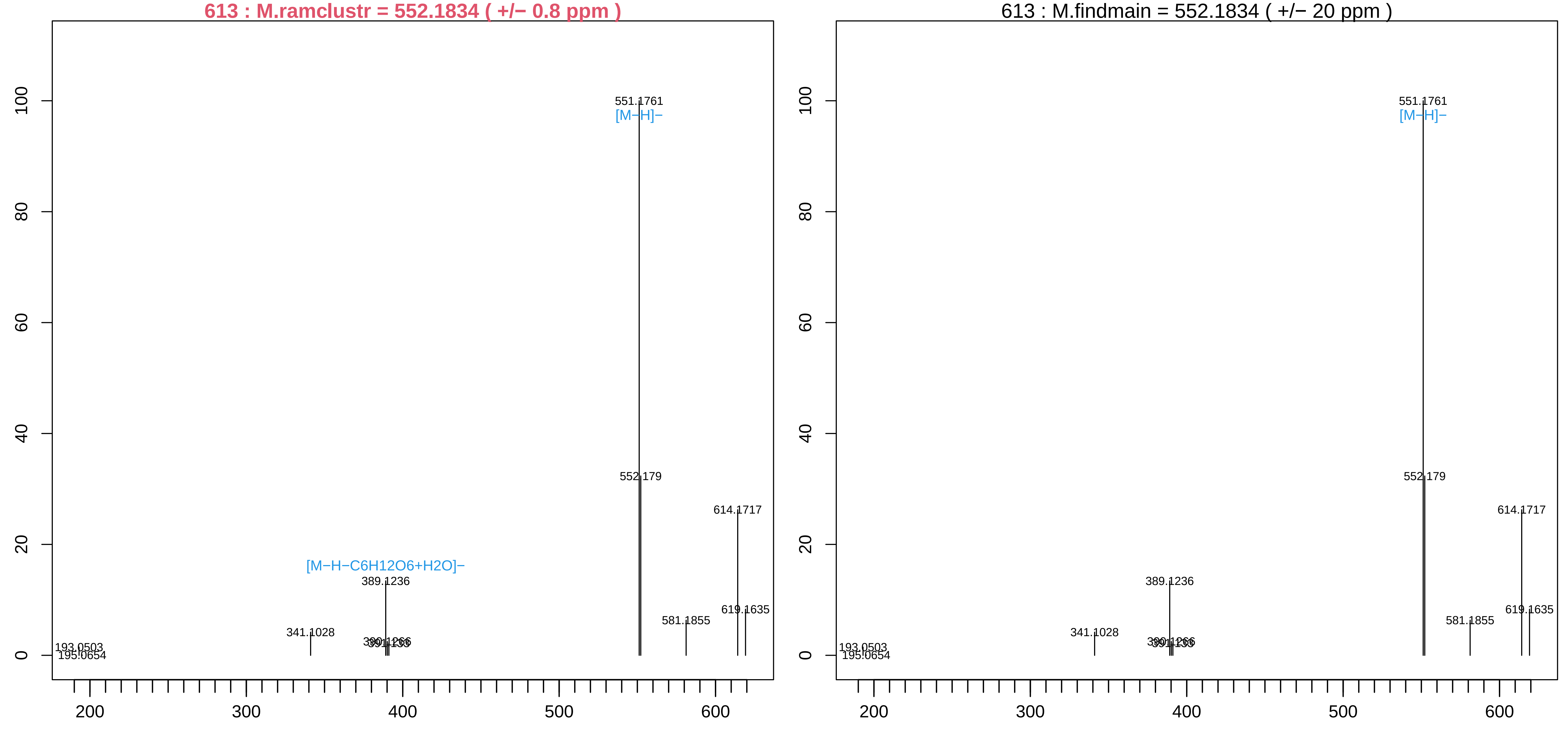

613 : M.ramclustr = 552.1834 (+/− 0.8 ppm)
551.1761
[M−H]−
552.179
614.1717
[M−H−C6H12O6+H2O]−
389.1236
619.1635
581.1855
341.1028
193.0503
195.0654
613 : M.findmain = 552.1834 (+/− 20 ppm)
551.1761
[M−H]−
552.179
614.1717
389.1236
619.1635
581.1855
341.1028
193.0503
195.0654
0
20
40
60
80
100
200
300
400
500
600

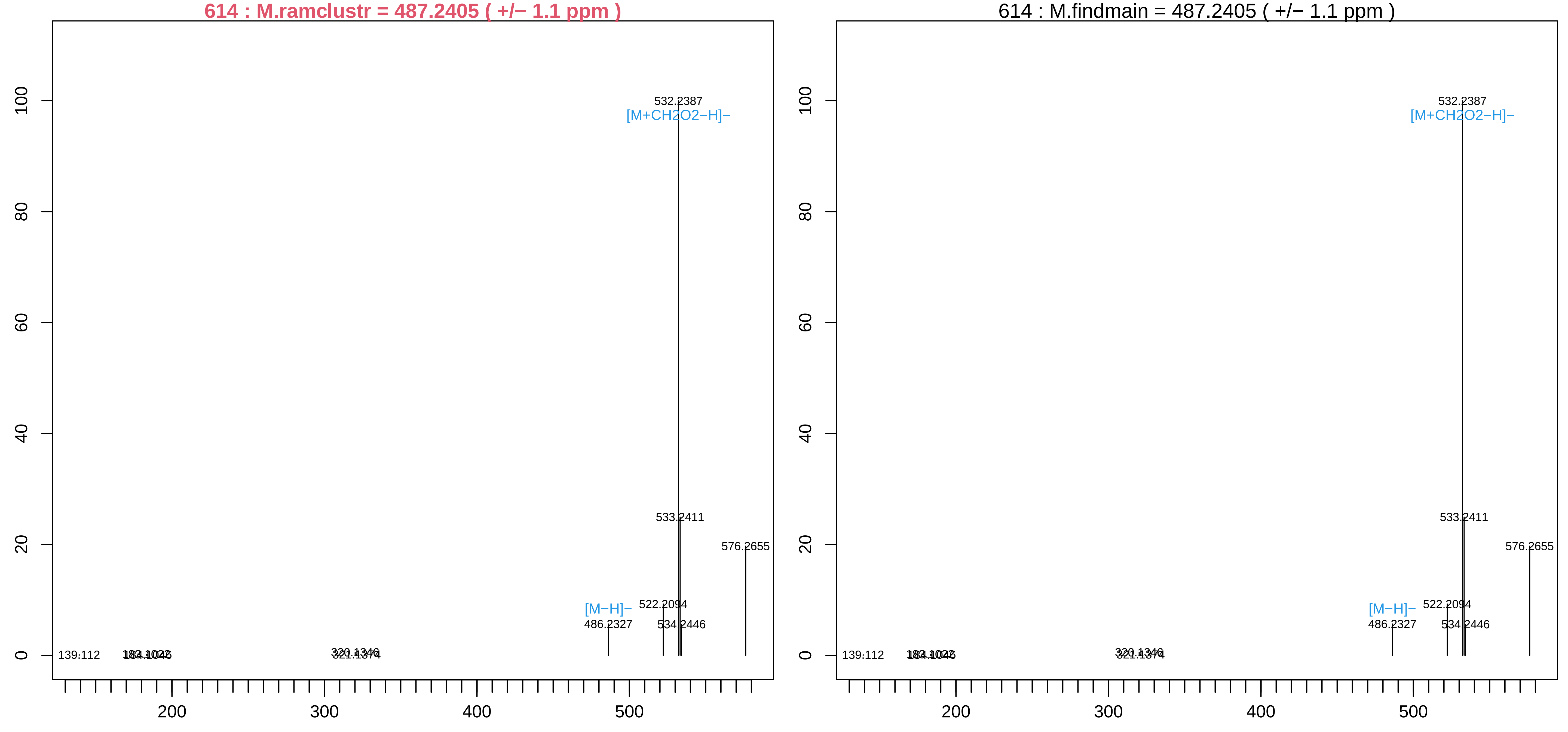
614 : M.ramclustr = 487.2405 (+/− 1.1 ppm)
614 : M.findmain = 487.2405 (+/− 1.1 ppm)
532.2387
[M+CH2O2−H]−
533.2411
576.2655
[M−H]−
522.2094
486.2327
534.2446
139:112
320.1346
321.1374
0
20
40
60
80
100
200
300
400
500

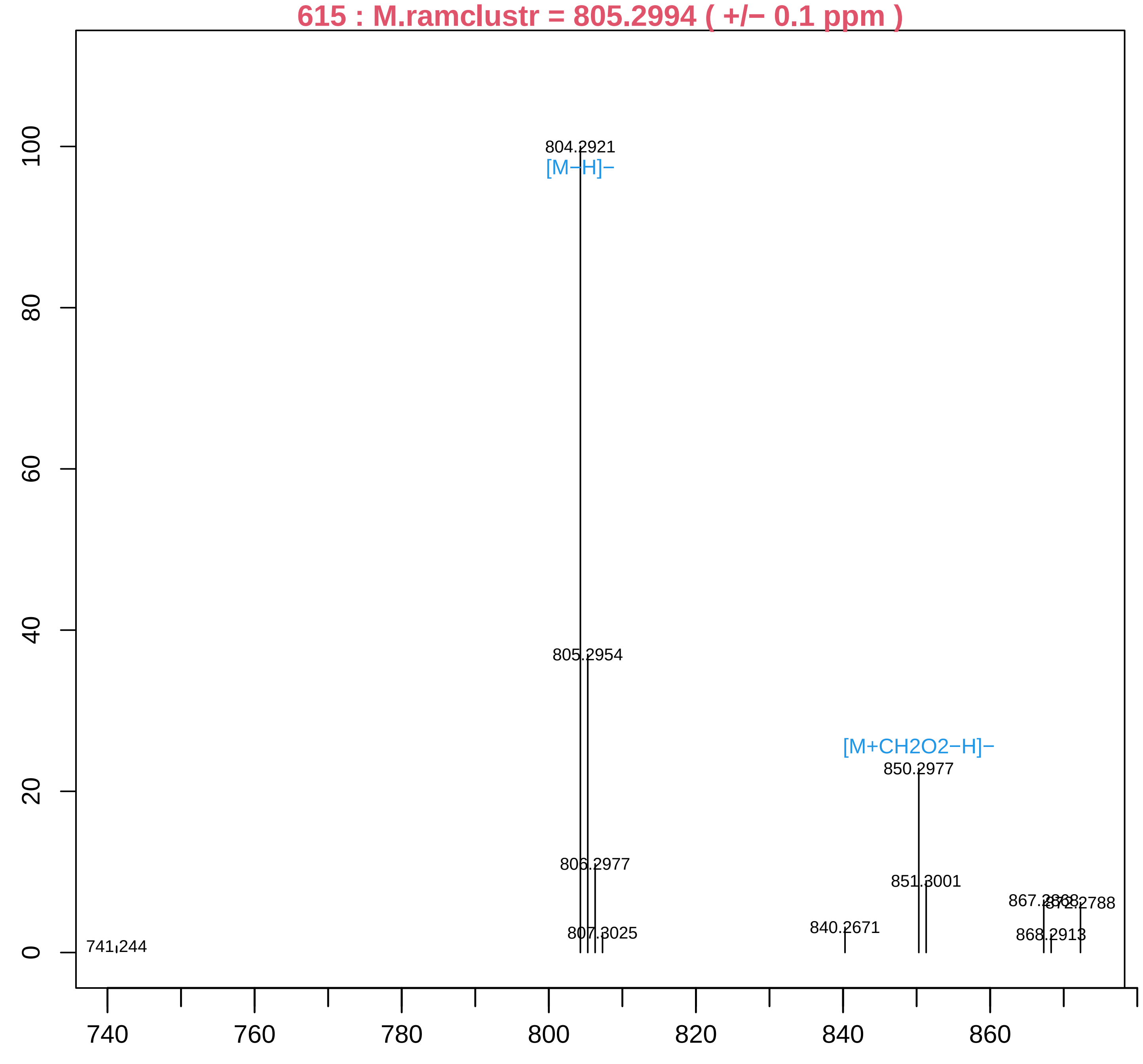

615 : M.ramclustr = 805.2994 (+/− 0.1 ppm)
804.2921
[M−H]−
805.2954
806.2977
807.3025
[M+CH2O2−H]−
850.2977
851.3001
840.2671
867.2868
872.2788
868.2913
741.244
0
20
40
60
80
100
740
760
780
800
820
840
860

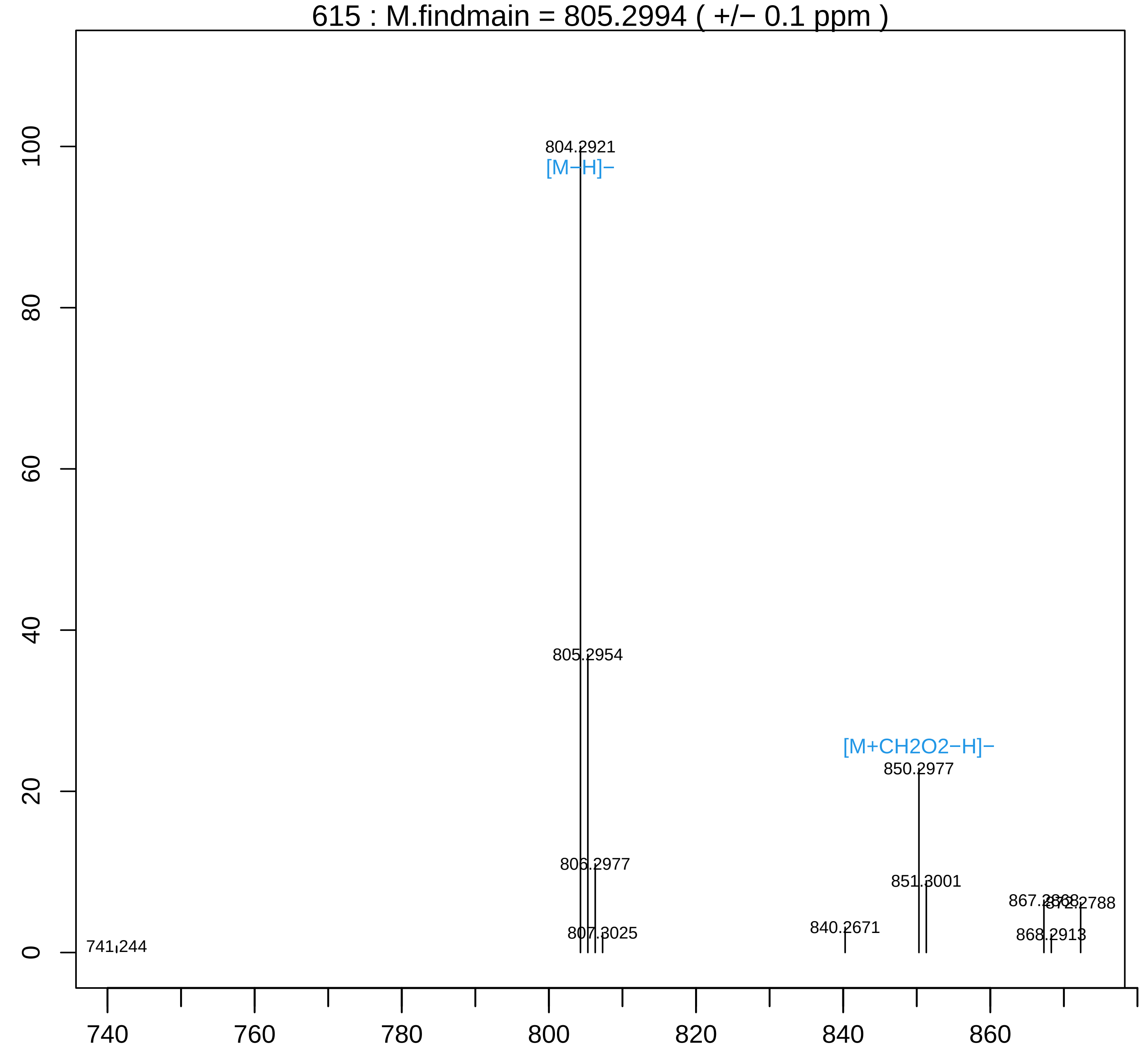

615 : M.findmain = 805.2994 (+/− 0.1 ppm)
804.2921
[M−H]−
805.2954
806.2977
807.3025
[M+CH2O2−H]−
850.2977
851.3001
840.2671
867.2868
872.2788
868.2913
741.244
0
20
40
60
80
100
740
760
780
800
820
840
860

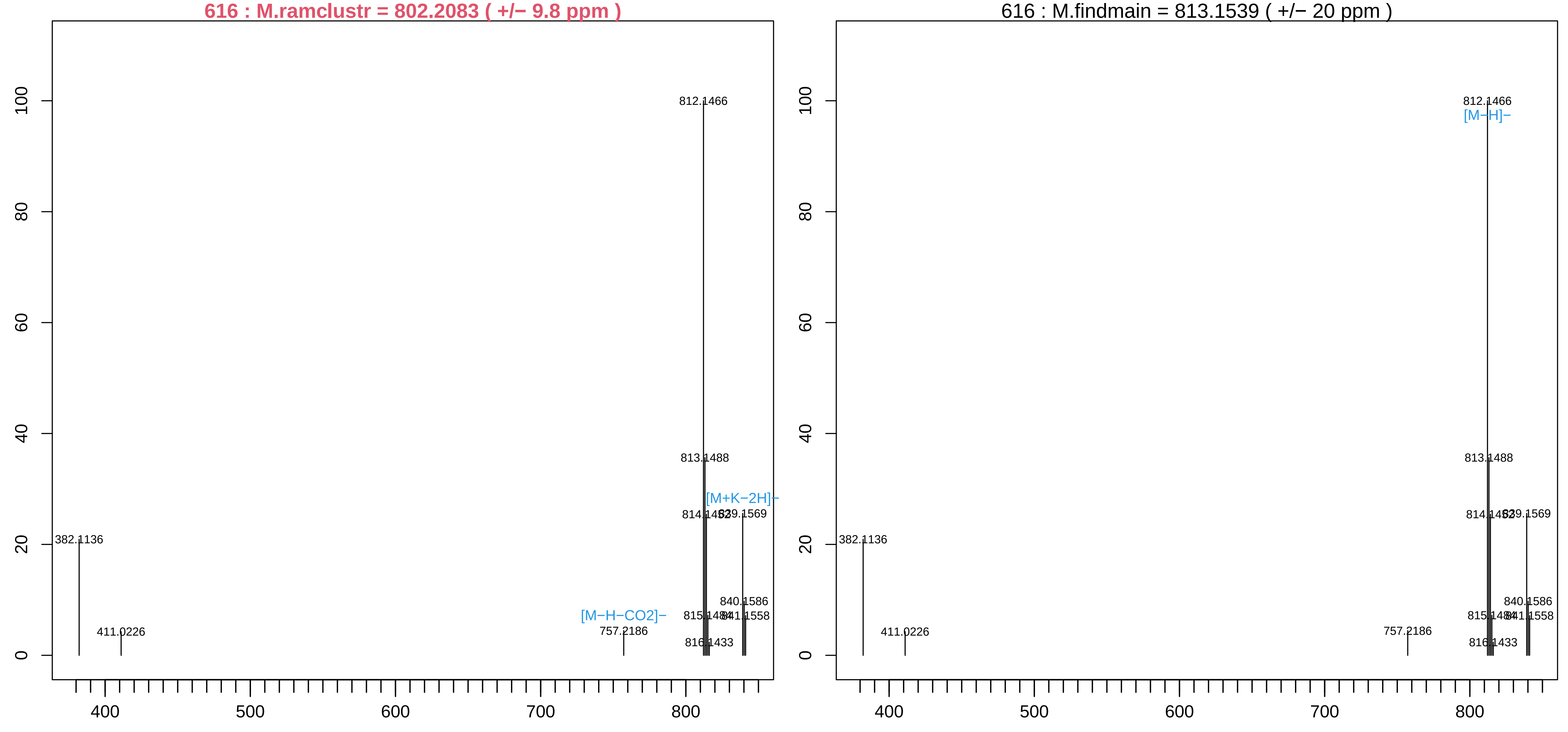
616 : M.ramclustr = 802.2083 (+/− 9.8 ppm)
812.1466
813.1488
[M+K−2H]−
814.1482
839.1569
382.1136
840.1586
[M−H−CO2]−
815.1484
841.1558
411.0226
757.2186
816.1433
616 : M.findmain = 813.1539 (+/− 20 ppm)
812.1466
[M−H]−
813.1488
814.1482
839.1569
382.1136
840.1586
815.1484
841.1558
411.0226
757.2186
816.1433
0
20
40
60
80
100
400
500
600
700
800

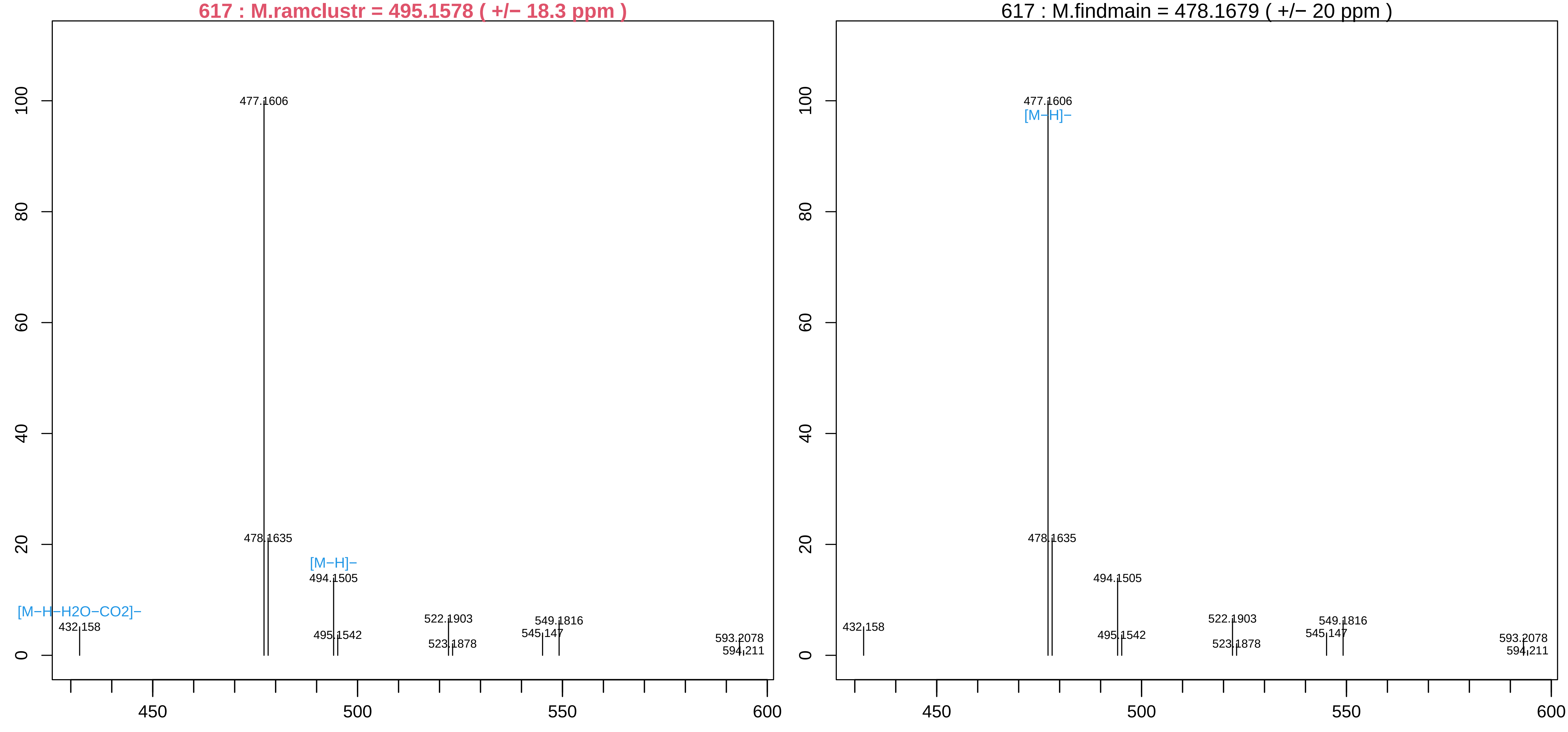
617 : M.ramclustr = 495.1578 (+/− 18.3 ppm)
477.1606
478.1635
[M−H]−
494.1505
[M−H−H2O−CO2]−
432.158
495.1542
522.1903
523.1878
549.1816
545.147
593.2078
594.211
617 : M.findmain = 478.1679 (+/− 20 ppm)
477.1606
[M−H]−
478.1635
494.1505
432.158
495.1542
522.1903
523.1878
549.1816
545.147
593.2078
594.211

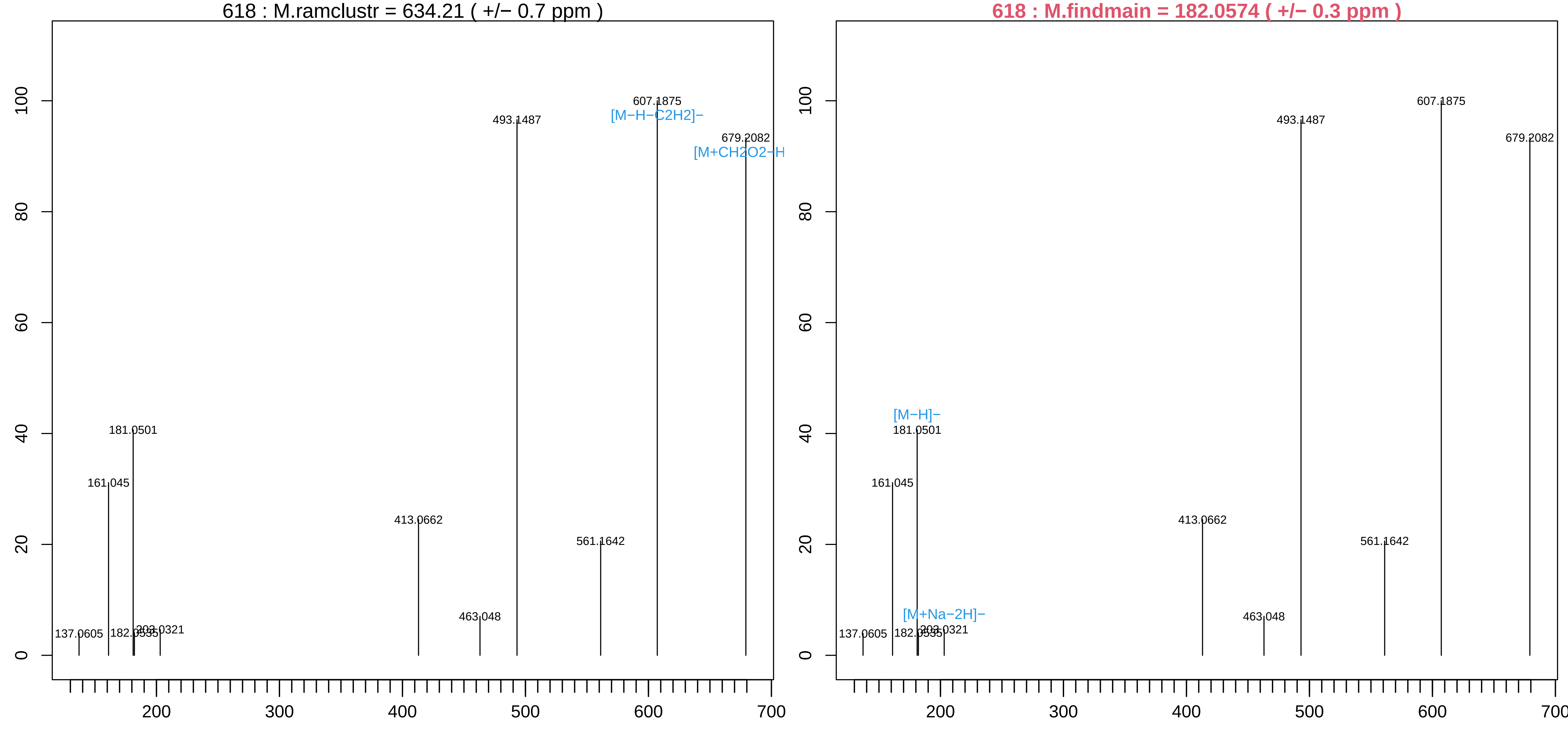
618 : M.ramclustr = 634.21 (+/− 0.7 ppm)
618 : M.findmain = 182.0574 (+/− 0.3 ppm)
607.1875
[M−H−C2H2]−
493.1487
679.2082
[M+CH2O2−H
181.0501
161.045
413.0662
561.1642
463.048
137.0605
182.0535
203.0321
[M−H]−
[M+Na−2H]−
0
20
40
60
80
100
200
300
400
500
600
700

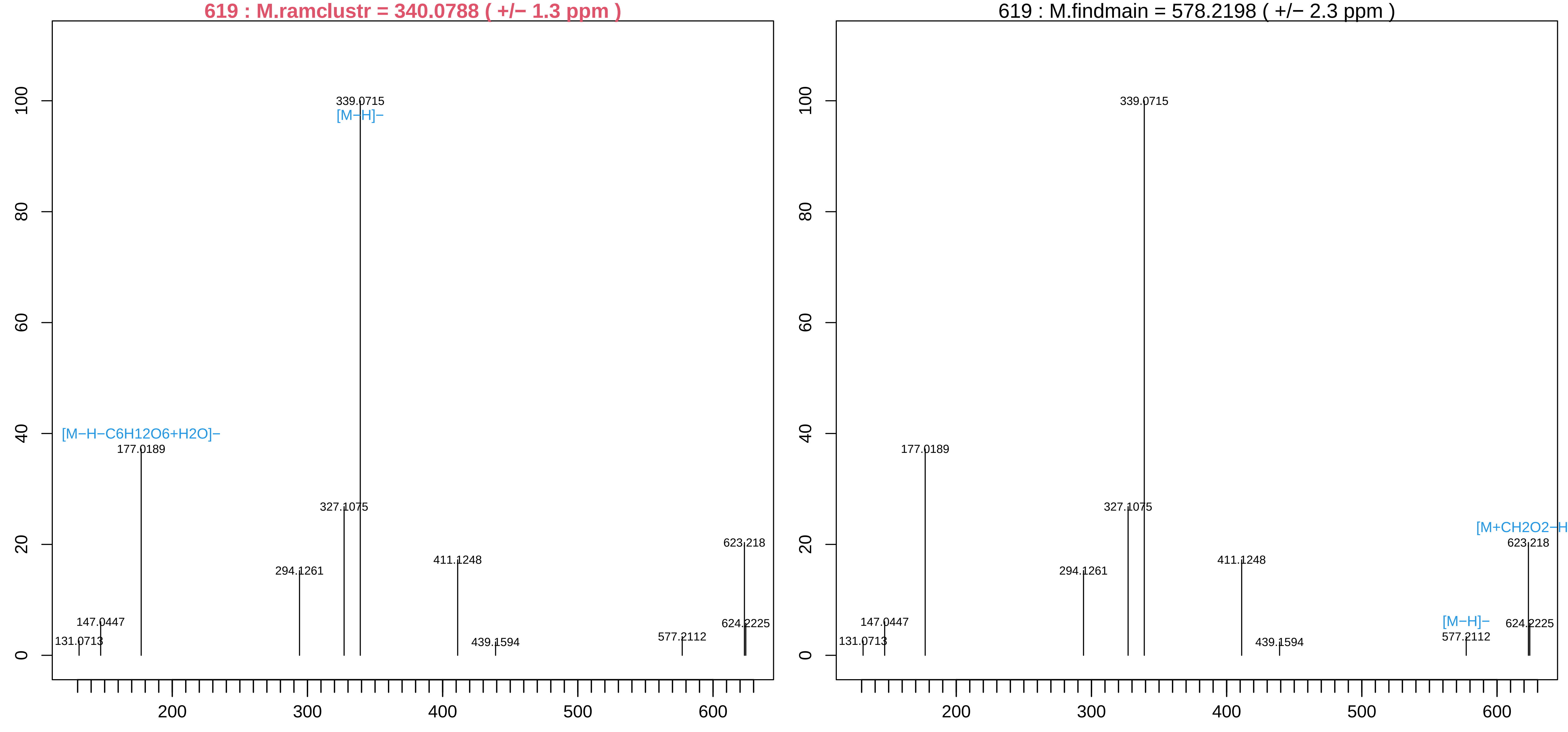

619 : M.ramclustr = 340.0788 (+/− 1.3 ppm)
339.0715
[M−H]−
[M−H−C6H12O6+H2O]−
177.0189
327.1075
294.1261
411.1248
623.218
147.0447
624.2225
131.0713
439.1594
577.2112
0
20
40
60
80
100
200
300
400
500
600
619 : M.findmain = 578.2198 (+/− 2.3 ppm)
339.0715
177.0189
327.1075
[M+CH2O2−H
623.218
411.1248
294.1261
147.0447
[M−H]−
624.2225
131.0713
439.1594
577.2112
0
20
40
60
80
100
200
300
400
500
600

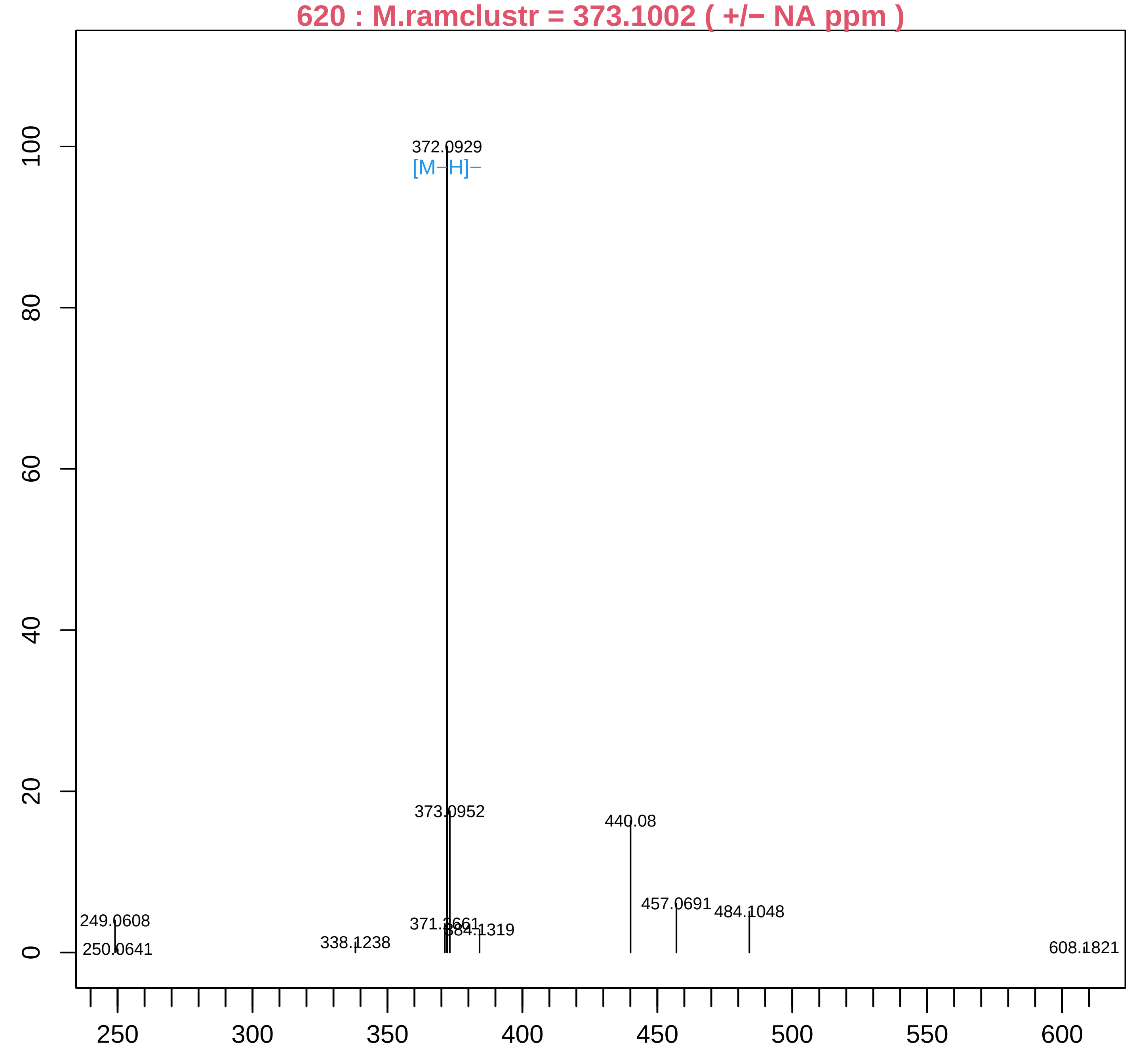
620 : M.ramclustr = 373.1002 (+/− NA ppm)
372.0929
[M−H]−
373.0952
440.08
457.0691
484.1048
249.0608
250.0641
338.1238
371.3661
384.1319
608.1821
0
20
40
60
80
100
250
300
350
400
450
500
550
600

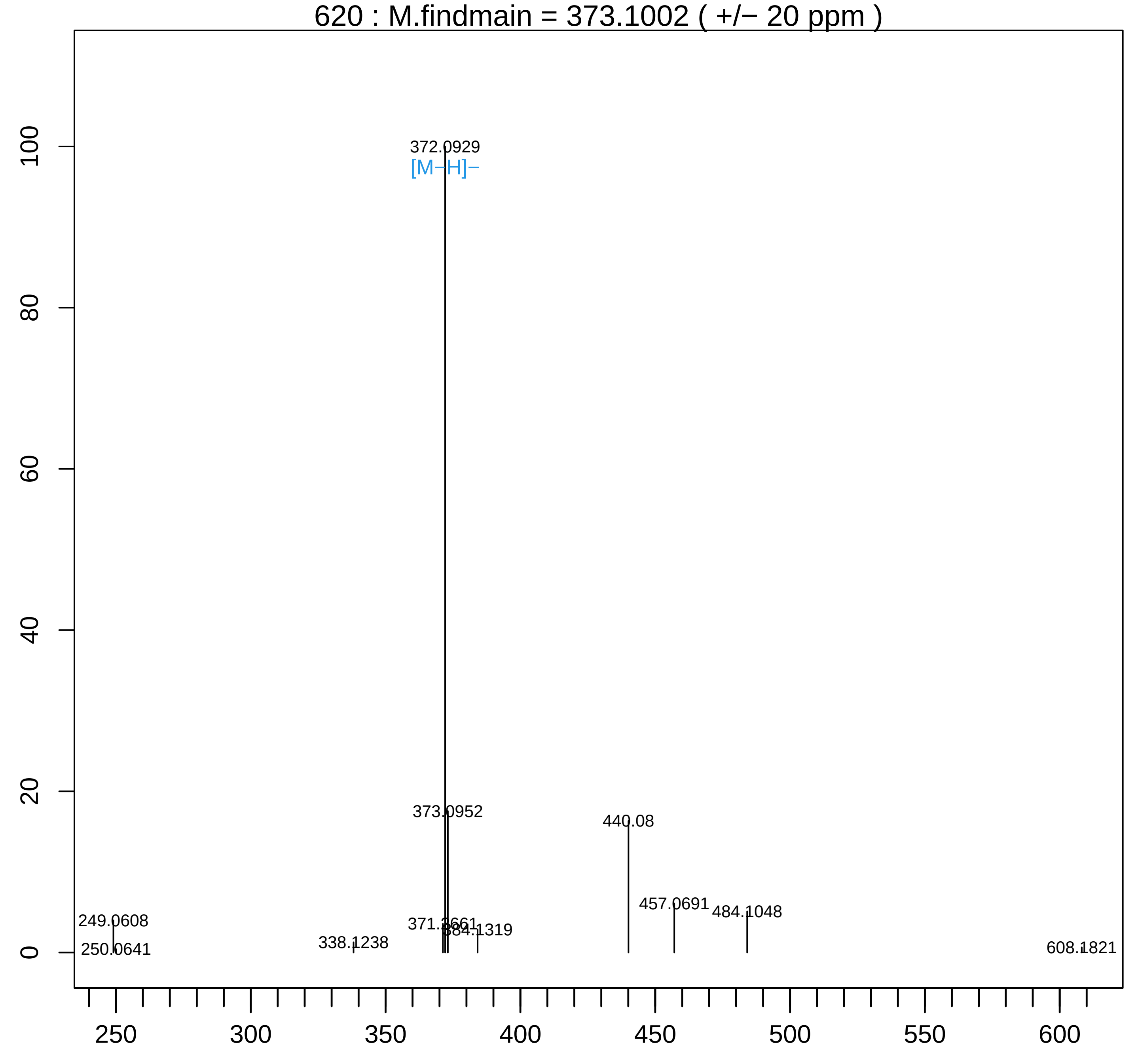
620 : M.findmain = 373.1002 (+/− 20 ppm)
372.0929
[M−H]−
373.0952
440.08
457.0691
484.1048
249.0608
250.0641
338.1238
371.3661
384.1319
608.1821
0
20
40
60
80
100
250
300
350
400
450
500
550
600

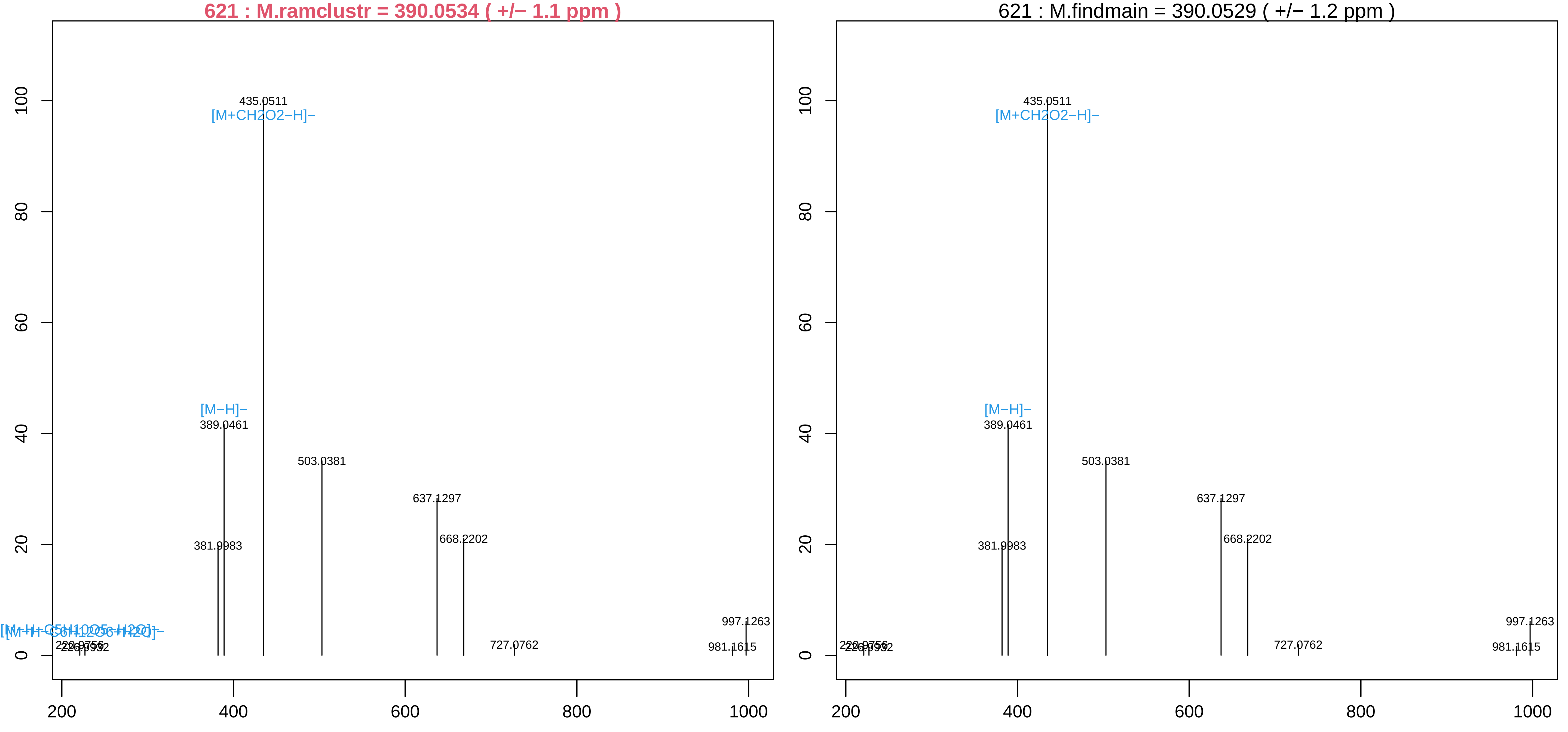

621 : M.ramclustr = 390.0534 (+/− 1.1 ppm)
435.0511
[M+CH2O2−H]−
[M−H]−
389.0461
503.0381
637.1297
668.2202
381.9983
727.0762
997.1263
981.1615
621 : M.findmain = 390.0529 (+/− 1.2 ppm)
435.0511
[M+CH2O2−H]−
[M−H]−
389.0461
503.0381
637.1297
668.2202
381.9983
727.0762
997.1263
981.1615
0
20
40
60
80
100
200
400
600
800
1000

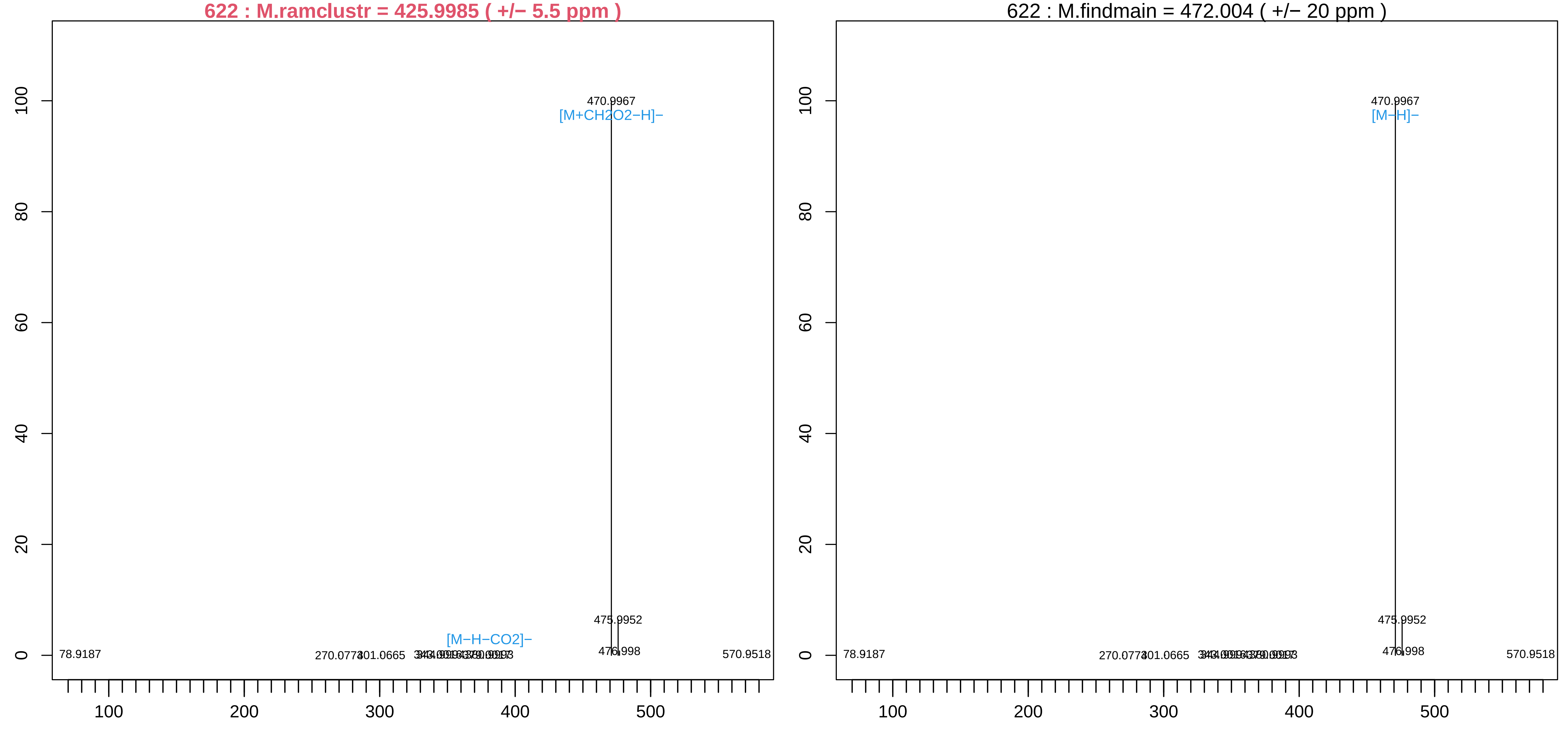
622 : M.ramclustr = 425.9985 (+/− 5.5 ppm)
470.9967
[M+CH2O2−H]−
475.9952
[M−H−CO2]−
476.998
78.9187
270.0774
301.0665
570.9518
622 : M.findmain = 472.004 (+/− 20 ppm)
470.9967
[M−H]−
475.9952
476.998
78.9187
270.0774
301.0665
570.9518
0
20
40
60
80
100
100
200
300
400
500

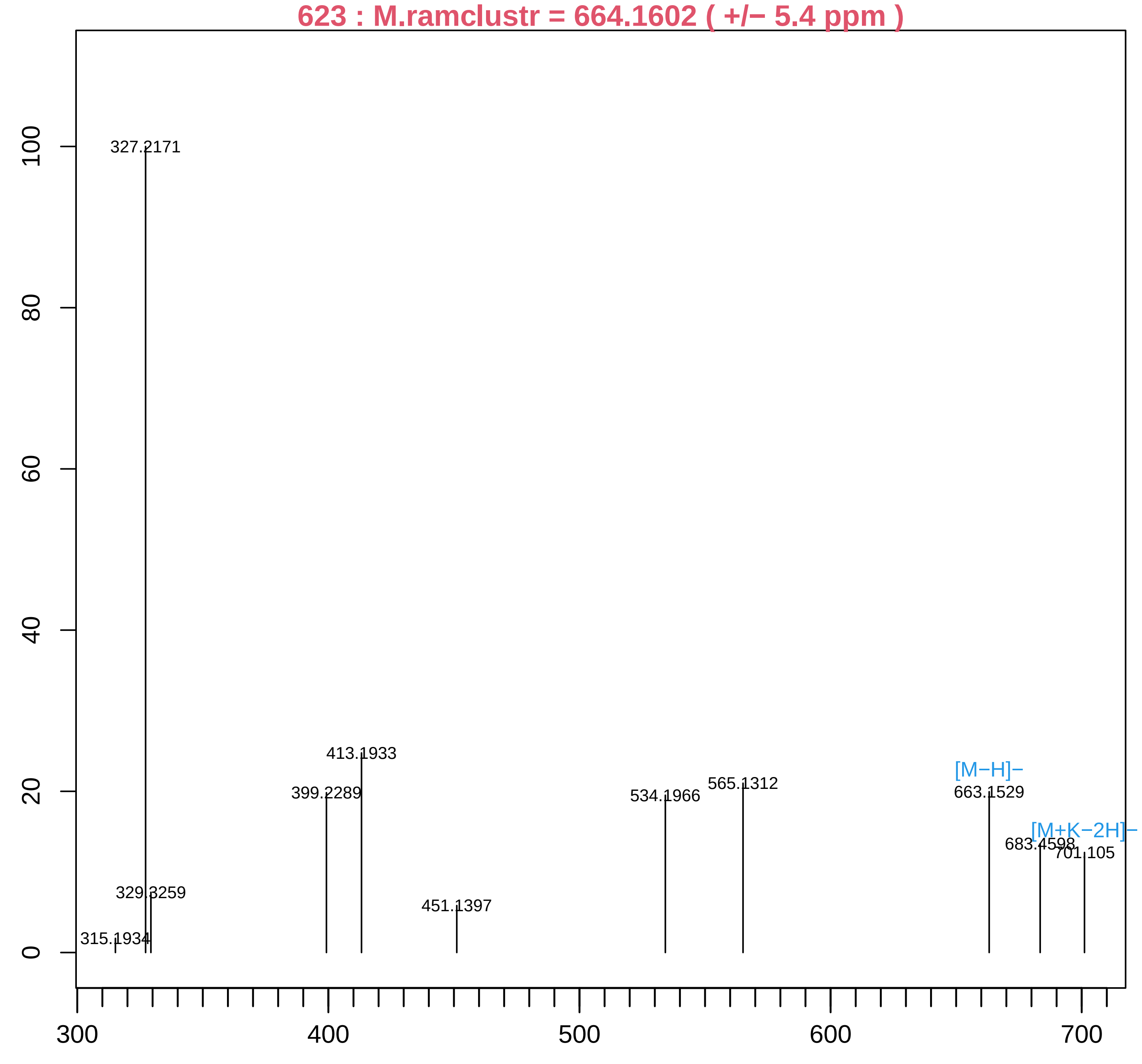
623 : M.ramclustr = 664.1602 (+/− 5.4 ppm)
327.2171
329.3259
315.1934
399.2289
413.1933
451.1397
534.1966
565.1312
[M−H]−
663.1529
[M+K−2H]−
683.4598
701.105
0
20
40
60
80
100
300
400
500
600
700

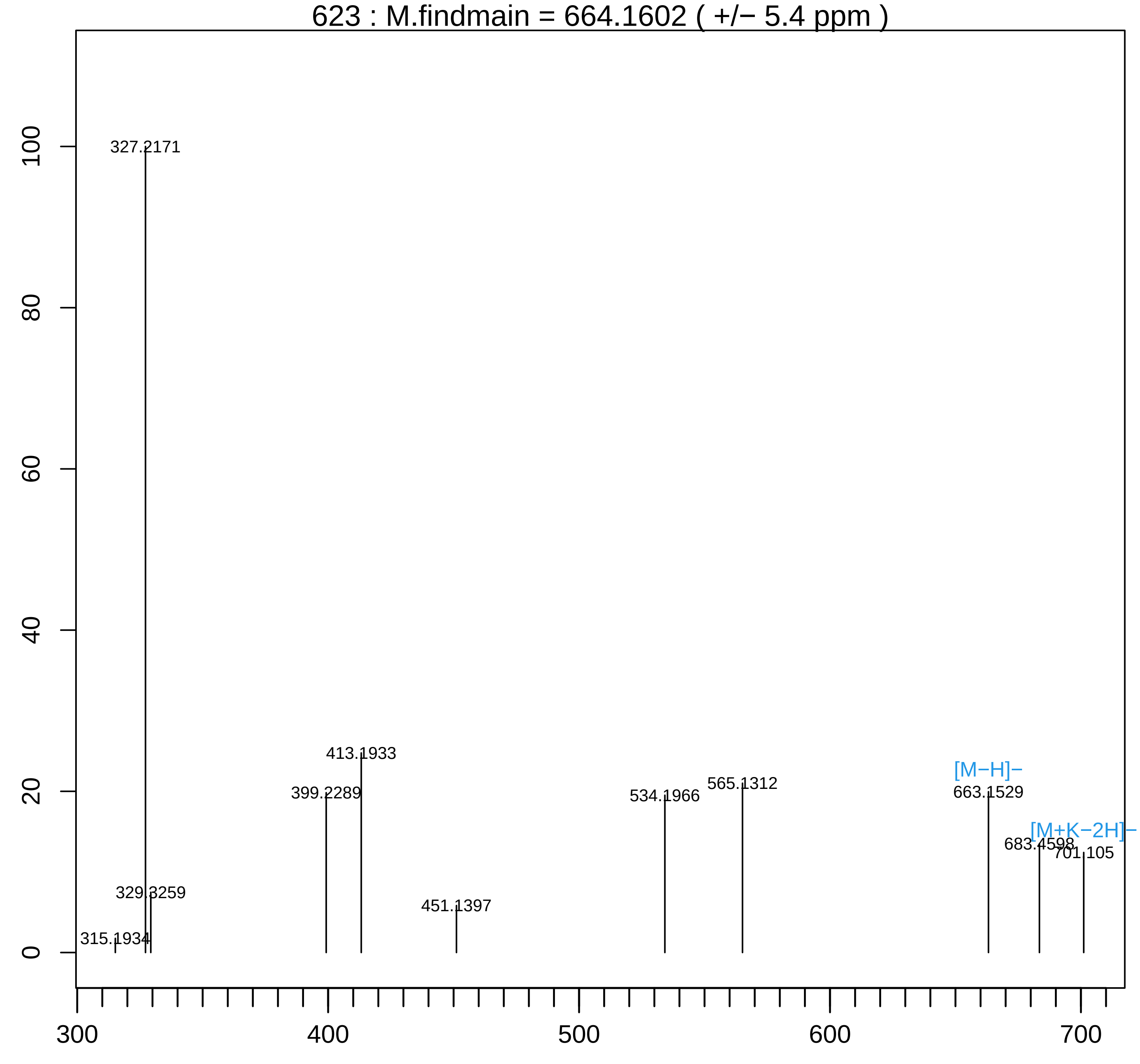
623 : M.findmain = 664.1602 (+/− 5.4 ppm)
327.2171
329.3259
315.1934
399.2289
413.1933
451.1397
534.1966
565.1312
[M−H]−
663.1529
[M+K−2H]−
683.4598
701.105
0
20
40
60
80
100
300
400
500
600
700

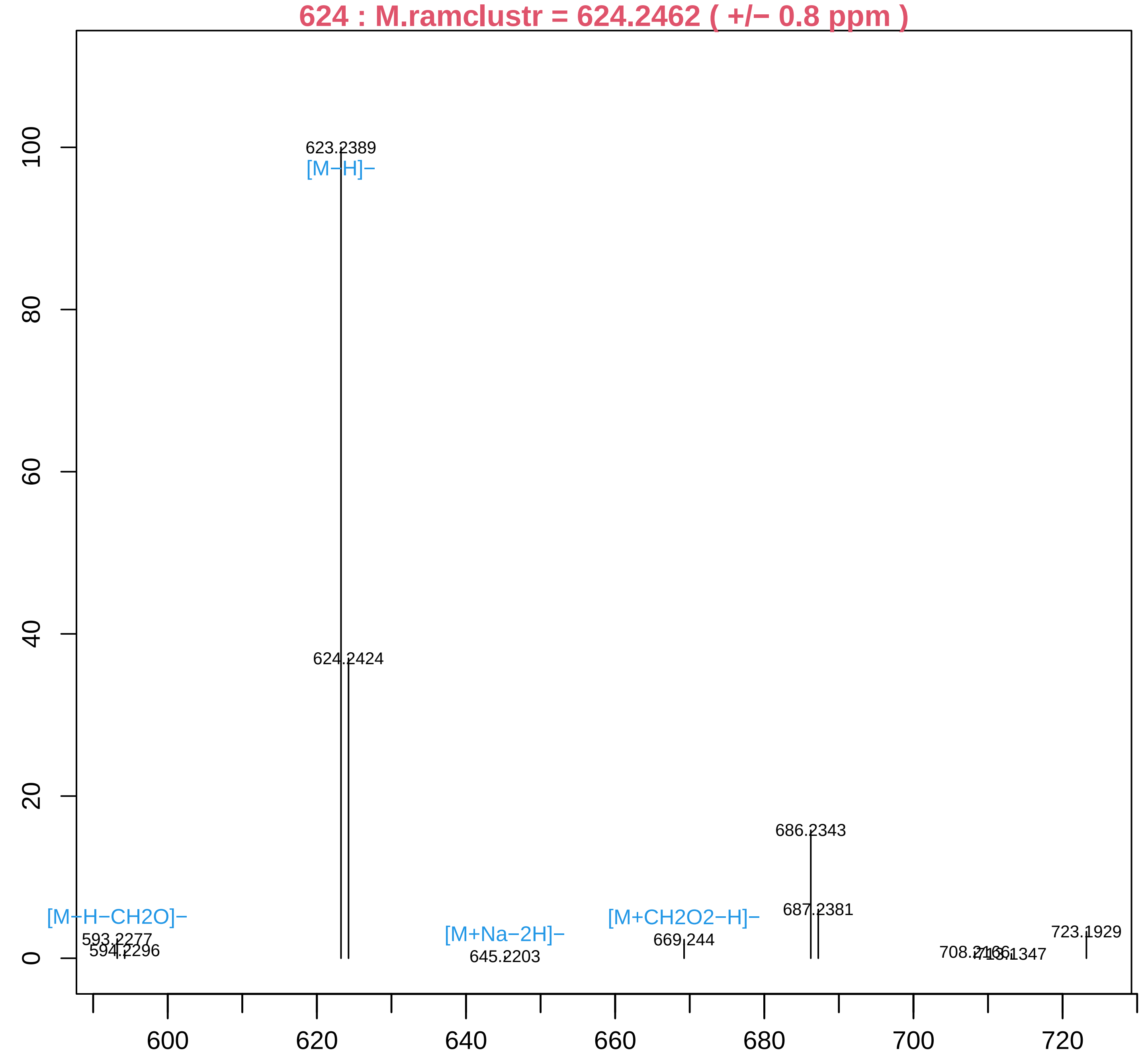

624 : M.ramclustr = 624.2462 (+/− 0.8 ppm)
623.2389
[M−H]−
624.2424
686.2343
687.2381
[M−H−CH2O]−
593.2277
594.2296
[M+Na−2H]−
645.2203
[M+CH2O2−H]−
669.244
708.2136
713.1347
723.1929
0
20
40
60
80
100
600
620
640
660
680
700
720

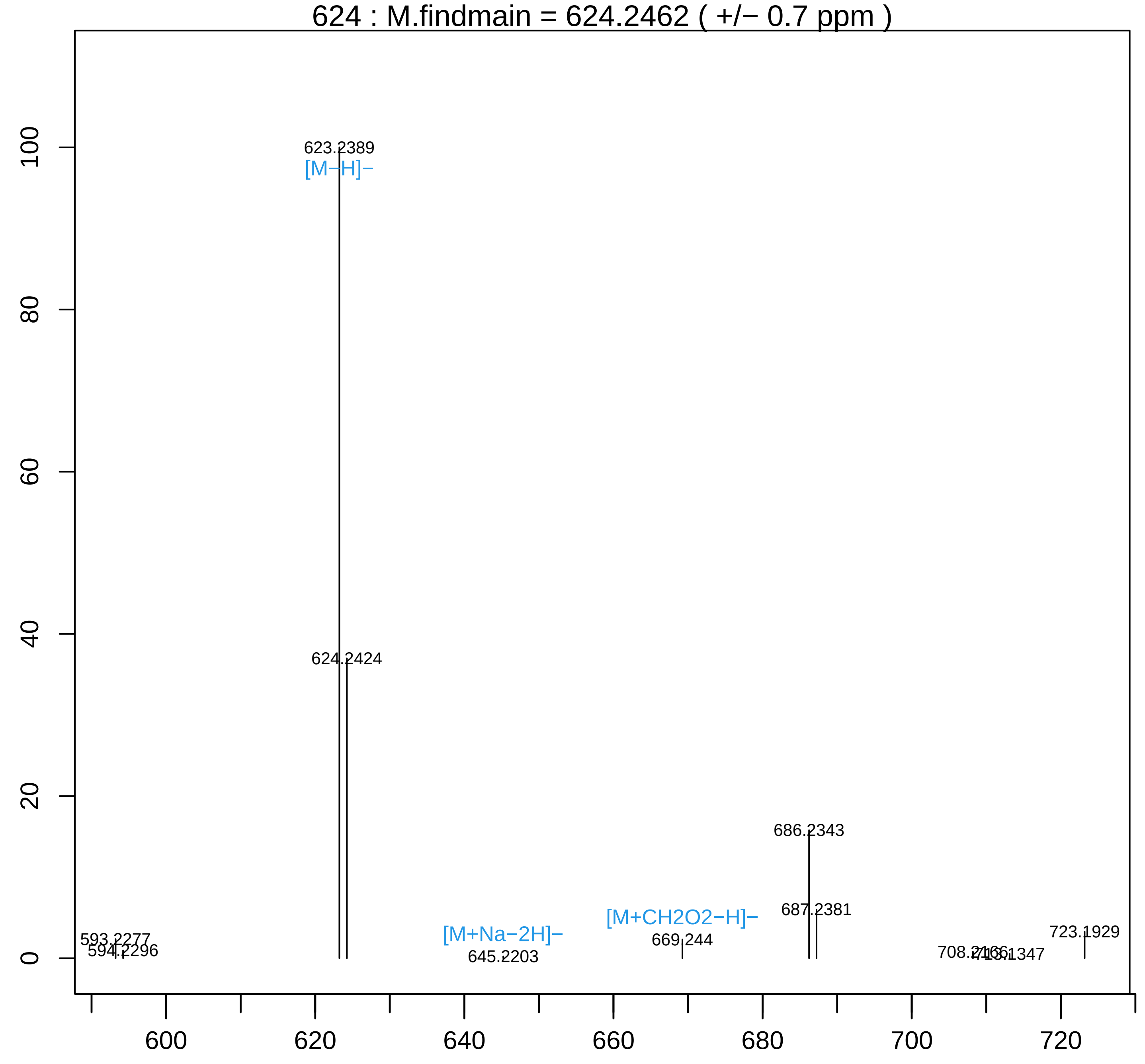

624 : M.findmain = 624.2462 (+/− 0.7 ppm)
623.2389
[M−H]−
624.2424
686.2343
687.2381
593.2277
594.2296
[M+Na−2H]−
645.2203
[M+CH2O2−H]−
669.244
708.2136
713.1347
723.1929
0
20
40
60
80
100
600
620
640
660
680
700
720

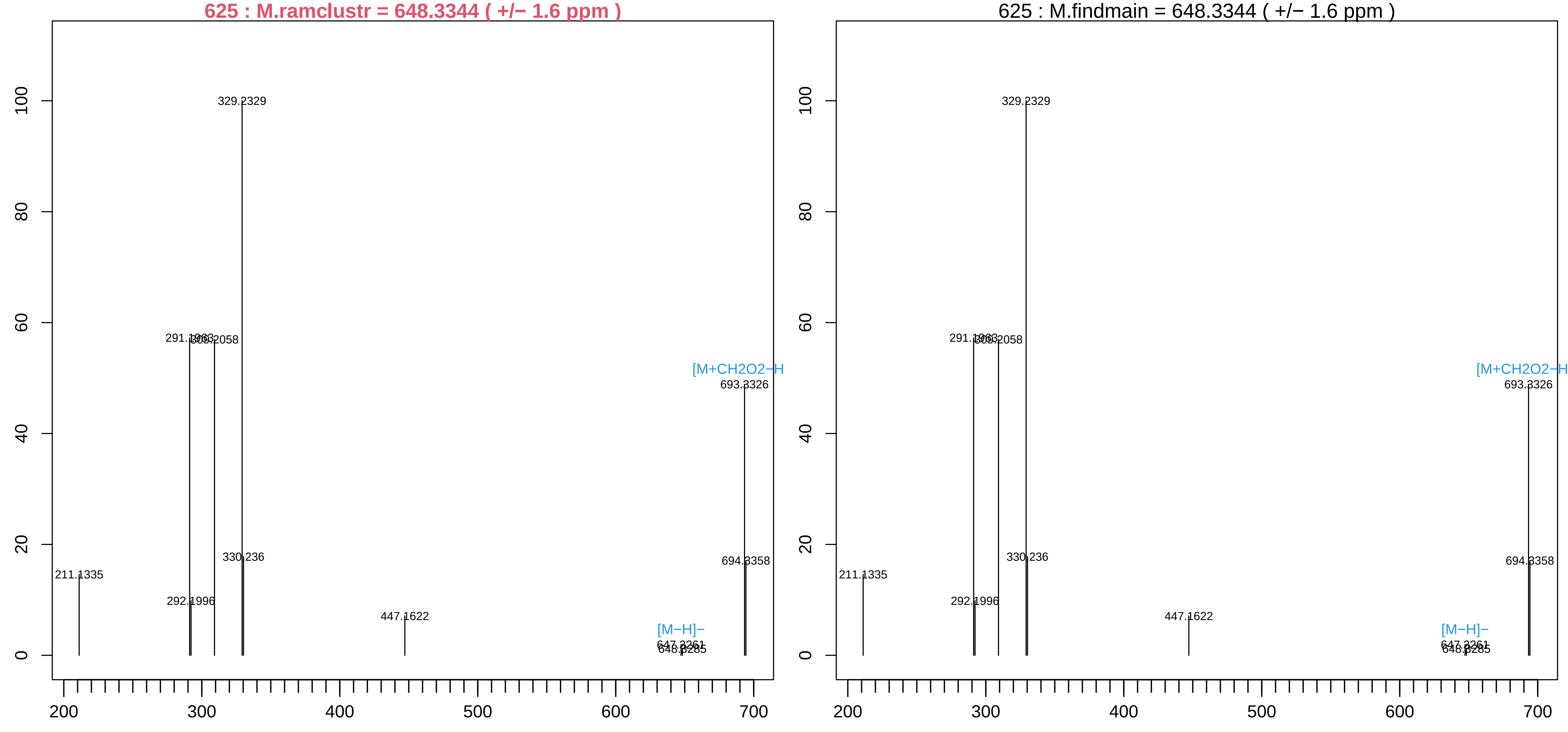
625 : M.ramclustr = 648.3344 (+/− 1.6 ppm)
625 : M.findmain = 648.3344 (+/− 1.6 ppm)
329.2329
291.1963
309.2058
[M+CH2O2−H
693.3326
330.236
694.3358
211.1335
292.1996
447.1622
[M−H]−
647.3261
648.3285
0
20
40
60
80
100
200
300
400
500
600
700

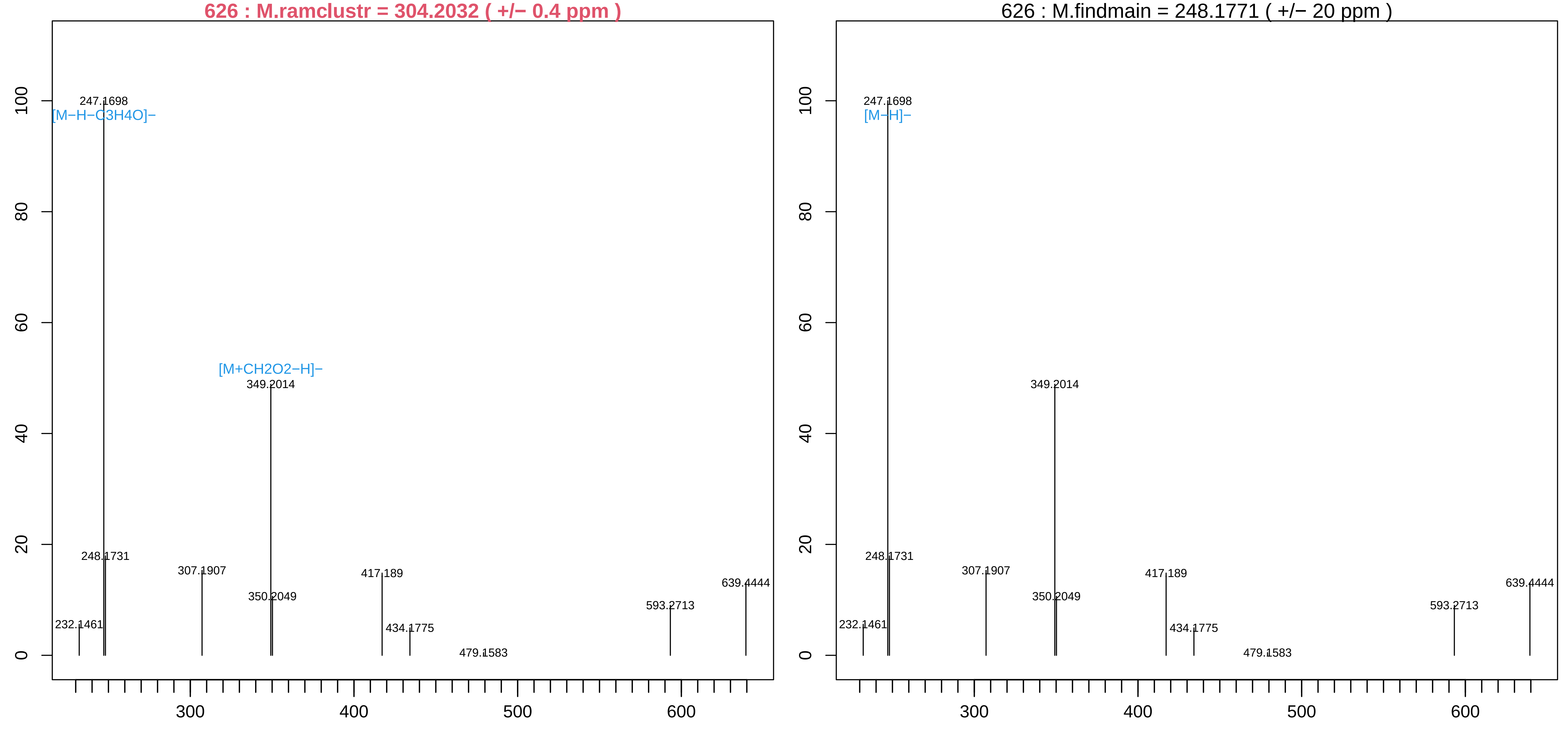
626 : M.ramclustr = 304.2032 (+/− 0.4 ppm)
247.1698
[M−H−C3H4O]−
[M+CH2O2−H]−
349.2014
248.1731
307.1907
350.2049
417.189
434.1775
479.1583
593.2713
639.4444
232.1461
0
20
40
60
80
100
300
400
500
600
626 : M.findmain = 248.1771 (+/− 20 ppm)
247.1698
[M−H]−
349.2014
248.1731
307.1907
350.2049
417.189
434.1775
479.1583
593.2713
639.4444
232.1461
0
20
40
60
80
100
300
400
500
600

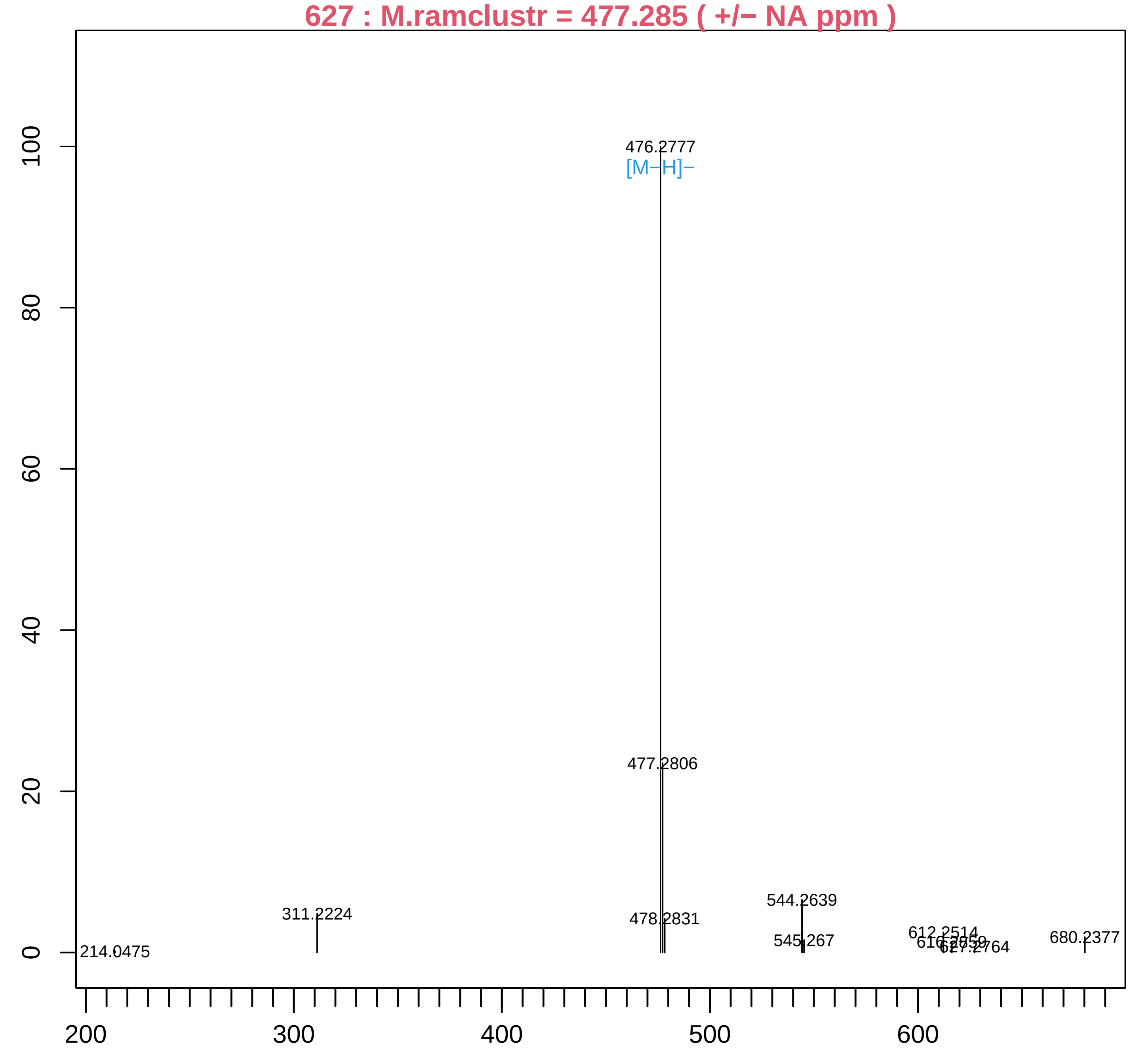
627 : M.ramclustr = 477.285 (+/− NA ppm)
476.2777
[M−H]−
477.2806
478.2831
311.2224
214.0475
544.2639
545.267
612.2514
616.2859
627.2764
680.2377
0
20
40
60
80
100
200
300
400
500
600
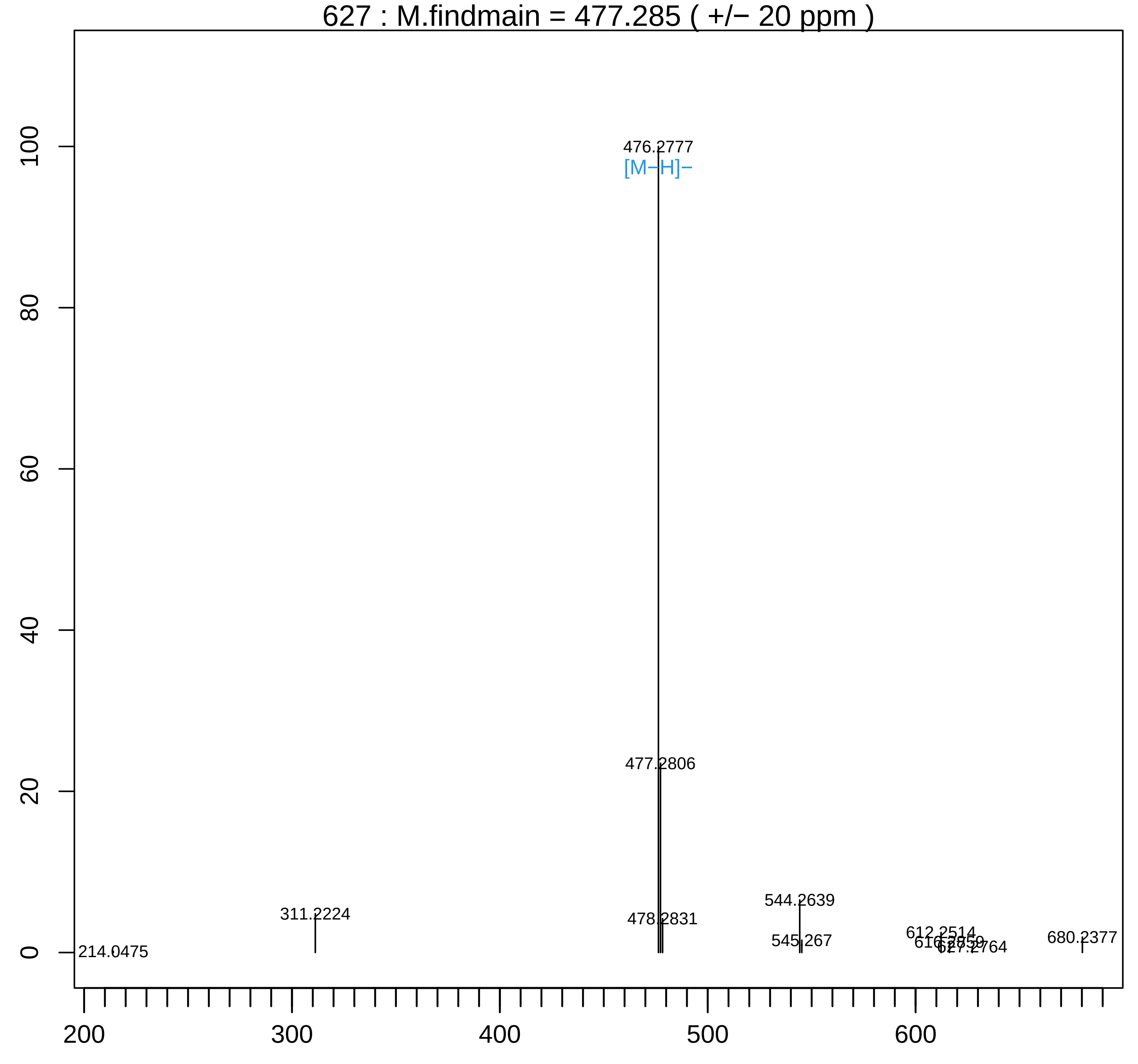
627 : M.findmain = 477.285 (+/− 20 ppm)
476.2777
[M−H]−
477.2806
478.2831
311.2224
214.0475
544.2639
545.267
612.2514
616.2859
627.2764
680.2377
0
20
40
60
80
100
200
300
400
500
600

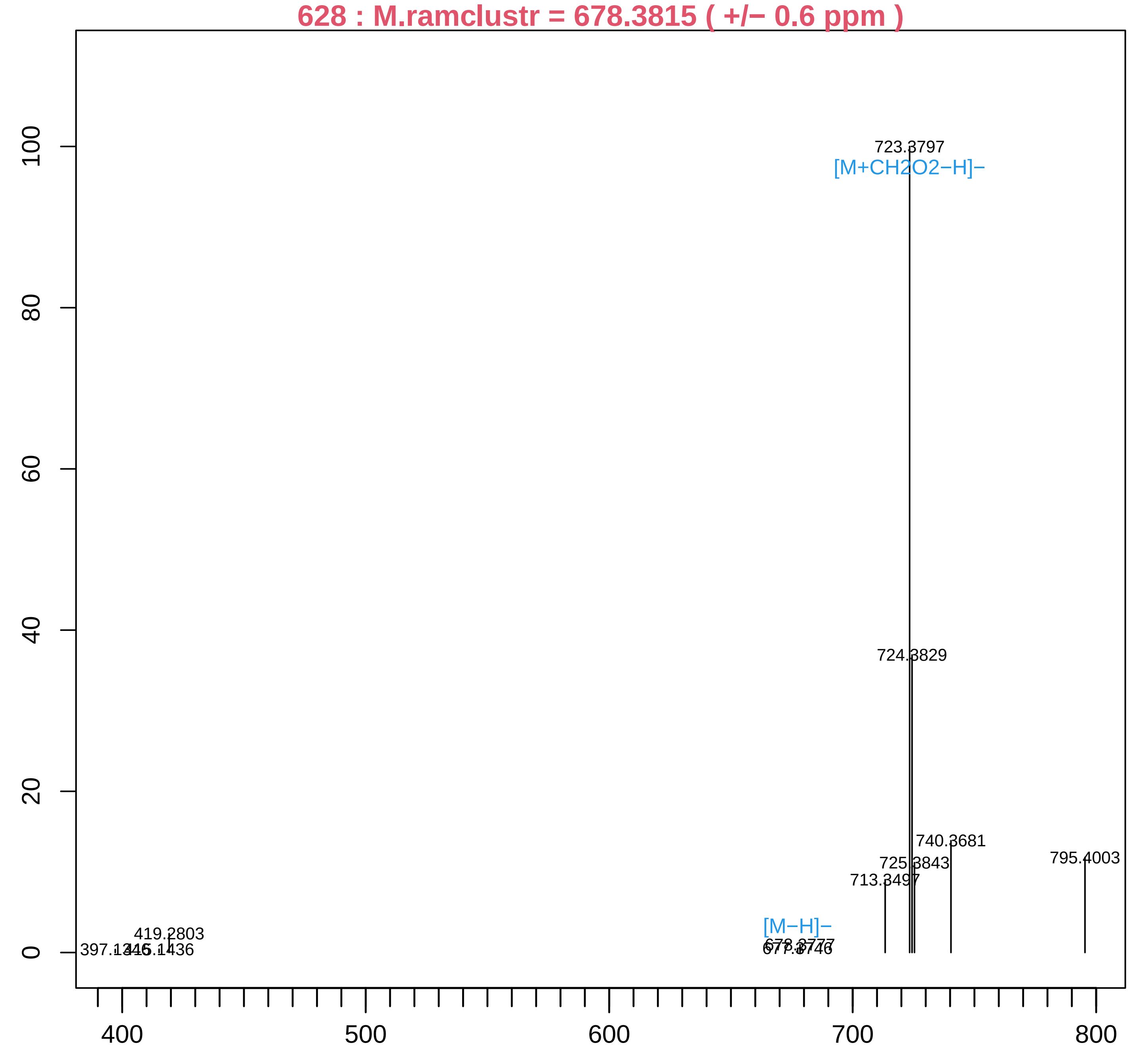
628 : M.ramclustr = 678.3815 (+/− 0.6 ppm)
723.3797
[M+CH2O2−H]−
724.3829
740.3681
725.3843
795.4003
713.3497
[M−H]−
419.2803
397.1345
415.1436
678.3777
677.3746
0
20
40
60
80
100
400
500
600
700
800

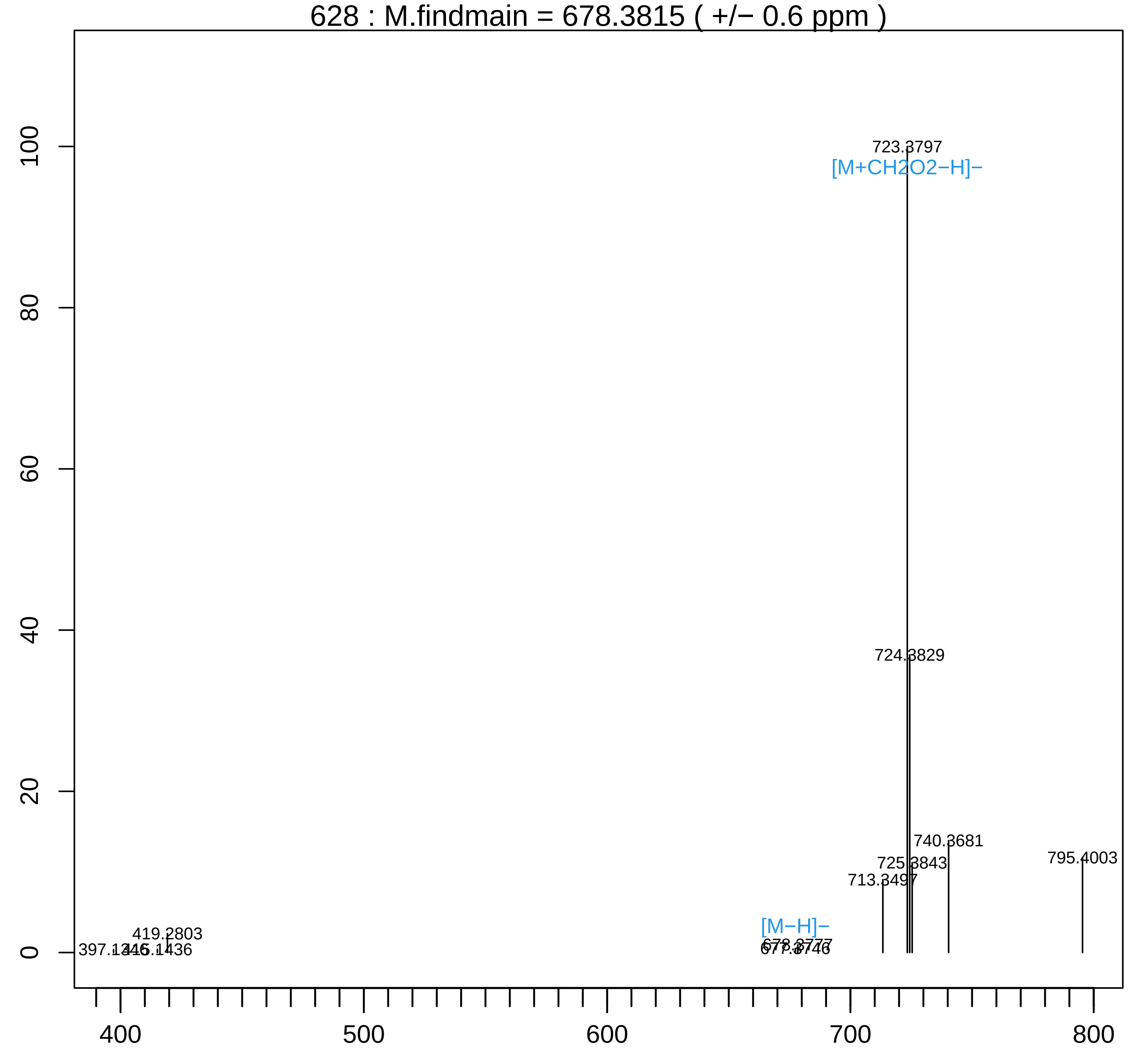
628 : M.findmain = 678.3815 (+/− 0.6 ppm)
723.3797
[M+CH2O2−H]−
724.3829
740.3681
725.3843
795.4003
713.3497
[M−H]−
419.2803
397.1345
415.1436
678.3777
677.3746
0
20
40
60
80
100
400
500
600
700
800

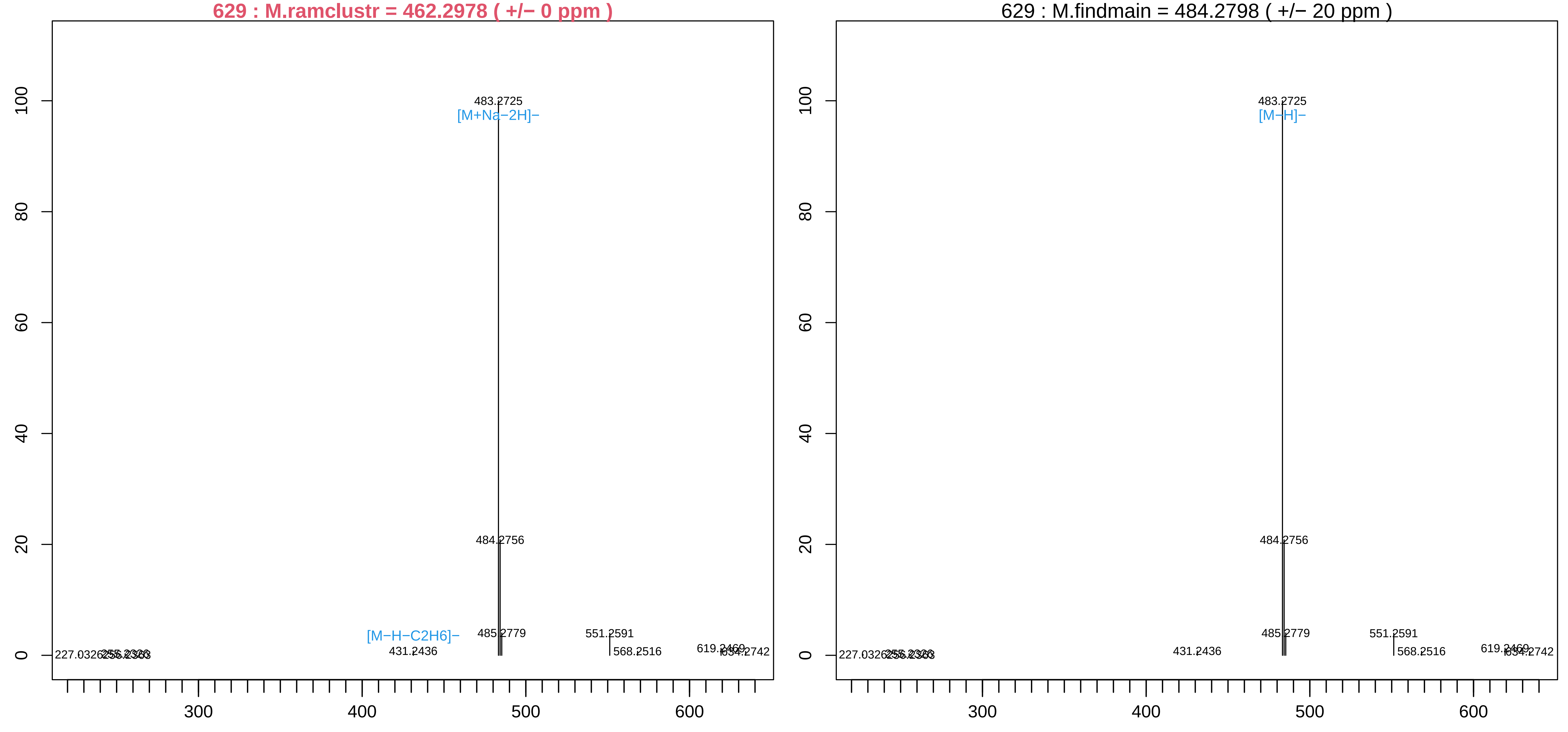
629 : M.ramclustr = 462.2978 (+/− 0 ppm)
483.2725
[M+Na−2H]−
484.2756
485.2779
[M−H−C2H6]−
431.2436
551.2591
568.2516
619.2469
634.2742
227.0326
255.2323
256.2363
629 : M.findmain = 484.2798 (+/− 20 ppm)
[M−H]−
0
20
40
60
80
100
300
400
500
600

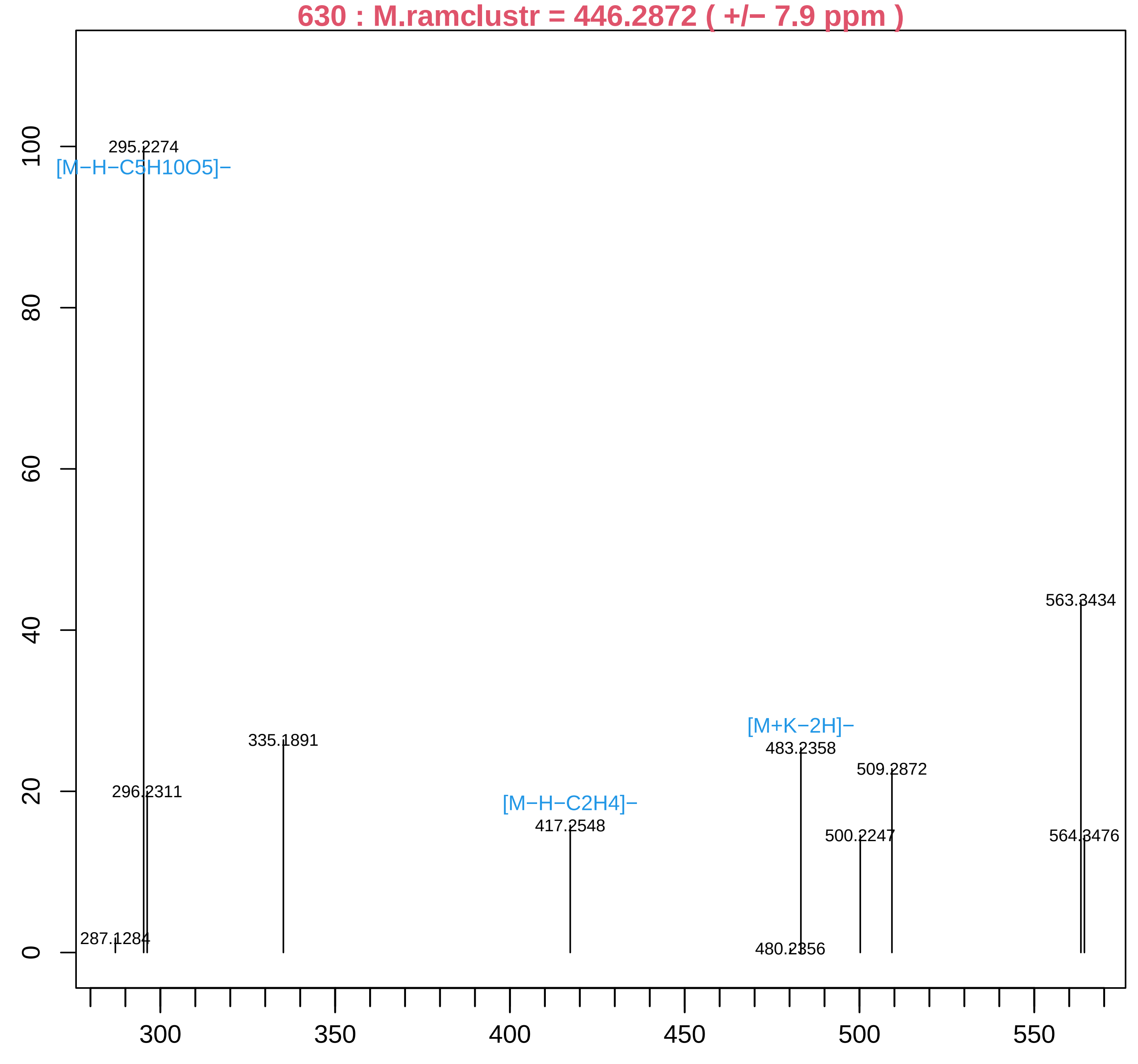
630 : M.ramclustr = 446.2872 (+/− 7.9 ppm)
295.2274
[M−H−C5H10O5]−
563.3434
335.1891
[M+K−2H]−
483.2358
509.2872
296.2311
[M−H−C2H4]−
417.2548
500.2247
564.3476
287.1284
480.2356
0
20
40
60
80
100
300
350
400
450
500
550

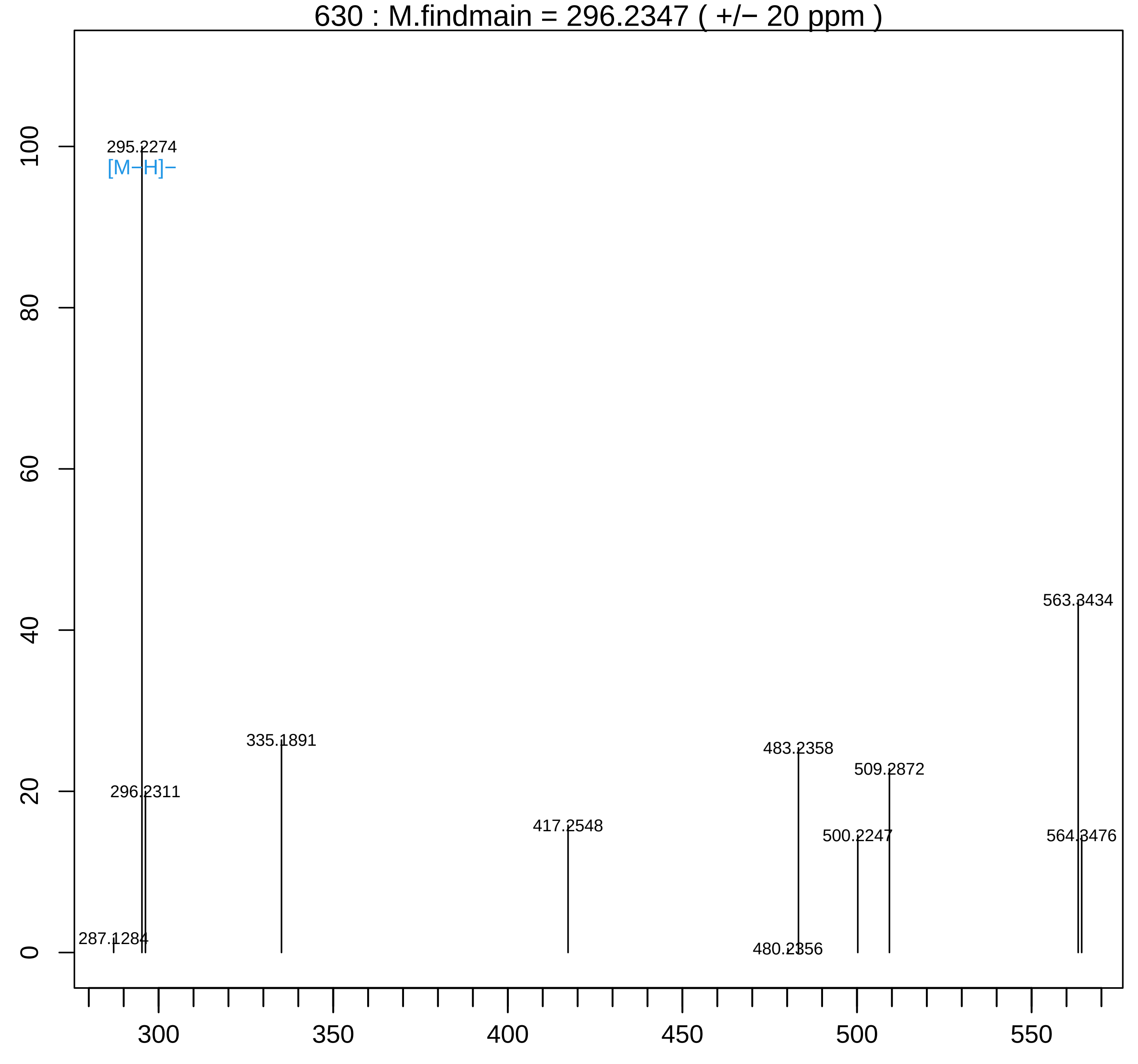
630 : M.findmain = 296.2347 (+/− 20 ppm)
295.2274
[M−H]−
563.3434
335.1891
483.2358
509.2872
296.2311
417.2548
500.2247
564.3476
287.1284
480.2356
0
20
40
60
80
100
300
350
400
450
500
550

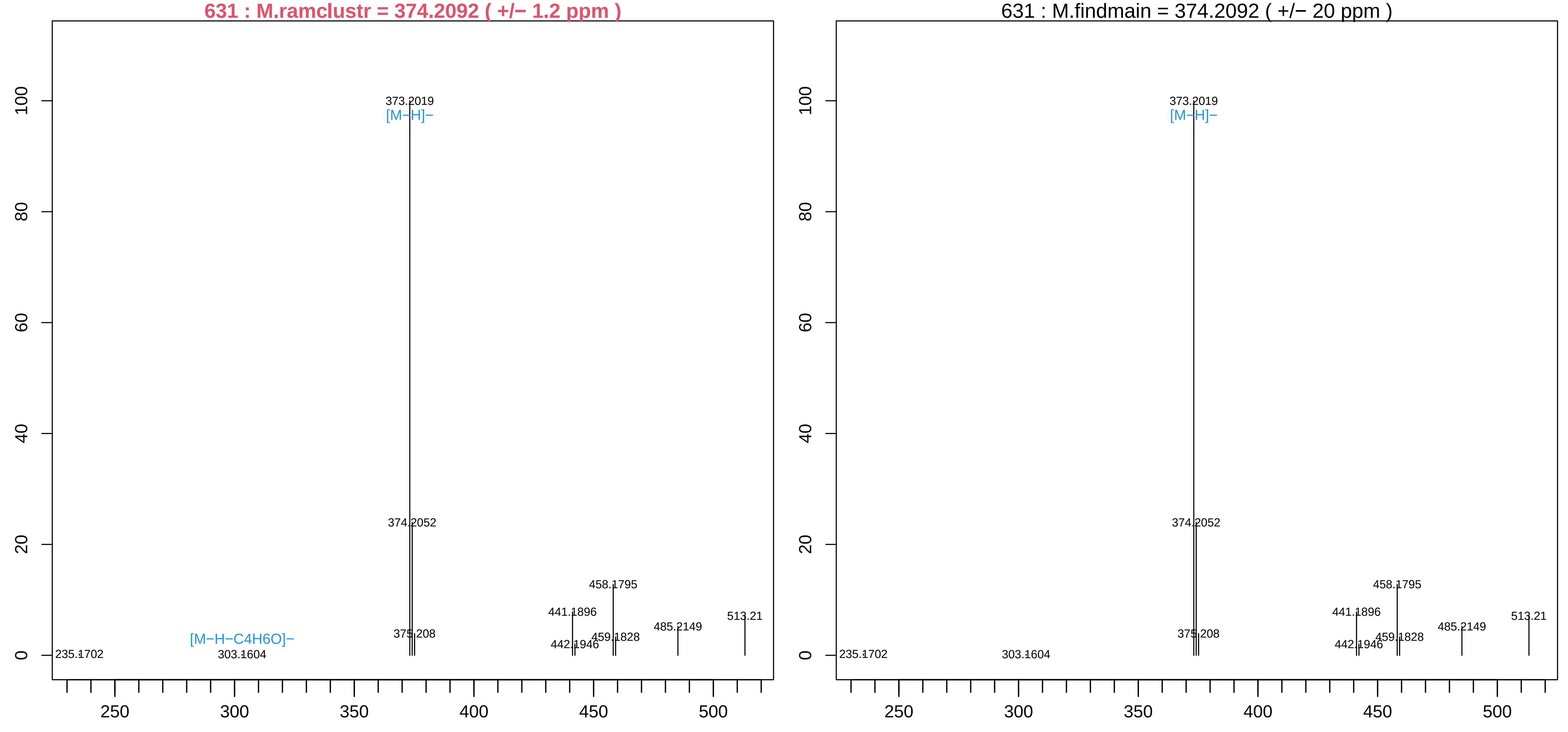

631 : M.ramclustr = 374.2092 (+/− 1.2 ppm)
373.2019
[M−H]−
374.2052
458.1795
441.1896
485.2149
513.21
375.208
459.1828
442.1946
[M−H−C4H6O]−
235.1702
303.1604
631 : M.findmain = 374.2092 (+/− 20 ppm)
373.2019
[M−H]−
374.2052
458.1795
441.1896
485.2149
513.21
375.208
459.1828
442.1946
235.1702
303.1604

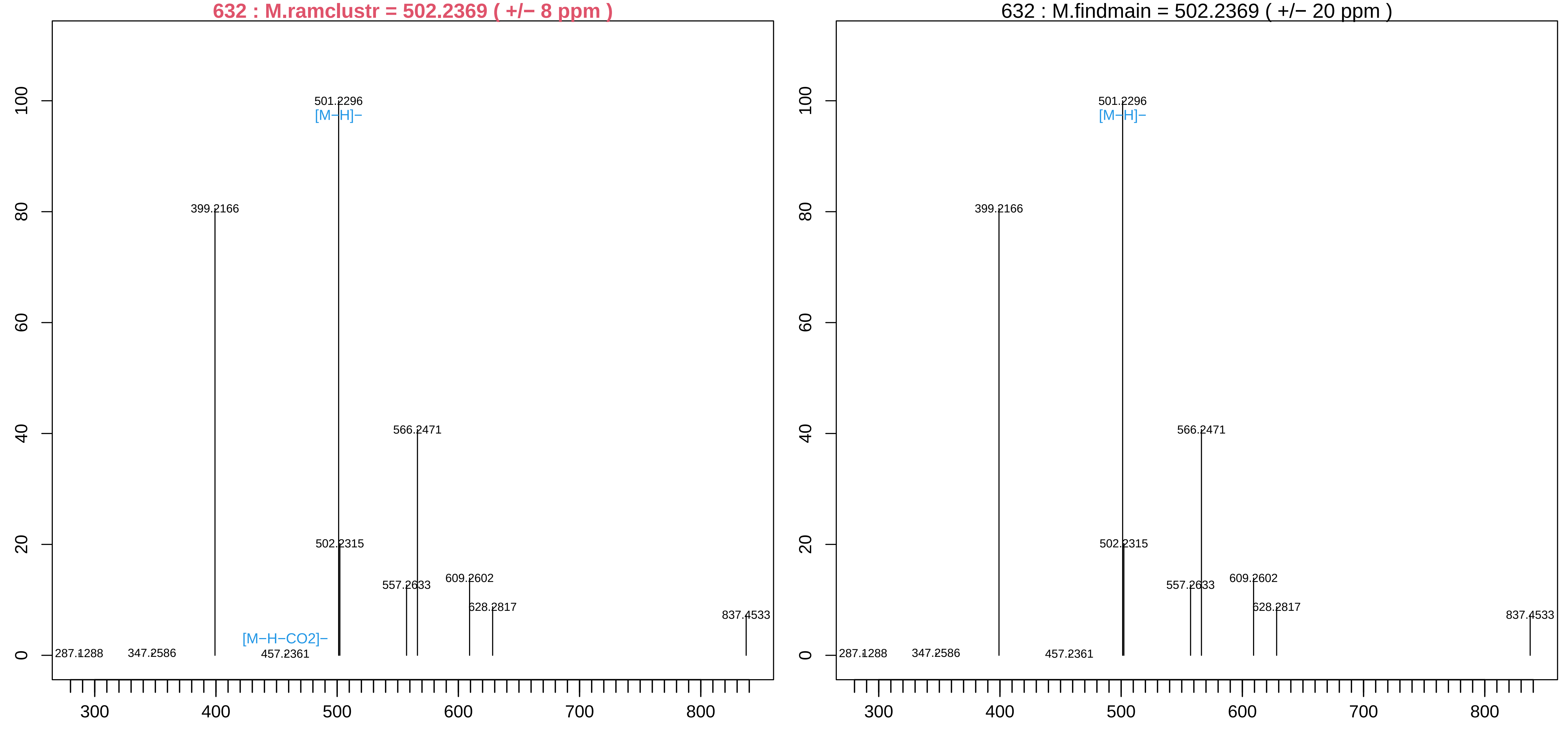
632 : M.ramclustr = 502.2369 (+/− 8 ppm)
501.2296
[M−H]−
399.2166
566.2471
502.2315
609.2602
557.2633
628.2817
837.4533
[M−H−CO2]−
287.1288
347.2586
457.2361
632 : M.findmain = 502.2369 (+/− 20 ppm)
501.2296
[M−H]−
399.2166
566.2471
502.2315
609.2602
557.2633
628.2817
837.4533
287.1288
347.2586
457.2361
0
20
40
60
80
100
300
400
500
600
700
800

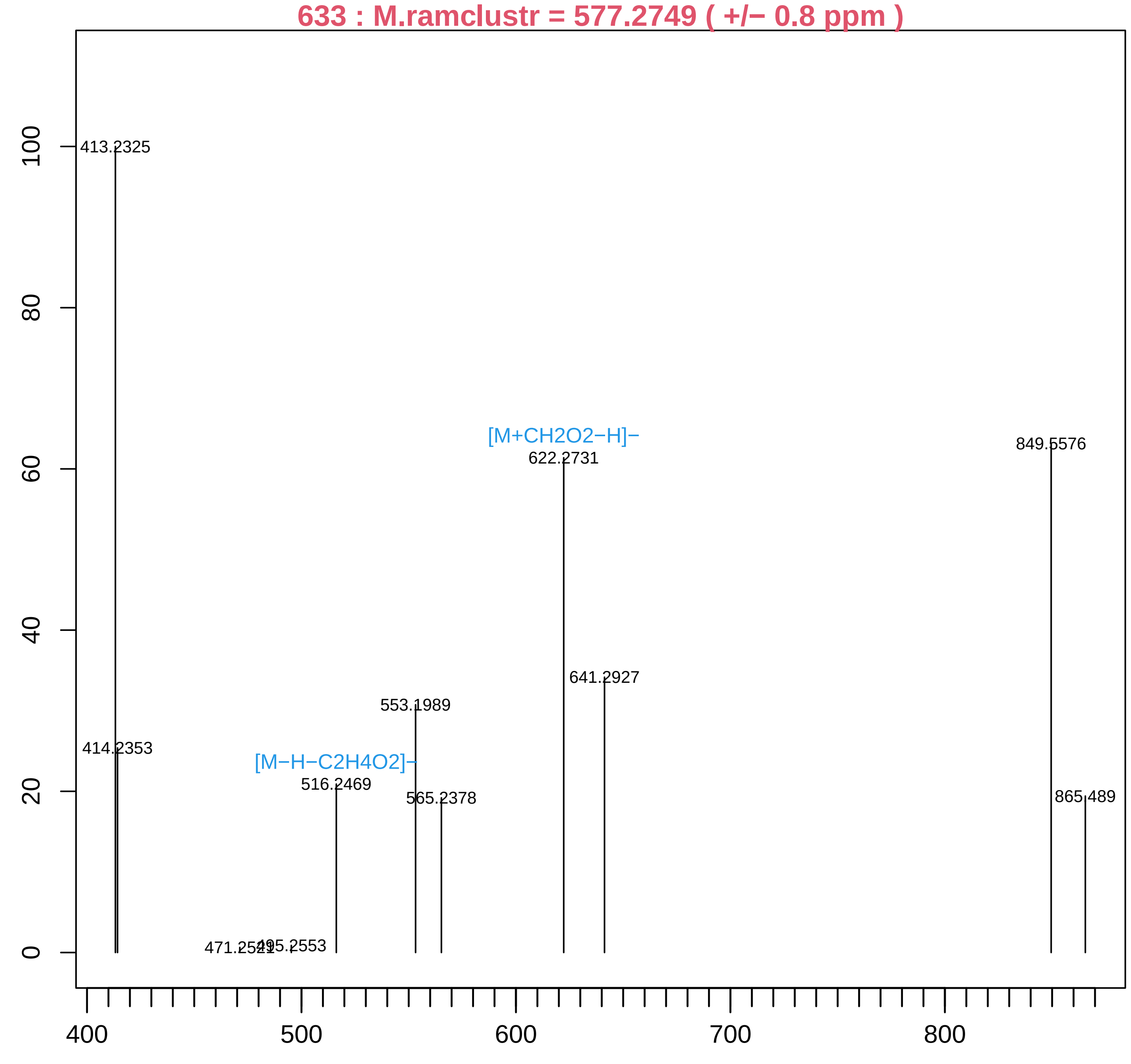
633 : M.ramclustr = 577.2749 (+/− 0.8 ppm)
413.2325
414.2353
471.2541
495.2553
516.2469
[M−H−C2H4O2]−
553.1989
565.2378
622.2731
[M+CH2O2−H]−
641.2927
849.5576
865.489
0
20
40
60
80
100
400
500
600
700
800

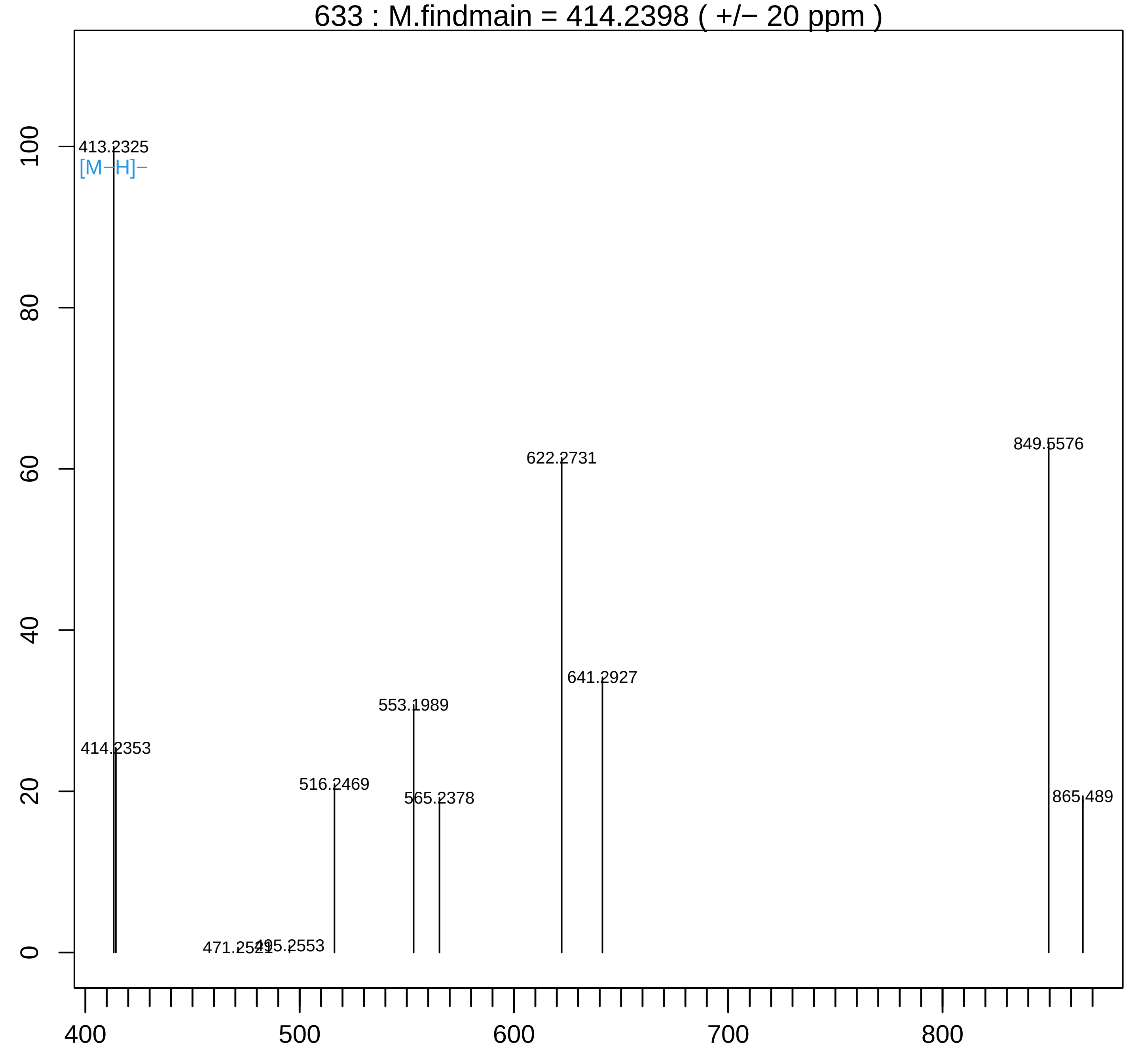
633 : M.findmain = 414.2398 (+/− 20 ppm)
413.2325
[M−H]−
414.2353
471.2541
495.2553
516.2469
553.1989
565.2378
622.2731
641.2927
849.5576
865.489
0
20
40
60
80
100
400
500
600
700
800

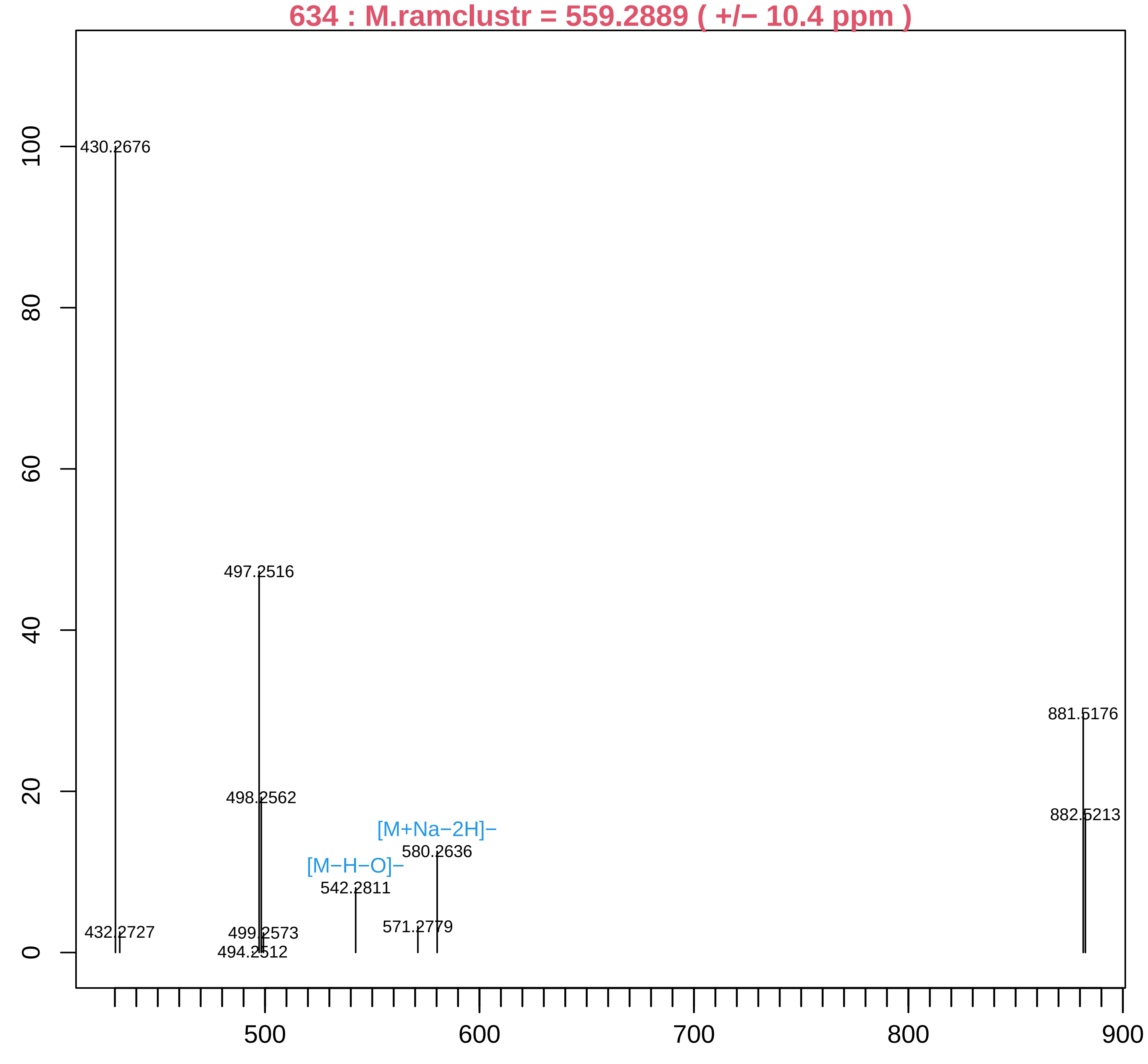
634 : M.ramclustr = 559.2889 (+/− 10.4 ppm)
430.2676
497.2516
498.2562
[M+Na−2H]−
580.2636
[M−H−O]−
542.2811
432.2727
499.2573
494.2512
571.2779
881.5176
882.5213
0
20
40
60
80
100
500
600
700
800
900

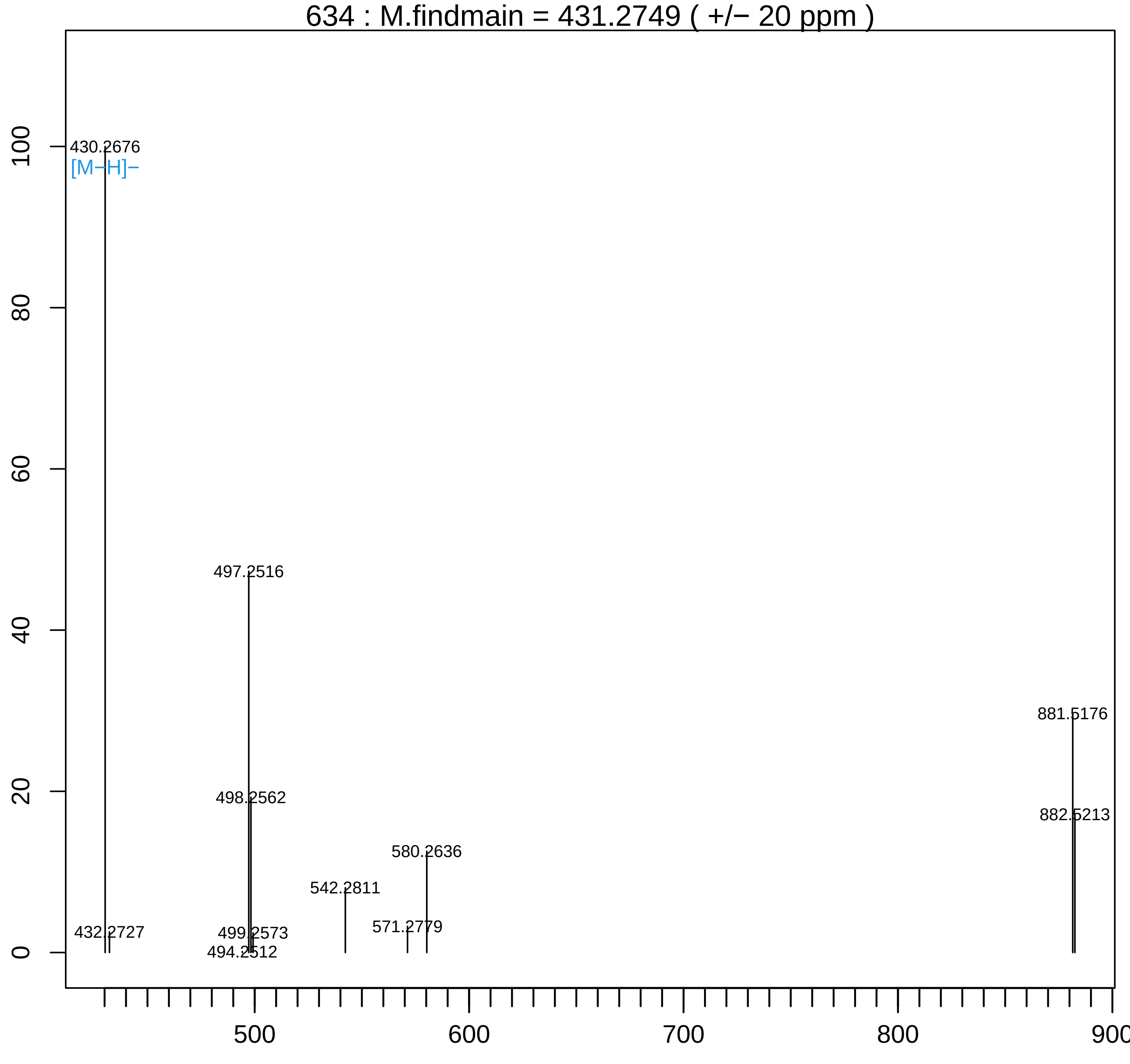
634 : M.findmain = 431.2749 (+/− 20 ppm)
430.2676
[M−H]−
497.2516
498.2562
580.2636
542.2811
432.2727
499.2573
494.2512
571.2779
881.5176
882.5213
0
20
40
60
80
100
500
600
700
800
900

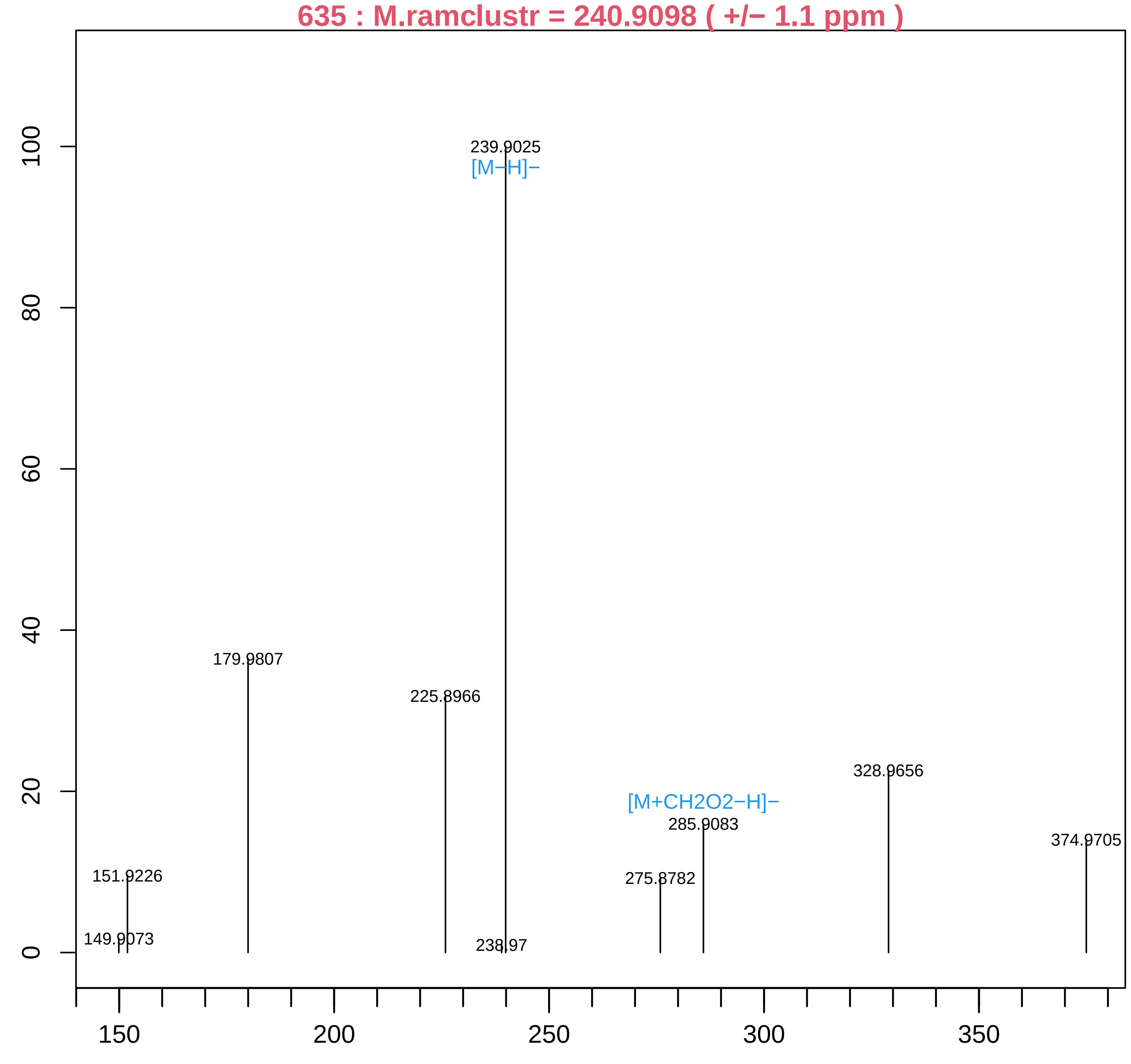
635 : M.ramclustr = 240.9098 (+/− 1.1 ppm)
239.9025
[M−H]−
179.9807
225.8966
328.9656
[M+CH2O2−H]−
285.9083
374.9705
151.9226
275.8782
149.9073
238.97
0
20
40
60
80
100
150
200
250
300
350

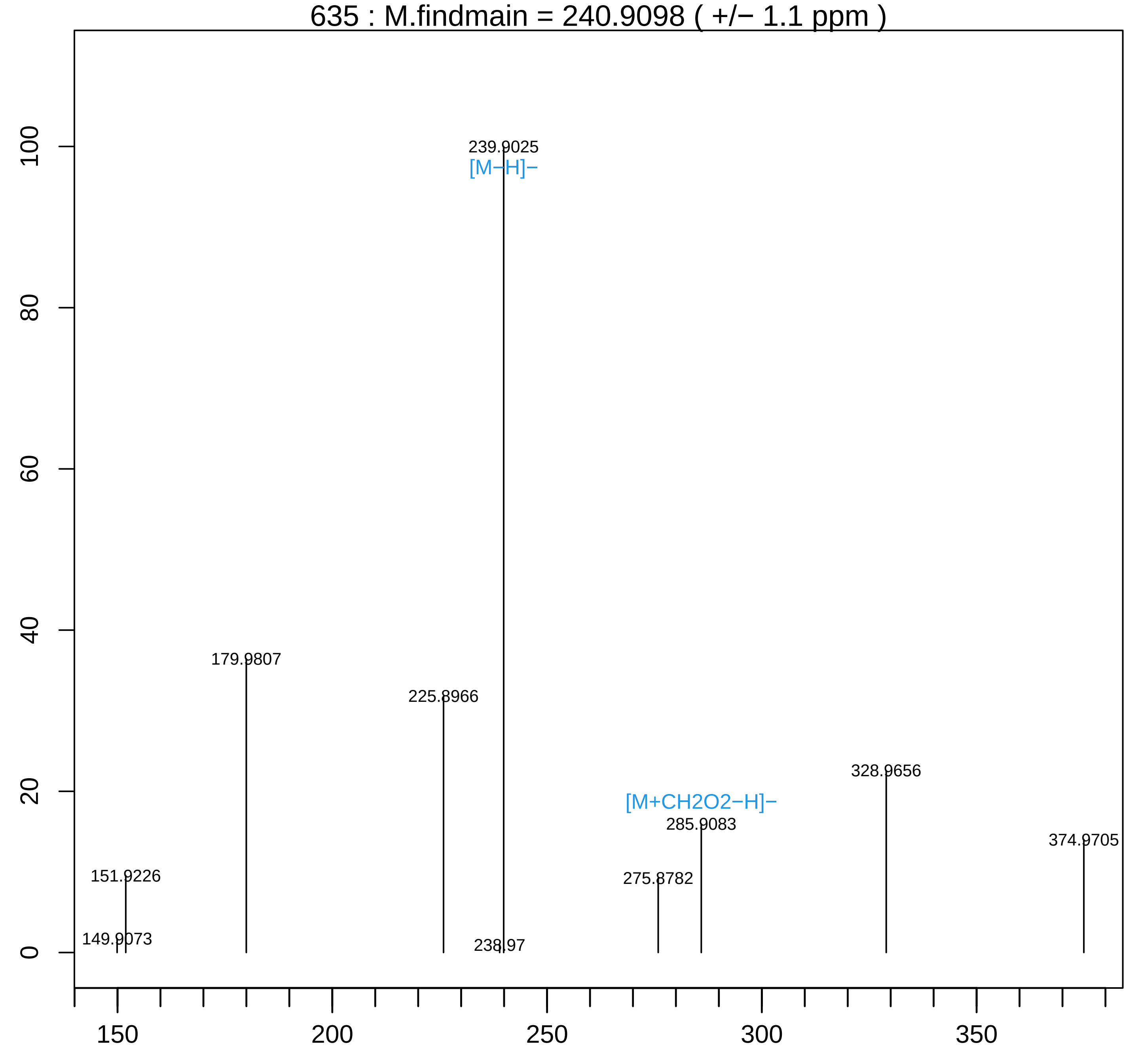
635 : M.findmain = 240.9098 (+/− 1.1 ppm)
239.9025
[M−H]−
179.9807
225.8966
328.9656
[M+CH2O2−H]−
285.9083
374.9705
151.9226
275.8782
149.9073
238.97
0
20
40
60
80
100
150
200
250
300
350

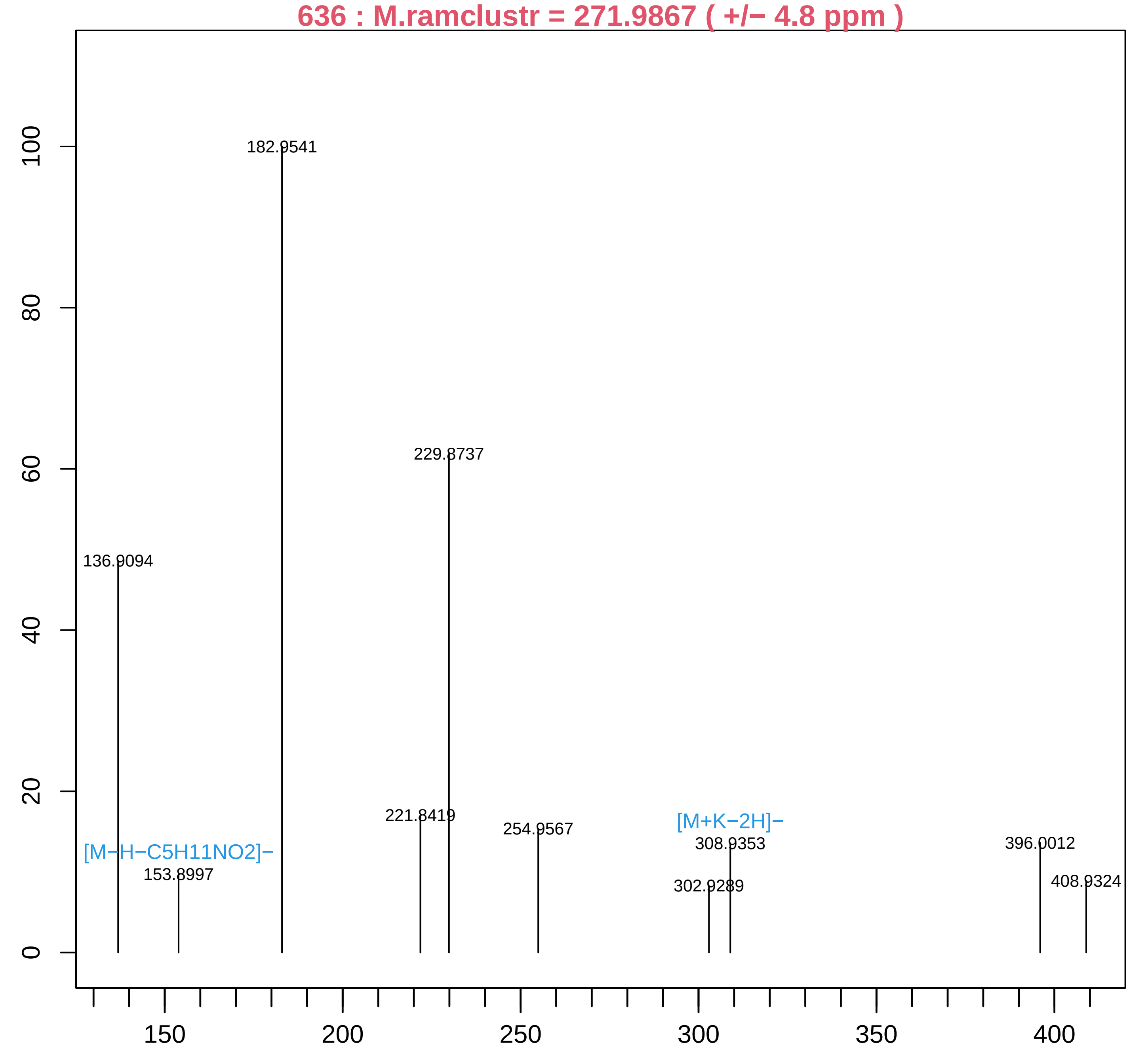
636 : M.ramclustr = 271.9867 (+/− 4.8 ppm)
182.9541
229.8737
136.9094
221.8419
254.9567
[M+K−2H]−
308.9353
396.0012
[M−H−C5H11NO2]−
153.8997
408.9324
302.9289

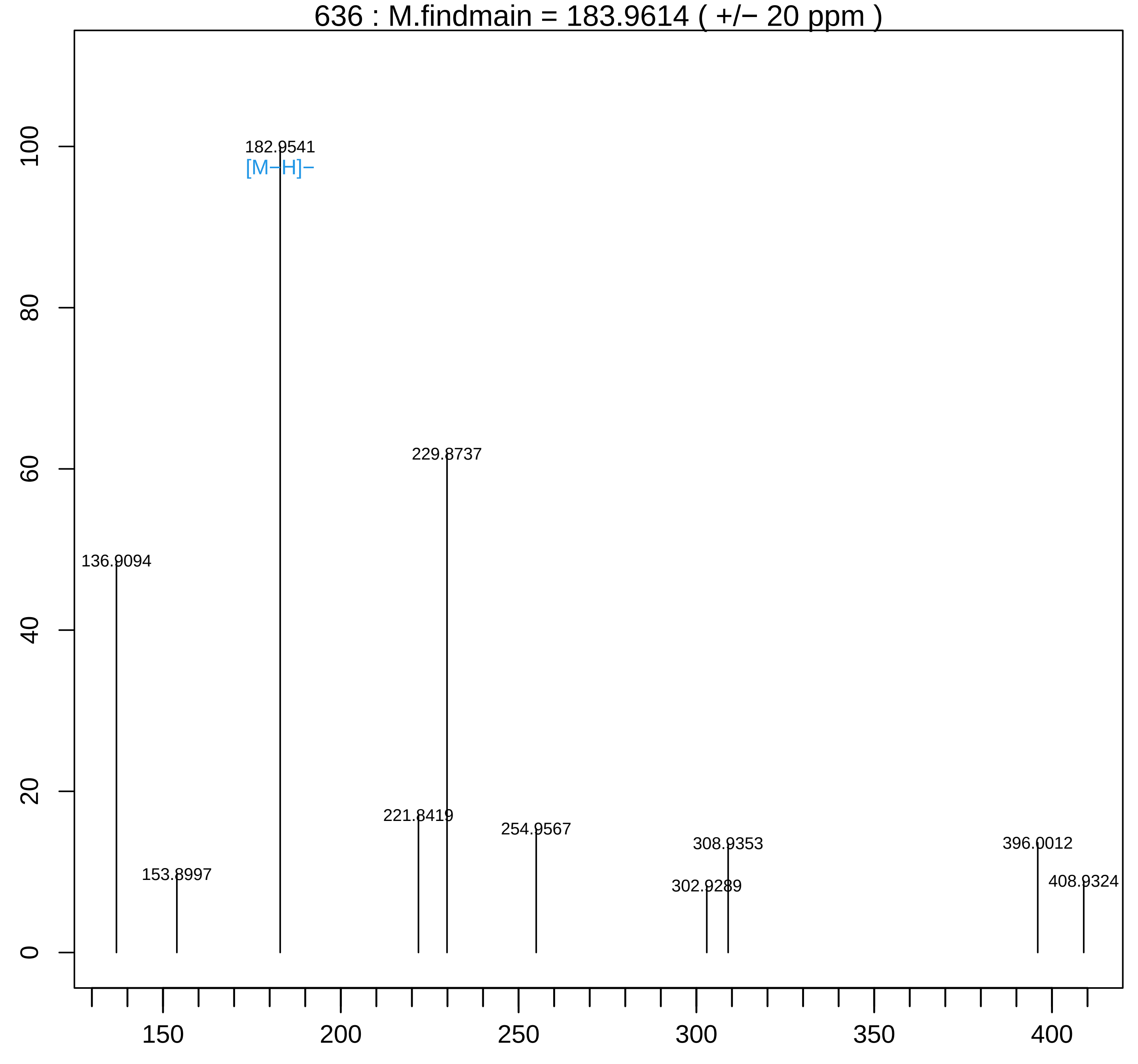
636 : M.findmain = 183.9614 (+/− 20 ppm)
182.9541
[M−H]−
229.8737
136.9094
221.8419
254.9567
308.9353
396.0012
153.8997
408.9324
302.9289

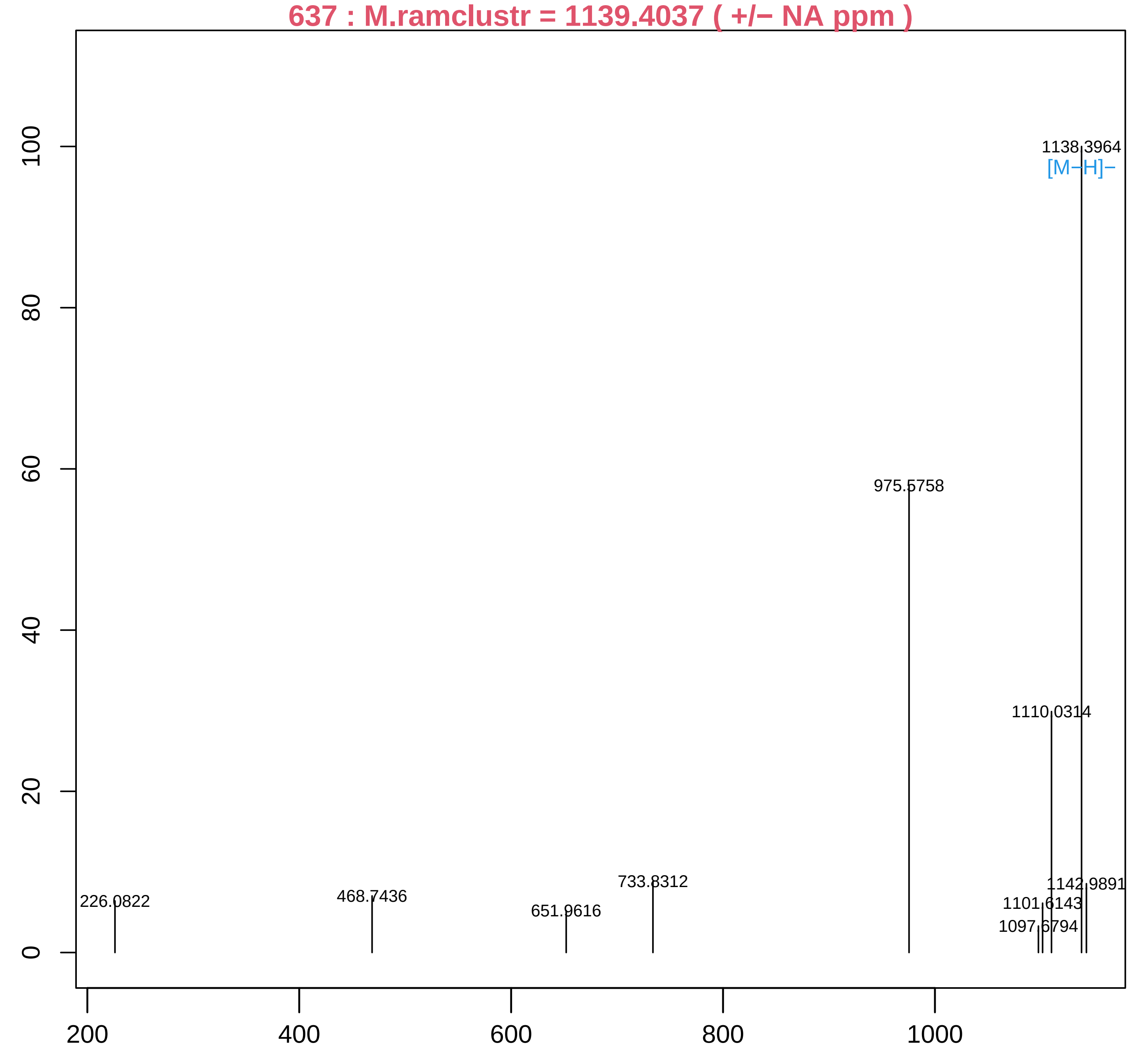
637 : M.ramclustr = 1139.4037 (+/− NA ppm)
1138.3964
[M−H]−
975.5758
1110.0314
1142.9891
1101.6143
1097.6794
733.8312
468.7436
226.0822
651.9616

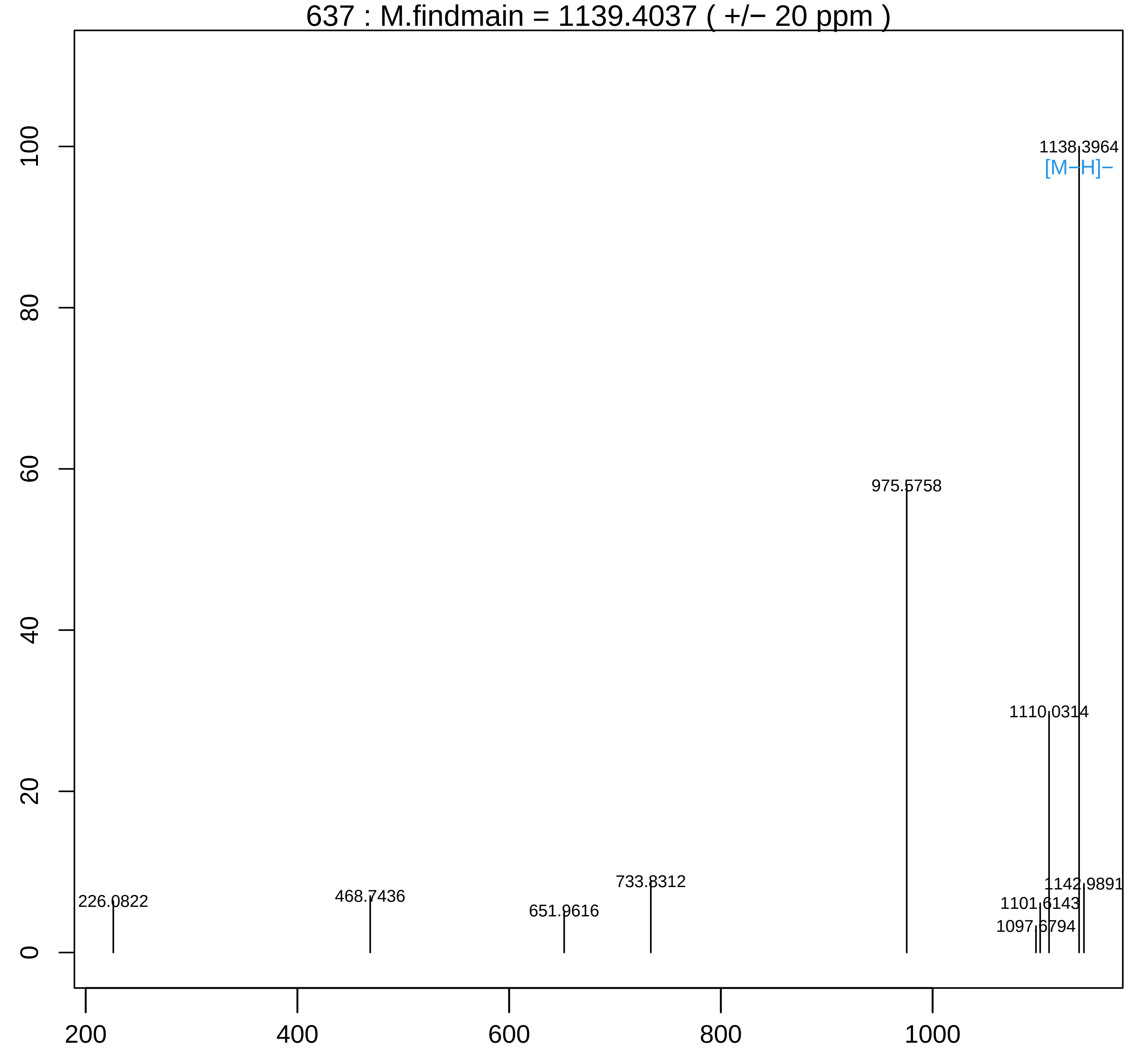
637 : M.findmain = 1139.4037 (+/− 20 ppm)
1138.3964
[M−H]−
975.5758
1110.0314
1142.9891
1101.6143
1097.6794
733.8312
468.7436
226.0822
651.9616

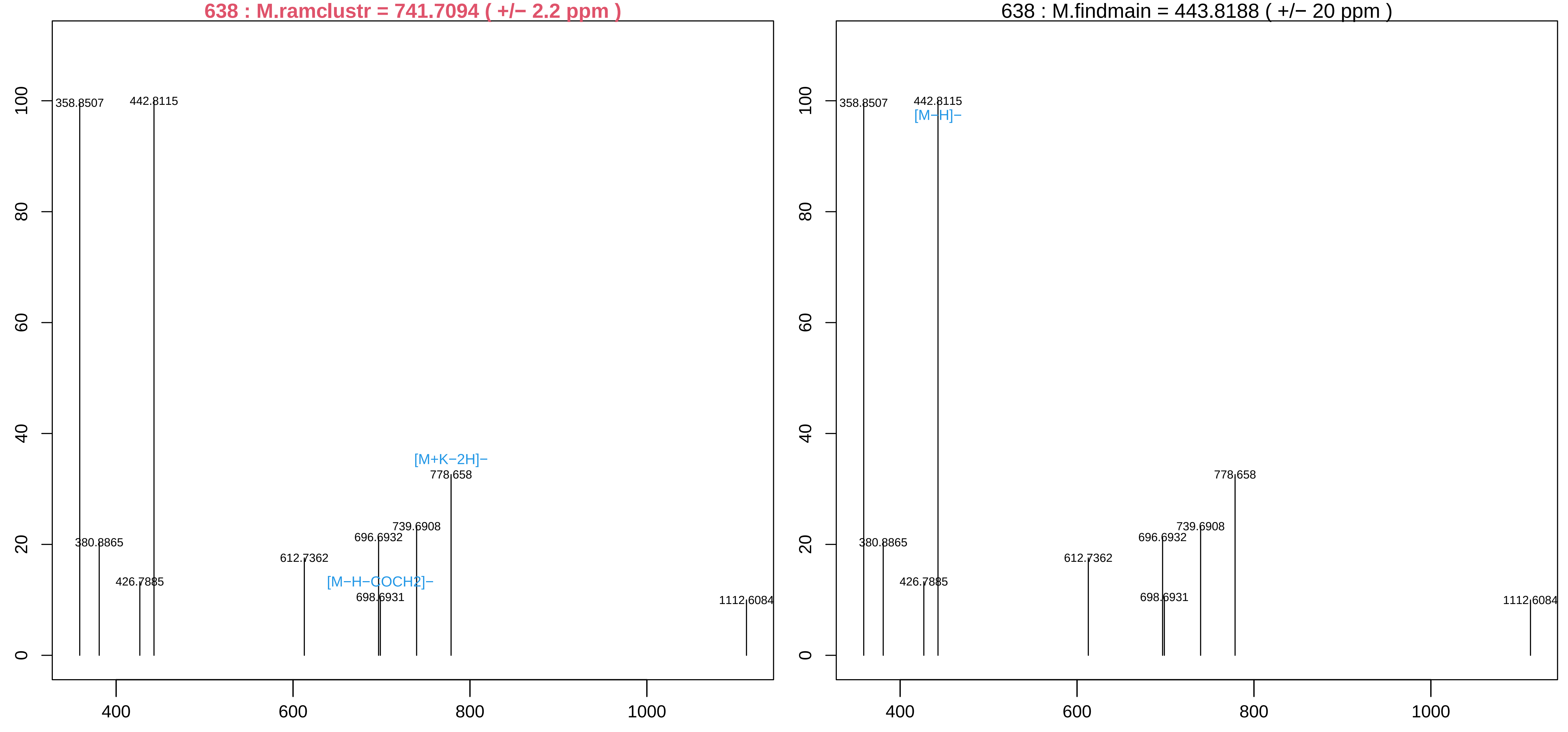
638 : M.ramclustr = 741.7094 (+/− 2.2 ppm)
358.8507
442.8115
380.8865
426.7885
612.7362
696.6932
739.6908
[M+K−2H]−
778.658
[M−H−COCH2]−
698.6931
1112.6084
638 : M.findmain = 443.8188 (+/− 20 ppm)
[M−H]−
0
20
40
60
80
100
400
600
800
1000

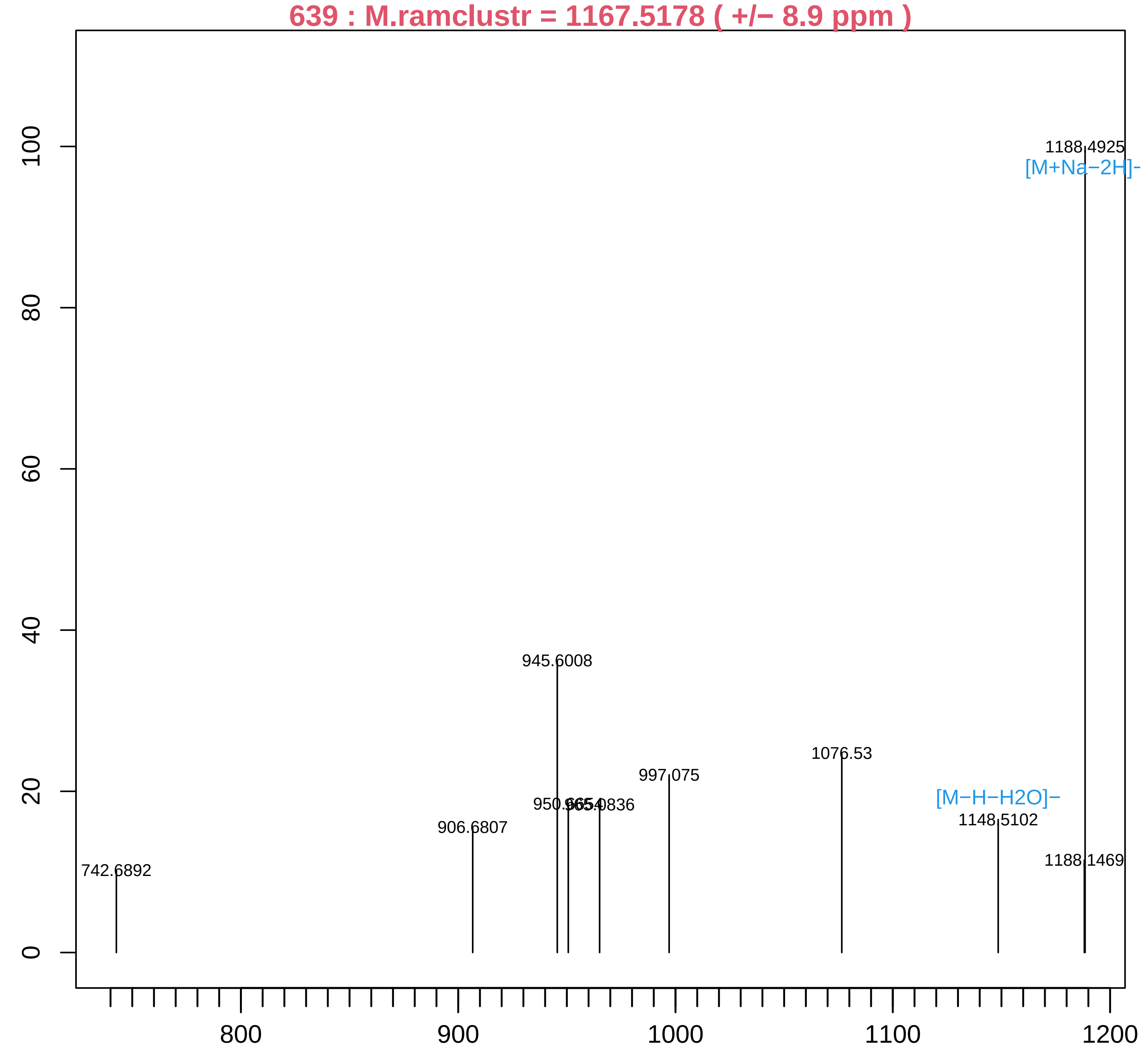
639 : M.ramclustr = 1167.5178 (+/− 8.9 ppm)
1188.4925
[M+Na−2H]-
945.6008
1076.53
997.075
[M−H−H2O]−
950.965
965.0836
1148.5102
906.6807
1188.1469
742.6892
0
20
40
60
80
100
800
900
1000
1100
1200

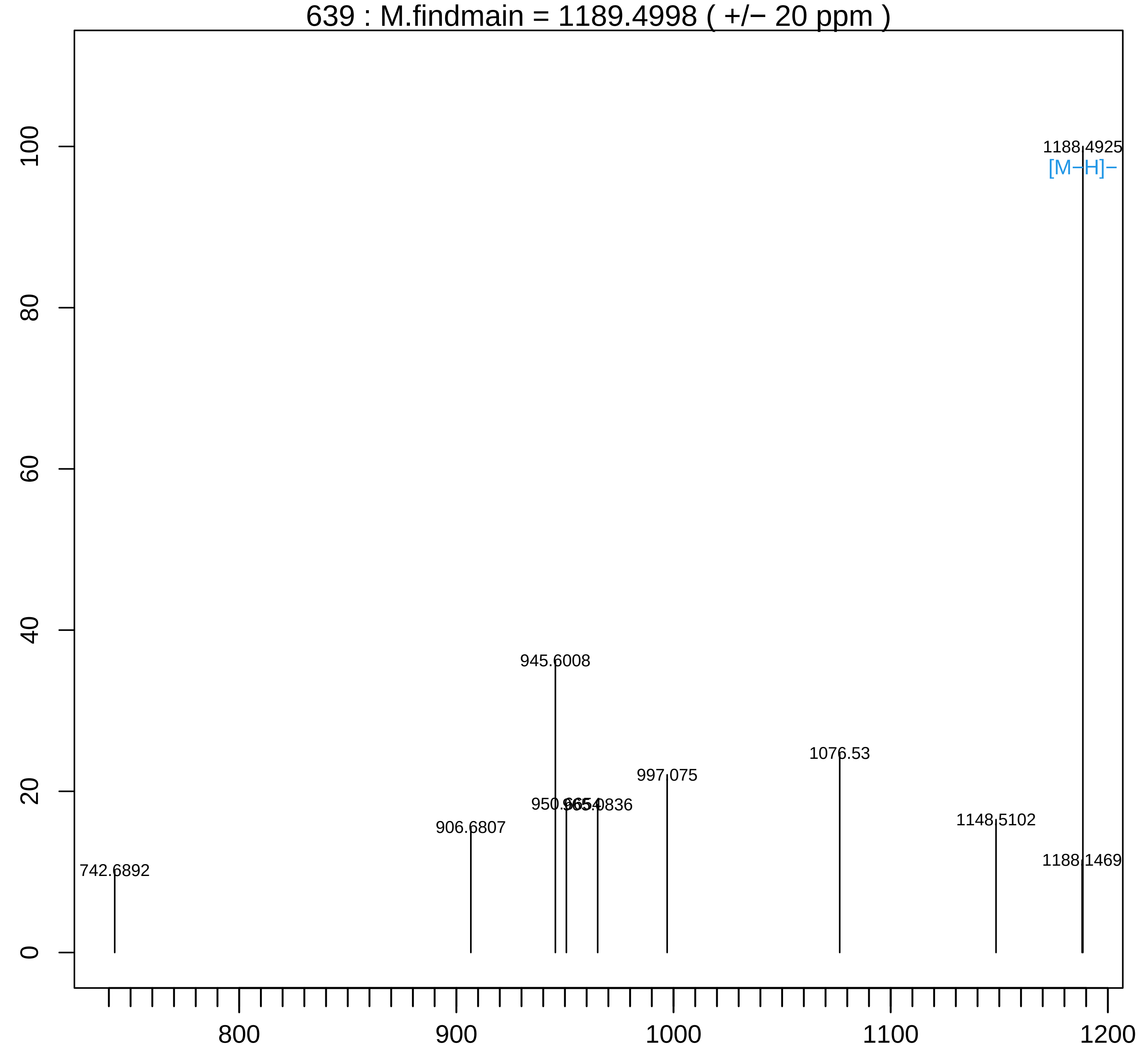
639 : M.findmain = 1189.4998 (+/− 20 ppm)
1188.4925
[M−H]−
945.6008
1076.53
997.075
950.965
965.0836
1148.5102
906.6807
1188.1469
742.6892
0
20
40
60
80
100
800
900
1000
1100
1200

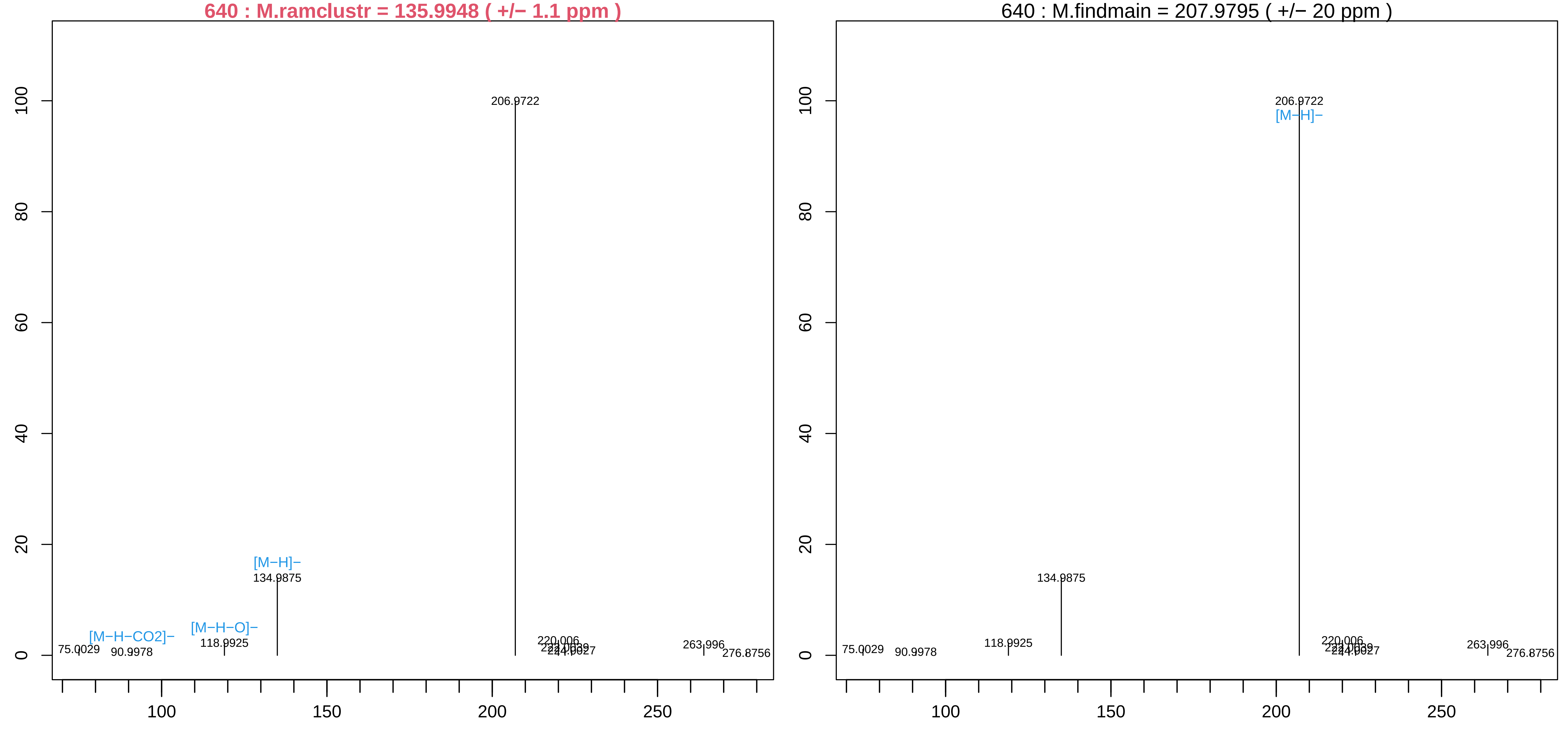
640 : M.ramclustr = 135.9948 (+/− 1.1 ppm)
206.9722
[M−H]−
134.9875
[M−H−CO2]−
[M−H−O]−
118.9925
75.0029
90.9978
220.006
224.0027
263.996
276.8756
640 : M.findmain = 207.9795 (+/− 20 ppm)
206.9722
[M−H]−
134.9875
118.9925
75.0029
90.9978
220.006
224.0027
263.996
276.8756
0
20
40
60
80
100
100
150
200
250

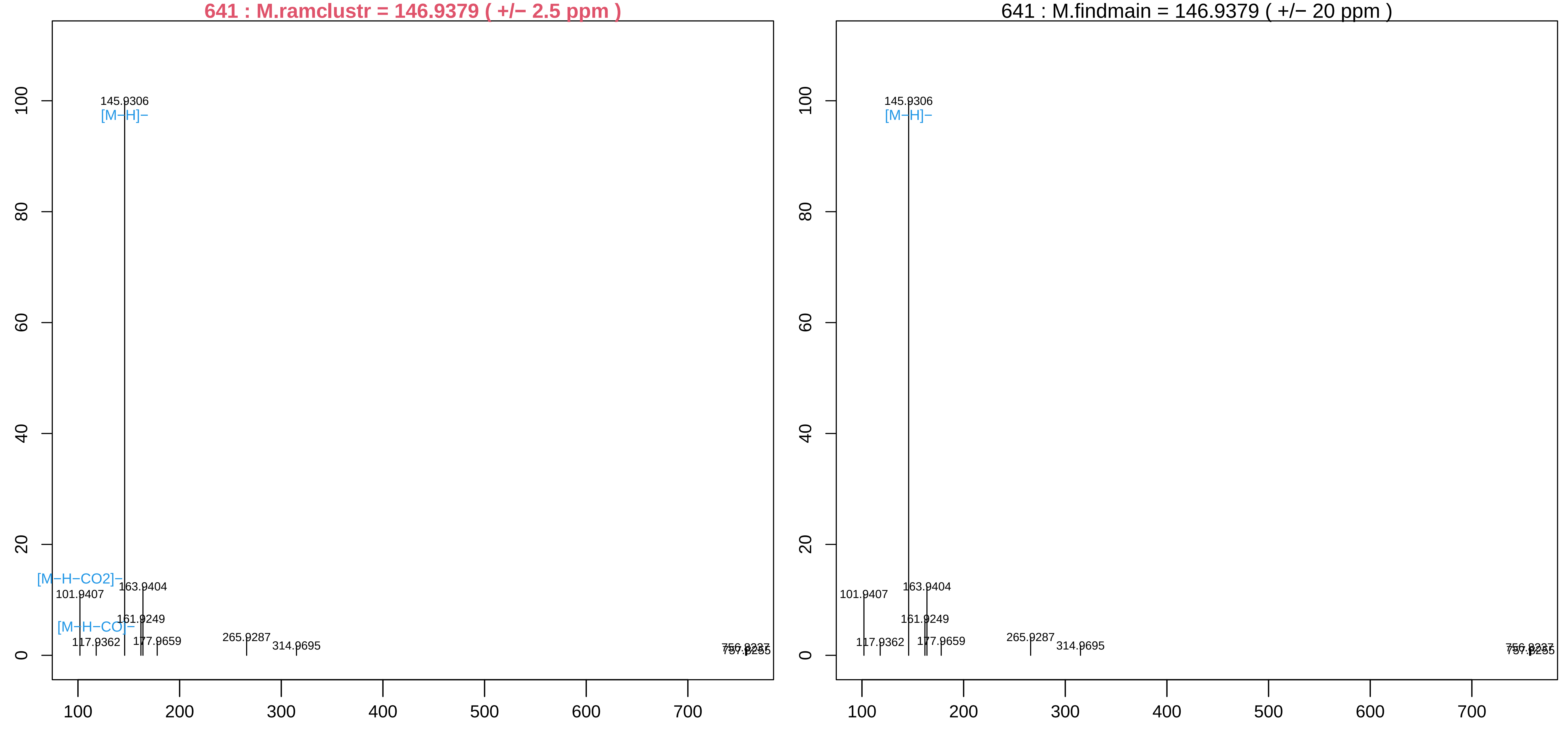
641 : M.ramclustr = 146.9379 (+/− 2.5 ppm)
145.9306
[M−H]−
[M−H−CO2]−
101.9407
163.9404
[M−H−CO]−
161.9249
117.9362
177.9659
265.9287
314.9695
756.8237
757.8255
641 : M.findmain = 146.9379 (+/− 20 ppm)
145.9306
[M−H]−
101.9407
163.9404
161.9249
117.9362
177.9659
265.9287
314.9695
756.8237
757.8255

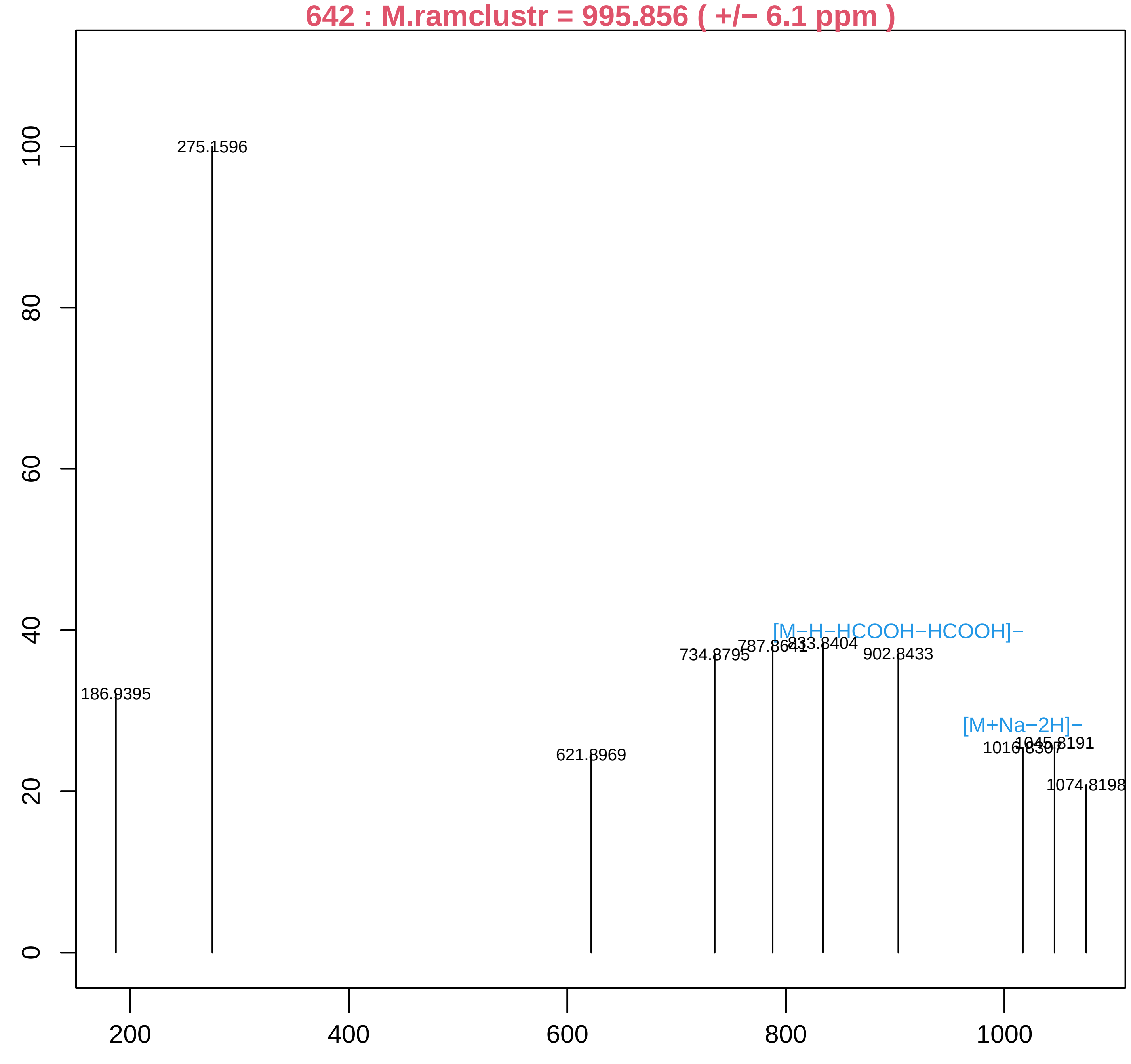

642 : M.ramclustr = 995.856 (+/− 6.1 ppm)
275.1596
186.9395
621.8969
734.8795
787.8641
833.8404
902.8433
[M−H−HCOOH−HCOOH]−
[M+Na−2H]−
1016.8307
1045.8191
1074.8198
0
20
40
60
80
100
200
400
600
800
1000

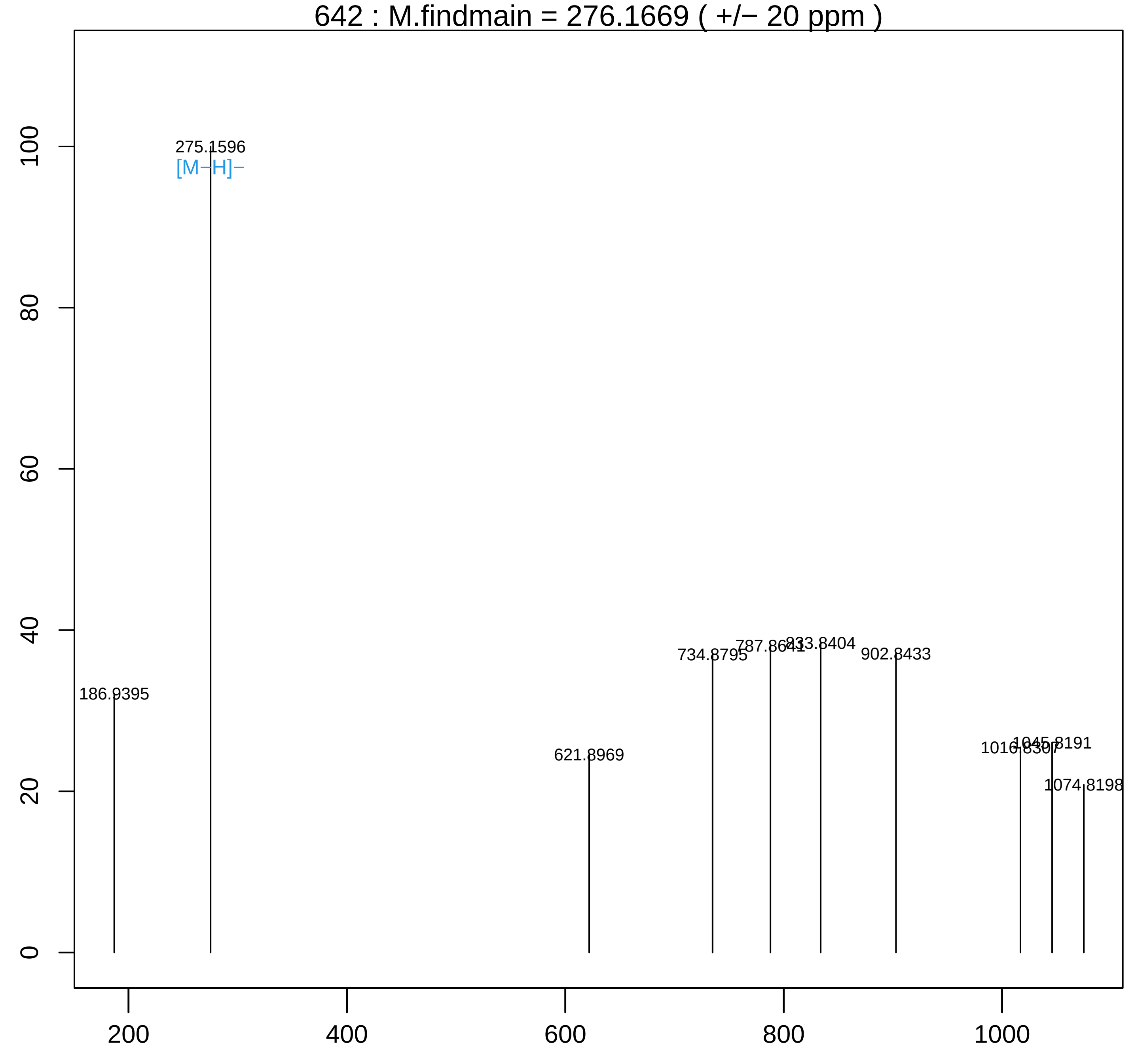

642 : M.findmain = 276.1669 (+/− 20 ppm)
275.1596
[M−H]−
186.9395
621.8969
734.8795
787.8641
833.8404
902.8433
1016.8307
1045.8191
1074.8198
0
20
40
60
80
100
200
400
600
800
1000

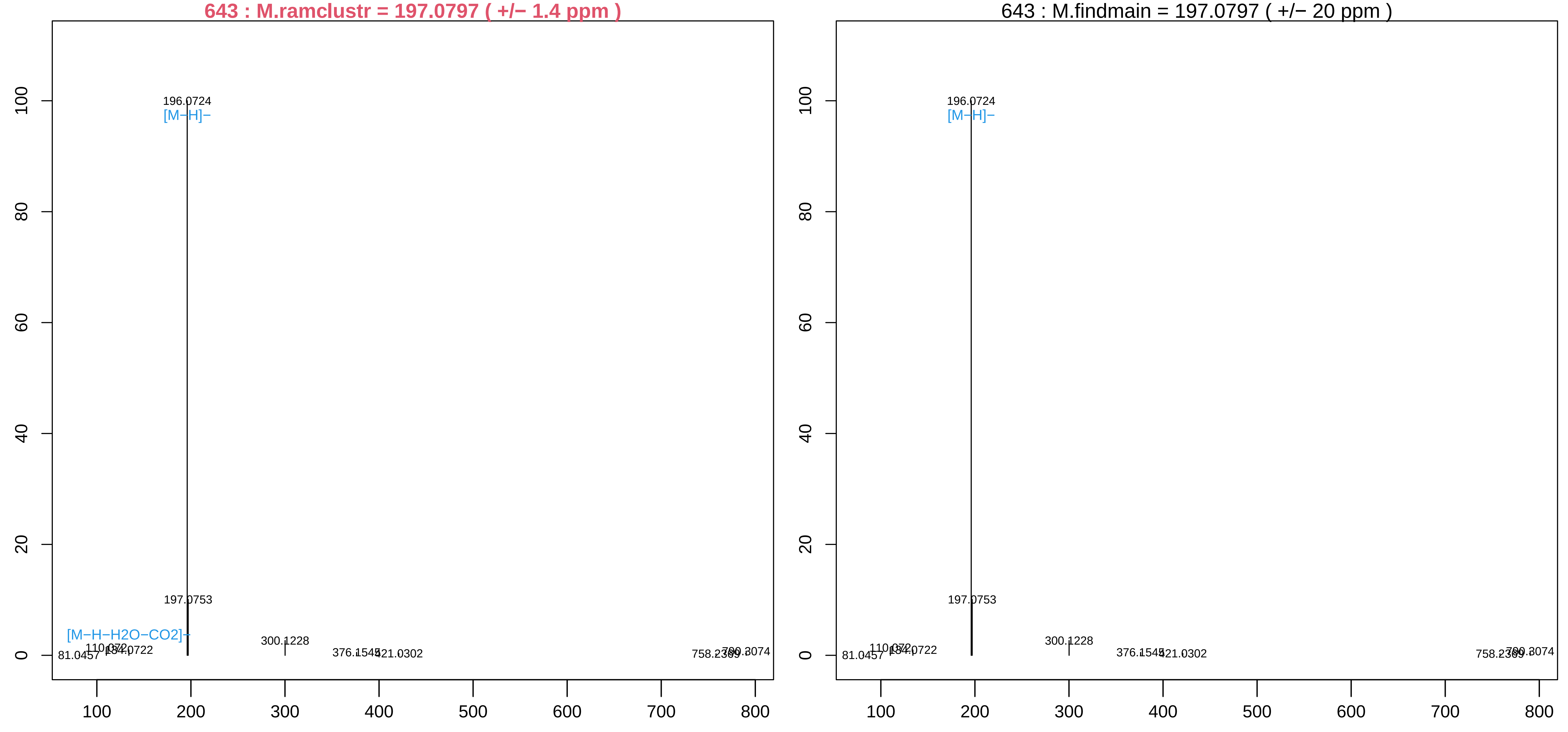
643 : M.ramclustr = 197.0797 (+/− 1.4 ppm)
196.0724
[M−H]−
197.0753
[M−H−H2O−CO2]−
110.072
81.0457
134.0722
300.1228
376.1545
421.0302
758.2389
790.3074
643 : M.findmain = 197.0797 (+/− 20 ppm)
196.0724
[M−H]−
197.0753
110.072
81.0457
134.0722
300.1228
376.1545
421.0302
758.2389
790.3074

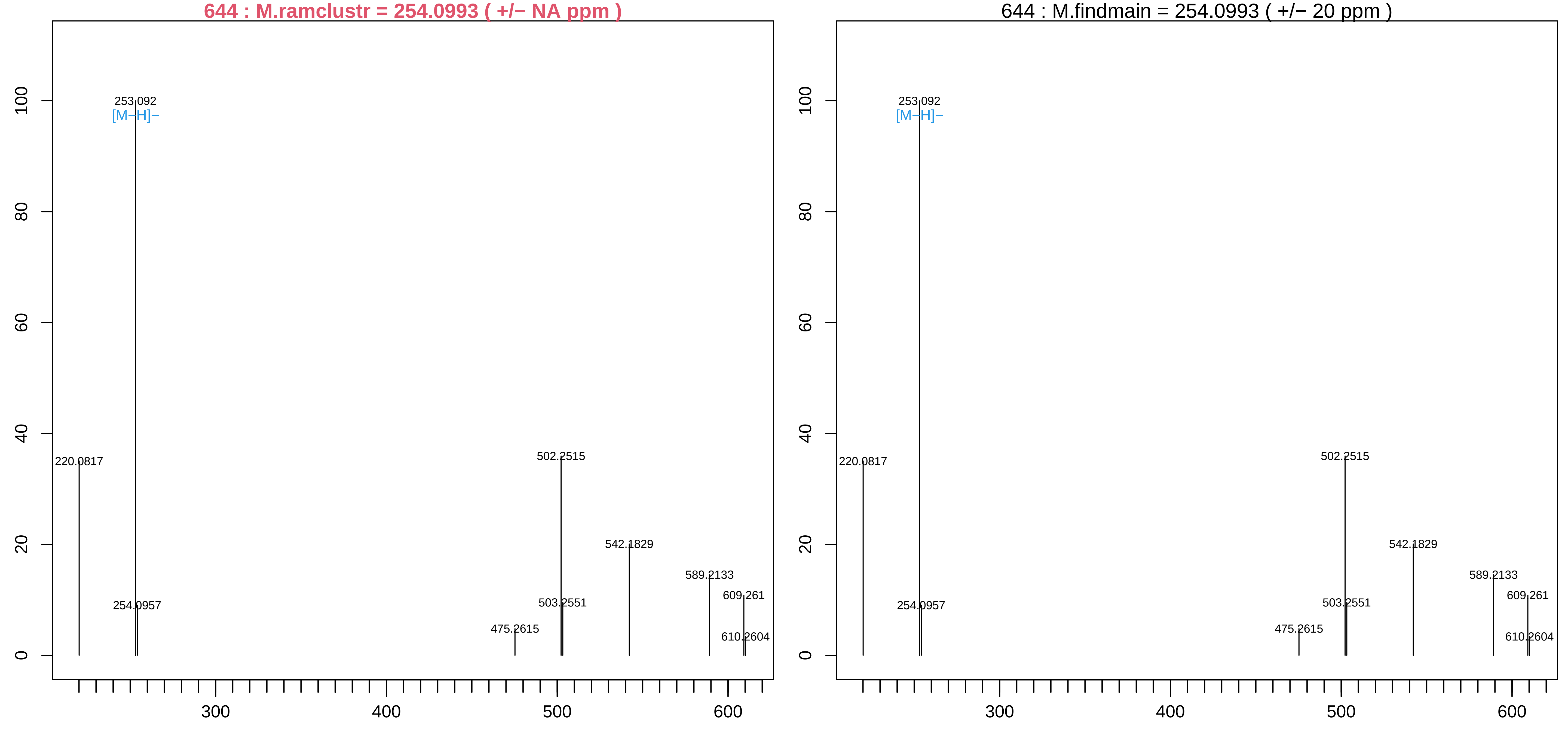

644 : M.ramclustr = 254.0993 (+/− NA ppm)
253.092
[M−H]−
220.0817
254.0957
475.2615
502.2515
503.2551
542.1829
589.2133
609.261
610.2604
644 : M.findmain = 254.0993 (+/− 20 ppm)
253.092
[M−H]−
220.0817
254.0957
475.2615
502.2515
503.2551
542.1829
589.2133
609.261
610.2604
0
20
40
60
80
100
300
400
500
600

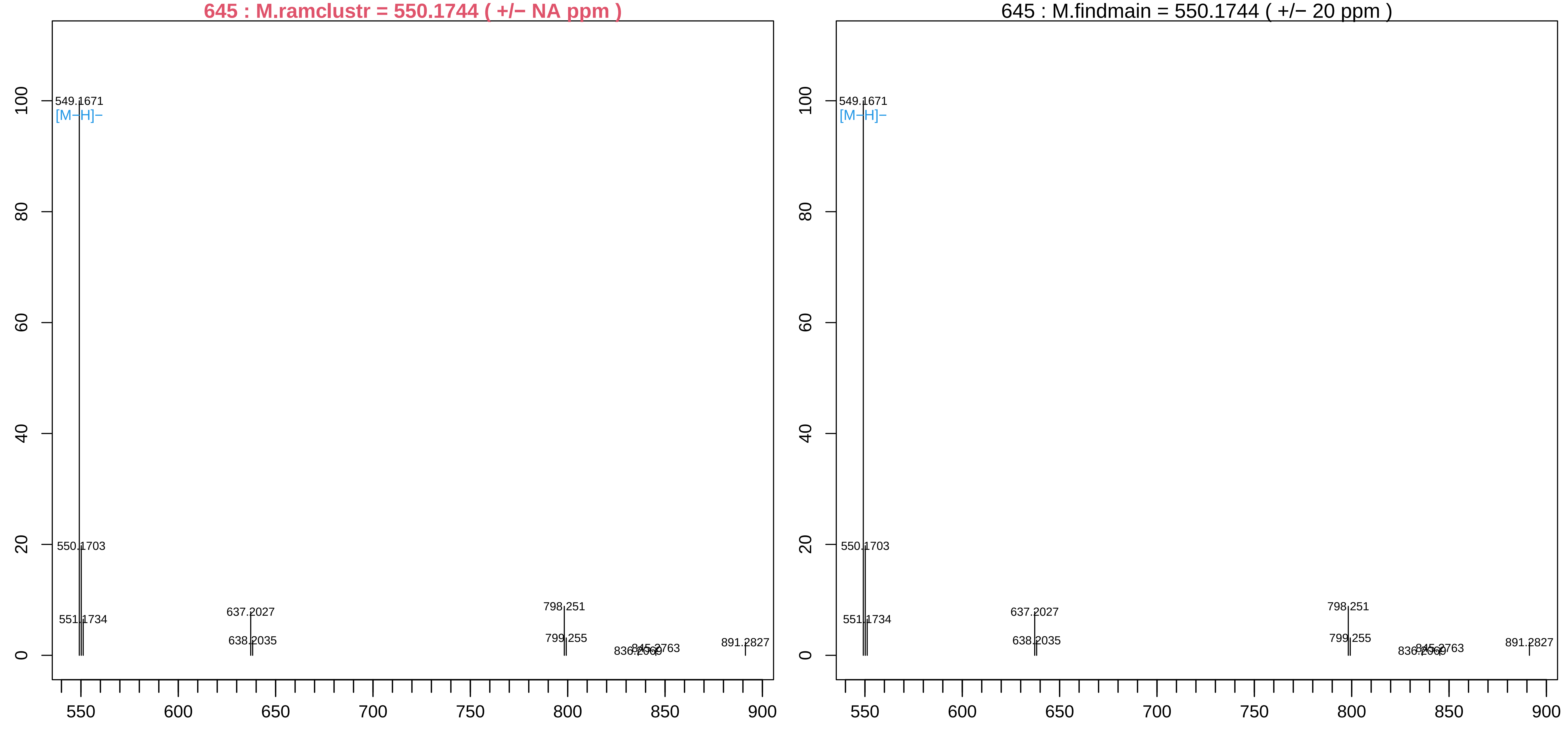
645 : M.ramclustr = 550.1744 (+/− NA ppm)
549.1671
[M−H]−
550.1703
551.1734
637.2027
638.2035
798.251
799.255
836.2069
845.2763
891.2827
645 : M.findmain = 550.1744 (+/− 20 ppm)
549.1671
[M−H]−
550.1703
551.1734
637.2027
638.2035
798.251
799.255
836.2069
845.2763
891.2827
0
20
40
60
80
100
550
600
650
700
750
800
850
900

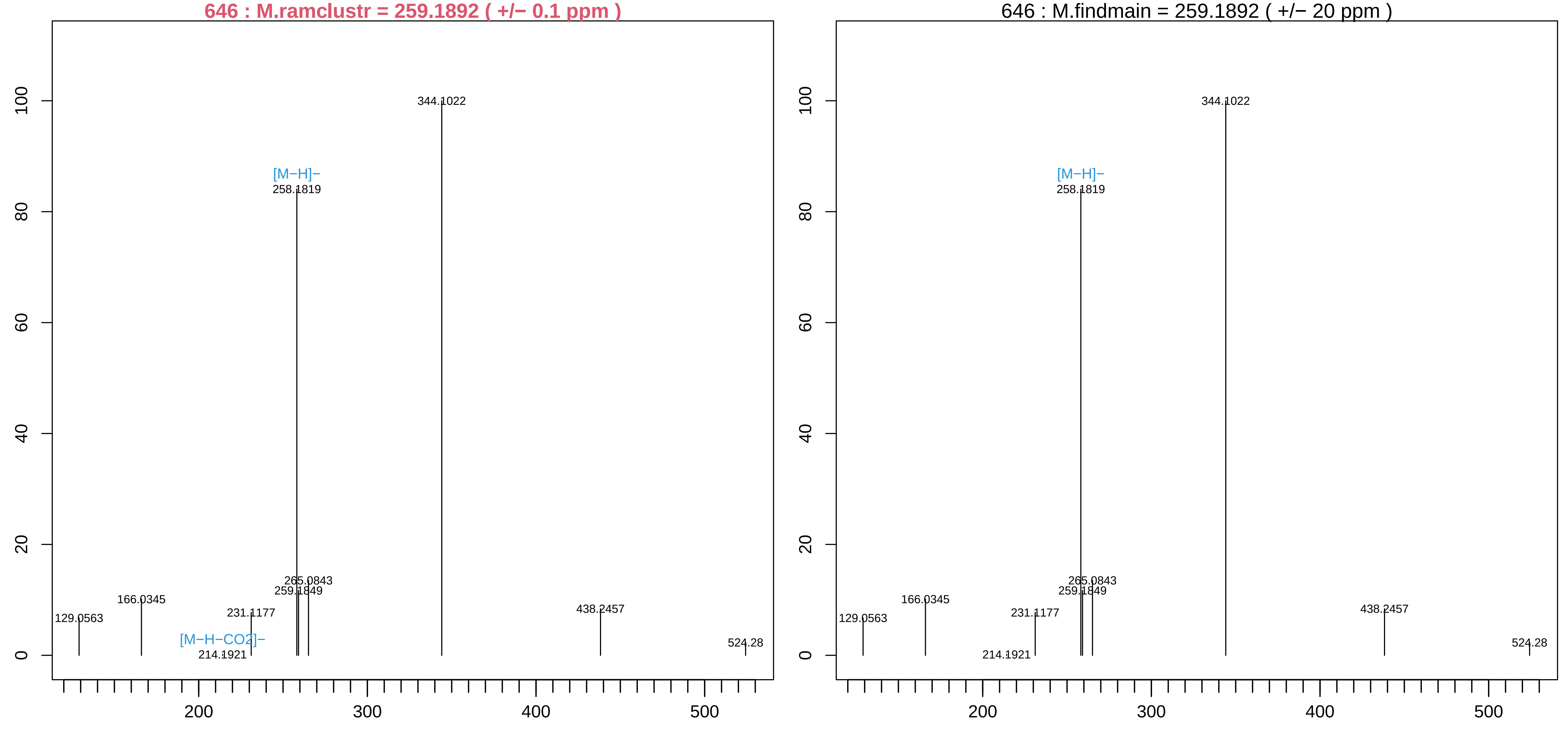
646 : M.ramclustr = 259.1892 (+/− 0.1 ppm)
344.1022
[M−H]−
258.1819
265.0843
259.1849
166.0345
129.0563
231.1177
[M−H−CO2]−
214.1921
438.2457
524.28
0
20
40
60
80
100
200
300
400
500
646 : M.findmain = 259.1892 (+/− 20 ppm)
344.1022
[M−H]−
258.1819
265.0843
259.1849
166.0345
129.0563
231.1177
214.1921
438.2457
524.28
0
20
40
60
80
100
200
300
400
500

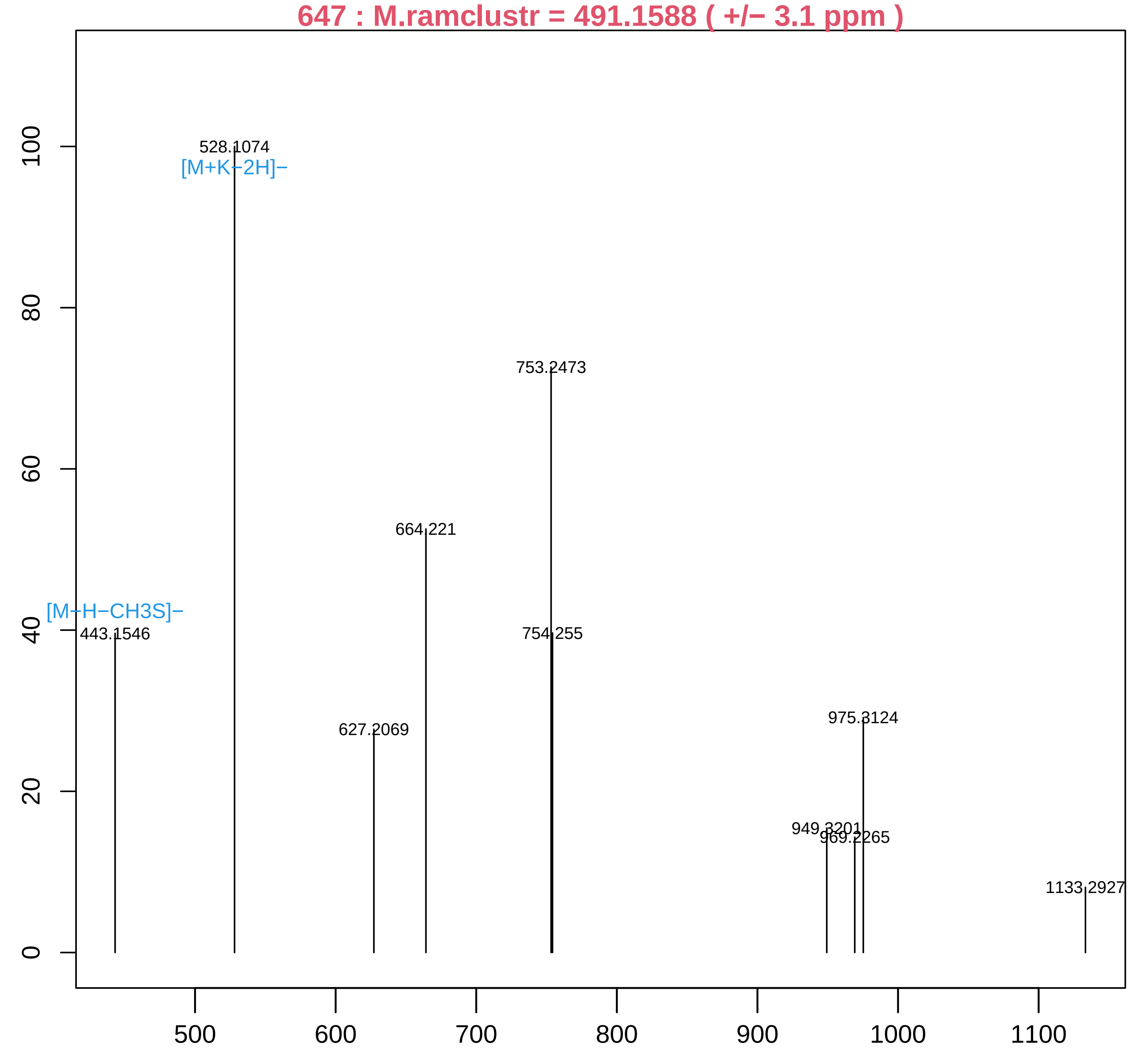
647 : M.ramclustr = 491.1588 (+/− 3.1 ppm)
528.1074
[M+K−2H]−
753.2473
664.221
[M−H−CH3S]−
443.1546
754.255
627.2069
975.3124
949.3201
969.2265
1133.2927
0
20
40
60
80
100
500
600
700
800
900
1000
1100

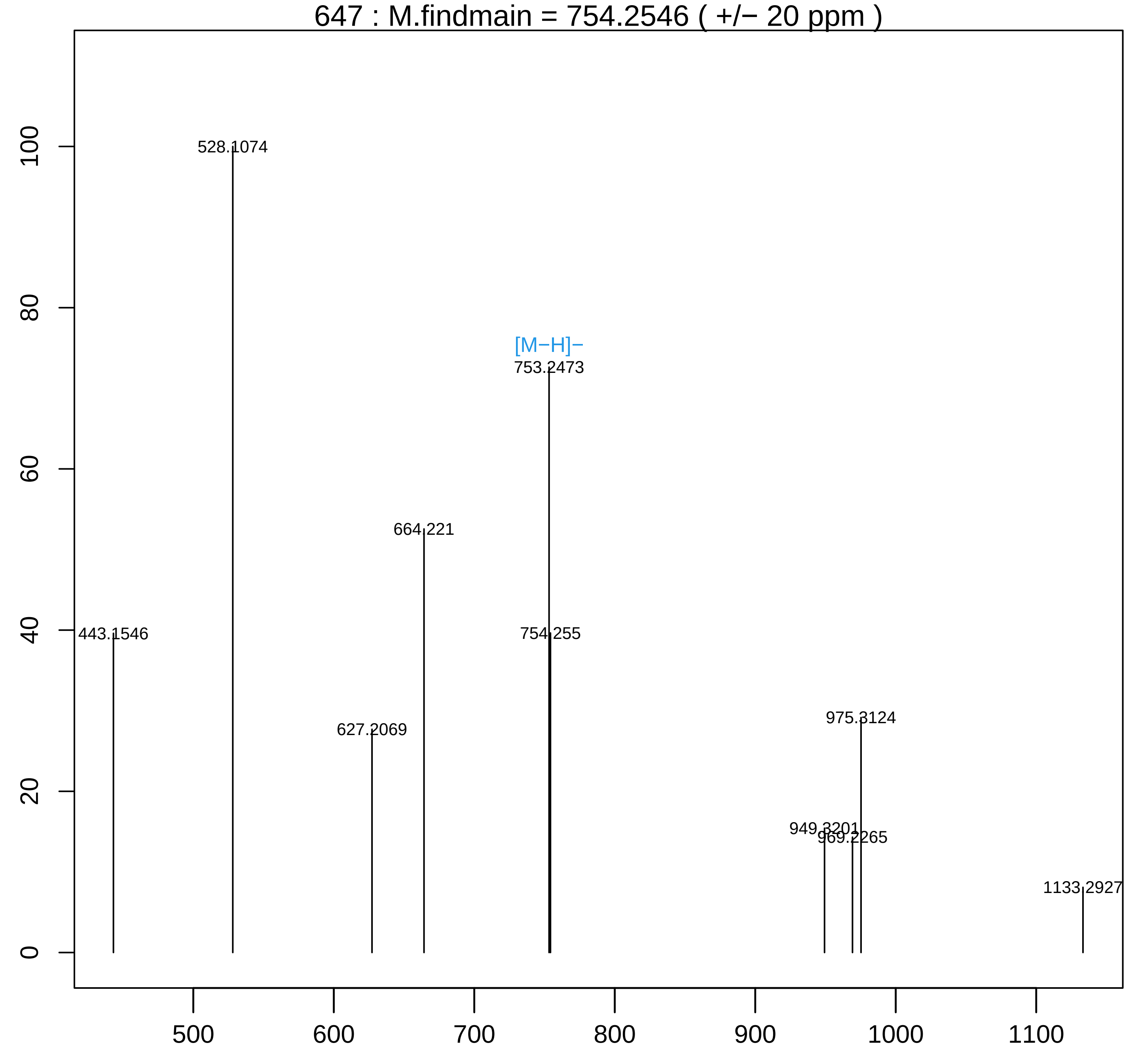
647 : M.findmain = 754.2546 (+/− 20 ppm)
528.1074
[M−H]−
753.2473
664.221
443.1546
754.255
627.2069
975.3124
949.3201
969.2265
1133.2927
0
20
40
60
80
100
500
600
700
800
900
1000
1100

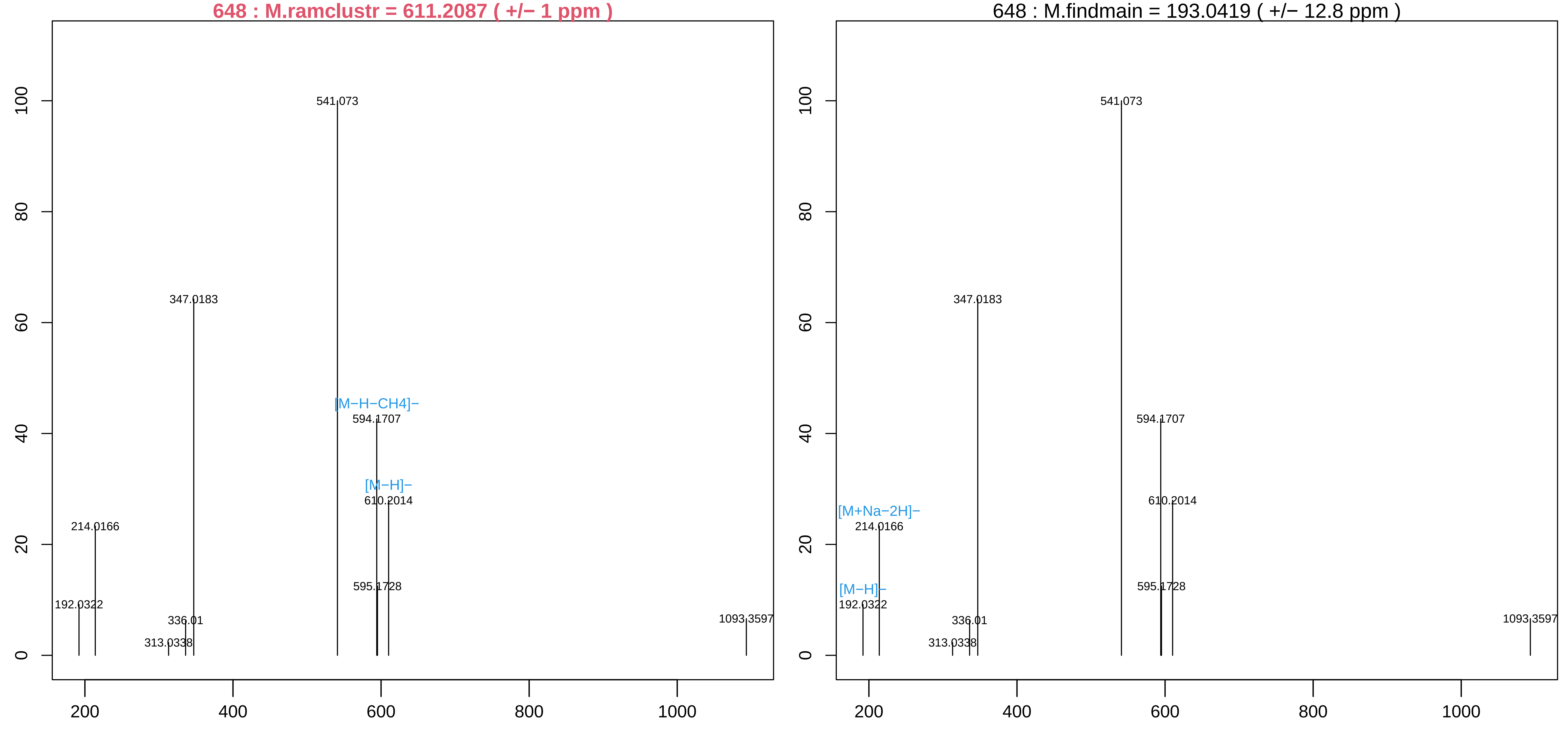

648 : M.ramclustr = 611.2087 (+/− 1 ppm)
541.073
347.0183
[M−H−CH4]−
594.1707
[M−H]−
610.2014
214.0166
595.1728
192.0322
336.01
313.0338
1093.3597
648 : M.findmain = 193.0419 (+/− 12.8 ppm)
541.073
347.0183
594.1707
610.2014
[M+Na−2H]−
214.0166
[M−H]−
192.0322
595.1728
336.01
313.0338
1093.3597
0
20
40
60
80
100
200
400
600
800
1000

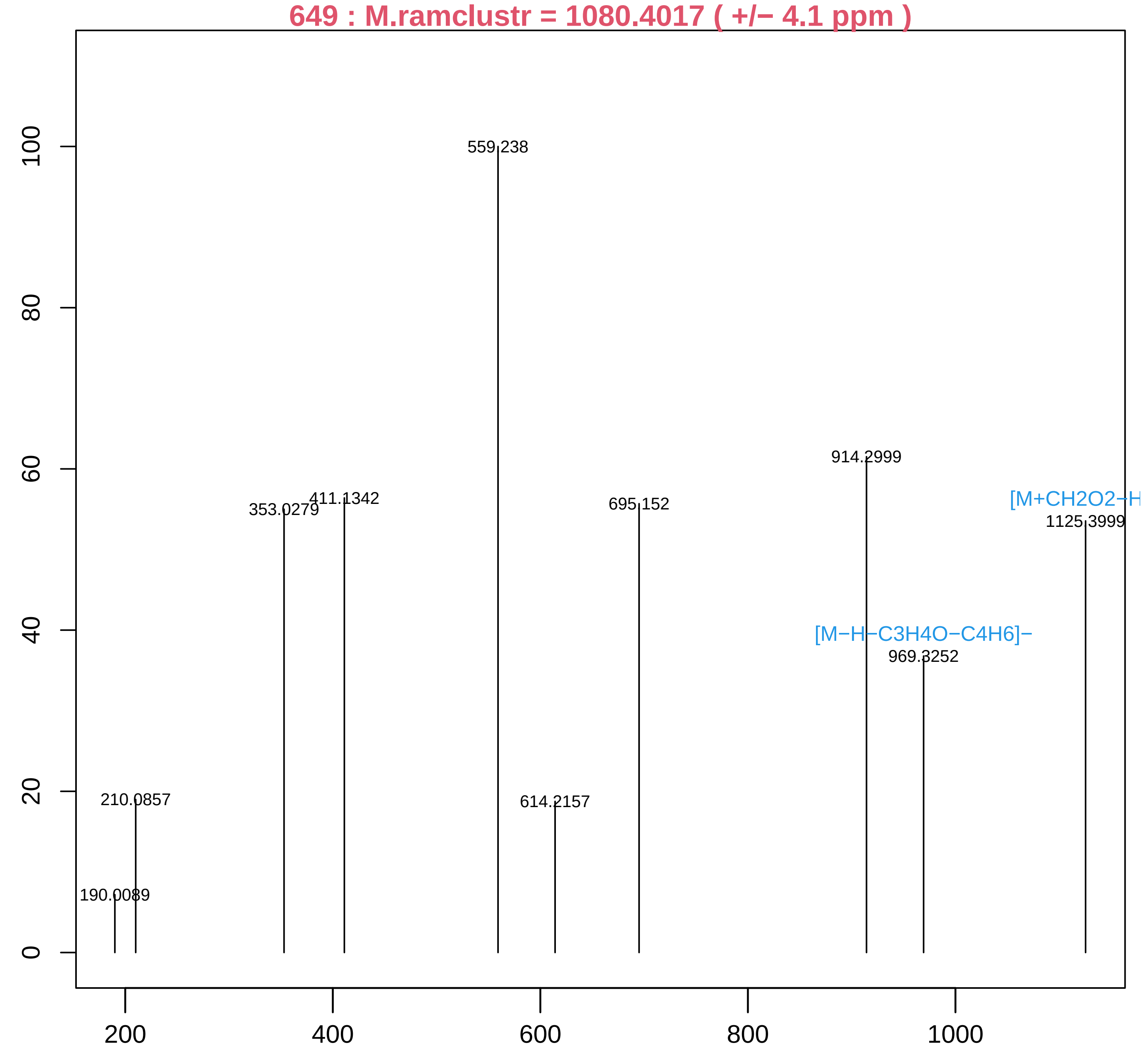

649 : M.ramclustr = 1080.4017 (+/− 4.1 ppm)
559.238
914.2999
[M+CH2O2−H
411.1342
353.0279
695.152
1125.3999
[M−H−C3H4O−C4H6]−
969.3252
210.0857
614.2157
190.0089
0
20
40
60
80
100
200
400
600
800
1000

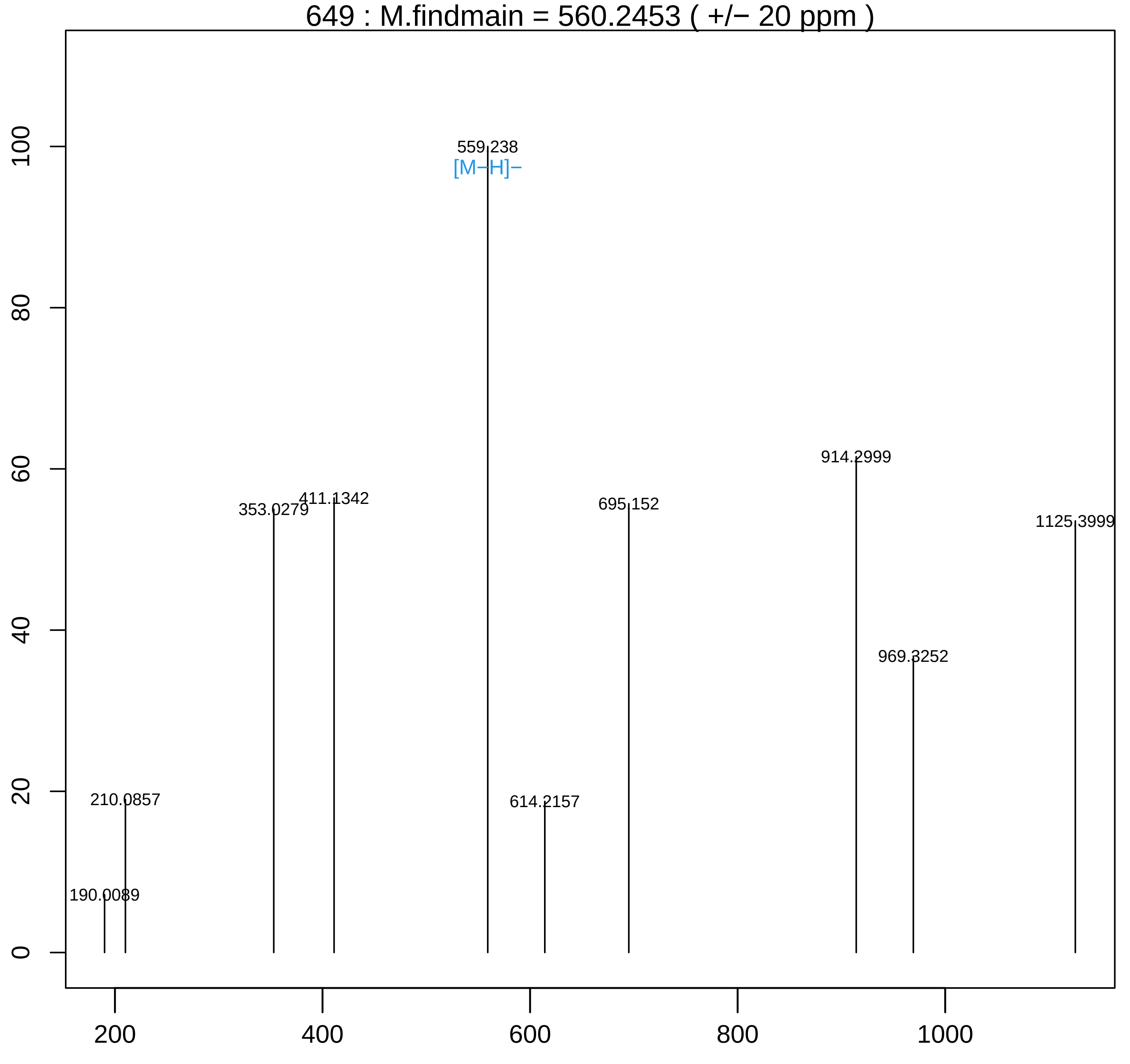

649 : M.findmain = 560.2453 (+/− 20 ppm)
559.238
[M−H]−
914.2999
411.1342
353.0279
695.152
1125.3999
969.3252
210.0857
614.2157
190.0089
0
20
40
60
80
100
200
400
600
800
1000

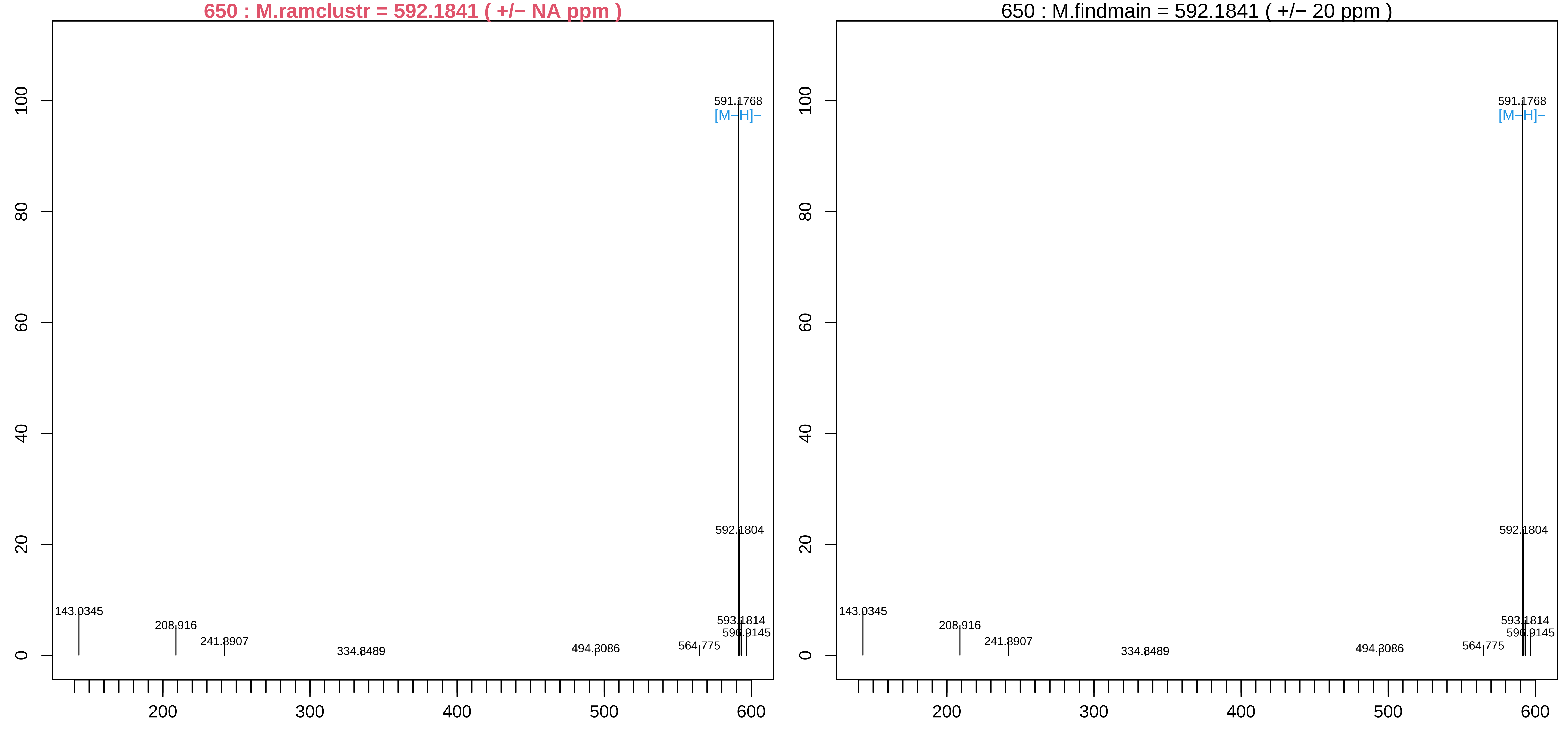
650 : M.ramclustr = 592.1841 (+/− NA ppm)
650 : M.findmain = 592.1841 (+/− 20 ppm)
591.1768
[M−H]−
592.1804
593.1814
596.9145
564.775
494.3086
334.8489
241.8907
208.916
143.0345
0
20
40
60
80
100
200
300
400
500
600

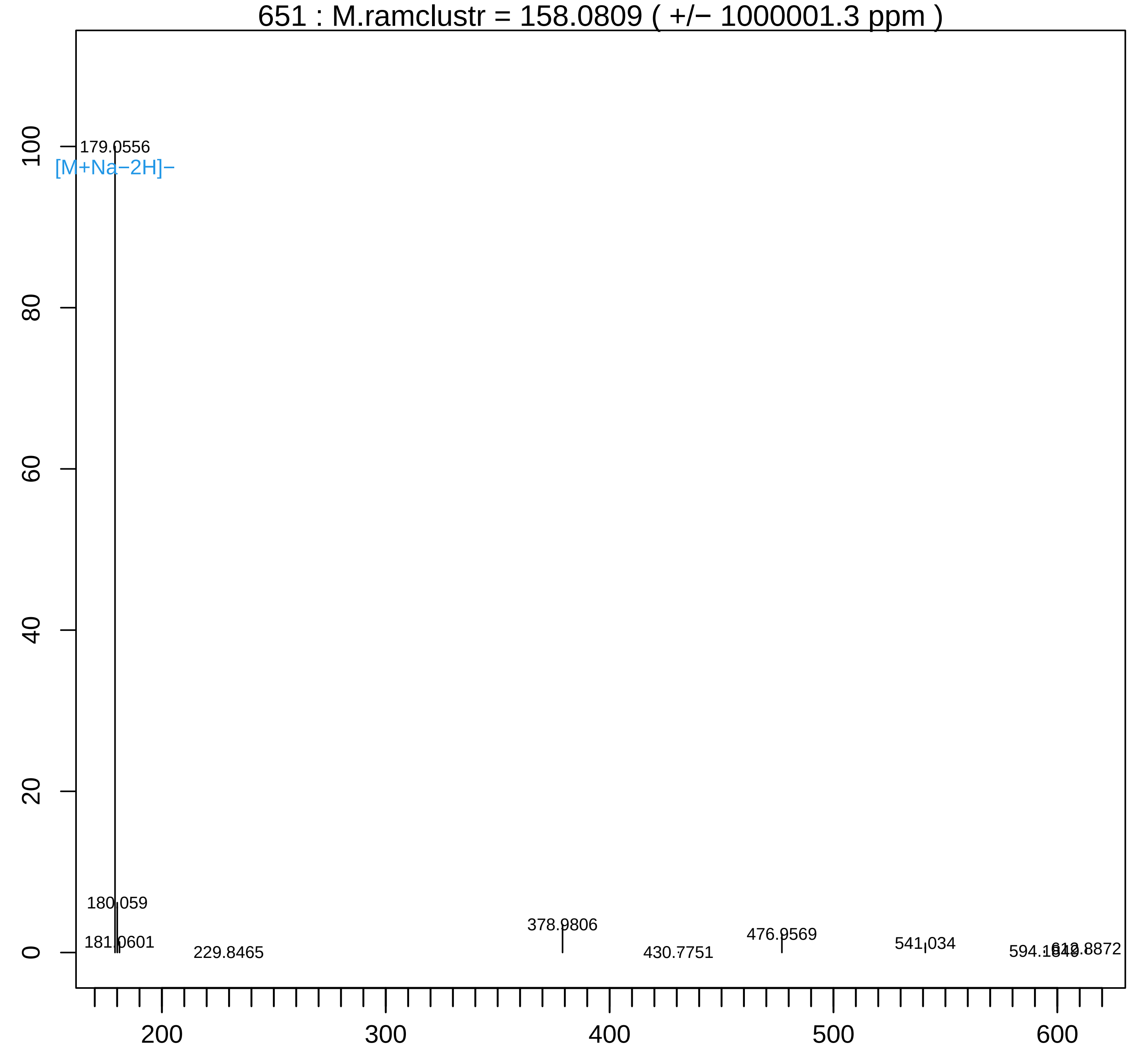
651 : M.ramclustr = 158.0809 (+/− 1000001.3 ppm)
179.0556
[M+Na−2H]−
180.059
181.0601
229.8465
378.9806
430.7751
476.9569
541.034
594.1648
612.8872
0
20
40
60
80
100
200
300
400
500
600

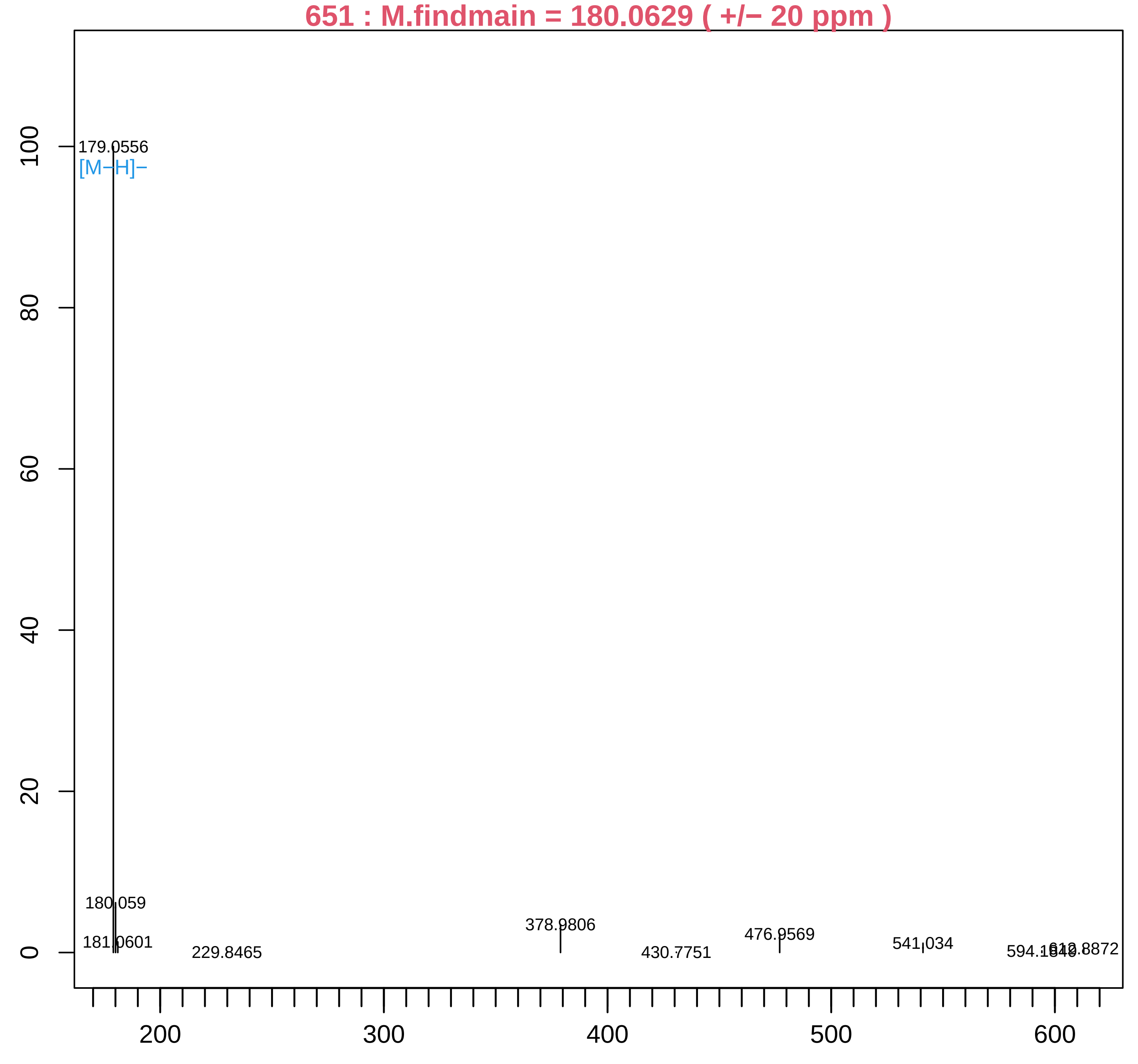
651 : M.findmain = 180.0629 (+/− 20 ppm)
179.0556
[M−H]−
180.059
181.0601
229.8465
378.9806
430.7751
476.9569
541.034
594.1648
612.8872
0
20
40
60
80
100
200
300
400
500
600

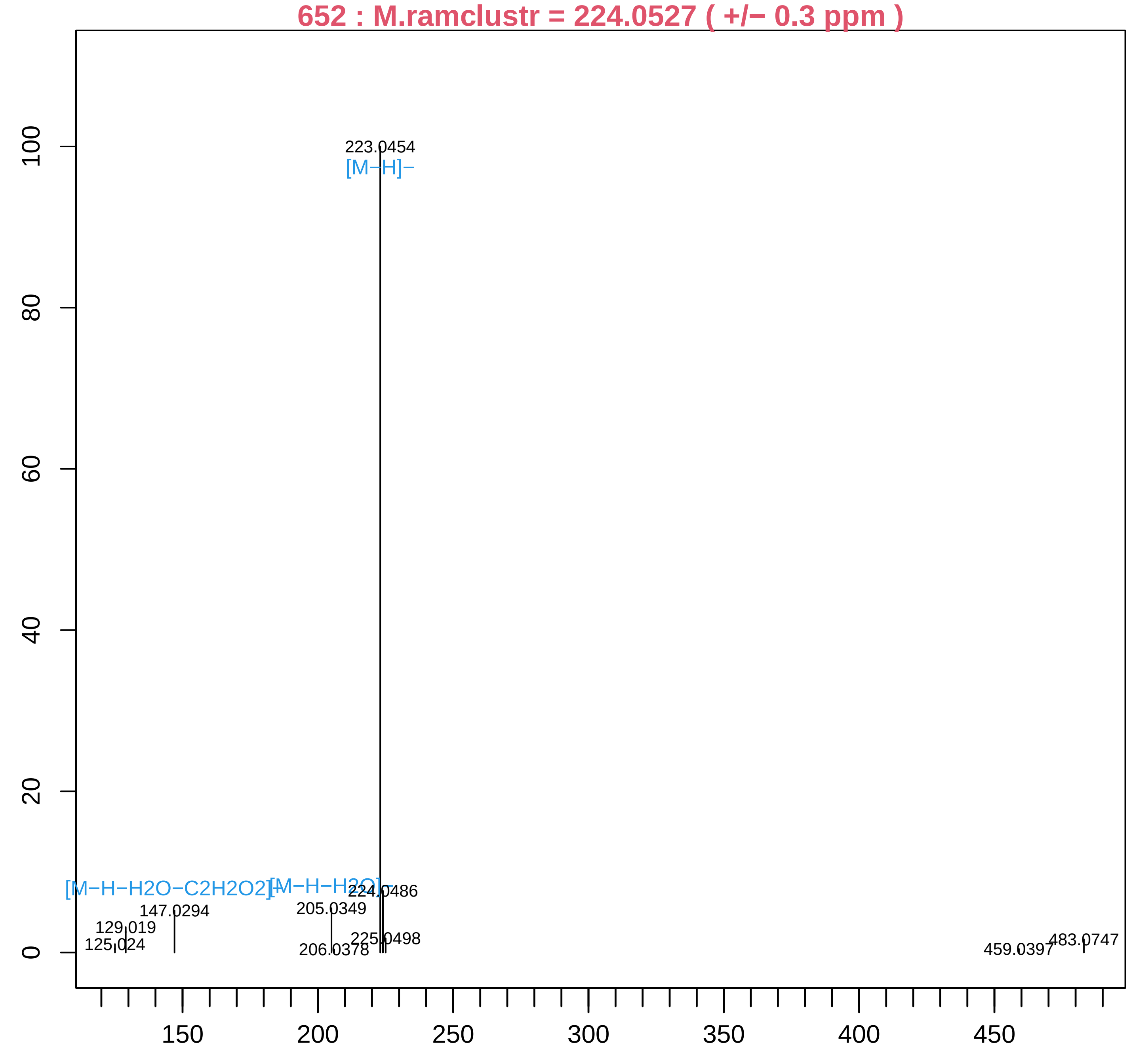
652 : M.ramclustr = 224.0527 (+/− 0.3 ppm)
223.0454
[M−H]−
[M−H−H2O−C2H2O2]−
[M−H−H2O]−
224.0486
147.0294
205.0349
129.019
125.024
225.0498
206.0378
459.0397
483.0747
0
20
40
60
80
100
150
200
250
300
350
400
450

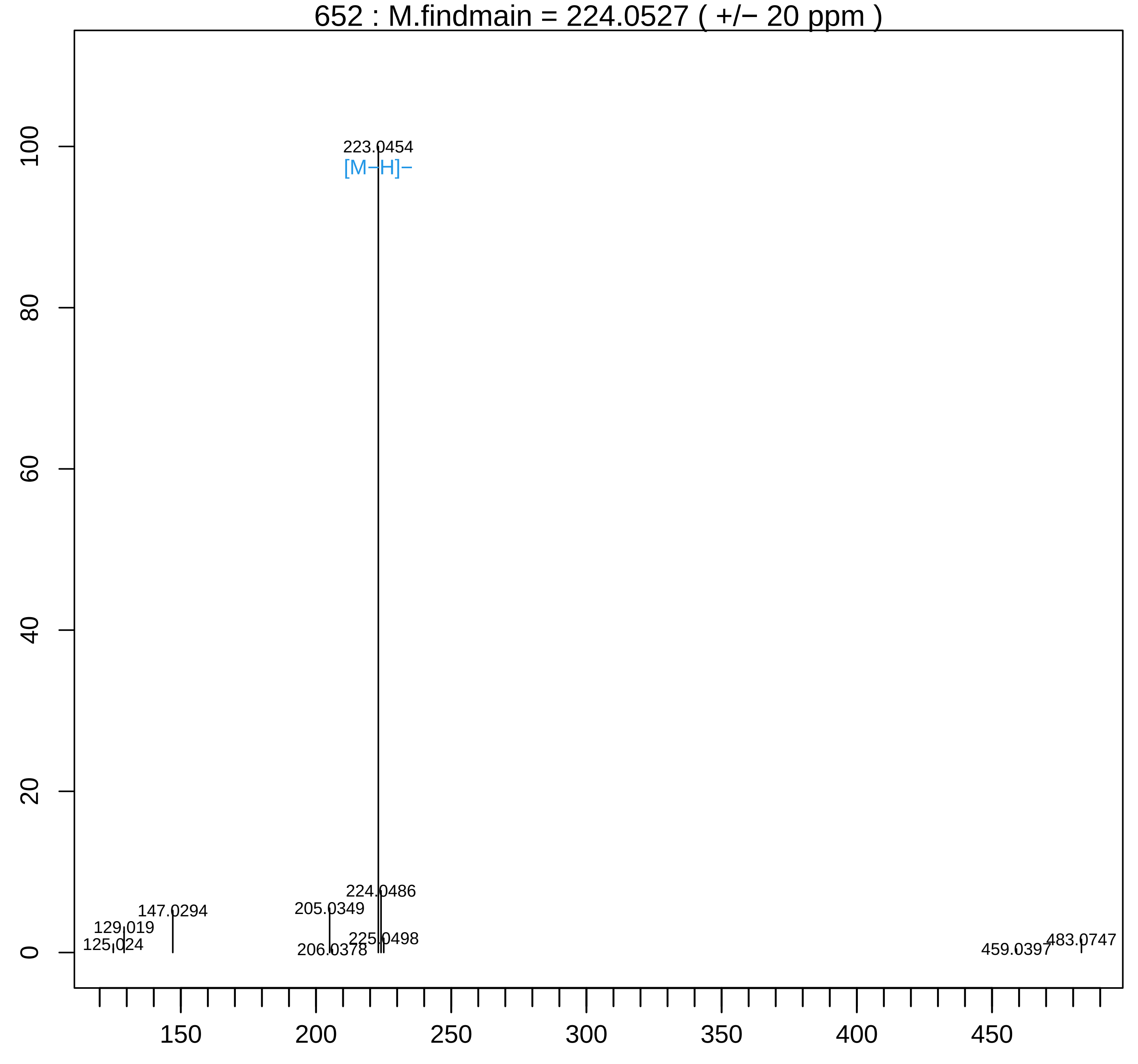
652 : M.findmain = 224.0527 (+/− 20 ppm)
223.0454
[M−H]−
224.0486
147.0294
205.0349
129.019
125.024
225.0498
206.0378
459.0397
483.0747
0
20
40
60
80
100
150
200
250
300
350
400
450

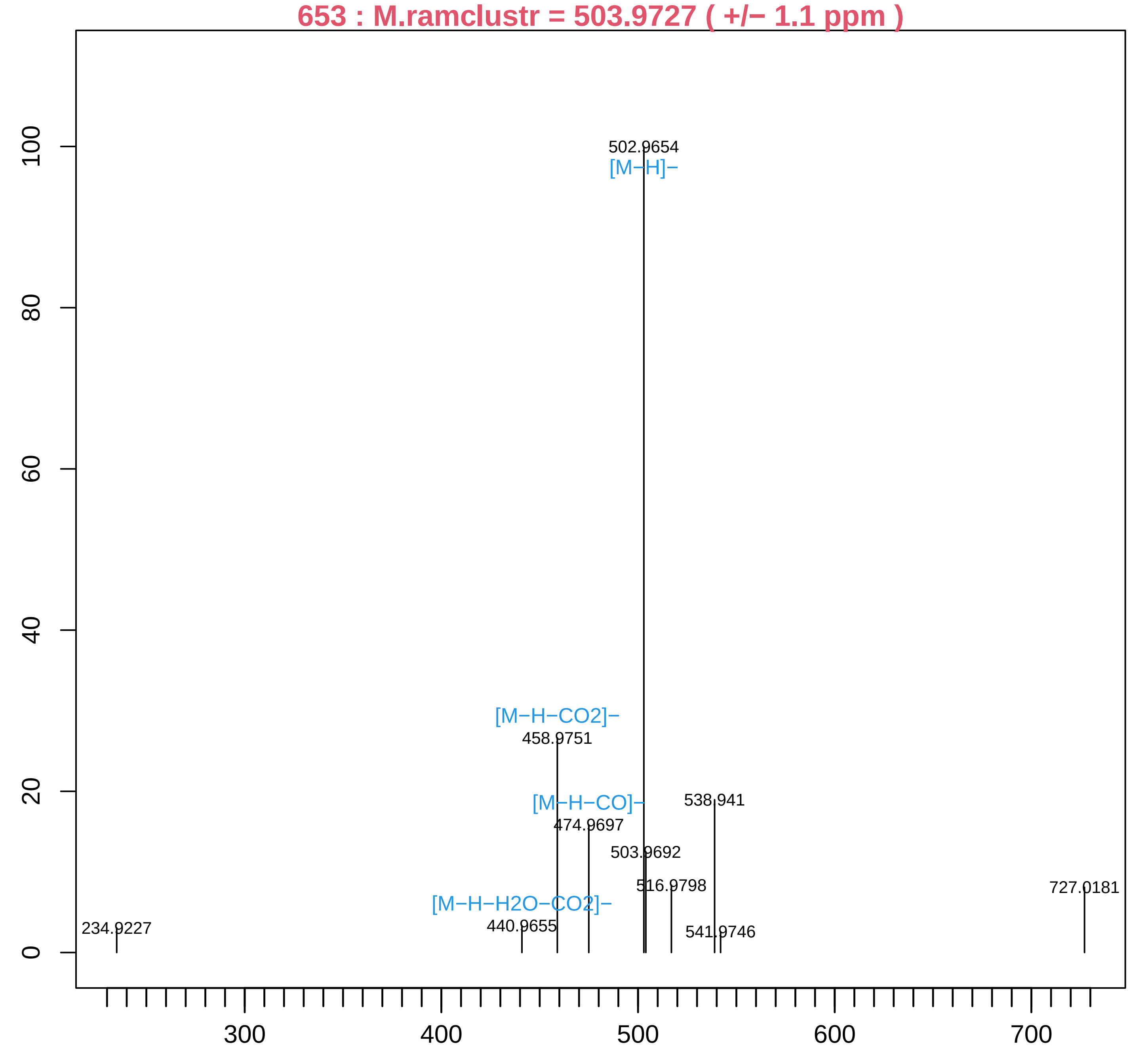
653 : M.ramclustr = 503.9727 (+/− 1.1 ppm)
502.9654
[M−H]−
[M−H−CO2]−
458.9751
[M−H−CO]−
474.9697
538.941
503.9692
516.9798
727.0181
[M−H−H2O−CO2]−
440.9655
234.9227
541.9746
0
20
40
60
80
100
300
400
500
600
700

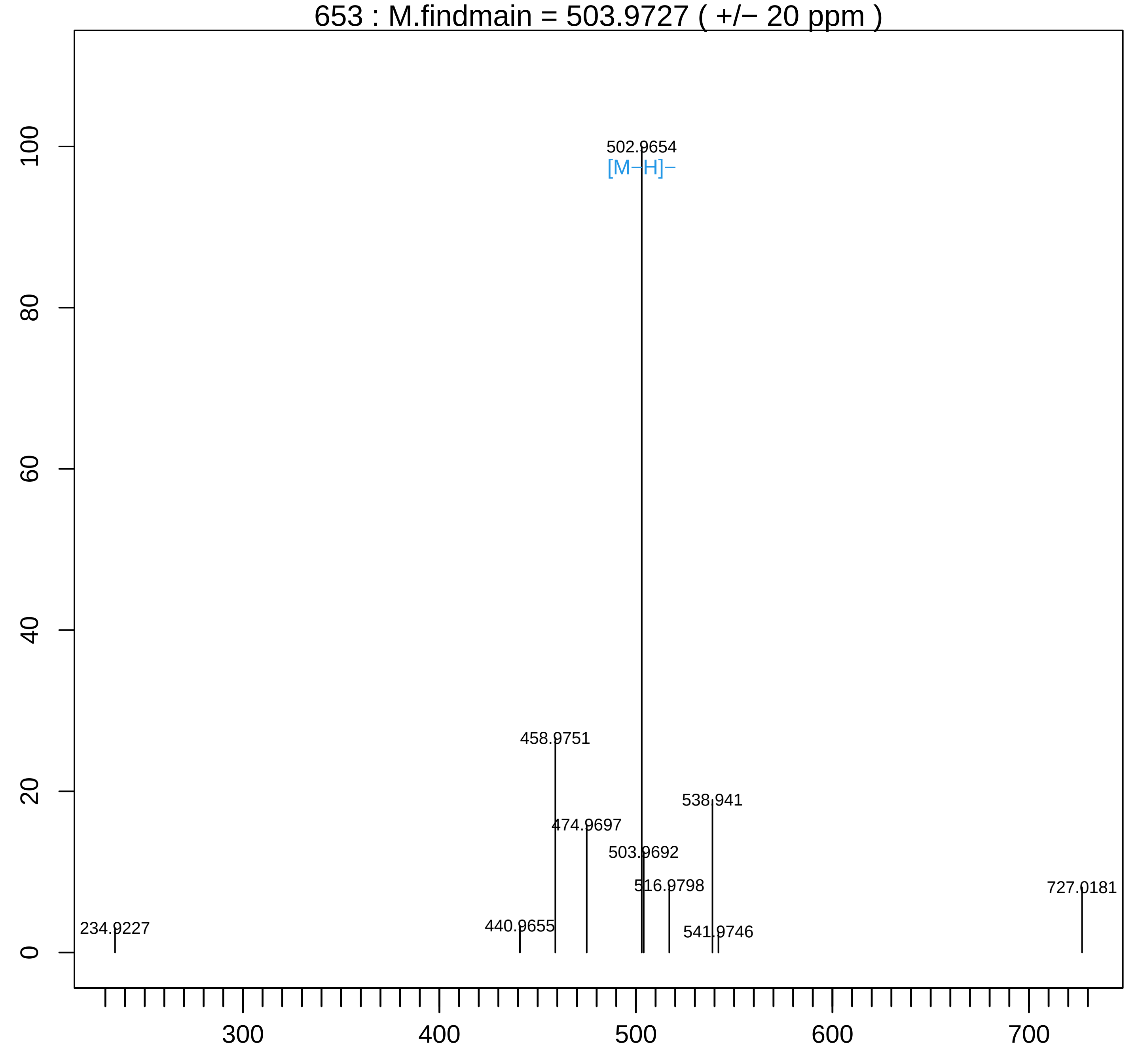
653 : M.findmain = 503.9727 (+/− 20 ppm)
502.9654
[M−H]−
458.9751
538.941
474.9697
503.9692
516.9798
727.0181
440.9655
234.9227
541.9746
0
20
40
60
80
100
300
400
500
600
700

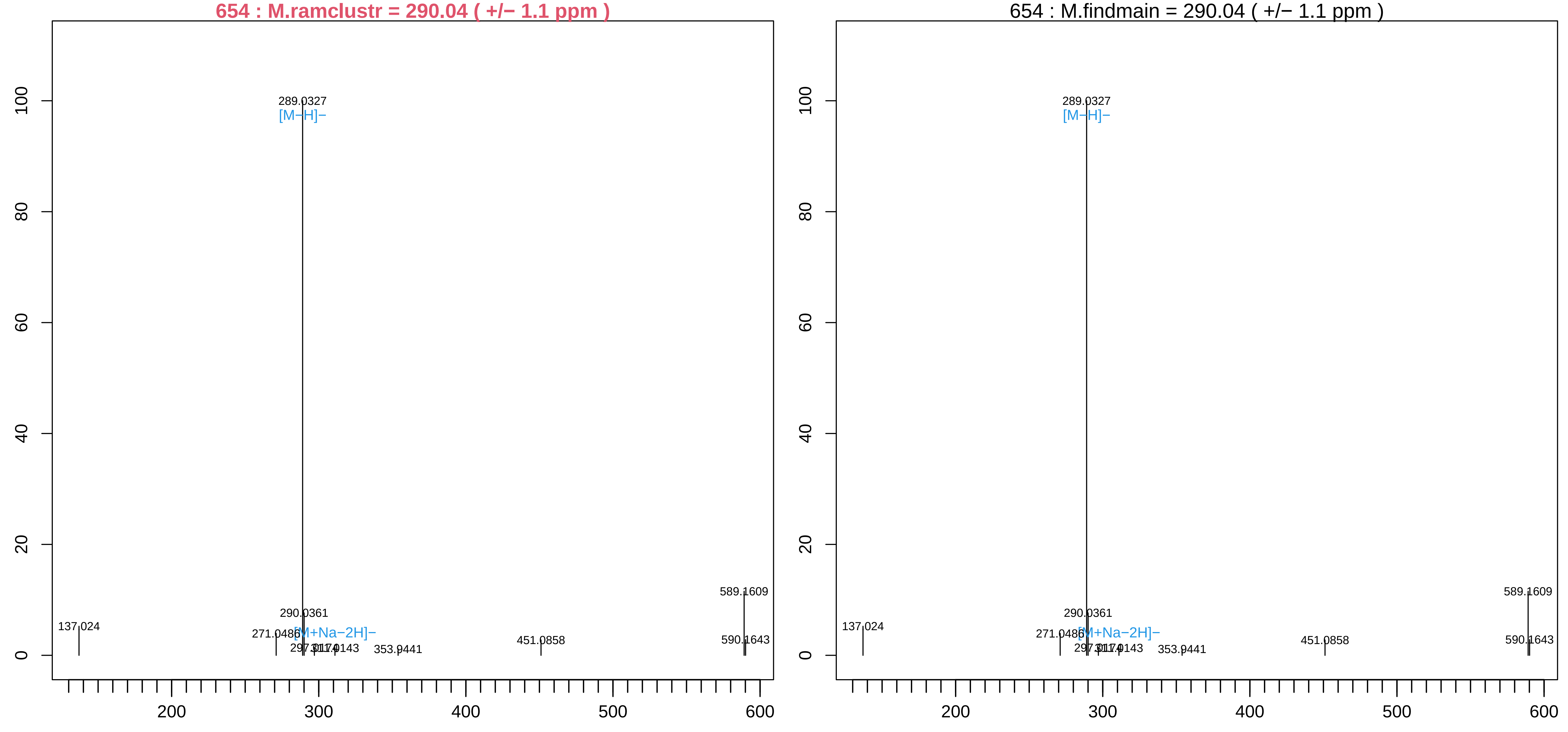

654 : M.ramclustr = 290.04 (+/− 1.1 ppm)
654 : M.findmain = 290.04 (+/− 1.1 ppm)
289.0327
[M−H]−
290.0361
[M+Na−2H]−
137.024
271.0486
297.3117
311.0143
353.9441
451.0858
589.1609
590.1643
0
20
40
60
80
100
200
300
400
500
600

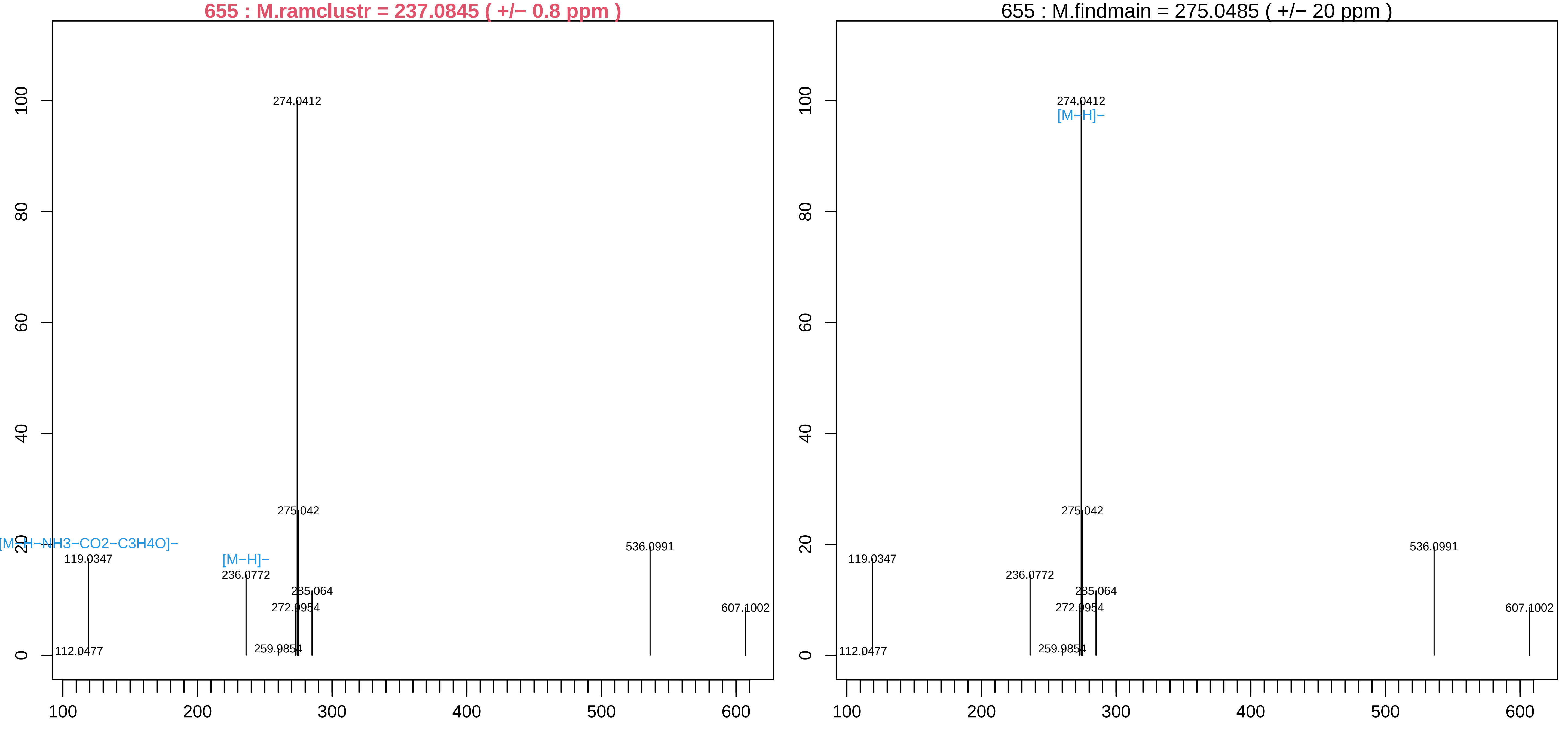

655 : M.ramclustr = 237.0845 (+/− 0.8 ppm)
274.0412
275.042
[M−H−NH3−CO2−C3H4O]−
119.0347
[M−H]−
236.0772
285.064
272.9954
536.0991
607.1002
112.0477
259.9854
0
20
40
60
80
100
100
200
300
400
500
600
655 : M.findmain = 275.0485 (+/− 20 ppm)
274.0412
[M−H]−
275.042
119.0347
236.0772
285.064
272.9954
536.0991
607.1002
112.0477
259.9854
0
20
40
60
80
100
100
200
300
400
500
600

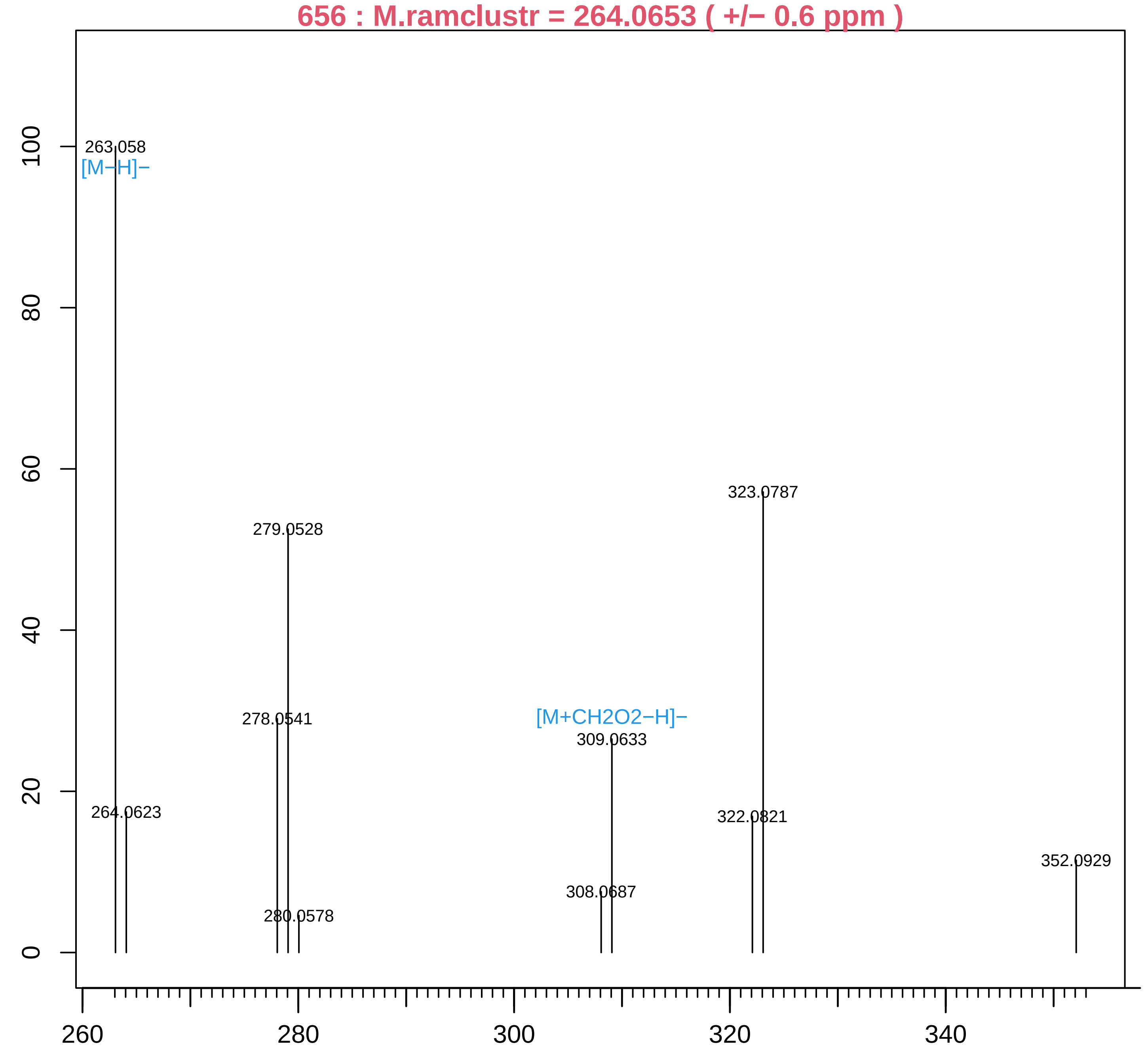
656 : M.ramclustr = 264.0653 (+/− 0.6 ppm)
263.058
[M−H]−
264.0623
279.0528
278.0541
280.0578
[M+CH2O2−H]−
309.0633
308.0687
323.0787
322.0821
352.0929
0
20
40
60
80
100
260
280
300
320
340
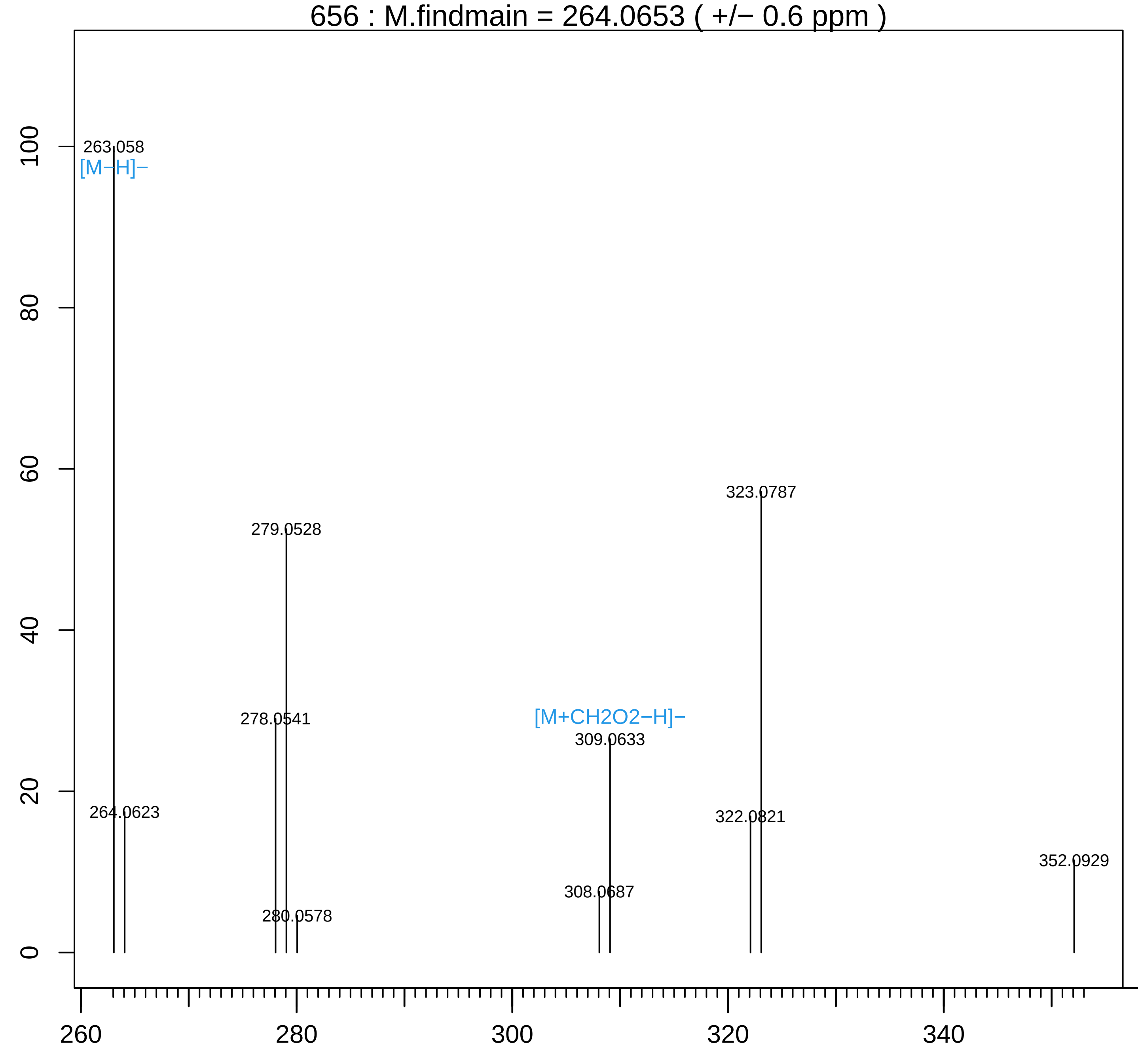
656 : M.findmain = 264.0653 (+/− 0.6 ppm)
263.058
[M−H]−
264.0623
279.0528
278.0541
280.0578
[M+CH2O2−H]−
309.0633
308.0687
323.0787
322.0821
352.0929
0
20
40
60
80
100
260
280
300
320
340

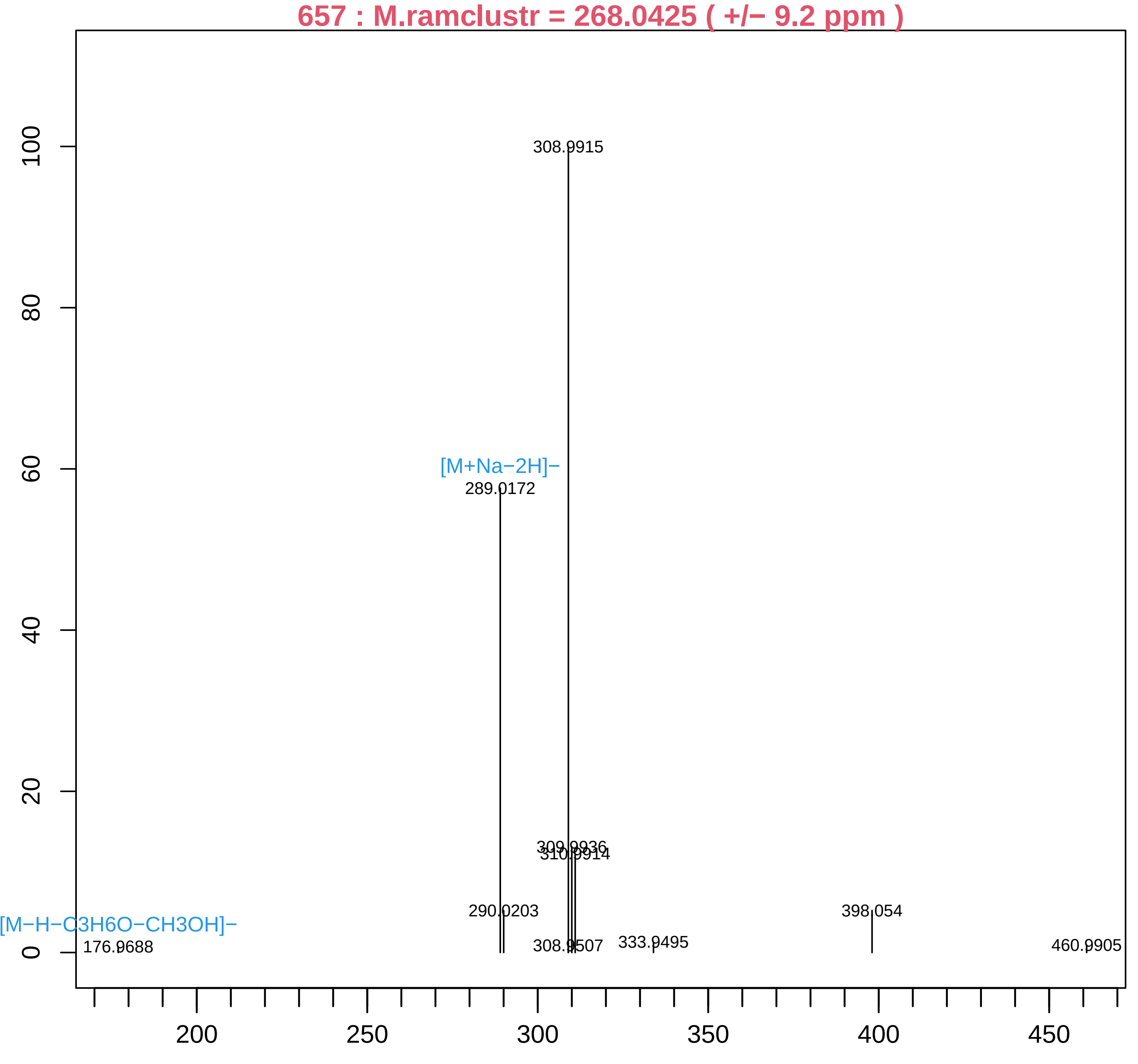

657 : M.ramclustr = 268.0425 (+/− 9.2 ppm)
308.9915
[M+Na−2H]−
289.0172
309.9936
310.9914
290.0203
398.054
[M−H−C3H6O−CH3OH]−
176.9688
308.9507
333.9495
460.9905
0
20
40
60
80
100
200
250
300
350
400
450

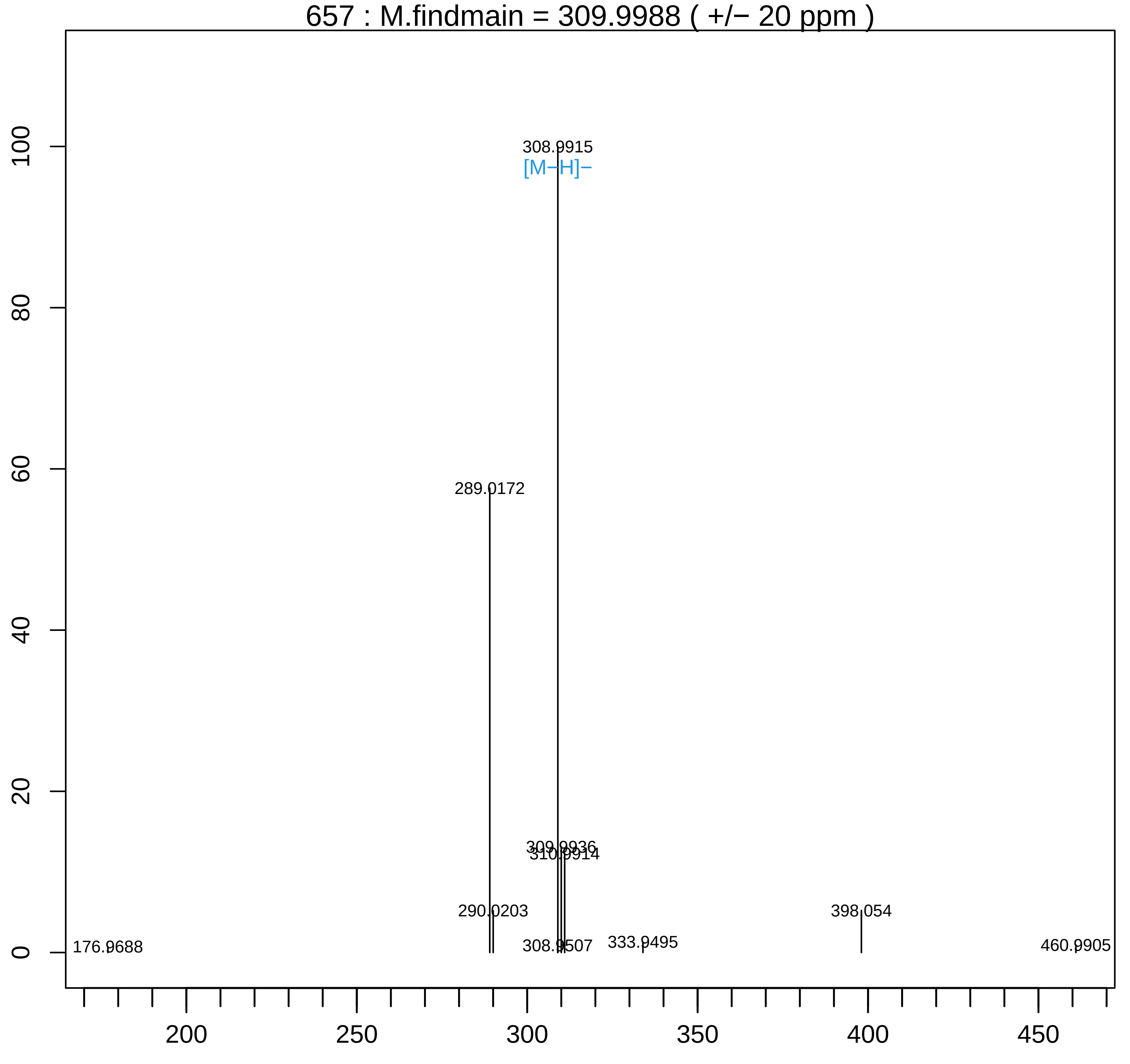

657 : M.findmain = 309.9988 (+/− 20 ppm)
308.9915
[M−H]−
289.0172
309.9936
310.9914
290.0203
398.054
176.9688
308.9507
333.9495
460.9905
0
20
40
60
80
100
200
250
300
350
400
450

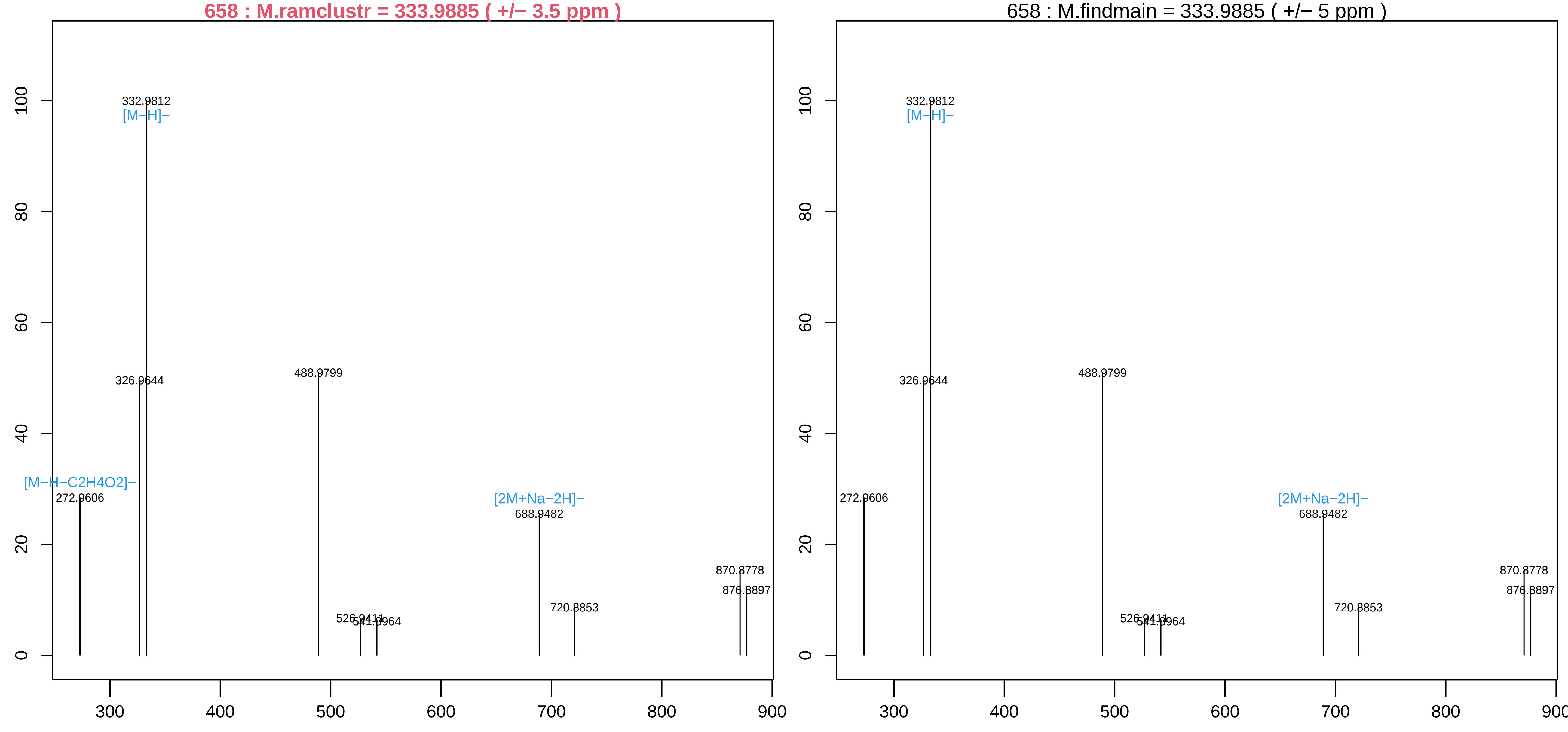
658 : M.ramclustr = 333.9885 (+/− 3.5 ppm)
332.9812
[M−H]−
326.9644
488.9799
[M−H−C2H4O2]−
272.9606
[2M+Na−2H]−
688.9482
870.8778
876.8897
720.8853
526.9411
541.8964
658 : M.findmain = 333.9885 (+/− 5 ppm)
332.9812
[M−H]−
326.9644
488.9799
272.9606
[2M+Na−2H]−
688.9482
870.8778
876.8897
720.8853
526.9411
541.8964

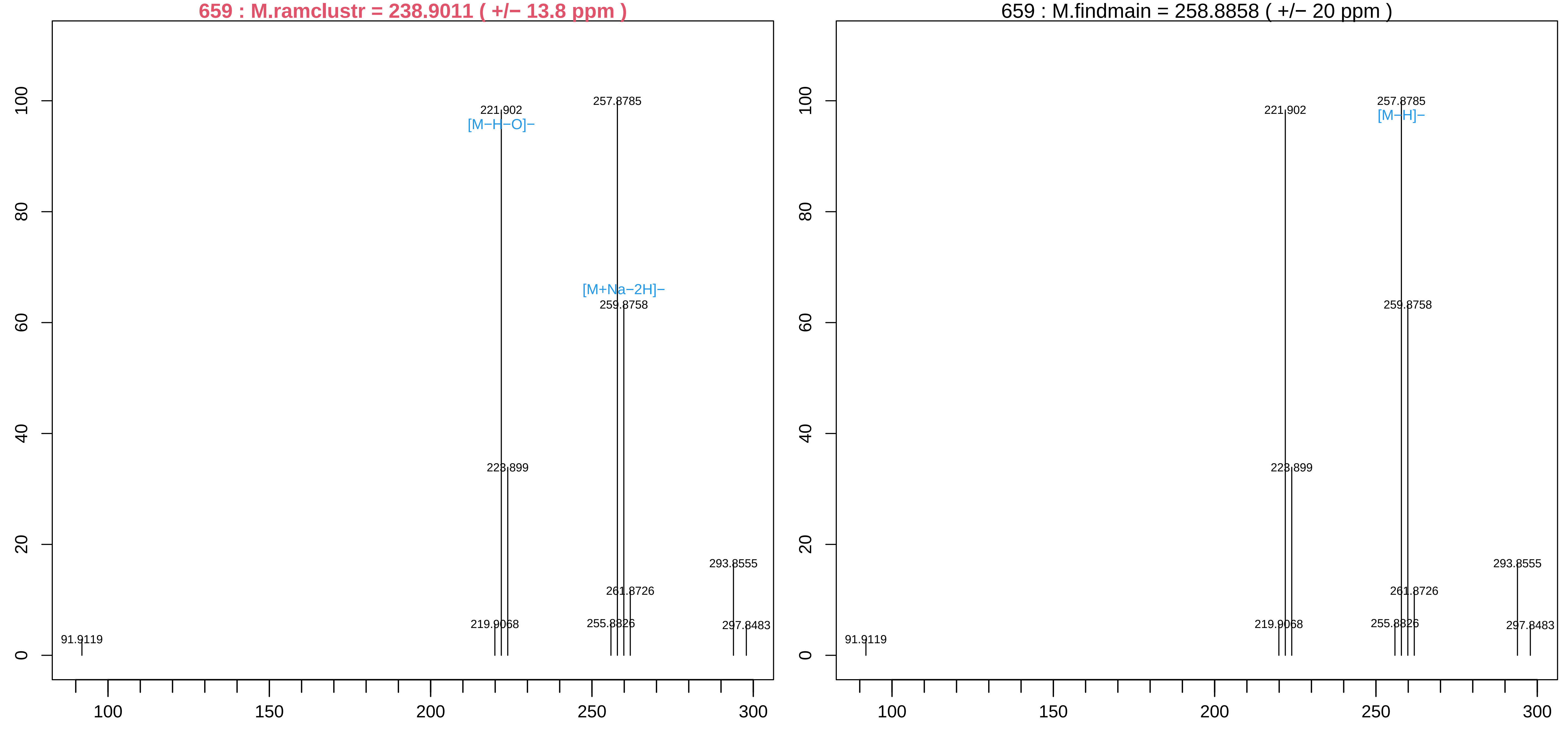

659 : M.ramclustr = 238.9011 (+/− 13.8 ppm)
[M−H−O]−
[M+Na−2H]−
221.902
257.8785
259.8758
223.899
293.8555
261.8726
219.9068
255.8826
297.8483
91.9119
659 : M.findmain = 258.8858 (+/− 20 ppm)
[M−H]−

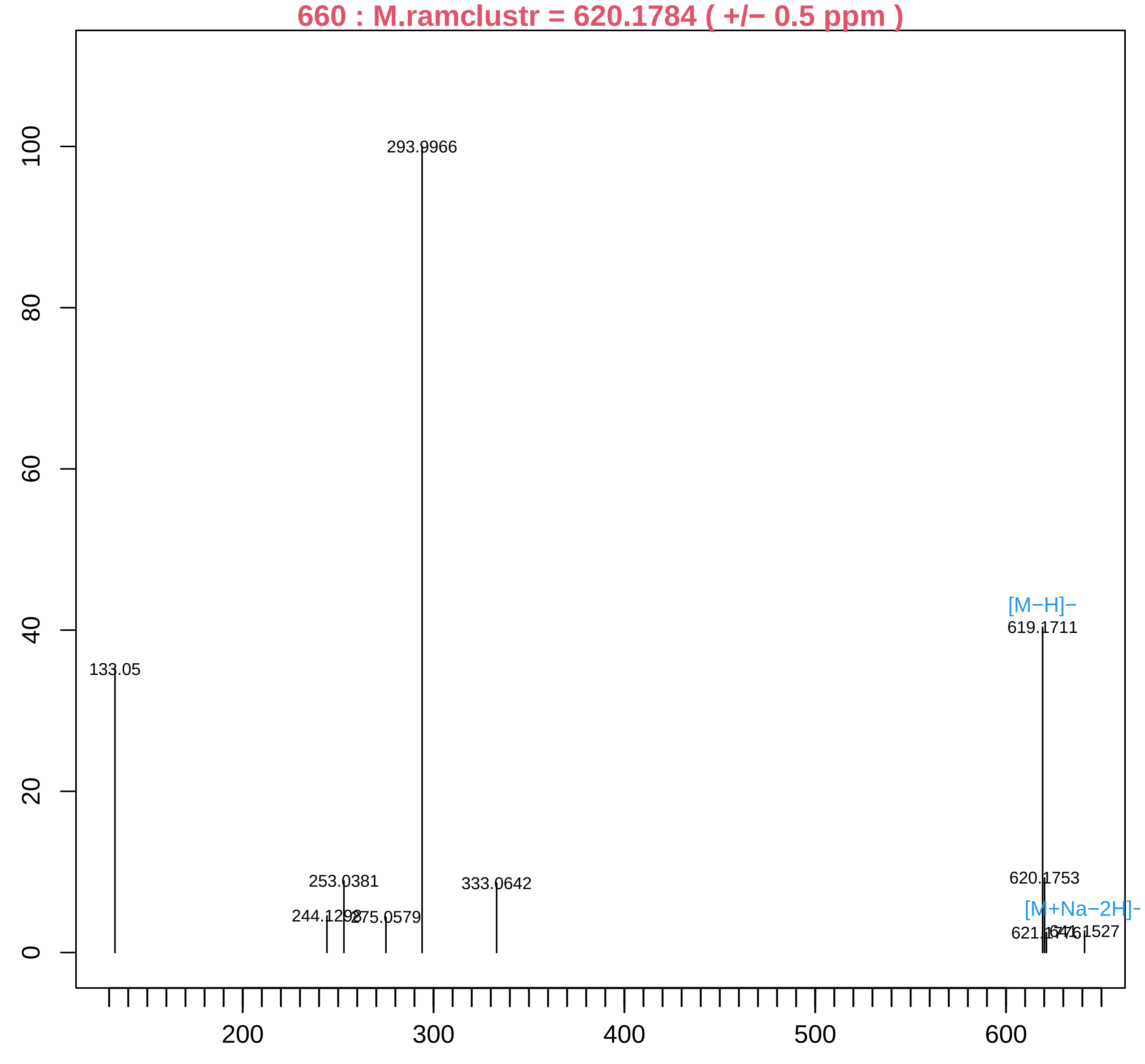
660 : M.ramclustr = 620.1784 (+/− 0.5 ppm)
293.9966
133.05
253.0381
244.1298
275.0579
333.0642
[M−H]−
619.1711
620.1753
[M+Na−2H]−
621.1741
641.1527
0
20
40
60
80
100
200
300
400
500
600

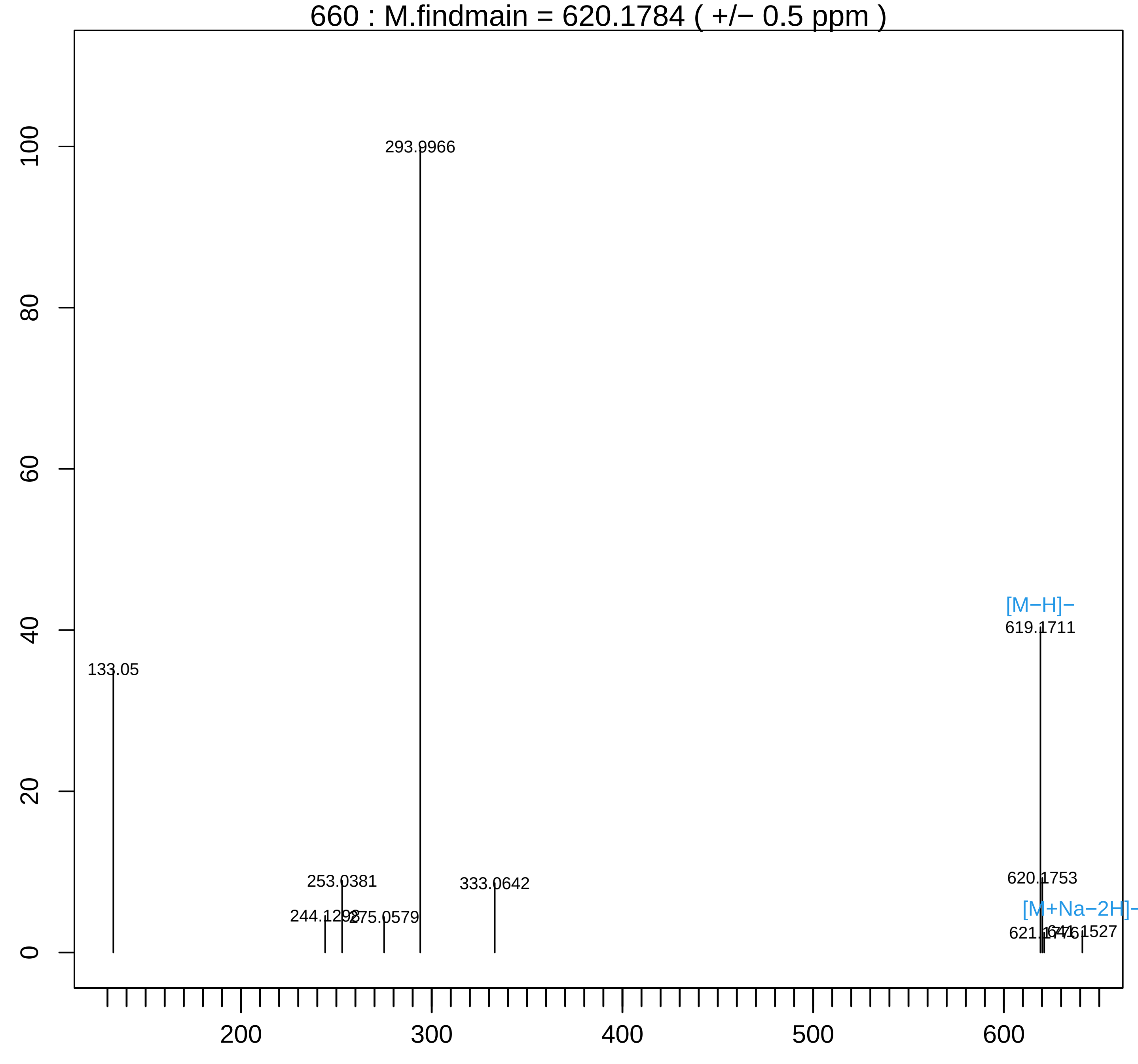
660 : M.findmain = 620.1784 (+/− 0.5 ppm)
293.9966
133.05
253.0381
244.1298
275.0579
333.0642
[M−H]−
619.1711
620.1753
[M+Na−2H]−
621.1741
641.1527
0
20
40
60
80
100
200
300
400
500
600

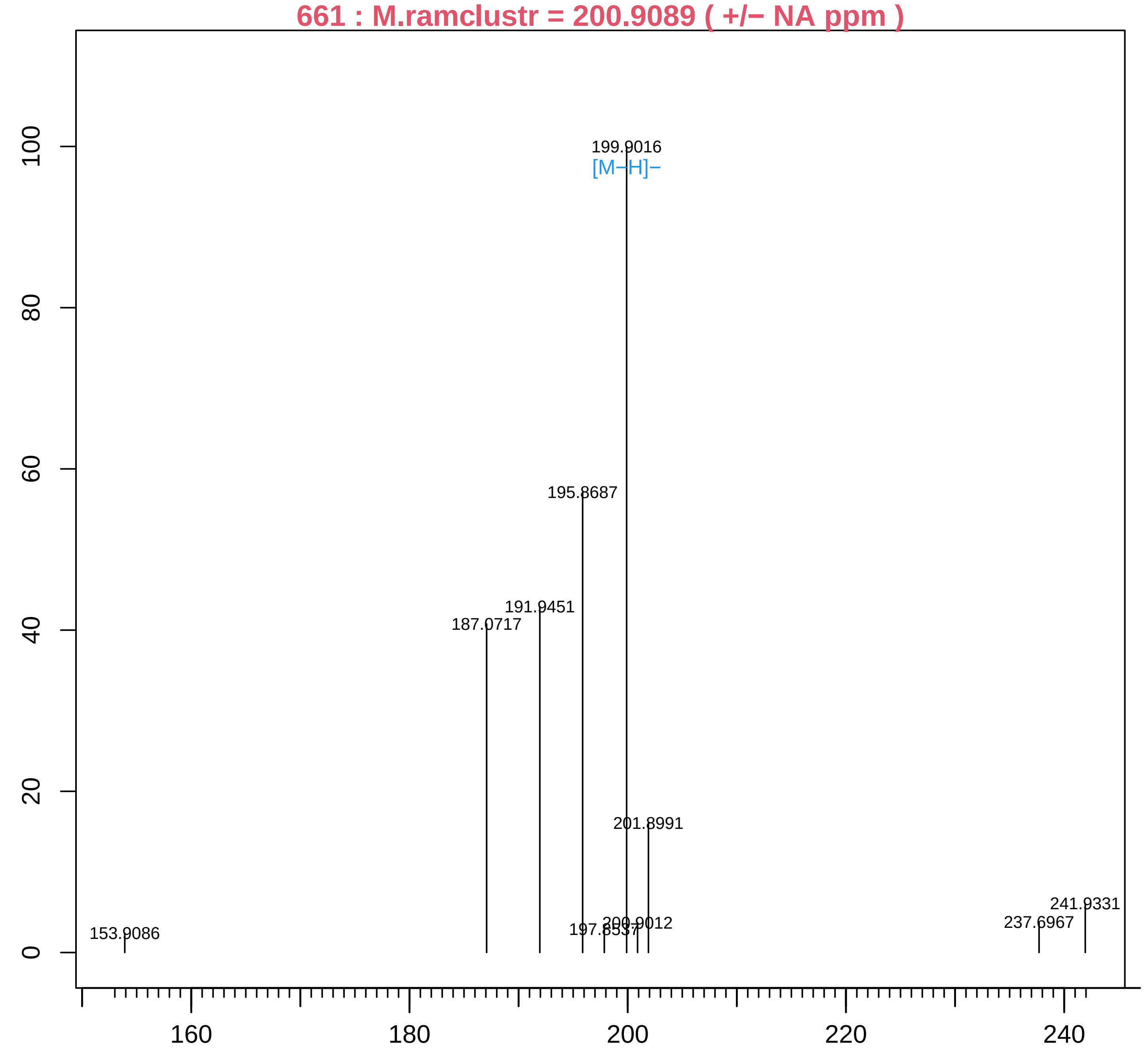
661 : M.ramclustr = 200.9089 (+/− NA ppm)
199.9016
[M−H]−
195.8687
191.9451
187.0717
201.8991
153.9086
197.8537
200.9012
237.6967
241.9331
0
20
40
60
80
100
160
180
200
220
240

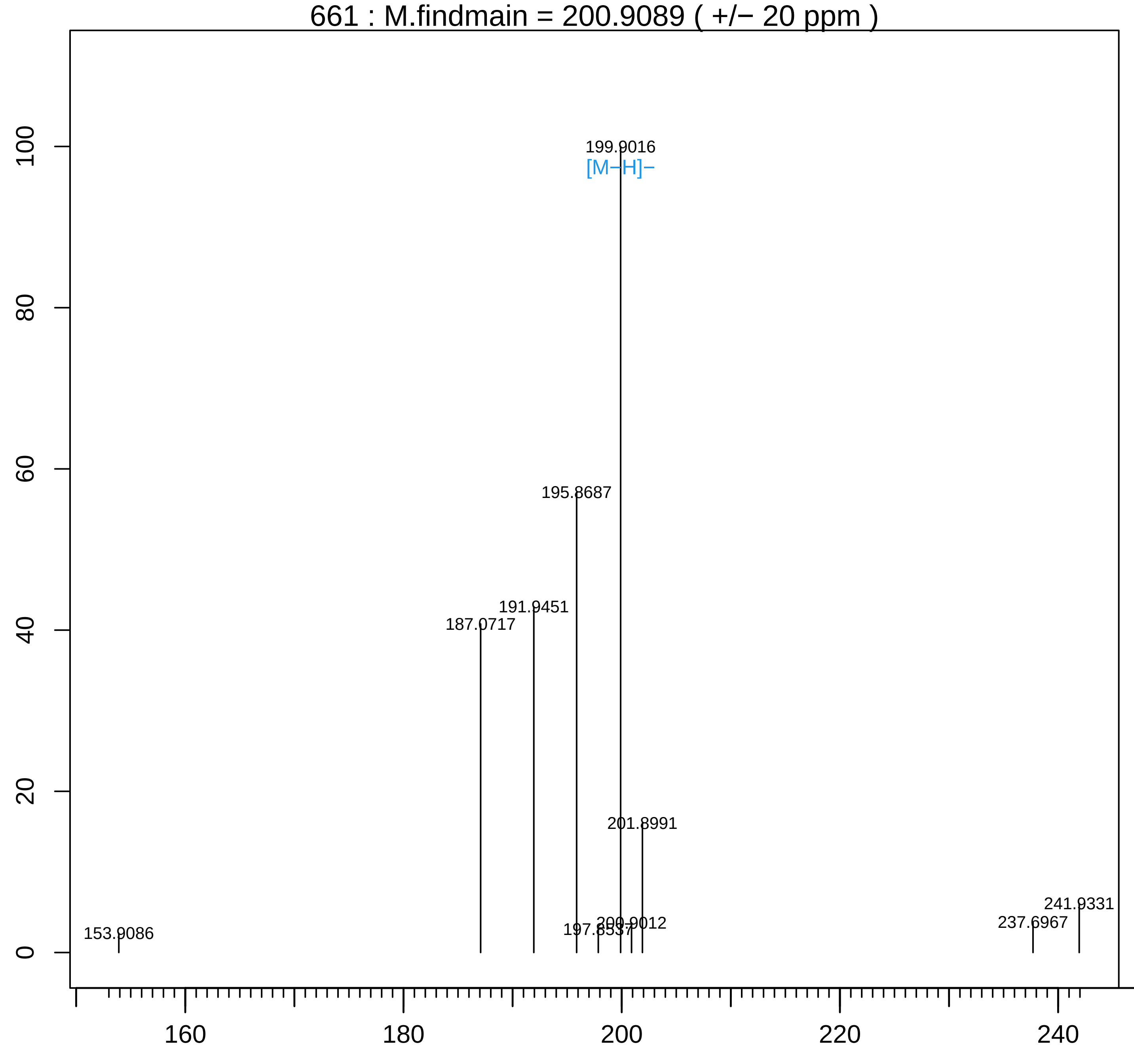
661 : M.findmain = 200.9089 (+/− 20 ppm)
199.9016
[M−H]−
195.8687
191.9451
187.0717
201.8991
153.9086
197.8537
200.9012
237.6967
241.9331
0
20
40
60
80
100
160
180
200
220
240

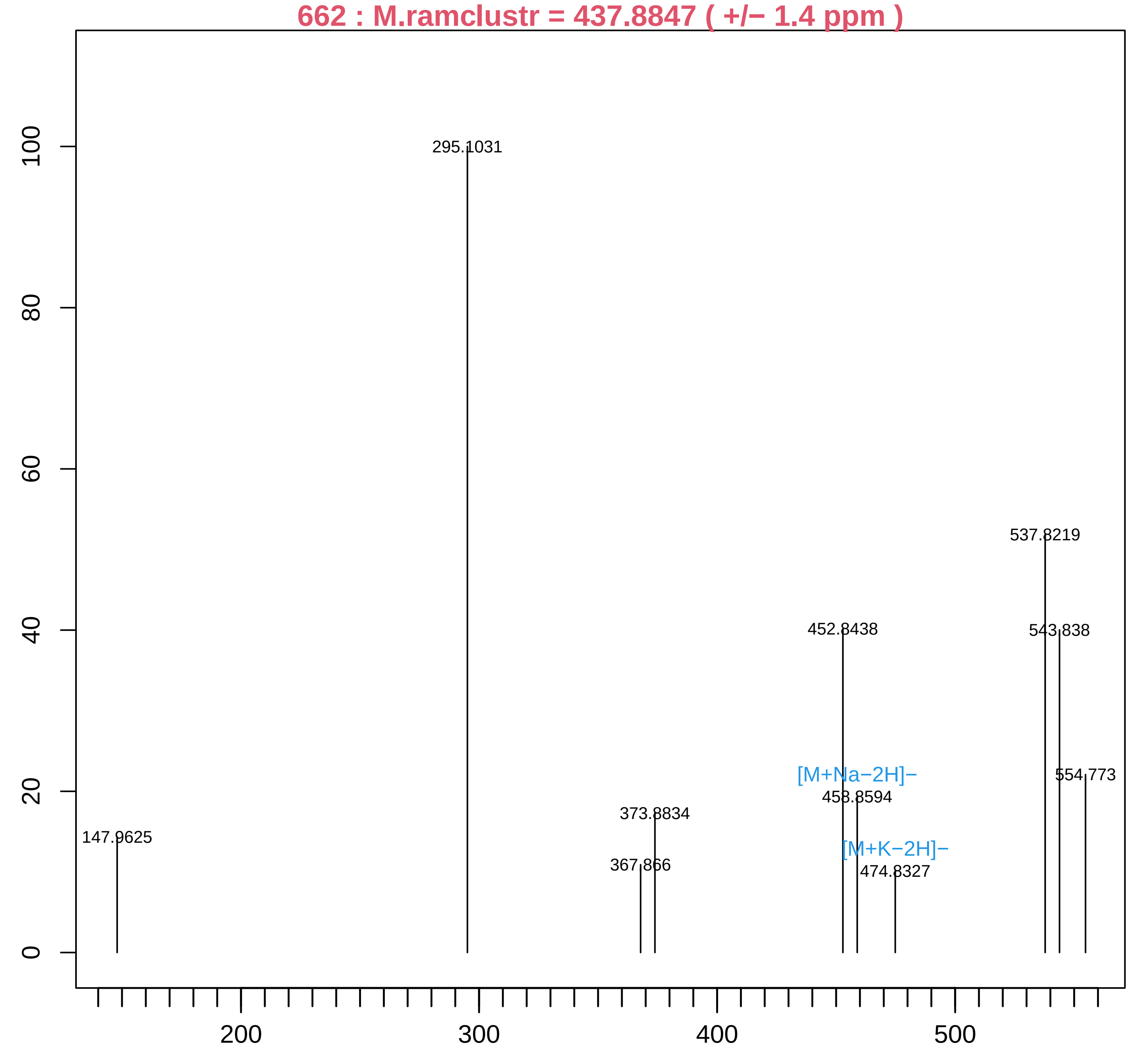
662 : M.ramclustr = 437.8847 (+/− 1.4 ppm)
295.1031
537.8219
452.8438
543.838
[M+Na−2H]−
554.773
453.8594
373.8834
[M+K−2H]−
147.9625
367.866
474.8327
0
20
40
60
80
100
200
300
400
500
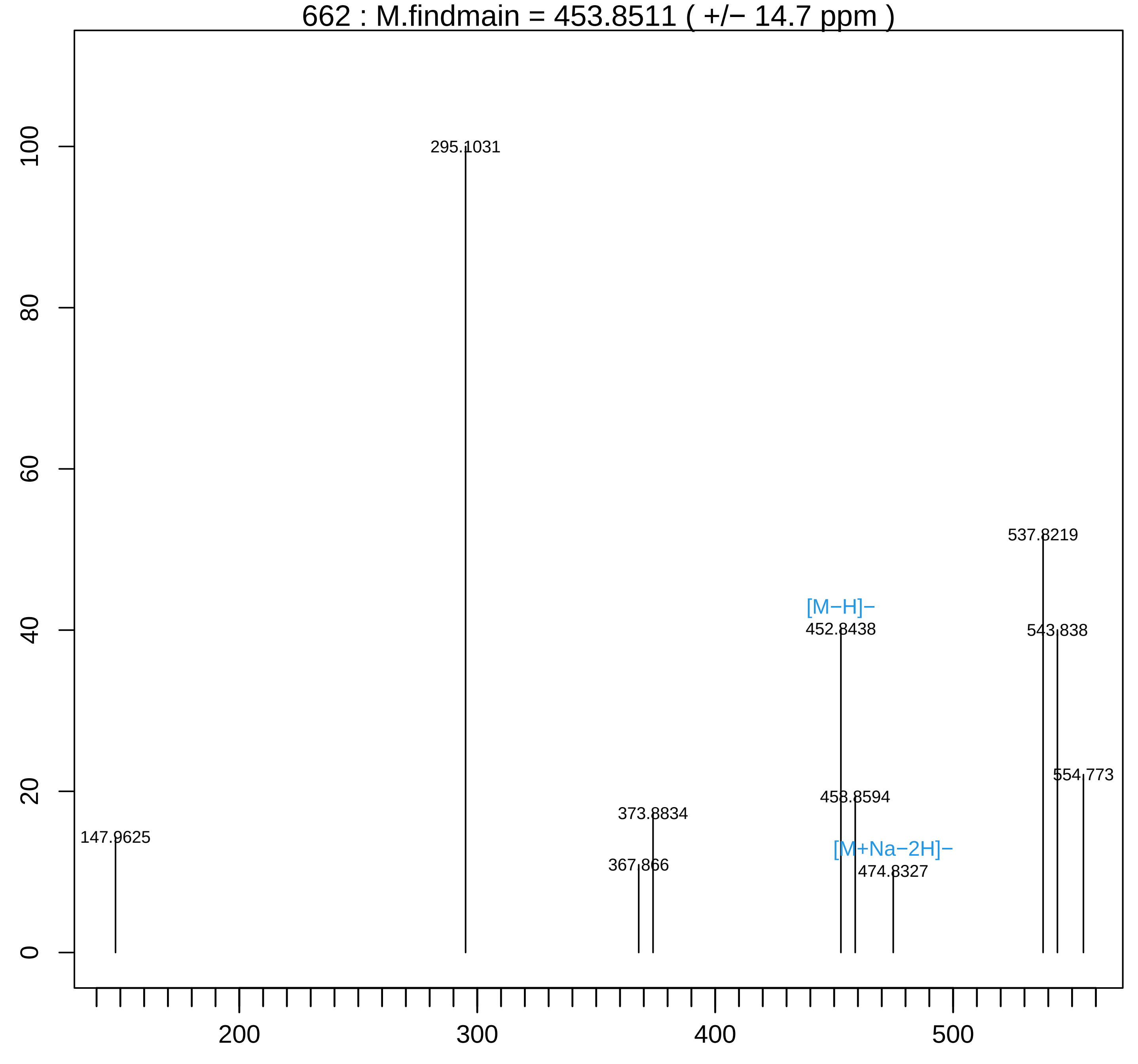
662 : M.findmain = 453.8511 (+/− 14.7 ppm)
295.1031
537.8219
[M−H]−
452.8438
543.838
554.773
453.8594
373.8834
[M+Na−2H]−
147.9625
367.866
474.8327
0
20
40
60
80
100
200
300
400
500

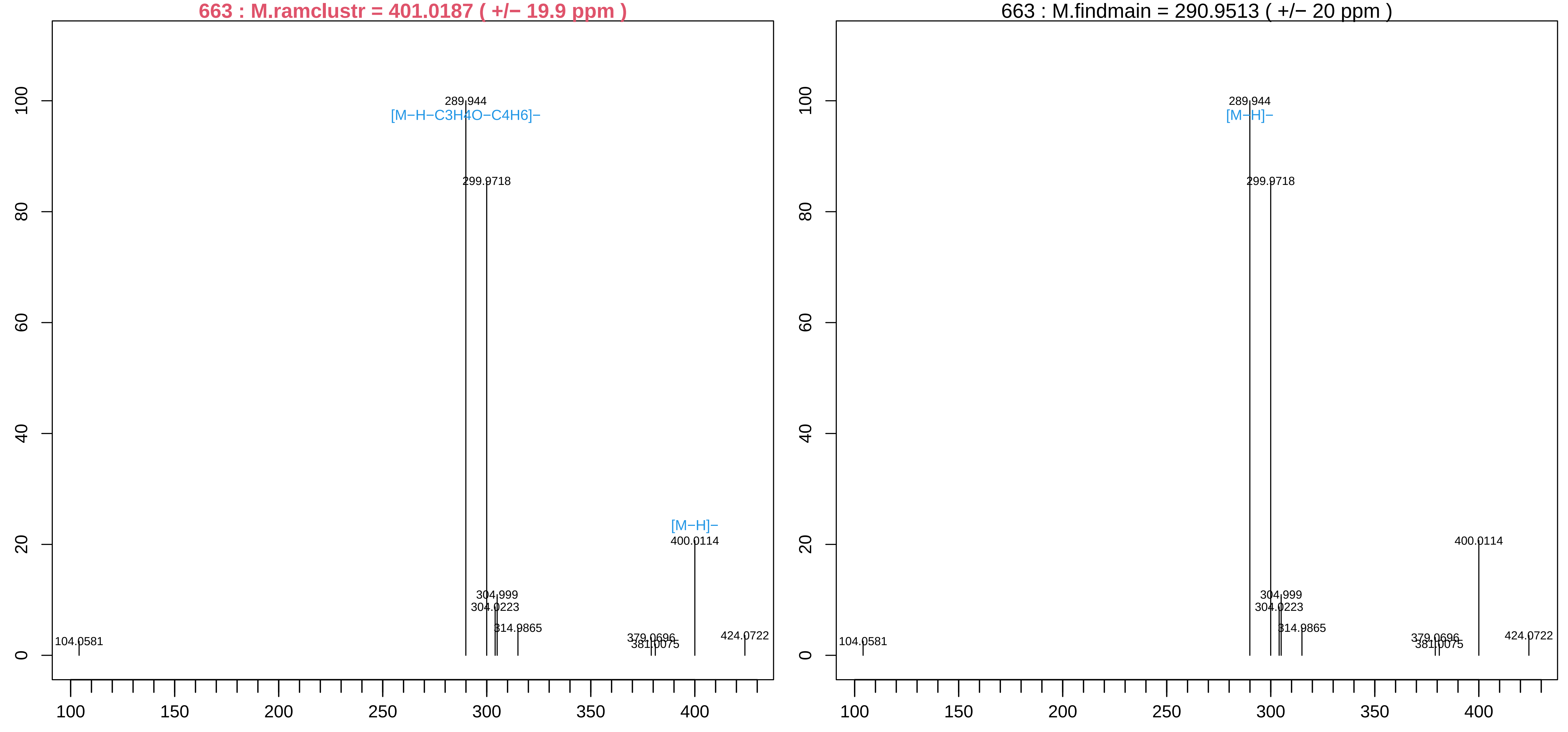
663 : M.ramclustr = 401.0187 (+/− 19.9 ppm)
289.944
[M−H−C3H4O−C4H6]−
299.9718
304.999
304.0223
314.9865
104.0581
379.0696
381.0075
[M−H]−
400.0114
424.0722
663 : M.findmain = 290.9513 (+/− 20 ppm)
289.944
[M−H]−
299.9718
304.999
304.0223
314.9865
104.0581
379.0696
381.0075
400.0114
424.0722

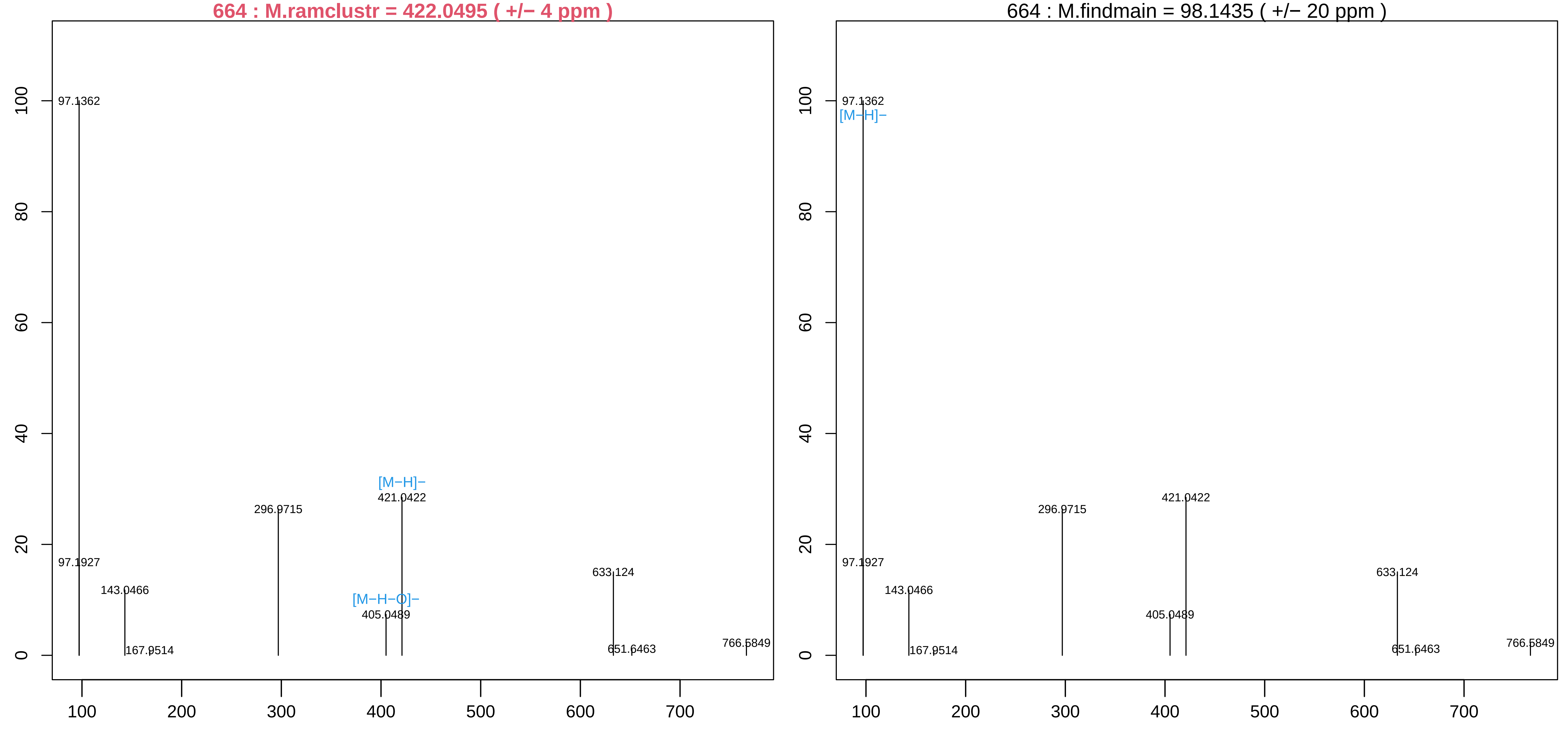

664 : M.ramclustr = 422.0495 (+/− 4 ppm)
97.1362
97.1927
143.0466
167.9514
296.9715
[M−H]−
421.0422
[M−H−O]−
405.0489
633.124
651.6463
766.5849
664 : M.findmain = 98.1435 (+/− 20 ppm)
97.1362
[M−H]−
97.1927
143.0466
167.9514
296.9715
421.0422
405.0489
633.124
651.6463
766.5849

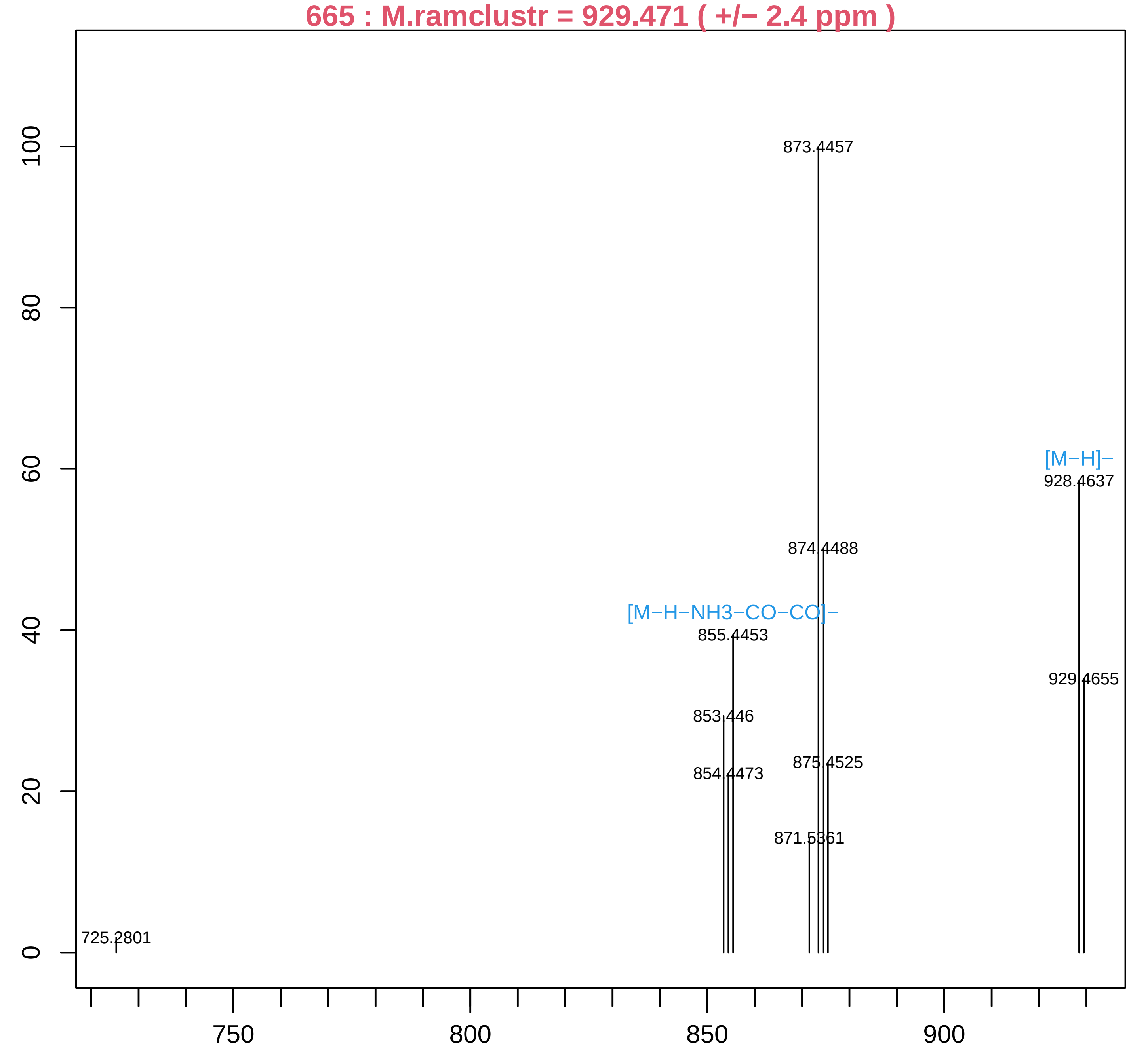
665 : M.ramclustr = 929.471 (+/− 2.4 ppm)
873.4457
874.4488
[M−H−NH3−CO−CO]−
855.4453
853.446
854.4473
875.4525
871.5361
[M−H]−
928.4637
929.4655
725.2801
0
20
40
60
80
100
750
800
850
900

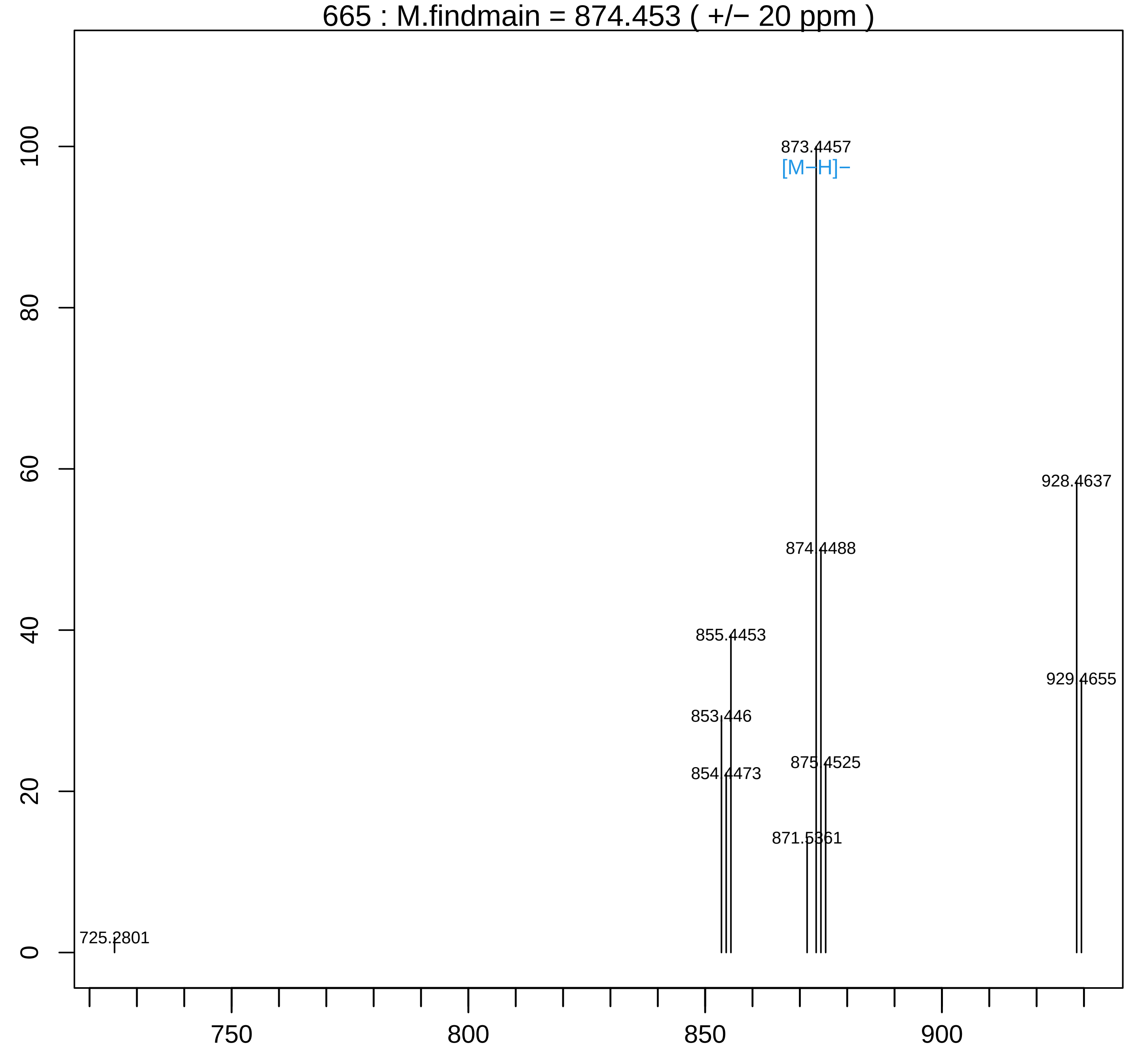
665 : M.findmain = 874.453 (+/− 20 ppm)
873.4457
[M−H]−
874.4488
855.4453
853.446
854.4473
875.4525
871.5361
928.4637
929.4655
725.2801
0
20
40
60
80
100
750
800
850
900

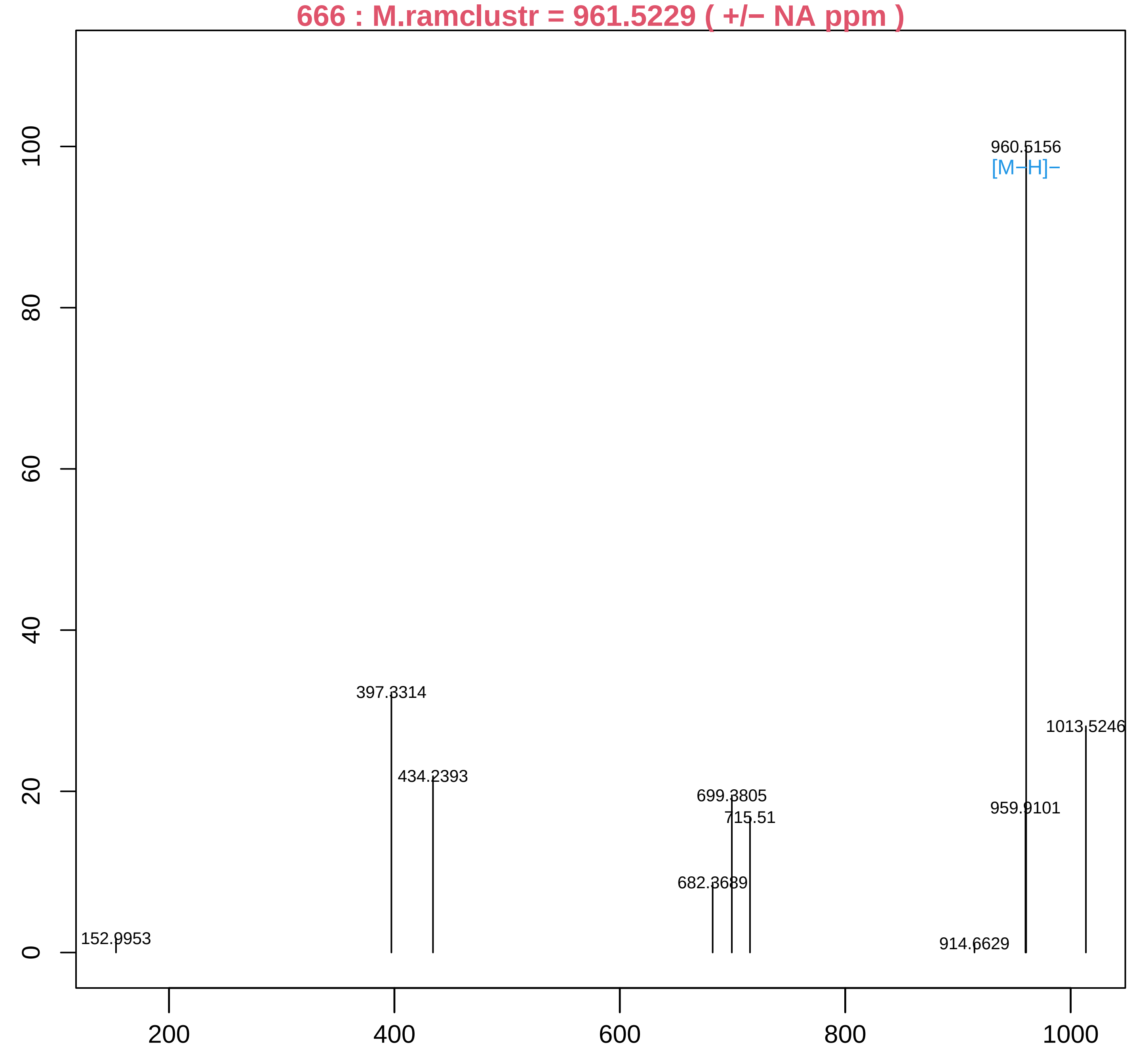
666 : M.ramclustr = 961.5229 (+/− NA ppm)
960.5156
[M−H]−
397.3314
434.2393
699.3805
715.51
682.3689
959.9101
1013.5246
152.9953
914.6629
0
20
40
60
80
100
200
400
600
800
1000

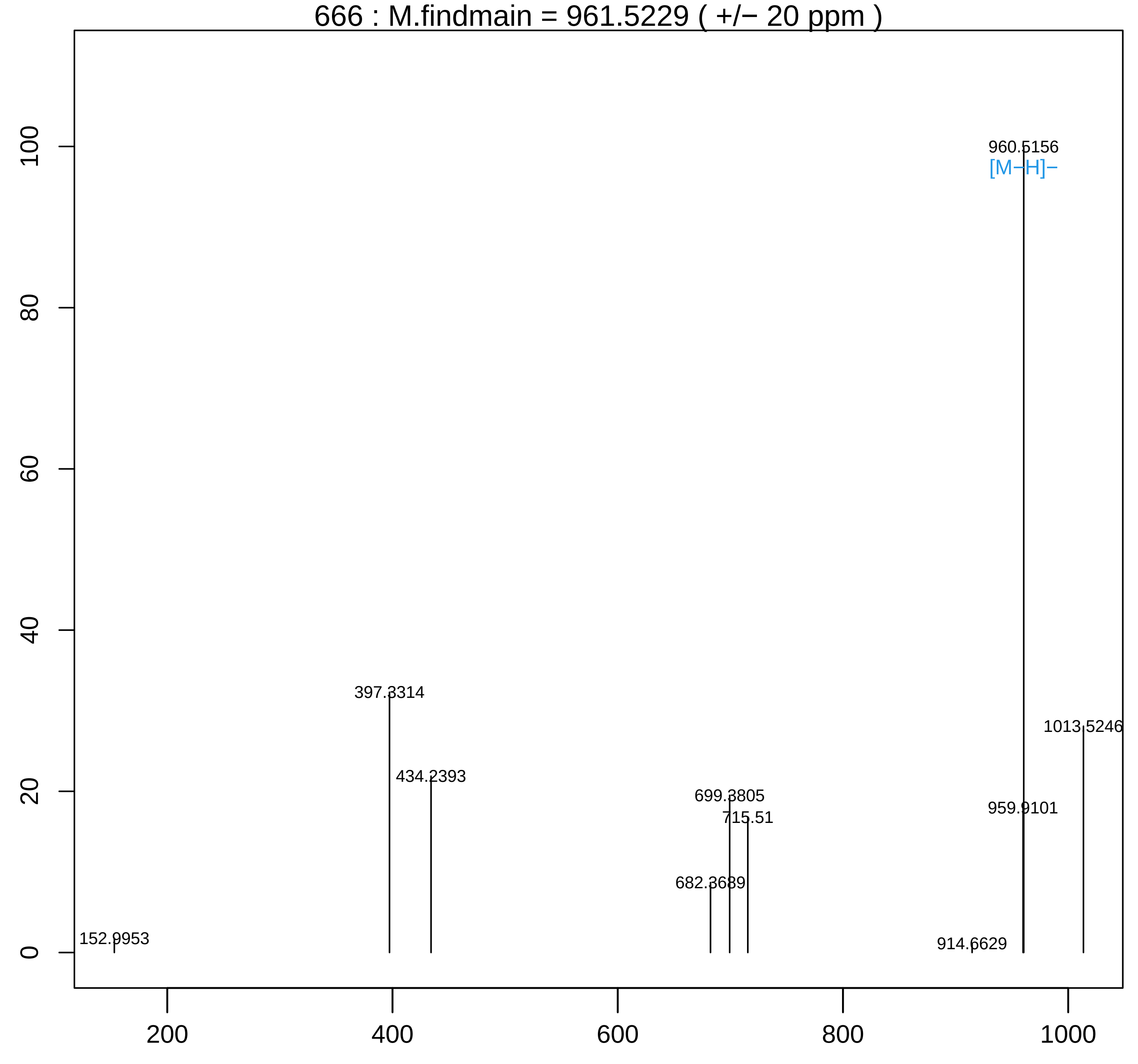
666 : M.findmain = 961.5229 (+/− 20 ppm)
960.5156
[M−H]−
397.3314
434.2393
699.3805
715.51
682.3689
959.9101
1013.5246
152.9953
914.6629
0
20
40
60
80
100
200
400
600
800
1000

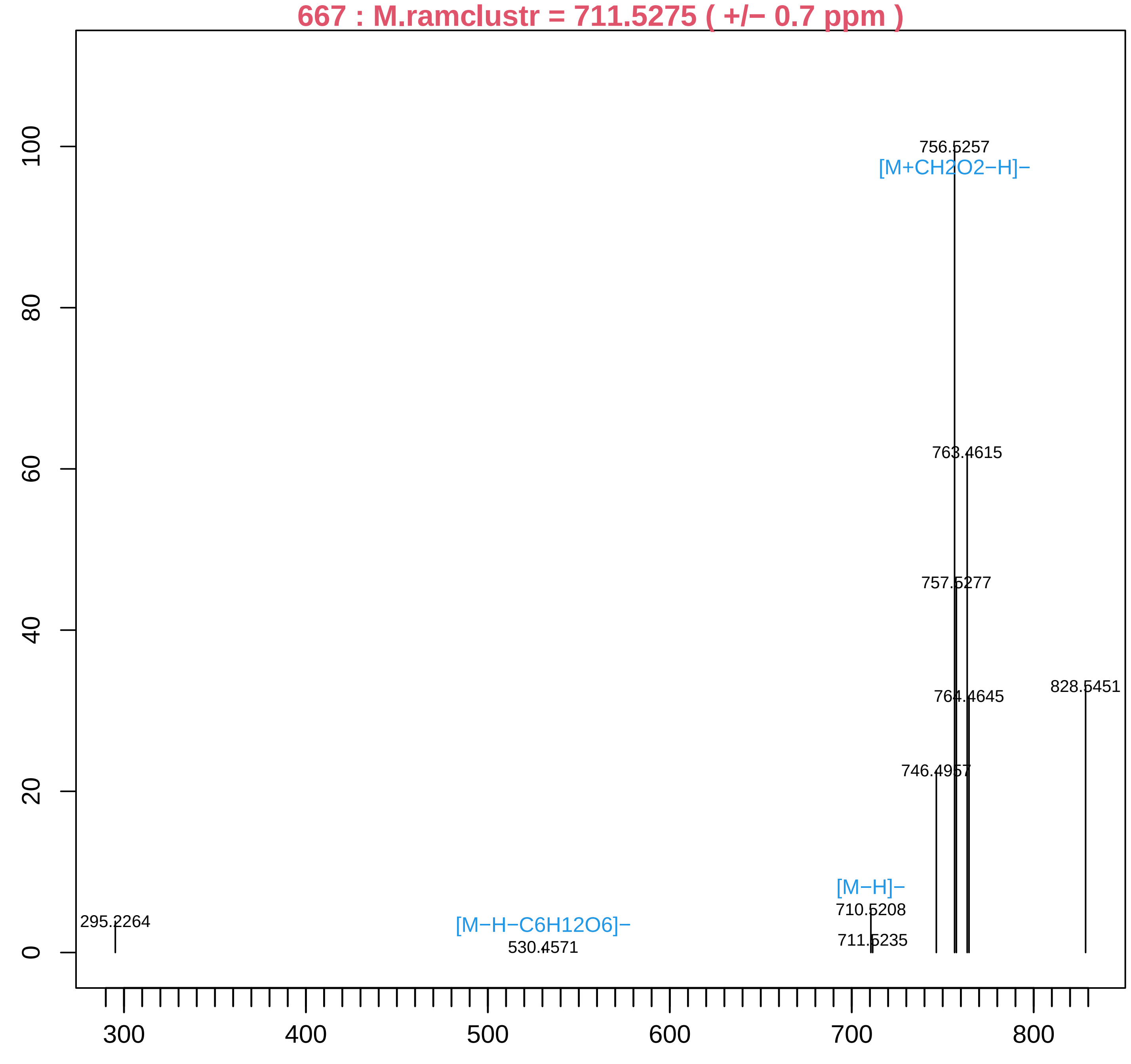
667 : M.ramclustr = 711.5275 (+/− 0.7 ppm)
756.5257
[M+CH2O2−H]−
763.4615
757.5277
828.5451
764.4645
746.4957
[M−H]−
710.5208
711.5235
295.2264
[M−H−C6H12O6]−
530.4571
0
20
40
60
80
100
300
400
500
600
700
800

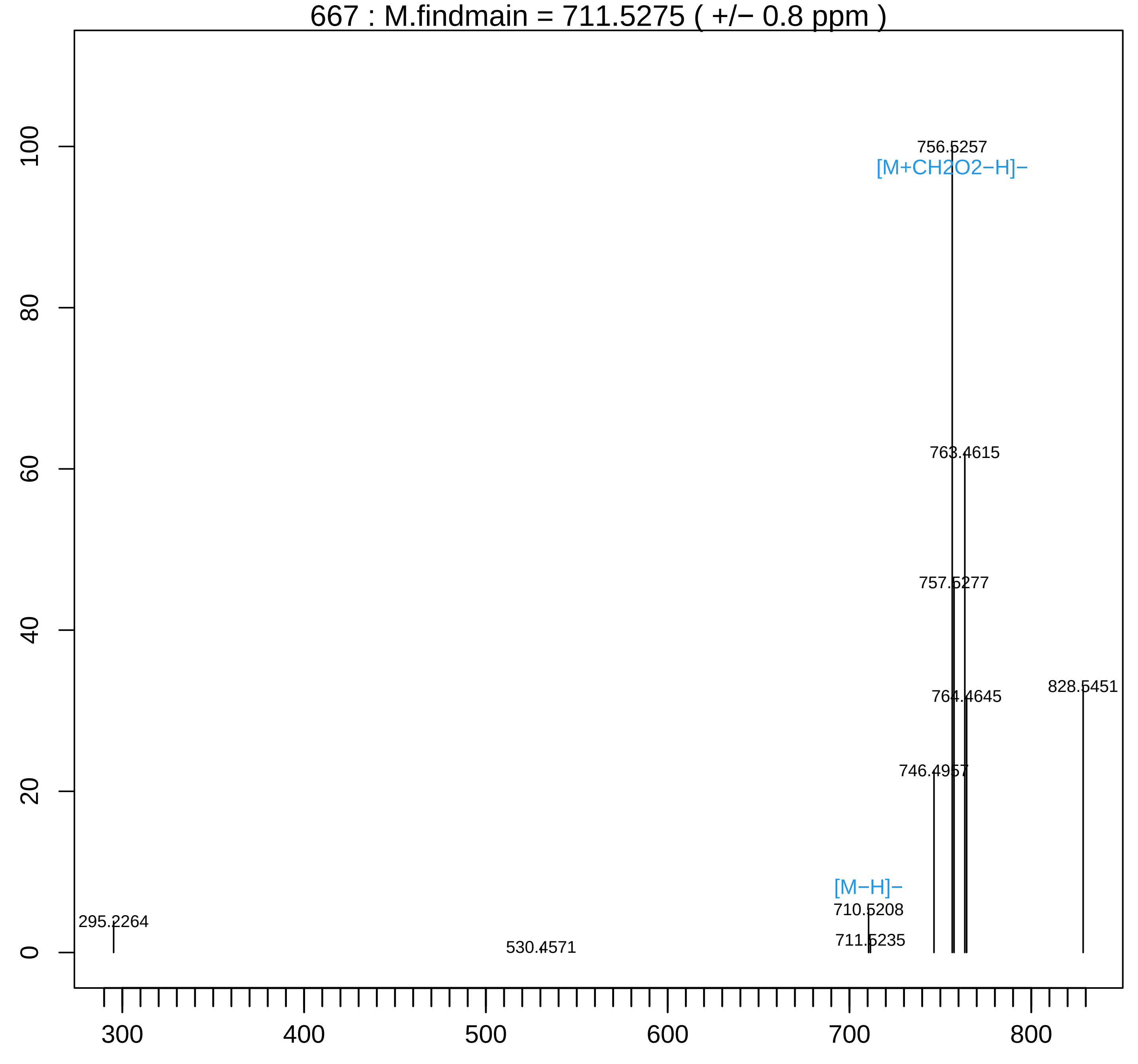
667 : M.findmain = 711.5275 (+/− 0.8 ppm)
756.5257
[M+CH2O2−H]−
763.4615
757.5277
828.5451
764.4645
746.4957
[M−H]−
710.5208
711.5235
295.2264
530.4571
0
20
40
60
80
100
300
400
500
600
700
800

668 : M.ramclustr = 682.3025 (+/− NA ppm)

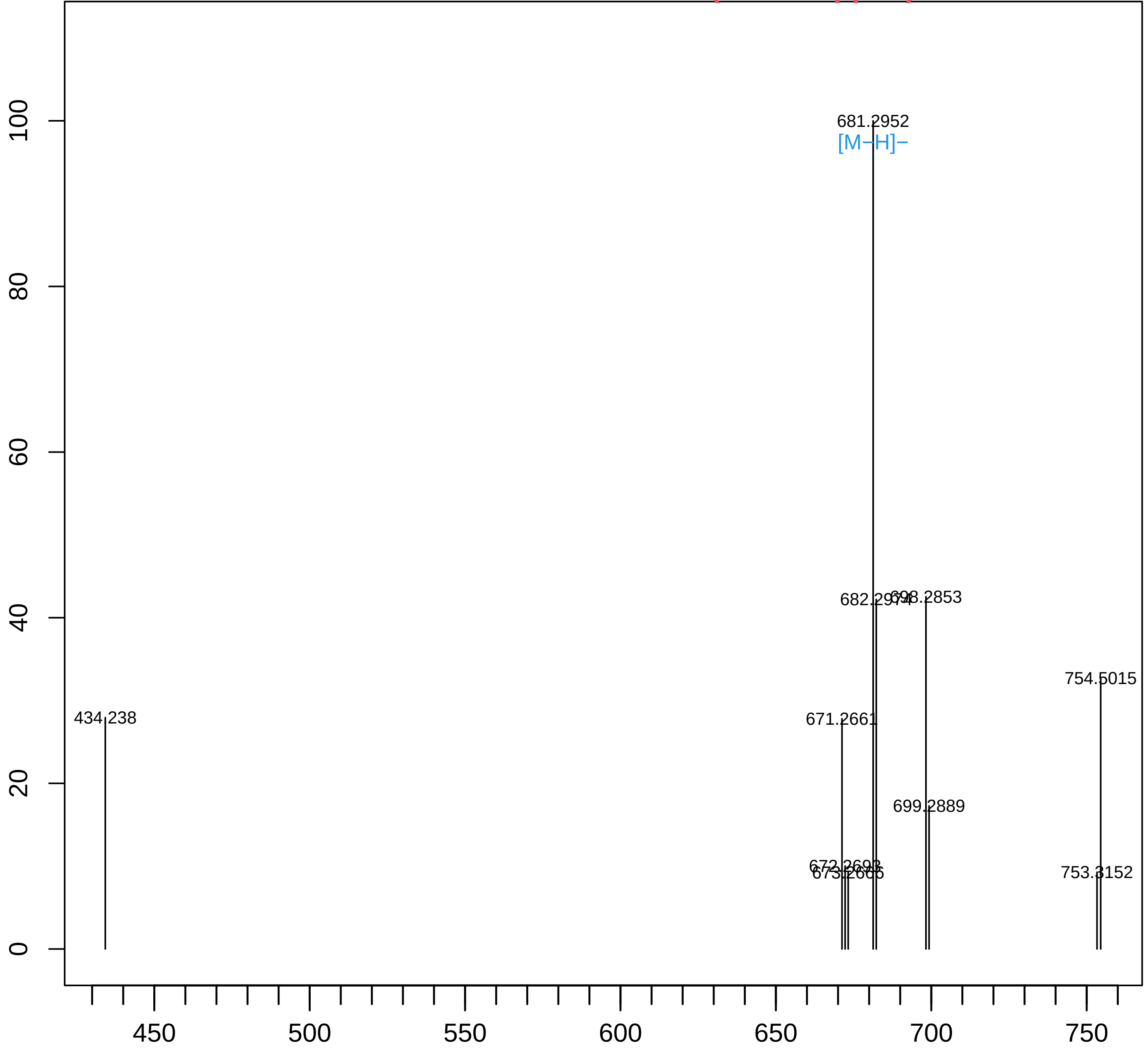

668 : M.findmain = 682.3025 (+/− 20 ppm)

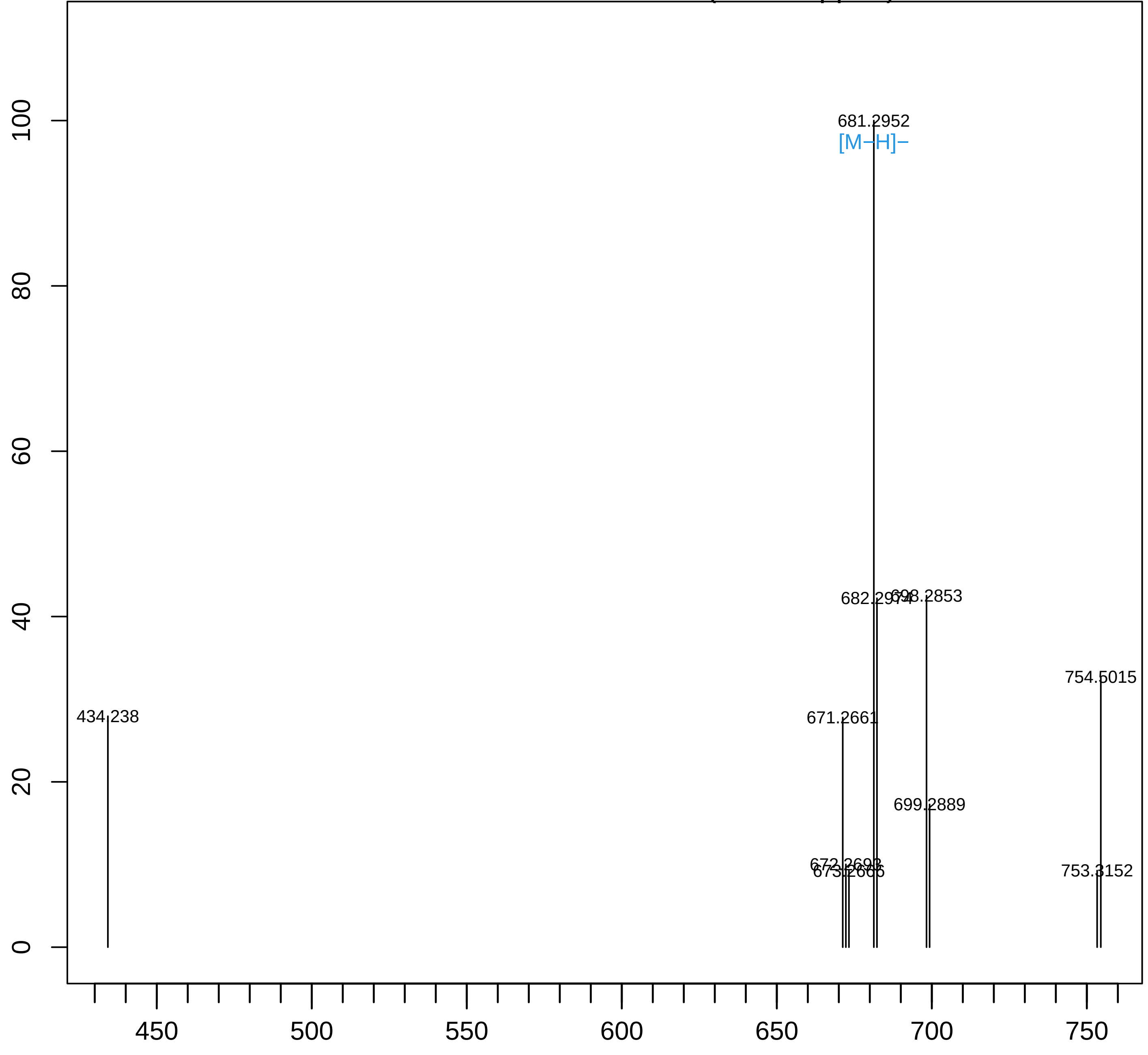

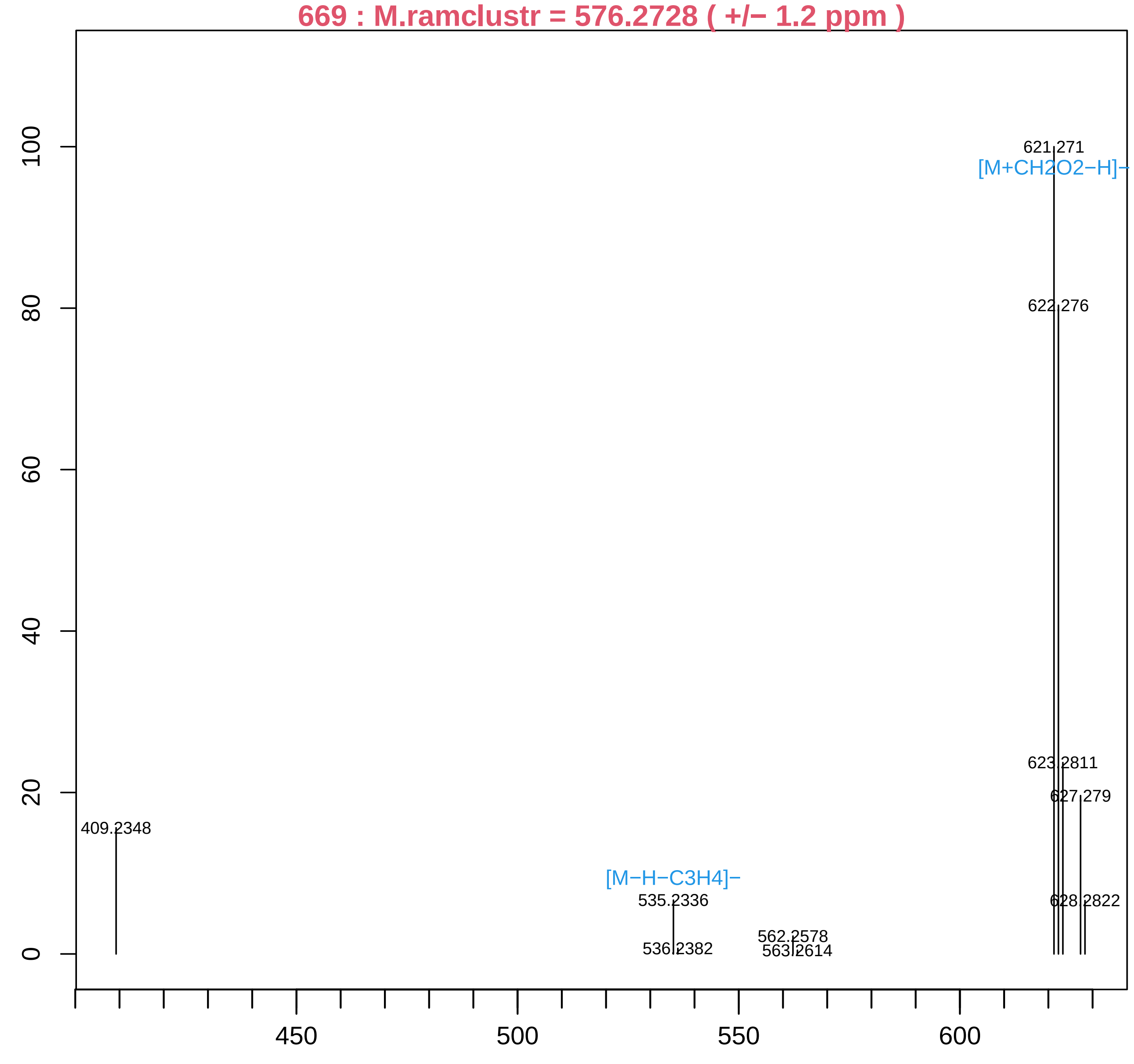
669 : M.ramclustr = 576.2728 (+/− 1.2 ppm)
621.271
[M+CH2O2−H]−
622.276
623.2811
627.279
628.2822
409.2348
[M−H−C3H4]−
535.2336
536.2382
562.2578
563.2614
0
20
40
60
80
100
450
500
550
600

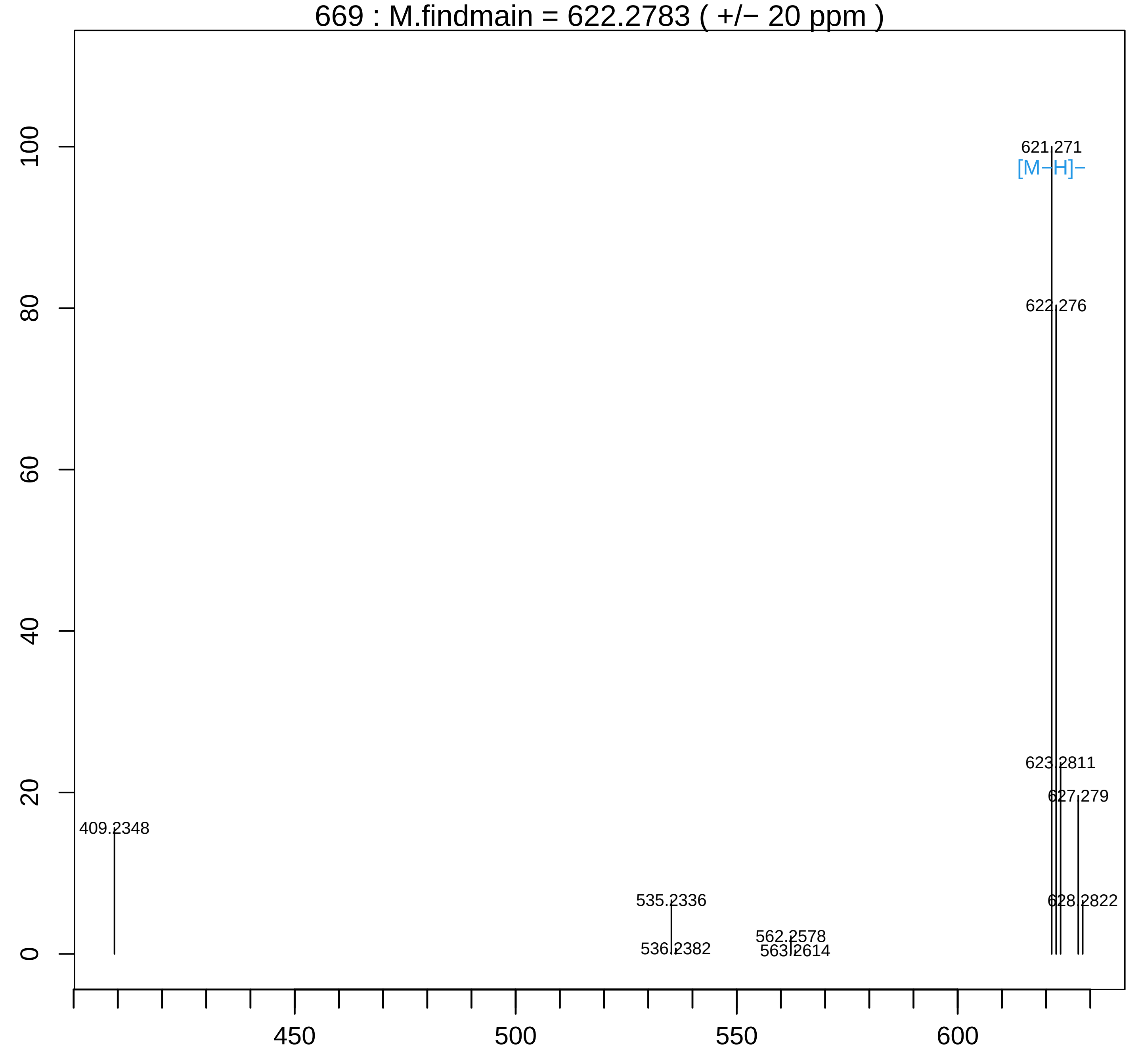
669 : M.findmain = 622.2783 (+/− 20 ppm)
621.271
[M−H]−
622.276
623.2811
627.279
628.2822
409.2348
535.2336
536.2382
562.2578
563.2614
0
20
40
60
80
100
450
500
550
600

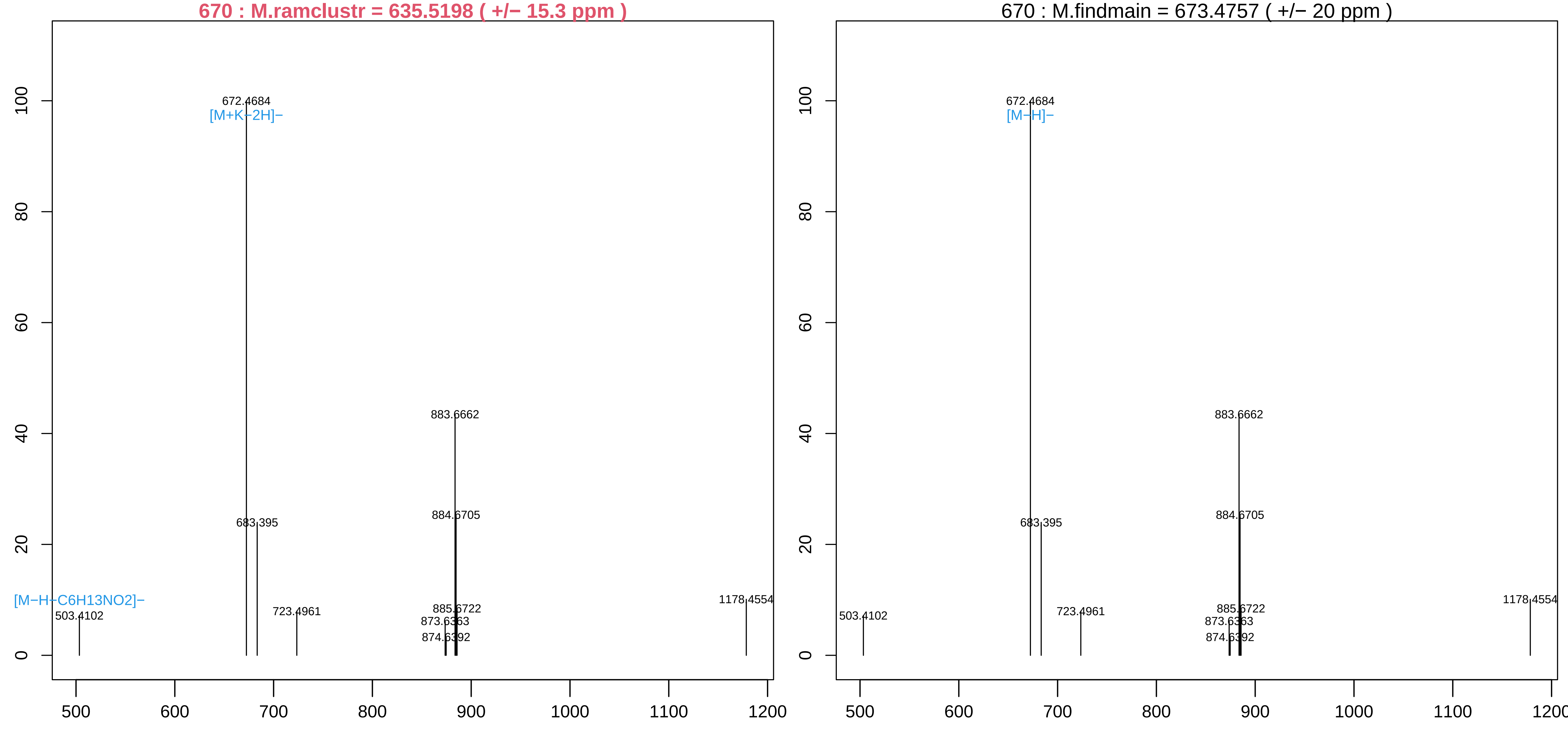
670 : M.ramclustr = 635.5198 (+/− 15.3 ppm)
672.4684
[M+K−2H]−
683.395
723.4961
883.6662
884.6705
885.6722
873.6363
874.6392
1178.4554
[M−H−C6H13NO2]−
503.4102
670 : M.findmain = 673.4757 (+/− 20 ppm)
672.4684
[M−H]−
683.395
723.4961
883.6662
884.6705
885.6722
873.6363
874.6392
1178.4554
503.4102
0
20
40
60
80
100
500
600
700
800
900
1000
1100
1200

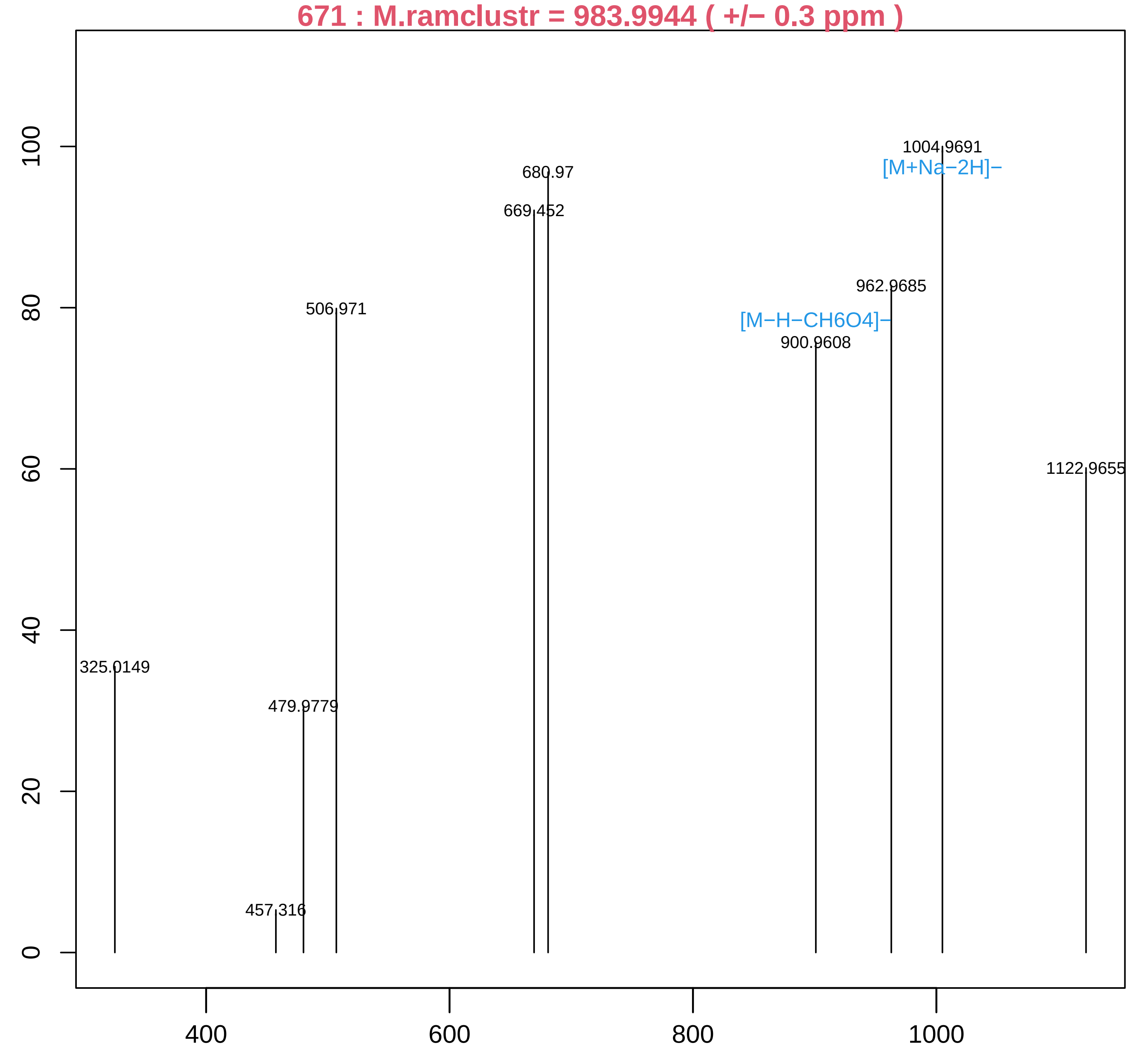
671 : M.ramclustr = 983.9944 (+/− 0.3 ppm)
325.0149
457.316
479.9779
506.971
669.452
680.97
[M−H−CH6O4]−
900.9608
962.9685
1004.9691
[M+Na−2H]−
1122.9655
0
20
40
60
80
100
400
600
800
1000

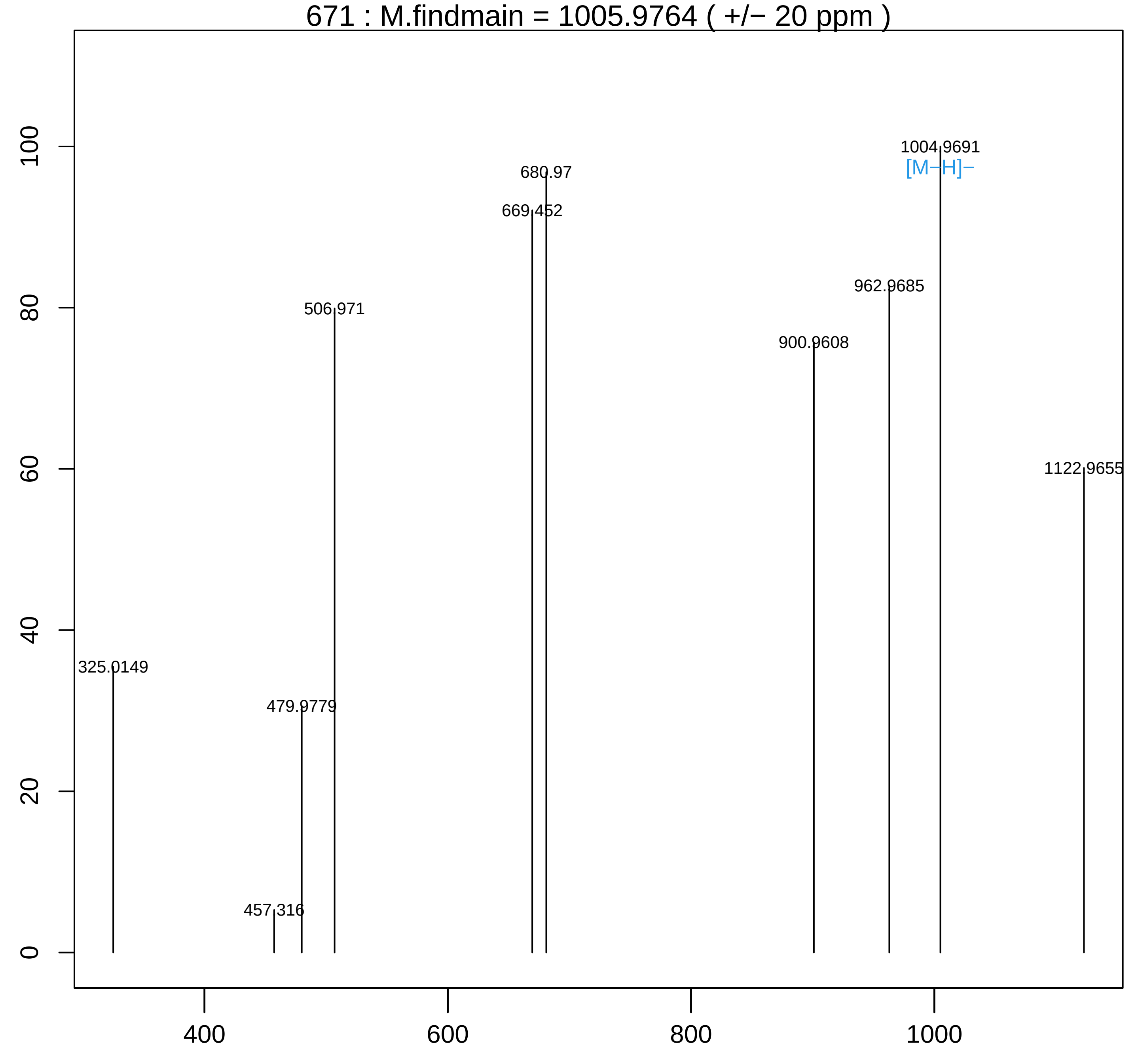
671 : M.findmain = 1005.9764 (+/− 20 ppm)
325.0149
457.316
479.9779
506.971
669.452
680.97
900.9608
962.9685
1004.9691
[M−H]−
1122.9655
0
20
40
60
80
100
400
600
800
1000

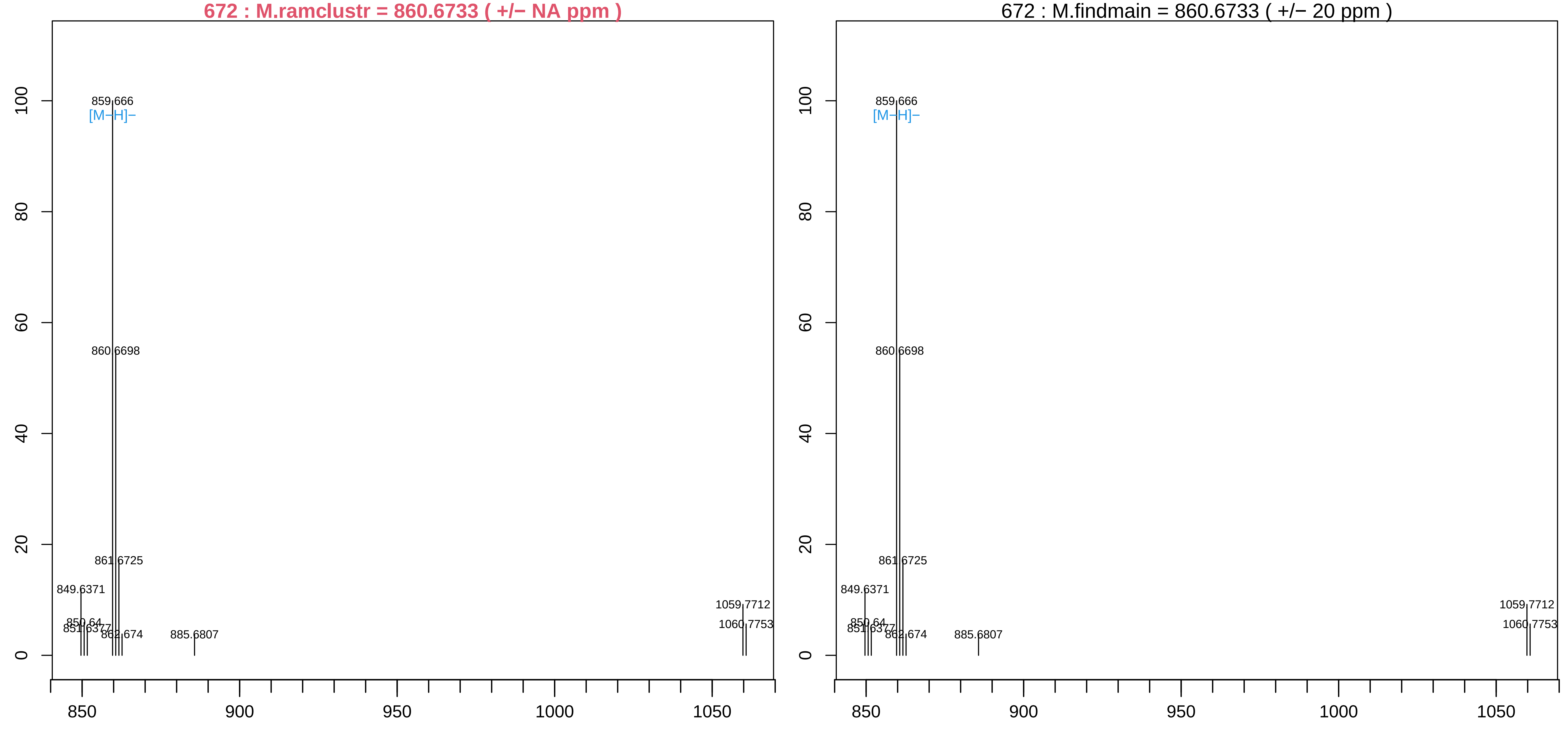

672 : M.ramclustr = 860.6733 (+/− NA ppm)
859.666
[M−H]−
860.6698
861.6725
849.6371
850.64
851.6377
862.674
885.6807
1059.7712
1060.7753
672 : M.findmain = 860.6733 (+/− 20 ppm)
859.666
[M−H]−
860.6698
861.6725
849.6371
850.64
851.6377
862.674
885.6807
1059.7712
1060.7753

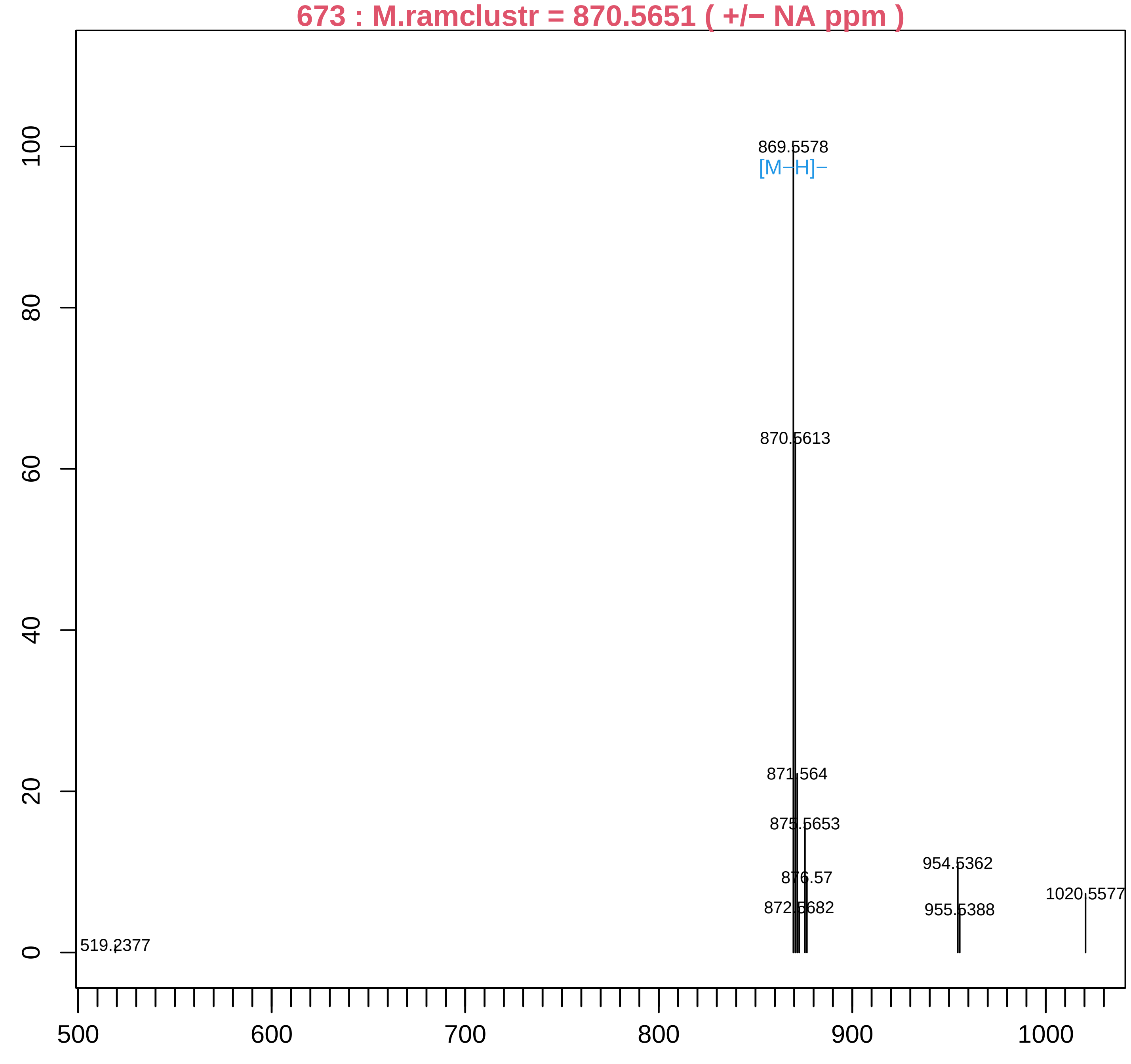

673 : M.ramclustr = 870.5651 (+/− NA ppm)
869.5578
[M−H]−
870.5613
871.564
875.5653
954.5362
876.57
872.5682
955.5388
1020.5577
519.2377
0
20
40
60
80
100
500
600
700
800
900
1000

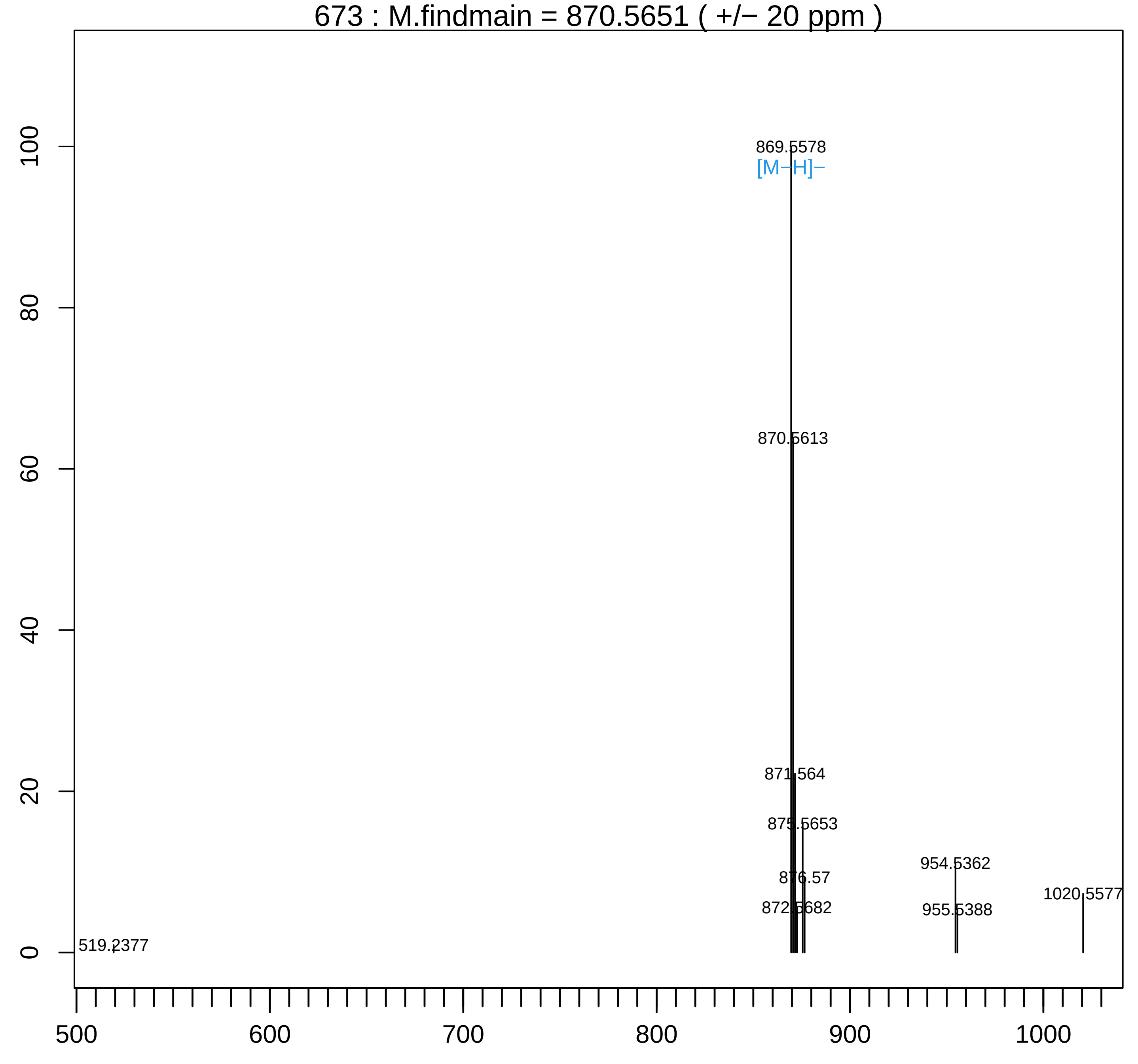

673 : M.findmain = 870.5651 (+/− 20 ppm)
869.5578
[M−H]−
870.5613
871.564
875.5653
954.5362
876.57
872.5682
955.5388
1020.5577
519.2377
0
20
40
60
80
100
500
600
700
800
900
1000

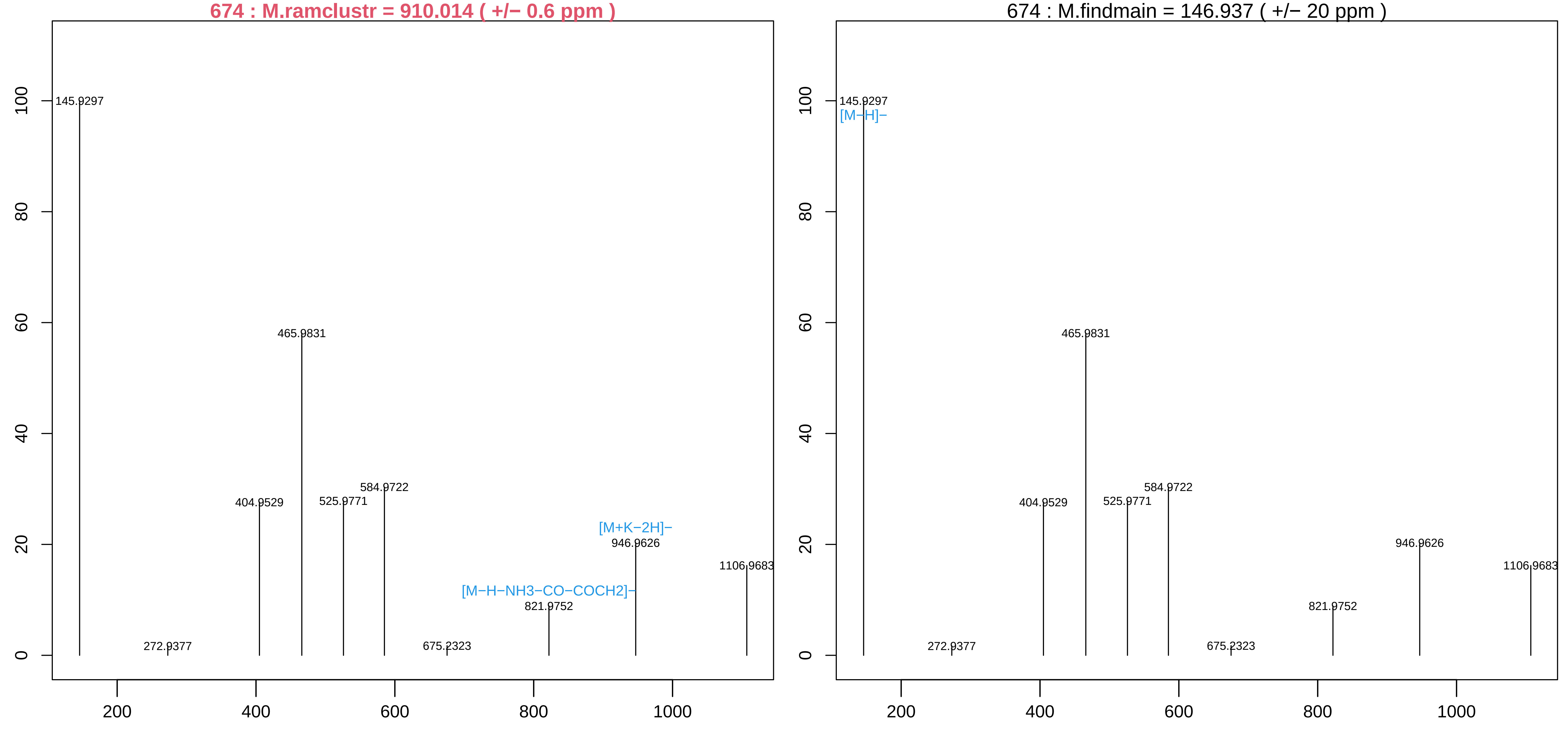

674 : M.ramclustr = 910.014 (+/− 0.6 ppm)
674 : M.findmain = 146.937 (+/− 20 ppm)
145.9297
[M−H]−
465.9831
404.9529
525.9771
584.9722
[M+K−2H]−
946.9626
1106.9683
[M−H−NH3−CO−COCH2]−
821.9752
272.9377
675.2323
0
20
40
60
80
100
200
400
600
800
1000

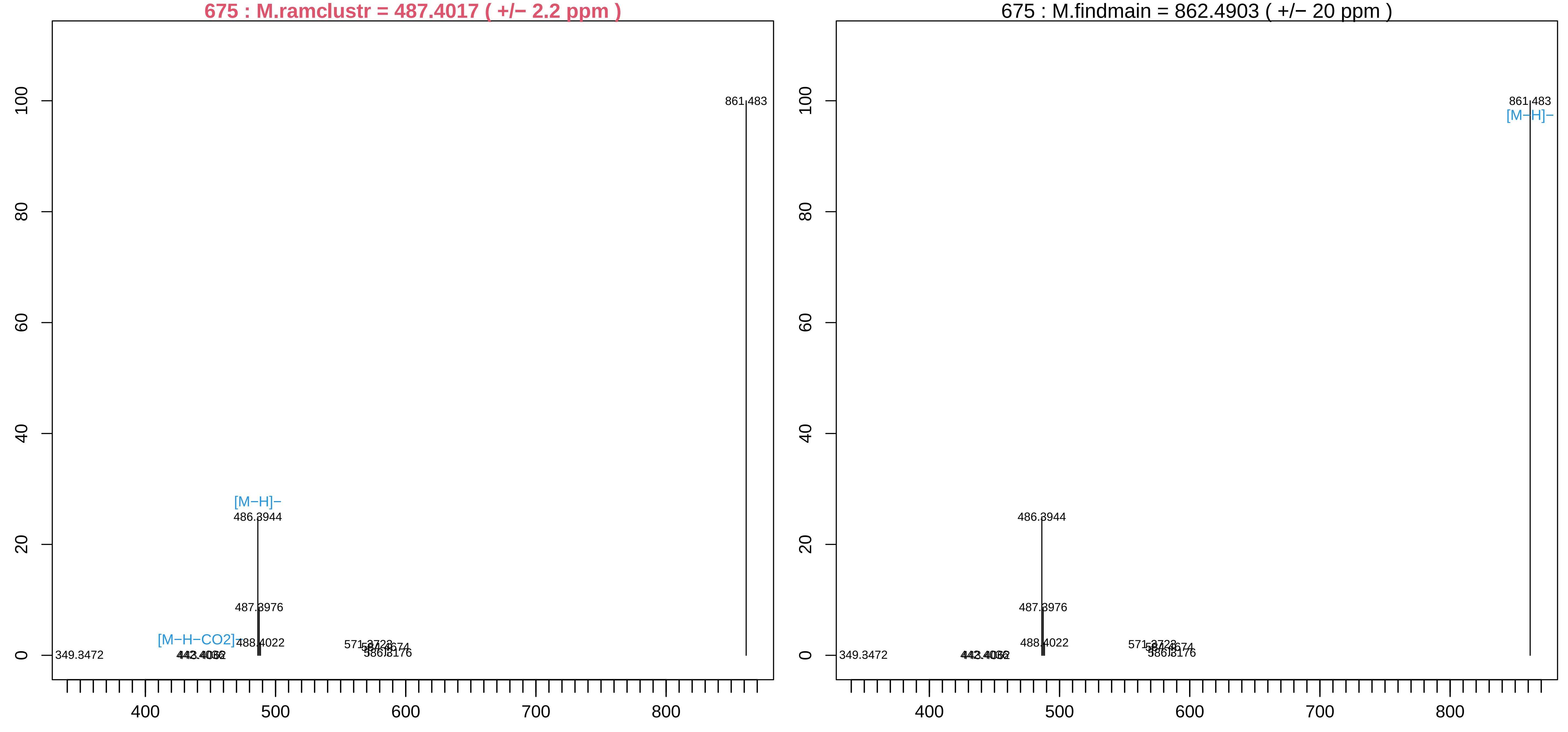
675 : M.ramclustr = 487.4017 (+/− 2.2 ppm)
675 : M.findmain = 862.4903 (+/− 20 ppm)
861.483
[M−H]−
486.3944
487.3976
488.4022
[M−H−CO2]−
349.3472
571.3723
586.3176
0
20
40
60
80
100
400
500
600
700
800

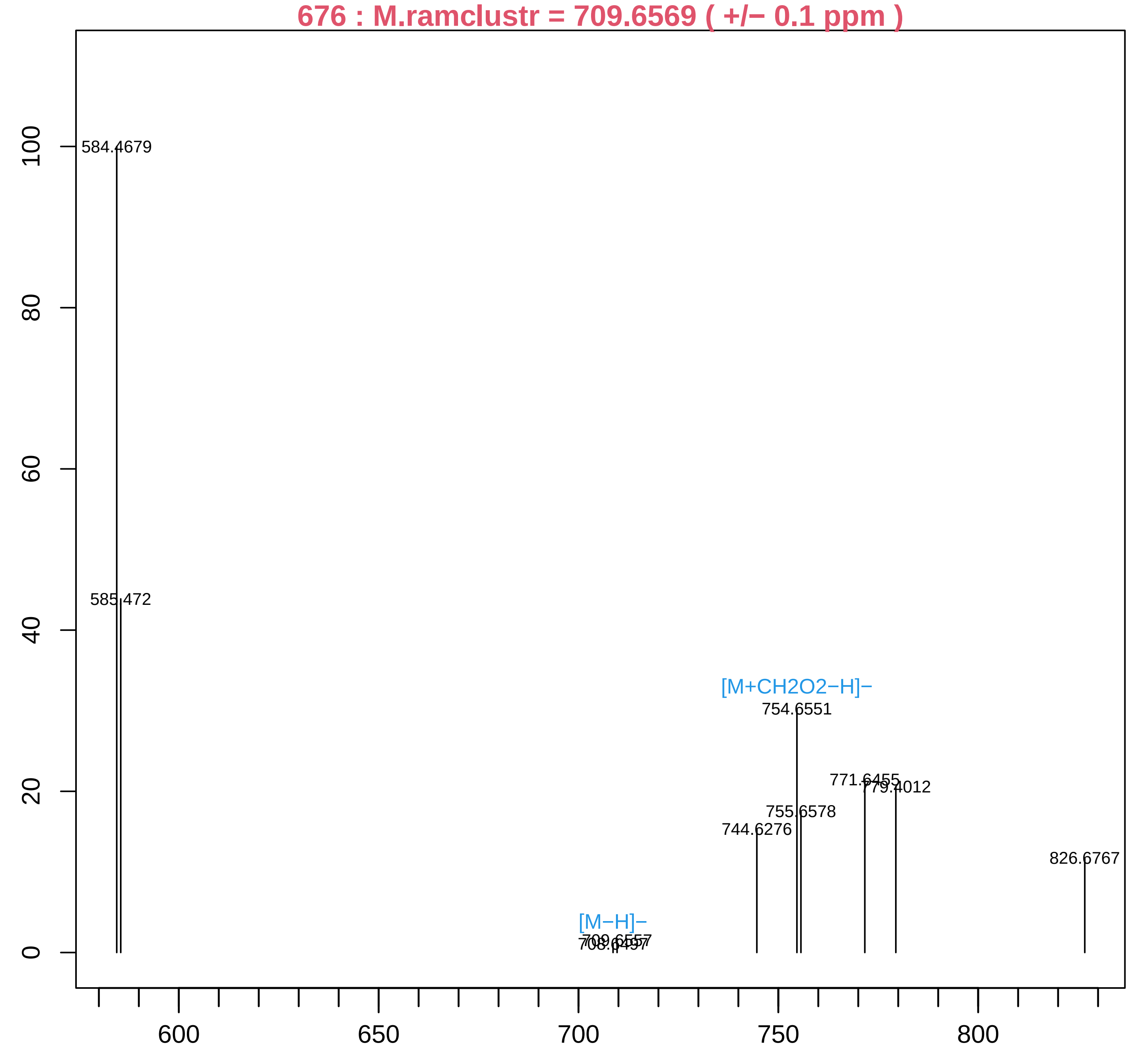
676 : M.ramclustr = 709.6569 (+/− 0.1 ppm)
584.4679
585.472
[M+CH2O2−H]−
754.6551
771.6455
779.4012
755.6578
744.6276
826.6767
[M−H]−
709.6557
708.6497
0
20
40
60
80
100
600
650
700
750
800

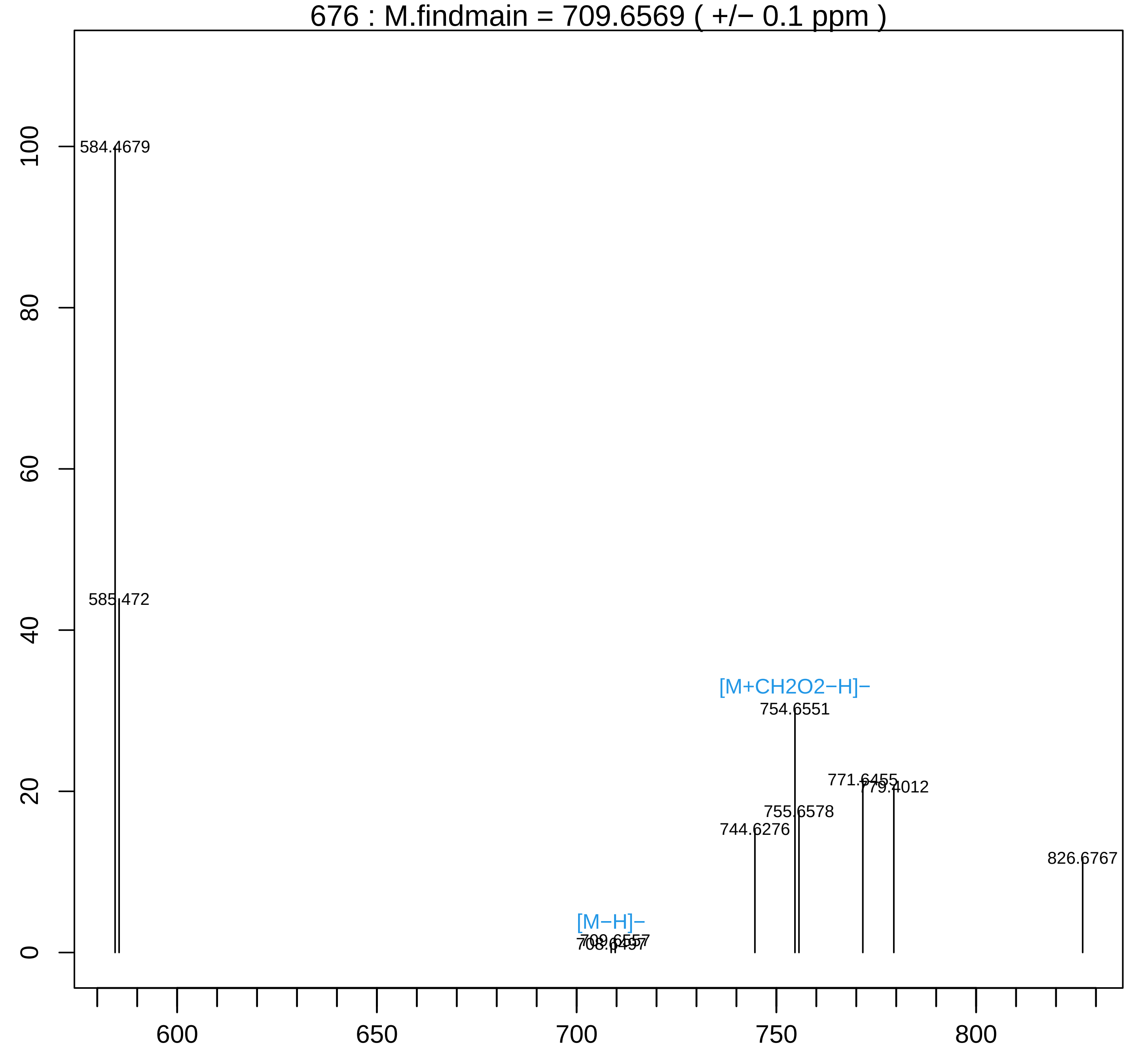
676 : M.findmain = 709.6569 (+/− 0.1 ppm)
584.4679
585.472
[M+CH2O2−H]−
754.6551
771.6455
779.4012
755.6578
744.6276
826.6767
[M−H]−
709.6557
708.6497
0
20
40
60
80
100
600
650
700
750
800

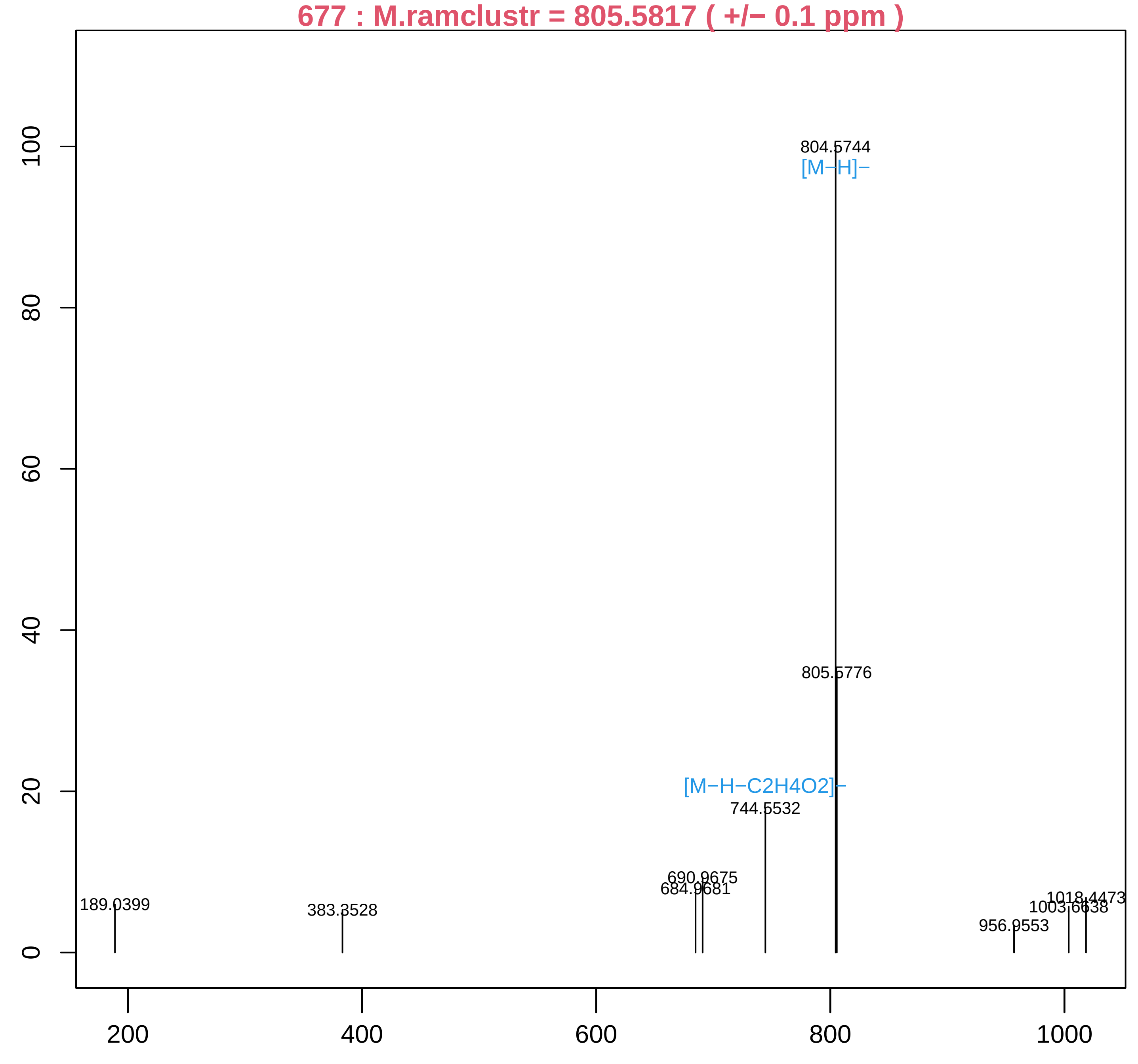
677 : M.ramclustr = 805.5817 (+/− 0.1 ppm)
804.5744
[M−H]−
805.5776
[M−H−C2H4O2]−
744.5532
690.9675
684.9681
189.0399
383.3528
956.9553
1018.4473
1003.6638
0
20
40
60
80
100
200
400
600
800
1000

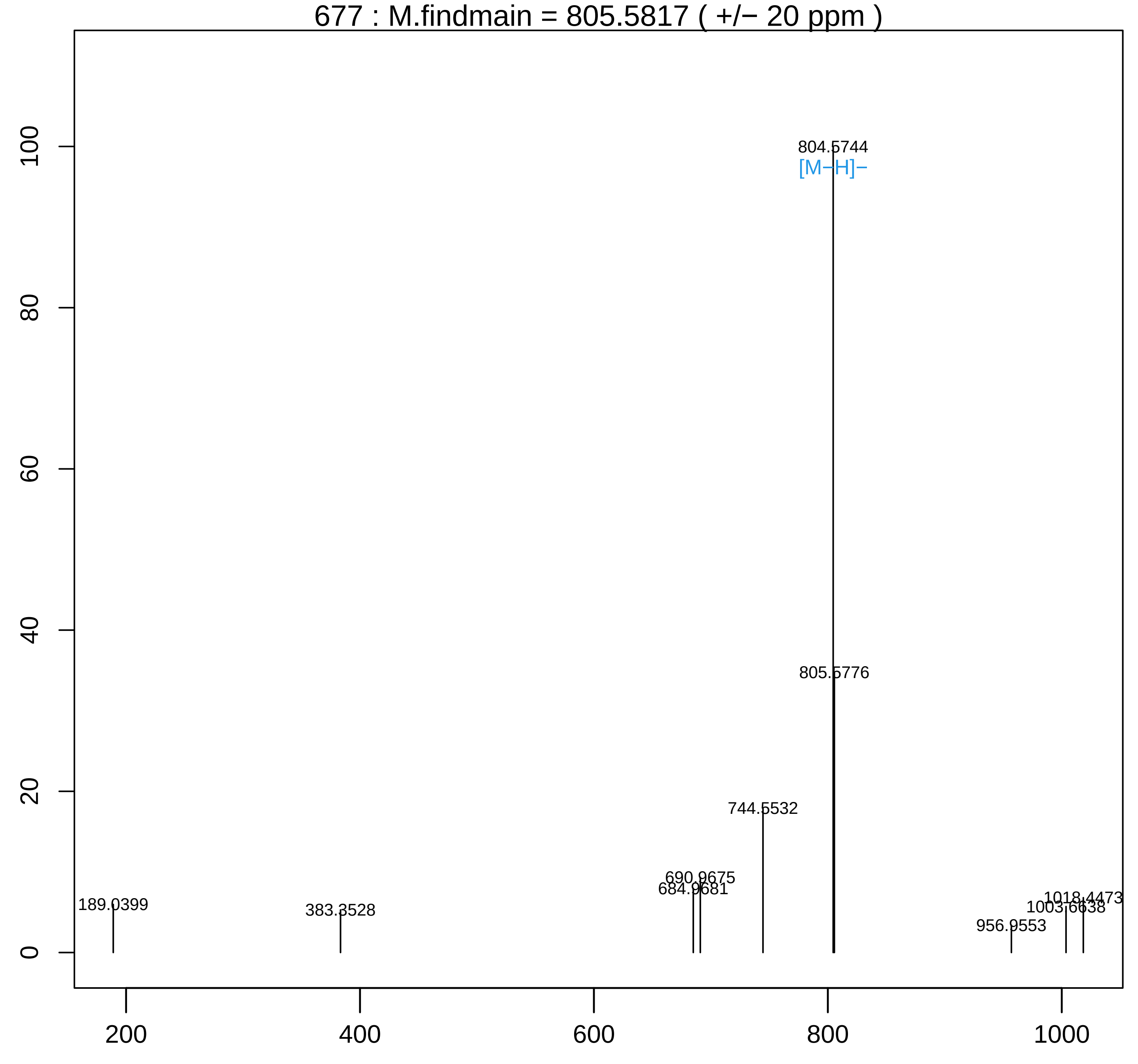
677 : M.findmain = 805.5817 (+/− 20 ppm)
804.5744
[M−H]−
805.5776
744.5532
690.9675
684.9681
189.0399
383.3528
956.9553
1018.4473
1003.6638
0
20
40
60
80
100
200
400
600
800
1000

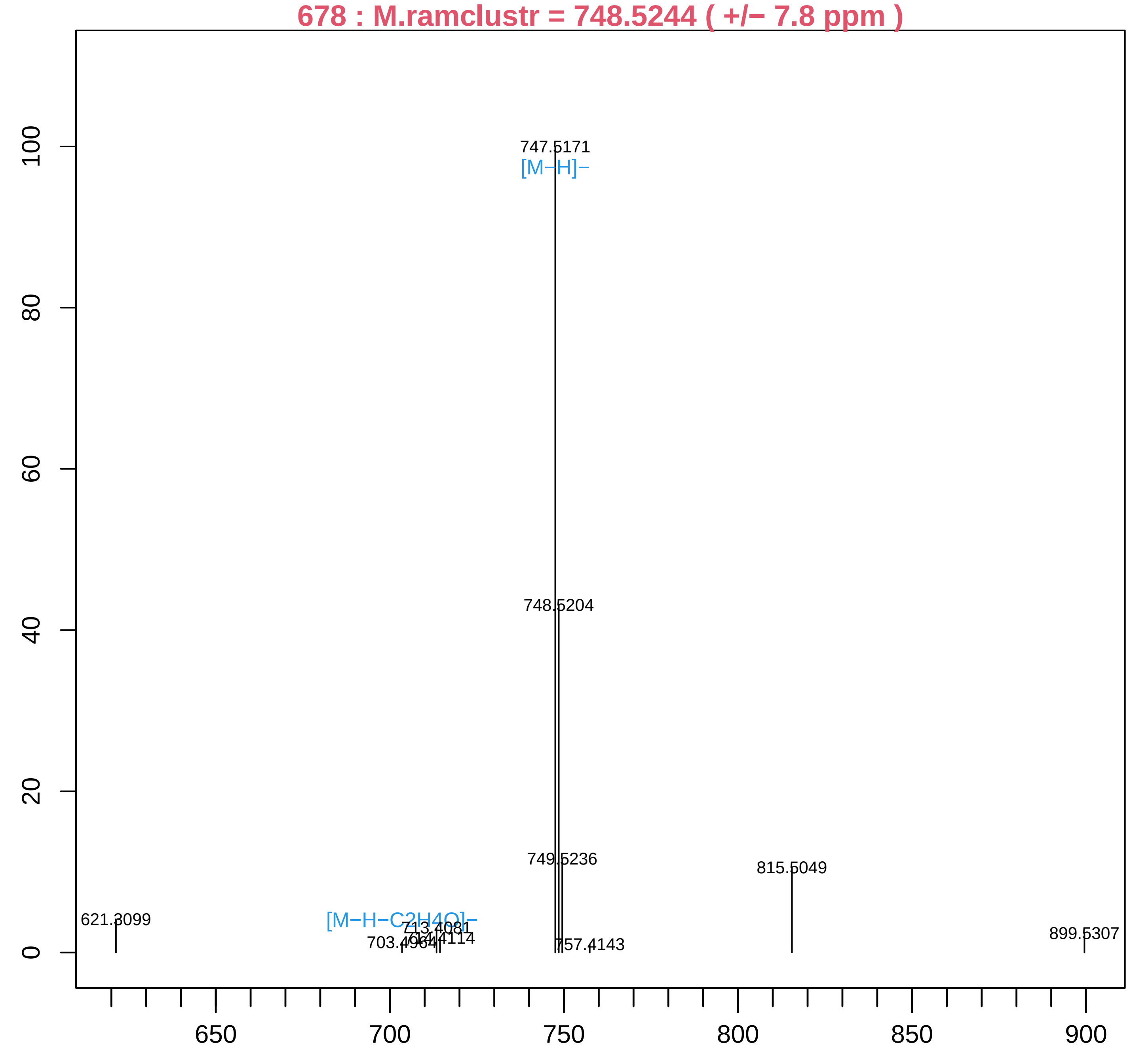
678 : M.ramclustr = 748.5244 (+/− 7.8 ppm)
747.5171
[M−H]−
748.5204
749.5236
815.5049
621.3099
[M−H−C2H4O]−
713.4081
703.4964
714.4114
757.4143
899.5307
0
20
40
60
80
100
650
700
750
800
850
900

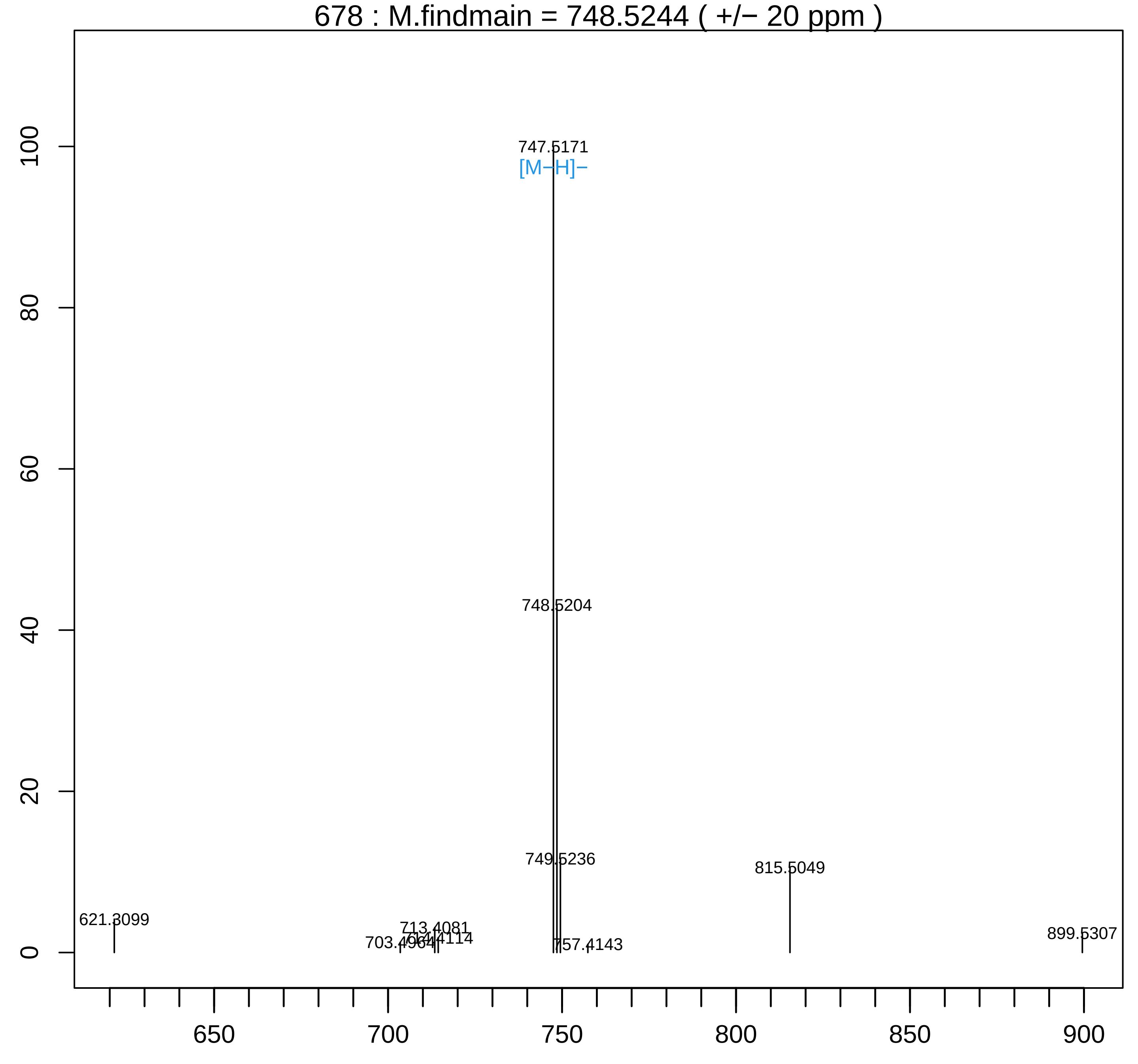
678 : M.findmain = 748.5244 (+/− 20 ppm)
747.5171
[M−H]−
748.5204
749.5236
815.5049
621.3099
713.4081
703.4964
714.4114
757.4143
899.5307
0
20
40
60
80
100
650
700
750
800
850
900

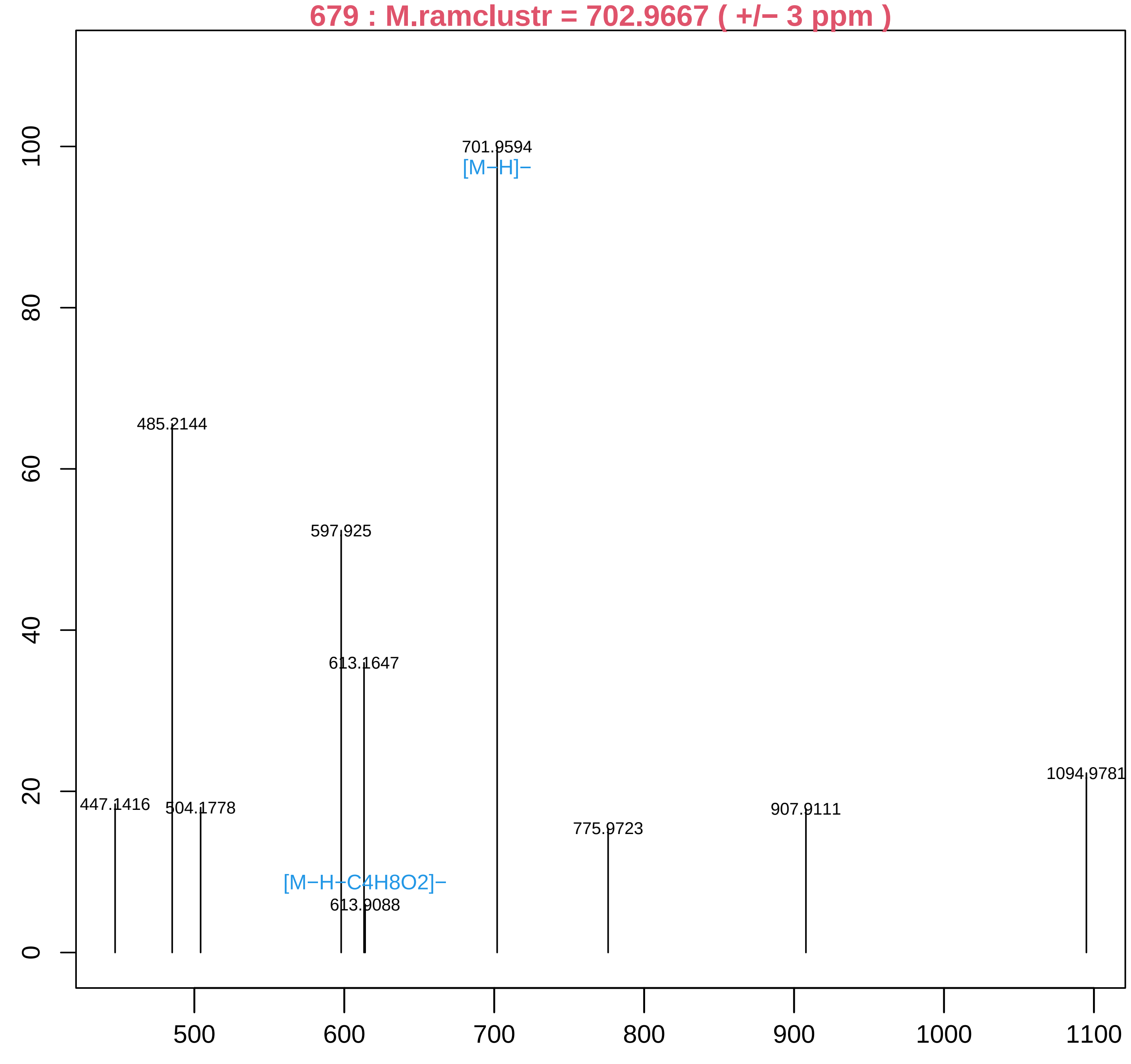

679 : M.ramclustr = 702.9667 (+/− 3 ppm)
701.9594
[M−H]−
485.2144
597.925
613.1647
447.1416
504.1778
[M−H−C4H8O2]−
613.9088
775.9723
907.9111
1094.9781
0
20
40
60
80
100
500
600
700
800
900
1000
1100

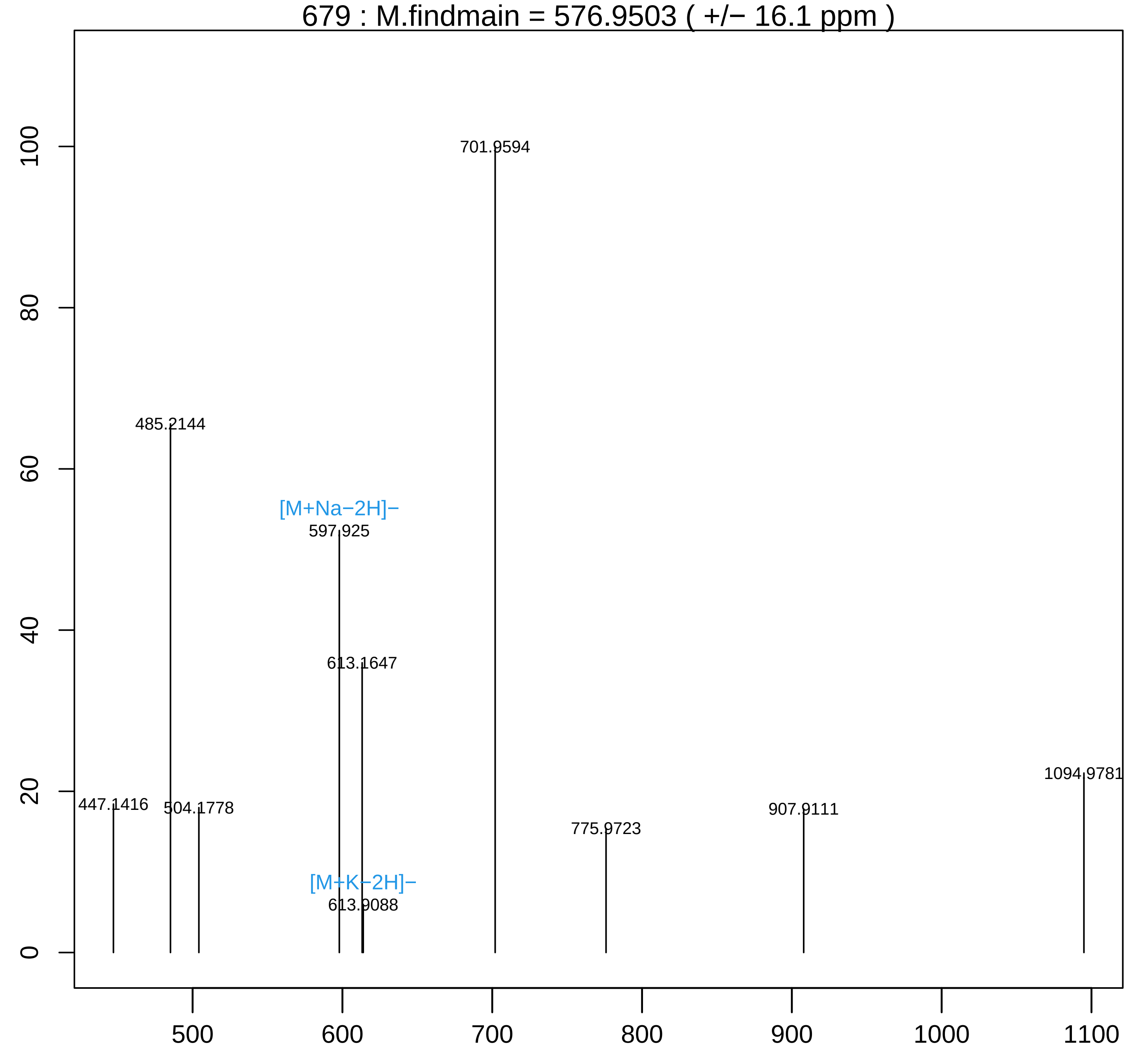

679 : M.findmain = 576.9503 (+/− 16.1 ppm)
701.9594
485.2144
[M+Na−2H]−
597.925
613.1647
447.1416
504.1778
[M+K−2H]−
613.9088
775.9723
907.9111
1094.9781
0
20
40
60
80
100
500
600
700
800
900
1000
1100

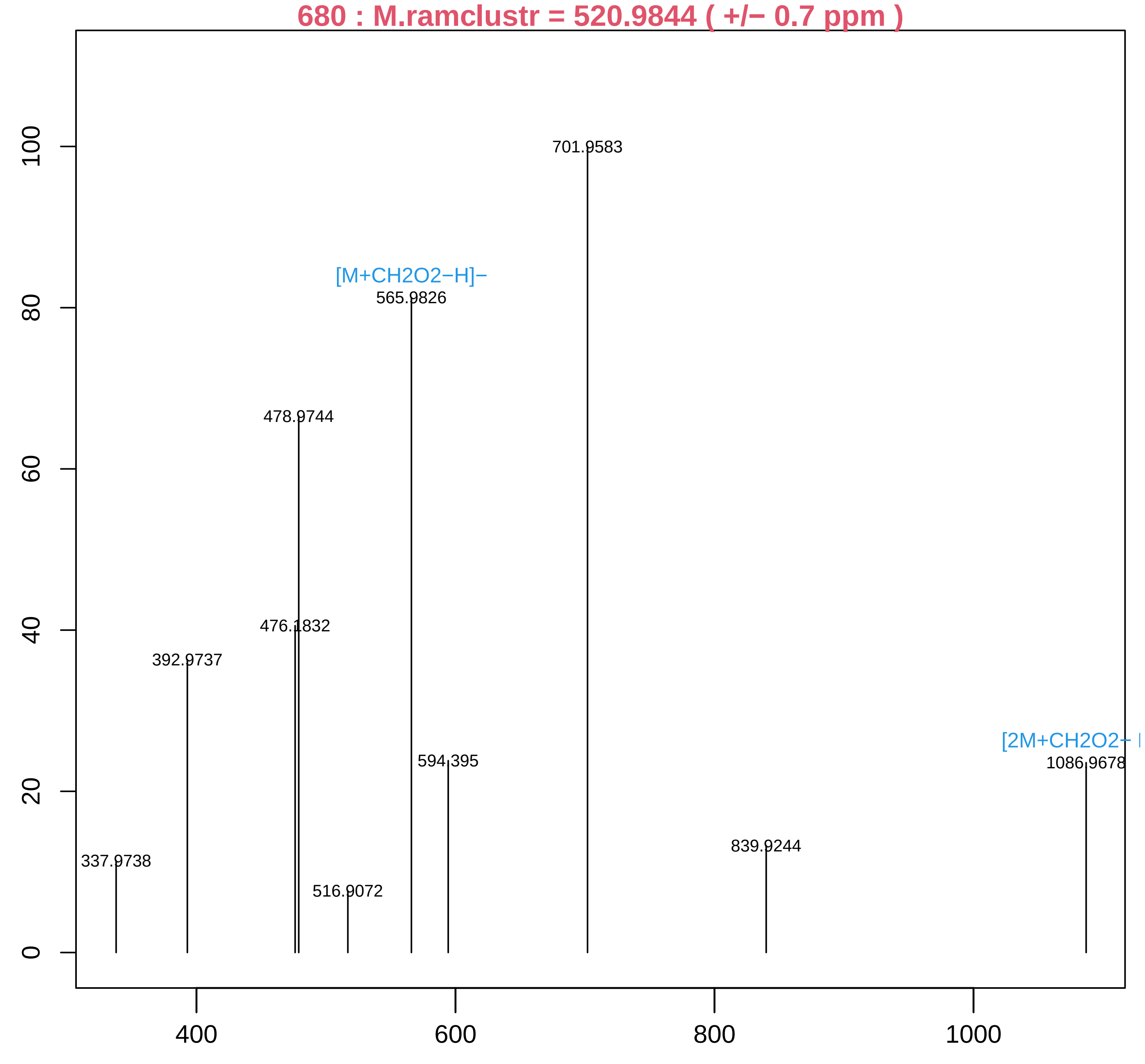
680 : M.ramclustr = 520.9844 (+/− 0.7 ppm)
701.9583
[M+CH2O2−H]−
565.9826
478.9744
476.1832
392.9737
594.395
[2M+CH2O2−
1086.9678
337.9738
839.9244
516.9072
0
20
40
60
80
100
400
600
800
1000

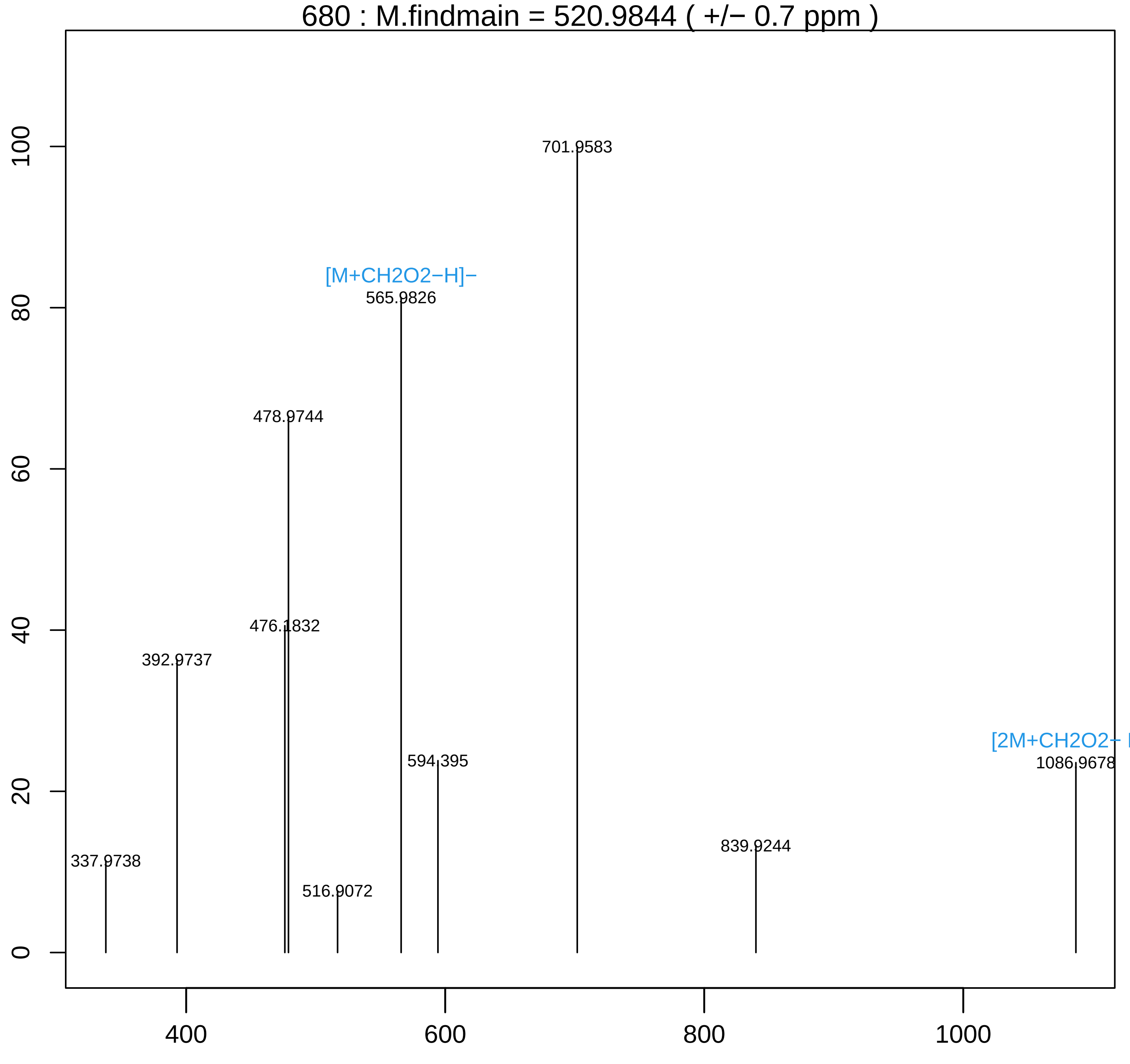
680 : M.findmain = 520.9844 (+/− 0.7 ppm)
701.9583
[M+CH2O2−H]−
565.9826
478.9744
476.1832
392.9737
594.395
[2M+CH2O2−
1086.9678
337.9738
839.9244
516.9072
0
20
40
60
80
100
400
600
800
1000

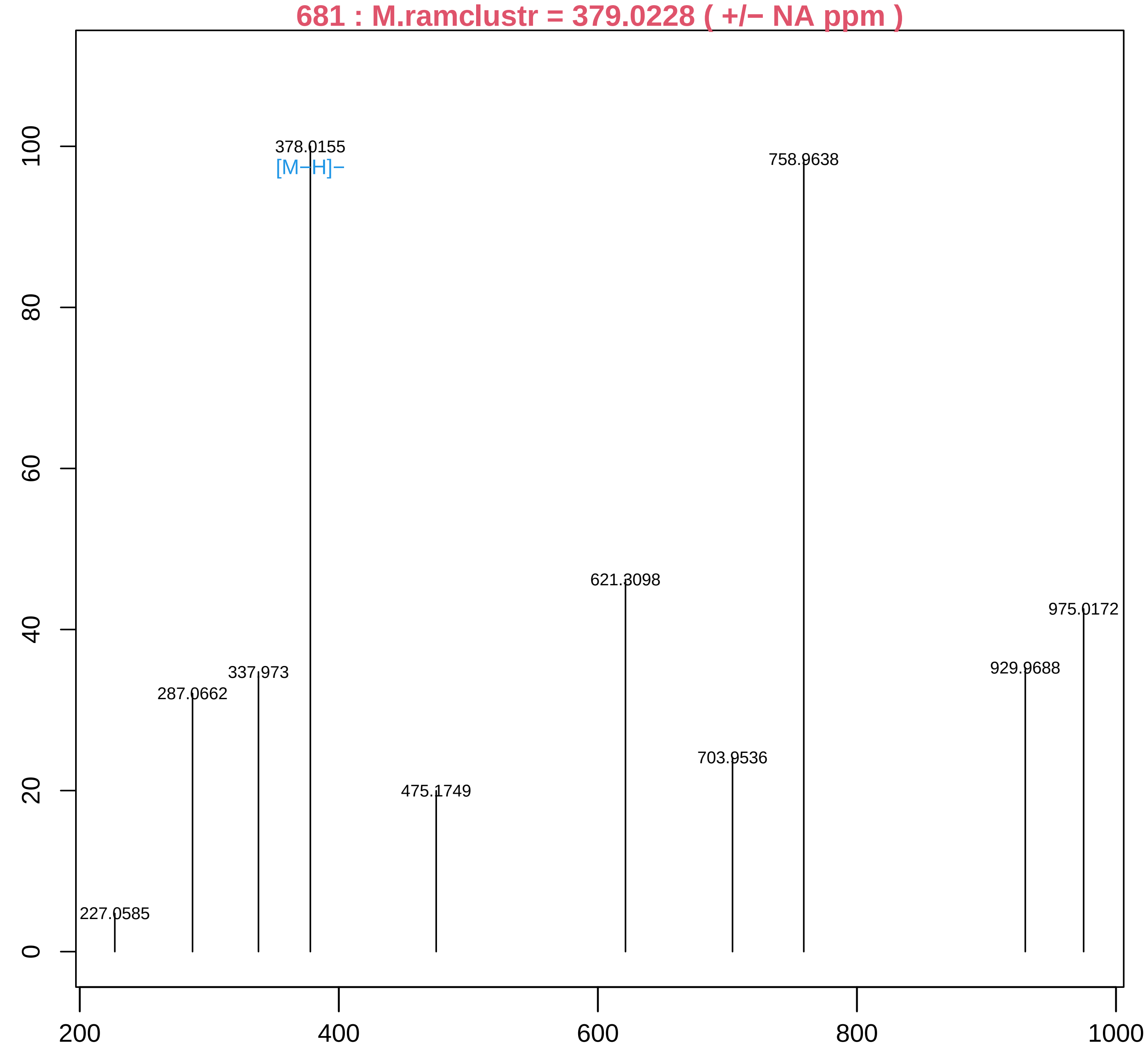
681 : M.ramclustr = 379.0228 (+/− NA ppm)
378.0155
[M−H]−
758.9638
621.3098
975.0172
929.9688
337.973
287.0662
703.9536
475.1749
227.0585

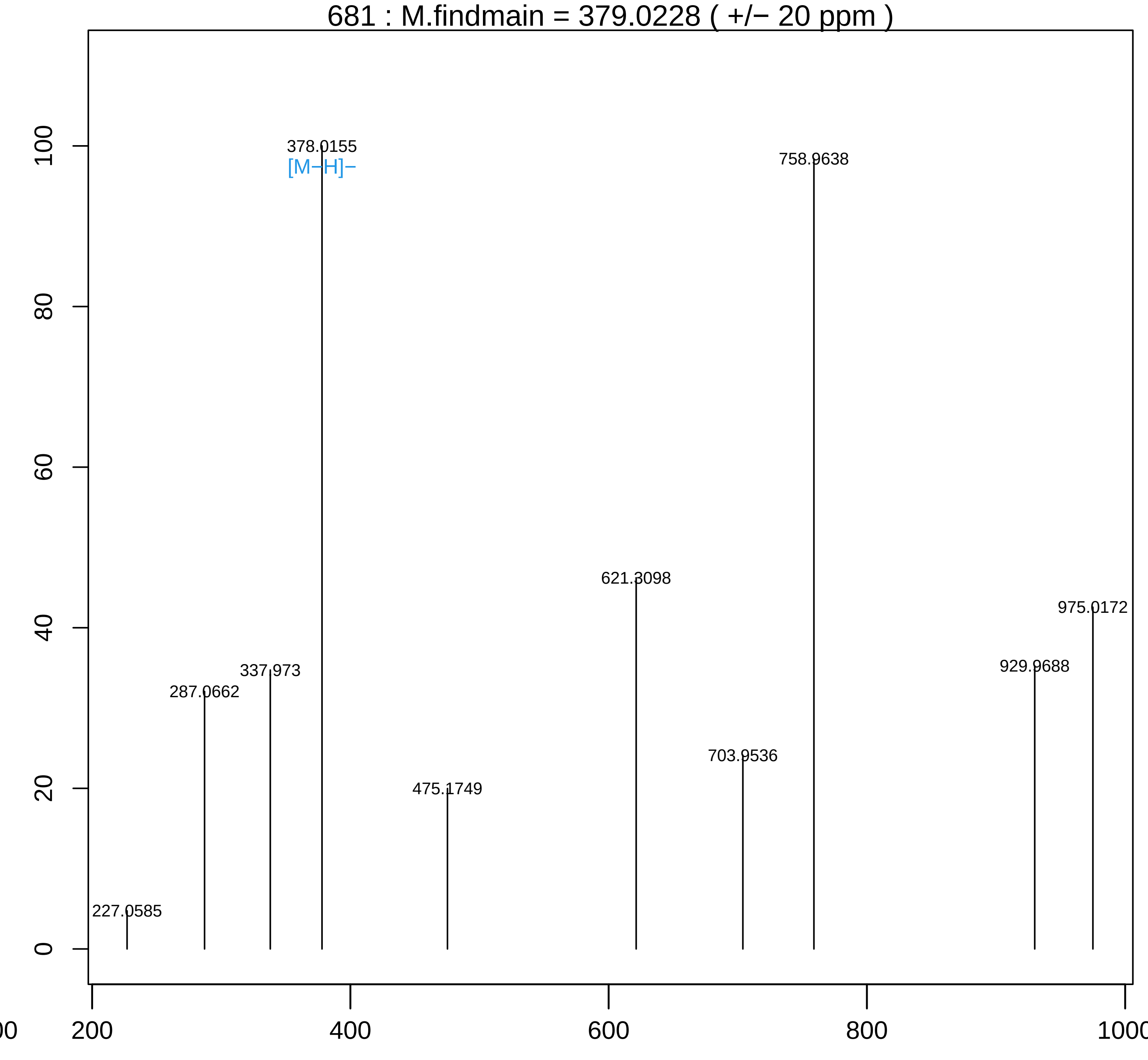
681 : M.findmain = 379.0228 (+/− 20 ppm)
378.0155
[M−H]−
758.9638
621.3098
975.0172
929.9688
337.973
287.0662
703.9536
475.1749
227.0585

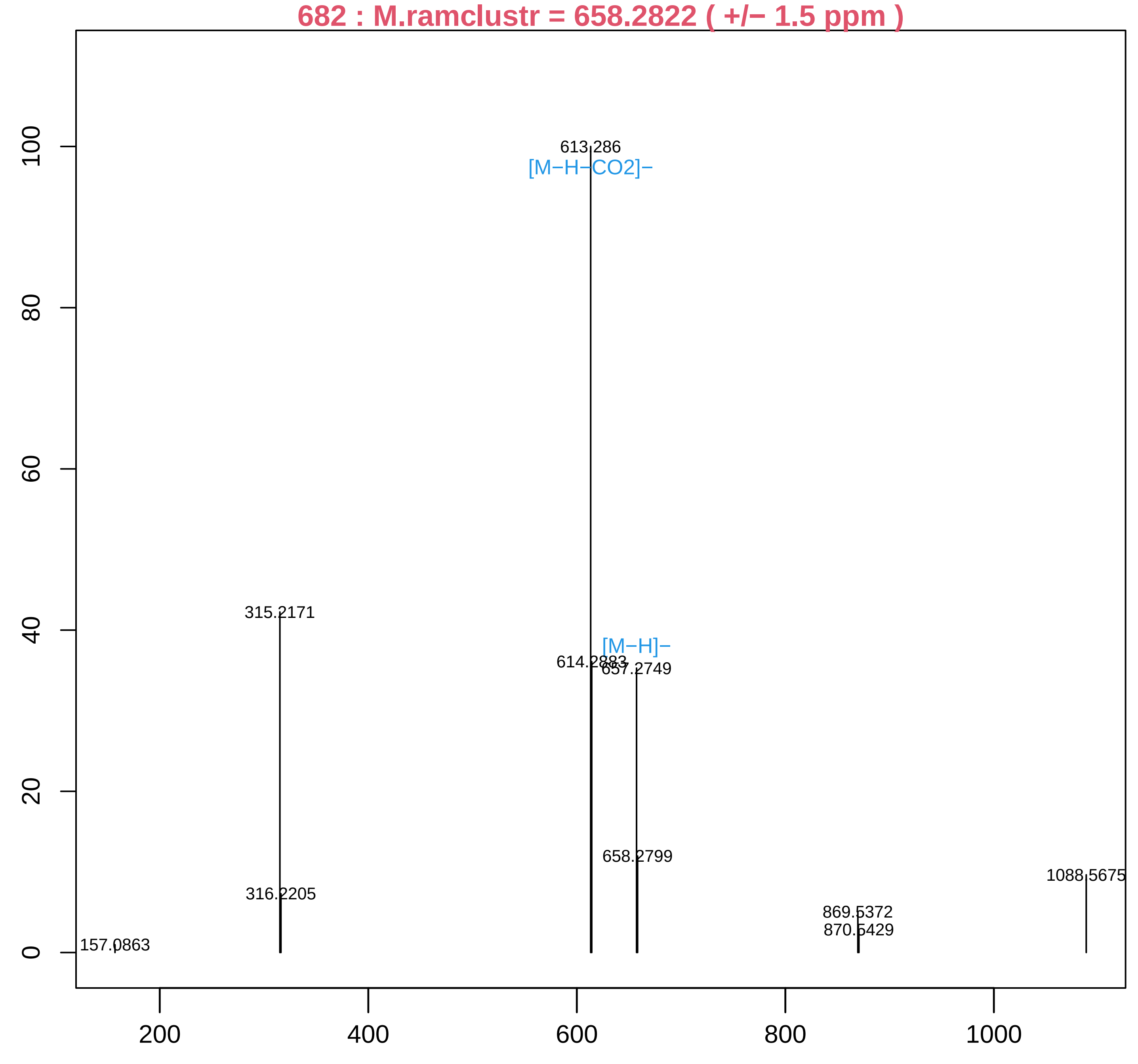
682 : M.ramclustr = 658.2822 (+/− 1.5 ppm)
613.286
[M−H−CO2]−
315.2171
[M−H]−
614.2883
657.2749
658.2799
316.2205
1088.5675
869.5372
870.5429
157.0863
0
20
40
60
80
100
200
400
600
800
1000

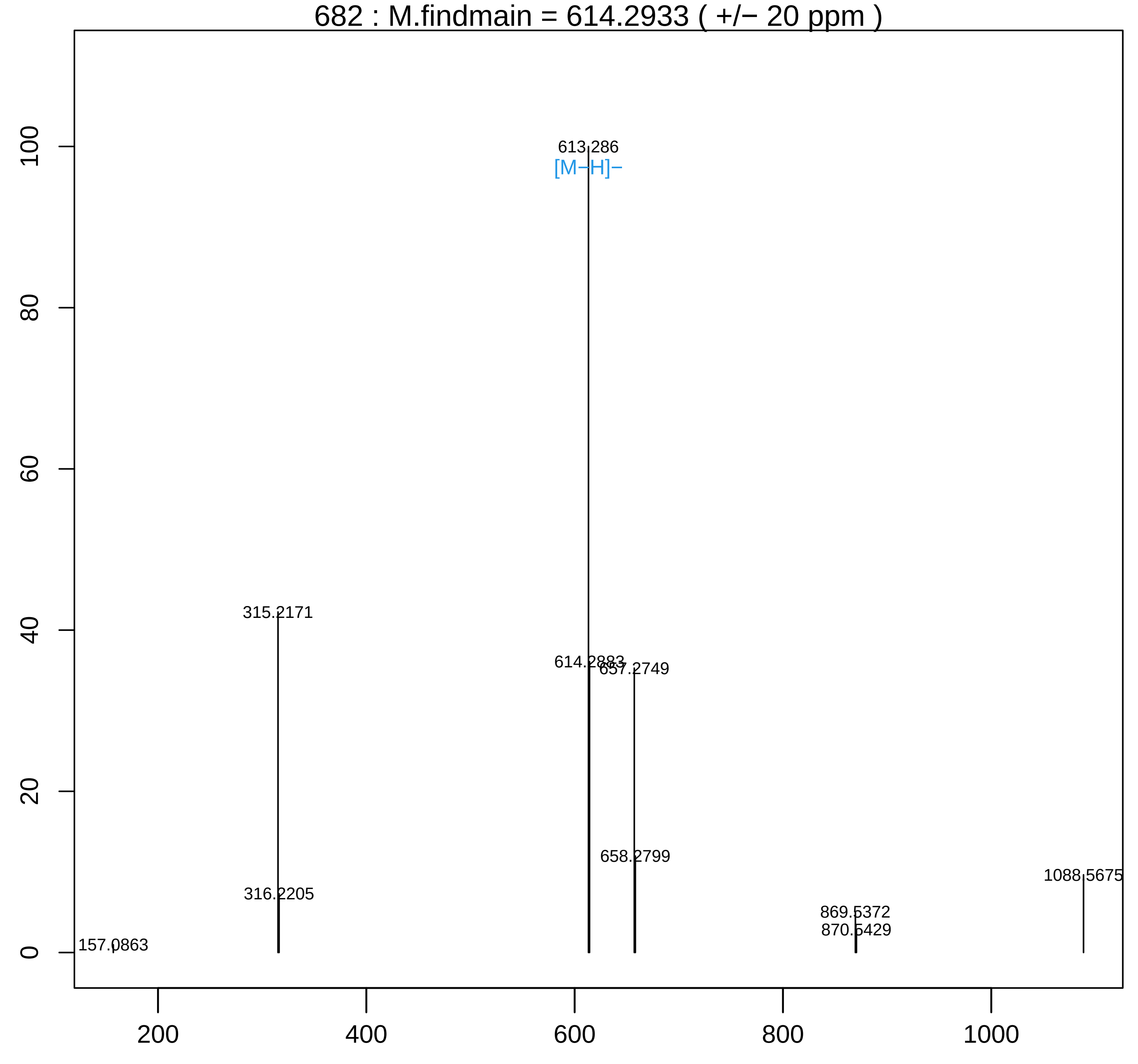
682 : M.findmain = 614.2933 (+/− 20 ppm)
613.286
[M−H]−
315.2171
614.2883
657.2749
658.2799
316.2205
1088.5675
869.5372
870.5429
157.0863
0
20
40
60
80
100
200
400
600
800
1000

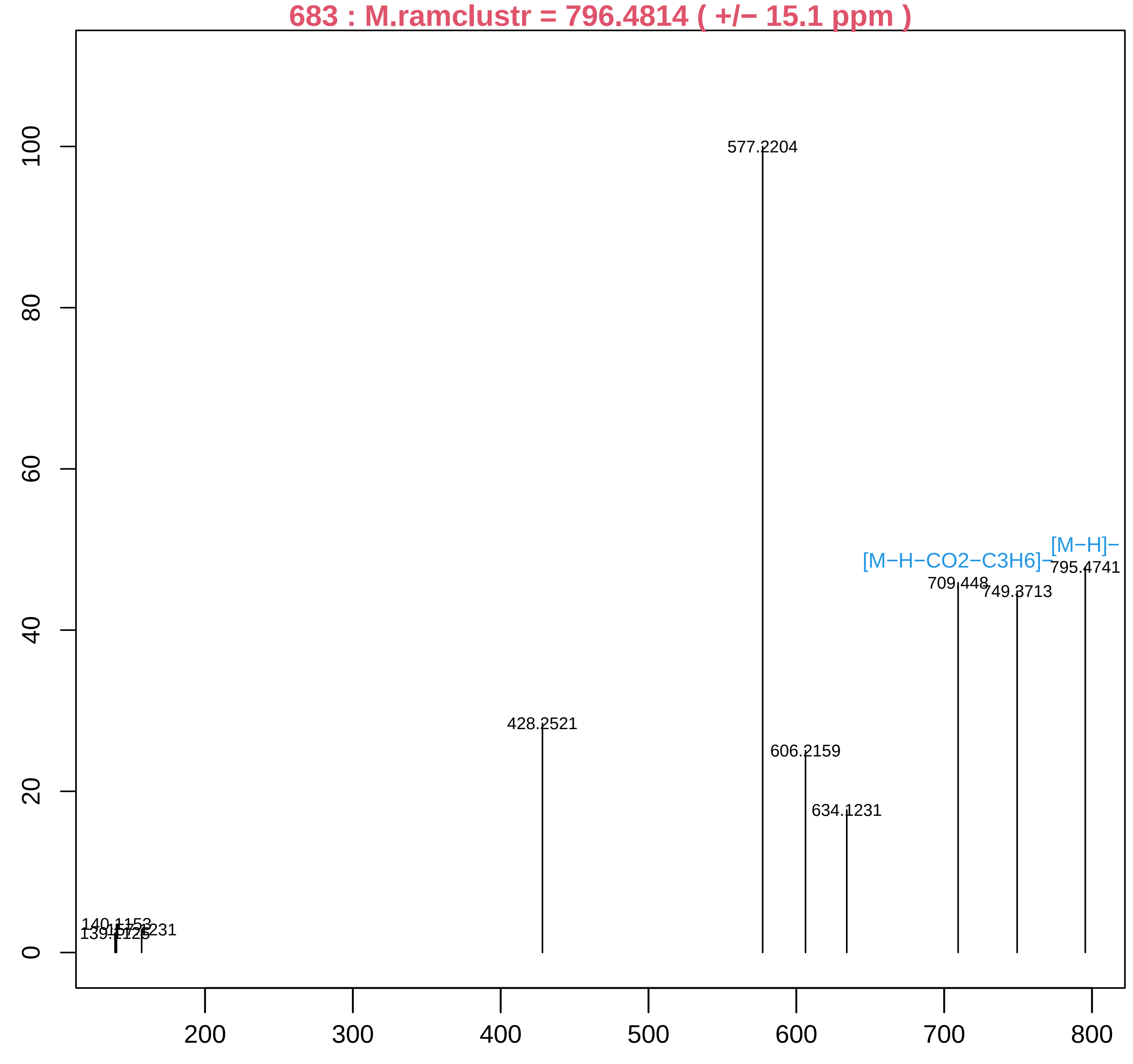
683 : M.ramclustr = 796.4814 (+/− 15.1 ppm)
577.2204
428.2521
606.2159
634.1231
709.448
749.3713
795.4741
[M−H−CO2−C3H6]−
[M−H]−
140.1153
0
20
40
60
80
100
200
300
400
500
600
700
800

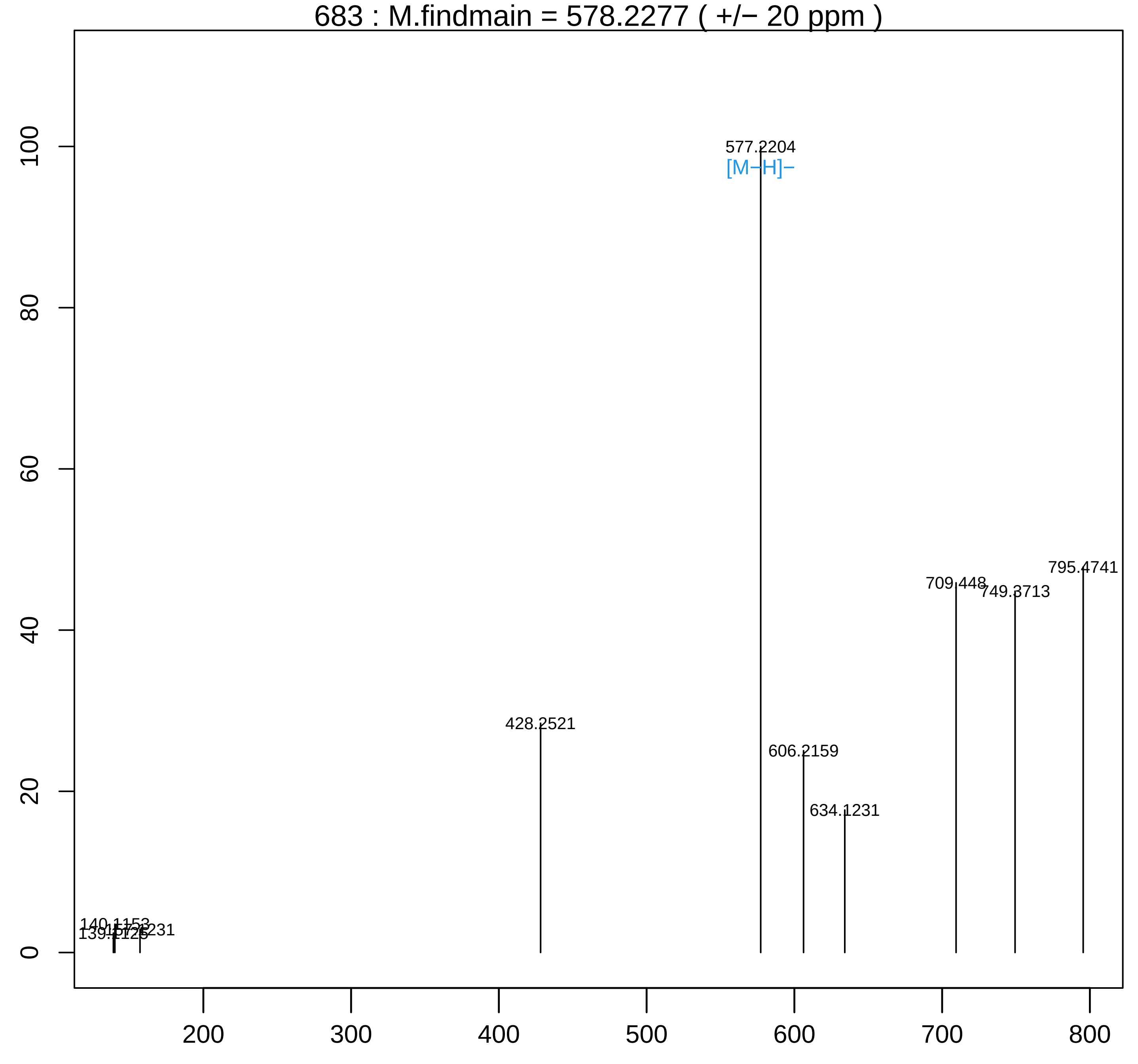
683 : M.findmain = 578.2277 (+/− 20 ppm)
577.2204
[M−H]−
428.2521
606.2159
634.1231
709.448
749.3713
795.4741
140.1153
0
20
40
60
80
100
200
300
400
500
600
700
800

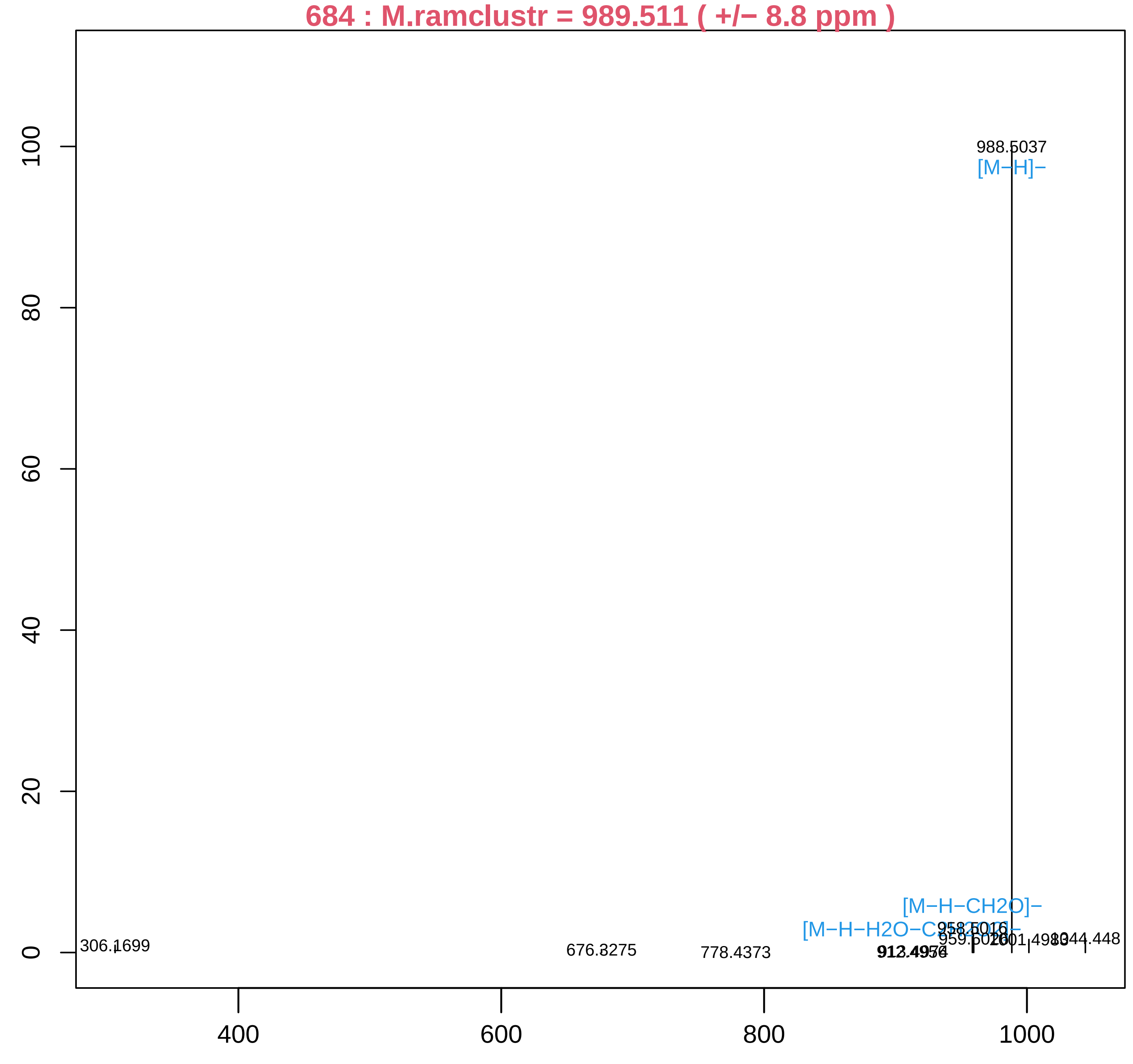

684 : M.ramclustr = 989.511 (+/− 8.8 ppm)
988.5037
[M−H]−
[M−H−CH2O]−
[M−H−H2O−C...]−
958.5016
306.1699
676.8275
778.4373
912.4974
1044.448
0
20
40
60
80
100
400
600
800
1000

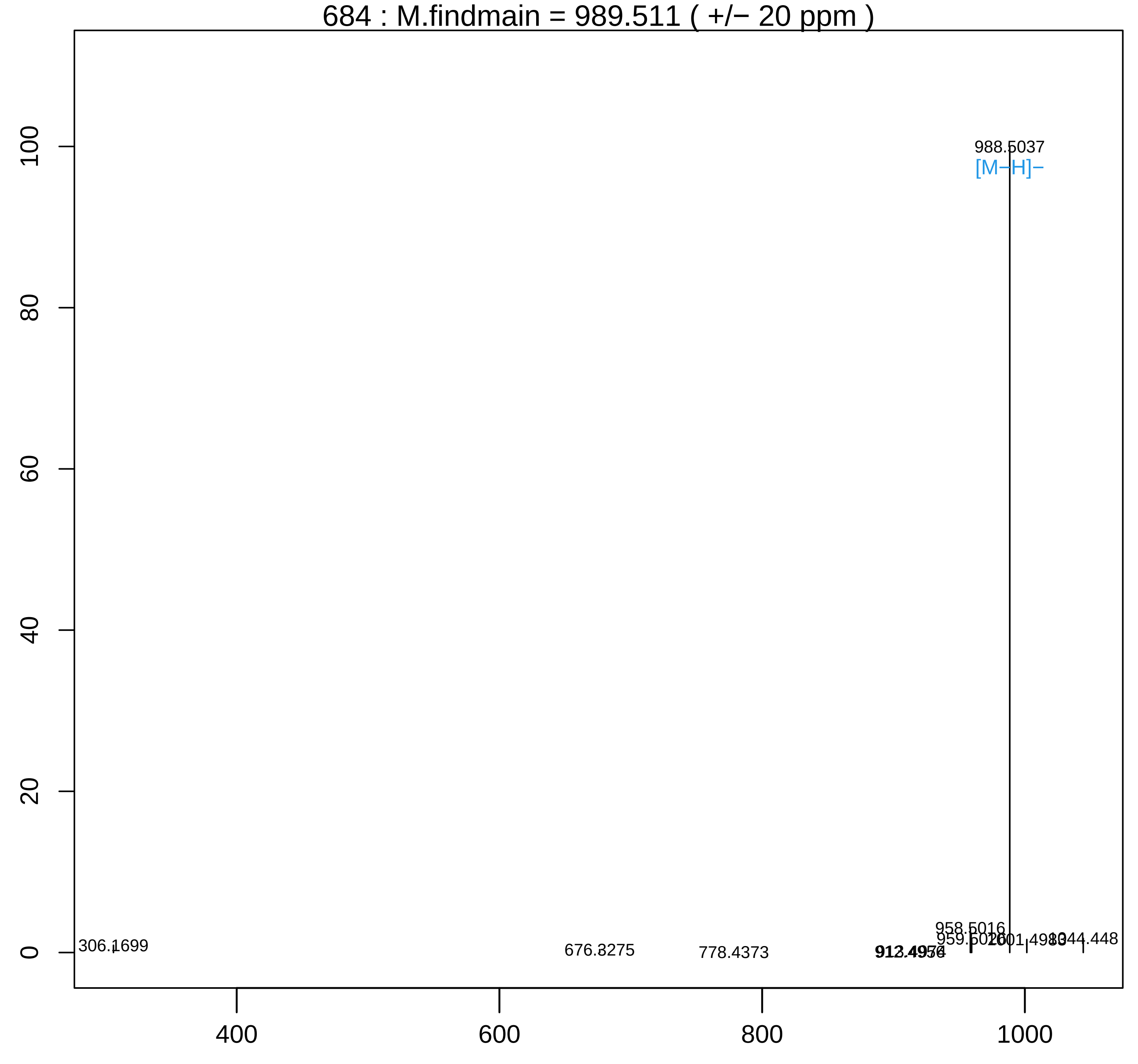

684 : M.findmain = 989.511 (+/− 20 ppm)
988.5037
[M−H]−
958.5016
306.1699
676.8275
778.4373
912.4974
1044.448
0
20
40
60
80
100
400
600
800
1000

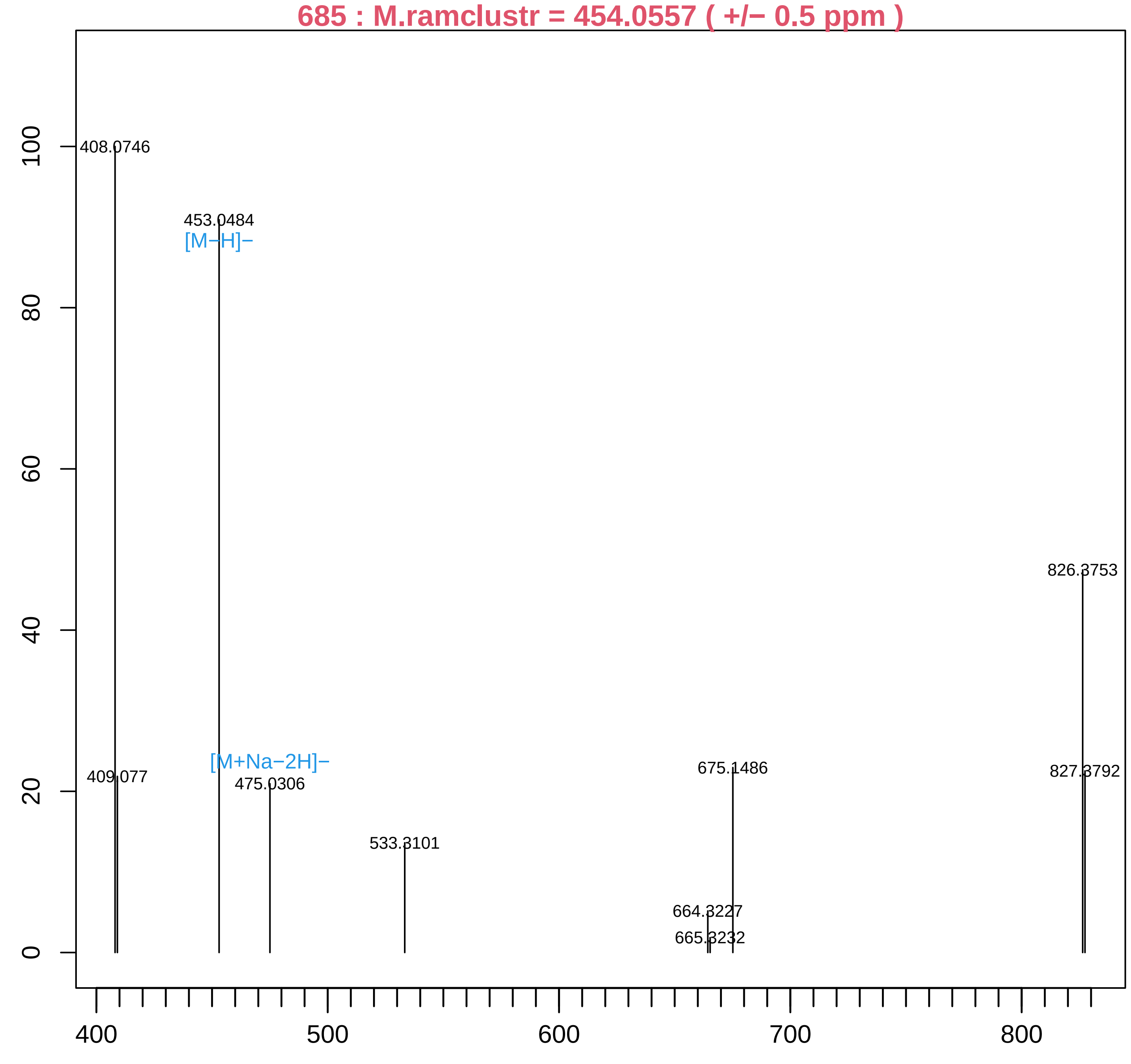
685 : M.ramclustr = 454.0557 (+/− 0.5 ppm)
408.0746
453.0484
[M−H]−
409.077
[M+Na−2H]−
475.0306
533.3101
664.3227
665.3232
675.1486
826.3753
827.3792
0
20
40
60
80
100
400
500
600
700
800

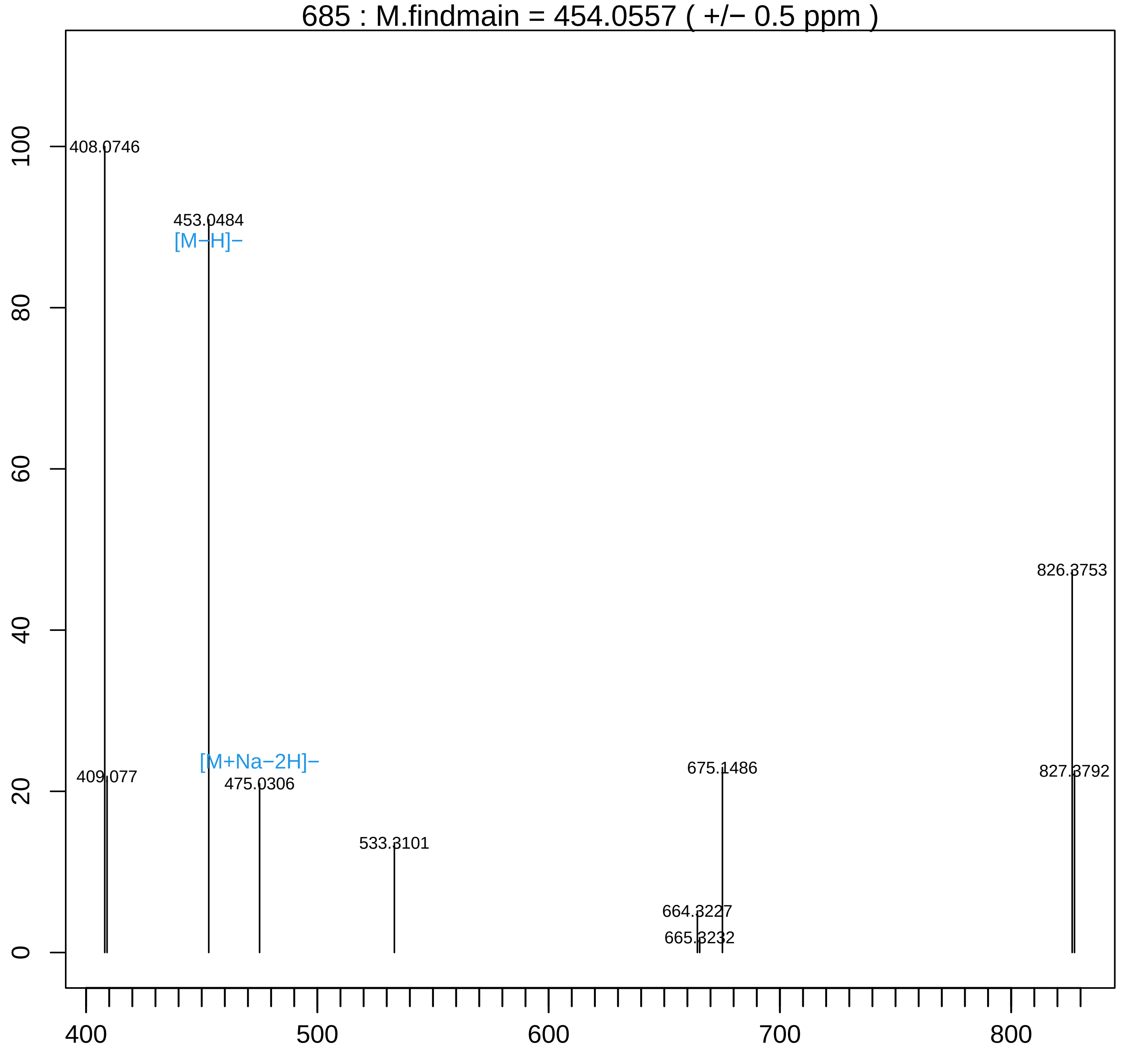
685 : M.findmain = 454.0557 (+/− 0.5 ppm)
408.0746
453.0484
[M−H]−
409.077
[M+Na−2H]−
475.0306
533.3101
664.3227
665.3232
675.1486
826.3753
827.3792
0
20
40
60
80
100
400
500
600
700
800

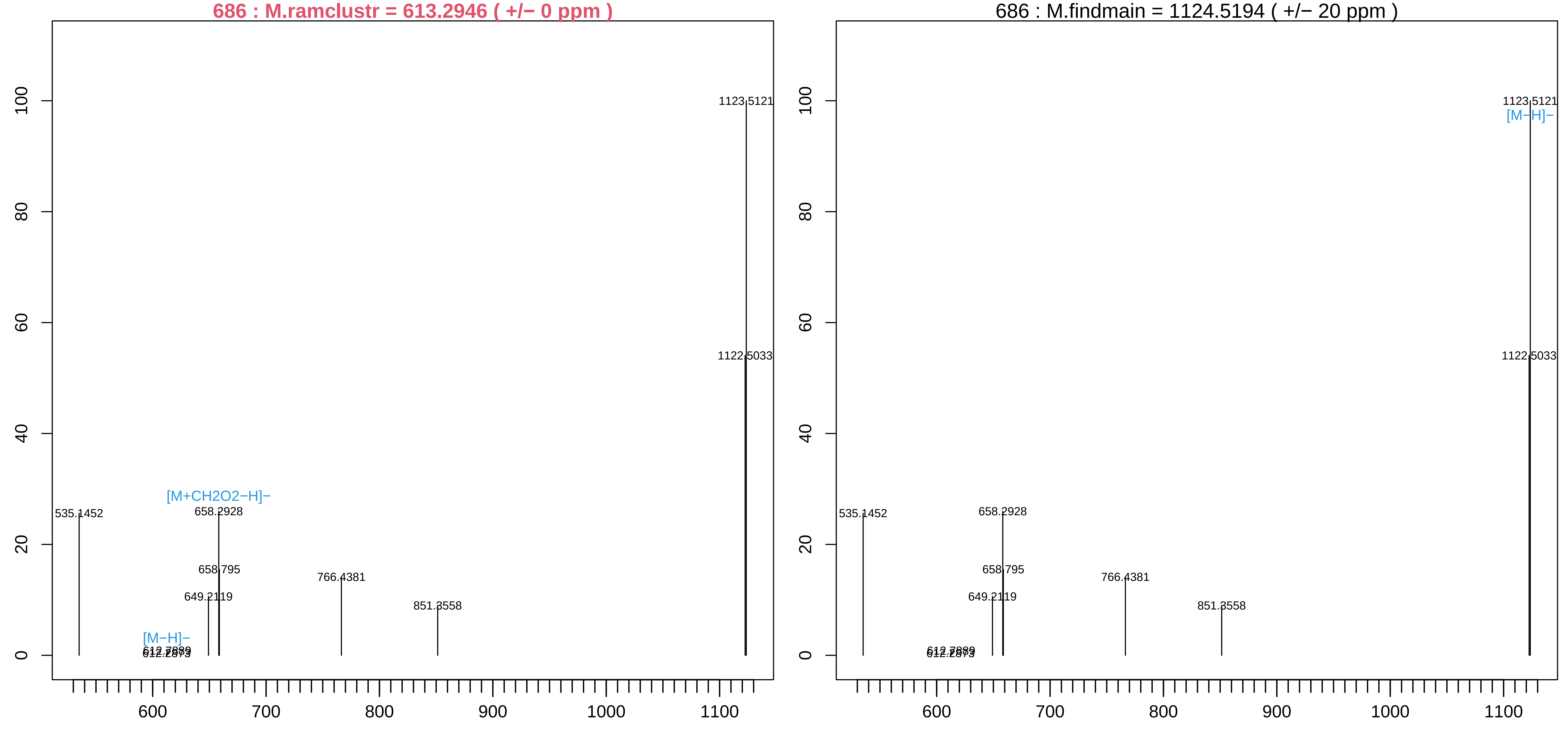
686 : M.ramclustr = 613.2946 (+/− 0 ppm)
[M+CH2O2−H]−
[M−H]−
535.1452
658.2928
658.795
649.2119
612.7889
612.2873
766.4381
851.3558
1123.5121
1122.5033
686 : M.findmain = 1124.5194 (+/− 20 ppm)
[M−H]−
0
20
40
60
80
100
600
700
800
900
1000
1100

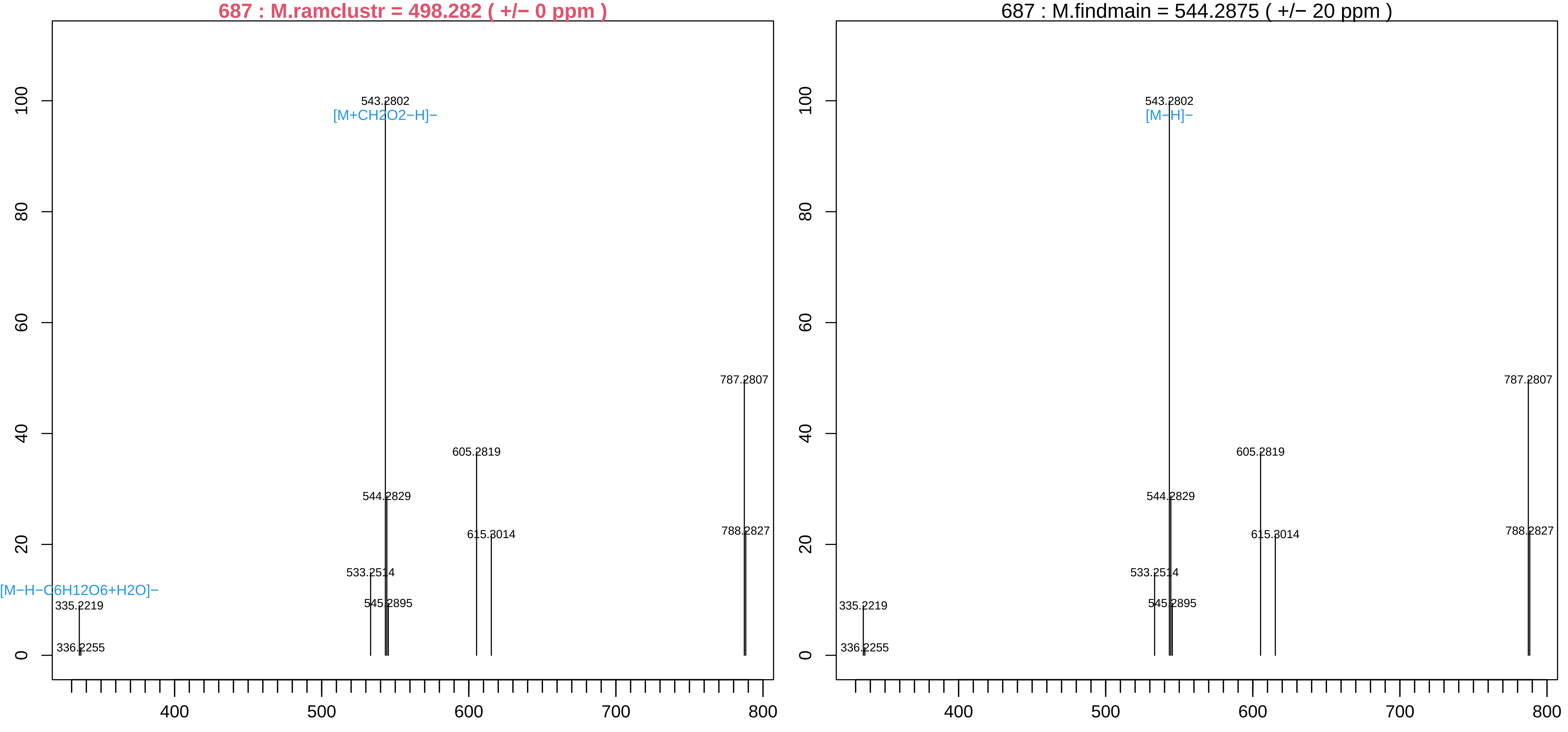

687 : M.ramclustr = 498.282 (+/− 0 ppm)
543.2802
[M+CH2O2−H]−
787.2807
605.2819
544.2829
615.3014
788.2827
533.2514
[M−H−C6H12O6+H2O]−
545.2895
335.2219
336.2255
687 : M.findmain = 544.2875 (+/− 20 ppm)
543.2802
[M−H]−
787.2807
605.2819
544.2829
615.3014
788.2827
533.2514
545.2895
335.2219
336.2255
0
20
40
60
80
100
400
500
600
700
800

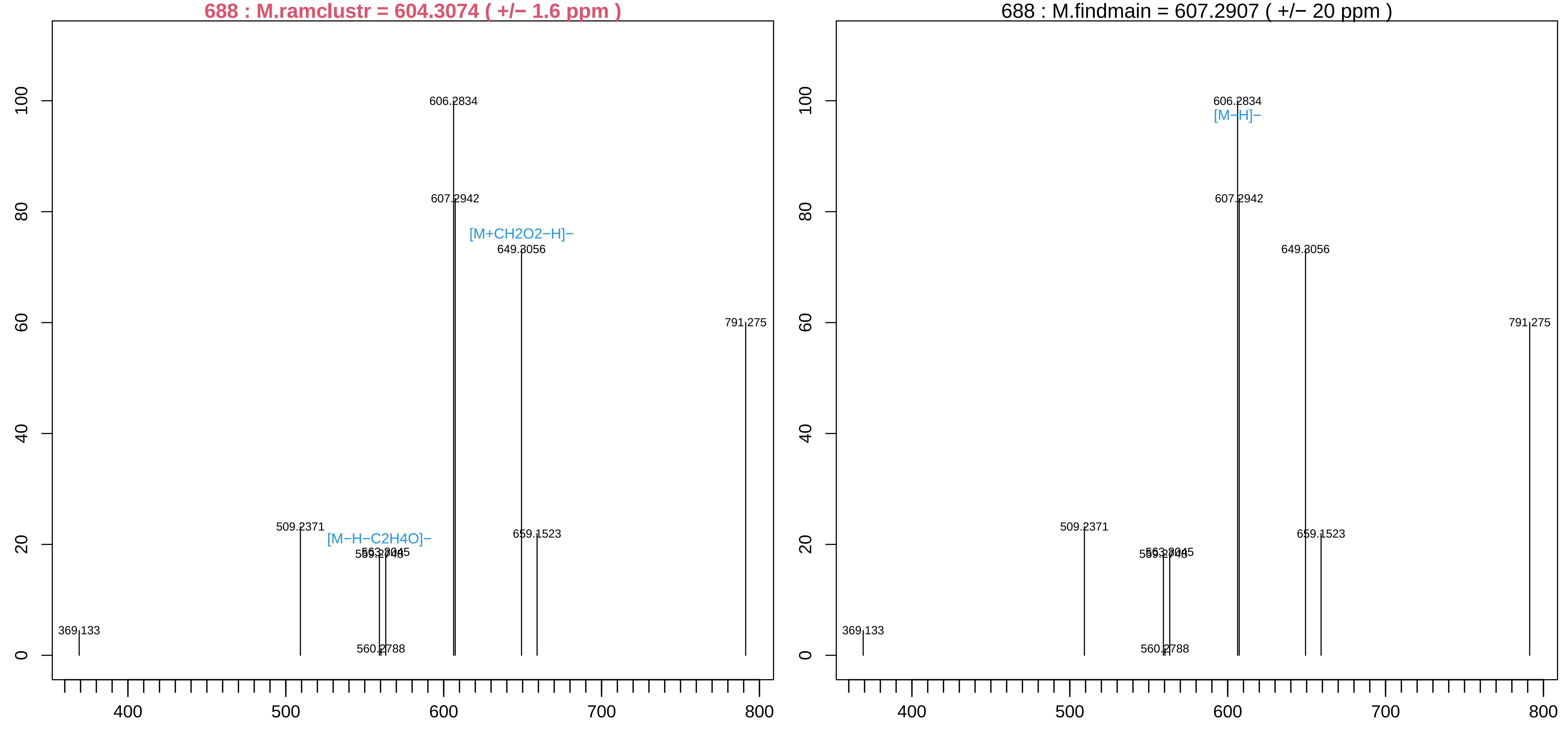
688 : M.ramclustr = 604.3074 (+/− 1.6 ppm)
606.2834
607.2942
[M+CH2O2−H]−
649.3056
791.275
509.2371
[M−H−C2H4O]−
563.3045
559.2746
659.1523
369.133
560.2788
688 : M.findmain = 607.2907 (+/− 20 ppm)
606.2834
[M−H]−
607.2942
649.3056
791.275
509.2371
563.3045
559.2746
659.1523
369.133
560.2788
0
20
40
60
80
100
400
500
600
700
800

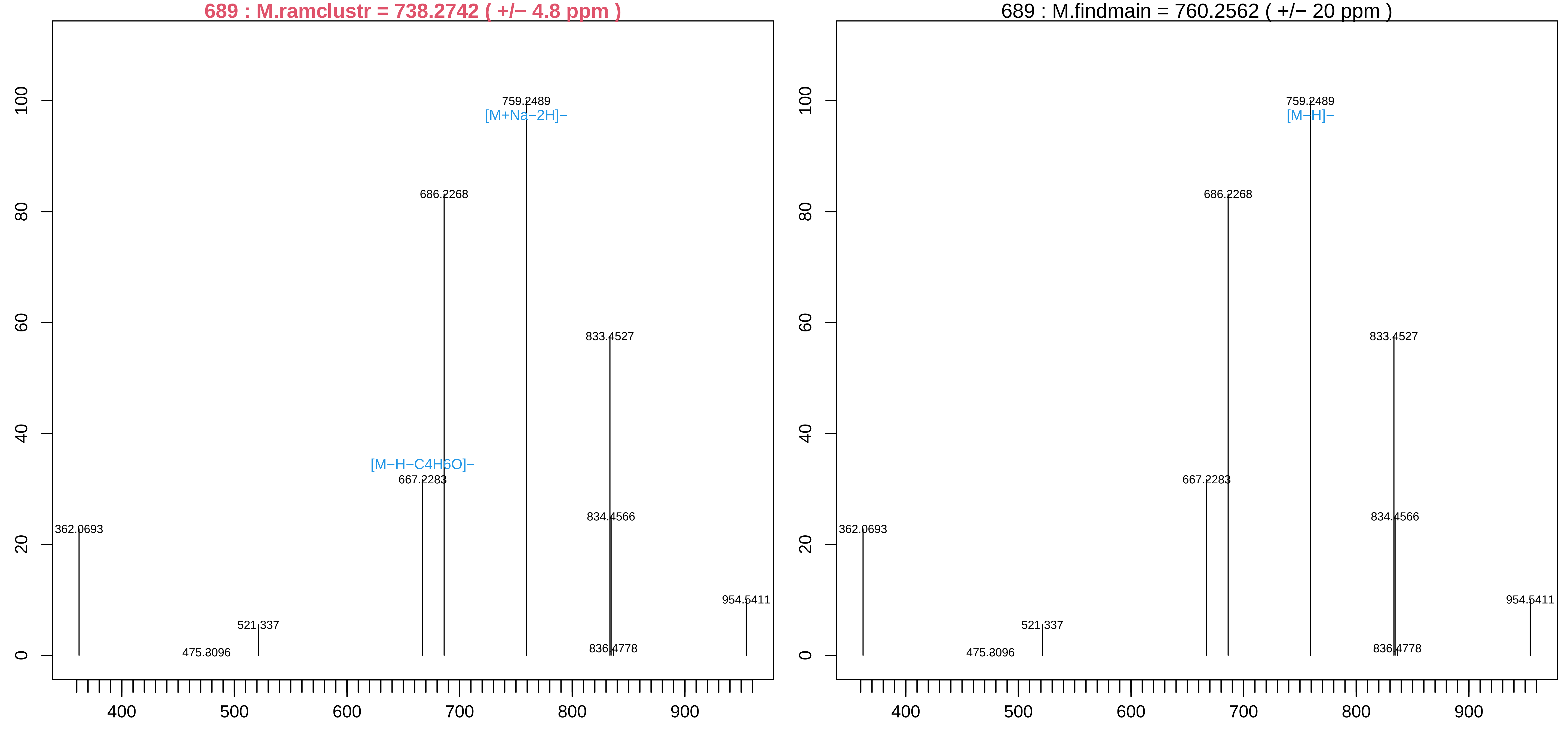

689 : M.ramclustr = 738.2742 (+/− 4.8 ppm)
759.2489
[M+Na−2H]−
686.2268
833.4527
[M−H−C4H6O]−
667.2283
362.0693
834.4566
954.6411
521.337
475.3096
836.4778
0
20
40
60
80
100
400
500
600
700
800
900
689 : M.findmain = 760.2562 (+/− 20 ppm)
759.2489
[M−H]−
686.2268
833.4527
667.2283
362.0693
834.4566
954.6411
521.337
475.3096
836.4778

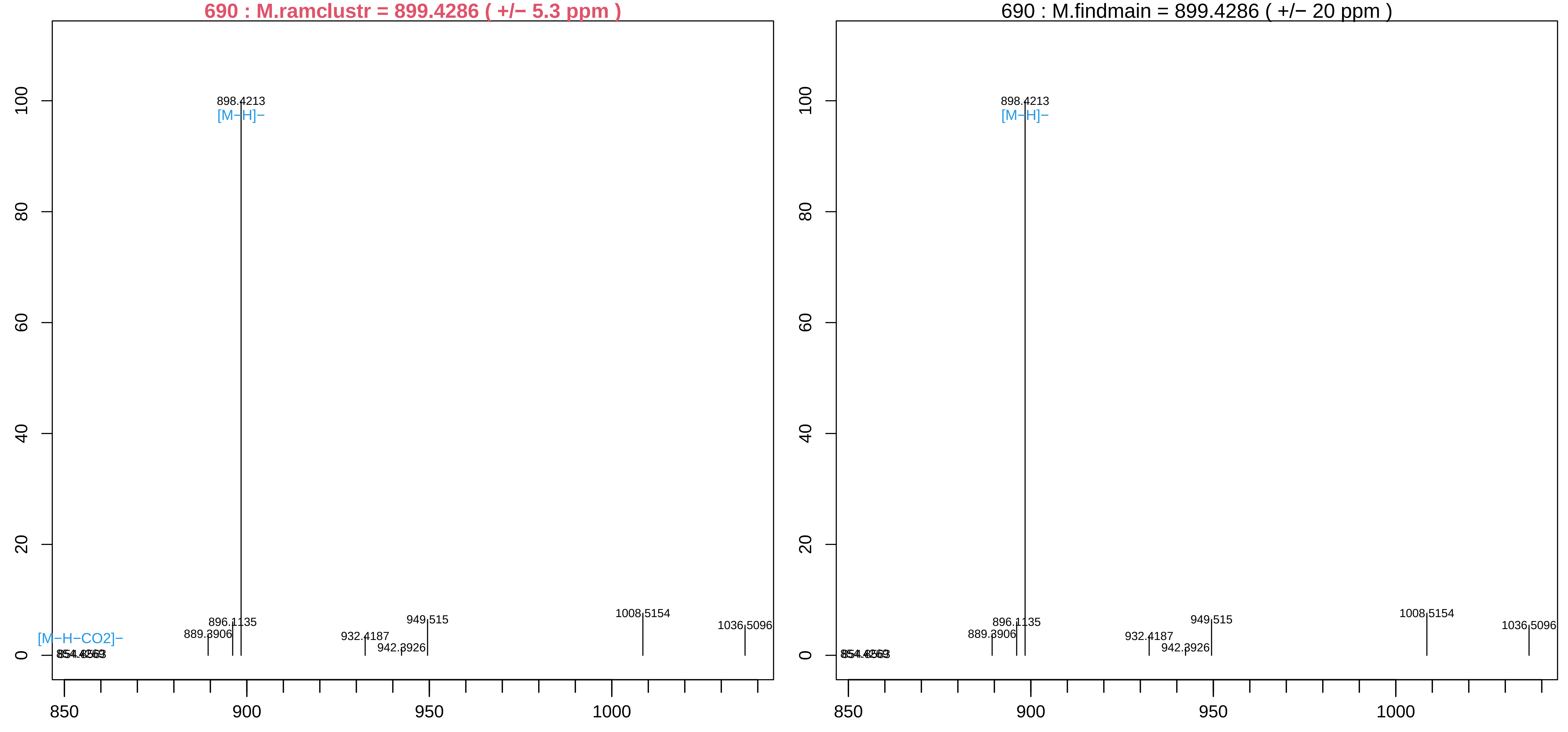
690 : M.ramclustr = 899.4286 (+/− 5.3 ppm)
898.4213
[M−H]−
[M−H−CO2]−
889.3906
896.1135
932.4187
942.3926
949.515
1008.5154
1036.5096
690 : M.findmain = 899.4286 (+/− 20 ppm)
898.4213
[M−H]−
889.3906
896.1135
932.4187
942.3926
949.515
1008.5154
1036.5096
0
20
40
60
80
100
850
900
950
1000

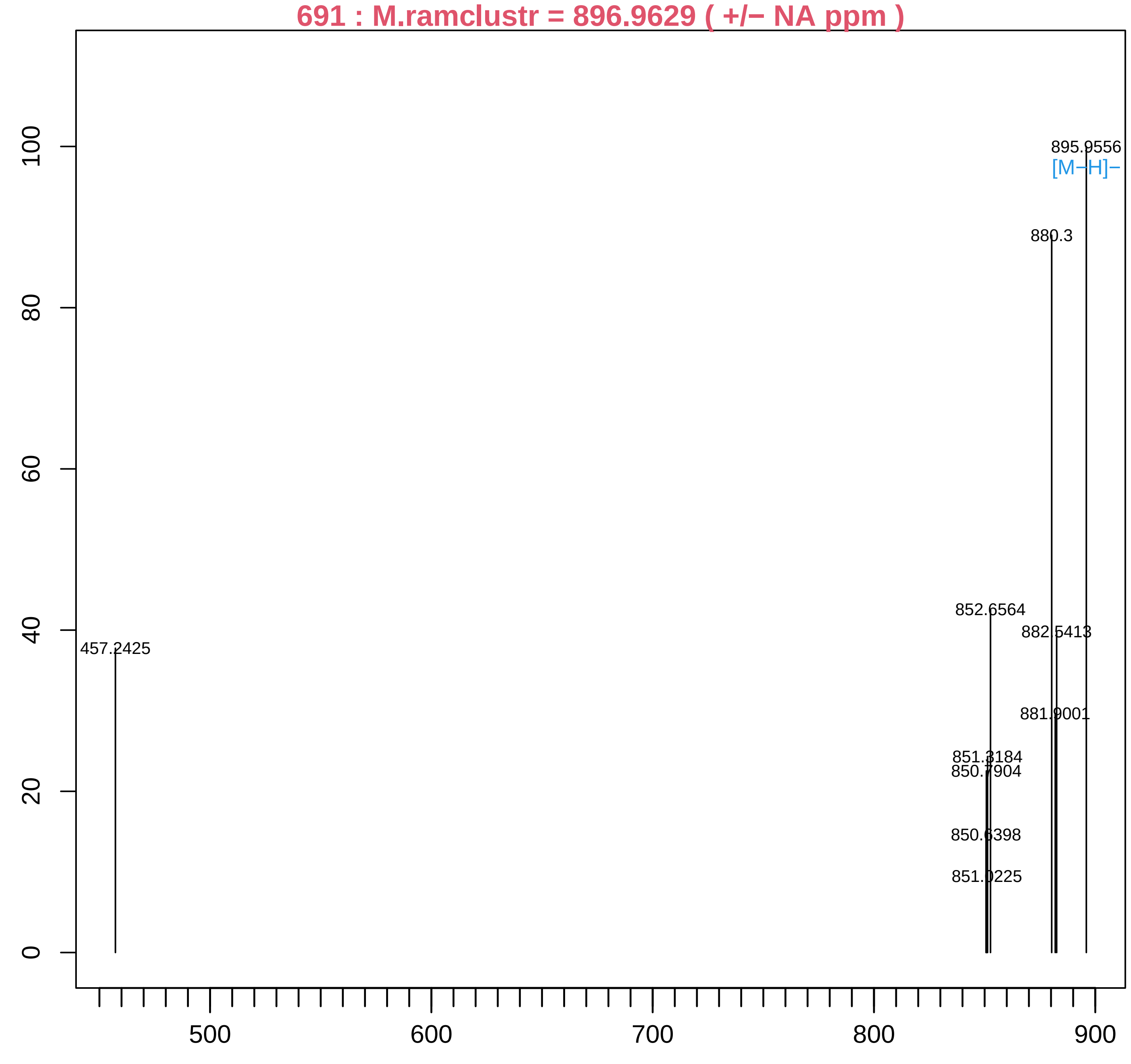
691 : M.ramclustr = 896.9629 (+/− NA ppm)
0
20
40
60
80
100
500
600
700
800
900
457.2425
852.6564
882.5413
881.9001
851.3184
850.7904
850.6398
851.0225
880.3
895.9556
[M−H]−

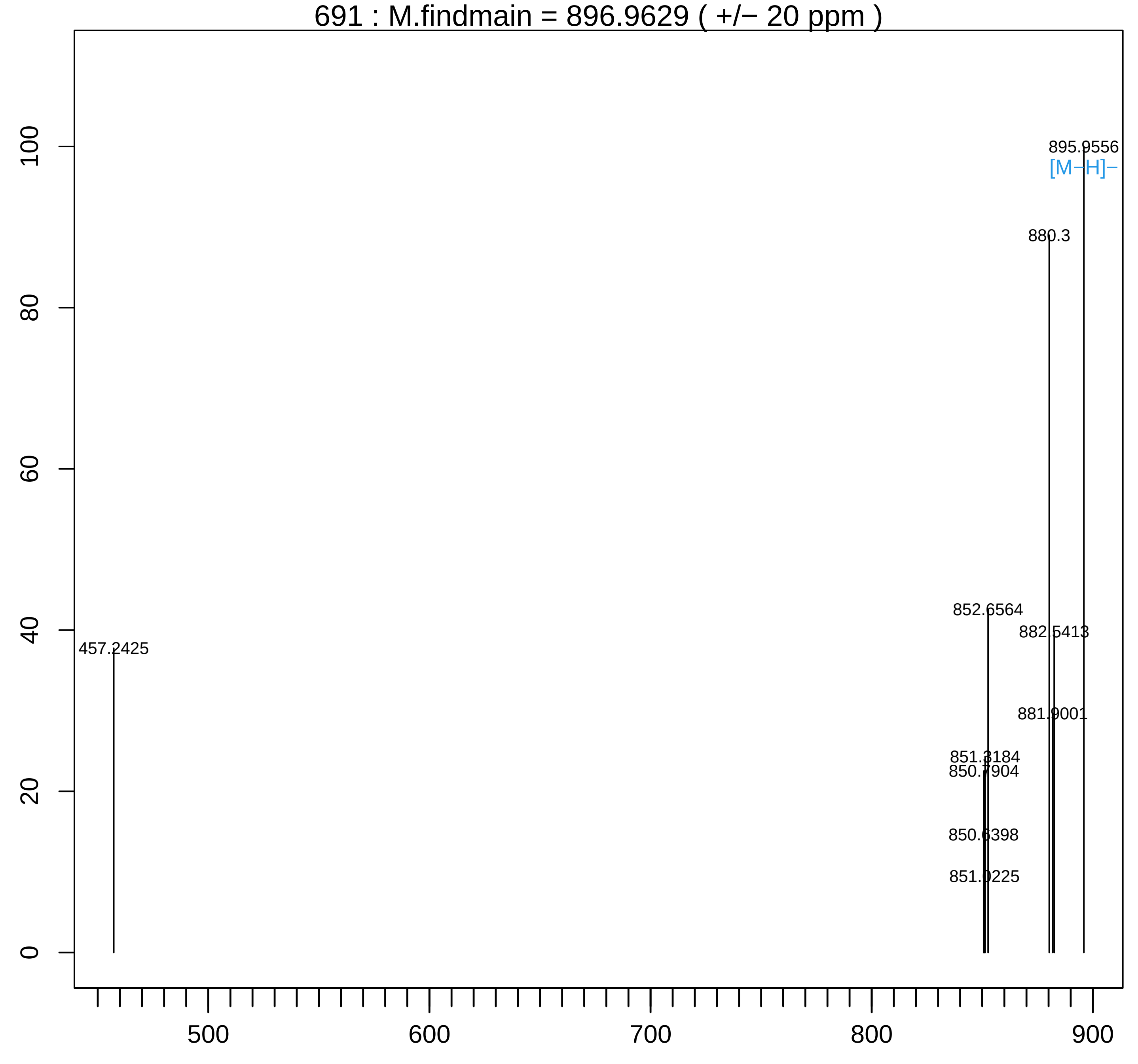
691 : M.findmain = 896.9629 (+/− 20 ppm)
0
20
40
60
80
100
500
600
700
800
900
457.2425
852.6564
882.5413
881.9001
851.3184
850.7904
850.6398
851.0225
880.3
895.9556
[M−H]−

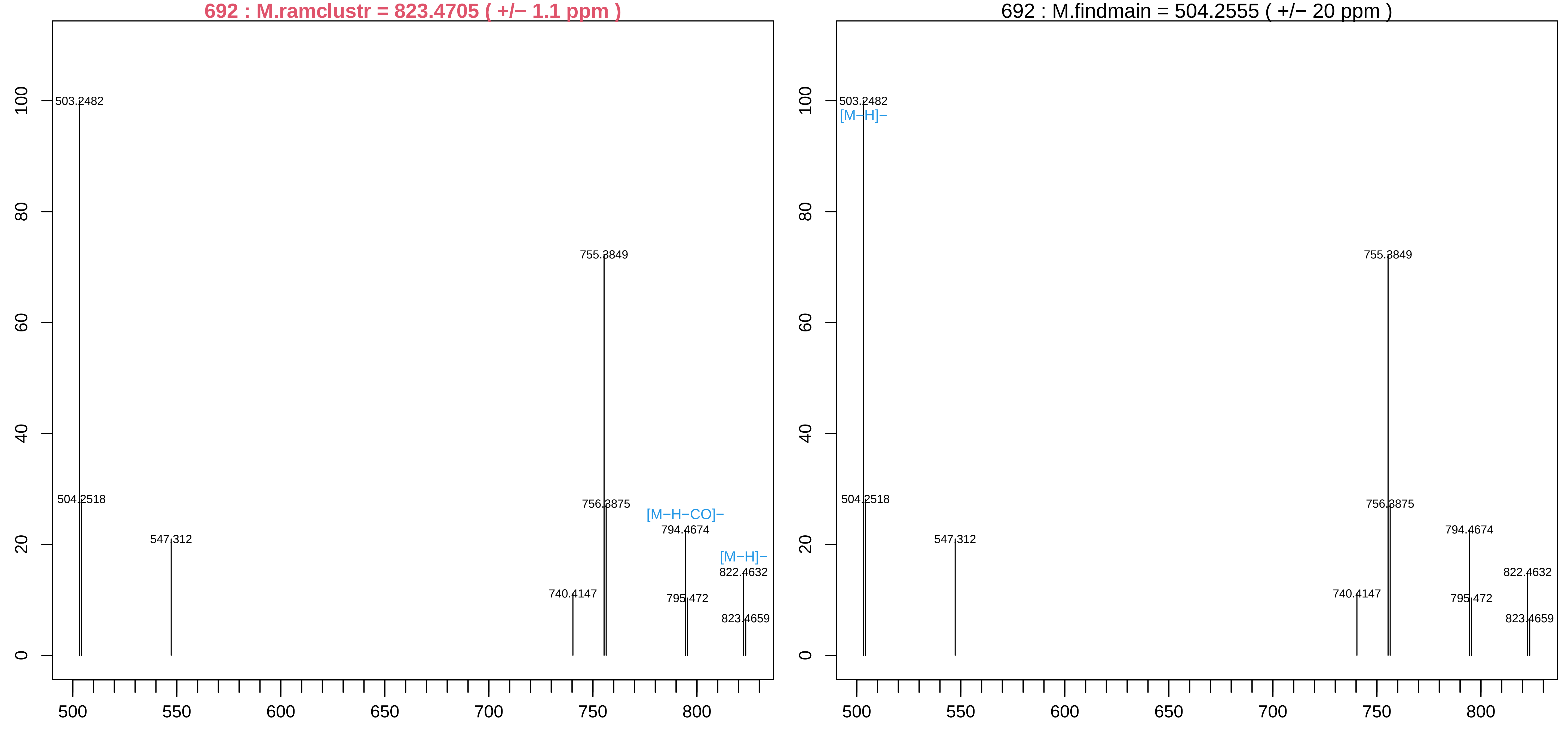
692 : M.ramclustr = 823.4705 (+/− 1.1 ppm)
503.2482
504.2518
547.312
740.4147
755.3849
756.3875
[M−H−CO]−
794.4674
795.472
[M−H]−
822.4632
823.4659
692 : M.findmain = 504.2555 (+/− 20 ppm)
503.2482
[M−H]−
504.2518
547.312
740.4147
755.3849
756.3875
794.4674
795.472
822.4632
823.4659

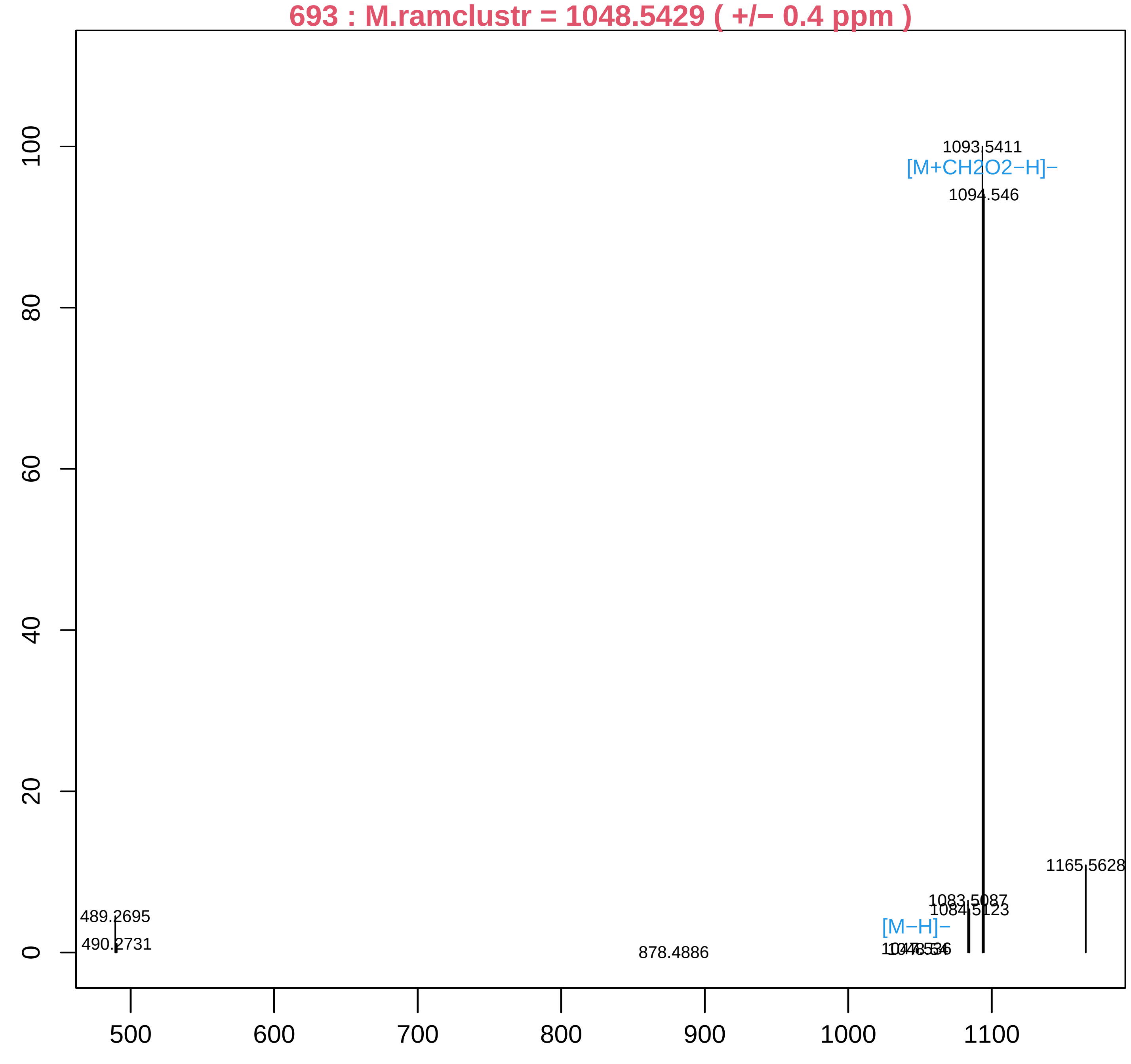
693 : M.ramclustr = 1048.5429 (+/− 0.4 ppm)
1093.5411
[M+CH2O2−H]−
1094.546
1165.5628
1083.5087
1084.5123
489.2695
[M−H]−
490.2731
878.4886
0
20
40
60
80
100
500
600
700
800
900
1000
1100

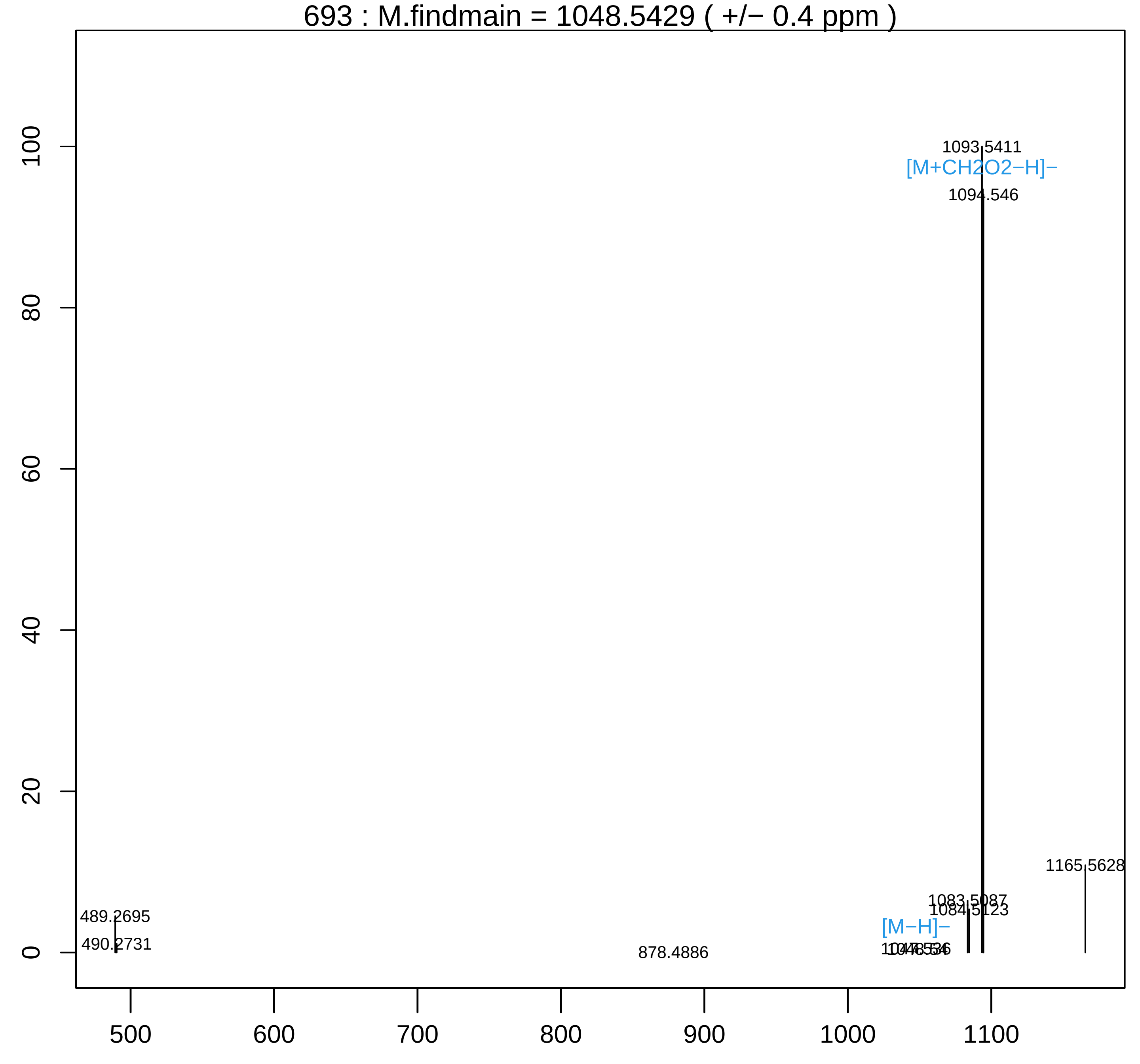
693 : M.findmain = 1048.5429 (+/− 0.4 ppm)
1093.5411
[M+CH2O2−H]−
1094.546
1165.5628
1083.5087
1084.5123
489.2695
[M−H]−
490.2731
878.4886
0
20
40
60
80
100
500
600
700
800
900
1000
1100

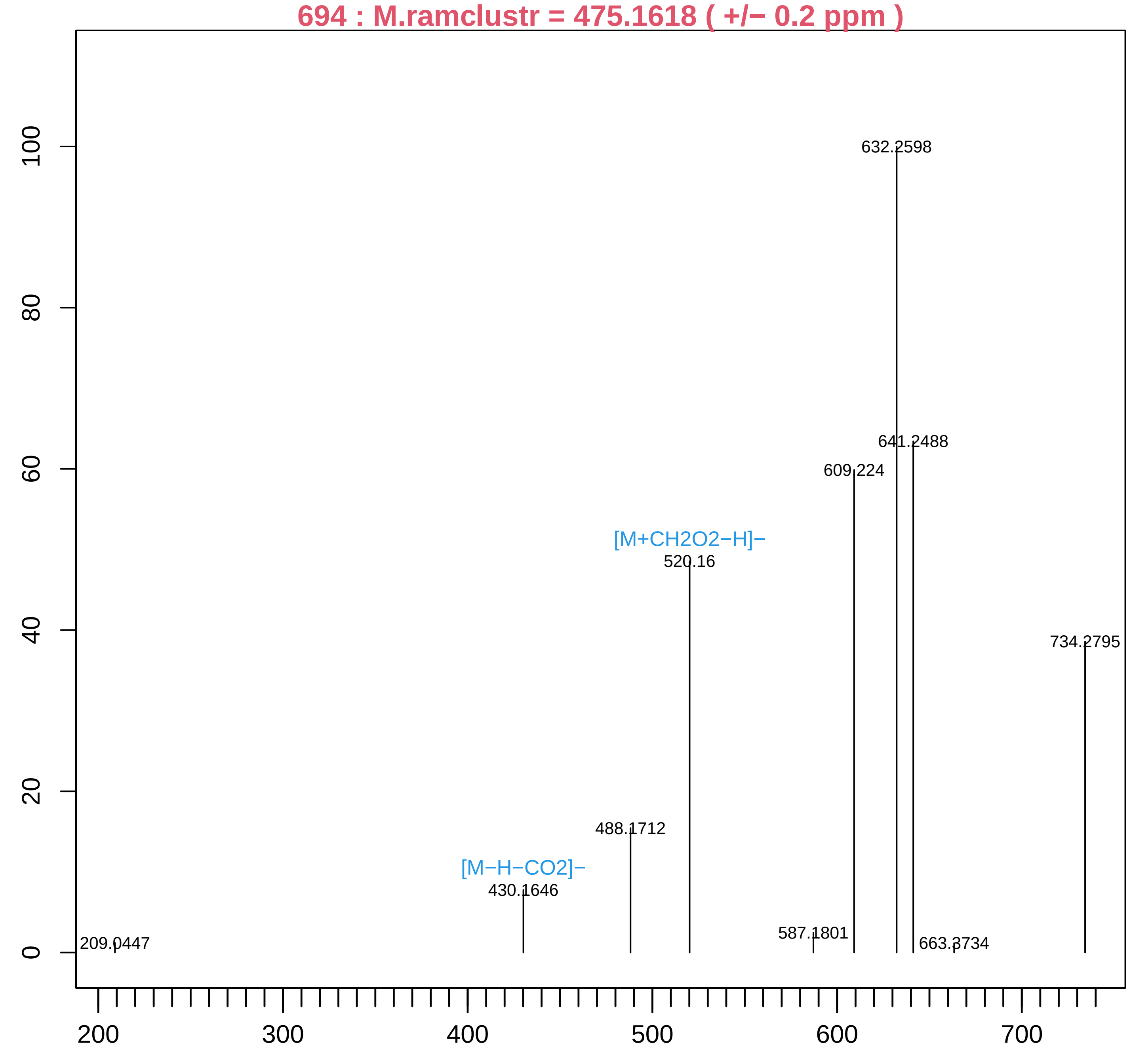
694 : M.ramclustr = 475.1618 (+/− 0.2 ppm)
632.2598
641.2488
609.224
[M+CH2O2−H]−
520.16
734.2795
488.1712
[M−H−CO2]−
430.1646
587.1801
209.0447
663.3734
0
20
40
60
80
100
200
300
400
500
600
700

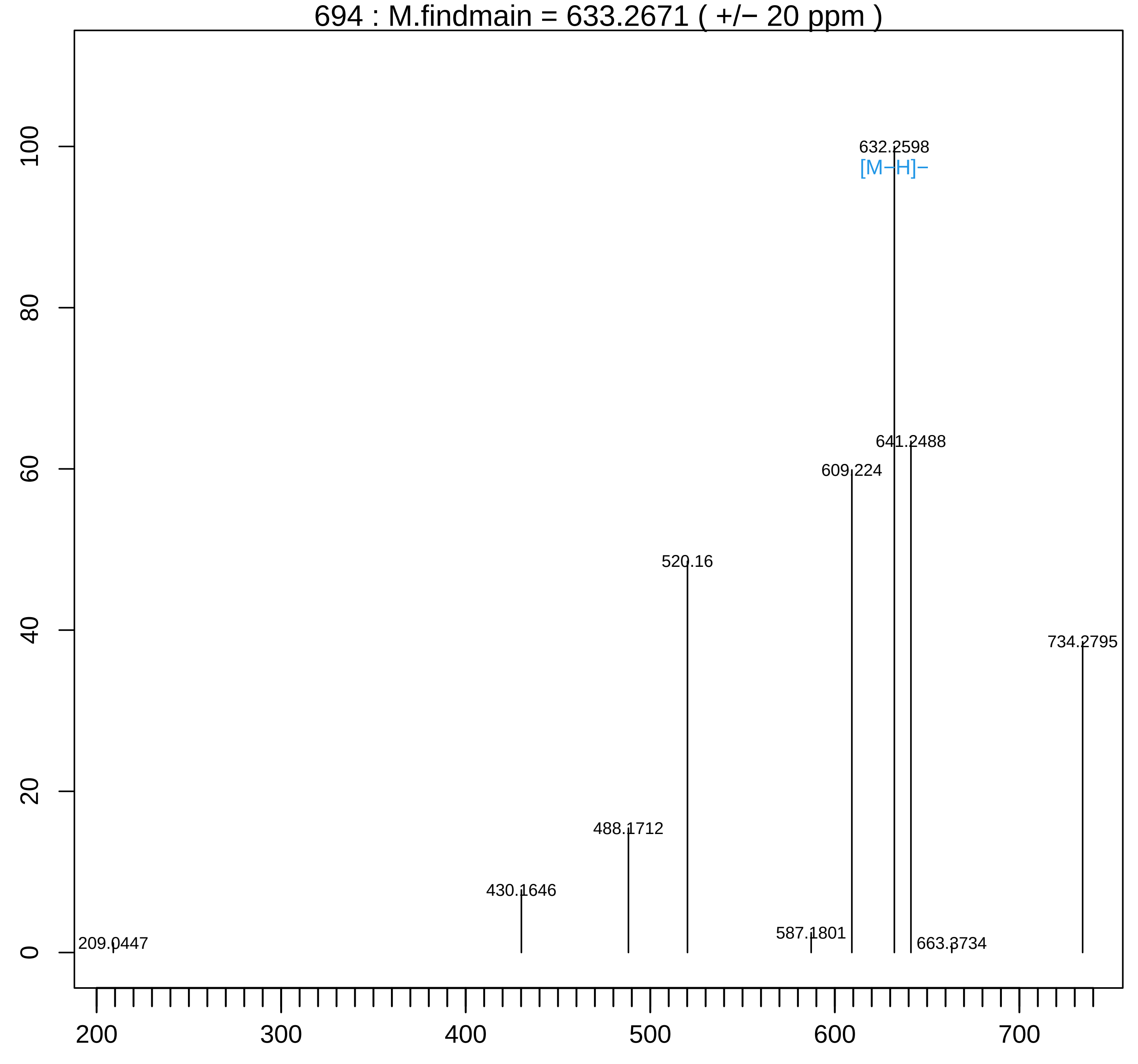
694 : M.findmain = 633.2671 (+/− 20 ppm)
632.2598
[M−H]−
641.2488
609.224
520.16
734.2795
488.1712
430.1646
587.1801
209.0447
663.3734
0
20
40
60
80
100
200
300
400
500
600
700

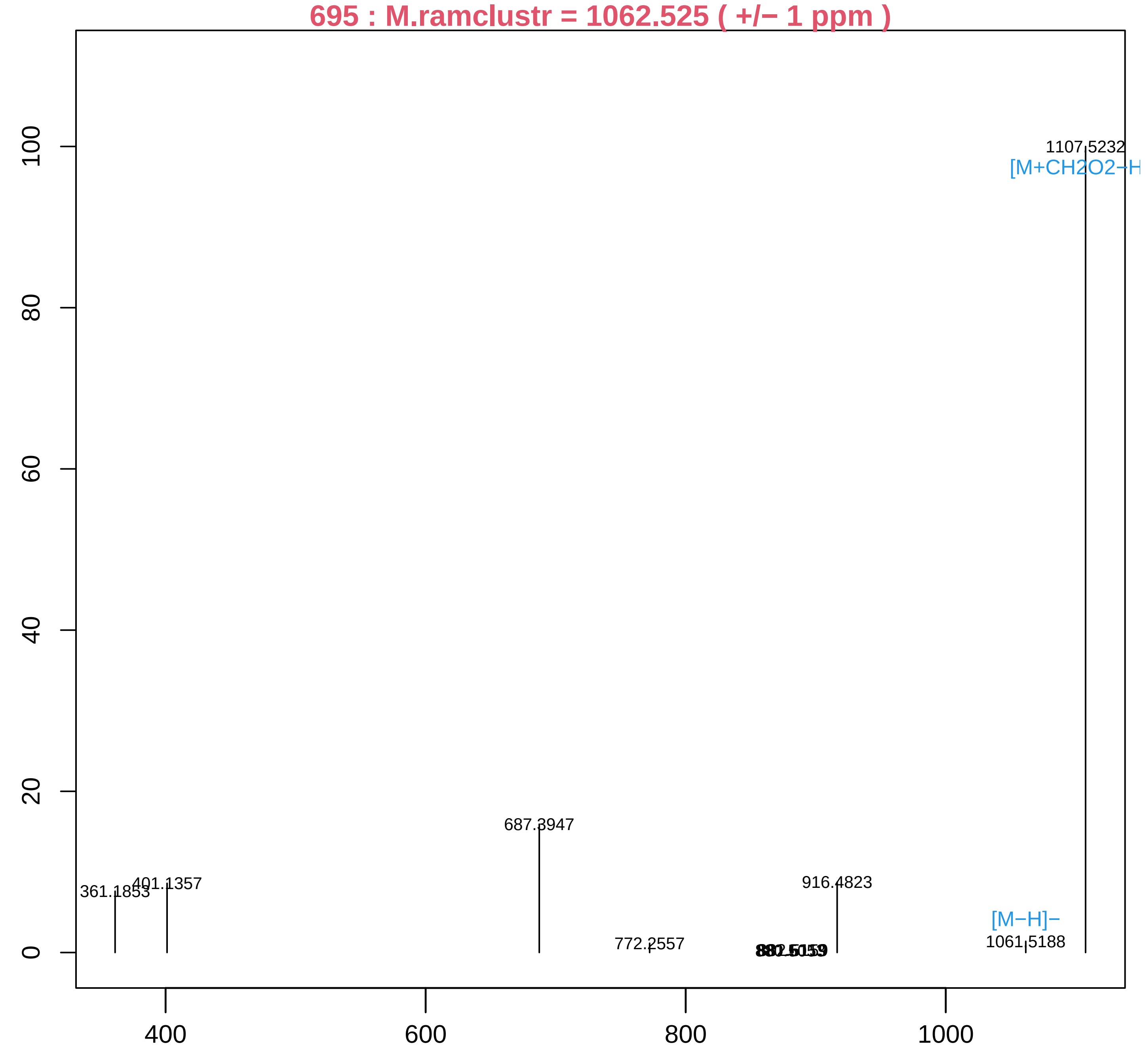
695 : M.ramclustr = 1062.525 (+/− 1 ppm)
1107.5232
[M+CH2O2−H
687.3947
361.1853
401.1357
916.4823
772.2557
[M−H]−
1061.5188
0
20
40
60
80
100
400
600
800
1000

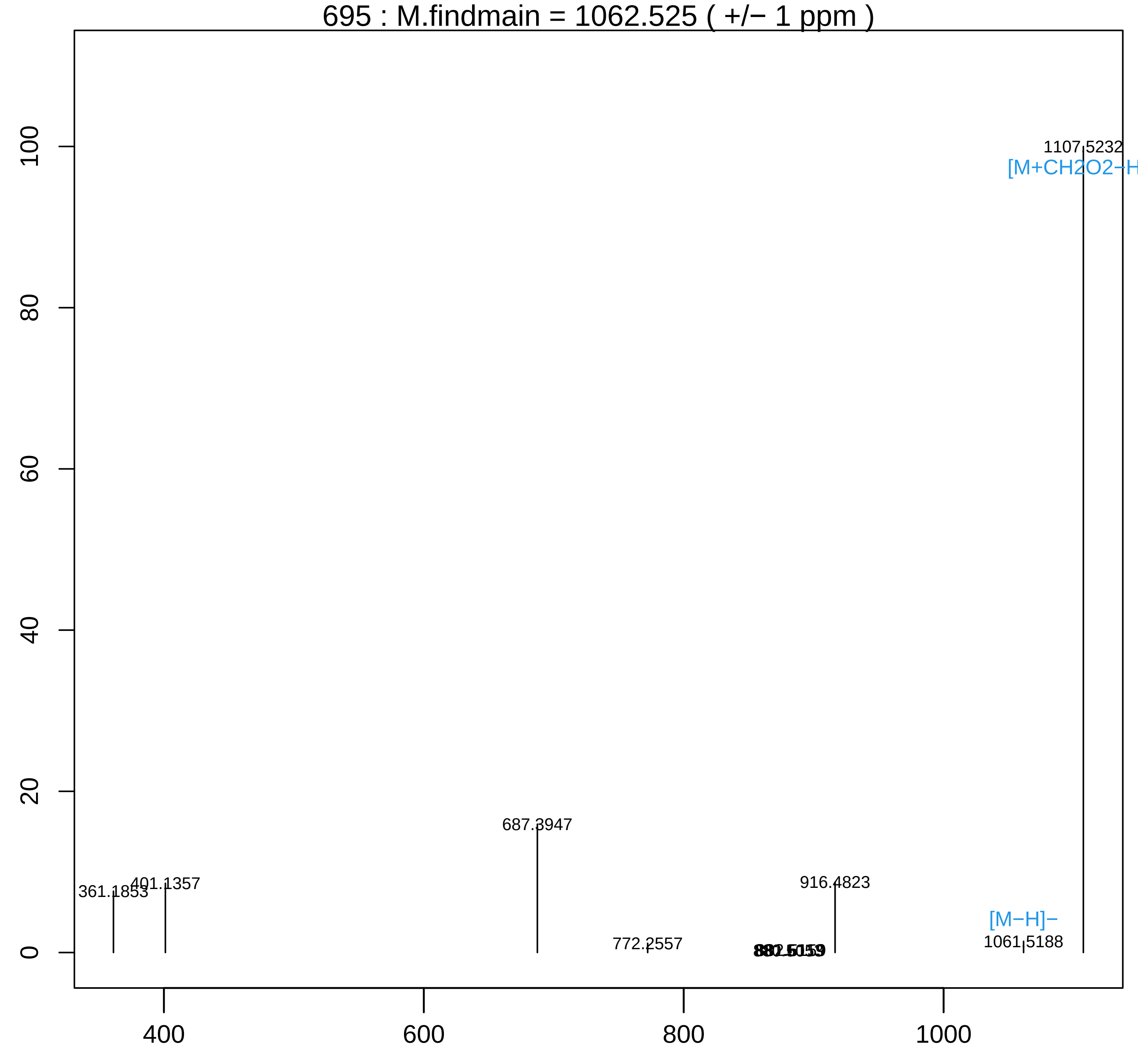
695 : M.findmain = 1062.525 (+/− 1 ppm)
1107.5232
[M+CH2O2−H
687.3947
361.1853
401.1357
916.4823
772.2557
[M−H]−
1061.5188
0
20
40
60
80
100
400
600
800
1000

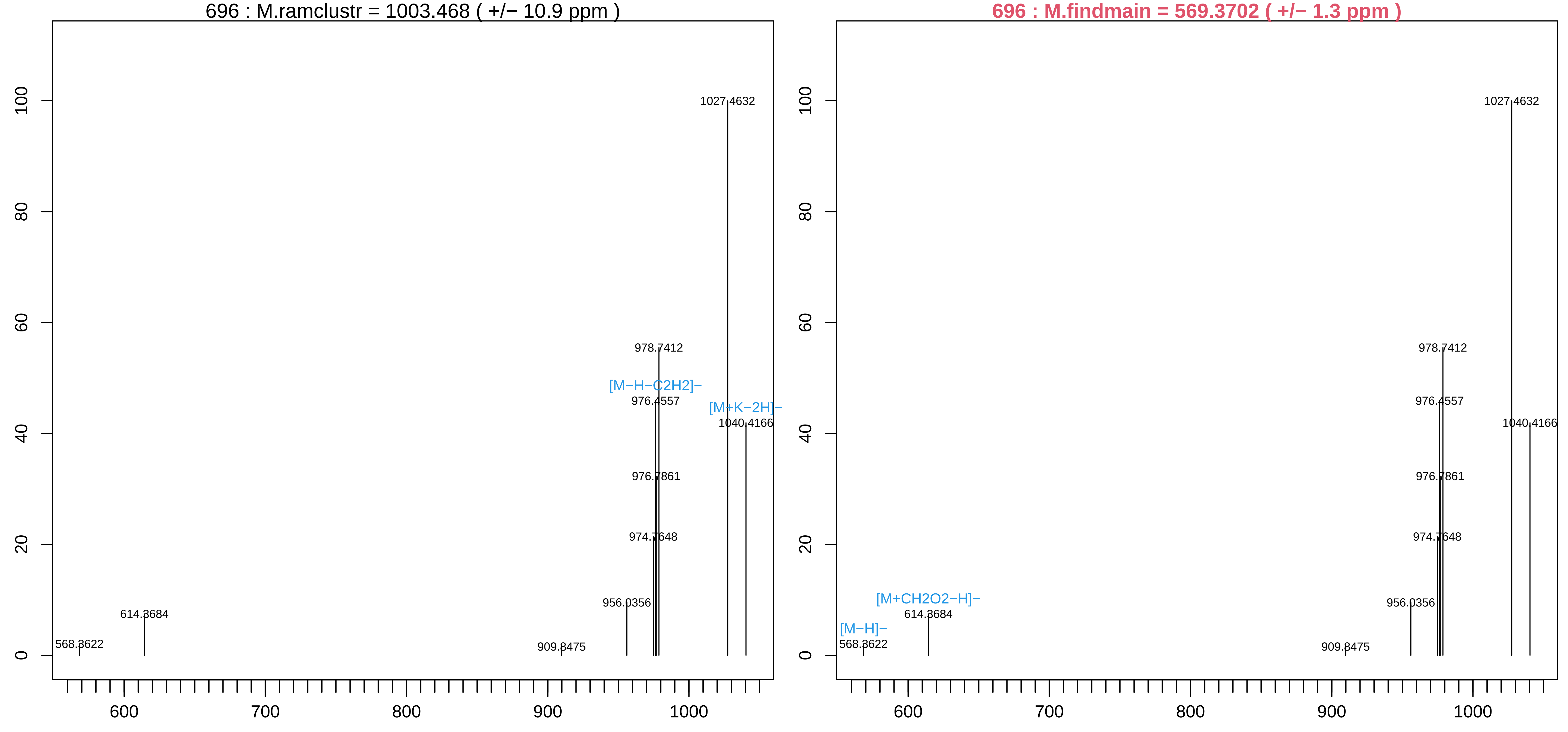
696 : M.ramclustr = 1003.468 (+/− 10.9 ppm)
1027.4632
978.7412
[M−H−C2H2]−
976.4557
[M+K−2H]−
1040.4166
976.7861
974.7648
956.0356
614.3684
568.3622
909.8475
0
20
40
60
80
100
600
700
800
900
1000
696 : M.findmain = 569.3702 (+/− 1.3 ppm)
1027.4632
978.7412
976.4557
1040.4166
976.7861
974.7648
[M+CH2O2−H]−
956.0356
614.3684
[M−H]−
568.3622
909.8475

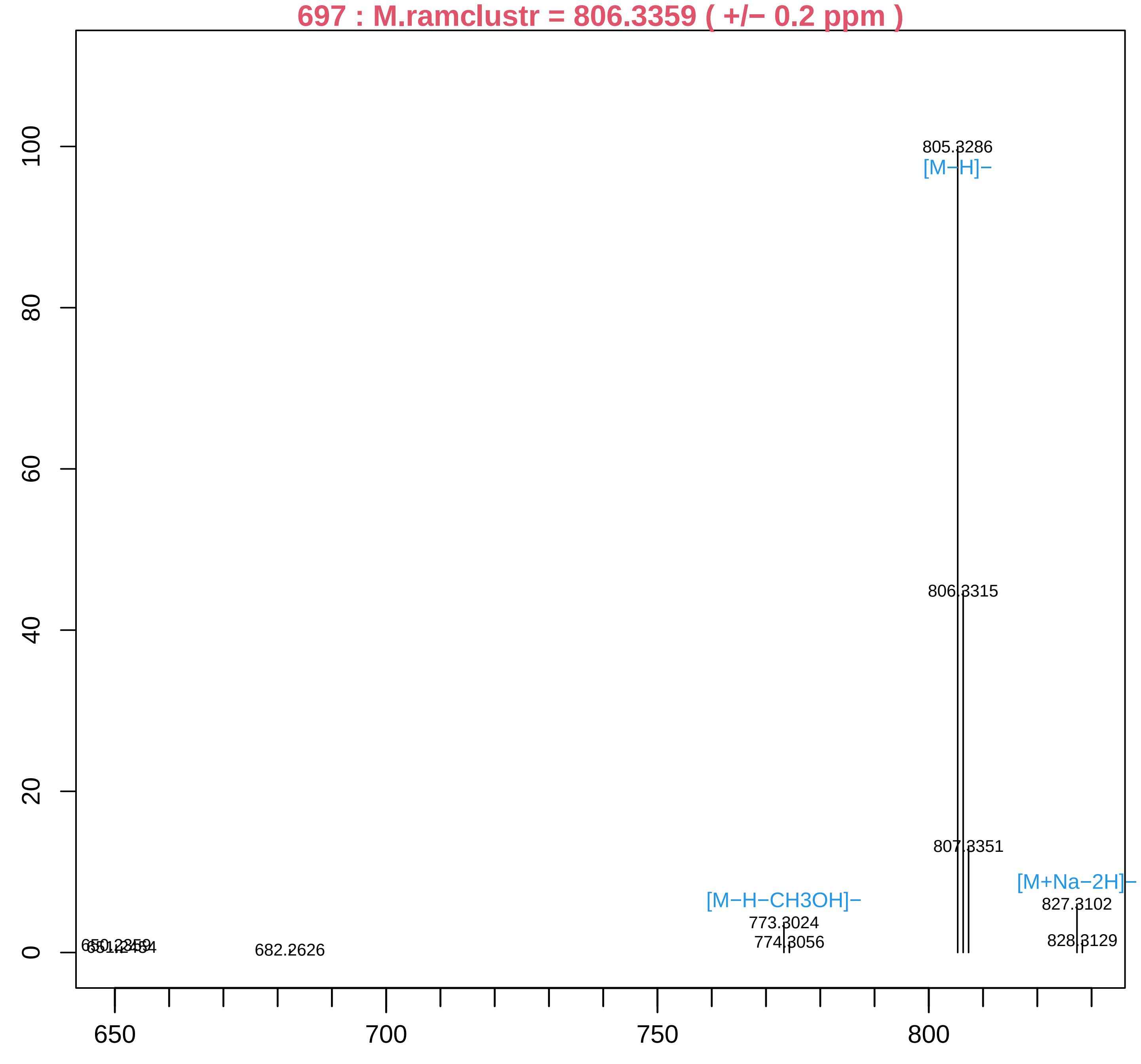
697 : M.ramclustr = 806.3359 (+/− 0.2 ppm)
805.3286
[M−H]−
806.3315
807.3351
[M+Na−2H]−
827.3102
828.3129
[M−H−CH3OH]−
773.3024
774.3056
682.2626
650.2259
651.2454
0
20
40
60
80
100
650
700
750
800

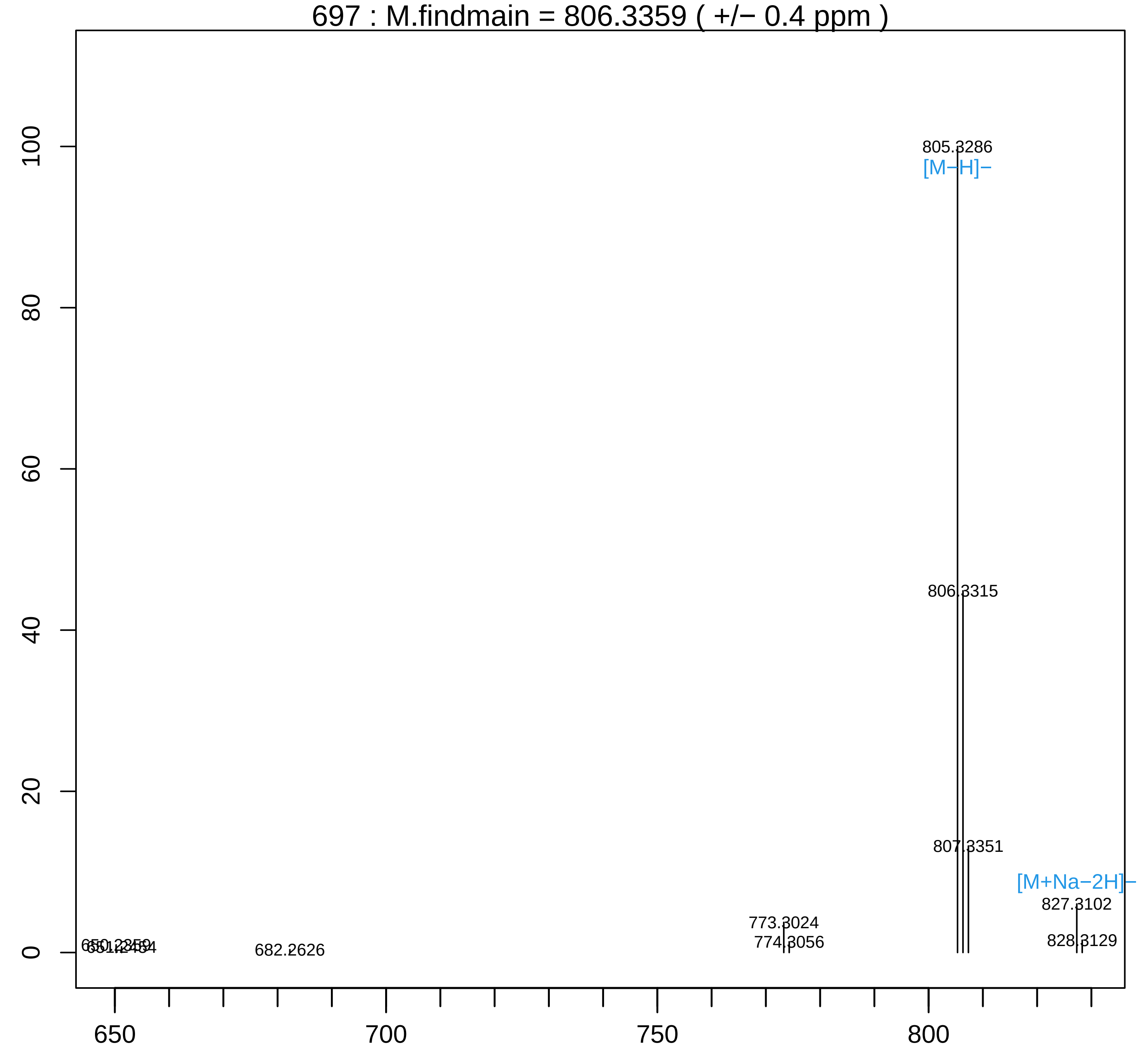
697 : M.findmain = 806.3359 (+/− 0.4 ppm)
805.3286
[M−H]−
806.3315
807.3351
[M+Na−2H]−
827.3102
828.3129
773.3024
774.3056
682.2626
650.2259
651.2454
0
20
40
60
80
100
650
700
750
800

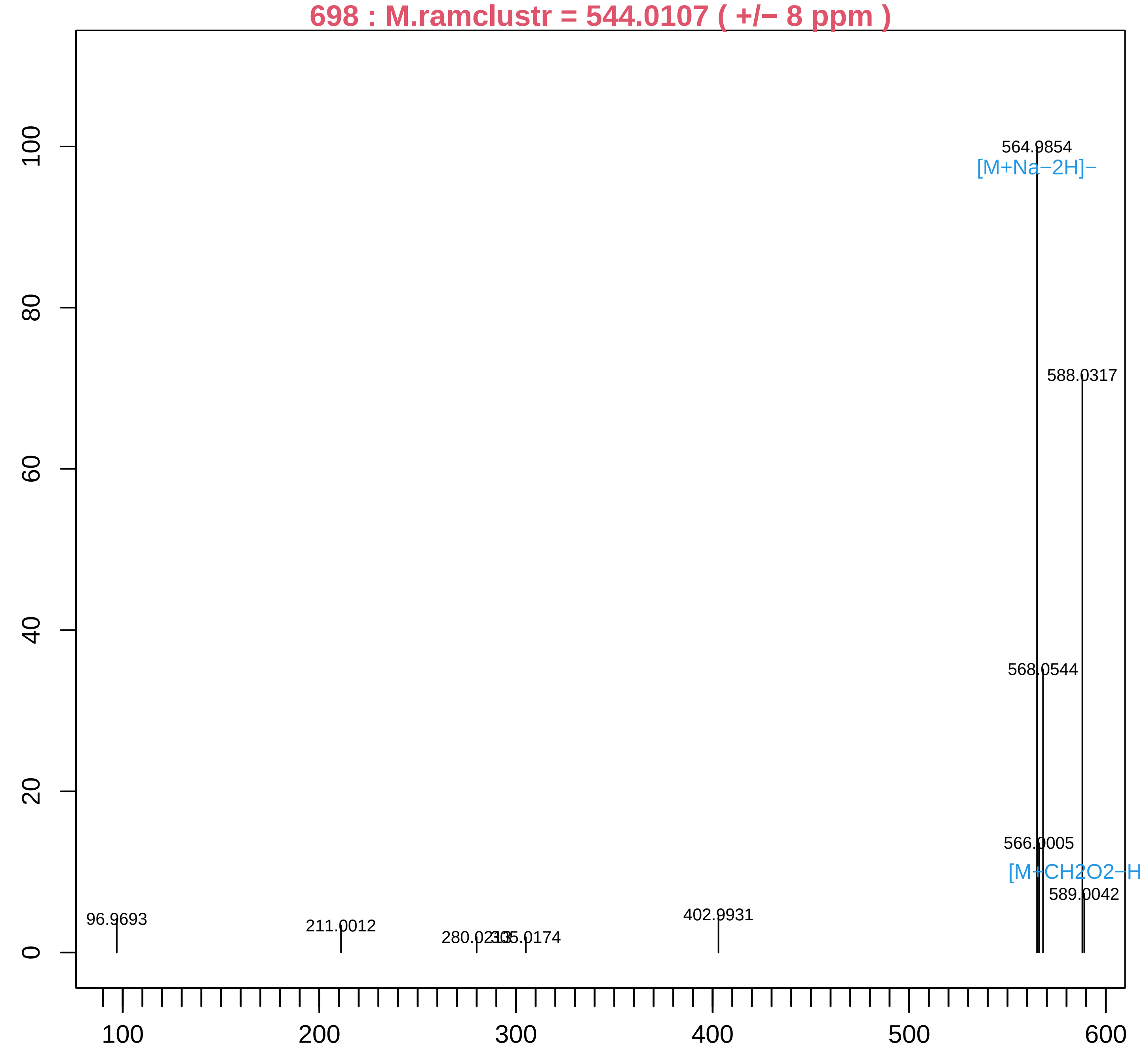
698 : M.ramclustr = 544.0107 (+/− 8 ppm)
564.9854
[M+Na−2H]−
588.0317
568.0544
566.0005
[M+CH2O2−H
589.0042
96.9693
211.0012
280.0233
305.0174
402.9931
0
20
40
60
80
100
100
200
300
400
500
600

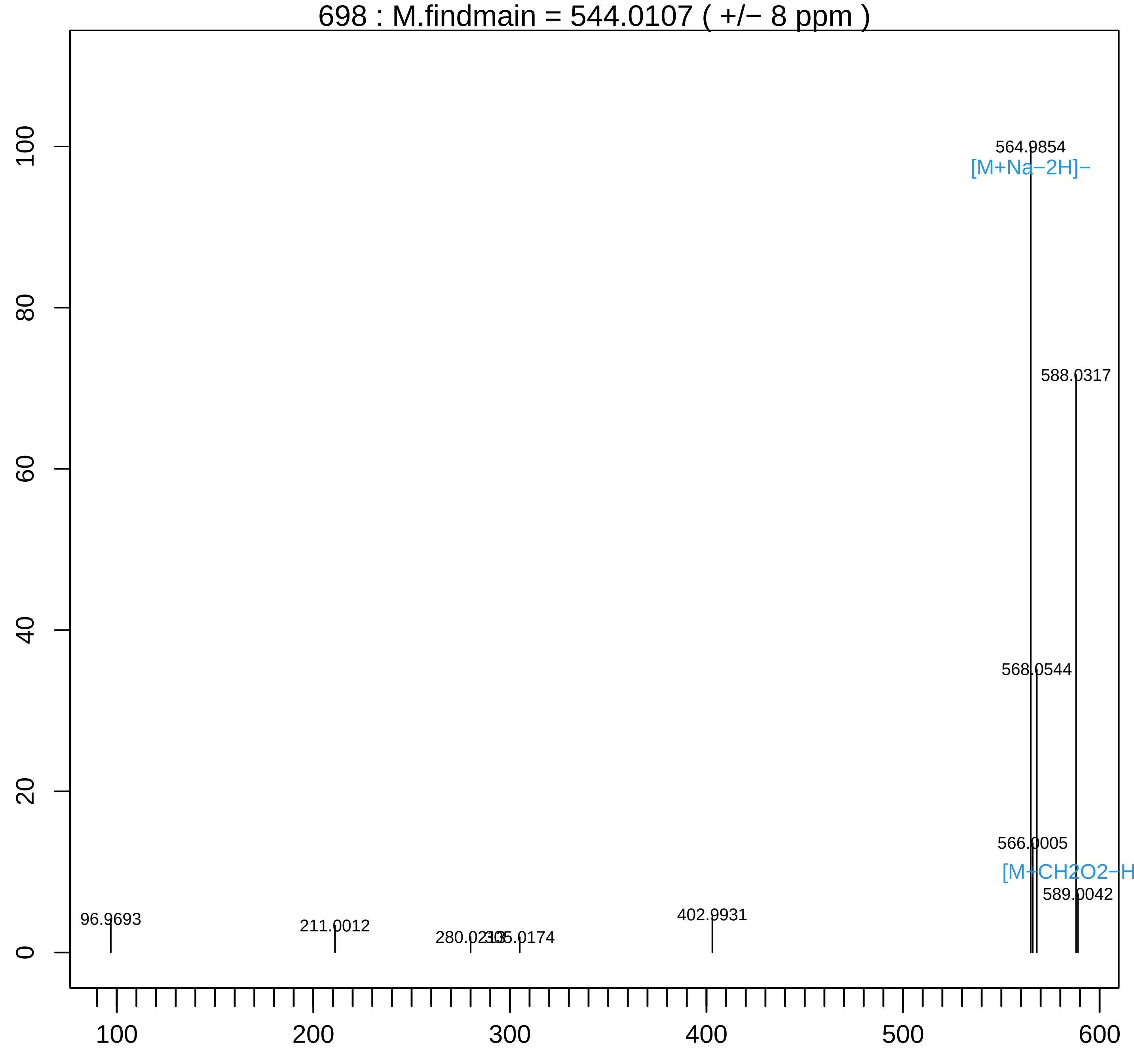
698 : M.findmain = 544.0107 (+/− 8 ppm)
564.9854
[M+Na−2H]−
588.0317
568.0544
566.0005
[M+CH2O2−H
589.0042
96.9693
211.0012
280.0233
305.0174
402.9931
0
20
40
60
80
100
100
200
300
400
500
600

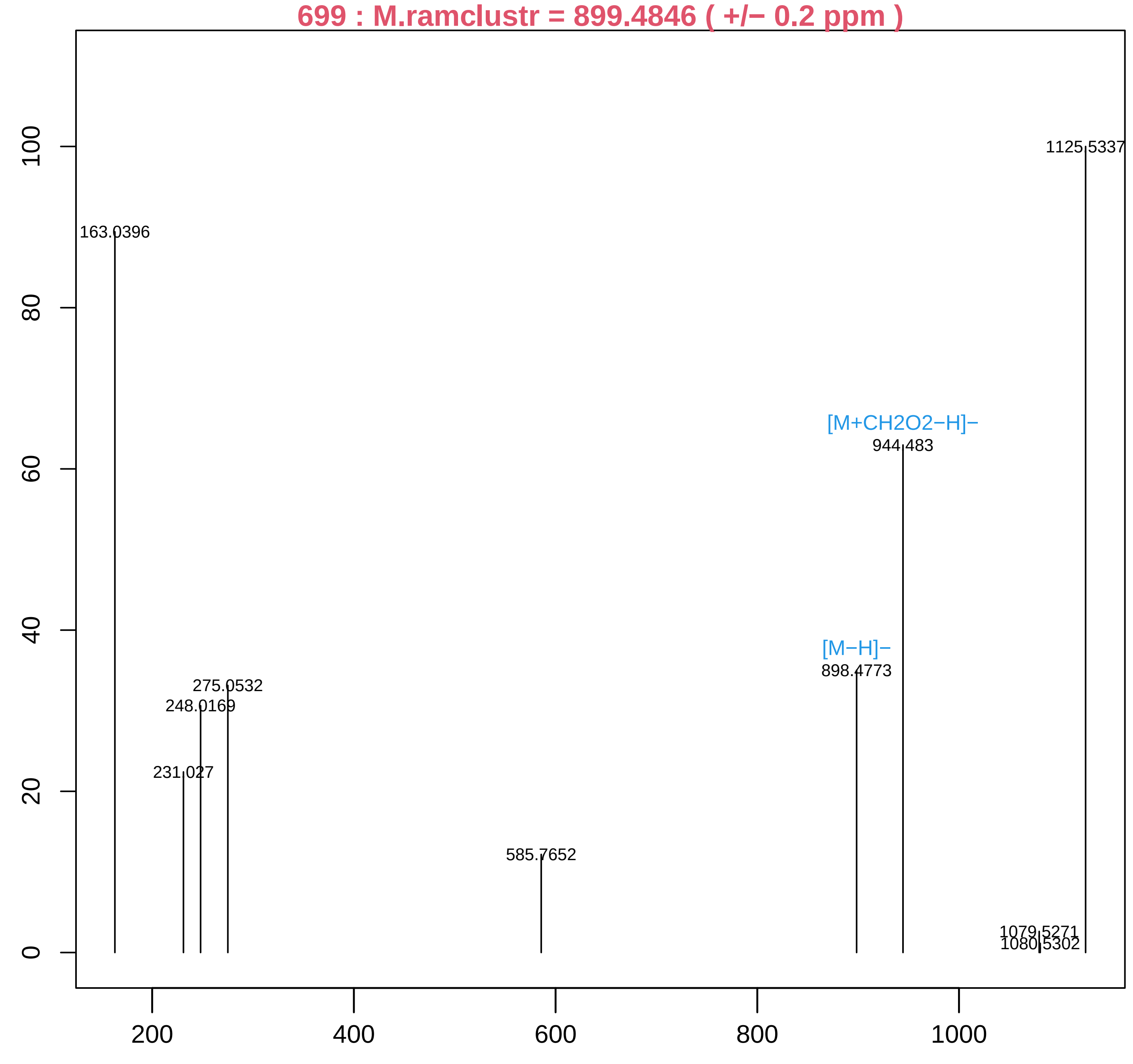

699 : M.ramclustr = 899.4846 (+/− 0.2 ppm)
163.0396
231.027
248.0169
275.0532
585.7652
[M−H]−
898.4773
[M+CH2O2−H]−
944.483
1079.5271
1080.5302
1125.5337
0
20
40
60
80
100
200
400
600
800
1000

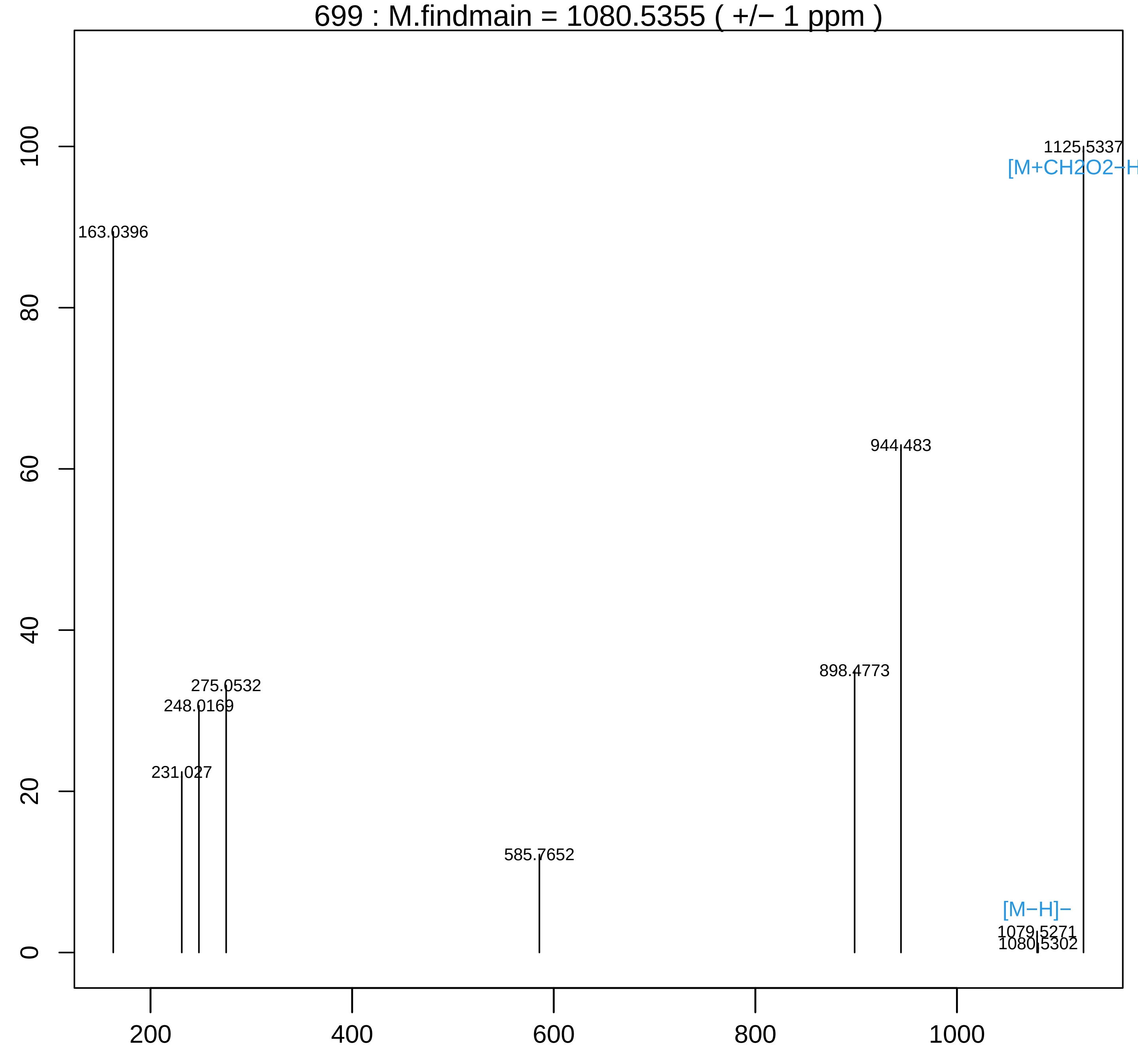

699 : M.findmain = 1080.5355 (+/− 1 ppm)
163.0396
231.027
248.0169
275.0532
585.7652
898.4773
944.483
[M−H]−
1079.5271
1080.5302
1125.5337
0
20
40
60
80
100
200
400
600
800
1000

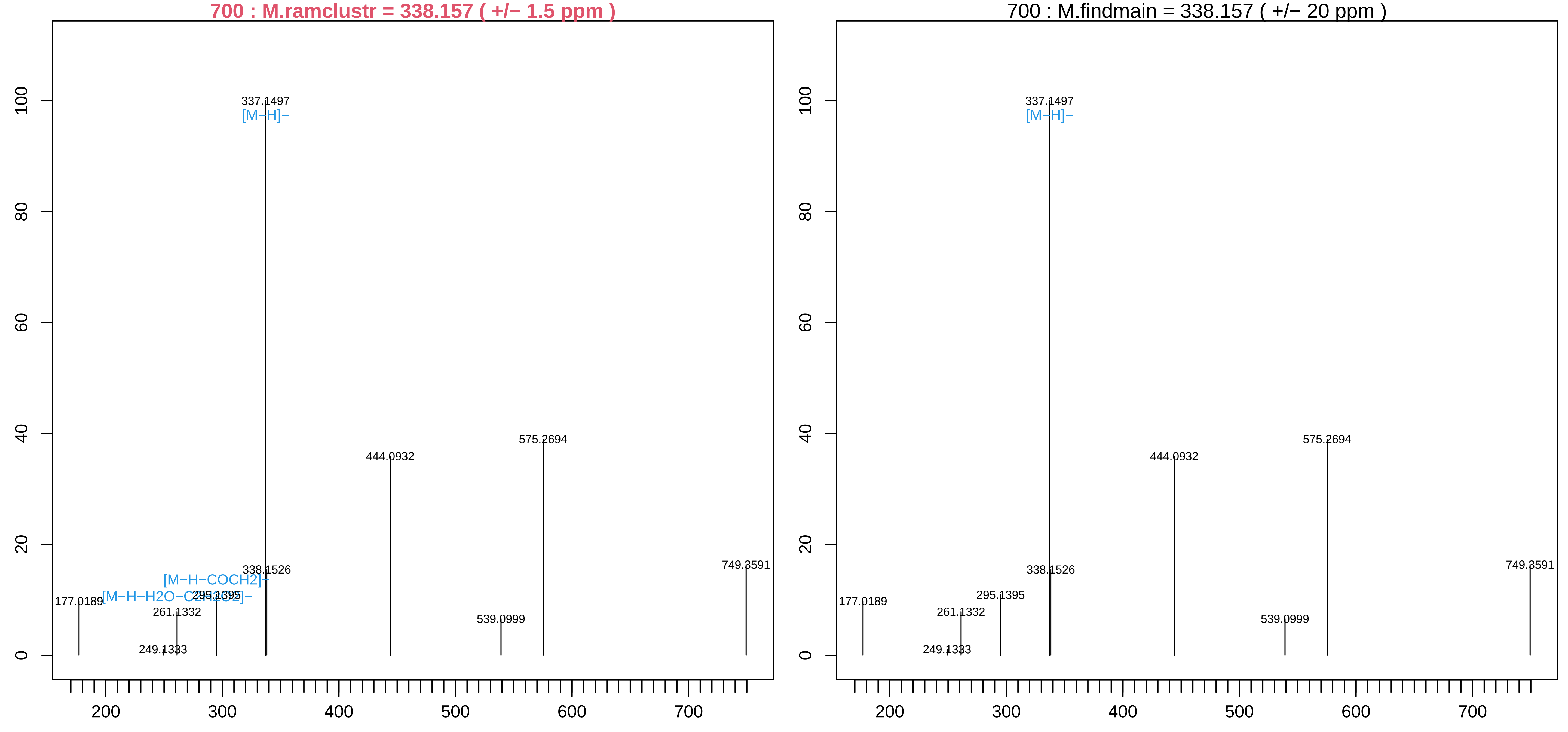
700 : M.ramclustr = 338.157 (+/− 1.5 ppm)
337.1497
[M−H]−
338.1526
[M−H−COCH2]−
[M−H−H2O−C2H2O2]−
177.0189
295.1395
261.1332
249.1333
444.0932
575.2694
539.0999
749.3591
700 : M.findmain = 338.157 (+/− 20 ppm)
337.1497
[M−H]−
338.1526
177.0189
295.1395
261.1332
249.1333
444.0932
575.2694
539.0999
749.3591
0
20
40
60
80
100
200
300
400
500
600
700

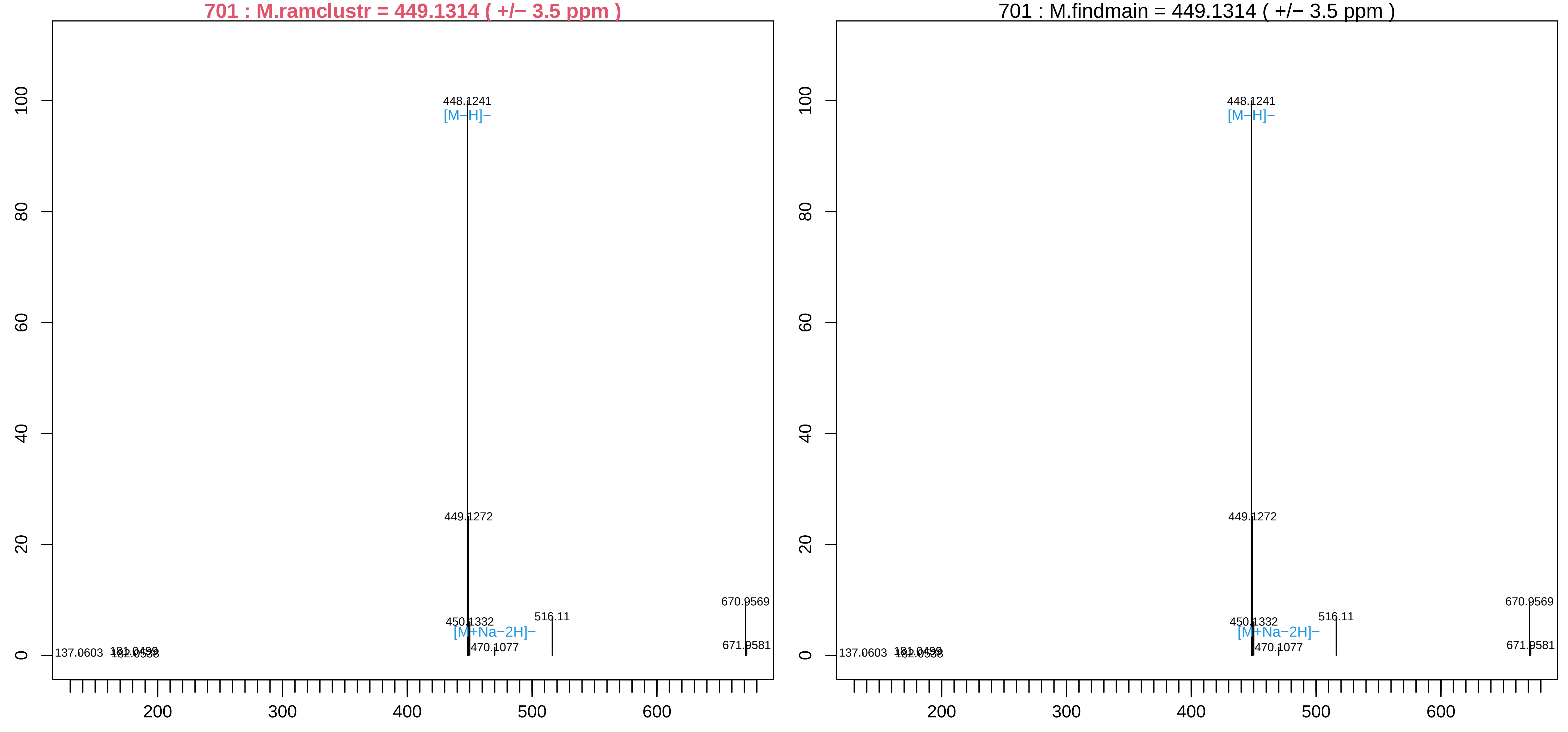
701 : M.ramclustr = 449.1314 (+/− 3.5 ppm)
701 : M.findmain = 449.1314 (+/− 3.5 ppm)
448.1241
[M−H]−
449.1272
450.1332
[M+Na−2H]−
470.1077
516.11
670.9569
671.9581
137.0603
181.0499
182.0538
0
20
40
60
80
100
200
300
400
500
600

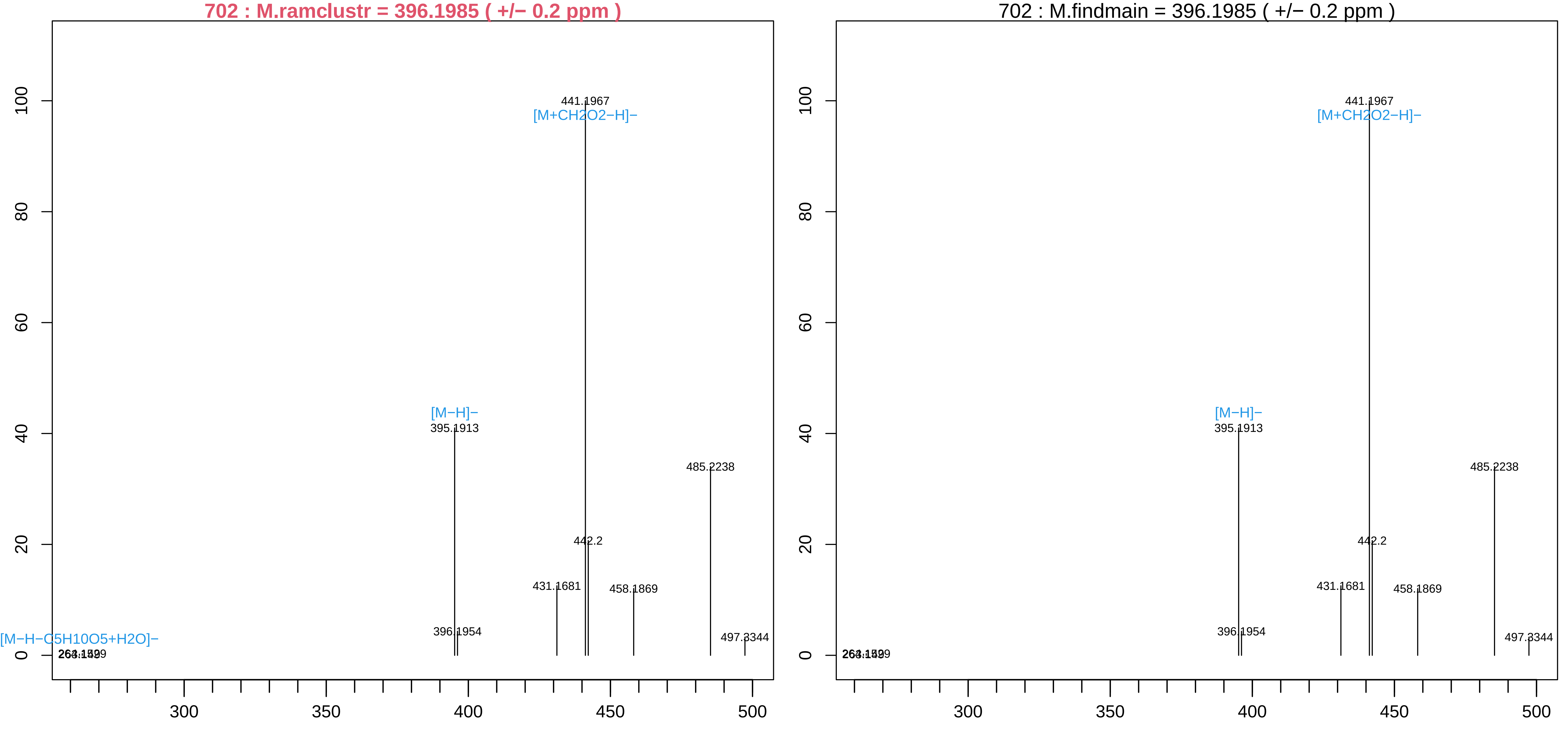

702 : M.ramclustr = 396.1985 (+/− 0.2 ppm)
441.1967
[M+CH2O2−H]−
[M−H]−
395.1913
485.2238
442.2
431.1681
458.1869
396.1954
497.3344
[M−H−C5H10O5+H2O]−
0
20
40
60
80
100
300
350
400
450
500
702 : M.findmain = 396.1985 (+/− 0.2 ppm)
441.1967
[M+CH2O2−H]−
[M−H]−
395.1913
485.2238
442.2
431.1681
458.1869
396.1954
497.3344
0
20
40
60
80
100
300
350
400
450
500

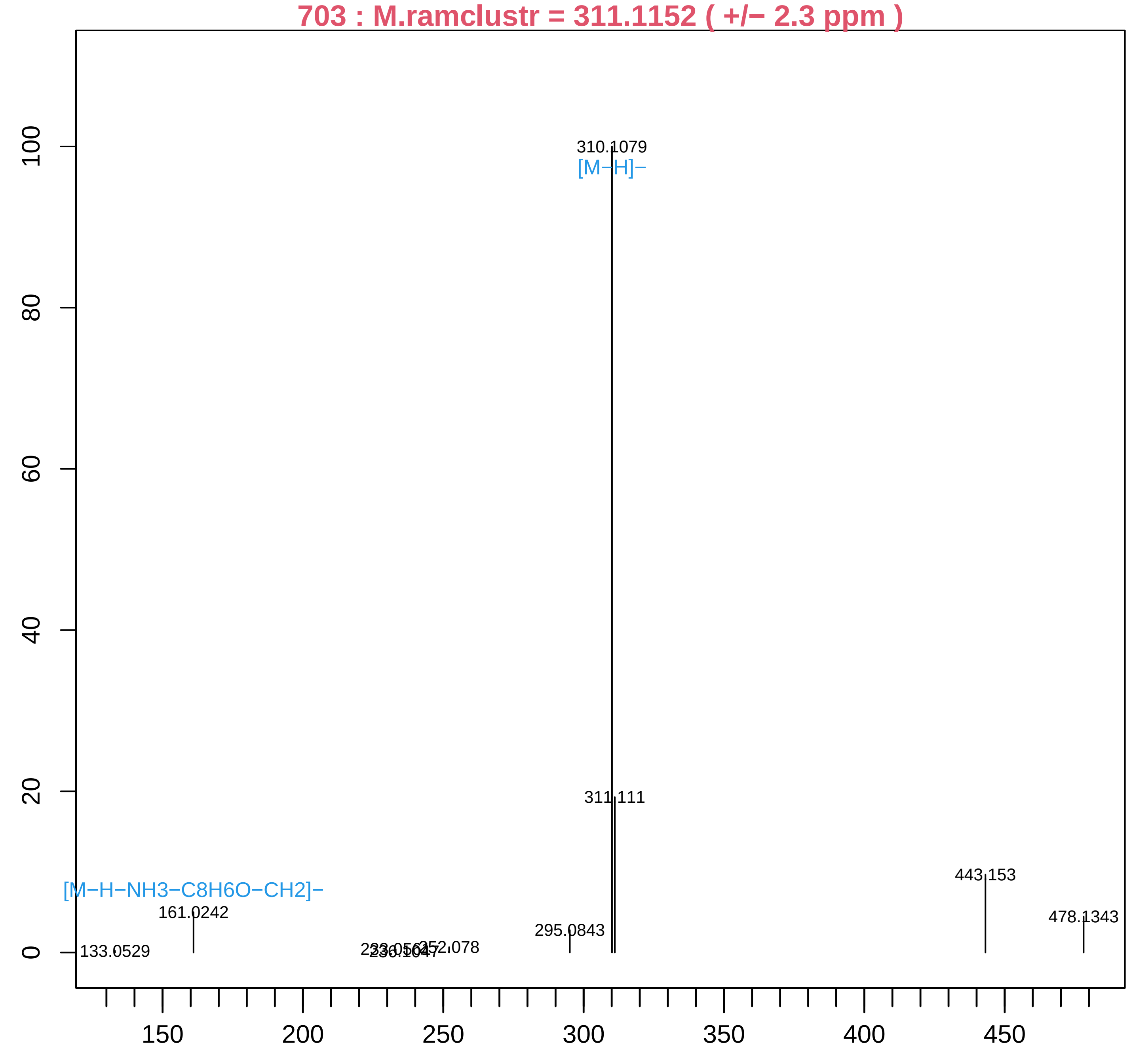

703 : M.ramclustr = 311.1152 (+/− 2.3 ppm)
310.1079
[M−H]−
311.111
[M−H−NH3−C8H6O−CH2]−
161.0242
133.0529
295.0843
443.153
478.1343
252.078
236.1047

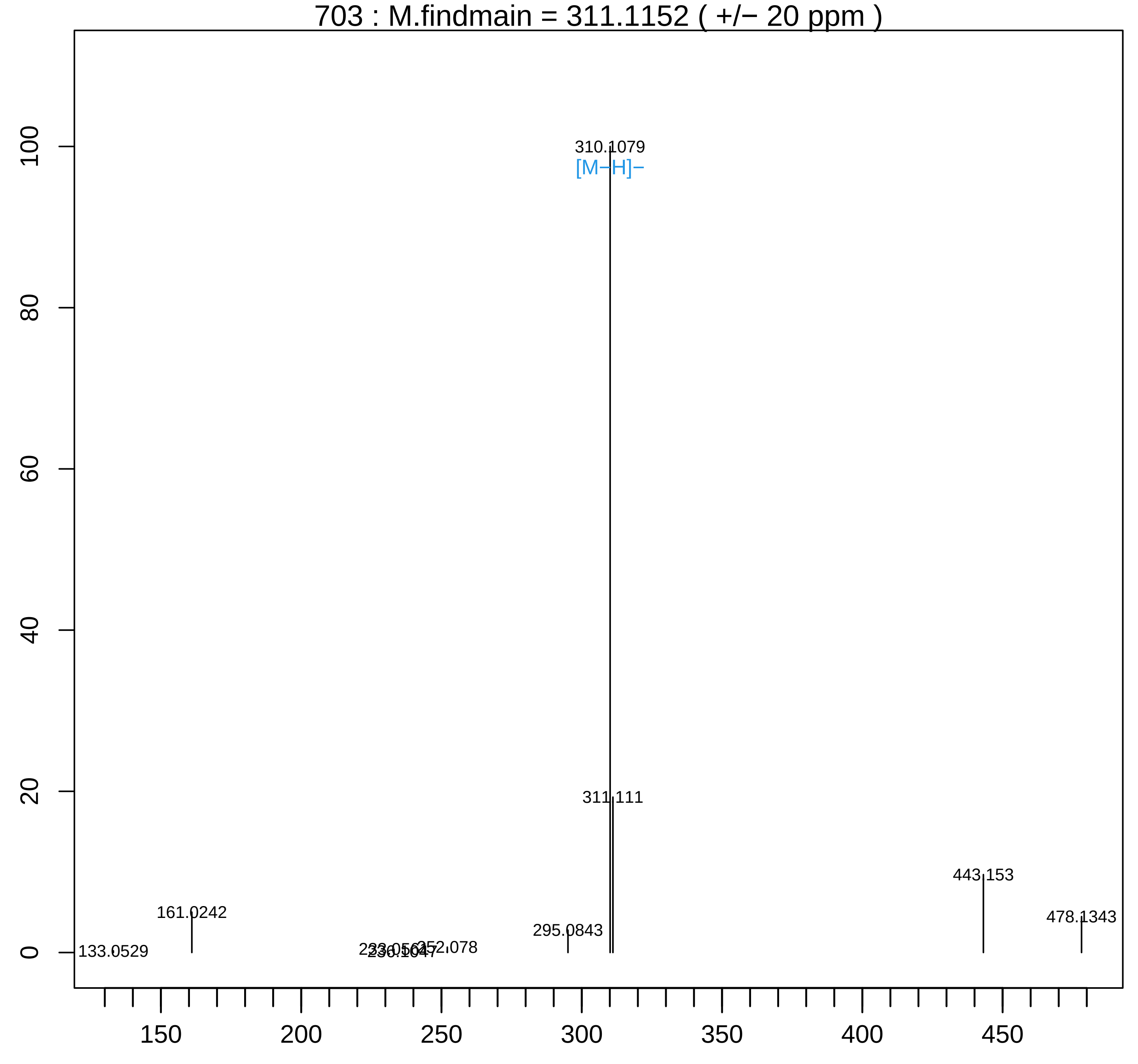

703 : M.findmain = 311.1152 (+/− 20 ppm)
310.1079
[M−H]−
311.111
161.0242
133.0529
295.0843
443.153
478.1343
252.078
236.1047

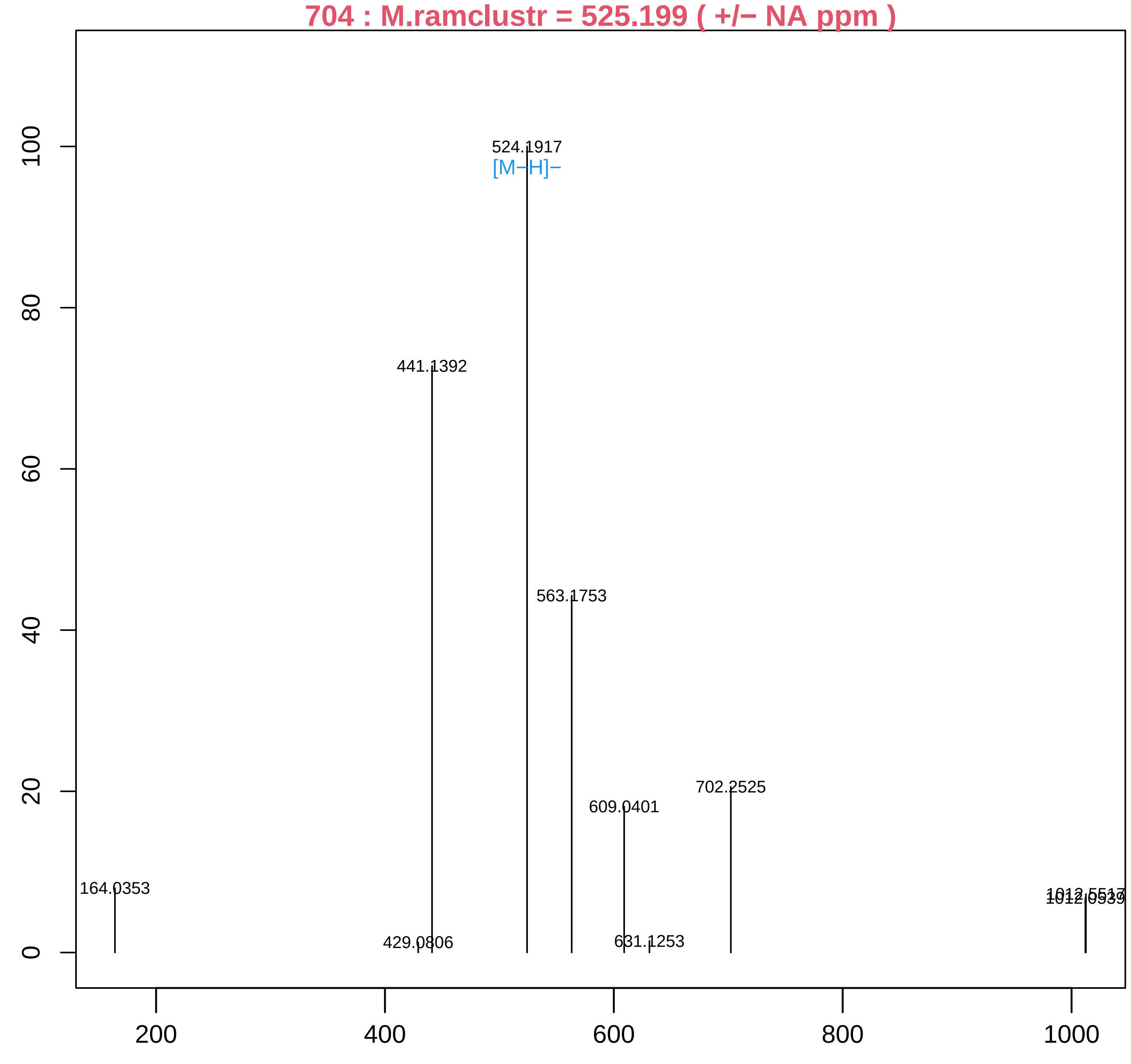
704 : M.ramclustr = 525.199 (+/− NA ppm)
524.1917
[M−H]−
441.1392
563.1753
702.2525
609.0401
164.0353
1012.5517
1012.0539
429.0806
631.1253
0
20
40
60
80
100
200
400
600
800
1000

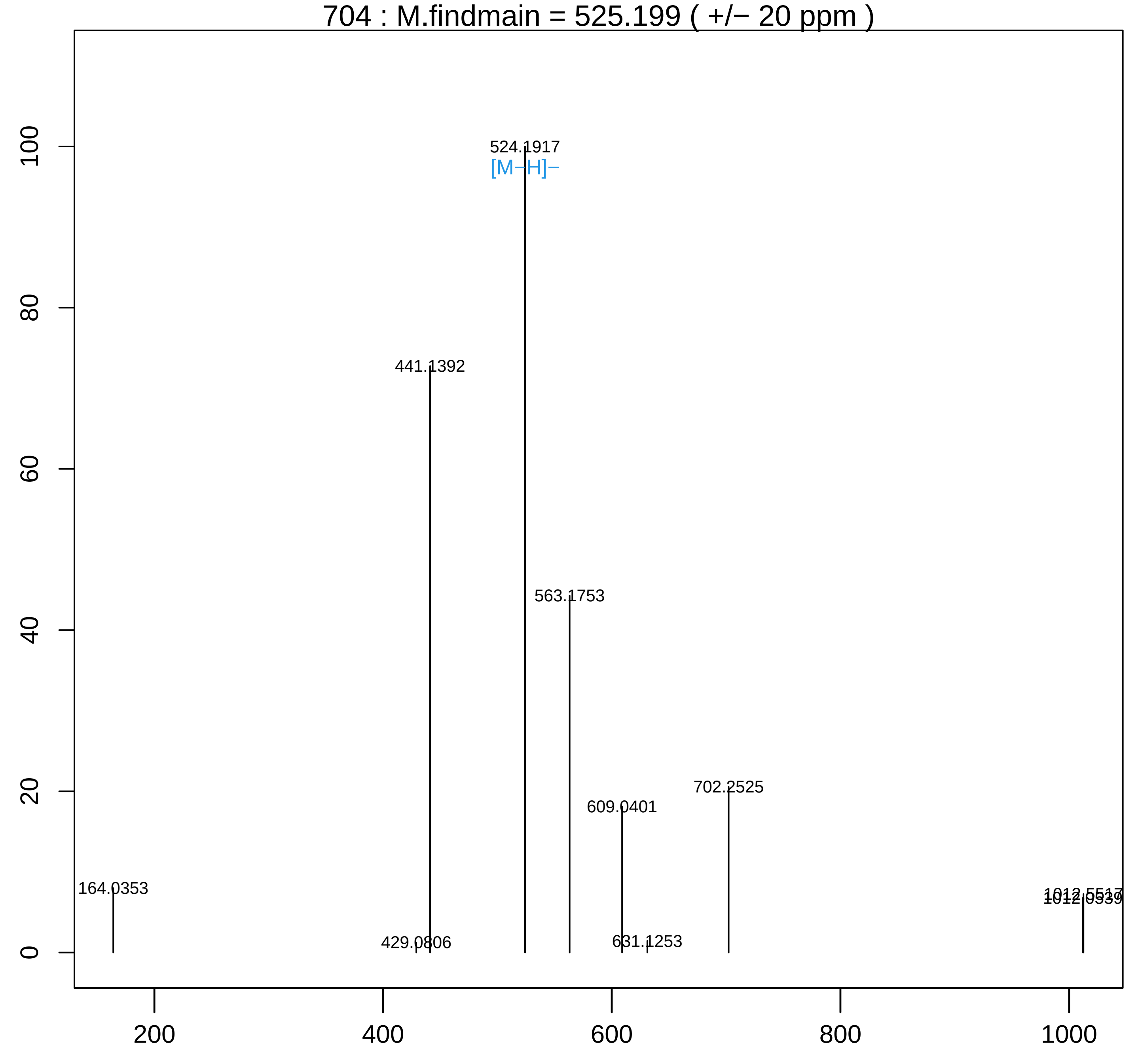
704 : M.findmain = 525.199 (+/− 20 ppm)
524.1917
[M−H]−
441.1392
563.1753
702.2525
609.0401
164.0353
1012.5517
1012.0539
429.0806
631.1253
0
20
40
60
80
100
200
400
600
800
1000

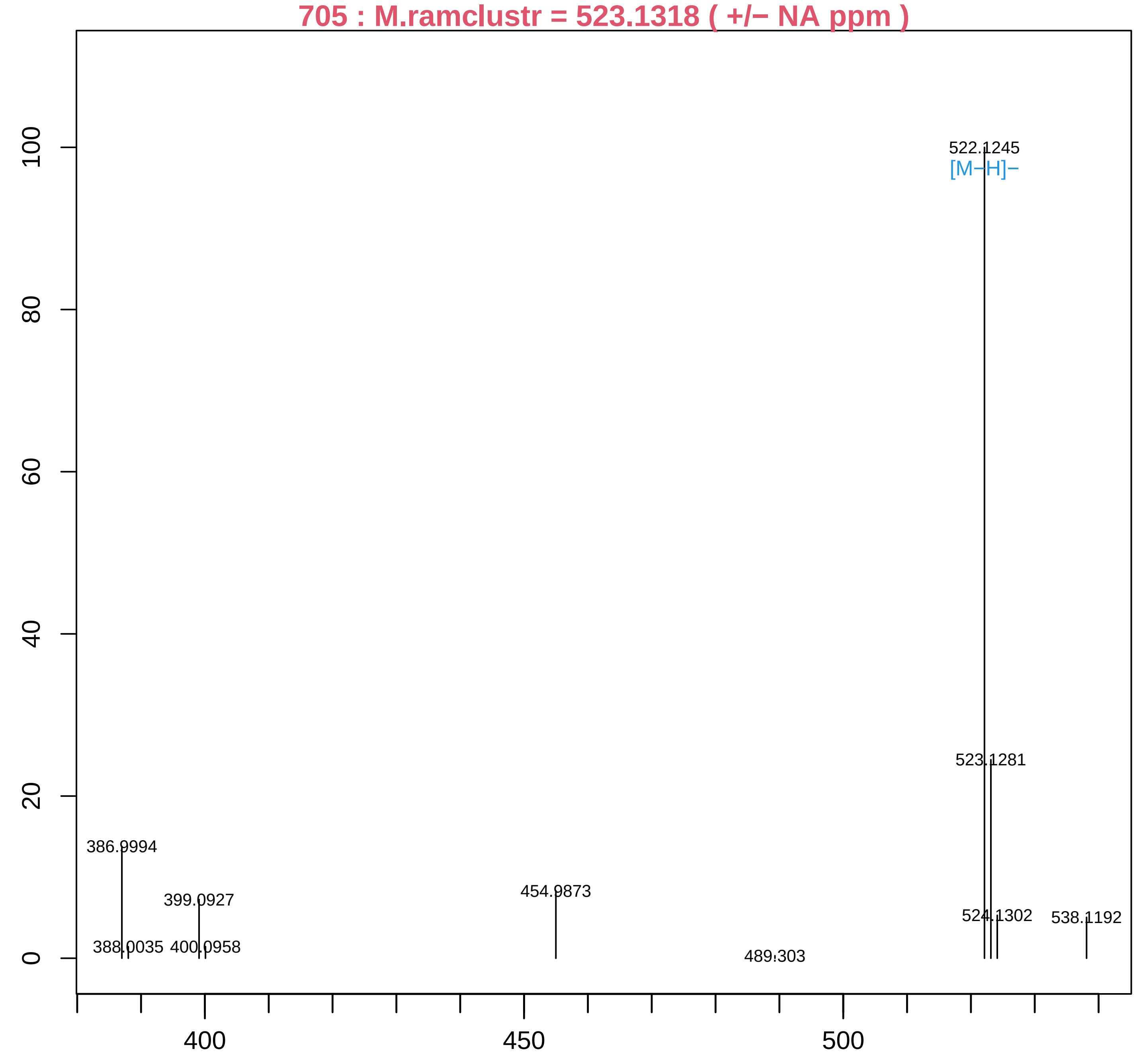
705 : M.ramclustr = 523.1318 (+/− NA ppm)
522.1245
[M−H]−
523.1281
386.9994
399.0927
454.9873
524.1302
538.1192
388.0035
400.0958
489.303
0
20
40
60
80
100
400
450
500

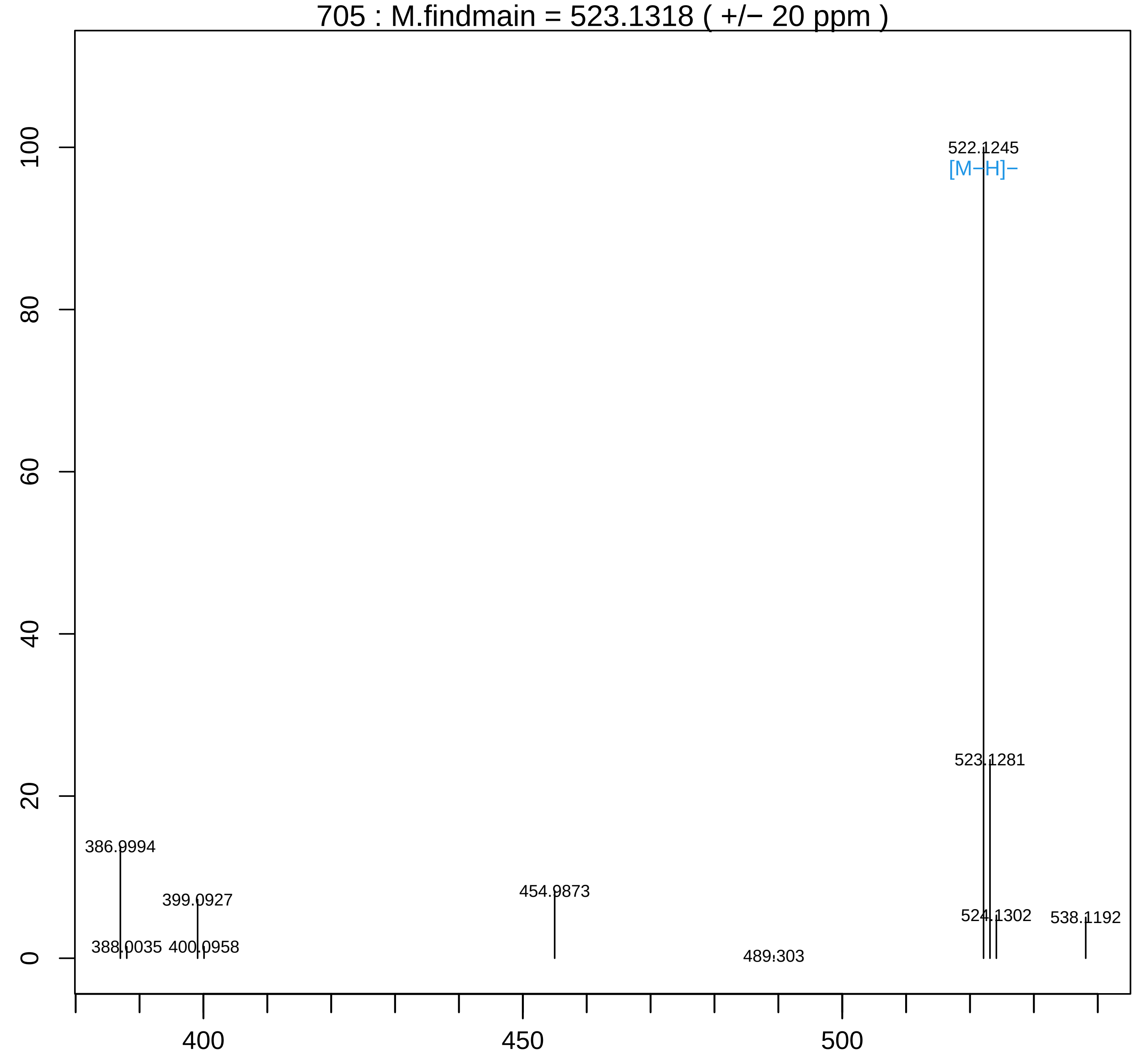
705 : M.findmain = 523.1318 (+/− 20 ppm)
522.1245
[M−H]−
523.1281
386.9994
399.0927
454.9873
524.1302
538.1192
388.0035
400.0958
489.303
0
20
40
60
80
100
400
450
500

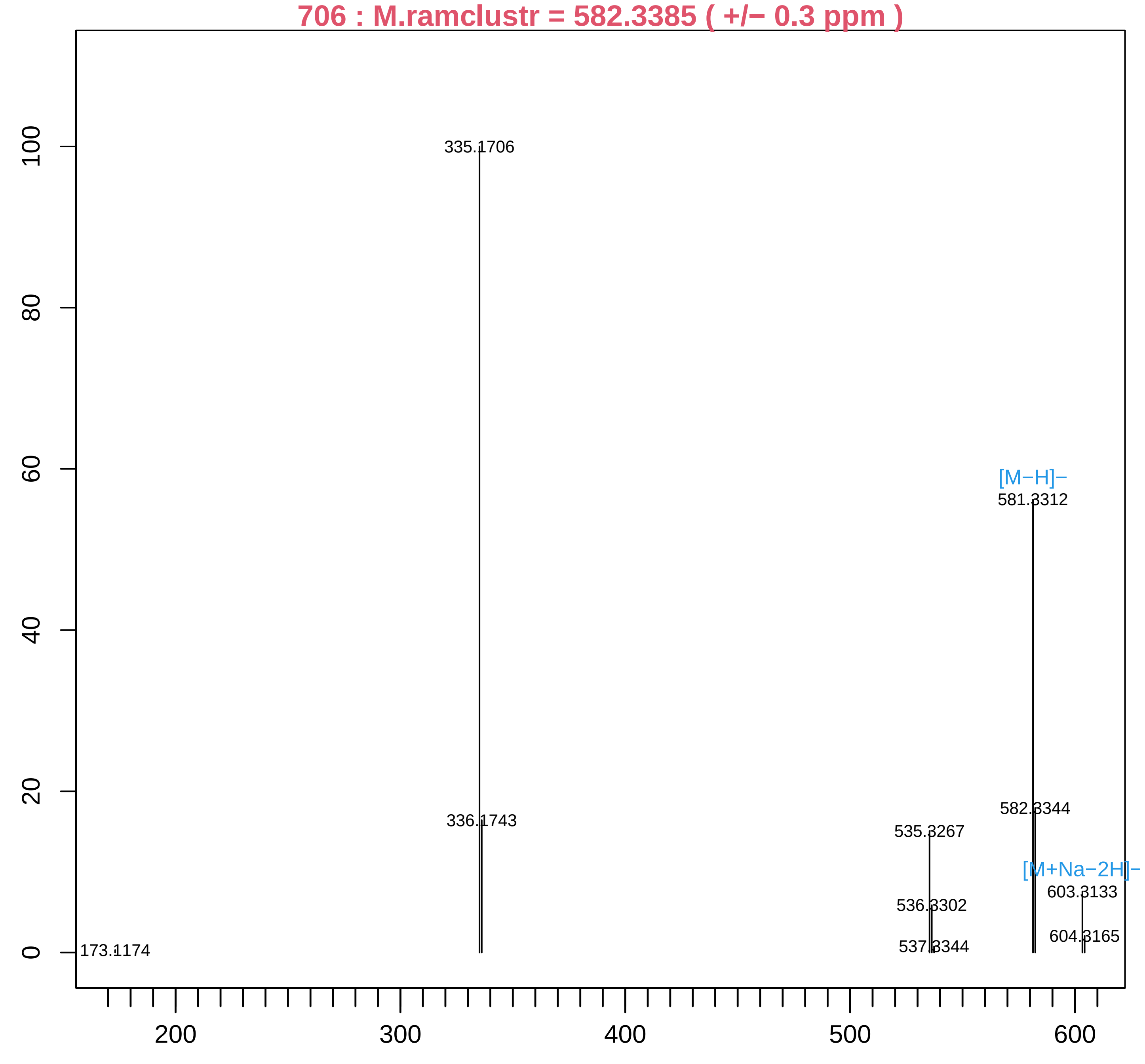
706 : M.ramclustr = 582.3385 (+/− 0.3 ppm)
335.1706
336.1743
[M−H]−
581.3312
582.3344
535.3267
536.3302
537.3344
[M+Na−2H]−
603.3133
604.3165
173.1174
0
20
40
60
80
100
200
300
400
500
600

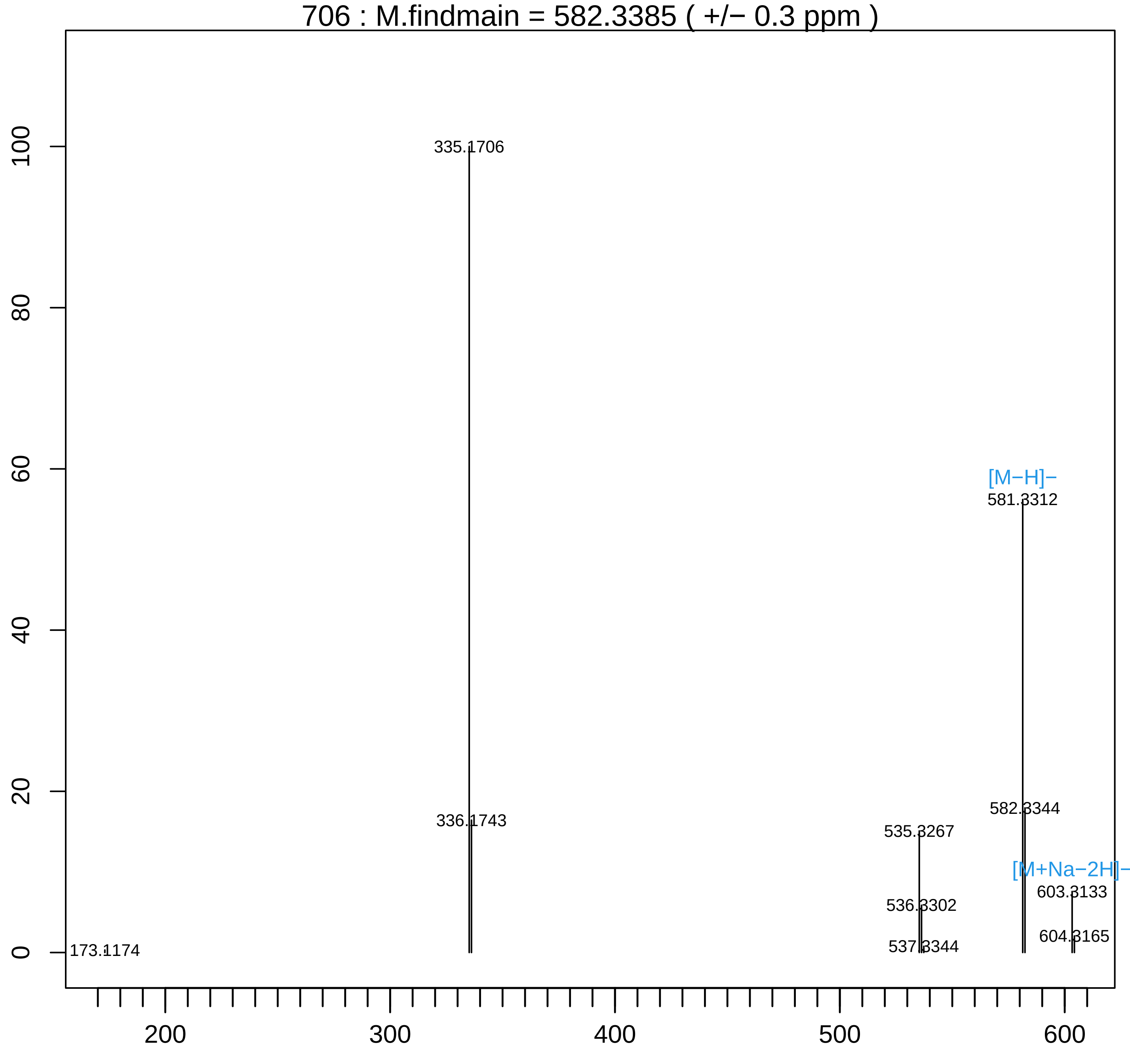
706 : M.findmain = 582.3385 (+/− 0.3 ppm)
335.1706
336.1743
[M−H]−
581.3312
582.3344
535.3267
536.3302
537.3344
[M+Na−2H]−
603.3133
604.3165
173.1174
0
20
40
60
80
100
200
300
400
500
600

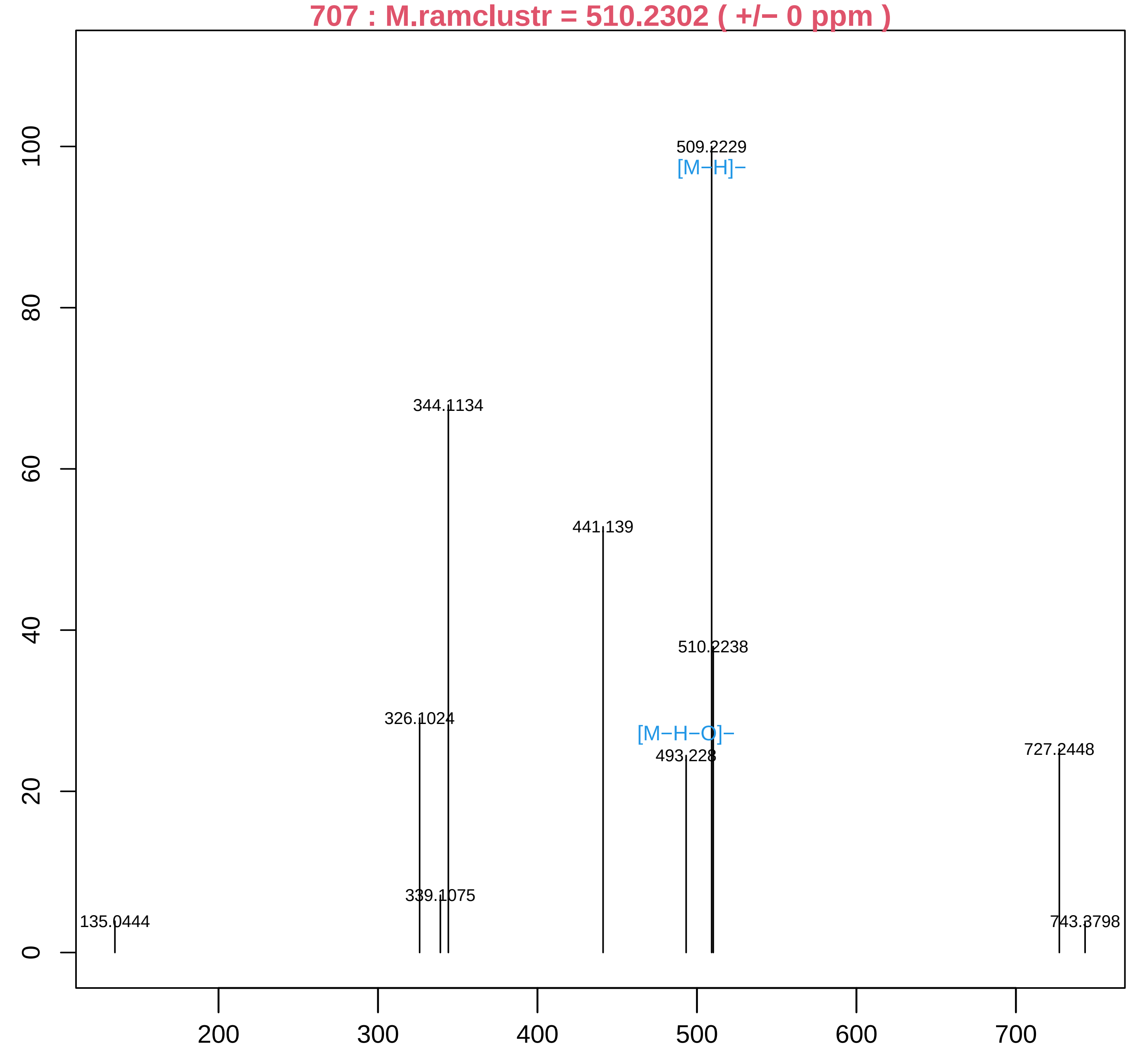

707 : M.ramclustr = 510.2302 (+/− 0 ppm)
509.2229
[M−H]−
344.1134
441.139
510.2238
326.1024
[M−H−O]−
493.228
727.2448
339.1075
135.0444
743.3798
0
20
40
60
80
100
200
300
400
500
600
700

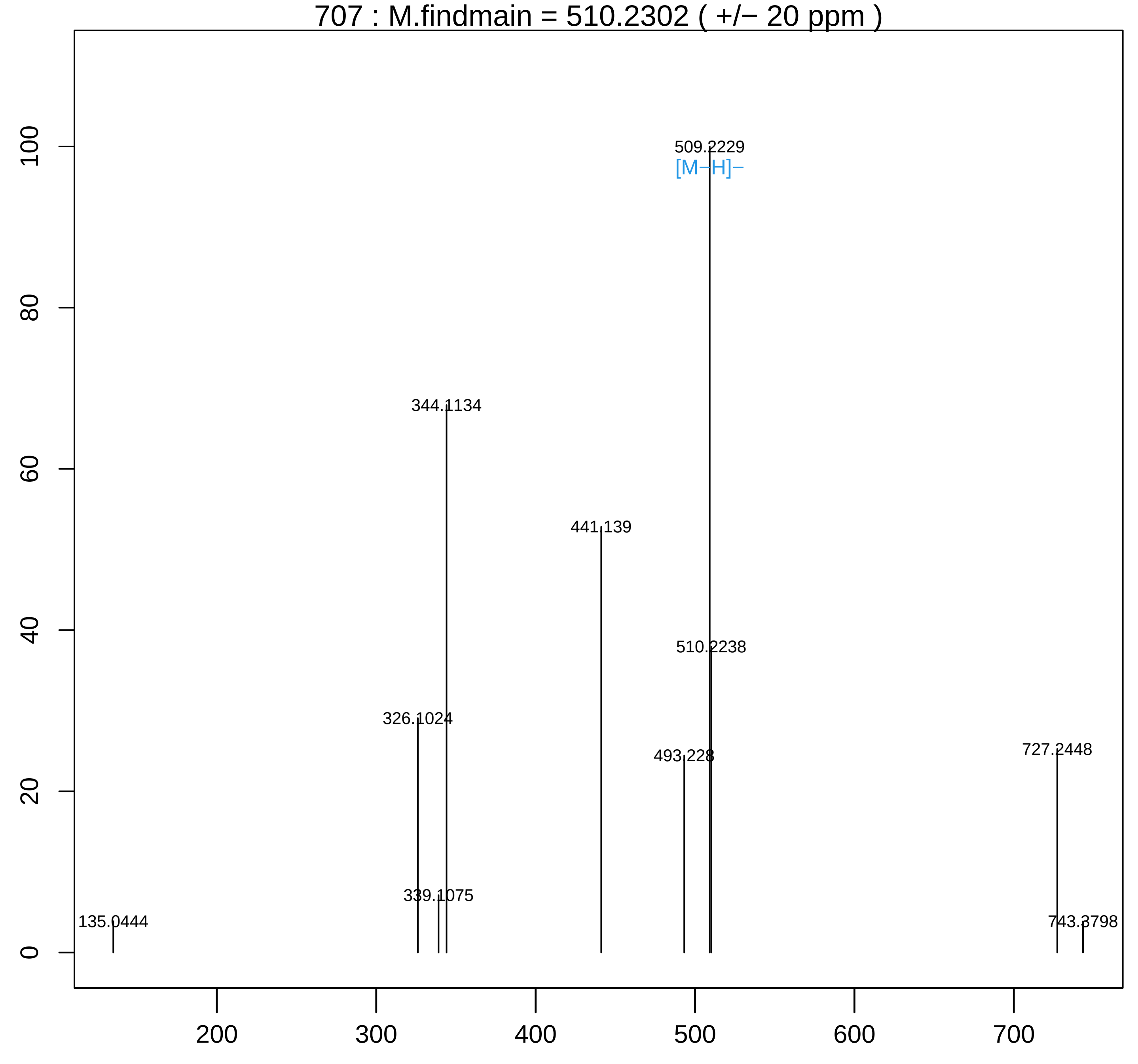

707 : M.findmain = 510.2302 (+/− 20 ppm)
509.2229
[M−H]−
344.1134
441.139
510.2238
326.1024
493.228
727.2448
339.1075
135.0444
743.3798
0
20
40
60
80
100
200
300
400
500
600
700

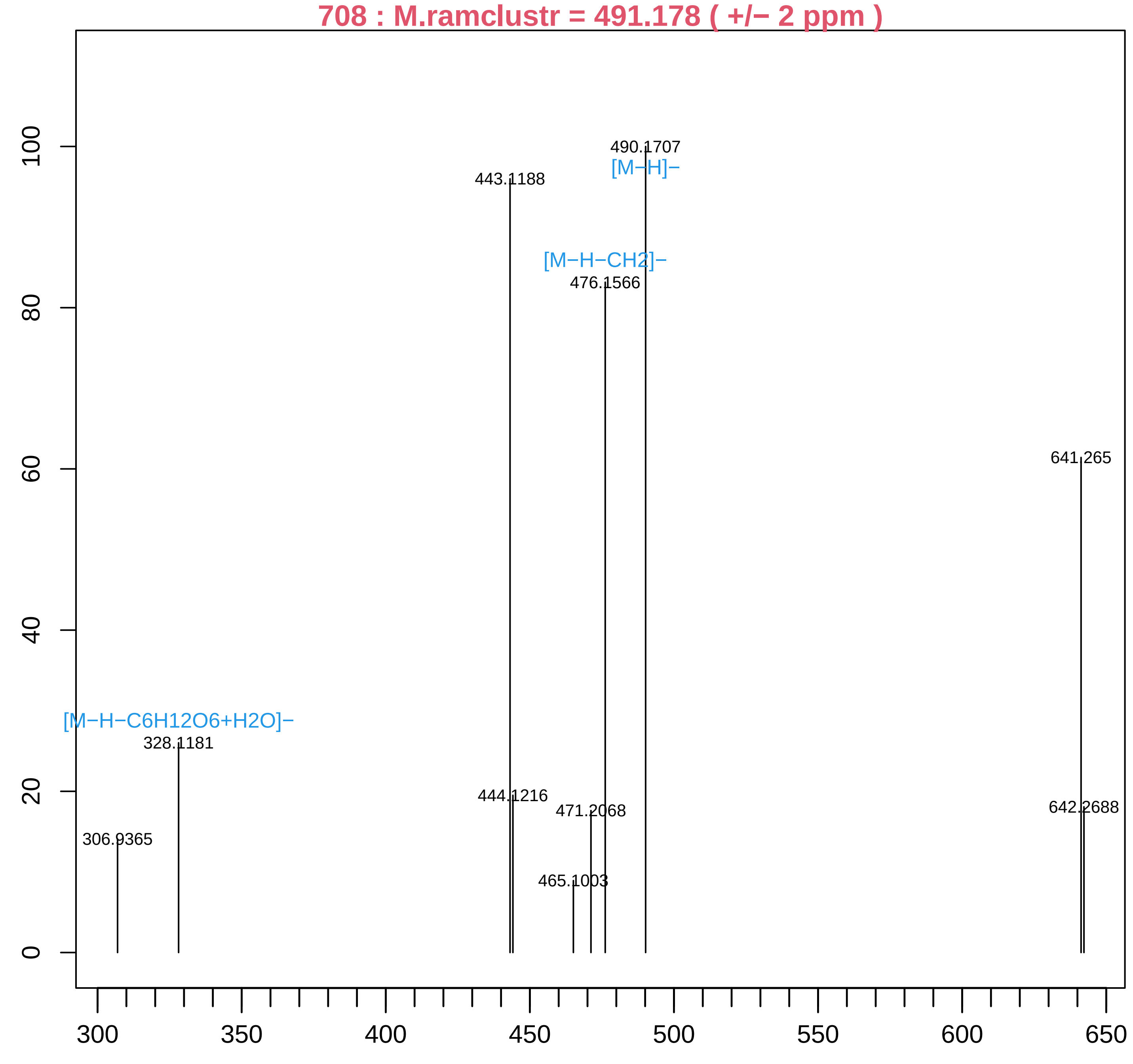

708 : M.ramclustr = 491.178 (+/− 2 ppm)
490.1707
[M−H]−
443.1188
[M−H−CH2]−
476.1566
641.265
[M−H−C6H12O6+H2O]−
328.1181
444.1216
471.2068
642.2688
306.9365
465.1003
0
20
40
60
80
100
300
350
400
450
500
550
600
650

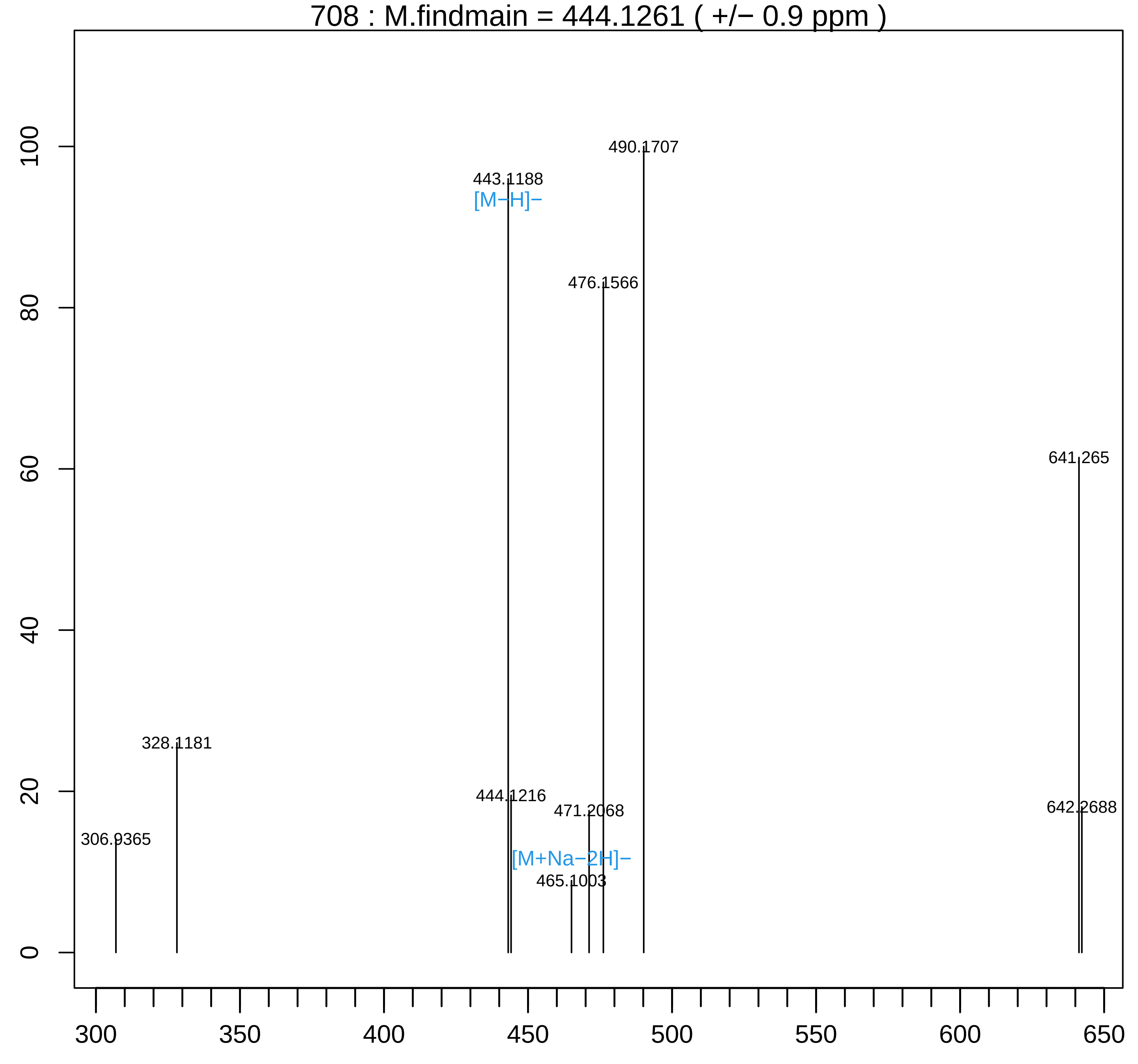

708 : M.findmain = 444.1261 (+/− 0.9 ppm)
490.1707
443.1188
[M−H]−
476.1566
641.265
328.1181
444.1216
471.2068
642.2688
306.9365
[M+Na−2H]−
465.1003
0
20
40
60
80
100
300
350
400
450
500
550
600
650

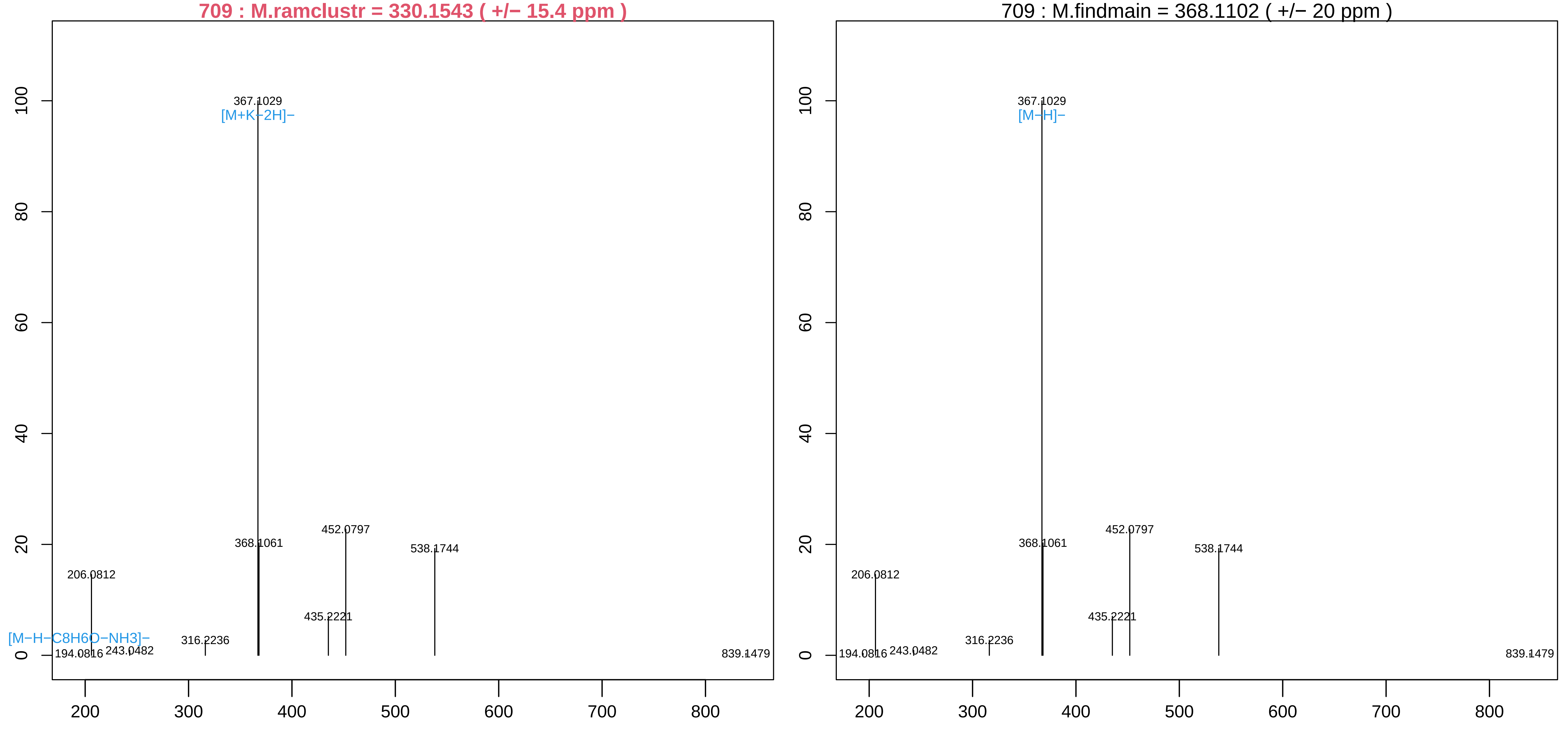

709 : M.ramclustr = 330.1543 (+/− 15.4 ppm)
367.1029
[M+K−2H]−
368.1061
452.0797
538.1744
206.0812
435.2221
[M−H−C8H6O−NH3]−
316.2236
194.0816
243.0482
839.1479
709 : M.findmain = 368.1102 (+/− 20 ppm)
367.1029
[M−H]−
368.1061
452.0797
538.1744
206.0812
435.2221
316.2236
194.0816
243.0482
839.1479
0
20
40
60
80
100
200
300
400
500
600
700
800

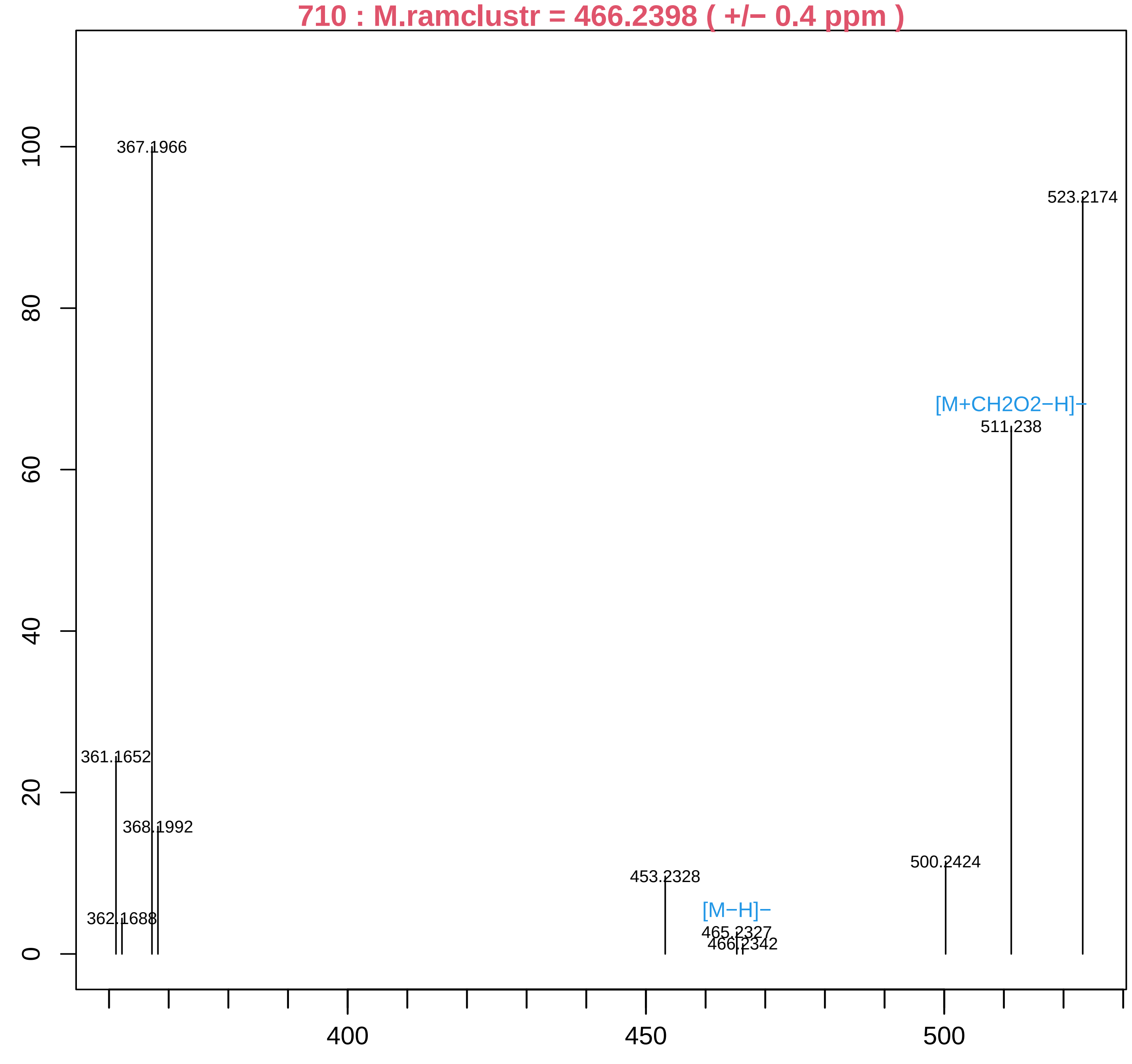
710 : M.ramclustr = 466.2398 (+/− 0.4 ppm)
367.1966
523.2174
[M+CH2O2−H]−
511.238
361.1652
368.1992
500.2424
453.2328
[M−H]−
362.1688
465.2327
466.2342
0
20
40
60
80
100
400
450
500
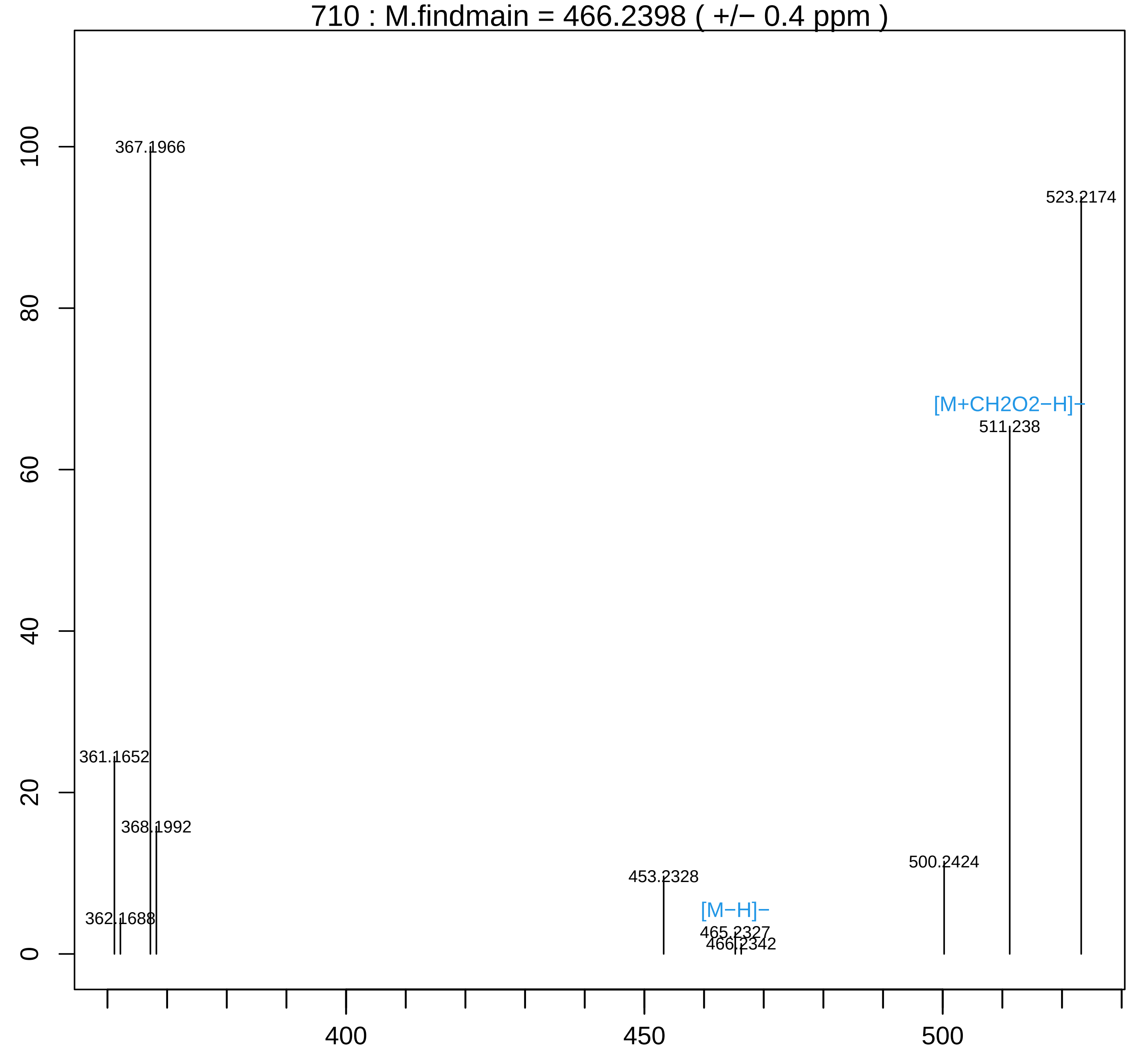
710 : M.findmain = 466.2398 (+/− 0.4 ppm)
367.1966
523.2174
[M+CH2O2−H]−
511.238
361.1652
368.1992
500.2424
453.2328
[M−H]−
362.1688
465.2327
466.2342
0
20
40
60
80
100
400
450
500

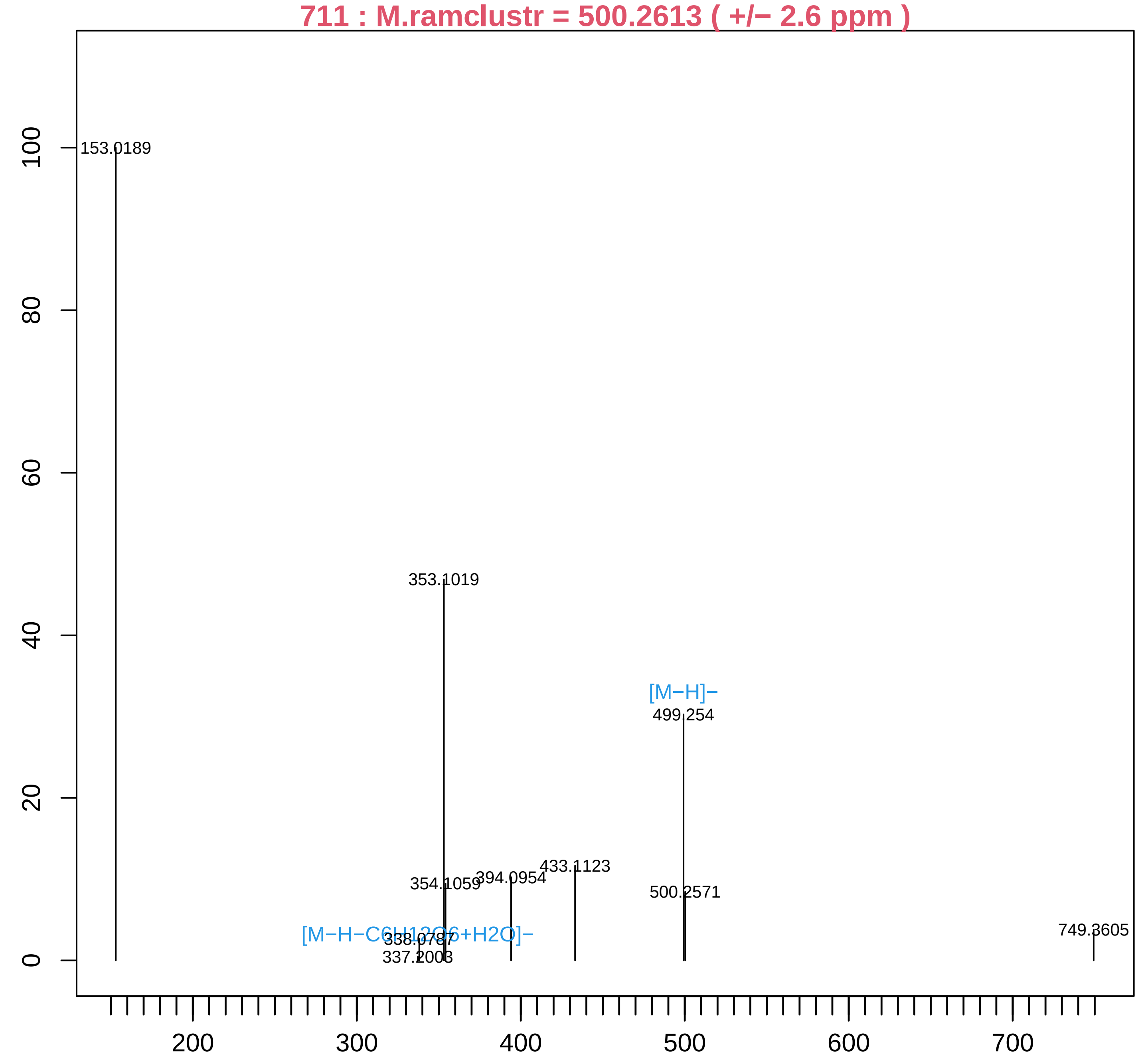
711 : M.ramclustr = 500.2613 (+/− 2.6 ppm)
153.0189
353.1019
[M−H]−
499.254
354.1059
394.0954
433.1123
500.2571
[M−H−C6H12O6+H2O]−
338.0787
337.2003
749.3605
0
20
40
60
80
100
200
300
400
500
600
700

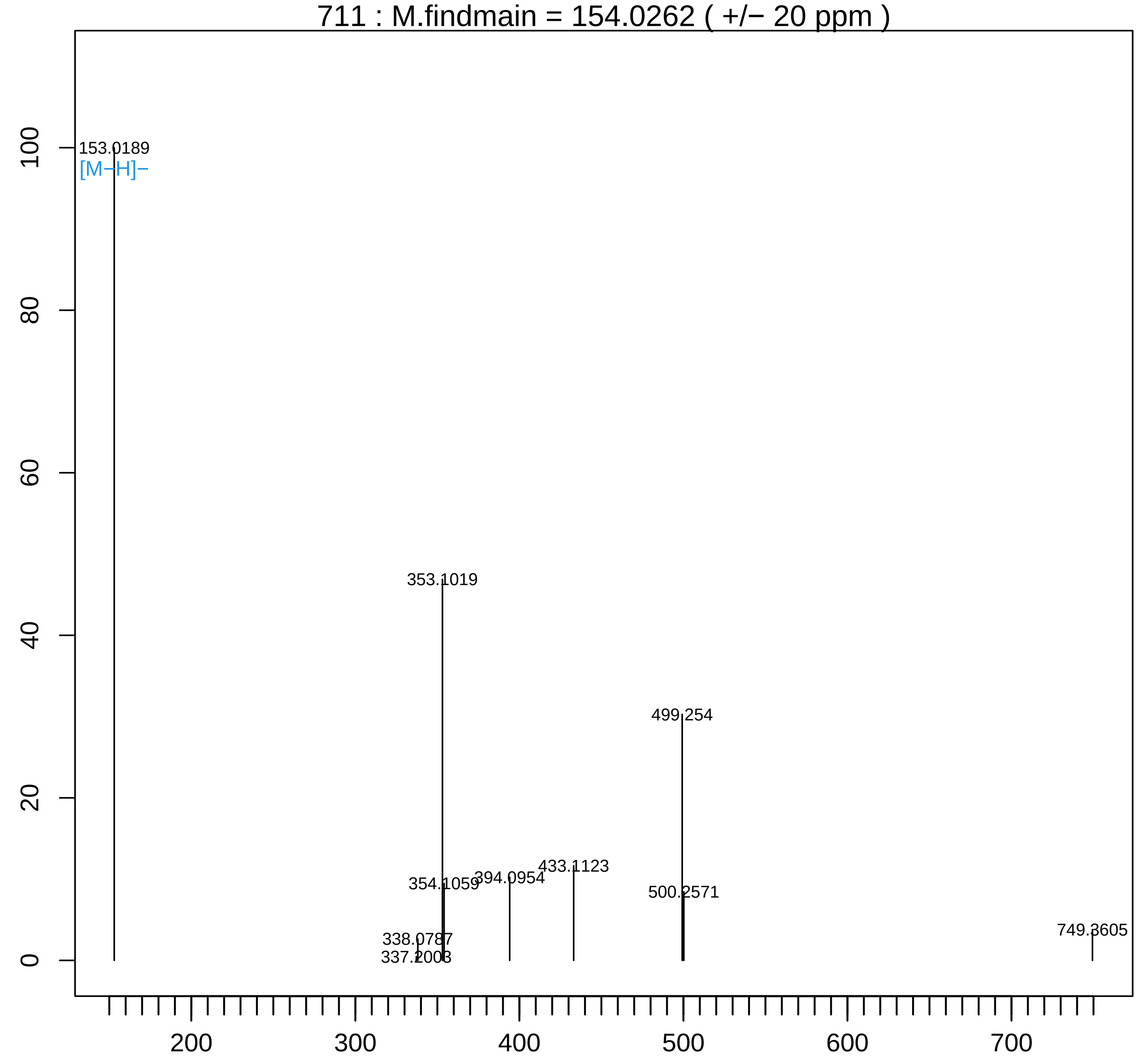
711 : M.findmain = 154.0262 (+/− 20 ppm)
153.0189
[M−H]−
353.1019
499.254
354.1059
394.0954
433.1123
500.2571
338.0787
337.2003
749.3605
0
20
40
60
80
100
200
300
400
500
600
700

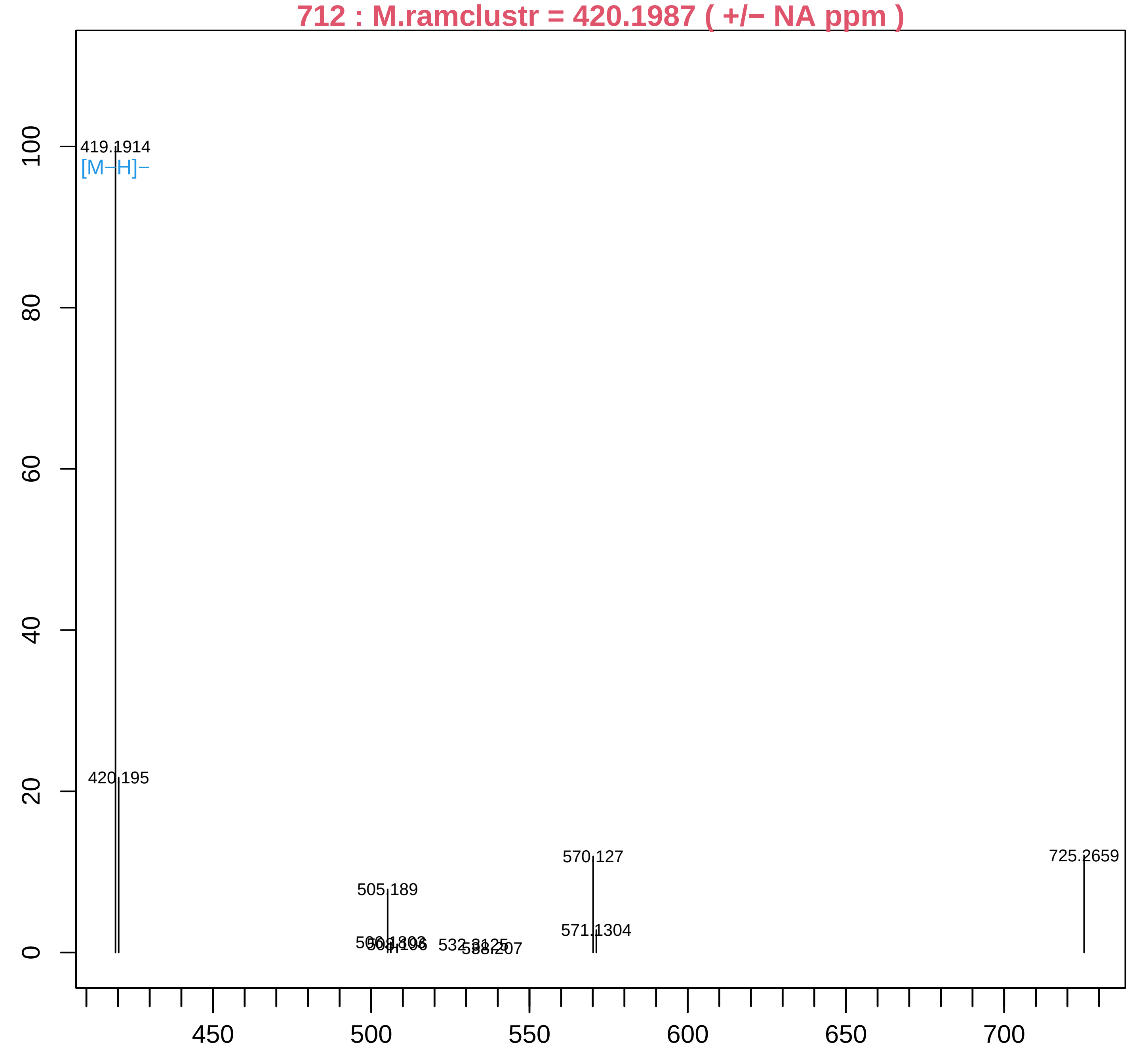

712 : M.ramclustr = 420.1987 (+/− NA ppm)
419.1914
[M−H]−
420.195
505.189
570.127
571.1304
725.2659
0
20
40
60
80
100
450
500
550
600
650
700

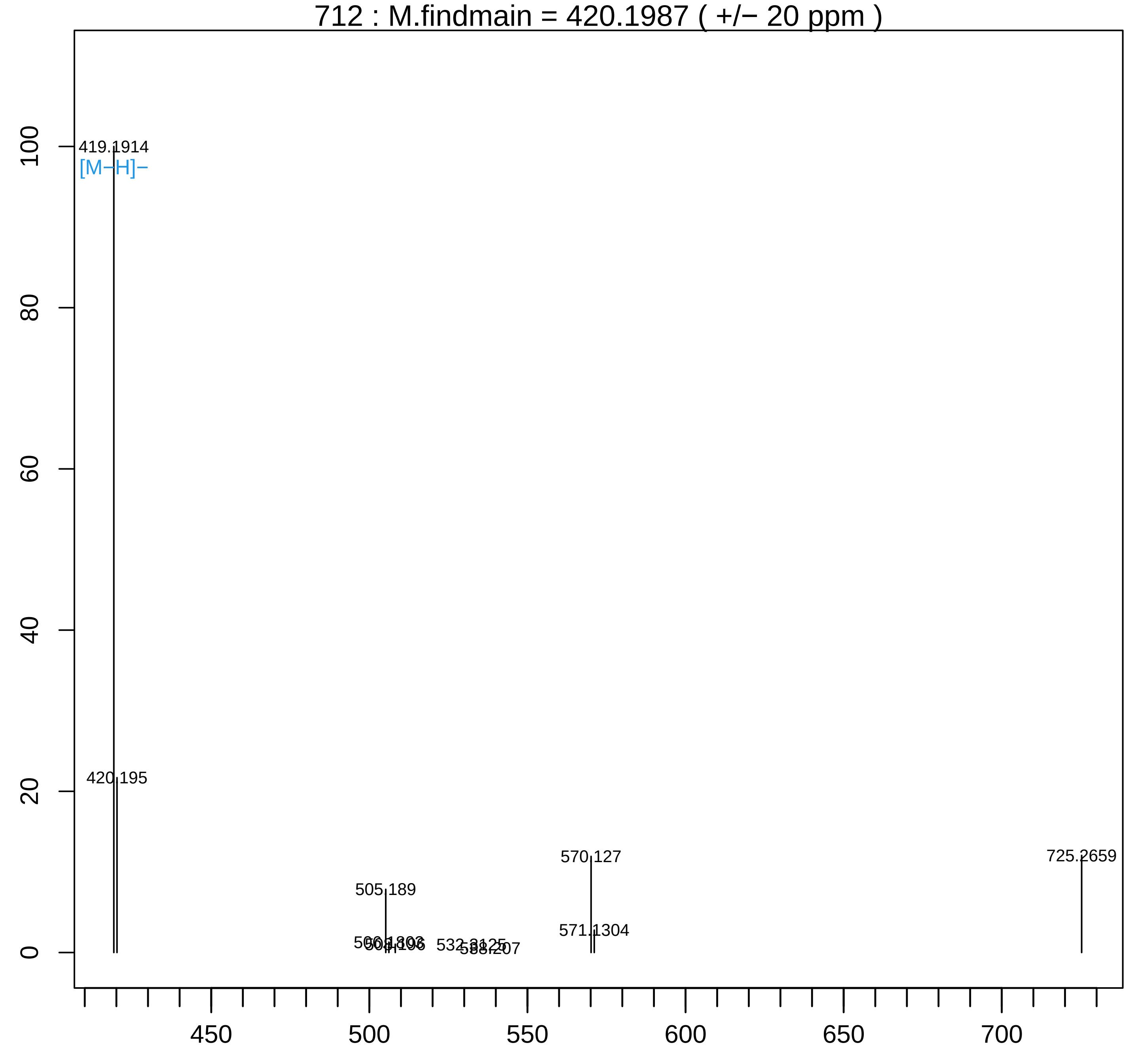

712 : M.findmain = 420.1987 (+/− 20 ppm)
419.1914
[M−H]−
420.195
505.189
570.127
571.1304
725.2659
0
20
40
60
80
100
450
500
550
600
650
700

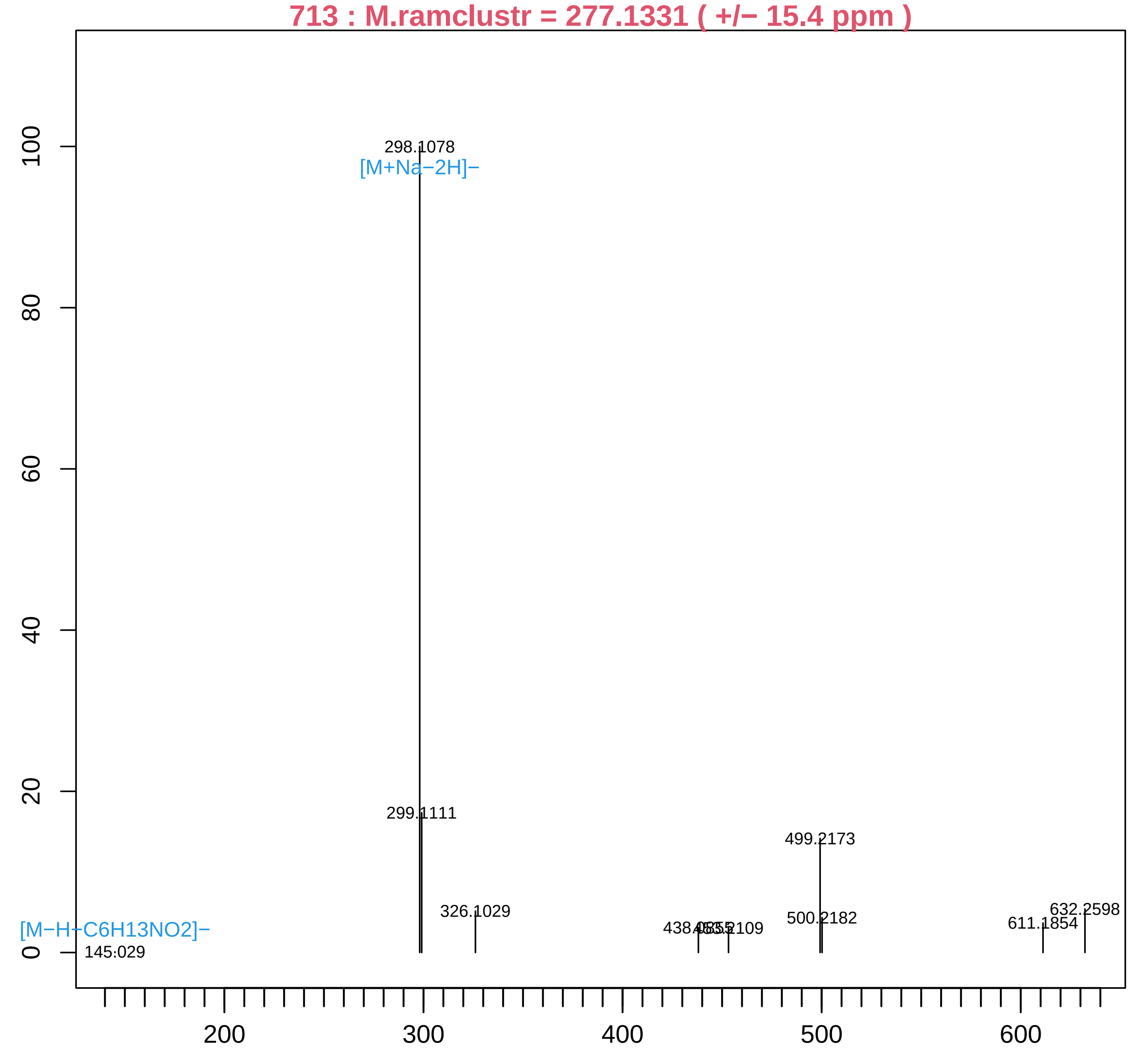

713 : M.ramclustr = 277.1331 (+/− 15.4 ppm)
298.1078
[M+Na−2H]−
299.1111
499.2173
326.1029
500.2182
632.2598
611.1854
[M−H−C6H13NO2]−
145:029
0
20
40
60
80
100
200
300
400
500
600

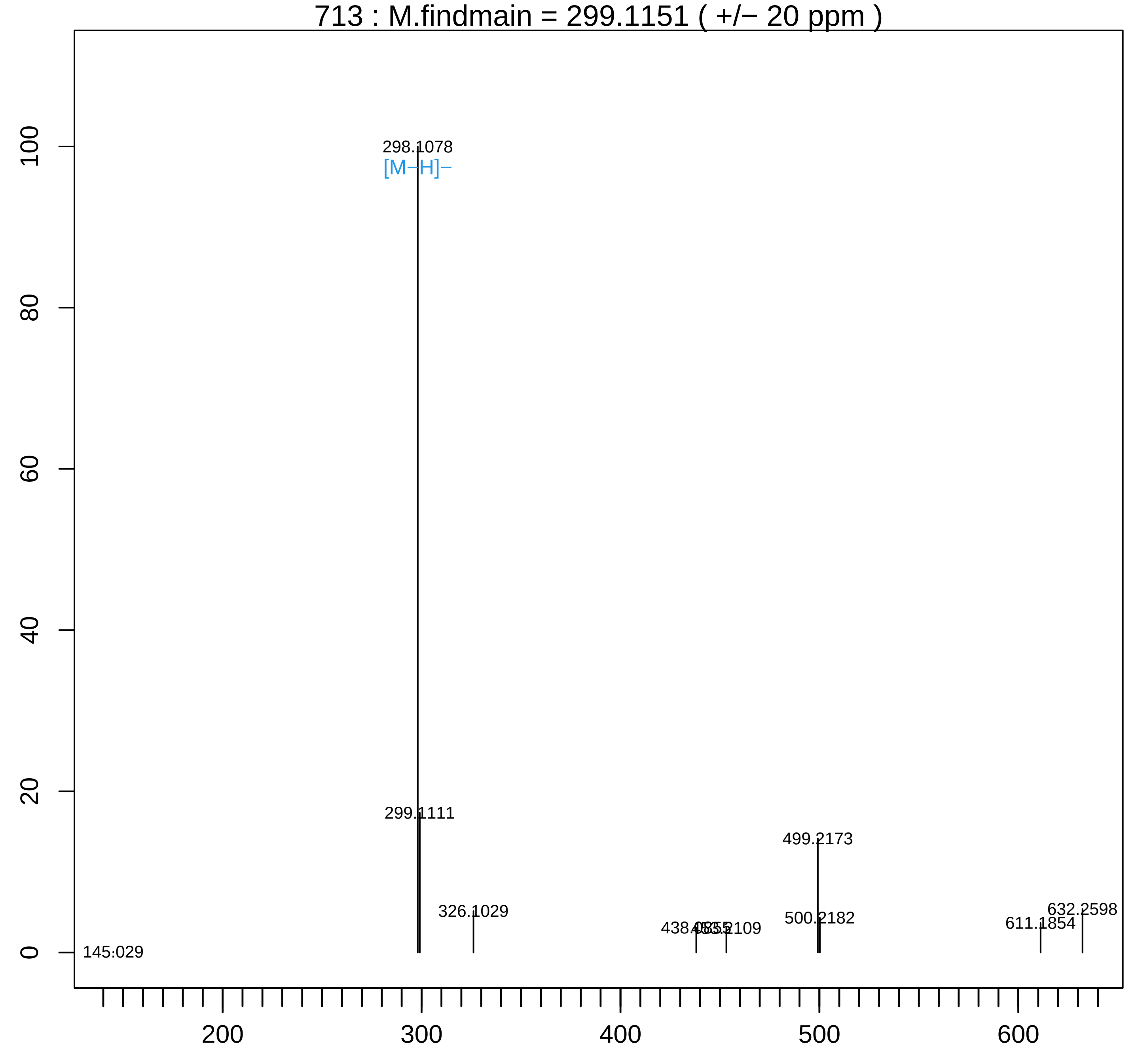

713 : M.findmain = 299.1151 (+/− 20 ppm)
298.1078
[M−H]−
299.1111
499.2173
326.1029
500.2182
632.2598
611.1854
145:029
0
20
40
60
80
100
200
300
400
500
600

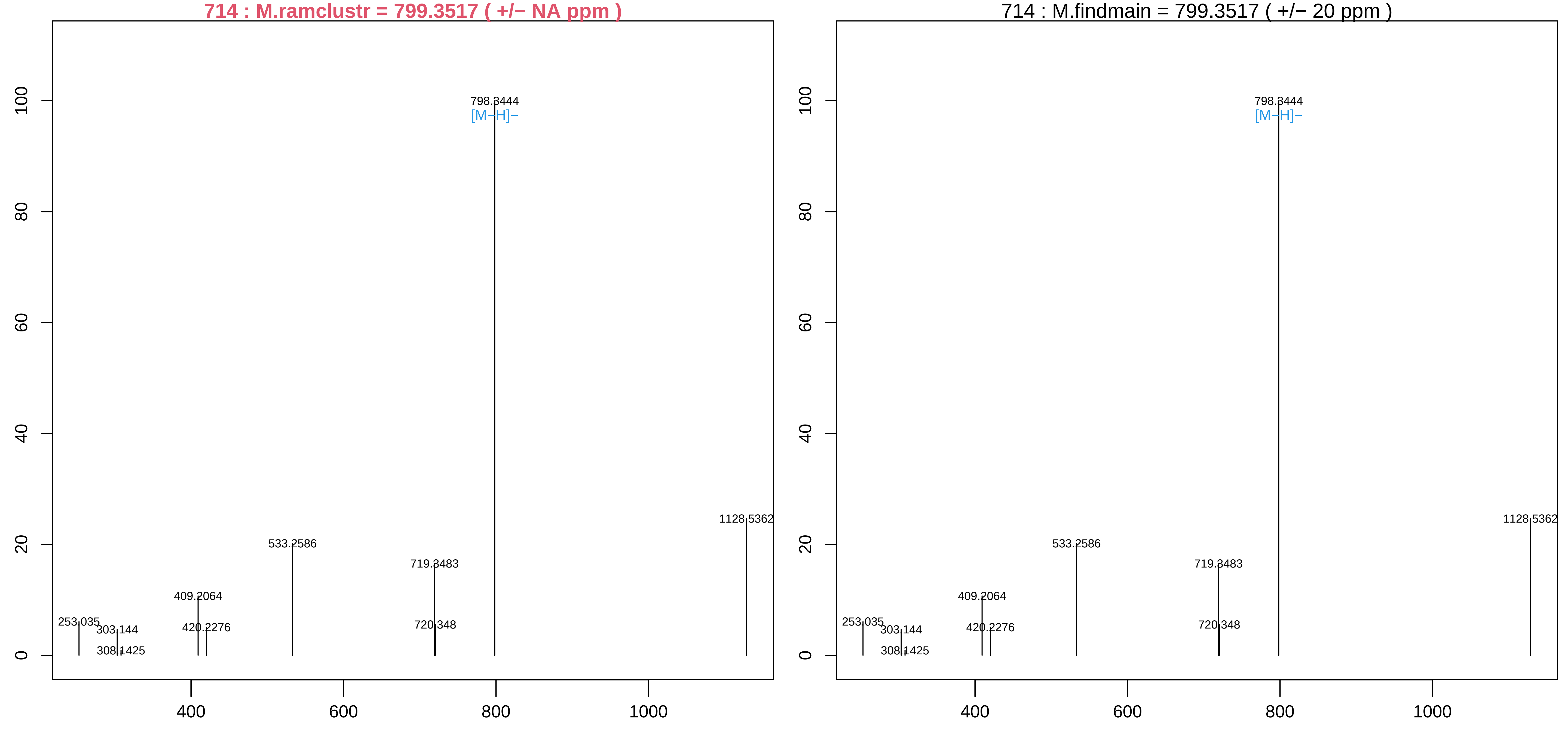
714 : M.ramclustr = 799.3517 (+/− NA ppm)
798.3444
[M−H]−
1128.5362
533.2586
719.3483
409.2064
253.035
303.144
420.2276
720.348
308.1425
0
20
40
60
80
100
400
600
800
1000
714 : M.findmain = 799.3517 (+/− 20 ppm)
798.3444
[M−H]−
1128.5362
533.2586
719.3483
409.2064
253.035
303.144
420.2276
720.348
308.1425
0
20
40
60
80
100
400
600
800
1000

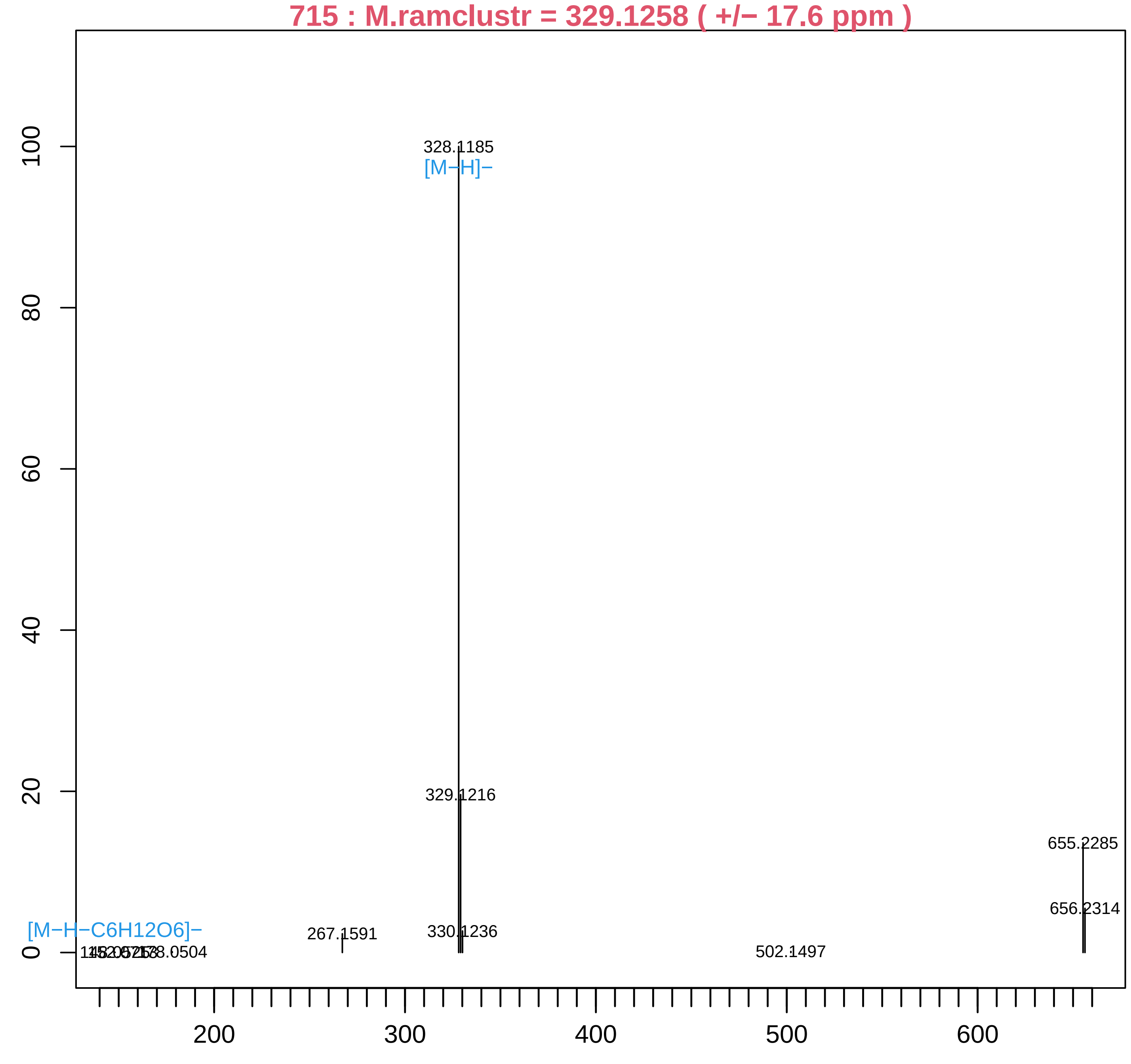
715 : M.ramclustr = 329.1258 (+/− 17.6 ppm)
328.1185
[M−H]−
329.1216
655.2285
656.2314
[M−H−C6H12O6]−
267.1591
330.1236
502.1497
3.0504
0
20
40
60
80
100
200
300
400
500
600
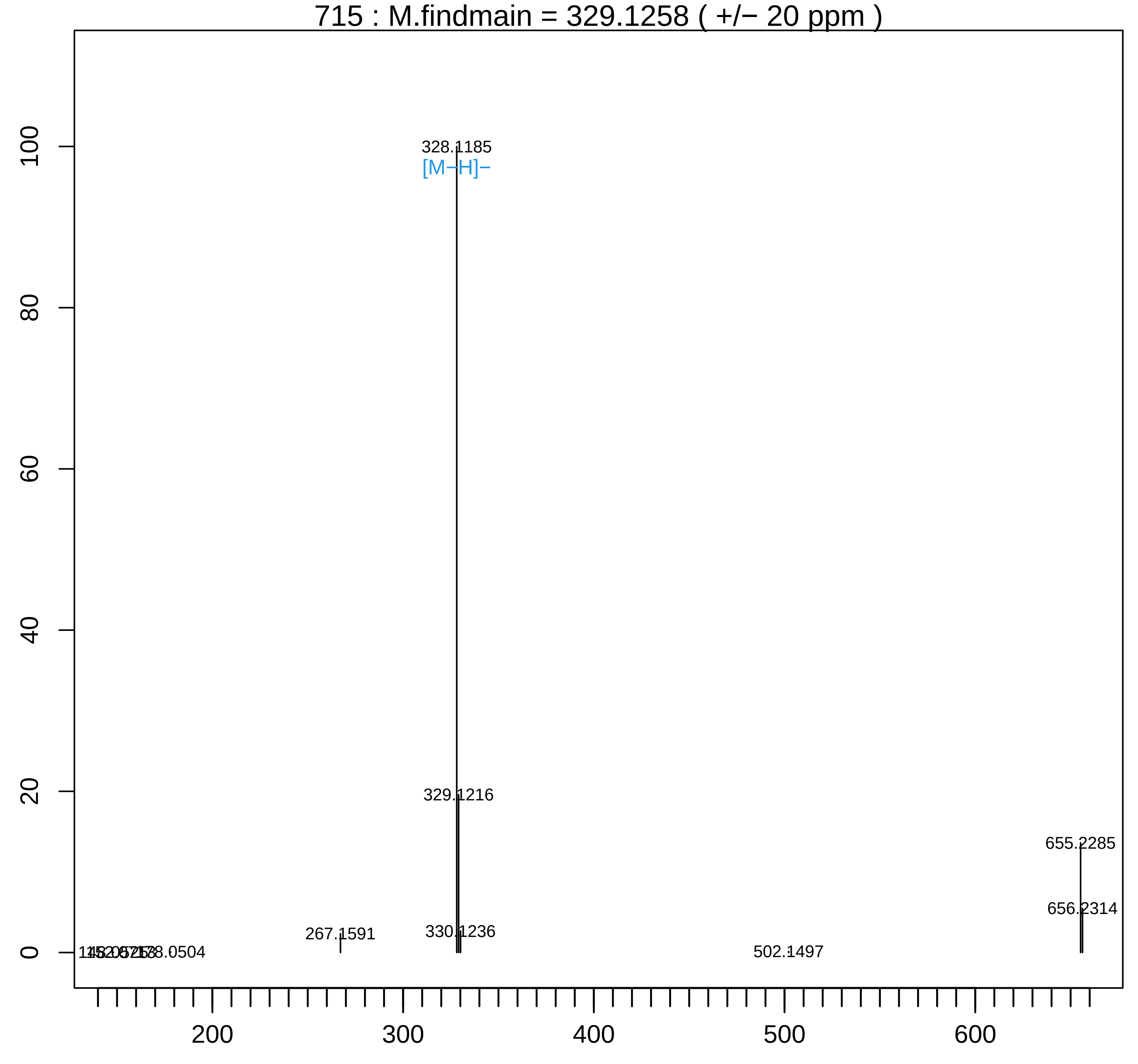
715 : M.findmain = 329.1258 (+/− 20 ppm)
328.1185
[M−H]−
329.1216
655.2285
656.2314
267.1591
330.1236
502.1497
3.0504
0
20
40
60
80
100
200
300
400
500
600

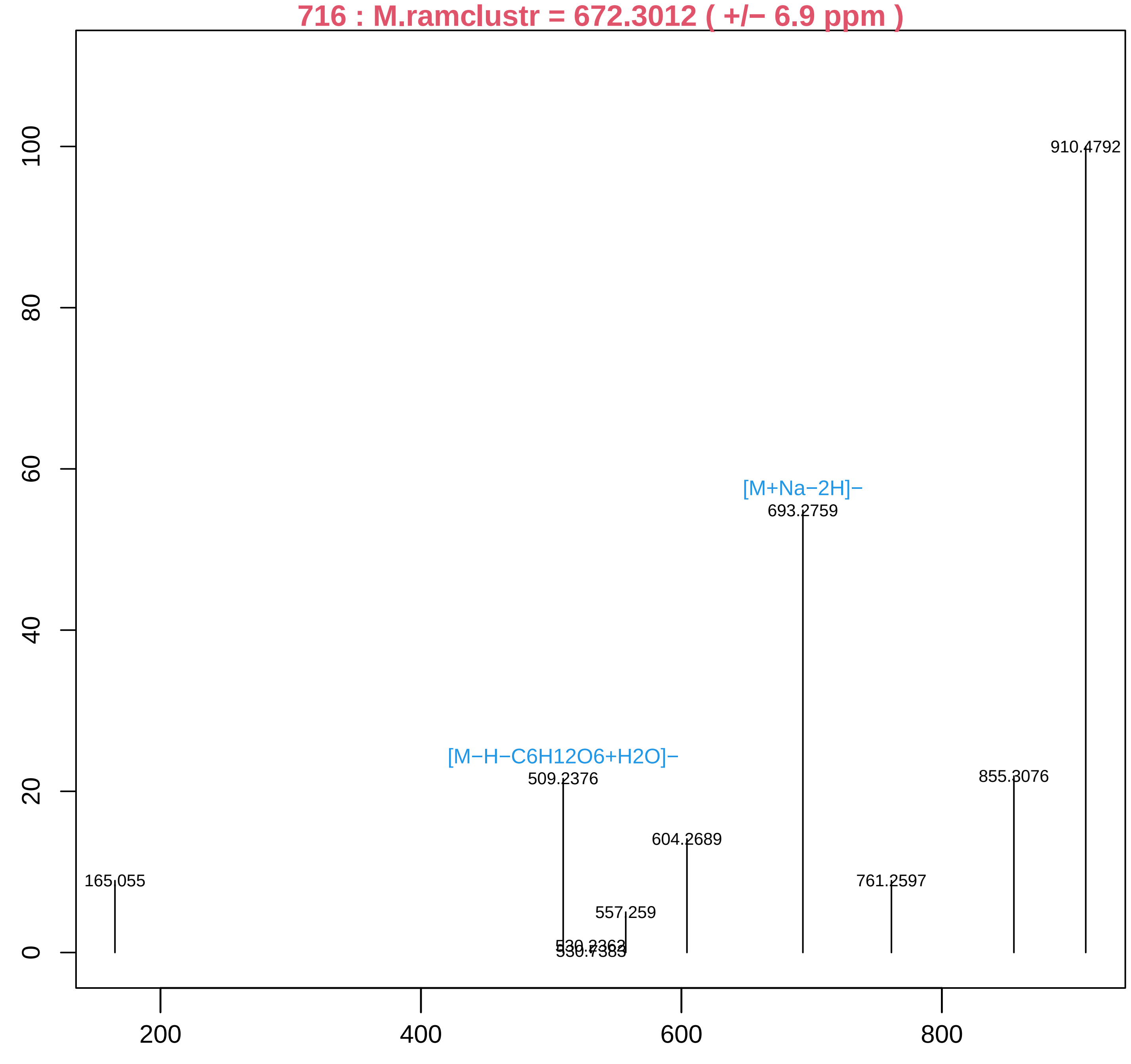

716 : M.ramclustr = 672.3012 (+/− 6.9 ppm)
910.4792
[M+Na−2H]−
693.2759
[M−H−C6H12O6+H2O]−
509.2376
855.3076
604.2689
165.055
761.2597
557.259
530.2362
530.7383
0
20
40
60
80
100
200
400
600
800

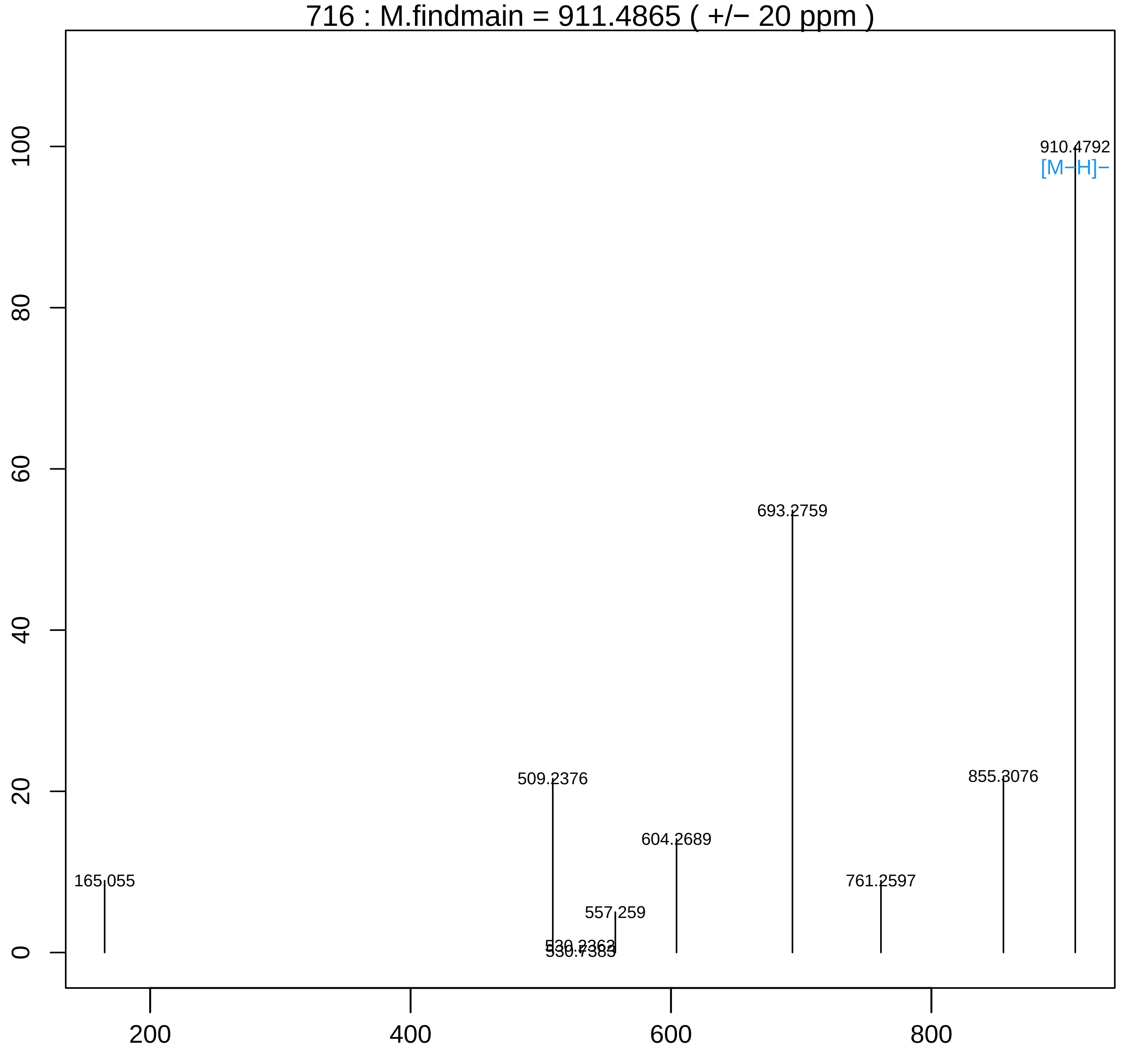

716 : M.findmain = 911.4865 (+/− 20 ppm)
910.4792
[M−H]−
693.2759
509.2376
855.3076
604.2689
165.055
761.2597
557.259
530.2362
530.7383
0
20
40
60
80
100
200
400
600
800

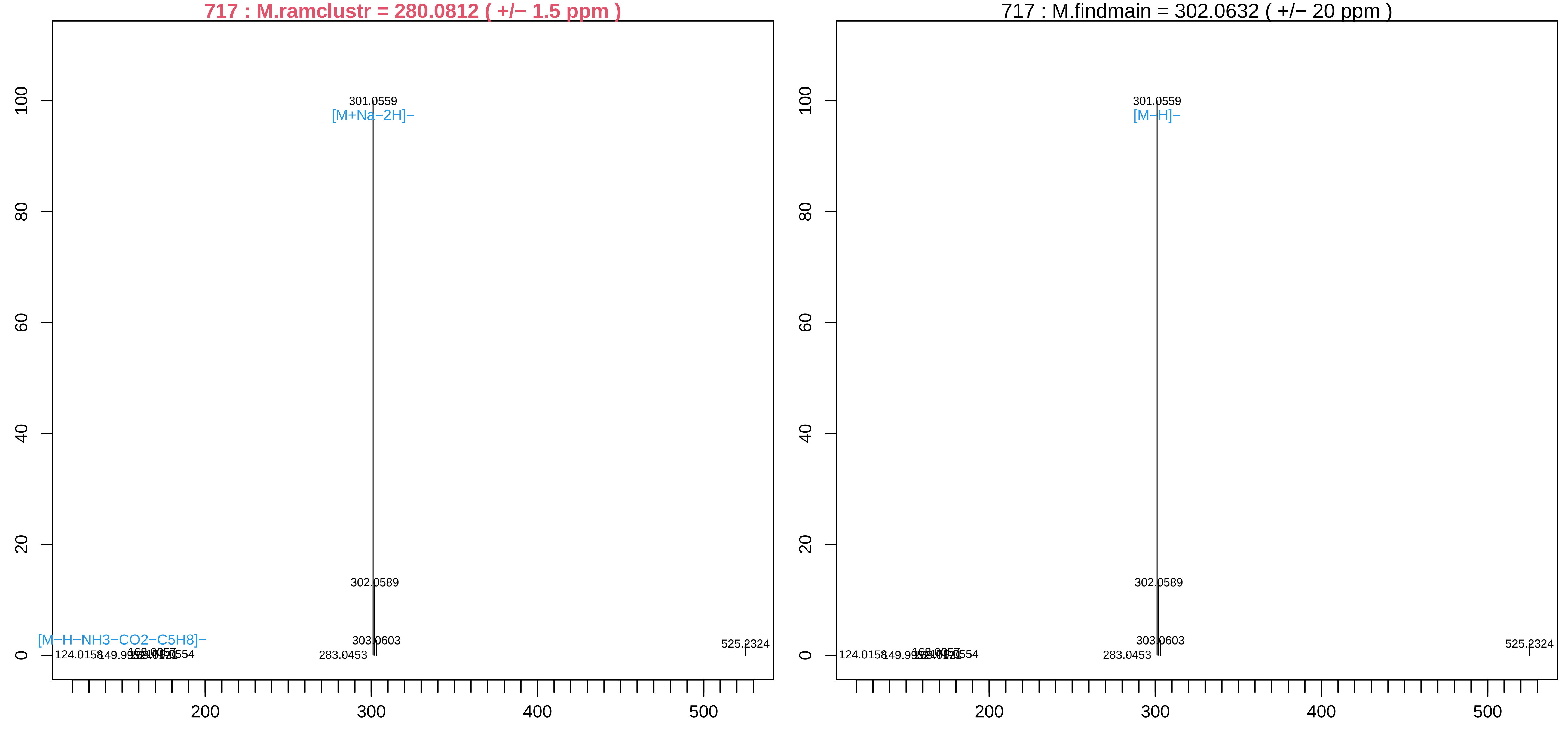
717 : M.ramclustr = 280.0812 (+/− 1.5 ppm)
301.0559
[M+Na−2H]−
302.0589
303.0603
283.0453
525.2324
[M−H−NH3−CO2−C5H8]−
124.0158
717 : M.findmain = 302.0632 (+/− 20 ppm)
301.0559
[M−H]−
302.0589
303.0603
283.0453
525.2324
124.0158
0
20
40
60
80
100
200
300
400
500

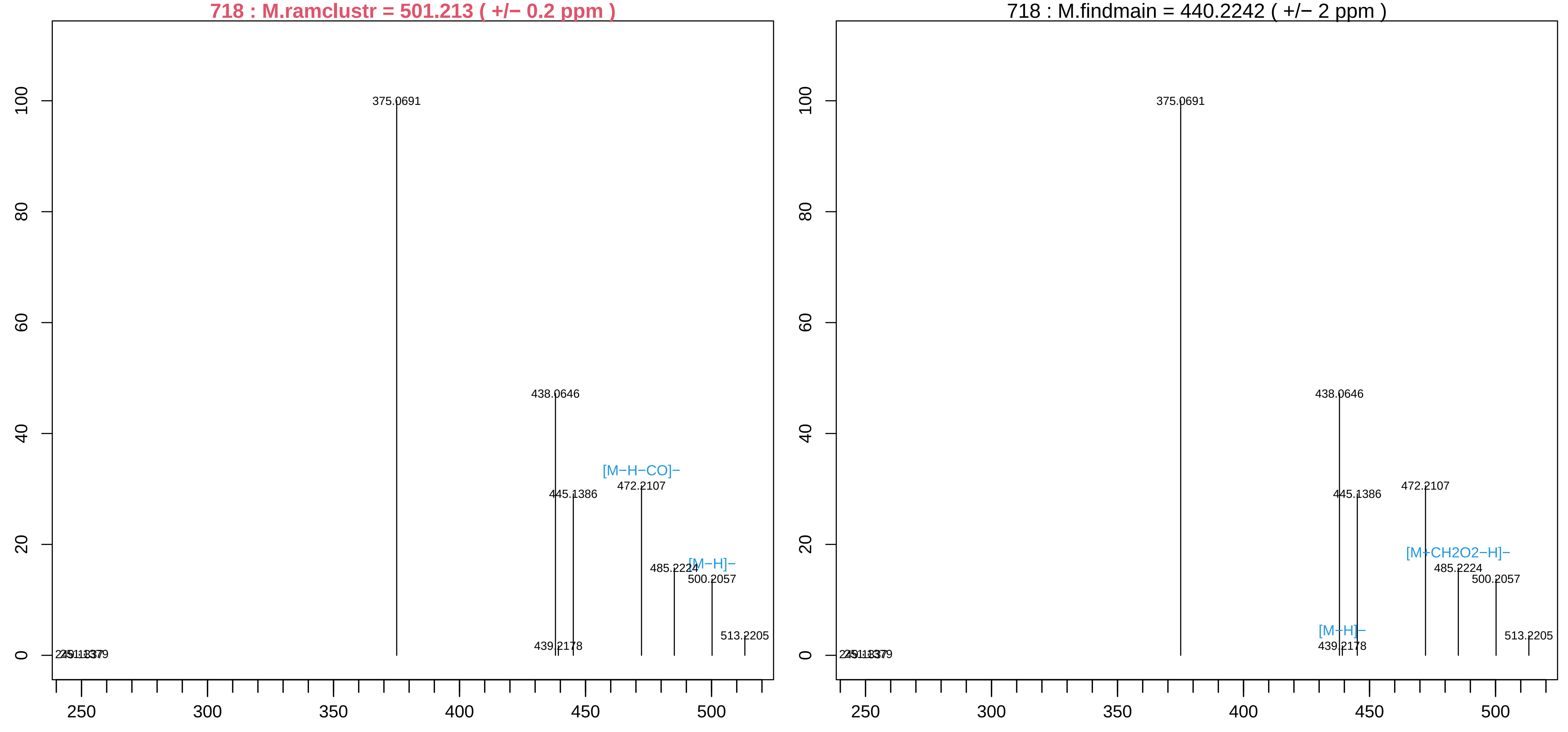

718 : M.ramclustr = 501.213 (+/− 0.2 ppm)
375.0691
438.0646
[M−H−CO]−
472.2107
445.1386
485.2224
[M−H]−
500.2057
513.2205
439.2178
718 : M.findmain = 440.2242 (+/− 2 ppm)
375.0691
438.0646
472.2107
445.1386
[M+CH2O2−H]−
485.2224
500.2057
[M−H]−
439.2178
513.2205
0
20
40
60
80
100
250
300
350
400
450
500

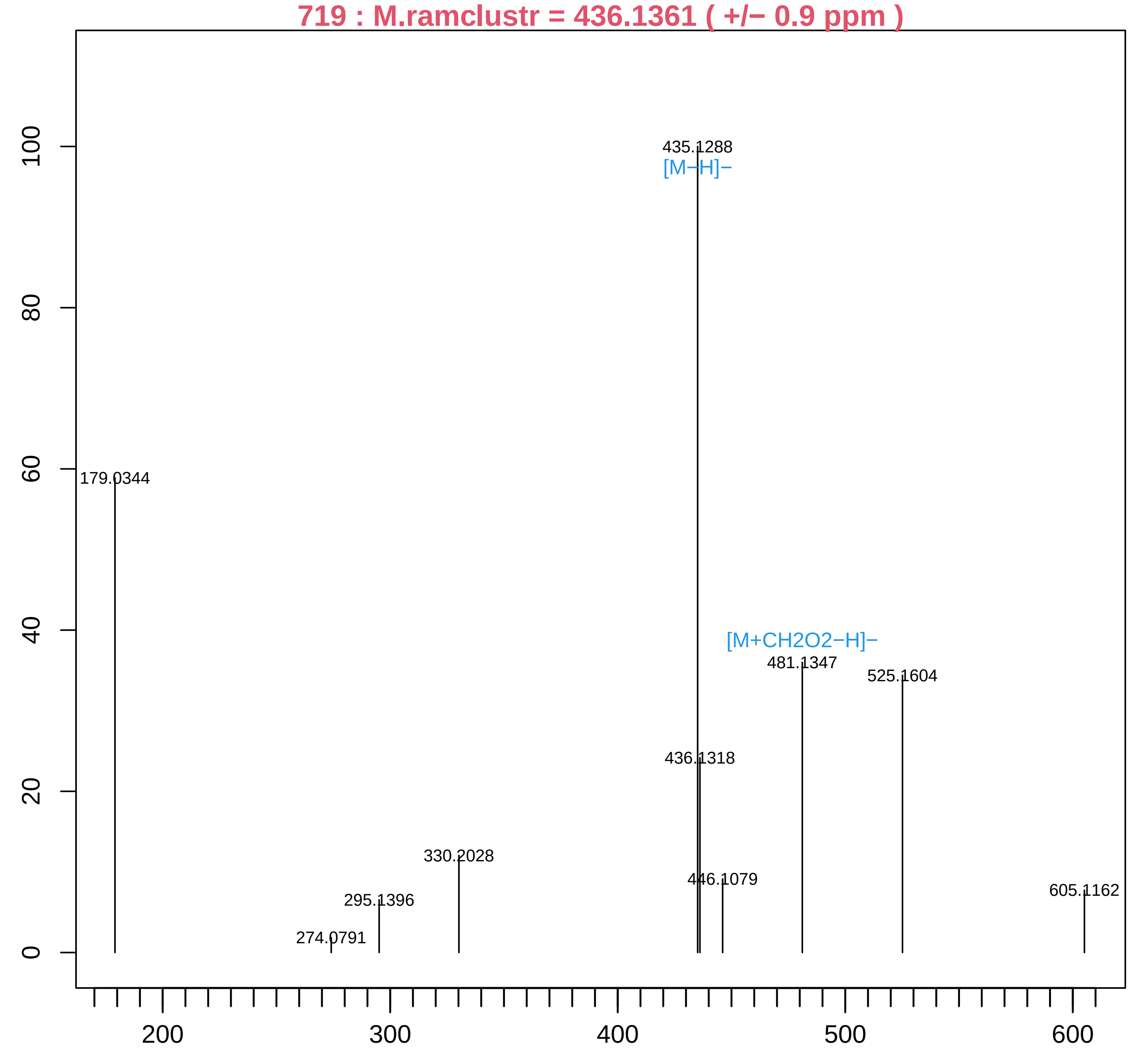

719 : M.ramclustr = 436.1361 (+/− 0.9 ppm)
435.1288
[M−H]−
179.0344
[M+CH2O2−H]−
481.1347
525.1604
436.1318
330.2028
446.1079
605.1162
295.1396
274.0791
0
20
40
60
80
100
200
300
400
500
600

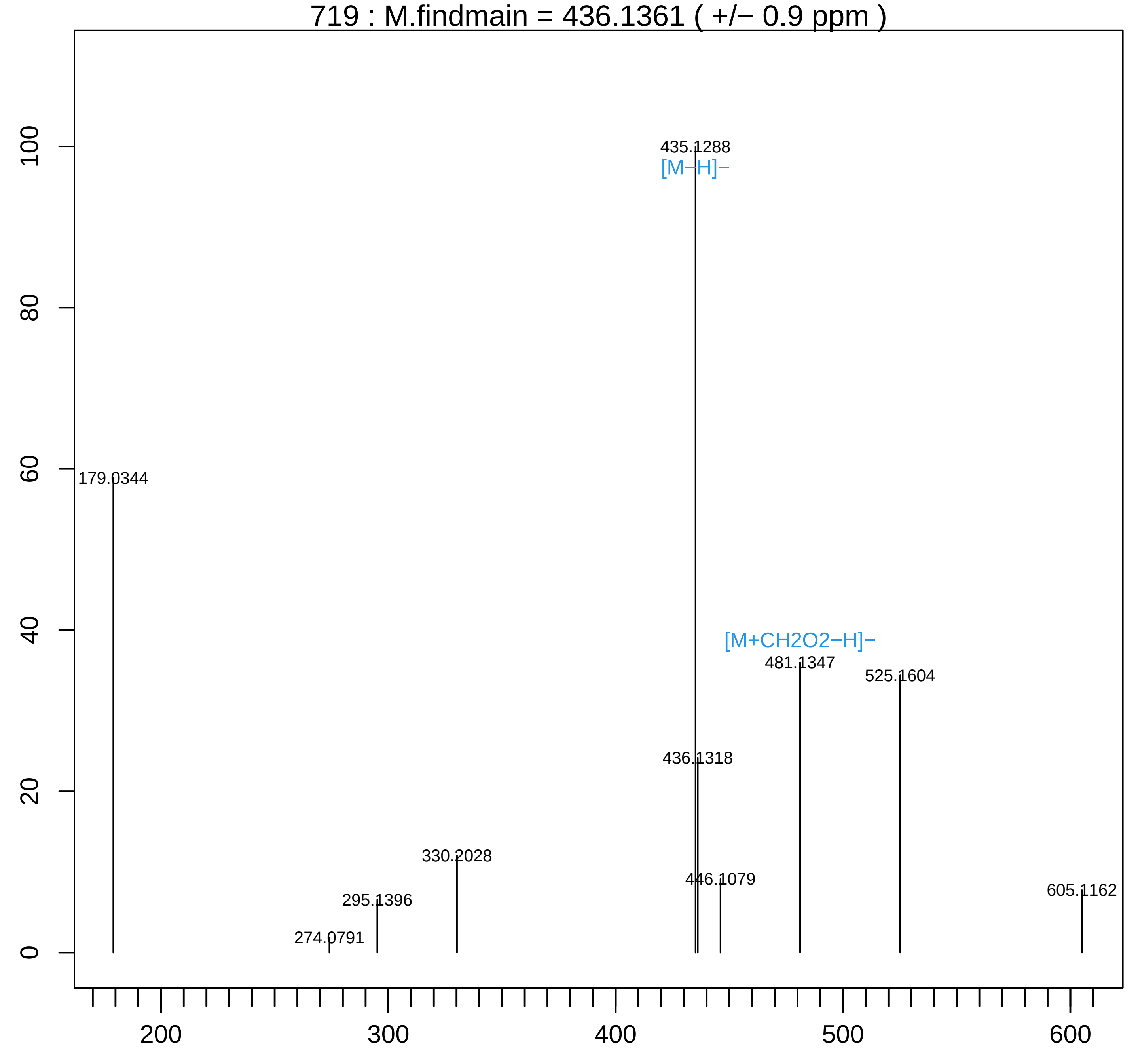

719 : M.findmain = 436.1361 (+/− 0.9 ppm)
435.1288
[M−H]−
179.0344
[M+CH2O2−H]−
481.1347
525.1604
436.1318
330.2028
446.1079
605.1162
295.1396
274.0791
0
20
40
60
80
100
200
300
400
500
600

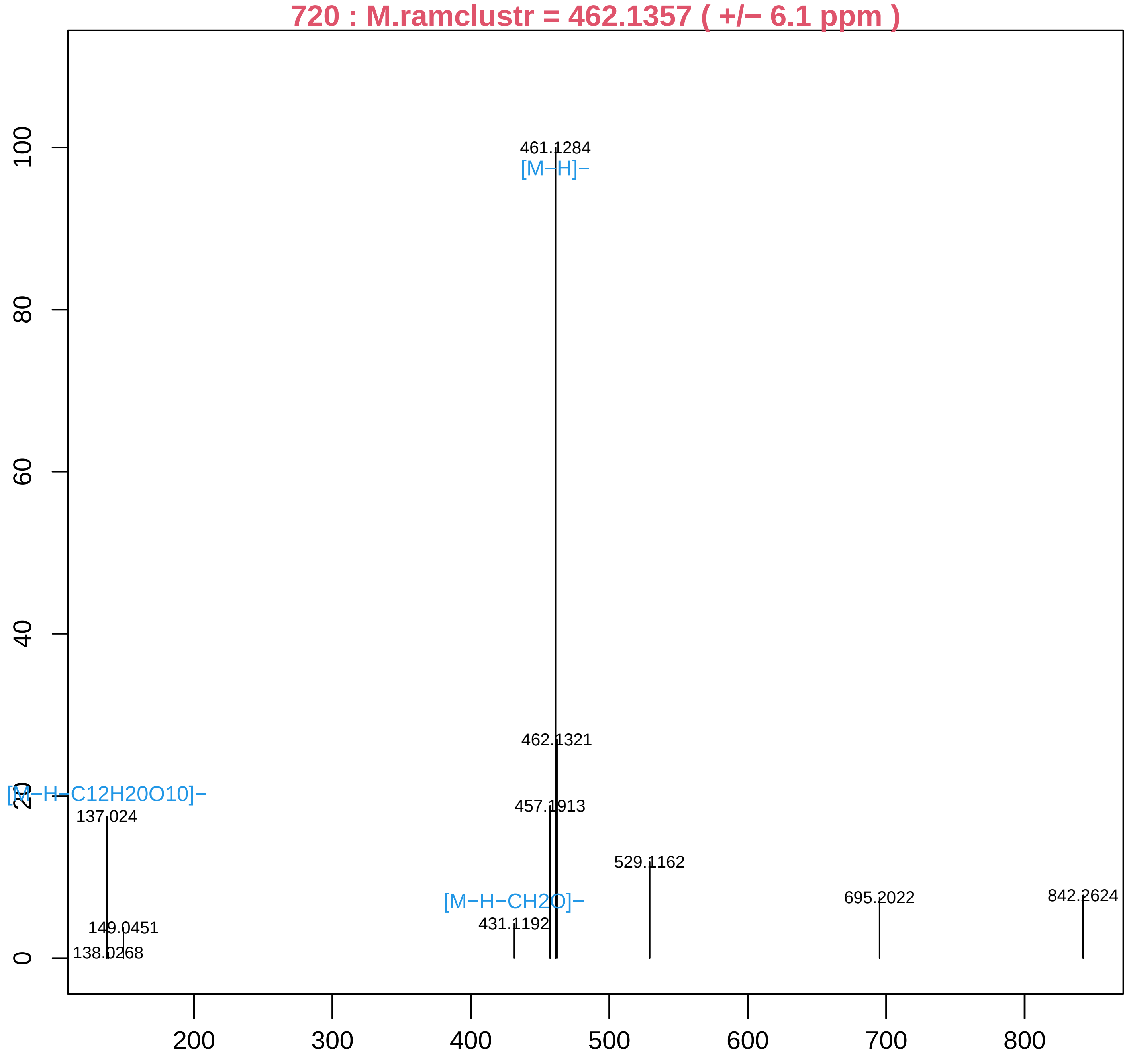

720 : M.ramclustr = 462.1357 (+/− 6.1 ppm)
461.1284
[M−H]−
462.1321
[M−H−C12H20O10]−
137.024
457.1913
529.1162
[M−H−CH2O]−
695.2022
842.2624
431.1192
149.0451
138.0268
0
20
40
60
80
100
200
300
400
500
600
700
800

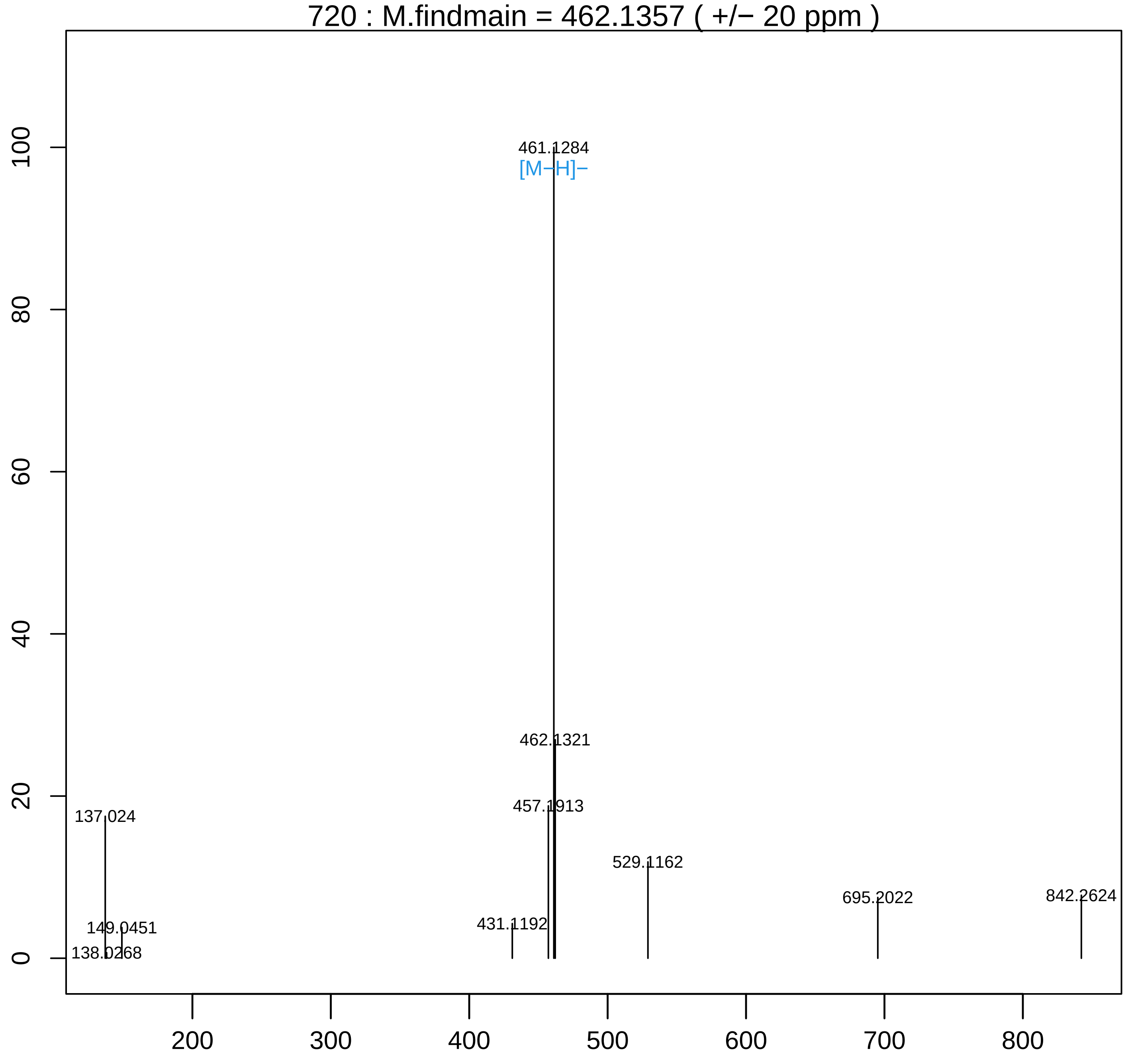

720 : M.findmain = 462.1357 (+/− 20 ppm)
461.1284
[M−H]−
462.1321
137.024
457.1913
529.1162
695.2022
842.2624
431.1192
149.0451
138.0268
0
20
40
60
80
100
200
300
400
500
600
700
800

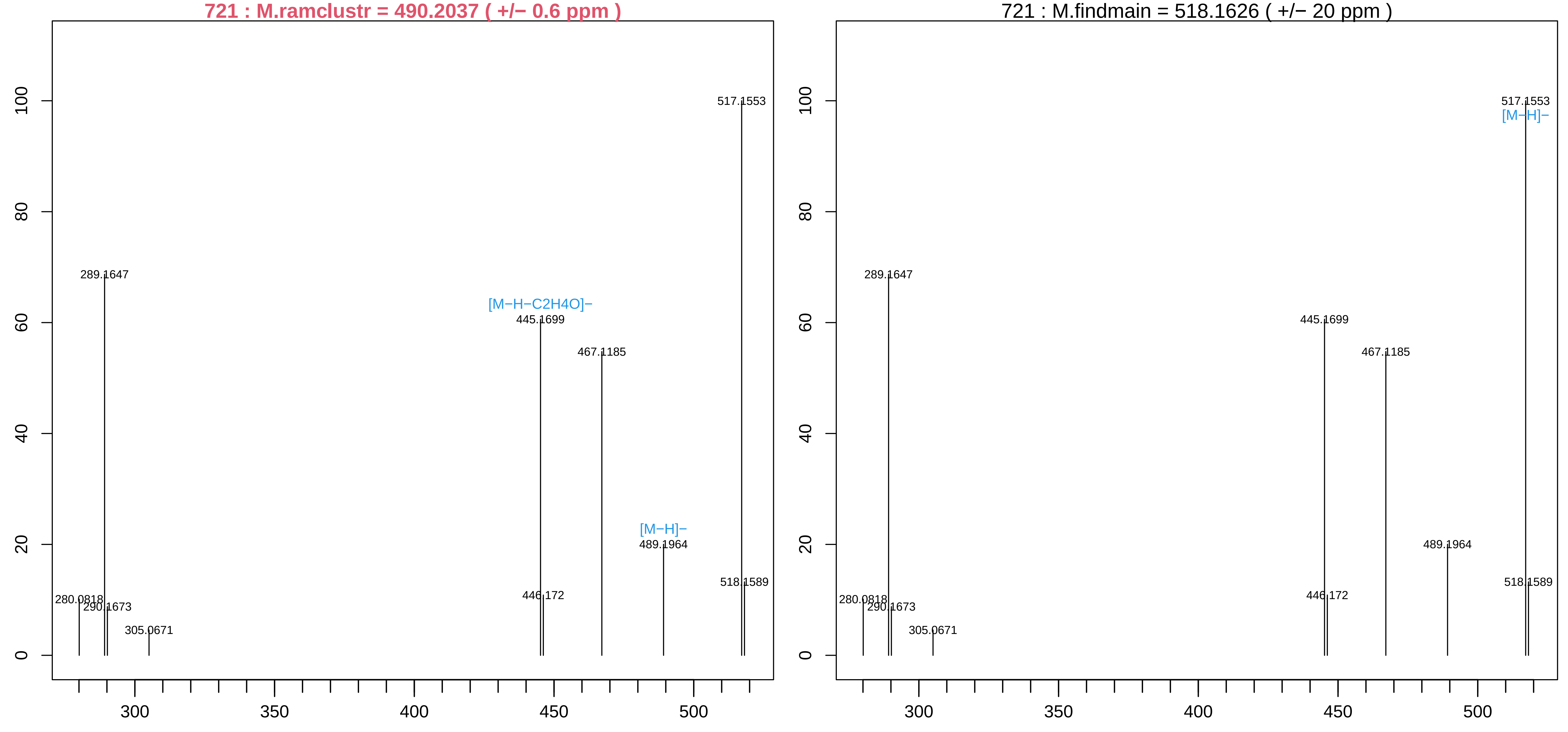

721 : M.ramclustr = 490.2037 (+/− 0.6 ppm)
517.1553
289.1647
[M−H−C2H4O]−
445.1699
467.1185
[M−H]−
489.1964
518.1589
446.172
280.0818
290.1673
305.0671
721 : M.findmain = 518.1626 (+/− 20 ppm)
517.1553
[M−H]−
289.1647
445.1699
467.1185
489.1964
518.1589
446.172
280.0818
290.1673
305.0671
0
20
40
60
80
100
300
350
400
450
500

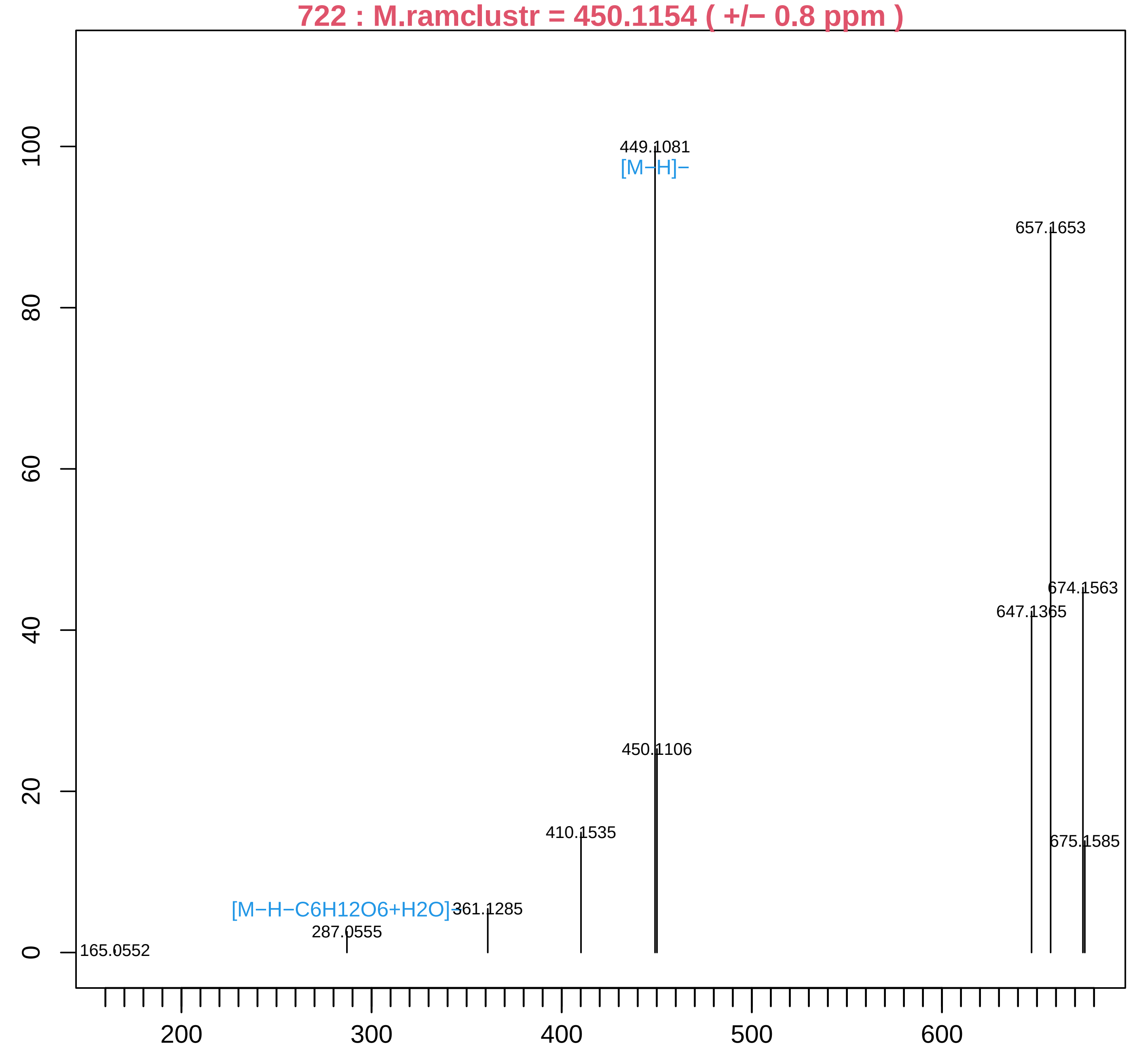

722 : M.ramclustr = 450.1154 (+/− 0.8 ppm)
449.1081
[M−H]−
657.1653
674.1563
647.1365
450.1106
410.1535
675.1585
[M−H−C6H12O6+H2O]-
361.1285
287.0555
165.0552
0
20
40
60
80
100
200
300
400
500
600

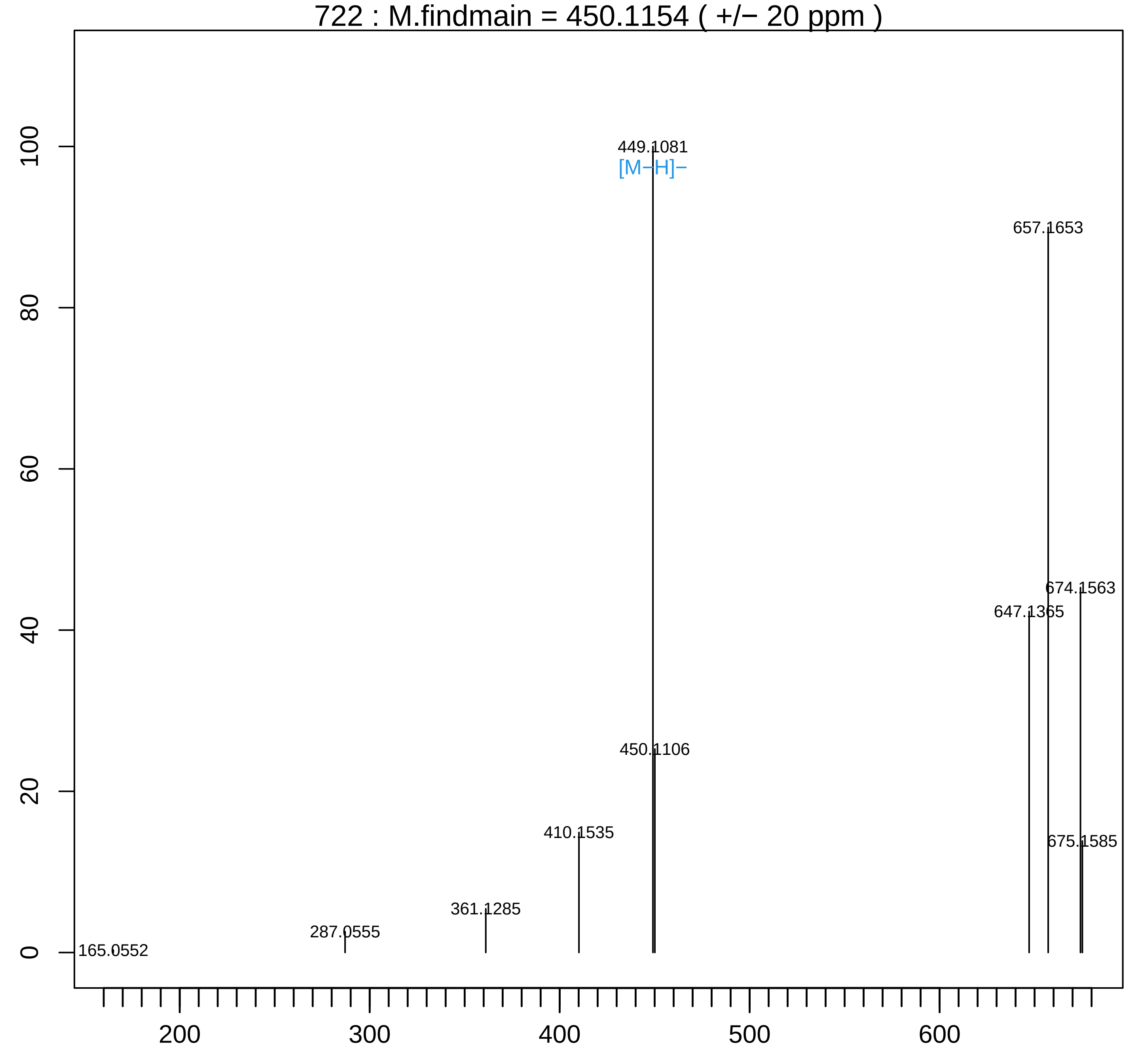

722 : M.findmain = 450.1154 (+/− 20 ppm)
449.1081
[M−H]−
657.1653
674.1563
647.1365
450.1106
410.1535
675.1585
361.1285
287.0555
165.0552
0
20
40
60
80
100
200
300
400
500
600

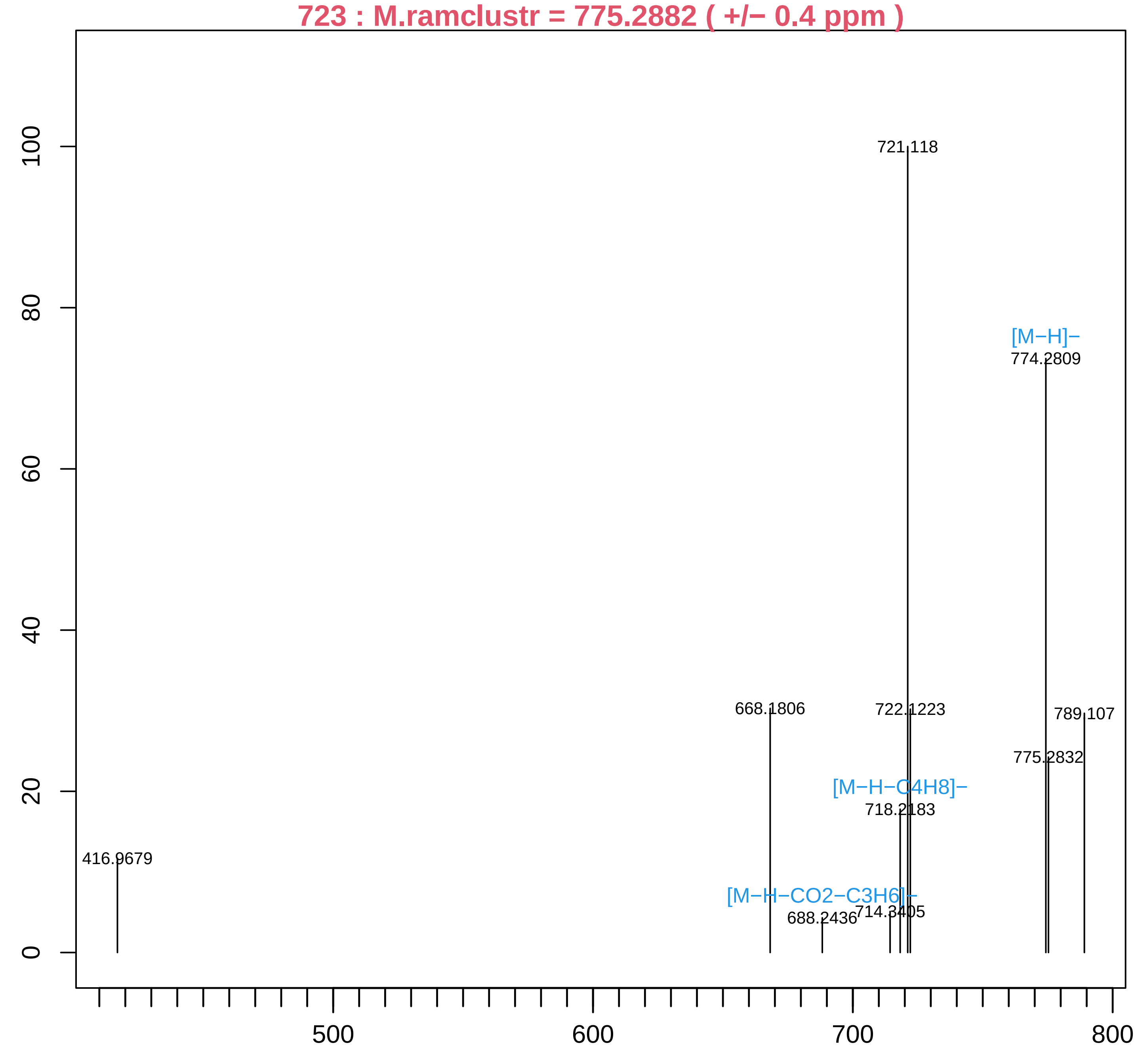

723 : M.ramclustr = 775.2882 (+/− 0.4 ppm)
721.118
[M−H]−
774.2809
668.1806
722.1223
789.107
775.2832
[M−H−C4H8]−
718.2183
416.9679
[M−H−CO2−C3H6]−
688.2436
714.3405
0
20
40
60
80
100
500
600
700
800

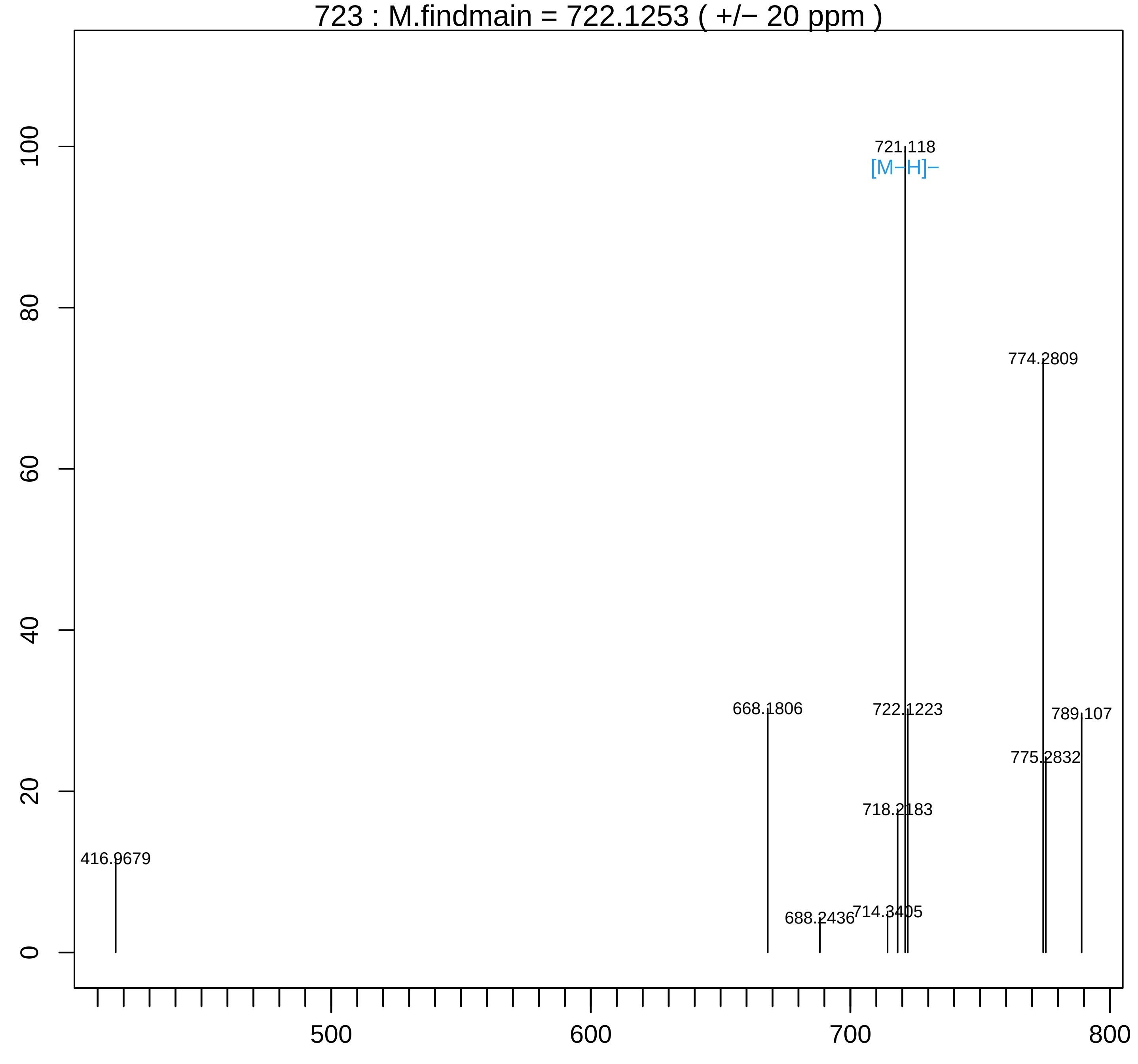

723 : M.findmain = 722.1253 (+/− 20 ppm)
721.118
[M−H]−
774.2809
668.1806
722.1223
789.107
775.2832
718.2183
416.9679
688.2436
714.3405
0
20
40
60
80
100
500
600
700
800

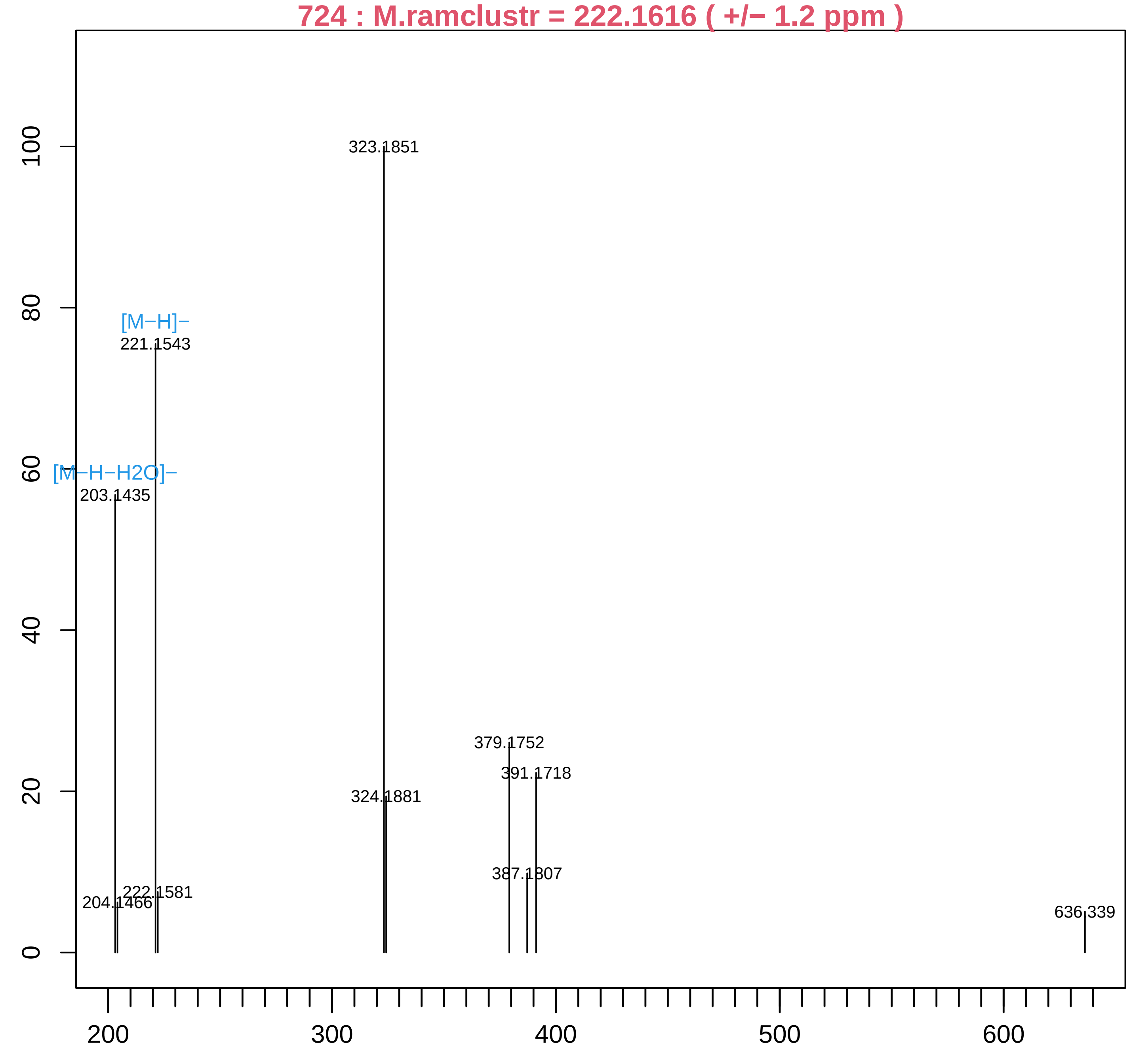

724 : M.ramclustr = 222.1616 (+/− 1.2 ppm)
323.1851
[M−H]−
221.1543
[M−H−H2O]−
203.1435
379.1752
391.1718
324.1881
387.1807
222.1581
204.1466
636.339
0
20
40
60
80
100
200
300
400
500
600

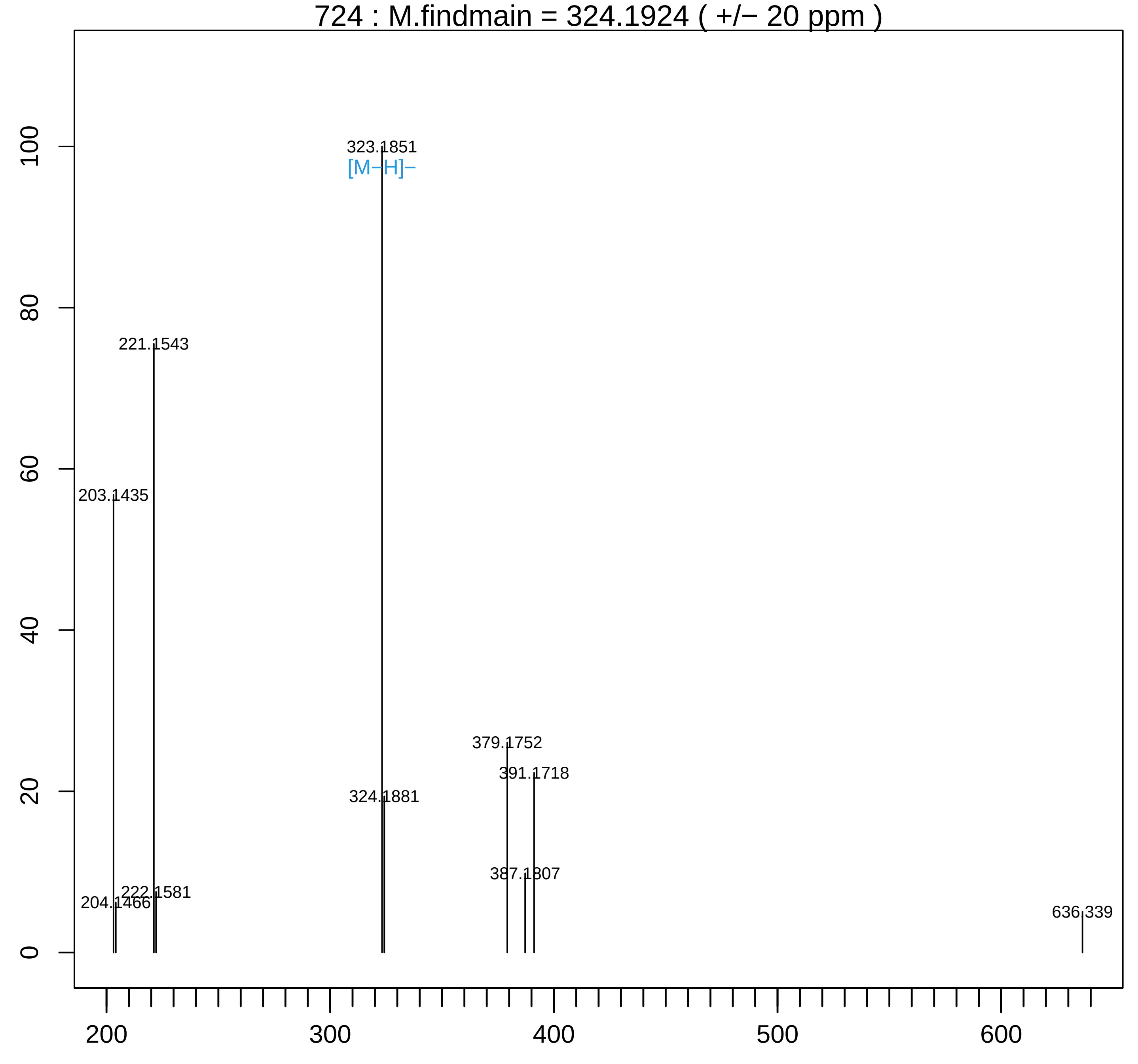

724 : M.findmain = 324.1924 (+/− 20 ppm)
323.1851
[M−H]−
221.1543
203.1435
379.1752
391.1718
324.1881
387.1807
222.1581
204.1466
636.339
0
20
40
60
80
100
200
300
400
500
600

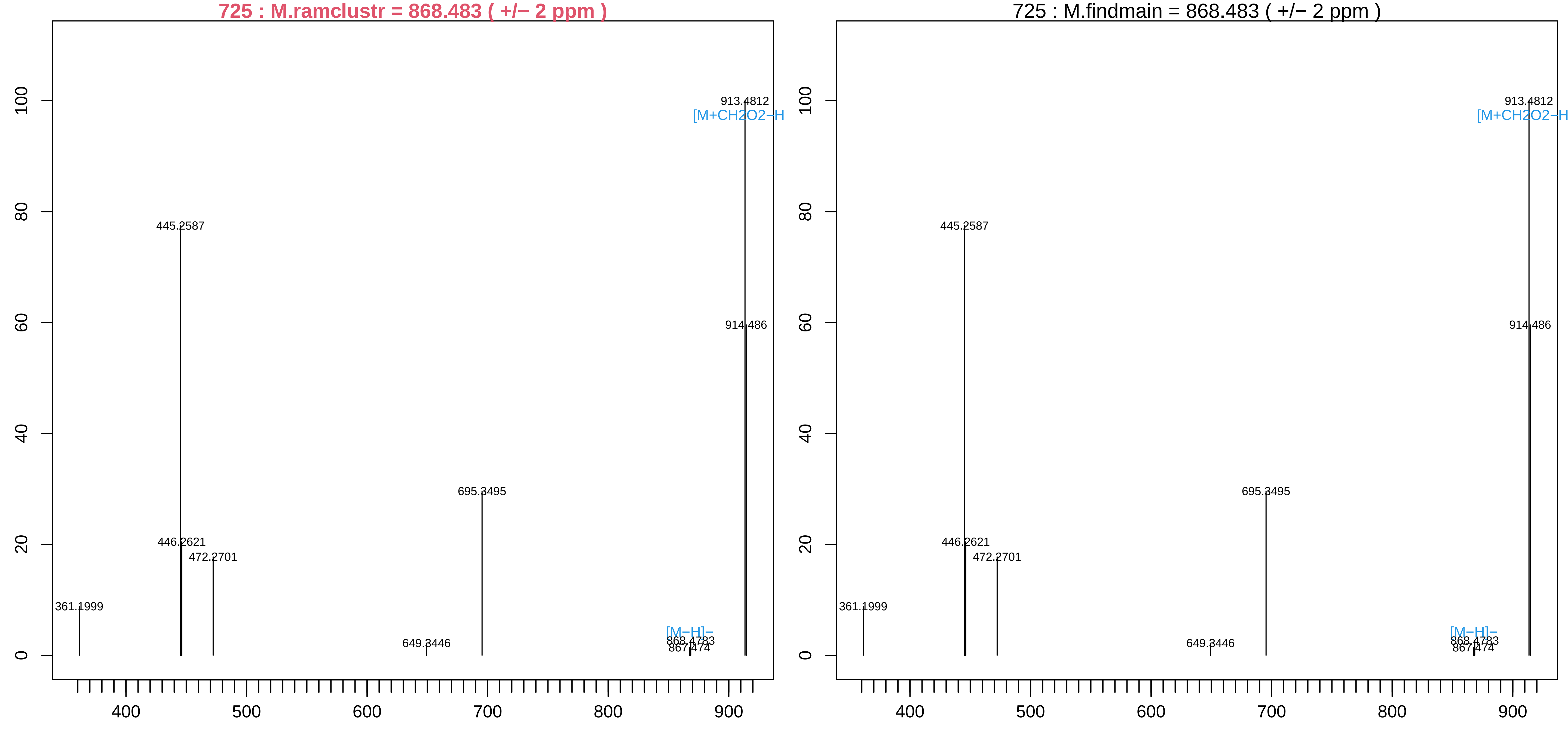

725 : M.ramclustr = 868.483 (+/− 2 ppm)
725 : M.findmain = 868.483 (+/− 2 ppm)
913.4812
[M+CH2O2−H
914.486
445.2587
446.2621
472.2701
361.1999
649.3446
695.3495
[M−H]−
868.4783
867.474
0
20
40
60
80
100
400
500
600
700
800
900

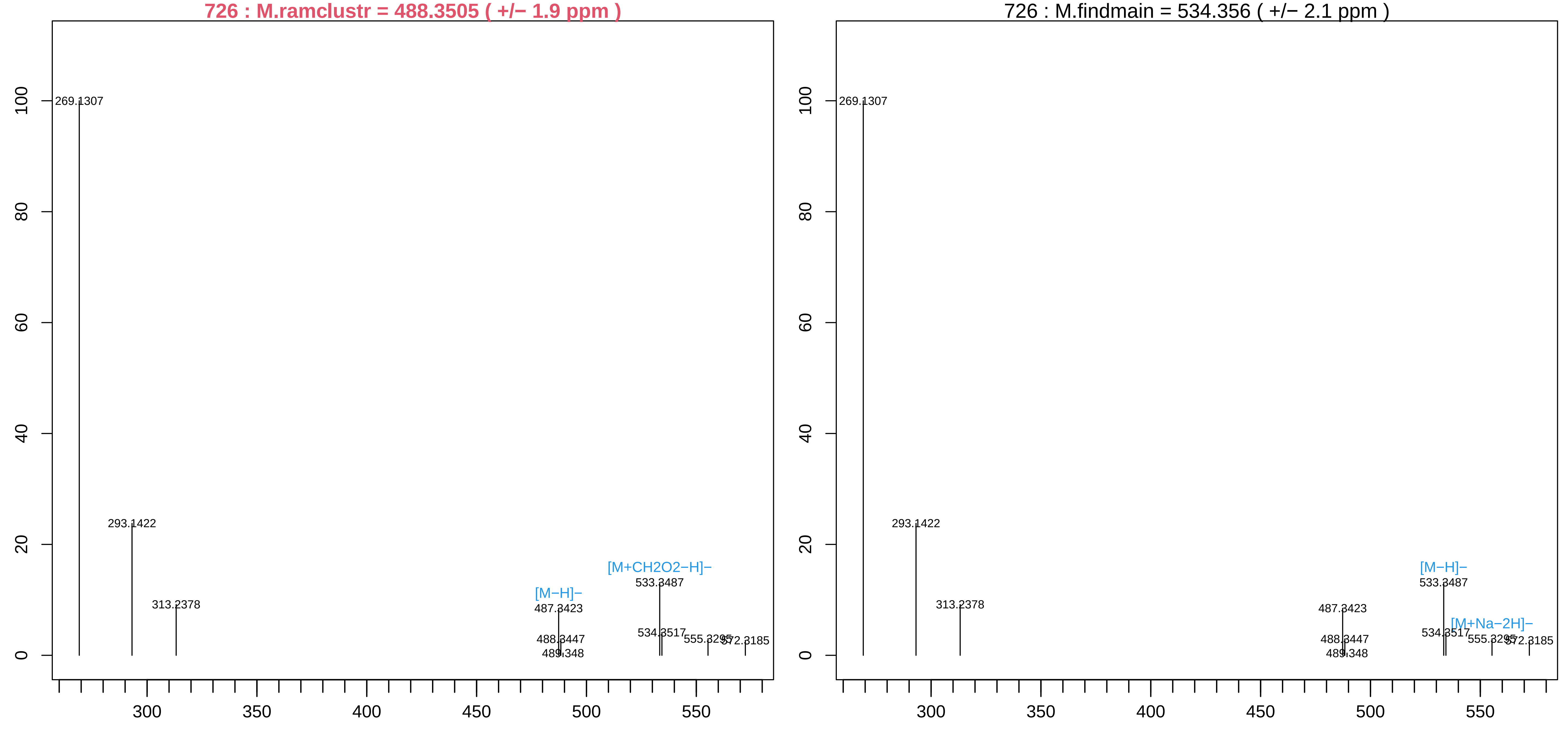
726 : M.ramclustr = 488.3505 (+/− 1.9 ppm)
726 : M.findmain = 534.356 (+/− 2.1 ppm)
269.1307
293.1422
313.2378
[M−H]−
487.3423
488.3447
489.348
[M+CH2O2−H]−
533.3487
534.3517
555.3295
572.3185
[M+Na−2H]−
0
20
40
60
80
100
300
350
400
450
500
550

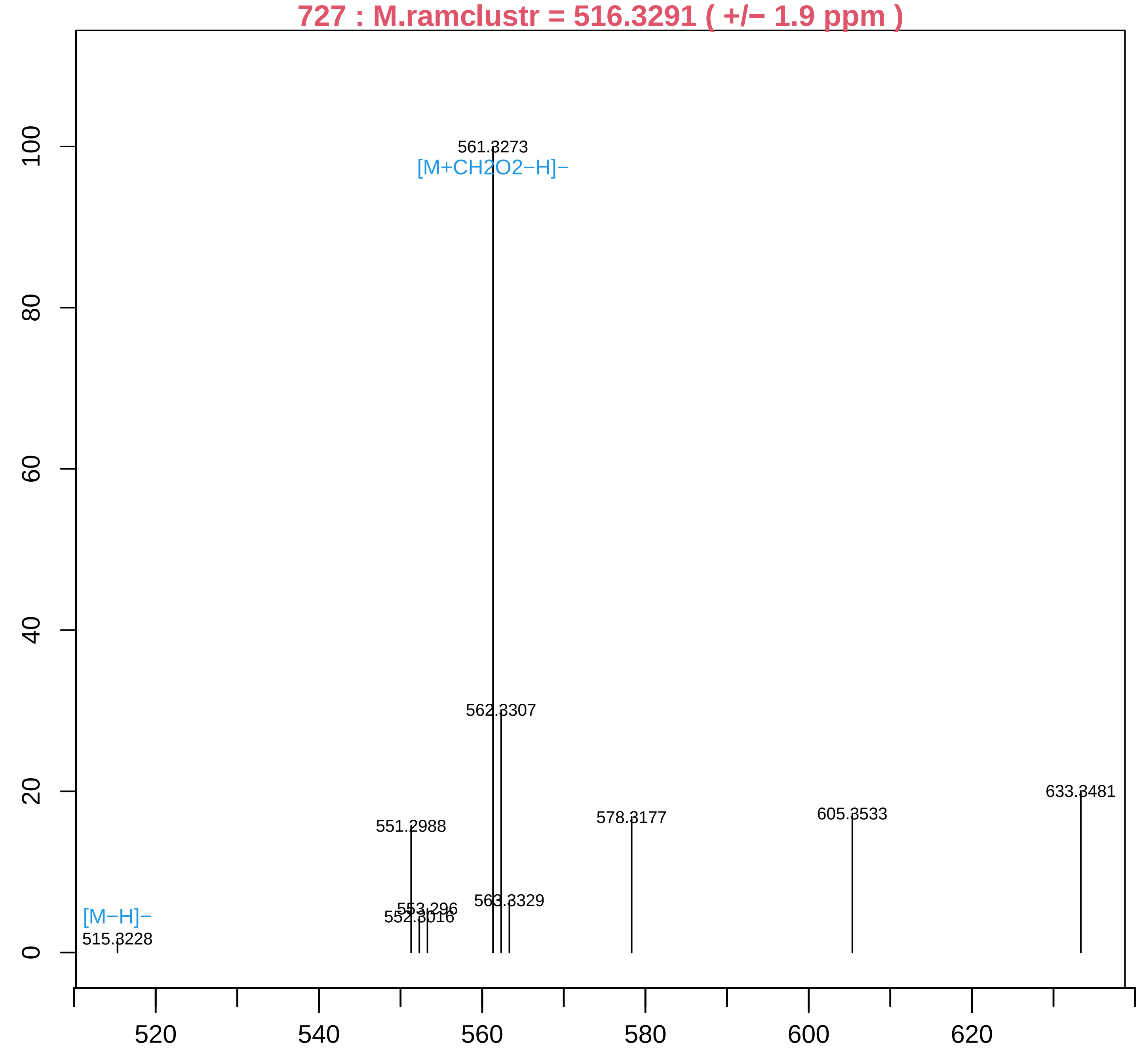
727 : M.ramclustr = 516.3291 (+/− 1.9 ppm)
561.3273
[M+CH2O2−H]−
562.3307
551.2988
578.3177
605.3533
633.3481
553.296
552.3016
563.3329
[M−H]−
515.3228
0
20
40
60
80
100
520
540
560
580
600
620

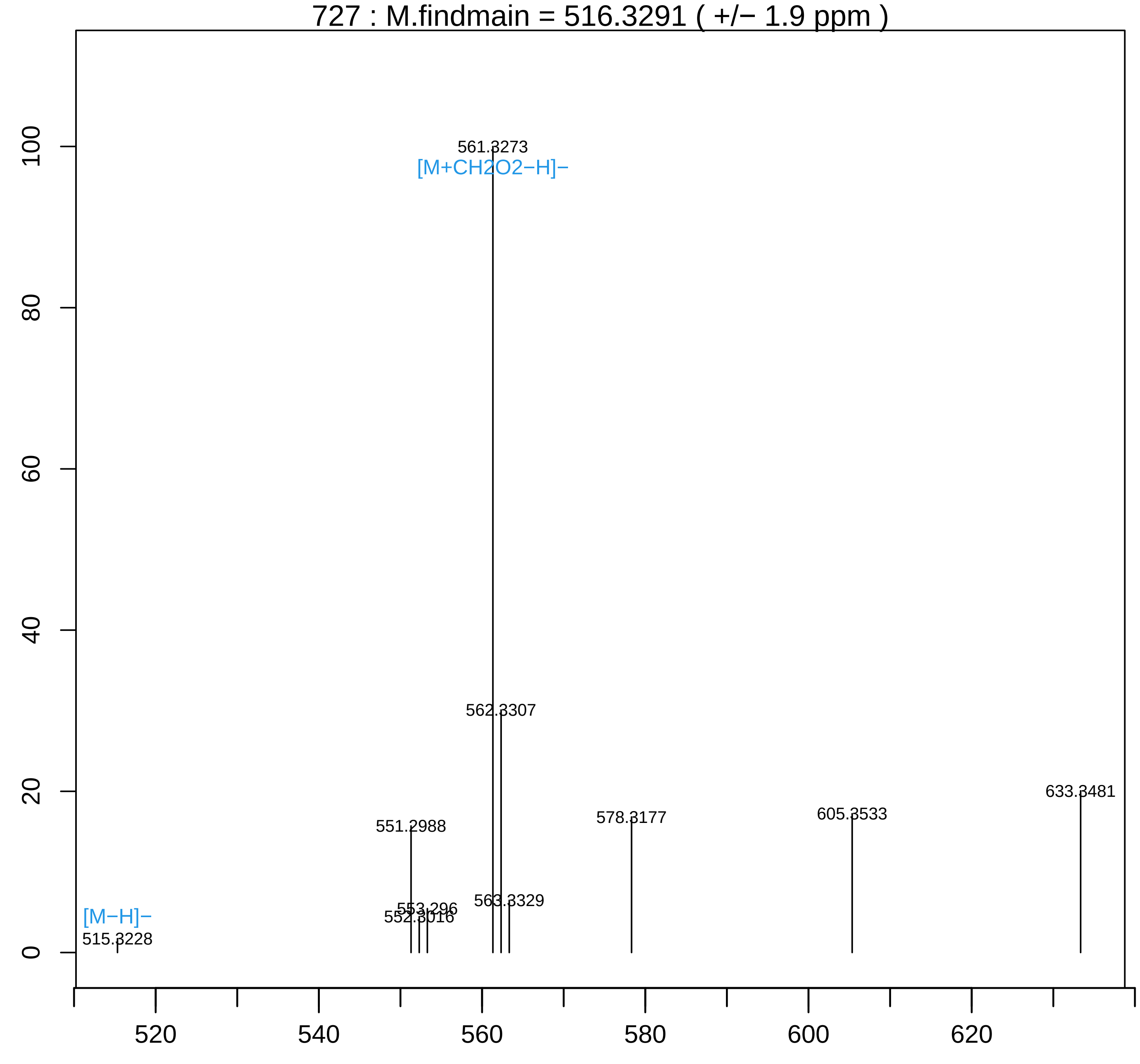
727 : M.findmain = 516.3291 (+/− 1.9 ppm)
561.3273
[M+CH2O2−H]−
562.3307
551.2988
578.3177
605.3533
633.3481
553.296
552.3016
563.3329
[M−H]−
515.3228
0
20
40
60
80
100
520
540
560
580
600
620

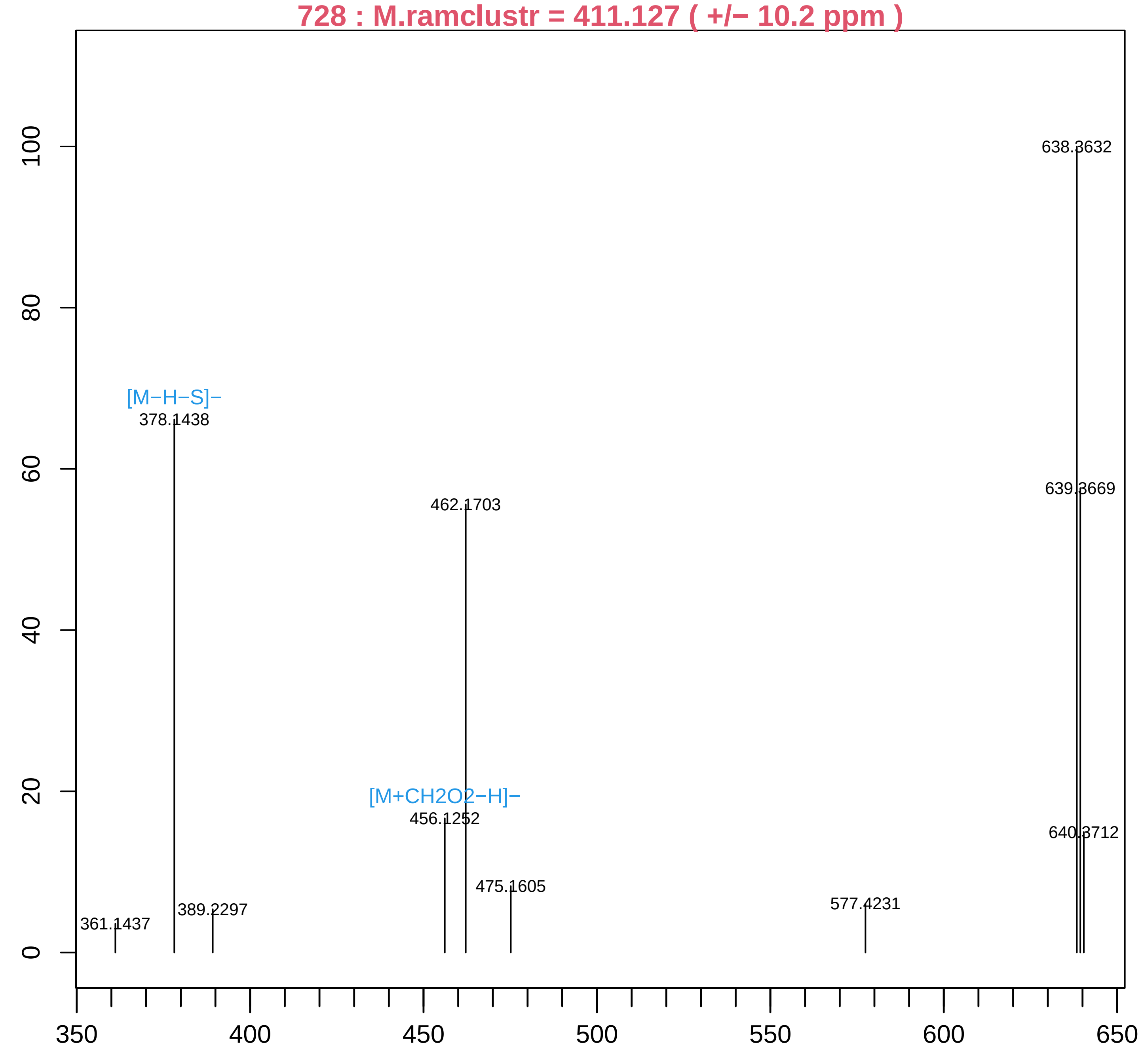

728 : M.ramclustr = 411.127 (+/− 10.2 ppm)
[M−H−S]−
378.1438
[M+CH2O2−H]−
456.1252
462.1703
475.1605
361.1437
389.2297
577.4231
638.3632
639.3669
640.3712
0
20
40
60
80
100
350
400
450
500
550
600
650

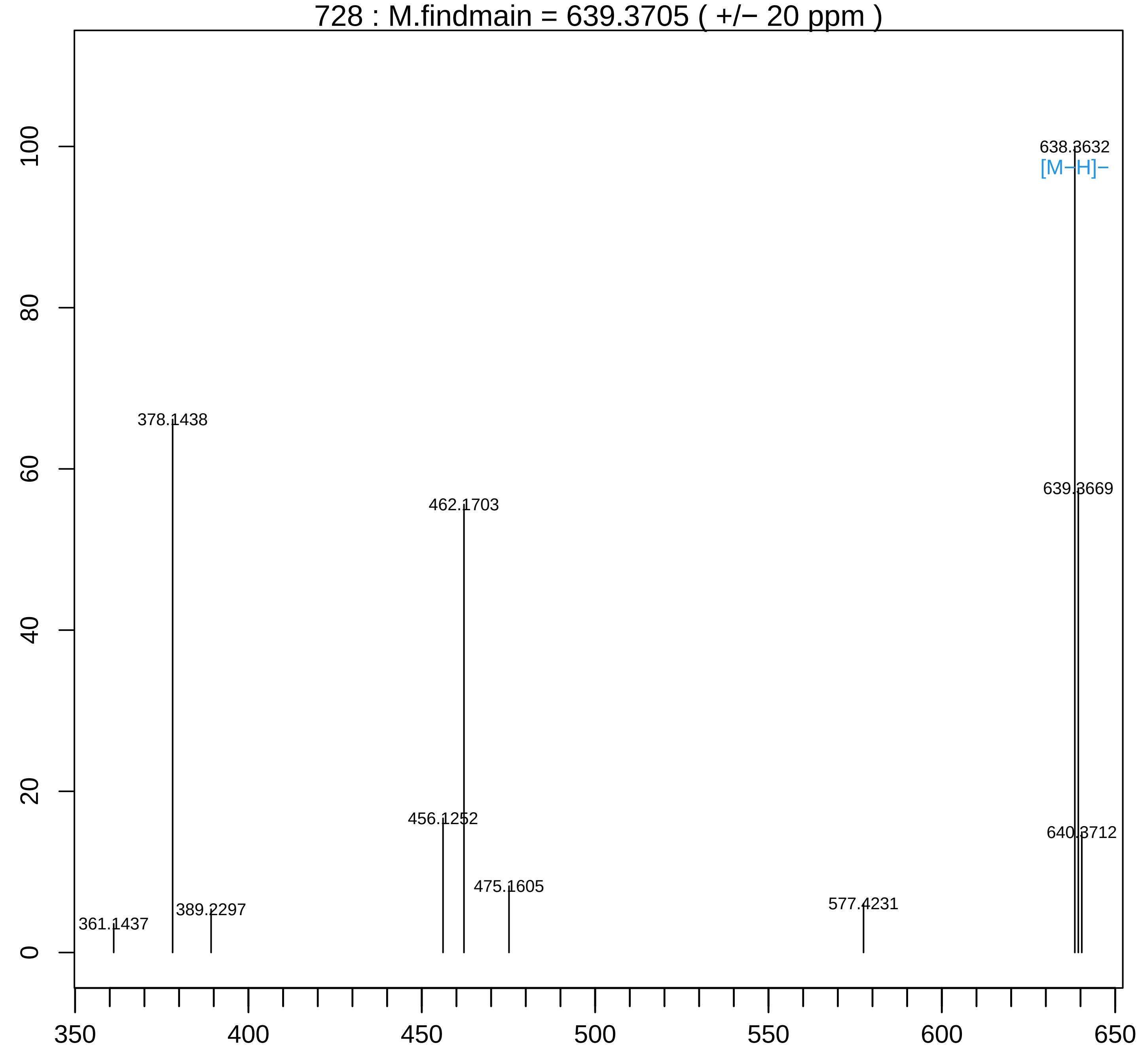

728 : M.findmain = 639.3705 (+/− 20 ppm)
638.3632
[M−H]−
639.3669
640.3712
378.1438
462.1703
456.1252
475.1605
361.1437
389.2297
577.4231
0
20
40
60
80
100
350
400
450
500
550
600
650

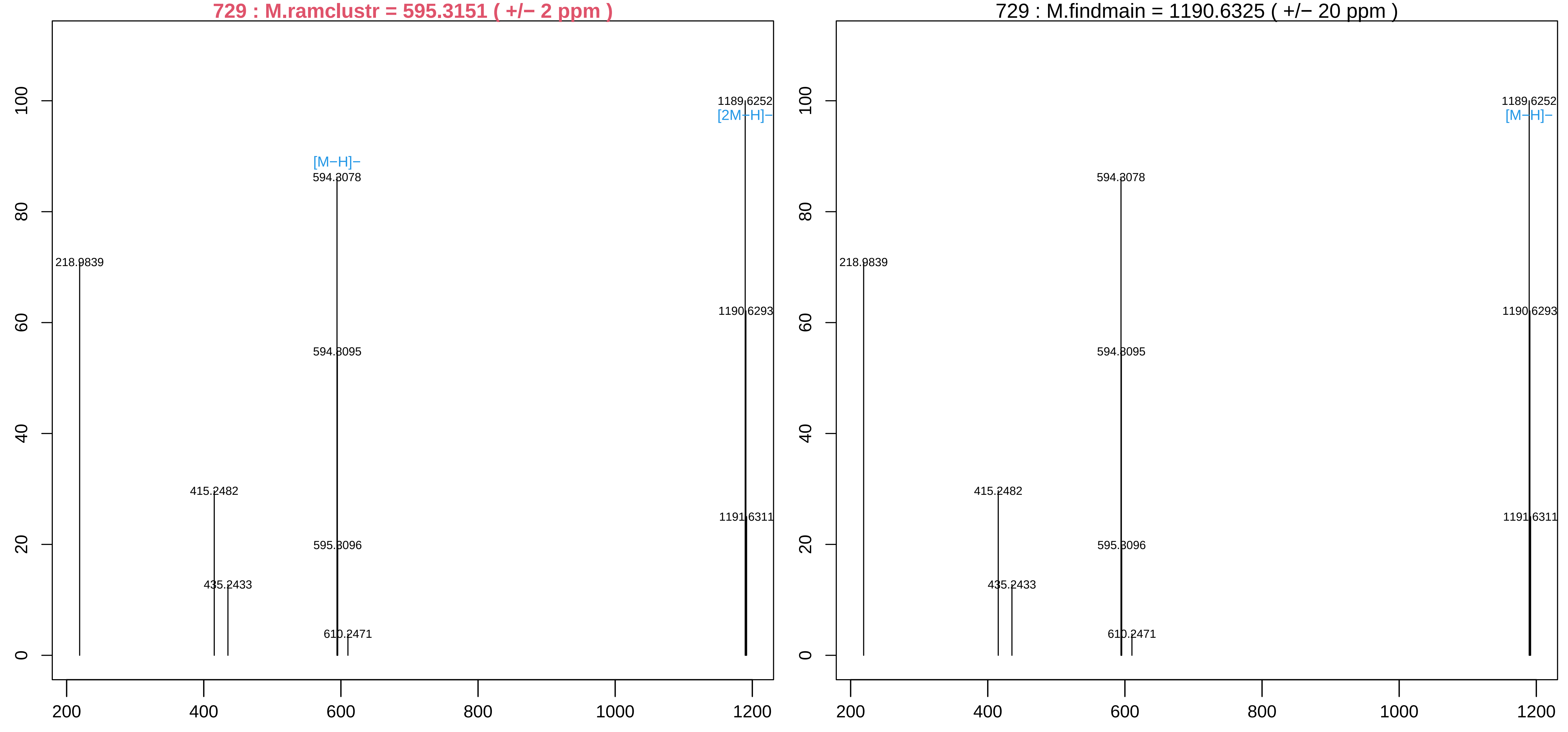
729 : M.ramclustr = 595.3151 (+/− 2 ppm)
1189.6252
[2M−H]−
[M−H]−
594.3078
218.9839
1190.6293
594.3095
415.2482
1191.6311
595.3096
435.2433
610.2471
729 : M.findmain = 1190.6325 (+/− 20 ppm)
1189.6252
[M−H]−
594.3078
218.9839
1190.6293
594.3095
415.2482
1191.6311
595.3096
435.2433
610.2471

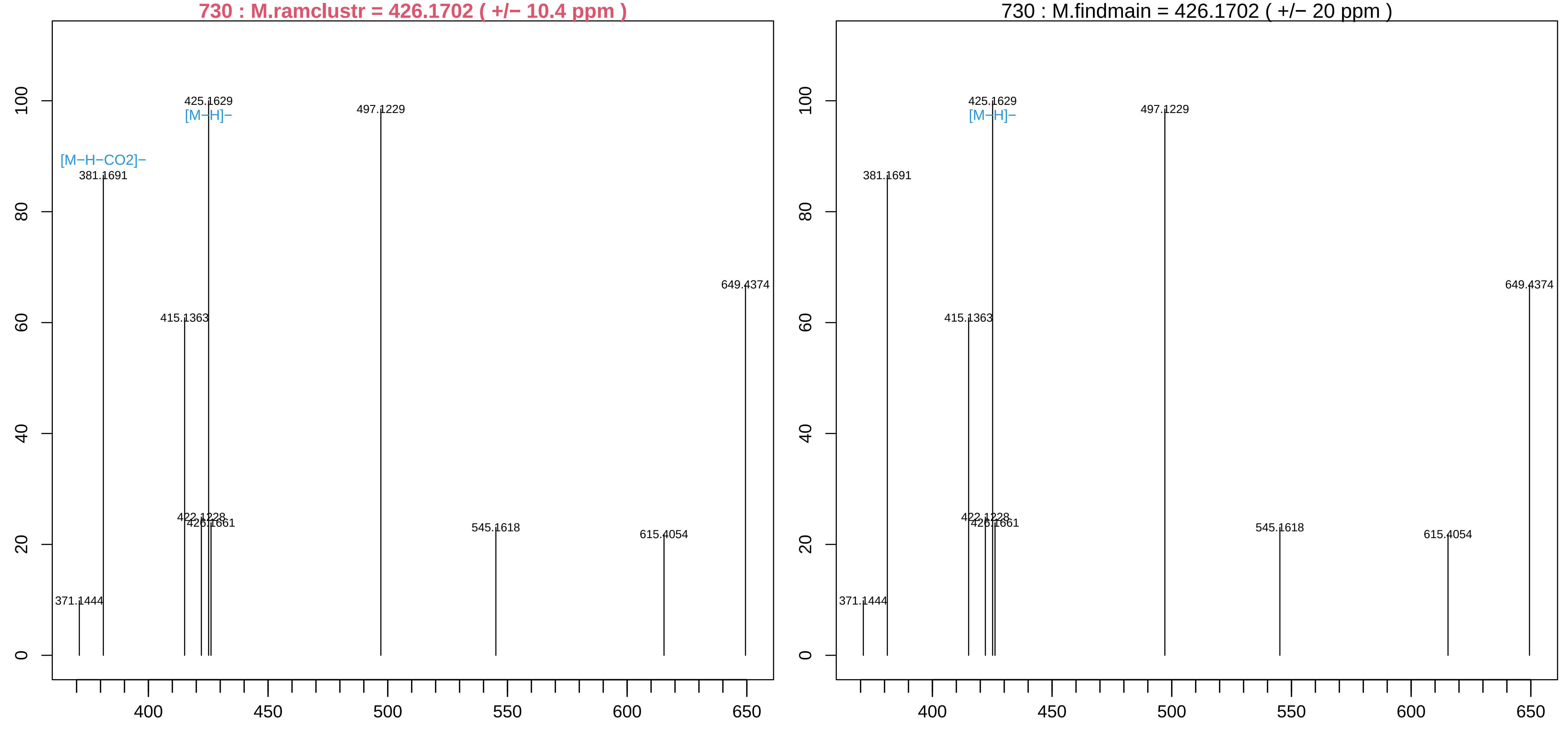

730 : M.ramclustr = 426.1702 (+/− 10.4 ppm)
730 : M.findmain = 426.1702 (+/− 20 ppm)
[M−H]−
[M−H−CO2]−
371.1444
381.1691
415.1363
422.1228
425.1629
426.1661
497.1229
545.1618
615.4054
649.4374
0
20
40
60
80
100
400
450
500
550
600
650

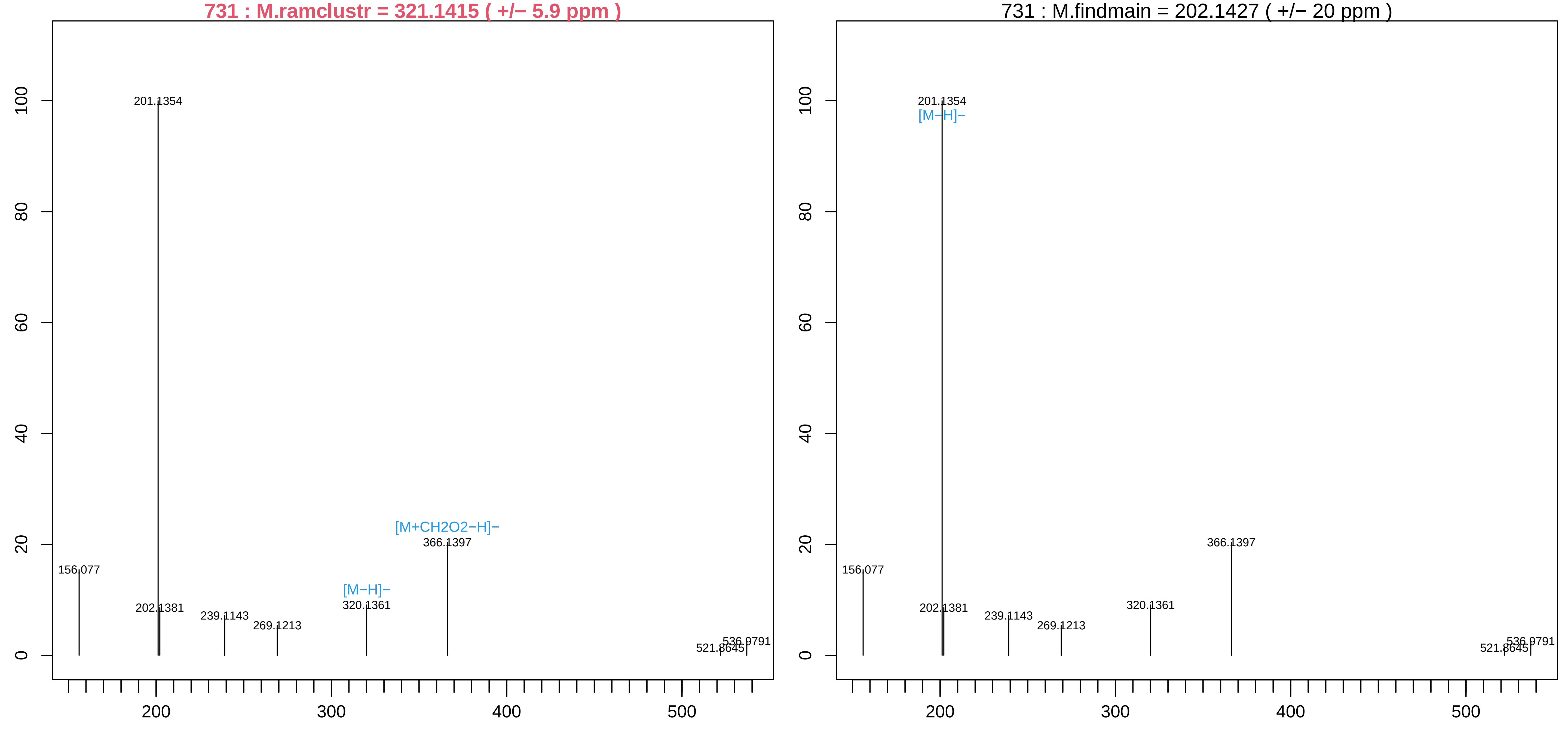
731 : M.ramclustr = 321.1415 (+/− 5.9 ppm)
201.1354
156.077
202.1381
239.1143
269.1213
[M−H]−
320.1361
[M+CH2O2−H]−
366.1397
521.8645
536.9791
731 : M.findmain = 202.1427 (+/− 20 ppm)
201.1354
[M−H]−
156.077
202.1381
239.1143
269.1213
320.1361
366.1397
521.8645
536.9791

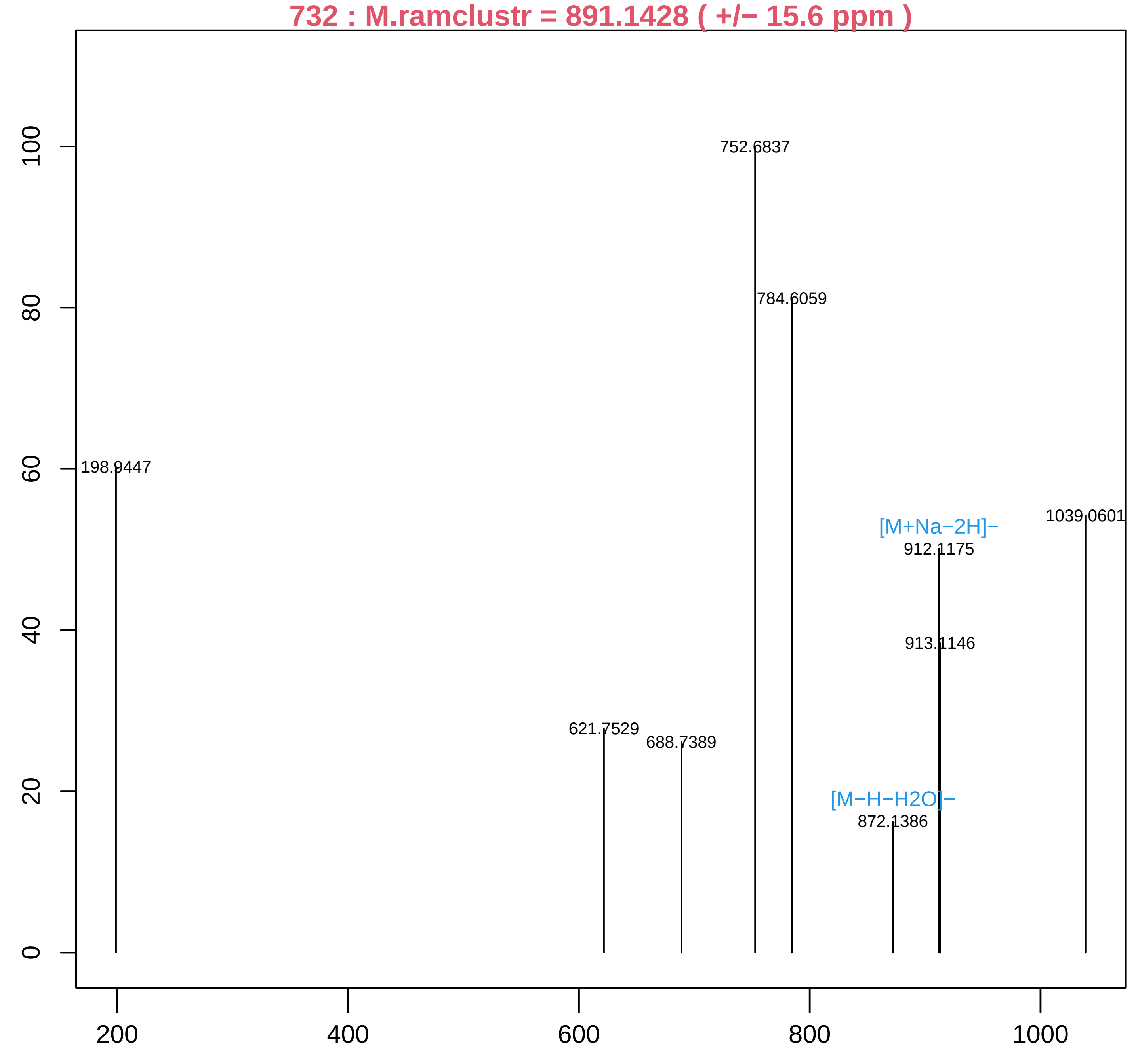

732 : M.ramclustr = 891.1428 (+/− 15.6 ppm)
752.6837
784.6059
198.9447
[M+Na−2H]−
912.1175
1039.0601
913.1146
621.7529
688.7389
[M−H−H2O]−
872.1386
0
20
40
60
80
100
200
400
600
800
1000

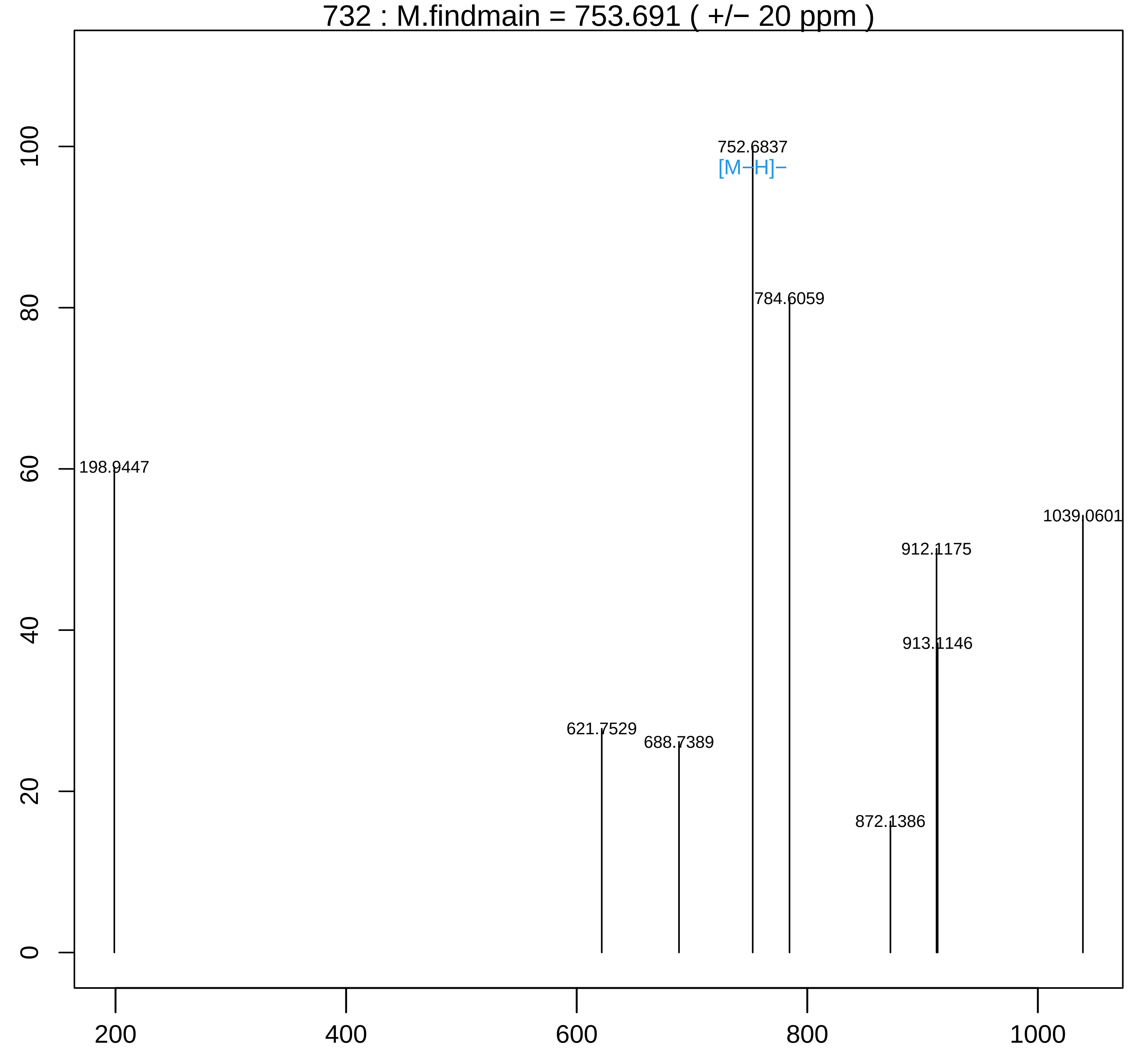

732 : M.findmain = 753.691 (+/− 20 ppm)
752.6837
[M−H]−
784.6059
198.9447
1039.0601
912.1175
913.1146
621.7529
688.7389
872.1386
0
20
40
60
80
100
200
400
600
800
1000

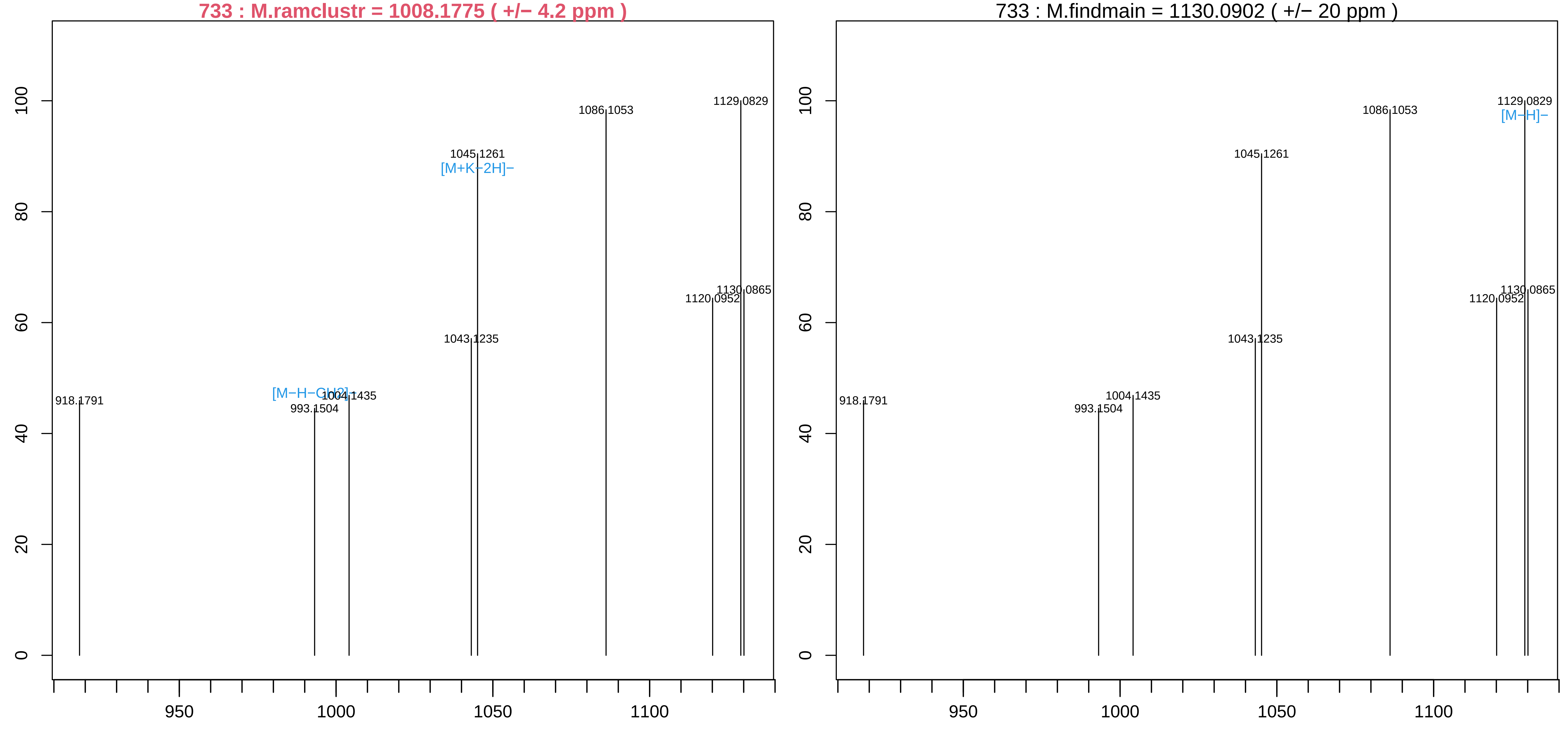
733 : M.ramclustr = 1008.1775 (+/− 4.2 ppm)
733 : M.findmain = 1130.0902 (+/− 20 ppm)
918.1791
993.1504
1004.1435
1043.1235
1045.1261
1086.1053
1120.0952
1129.0829
1130.0865
[M+K−2H]−
[M−H−CH2]−
[M−H]−
0
20
40
60
80
100
950
1000
1050
1100

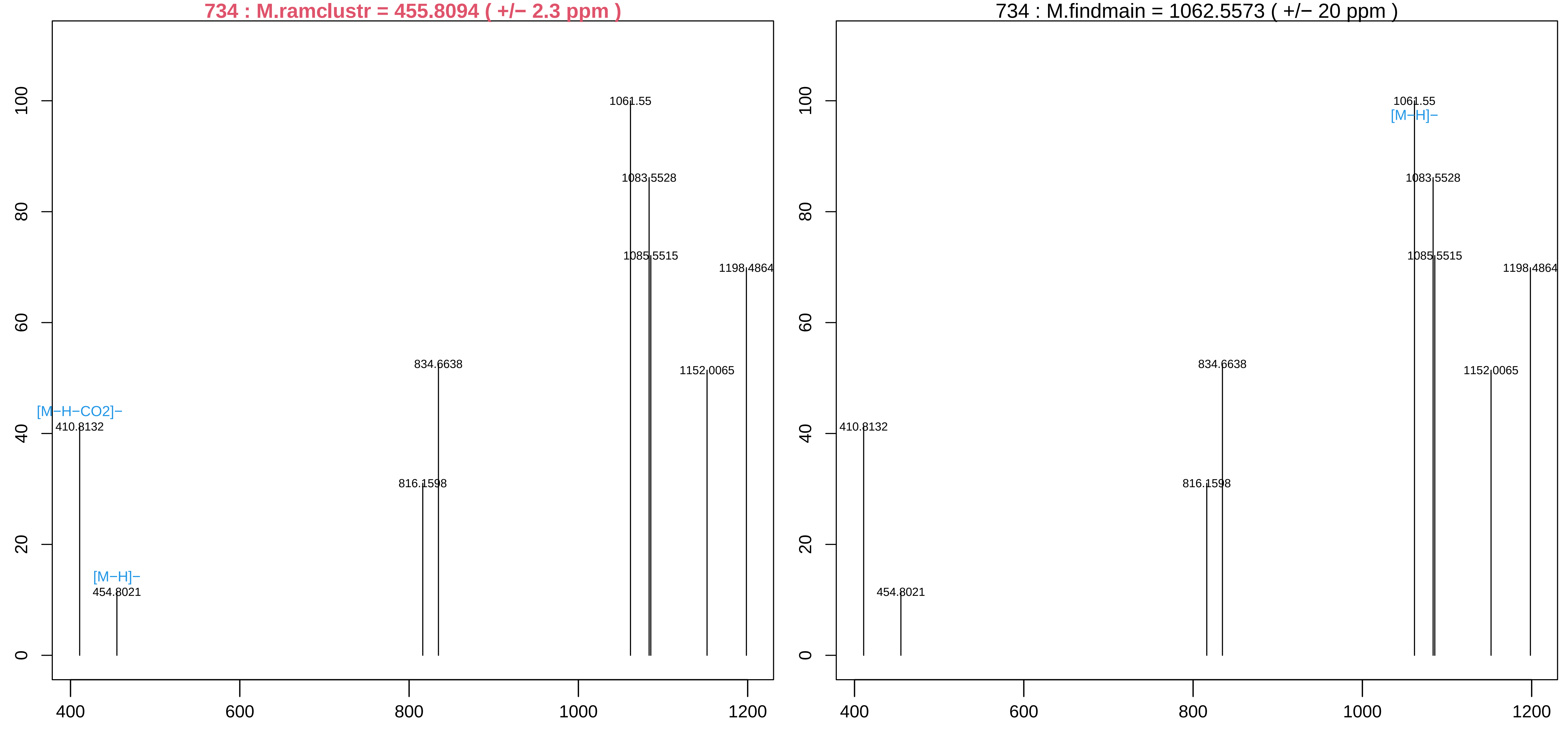
734 : M.ramclustr = 455.8094 (+/− 2.3 ppm)
[M−H−CO2]−
410.8132
[M−H]−
454.8021
816.1598
834.6638
1061.55
1083.5528
1085.5515
1152.0065
1198.4864
734 : M.findmain = 1062.5573 (+/− 20 ppm)
410.8132
454.8021
816.1598
834.6638
1061.55
[M−H]−
1083.5528
1085.5515
1152.0065
1198.4864
0
20
40
60
80
100
400
600
800
1000
1200

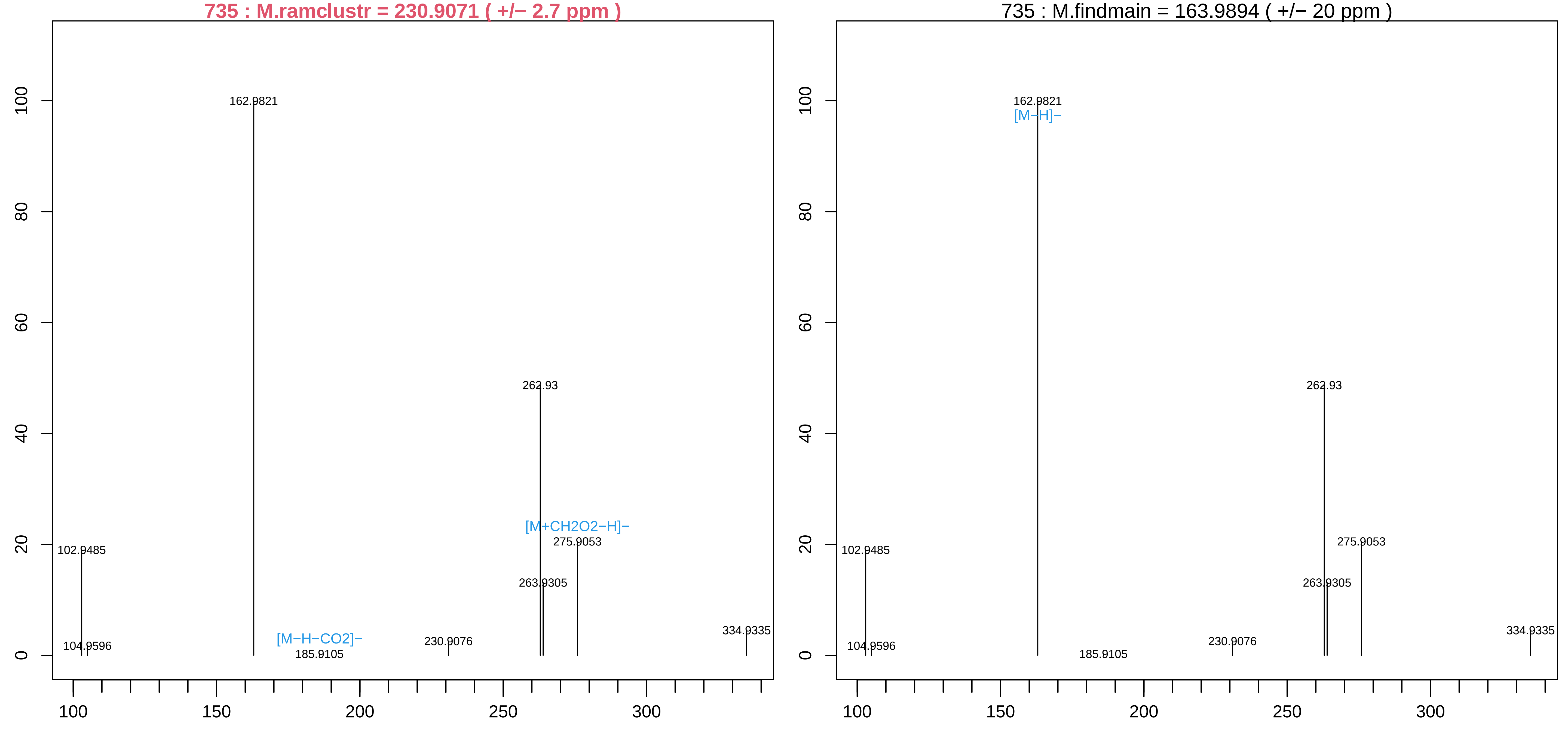

735 : M.ramclustr = 230.9071 (+/− 2.7 ppm)
162.9821
262.93
[M+CH2O2−H]−
275.9053
102.9485
263.9305
334.9335
[M−H−CO2]−
230.9076
104.9596
185.9105
735 : M.findmain = 163.9894 (+/− 20 ppm)
162.9821
[M−H]−
262.93
275.9053
102.9485
263.9305
334.9335
230.9076
104.9596
185.9105
0
20
40
60
80
100
150
200
250
300

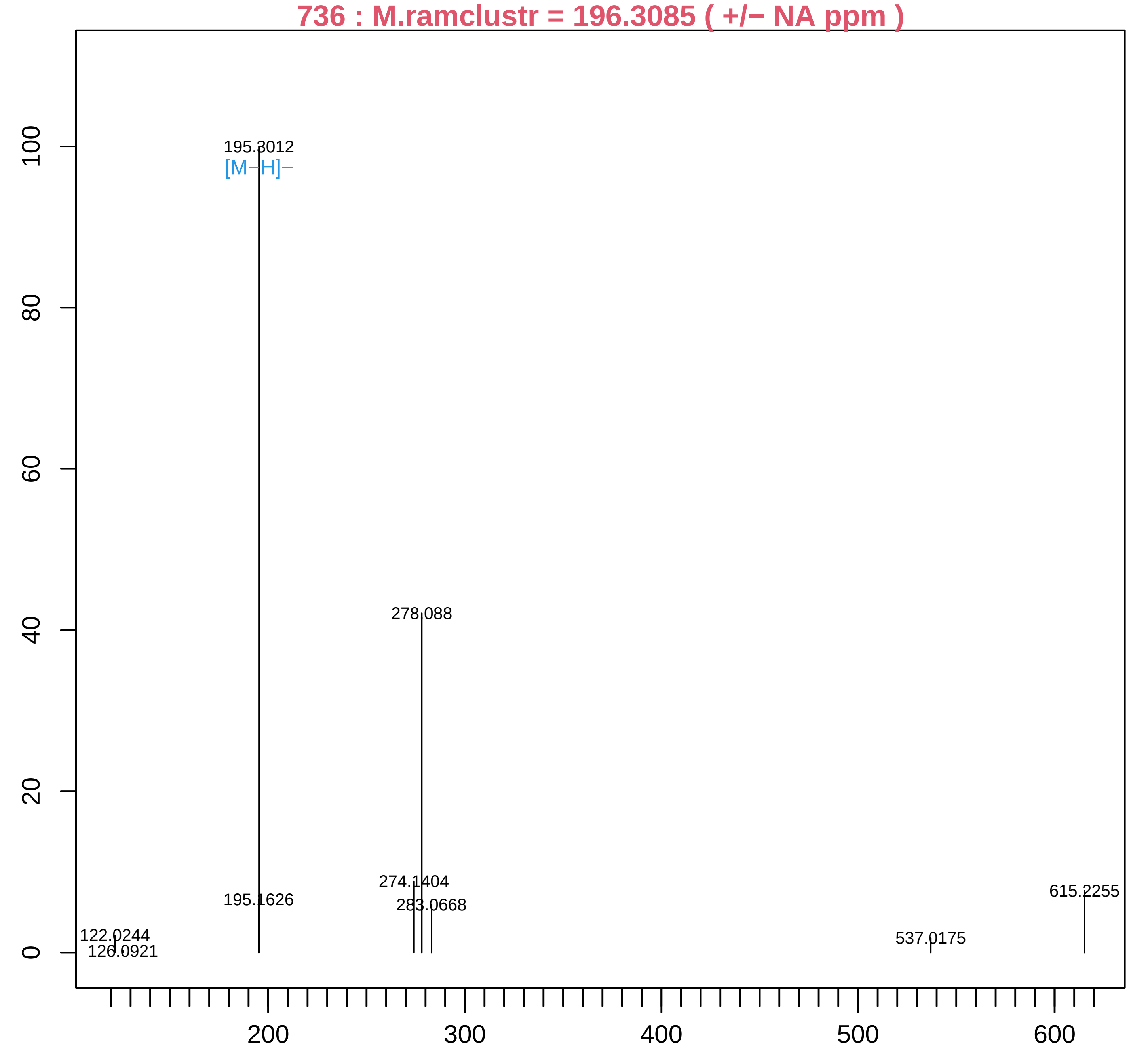

736 : M.ramclustr = 196.3085 (+/− NA ppm)
195.3012
[M−H]−
278.088
274.1404
283.0668
195.1626
122.0244
126.0921
537.0175
615.2255
0
20
40
60
80
100
200
300
400
500
600

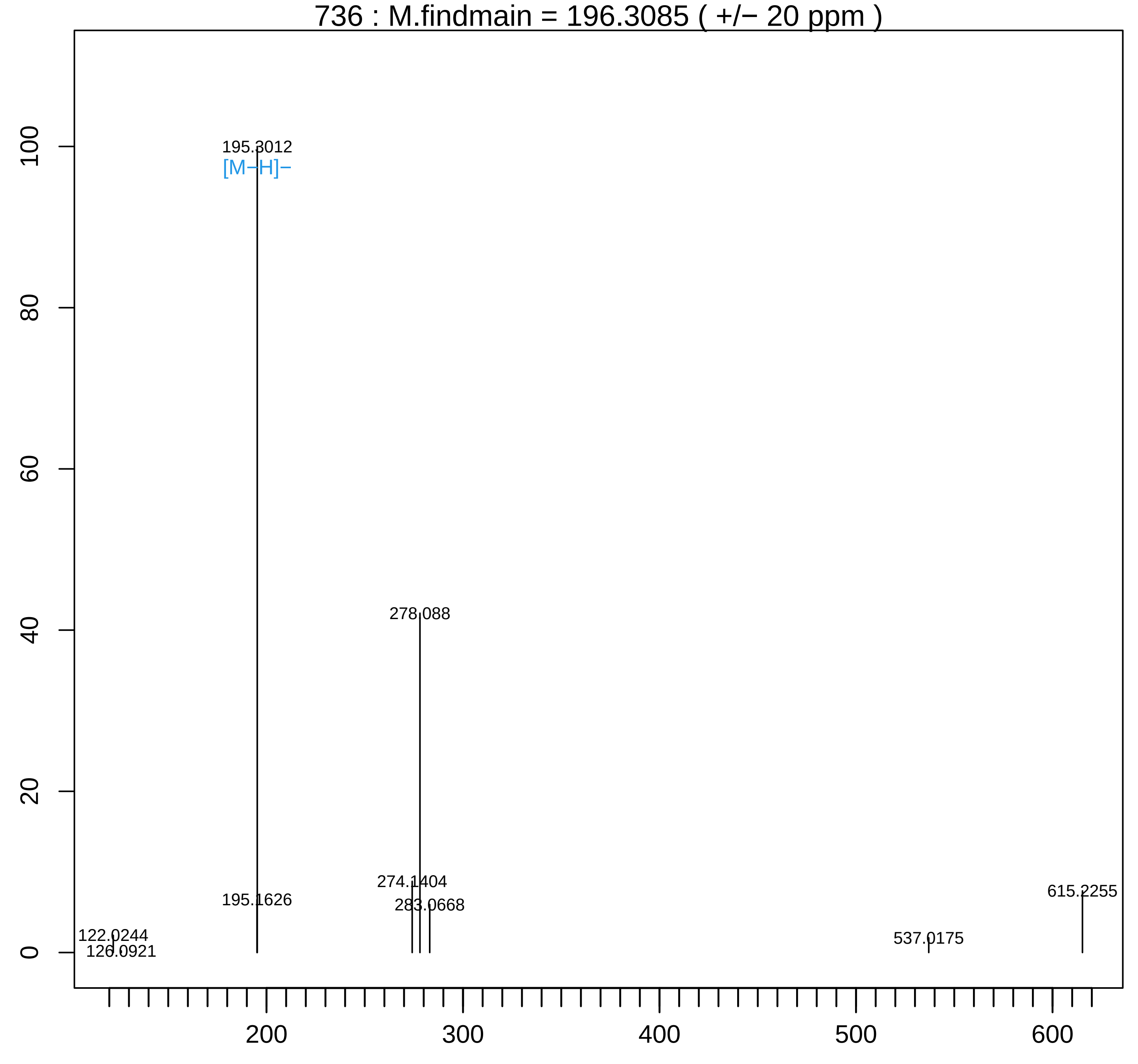

736 : M.findmain = 196.3085 (+/− 20 ppm)
195.3012
[M−H]−
278.088
274.1404
283.0668
195.1626
122.0244
126.0921
537.0175
615.2255
0
20
40
60
80
100
200
300
400
500
600

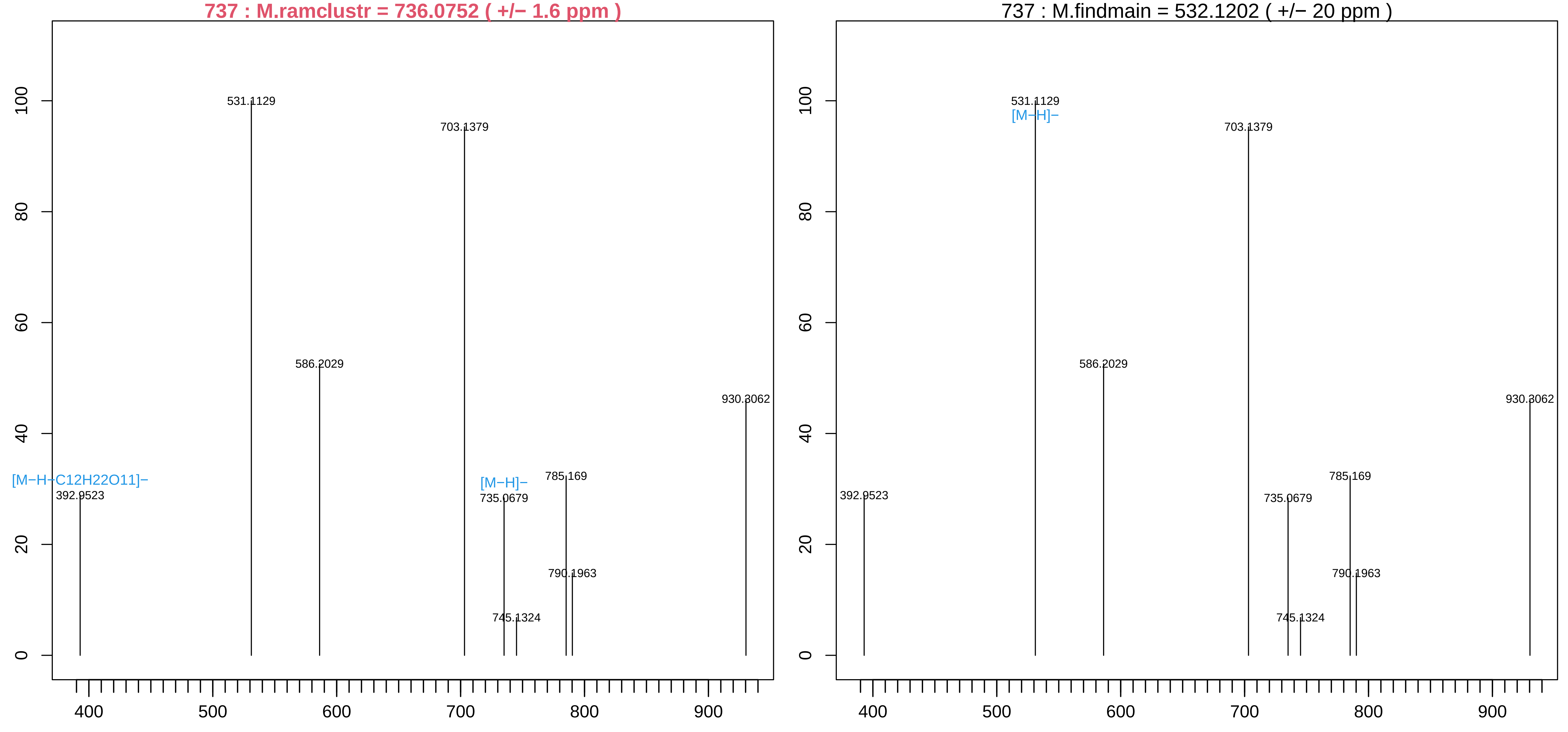

737 : M.ramclustr = 736.0752 (+/− 1.6 ppm)
737 : M.findmain = 532.1202 (+/− 20 ppm)
531.1129
703.1379
586.2029
930.3062
[M−H−C12H22O11]−
392.9523
[M−H]−
735.0679
785.169
790.1963
745.1324
0
20
40
60
80
100
400
500
600
700
800
900

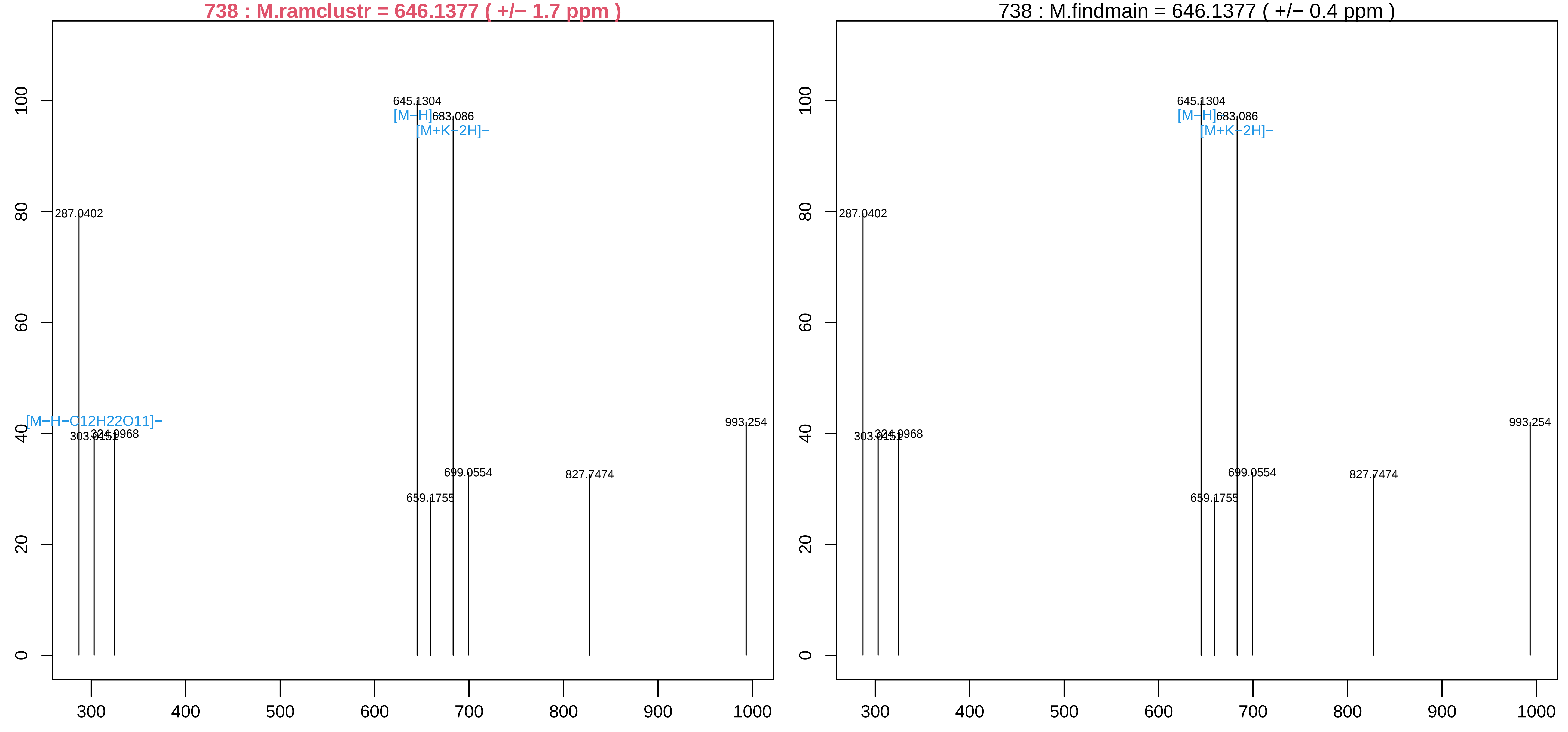
738 : M.ramclustr = 646.1377 (+/− 1.7 ppm)
738 : M.findmain = 646.1377 (+/− 0.4 ppm)
645.1304
[M−H]−
683.086
[M+K−2H]−
287.0402
[M−H−C12H22O11]−
303.0151
324.9968
699.0554
659.1755
827.7474
993.254
0
20
40
60
80
100
300
400
500
600
700
800
900
1000

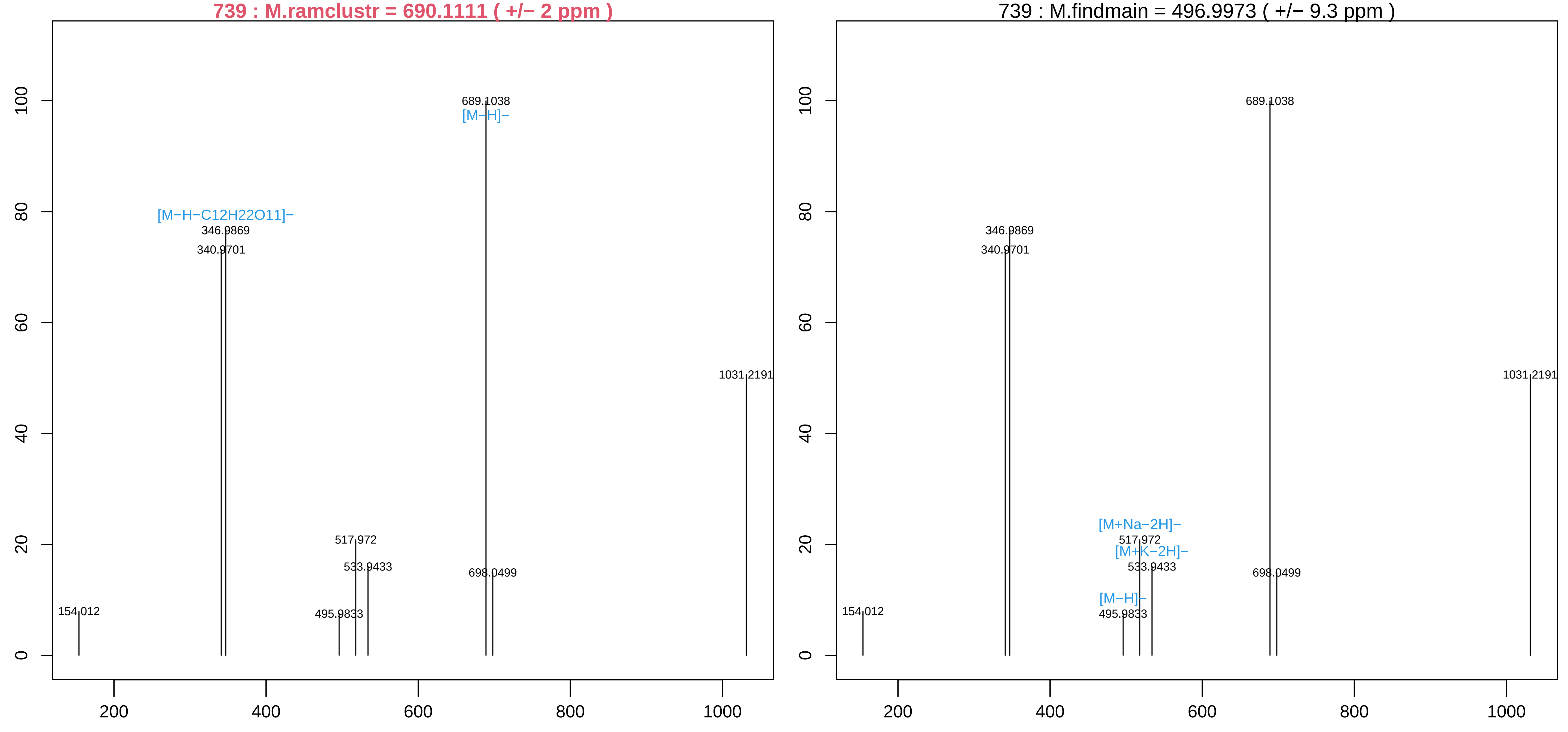
739 : M.ramclustr = 690.1111 (+/− 2 ppm)
[M−H−C12H22O11]−
346.9869
340.9701
689.1038
[M−H]−
1031.2191
517.972
533.9433
698.0499
154.012
495.9833
739 : M.findmain = 496.9973 (+/− 9.3 ppm)
346.9869
340.9701
689.1038
1031.2191
[M+Na−2H]−
517.972
[M+K−2H]−
533.9433
698.0499
[M−H]−
154.012
495.9833
0
20
40
60
80
100
200
400
600
800
1000

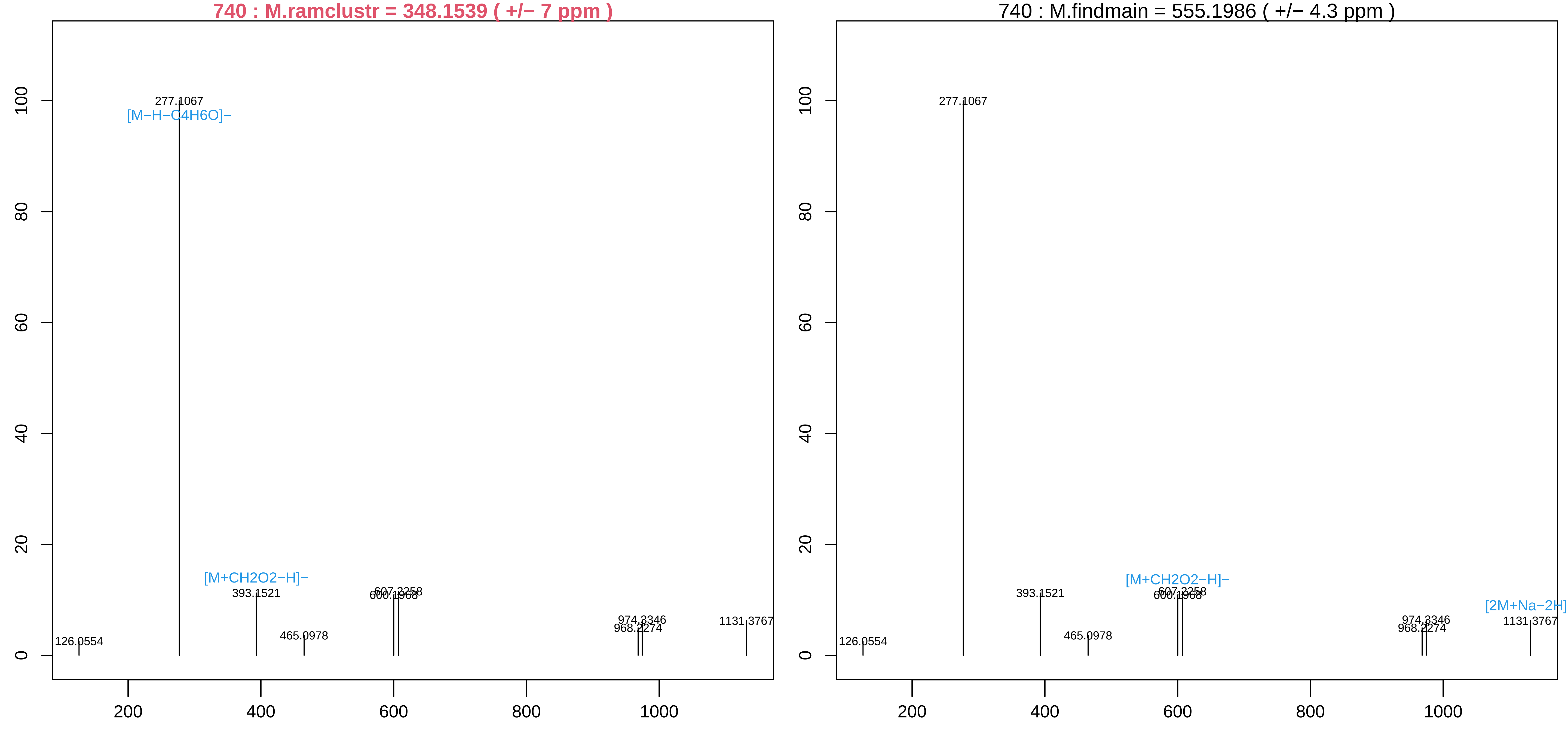

740 : M.ramclustr = 348.1539 (+/− 7 ppm)
277.1067
[M−H−C4H6O]−
[M+CH2O2−H]−
393.1521
465.0978
607.2258
600.1968
974.3346
968.2274
1131.3767
126.0554
740 : M.findmain = 555.1986 (+/− 4.3 ppm)
[2M+Na−2H]
0
20
40
60
80
100
200
400
600
800
1000

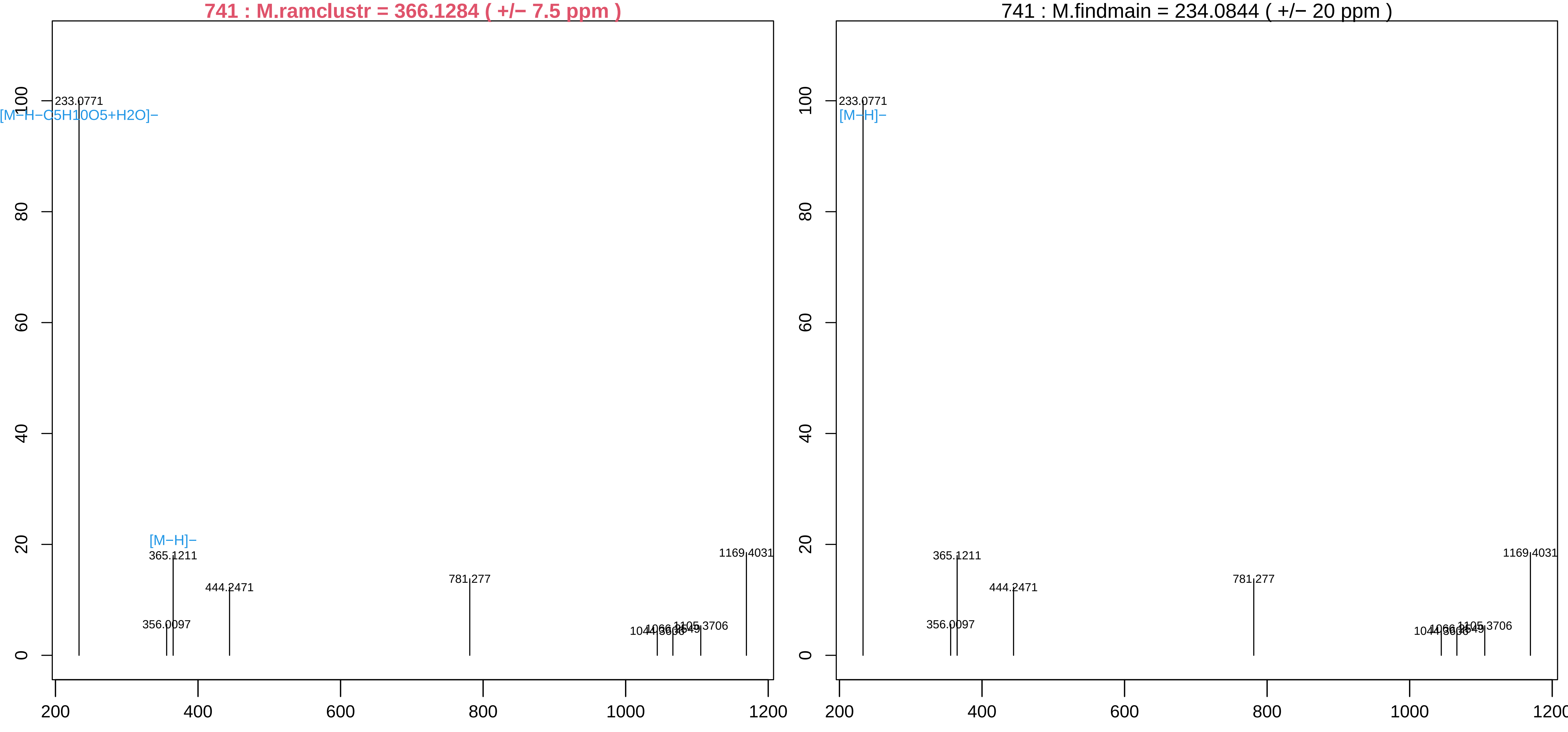

741 : M.ramclustr = 366.1284 (+/− 7.5 ppm)
233.0771
[M−H−C5H10O5+H2O]−
[M−H]−
365.1211
356.0097
444.2471
781.277
1044.3636
1066.3649
1105.3706
1169.4031
741 : M.findmain = 234.0844 (+/− 20 ppm)
233.0771
[M−H]−
365.1211
356.0097
444.2471
781.277
1044.3636
1066.3649
1105.3706
1169.4031

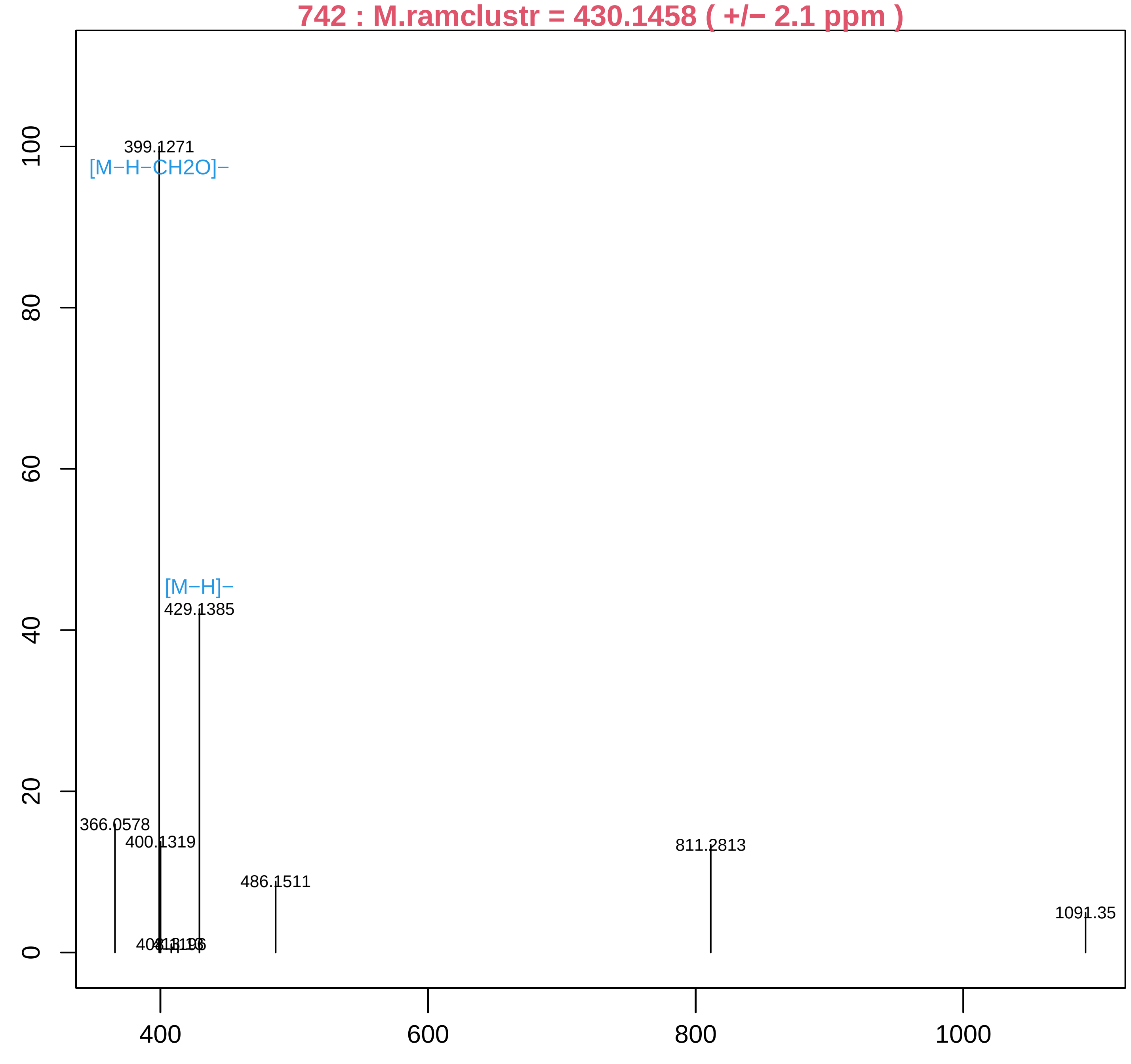

742 : M.ramclustr = 430.1458 (+/− 2.1 ppm)
399.1271
[M−H−CH2O]−
[M−H]−
429.1385
366.0578
400.1319
486.1511
811.2813
1091.35
0
20
40
60
80
100
400
600
800
1000

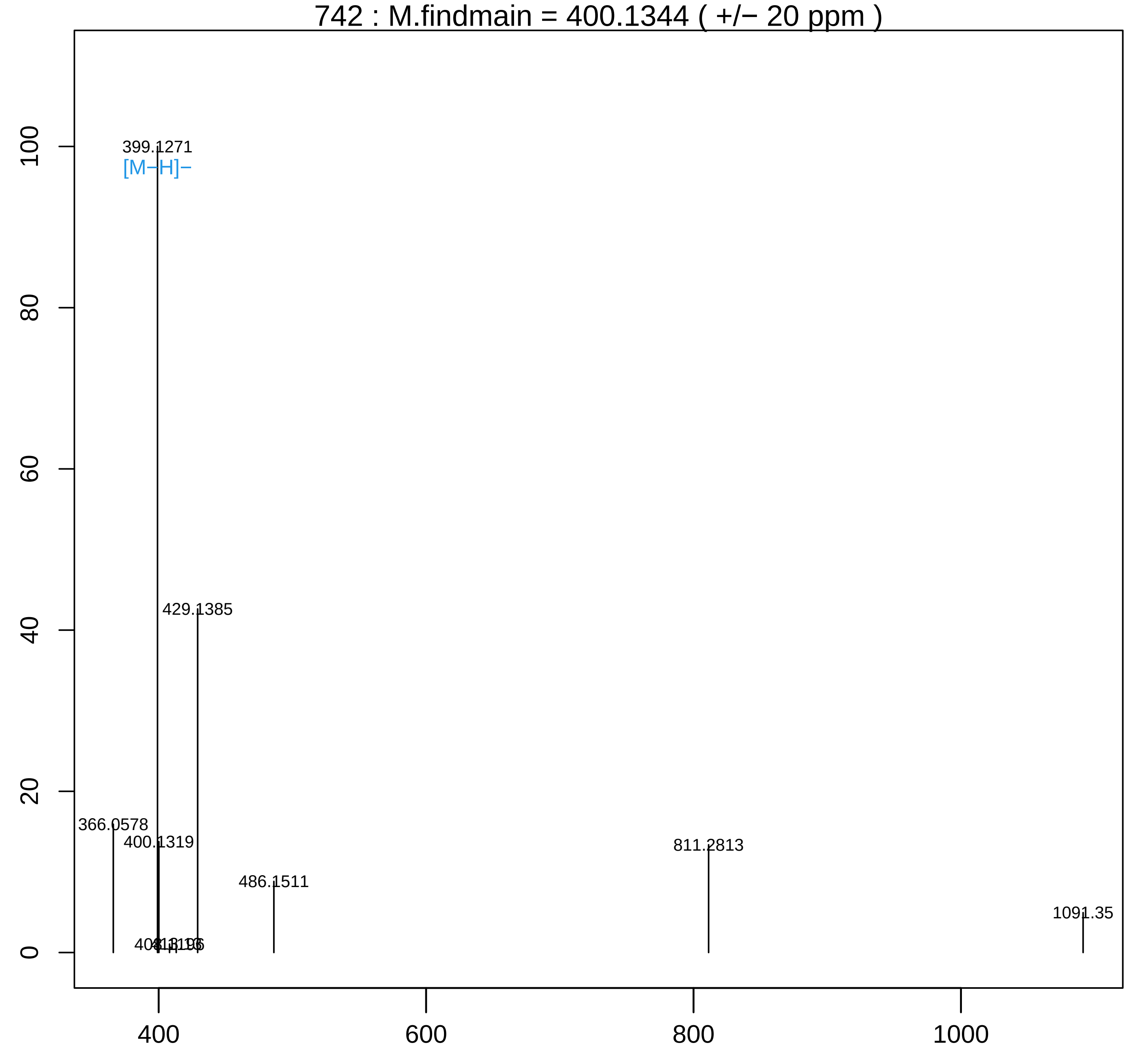

742 : M.findmain = 400.1344 (+/− 20 ppm)
399.1271
[M−H]−
429.1385
366.0578
400.1319
486.1511
811.2813
1091.35
0
20
40
60
80
100
400
600
800
1000

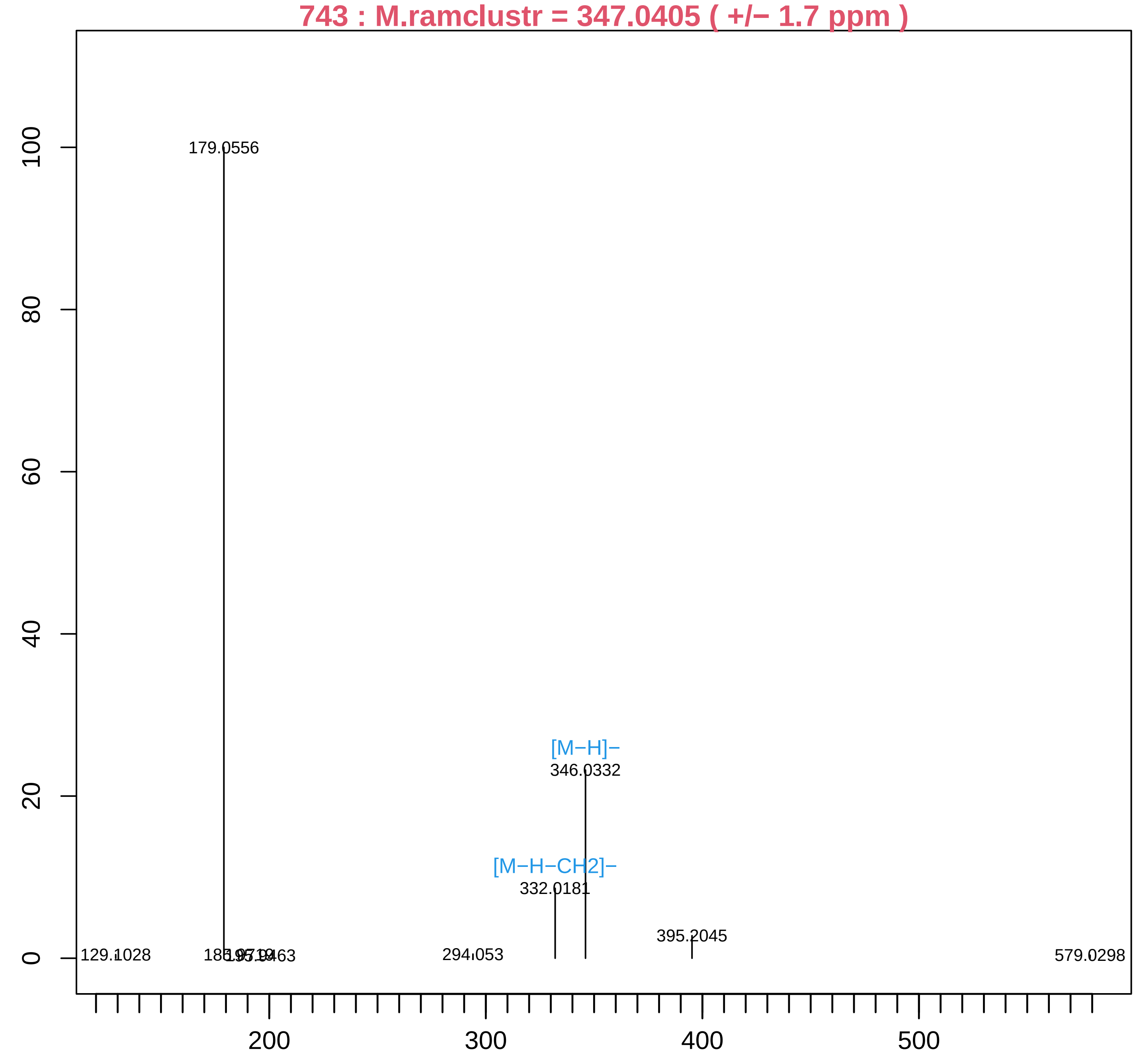
743 : M.ramclustr = 347.0405 (+/− 1.7 ppm)
179.0556
[M−H]−
346.0332
[M−H−CH2]−
332.0181
395.2045
129.1028
294.053
579.0298
0
20
40
60
80
100
200
300
400
500
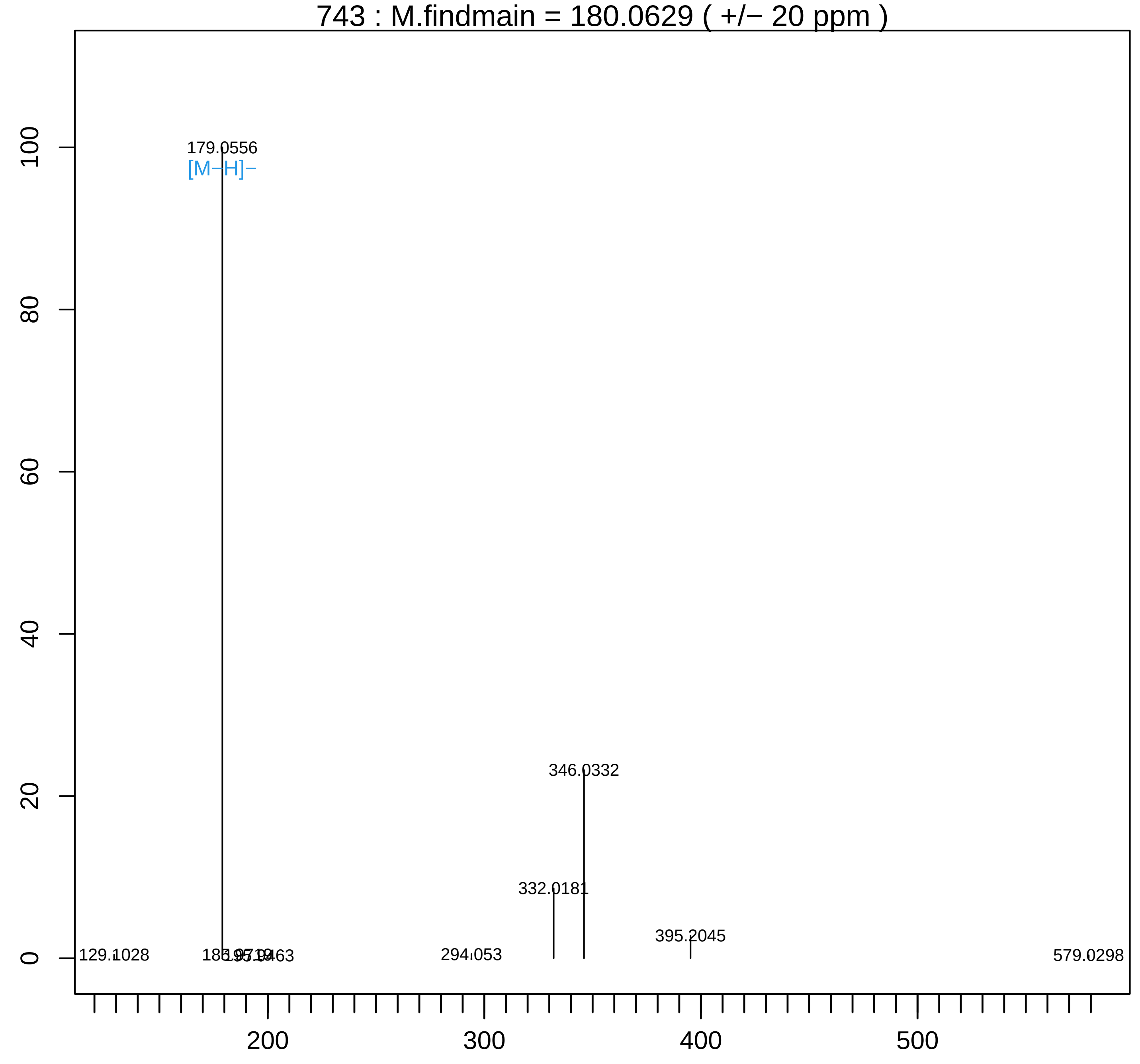
743 : M.findmain = 180.0629 (+/− 20 ppm)
179.0556
[M−H]−
346.0332
332.0181
395.2045
129.1028
294.053
579.0298
0
20
40
60
80
100
200
300
400
500

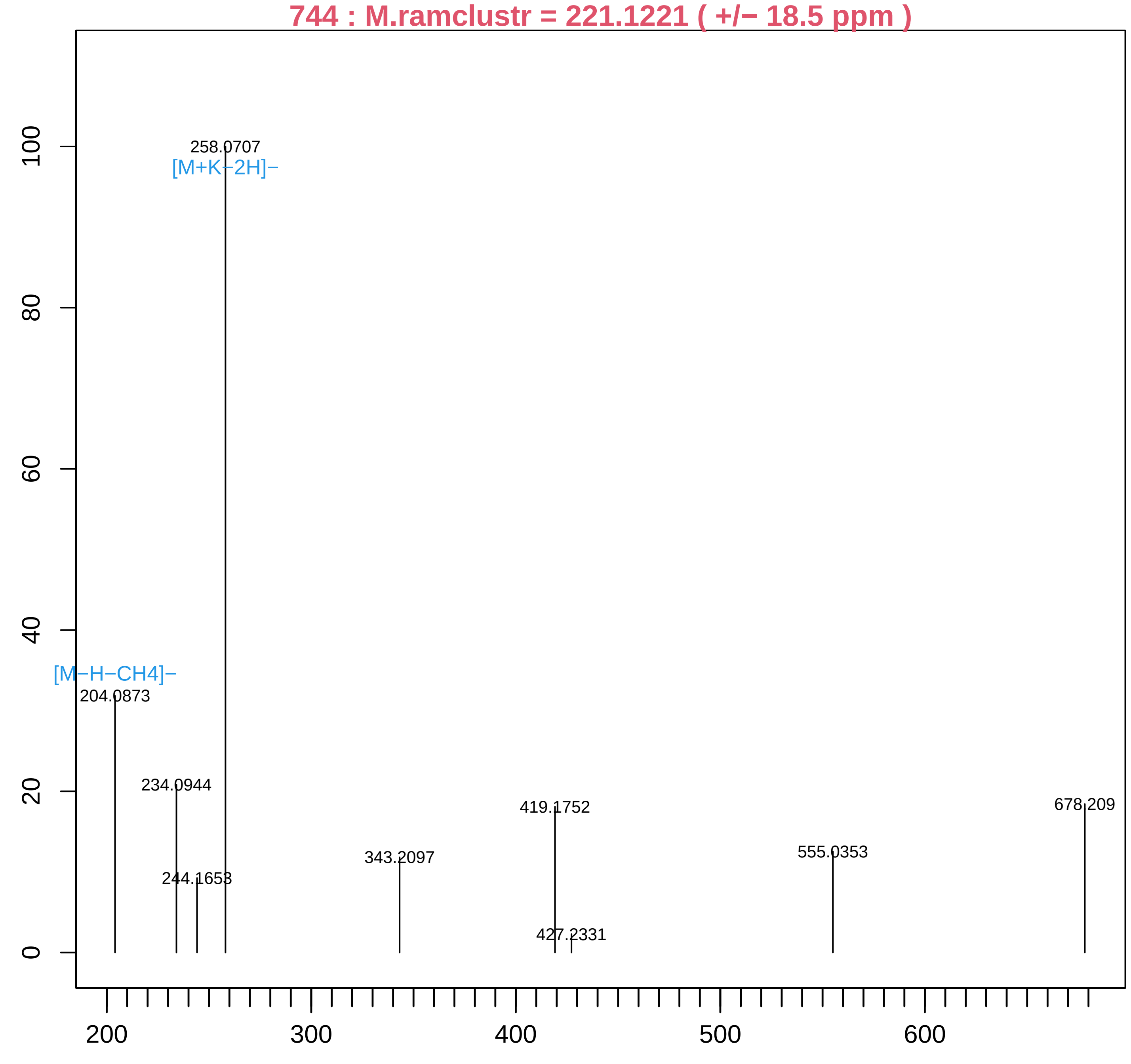
744 : M.ramclustr = 221.1221 (+/− 18.5 ppm)
258.0707
[M+K−2H]−
[M−H−CH4]−
204.0873
234.0944
244.1653
343.2097
419.1752
427.2331
555.0353
678.209
0
20
40
60
80
100
200
300
400
500
600

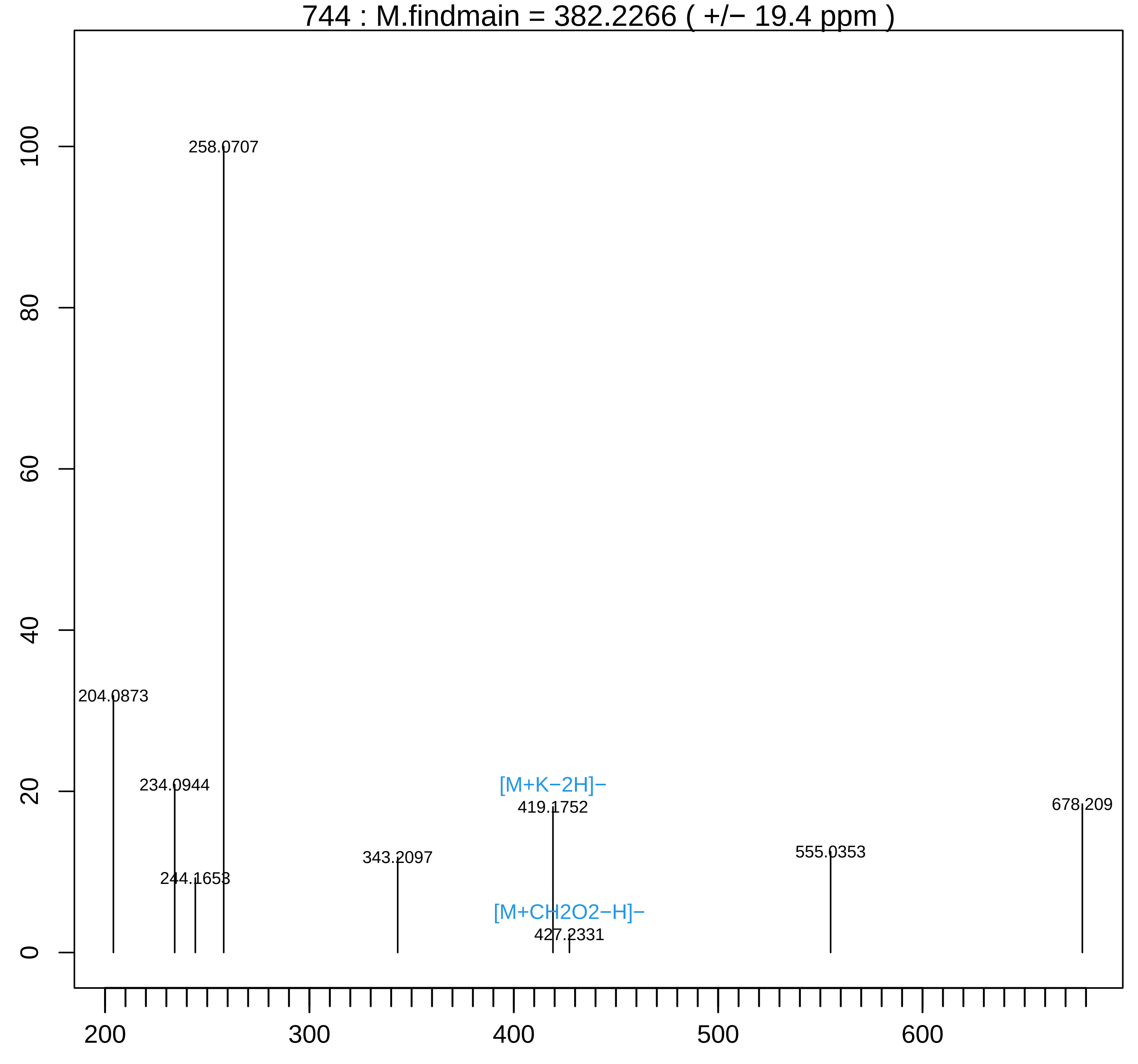
744 : M.findmain = 382.2266 (+/− 19.4 ppm)
258.0707
204.0873
234.0944
244.1653
343.2097
[M+K−2H]−
419.1752
[M+CH2O2−H]−
427.2331
555.0353
678.209
0
20
40
60
80
100
200
300
400
500
600

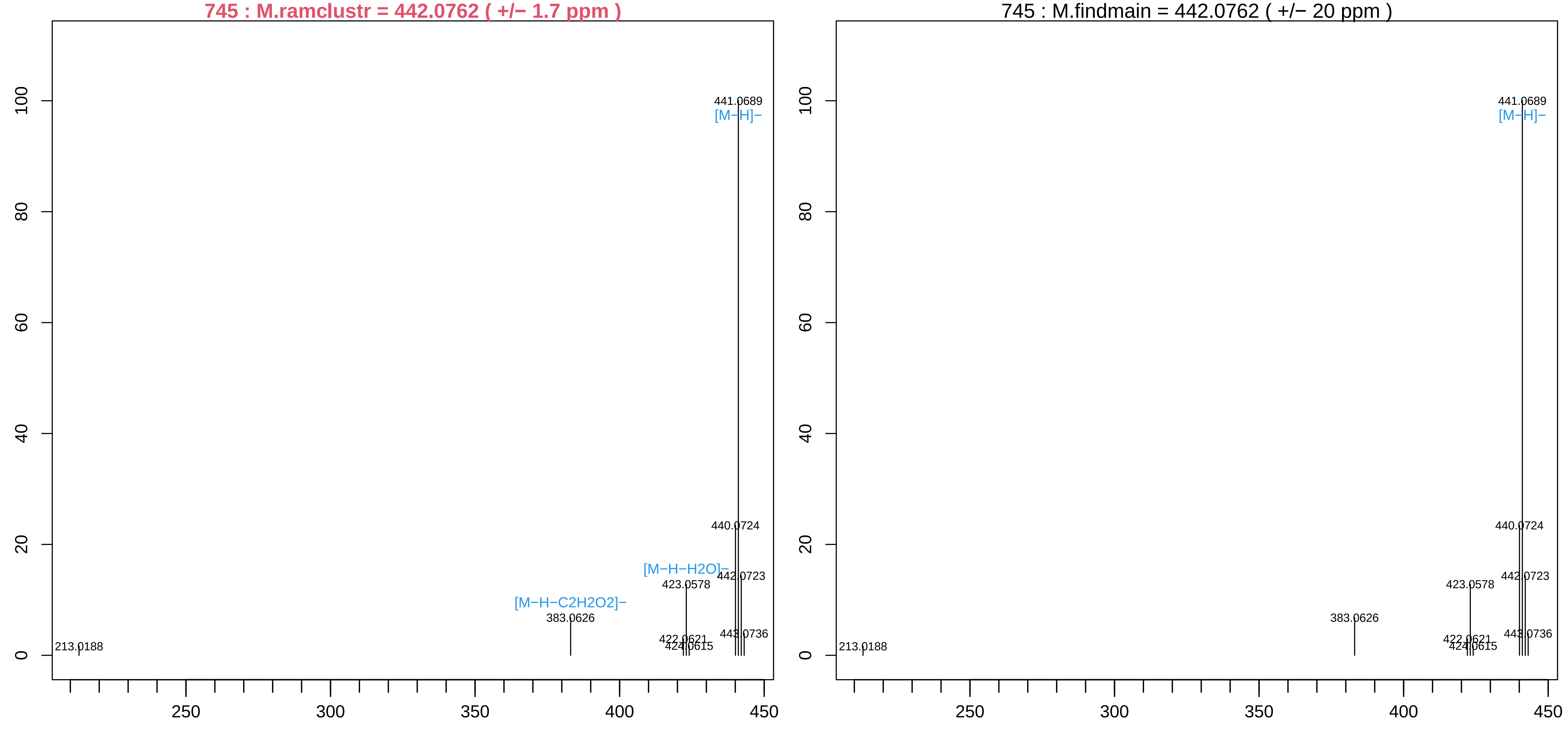
745 : M.ramclustr = 442.0762 (+/− 1.7 ppm)
441.0689
[M−H]−
440.0724
[M−H−H2O]−
442.0723
423.0578
[M−H−C2H2O2]−
383.0626
443.0736
422.0621
424.0615
213.0188
0
20
40
60
80
100
250
300
350
400
450
745 : M.findmain = 442.0762 (+/− 20 ppm)
441.0689
[M−H]−
440.0724
442.0723
423.0578
383.0626
443.0736
422.0621
424.0615
213.0188
0
20
40
60
80
100
250
300
350
400
450

746 : M.ramclustr = 382.0554 (+/− 0.8 ppm)

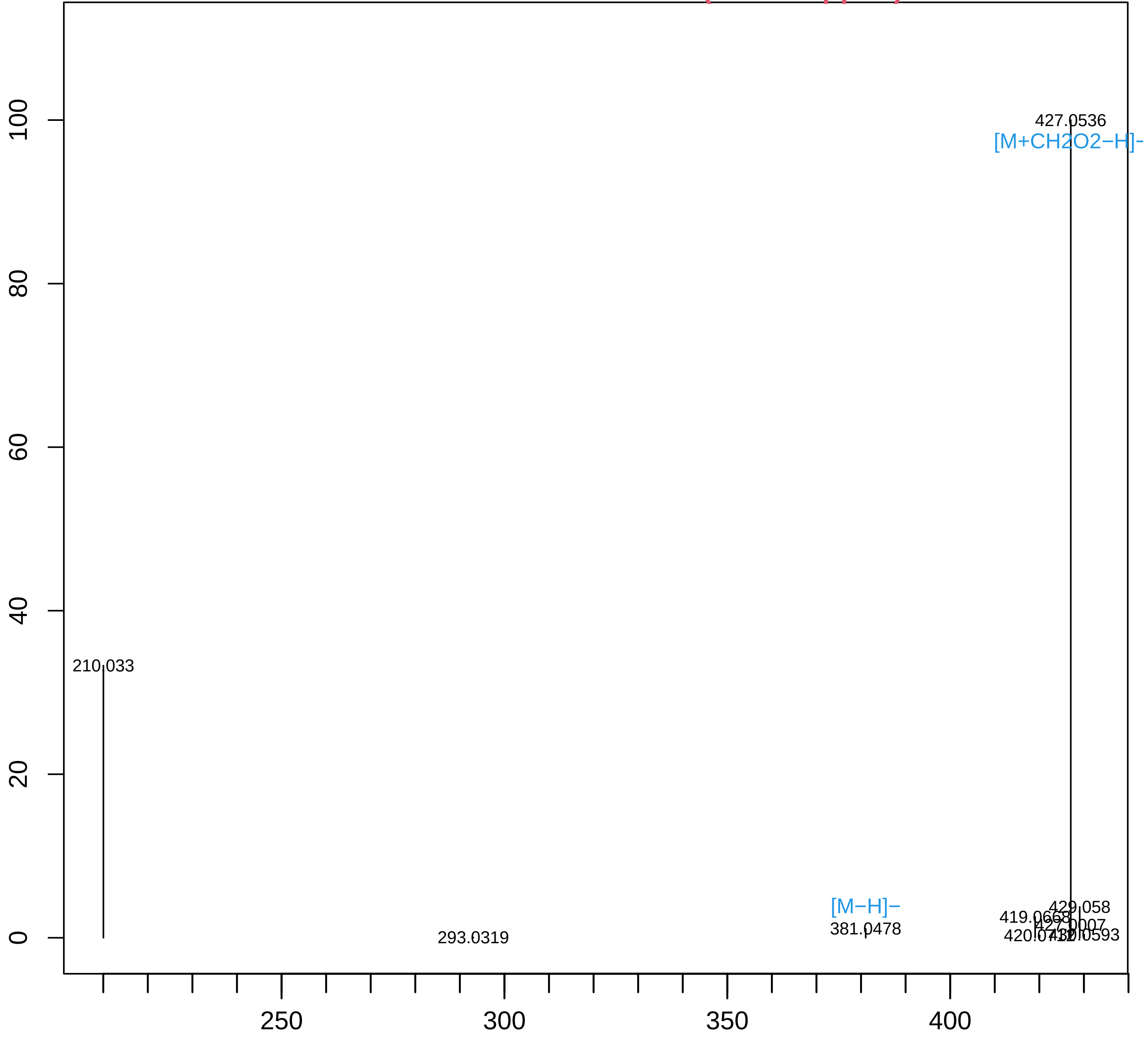

746 : M.findmain = 382.0554 (+/− 0.8 ppm)

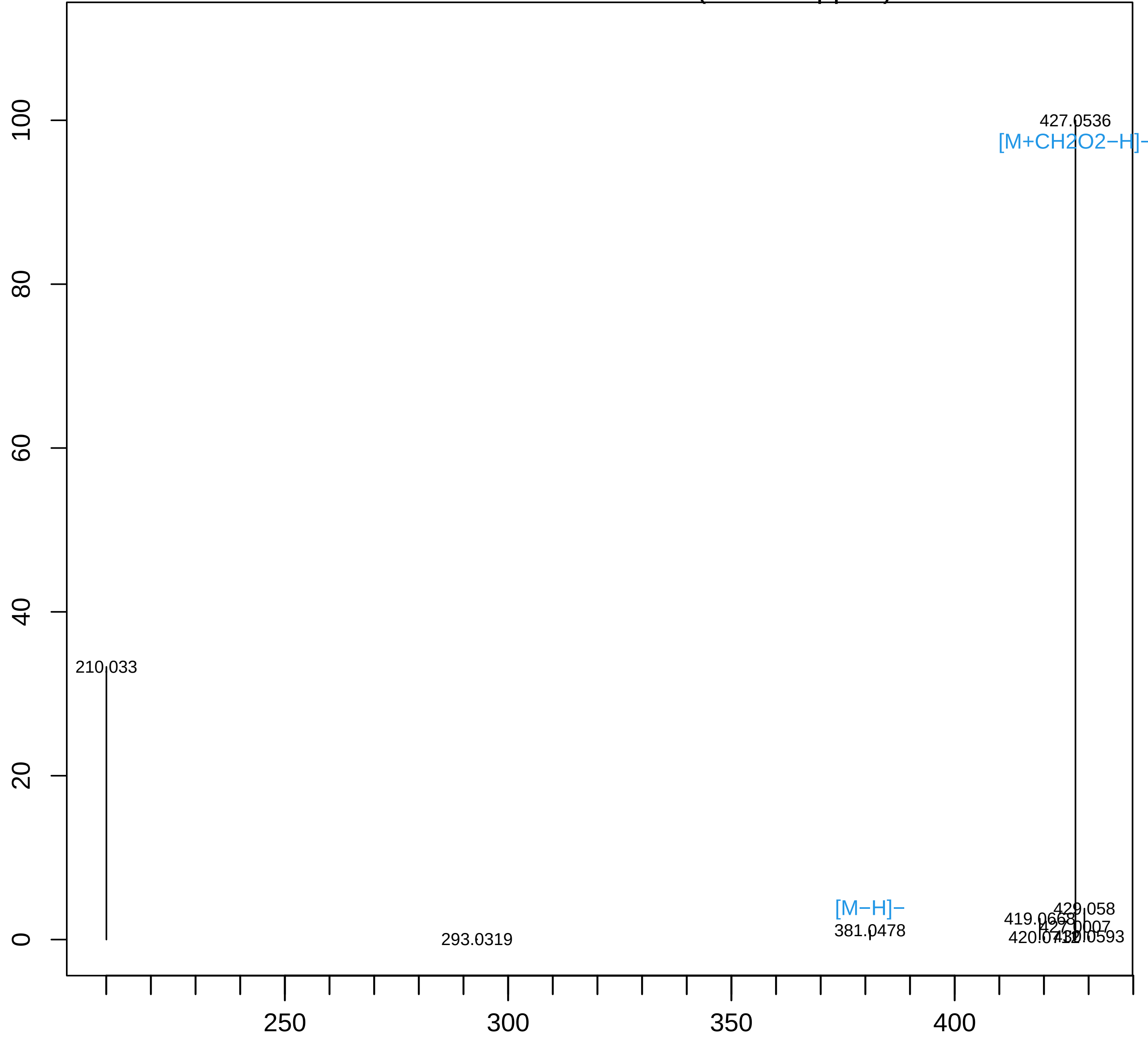

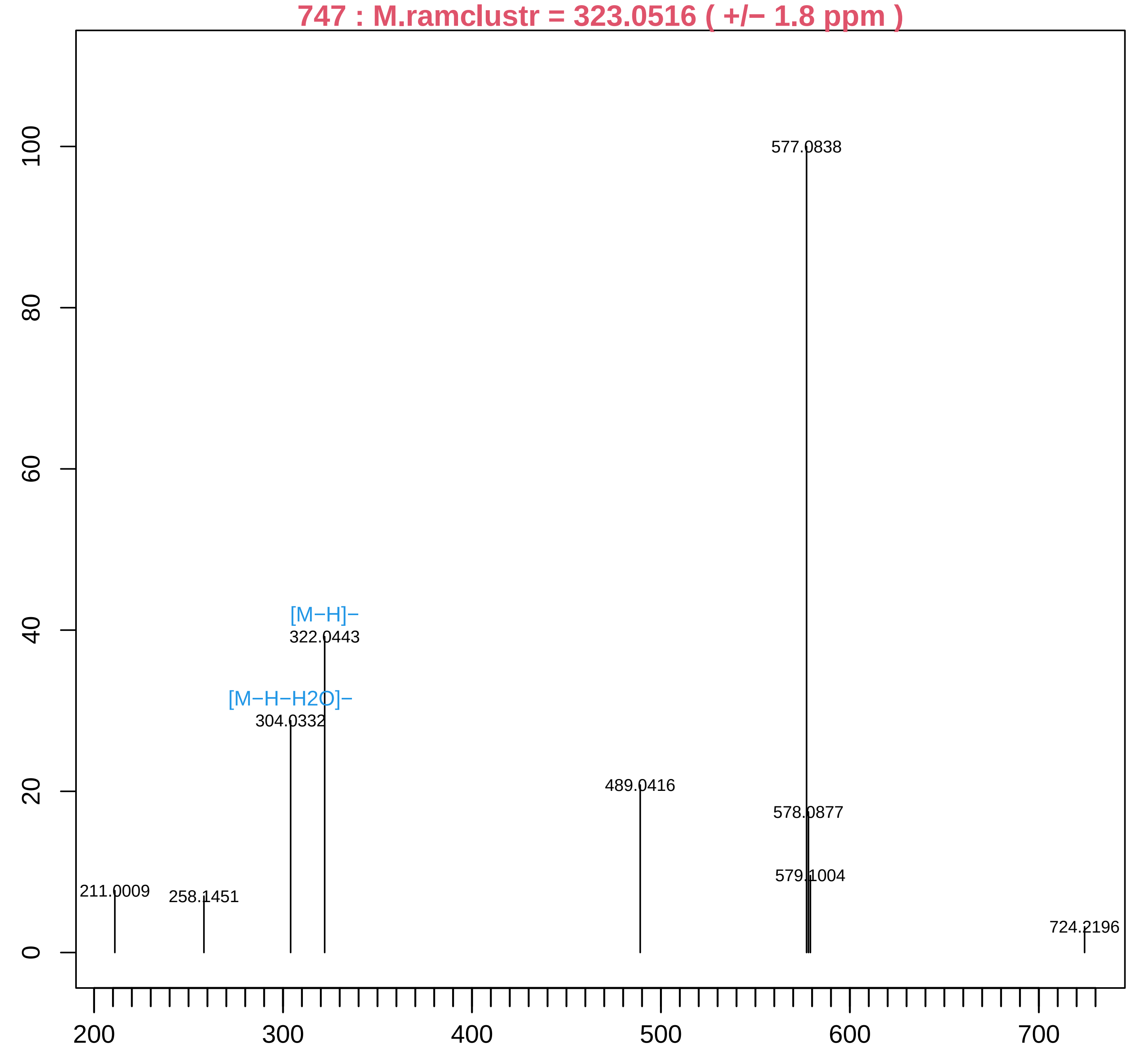

747 : M.ramclustr = 323.0516 (+/− 1.8 ppm)
577.0838
[M−H]−
322.0443
[M−H−H2O]−
304.0332
489.0416
578.0877
579.1004
211.0009
258.1451
724.2196
0
20
40
60
80
100
200
300
400
500
600
700

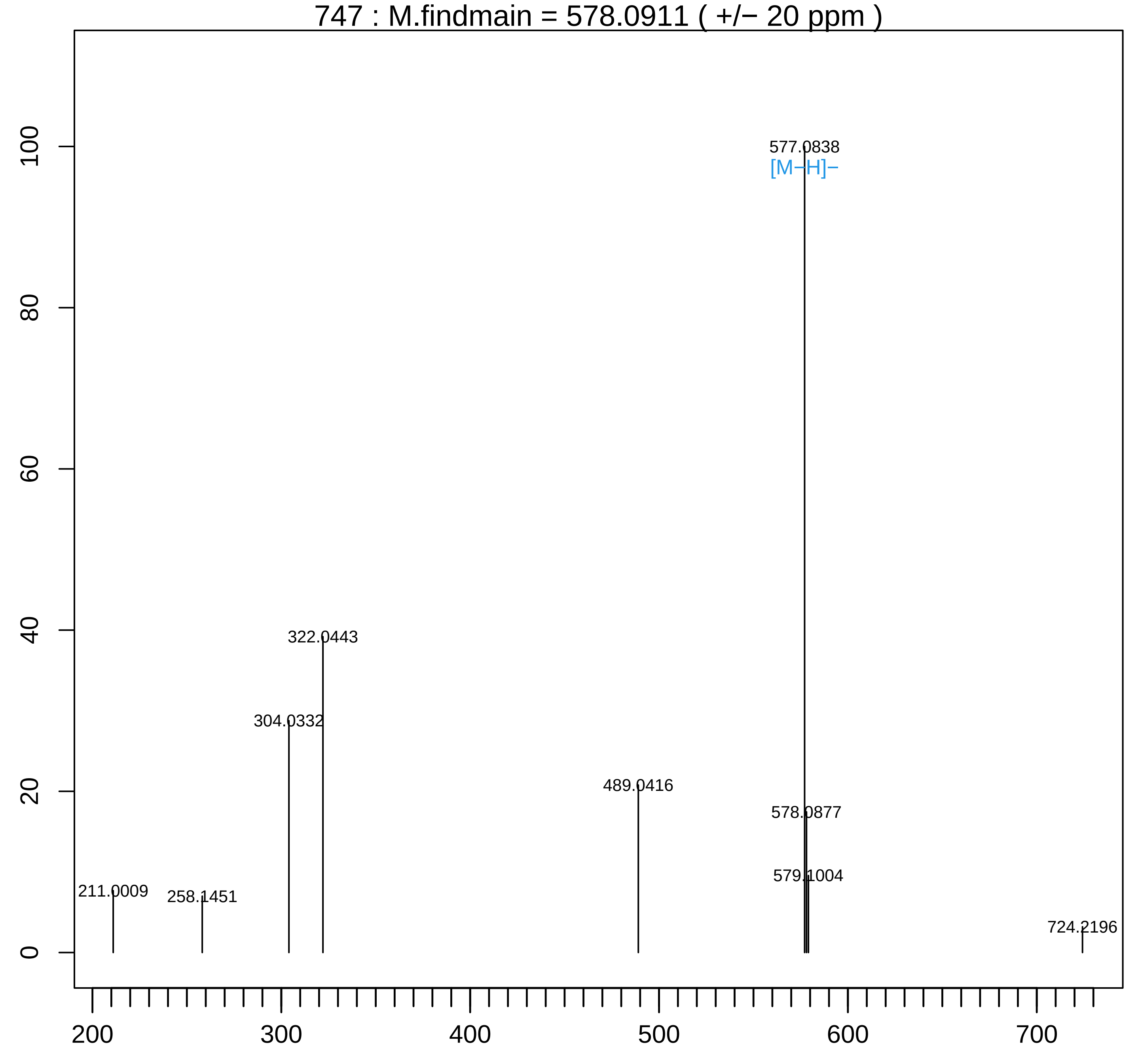

747 : M.findmain = 578.0911 (+/− 20 ppm)
577.0838
[M−H]−
322.0443
304.0332
489.0416
578.0877
579.1004
211.0009
258.1451
724.2196
0
20
40
60
80
100
200
300
400
500
600
700

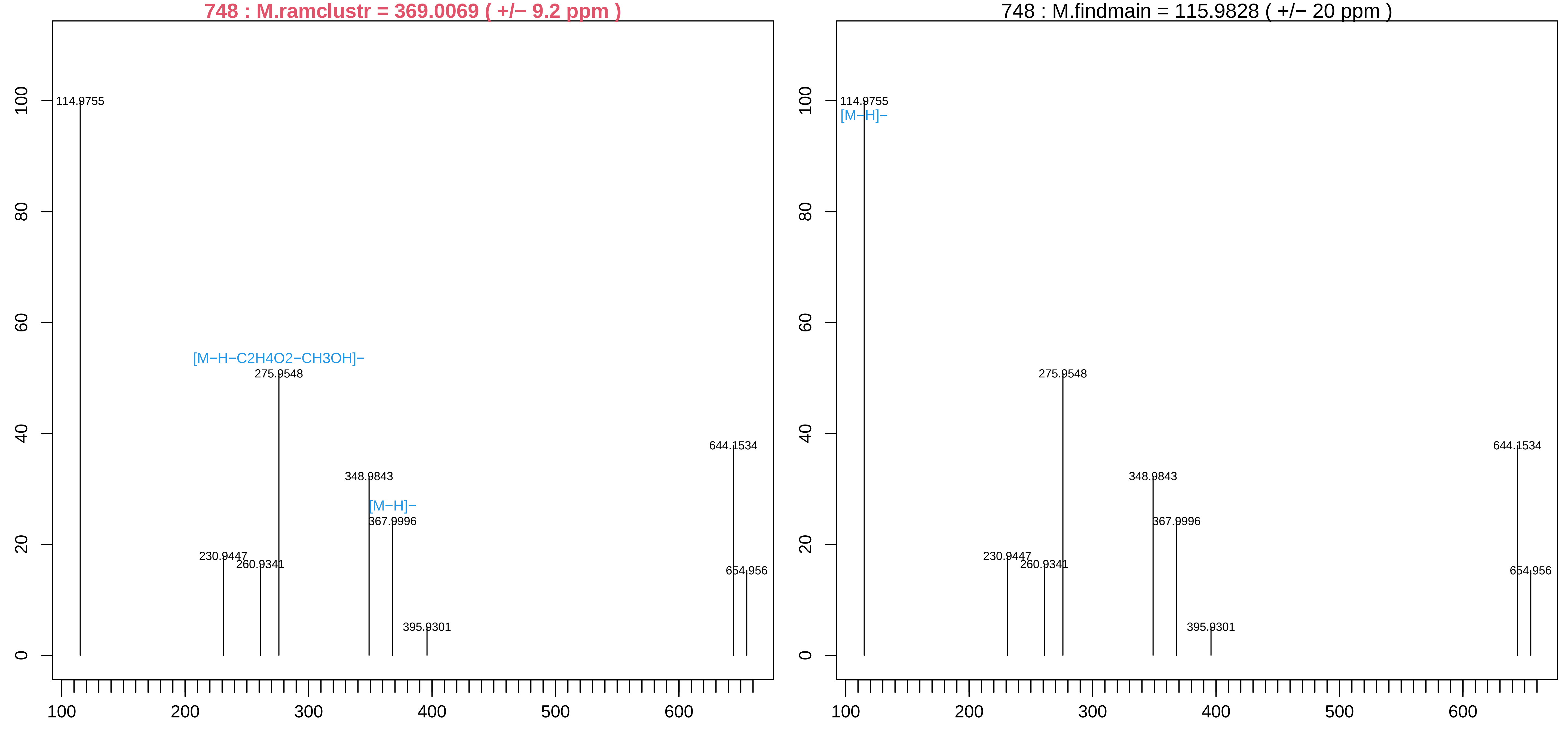
748 : M.ramclustr = 369.0069 (+/− 9.2 ppm)
114.9755
[M−H−C2H4O2−CH3OH]−
275.9548
230.9447
260.9341
348.9843
[M−H]−
367.9996
395.9301
644.1534
654.956
748 : M.findmain = 115.9828 (+/− 20 ppm)
114.9755
[M−H]−
275.9548
230.9447
260.9341
348.9843
367.9996
395.9301
644.1534
654.956
0
20
40
60
80
100
100
200
300
400
500
600

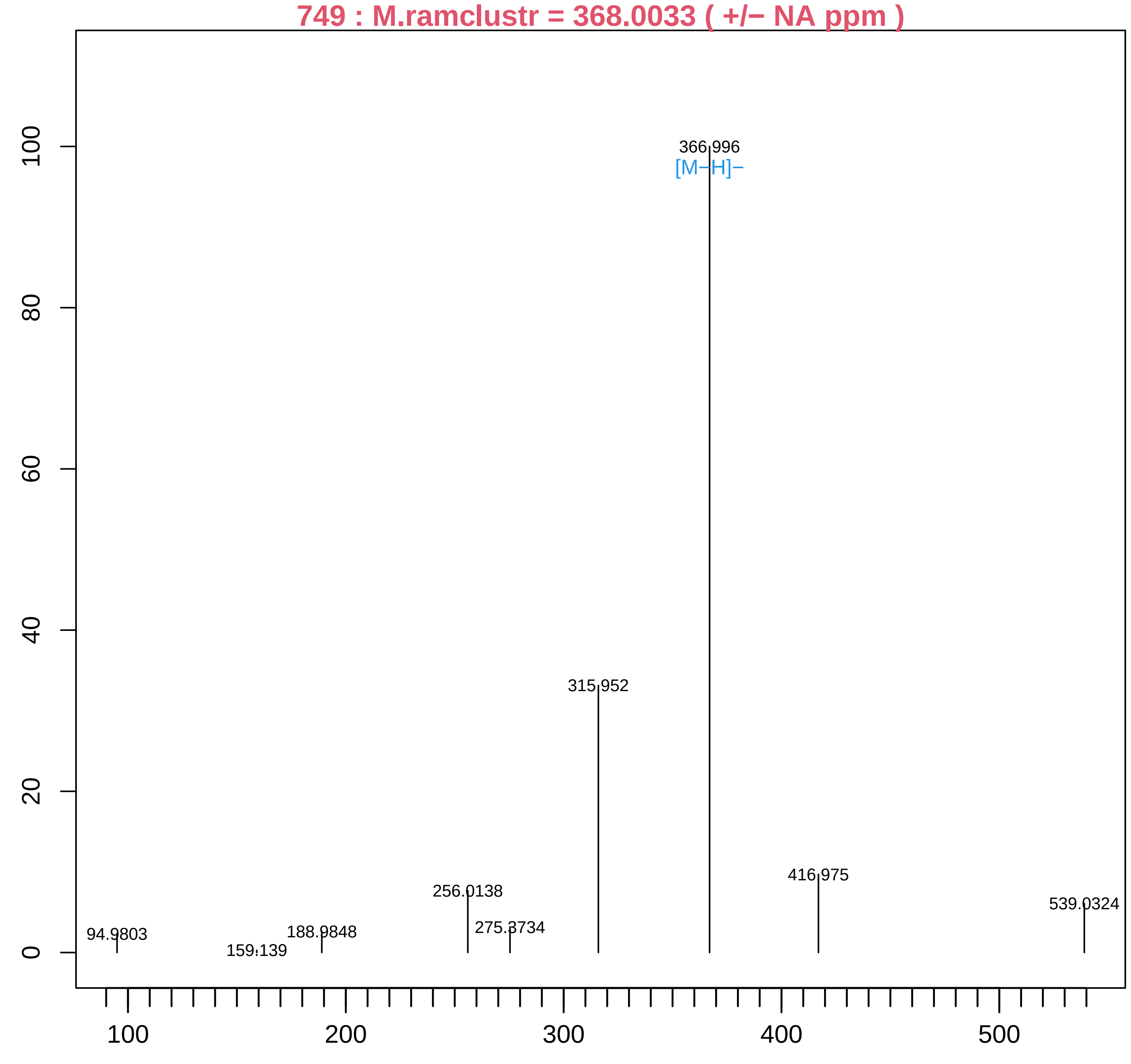
749 : M.ramclustr = 368.0033 (+/− NA ppm)
366.996
[M−H]−
315.952
256.0138
275.3734
416.975
539.0324
94.9803
159.139
188.9848
0
20
40
60
80
100
100
200
300
400
500

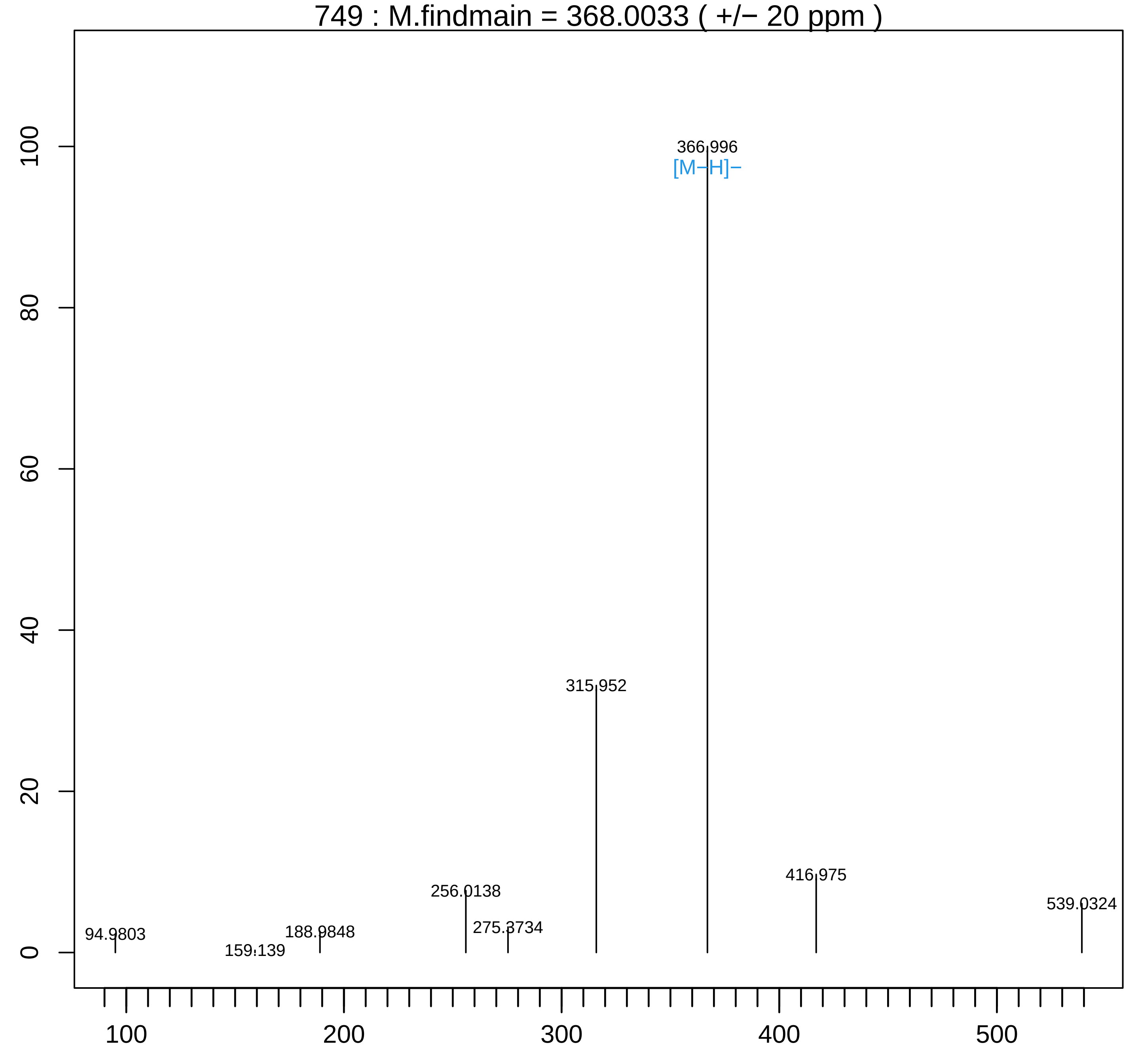
749 : M.findmain = 368.0033 (+/− 20 ppm)
366.996
[M−H]−
315.952
256.0138
275.3734
416.975
539.0324
94.9803
159.139
188.9848
0
20
40
60
80
100
100
200
300
400
500

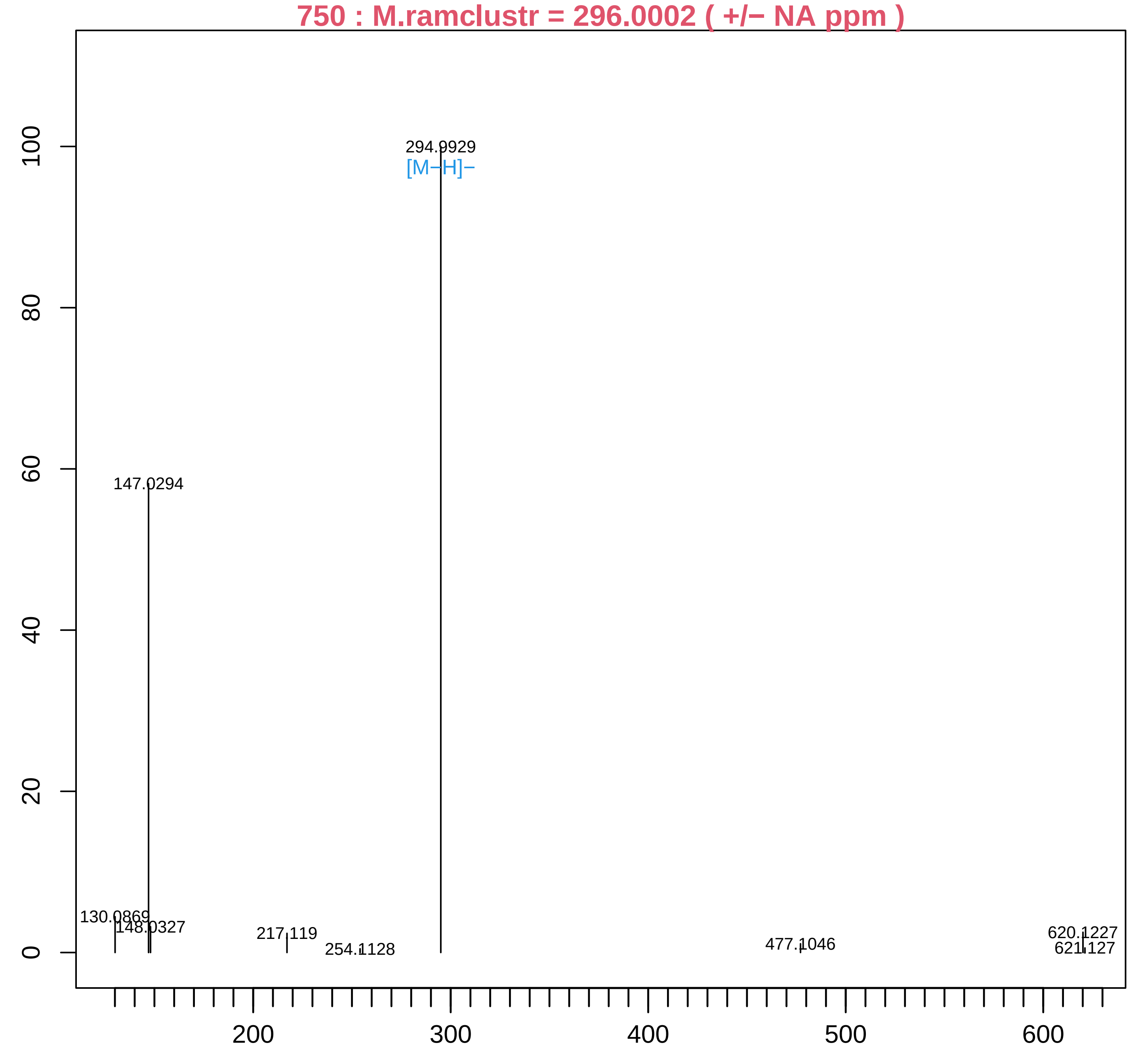
750 : M.ramclustr = 296.0002 (+/− NA ppm)
294.9929
[M−H]−
147.0294
130.0869
148.0327
217.119
254.1128
477.1046
620.1227
621.127
0
20
40
60
80
100
200
300
400
500
600

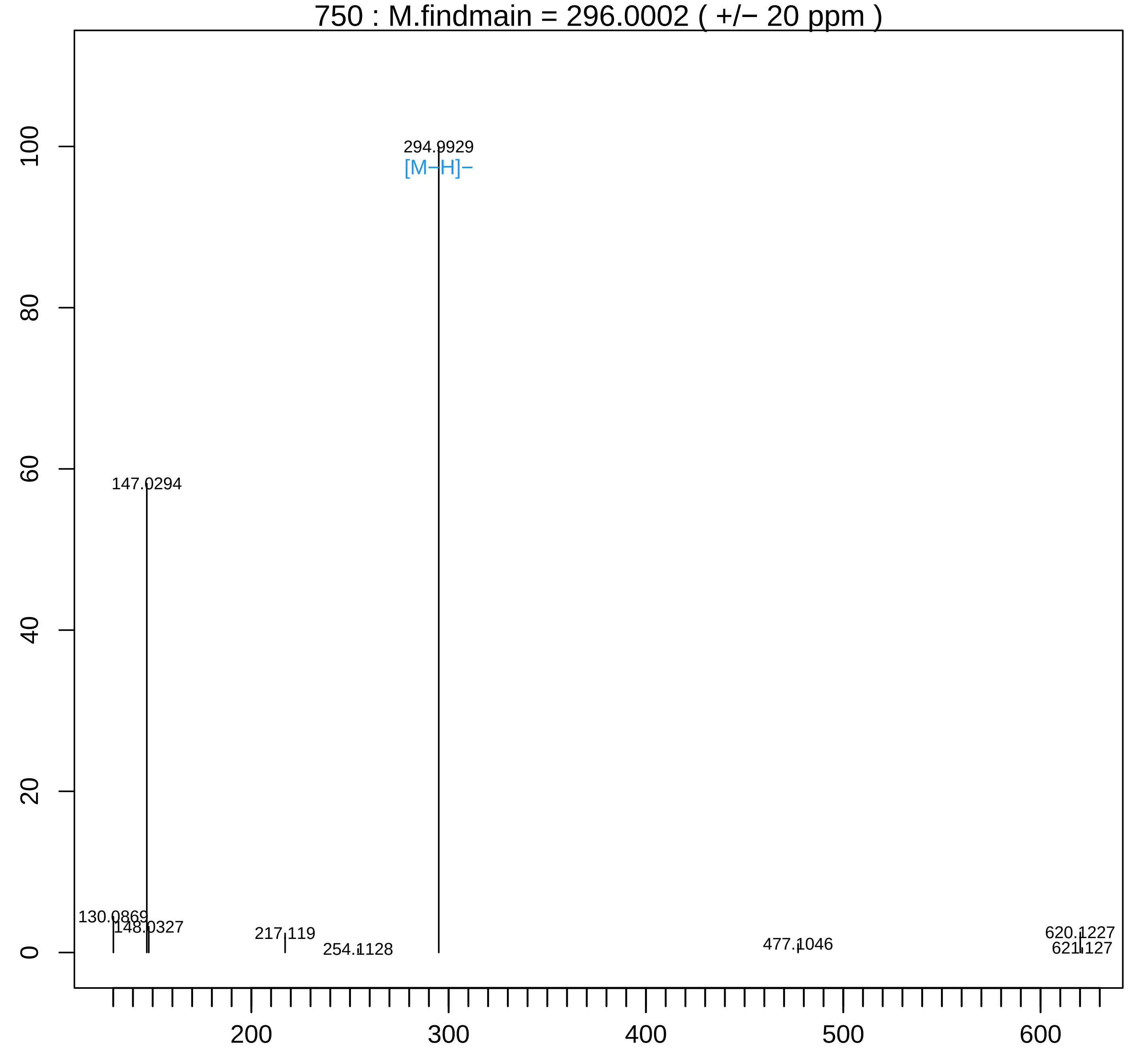
750 : M.findmain = 296.0002 (+/− 20 ppm)
294.9929
[M−H]−
147.0294
130.0869
148.0327
217.119
254.1128
477.1046
620.1227
621.127
0
20
40
60
80
100
200
300
400
500
600

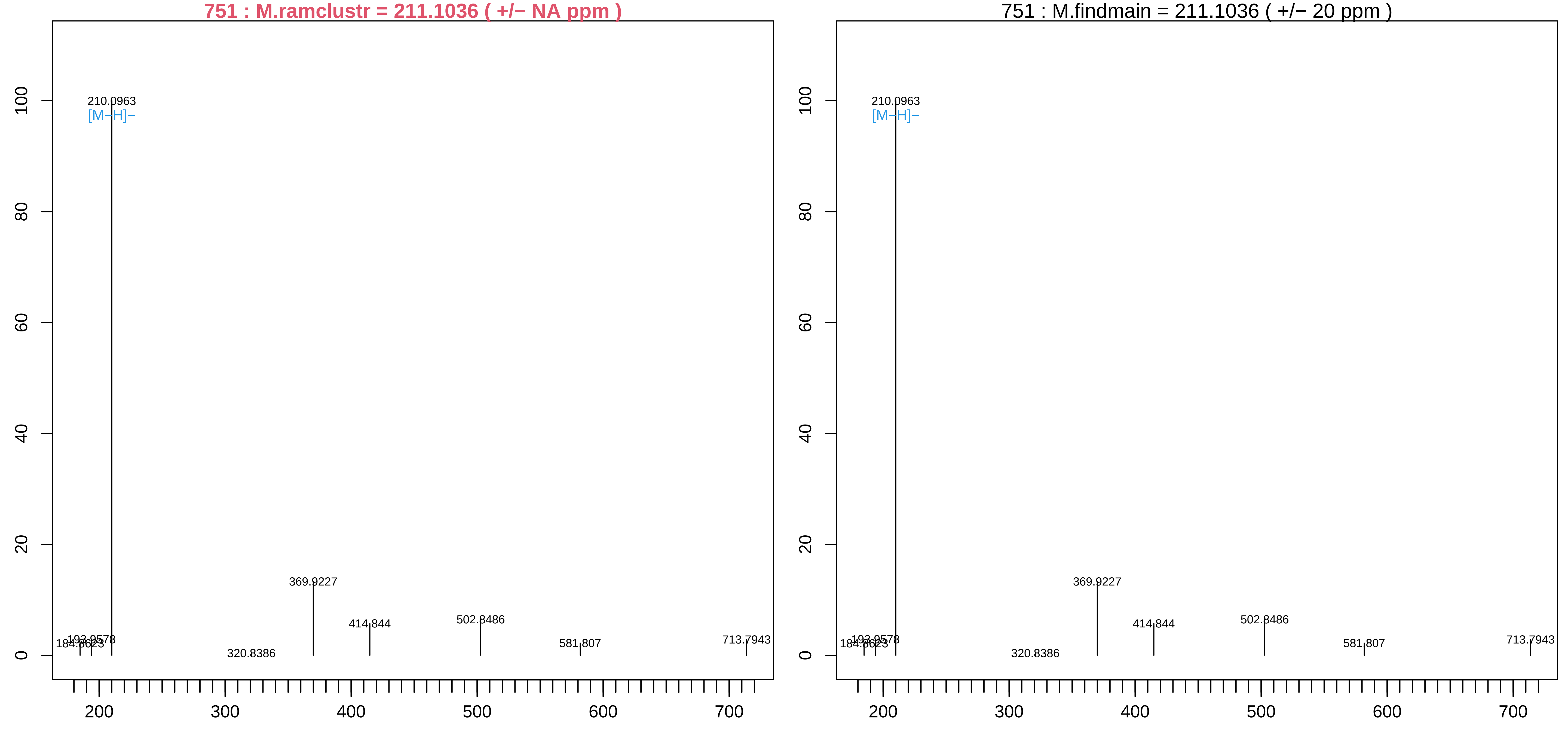
751 : M.ramclustr = 211.1036 (+/− NA ppm)
751 : M.findmain = 211.1036 (+/− 20 ppm)
210.0963
[M−H]−
184.8623
193.9578
320.8386
369.9227
414.844
502.8486
581.807
713.7943
0
20
40
60
80
100
200
300
400
500
600
700

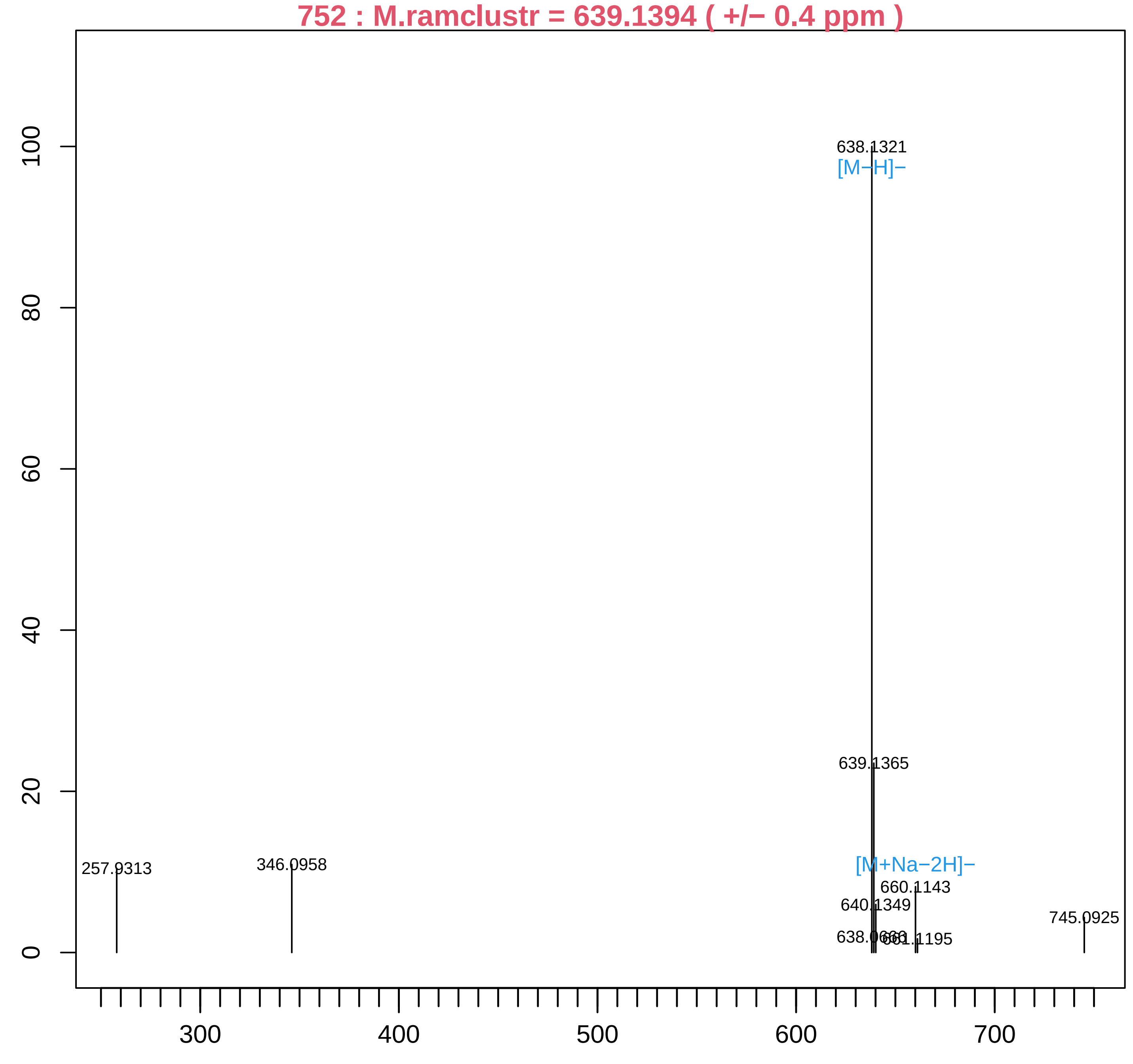

752 : M.ramclustr = 639.1394 (+/− 0.4 ppm)
638.1321
[M−H]−
639.1365
[M+Na−2H]−
660.1143
640.1349
638.0668
661.1195
257.9313
346.0958
745.0925
0
20
40
60
80
100
300
400
500
600
700

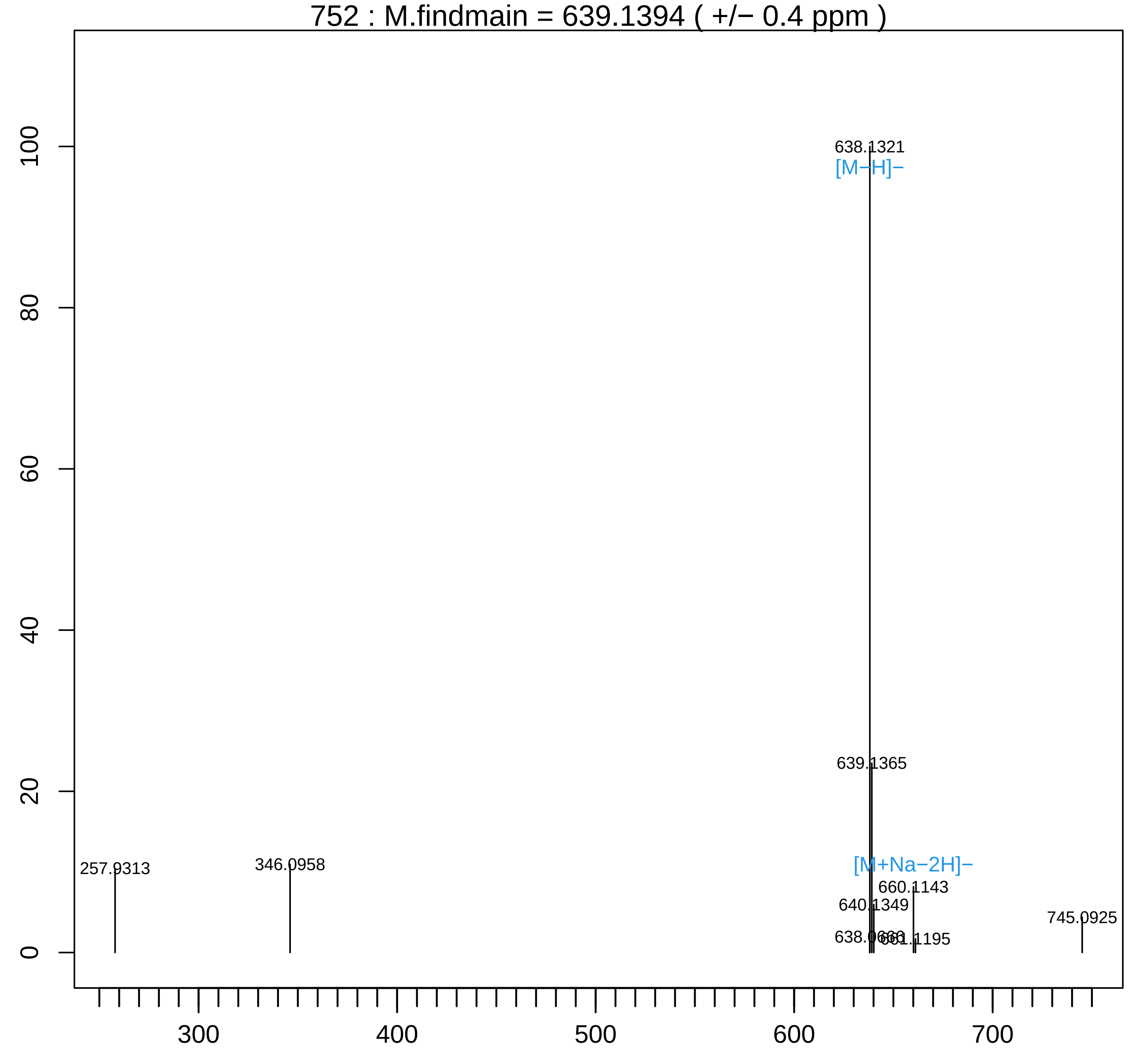

752 : M.findmain = 639.1394 (+/− 0.4 ppm)
638.1321
[M−H]−
639.1365
[M+Na−2H]−
660.1143
640.1349
638.0668
661.1195
257.9313
346.0958
745.0925
0
20
40
60
80
100
300
400
500
600
700

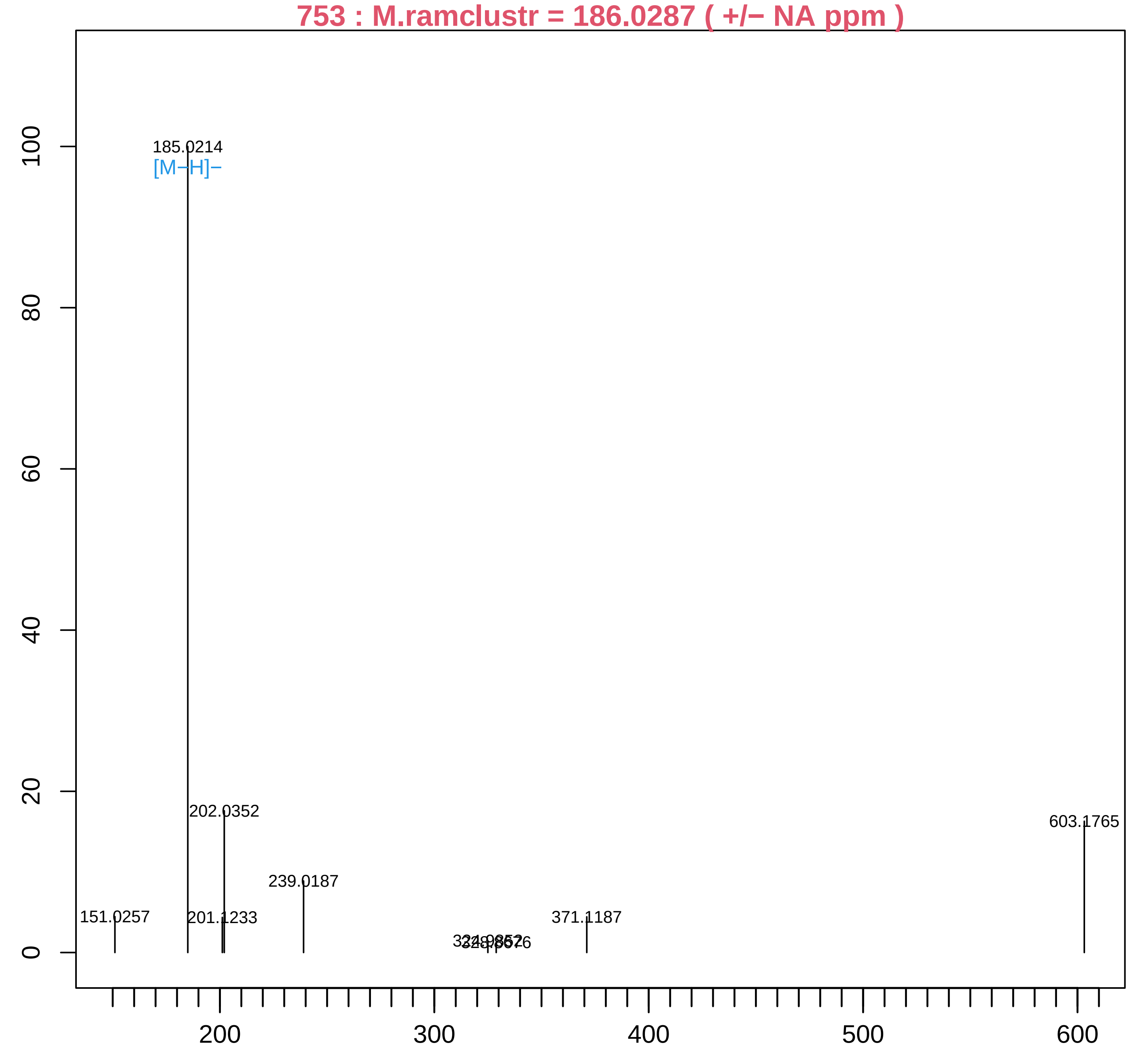

753 : M.ramclustr = 186.0287 (+/− NA ppm)
185.0214
[M−H]−
202.0352
603.1765
239.0187
151.0257
201.1233
371.1187
0
20
40
60
80
100
200
300
400
500
600

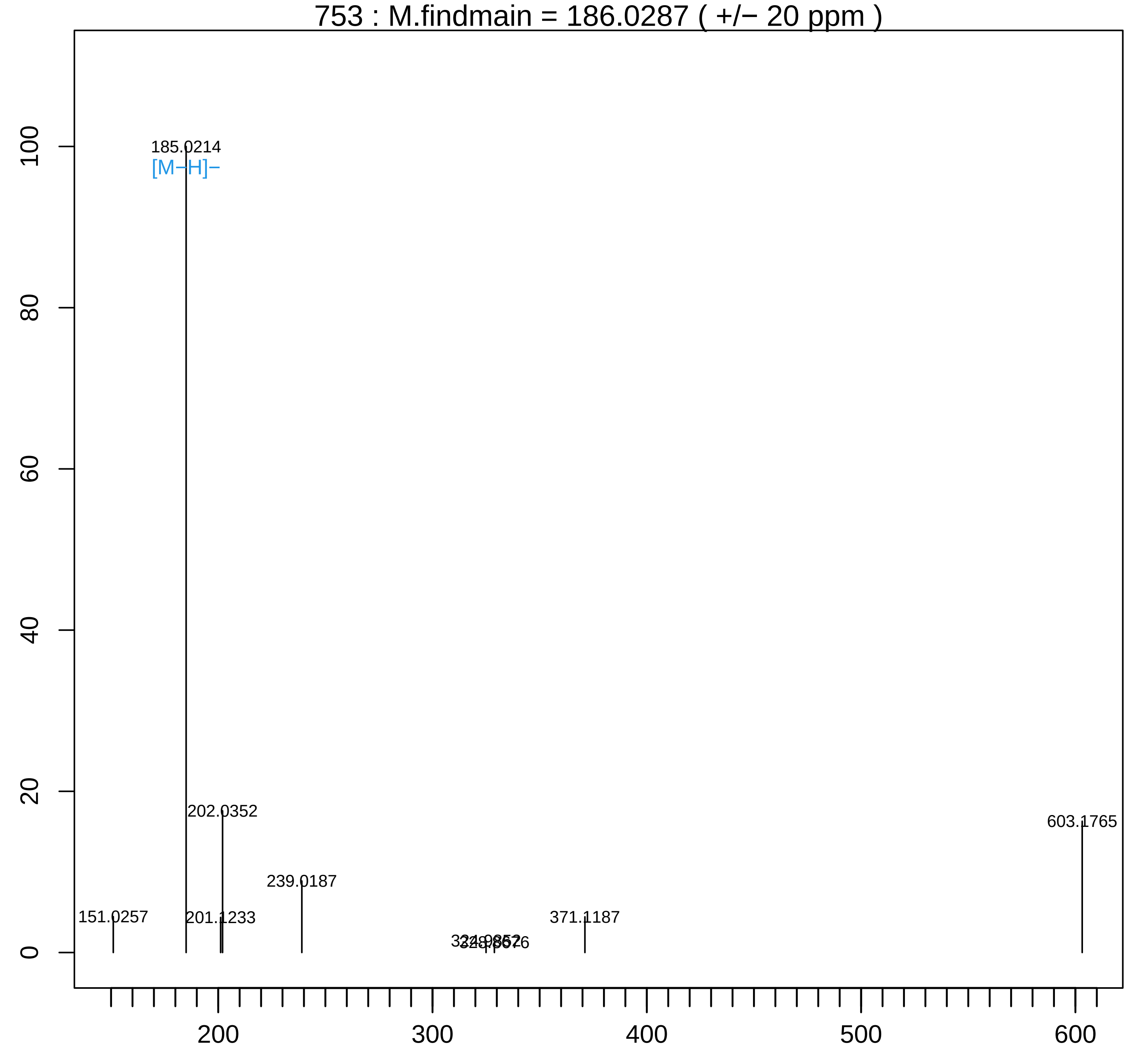

753 : M.findmain = 186.0287 (+/− 20 ppm)
185.0214
[M−H]−
202.0352
603.1765
239.0187
151.0257
201.1233
371.1187
0
20
40
60
80
100
200
300
400
500
600

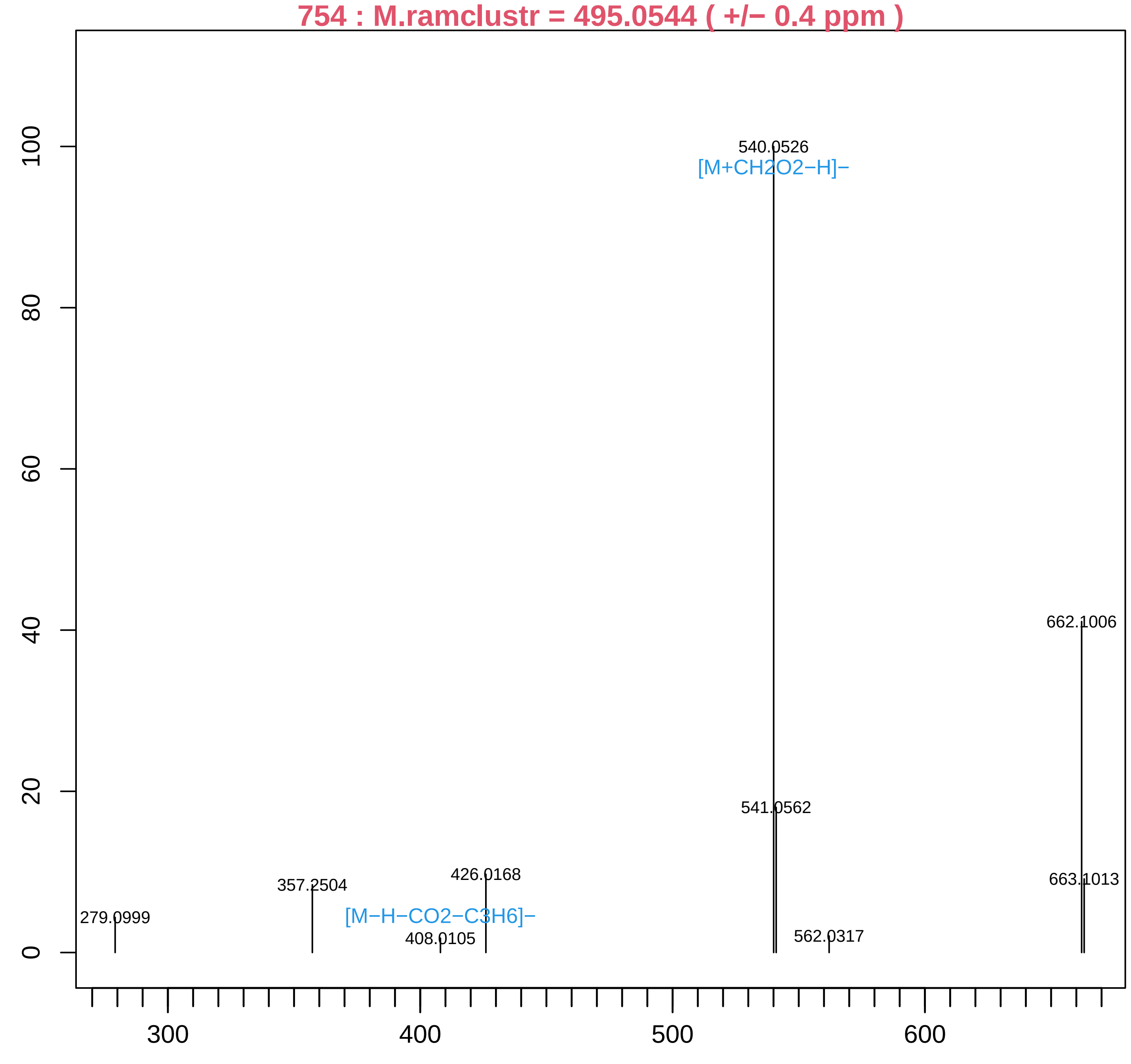
754 : M.ramclustr = 495.0544 (+/− 0.4 ppm)
540.0526
[M+CH2O2−H]−
662.1006
541.0562
426.0168
357.2504
663.1013
279.0999
[M−H−CO2−C3H6]−
408.0105
562.0317
0
20
40
60
80
100
300
400
500
600

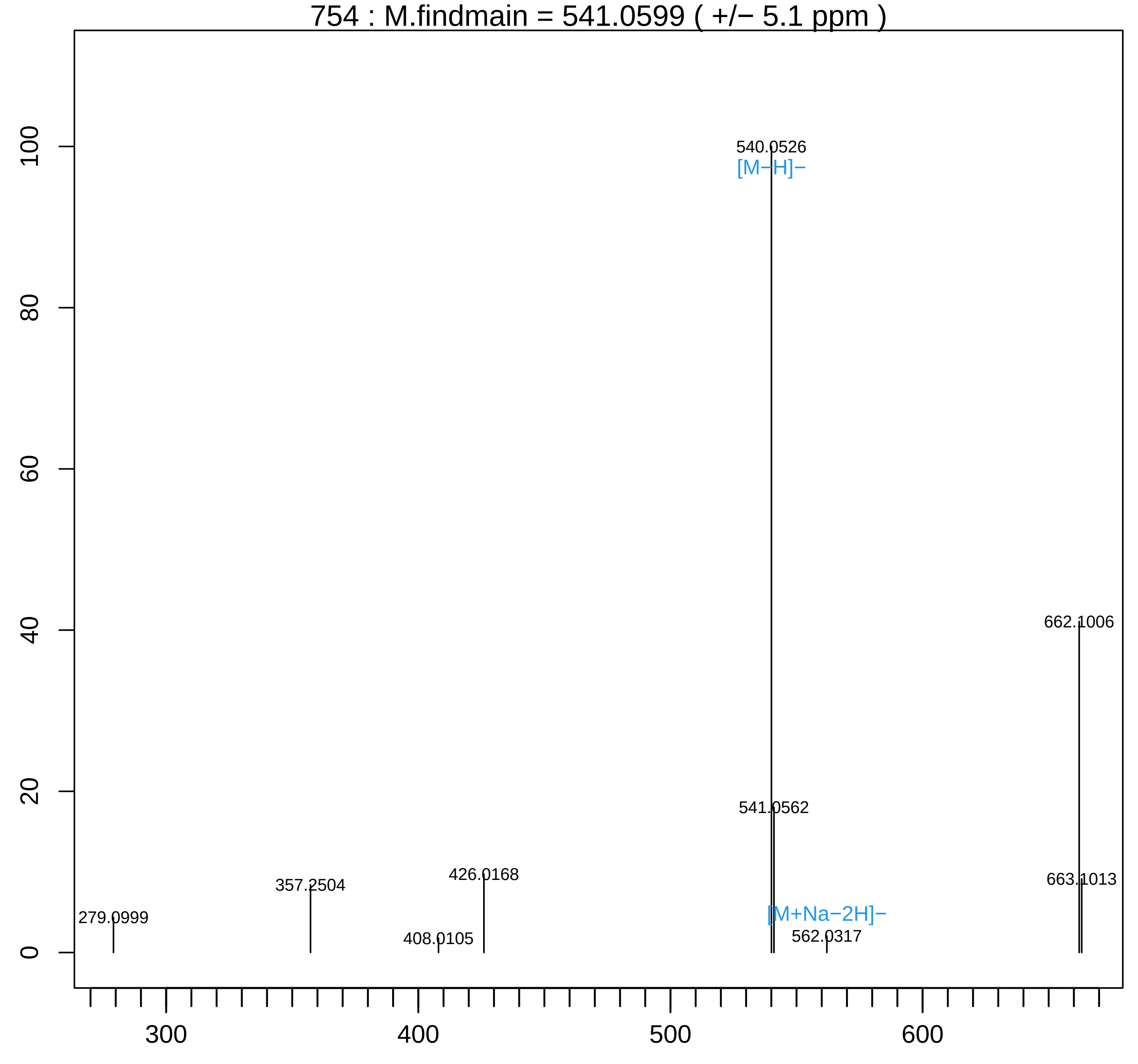
754 : M.findmain = 541.0599 (+/− 5.1 ppm)
540.0526
[M−H]−
662.1006
541.0562
426.0168
357.2504
663.1013
279.0999
[M+Na−2H]−
408.0105
562.0317
0
20
40
60
80
100
300
400
500
600

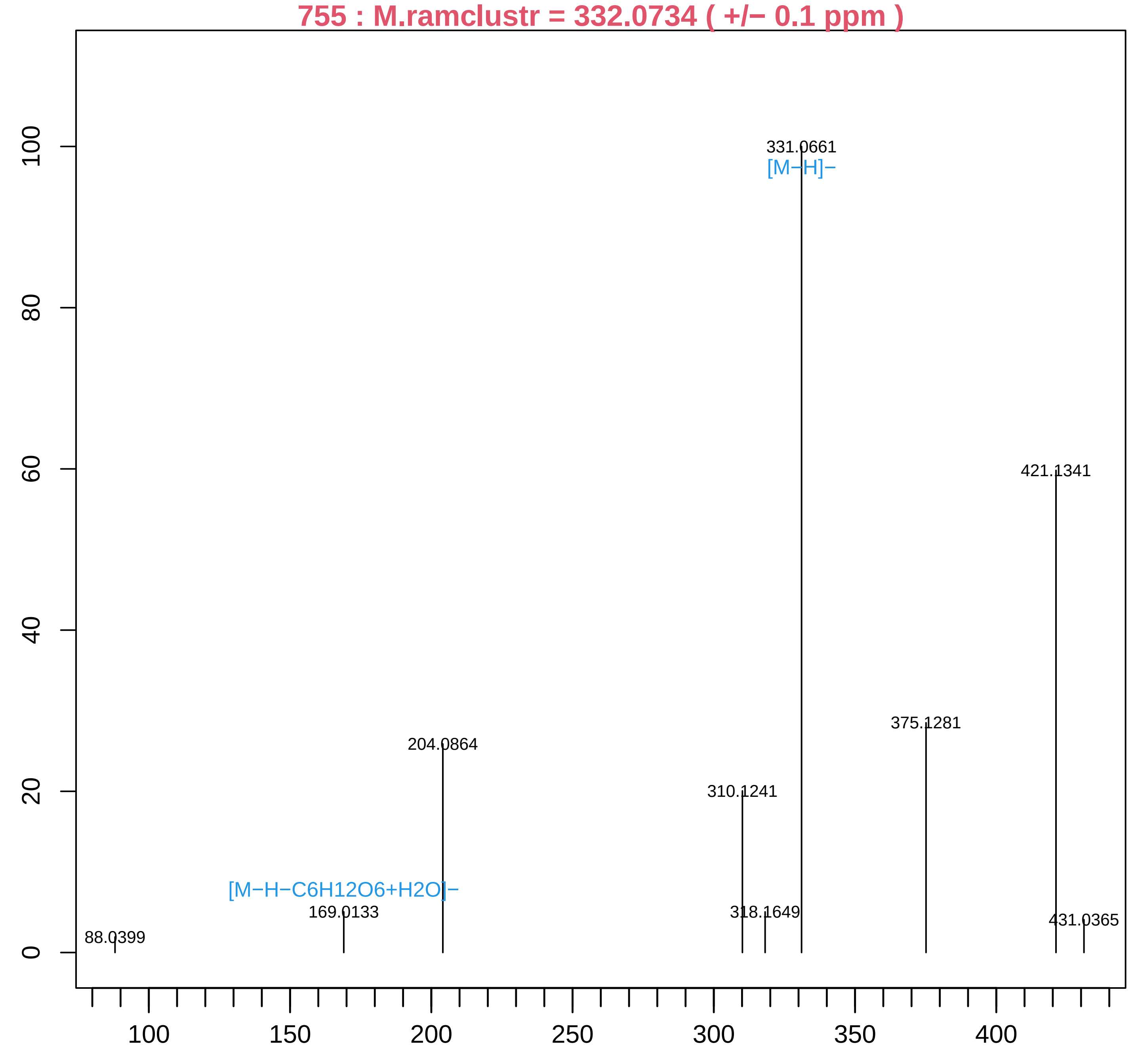

755 : M.ramclustr = 332.0734 (+/− 0.1 ppm)
0
20
40
60
80
100
100
150
200
250
300
350
400
331.0661
[M−H]−
421.1341
375.1281
204.0864
310.1241
[M−H−C6H12O6+H2O]−
169.0133
318.1649
431.0365
88.0399

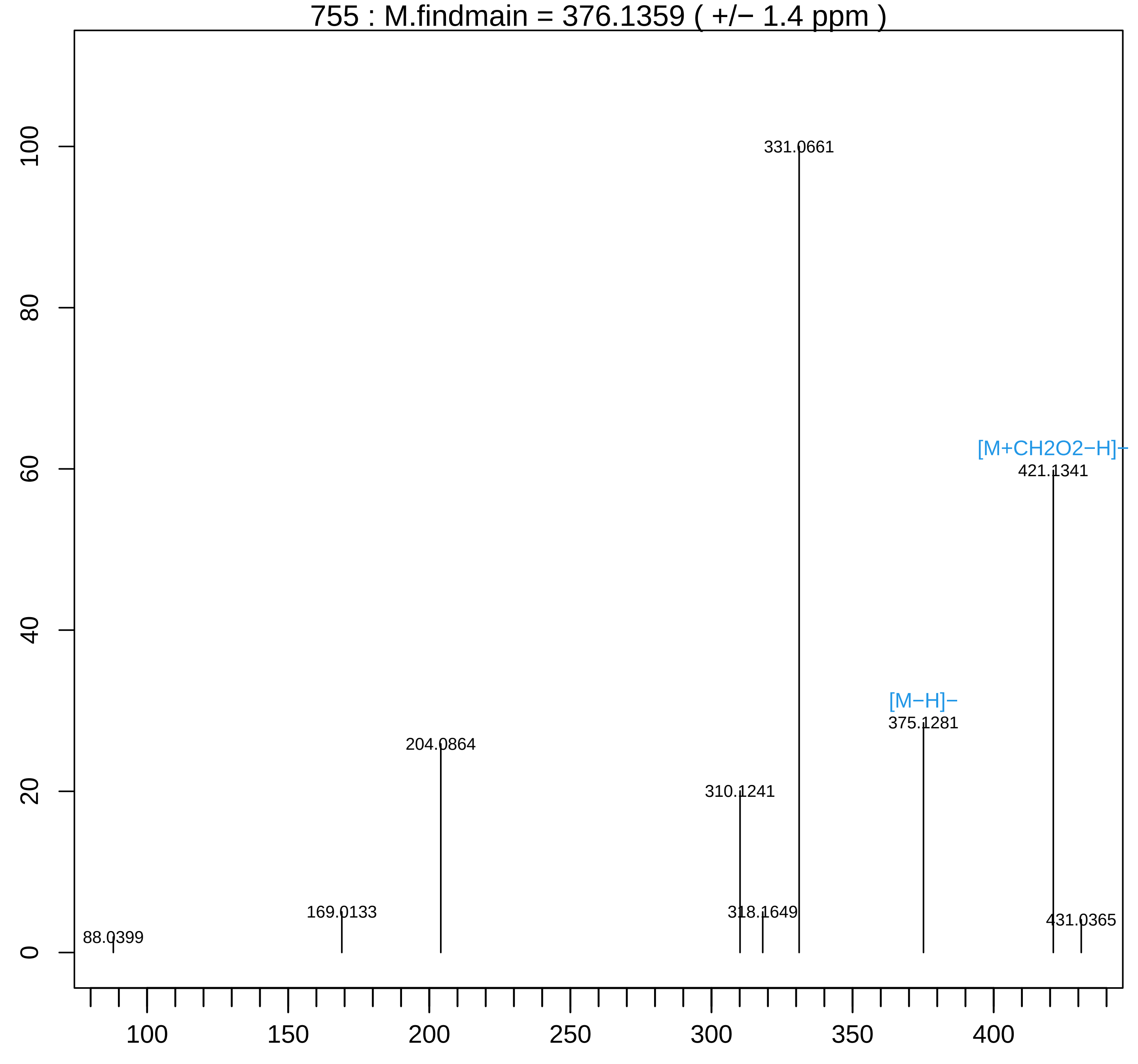

755 : M.findmain = 376.1359 (+/− 1.4 ppm)
0
20
40
60
80
100
100
150
200
250
300
350
400
331.0661
[M+CH2O2−H]−
421.1341
[M−H]−
375.1281
204.0864
310.1241
169.0133
318.1649
431.0365
88.0399

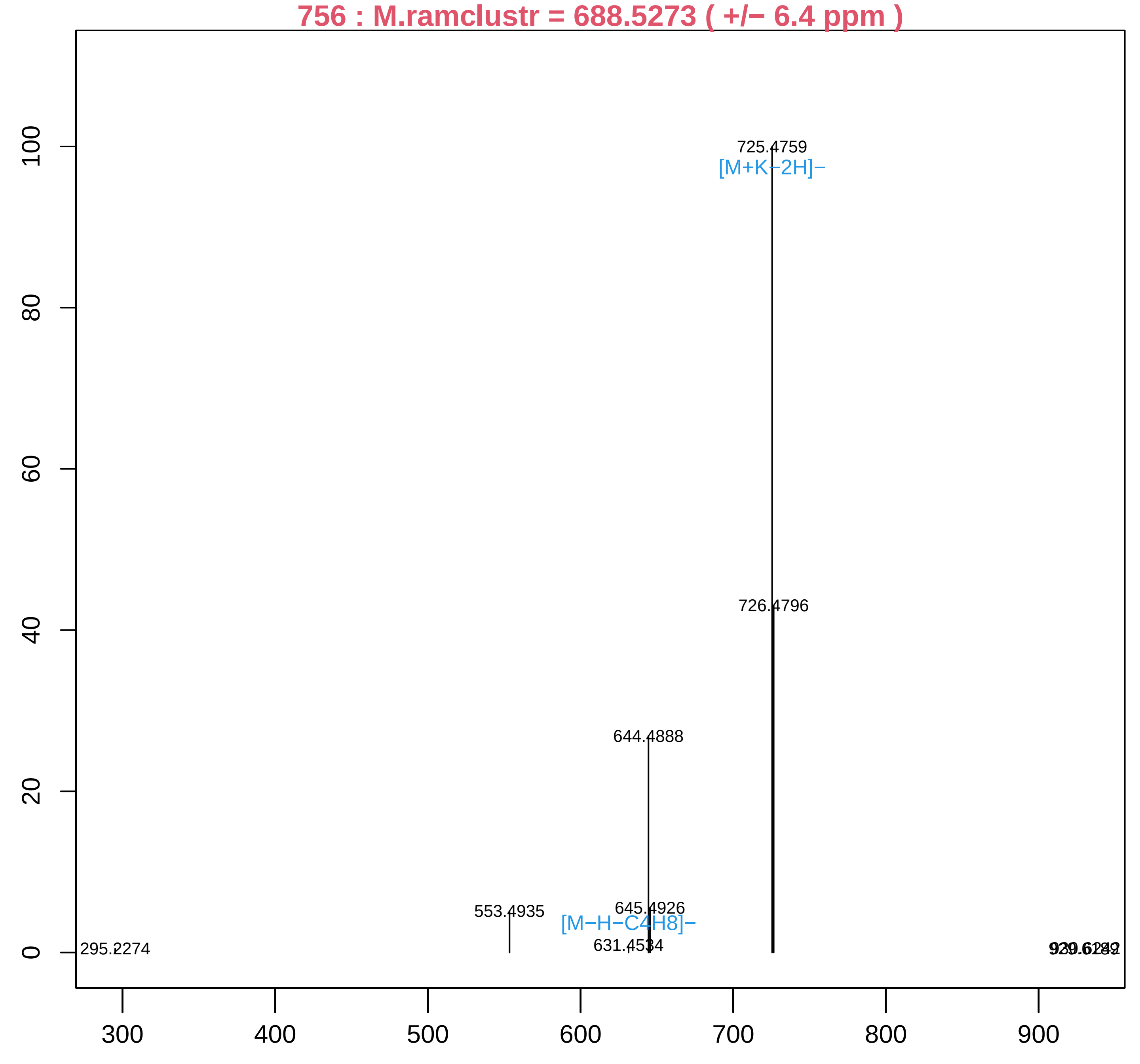
756 : M.ramclustr = 688.5273 (+/− 6.4 ppm)
725.4759
[M+K−2H]−
726.4796
644.4888
553.4935
645.4926
[M−H−C4H8]−
631.4534
295.2274
0
20
40
60
80
100
300
400
500
600
700
800
900

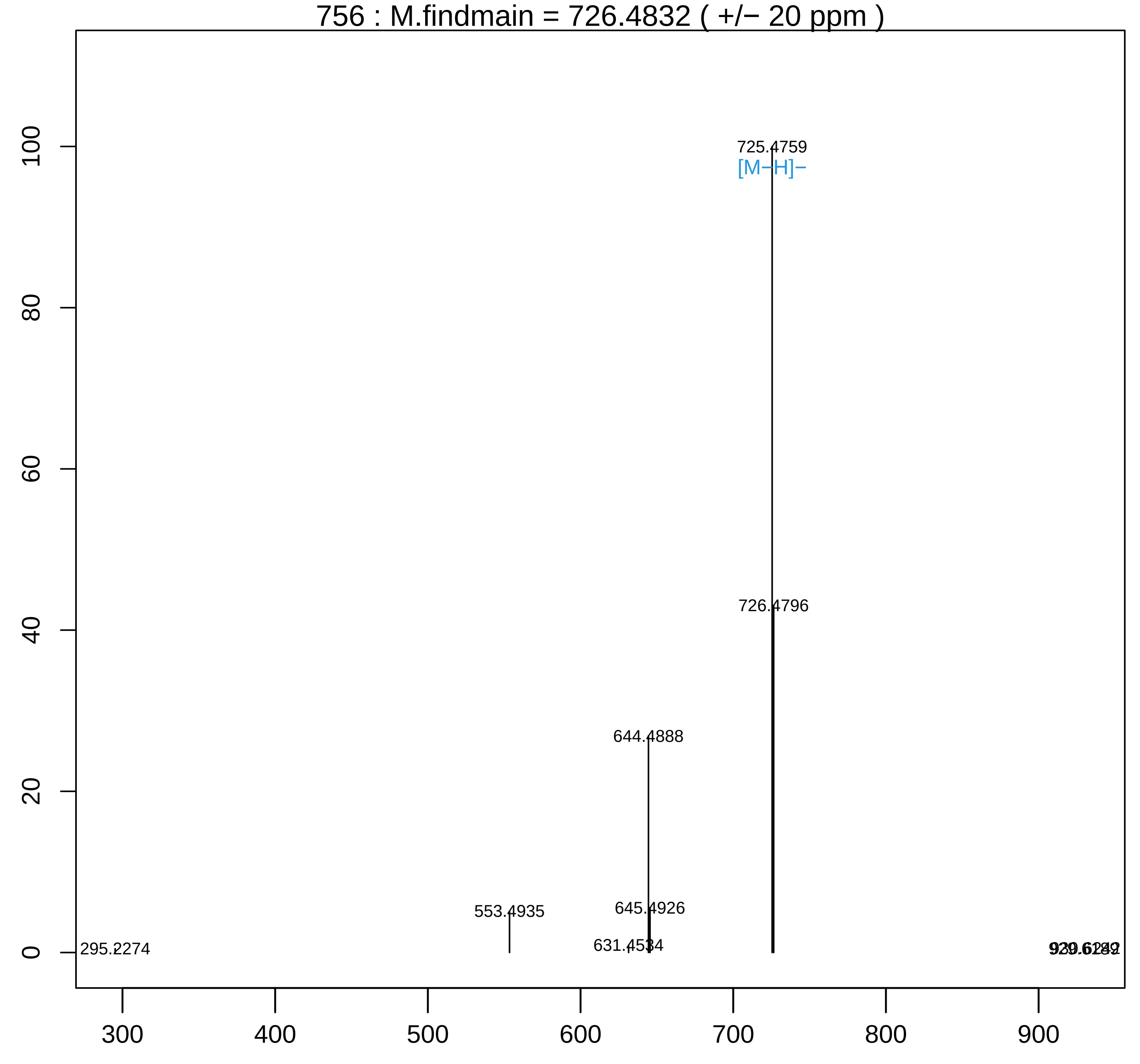
756 : M.findmain = 726.4832 (+/− 20 ppm)
725.4759
[M−H]−
726.4796
644.4888
553.4935
645.4926
631.4534
295.2274
0
20
40
60
80
100
300
400
500
600
700
800
900

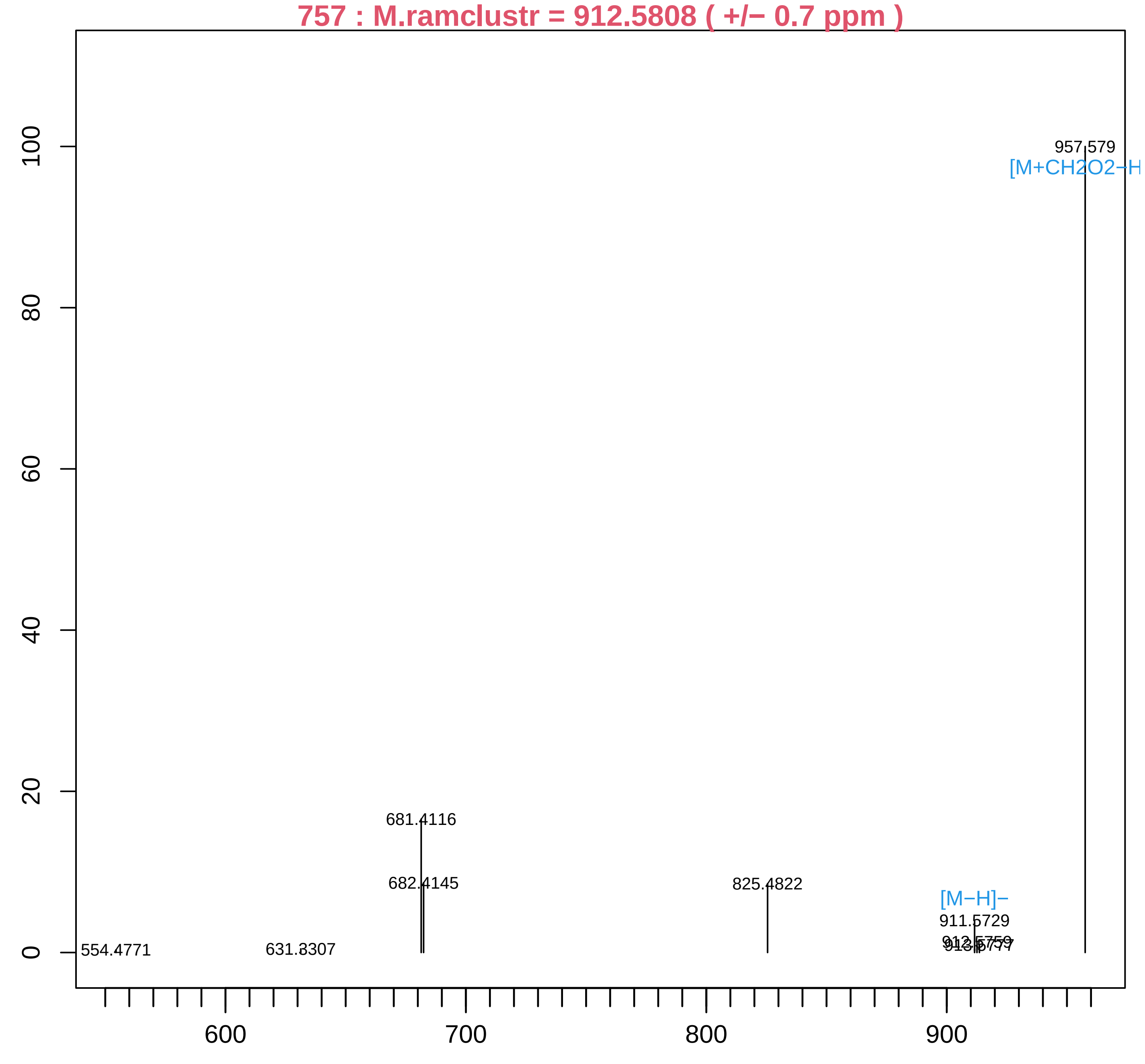
757 : M.ramclustr = 912.5808 (+/− 0.7 ppm)
957.579
[M+CH2O2−H
681.4116
682.4145
825.4822
[M−H]−
911.5729
554.4771
631.8307
0
20
40
60
80
100
600
700
800
900

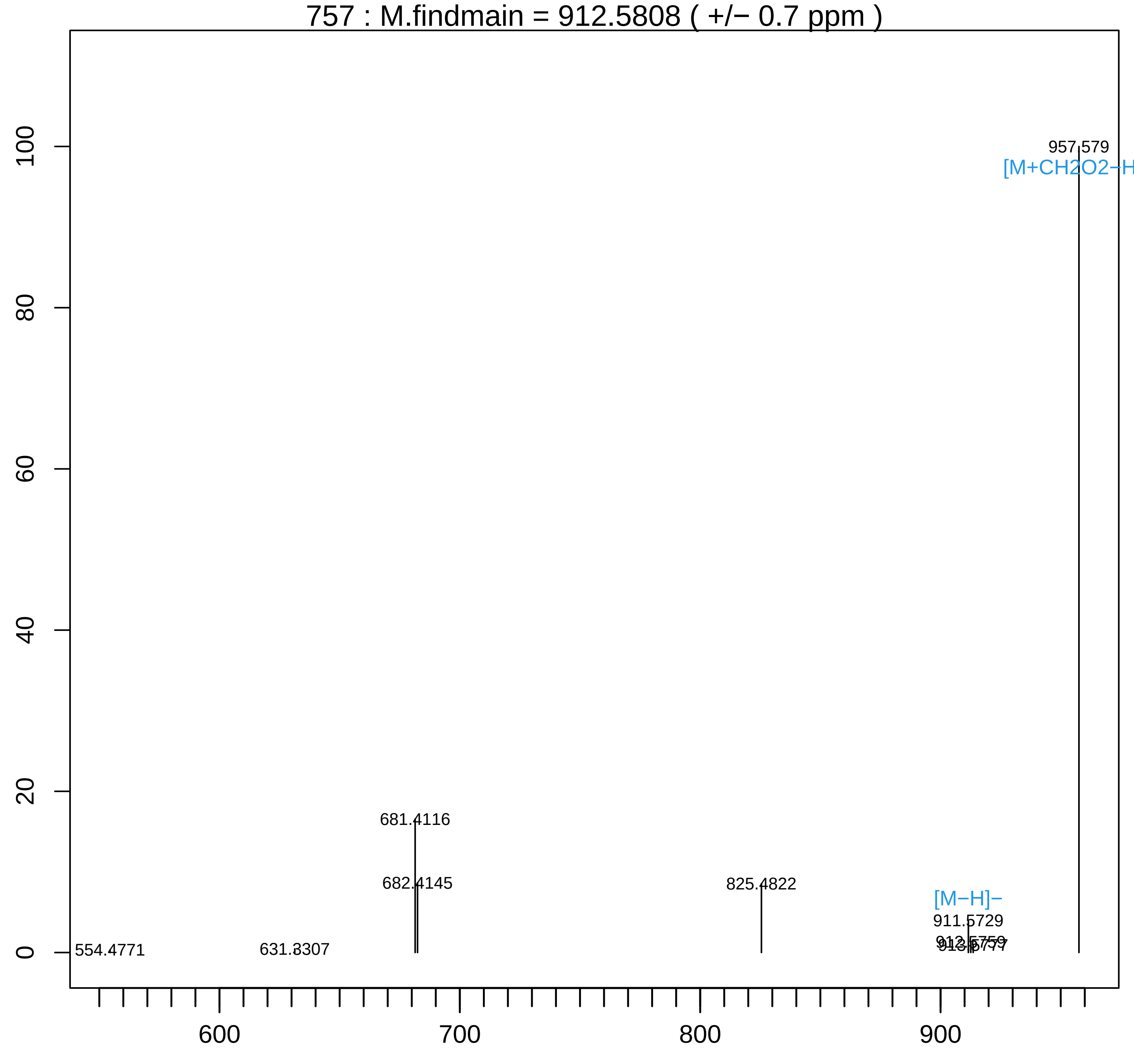
757 : M.findmain = 912.5808 (+/− 0.7 ppm)
957.579
[M+CH2O2−H
681.4116
682.4145
825.4822
[M−H]−
911.5729
554.4771
631.8307
0
20
40
60
80
100
600
700
800
900

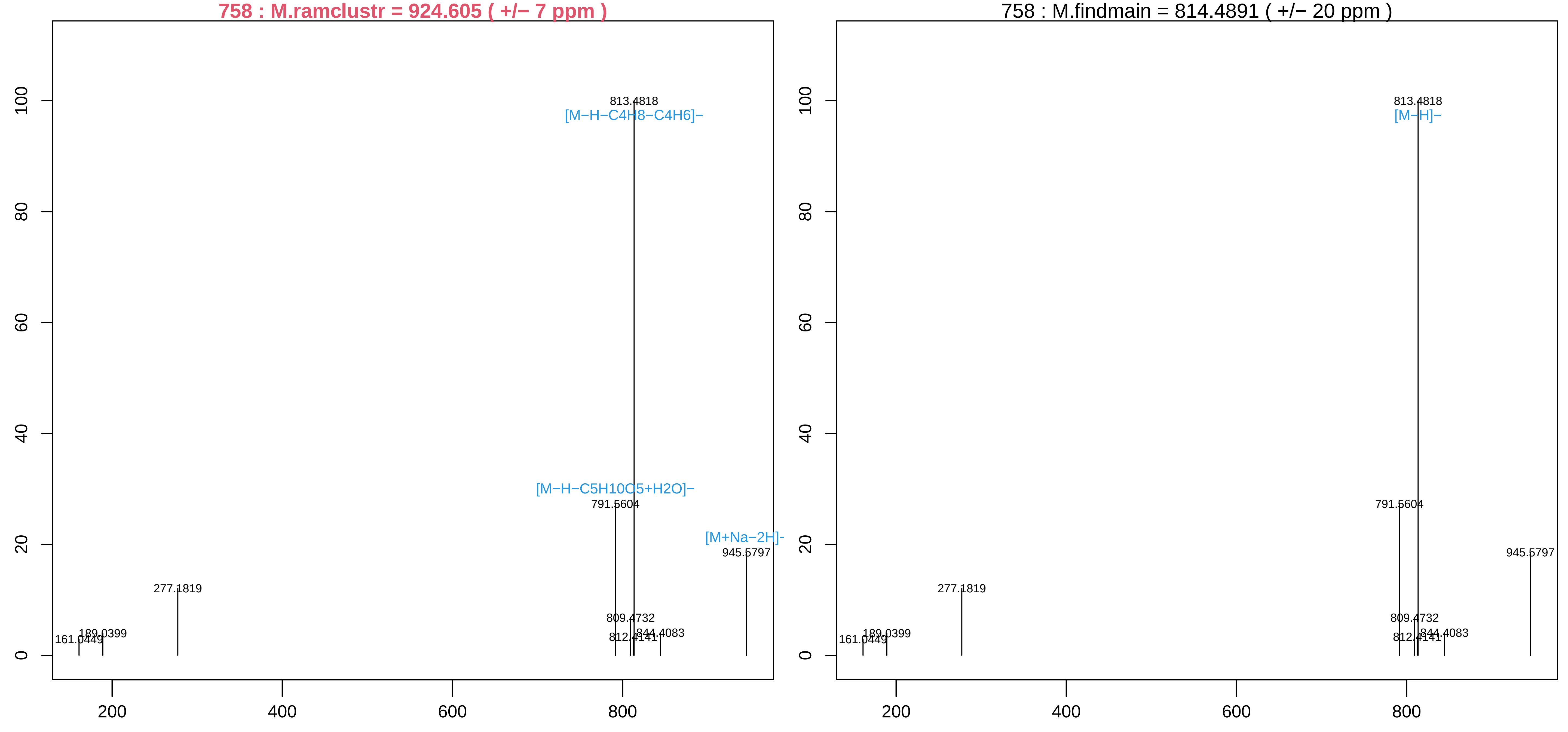
758 : M.ramclustr = 924.605 (+/− 7 ppm)
813.4818
[M−H−C4H8−C4H6]−
[M−H−C5H10O5+H2O]−
791.5604
[M+Na−2H]-
945.5797
277.1819
809.4732
189.0399
161.0449
812.4141
844.4083
758 : M.findmain = 814.4891 (+/− 20 ppm)
813.4818
[M−H]−
791.5604
945.5797
277.1819
809.4732
189.0399
161.0449
812.4141
844.4083
0
20
40
60
80
100
200
400
600
800

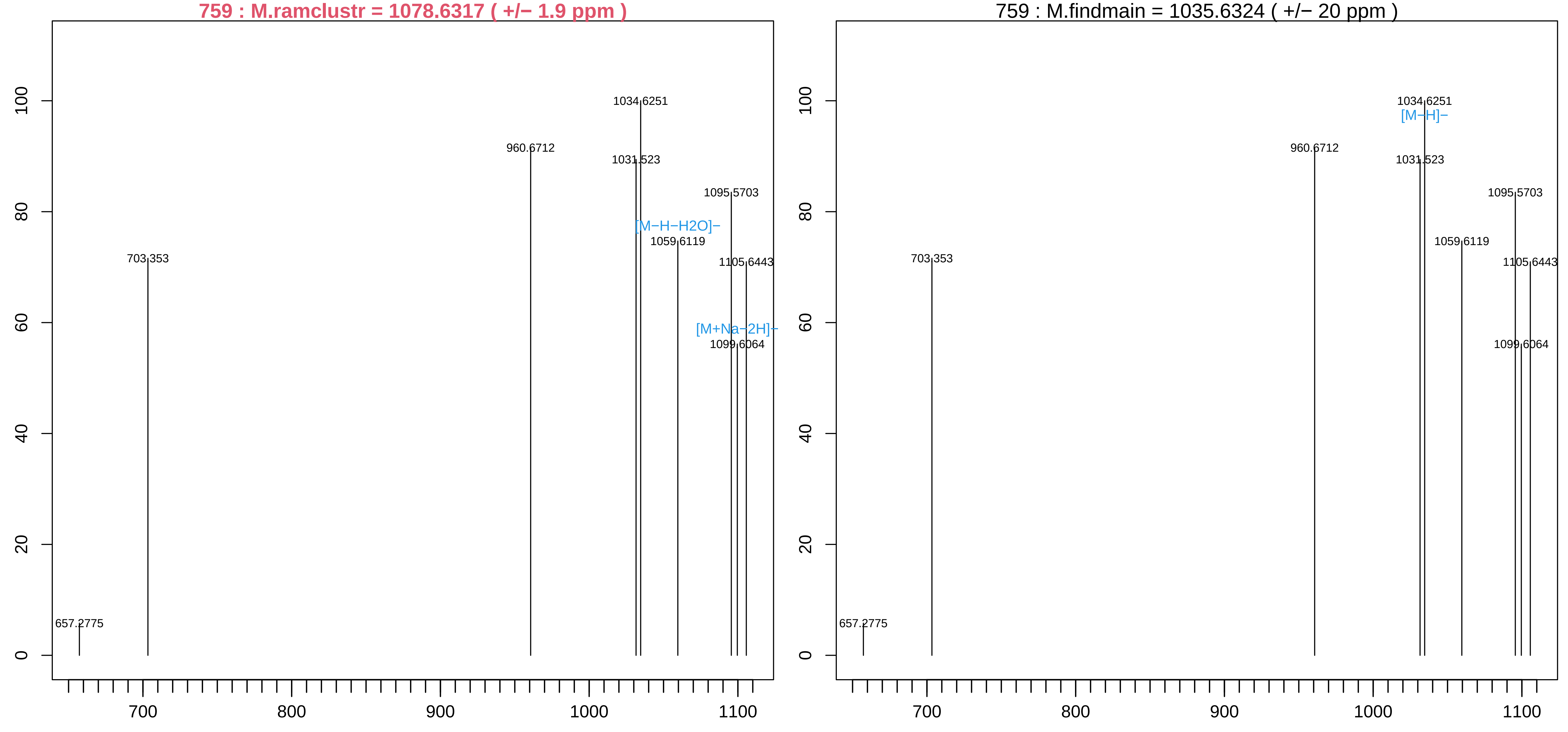
759 : M.ramclustr = 1078.6317 (+/− 1.9 ppm)
657.2775
703.353
960.6712
1034.6251
1031.523
[M−H−H2O]−
1059.6119
1095.5703
1105.6443
[M+Na−2H]−
1099.6064
759 : M.findmain = 1035.6324 (+/− 20 ppm)
[M−H]−
0
20
40
60
80
100
700
800
900
1000
1100

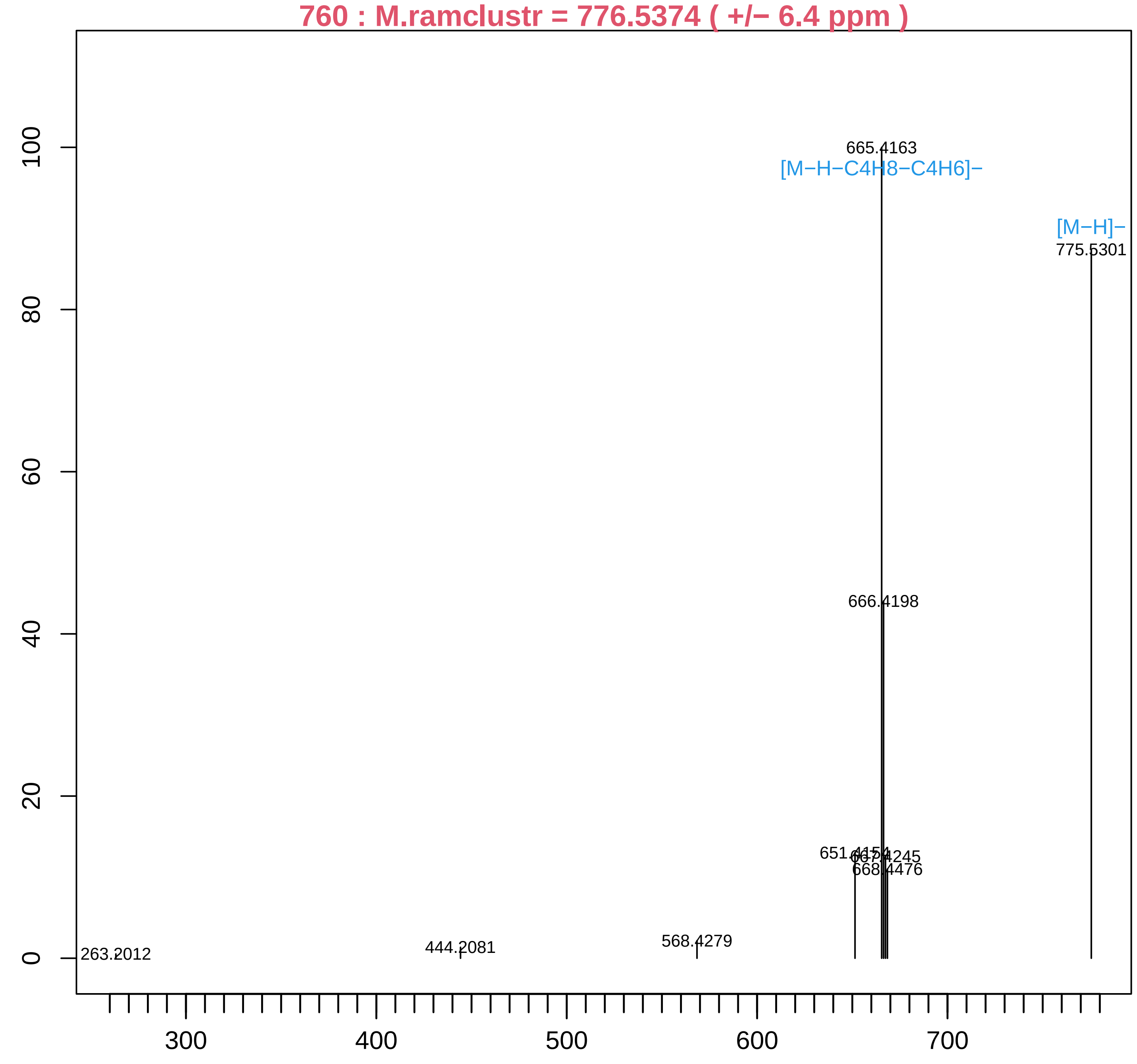

760 : M.ramclustr = 776.5374 (+/− 6.4 ppm)
665.4163
[M−H−C4H8−C4H6]−
[M−H]−
775.5301
666.4198
651.4154
667.4245
668.4476
263.2012
444.2081
568.4279
0
20
40
60
80
100
300
400
500
600
700

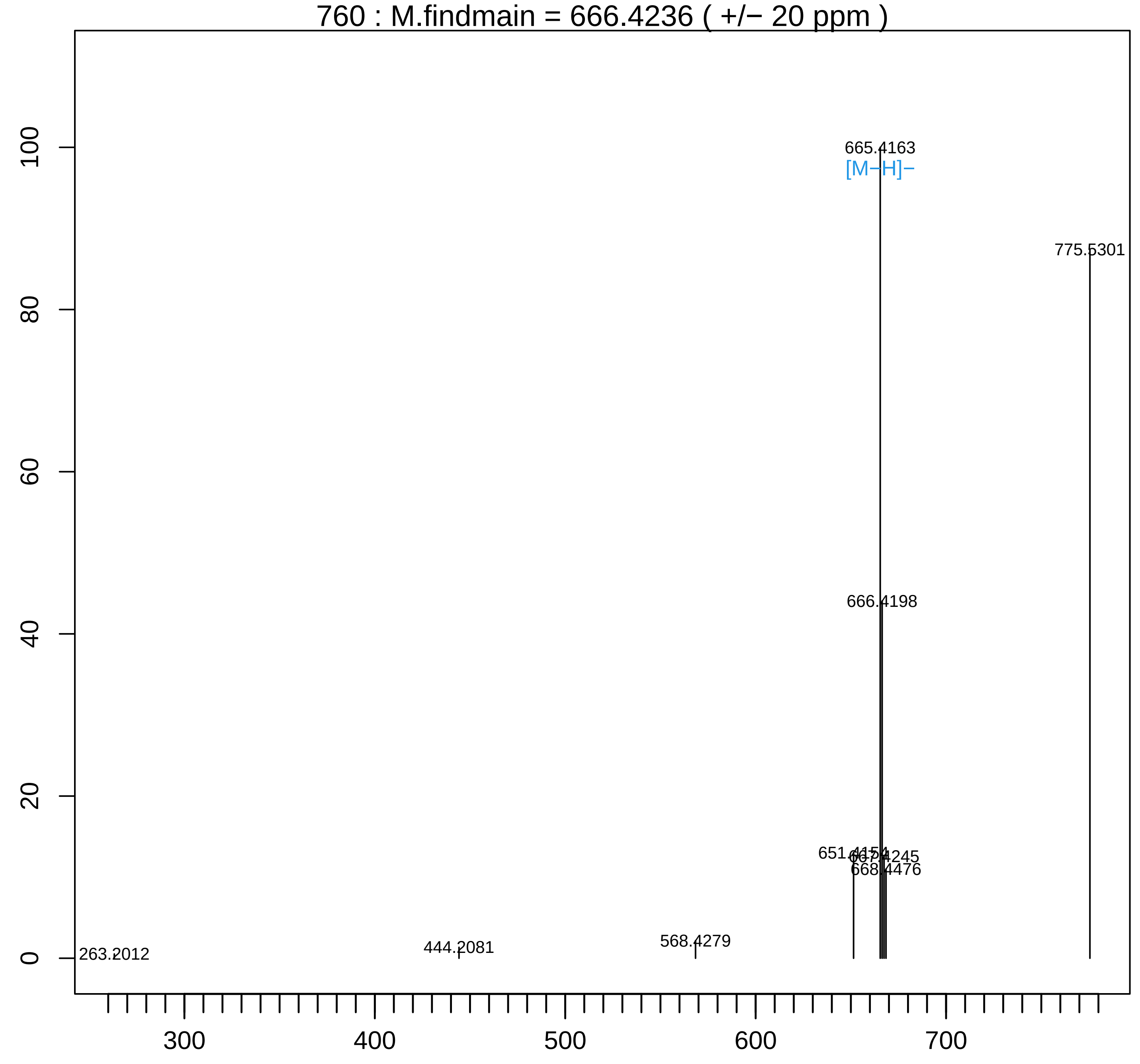

760 : M.findmain = 666.4236 (+/− 20 ppm)
665.4163
[M−H]−
775.5301
666.4198
651.4154
667.4245
668.4476
263.2012
444.2081
568.4279
0
20
40
60
80
100
300
400
500
600
700

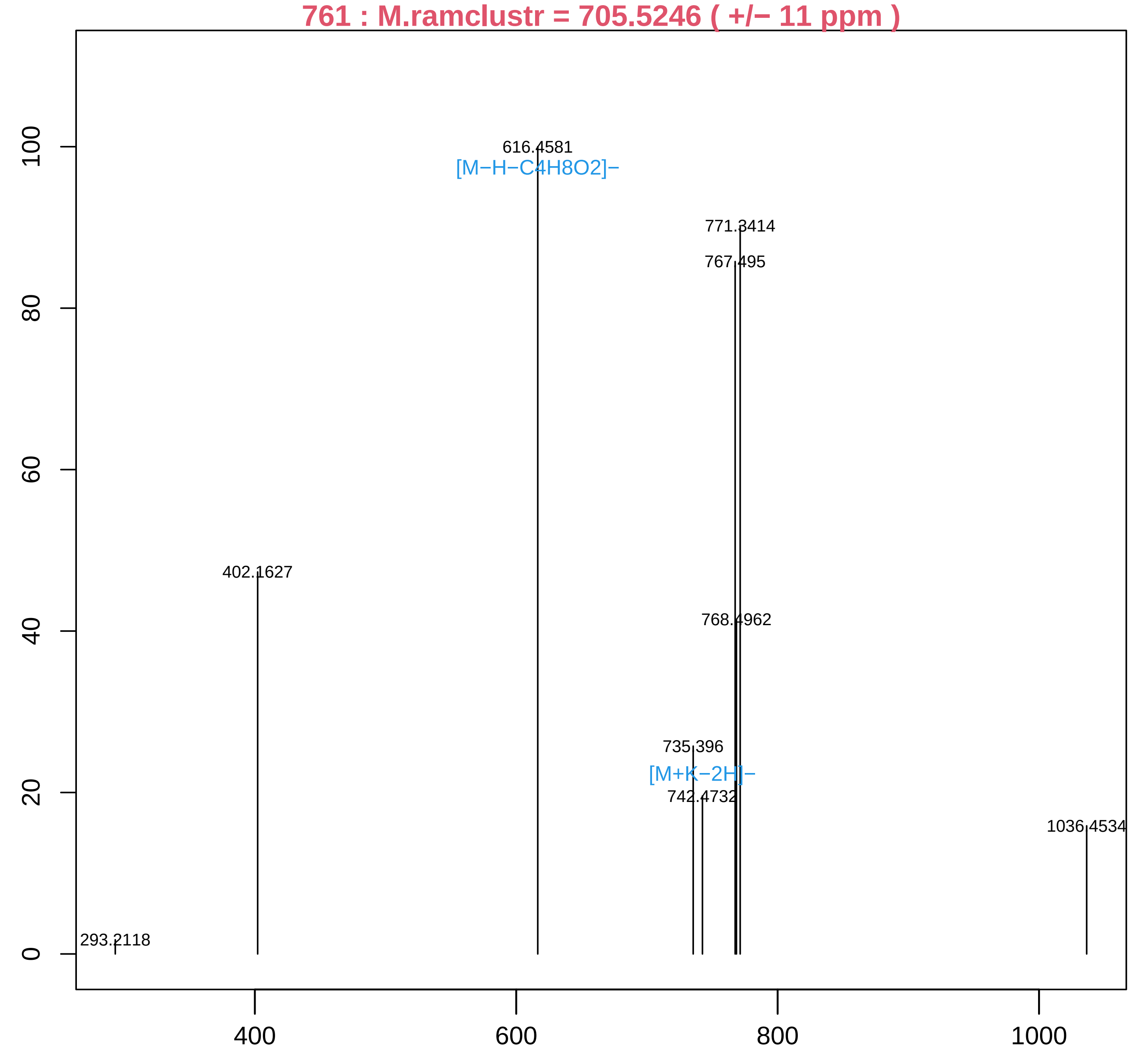
761 : M.ramclustr = 705.5246 (+/− 11 ppm)
616.4581
[M−H−C4H8O2]−
771.3414
767.495
402.1627
768.4962
735.396
[M+K−2H]−
742.4732
1036.4534
293.2118
0
20
40
60
80
100
400
600
800
1000

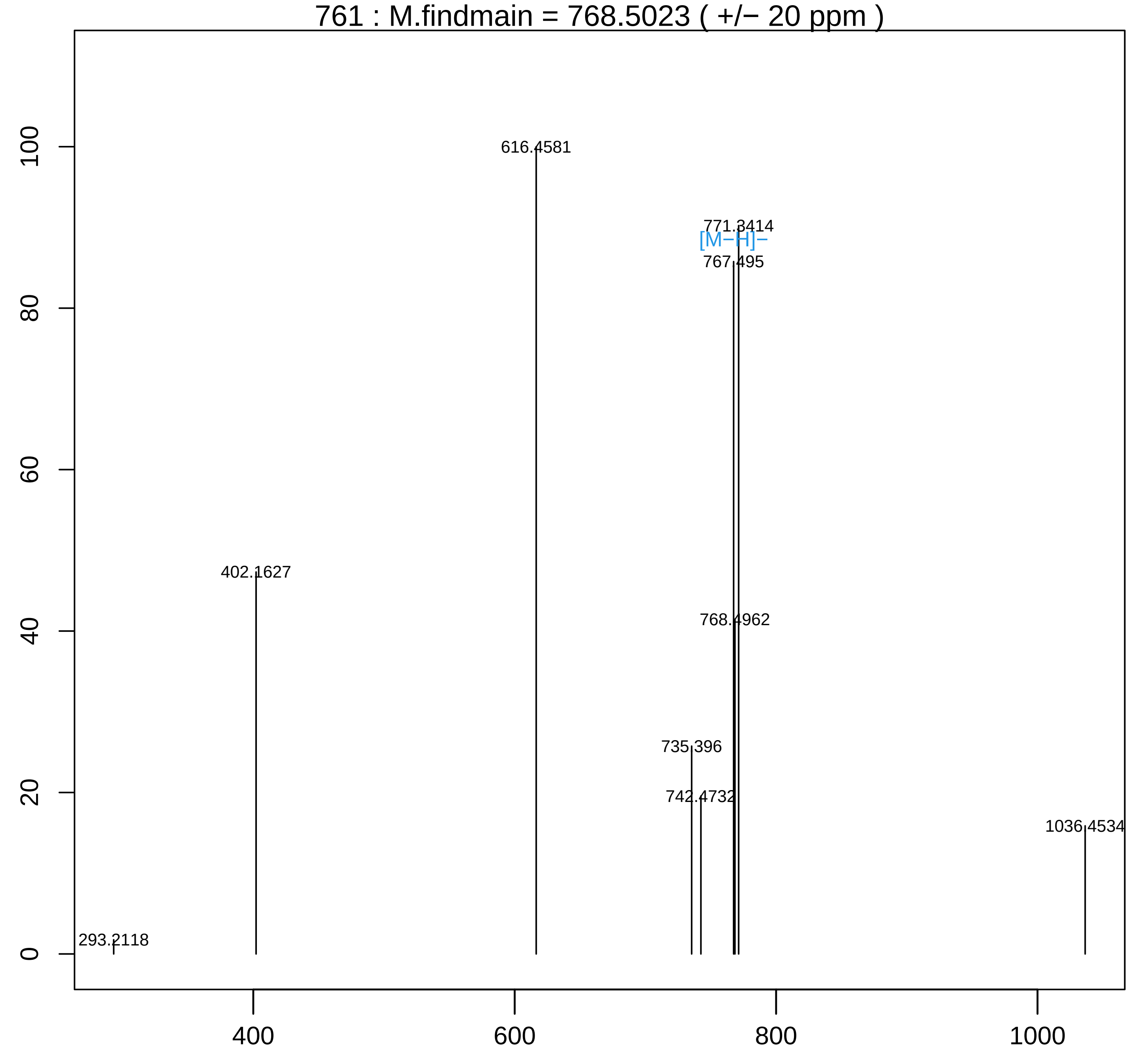
761 : M.findmain = 768.5023 (+/− 20 ppm)
616.4581
771.3414
[M−H]−
767.495
402.1627
768.4962
735.396
742.4732
1036.4534
293.2118
0
20
40
60
80
100
400
600
800
1000

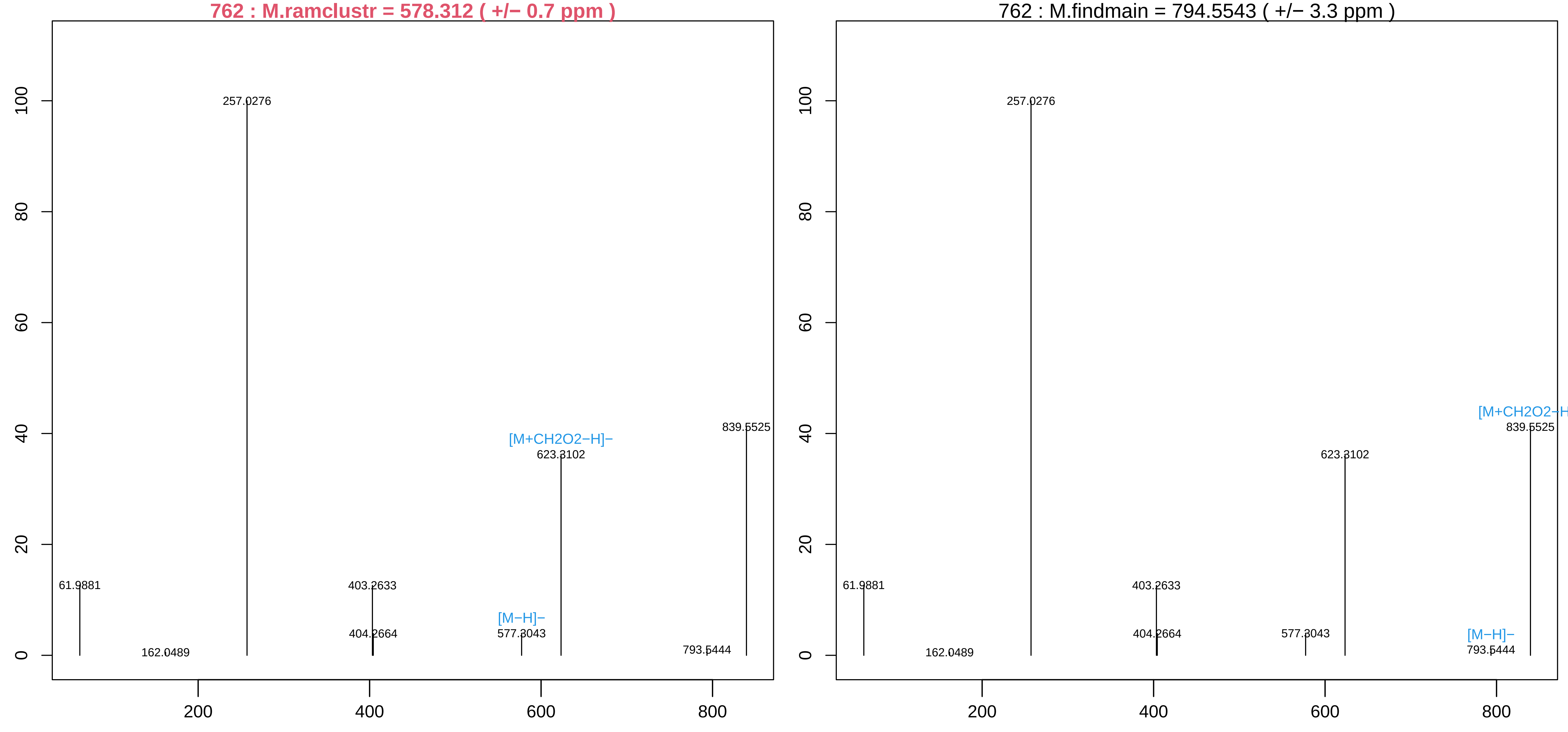

762 : M.ramclustr = 578.312 (+/− 0.7 ppm)
257.0276
839.5525
[M+CH2O2−H]−
623.3102
61.9881
403.2633
[M−H]−
577.3043
404.2664
162.0489
793.5444
0
20
40
60
80
100
200
400
600
800
762 : M.findmain = 794.5543 (+/− 3.3 ppm)
257.0276
[M+CH2O2−H
839.5525
623.3102
61.9881
403.2633
404.2664
577.3043
[M−H]−
793.5444
162.0489
0
20
40
60
80
100
200
400
600
800

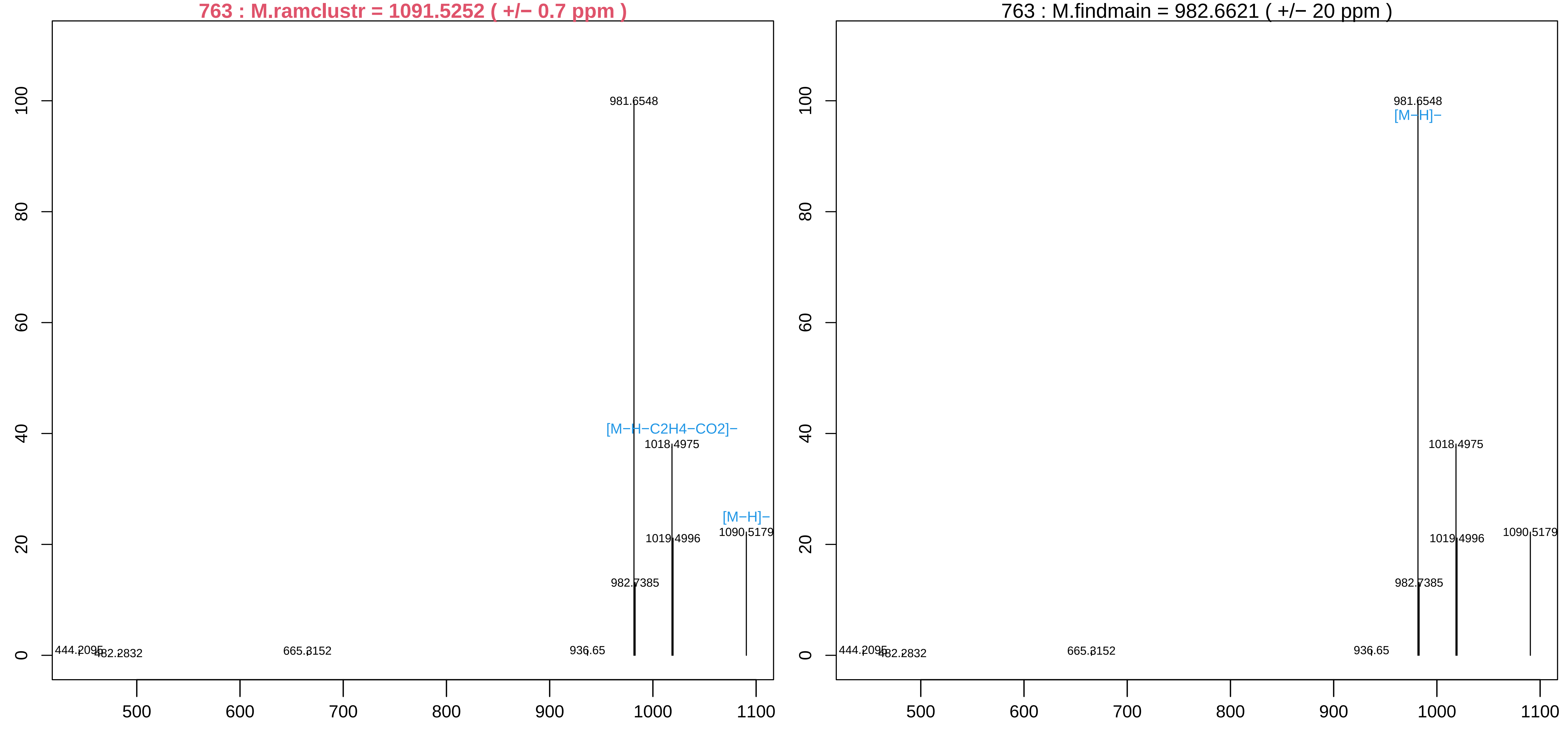
763 : M.ramclustr = 1091.5252 (+/− 0.7 ppm)
981.6548
[M−H−C2H4−CO2]−
1018.4975
[M−H]−
1090.5179
1019.4996
982.7385
444.2095
482.2832
665.3152
936.65
763 : M.findmain = 982.6621 (+/− 20 ppm)
981.6548
[M−H]−
1018.4975
1090.5179
1019.4996
982.7385
444.2095
482.2832
665.3152
936.65
0
20
40
60
80
100
500
600
700
800
900
1000
1100

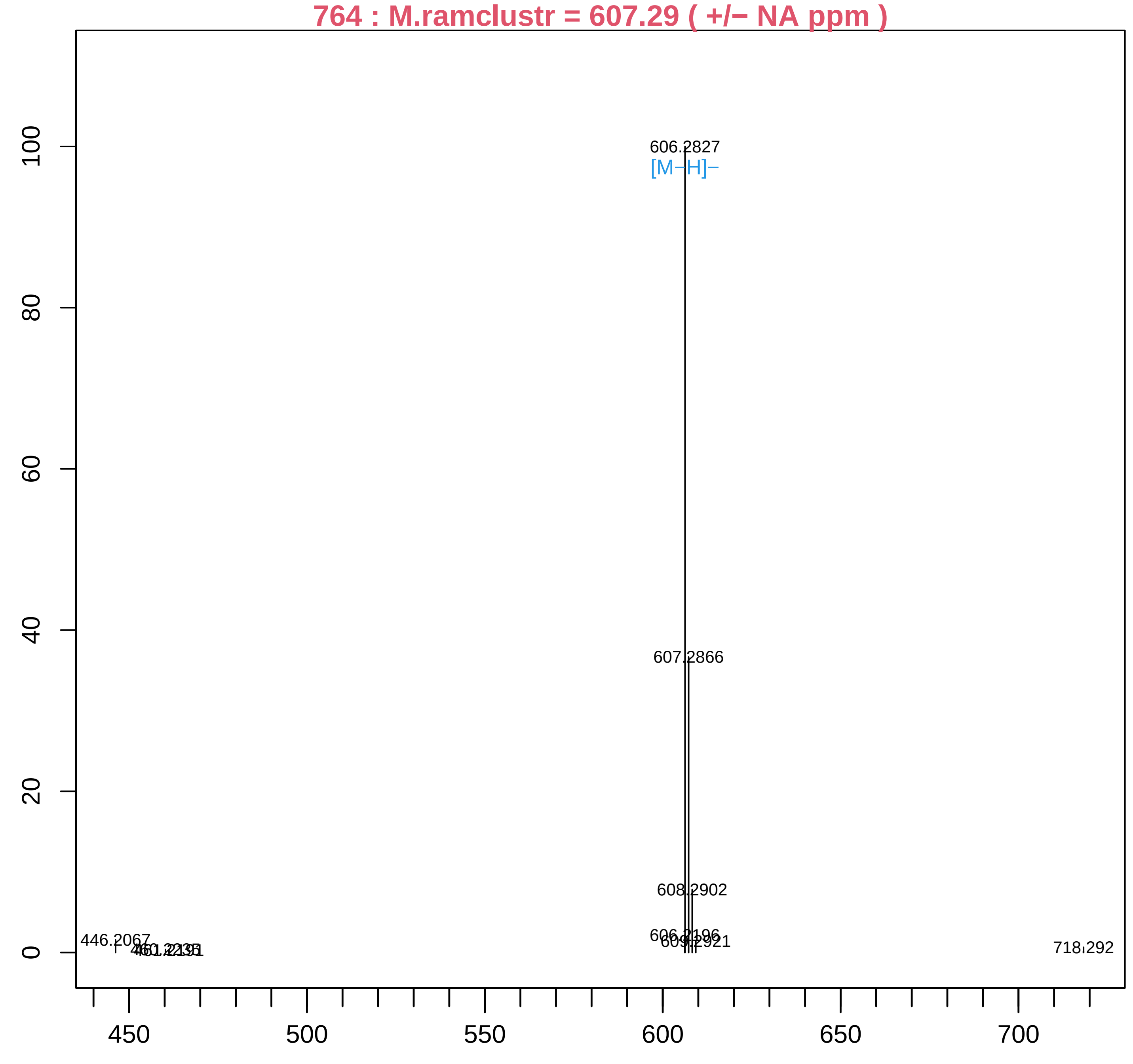
764 : M.ramclustr = 607.29 (+/− NA ppm)
606.2827
[M−H]−
607.2866
608.2902
606.2196
609.2921
446.2067
718.292
0
20
40
60
80
100
450
500
550
600
650
700

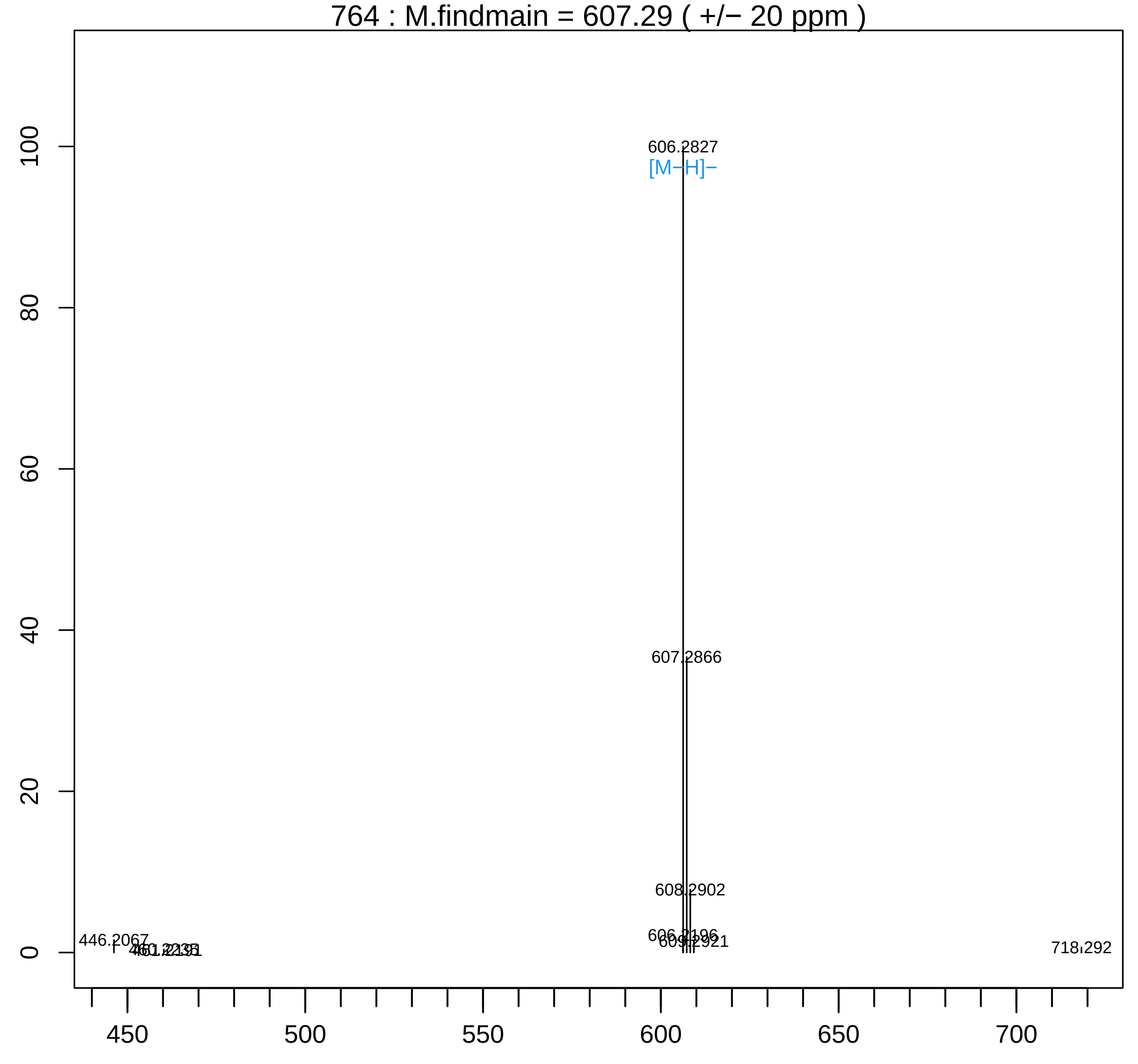
764 : M.findmain = 607.29 (+/− 20 ppm)
606.2827
[M−H]−
607.2866
608.2902
606.2196
609.2921
446.2067
718.292
0
20
40
60
80
100
450
500
550
600
650
700

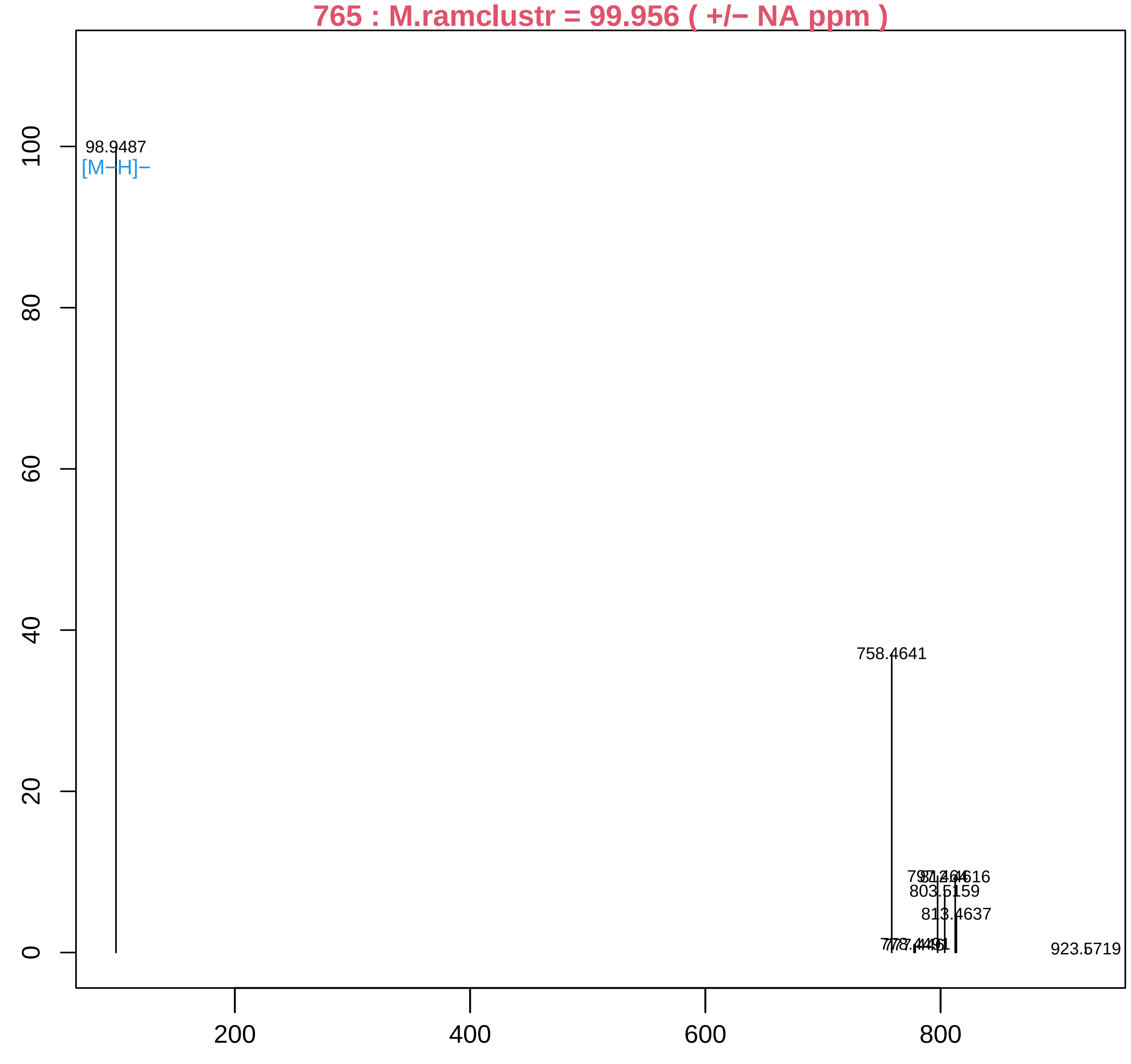

765 : M.ramclustr = 99.956 (+/− NA ppm)
98.9487
[M−H]−
758.4641
803.5159
813.4637
923.5719
0
20
40
60
80
100
200
400
600
800

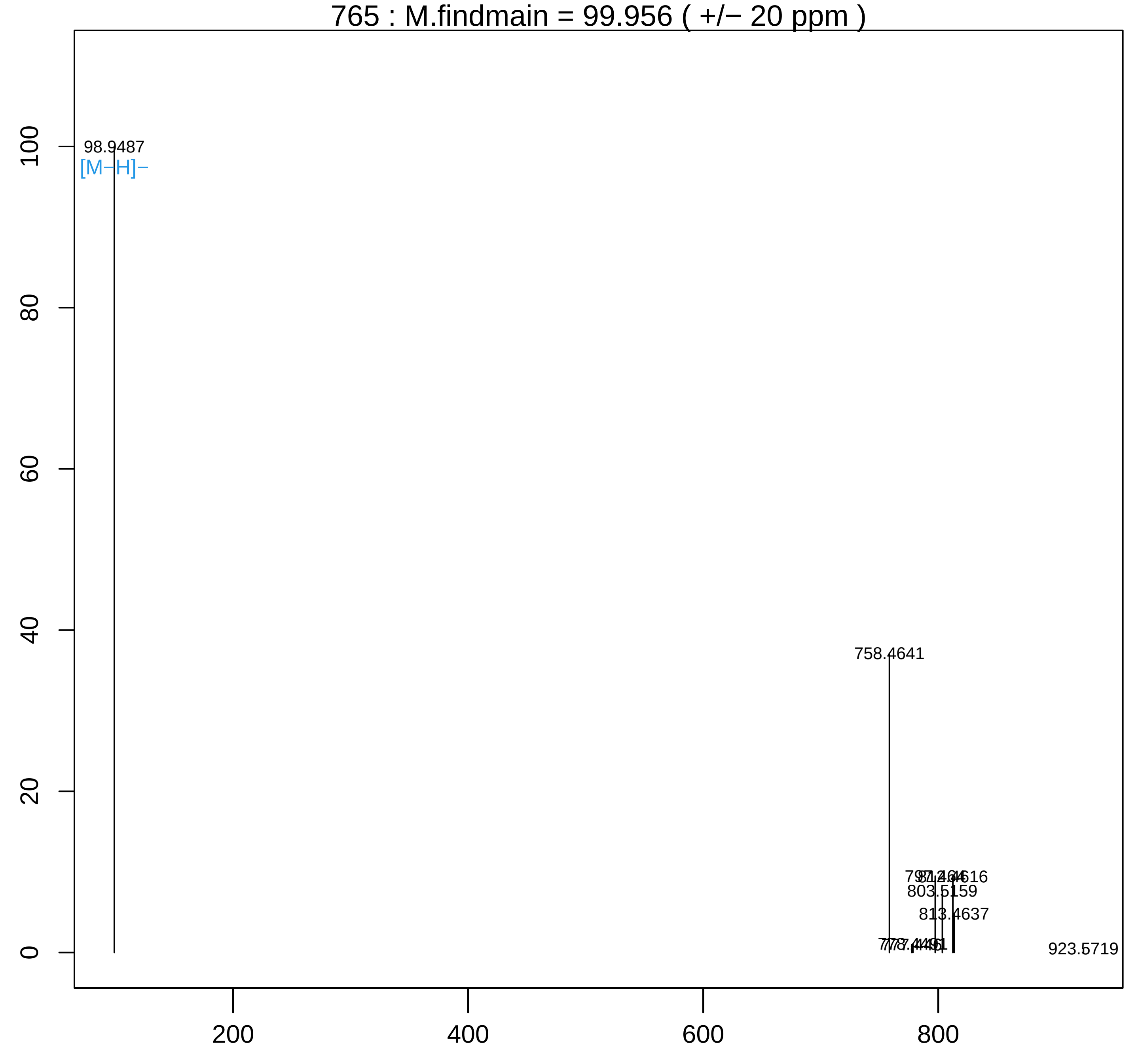

765 : M.findmain = 99.956 (+/− 20 ppm)
98.9487
[M−H]−
758.4641
803.5159
813.4637
923.5719
0
20
40
60
80
100
200
400
600
800

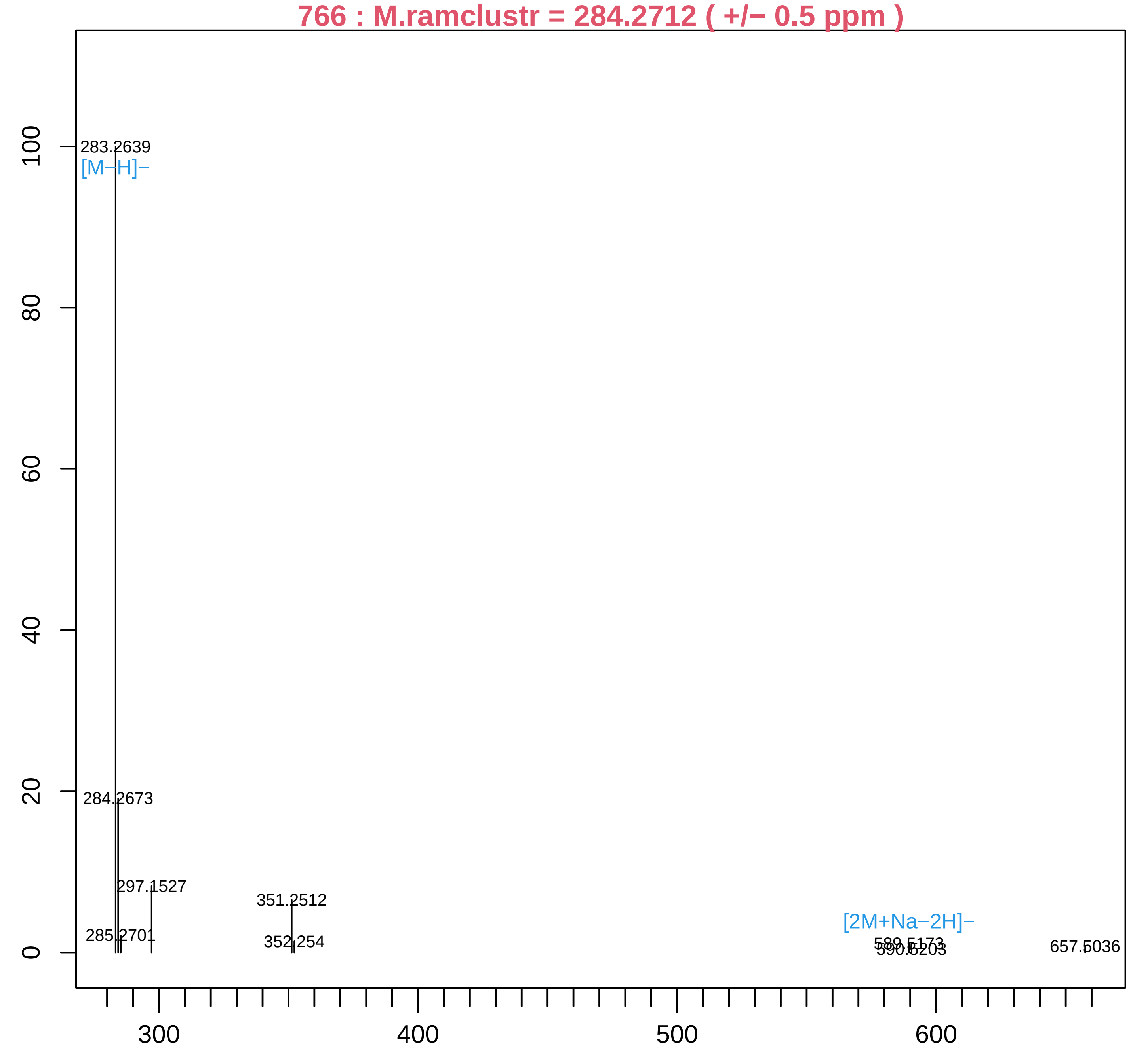
766 : M.ramclustr = 284.2712 (+/− 0.5 ppm)
283.2639
[M−H]−
284.2673
297.1527
351.2512
285.2701
352.254
[2M+Na−2H]−
589.5173
590.5203
657.5036

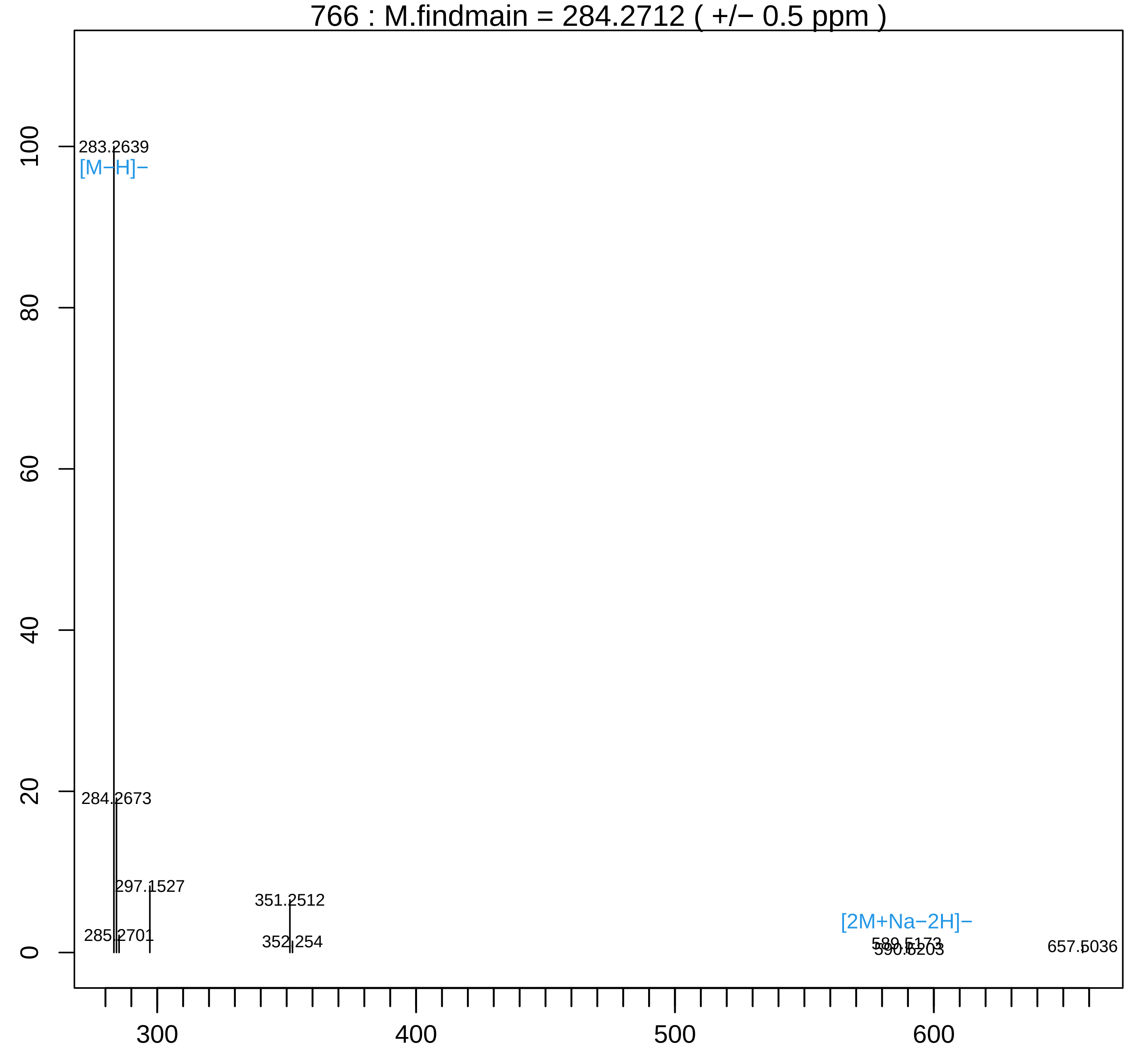
766 : M.findmain = 284.2712 (+/− 0.5 ppm)
283.2639
[M−H]−
284.2673
297.1527
351.2512
285.2701
352.254
[2M+Na−2H]−
589.5173
590.5203
657.5036

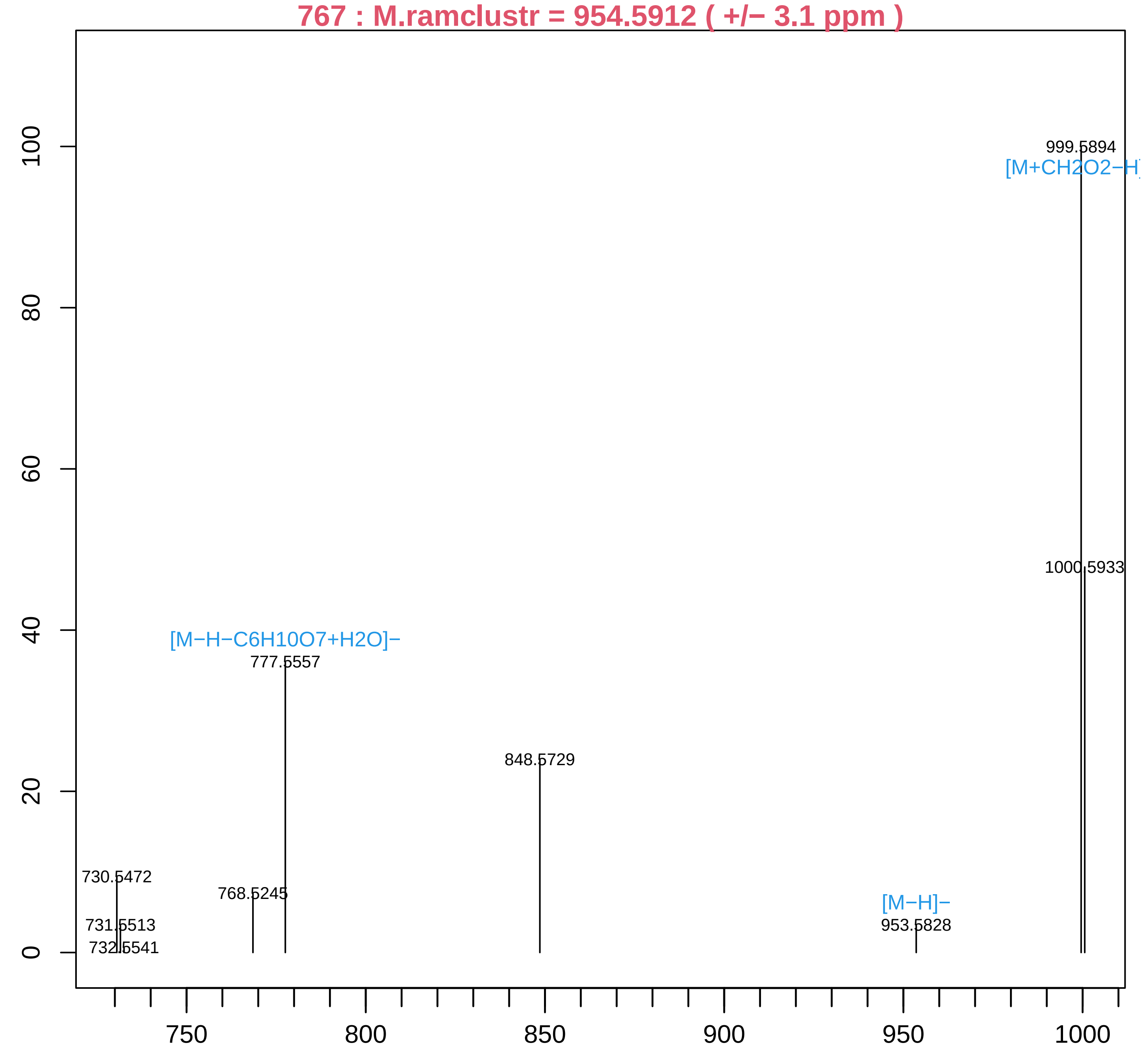

767 : M.ramclustr = 954.5912 (+/− 3.1 ppm)
999.5894
[M+CH2O2−H]
1000.5933
[M−H−C6H10O7+H2O]−
777.5557
848.5729
730.5472
768.5245
[M−H]−
953.5828
731.5513
732.5541
0
20
40
60
80
100
750
800
850
900
950
1000

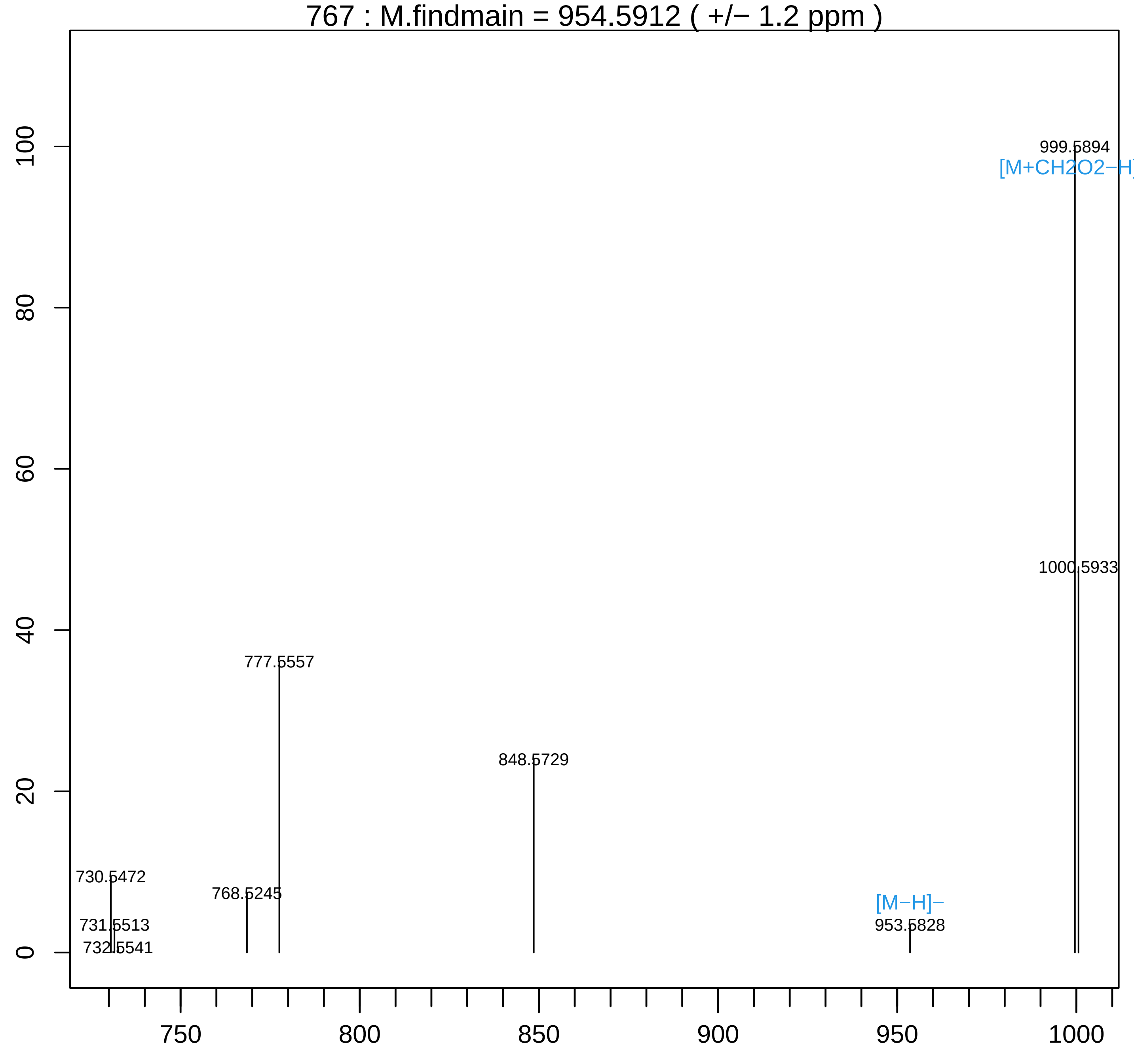

767 : M.findmain = 954.5912 (+/− 1.2 ppm)
999.5894
[M+CH2O2−H]
1000.5933
777.5557
848.5729
730.5472
768.5245
[M−H]−
953.5828
731.5513
732.5541
0
20
40
60
80
100
750
800
850
900
950
1000

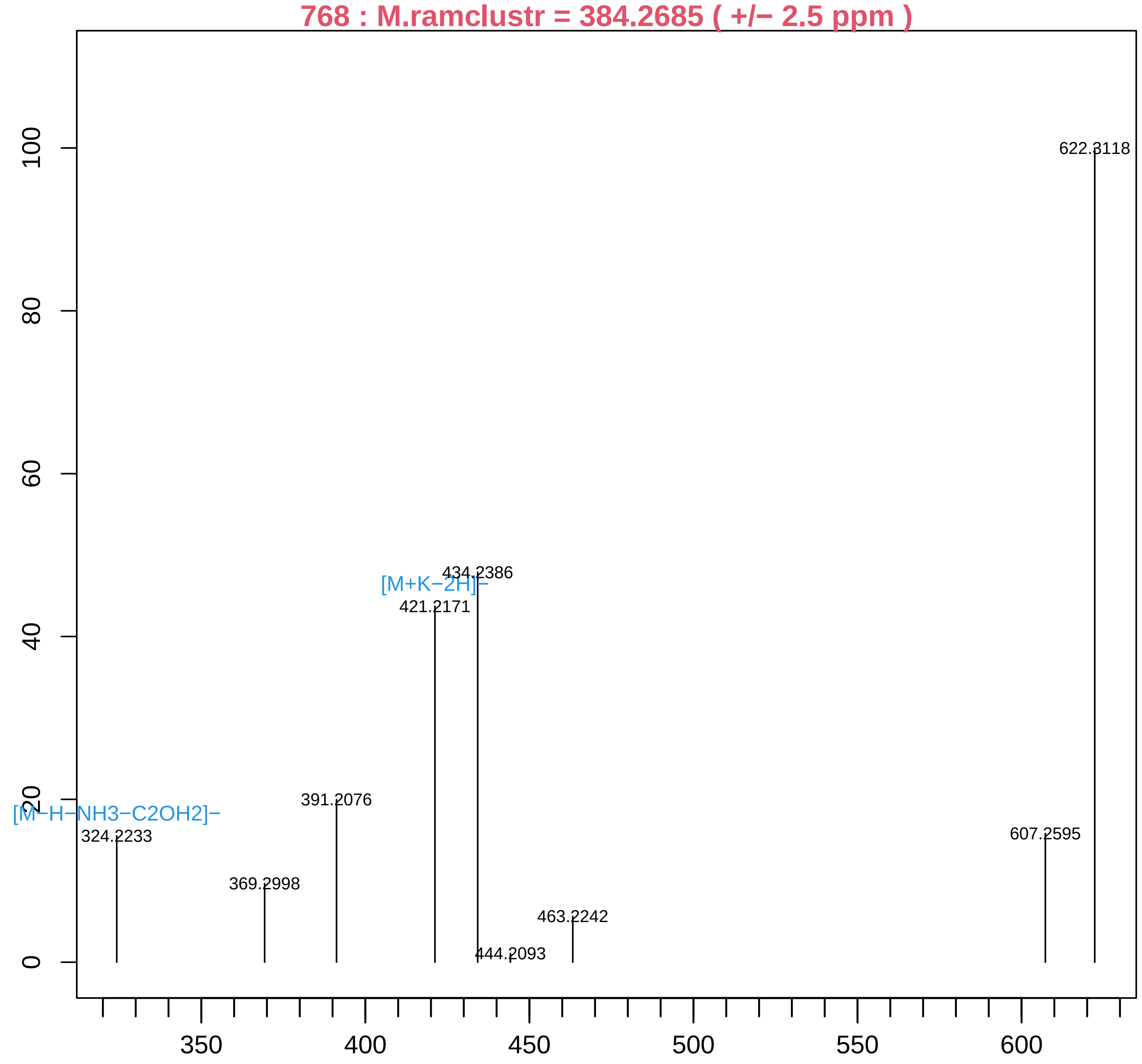
768 : M.ramclustr = 384.2685 (+/− 2.5 ppm)
622.3118
434.2386
[M+K−2H]−
421.2171
391.2076
[M−H−NH3−C2OH2]−
324.2233
369.2998
463.2242
444.2093
607.2595
0
20
40
60
80
100
350
400
450
500
550
600

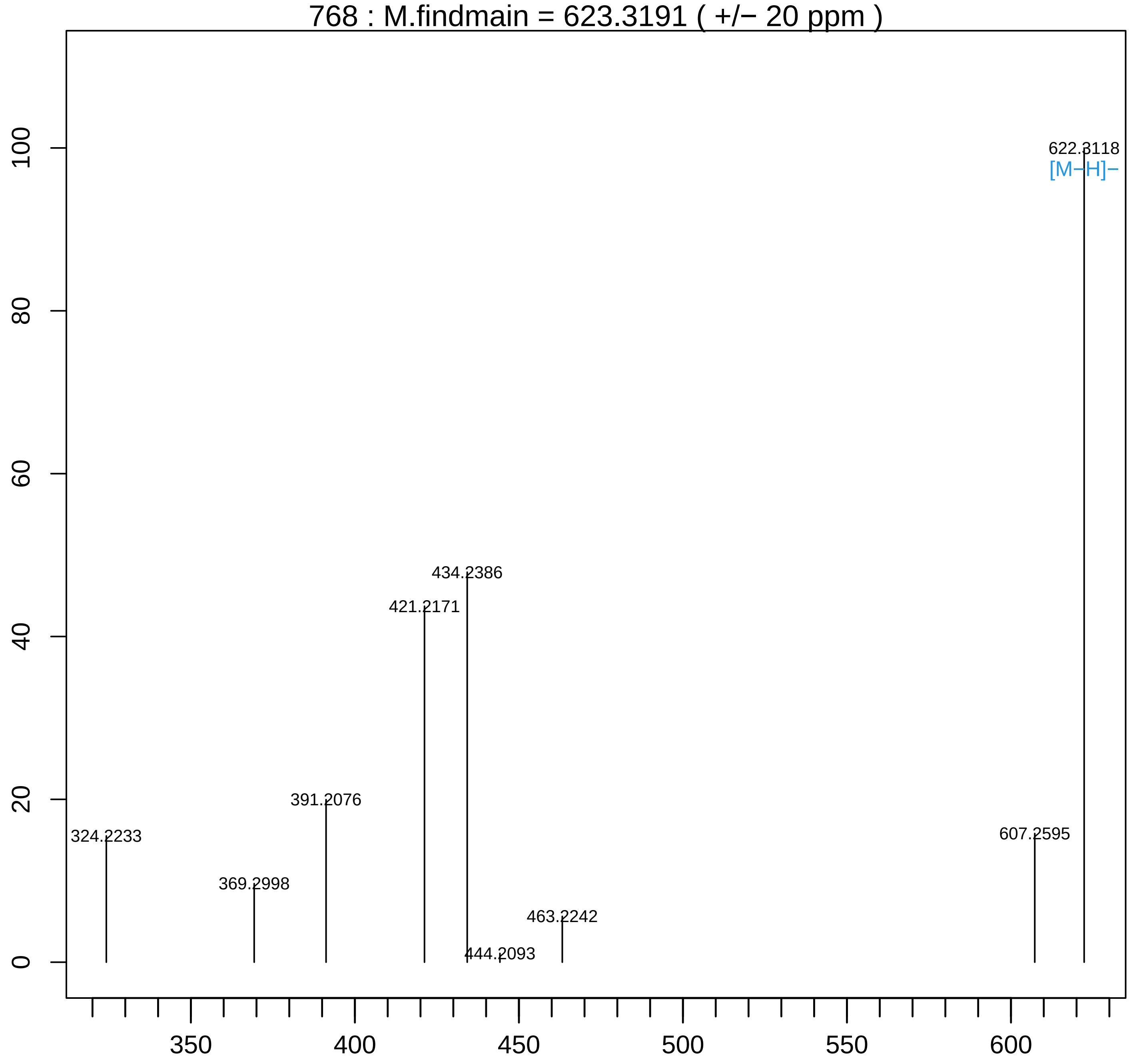
768 : M.findmain = 623.3191 (+/− 20 ppm)
622.3118
[M−H]−
434.2386
421.2171
391.2076
324.2233
369.2998
463.2242
444.2093
607.2595
0
20
40
60
80
100
350
400
450
500
550
600

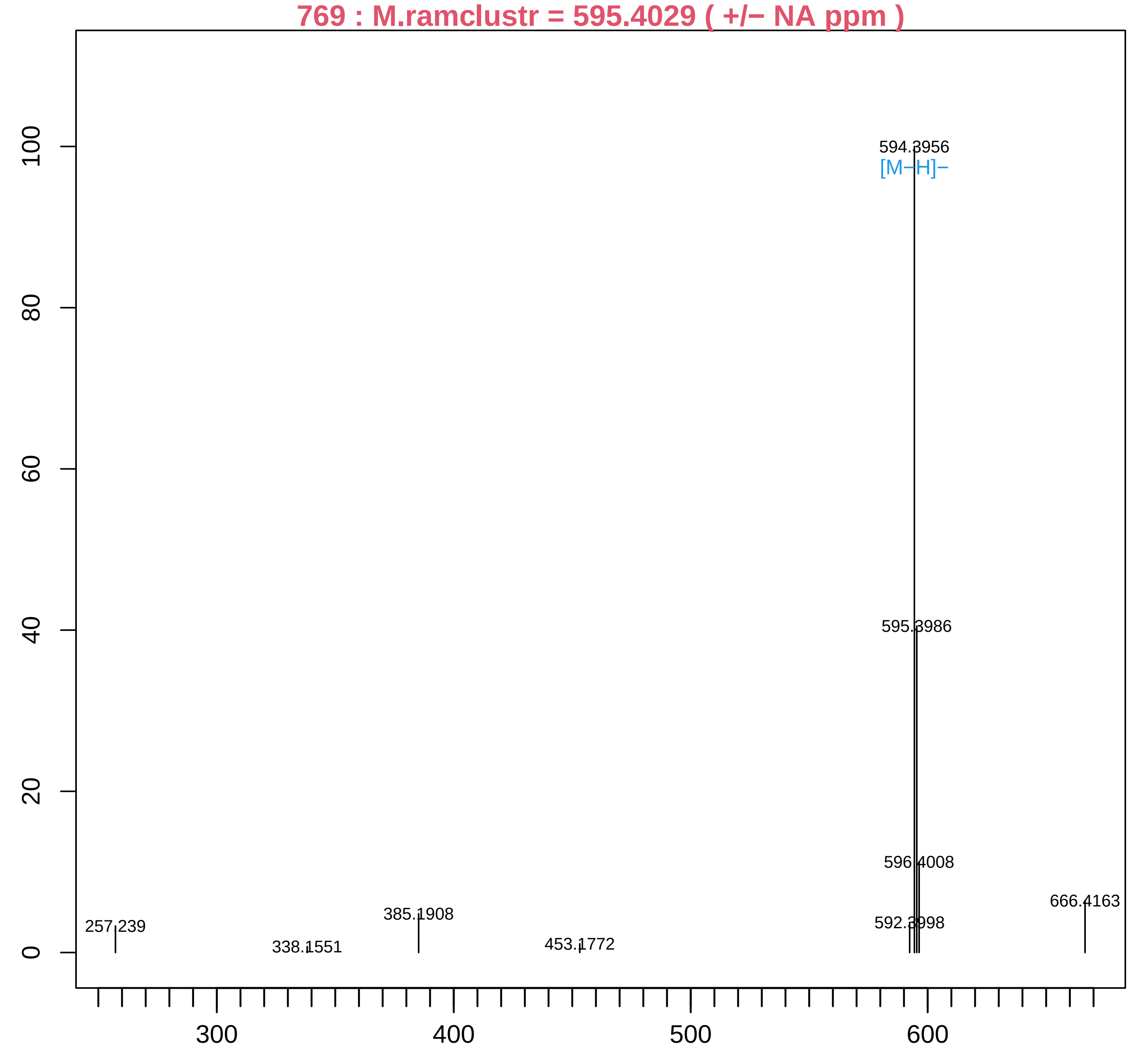
769 : M.ramclustr = 595.4029 (+/− NA ppm)
594.3956
[M−H]−
595.3986
596.4008
592.3998
666.4163
257.239
338.1551
385.1908
453.1772
0
20
40
60
80
100
300
400
500
600

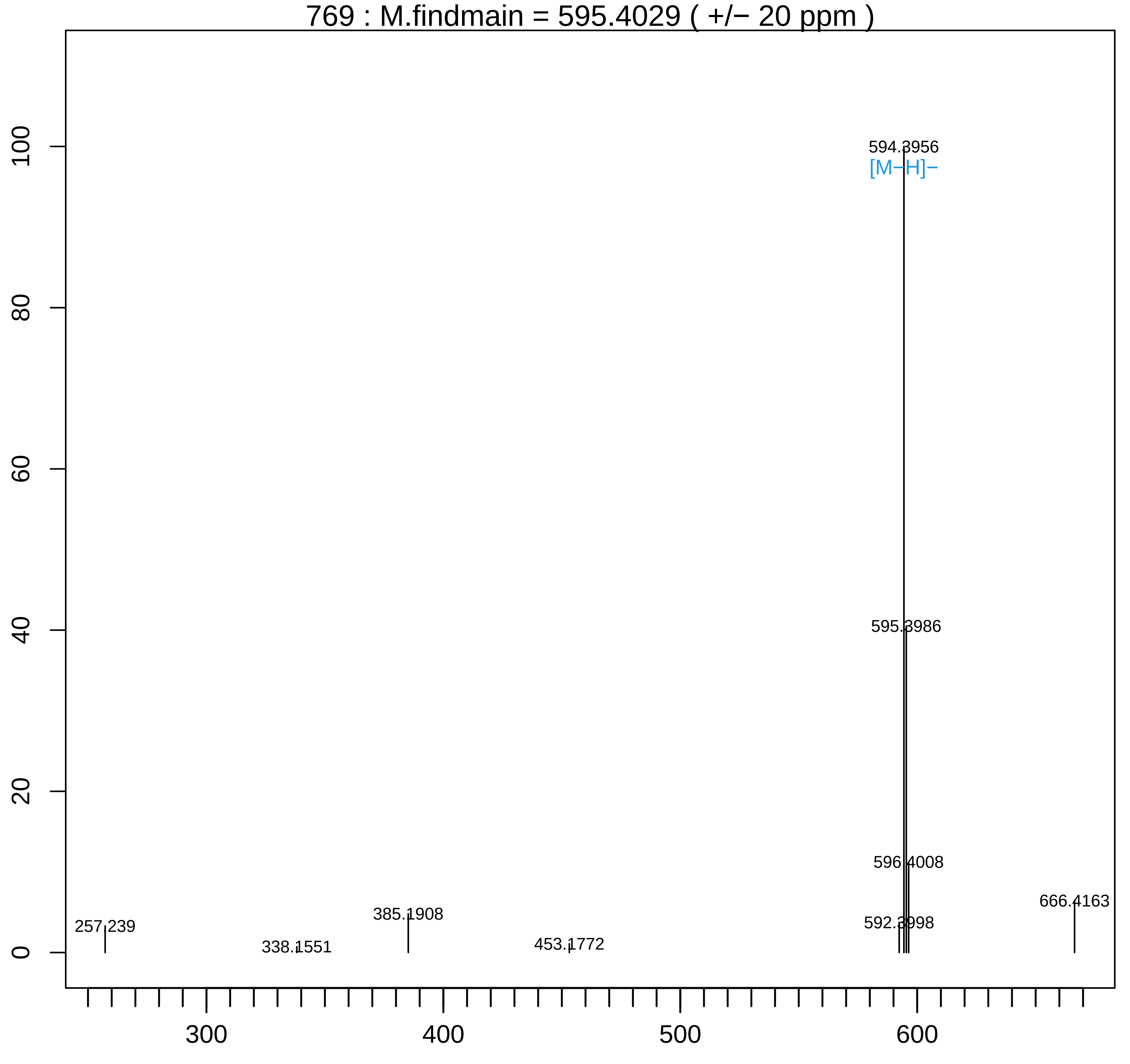
769 : M.findmain = 595.4029 (+/− 20 ppm)
594.3956
[M−H]−
595.3986
596.4008
592.3998
666.4163
257.239
338.1551
385.1908
453.1772
0
20
40
60
80
100
300
400
500
600

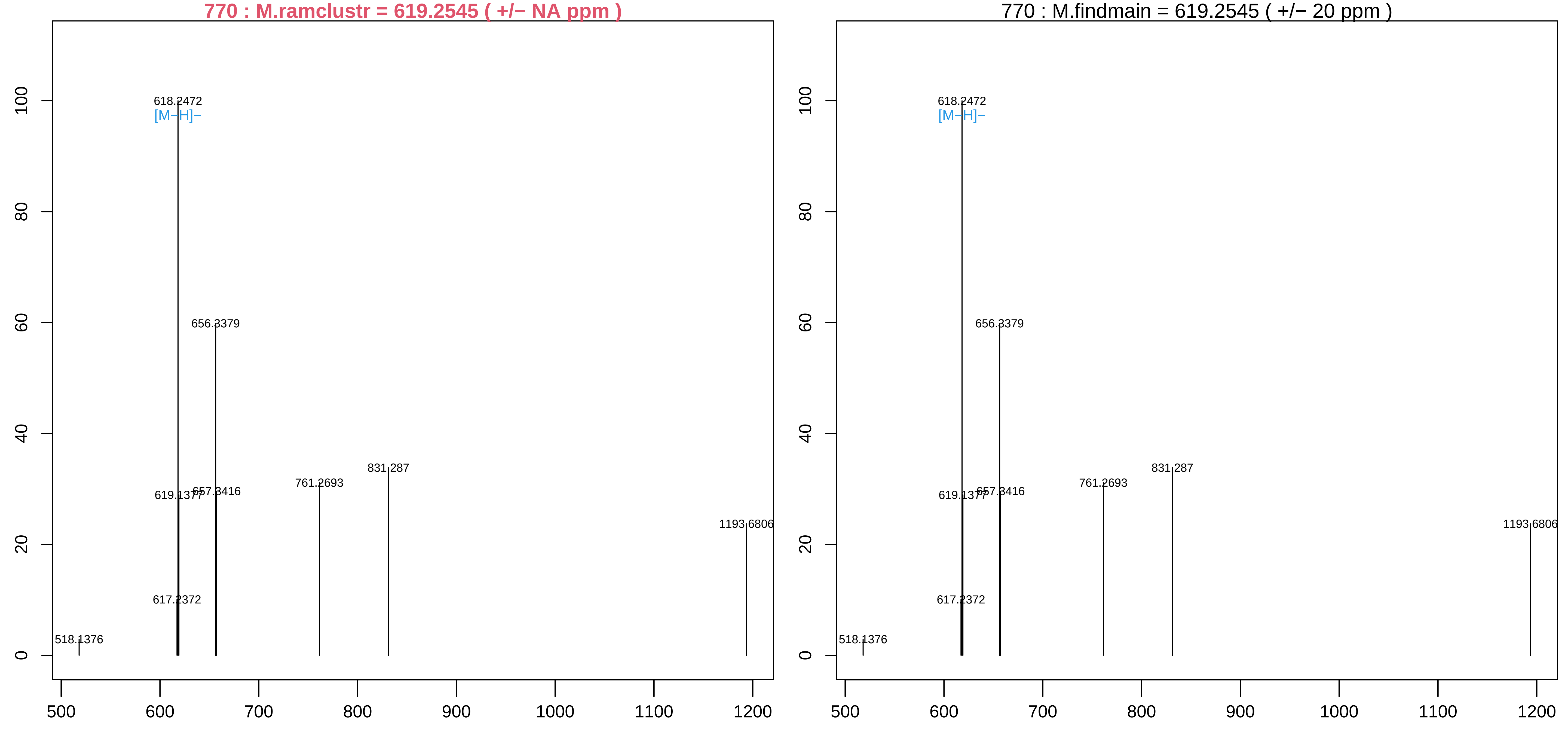
770 : M.ramclustr = 619.2545 (+/− NA ppm)
770 : M.findmain = 619.2545 (+/− 20 ppm)
618.2472
[M−H]−
656.3379
831.287
761.2693
619.1377
657.3416
1193.6806
617.2372
518.1376
0
20
40
60
80
100
500
600
700
800
900
1000
1100
1200

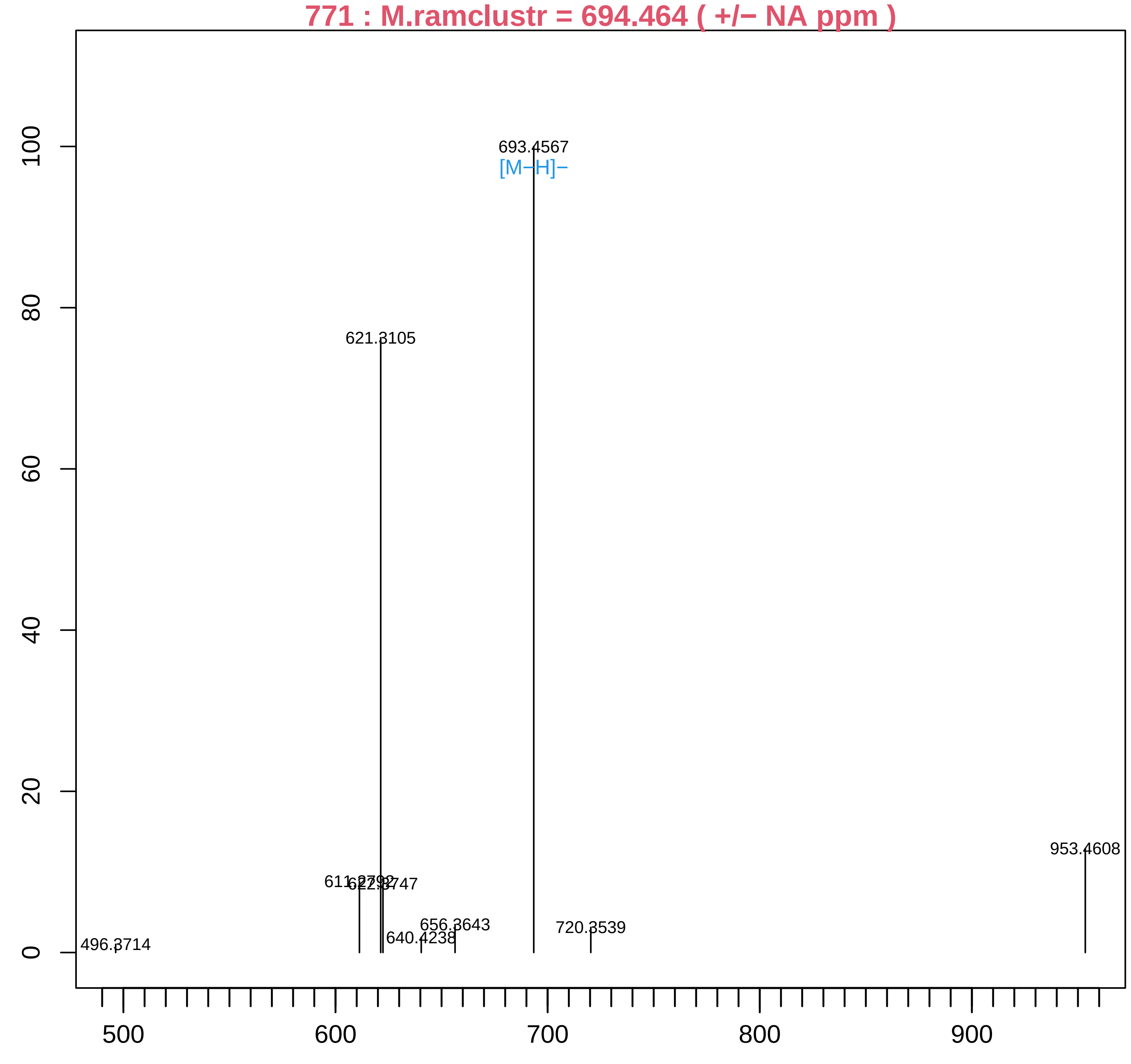
771 : M.ramclustr = 694.464 (+/− NA ppm)
693.4567
[M−H]−
621.3105
611.2792
622.3747
656.3643
640.4238
720.3539
953.4608
496.3714
0
20
40
60
80
100
500
600
700
800
900

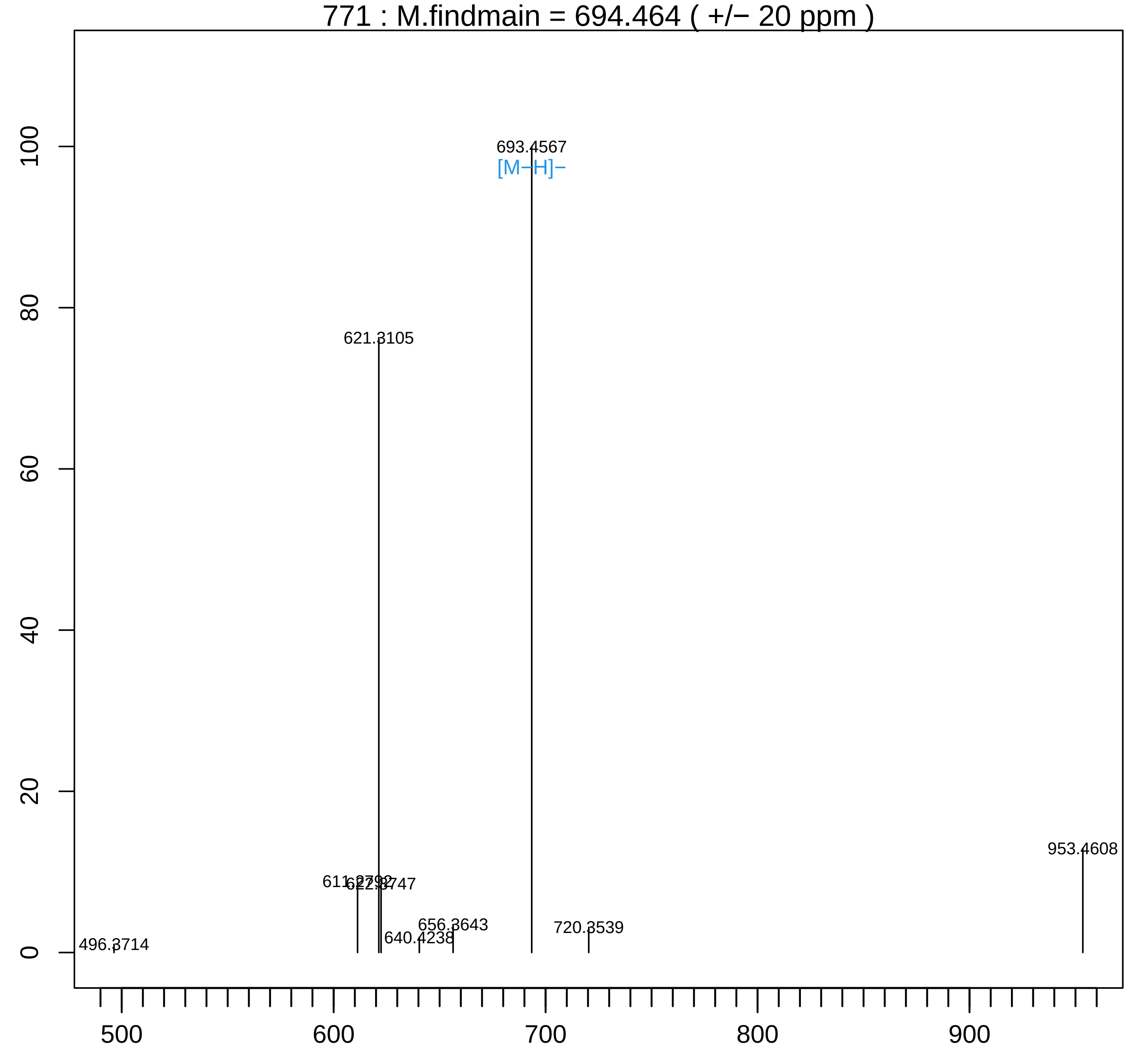
771 : M.findmain = 694.464 (+/− 20 ppm)
693.4567
[M−H]−
621.3105
611.2792
622.3747
656.3643
640.4238
720.3539
953.4608
496.3714
0
20
40
60
80
100
500
600
700
800
900

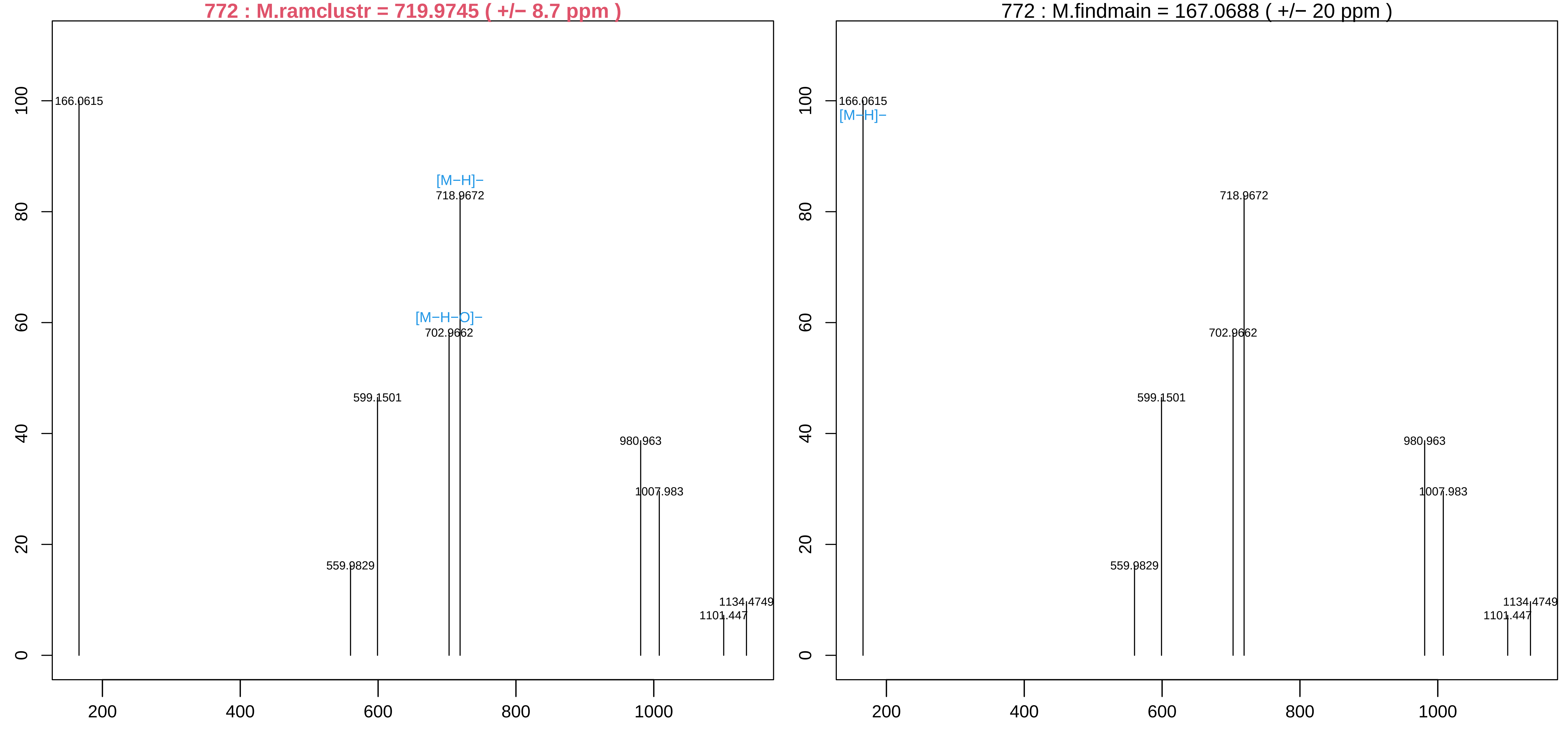

772 : M.ramclustr = 719.9745 (+/− 8.7 ppm)
166.0615
[M−H]−
718.9672
[M−H−O]−
702.9662
599.1501
559.9829
980.963
1007.983
1134.4749
1101.447
772 : M.findmain = 167.0688 (+/− 20 ppm)
166.0615
[M−H]−
718.9672
702.9662
599.1501
559.9829
980.963
1007.983
1134.4749
1101.447
0
20
40
60
80
100
200
400
600
800
1000

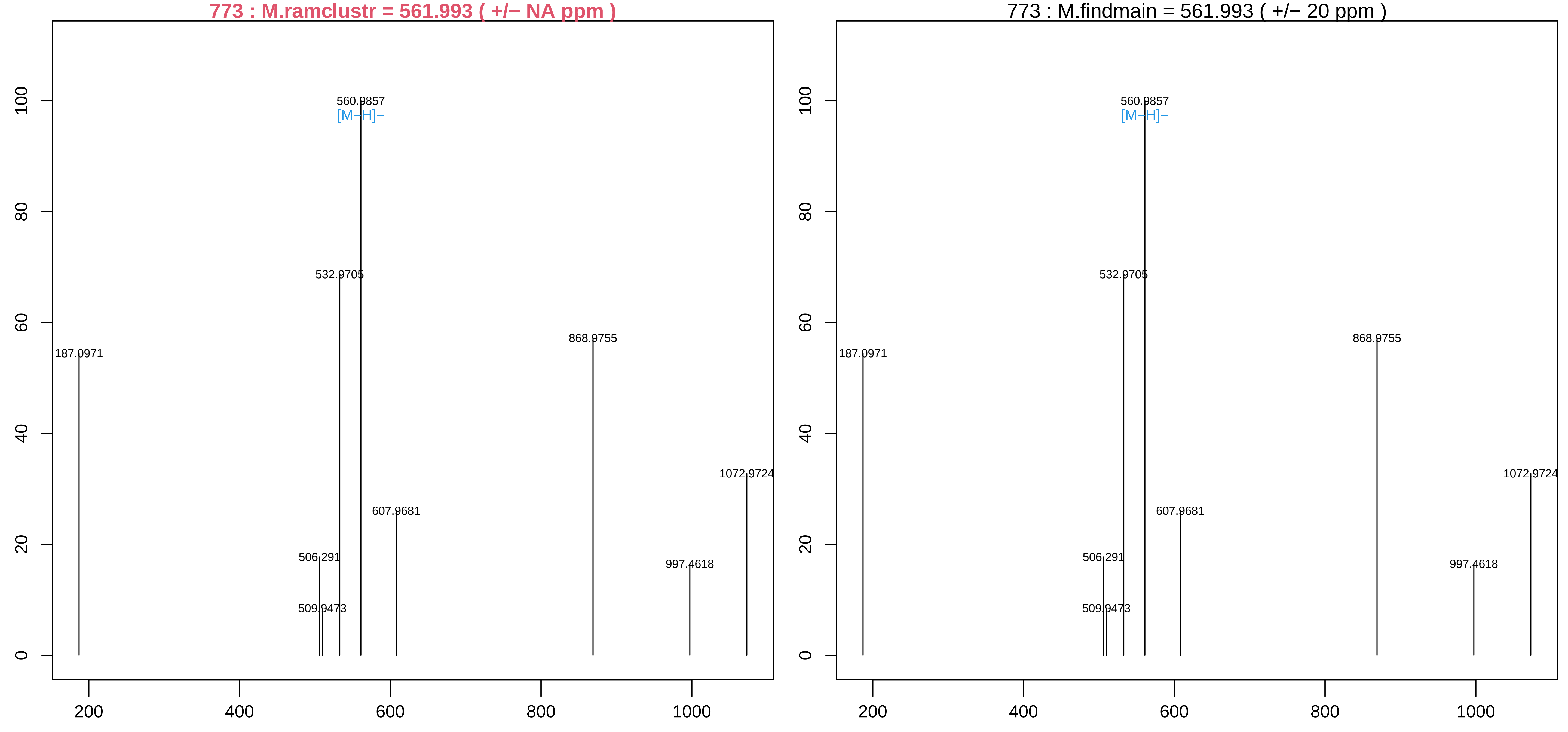
773 : M.ramclustr = 561.993 (+/− NA ppm)
773 : M.findmain = 561.993 (+/− 20 ppm)
560.9857
[M−H]−
532.9705
868.9755
187.0971
1072.9724
607.9681
506.291
997.4618
509.9473
0
20
40
60
80
100
200
400
600
800
1000

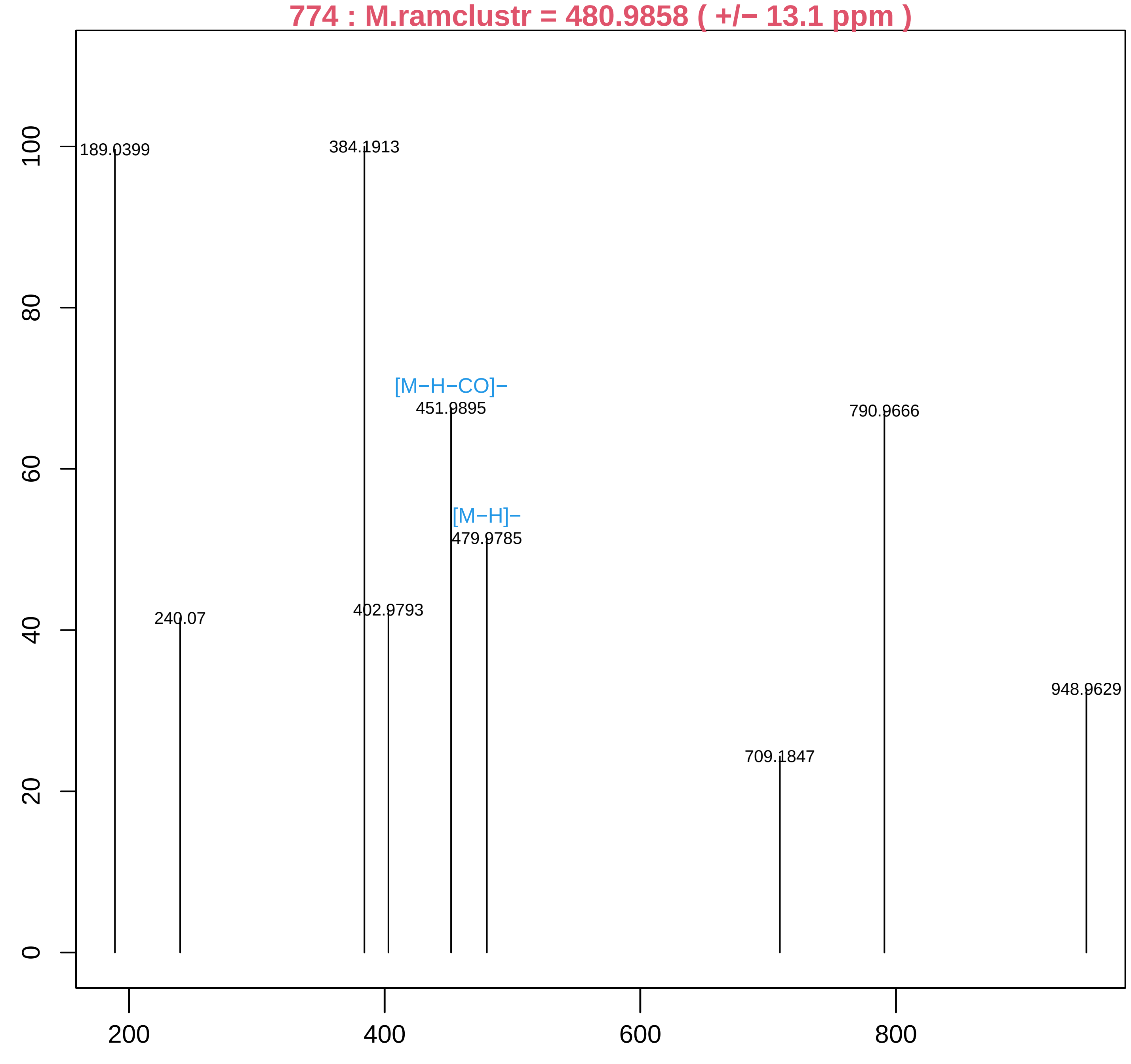

774 : M.ramclustr = 480.9858 (+/− 13.1 ppm)
189.0399
240.07
384.1913
402.9793
[M−H−CO]−
451.9895
[M−H]−
479.9785
709.1847
790.9666
948.9629
0
20
40
60
80
100
200
400
600
800

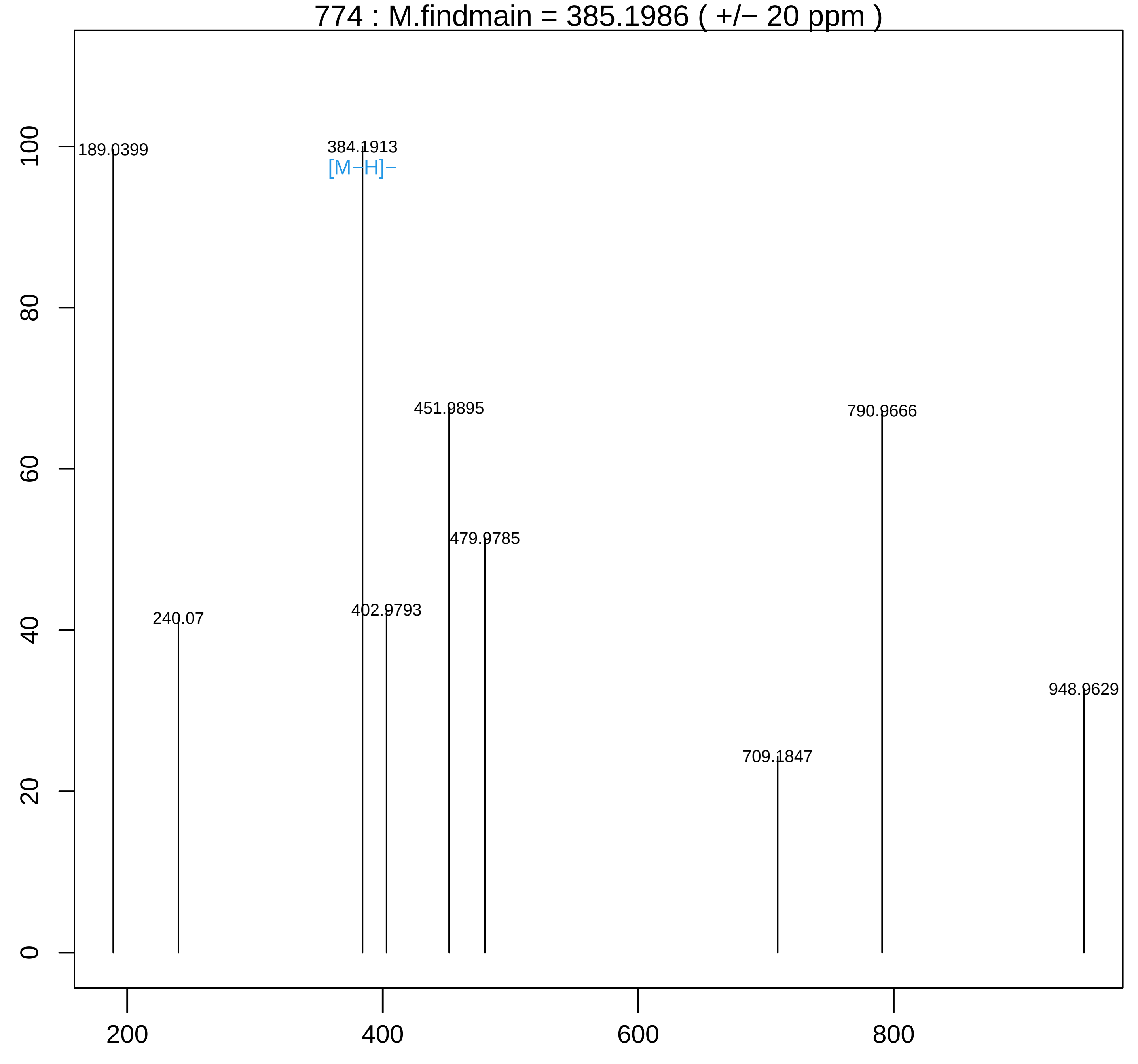

774 : M.findmain = 385.1986 (+/− 20 ppm)
189.0399
240.07
384.1913
[M−H]−
402.9793
451.9895
479.9785
709.1847
790.9666
948.9629
0
20
40
60
80
100
200
400
600
800

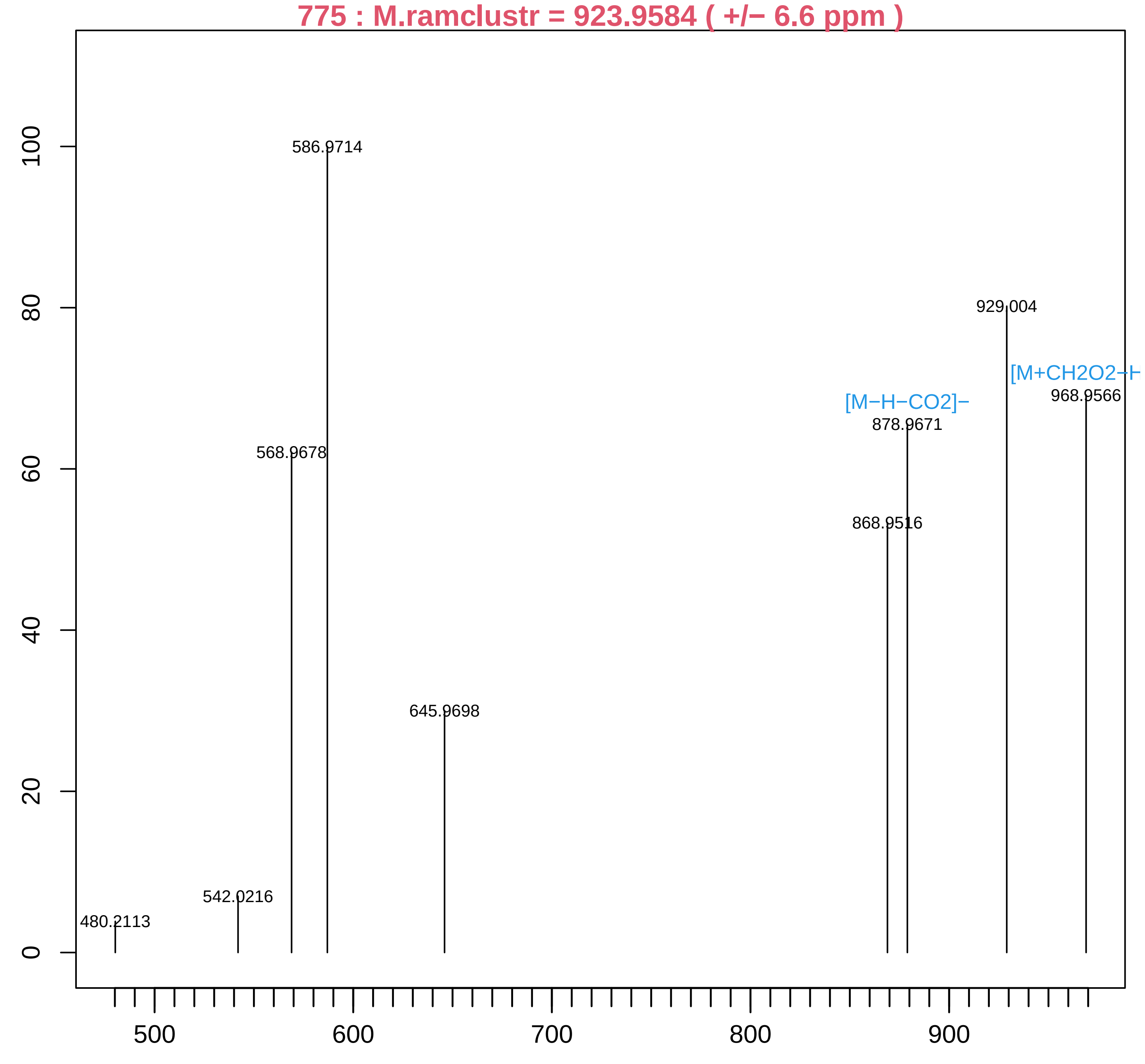

775 : M.ramclustr = 923.9584 (+/− 6.6 ppm)
586.9714
929.004
[M+CH2O2−H
[M−H−CO2]−
968.9566
878.9671
568.9678
868.9516
645.9698
542.0216
480.2113
0
20
40
60
80
100
500
600
700
800
900

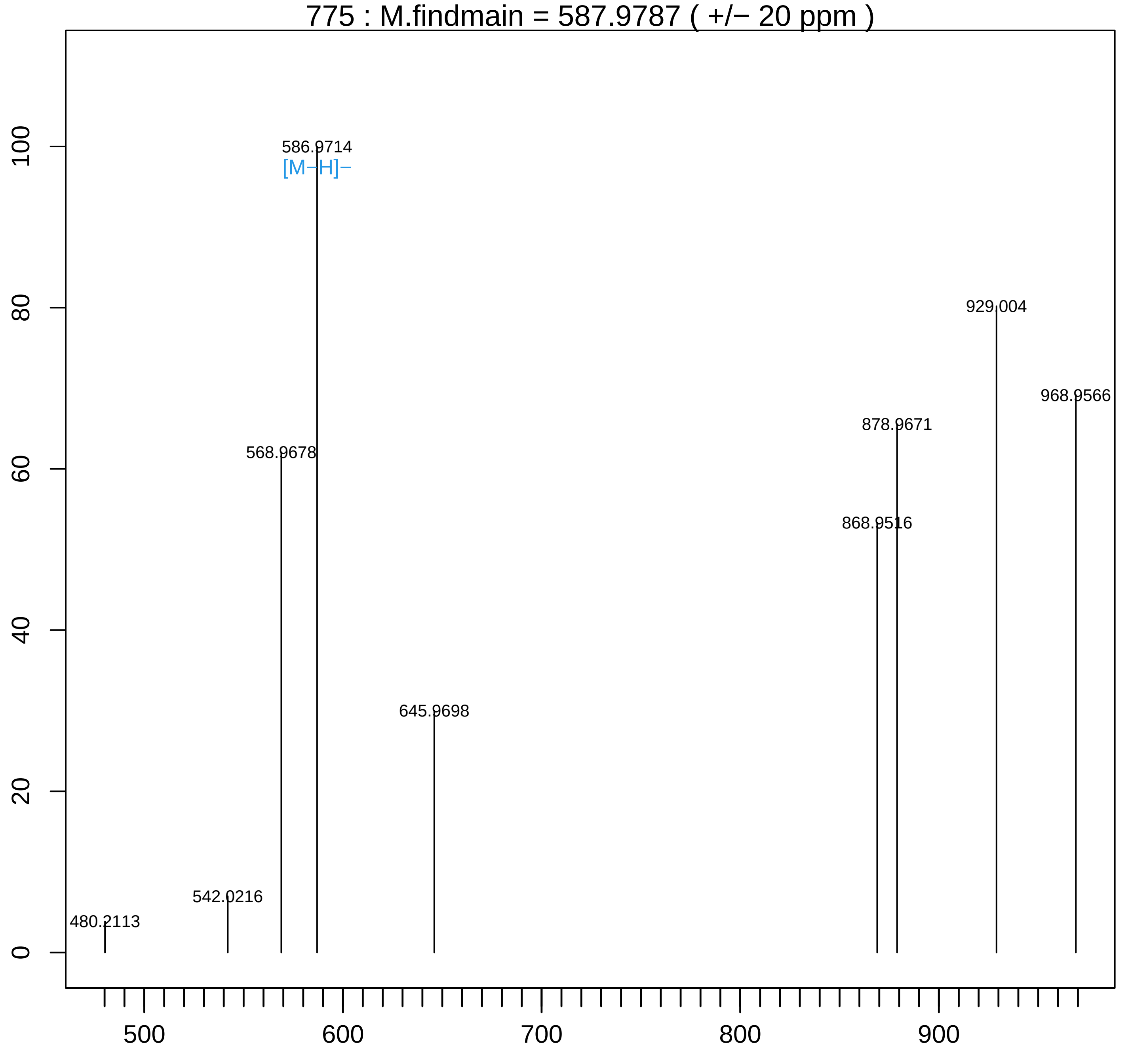

775 : M.findmain = 587.9787 (+/− 20 ppm)
586.9714
[M−H]−
929.004
968.9566
878.9671
568.9678
868.9516
645.9698
542.0216
480.2113
0
20
40
60
80
100
500
600
700
800
900

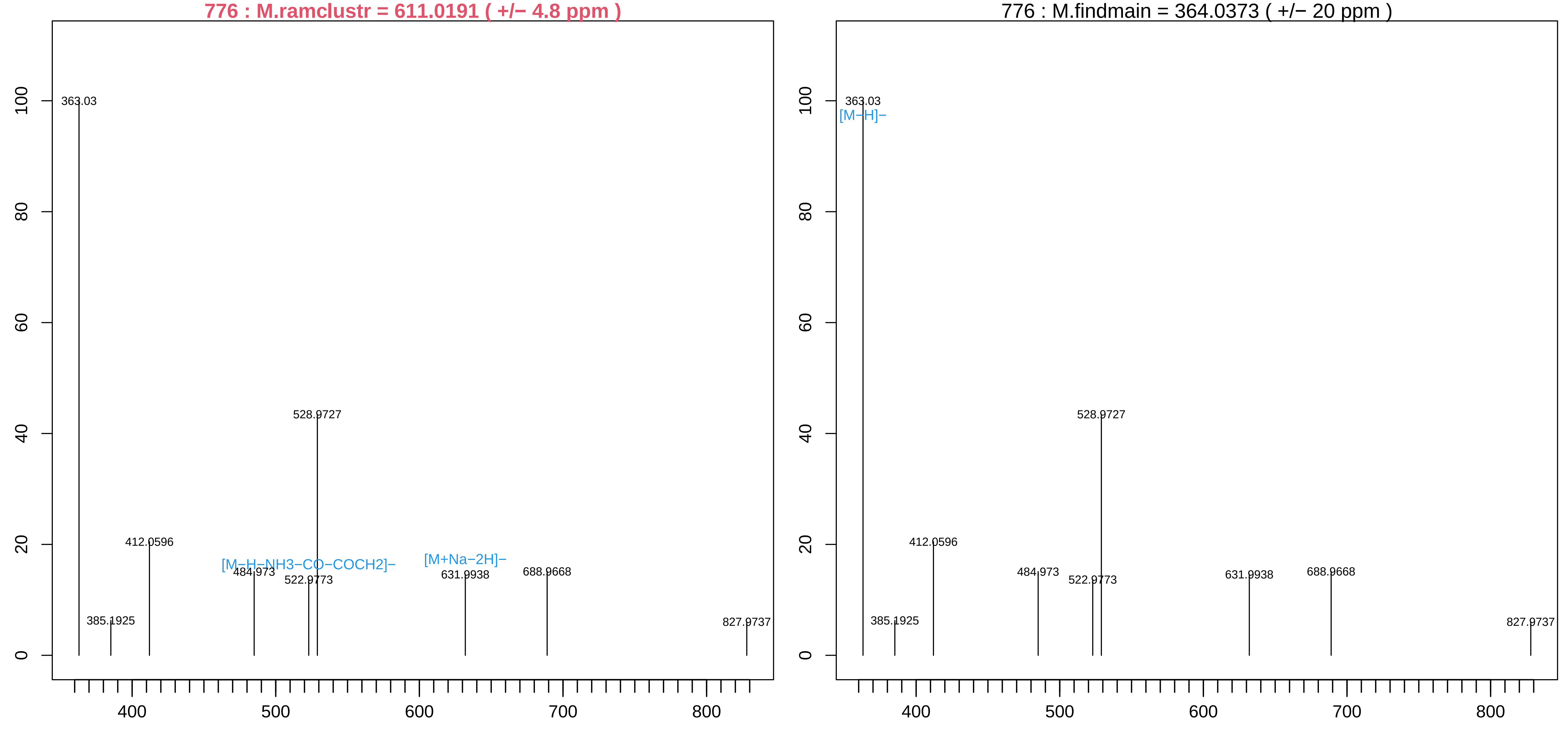

776 : M.ramclustr = 611.0191 (+/− 4.8 ppm)
363.03
385.1925
412.0596
[M−H−NH3−CO−COCH2]−
484.973
522.9773
528.9727
[M+Na−2H]−
631.9938
688.9668
827.9737
776 : M.findmain = 364.0373 (+/− 20 ppm)
363.03
[M−H]−
385.1925
412.0596
484.973
522.9773
528.9727
631.9938
688.9668
827.9737

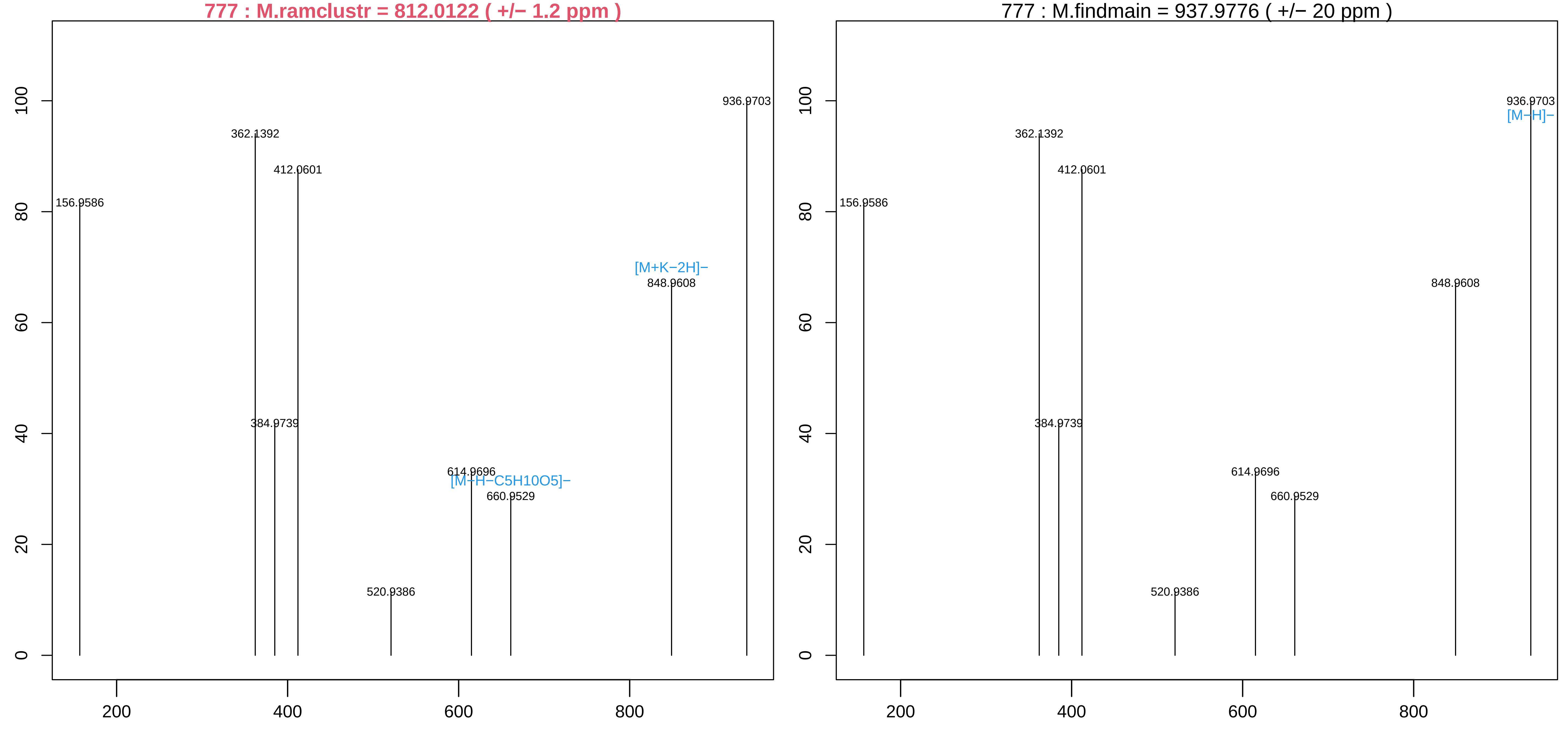
777 : M.ramclustr = 812.0122 (+/− 1.2 ppm)
156.9586
362.1392
412.0601
384.9739
520.9386
614.9696
[M−H−C5H10O5]−
660.9529
[M+K−2H]−
848.9608
936.9703
0
20
40
60
80
100
200
400
600
800
777 : M.findmain = 937.9776 (+/− 20 ppm)
156.9586
362.1392
412.0601
384.9739
520.9386
614.9696
660.9529
848.9608
936.9703
[M−H]−
0
20
40
60
80
100
200
400
600
800

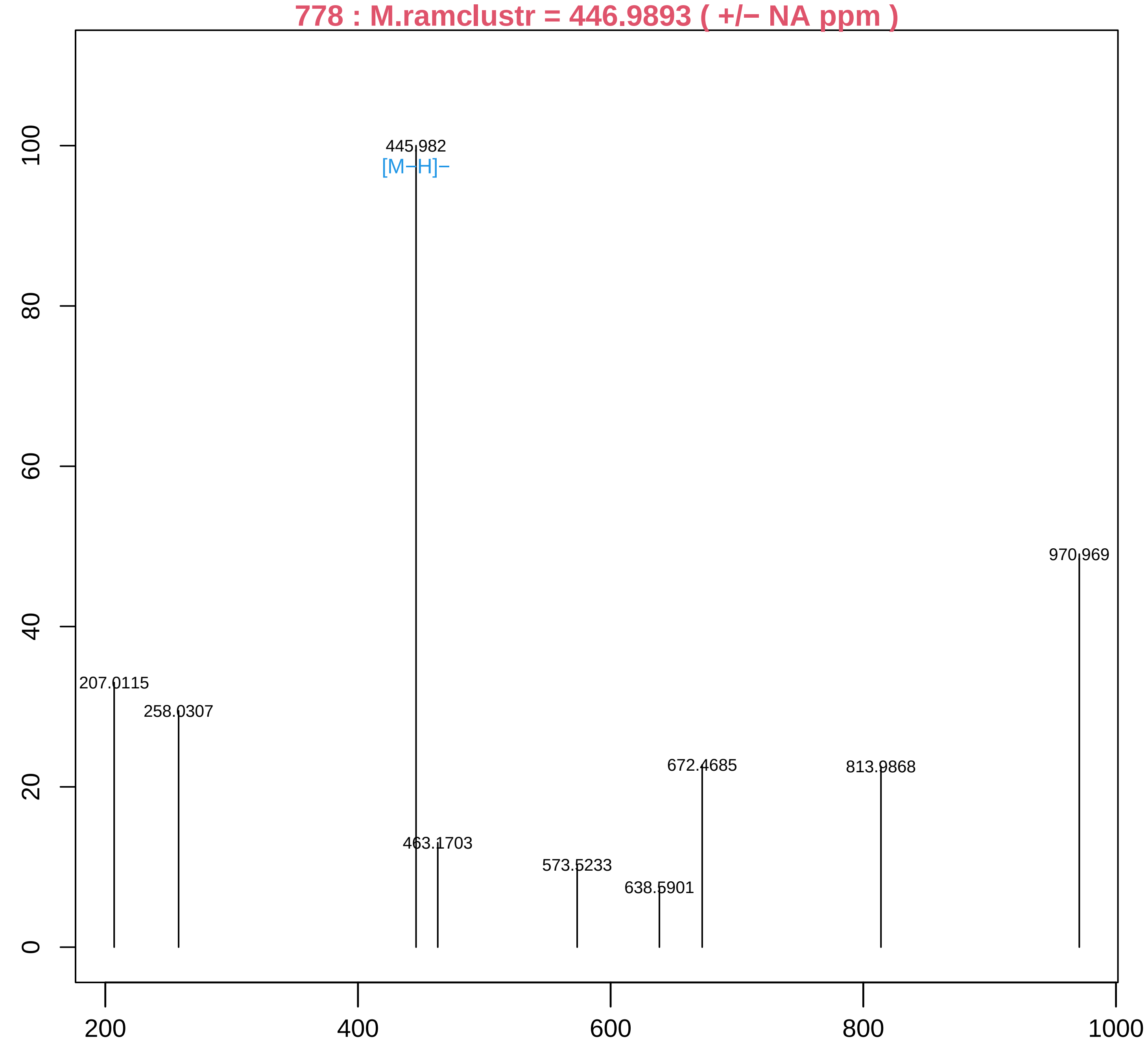

778 : M.ramclustr = 446.9893 (+/− NA ppm)
445.982
[M−H]−
970.969
207.0115
258.0307
672.4685
813.9868
463.1703
573.5233
638.5901
0
20
40
60
80
100
200
400
600
800
1000

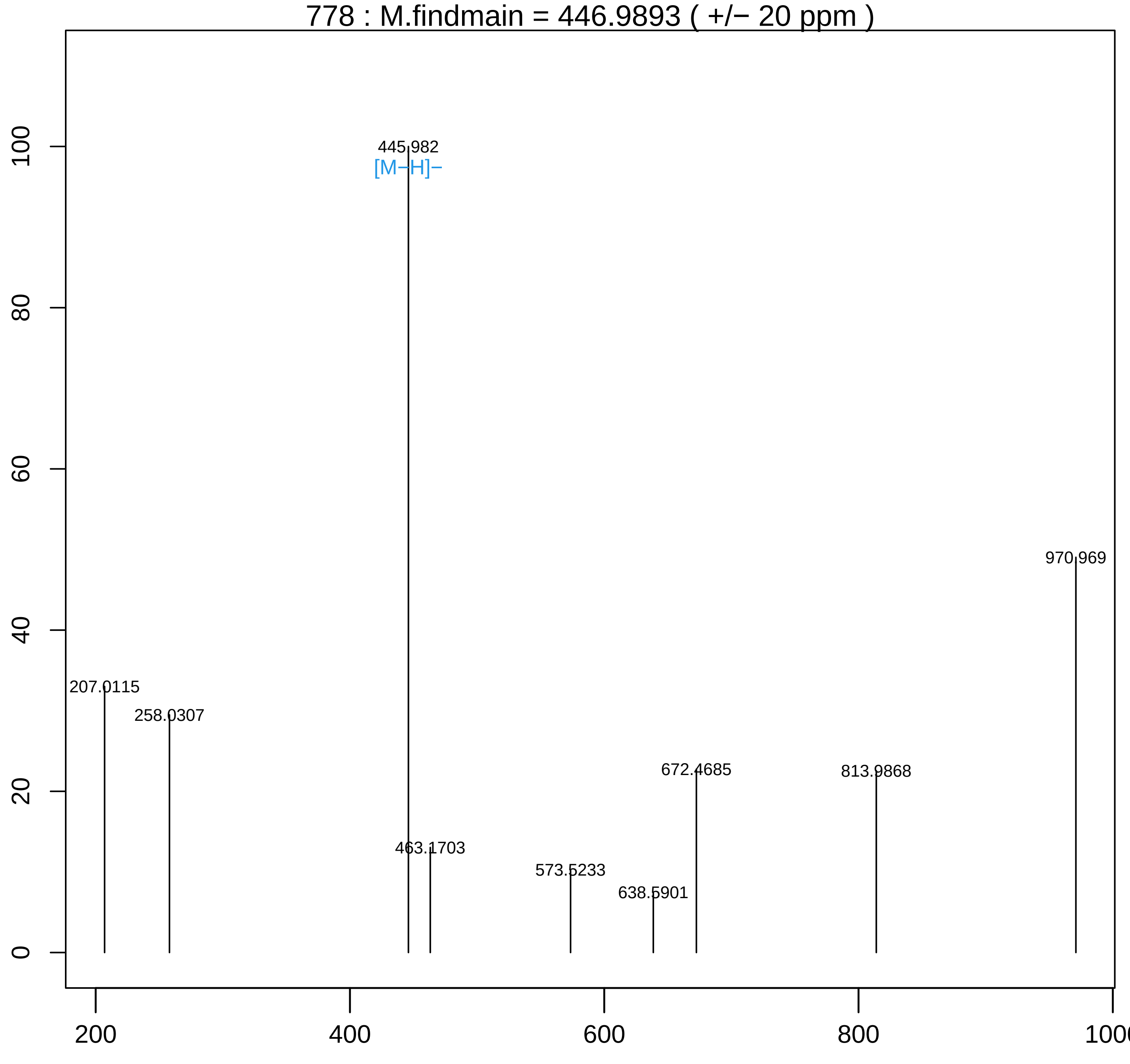

778 : M.findmain = 446.9893 (+/− 20 ppm)
445.982
[M−H]−
970.969
207.0115
258.0307
672.4685
813.9868
463.1703
573.5233
638.5901
0
20
40
60
80
100
200
400
600
800

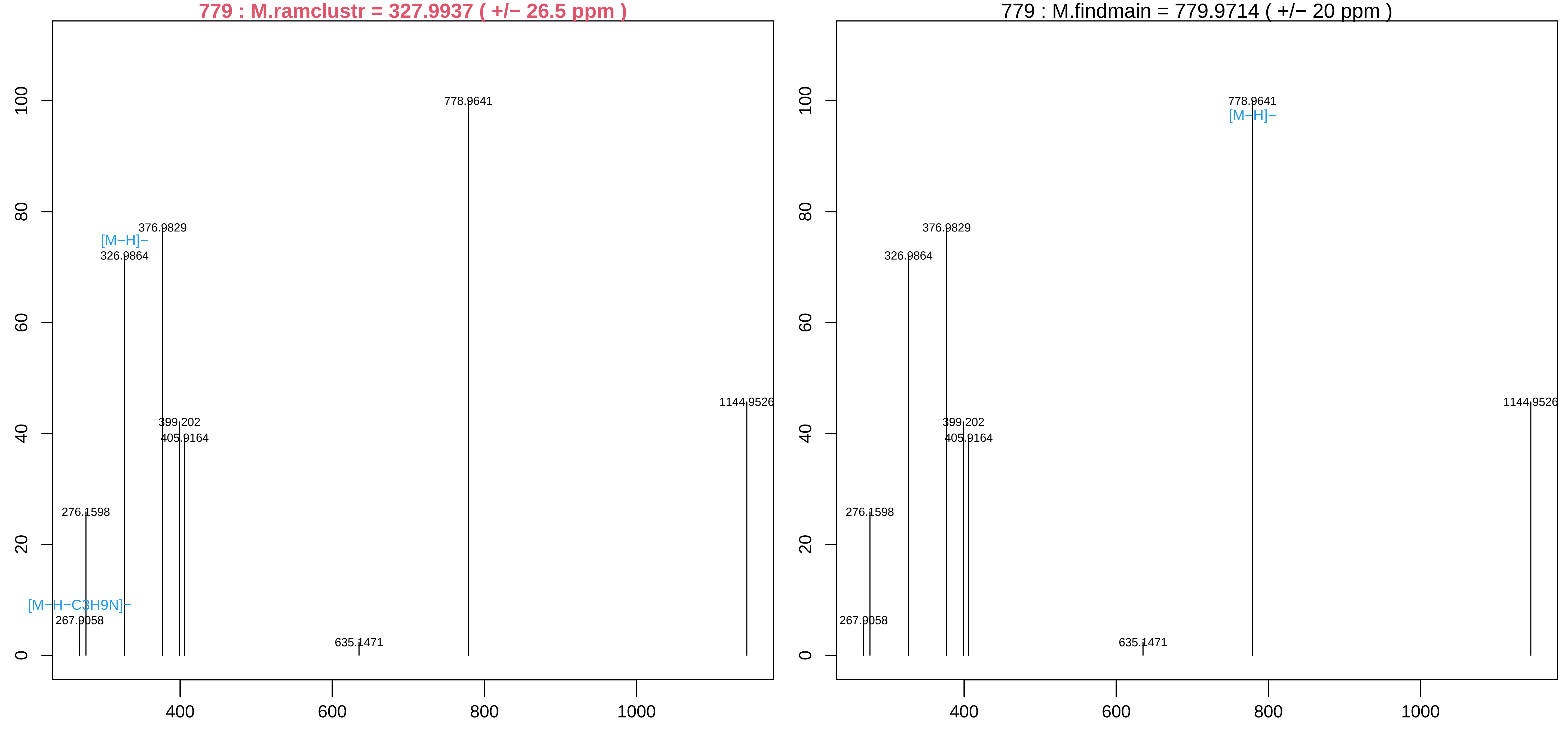
779 : M.ramclustr = 327.9937 (+/− 26.5 ppm)
778.9641
376.9829
[M−H]−
326.9864
399.202
405.9164
276.1598
[M−H−C3H9N]−
267.9058
635.1471
1144.9526
779 : M.findmain = 779.9714 (+/− 20 ppm)
778.9641
[M−H]−
376.9829
326.9864
399.202
405.9164
276.1598
267.9058
635.1471
1144.9526

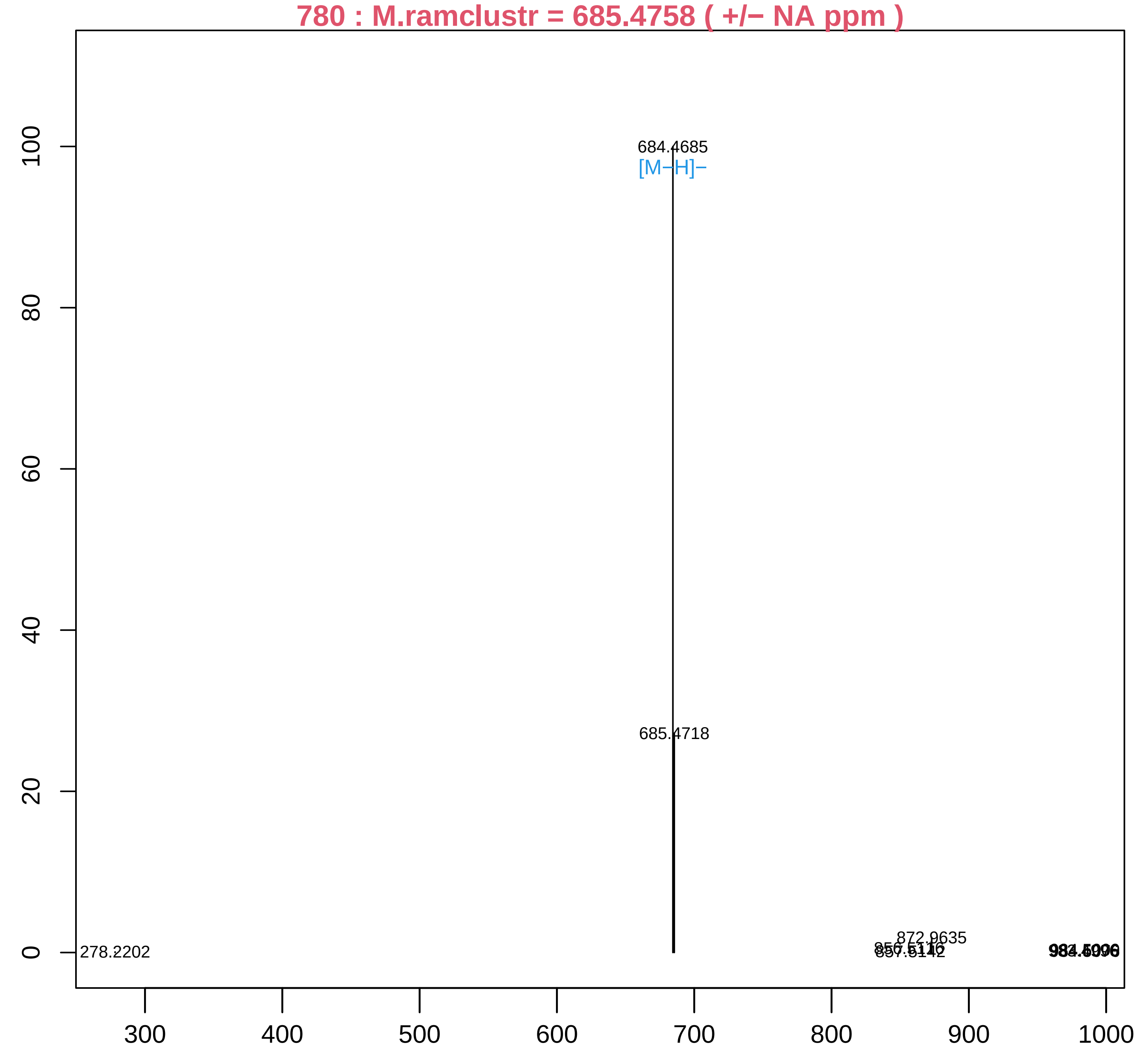
780 : M.ramclustr = 685.4758 (+/− NA ppm)
684.4685
[M−H]−
685.4718
278.2202
872.9635
856.5116
857.5142
984.5096
300
400
500
600
700
800
900
1000
0
20
40
60
80
100

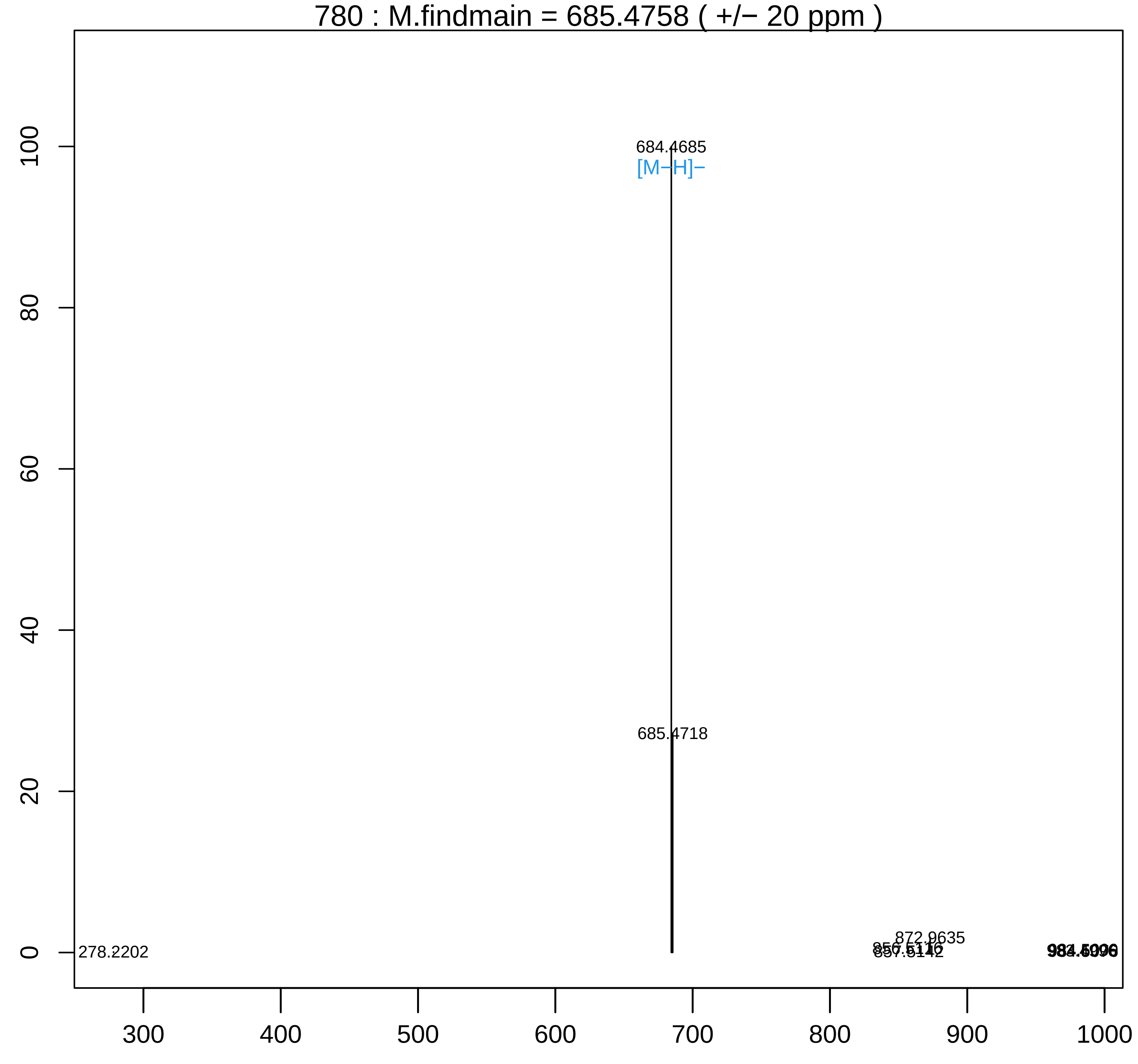
780 : M.findmain = 685.4758 (+/− 20 ppm)
684.4685
[M−H]−
685.4718
278.2202
872.9635
856.5116
857.5142
984.5096
300
400
500
600
700
800
900
1000
0
20
40
60
80
100

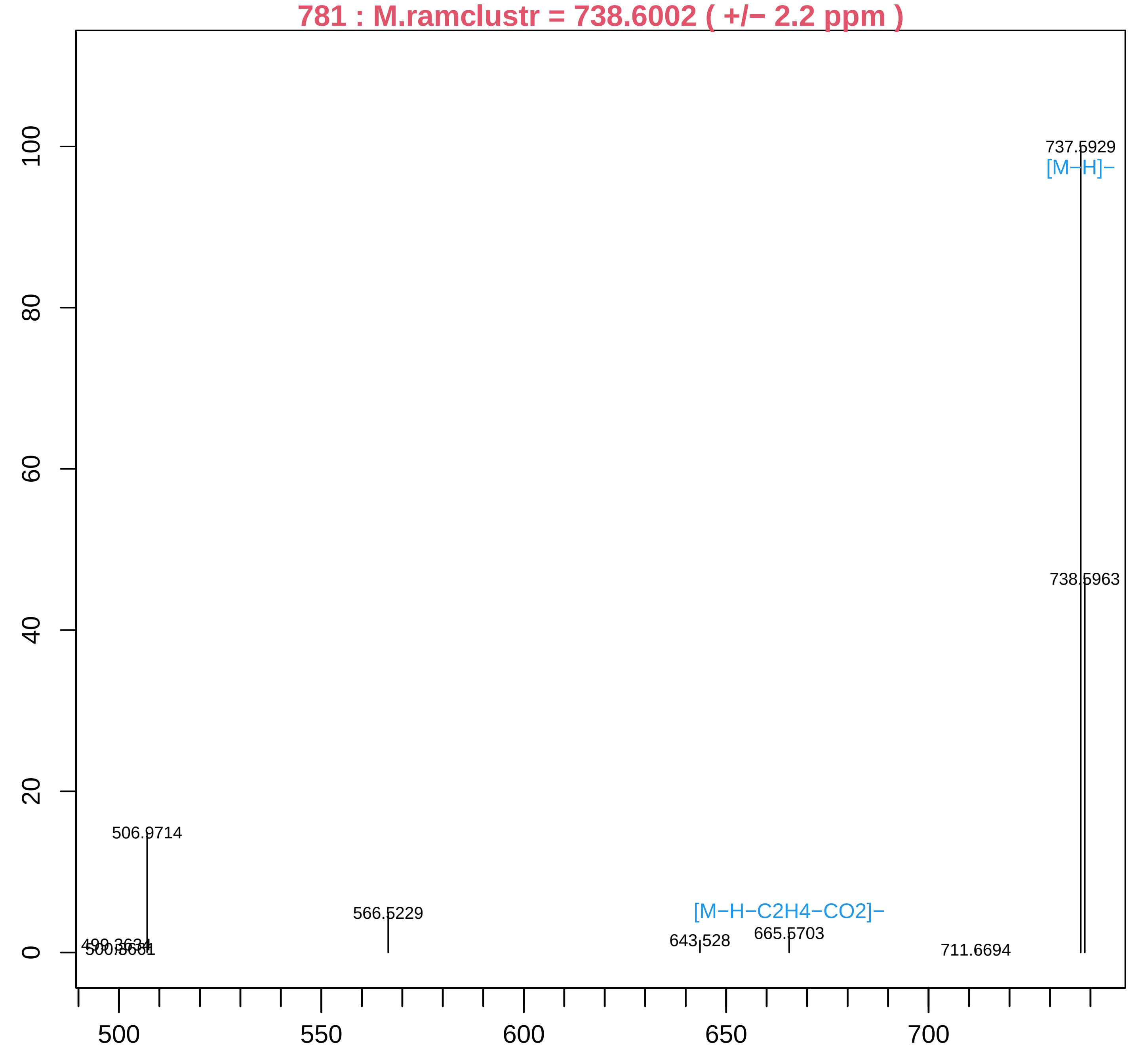
781 : M.ramclustr = 738.6002 (+/− 2.2 ppm)
737.5929
[M−H]−
738.5963
506.9714
566.5229
[M−H−C2H4−CO2]−
643.528
665.5703
711.6694
0
20
40
60
80
100
500
550
600
650
700

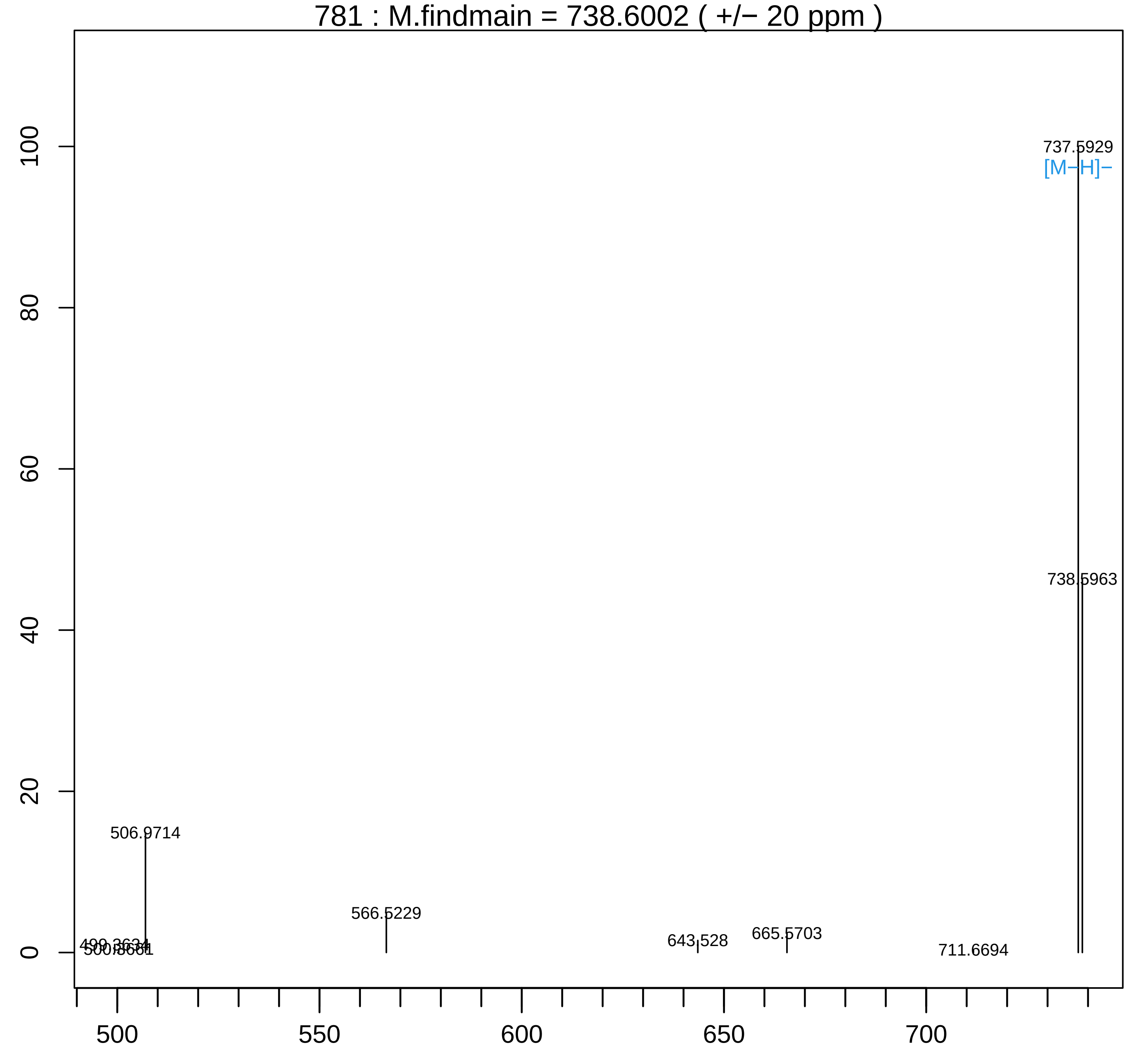
781 : M.findmain = 738.6002 (+/− 20 ppm)
737.5929
[M−H]−
738.5963
506.9714
566.5229
643.528
665.5703
711.6694
0
20
40
60
80
100
500
550
600
650
700

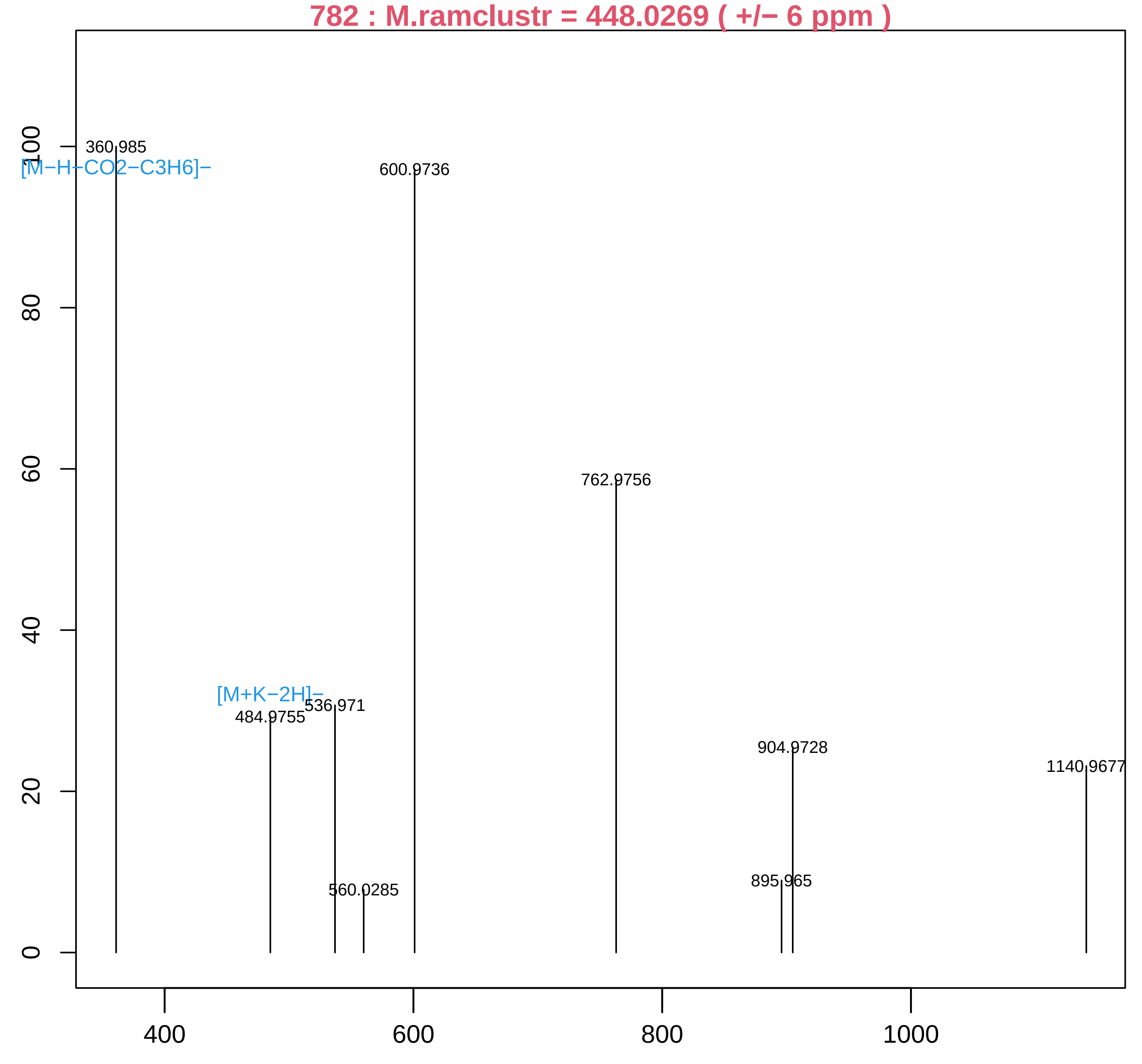
782 : M.ramclustr = 448.0269 (+/− 6 ppm)
360.985
[M−H−CO2−C3H6]−
600.9736
762.9756
[M+K−2H]−
484.9755
536.971
904.9728
1140.9677
560.0285
895.965
0
20
40
60
80
100
400
600
800
1000
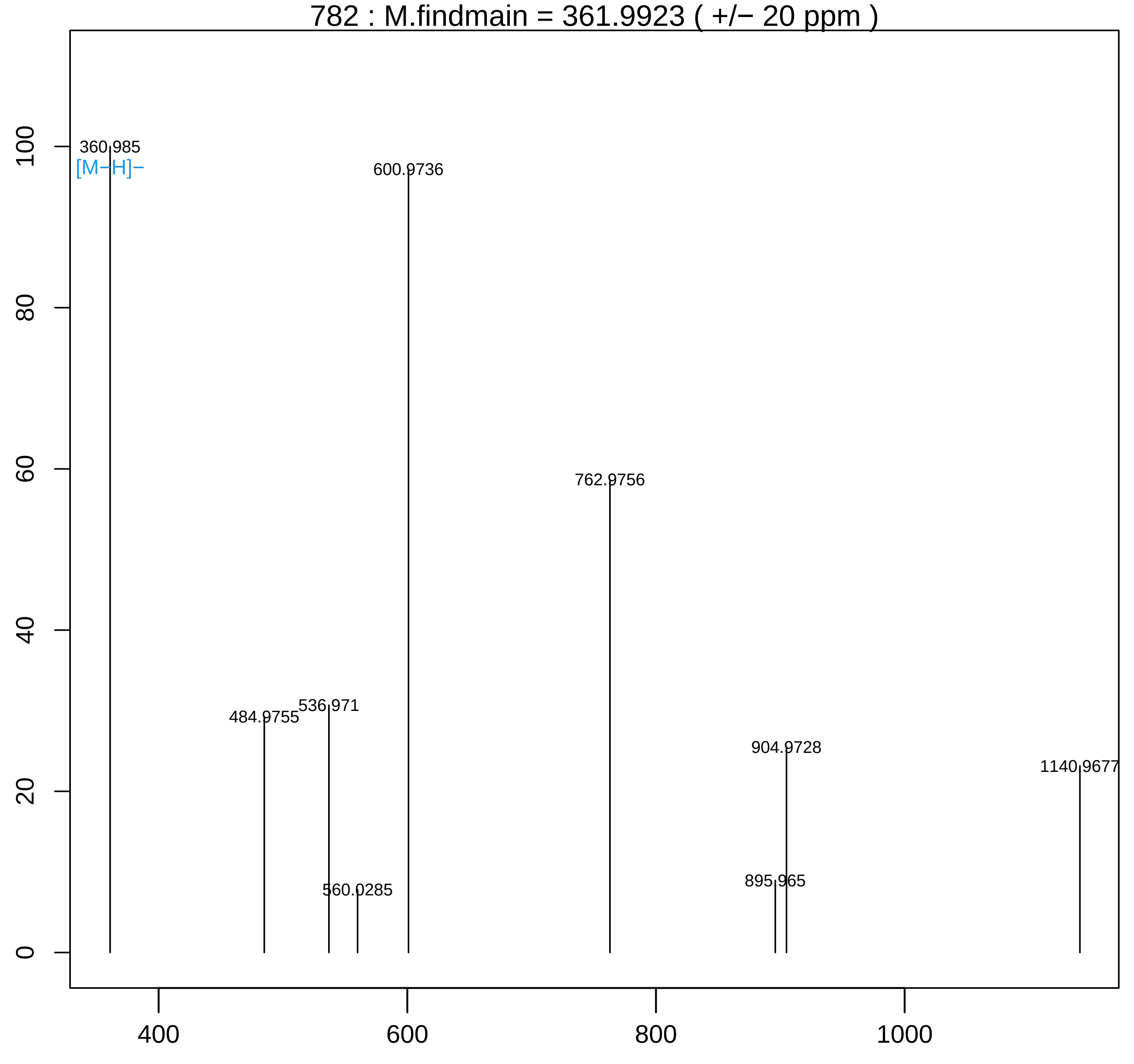
782 : M.findmain = 361.9923 (+/− 20 ppm)
360.985
[M−H]−
600.9736
762.9756
484.9755
536.971
904.9728
1140.9677
560.0285
895.965
0
20
40
60
80
100
400
600
800
1000

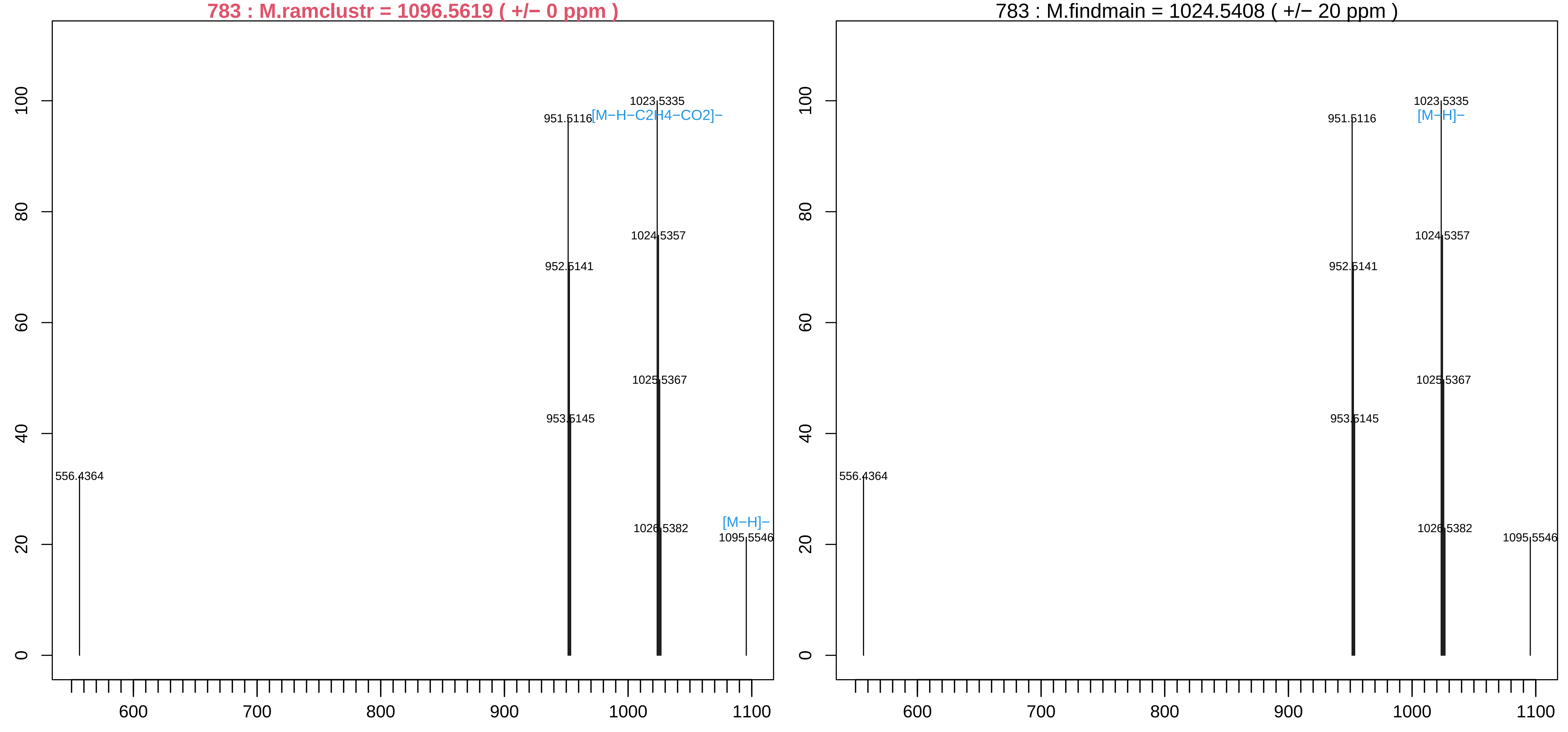

783 : M.ramclustr = 1096.5619 (+/− 0 ppm)
1023.5335
[M−H−C2H4−CO2]−
951.5116
1024.5357
952.5141
1025.5367
953.5145
556.4364
[M−H]−
1026.5382
1095.5546
783 : M.findmain = 1024.5408 (+/− 20 ppm)
1023.5335
[M−H]−
951.5116
1024.5357
952.5141
1025.5367
953.5145
556.4364
1026.5382
1095.5546
0
20
40
60
80
100
600
700
800
900
1000
1100

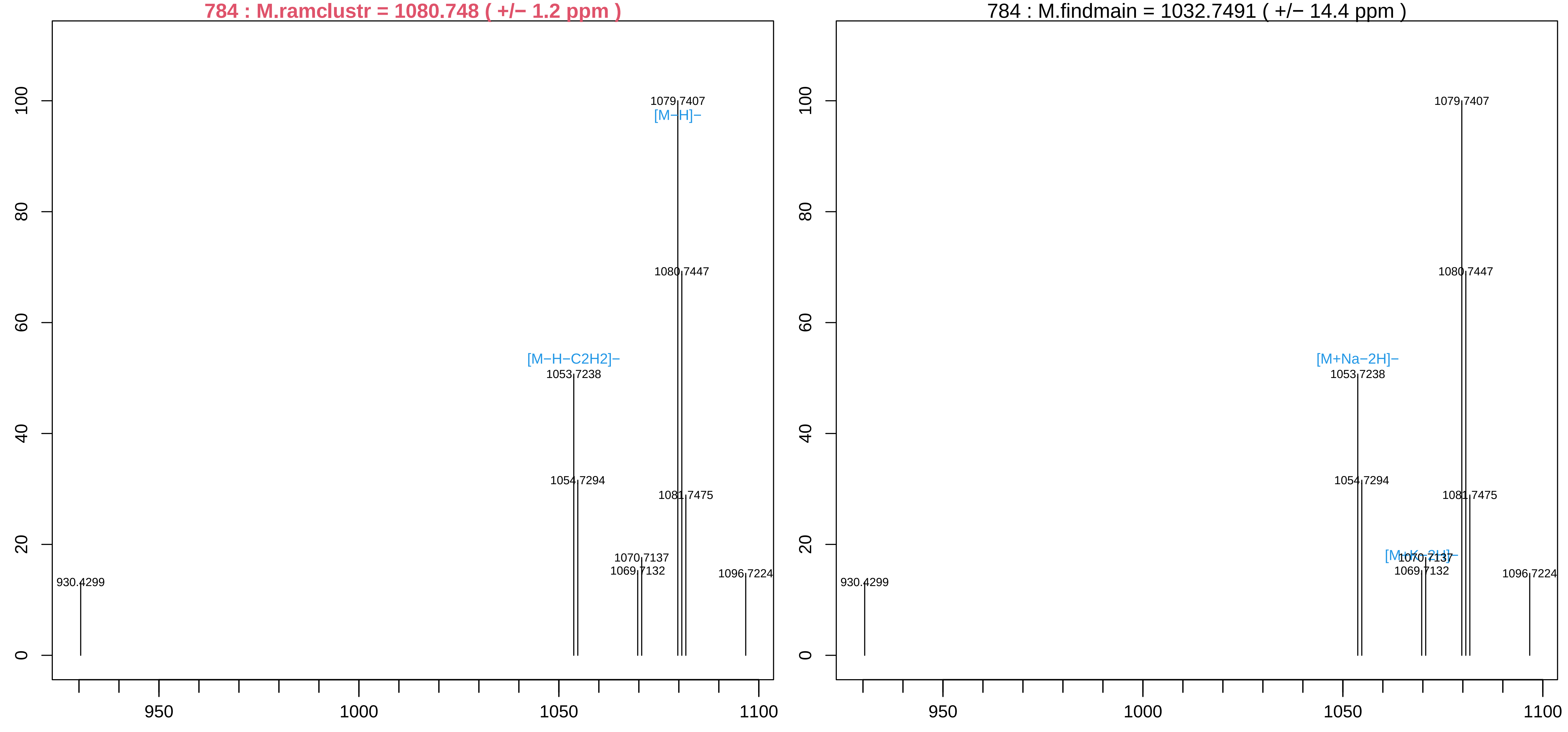
784 : M.ramclustr = 1080.748 (+/− 1.2 ppm)
784 : M.findmain = 1032.7491 (+/− 14.4 ppm)
[M−H]−
[M−H−C2H2]−
[M+Na−2H]−
[M+K−2H]−
1079.7407
1080.7447
1053.7238
1054.7294
1081.7475
1070.7137
1069.7132
1096.7224
930.4299
0
20
40
60
80
100
950
1000
1050
1100

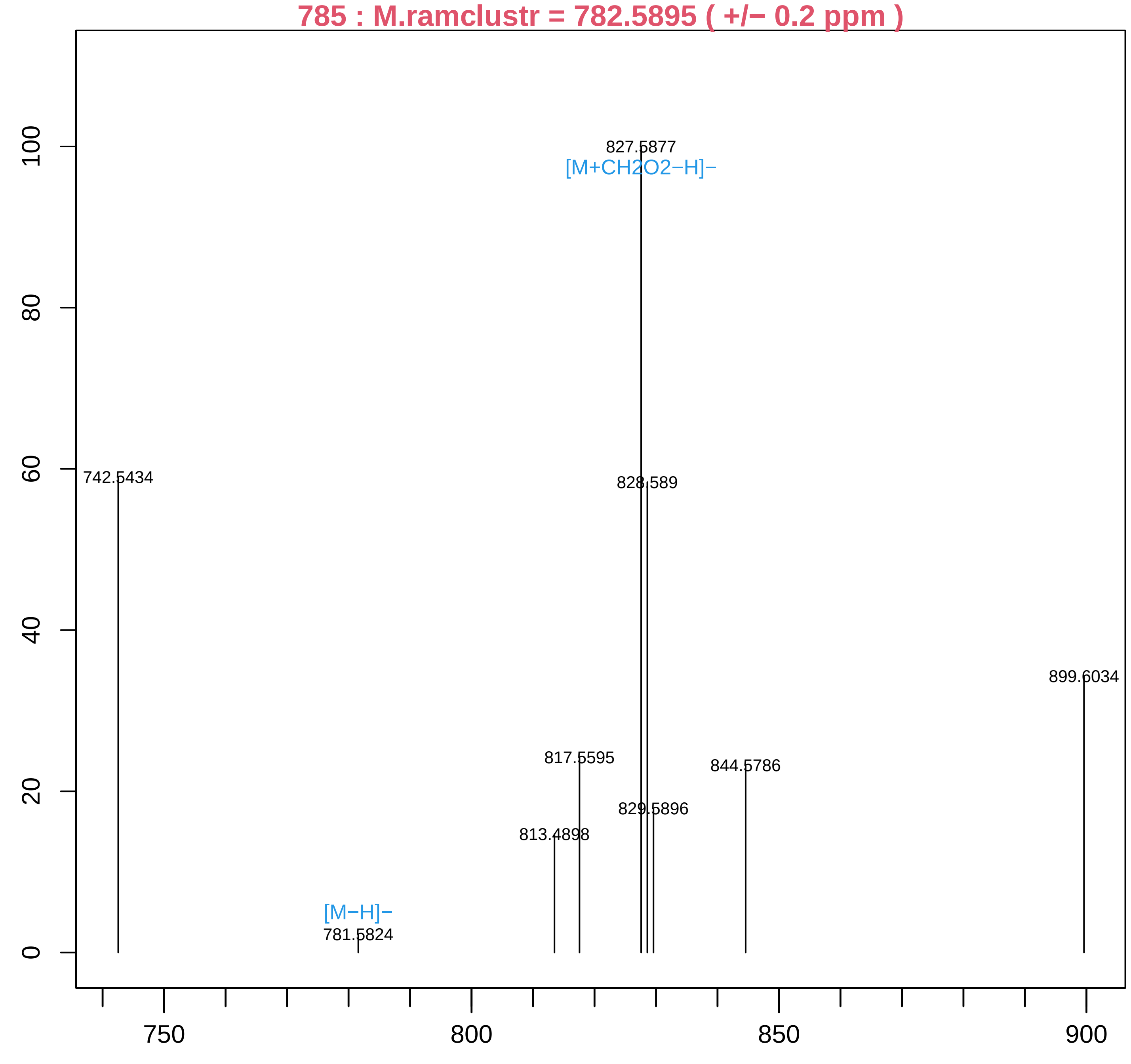
785 : M.ramclustr = 782.5895 (+/− 0.2 ppm)
827.5877
[M+CH2O2−H]−
742.5434
828.589
899.6034
817.5595
844.5786
829.5896
813.4898
[M−H]−
781.5824
0
20
40
60
80
100
750
800
850
900
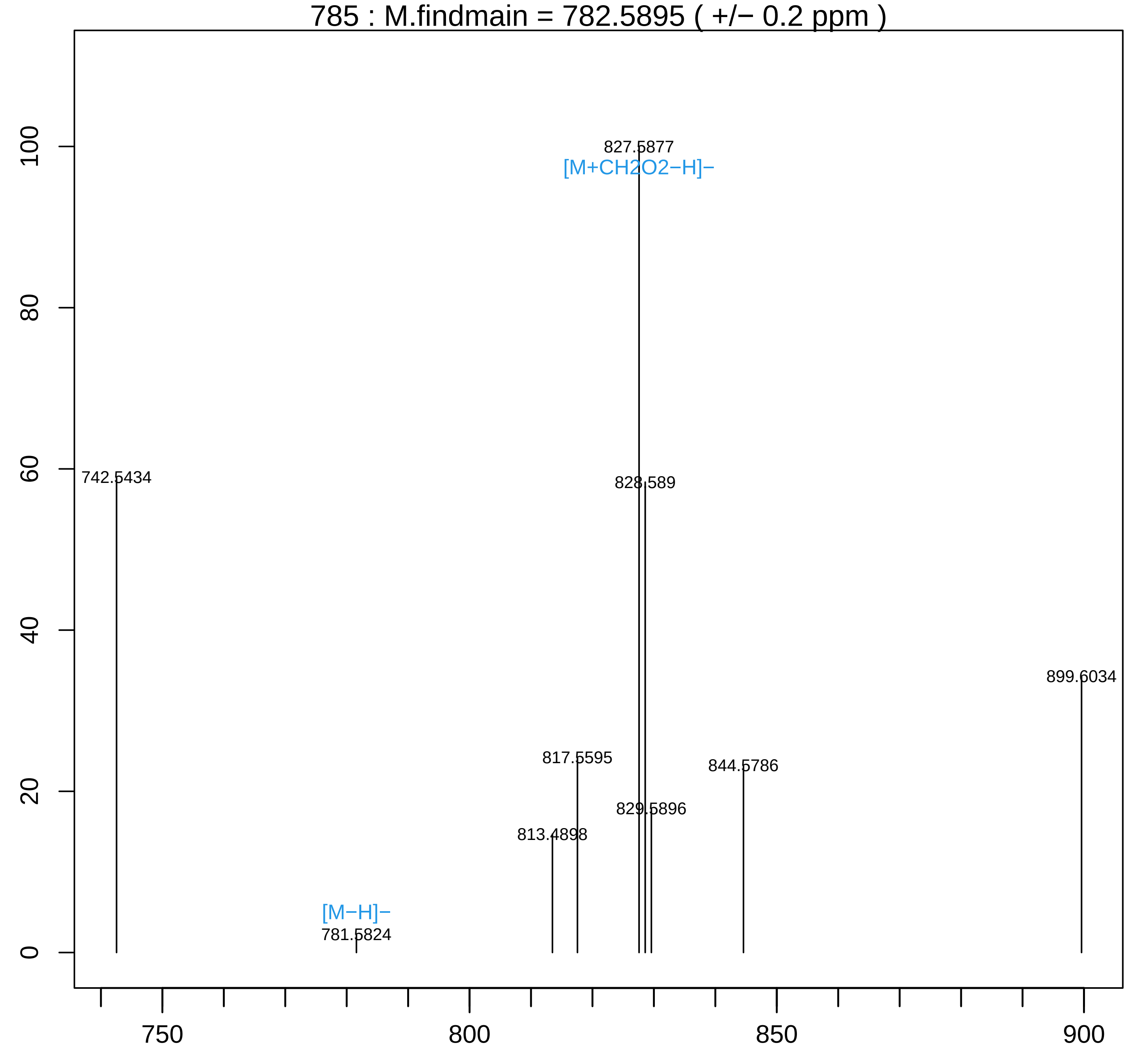
785 : M.findmain = 782.5895 (+/− 0.2 ppm)
827.5877
[M+CH2O2−H]−
742.5434
828.589
899.6034
817.5595
844.5786
829.5896
813.4898
[M−H]−
781.5824
0
20
40
60
80
100
750
800
850
900

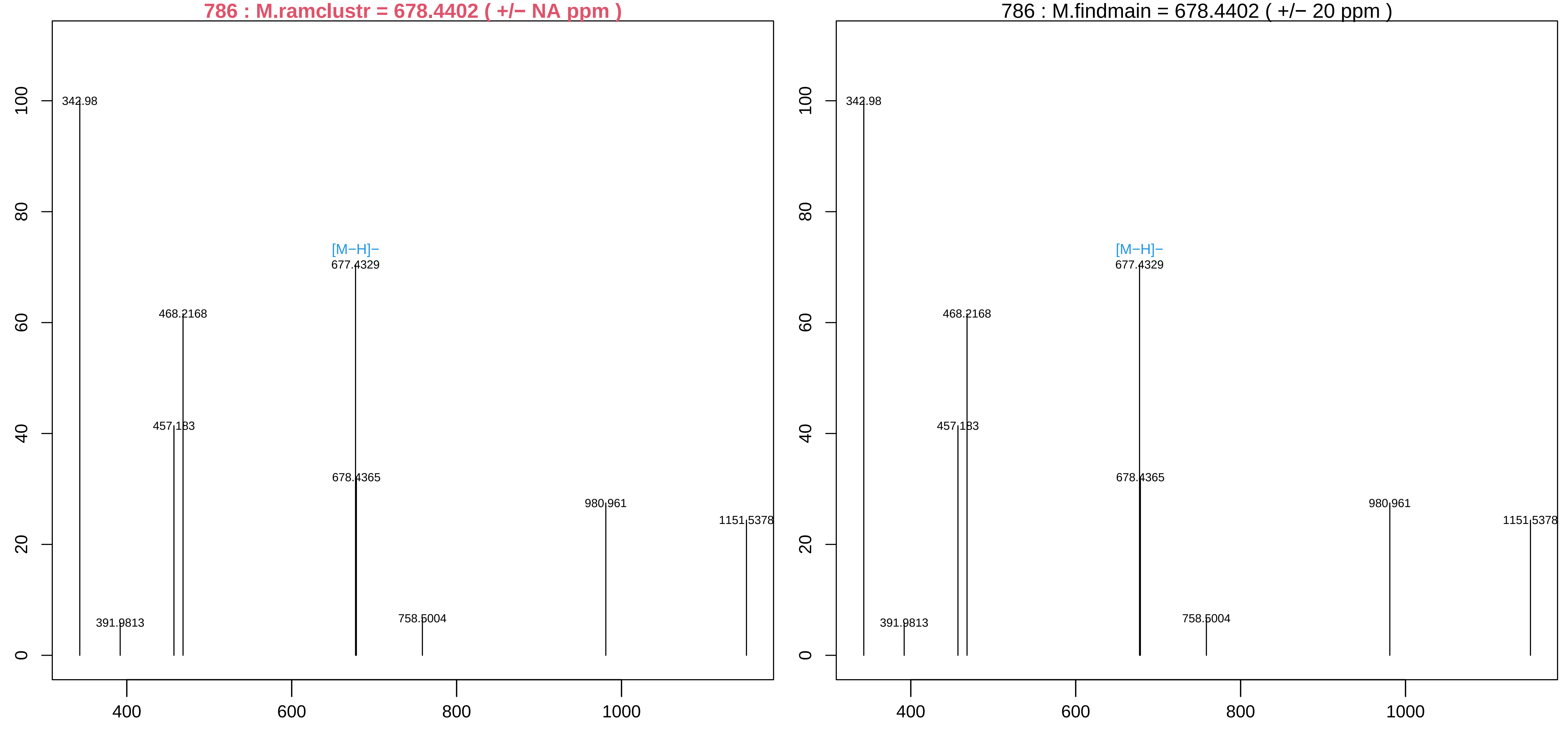
786 : M.ramclustr = 678.4402 (+/− NA ppm)
786 : M.findmain = 678.4402 (+/− 20 ppm)
[M−H]−
342.98
391.9813
457.133
468.2168
677.4329
678.4365
758.5004
980.961
1151.5378
0
20
40
60
80
100
400
600
800
1000

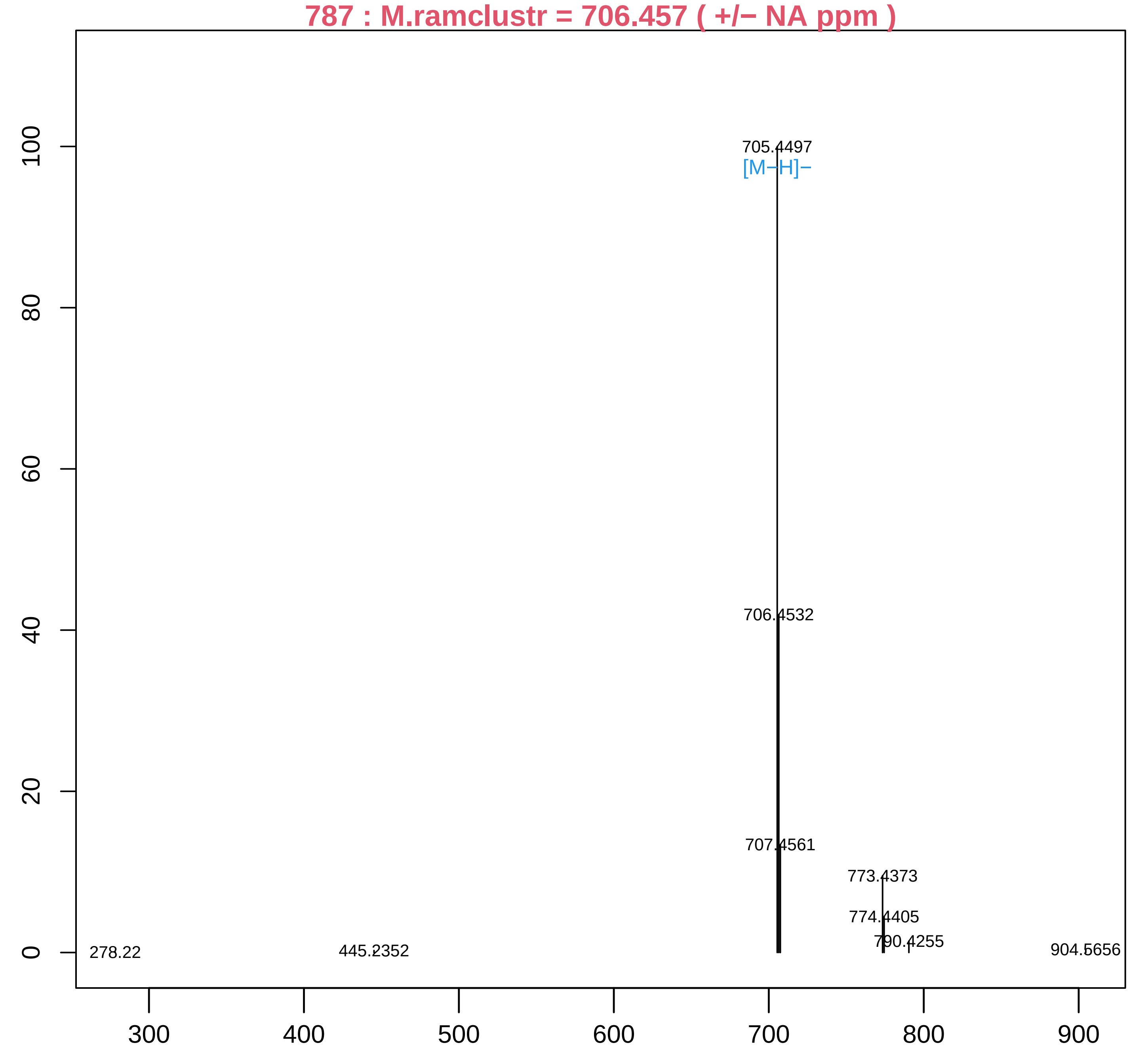

787 : M.ramclustr = 706.457 (+/− NA ppm)
705.4497
[M−H]−
706.4532
707.4561
773.4373
774.4405
790.4255
278.22
445.2352
904.5656
0
20
40
60
80
100
300
400
500
600
700
800
900

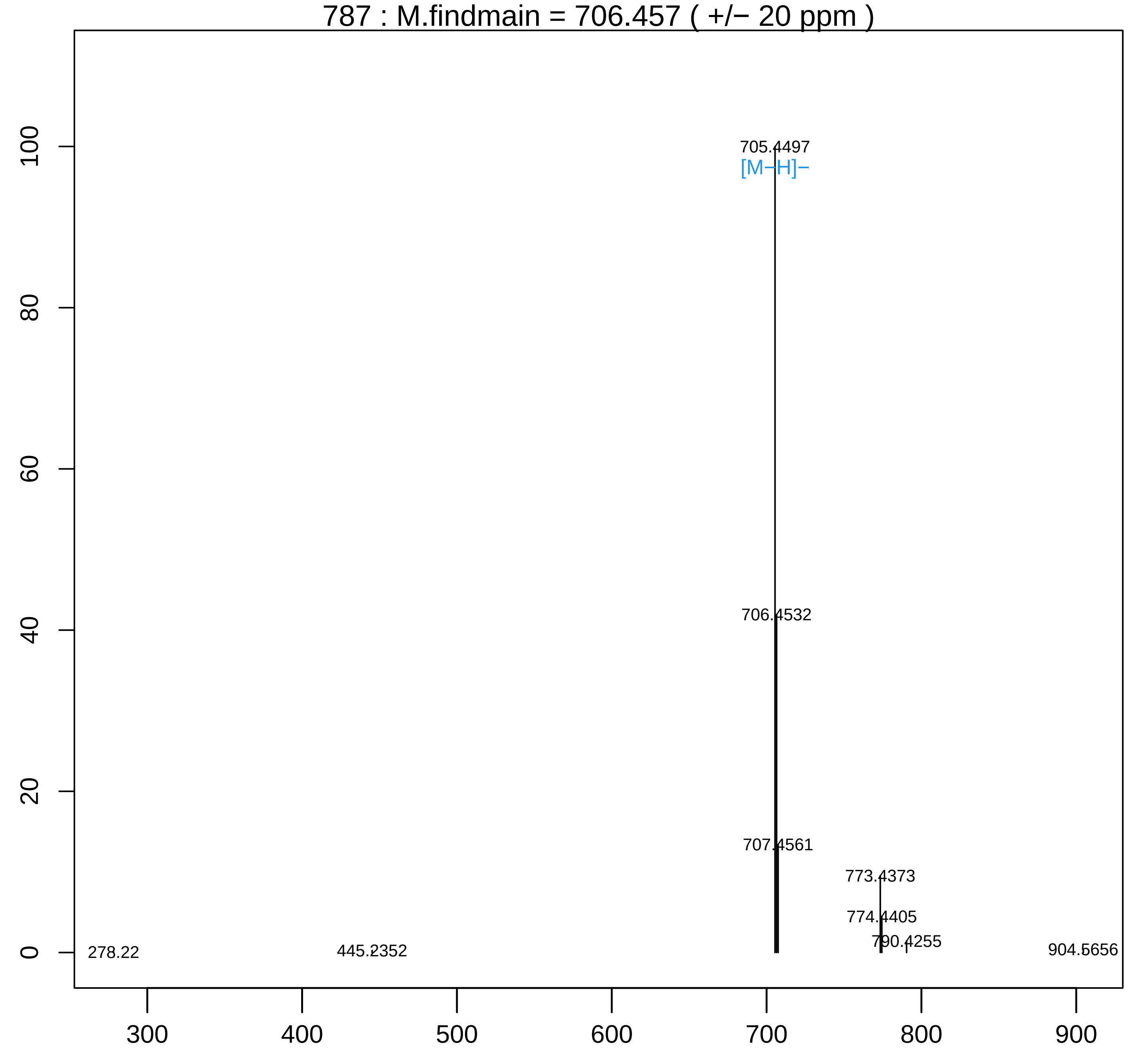

787 : M.findmain = 706.457 (+/− 20 ppm)
705.4497
[M−H]−
706.4532
707.4561
773.4373
774.4405
790.4255
278.22
445.2352
904.5656
0
20
40
60
80
100
300
400
500
600
700
800
900

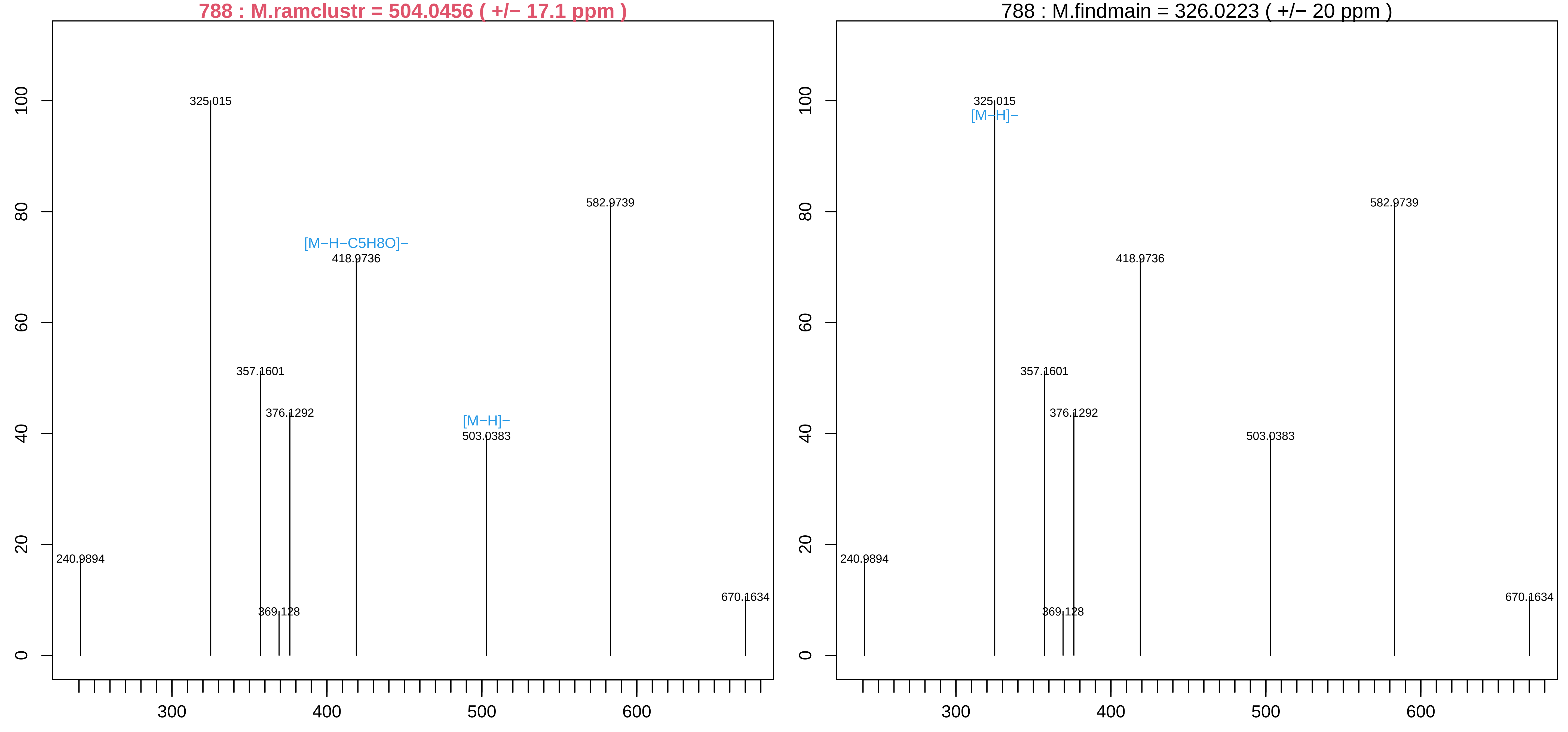

788 : M.ramclustr = 504.0456 (+/− 17.1 ppm)
325.015
582.9739
[M−H−C5H8O]−
418.9736
357.1601
376.1292
[M−H]−
503.0383
240.9894
369.128
670.1634
0
20
40
60
80
100
300
400
500
600
788 : M.findmain = 326.0223 (+/− 20 ppm)
325.015
[M−H]−
582.9739
418.9736
357.1601
376.1292
503.0383
240.9894
369.128
670.1634
0
20
40
60
80
100
300
400
500
600

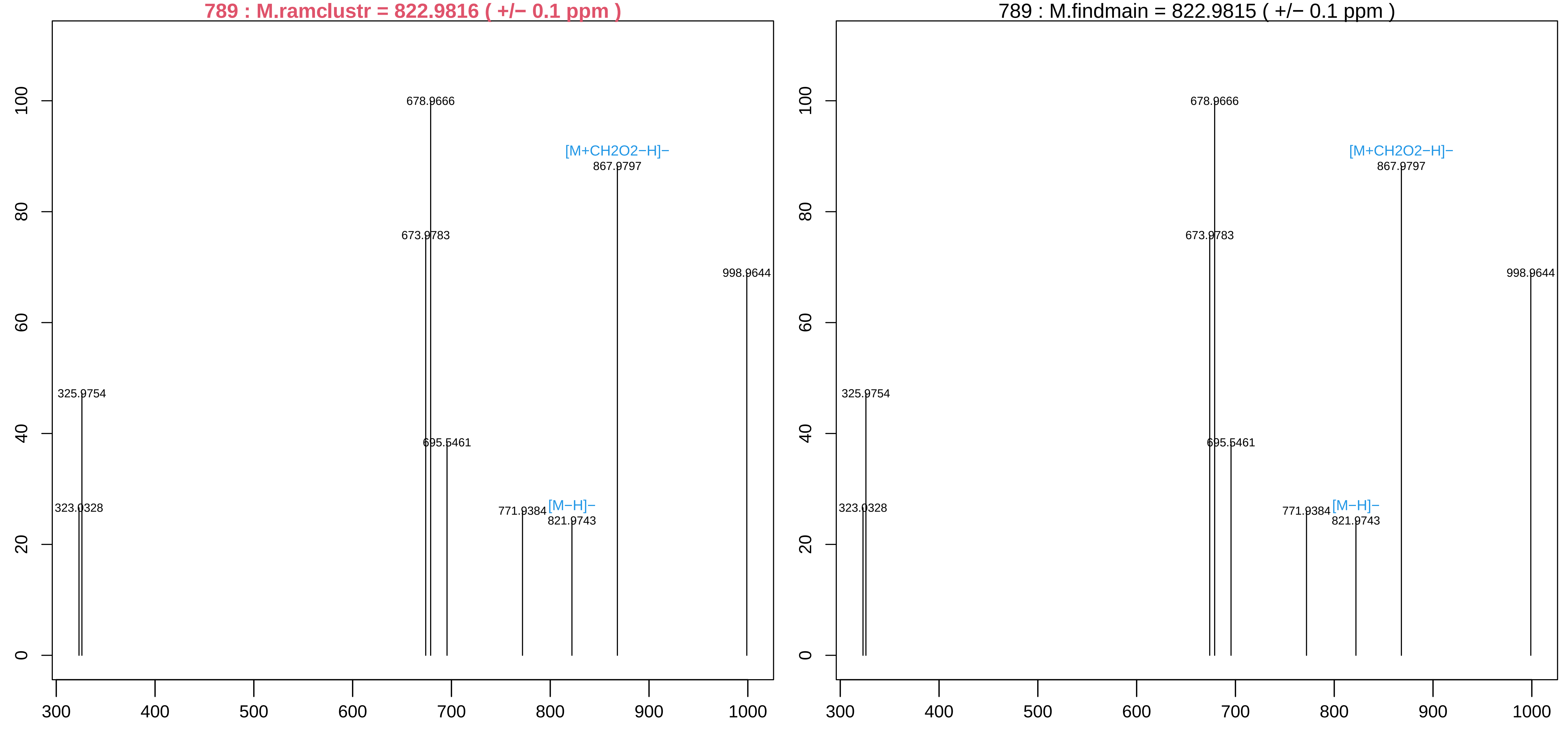
789 : M.ramclustr = 822.9816 (+/− 0.1 ppm)
789 : M.findmain = 822.9815 (+/− 0.1 ppm)
678.9666
[M+CH2O2−H]−
867.9797
673.9783
998.9644
325.9754
695.5461
323.0328
771.9384
[M−H]−
821.9743
0
20
40
60
80
100
300
400
500
600
700
800
900
1000

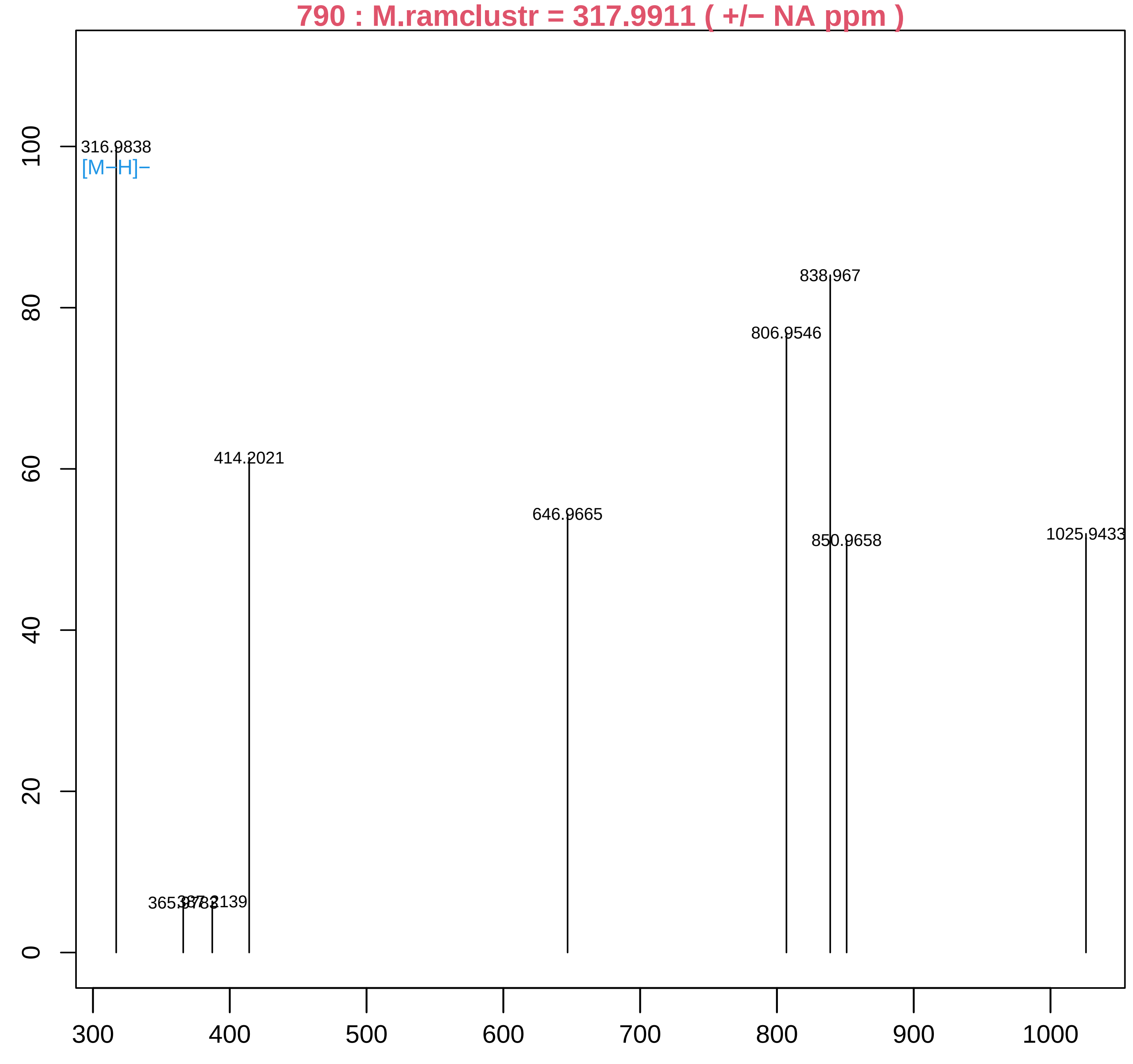
790 : M.ramclustr = 317.9911 (+/− NA ppm)
316.9838
[M−H]−
838.967
806.9546
414.2021
646.9665
850.9658
1025.9433
365.9783
387.2139
0
20
40
60
80
100
300
400
500
600
700
800
900
1000

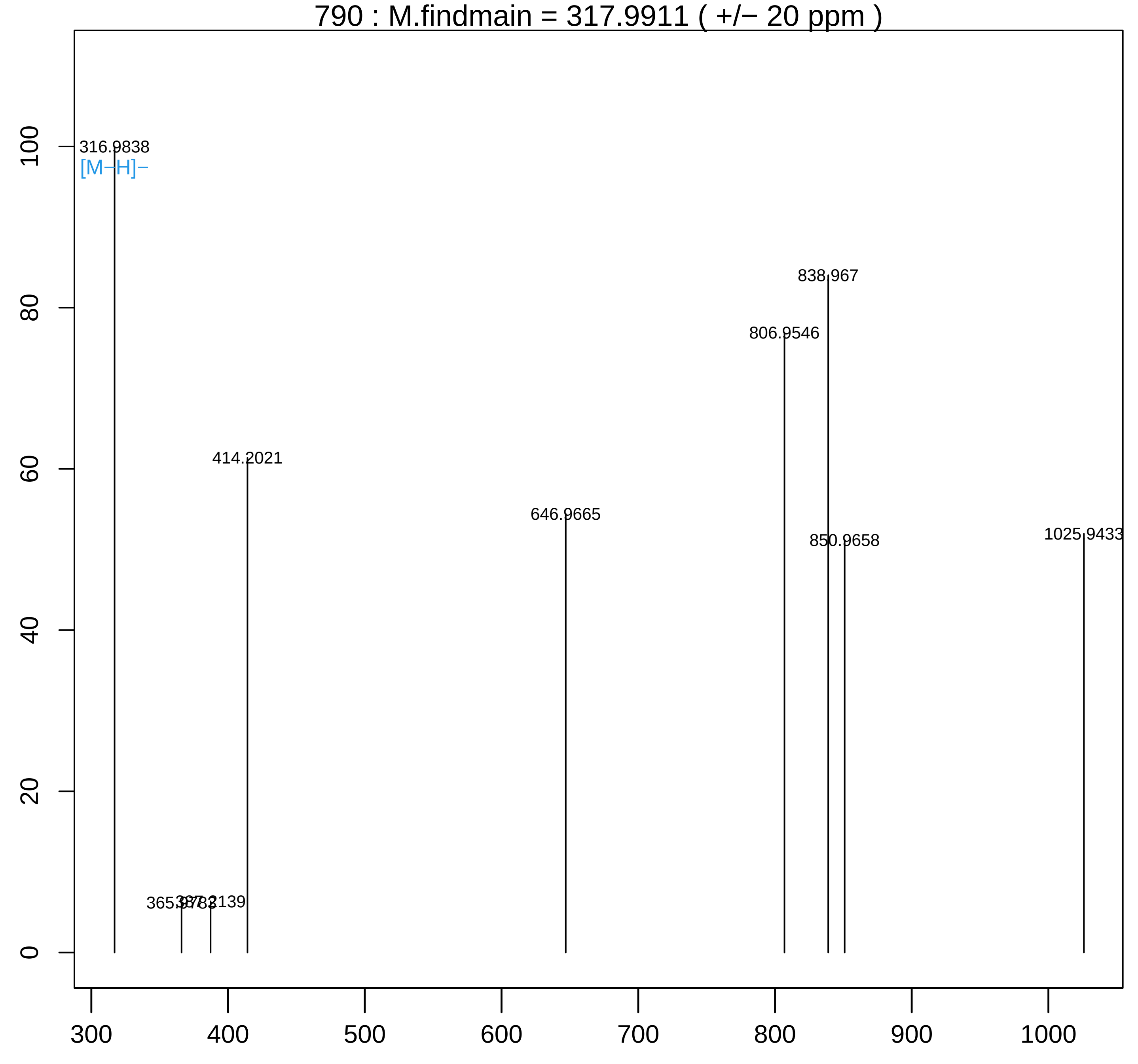
790 : M.findmain = 317.9911 (+/− 20 ppm)
316.9838
[M−H]−
838.967
806.9546
414.2021
646.9665
850.9658
1025.9433
365.9783
387.2139
0
20
40
60
80
100
300
400
500
600
700
800
900
1000

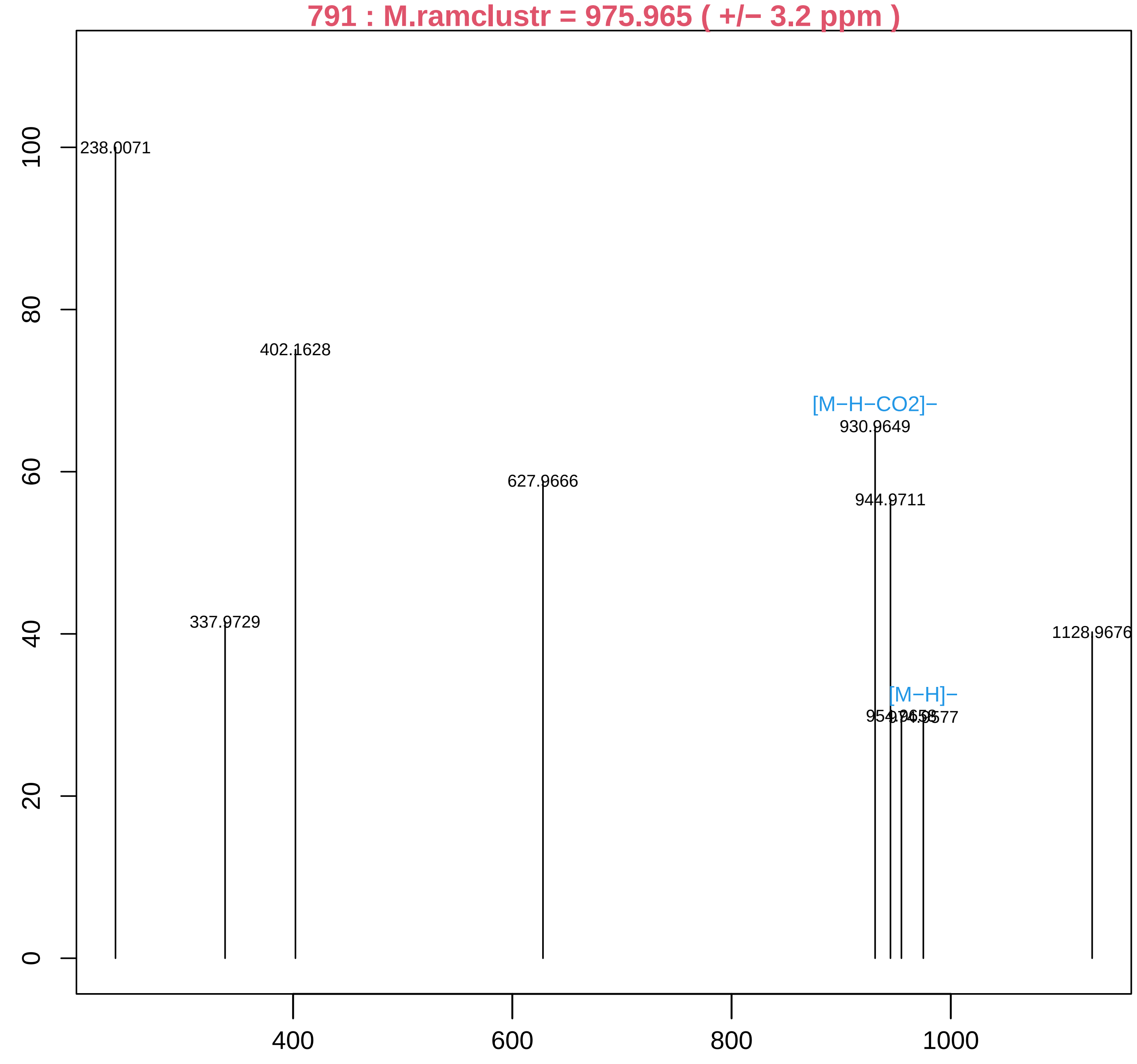
791 : M.ramclustr = 975.965 (+/− 3.2 ppm)
238.0071
337.9729
402.1628
627.9666
[M−H−CO2]−
930.9649
944.9711
[M−H]−
1128.9676
0
20
40
60
80
100
400
600
800
1000

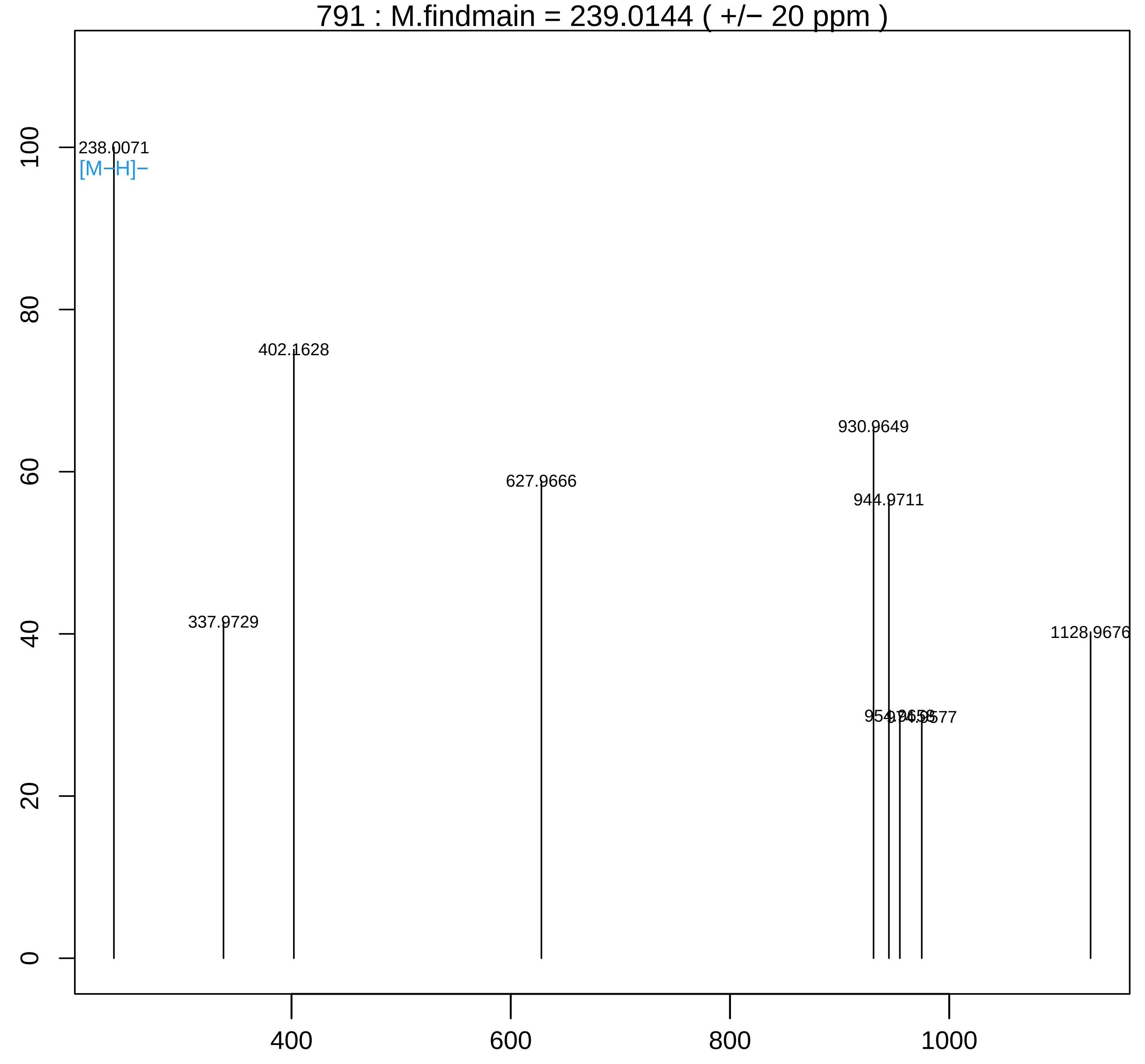
791 : M.findmain = 239.0144 (+/− 20 ppm)
238.0071
[M−H]−
337.9729
402.1628
627.9666
930.9649
944.9711
1128.9676
0
20
40
60
80
100
400
600
800
1000

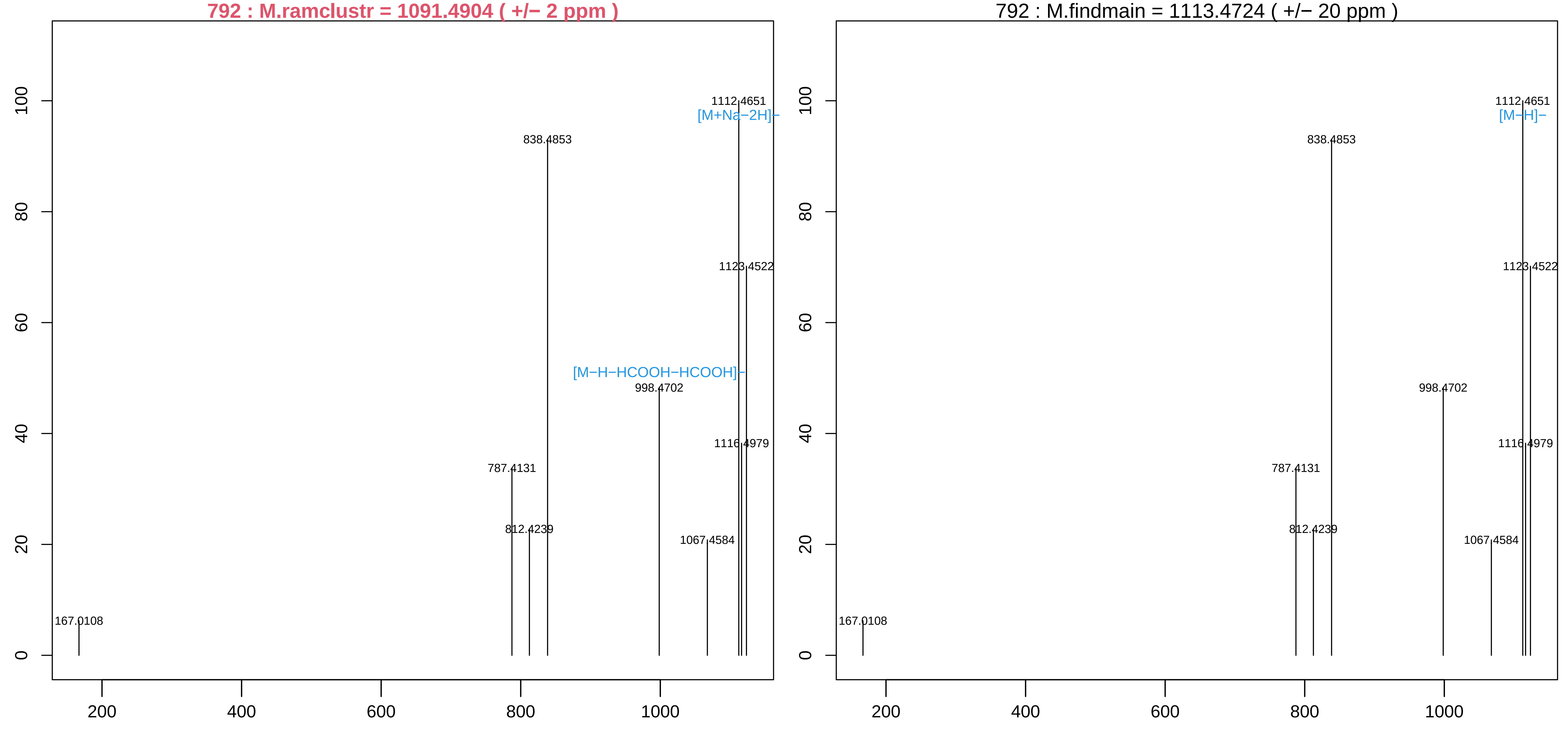

792 : M.ramclustr = 1091.4904 (+/− 2 ppm)
1112.4651
[M+Na−2H]−
838.4853
1123.4522
[M−H−HCOOH−HCOOH]−
998.4702
1116.4979
787.4131
812.4239
1067.4584
167.0108
0
20
40
60
80
100
200
400
600
800
1000
792 : M.findmain = 1113.4724 (+/− 20 ppm)
1112.4651
[M−H]−
838.4853
1123.4522
998.4702
1116.4979
787.4131
812.4239
1067.4584
167.0108
0
20
40
60
80
100
200
400
600
800
1000

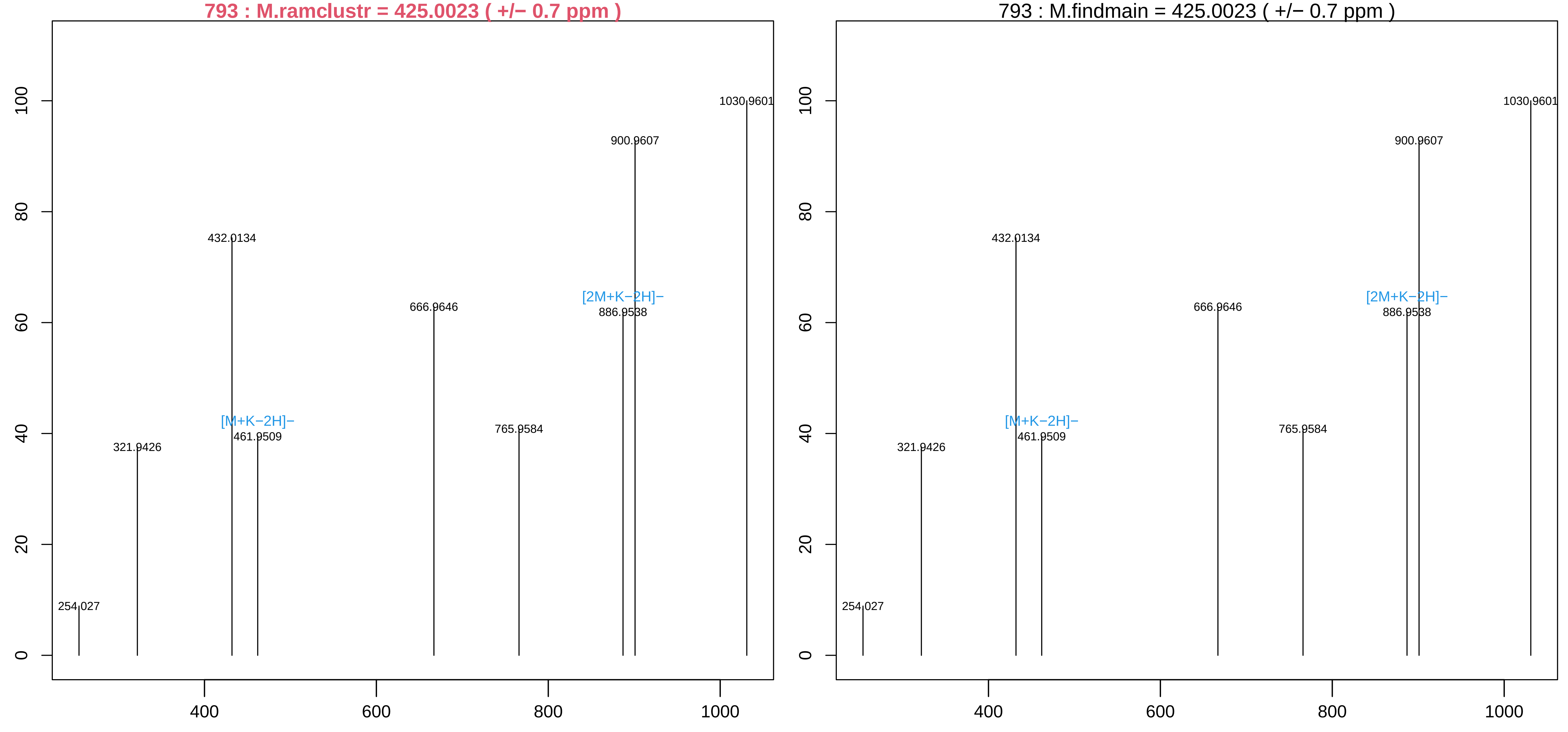

793 : M.ramclustr = 425.0023 (+/− 0.7 ppm)
793 : M.findmain = 425.0023 (+/− 0.7 ppm)
1030.9601
900.9607
432.0134
666.9646
[2M+K−2H]−
886.9538
[M+K−2H]−
461.9509
765.9584
321.9426
254.027
0
20
40
60
80
100
400
600
800
1000

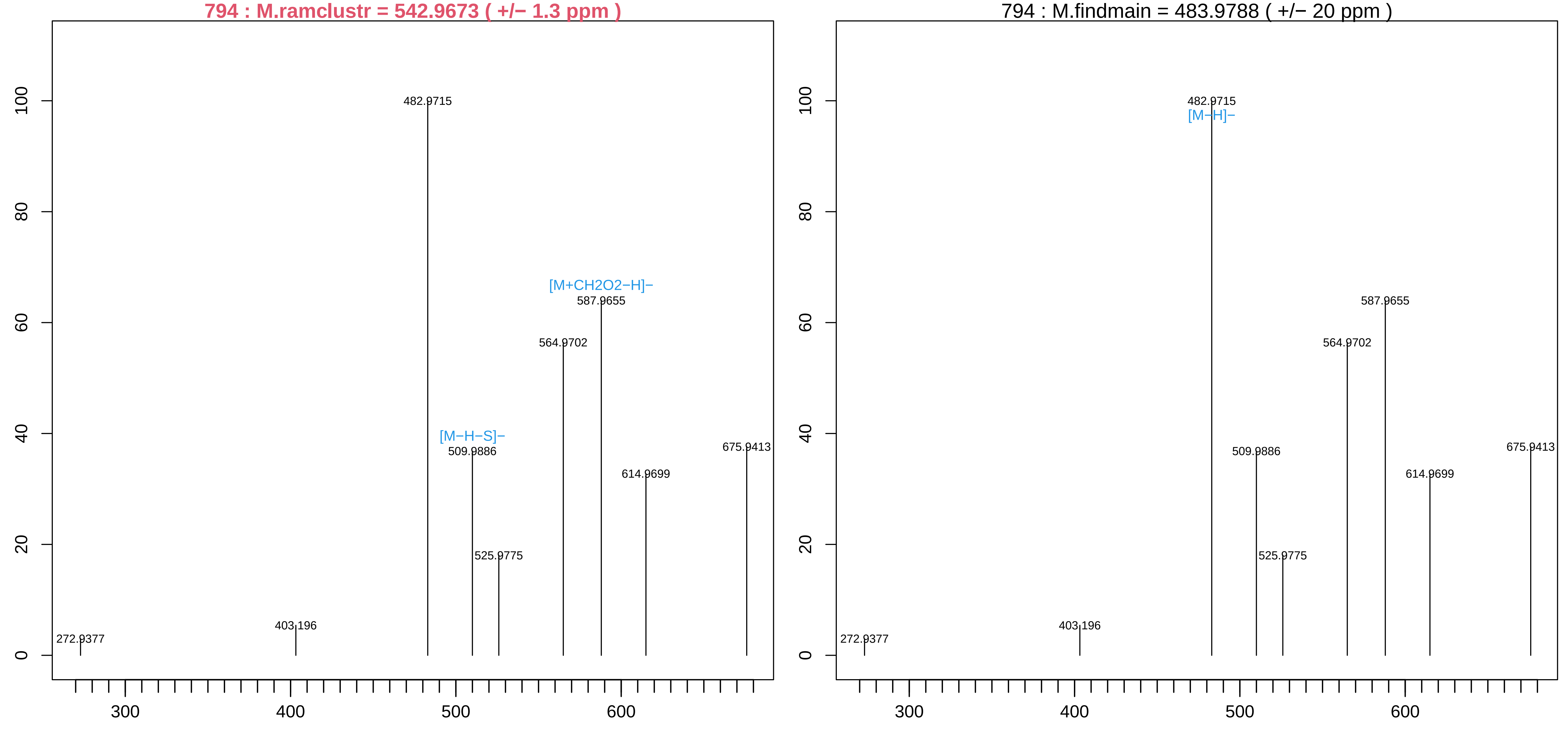

794 : M.ramclustr = 542.9673 (+/− 1.3 ppm)
482.9715
[M−H−S]−
509.9886
525.9775
564.9702
[M+CH2O2−H]−
587.9655
614.9699
675.9413
272.9377
403.196
794 : M.findmain = 483.9788 (+/− 20 ppm)
482.9715
[M−H]−
509.9886
525.9775
564.9702
587.9655
614.9699
675.9413
272.9377
403.196
0
20
40
60
80
100
300
400
500
600

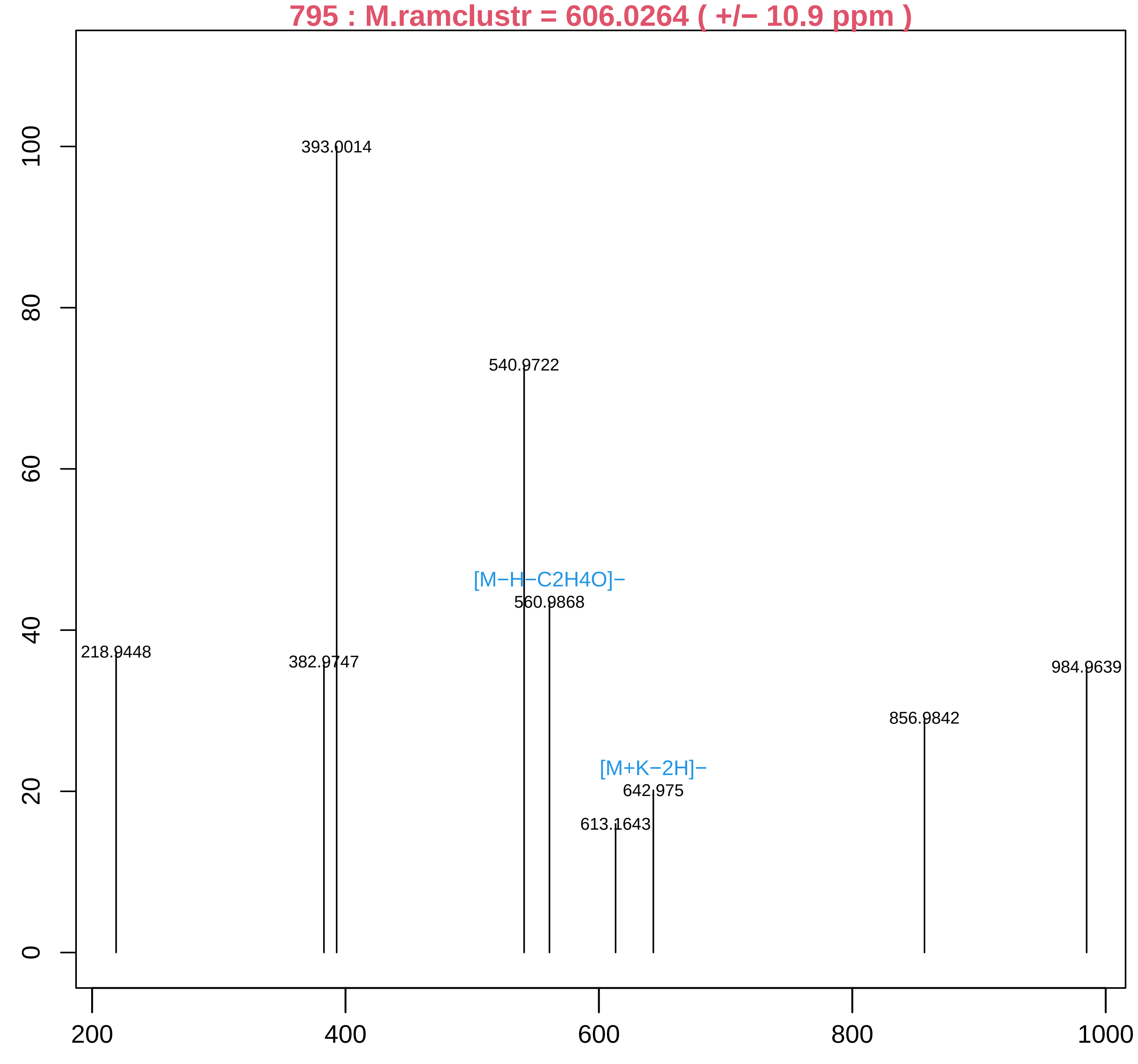

795 : M.ramclustr = 606.0264 (+/− 10.9 ppm)
393.0014
540.9722
[M−H−C2H4O]−
560.9868
218.9448
382.9747
984.9639
856.9842
[M+K−2H]−
642.975
613.1643
0
20
40
60
80
100
200
400
600
800
1000

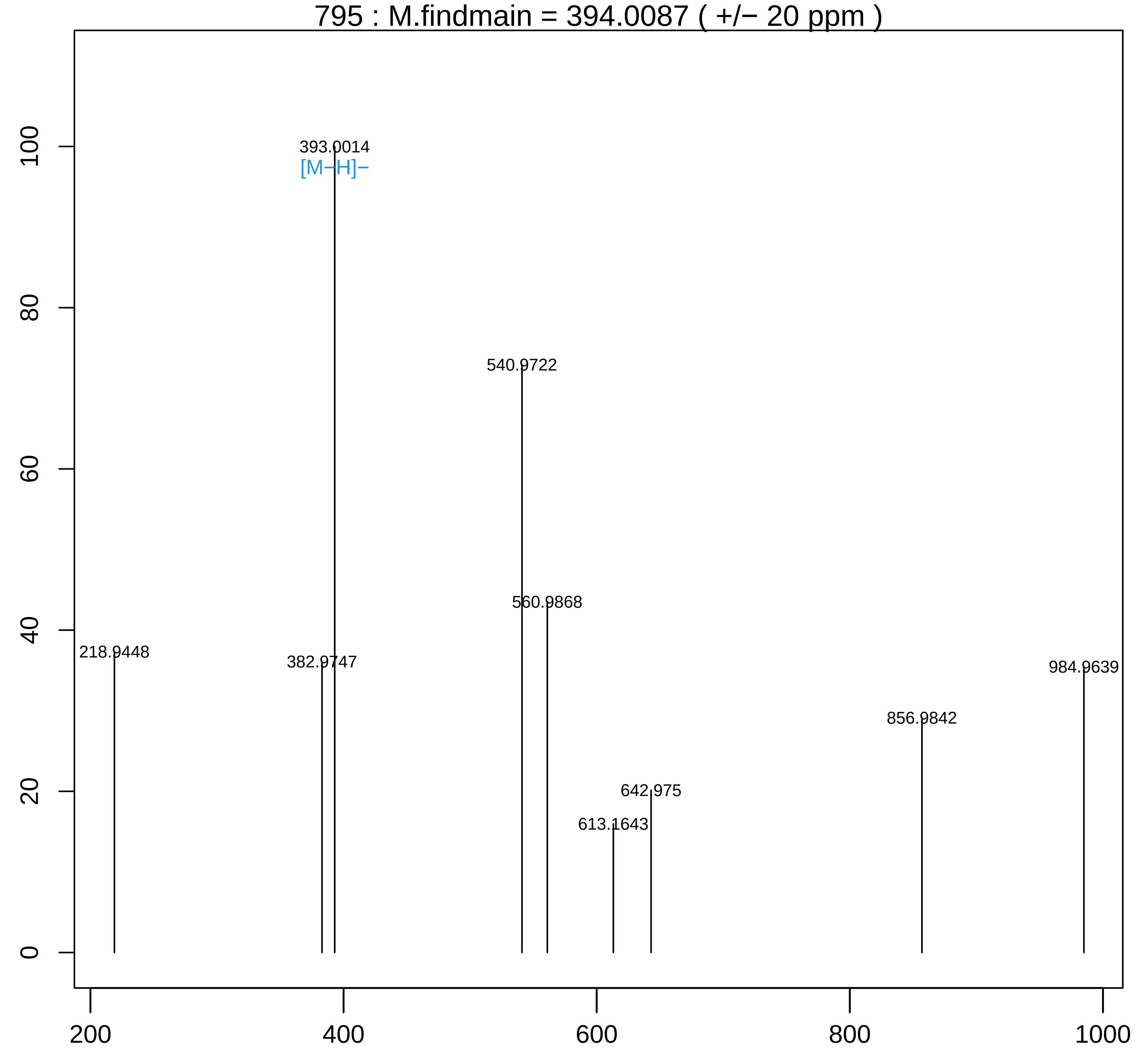

795 : M.findmain = 394.0087 (+/− 20 ppm)
393.0014
[M−H]−
540.9722
560.9868
218.9448
382.9747
984.9639
856.9842
642.975
613.1643
0
20
40
60
80
100
200
400
600
800
1000

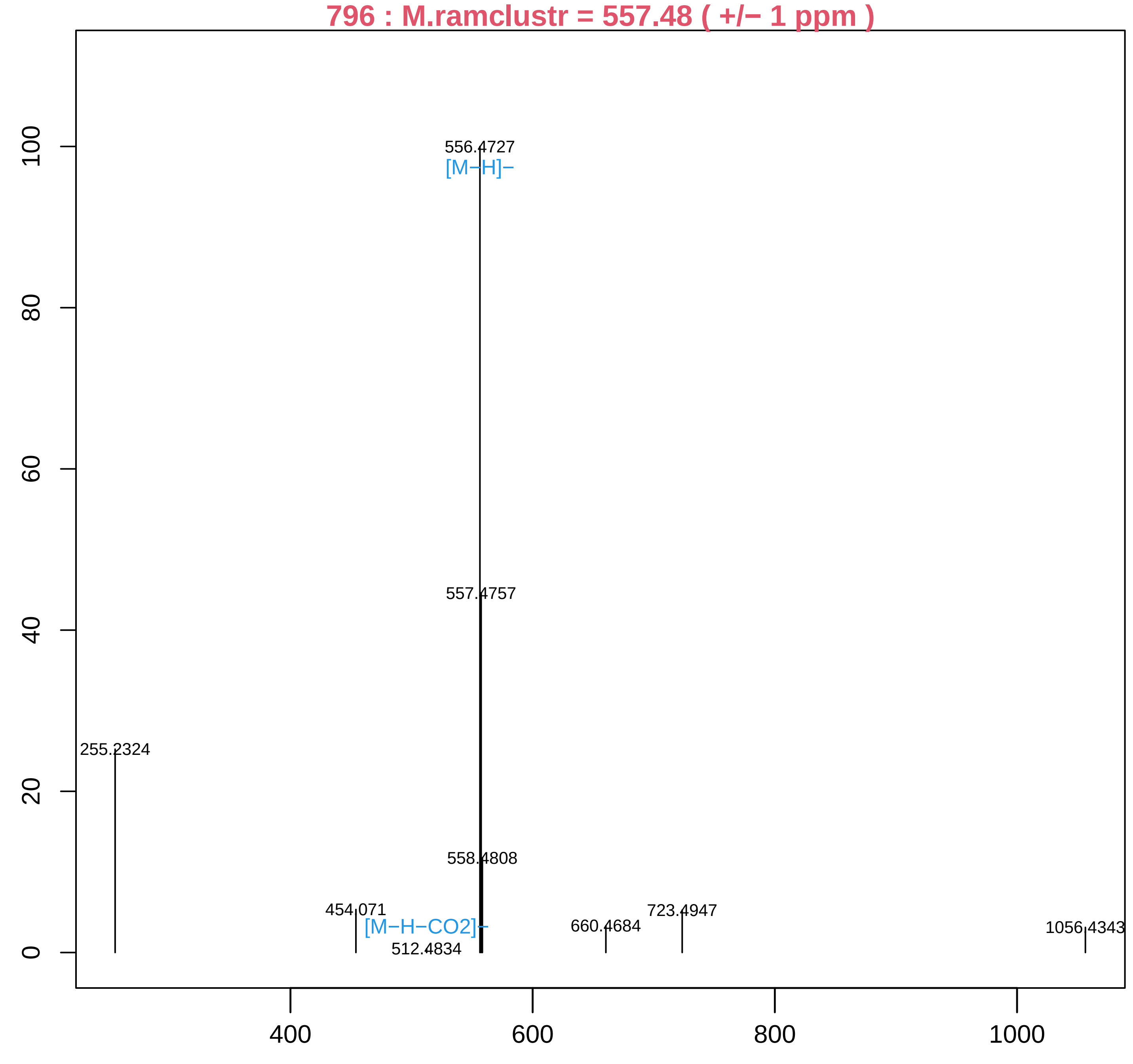
796 : M.ramclustr = 557.48 (+/− 1 ppm)
556.4727
[M−H]−
557.4757
255.2324
558.4808
454.071
[M−H−CO2]−
512.4834
660.4684
723.4947
1056.4343
0
20
40
60
80
100
400
600
800
1000
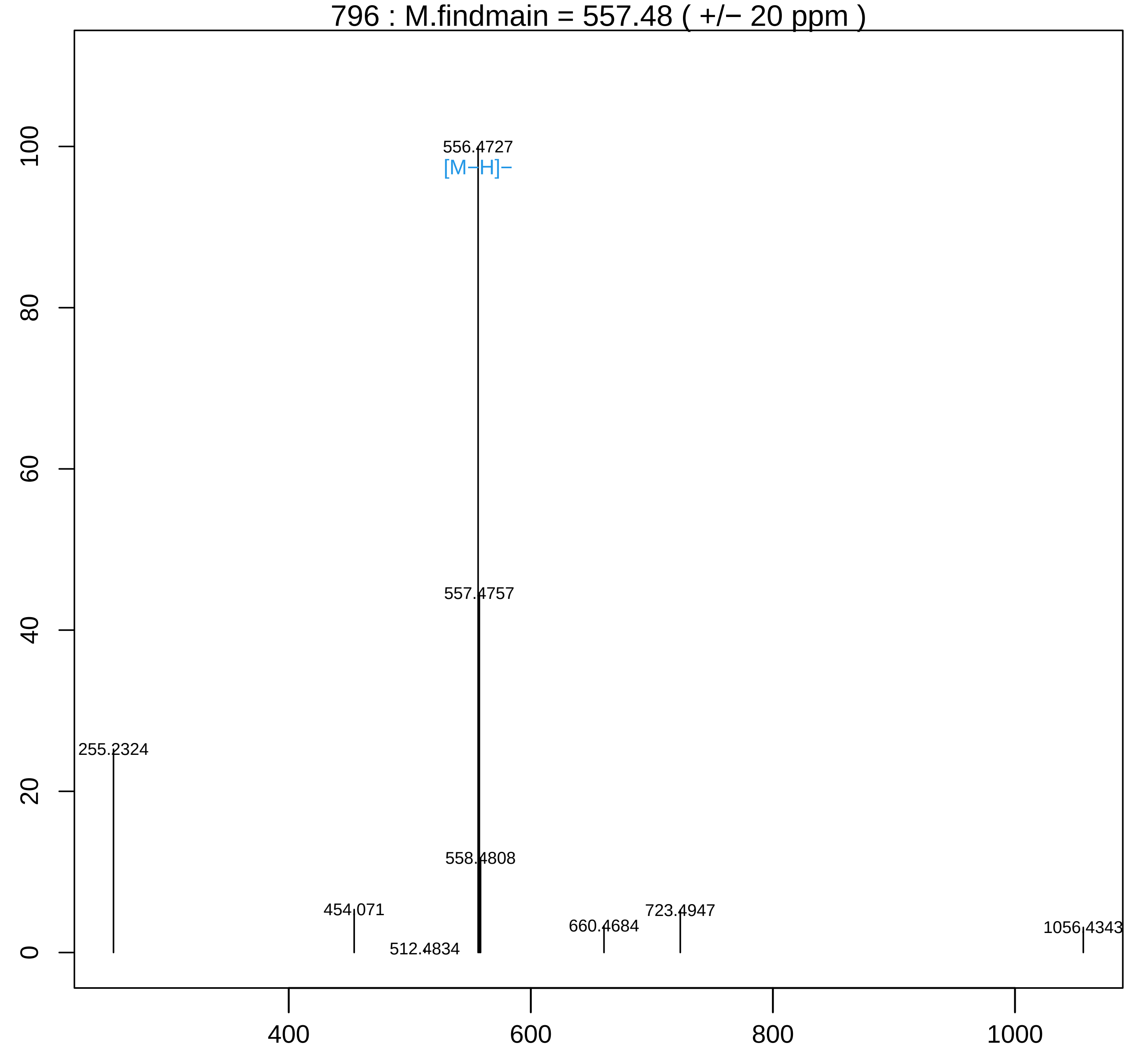
796 : M.findmain = 557.48 (+/− 20 ppm)
556.4727
[M−H]−
557.4757
255.2324
558.4808
454.071
512.4834
660.4684
723.4947
1056.4343
0
20
40
60
80
100
400
600
800
1000

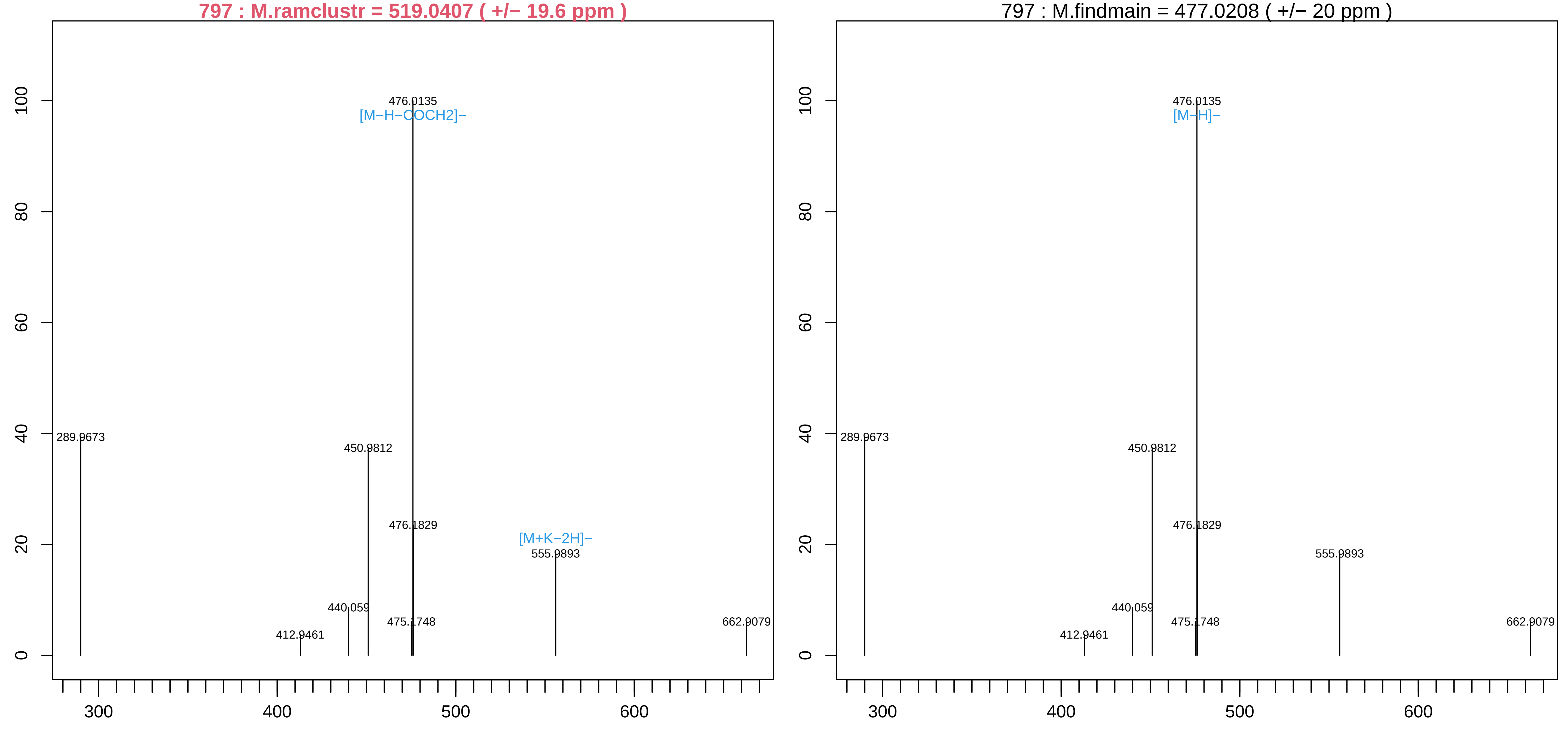

797 : M.ramclustr = 519.0407 (+/− 19.6 ppm)
476.0135
[M−H−COCH2]−
289.9673
450.9812
476.1829
[M+K−2H]−
555.9893
440.059
475.1748
662.9079
412.9461
797 : M.findmain = 477.0208 (+/− 20 ppm)
476.0135
[M−H]−
289.9673
450.9812
476.1829
555.9893
440.059
475.1748
662.9079
412.9461
0
20
40
60
80
100
300
400
500
600

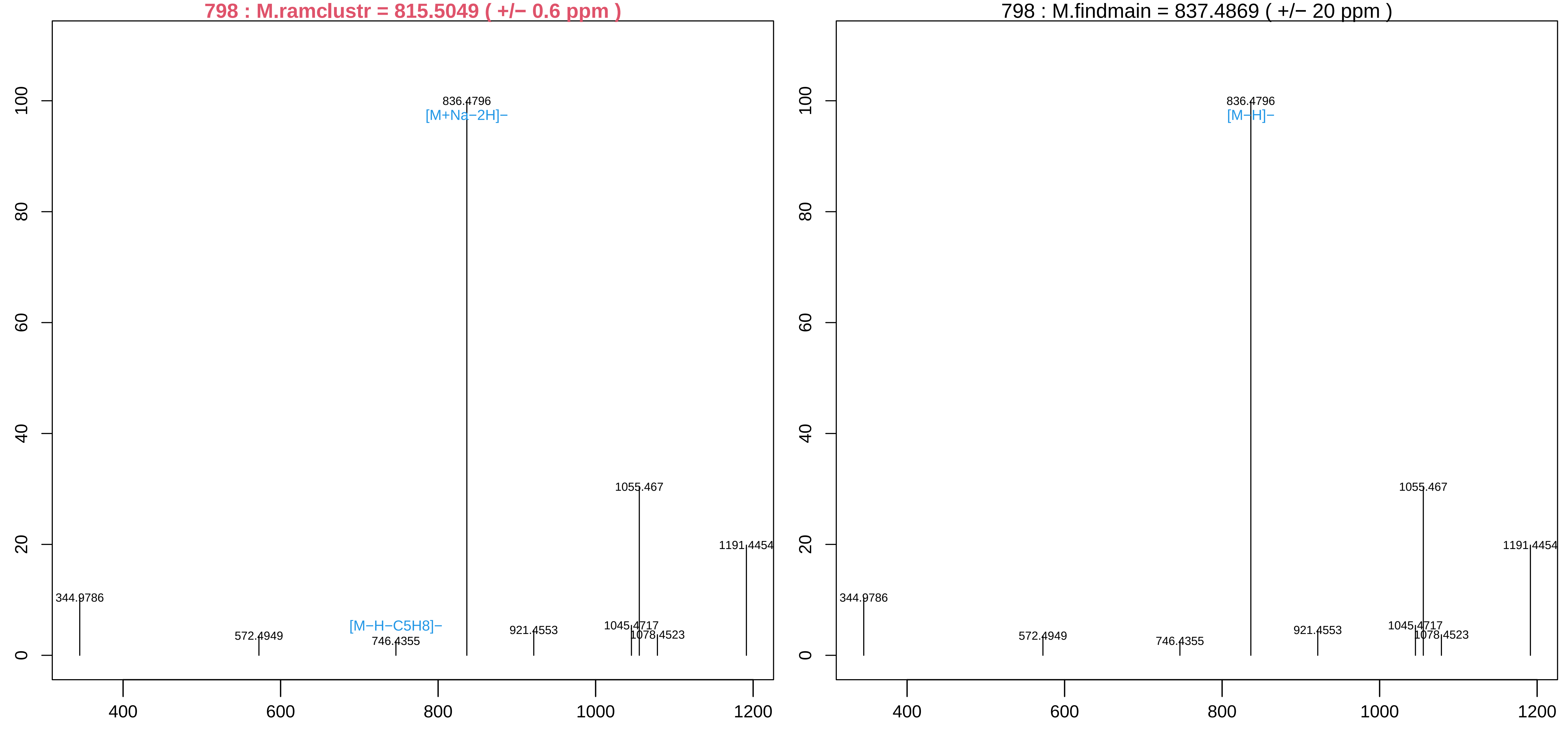
798 : M.ramclustr = 815.5049 (+/− 0.6 ppm)
836.4796
[M+Na−2H]−
1055.467
1191.4454
344.9786
[M−H−C5H8]−
572.4949
746.4355
921.4553
1045.4717
1078.4523
798 : M.findmain = 837.4869 (+/− 20 ppm)
836.4796
[M−H]−
1055.467
1191.4454
344.9786
572.4949
746.4355
921.4553
1045.4717
1078.4523
0
20
40
60
80
100
400
600
800
1000
1200

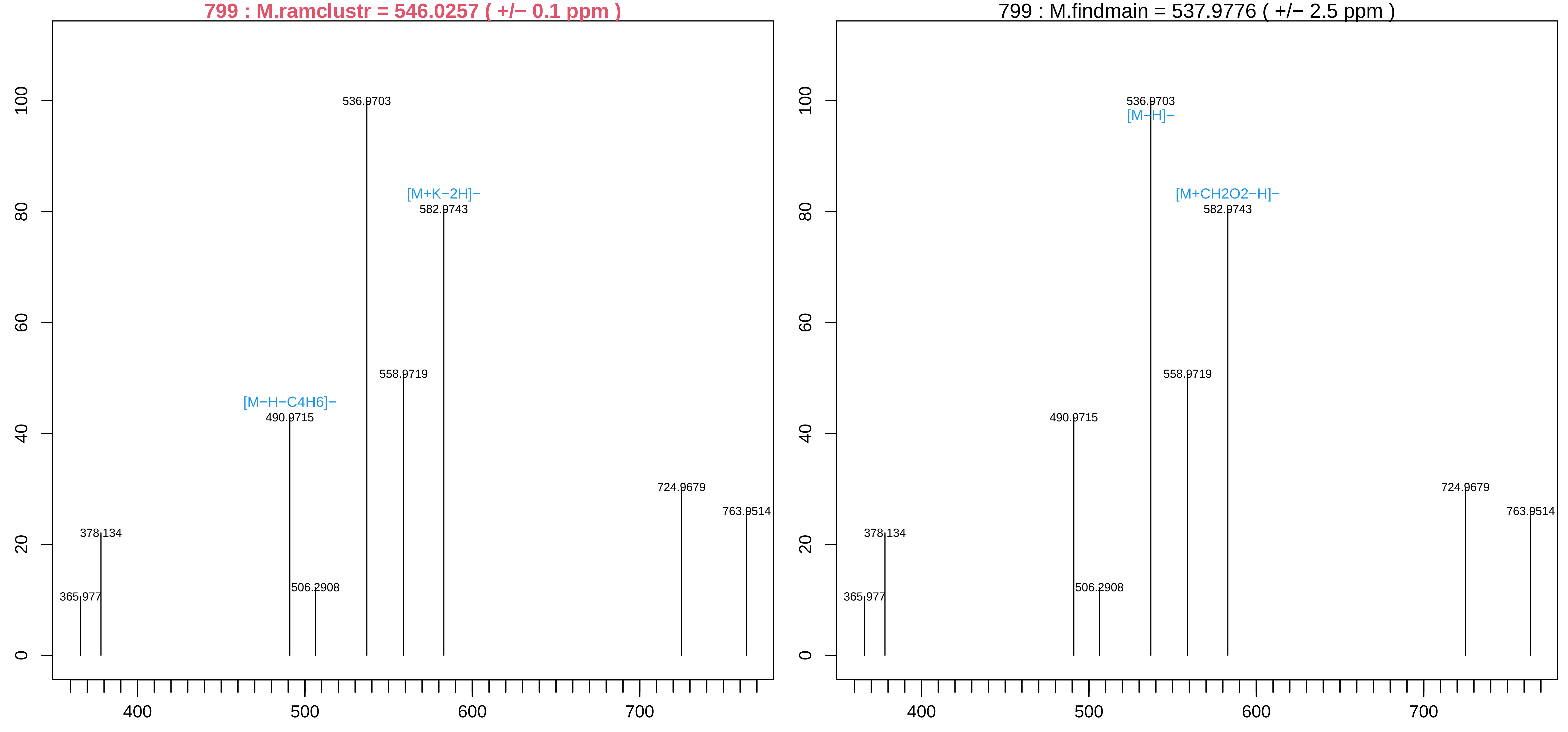
799 : M.ramclustr = 546.0257 (+/− 0.1 ppm)
536.9703
[M+K−2H]−
582.9743
558.9719
[M−H−C4H6]−
490.9715
724.9679
763.9514
378.134
365.977
506.2908
799 : M.findmain = 537.9776 (+/− 2.5 ppm)
[M−H]−
[M+CH2O2−H]−
0
20
40
60
80
100
400
500
600
700

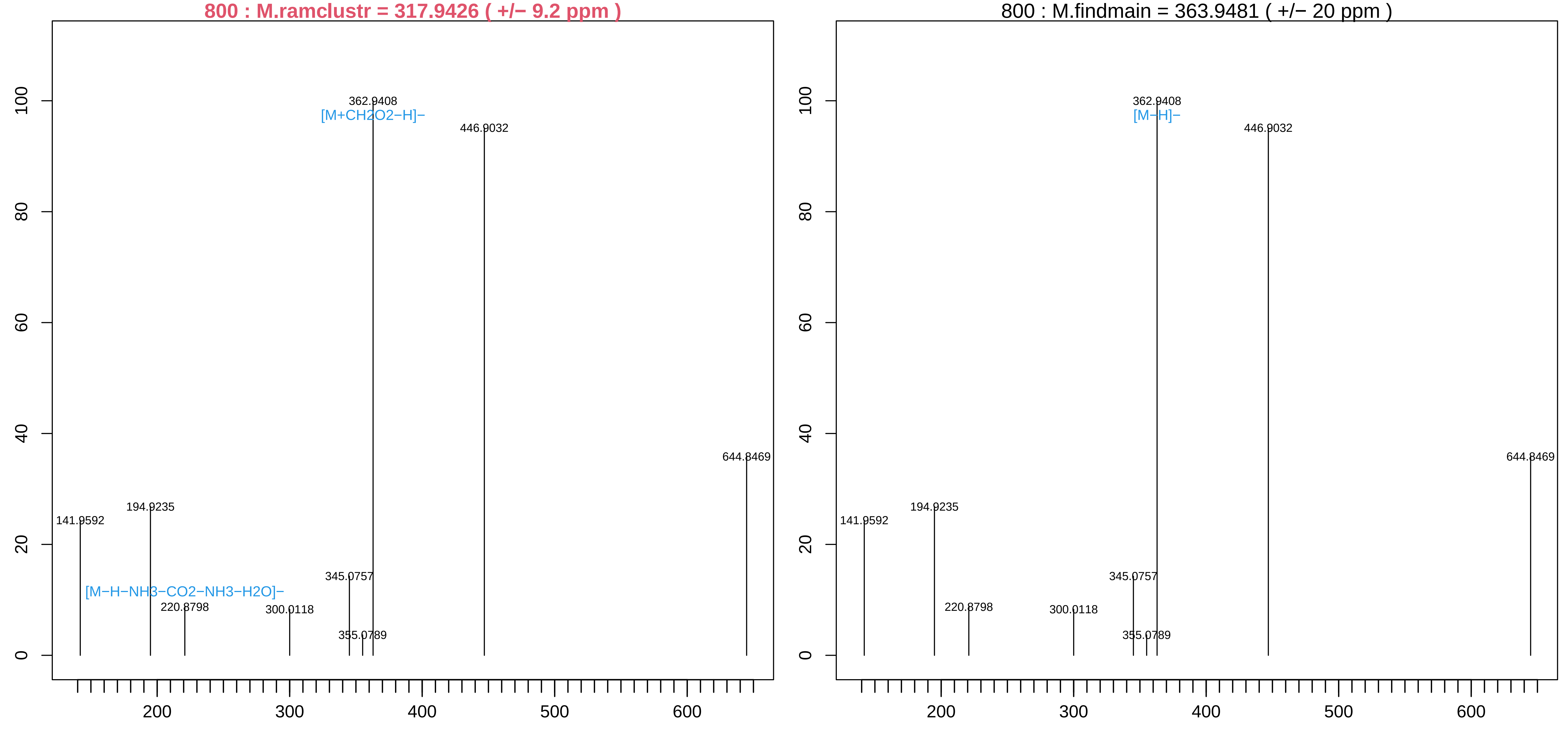
800 : M.ramclustr = 317.9426 (+/− 9.2 ppm)
362.9408
[M+CH2O2−H]−
446.9032
644.8469
141.9592
194.9235
[M−H−NH3−CO2−NH3−H2O]−
220.8798
300.0118
345.0757
355.0789
0
20
40
60
80
100
200
300
400
500
600
800 : M.findmain = 363.9481 (+/− 20 ppm)
362.9408
[M−H]−
446.9032
644.8469
141.9592
194.9235
220.8798
300.0118
345.0757
355.0789
0
20
40
60
80
100
200
300
400
500
600

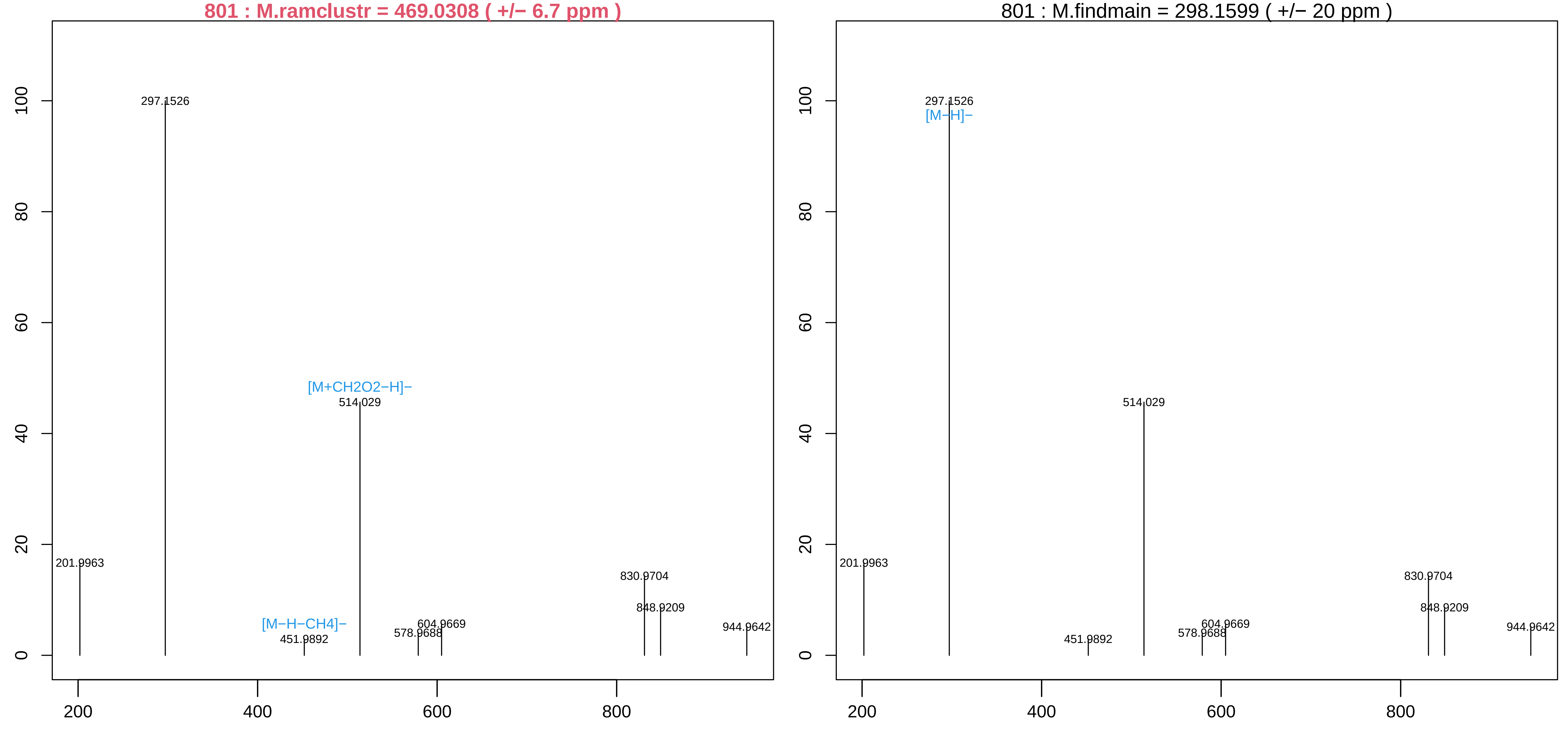
801 : M.ramclustr = 469.0308 (+/− 6.7 ppm)
297.1526
[M+CH2O2−H]−
514.029
201.9963
[M−H−CH4]−
451.9892
578.9688
604.9669
830.9704
848.9209
944.9642
801 : M.findmain = 298.1599 (+/− 20 ppm)
297.1526
[M−H]−
514.029
201.9963
451.9892
578.9688
604.9669
830.9704
848.9209
944.9642
0
20
40
60
80
100
200
400
600
800

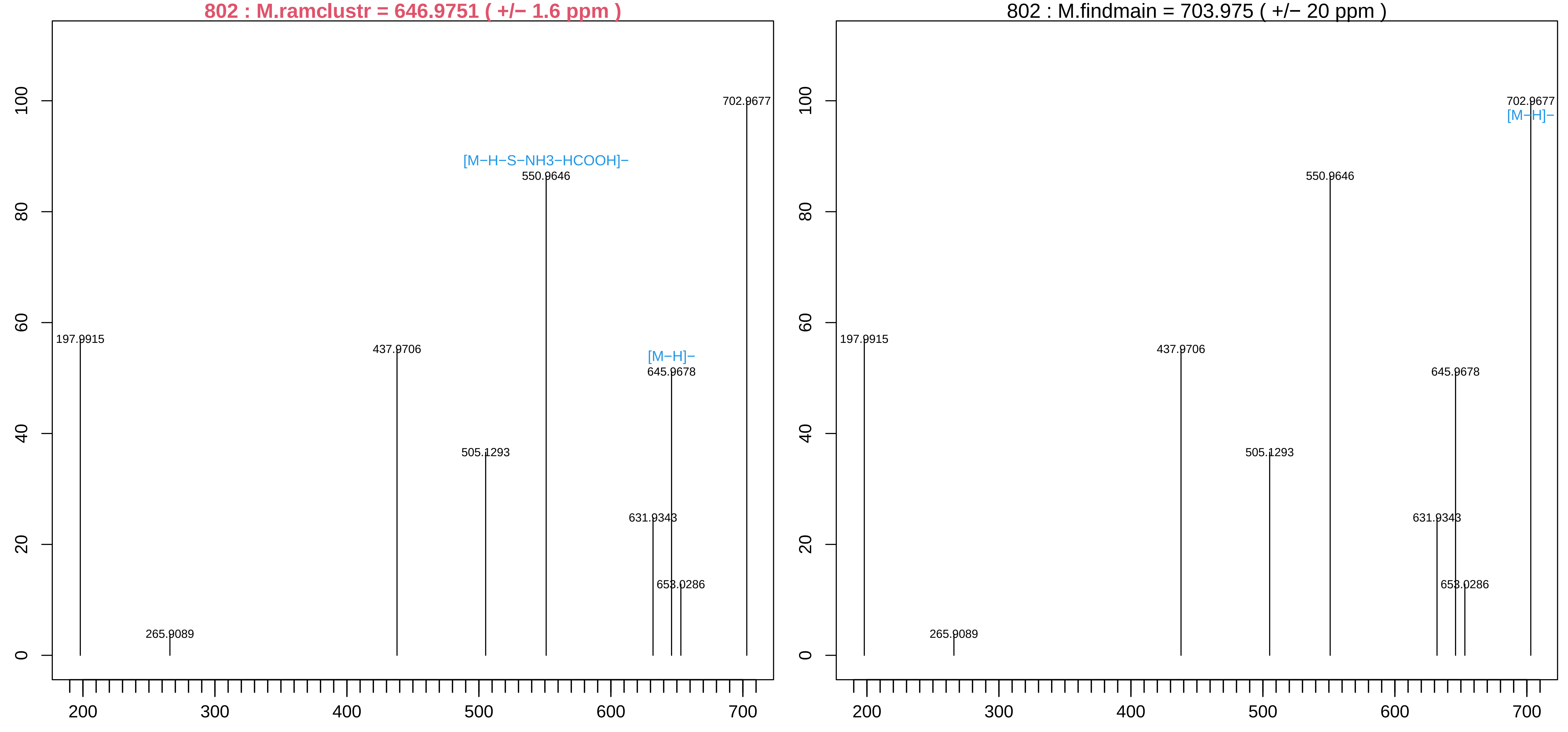
802 : M.ramclustr = 646.9751 (+/− 1.6 ppm)
[M−H−S−NH3−HCOOH]−
[M−H]−
197.9915
265.9089
437.9706
505.1293
550.9646
631.9343
645.9678
653.0286
702.9677
802 : M.findmain = 703.975 (+/− 20 ppm)
[M−H]−
197.9915
265.9089
437.9706
505.1293
550.9646
631.9343
645.9678
653.0286
702.9677

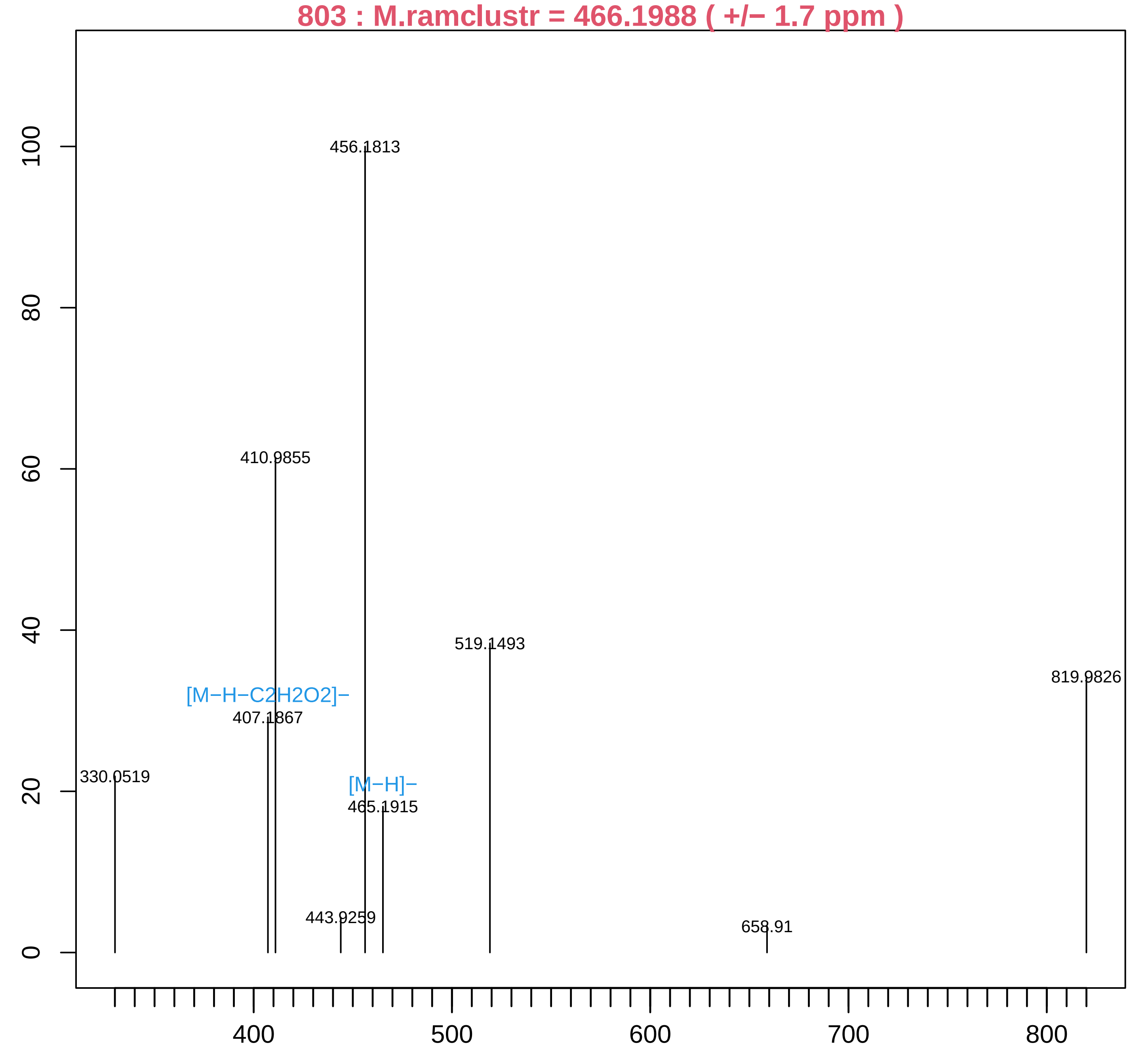

803 : M.ramclustr = 466.1988 (+/− 1.7 ppm)
456.1813
410.9855
519.1493
819.9826
[M−H−C2H2O2]−
407.1867
330.0519
[M−H]−
465.1915
443.9259
658.91
0
20
40
60
80
100
400
500
600
700
800

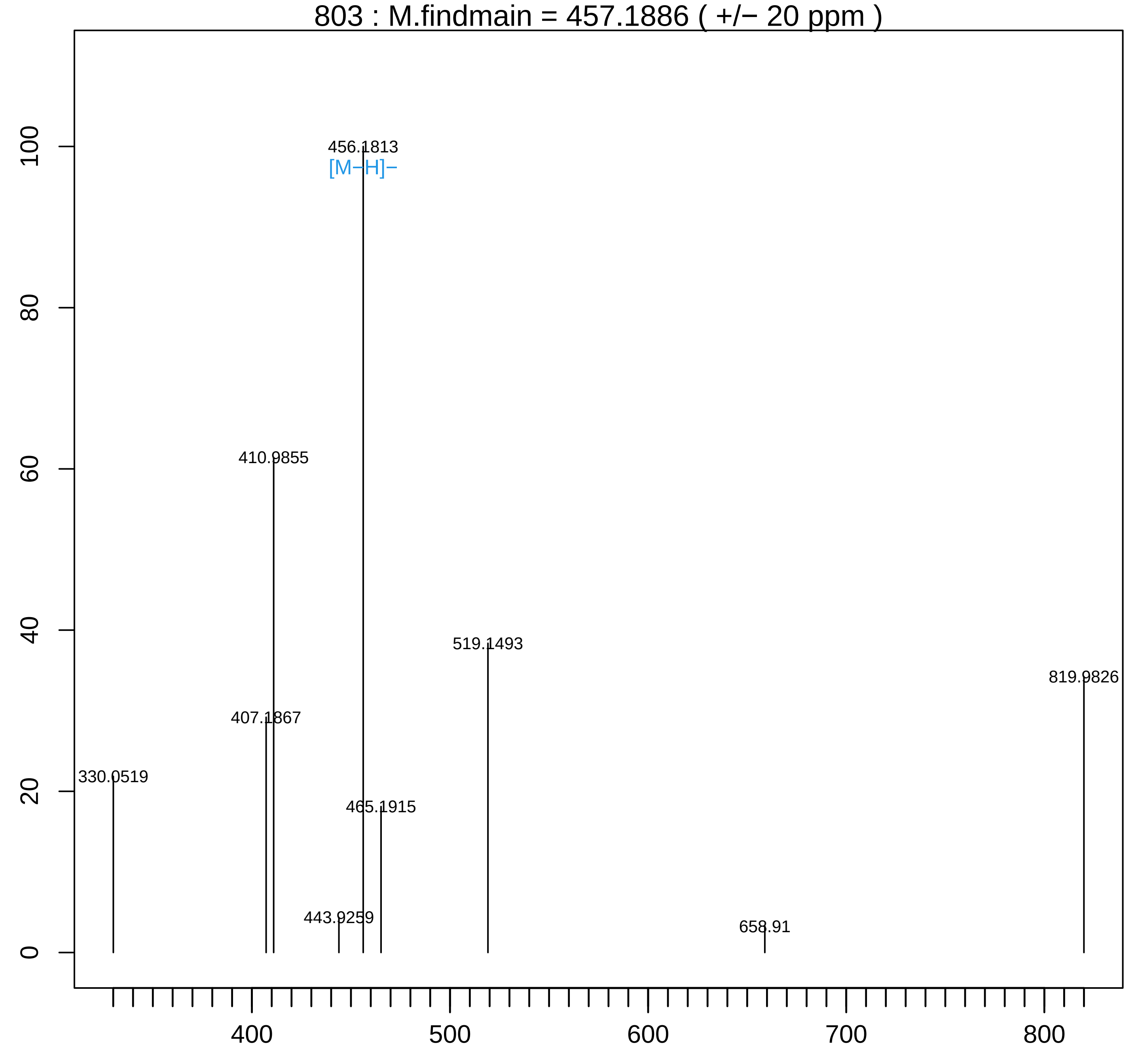

803 : M.findmain = 457.1886 (+/− 20 ppm)
456.1813
[M−H]−
410.9855
519.1493
819.9826
407.1867
330.0519
465.1915
443.9259
658.91
0
20
40
60
80
100
400
500
600
700
800

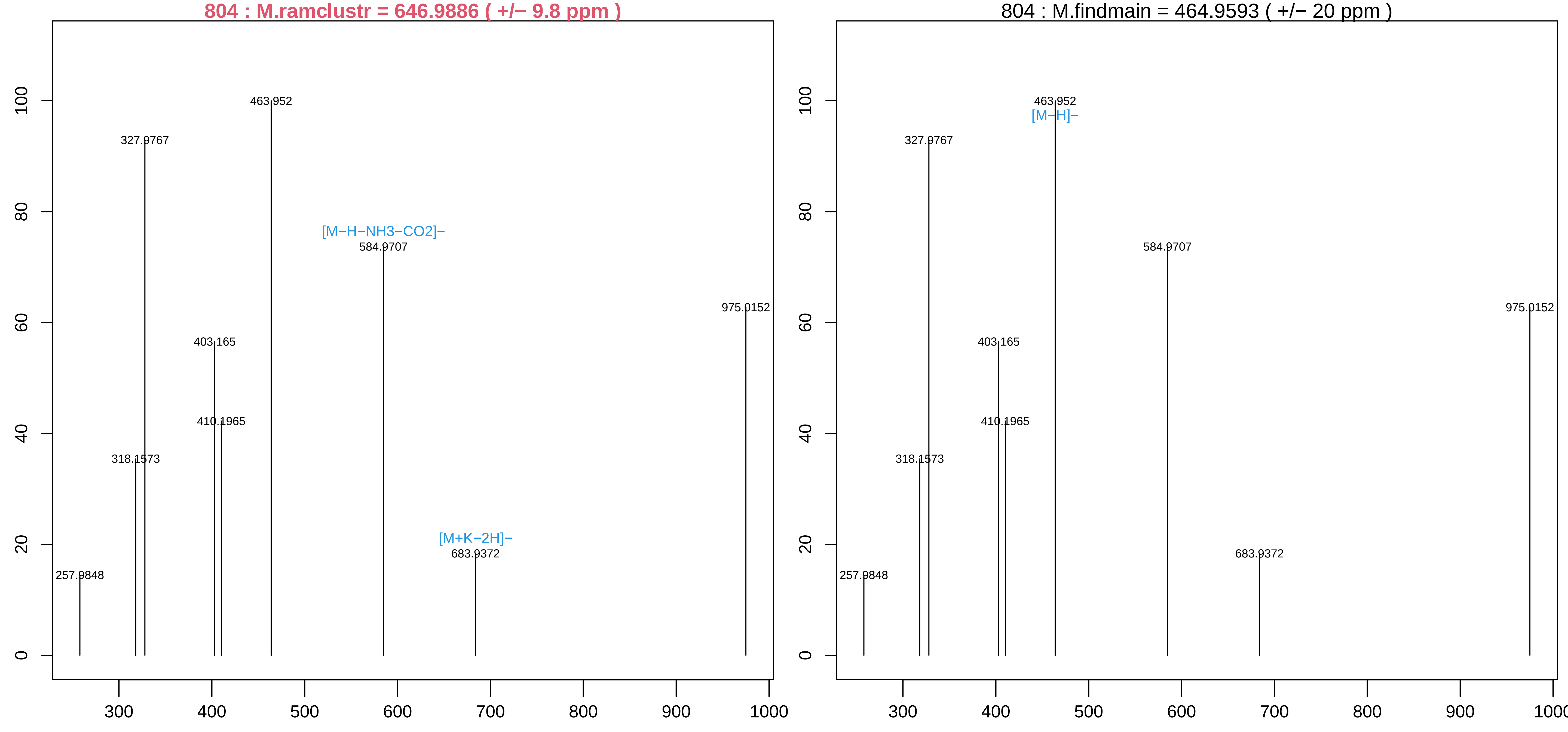
804 : M.ramclustr = 646.9886 (+/− 9.8 ppm)
463.952
327.9767
[M−H−NH3−CO2]−
584.9707
975.0152
403.165
410.1965
318.1573
[M+K−2H]−
683.9372
257.9848
0
20
40
60
80
100
300
400
500
600
700
800
900
1000
804 : M.findmain = 464.9593 (+/− 20 ppm)
463.952
[M−H]−
327.9767
584.9707
975.0152
403.165
410.1965
318.1573
683.9372
257.9848
0
20
40
60
80
100
300
400
500
600
700
800
900
1000

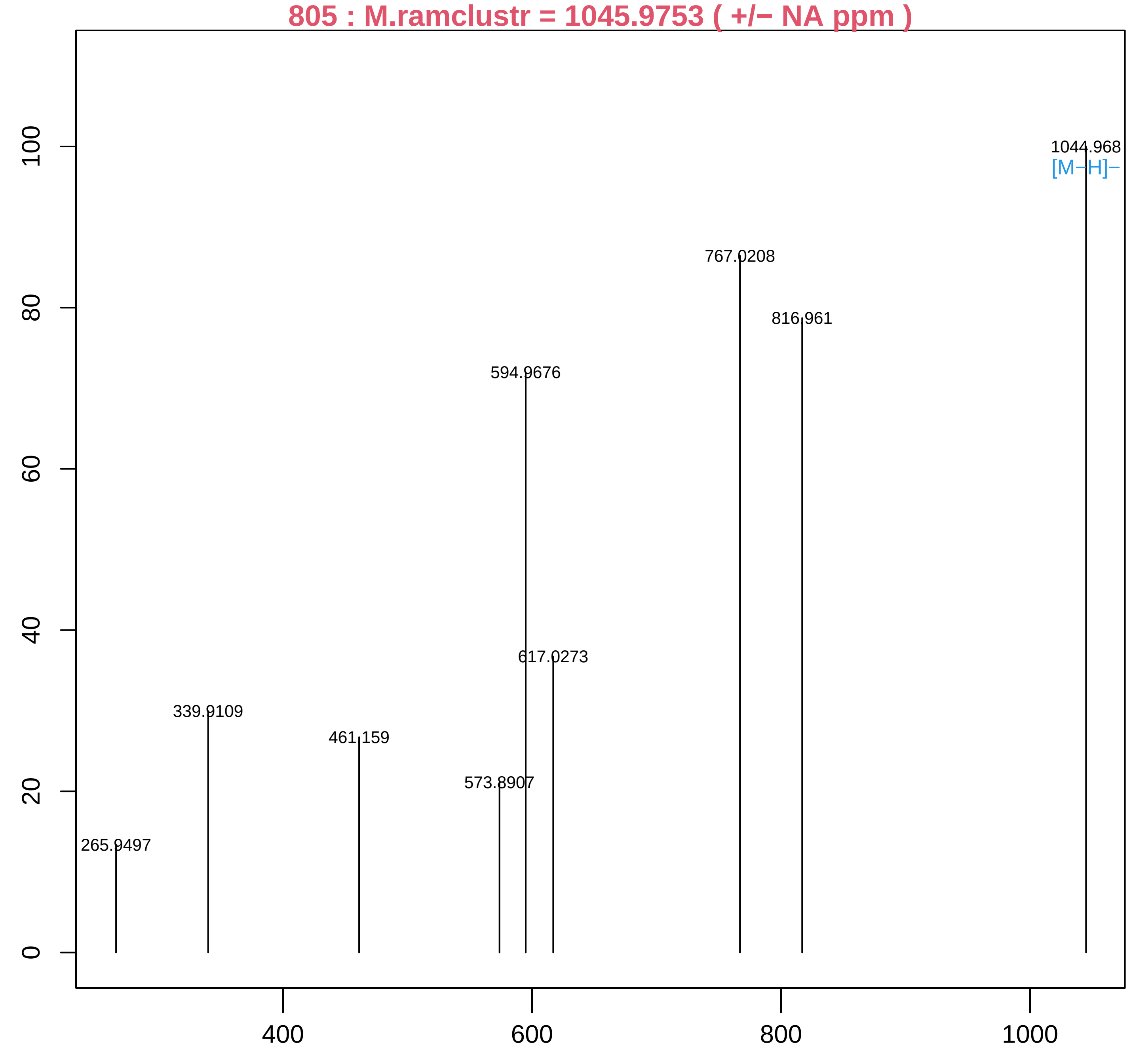
805 : M.ramclustr = 1045.9753 (+/− NA ppm)
1044.968
[M−H]−
767.0208
816.961
594.9676
617.0273
339.9109
461.159
573.8907
265.9497
0
20
40
60
80
100
400
600
800
1000

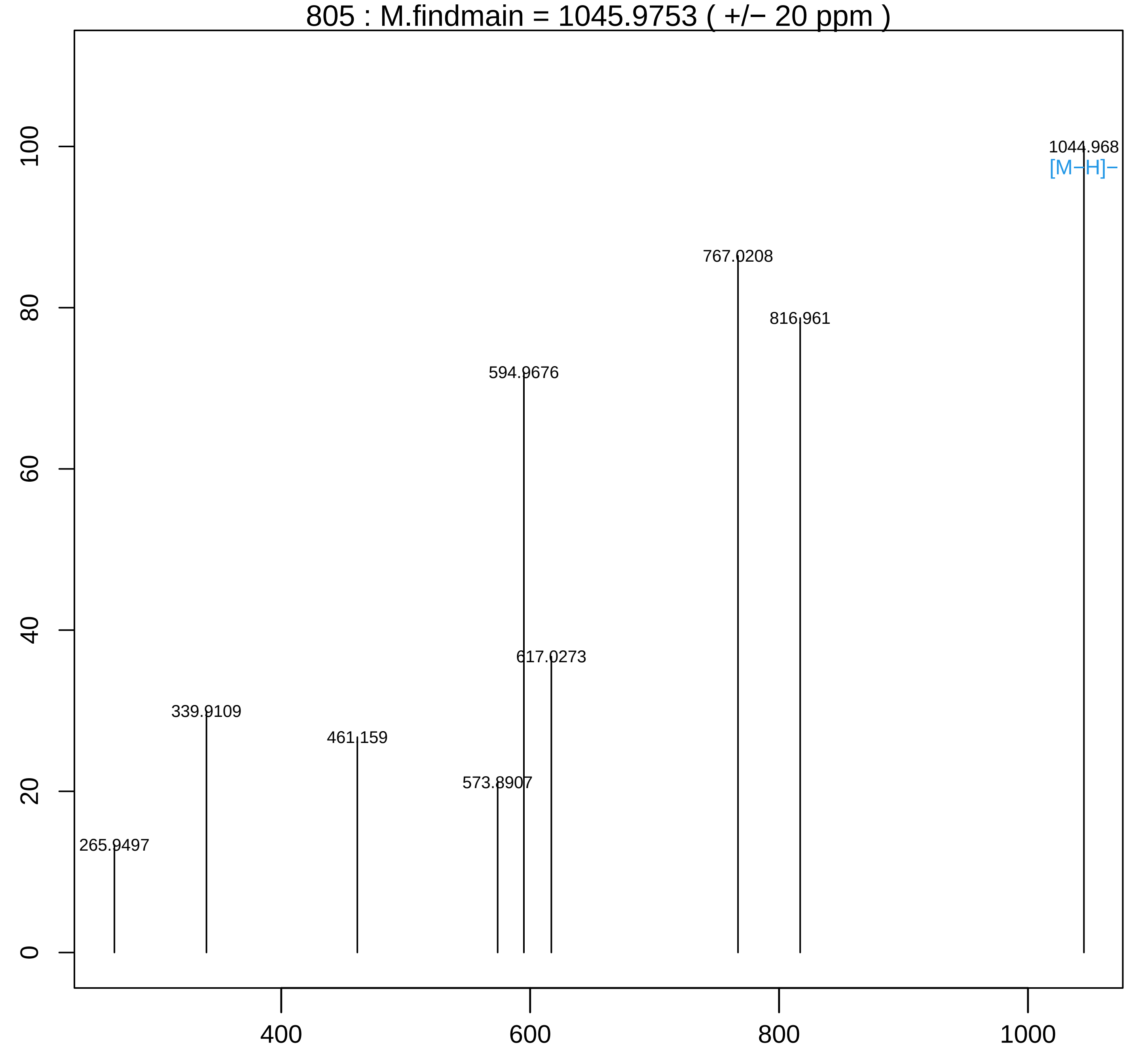
805 : M.findmain = 1045.9753 (+/− 20 ppm)
1044.968
[M−H]−
767.0208
816.961
594.9676
617.0273
339.9109
461.159
573.8907
265.9497
0
20
40
60
80
100
400
600
800
1000

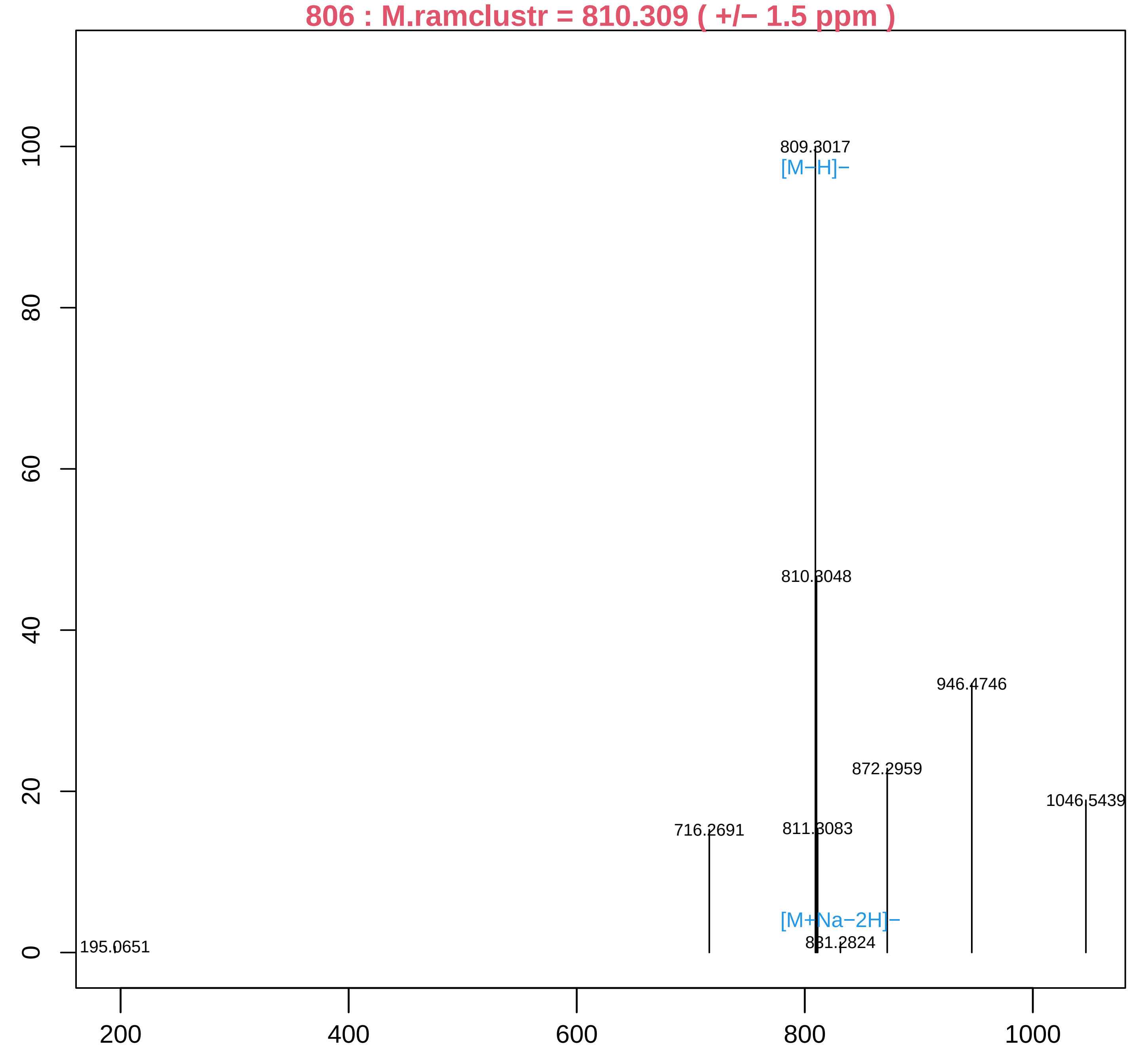

806 : M.ramclustr = 810.309 (+/− 1.5 ppm)
809.3017
[M−H]−
810.3048
946.4746
872.2959
1046.5439
716.2691
811.3083
[M+Na−2H]−
831.2824
195.0651
0
20
40
60
80
100
200
400
600
800
1000

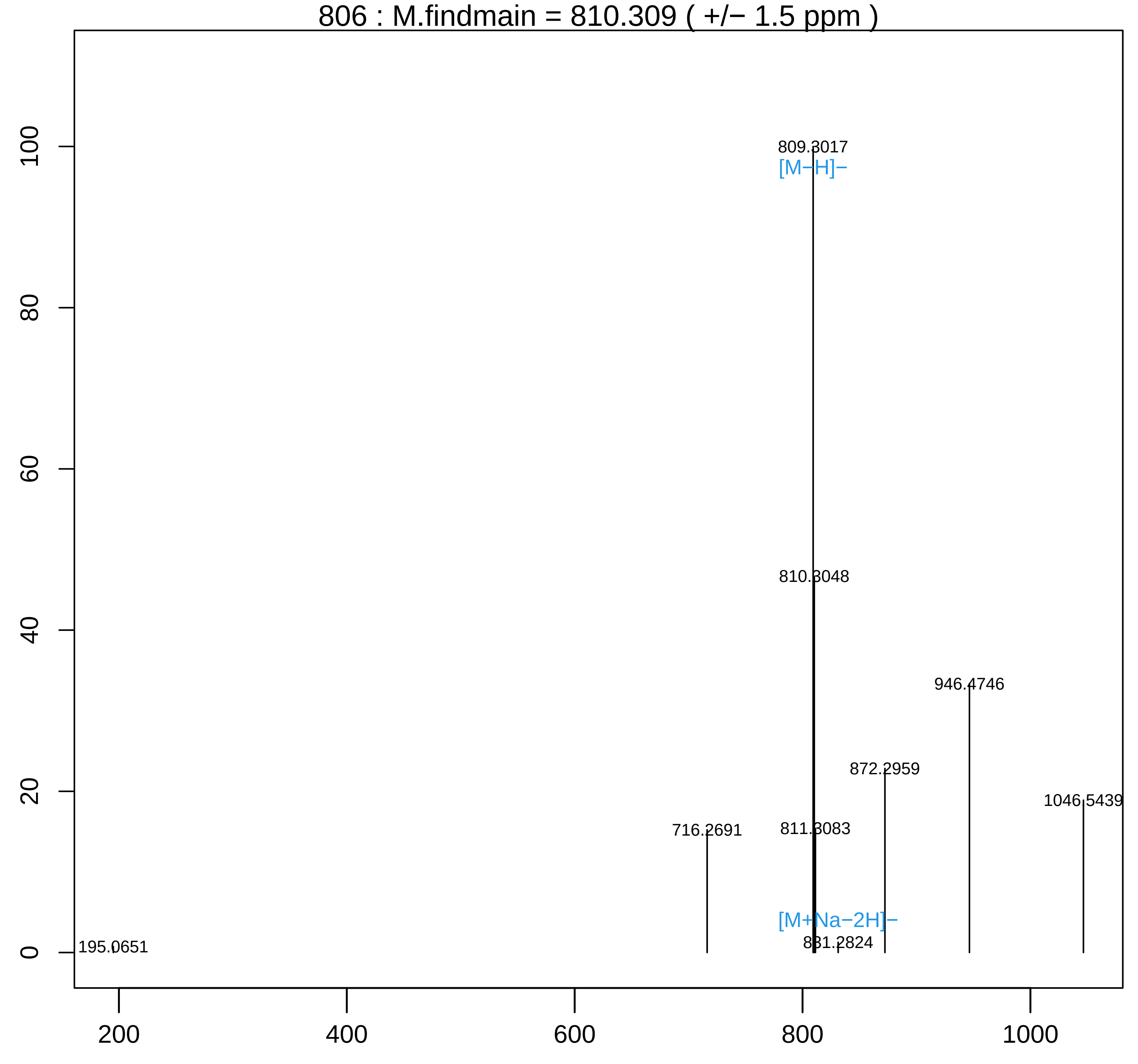

806 : M.findmain = 810.309 (+/− 1.5 ppm)
809.3017
[M−H]−
810.3048
946.4746
872.2959
1046.5439
716.2691
811.3083
[M+Na−2H]−
831.2824
195.0651
0
20
40
60
80
100
200
400
600
800
1000

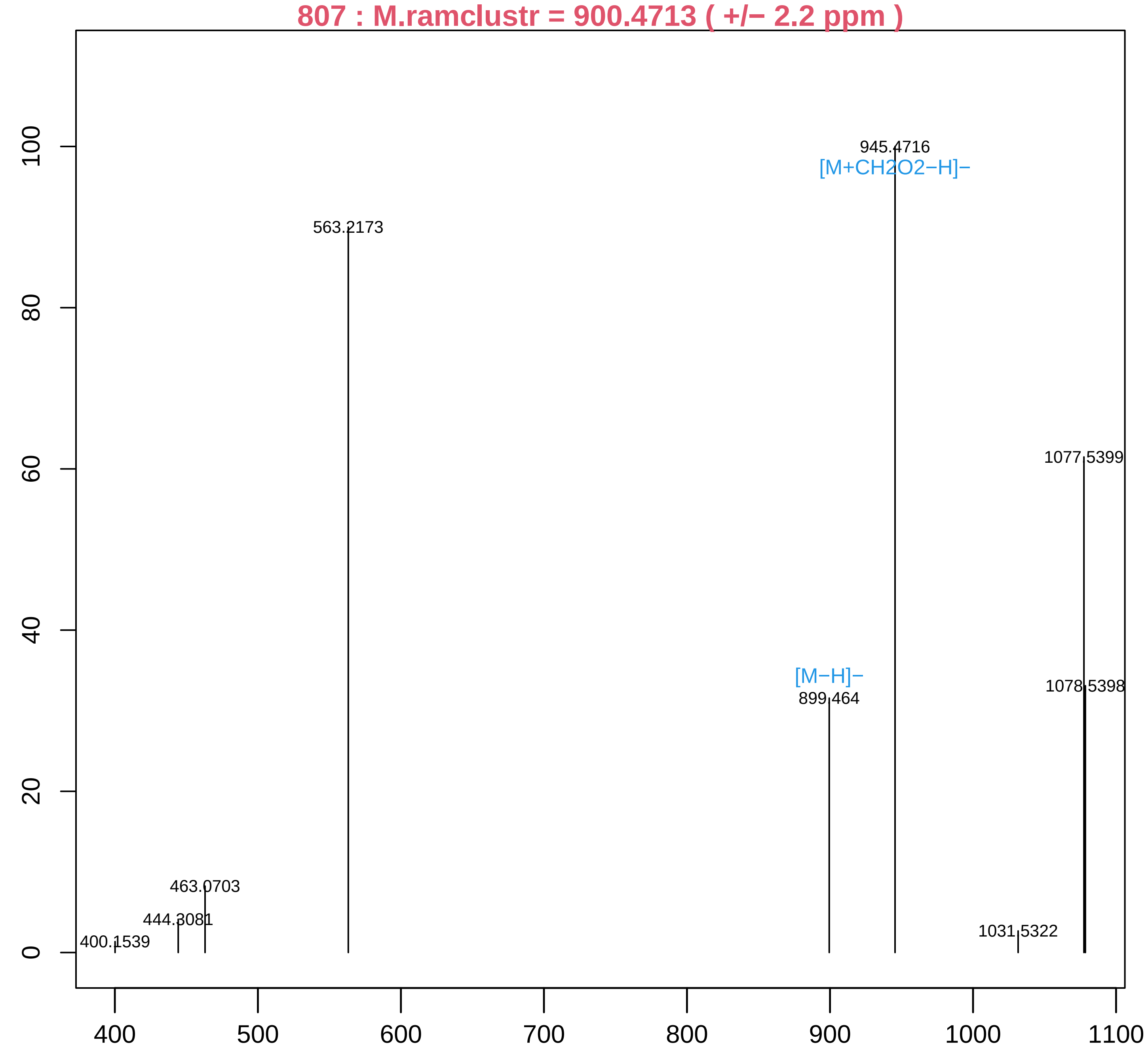
807 : M.ramclustr = 900.4713 (+/− 2.2 ppm)
945.4716
[M+CH2O2−H]−
563.2173
1077.5399
[M−H]−
899.464
1078.5398
463.0703
444.3081
400.1539
1031.5322
0
20
40
60
80
100
400
500
600
700
800
900
1000
1100

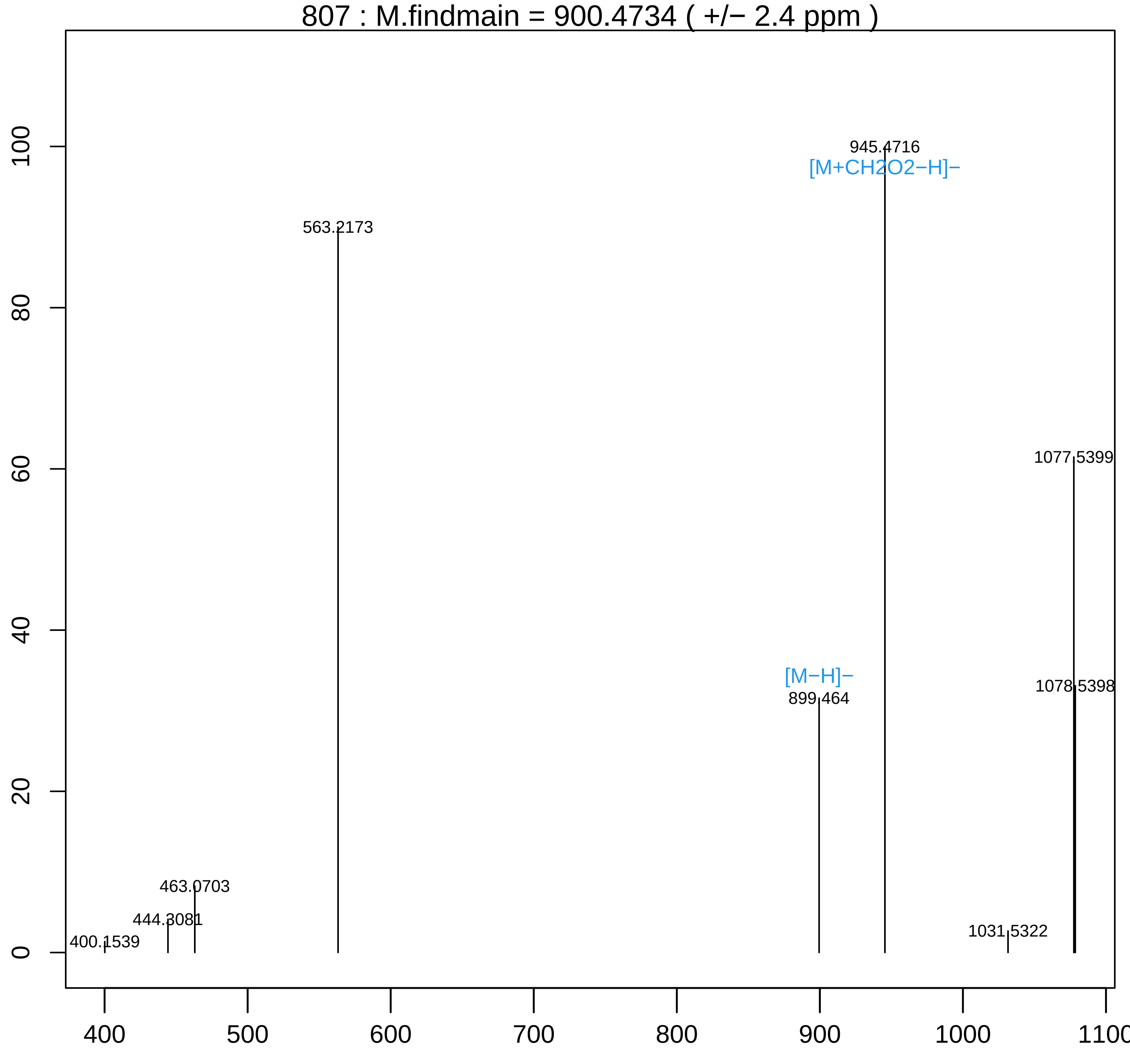
807 : M.findmain = 900.4734 (+/− 2.4 ppm)
945.4716
[M+CH2O2−H]−
563.2173
1077.5399
[M−H]−
899.464
1078.5398
463.0703
444.3081
400.1539
1031.5322
0
20
40
60
80
100
400
500
600
700
800
900
1000
1100

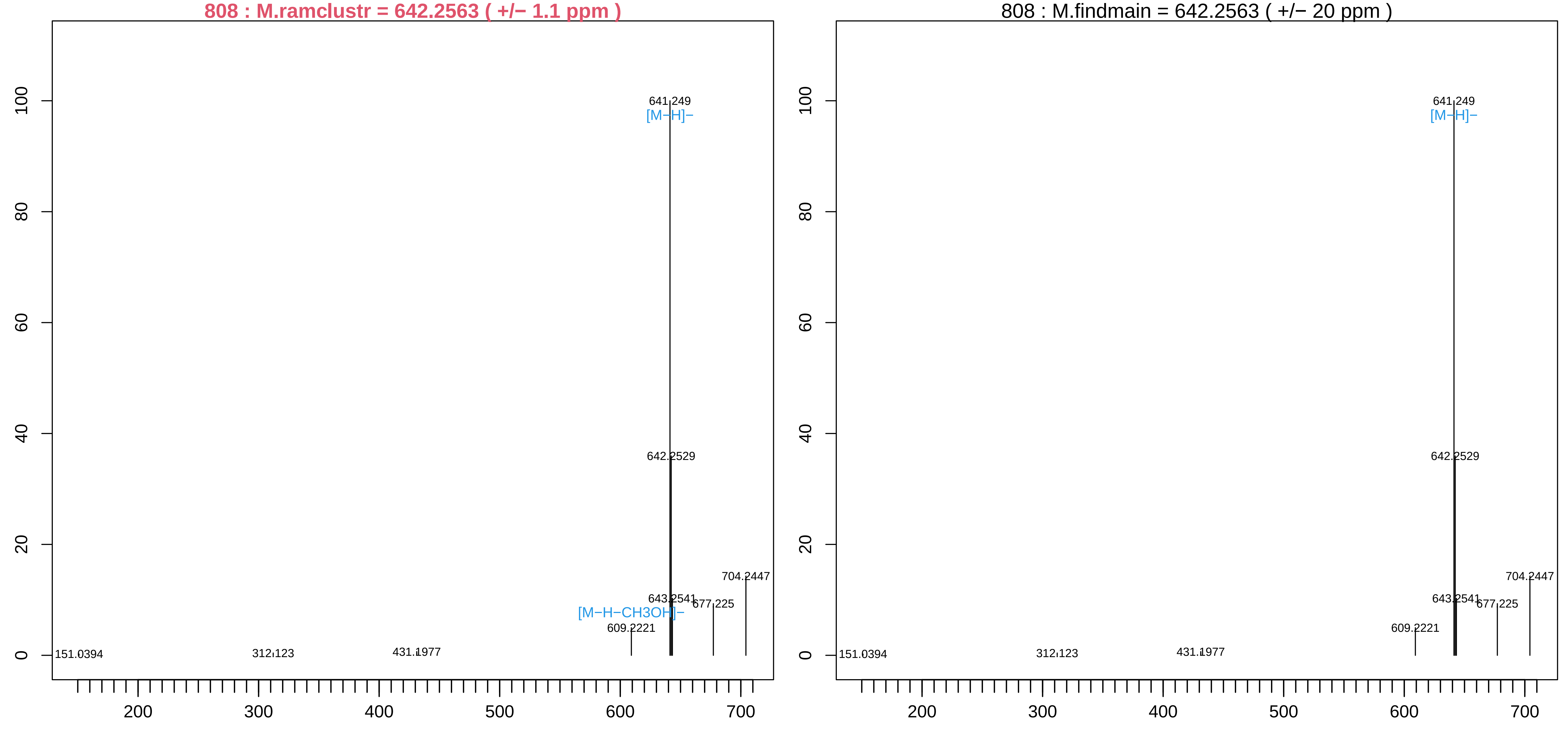
808 : M.ramclustr = 642.2563 (+/− 1.1 ppm)
808 : M.findmain = 642.2563 (+/− 20 ppm)
641.249
[M−H]−
642.2529
643.2541
677.225
704.2447
609.2221
[M−H−CH3OH]−
151.0394
312.123
431.1977
0
20
40
60
80
100
200
300
400
500
600
700

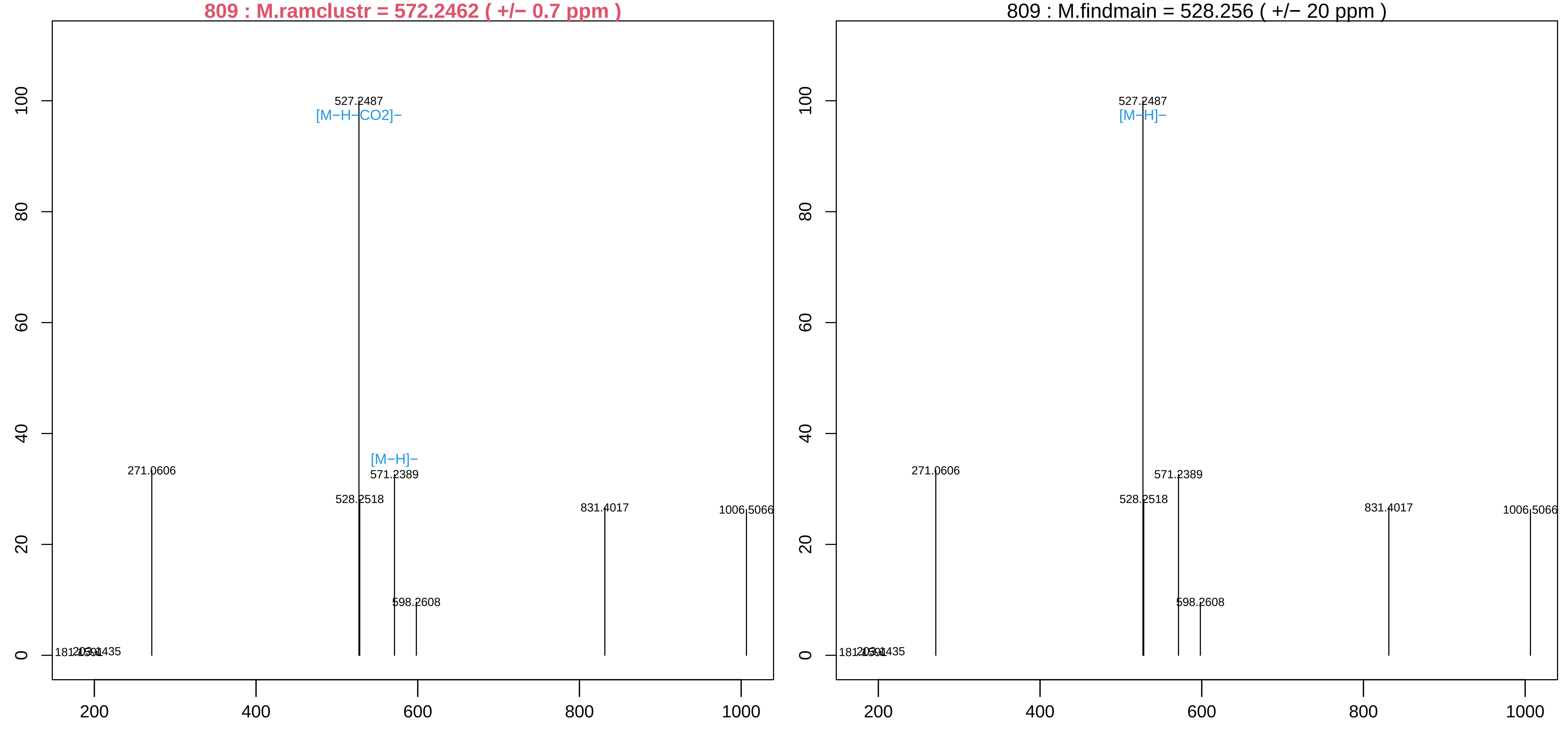
809 : M.ramclustr = 572.2462 (+/− 0.7 ppm)
527.2487
[M−H−CO2]−
[M−H]−
571.2389
271.0606
528.2518
831.4017
1006.5066
598.2608
0
20
40
60
80
100
200
400
600
800
1000
809 : M.findmain = 528.256 (+/− 20 ppm)
527.2487
[M−H]−
271.0606
571.2389
528.2518
831.4017
1006.5066
598.2608

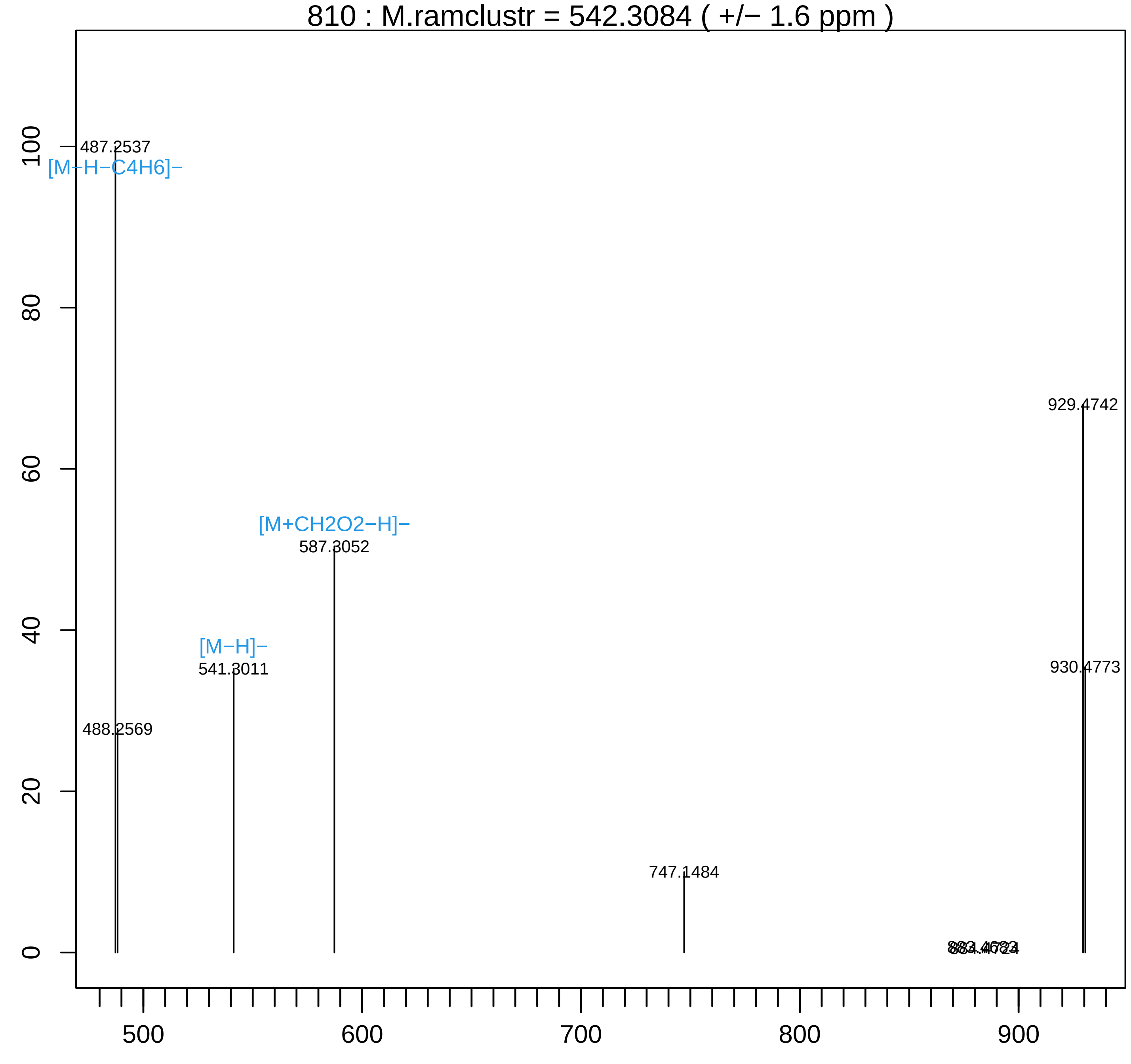

810 : M.ramclustr = 542.3084 (+/− 1.6 ppm)
487.2537
[M−H−C4H6]−
488.2569
[M−H]−
541.3011
[M+CH2O2−H]−
587.3052
747.1484
929.4742
930.4773
0
20
40
60
80
100
500
600
700
800
900

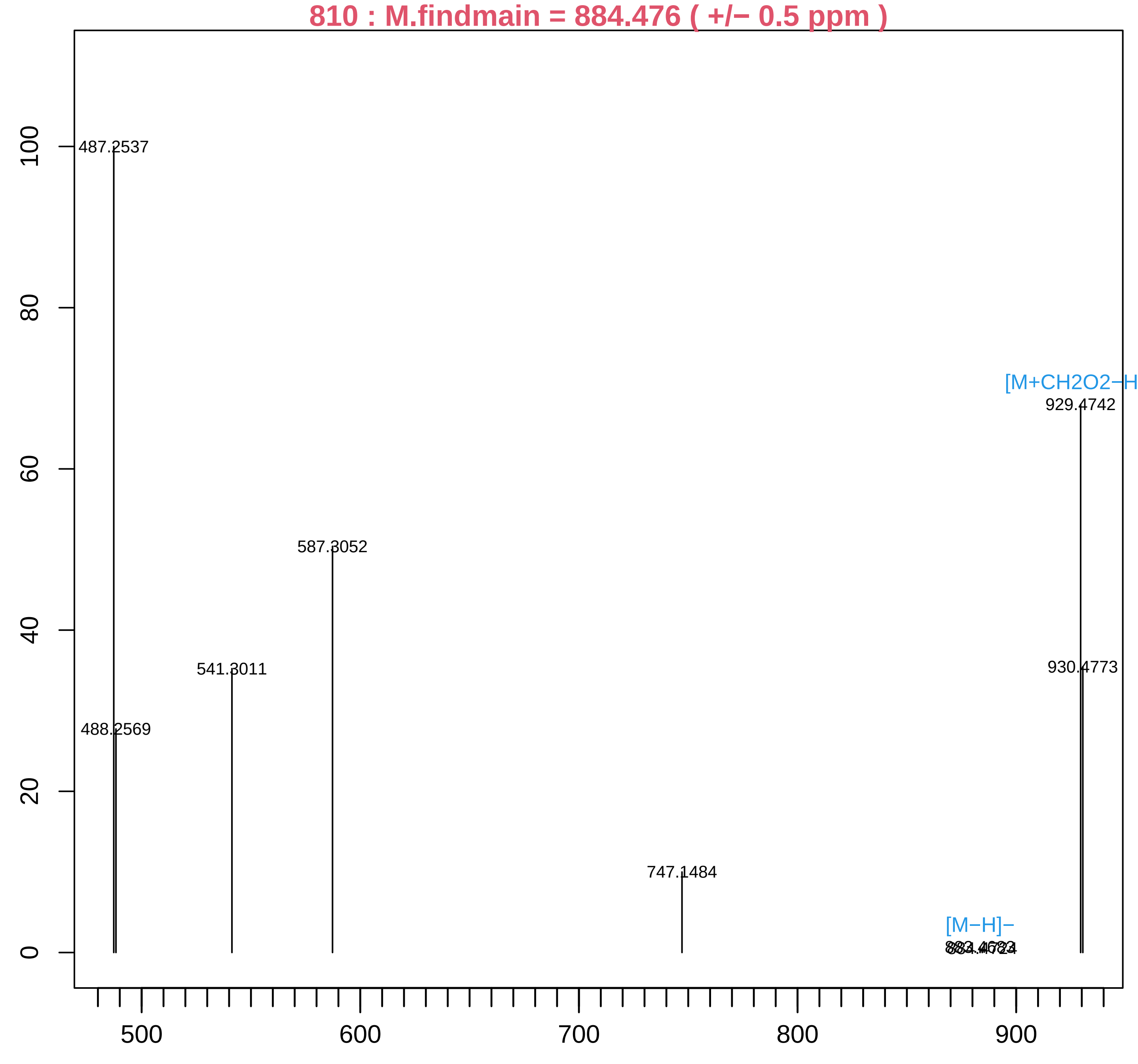

810 : M.findmain = 884.476 (+/− 0.5 ppm)
487.2537
488.2569
541.3011
587.3052
747.1484
[M+CH2O2−H
929.4742
930.4773
[M−H]−
0
20
40
60
80
100
500
600
700
800
900

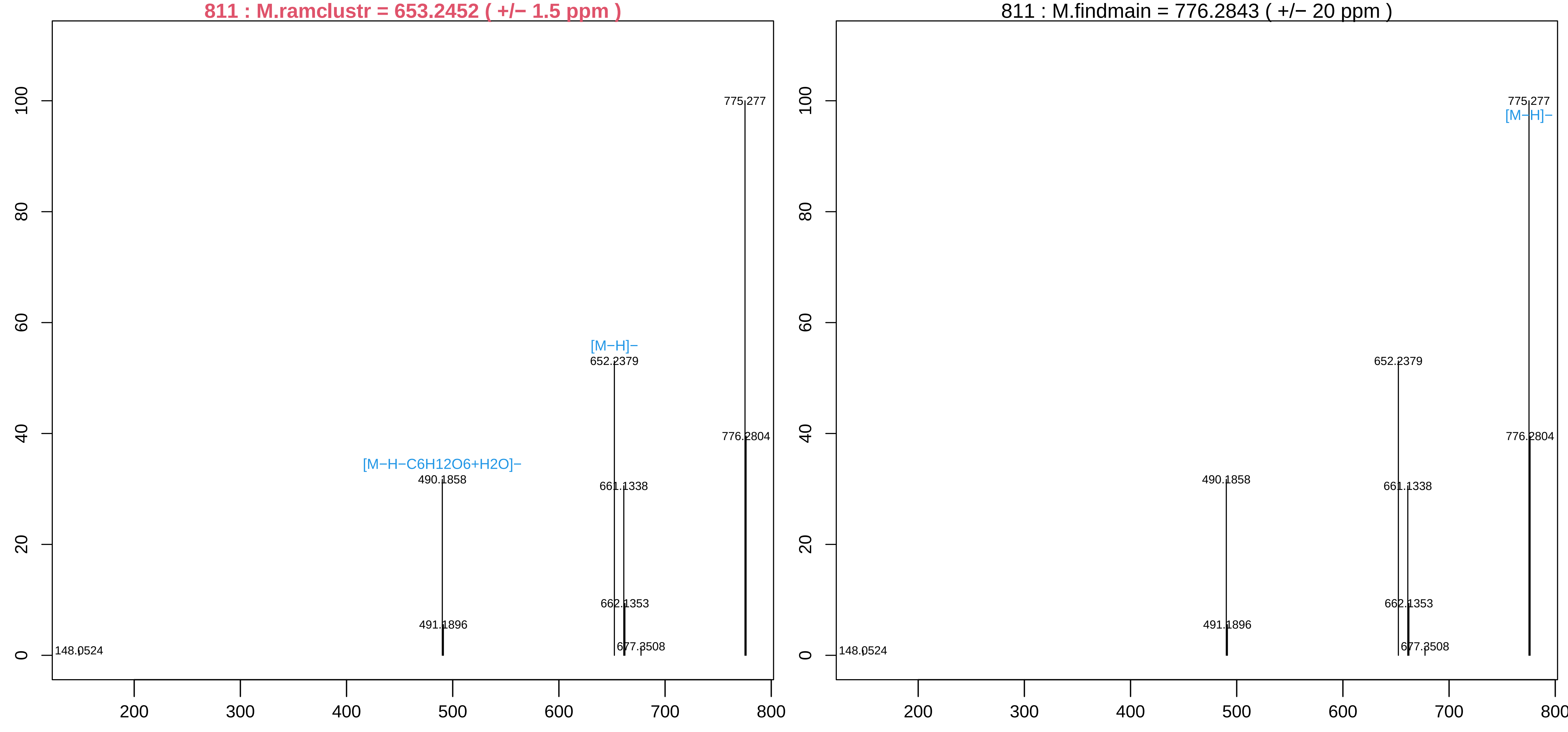

811 : M.ramclustr = 653.2452 (+/− 1.5 ppm)
[M−H]−
652.2379
775.277
776.2804
[M−H−C6H12O6+H2O]−
490.1858
661.1338
662.1353
491.1896
677.3508
148.0524
811 : M.findmain = 776.2843 (+/− 20 ppm)
775.277
[M−H]−
652.2379
776.2804
490.1858
661.1338
662.1353
491.1896
677.3508
148.0524
0
20
40
60
80
100
200
300
400
500
600
700
800

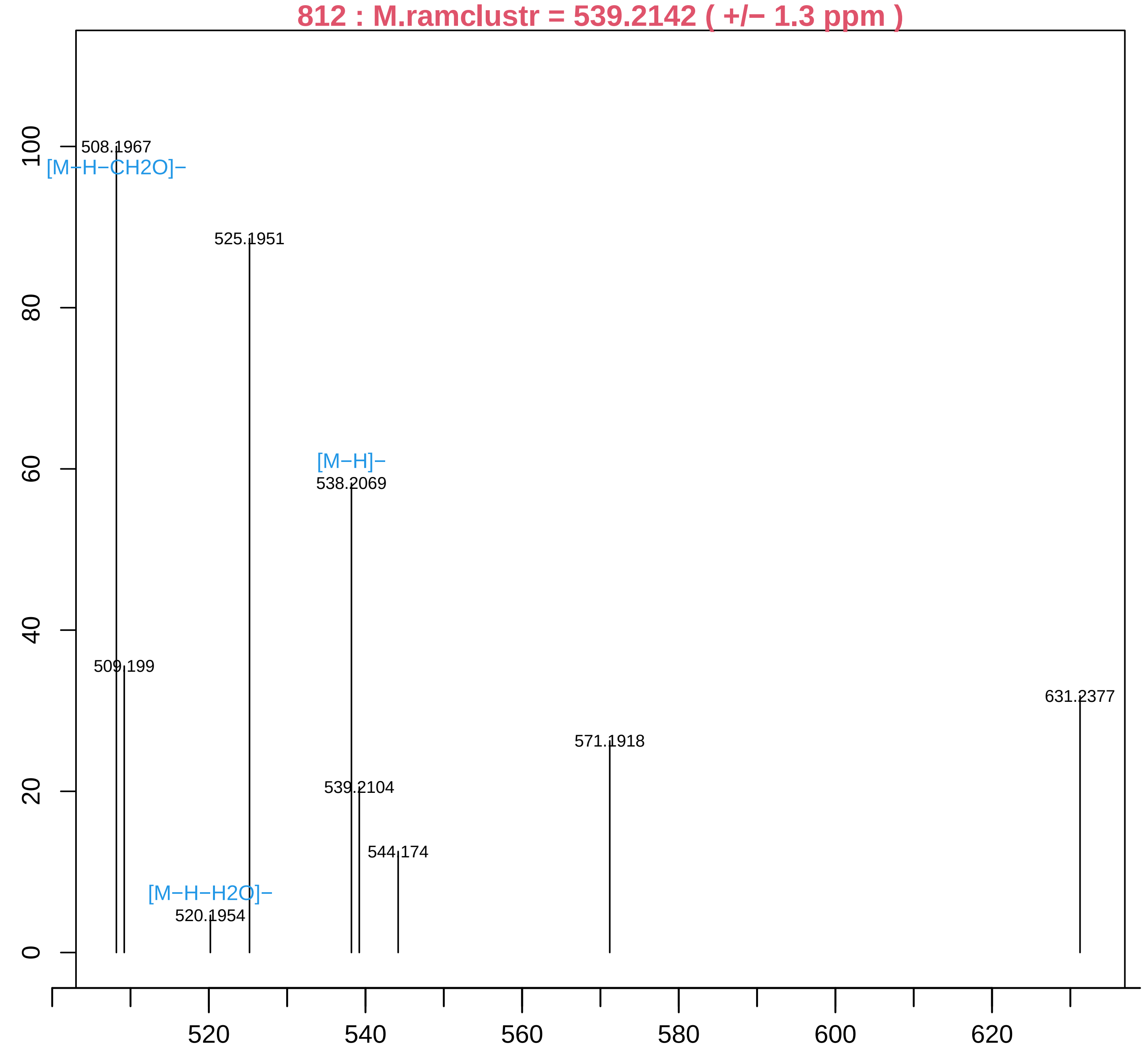

812 : M.ramclustr = 539.2142 (+/− 1.3 ppm)
508.1967
[M−H−CH2O]−
525.1951
[M−H]−
538.2069
509.199
631.2377
571.1918
539.2104
544.174
[M−H−H2O]−
520.1954
0
20
40
60
80
100
520
540
560
580
600
620

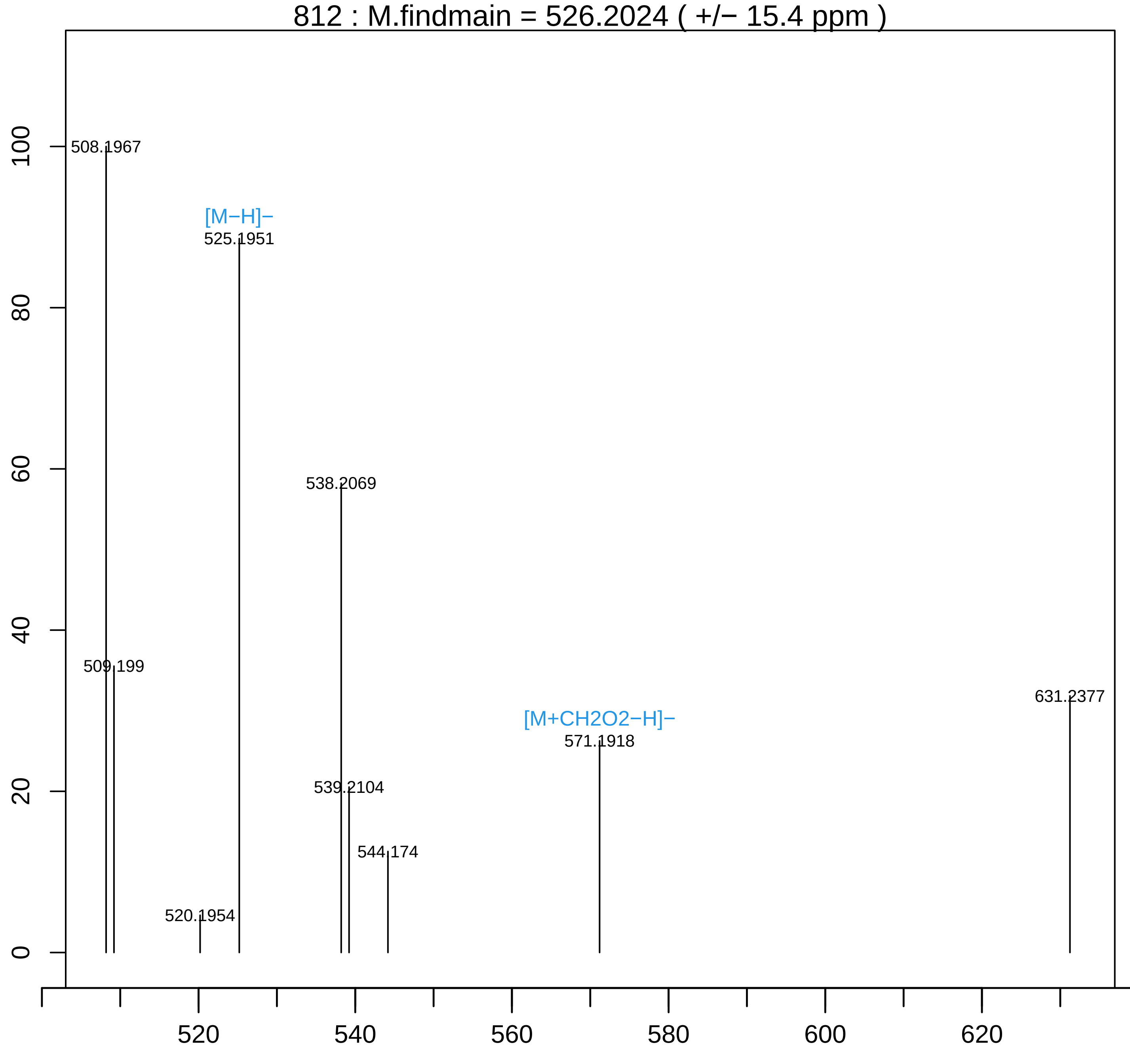

812 : M.findmain = 526.2024 (+/− 15.4 ppm)
508.1967
[M−H]−
525.1951
538.2069
509.199
631.2377
[M+CH2O2−H]−
571.1918
539.2104
544.174
520.1954
0
20
40
60
80
100
520
540
560
580
600
620

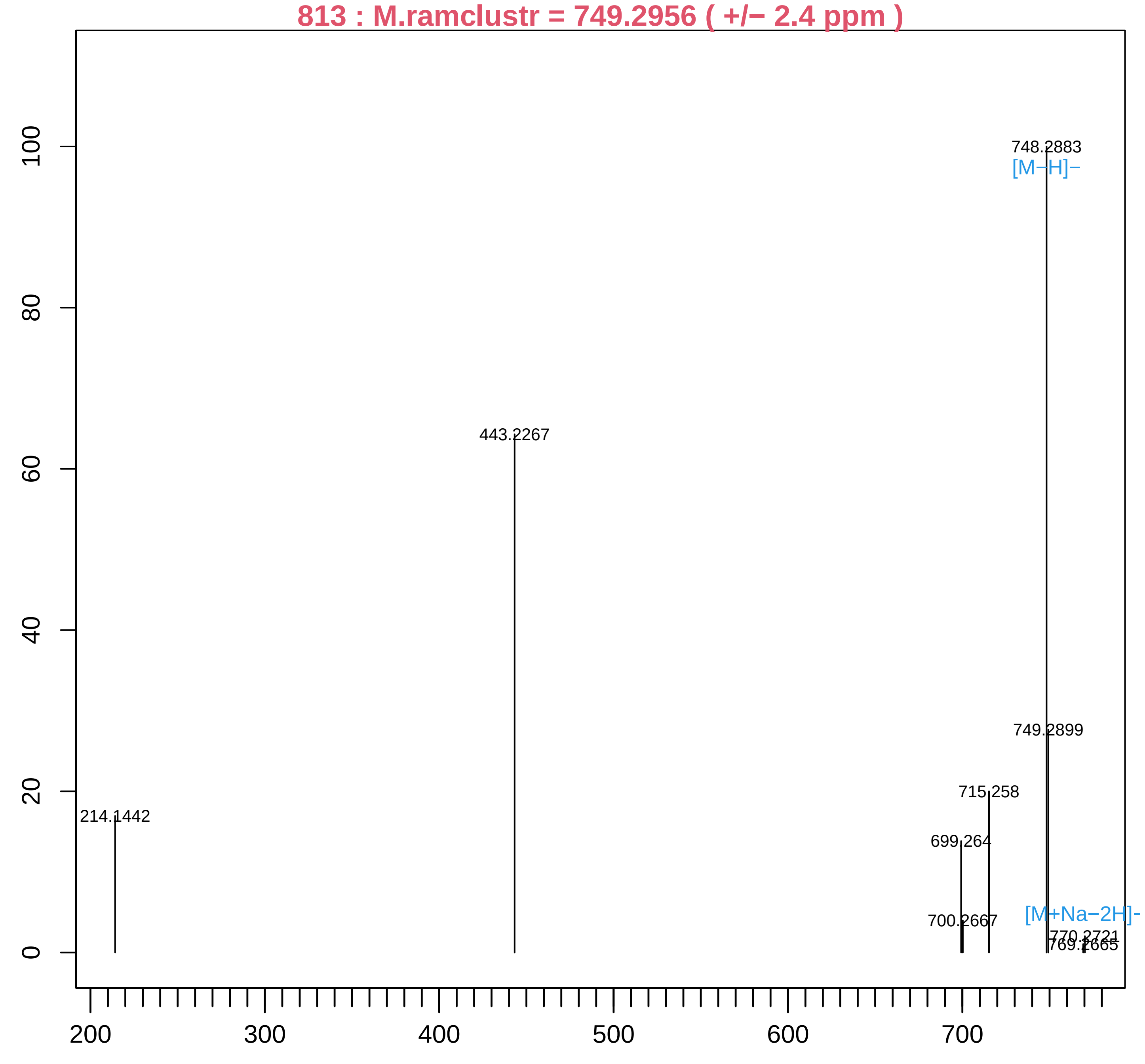

813 : M.ramclustr = 749.2956 (+/− 2.4 ppm)
748.2883
[M−H]−
443.2267
749.2899
715.258
214.1442
699.264
700.2667
[M+Na−2H]−
770.2721
769.2665

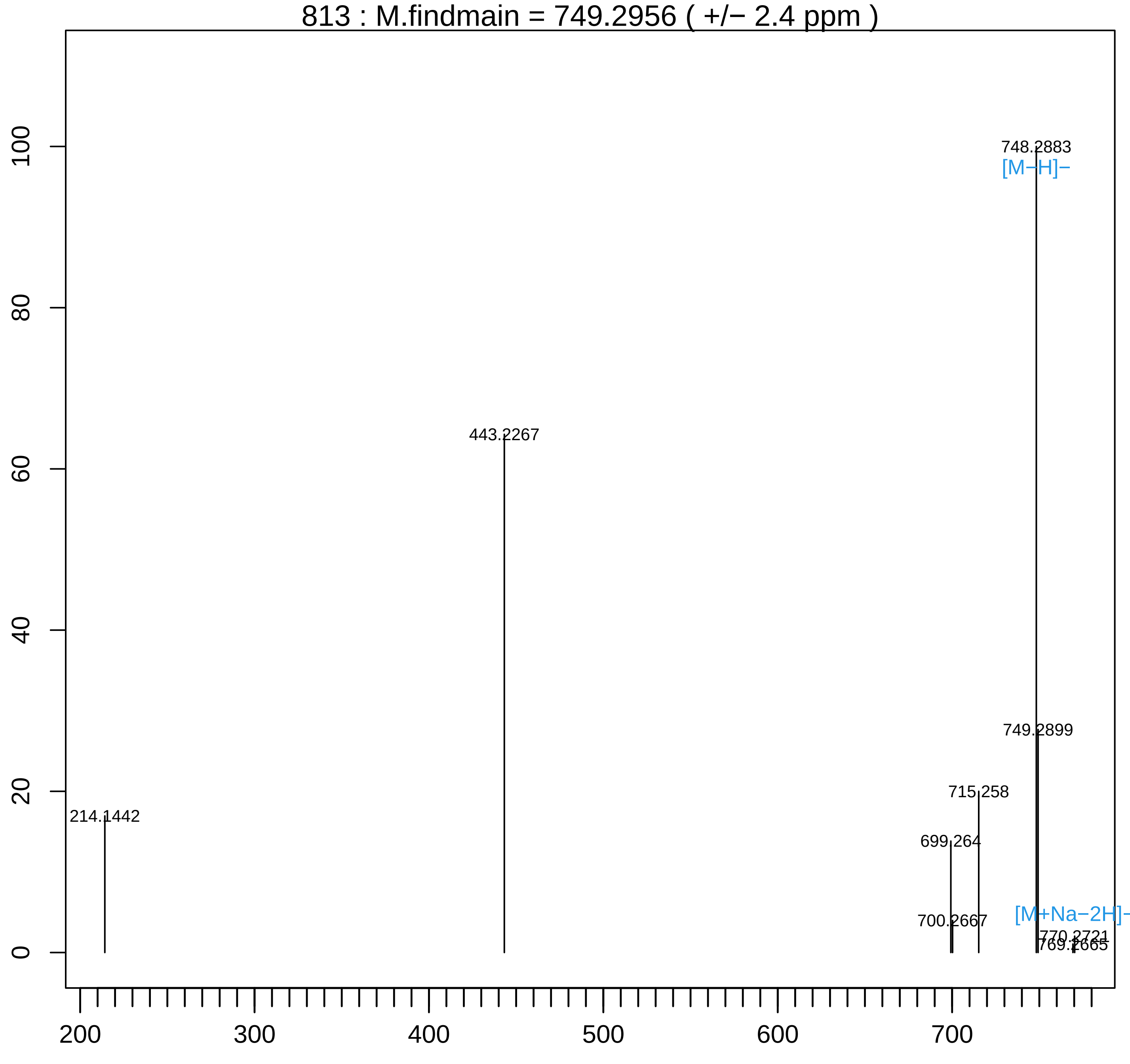

813 : M.findmain = 749.2956 (+/− 2.4 ppm)
748.2883
[M−H]−
443.2267
749.2899
715.258
214.1442
699.264
700.2667
[M+Na−2H]−
770.2721
769.2665

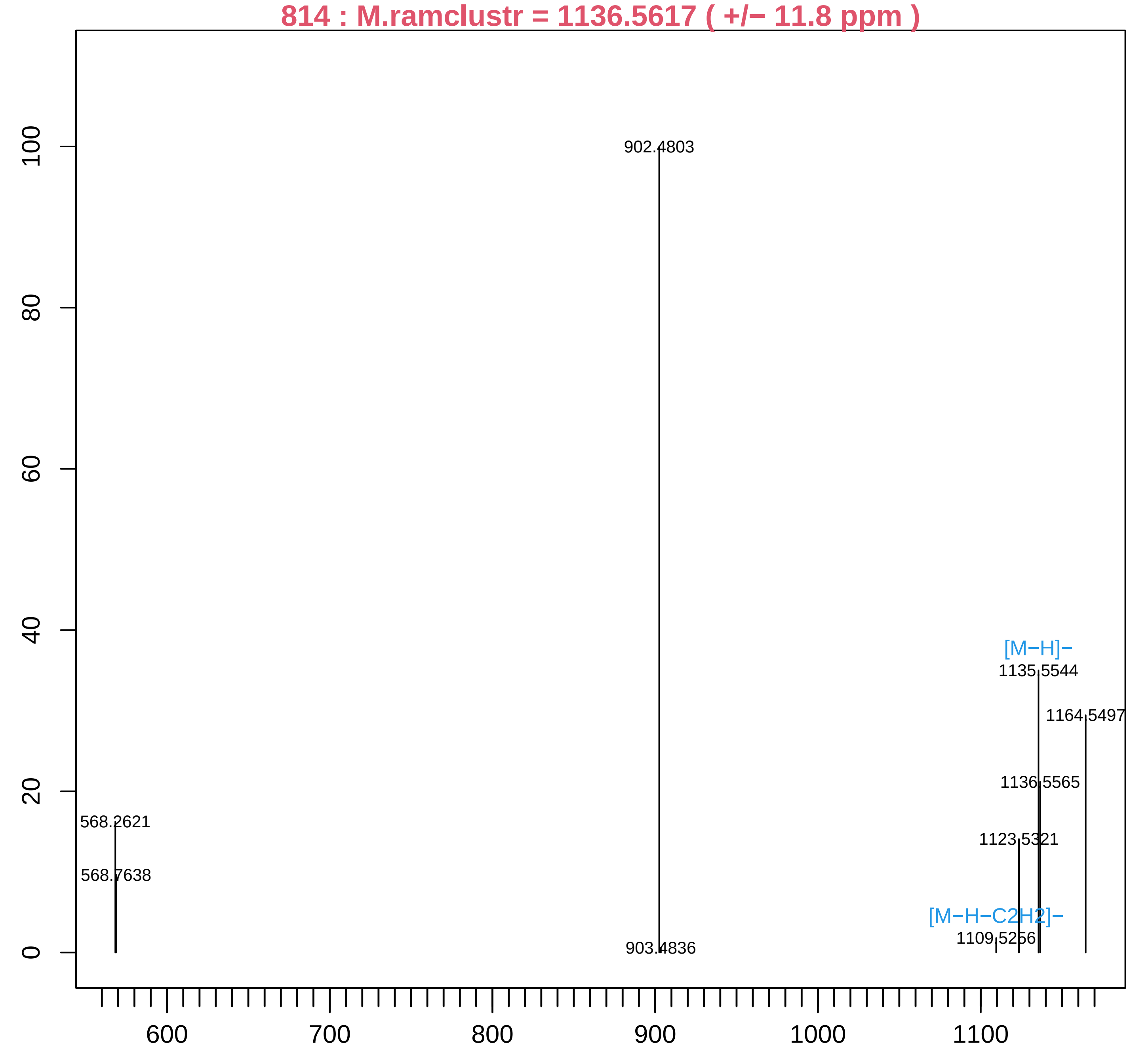
814 : M.ramclustr = 1136.5617 (+/− 11.8 ppm)
902.4803
[M−H]−
1135.5544
1164.5497
1136.5565
568.2621
1123.5321
568.7638
[M−H−C2H2]−
1109.5256
903.4836
0
20
40
60
80
100
600
700
800
900
1000
1100

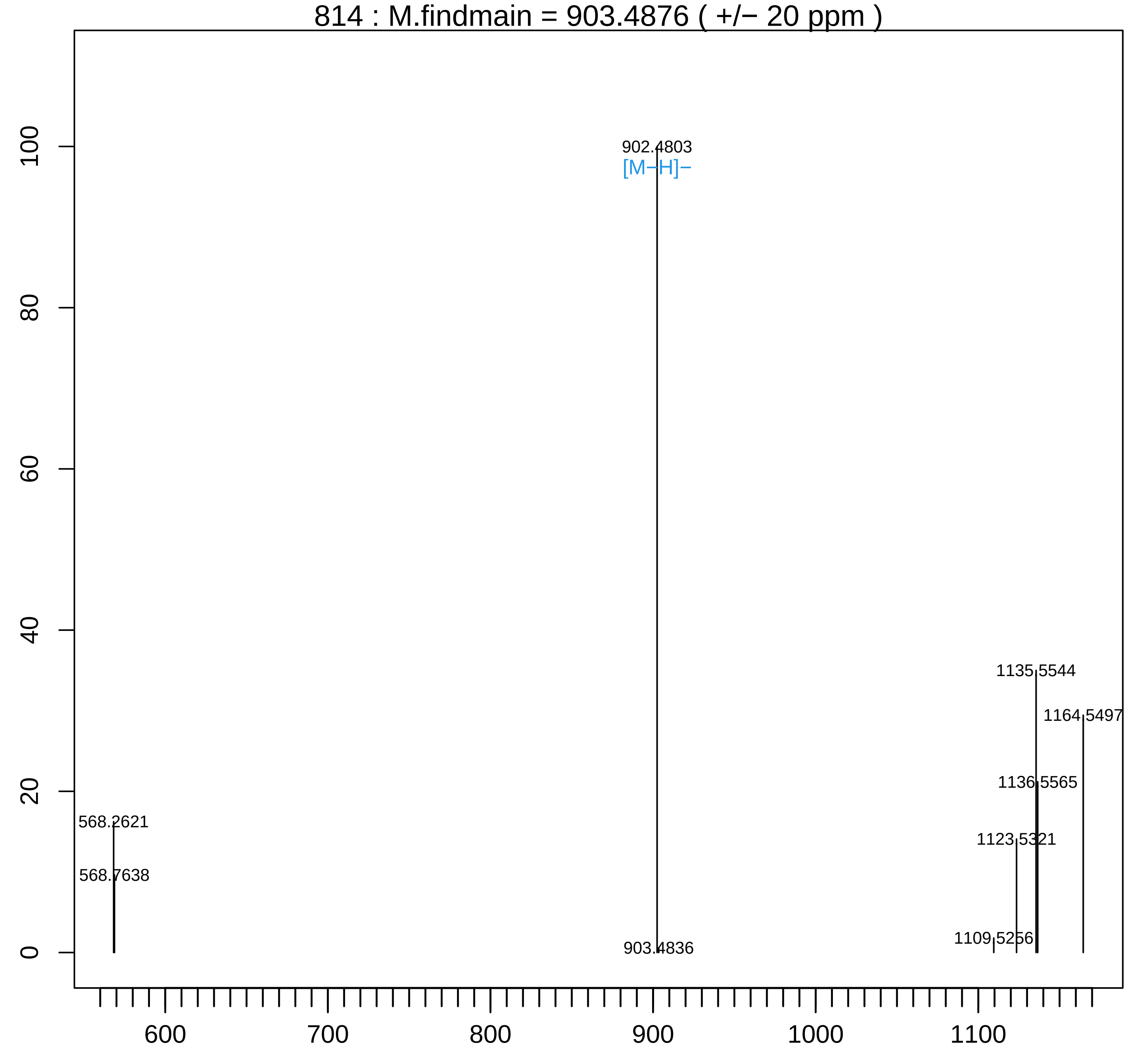
814 : M.findmain = 903.4876 (+/− 20 ppm)
902.4803
[M−H]−
1135.5544
1164.5497
1136.5565
568.2621
1123.5321
568.7638
1109.5256
903.4836
0
20
40
60
80
100
600
700
800
900
1000
1100

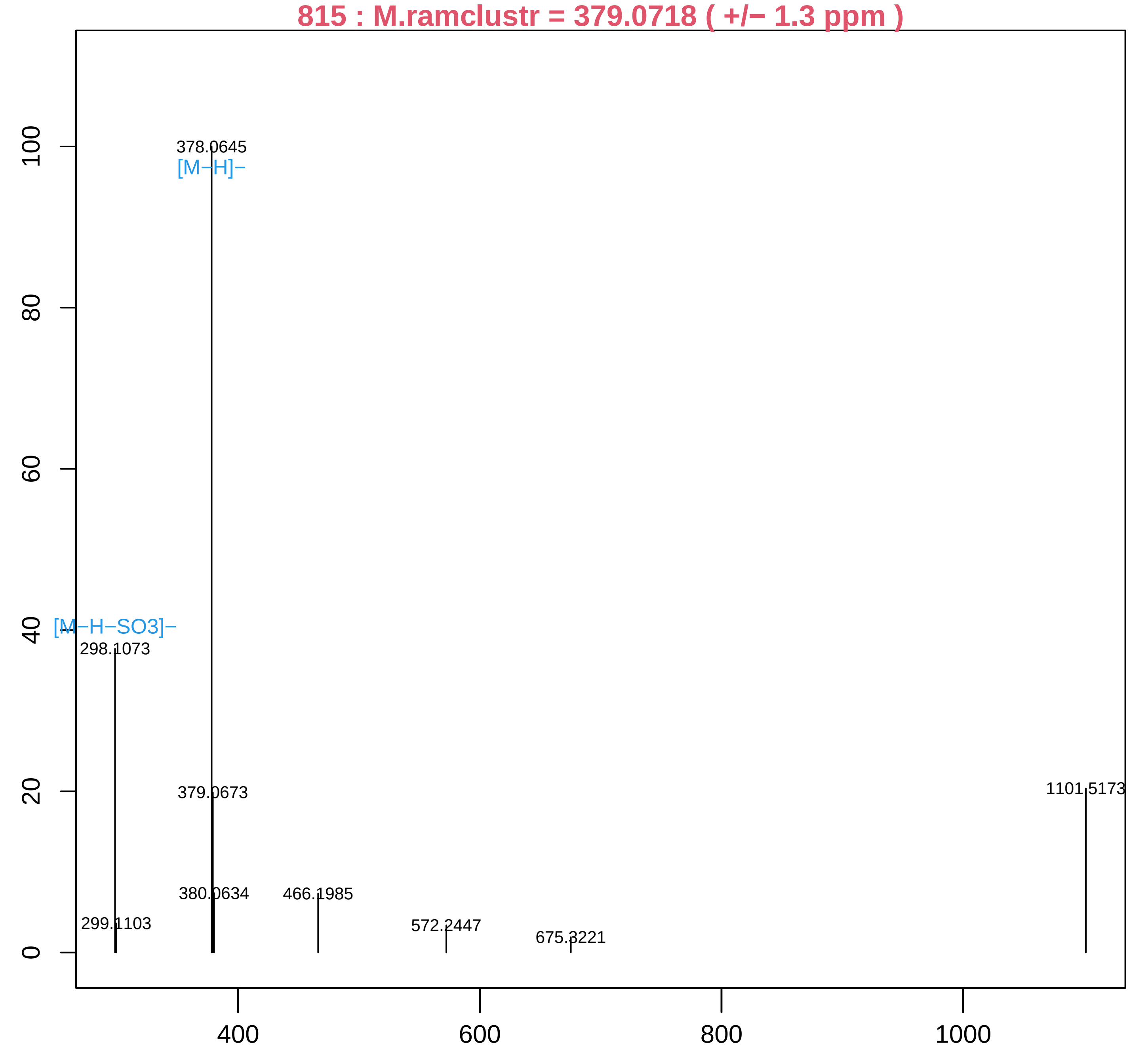

815 : M.ramclustr = 379.0718 (+/− 1.3 ppm)
378.0645
[M−H]−
[M−H−SO3]−
298.1073
379.0673
380.0634
466.1985
299.1103
572.2447
675.3221
1101.5173
0
20
40
60
80
100
400
600
800
1000

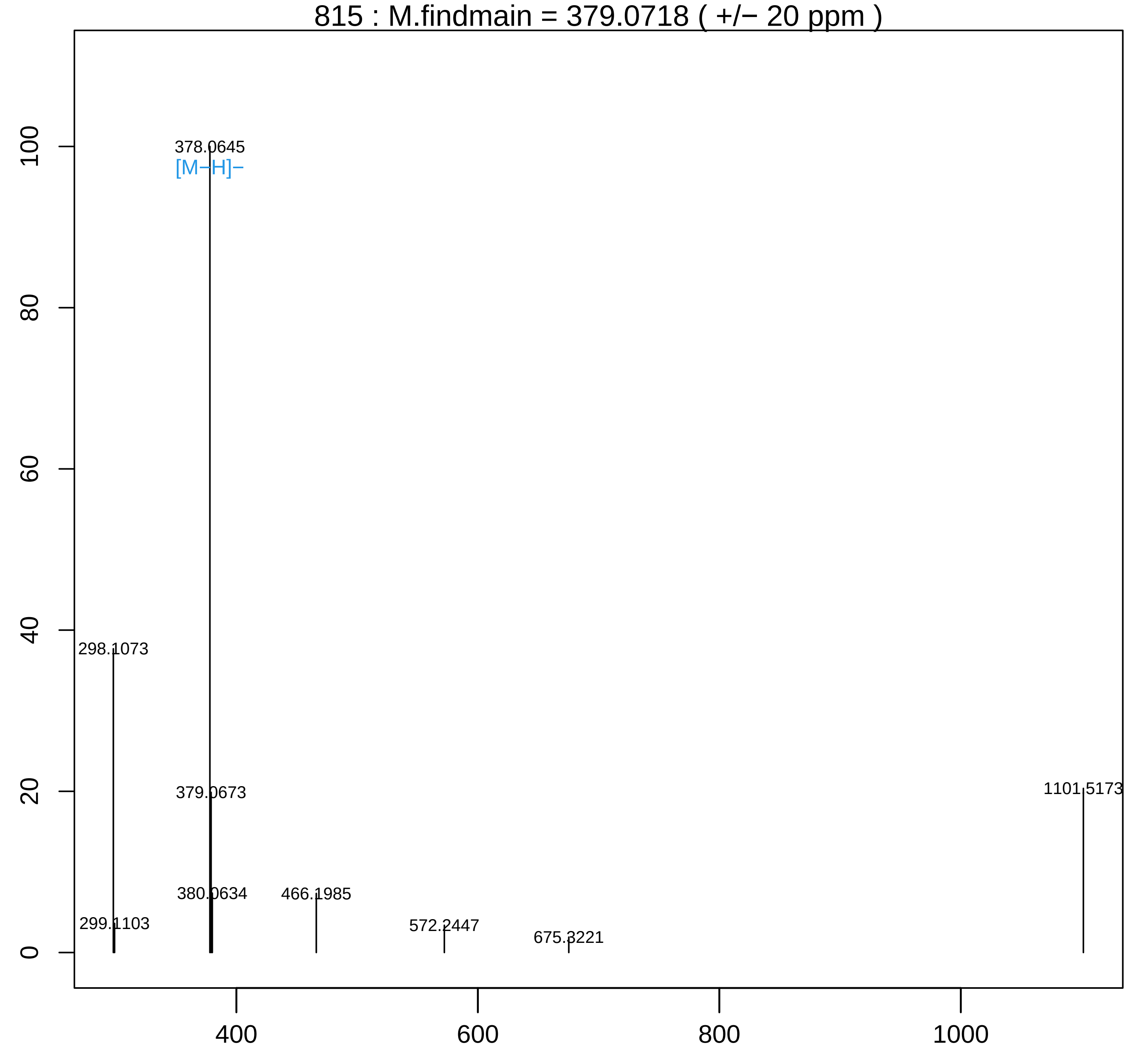

815 : M.findmain = 379.0718 (+/− 20 ppm)
378.0645
[M−H]−
298.1073
379.0673
380.0634
466.1985
299.1103
572.2447
675.3221
1101.5173
0
20
40
60
80
100
400
600
800
1000

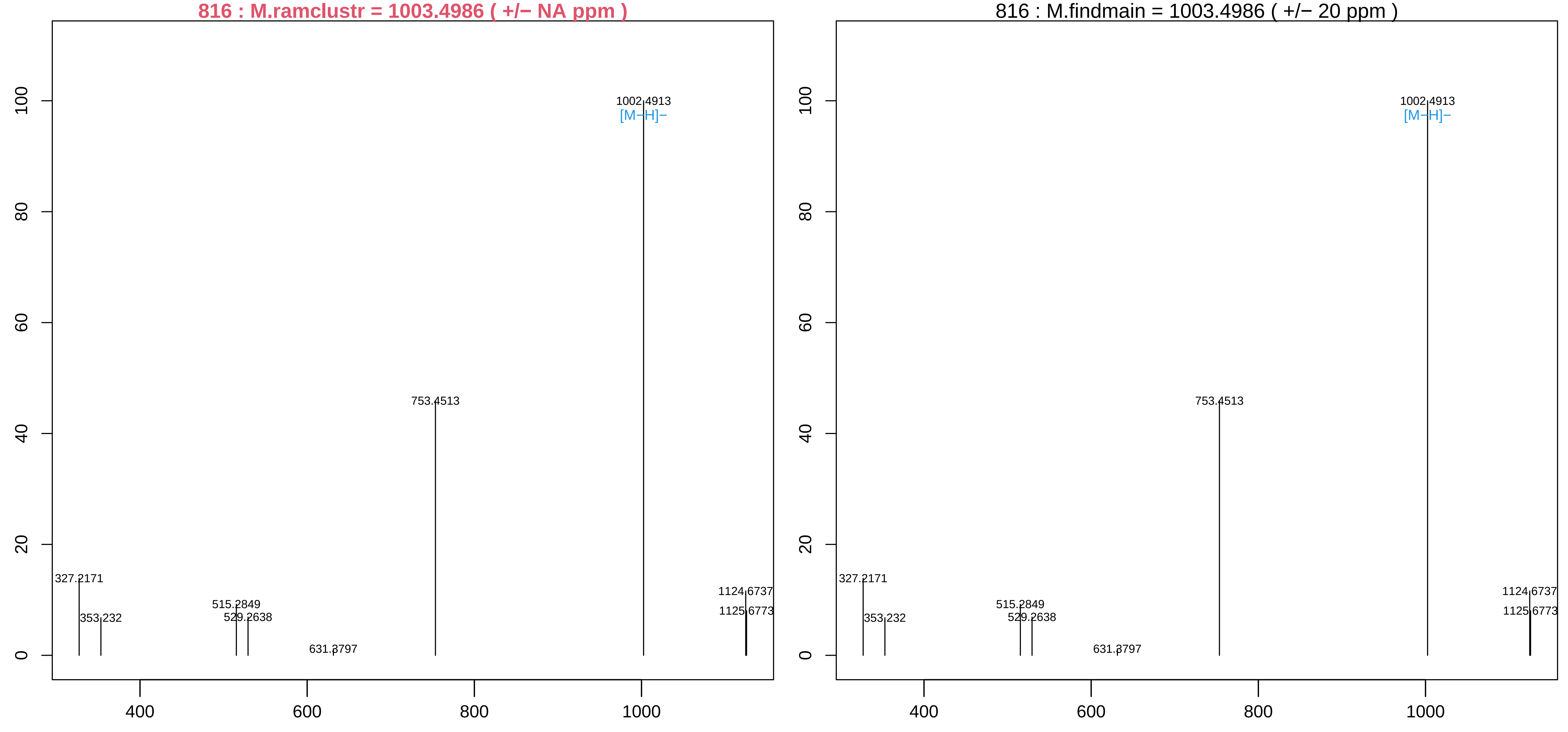
816 : M.ramclustr = 1003.4986 (+/− NA ppm)
816 : M.findmain = 1003.4986 (+/− 20 ppm)
1002.4913
[M−H]−
753.4513
327.2171
353.232
515.2849
529.2638
631.3797
1124.6737
1125.6773
0
20
40
60
80
100
400
600
800
1000

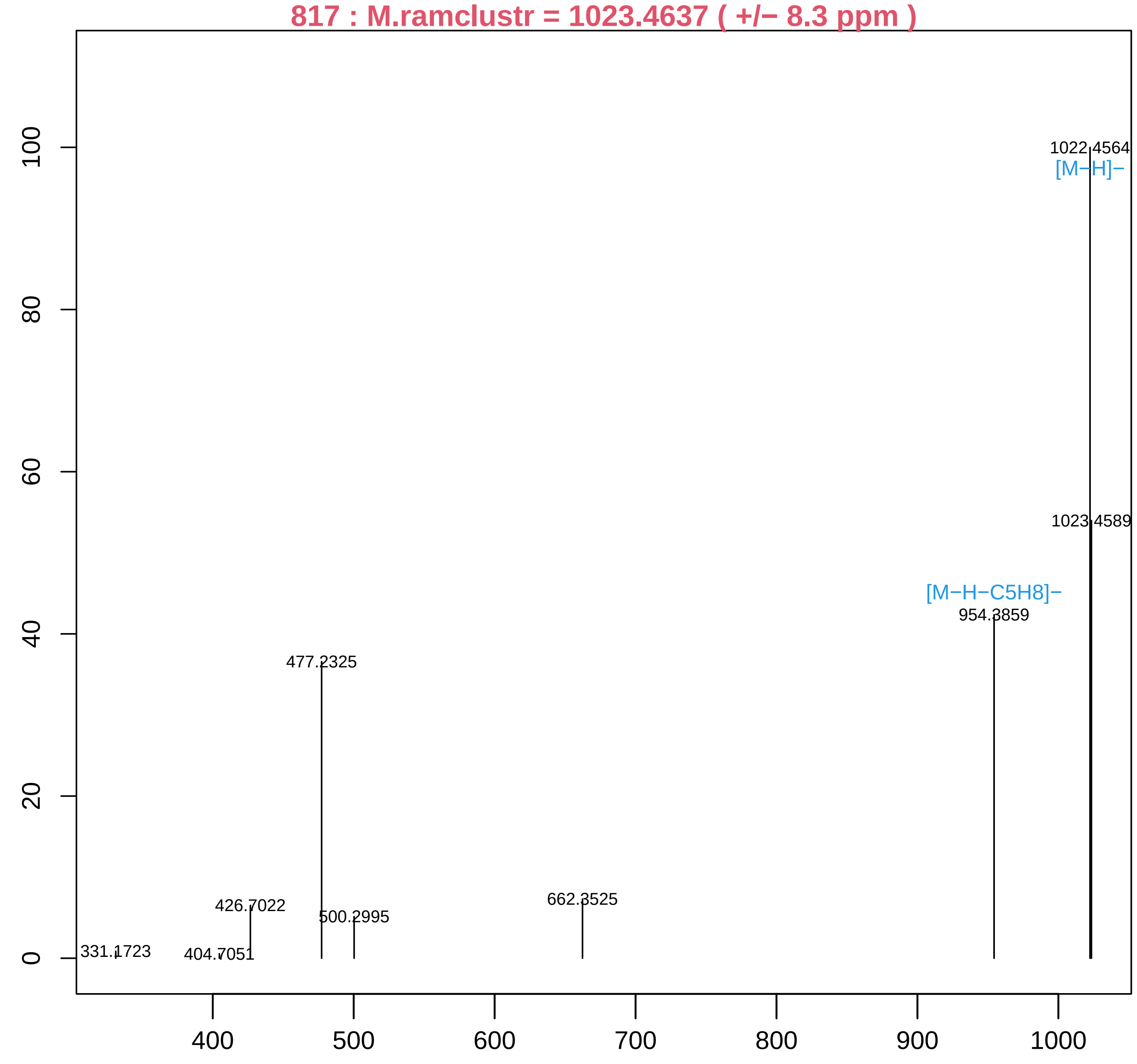
817 : M.ramclustr = 1023.4637 (+/− 8.3 ppm)
1022.4564
[M−H]−
1023.4589
[M−H−C5H8]−
954.3859
477.2325
426.7022
500.2995
662.3525
331.1723
404.7051
0
20
40
60
80
100
400
500
600
700
800
900
1000

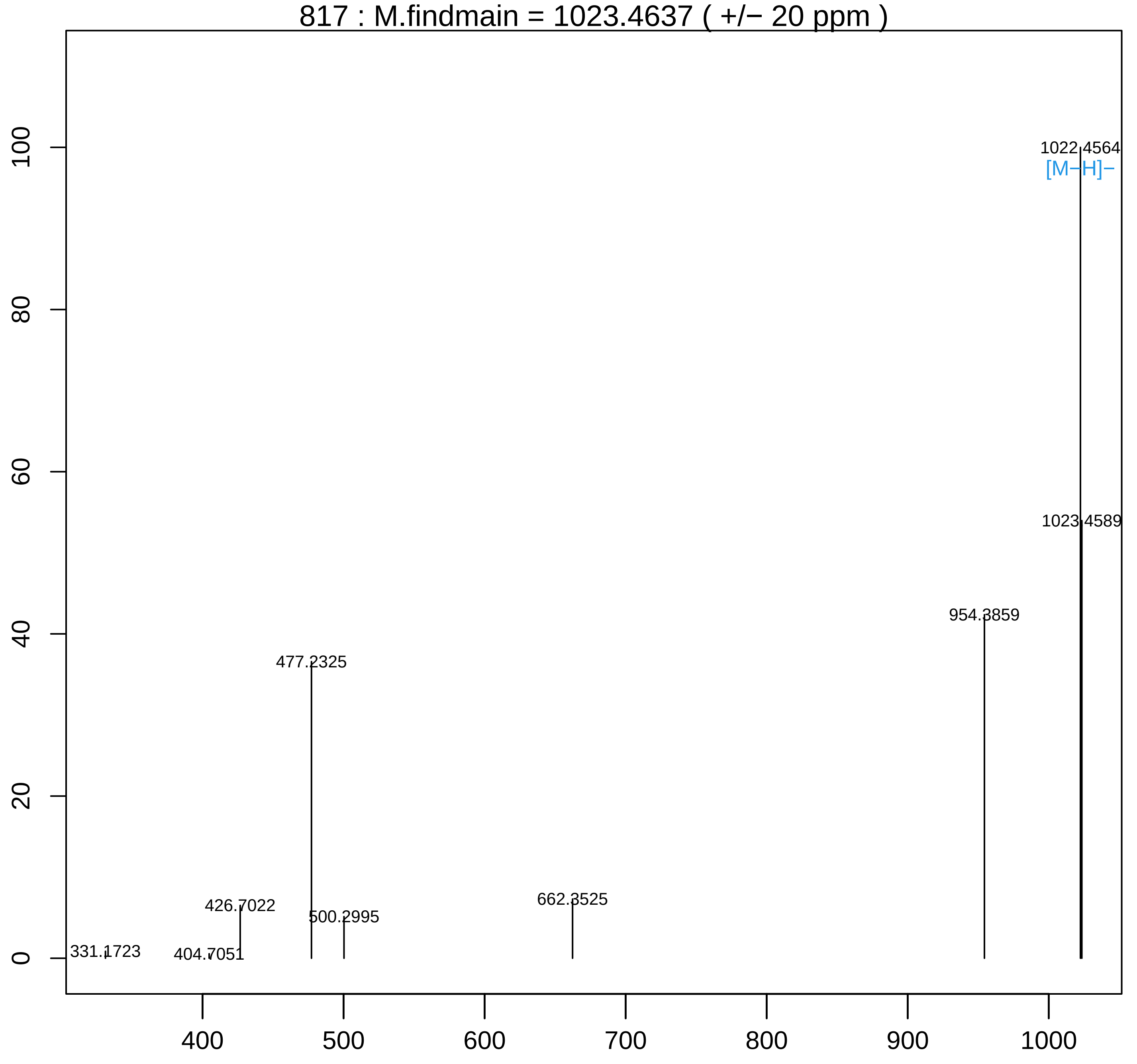
817 : M.findmain = 1023.4637 (+/− 20 ppm)
1022.4564
[M−H]−
1023.4589
954.3859
477.2325
426.7022
500.2995
662.3525
331.1723
404.7051
0
20
40
60
80
100
400
500
600
700
800
900
1000

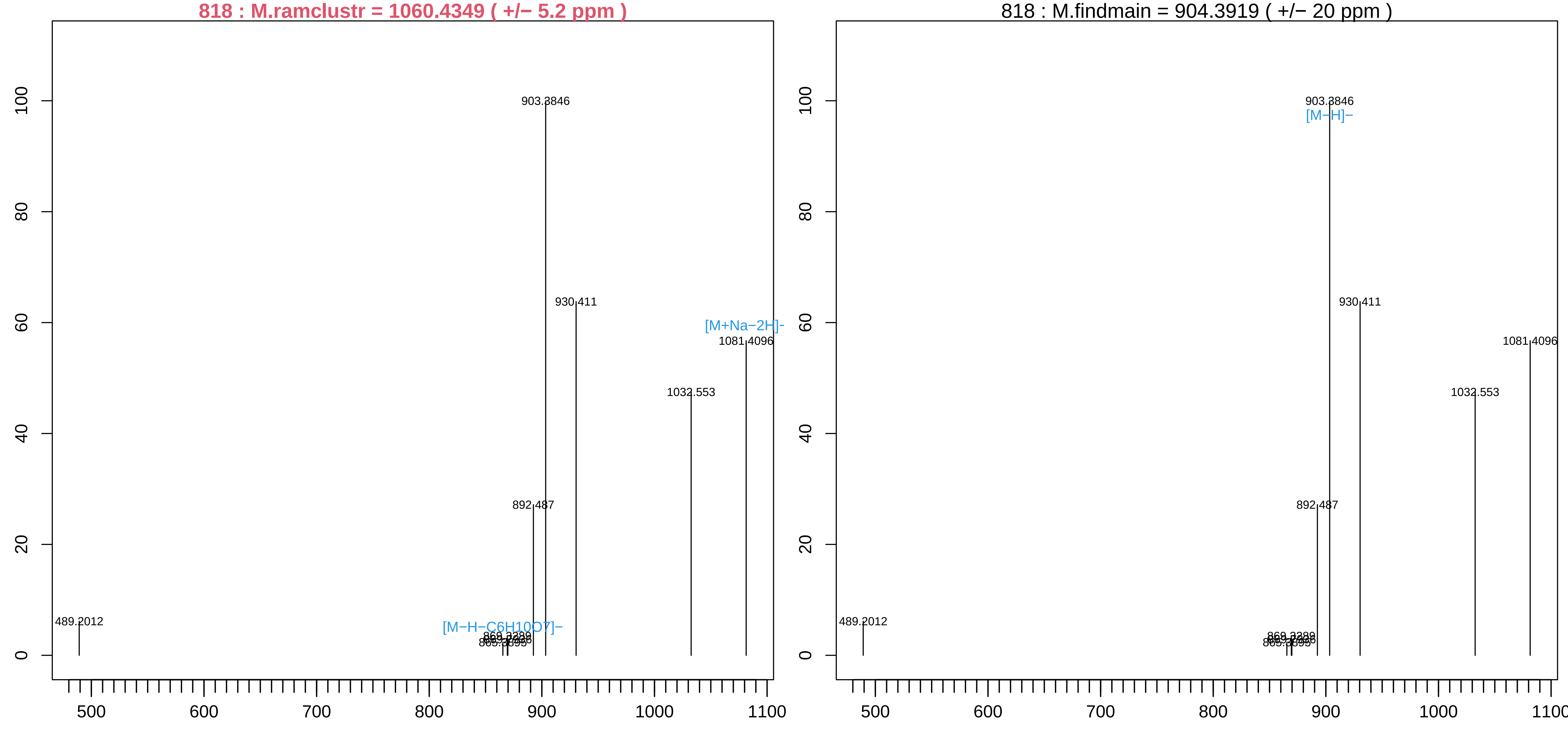
818 : M.ramclustr = 1060.4349 (+/− 5.2 ppm)
903.3846
930.411
[M+Na−2H]-
1081.4096
1032.553
892.487
489.2012
[M−H−C6H10O7]−
0
20
40
60
80
100
500
600
700
800
900
1000
1100
818 : M.findmain = 904.3919 (+/− 20 ppm)
903.3846
[M−H]−
930.411
1081.4096
1032.553
892.487
489.2012
0
20
40
60
80
100
500
600
700
800
900
1000
1100

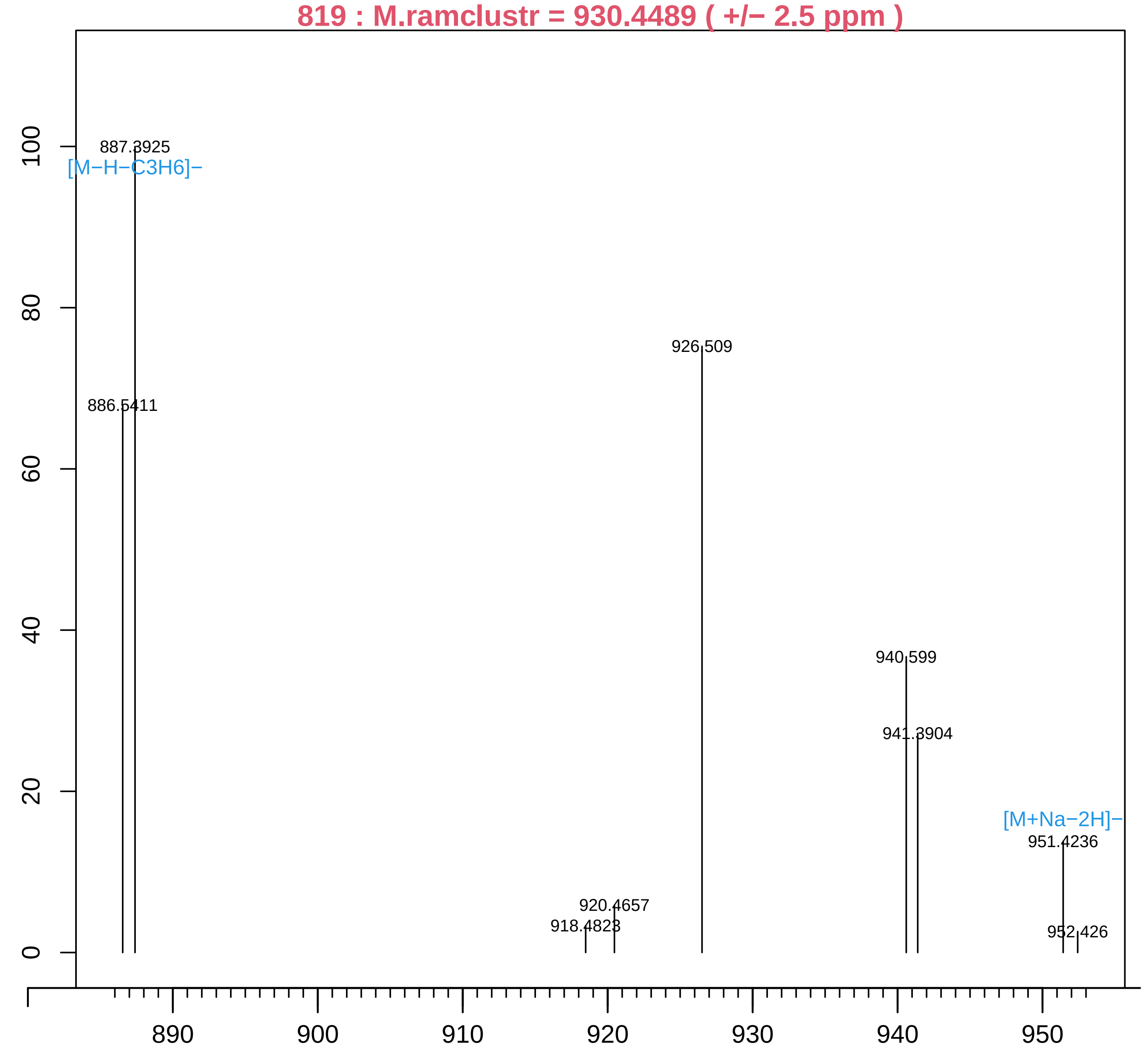

819 : M.ramclustr = 930.4489 (+/− 2.5 ppm)
887.3925
[M−H−C3H6]−
886.5411
926.509
940.599
941.3904
[M+Na−2H]−
951.4236
920.4657
918.4823
952.426

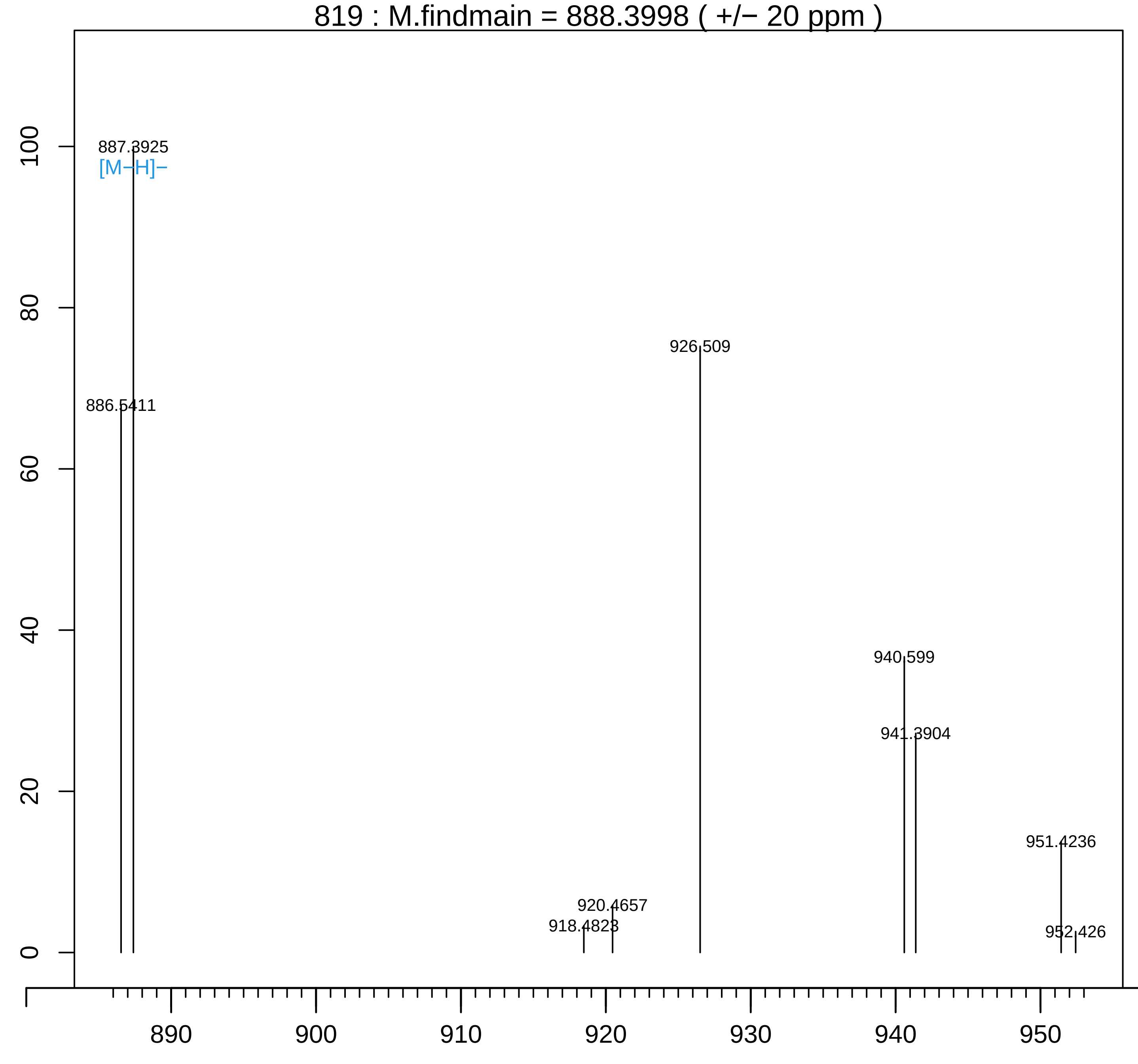

819 : M.findmain = 888.3998 (+/− 20 ppm)
887.3925
[M−H]−
886.5411
926.509
940.599
941.3904
951.4236
920.4657
918.4823
952.426

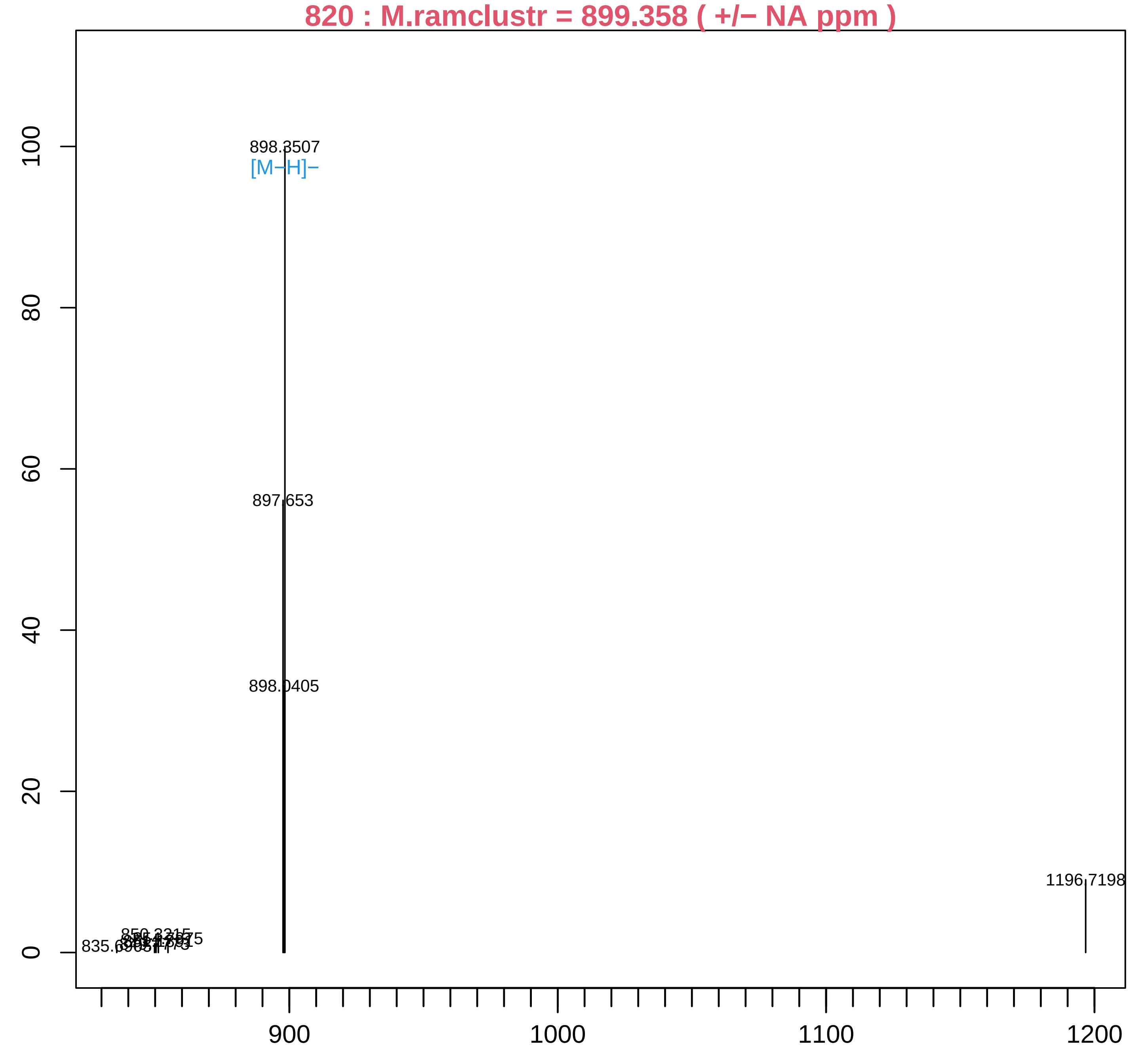
820 : M.ramclustr = 899.358 (+/− NA ppm)
898.3507
[M−H]−
897.653
898.0405
1196.7198
835.6065
0
20
40
60
80
100
900
1000
1100
1200

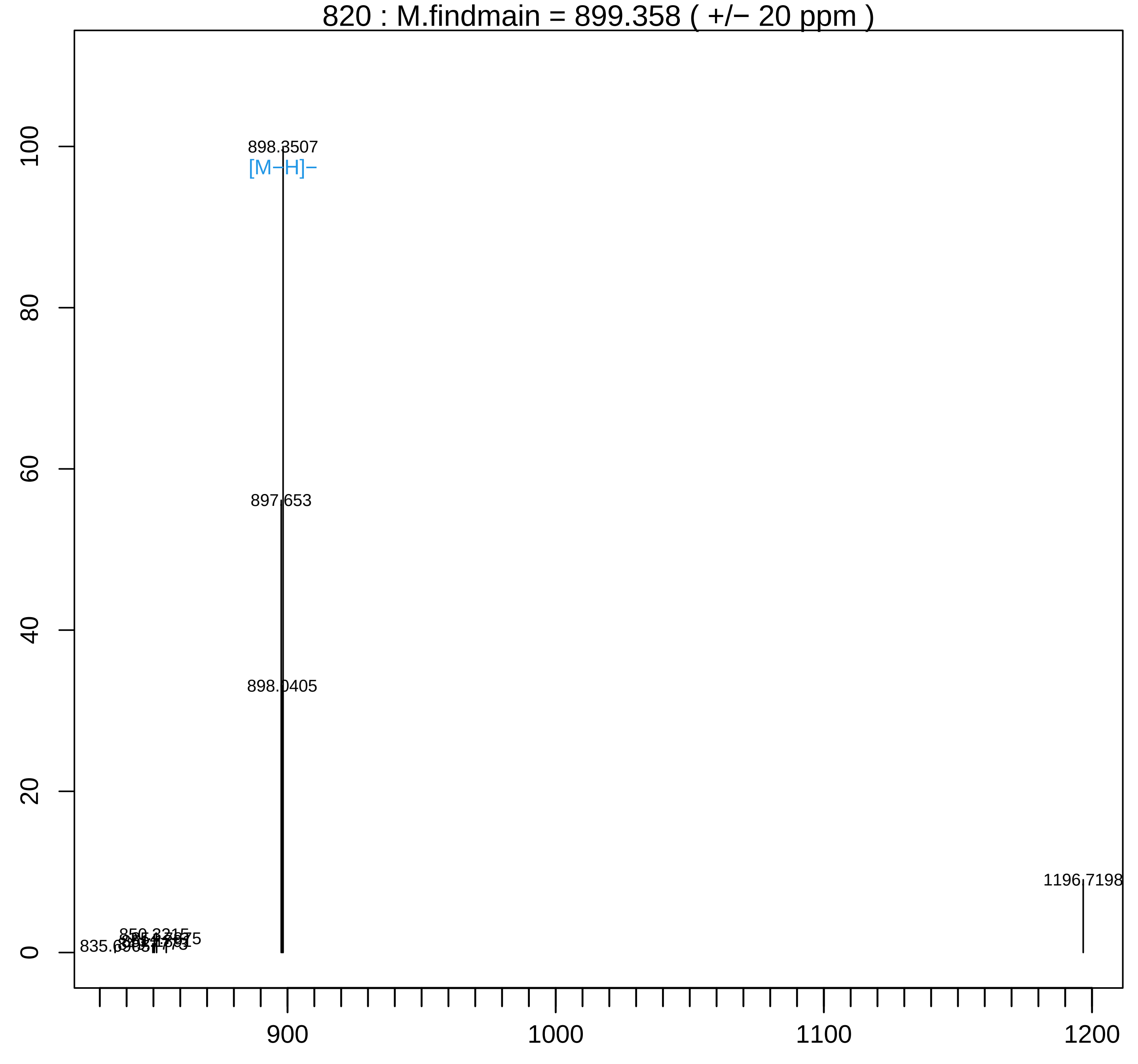
820 : M.findmain = 899.358 (+/− 20 ppm)
898.3507
[M−H]−
897.653
898.0405
1196.7198
835.6065
0
20
40
60
80
100
900
1000
1100
1200

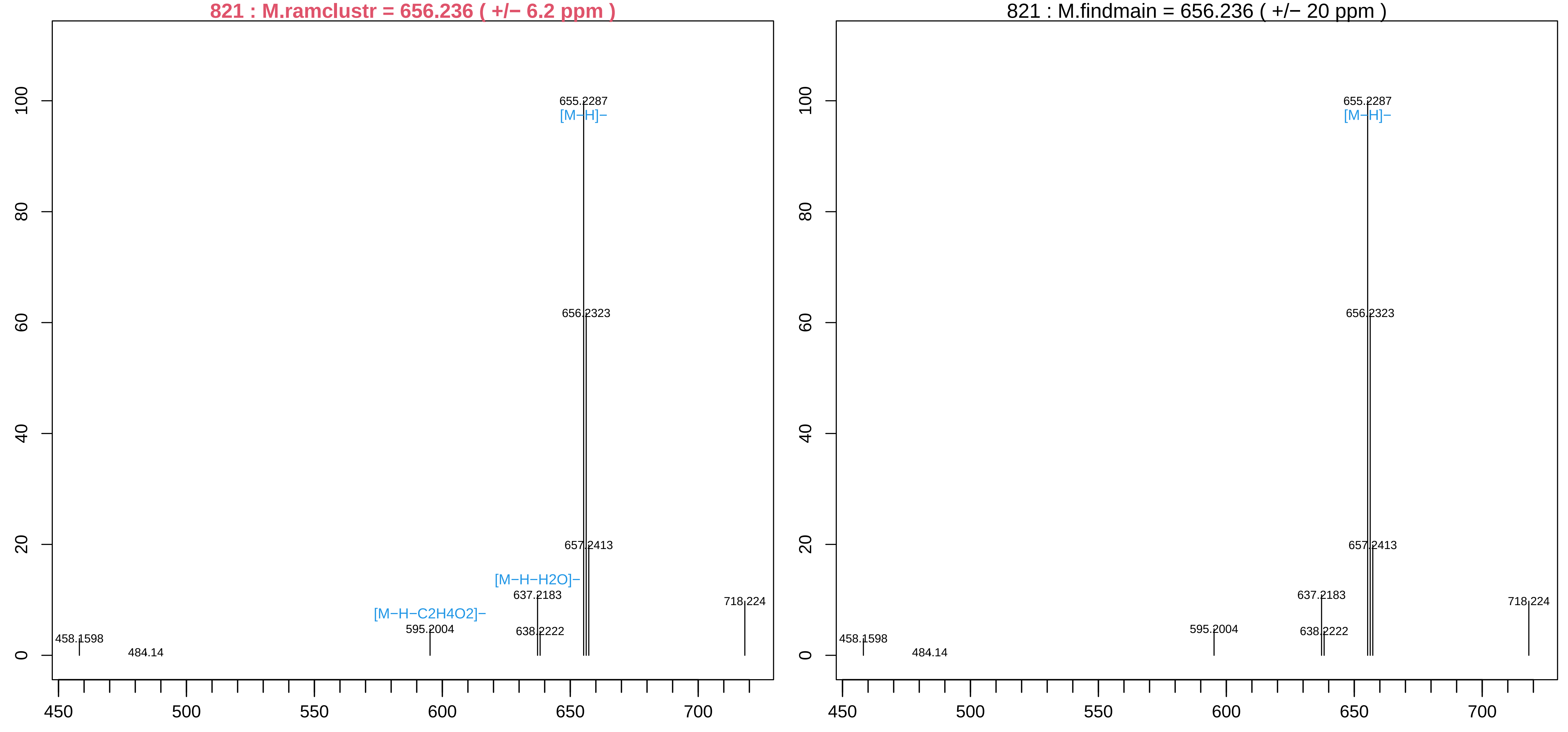
821 : M.ramclustr = 656.236 (+/− 6.2 ppm)
655.2287
[M−H]−
656.2323
657.2413
[M−H−H2O]−
637.2183
718.224
[M−H−C2H4O2]−
595.2004
638.2222
458.1598
484.14
821 : M.findmain = 656.236 (+/− 20 ppm)
655.2287
[M−H]−
656.2323
657.2413
637.2183
718.224
595.2004
638.2222
458.1598
484.14
0
20
40
60
80
100
450
500
550
600
650
700

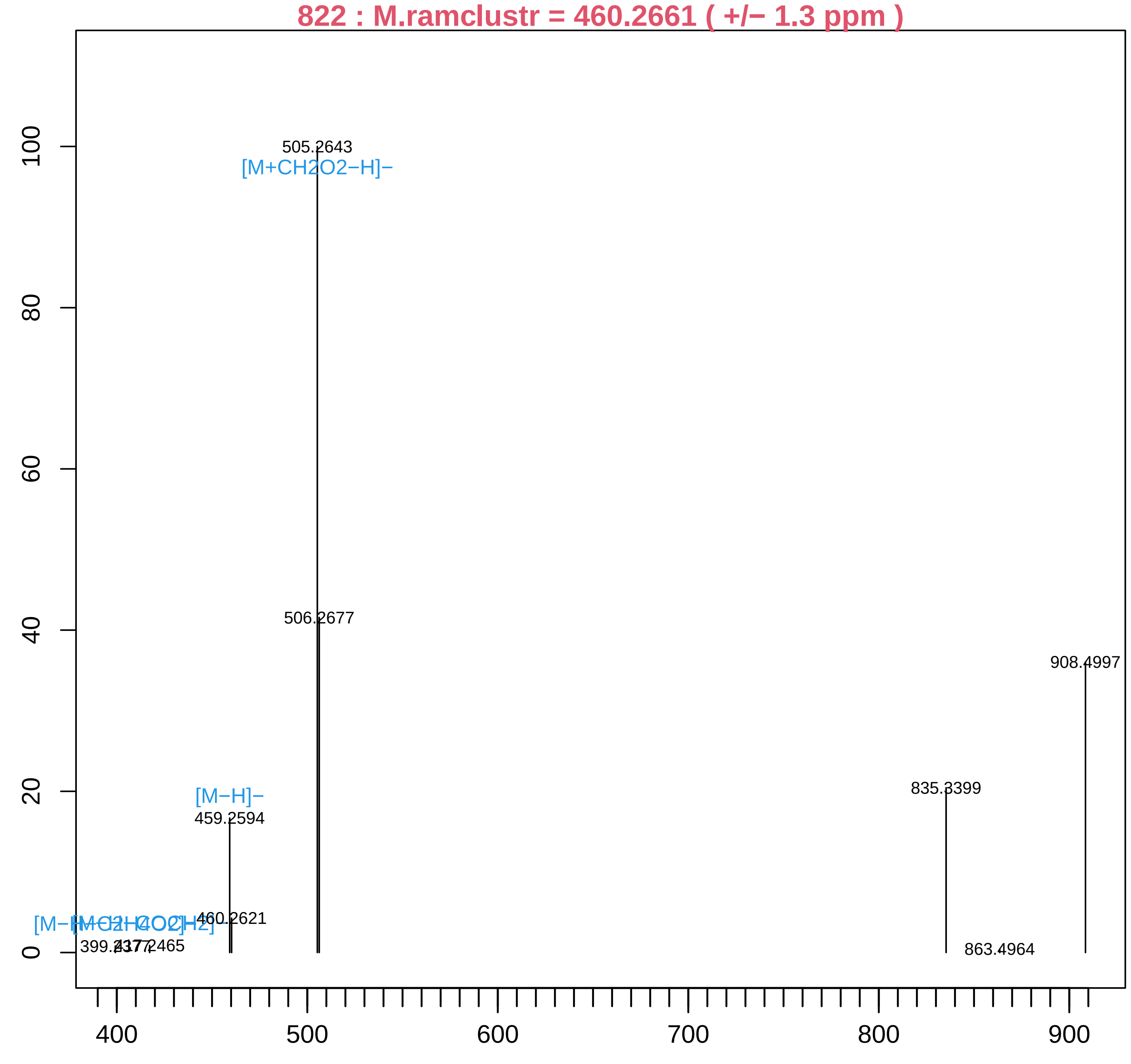

822 : M.ramclustr = 460.2661 (+/− 1.3 ppm)
505.2643
[M+CH2O2−H]−
506.2677
908.4997
835.3399
863.4964
[M−H]−
459.2594
460.2621
399.2377
437.2465
0
20
40
60
80
100
400
500
600
700
800
900

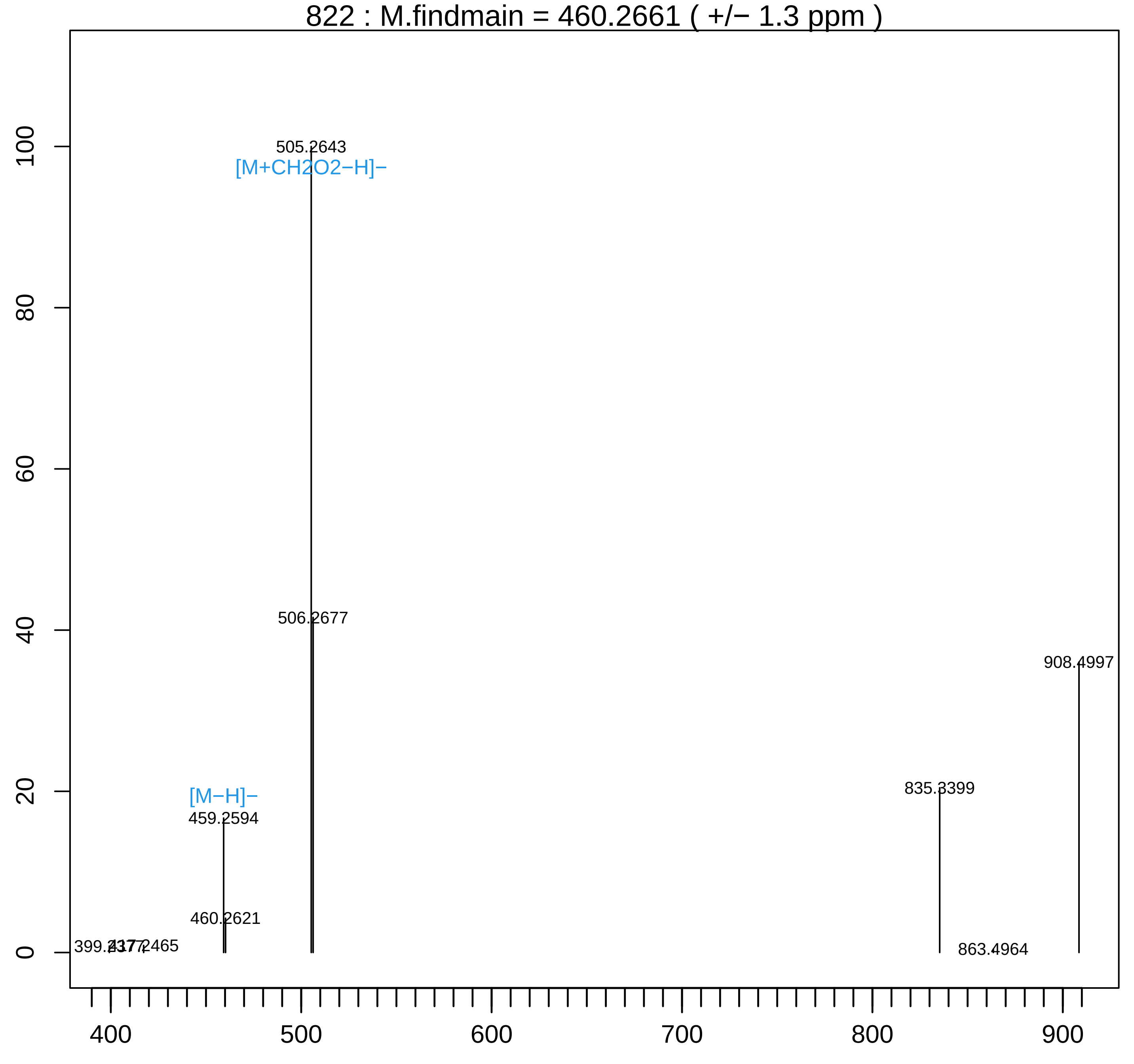

822 : M.findmain = 460.2661 (+/− 1.3 ppm)
505.2643
[M+CH2O2−H]−
506.2677
908.4997
835.3399
863.4964
[M−H]−
459.2594
460.2621
399.2377
437.2465
0
20
40
60
80
100
400
500
600
700
800
900

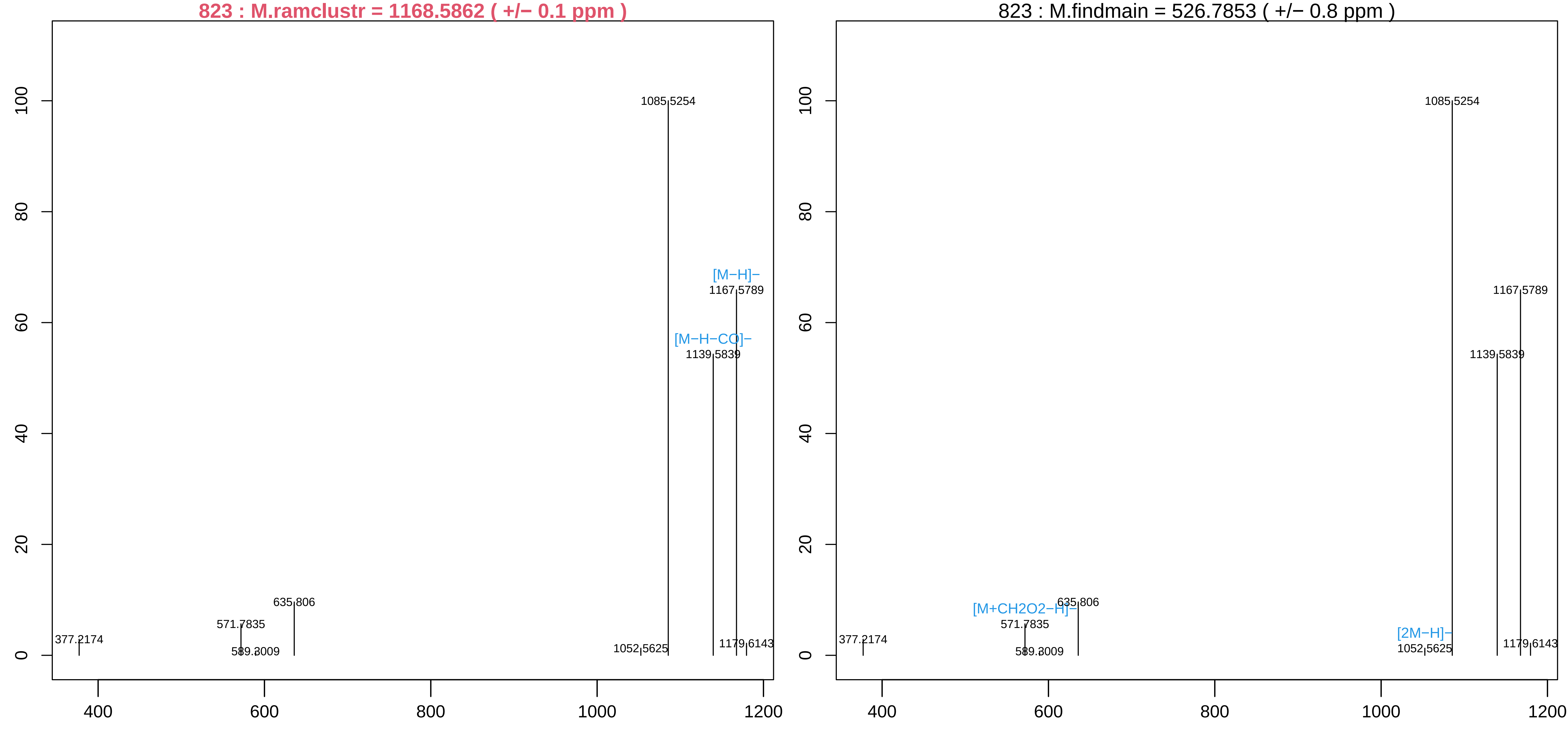

823 : M.ramclustr = 1168.5862 (+/− 0.1 ppm)
1085.5254
[M−H]−
1167.5789
[M−H−CO]−
1139.5839
635.806
571.7835
377.2174
589.3009
1052.5625
1179.6143
823 : M.findmain = 526.7853 (+/− 0.8 ppm)
1085.5254
1167.5789
1139.5839
[M+CH2O2−H]−
635.806
571.7835
377.2174
589.3009
[2M−H]−
1052.5625
1179.6143
0
20
40
60
80
100
400
600
800
1000
1200

824 : M.ramclustr = 781.4605 (+/− NA ppm)

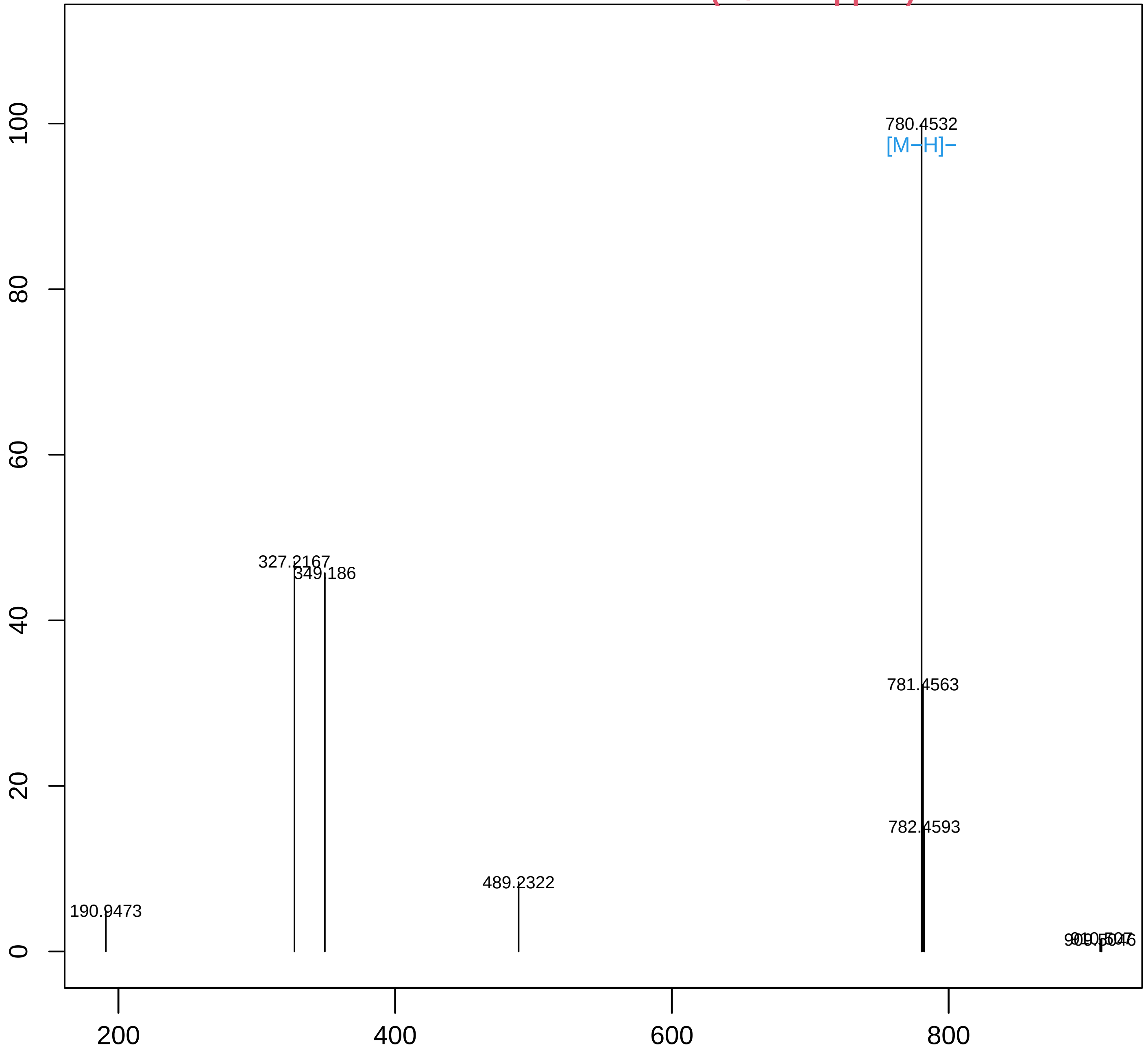

824 : M.findmain = 781.4605 (+/− 20 ppm)

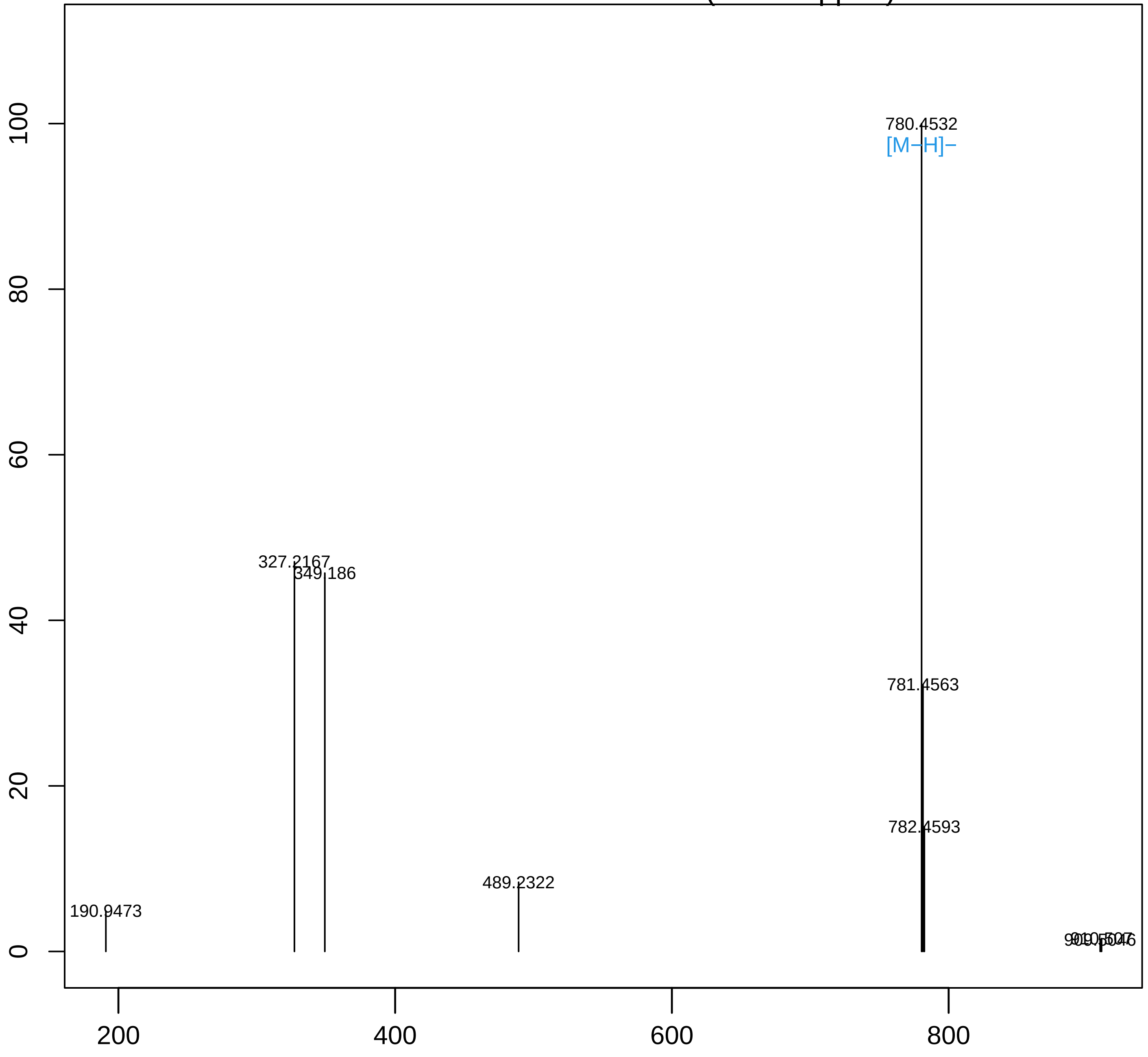

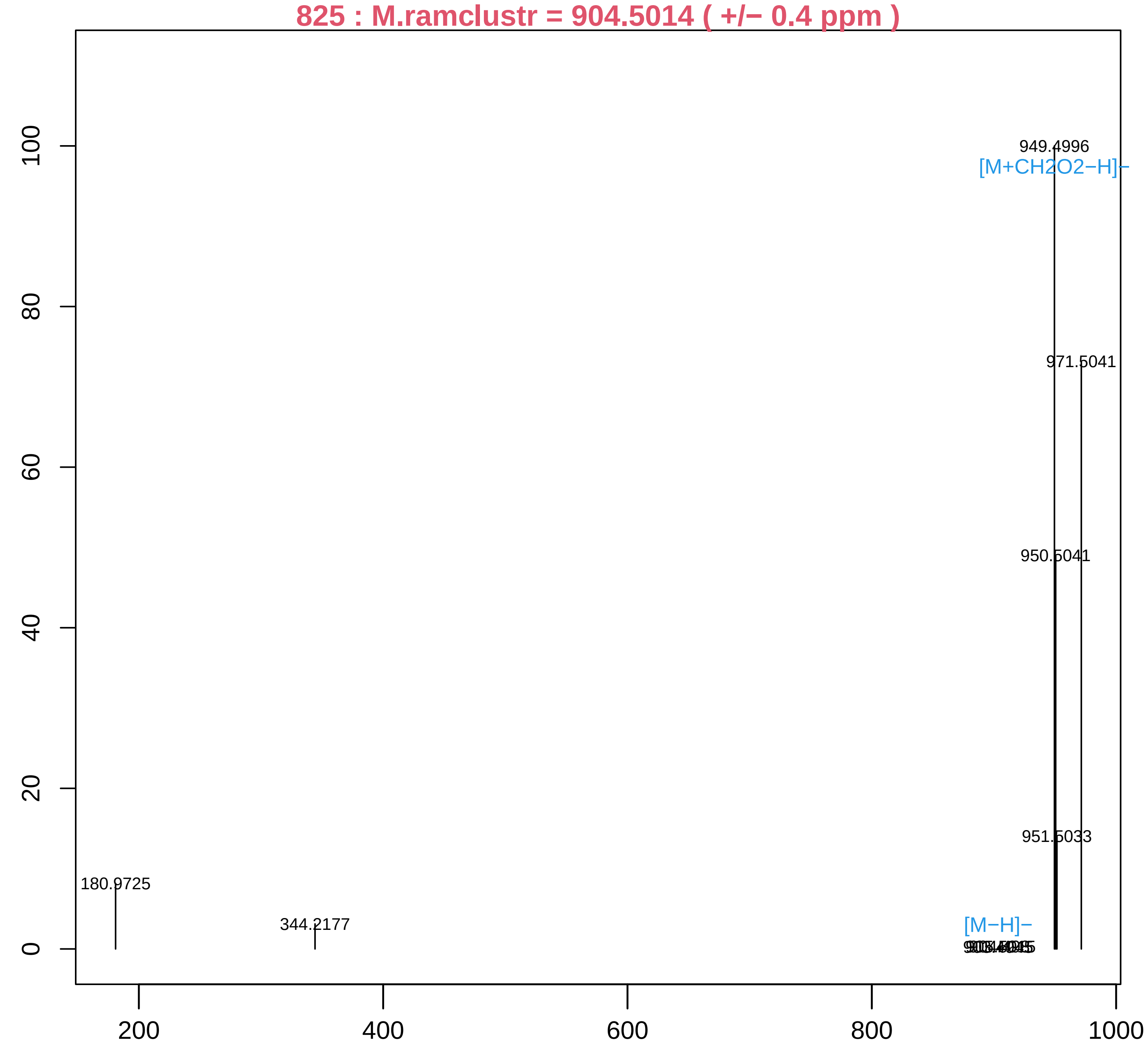

825 : M.ramclustr = 904.5014 (+/− 0.4 ppm)
949.4996
[M+CH2O2−H]−
971.5041
950.5041
951.5033
180.9725
344.2177
[M−H]−
0
20
40
60
80
100
200
400
600
800
1000

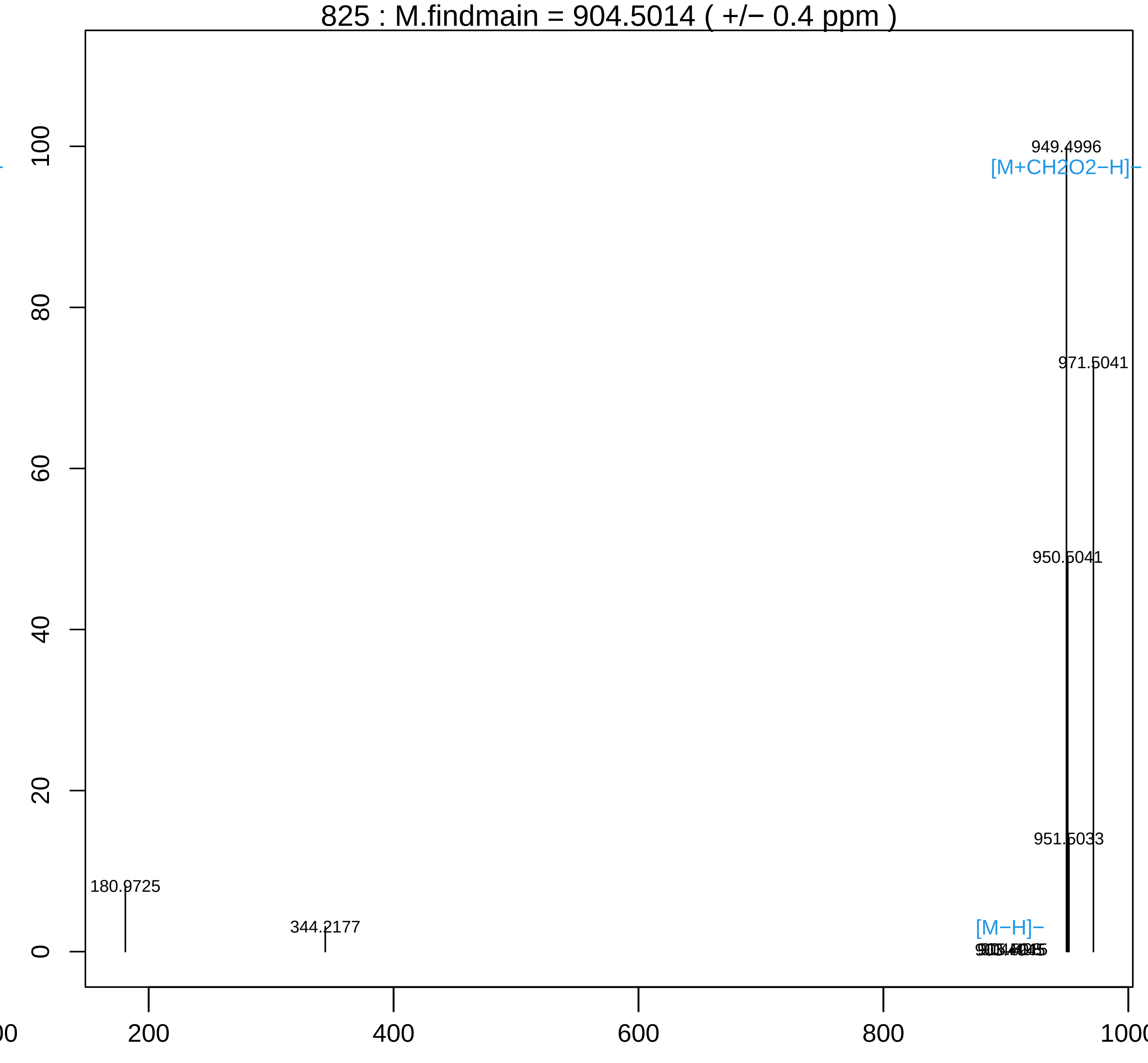

825 : M.findmain = 904.5014 (+/− 0.4 ppm)
949.4996
[M+CH2O2−H]−
971.5041
950.5041
951.5033
180.9725
344.2177
[M−H]−
0
20
40
60
80
100
200
400
600
800
1000

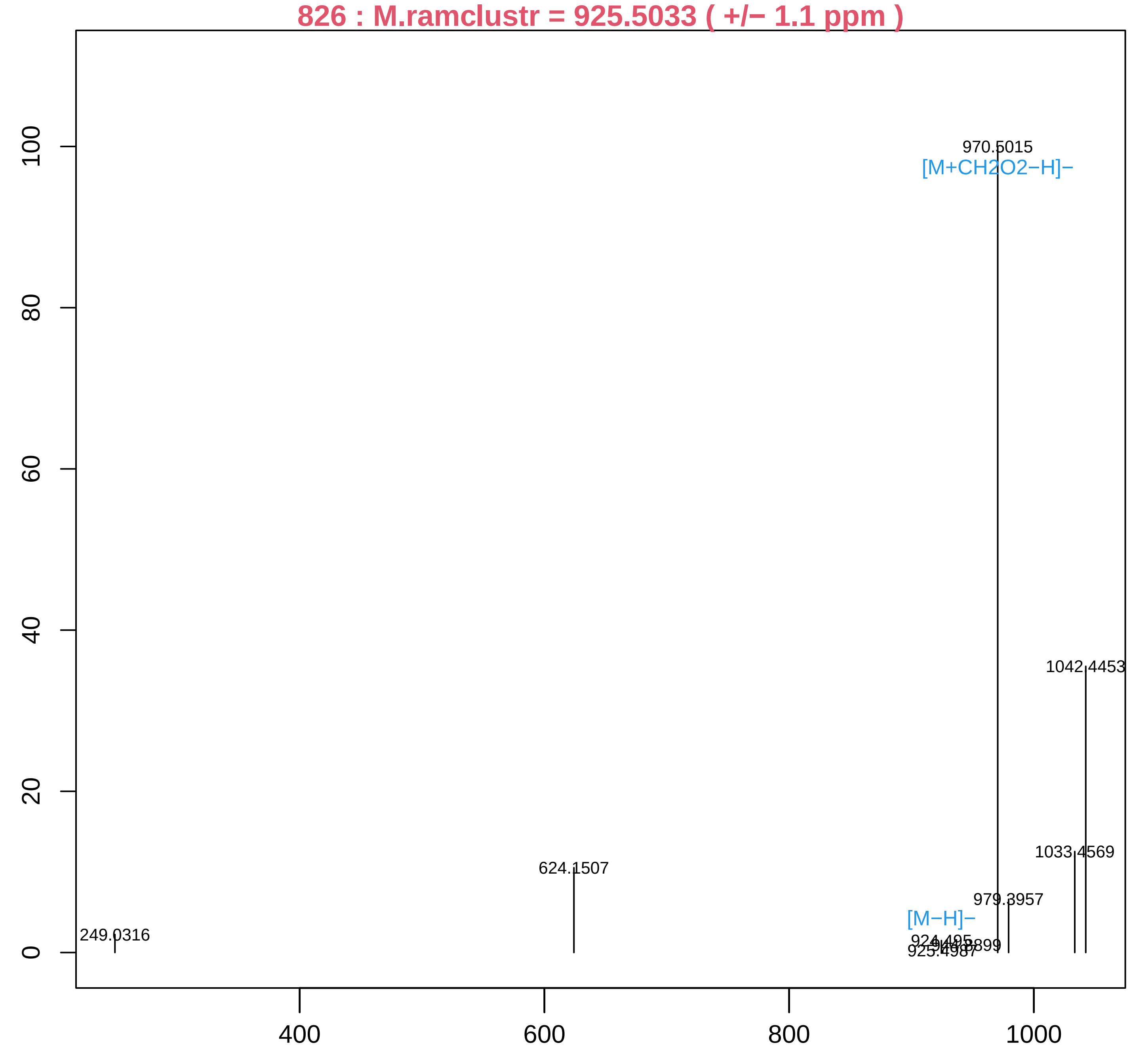

826 : M.ramclustr = 925.5033 (+/− 1.1 ppm)
970.5015
[M+CH2O2−H]−
1042.4453
1033.4569
624.1507
979.3957
[M−H]−
249.0316
924.495
925.4987
0
20
40
60
80
100
400
600
800
1000

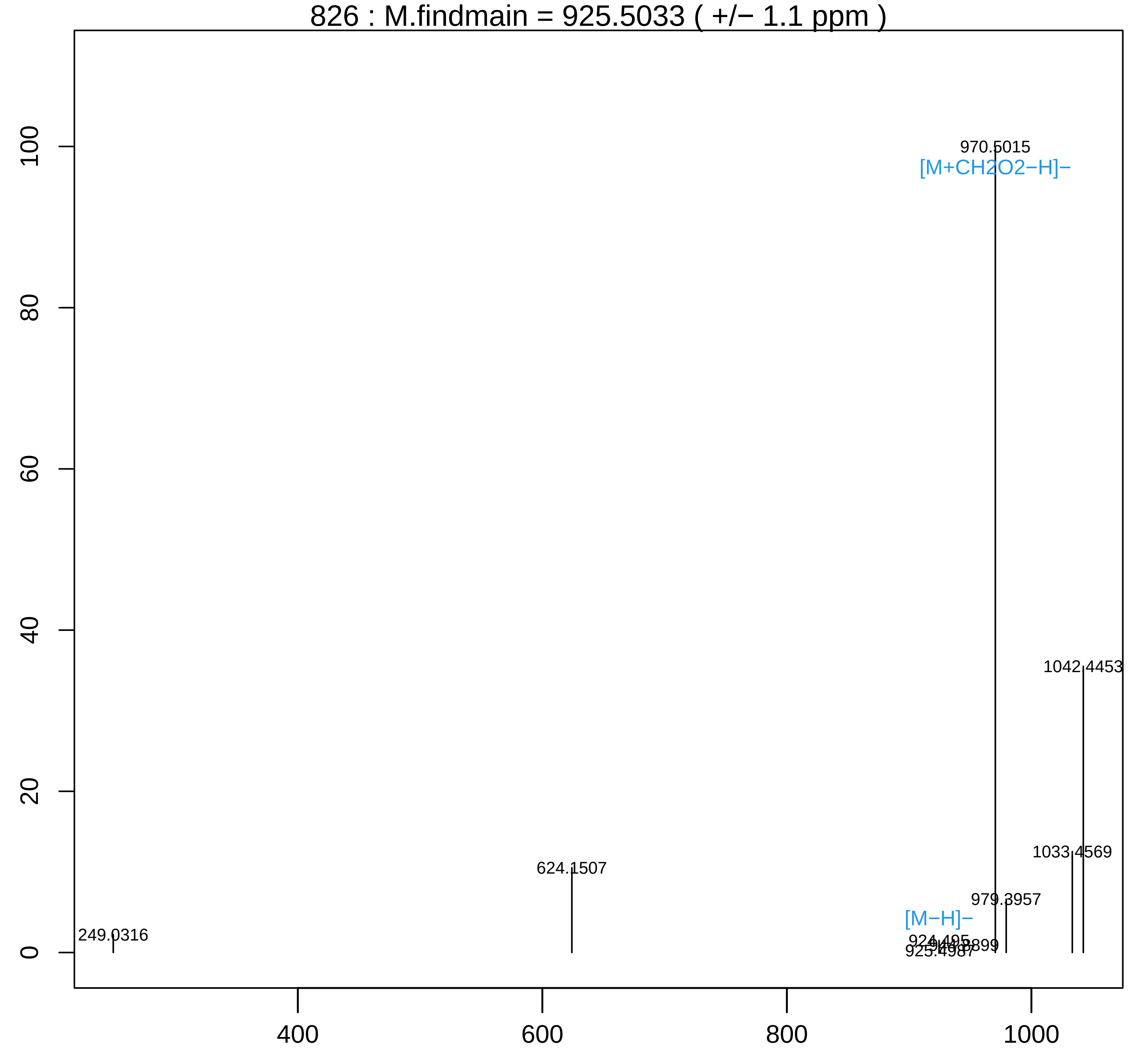

826 : M.findmain = 925.5033 (+/− 1.1 ppm)
970.5015
[M+CH2O2−H]−
1042.4453
1033.4569
624.1507
979.3957
[M−H]−
249.0316
924.495
925.4987
0
20
40
60
80
100
400
600
800
1000

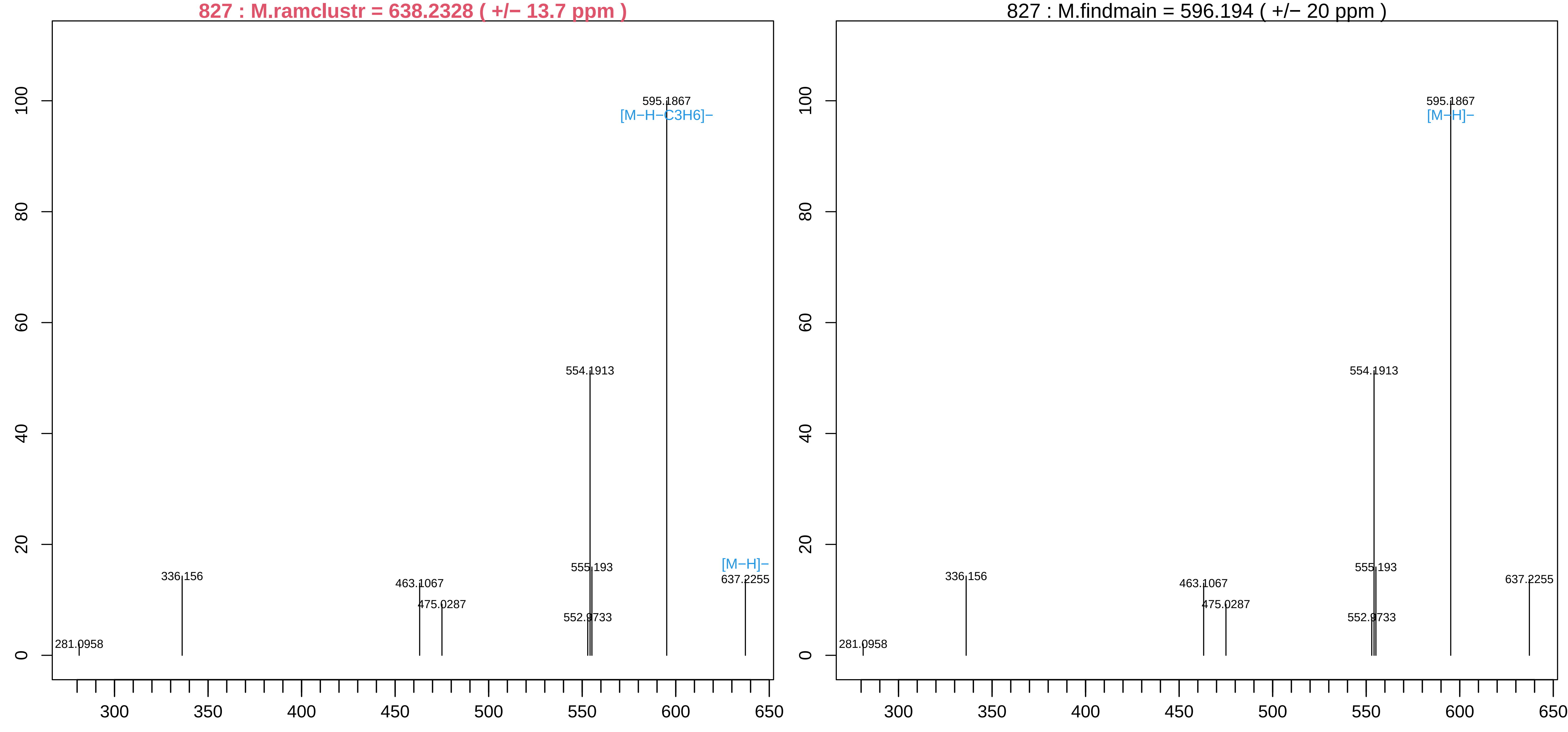
827 : M.ramclustr = 638.2328 (+/− 13.7 ppm)
595.1867
[M−H−C3H6]−
554.1913
555.193
552.9733
463.1067
475.0287
336.156
281.0958
[M−H]−
637.2255
827 : M.findmain = 596.194 (+/− 20 ppm)
595.1867
[M−H]−
554.1913
555.193
552.9733
463.1067
475.0287
336.156
281.0958
637.2255
0
20
40
60
80
100
300
350
400
450
500
550
600
650

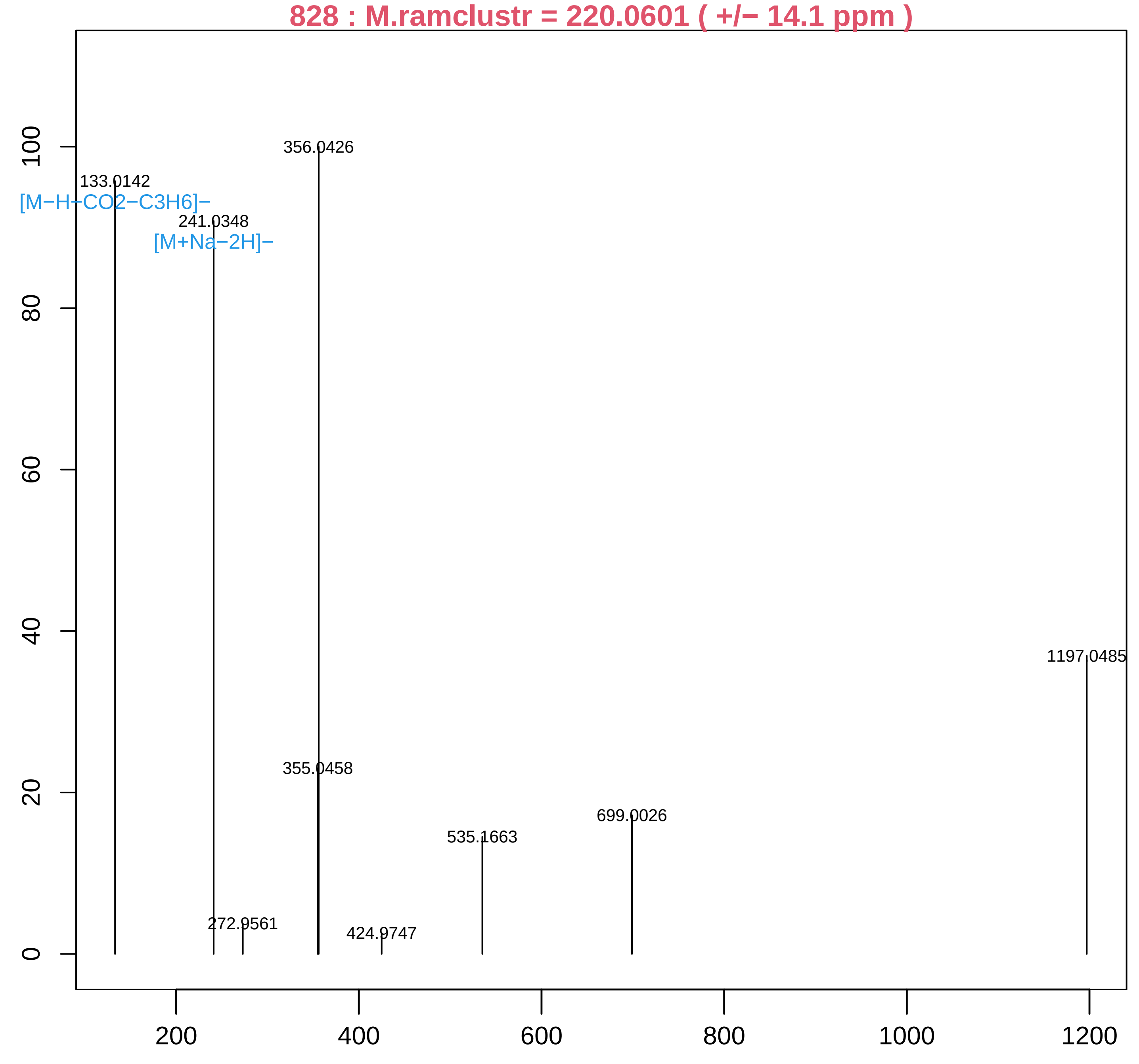
828 : M.ramclustr = 220.0601 (+/− 14.1 ppm)
356.0426
133.0142
[M−H−CO2−C3H6]−
241.0348
[M+Na−2H]−
1197.0485
355.0458
699.0026
535.1663
272.9561
424.9747
0
20
40
60
80
100
200
400
600
800
1000
1200

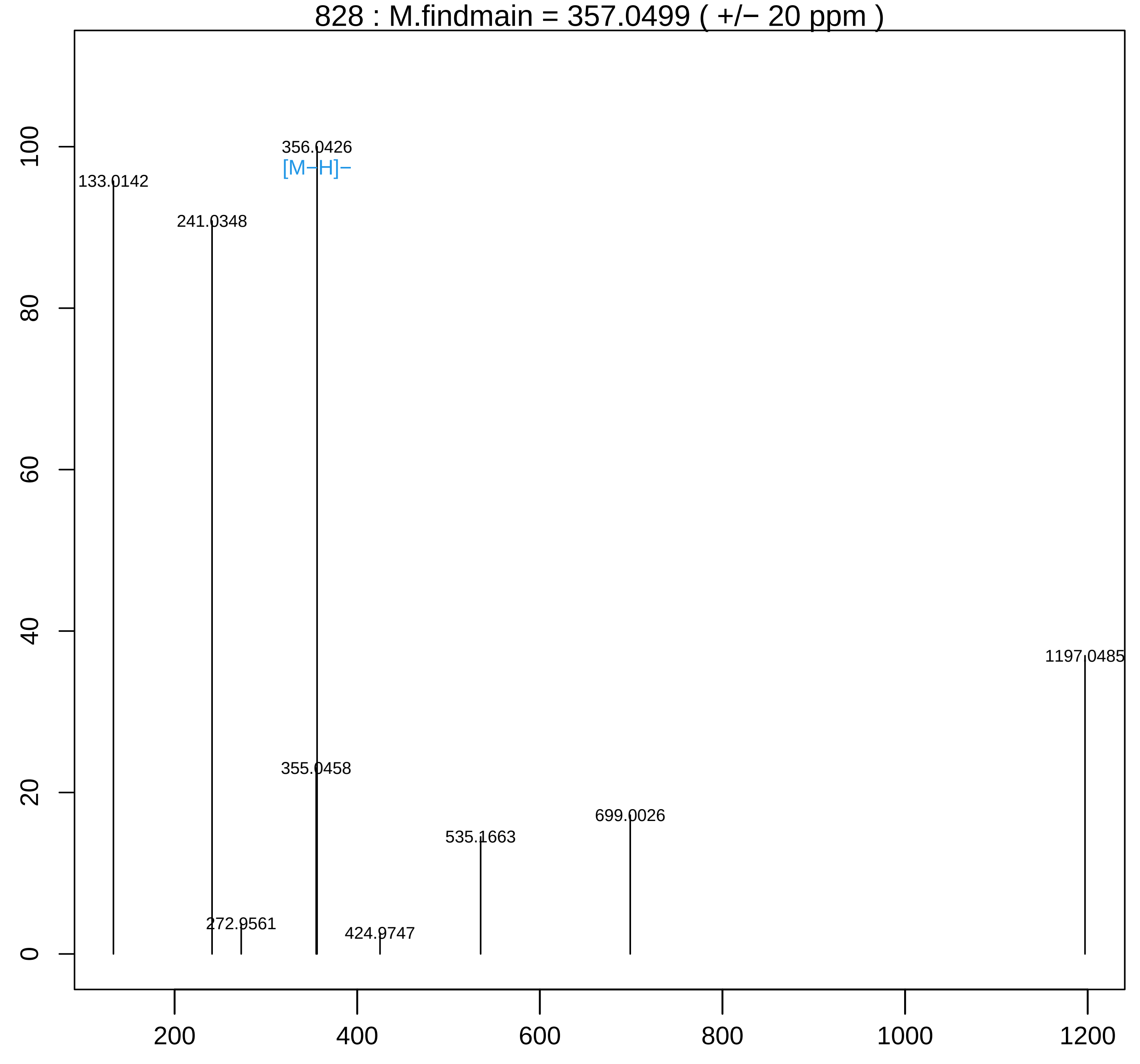
828 : M.findmain = 357.0499 (+/− 20 ppm)
356.0426
[M−H]−
133.0142
241.0348
1197.0485
355.0458
699.0026
535.1663
272.9561
424.9747
0
20
40
60
80
100
200
400
600
800
1000
1200

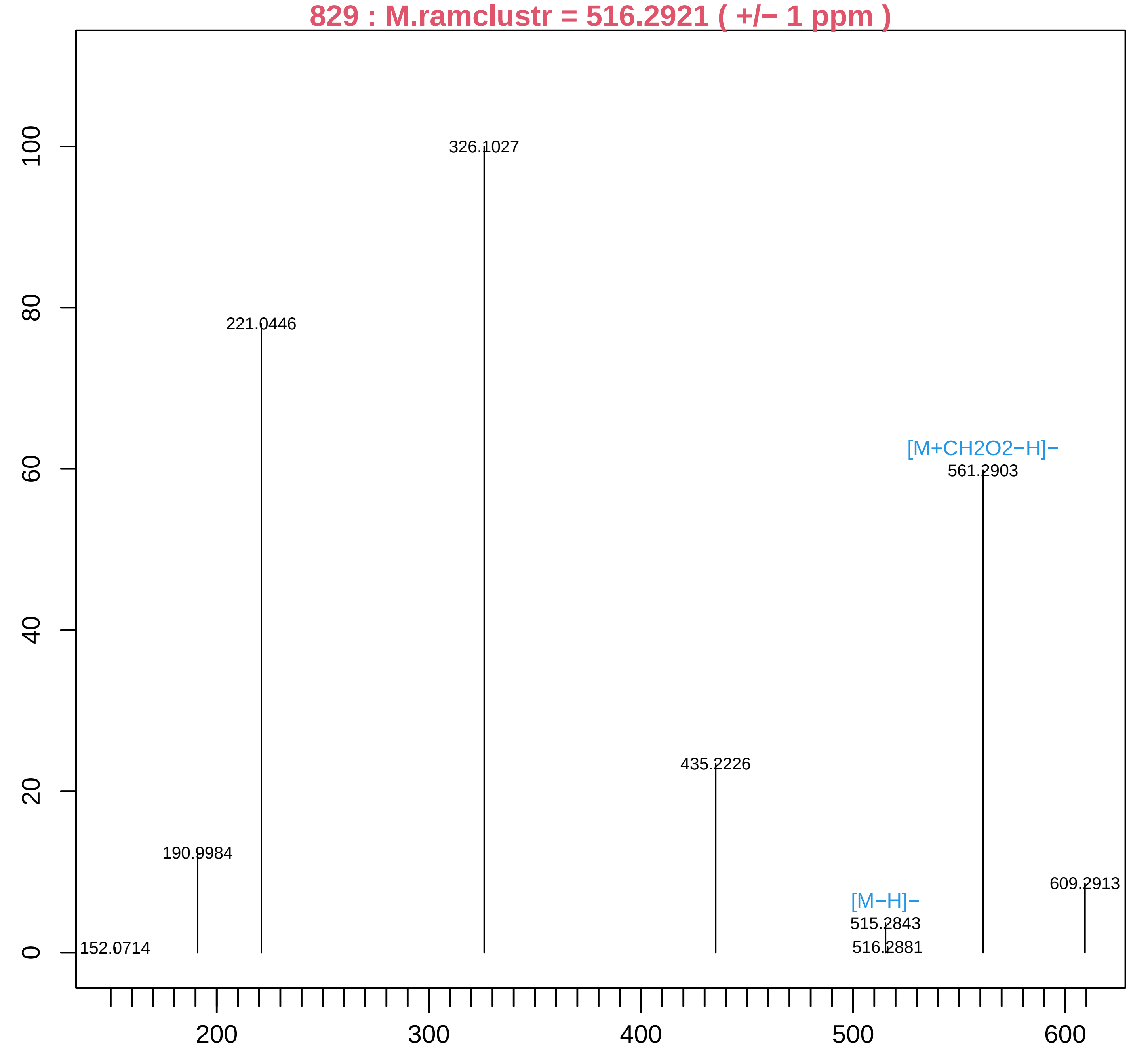
829 : M.ramclustr = 516.2921 (+/− 1 ppm)
326.1027
221.0446
[M+CH2O2−H]−
561.2903
435.2226
190.9984
609.2913
[M−H]−
515.2843
516.2881
152.0714
0
20
40
60
80
100
200
300
400
500
600

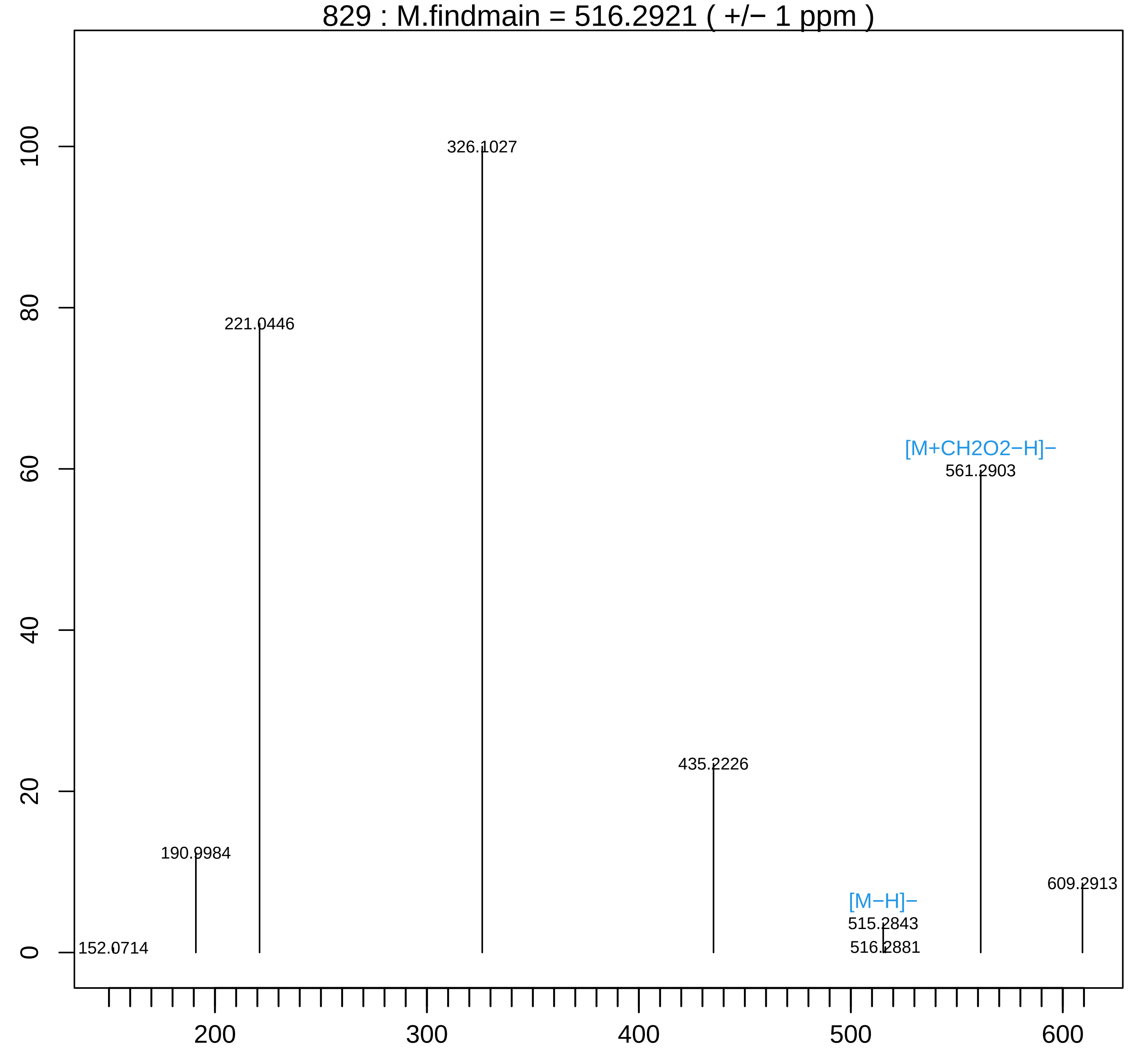
829 : M.findmain = 516.2921 (+/− 1 ppm)
326.1027
221.0446
[M+CH2O2−H]−
561.2903
435.2226
190.9984
609.2913
[M−H]−
515.2843
516.2881
152.0714
0
20
40
60
80
100
200
300
400
500
600

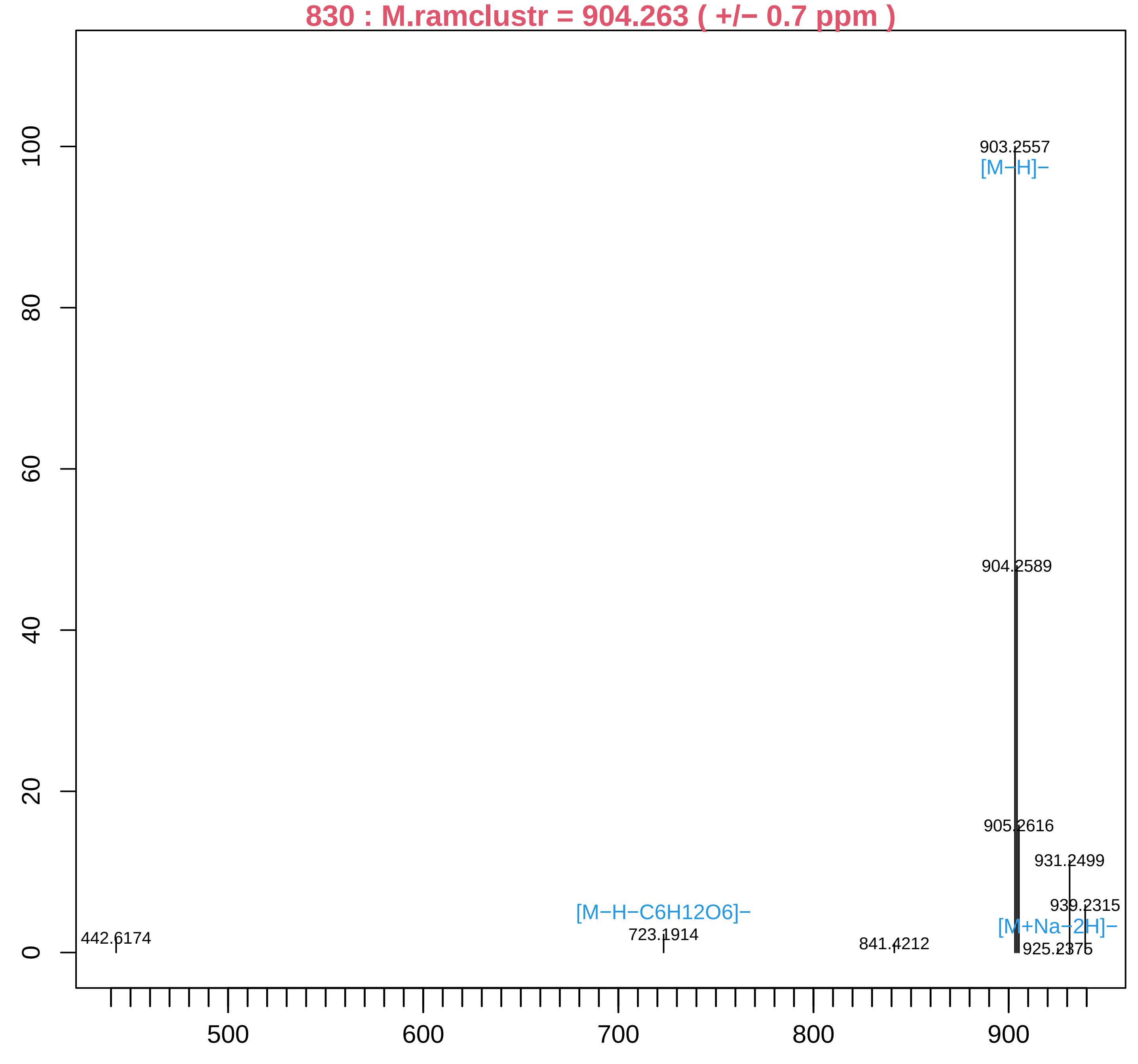

830 : M.ramclustr = 904.263 (+/− 0.7 ppm)
0
20
40
60
80
100
500
600
700
800
900
903.2557
[M−H]−
904.2589
905.2616
931.2499
939.2315
[M+Na−2H]−
925.2375
[M−H−C6H12O6]−
723.1914
841.4212
442.6174

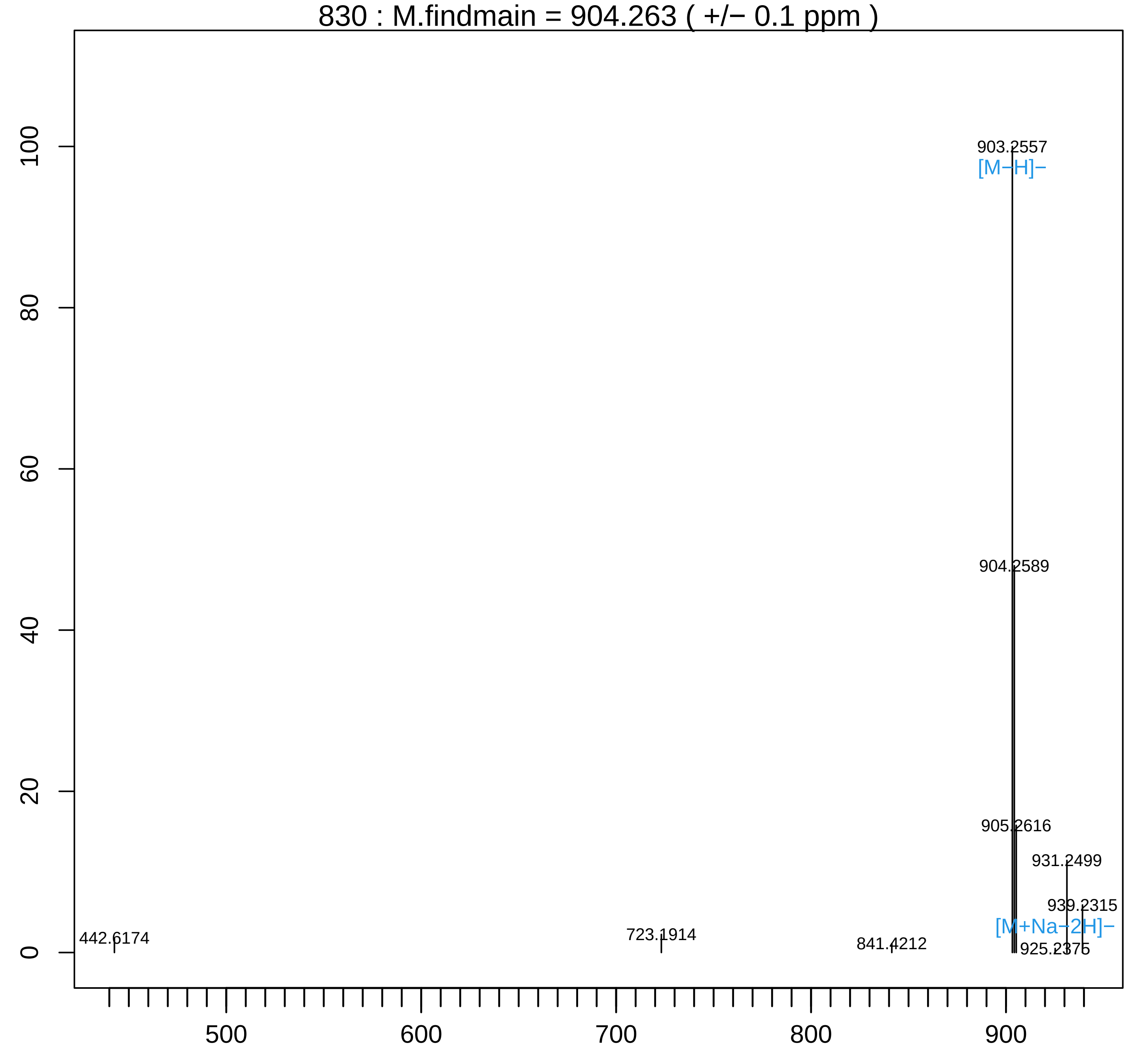

830 : M.findmain = 904.263 (+/− 0.1 ppm)
0
20
40
60
80
100
500
600
700
800
900
903.2557
[M−H]−
904.2589
905.2616
931.2499
939.2315
[M+Na−2H]−
925.2375
723.1914
841.4212
442.6174

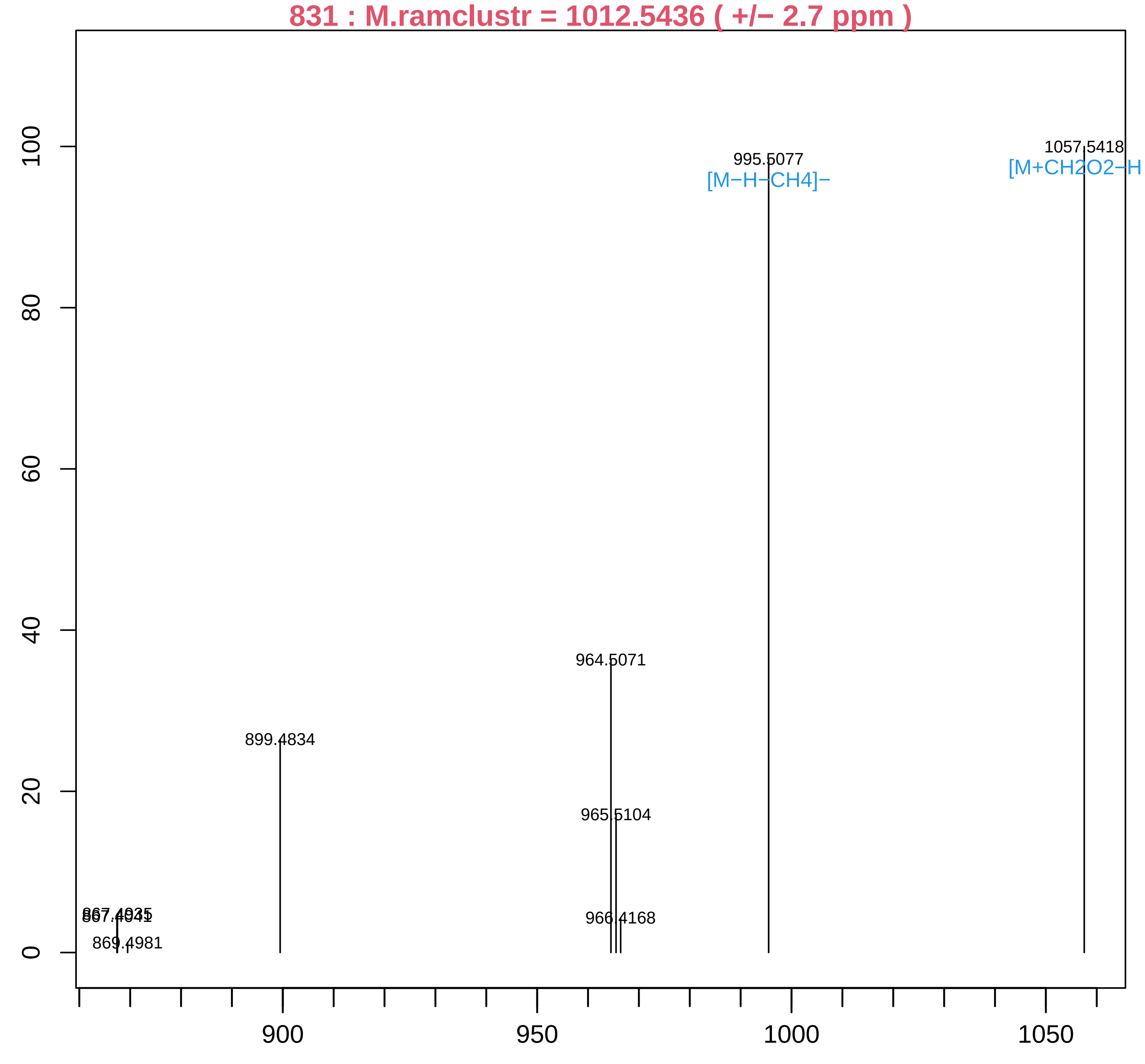

831 : M.ramclustr = 1012.5436 (+/− 2.7 ppm)
1057.5418
[M+CH2O2−H
995.5077
[M−H−CH4]−
964.5071
965.5104
966.4168
899.4834
867.4935
867.4041
869.4981
0
20
40
60
80
100
900
950
1000
1050

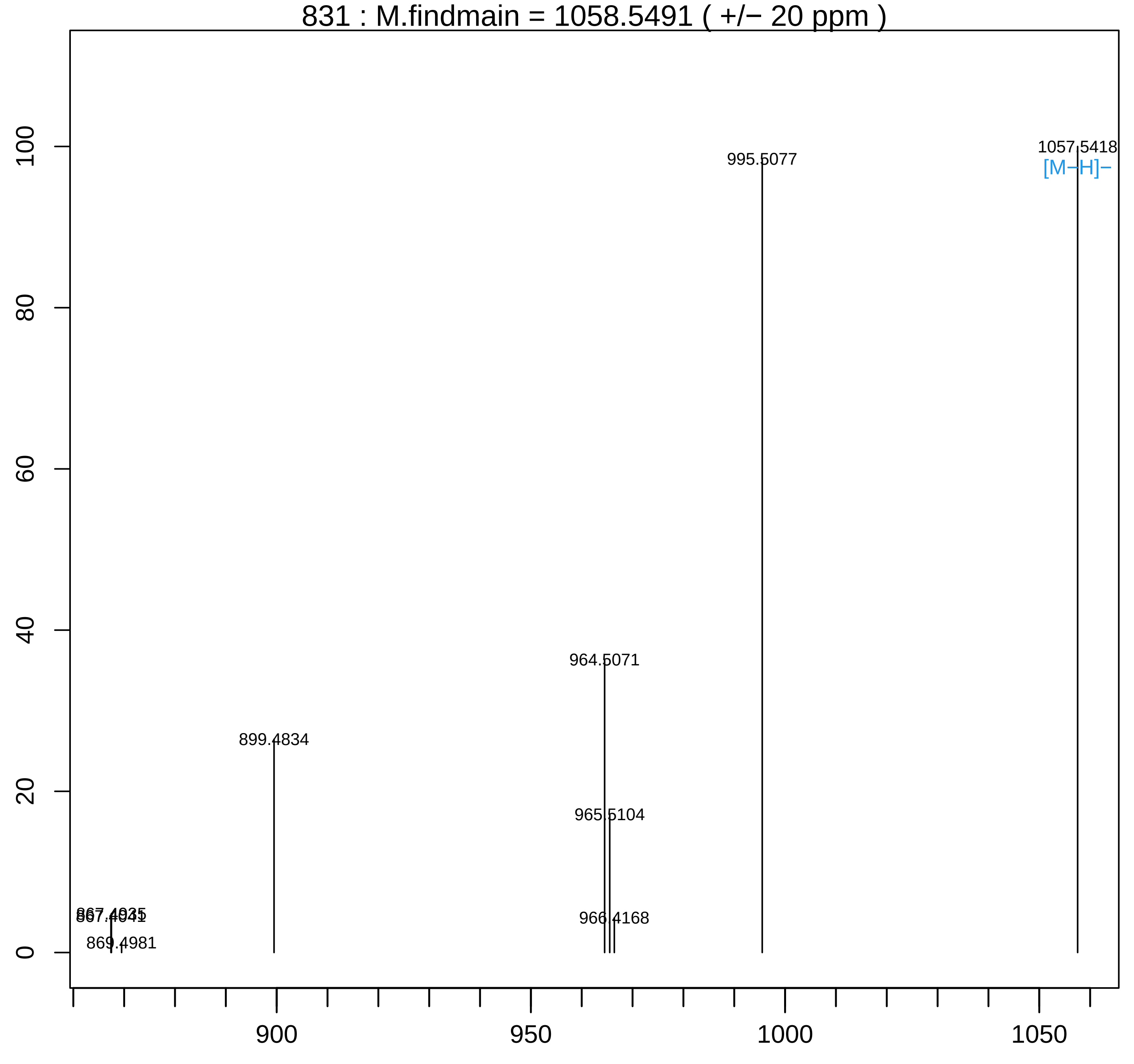

831 : M.findmain = 1058.5491 (+/− 20 ppm)
1057.5418
[M−H]−
995.5077
964.5071
965.5104
966.4168
899.4834
867.4935
867.4041
869.4981
0
20
40
60
80
100
900
950
1000
1050

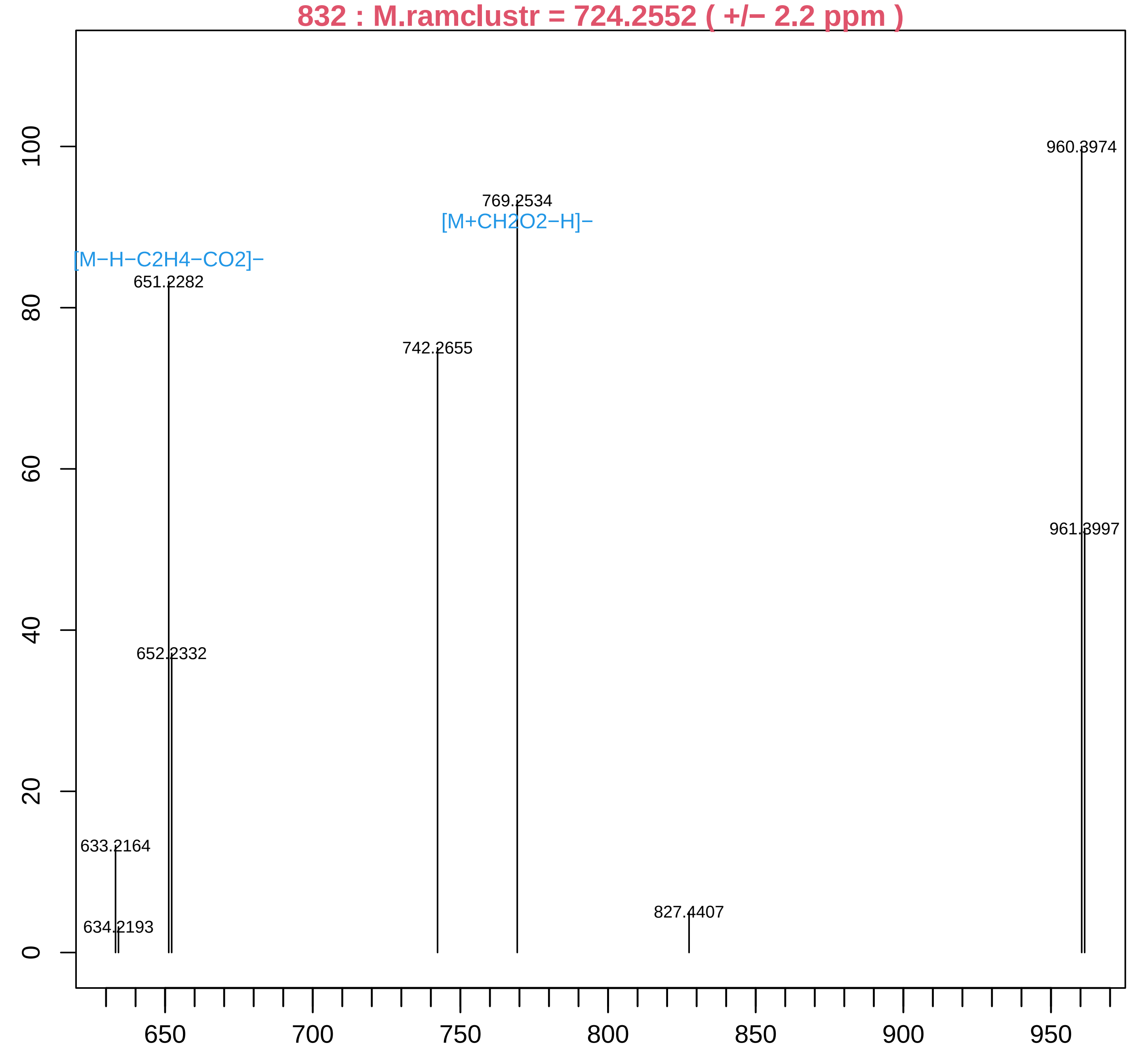
832 : M.ramclustr = 724.2552 (+/− 2.2 ppm)
960.3974
769.2534
[M+CH2O2−H]−
[M−H−C2H4−CO2]−
651.2282
742.2655
961.3997
652.2332
633.2164
634.2193
827.4407
0
20
40
60
80
100
650
700
750
800
850
900
950

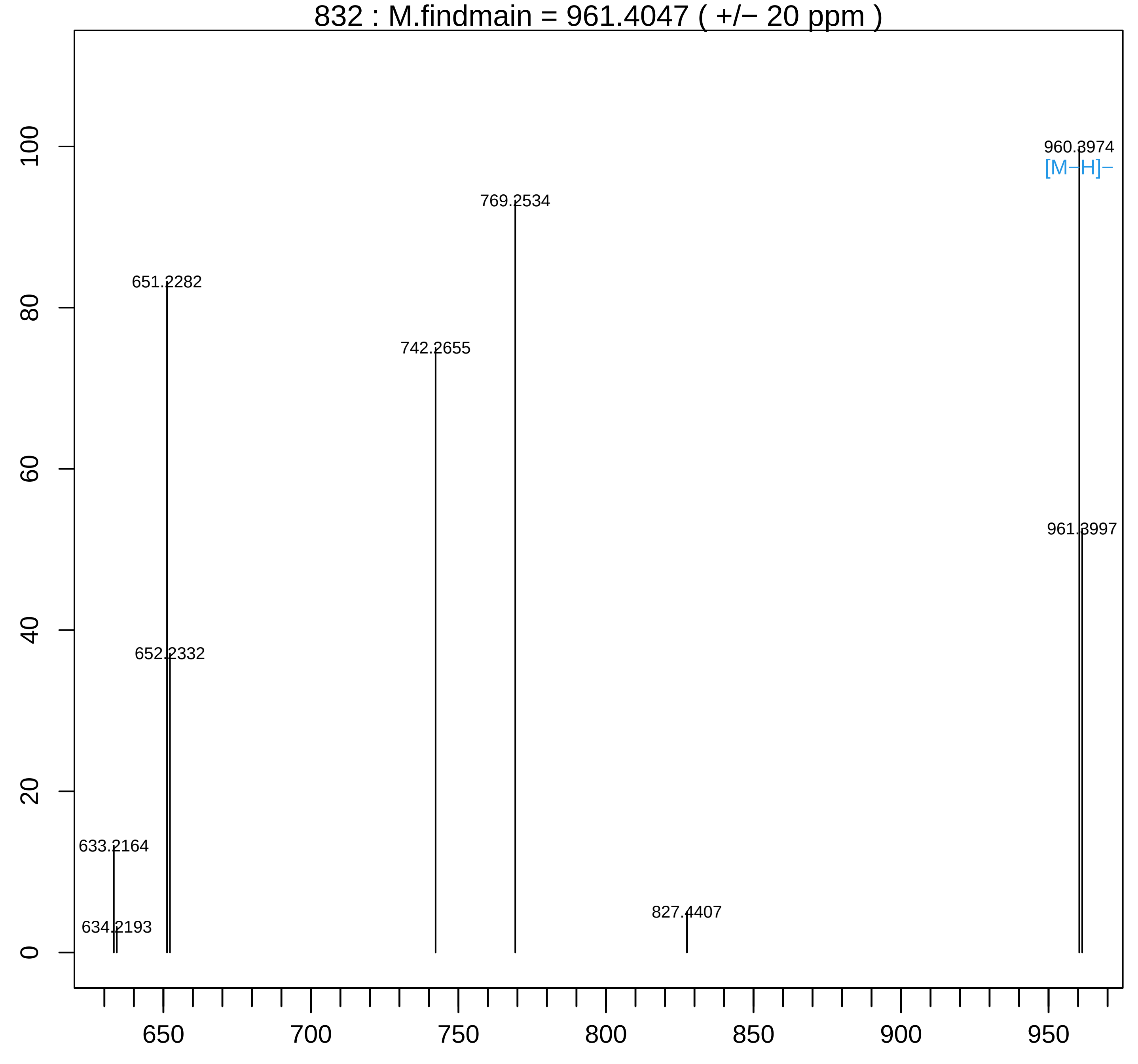
832 : M.findmain = 961.4047 (+/− 20 ppm)
960.3974
[M−H]−
769.2534
651.2282
742.2655
961.3997
652.2332
633.2164
634.2193
827.4407
0
20
40
60
80
100
650
700
750
800
850
900
950

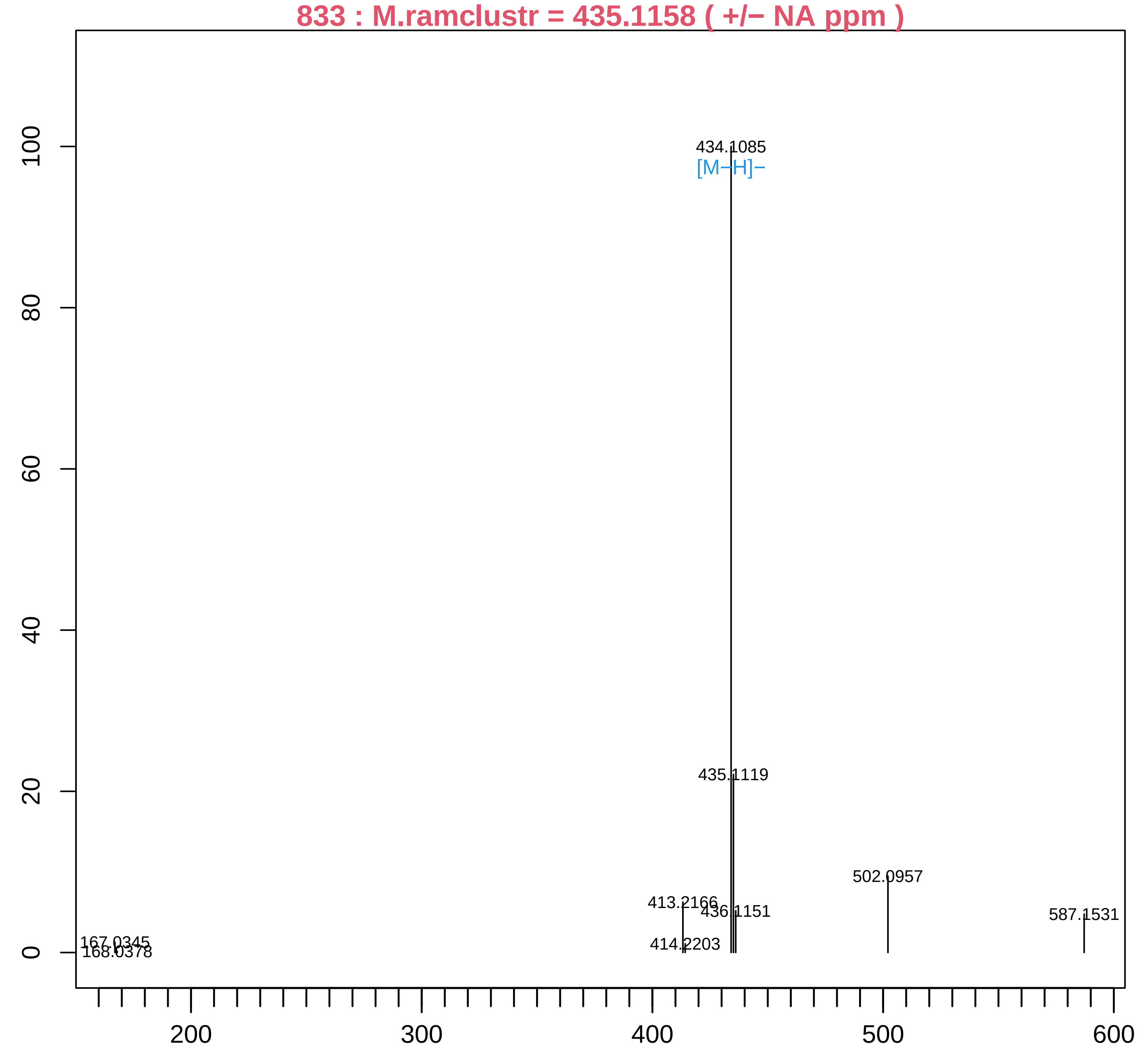

833 : M.ramclustr = 435.1158 (+/− NA ppm)
434.1085
[M−H]−
435.1119
502.0957
413.2166
436.1151
587.1531
167.0345
168.0378
414.2203
0
20
40
60
80
100
200
300
400
500
600

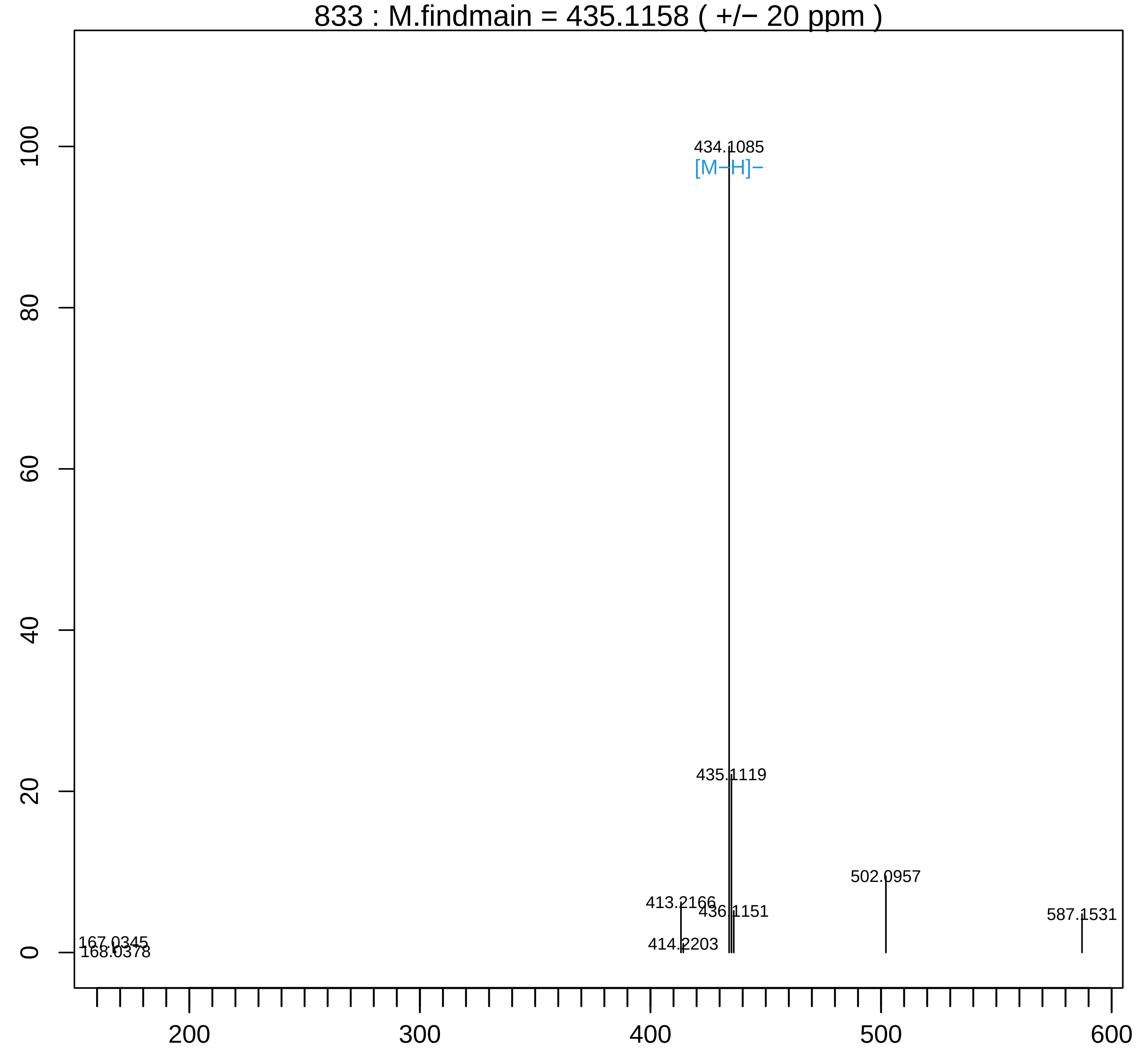

833 : M.findmain = 435.1158 (+/− 20 ppm)
434.1085
[M−H]−
435.1119
502.0957
413.2166
436.1151
587.1531
167.0345
168.0378
414.2203
0
20
40
60
80
100
200
300
400
500
600

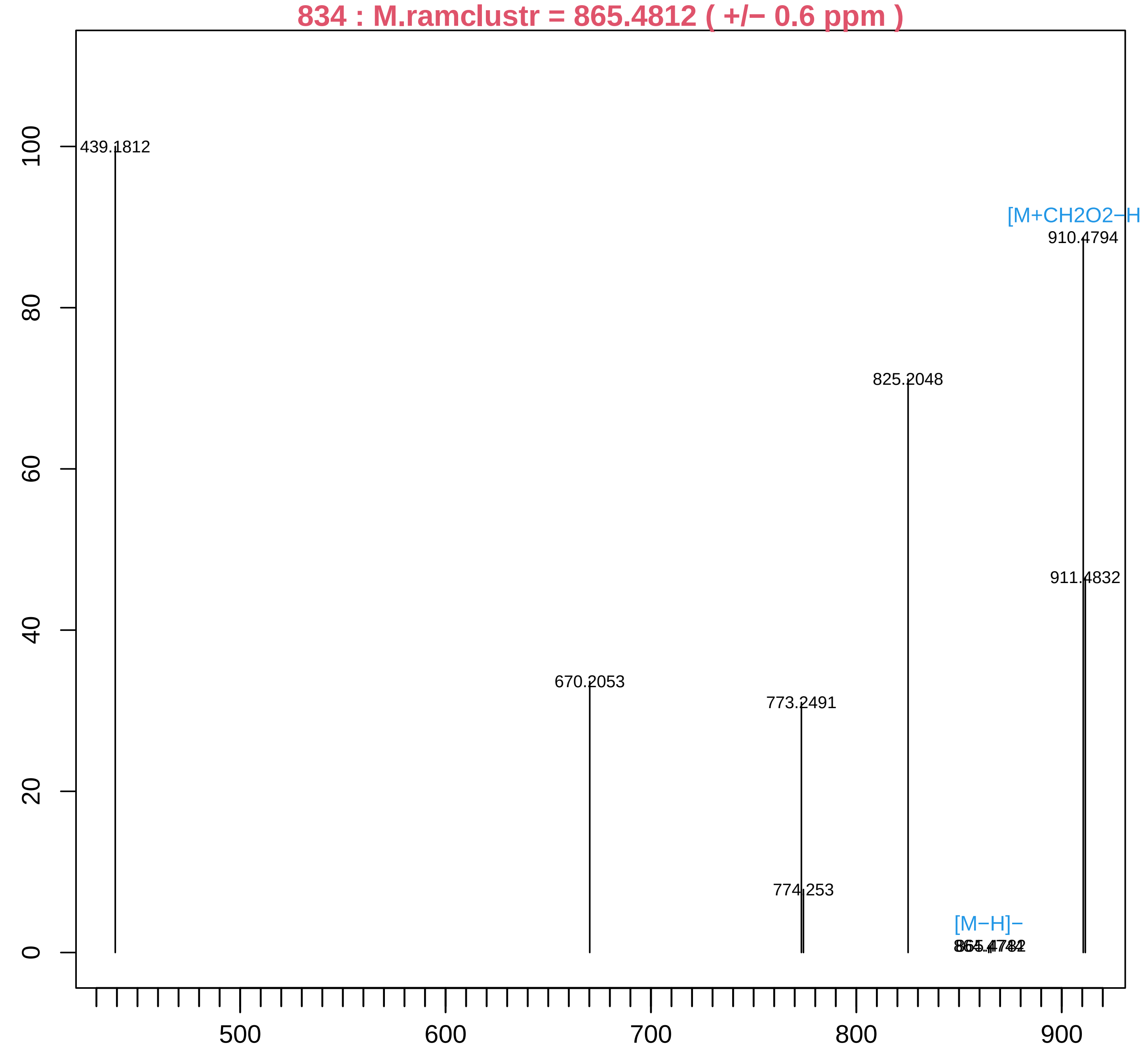

834 : M.ramclustr = 865.4812 (+/− 0.6 ppm)
439.1812
[M+CH2O2−H
910.4794
825.2048
911.4832
670.2053
773.2491
774.253
[M−H]−
865.4782
0
20
40
60
80
100
500
600
700
800
900

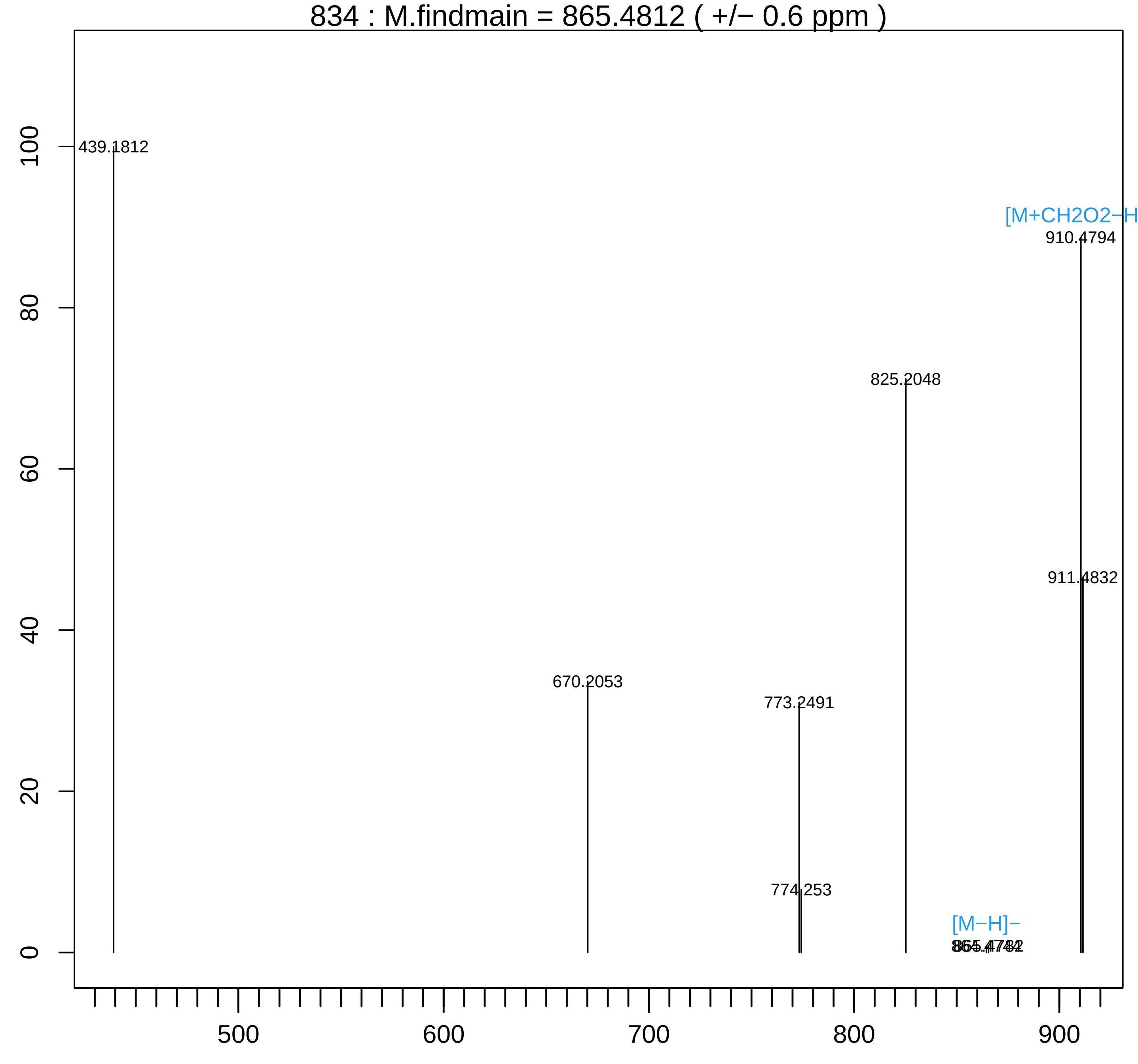

834 : M.findmain = 865.4812 (+/− 0.6 ppm)
439.1812
[M+CH2O2−H
910.4794
825.2048
911.4832
670.2053
773.2491
774.253
[M−H]−
865.4782
0
20
40
60
80
100
500
600
700
800
900

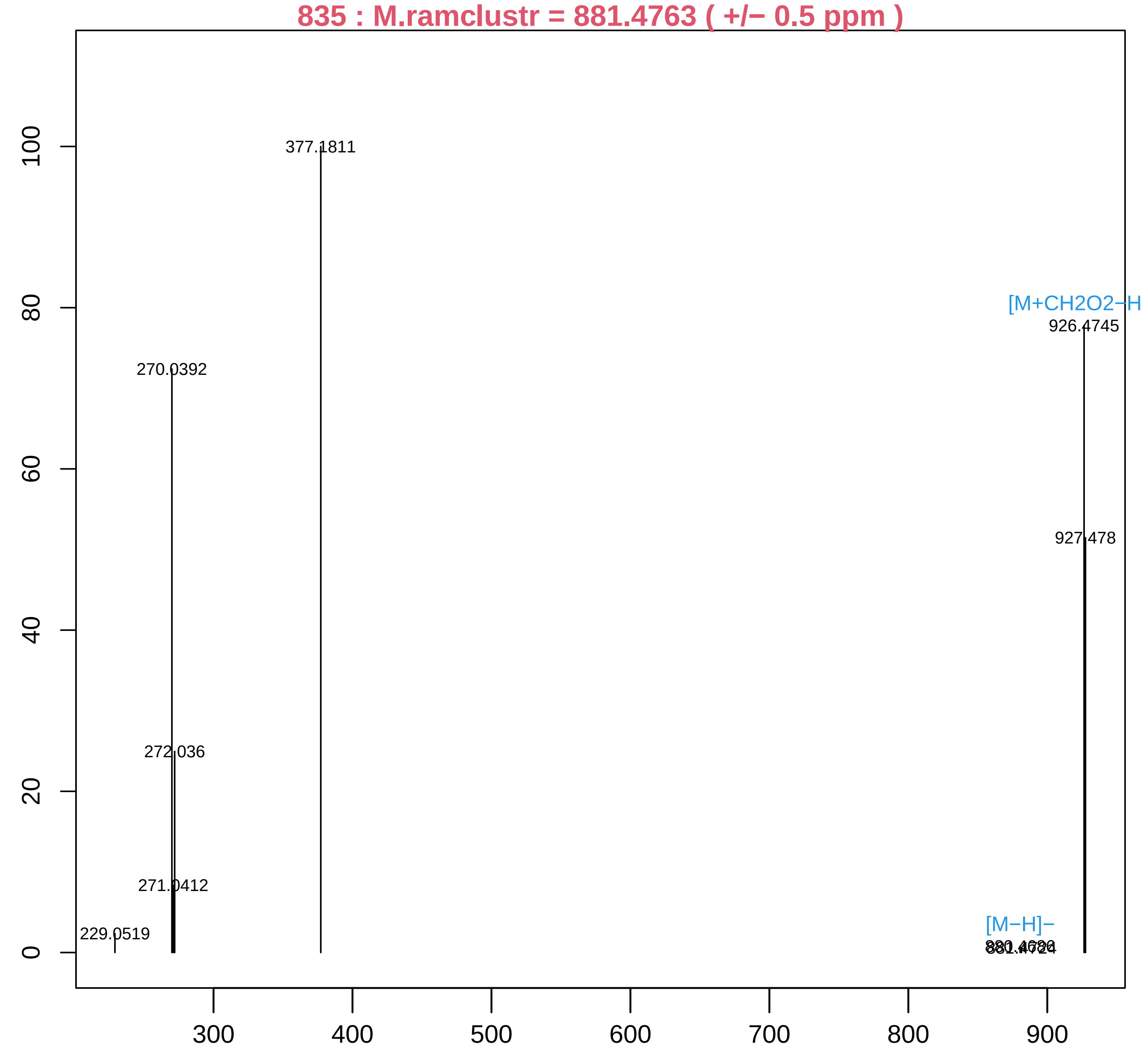

835 : M.ramclustr = 881.4763 (+/− 0.5 ppm)
377.1811
[M+CH2O2−H
926.4745
270.0392
927.478
272.036
271.0412
229.0519
[M−H]−
881.4724
0
20
40
60
80
100
300
400
500
600
700
800
900

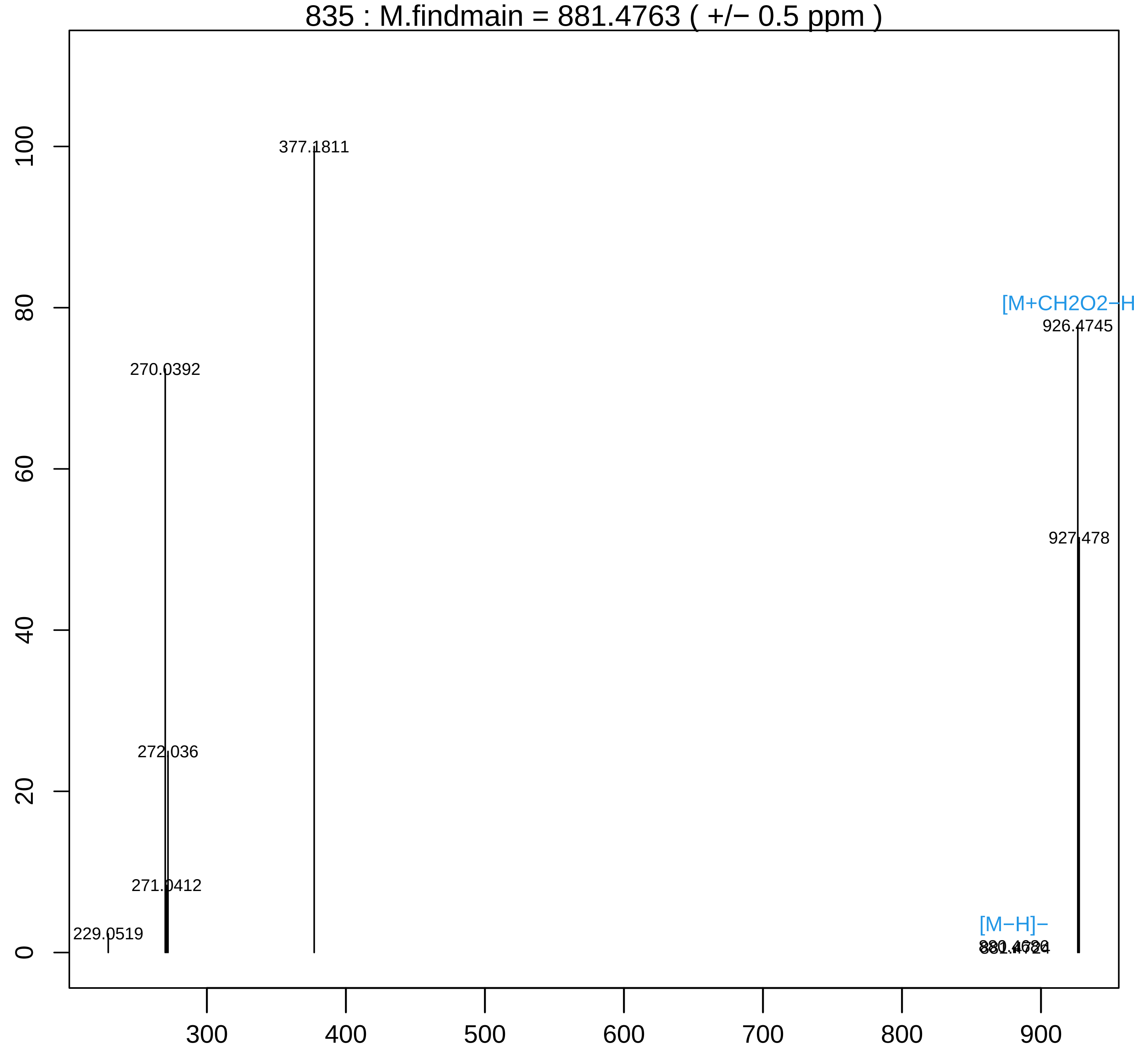

835 : M.findmain = 881.4763 (+/− 0.5 ppm)
377.1811
[M+CH2O2−H
926.4745
270.0392
927.478
272.036
271.0412
229.0519
[M−H]−
881.4724
0
20
40
60
80
100
300
400
500
600
700
800
900

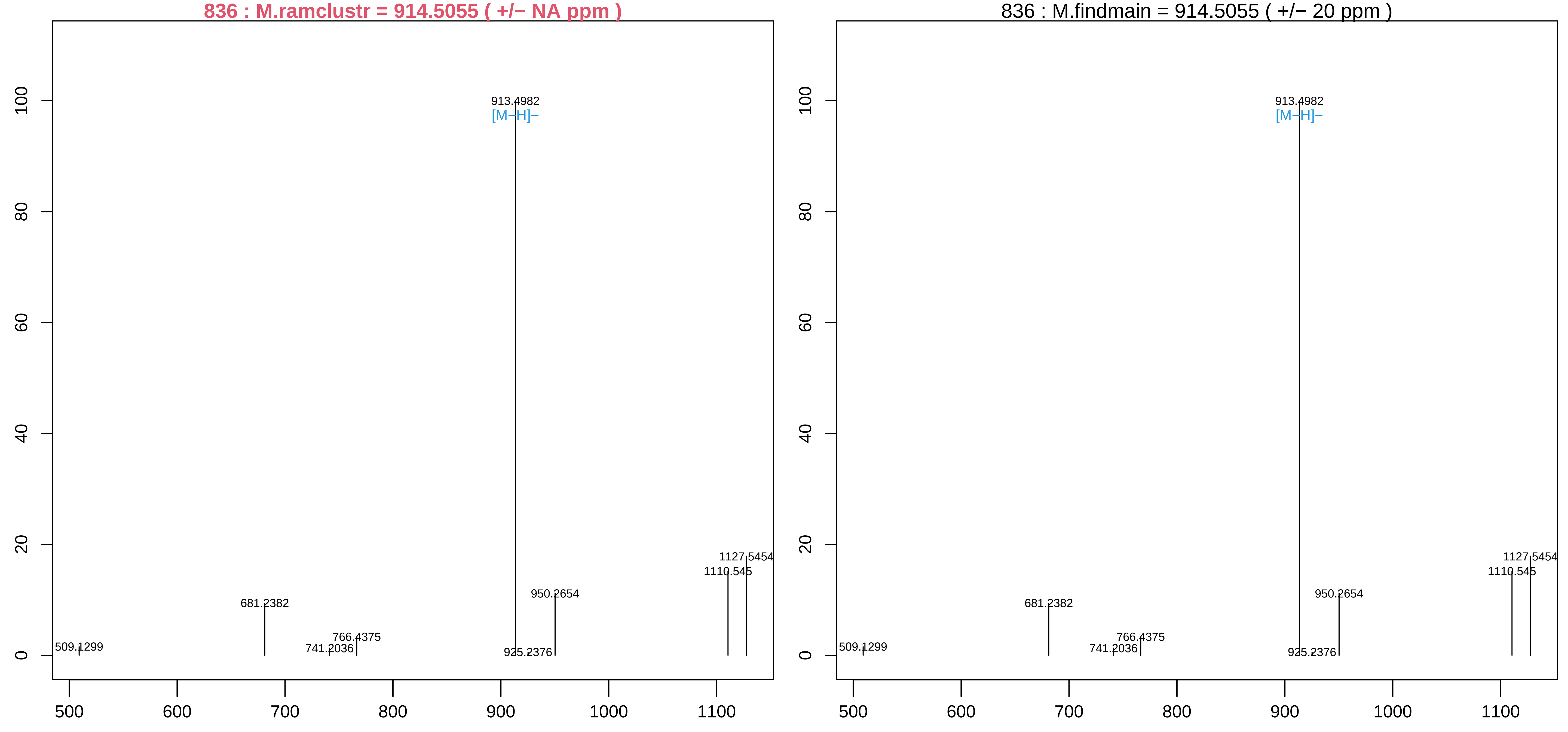
836 : M.ramclustr = 914.5055 (+/− NA ppm)
836 : M.findmain = 914.5055 (+/− 20 ppm)
913.4982
[M−H]−
1127.5454
1110.545
950.2654
681.2382
766.4375
509.1299
741.2036
925.2376
0
20
40
60
80
100
500
600
700
800
900
1000
1100

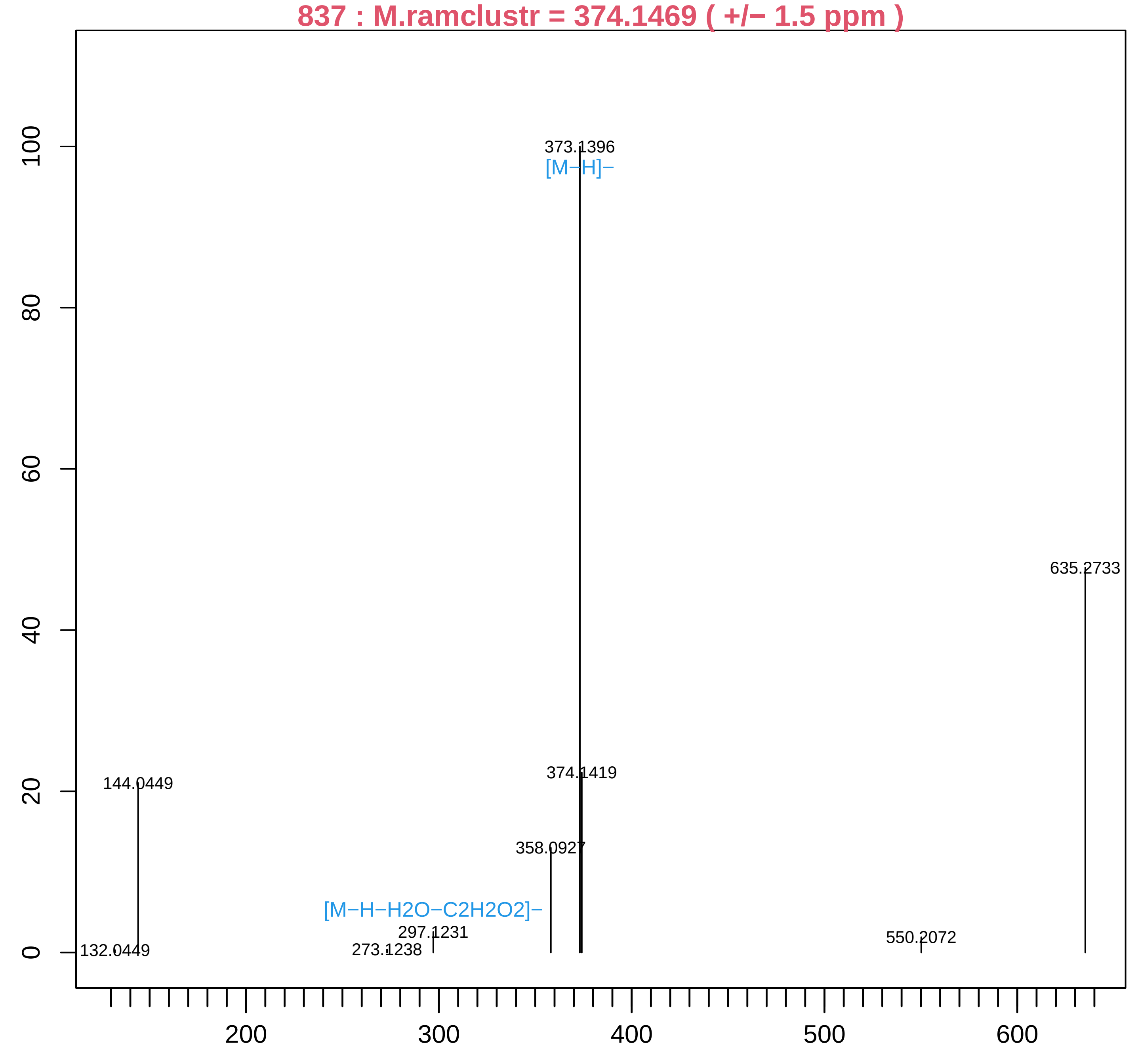
837 : M.ramclustr = 374.1469 (+/− 1.5 ppm)
373.1396
[M−H]−
635.2733
144.0449
374.1419
358.0927
[M−H−H2O−C2H2O2]−
297.1231
550.2072
132.0449
273.1238
0
20
40
60
80
100
200
300
400
500
600

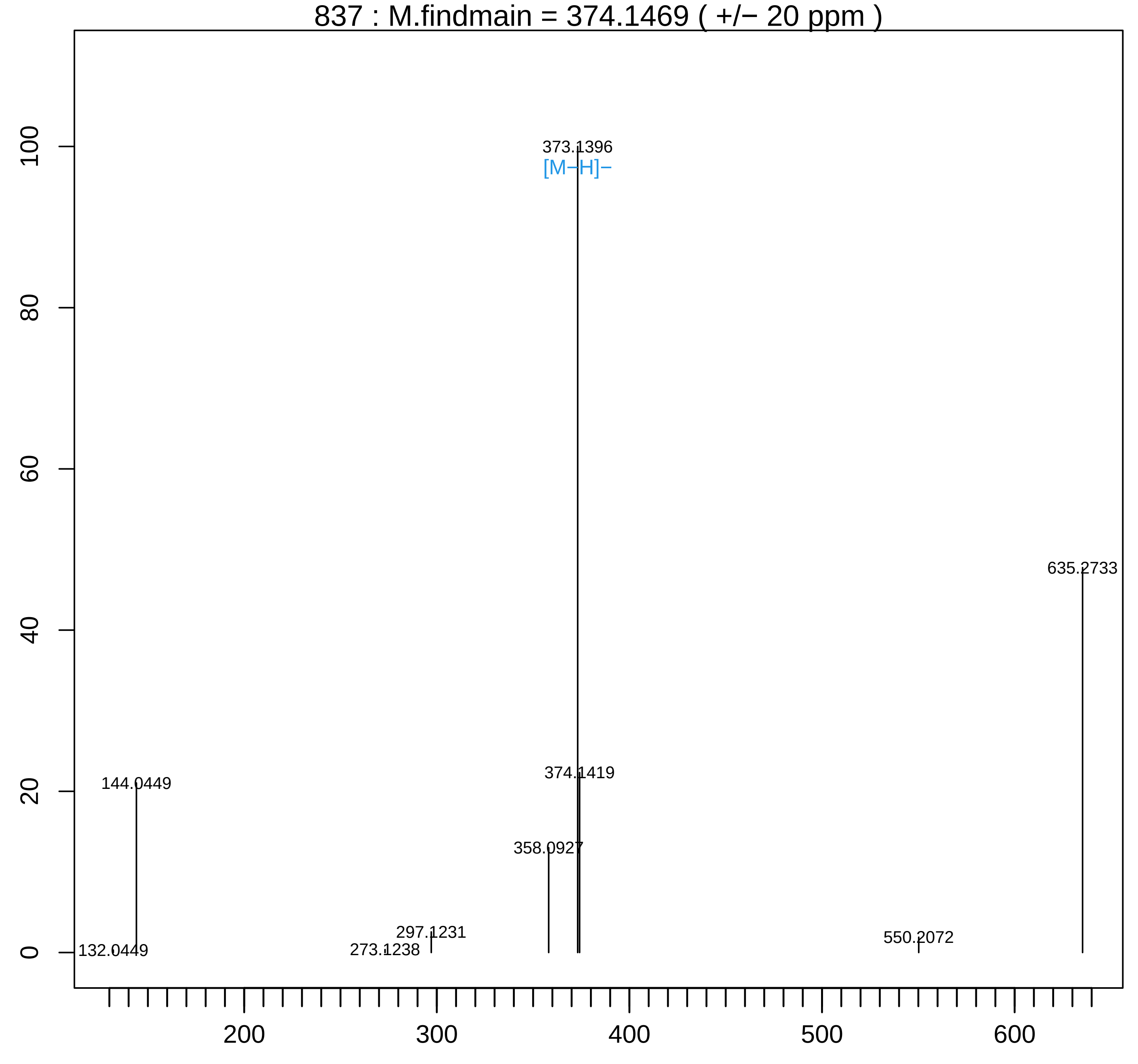
837 : M.findmain = 374.1469 (+/− 20 ppm)
373.1396
[M−H]−
635.2733
144.0449
374.1419
358.0927
297.1231
550.2072
132.0449
273.1238
0
20
40
60
80
100
200
300
400
500
600

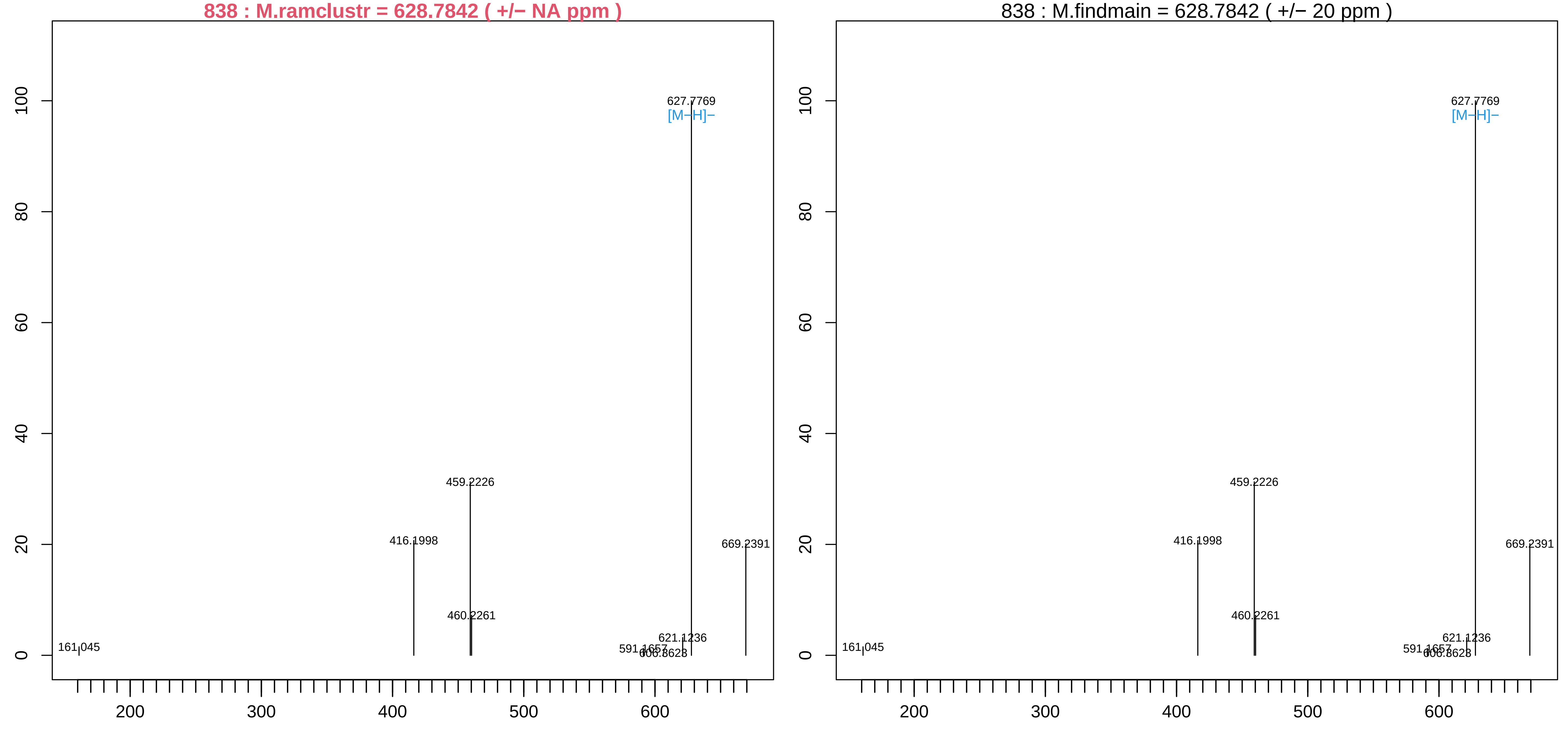
838 : M.ramclustr = 628.7842 (+/− NA ppm)
838 : M.findmain = 628.7842 (+/− 20 ppm)
627.7769
[M−H]−
459.2226
416.1998
669.2391
460.2261
621.1236
591.1657
606.8628
161.045
0
20
40
60
80
100
200
300
400
500
600

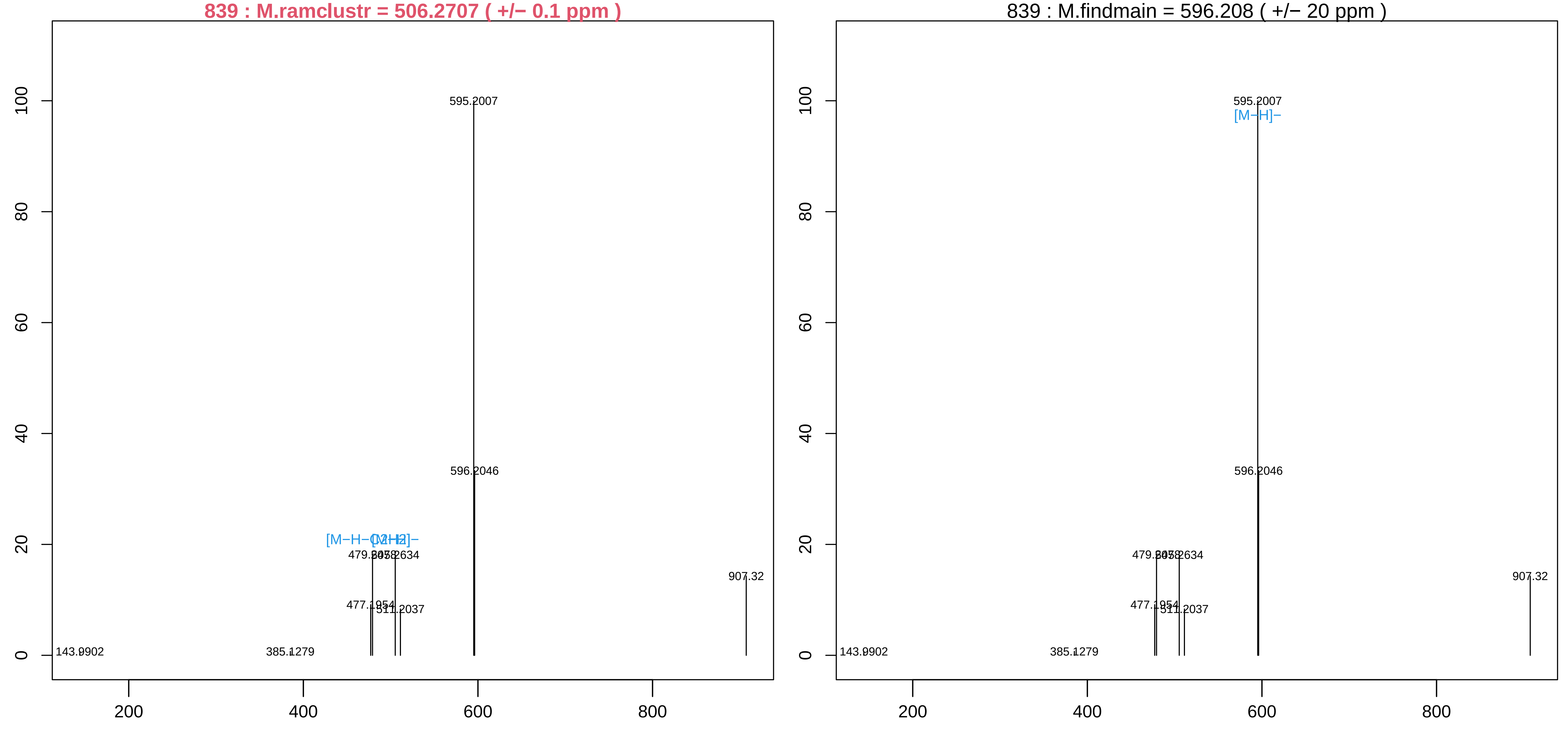
839 : M.ramclustr = 506.2707 (+/− 0.1 ppm)
595.2007
596.2046
[M−H−CO2−H2]−
479.2058
505.2634
477.1954
511.2037
907.32
143.0902
385.1279
839 : M.findmain = 596.208 (+/− 20 ppm)
595.2007
[M−H]−
596.2046
479.2058
505.2634
477.1954
511.2037
907.32
143.0902
385.1279
0
20
40
60
80
100
200
400
600
800

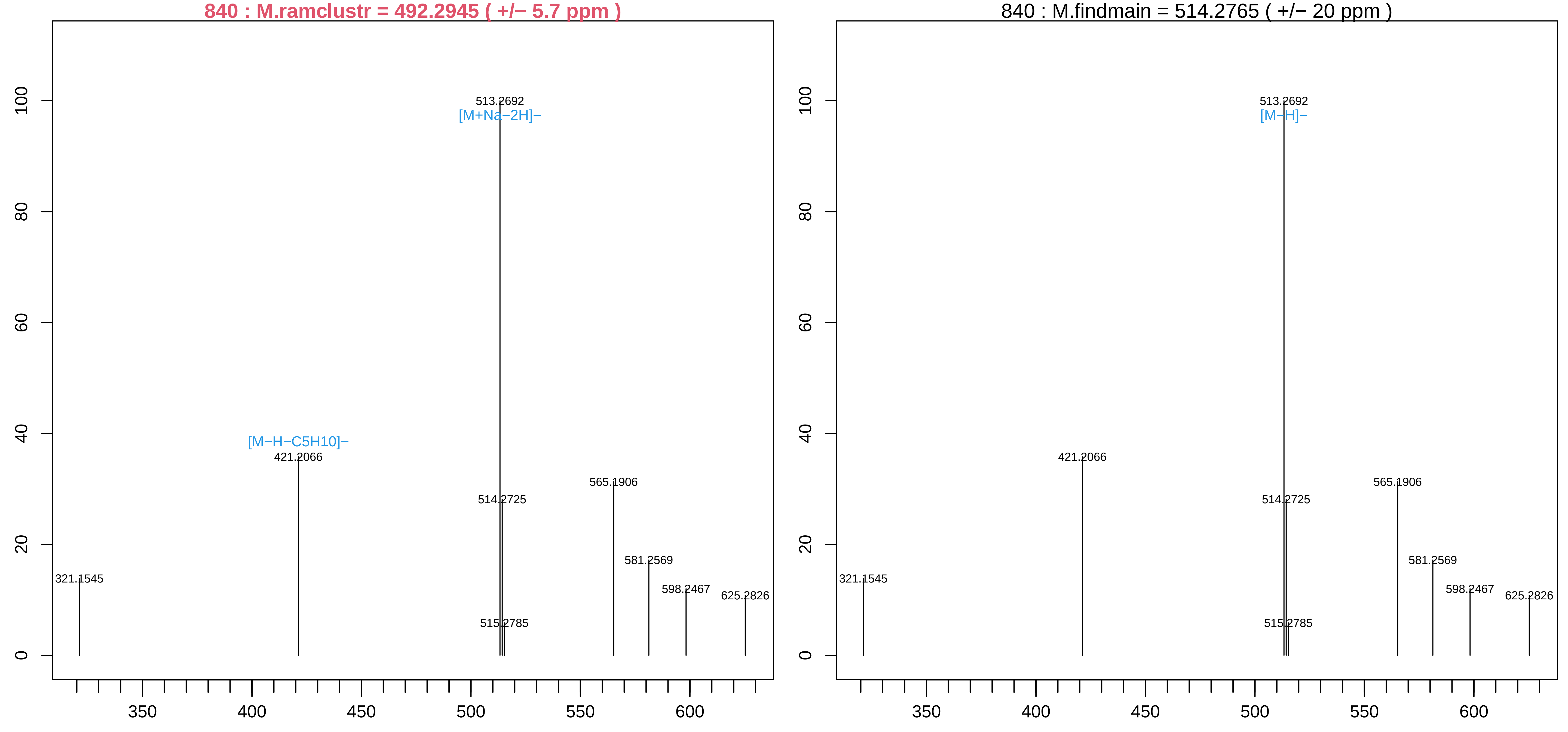

840 : M.ramclustr = 492.2945 (+/− 5.7 ppm)
513.2692
[M+Na−2H]−
[M−H−C5H10]−
421.2066
565.1906
514.2725
581.2569
321.1545
598.2467
625.2826
515.2785
840 : M.findmain = 514.2765 (+/− 20 ppm)
513.2692
[M−H]−
421.2066
565.1906
514.2725
581.2569
321.1545
598.2467
625.2826
515.2785

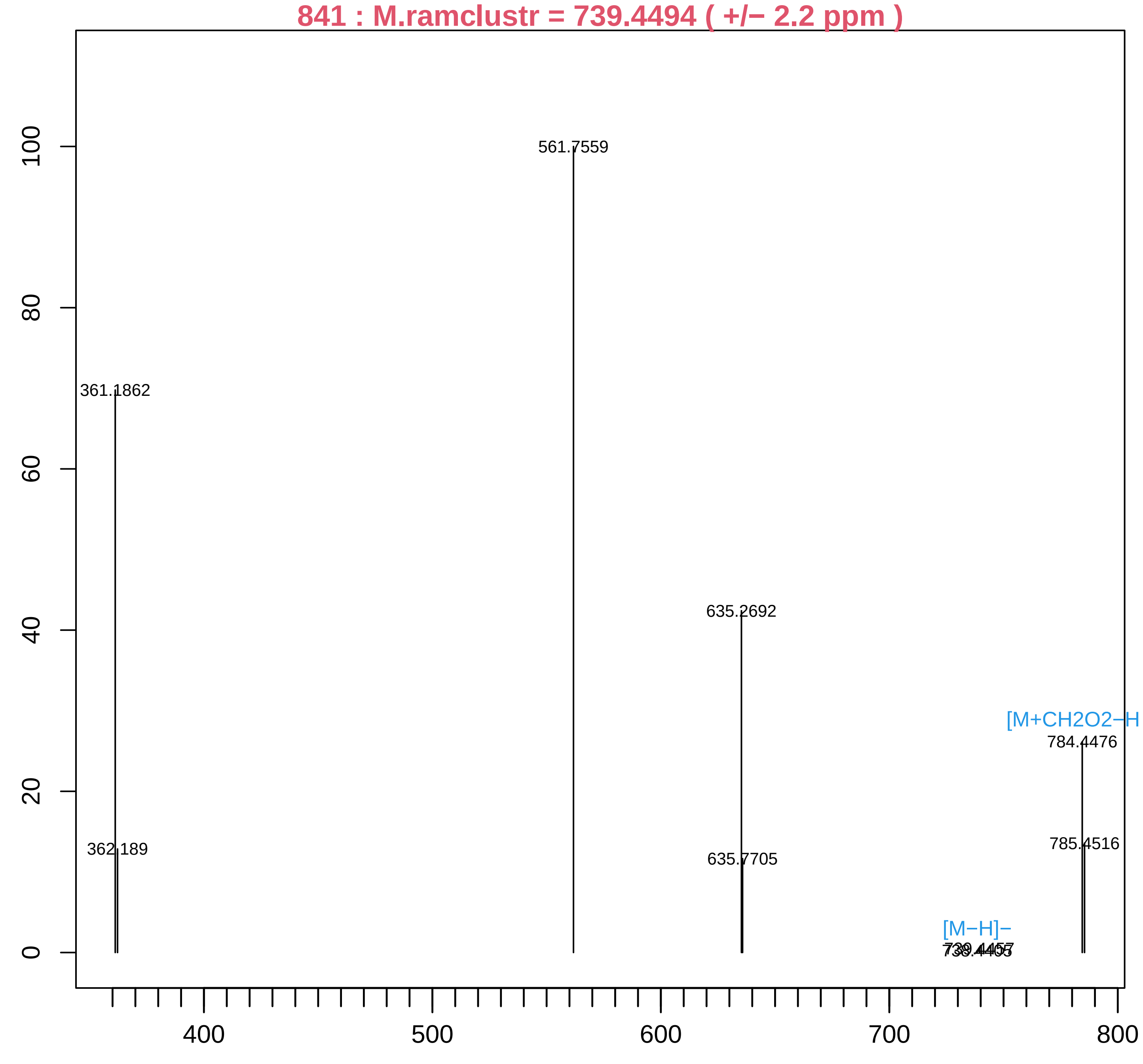

841 : M.ramclustr = 739.4494 (+/− 2.2 ppm)
361.1862
362.189
561.7559
635.2692
635.7705
[M+CH2O2−H
784.4476
785.4516
[M−H]−
739.4457
738.4403
0
20
40
60
80
100
400
500
600
700
800

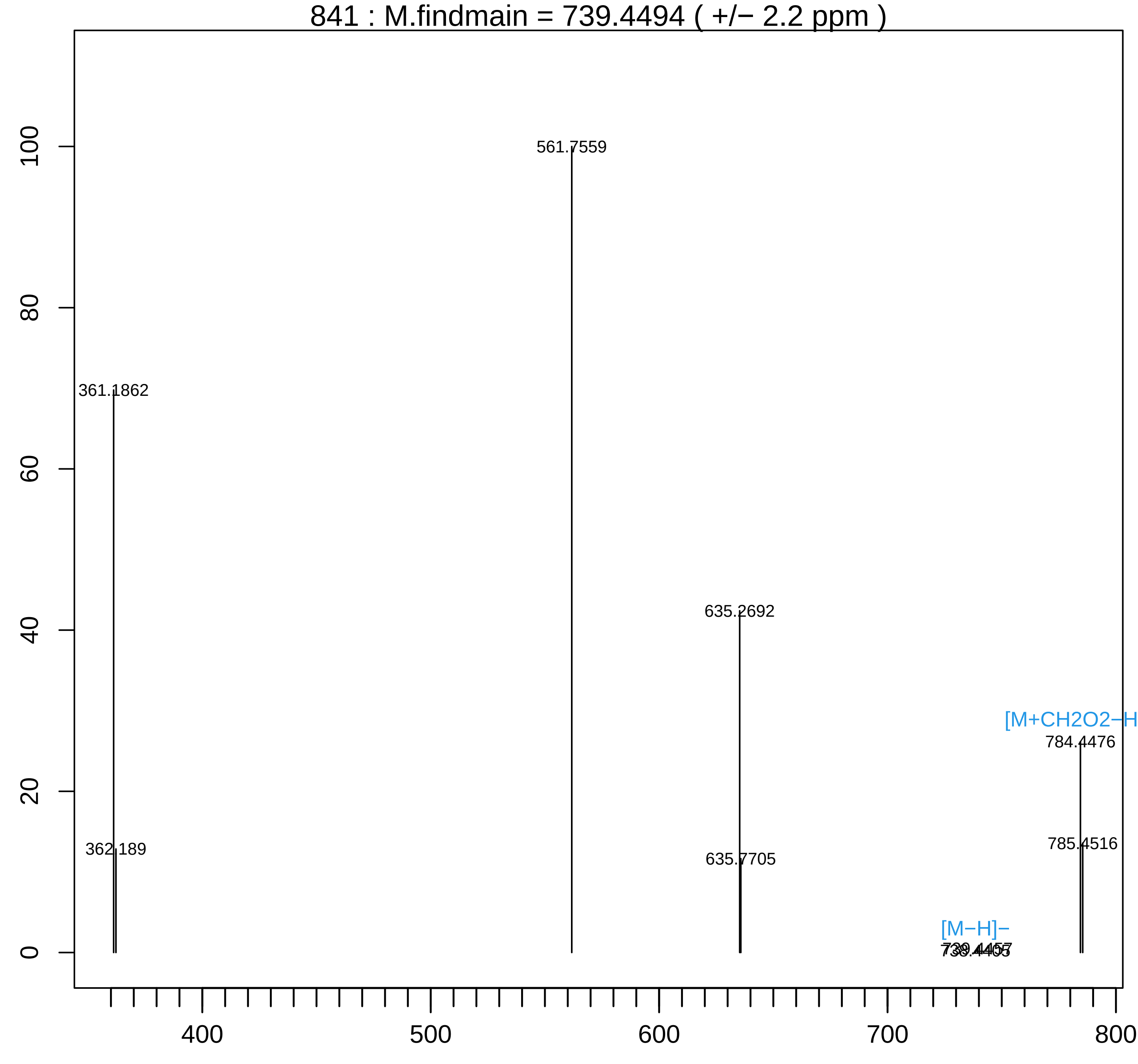

841 : M.findmain = 739.4494 (+/− 2.2 ppm)
361.1862
362.189
561.7559
635.2692
635.7705
[M+CH2O2−H
784.4476
785.4516
[M−H]−
739.4457
738.4403
0
20
40
60
80
100
400
500
600
700
800

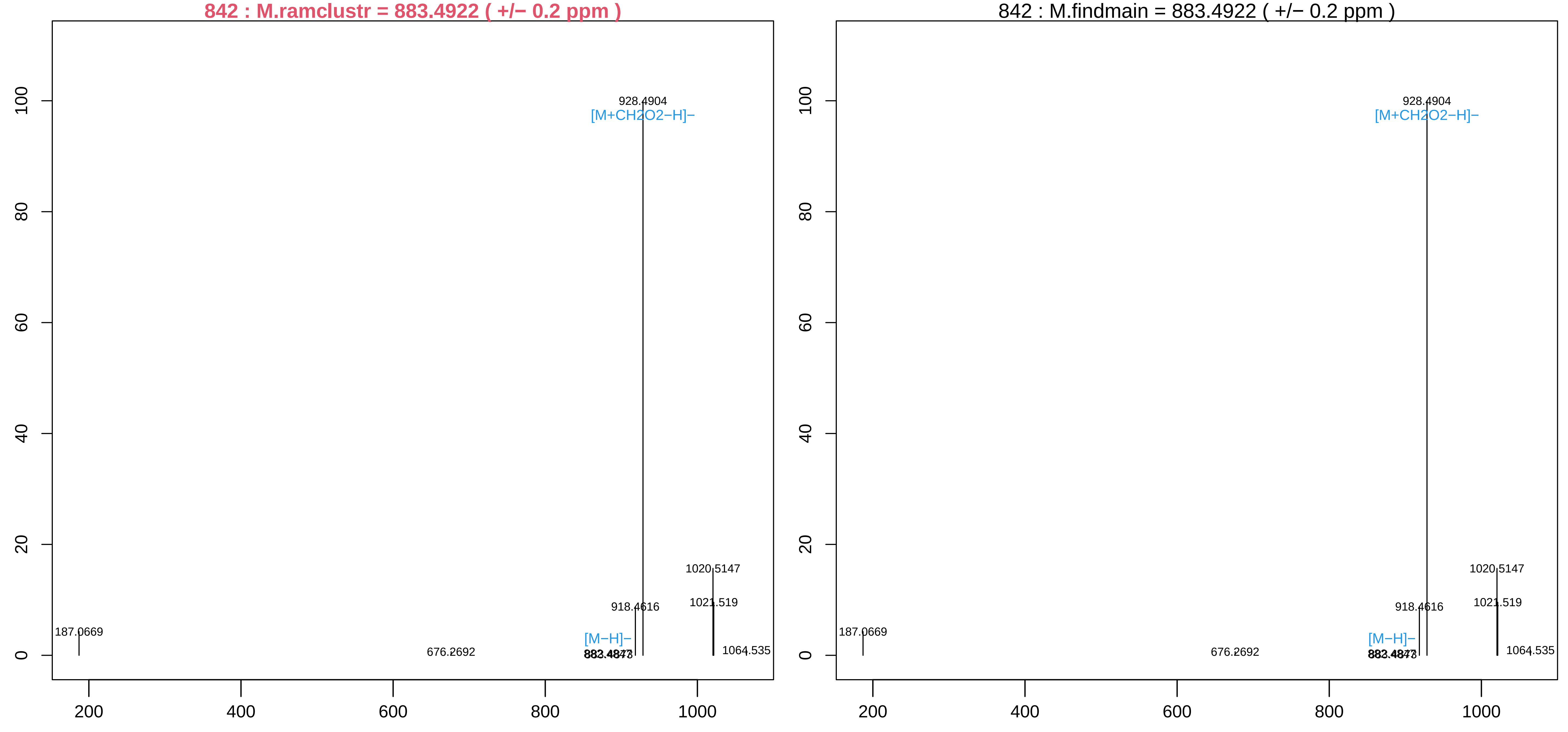
842 : M.ramclustr = 883.4922 (+/− 0.2 ppm)
928.4904
[M+CH2O2−H]−
1020.5147
918.4616
1021.519
187.0669
[M−H]−
676.2692
883.4873
1064.535
842 : M.findmain = 883.4922 (+/− 0.2 ppm)
928.4904
[M+CH2O2−H]−
1020.5147
918.4616
1021.519
187.0669
[M−H]−
676.2692
883.4873
1064.535

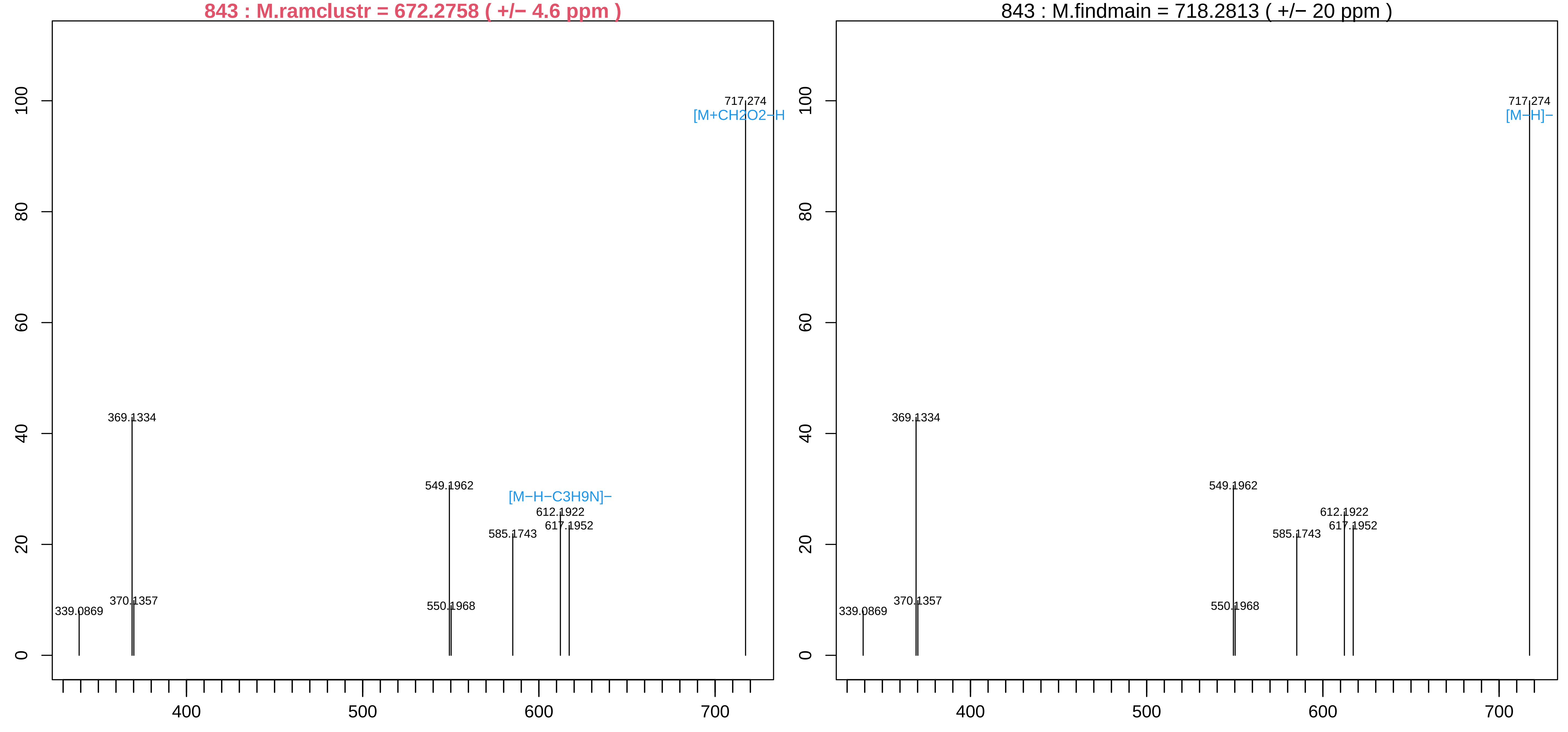
843 : M.ramclustr = 672.2758 (+/− 4.6 ppm)
717.274
[M+CH2O2−H
369.1334
549.1962
[M−H−C3H9N]−
612.1922
617.1952
585.1743
370.1357
550.1968
339.0869
843 : M.findmain = 718.2813 (+/− 20 ppm)
717.274
[M−H]−
369.1334
549.1962
612.1922
617.1952
585.1743
370.1357
550.1968
339.0869
0
20
40
60
80
100
400
500
600
700

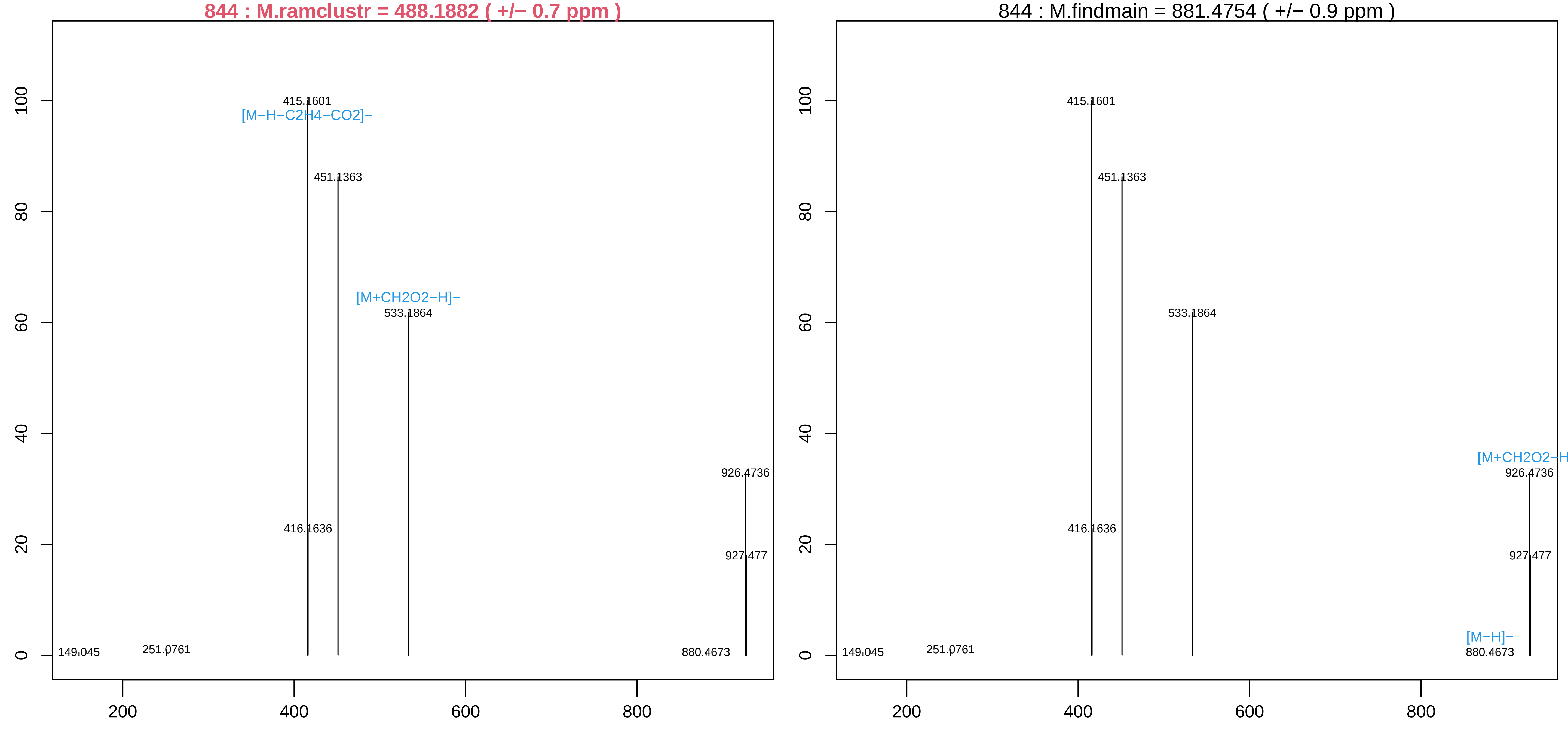
844 : M.ramclustr = 488.1882 (+/− 0.7 ppm)
[M−H−C2H4−CO2]−
[M+CH2O2−H]−
415.1601
451.1363
533.1864
416.1636
926.4736
927.477
880.4673
149.045
251.0761
844 : M.findmain = 881.4754 (+/− 0.9 ppm)
[M−H]−
0
20
40
60
80
100
200
400
600
800

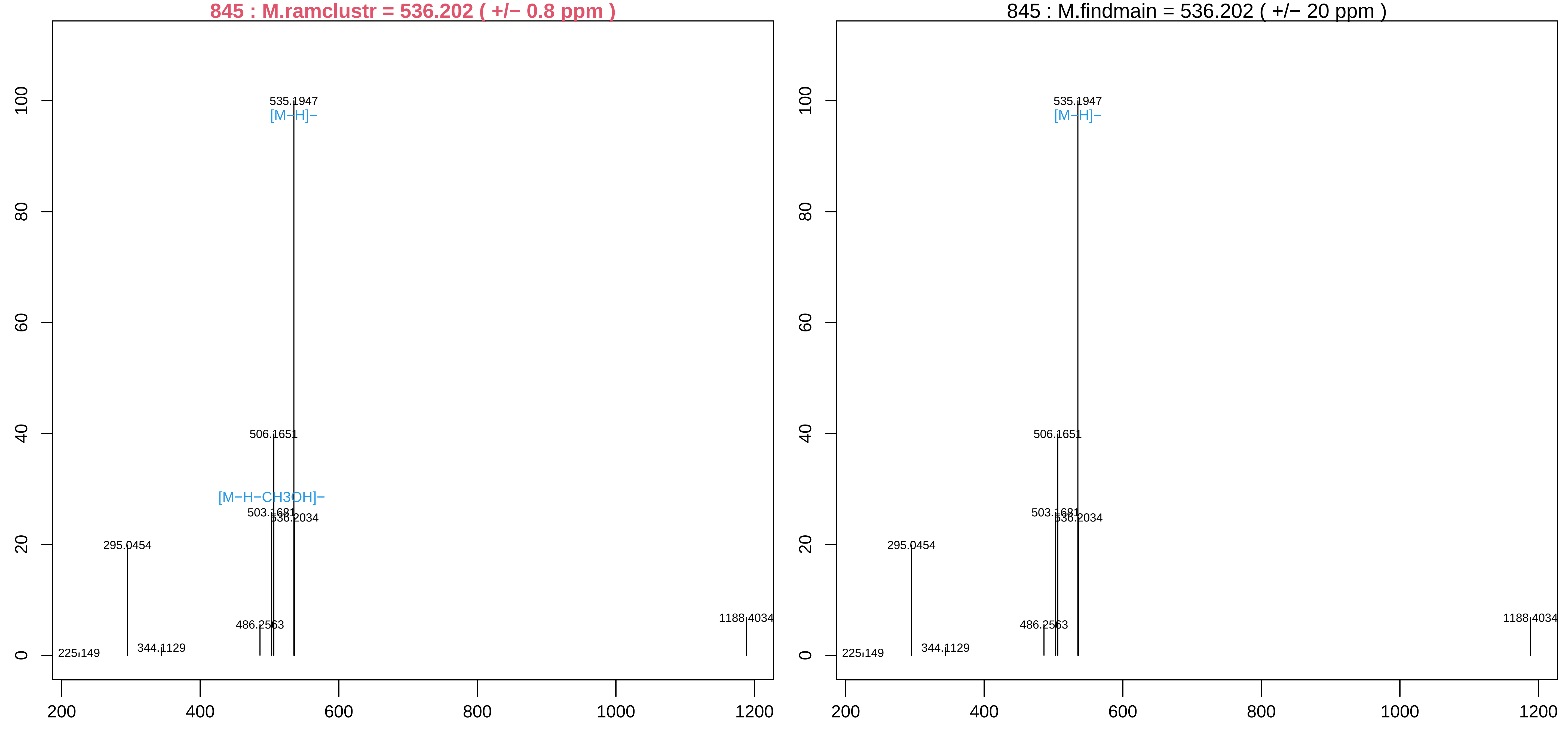
845 : M.ramclustr = 536.202 (+/− 0.8 ppm)
535.1947
[M−H]−
506.1651
[M−H−CH3OH]−
503.1681
536.2034
295.0454
486.2563
344.1129
225.149
1188.4034
845 : M.findmain = 536.202 (+/− 20 ppm)
0
20
40
60
80
100
200
400
600
800
1000
1200

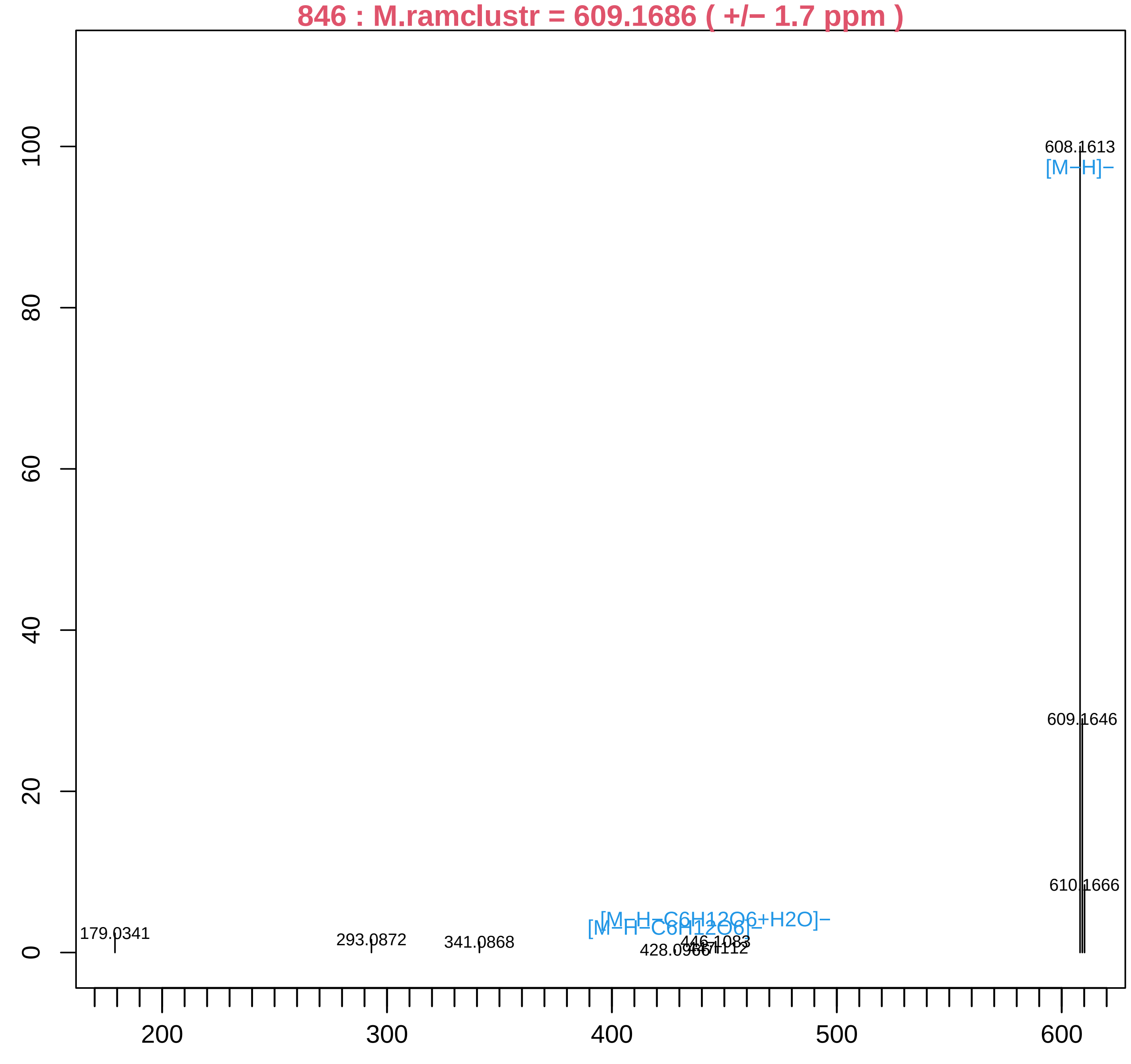

846 : M.ramclustr = 609.1686 (+/− 1.7 ppm)
608.1613
[M−H]−
609.1646
610.1666
179.0341
293.0872
341.0868
[M−H−C6H12O6+H2O]−
[M−H−C6H12O6]−
446.1083
428.0966
0
20
40
60
80
100
200
300
400
500
600

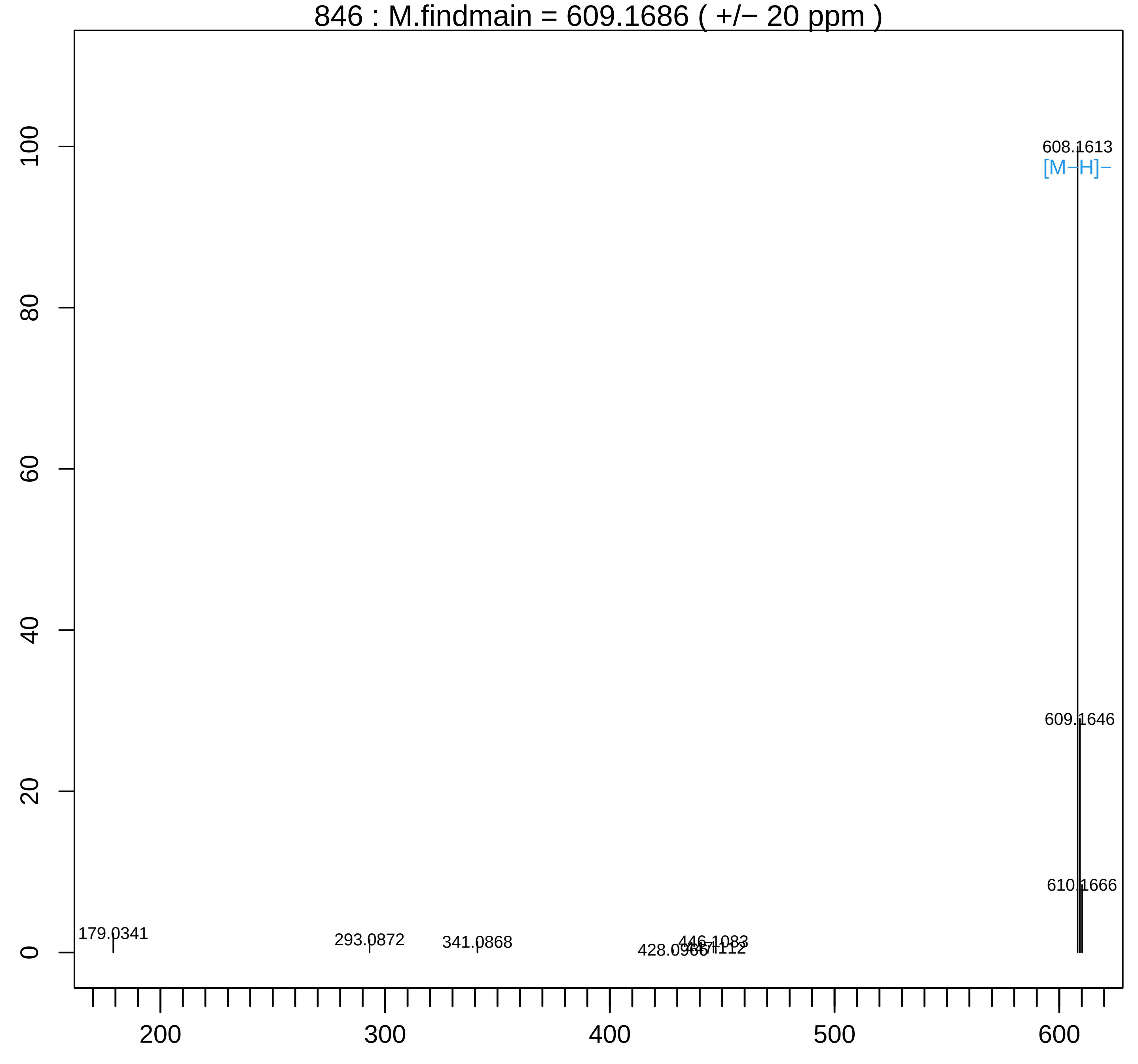

846 : M.findmain = 609.1686 (+/− 20 ppm)
608.1613
[M−H]−
609.1646
610.1666
179.0341
293.0872
341.0868
446.1083
428.0966
0
20
40
60
80
100
200
300
400
500
600

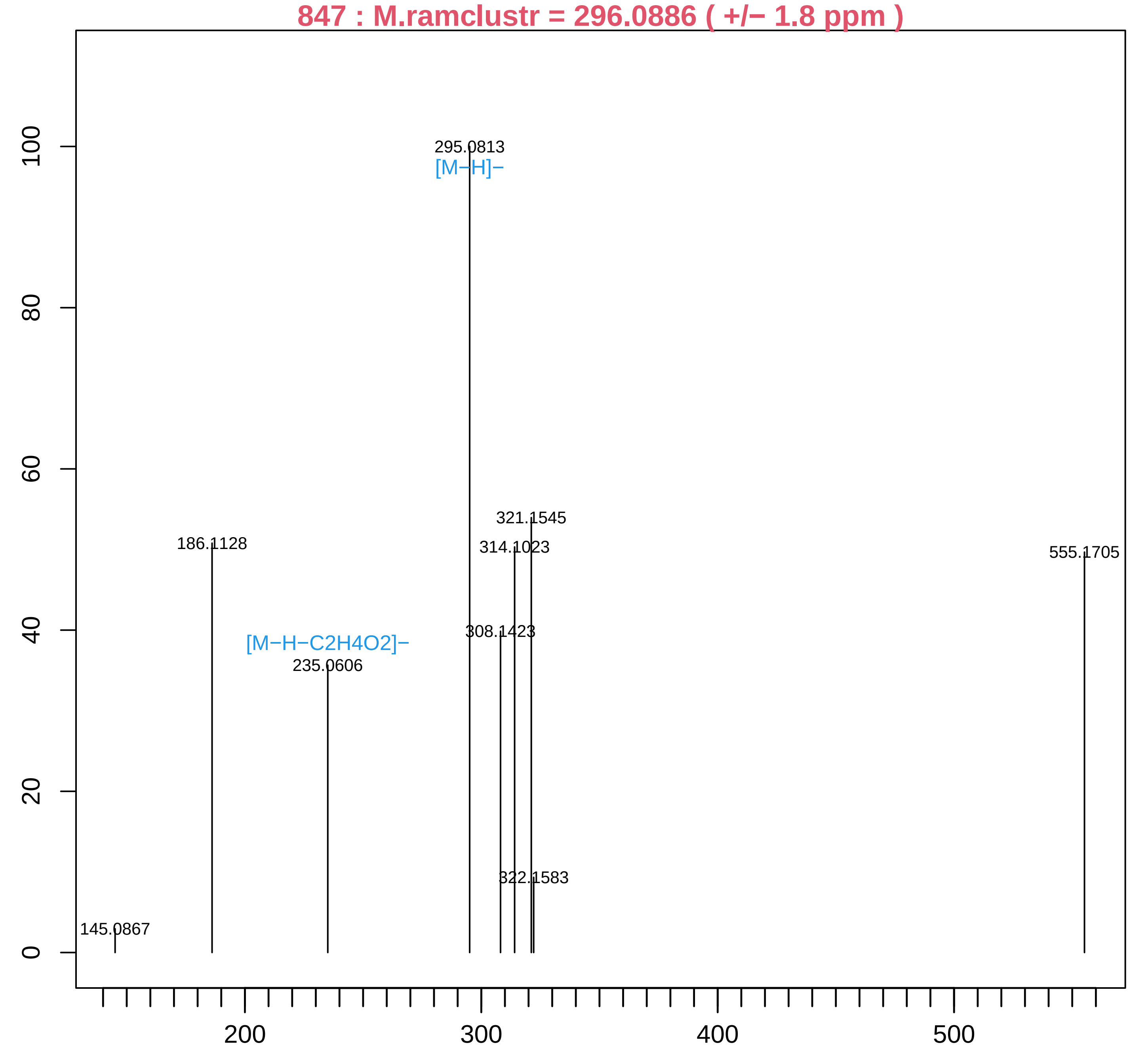
847 : M.ramclustr = 296.0886 (+/− 1.8 ppm)
295.0813
[M−H]−
321.1545
186.1128
314.1023
555.1705
308.1423
[M−H−C2H4O2]−
235.0606
322.1583
145.0867
0
20
40
60
80
100
200
300
400
500

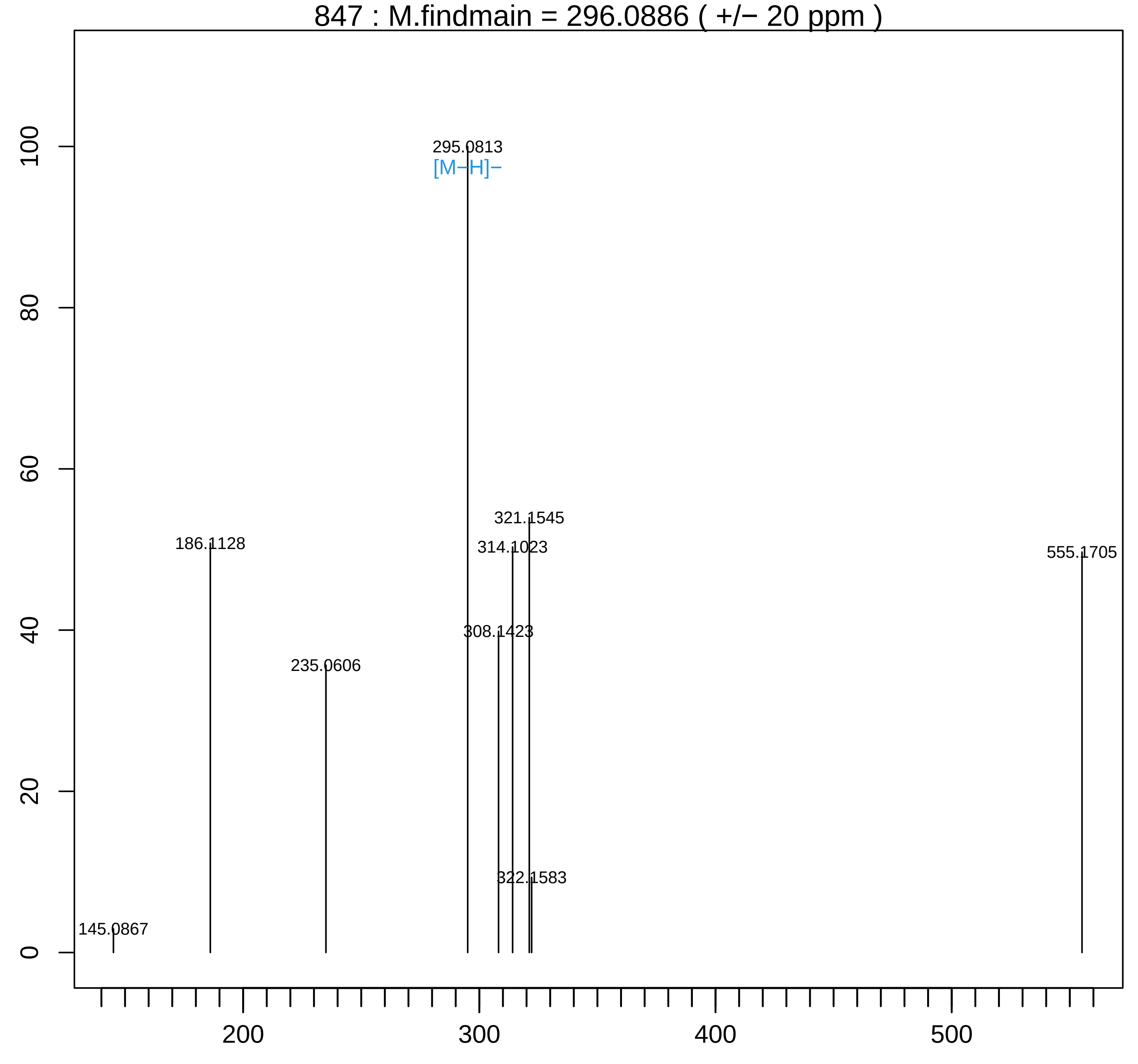
847 : M.findmain = 296.0886 (+/− 20 ppm)
295.0813
[M−H]−
321.1545
186.1128
314.1023
555.1705
308.1423
235.0606
322.1583
145.0867
0
20
40
60
80
100
200
300
400
500

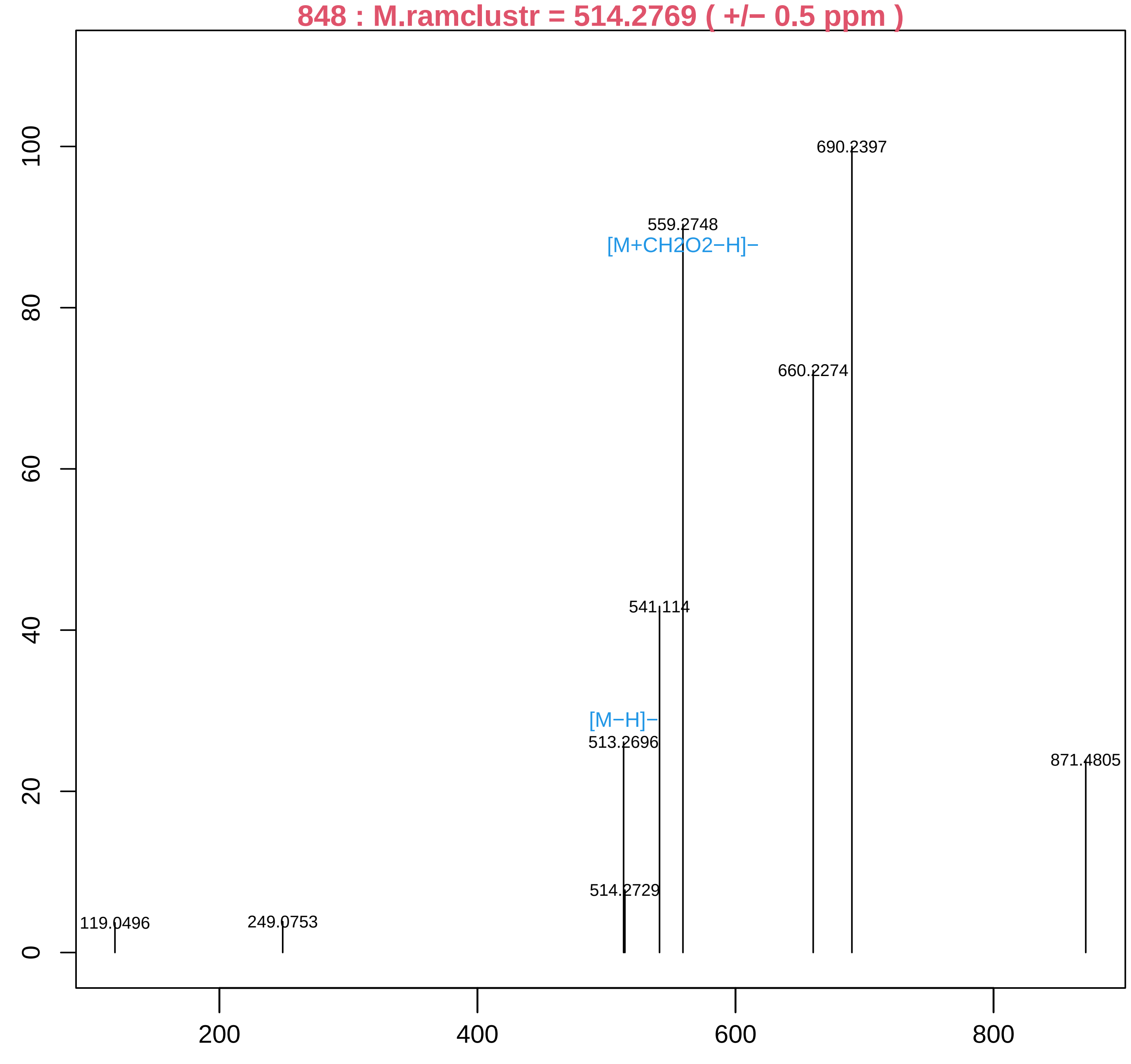

848 : M.ramclustr = 514.2769 (+/− 0.5 ppm)
690.2397
559.2748
[M+CH2O2−H]−
660.2274
541.114
[M−H]−
513.2696
871.4805
514.2729
119.0496
249.0753
0
20
40
60
80
100
200
400
600
800

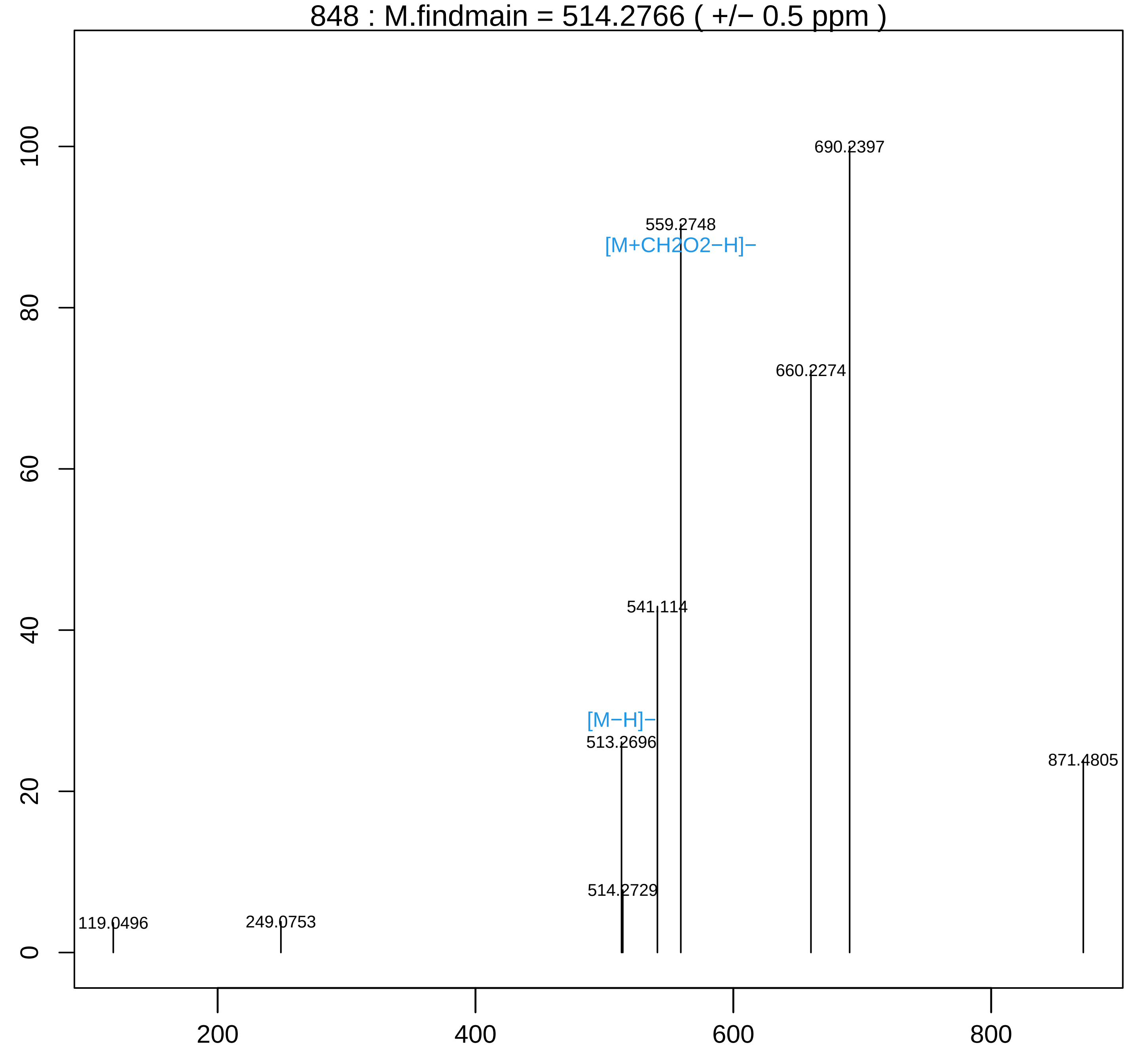

848 : M.findmain = 514.2766 (+/− 0.5 ppm)
690.2397
559.2748
[M+CH2O2−H]−
660.2274
541.114
[M−H]−
513.2696
871.4805
514.2729
119.0496
249.0753
0
20
40
60
80
100
200
400
600
800

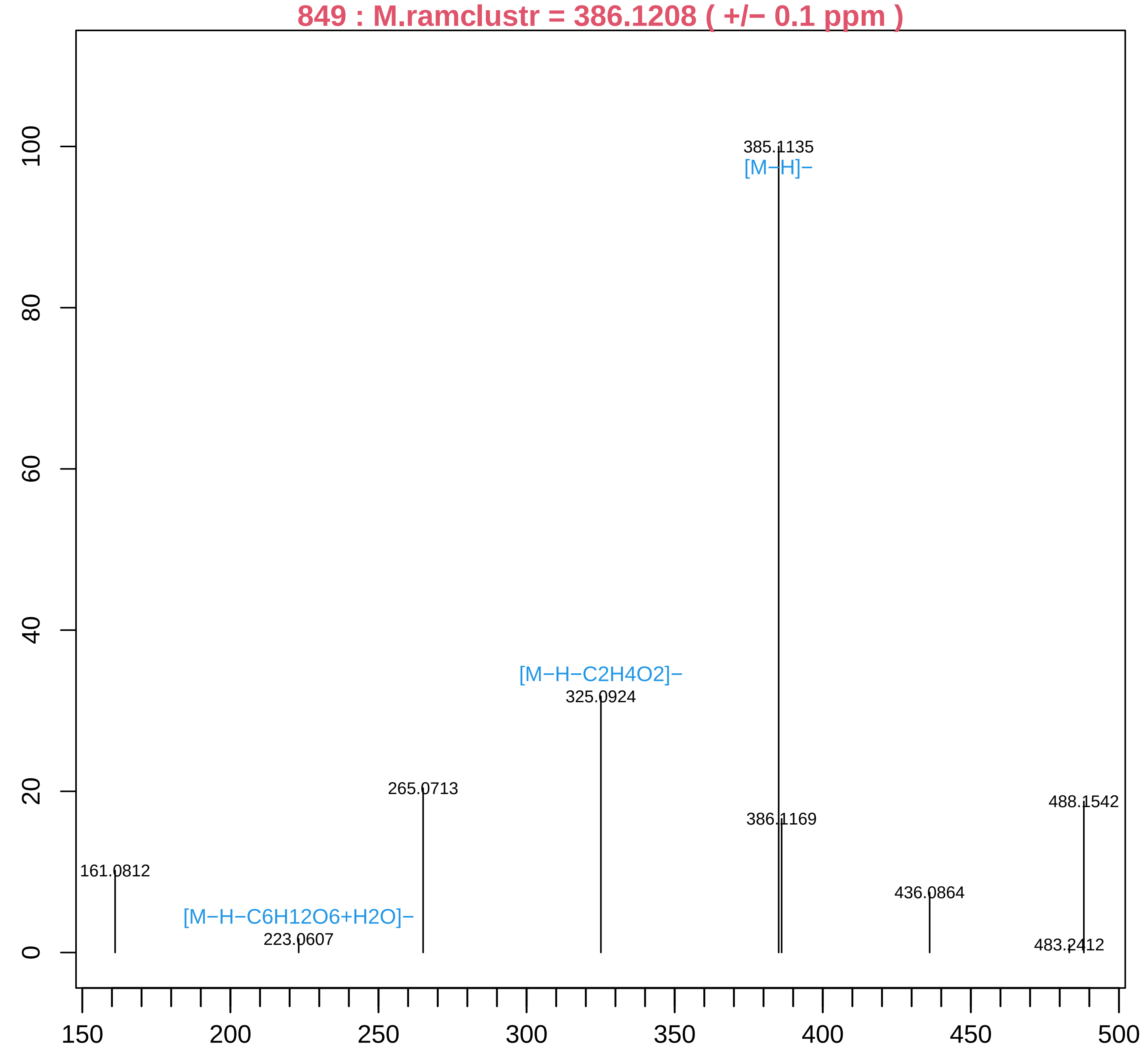
849 : M.ramclustr = 386.1208 (+/− 0.1 ppm)
385.1135
[M−H]−
[M−H−C2H4O2]−
325.0924
265.0713
386.1169
488.1542
161.0812
436.0864
[M−H−C6H12O6+H2O]−
223.0607
483.2412
0
20
40
60
80
100
150
200
250
300
350
400
450
500

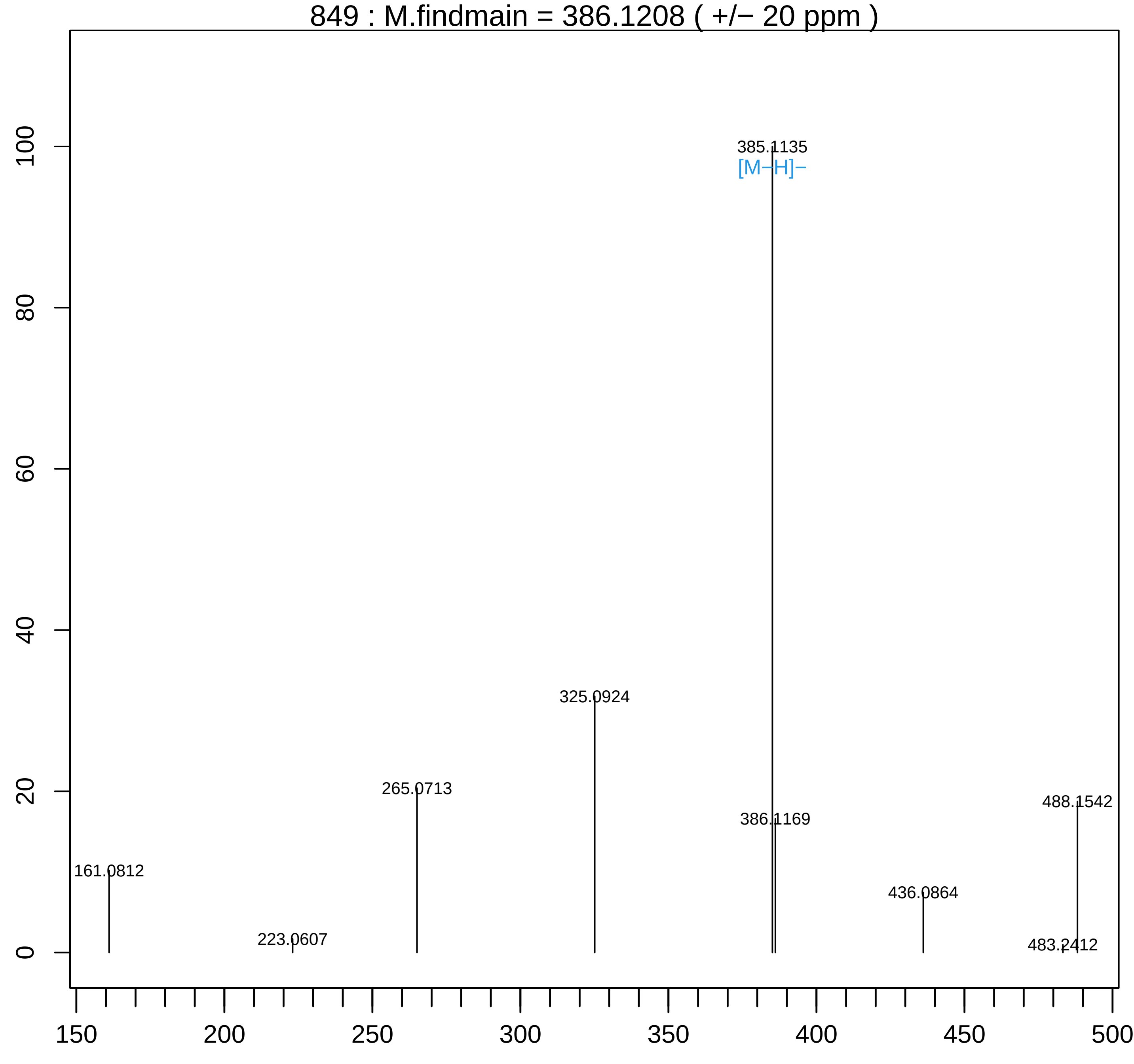
849 : M.findmain = 386.1208 (+/− 20 ppm)
385.1135
[M−H]−
325.0924
265.0713
386.1169
488.1542
161.0812
436.0864
223.0607
483.2412
0
20
40
60
80
100
150
200
250
300
350
400
450
500

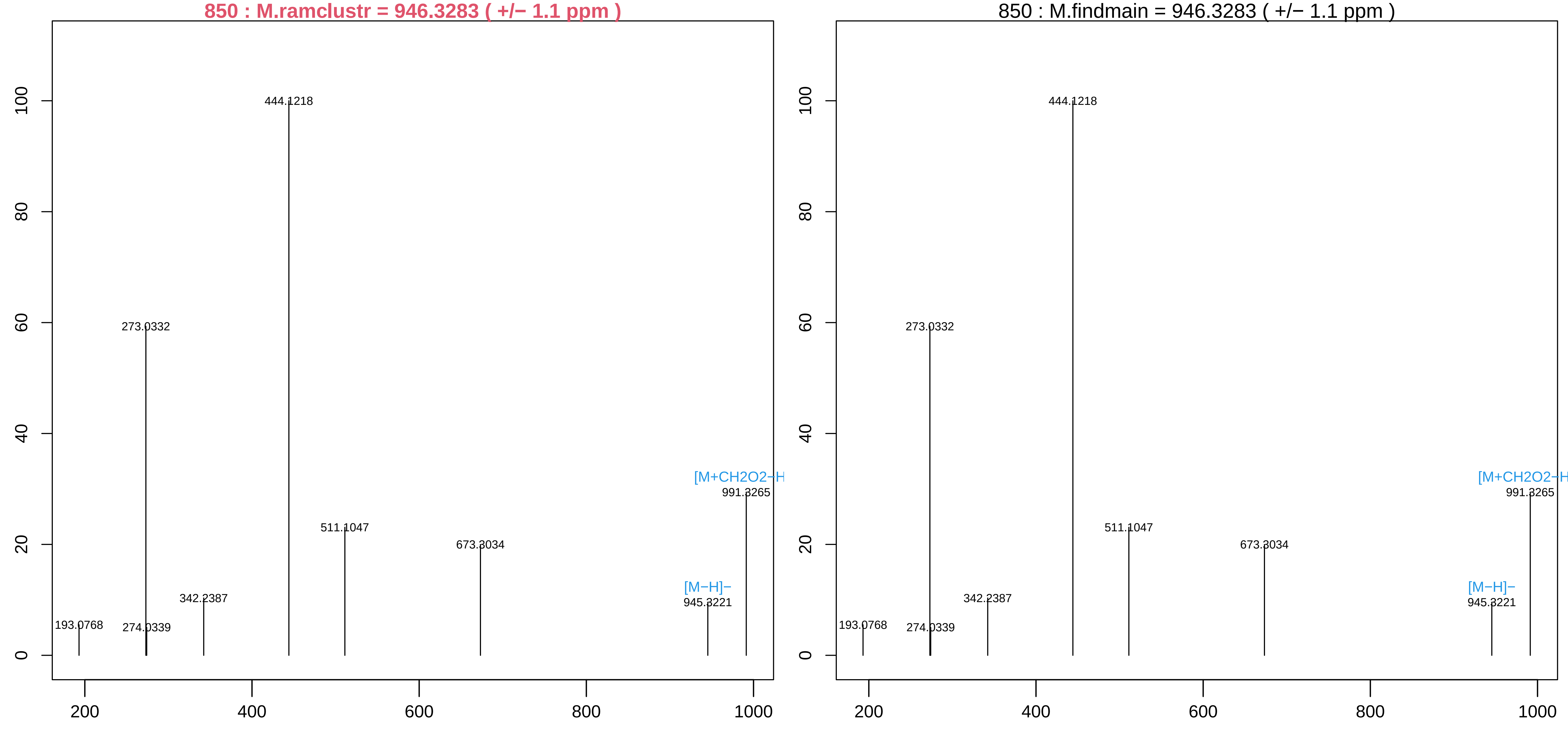

850 : M.ramclustr = 946.3283 (+/− 1.1 ppm)
444.1218
273.0332
511.1047
673.3034
342.2387
193.0768
274.0339
[M−H]−
945.3221
[M+CH2O2−H
991.3265
850 : M.findmain = 946.3283 (+/− 1.1 ppm)
444.1218
273.0332
511.1047
673.3034
342.2387
193.0768
274.0339
[M−H]−
945.3221
[M+CH2O2−H
991.3265
0
20
40
60
80
100
200
400
600
800
1000

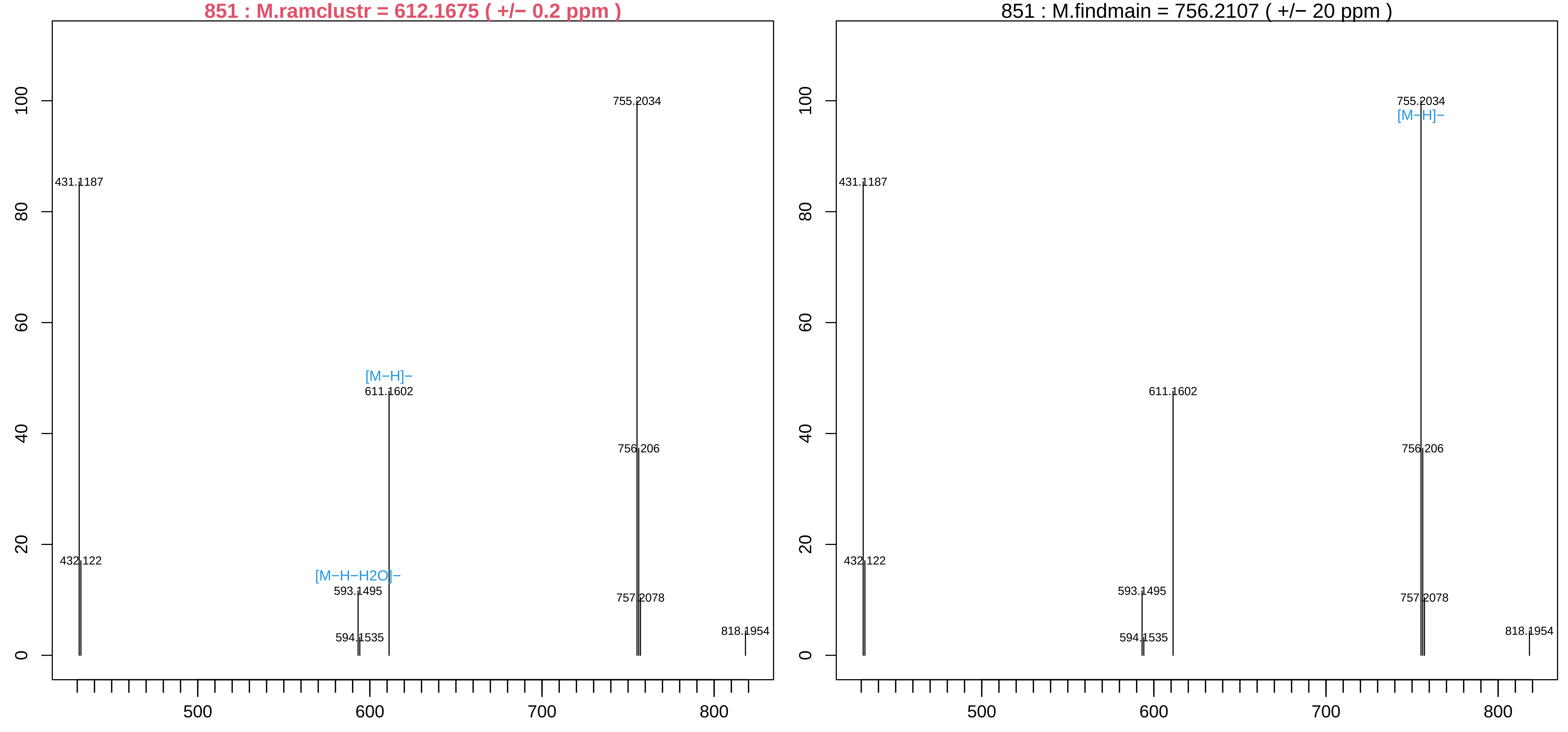

851 : M.ramclustr = 612.1675 (+/− 0.2 ppm)
755.2034
431.1187
[M−H]−
611.1602
756.206
432.122
[M−H−H2O]−
593.1495
757.2078
594.1535
818.1954
851 : M.findmain = 756.2107 (+/− 20 ppm)
755.2034
[M−H]−
431.1187
611.1602
756.206
432.122
593.1495
757.2078
594.1535
818.1954

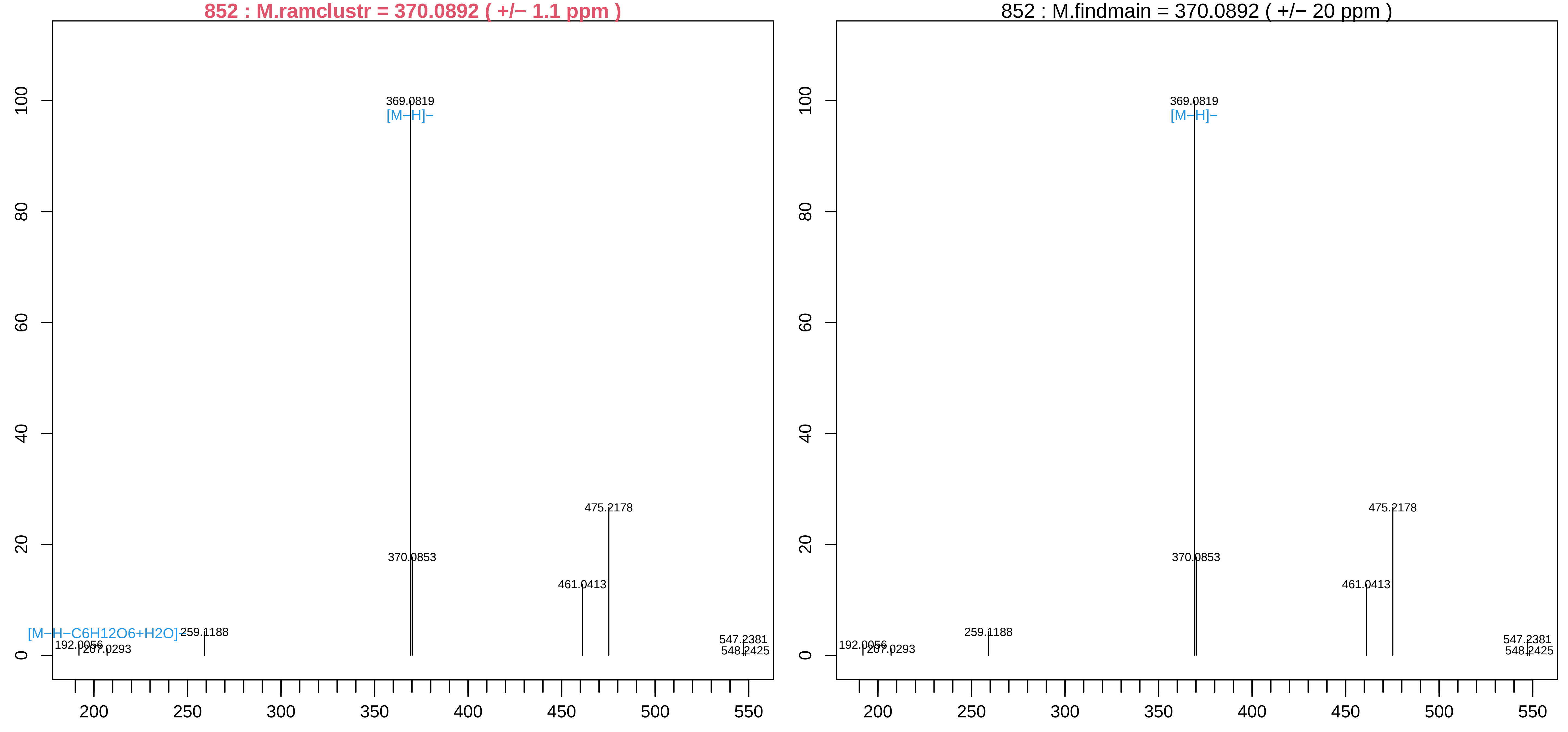
852 : M.ramclustr = 370.0892 (+/− 1.1 ppm)
369.0819
[M−H]−
370.0853
461.0413
475.2178
[M−H−C6H12O6+H2O]−
259.1188
192.0056
207.0293
547.2381
548.2425
852 : M.findmain = 370.0892 (+/− 20 ppm)
369.0819
[M−H]−
370.0853
461.0413
475.2178
259.1188
192.0056
207.0293
547.2381
548.2425

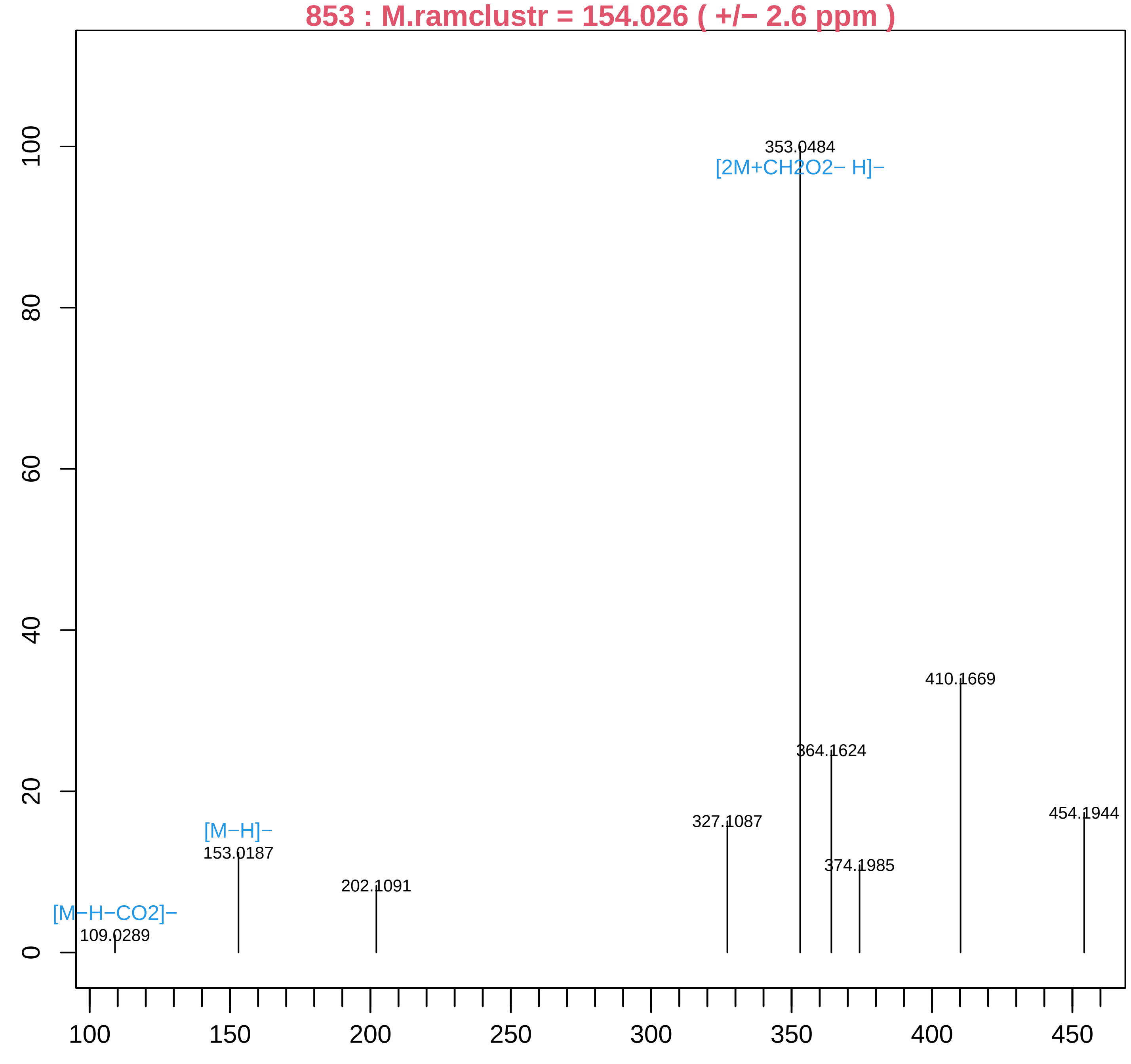

853 : M.ramclustr = 154.026 (+/− 2.6 ppm)
353.0484
[2M+CH2O2− H]−
410.1669
364.1624
327.1087
454.1944
[M−H]−
153.0187
374.1985
202.1091
[M−H−CO2]−
109.0289
0
20
40
60
80
100
100
150
200
250
300
350
400
450

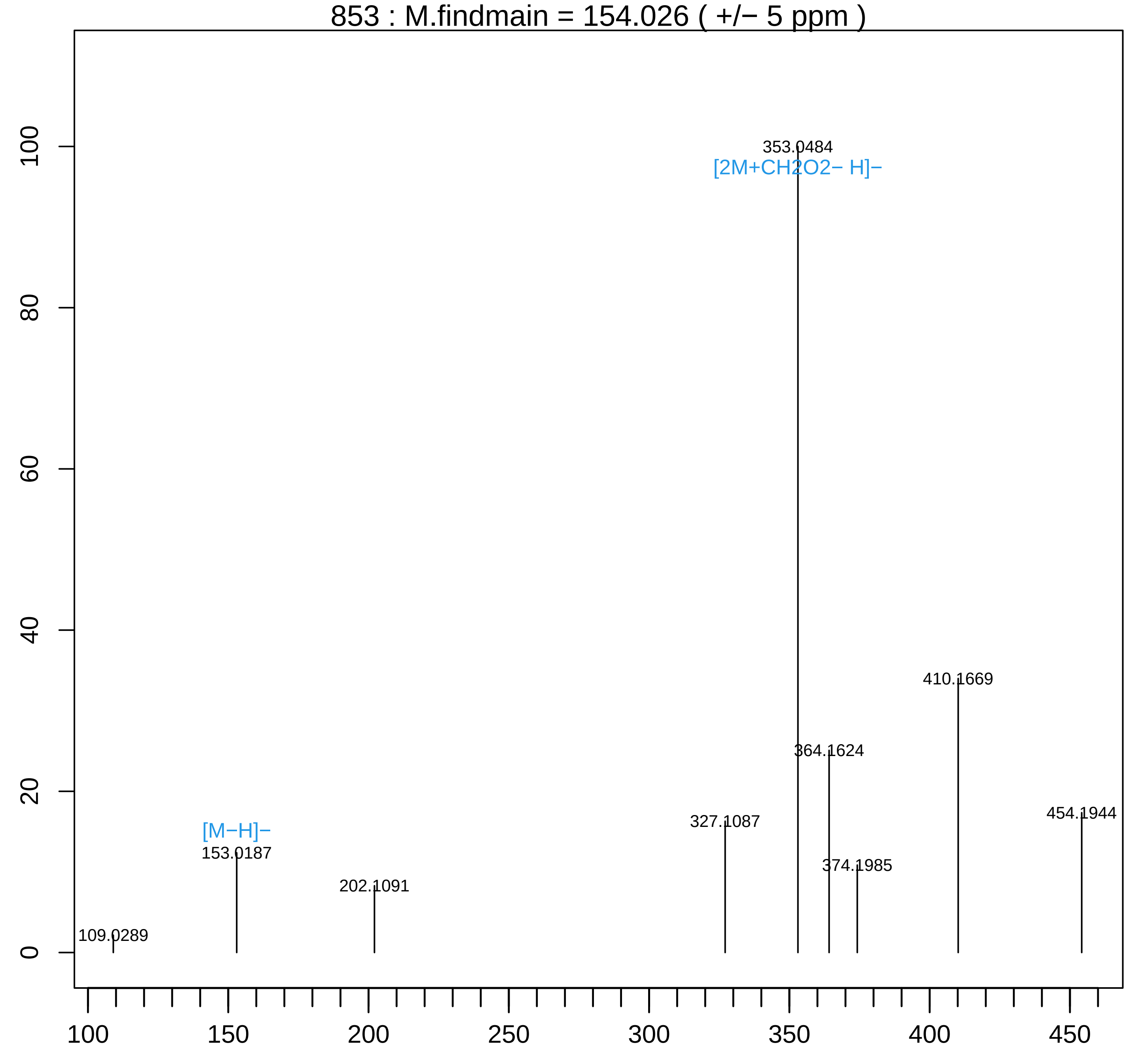

853 : M.findmain = 154.026 (+/− 5 ppm)
353.0484
[2M+CH2O2− H]−
410.1669
364.1624
327.1087
454.1944
[M−H]−
153.0187
374.1985
202.1091
109.0289
0
20
40
60
80
100
100
150
200
250
300
350
400
450

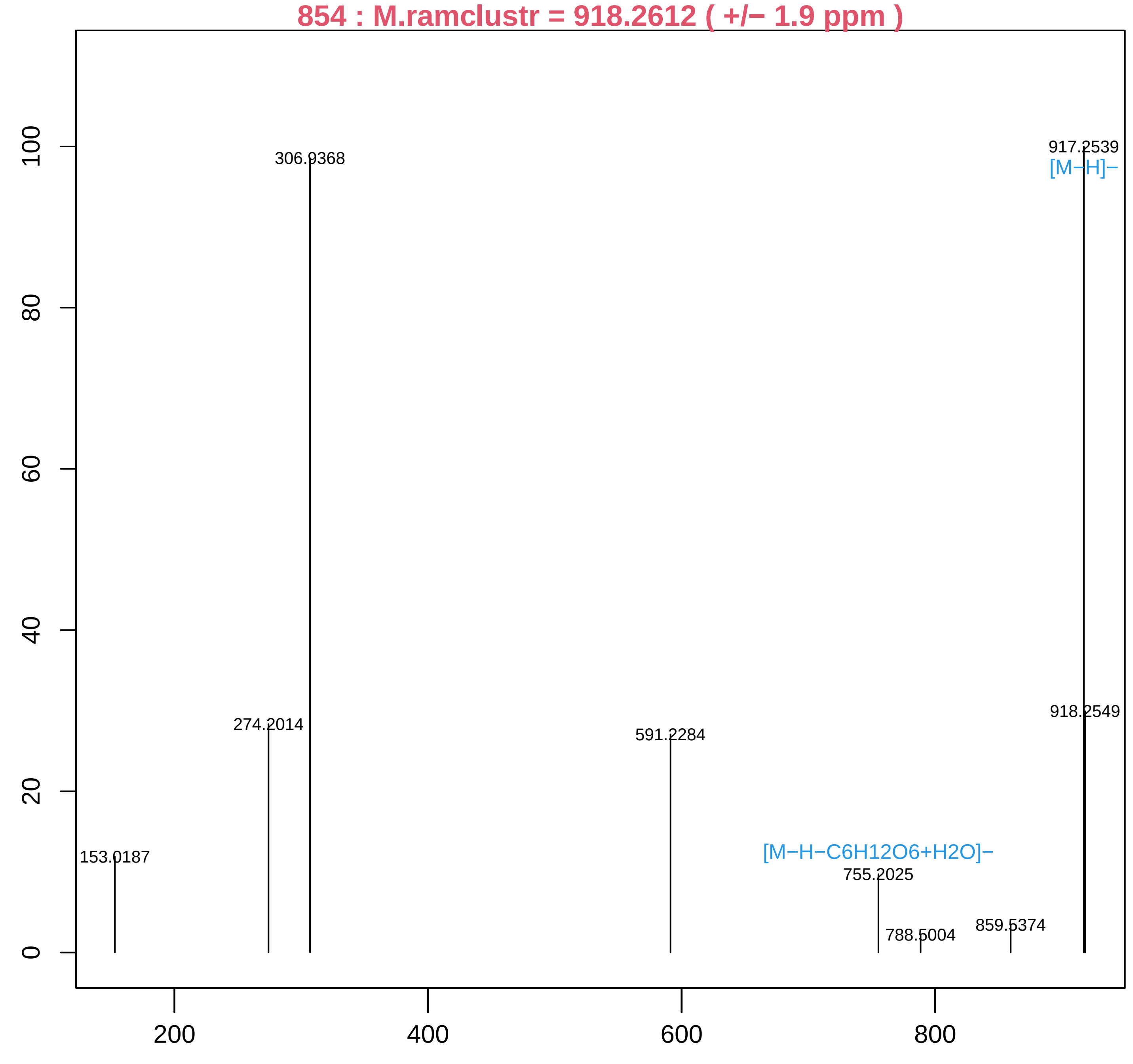

854 : M.ramclustr = 918.2612 (+/− 1.9 ppm)
917.2539
[M−H]−
306.9368
274.2014
591.2284
918.2549
153.0187
[M−H−C6H12O6+H2O]−
755.2025
788.5004
859.5374
0
20
40
60
80
100
200
400
600
800

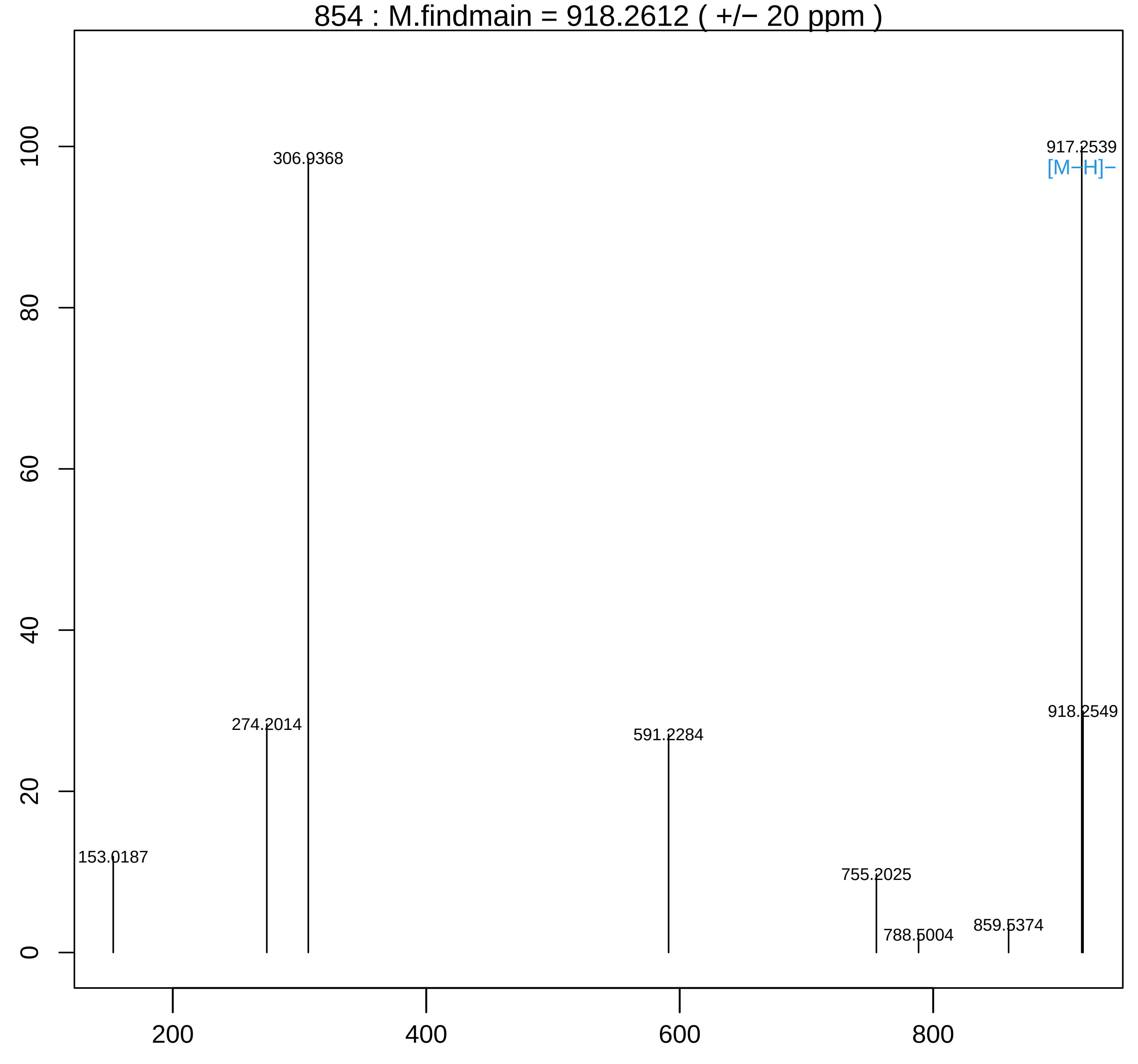

854 : M.findmain = 918.2612 (+/− 20 ppm)
917.2539
[M−H]−
306.9368
274.2014
591.2284
918.2549
153.0187
755.2025
788.5004
859.5374
0
20
40
60
80
100
200
400
600
800

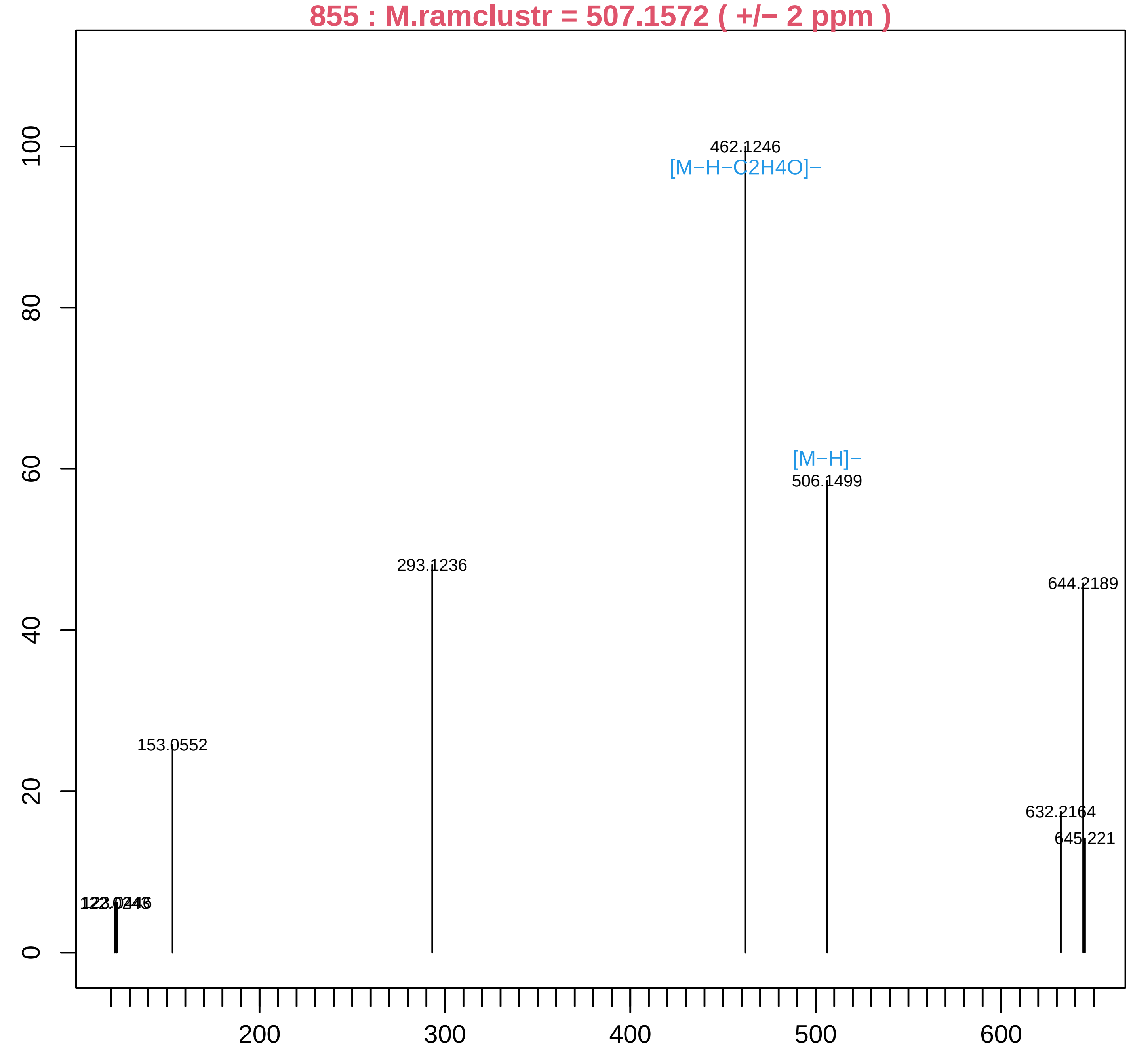
855 : M.ramclustr = 507.1572 (+/− 2 ppm)
462.1246
[M−H−C2H4O]−
[M−H]−
506.1499
293.1236
644.2189
153.0552
632.2164
645.221
0
20
40
60
80
100
200
300
400
500
600

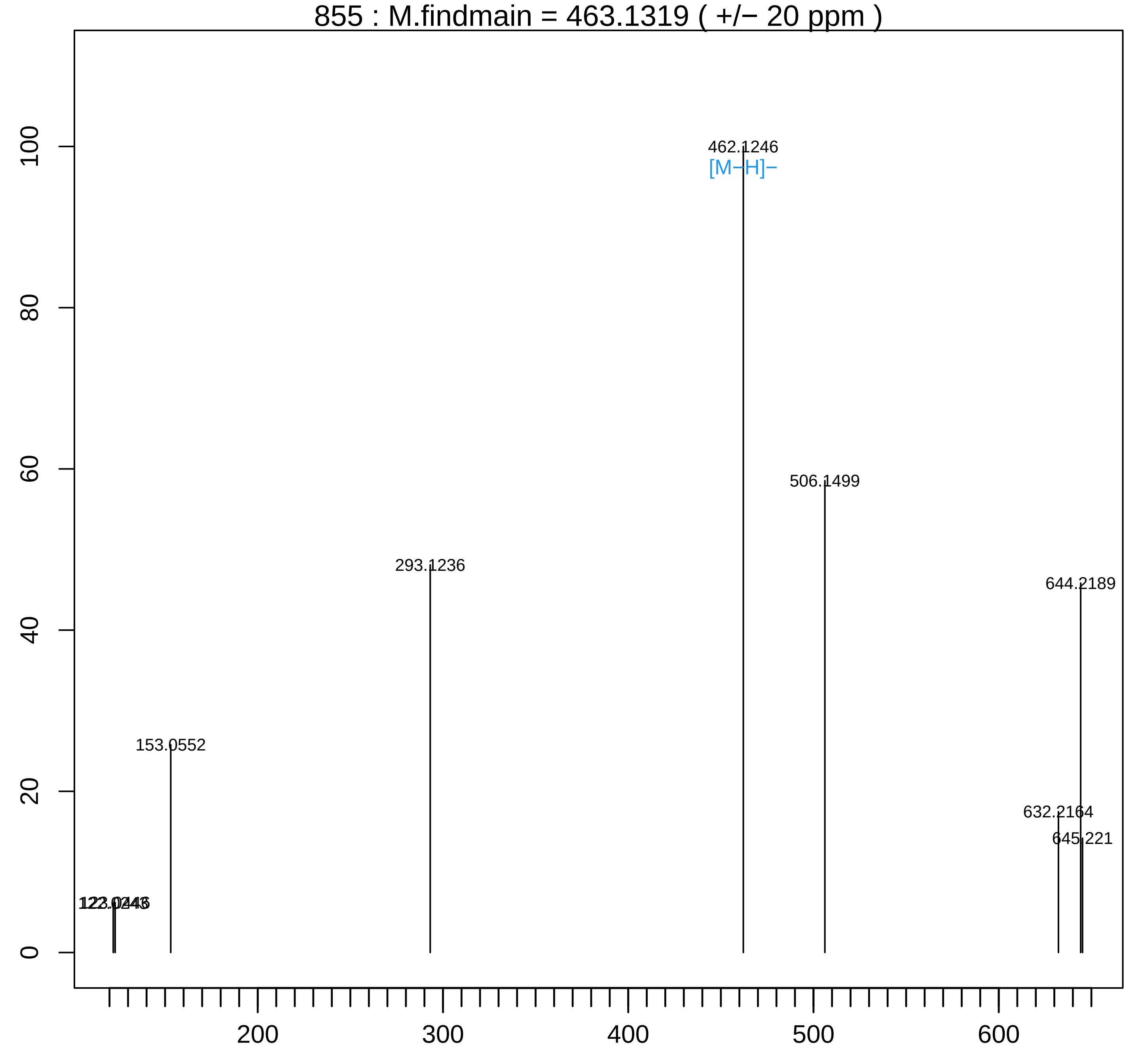
855 : M.findmain = 463.1319 (+/− 20 ppm)
462.1246
[M−H]−
506.1499
293.1236
644.2189
153.0552
632.2164
645.221
0
20
40
60
80
100
200
300
400
500
600

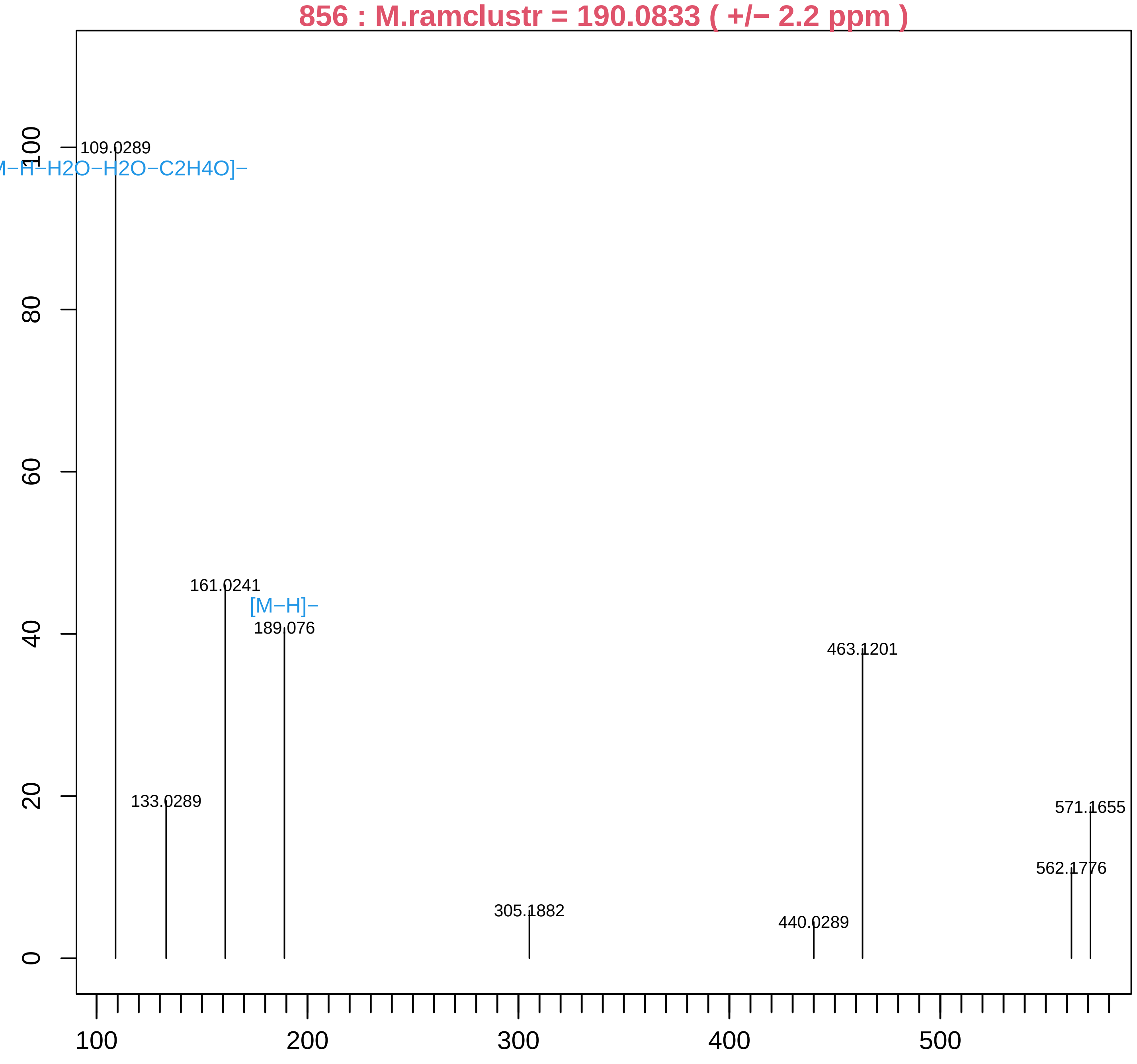

856 : M.ramclustr = 190.0833 (+/− 2.2 ppm)
109.0289
[M−H−H2O−H2O−C2H4O]−
161.0241
[M−H]−
189.076
133.0289
305.1882
440.0289
463.1201
562.1776
571.1655
0
20
40
60
80
100
100
200
300
400
500

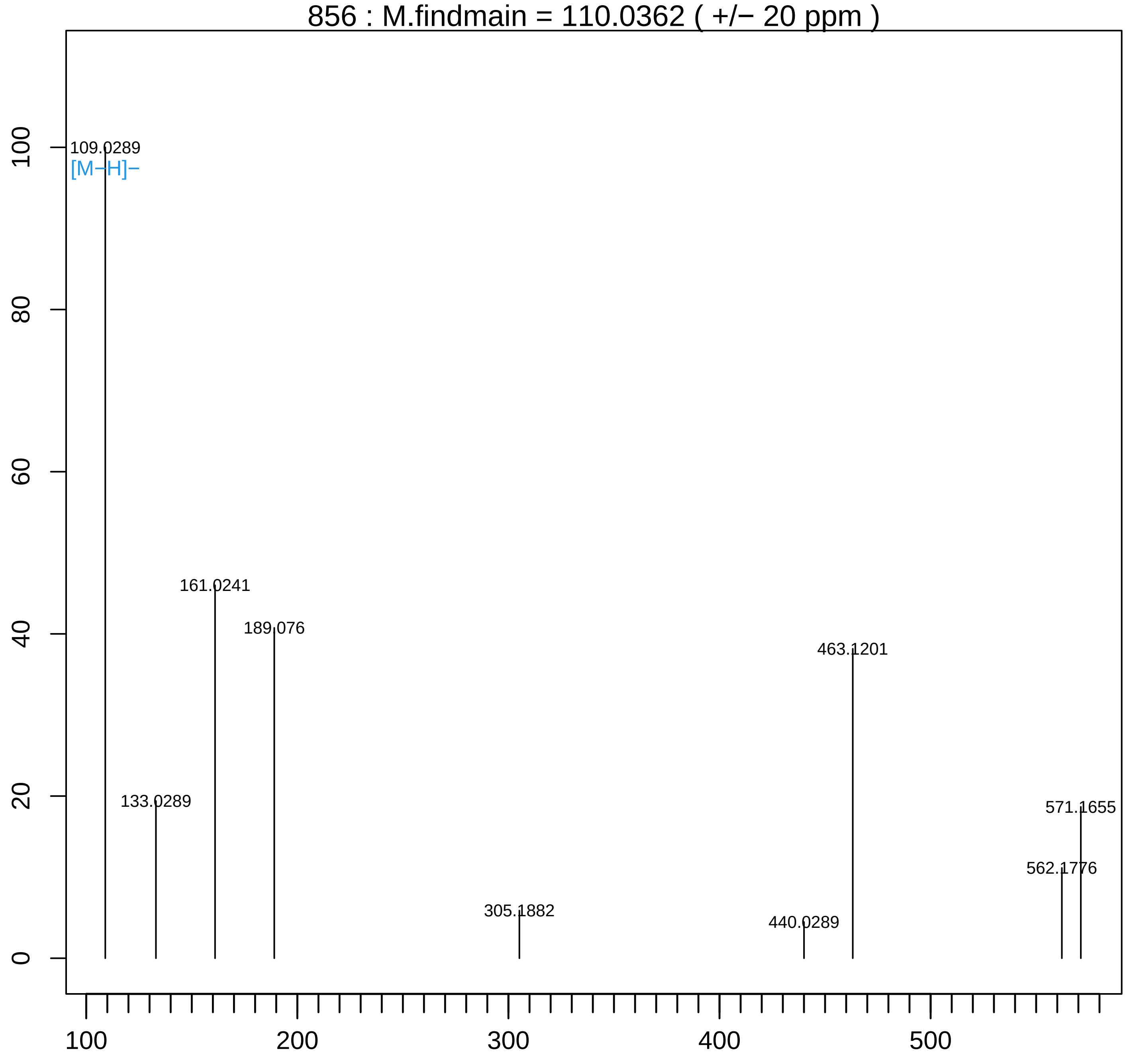

856 : M.findmain = 110.0362 (+/− 20 ppm)
109.0289
[M−H]−
161.0241
189.076
133.0289
305.1882
440.0289
463.1201
562.1776
571.1655
0
20
40
60
80
100
100
200
300
400
500

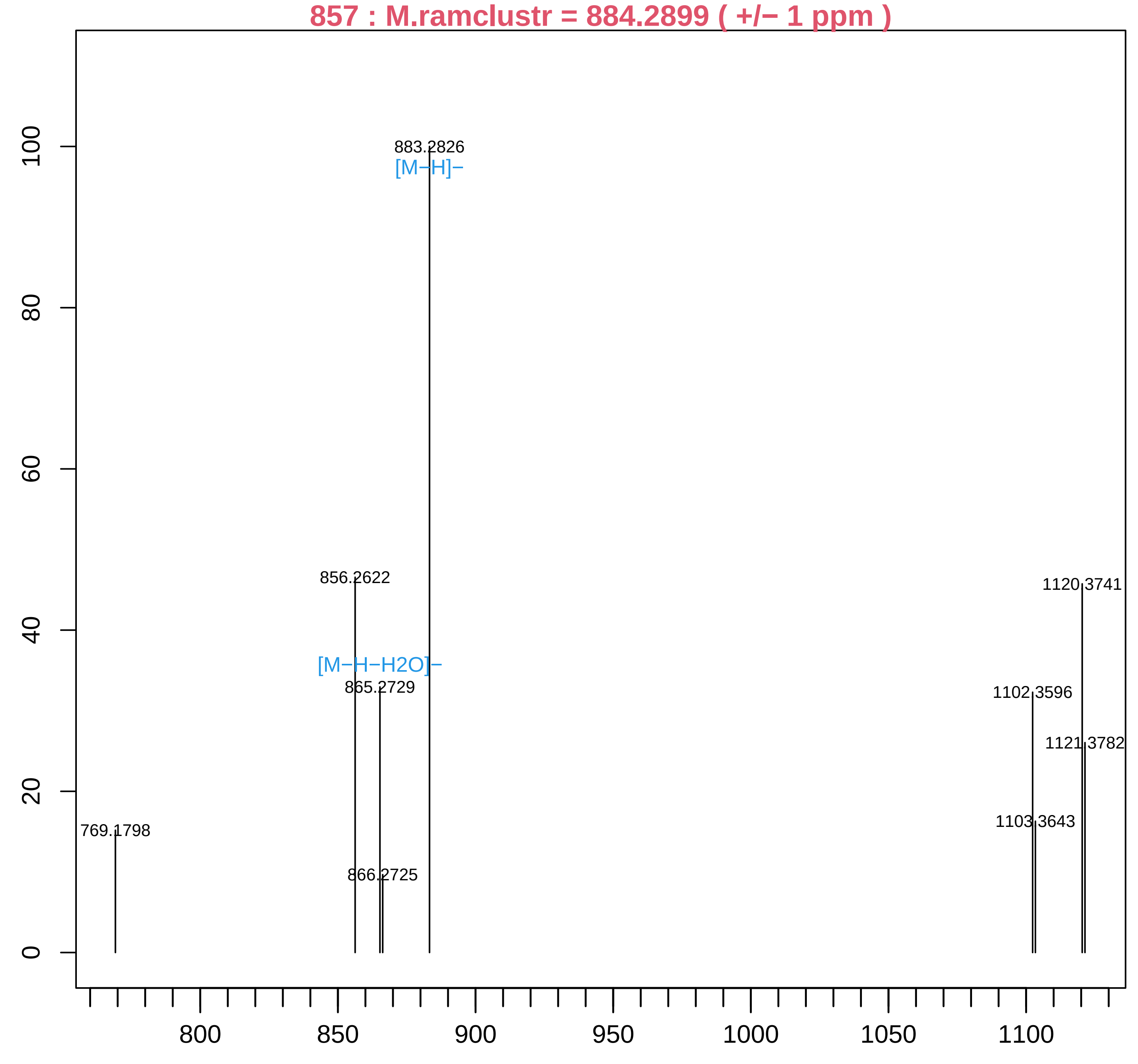

857 : M.ramclustr = 884.2899 (+/− 1 ppm)
883.2826
[M−H]−
856.2622
1120.3741
[M−H−H2O]−
865.2729
1102.3596
1121.3782
769.1798
1103.3643
866.2725
0
20
40
60
80
100
800
850
900
950
1000
1050
1100

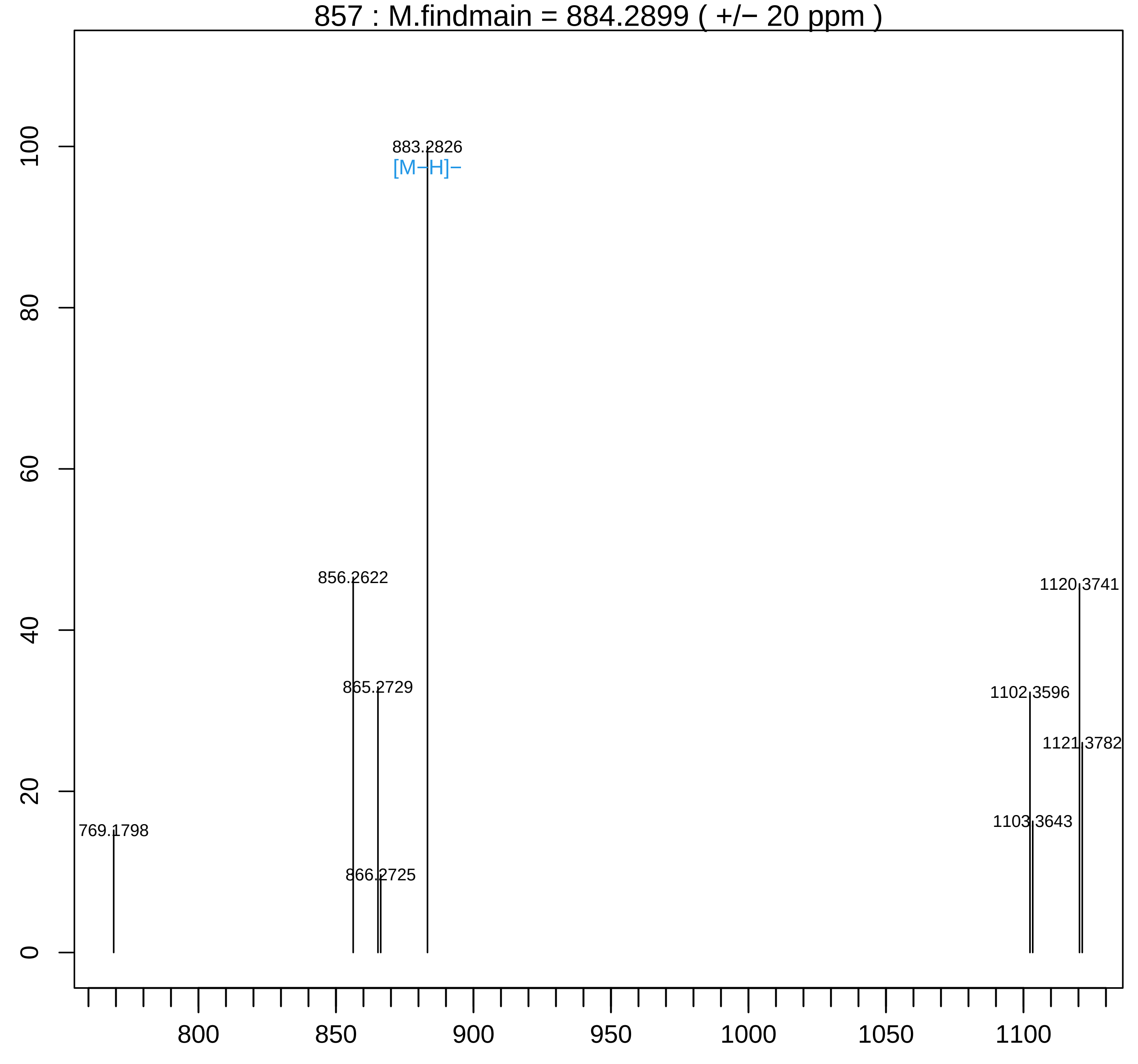

857 : M.findmain = 884.2899 (+/− 20 ppm)
883.2826
[M−H]−
856.2622
1120.3741
865.2729
1102.3596
1121.3782
769.1798
1103.3643
866.2725
0
20
40
60
80
100
800
850
900
950
1000
1050
1100

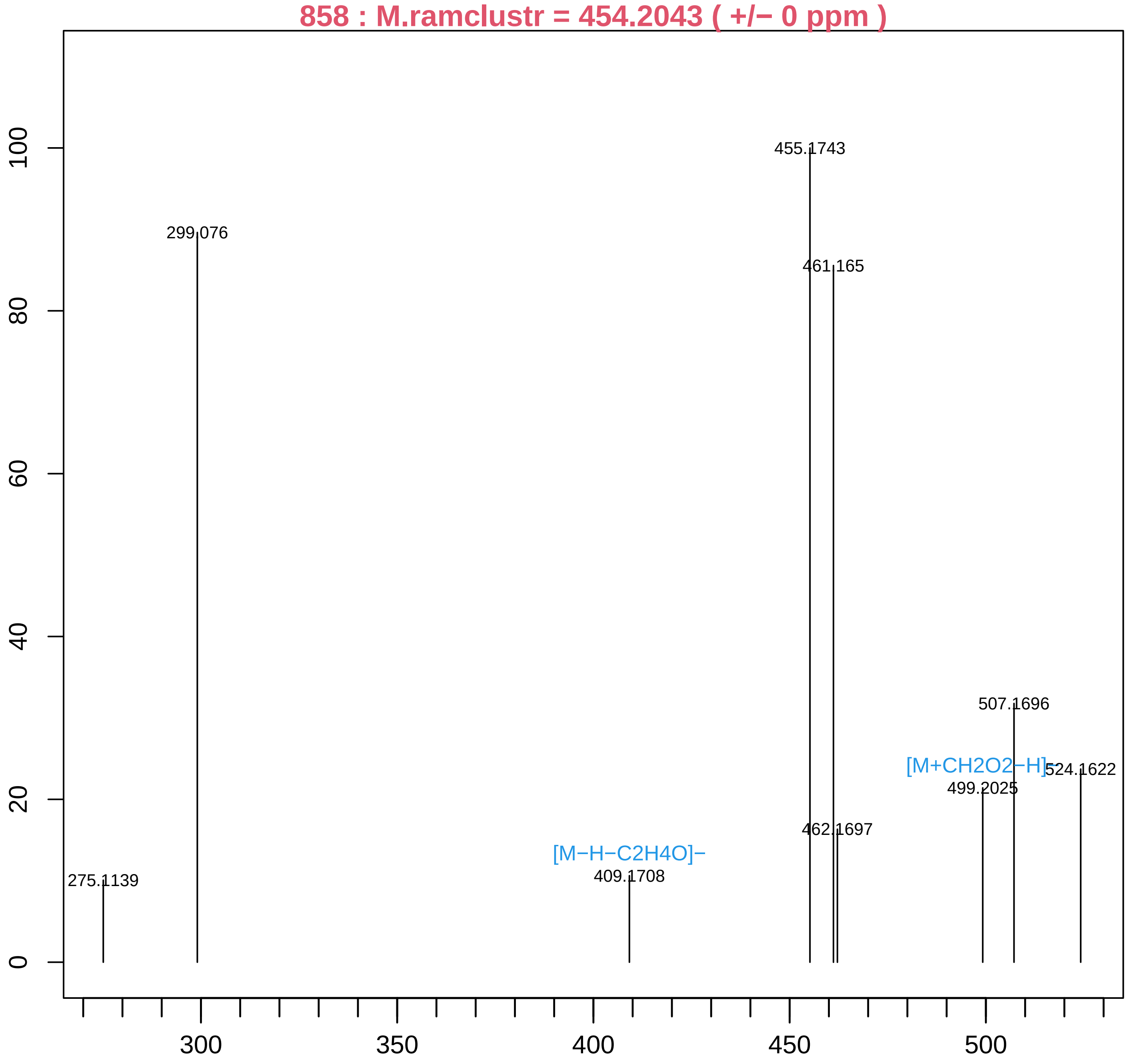
858 : M.ramclustr = 454.2043 (+/− 0 ppm)
455.1743
299.076
461.165
507.1696
[M+CH2O2−H]−
524.1622
499.2025
462.1697
[M−H−C2H4O]−
409.1708
275.1139
0
20
40
60
80
100
300
350
400
450
500

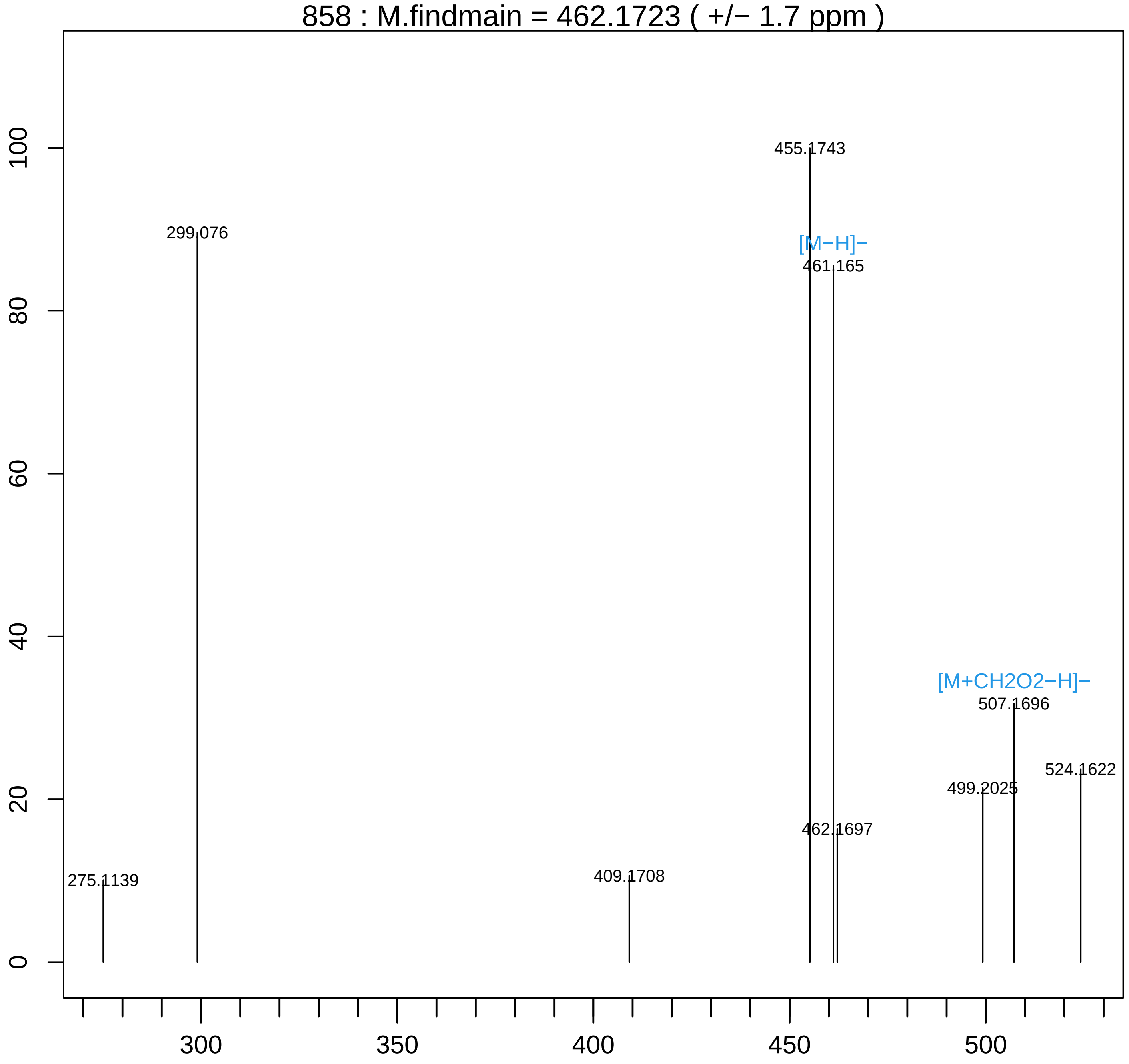
858 : M.findmain = 462.1723 (+/− 1.7 ppm)
455.1743
299.076
[M−H]−
461.165
[M+CH2O2−H]−
507.1696
524.1622
499.2025
462.1697
409.1708
275.1139
0
20
40
60
80
100
300
350
400
450
500

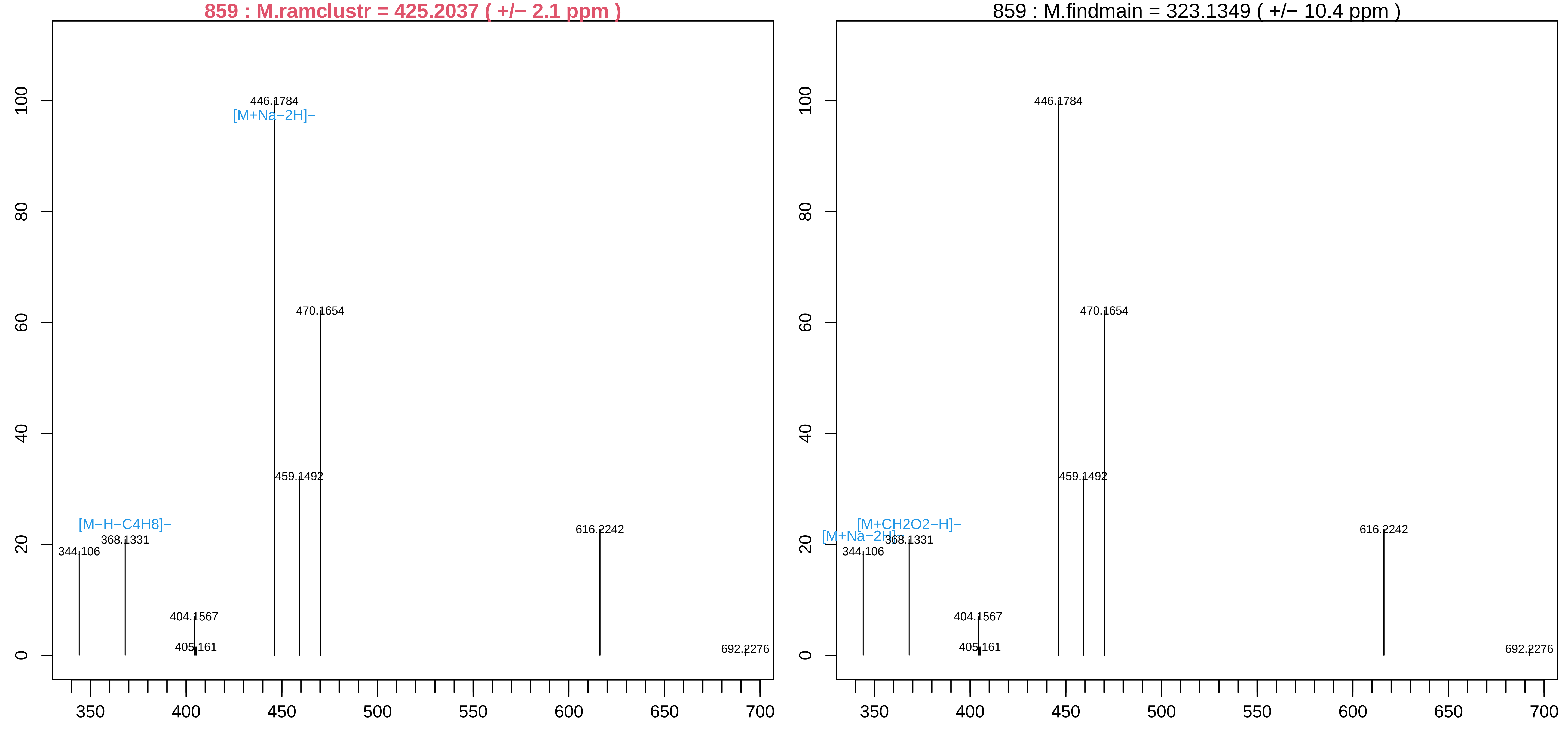
859 : M.ramclustr = 425.2037 (+/− 2.1 ppm)
446.1784
[M+Na−2H]−
470.1654
459.1492
[M−H−C4H8]−
368.1331
344.106
404.1567
405.161
616.2242
692.2276
859 : M.findmain = 323.1349 (+/− 10.4 ppm)
446.1784
470.1654
459.1492
[M+CH2O2−H]−
[M+Na−2H]−
368.1331
344.106
404.1567
405.161
616.2242
692.2276
0
20
40
60
80
100
350
400
450
500
550
600
650
700

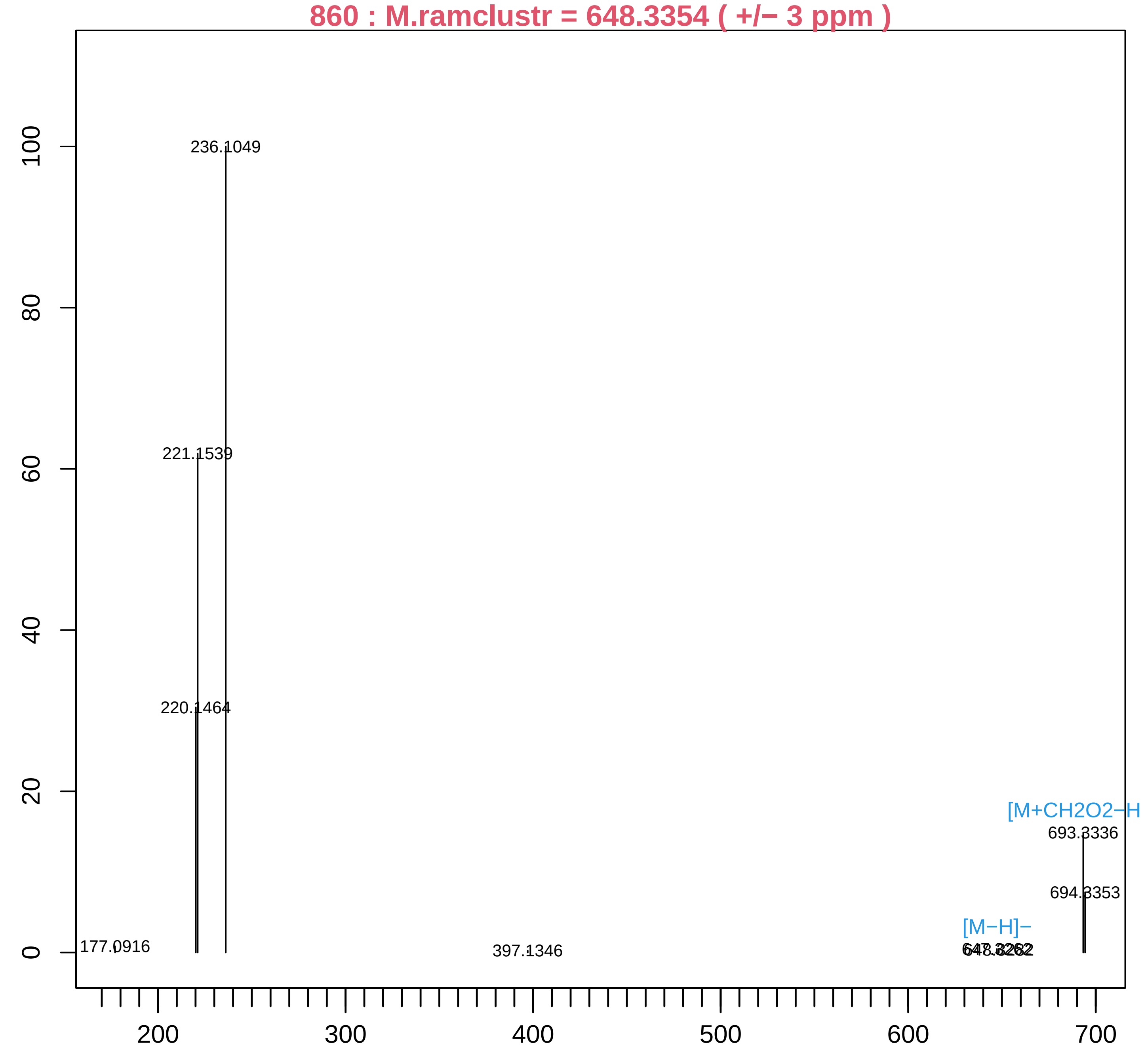
860 : M.ramclustr = 648.3354 (+/− 3 ppm)
236.1049
221.1539
220.1464
177.0916
397.1346
[M+CH2O2−H
693.3336
694.3353
[M−H]−
647.3262
648.3282
0
20
40
60
80
100
200
300
400
500
600
700

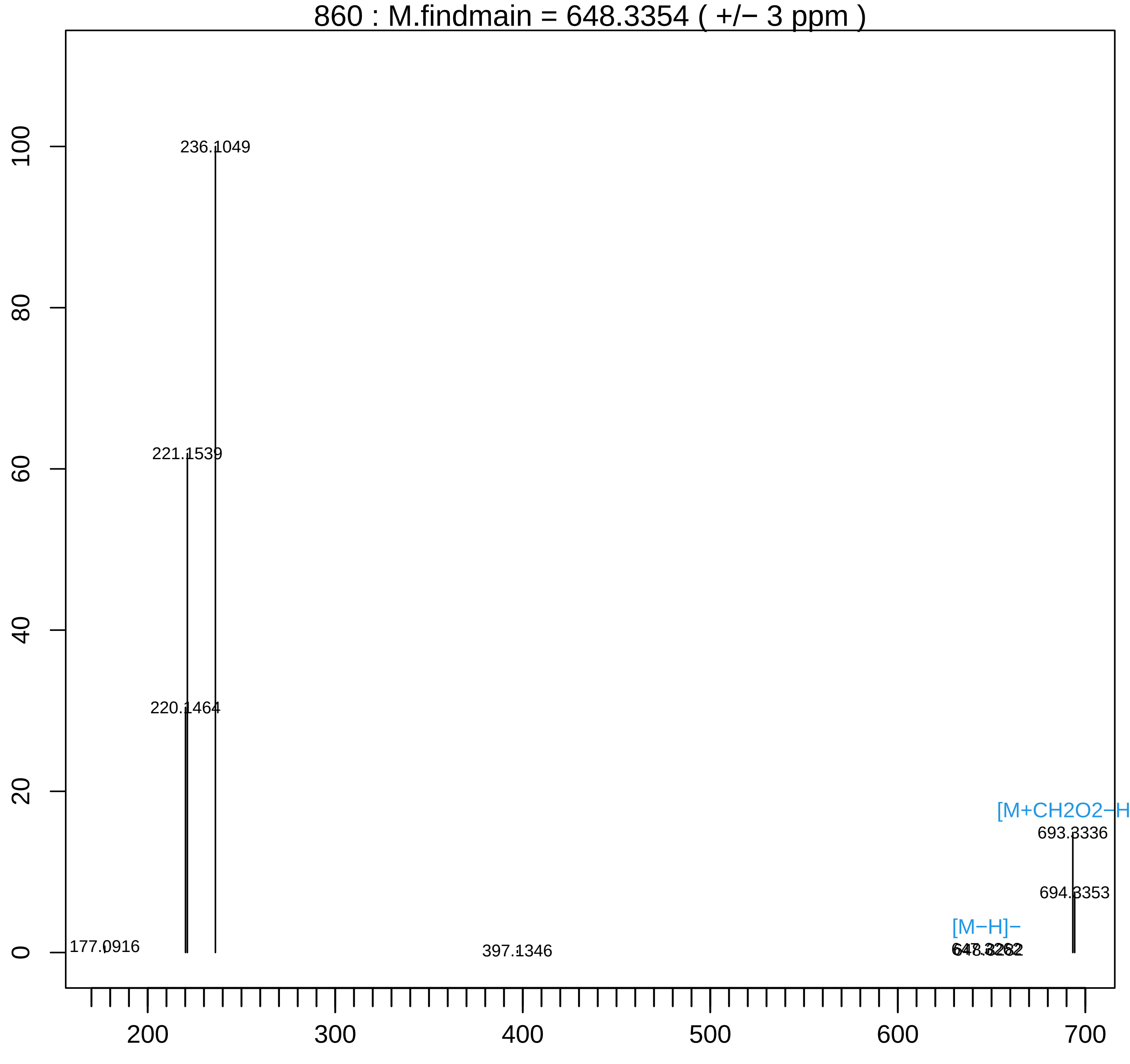
860 : M.findmain = 648.3354 (+/− 3 ppm)
236.1049
221.1539
220.1464
177.0916
397.1346
[M+CH2O2−H
693.3336
694.3353
[M−H]−
647.3262
648.3282
0
20
40
60
80
100
200
300
400
500
600
700

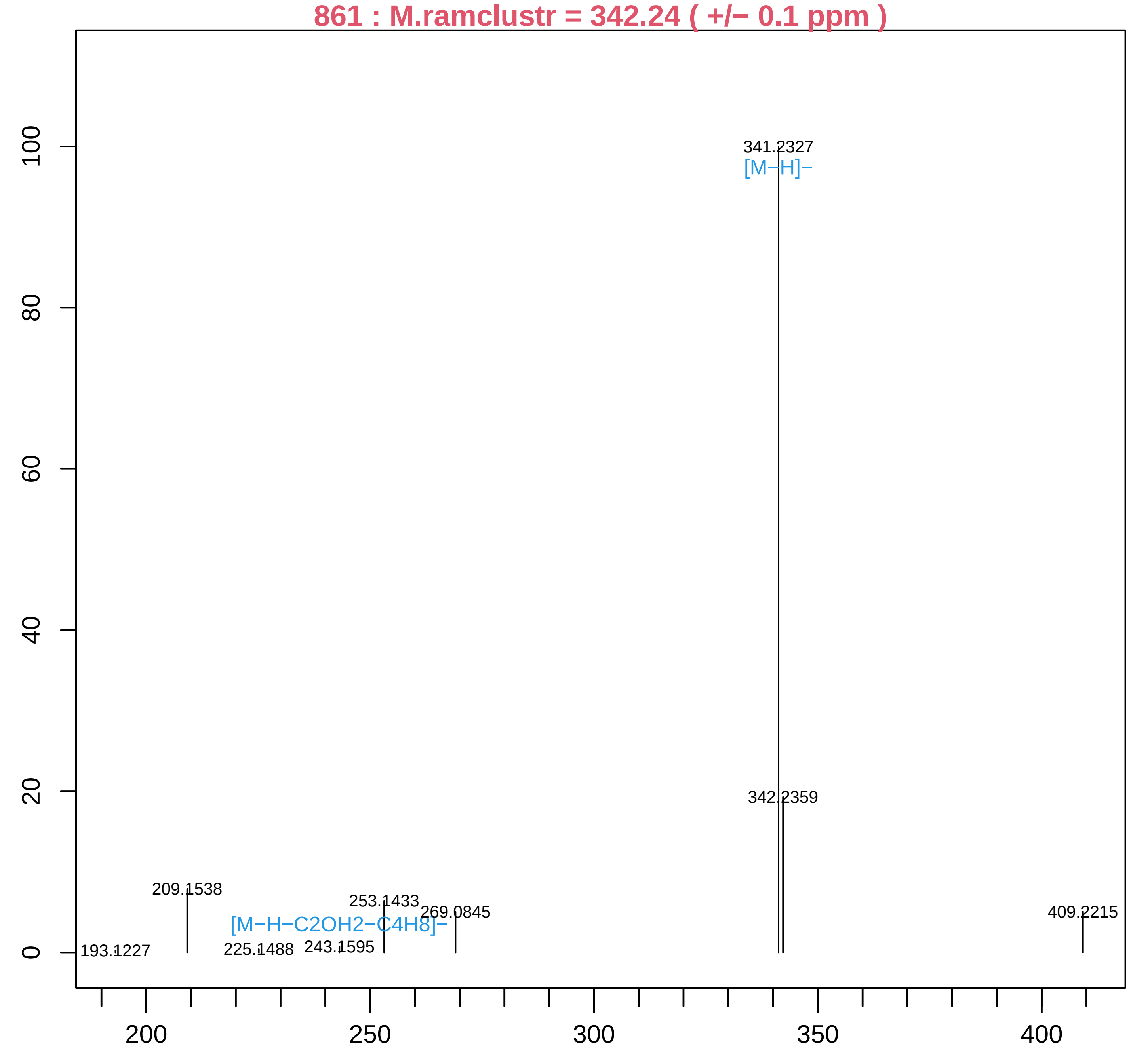
861 : M.ramclustr = 342.24 (+/− 0.1 ppm)
341.2327
[M−H]−
342.2359
209.1538
253.1433
269.0845
409.2215
[M−H−C2OH2−C4H8]−
193.1227
225.1488
243.1595
0
20
40
60
80
100
200
250
300
350
400

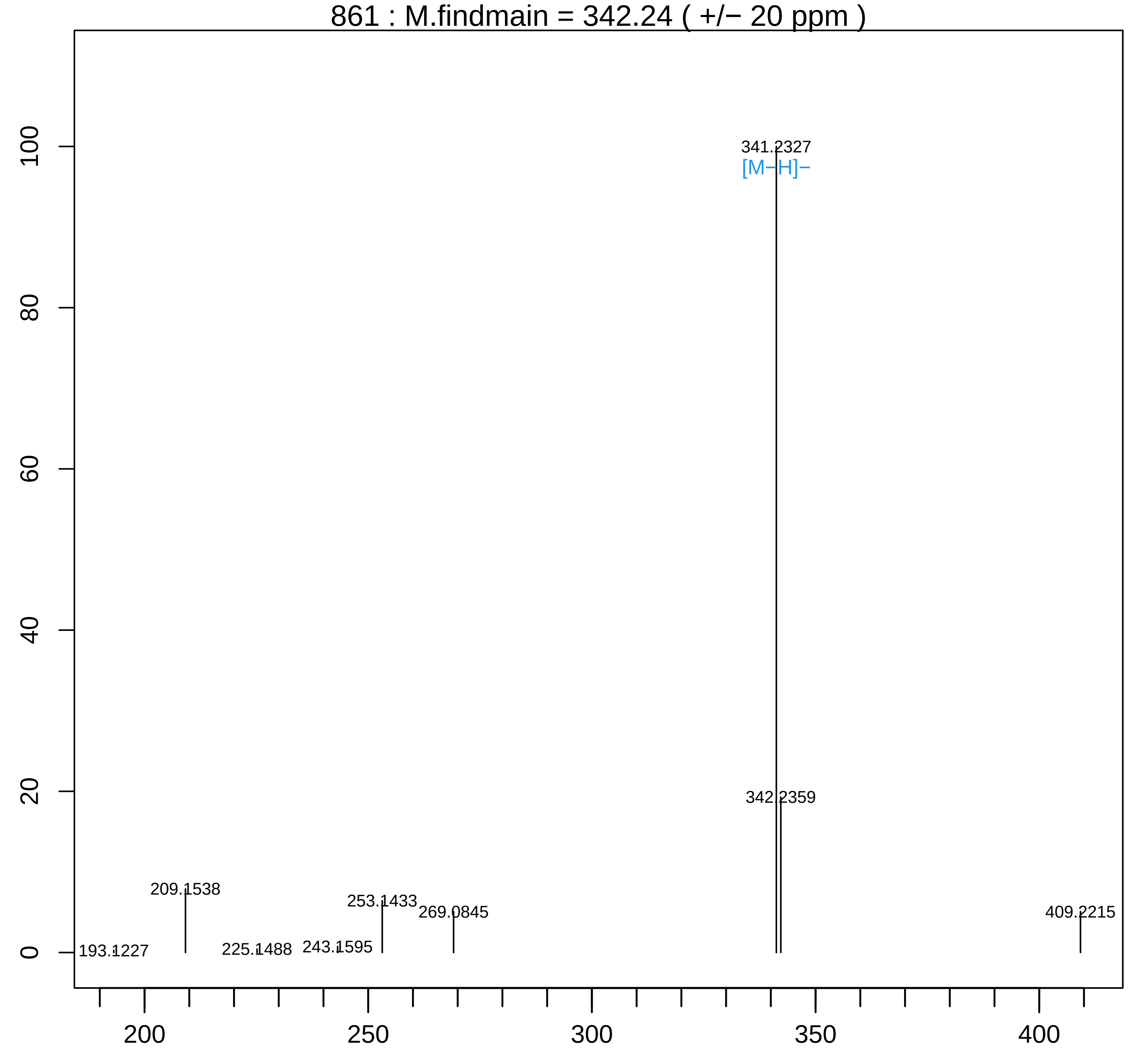
861 : M.findmain = 342.24 (+/− 20 ppm)
341.2327
[M−H]−
342.2359
209.1538
253.1433
269.0845
409.2215
193.1227
225.1488
243.1595
0
20
40
60
80
100
200
250
300
350
400

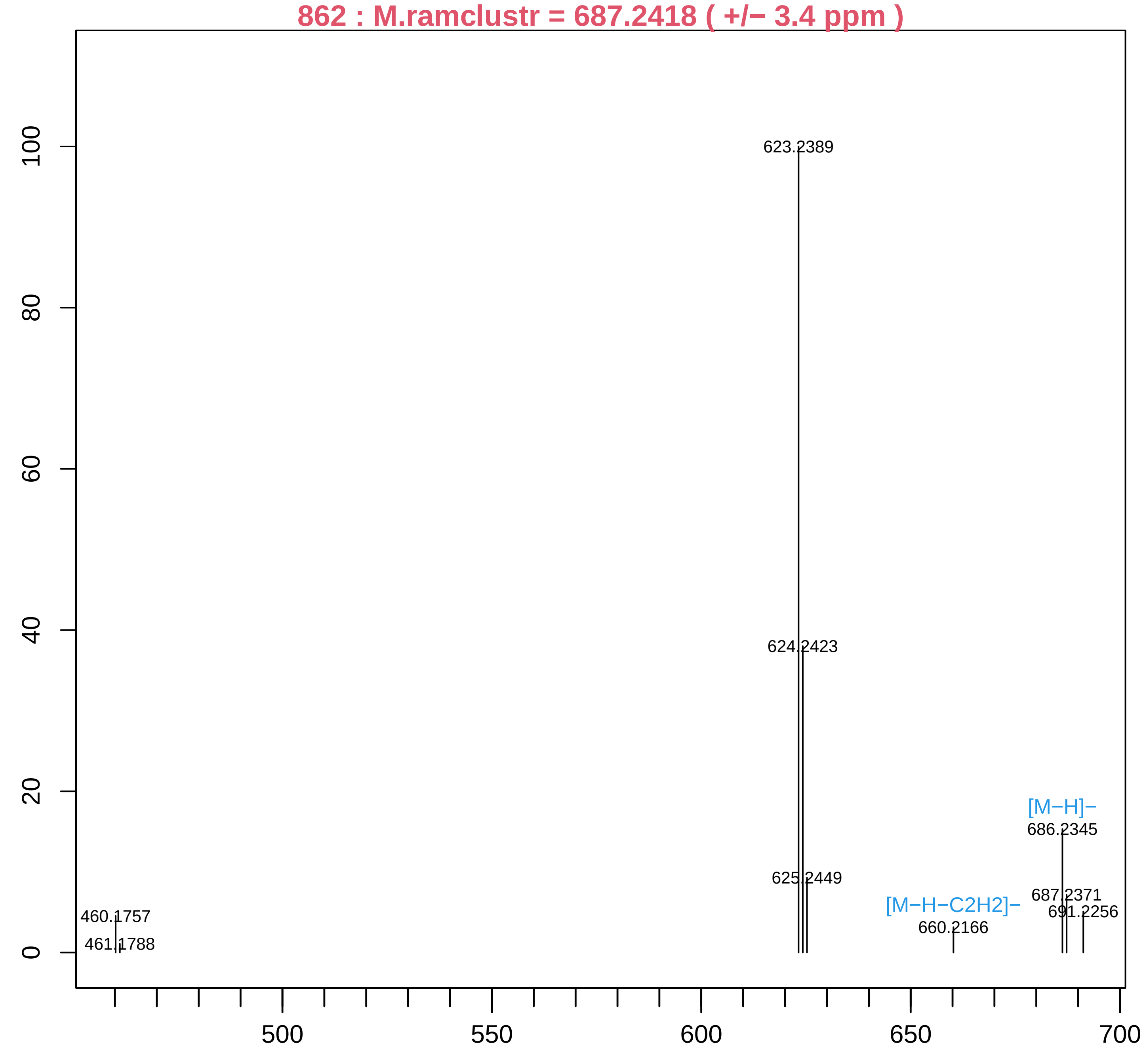
862 : M.ramclustr = 687.2418 (+/− 3.4 ppm)
623.2389
624.2423
625.2449
[M−H]−
686.2345
687.2371
691.2256
[M−H−C2H2]−
660.2166
460.1757
461.1788
0
20
40
60
80
100
500
550
600
650
700

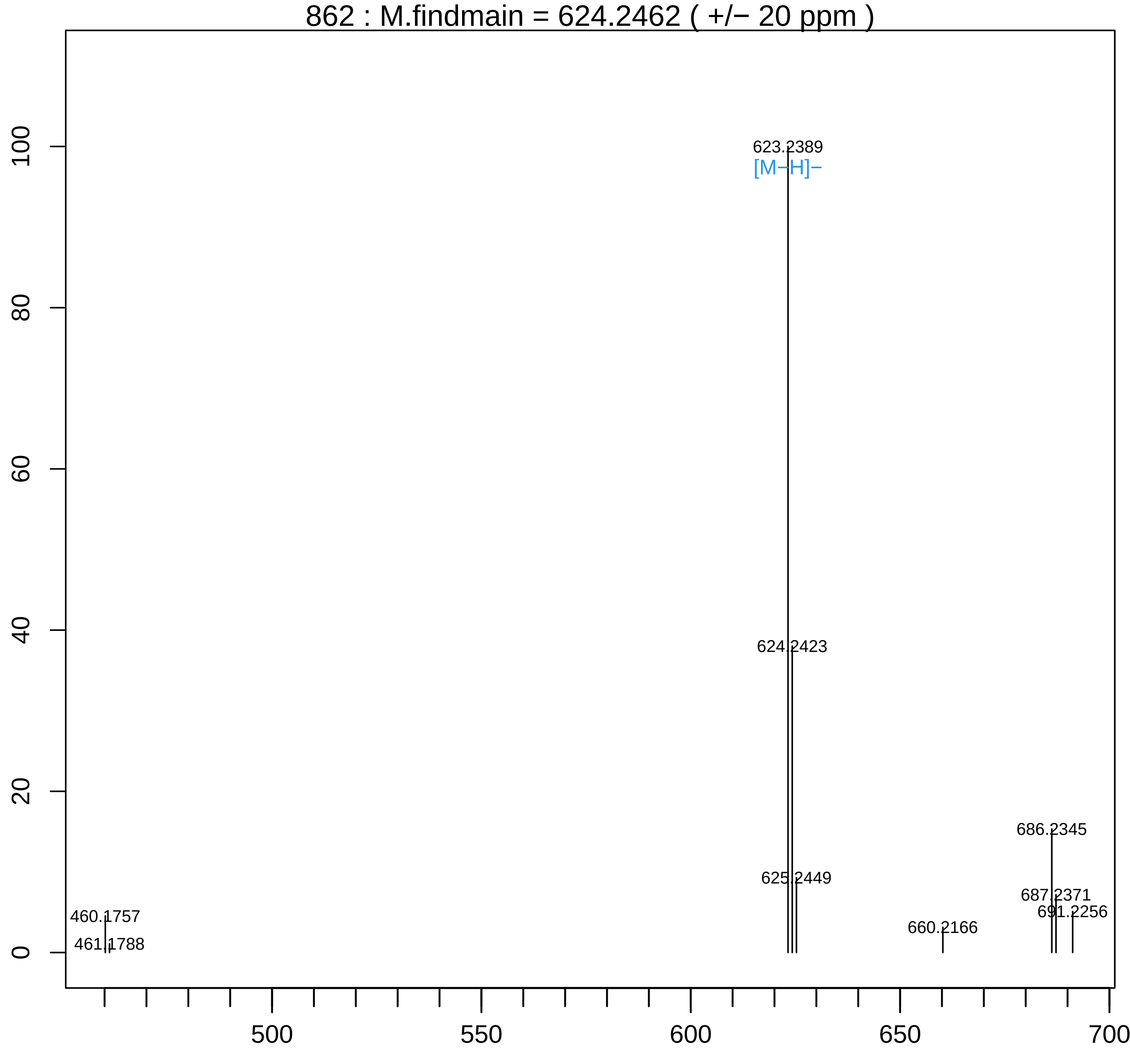
862 : M.findmain = 624.2462 (+/− 20 ppm)
623.2389
[M−H]−
624.2423
625.2449
686.2345
687.2371
691.2256
660.2166
460.1757
461.1788
0
20
40
60
80
100
500
550
600
650
700

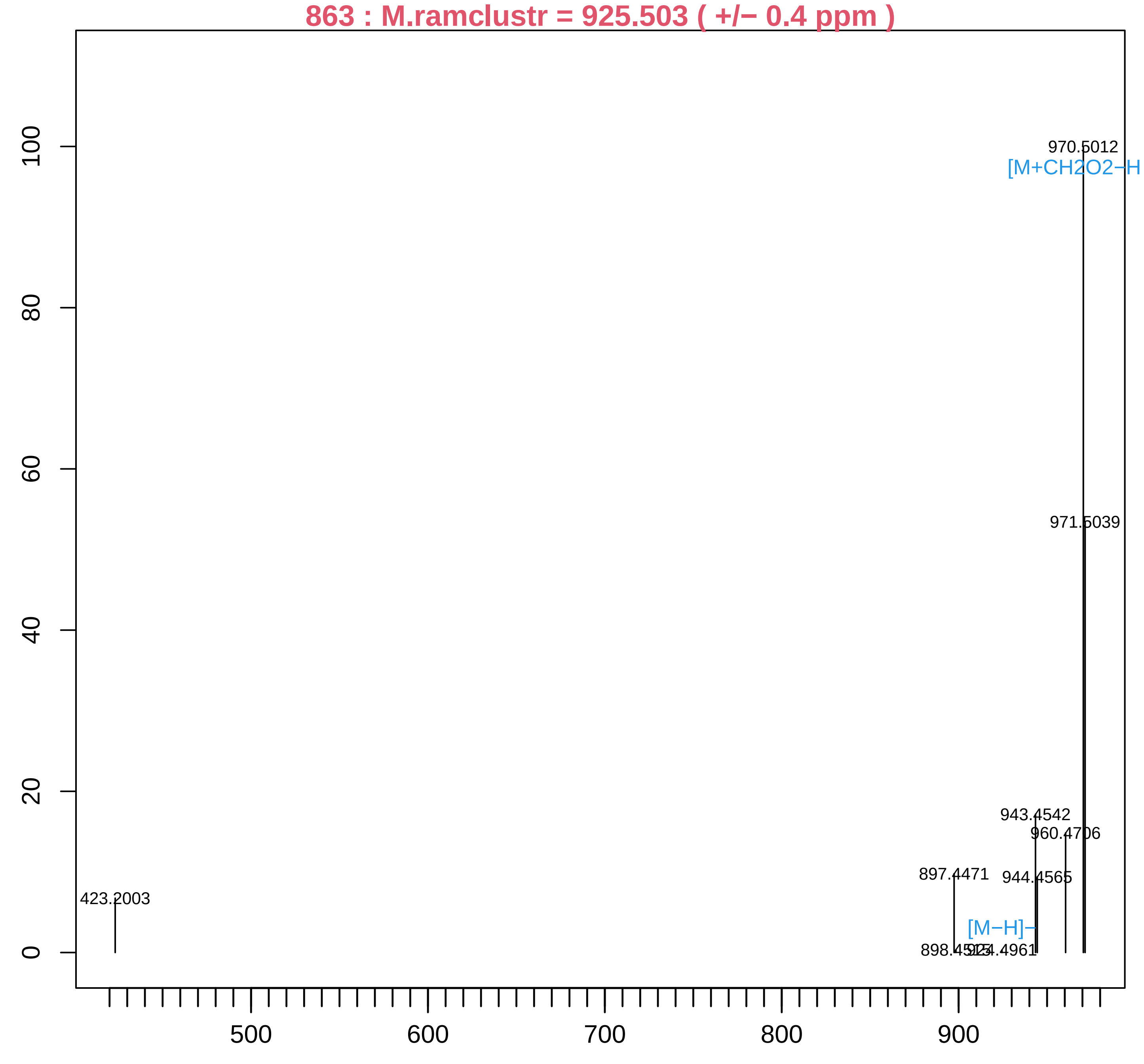
863 : M.ramclustr = 925.503 (+/− 0.4 ppm)
970.5012
[M+CH2O2−H
971.5039
943.4542
960.4706
897.4471
944.4565
423.2003
[M−H]−
898.4503
924.4961
0
20
40
60
80
100
500
600
700
800
900

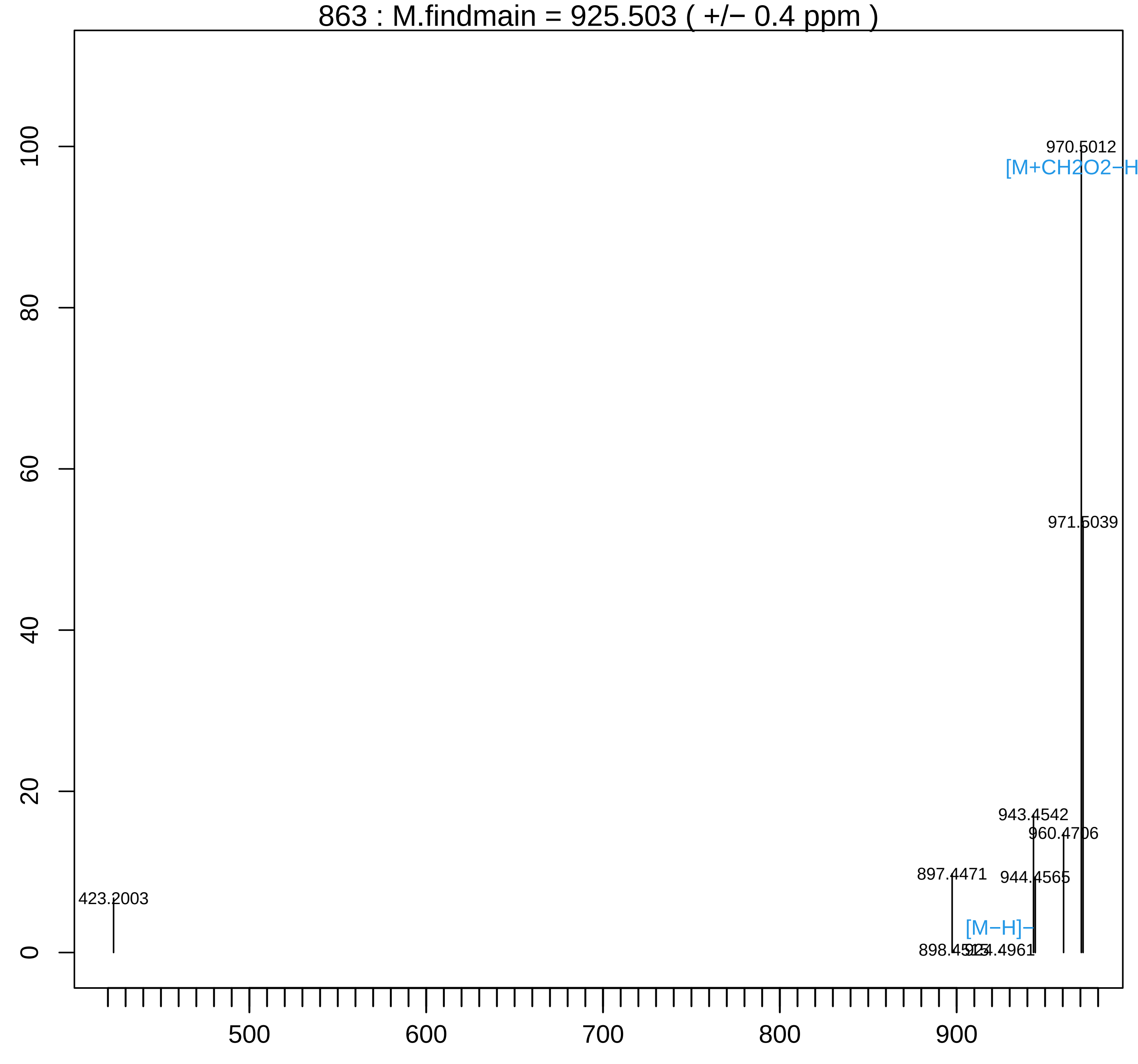
863 : M.findmain = 925.503 (+/− 0.4 ppm)
970.5012
[M+CH2O2−H
971.5039
943.4542
960.4706
897.4471
944.4565
423.2003
[M−H]−
898.4503
924.4961
0
20
40
60
80
100
500
600
700
800
900

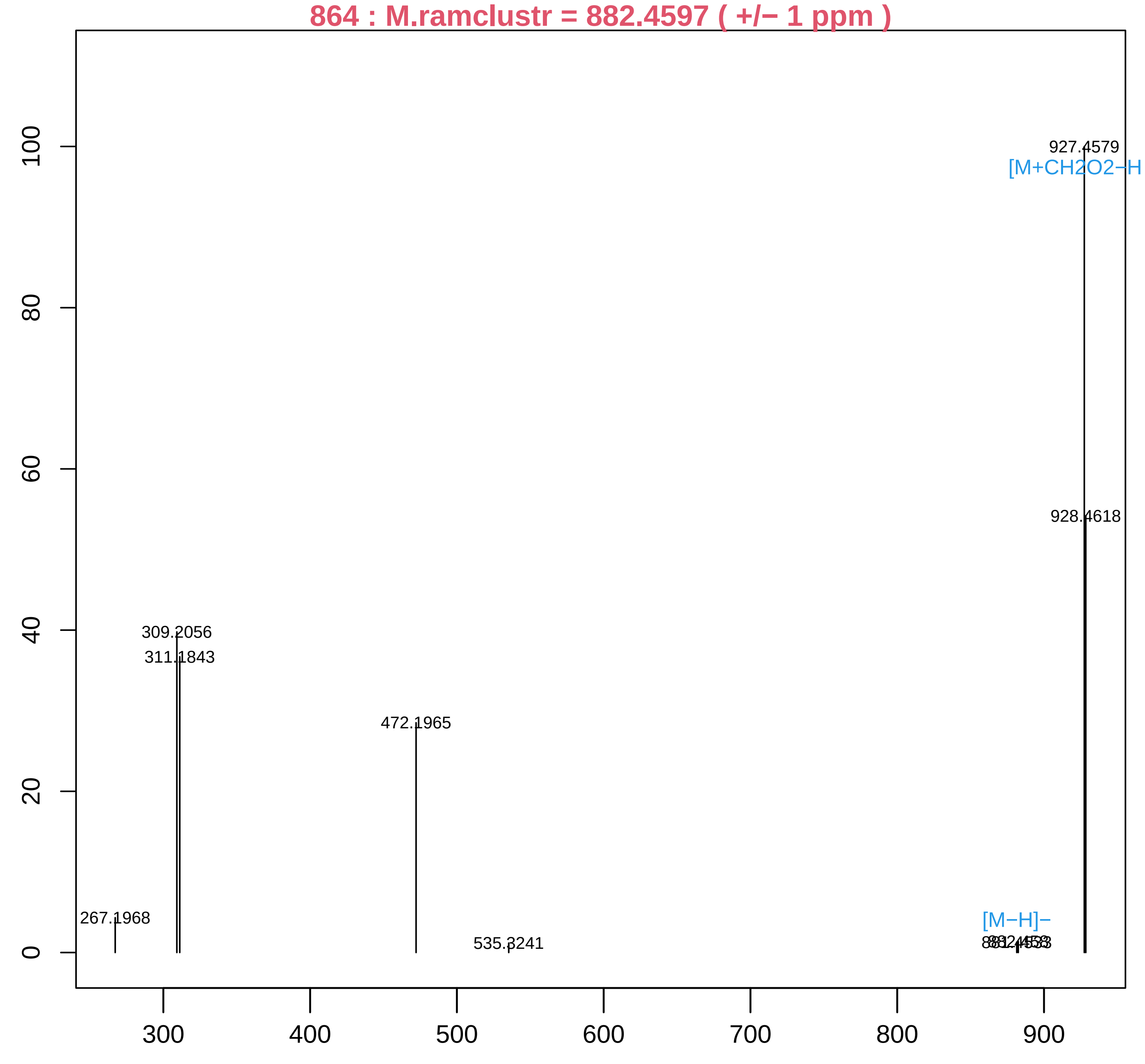
864 : M.ramclustr = 882.4597 (+/− 1 ppm)
927.4579
[M+CH2O2−H
928.4618
309.2056
311.1843
472.1965
267.1968
535.3241
[M−H]−
0
20
40
60
80
100
300
400
500
600
700
800
900

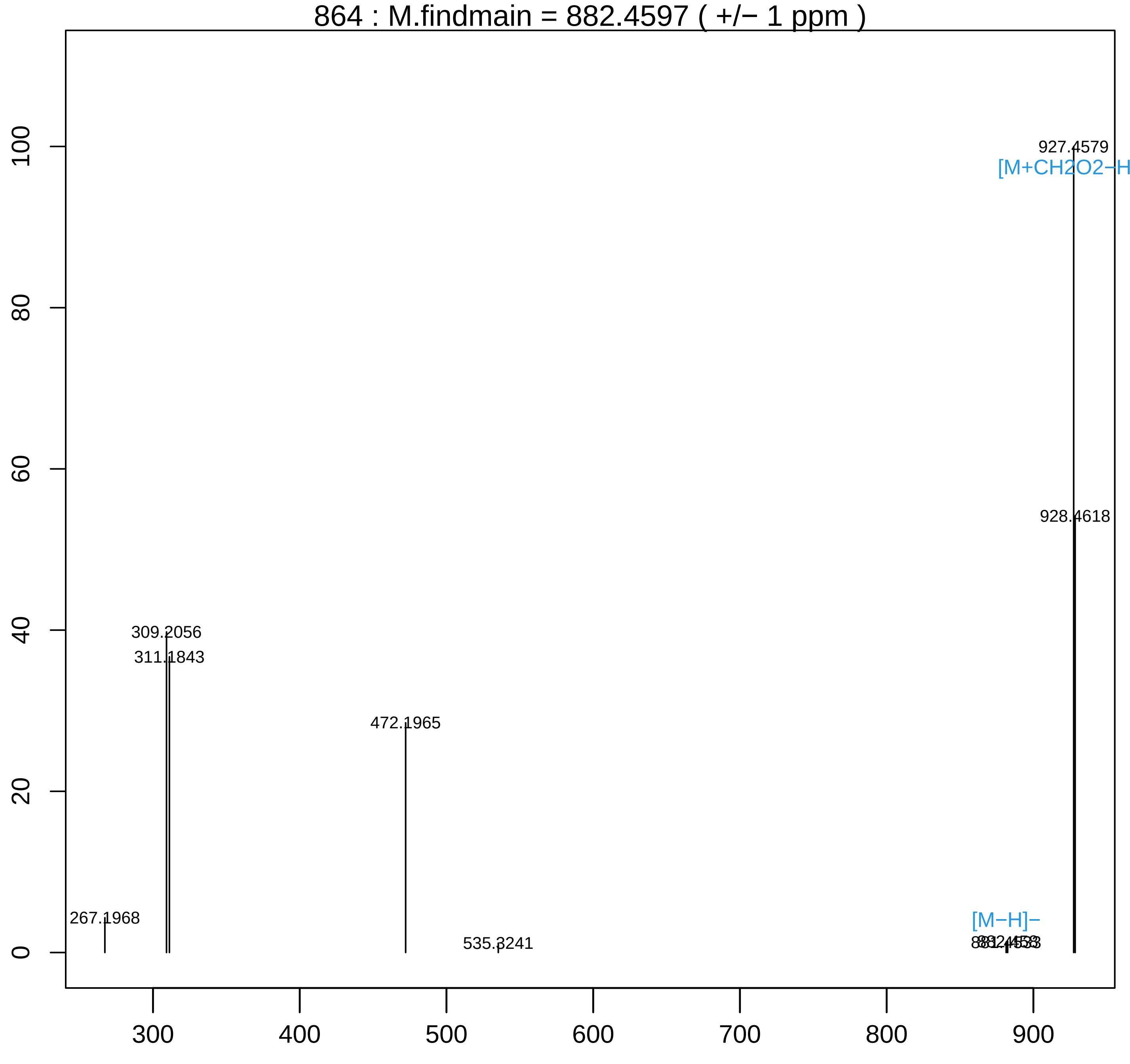
864 : M.findmain = 882.4597 (+/− 1 ppm)
927.4579
[M+CH2O2−H
928.4618
309.2056
311.1843
472.1965
267.1968
535.3241
[M−H]−
0
20
40
60
80
100
300
400
500
600
700
800
900

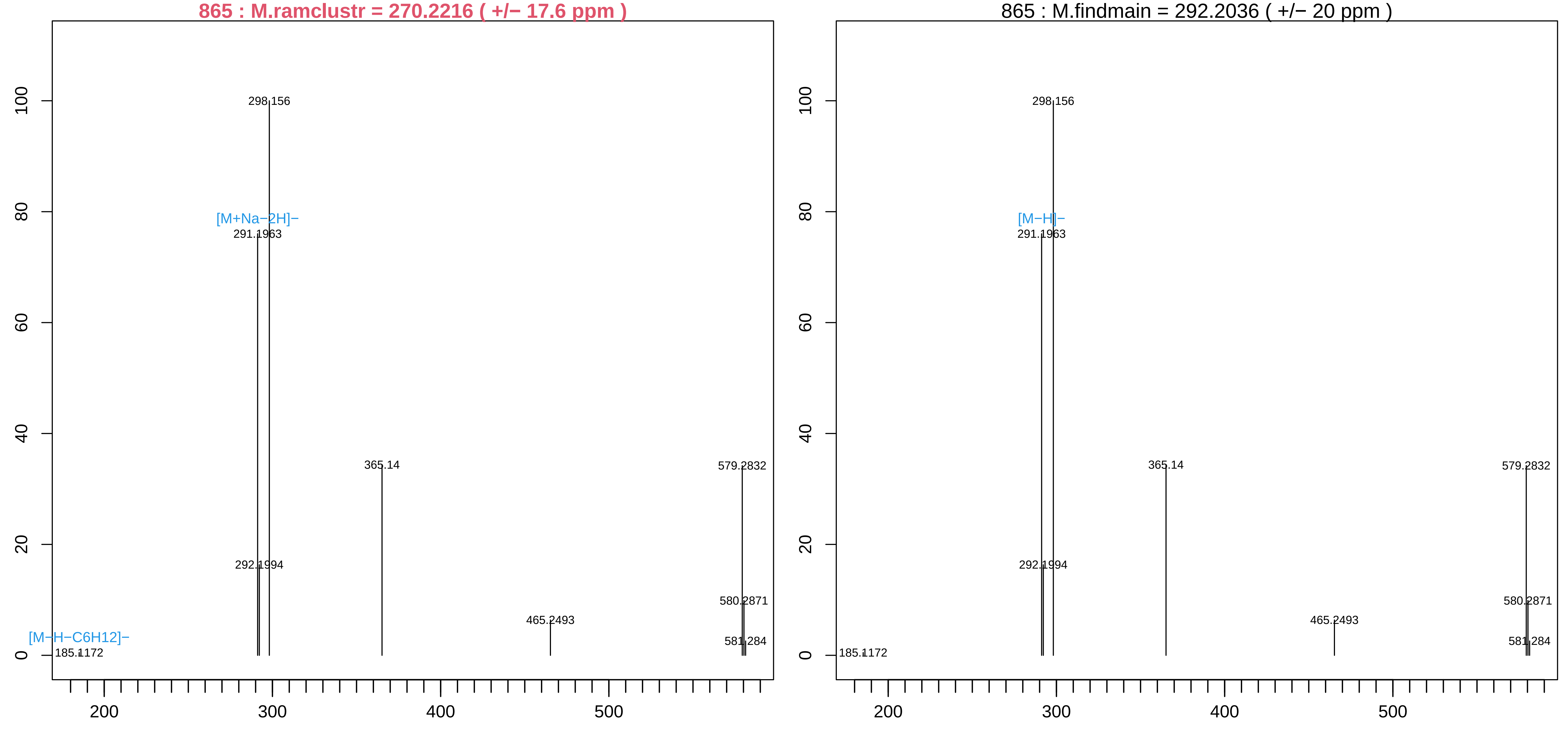
865 : M.ramclustr = 270.2216 (+/− 17.6 ppm)
[M+Na−2H]−
[M−H−C6H12]−
298.156
291.1963
292.1994
365.14
465.2493
579.2832
580.2871
581.284
185.1172
865 : M.findmain = 292.2036 (+/− 20 ppm)
[M−H]−
0
20
40
60
80
100
200
300
400
500

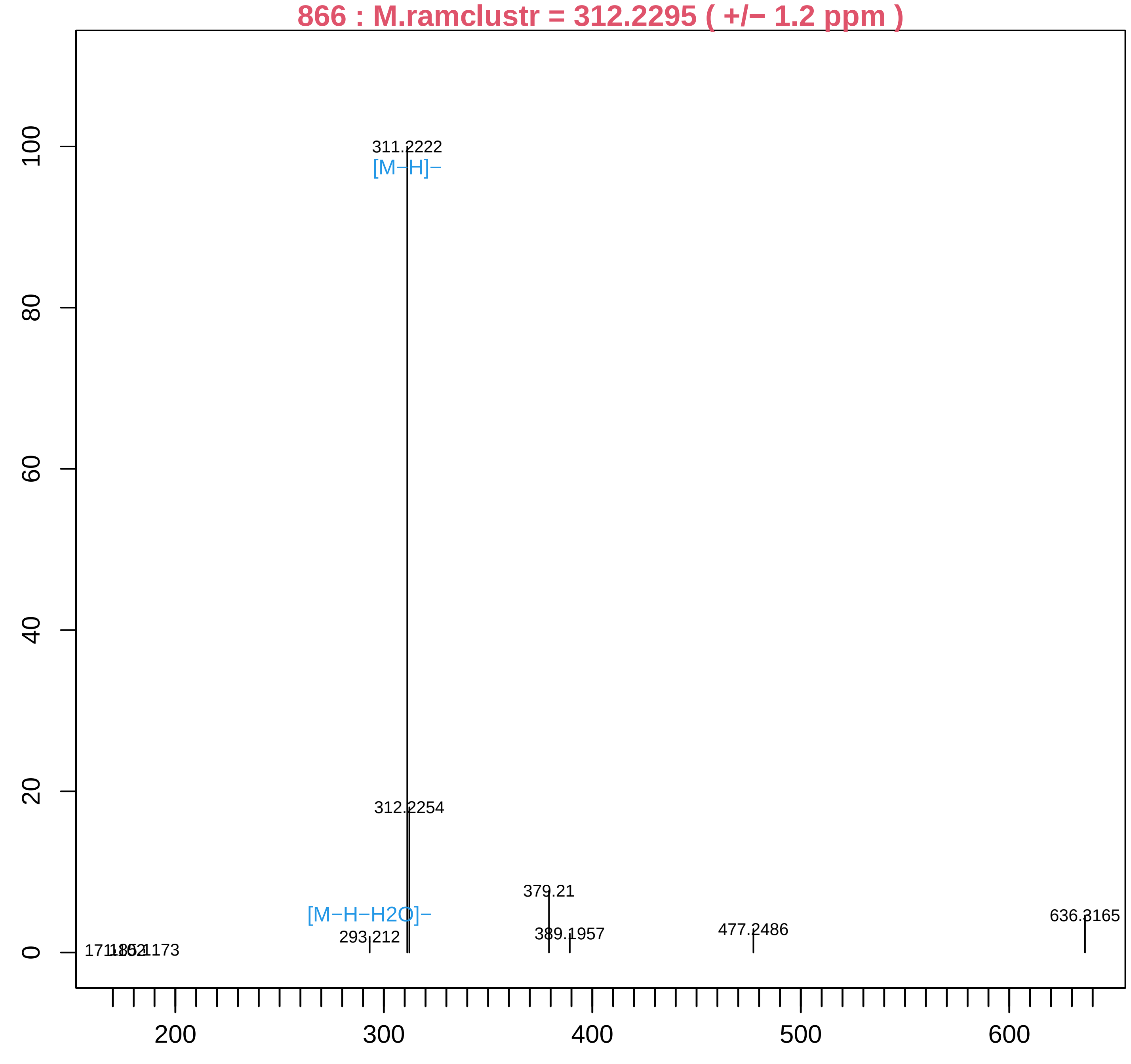

866 : M.ramclustr = 312.2295 (+/− 1.2 ppm)
311.2222
[M−H]−
312.2254
379.21
[M−H−H2O]−
636.3165
293.212
389.1957
477.2486
0
20
40
60
80
100
200
300
400
500
600

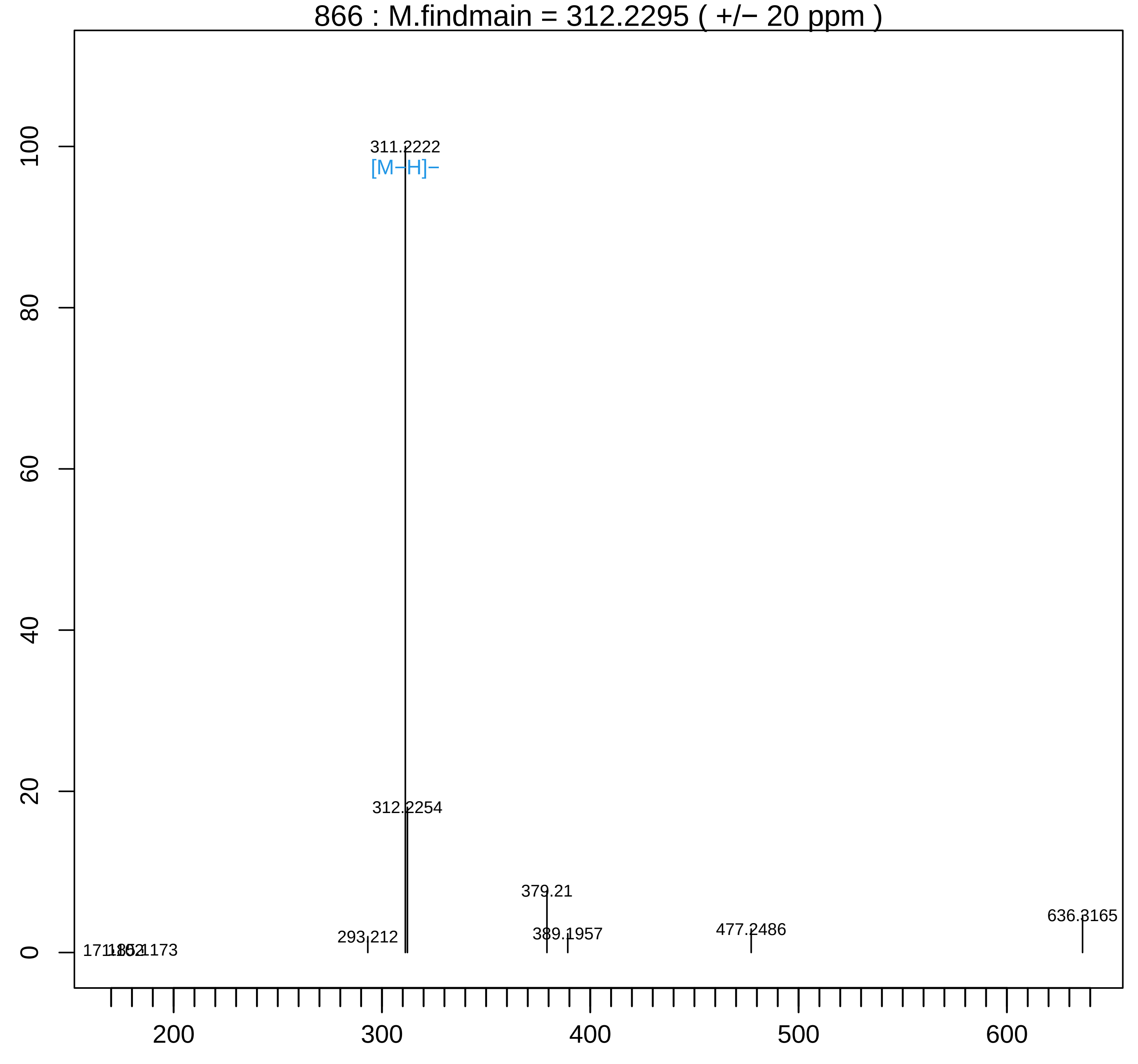

866 : M.findmain = 312.2295 (+/− 20 ppm)
311.2222
[M−H]−
312.2254
379.21
636.3165
293.212
389.1957
477.2486
0
20
40
60
80
100
200
300
400
500
600

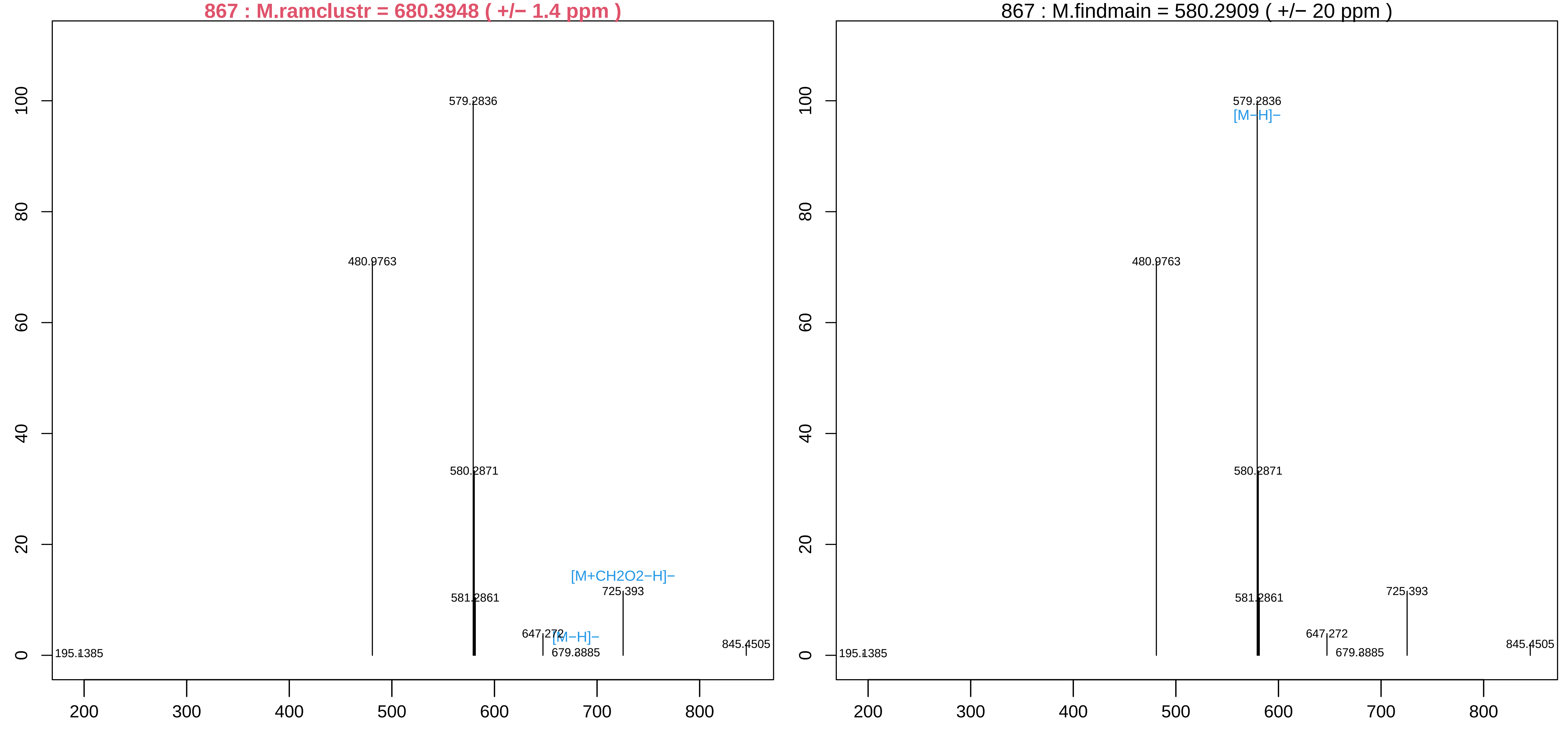

867 : M.ramclustr = 680.3948 (+/− 1.4 ppm)
579.2836
480.9763
580.2871
581.2861
[M+CH2O2−H]−
725.393
647.272
[M−H]−
679.8885
845.4505
195.1385
867 : M.findmain = 580.2909 (+/− 20 ppm)
579.2836
[M−H]−
480.9763
580.2871
581.2861
725.393
647.272
679.8885
845.4505
195.1385

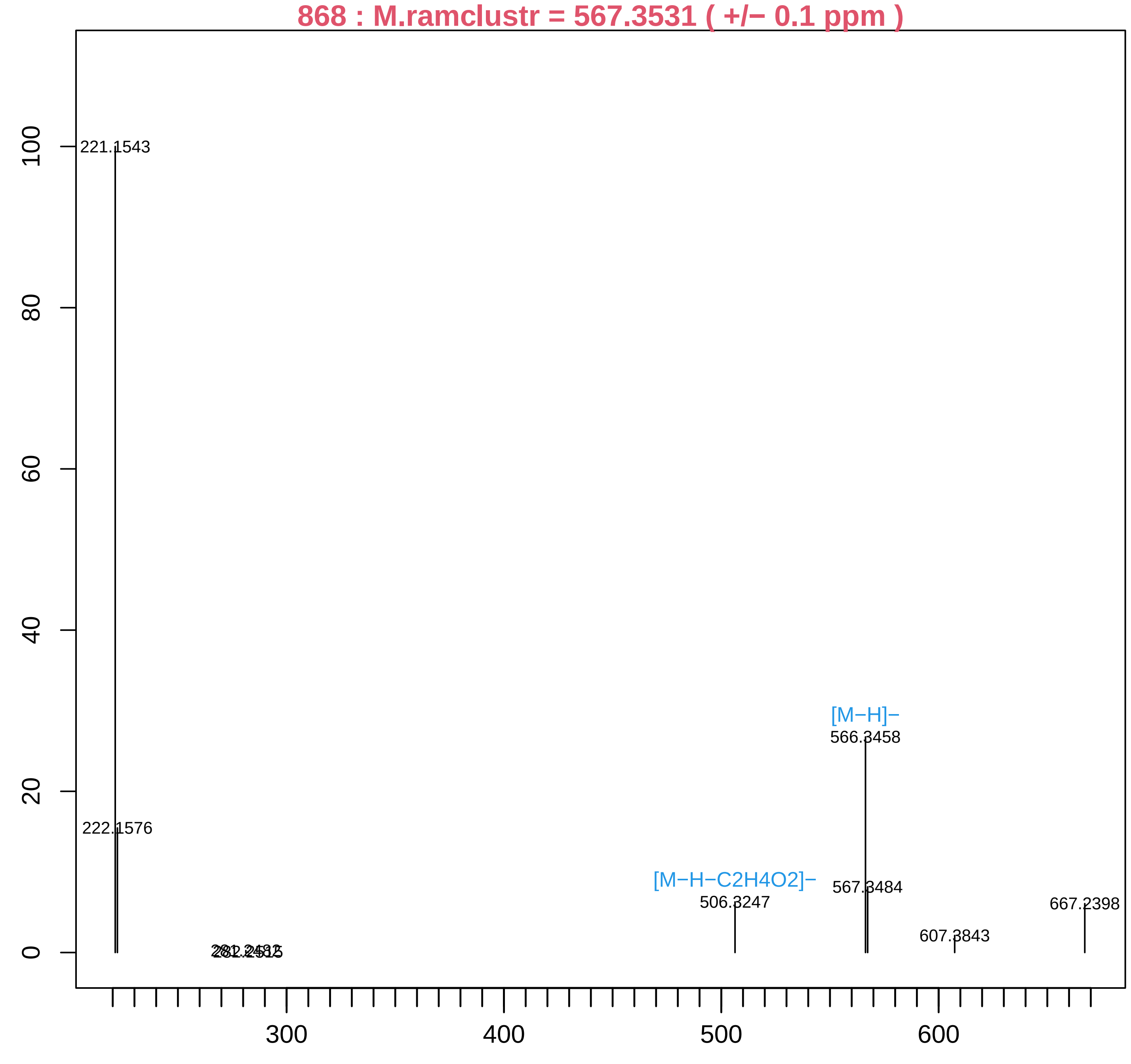
868 : M.ramclustr = 567.3531 (+/− 0.1 ppm)
221.1543
222.1576
[M−H]−
566.3458
[M−H−C2H4O2]−
506.3247
567.3484
607.3843
667.2398
0
20
40
60
80
100
300
400
500
600

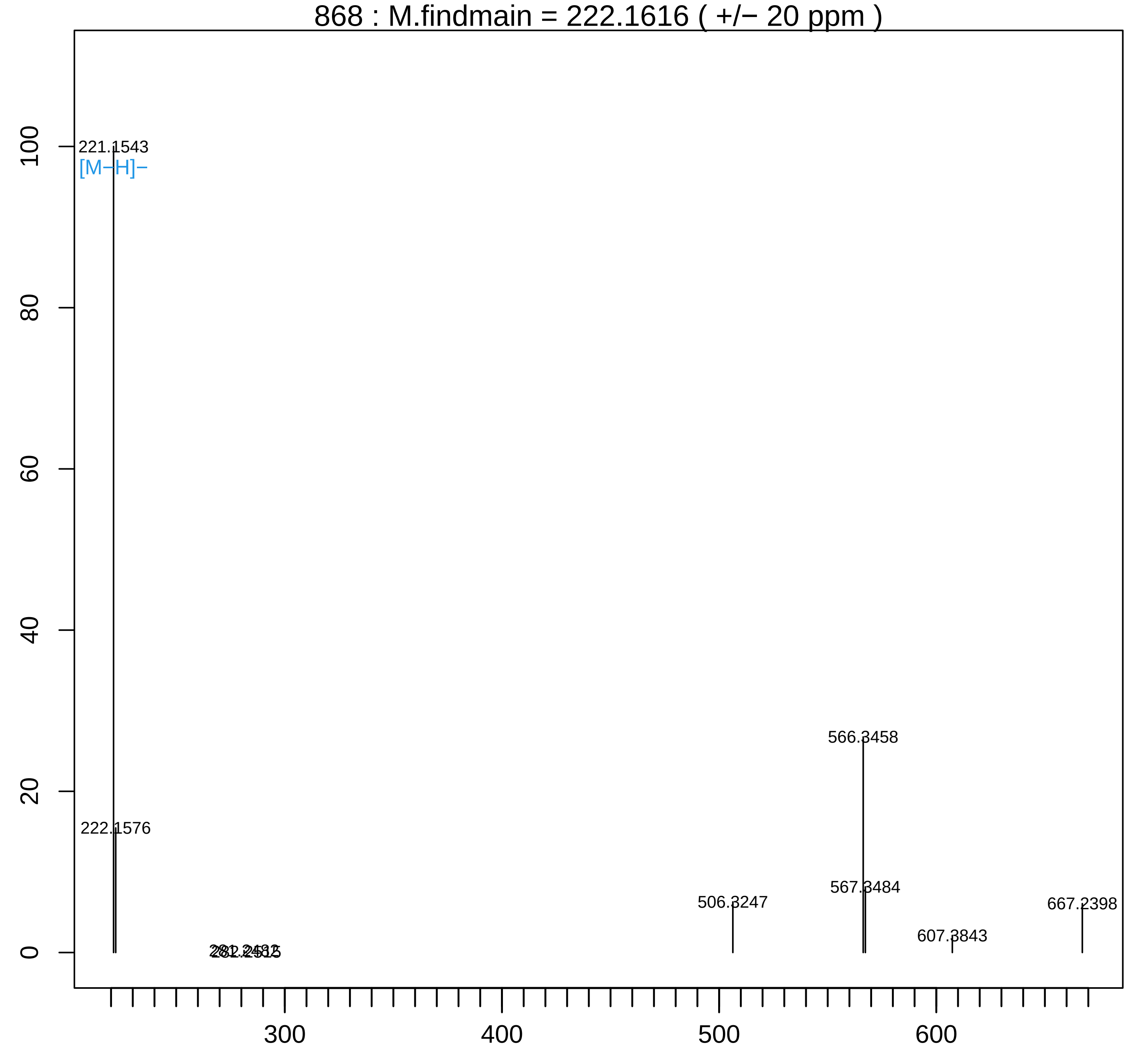
868 : M.findmain = 222.1616 (+/− 20 ppm)
221.1543
[M−H]−
222.1576
566.3458
506.3247
567.3484
607.3843
667.2398
0
20
40
60
80
100
300
400
500
600

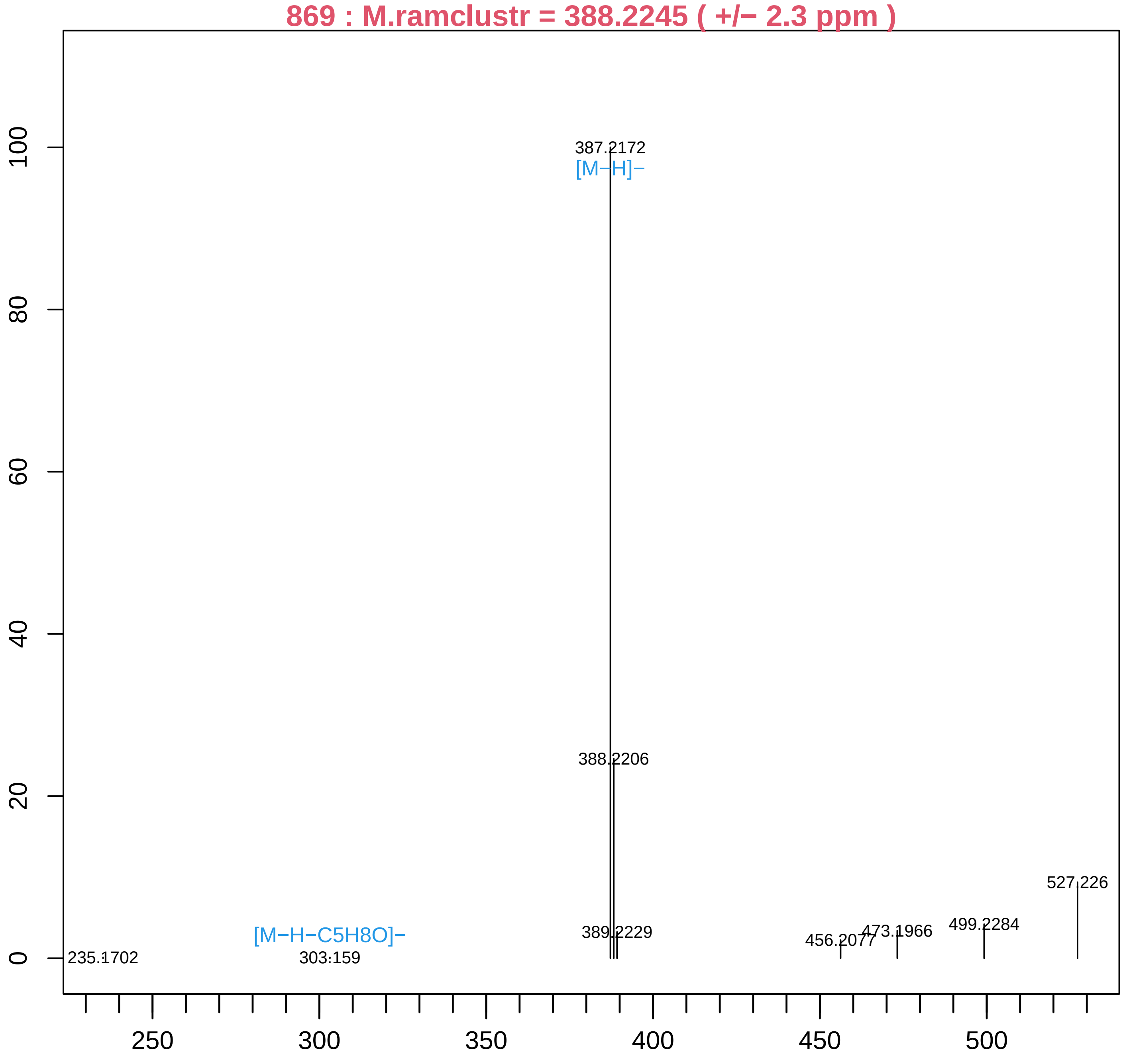
869 : M.ramclustr = 388.2245 (+/− 2.3 ppm)
387.2172
[M−H]−
388.2206
389.2229
[M−H−C5H8O]−
235.1702
303:159
456.2077
473.1966
499.2284
527.226
0
20
40
60
80
100
250
300
350
400
450
500
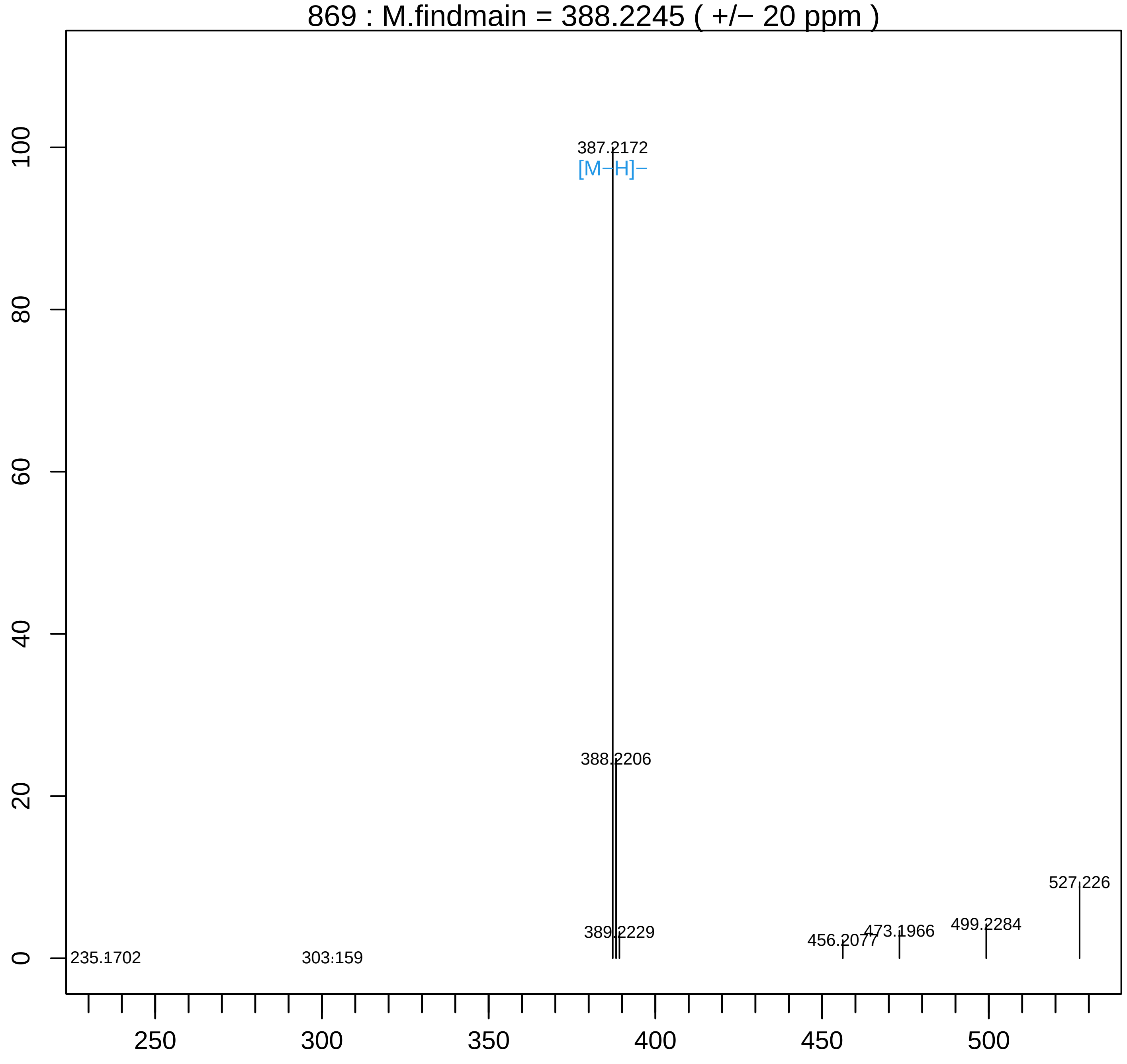
869 : M.findmain = 388.2245 (+/− 20 ppm)
387.2172
[M−H]−
388.2206
389.2229
235.1702
303:159
456.2077
473.1966
499.2284
527.226
0
20
40
60
80
100
250
300
350
400
450
500

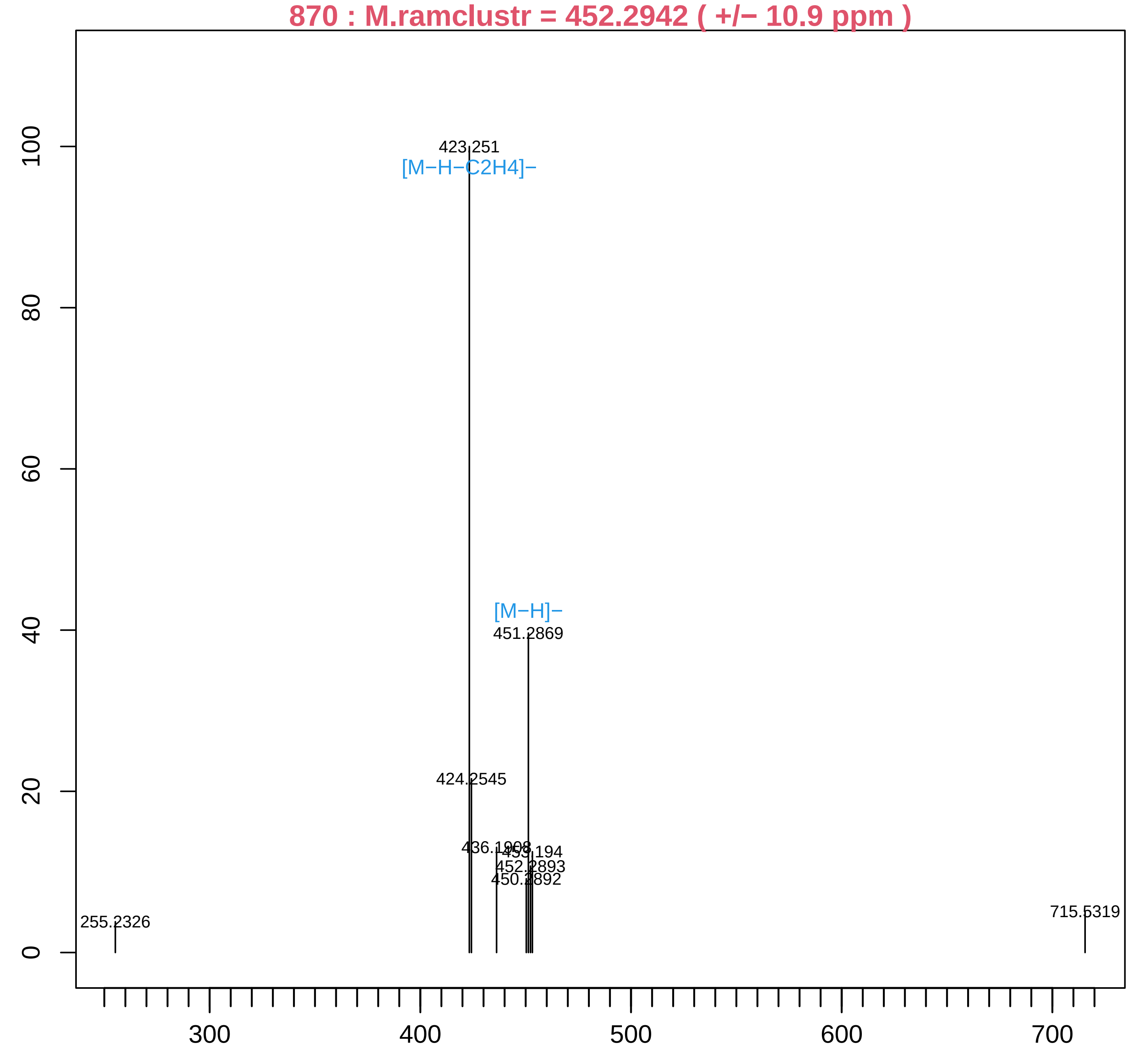

870 : M.ramclustr = 452.2942 (+/− 10.9 ppm)
423.251
[M−H−C2H4]−
[M−H]−
451.2869
424.2545
436.1908
453.3194
452.2893
450.2892
255.2326
715.5319
0
20
40
60
80
100
300
400
500
600
700

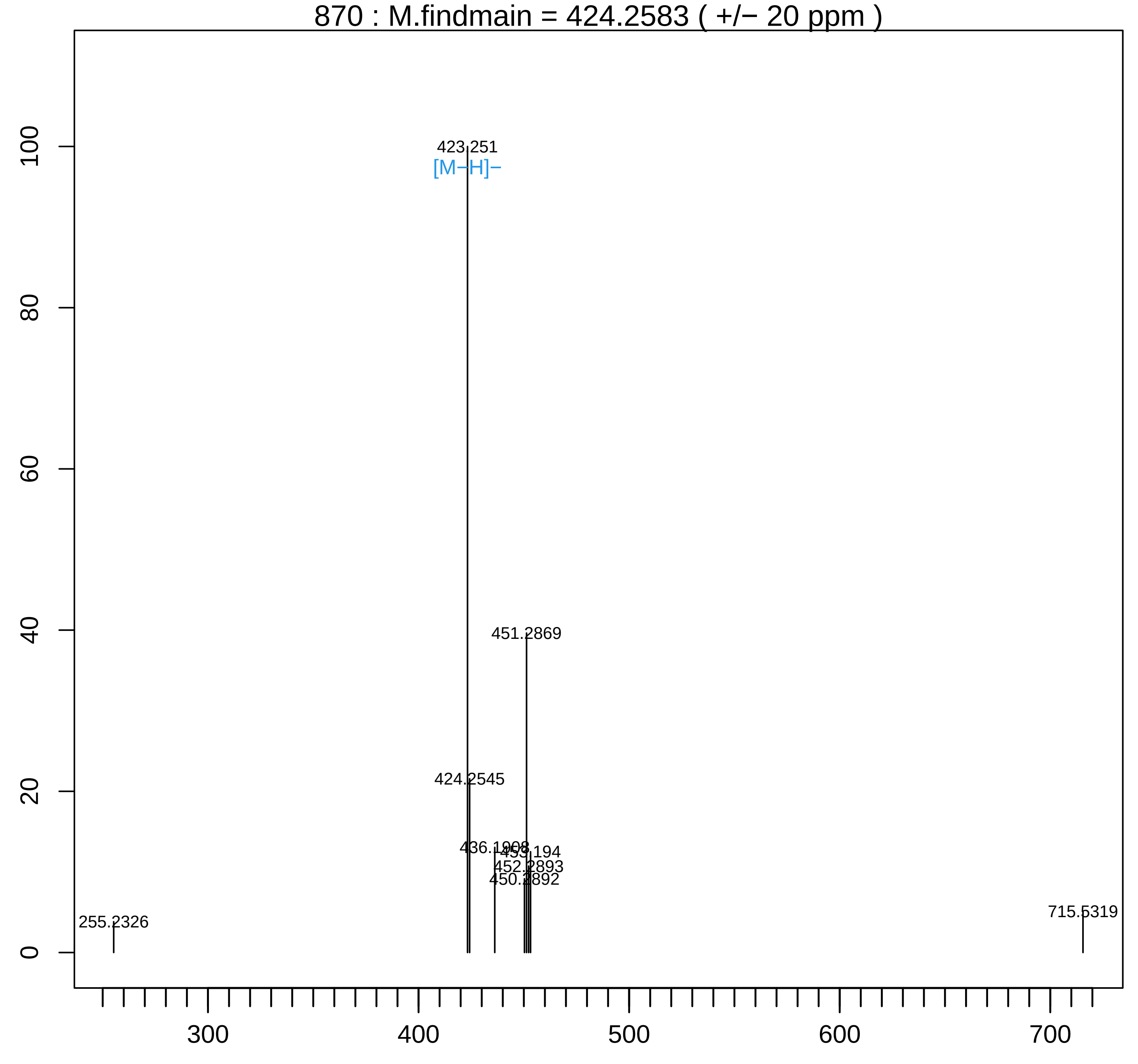

870 : M.findmain = 424.2583 (+/− 20 ppm)
423.251
[M−H]−
451.2869
424.2545
436.1908
453.3194
452.2893
450.2892
255.2326
715.5319
0
20
40
60
80
100
300
400
500
600
700

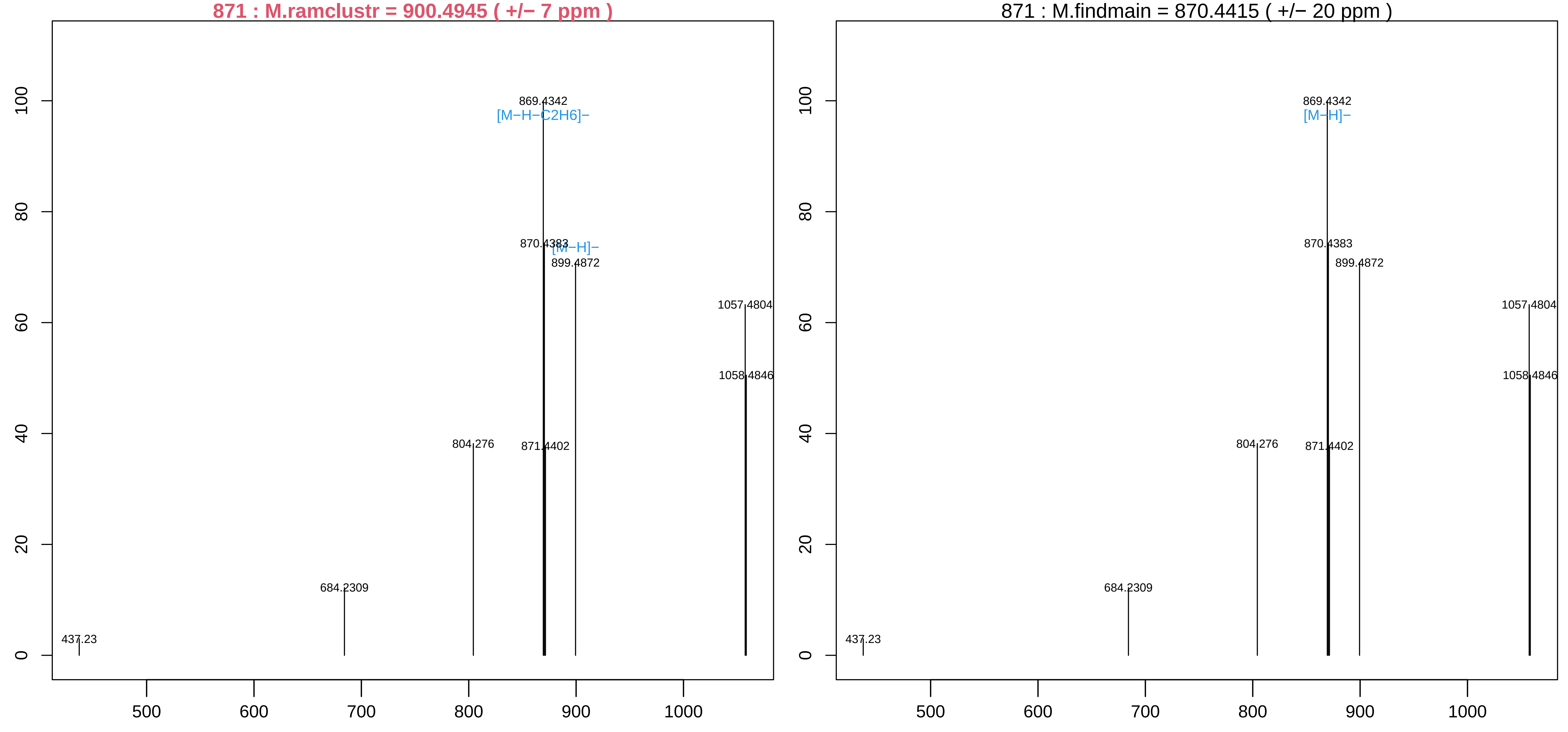

871 : M.ramclustr = 900.4945 (+/− 7 ppm)
869.4342
[M−H−C2H6]−
870.4383
[M−H]−
899.4872
1057.4804
1058.4846
804.276
871.4402
684.2309
437.23
0
20
40
60
80
100
500
600
700
800
900
1000
871 : M.findmain = 870.4415 (+/− 20 ppm)
869.4342
[M−H]−
870.4383
899.4872
1057.4804
1058.4846
804.276
871.4402
684.2309
437.23
0
20
40
60
80
100
500
600
700
800
900
1000

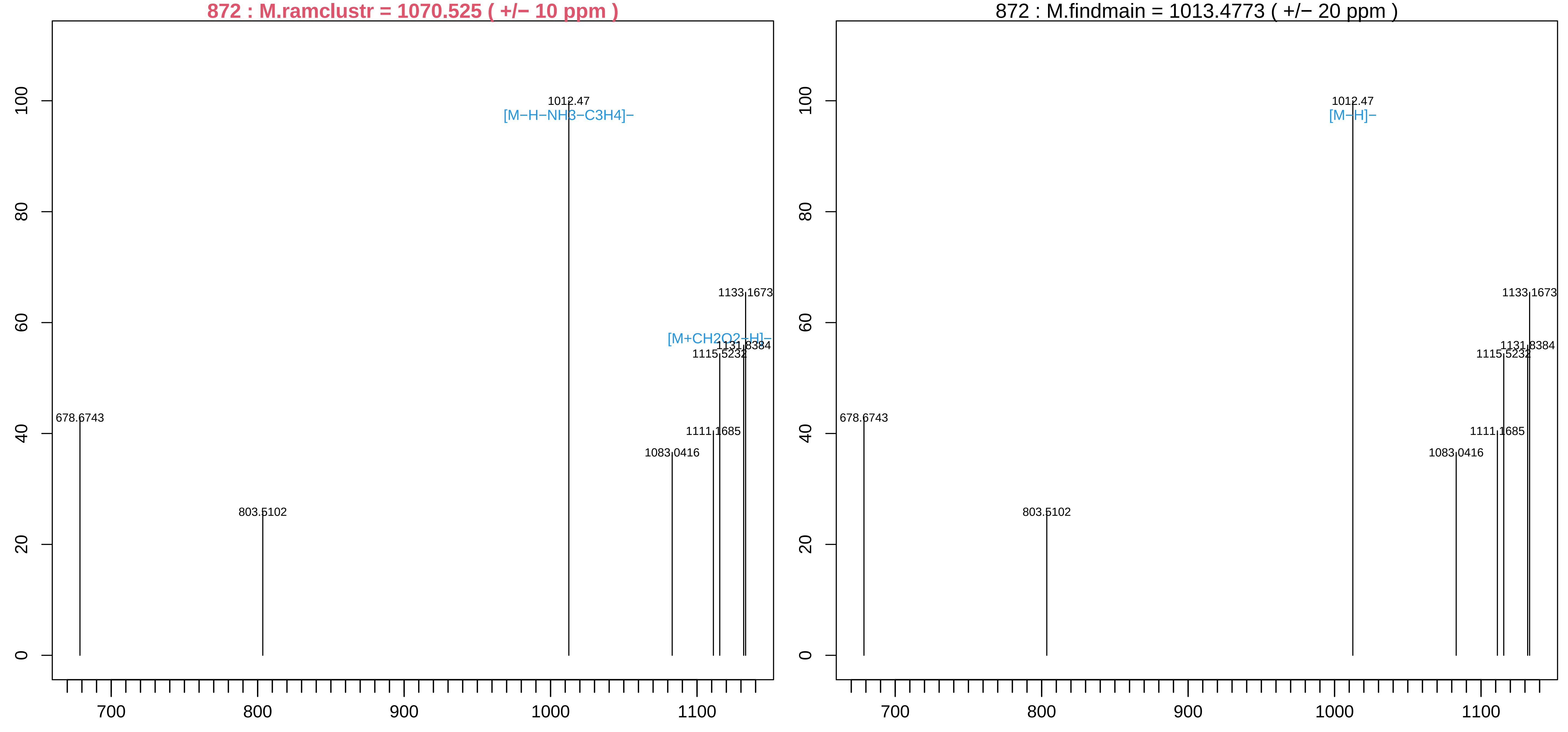

872 : M.ramclustr = 1070.525 (+/− 10 ppm)
872 : M.findmain = 1013.4773 (+/− 20 ppm)
1012.47
[M−H−NH3−C3H4]−
[M−H]−
1133.1673
[M+CH2O2−H]−
1131.8384
1115.5232
1111.1685
1083.0416
678.6743
803.5102
0
20
40
60
80
100
700
800
900
1000
1100

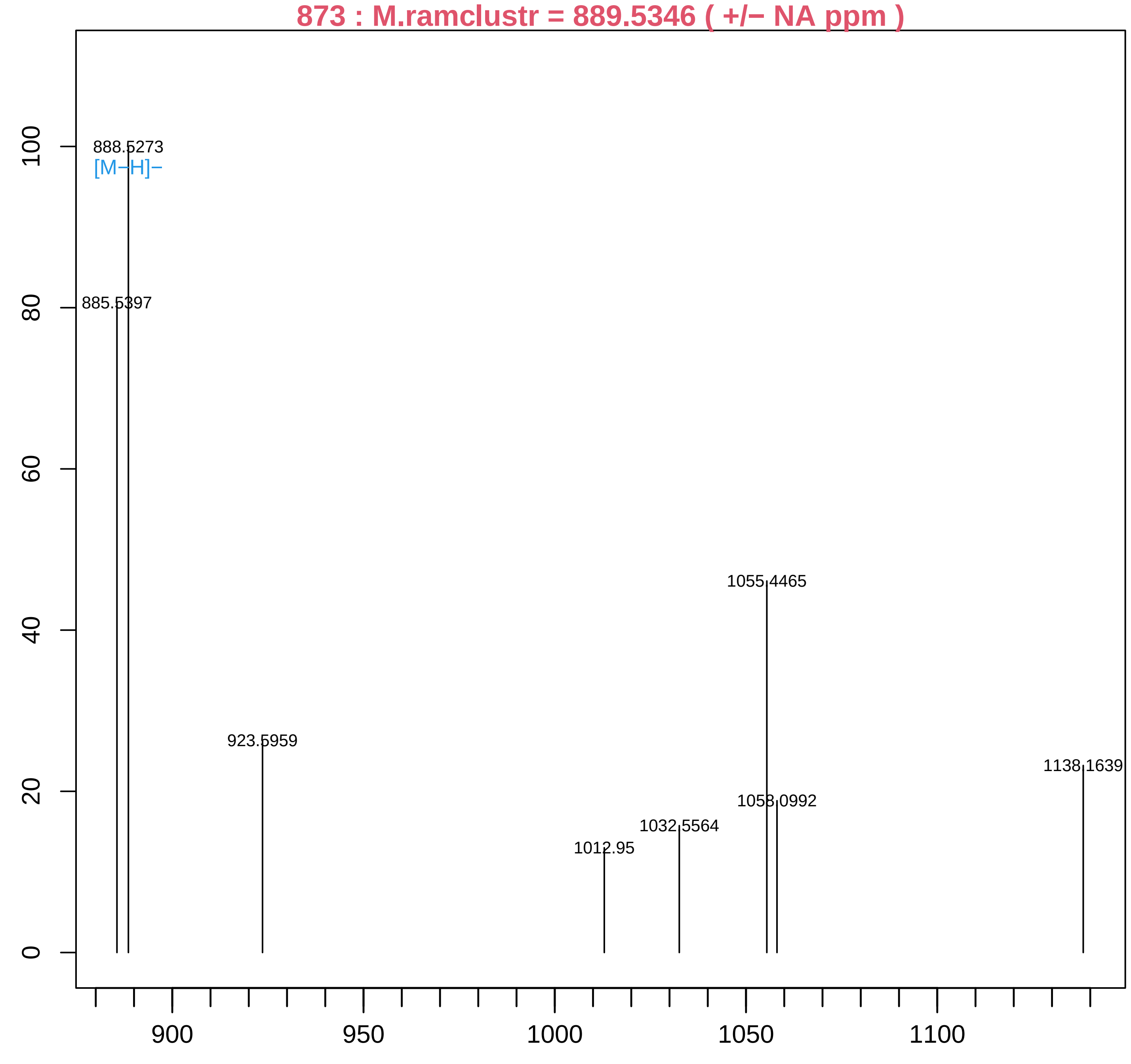
873 : M.ramclustr = 889.5346 (+/− NA ppm)
888.5273
[M−H]−
885.5397
923.5959
1055.4465
1058.0992
1032.5564
1012.95
1138.1639
0
20
40
60
80
100
900
950
1000
1050
1100
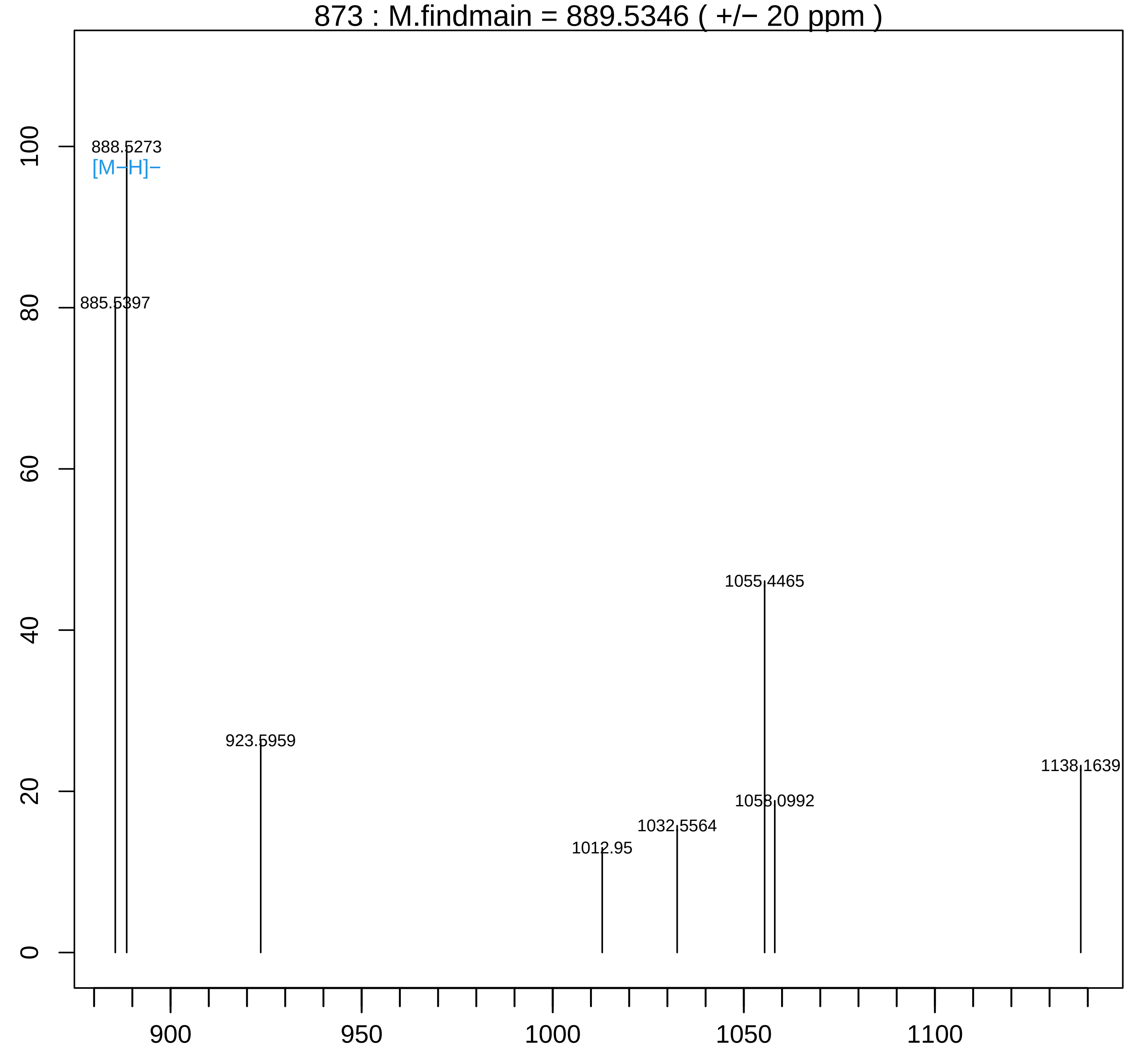
873 : M.findmain = 889.5346 (+/− 20 ppm)
888.5273
[M−H]−
885.5397
923.5959
1055.4465
1058.0992
1032.5564
1012.95
1138.1639
0
20
40
60
80
100
900
950
1000
1050
1100

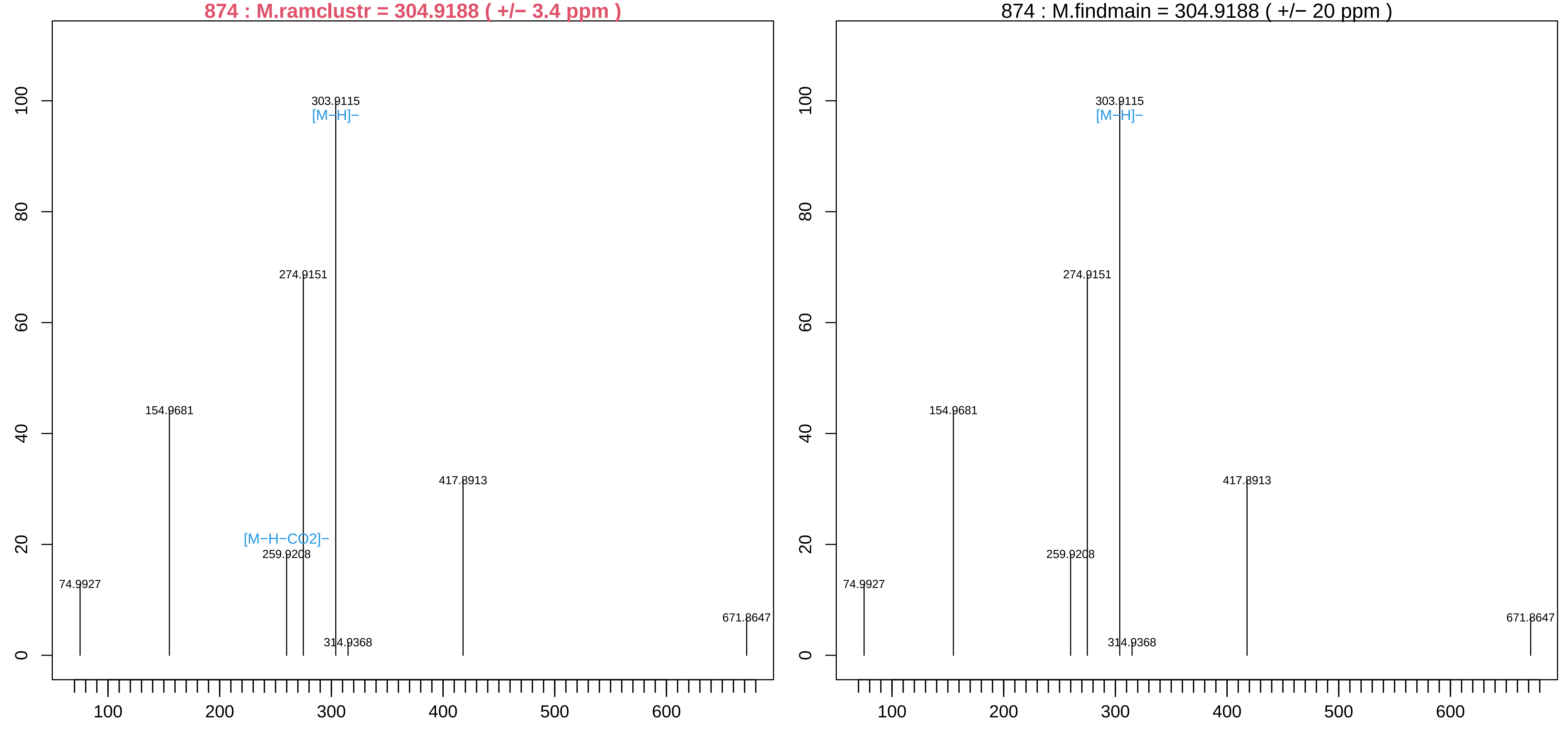
874 : M.ramclustr = 304.9188 (+/− 3.4 ppm)
303.9115
[M−H]−
274.9151
154.9681
417.8913
[M−H−CO2]−
259.9208
74.9927
671.8647
314.9368
0
20
40
60
80
100
100
200
300
400
500
600
874 : M.findmain = 304.9188 (+/− 20 ppm)
303.9115
[M−H]−
274.9151
154.9681
417.8913
259.9208
74.9927
671.8647
314.9368
0
20
40
60
80
100
100
200
300
400
500
600

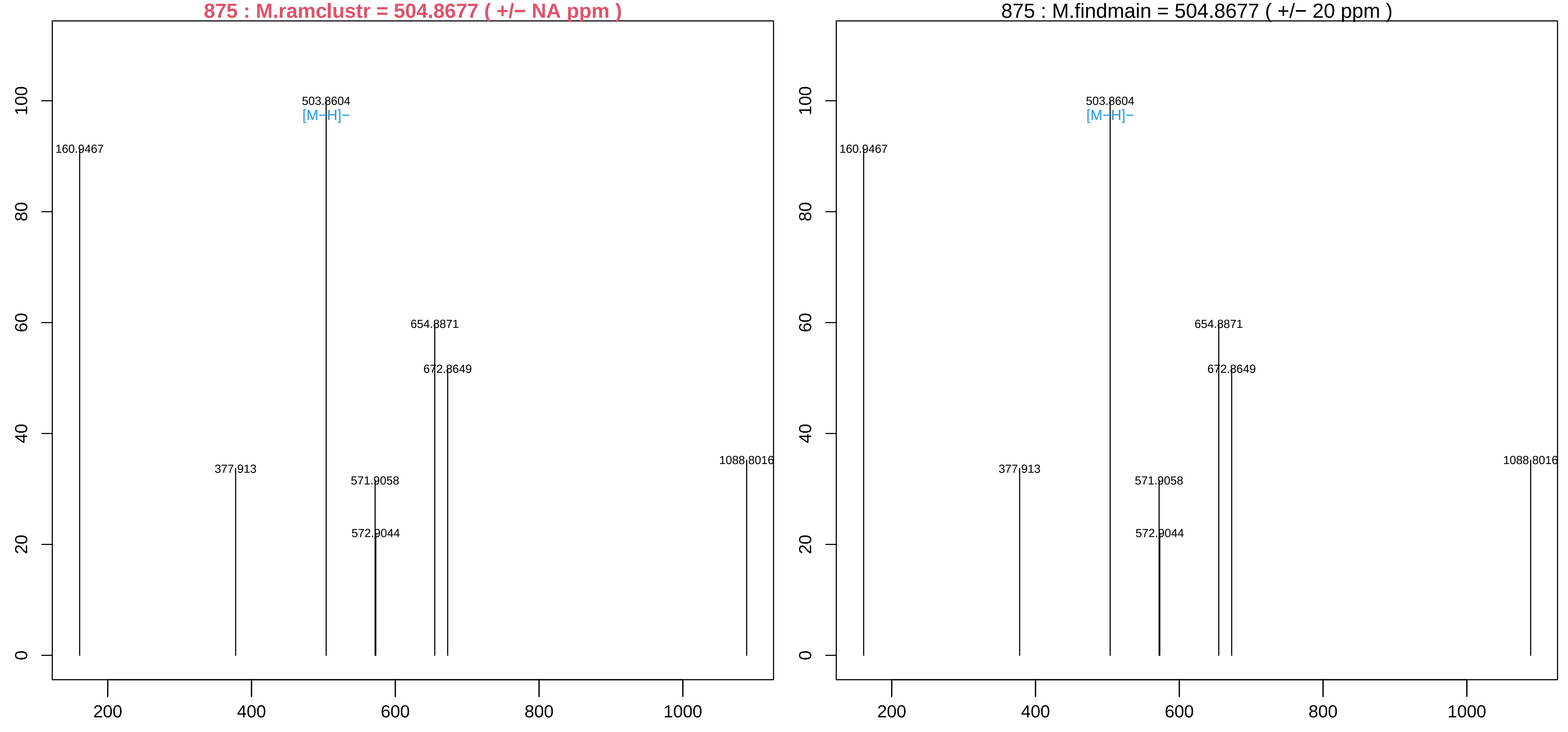
875 : M.ramclustr = 504.8677 (+/− NA ppm)
875 : M.findmain = 504.8677 (+/− 20 ppm)
503.8604
[M−H]−
160.9467
654.8871
672.8649
377.913
571.9058
572.9044
1088.8016
0
20
40
60
80
100
200
400
600
800
1000

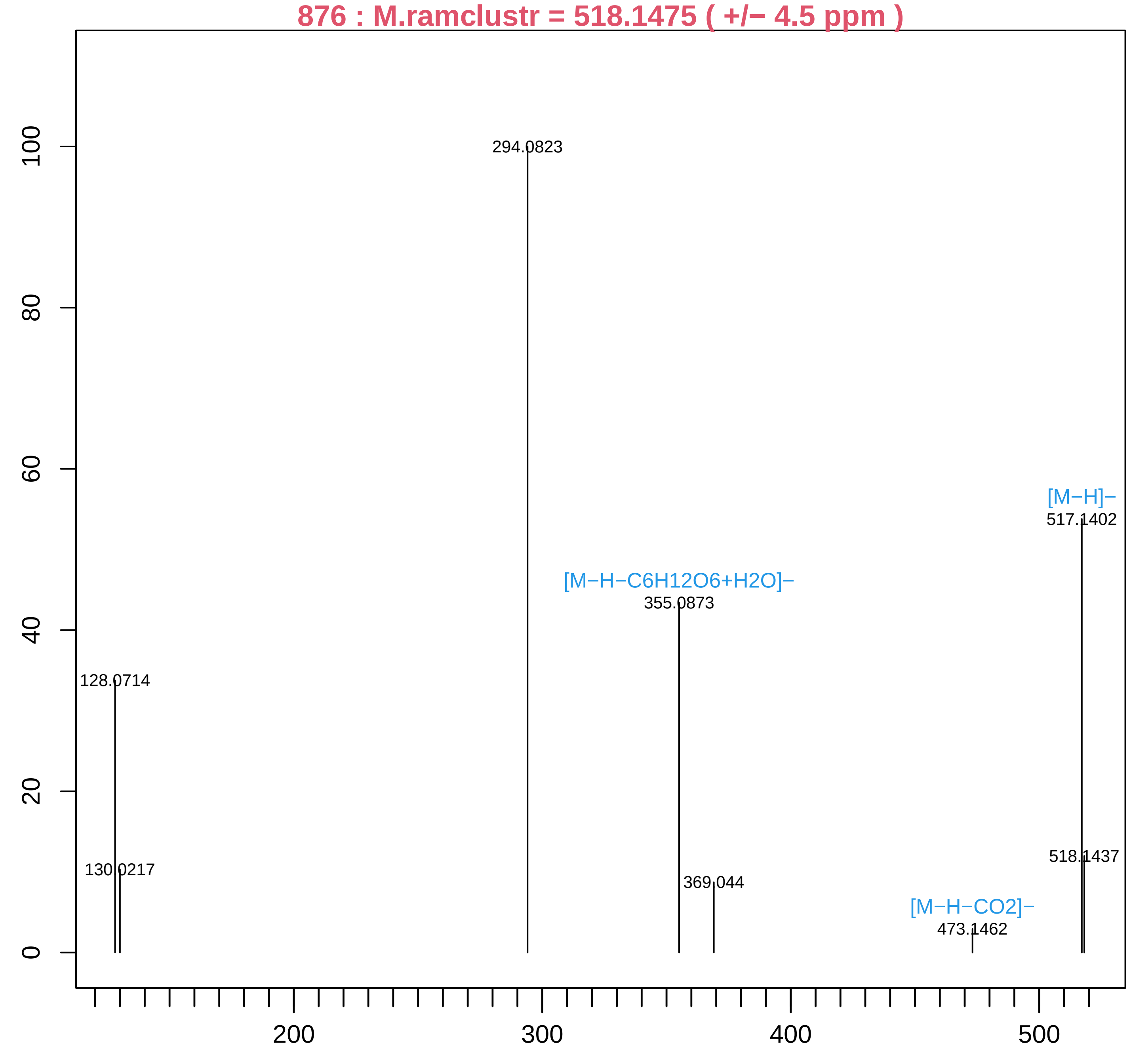

876 : M.ramclustr = 518.1475 (+/− 4.5 ppm)
294.0823
[M−H]−
517.1402
[M−H−C6H12O6+H2O]−
355.0873
128.0714
130.0217
369.044
[M−H−CO2]−
473.1462
518.1437
0
20
40
60
80
100
200
300
400
500

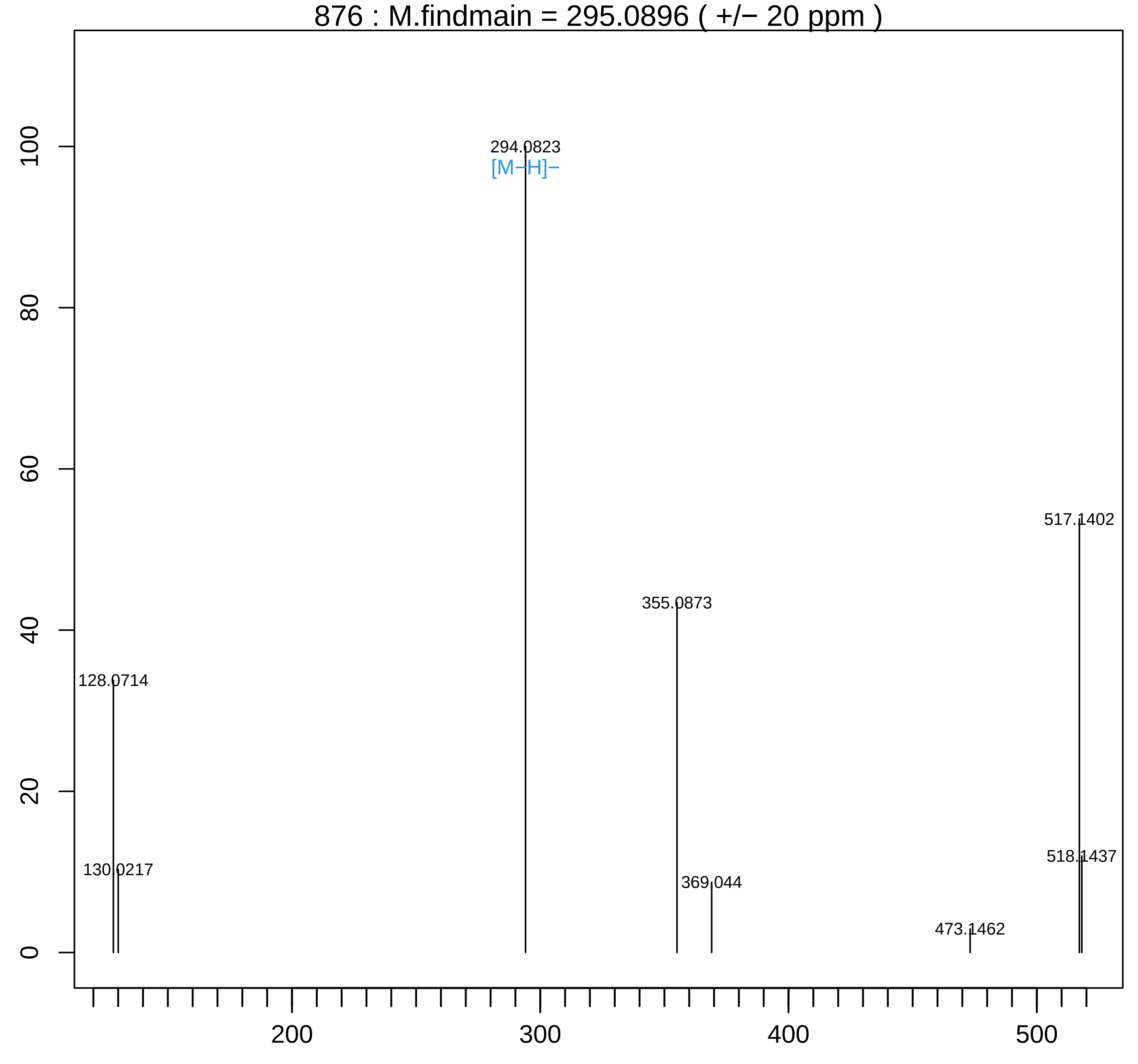

876 : M.findmain = 295.0896 (+/− 20 ppm)
294.0823
[M−H]−
517.1402
355.0873
128.0714
130.0217
369.044
473.1462
518.1437
0
20
40
60
80
100
200
300
400
500

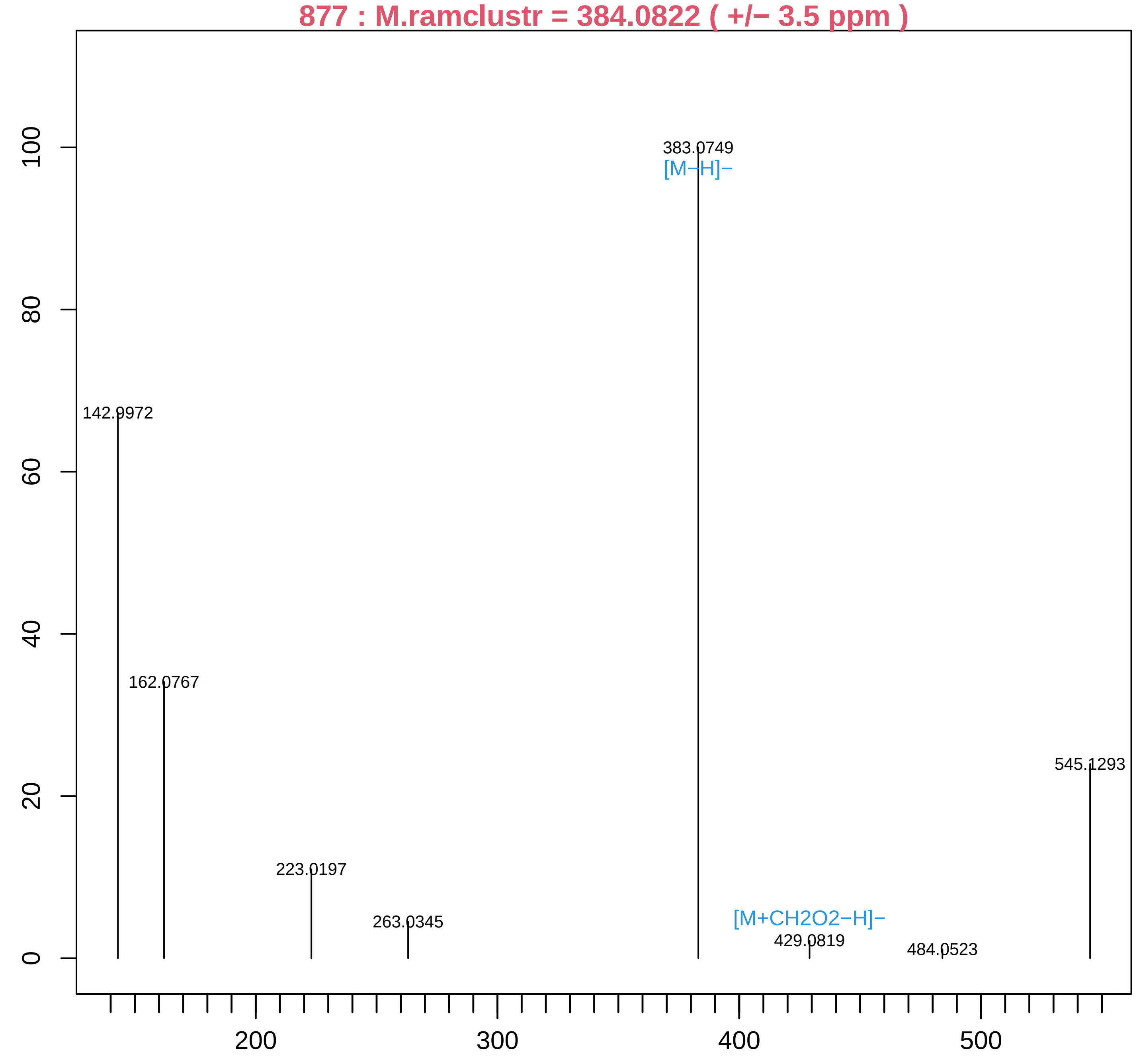
877 : M.ramclustr = 384.0822 (+/− 3.5 ppm)
383.0749
[M−H]−
142.9972
162.0767
223.0197
263.0345
[M+CH2O2−H]−
429.0819
484.0523
545.1293
0
20
40
60
80
100
200
300
400
500
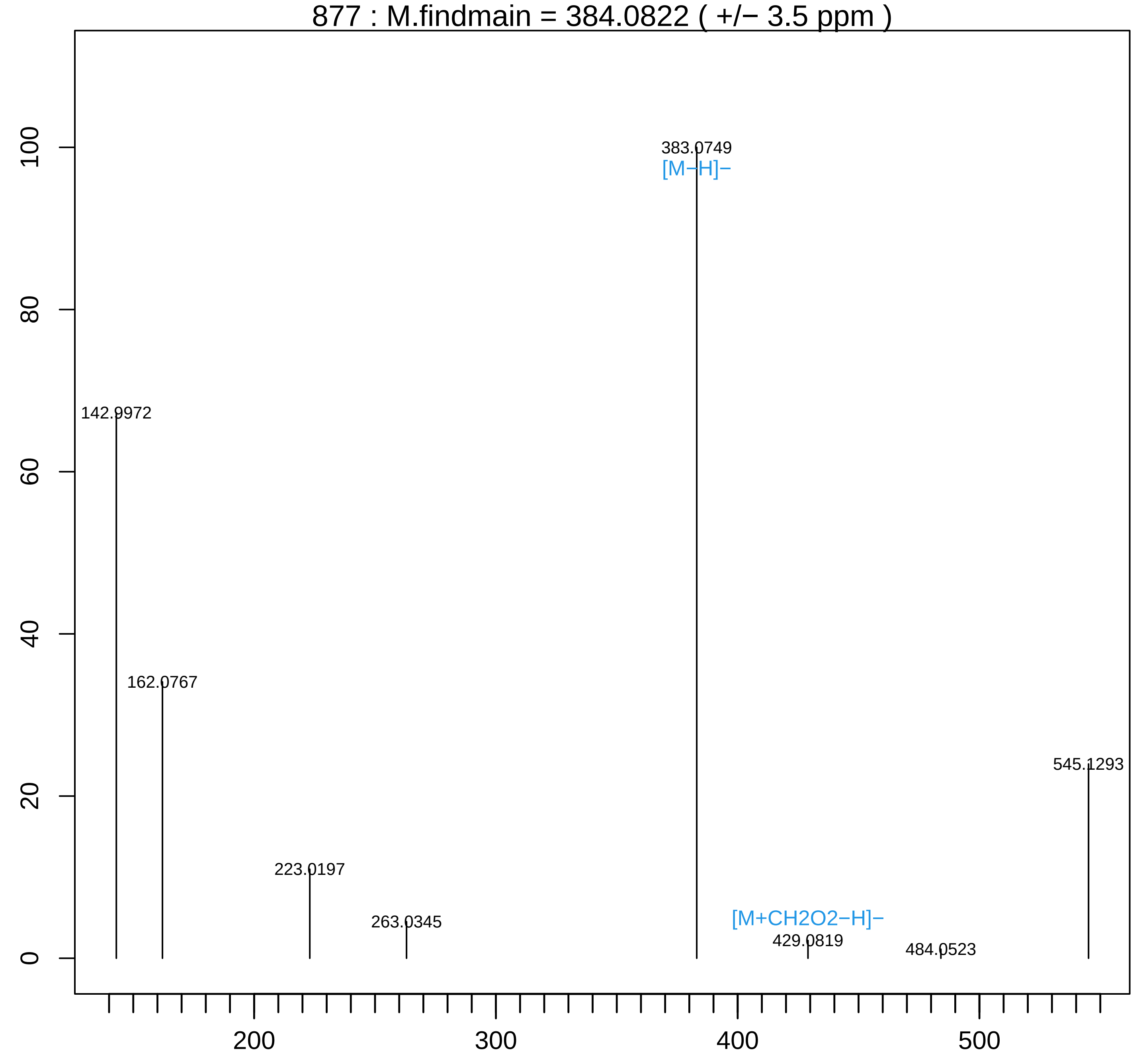
877 : M.findmain = 384.0822 (+/− 3.5 ppm)
383.0749
[M−H]−
142.9972
162.0767
223.0197
263.0345
[M+CH2O2−H]−
429.0819
484.0523
545.1293
0
20
40
60
80
100
200
300
400
500

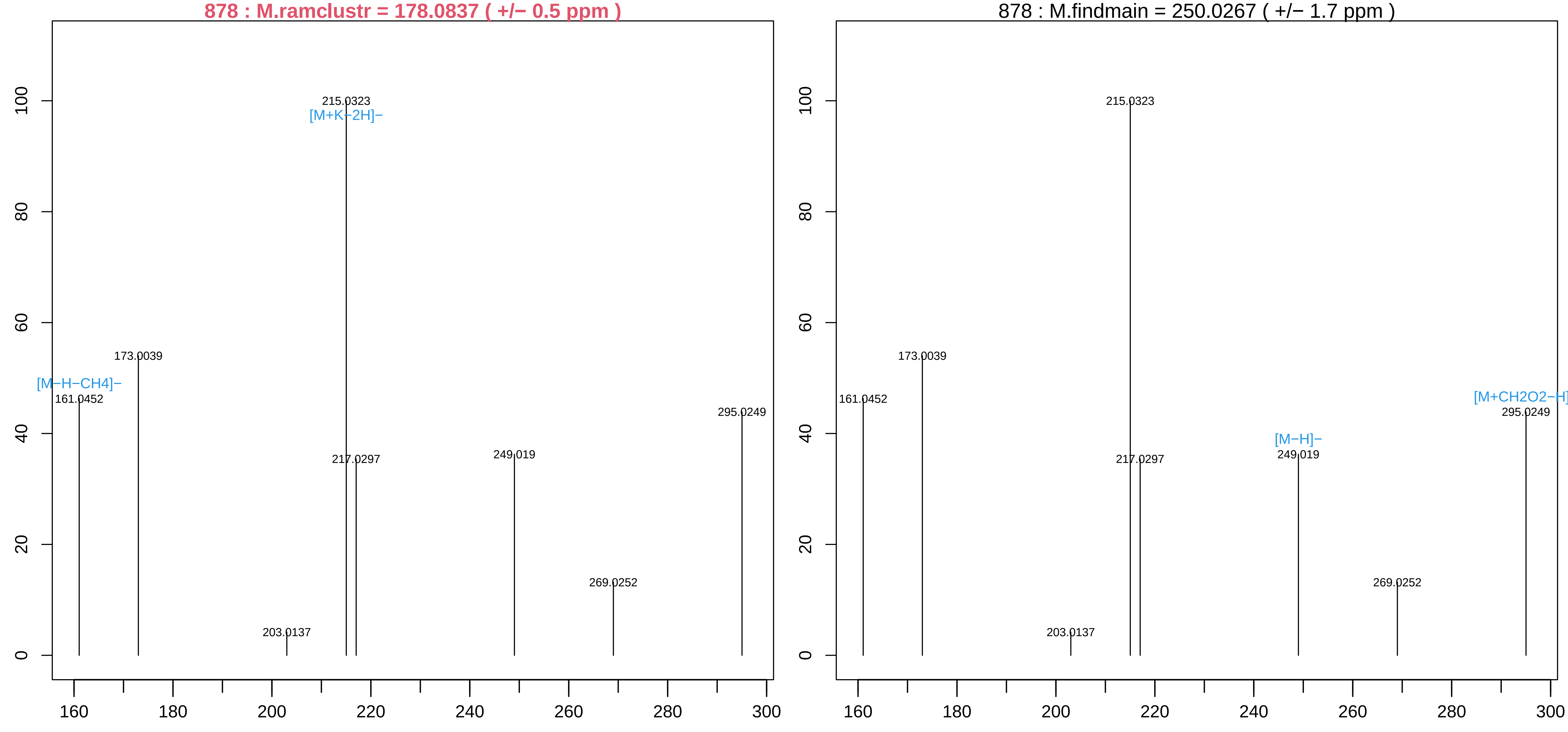

878 : M.ramclustr = 178.0837 (+/− 0.5 ppm)
215.0323
[M+K−2H]−
173.0039
[M−H−CH4]−
161.0452
217.0297
249.019
295.0249
269.0252
203.0137
878 : M.findmain = 250.0267 (+/− 1.7 ppm)
215.0323
173.0039
161.0452
[M+CH2O2−H]
295.0249
[M−H]−
249.019
217.0297
269.0252
203.0137

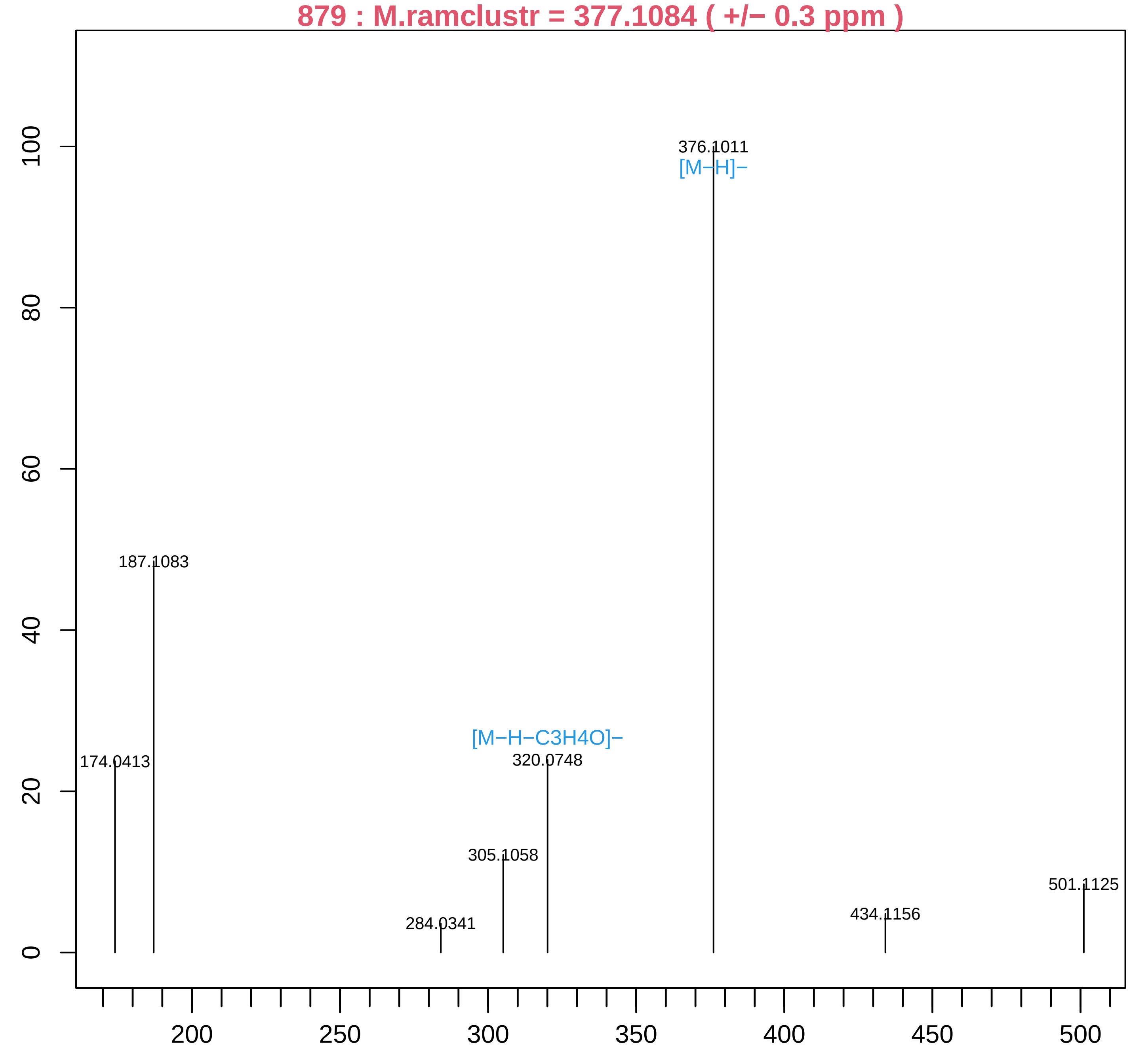
879 : M.ramclustr = 377.1084 (+/− 0.3 ppm)
376.1011
[M−H]−
187.1083
174.0413
[M−H−C3H4O]−
320.0748
305.1058
284.0341
434.1156
501.1125
0
20
40
60
80
100
200
250
300
350
400
450
500

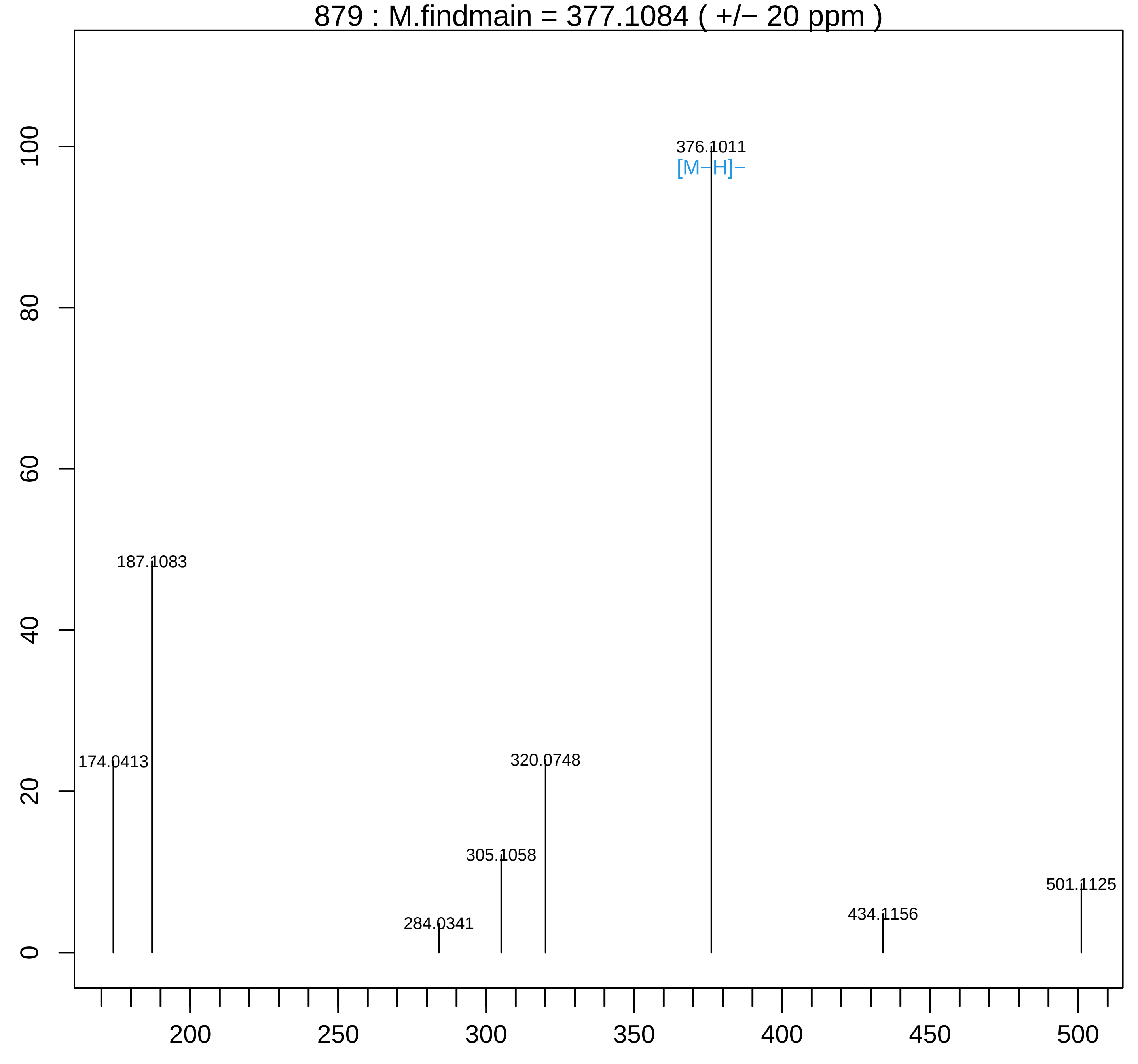
879 : M.findmain = 377.1084 (+/− 20 ppm)
376.1011
[M−H]−
187.1083
174.0413
320.0748
305.1058
284.0341
434.1156
501.1125
0
20
40
60
80
100
200
250
300
350
400
450
500

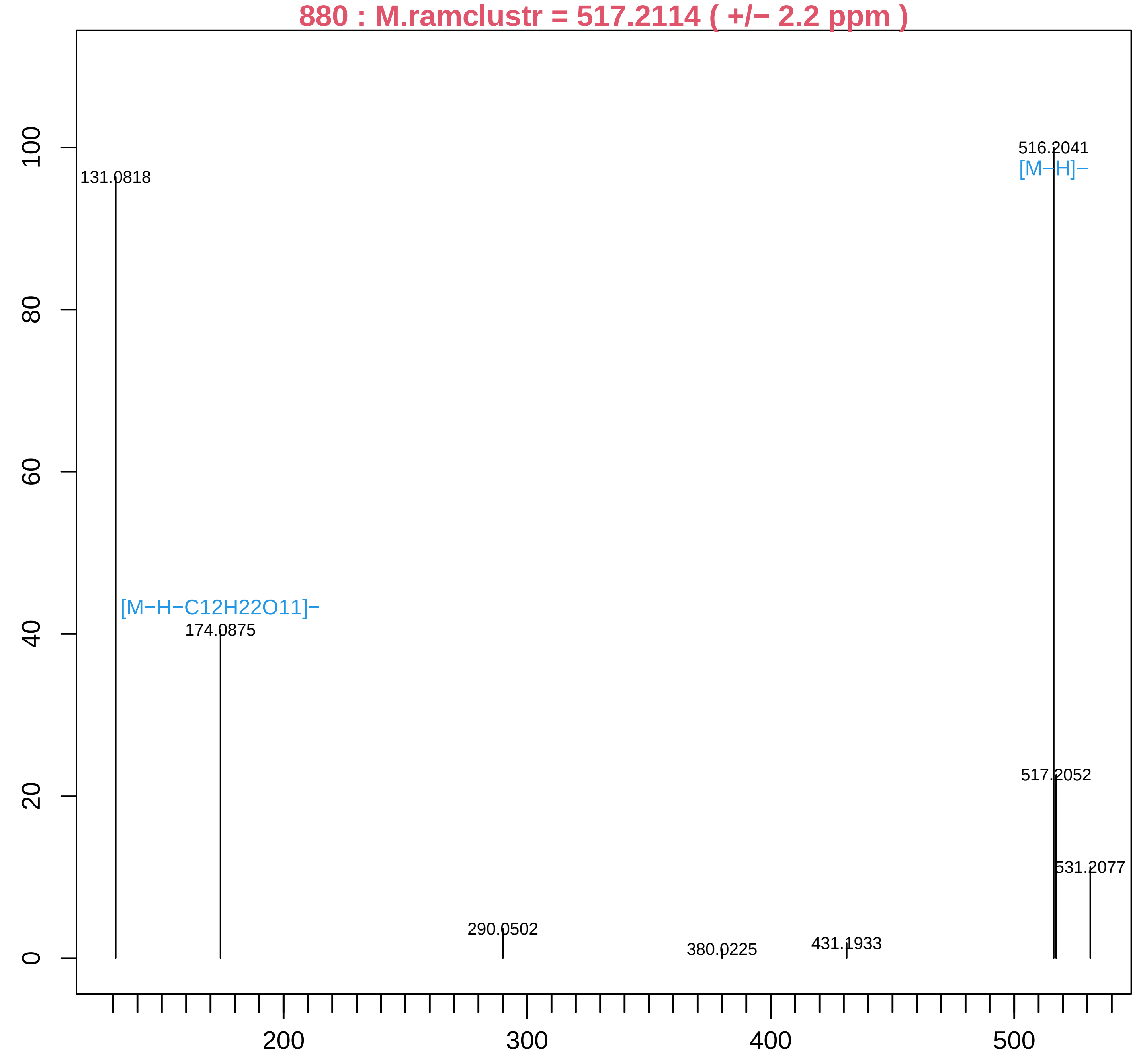
880 : M.ramclustr = 517.2114 (+/− 2.2 ppm)
131.0818
174.0875
[M−H−C12H22O11]−
290.0502
380.0225
431.1933
516.2041
[M−H]−
517.2052
531.2077
0
20
40
60
80
100
200
300
400
500

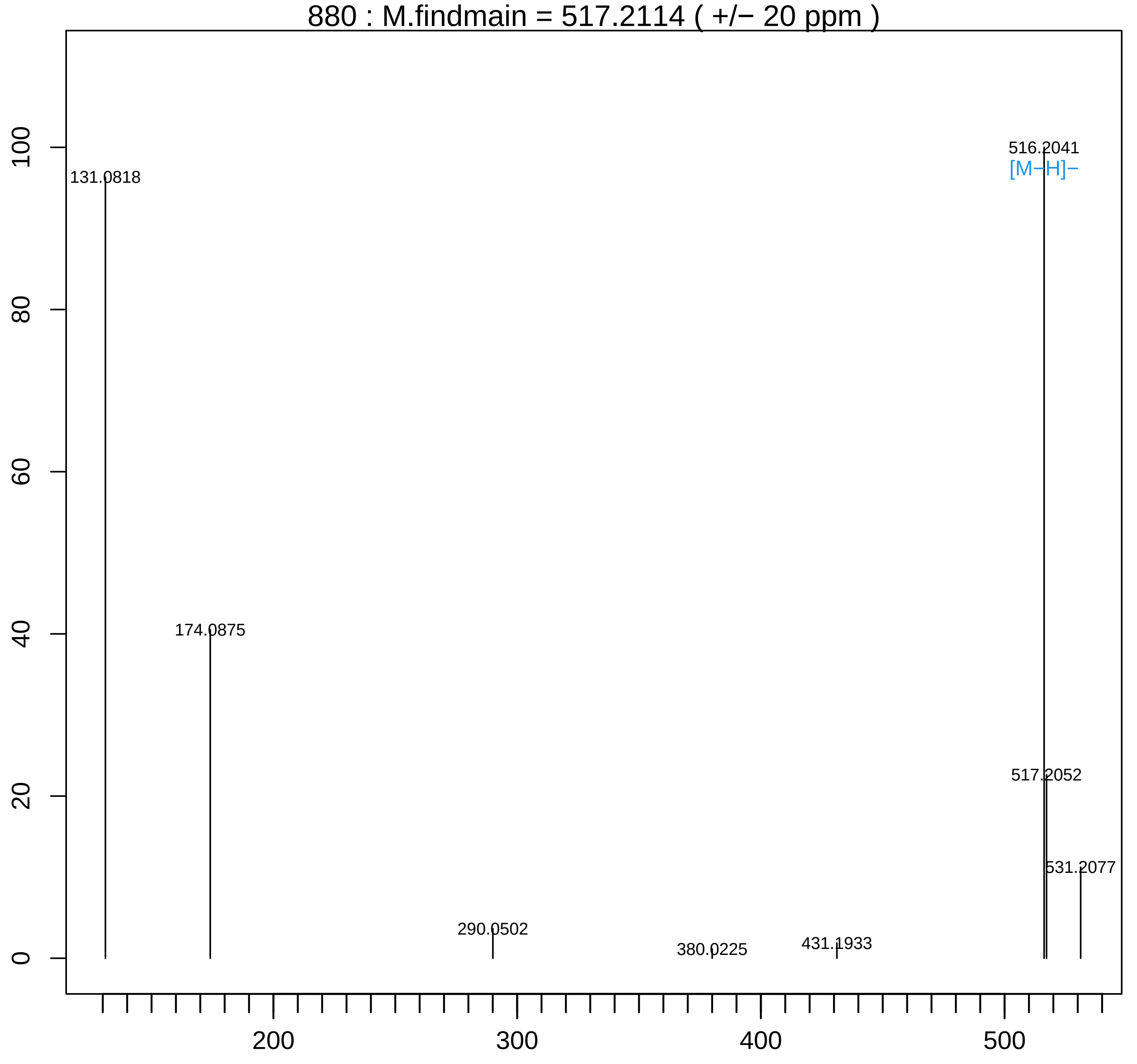
880 : M.findmain = 517.2114 (+/− 20 ppm)
131.0818
174.0875
290.0502
380.0225
431.1933
516.2041
[M−H]−
517.2052
531.2077
0
20
40
60
80
100
200
300
400
500

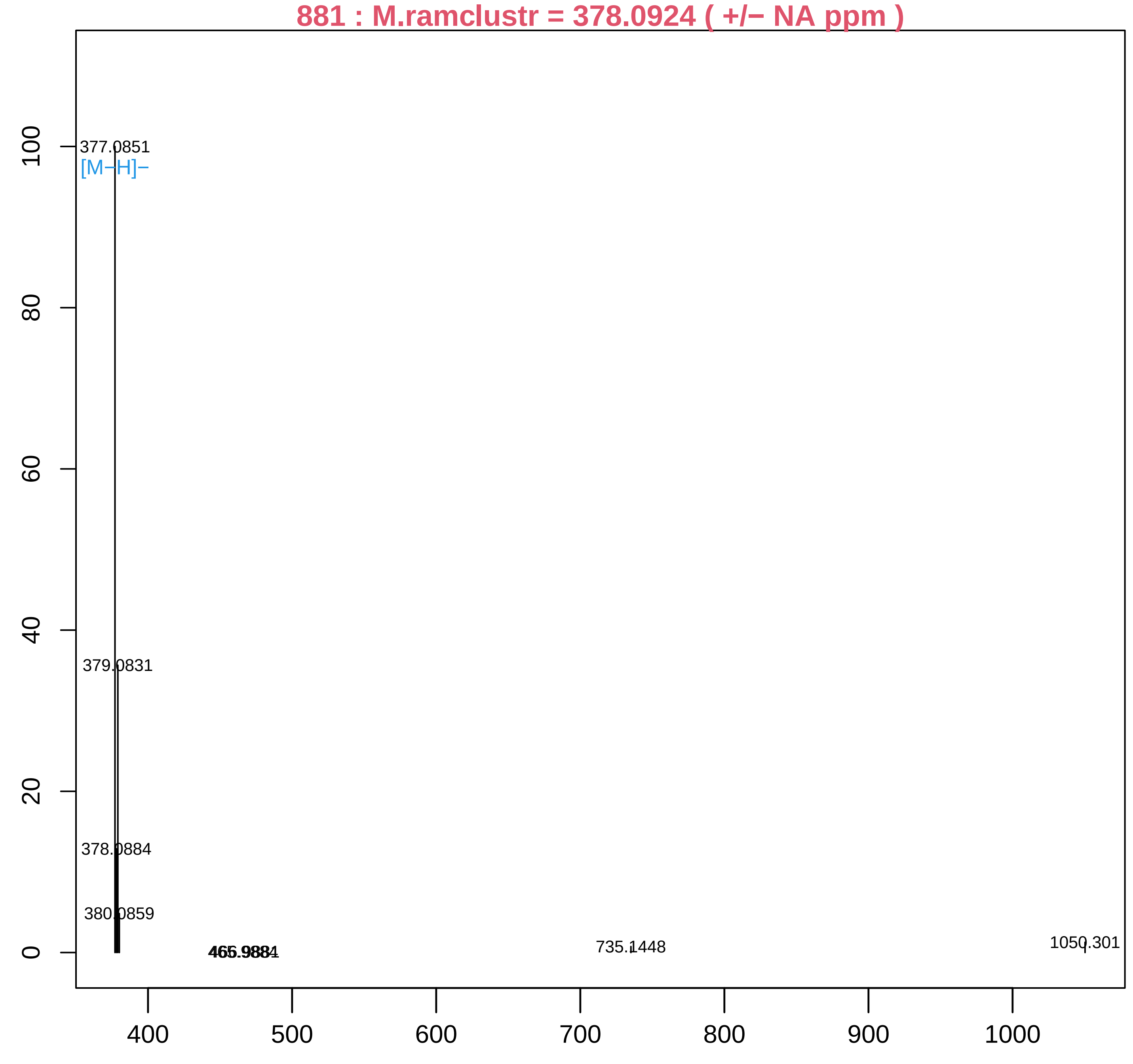

881 : M.ramclustr = 378.0924 (+/− NA ppm)
377.0851
[M−H]−
379.0831
378.0884
380.0859
465.9884
735.1448
1050.301
0
20
40
60
80
100
400
500
600
700
800
900
1000

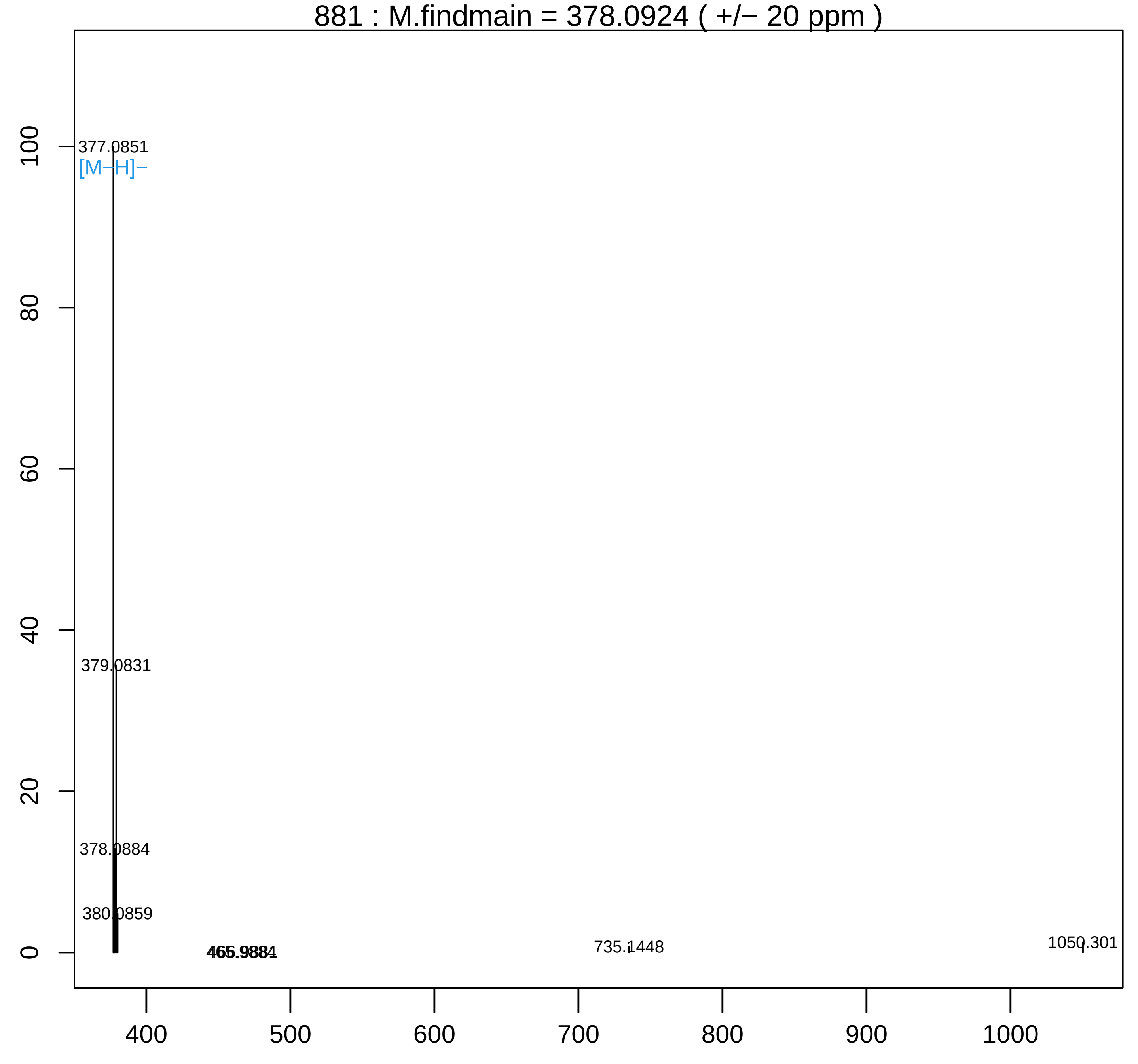

881 : M.findmain = 378.0924 (+/− 20 ppm)
377.0851
[M−H]−
379.0831
378.0884
380.0859
465.9884
735.1448
1050.301
0
20
40
60
80
100
400
500
600
700
800
900
1000

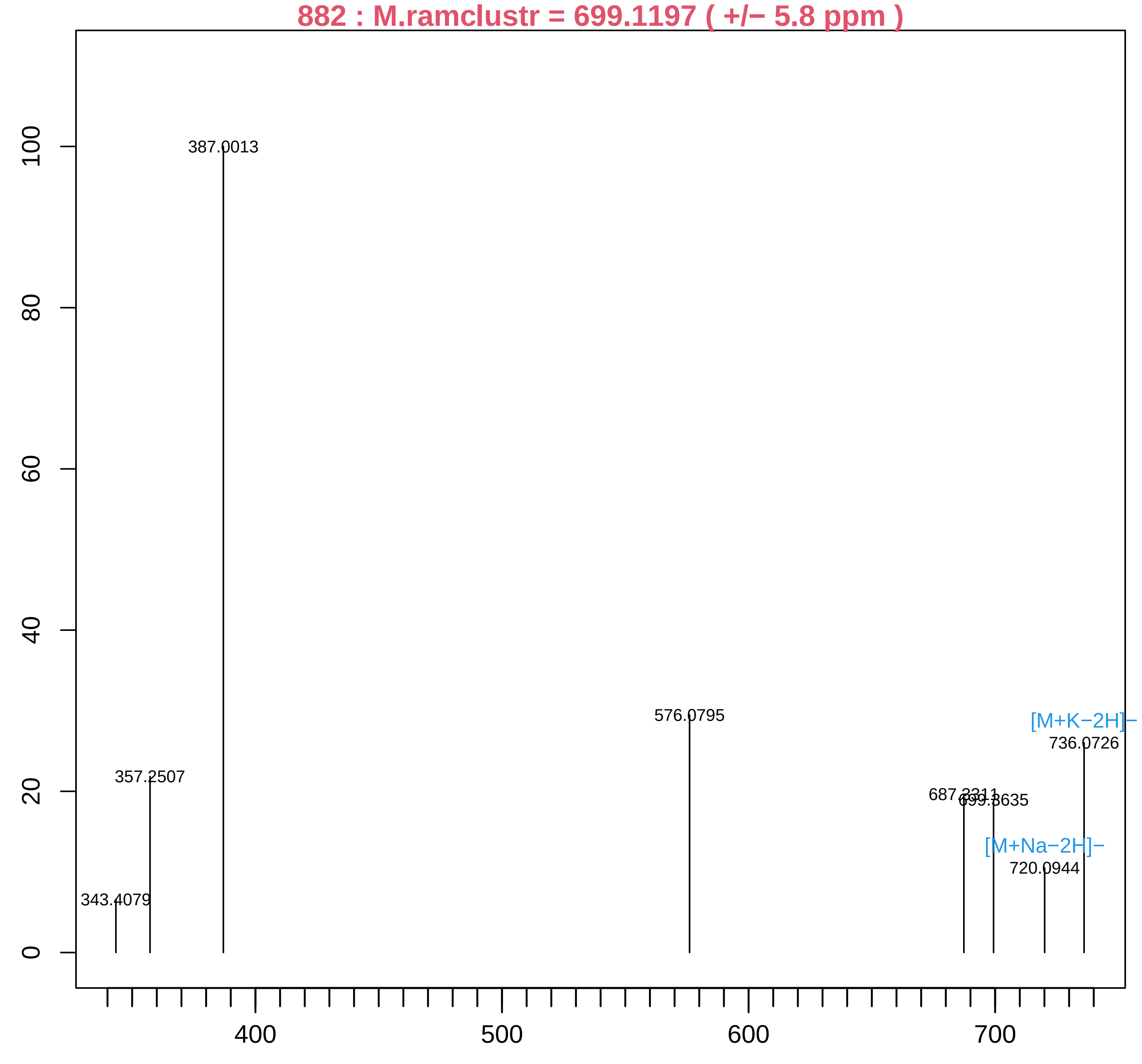
882 : M.ramclustr = 699.1197 (+/− 5.8 ppm)
387.0013
576.0795
[M+K−2H]−
736.0726
357.2507
687.2311
699.3635
[M+Na−2H]−
720.0944
343.4079
0
20
40
60
80
100
400
500
600
700

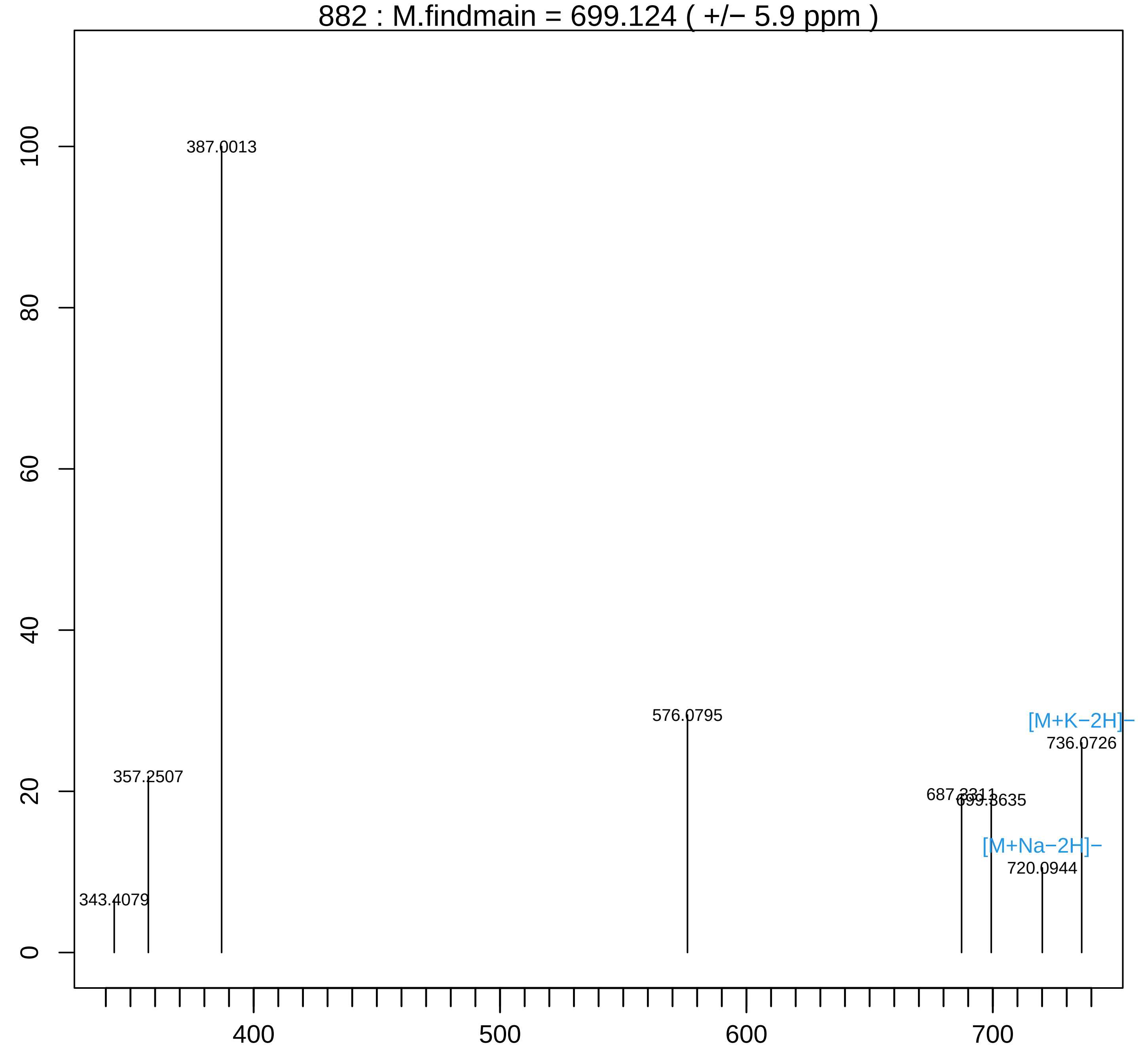
882 : M.findmain = 699.124 (+/− 5.9 ppm)
387.0013
576.0795
[M+K−2H]−
736.0726
357.2507
687.2311
699.3635
[M+Na−2H]−
720.0944
343.4079
0
20
40
60
80
100
400
500
600
700

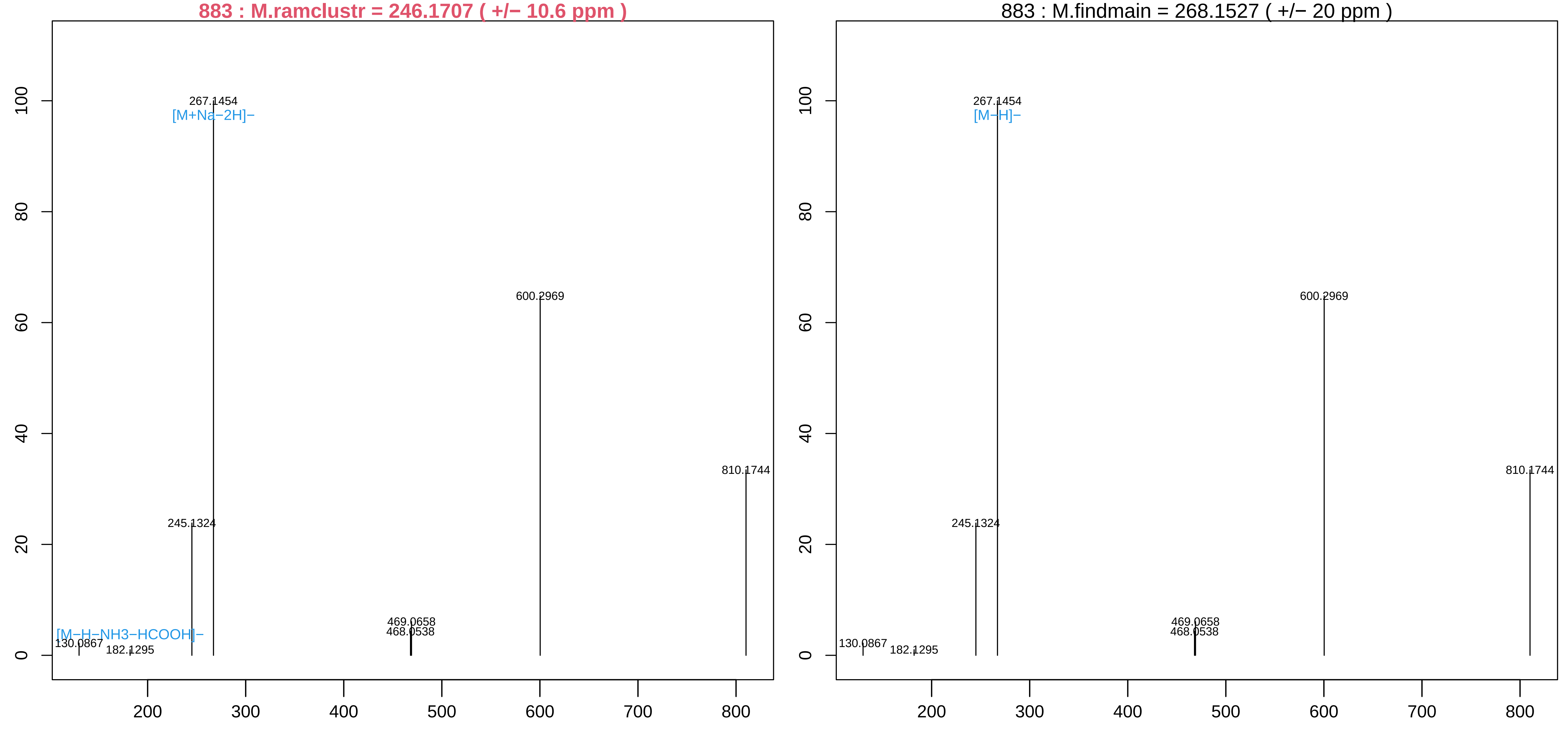
883 : M.ramclustr = 246.1707 (+/− 10.6 ppm)
267.1454
[M+Na−2H]−
600.2969
810.1744
245.1324
469.0658
468.0538
[M−H−NH3−HCOOH]−
130.0867
182.1295
0
20
40
60
80
100
200
300
400
500
600
700
800
883 : M.findmain = 268.1527 (+/− 20 ppm)
267.1454
[M−H]−
600.2969
810.1744
245.1324
469.0658
468.0538
130.0867
182.1295
0
20
40
60
80
100
200
300
400
500
600
700
800

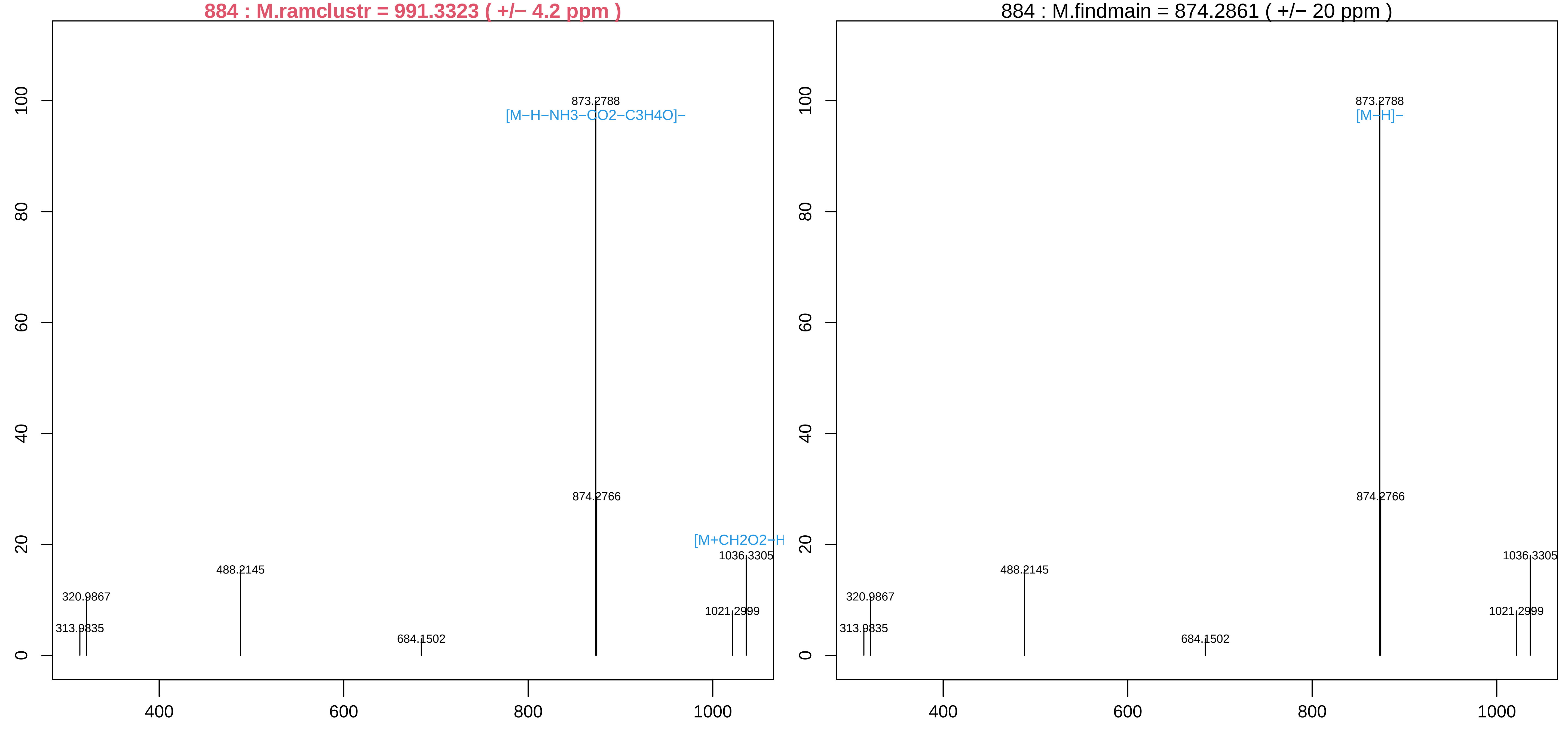
884 : M.ramclustr = 991.3323 (+/− 4.2 ppm)
884 : M.findmain = 874.2861 (+/− 20 ppm)
873.2788
[M−H−NH3−CO2−C3H4O]−
[M−H]−
874.2766
[M+CH2O2−H
1036.3305
1021.2999
488.2145
320.9867
313.9835
684.1502
0
20
40
60
80
100
400
600
800
1000

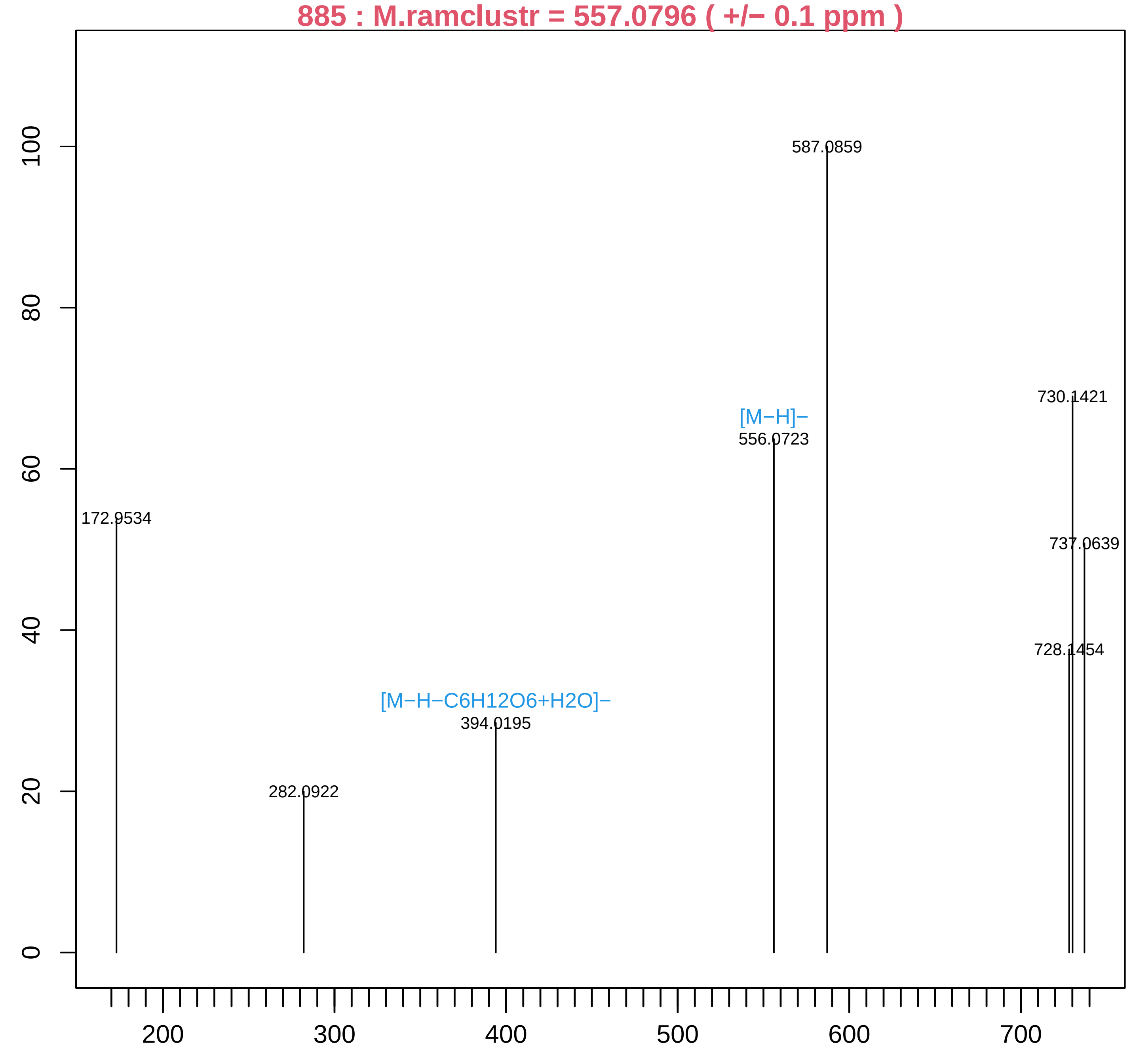

885 : M.ramclustr = 557.0796 (+/− 0.1 ppm)
587.0859
730.1421
[M−H]−
556.0723
172.9534
737.0639
728.1454
[M−H−C6H12O6+H2O]−
394.0195
282.0922
0
20
40
60
80
100
200
300
400
500
600
700

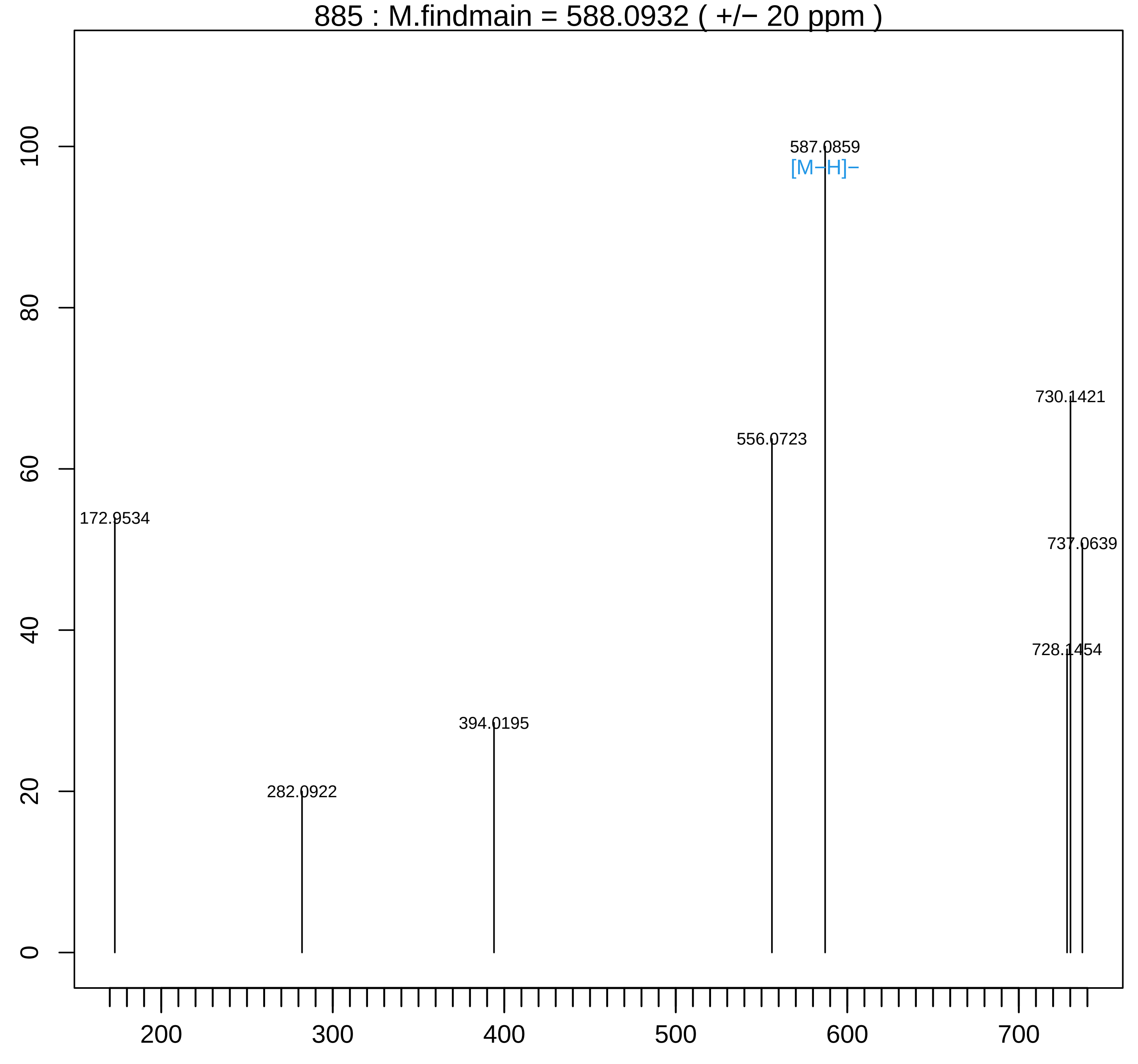

885 : M.findmain = 588.0932 (+/− 20 ppm)
587.0859
[M−H]−
730.1421
556.0723
172.9534
737.0639
728.1454
394.0195
282.0922
0
20
40
60
80
100
200
300
400
500
600
700

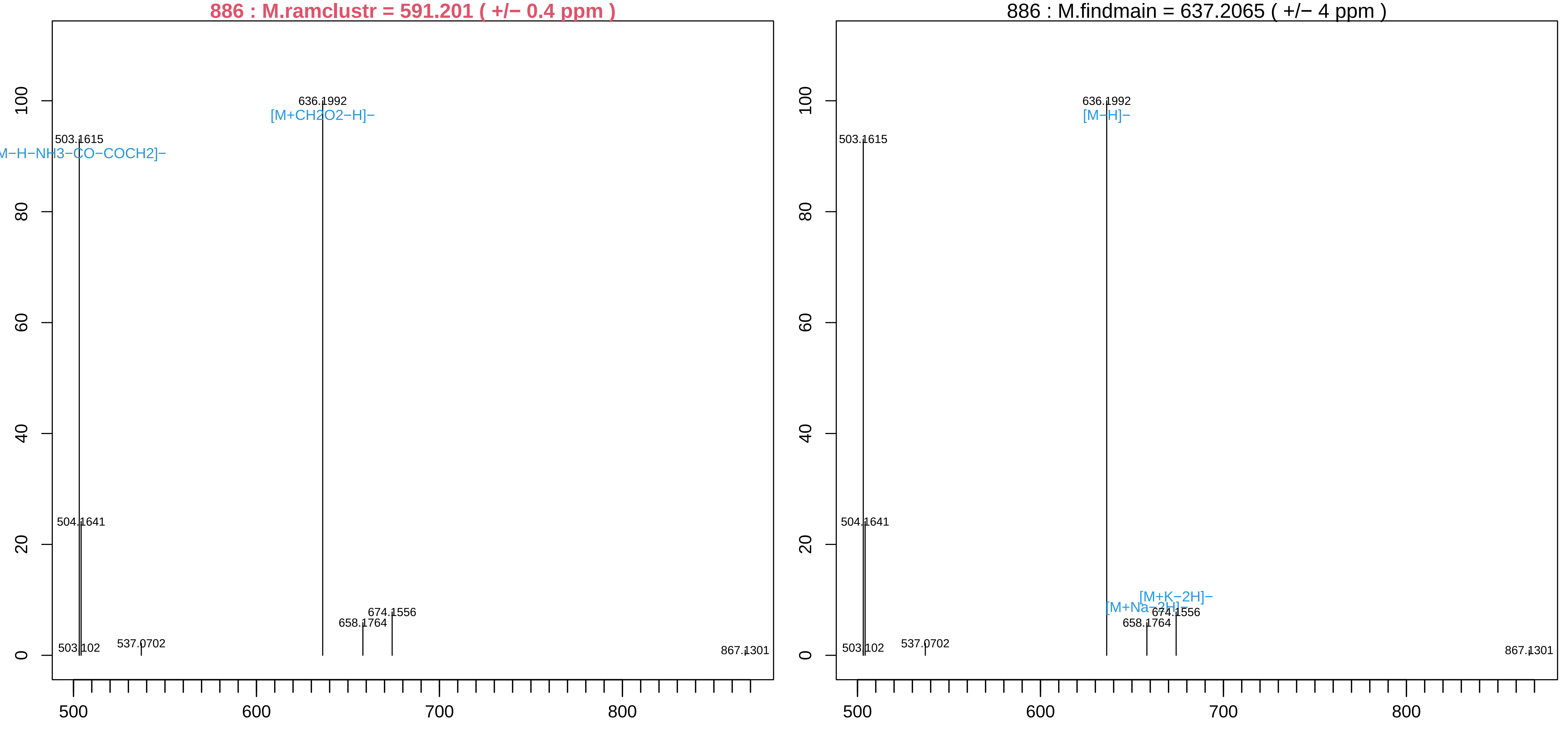
886 : M.ramclustr = 591.201 (+/− 0.4 ppm)
636.1992
[M+CH2O2−H]−
503.1615
M−H−NH3−CO−COCH2]−
504.1641
674.1556
658.1764
537.0702
503.102
867.1301
886 : M.findmain = 637.2065 (+/− 4 ppm)
636.1992
[M−H]−
503.1615
504.1641
[M+K−2H]−
[M+Na−2H]−
674.1556
658.1764
537.0702
503.102
867.1301
0
20
40
60
80
100
500
600
700
800

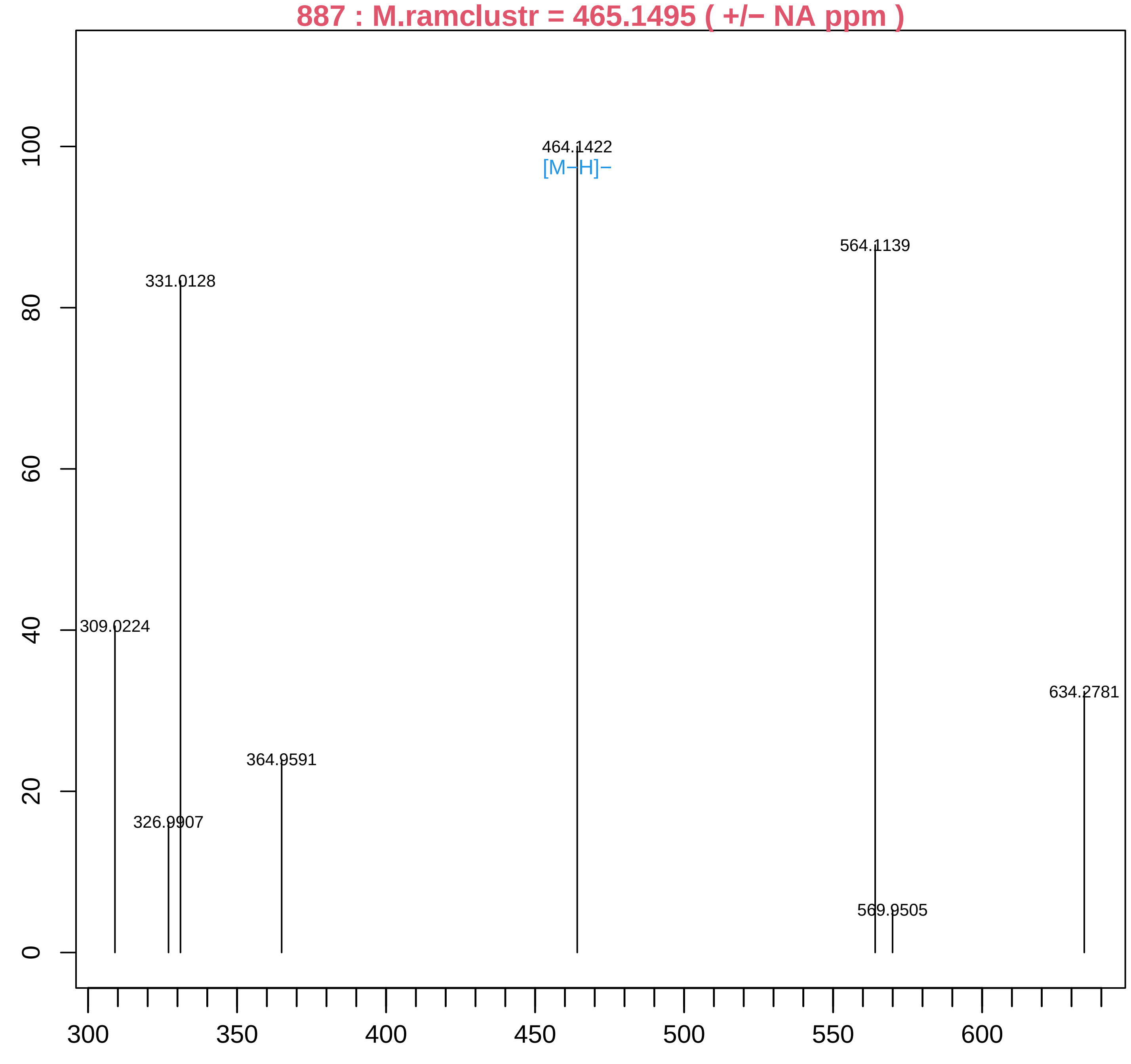
887 : M.ramclustr = 465.1495 (+/− NA ppm)
464.1422
[M−H]−
564.1139
331.0128
309.0224
634.2781
364.9591
326.9907
569.9505
0
20
40
60
80
100
300
350
400
450
500
550
600

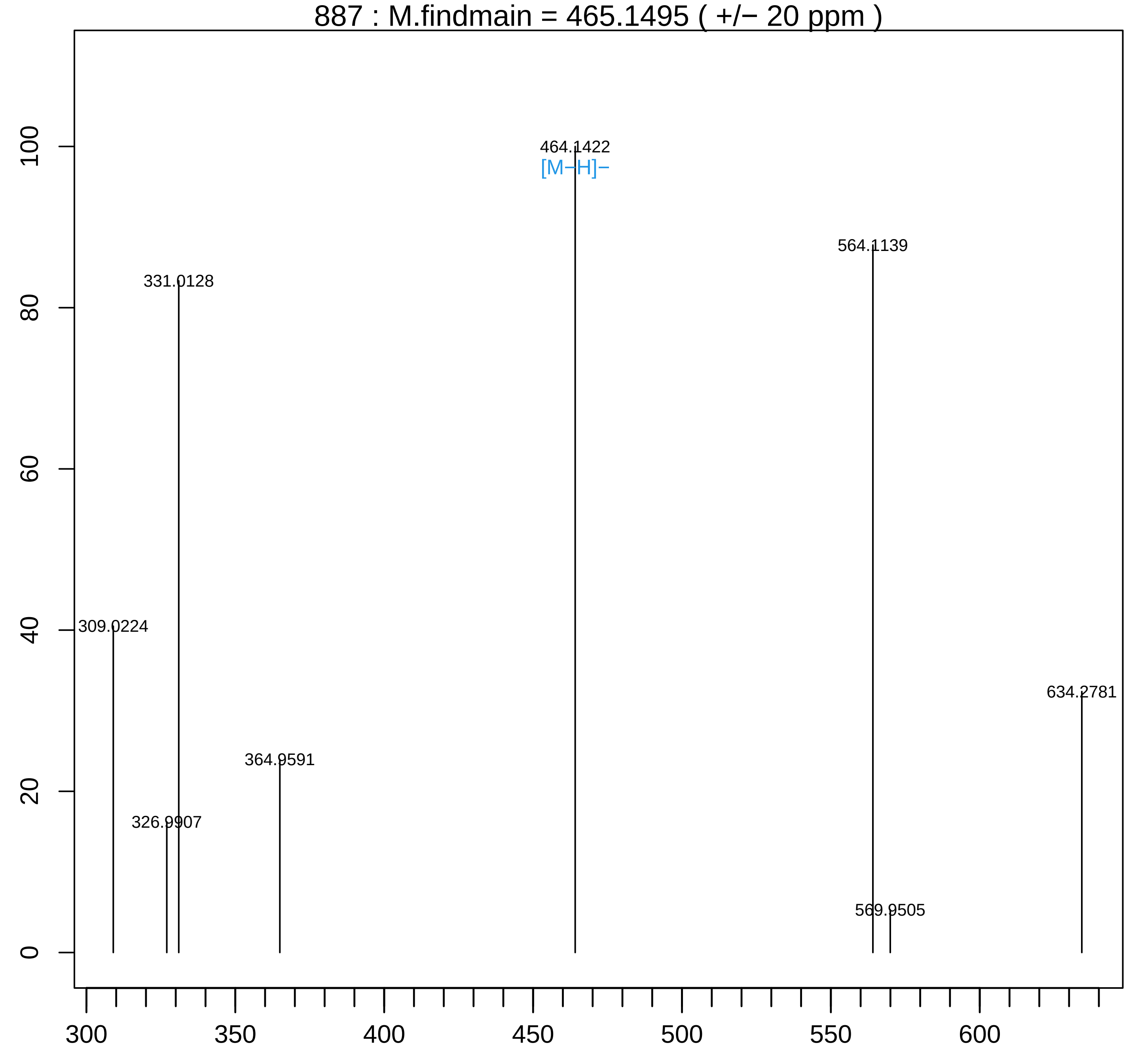
887 : M.findmain = 465.1495 (+/− 20 ppm)
464.1422
[M−H]−
564.1139
331.0128
309.0224
634.2781
364.9591
326.9907
569.9505
0
20
40
60
80
100
300
350
400
450
500
550
600

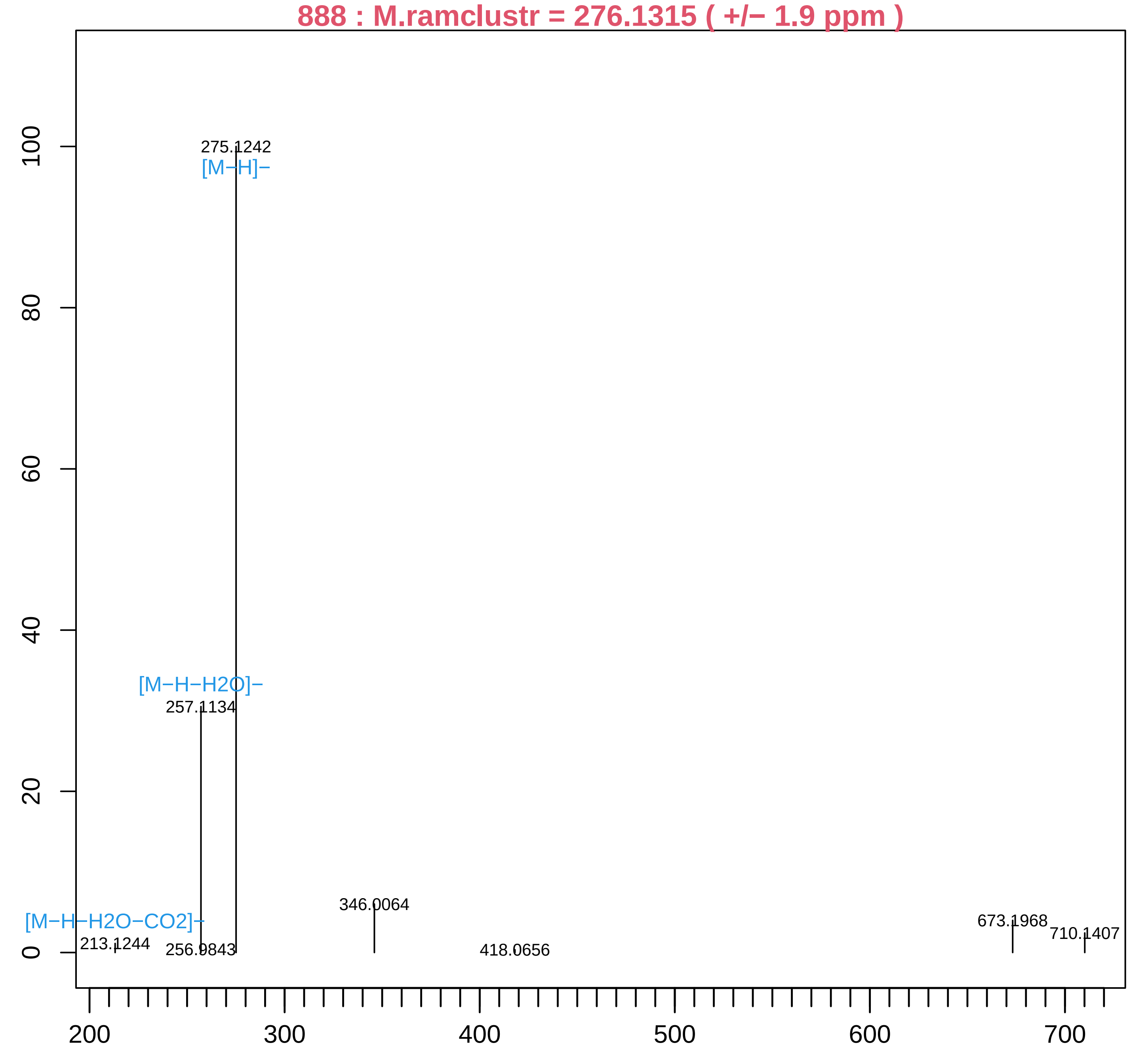
888 : M.ramclustr = 276.1315 (+/− 1.9 ppm)
275.1242
[M−H]−
[M−H−H2O]−
257.1134
[M−H−H2O−CO2]−
213.1244
256.9843
346.0064
418.0656
673.1968
710.1407

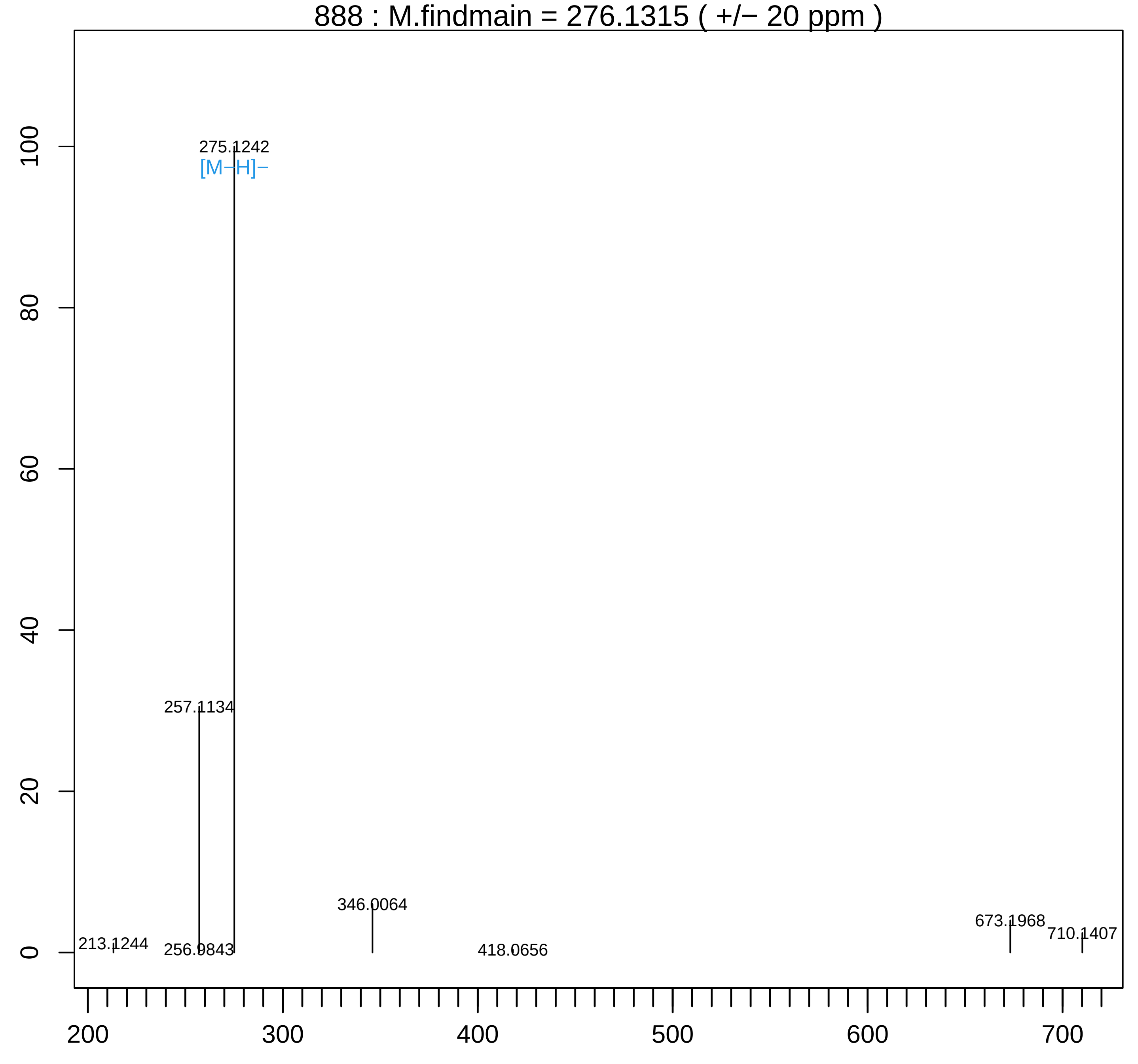
888 : M.findmain = 276.1315 (+/− 20 ppm)
275.1242
[M−H]−
257.1134
213.1244
256.9843
346.0064
418.0656
673.1968
710.1407

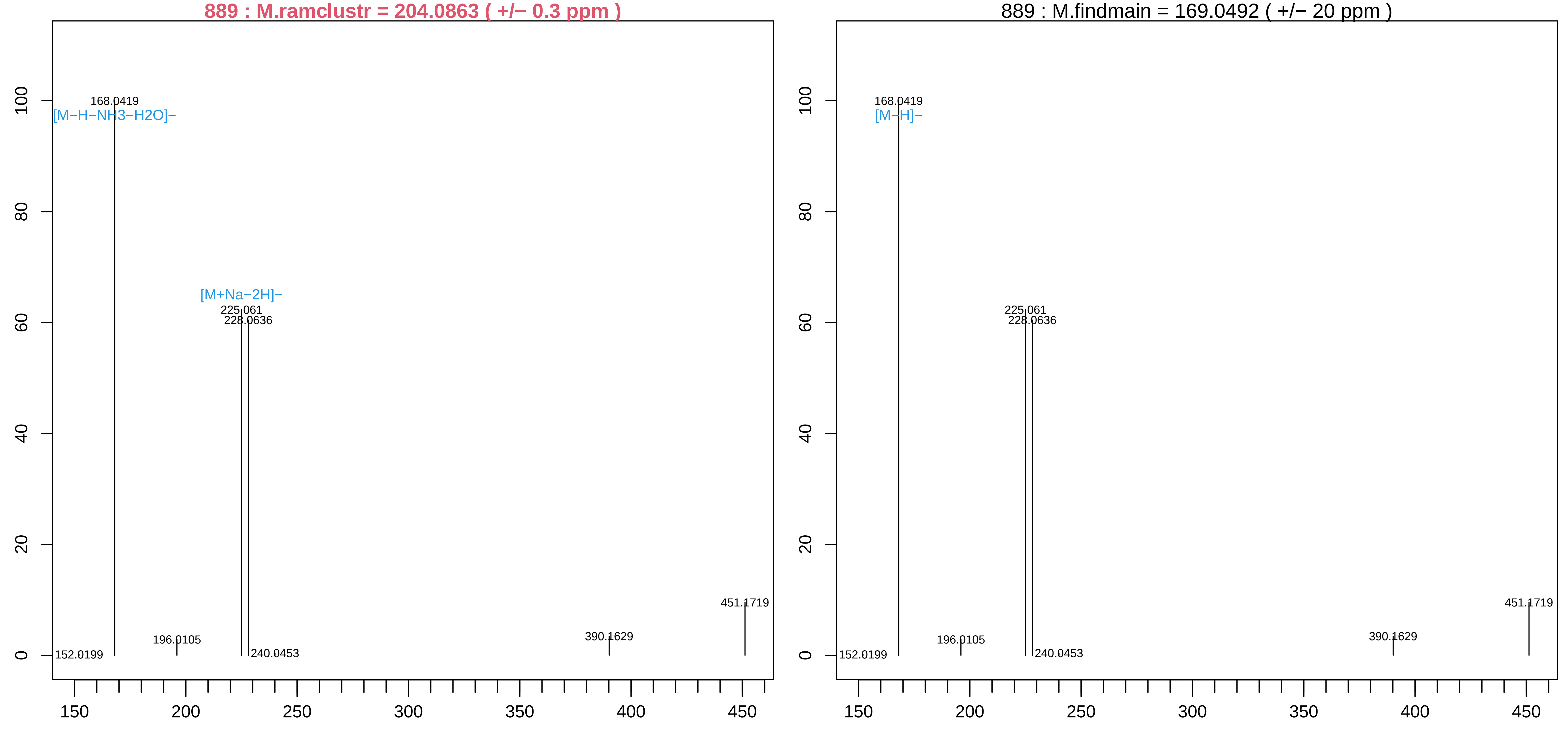
889 : M.ramclustr = 204.0863 (+/− 0.3 ppm)
[M−H−NH3−H2O]−
168.0419
[M+Na−2H]−
225.061
228.0636
152.0199
196.0105
240.0453
390.1629
451.1719
889 : M.findmain = 169.0492 (+/− 20 ppm)
[M−H]−
168.0419
225.061
228.0636
152.0199
196.0105
240.0453
390.1629
451.1719
0
20
40
60
80
100
150
200
250
300
350
400
450

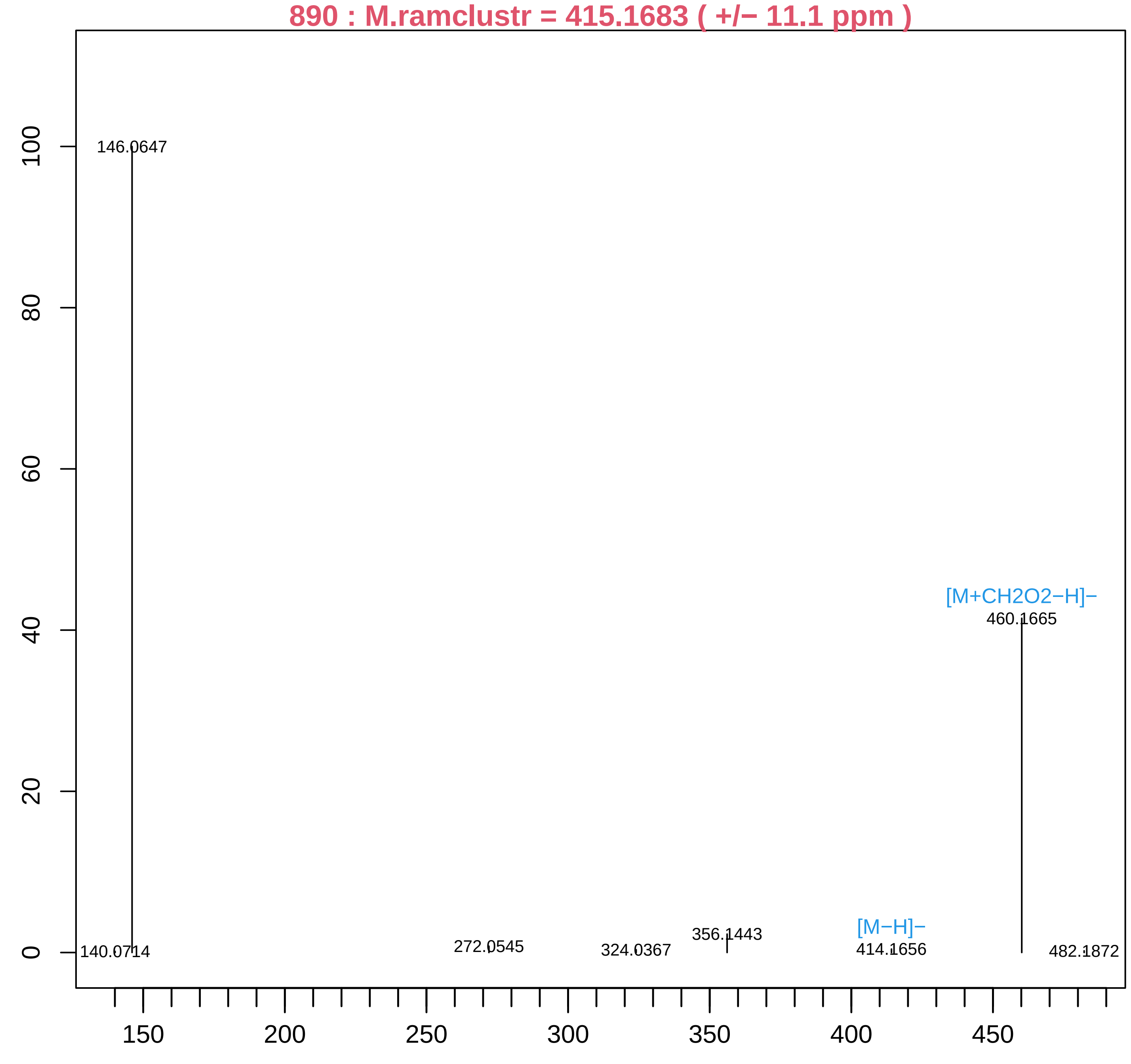
890 : M.ramclustr = 415.1683 (+/− 11.1 ppm)
146.0647
140.0714
272.0545
324.0367
356.1443
[M−H]−
414.1656
[M+CH2O2−H]−
460.1665
482.1872
0
20
40
60
80
100
150
200
250
300
350
400
450

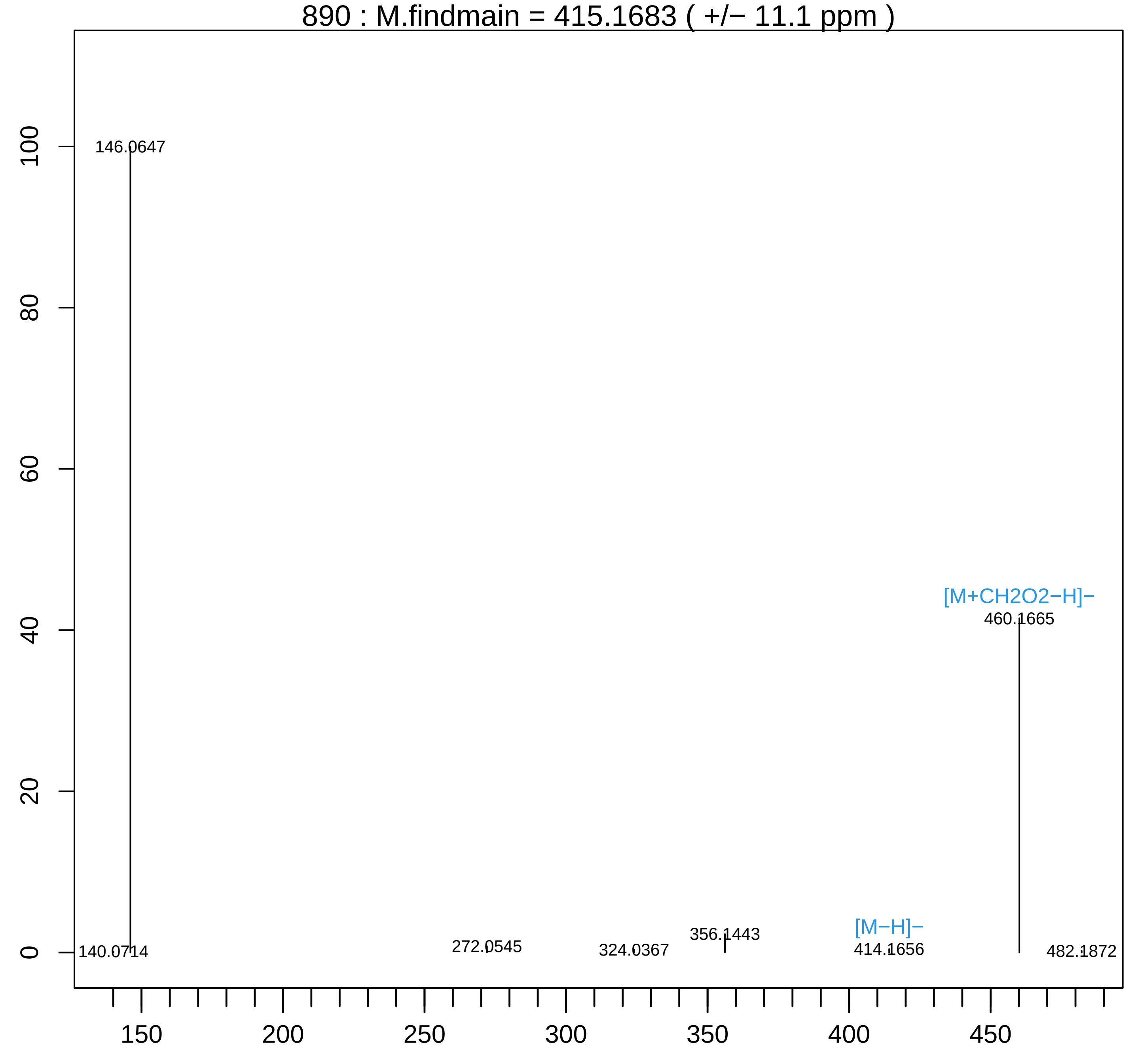
890 : M.findmain = 415.1683 (+/− 11.1 ppm)
146.0647
140.0714
272.0545
324.0367
356.1443
[M−H]−
414.1656
[M+CH2O2−H]−
460.1665
482.1872
0
20
40
60
80
100
150
200
250
300
350
400
450

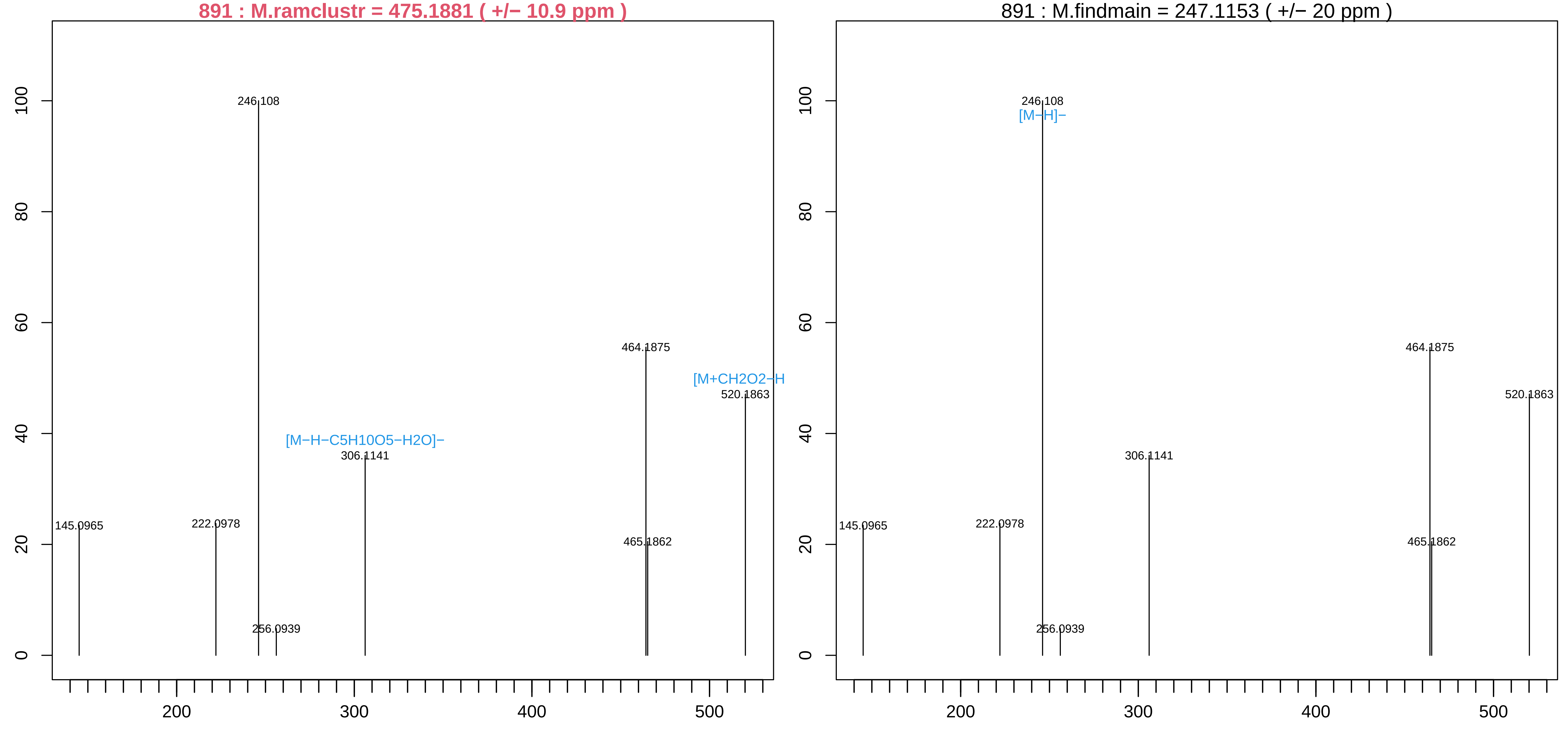

891 : M.ramclustr = 475.1881 (+/− 10.9 ppm)
246.108
464.1875
[M+CH2O2−H
520.1863
[M−H−C5H10O5−H2O]−
306.1141
145.0965
222.0978
465.1862
256.0939
891 : M.findmain = 247.1153 (+/− 20 ppm)
246.108
[M−H]−
464.1875
520.1863
306.1141
145.0965
222.0978
465.1862
256.0939

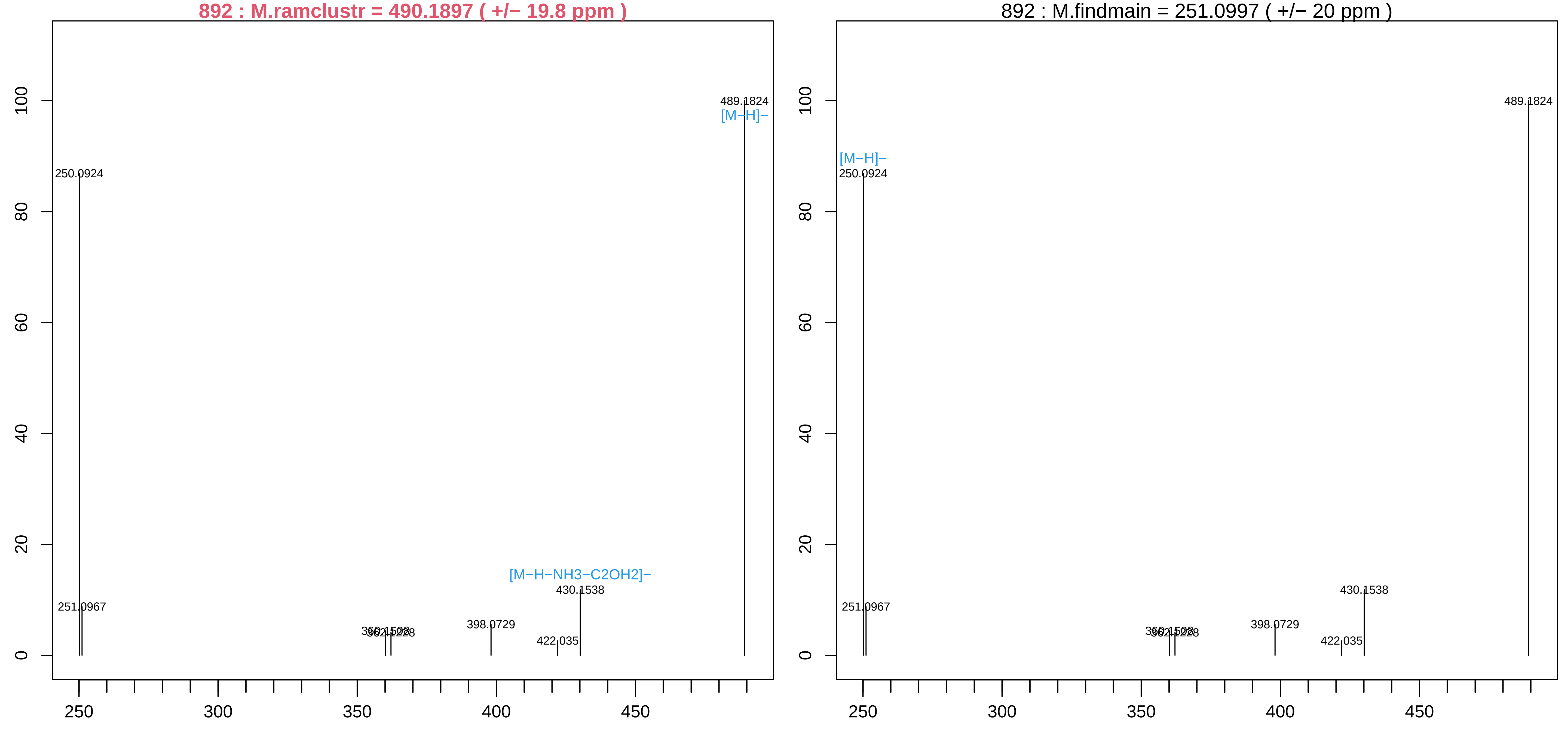
892 : M.ramclustr = 490.1897 (+/− 19.8 ppm)
489.1824
[M−H]−
250.0924
[M−H−NH3−C2OH2]−
430.1538
251.0967
398.0729
422.035
0
20
40
60
80
100
250
300
350
400
450
892 : M.findmain = 251.0997 (+/− 20 ppm)
[M−H]−
250.0924
489.1824
430.1538
251.0967
398.0729
422.035

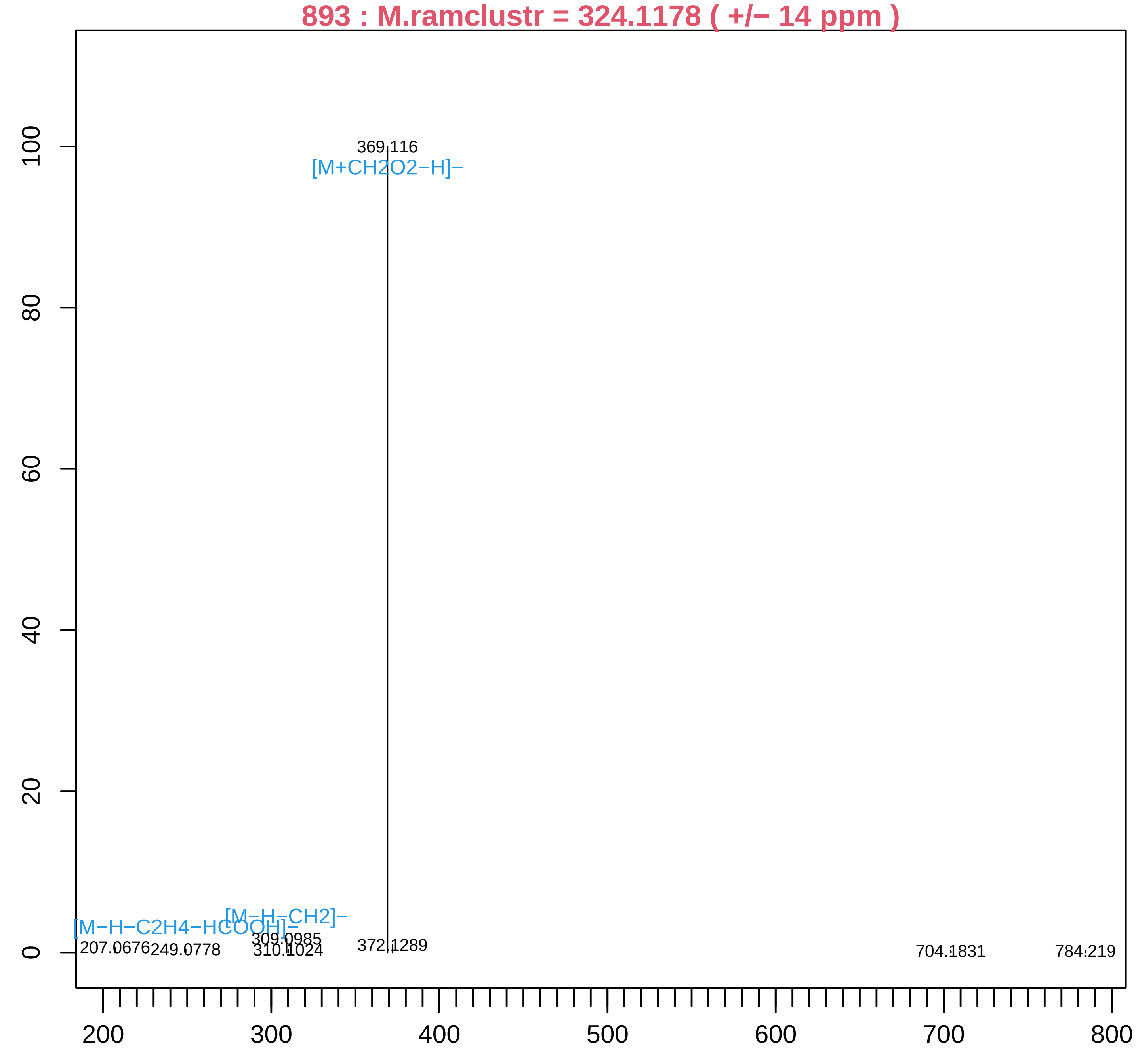

893 : M.ramclustr = 324.1178 (+/− 14 ppm)
369.116
[M+CH2O2−H]−
[M−H−CH2]−
[M−H−C2H4−HCOOH]−
207.0676
249.0778
309.0985
310.1024
372.1289
704.1831
784.219

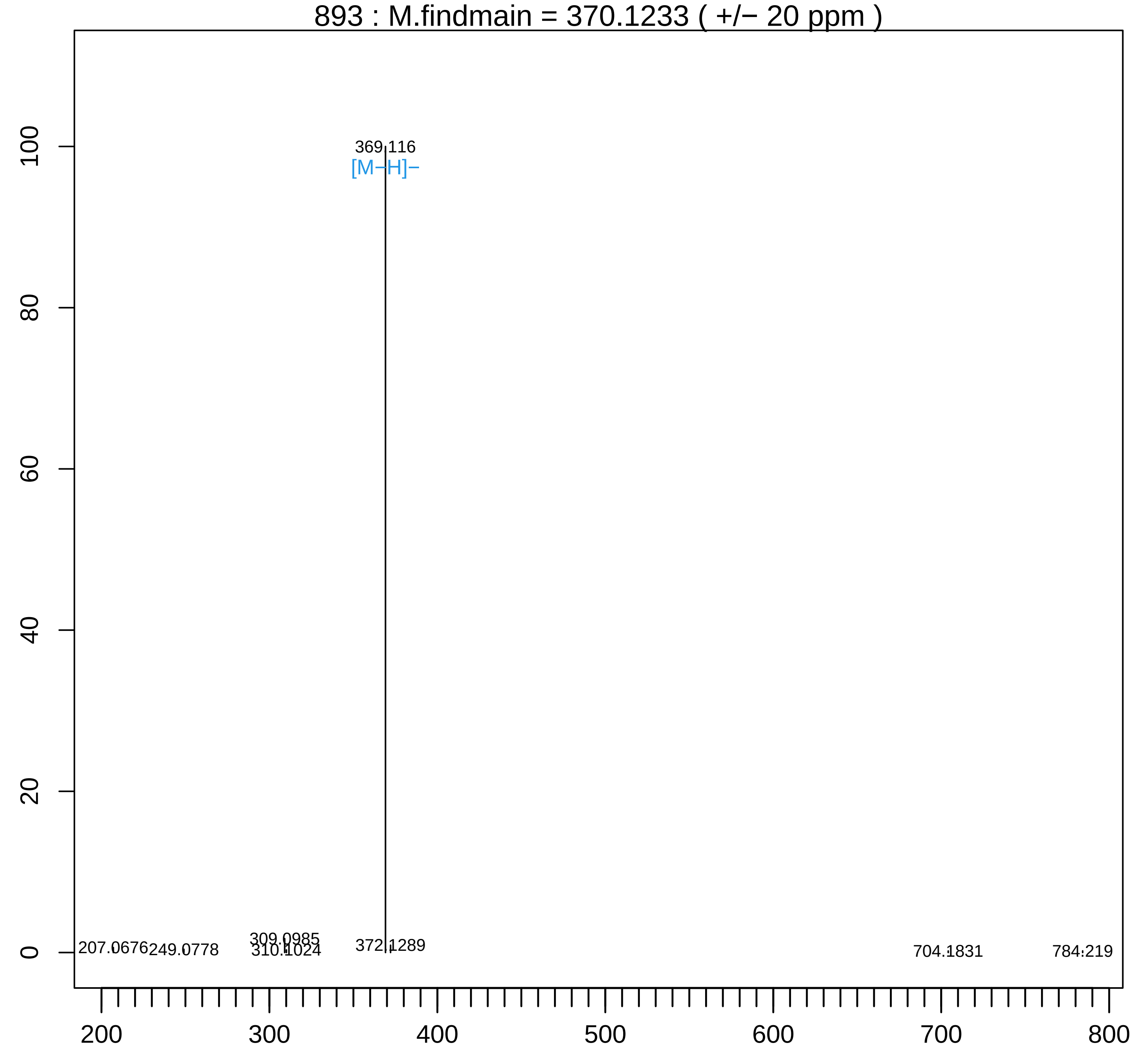

893 : M.findmain = 370.1233 (+/− 20 ppm)
369.116
[M−H]−
207.0676
249.0778
309.0985
310.1024
372.1289
704.1831
784.219

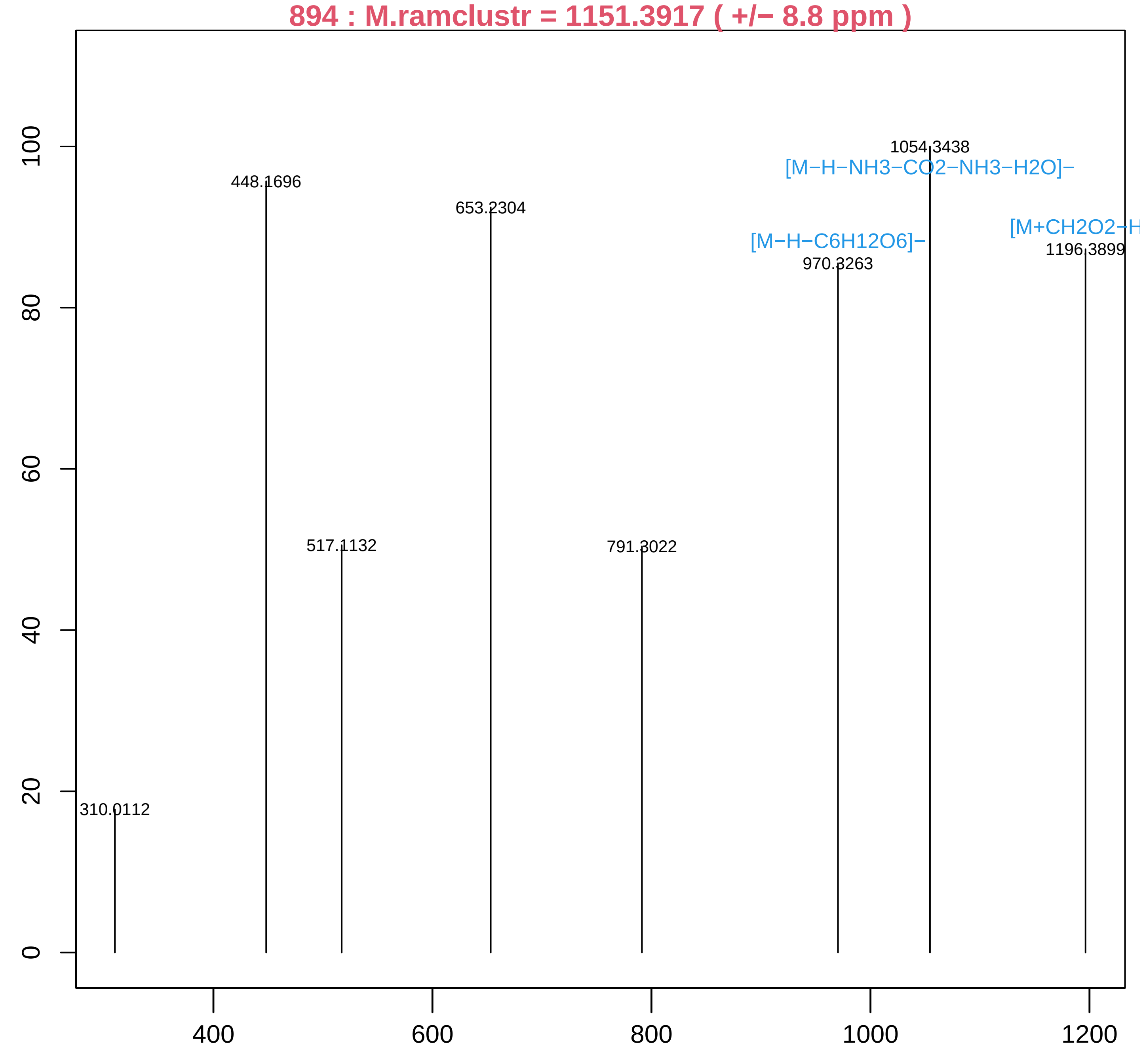

894 : M.ramclustr = 1151.3917 (+/− 8.8 ppm)
100
80
60
40
20
0
400
600
800
1000
1200
310.0112
448.1696
517.1132
653.2304
791.3022
970.3263
1054.3438
1196.3899
[M−H−NH3−CO2−NH3−H2O]−
[M−H−C6H12O6]−
[M+CH2O2−H

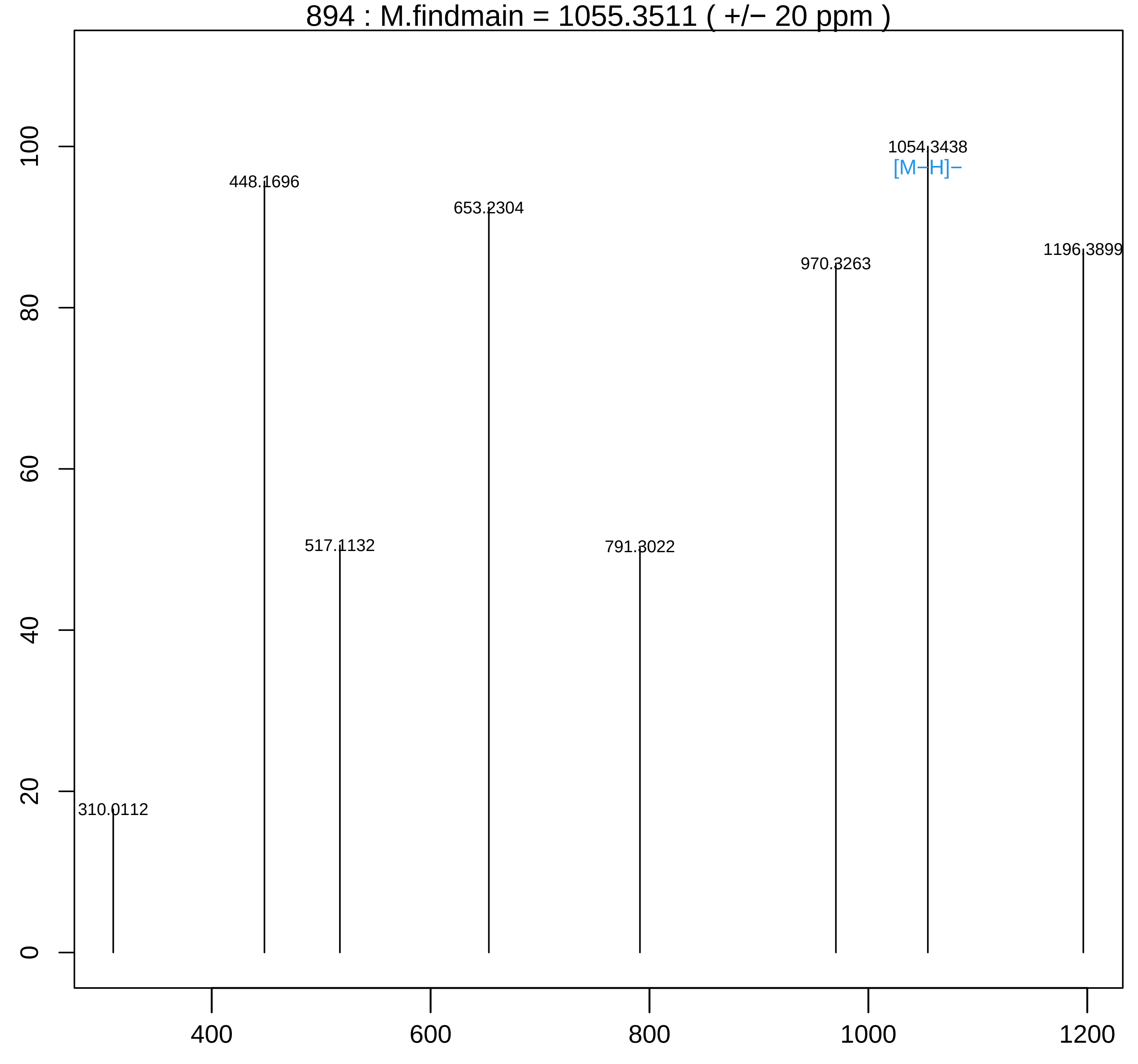

894 : M.findmain = 1055.3511 (+/− 20 ppm)
100
80
60
40
20
0
400
600
800
1000
1200
310.0112
448.1696
517.1132
653.2304
791.3022
970.3263
1054.3438
1196.3899
[M−H]−

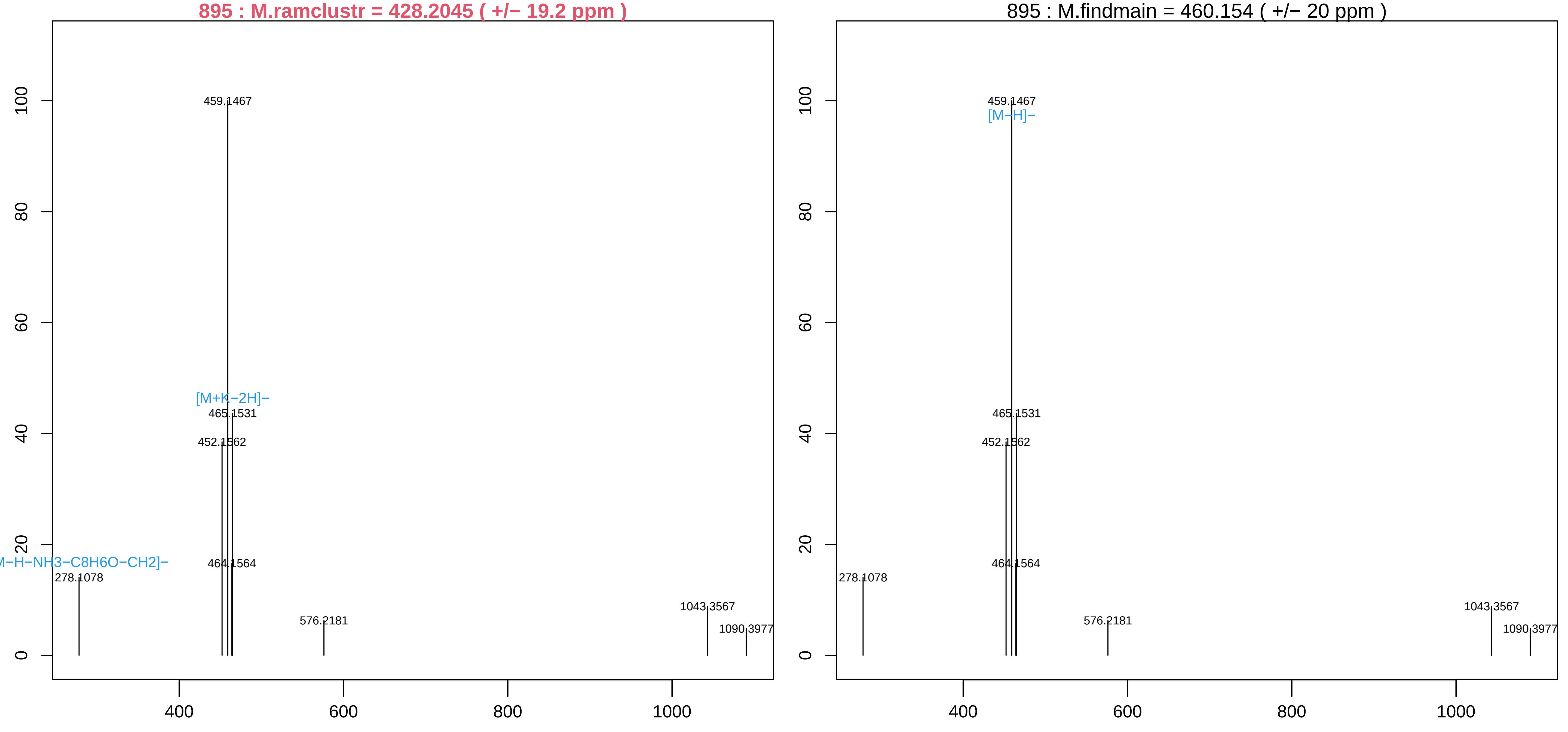

895 : M.ramclustr = 428.2045 (+/− 19.2 ppm)
459.1467
[M+K−2H]−
465.1531
452.1562
464.1564
M−H−NH3−C8H6O−CH2]−
278.1078
576.2181
1043.3567
1090.3977
895 : M.findmain = 460.154 (+/− 20 ppm)
459.1467
[M−H]−
465.1531
452.1562
464.1564
278.1078
576.2181
1043.3567
1090.3977
0
20
40
60
80
100
400
600
800
1000

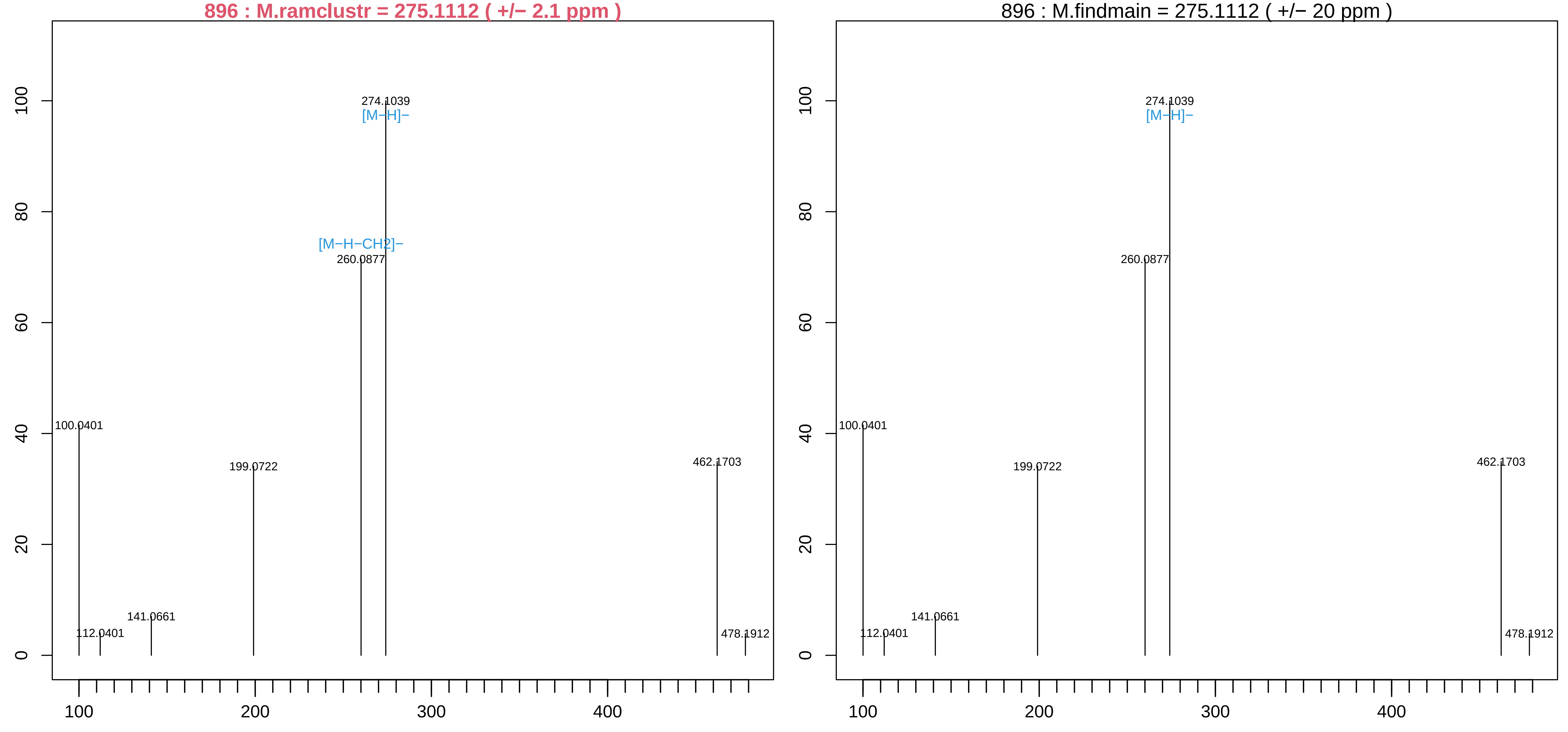

896 : M.ramclustr = 275.1112 (+/− 2.1 ppm)
274.1039
[M−H]−
[M−H−CH2]−
260.0877
100.0401
199.0722
462.1703
141.0661
112.0401
478.1912
0
20
40
60
80
100
100
200
300
400
896 : M.findmain = 275.1112 (+/− 20 ppm)
274.1039
[M−H]−
260.0877
100.0401
199.0722
462.1703
141.0661
112.0401
478.1912
0
20
40
60
80
100
100
200
300
400

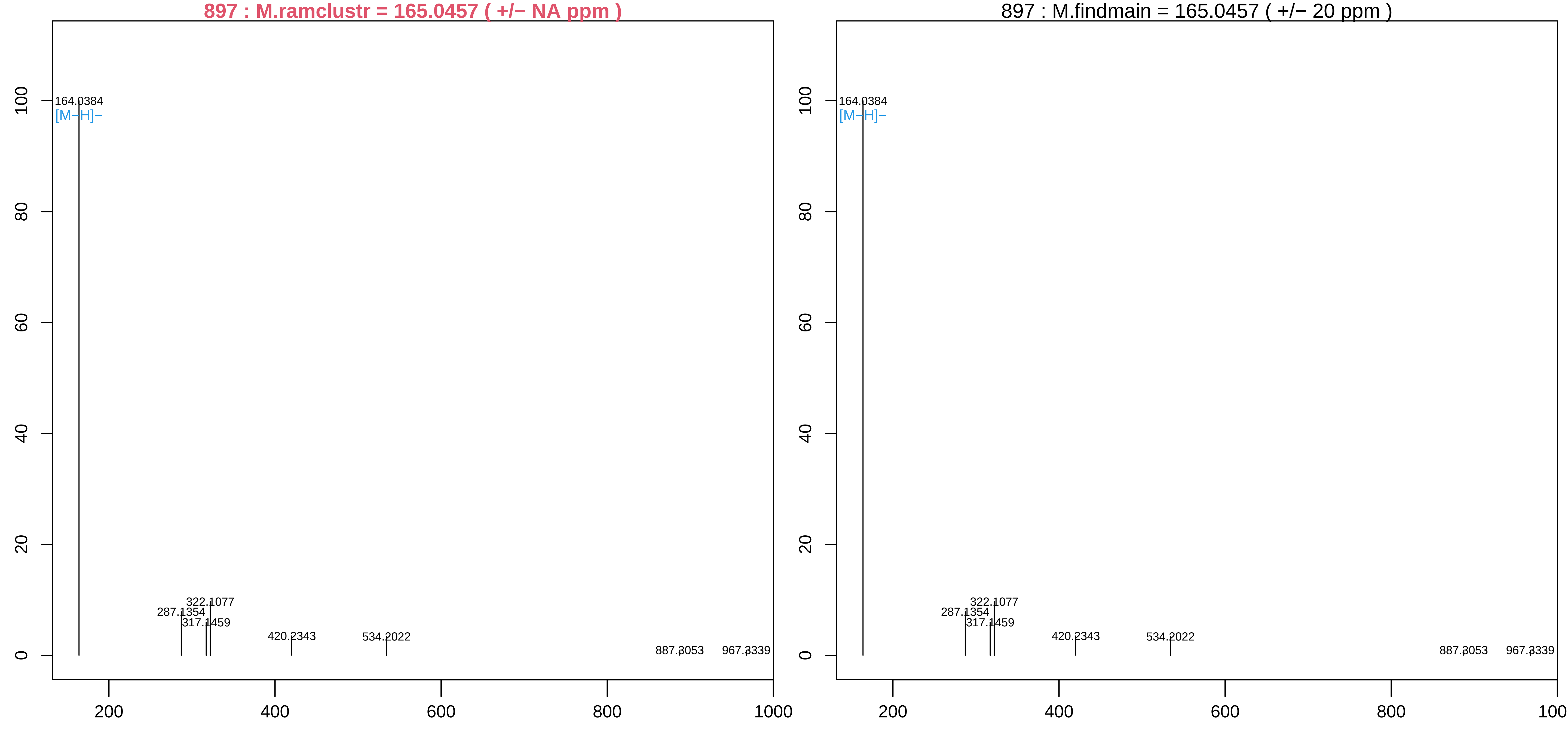
897 : M.ramclustr = 165.0457 (+/− NA ppm)
164.0384
[M−H]−
287.1354
322.1077
317.1459
420.2343
534.2022
887.3053
967.3339
897 : M.findmain = 165.0457 (+/− 20 ppm)
164.0384
[M−H]−
287.1354
322.1077
317.1459
420.2343
534.2022
887.3053
967.3339

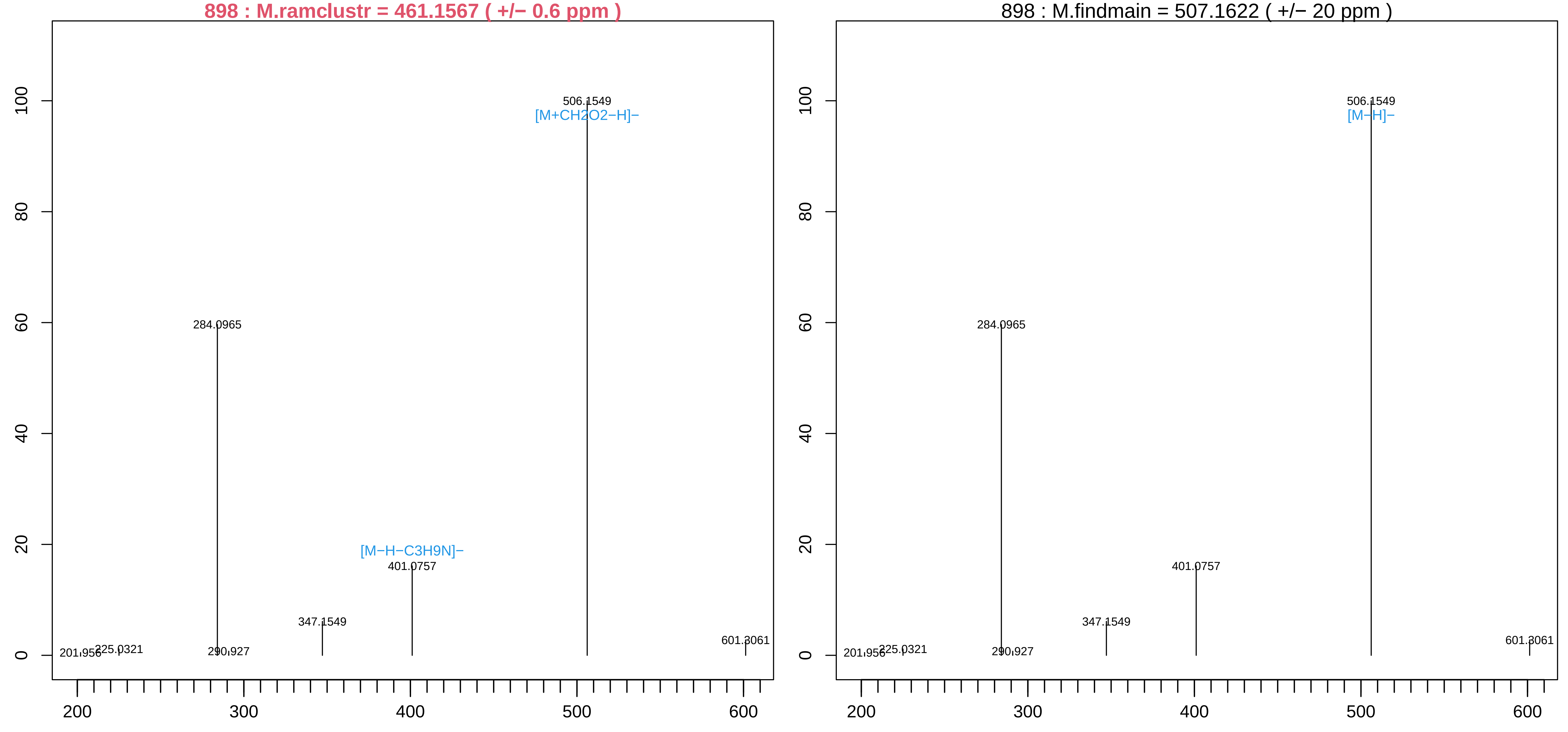

898 : M.ramclustr = 461.1567 (+/− 0.6 ppm)
506.1549
[M+CH2O2−H]−
284.0965
[M−H−C3H9N]−
401.0757
347.1549
601.3061
201.956
225.0321
290.927
898 : M.findmain = 507.1622 (+/− 20 ppm)
506.1549
[M−H]−
284.0965
401.0757
347.1549
601.3061
201.956
225.0321
290.927
0
20
40
60
80
100
200
300
400
500
600

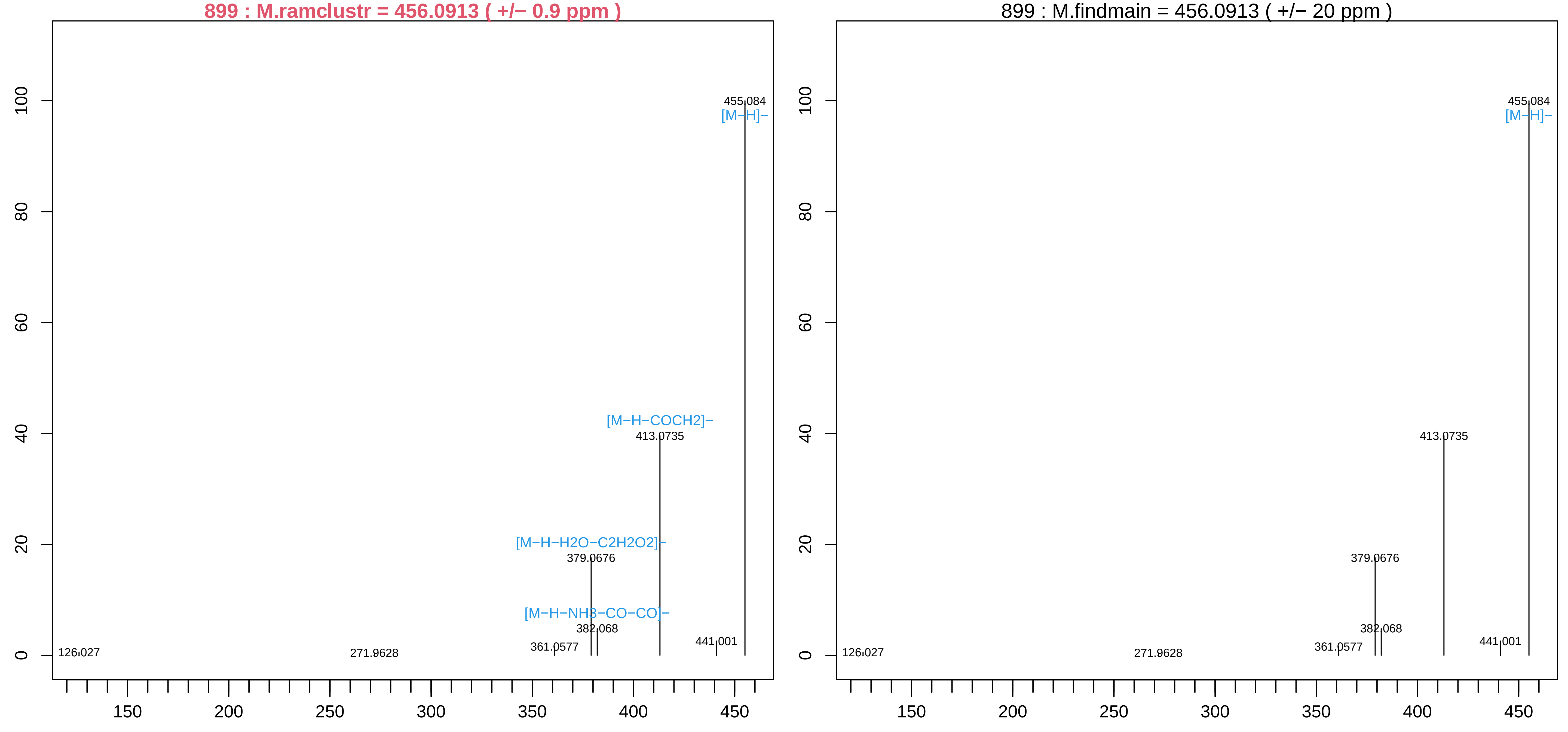
899 : M.ramclustr = 456.0913 (+/− 0.9 ppm)
455.084
[M−H]−
[M−H−COCH2]−
413.0735
[M−H−H2O−C2H2O2]−
379.0676
[M−H−NH3−CO−CO]−
382.068
361.0577
441.001
126.027
271.9628
899 : M.findmain = 456.0913 (+/− 20 ppm)
455.084
[M−H]−
413.0735
379.0676
382.068
361.0577
441.001
126.027
271.9628

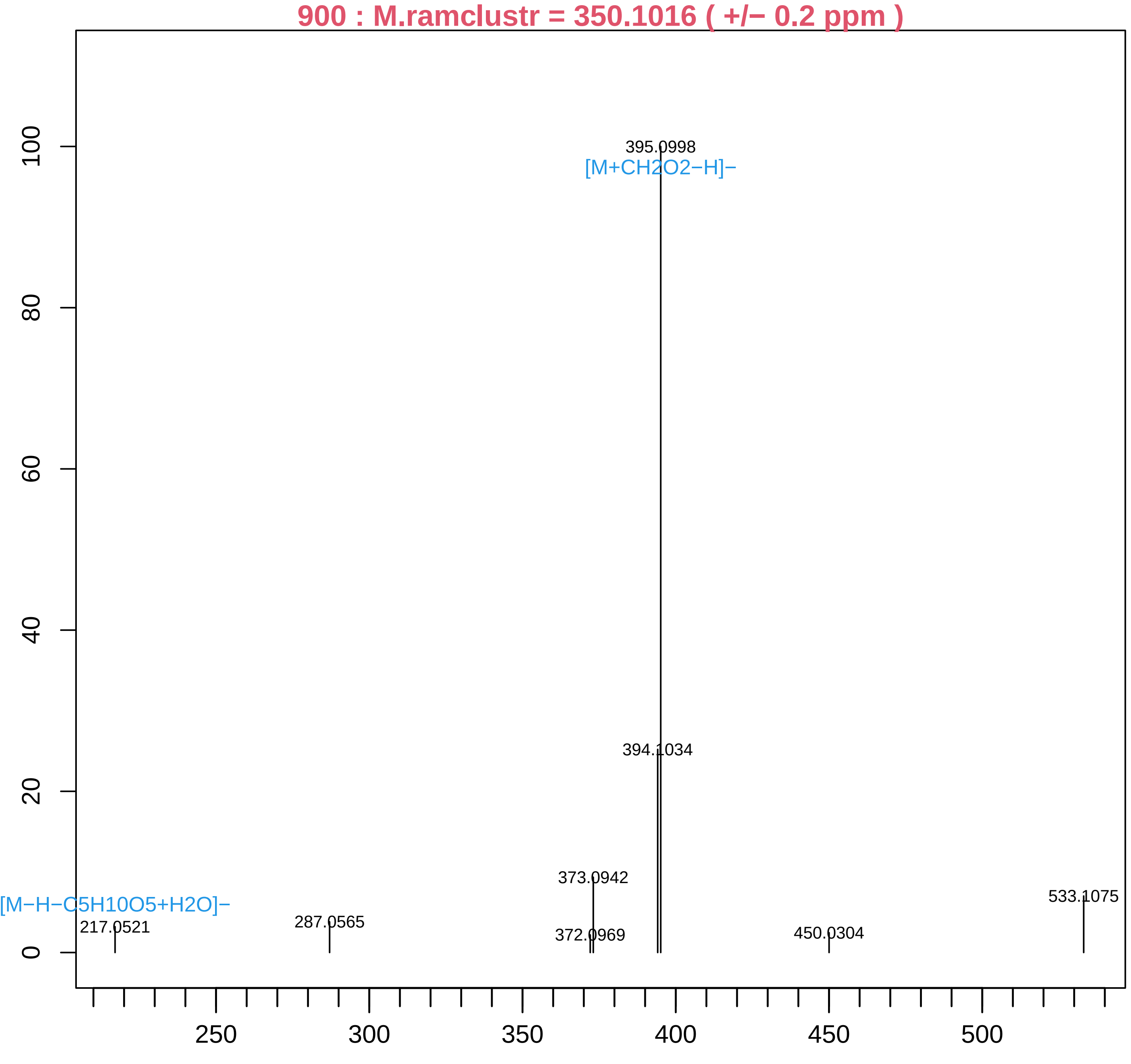
900 : M.ramclustr = 350.1016 (+/− 0.2 ppm)
395.0998
[M+CH2O2−H]−
394.1034
373.0942
372.0969
287.0565
217.0521
[M−H−C5H10O5+H2O]−
450.0304
533.1075
0
20
40
60
80
100
250
300
350
400
450
500
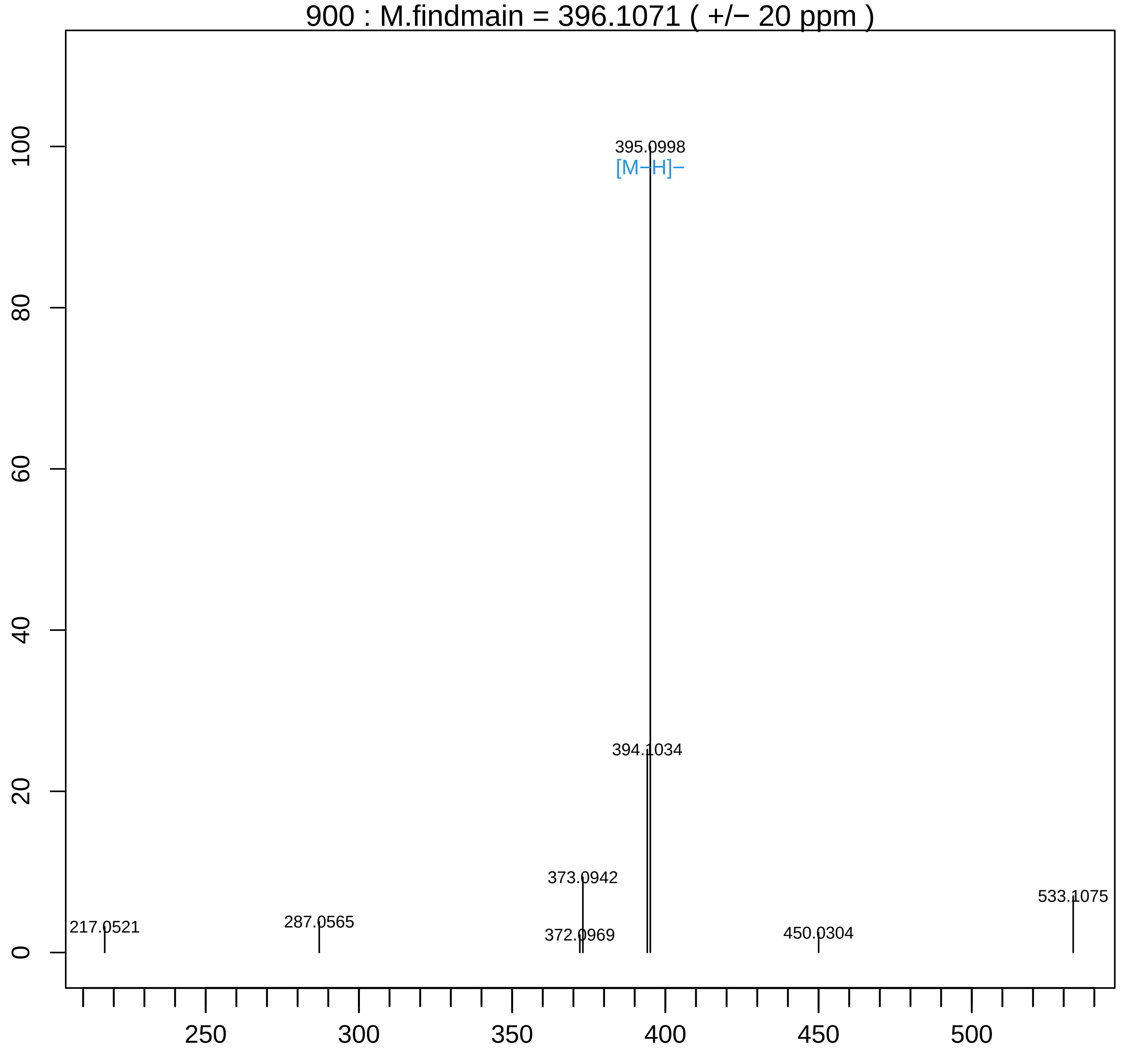
900 : M.findmain = 396.1071 (+/− 20 ppm)
395.0998
[M−H]−
394.1034
373.0942
372.0969
287.0565
217.0521
450.0304
533.1075
0
20
40
60
80
100
250
300
350
400
450
500

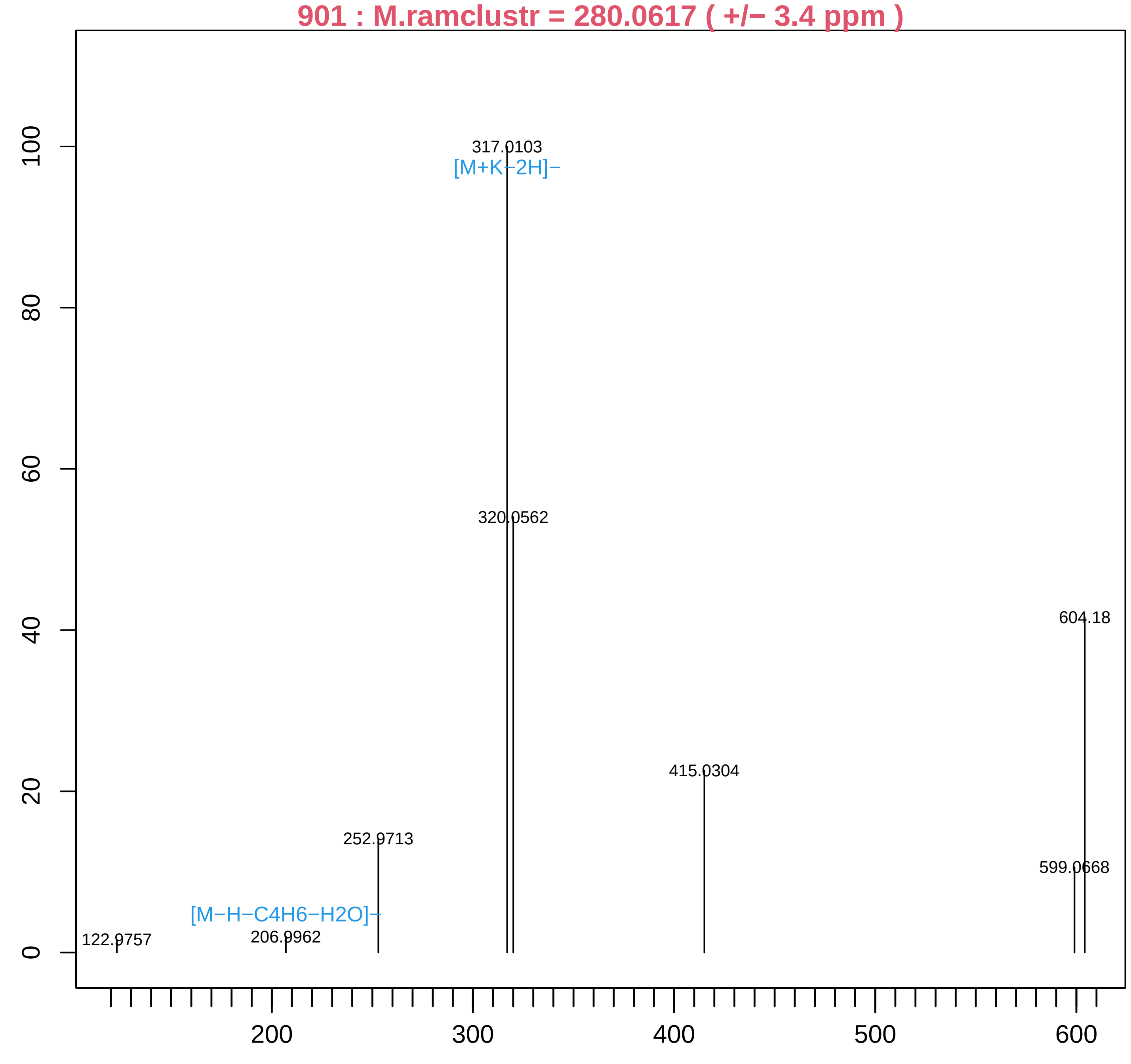

901 : M.ramclustr = 280.0617 (+/− 3.4 ppm)
317.0103
[M+K−2H]−
320.0562
604.18
415.0304
252.9713
599.0668
[M−H−C4H6−H2O]−
122.9757
206.9962
0
20
40
60
80
100
200
300
400
500
600

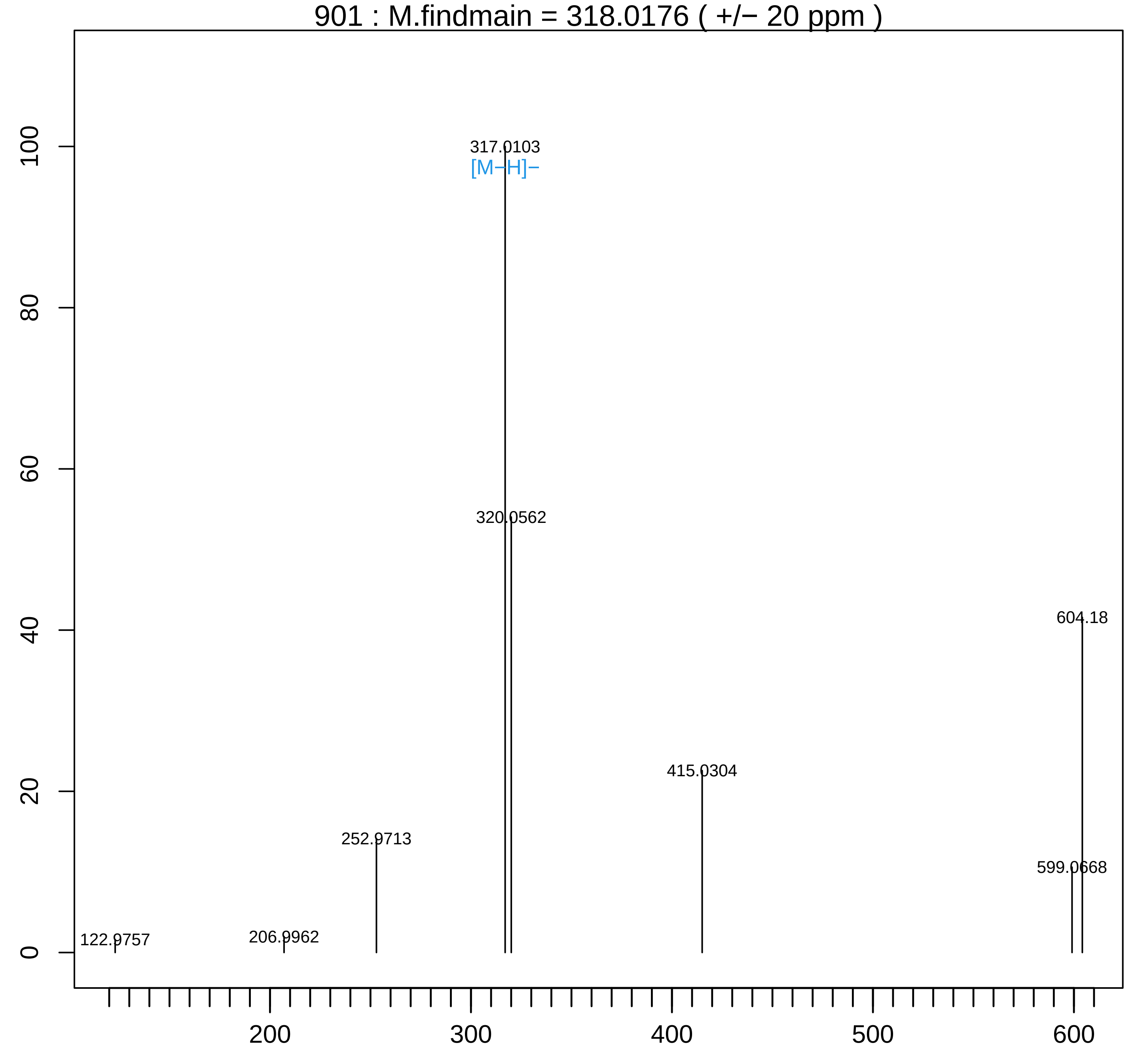

901 : M.findmain = 318.0176 (+/− 20 ppm)
317.0103
[M−H]−
320.0562
604.18
415.0304
252.9713
599.0668
122.9757
206.9962
0
20
40
60
80
100
200
300
400
500
600

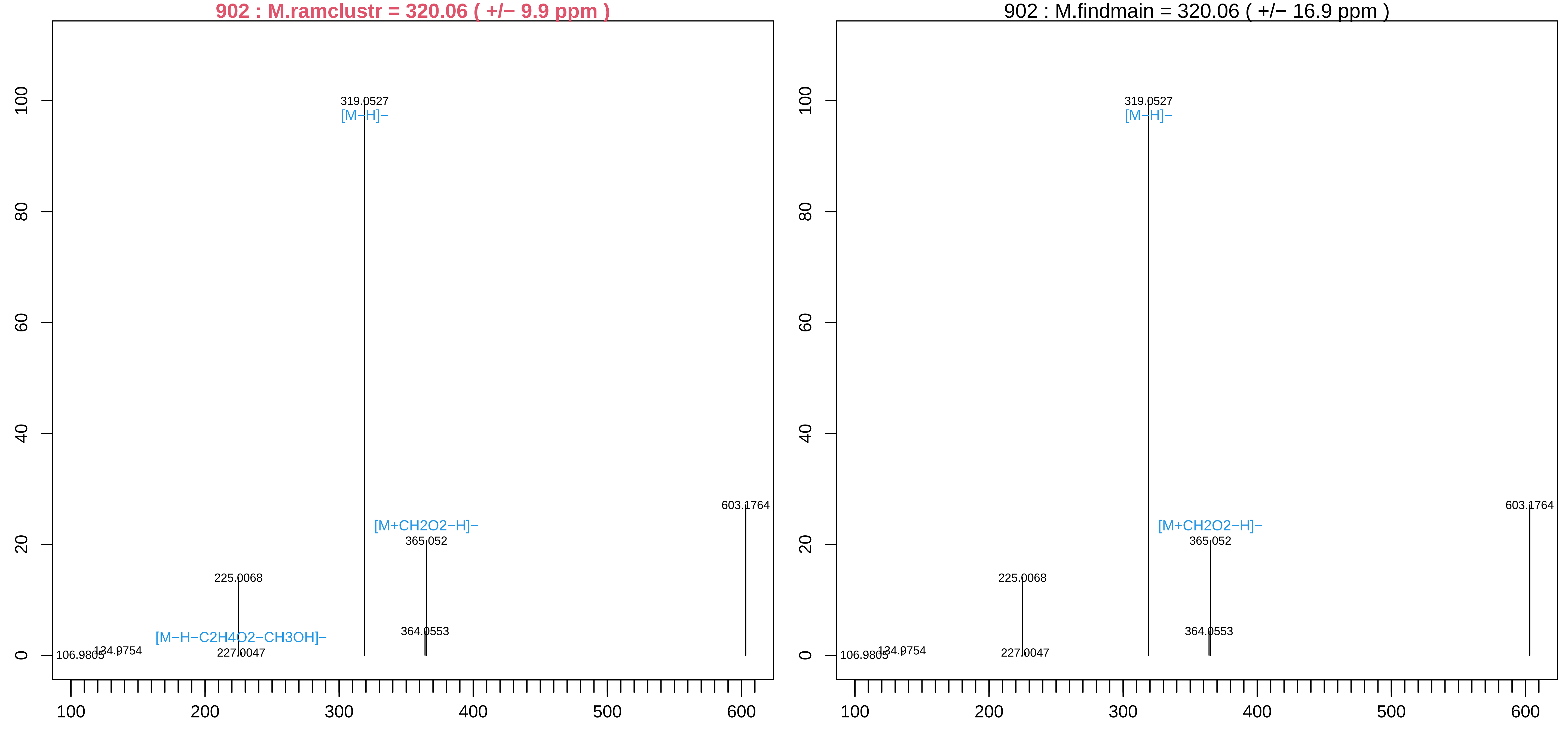
902 : M.ramclustr = 320.06 (+/− 9.9 ppm)
319.0527
[M−H]−
[M+CH2O2−H]−
365.052
603.1764
225.0068
364.0553
[M−H−C2H4O2−CH3OH]−
106.9805
134.9754
227.0047
0
20
40
60
80
100
100
200
300
400
500
600
902 : M.findmain = 320.06 (+/− 16.9 ppm)
319.0527
[M−H]−
[M+CH2O2−H]−
365.052
603.1764
225.0068
364.0553
106.9805
134.9754
227.0047
0
20
40
60
80
100
100
200
300
400
500
600

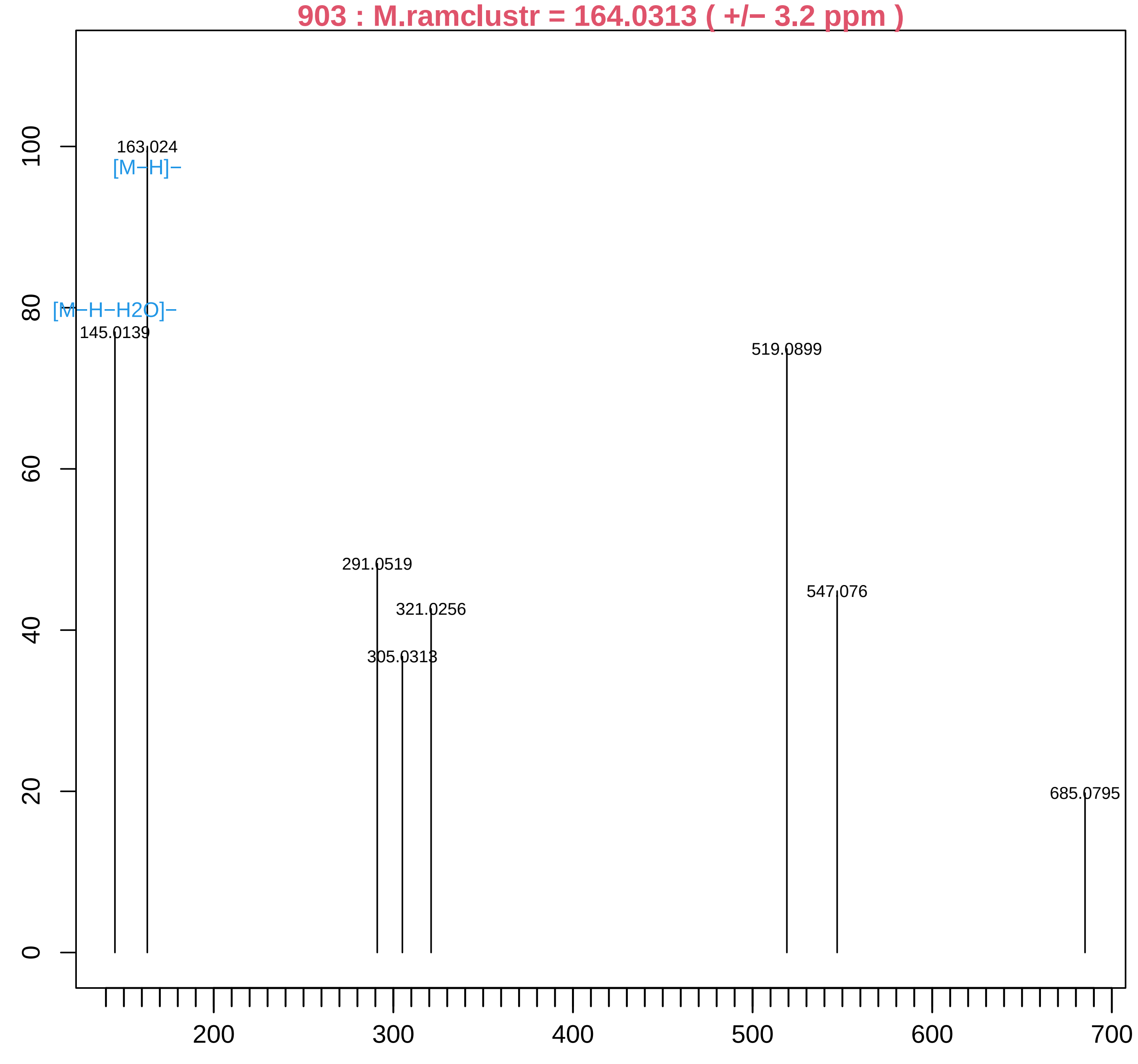

903 : M.ramclustr = 164.0313 (+/− 3.2 ppm)
163.024
[M−H]−
[M−H−H2O]−
145.0139
519.0899
291.0519
321.0256
305.0313
547.076
685.0795
0
20
40
60
80
100
200
300
400
500
600
700

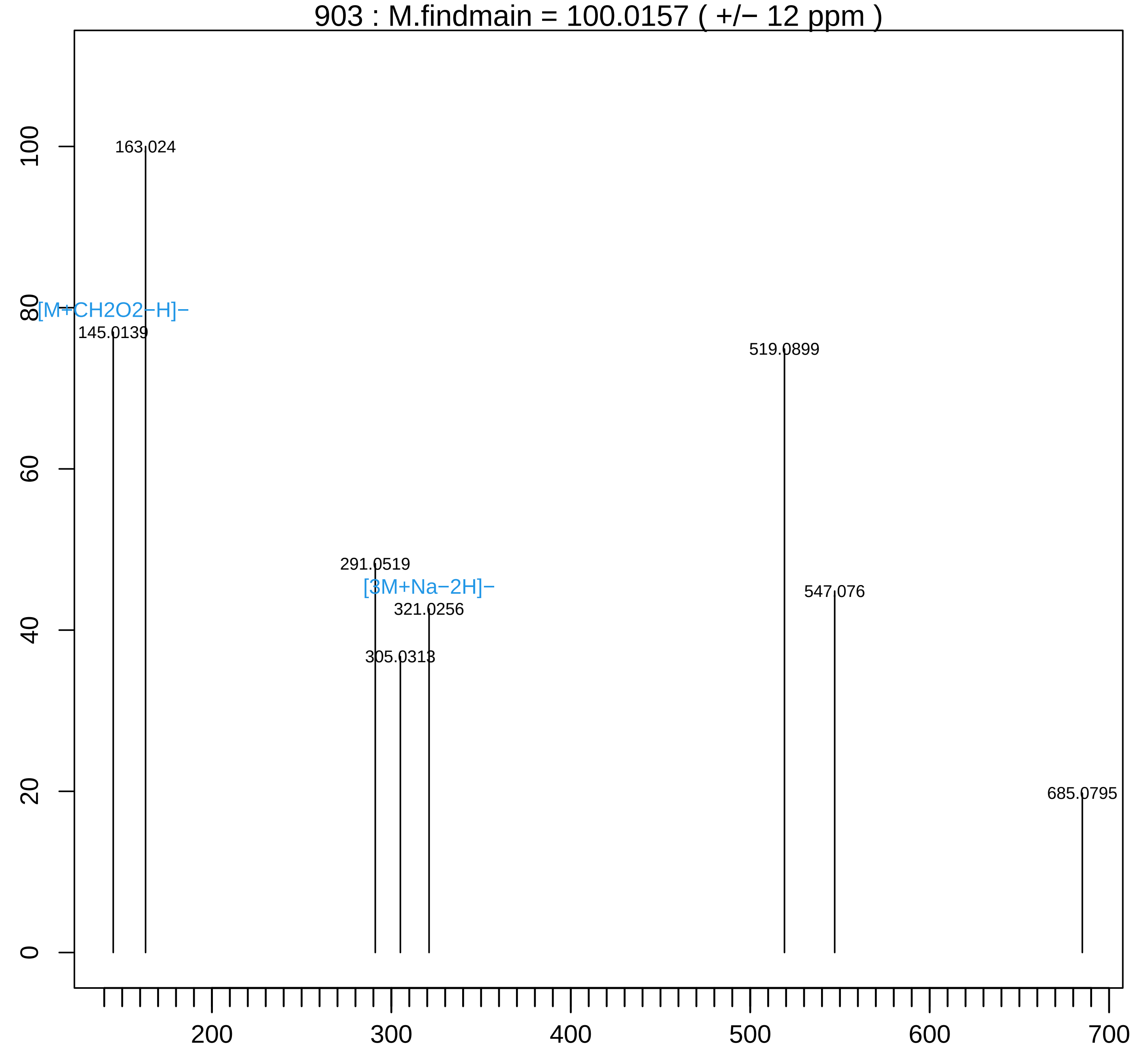

903 : M.findmain = 100.0157 (+/− 12 ppm)
163.024
[M+CH2O2−H]−
145.0139
519.0899
291.0519
[3M+Na−2H]−
321.0256
305.0313
547.076
685.0795
0
20
40
60
80
100
200
300
400
500
600
700

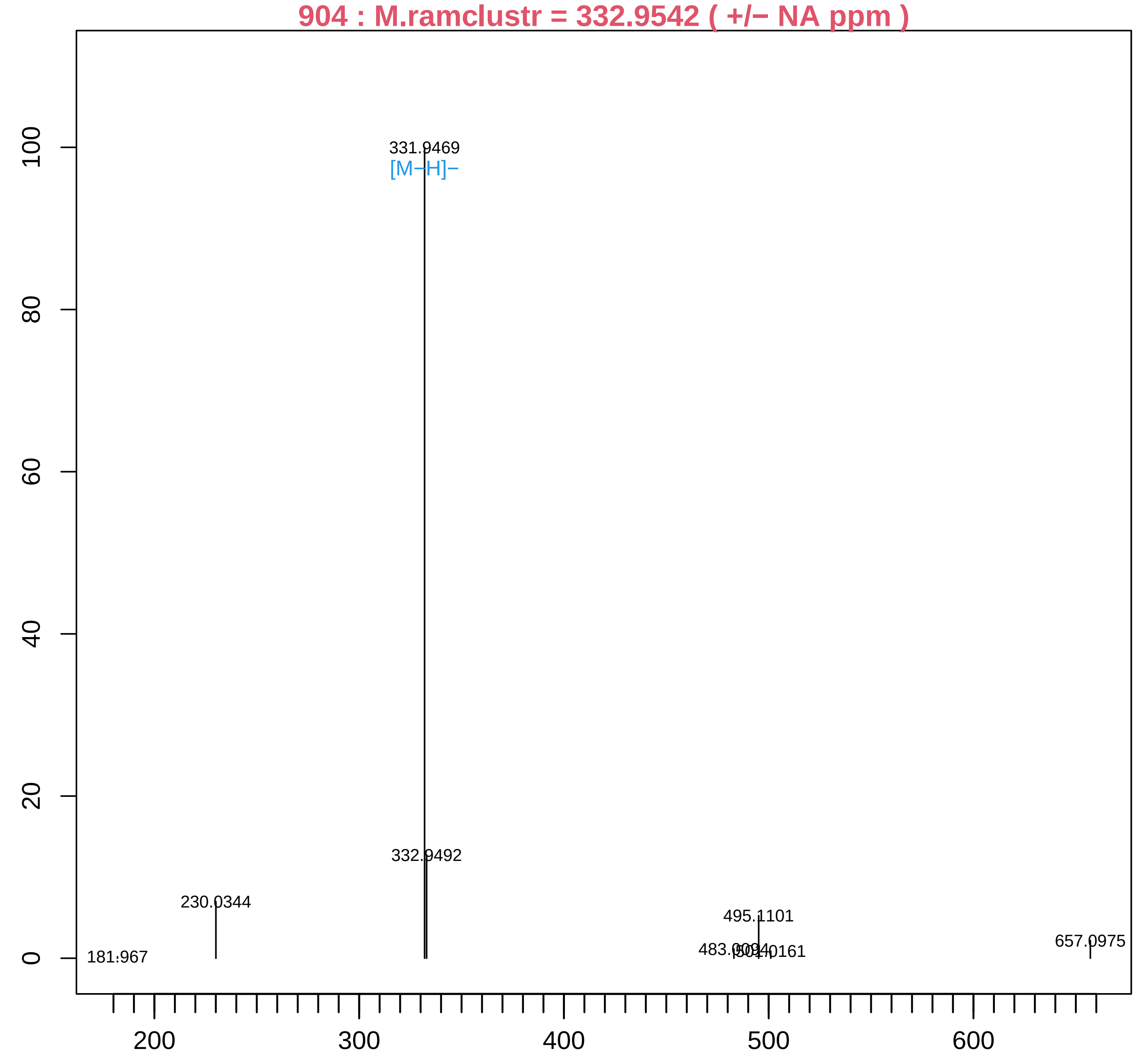
904 : M.ramclustr = 332.9542 (+/− NA ppm)
331.9469
[M−H]−
332.9492
230.0344
181.967
495.1101
483.0094
501.0161
657.0975
0
20
40
60
80
100
200
300
400
500
600

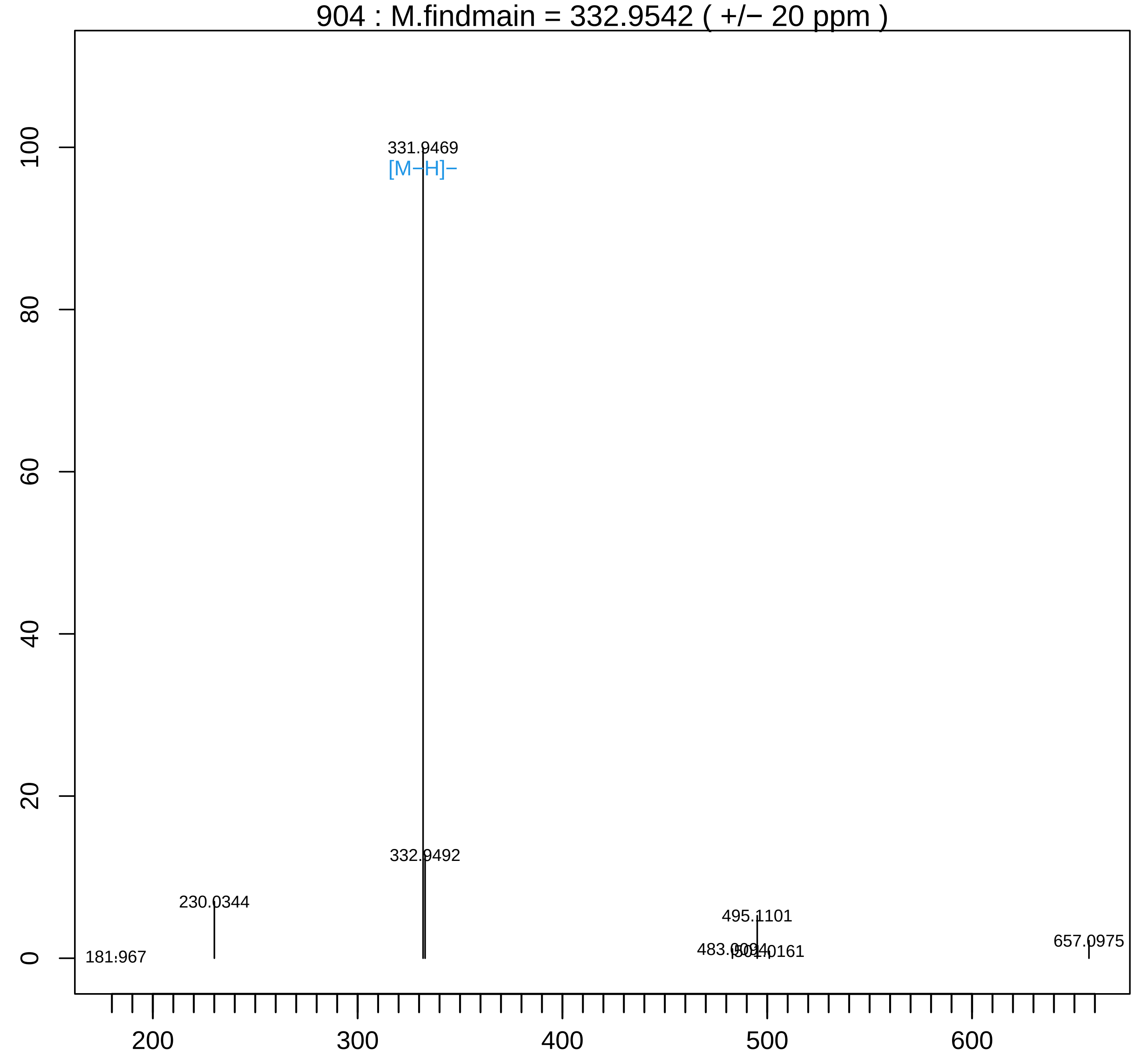
904 : M.findmain = 332.9542 (+/− 20 ppm)
331.9469
[M−H]−
332.9492
230.0344
181.967
495.1101
483.0094
501.0161
657.0975
0
20
40
60
80
100
200
300
400
500
600

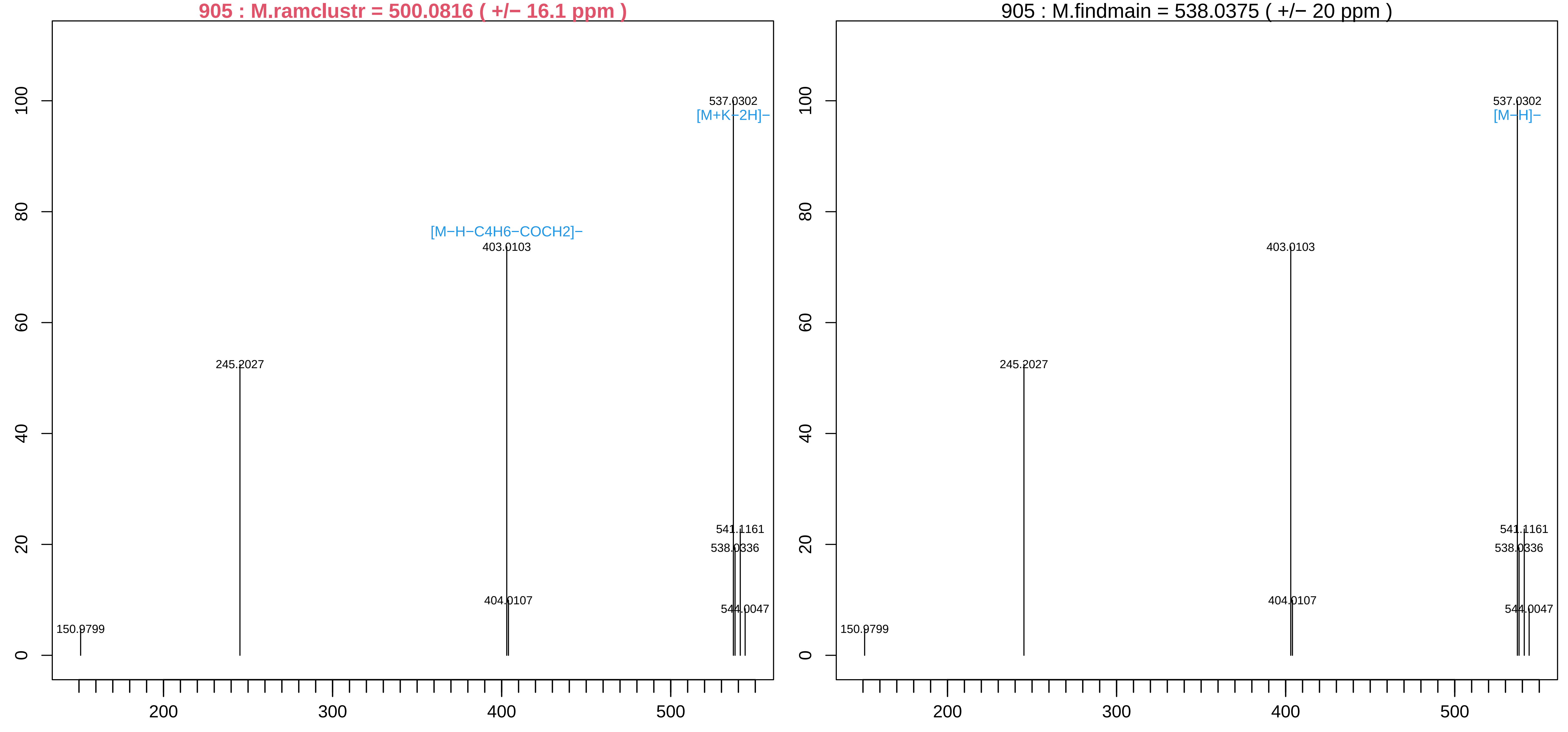
905 : M.ramclustr = 500.0816 (+/− 16.1 ppm)
537.0302
[M+K−2H]−
[M−H−C4H6−COCH2]−
403.0103
245.2027
541.1161
538.0336
404.0107
544.0047
150.9799
905 : M.findmain = 538.0375 (+/− 20 ppm)
537.0302
[M−H]−
403.0103
245.2027
541.1161
538.0336
404.0107
544.0047
150.9799

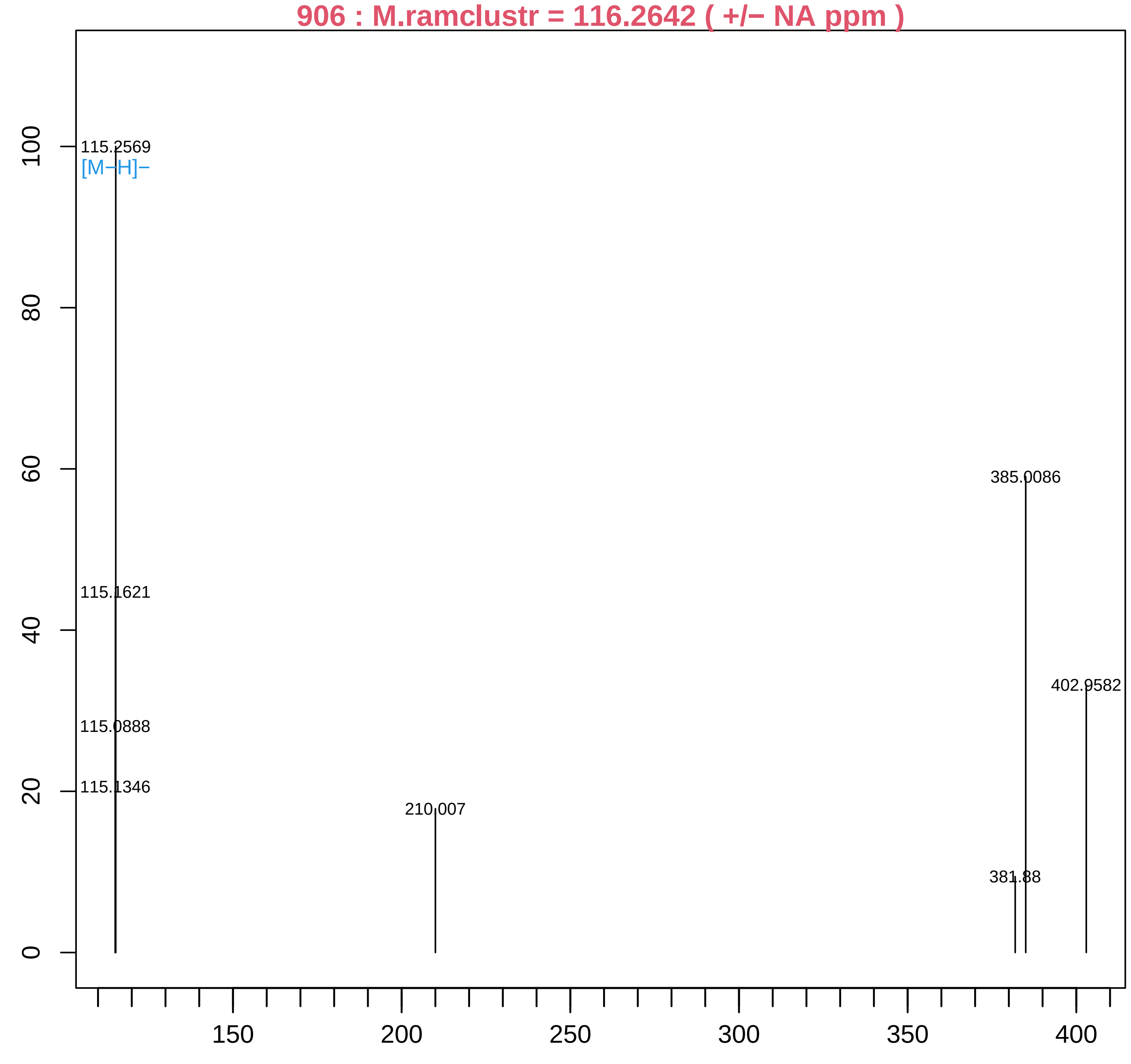
906 : M.ramclustr = 116.2642 (+/− NA ppm)
115.2569
[M−H]−
115.1621
115.0888
115.1346
210.007
385.0086
381.88
402.9582
0
20
40
60
80
100
150
200
250
300
350
400

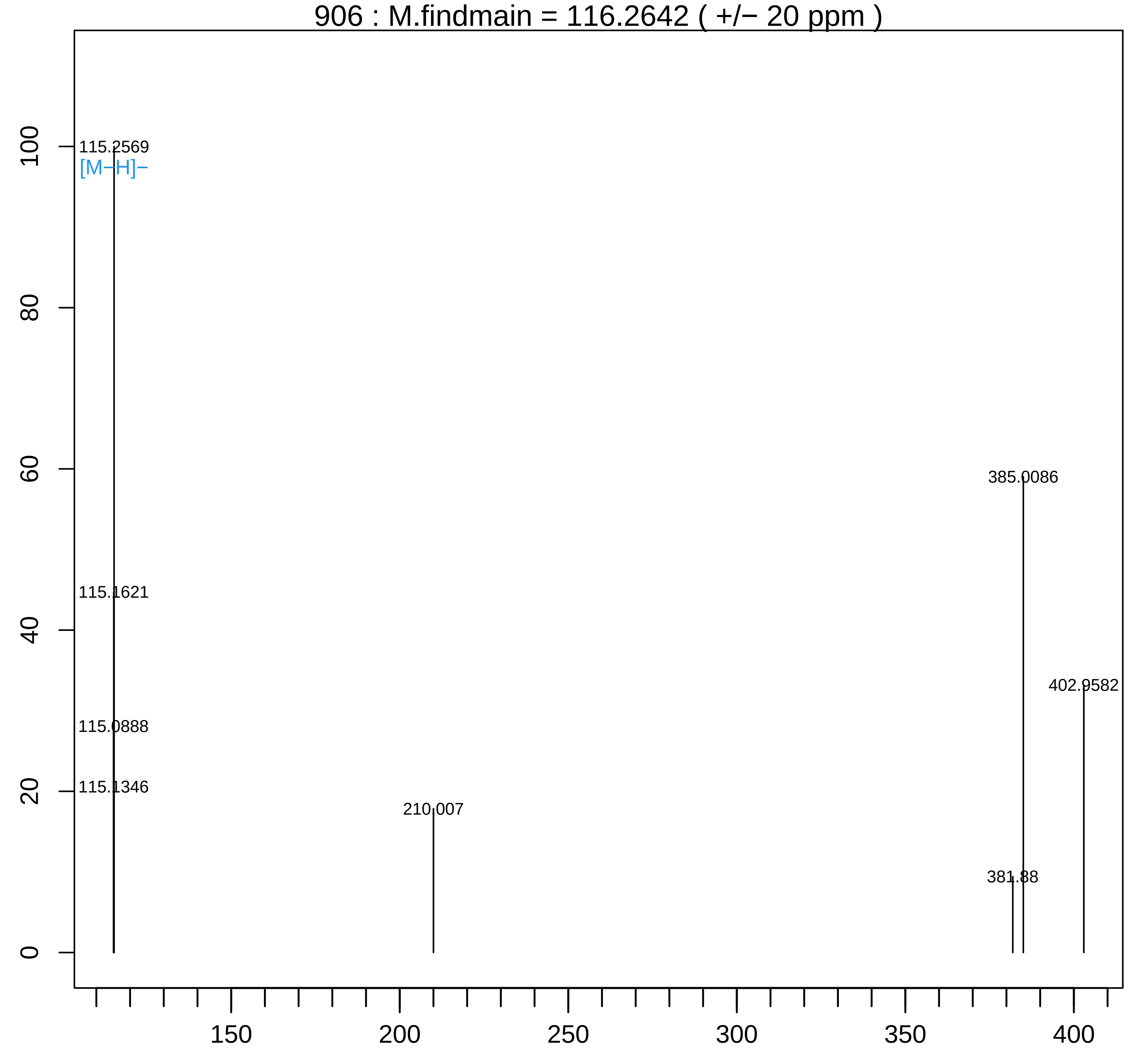
906 : M.findmain = 116.2642 (+/− 20 ppm)
115.2569
[M−H]−
115.1621
115.0888
115.1346
210.007
385.0086
381.88
402.9582
0
20
40
60
80
100
150
200
250
300
350
400

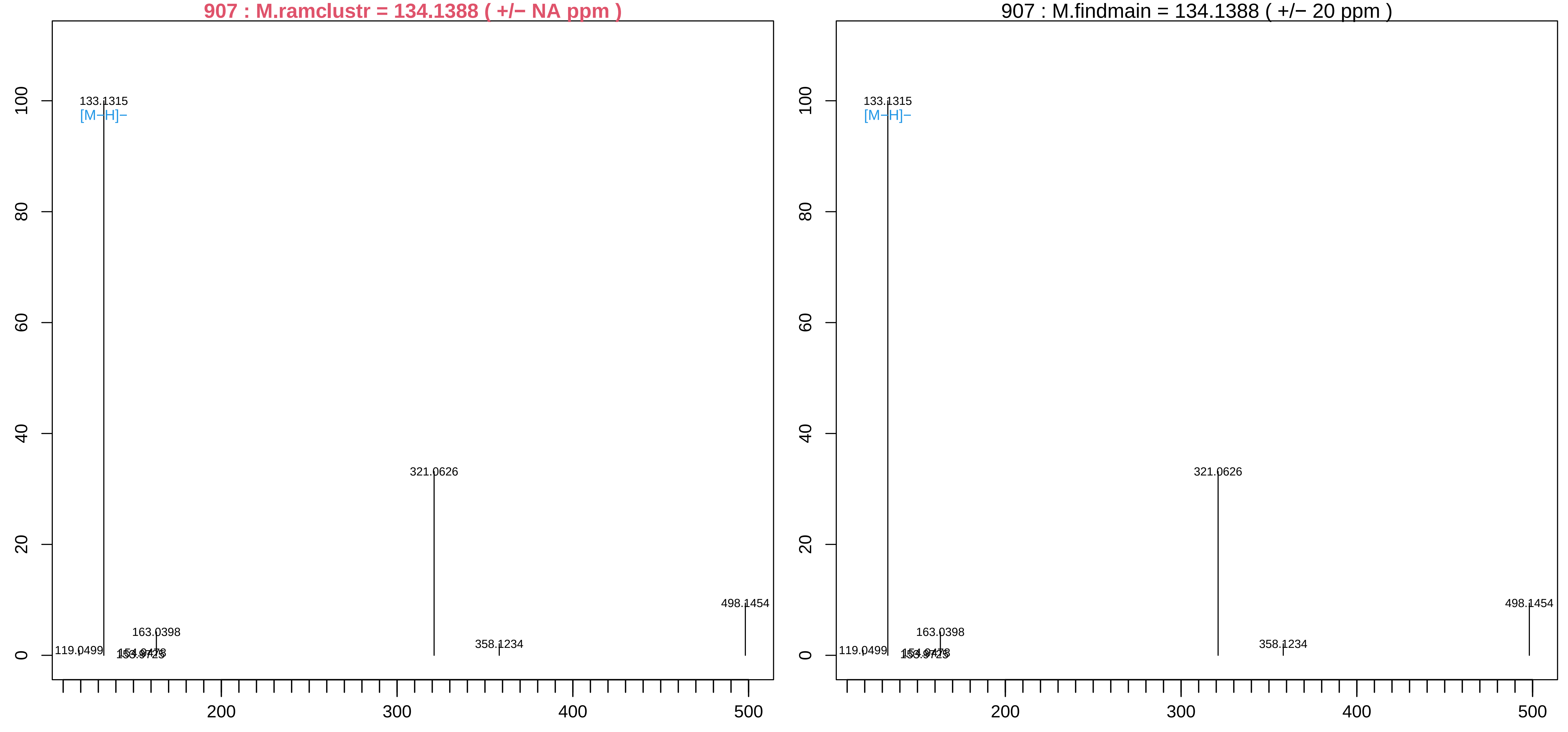
907 : M.ramclustr = 134.1388 (+/− NA ppm)
133.1315
[M−H]−
321.0626
498.1454
163.0398
358.1234
119.0499
154.9478
153.9723
907 : M.findmain = 134.1388 (+/− 20 ppm)
133.1315
[M−H]−
321.0626
498.1454
163.0398
358.1234
119.0499
154.9478
153.9723

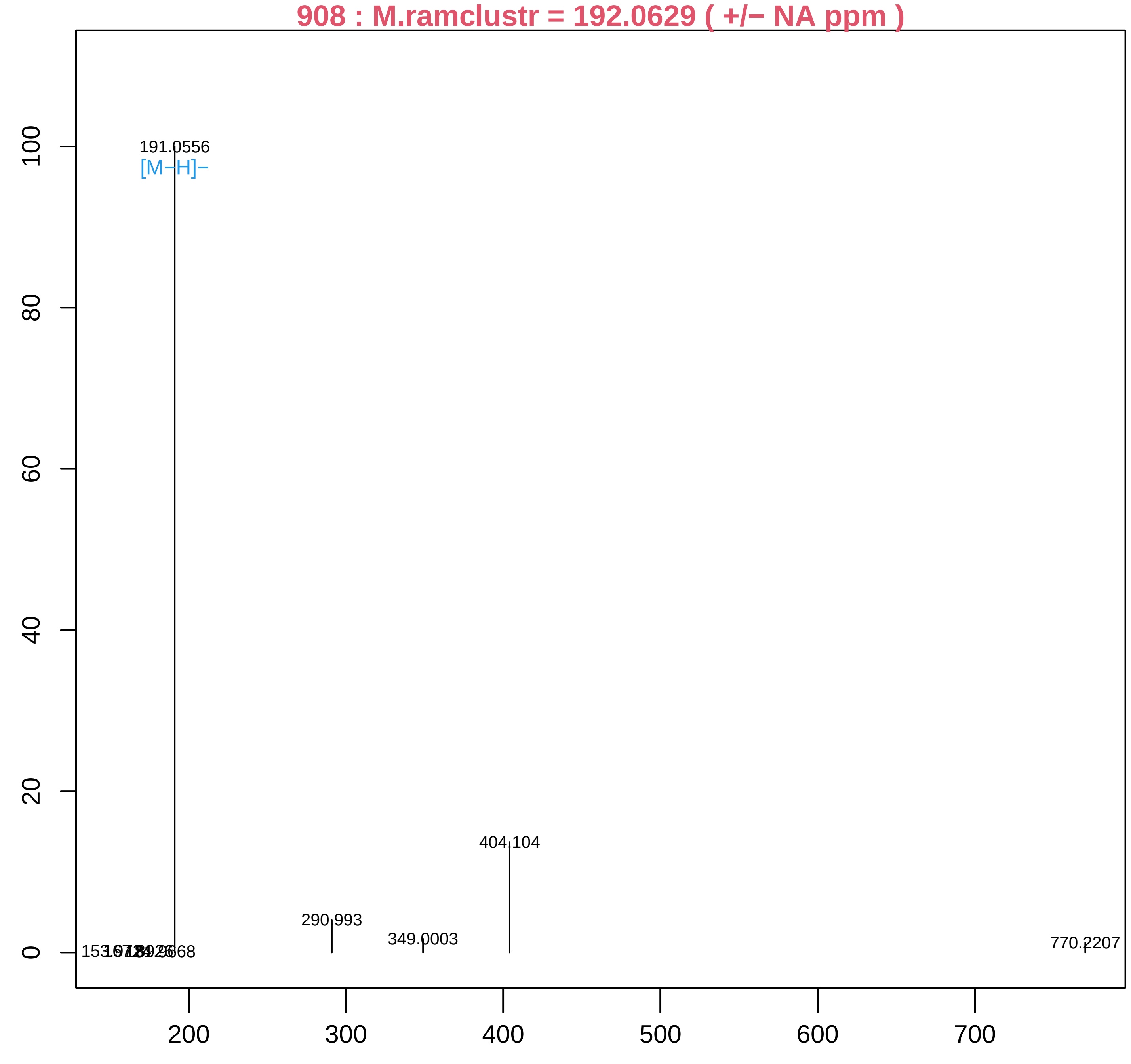
908 : M.ramclustr = 192.0629 (+/− NA ppm)
191.0556
[M−H]−
404.104
290.993
349.0003
770.2207
0
20
40
60
80
100
200
300
400
500
600
700

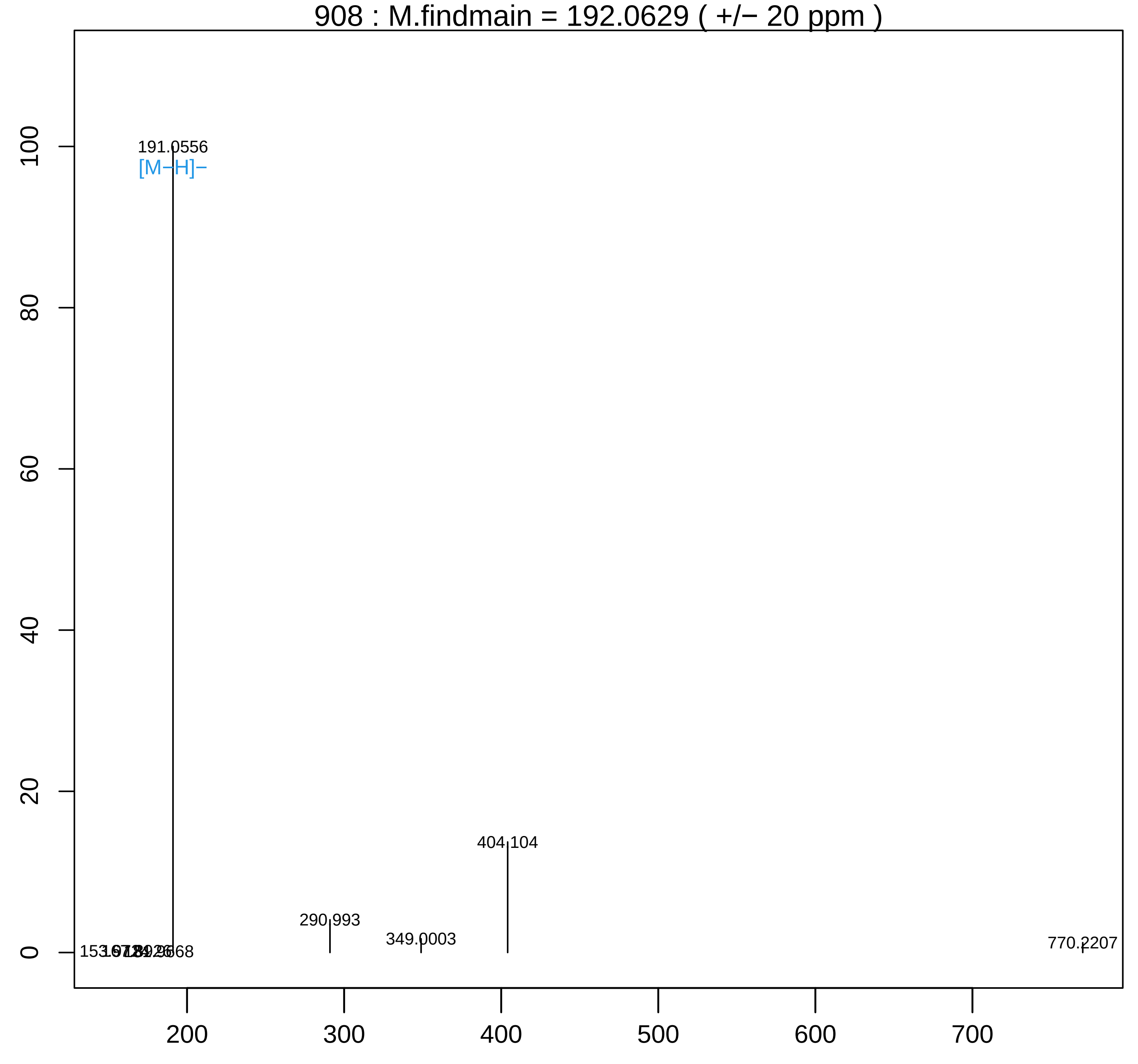
908 : M.findmain = 192.0629 (+/− 20 ppm)
191.0556
[M−H]−
404.104
290.993
349.0003
770.2207
0
20
40
60
80
100
200
300
400
500
600
700

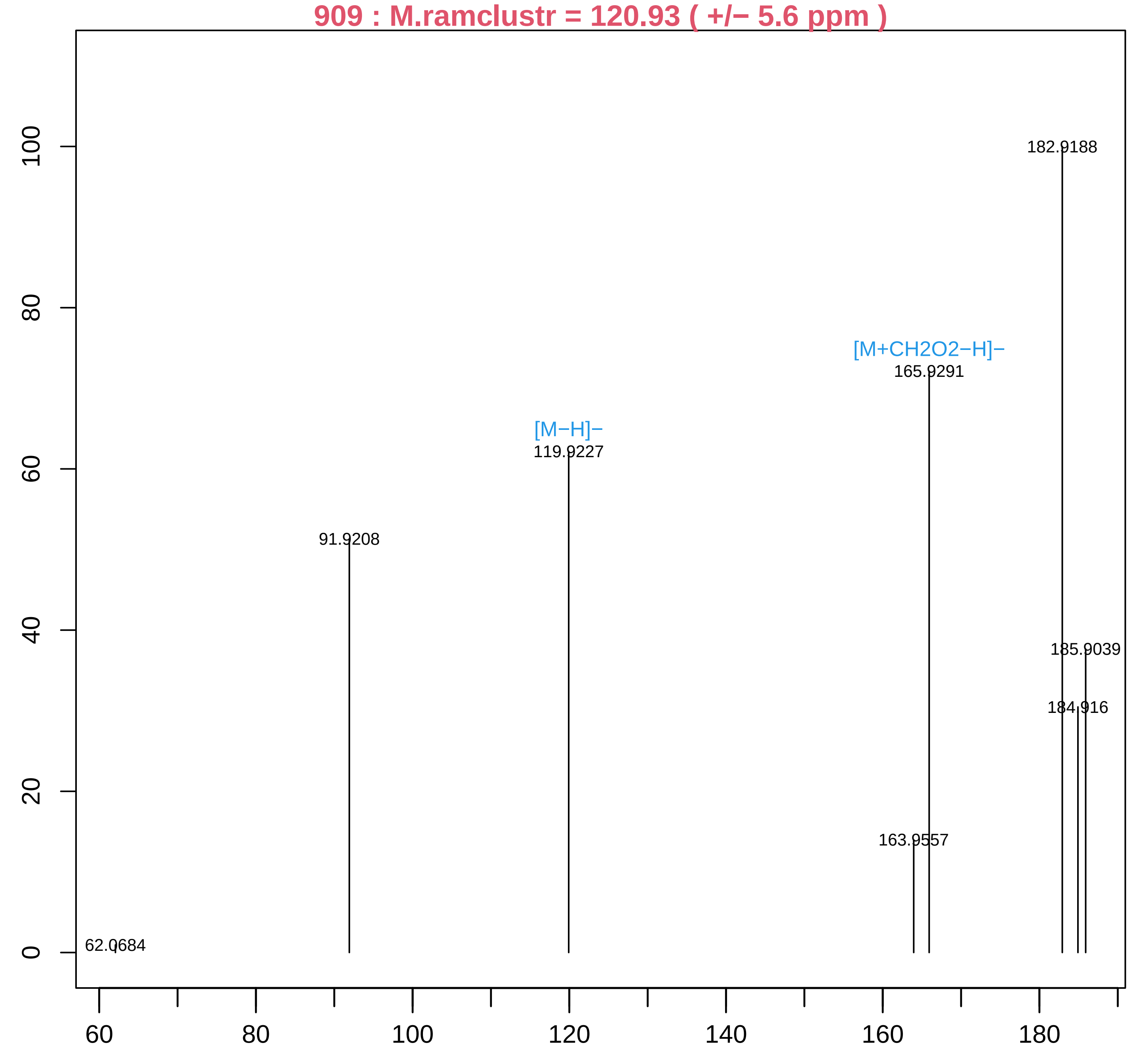
909 : M.ramclustr = 120.93 (+/− 5.6 ppm)
[M−H]−
119.9227
[M+CH2O2−H]−
165.9291
182.9188
91.9208
185.9039
184.916
163.9557
62.0684

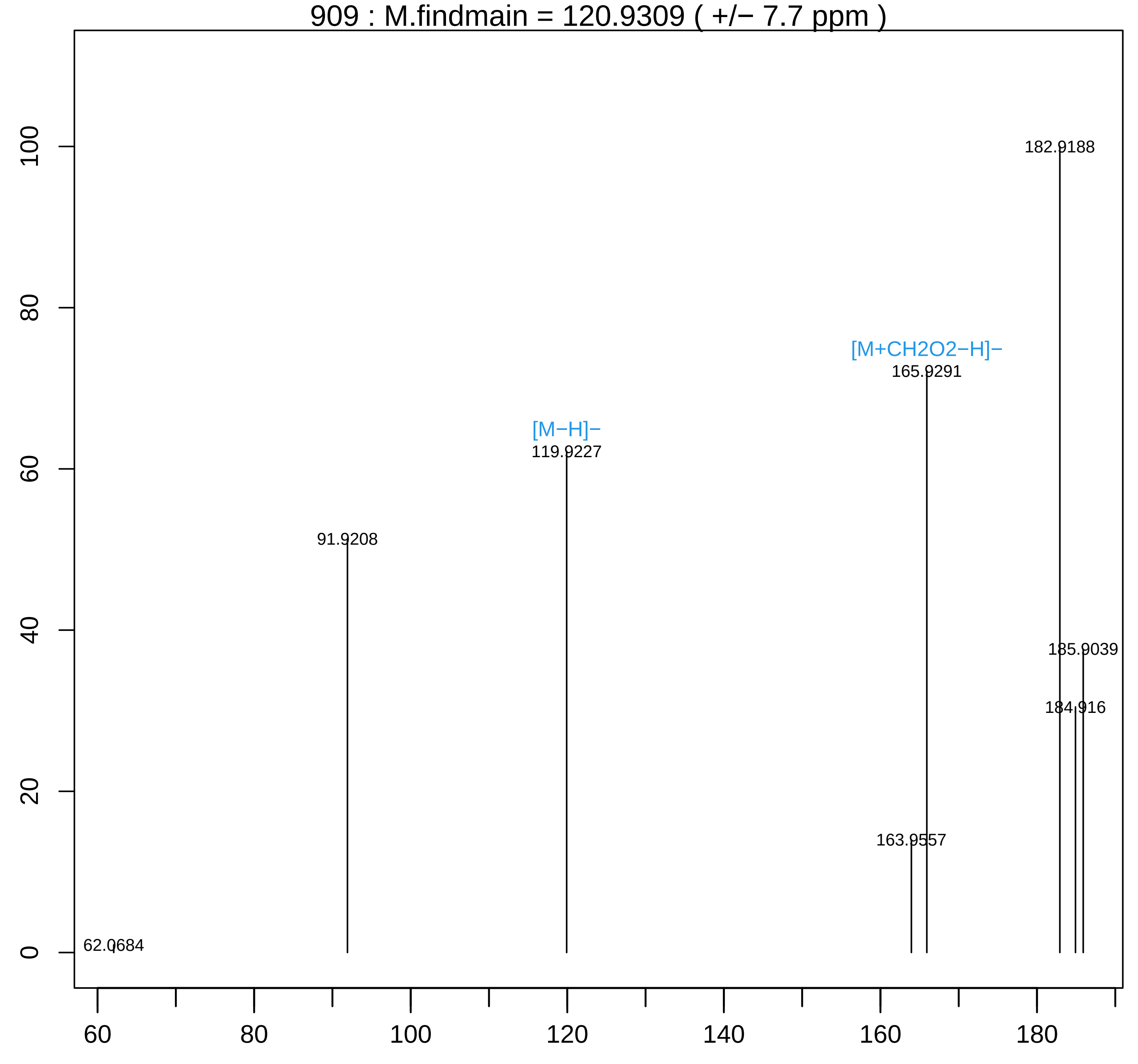
909 : M.findmain = 120.9309 (+/− 7.7 ppm)
[M−H]−
119.9227
[M+CH2O2−H]−
165.9291
182.9188
91.9208
185.9039
184.916
163.9557
62.0684

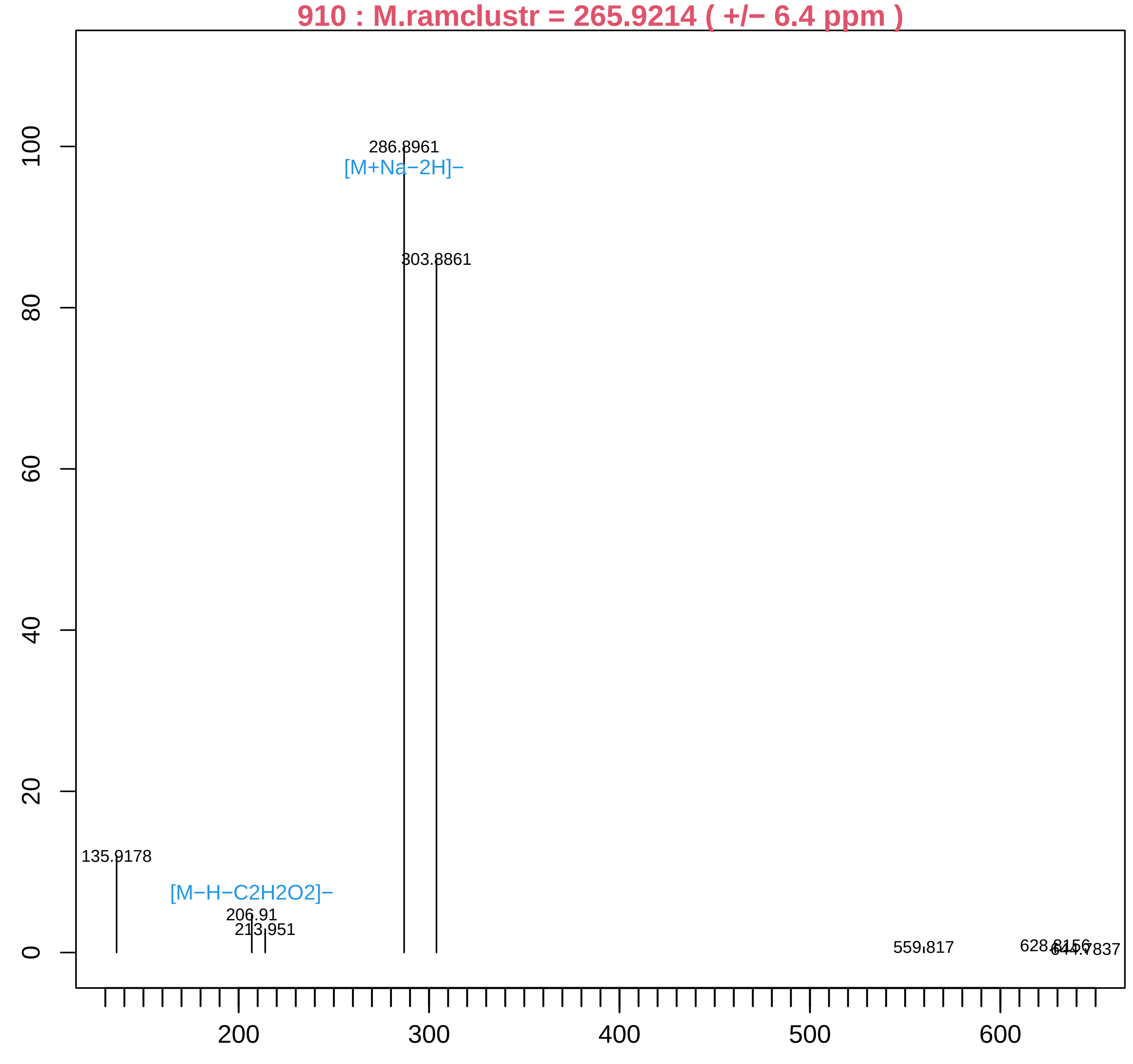

910 : M.ramclustr = 265.9214 (+/− 6.4 ppm)
286.8961
[M+Na−2H]−
303.8861
135.9178
[M−H−C2H2O2]−
206.91
213.951
559.817
628.8156
644.7837
0
20
40
60
80
100
200
300
400
500
600

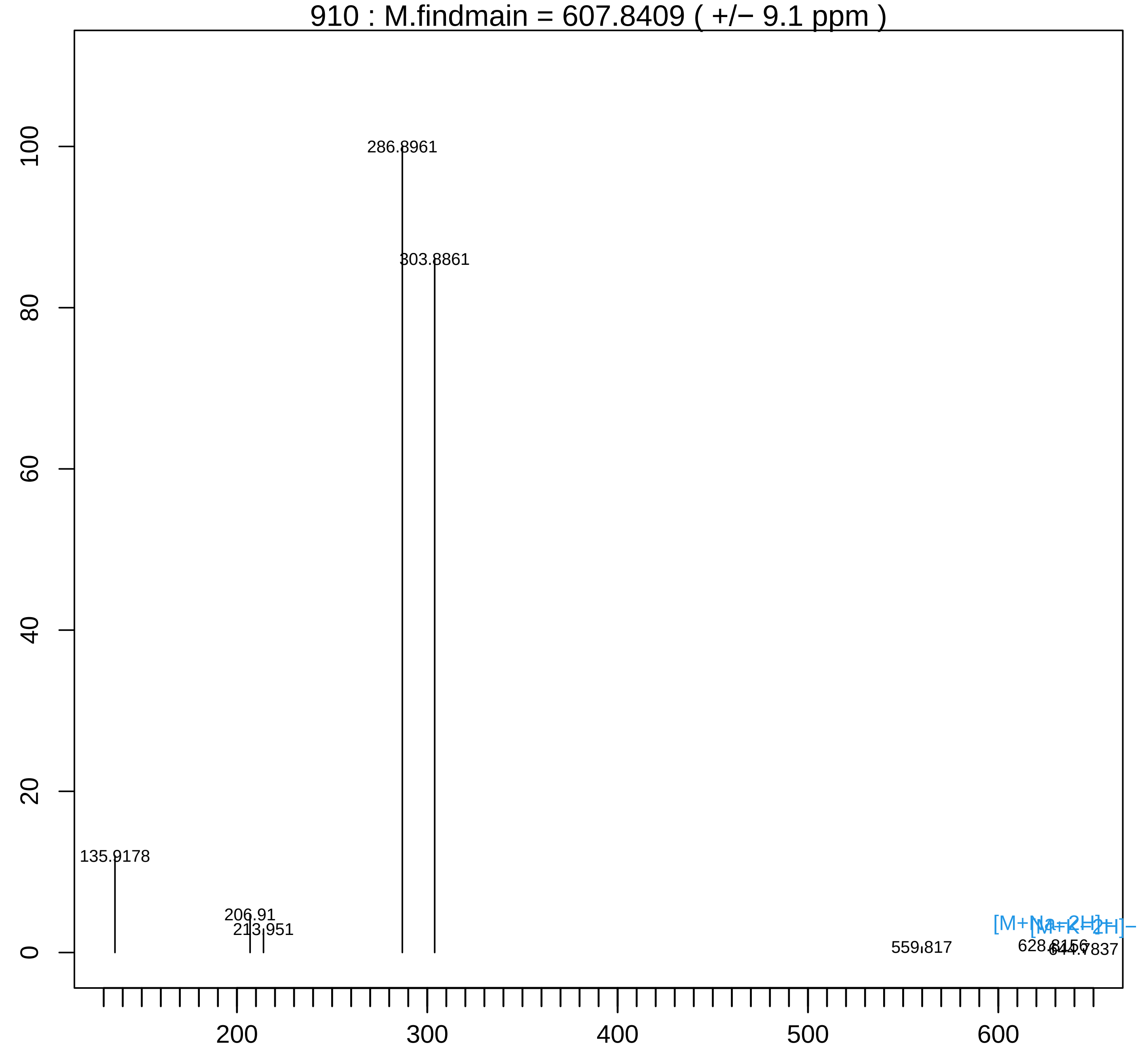

910 : M.findmain = 607.8409 (+/− 9.1 ppm)
286.8961
303.8861
135.9178
206.91
213.951
559.817
628.8156
644.7837
0
20
40
60
80
100
200
300
400
500
600

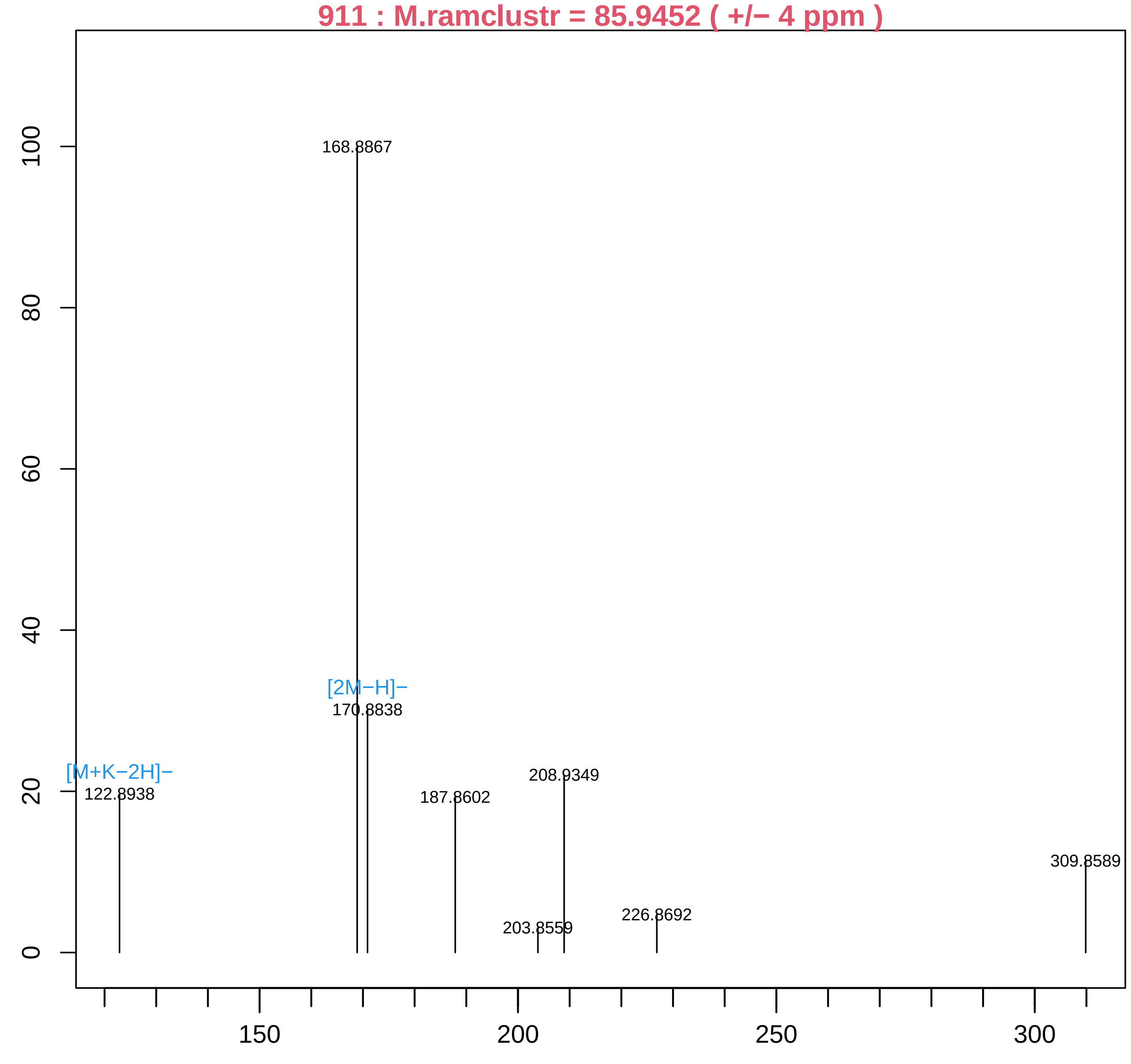
911 : M.ramclustr = 85.9452 (+/− 4 ppm)
168.8867
[2M−H]−
170.8838
[M+K−2H]−
122.8938
187.8602
208.9349
203.8559
226.8692
309.8589
0
20
40
60
80
100
150
200
250
300

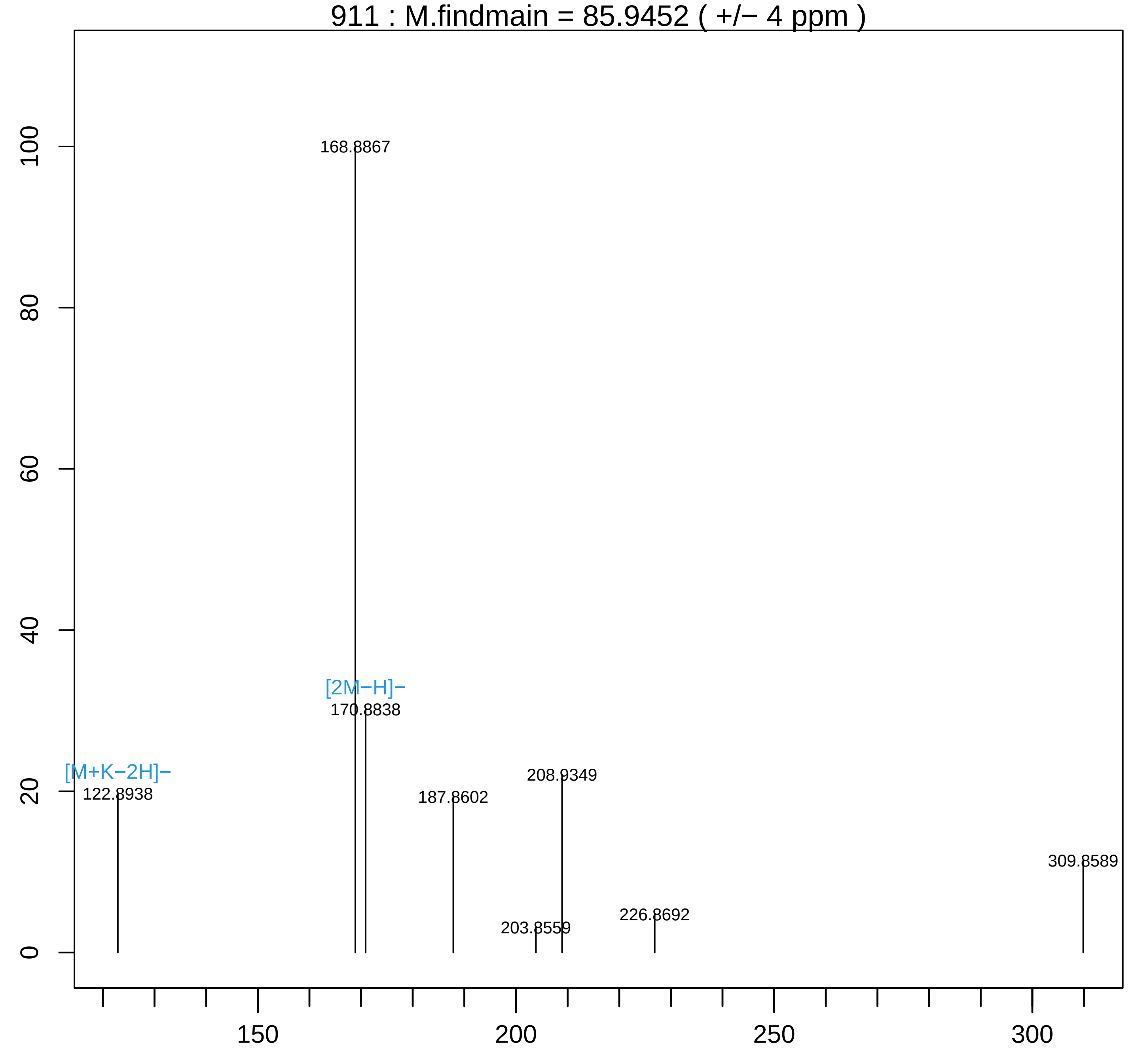
911 : M.findmain = 85.9452 (+/− 4 ppm)
168.8867
[2M−H]−
170.8838
[M+K−2H]−
122.8938
187.8602
208.9349
203.8559
226.8692
309.8589
0
20
40
60
80
100
150
200
250
300

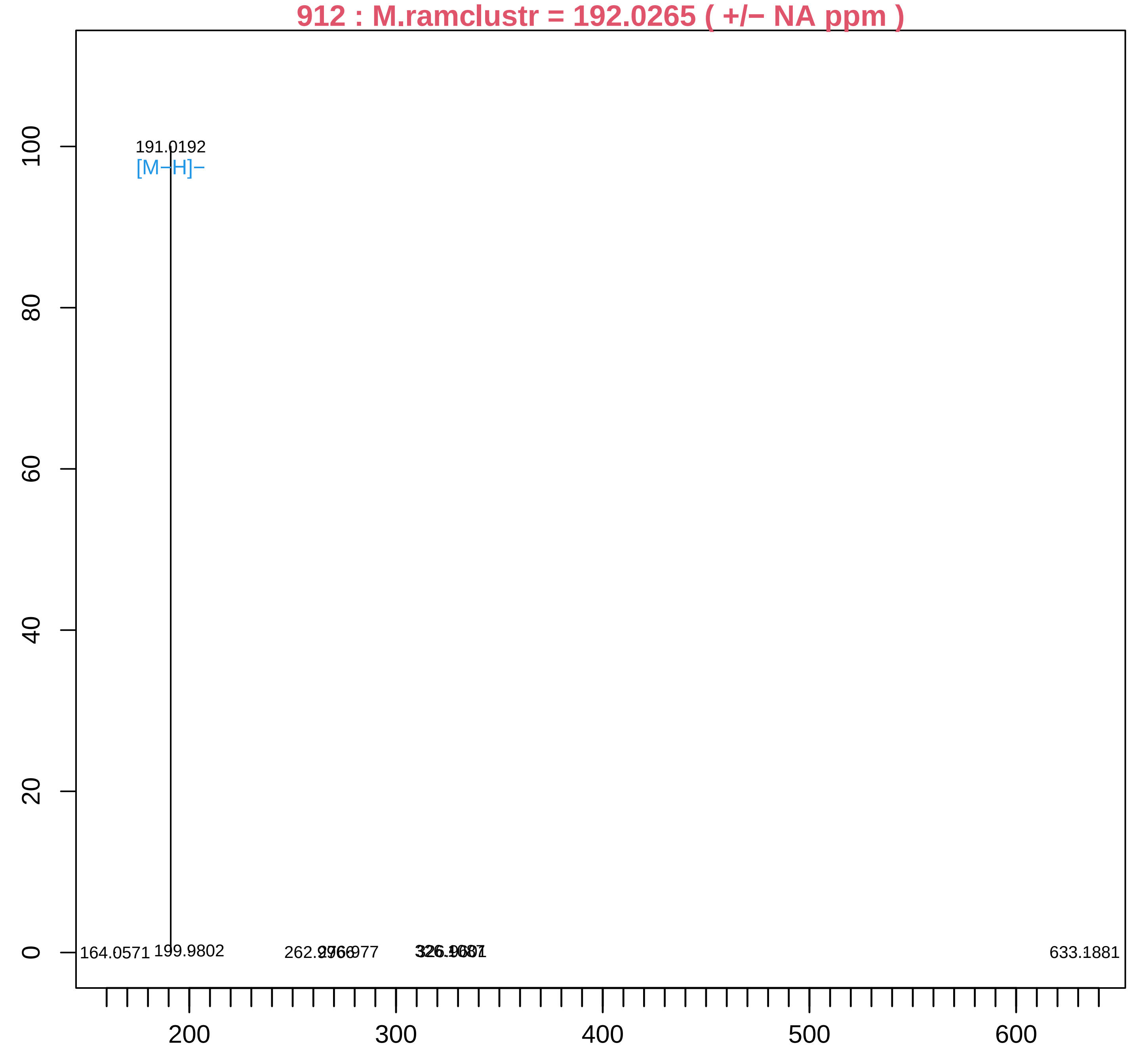
912 : M.ramclustr = 192.0265 (+/− NA ppm)
191.0192
[M−H]−
164.0571
199.9802
262.9766
276.977
326.9681
326.9687
633.1881
0
20
40
60
80
100
200
300
400
500
600

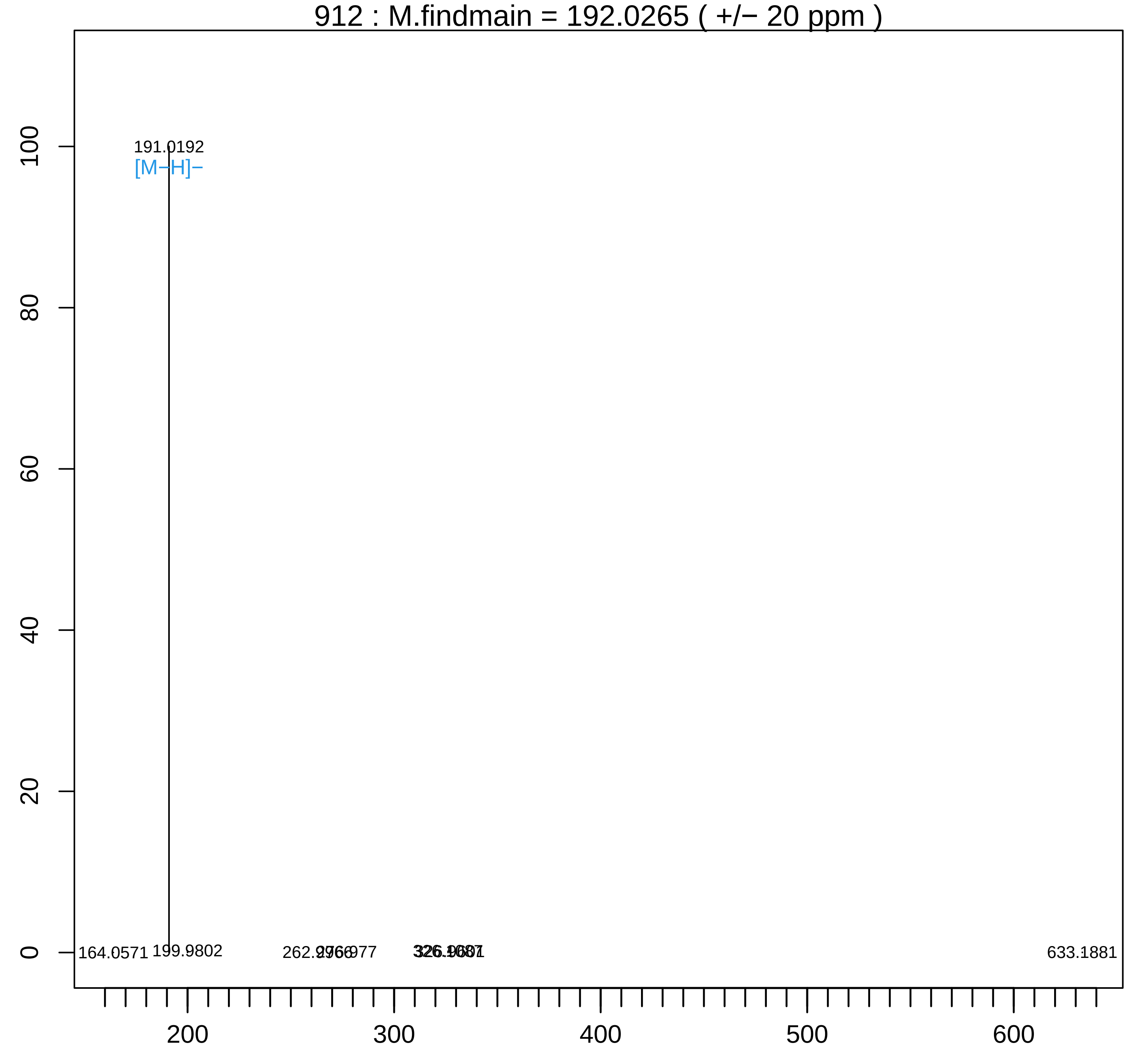
912 : M.findmain = 192.0265 (+/− 20 ppm)
191.0192
[M−H]−
164.0571
199.9802
262.9766
276.977
326.9681
326.9687
633.1881
0
20
40
60
80
100
200
300
400
500
600

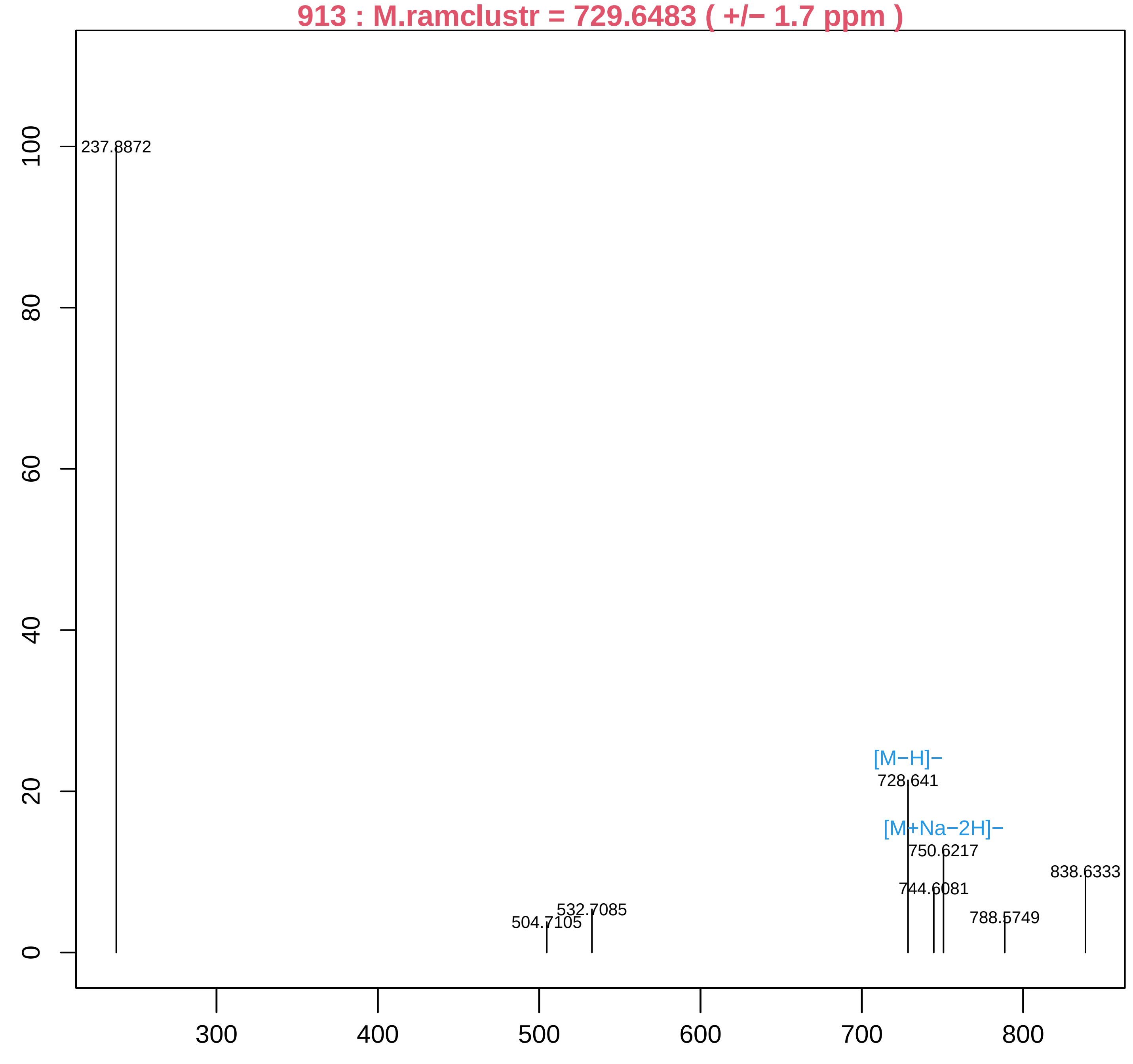
913 : M.ramclustr = 729.6483 (+/− 1.7 ppm)
237.8872
504.7105
532.7085
[M−H]−
728.641
[M+Na−2H]−
750.6217
744.6081
788.5749
838.6333
0
20
40
60
80
100
300
400
500
600
700
800

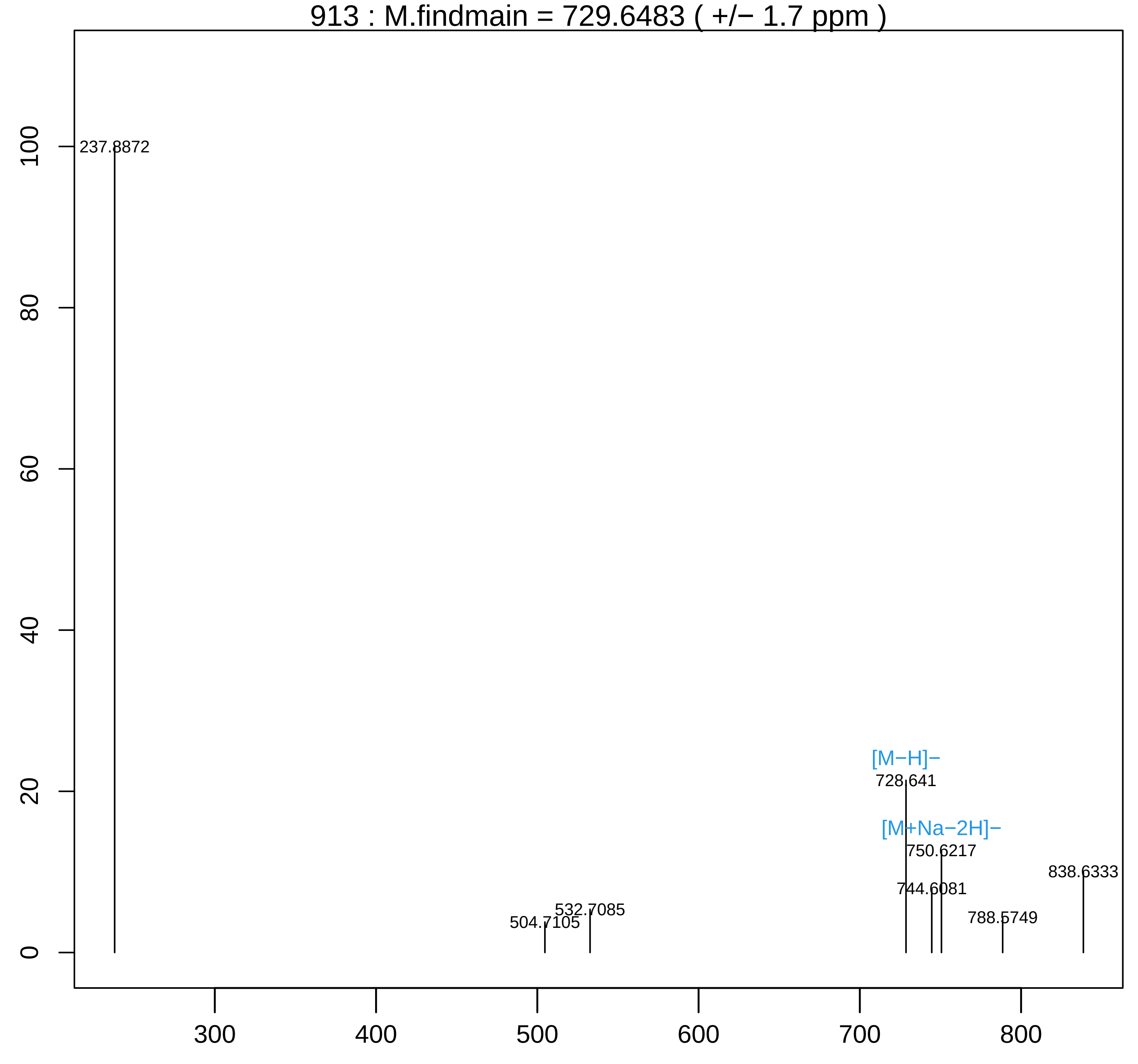
913 : M.findmain = 729.6483 (+/− 1.7 ppm)
237.8872
504.7105
532.7085
[M−H]−
728.641
[M+Na−2H]−
750.6217
744.6081
788.5749
838.6333
0
20
40
60
80
100
300
400
500
600
700
800

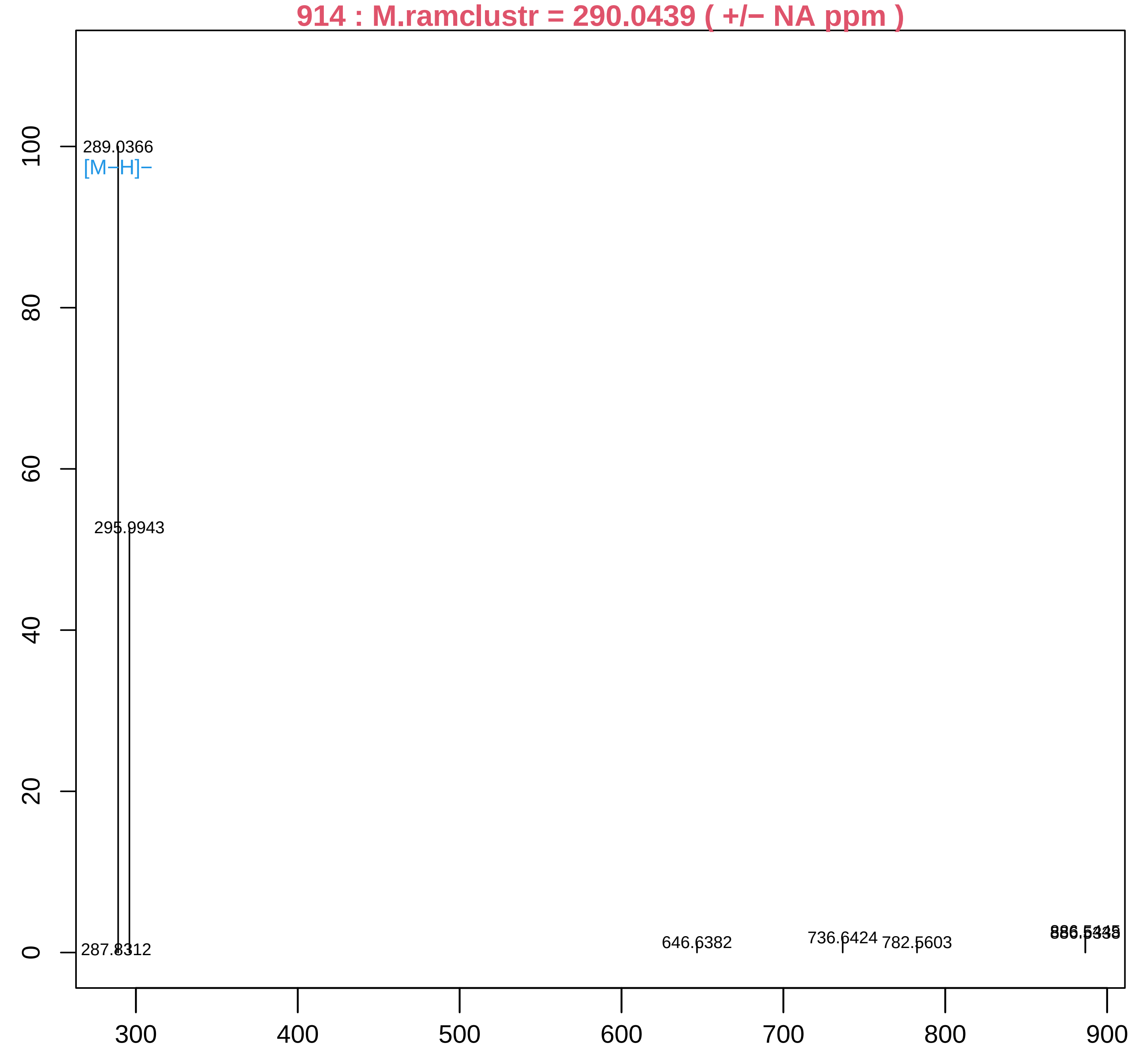
914 : M.ramclustr = 290.0439 (+/− NA ppm)
289.0366
[M−H]−
295.9943
287.8312
646.6382
736.6424
782.5603
0
20
40
60
80
100
300
400
500
600
700
800
900

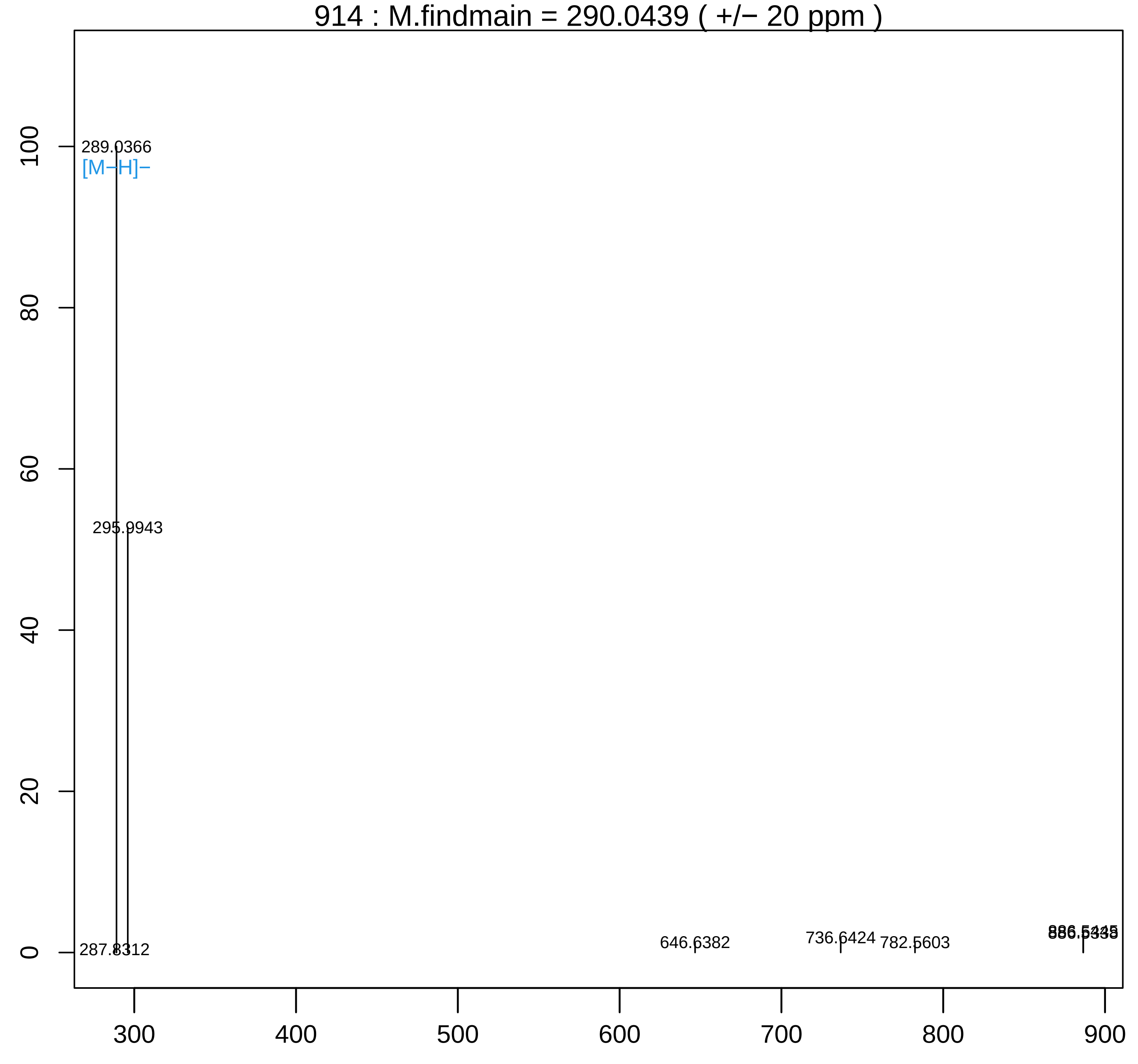
914 : M.findmain = 290.0439 (+/− 20 ppm)
289.0366
[M−H]−
295.9943
287.8312
646.6382
736.6424
782.5603
0
20
40
60
80
100
300
400
500
600
700
800
900

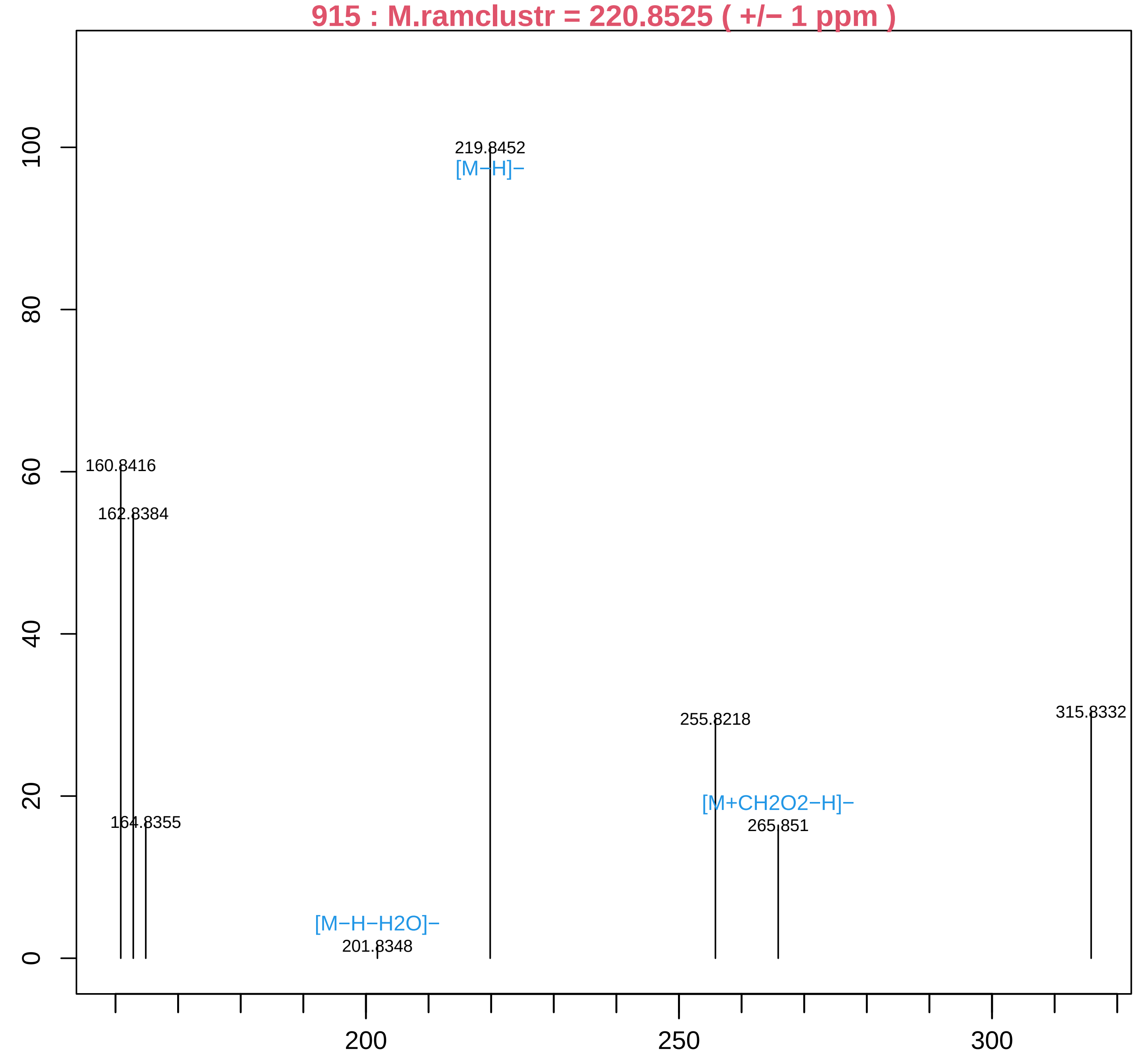
915 : M.ramclustr = 220.8525 (+/− 1 ppm)
219.8452
[M−H]−
160.8416
162.8384
164.8355
[M−H−H2O]−
201.8348
255.8218
[M+CH2O2−H]−
265.851
315.8332
0
20
40
60
80
100
200
250
300

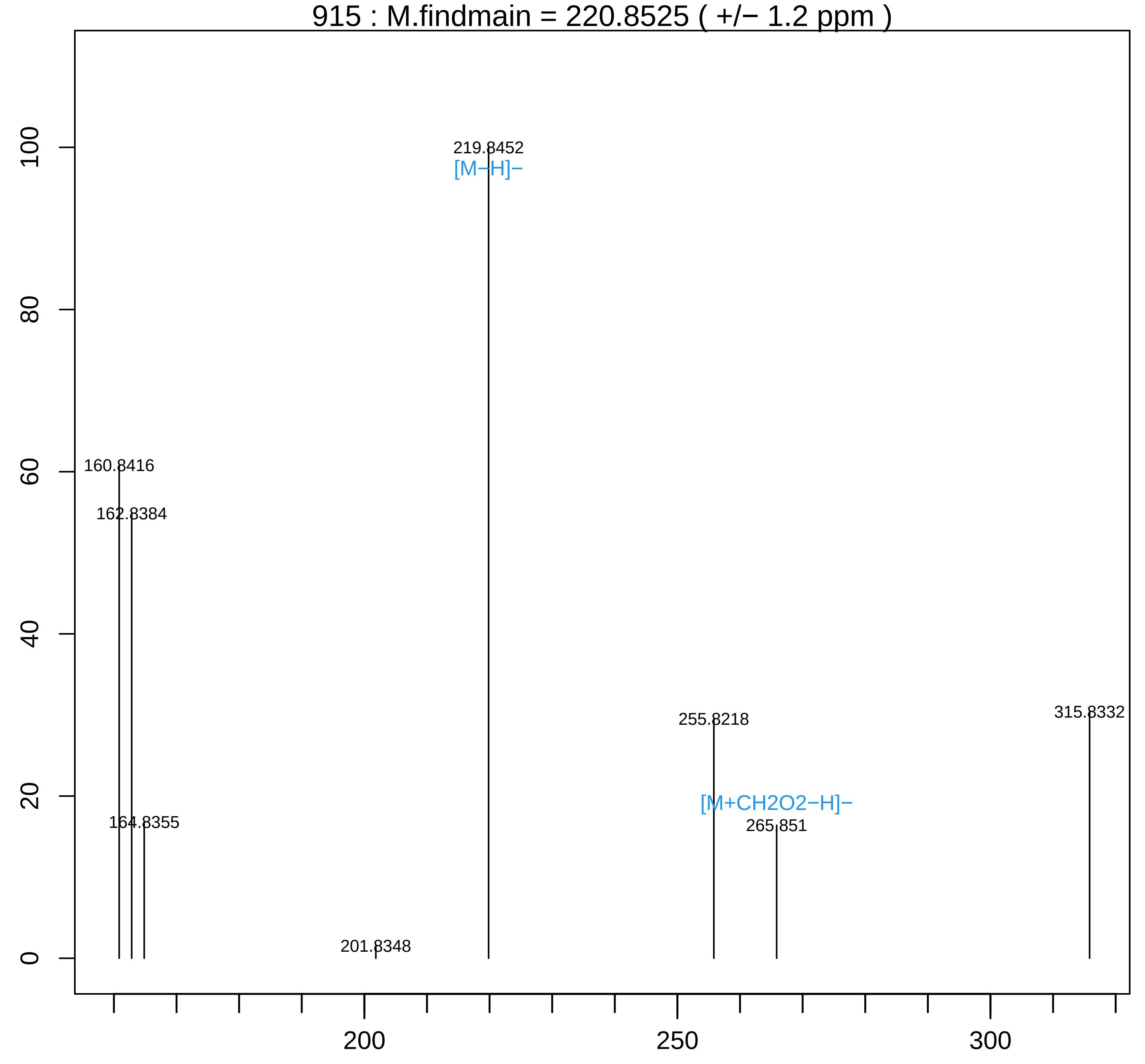
915 : M.findmain = 220.8525 (+/− 1.2 ppm)
219.8452
[M−H]−
160.8416
162.8384
164.8355
201.8348
255.8218
[M+CH2O2−H]−
265.851
315.8332
0
20
40
60
80
100
200
250
300

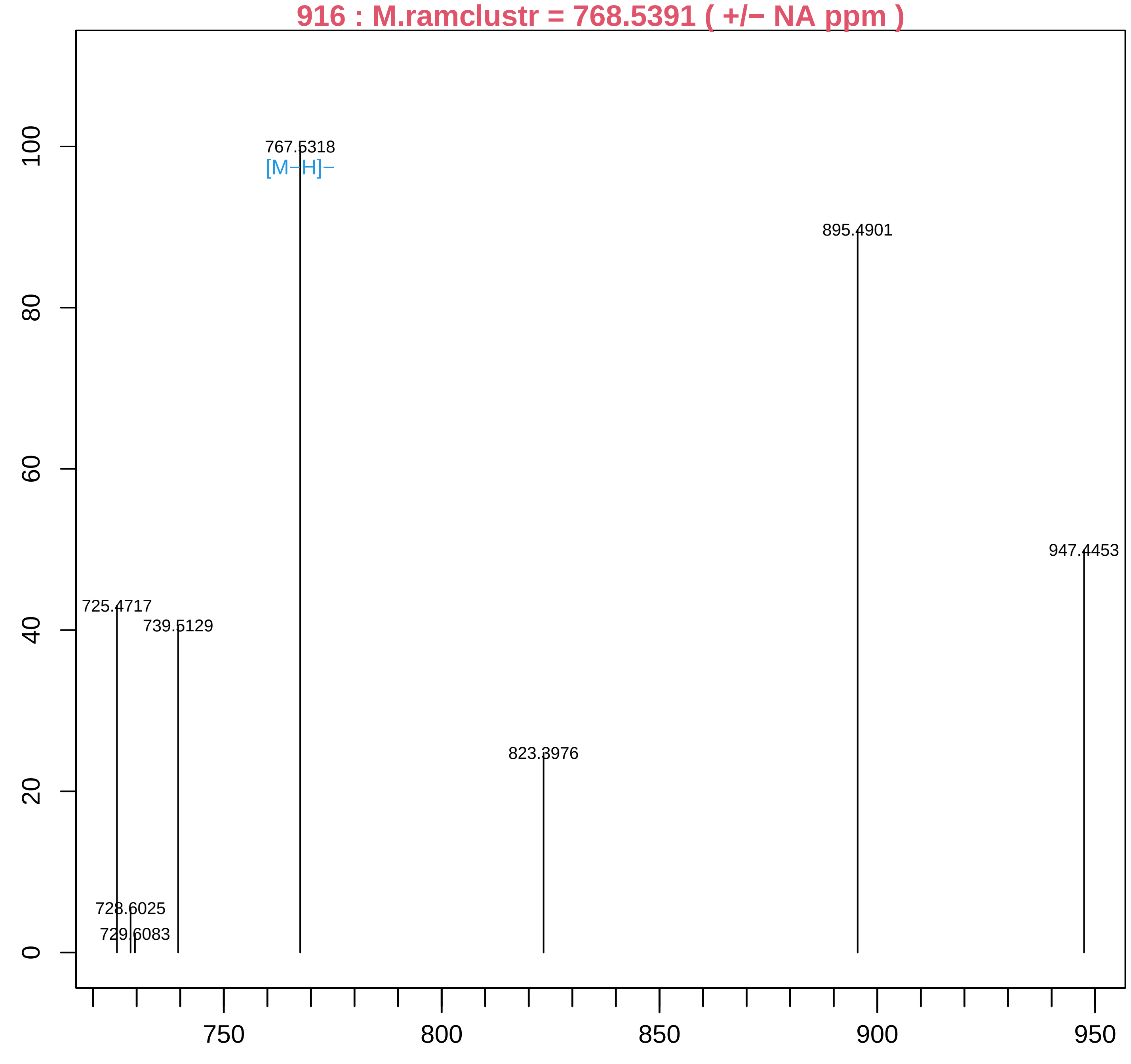
916 : M.ramclustr = 768.5391 (+/− NA ppm)
767.5318
[M−H]−
895.4901
947.4453
725.4717
739.5129
823.3976
728.6025
729.6083
0
20
40
60
80
100
750
800
850
900
950

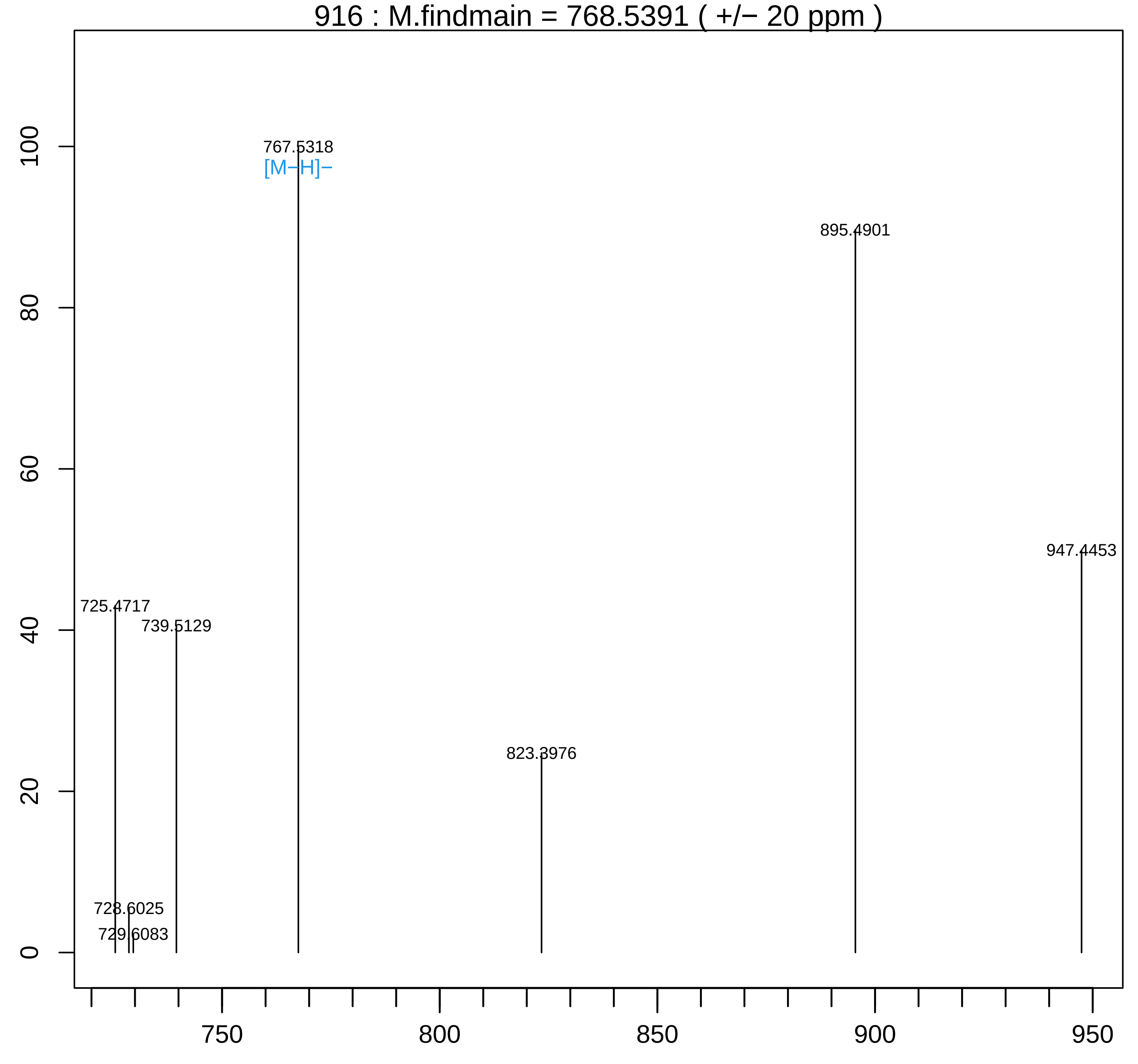
916 : M.findmain = 768.5391 (+/− 20 ppm)
767.5318
[M−H]−
895.4901
947.4453
725.4717
739.5129
823.3976
728.6025
729.6083
0
20
40
60
80
100
750
800
850
900
950

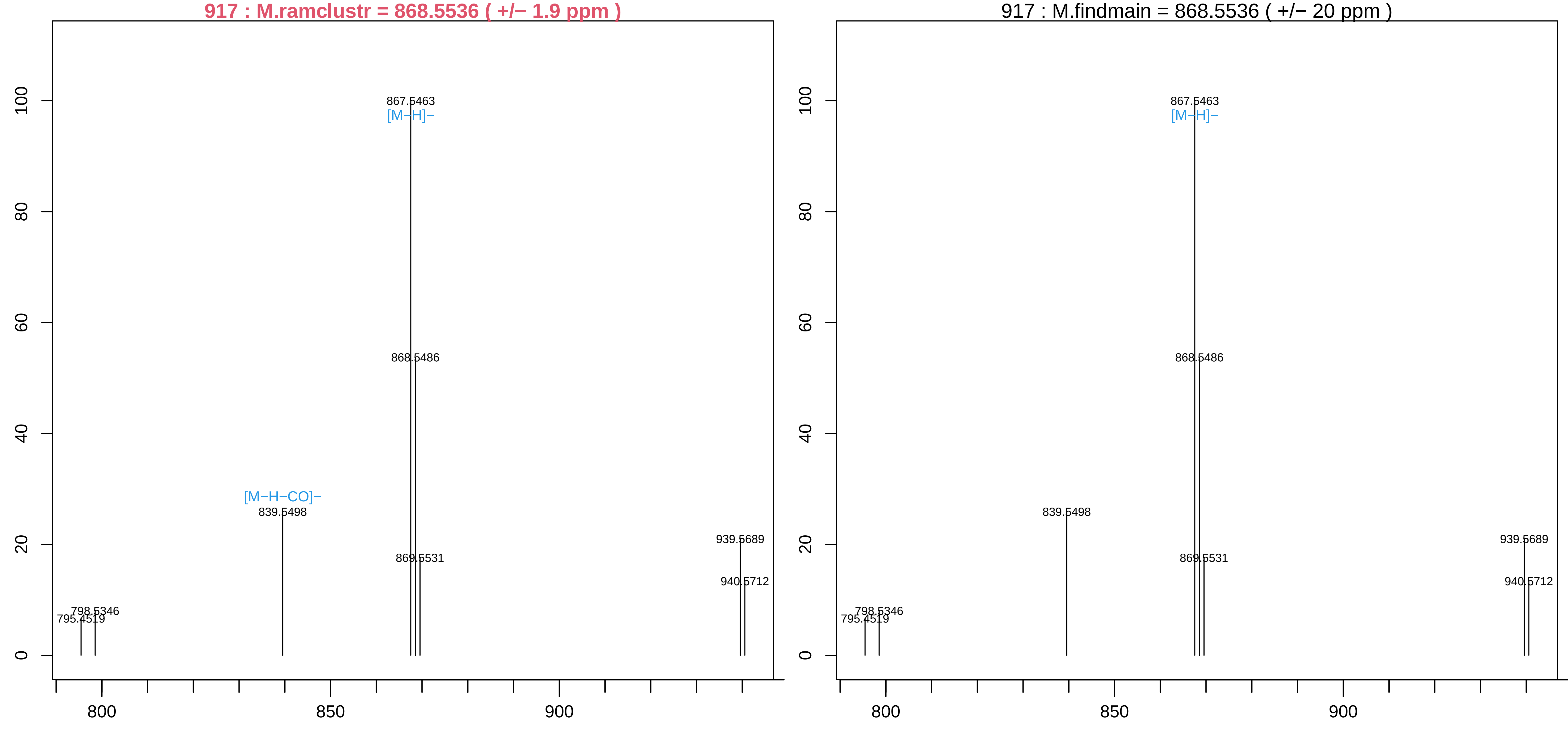

917 : M.ramclustr = 868.5536 (+/− 1.9 ppm)
867.5463
[M−H]−
868.5486
[M−H−CO]−
839.5498
869.5531
939.5689
940.5712
798.5346
795.4519
917 : M.findmain = 868.5536 (+/− 20 ppm)
867.5463
[M−H]−
868.5486
839.5498
869.5531
939.5689
940.5712
798.5346
795.4519
0
20
40
60
80
100
800
850
900

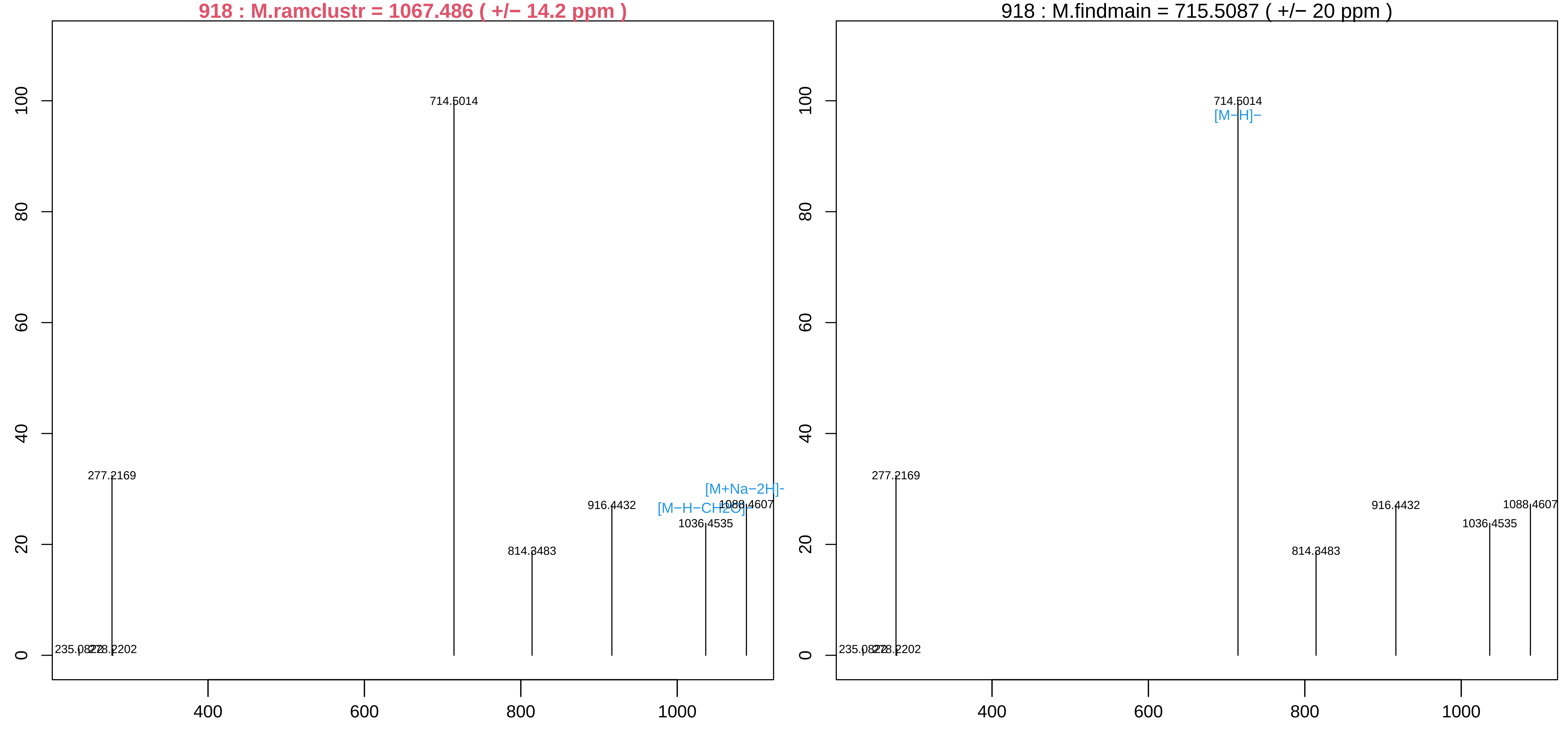

918 : M.ramclustr = 1067.486 (+/− 14.2 ppm)
714.5014
277.2169
[M+Na−2H]-
916.4432
[M−H−CH2O]-
1088.4607
1036.4535
814.3483
235.0877
278.2202
918 : M.findmain = 715.5087 (+/− 20 ppm)
714.5014
[M−H]−
277.2169
916.4432
1088.4607
1036.4535
814.3483
235.0877
278.2202

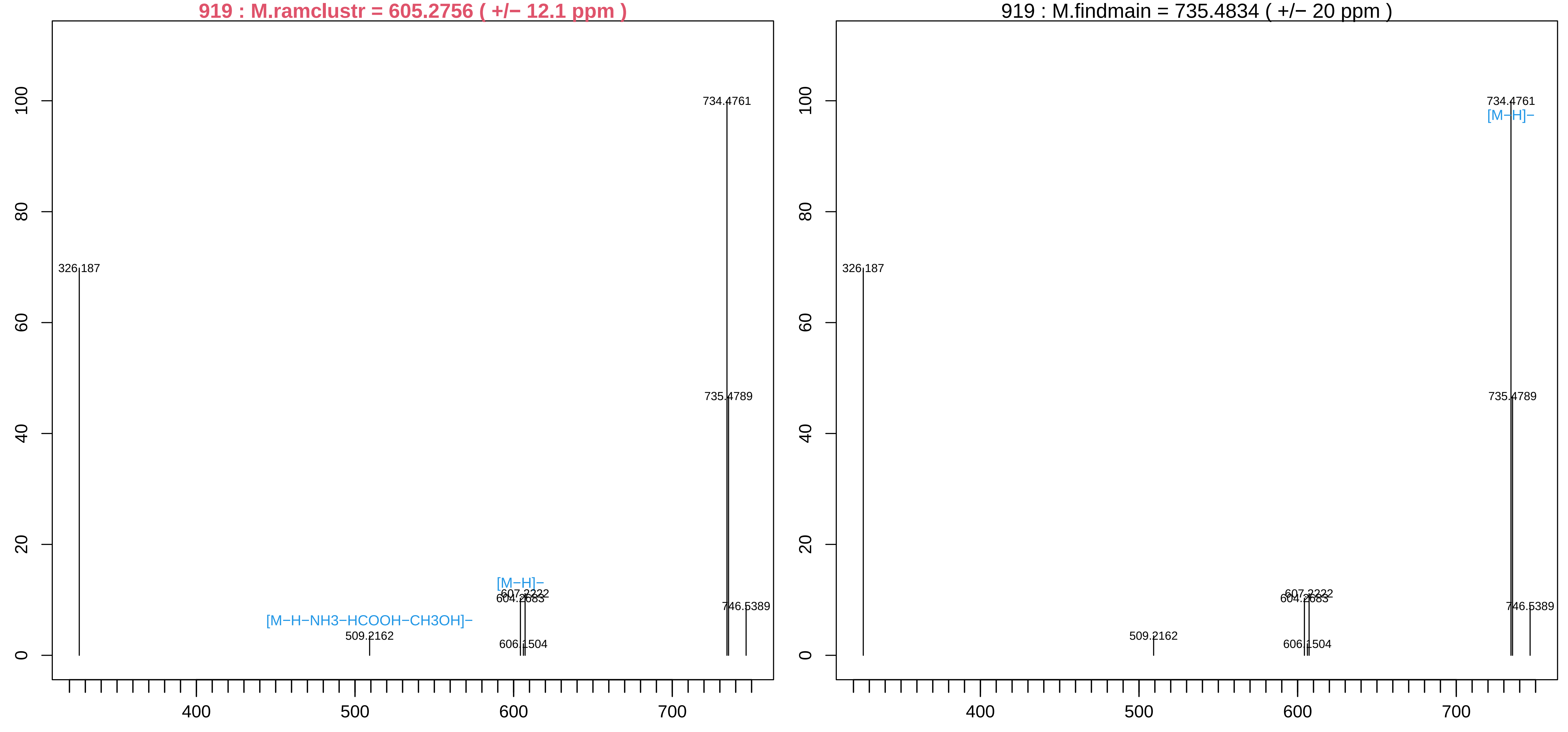

919 : M.ramclustr = 605.2756 (+/− 12.1 ppm)
734.4761
326.187
735.4789
[M−H]−
607.2222
604.2683
746.5389
[M−H−NH3−HCOOH−CH3OH]−
509.2162
606.1504
919 : M.findmain = 735.4834 (+/− 20 ppm)
734.4761
[M−H]−
326.187
735.4789
607.2222
604.2683
746.5389
509.2162
606.1504

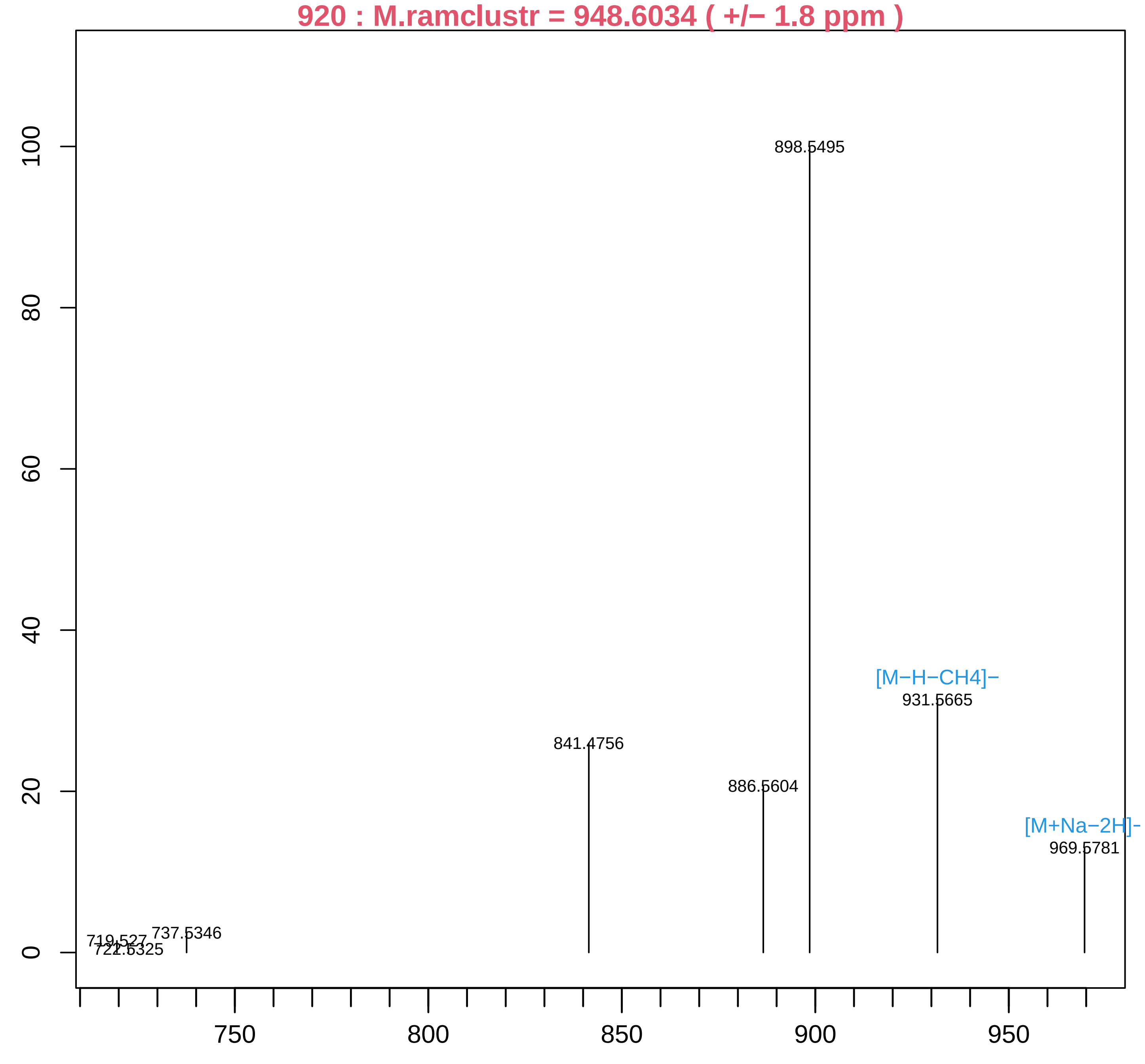

920 : M.ramclustr = 948.6034 (+/− 1.8 ppm)
898.5495
[M−H−CH4]−
931.5665
841.4756
886.5604
[M+Na−2H]−
969.5781
719.527
721.5325
737.5346
0
20
40
60
80
100
750
800
850
900
950

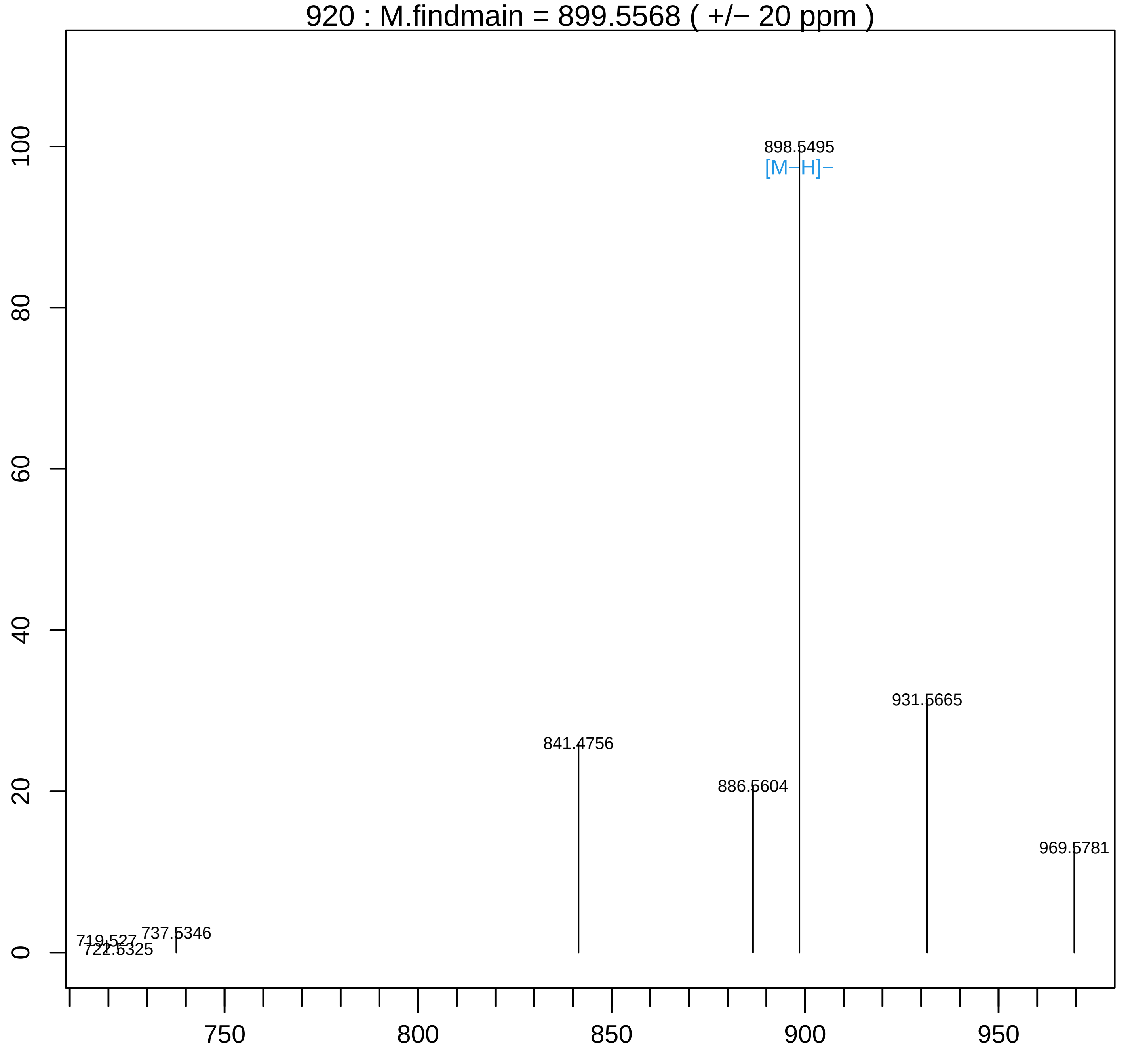

920 : M.findmain = 899.5568 (+/− 20 ppm)
898.5495
[M−H]−
931.5665
841.4756
886.5604
969.5781
719.527
721.5325
737.5346
0
20
40
60
80
100
750
800
850
900
950

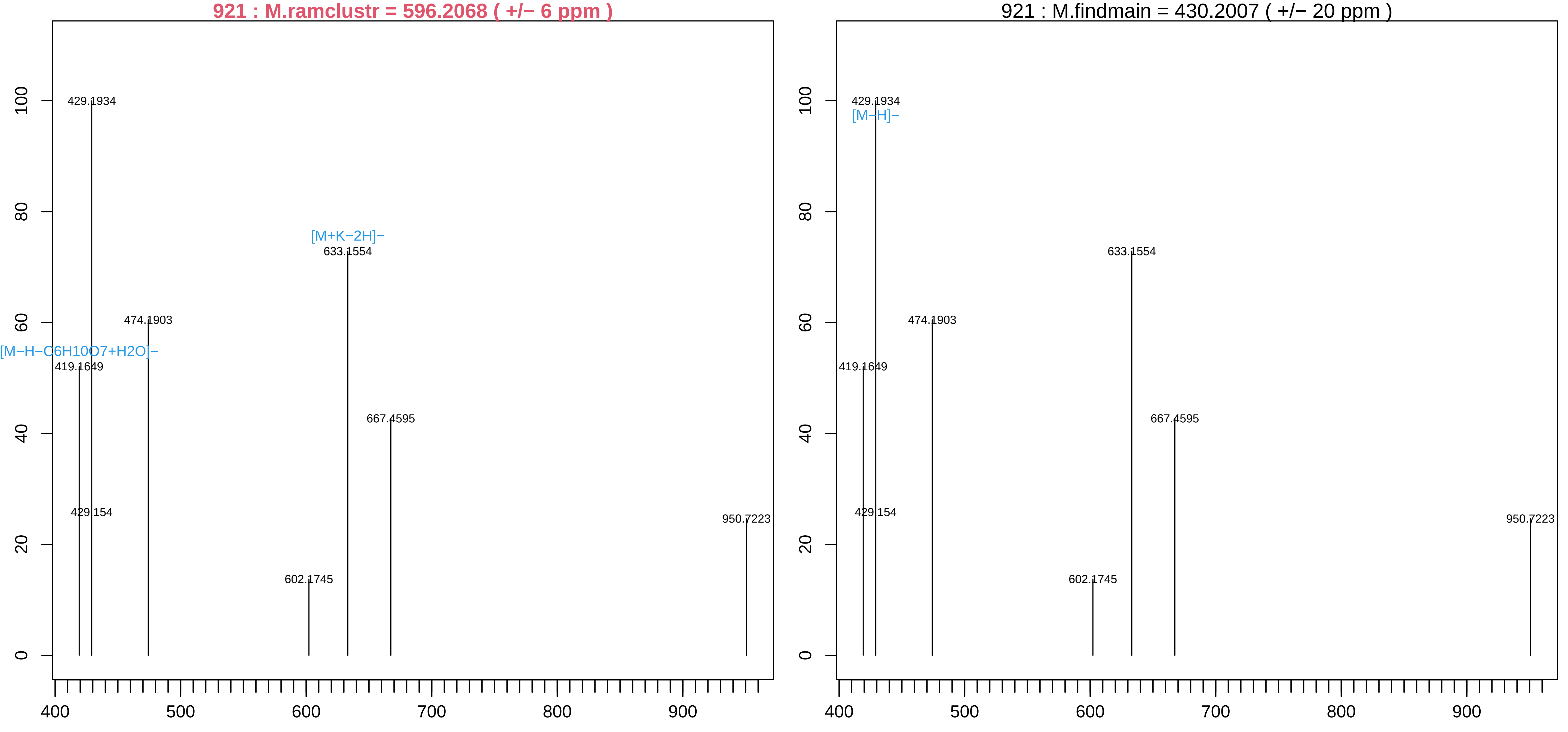
921 : M.ramclustr = 596.2068 (+/− 6 ppm)
429.1934
[M+K−2H]−
633.1554
474.1903
[M−H−C6H10O7+H2O]−
419.1649
667.4595
429.154
950.7223
602.1745
921 : M.findmain = 430.2007 (+/− 20 ppm)
429.1934
[M−H]−
633.1554
474.1903
419.1649
667.4595
429.154
950.7223
602.1745
0
20
40
60
80
100
400
500
600
700
800
900

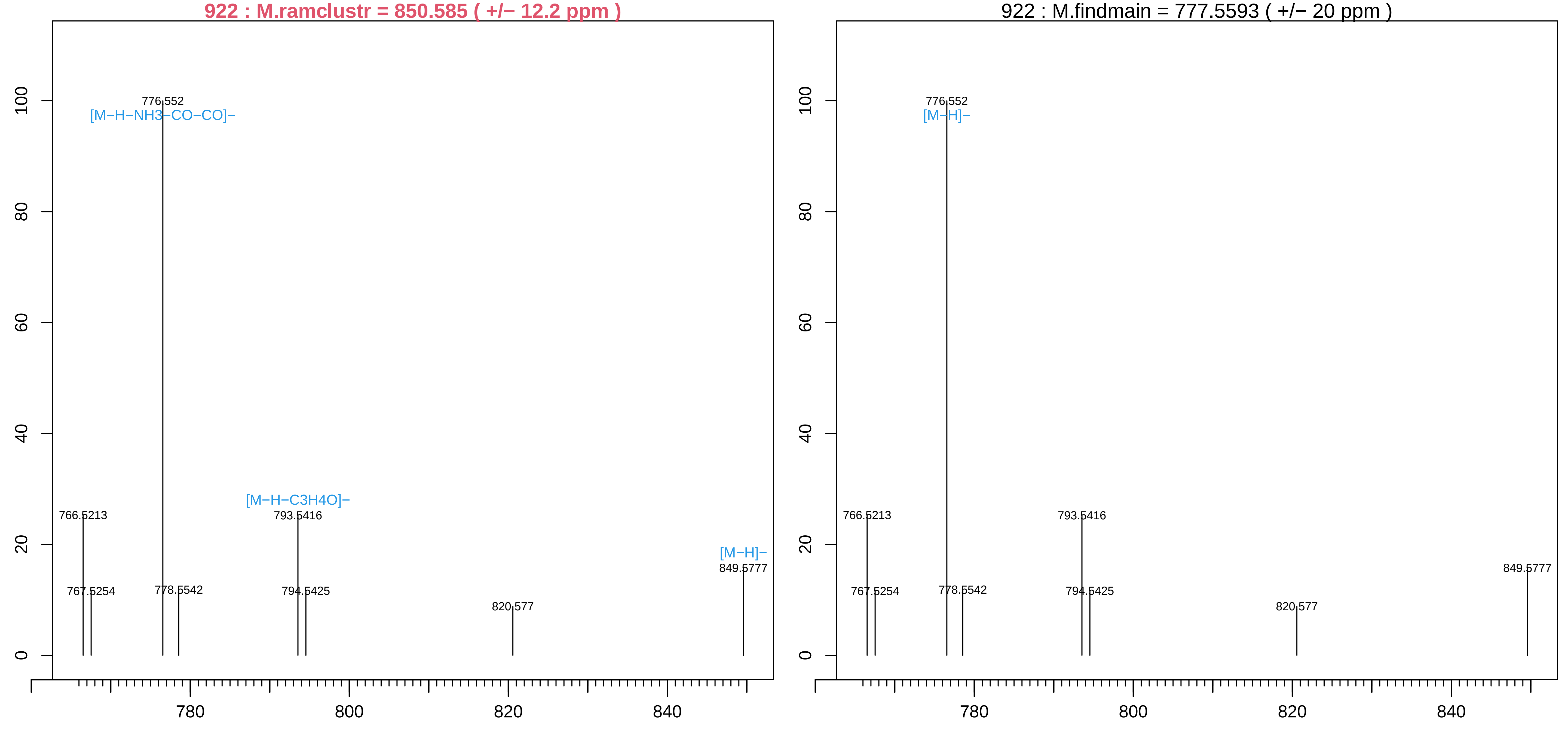
922 : M.ramclustr = 850.585 (+/− 12.2 ppm)
776.552
[M−H−NH3−CO−CO]−
[M−H−C3H4O]−
766.5213
793.5416
[M−H]−
849.5777
767.5254
778.5542
794.5425
820.577
922 : M.findmain = 777.5593 (+/− 20 ppm)
776.552
[M−H]−
766.5213
793.5416
849.5777
767.5254
778.5542
794.5425
820.577
0
20
40
60
80
100
780
800
820
840

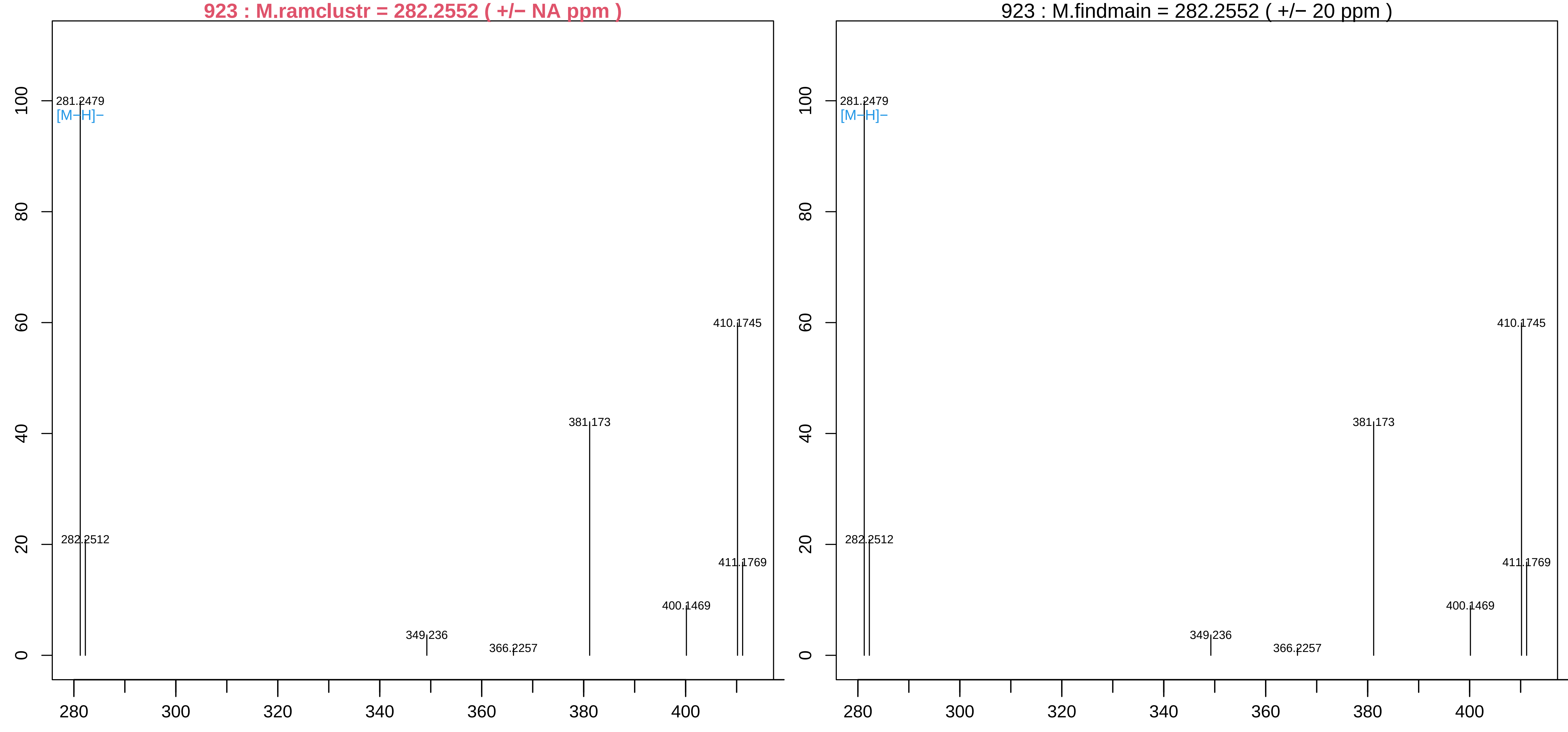

923 : M.ramclustr = 282.2552 (+/− NA ppm)
281.2479
[M−H]−
282.2512
349.236
366.2257
381.173
400.1469
410.1745
411.1769
0
20
40
60
80
100
280
300
320
340
360
380
400
923 : M.findmain = 282.2552 (+/− 20 ppm)
281.2479
[M−H]−
282.2512
349.236
366.2257
381.173
400.1469
410.1745
411.1769
0
20
40
60
80
100
280
300
320
340
360
380
400

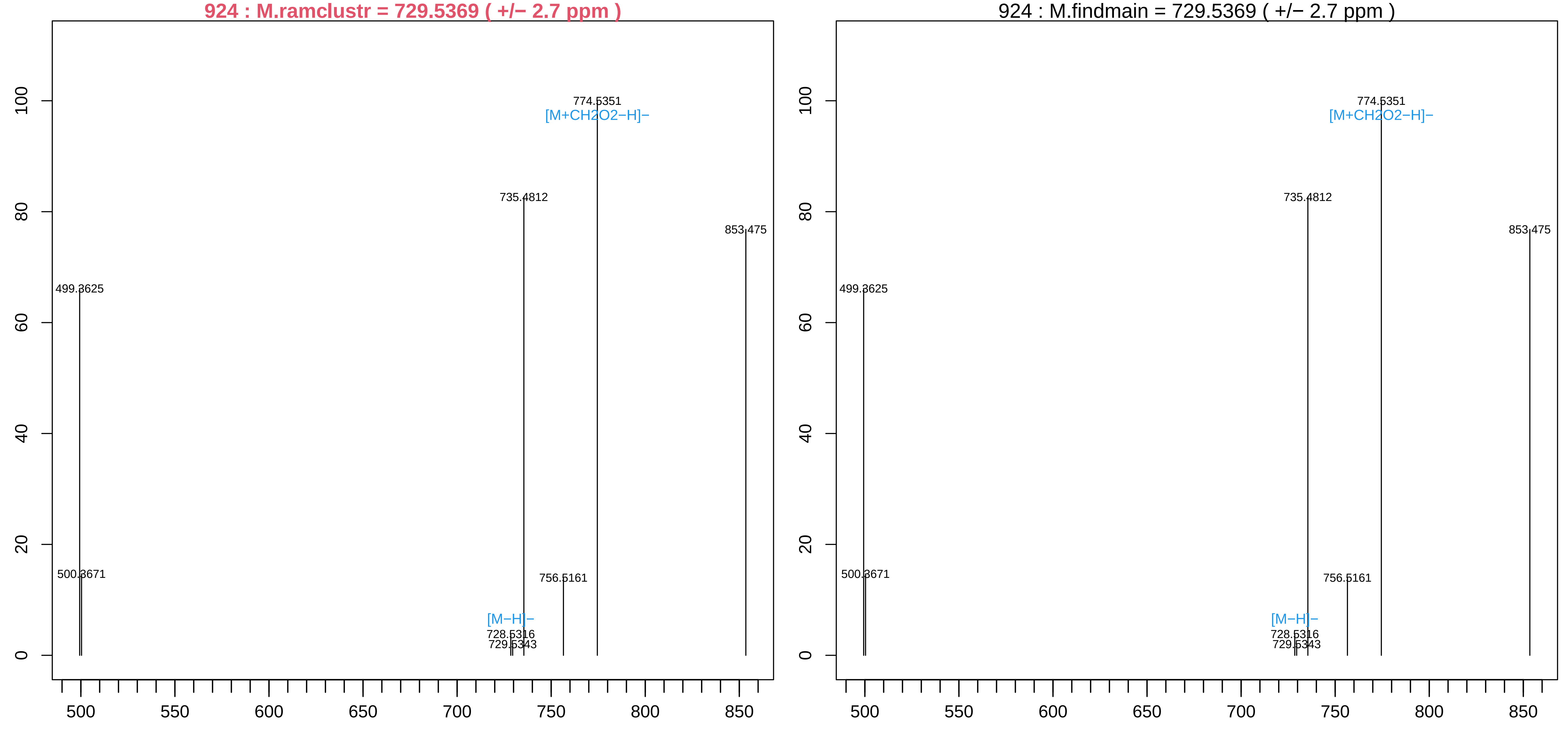
924 : M.ramclustr = 729.5369 (+/− 2.7 ppm)
774.5351
[M+CH2O2−H]−
735.4812
853.475
499.3625
500.3671
756.5161
[M−H]−
728.5316
729.5343
924 : M.findmain = 729.5369 (+/− 2.7 ppm)
774.5351
[M+CH2O2−H]−
735.4812
853.475
499.3625
500.3671
756.5161
[M−H]−
728.5316
729.5343

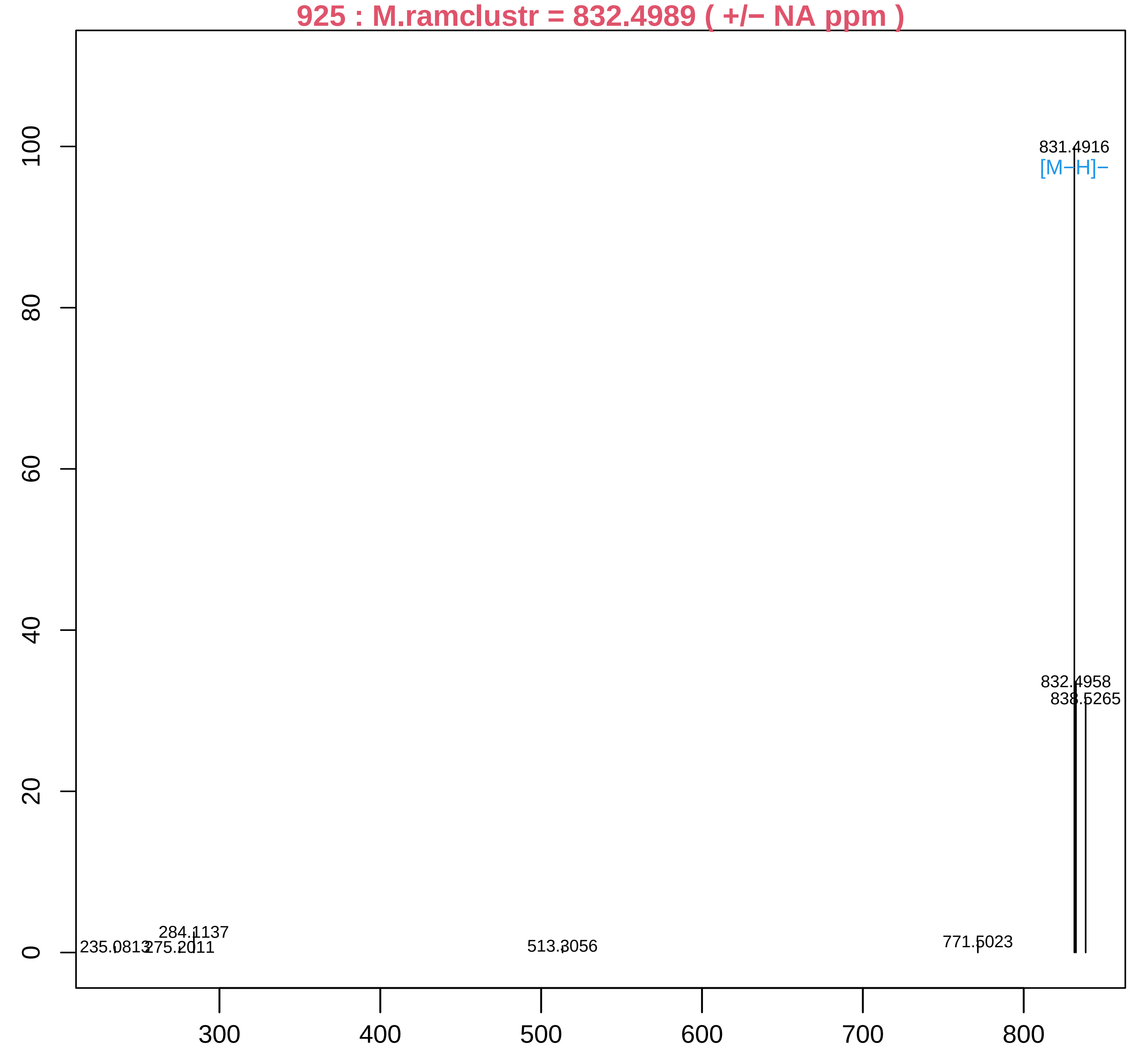

925 : M.ramclustr = 832.4989 (+/− NA ppm)
831.4916
[M−H]−
832.4958
838.5265
284.1137
235.0813
275.2011
513.3056
771.5023
0
20
40
60
80
100
300
400
500
600
700
800

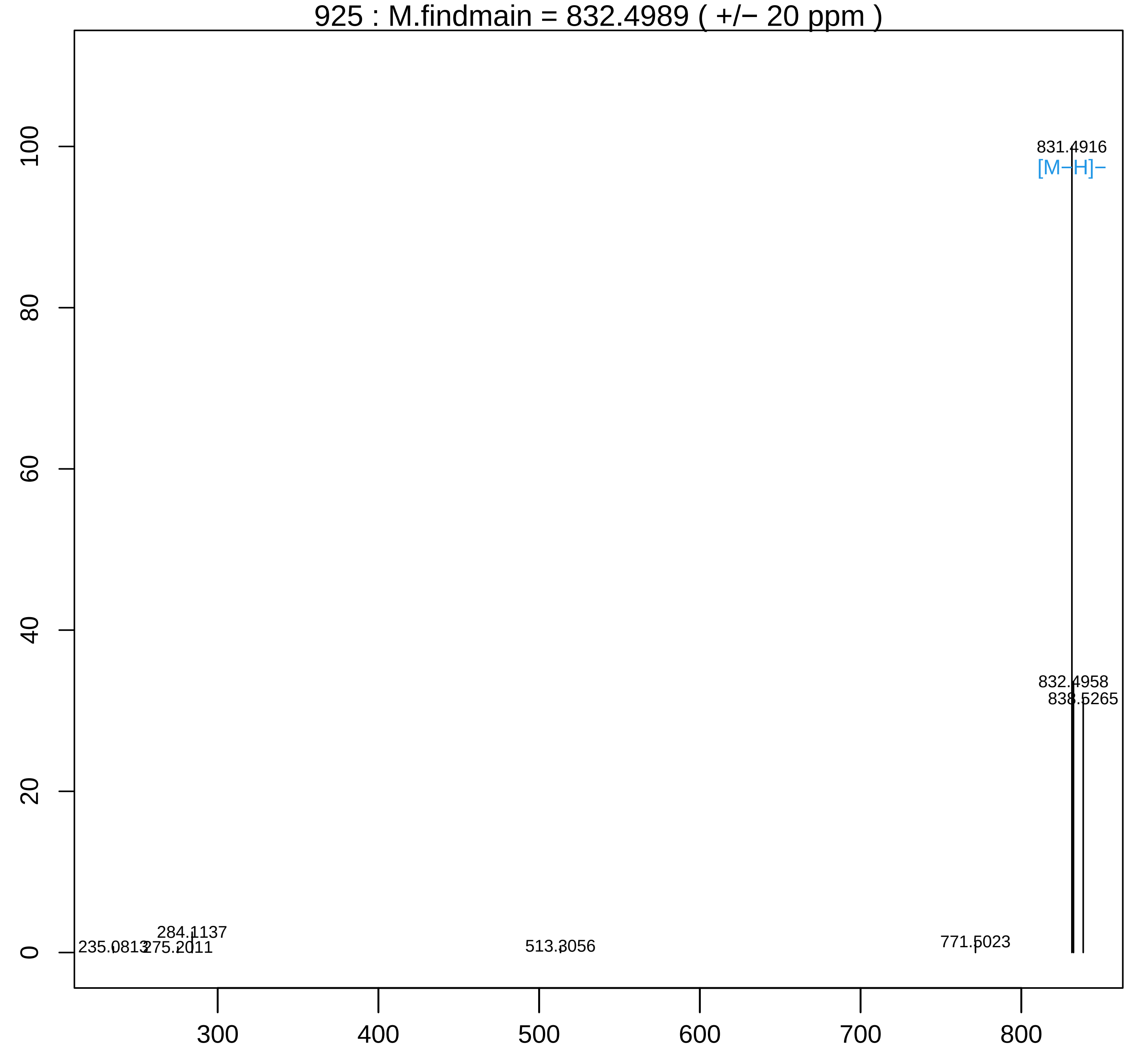

925 : M.findmain = 832.4989 (+/− 20 ppm)
831.4916
[M−H]−
832.4958
838.5265
284.1137
235.0813
275.2011
513.3056
771.5023
0
20
40
60
80
100
300
400
500
600
700
800

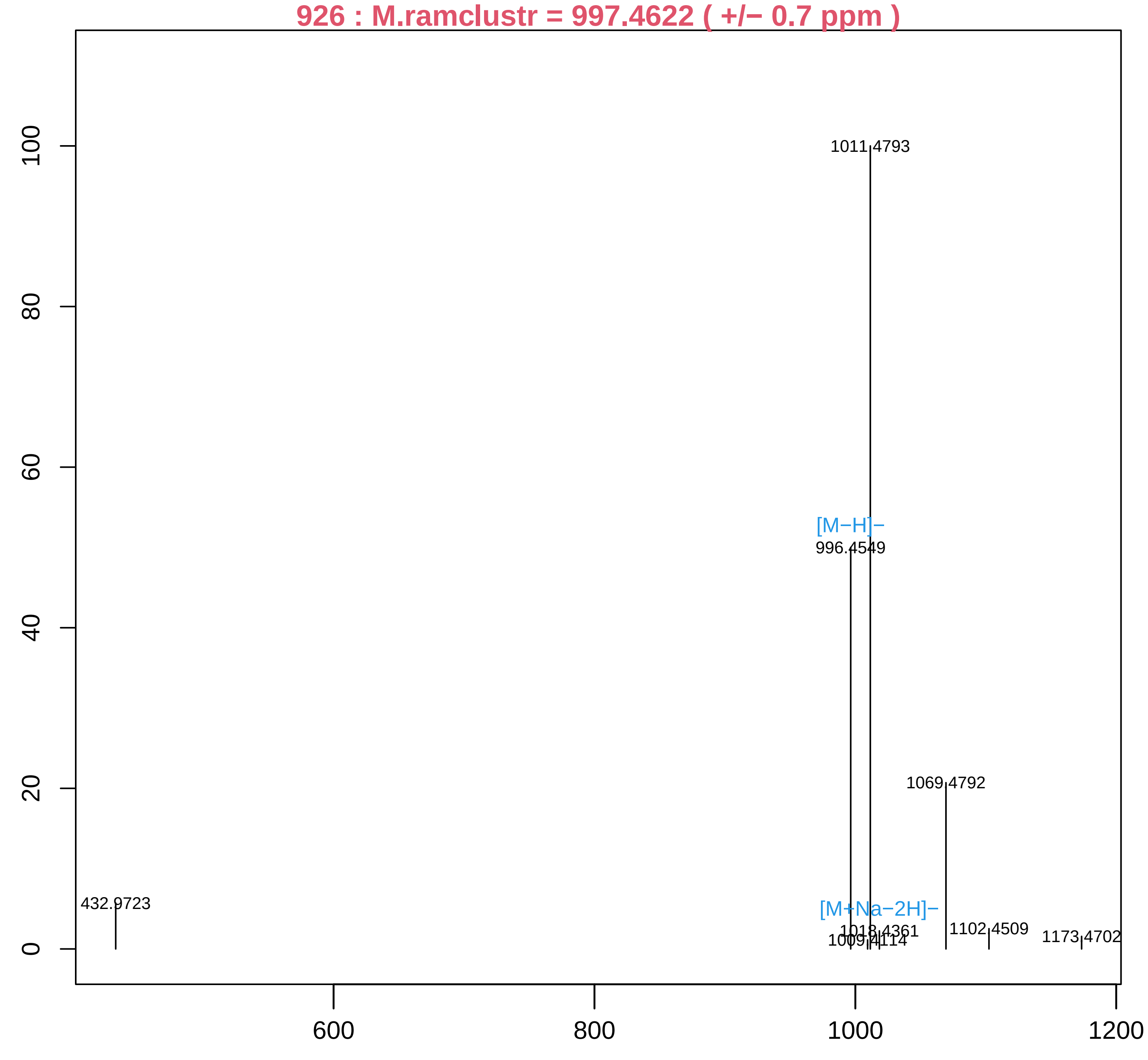

926 : M.ramclustr = 997.4622 (+/− 0.7 ppm)
1011.4793
[M−H]−
996.4549
1069.4792
432.9723
[M+Na−2H]−
1018.4361
1009.4114
1102.4509
1173.4702
0
20
40
60
80
100
600
800
1000
1200

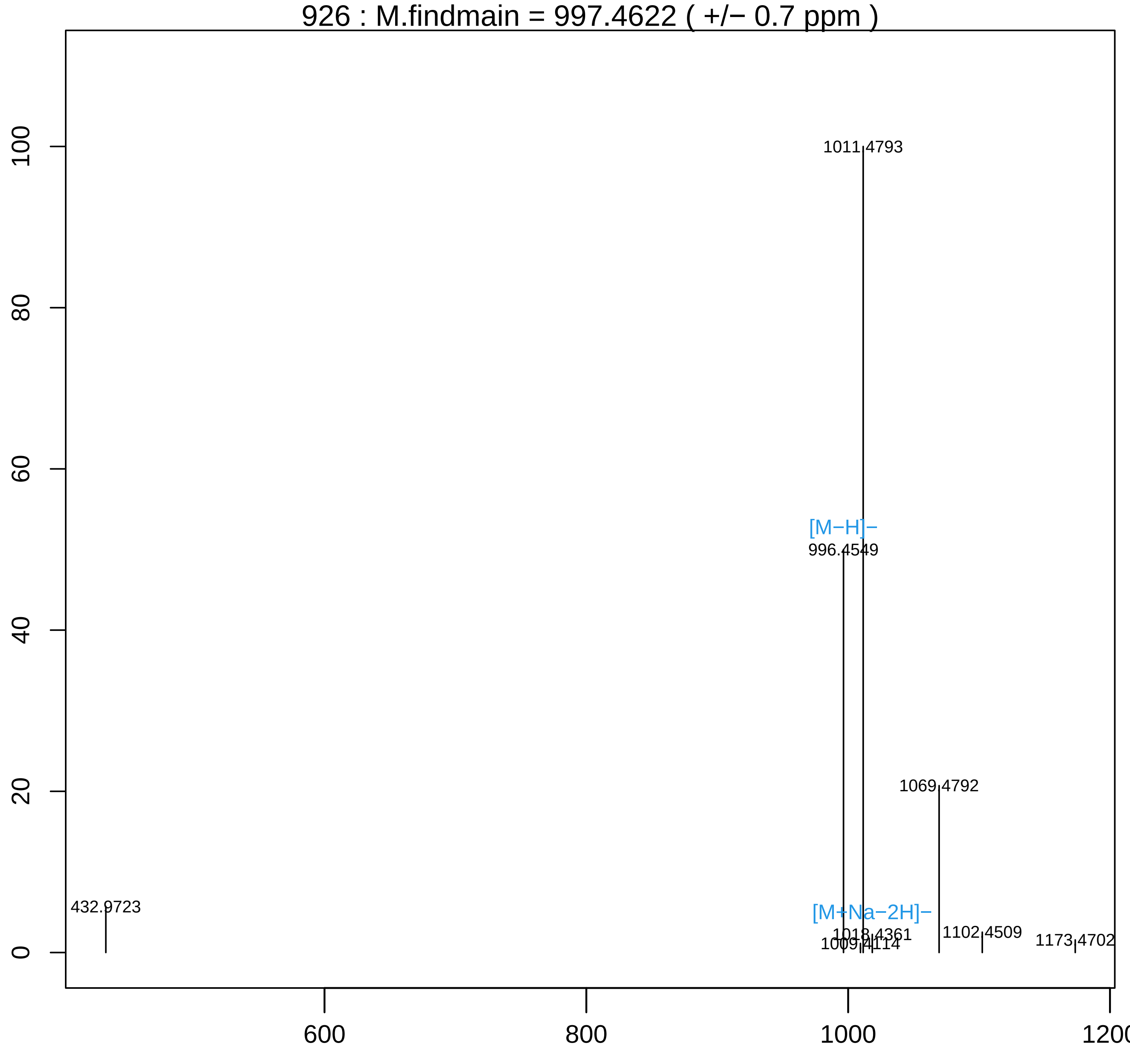

926 : M.findmain = 997.4622 (+/− 0.7 ppm)
1011.4793
[M−H]−
996.4549
1069.4792
432.9723
[M+Na−2H]−
1018.4361
1009.4114
1102.4509
1173.4702
0
20
40
60
80
100
600
800
1000
1200

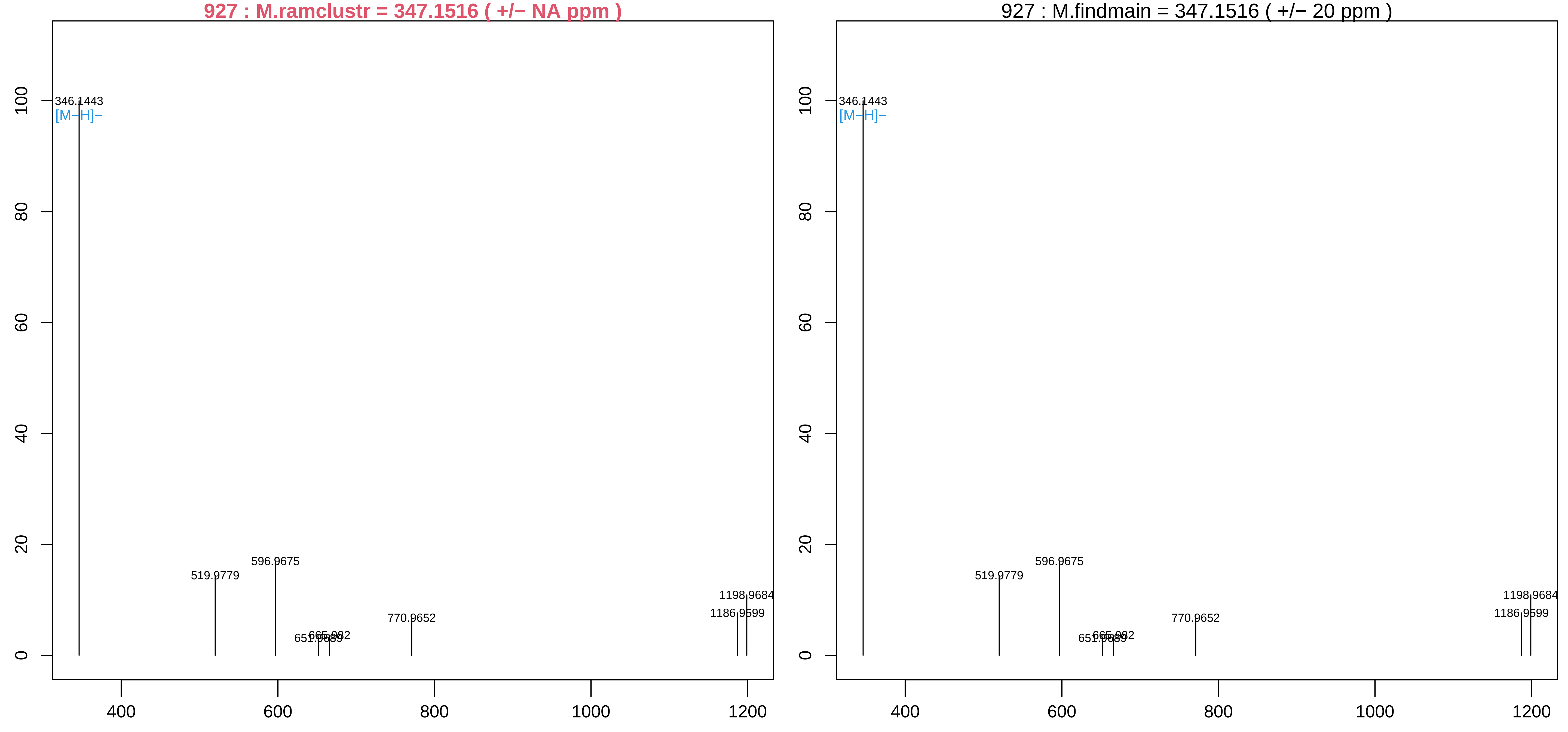

927 : M.ramclustr = 347.1516 (+/− NA ppm)
346.1443
[M−H]−
519.9779
596.9675
651.9689
665.982
770.9652
1186.9599
1198.9684
927 : M.findmain = 347.1516 (+/− 20 ppm)
346.1443
[M−H]−
519.9779
596.9675
651.9689
665.982
770.9652
1186.9599
1198.9684

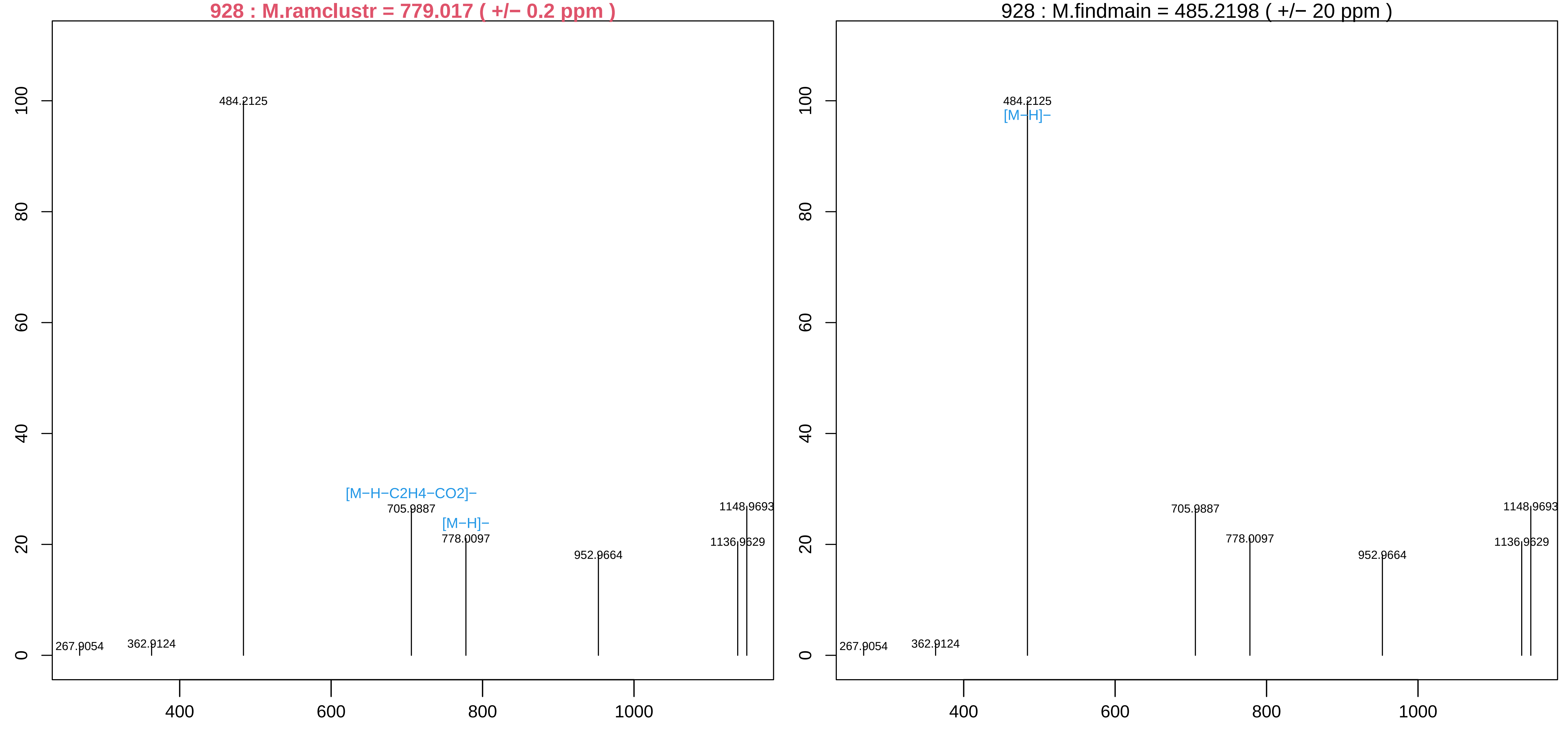
928 : M.ramclustr = 779.017 (+/− 0.2 ppm)
484.2125
[M−H−C2H4−CO2]−
705.9887
[M−H]−
778.0097
952.9664
1136.9629
1148.9693
267.9054
362.9124
0
20
40
60
80
100
400
600
800
1000
928 : M.findmain = 485.2198 (+/− 20 ppm)
484.2125
[M−H]−
705.9887
778.0097
952.9664
1136.9629
1148.9693
267.9054
362.9124
0
20
40
60
80
100
400
600
800
1000

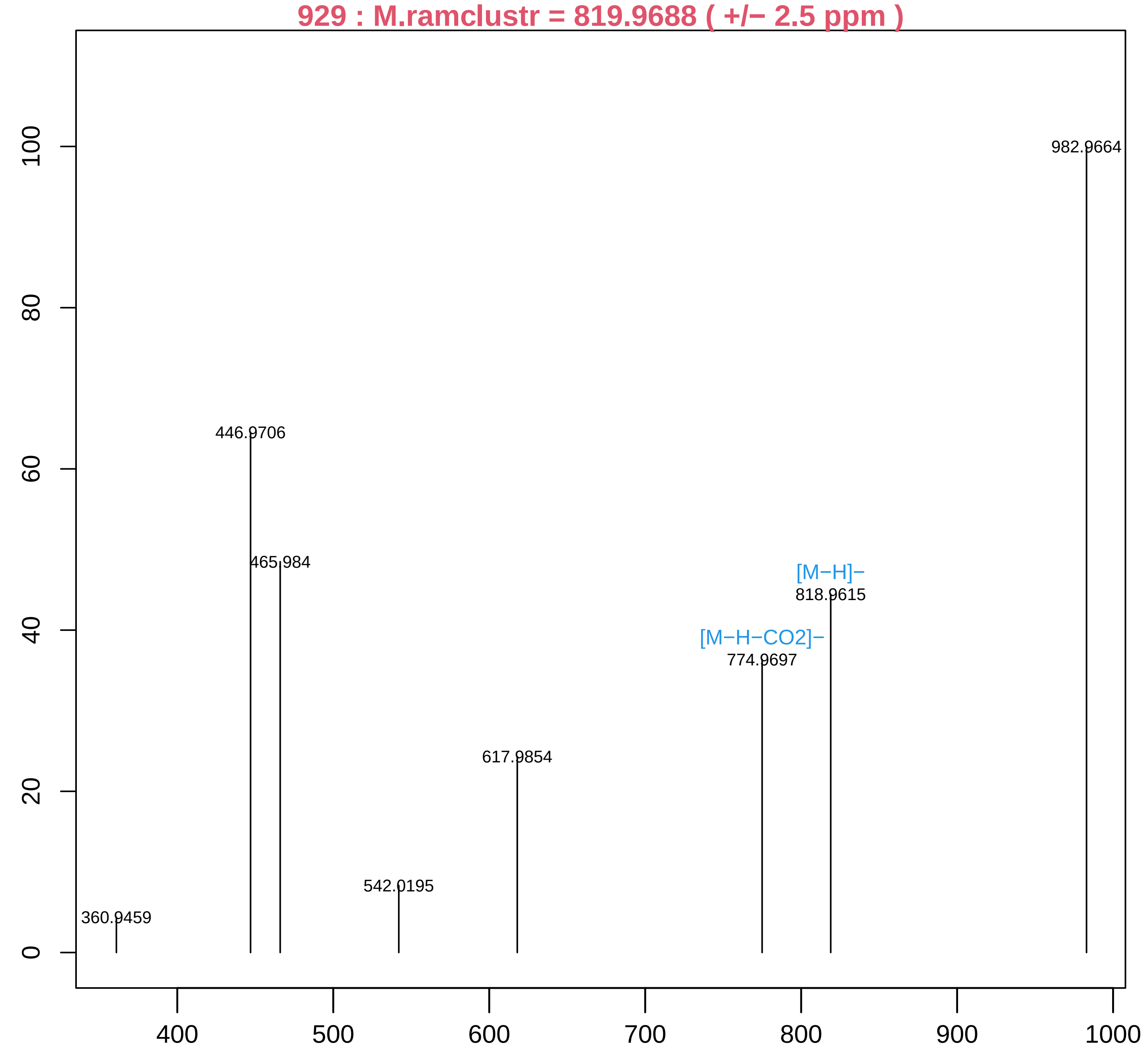
929 : M.ramclustr = 819.9688 (+/− 2.5 ppm)
982.9664
446.9706
465.984
[M−H]−
818.9615
[M−H−CO2]−
774.9697
617.9854
542.0195
360.9459
0
20
40
60
80
100
400
500
600
700
800
900
1000

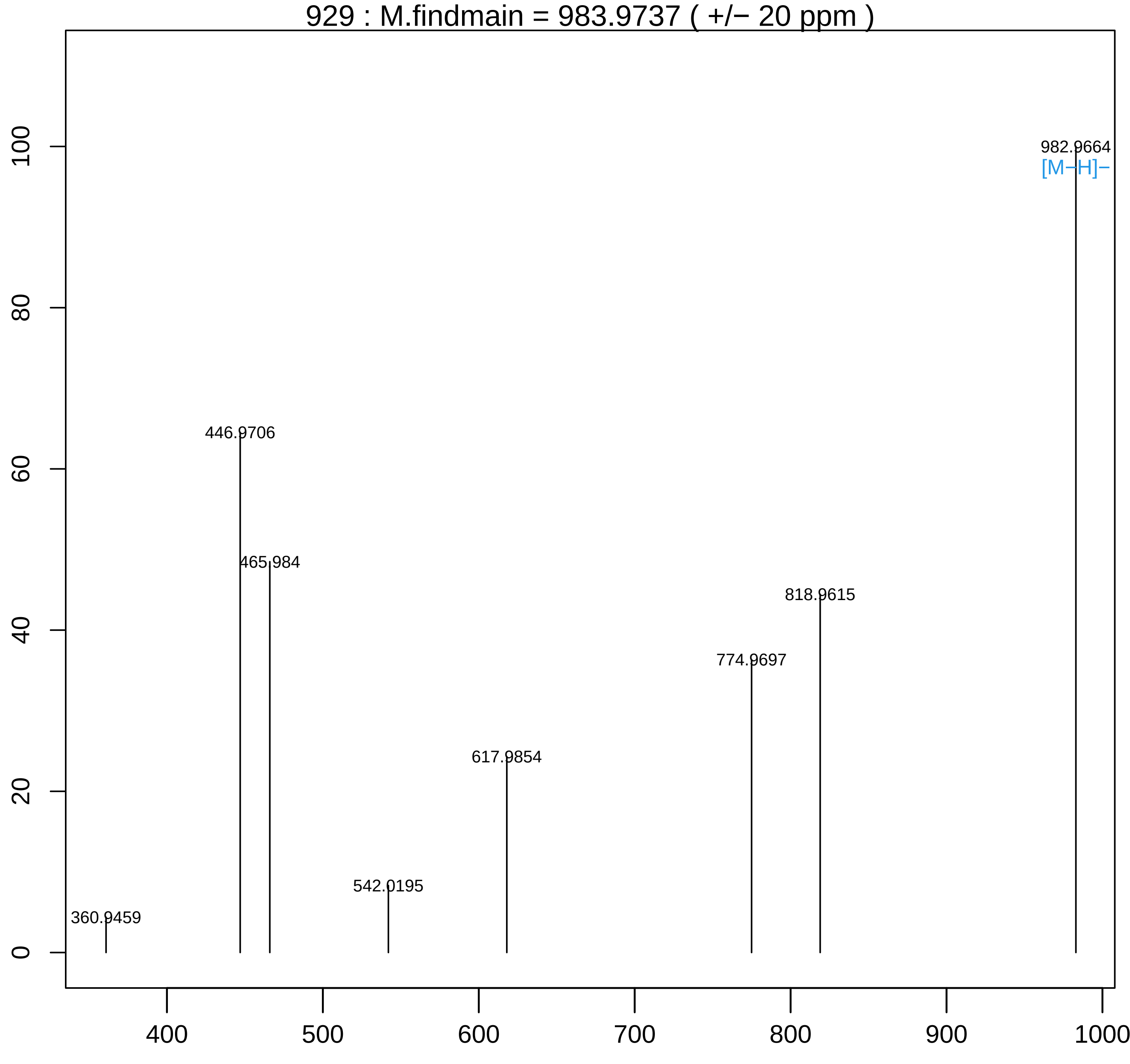
929 : M.findmain = 983.9737 (+/− 20 ppm)
982.9664
[M−H]−
446.9706
465.984
818.9615
774.9697
617.9854
542.0195
360.9459
0
20
40
60
80
100
400
500
600
700
800
900
1000

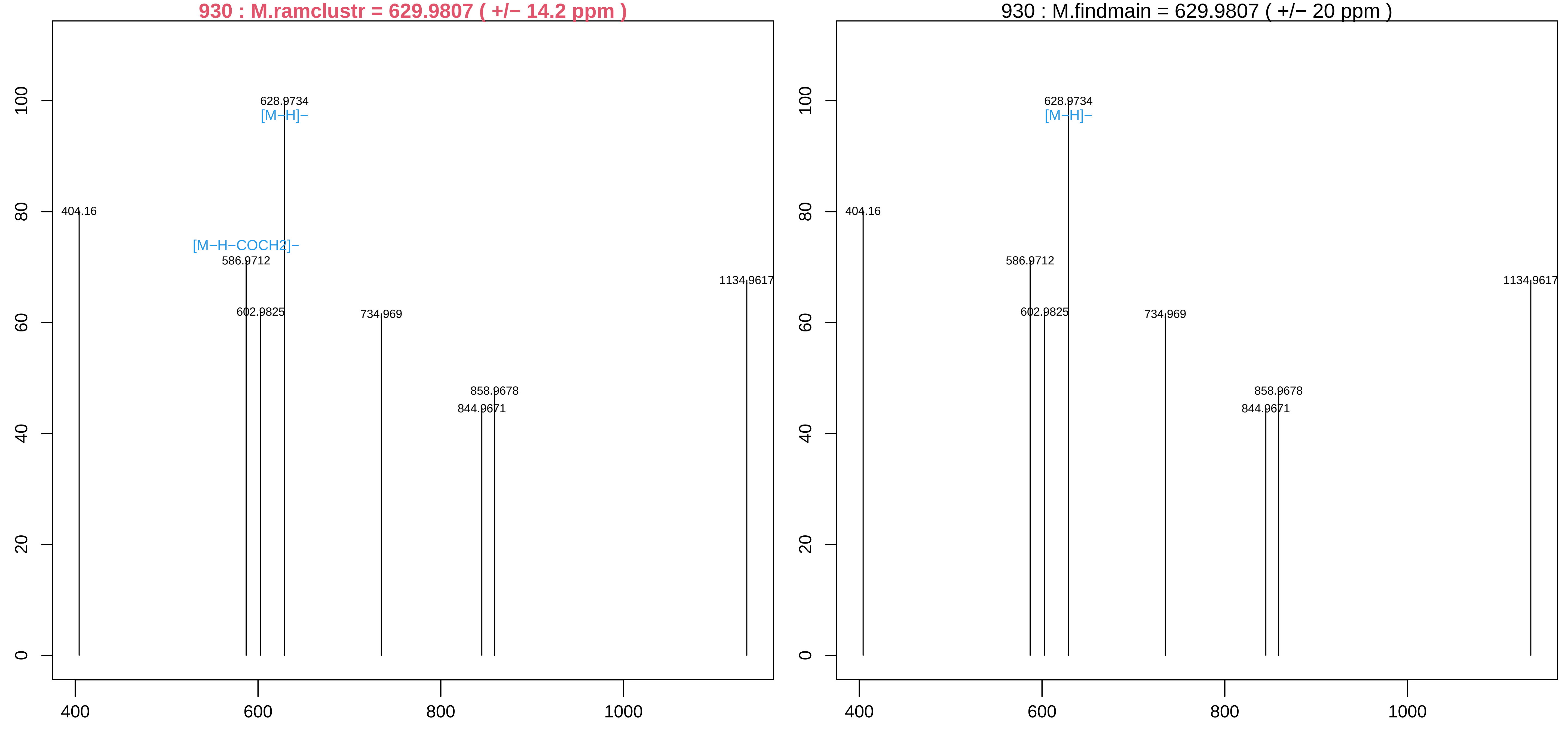

930 : M.ramclustr = 629.9807 (+/− 14.2 ppm)
628.9734
[M−H]−
404.16
[M−H−COCH2]−
586.9712
602.9825
734.969
858.9678
844.9671
1134.9617
930 : M.findmain = 629.9807 (+/− 20 ppm)
628.9734
[M−H]−
404.16
586.9712
602.9825
734.969
858.9678
844.9671
1134.9617

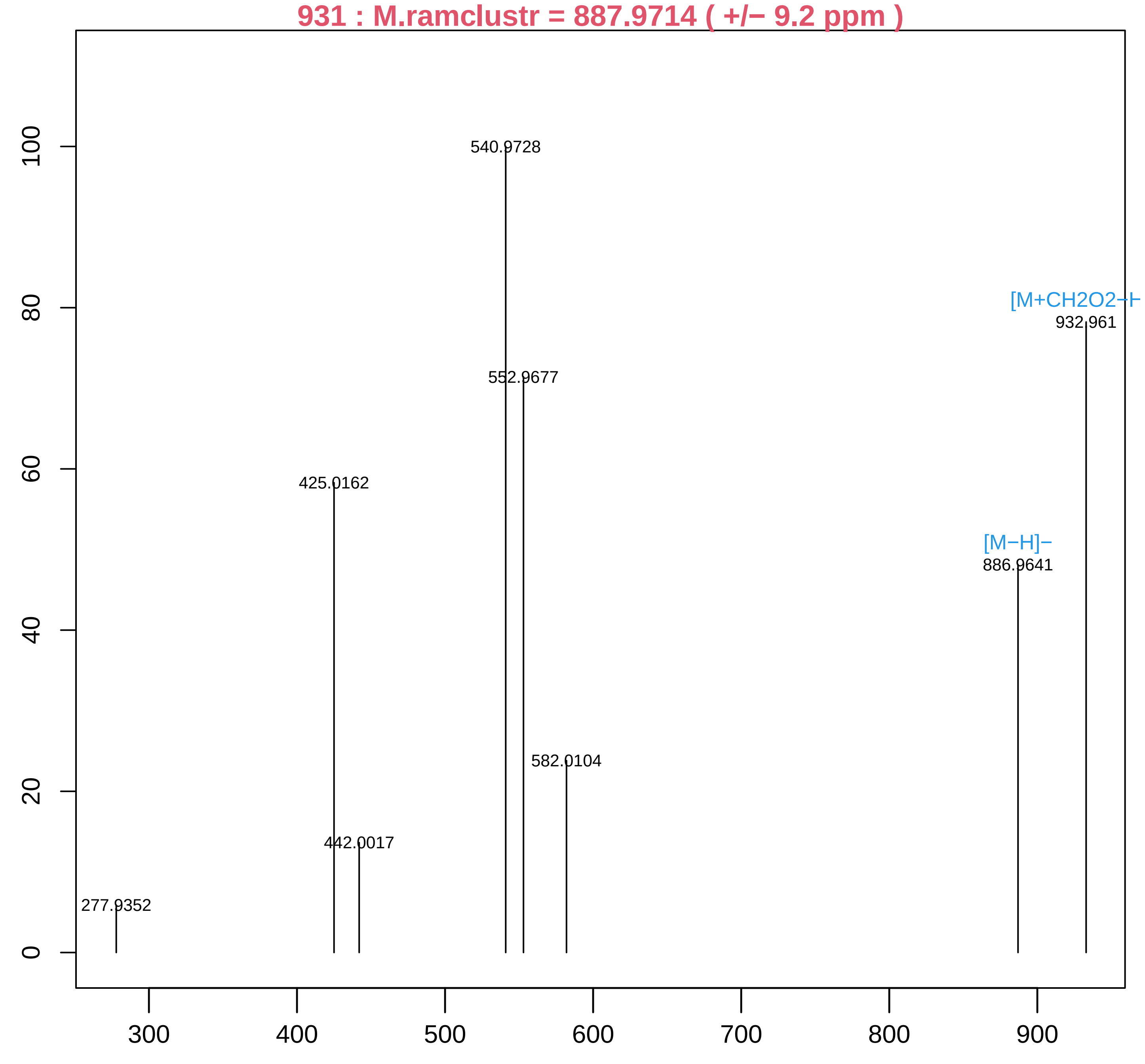

931 : M.ramclustr = 887.9714 (+/− 9.2 ppm)
540.9728
552.9677
425.0162
442.0017
277.9352
582.0104
[M−H]−
886.9641
[M+CH2O2−H
932.961
0
20
40
60
80
100
300
400
500
600
700
800
900

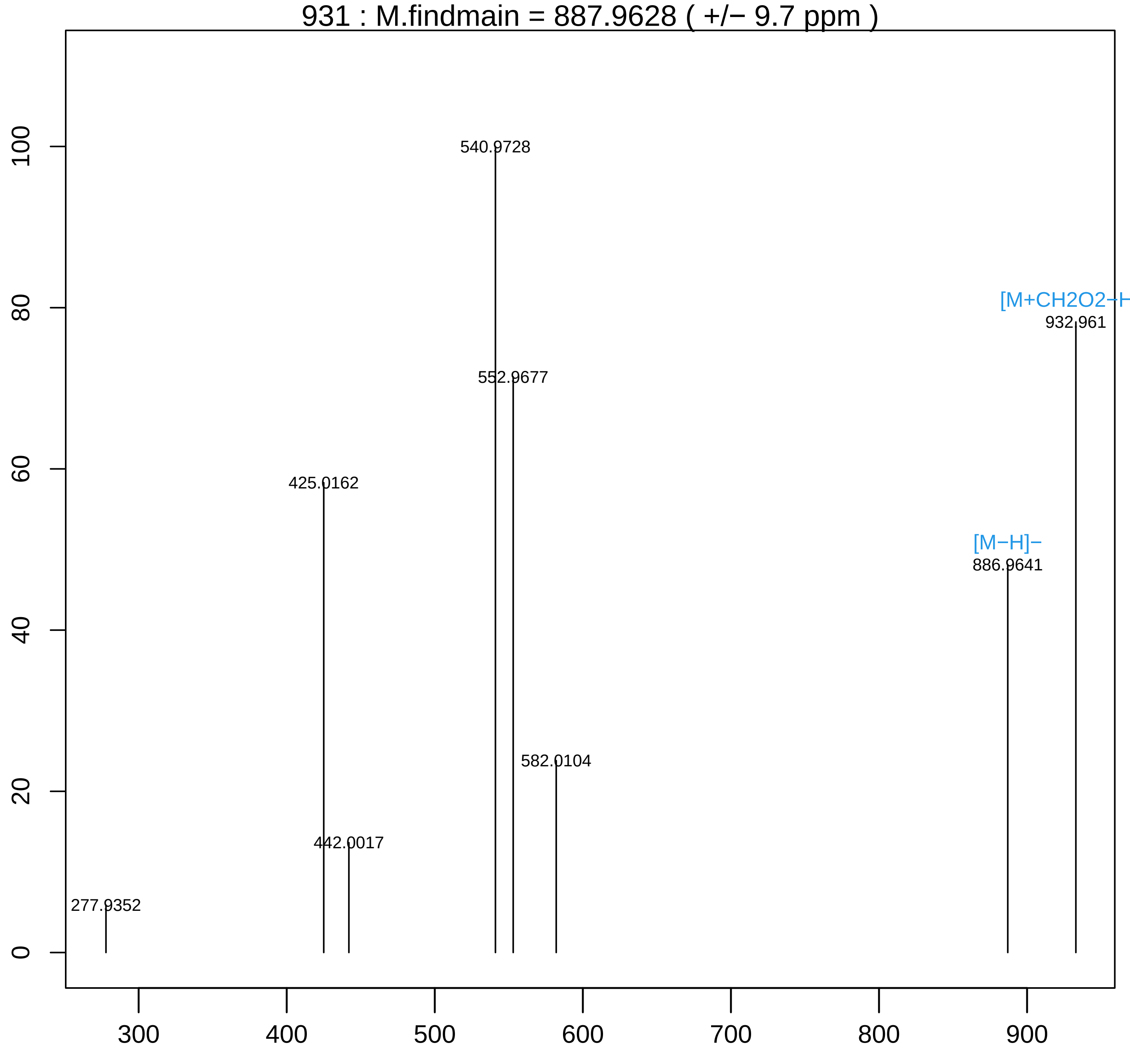

931 : M.findmain = 887.9628 (+/− 9.7 ppm)
540.9728
552.9677
425.0162
442.0017
277.9352
582.0104
[M−H]−
886.9641
[M+CH2O2−H
932.961
0
20
40
60
80
100
300
400
500
600
700
800
900

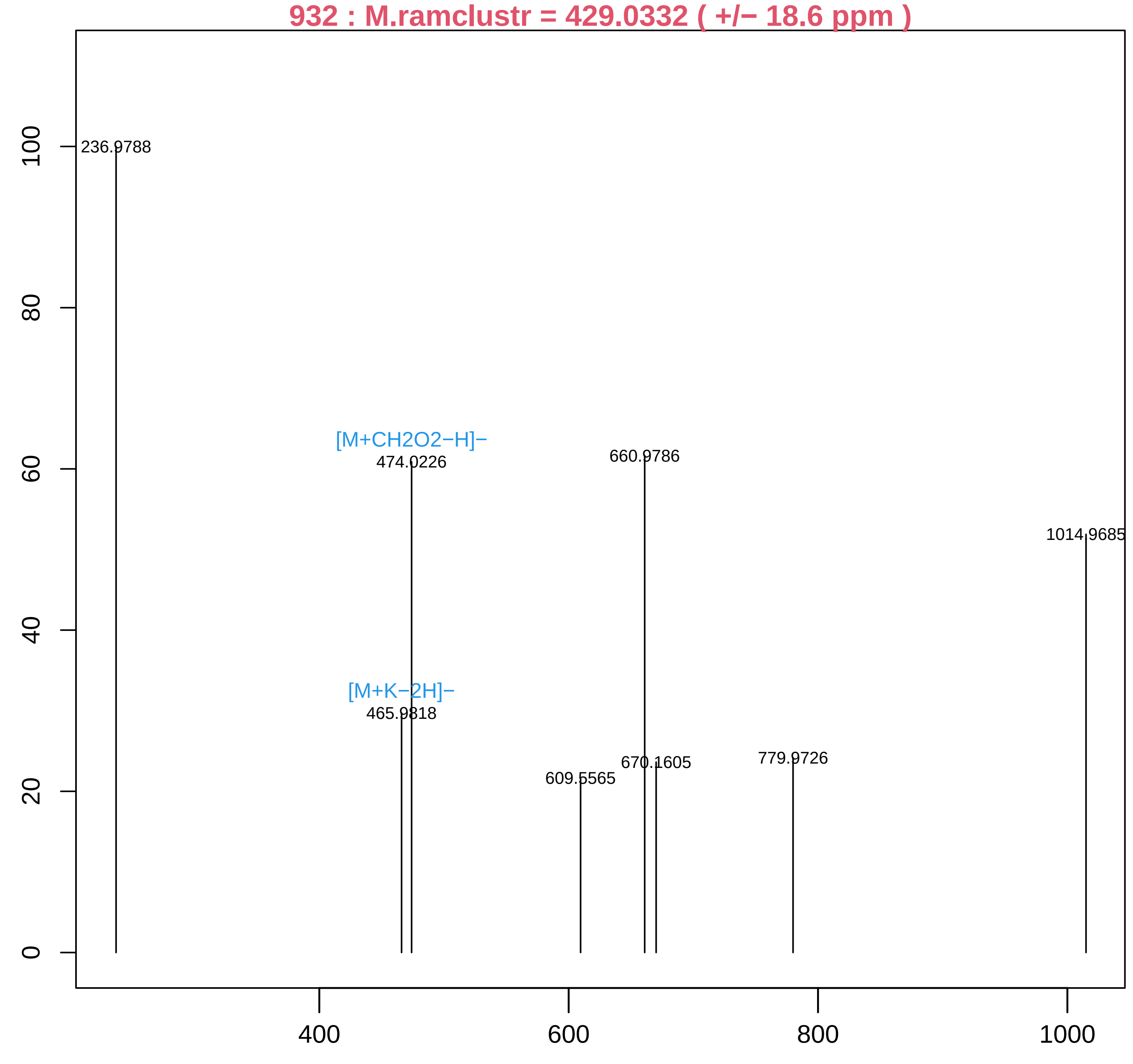
932 : M.ramclustr = 429.0332 (+/− 18.6 ppm)
236.9788
[M+CH2O2−H]−
474.0226
660.9786
1014.9685
[M+K−2H]−
465.9818
609.5565
670.1605
779.9726
0
20
40
60
80
100
400
600
800
1000

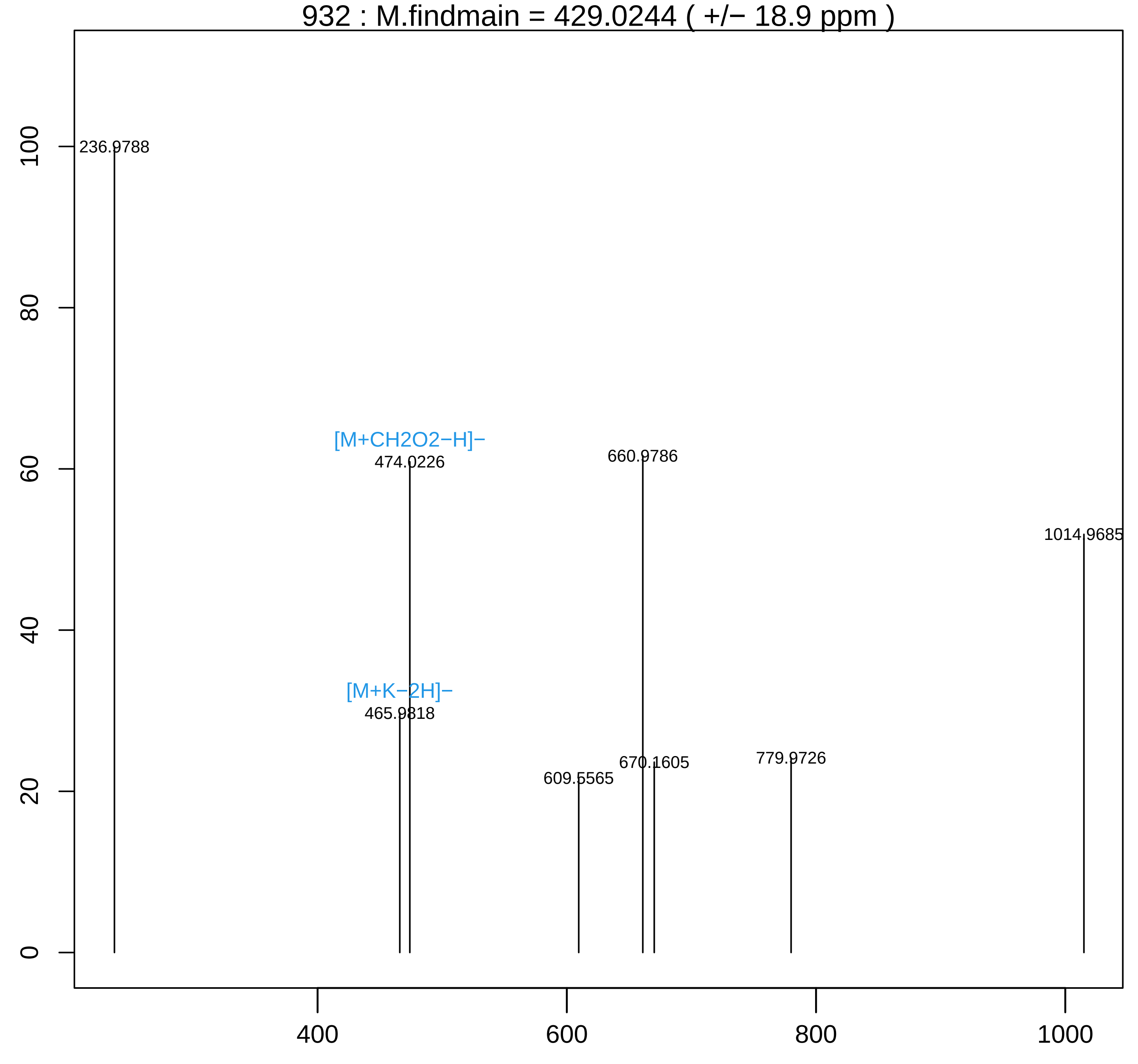
932 : M.findmain = 429.0244 (+/− 18.9 ppm)
236.9788
[M+CH2O2−H]−
474.0226
660.9786
1014.9685
[M+K−2H]−
465.9818
609.5565
670.1605
779.9726
0
20
40
60
80
100
400
600
800
1000

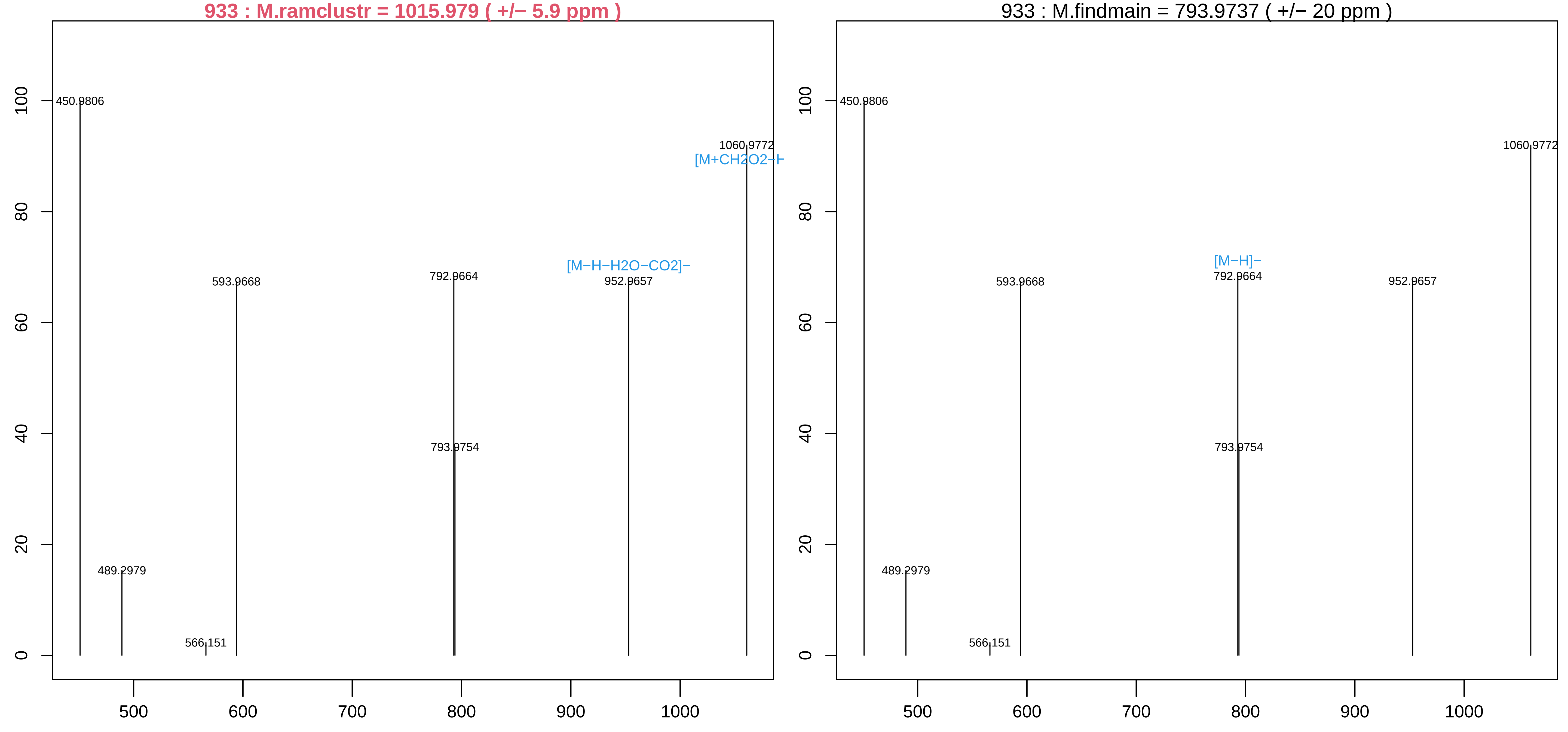

933 : M.ramclustr = 1015.979 (+/− 5.9 ppm)
450.9806
489.2979
566.151
593.9668
792.9664
793.9754
[M−H−H2O−CO2]−
952.9657
1060.9772
[M+CH2O2−H
0
20
40
60
80
100
500
600
700
800
900
1000
933 : M.findmain = 793.9737 (+/− 20 ppm)
450.9806
489.2979
566.151
593.9668
[M−H]−
792.9664
793.9754
952.9657
1060.9772
0
20
40
60
80
100
500
600
700
800
900
1000

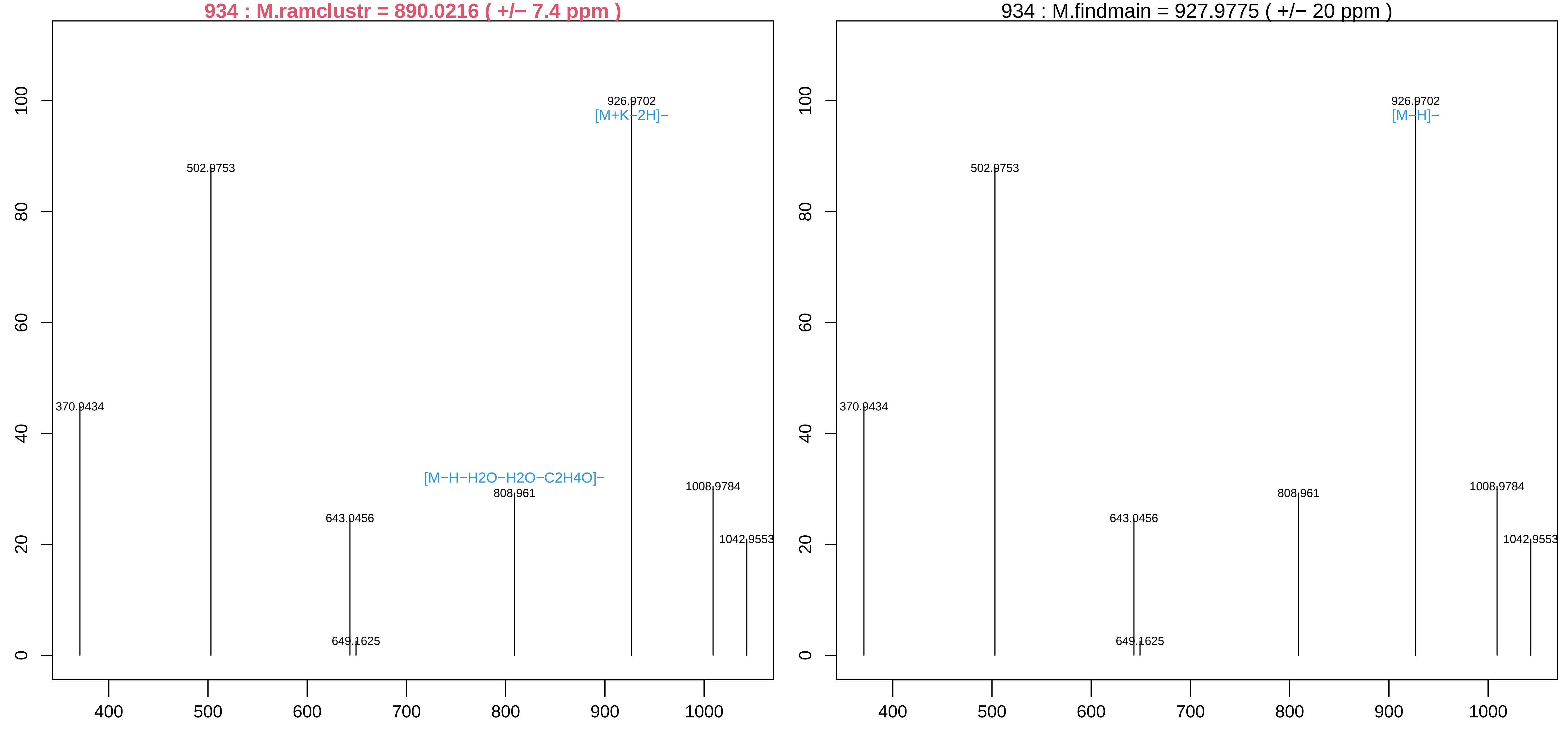
934 : M.ramclustr = 890.0216 (+/− 7.4 ppm)
926.9702
[M+K−2H]−
502.9753
370.9434
[M−H−H2O−H2O−C2H4O]−
808.961
1008.9784
643.0456
1042.9553
649.1625
0
20
40
60
80
100
400
500
600
700
800
900
1000
934 : M.findmain = 927.9775 (+/− 20 ppm)
926.9702
[M−H]−
502.9753
370.9434
808.961
1008.9784
643.0456
1042.9553
649.1625
0
20
40
60
80
100
400
500
600
700
800
900
1000

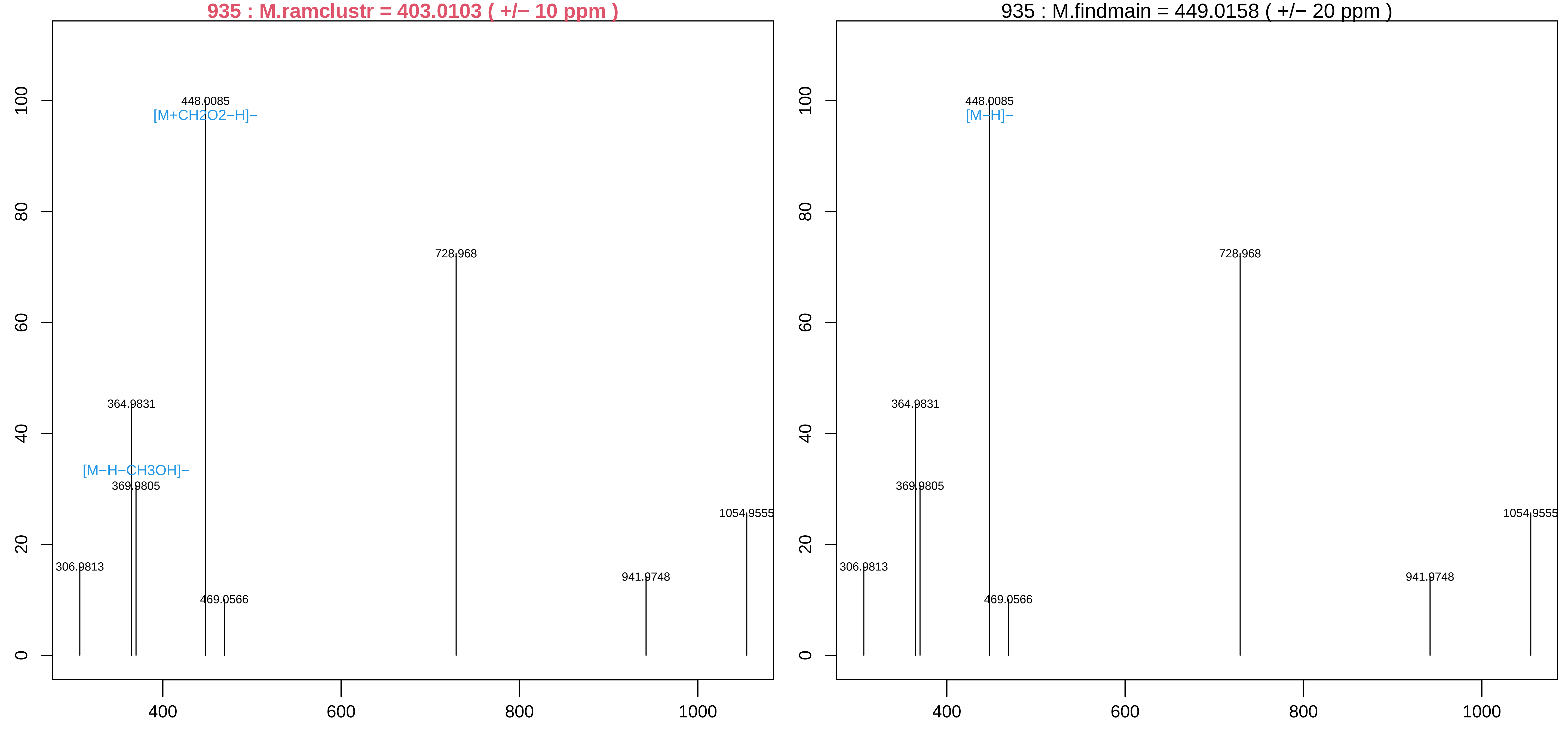

935 : M.ramclustr = 403.0103 (+/− 10 ppm)
448.0085
[M+CH2O2−H]−
728.968
364.9831
[M−H−CH3OH]−
369.9805
1054.9555
306.9813
941.9748
469.0566
935 : M.findmain = 449.0158 (+/− 20 ppm)
448.0085
[M−H]−
728.968
364.9831
369.9805
1054.9555
306.9813
941.9748
469.0566
0
20
40
60
80
100
400
600
800
1000

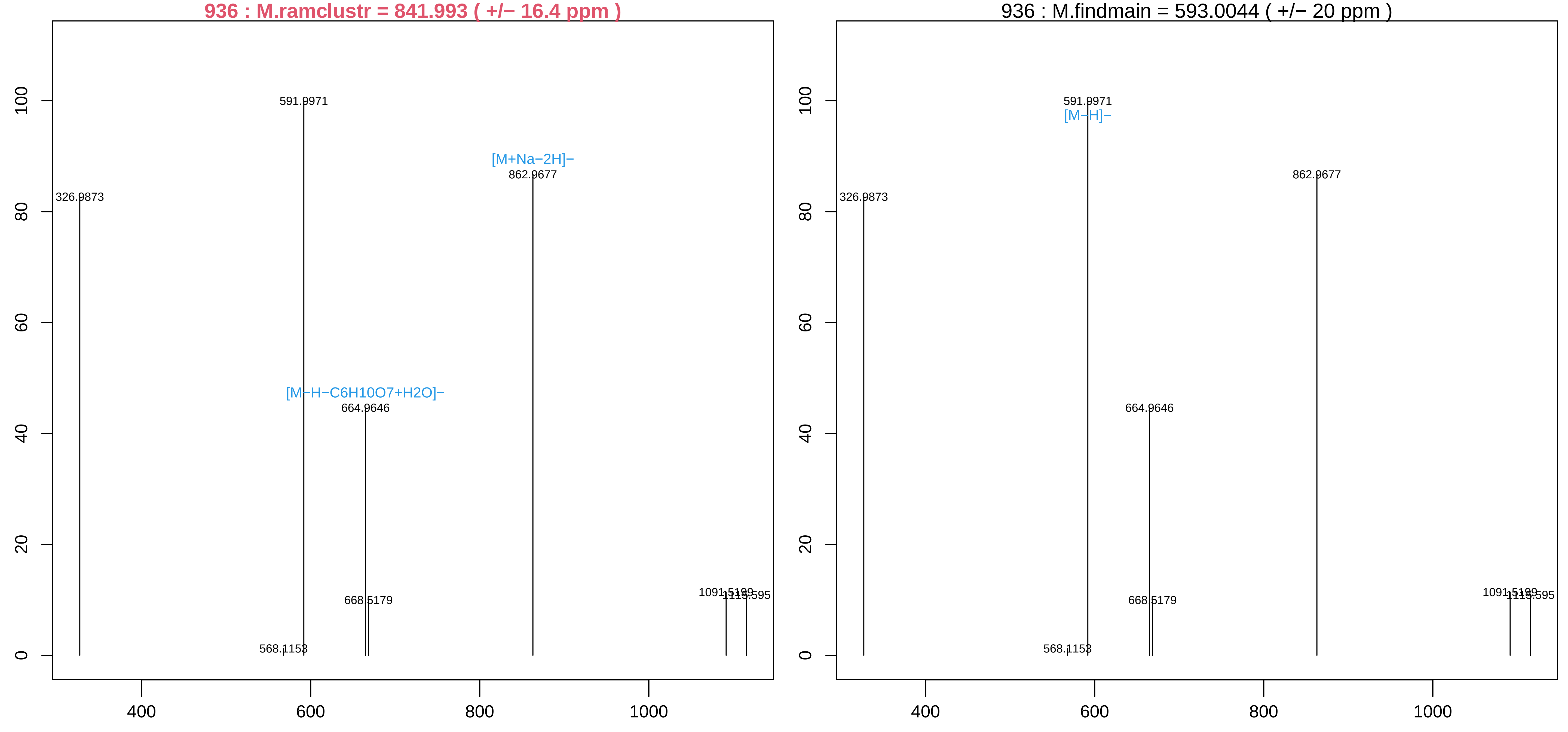
936 : M.ramclustr = 841.993 (+/− 16.4 ppm)
591.9971
[M+Na−2H]−
862.9677
326.9873
[M−H−C6H10O7+H2O]−
664.9646
668.5179
1091.5199
1115.595
568.1153
936 : M.findmain = 593.0044 (+/− 20 ppm)
591.9971
[M−H]−
862.9677
326.9873
664.9646
668.5179
1091.5199
1115.595
568.1153
0
20
40
60
80
100
400
600
800
1000

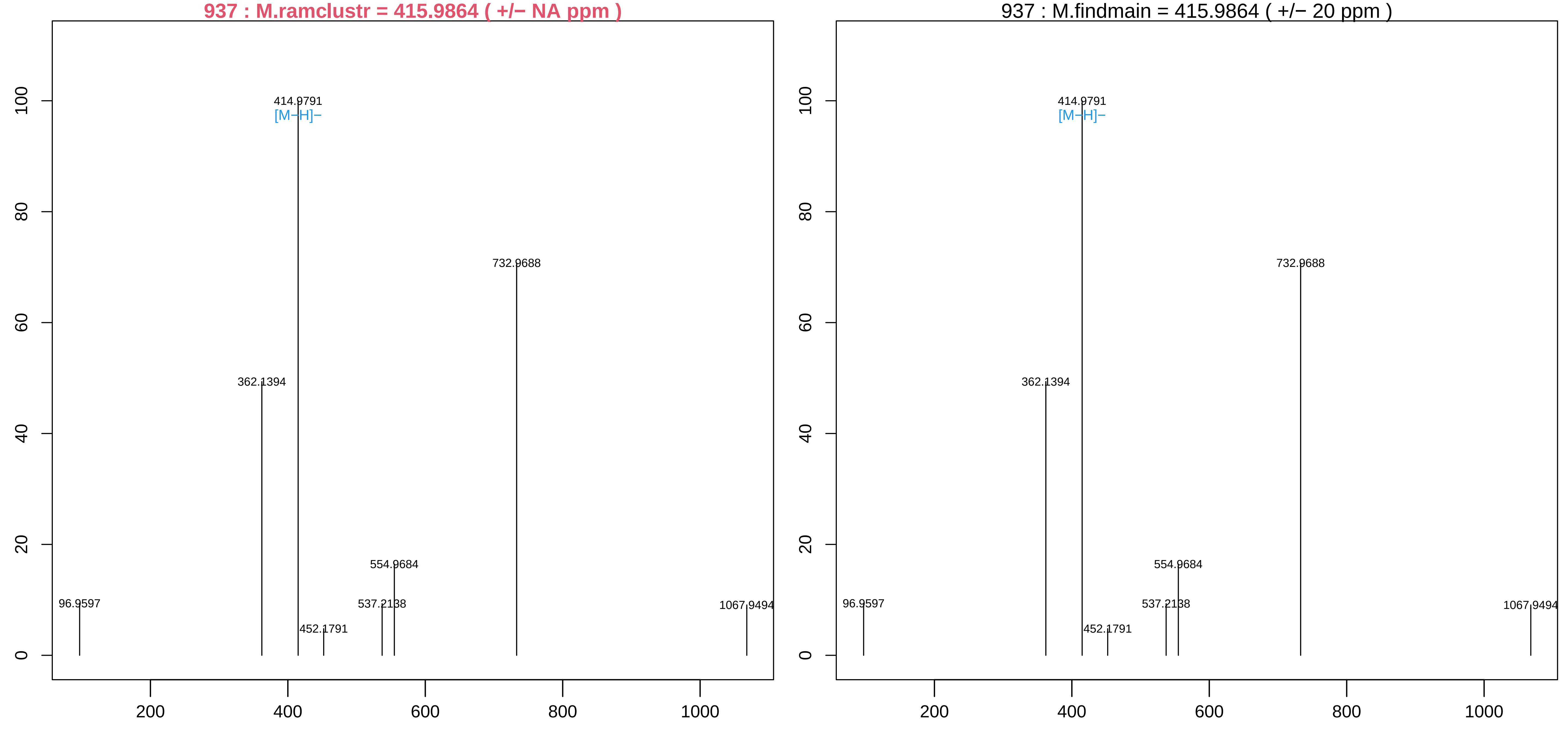

937 : M.ramclustr = 415.9864 (+/− NA ppm)
414.9791
[M−H]−
732.9688
362.1394
554.9684
96.9597
537.2138
1067.9494
452.1791
937 : M.findmain = 415.9864 (+/− 20 ppm)
414.9791
[M−H]−
732.9688
362.1394
554.9684
96.9597
537.2138
1067.9494
452.1791

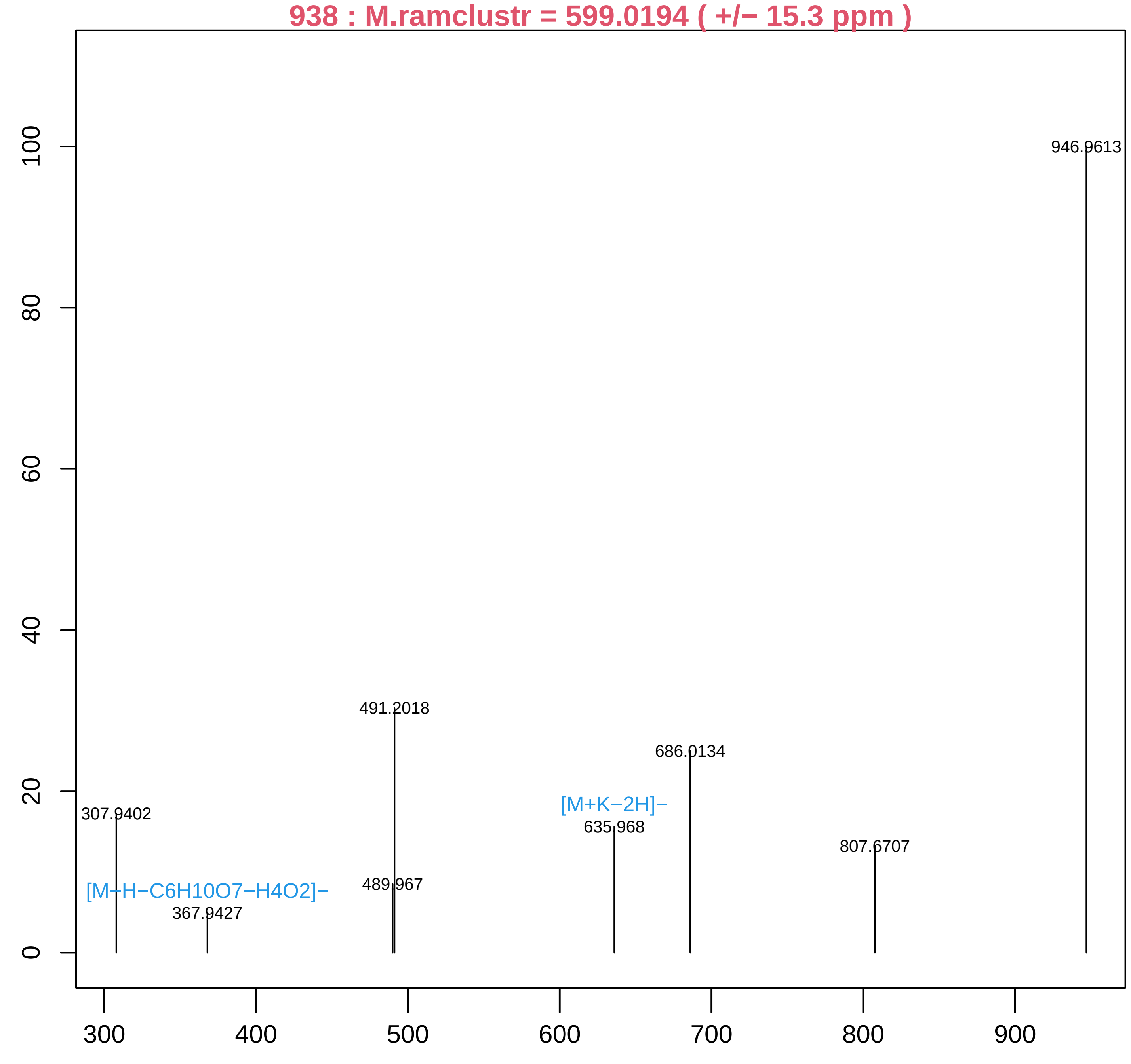
938 : M.ramclustr = 599.0194 (+/− 15.3 ppm)
946.9613
491.2018
686.0134
[M+K−2H]−
635.968
307.9402
807.6707
[M−H−C6H10O7−H4O2]−
489.967
367.9427
0
20
40
60
80
100
300
400
500
600
700
800
900

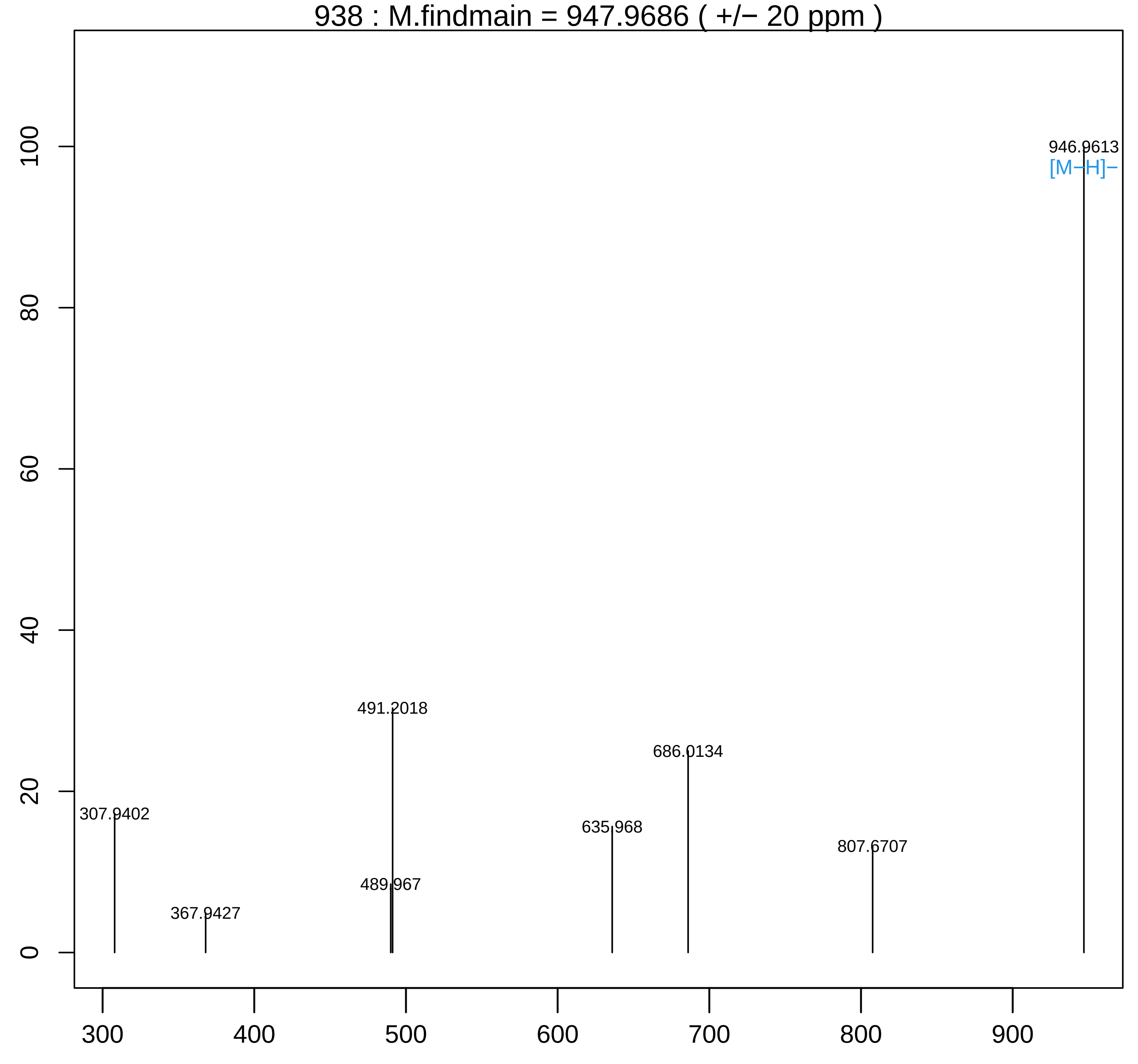
938 : M.findmain = 947.9686 (+/− 20 ppm)
946.9613
[M−H]−
491.2018
686.0134
307.9402
635.968
807.6707
489.967
367.9427
0
20
40
60
80
100
300
400
500
600
700
800
900

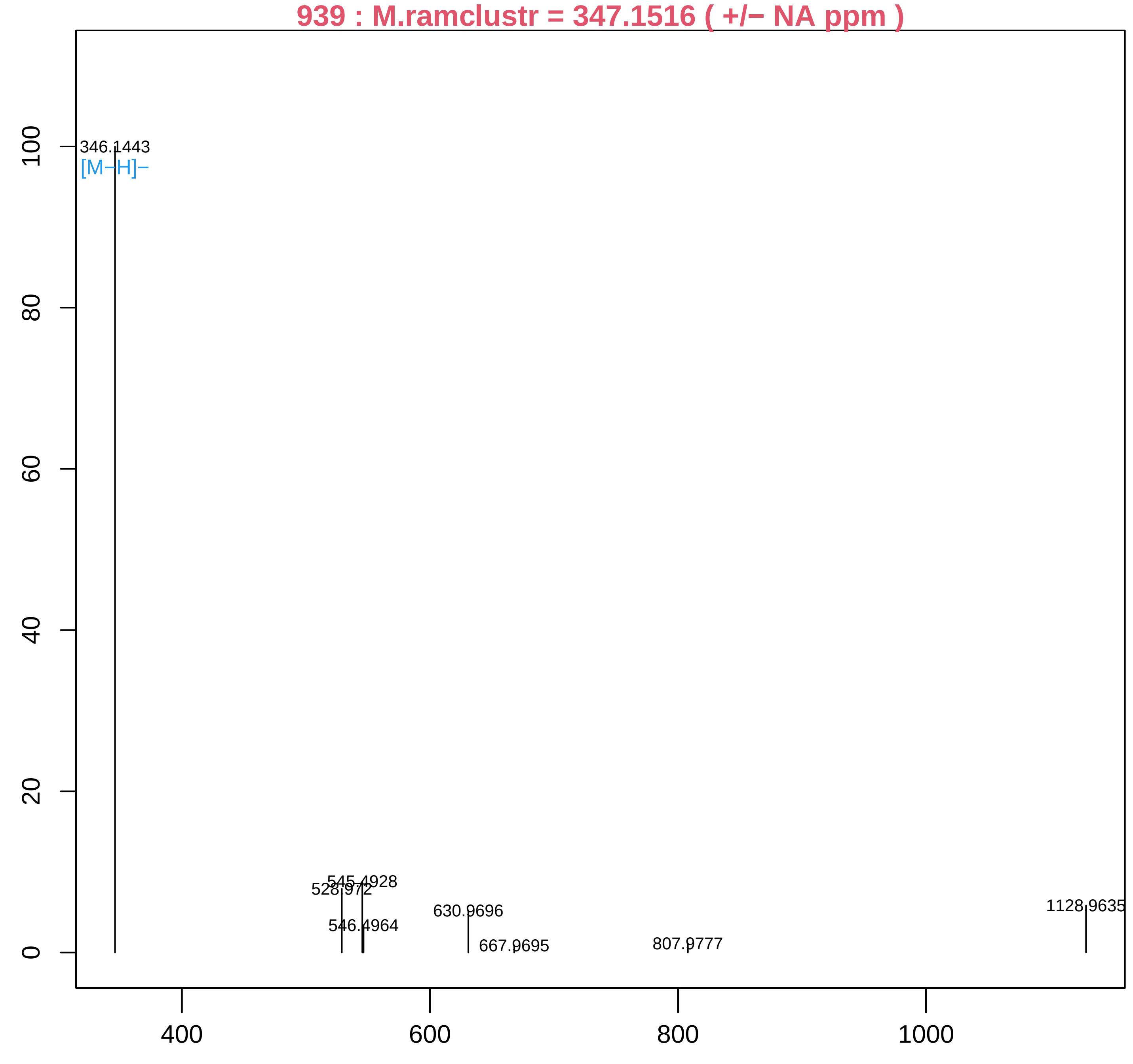
939 : M.ramclustr = 347.1516 (+/− NA ppm)
346.1443
[M−H]−
528.972
545.4928
546.4964
630.9696
667.9695
807.9777
1128.9635
0
20
40
60
80
100
400
600
800
1000

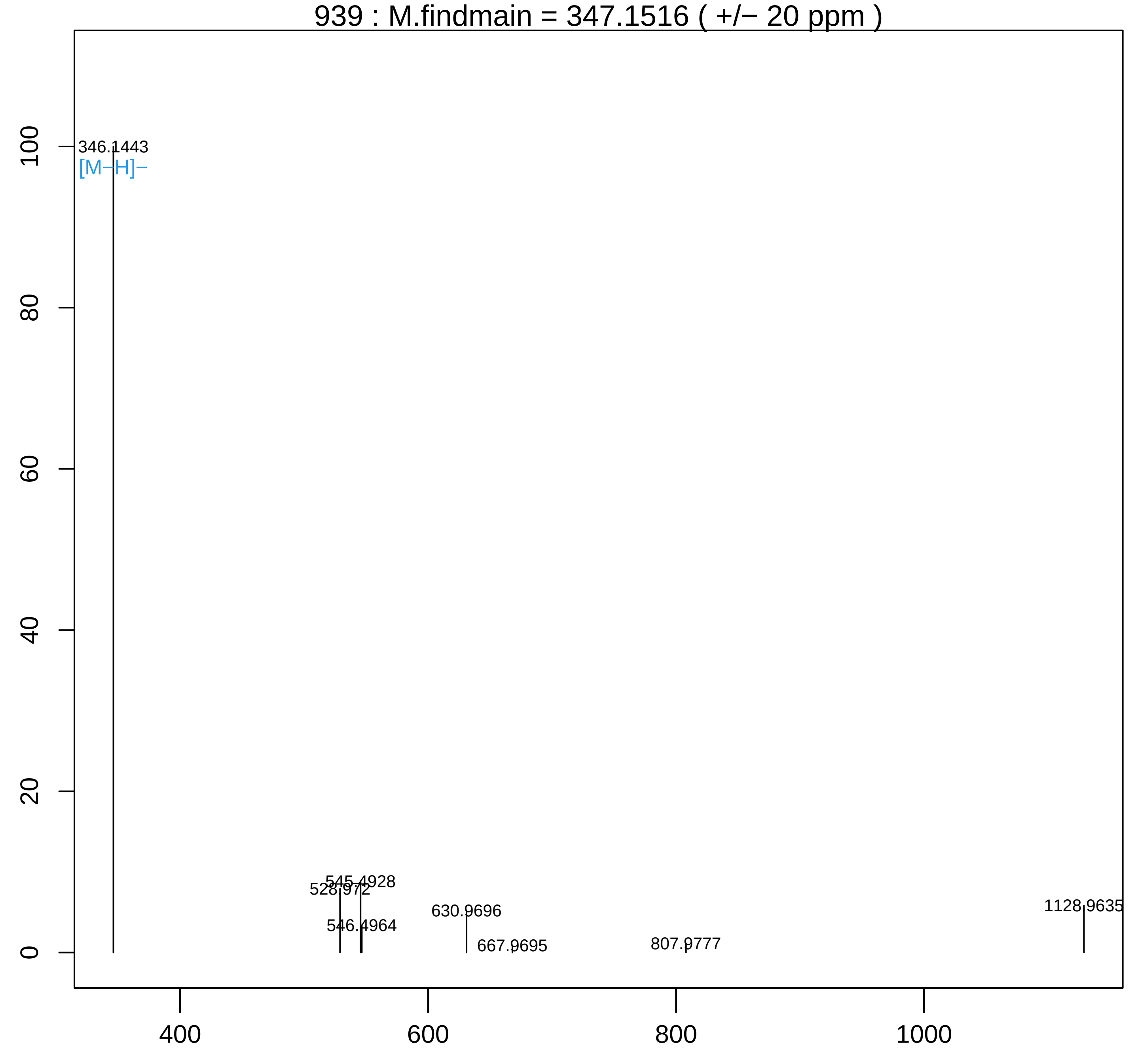
939 : M.findmain = 347.1516 (+/− 20 ppm)
346.1443
[M−H]−
528.972
545.4928
546.4964
630.9696
667.9695
807.9777
1128.9635
0
20
40
60
80
100
400
600
800
1000

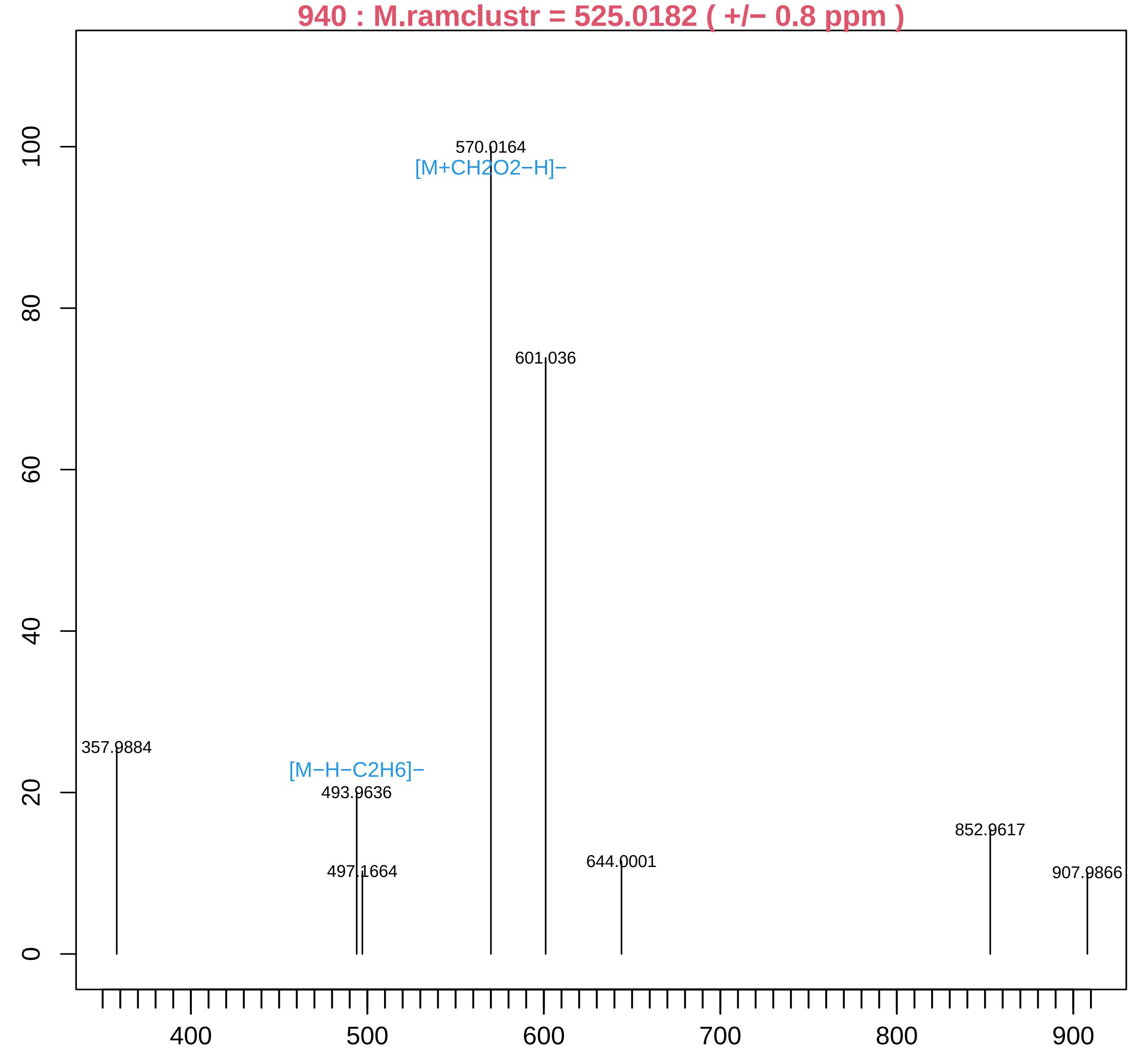

940 : M.ramclustr = 525.0182 (+/− 0.8 ppm)
570.0164
[M+CH2O2−H]−
601.036
357.9884
[M−H−C2H6]−
493.9636
497.1664
644.0001
852.9617
907.9866
0
20
40
60
80
100
400
500
600
700
800
900

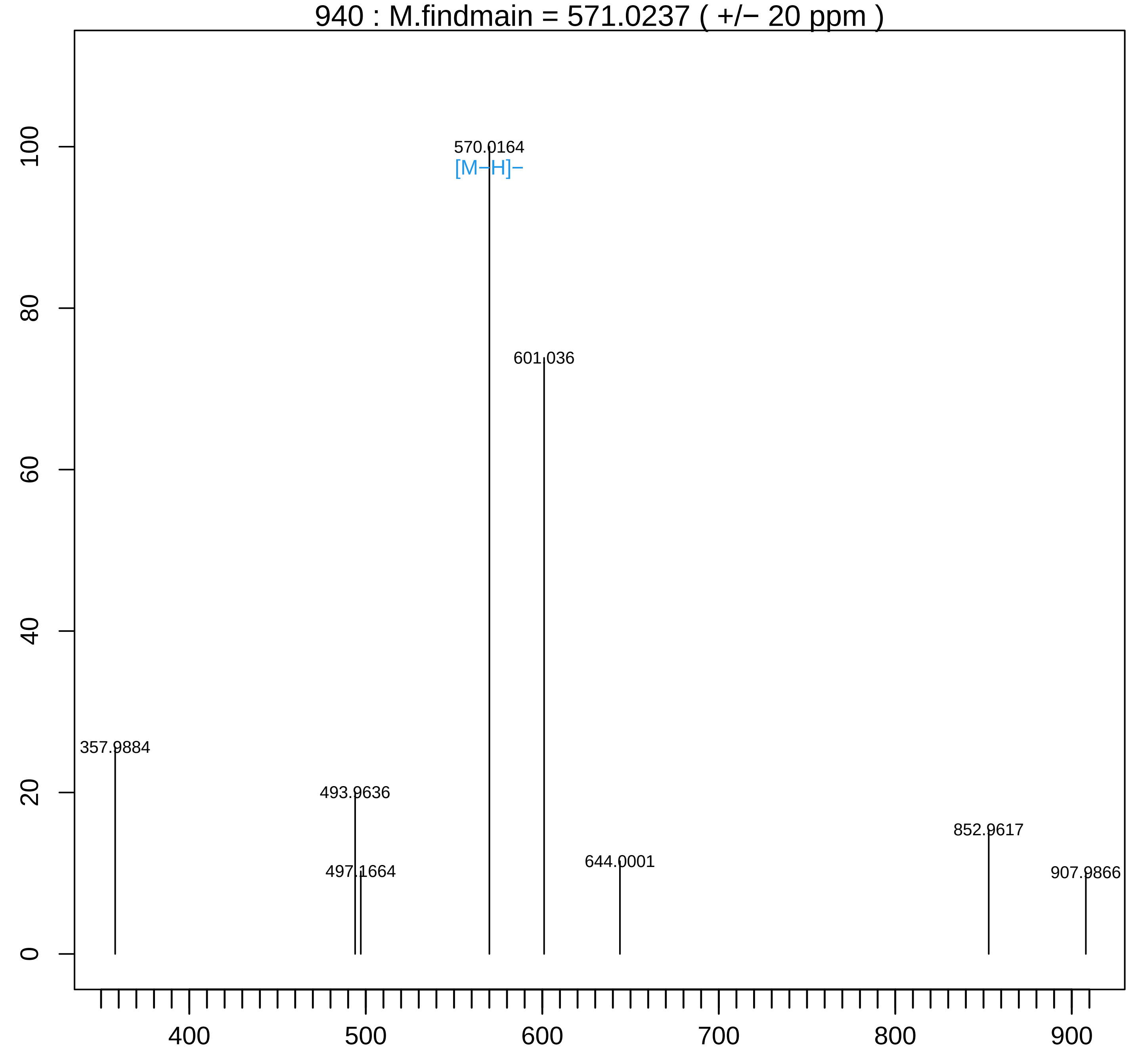

940 : M.findmain = 571.0237 (+/− 20 ppm)
570.0164
[M−H]−
601.036
357.9884
493.9636
497.1664
644.0001
852.9617
907.9866
0
20
40
60
80
100
400
500
600
700
800
900

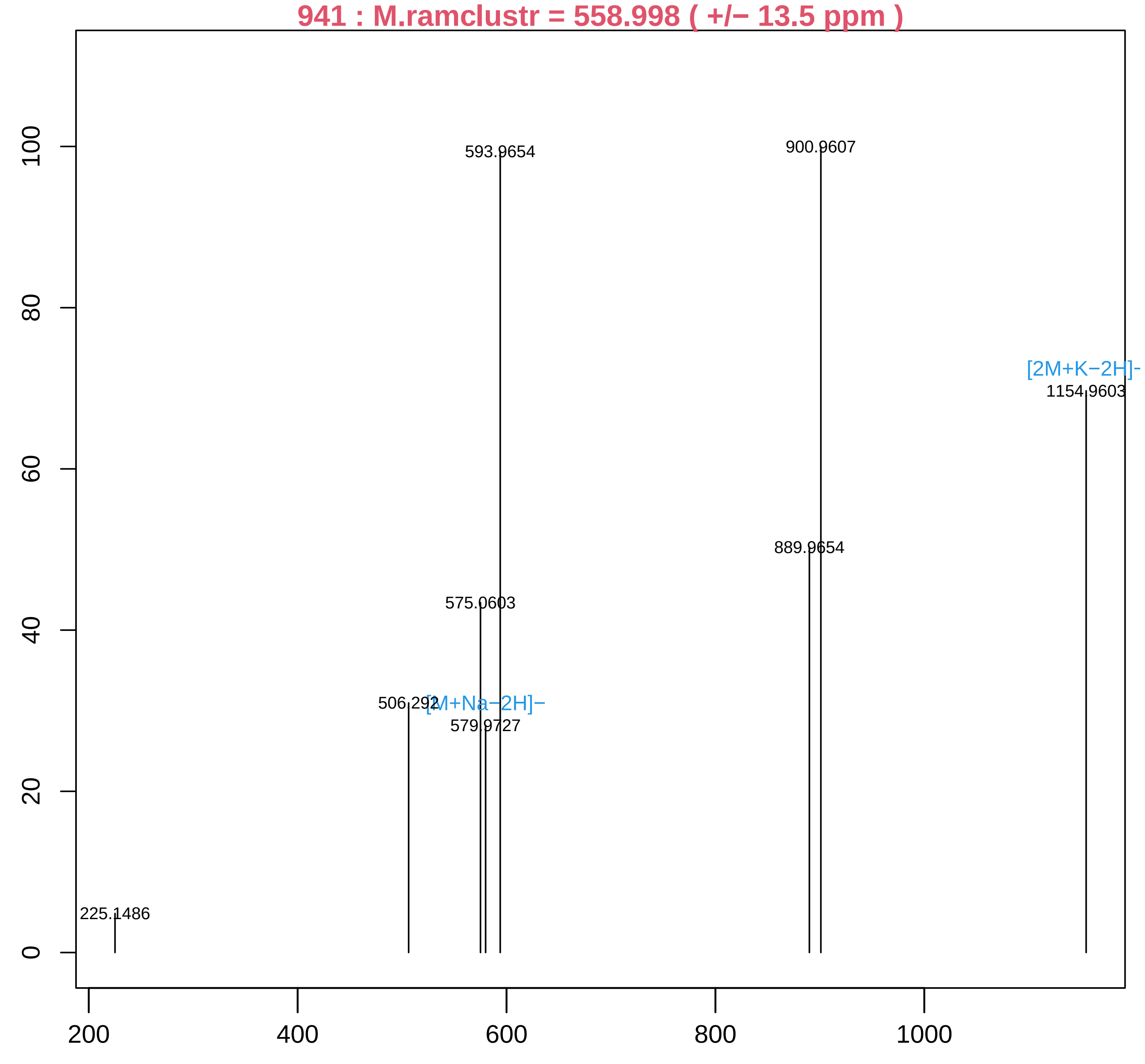
941 : M.ramclustr = 558.998 (+/− 13.5 ppm)
593.9654
900.9607
[2M+K−2H]-
1154.9603
889.9654
575.0603
506.292
[M+Na−2H]−
579.9727
225.1486
0
20
40
60
80
100
200
400
600
800
1000

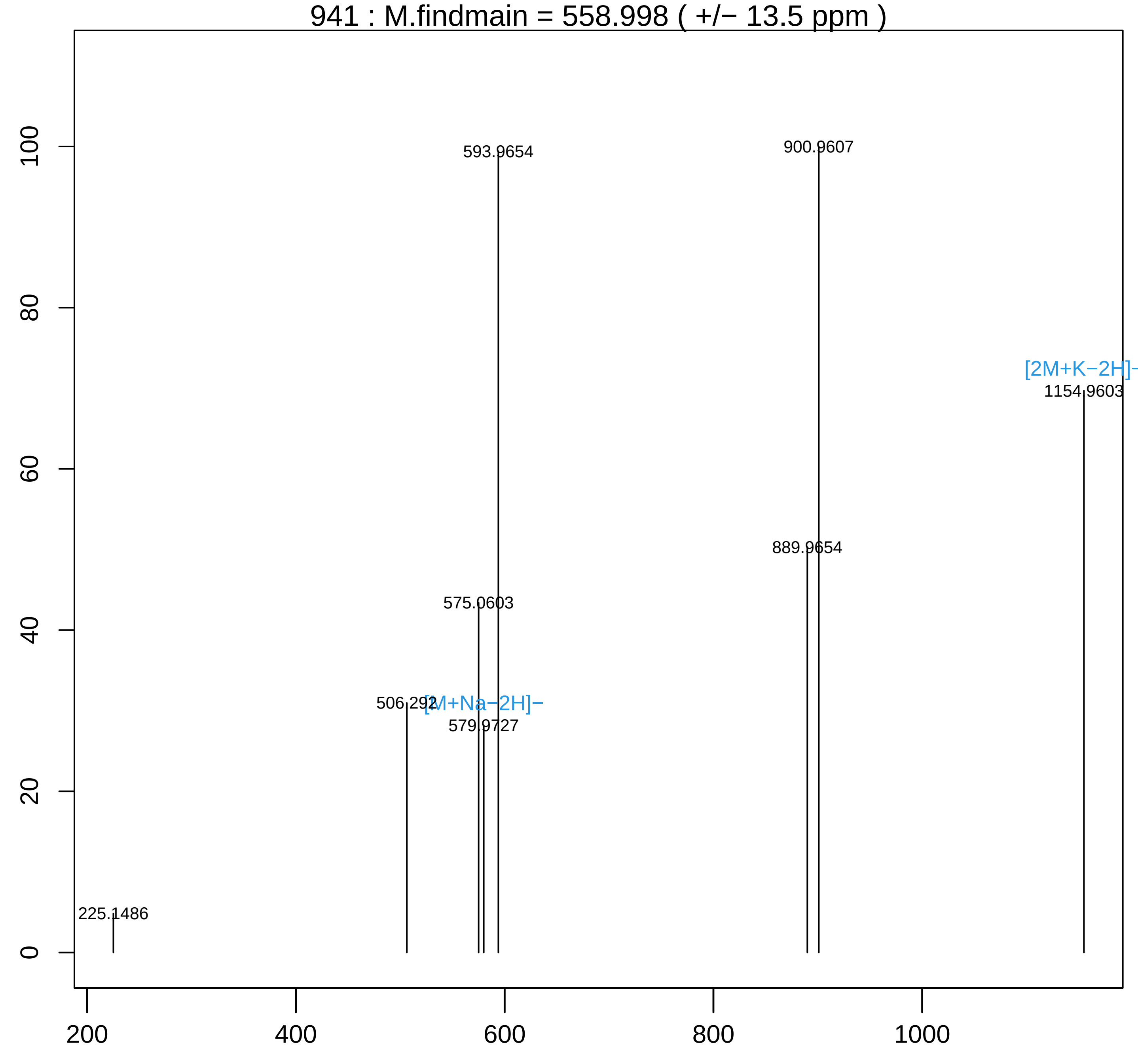
941 : M.findmain = 558.998 (+/− 13.5 ppm)
593.9654
900.9607
[2M+K−2H]-
1154.9603
889.9654
575.0603
506.292
[M+Na−2H]−
579.9727
225.1486
0
20
40
60
80
100
200
400
600
800
1000

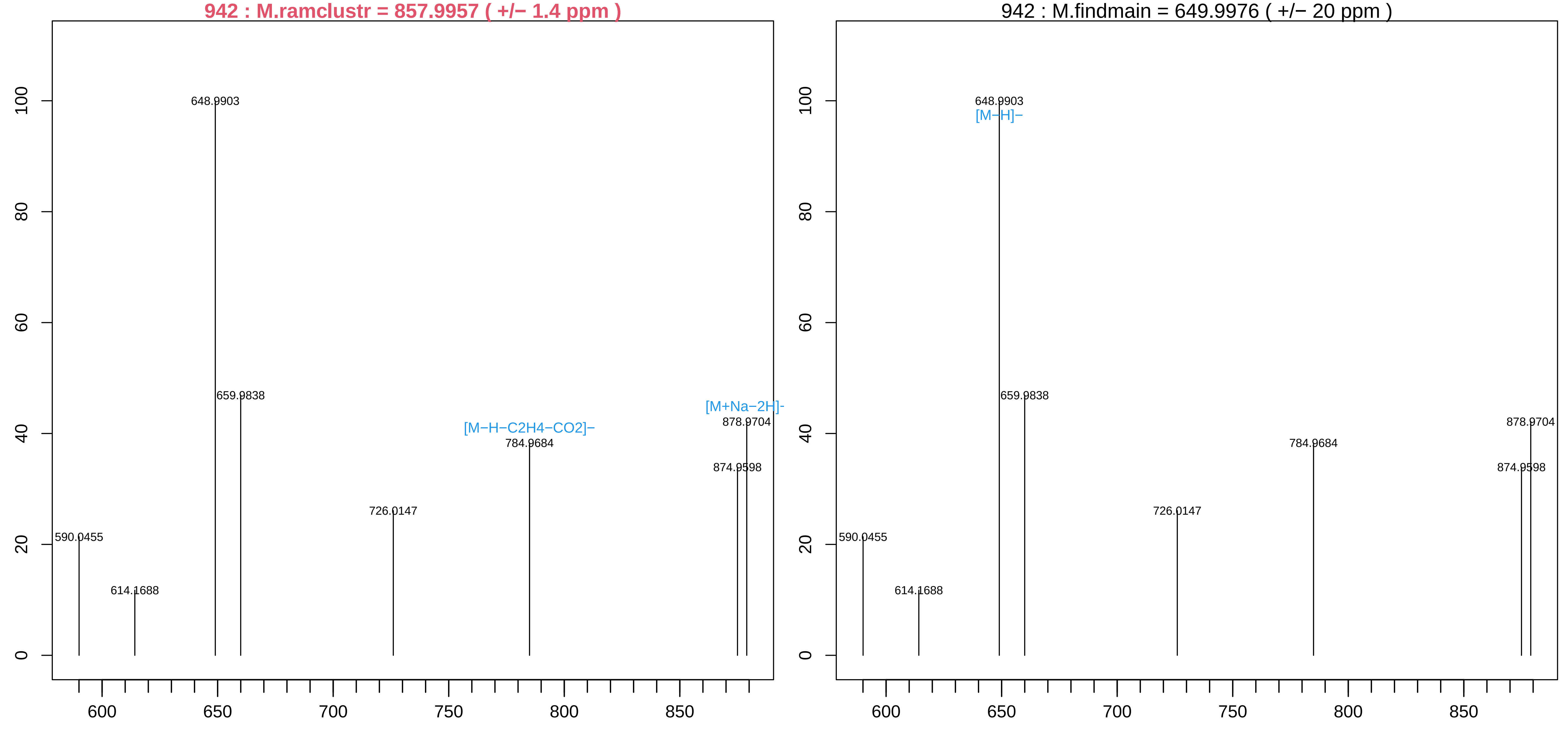
942 : M.ramclustr = 857.9957 (+/− 1.4 ppm)
648.9903
659.9838
[M+Na−2H]-
878.9704
[M−H−C2H4−CO2]−
784.9684
874.9598
726.0147
590.0455
614.1688
942 : M.findmain = 649.9976 (+/− 20 ppm)
648.9903
[M−H]−
659.9838
878.9704
784.9684
874.9598
726.0147
590.0455
614.1688
0
20
40
60
80
100
600
650
700
750
800
850

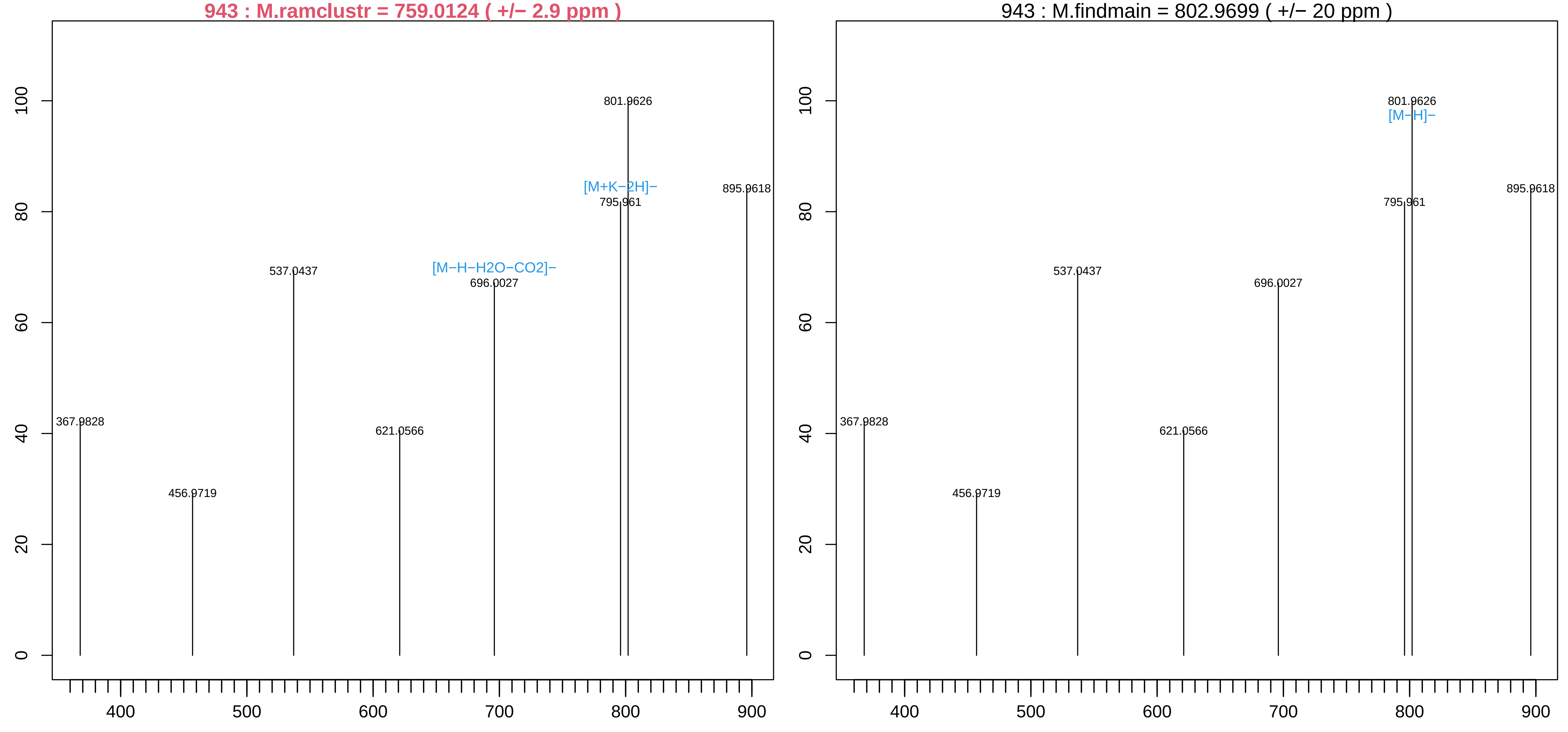
943 : M.ramclustr = 759.0124 (+/− 2.9 ppm)
801.9626
[M+K−2H]−
795.961
895.9618
537.0437
[M−H−H2O−CO2]−
696.0027
367.9828
621.0566
456.9719
0
20
40
60
80
100
400
500
600
700
800
900
943 : M.findmain = 802.9699 (+/− 20 ppm)
801.9626
[M−H]−
795.961
895.9618
537.0437
696.0027
367.9828
621.0566
456.9719
0
20
40
60
80
100
400
500
600
700
800
900

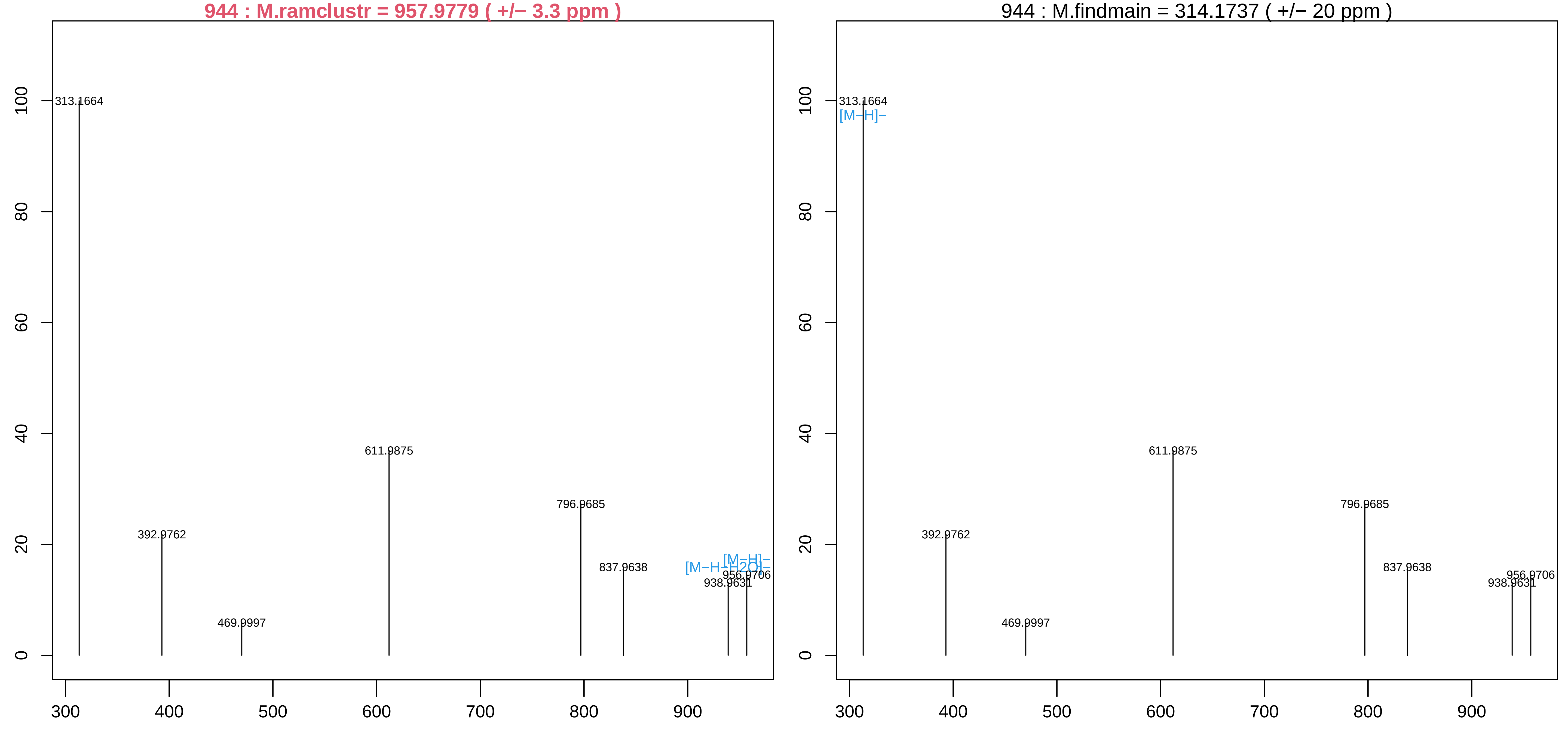
944 : M.ramclustr = 957.9779 (+/− 3.3 ppm)
313.1664
392.9762
469.9997
611.9875
796.9685
837.9638
938.9631
956.9706
[M−H]−
[M−H−H2O]−
0
20
40
60
80
100
300
400
500
600
700
800
900
944 : M.findmain = 314.1737 (+/− 20 ppm)
313.1664
[M−H]−
392.9762
469.9997
611.9875
796.9685
837.9638
938.9631
956.9706

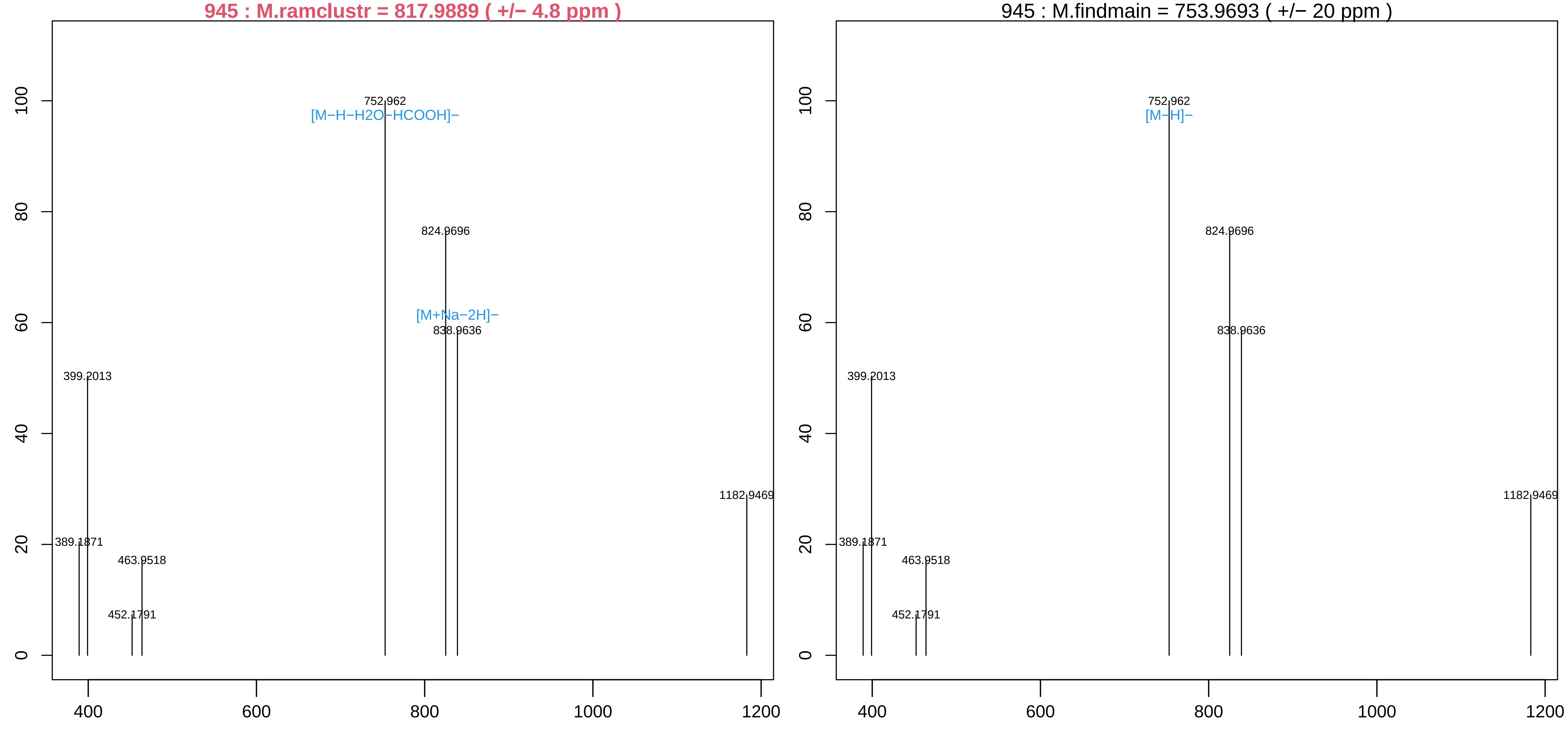

945 : M.ramclustr = 817.9889 (+/− 4.8 ppm)
752.962
[M−H−H2O−HCOOH]−
824.9696
[M+Na−2H]−
838.9636
399.2013
389.1871
463.9518
452.1791
1182.9469
945 : M.findmain = 753.9693 (+/− 20 ppm)
752.962
[M−H]−
824.9696
838.9636
399.2013
389.1871
463.9518
452.1791
1182.9469
0
20
40
60
80
100
400
600
800
1000
1200

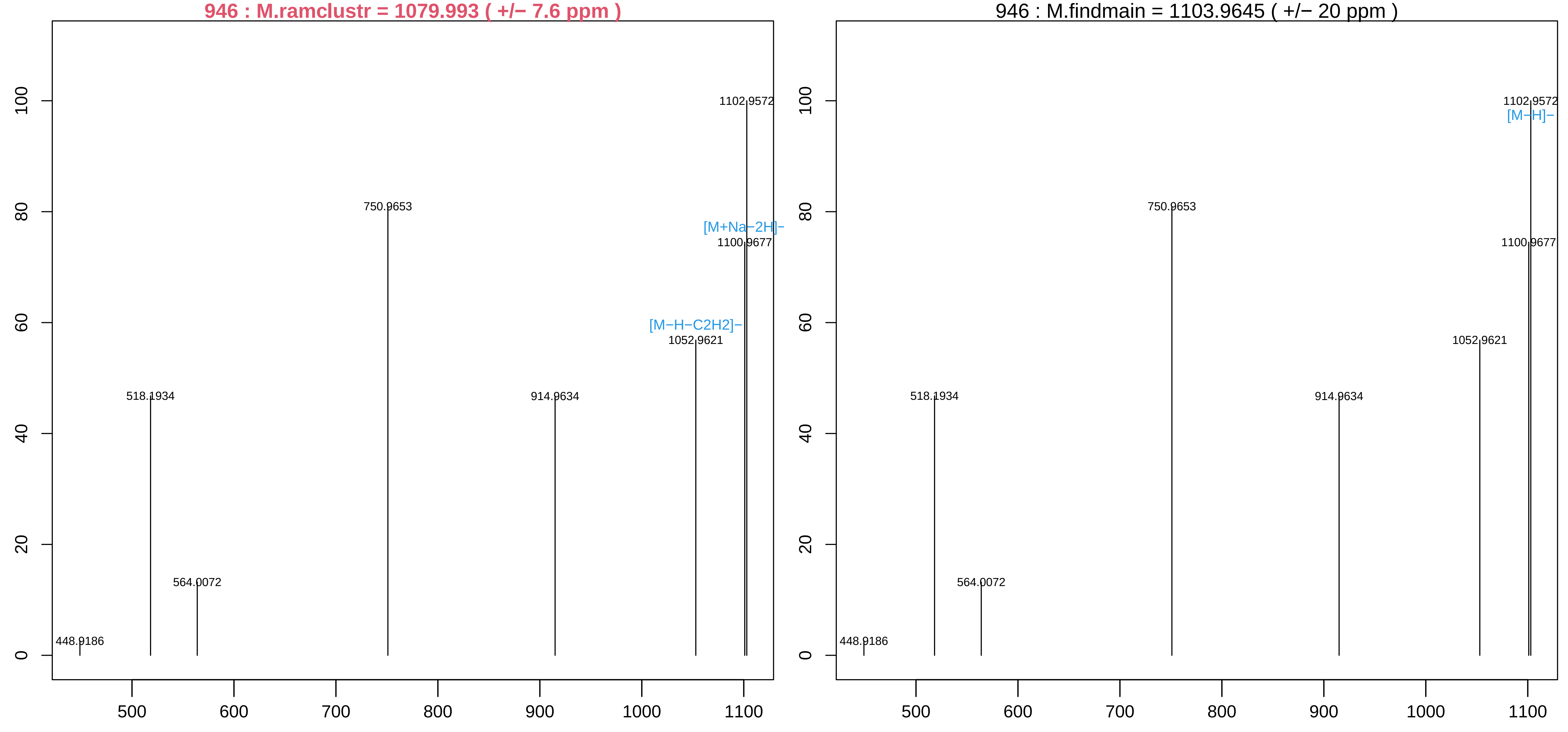

946 : M.ramclustr = 1079.993 (+/− 7.6 ppm)
1102.9572
750.9653
[M+Na−2H]−
1100.9677
[M−H−C2H2]−
1052.9621
518.1934
914.9634
564.0072
448.9186
946 : M.findmain = 1103.9645 (+/− 20 ppm)
1102.9572
[M−H]−
750.9653
1100.9677
1052.9621
518.1934
914.9634
564.0072
448.9186

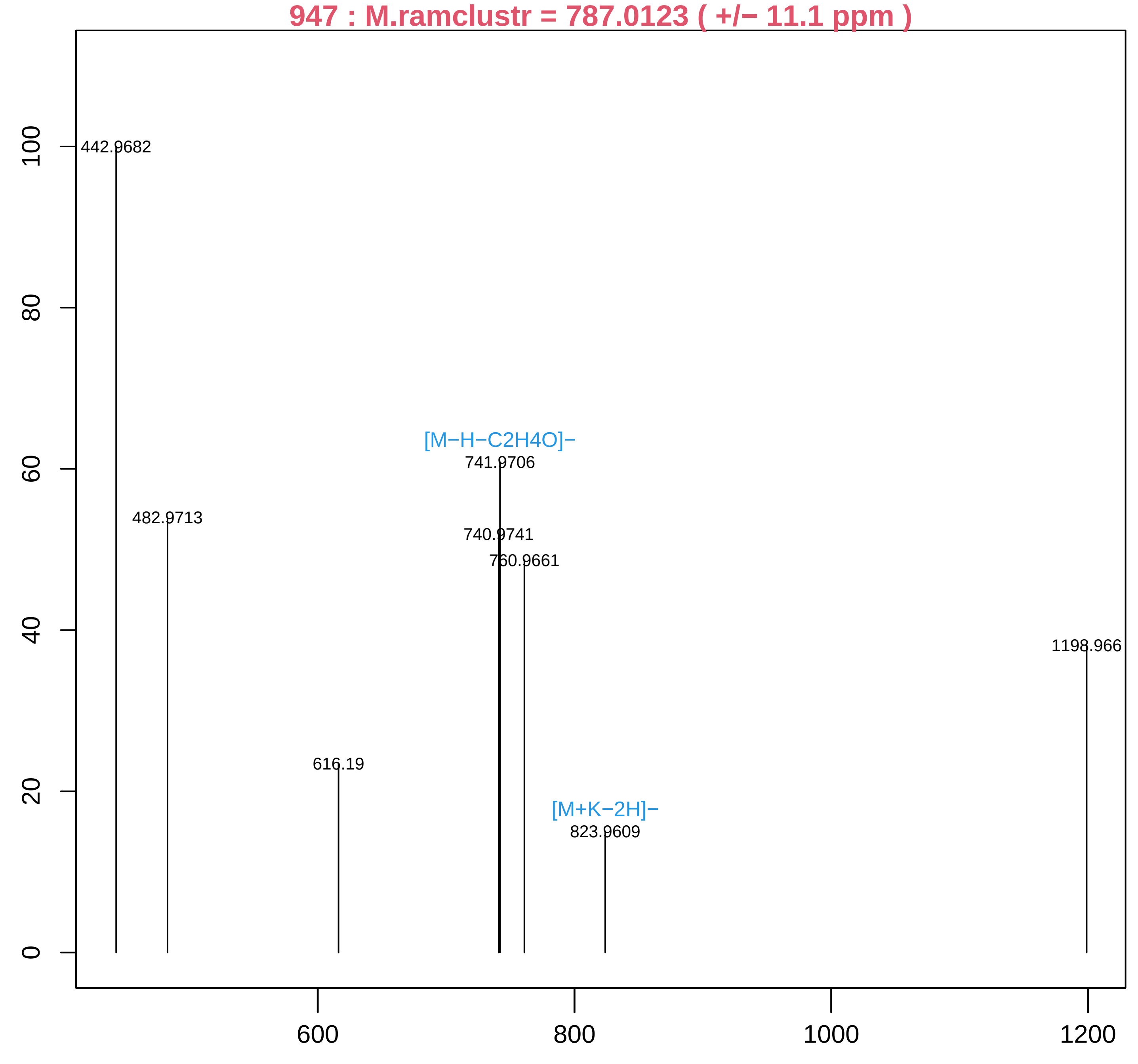
947 : M.ramclustr = 787.0123 (+/− 11.1 ppm)
0
20
40
60
80
100
600
800
1000
1200
442.9682
482.9713
616.19
[M−H−C2H4O]−
741.9706
740.9741
760.9661
[M+K−2H]−
823.9609
1198.966

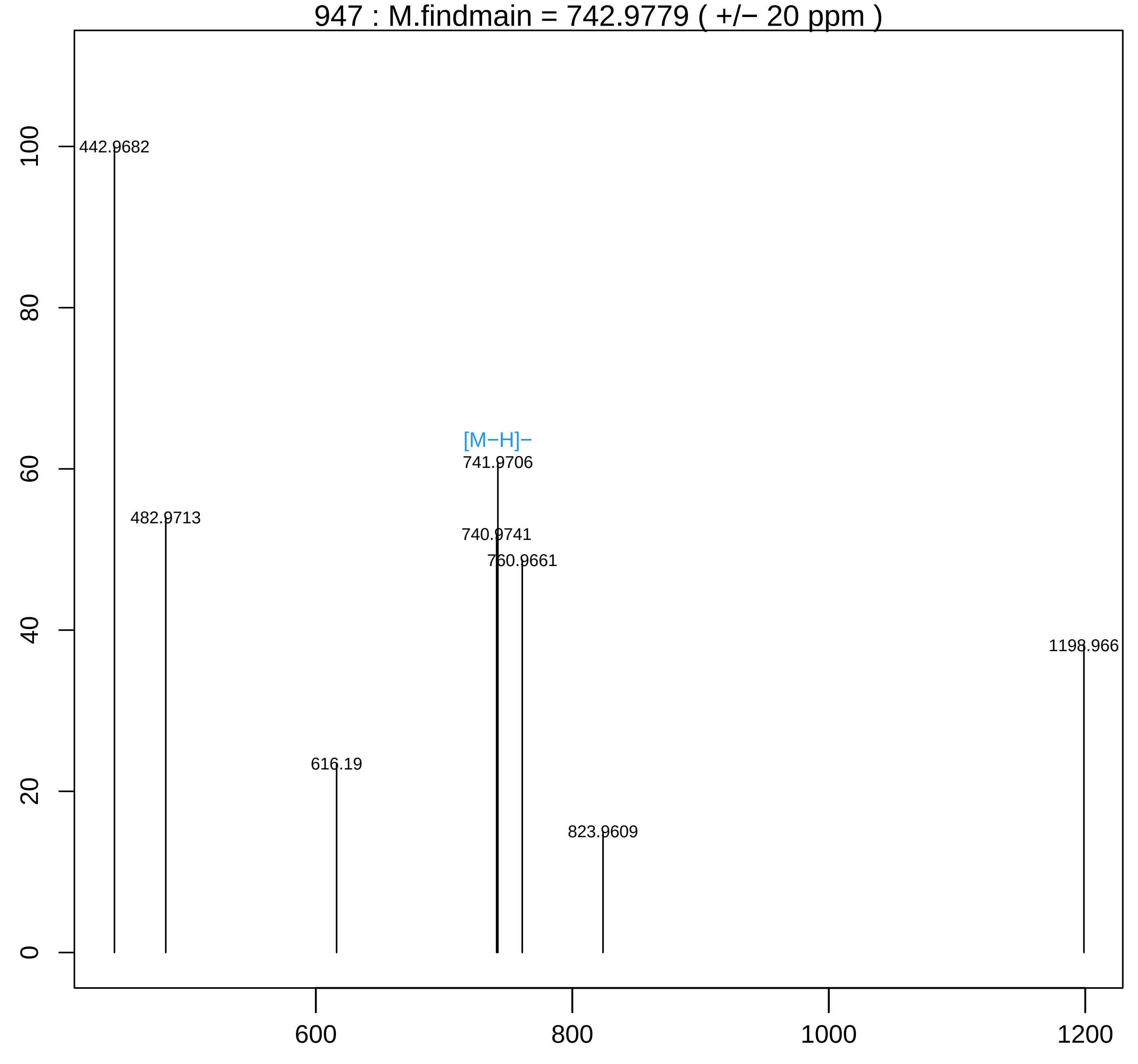
947 : M.findmain = 742.9779 (+/− 20 ppm)
0
20
40
60
80
100
600
800
1000
1200
442.9682
482.9713
616.19
[M−H]−
741.9706
740.9741
760.9661
823.9609
1198.966

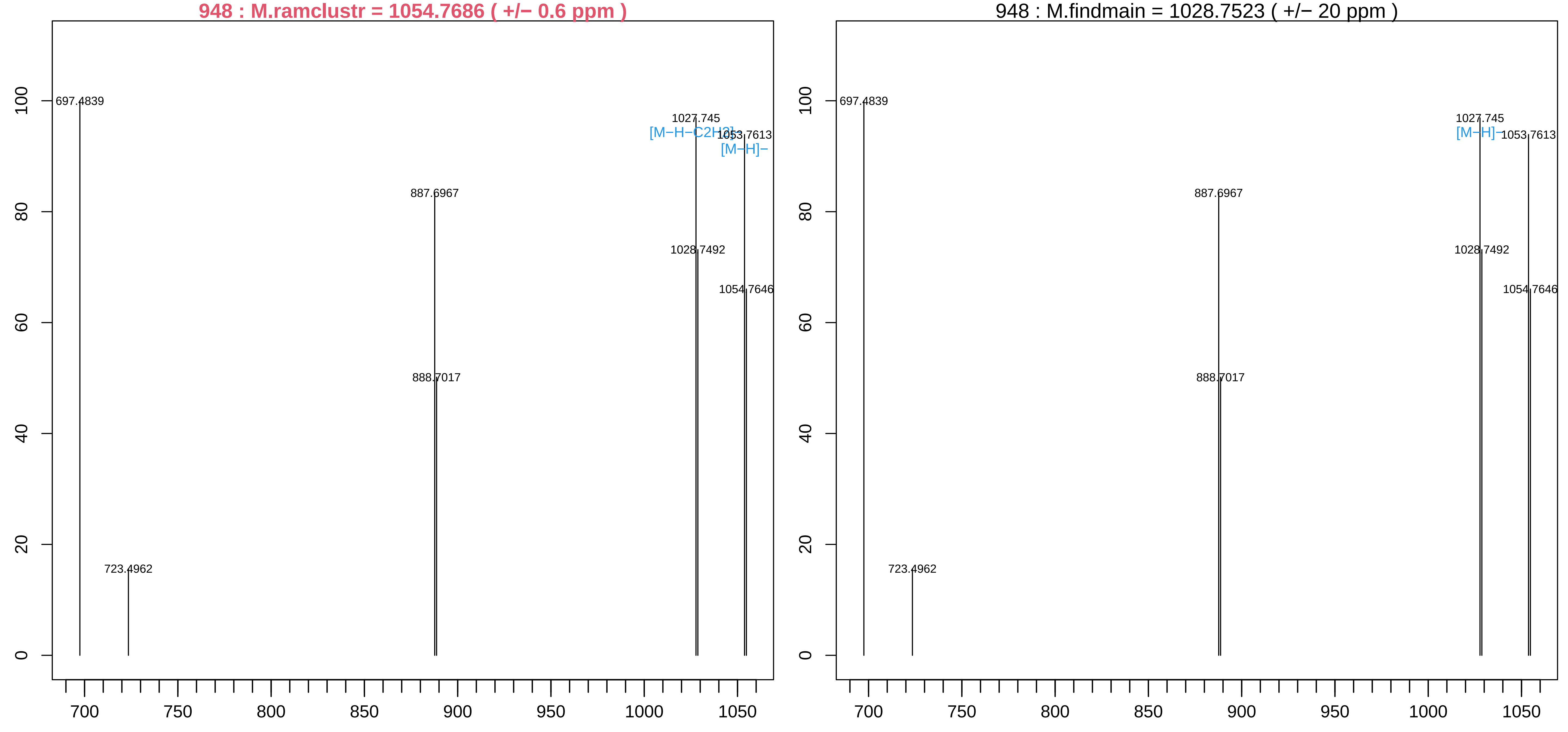
948 : M.ramclustr = 1054.7686 (+/− 0.6 ppm)
697.4839
723.4962
887.6967
888.7017
1027.745
[M−H−C2H2]−
1053.7613
[M−H]−
1028.7492
1054.7646
948 : M.findmain = 1028.7523 (+/− 20 ppm)
697.4839
723.4962
887.6967
888.7017
1027.745
[M−H]−
1053.7613
1028.7492
1054.7646
0
20
40
60
80
100
700
750
800
850
900
950
1000
1050

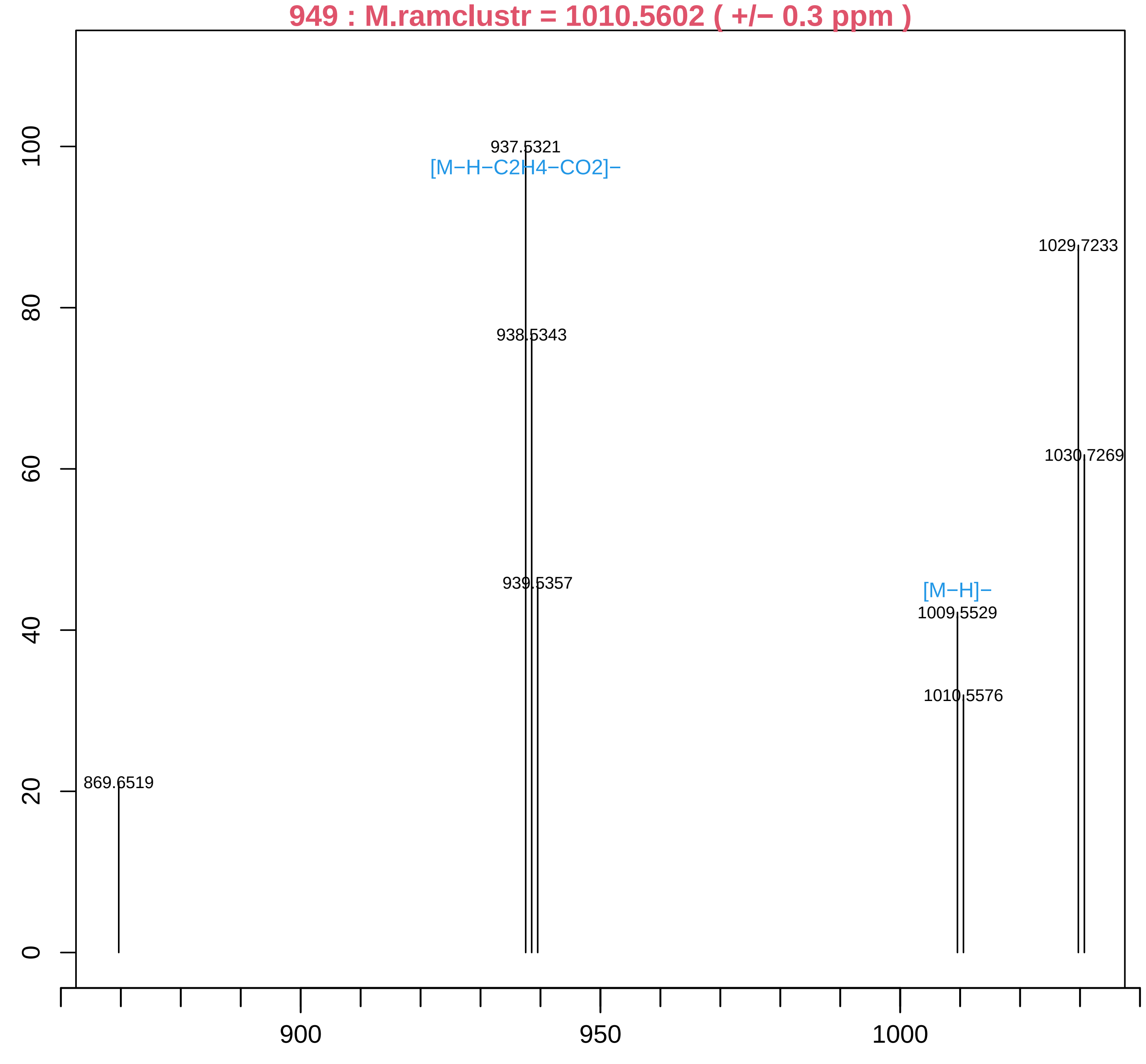

949 : M.ramclustr = 1010.5602 (+/− 0.3 ppm)
937.5321
[M−H−C2H4−CO2]−
1029.7233
938.5343
1030.7269
939.5357
[M−H]−
1009.5529
1010.5576
869.6519
0
20
40
60
80
100
900
950
1000

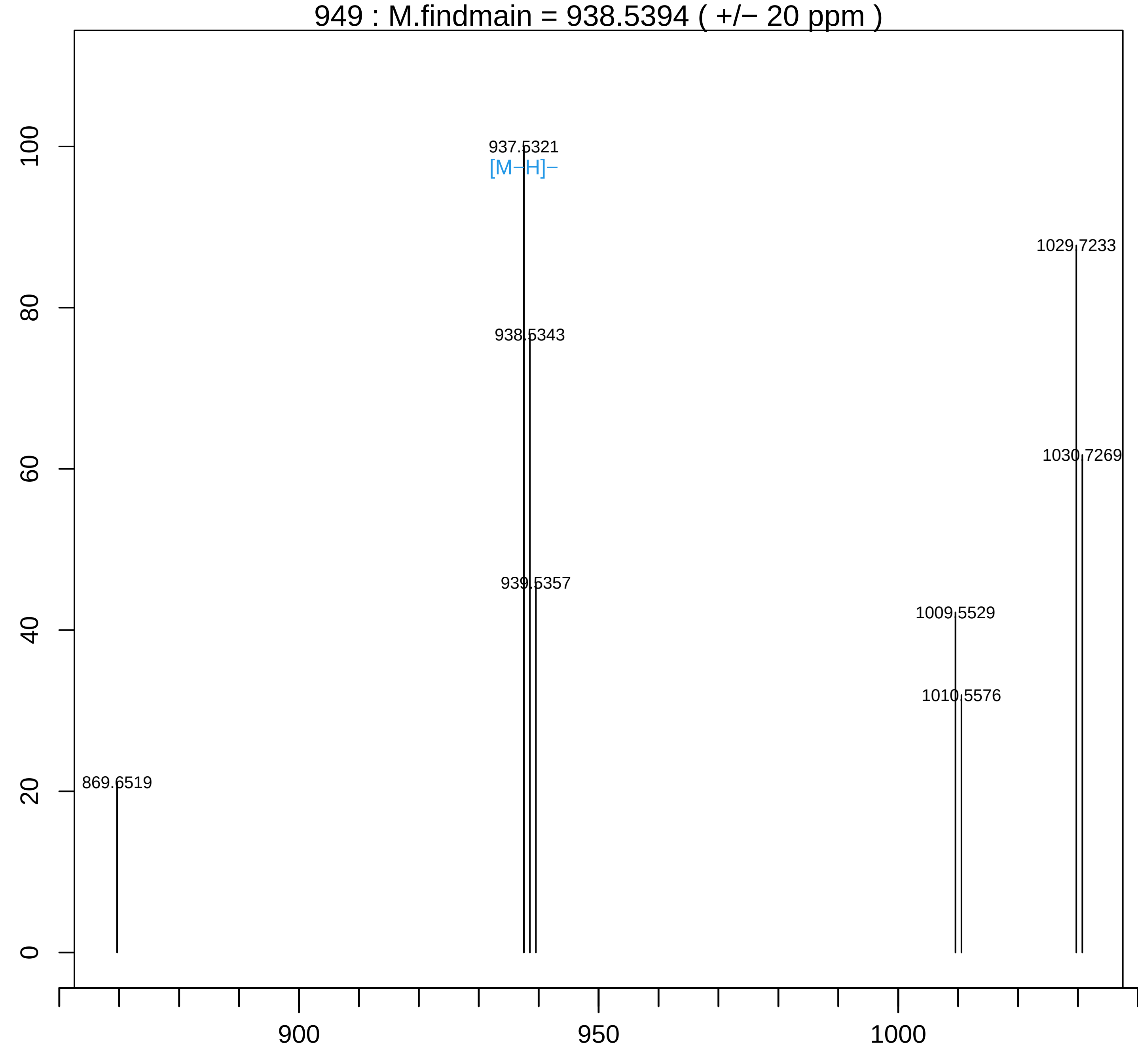

949 : M.findmain = 938.5394 (+/− 20 ppm)
937.5321
[M−H]−
1029.7233
938.5343
1030.7269
939.5357
1009.5529
1010.5576
869.6519
0
20
40
60
80
100
900
950
1000

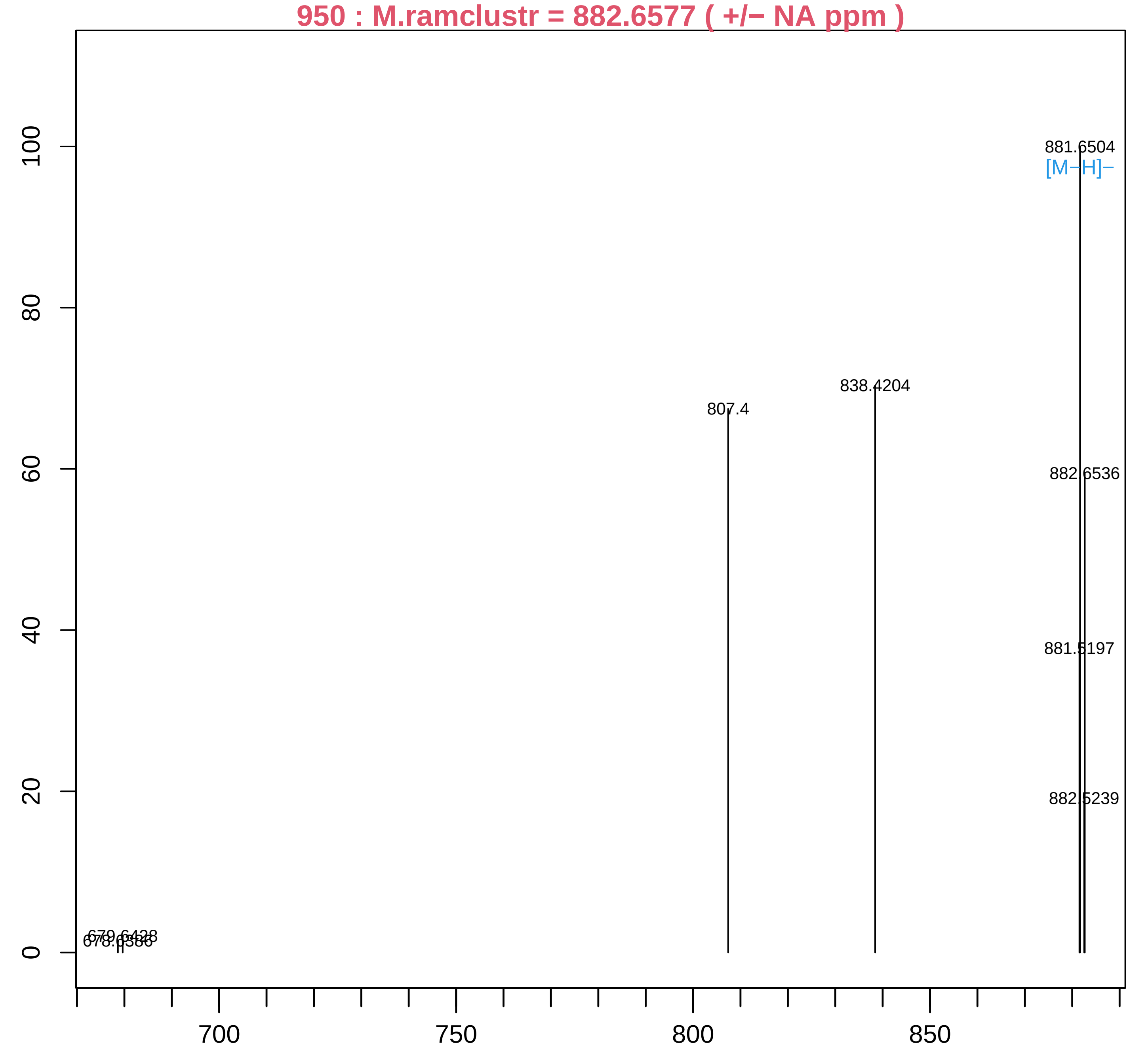

950 : M.ramclustr = 882.6577 (+/− NA ppm)
881.6504
[M−H]−
807.4
838.4204
882.6536
881.5197
882.5239
679.6428
678.6386
0
20
40
60
80
100
700
750
800
850

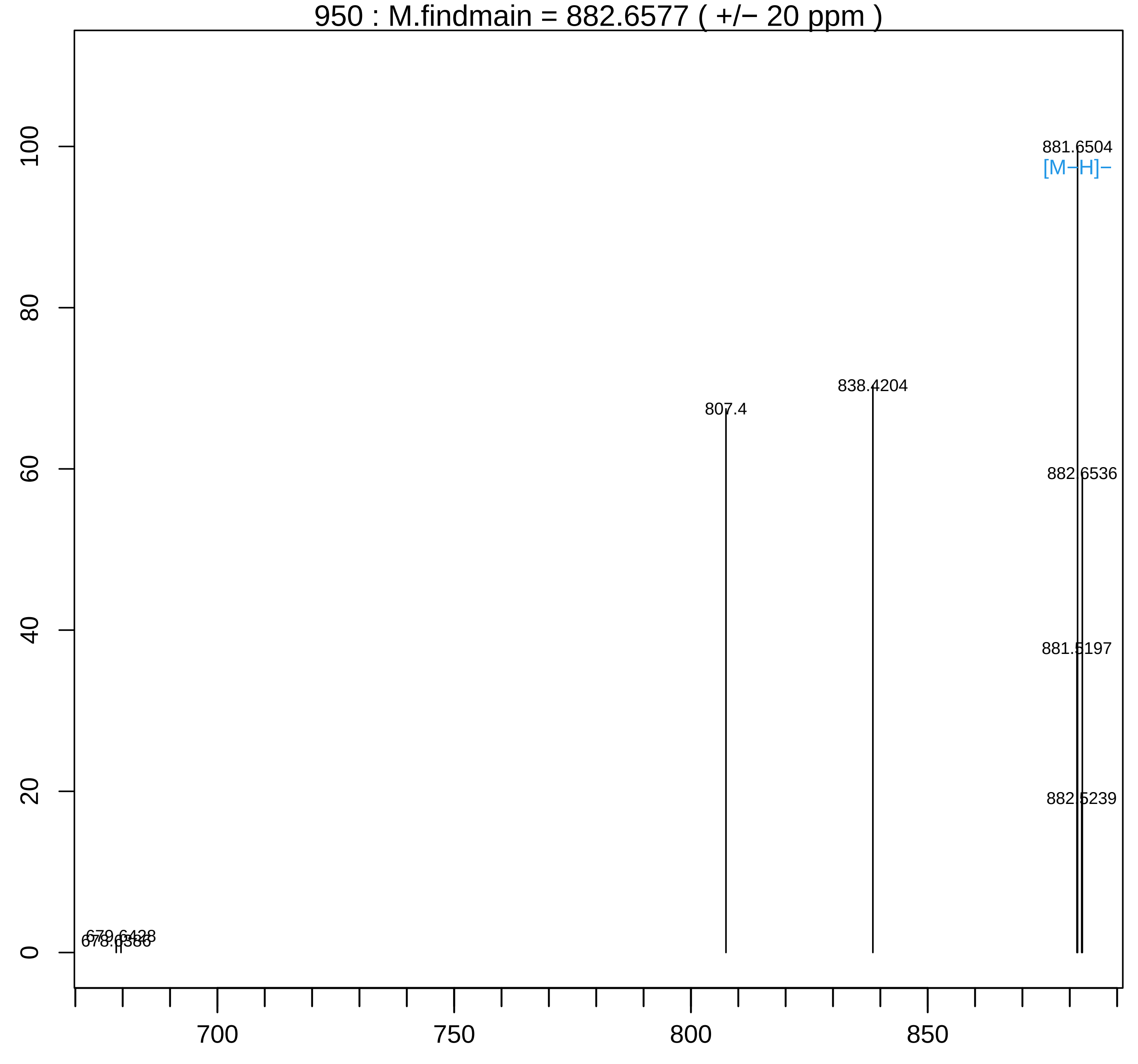

950 : M.findmain = 882.6577 (+/− 20 ppm)
881.6504
[M−H]−
807.4
838.4204
882.6536
881.5197
882.5239
679.6428
678.6386
0
20
40
60
80
100
700
750
800
850

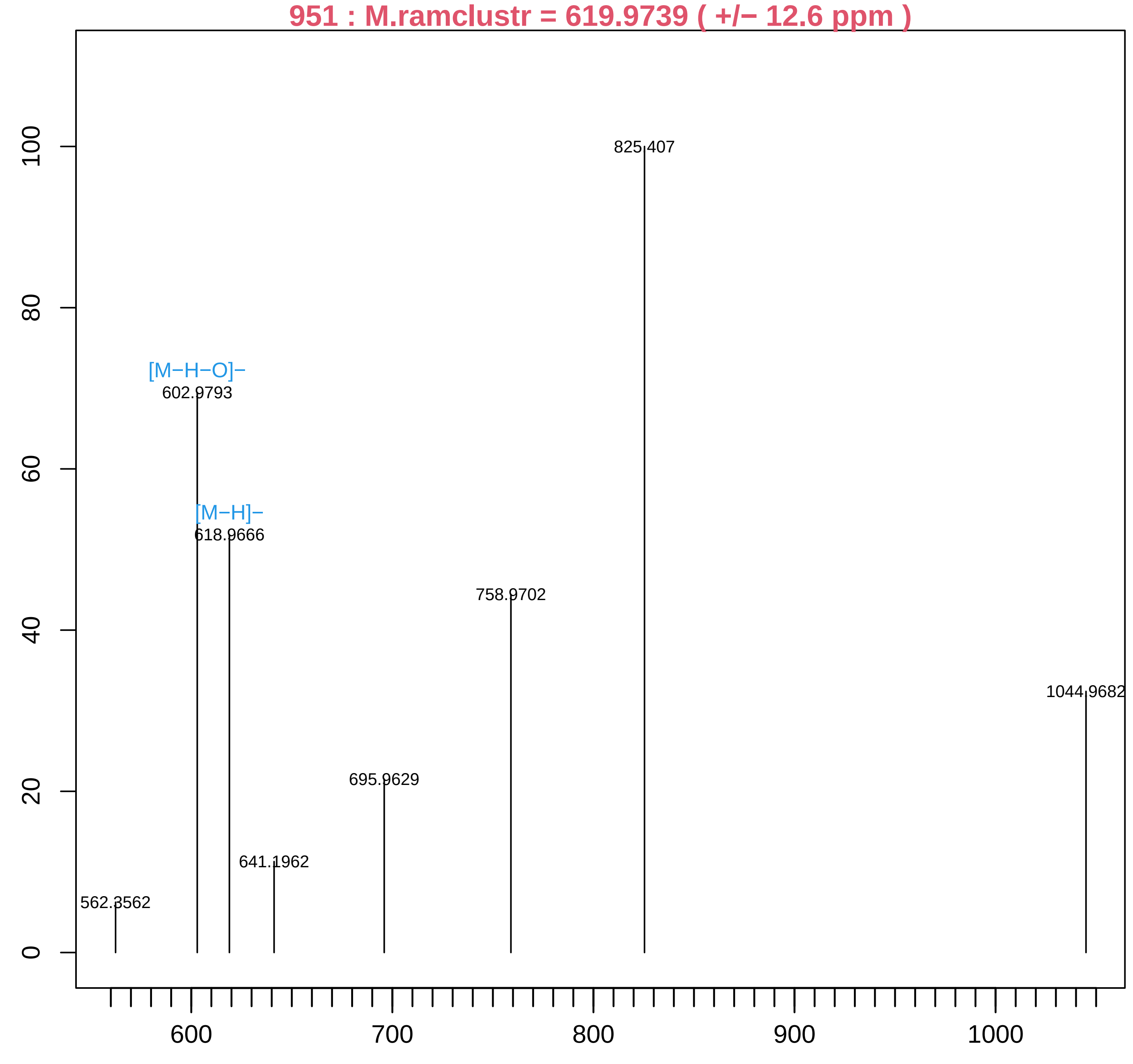
951 : M.ramclustr = 619.9739 (+/− 12.6 ppm)
[M−H−O]−
602.9793
[M−H]−
618.9666
825.407
758.9702
1044.9682
695.9629
641.1962
562.3562
0
20
40
60
80
100
600
700
800
900
1000

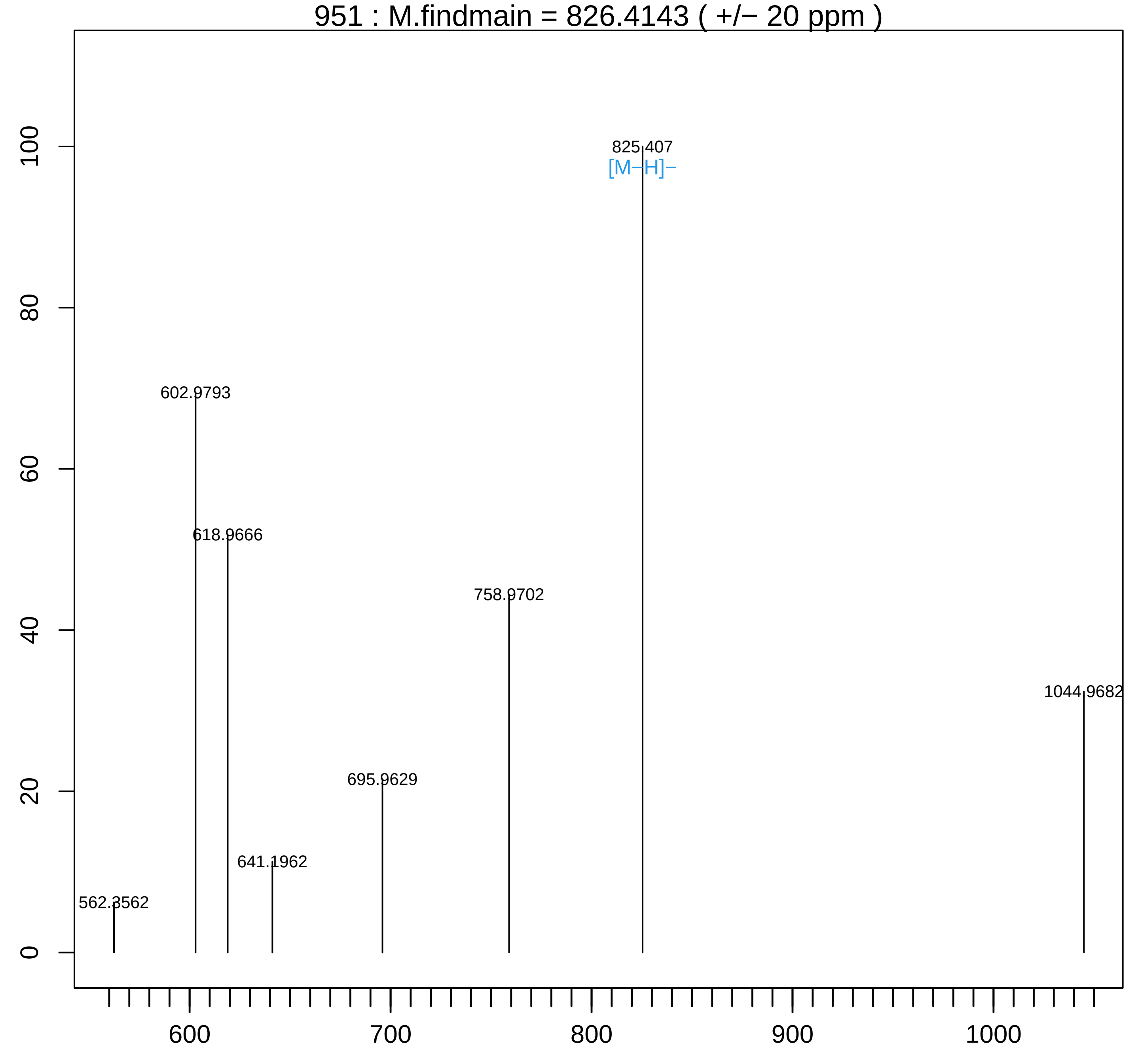
951 : M.findmain = 826.4143 (+/− 20 ppm)
825.407
[M−H]−
602.9793
618.9666
758.9702
1044.9682
695.9629
641.1962
562.3562
0
20
40
60
80
100
600
700
800
900
1000

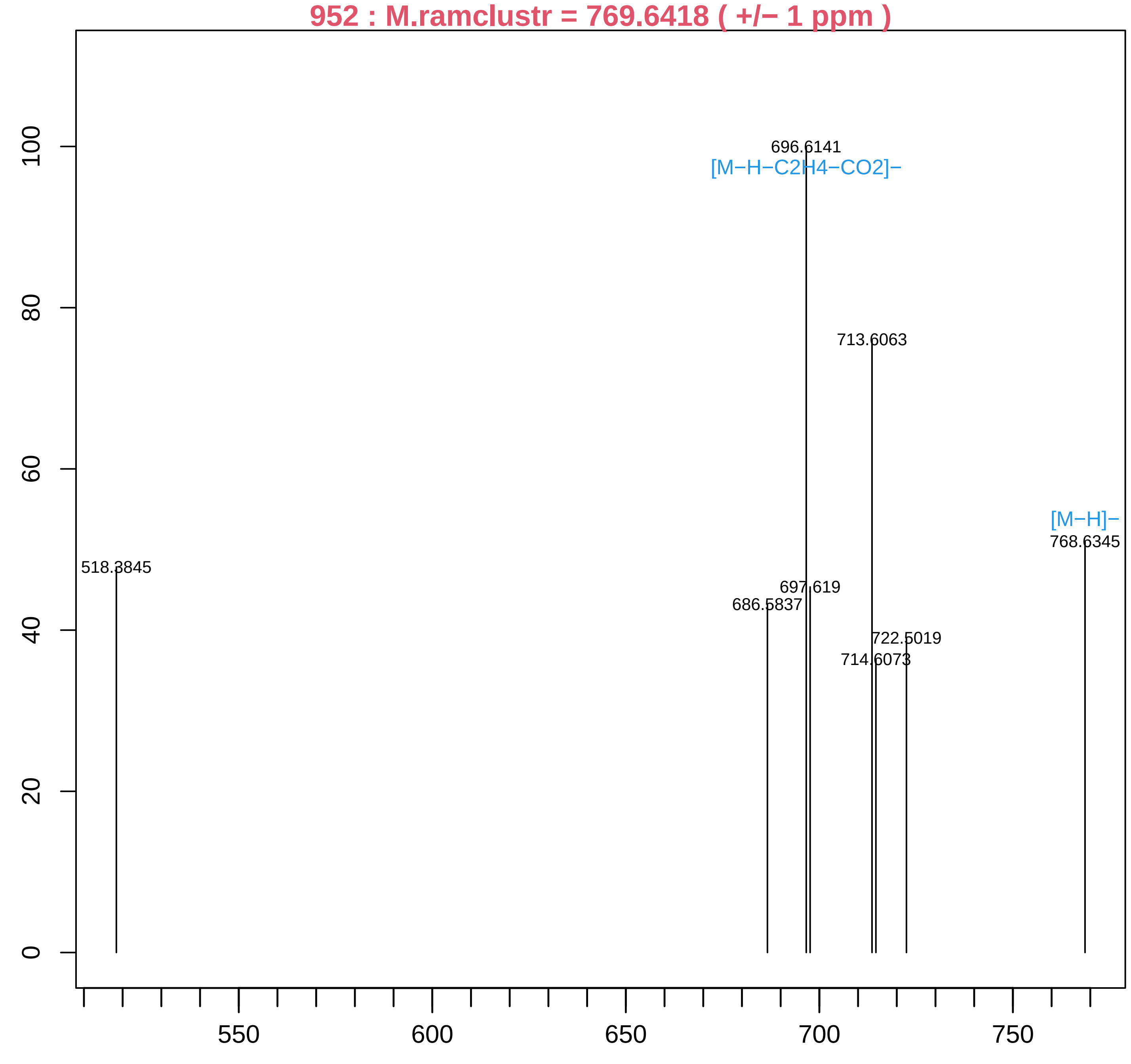

952 : M.ramclustr = 769.6418 (+/− 1 ppm)
696.6141
[M−H−C2H4−CO2]−
713.6063
[M−H]−
768.6345
518.3845
697.619
686.5837
722.5019
714.6073
0
20
40
60
80
100
550
600
650
700
750

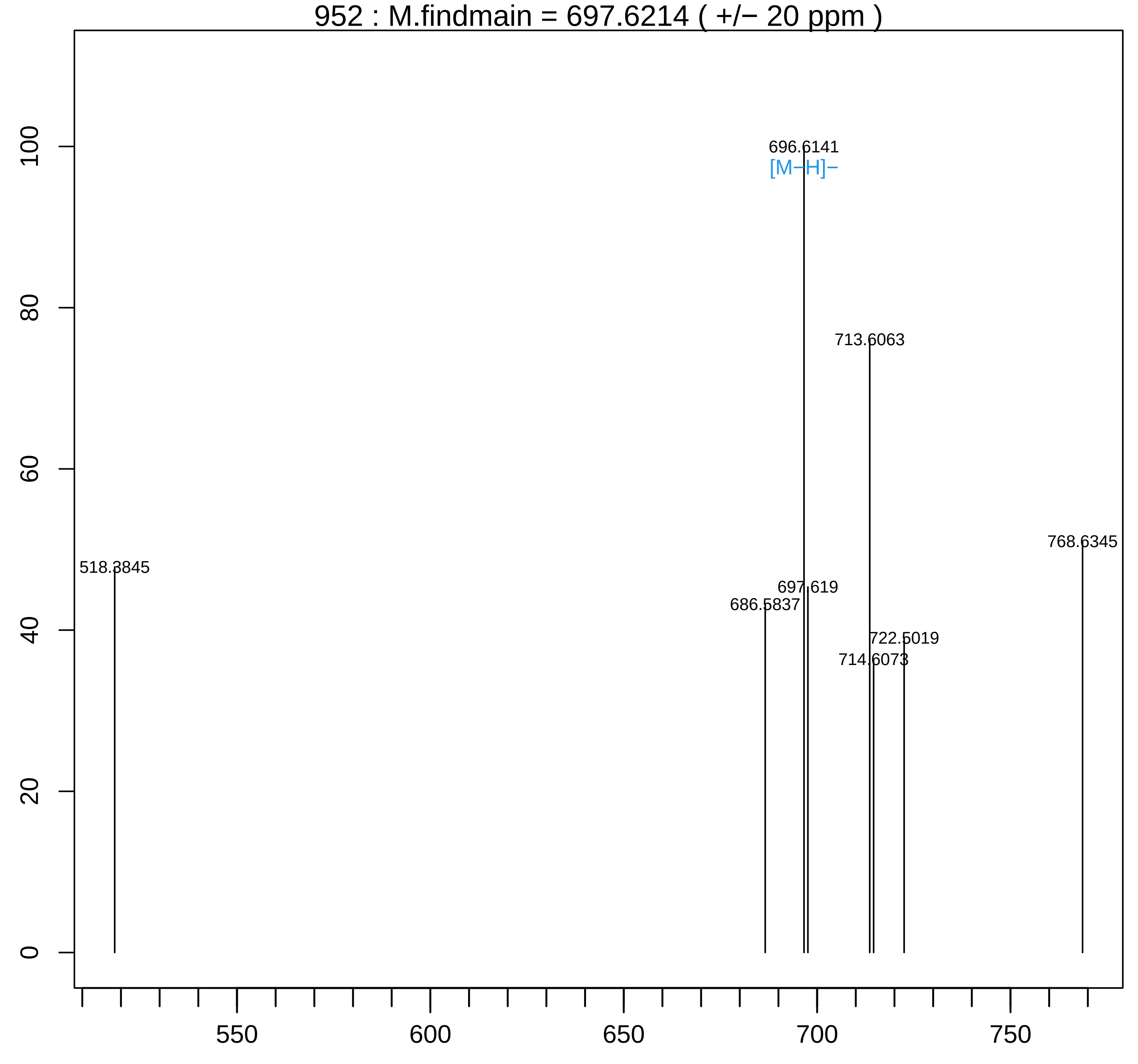

952 : M.findmain = 697.6214 (+/− 20 ppm)
696.6141
[M−H]−
713.6063
768.6345
518.3845
697.619
686.5837
722.5019
714.6073
0
20
40
60
80
100
550
600
650
700
750

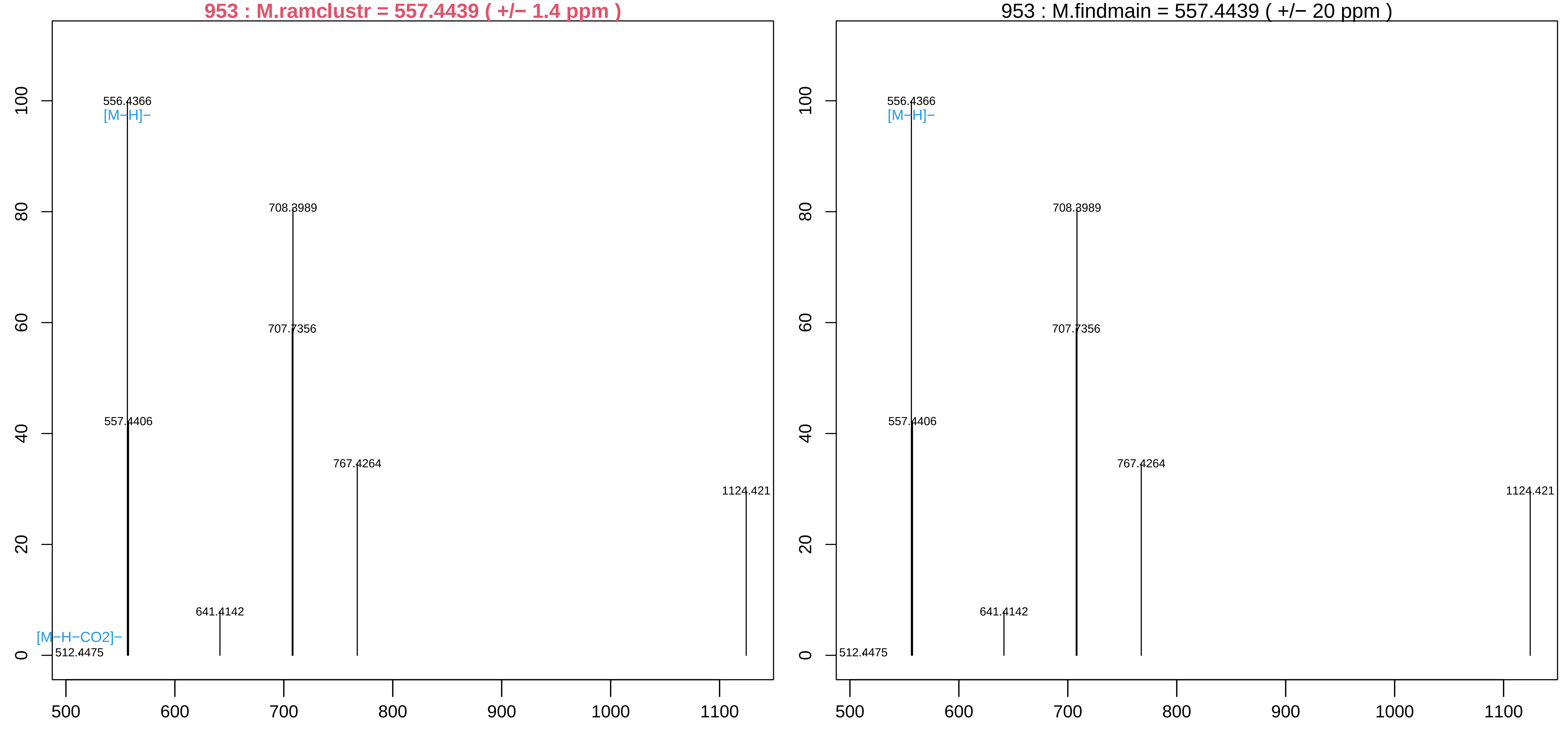
953 : M.ramclustr = 557.4439 (+/− 1.4 ppm)
556.4366
[M−H]−
708.3989
707.7356
557.4406
767.4264
1124.421
641.4142
[M−H−CO2]−
512.4475
953 : M.findmain = 557.4439 (+/− 20 ppm)
556.4366
[M−H]−
708.3989
707.7356
557.4406
767.4264
1124.421
641.4142
512.4475

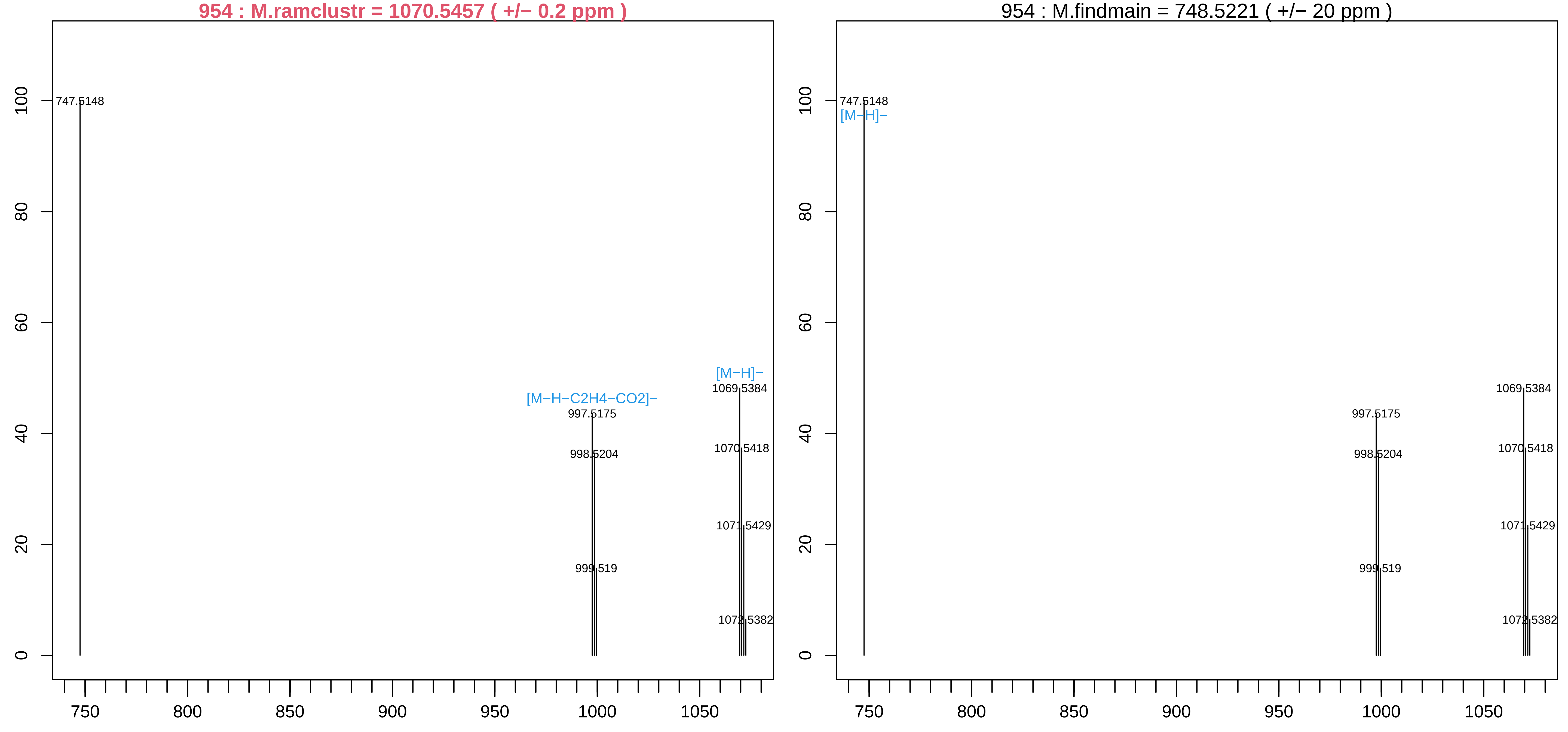

954 : M.ramclustr = 1070.5457 (+/− 0.2 ppm)
747.5148
[M−H−C2H4−CO2]−
997.5175
998.5204
999.519
[M−H]−
1069.5384
1070.5418
1071.5429
1072.5382
954 : M.findmain = 748.5221 (+/− 20 ppm)
747.5148
[M−H]−
997.5175
998.5204
999.519
1069.5384
1070.5418
1071.5429
1072.5382

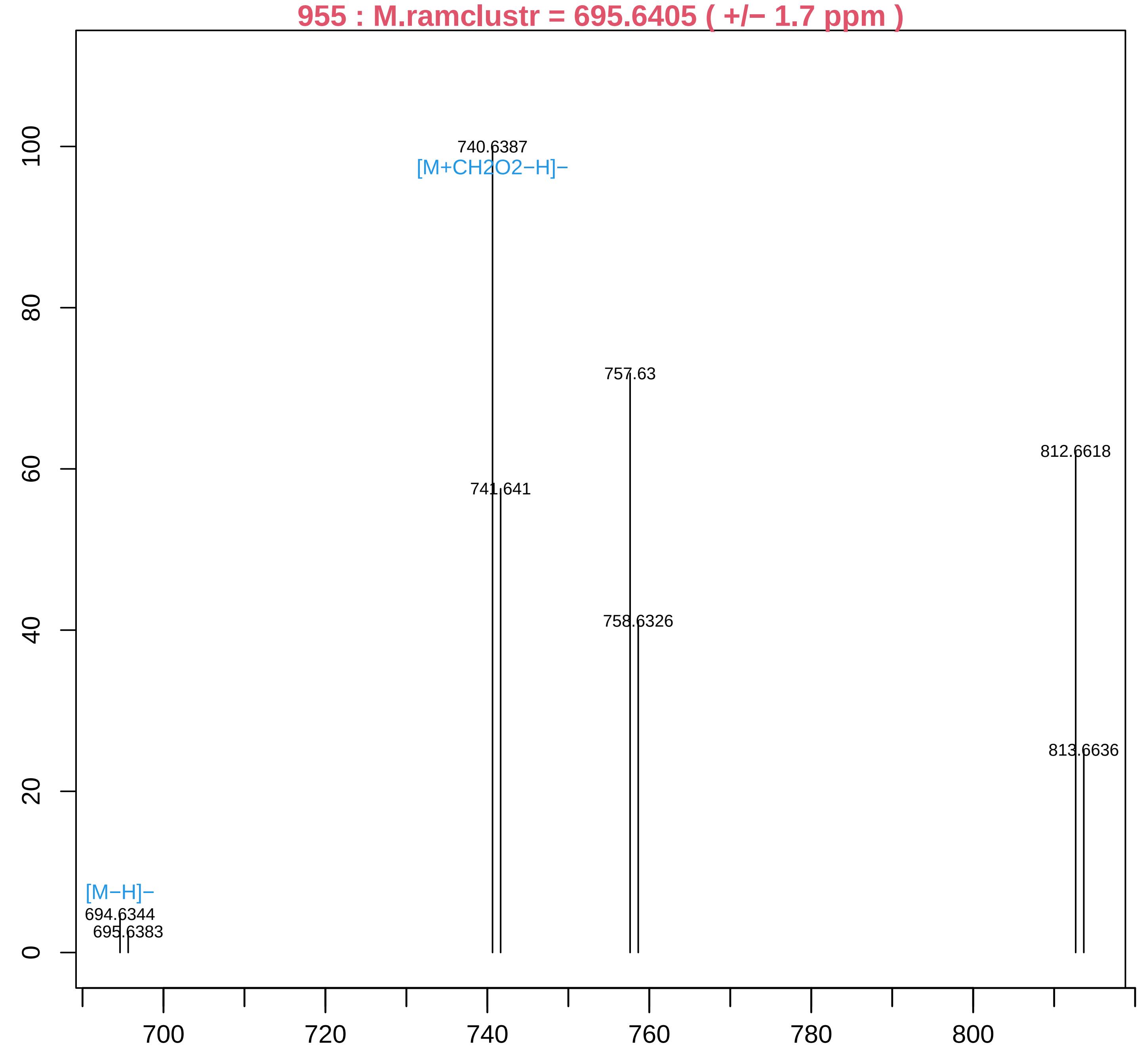
955 : M.ramclustr = 695.6405 (+/− 1.7 ppm)
740.6387
[M+CH2O2−H]−
757.63
812.6618
741.641
758.6326
813.6636
[M−H]−
694.6344
695.6383
0
20
40
60
80
100
700
720
740
760
780
800

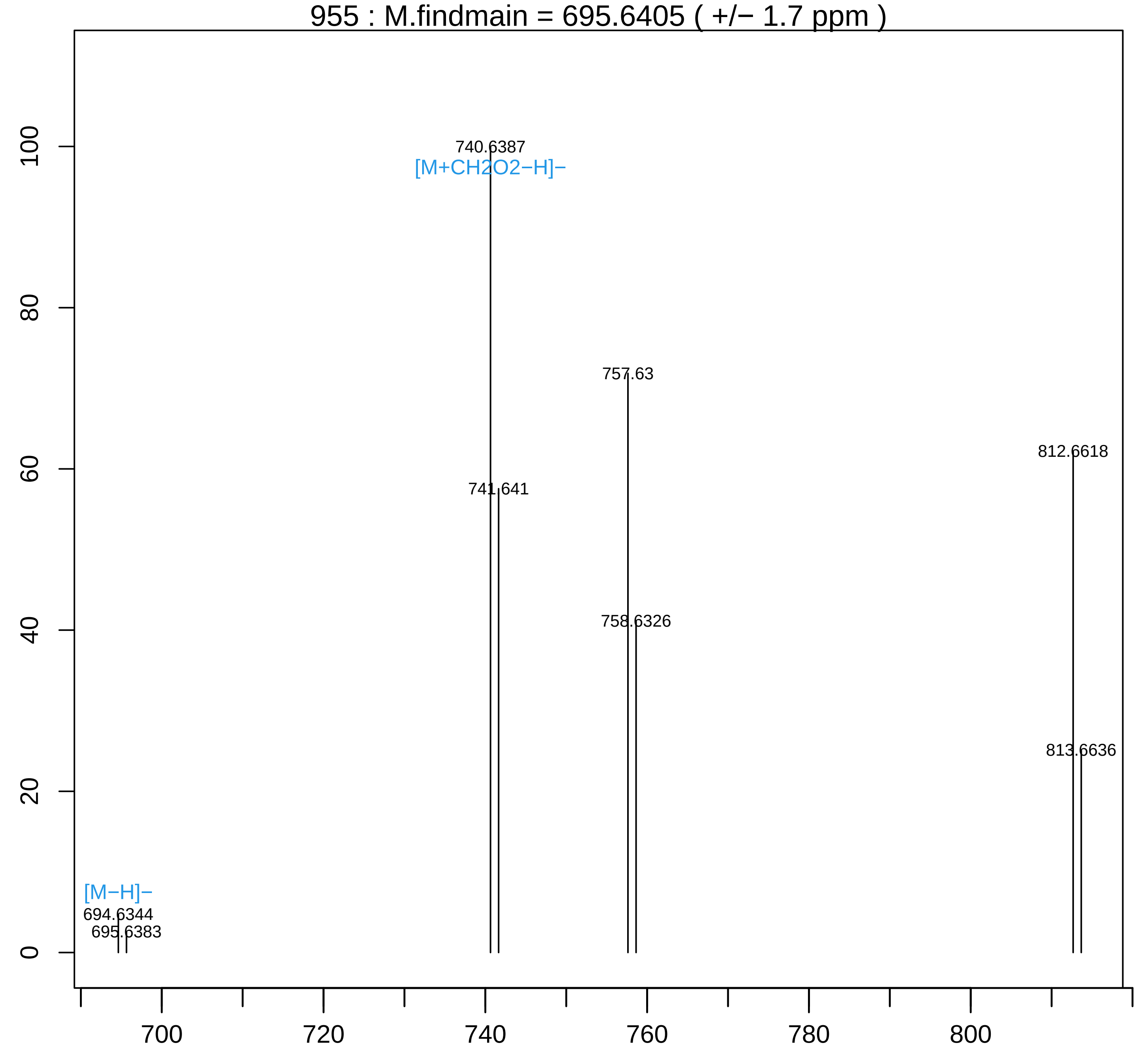
955 : M.findmain = 695.6405 (+/− 1.7 ppm)
740.6387
[M+CH2O2−H]−
757.63
812.6618
741.641
758.6326
813.6636
[M−H]−
694.6344
695.6383
0
20
40
60
80
100
700
720
740
760
780
800

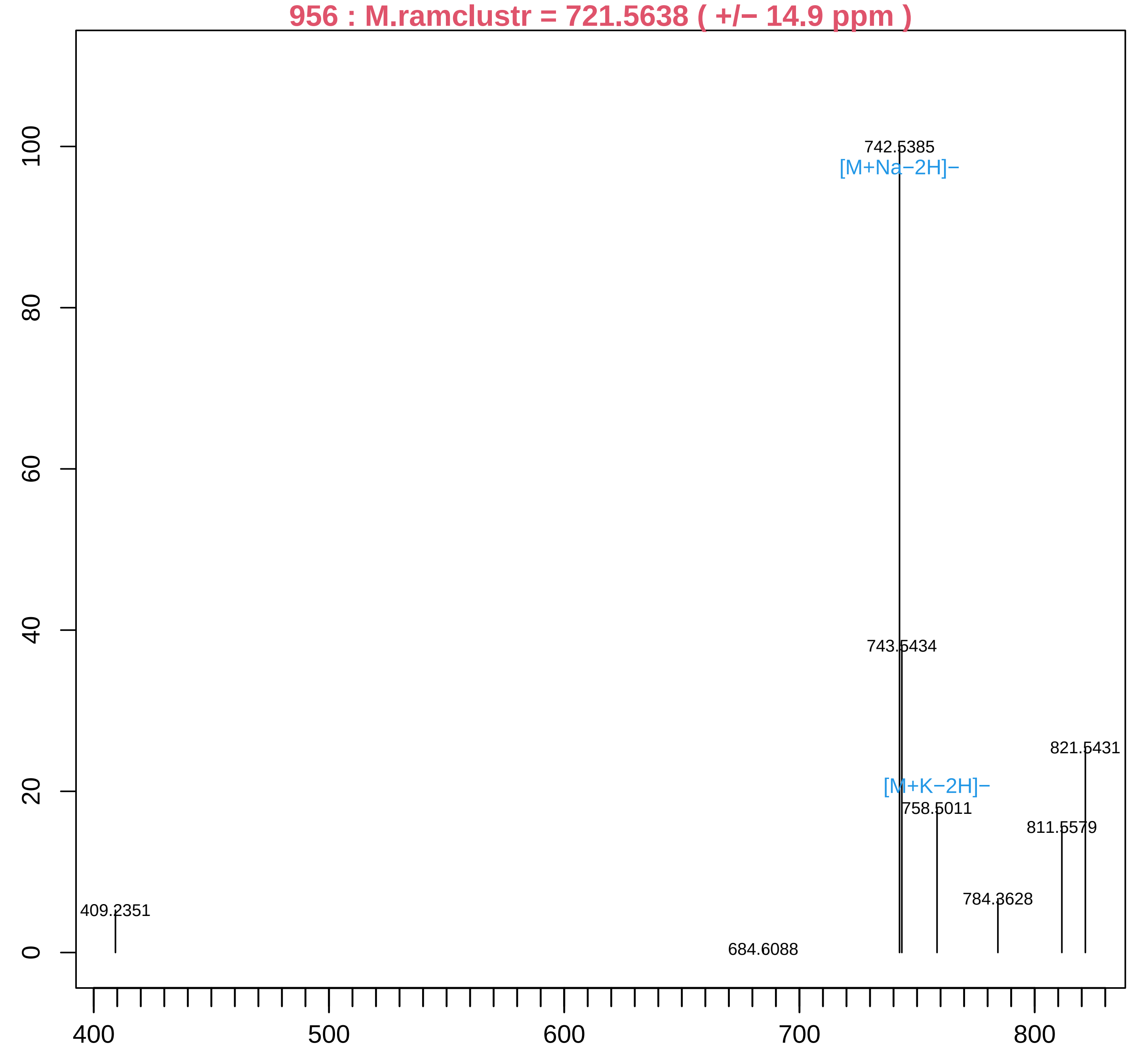
956 : M.ramclustr = 721.5638 (+/− 14.9 ppm)
742.5385
[M+Na−2H]−
743.5434
821.5431
[M+K−2H]−
758.5011
811.5579
784.3628
409.2351
684.6088
0
20
40
60
80
100
400
500
600
700
800

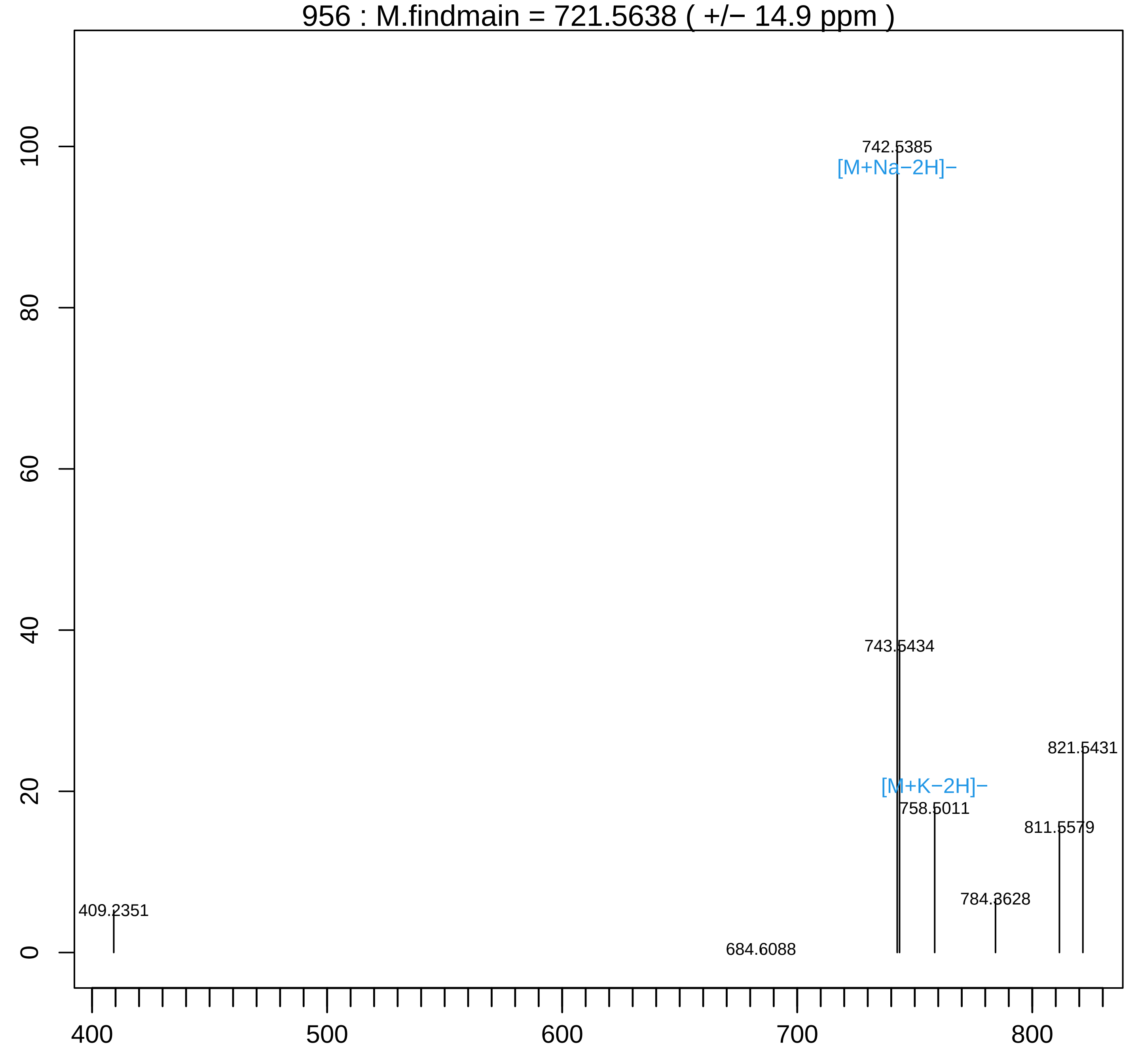
956 : M.findmain = 721.5638 (+/− 14.9 ppm)
742.5385
[M+Na−2H]−
743.5434
821.5431
[M+K−2H]−
758.5011
811.5579
784.3628
409.2351
684.6088
0
20
40
60
80
100
400
500
600
700
800

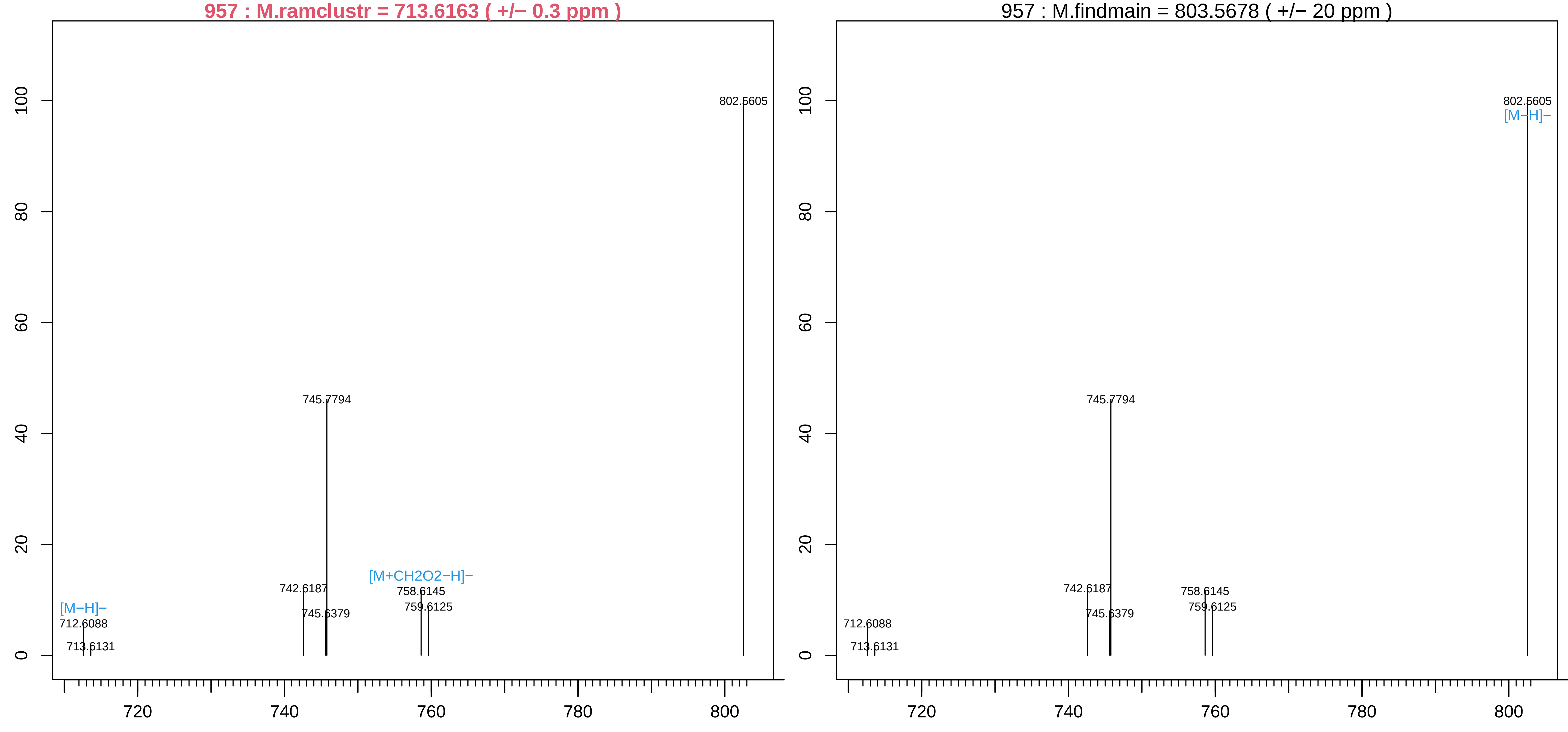
957 : M.ramclustr = 713.6163 (+/− 0.3 ppm)
802.5605
745.7794
[M+CH2O2−H]−
742.6187
758.6145
759.6125
745.6379
[M−H]−
712.6088
713.6131
957 : M.findmain = 803.5678 (+/− 20 ppm)
802.5605
[M−H]−
745.7794
742.6187
758.6145
759.6125
745.6379
712.6088
713.6131
0
20
40
60
80
100
720
740
760
780
800

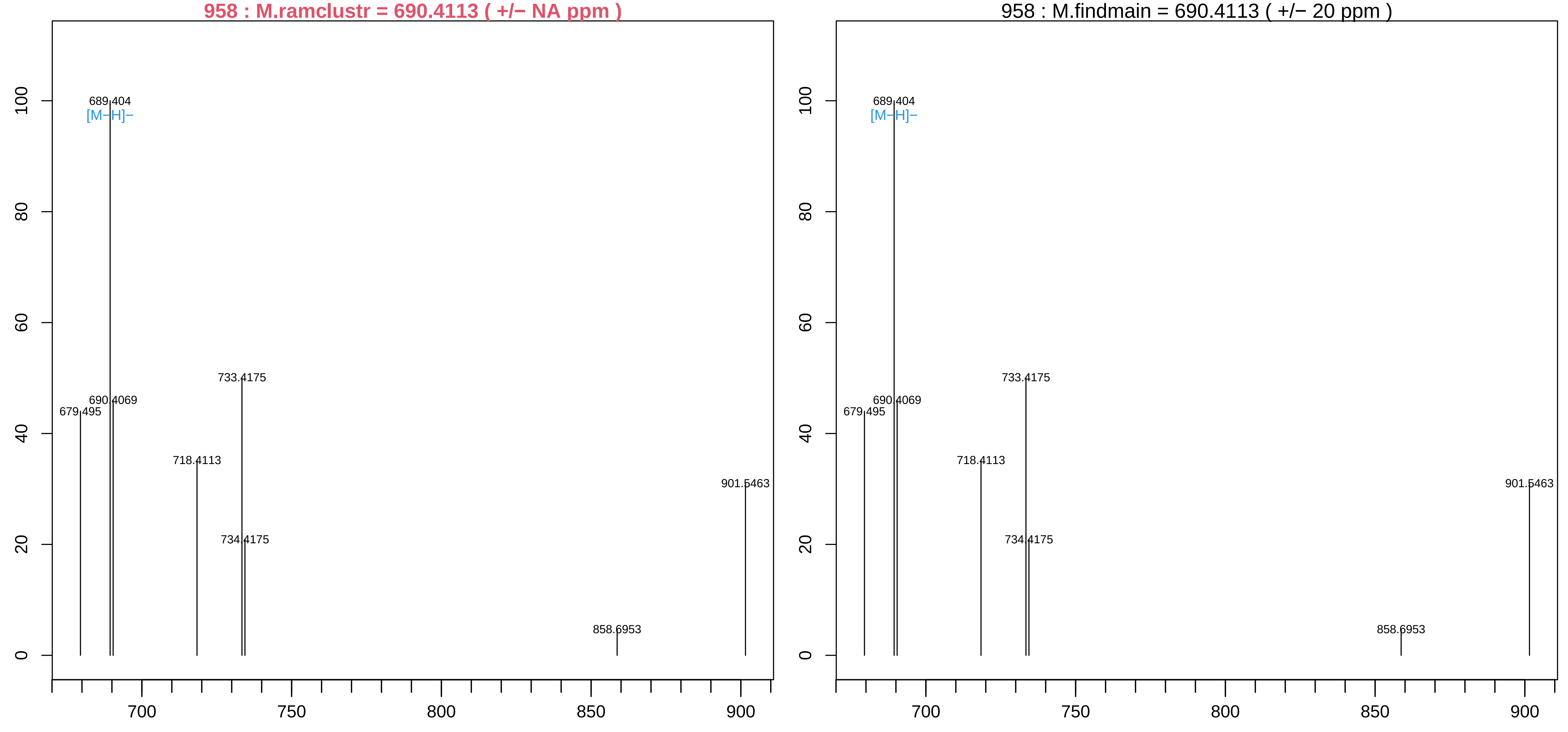
958 : M.ramclustr = 690.4113 (+/− NA ppm)
689.404
[M−H]−
733.4175
690.4069
679.495
718.4113
901.5463
734.4175
858.6953
958 : M.findmain = 690.4113 (+/− 20 ppm)
689.404
[M−H]−
733.4175
690.4069
679.495
718.4113
901.5463
734.4175
858.6953
0
20
40
60
80
100
700
750
800
850
900

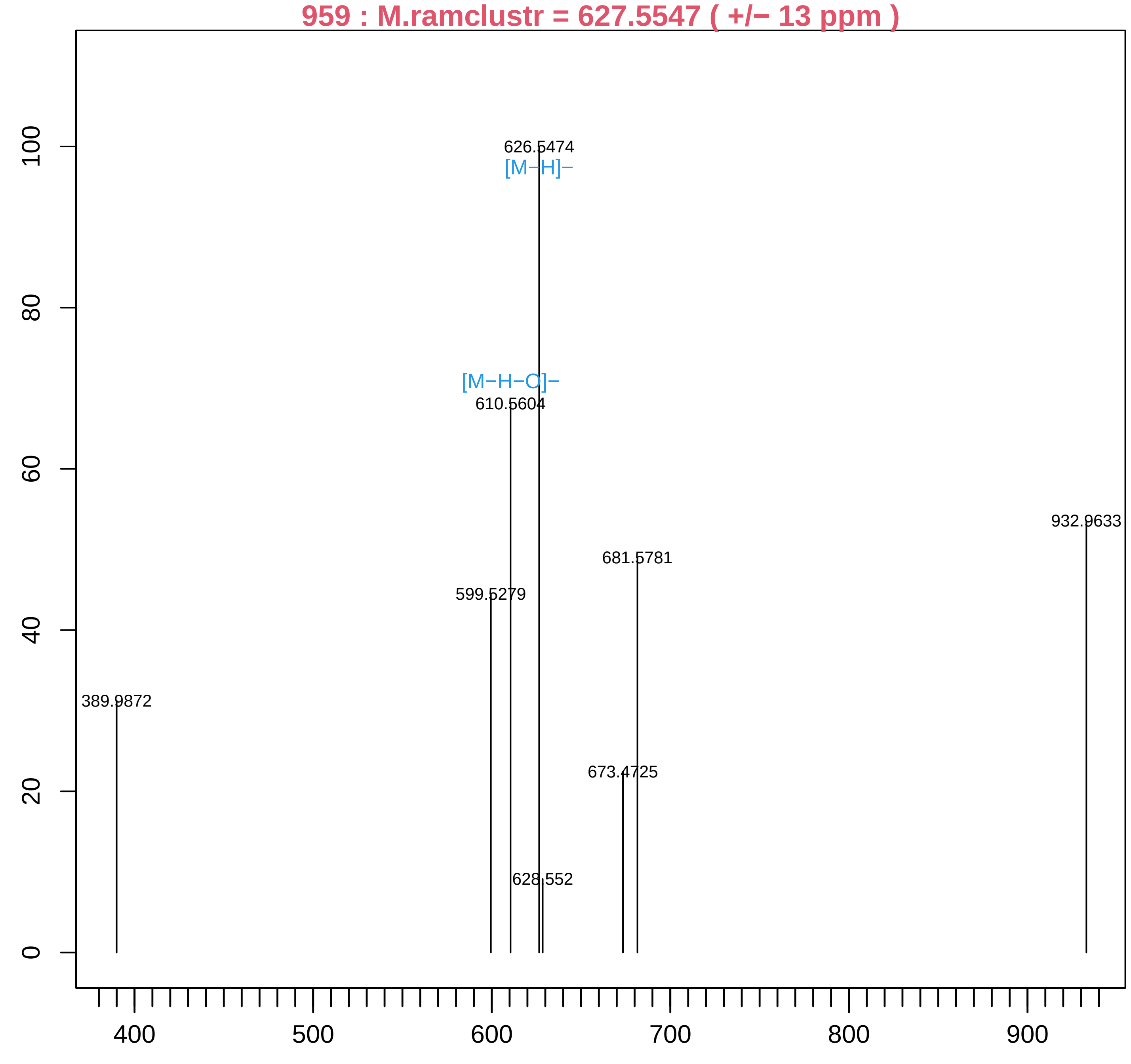

959 : M.ramclustr = 627.5547 (+/− 13 ppm)
626.5474
[M−H]−
[M−H−C]−
610.5604
932.9633
681.5781
599.5279
389.9872
673.4725
628.552
0
20
40
60
80
100
400
500
600
700
800
900

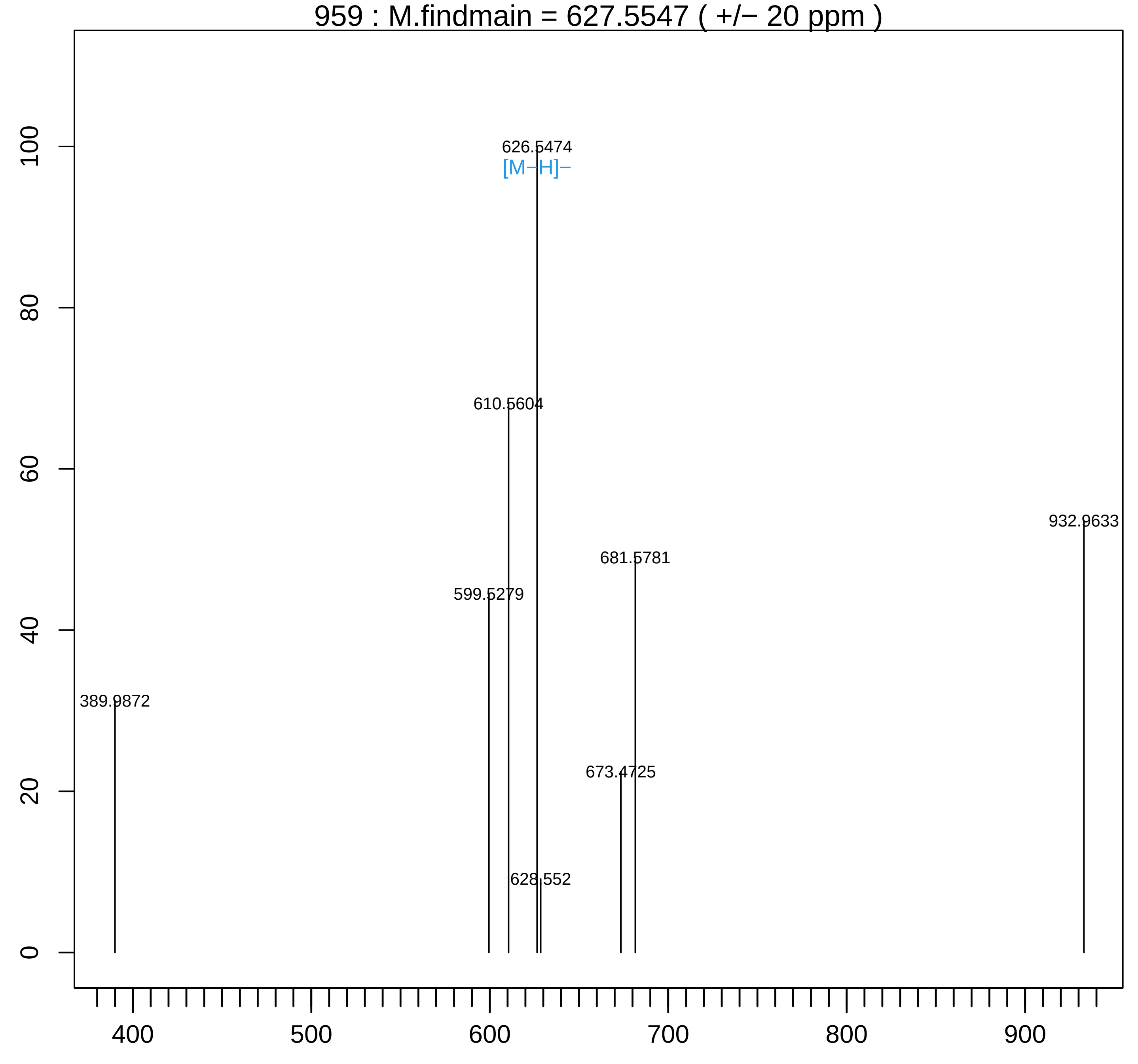

959 : M.findmain = 627.5547 (+/− 20 ppm)
626.5474
[M−H]−
610.5604
932.9633
681.5781
599.5279
389.9872
673.4725
628.552
0
20
40
60
80
100
400
500
600
700
800
900

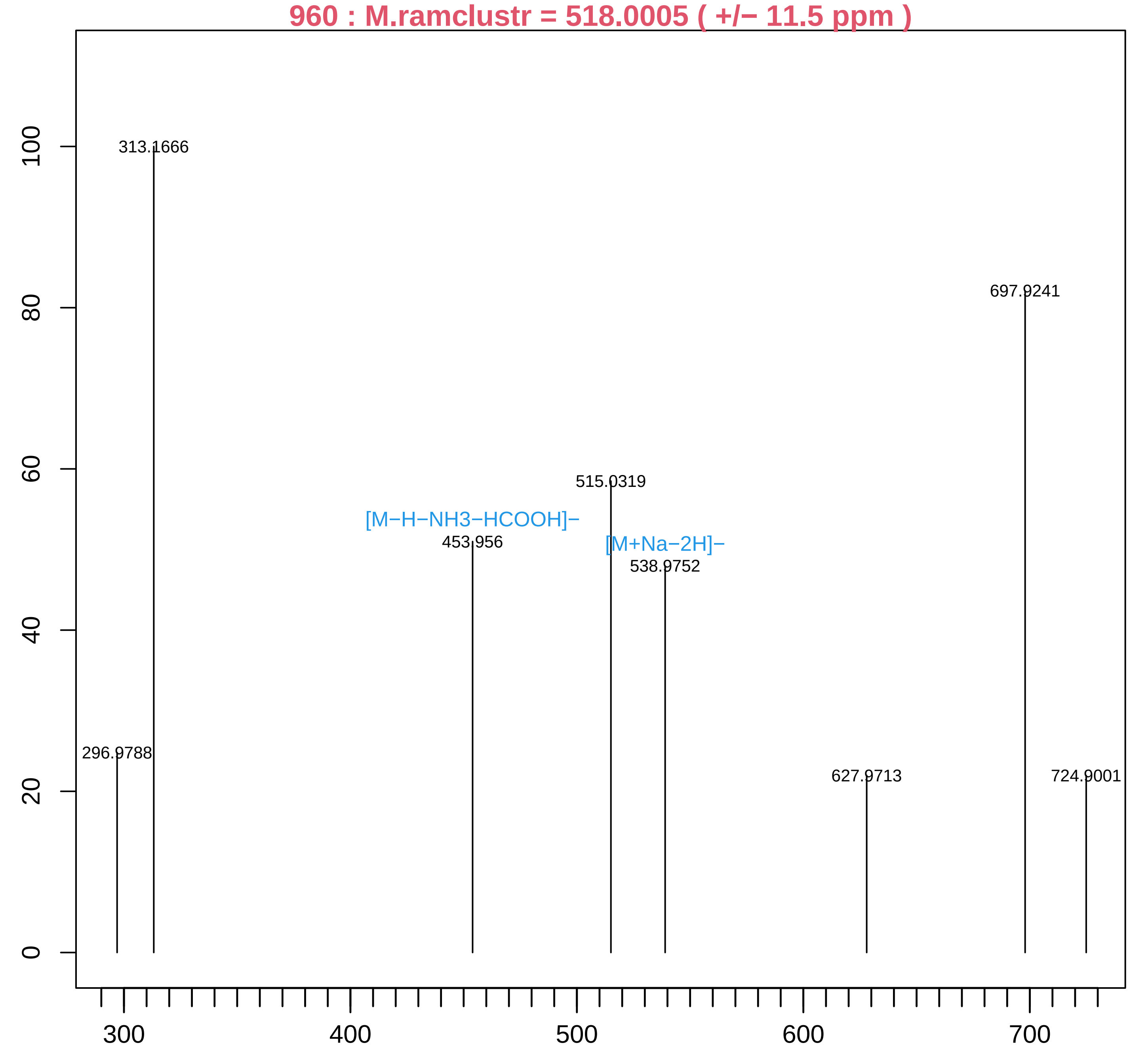

960 : M.ramclustr = 518.0005 (+/− 11.5 ppm)
313.1666
697.9241
515.0319
[M−H−NH3−HCOOH]−
453.956
[M+Na−2H]−
538.9752
296.9788
627.9713
724.9001
0
20
40
60
80
100
300
400
500
600
700

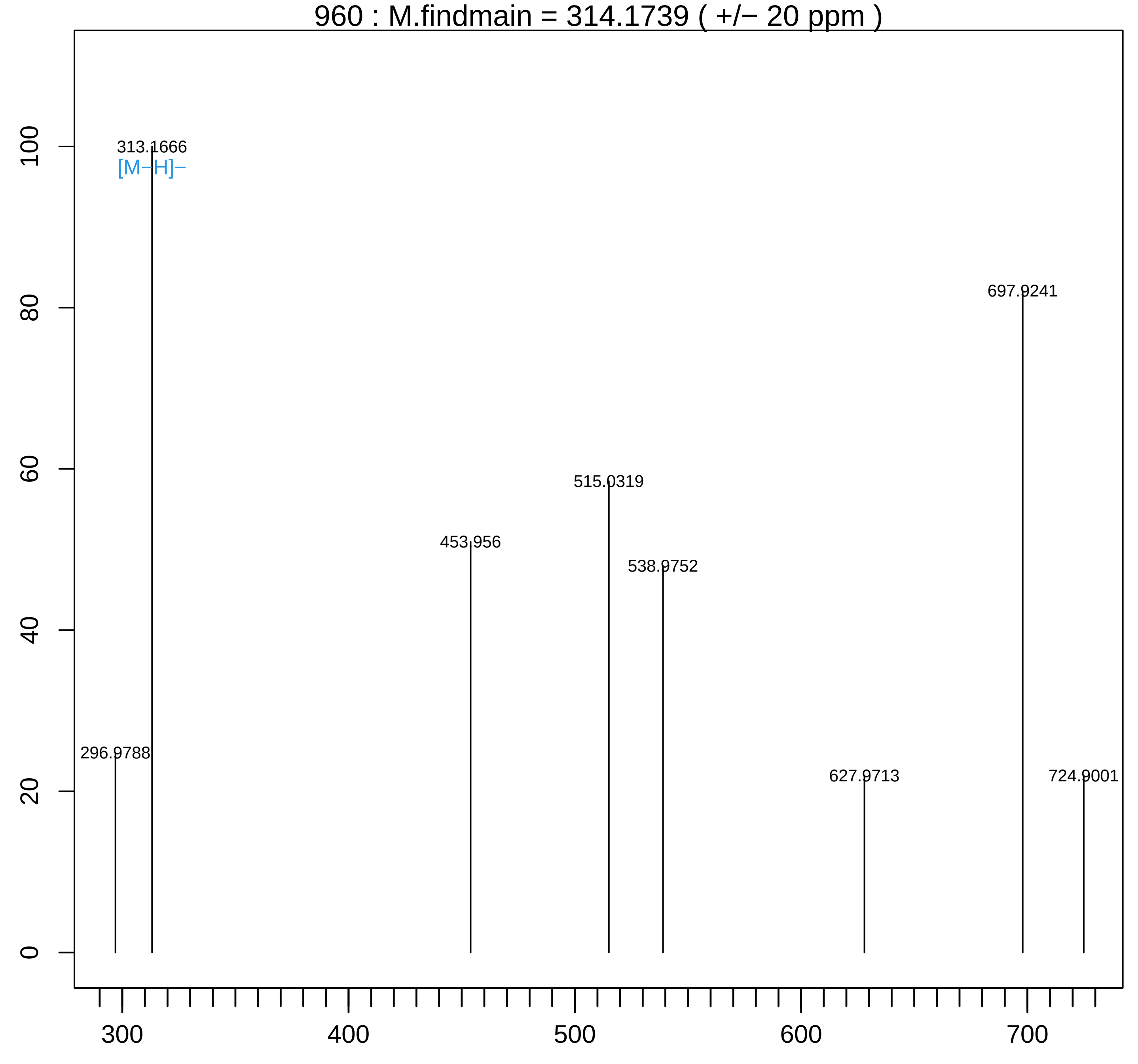

960 : M.findmain = 314.1739 (+/− 20 ppm)
313.1666
[M−H]−
697.9241
515.0319
453.956
538.9752
296.9788
627.9713
724.9001
0
20
40
60
80
100
300
400
500
600
700

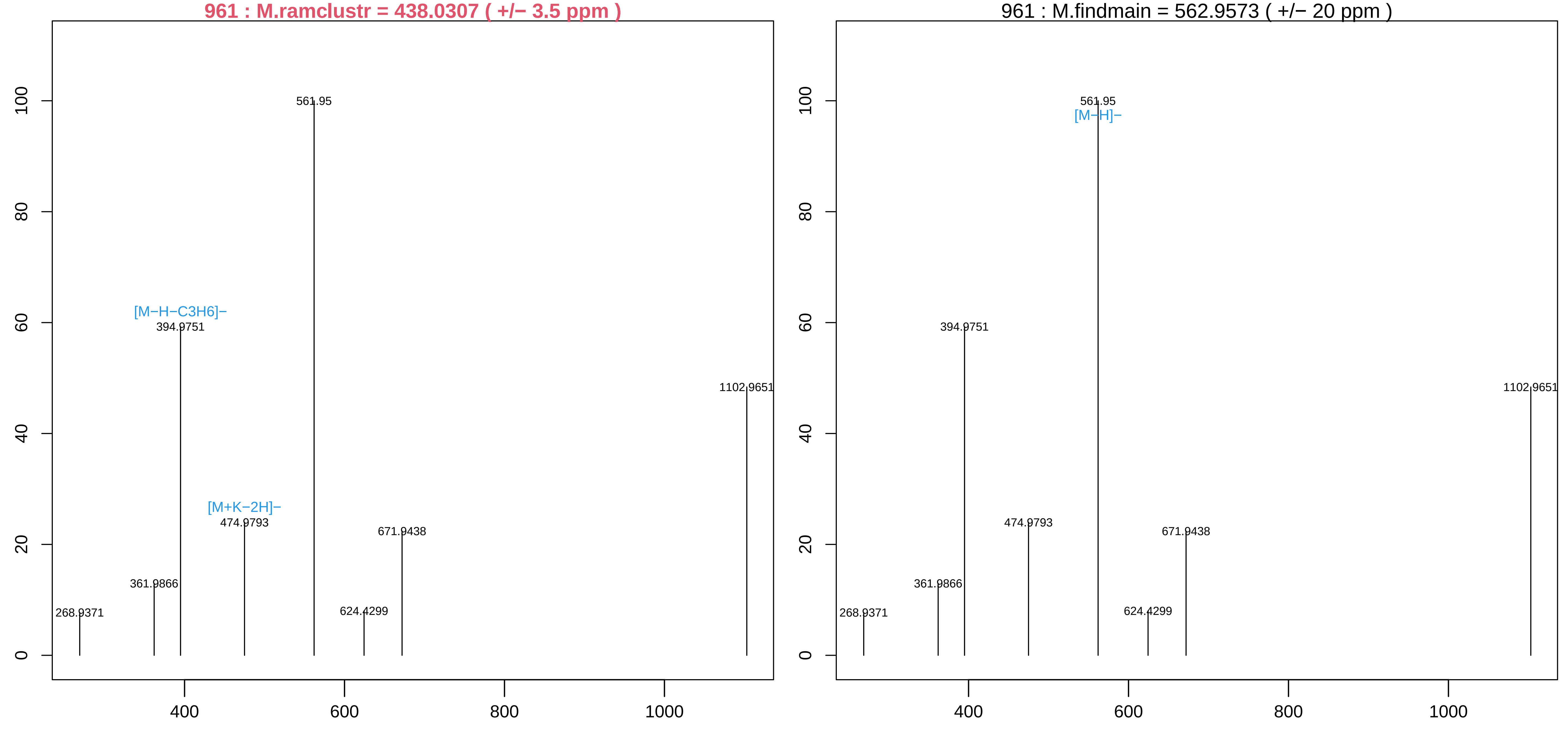

961 : M.ramclustr = 438.0307 (+/− 3.5 ppm)
561.95
[M−H−C3H6]−
394.9751
[M+K−2H]−
474.9793
671.9438
1102.9651
361.9866
268.9371
624.4299
961 : M.findmain = 562.9573 (+/− 20 ppm)
561.95
[M−H]−
394.9751
474.9793
671.9438
1102.9651
361.9866
268.9371
624.4299
0
20
40
60
80
100
400
600
800
1000

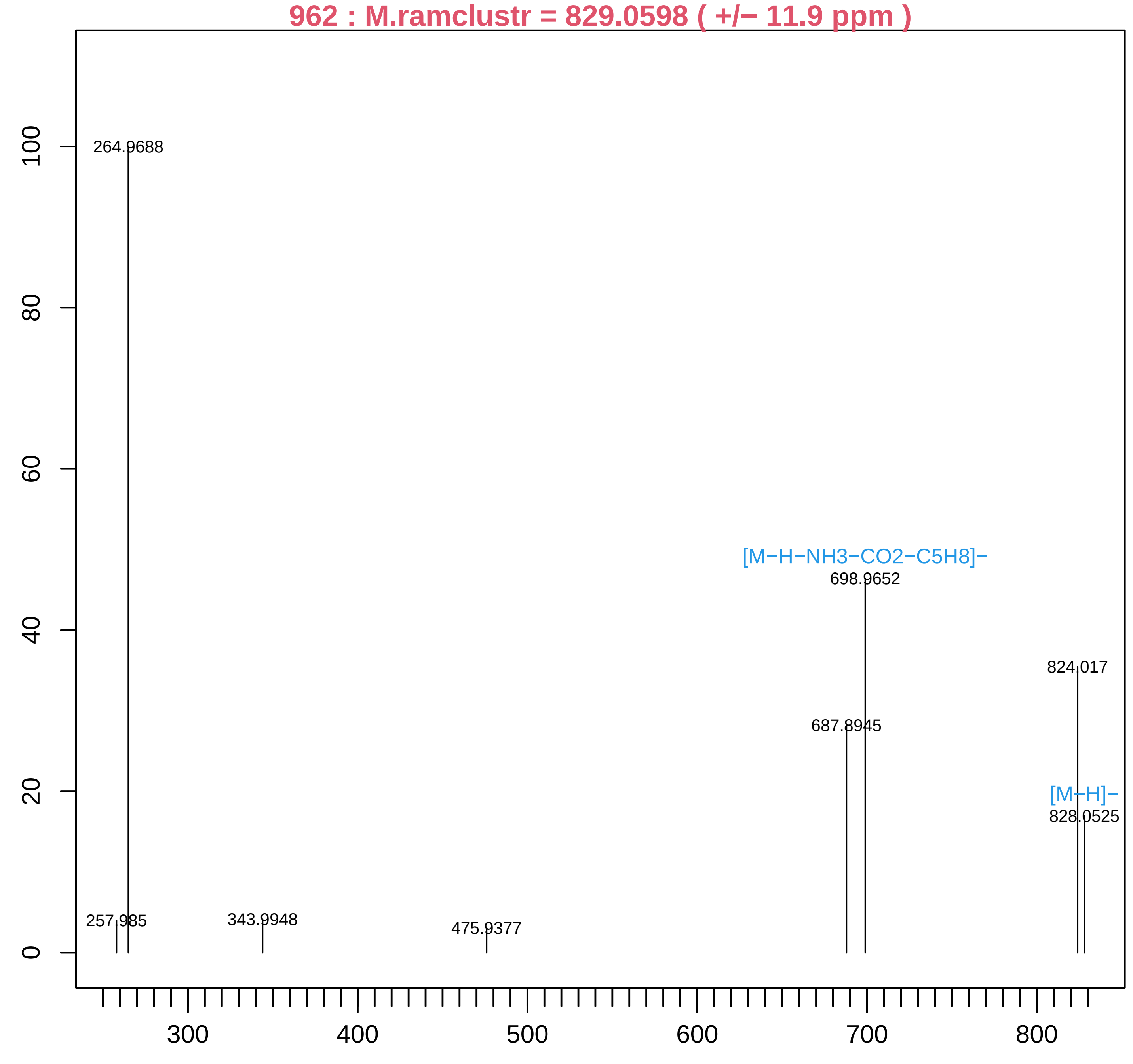

962 : M.ramclustr = 829.0598 (+/− 11.9 ppm)
264.9688
[M−H−NH3−CO2−C5H8]−
698.9652
824.017
687.8945
[M−H]−
828.0525
257.985
343.9948
475.9377

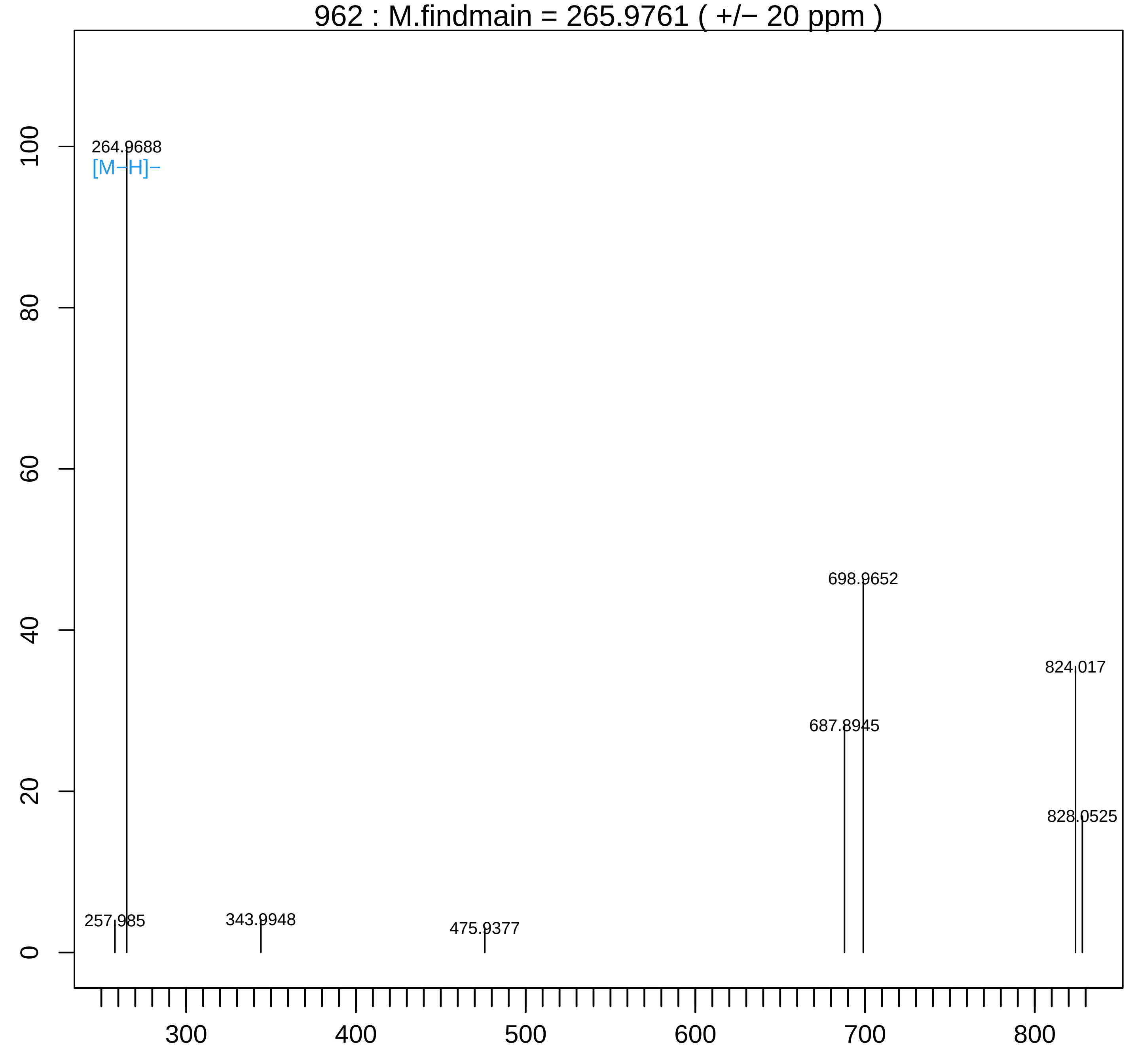

962 : M.findmain = 265.9761 (+/− 20 ppm)
264.9688
[M−H]−
698.9652
824.017
687.8945
828.0525
257.985
343.9948
475.9377

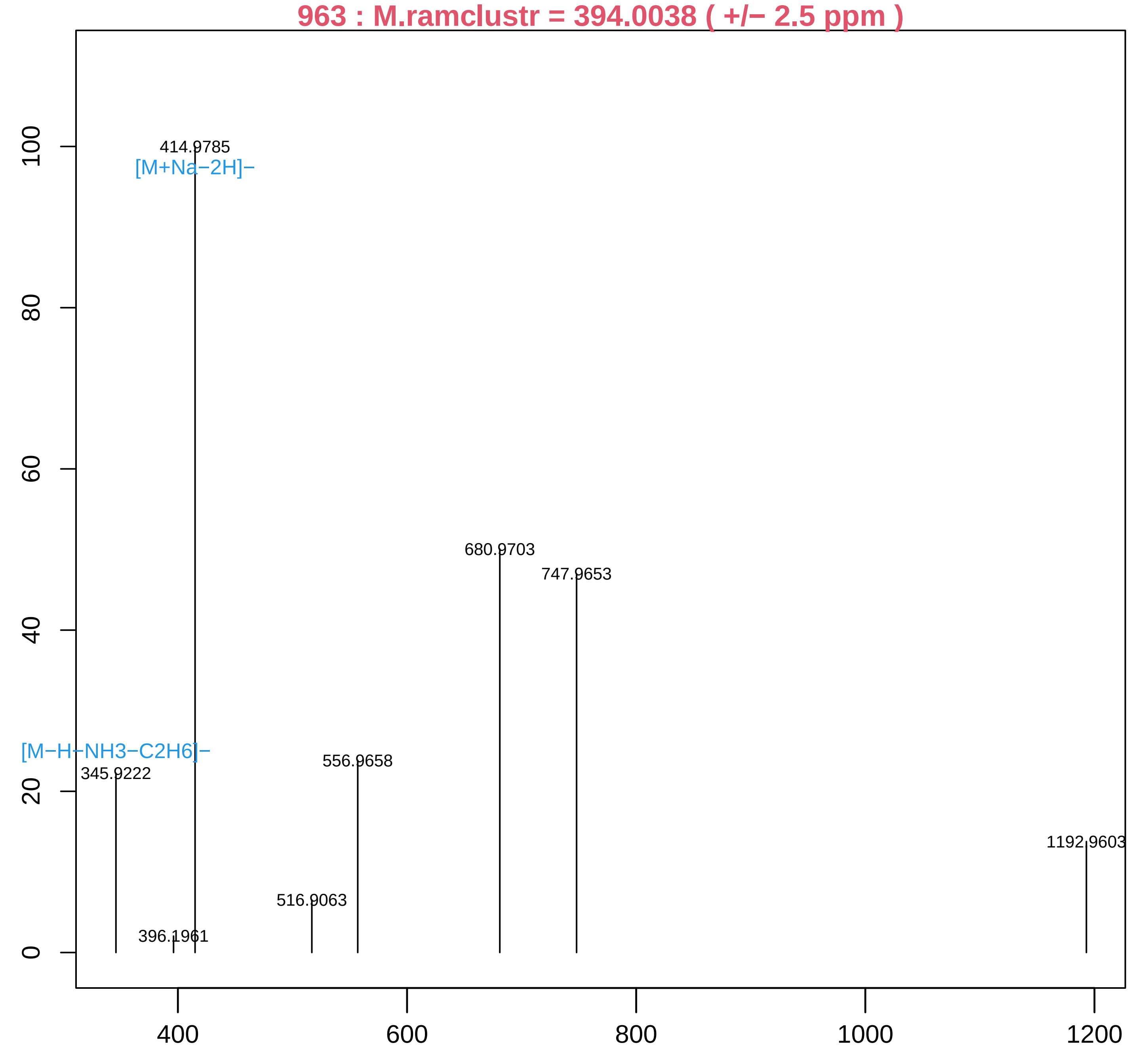
963 : M.ramclustr = 394.0038 (+/− 2.5 ppm)
414.9785
[M+Na−2H]−
[M−H−NH3−C2H6]−
345.9222
396.1961
516.9063
556.9658
680.9703
747.9653
1192.9603
0
20
40
60
80
100
400
600
800
1000
1200

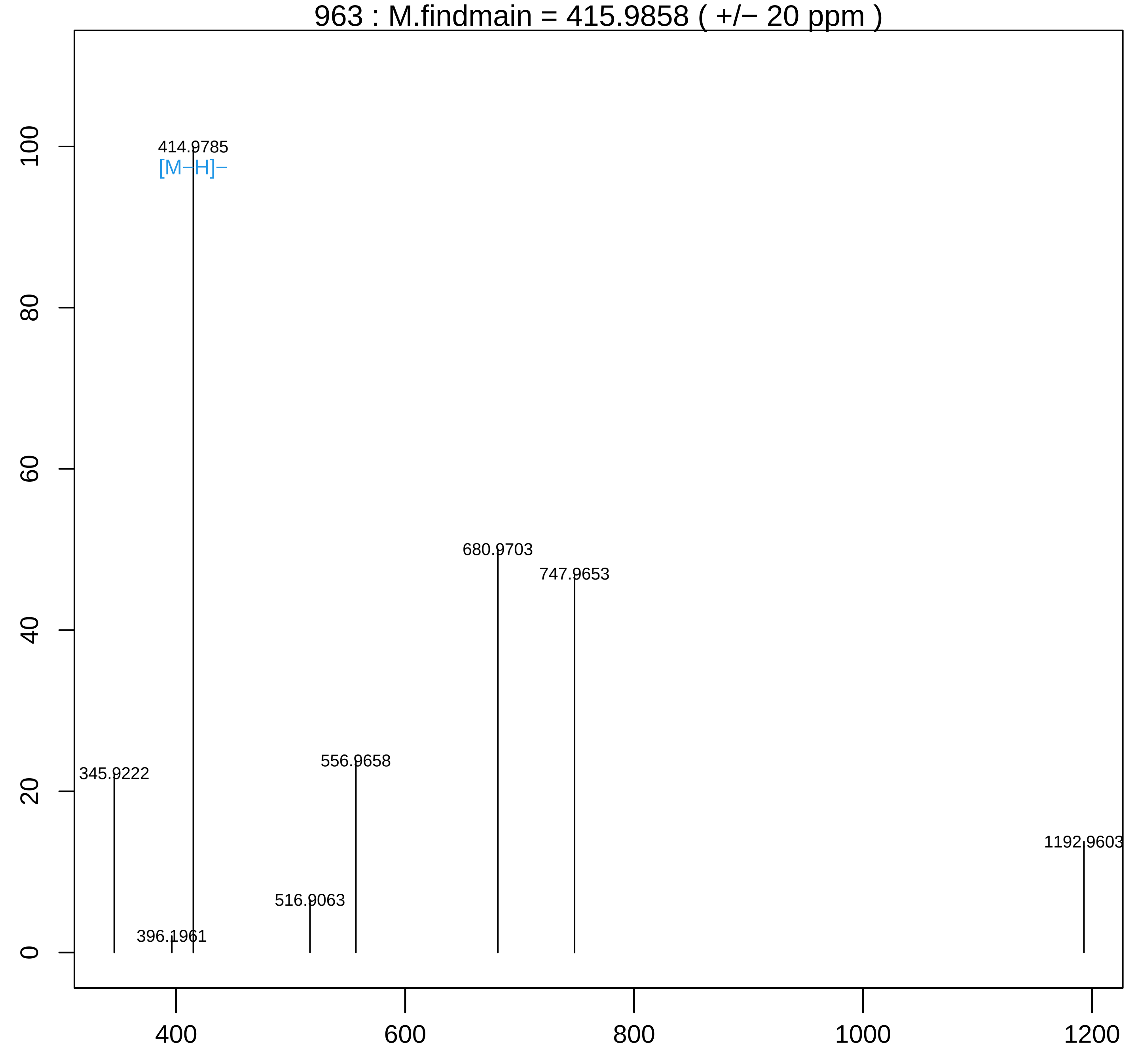
963 : M.findmain = 415.9858 (+/− 20 ppm)
414.9785
[M−H]−
345.9222
396.1961
516.9063
556.9658
680.9703
747.9653
1192.9603
0
20
40
60
80
100
400
600
800
1000
1200

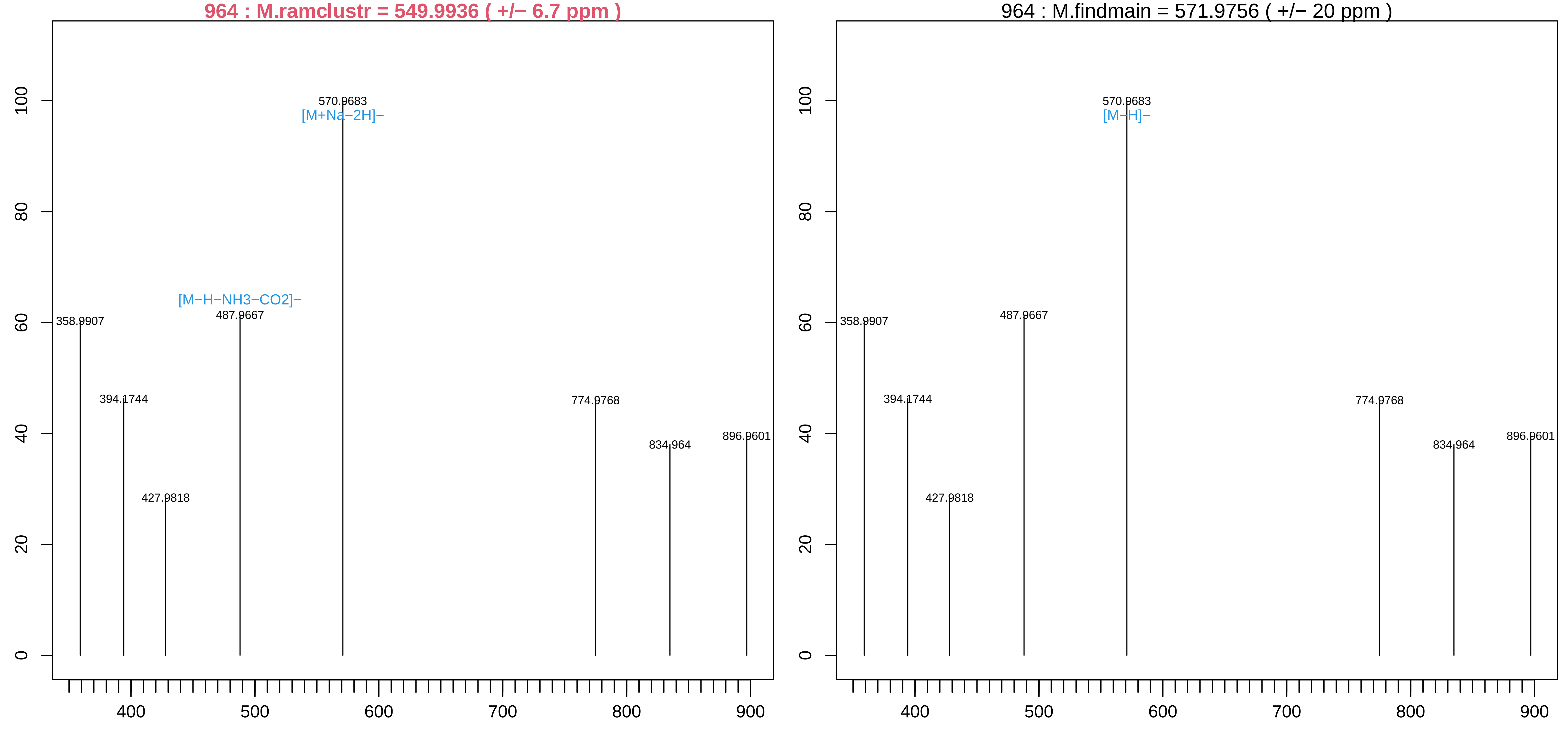
964 : M.ramclustr = 549.9936 (+/− 6.7 ppm)
570.9683
[M+Na−2H]−
[M−H−NH3−CO2]−
487.9667
358.9907
394.1744
427.9818
774.9768
834.964
896.9601
964 : M.findmain = 571.9756 (+/− 20 ppm)
570.9683
[M−H]−
358.9907
487.9667
394.1744
427.9818
774.9768
834.964
896.9601
0
20
40
60
80
100
400
500
600
700
800
900

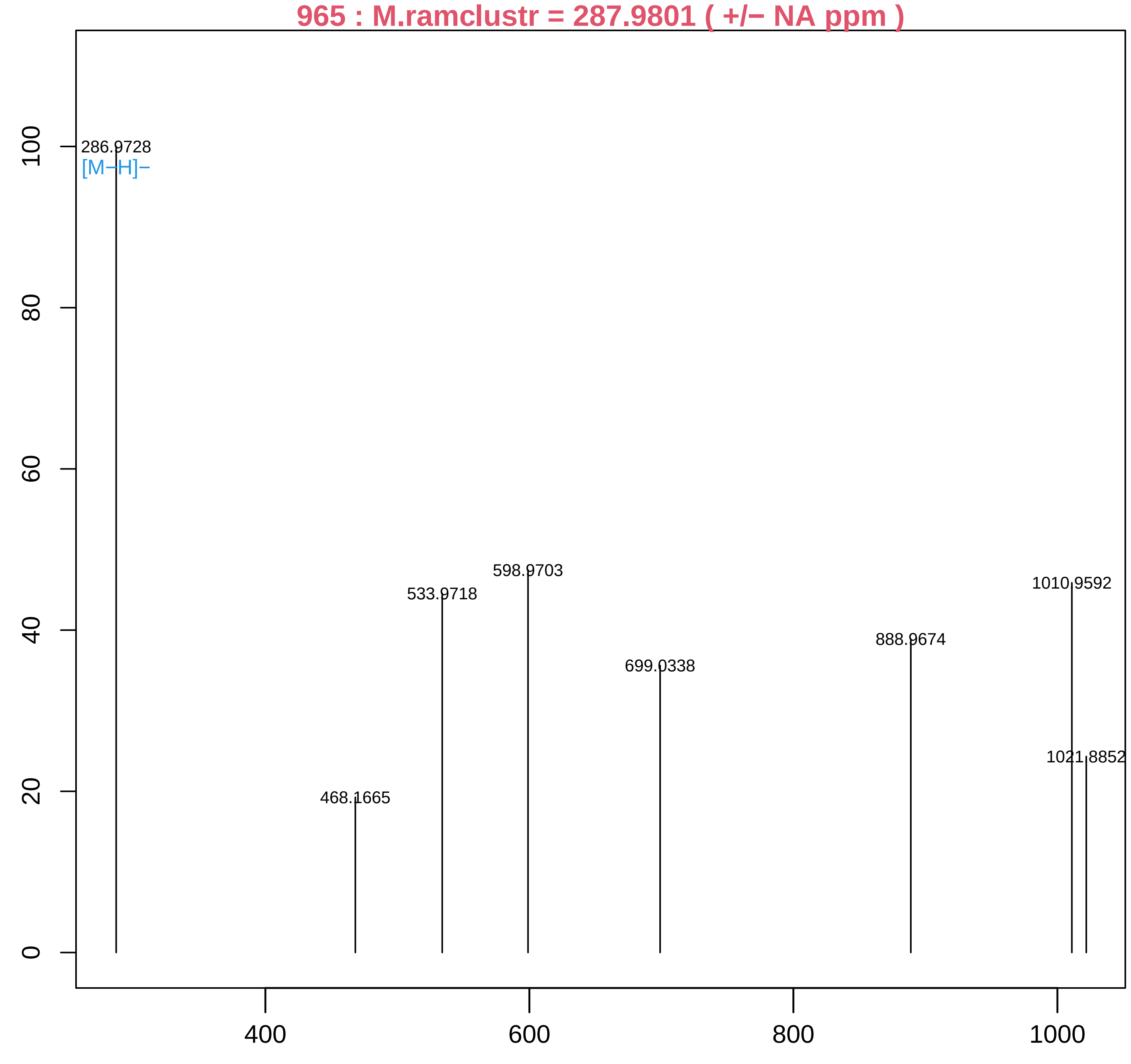
965 : M.ramclustr = 287.9801 (+/− NA ppm)
286.9728
[M−H]−
468.1665
533.9718
598.9703
699.0338
888.9674
1010.9592
1021.8852
0
20
40
60
80
100
400
600
800
1000

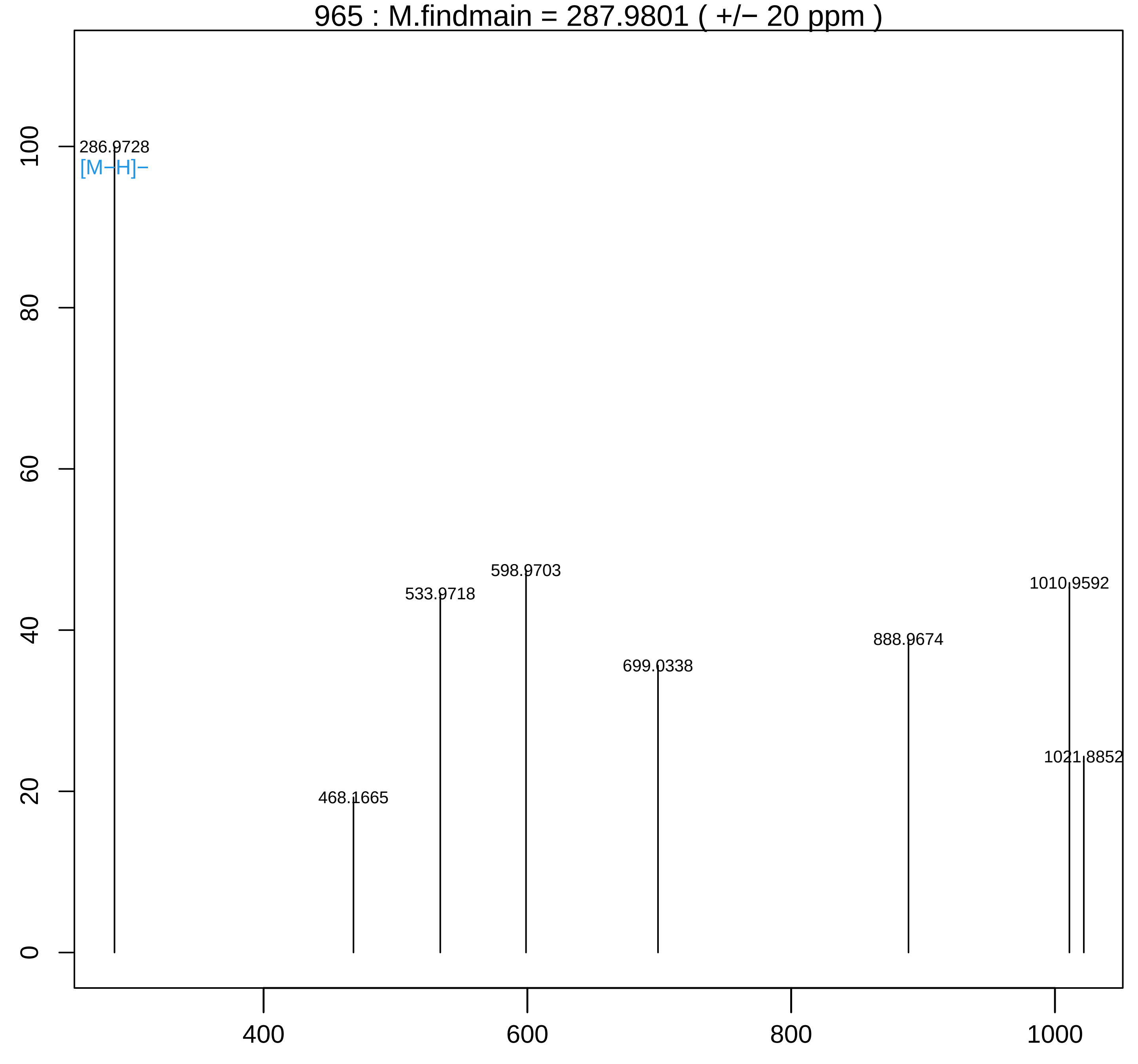
965 : M.findmain = 287.9801 (+/− 20 ppm)
286.9728
[M−H]−
468.1665
533.9718
598.9703
699.0338
888.9674
1010.9592
1021.8852
0
20
40
60
80
100
400
600
800
1000

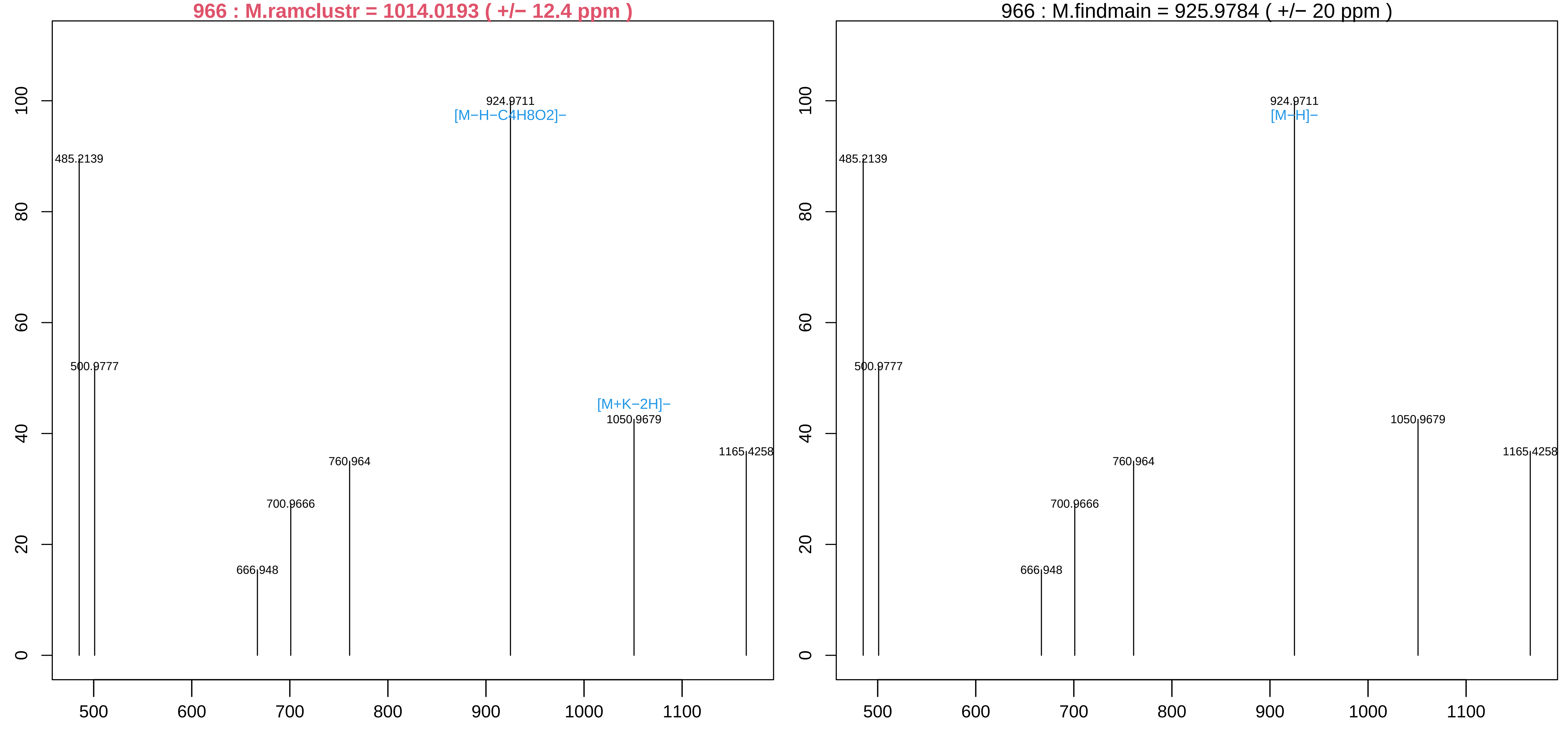

966 : M.ramclustr = 1014.0193 (+/− 12.4 ppm)
924.9711
[M−H−C4H8O2]−
485.2139
500.9777
[M+K−2H]−
1050.9679
1165.4258
760.964
700.9666
666.948
966 : M.findmain = 925.9784 (+/− 20 ppm)
924.9711
[M−H]−
485.2139
500.9777
1050.9679
1165.4258
760.964
700.9666
666.948

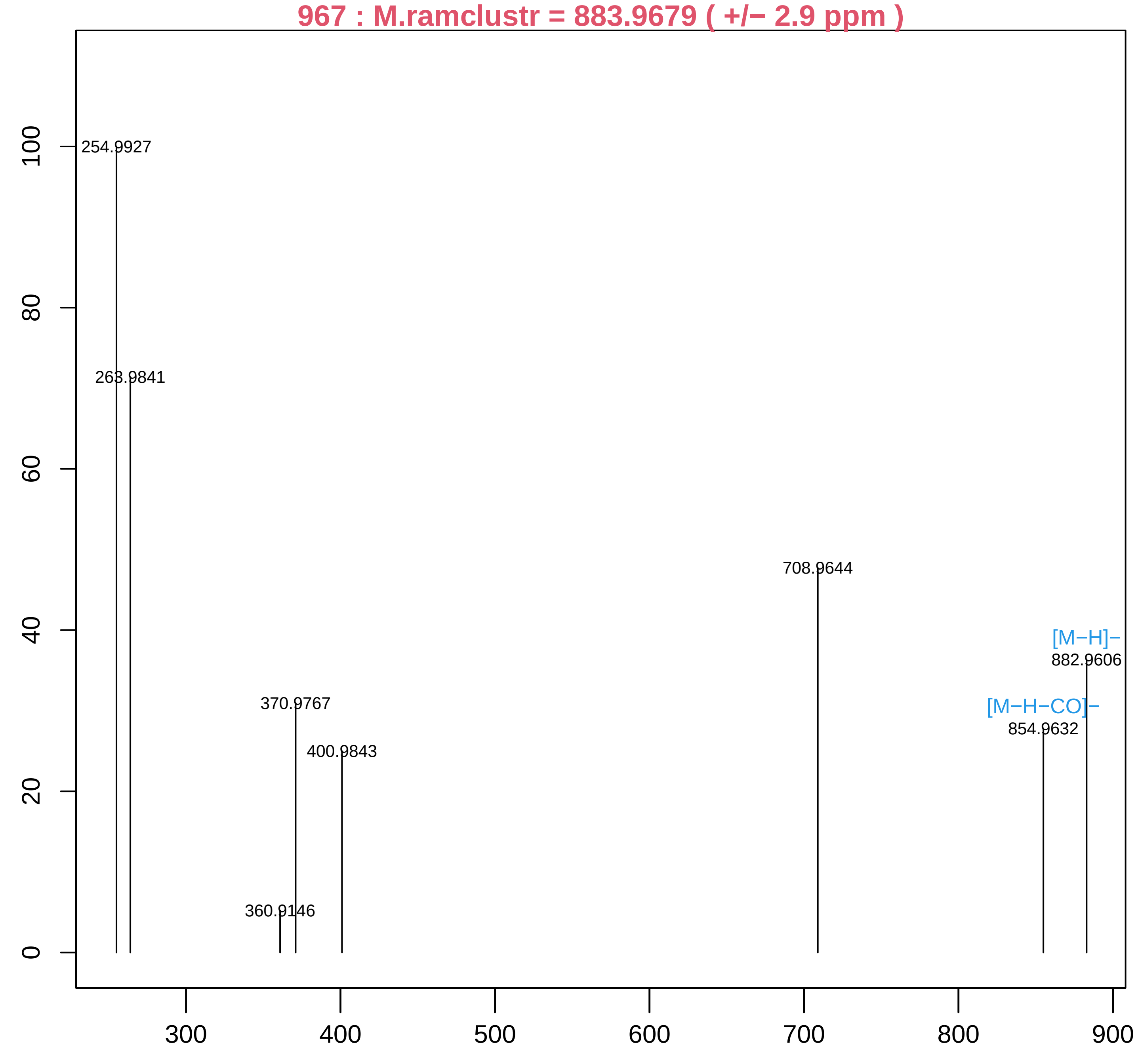
967 : M.ramclustr = 883.9679 (+/− 2.9 ppm)
254.9927
263.9841
360.9146
370.9767
400.9843
708.9644
[M−H−CO]−
854.9632
[M−H]−
882.9606
0
20
40
60
80
100
300
400
500
600
700
800
900

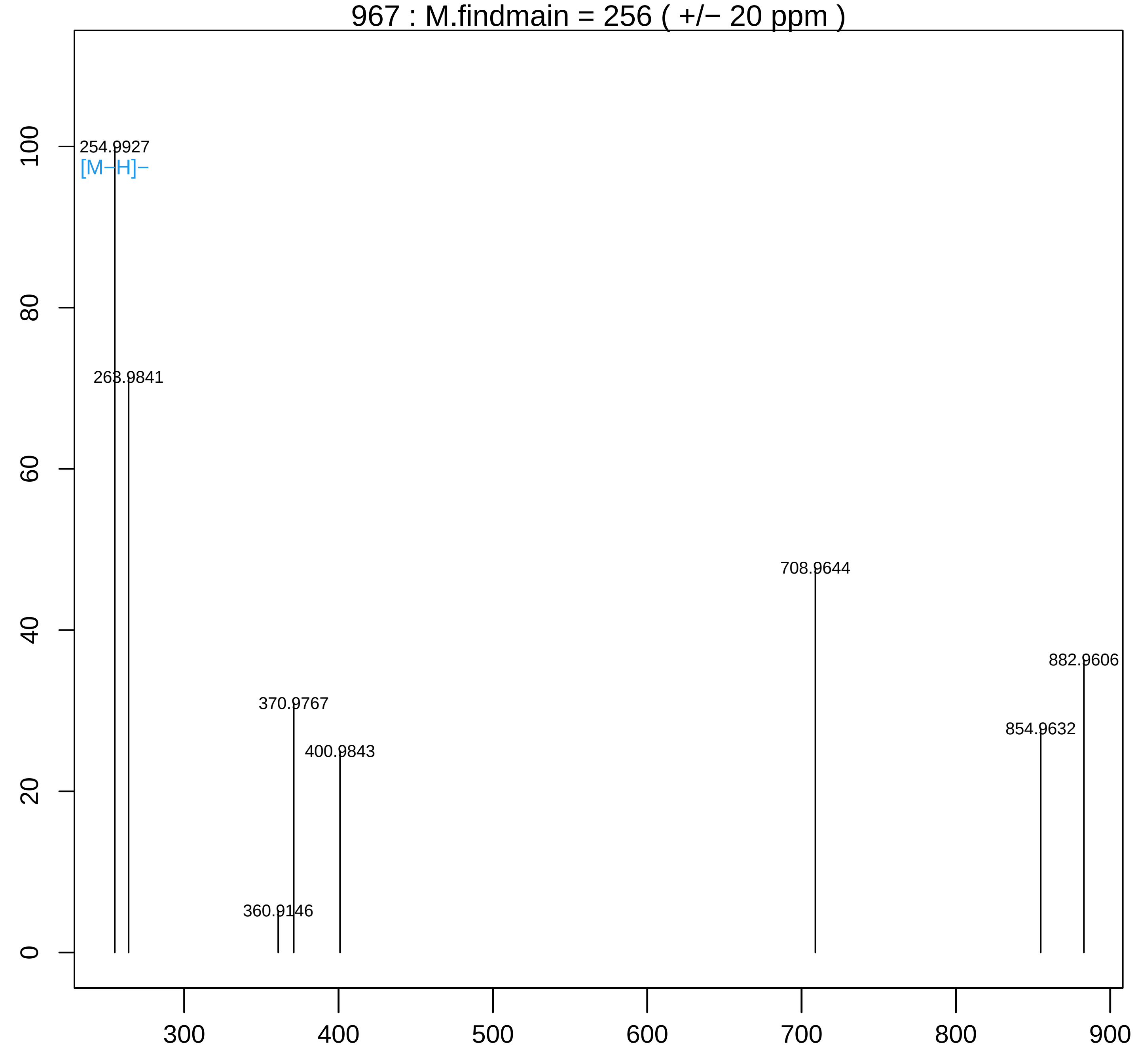
967 : M.findmain = 256 (+/− 20 ppm)
254.9927
[M−H]−
263.9841
360.9146
370.9767
400.9843
708.9644
854.9632
882.9606
0
20
40
60
80
100
300
400
500
600
700
800
900

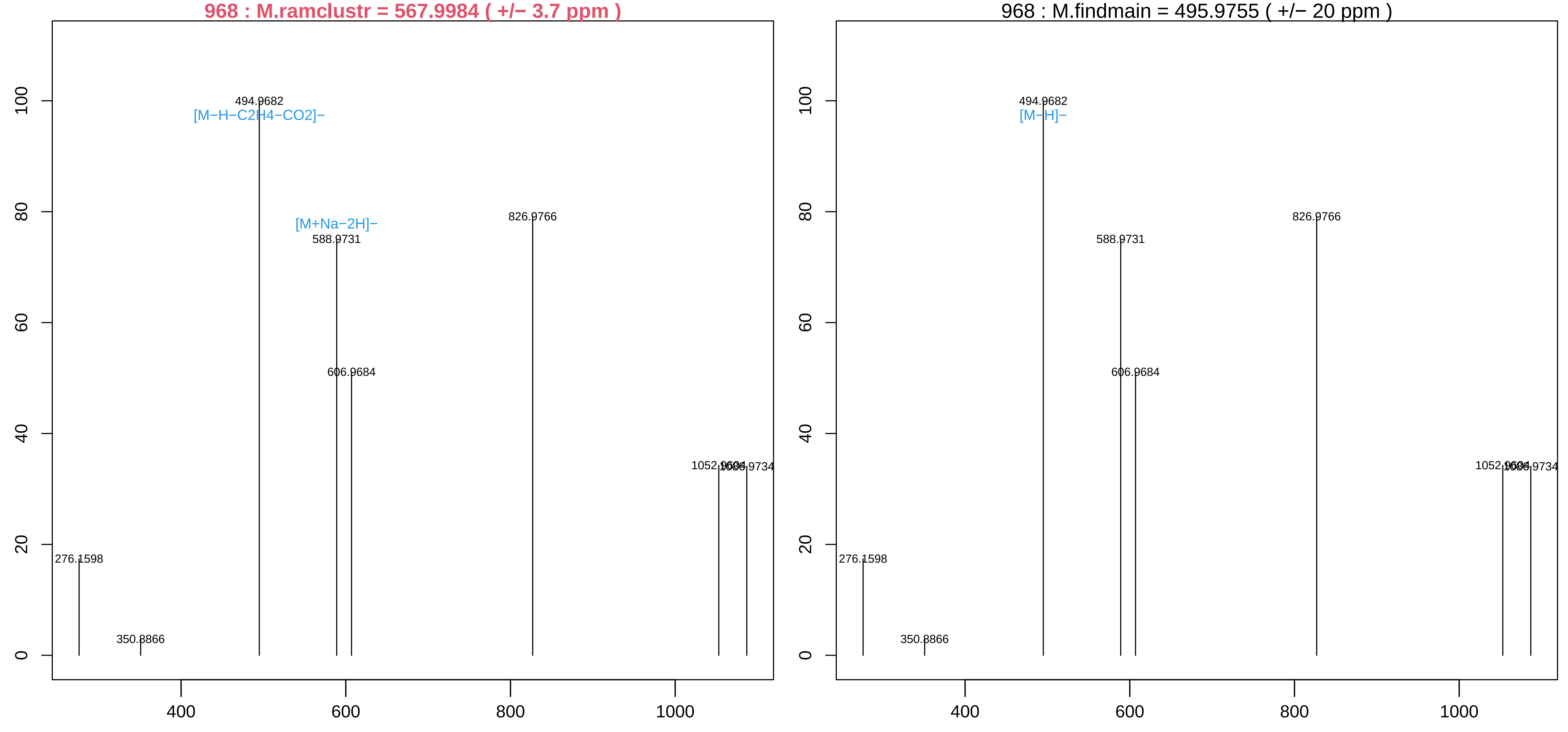

968 : M.ramclustr = 567.9984 (+/− 3.7 ppm)
494.9682
[M−H−C2H4−CO2]−
[M+Na−2H]−
588.9731
826.9766
606.9684
1052.9694
1086.9734
276.1598
350.8866
968 : M.findmain = 495.9755 (+/− 20 ppm)
494.9682
[M−H]−
588.9731
826.9766
606.9684
1052.9694
1086.9734
276.1598
350.8866

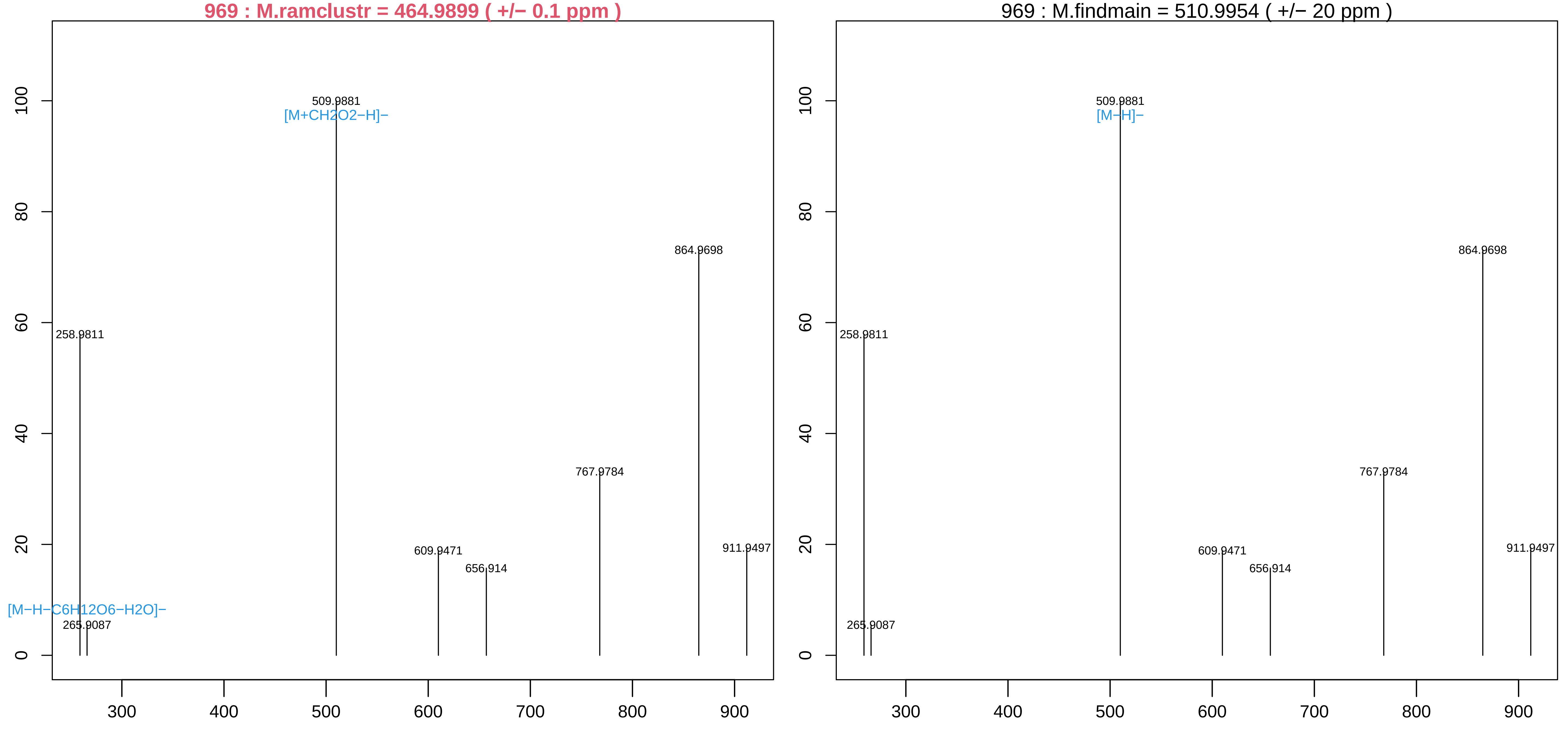
969 : M.ramclustr = 464.9899 (+/− 0.1 ppm)
509.9881
[M+CH2O2−H]−
864.9698
258.9811
767.9784
609.9471
656.914
911.9497
[M−H−C6H12O6−H2O]−
265.9087
969 : M.findmain = 510.9954 (+/− 20 ppm)
509.9881
[M−H]−
864.9698
258.9811
767.9784
609.9471
656.914
911.9497
265.9087

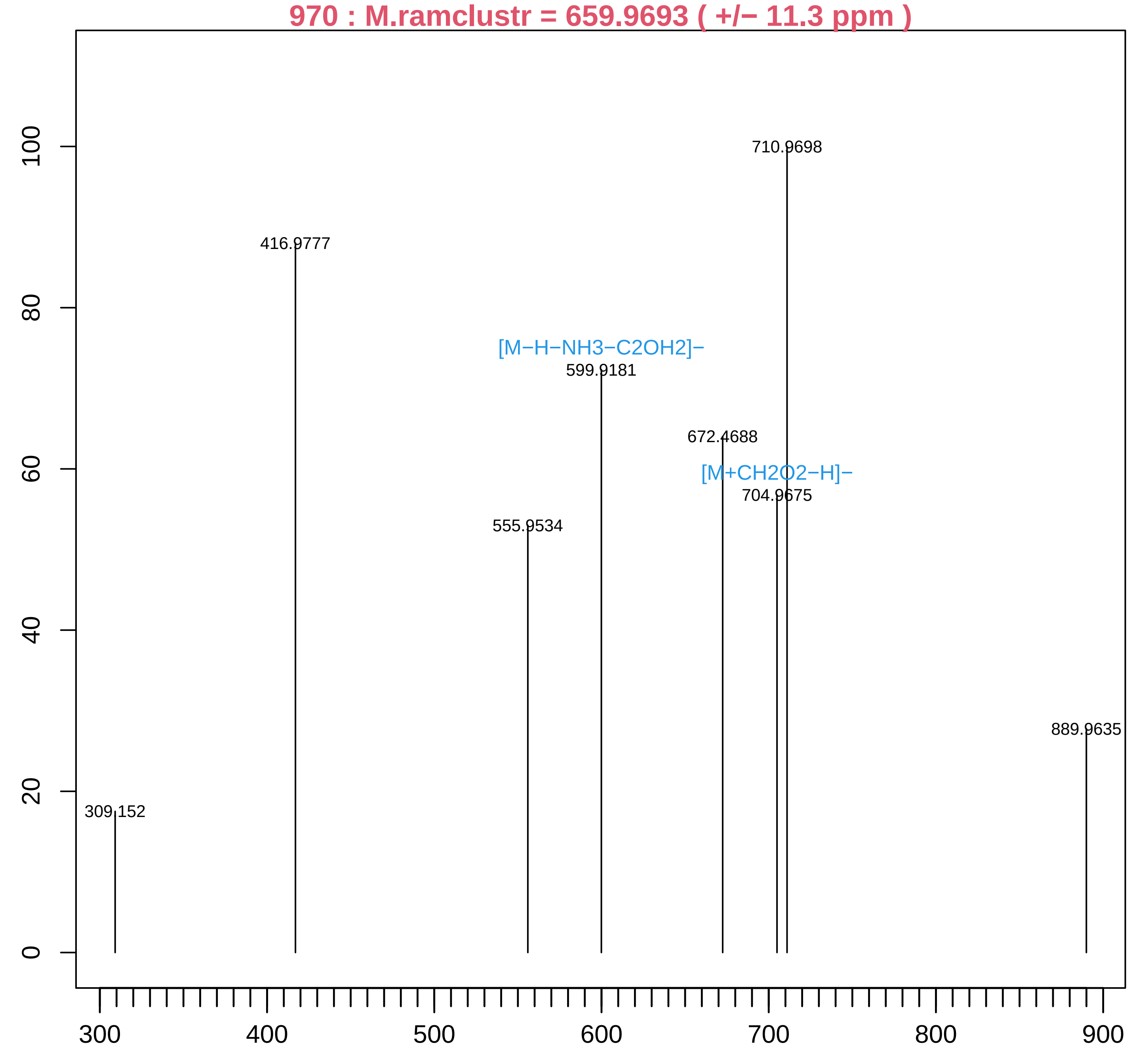
970 : M.ramclustr = 659.9693 (+/− 11.3 ppm)
710.9698
416.9777
[M−H−NH3−C2OH2]−
599.9181
672.4688
[M+CH2O2−H]−
704.9675
555.9534
889.9635
309.152
0
20
40
60
80
100
300
400
500
600
700
800
900

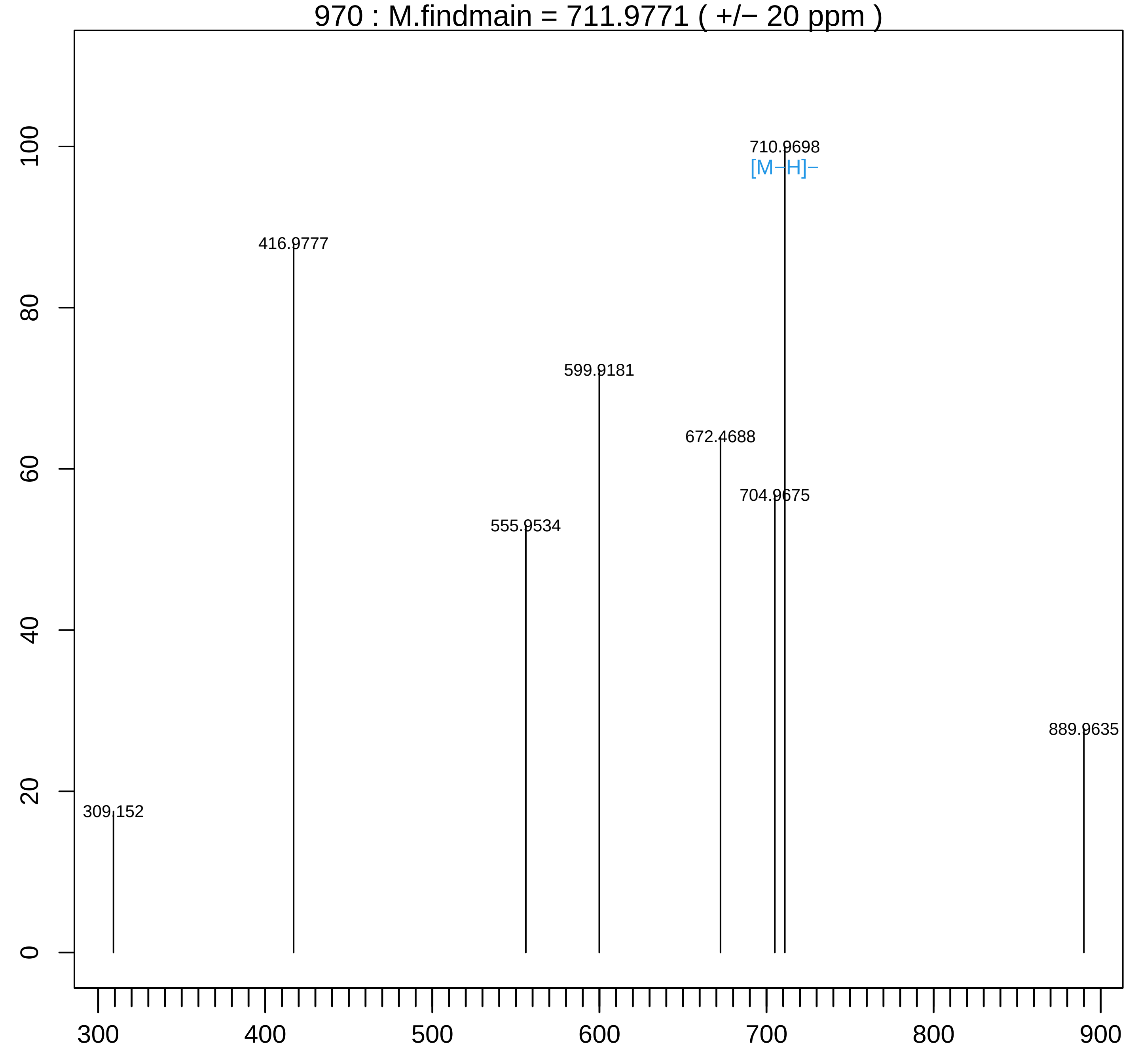
970 : M.findmain = 711.9771 (+/− 20 ppm)
710.9698
[M−H]−
416.9777
599.9181
672.4688
704.9675
555.9534
889.9635
309.152
0
20
40
60
80
100
300
400
500
600
700
800
900

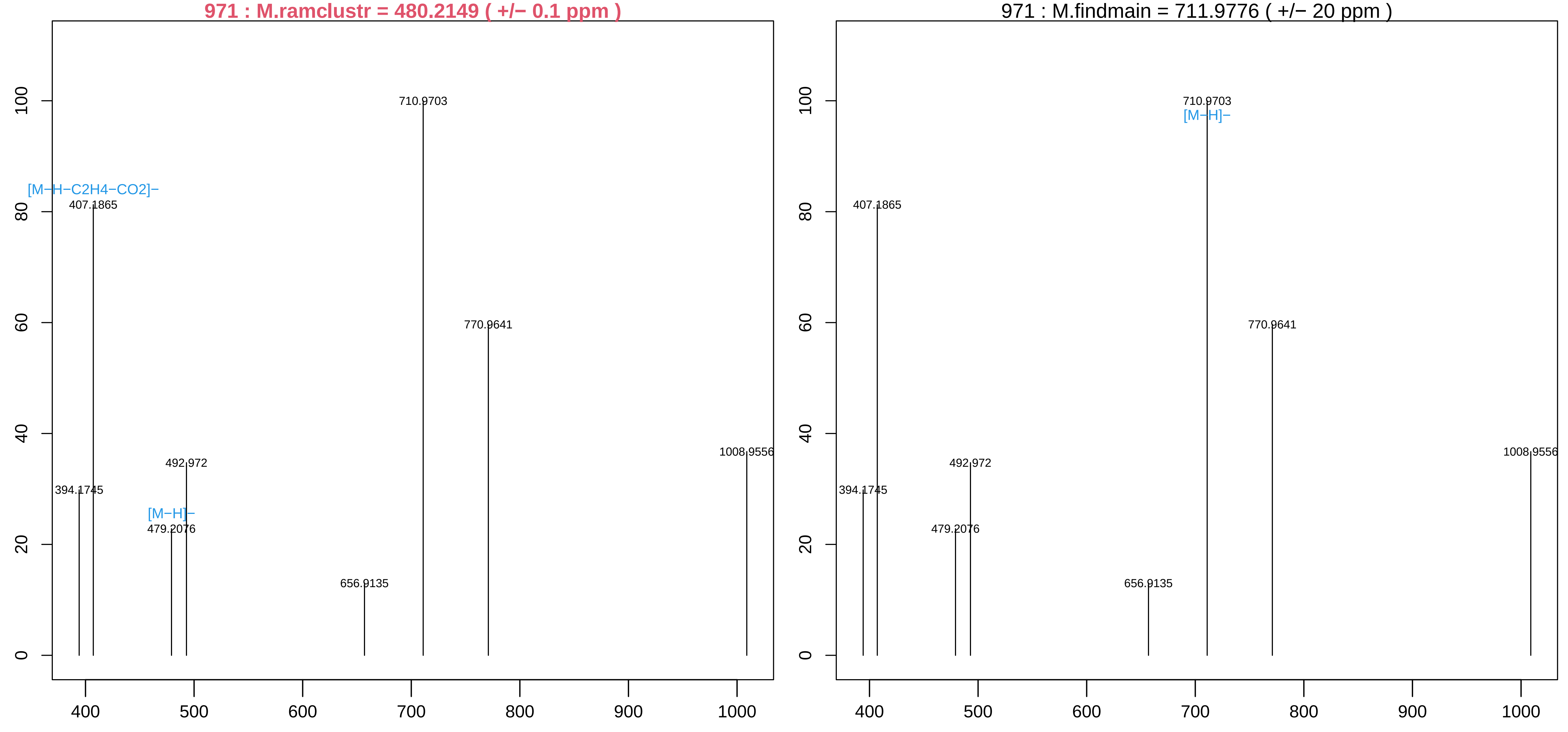

971 : M.ramclustr = 480.2149 (+/− 0.1 ppm)
[M−H−C2H4−CO2]−
407.1865
394.1745
[M−H]−
479.2076
492.972
656.9135
710.9703
770.9641
1008.9556
971 : M.findmain = 711.9776 (+/− 20 ppm)
710.9703
[M−H]−
407.1865
394.1745
479.2076
492.972
656.9135
770.9641
1008.9556
0
20
40
60
80
100
400
500
600
700
800
900
1000

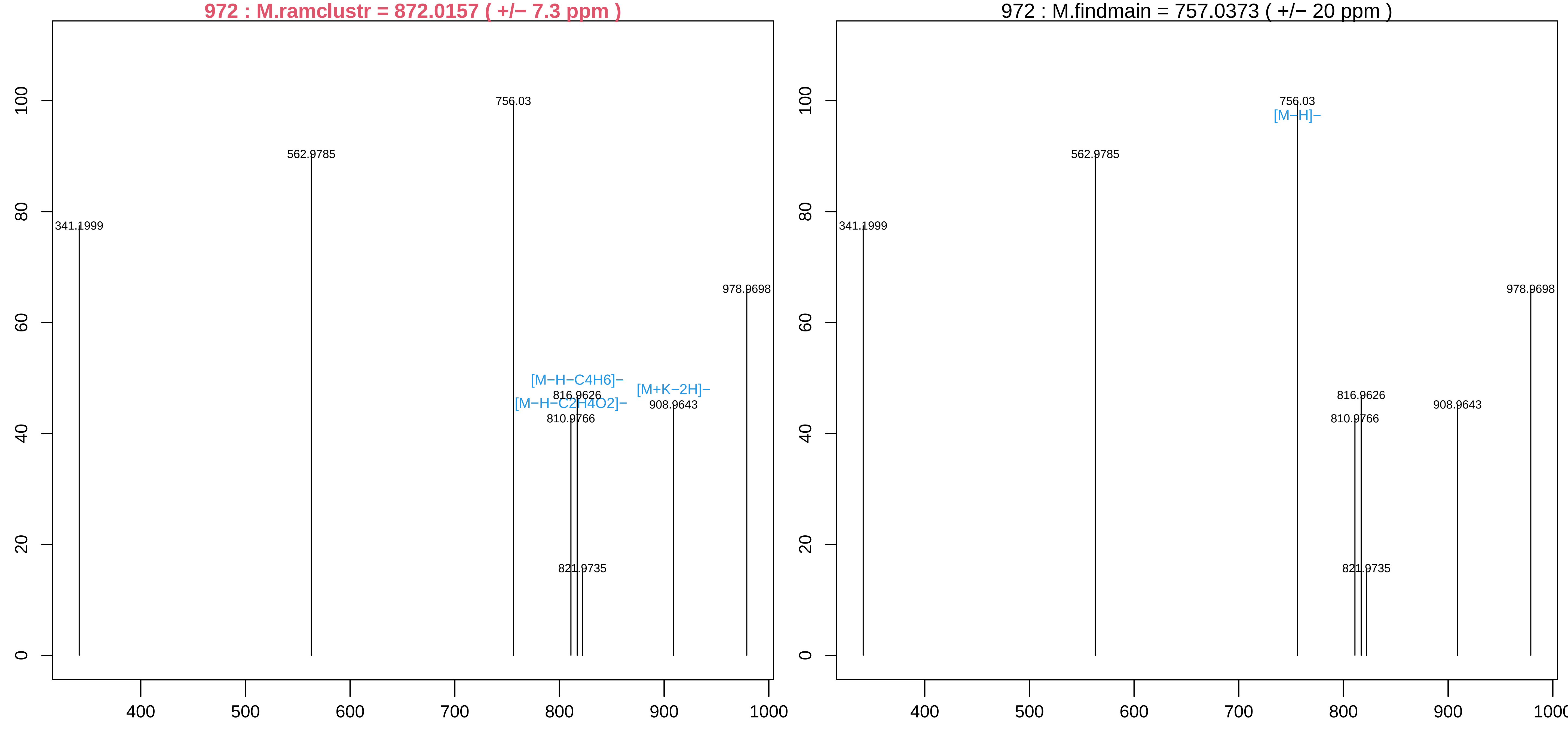

972 : M.ramclustr = 872.0157 (+/− 7.3 ppm)
341.1999
562.9785
756.03
[M−H−C4H6]−
816.9626
[M−H−C2H4O2]−
810.9766
821.9735
[M+K−2H]−
908.9643
978.9698
972 : M.findmain = 757.0373 (+/− 20 ppm)
341.1999
562.9785
756.03
[M−H]−
816.9626
810.9766
821.9735
908.9643
978.9698
0
20
40
60
80
100
400
500
600
700
800
900
1000

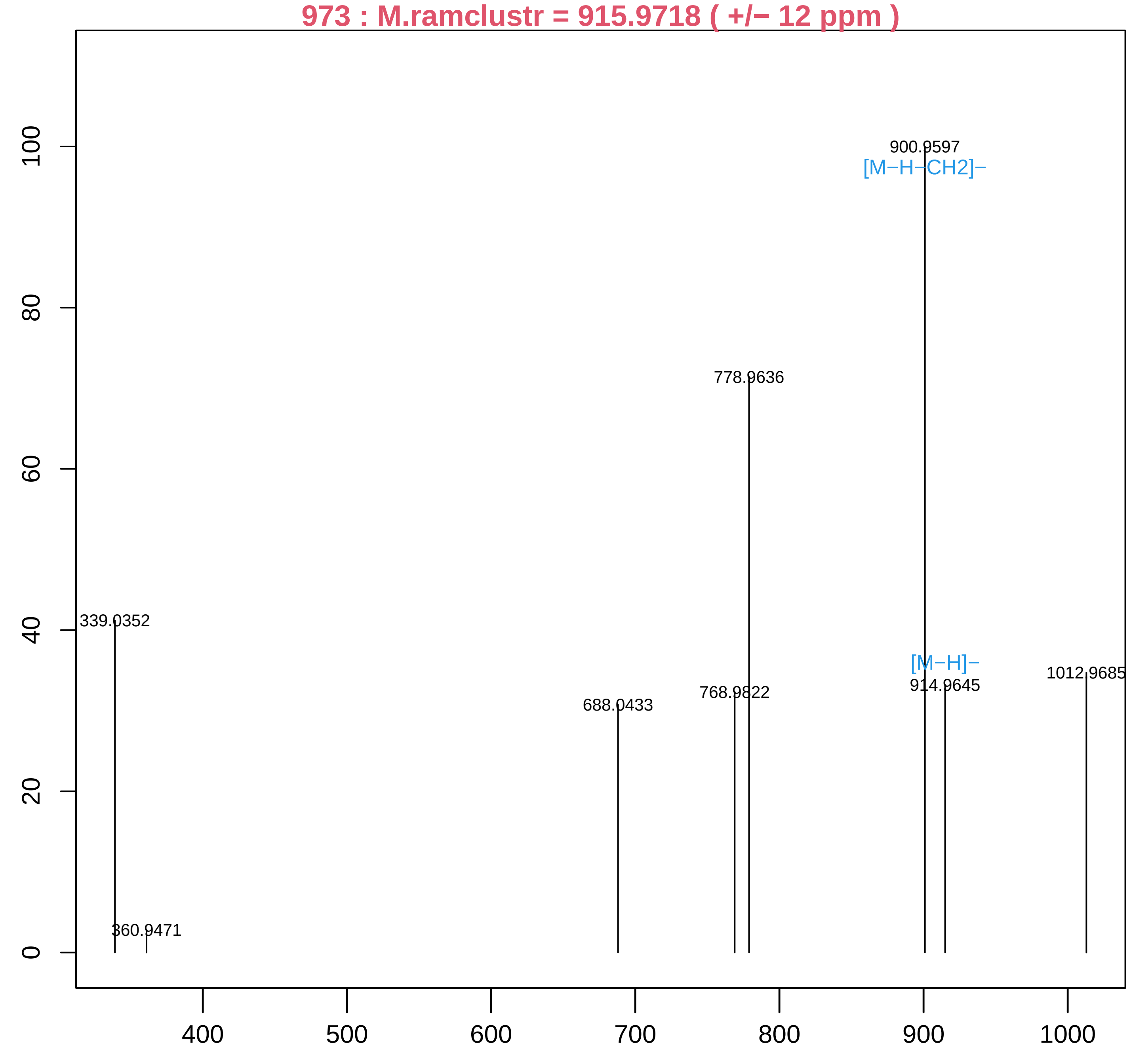

973 : M.ramclustr = 915.9718 (+/− 12 ppm)
900.9597
[M−H−CH2]−
778.9636
339.0352
[M−H]−
914.9645
1012.9685
688.0433
768.9822
360.9471
0
20
40
60
80
100
400
500
600
700
800
900
1000

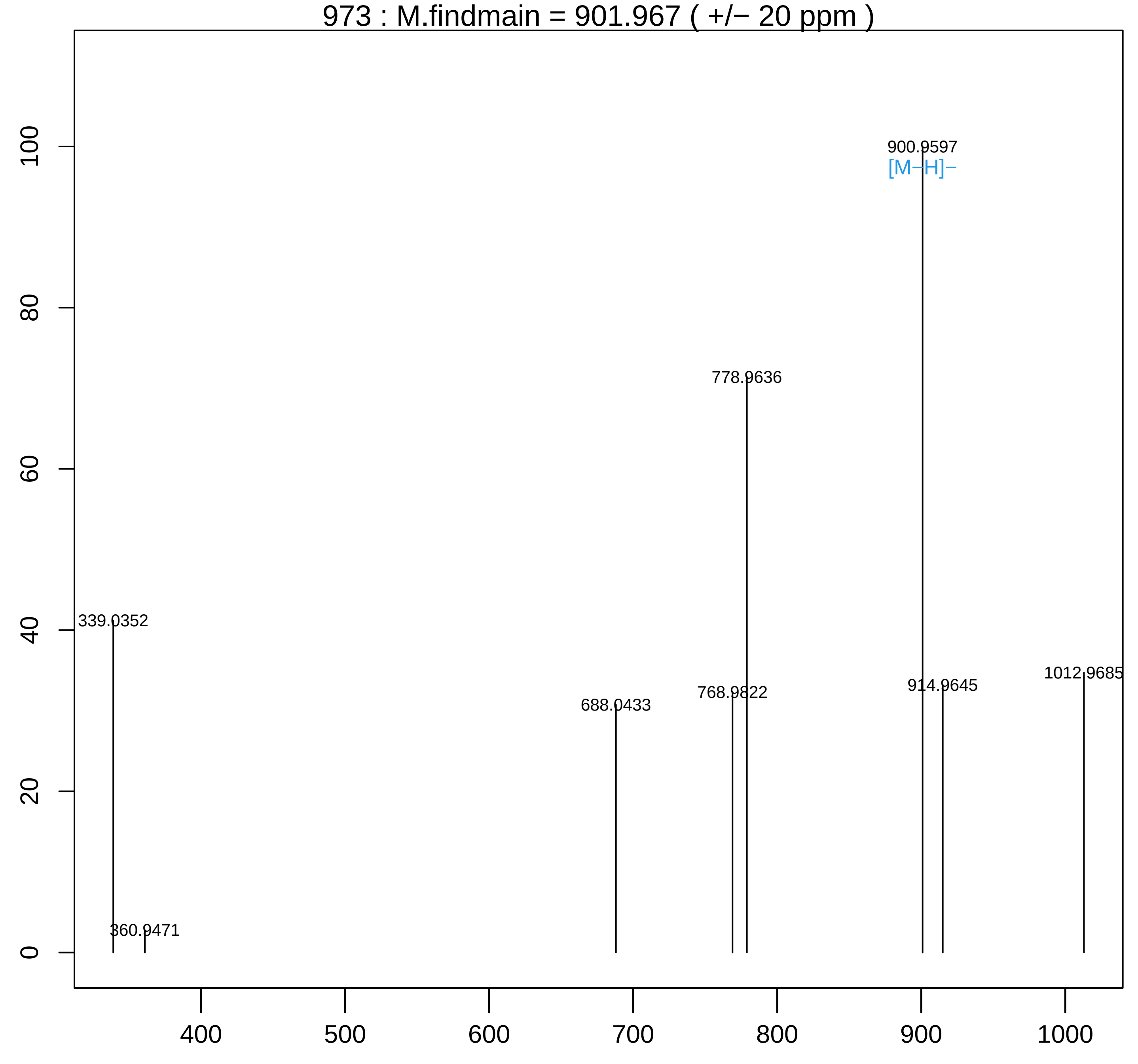

973 : M.findmain = 901.967 (+/− 20 ppm)
900.9597
[M−H]−
778.9636
339.0352
914.9645
1012.9685
688.0433
768.9822
360.9471
0
20
40
60
80
100
400
500
600
700
800
900
1000

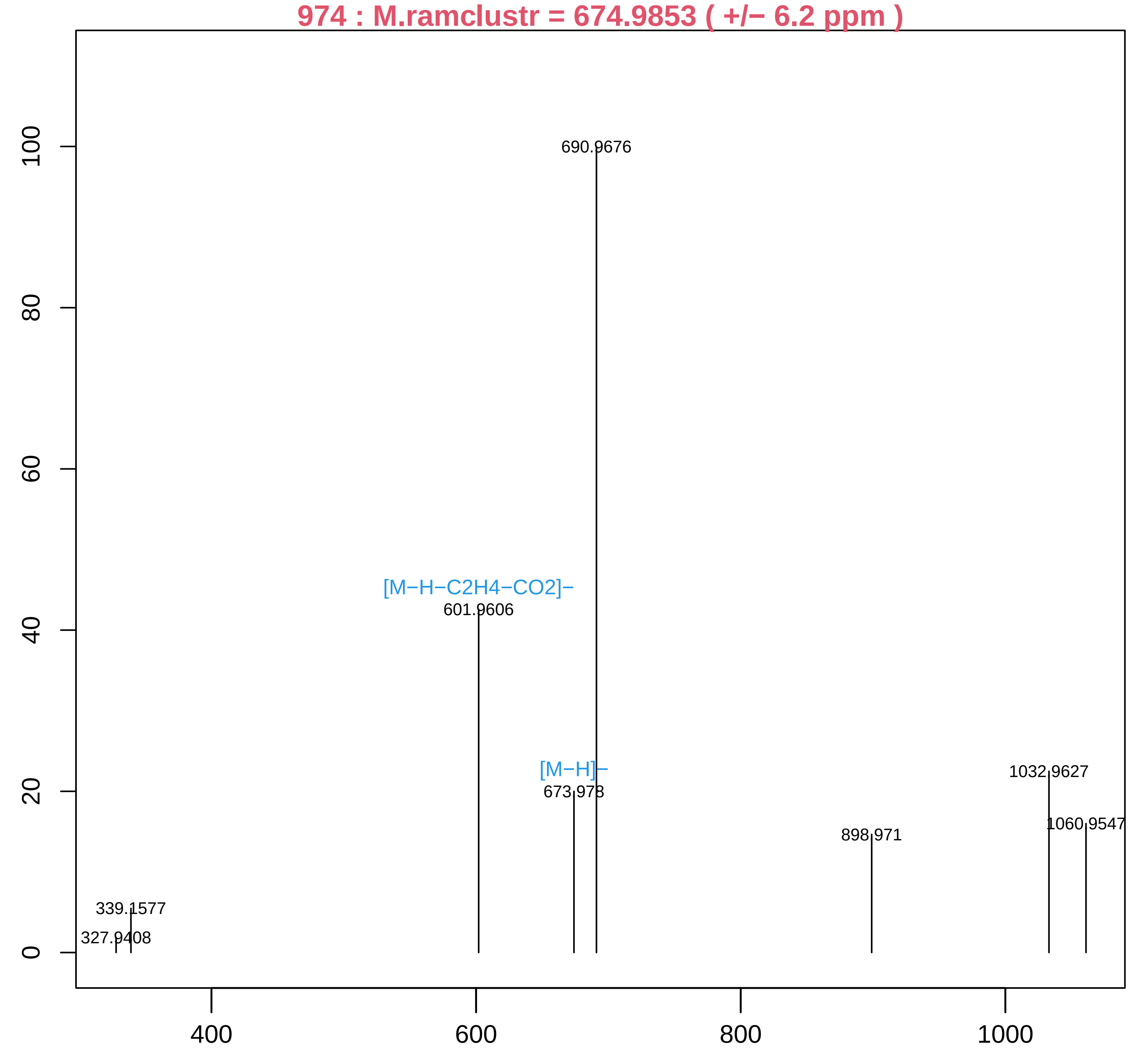
974 : M.ramclustr = 674.9853 (+/− 6.2 ppm)
[M−H−C2H4−CO2]−
601.9606
690.9676
[M−H]−
673.978
898.971
1032.9627
1060.9547
339.1577
327.9408
0
20
40
60
80
100
400
600
800
1000

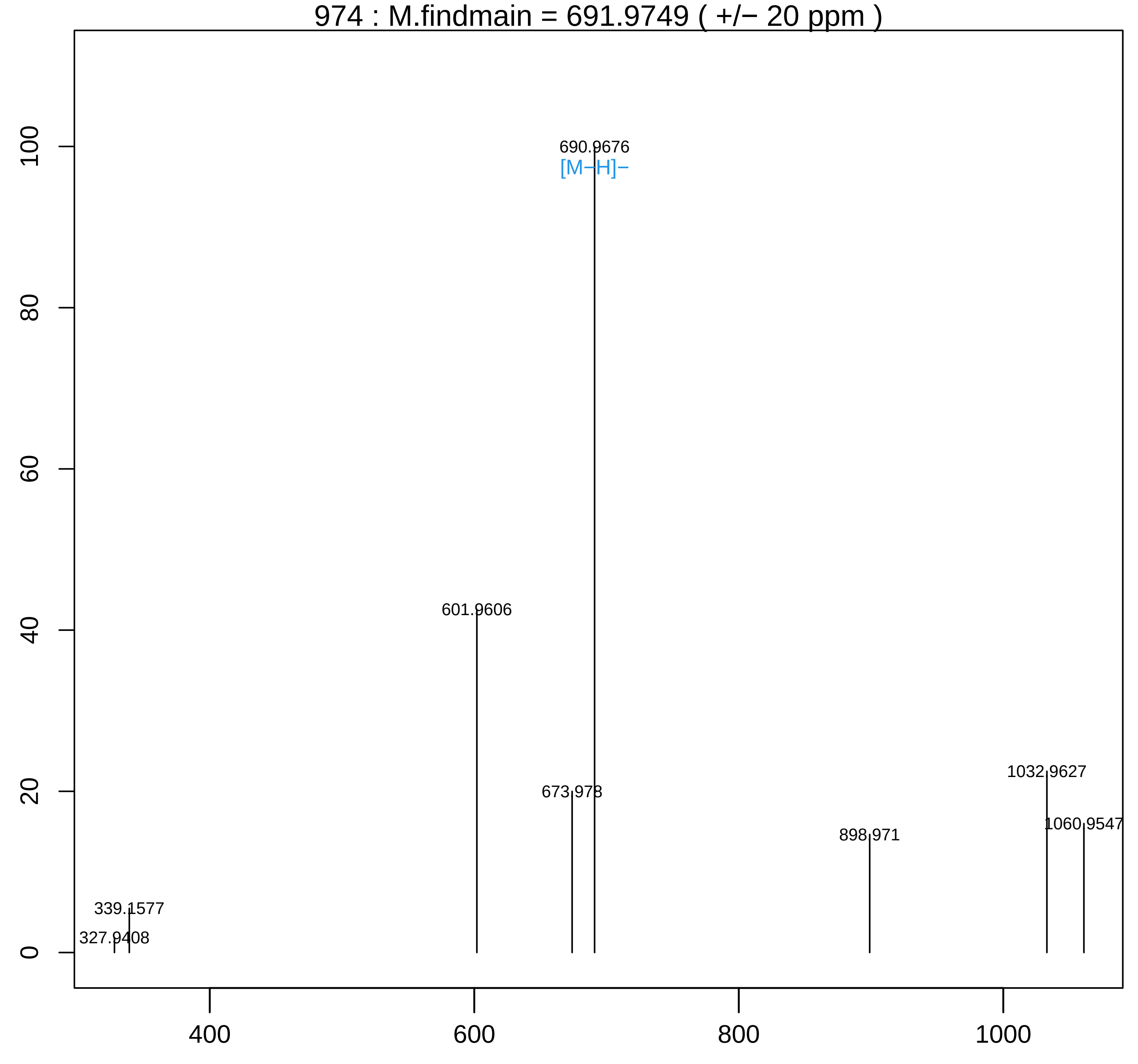
974 : M.findmain = 691.9749 (+/− 20 ppm)
690.9676
[M−H]−
601.9606
673.978
898.971
1032.9627
1060.9547
339.1577
327.9408
0
20
40
60
80
100
400
600
800
1000

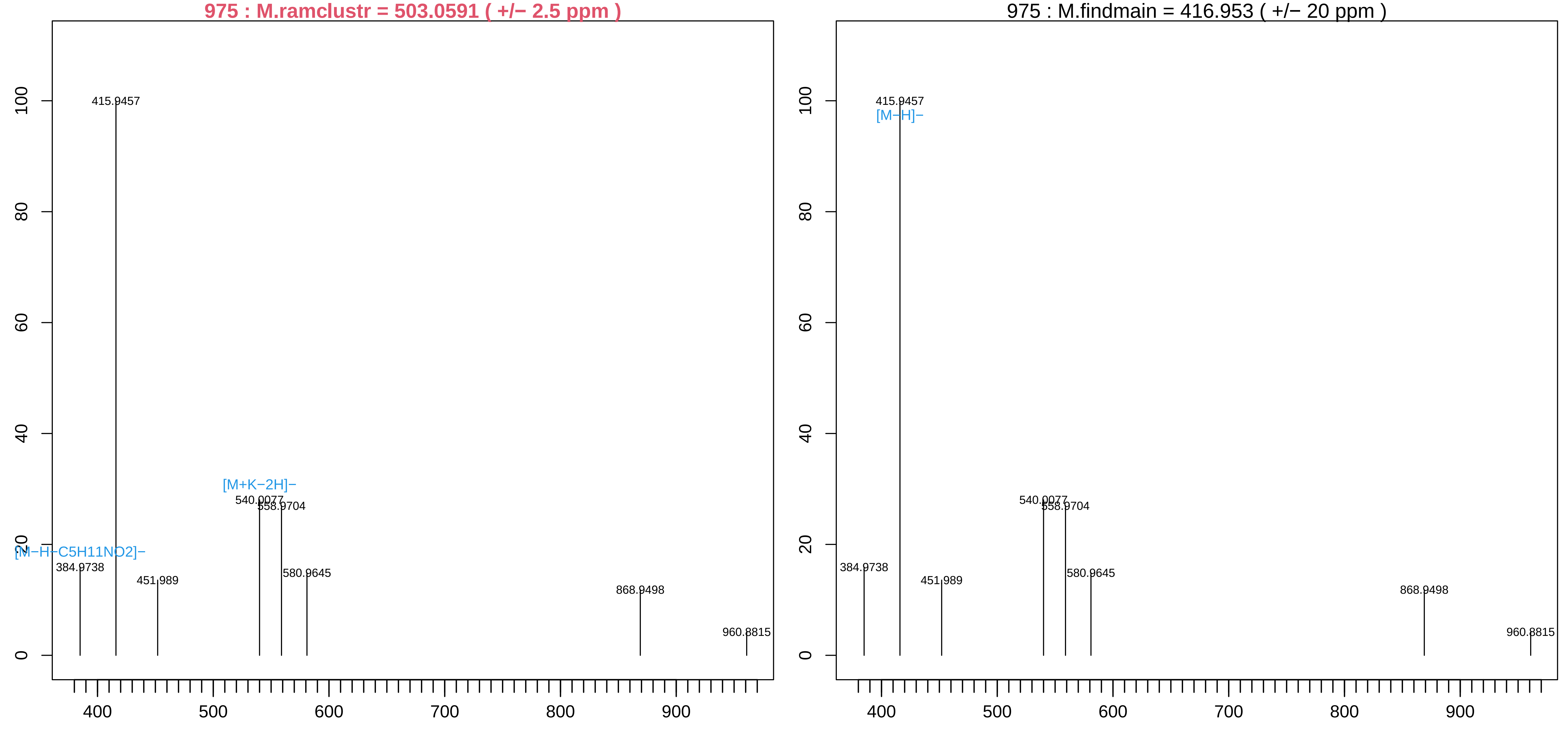

975 : M.ramclustr = 503.0591 (+/− 2.5 ppm)
415.9457
[M+K−2H]−
540.0077
558.9704
[M−H−C5H11NO2]−
384.9738
451.989
580.9645
868.9498
960.8815
975 : M.findmain = 416.953 (+/− 20 ppm)
415.9457
[M−H]−
540.0077
558.9704
384.9738
451.989
580.9645
868.9498
960.8815

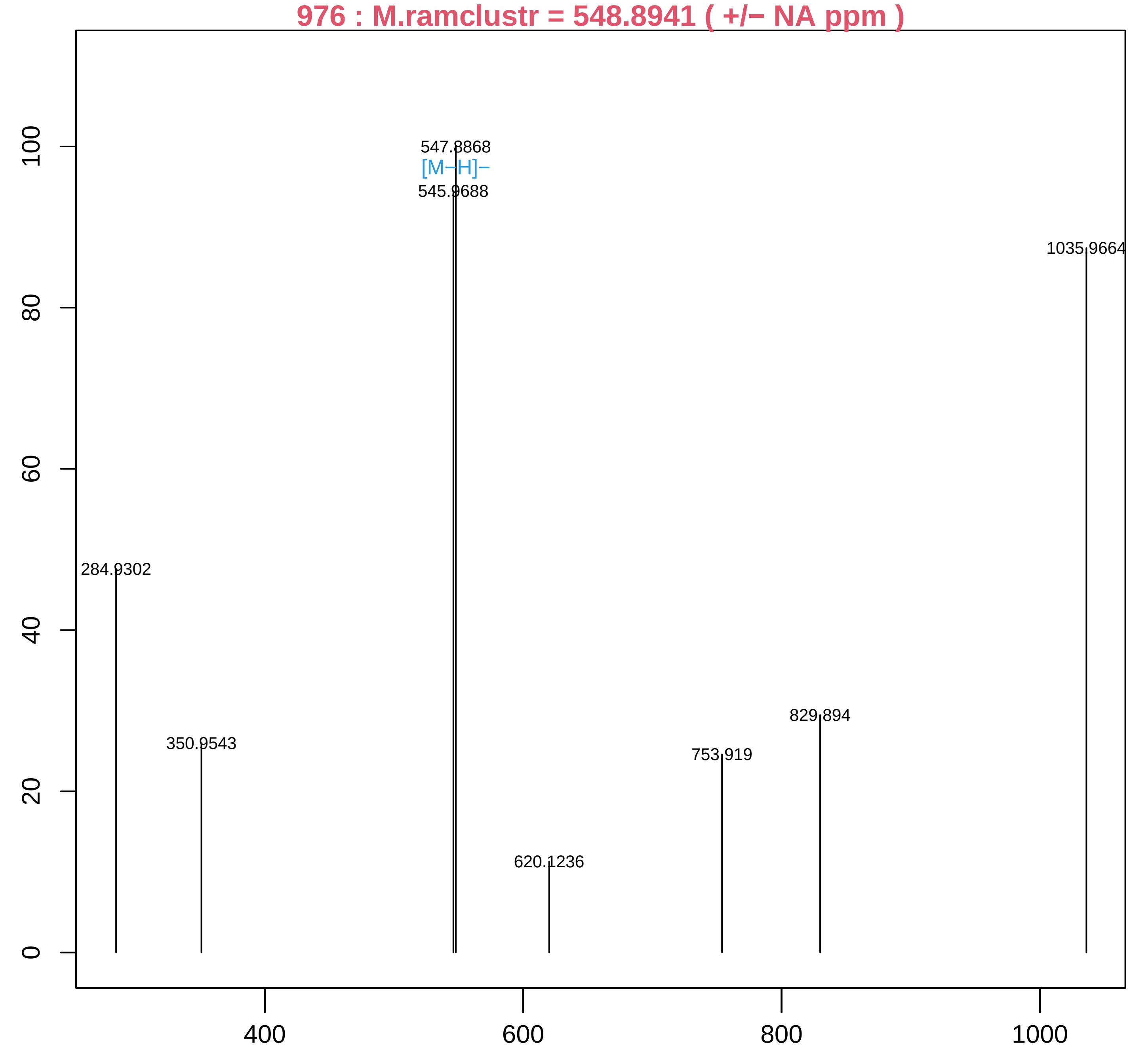

976 : M.ramclustr = 548.8941 (+/− NA ppm)
547.8868
[M−H]−
545.9688
1035.9664
284.9302
350.9543
620.1236
753.919
829.894
0
20
40
60
80
100
400
600
800
1000

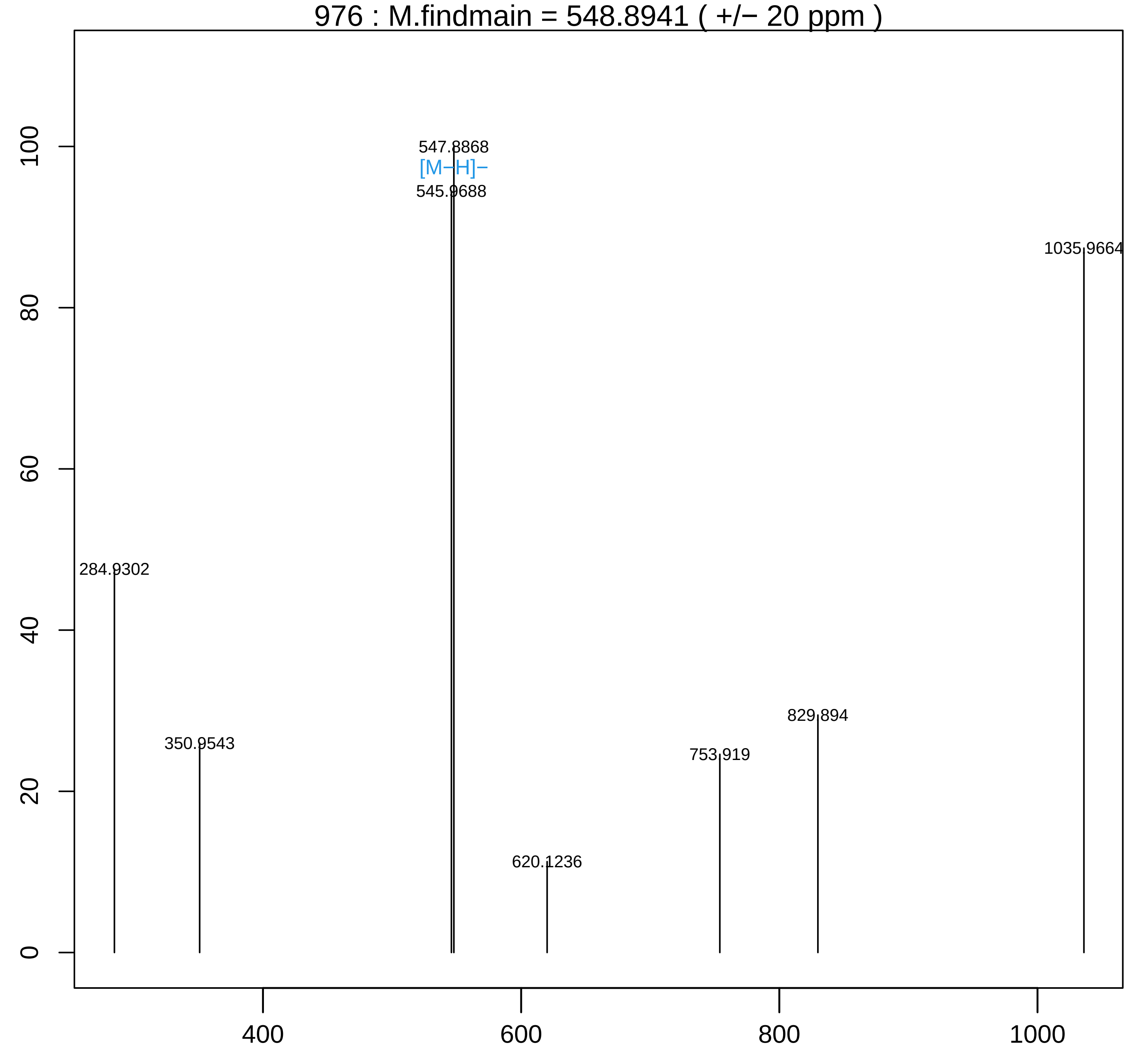

976 : M.findmain = 548.8941 (+/− 20 ppm)
547.8868
[M−H]−
545.9688
1035.9664
284.9302
350.9543
620.1236
753.919
829.894
0
20
40
60
80
100
400
600
800
1000

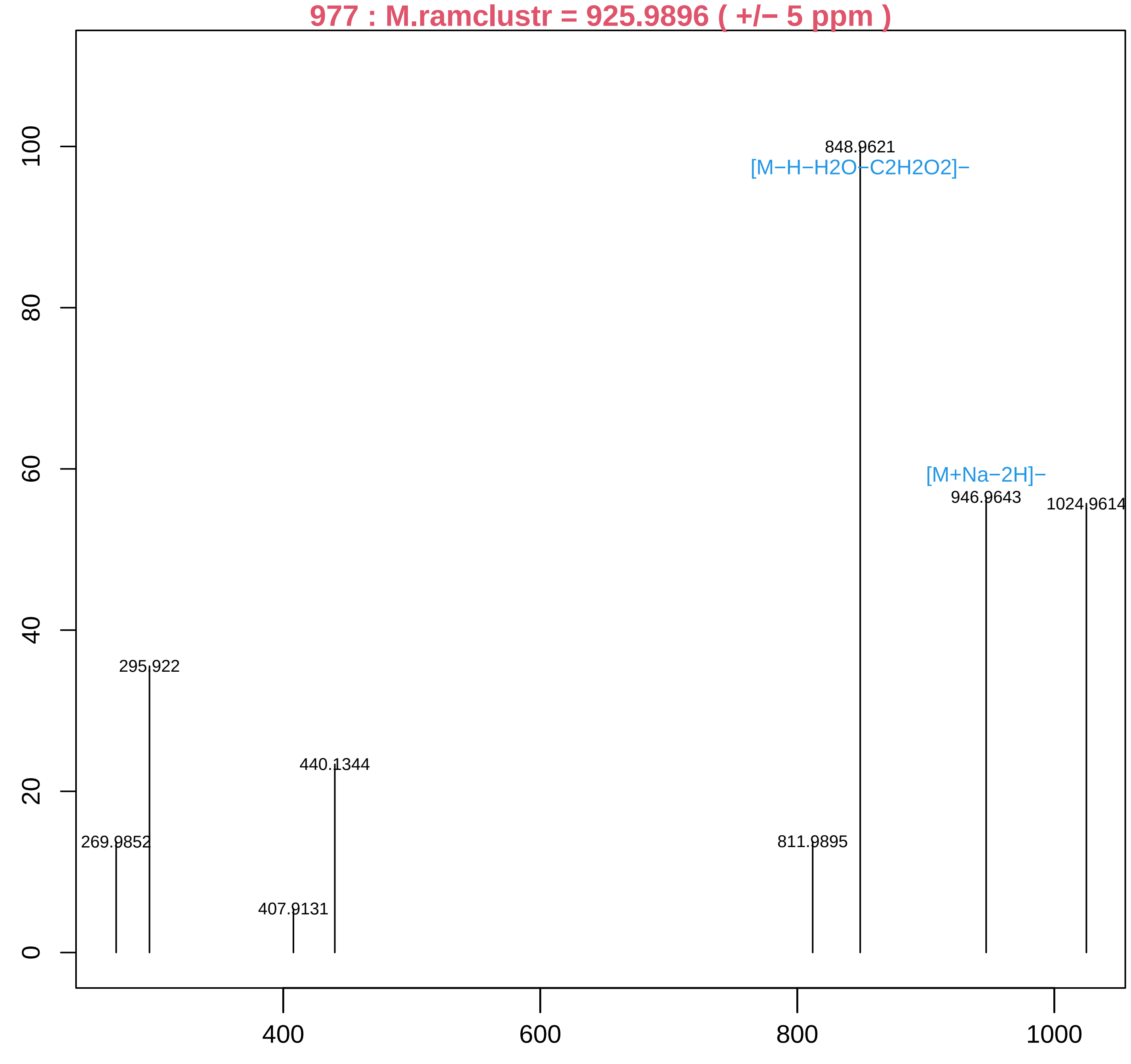
977 : M.ramclustr = 925.9896 (+/− 5 ppm)
848.9621
[M−H−H2O−C2H2O2]−
[M+Na−2H]−
946.9643
1024.9614
295.922
440.1344
269.9852
811.9895
407.9131
0
20
40
60
80
100
400
600
800
1000

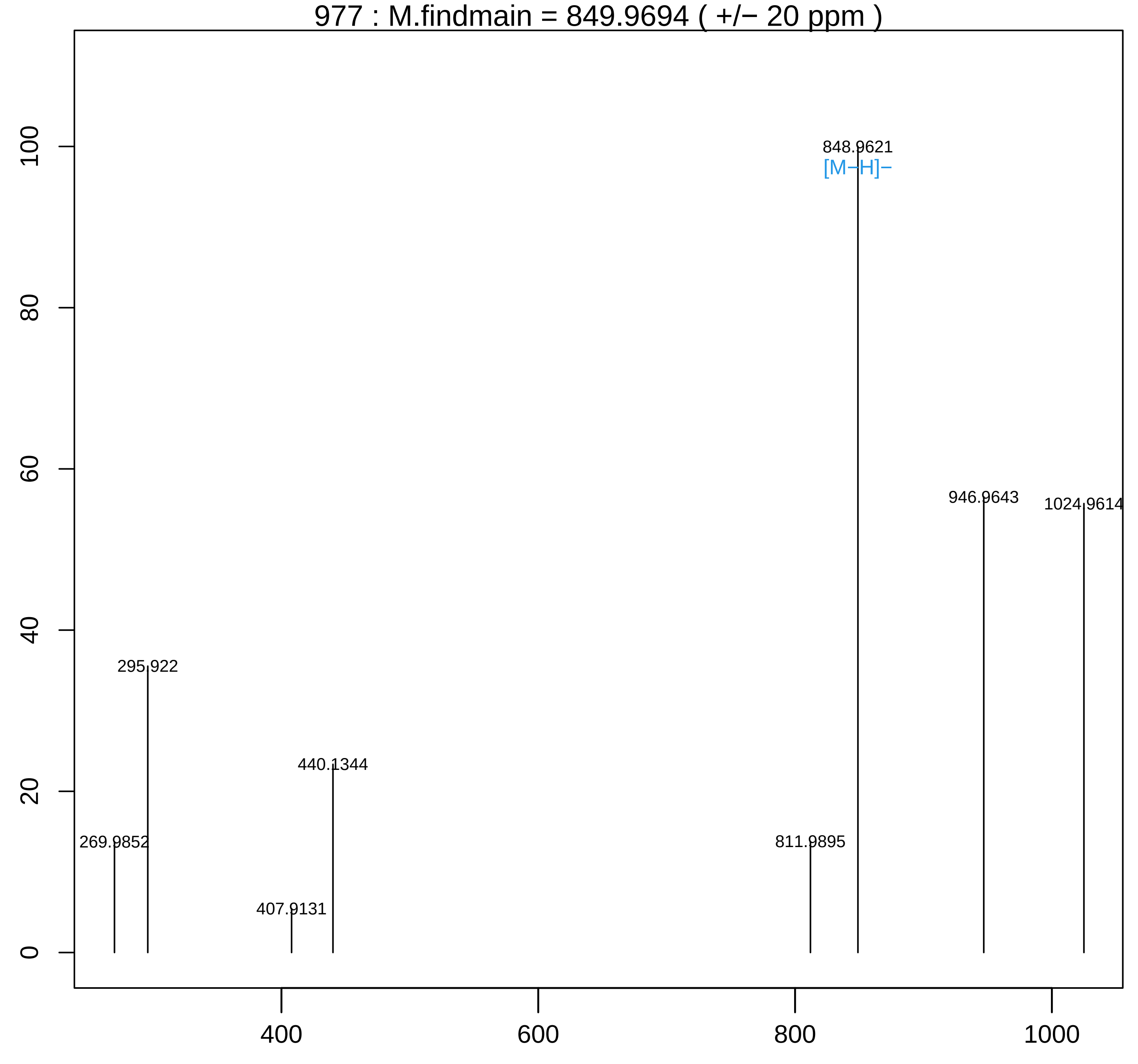
977 : M.findmain = 849.9694 (+/− 20 ppm)
848.9621
[M−H]−
946.9643
1024.9614
295.922
440.1344
269.9852
811.9895
407.9131
0
20
40
60
80
100
400
600
800
1000

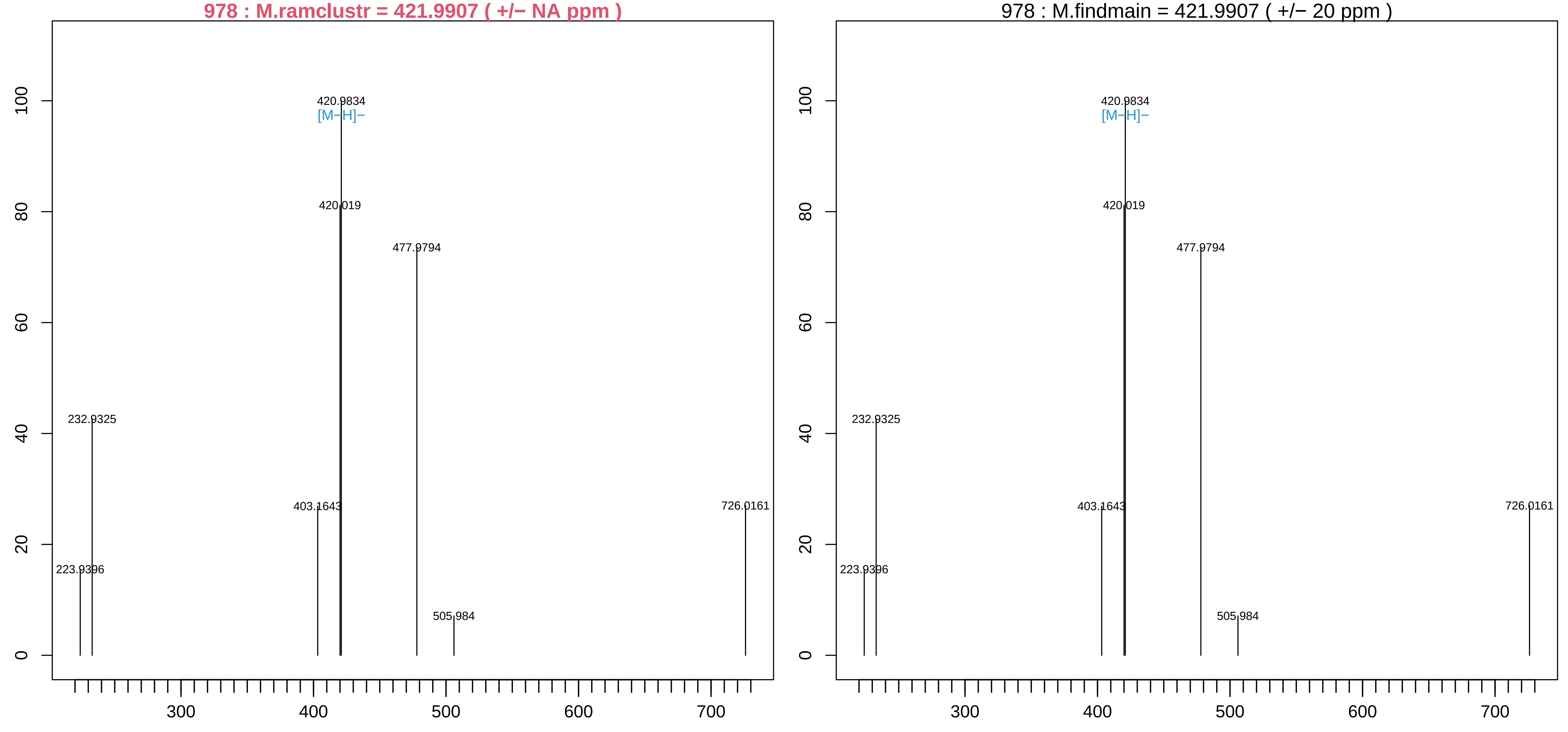
978 : M.ramclustr = 421.9907 (+/− NA ppm)
420.9834
[M−H]−
420.019
477.9794
232.9325
403.1643
726.0161
223.9396
505.984
0
20
40
60
80
100
300
400
500
600
700
978 : M.findmain = 421.9907 (+/− 20 ppm)
420.9834
[M−H]−
420.019
477.9794
232.9325
403.1643
726.0161
223.9396
505.984
0
20
40
60
80
100
300
400
500
600
700

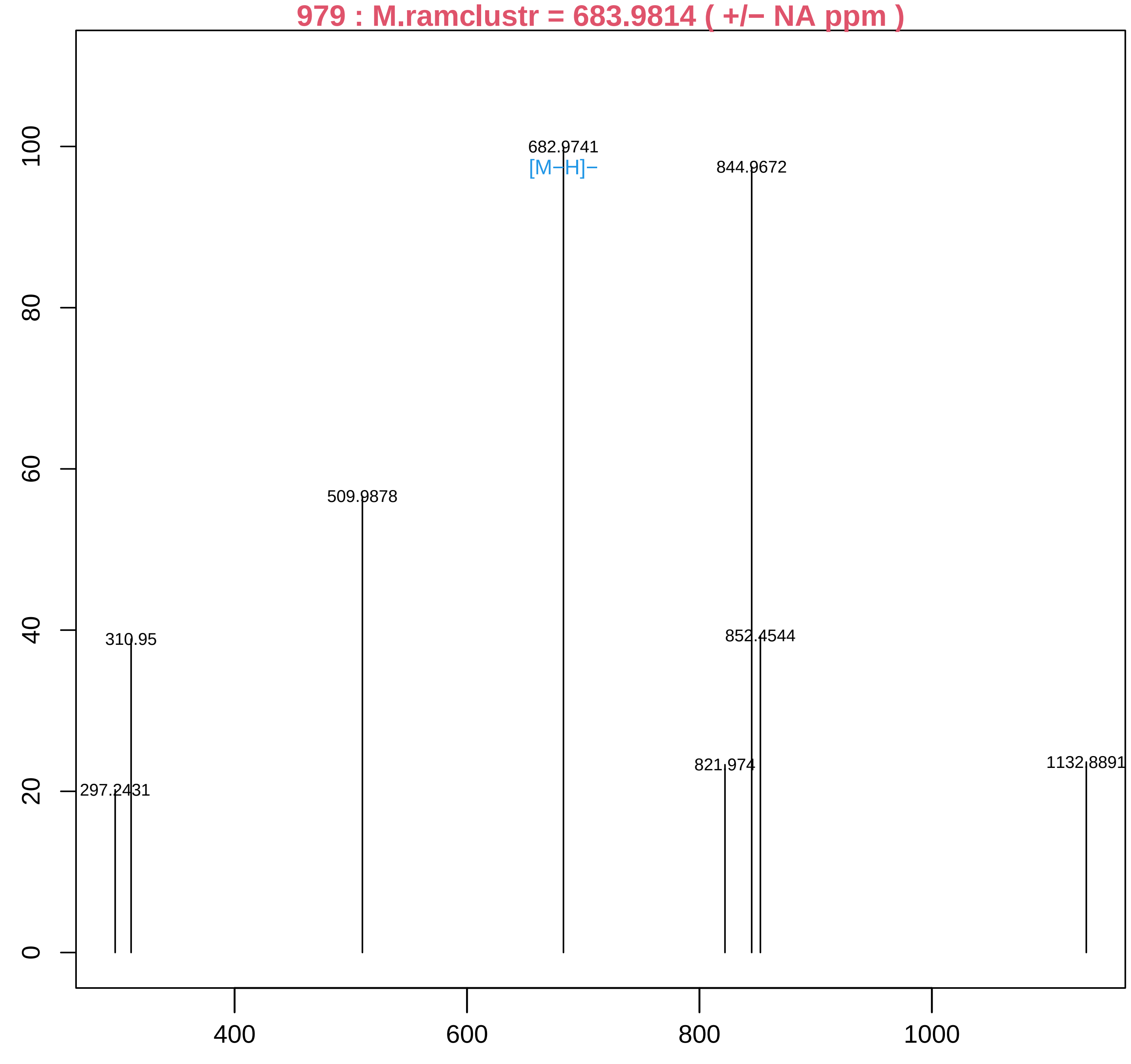
979 : M.ramclustr = 683.9814 (+/− NA ppm)
682.9741
[M−H]−
844.9672
509.9878
310.95
852.4544
821.974
1132.8891
297.2431
0
20
40
60
80
100
400
600
800
1000

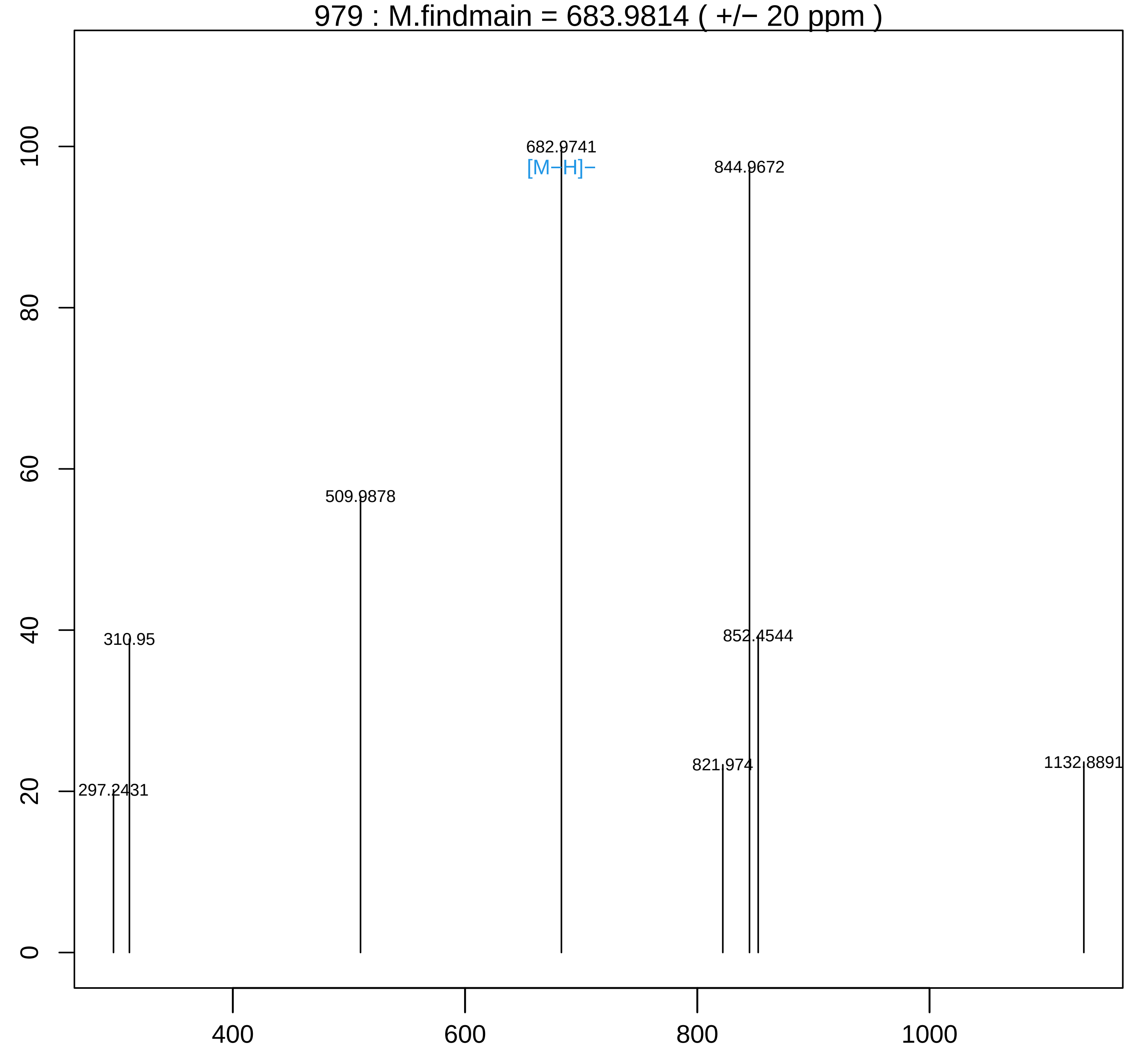
979 : M.findmain = 683.9814 (+/− 20 ppm)
682.9741
[M−H]−
844.9672
509.9878
310.95
852.4544
821.974
1132.8891
297.2431
0
20
40
60
80
100
400
600
800
1000

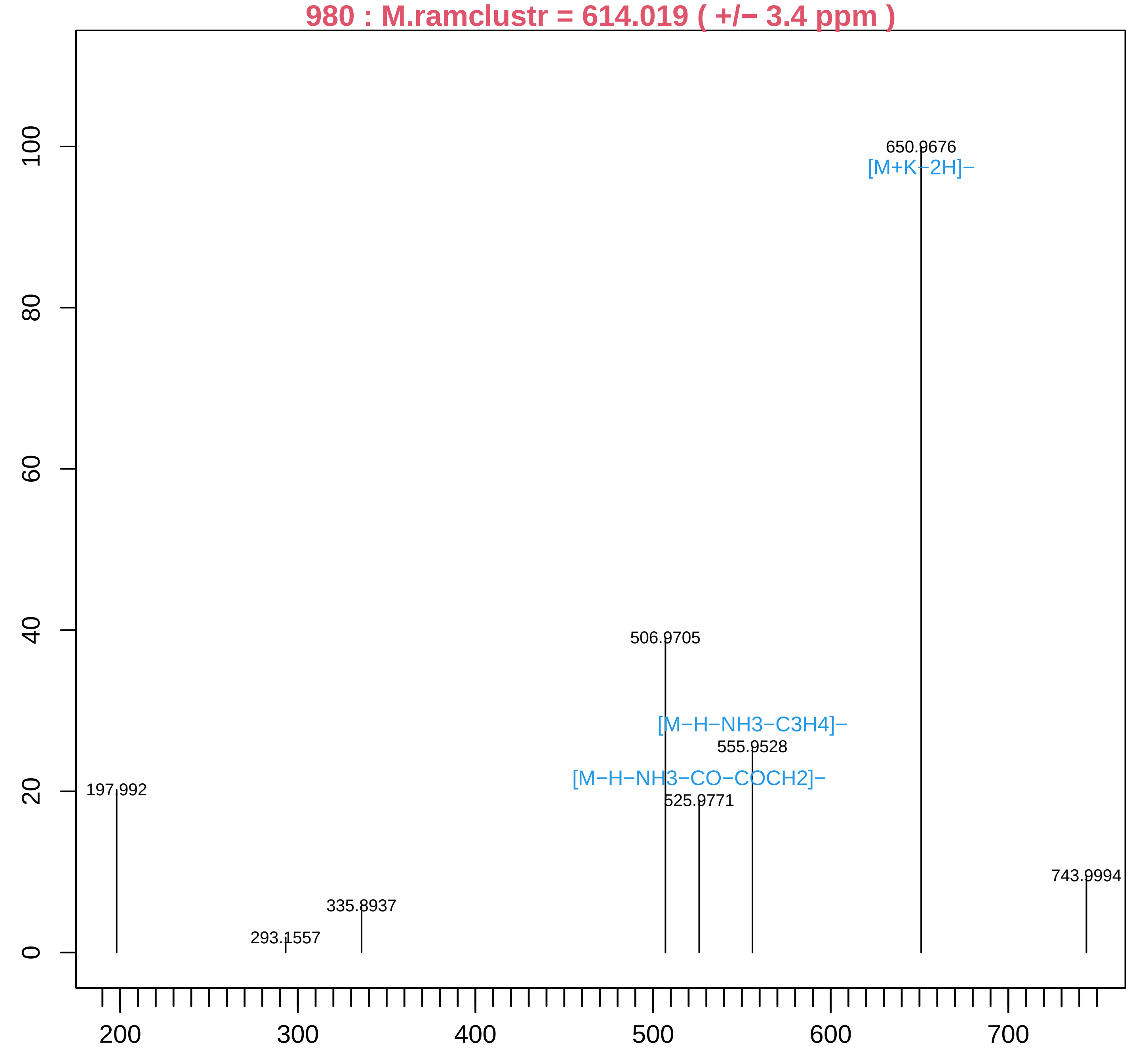

980 : M.ramclustr = 614.019 (+/− 3.4 ppm)
650.9676
[M+K−2H]−
506.9705
[M−H−NH3−C3H4]−
555.9528
[M−H−NH3−CO−COCH2]−
525.9771
197.992
743.9994
335.8937
293.1557
0
20
40
60
80
100
200
300
400
500
600
700

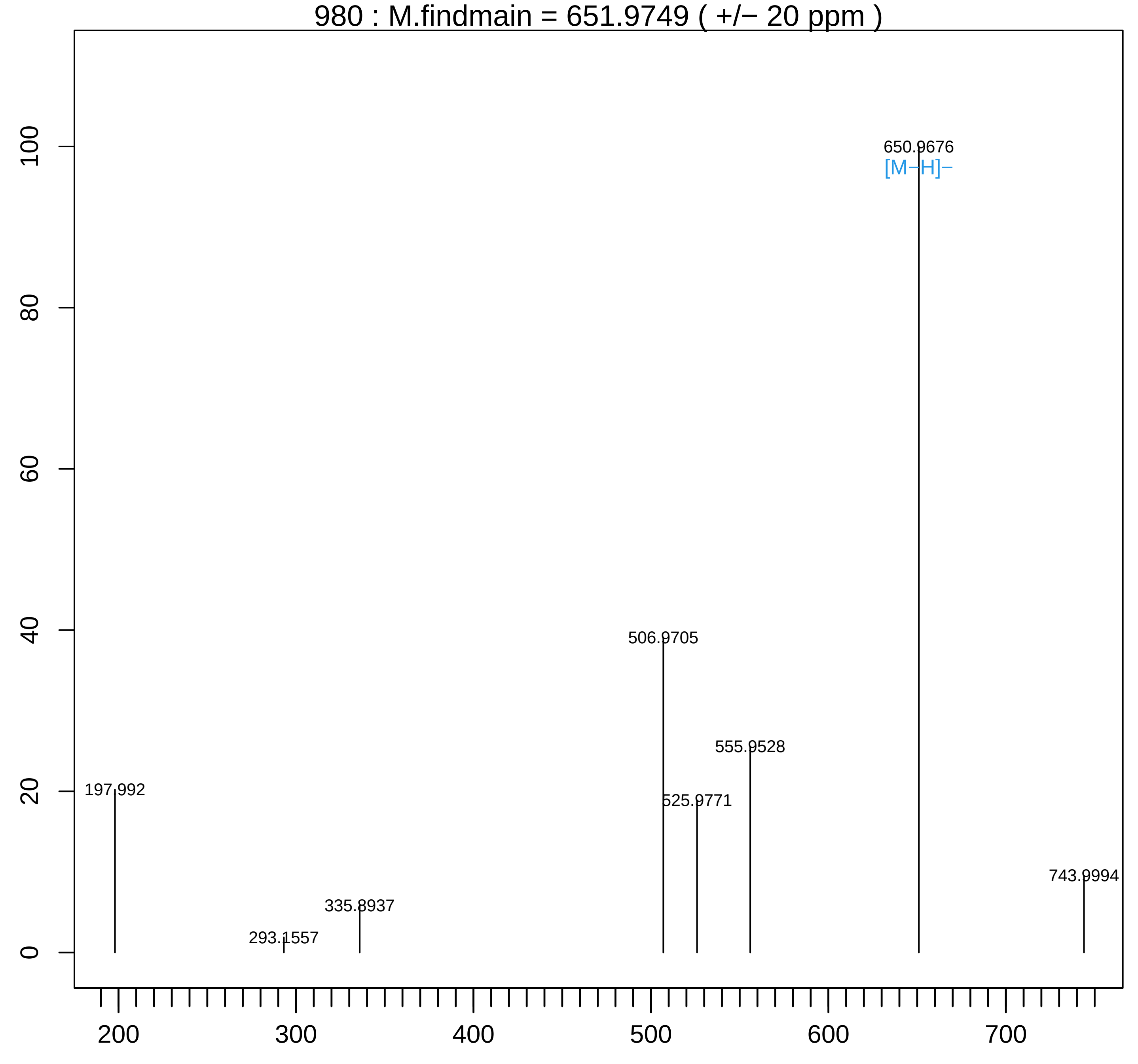

980 : M.findmain = 651.9749 (+/− 20 ppm)
650.9676
[M−H]−
506.9705
555.9528
525.9771
197.992
743.9994
335.8937
293.1557
0
20
40
60
80
100
200
300
400
500
600
700

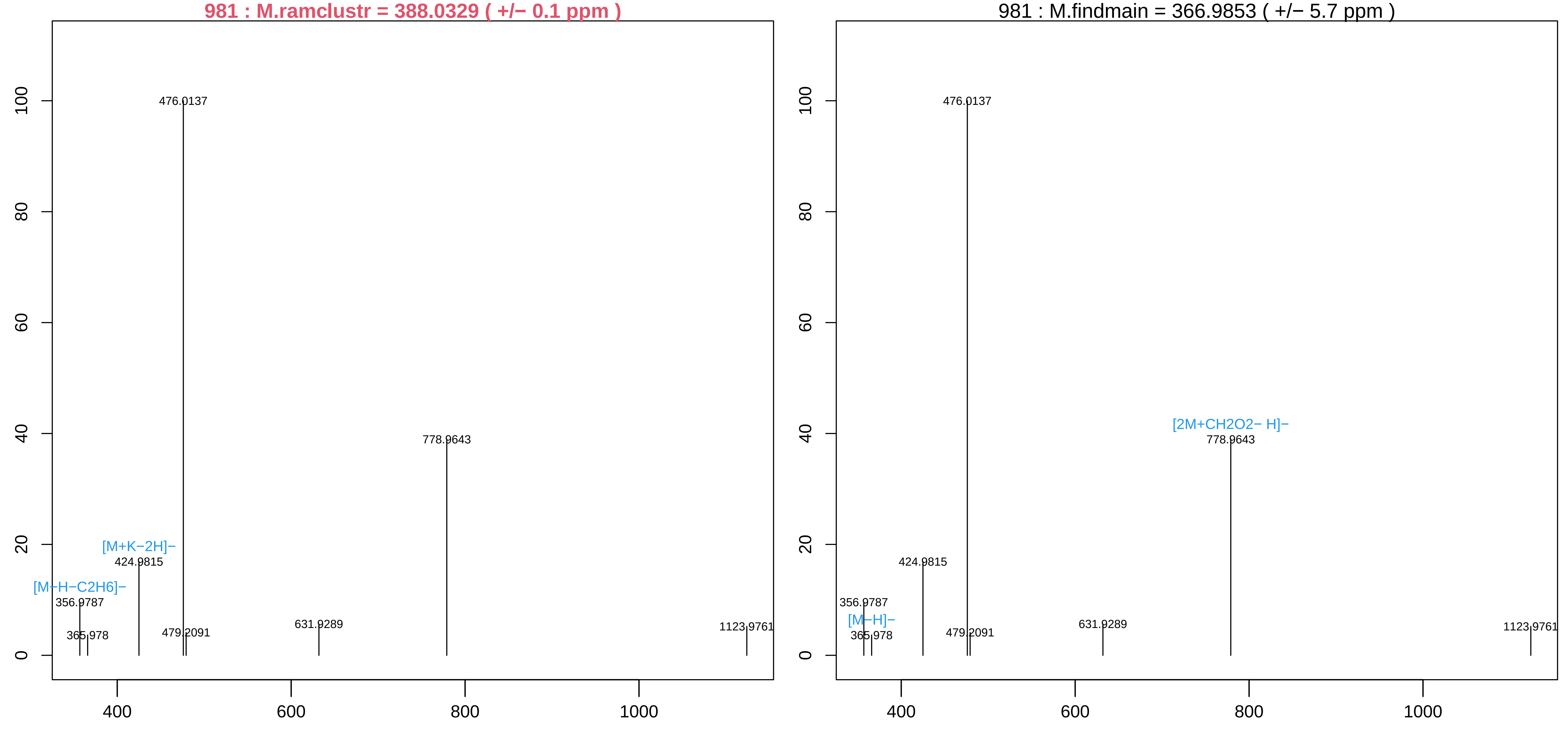

981 : M.ramclustr = 388.0329 (+/− 0.1 ppm)
476.0137
778.9643
[M+K−2H]−
424.9815
[M−H−C2H6]−
356.9787
365.978
479.2091
631.9289
1123.9761
0
20
40
60
80
100
400
600
800
1000
981 : M.findmain = 366.9853 (+/− 5.7 ppm)
476.0137
[2M+CH2O2− H]−
778.9643
424.9815
356.9787
[M−H]−
365.978
479.2091
631.9289
1123.9761
0
20
40
60
80
100
400
600
800
1000

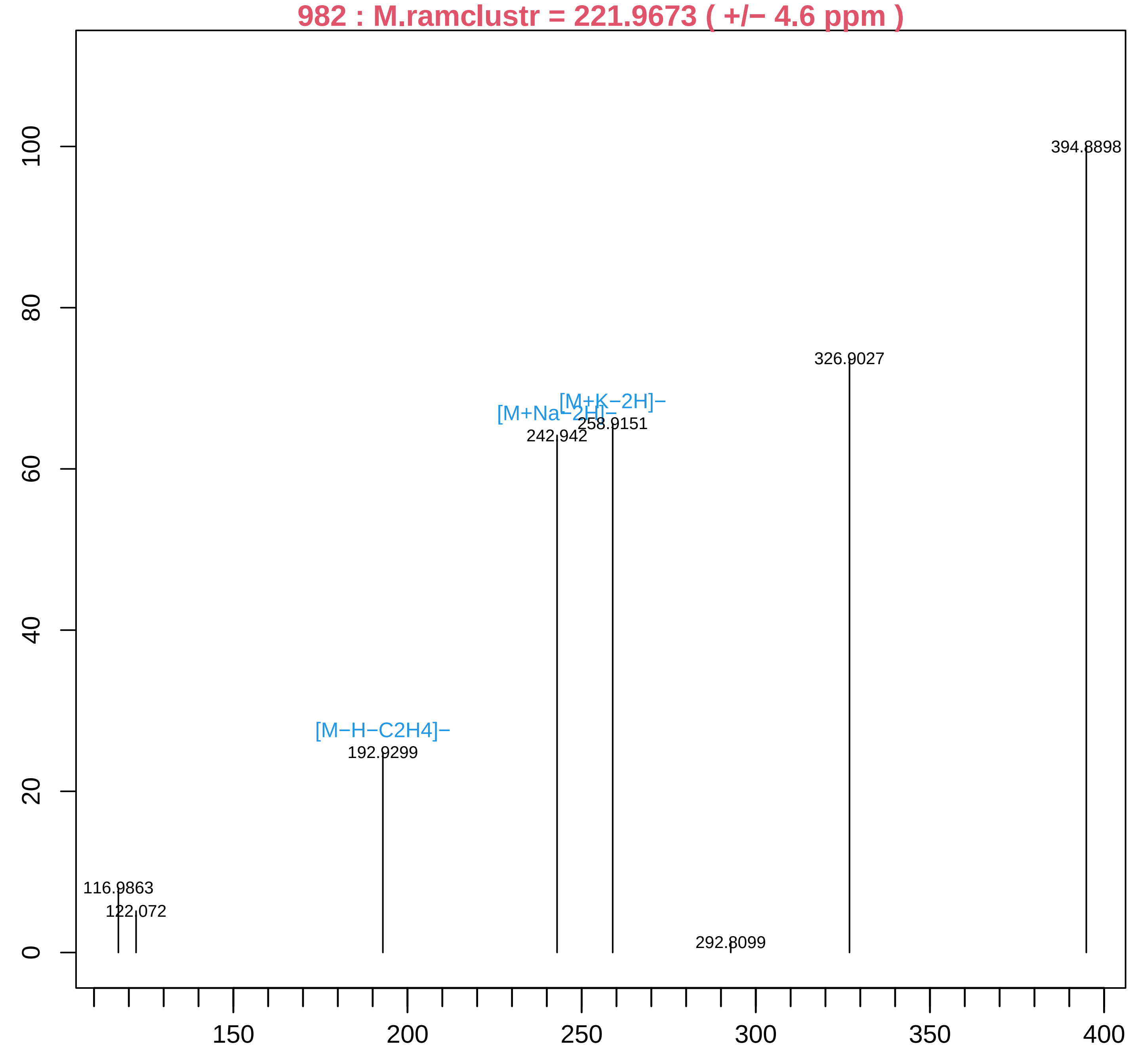

982 : M.ramclustr = 221.9673 (+/− 4.6 ppm)
394.8898
326.9027
[M+K−2H]−
[M+Na−2H]−
258.9151
242.942
[M−H−C2H4]−
192.9299
116.9863
122.072
292.8099
0
20
40
60
80
100
150
200
250
300
350
400

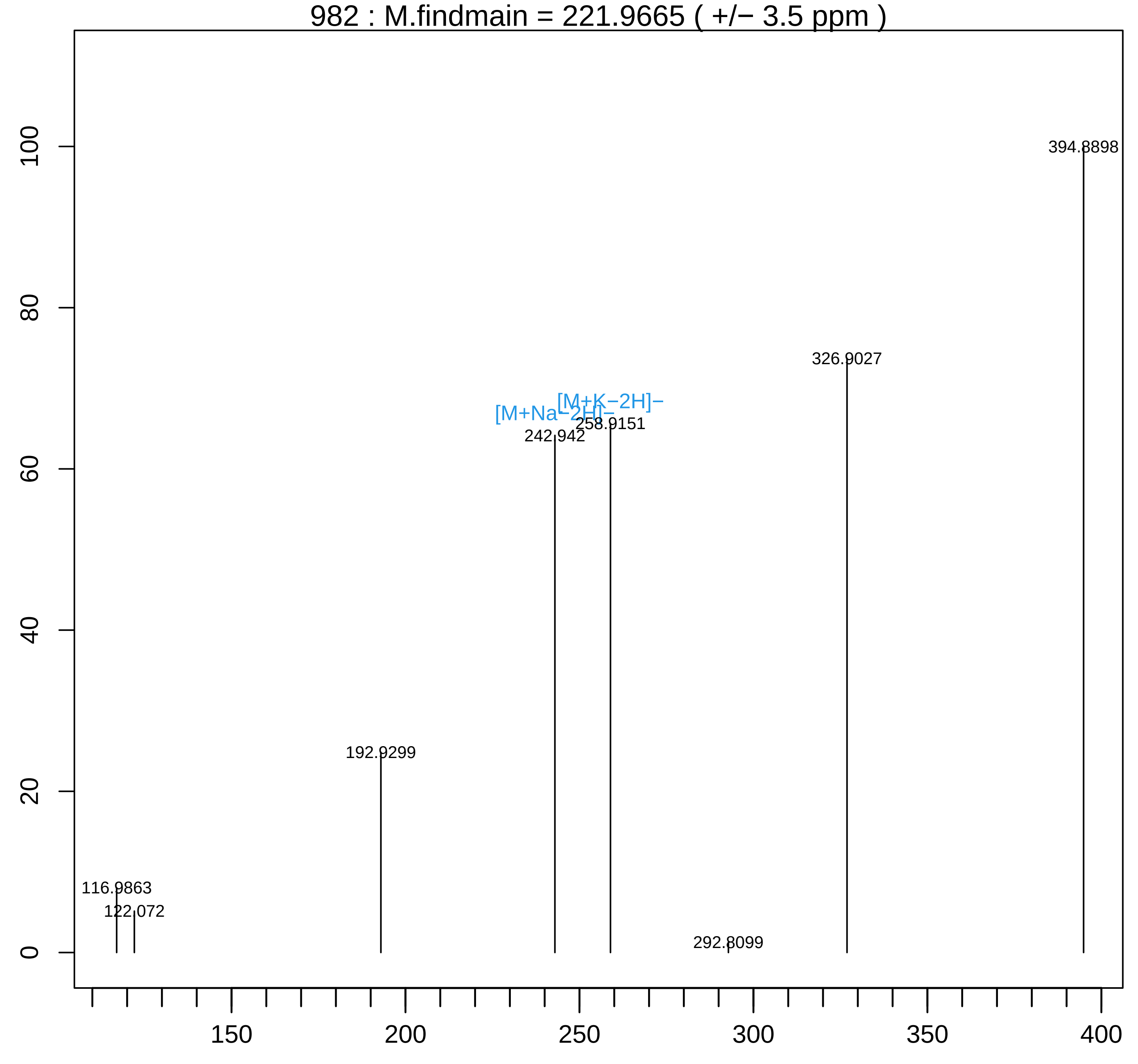

982 : M.findmain = 221.9665 (+/− 3.5 ppm)
394.8898
326.9027
[M+K−2H]−
[M+Na−2H]−
258.9151
242.942
192.9299
116.9863
122.072
292.8099
0
20
40
60
80
100
150
200
250
300
350
400

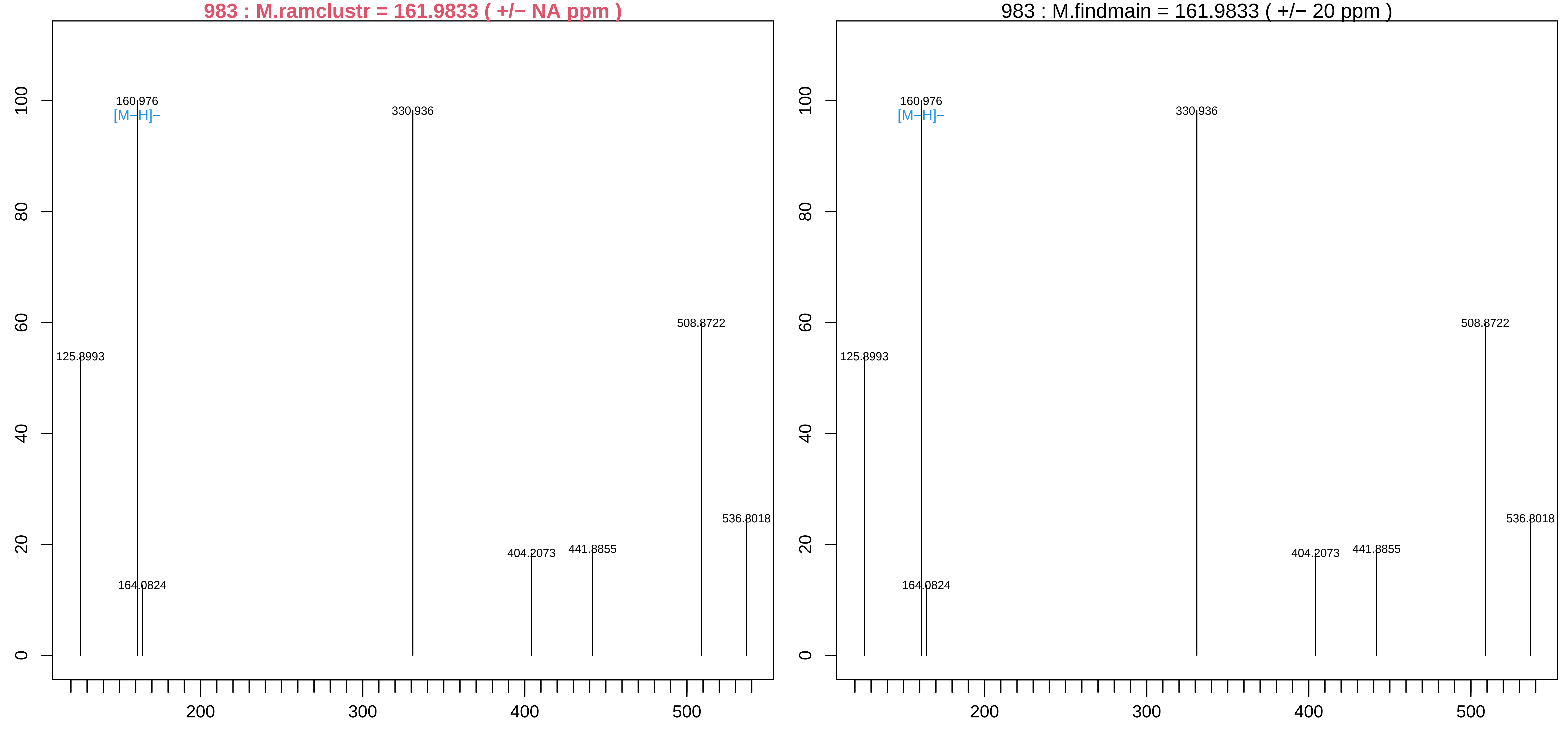

983 : M.ramclustr = 161.9833 (+/− NA ppm)
983 : M.findmain = 161.9833 (+/− 20 ppm)
160.976
[M−H]−
330.936
508.8722
125.8993
536.8018
404.2073
441.8855
164.0824
0
20
40
60
80
100
200
300
400
500

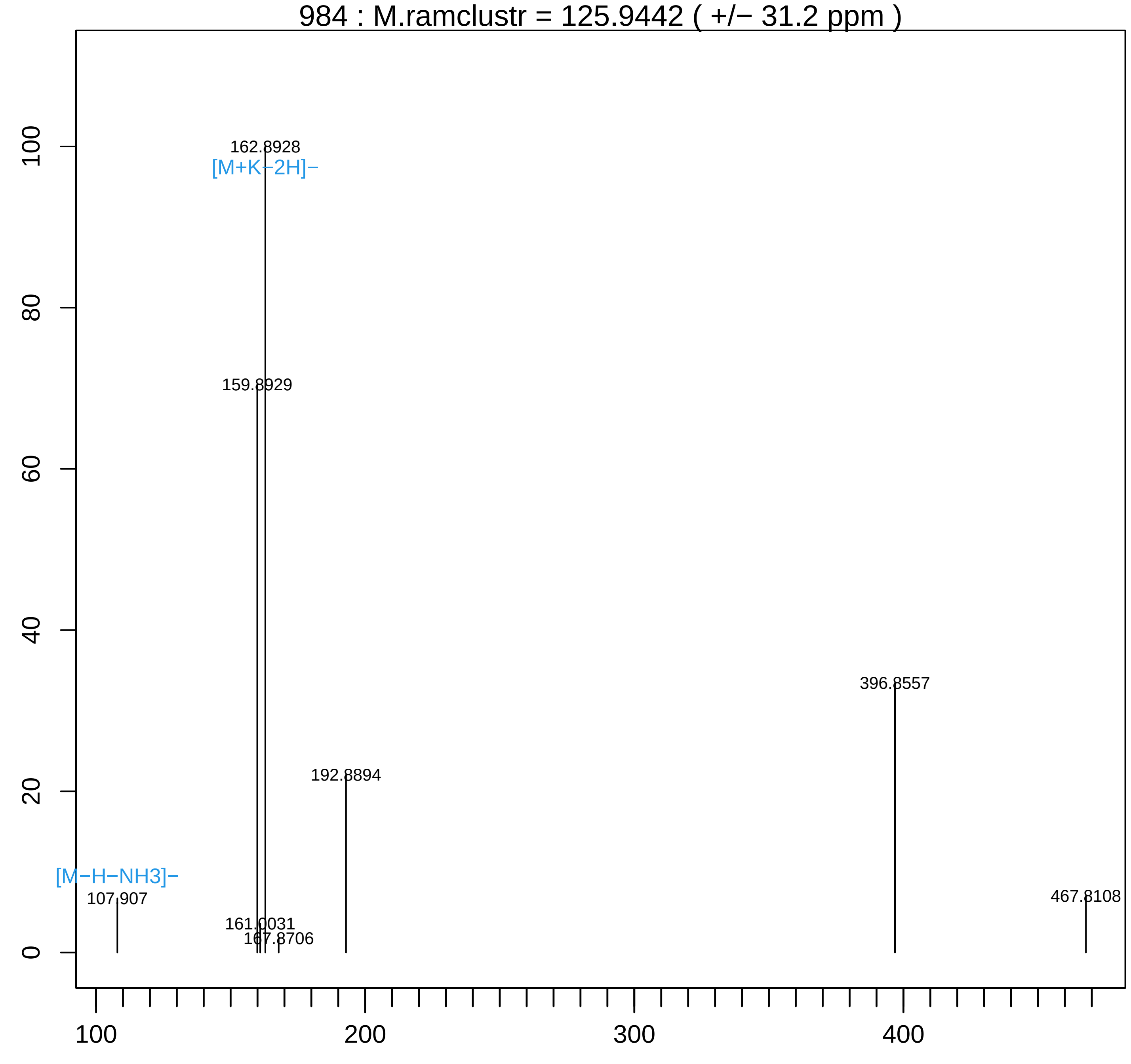
984 : M.ramclustr = 125.9442 (+/− 31.2 ppm)
162.8928
[M+K−2H]−
159.8929
396.8557
192.8894
[M−H−NH3]−
107.907
161.0031
167.8706
467.8108
0
20
40
60
80
100
100
200
300
400

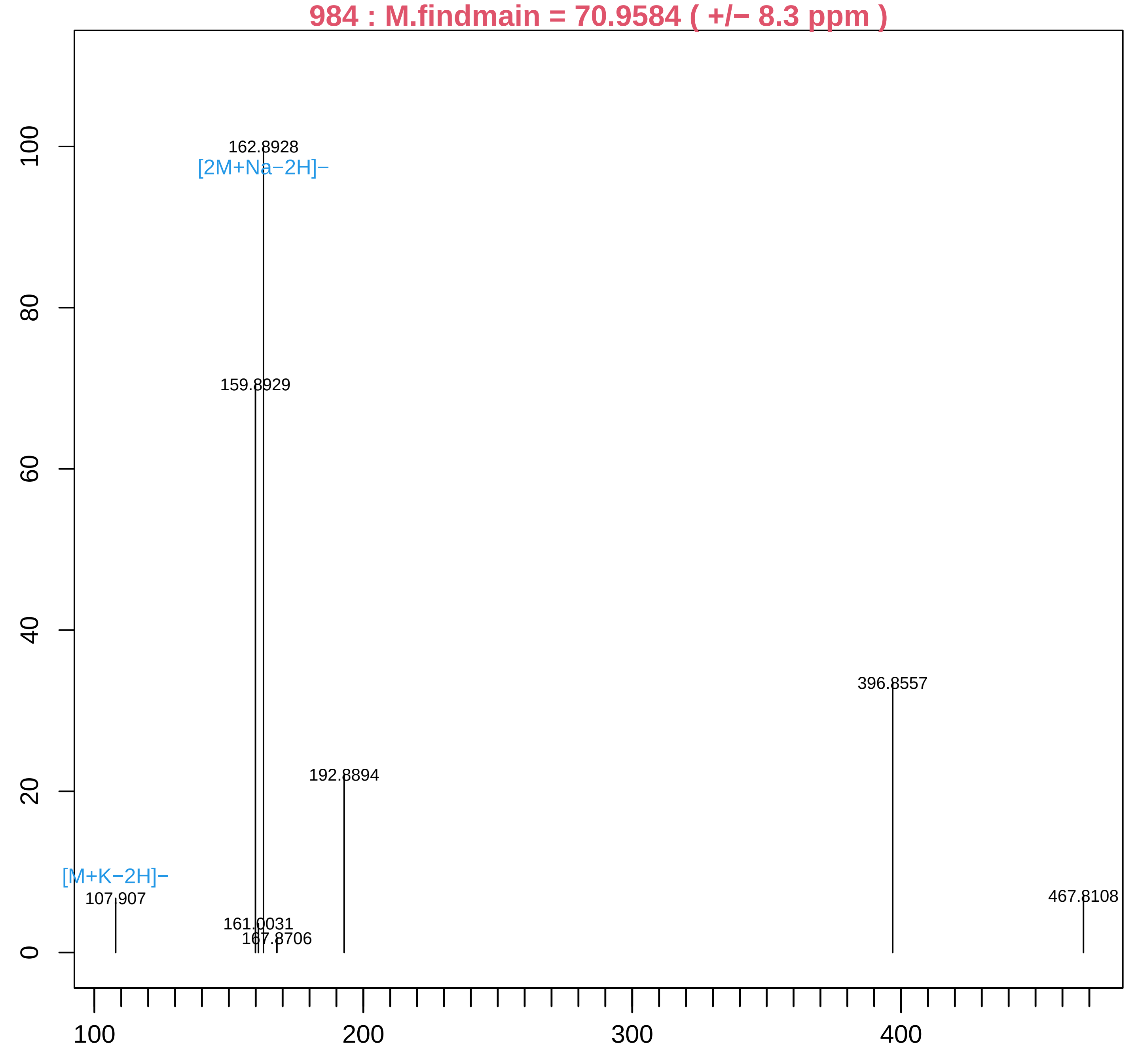
984 : M.findmain = 70.9584 (+/− 8.3 ppm)
162.8928
[2M+Na−2H]−
159.8929
396.8557
192.8894
[M+K−2H]−
107.907
161.0031
167.8706
467.8108
0
20
40
60
80
100
100
200
300
400

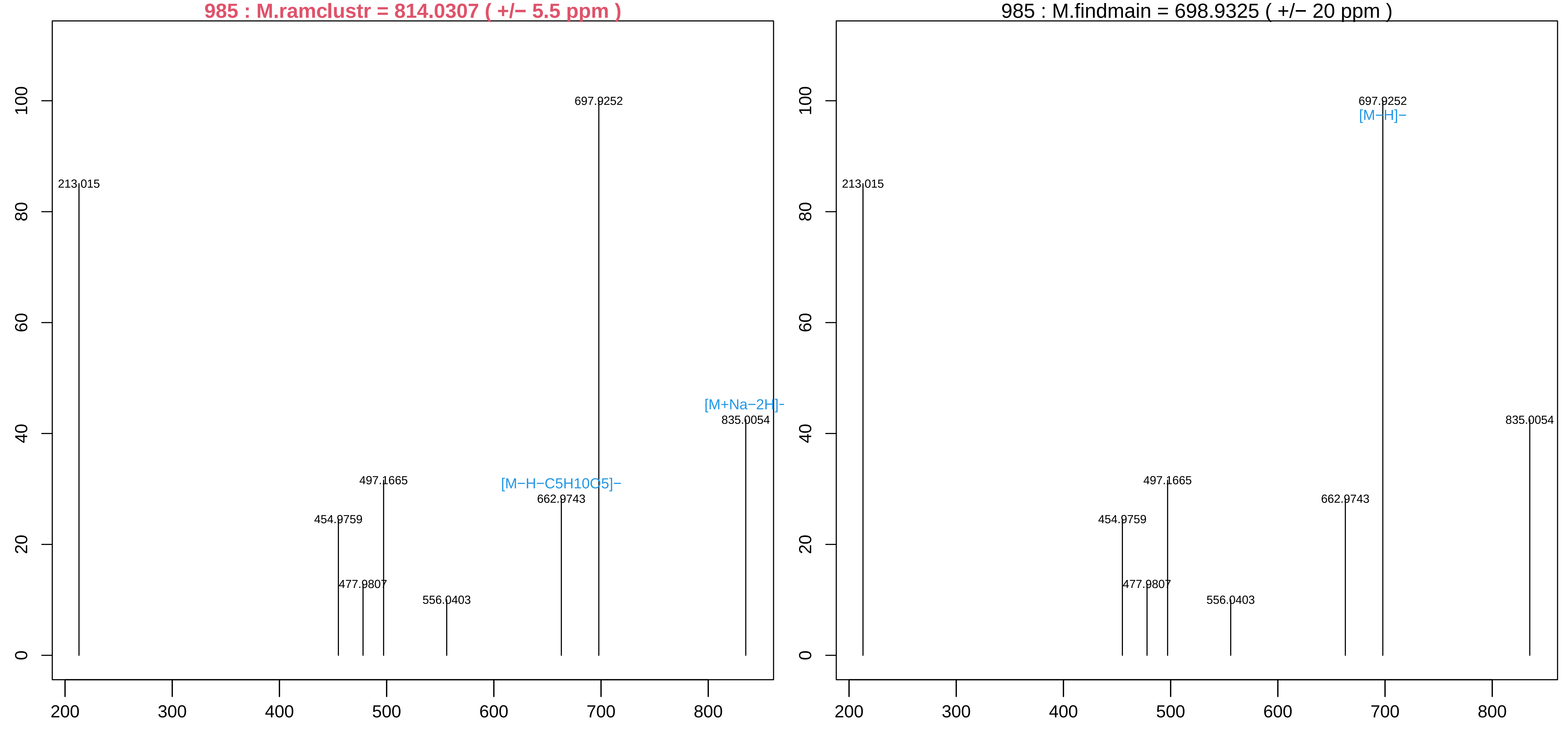
985 : M.ramclustr = 814.0307 (+/− 5.5 ppm)
213.015
454.9759
477.9807
497.1665
556.0403
662.9743
[M−H−C5H10O5]−
697.9252
835.0054
[M+Na−2H]-
985 : M.findmain = 698.9325 (+/− 20 ppm)
213.015
454.9759
477.9807
497.1665
556.0403
662.9743
697.9252
[M−H]−
835.0054
0
20
40
60
80
100
200
300
400
500
600
700
800

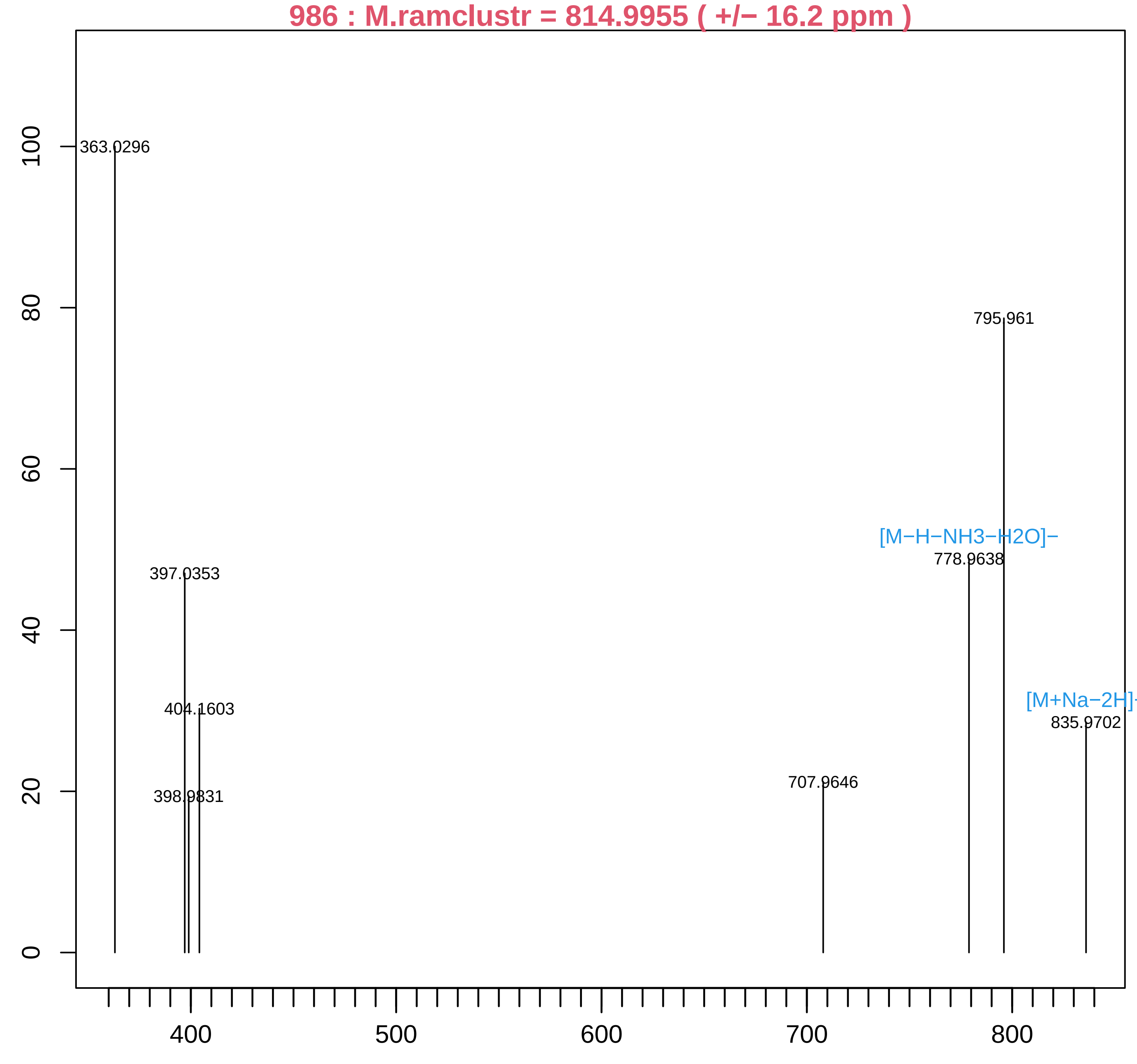

986 : M.ramclustr = 814.9955 (+/− 16.2 ppm)
363.0296
397.0353
404.1603
398.9831
707.9646
[M−H−NH3−H2O]−
778.9638
795.961
[M+Na−2H]-
835.9702
0
20
40
60
80
100
400
500
600
700
800

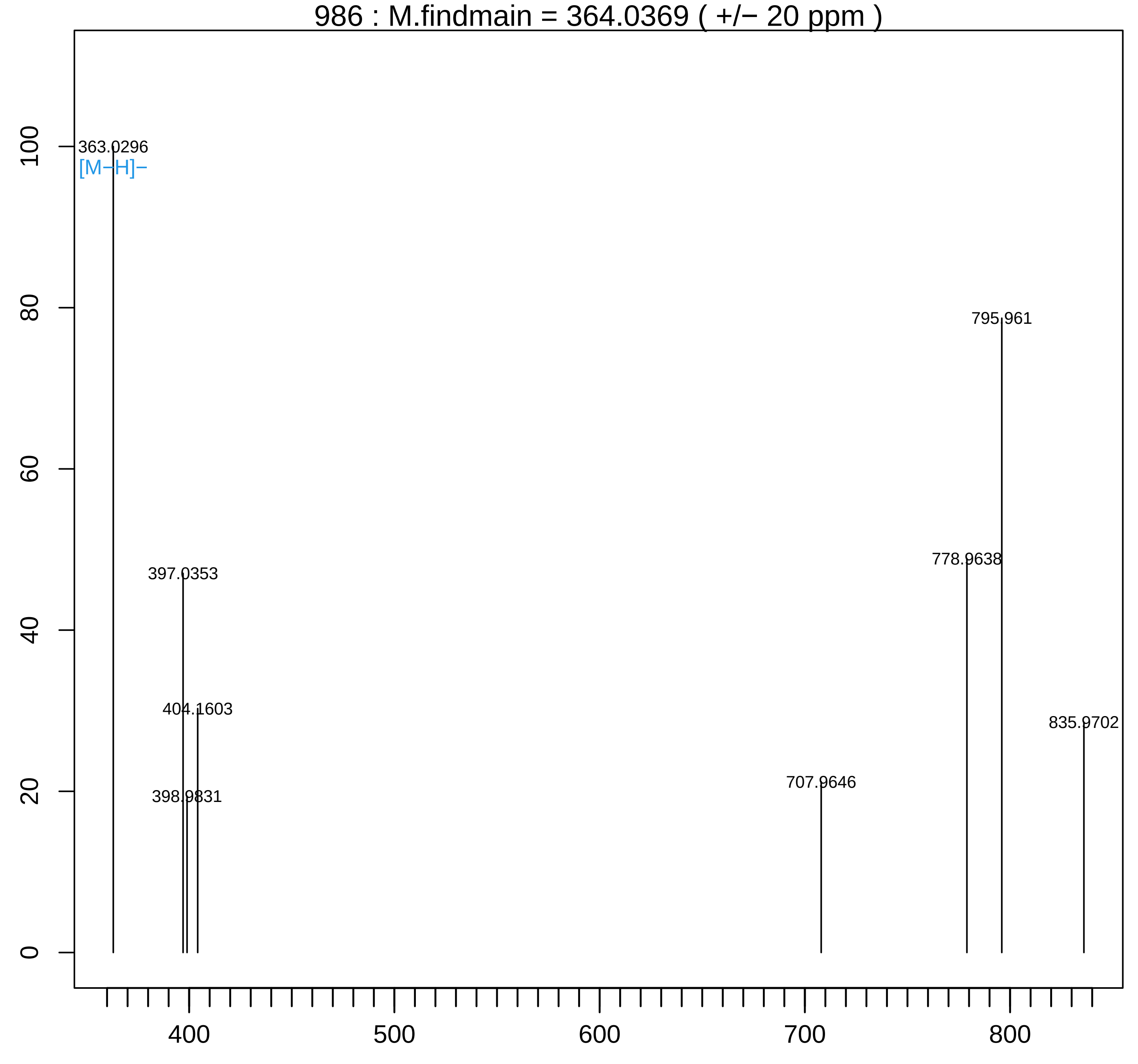

986 : M.findmain = 364.0369 (+/− 20 ppm)
363.0296
[M−H]−
397.0353
404.1603
398.9831
707.9646
778.9638
795.961
835.9702
0
20
40
60
80
100
400
500
600
700
800

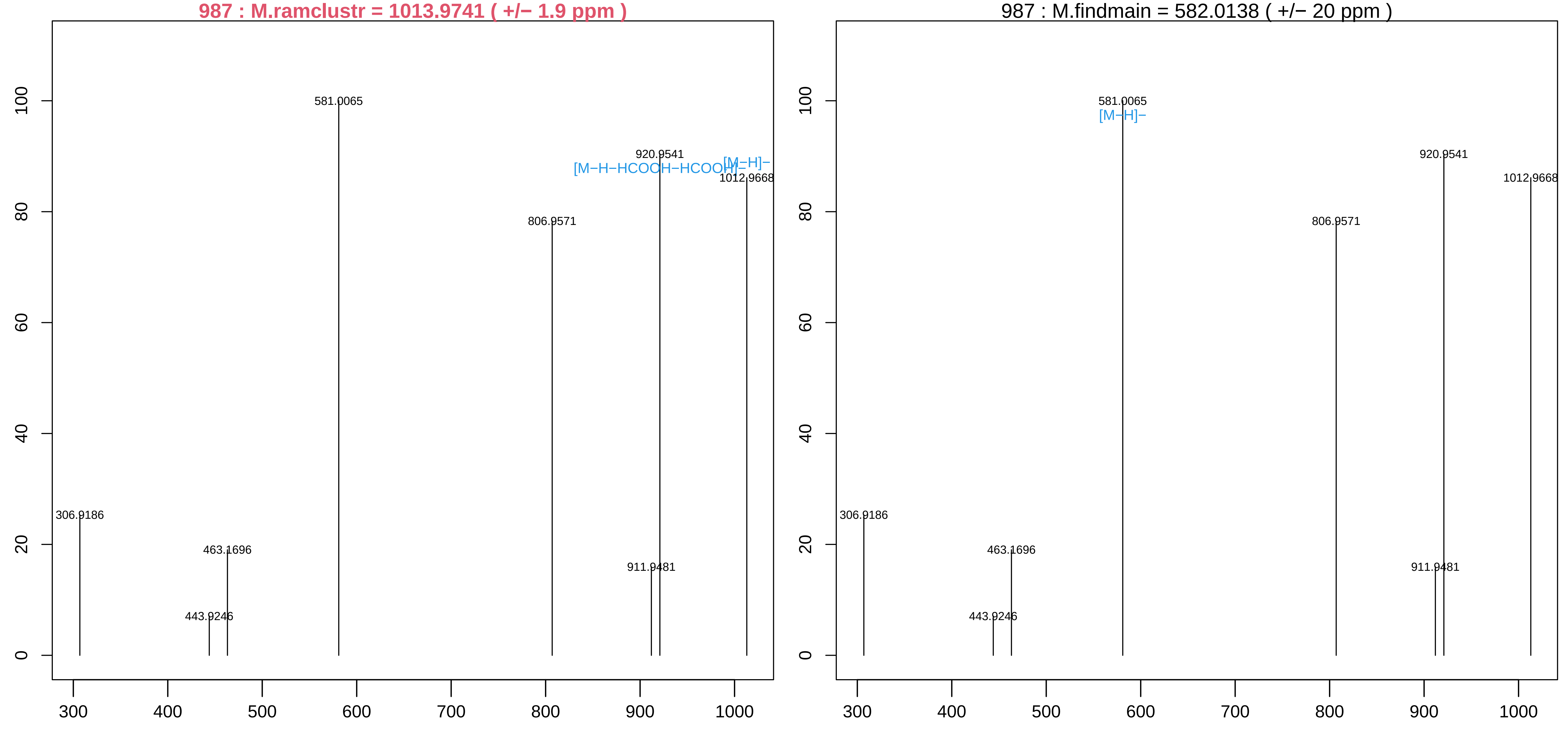
987 : M.ramclustr = 1013.9741 (+/− 1.9 ppm)
581.0065
920.9541
[M−H−HCOOH−HCOOH]−
[M−H]−
1012.9668
806.9571
306.9186
463.1696
443.9246
911.9481
987 : M.findmain = 582.0138 (+/− 20 ppm)
581.0065
[M−H]−
920.9541
1012.9668
806.9571
306.9186
463.1696
443.9246
911.9481

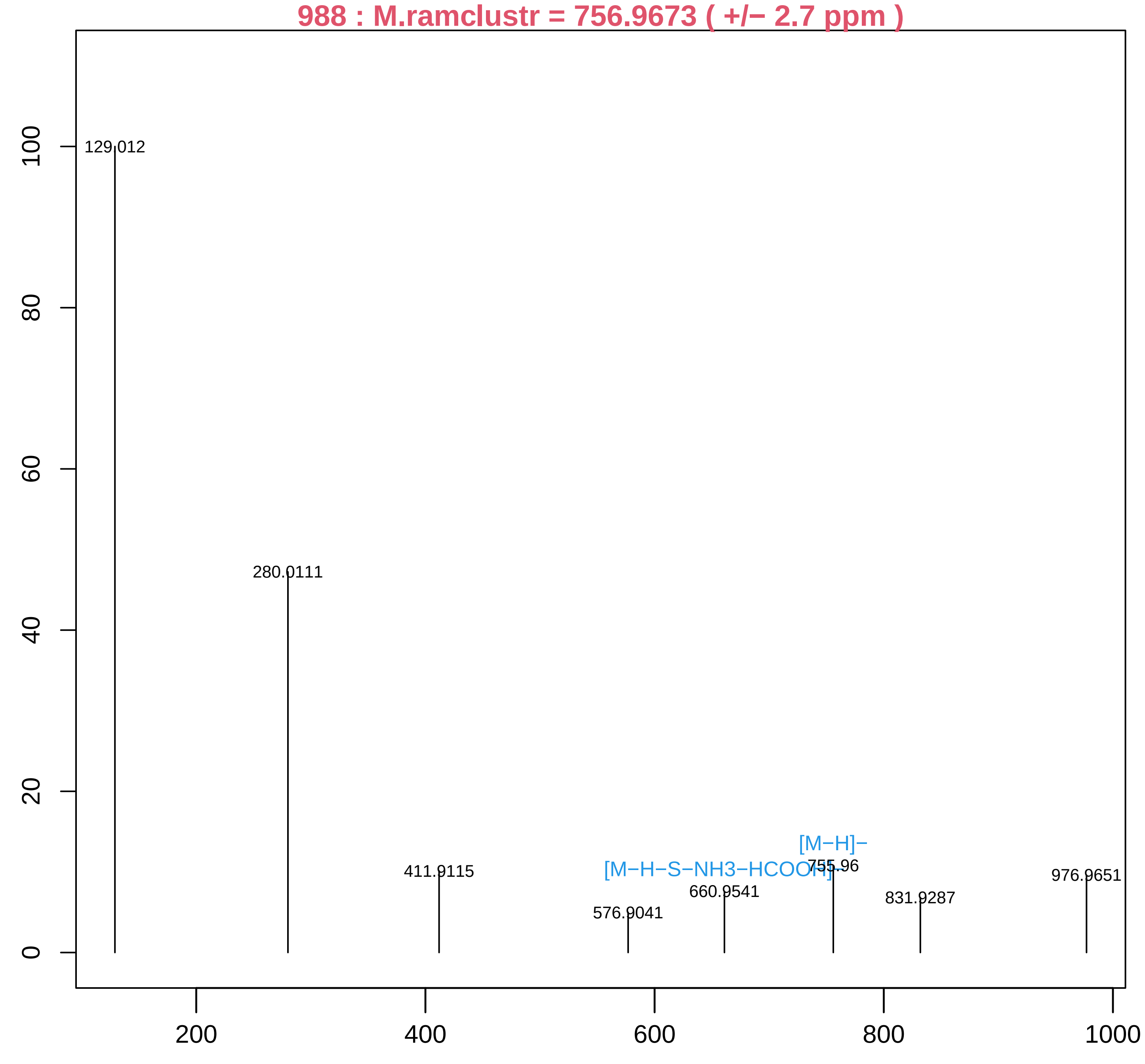
988 : M.ramclustr = 756.9673 (+/− 2.7 ppm)
129.012
280.0111
411.9115
576.9041
660.9541
[M−H−S−NH3−HCOOH]−
[M−H]−
755.96
831.9287
976.9651
0
20
40
60
80
100
200
400
600
800
1000

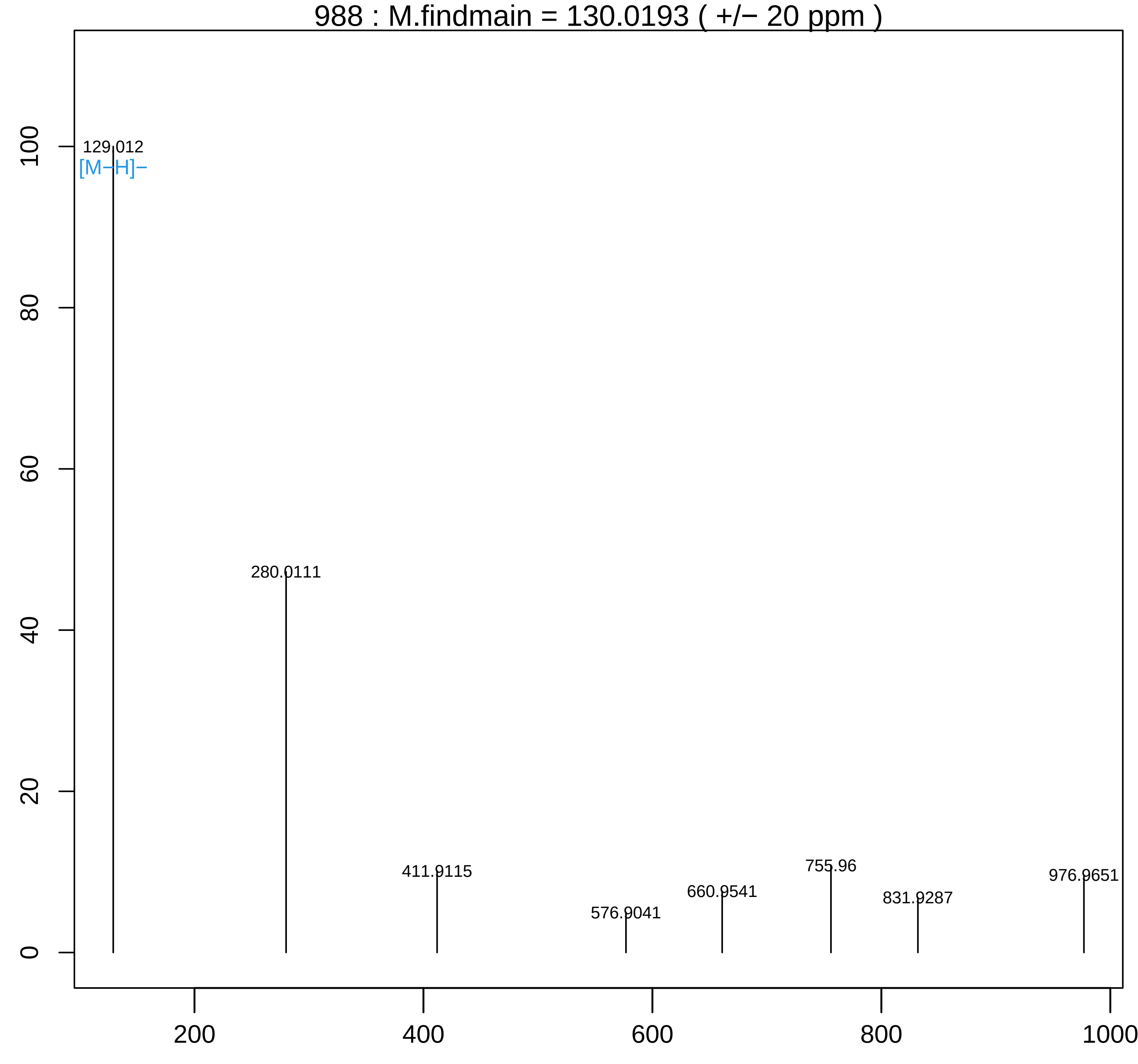
988 : M.findmain = 130.0193 (+/− 20 ppm)
129.012
[M−H]−
280.0111
411.9115
576.9041
660.9541
755.96
831.9287
976.9651
0
20
40
60
80
100
200
400
600
800
1000

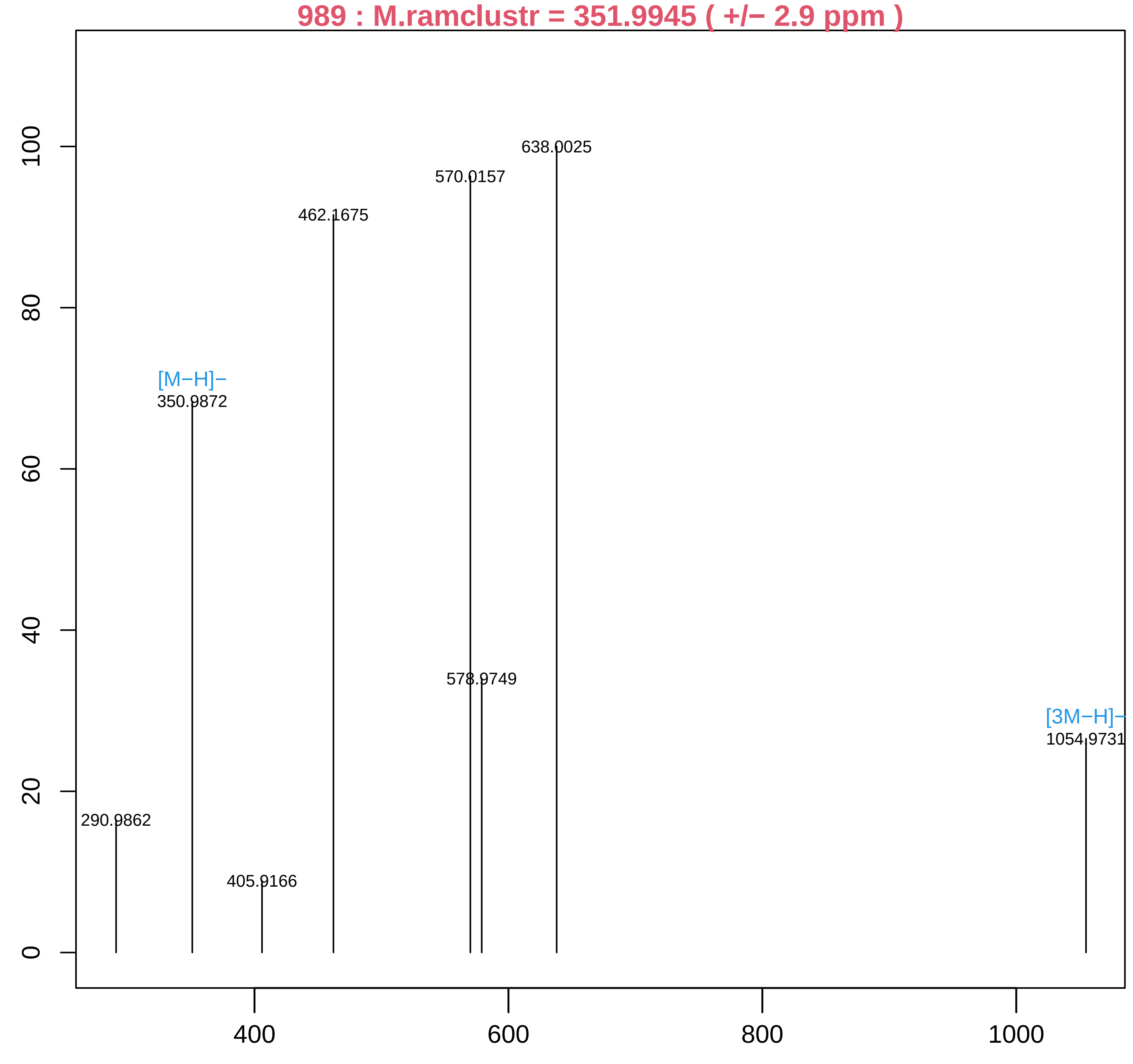

989 : M.ramclustr = 351.9945 (+/− 2.9 ppm)
0
20
40
60
80
100
400
600
800
1000
290.9862
[M−H]−
350.9872
405.9166
462.1675
570.0157
578.9749
638.0025
[3M−H]−
1054.9731

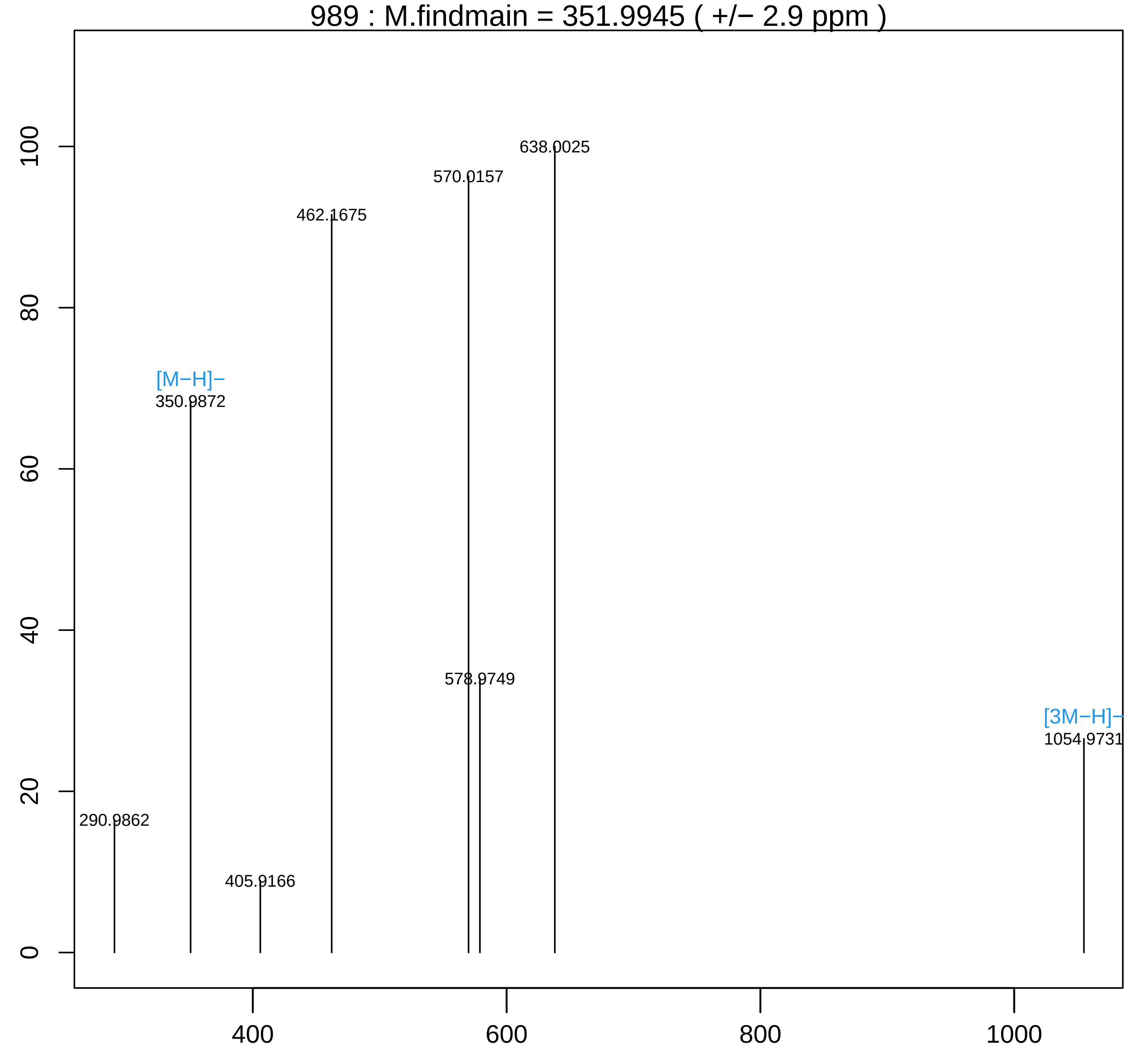

989 : M.findmain = 351.9945 (+/− 2.9 ppm)
0
20
40
60
80
100
400
600
800
1000
290.9862
[M−H]−
350.9872
405.9166
462.1675
570.0157
578.9749
638.0025
[3M−H]−
1054.9731

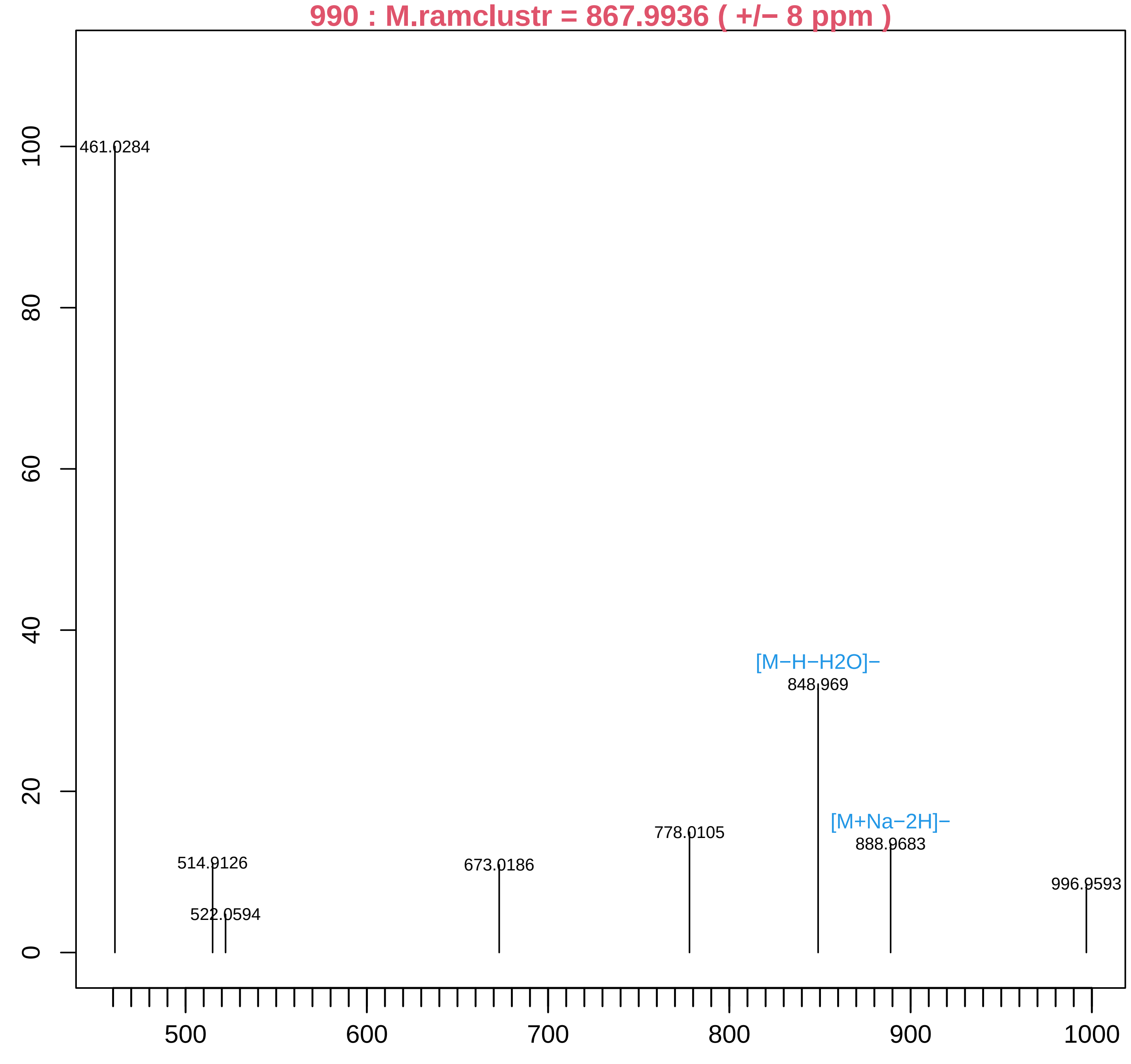

990 : M.ramclustr = 867.9936 (+/− 8 ppm)
461.0284
514.9126
522.0594
673.0186
778.0105
[M−H−H2O]−
848.969
[M+Na−2H]−
888.9683
996.9593
0
20
40
60
80
100
500
600
700
800
900
1000

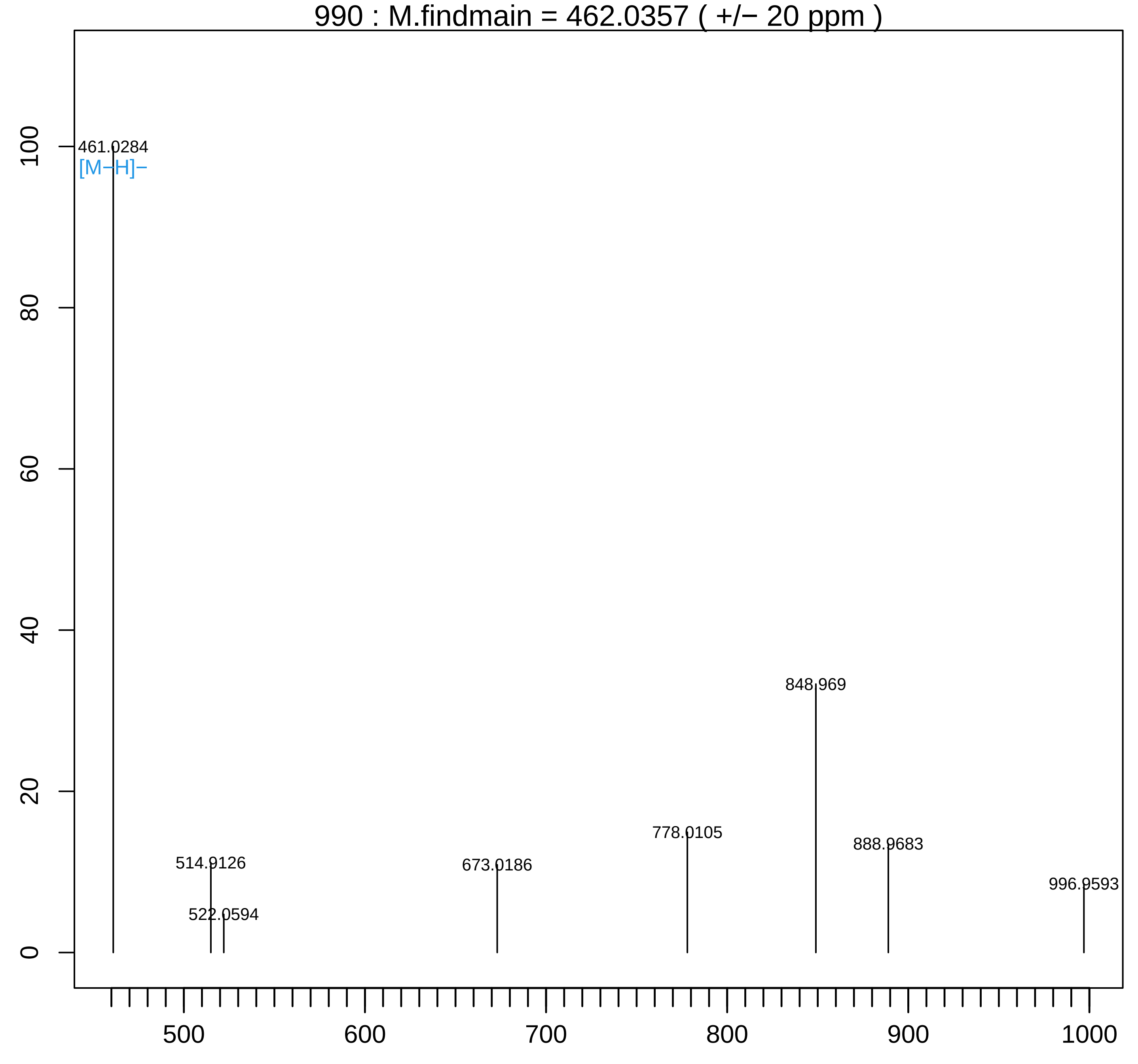

990 : M.findmain = 462.0357 (+/− 20 ppm)
461.0284
[M−H]−
514.9126
522.0594
673.0186
778.0105
848.969
888.9683
996.9593
0
20
40
60
80
100
500
600
700
800
900
1000

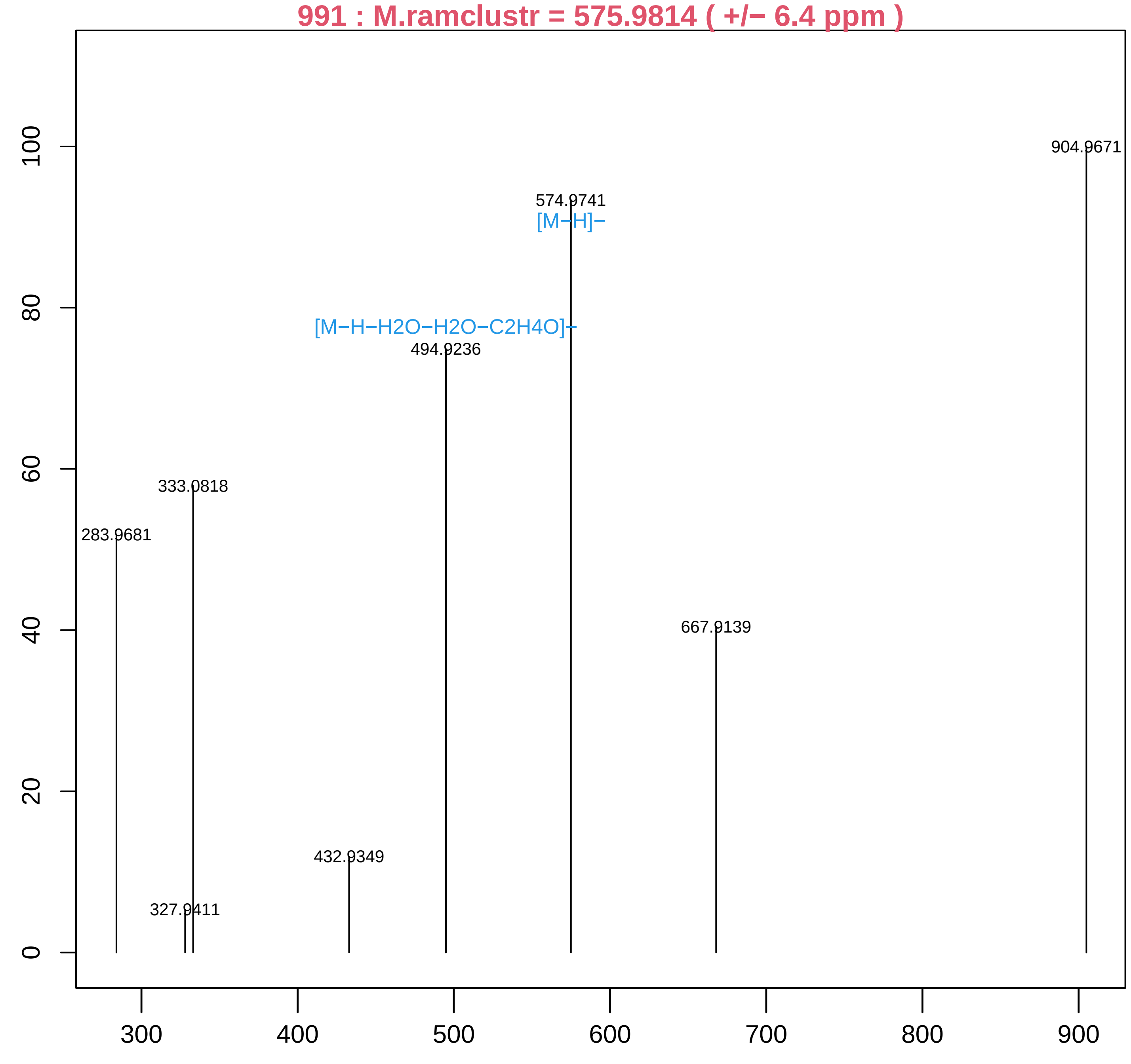

991 : M.ramclustr = 575.9814 (+/− 6.4 ppm)
904.9671
574.9741
[M−H]−
[M−H−H2O−H2O−C2H4O]−
494.9236
333.0818
283.9681
667.9139
432.9349
327.9411
0
20
40
60
80
100
300
400
500
600
700
800
900

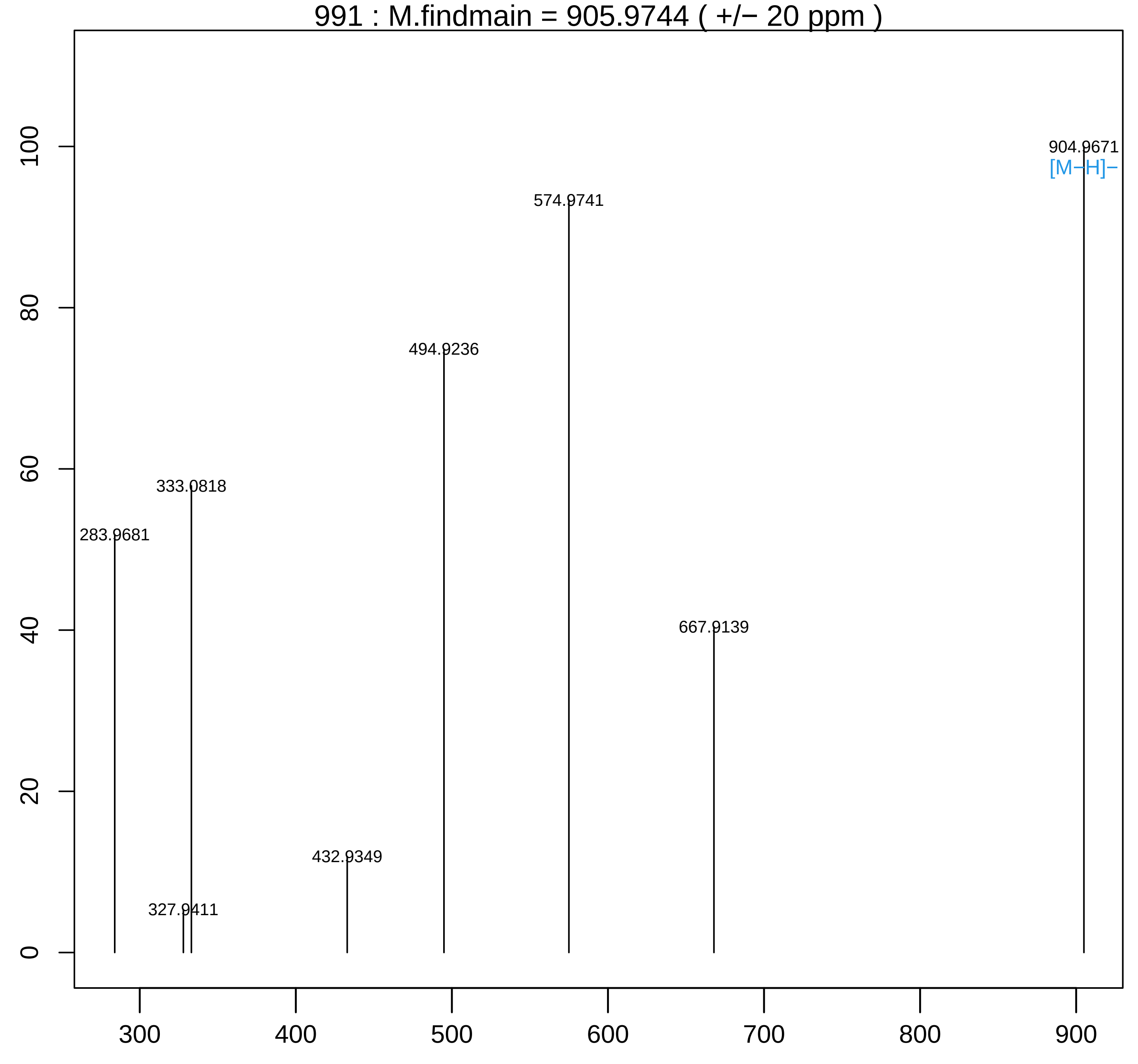

991 : M.findmain = 905.9744 (+/− 20 ppm)
904.9671
[M−H]−
574.9741
494.9236
333.0818
283.9681
667.9139
432.9349
327.9411
0
20
40
60
80
100
300
400
500
600
700
800
900

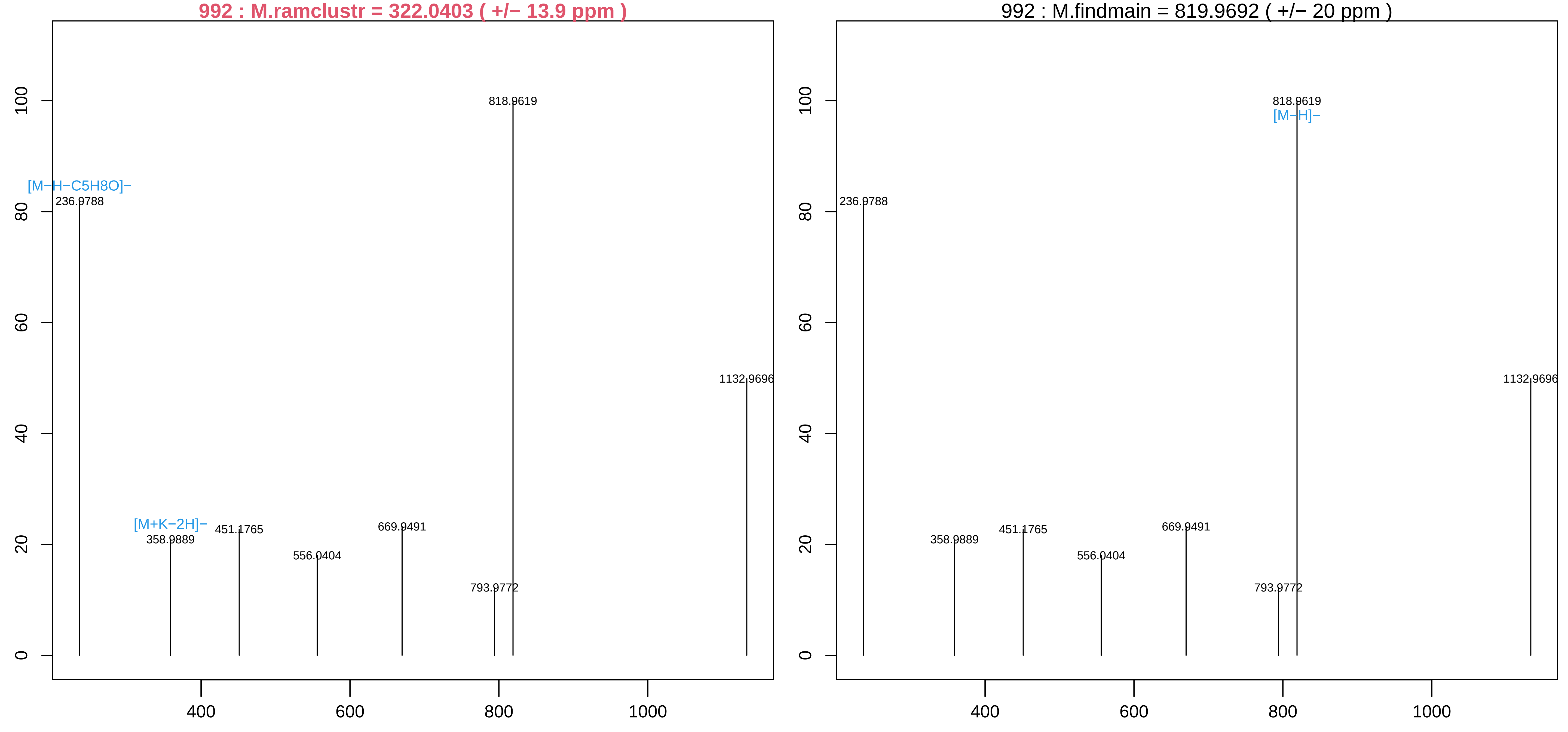
992 : M.ramclustr = 322.0403 (+/− 13.9 ppm)
[M−H−C5H8O]−
236.9788
[M+K−2H]−
358.9889
451.1765
556.0404
669.9491
793.9772
818.9619
1132.9696
992 : M.findmain = 819.9692 (+/− 20 ppm)
236.9788
358.9889
451.1765
556.0404
669.9491
793.9772
818.9619
[M−H]−
1132.9696
0
20
40
60
80
100
400
600
800
1000

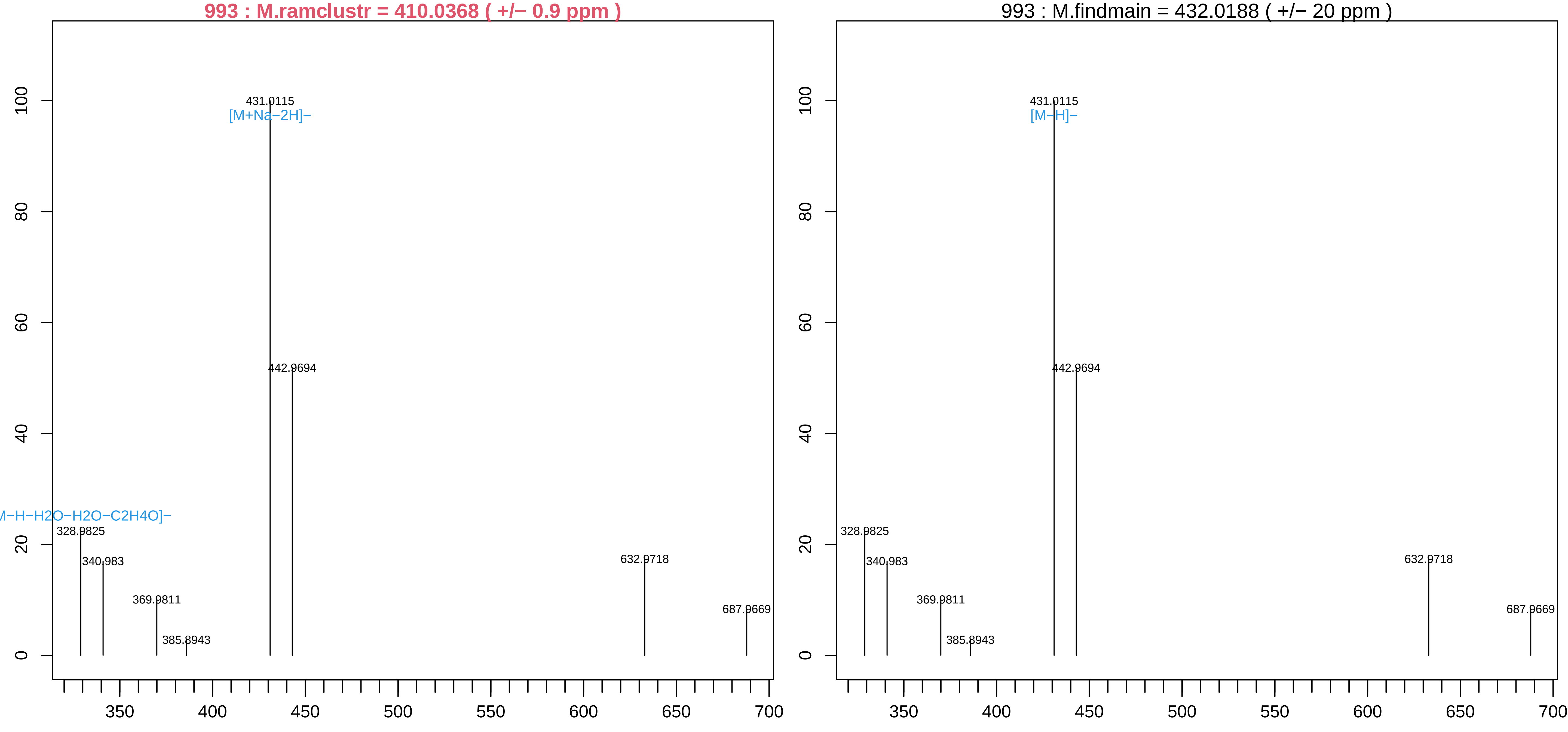

993 : M.ramclustr = 410.0368 (+/− 0.9 ppm)
431.0115
[M+Na−2H]−
442.9694
M−H−H2O−H2O−C2H4O]−
328.9825
340.983
369.9811
385.8943
632.9718
687.9669
993 : M.findmain = 432.0188 (+/− 20 ppm)
431.0115
[M−H]−
442.9694
328.9825
340.983
369.9811
385.8943
632.9718
687.9669

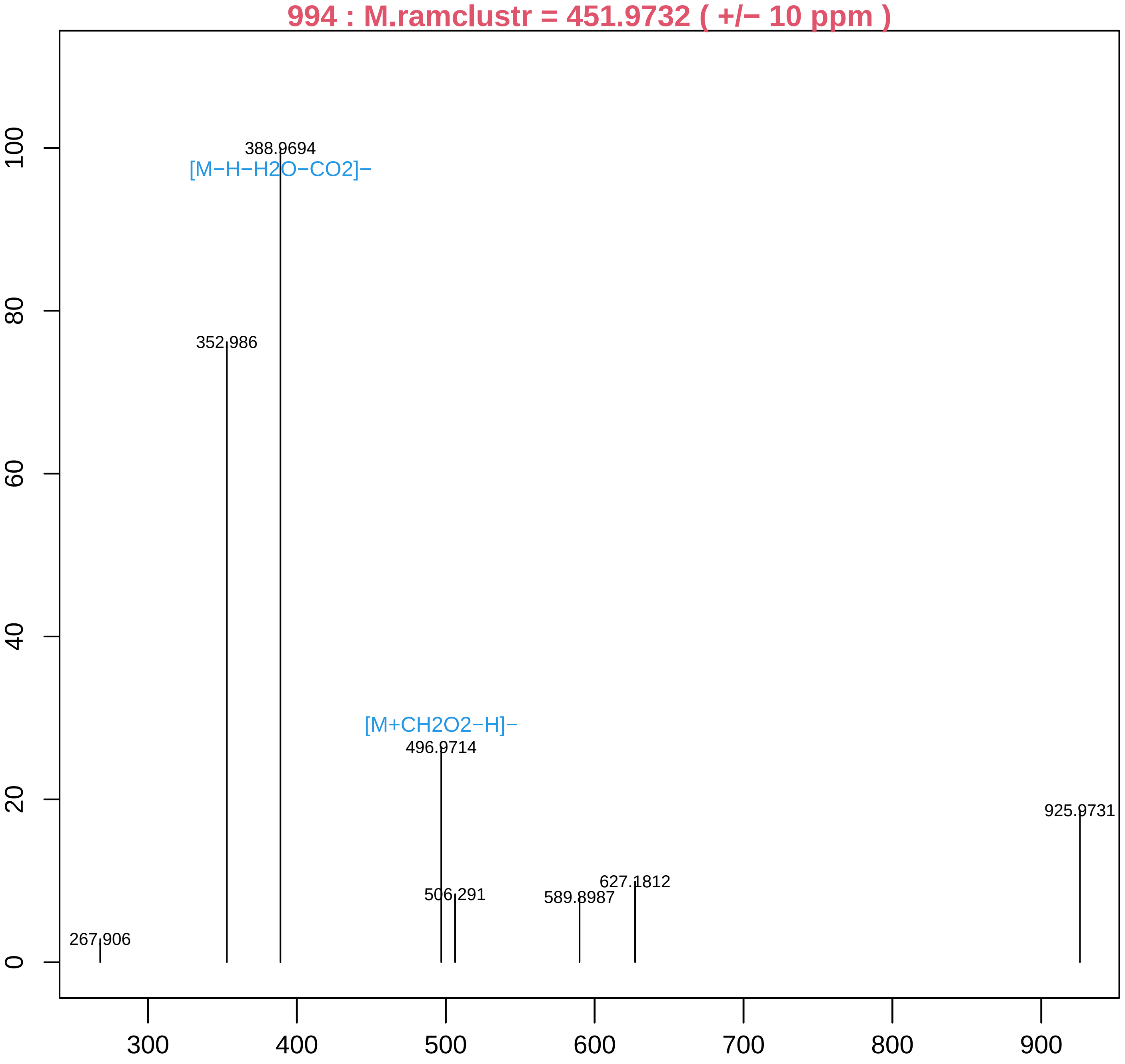

994 : M.ramclustr = 451.9732 (+/− 10 ppm)
388.9694
[M−H−H2O−CO2]−
352.986
[M+CH2O2−H]−
496.9714
506.291
589.8987
627.1812
925.9731
267.906
0
20
40
60
80
100
300
400
500
600
700
800
900

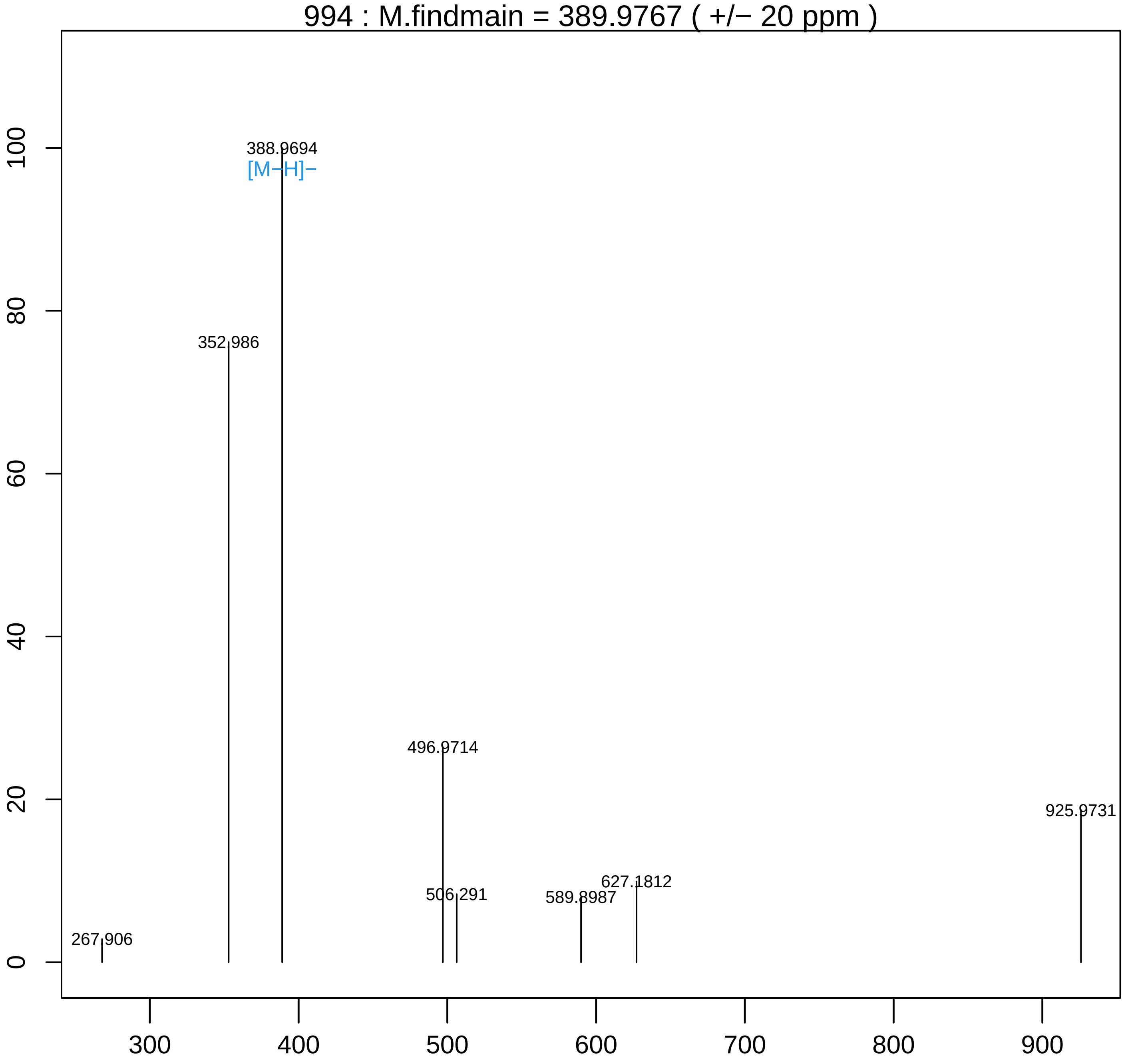

994 : M.findmain = 389.9767 (+/− 20 ppm)
388.9694
[M−H]−
352.986
496.9714
506.291
589.8987
627.1812
925.9731
267.906
0
20
40
60
80
100
300
400
500
600
700
800
900

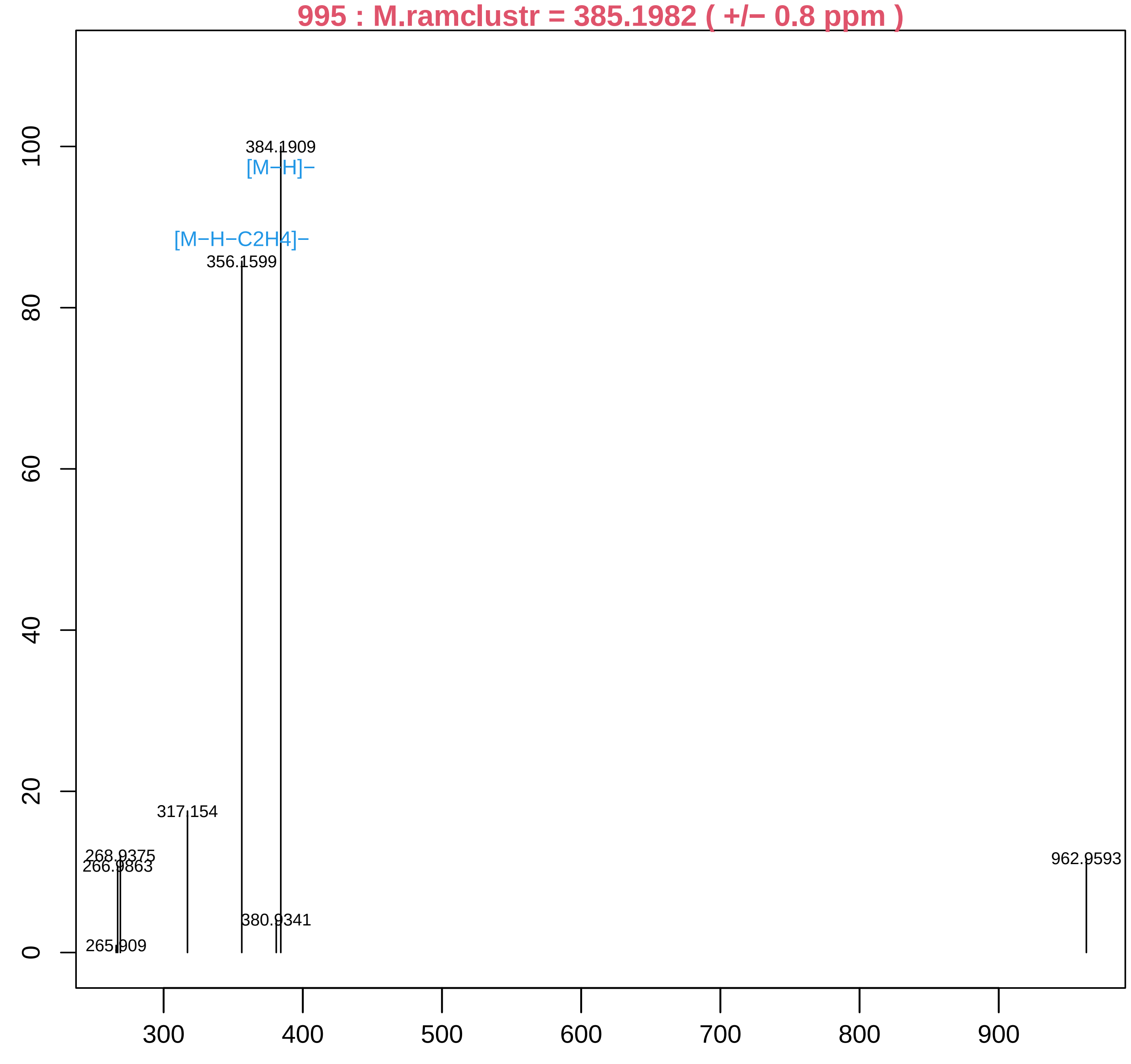
995 : M.ramclustr = 385.1982 (+/− 0.8 ppm)
384.1909
[M−H]−
[M−H−C2H4]−
356.1599
317.154
268.9375
266.9863
265.909
380.9341
962.9593
0
20
40
60
80
100
300
400
500
600
700
800
900

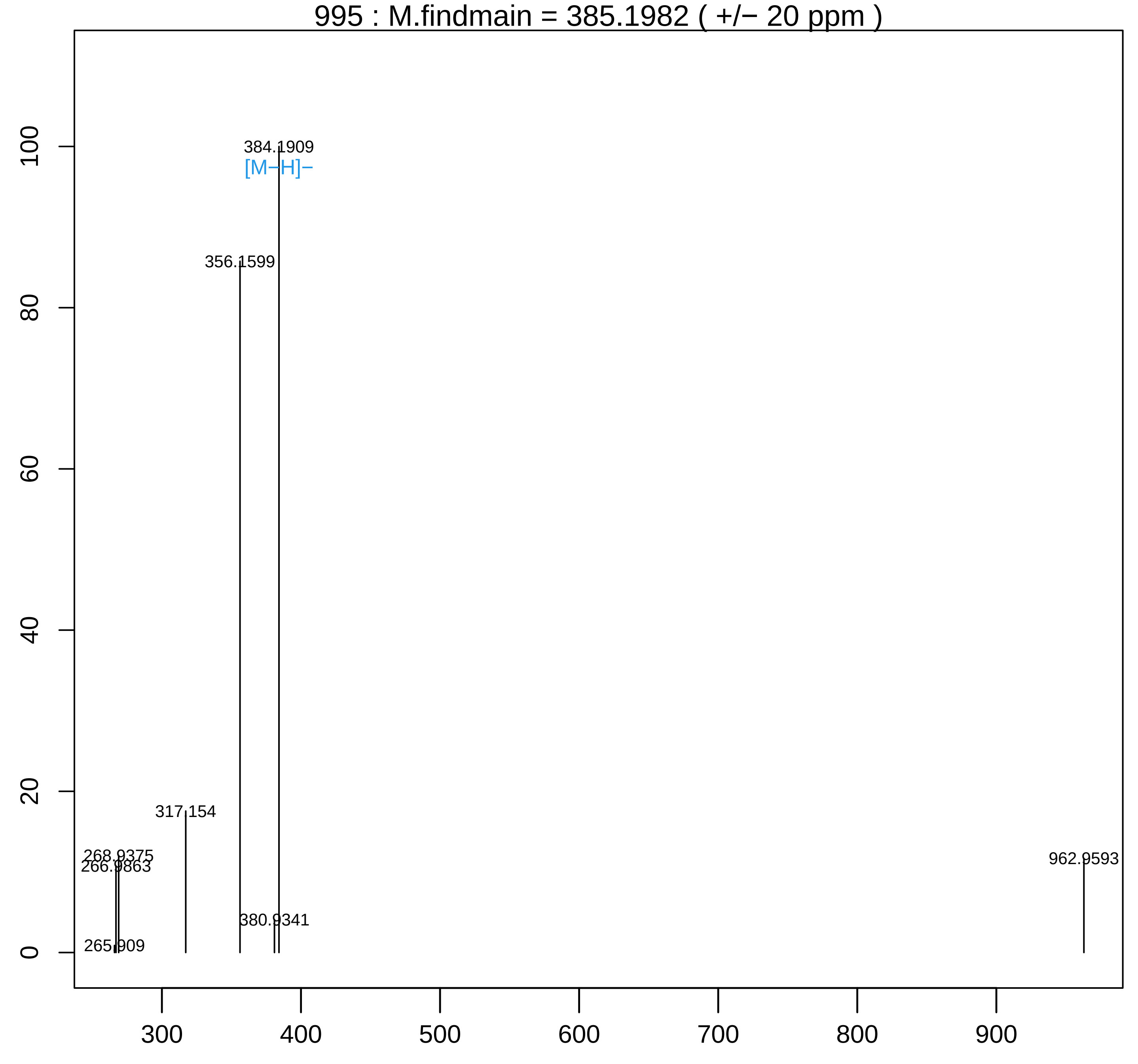
995 : M.findmain = 385.1982 (+/− 20 ppm)
384.1909
[M−H]−
356.1599
317.154
268.9375
266.9863
265.909
380.9341
962.9593
0
20
40
60
80
100
300
400
500
600
700
800
900

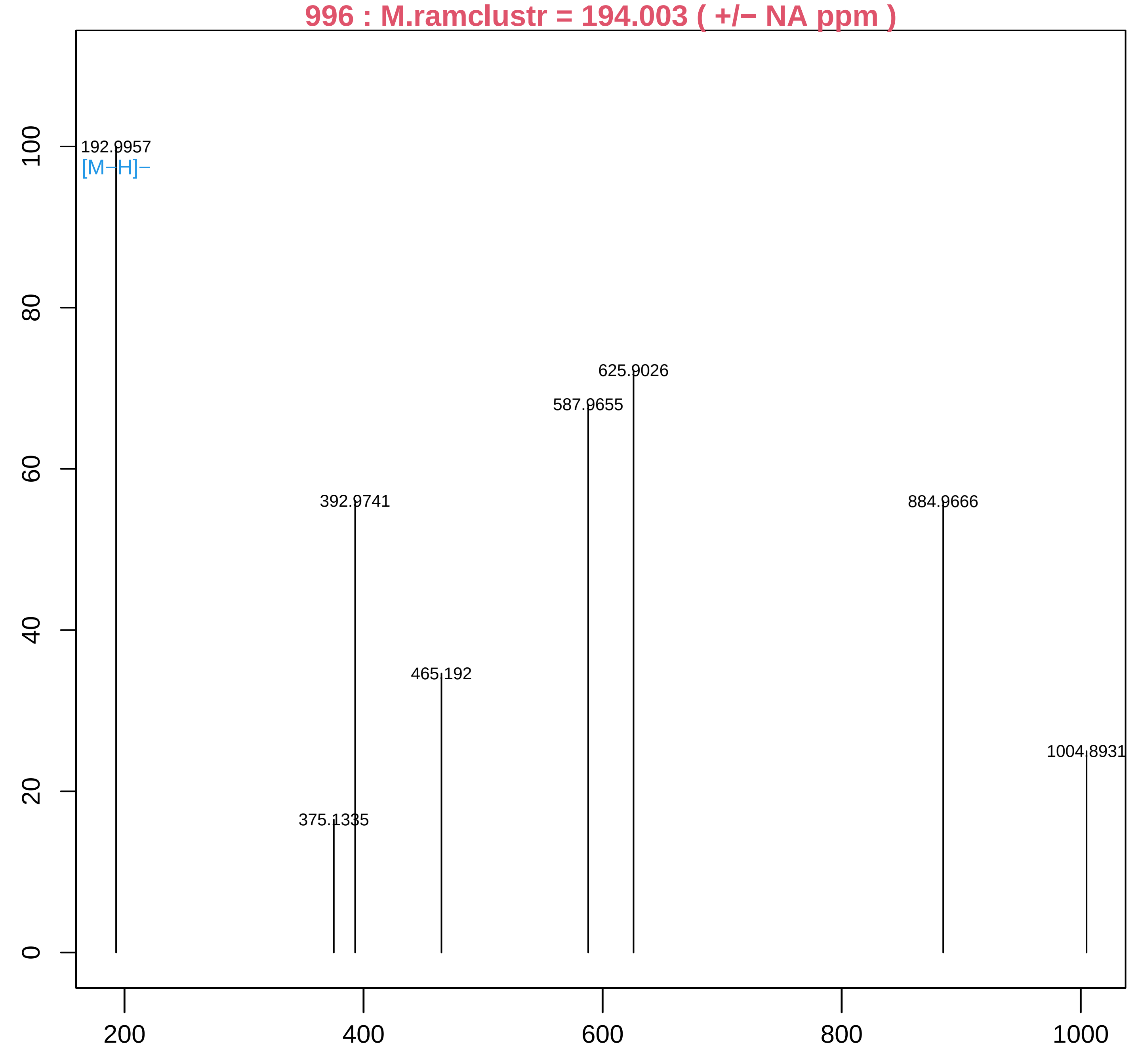

996 : M.ramclustr = 194.003 (+/− NA ppm)
192.9957
[M−H]−
625.9026
587.9655
392.9741
884.9666
465.192
1004.8931
375.1335
0
20
40
60
80
100
200
400
600
800
1000

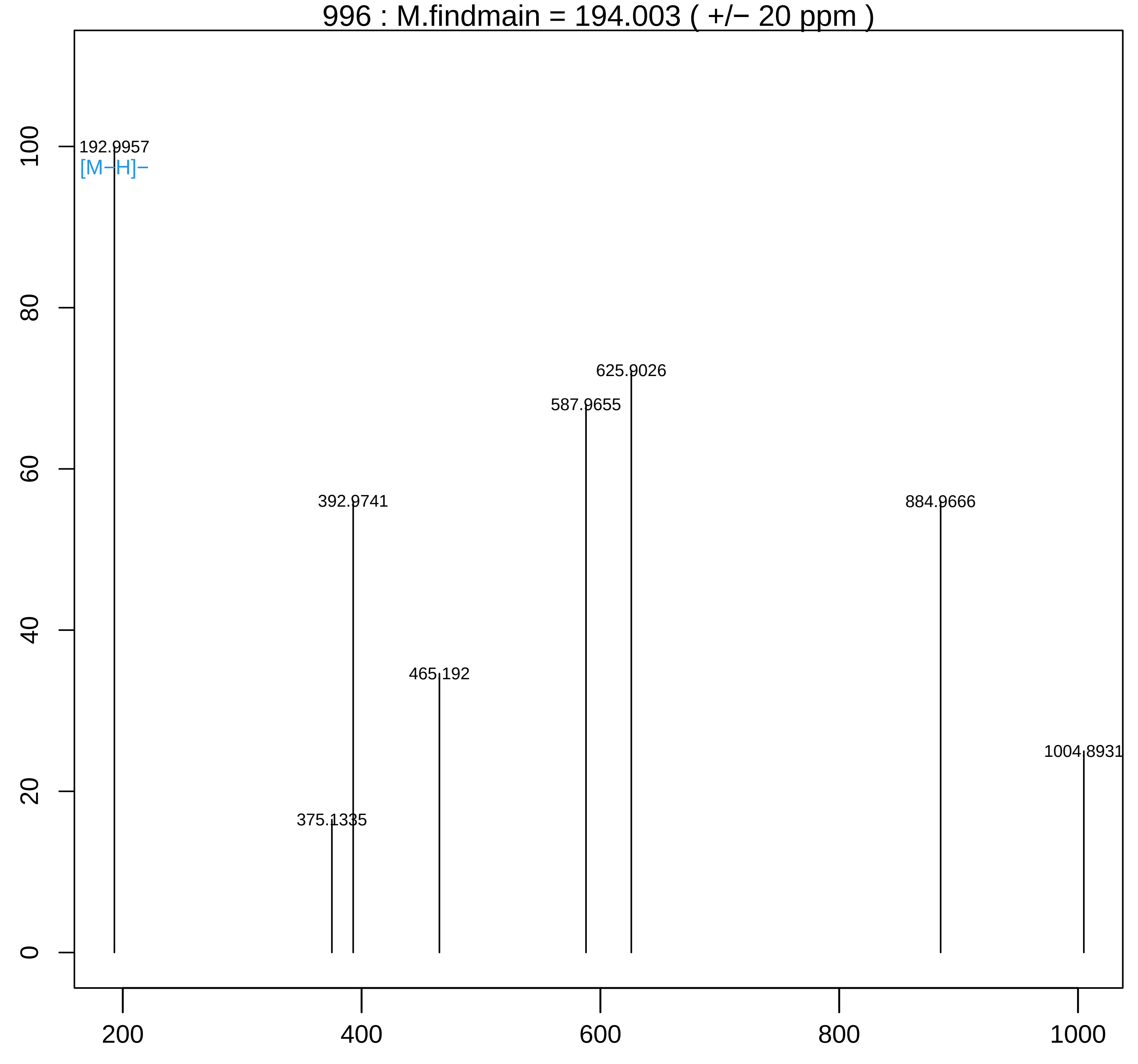

996 : M.findmain = 194.003 (+/− 20 ppm)
192.9957
[M−H]−
625.9026
587.9655
392.9741
884.9666
465.192
1004.8931
375.1335
0
20
40
60
80
100
200
400
600
800
1000

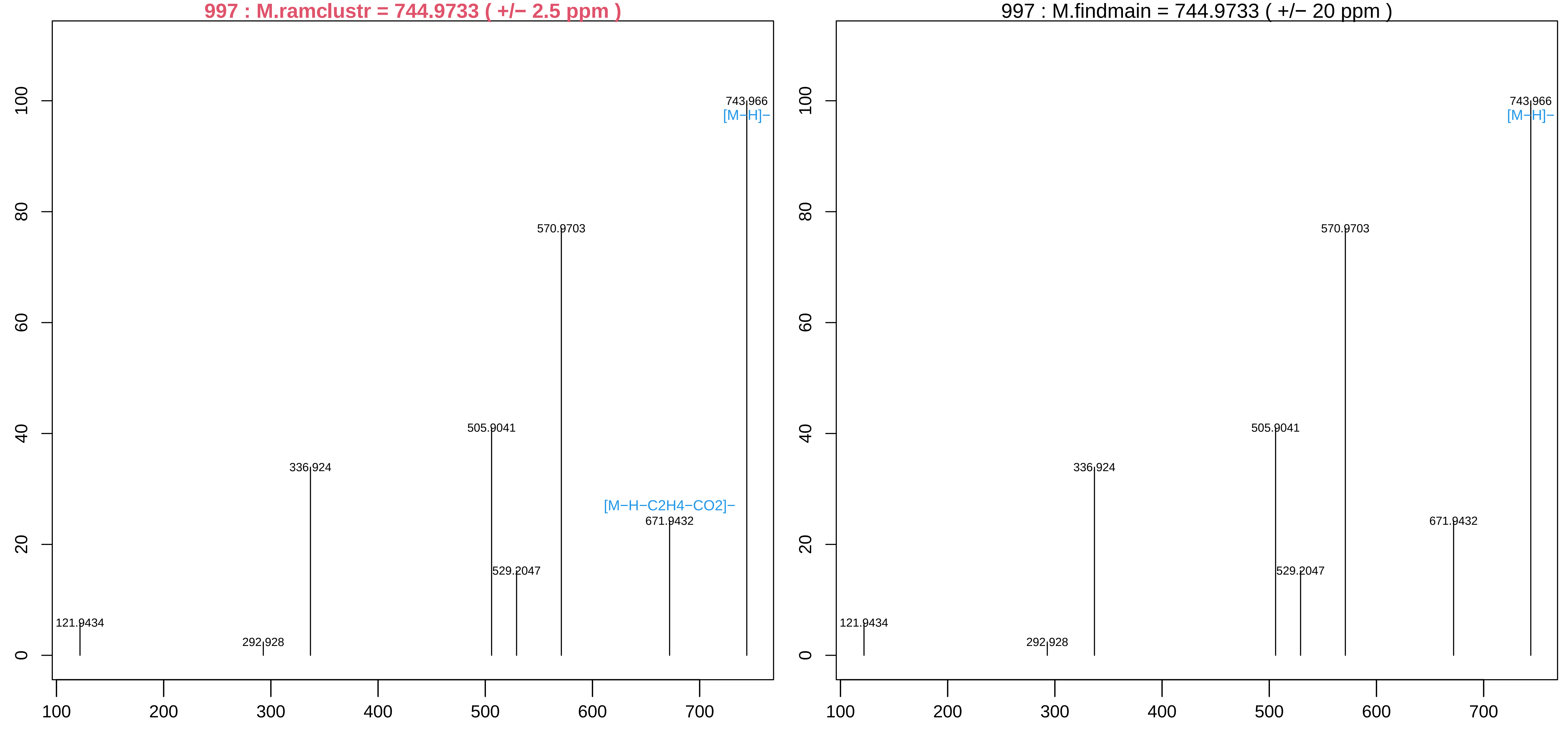
997 : M.ramclustr = 744.9733 (+/− 2.5 ppm)
997 : M.findmain = 744.9733 (+/− 20 ppm)
743.966
[M−H]−
570.9703
505.9041
336.924
[M−H−C2H4−CO2]−
671.9432
529.2047
121.9434
292.928
0
20
40
60
80
100
100
200
300
400
500
600
700

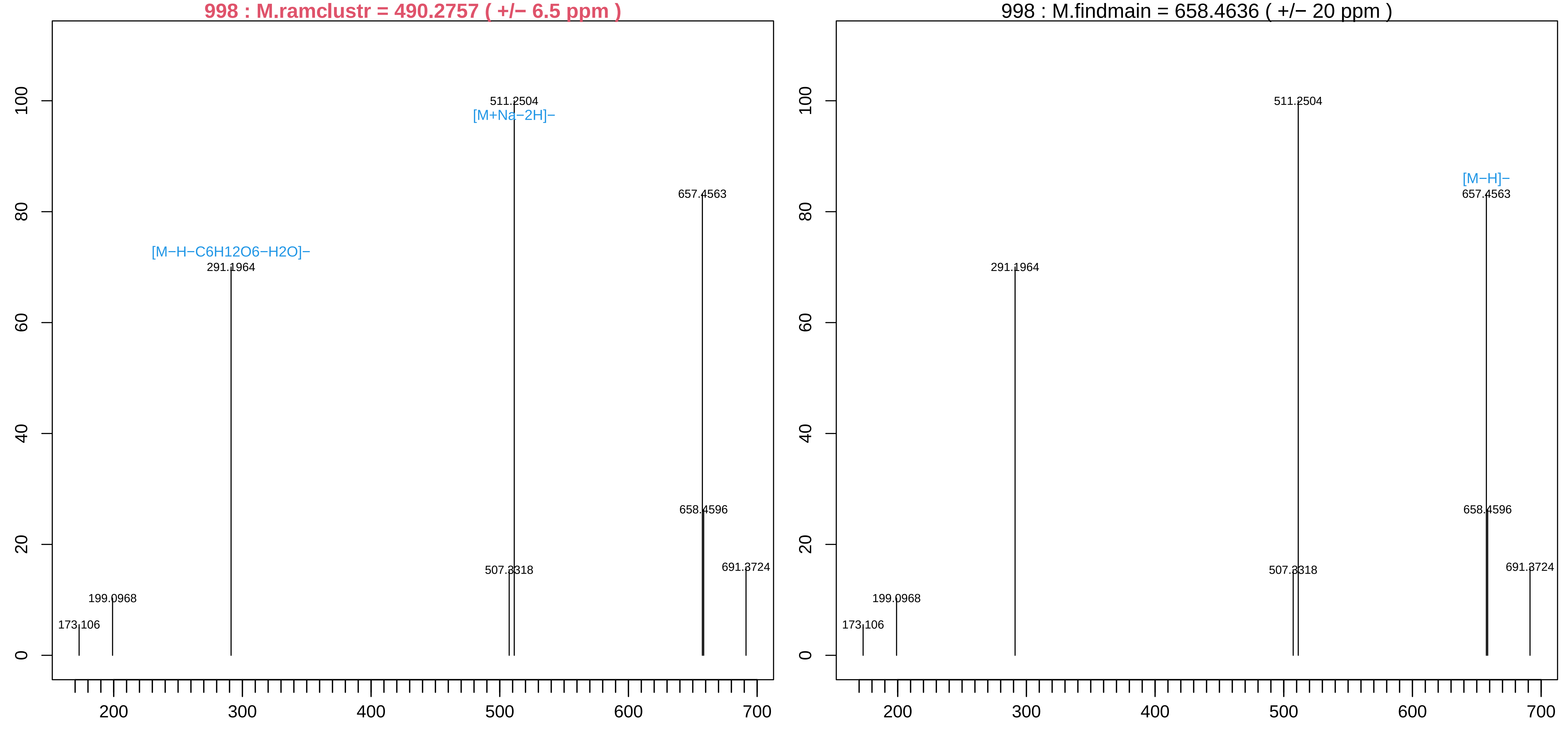

998 : M.ramclustr = 490.2757 (+/− 6.5 ppm)
511.2504
[M+Na−2H]−
657.4563
[M−H−C6H12O6−H2O]−
291.1964
658.4596
507.3318
691.3724
199.0968
173.106
0
20
40
60
80
100
200
300
400
500
600
700
998 : M.findmain = 658.4636 (+/− 20 ppm)
511.2504
[M−H]−
657.4563
291.1964
658.4596
507.3318
691.3724
199.0968
173.106
0
20
40
60
80
100
200
300
400
500
600
700

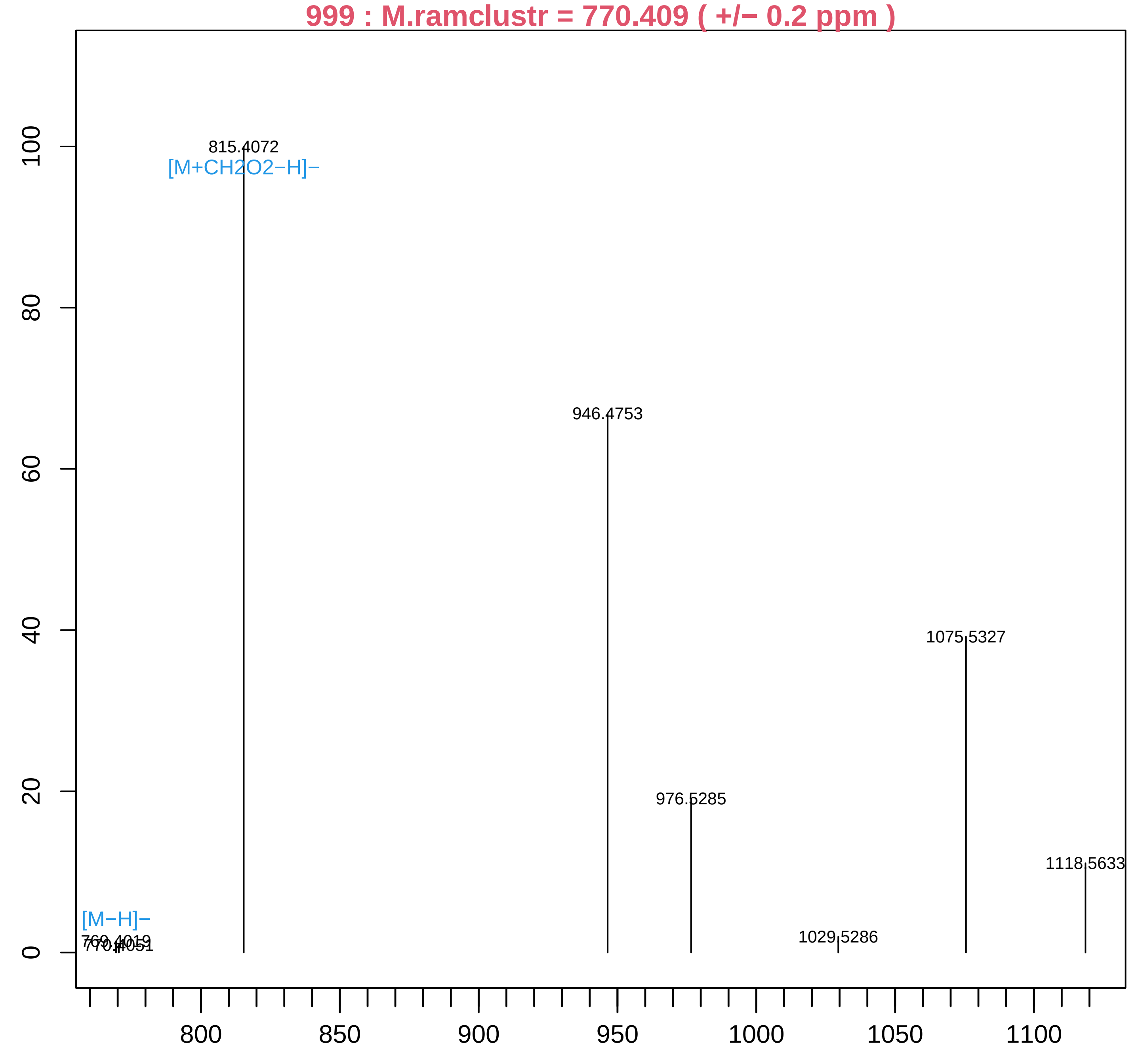
999 : M.ramclustr = 770.409 (+/− 0.2 ppm)
815.4072
[M+CH2O2−H]−
946.4753
976.5285
1029.5286
1075.5327
1118.5633
[M−H]−
769.4019
770.4051
0
20
40
60
80
100
800
850
900
950
1000
1050
1100

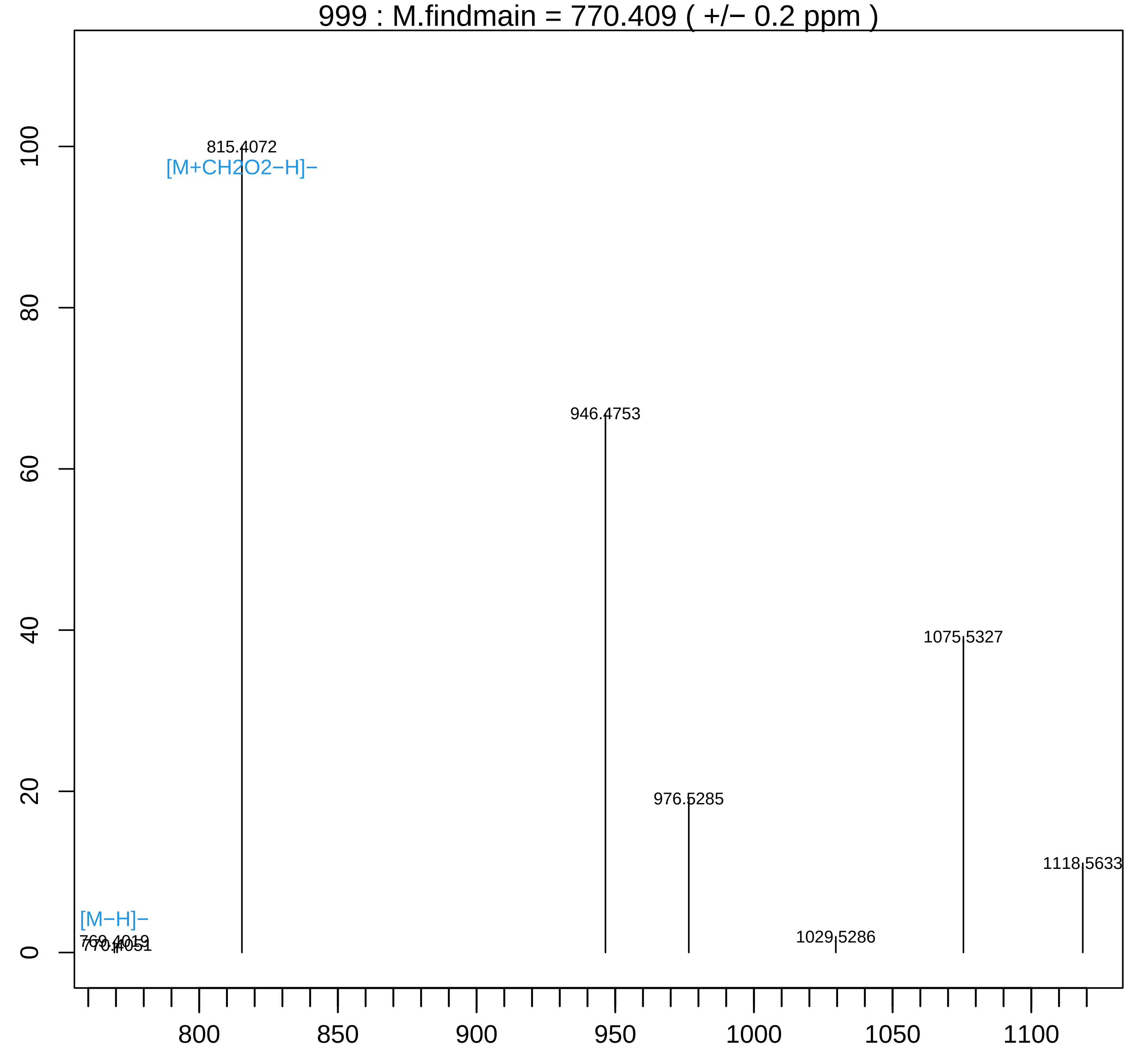
999 : M.findmain = 770.409 (+/− 0.2 ppm)
815.4072
[M+CH2O2−H]−
946.4753
976.5285
1029.5286
1075.5327
1118.5633
[M−H]−
769.4019
770.4051
0
20
40
60
80
100
800
850
900
950
1000
1050
1100

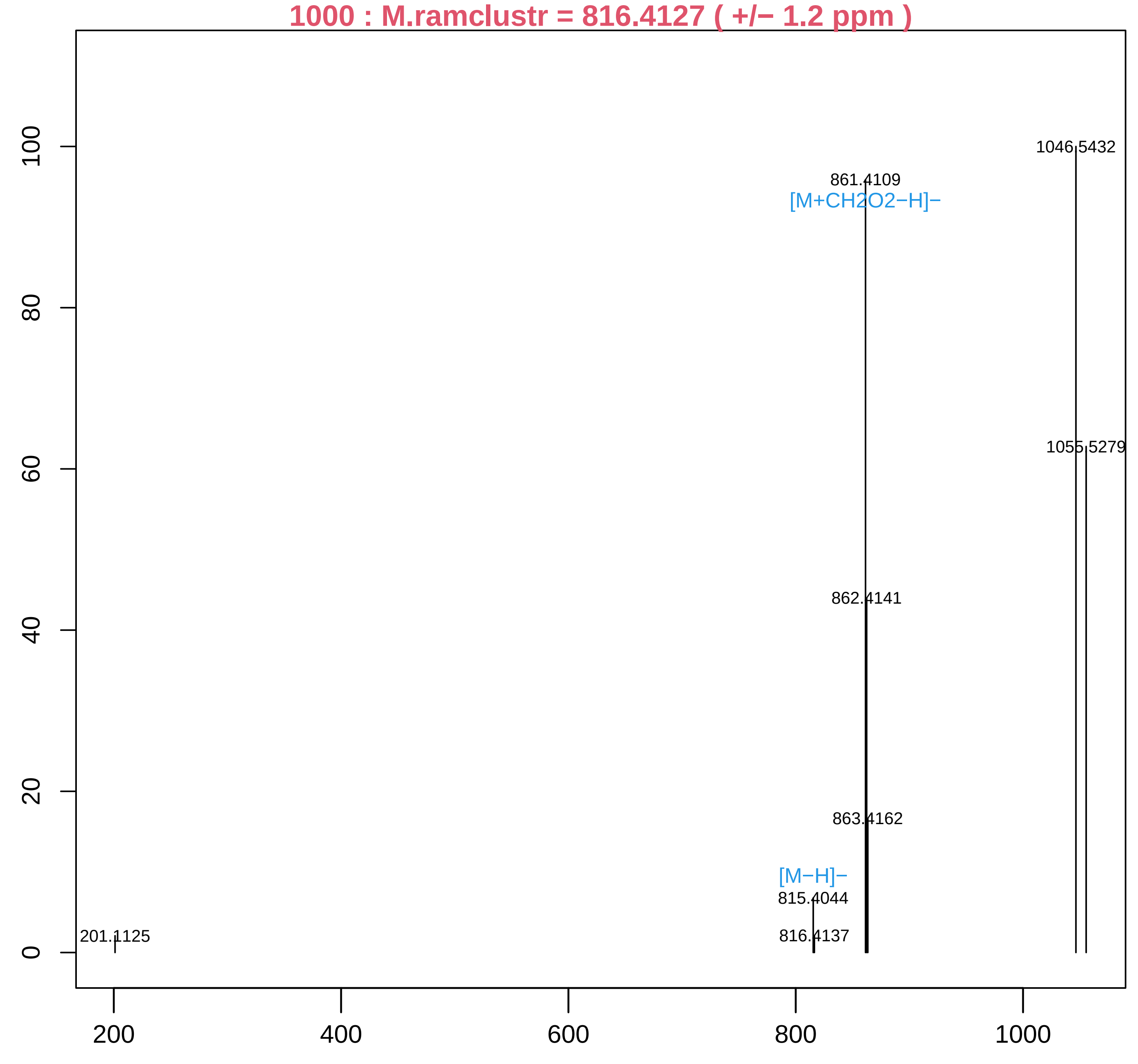
1000 : M.ramclustr = 816.4127 (+/− 1.2 ppm)
1046.5432
861.4109
[M+CH2O2−H]−
1055.5279
862.4141
863.4162
[M−H]−
815.4044
201.1125
816.4137
0
20
40
60
80
100
200
400
600
800
1000
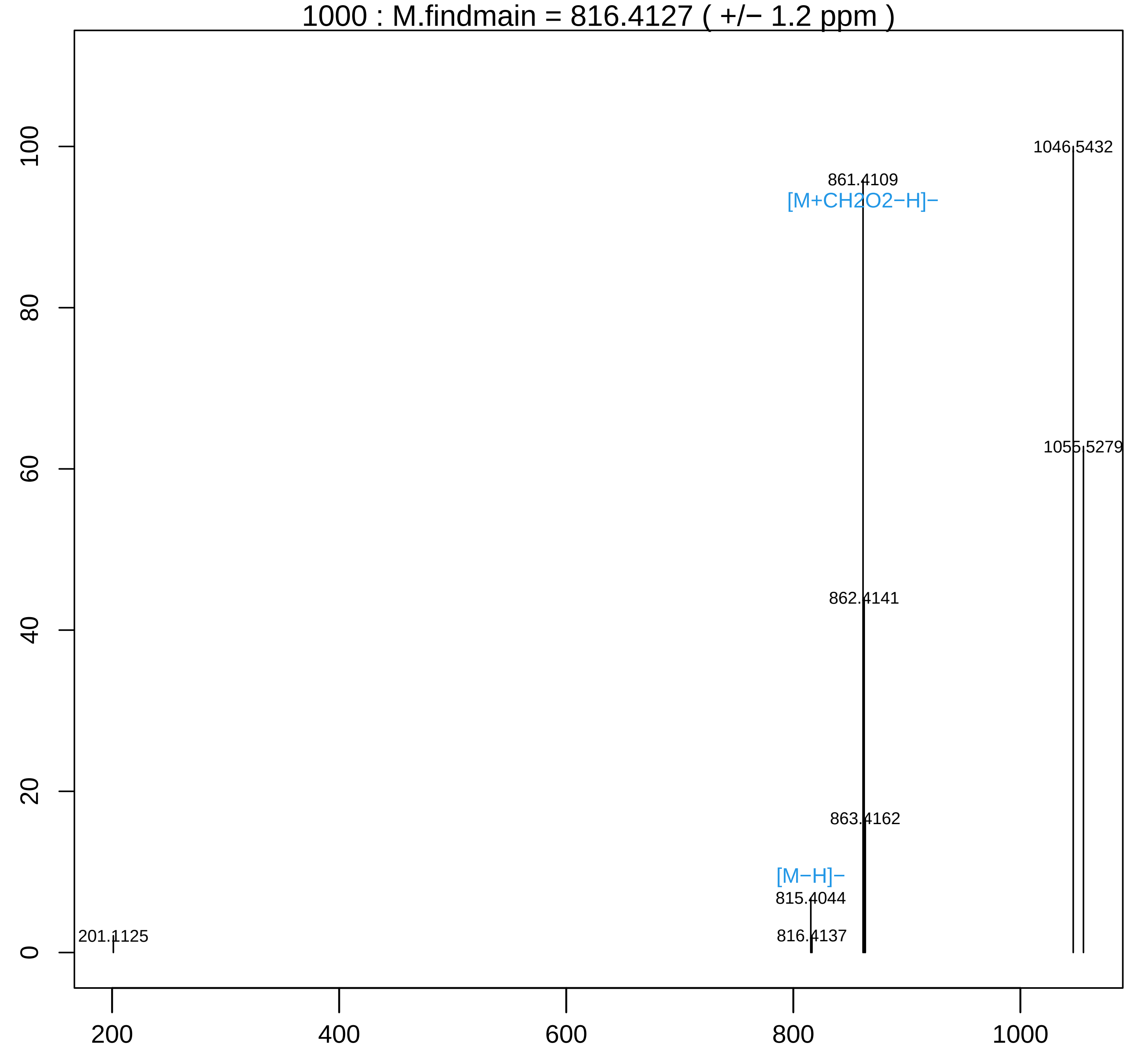
1000 : M.findmain = 816.4127 (+/− 1.2 ppm)
1046.5432
861.4109
[M+CH2O2−H]−
1055.5279
862.4141
863.4162
[M−H]−
815.4044
201.1125
816.4137
0
20
40
60
80
100
200
400
600
800
1000

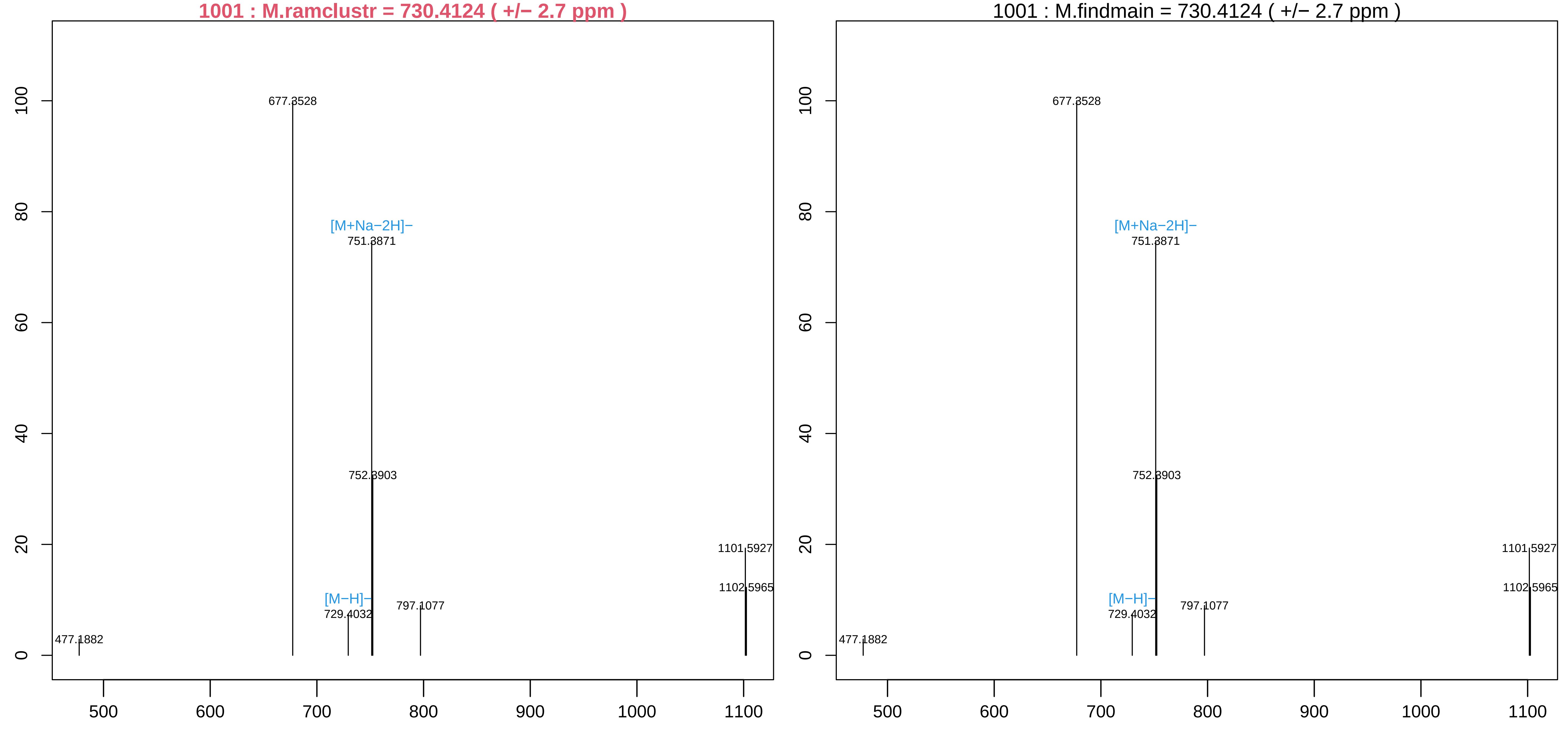
1001 : M.ramclustr = 730.4124 (+/− 2.7 ppm)
677.3528
[M+Na−2H]−
751.3871
752.3903
[M−H]−
729.4032
797.1077
477.1882
1101.5927
1102.5965
0
20
40
60
80
100
500
600
700
800
900
1000
1100
1001 : M.findmain = 730.4124 (+/− 2.7 ppm)
677.3528
[M+Na−2H]−
751.3871
752.3903
[M−H]−
729.4032
797.1077
477.1882
1101.5927
1102.5965
0
20
40
60
80
100
500
600
700
800
900
1000
1100

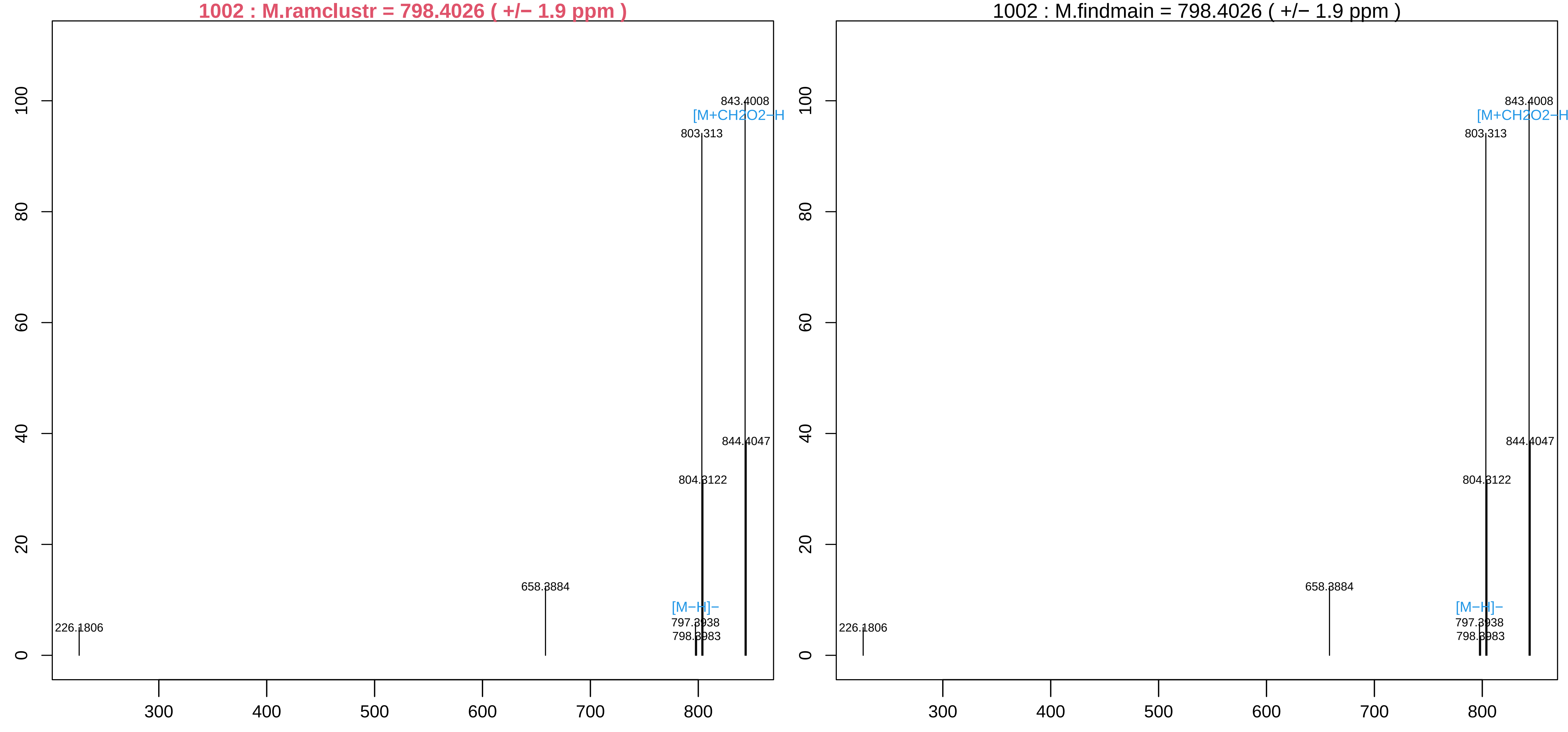
1002 : M.ramclustr = 798.4026 (+/− 1.9 ppm)
843.4008
[M+CH2O2−H
803.313
844.4047
804.3122
658.3884
[M−H]−
797.3938
798.3983
226.1806
0
20
40
60
80
100
300
400
500
600
700
800
1002 : M.findmain = 798.4026 (+/− 1.9 ppm)
843.4008
[M+CH2O2−H
803.313
844.4047
804.3122
658.3884
[M−H]−
797.3938
798.3983
226.1806
0
20
40
60
80
100
300
400
500
600
700
800

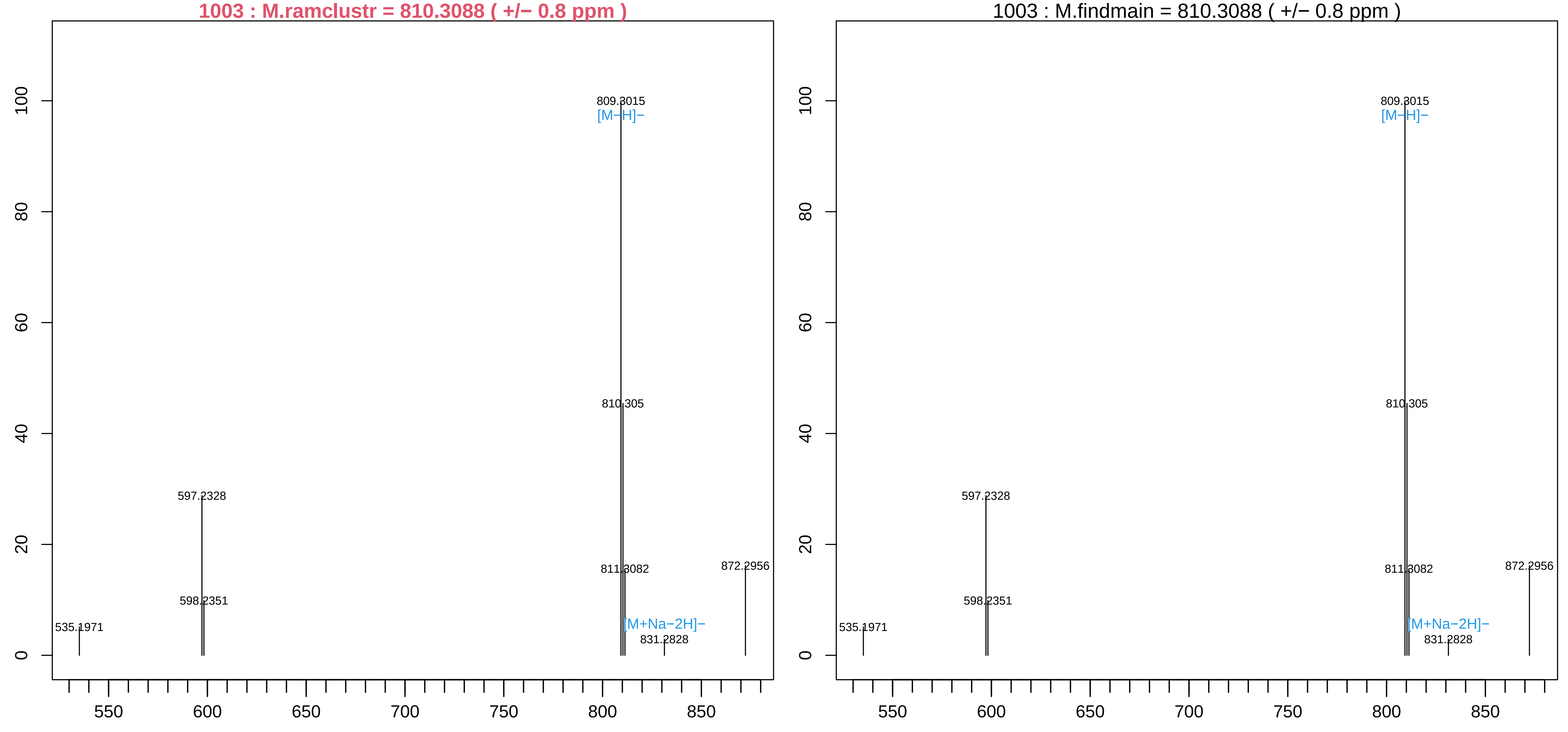
1003 : M.ramclustr = 810.3088 (+/− 0.8 ppm)
809.3015
[M−H]−
810.305
597.2328
598.2351
535.1971
811.3082
872.2956
[M+Na−2H]−
831.2828
1003 : M.findmain = 810.3088 (+/− 0.8 ppm)
809.3015
[M−H]−
810.305
597.2328
598.2351
535.1971
811.3082
872.2956
[M+Na−2H]−
831.2828
0
20
40
60
80
100
550
600
650
700
750
800
850

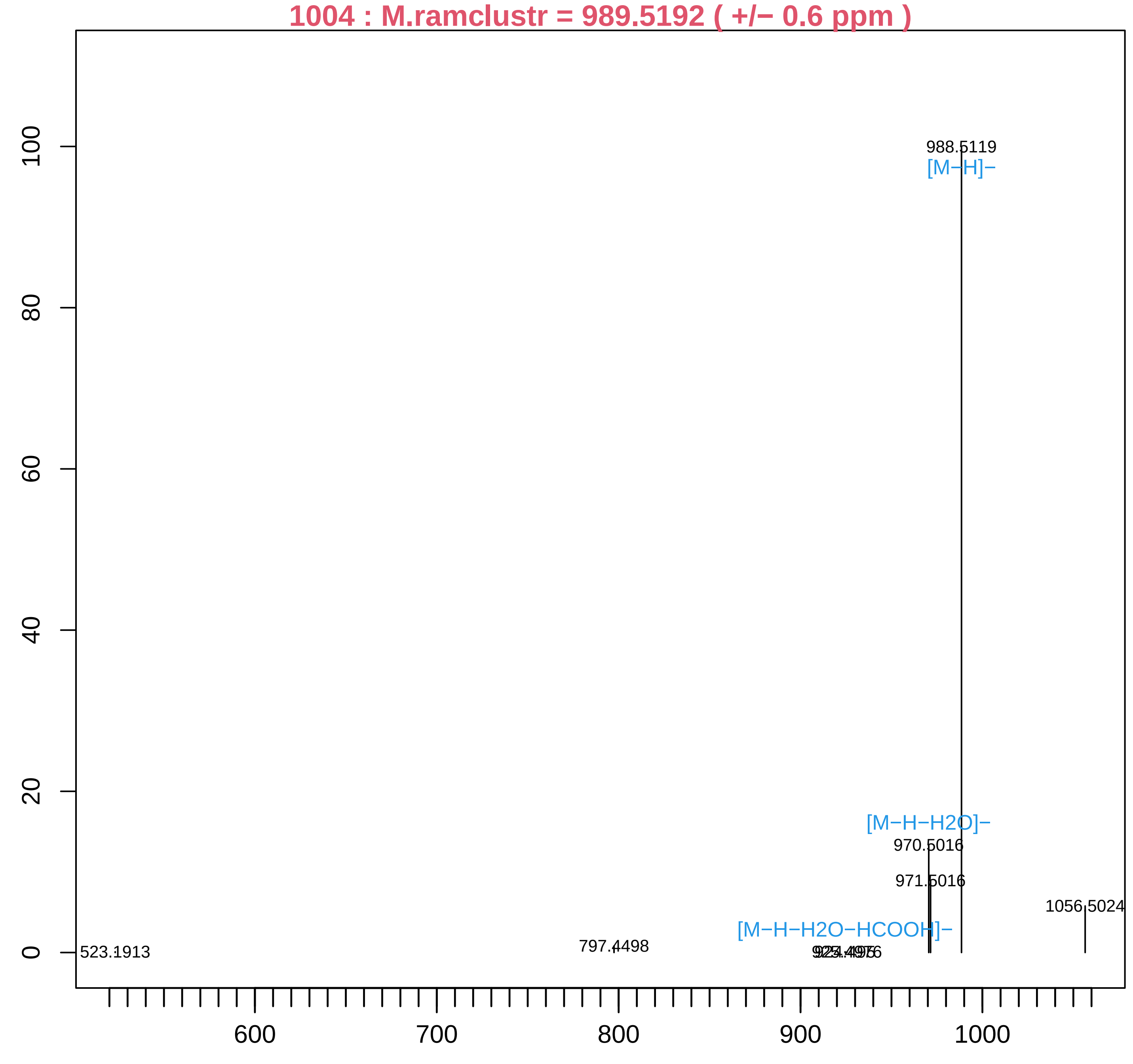
1004 : M.ramclustr = 989.5192 (+/− 0.6 ppm)
988.5119
[M−H]−
[M−H−H2O]−
970.5016
971.5016
1056.5024
[M−H−H2O−HCOOH]−
523.1913
797.4498
0
20
40
60
80
100
600
700
800
900
1000

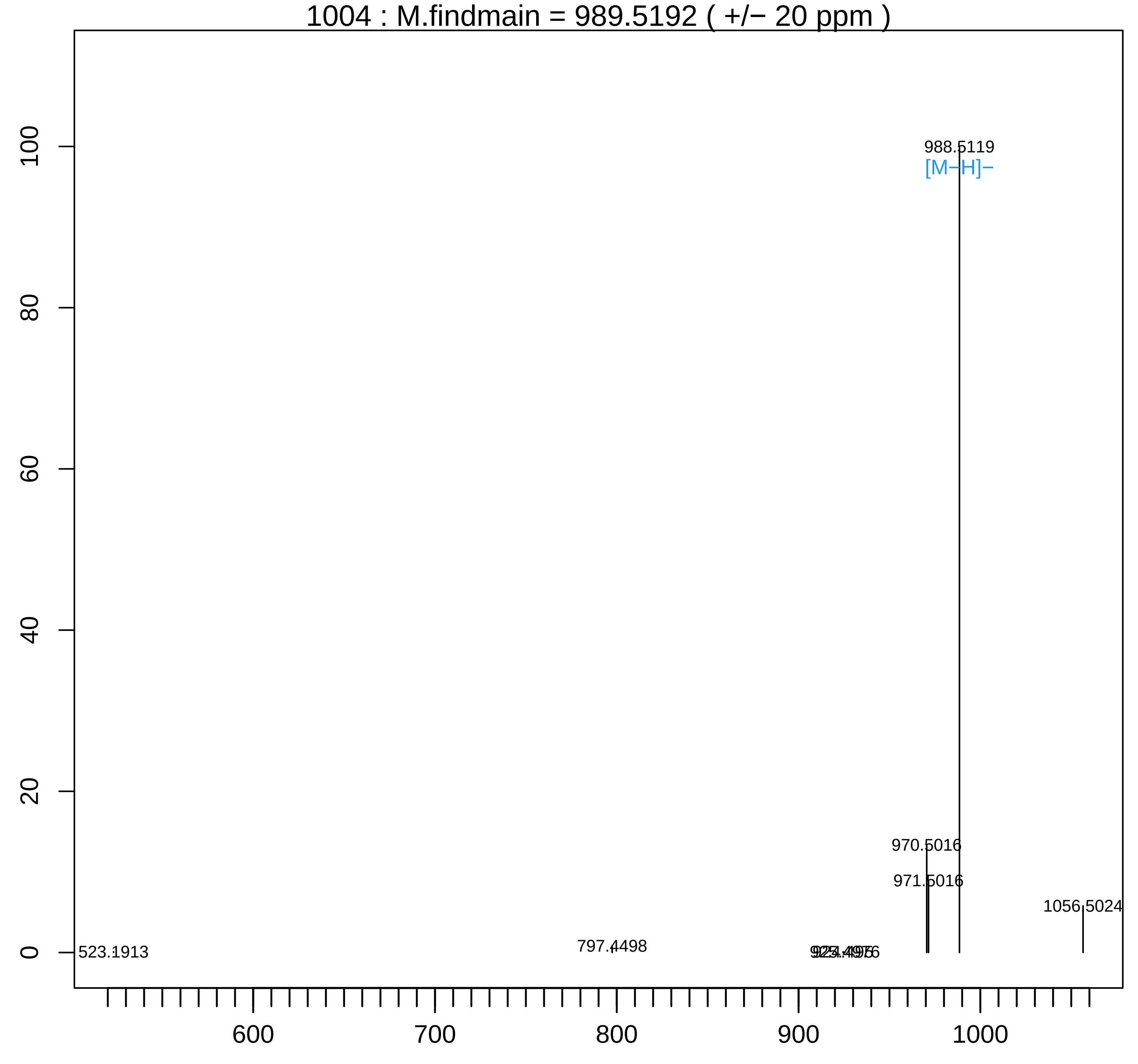
1004 : M.findmain = 989.5192 (+/− 20 ppm)
988.5119
[M−H]−
970.5016
971.5016
1056.5024
523.1913
797.4498
0
20
40
60
80
100
600
700
800
900
1000

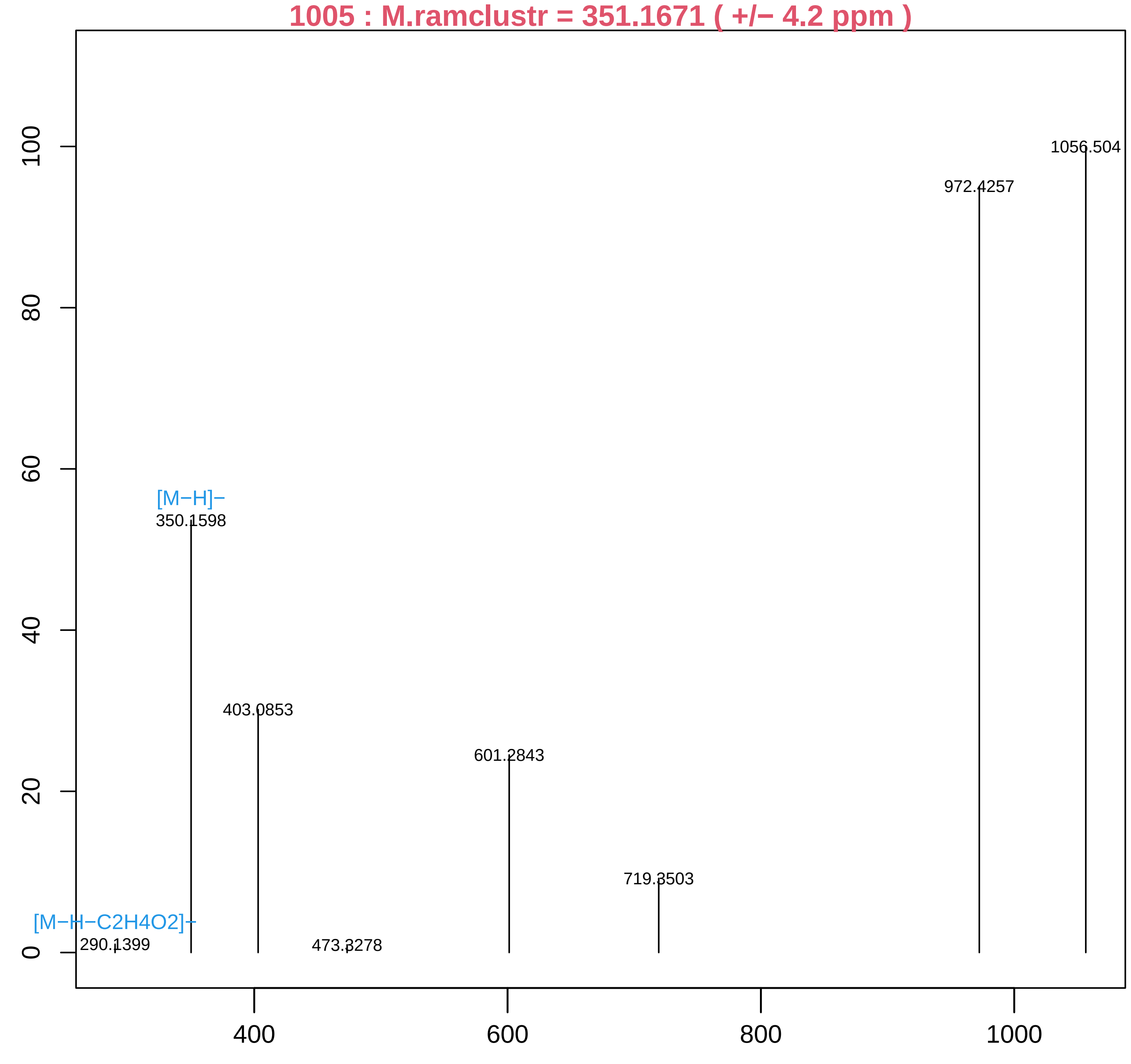

1005 : M.ramclustr = 351.1671 (+/− 4.2 ppm)
1056.504
972.4257
[M−H]−
350.1598
403.0853
601.2843
719.3503
[M−H−C2H4O2]−
290.1399
473.3278
0
20
40
60
80
100
400
600
800
1000

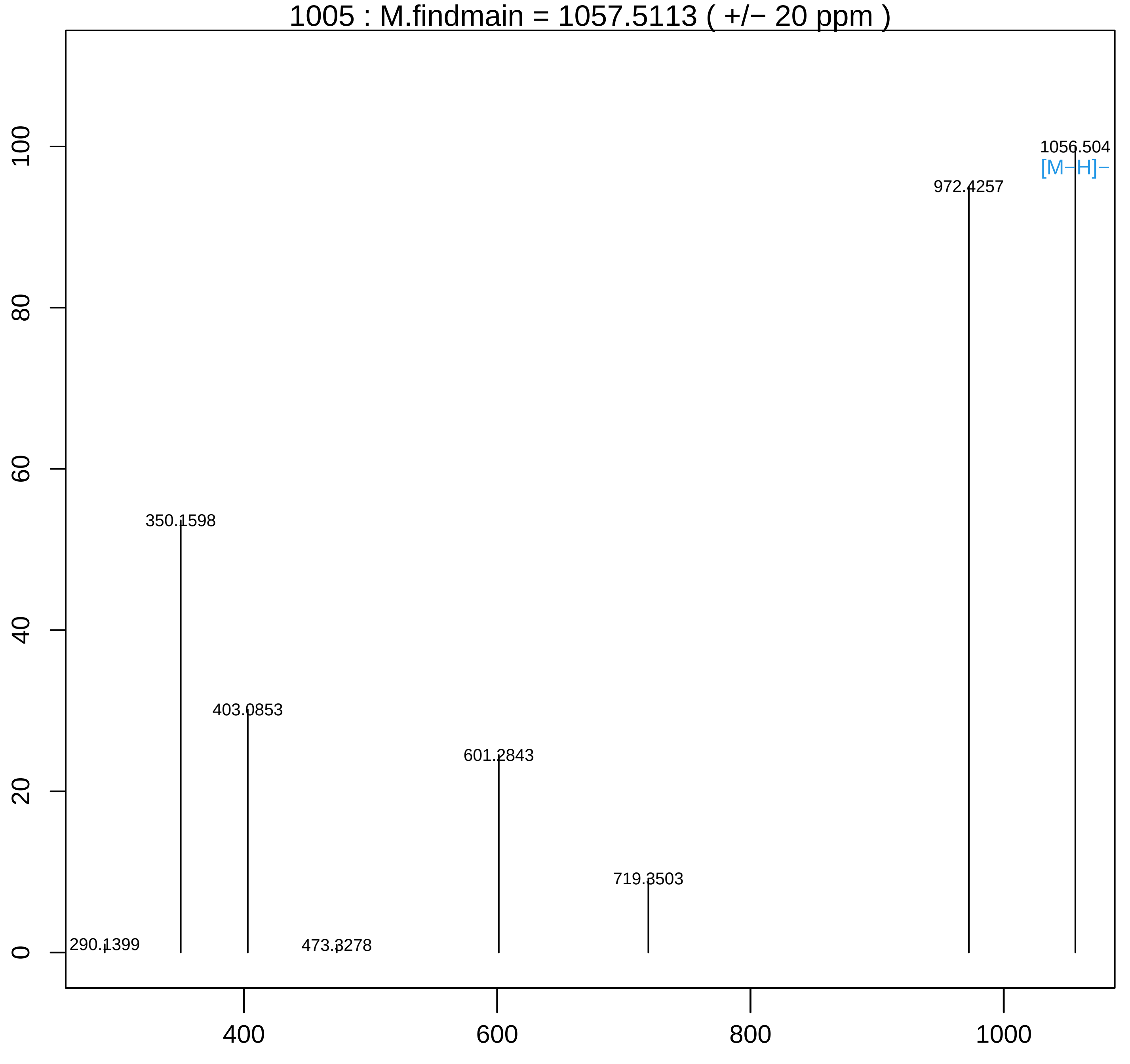

1005 : M.findmain = 1057.5113 (+/− 20 ppm)
1056.504
[M−H]−
972.4257
350.1598
403.0853
601.2843
719.3503
290.1399
473.3278
0
20
40
60
80
100
400
600
800
1000

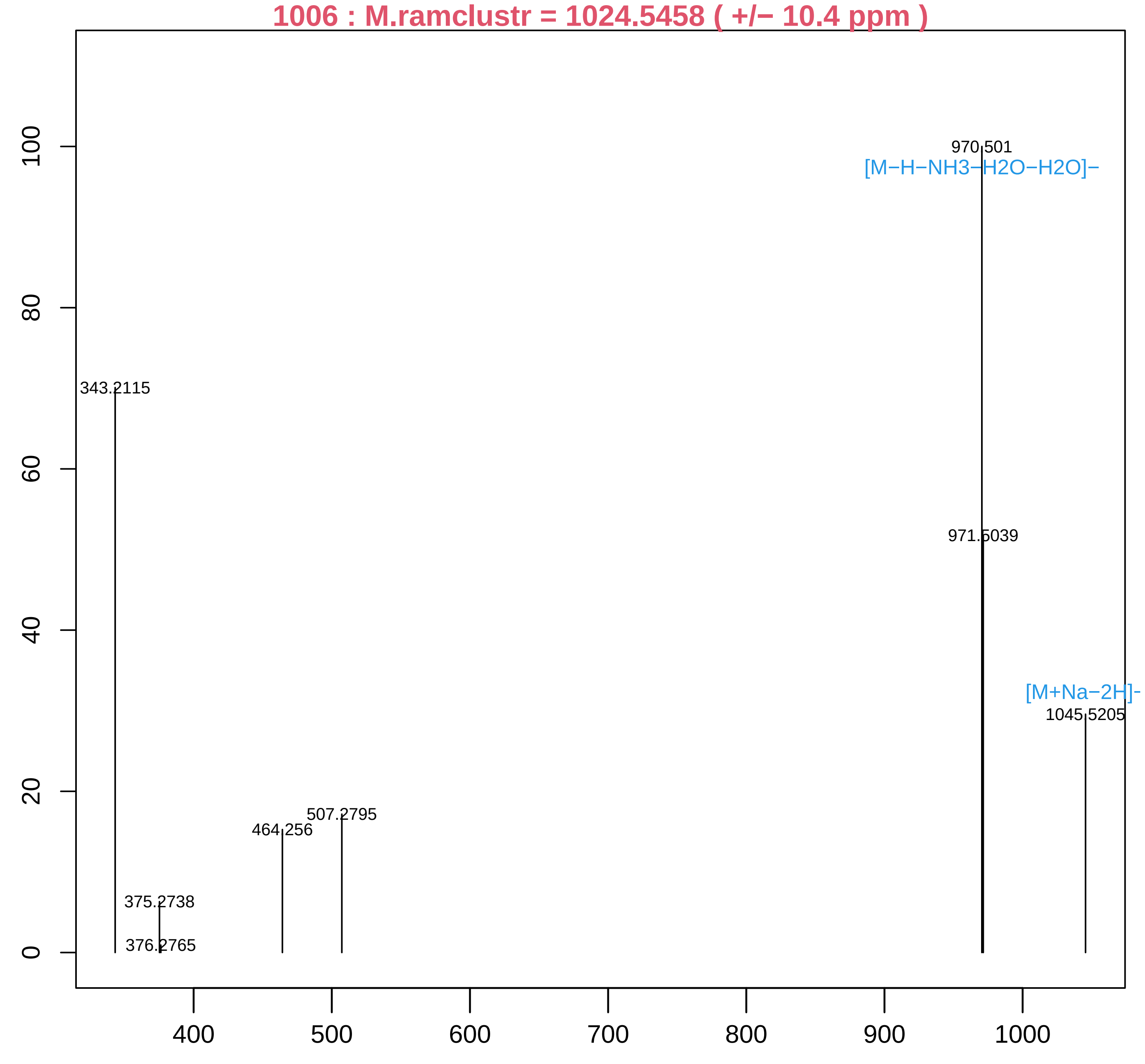

1006 : M.ramclustr = 1024.5458 (+/− 10.4 ppm)
970.501
[M−H−NH3−H2O−H2O]−
343.2115
971.5039
[M+Na−2H]-
1045.5205
507.2795
464.256
375.2738
376.2765
0
20
40
60
80
100
400
500
600
700
800
900
1000

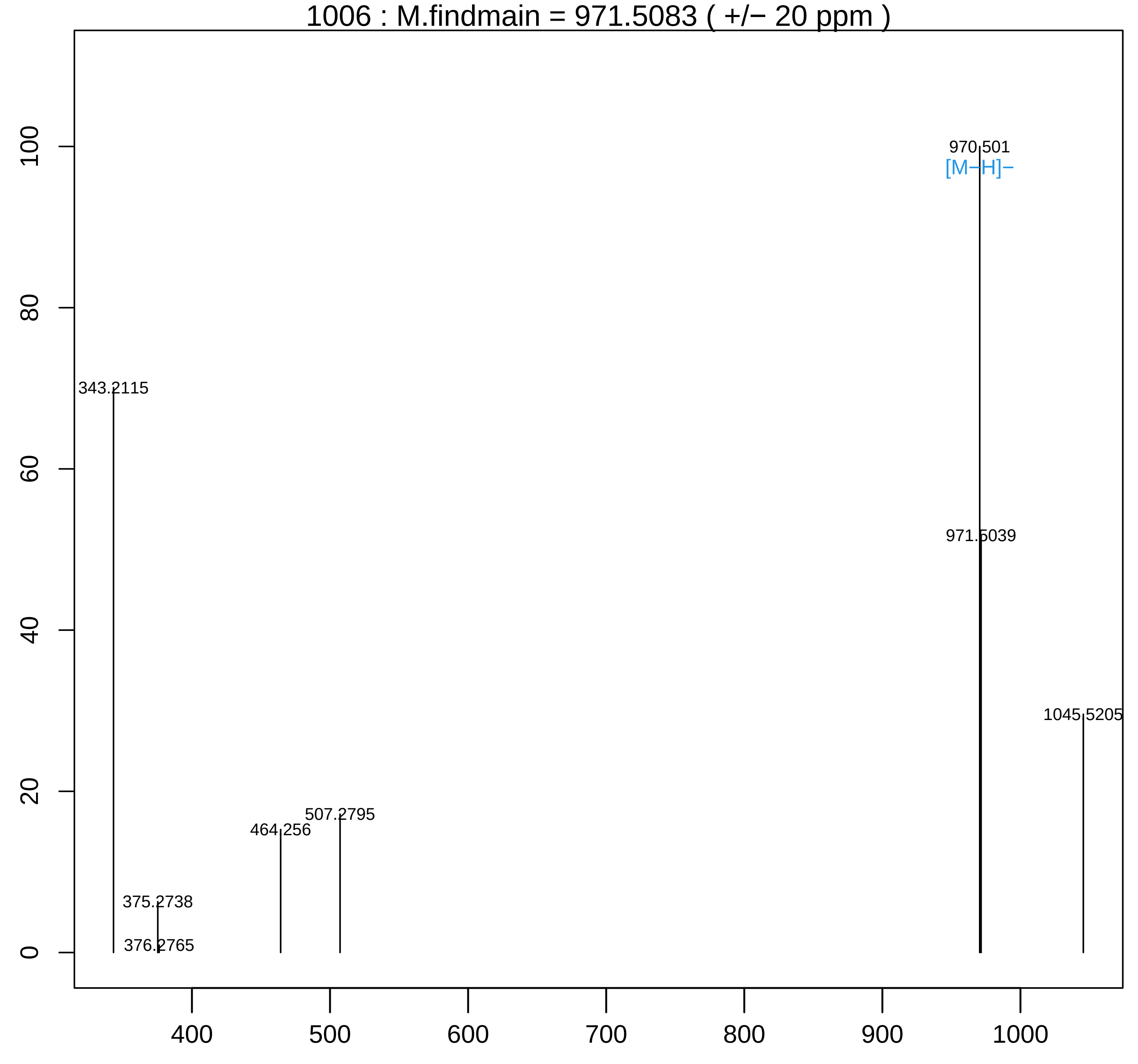

1006 : M.findmain = 971.5083 (+/− 20 ppm)
970.501
[M−H]−
343.2115
971.5039
1045.5205
507.2795
464.256
375.2738
376.2765
0
20
40
60
80
100
400
500
600
700
800
900
1000

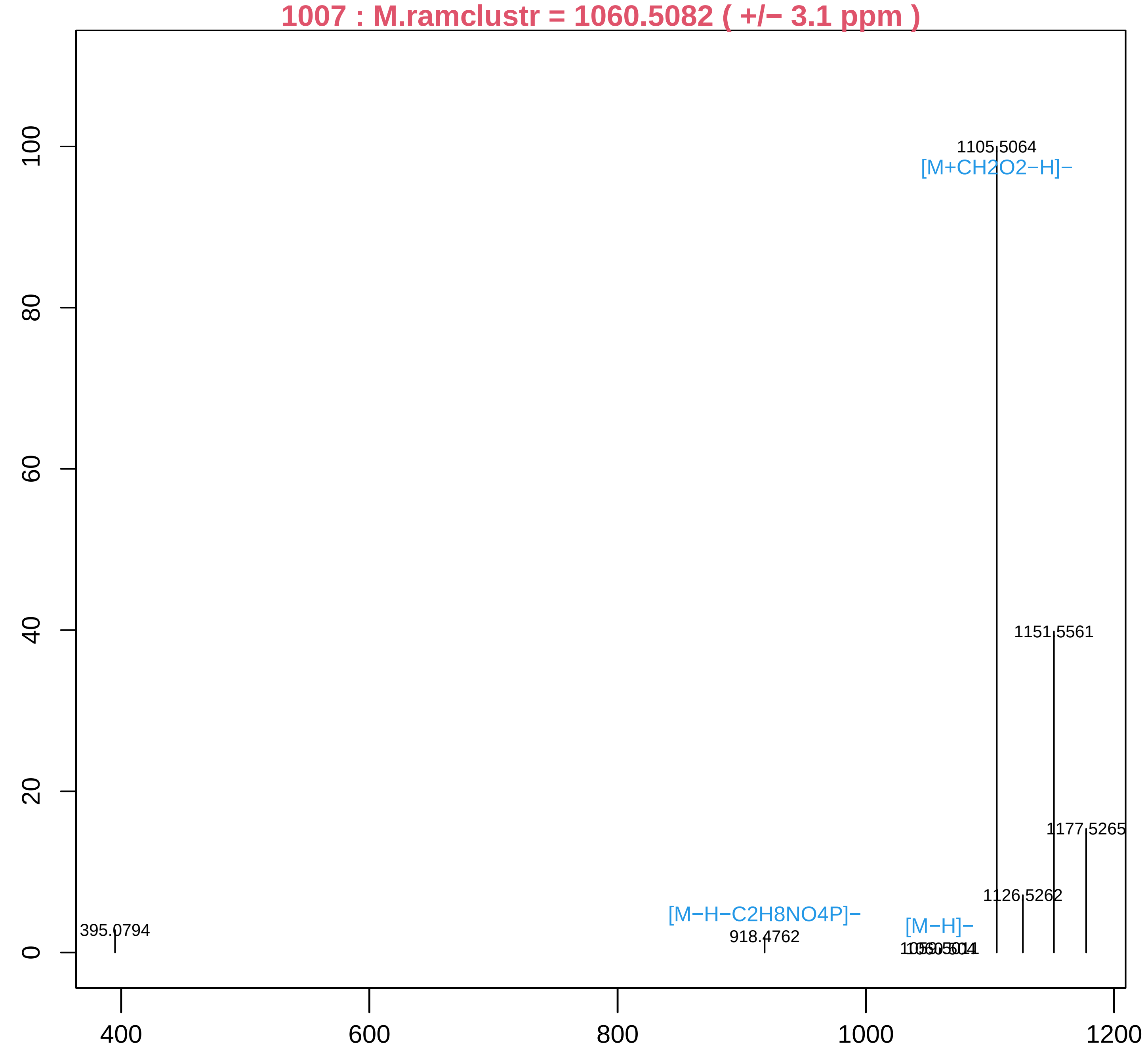
1007 : M.ramclustr = 1060.5082 (+/− 3.1 ppm)
1105.5064
[M+CH2O2−H]−
1151.5561
1177.5265
1126.5262
395.0794
[M−H−C2H8NO4P]−
918.4762
[M−H]−
0
20
40
60
80
100
400
600
800
1000
1200

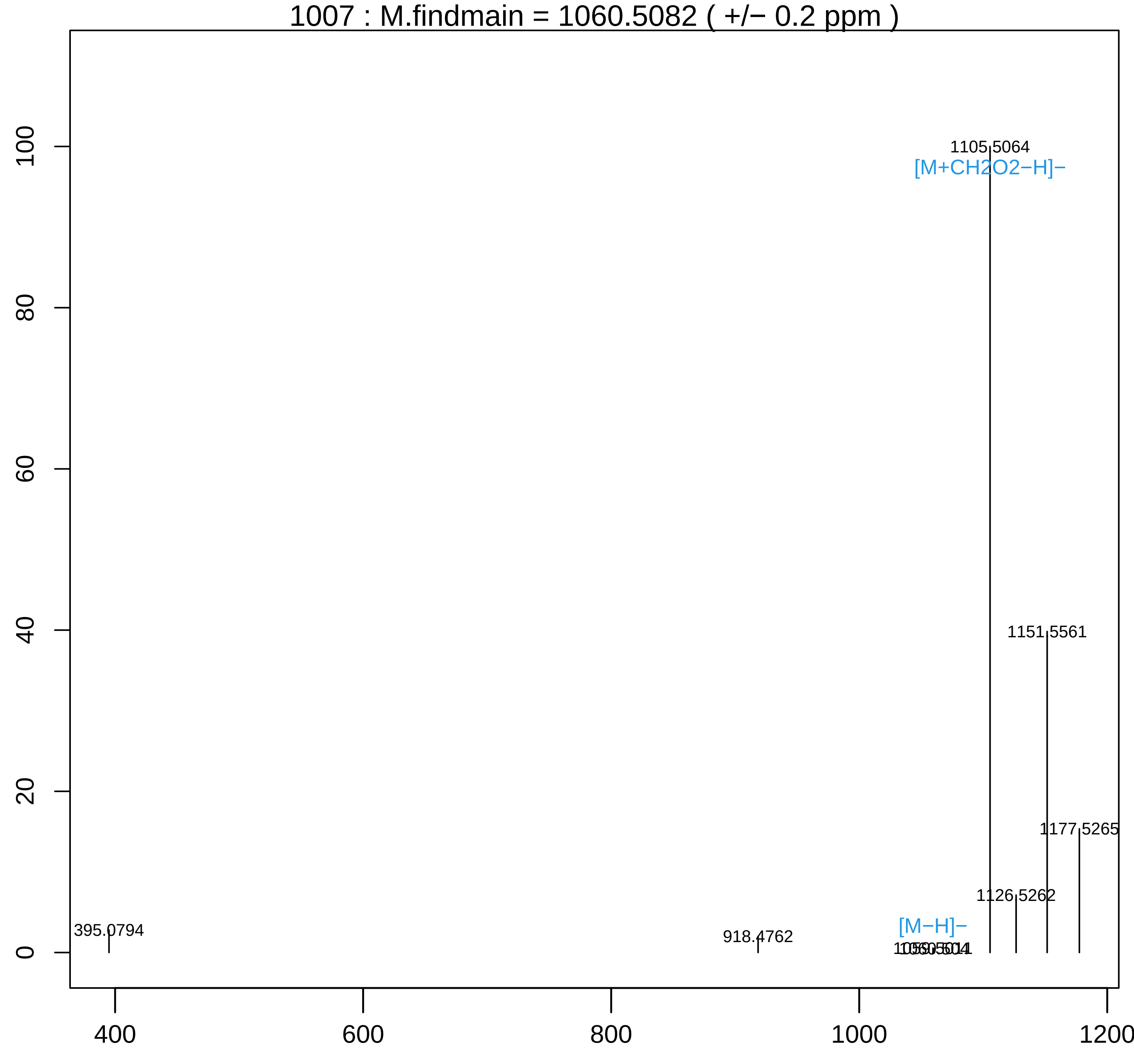
1007 : M.findmain = 1060.5082 (+/− 0.2 ppm)
1105.5064
[M+CH2O2−H]−
1151.5561
1177.5265
1126.5262
395.0794
918.4762
[M−H]−
0
20
40
60
80
100
400
600
800
1000
1200

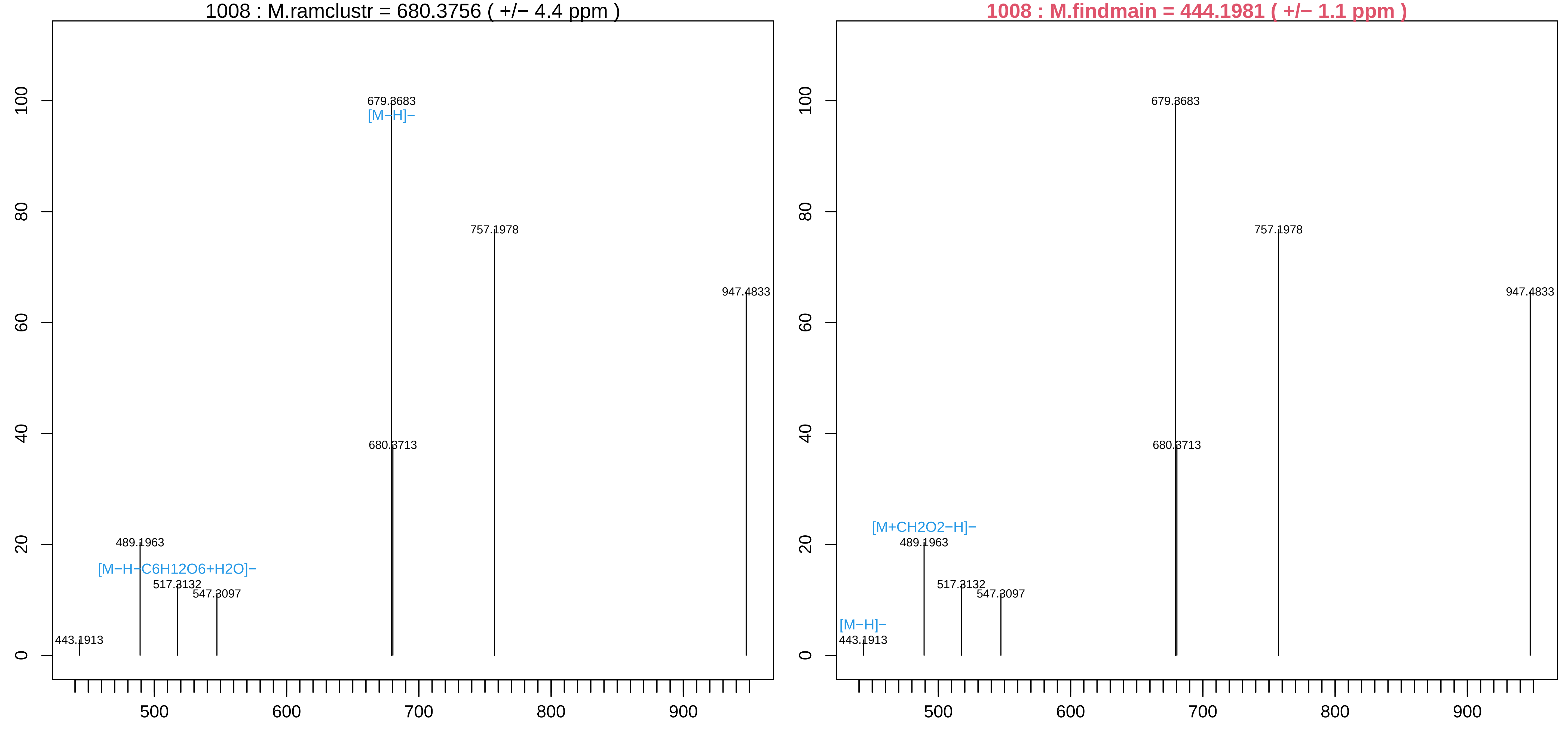

1008 : M.ramclustr = 680.3756 (+/− 4.4 ppm)
679.3683
[M−H]−
757.1978
947.4833
680.3713
489.1963
[M−H−C6H12O6+H2O]−
517.3132
547.3097
443.1913
1008 : M.findmain = 444.1981 (+/− 1.1 ppm)
679.3683
757.1978
947.4833
680.3713
[M+CH2O2−H]−
489.1963
517.3132
547.3097
[M−H]−
443.1913
0
20
40
60
80
100
500
600
700
800
900

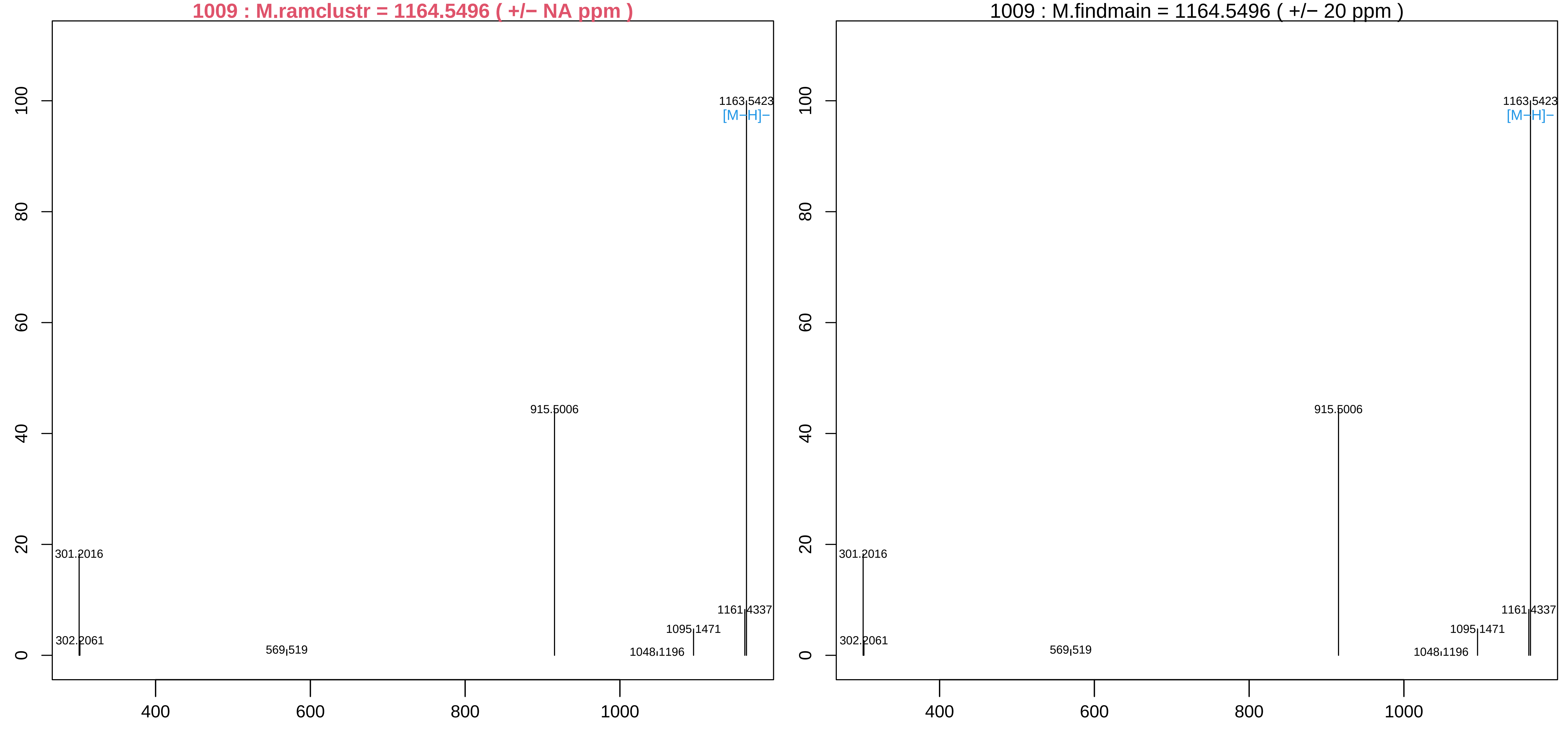
1009 : M.ramclustr = 1164.5496 (+/− NA ppm)
1163.5423
[M−H]−
915.5006
301.2016
302.2061
569.519
1048.1196
1095.1471
1161.4337
0
20
40
60
80
100
400
600
800
1000
1009 : M.findmain = 1164.5496 (+/− 20 ppm)
1163.5423
[M−H]−
915.5006
301.2016
302.2061
569.519
1048.1196
1095.1471
1161.4337
0
20
40
60
80
100
400
600
800
1000

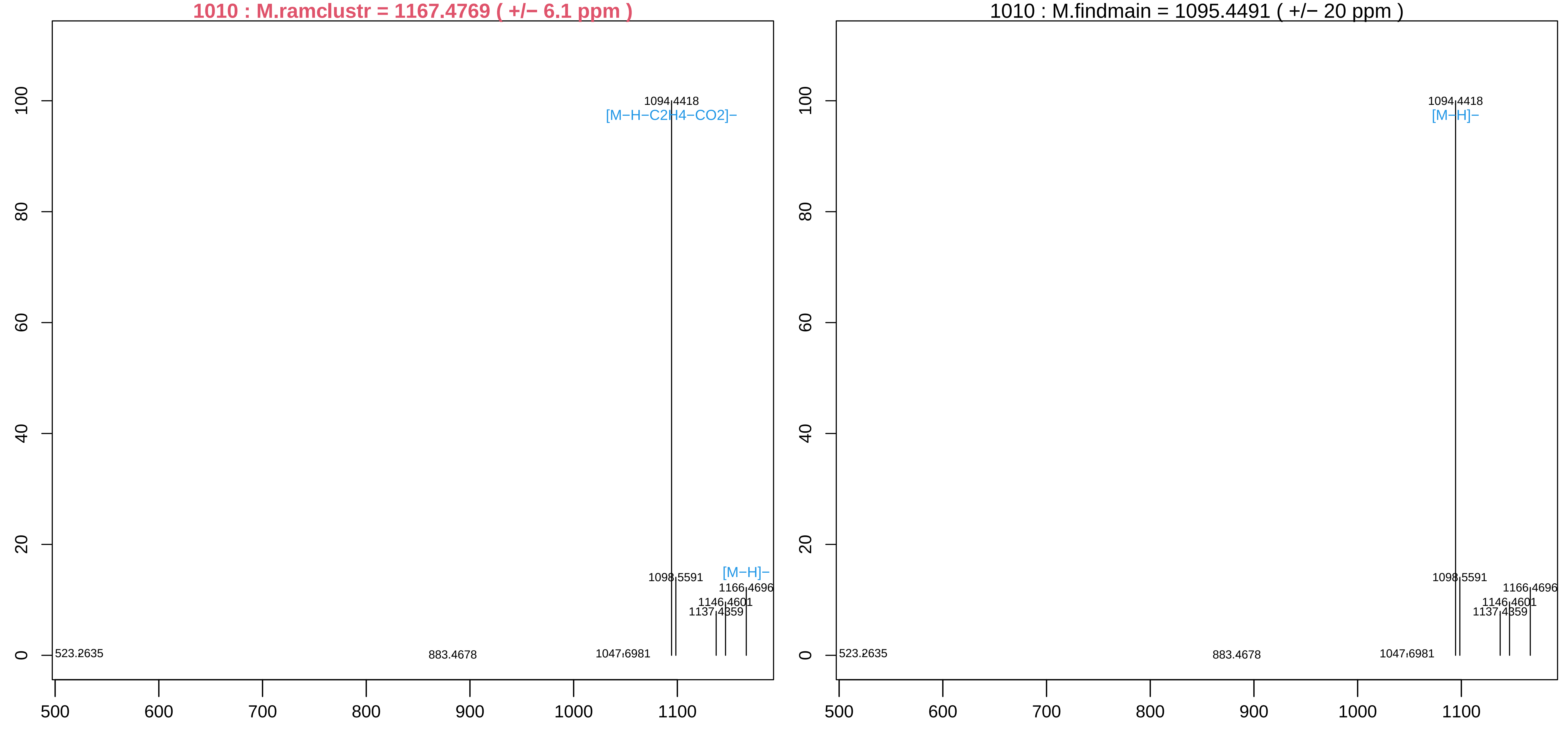
1010 : M.ramclustr = 1167.4769 (+/− 6.1 ppm)
1094.4418
[M−H−C2H4−CO2]−
1098.5591
[M−H]−
1166.4696
1146.4601
1137.4359
523.2635
883.4678
1047.6981
1010 : M.findmain = 1095.4491 (+/− 20 ppm)
1094.4418
[M−H]−
1098.5591
1166.4696
1146.4601
1137.4359
523.2635
883.4678
1047.6981
0
20
40
60
80
100
500
600
700
800
900
1000
1100

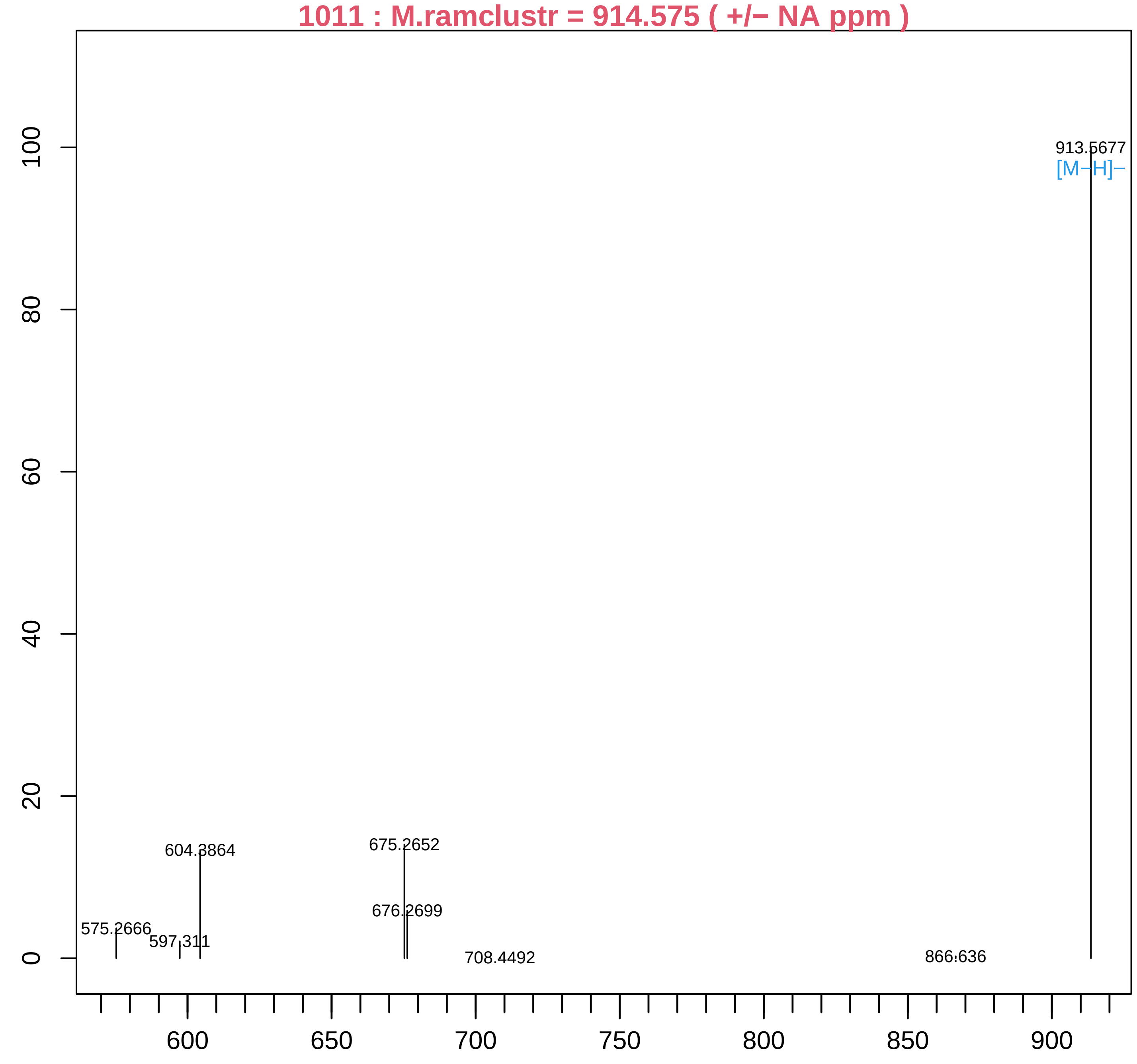
1011 : M.ramclustr = 914.575 (+/− NA ppm)
913.5677
[M−H]−
604.3864
675.2652
676.2699
575.2666
597.311
708.4492
866.636
0
20
40
60
80
100
600
650
700
750
800
850
900

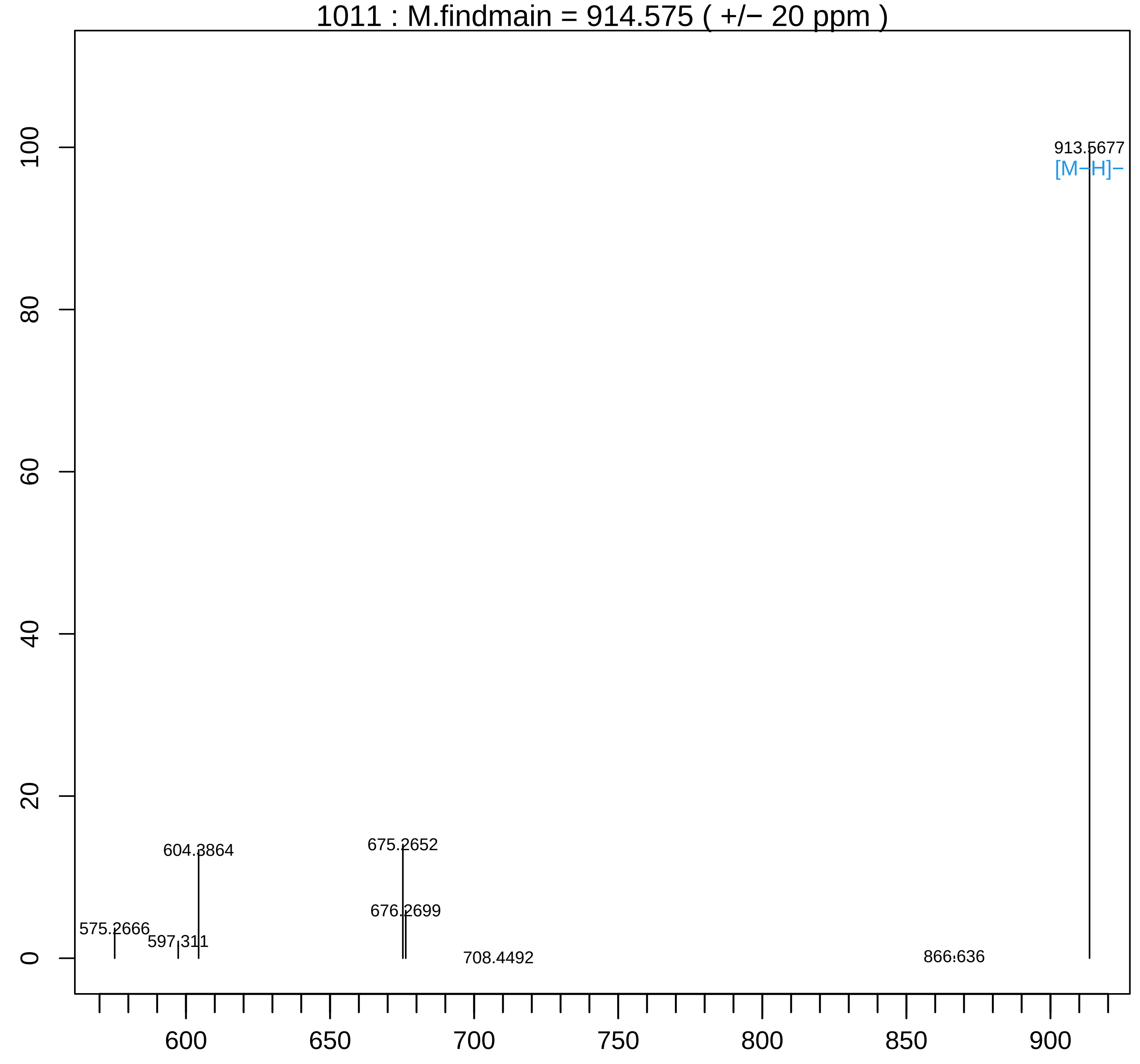
1011 : M.findmain = 914.575 (+/− 20 ppm)
913.5677
[M−H]−
604.3864
675.2652
676.2699
575.2666
597.311
708.4492
866.636
0
20
40
60
80
100
600
650
700
750
800
850
900

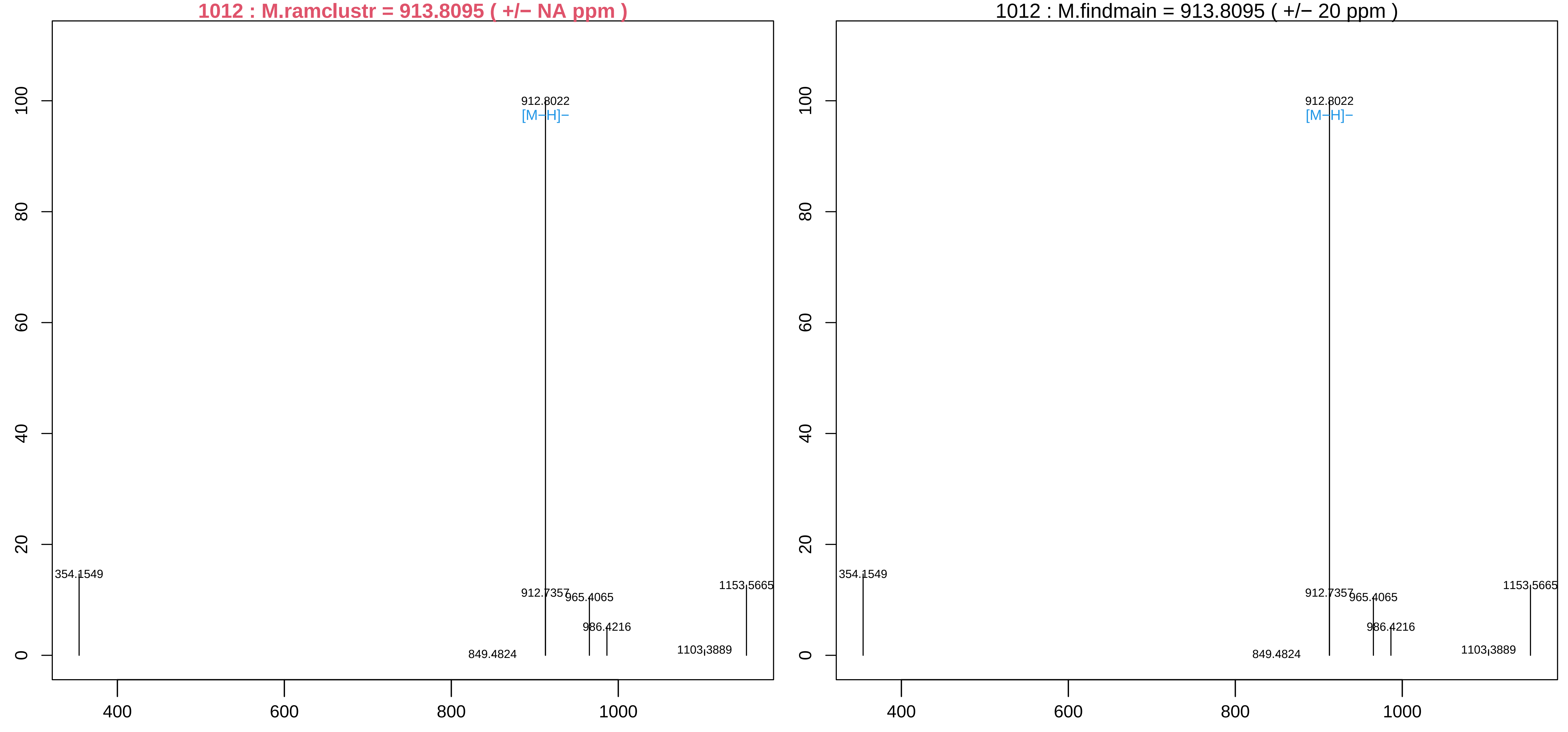
1012 : M.ramclustr = 913.8095 (+/− NA ppm)
912.8022
[M−H]−
354.1549
912.7357
965.4065
986.4216
1153.5665
1103.3889
849.4824
0
20
40
60
80
100
400
600
800
1000
1012 : M.findmain = 913.8095 (+/− 20 ppm)
912.8022
[M−H]−
354.1549
912.7357
965.4065
986.4216
1153.5665
1103.3889
849.4824
0
20
40
60
80
100
400
600
800
1000

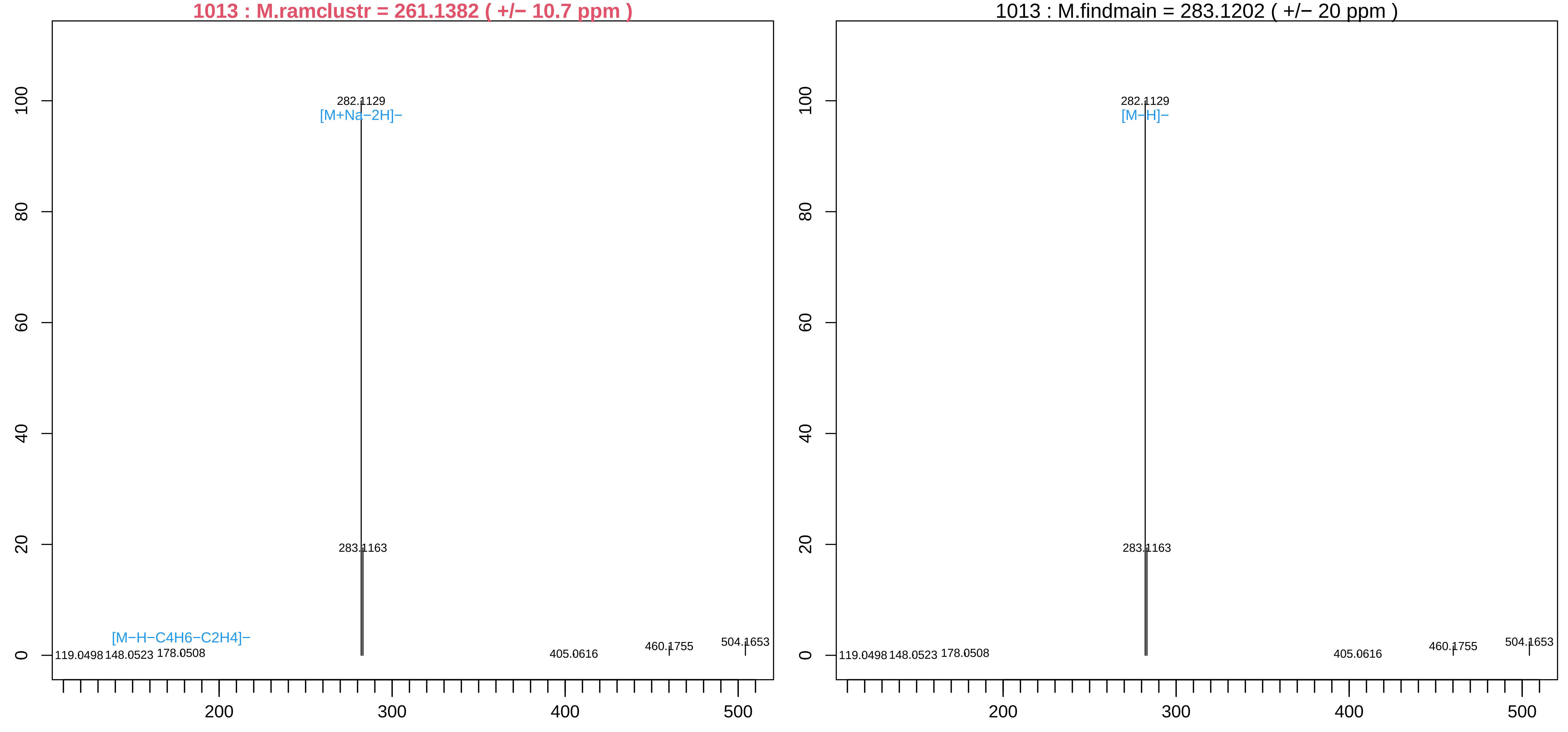
1013 : M.ramclustr = 261.1382 (+/− 10.7 ppm)
282.1129
[M+Na−2H]−
283.1163
[M−H−C4H6−C2H4]−
119.0498
148.0523
178.0508
405.0616
460.1755
504.1653
1013 : M.findmain = 283.1202 (+/− 20 ppm)
282.1129
[M−H]−
283.1163
119.0498
148.0523
178.0508
405.0616
460.1755
504.1653
0
20
40
60
80
100
200
300
400
500

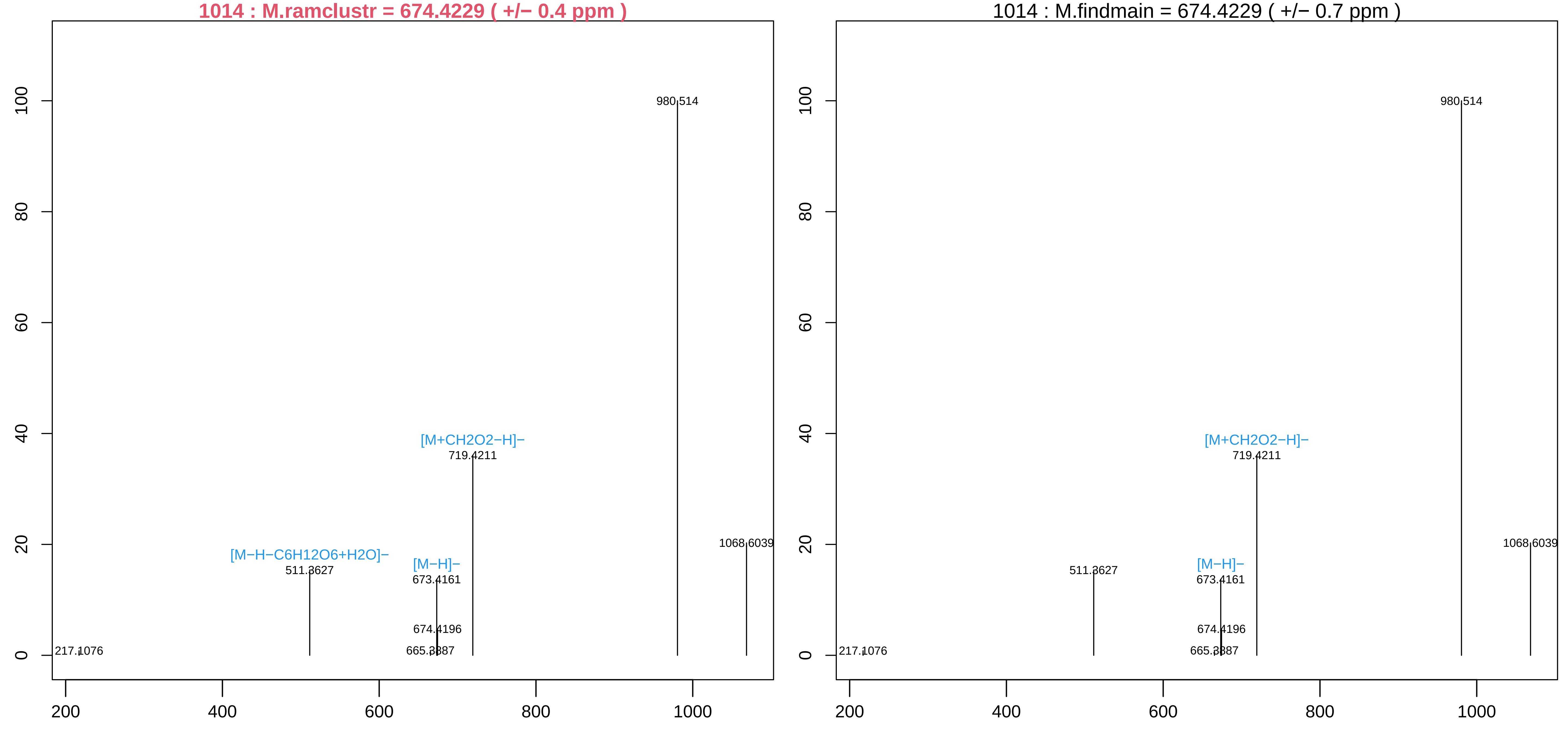
1014 : M.ramclustr = 674.4229 (+/− 0.4 ppm)
1014 : M.findmain = 674.4229 (+/− 0.7 ppm)
980.514
1068.6039
[M+CH2O2−H]−
719.4211
[M−H−C6H12O6+H2O]−
511.3627
[M−H]−
673.4161
674.4196
665.3887
217.1076
0
20
40
60
80
100
200
400
600
800
1000

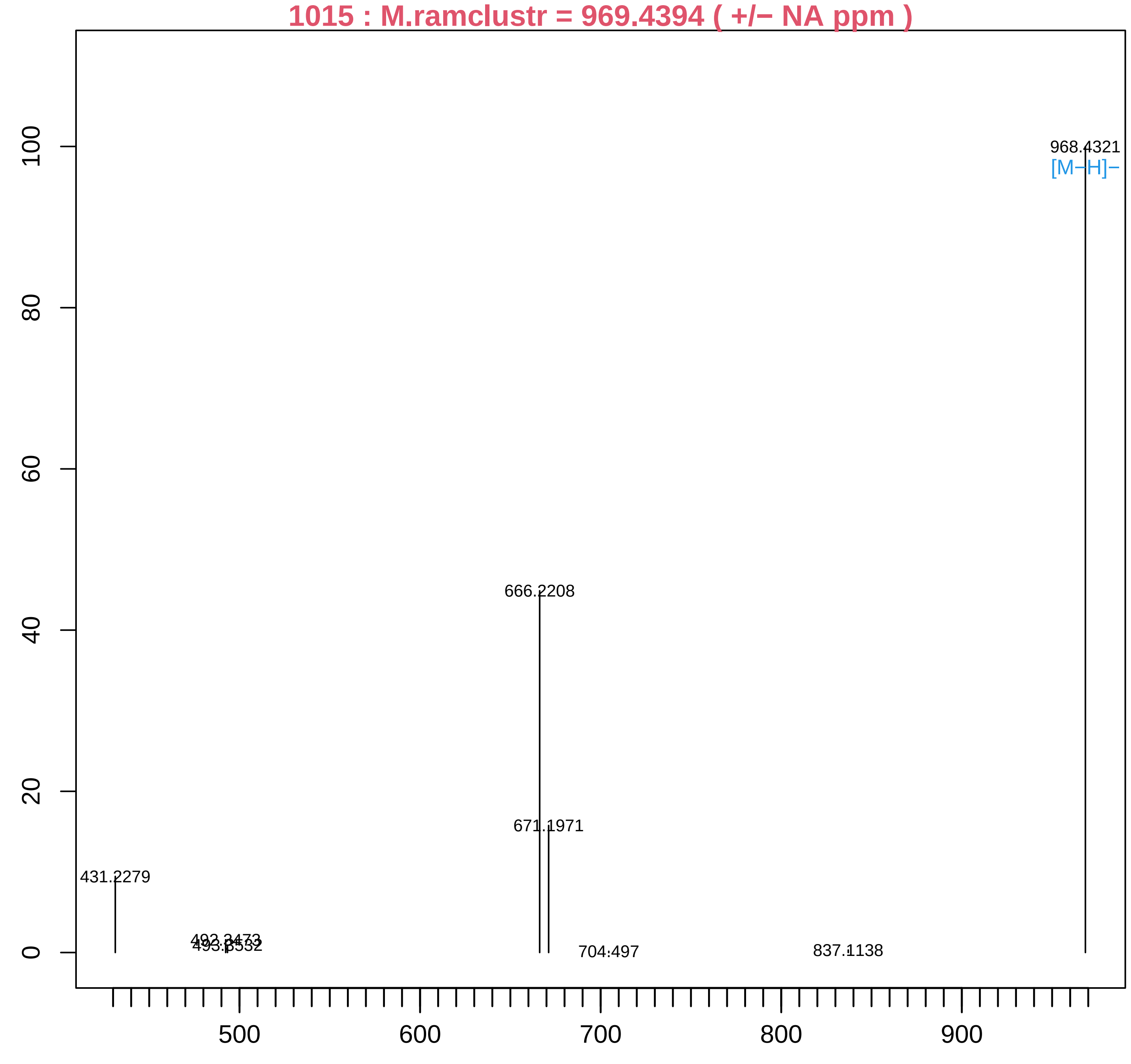
1015 : M.ramclustr = 969.4394 (+/− NA ppm)
968.4321
[M−H]−
666.2208
671.1971
431.2279
492.3473
493.3532
704.497
837.1138
0
20
40
60
80
100
500
600
700
800
900

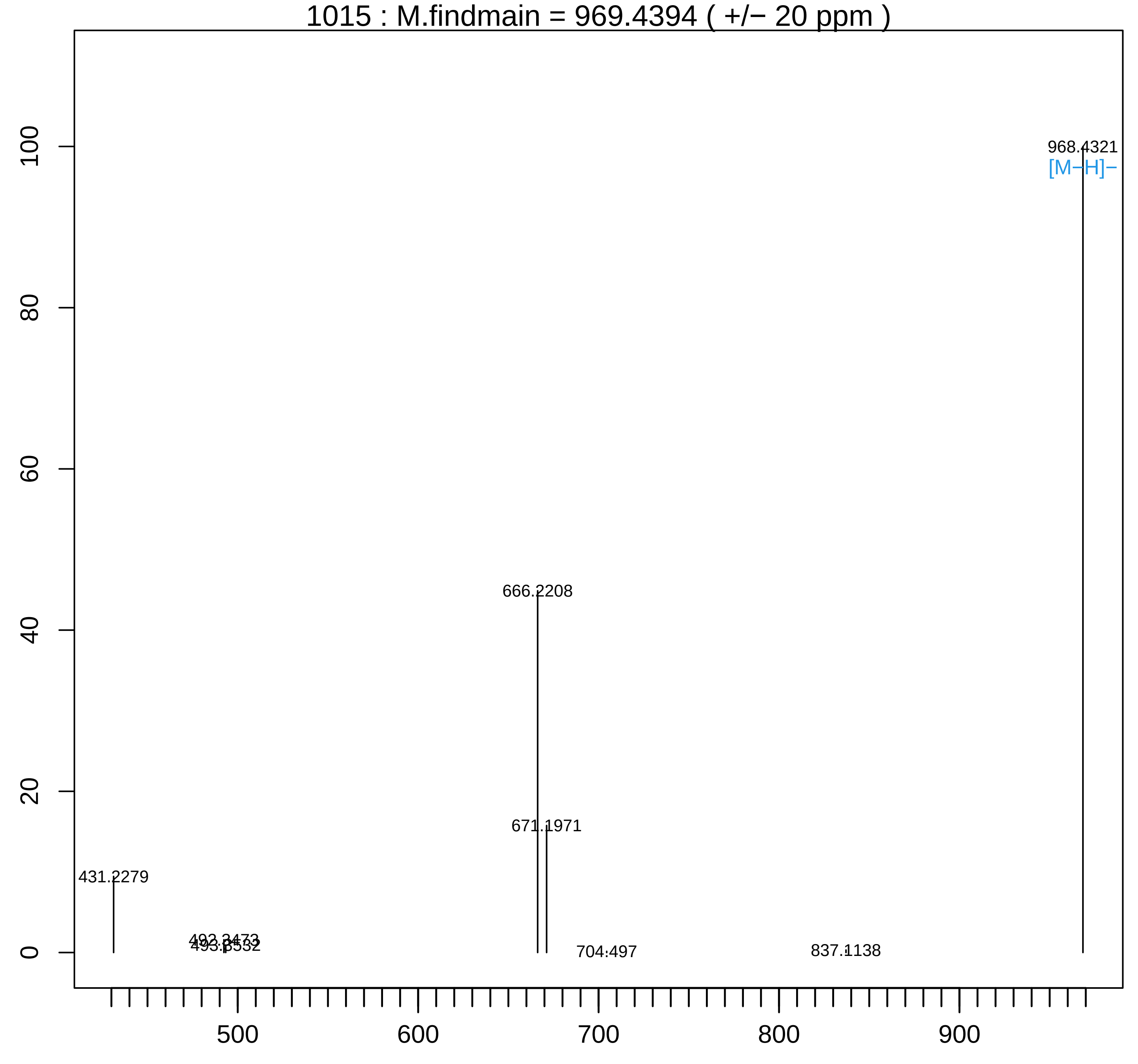
1015 : M.findmain = 969.4394 (+/− 20 ppm)
968.4321
[M−H]−
666.2208
671.1971
431.2279
492.3473
493.3532
704.497
837.1138
0
20
40
60
80
100
500
600
700
800
900

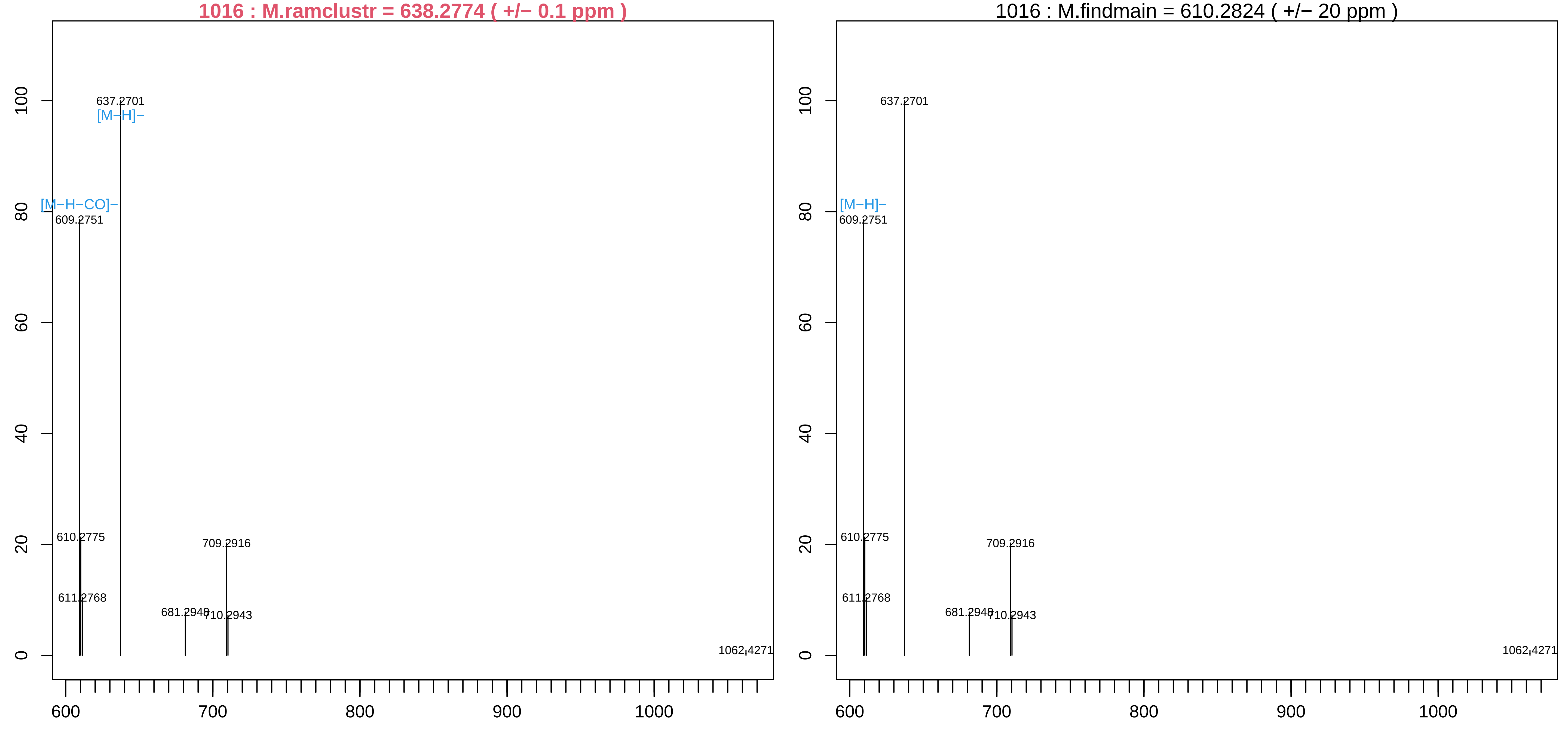
1016 : M.ramclustr = 638.2774 (+/− 0.1 ppm)
637.2701
[M−H]−
[M−H−CO]−
609.2751
610.2775
611.2768
681.2948
709.2916
710.2943
1062.4271
1016 : M.findmain = 610.2824 (+/− 20 ppm)
637.2701
[M−H]−
609.2751
610.2775
611.2768
681.2948
709.2916
710.2943
1062.4271
0
20
40
60
80
100
600
700
800
900
1000

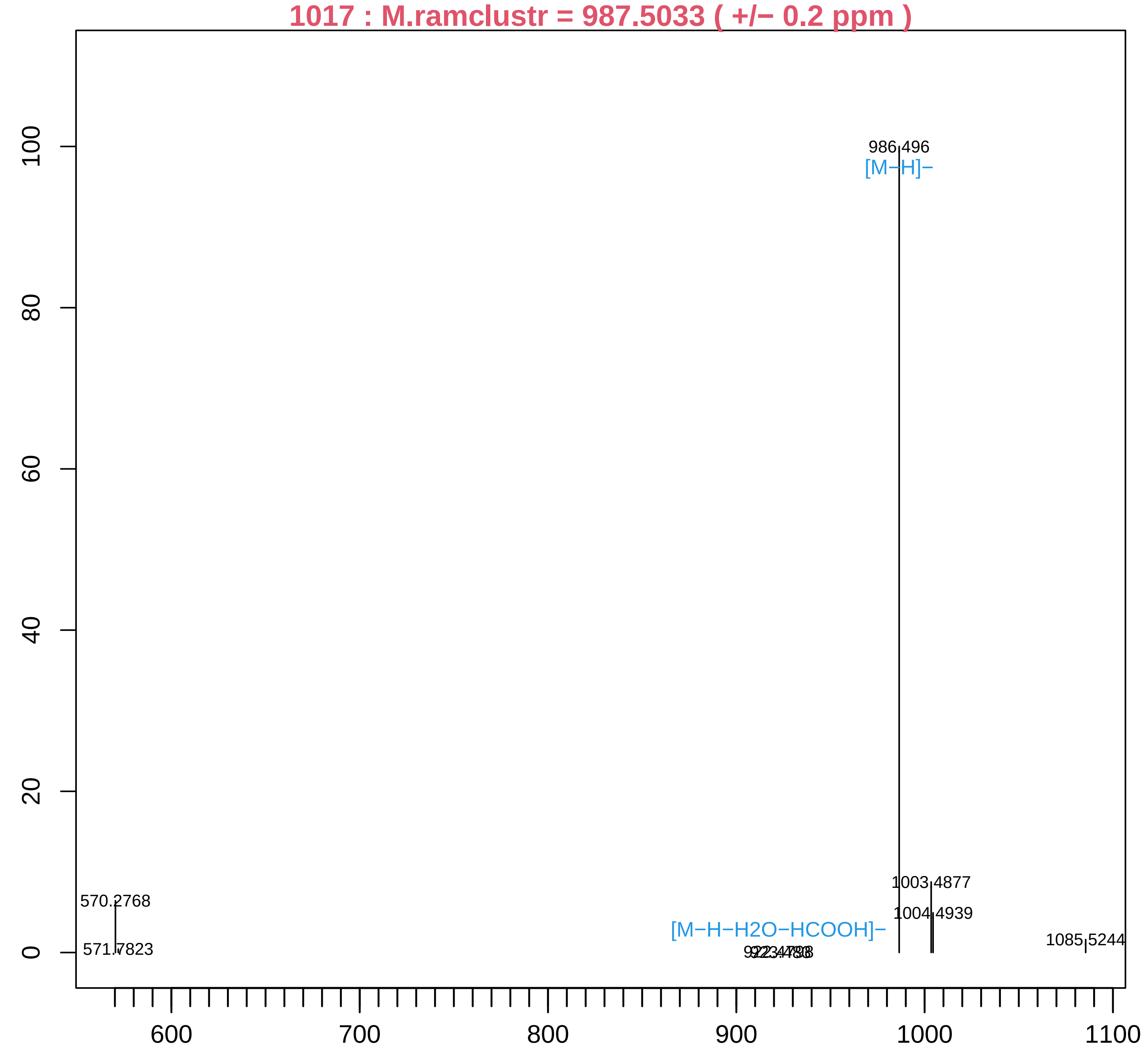
1017 : M.ramclustr = 987.5033 (+/− 0.2 ppm)
986.496
[M−H]−
1003.4877
1004.4939
570.2768
571.7823
[M−H−H2O−HCOOH]−
1085.5244
0
20
40
60
80
100
600
700
800
900
1000
1100

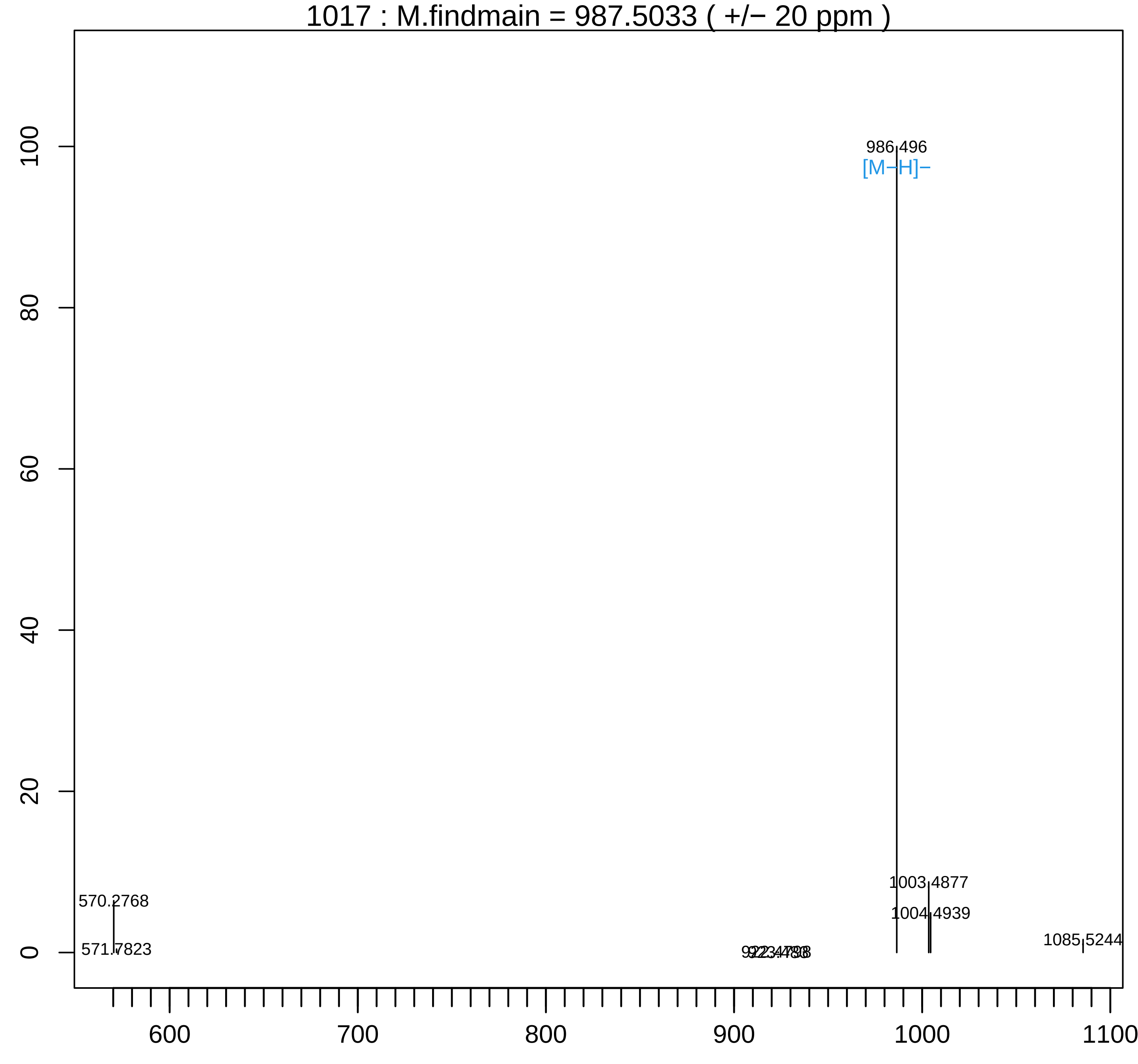
1017 : M.findmain = 987.5033 (+/− 20 ppm)
986.496
[M−H]−
1003.4877
1004.4939
570.2768
571.7823
1085.5244
0
20
40
60
80
100
600
700
800
900
1000
1100

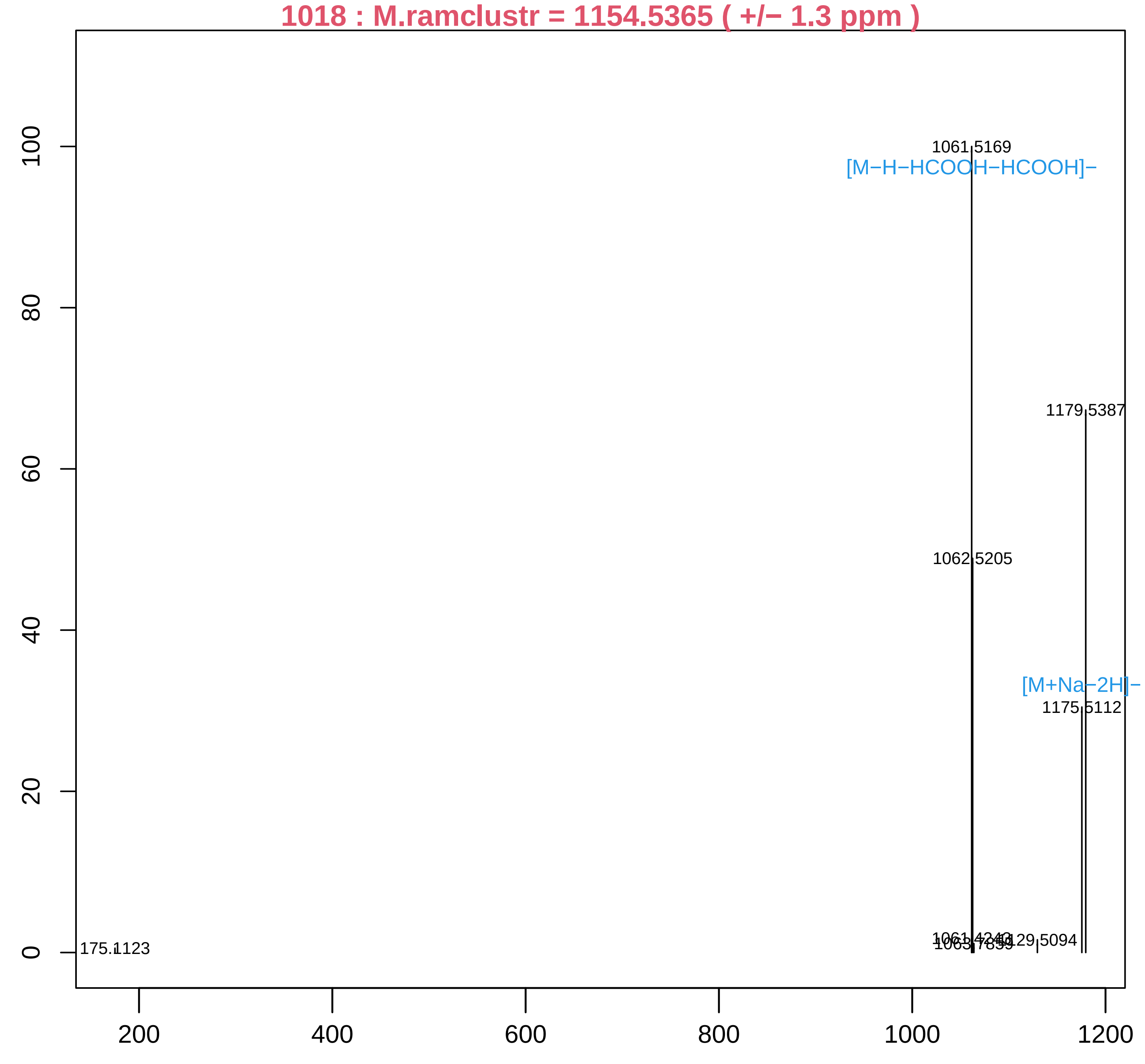
1018 : M.ramclustr = 1154.5365 (+/− 1.3 ppm)
1061.5169
[M−H−HCOOH−HCOOH]−
1179.5387
1062.5205
[M+Na−2H]−
1175.5112
1061.4243
1063.7659
1129.5094
175.1123
0
20
40
60
80
100
200
400
600
800
1000
1200

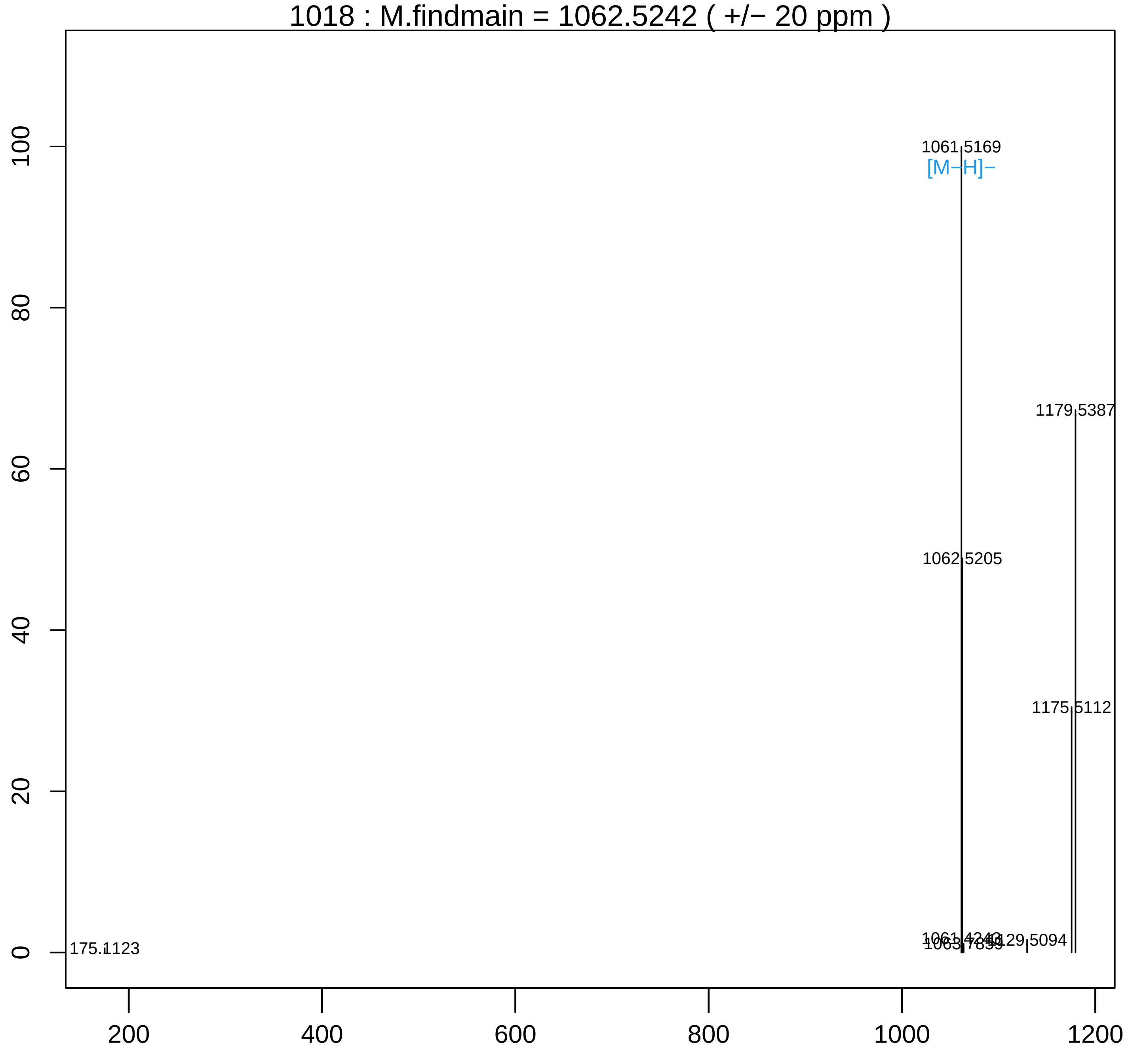
1018 : M.findmain = 1062.5242 (+/− 20 ppm)
1061.5169
[M−H]−
1179.5387
1062.5205
1175.5112
1061.4243
1063.7659
1129.5094
175.1123
0
20
40
60
80
100
200
400
600
800
1000
1200

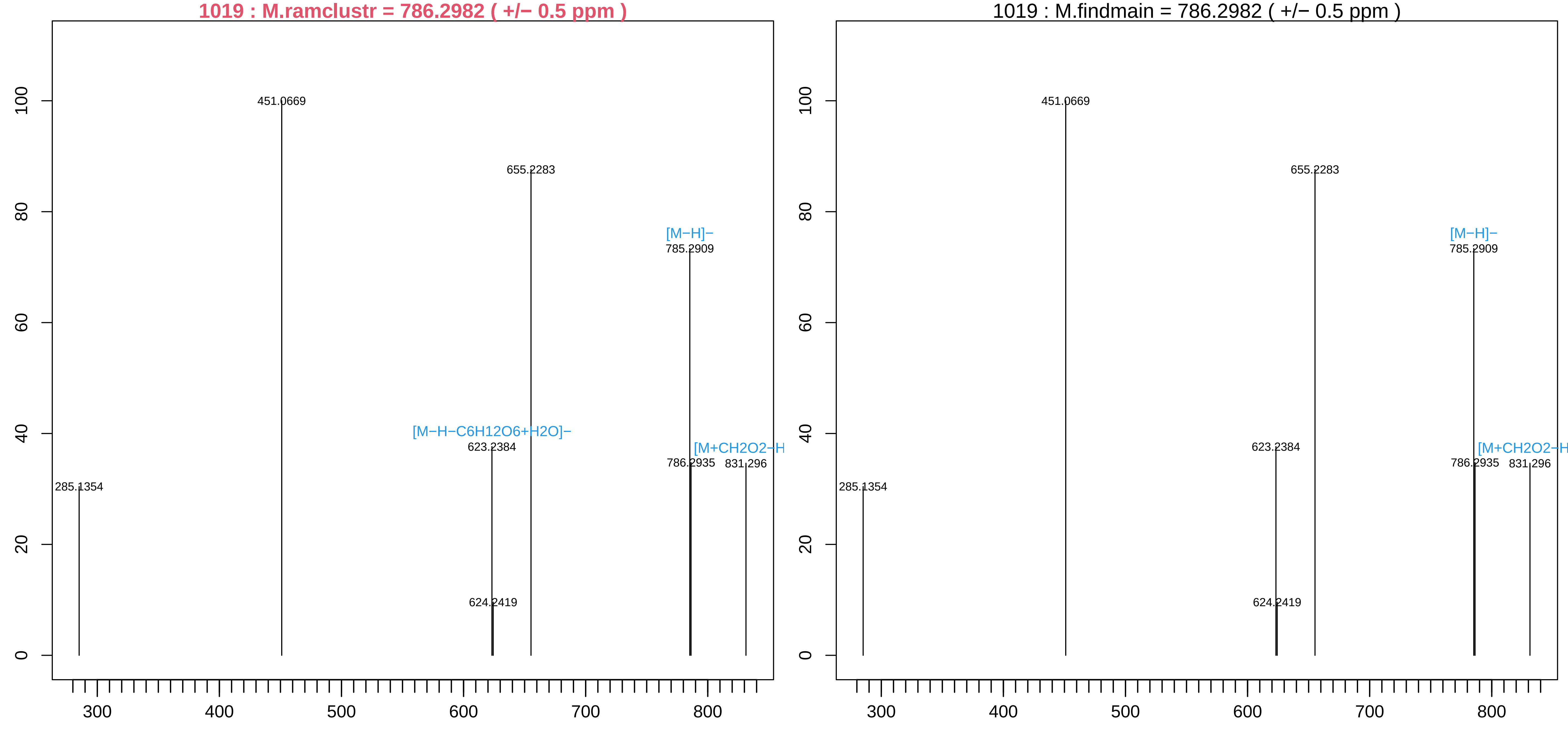
1019 : M.ramclustr = 786.2982 (+/− 0.5 ppm)
451.0669
655.2283
[M−H]−
785.2909
[M−H−C6H12O6+H2O]−
623.2384
[M+CH2O2−H
786.2935
831.296
285.1354
624.2419
1019 : M.findmain = 786.2982 (+/− 0.5 ppm)
451.0669
655.2283
[M−H]−
785.2909
623.2384
[M+CH2O2−H
786.2935
831.296
285.1354
624.2419
0
20
40
60
80
100
300
400
500
600
700
800

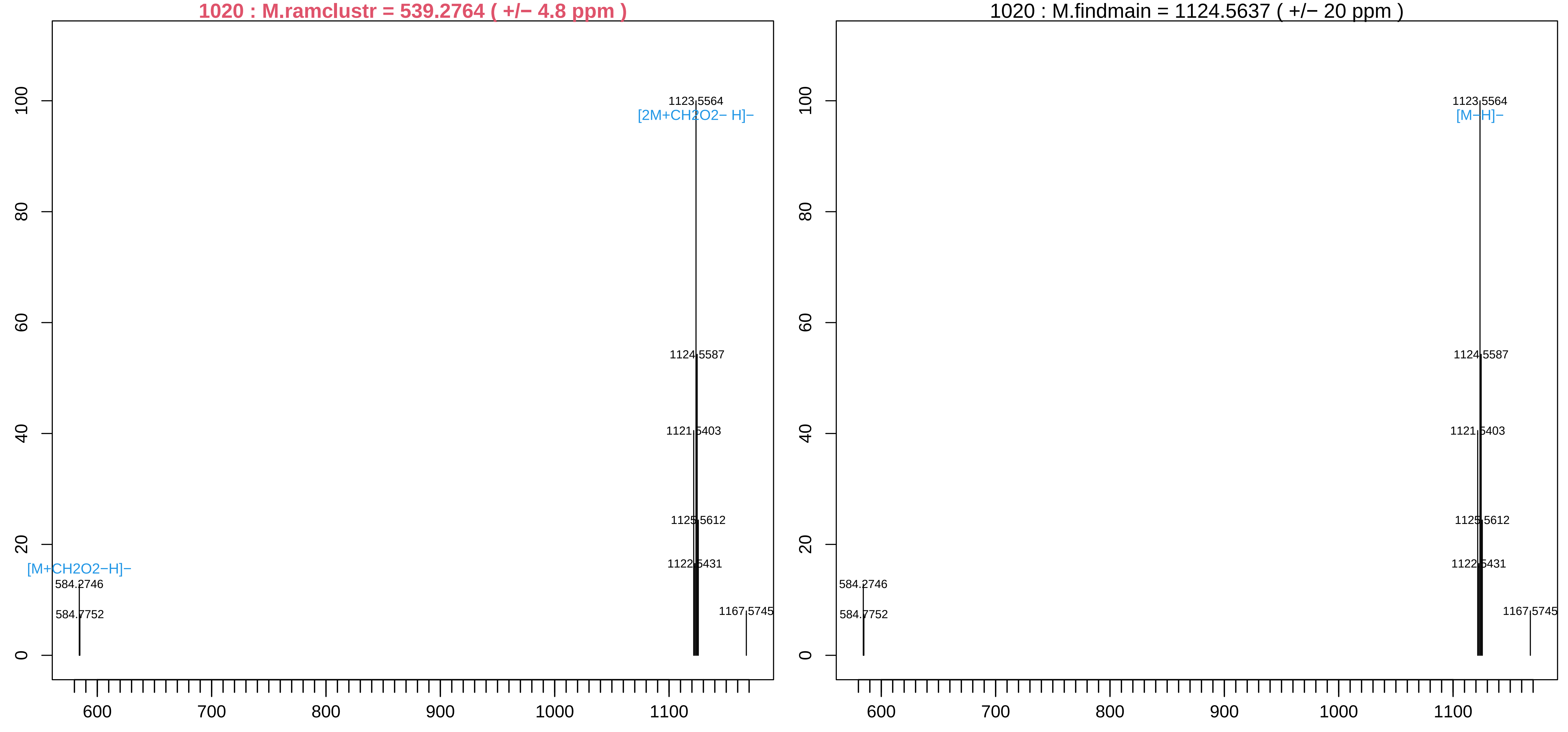
1020 : M.ramclustr = 539.2764 (+/− 4.8 ppm)
1123.5564
[2M+CH2O2− H]−
1124.5587
1121.5403
1125.5612
1122.5431
1167.5745
[M+CH2O2−H]−
584.2746
584.7752
1020 : M.findmain = 1124.5637 (+/− 20 ppm)
1123.5564
[M−H]−
1124.5587
1121.5403
1125.5612
1122.5431
1167.5745
584.2746
584.7752
0
20
40
60
80
100
600
700
800
900
1000
1100

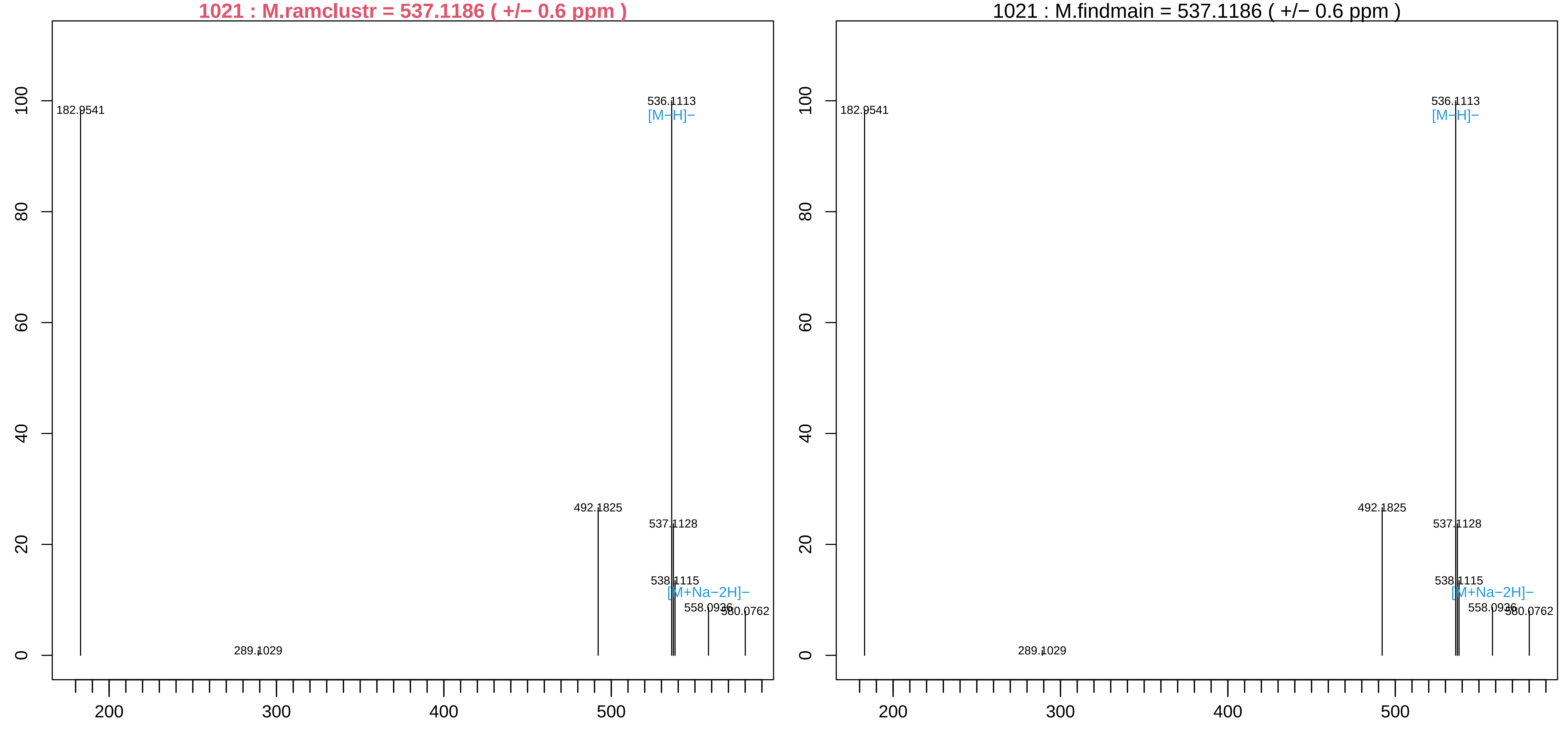
1021 : M.ramclustr = 537.1186 (+/− 0.6 ppm)
182.9541
536.1113
[M−H]−
492.1825
537.1128
538.1115
[M+Na−2H]−
558.0936
580.0762
289.1029
0
20
40
60
80
100
200
300
400
500
1021 : M.findmain = 537.1186 (+/− 0.6 ppm)
182.9541
536.1113
[M−H]−
492.1825
537.1128
538.1115
[M+Na−2H]−
558.0936
580.0762
289.1029
0
20
40
60
80
100
200
300
400
500

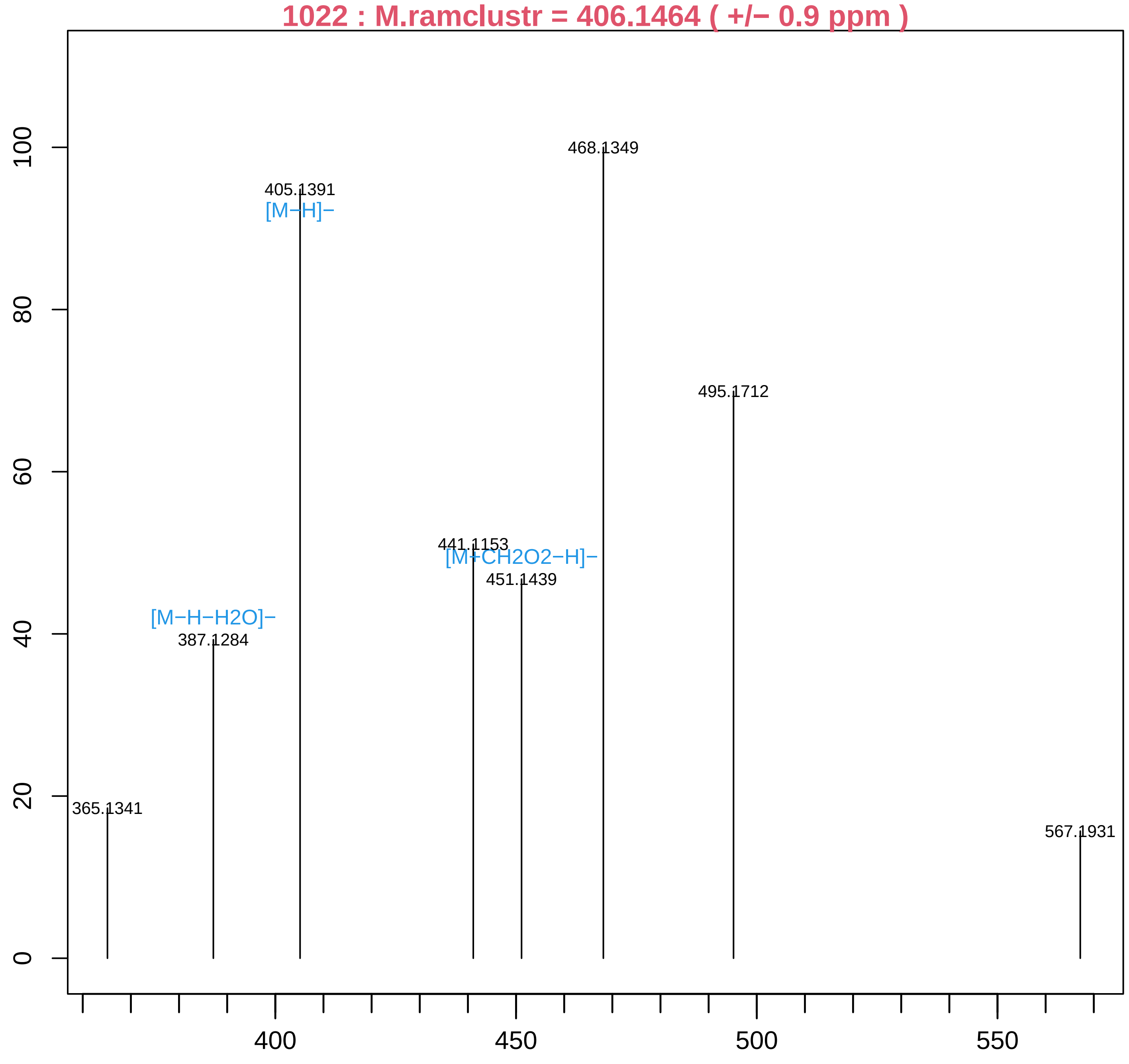
1022 : M.ramclustr = 406.1464 (+/− 0.9 ppm)
100
80
60
40
20
0
400
450
500
550
365.1341
[M−H−H2O]−
387.1284
405.1391
[M−H]−
441.1153
[M+CH2O2−H]−
451.1439
468.1349
495.1712
567.1931

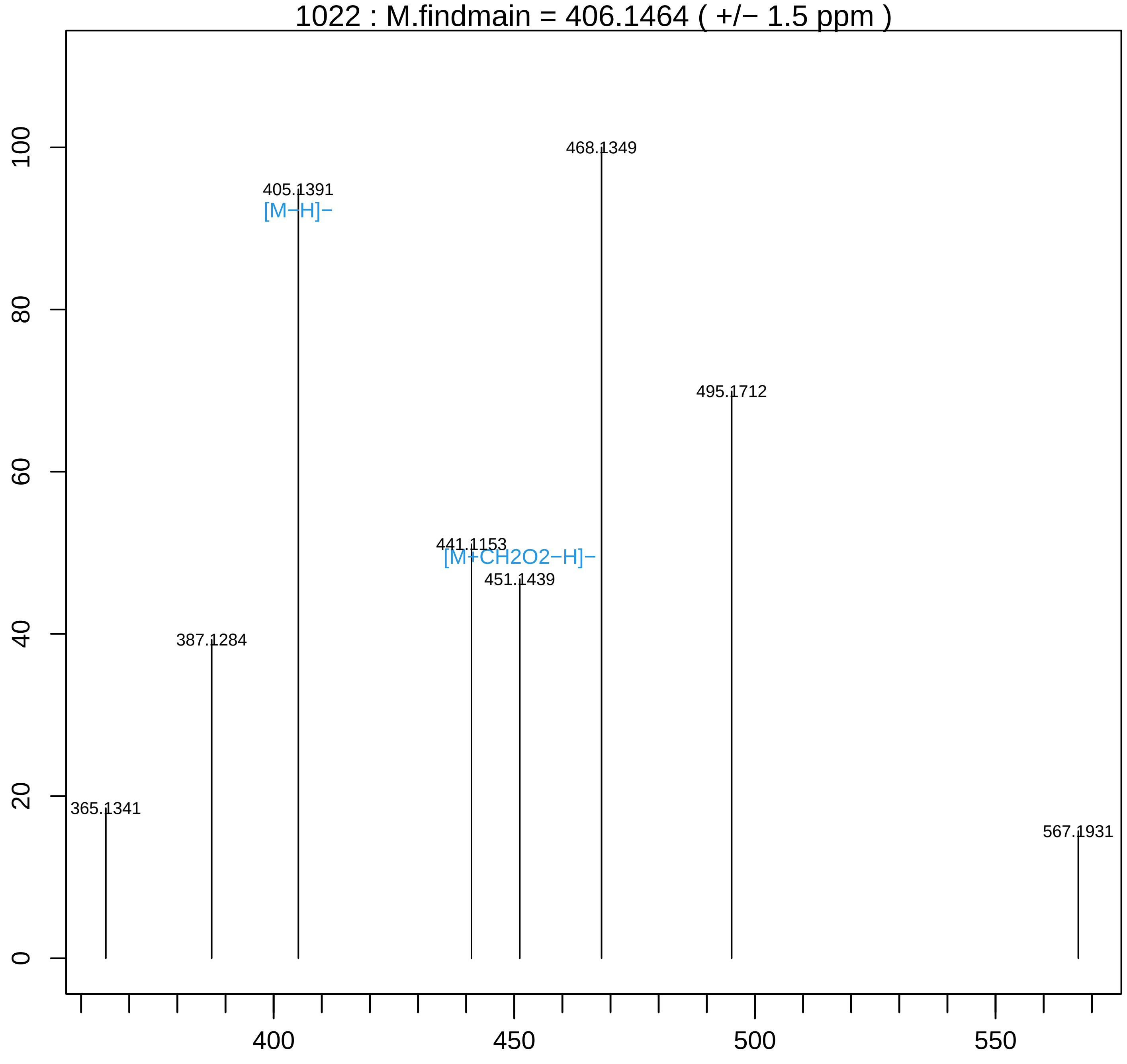
1022 : M.findmain = 406.1464 (+/− 1.5 ppm)
100
80
60
40
20
0
400
450
500
550
365.1341
387.1284
405.1391
[M−H]−
441.1153
[M+CH2O2−H]−
451.1439
468.1349
495.1712
567.1931

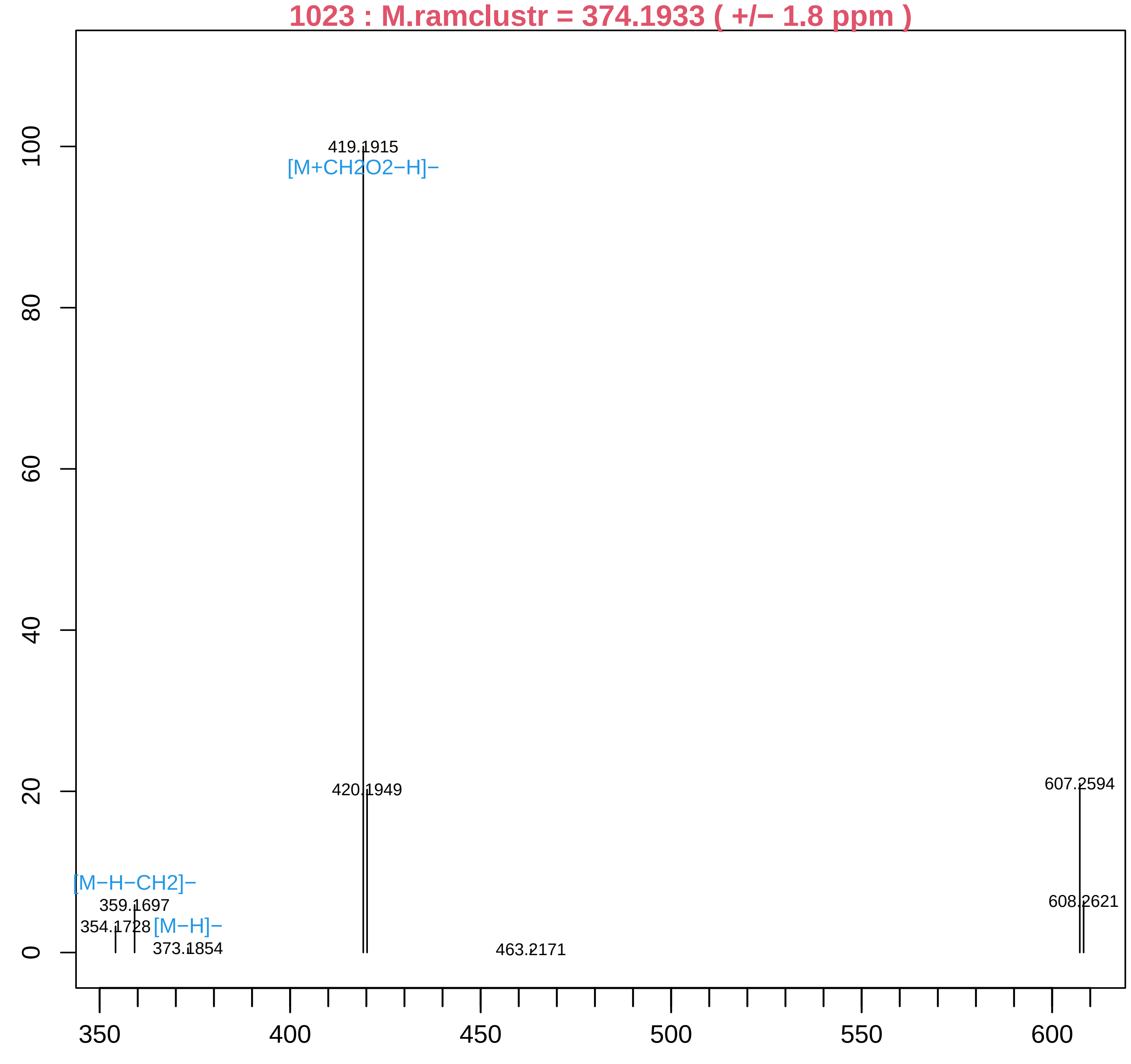
1023 : M.ramclustr = 374.1933 (+/− 1.8 ppm)
419.1915
[M+CH2O2−H]−
420.1949
607.2594
608.2621
[M−H−CH2]−
359.1697
354.1728
[M−H]−
373.1854
463.2171
0
20
40
60
80
100
350
400
450
500
550
600

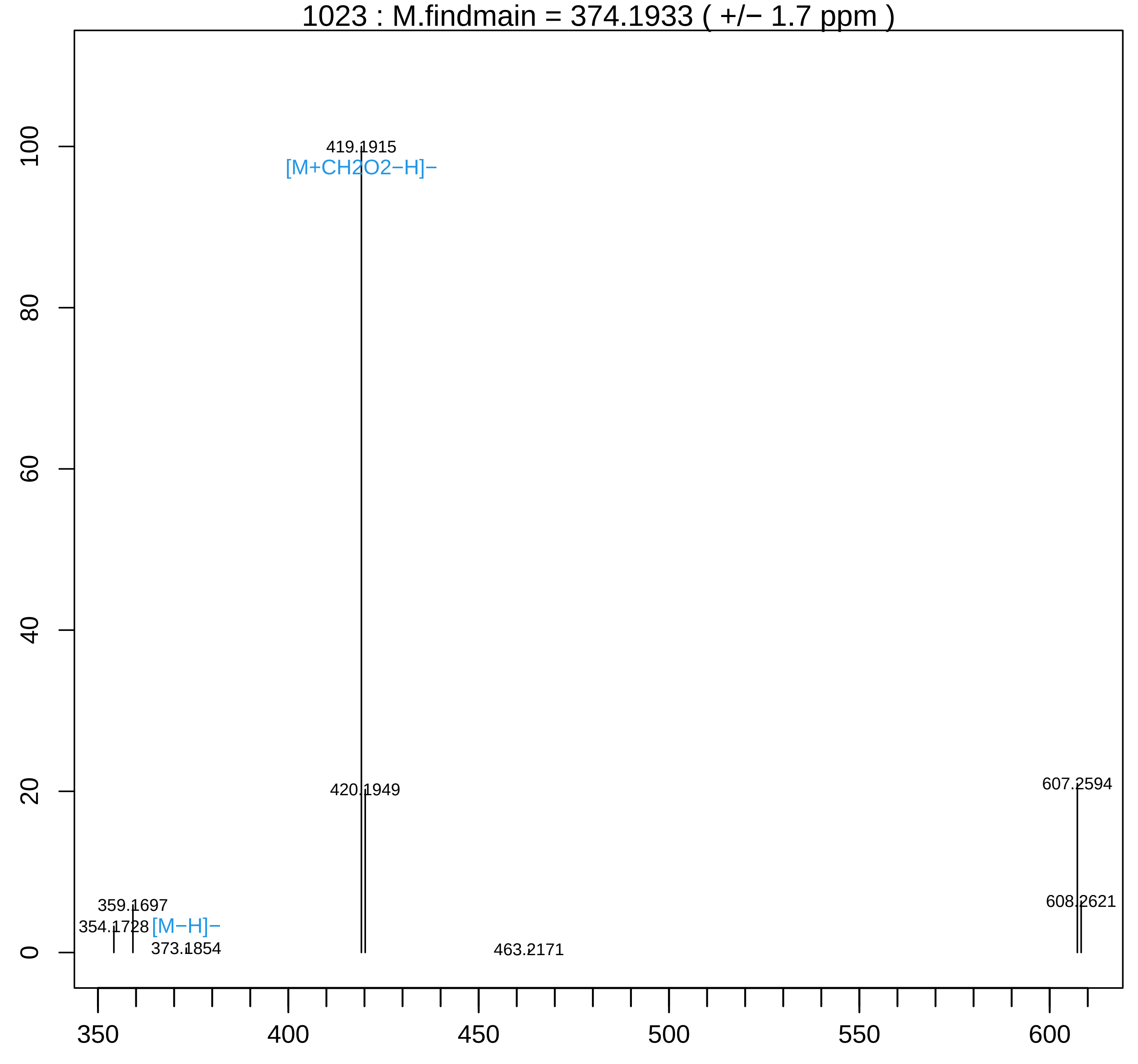
1023 : M.findmain = 374.1933 (+/− 1.7 ppm)
419.1915
[M+CH2O2−H]−
420.1949
607.2594
608.2621
359.1697
354.1728
[M−H]−
373.1854
463.2171
0
20
40
60
80
100
350
400
450
500
550
600

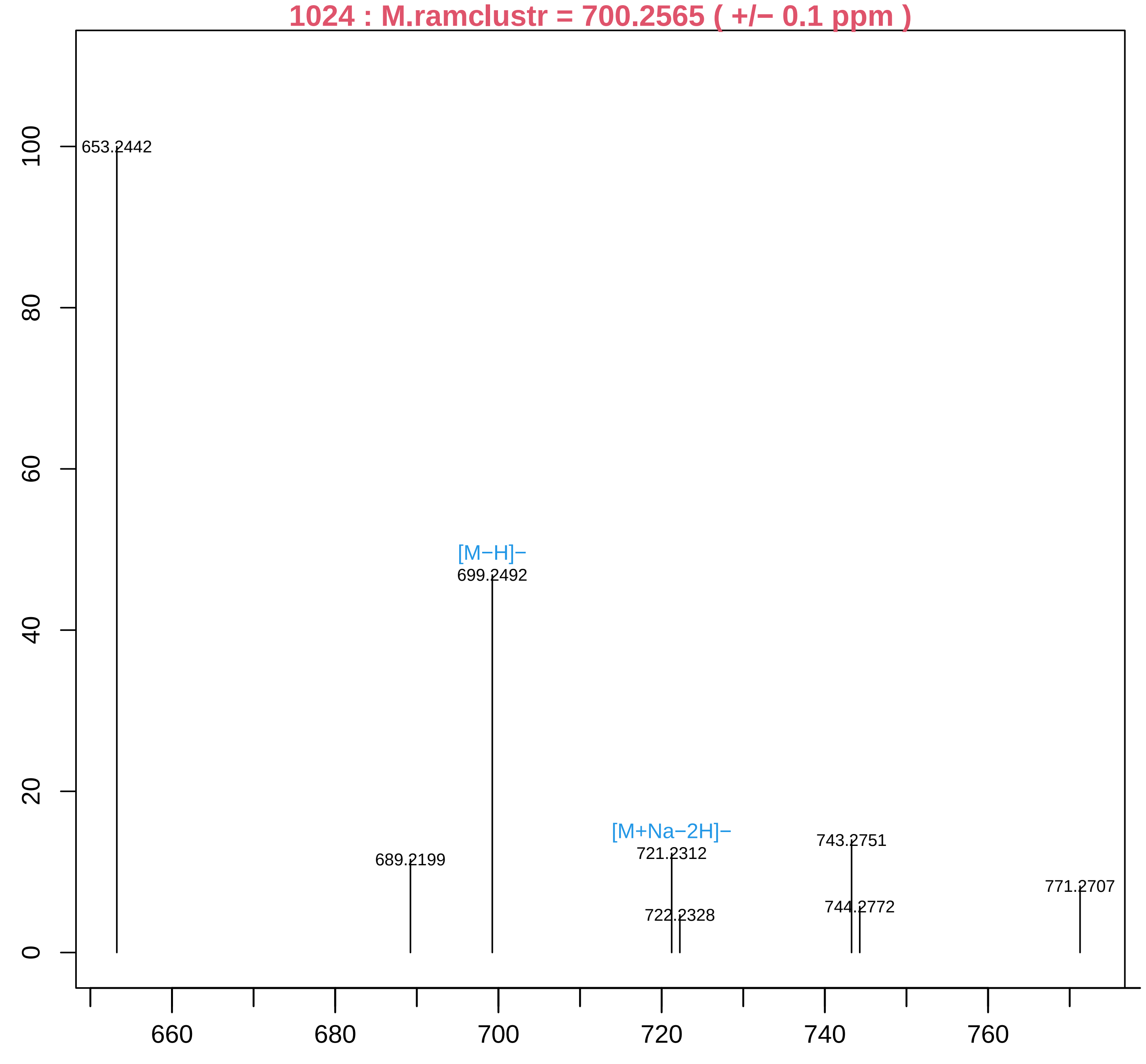

1024 : M.ramclustr = 700.2565 (+/− 0.1 ppm)
653.2442
[M−H]−
699.2492
689.2199
[M+Na−2H]−
721.2312
722.2328
743.2751
744.2772
771.2707
0
20
40
60
80
100
660
680
700
720
740
760

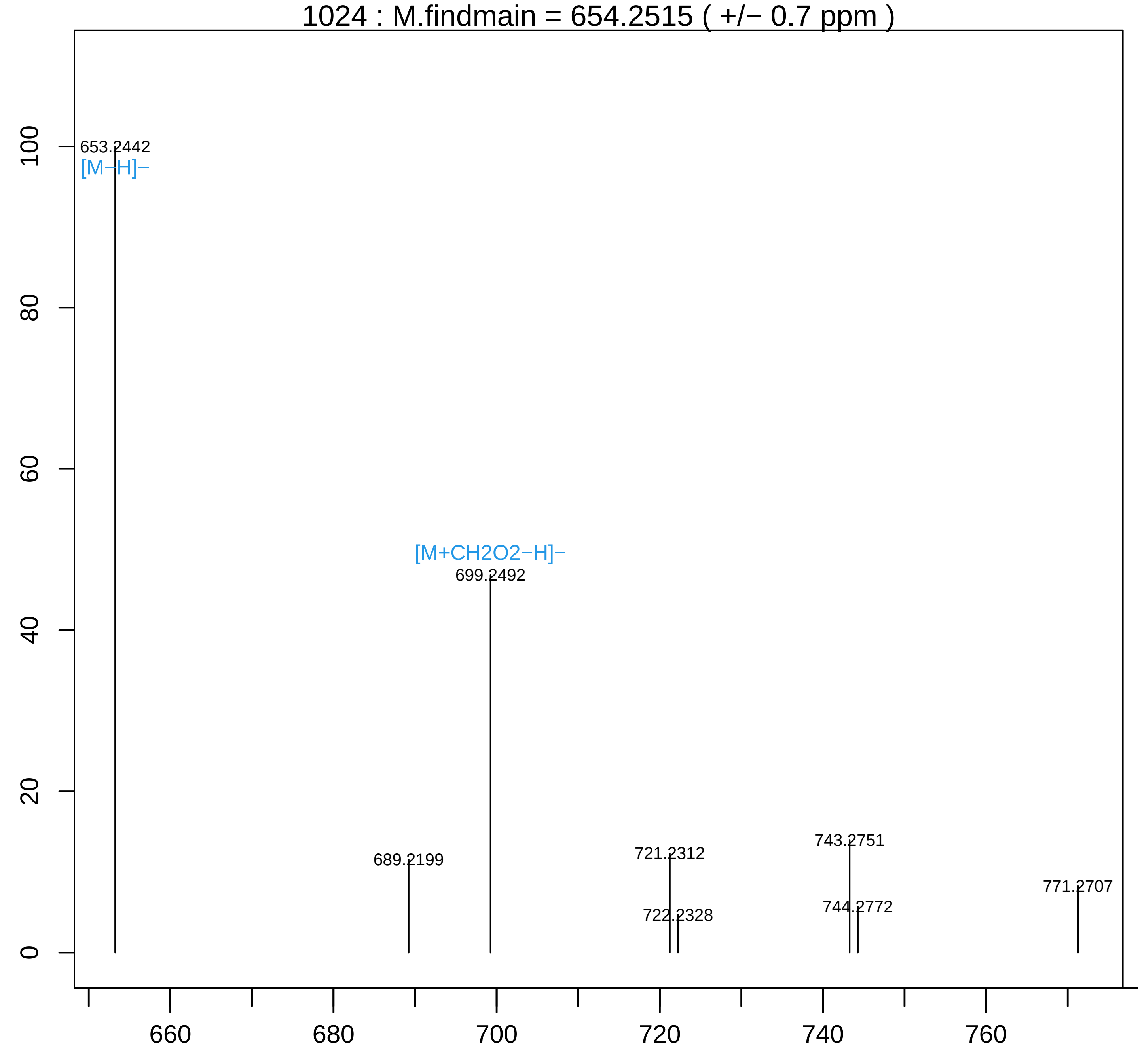

1024 : M.findmain = 654.2515 (+/− 0.7 ppm)
653.2442
[M−H]−
[M+CH2O2−H]−
699.2492
689.2199
721.2312
722.2328
743.2751
744.2772
771.2707
0
20
40
60
80
100
660
680
700
720
740
760

1025 : M.ramclustr = 491.1787 (+/− 2 ppm)

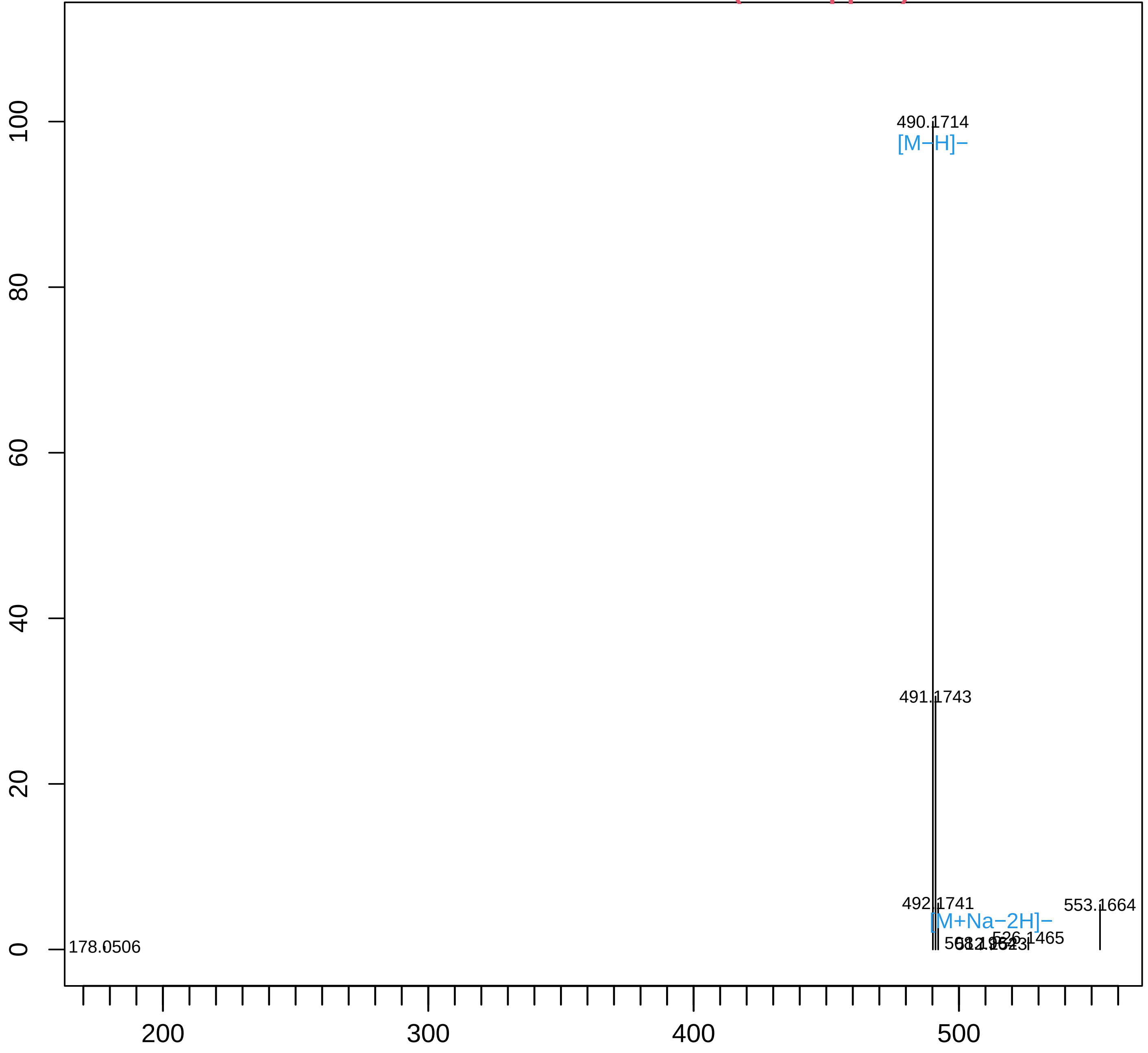

1025 : M.findmain = 491.1787 (+/− 2 ppm)

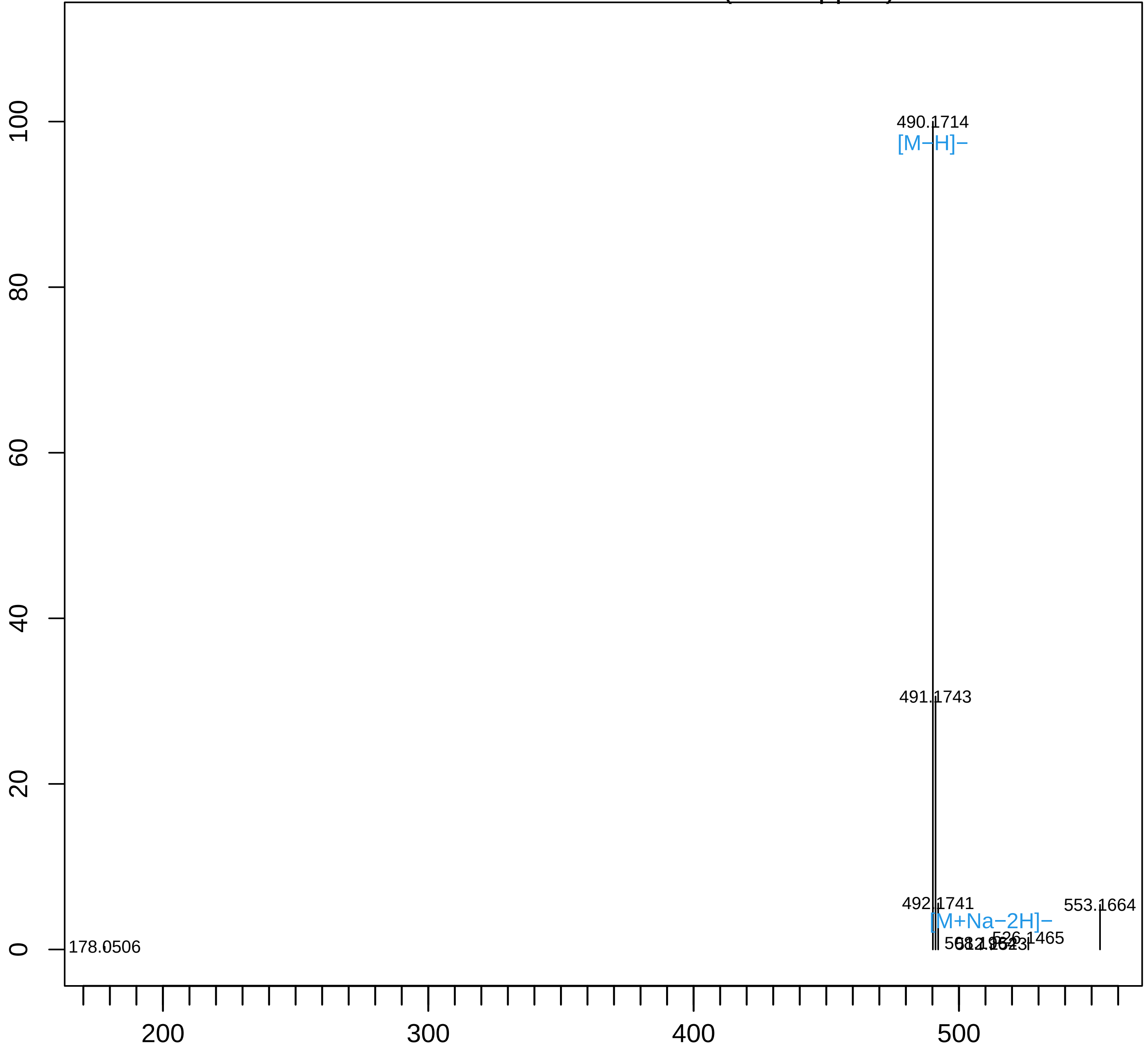

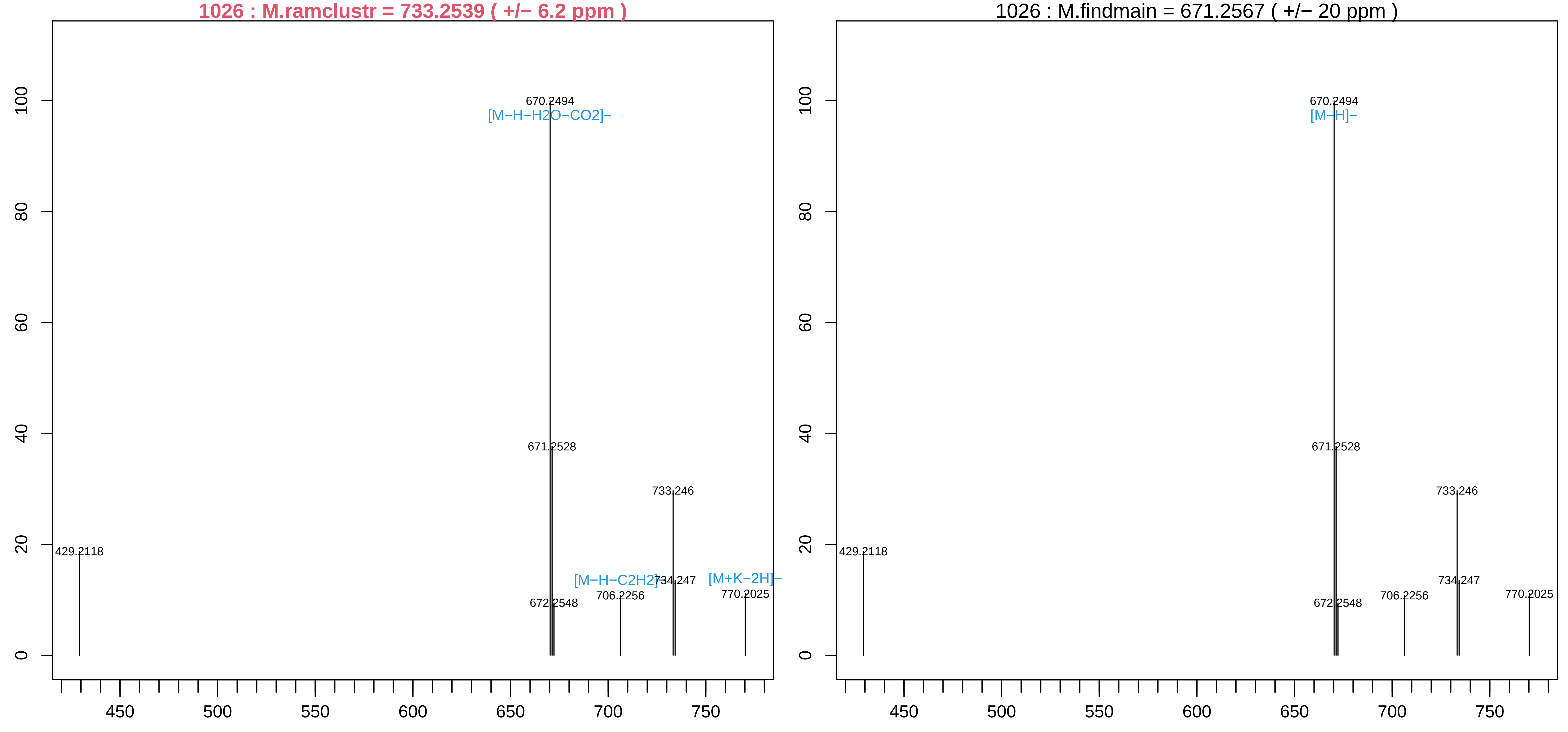
1026 : M.ramclustr = 733.2539 (+/− 6.2 ppm)
670.2494
[M−H−H2O−CO2]−
671.2528
733.246
429.2118
[M−H−C2H2]
734.247
[M+K−2H]−
770.2025
706.2256
672.2548
1026 : M.findmain = 671.2567 (+/− 20 ppm)
670.2494
[M−H]−
671.2528
733.246
429.2118
734.247
770.2025
706.2256
672.2548
0
20
40
60
80
100
450
500
550
600
650
700
750

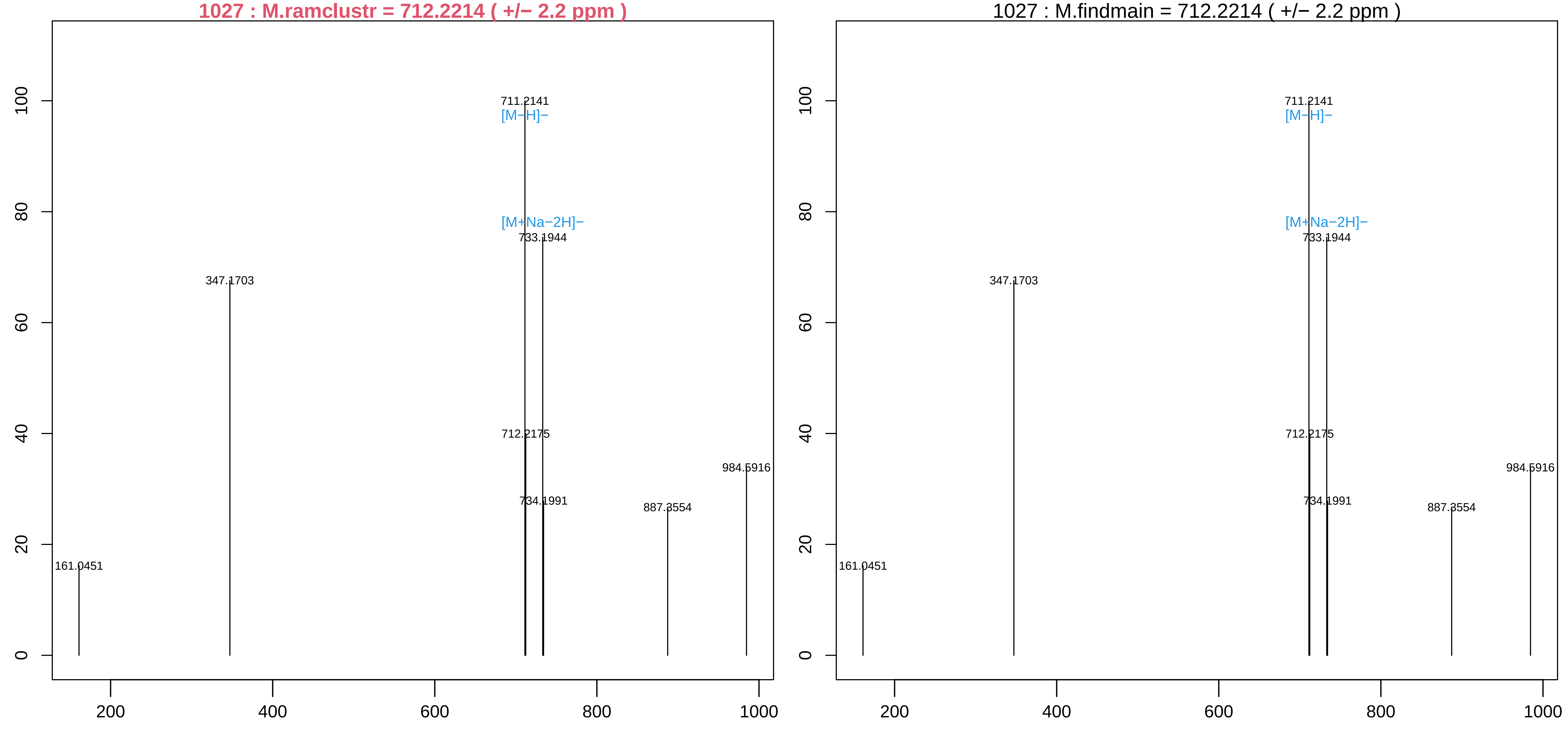
1027 : M.ramclustr = 712.2214 (+/− 2.2 ppm)
711.2141
[M−H]−
[M+Na−2H]−
733.1944
347.1703
712.2175
984.5916
734.1991
887.3554
161.0451
0
20
40
60
80
100
200
400
600
800
1000
1027 : M.findmain = 712.2214 (+/− 2.2 ppm)
711.2141
[M−H]−
[M+Na−2H]−
733.1944
347.1703
712.2175
984.5916
734.1991
887.3554
161.0451
0
20
40
60
80
100
200
400
600
800
1000

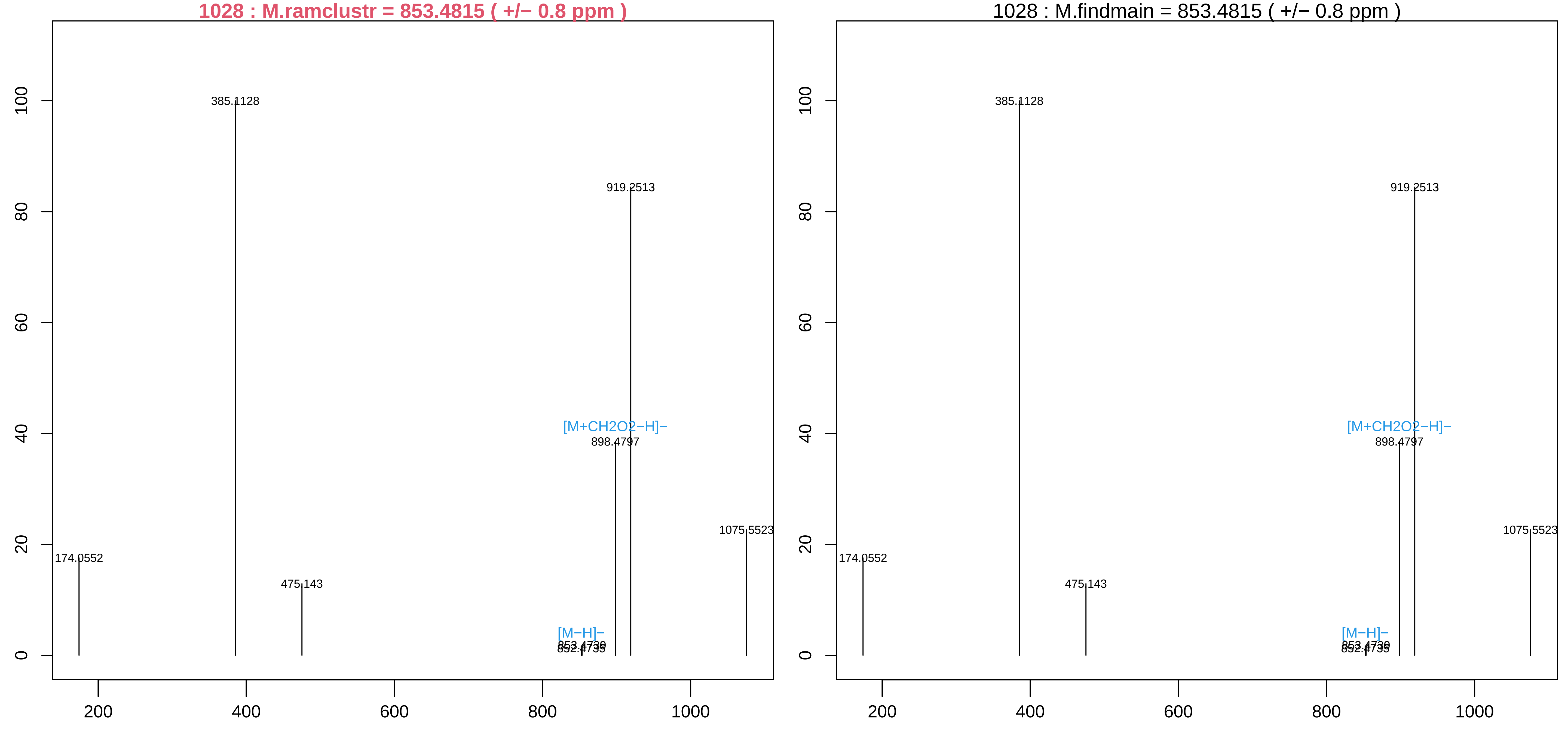
1028 : M.ramclustr = 853.4815 (+/− 0.8 ppm)
1028 : M.findmain = 853.4815 (+/− 0.8 ppm)
385.1128
919.2513
[M+CH2O2−H]−
898.4797
1075.5523
174.0552
475.143
[M−H]−
853.4739
852.4735
0
20
40
60
80
100
200
400
600
800
1000

1029 : M.ramclustr = 430.183 (+/− 1.9 ppm)

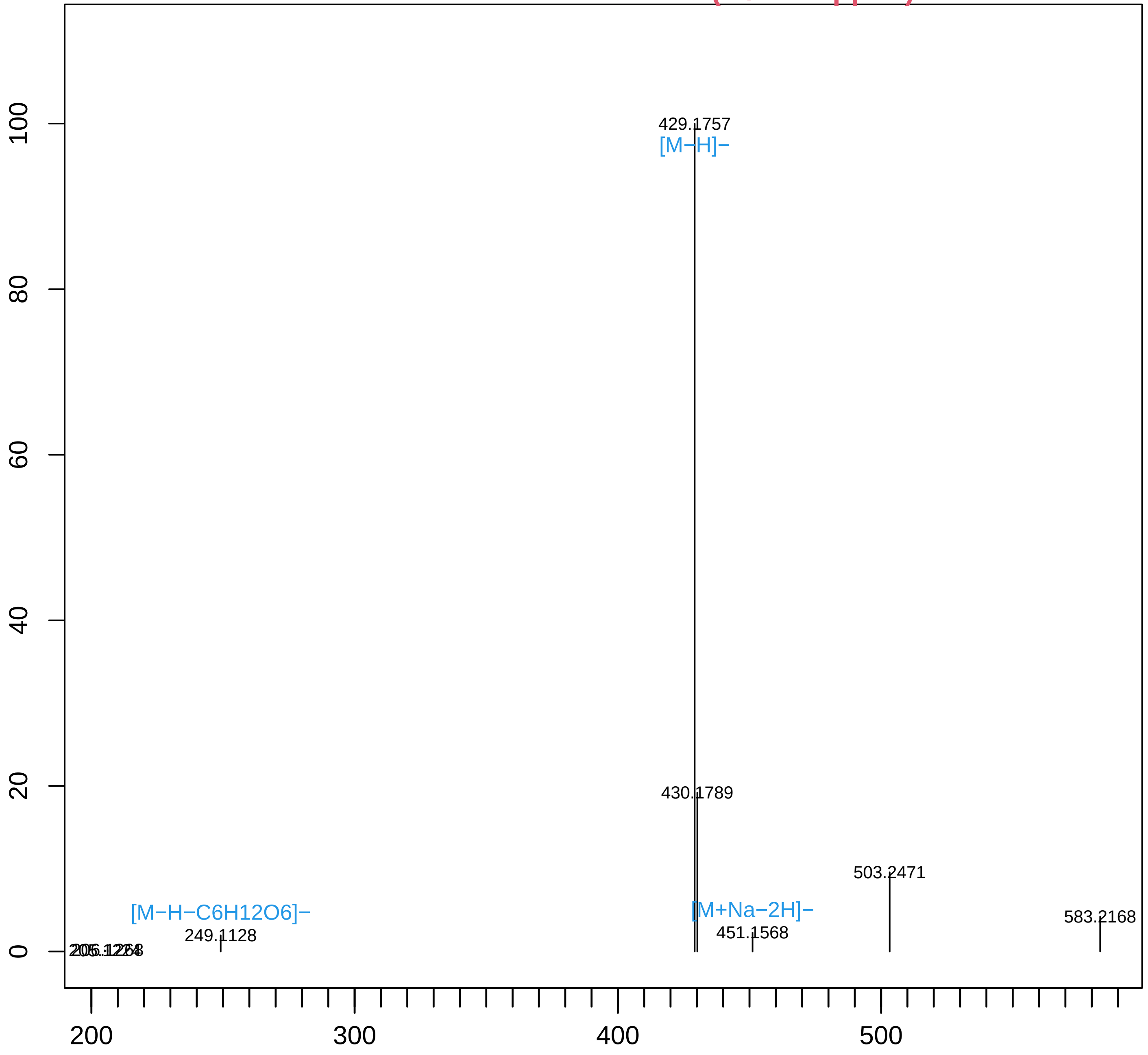

1029 : M.findmain = 430.183 (+/− 1.9 ppm)

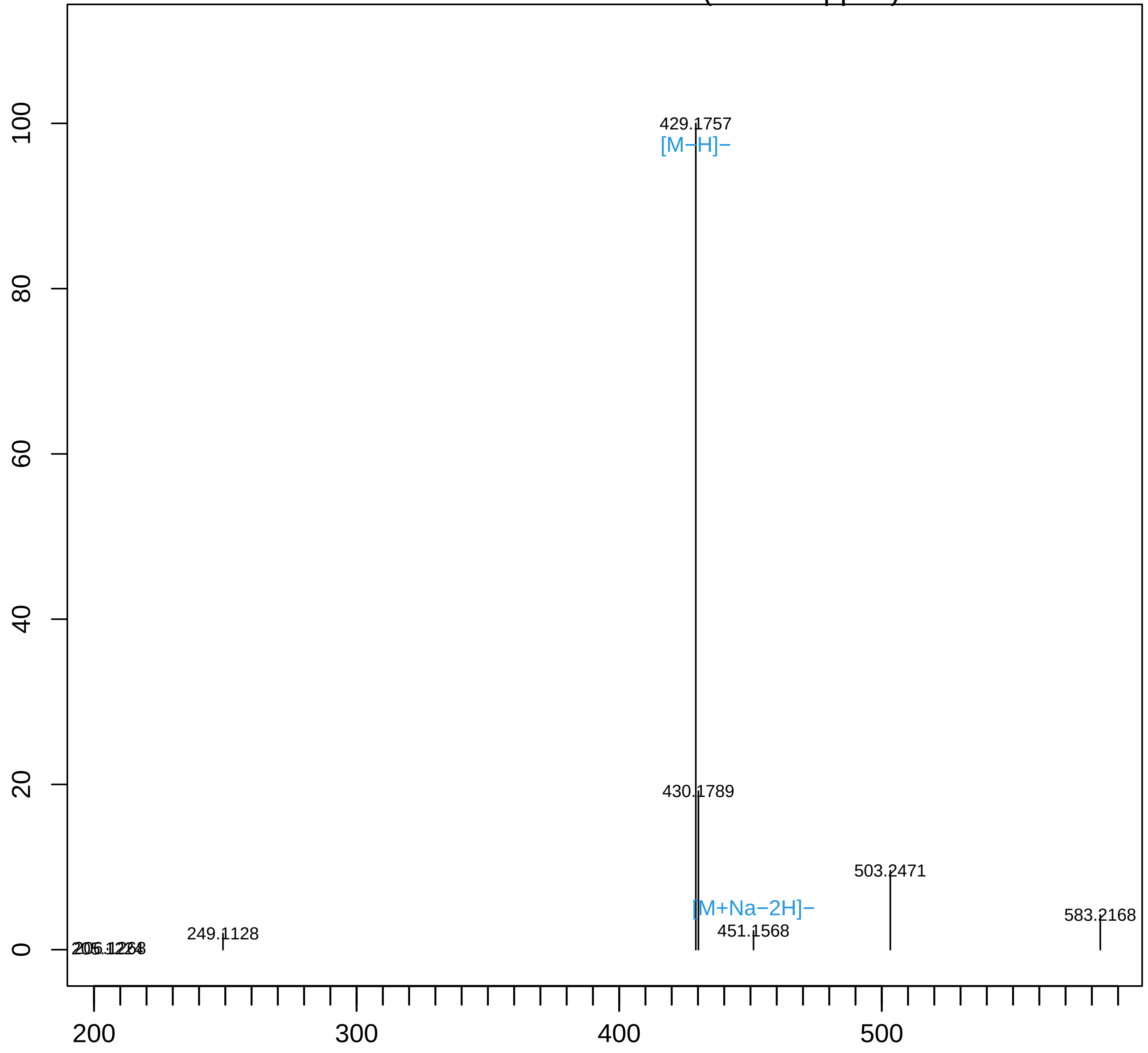

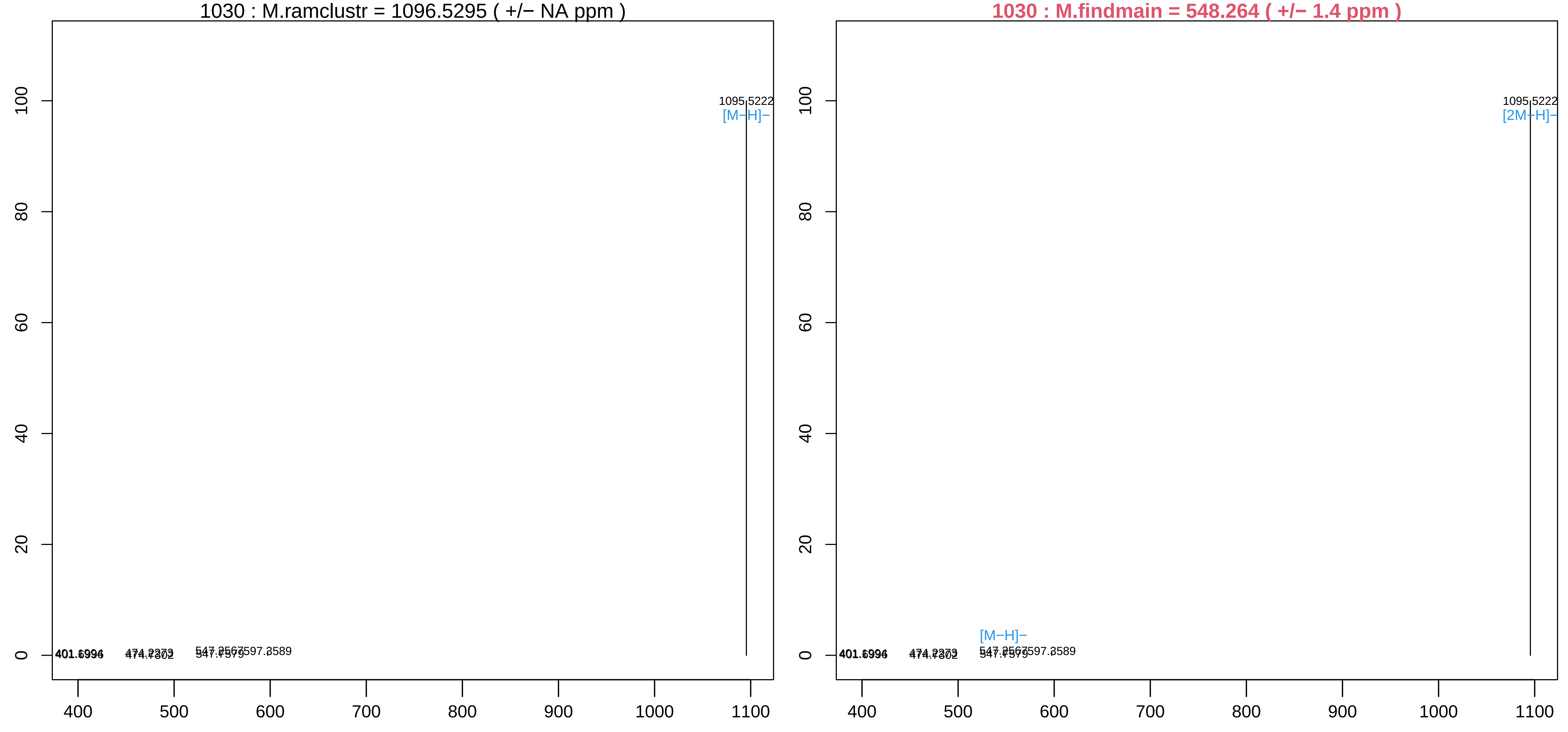
1030 : M.ramclustr = 1096.5295 (+/− NA ppm)
1095.5222
[M−H]−
401.1996
474.2273
474.7302
547.2567
597.3589
547.7575
1030 : M.findmain = 548.264 (+/− 1.4 ppm)
1095.5222
[2M−H]−
[M−H]−
401.1996
474.2273
474.7302
547.2567
597.3589
547.7575

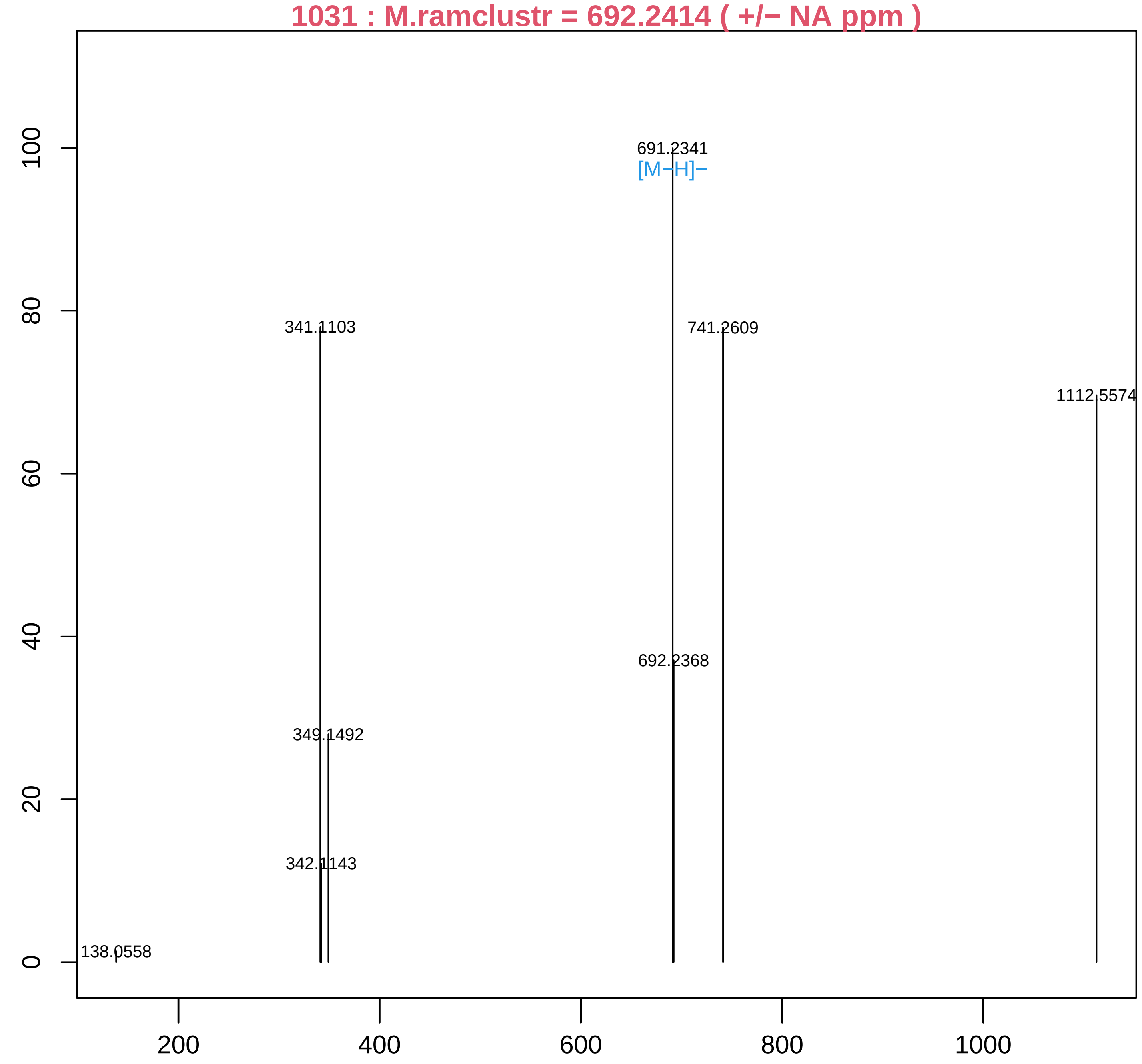
1031 : M.ramclustr = 692.2414 (+/− NA ppm)
691.2341
[M−H]−
341.1103
741.2609
1112.5574
692.2368
349.1492
342.1143
138.0558
0
20
40
60
80
100
200
400
600
800
1000

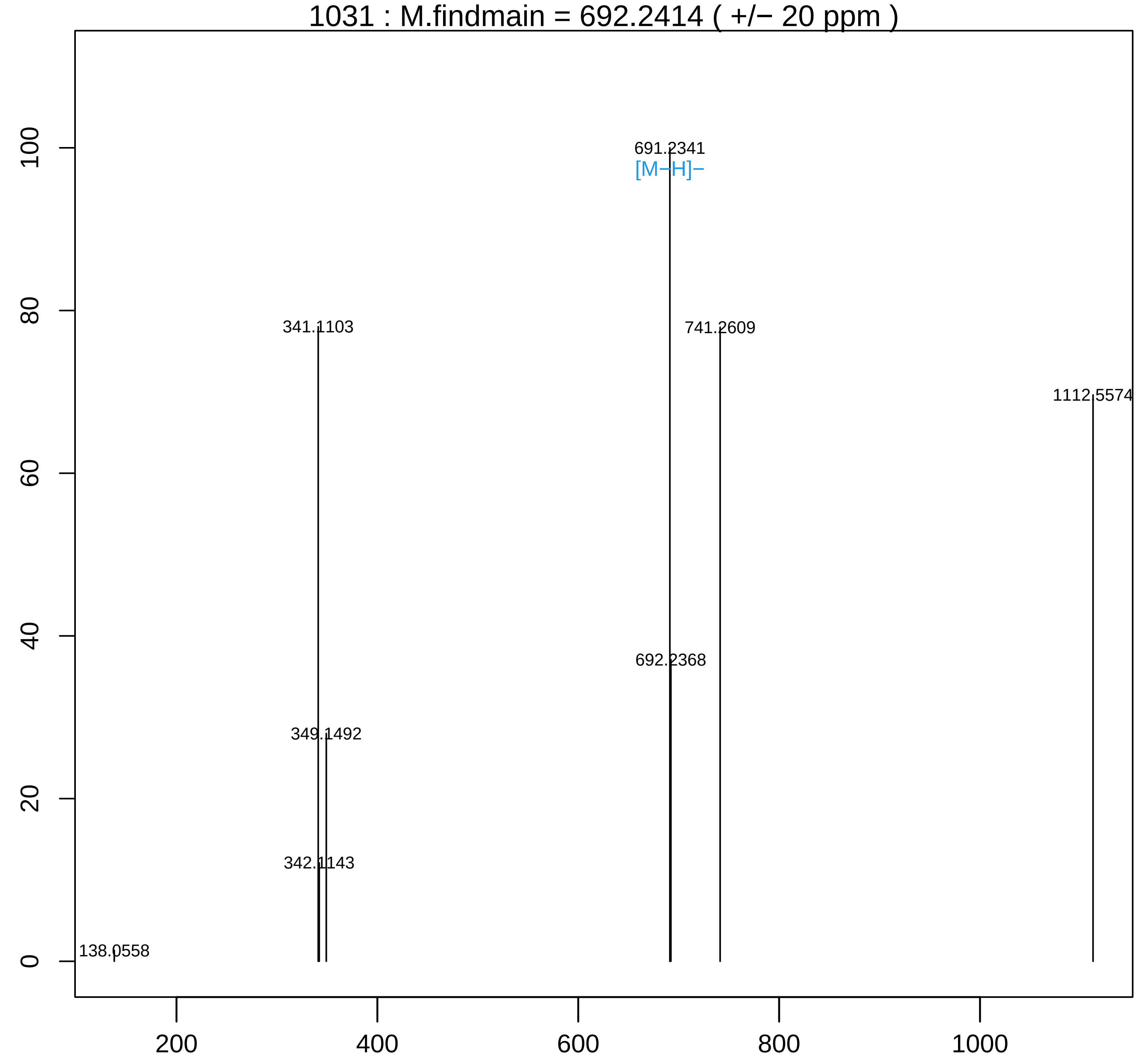
1031 : M.findmain = 692.2414 (+/− 20 ppm)
691.2341
[M−H]−
341.1103
741.2609
1112.5574
692.2368
349.1492
342.1143
138.0558
0
20
40
60
80
100
200
400
600
800
1000

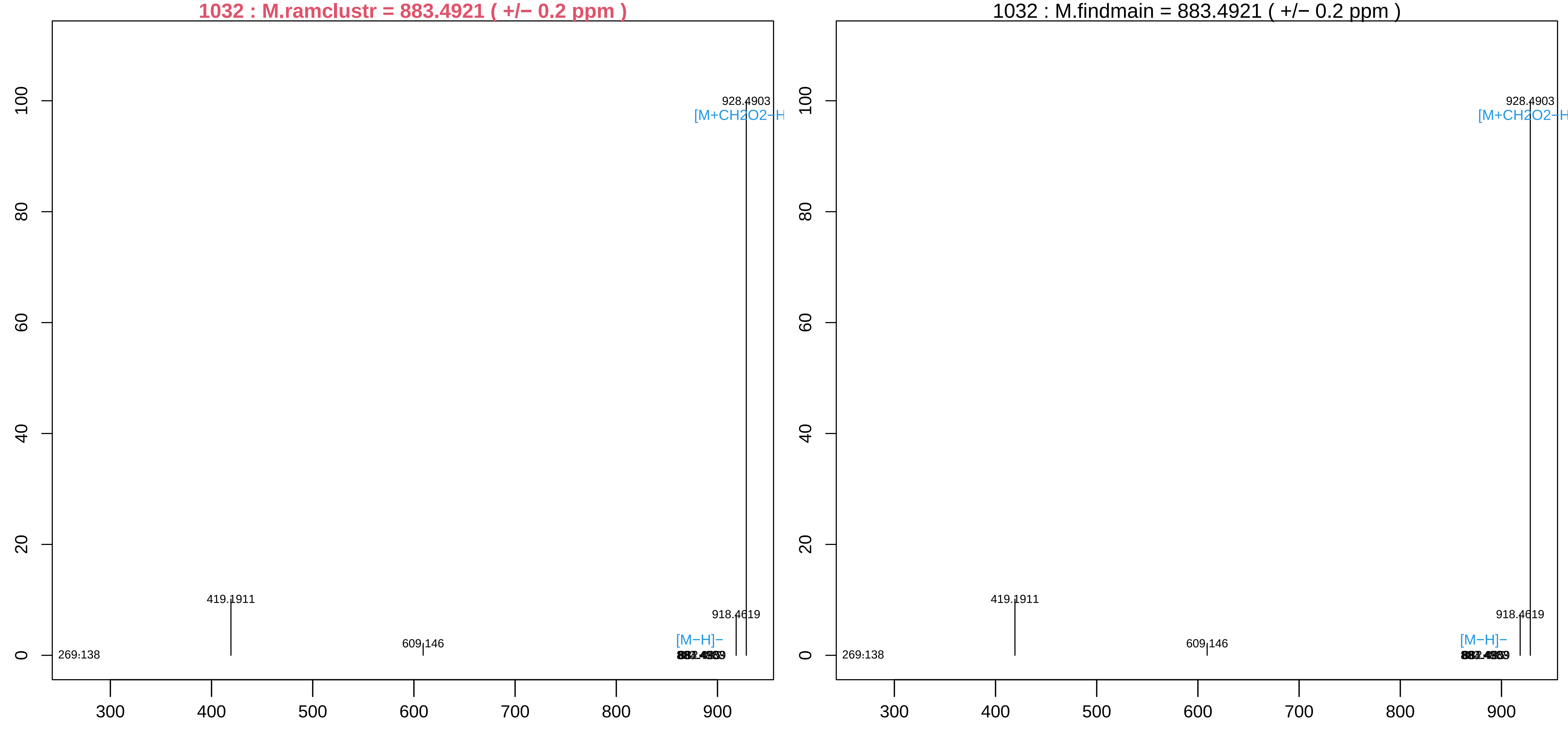
1032 : M.ramclustr = 883.4921 (+/− 0.2 ppm)
928.4903
[M+CH2O2−H
419.1911
609.146
918.4619
[M−H]−
269.138
1032 : M.findmain = 883.4921 (+/− 0.2 ppm)
928.4903
[M+CH2O2−H
419.1911
609.146
918.4619
[M−H]−
269.138

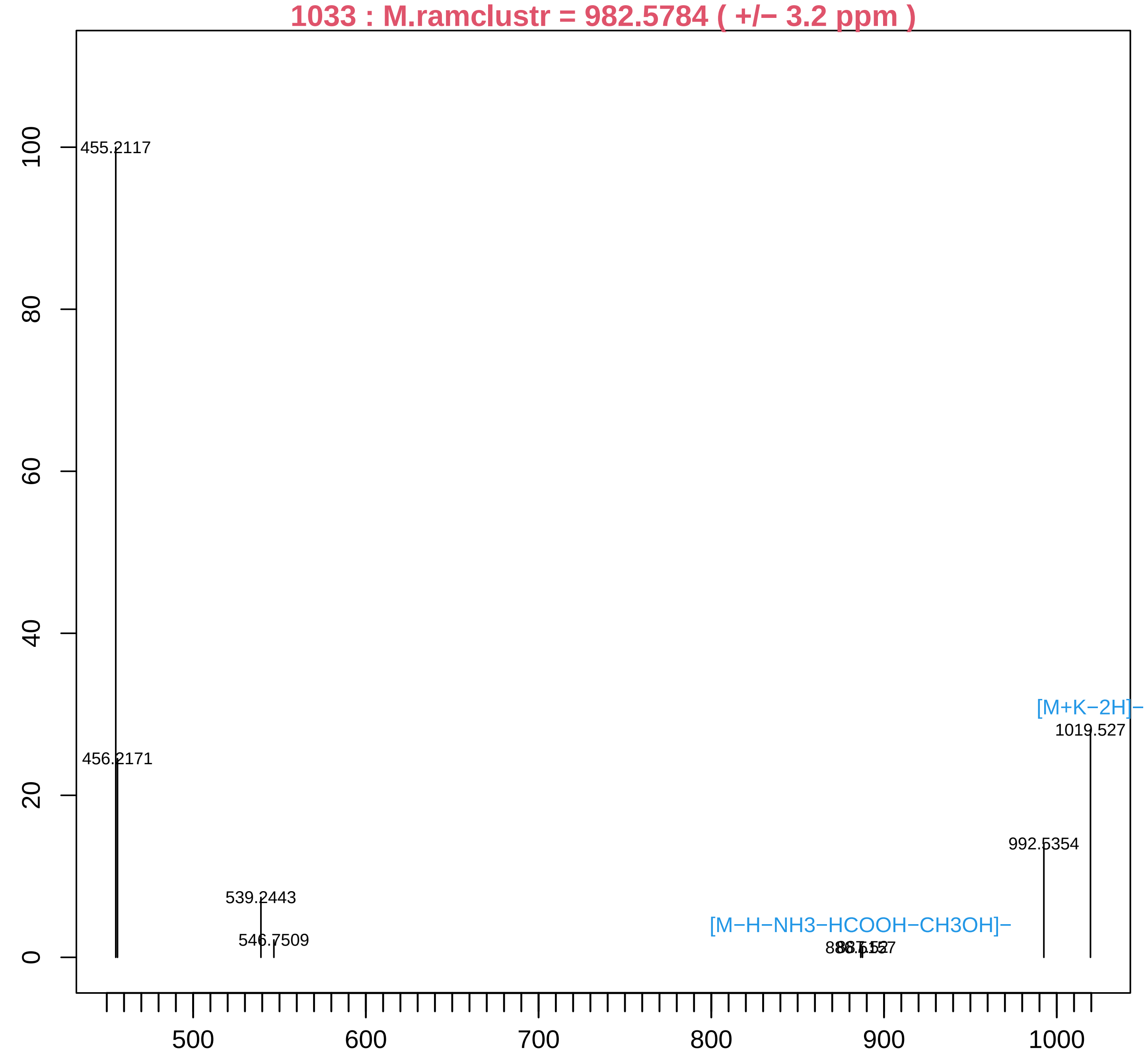
1033 : M.ramclustr = 982.5784 (+/− 3.2 ppm)
455.2117
456.2171
539.2443
546.7509
[M−H−NH3−HCOOH−CH3OH]−
992.5354
[M+K−2H]−
1019.527
0
20
40
60
80
100
500
600
700
800
900
1000

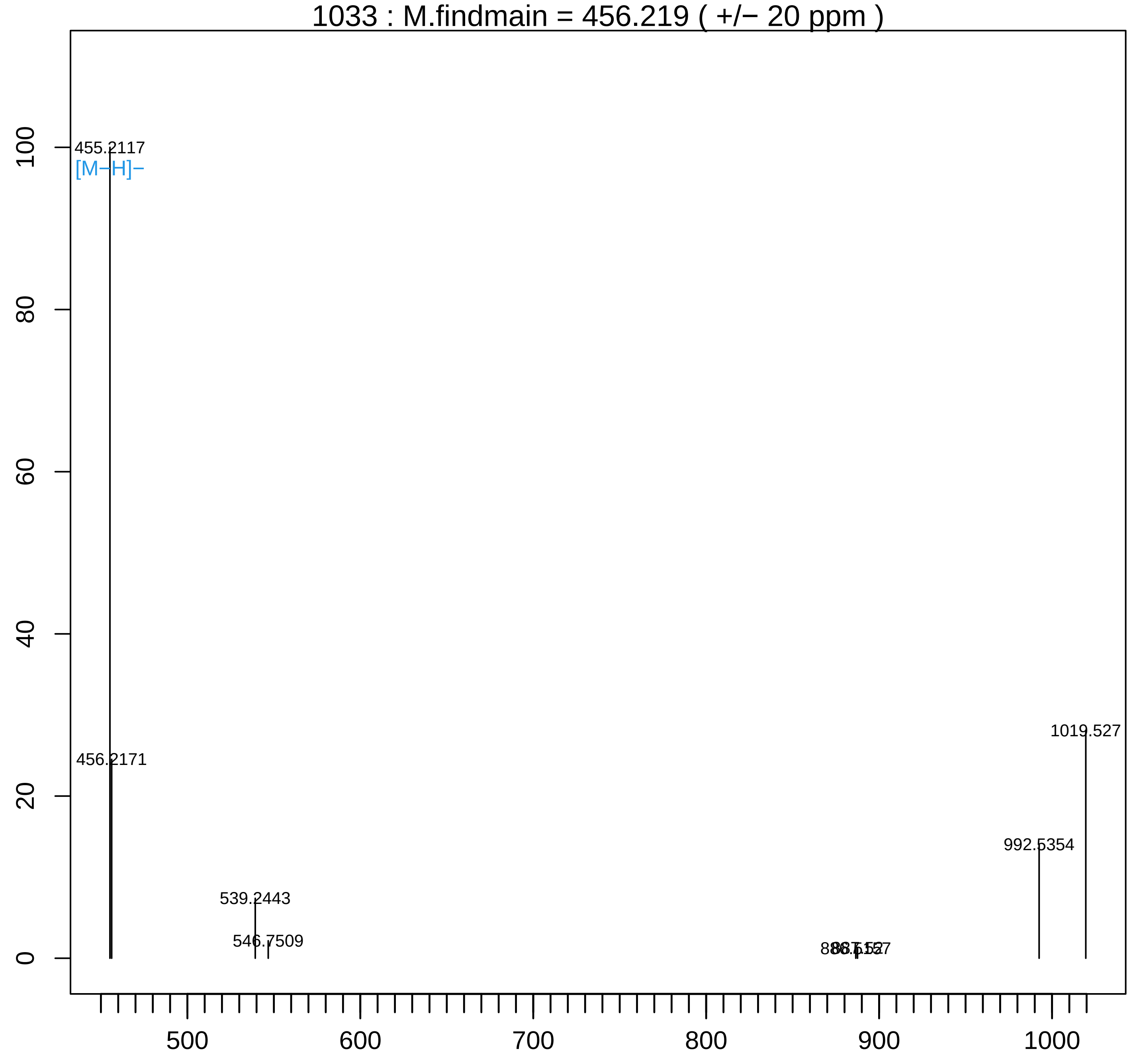
1033 : M.findmain = 456.219 (+/− 20 ppm)
455.2117
[M−H]−
456.2171
539.2443
546.7509
992.5354
1019.527
0
20
40
60
80
100
500
600
700
800
900
1000

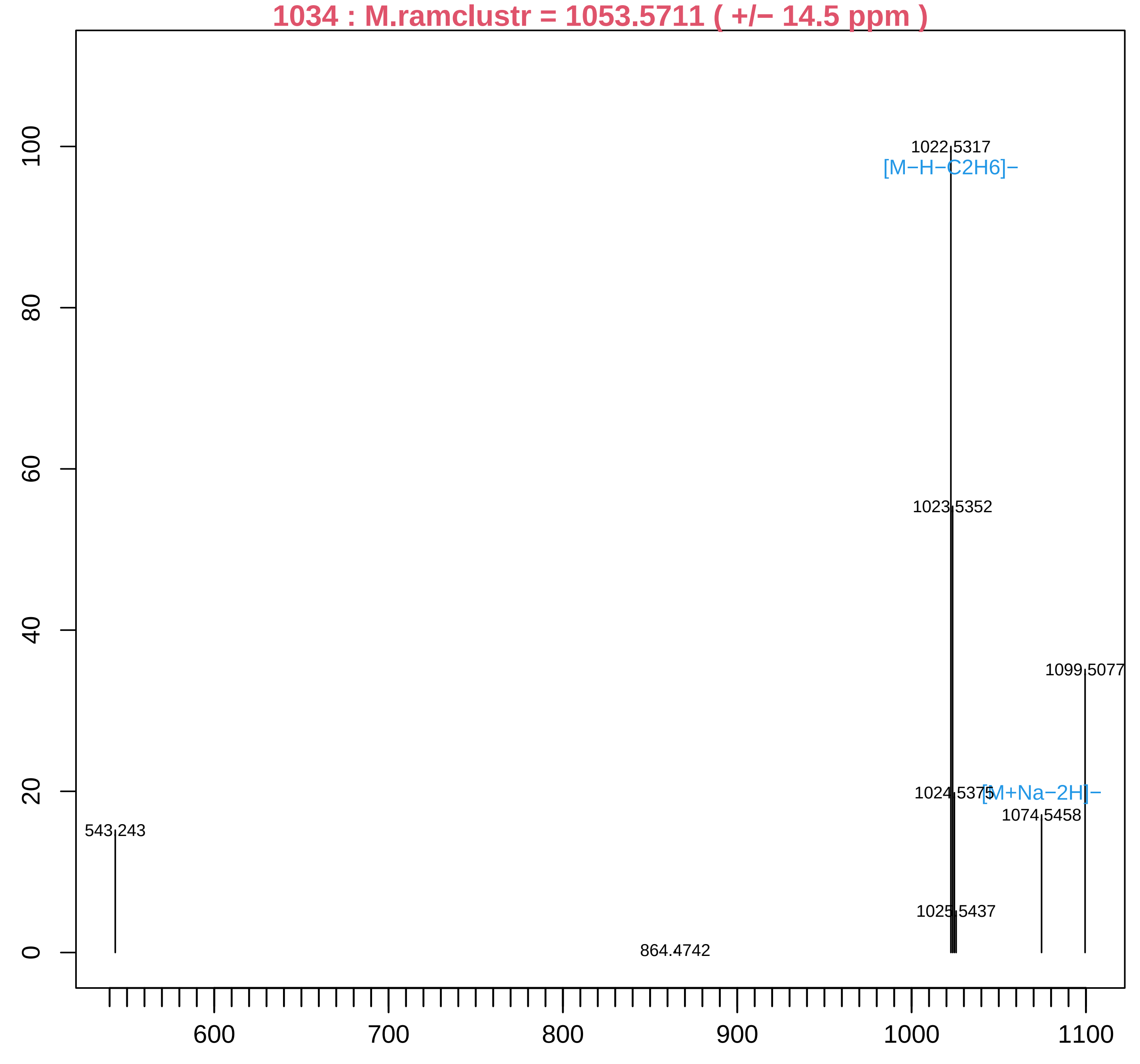
1034 : M.ramclustr = 1053.5711 (+/− 14.5 ppm)
1022.5317
[M−H−C2H6]−
1023.5352
1099.5077
1024.5375
[M+Na−2H]−
1074.5458
543.243
1025.5437
864.4742
0
20
40
60
80
100
600
700
800
900
1000
1100

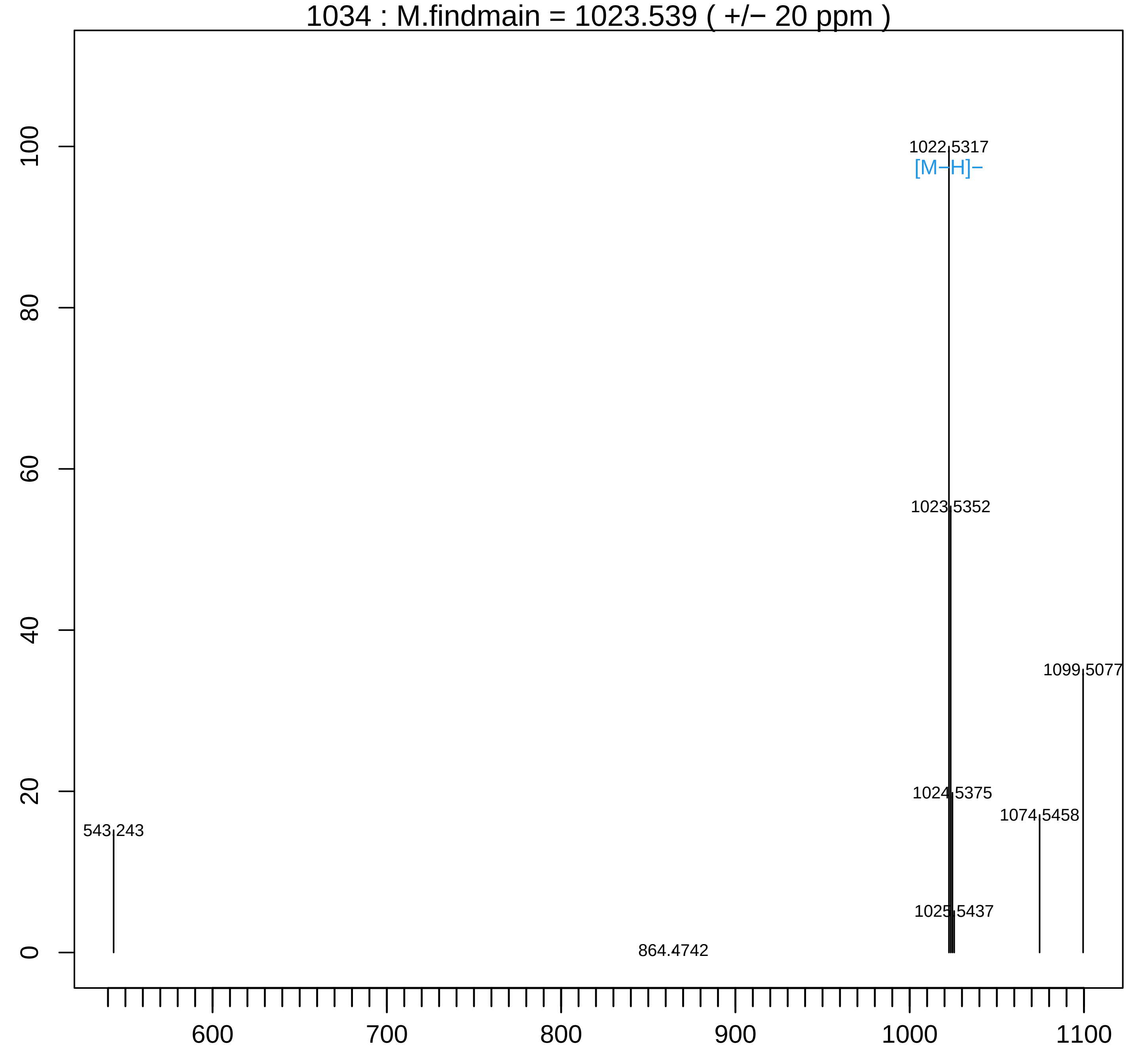
1034 : M.findmain = 1023.539 (+/− 20 ppm)
1022.5317
[M−H]−
1023.5352
1099.5077
1024.5375
1074.5458
543.243
1025.5437
864.4742
0
20
40
60
80
100
600
700
800
900
1000
1100

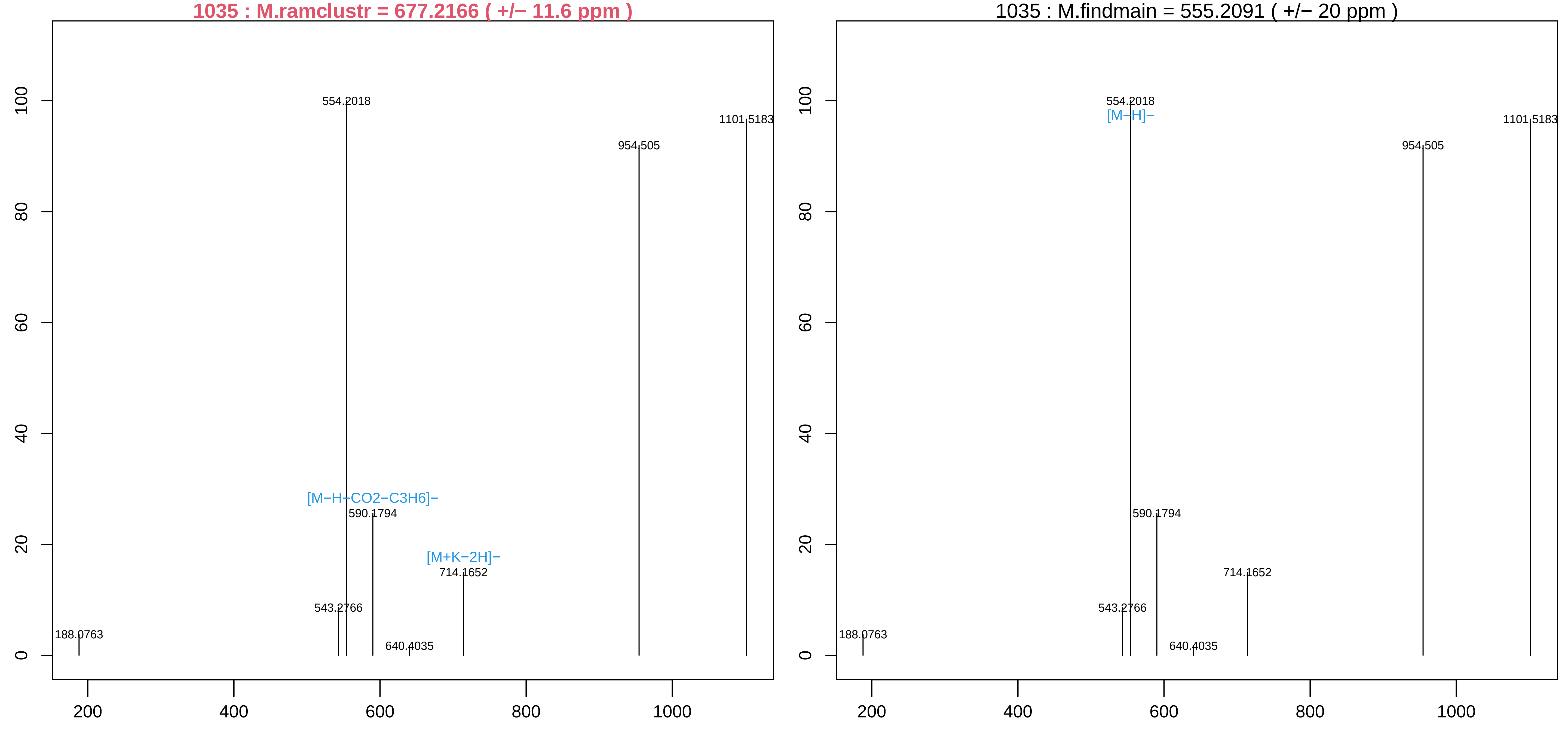

1035 : M.ramclustr = 677.2166 (+/− 11.6 ppm)
554.2018
1101.5183
954.505
[M−H−CO2−C3H6]−
590.1794
[M+K−2H]−
714.1652
543.2766
188.0763
640.4035
0
20
40
60
80
100
200
400
600
800
1000
1035 : M.findmain = 555.2091 (+/− 20 ppm)
554.2018
[M−H]−
1101.5183
954.505
590.1794
714.1652
543.2766
188.0763
640.4035

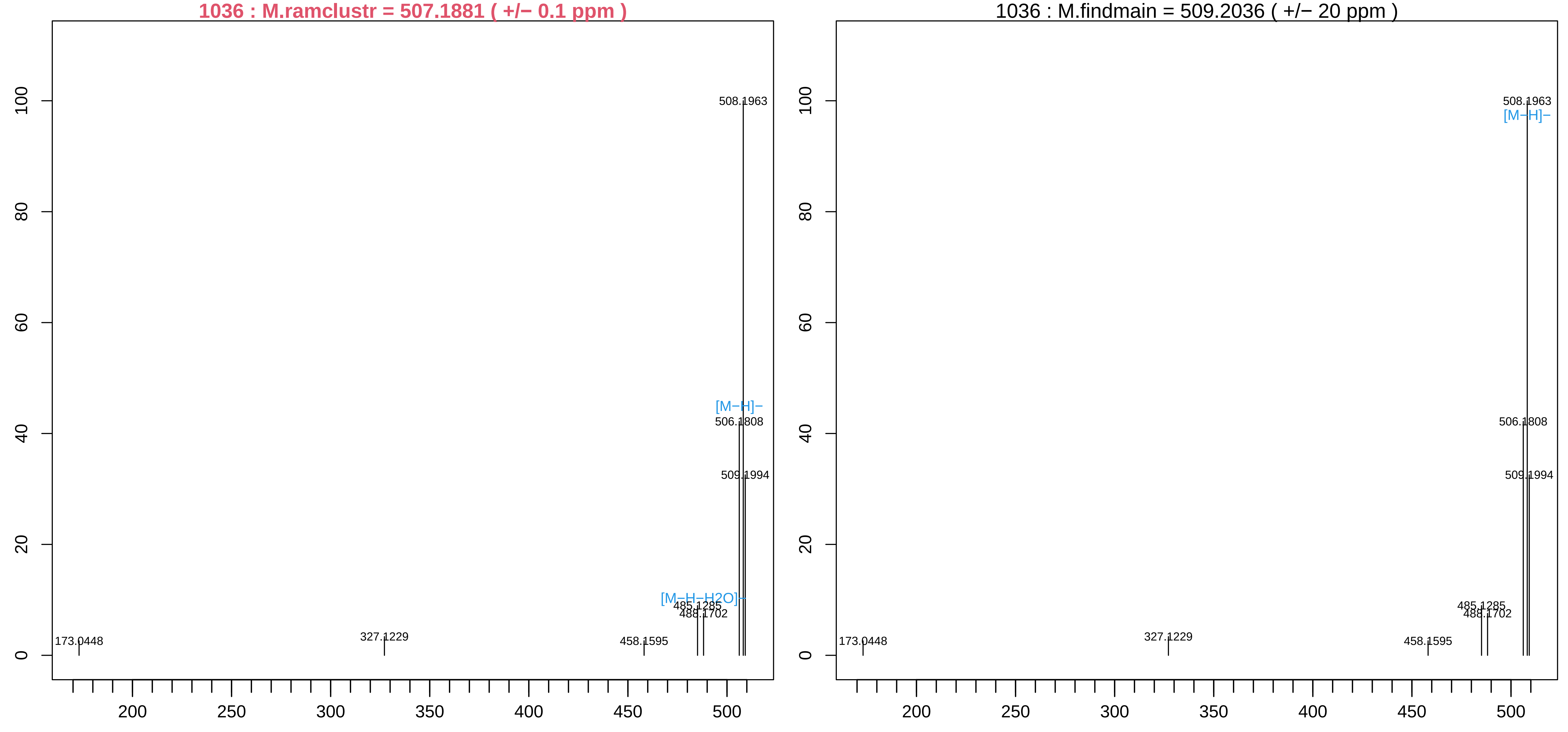

1036 : M.ramclustr = 507.1881 (+/− 0.1 ppm)
508.1963
[M−H]−
506.1808
509.1994
[M−H−H2O]−
485.1285
488.1702
173.0448
327.1229
458.1595
0
20
40
60
80
100
200
250
300
350
400
450
500
1036 : M.findmain = 509.2036 (+/− 20 ppm)
508.1963
[M−H]−
506.1808
509.1994
485.1285
488.1702
173.0448
327.1229
458.1595
0
20
40
60
80
100
200
250
300
350
400
450
500

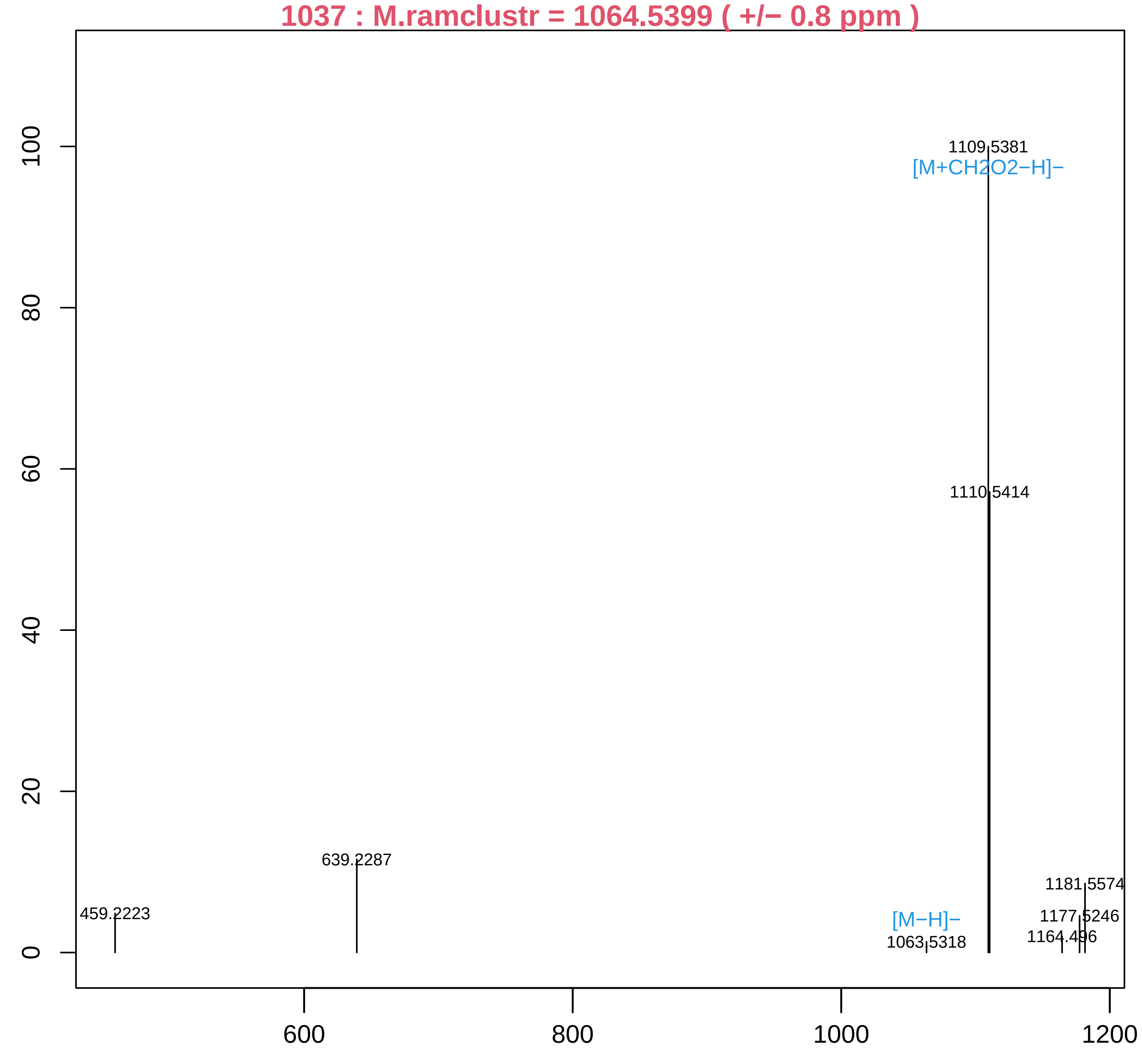

1037 : M.ramclustr = 1064.5399 (+/− 0.8 ppm)
1109.5381
[M+CH2O2−H]−
1110.5414
639.2287
459.2223
[M−H]−
1063.5318
1181.5574
1177.5246
1164.496
0
20
40
60
80
100
600
800
1000
1200

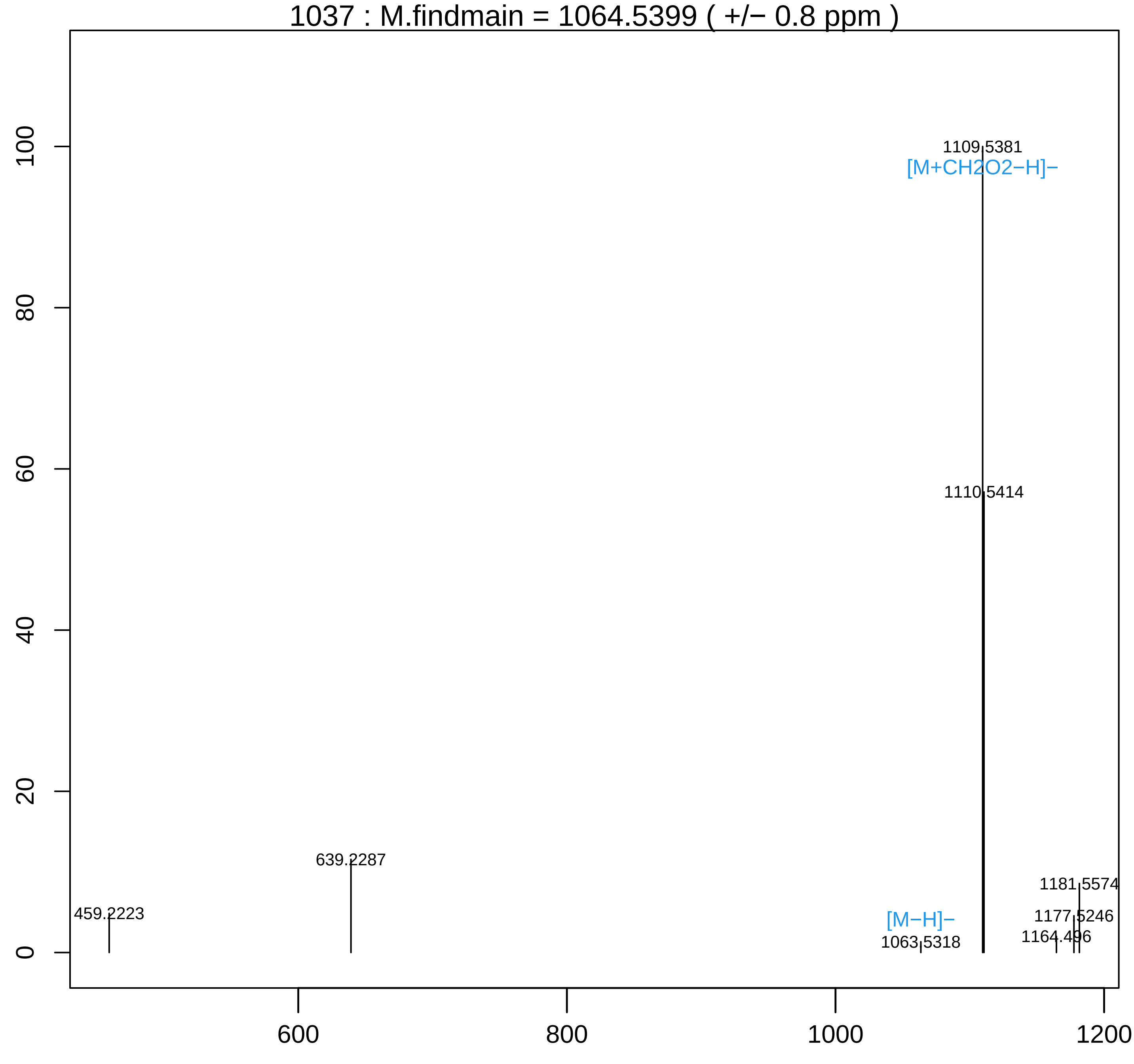

1037 : M.findmain = 1064.5399 (+/− 0.8 ppm)
1109.5381
[M+CH2O2−H]−
1110.5414
639.2287
459.2223
[M−H]−
1063.5318
1181.5574
1177.5246
1164.496
0
20
40
60
80
100
600
800
1000
1200

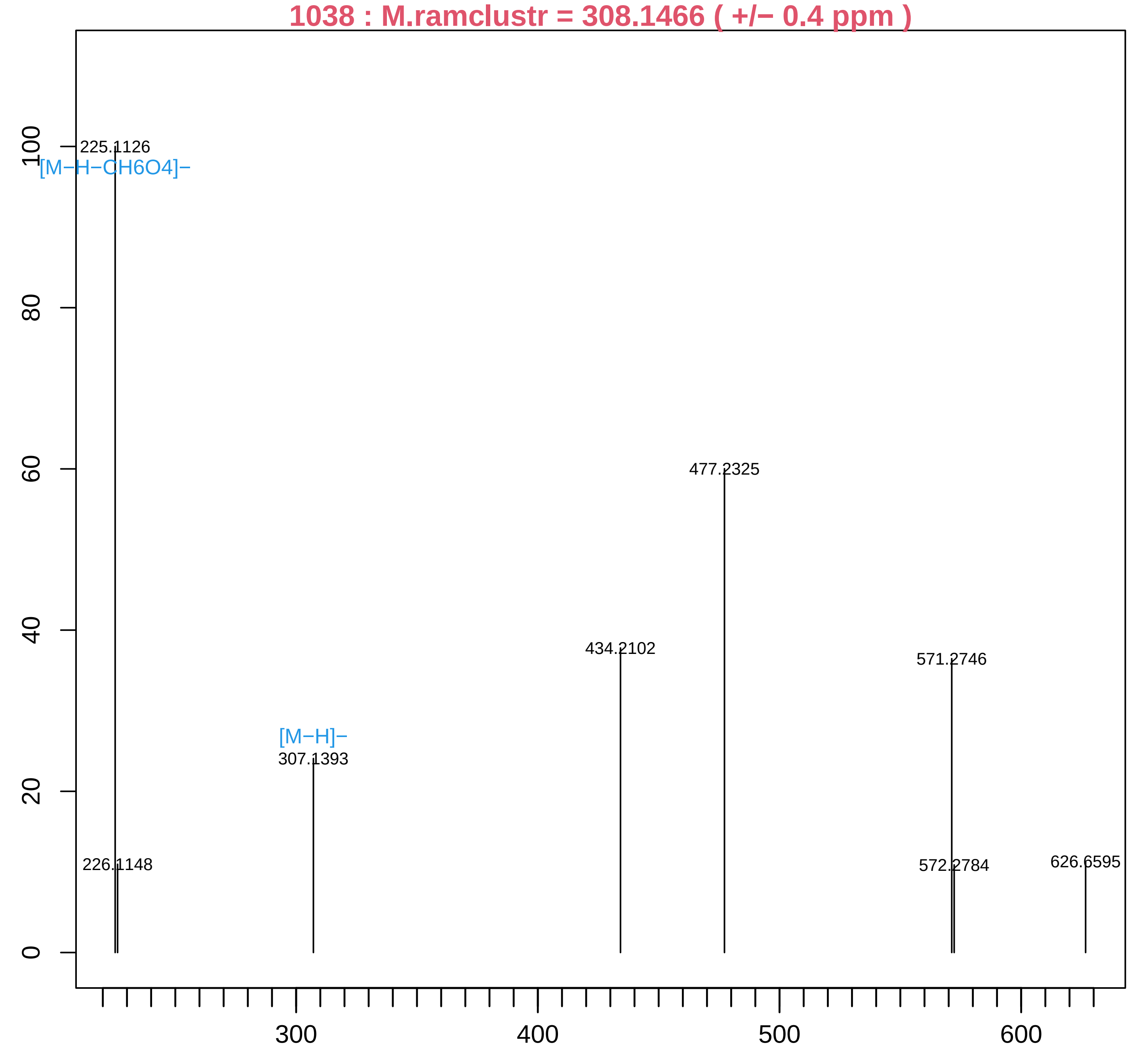
1038 : M.ramclustr = 308.1466 (+/− 0.4 ppm)
225.1126
[M−H−CH6O4]−
226.1148
[M−H]−
307.1393
434.2102
477.2325
571.2746
572.2784
626.6595
0
20
40
60
80
100
300
400
500
600

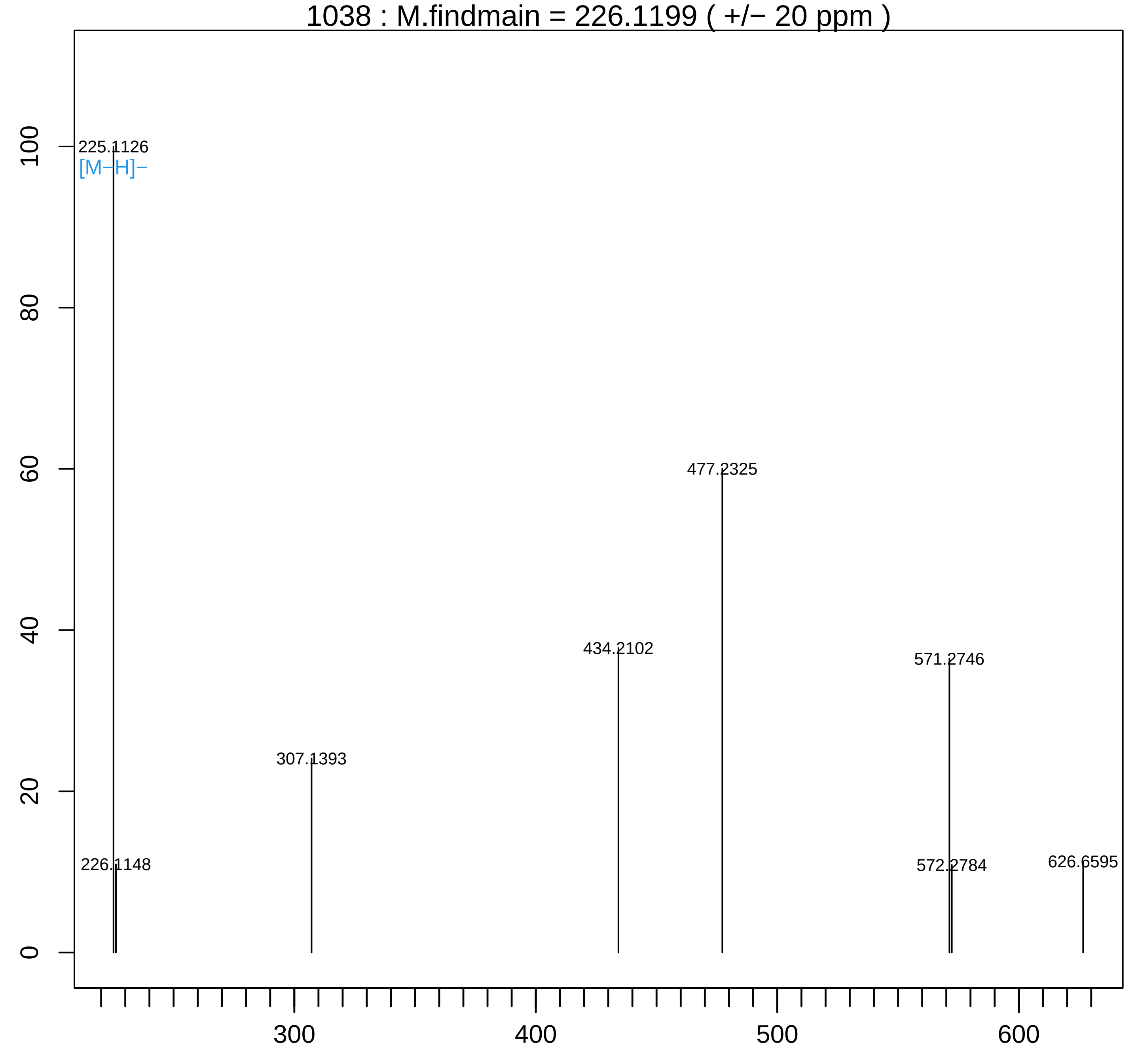
1038 : M.findmain = 226.1199 (+/− 20 ppm)
225.1126
[M−H]−
226.1148
307.1393
434.2102
477.2325
571.2746
572.2784
626.6595
0
20
40
60
80
100
300
400
500
600

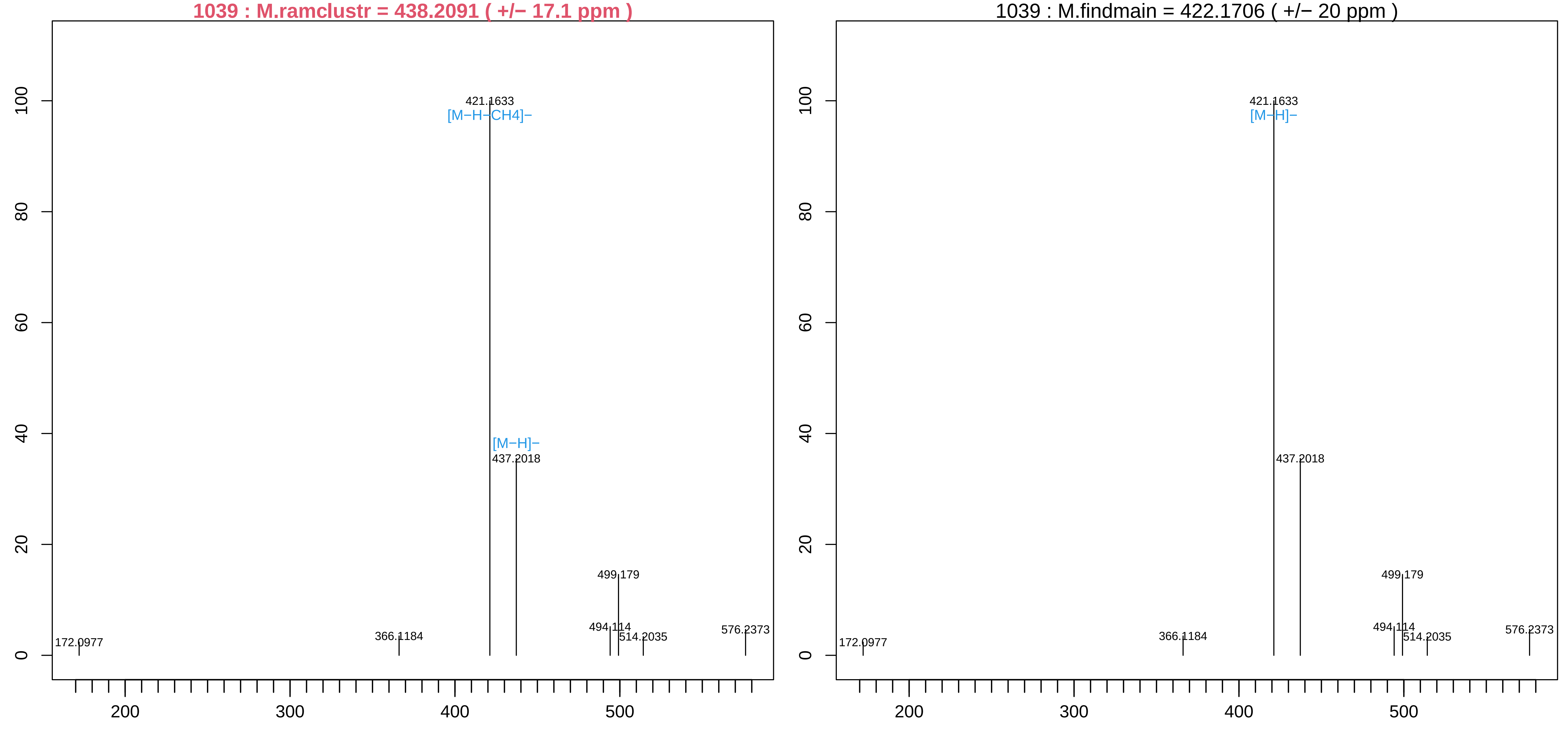

1039 : M.ramclustr = 438.2091 (+/− 17.1 ppm)
421.1633
[M−H−CH4]−
[M−H]−
437.2018
499.179
494.114
514.2035
576.2373
172.0977
366.1184
1039 : M.findmain = 422.1706 (+/− 20 ppm)
421.1633
[M−H]−
437.2018
499.179
494.114
514.2035
576.2373
172.0977
366.1184

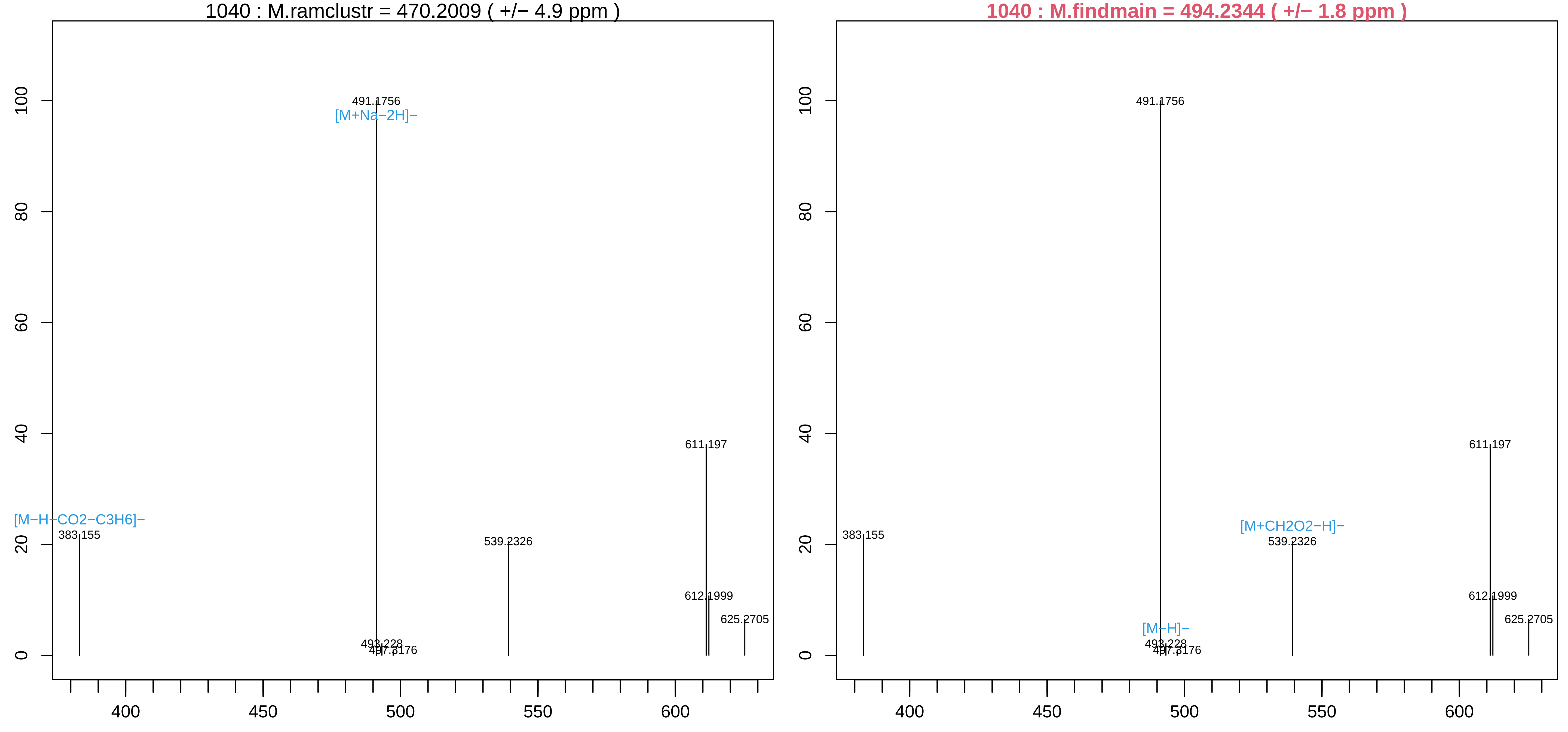
1040 : M.ramclustr = 470.2009 (+/− 4.9 ppm)
491.1756
[M+Na−2H]−
[M−H−CO2−C3H6]−
383.155
539.2326
611.197
612.1999
625.2705
493.228
497.3176
0
20
40
60
80
100
400
450
500
550
600
1040 : M.findmain = 494.2344 (+/− 1.8 ppm)
491.1756
611.197
[M+CH2O2−H]−
383.155
539.2326
612.1999
625.2705
[M−H]−
493.228
497.3176
0
20
40
60
80
100
400
450
500
550
600

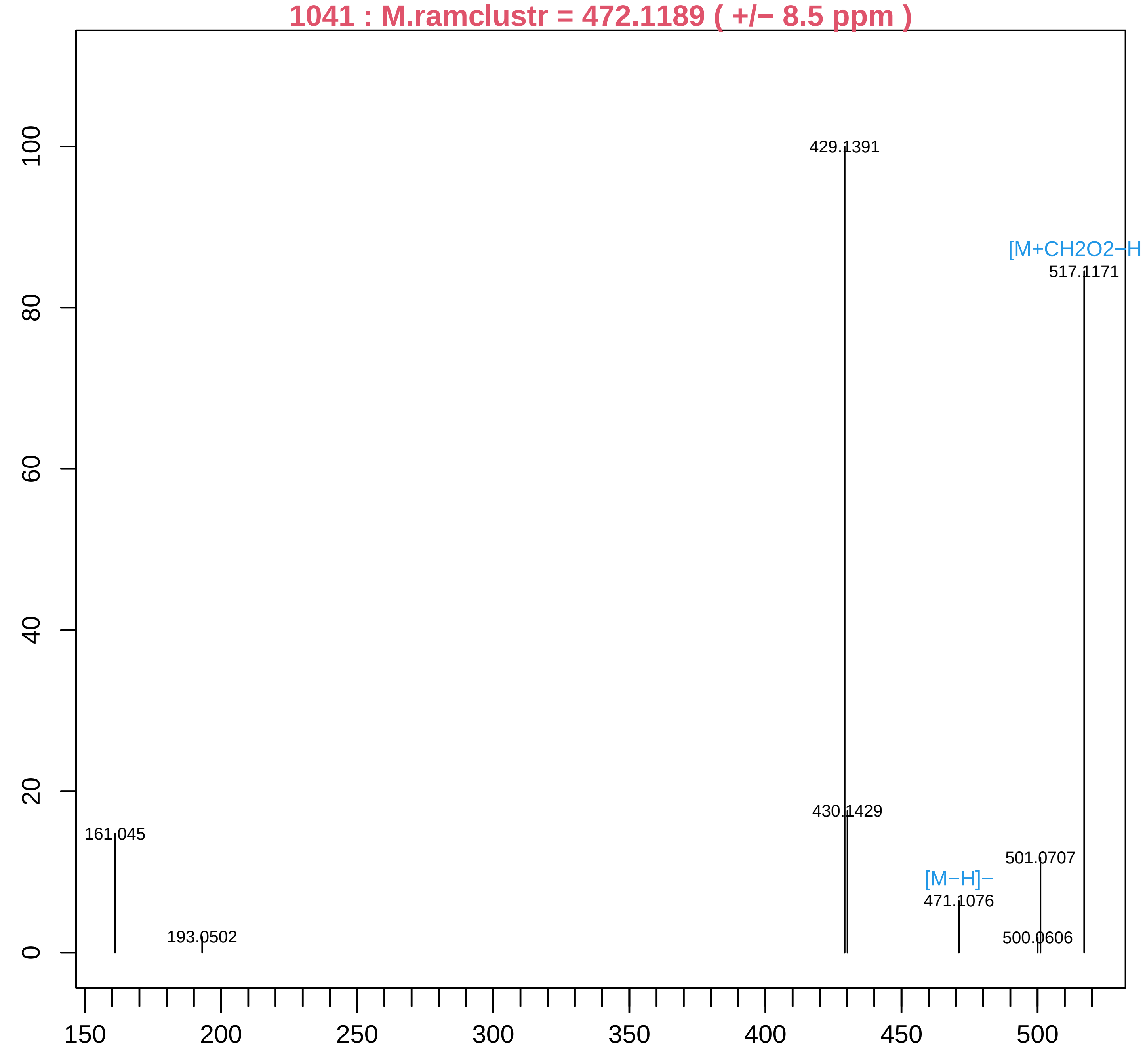
1041 : M.ramclustr = 472.1189 (+/− 8.5 ppm)
429.1391
[M+CH2O2−H
517.1171
430.1429
161.045
501.0707
[M−H]−
471.1076
193.0502
500.0606
0
20
40
60
80
100
150
200
250
300
350
400
450
500

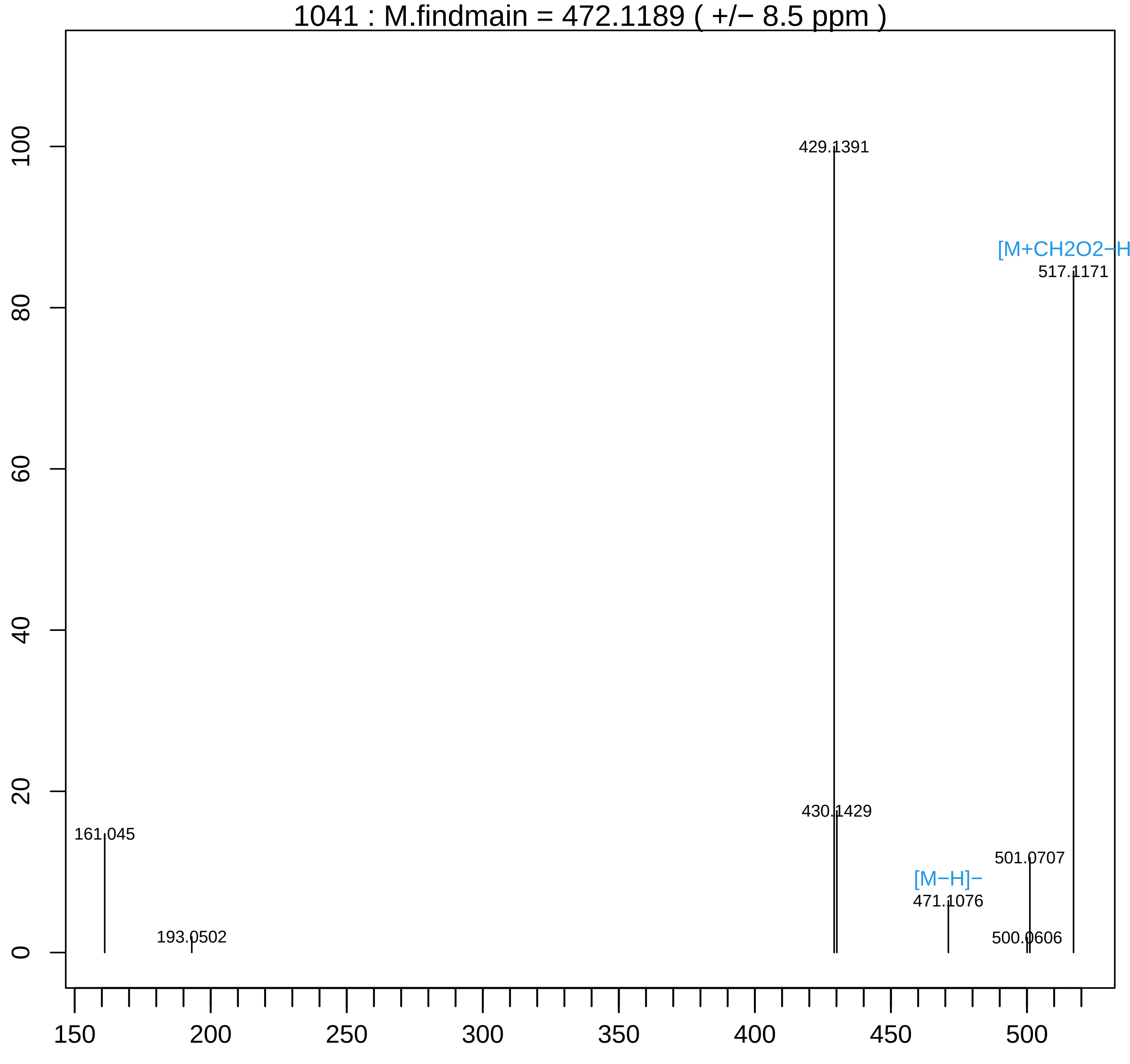
1041 : M.findmain = 472.1189 (+/− 8.5 ppm)
429.1391
[M+CH2O2−H
517.1171
430.1429
161.045
501.0707
[M−H]−
471.1076
193.0502
500.0606
0
20
40
60
80
100
150
200
250
300
350
400
450
500

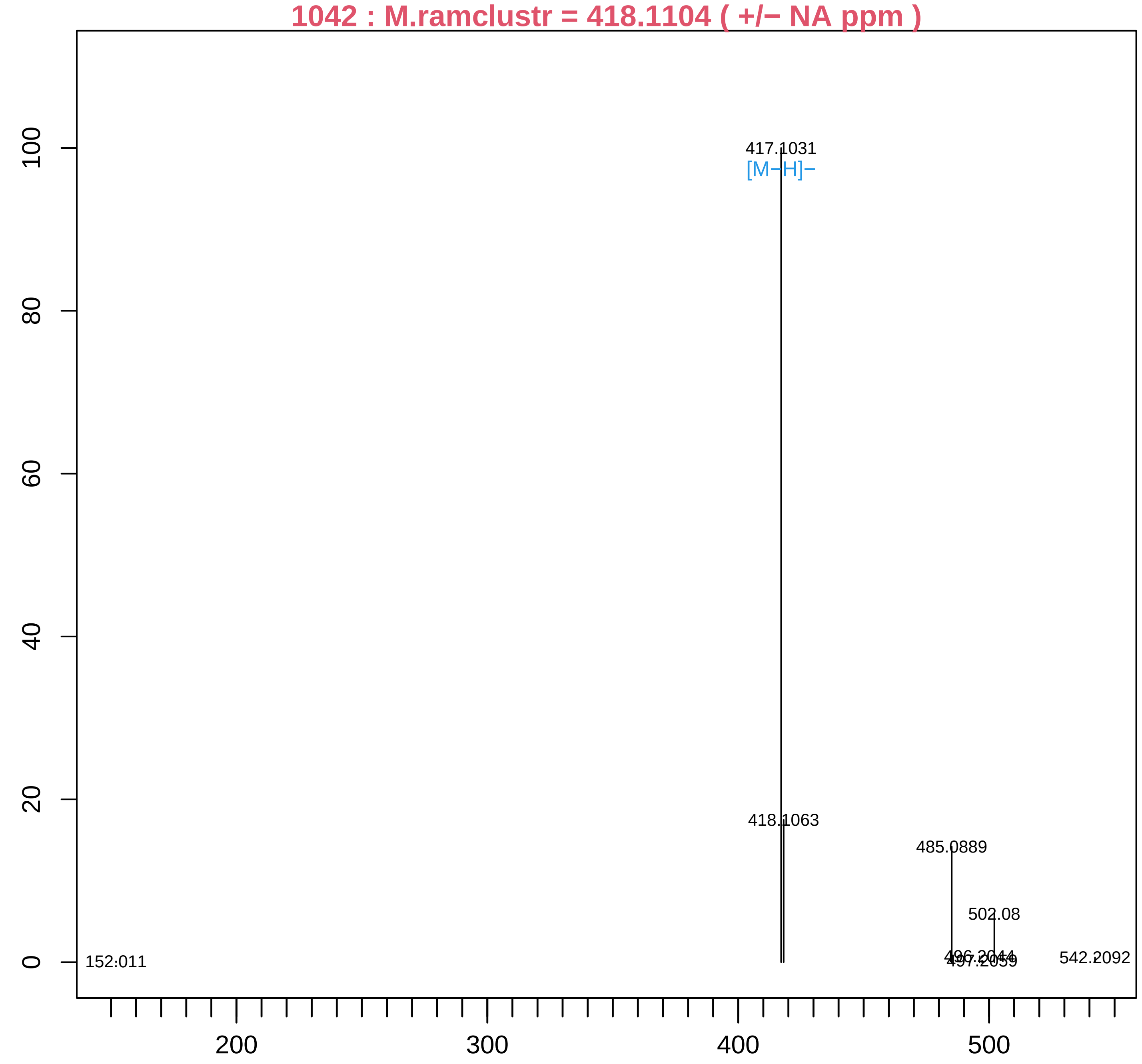
1042 : M.ramclustr = 418.1104 (+/− NA ppm)
417.1031
[M−H]−
418.1063
485.0889
502.08
496.2044
497.2059
542.2092
152.011
0
20
40
60
80
100
200
300
400
500

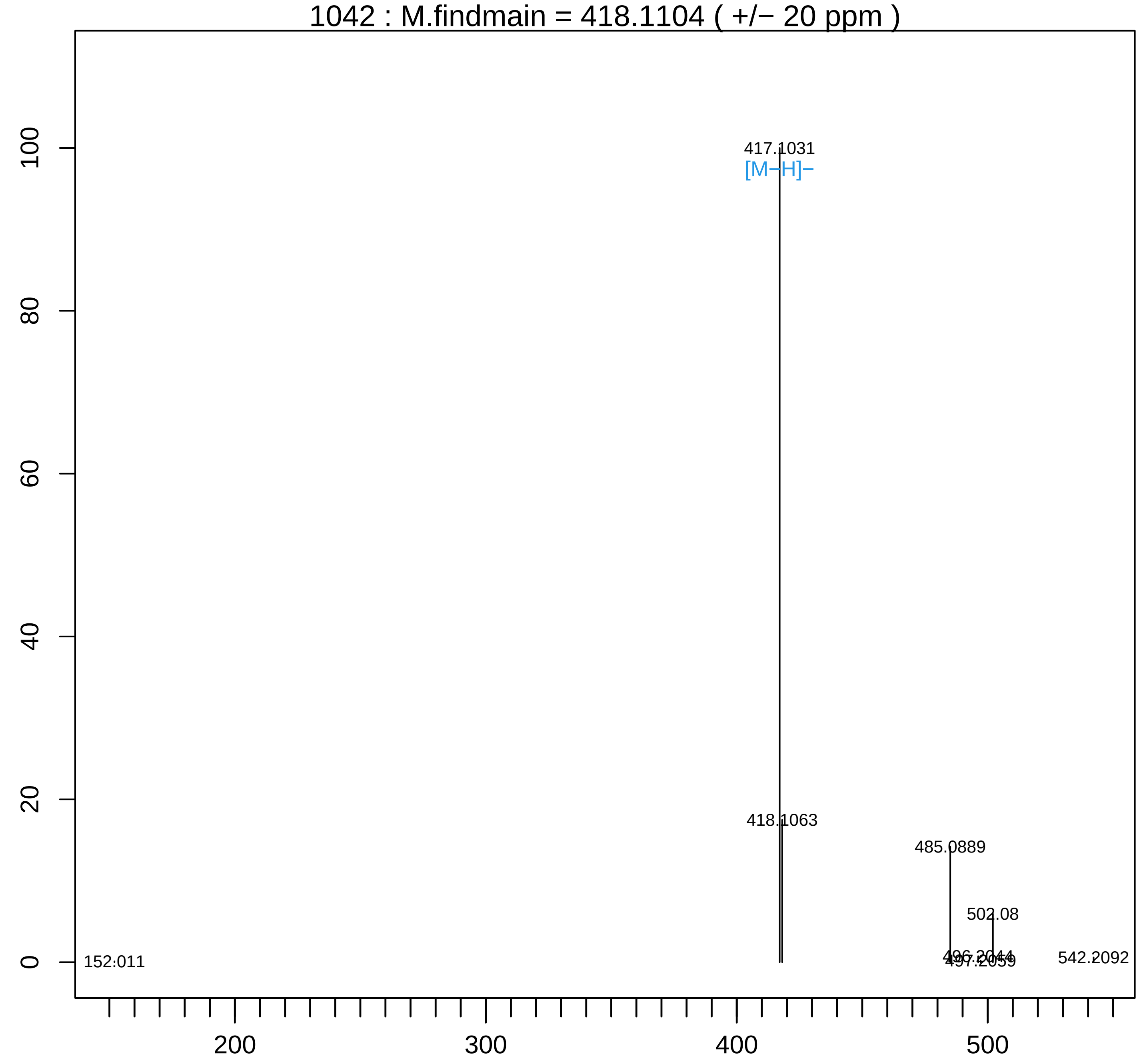
1042 : M.findmain = 418.1104 (+/− 20 ppm)
417.1031
[M−H]−
418.1063
485.0889
502.08
496.2044
497.2059
542.2092
152.011
0
20
40
60
80
100
200
300
400
500

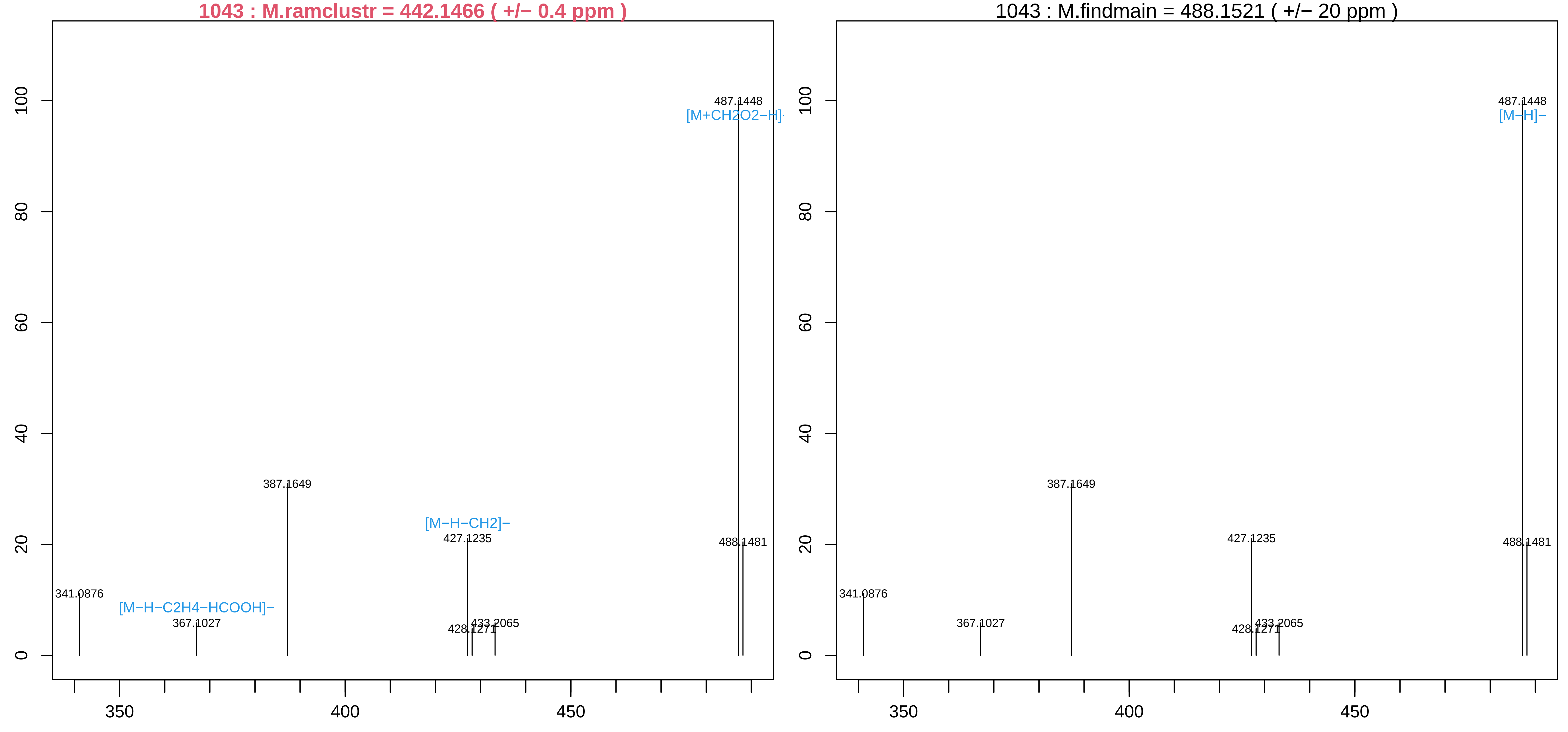

1043 : M.ramclustr = 442.1466 (+/− 0.4 ppm)
487.1448
[M+CH2O2−H]−
387.1649
[M−H−CH2]−
427.1235
488.1481
341.0876
[M−H−C2H4−HCOOH]−
367.1027
428.1271
433.2065
1043 : M.findmain = 488.1521 (+/− 20 ppm)
487.1448
[M−H]−
387.1649
427.1235
488.1481
341.0876
367.1027
428.1271
433.2065
0
20
40
60
80
100
350
400
450

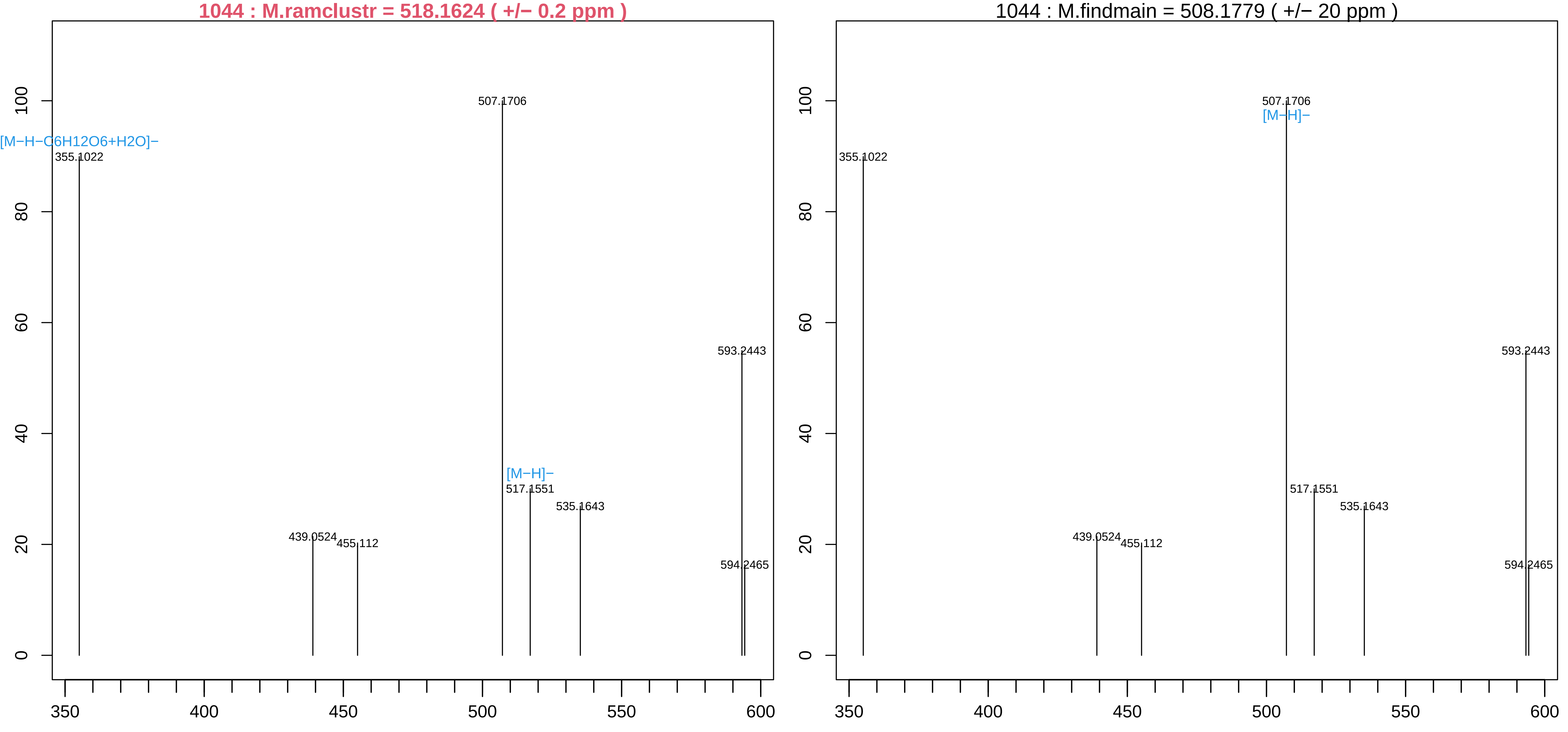
1044 : M.ramclustr = 518.1624 (+/− 0.2 ppm)
[M−H−C6H12O6+H2O]−
355.1022
439.0524
455.112
507.1706
[M−H]−
517.1551
535.1643
593.2443
594.2465
0
20
40
60
80
100
350
400
450
500
550
600
1044 : M.findmain = 508.1779 (+/− 20 ppm)
355.1022
439.0524
455.112
507.1706
[M−H]−
517.1551
535.1643
593.2443
594.2465
0
20
40
60
80
100
350
400
450
500
550
600

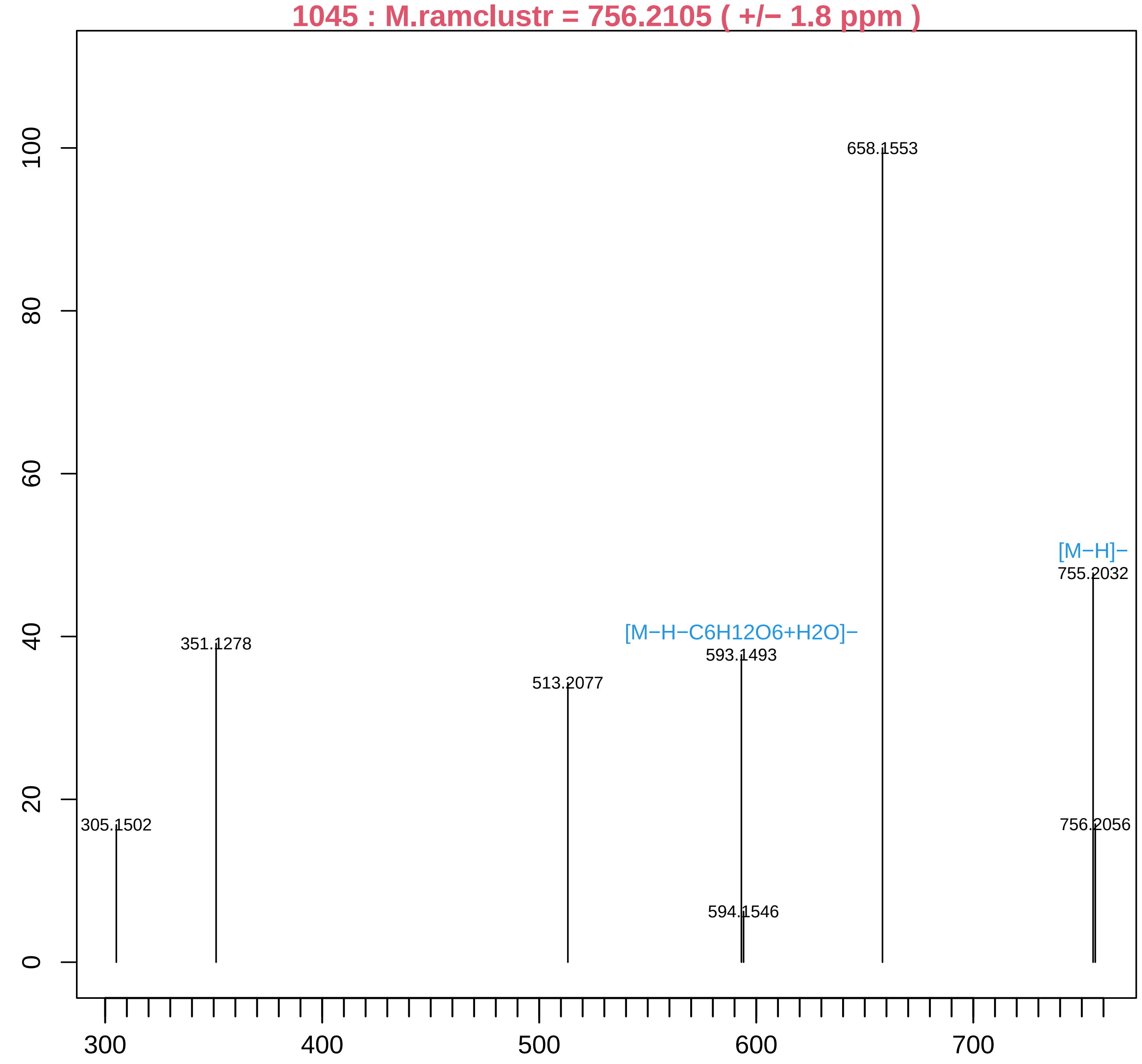

1045 : M.ramclustr = 756.2105 (+/− 1.8 ppm)
658.1553
[M−H]−
755.2032
[M−H−C6H12O6+H2O]−
593.1493
351.1278
513.2077
305.1502
756.2056
594.1546
0
20
40
60
80
100
300
400
500
600
700

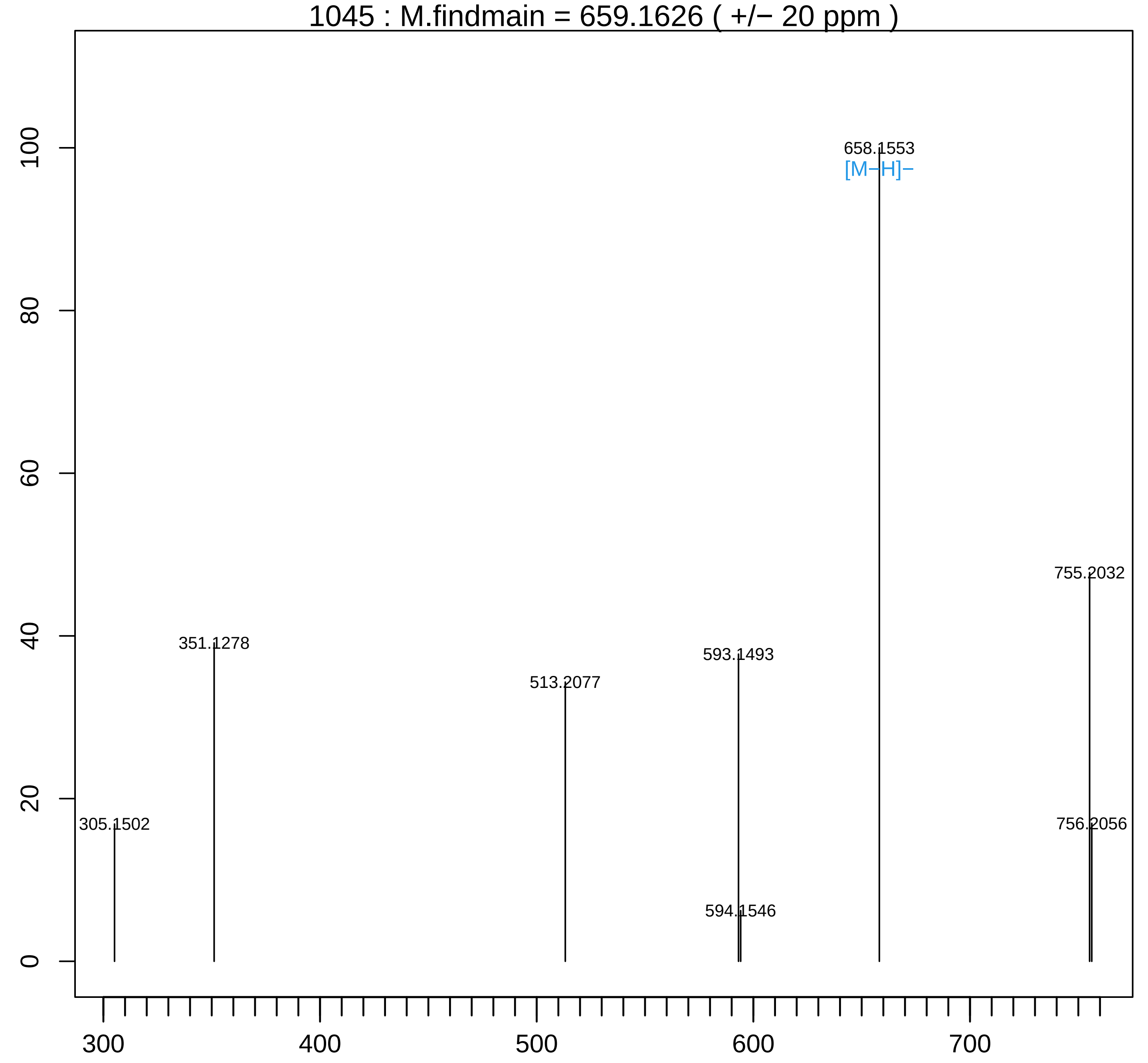

1045 : M.findmain = 659.1626 (+/− 20 ppm)
658.1553
[M−H]−
755.2032
351.1278
593.1493
513.2077
305.1502
756.2056
594.1546
0
20
40
60
80
100
300
400
500
600
700

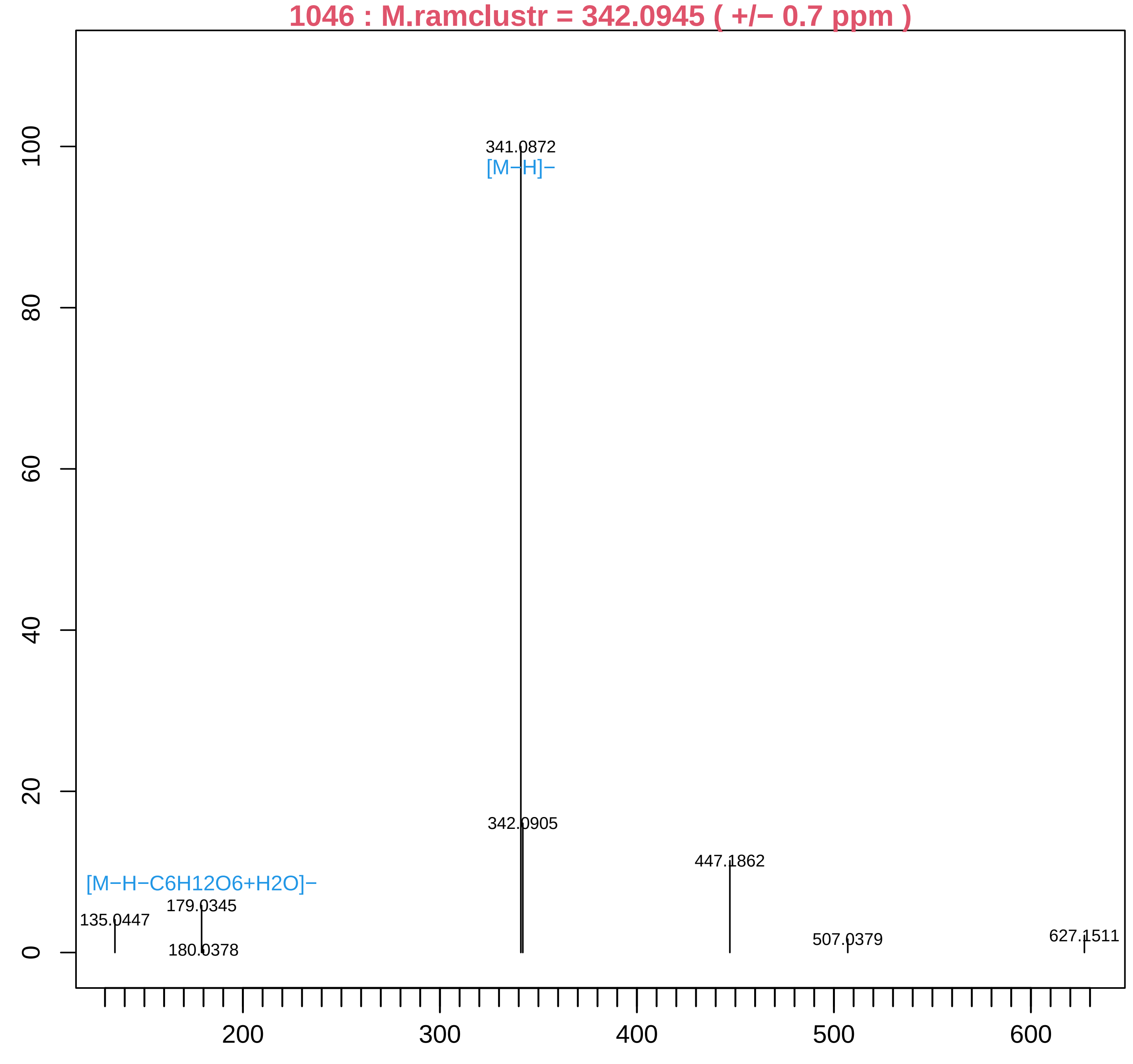
1046 : M.ramclustr = 342.0945 (+/− 0.7 ppm)
341.0872
[M−H]−
342.0905
447.1862
[M−H−C6H12O6+H2O]−
179.0345
135.0447
180.0378
507.0379
627.1511
0
20
40
60
80
100
200
300
400
500
600

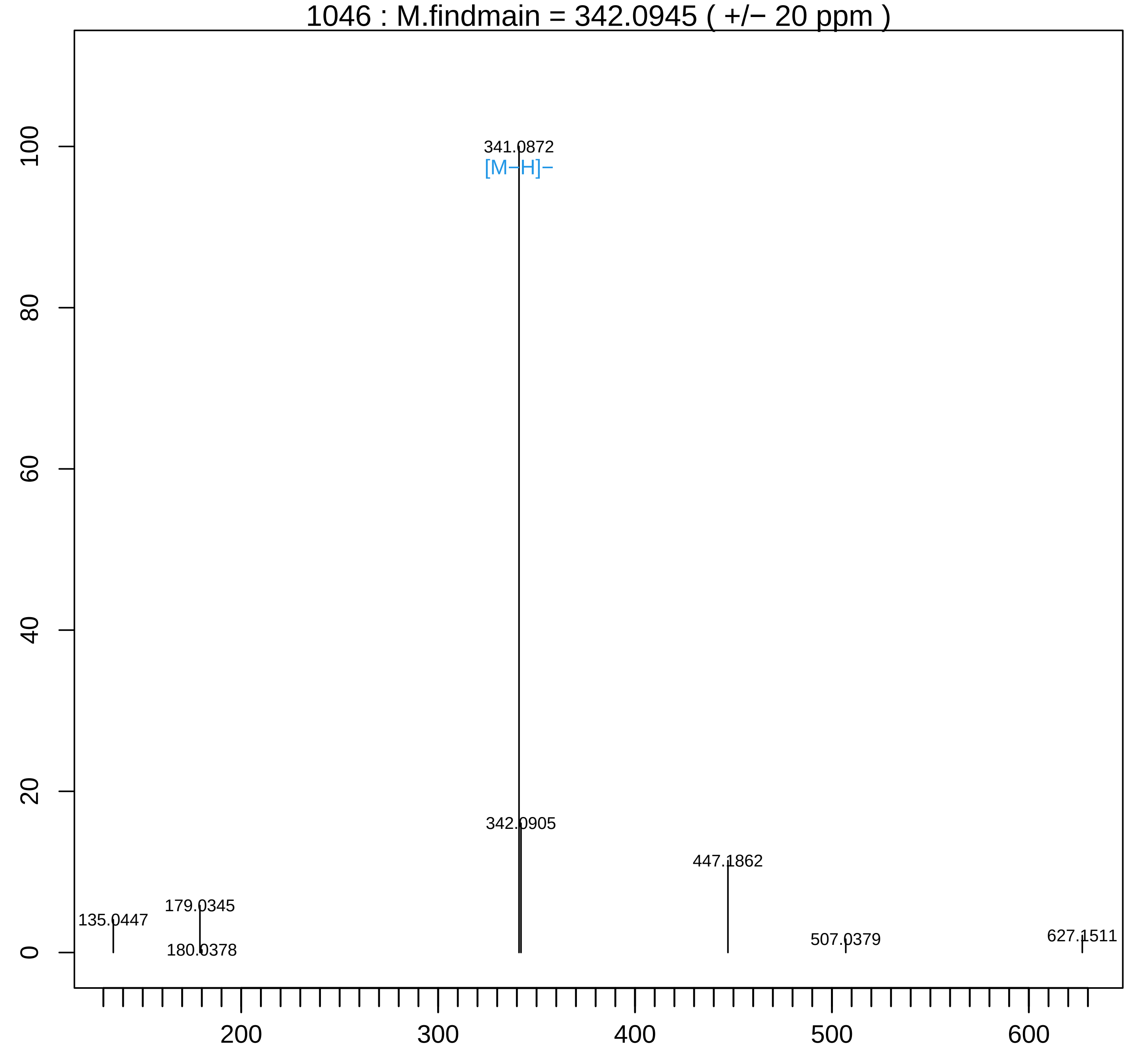
1046 : M.findmain = 342.0945 (+/− 20 ppm)
341.0872
[M−H]−
342.0905
447.1862
179.0345
135.0447
180.0378
507.0379
627.1511
0
20
40
60
80
100
200
300
400
500
600

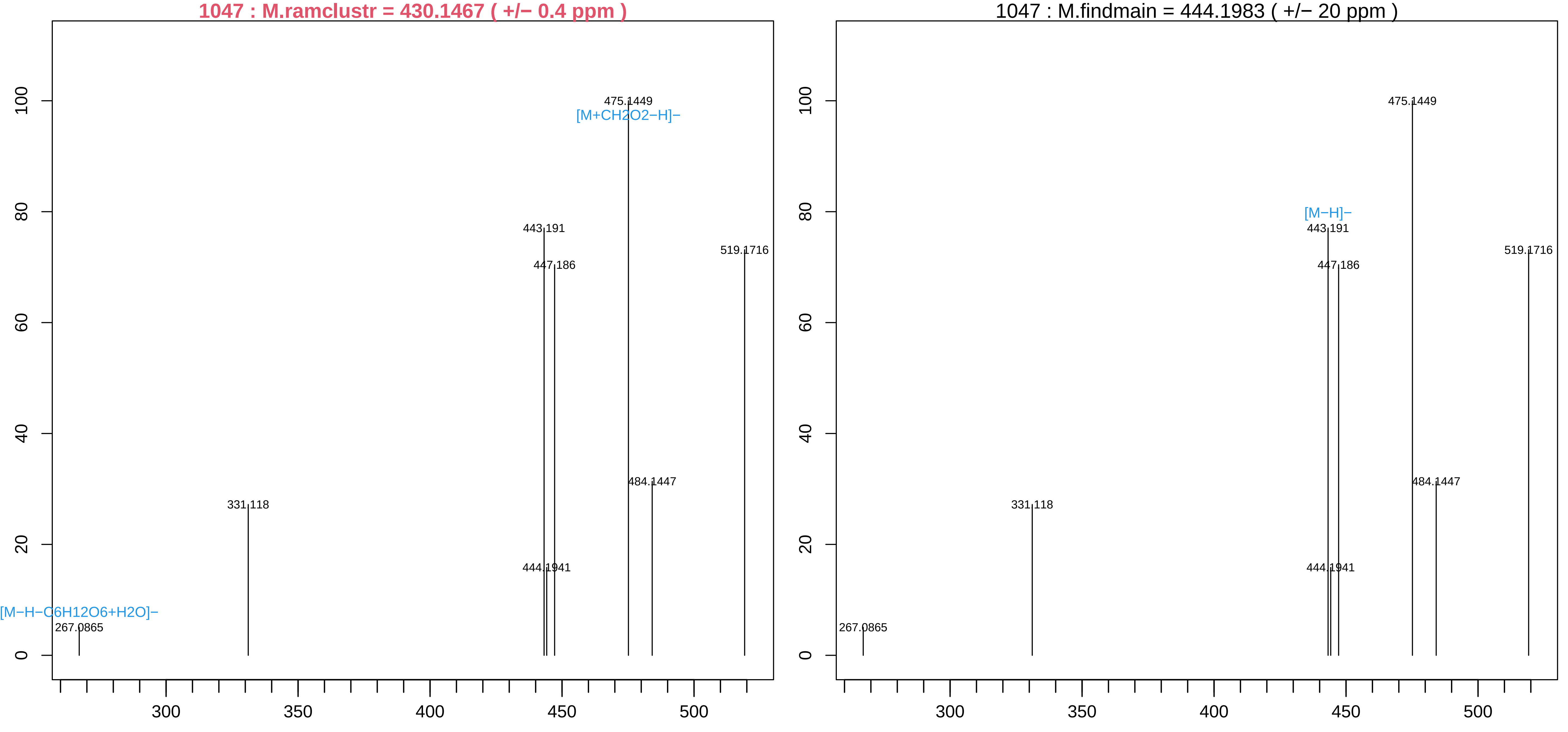
1047 : M.ramclustr = 430.1467 (+/− 0.4 ppm)
1047 : M.findmain = 444.1983 (+/− 20 ppm)
475.1449
[M+CH2O2−H]−
443.191
447.186
519.1716
484.1447
331.118
444.1941
[M−H−C6H12O6+H2O]−
267.0865
[M−H]−
0
20
40
60
80
100
300
350
400
450
500

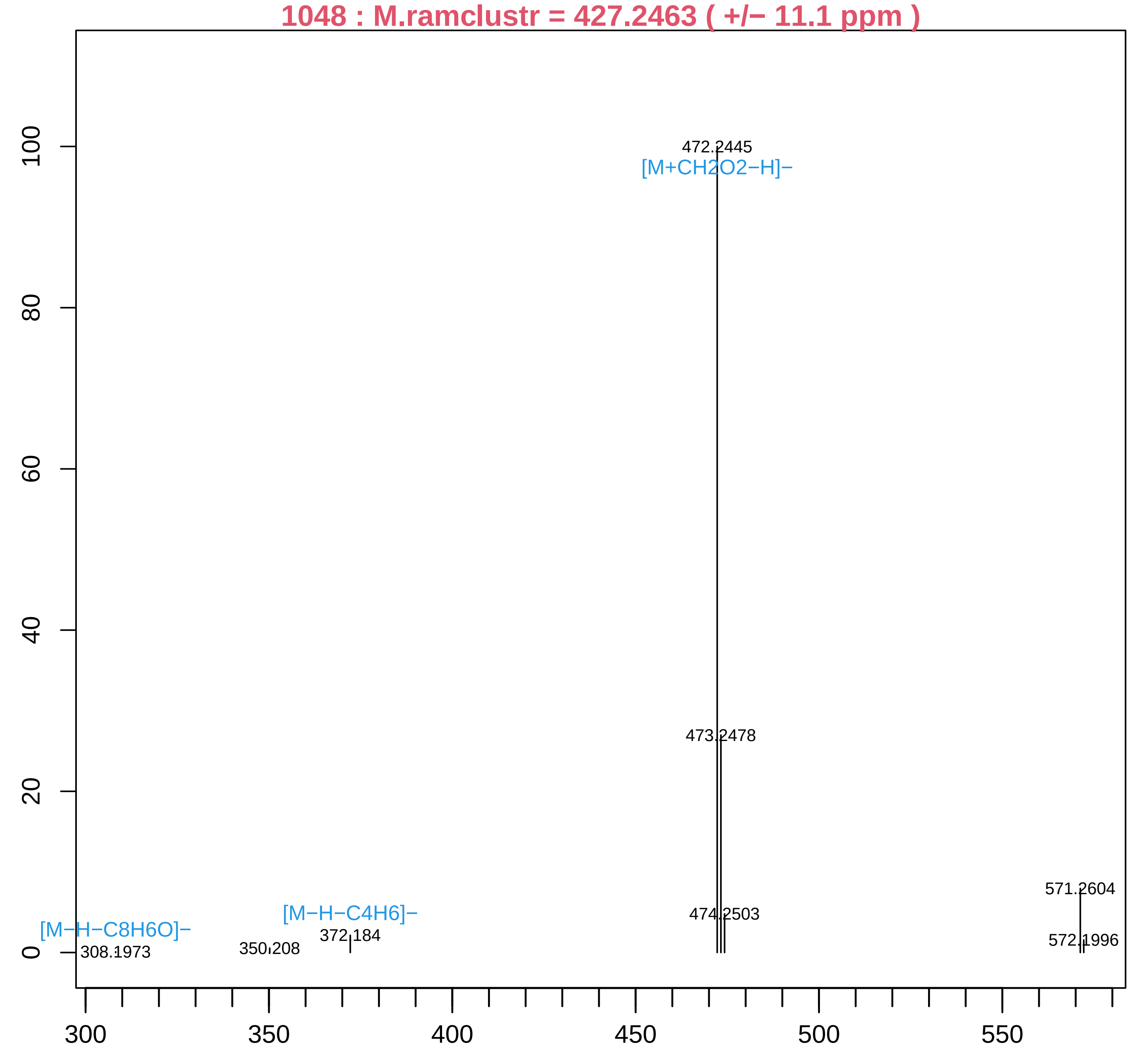
1048 : M.ramclustr = 427.2463 (+/− 11.1 ppm)
472.2445
[M+CH2O2−H]−
473.2478
474.2503
571.2604
572.1996
[M−H−C8H6O]−
308.1973
350.208
[M−H−C4H6]−
372.184
0
20
40
60
80
100
300
350
400
450
500
550

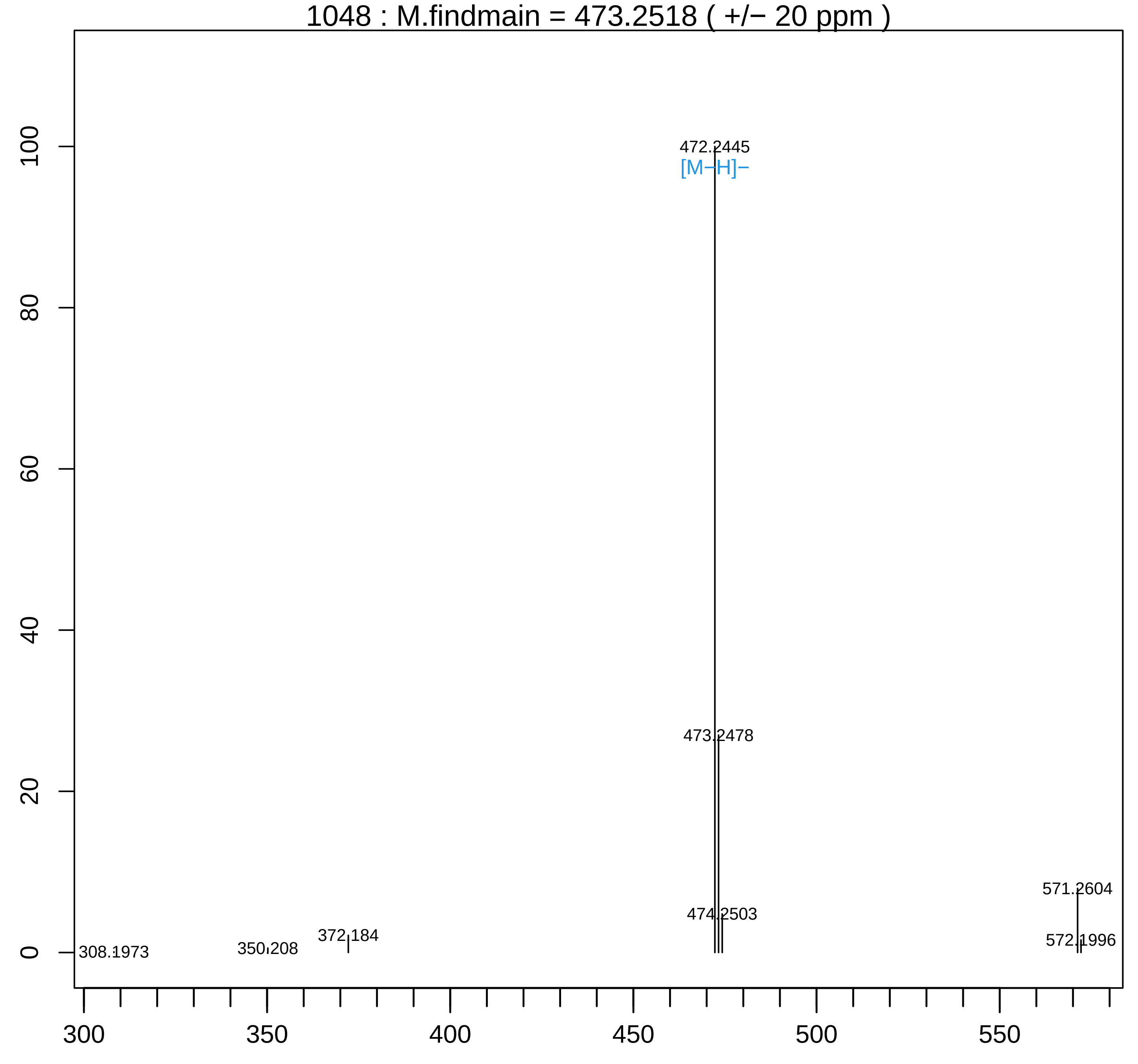
1048 : M.findmain = 473.2518 (+/− 20 ppm)
472.2445
[M−H]−
473.2478
474.2503
571.2604
572.1996
308.1973
350.208
372.184
0
20
40
60
80
100
300
350
400
450
500
550

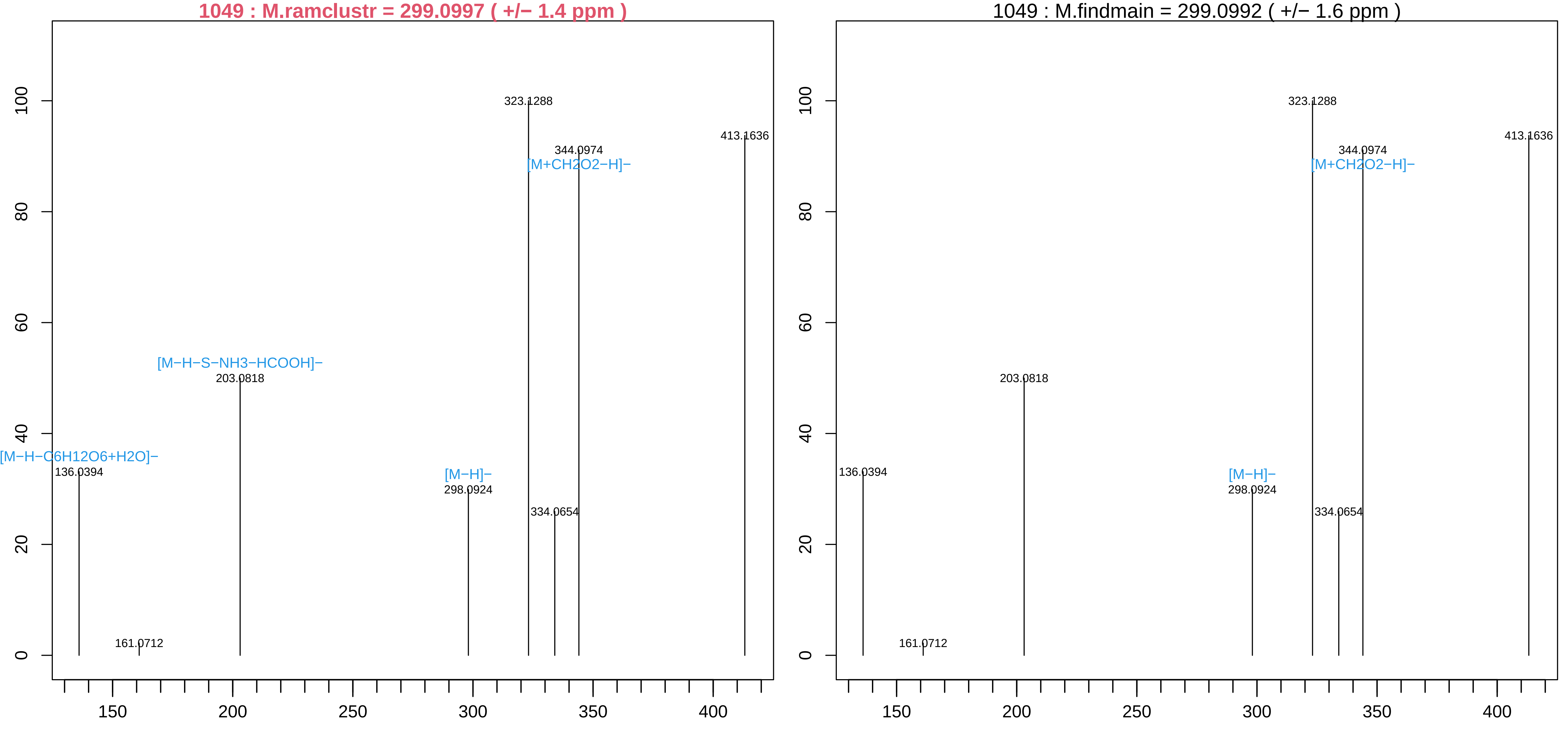
1049 : M.ramclustr = 299.0997 (+/− 1.4 ppm)
1049 : M.findmain = 299.0992 (+/− 1.6 ppm)
[M−H−C6H12O6+H2O]−
136.0394
161.0712
[M−H−S−NH3−HCOOH]−
203.0818
[M−H]−
298.0924
323.1288
334.0654
344.0974
[M+CH2O2−H]−
413.1636
136.0394
161.0712
203.0818
[M−H]−
298.0924
323.1288
334.0654
344.0974
[M+CH2O2−H]−
413.1636

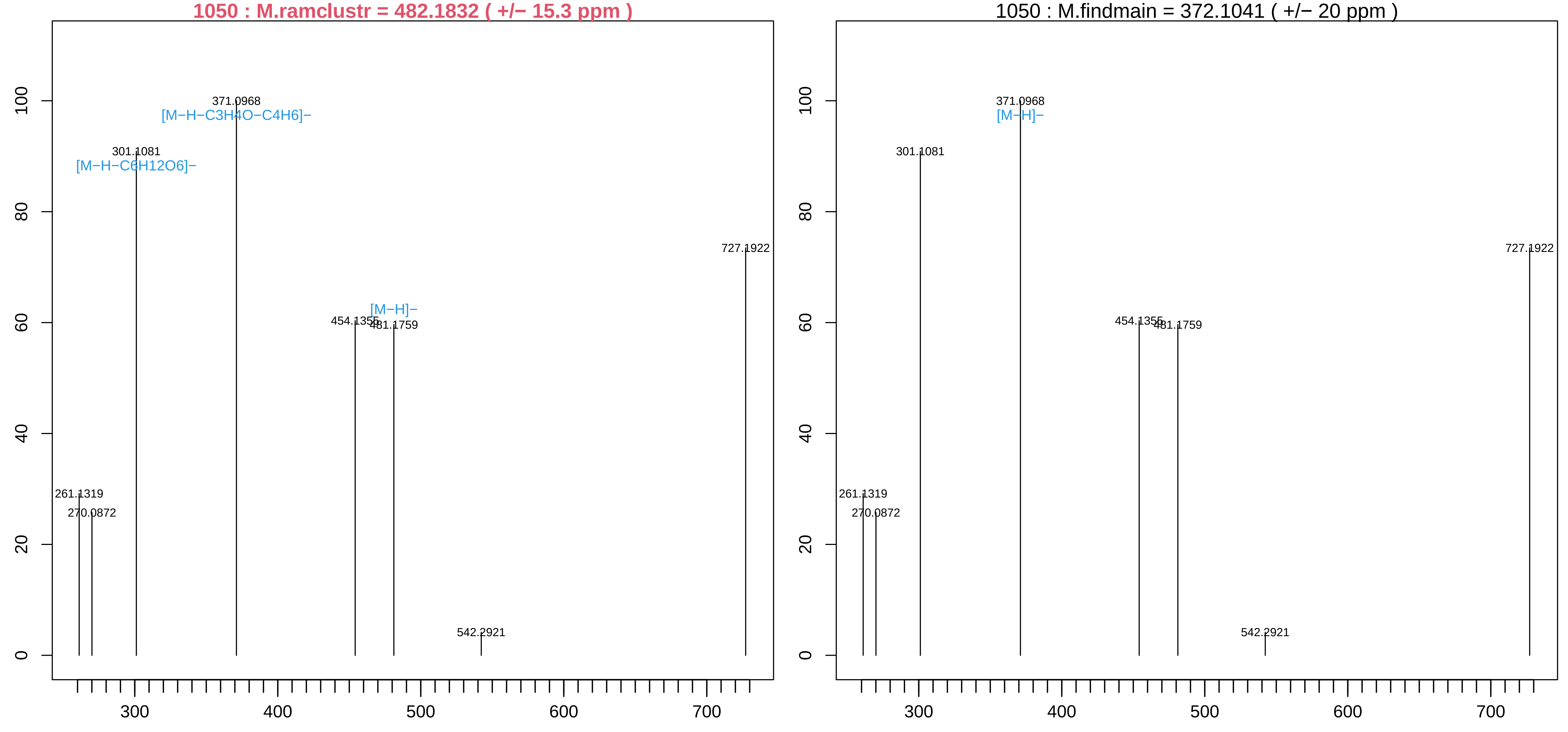

1050 : M.ramclustr = 482.1832 (+/− 15.3 ppm)
371.0968
[M−H−C3H4O−C4H6]−
301.1081
[M−H−C6H12O6]−
727.1922
[M−H]−
454.1355
481.1759
261.1319
270.0872
542.2921
1050 : M.findmain = 372.1041 (+/− 20 ppm)
371.0968
[M−H]−
301.1081
727.1922
454.1355
481.1759
261.1319
270.0872
542.2921
0
20
40
60
80
100
300
400
500
600
700

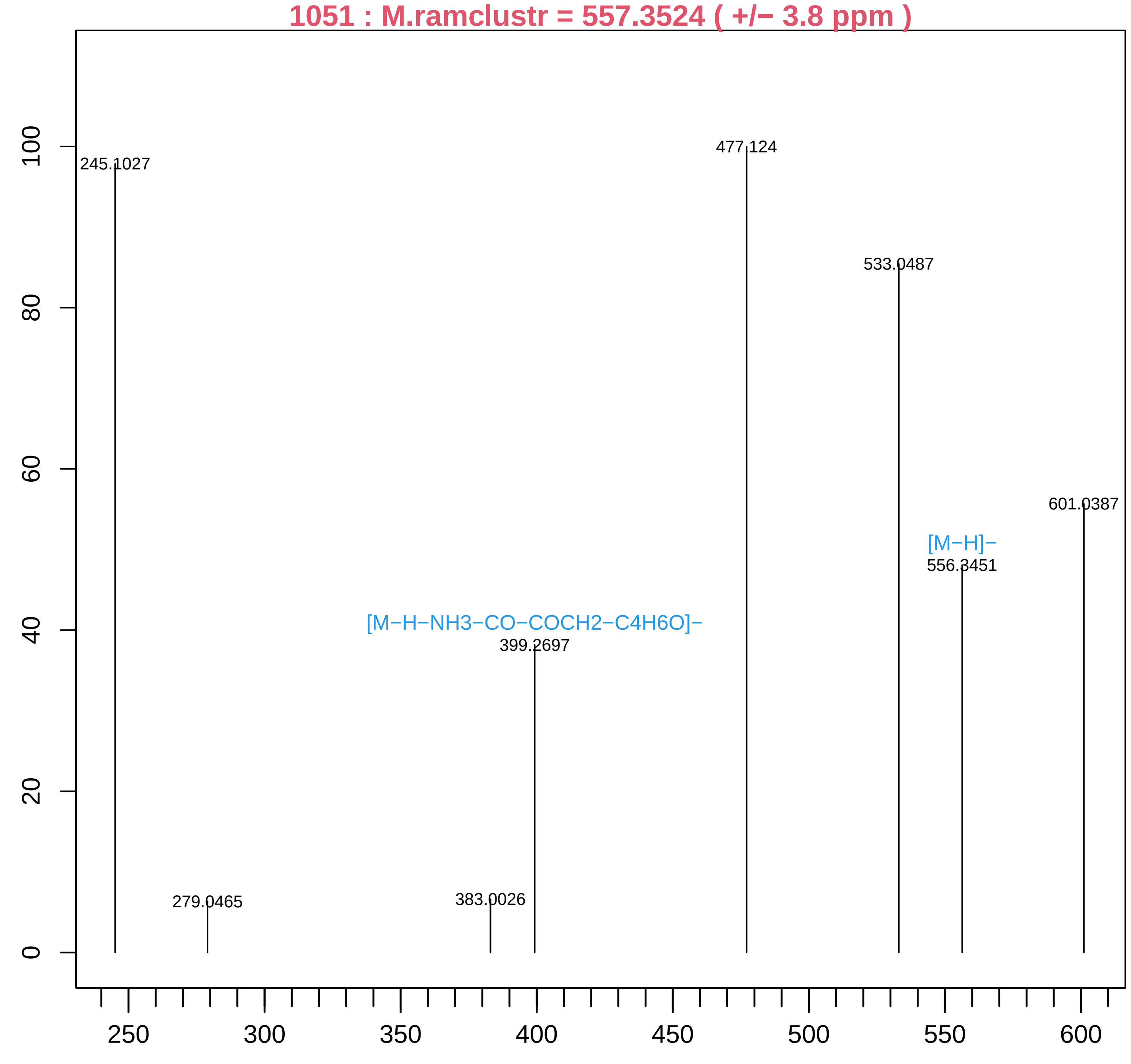
1051 : M.ramclustr = 557.3524 (+/− 3.8 ppm)
245.1027
279.0465
383.0026
399.2697
[M−H−NH3−CO−COCH2−C4H6O]−
477.124
533.0487
556.3451
[M−H]−
601.0387
0
20
40
60
80
100
250
300
350
400
450
500
550
600
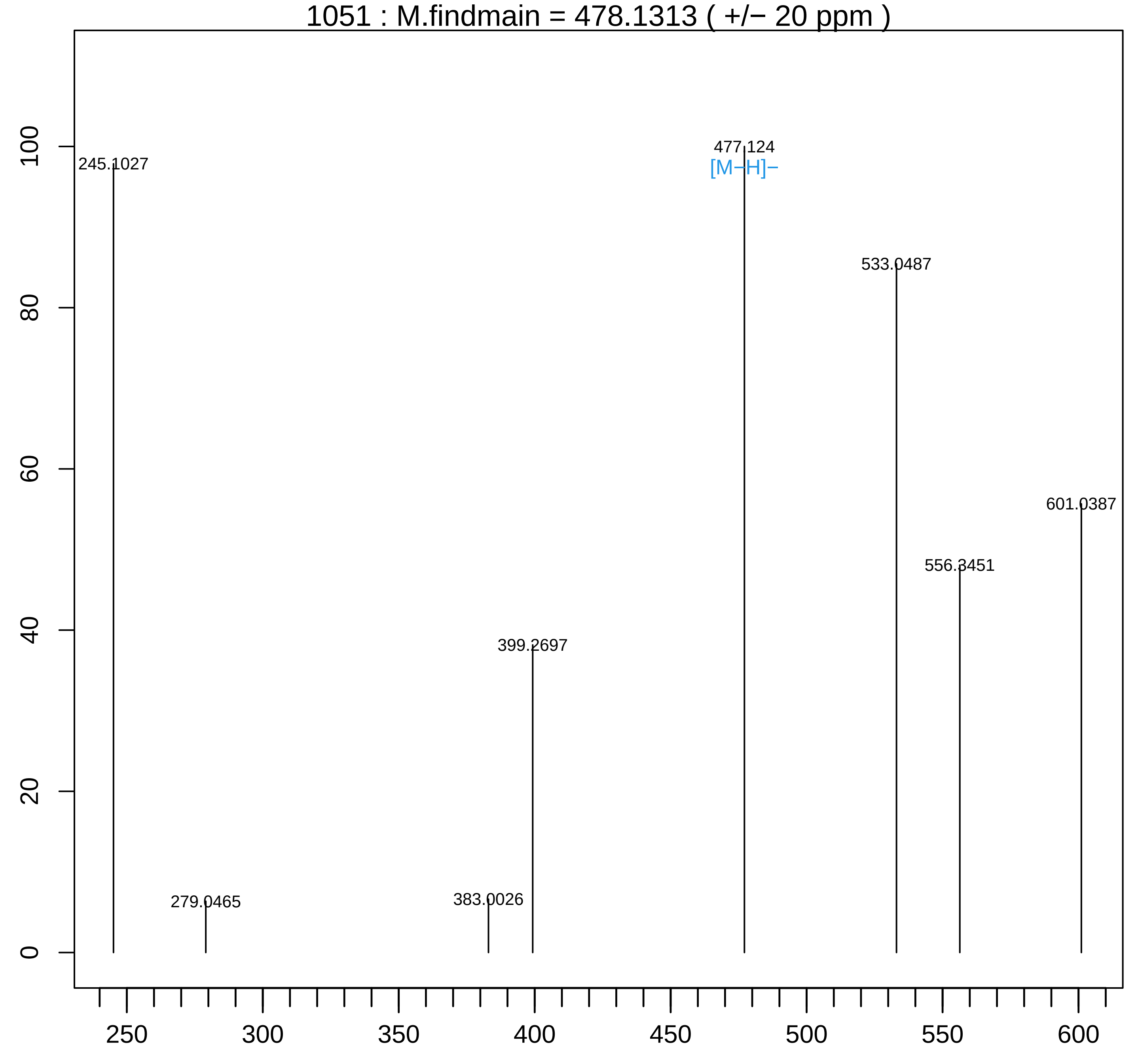
1051 : M.findmain = 478.1313 (+/− 20 ppm)
245.1027
279.0465
383.0026
399.2697
477.124
[M−H]−
533.0487
556.3451
601.0387
0
20
40
60
80
100
250
300
350
400
450
500
550
600

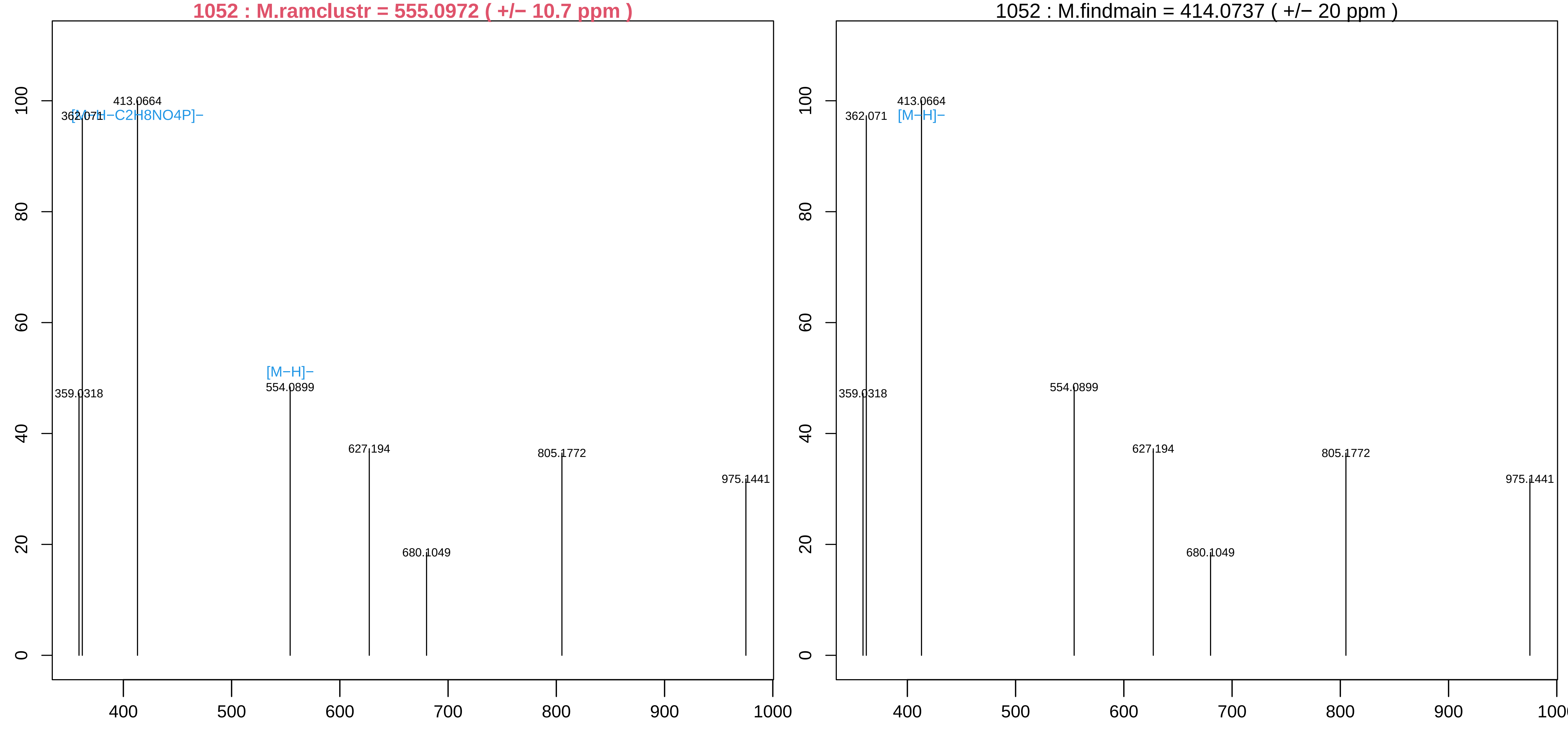
1052 : M.ramclustr = 555.0972 (+/− 10.7 ppm)
[M−H−C2H8NO4P]−
[M−H]−
413.0664
362.071
359.0318
554.0899
627.194
680.1049
805.1772
975.1441
1052 : M.findmain = 414.0737 (+/− 20 ppm)
[M−H]−
413.0664
362.071
359.0318
554.0899
627.194
680.1049
805.1772
975.1441
0
20
40
60
80
100
400
500
600
700
800
900
1000

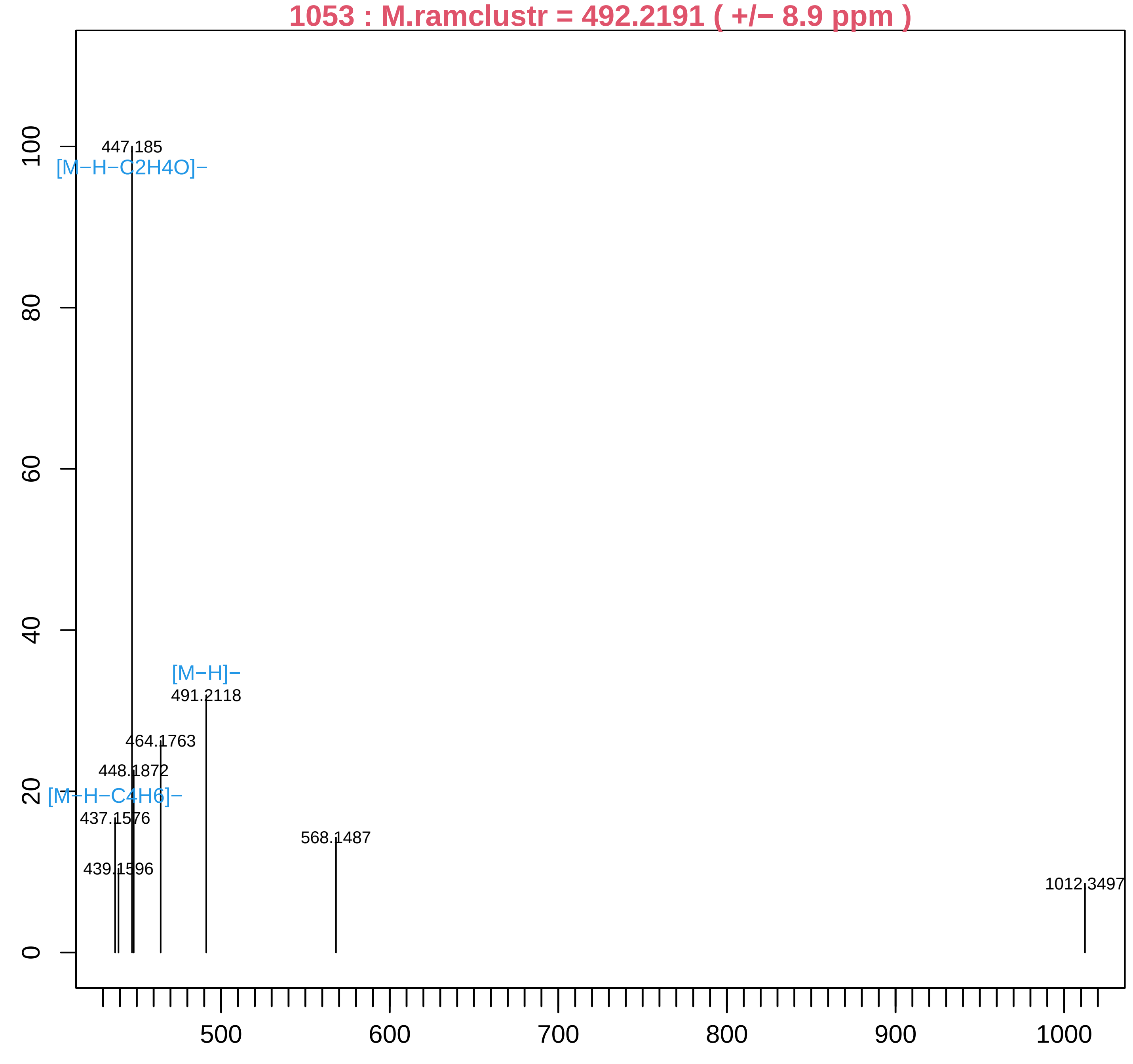

1053 : M.ramclustr = 492.2191 (+/− 8.9 ppm)
447.185
[M−H−C2H4O]−
[M−H]−
491.2118
464.1763
448.1872
[M−H−C4H6]−
437.1576
568.1487
439.1596
1012.3497
0
20
40
60
80
100
500
600
700
800
900
1000

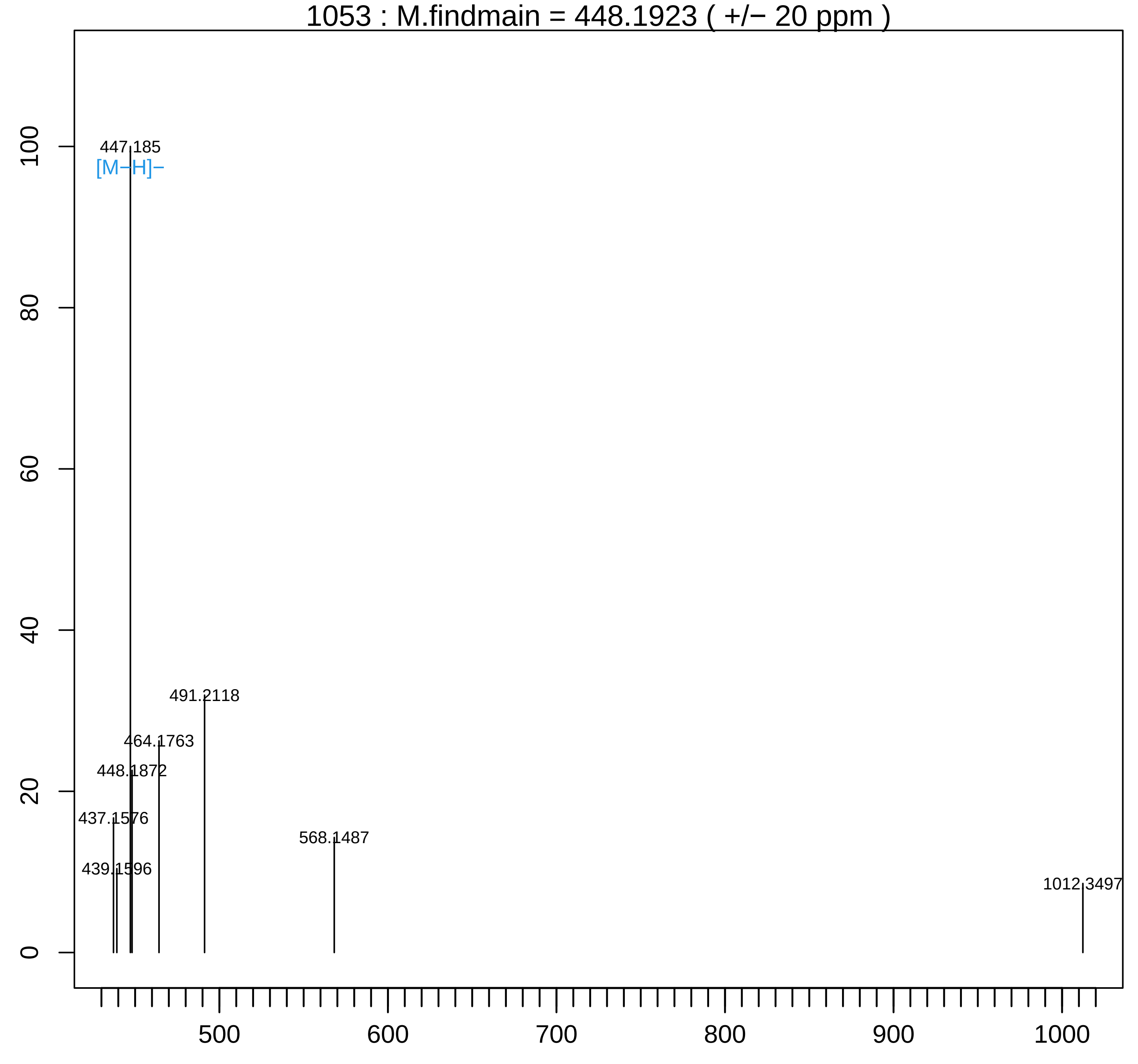

1053 : M.findmain = 448.1923 (+/− 20 ppm)
447.185
[M−H]−
491.2118
464.1763
448.1872
437.1576
568.1487
439.1596
1012.3497
0
20
40
60
80
100
500
600
700
800
900
1000

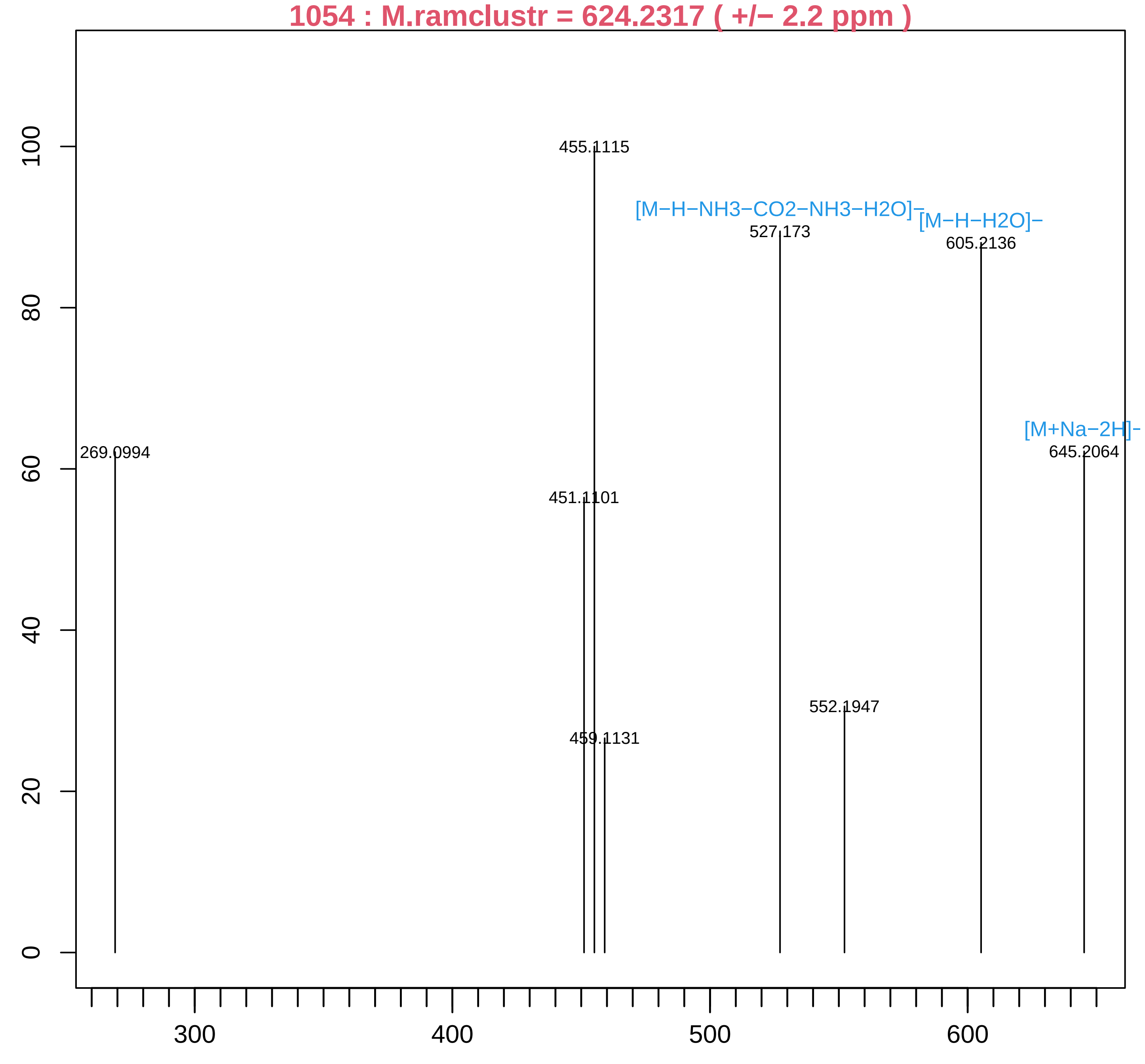
1054 : M.ramclustr = 624.2317 (+/− 2.2 ppm)
455.1115
[M−H−NH3−CO2−NH3−H2O]−
527.173
[M−H−H2O]−
605.2136
[M+Na−2H]−
645.2064
269.0994
451.1101
552.1947
459.1131
0
20
40
60
80
100
300
400
500
600

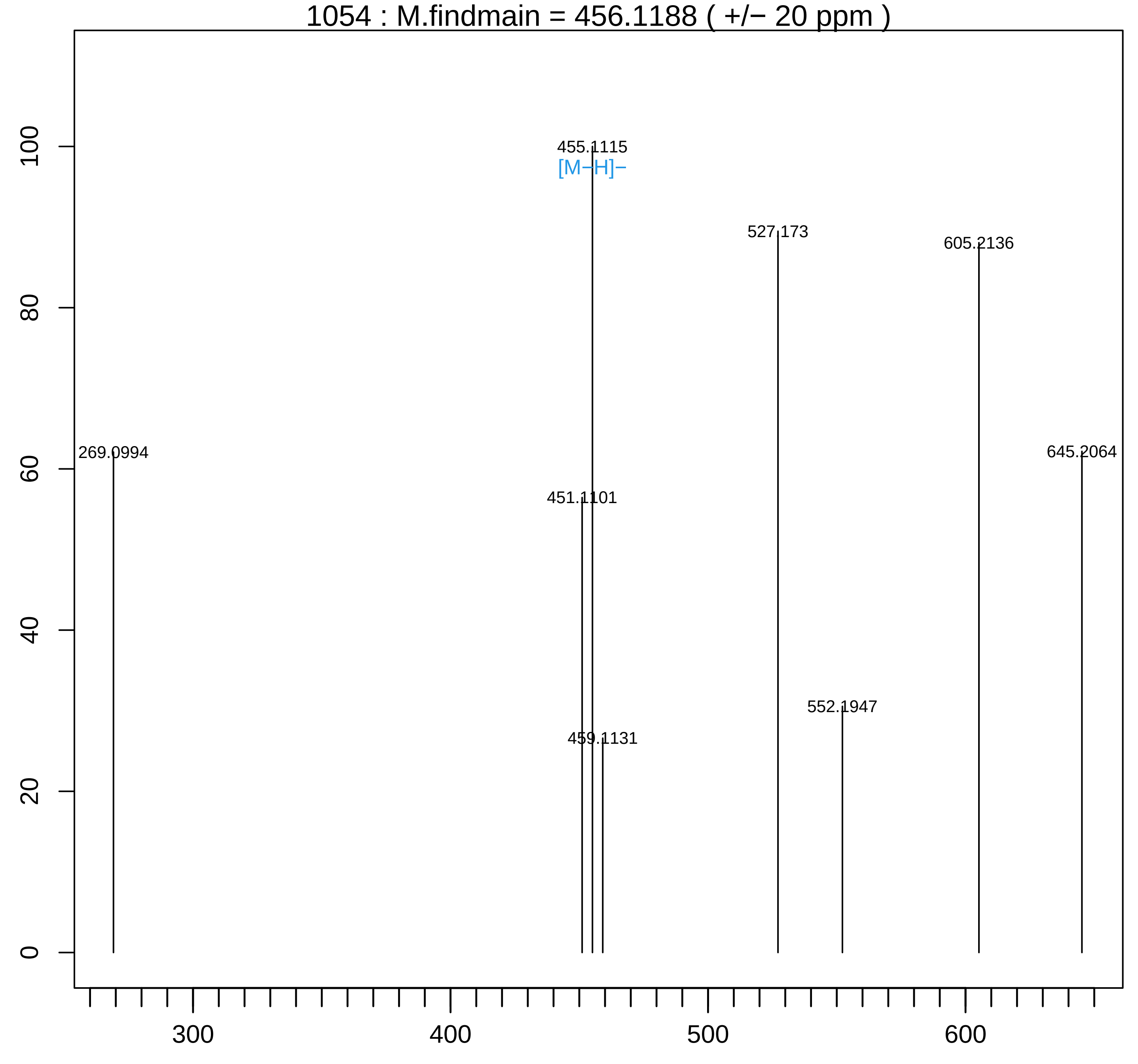
1054 : M.findmain = 456.1188 (+/− 20 ppm)
455.1115
[M−H]−
527.173
605.2136
269.0994
645.2064
451.1101
552.1947
459.1131
0
20
40
60
80
100
300
400
500
600

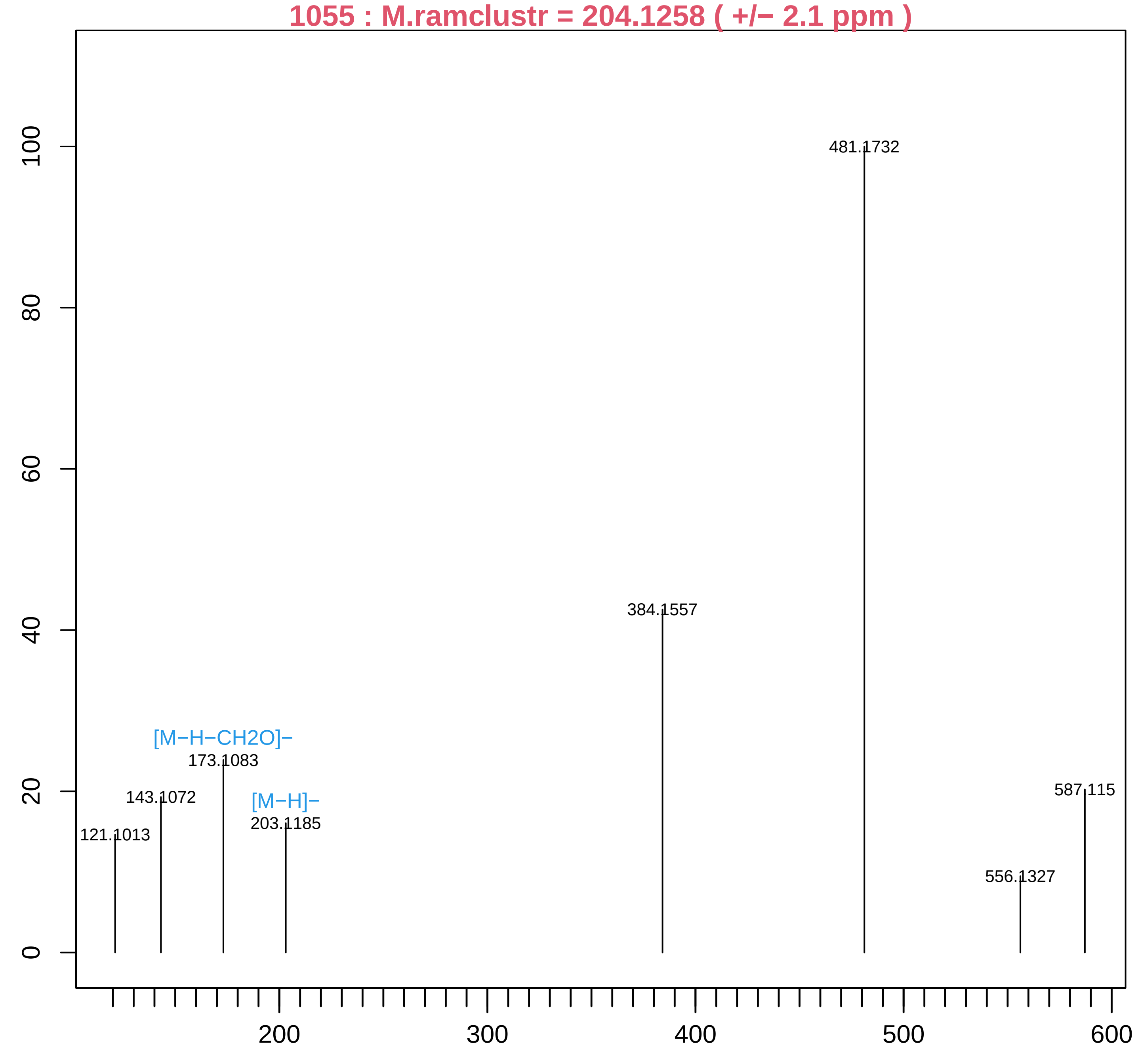
1055 : M.ramclustr = 204.1258 (+/− 2.1 ppm)
481.1732
384.1557
[M−H−CH2O]−
173.1083
143.1072
[M−H]−
203.1185
121.1013
556.1327
587.115
0
20
40
60
80
100
200
300
400
500
600

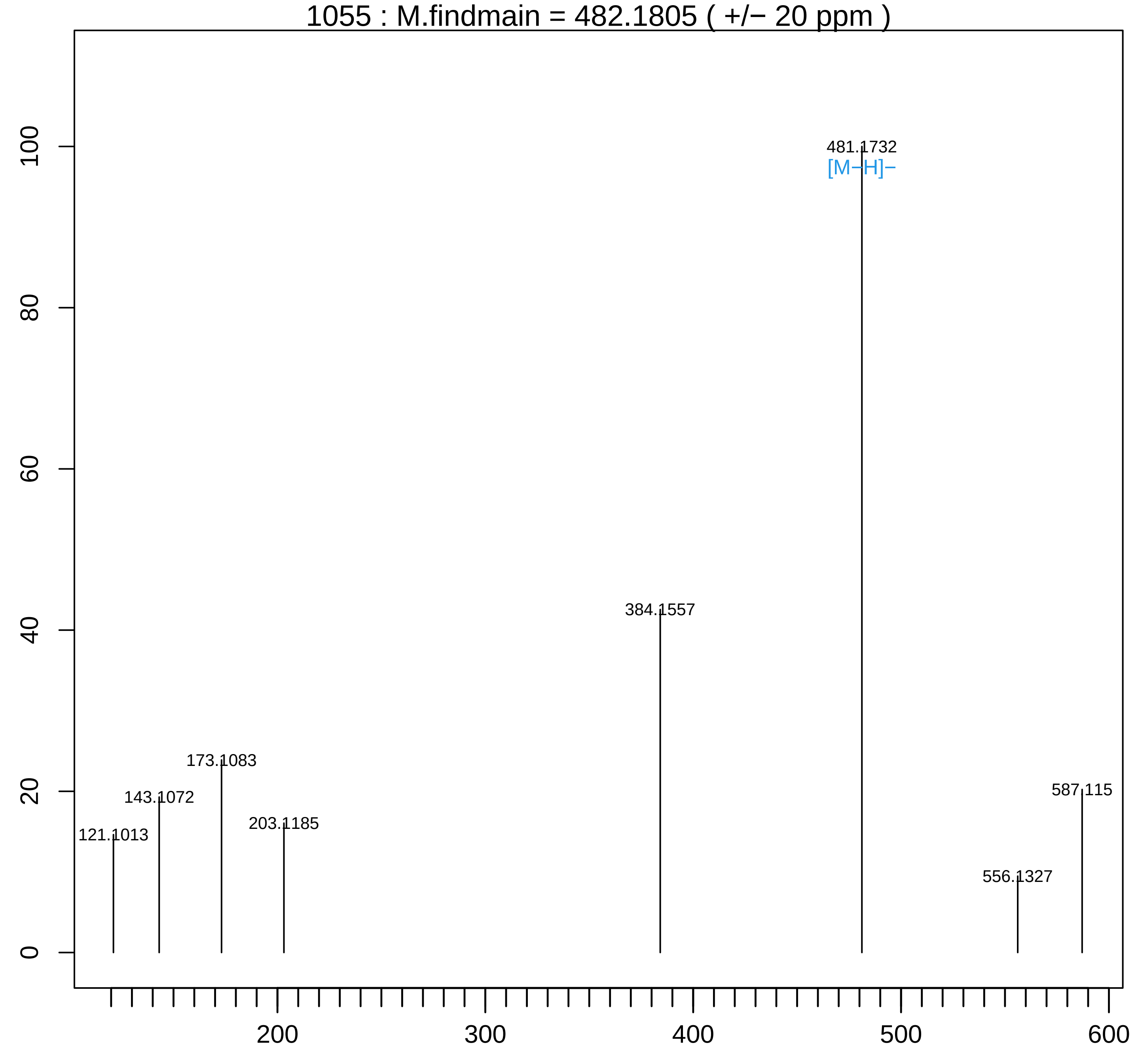
1055 : M.findmain = 482.1805 (+/− 20 ppm)
481.1732
[M−H]−
384.1557
173.1083
143.1072
203.1185
121.1013
556.1327
587.115
0
20
40
60
80
100
200
300
400
500
600

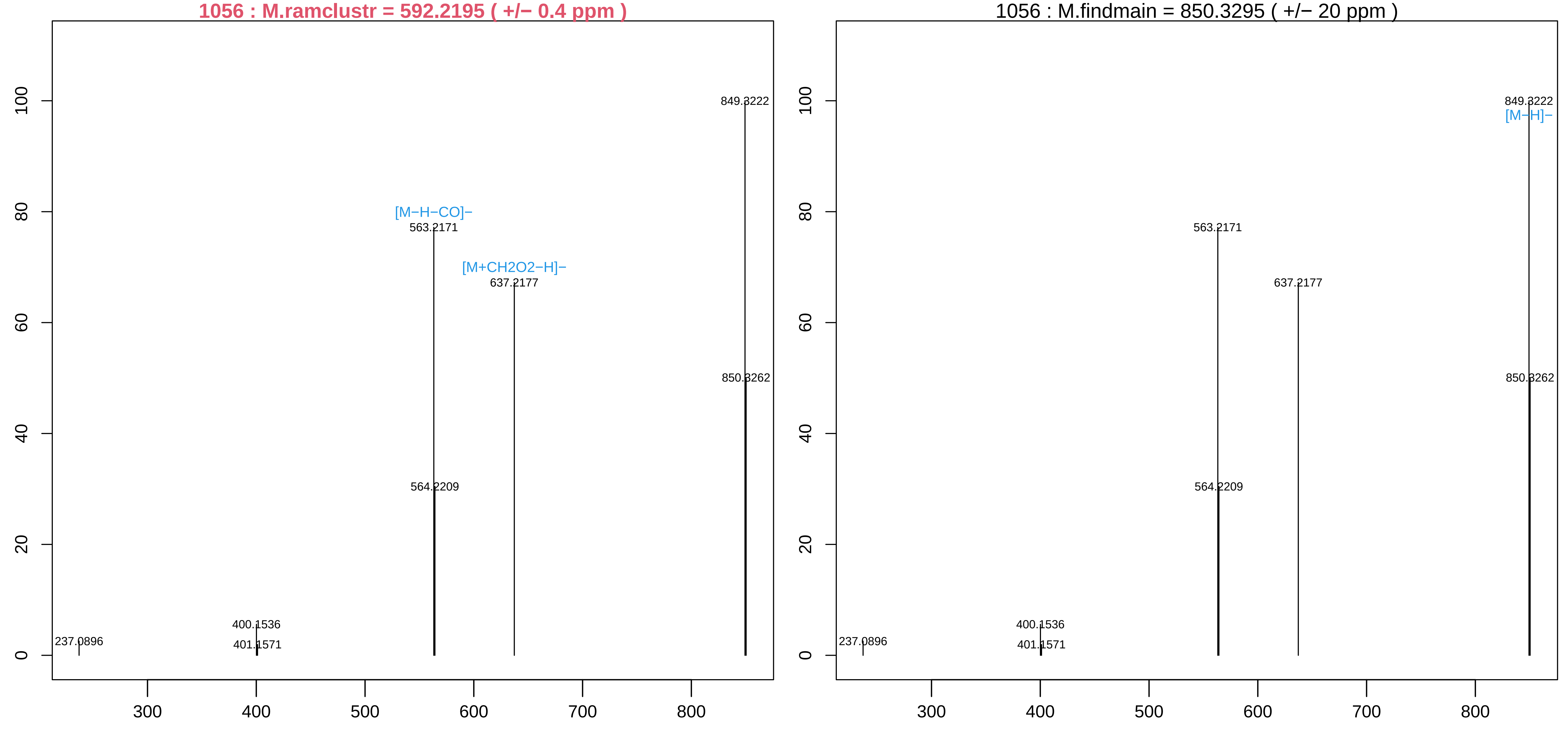
1056 : M.ramclustr = 592.2195 (+/− 0.4 ppm)
[M−H−CO]−
563.2171
[M+CH2O2−H]−
637.2177
849.3222
850.3262
564.2209
400.1536
401.1571
237.0896
1056 : M.findmain = 850.3295 (+/− 20 ppm)
849.3222
[M−H]−
850.3262
563.2171
637.2177
564.2209
400.1536
401.1571
237.0896

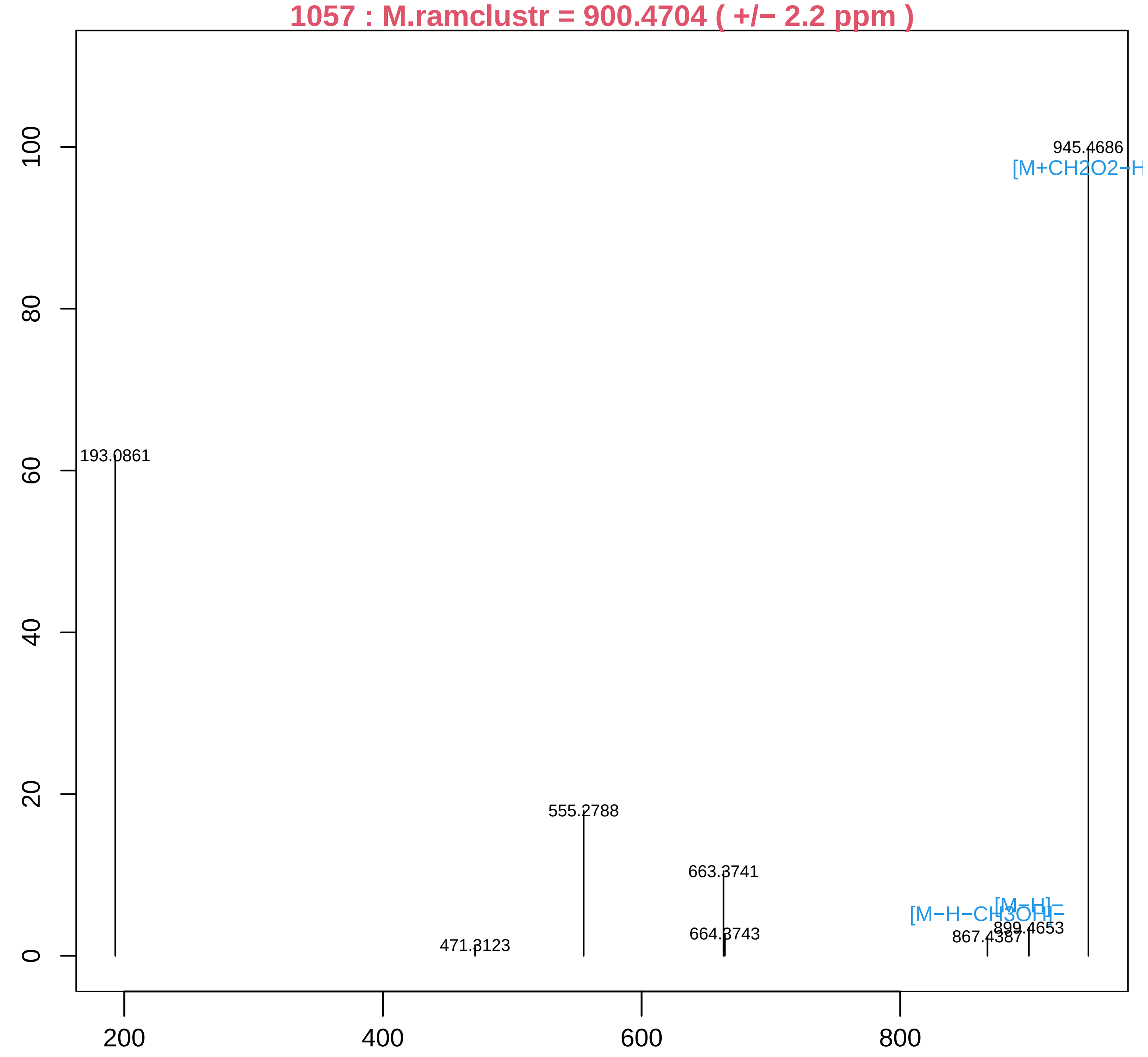
1057 : M.ramclustr = 900.4704 (+/− 2.2 ppm)
945.4686
[M+CH2O2−H
193.0861
555.2788
663.3741
664.3743
471.3123
867.4387
899.4653
[M−H]−
[M−H−CH3OH]−
0
20
40
60
80
100
200
400
600
800

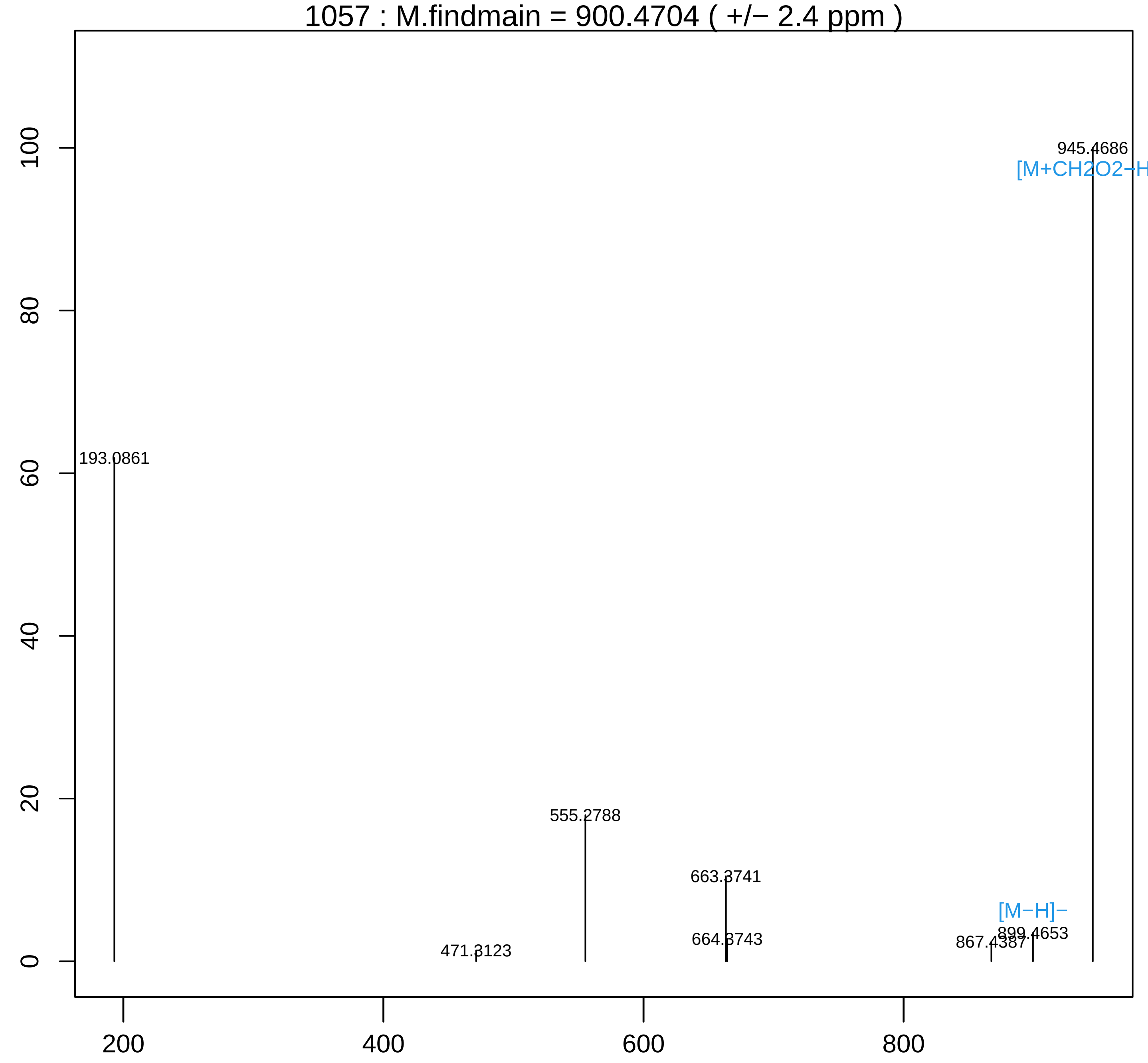
1057 : M.findmain = 900.4704 (+/− 2.4 ppm)
945.4686
[M+CH2O2−H
193.0861
555.2788
663.3741
664.3743
471.3123
867.4387
899.4653
[M−H]−
0
20
40
60
80
100
200
400
600
800

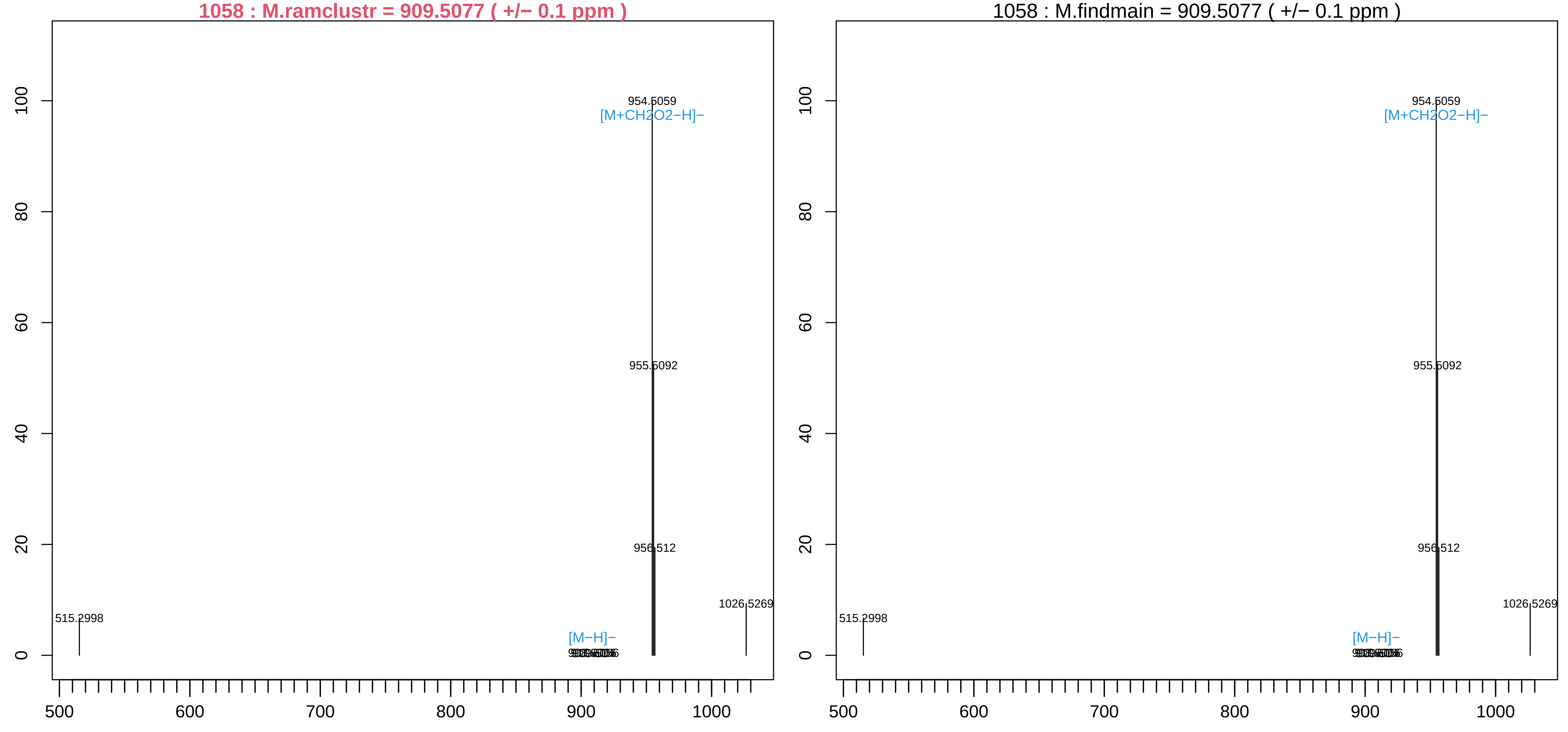
1058 : M.ramclustr = 909.5077 (+/− 0.1 ppm)
954.5059
[M+CH2O2−H]−
955.5092
956.512
1026.5269
515.2998
[M−H]−
1058 : M.findmain = 909.5077 (+/− 0.1 ppm)
954.5059
[M+CH2O2−H]−
955.5092
956.512
1026.5269
515.2998
[M−H]−
0
20
40
60
80
100
500
600
700
800
900
1000

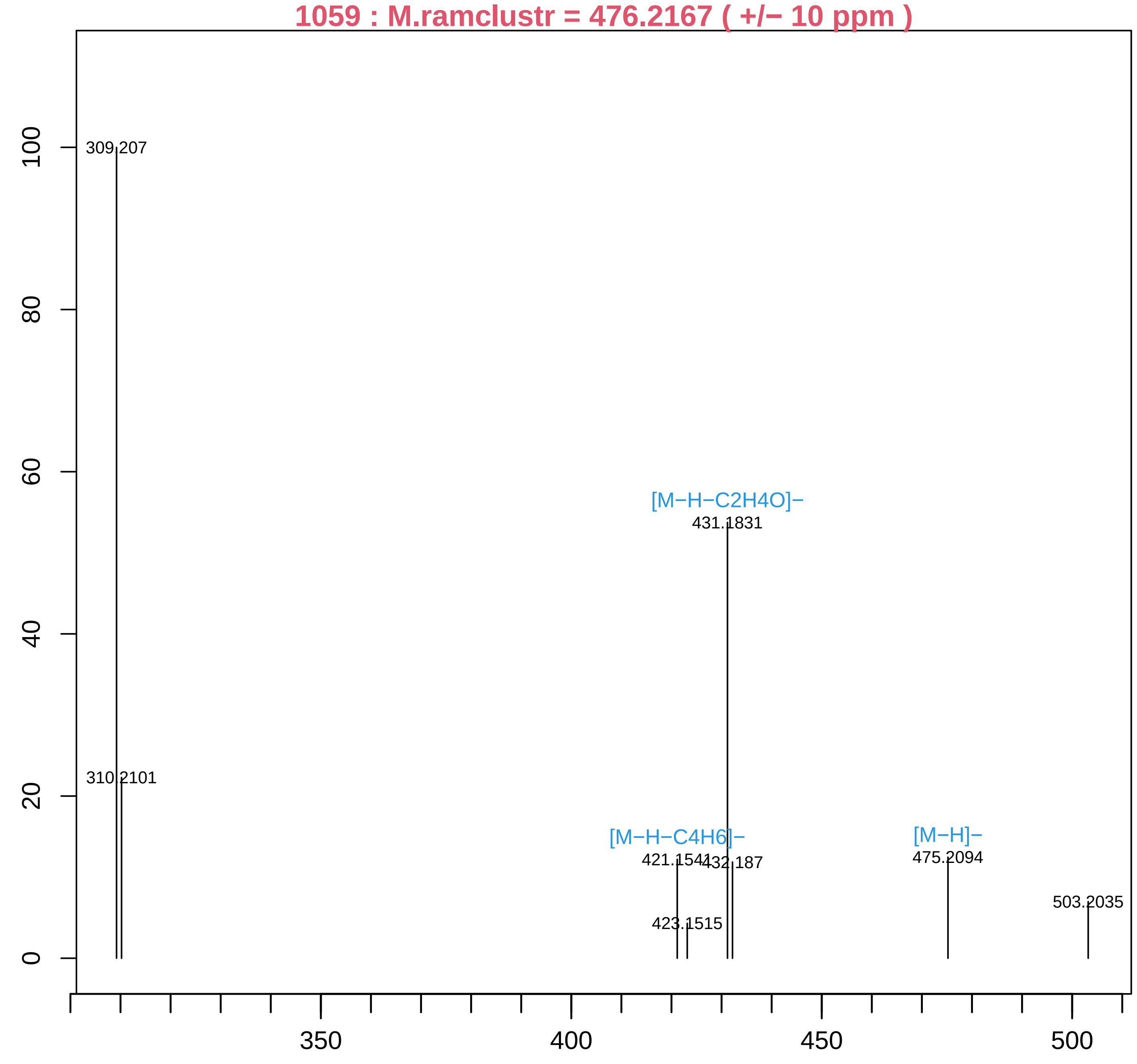

1059 : M.ramclustr = 476.2167 (+/− 10 ppm)
309.207
310.2101
[M−H−C2H4O]−
431.1831
[M−H−C4H6]−
421.1541
432.187
423.1515
[M−H]−
475.2094
503.2035
0
20
40
60
80
100
350
400
450
500

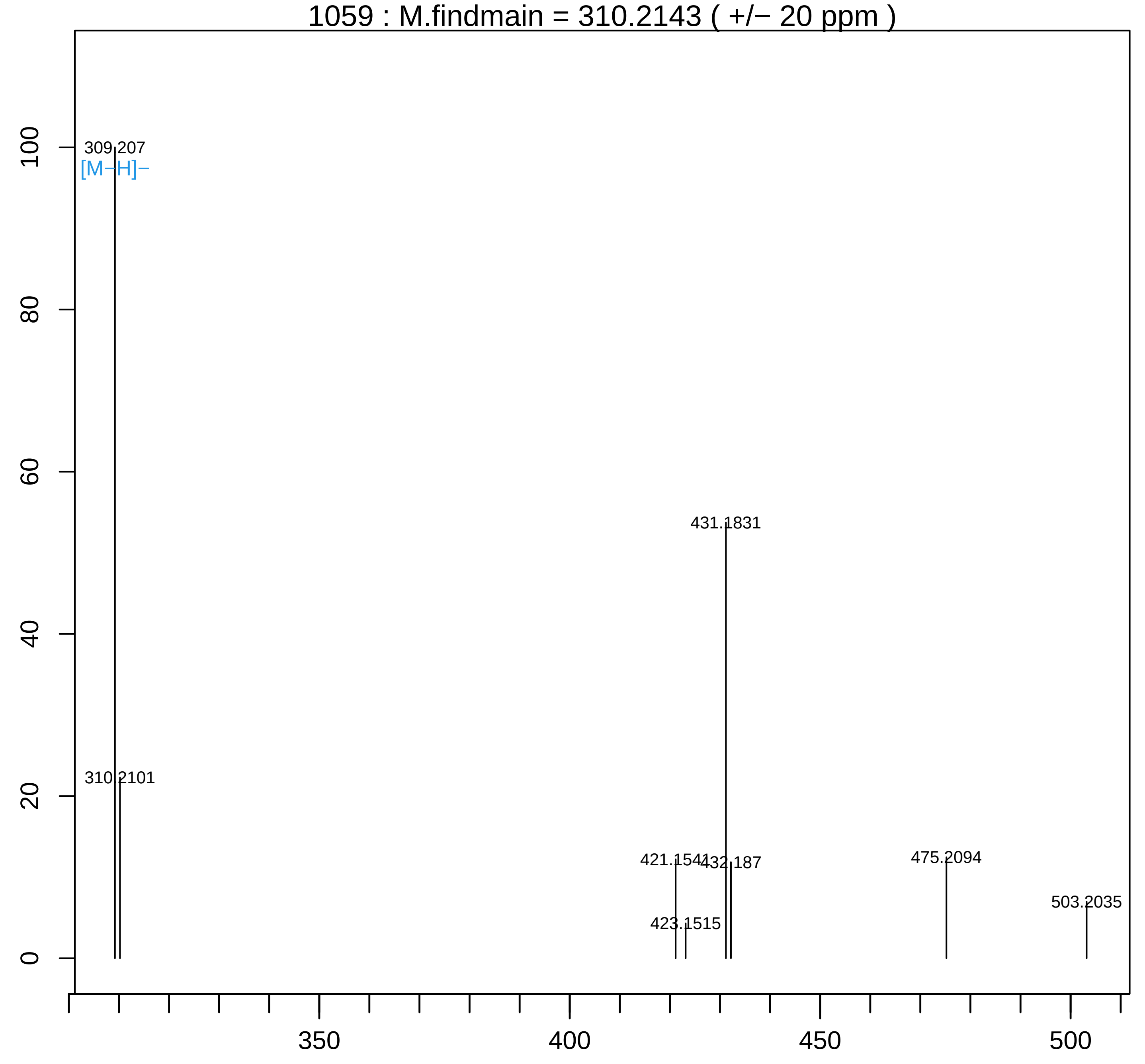

1059 : M.findmain = 310.2143 (+/− 20 ppm)
309.207
[M−H]−
310.2101
431.1831
421.1541
432.187
423.1515
475.2094
503.2035
0
20
40
60
80
100
350
400
450
500

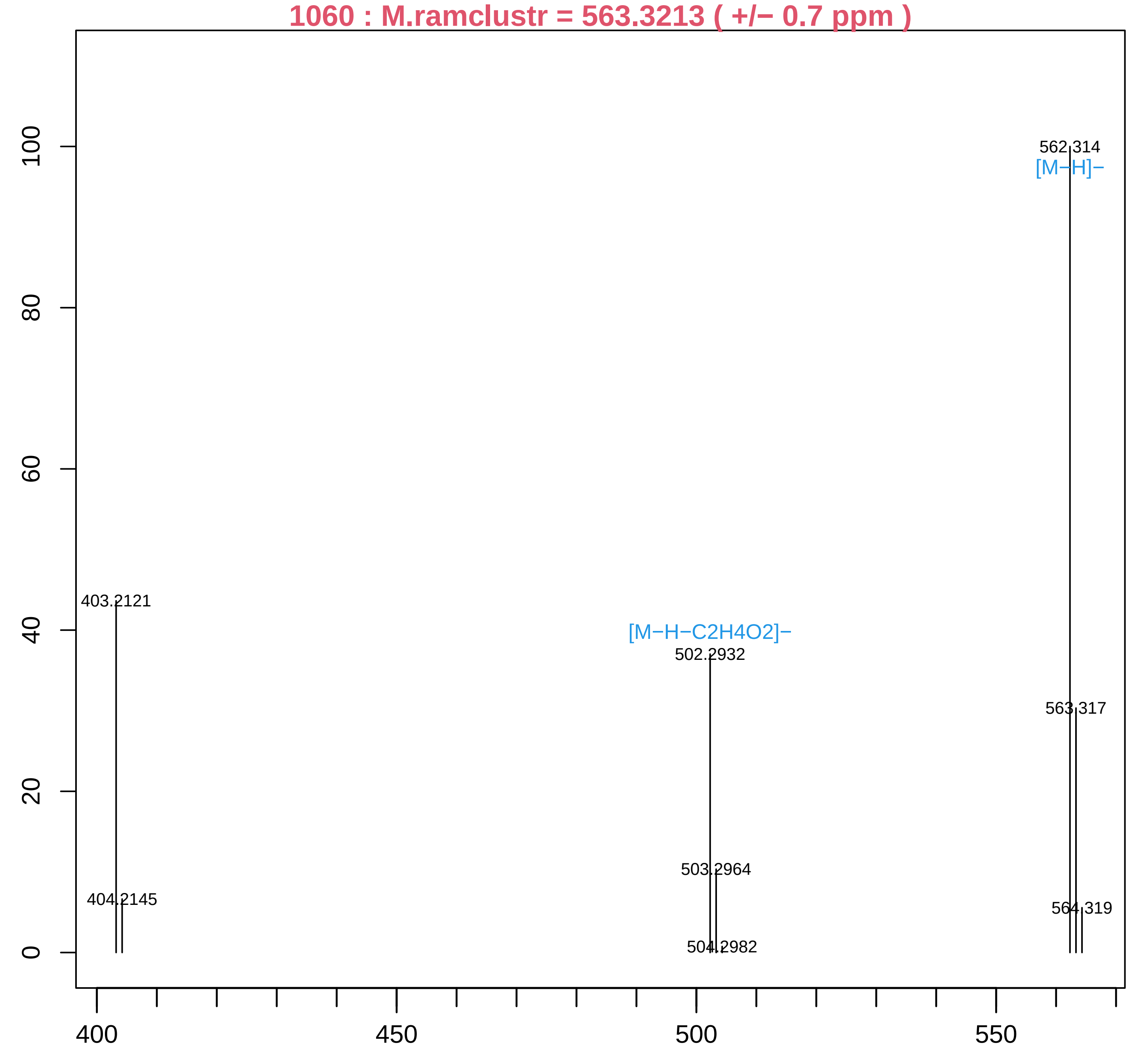
1060 : M.ramclustr = 563.3213 (+/− 0.7 ppm)
403.2121
404.2145
[M−H−C2H4O2]−
502.2932
503.2964
504.2982
562.314
[M−H]−
563.317
564.319
0
20
40
60
80
100
400
450
500
550

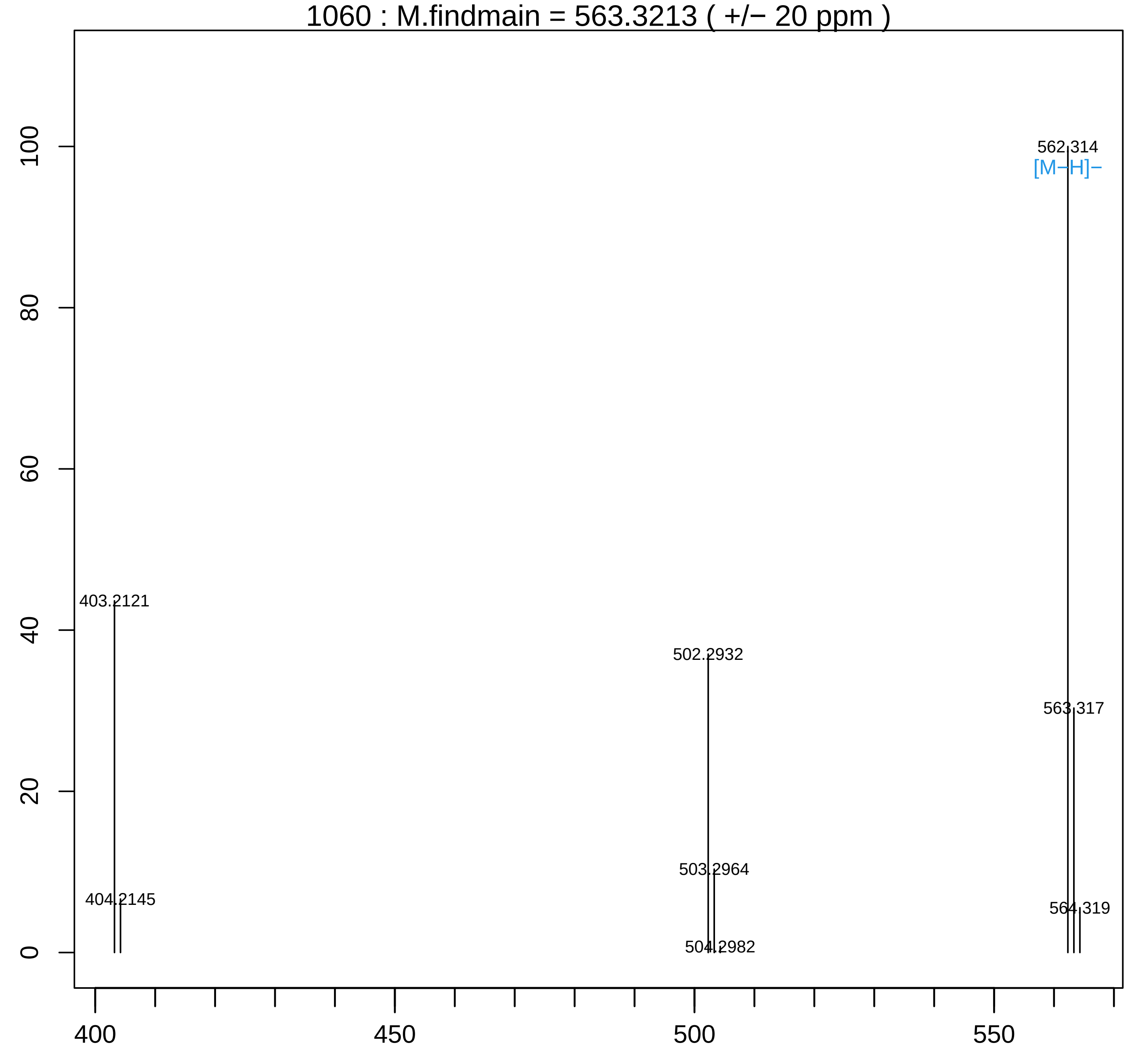
1060 : M.findmain = 563.3213 (+/− 20 ppm)
403.2121
404.2145
502.2932
503.2964
504.2982
562.314
[M−H]−
563.317
564.319
0
20
40
60
80
100
400
450
500
550

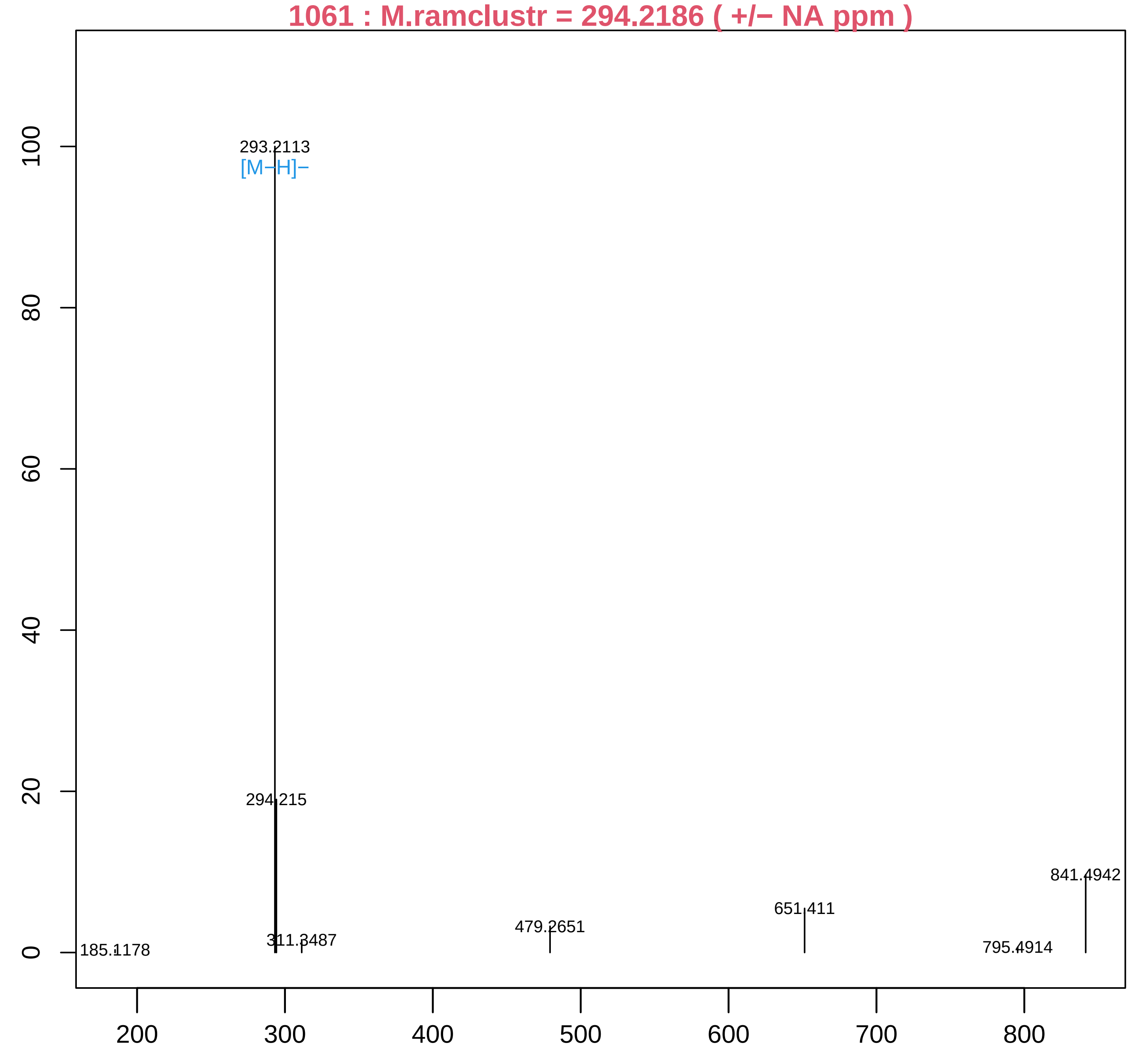
1061 : M.ramclustr = 294.2186 (+/− NA ppm)
293.2113
[M−H]−
294.215
311.3487
185.1178
479.2651
651.411
795.4914
841.4942
0
20
40
60
80
100
200
300
400
500
600
700
800

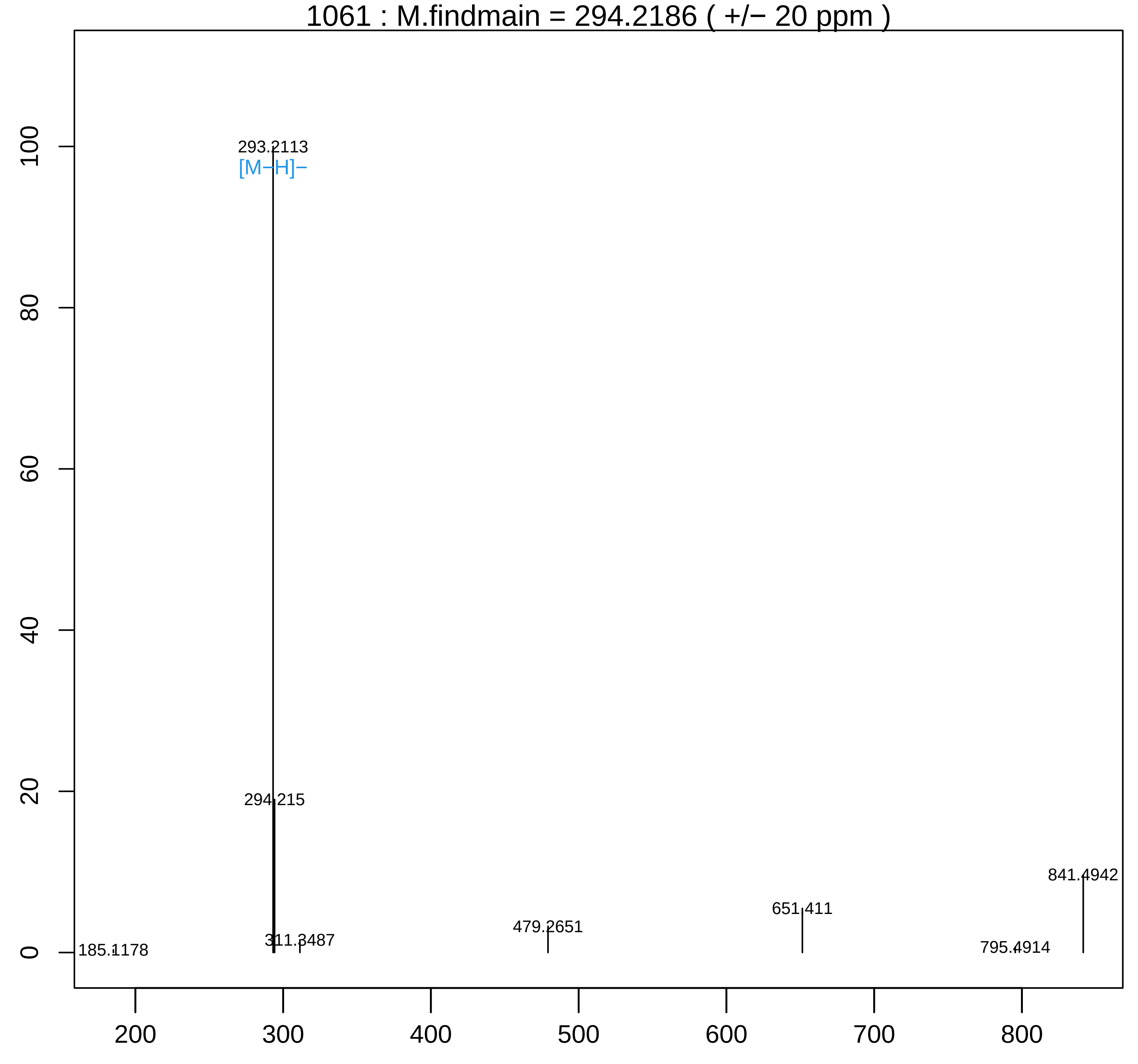
1061 : M.findmain = 294.2186 (+/− 20 ppm)
293.2113
[M−H]−
294.215
311.3487
185.1178
479.2651
651.411
795.4914
841.4942
0
20
40
60
80
100
200
300
400
500
600
700
800

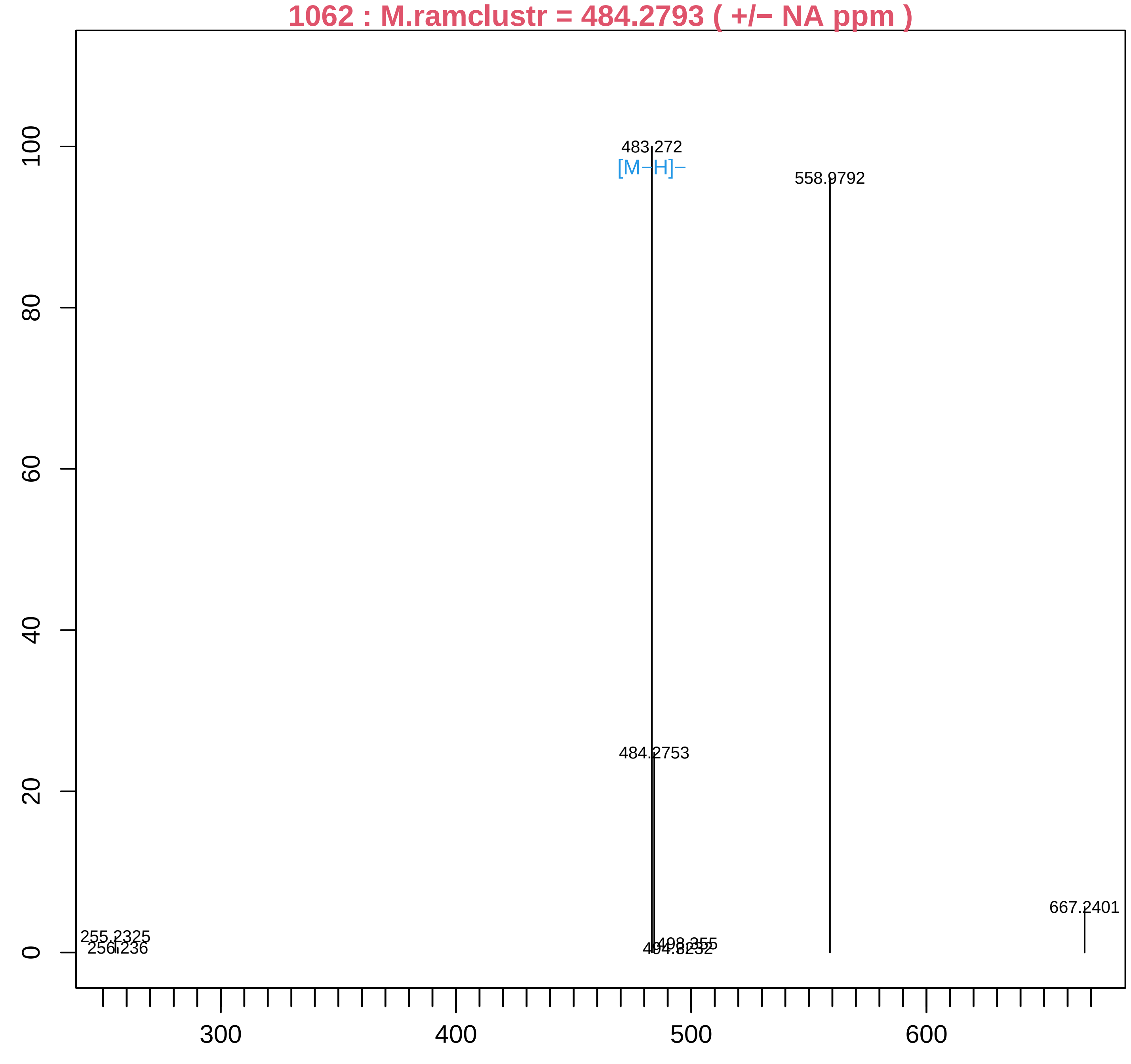
1062 : M.ramclustr = 484.2793 (+/− NA ppm)
483.272
[M−H]−
558.9792
484.2753
667.2401
255.2325
256.236
498.355
0
20
40
60
80
100
300
400
500
600

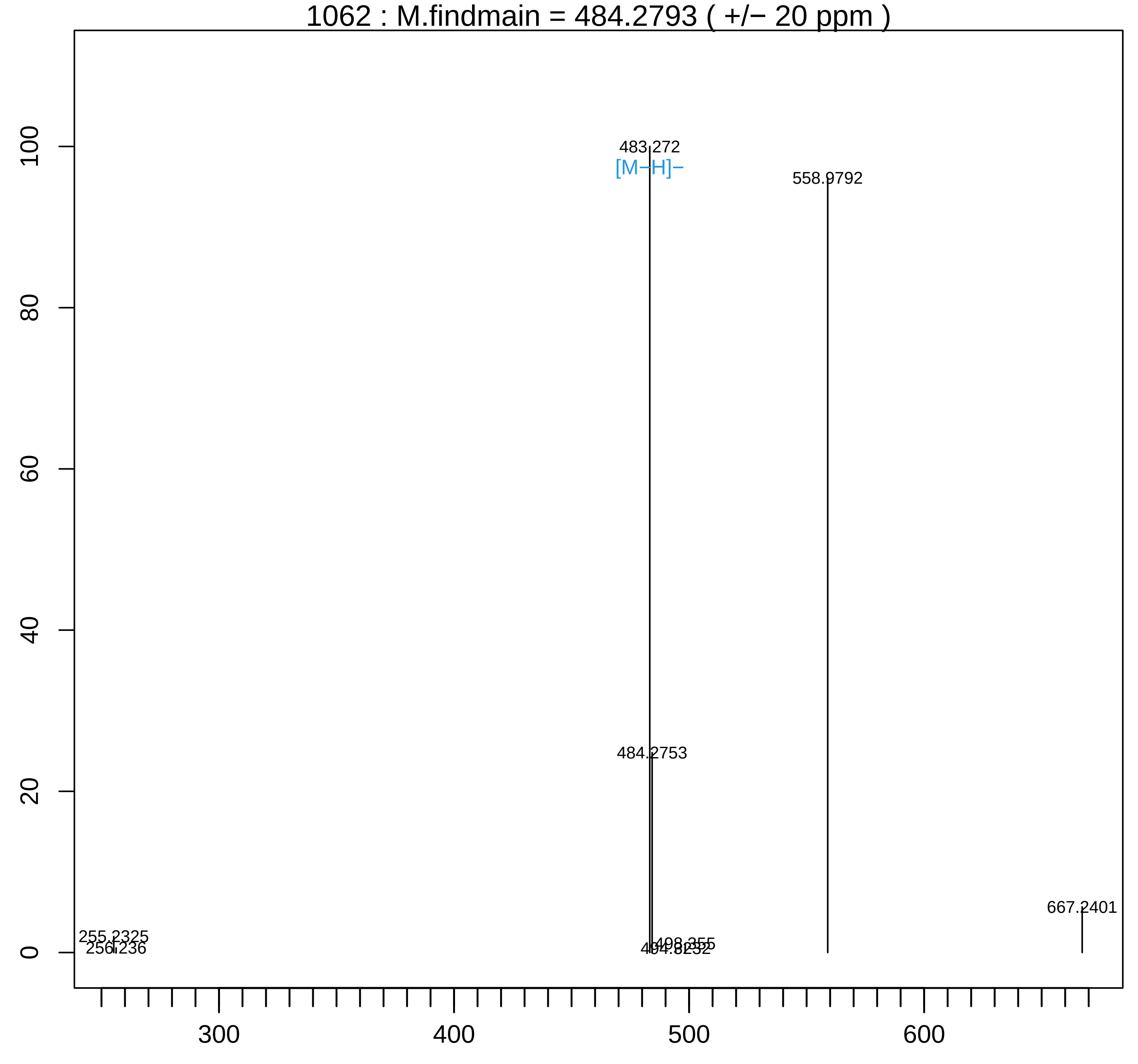
1062 : M.findmain = 484.2793 (+/− 20 ppm)
483.272
[M−H]−
558.9792
484.2753
667.2401
255.2325
256.236
498.355
0
20
40
60
80
100
300
400
500
600

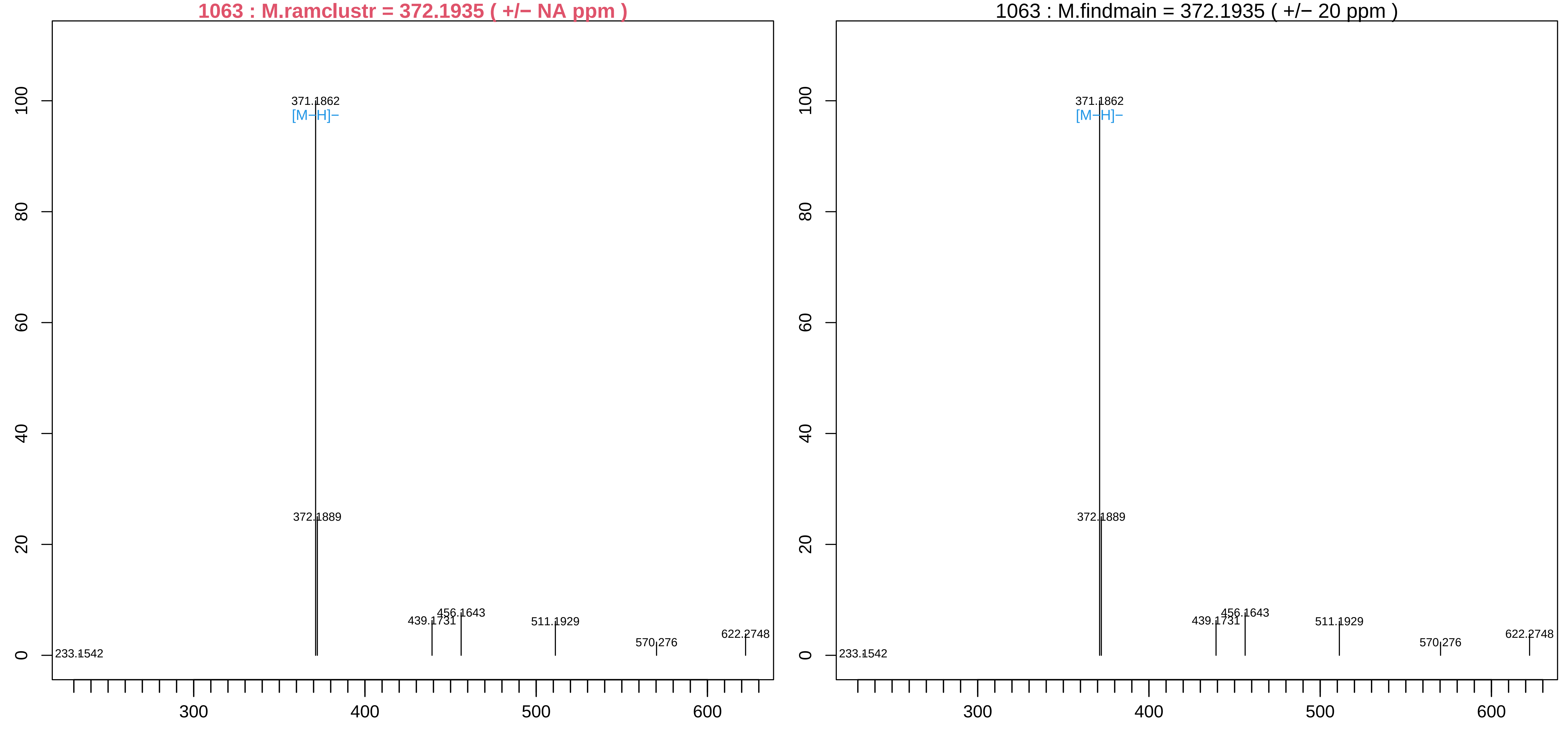
1063 : M.ramclustr = 372.1935 (+/− NA ppm)
371.1862
[M−H]−
372.1889
439.1731
456.1643
511.1929
570.276
622.2748
233.1542
1063 : M.findmain = 372.1935 (+/− 20 ppm)
371.1862
[M−H]−
372.1889
439.1731
456.1643
511.1929
570.276
622.2748
233.1542

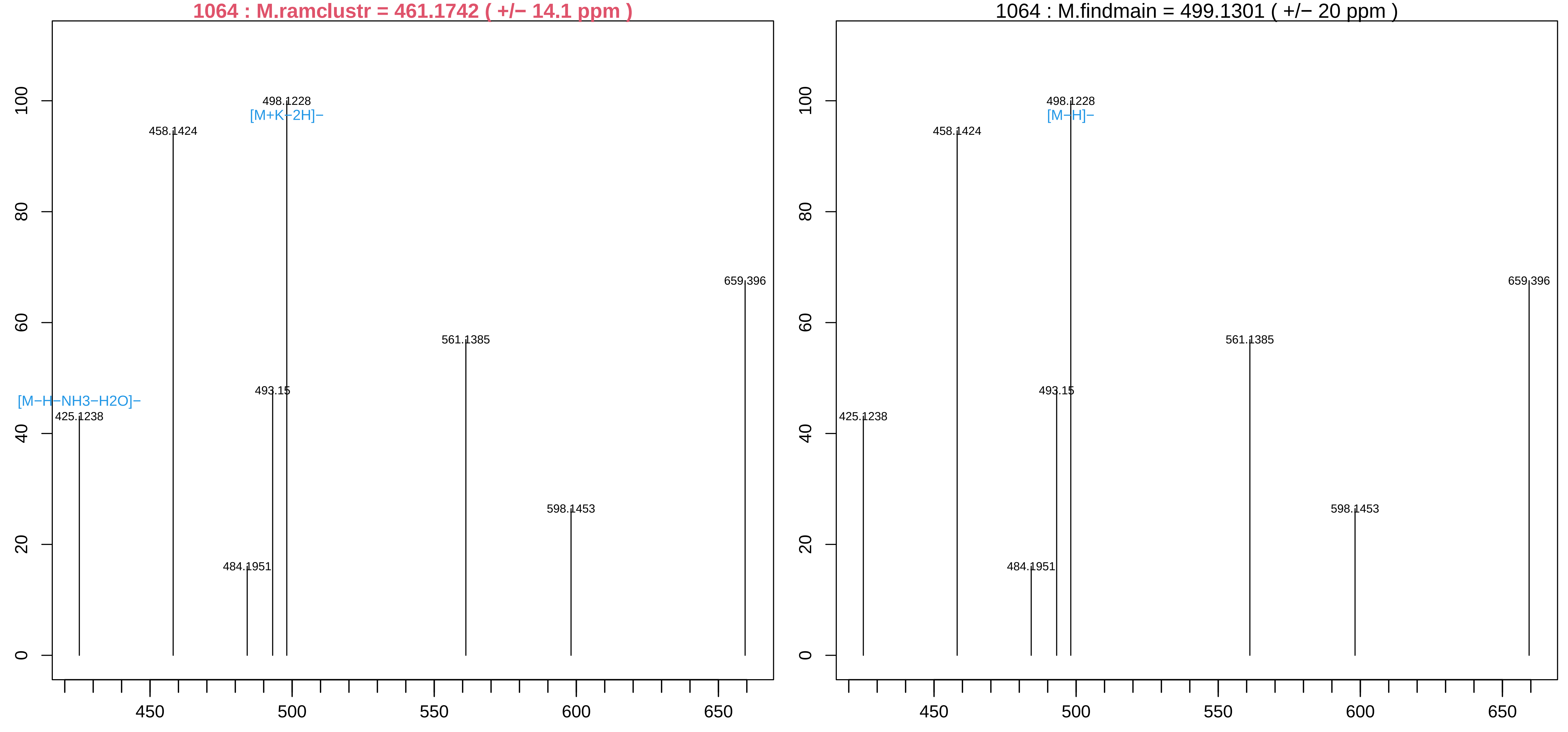
1064 : M.ramclustr = 461.1742 (+/− 14.1 ppm)
[M−H−NH3−H2O]−
425.1238
458.1424
484.1951
493.15
498.1228
[M+K−2H]−
561.1385
598.1453
659.396
1064 : M.findmain = 499.1301 (+/− 20 ppm)
425.1238
458.1424
484.1951
493.15
498.1228
[M−H]−
561.1385
598.1453
659.396
0
20
40
60
80
100
450
500
550
600
650

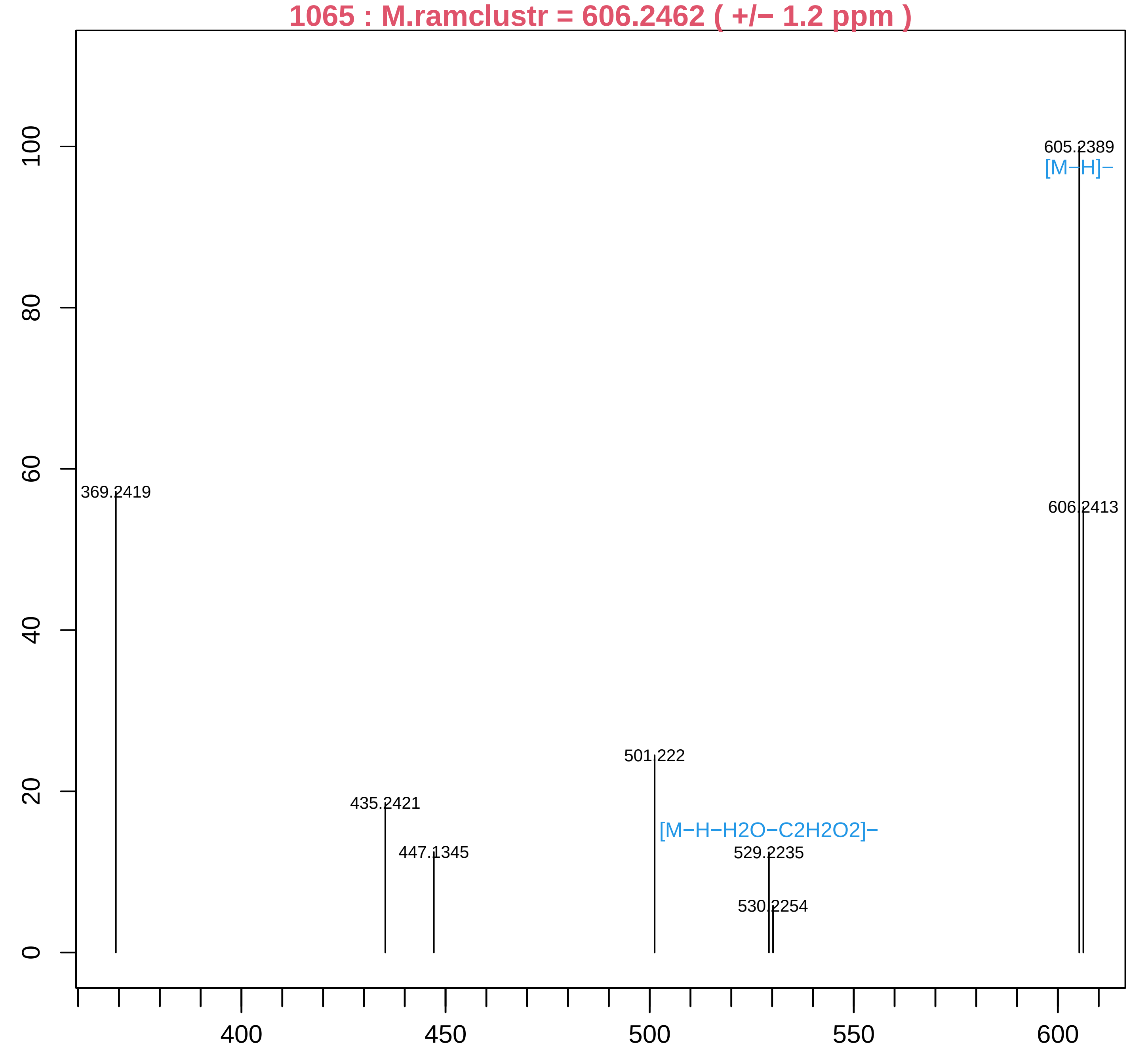

1065 : M.ramclustr = 606.2462 (+/− 1.2 ppm)
605.2389
[M−H]−
369.2419
606.2413
501.222
435.2421
447.1345
[M−H−H2O−C2H2O2]−
529.2235
530.2254
0
20
40
60
80
100
400
450
500
550
600

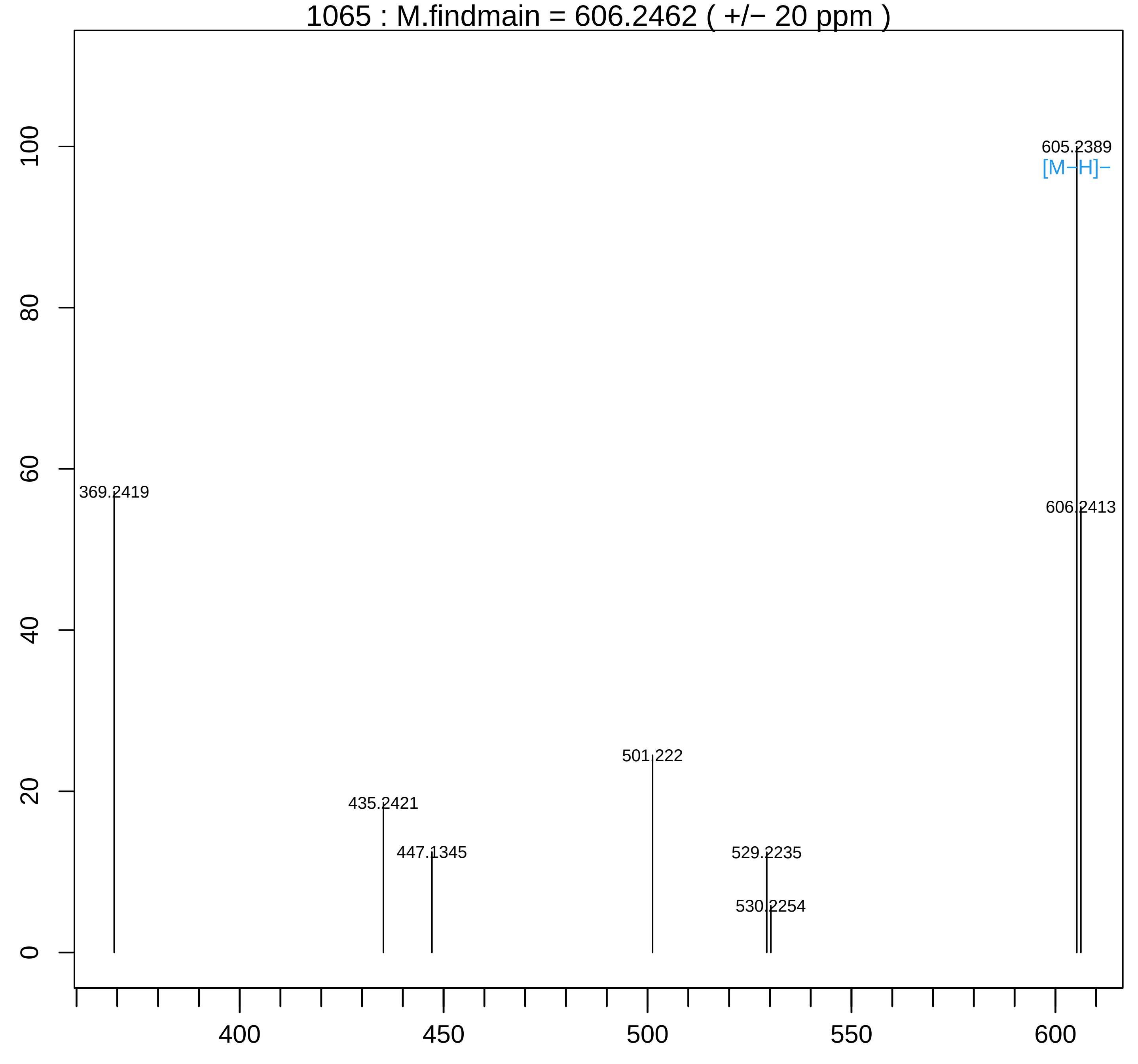

1065 : M.findmain = 606.2462 (+/− 20 ppm)
605.2389
[M−H]−
369.2419
606.2413
501.222
435.2421
447.1345
529.2235
530.2254
0
20
40
60
80
100
400
450
500
550
600

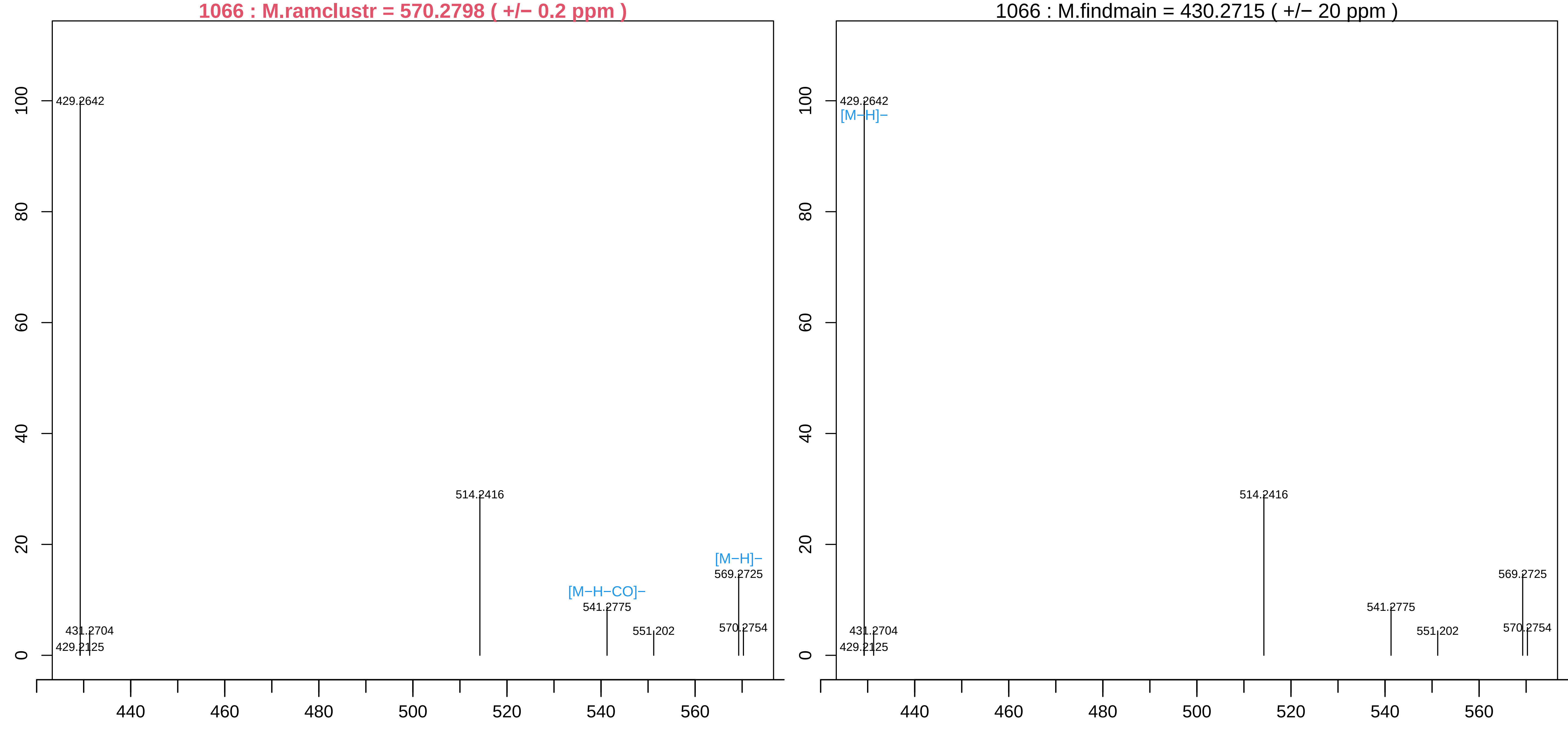

1066 : M.ramclustr = 570.2798 (+/− 0.2 ppm)
429.2642
431.2704
429.2125
514.2416
[M−H−CO]−
541.2775
551.202
[M−H]−
569.2725
570.2754
1066 : M.findmain = 430.2715 (+/− 20 ppm)
429.2642
[M−H]−
431.2704
429.2125
514.2416
541.2775
551.202
569.2725
570.2754

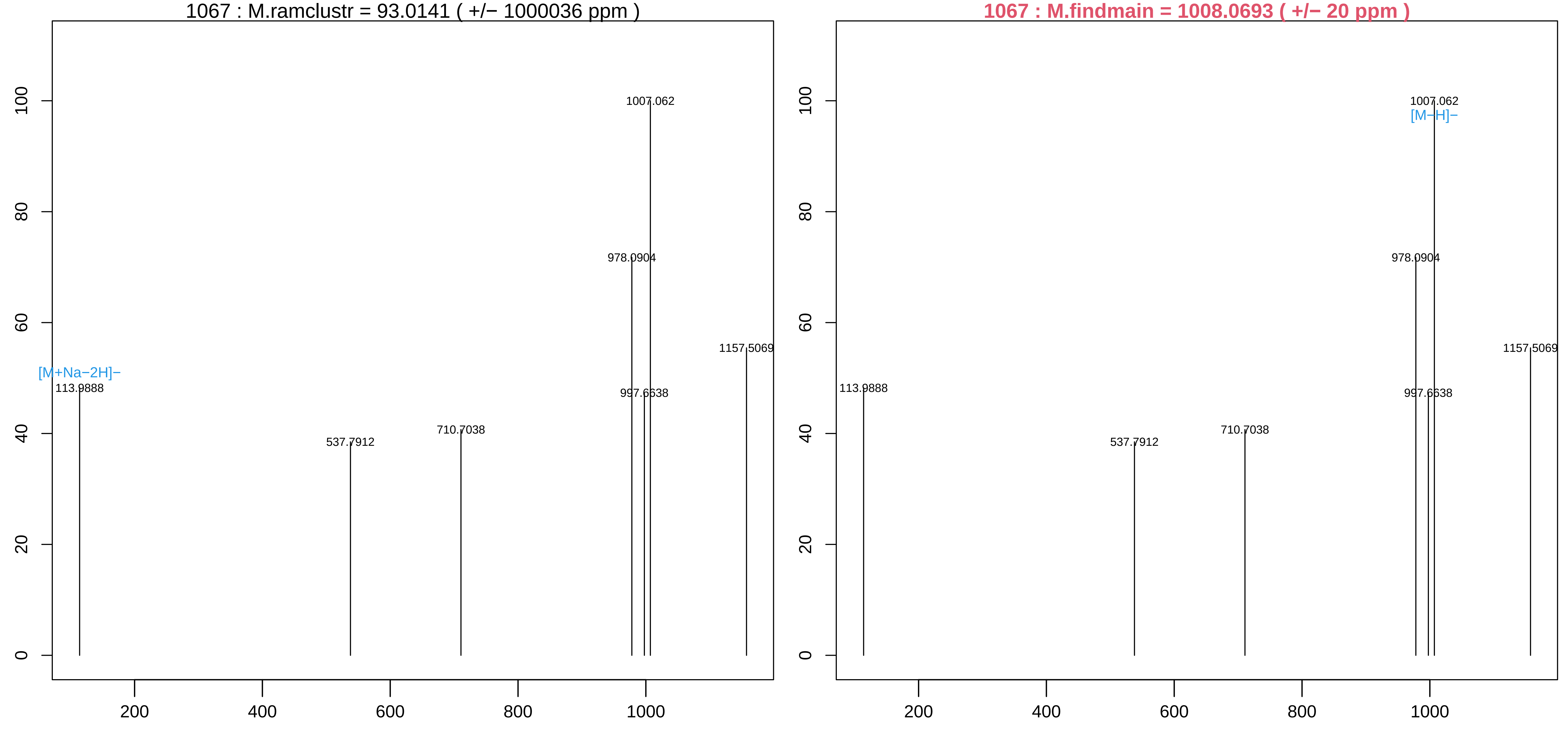
1067 : M.ramclustr = 93.0141 (+/− 1000036 ppm)
1067 : M.findmain = 1008.0693 (+/− 20 ppm)
1007.062
[M−H]−
978.0904
1157.5069
[M+Na−2H]−
113.9888
997.6638
710.7038
537.7912
0
20
40
60
80
100
200
400
600
800
1000

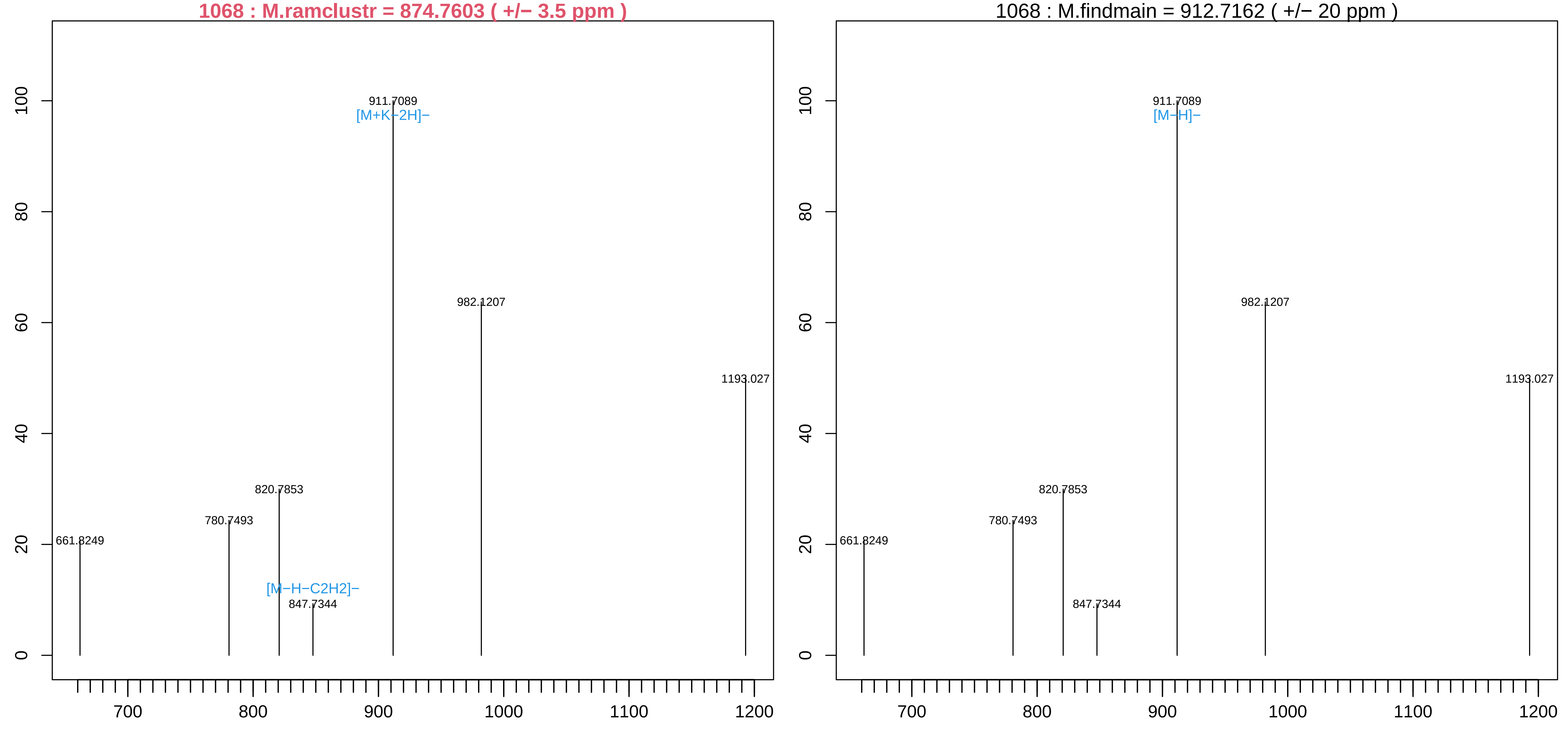
1068 : M.ramclustr = 874.7603 (+/− 3.5 ppm)
911.7089
[M+K−2H]−
982.1207
1193.027
820.7853
780.7493
661.8249
[M−H−C2H2]−
847.7344
0
20
40
60
80
100
700
800
900
1000
1100
1200
1068 : M.findmain = 912.7162 (+/− 20 ppm)
911.7089
[M−H]−
982.1207
1193.027
820.7853
780.7493
661.8249
847.7344
0
20
40
60
80
100
700
800
900
1000
1100
1200

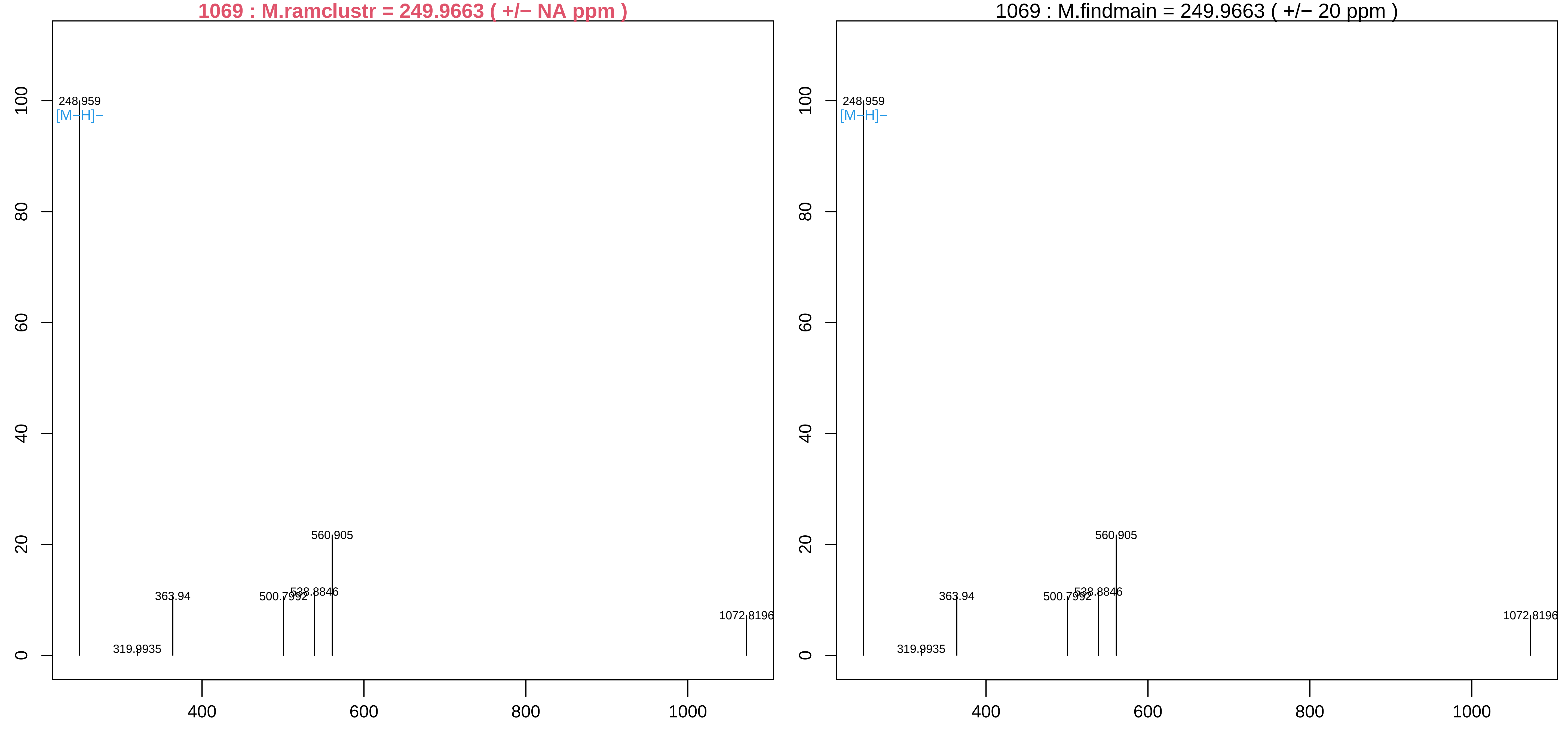
1069 : M.ramclustr = 249.9663 (+/− NA ppm)
248.959
[M−H]−
319.9935
363.94
500.7992
538.8846
560.905
1072.8196
0
20
40
60
80
100
400
600
800
1000
1069 : M.findmain = 249.9663 (+/− 20 ppm)
248.959
[M−H]−
319.9935
363.94
500.7992
538.8846
560.905
1072.8196
0
20
40
60
80
100
400
600
800
1000

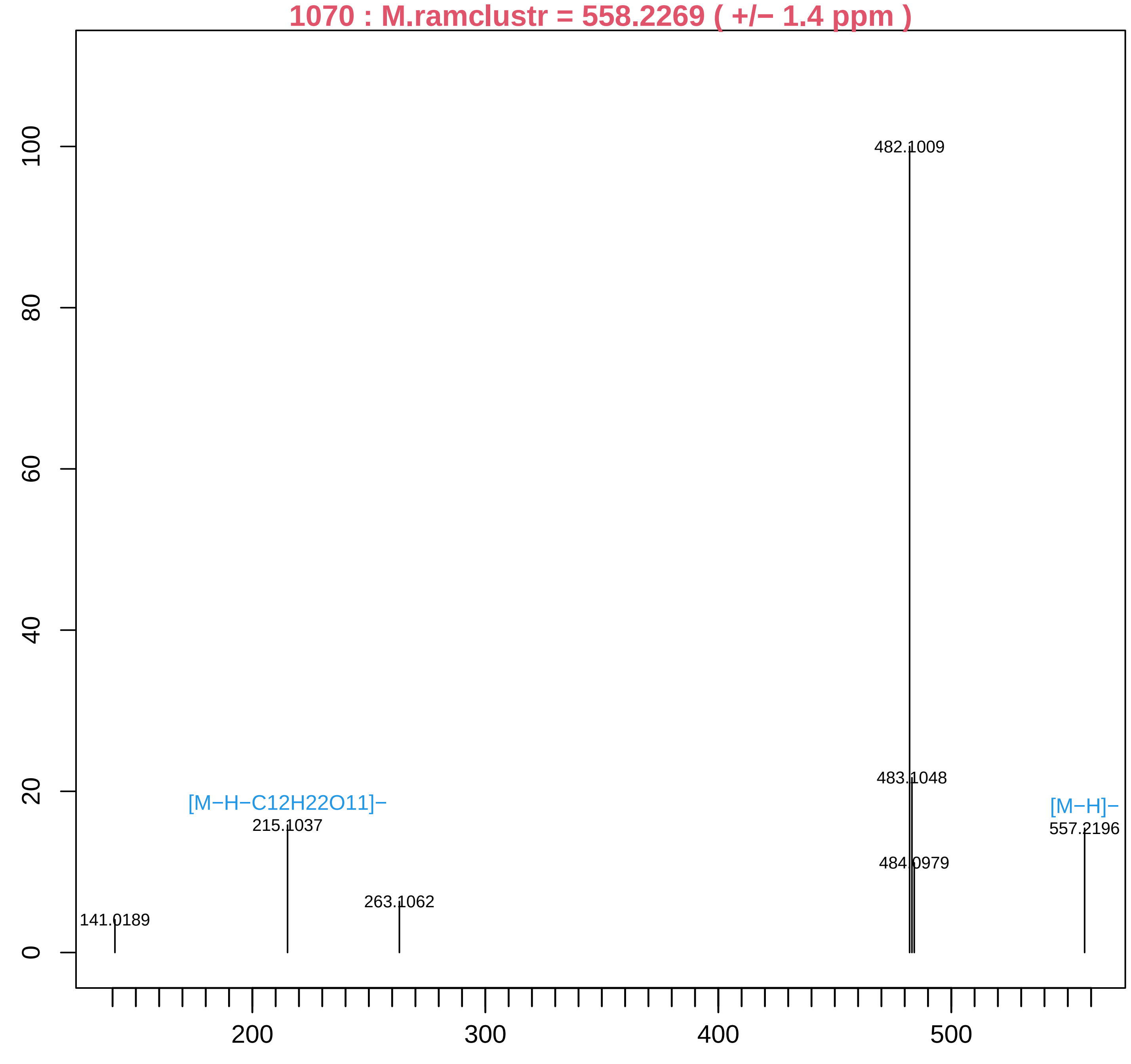

1070 : M.ramclustr = 558.2269 (+/− 1.4 ppm)
482.1009
483.1048
484.0979
[M−H−C12H22O11]−
215.1037
263.1062
141.0189
[M−H]−
557.2196
0
20
40
60
80
100
200
300
400
500

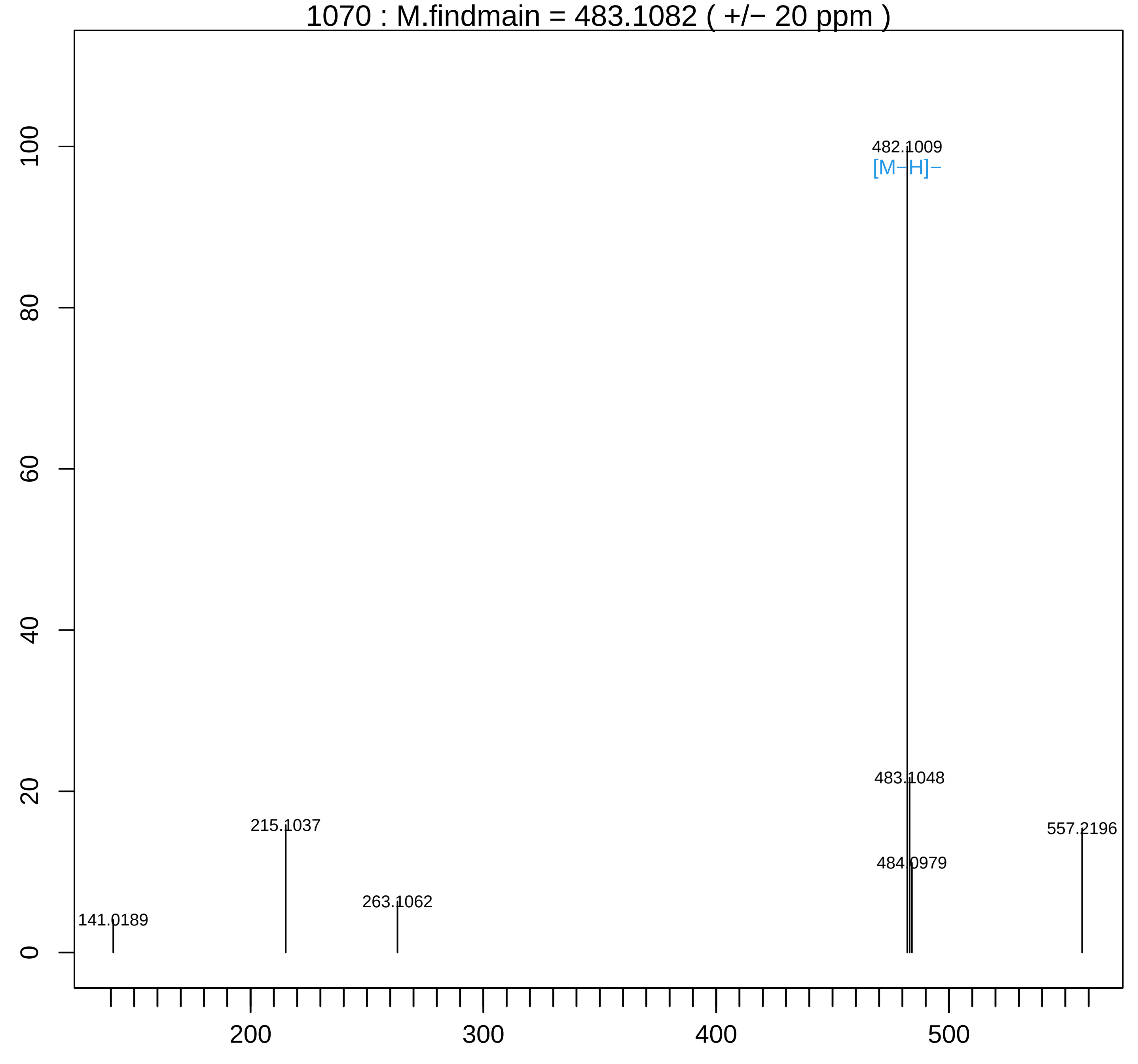

1070 : M.findmain = 483.1082 (+/− 20 ppm)
482.1009
[M−H]−
483.1048
484.0979
215.1037
263.1062
141.0189
557.2196
0
20
40
60
80
100
200
300
400
500

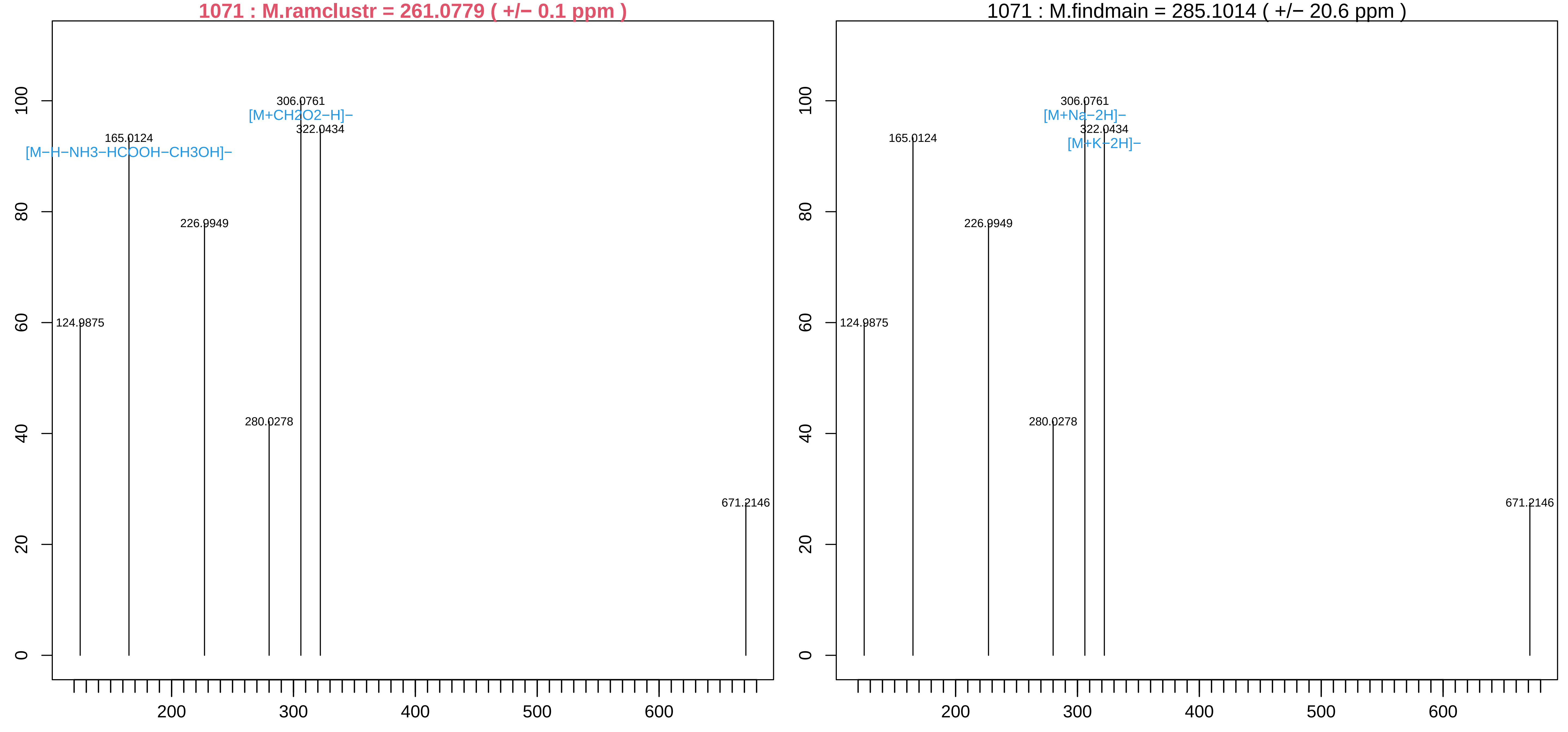
1071 : M.ramclustr = 261.0779 (+/− 0.1 ppm)
[M−H−NH3−HCOOH−CH3OH]−
[M+CH2O2−H]−
124.9875
165.0124
226.9949
280.0278
306.0761
322.0434
671.2146
1071 : M.findmain = 285.1014 (+/− 20.6 ppm)
[M+Na−2H]−
[M+K−2H]−
124.9875
165.0124
226.9949
280.0278
306.0761
322.0434
671.2146
0
20
40
60
80
100
200
300
400
500
600

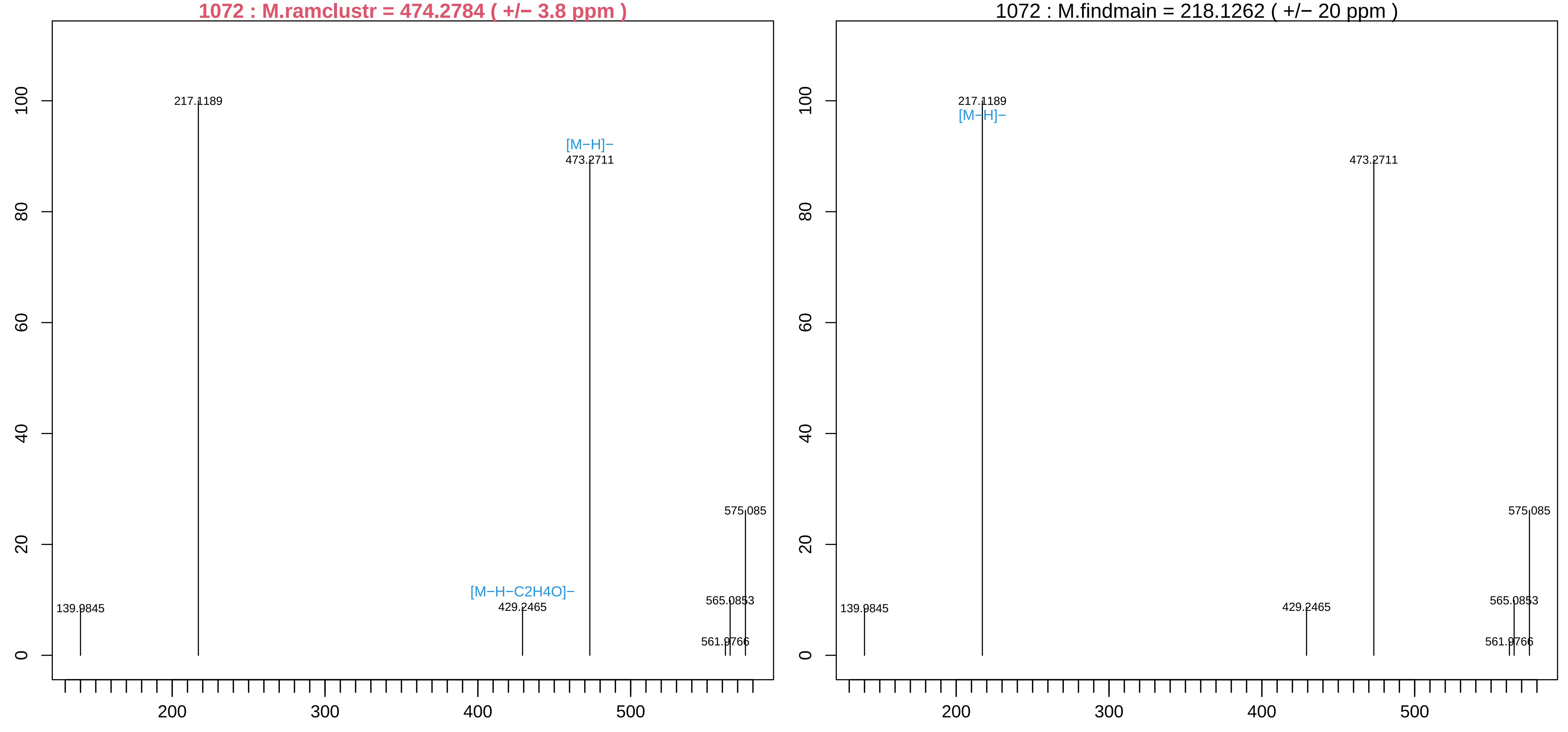

1072 : M.ramclustr = 474.2784 (+/− 3.8 ppm)
217.1189
[M−H]−
473.2711
575.085
565.0853
561.9766
[M−H−C2H4O]−
429.2465
139.9845
1072 : M.findmain = 218.1262 (+/− 20 ppm)
217.1189
[M−H]−
473.2711
575.085
565.0853
561.9766
429.2465
139.9845
0
20
40
60
80
100
200
300
400
500

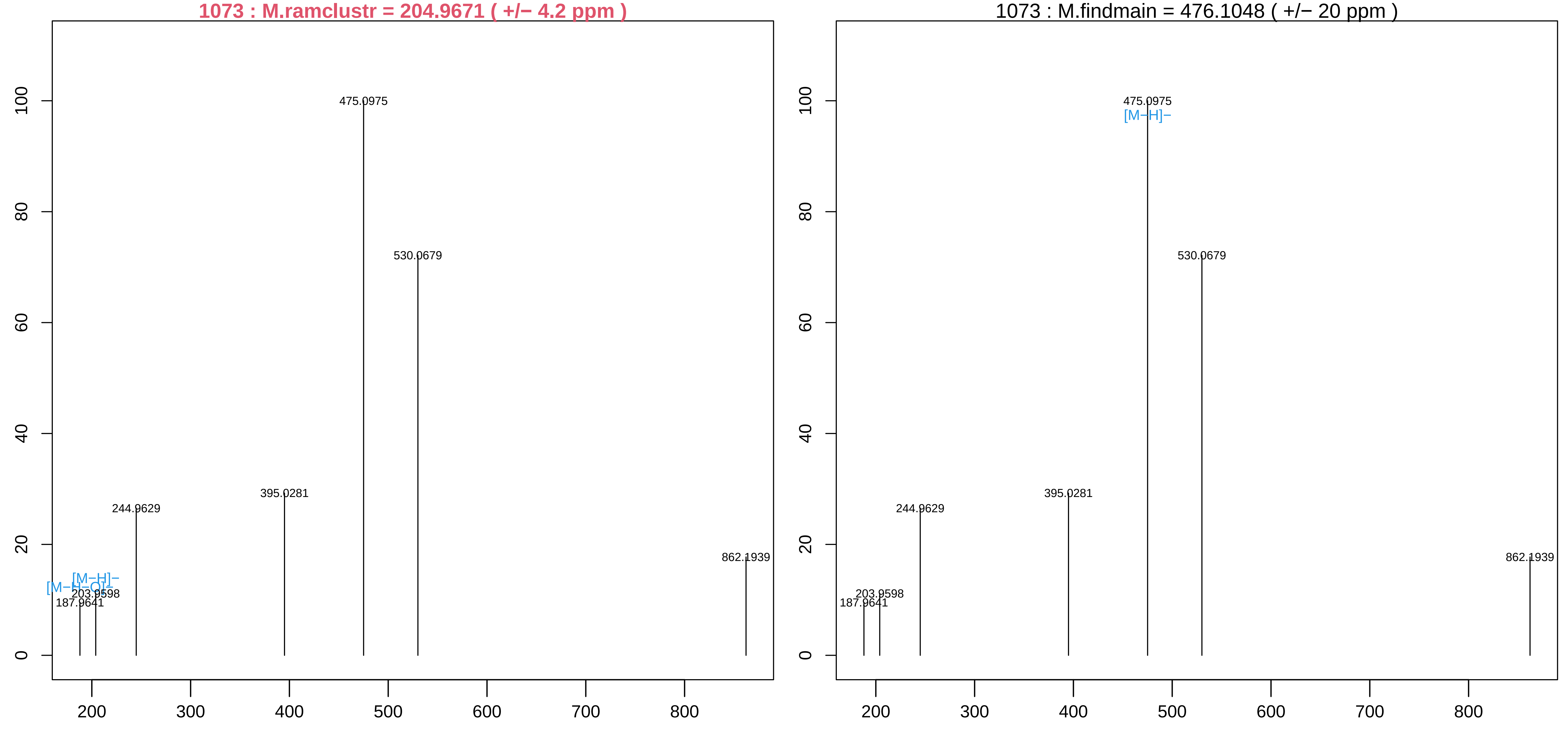
1073 : M.ramclustr = 204.9671 (+/− 4.2 ppm)
475.0975
530.0679
395.0281
244.9629
862.1939
[M−H]−
[M−H−O]−
203.9598
187.9641
1073 : M.findmain = 476.1048 (+/− 20 ppm)
475.0975
[M−H]−
530.0679
395.0281
244.9629
862.1939
203.9598
187.9641
0
20
40
60
80
100
200
300
400
500
600
700
800

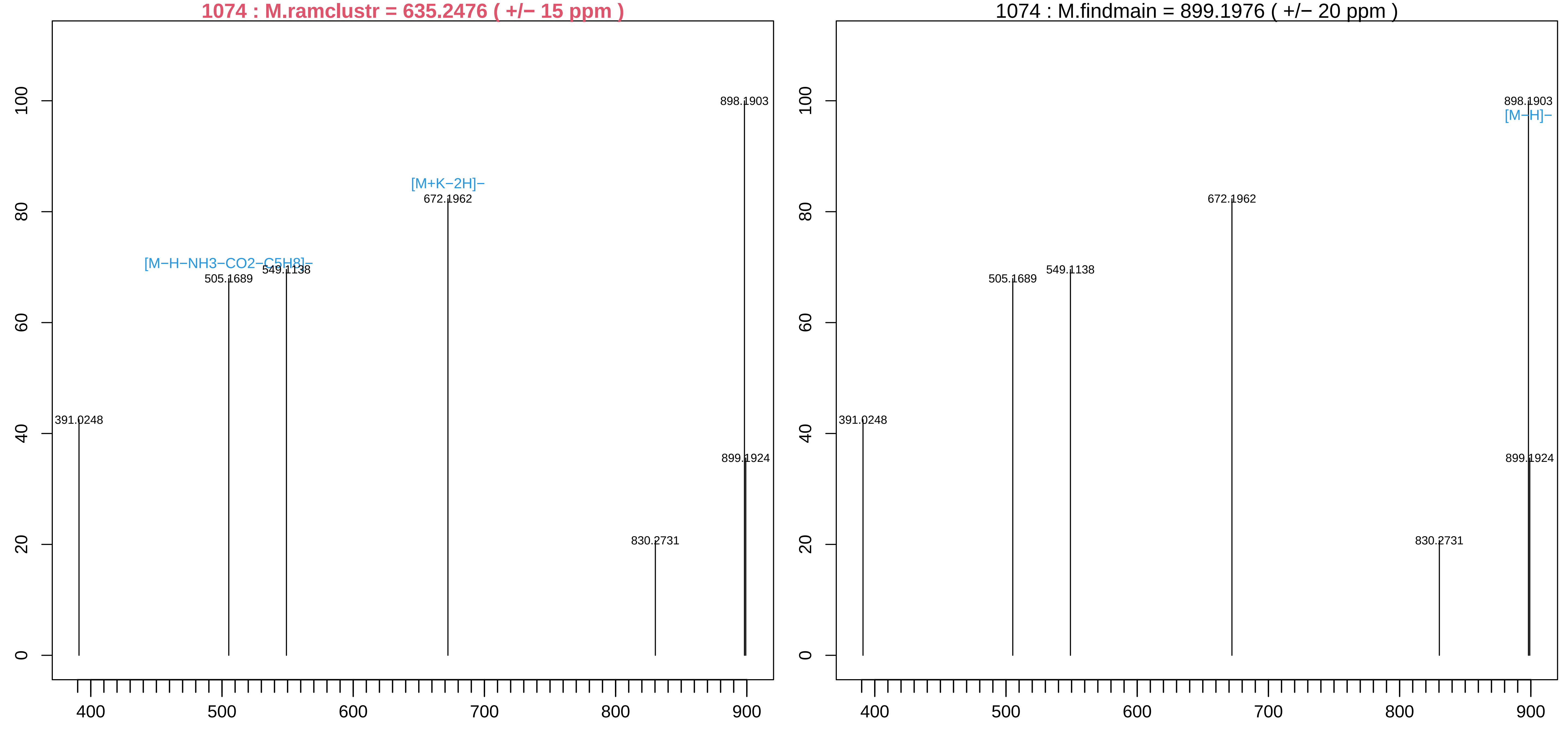
1074 : M.ramclustr = 635.2476 (+/− 15 ppm)
[M−H−NH3−CO2−C5H8]−
[M+K−2H]−
391.0248
505.1689
549.1138
672.1962
830.2731
898.1903
899.1924
1074 : M.findmain = 899.1976 (+/− 20 ppm)
[M−H]−
0
20
40
60
80
100
400
500
600
700
800
900

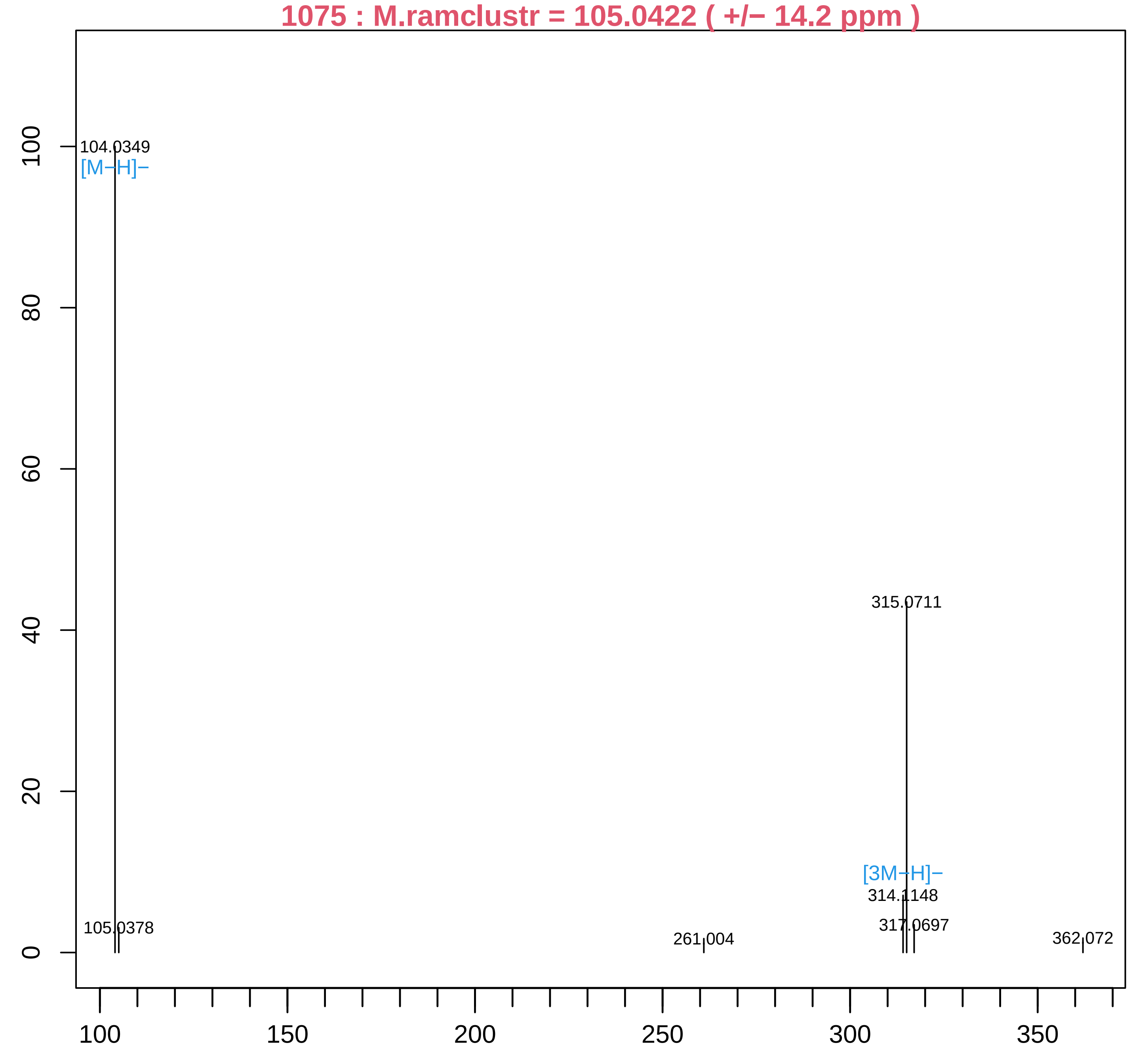
1075 : M.ramclustr = 105.0422 (+/− 14.2 ppm)
104.0349
[M−H]−
315.0711
[3M−H]−
314.1148
317.0697
105.0378
261.004
362.072
0
20
40
60
80
100
100
150
200
250
300
350

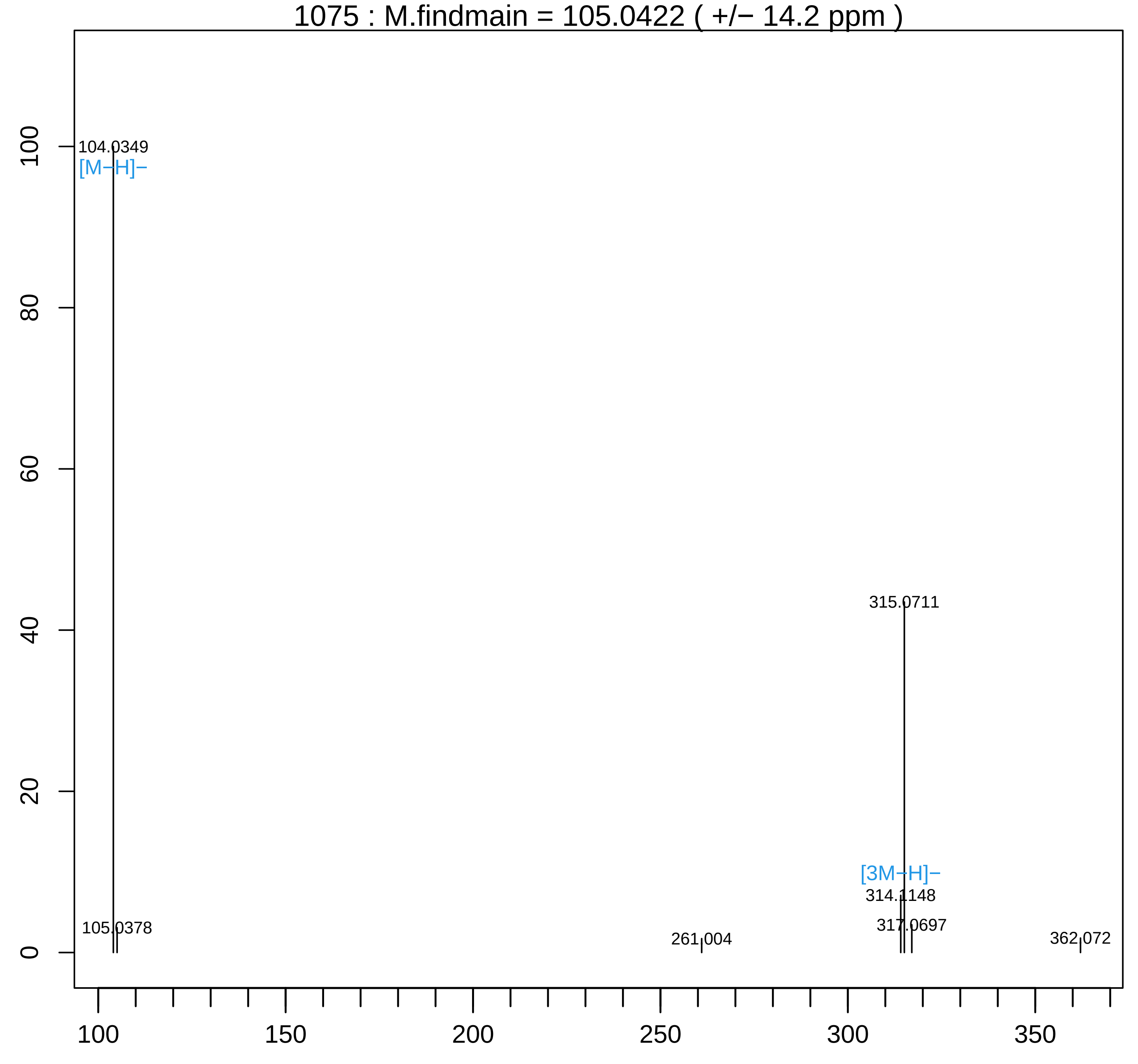
1075 : M.findmain = 105.0422 (+/− 14.2 ppm)
104.0349
[M−H]−
315.0711
[3M−H]−
314.1148
317.0697
105.0378
261.004
362.072
0
20
40
60
80
100
100
150
200
250
300
350

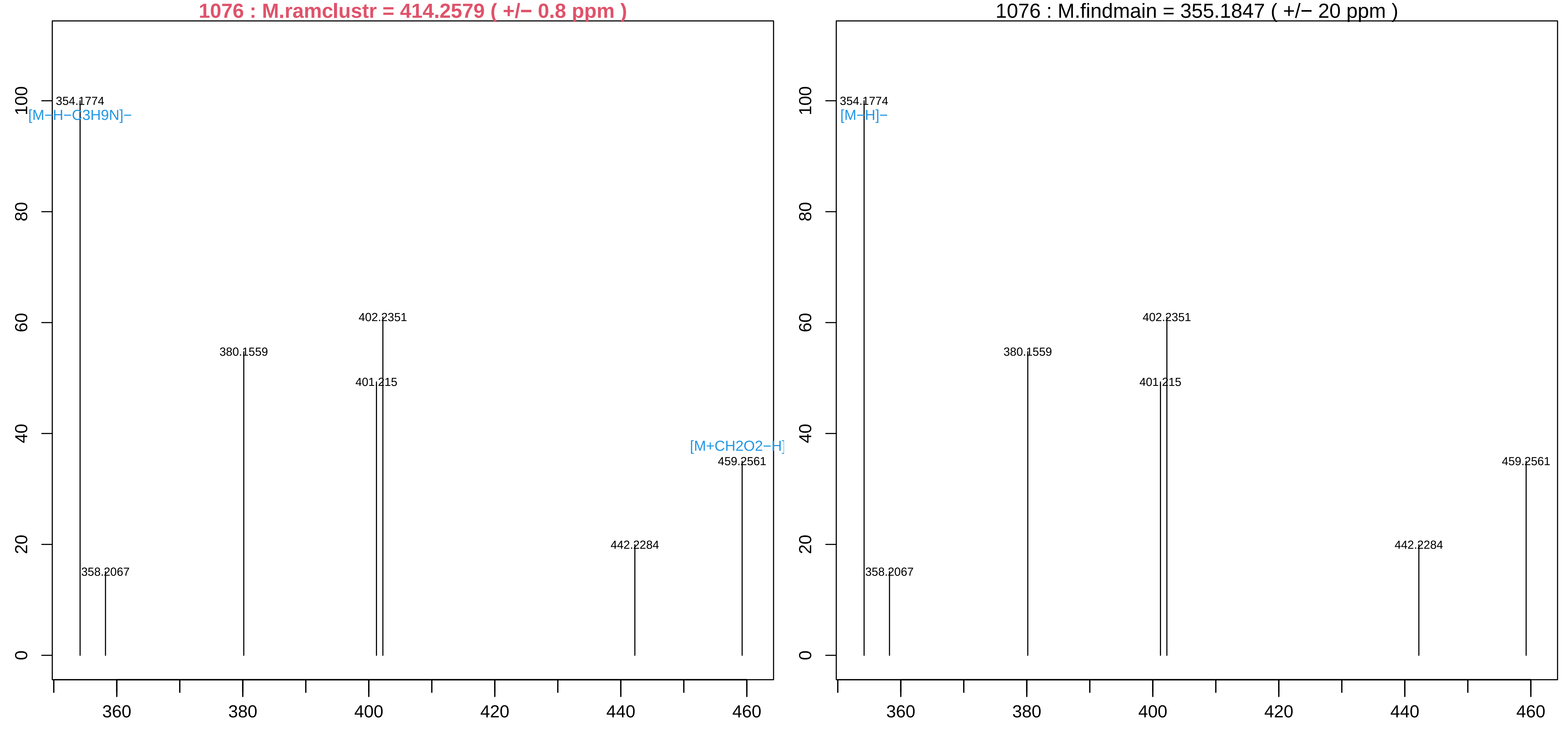

1076 : M.ramclustr = 414.2579 (+/− 0.8 ppm)
354.1774
[M−H−C3H9N]−
358.2067
380.1559
401.215
402.2351
442.2284
459.2561
[M+CH2O2−H]
0
20
40
60
80
100
360
380
400
420
440
460
1076 : M.findmain = 355.1847 (+/− 20 ppm)
354.1774
[M−H]−
358.2067
380.1559
401.215
402.2351
442.2284
459.2561
0
20
40
60
80
100
360
380
400
420
440
460

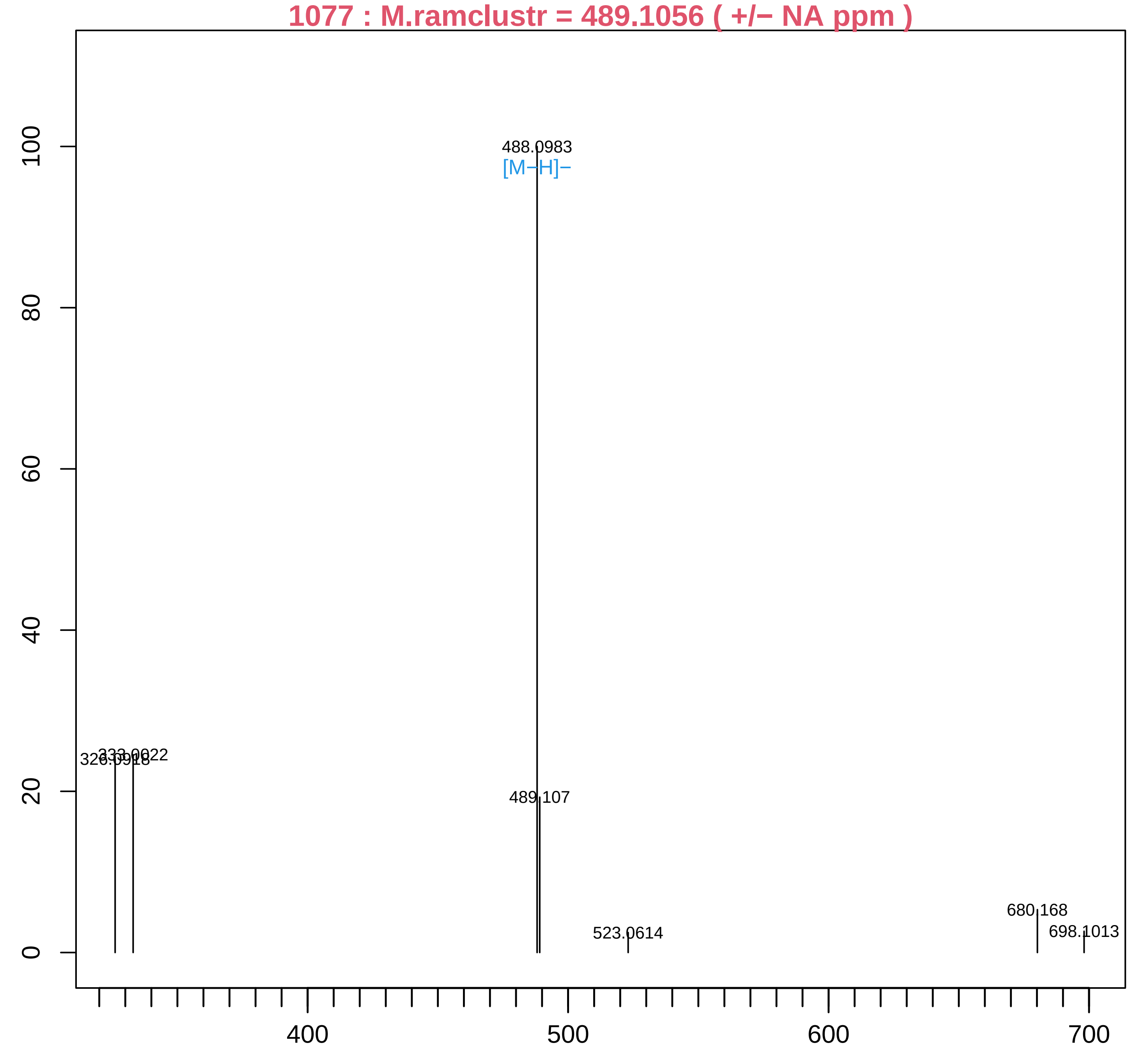

1077 : M.ramclustr = 489.1056 (+/− NA ppm)
488.0983
[M−H]−
489.107
326.0918
333.0022
523.0614
680.168
698.1013
0
20
40
60
80
100
400
500
600
700

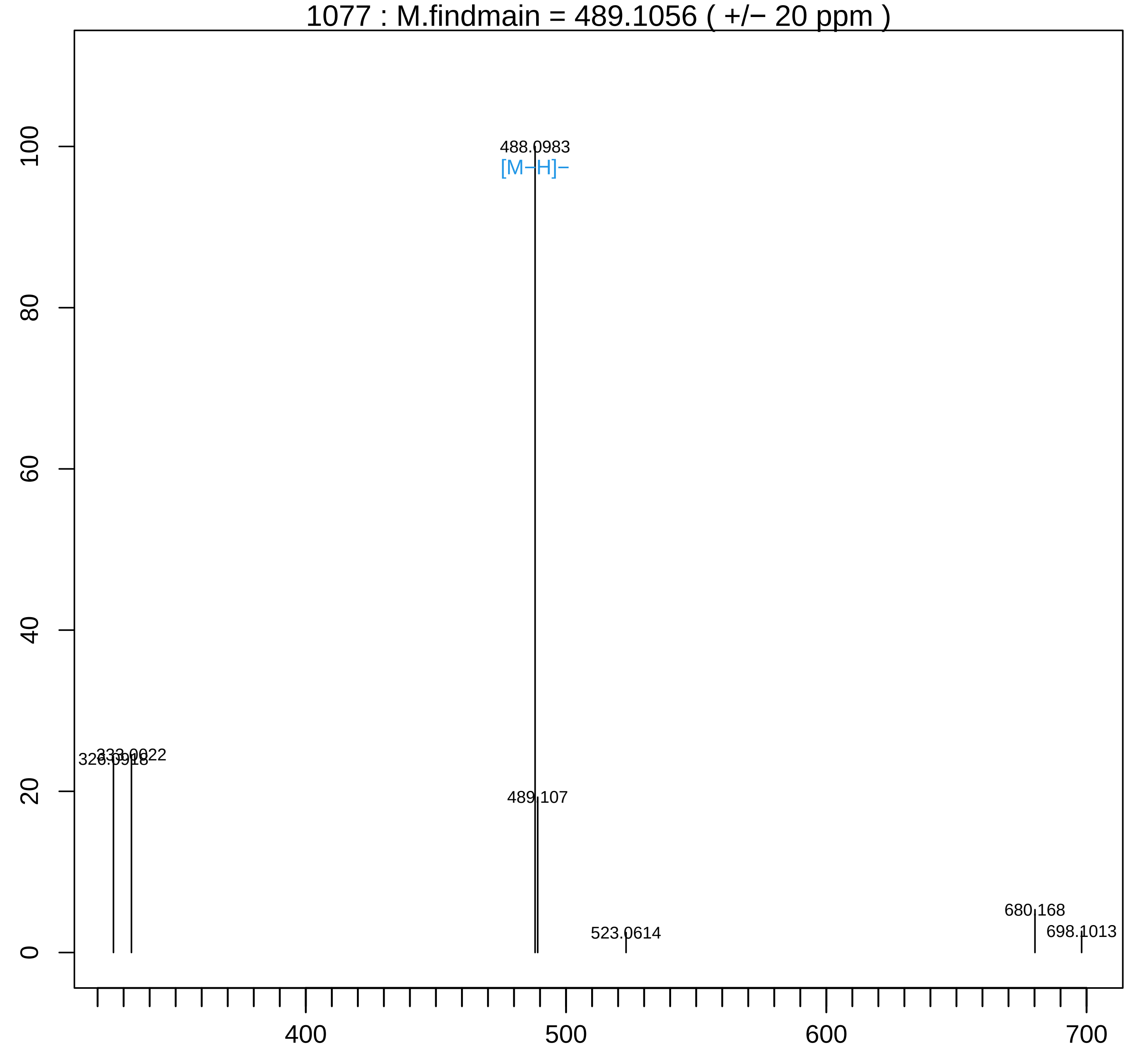

1077 : M.findmain = 489.1056 (+/− 20 ppm)
488.0983
[M−H]−
489.107
326.0918
333.0022
523.0614
680.168
698.1013
0
20
40
60
80
100
400
500
600
700

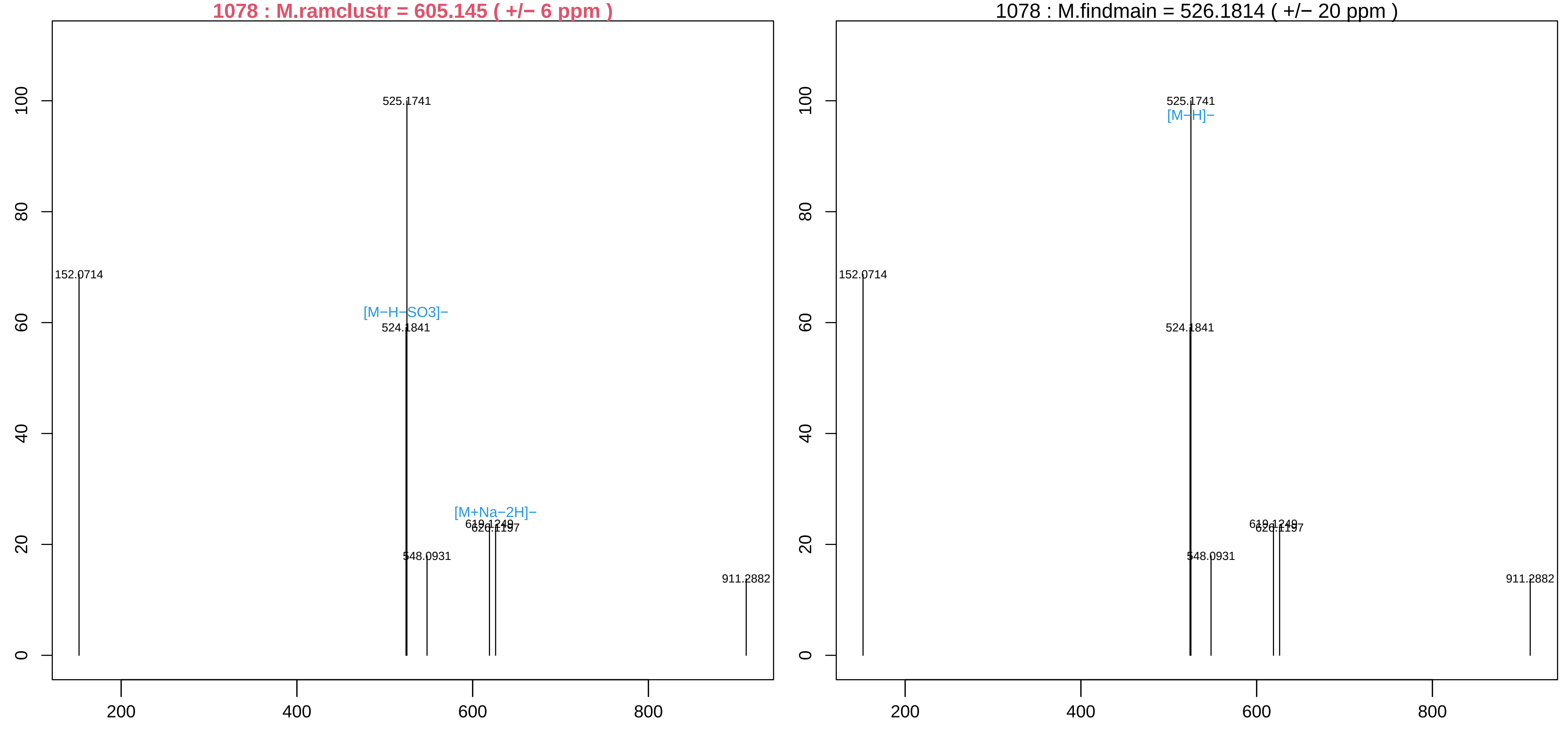
1078 : M.ramclustr = 605.145 (+/− 6 ppm)
525.1741
152.0714
[M−H−SO3]−
524.1841
[M+Na−2H]−
619.1249
626.1197
548.0931
911.2882
1078 : M.findmain = 526.1814 (+/− 20 ppm)
525.1741
[M−H]−
152.0714
524.1841
619.1249
626.1197
548.0931
911.2882
0
20
40
60
80
100
200
400
600
800

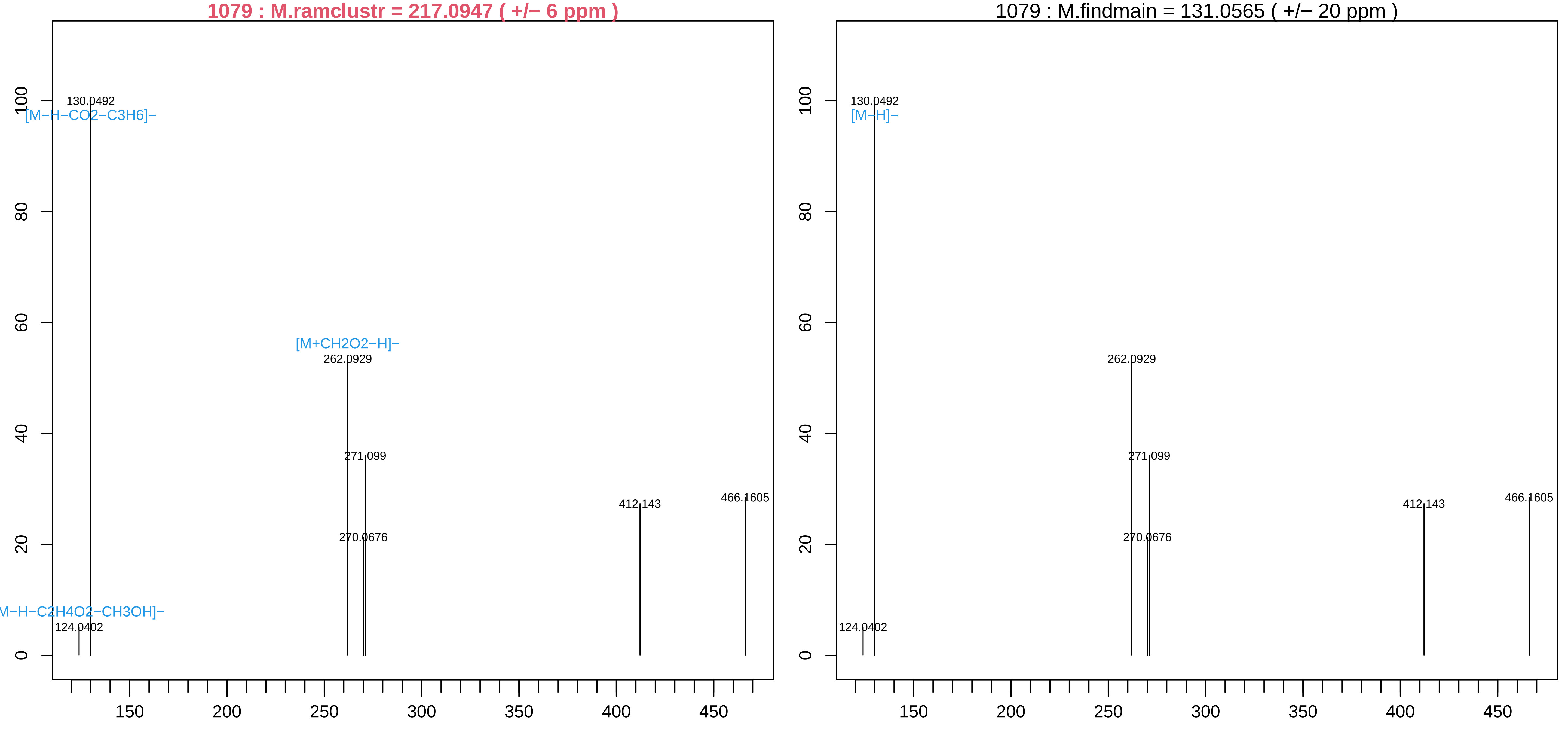
1079 : M.ramclustr = 217.0947 (+/− 6 ppm)
130.0492
[M−H−CO2−C3H6]−
[M+CH2O2−H]−
262.0929
271.099
270.0676
412.143
466.1605
M−H−C2H4O2−CH3OH]−
124.0402
1079 : M.findmain = 131.0565 (+/− 20 ppm)
130.0492
[M−H]−
262.0929
271.099
270.0676
412.143
466.1605
124.0402

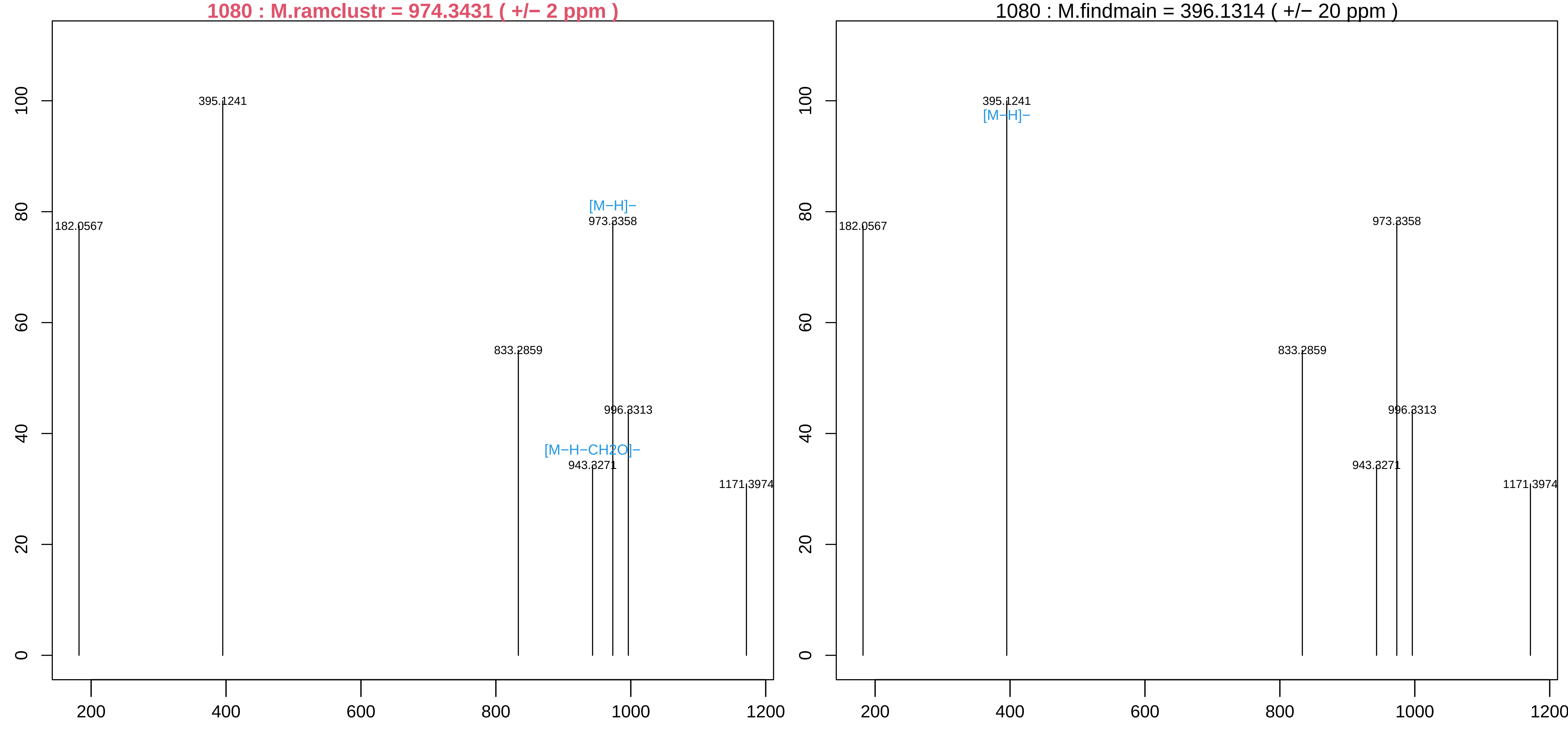
1080 : M.ramclustr = 974.3431 (+/− 2 ppm)
182.0567
395.1241
833.2859
[M−H]−
973.3358
996.3313
[M−H−CH2O]−
943.3271
1171.3974
1080 : M.findmain = 396.1314 (+/− 20 ppm)
182.0567
395.1241
[M−H]−
833.2859
973.3358
996.3313
943.3271
1171.3974

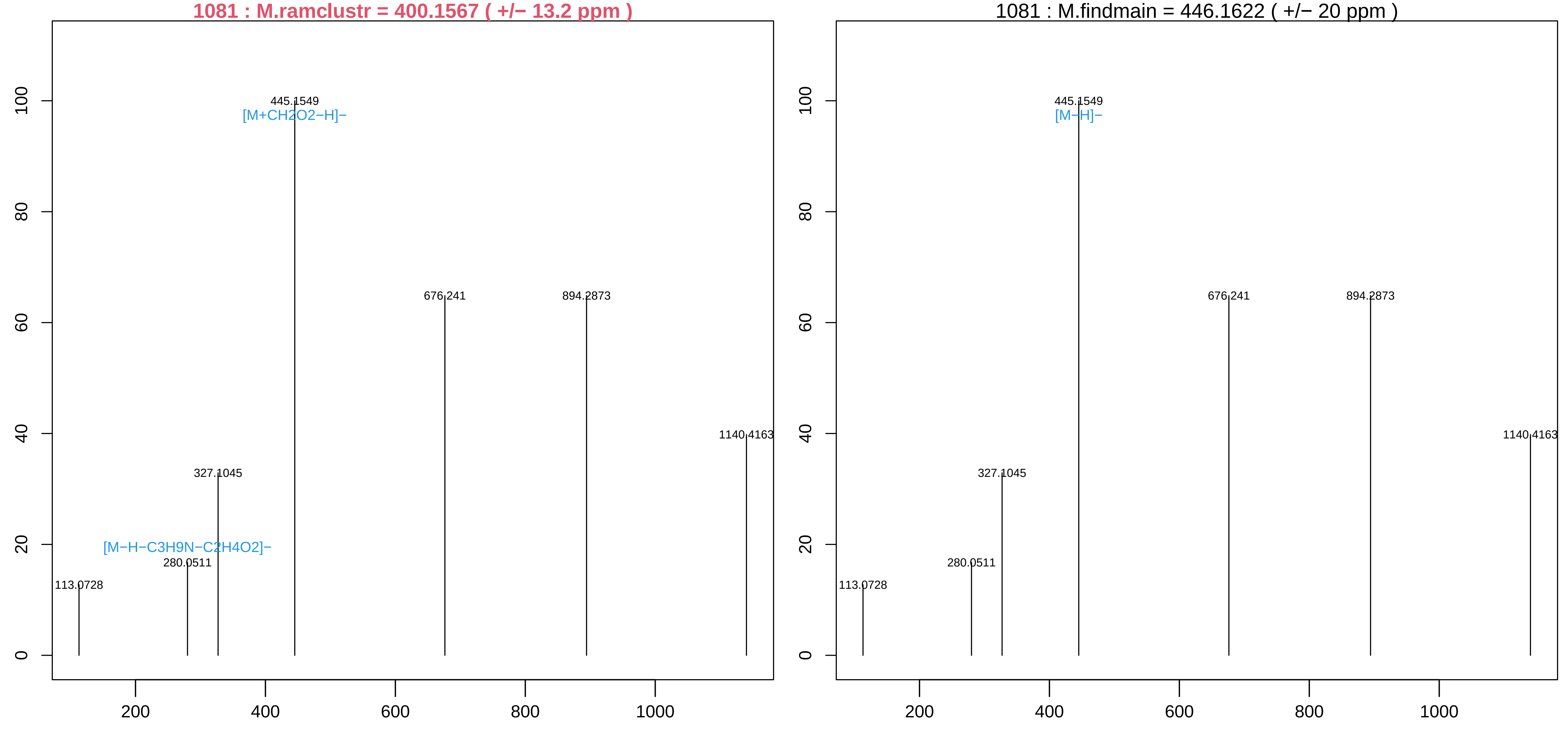
1081 : M.ramclustr = 400.1567 (+/− 13.2 ppm)
445.1549
[M+CH2O2−H]−
676.241
894.2873
1140.4163
327.1045
[M−H−C3H9N−C2H4O2]−
280.0511
113.0728
1081 : M.findmain = 446.1622 (+/− 20 ppm)
445.1549
[M−H]−
676.241
894.2873
1140.4163
327.1045
280.0511
113.0728
0
20
40
60
80
100
200
400
600
800
1000

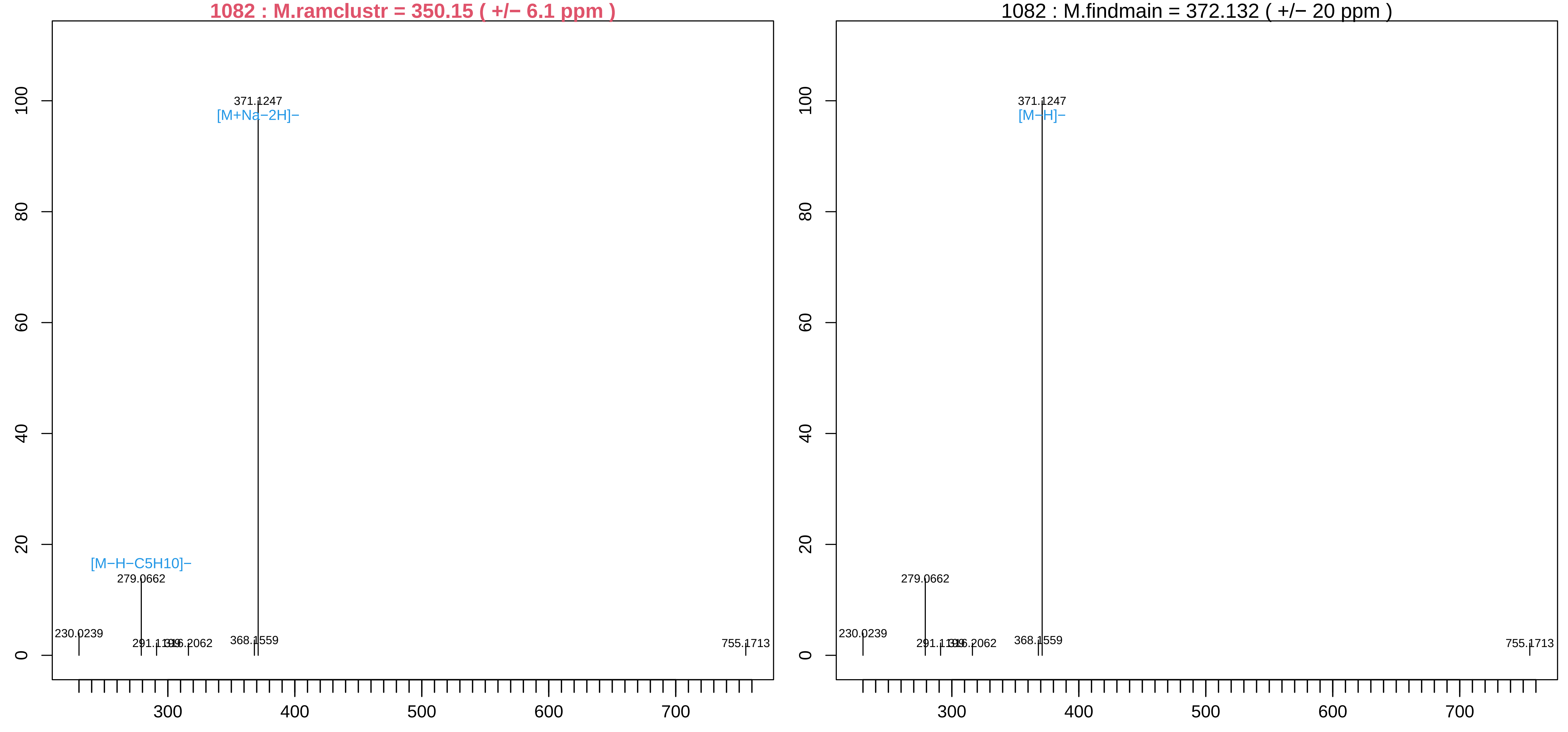

1082 : M.ramclustr = 350.15 (+/− 6.1 ppm)
371.1247
[M+Na−2H]−
[M−H−C5H10]−
279.0662
230.0239
291.1399
316.2062
368.1559
755.1713
1082 : M.findmain = 372.132 (+/− 20 ppm)
371.1247
[M−H]−
279.0662
230.0239
291.1399
316.2062
368.1559
755.1713
0
20
40
60
80
100
300
400
500
600
700

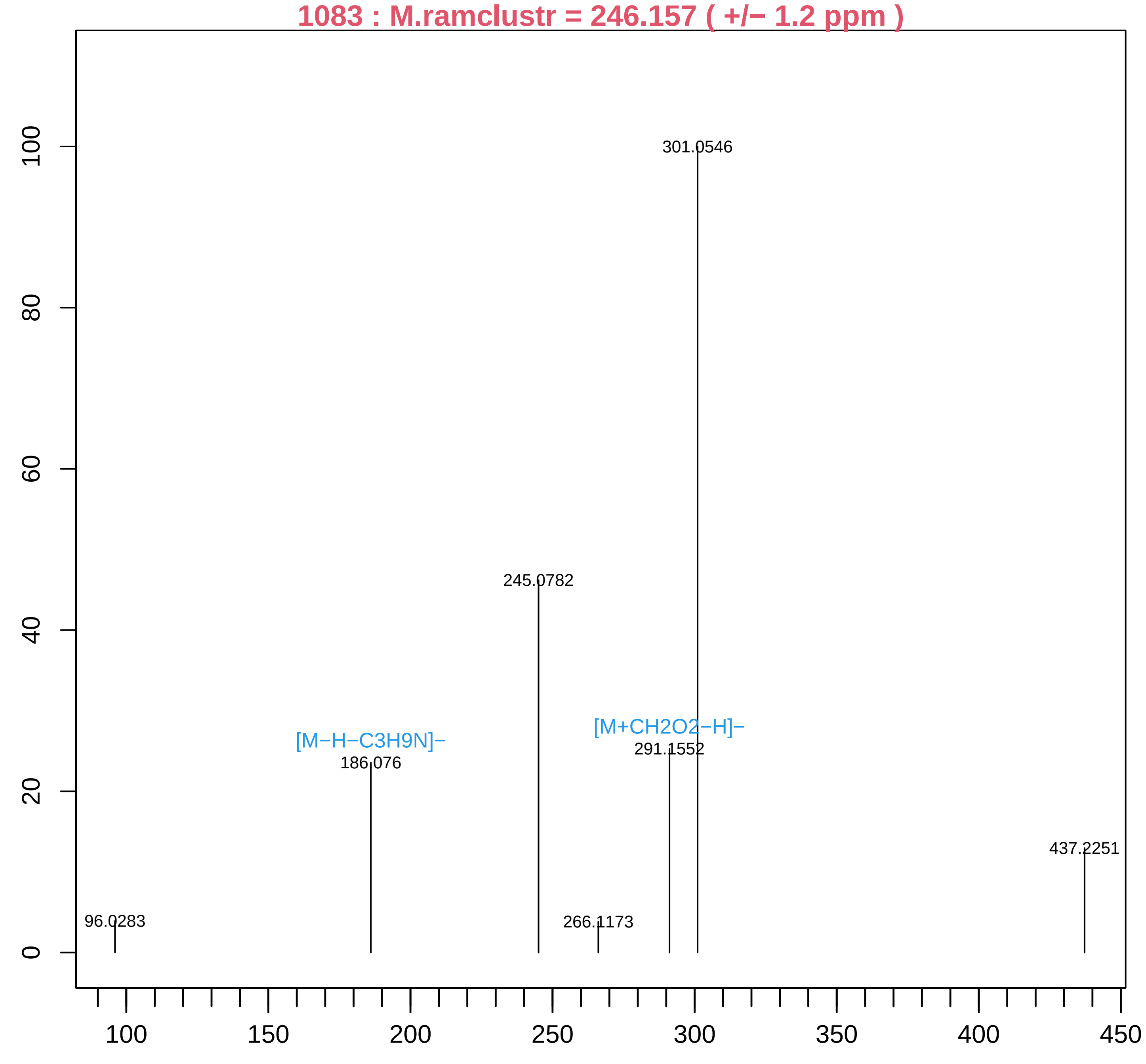
1083 : M.ramclustr = 246.157 (+/− 1.2 ppm)
301.0546
245.0782
[M−H−C3H9N]−
186.076
[M+CH2O2−H]−
291.1552
437.2251
96.0283
266.1173
0
20
40
60
80
100
100
150
200
250
300
350
400
450

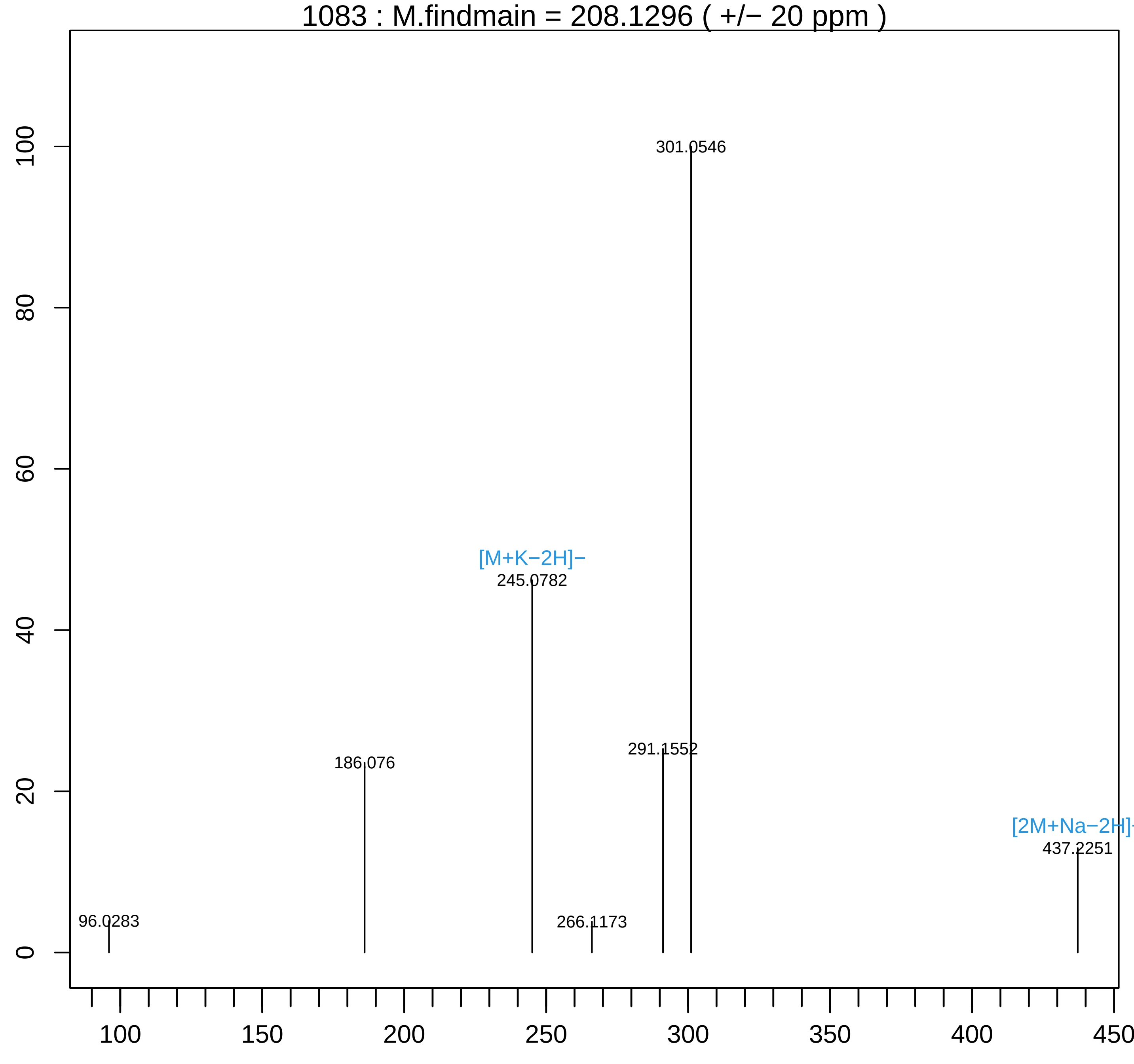
1083 : M.findmain = 208.1296 (+/− 20 ppm)
301.0546
[M+K−2H]−
245.0782
186.076
291.1552
[2M+Na−2H]
437.2251
96.0283
266.1173
0
20
40
60
80
100
100
150
200
250
300
350
400
450

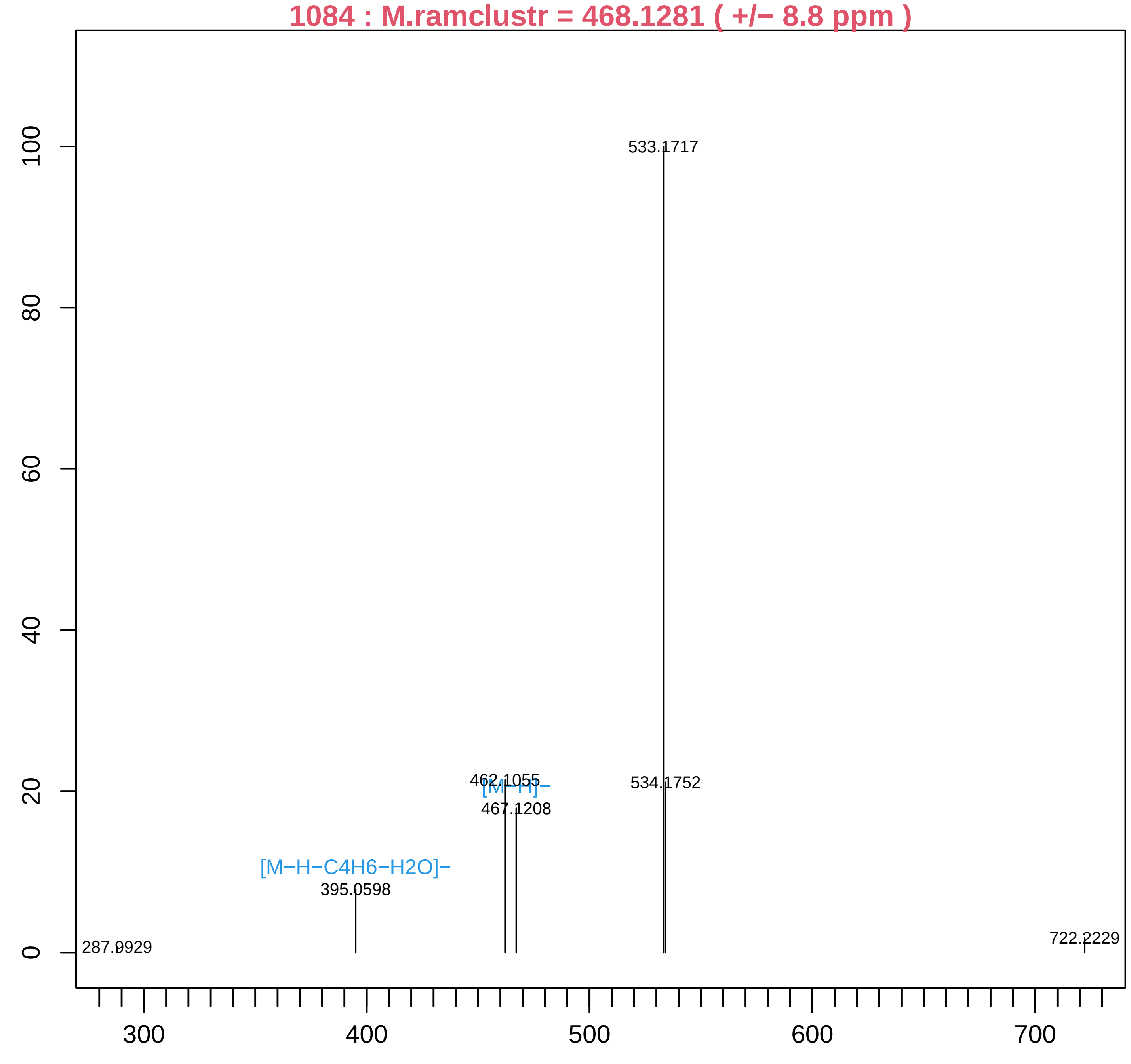
1084 : M.ramclustr = 468.1281 (+/− 8.8 ppm)
533.1717
462.1055
[M−H]−
467.1208
534.1752
[M−H−C4H6−H2O]−
395.0598
287.9929
722.2229
0
20
40
60
80
100
300
400
500
600
700

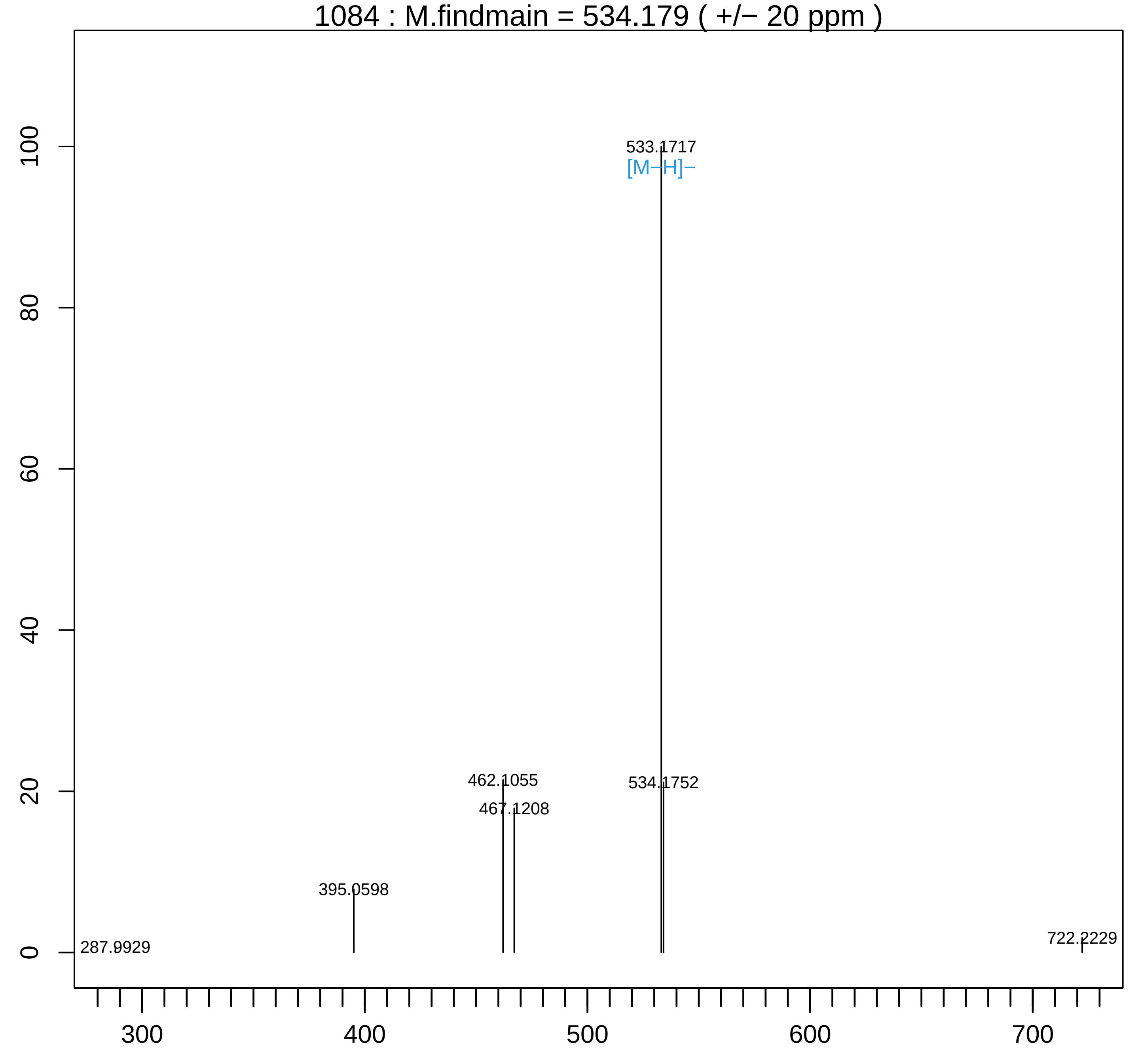
1084 : M.findmain = 534.179 (+/− 20 ppm)
533.1717
[M−H]−
462.1055
467.1208
534.1752
395.0598
287.9929
722.2229
0
20
40
60
80
100
300
400
500
600
700

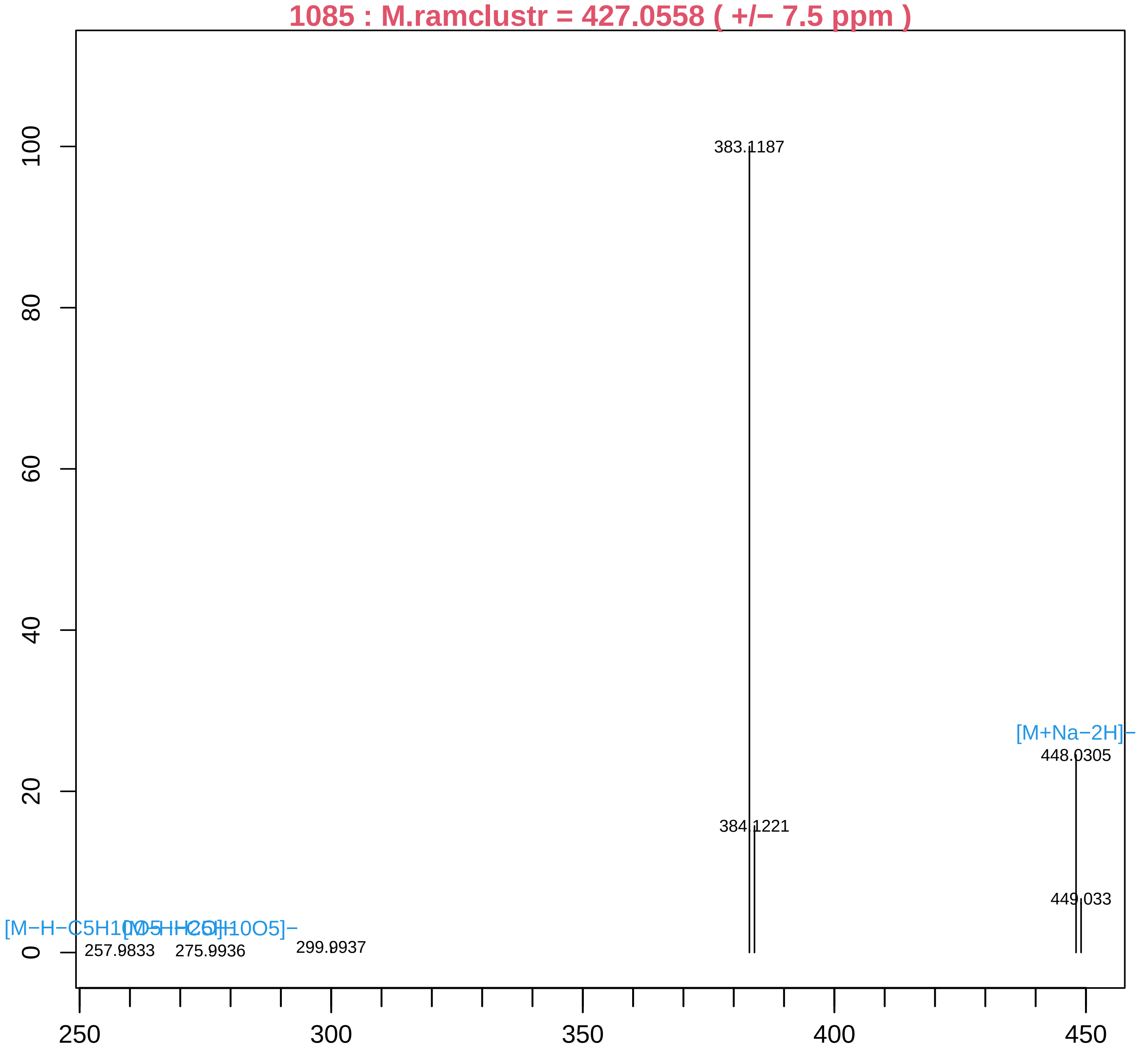
1085 : M.ramclustr = 427.0558 (+/− 7.5 ppm)
383.1187
[M+Na−2H]−
448.0305
384.1221
449.033
[M−H−C5H10O5−H2O]−
[M−H−C5H10O5]−
257.9833
275.9936
299.9937
0
20
40
60
80
100
250
300
350
400
450
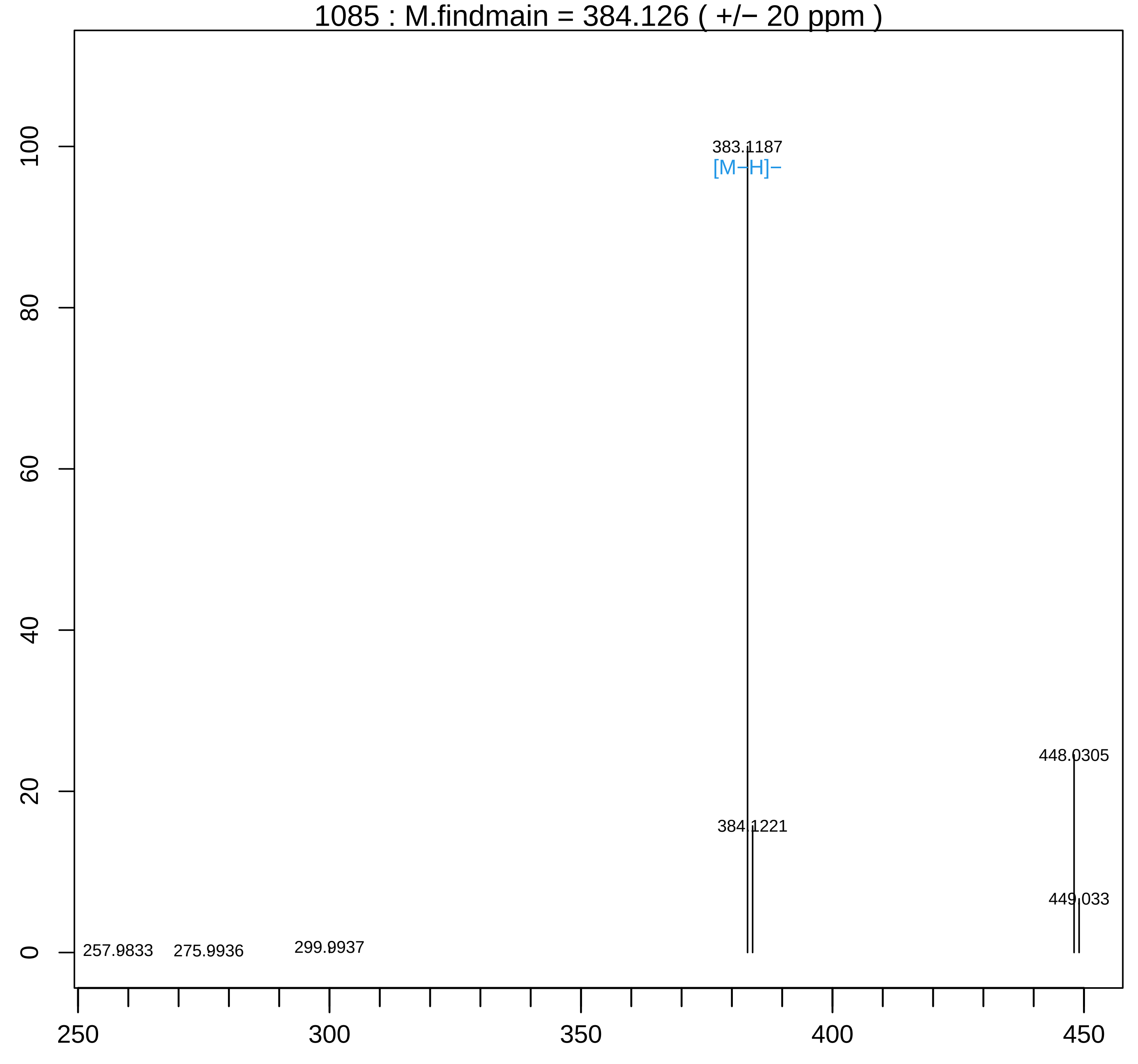
1085 : M.findmain = 384.126 (+/− 20 ppm)
383.1187
[M−H]−
448.0305
384.1221
449.033
257.9833
275.9936
299.9937
0
20
40
60
80
100
250
300
350
400
450

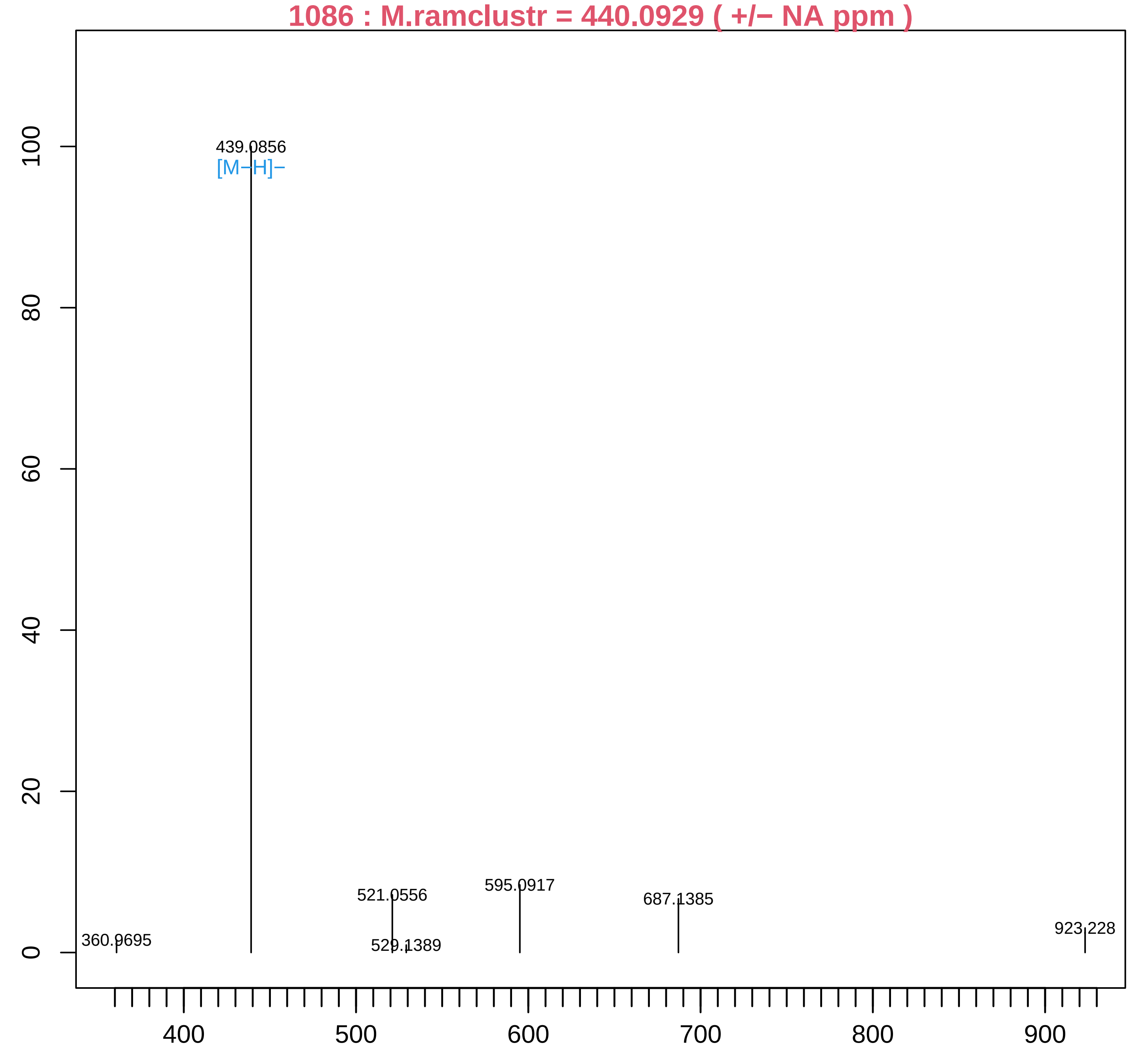
1086 : M.ramclustr = 440.0929 (+/− NA ppm)
439.0856
[M−H]−
521.0556
595.0917
687.1385
360.9695
529.1389
923.228
0
20
40
60
80
100
400
500
600
700
800
900

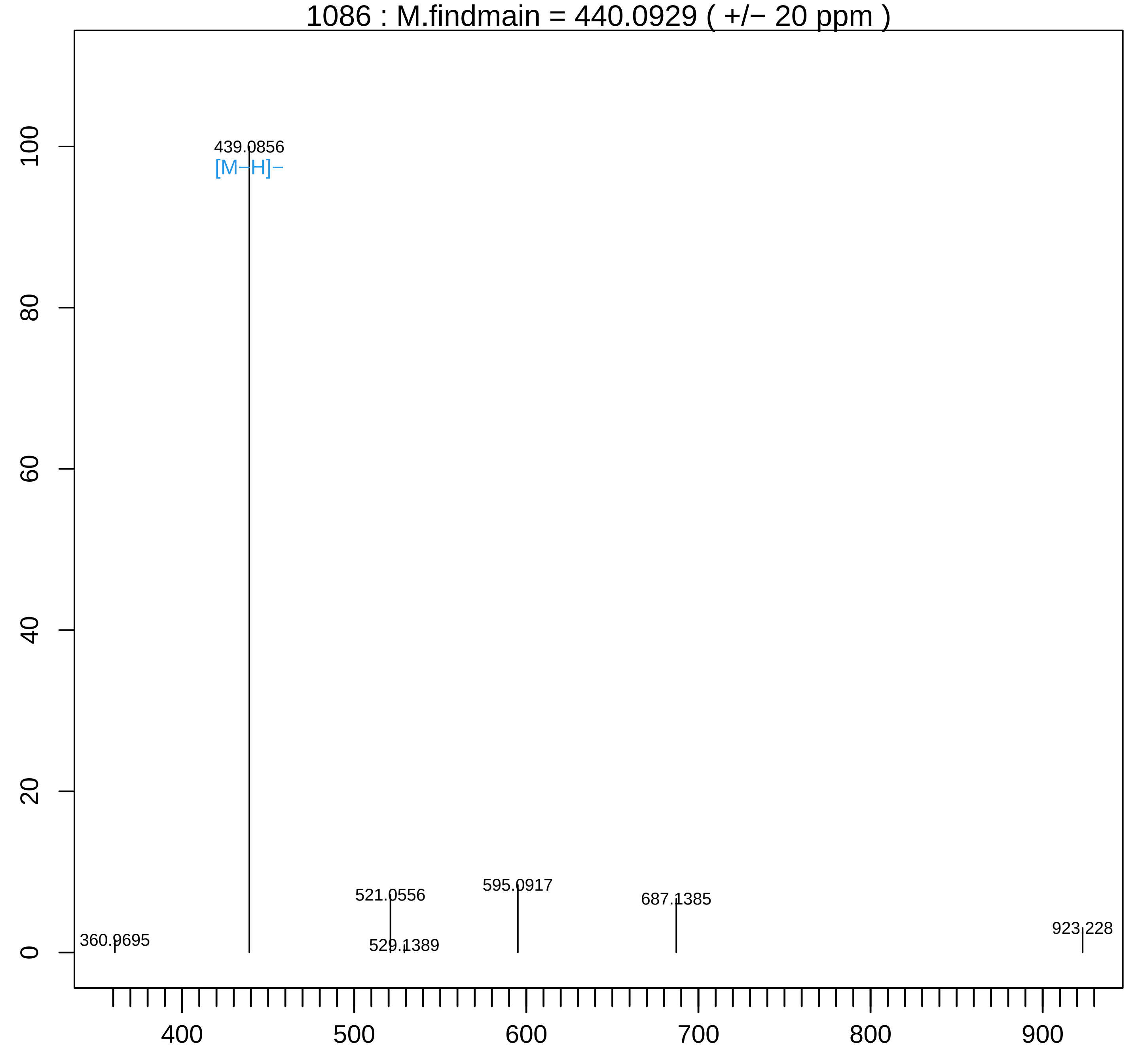
1086 : M.findmain = 440.0929 (+/− 20 ppm)
439.0856
[M−H]−
521.0556
595.0917
687.1385
360.9695
529.1389
923.228
0
20
40
60
80
100
400
500
600
700
800
900

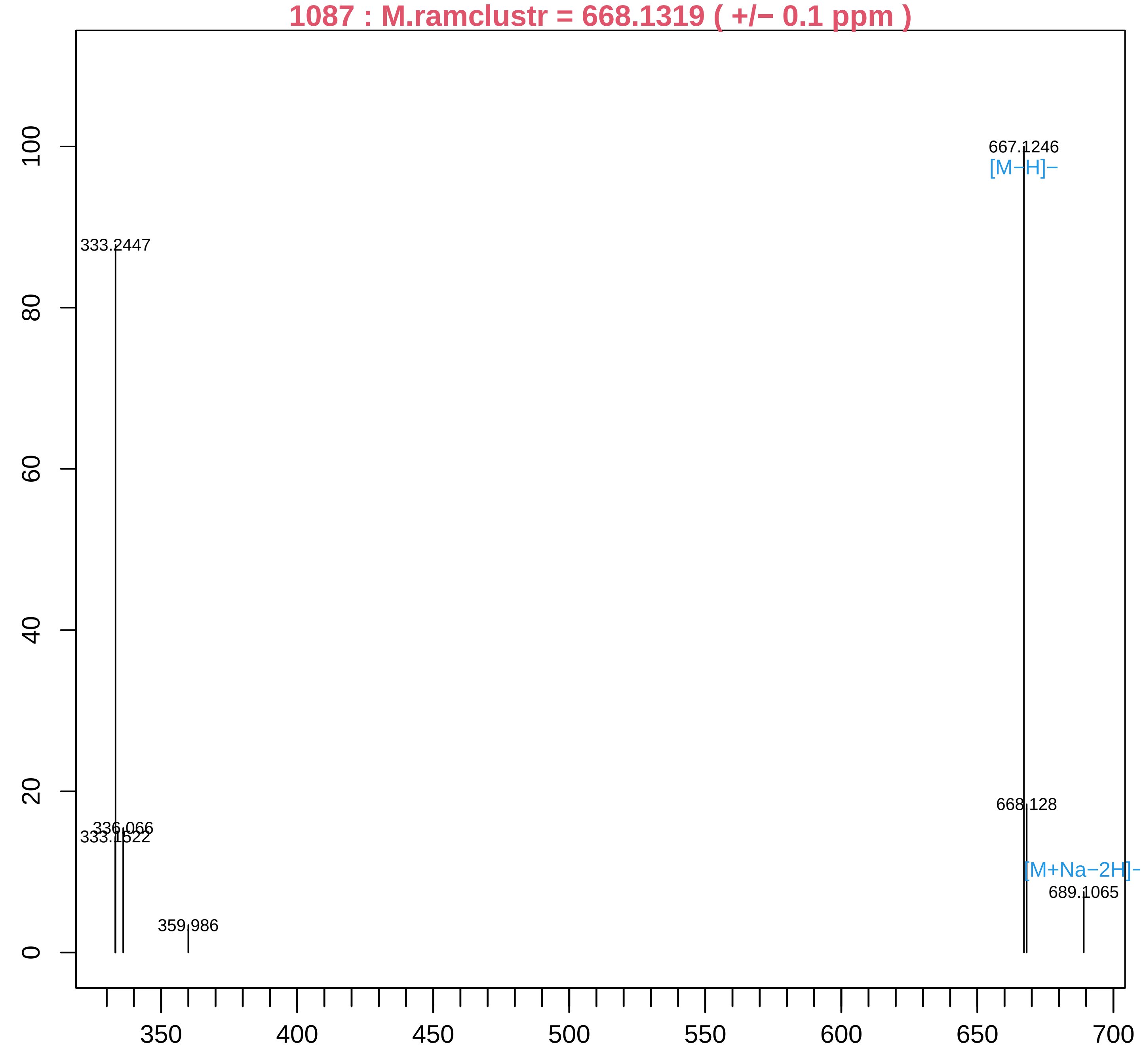
1087 : M.ramclustr = 668.1319 (+/− 0.1 ppm)
667.1246
[M−H]−
333.2447
336.066
333.1522
359.986
668.128
[M+Na−2H]−
689.1065
0
20
40
60
80
100
350
400
450
500
550
600
650
700

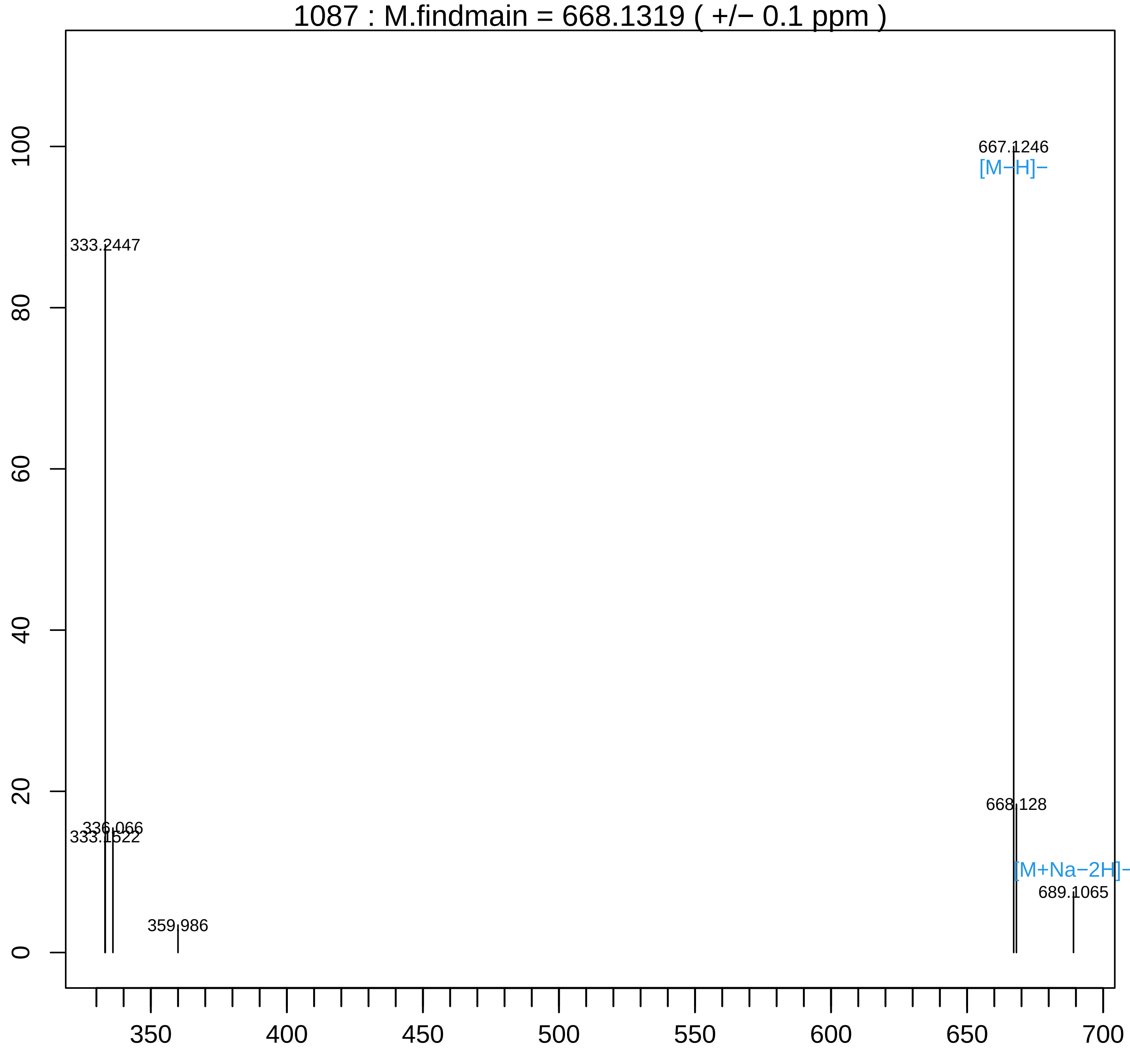
1087 : M.findmain = 668.1319 (+/− 0.1 ppm)
667.1246
[M−H]−
333.2447
336.066
333.1522
359.986
668.128
[M+Na−2H]−
689.1065
0
20
40
60
80
100
350
400
450
500
550
600
650
700

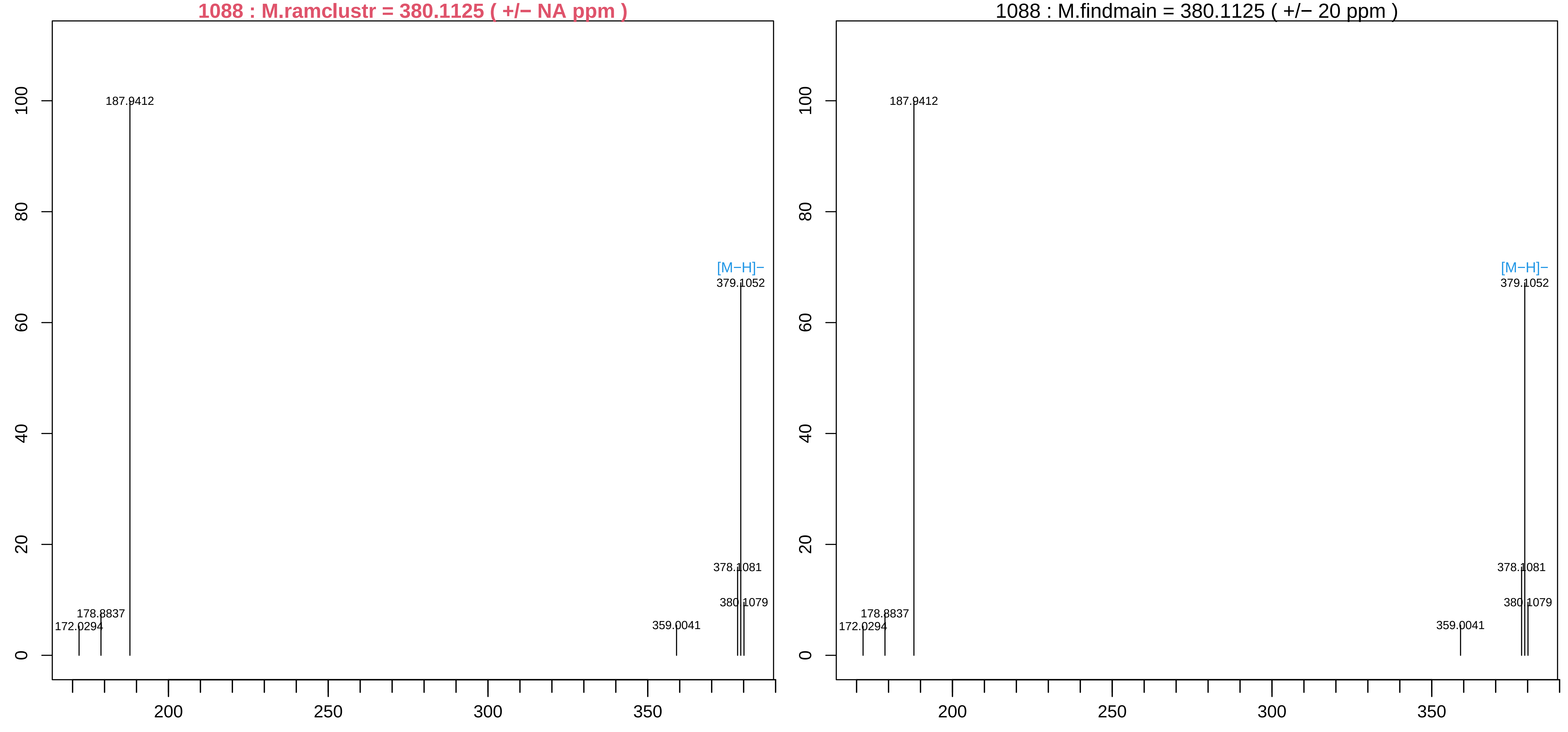
1088 : M.ramclustr = 380.1125 (+/− NA ppm)
187.9412
[M−H]−
379.1052
378.1081
380.1079
178.8837
172.0294
359.0041
1088 : M.findmain = 380.1125 (+/− 20 ppm)
187.9412
[M−H]−
379.1052
378.1081
380.1079
178.8837
172.0294
359.0041
0
20
40
60
80
100
200
250
300
350

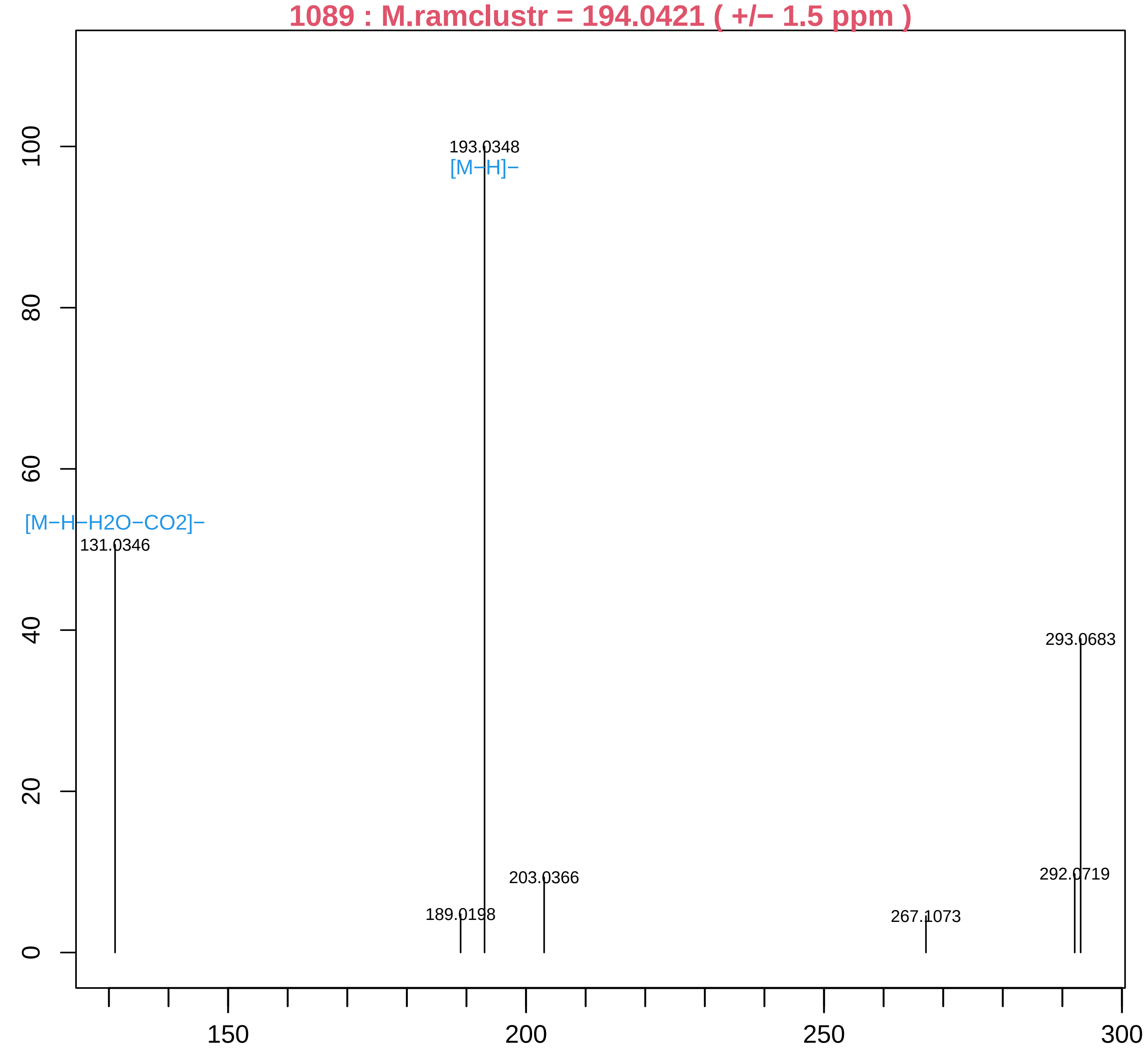
1089 : M.ramclustr = 194.0421 (+/− 1.5 ppm)
193.0348
[M−H]−
[M−H−H2O−CO2]−
131.0346
293.0683
292.0719
203.0366
189.0198
267.1073
0
20
40
60
80
100
150
200
250
300

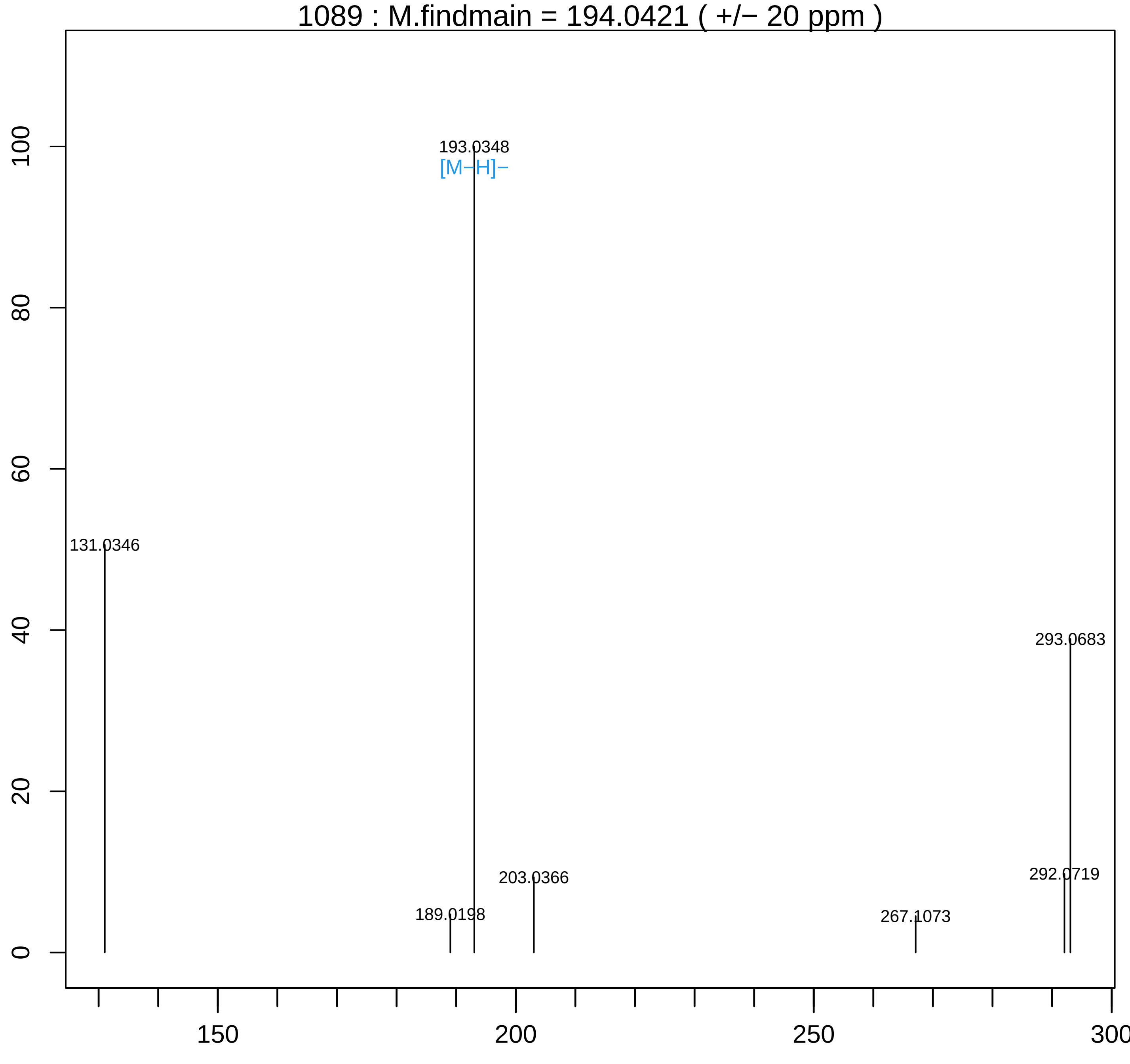
1089 : M.findmain = 194.0421 (+/− 20 ppm)
193.0348
[M−H]−
131.0346
293.0683
292.0719
203.0366
189.0198
267.1073
0
20
40
60
80
100
150
200
250
300

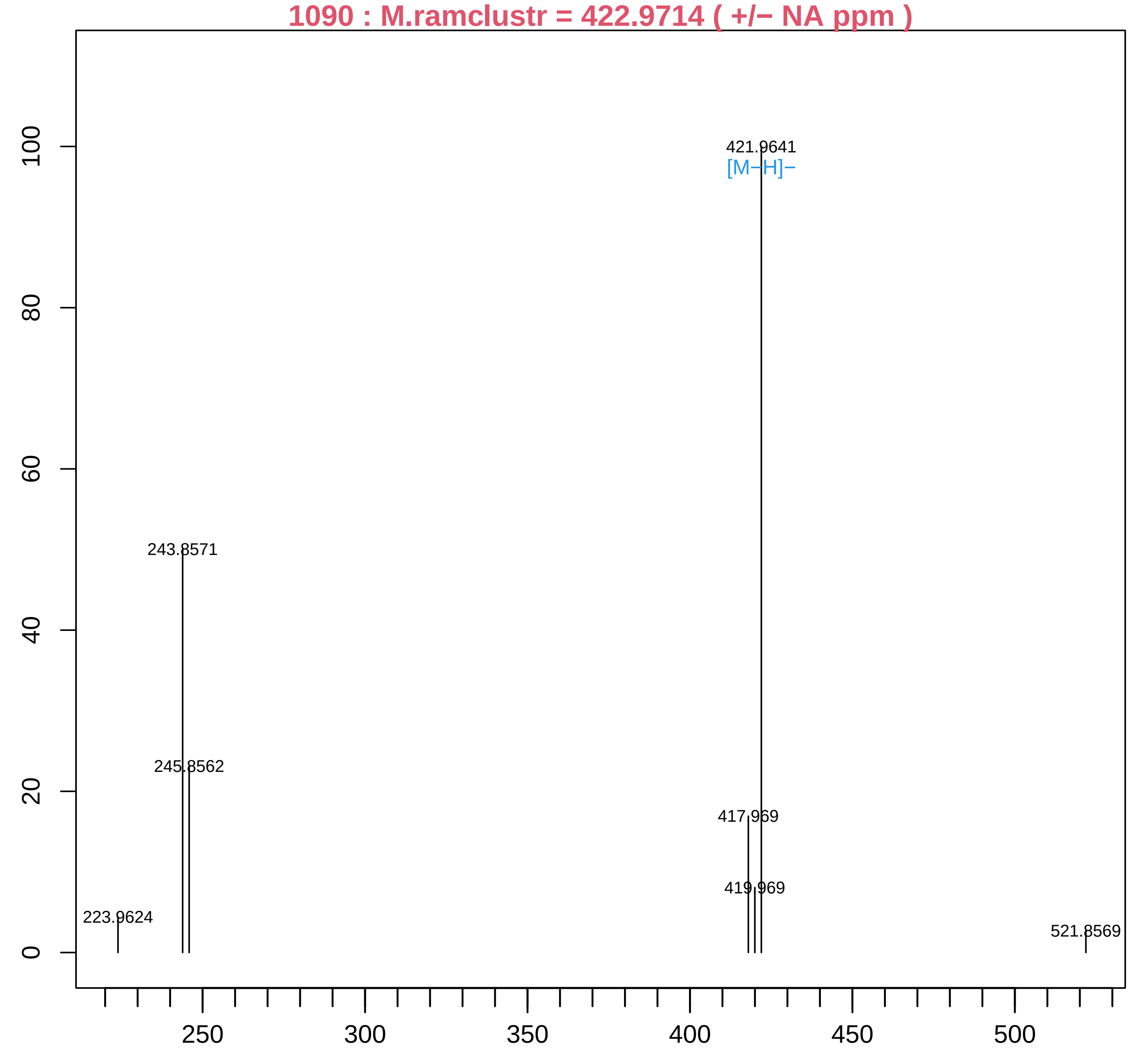
1090 : M.ramclustr = 422.9714 (+/− NA ppm)
421.9641
[M−H]−
243.8571
245.8562
417.969
419.969
223.9624
521.8569
0
20
40
60
80
100
250
300
350
400
450
500

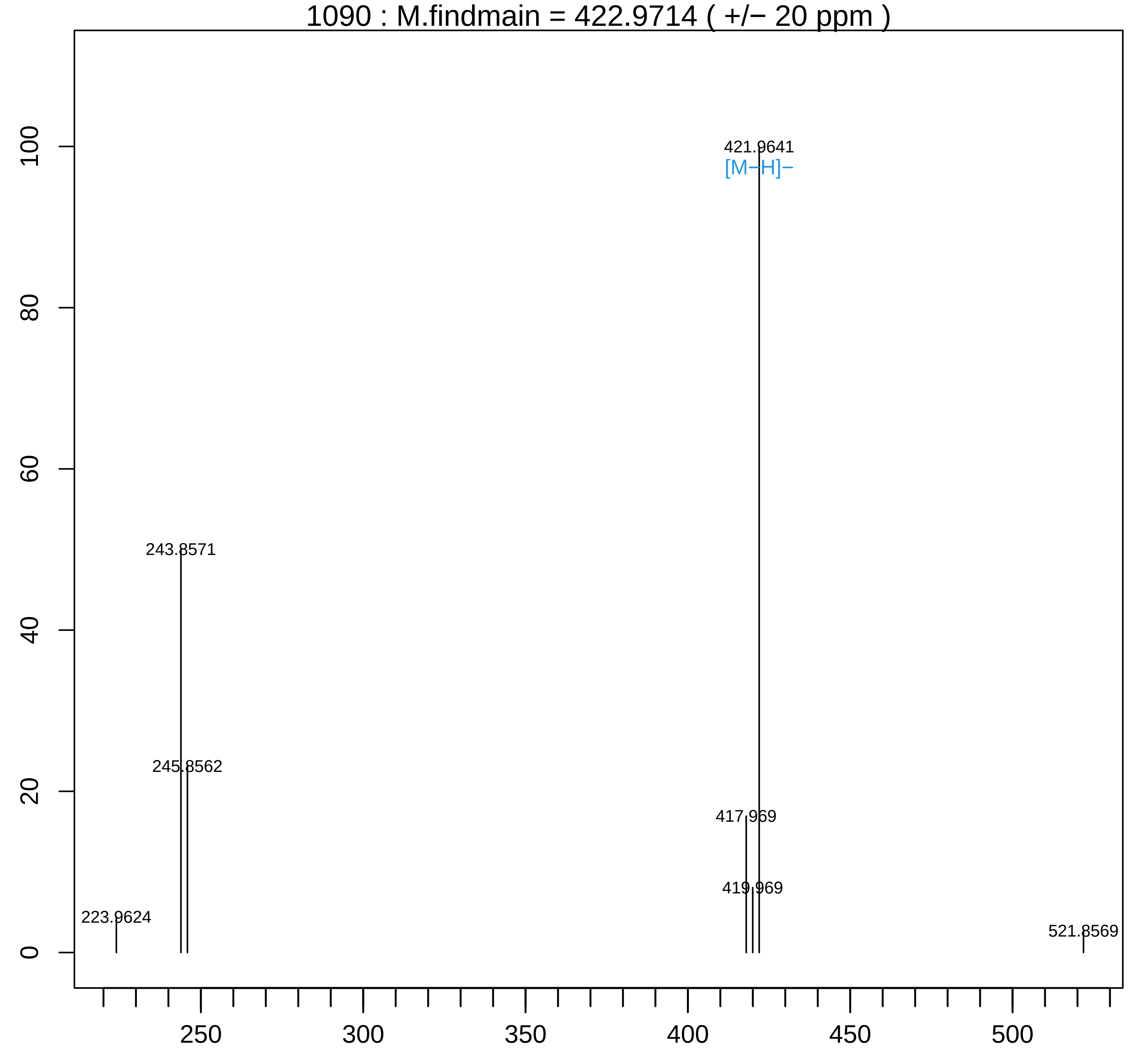
1090 : M.findmain = 422.9714 (+/− 20 ppm)
421.9641
[M−H]−
243.8571
245.8562
417.969
419.969
223.9624
521.8569
0
20
40
60
80
100
250
300
350
400
450
500

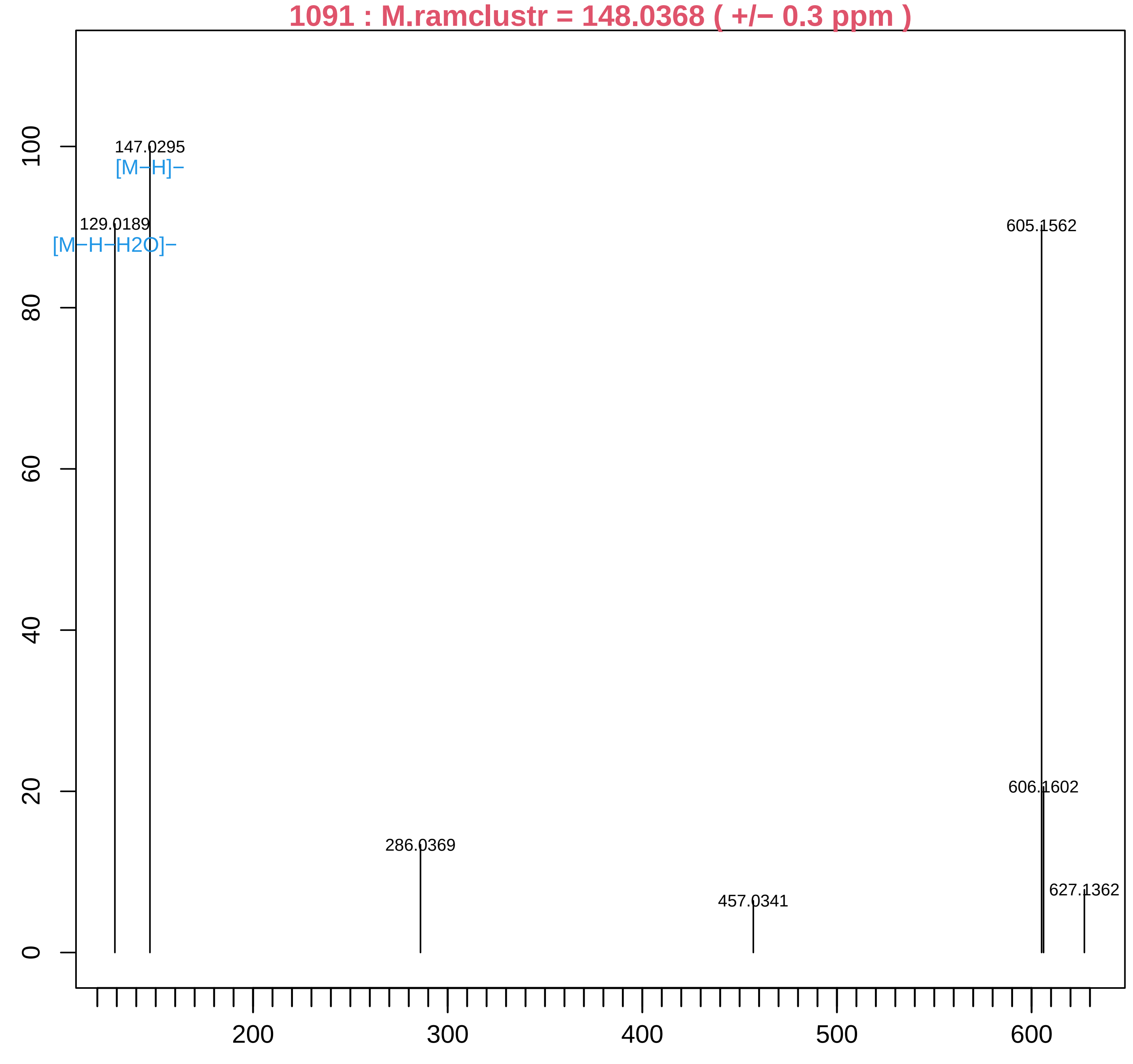
1091 : M.ramclustr = 148.0368 (+/− 0.3 ppm)
147.0295
[M−H]−
129.0189
[M−H−H2O]−
605.1562
606.1602
286.0369
457.0341
627.1362
0
20
40
60
80
100
200
300
400
500
600

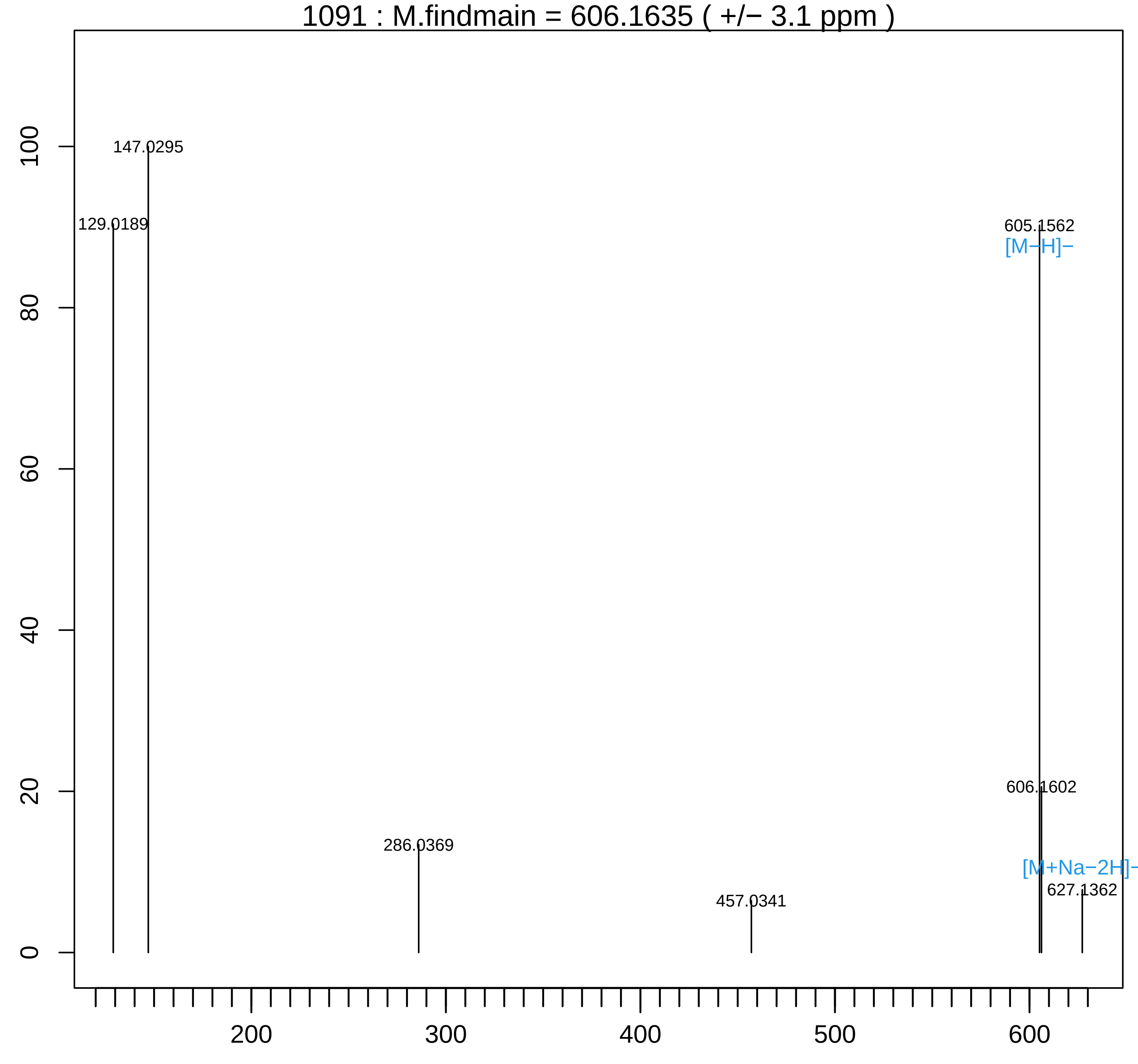
1091 : M.findmain = 606.1635 (+/− 3.1 ppm)
147.0295
129.0189
605.1562
[M−H]−
606.1602
[M+Na−2H]−
286.0369
457.0341
627.1362
0
20
40
60
80
100
200
300
400
500
600

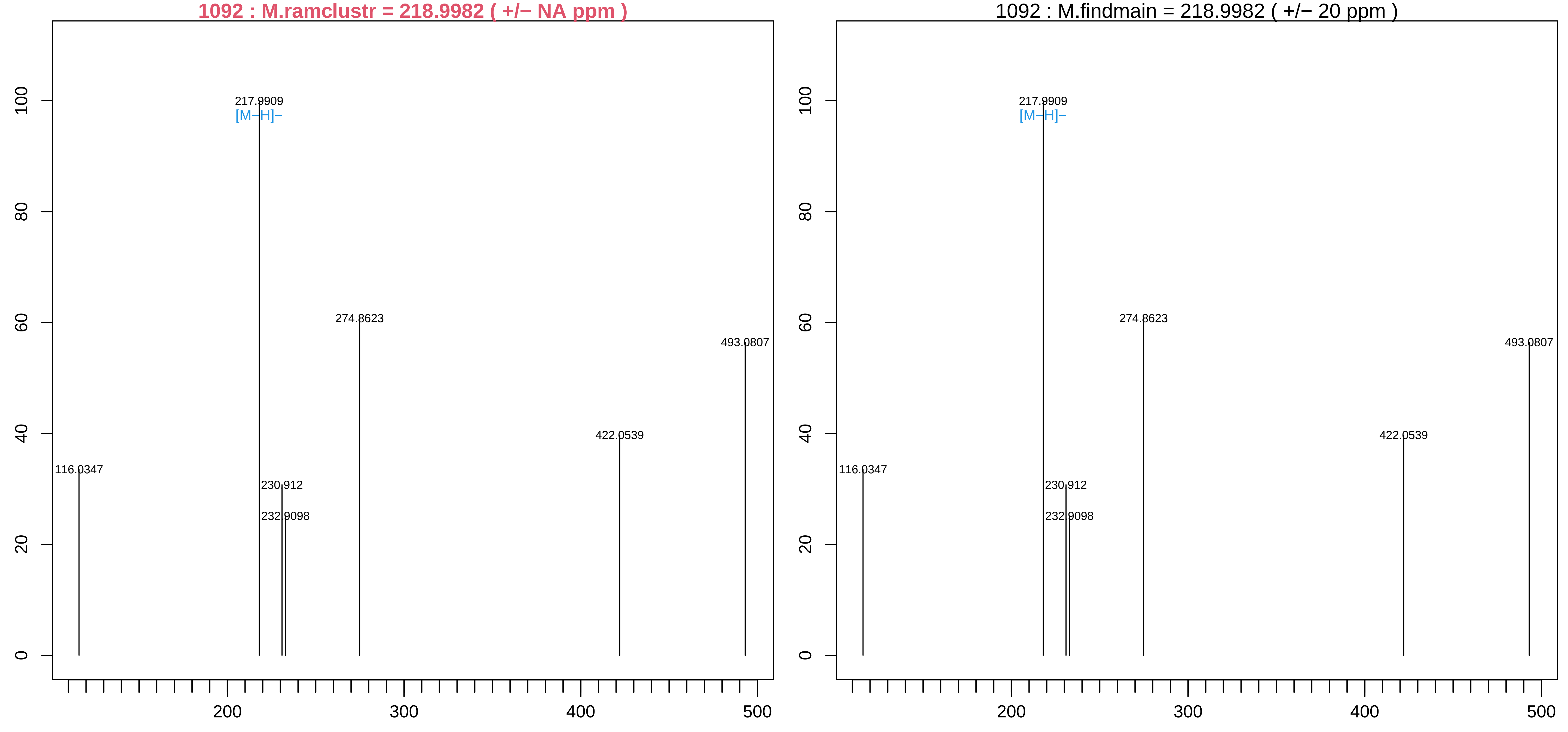

1092 : M.ramclustr = 218.9982 (+/− NA ppm)
1092 : M.findmain = 218.9982 (+/− 20 ppm)
217.9909
[M−H]−
274.8623
493.0807
422.0539
116.0347
230.912
232.9098
0
20
40
60
80
100
200
300
400
500

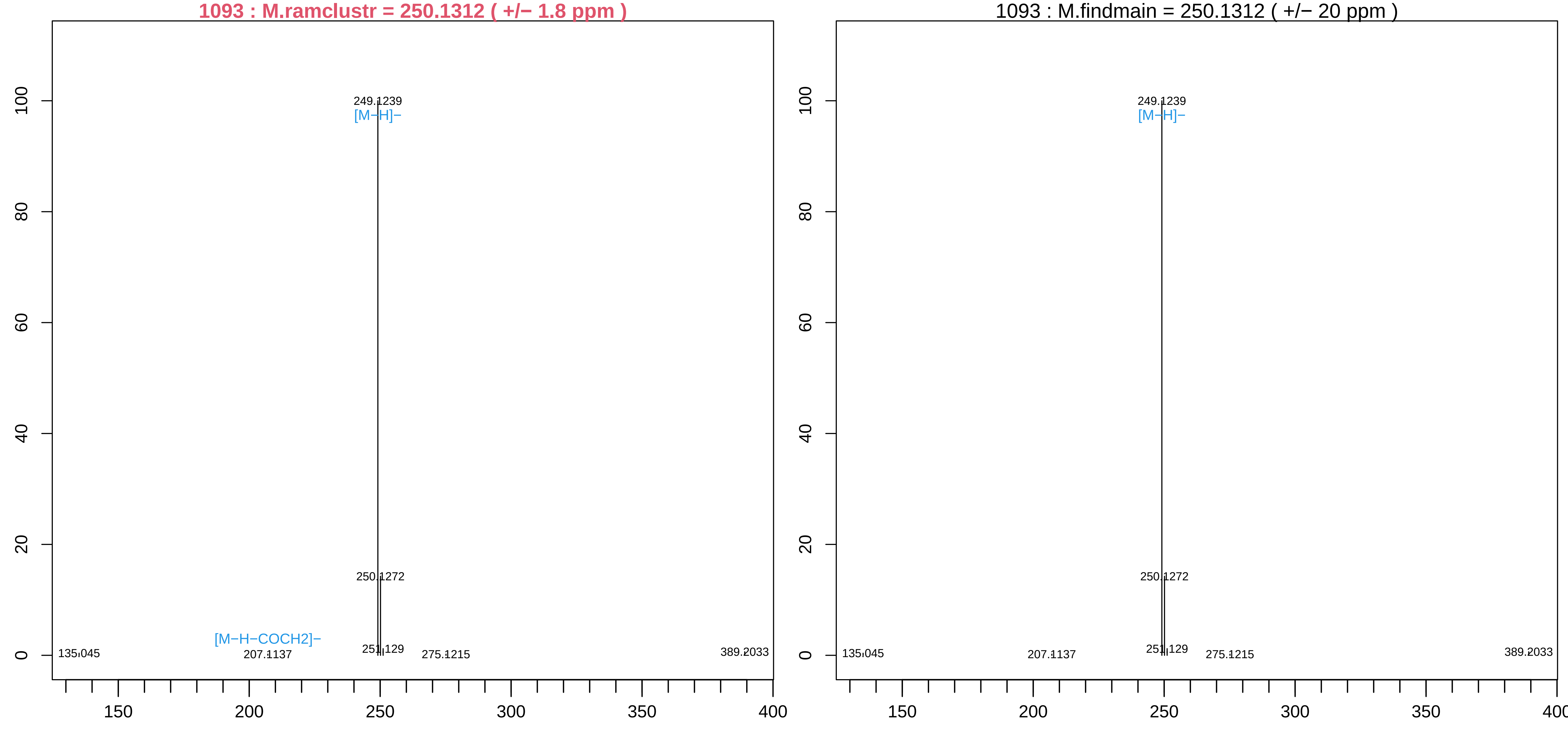

1093 : M.ramclustr = 250.1312 (+/− 1.8 ppm)
249.1239
[M−H]−
250.1272
[M−H−COCH2]−
135.045
207.1137
251.129
275.1215
389.2033
1093 : M.findmain = 250.1312 (+/− 20 ppm)
249.1239
[M−H]−
250.1272
135.045
207.1137
251.129
275.1215
389.2033

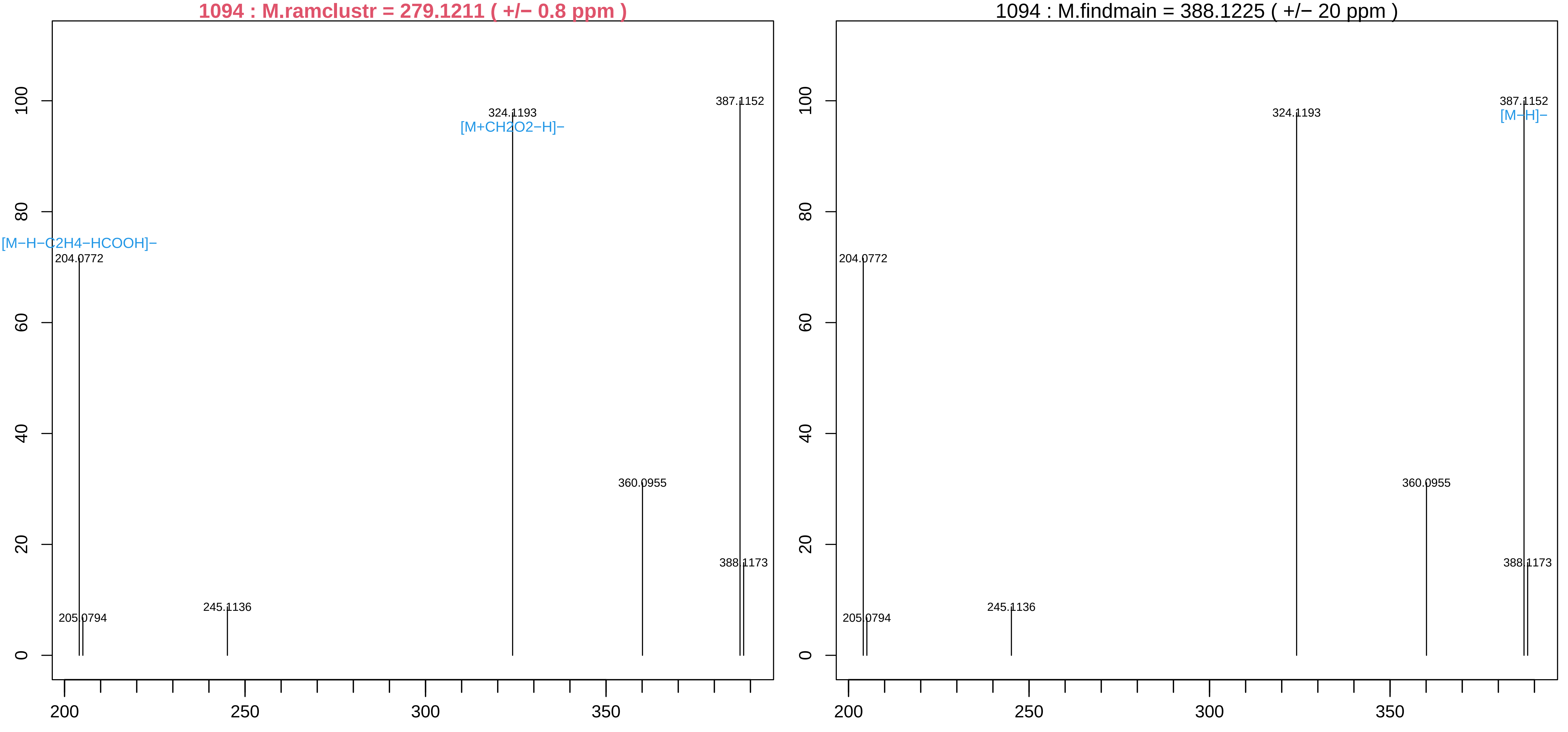
1094 : M.ramclustr = 279.1211 (+/− 0.8 ppm)
[M−H−C2H4−HCOOH]−
204.0772
205.0794
245.1136
324.1193
[M+CH2O2−H]−
360.0955
387.1152
388.1173
1094 : M.findmain = 388.1225 (+/− 20 ppm)
204.0772
205.0794
245.1136
324.1193
360.0955
387.1152
[M−H]−
388.1173
0
20
40
60
80
100
200
250
300
350

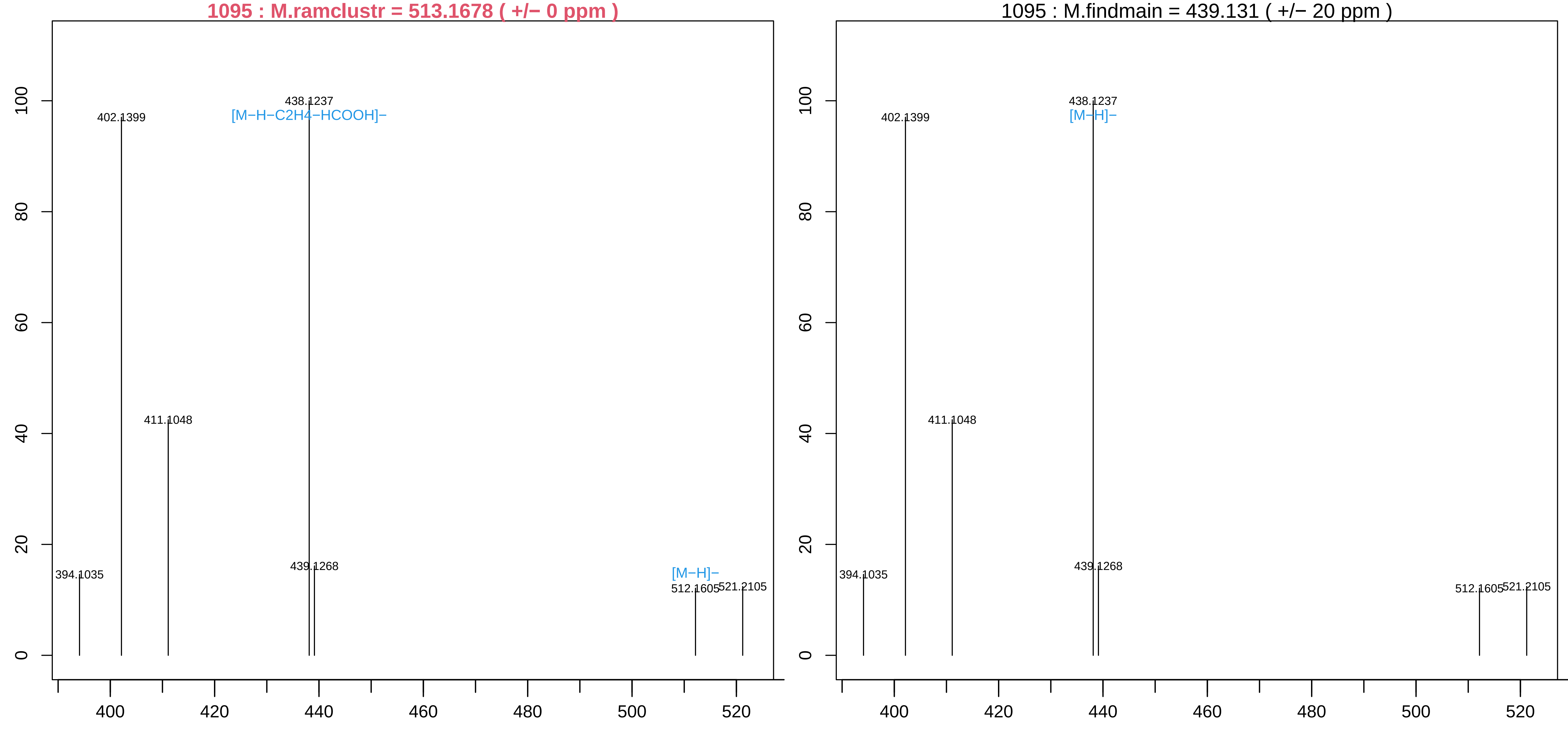

1095 : M.ramclustr = 513.1678 (+/− 0 ppm)
402.1399
438.1237
[M−H−C2H4−HCOOH]−
411.1048
394.1035
439.1268
[M−H]−
512.1605
521.2105
1095 : M.findmain = 439.131 (+/− 20 ppm)
402.1399
438.1237
[M−H]−
411.1048
394.1035
439.1268
512.1605
521.2105
0
20
40
60
80
100
400
420
440
460
480
500
520

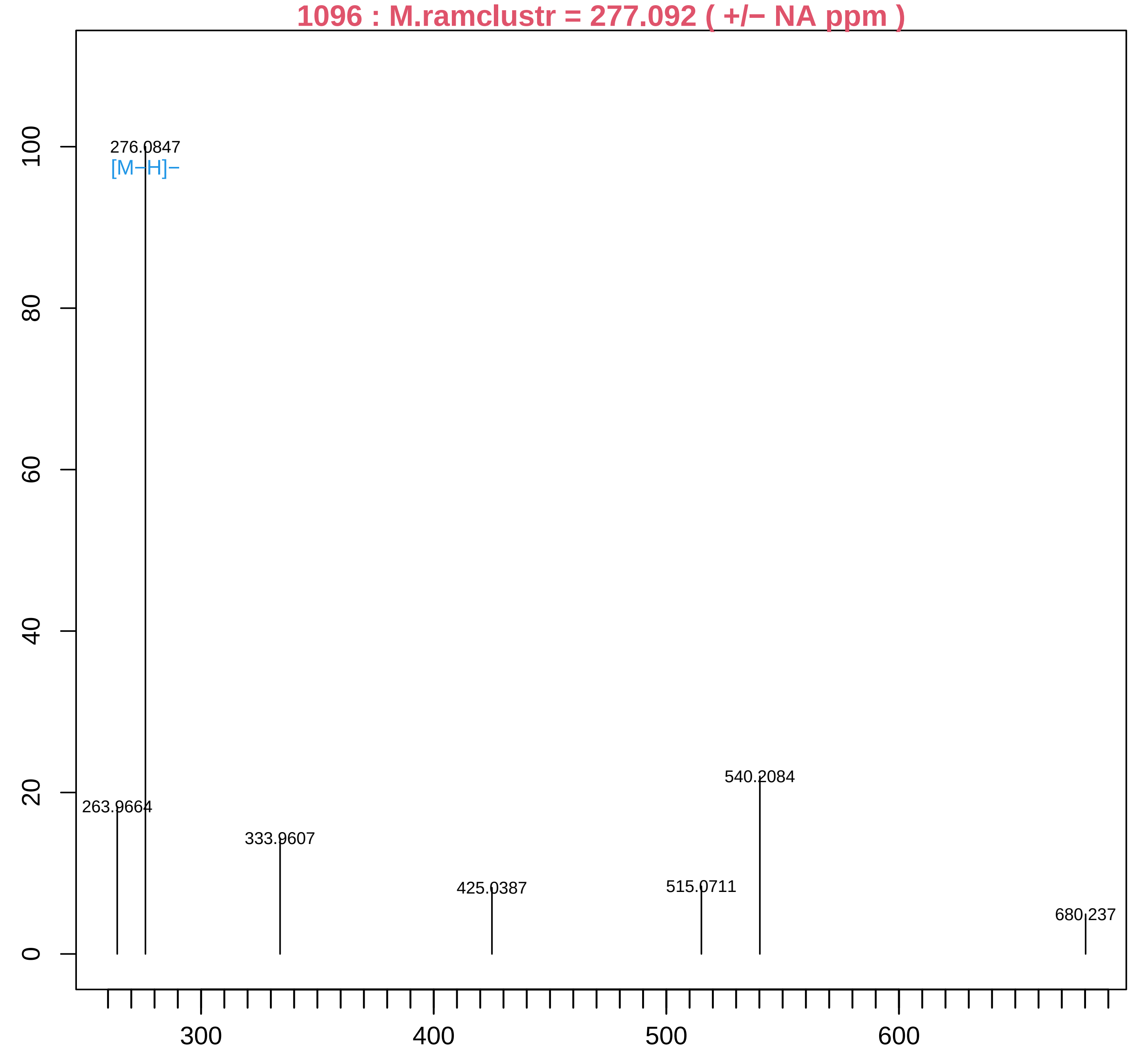
1096 : M.ramclustr = 277.092 (+/− NA ppm)
276.0847
[M−H]−
263.9664
333.9607
425.0387
515.0711
540.2084
680.237
0
20
40
60
80
100
300
400
500
600

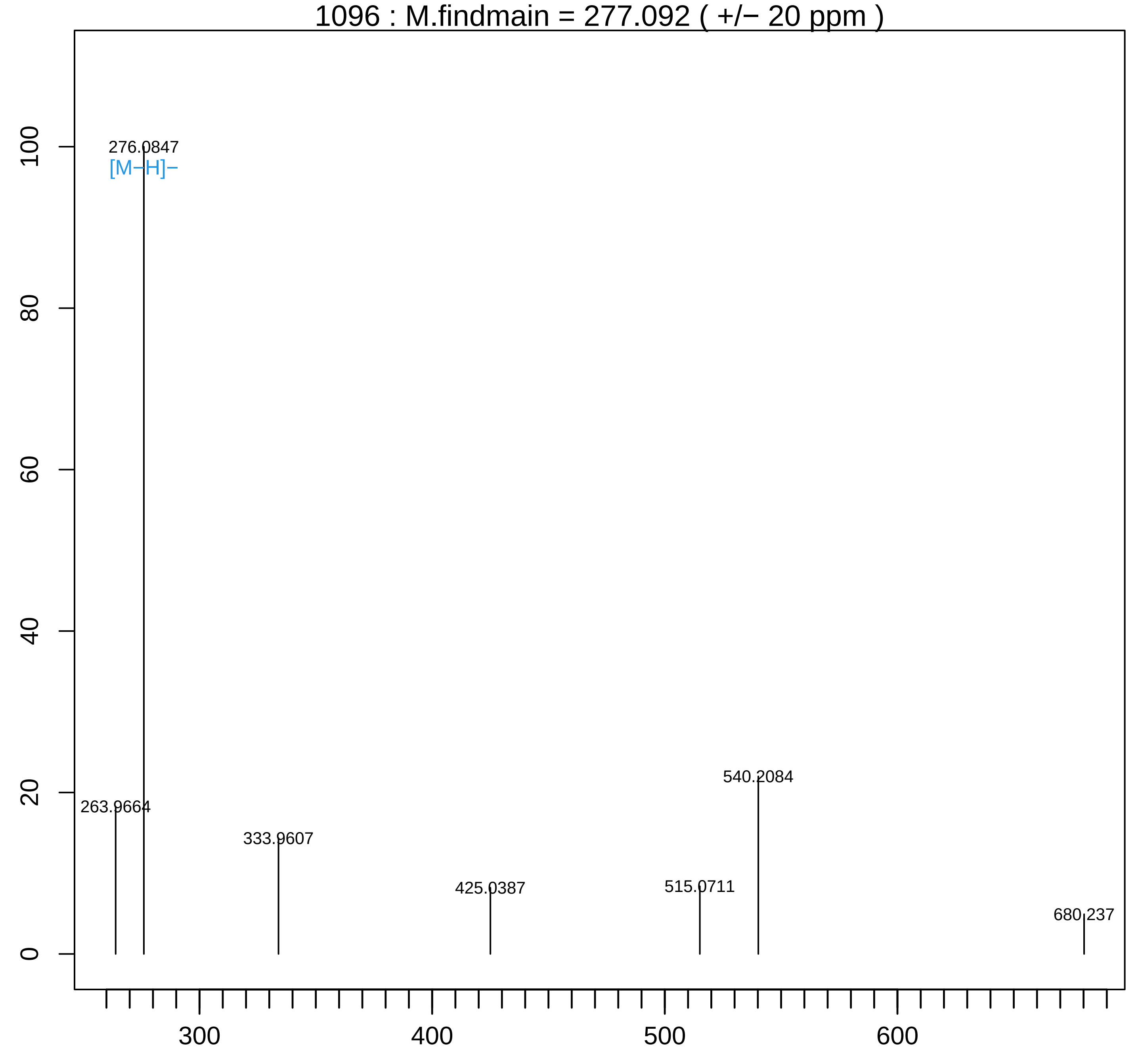
1096 : M.findmain = 277.092 (+/− 20 ppm)
276.0847
[M−H]−
263.9664
333.9607
425.0387
515.0711
540.2084
680.237
0
20
40
60
80
100
300
400
500
600

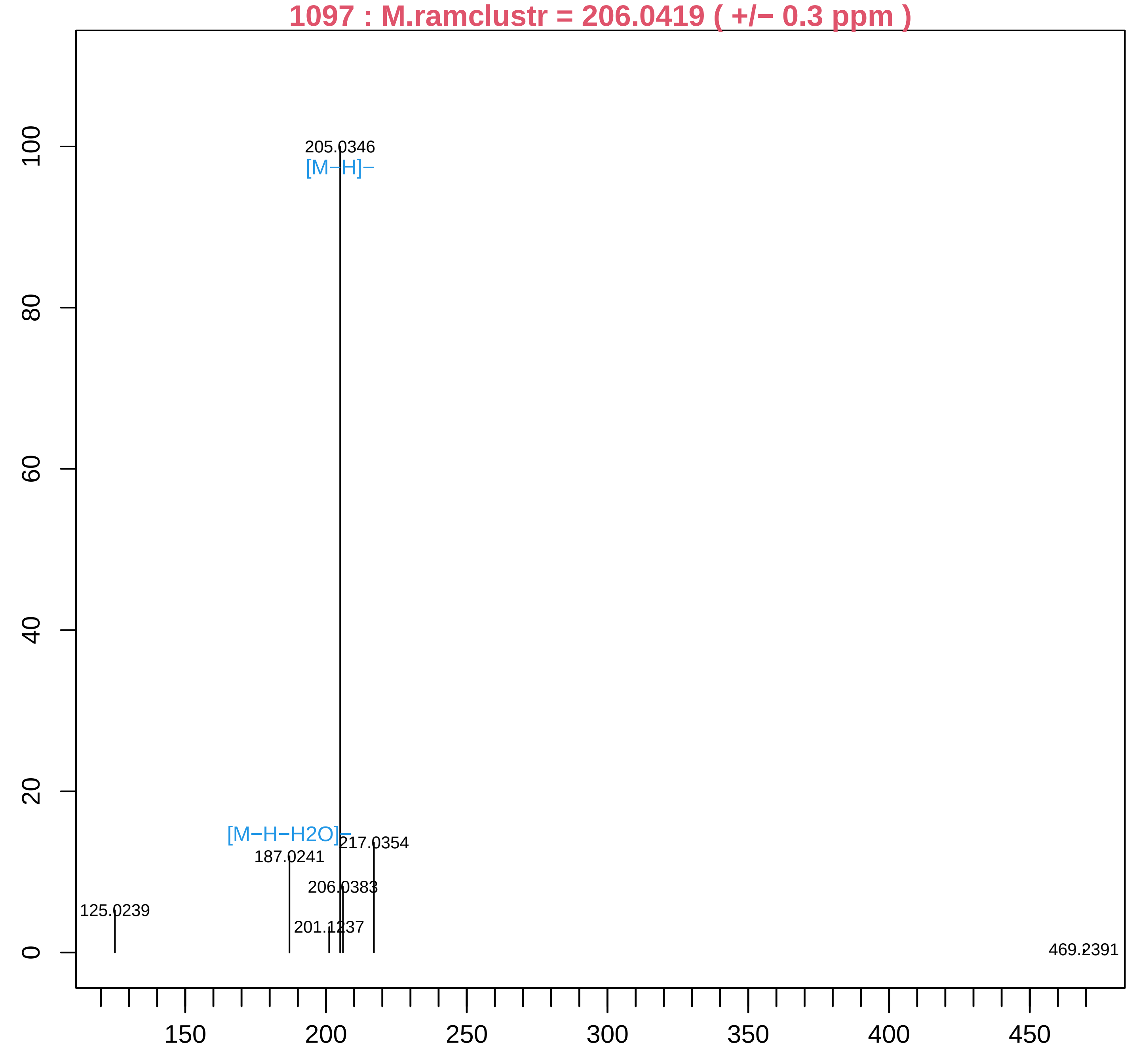
1097 : M.ramclustr = 206.0419 (+/− 0.3 ppm)
205.0346
[M−H]−
[M−H−H2O]−
187.0241
217.0354
206.0383
125.0239
201.1237
469.2391
0
20
40
60
80
100
150
200
250
300
350
400
450

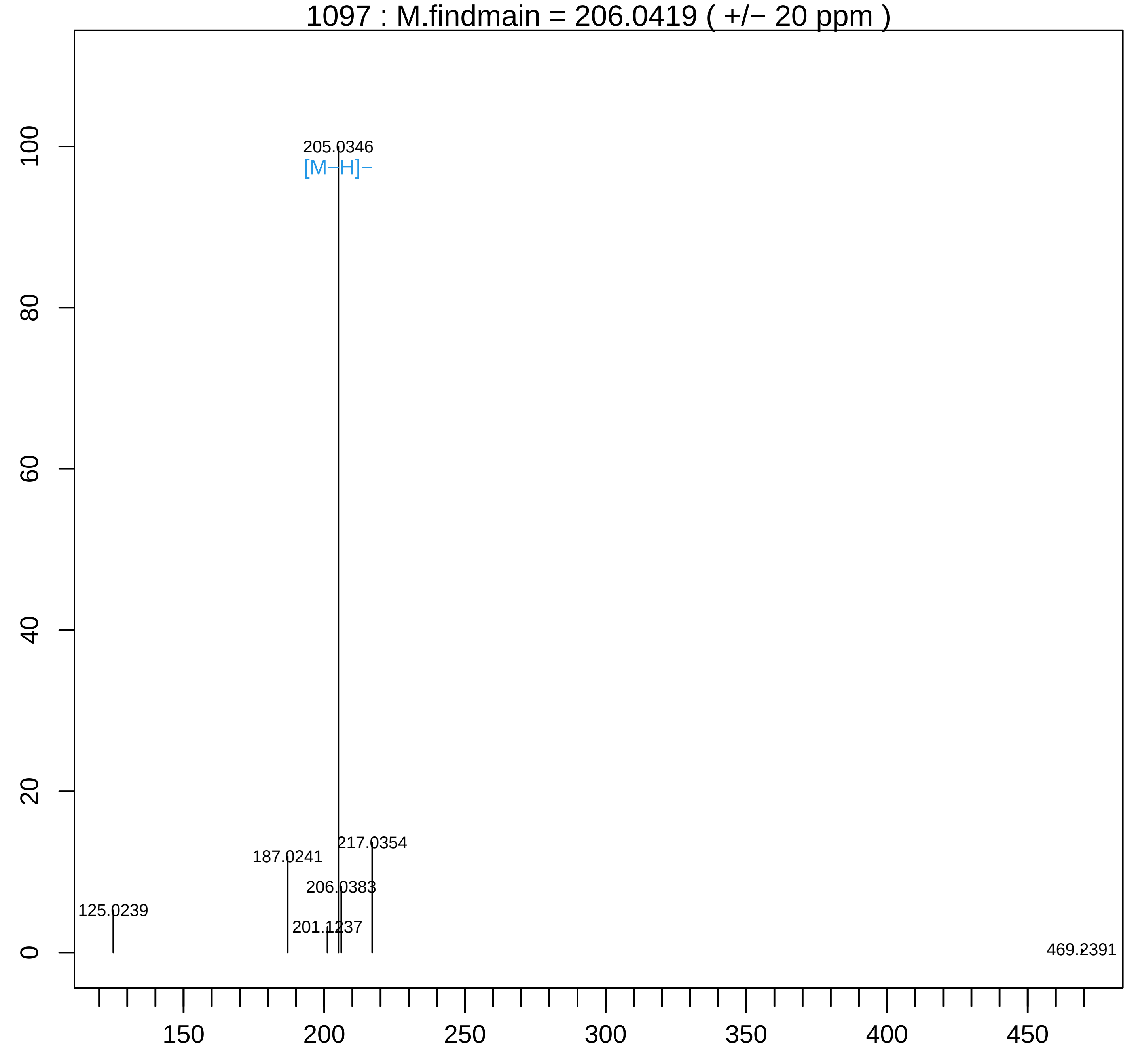
1097 : M.findmain = 206.0419 (+/− 20 ppm)
205.0346
[M−H]−
187.0241
217.0354
206.0383
125.0239
201.1237
469.2391
0
20
40
60
80
100
150
200
250
300
350
400
450

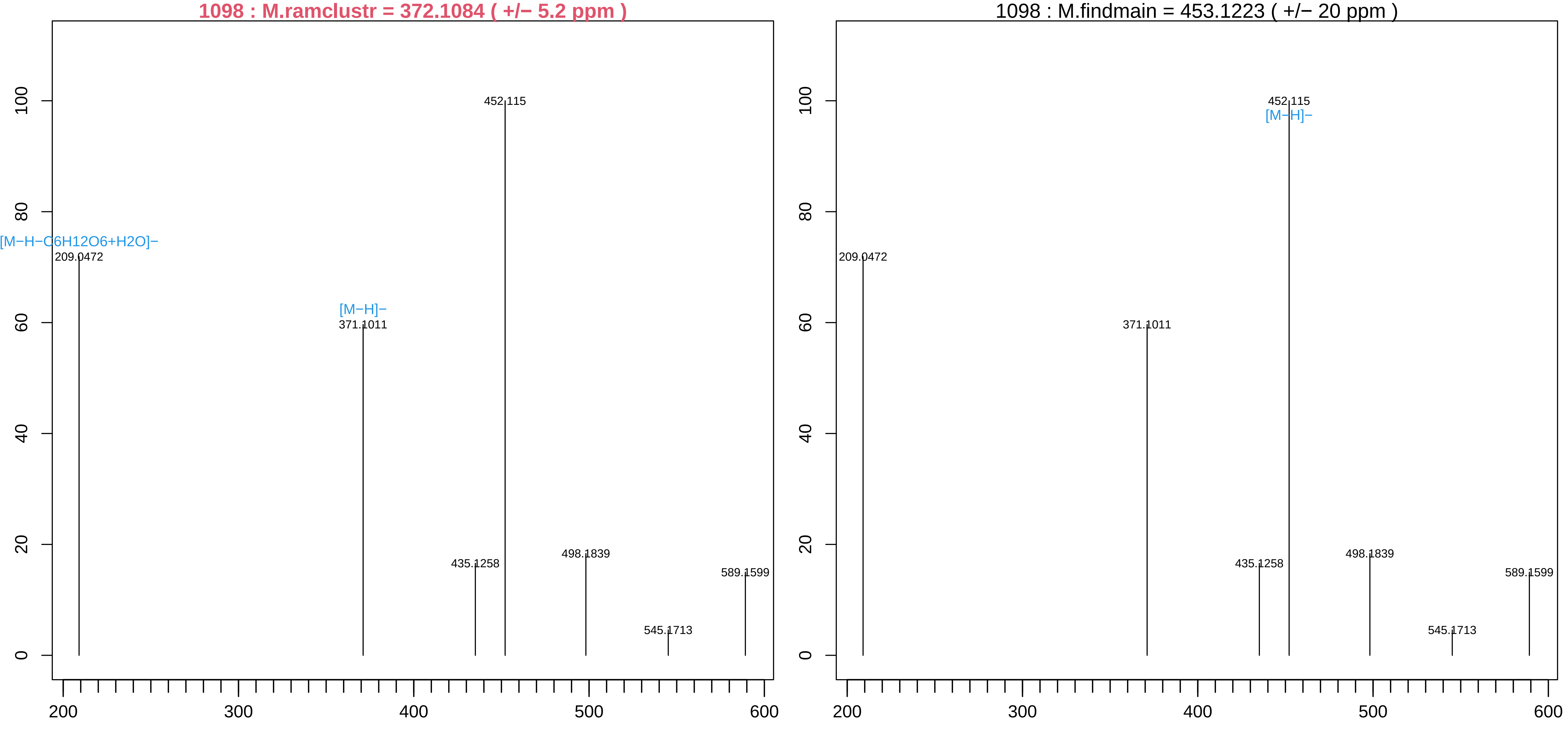
1098 : M.ramclustr = 372.1084 (+/− 5.2 ppm)
[M−H−C6H12O6+H2O]−
209.0472
[M−H]−
371.1011
452.115
435.1258
498.1839
545.1713
589.1599
1098 : M.findmain = 453.1223 (+/− 20 ppm)
209.0472
371.1011
452.115
[M−H]−
435.1258
498.1839
545.1713
589.1599
0
20
40
60
80
100
200
300
400
500
600

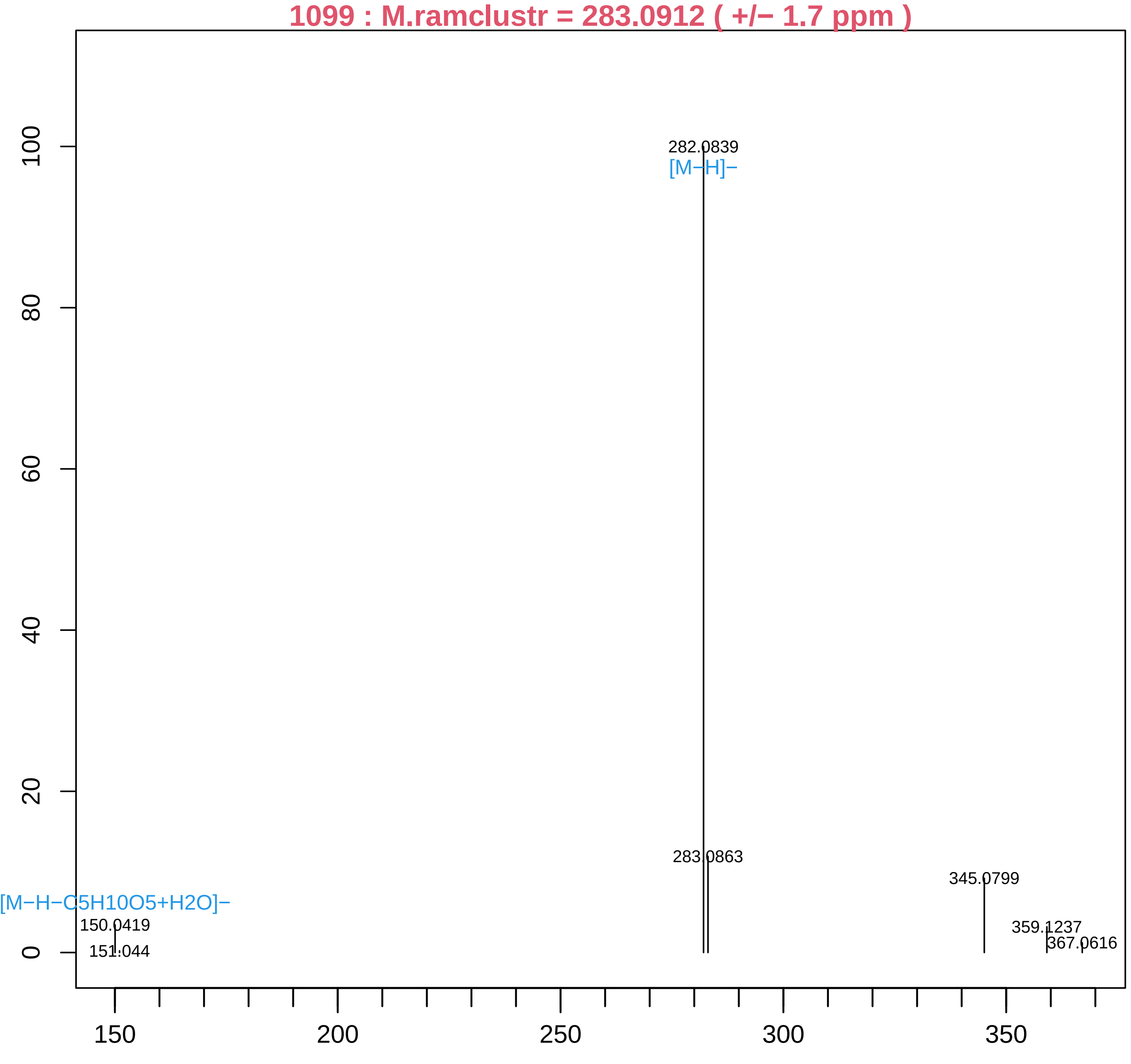
1099 : M.ramclustr = 283.0912 (+/− 1.7 ppm)
282.0839
[M−H]−
283.0863
345.0799
[M−H−C5H10O5+H2O]−
150.0419
151.044
359.1237
367.0616
0
20
40
60
80
100
150
200
250
300
350

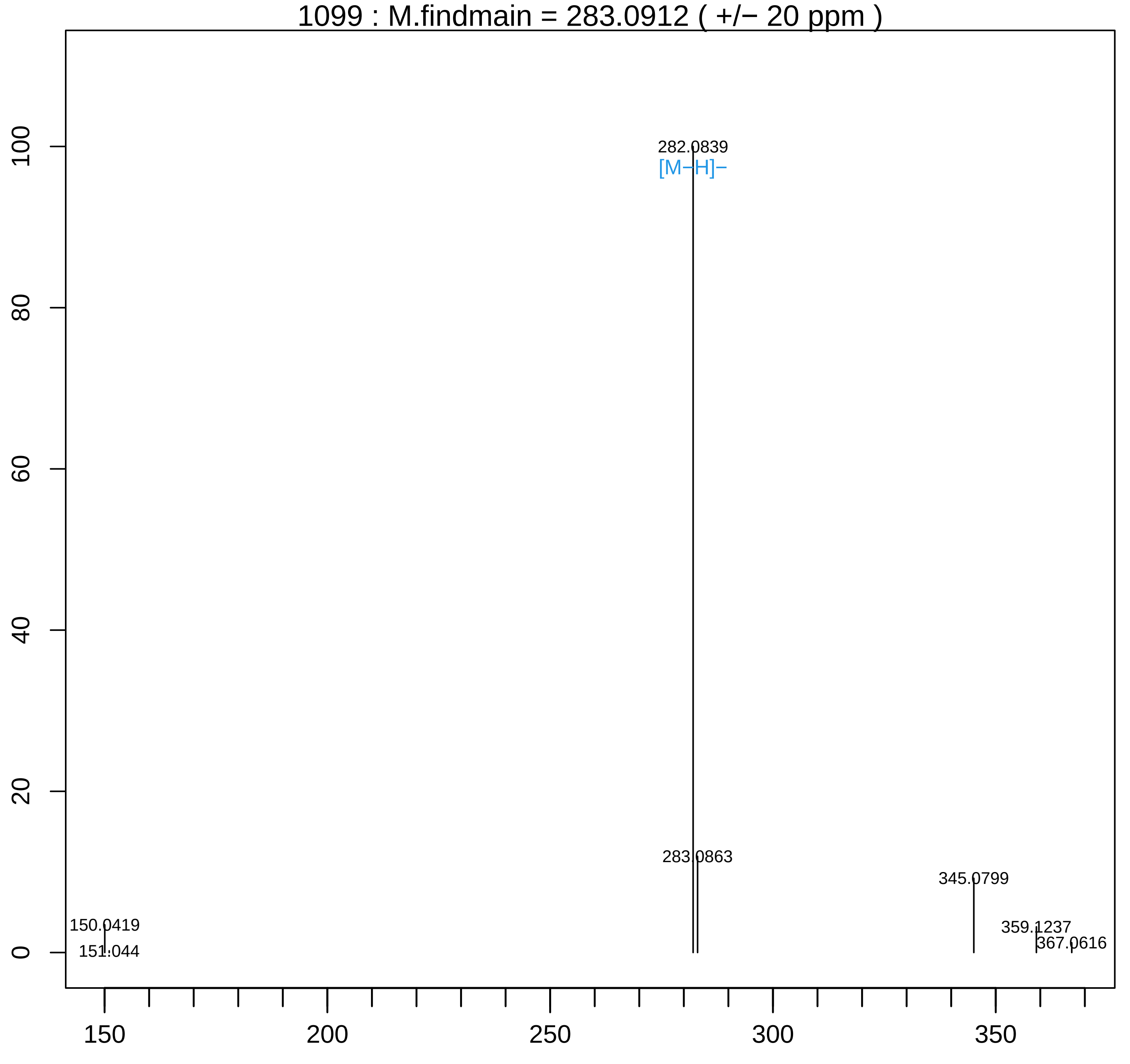
1099 : M.findmain = 283.0912 (+/− 20 ppm)
282.0839
[M−H]−
283.0863
345.0799
150.0419
151.044
359.1237
367.0616
0
20
40
60
80
100
150
200
250
300
350

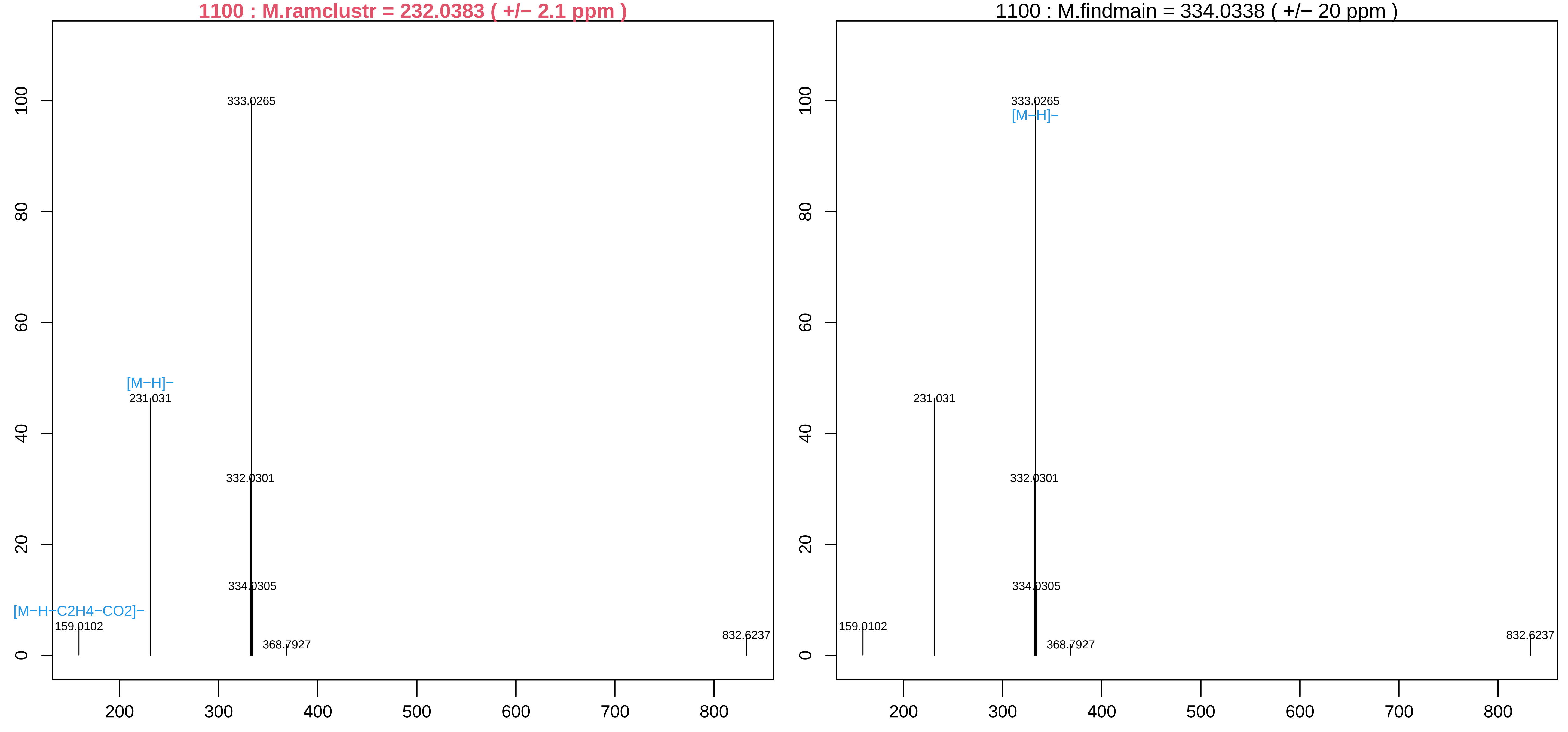
1100 : M.ramclustr = 232.0383 (+/− 2.1 ppm)
333.0265
[M−H]−
231.031
332.0301
334.0305
[M−H−C2H4−CO2]−
159.0102
368.7927
832.6237
0
20
40
60
80
100
200
300
400
500
600
700
800
1100 : M.findmain = 334.0338 (+/− 20 ppm)
333.0265
[M−H]−
231.031
332.0301
334.0305
159.0102
368.7927
832.6237

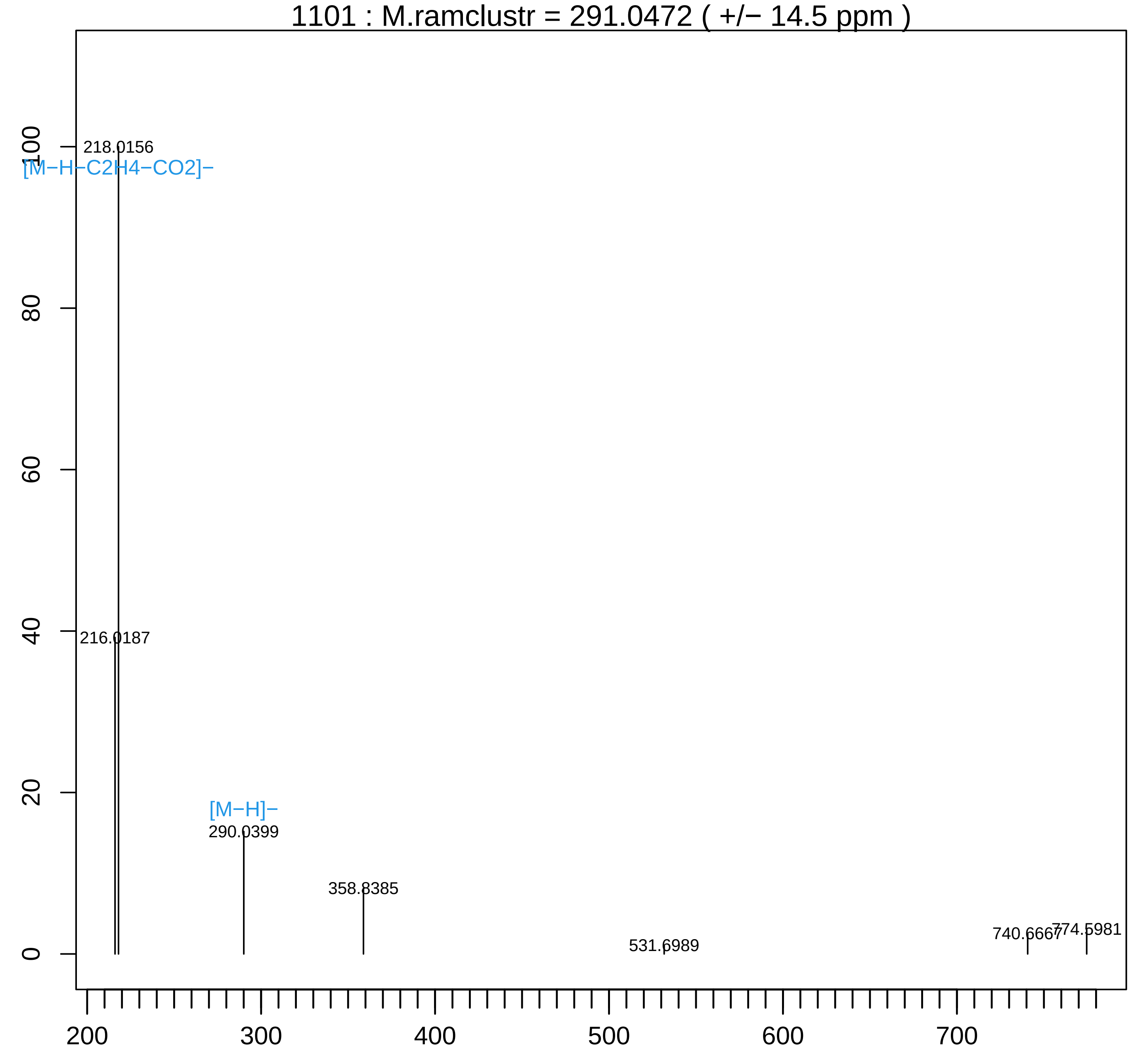
1101 : M.ramclustr = 291.0472 (+/− 14.5 ppm)
218.0156
[M−H−C2H4−CO2]−
216.0187
[M−H]−
290.0399
358.8385
531.6989
740.6667
774.5981
0
20
40
60
80
100
200
300
400
500
600
700

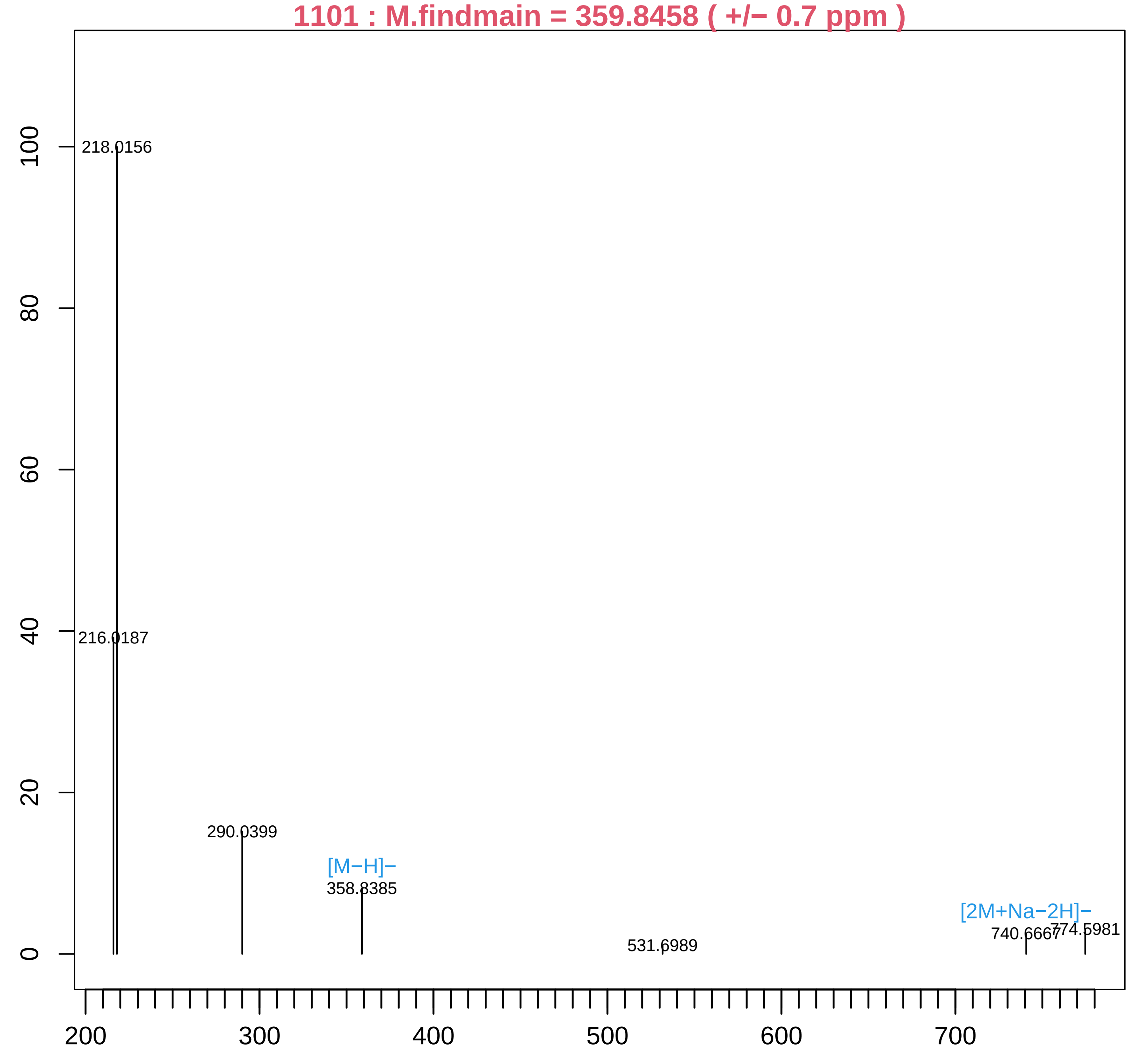
1101 : M.findmain = 359.8458 (+/− 0.7 ppm)
218.0156
216.0187
290.0399
[M−H]−
358.8385
531.6989
[2M+Na−2H]−
740.6667
774.5981
0
20
40
60
80
100
200
300
400
500
600
700

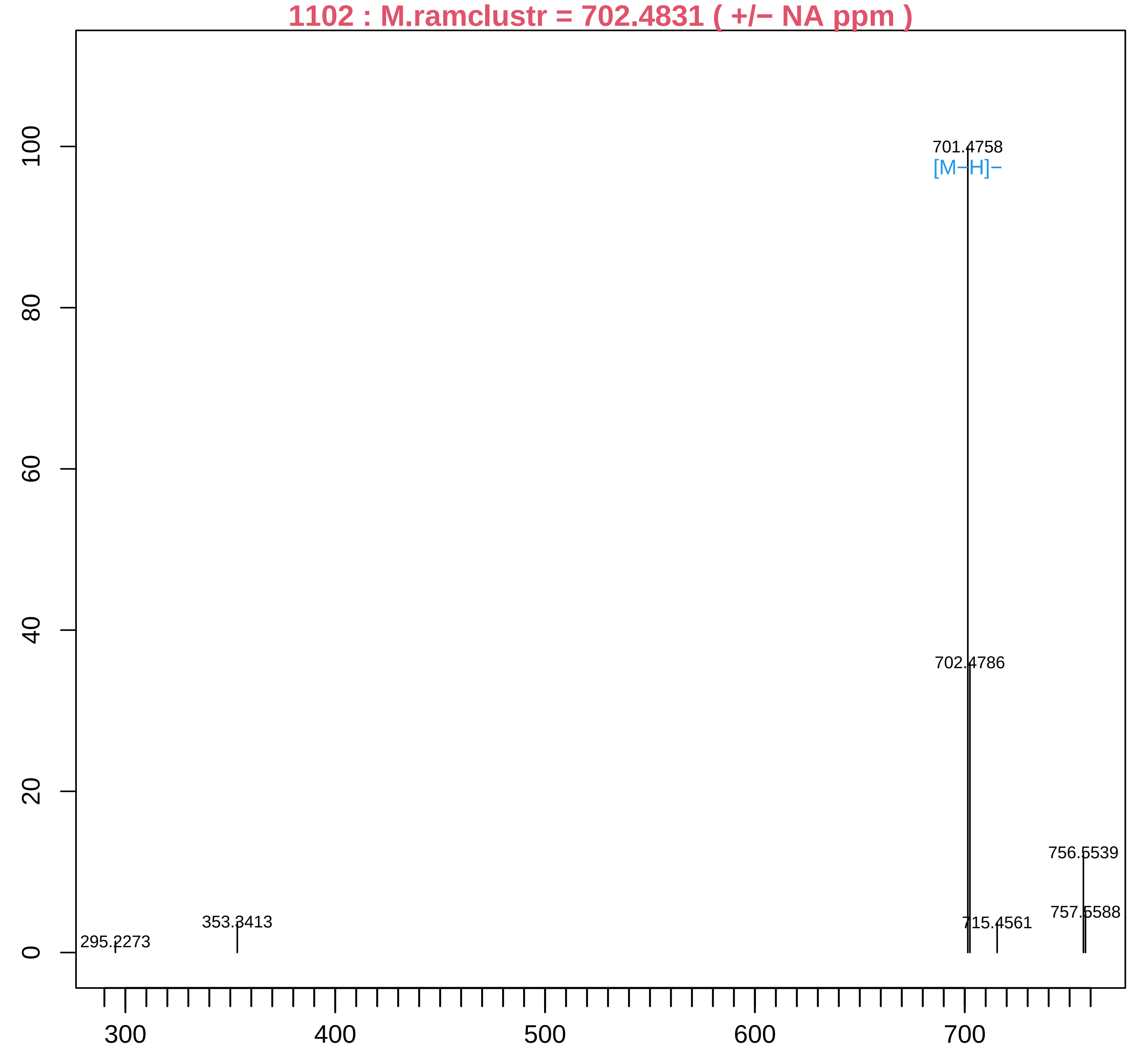
1102 : M.ramclustr = 702.4831 (+/− NA ppm)
701.4758
[M−H]−
702.4786
756.5539
757.5588
715.4561
353.3413
295.2273
0
20
40
60
80
100
300
400
500
600
700

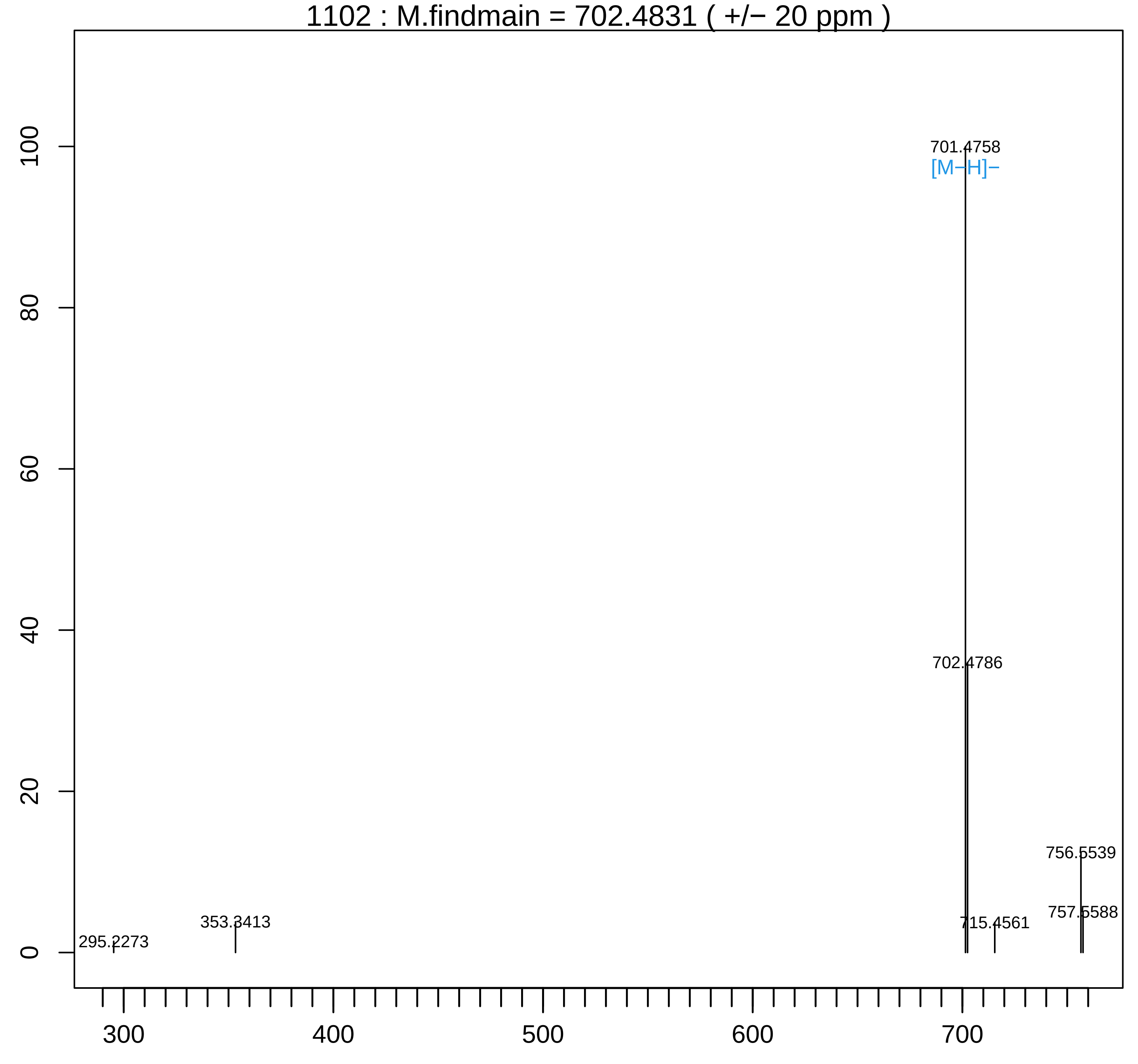
1102 : M.findmain = 702.4831 (+/− 20 ppm)
701.4758
[M−H]−
702.4786
756.5539
757.5588
715.4561
353.3413
295.2273
0
20
40
60
80
100
300
400
500
600
700

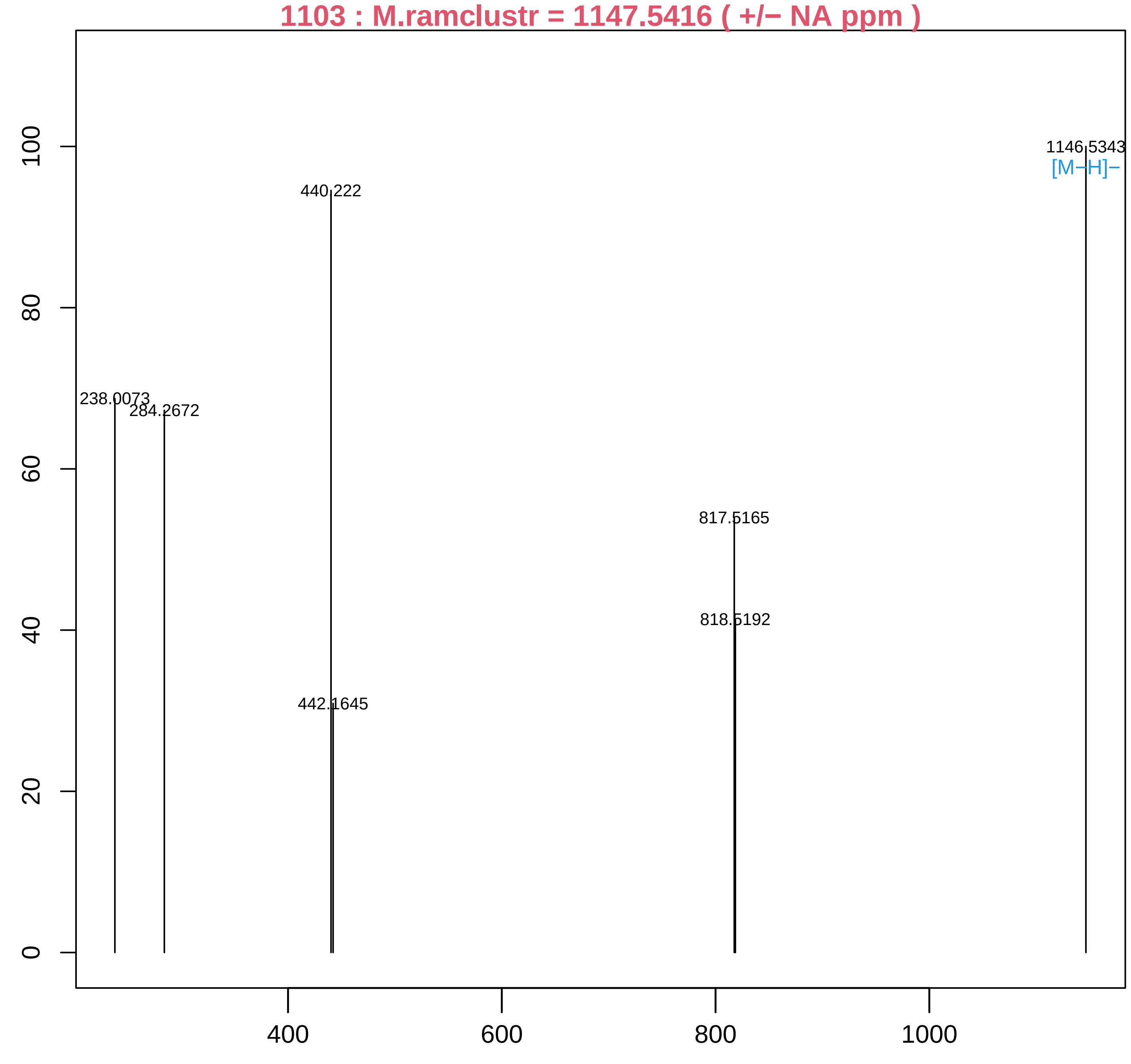
1103 : M.ramclustr = 1147.5416 (+/− NA ppm)
1146.5343
[M−H]−
440.222
238.0073
284.2672
817.5165
818.5192
442.1645
0
20
40
60
80
100
400
600
800
1000

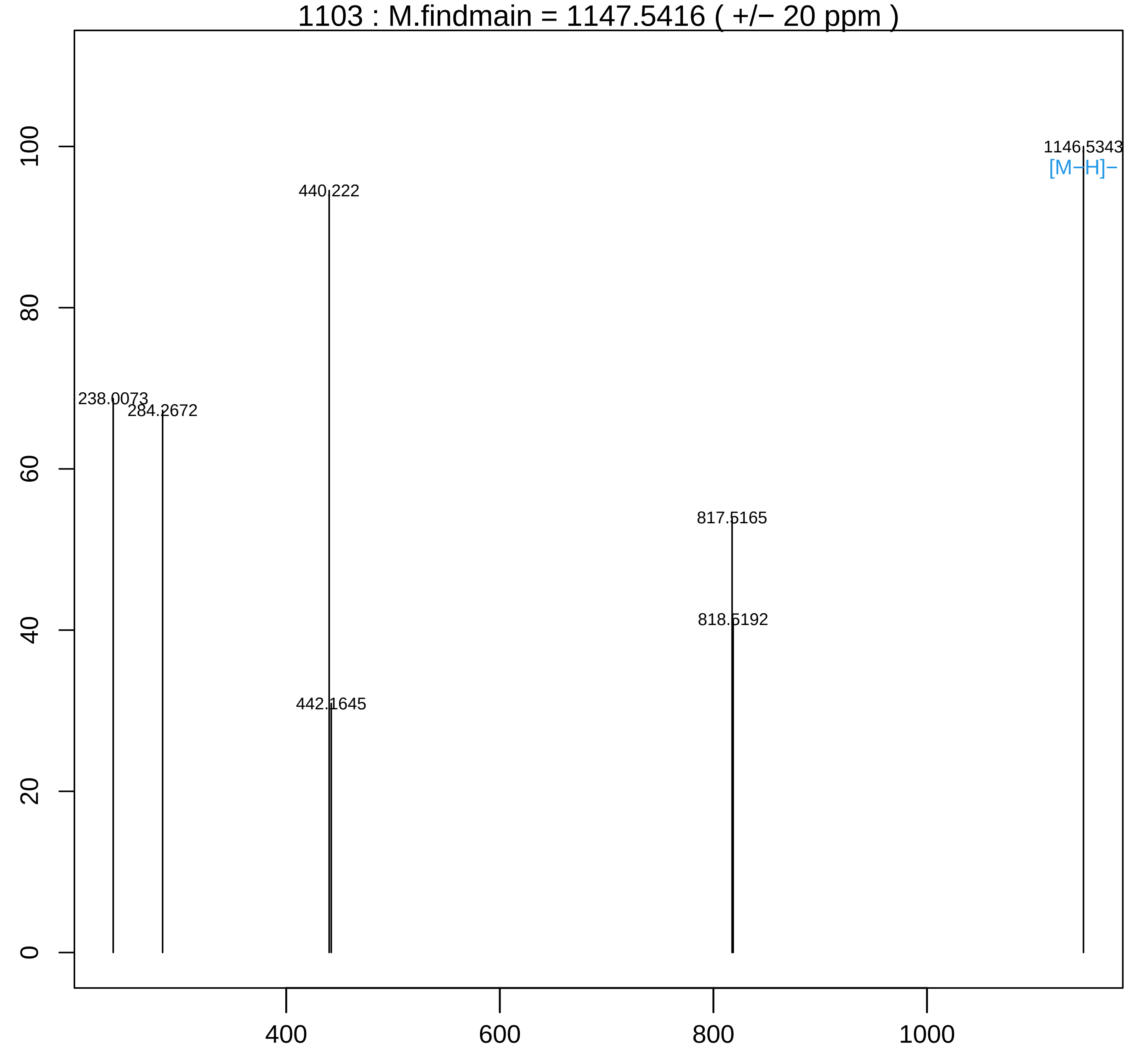
1103 : M.findmain = 1147.5416 (+/− 20 ppm)
1146.5343
[M−H]−
440.222
238.0073
284.2672
817.5165
818.5192
442.1645
0
20
40
60
80
100
400
600
800
1000

1104 : M.ramclustr = 788.4737 (+/− 14.6 ppm)

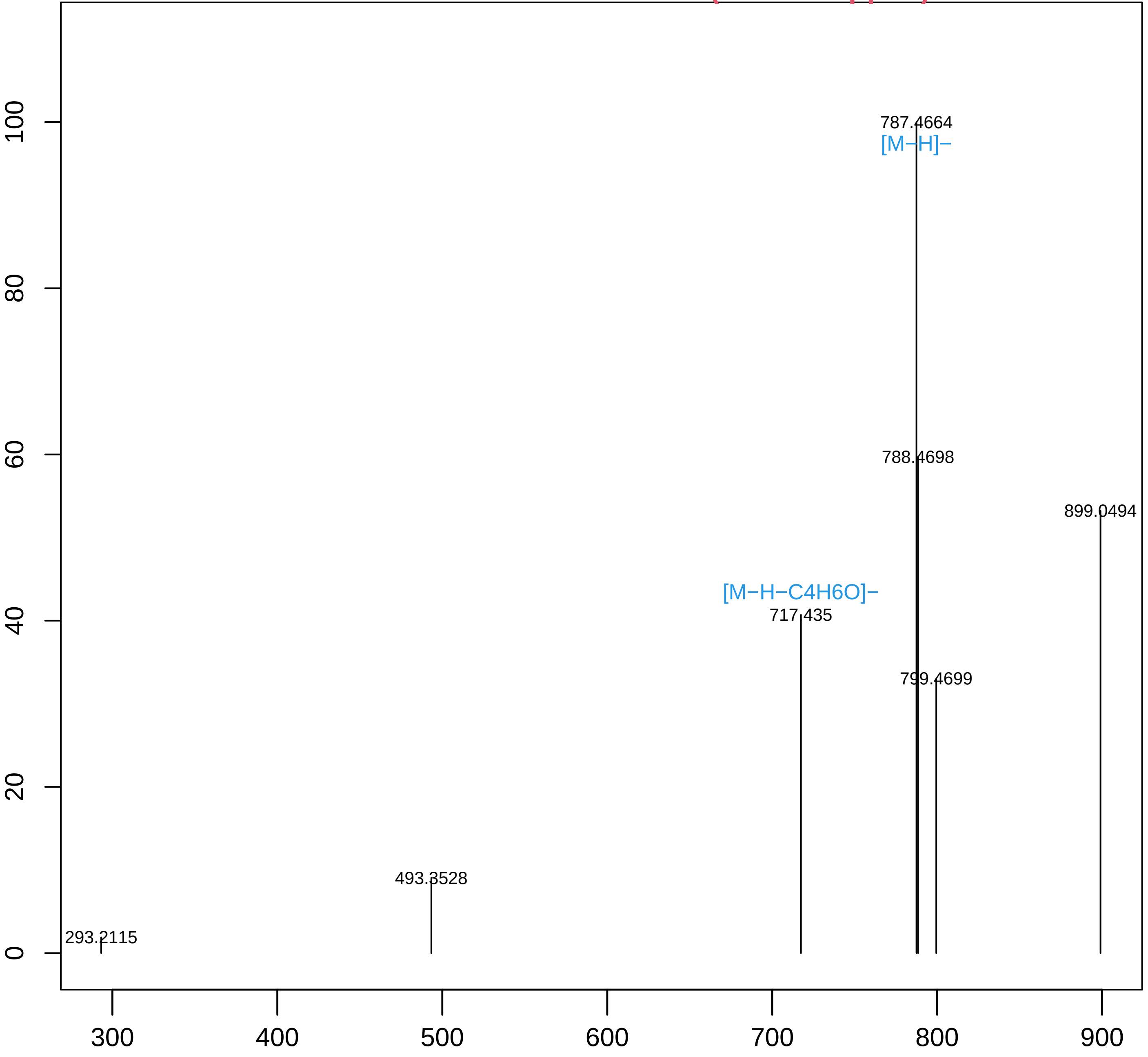

1104 : M.findmain = 788.4737 (+/− 20 ppm)

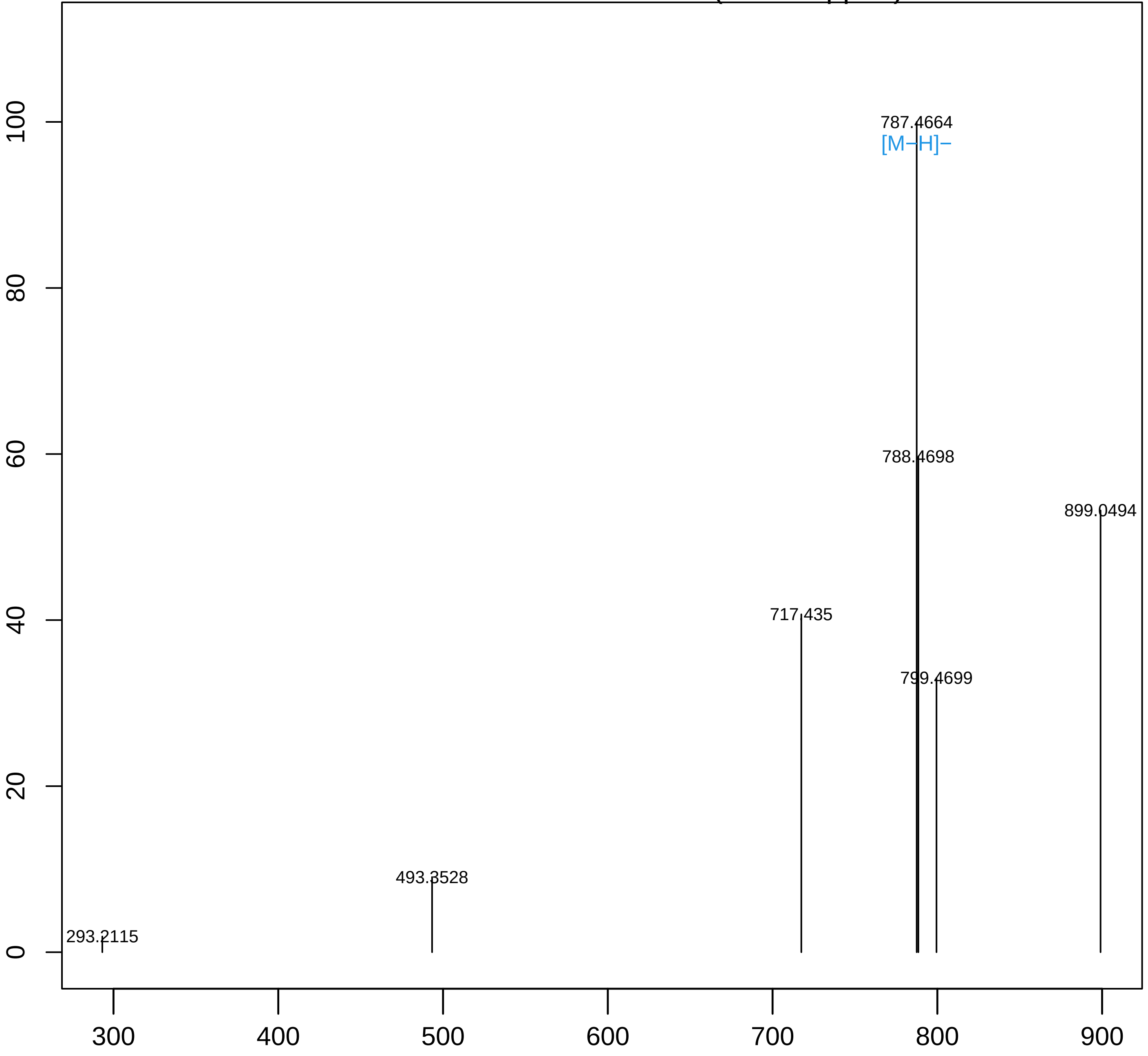

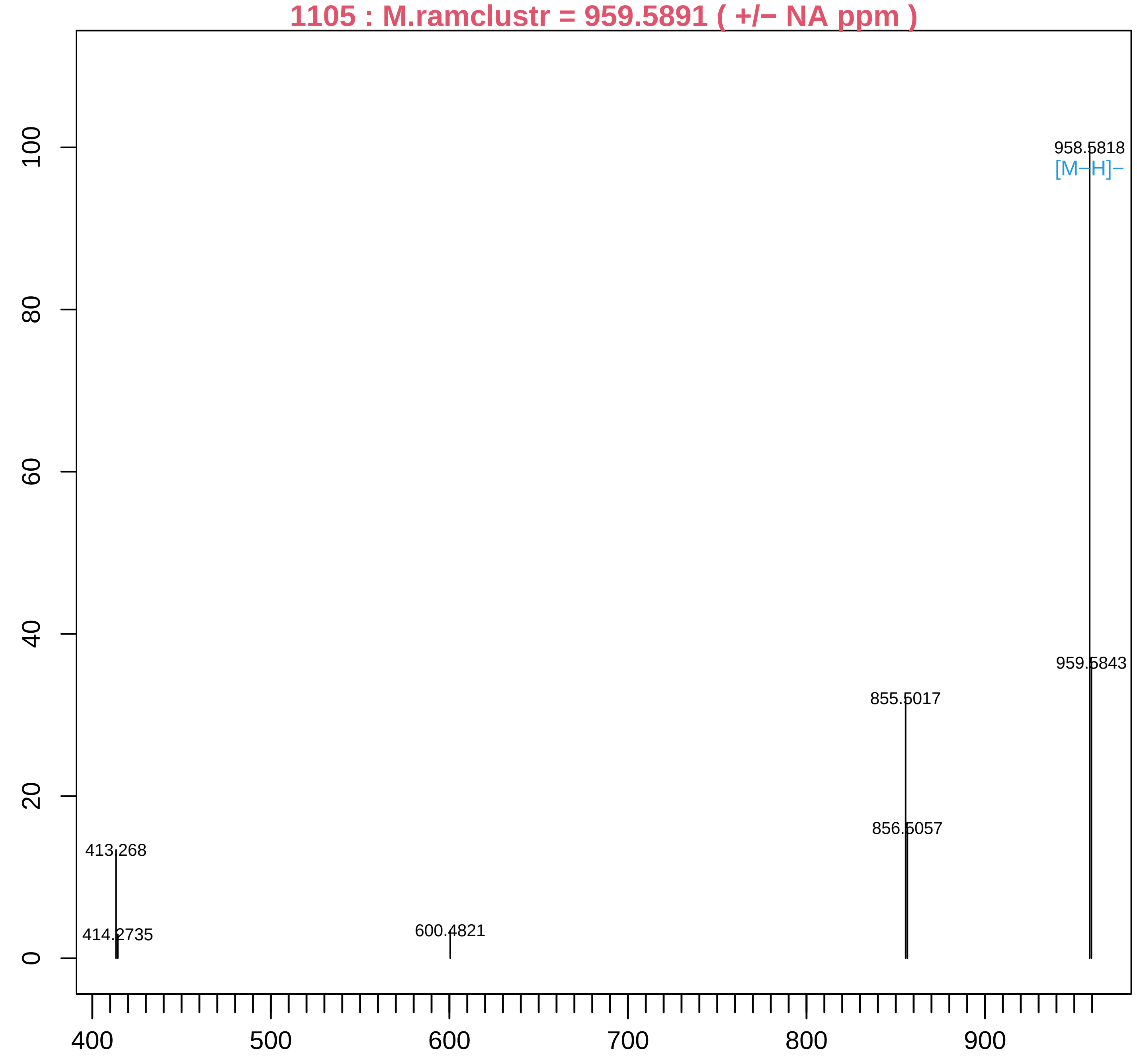

1105 : M.ramclustr = 959.5891 (+/− NA ppm)
958.5818
[M−H]−
959.5843
855.5017
856.5057
413.268
414.2735
600.4821
0
20
40
60
80
100
400
500
600
700
800
900

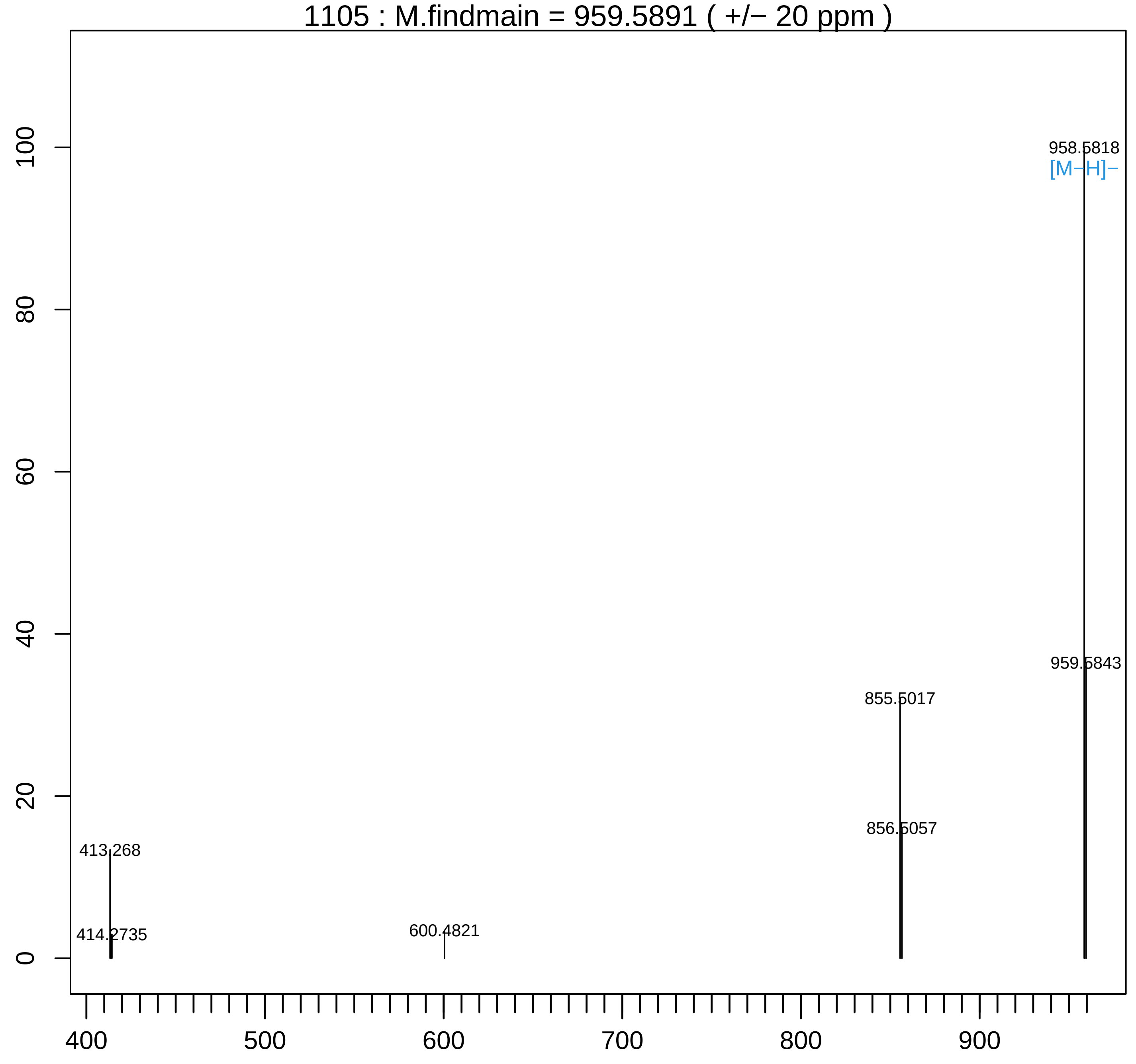

1105 : M.findmain = 959.5891 (+/− 20 ppm)
958.5818
[M−H]−
959.5843
855.5017
856.5057
413.268
414.2735
600.4821
0
20
40
60
80
100
400
500
600
700
800
900

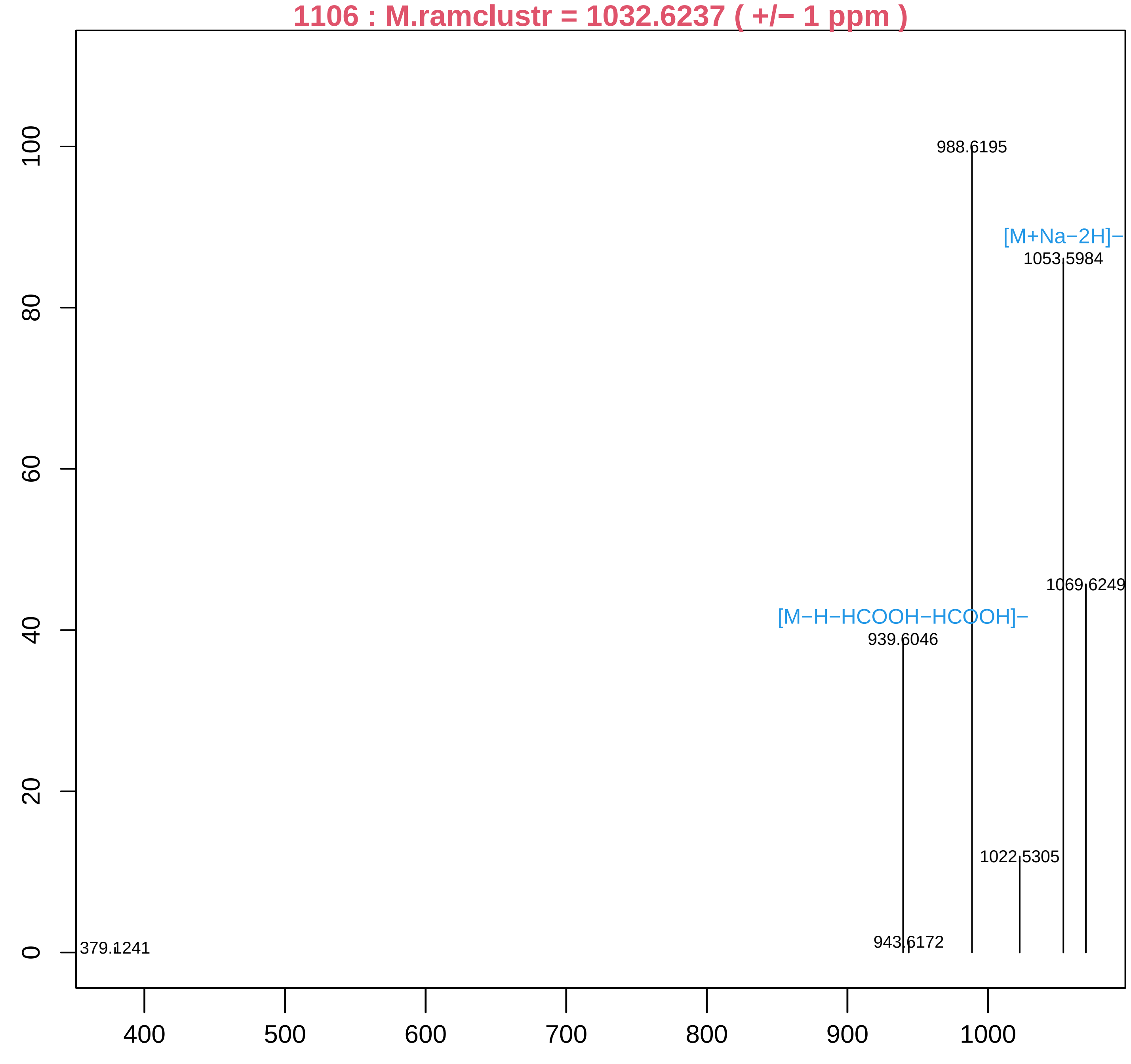
1106 : M.ramclustr = 1032.6237 (+/− 1 ppm)
988.6195
[M+Na−2H]−
1053.5984
1069.6249
[M−H−HCOOH−HCOOH]−
939.6046
1022.5305
943.6172
379.1241
0
20
40
60
80
100
400
500
600
700
800
900
1000

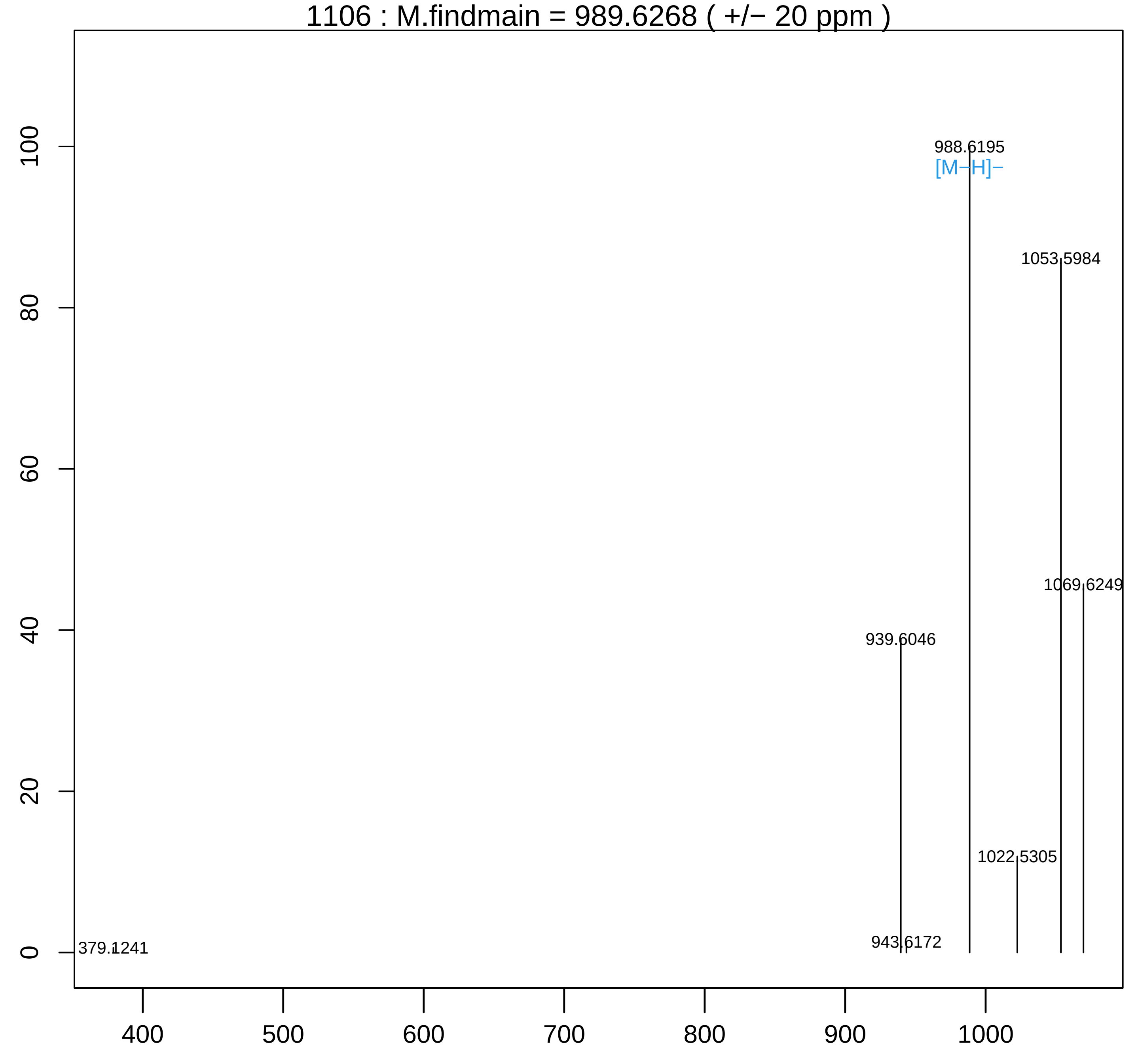
1106 : M.findmain = 989.6268 (+/− 20 ppm)
988.6195
[M−H]−
1053.5984
1069.6249
939.6046
1022.5305
943.6172
379.1241
0
20
40
60
80
100
400
500
600
700
800
900
1000

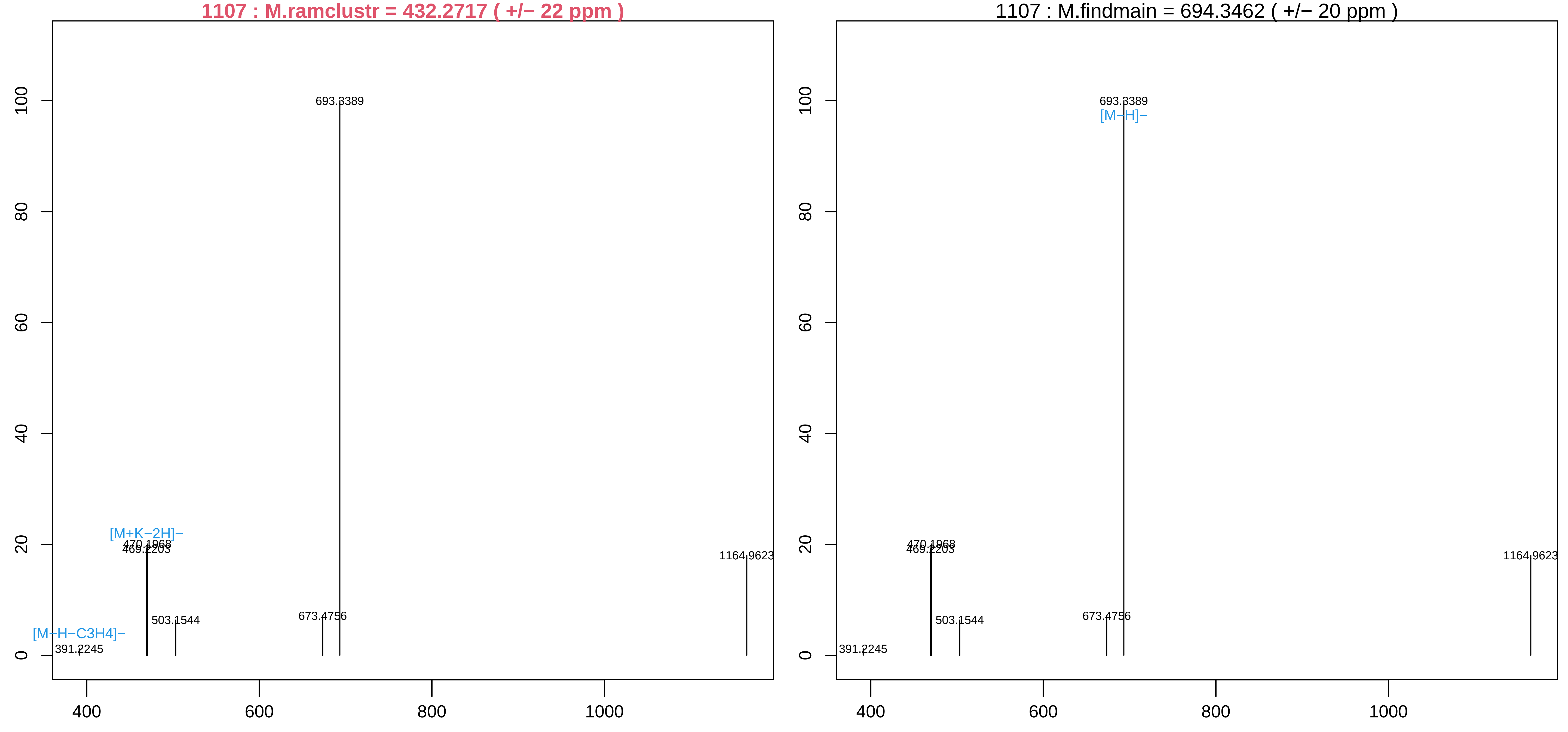
1107 : M.ramclustr = 432.2717 (+/− 22 ppm)
693.3389
[M+K−2H]−
470.1968
469.2203
1164.9623
503.1544
673.4756
[M−H−C3H4]−
391.2245
1107 : M.findmain = 694.3462 (+/− 20 ppm)
693.3389
[M−H]−
470.1968
469.2203
1164.9623
503.1544
673.4756
391.2245
0
20
40
60
80
100
400
600
800
1000

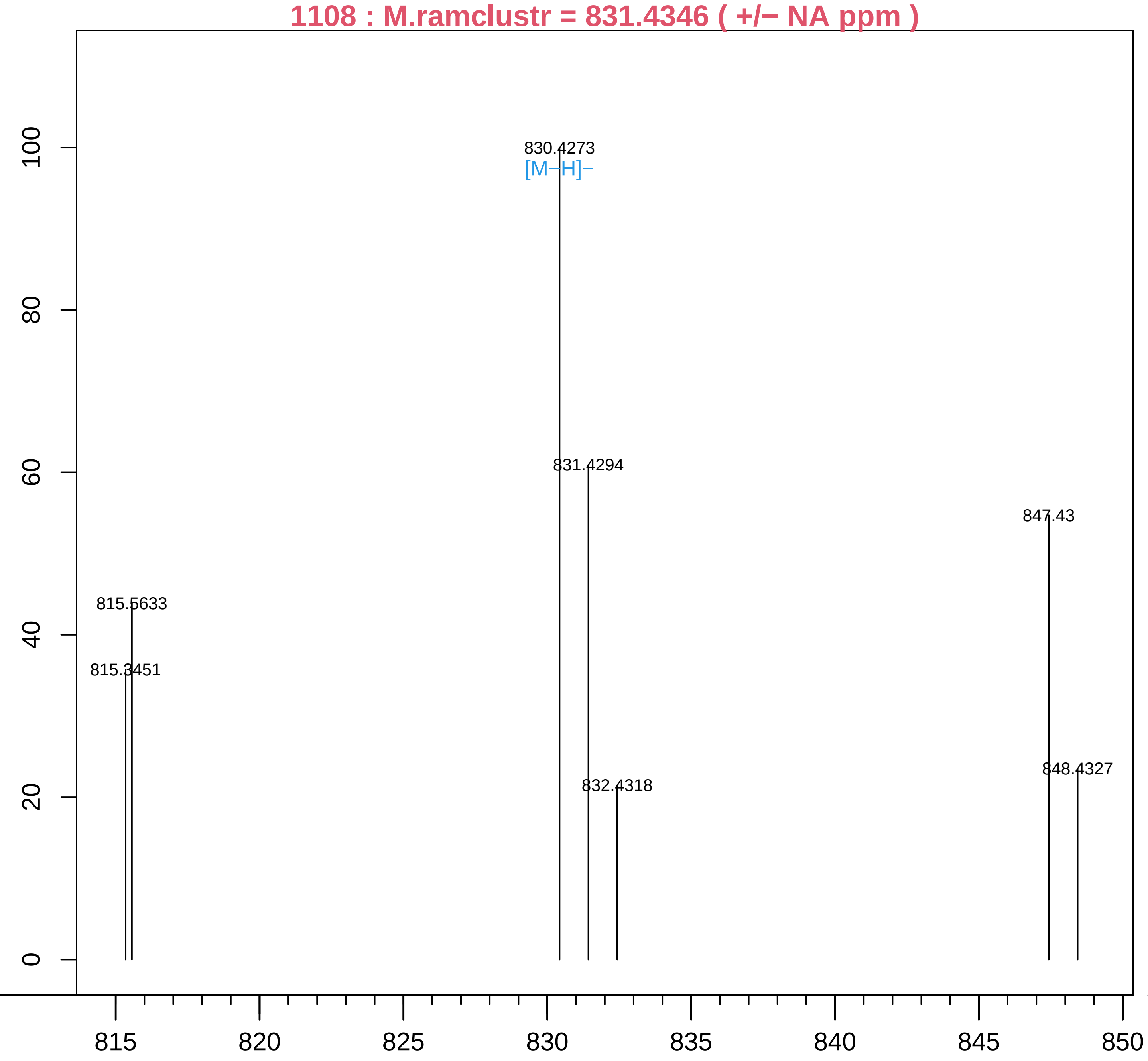
1108 : M.ramclustr = 831.4346 (+/− NA ppm)
830.4273
[M−H]−
831.4294
847.43
815.5633
815.3451
848.4327
832.4318
0
20
40
60
80
100
815
820
825
830
835
840
845
850

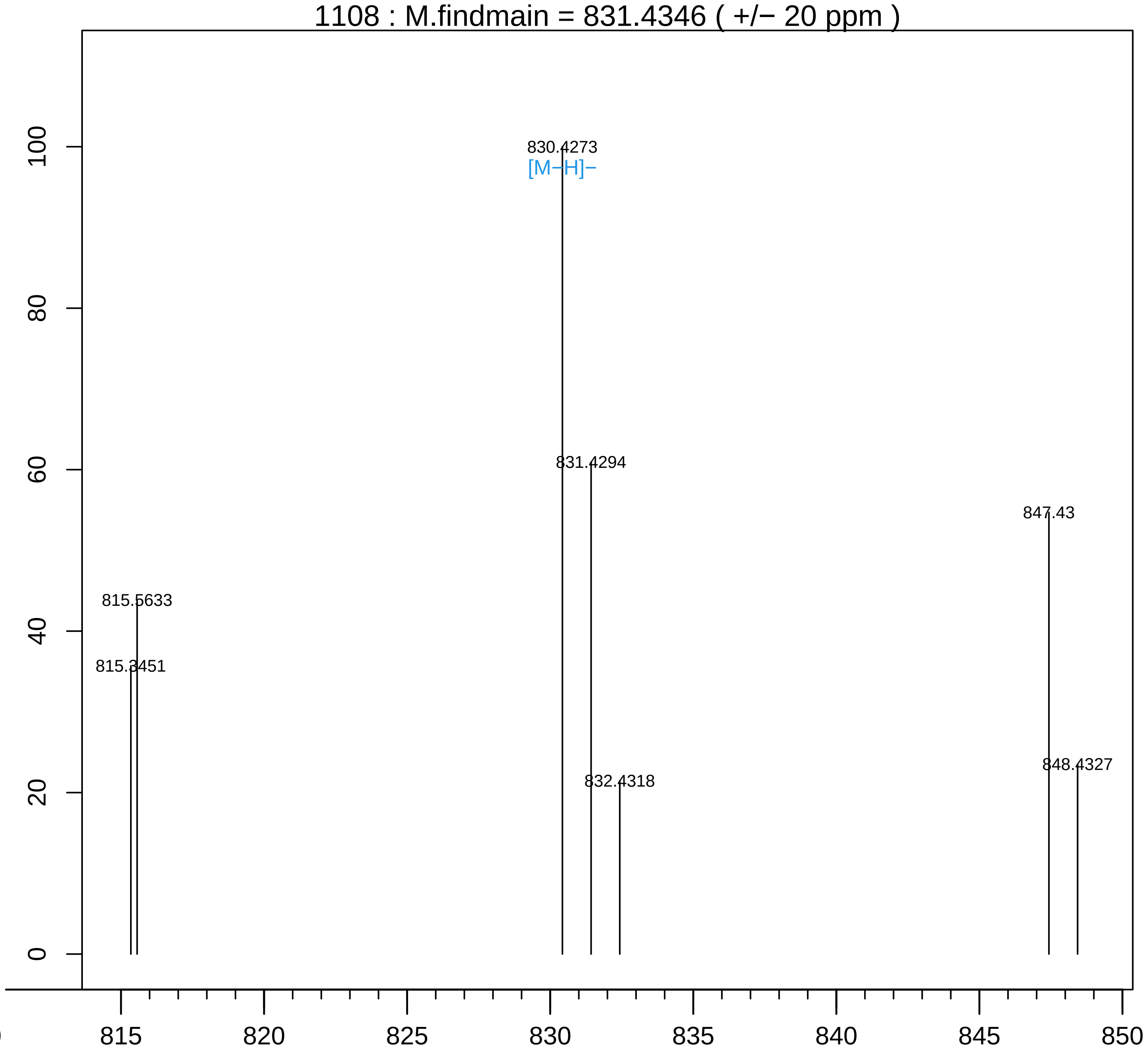
1108 : M.findmain = 831.4346 (+/− 20 ppm)
830.4273
[M−H]−
831.4294
847.43
815.5633
815.3451
848.4327
832.4318
0
20
40
60
80
100
815
820
825
830
835
840
845
850

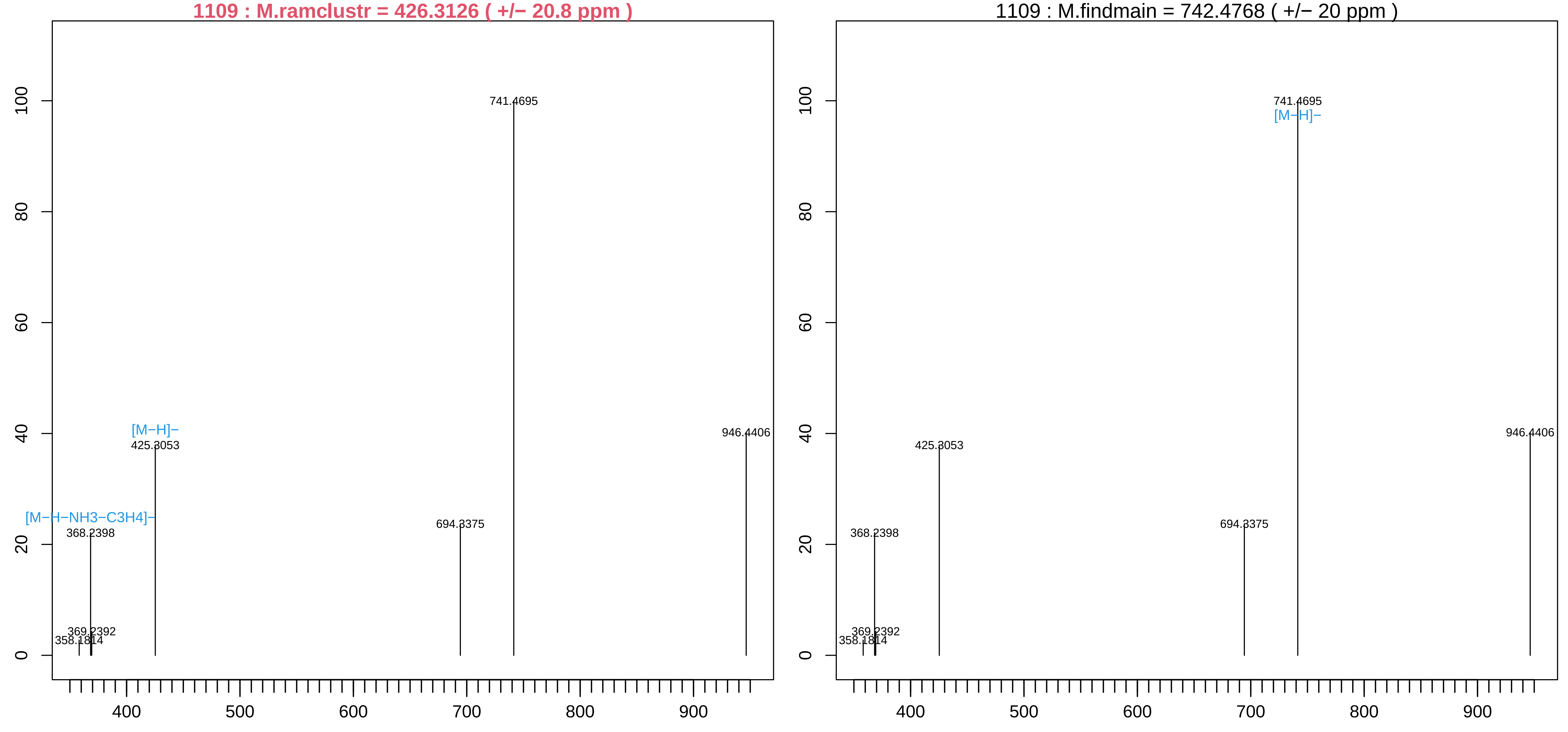
1109 : M.ramclustr = 426.3126 (+/− 20.8 ppm)
741.4695
946.4406
[M−H]−
425.3053
[M−H−NH3−C3H4]−
368.2398
694.3375
369.2392
358.1814
1109 : M.findmain = 742.4768 (+/− 20 ppm)
741.4695
[M−H]−
425.3053
946.4406
368.2398
694.3375
369.2392
358.1814
0
20
40
60
80
100
400
500
600
700
800
900

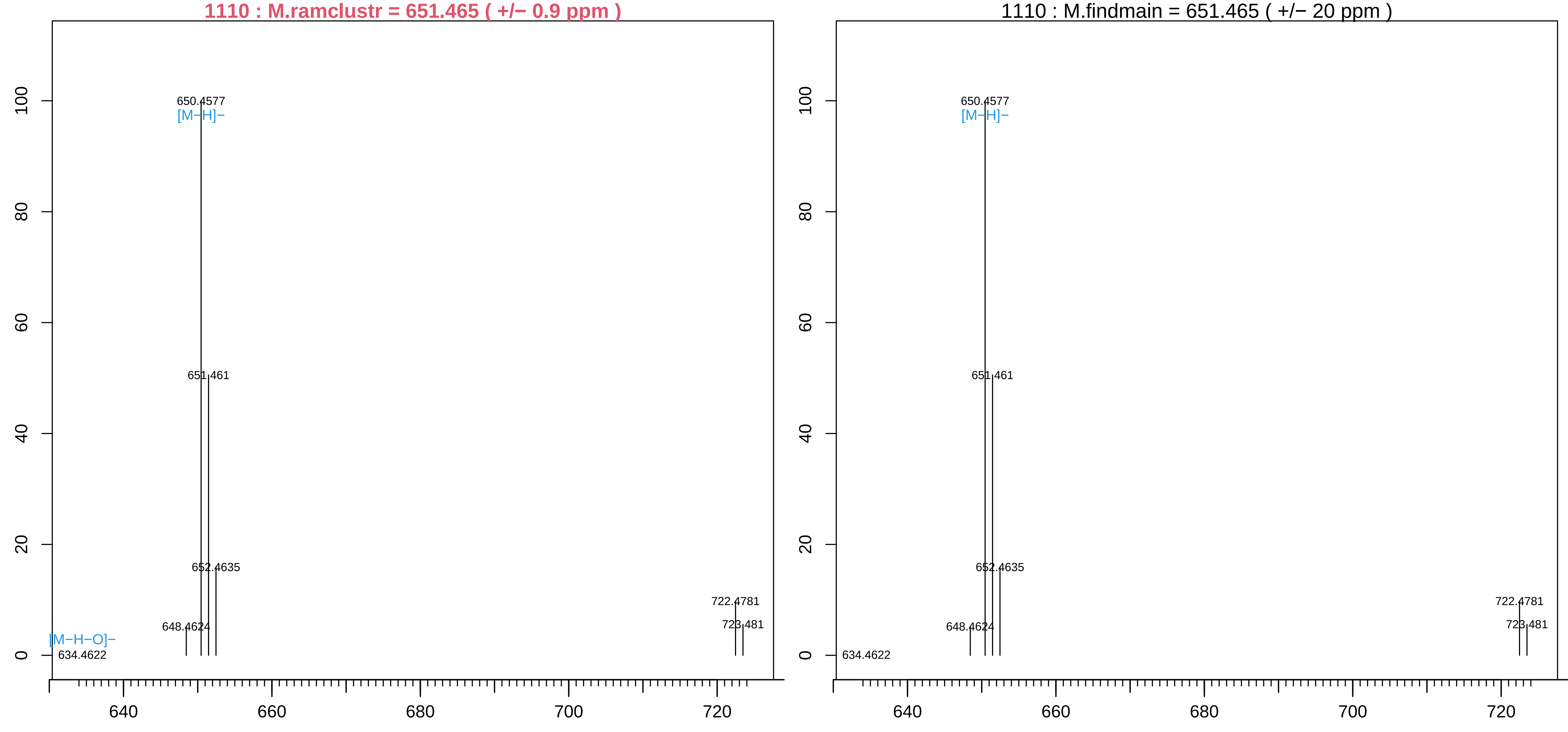

1110 : M.ramclustr = 651.465 (+/− 0.9 ppm)
650.4577
[M−H]−
651.461
652.4635
648.4624
[M−H−O]−
634.4622
722.4781
723.481
1110 : M.findmain = 651.465 (+/− 20 ppm)
650.4577
[M−H]−
651.461
652.4635
648.4624
634.4622
722.4781
723.481

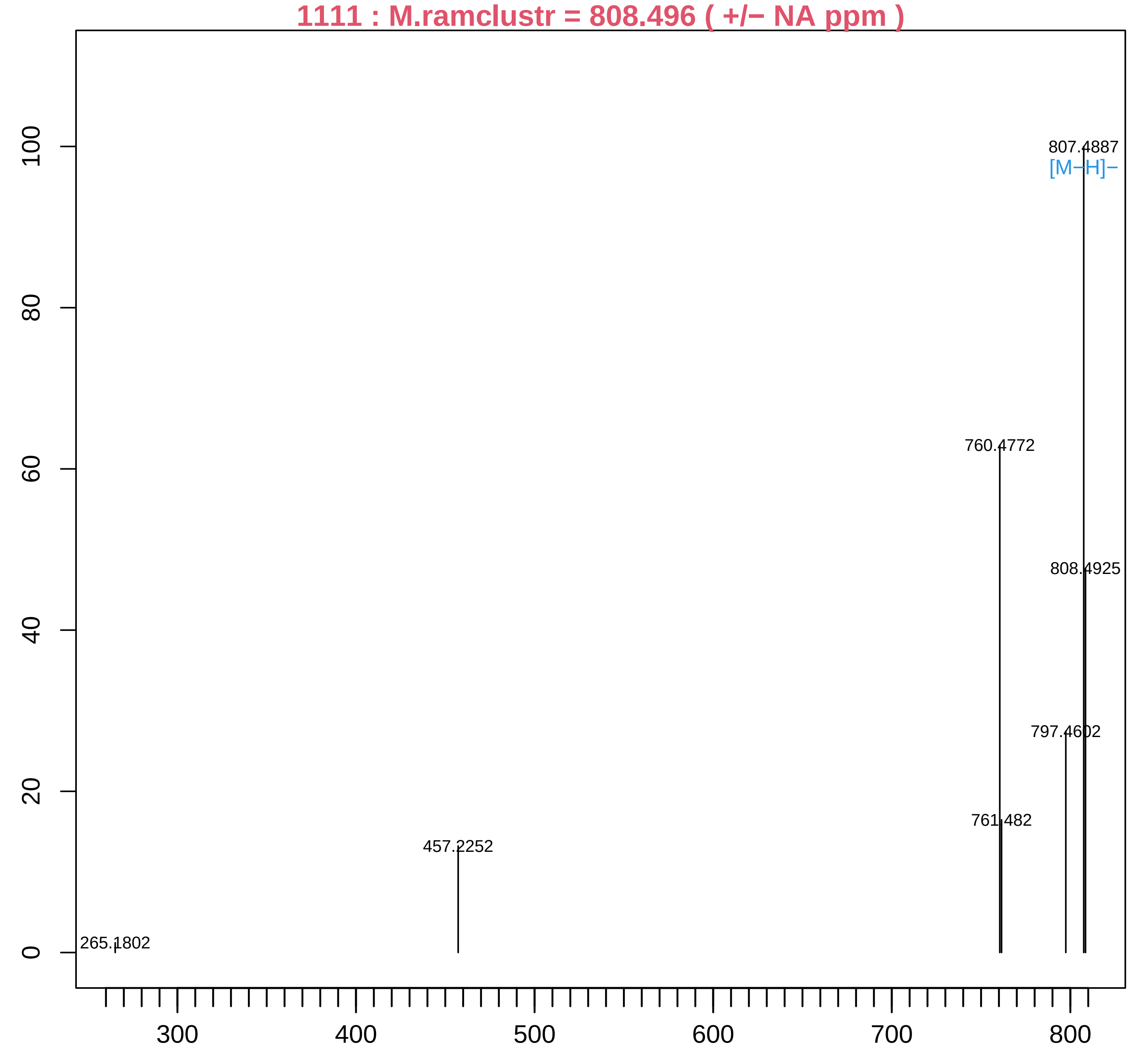
1111 : M.ramclustr = 808.496 (+/− NA ppm)
807.4887
[M−H]−
760.4772
808.4925
797.4602
761.482
457.2252
265.1802
0
20
40
60
80
100
300
400
500
600
700
800

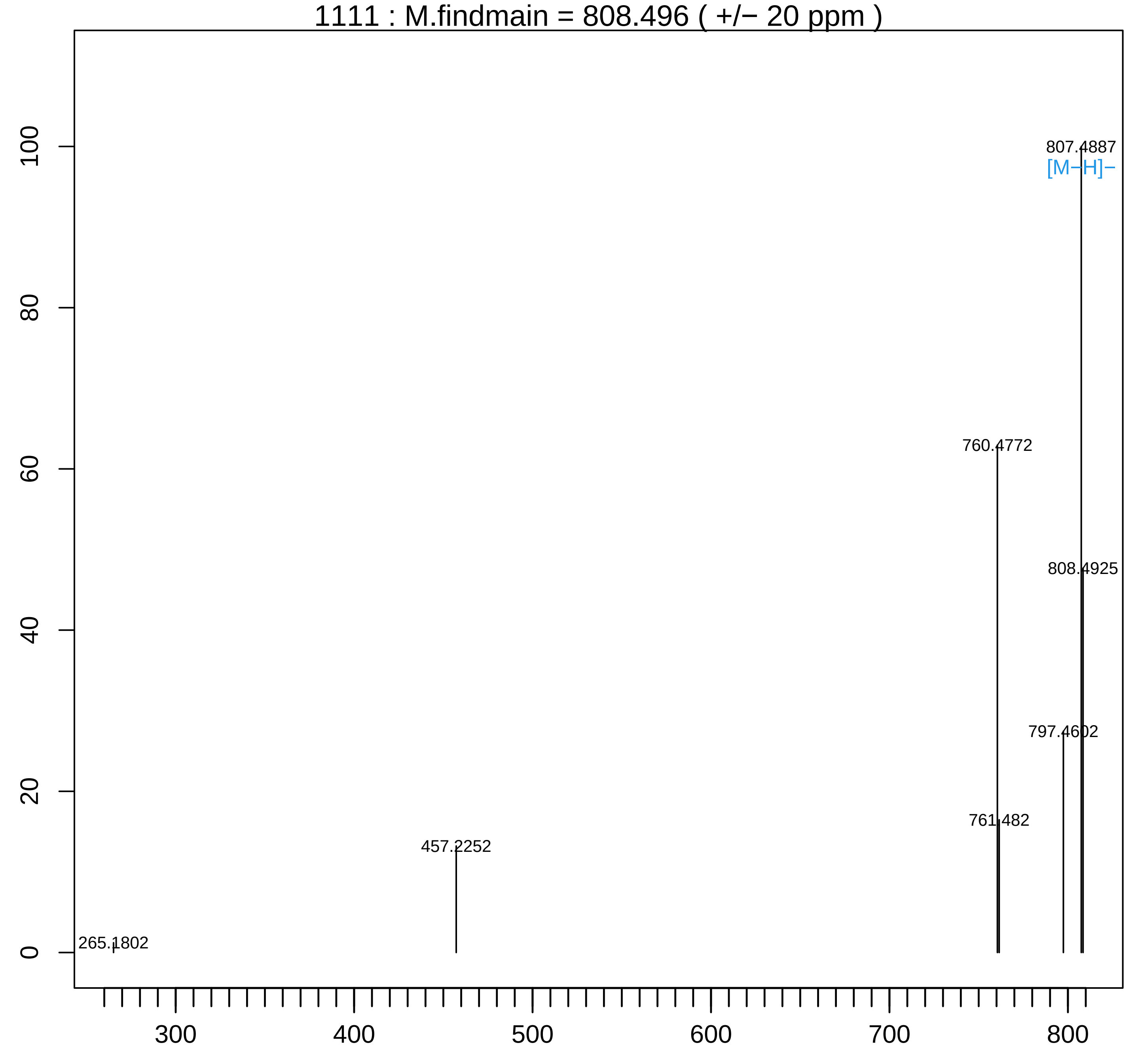
1111 : M.findmain = 808.496 (+/− 20 ppm)
807.4887
[M−H]−
760.4772
808.4925
797.4602
761.482
457.2252
265.1802
0
20
40
60
80
100
300
400
500
600
700
800

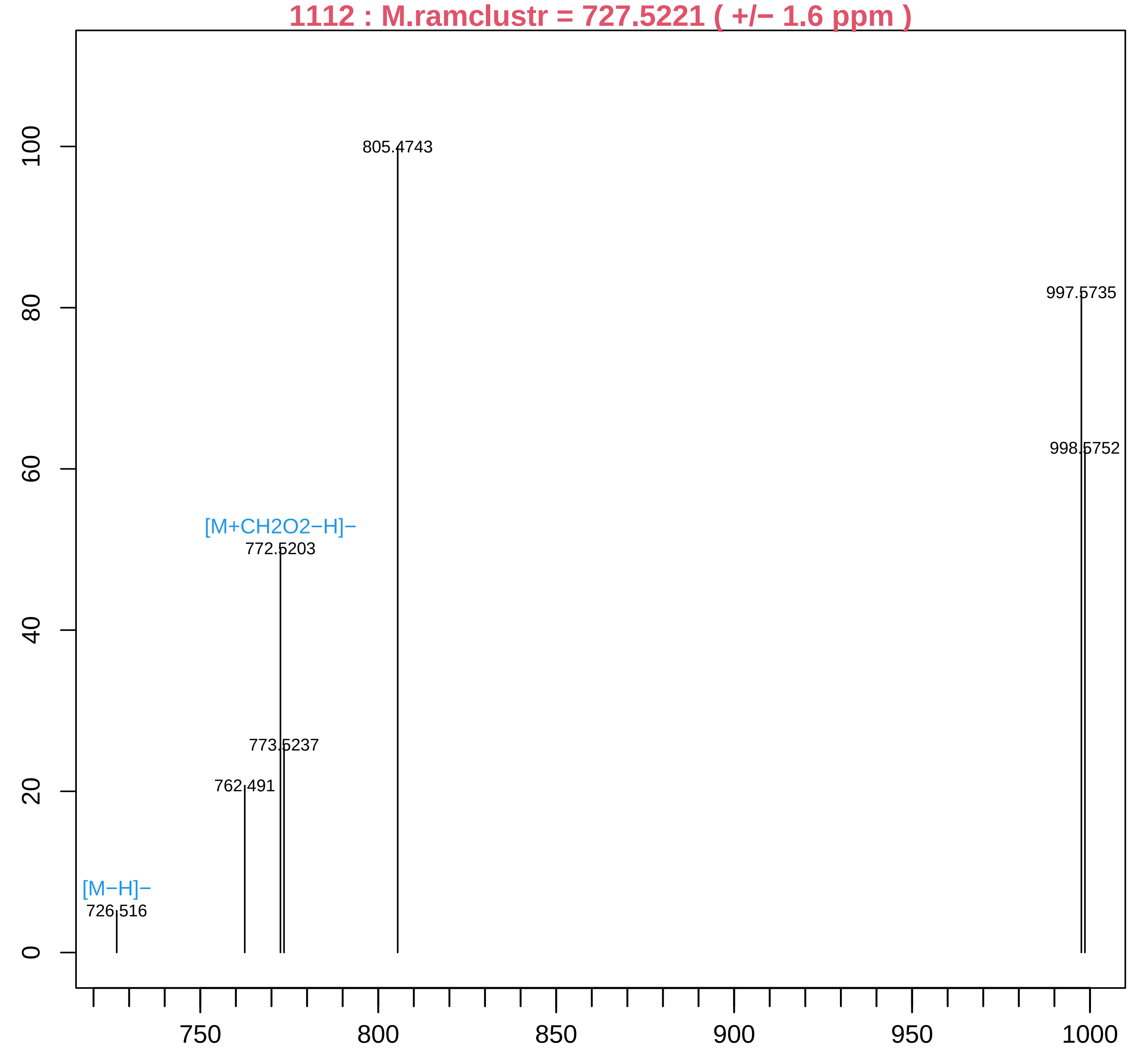
1112 : M.ramclustr = 727.5221 (+/− 1.6 ppm)
805.4743
997.5735
998.5752
[M+CH2O2−H]−
772.5203
773.5237
762.491
[M−H]−
726.516
0
20
40
60
80
100
750
800
850
900
950
1000

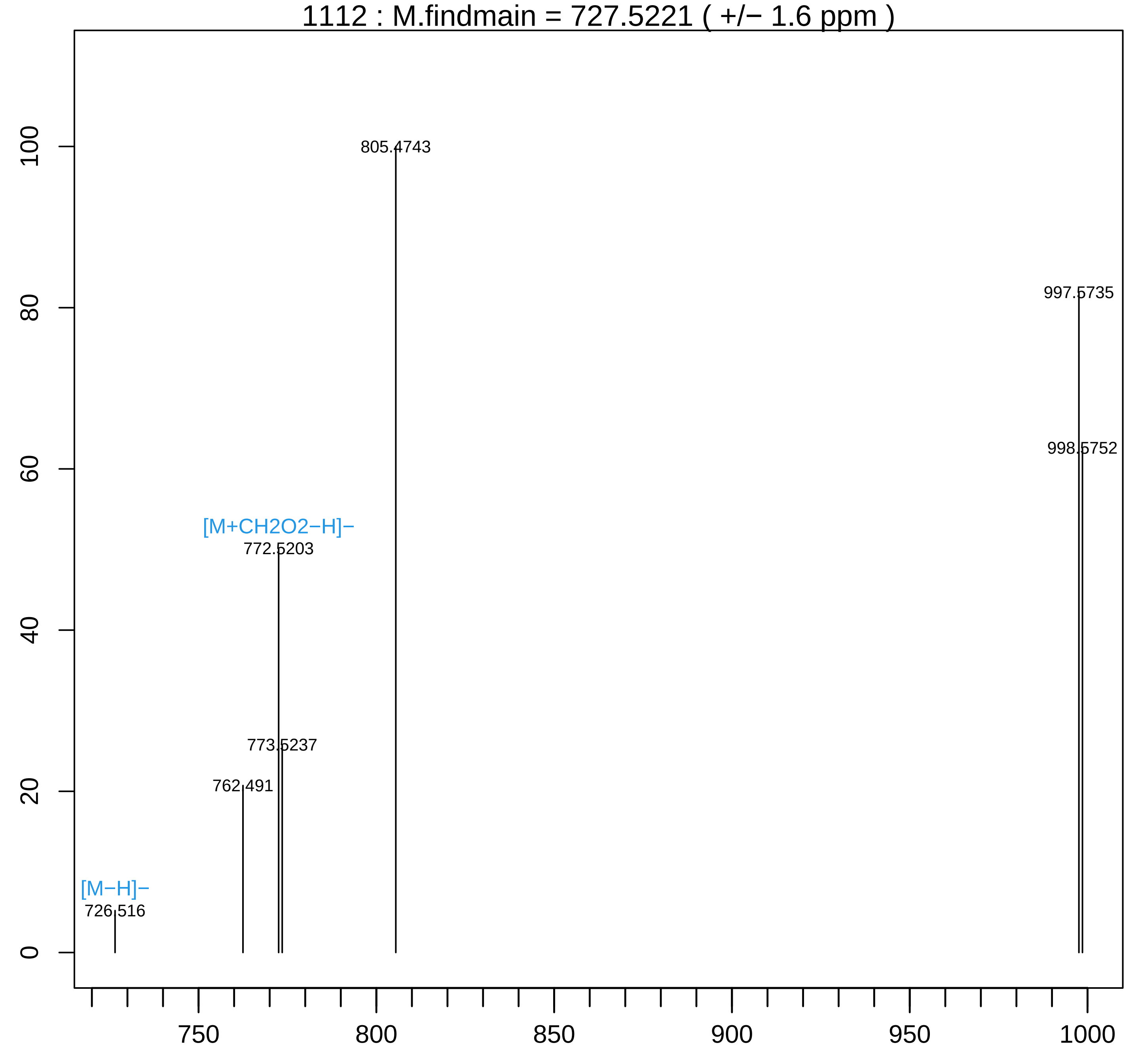
1112 : M.findmain = 727.5221 (+/− 1.6 ppm)
805.4743
997.5735
998.5752
[M+CH2O2−H]−
772.5203
773.5237
762.491
[M−H]−
726.516
0
20
40
60
80
100
750
800
850
900
950
1000

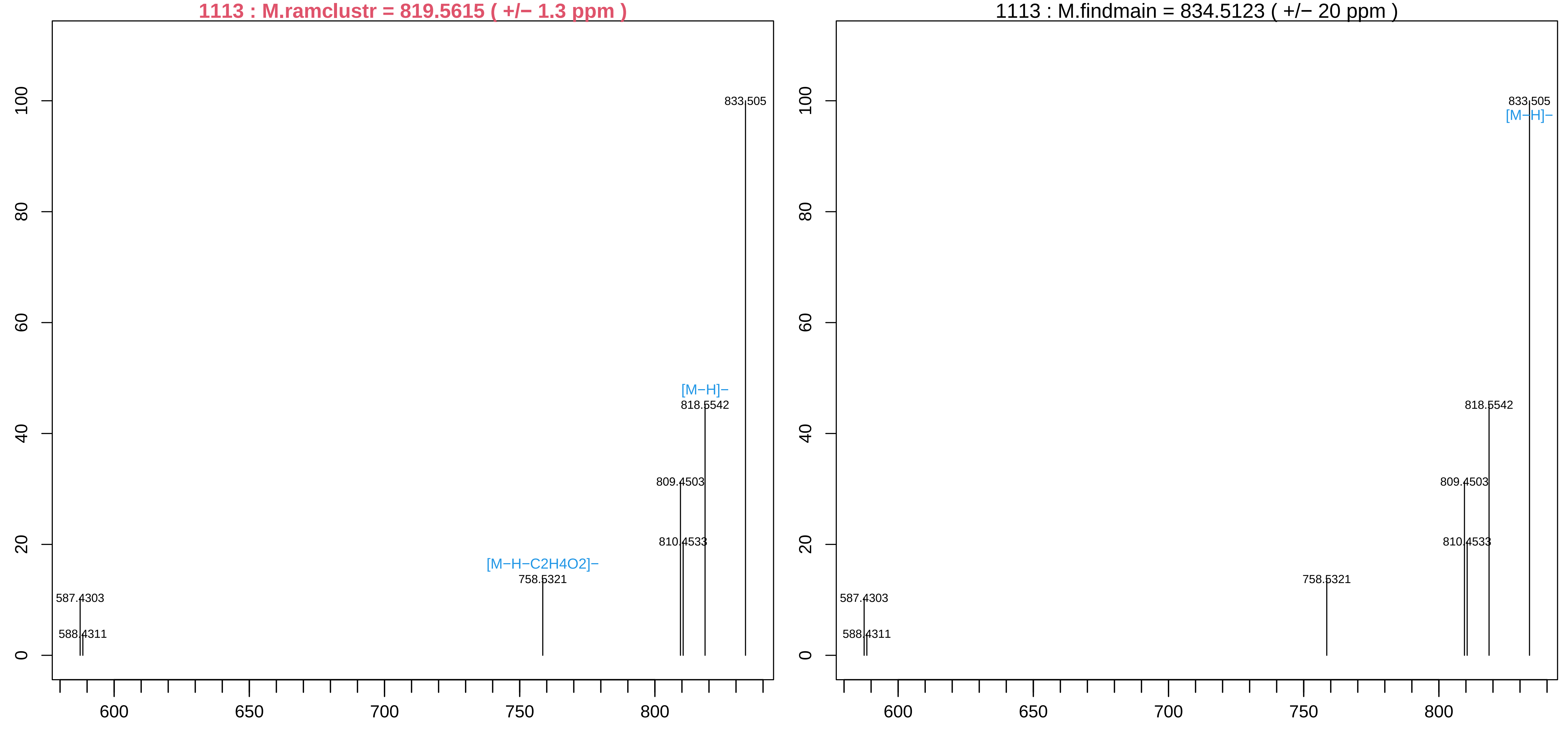

1113 : M.ramclustr = 819.5615 (+/− 1.3 ppm)
833.505
[M−H]−
818.5542
809.4503
810.4533
[M−H−C2H4O2]−
758.5321
587.4303
588.4311
0
20
40
60
80
100
600
650
700
750
800
1113 : M.findmain = 834.5123 (+/− 20 ppm)
833.505
[M−H]−
818.5542
809.4503
810.4533
758.5321
587.4303
588.4311
0
20
40
60
80
100
600
650
700
750
800

1114 : M.ramclustr = 858.6574 (+/− NA ppm)

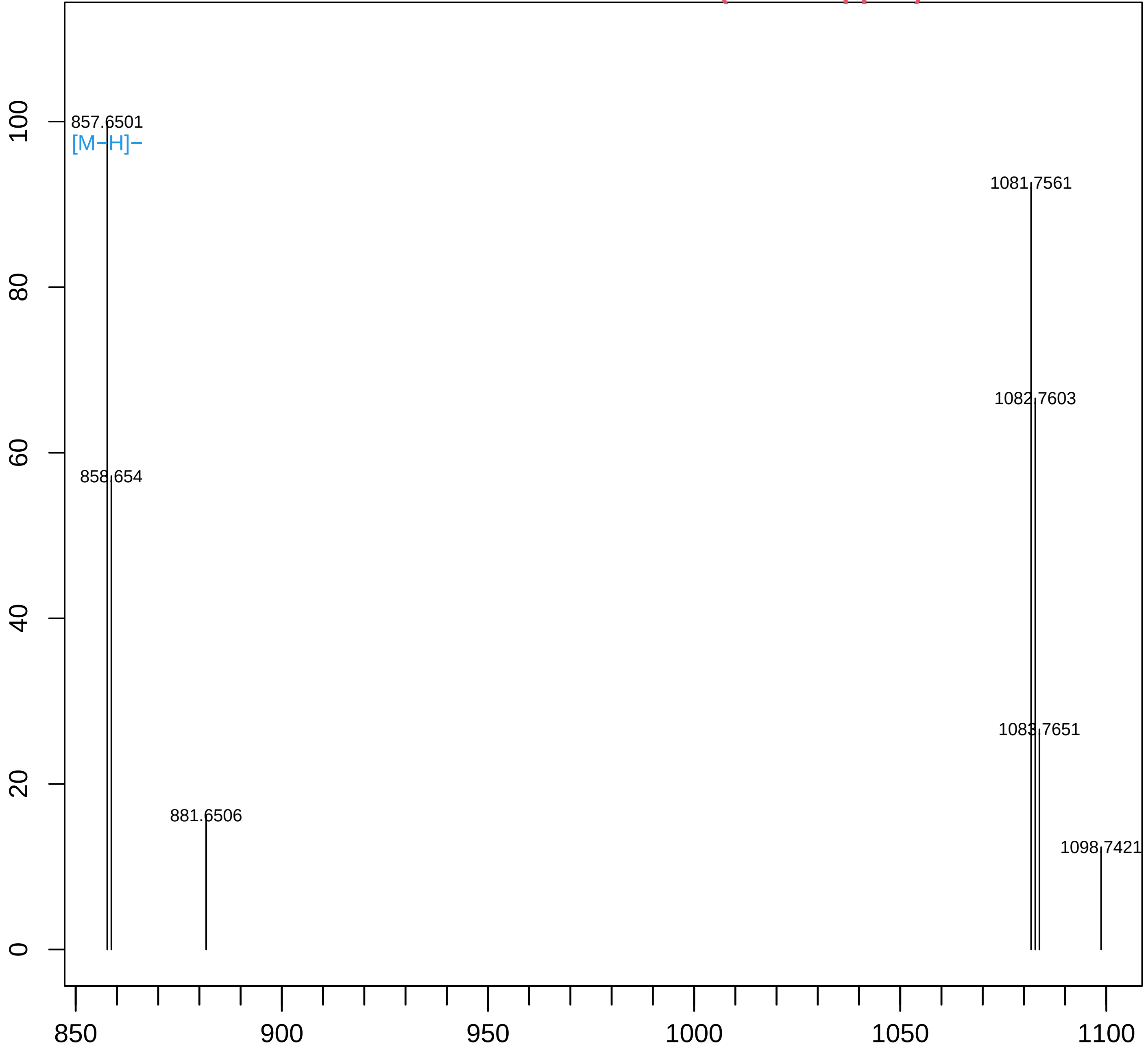

1114 : M.findmain = 858.6574 (+/− 20 ppm)

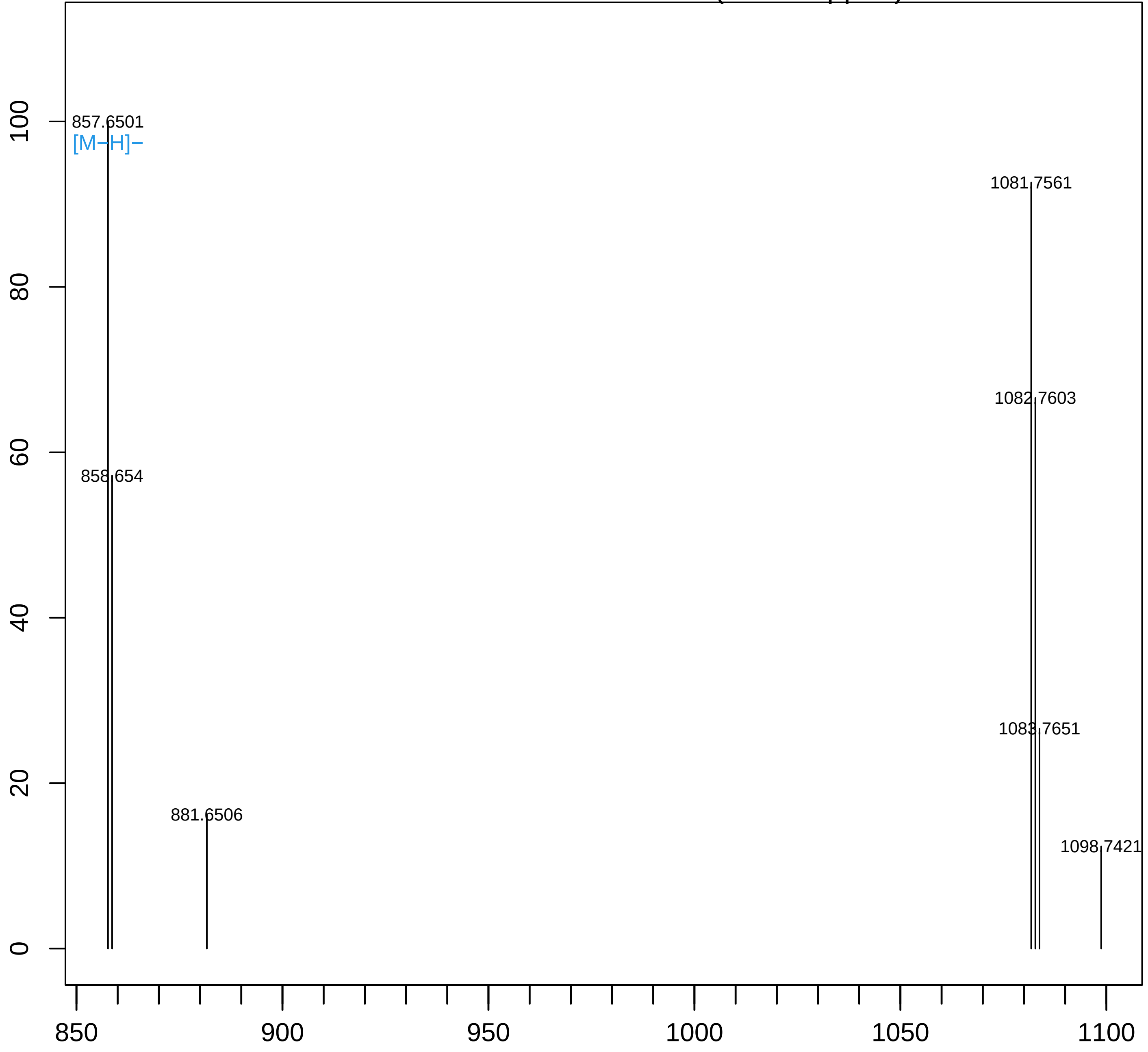

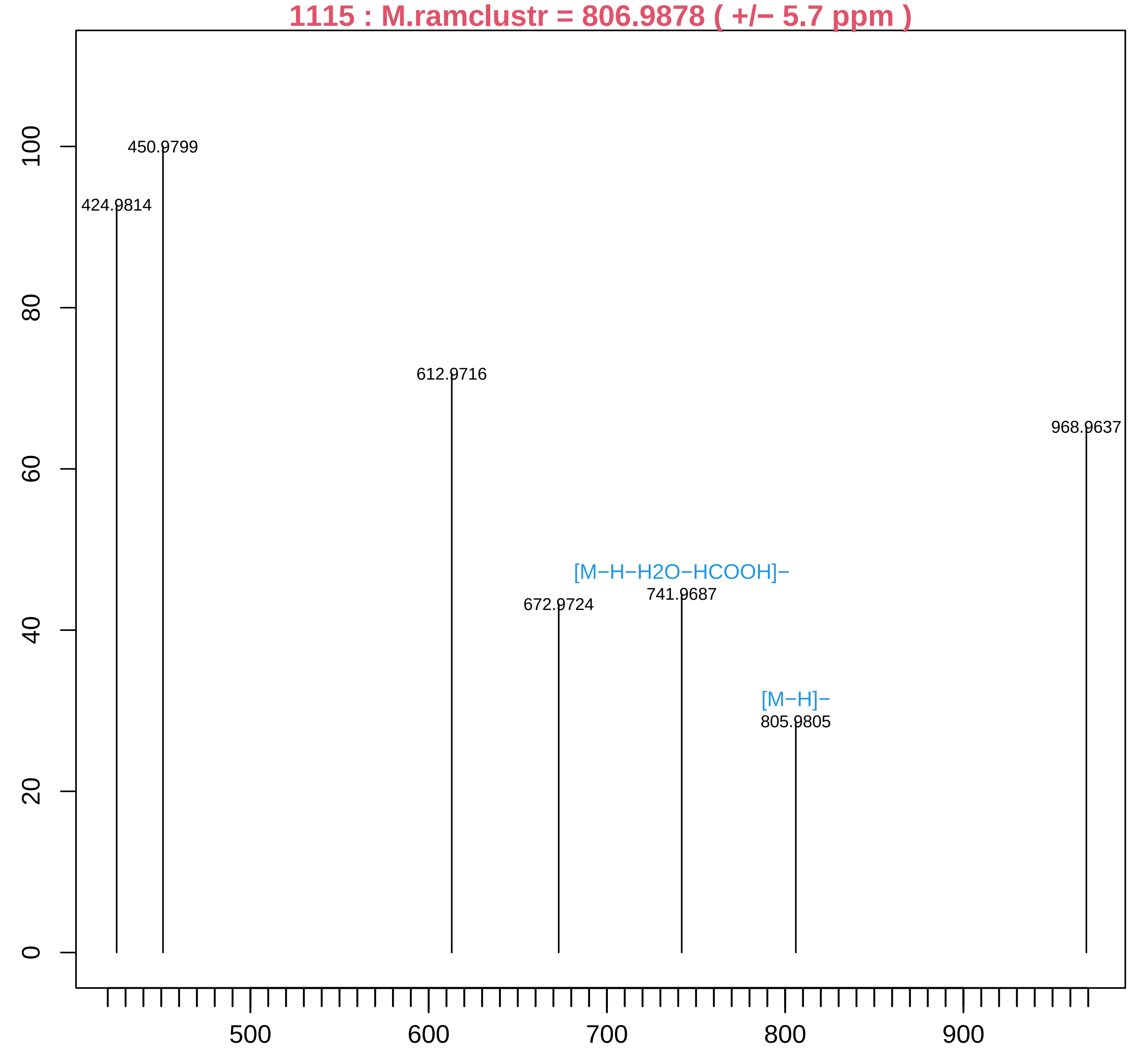

1115 : M.ramclustr = 806.9878 (+/− 5.7 ppm)
100
80
60
40
20
0
450.9799
424.9814
612.9716
968.9637
[M−H−H2O−HCOOH]−
741.9687
672.9724
[M−H]−
805.9805
500
600
700
800
900

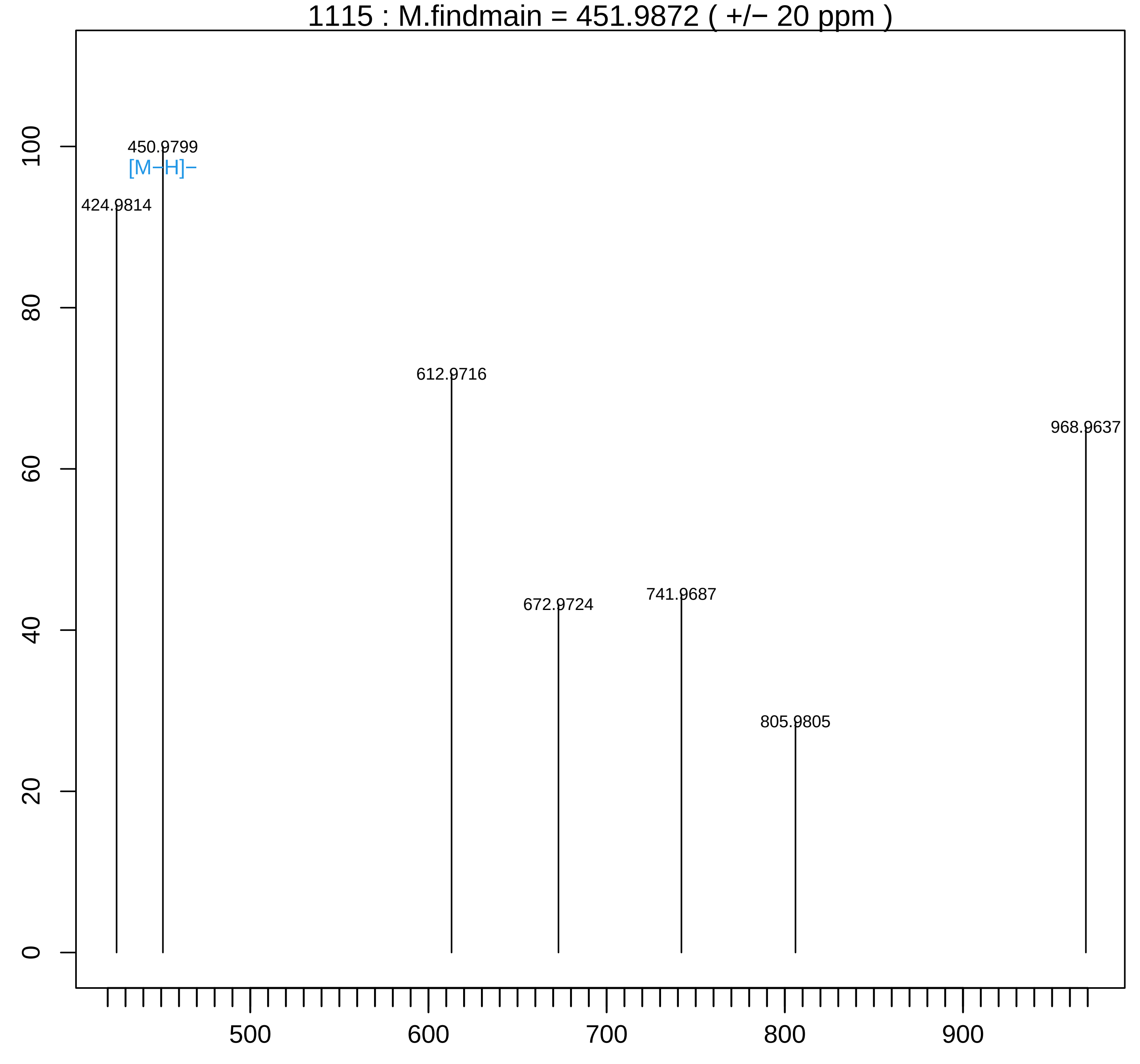

1115 : M.findmain = 451.9872 (+/− 20 ppm)
100
80
60
40
20
0
450.9799
[M−H]−
424.9814
612.9716
968.9637
672.9724
741.9687
805.9805
500
600
700
800
900

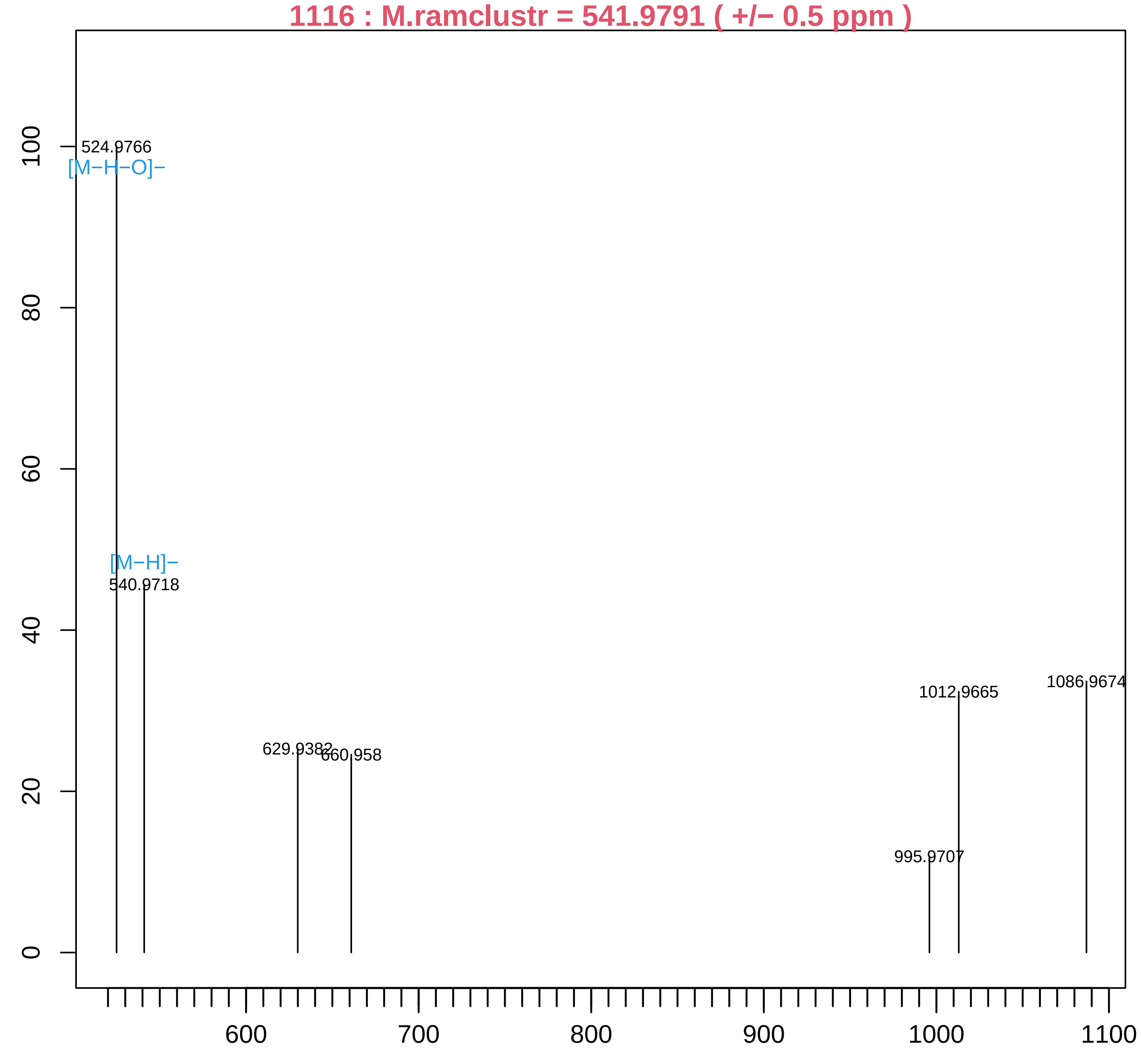
1116 : M.ramclustr = 541.9791 (+/− 0.5 ppm)
524.9766
[M−H−O]−
[M−H]−
540.9718
629.9382
660.958
995.9707
1012.9665
1086.9674
0
20
40
60
80
100
600
700
800
900
1000
1100
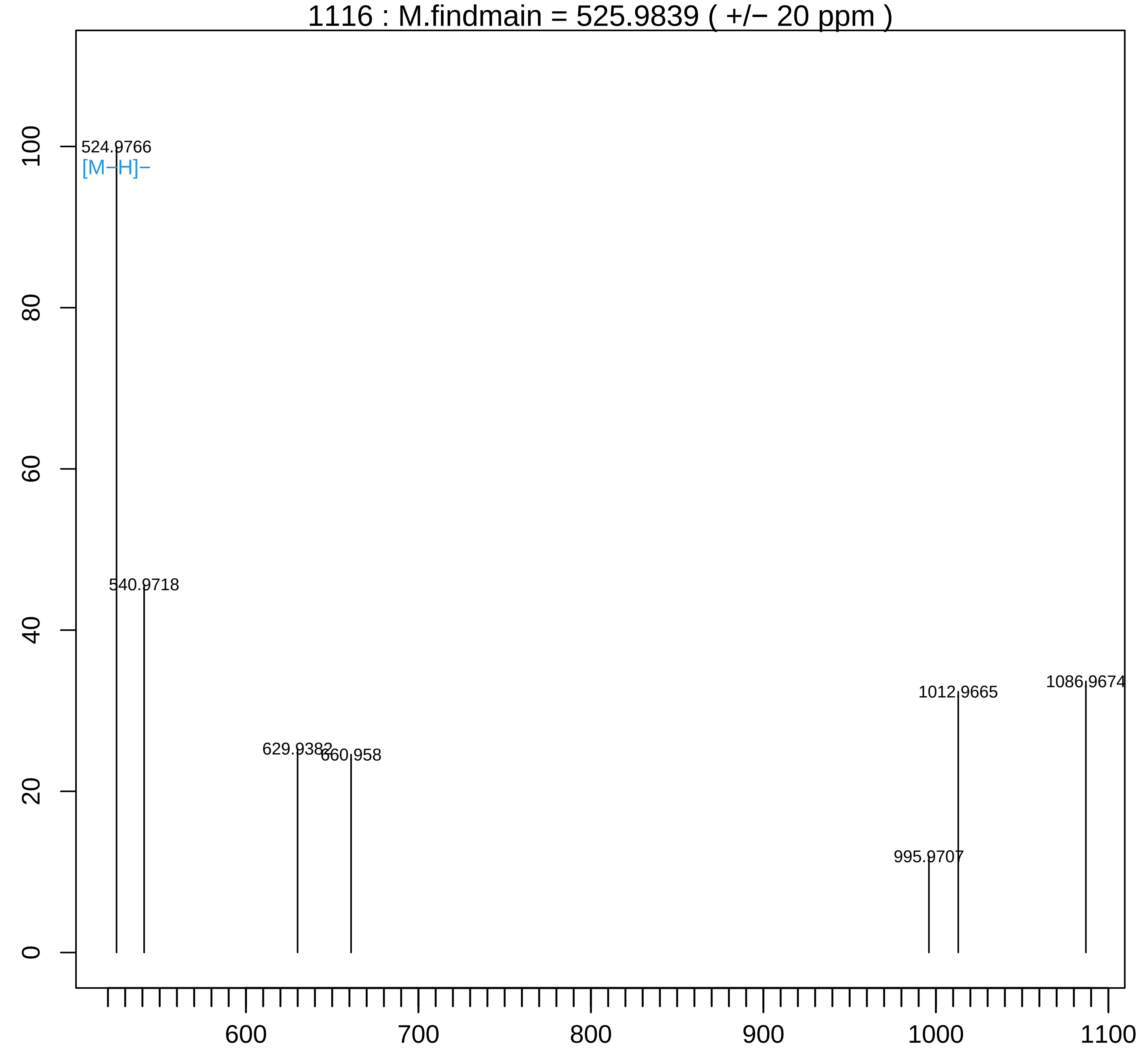
1116 : M.findmain = 525.9839 (+/− 20 ppm)
524.9766
[M−H]−
540.9718
629.9382
660.958
995.9707
1012.9665
1086.9674
0
20
40
60
80
100
600
700
800
900
1000
1100

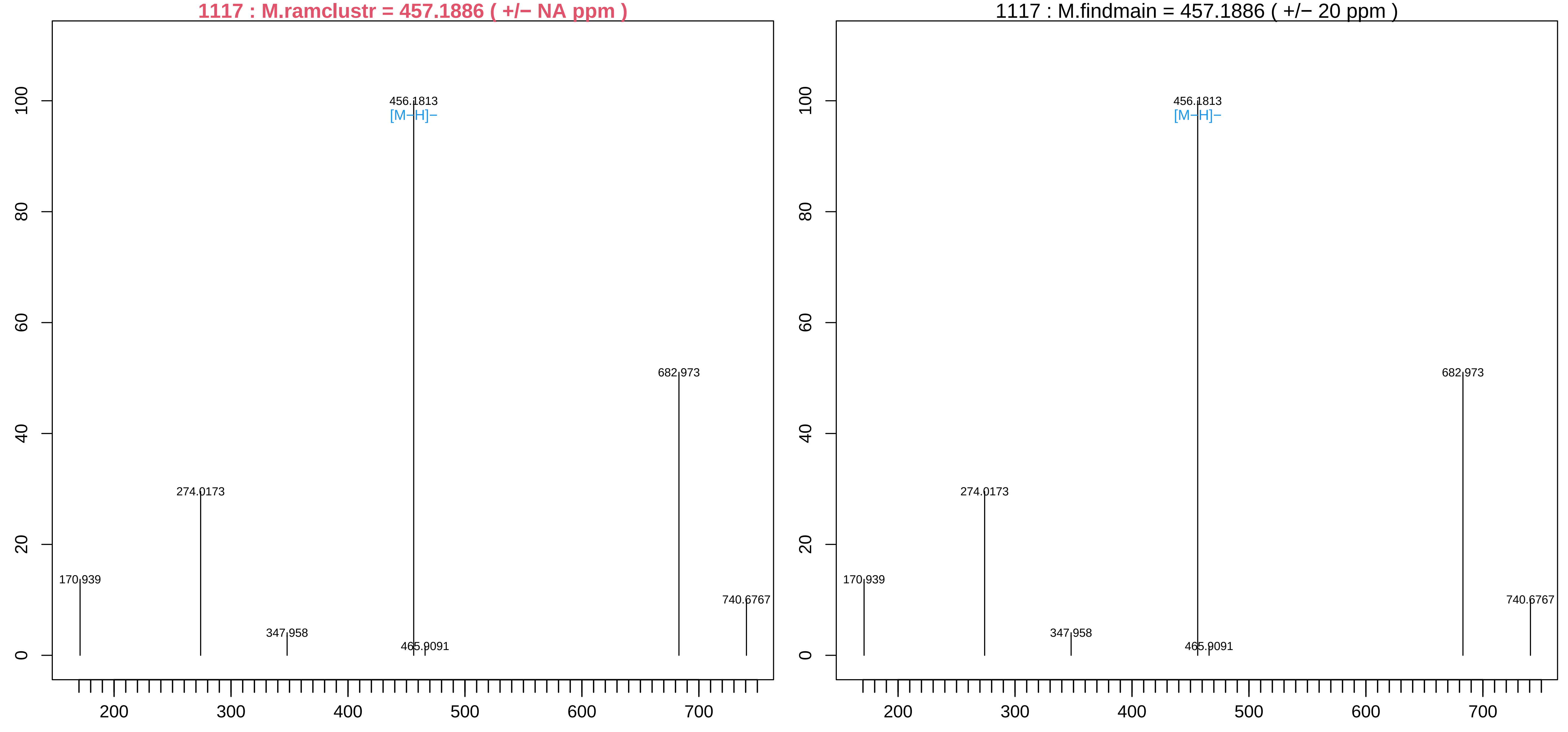

1117 : M.ramclustr = 457.1886 (+/− NA ppm)
456.1813
[M−H]−
682.973
274.0173
170.939
740.6767
347.958
465.9091
1117 : M.findmain = 457.1886 (+/− 20 ppm)
456.1813
[M−H]−
682.973
274.0173
170.939
740.6767
347.958
465.9091

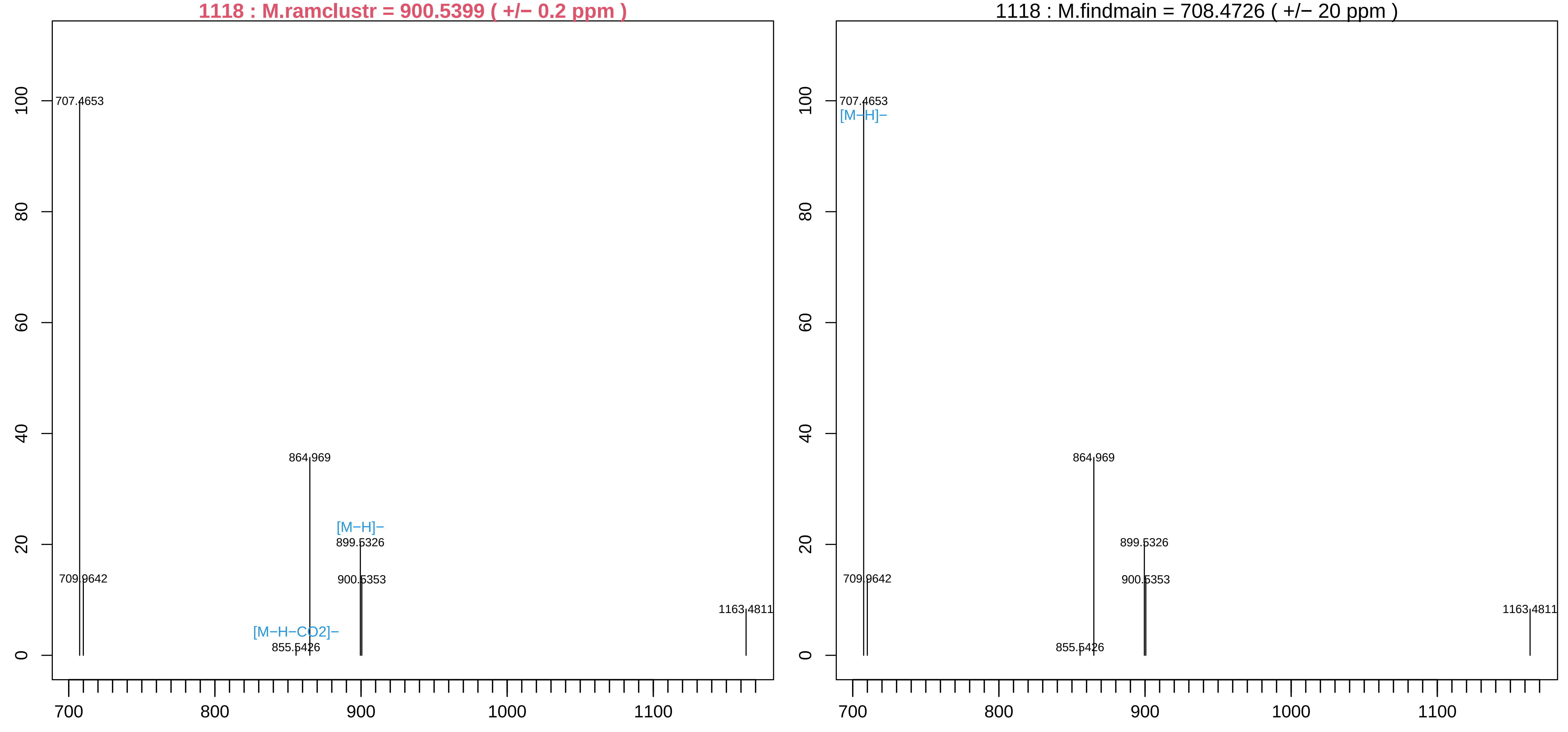

1118 : M.ramclustr = 900.5399 (+/− 0.2 ppm)
707.4653
709.9642
864.969
[M−H]−
899.5326
900.5353
[M−H−CO2]−
855.5426
1163.4811
0
20
40
60
80
100
700
800
900
1000
1100
1118 : M.findmain = 708.4726 (+/− 20 ppm)
707.4653
[M−H]−
709.9642
864.969
899.5326
900.5353
855.5426
1163.4811

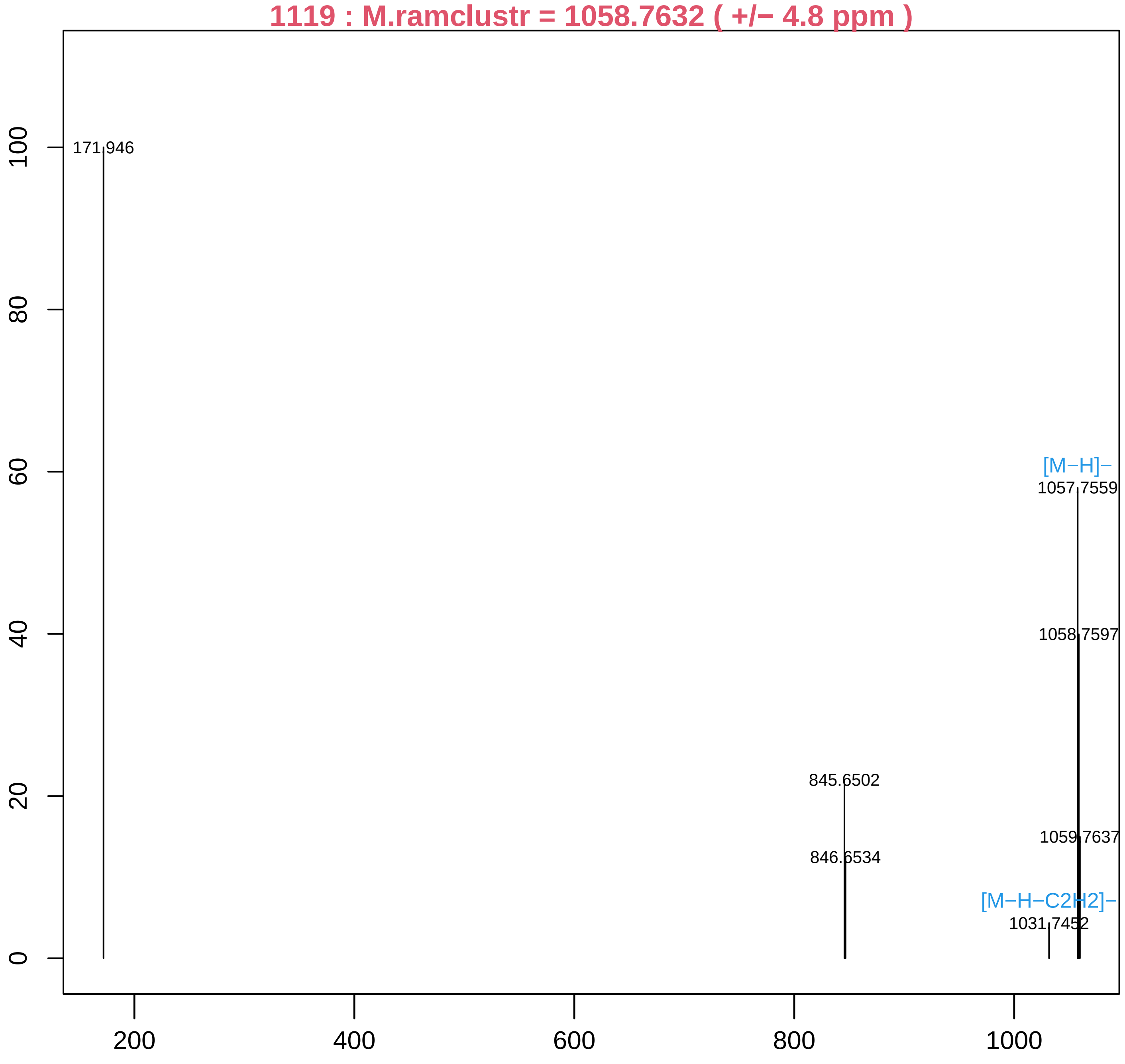
1119 : M.ramclustr = 1058.7632 (+/− 4.8 ppm)
171.946
[M−H]−
1057.7559
1058.7597
845.6502
846.6534
1059.7637
[M−H−C2H2]−
1031.7452
0
20
40
60
80
100
200
400
600
800
1000

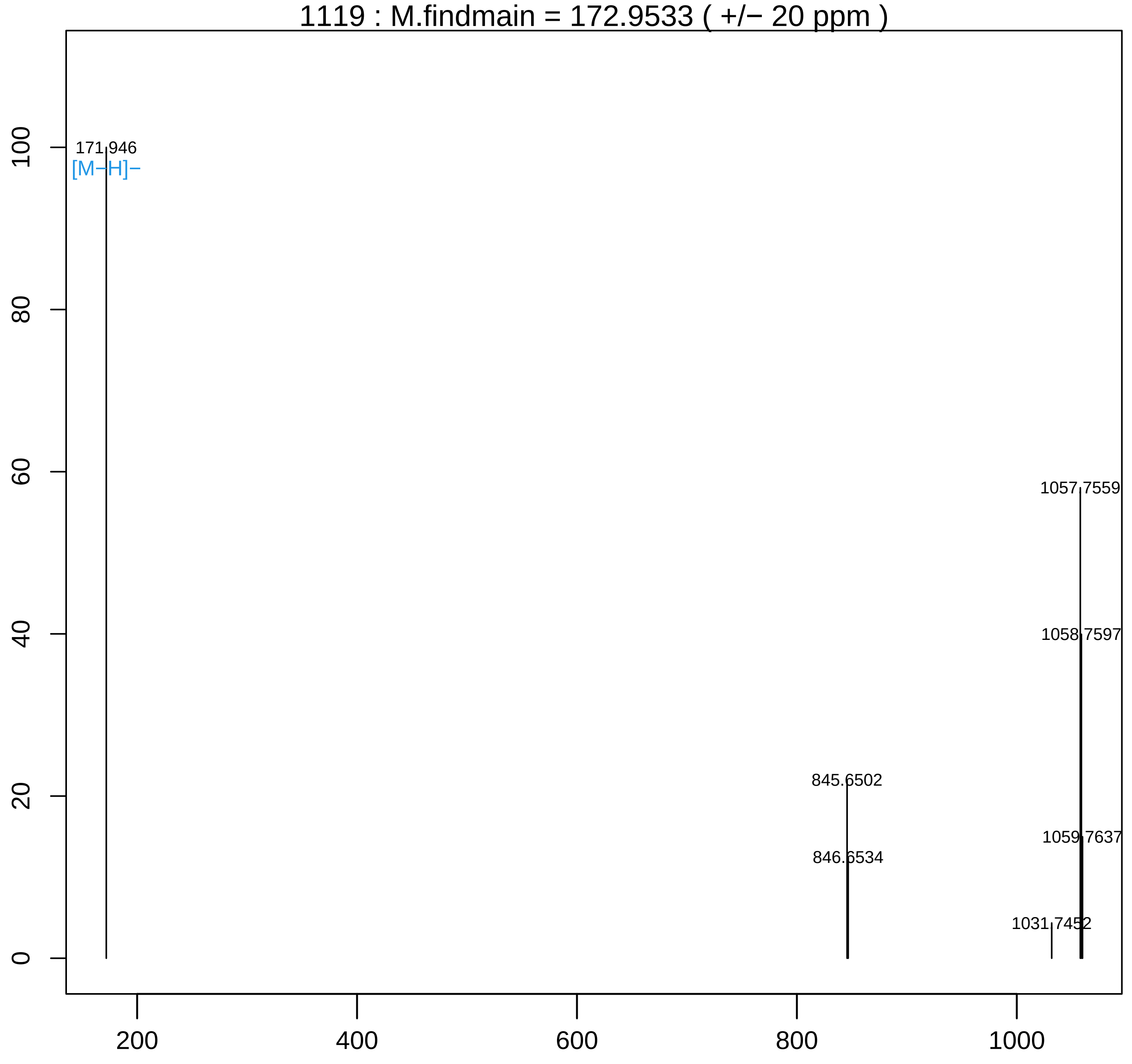
1119 : M.findmain = 172.9533 (+/− 20 ppm)
171.946
[M−H]−
1057.7559
1058.7597
845.6502
846.6534
1059.7637
1031.7452
0
20
40
60
80
100
200
400
600
800
1000

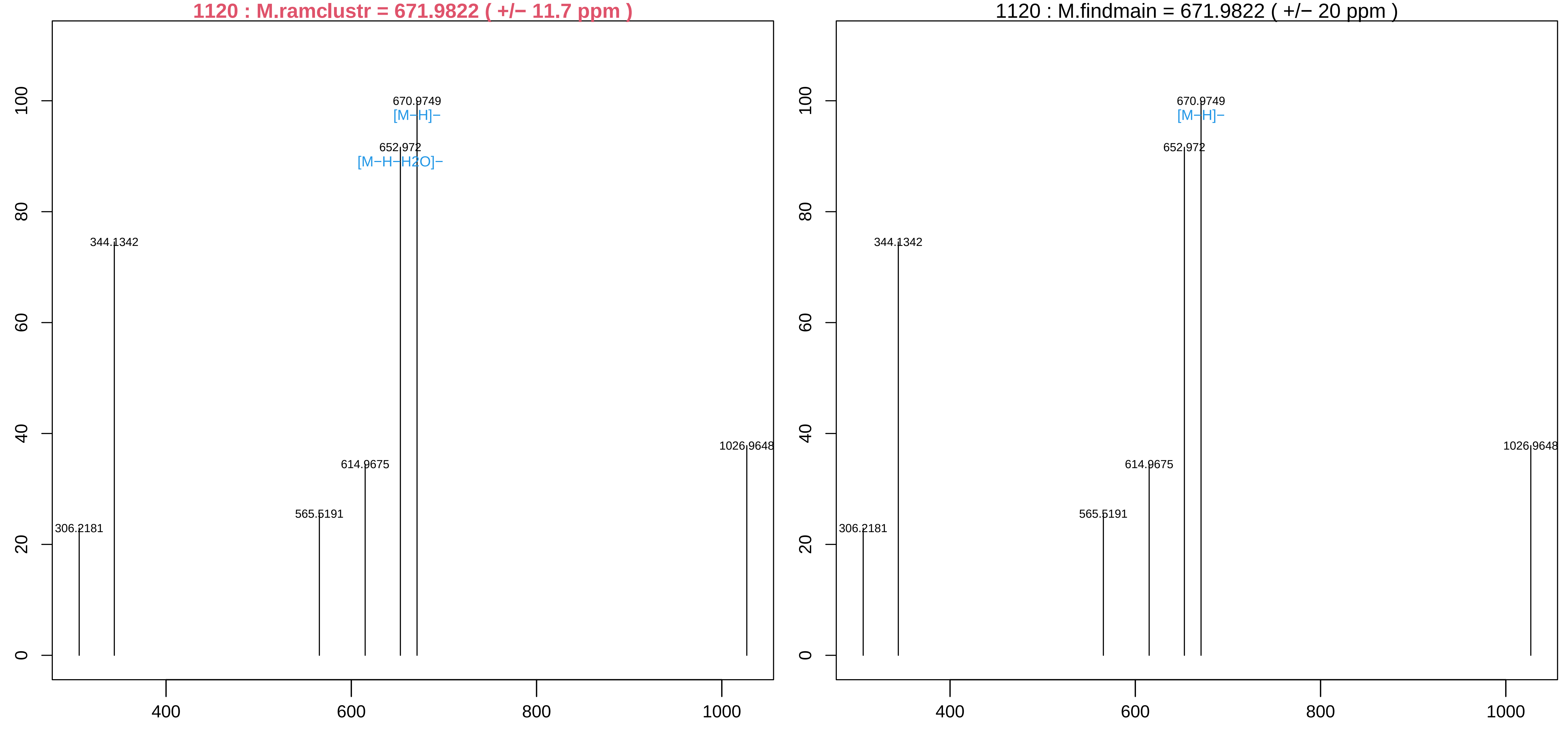
1120 : M.ramclustr = 671.9822 (+/− 11.7 ppm)
670.9749
[M−H]−
652.972
[M−H−H2O]−
344.1342
306.2181
565.5191
614.9675
1026.9648
1120 : M.findmain = 671.9822 (+/− 20 ppm)
670.9749
[M−H]−
652.972
344.1342
306.2181
565.5191
614.9675
1026.9648
0
20
40
60
80
100
400
600
800
1000

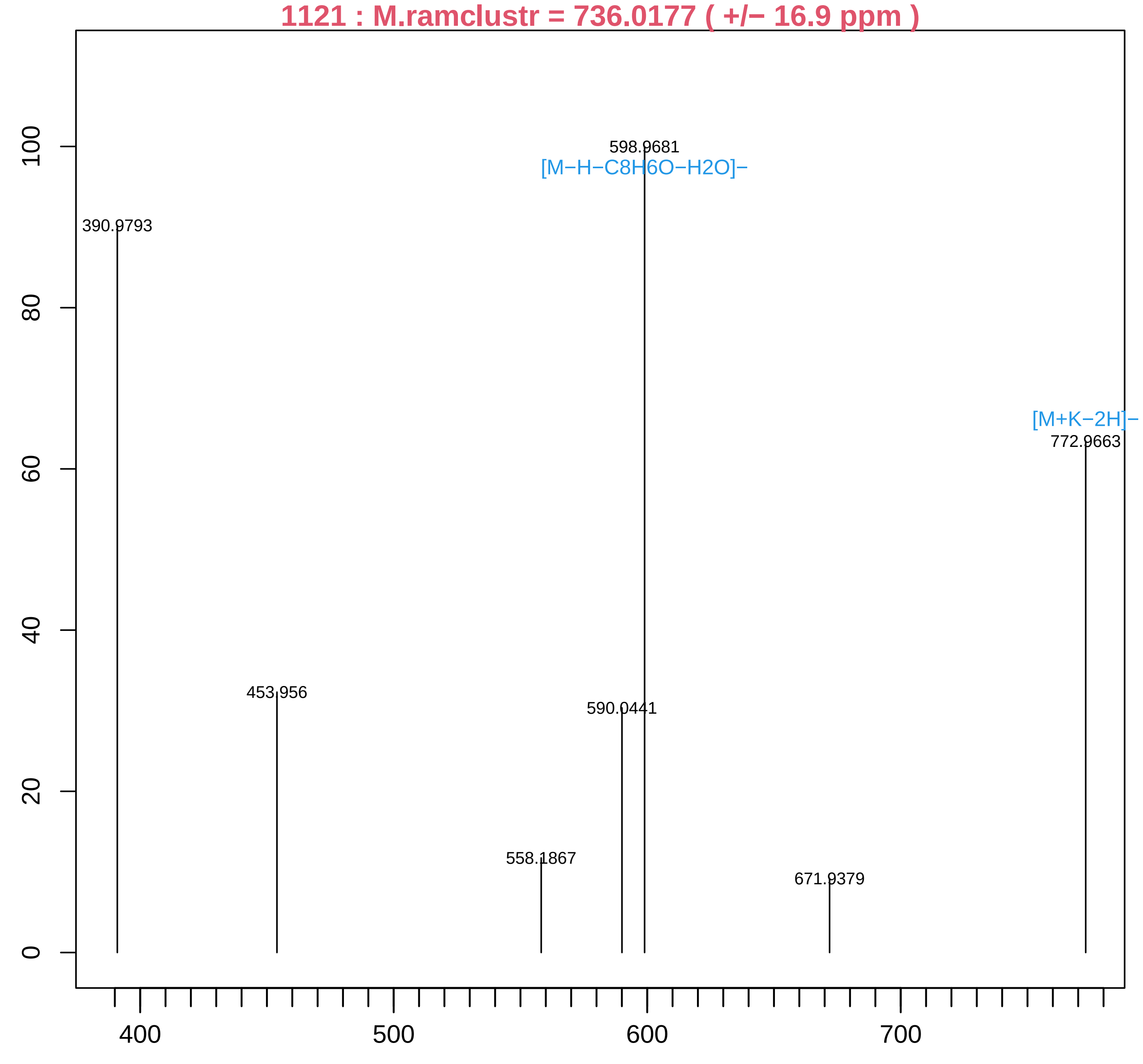
1121 : M.ramclustr = 736.0177 (+/− 16.9 ppm)
598.9681
[M−H−C8H6O−H2O]−
390.9793
[M+K−2H]−
772.9663
453.956
590.0441
558.1867
671.9379
0
20
40
60
80
100
400
500
600
700

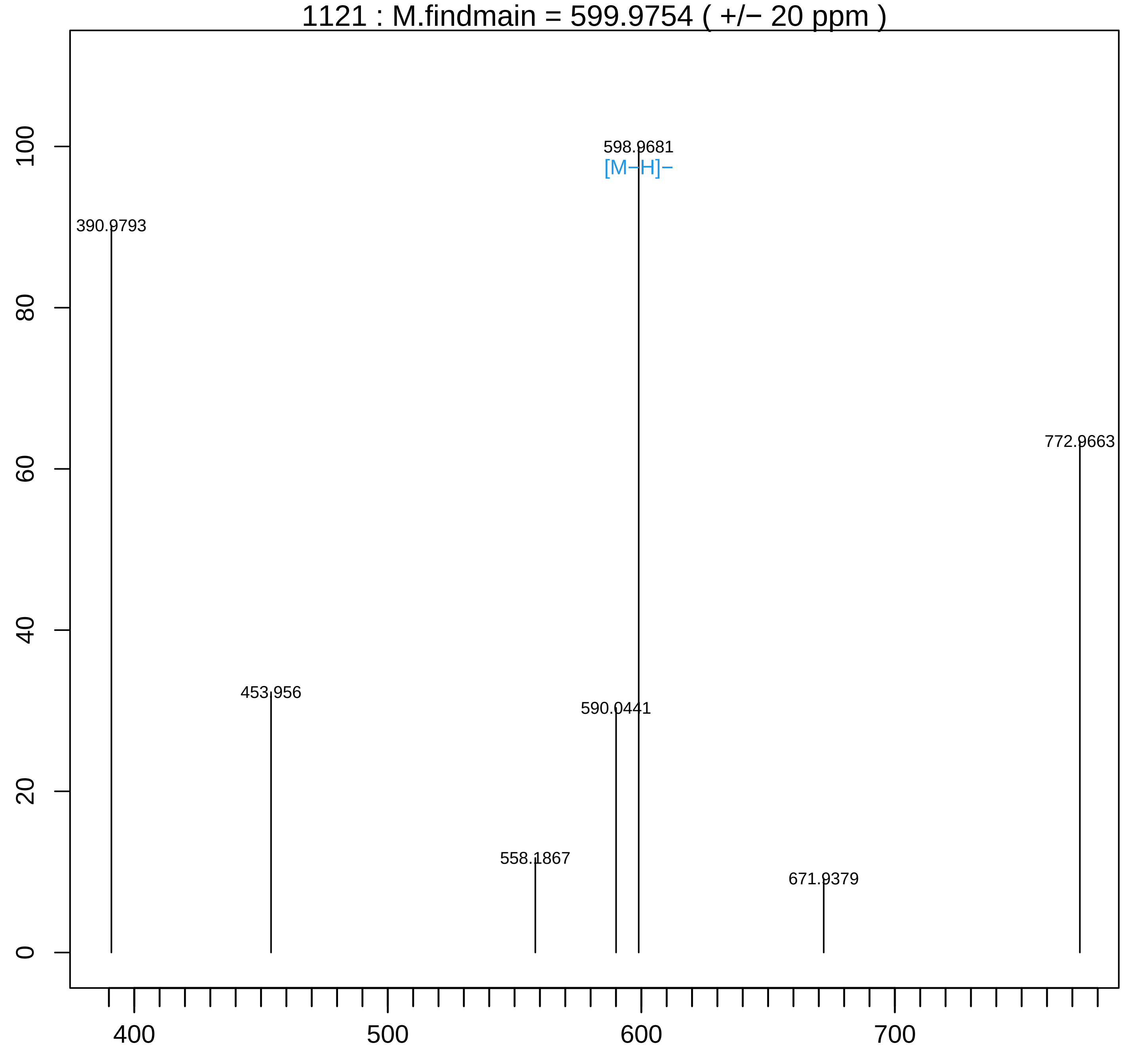
1121 : M.findmain = 599.9754 (+/− 20 ppm)
598.9681
[M−H]−
390.9793
772.9663
453.956
590.0441
558.1867
671.9379
0
20
40
60
80
100
400
500
600
700

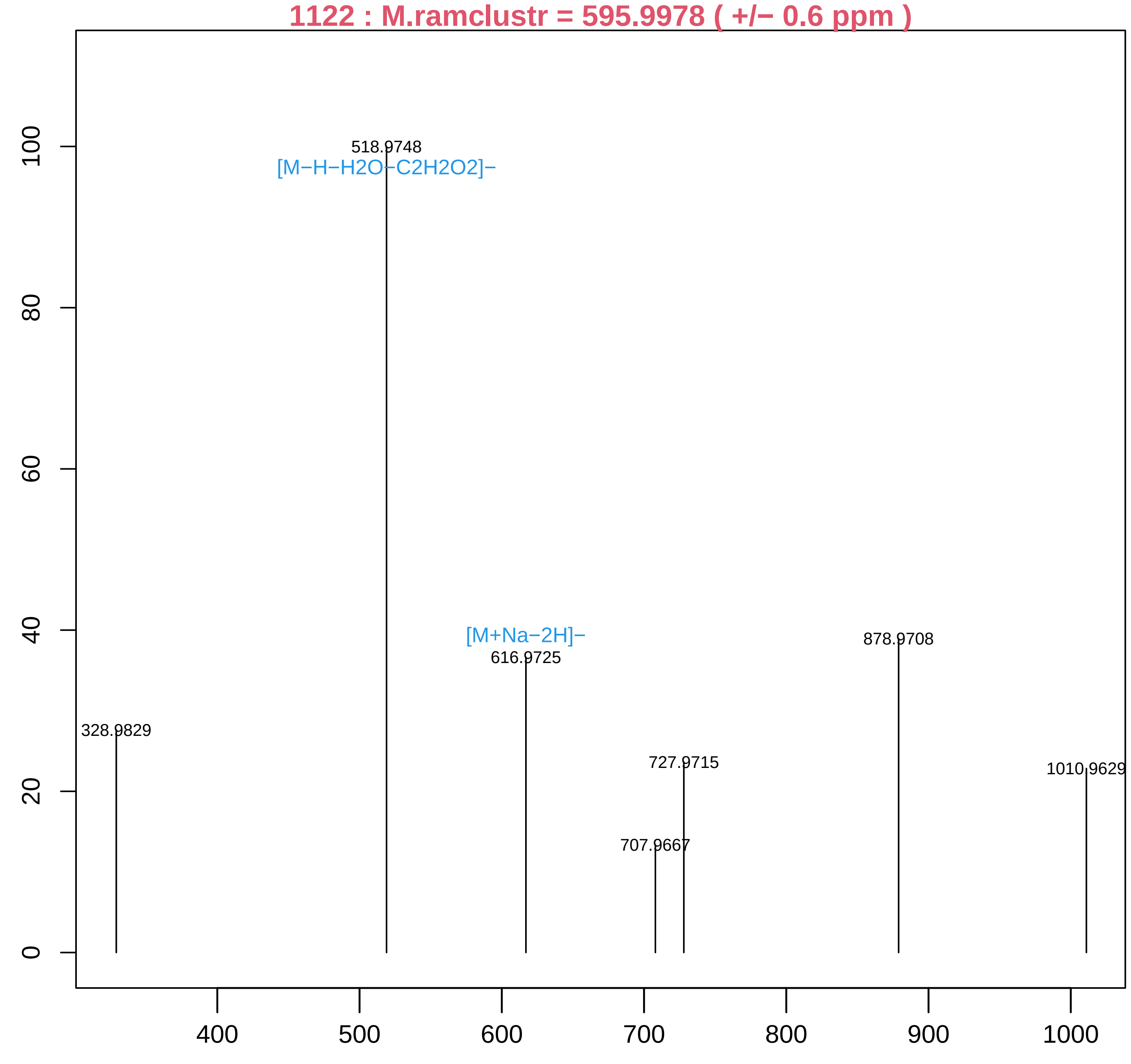
1122 : M.ramclustr = 595.9978 (+/− 0.6 ppm)
518.9748
[M−H−H2O−C2H2O2]−
[M+Na−2H]−
616.9725
878.9708
328.9829
727.9715
707.9667
1010.9629
0
20
40
60
80
100
400
500
600
700
800
900
1000

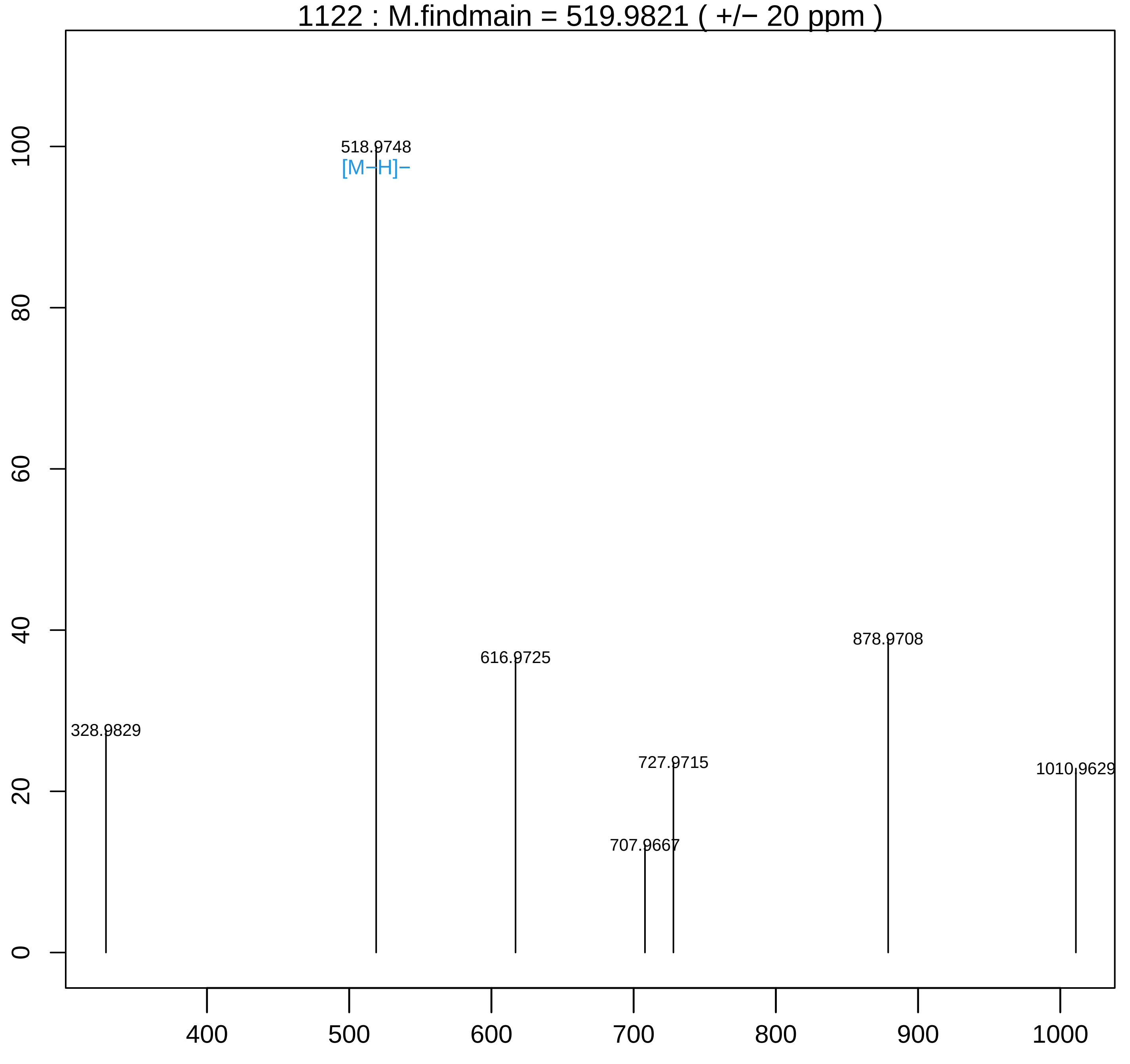
1122 : M.findmain = 519.9821 (+/− 20 ppm)
518.9748
[M−H]−
616.9725
878.9708
328.9829
727.9715
707.9667
1010.9629
0
20
40
60
80
100
400
500
600
700
800
900
1000

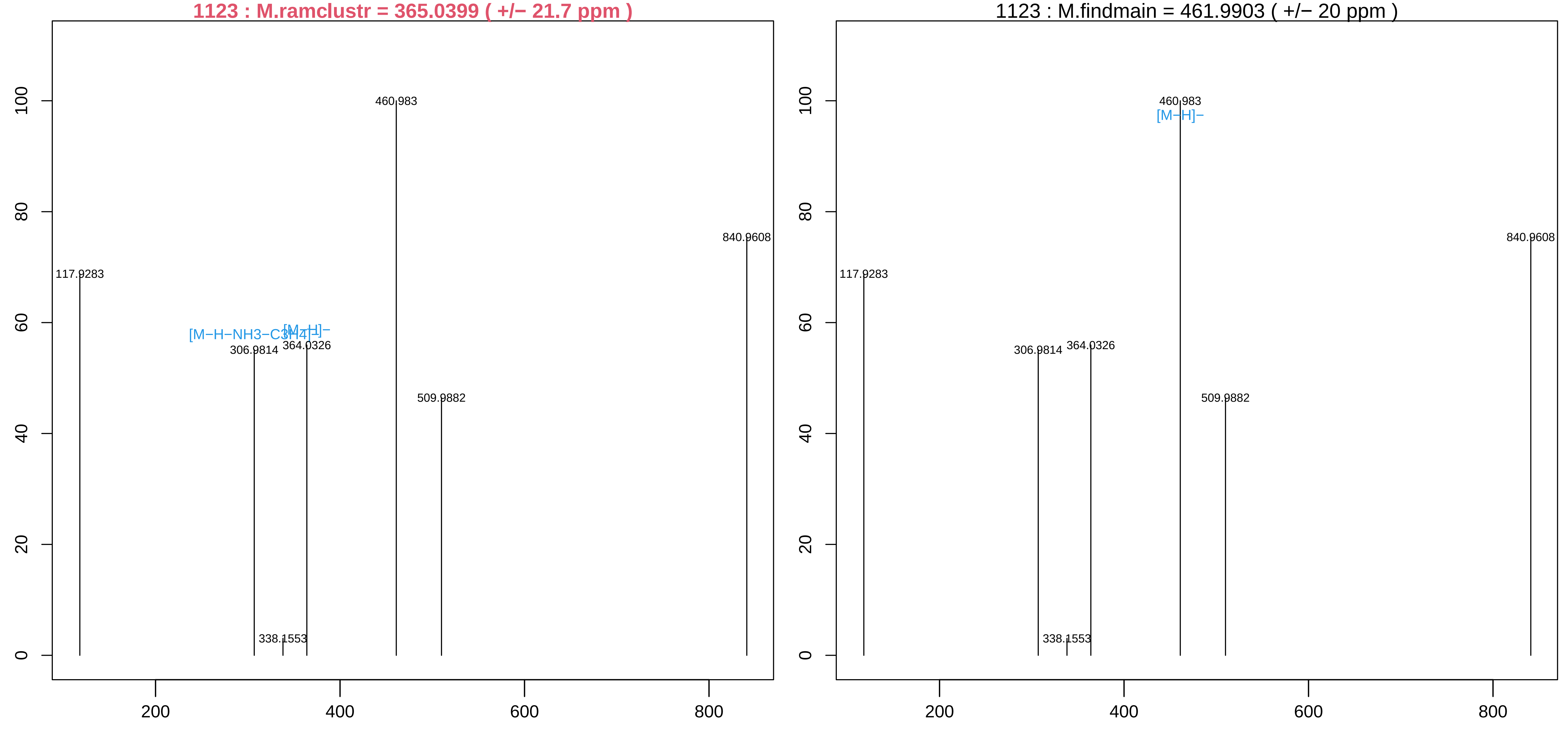
1123 : M.ramclustr = 365.0399 (+/− 21.7 ppm)
460.983
840.9608
117.9283
[M−H−NH3−C3H4]−
[M−H]−
306.9814
364.0326
509.9882
338.1553
1123 : M.findmain = 461.9903 (+/− 20 ppm)
460.983
[M−H]−
840.9608
117.9283
306.9814
364.0326
509.9882
338.1553
0
20
40
60
80
100
200
400
600
800

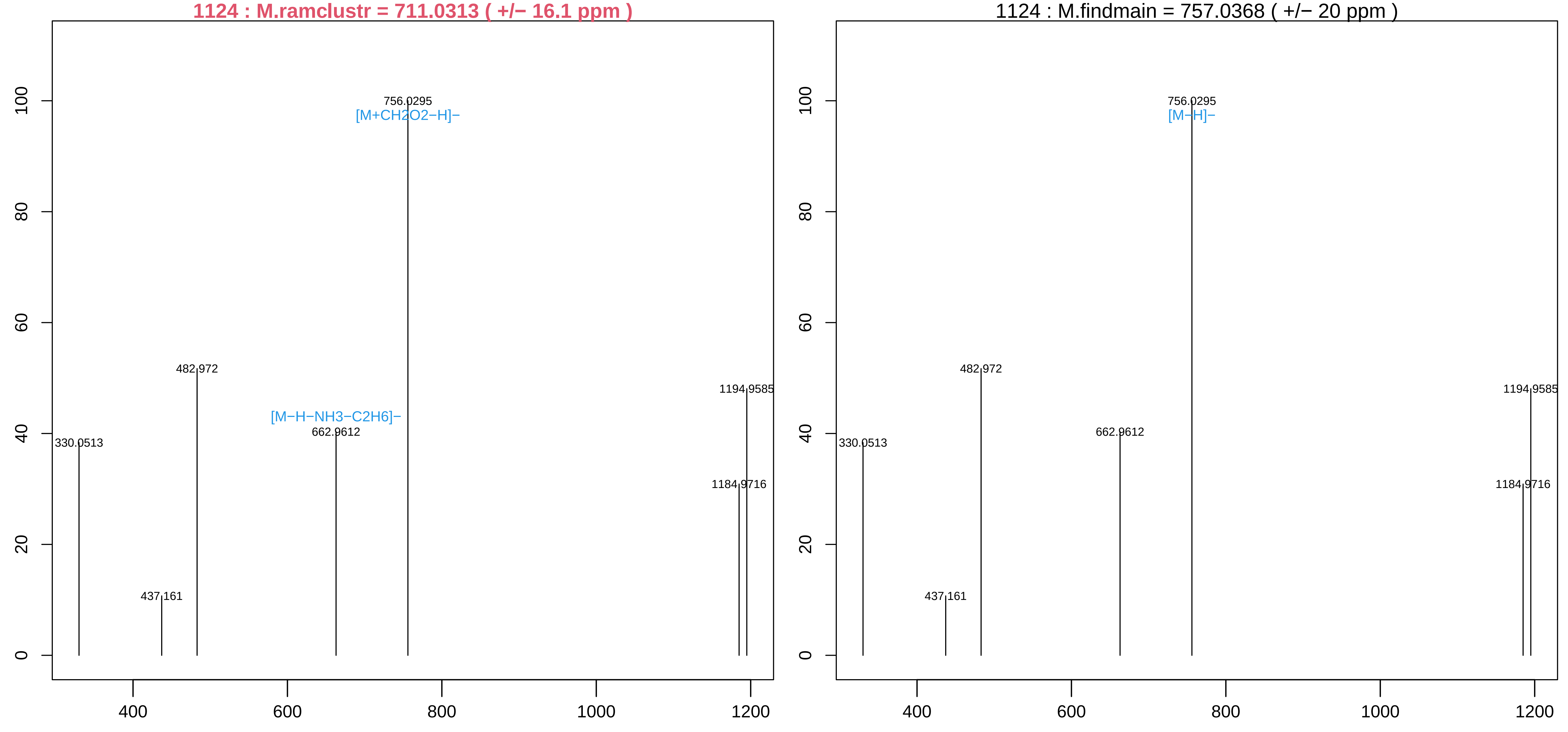
1124 : M.ramclustr = 711.0313 (+/− 16.1 ppm)
756.0295
[M+CH2O2−H]−
482.972
1194.9585
[M−H−NH3−C2H6]−
662.9612
330.0513
1184.9716
437.161
1124 : M.findmain = 757.0368 (+/− 20 ppm)
756.0295
[M−H]−
482.972
1194.9585
662.9612
330.0513
1184.9716
437.161

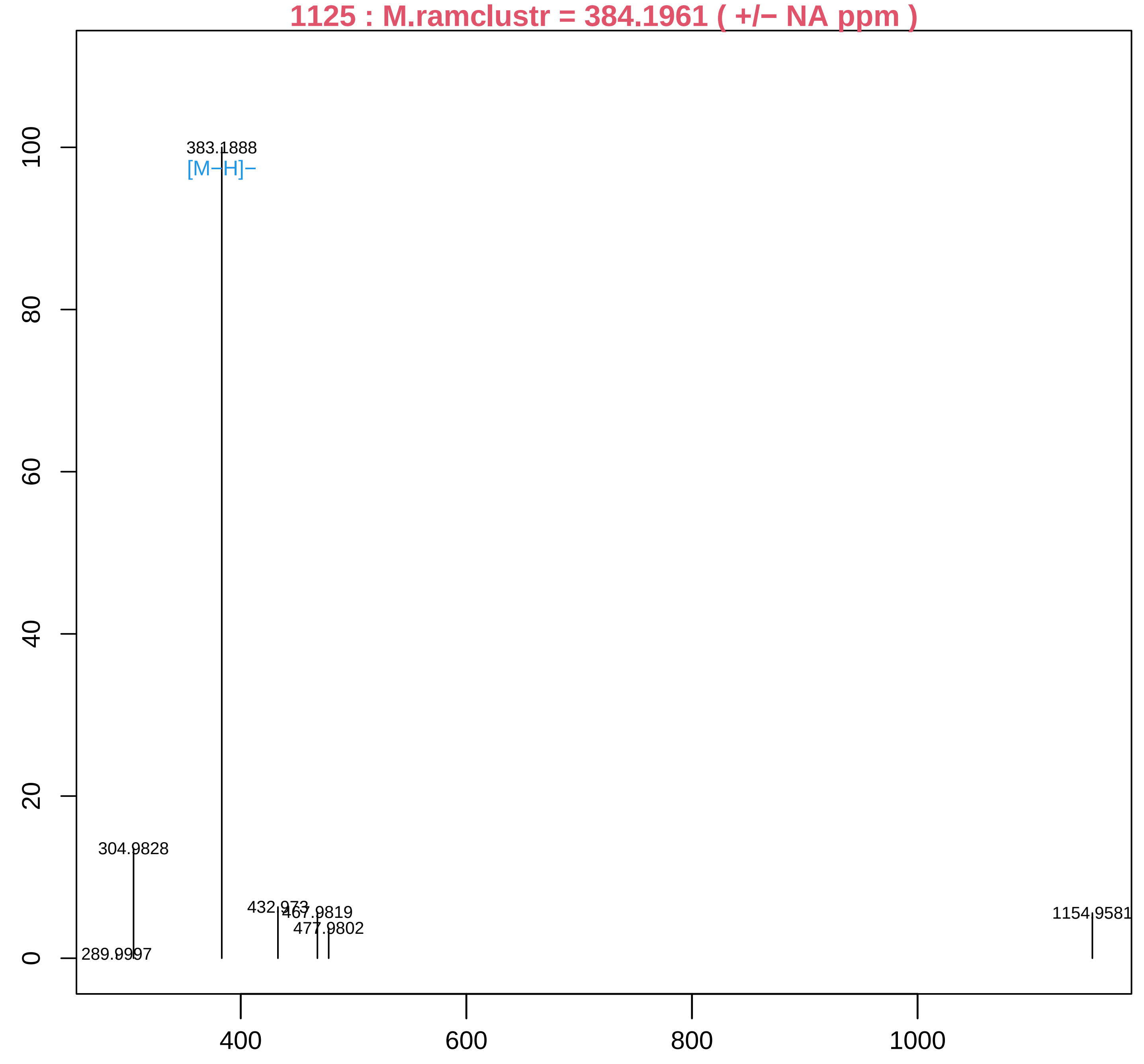
1125 : M.ramclustr = 384.1961 (+/− NA ppm)
383.1888
[M−H]−
304.9828
432.973
467.9819
477.9802
289.9997
1154.9581
0
20
40
60
80
100
400
600
800
1000

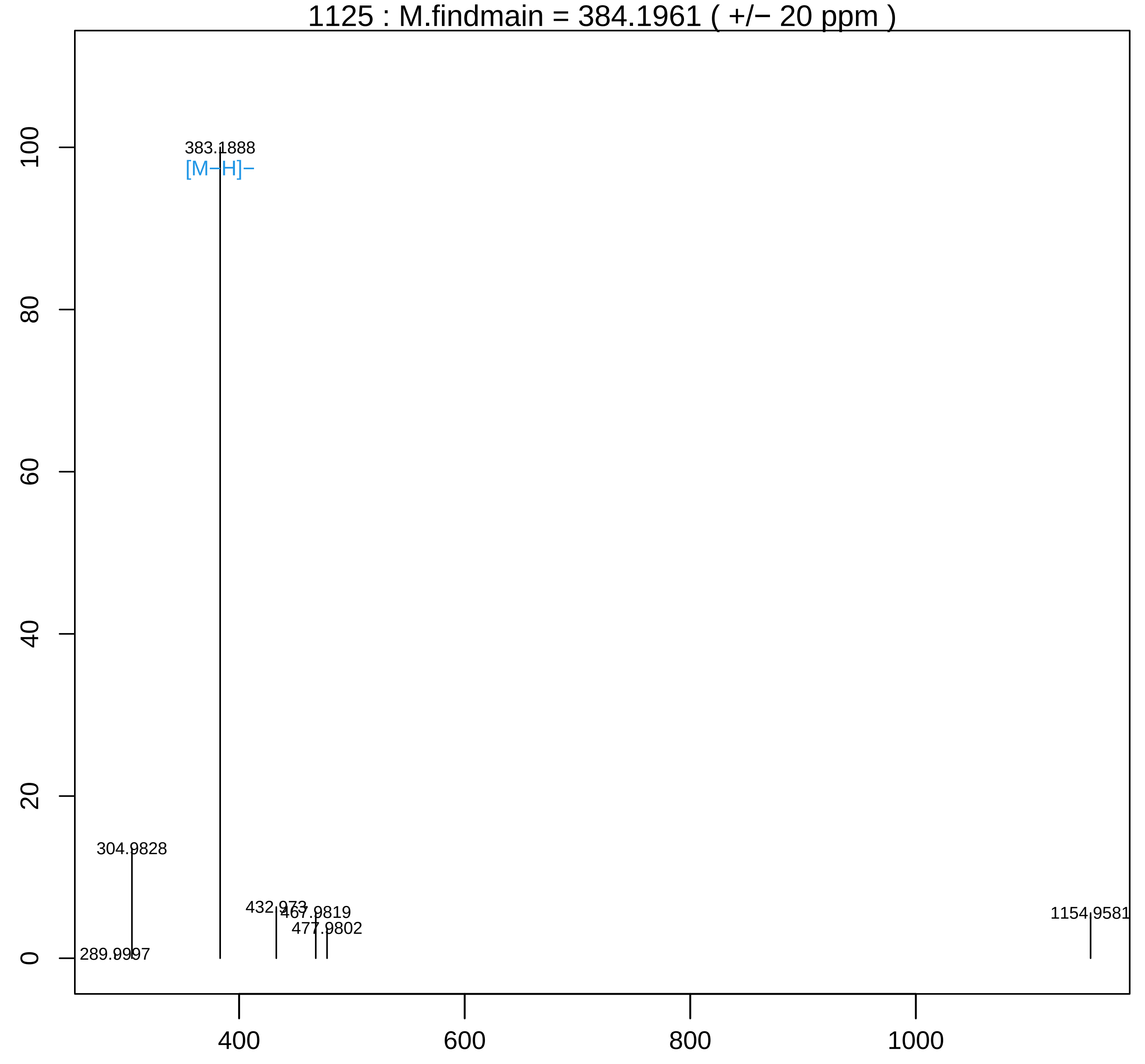
1125 : M.findmain = 384.1961 (+/− 20 ppm)
383.1888
[M−H]−
304.9828
432.973
467.9819
477.9802
289.9997
1154.9581
0
20
40
60
80
100
400
600
800
1000

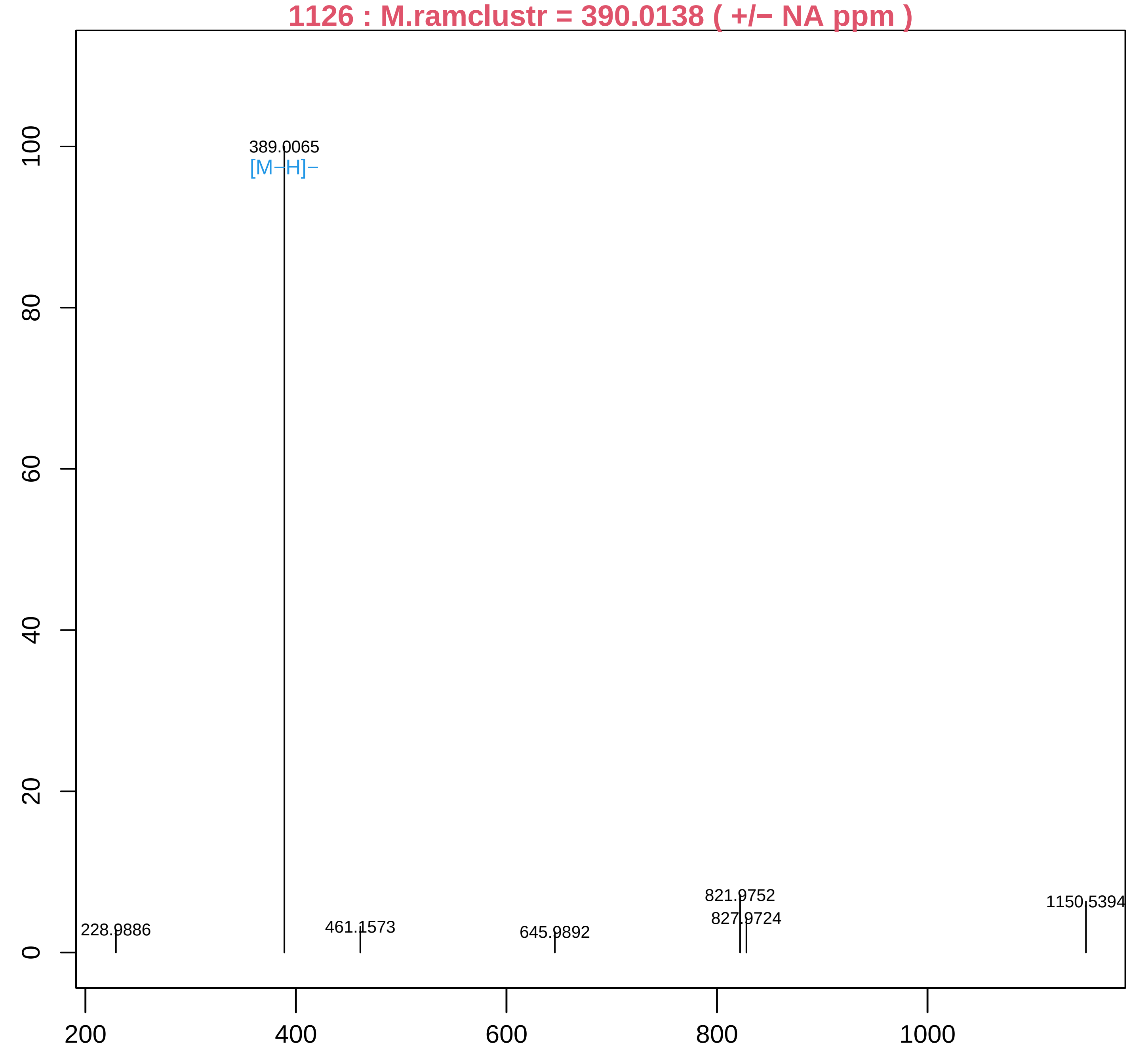
1126 : M.ramclustr = 390.0138 (+/− NA ppm)
389.0065
[M−H]−
228.9886
461.1573
645.9892
821.9752
827.9724
1150.5394
0
20
40
60
80
100
200
400
600
800
1000

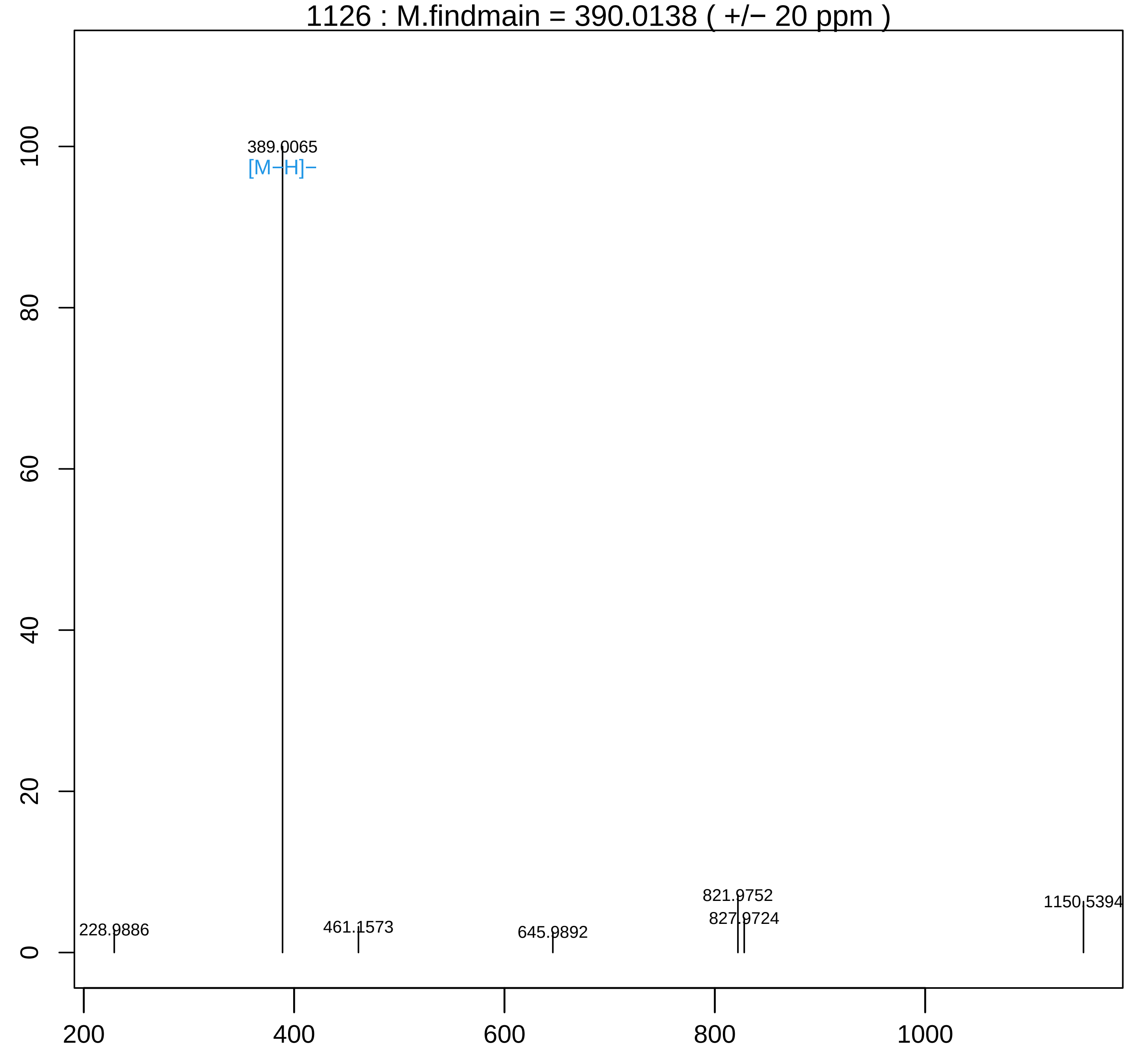
1126 : M.findmain = 390.0138 (+/− 20 ppm)
389.0065
[M−H]−
228.9886
461.1573
645.9892
821.9752
827.9724
1150.5394
0
20
40
60
80
100
200
400
600
800
1000

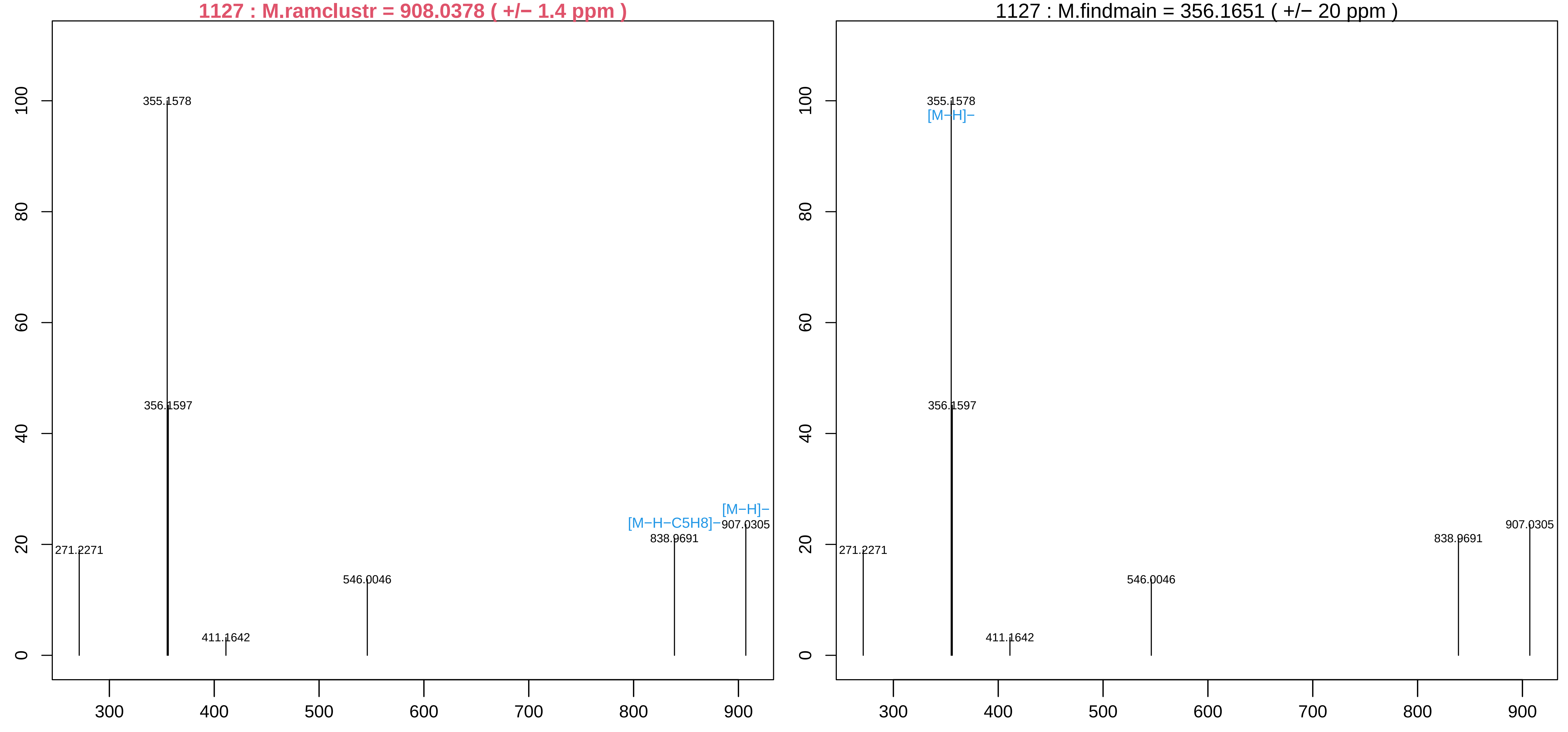

1127 : M.ramclustr = 908.0378 (+/− 1.4 ppm)
355.1578
356.1597
271.2271
411.1642
546.0046
838.9691
907.0305
[M−H−C5H8]−
[M−H]−
0
20
40
60
80
100
300
400
500
600
700
800
900
1127 : M.findmain = 356.1651 (+/− 20 ppm)
355.1578
[M−H]−
356.1597
271.2271
411.1642
546.0046
838.9691
907.0305

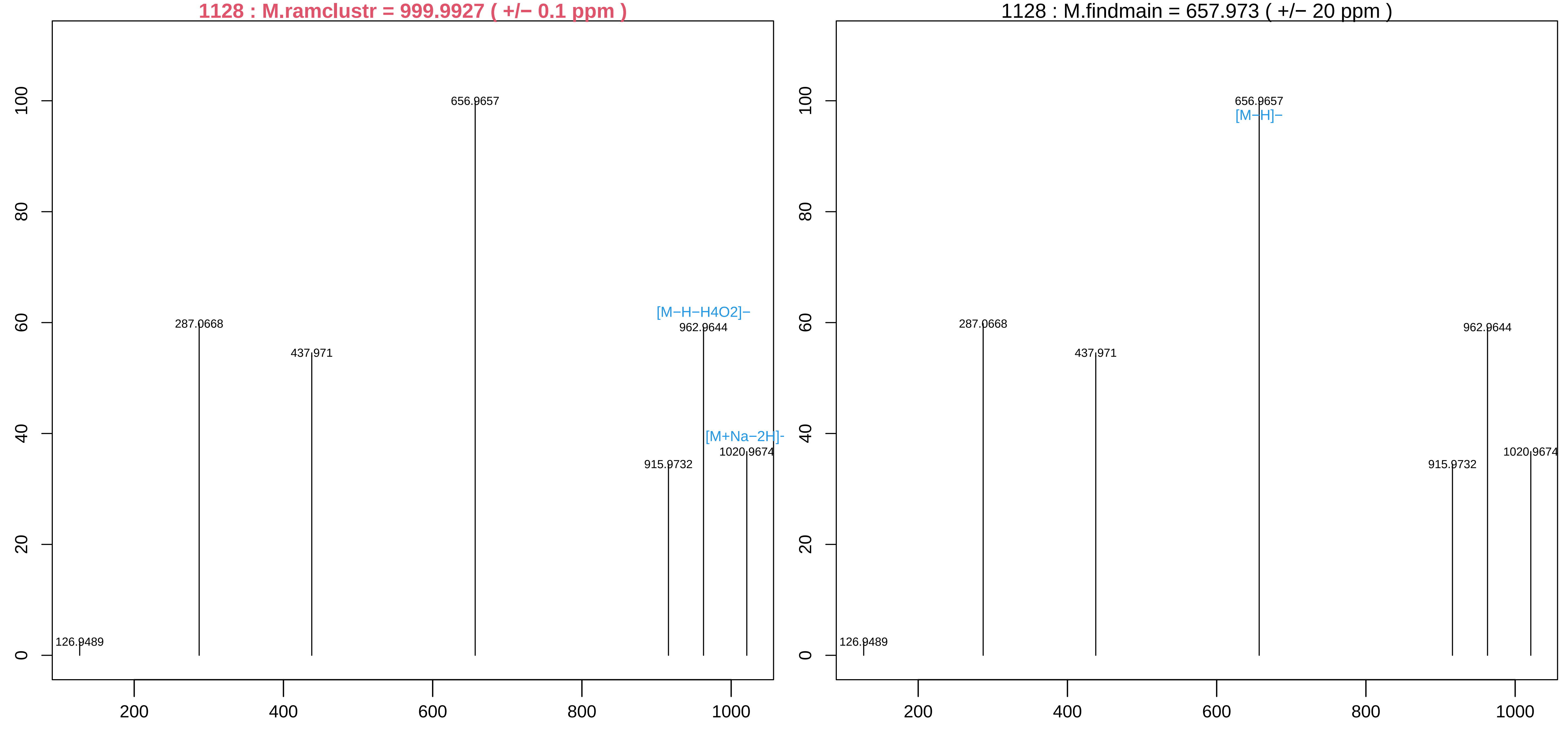

1128 : M.ramclustr = 999.9927 (+/− 0.1 ppm)
656.9657
287.0668
437.971
[M−H−H4O2]−
962.9644
[M+Na−2H]-
1020.9674
915.9732
126.9489
0
20
40
60
80
100
200
400
600
800
1000
1128 : M.findmain = 657.973 (+/− 20 ppm)
656.9657
[M−H]−
287.0668
437.971
962.9644
1020.9674
915.9732
126.9489

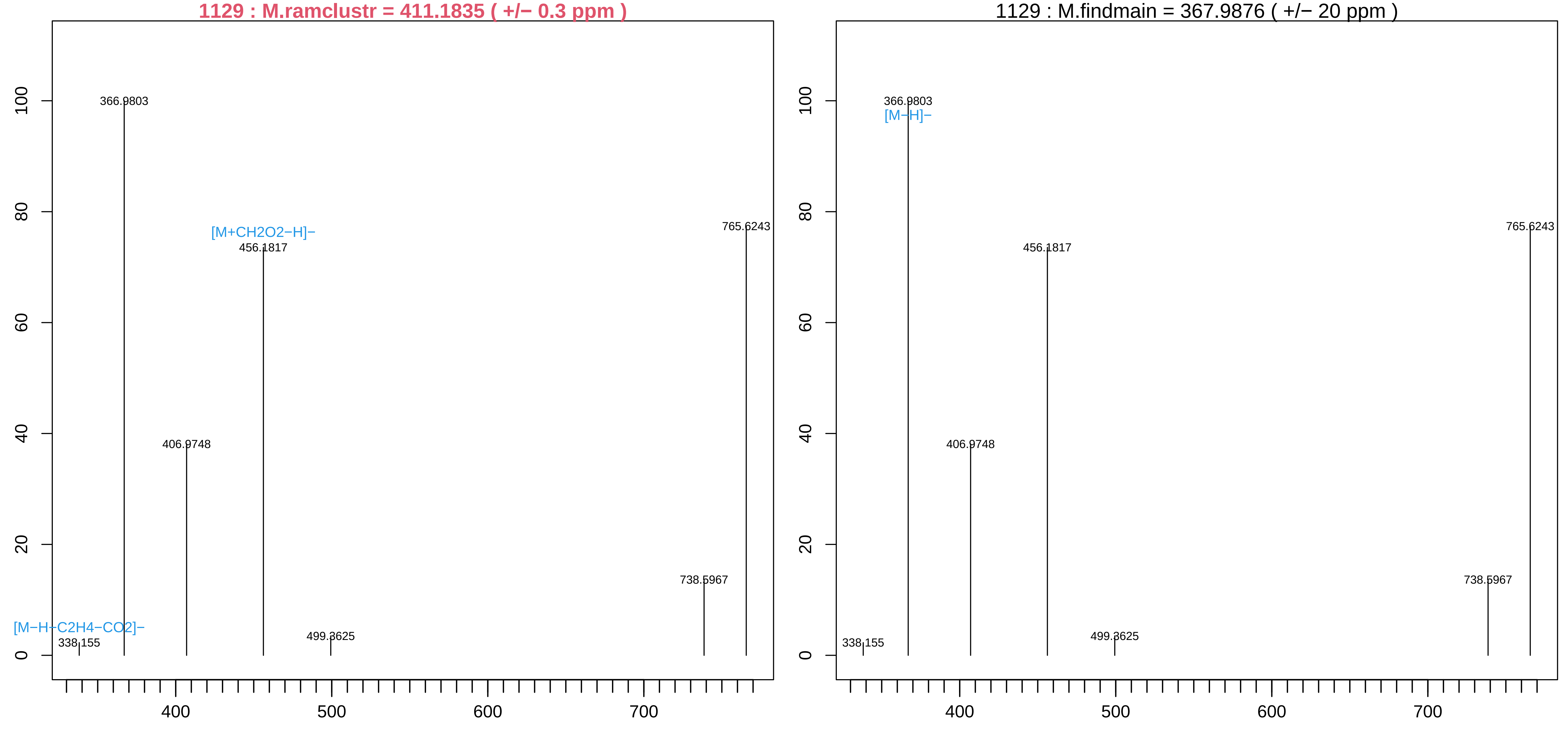
1129 : M.ramclustr = 411.1835 (+/− 0.3 ppm)
366.9803
406.9748
[M+CH2O2−H]−
456.1817
499.3625
738.5967
765.6243
[M−H−C2H4−CO2]−
338.155
1129 : M.findmain = 367.9876 (+/− 20 ppm)
366.9803
[M−H]−
406.9748
456.1817
499.3625
738.5967
765.6243
338.155
0
20
40
60
80
100
400
500
600
700

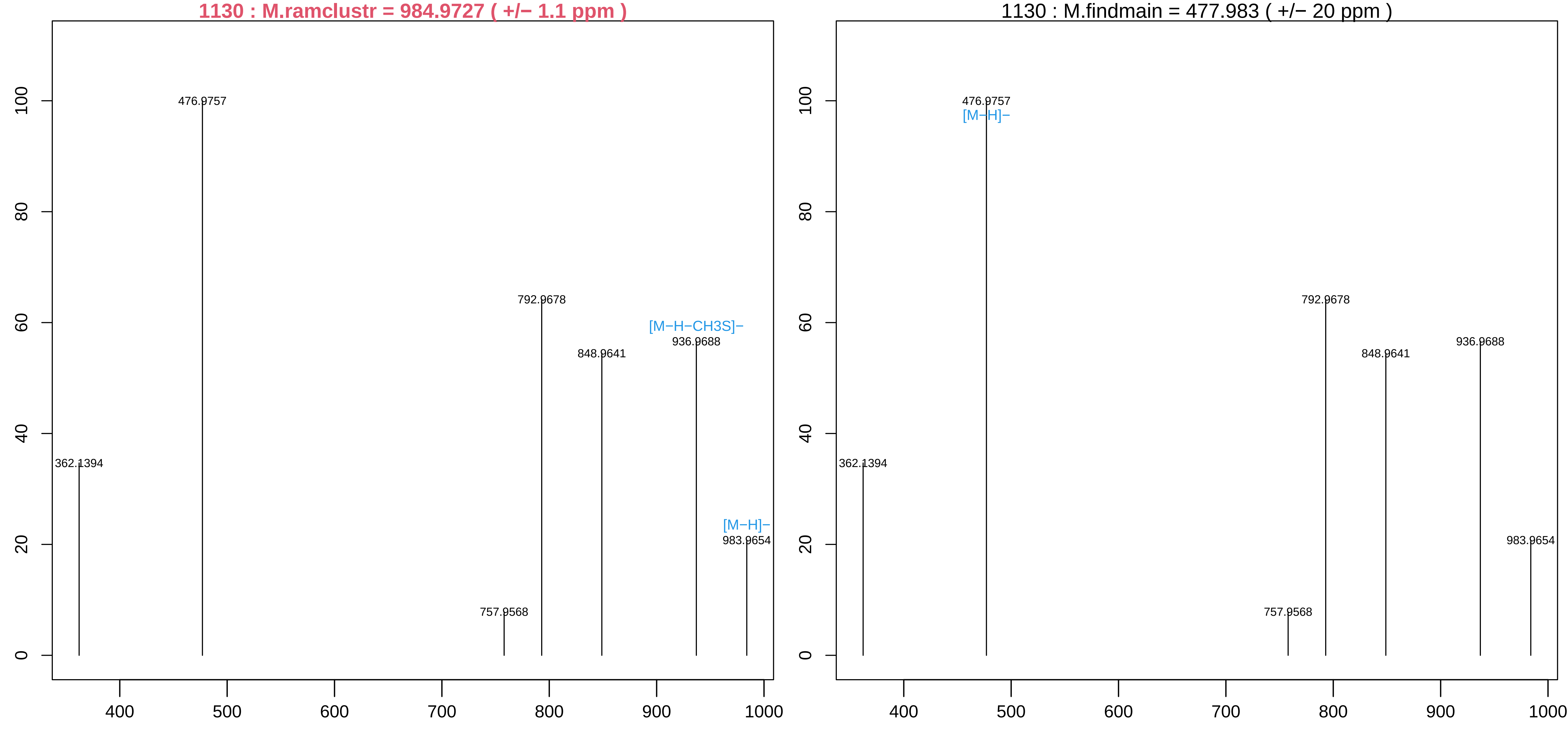
1130 : M.ramclustr = 984.9727 (+/− 1.1 ppm)
476.9757
792.9678
[M−H−CH3S]−
936.9688
848.9641
362.1394
[M−H]−
983.9654
757.9568
0
20
40
60
80
100
400
500
600
700
800
900
1000
1130 : M.findmain = 477.983 (+/− 20 ppm)
476.9757
[M−H]−
792.9678
936.9688
848.9641
362.1394
983.9654
757.9568

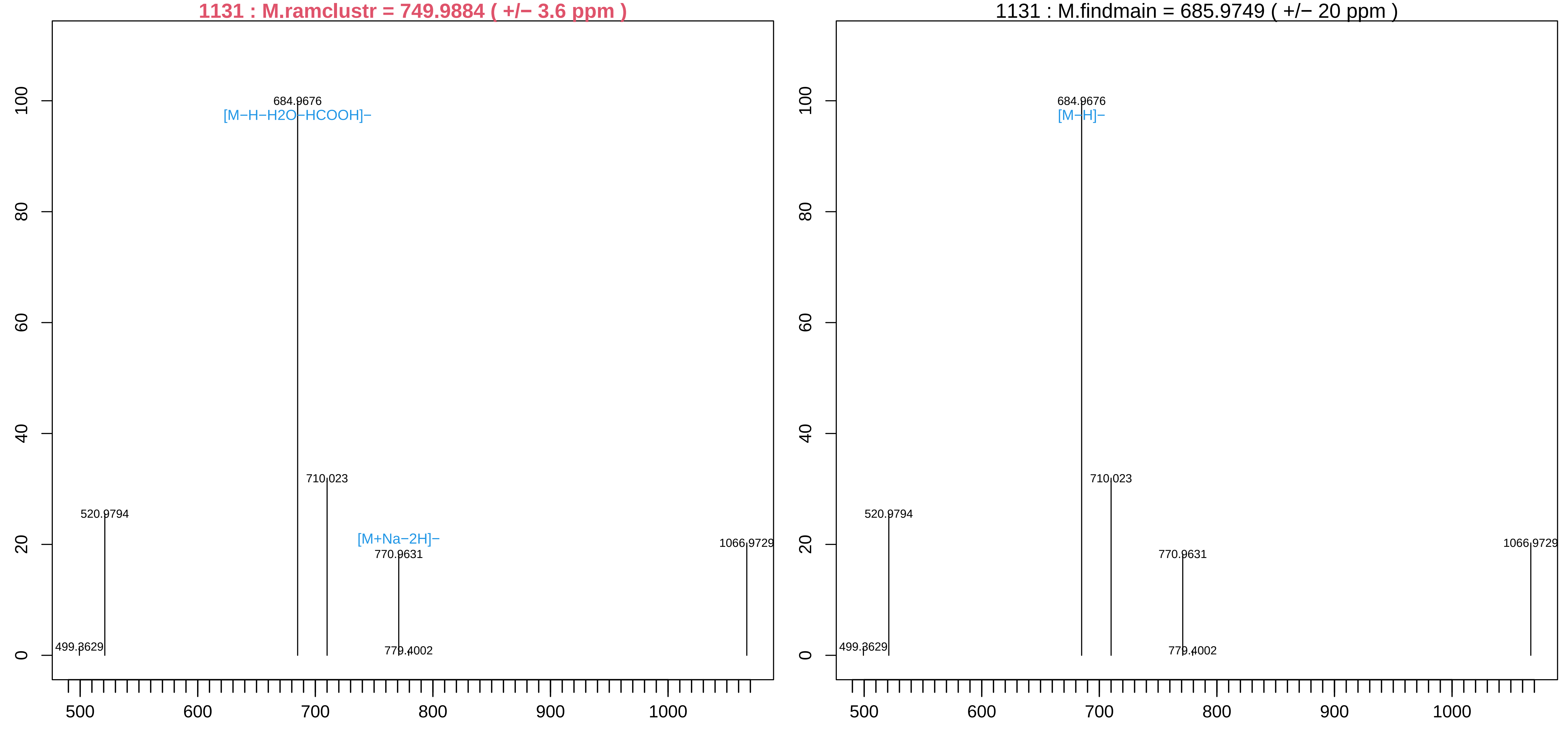

1131 : M.ramclustr = 749.9884 (+/− 3.6 ppm)
684.9676
[M−H−H2O−HCOOH]−
710.023
520.9794
[M+Na−2H]−
770.9631
1066.9729
499.3629
779.4002
1131 : M.findmain = 685.9749 (+/− 20 ppm)
684.9676
[M−H]−
710.023
520.9794
770.9631
1066.9729
499.3629
779.4002
0
20
40
60
80
100
500
600
700
800
900
1000

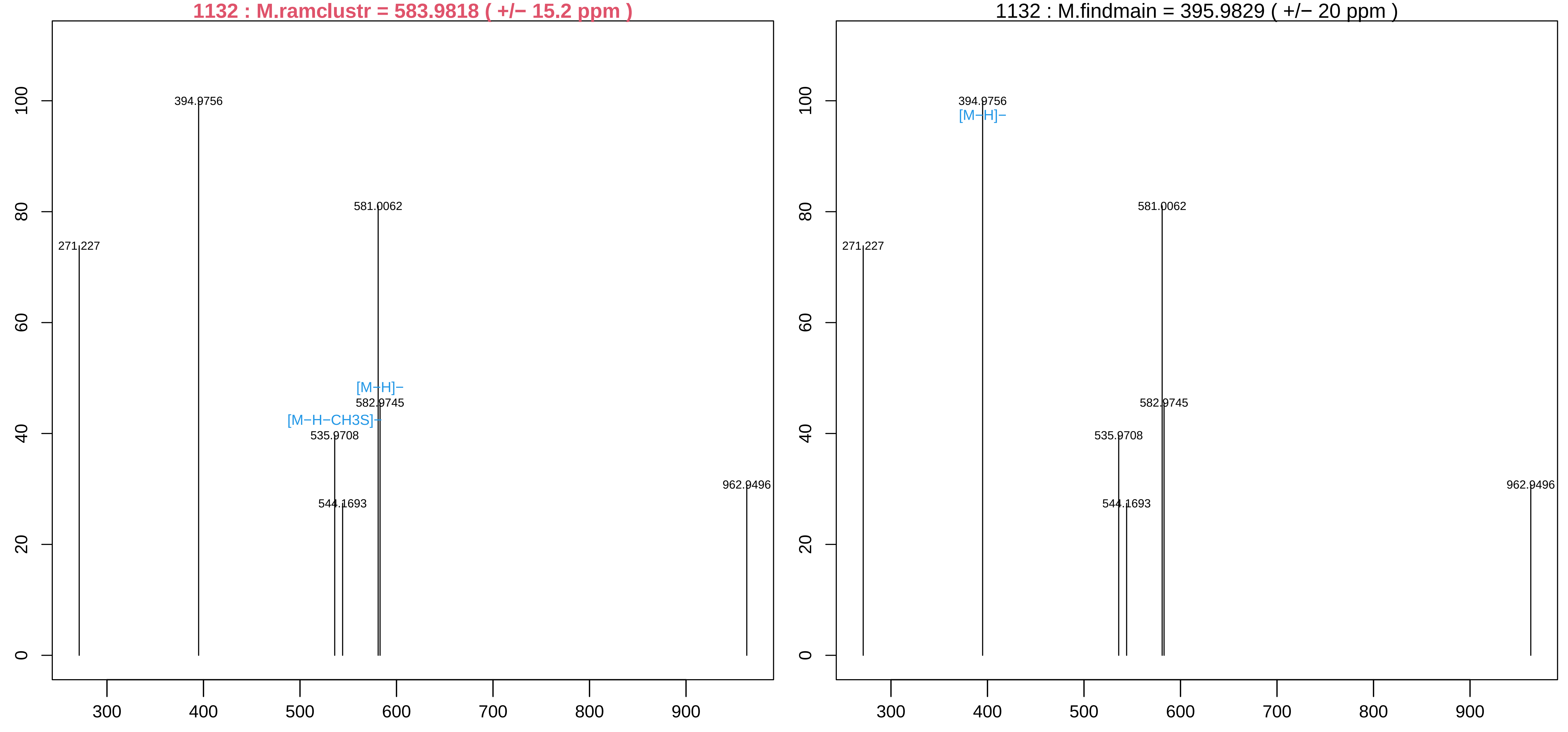

1132 : M.ramclustr = 583.9818 (+/− 15.2 ppm)
394.9756
581.0062
271.227
[M−H]−
582.9745
[M−H−CH3S]−
535.9708
544.1693
962.9496
1132 : M.findmain = 395.9829 (+/− 20 ppm)
394.9756
[M−H]−
581.0062
271.227
582.9745
535.9708
544.1693
962.9496

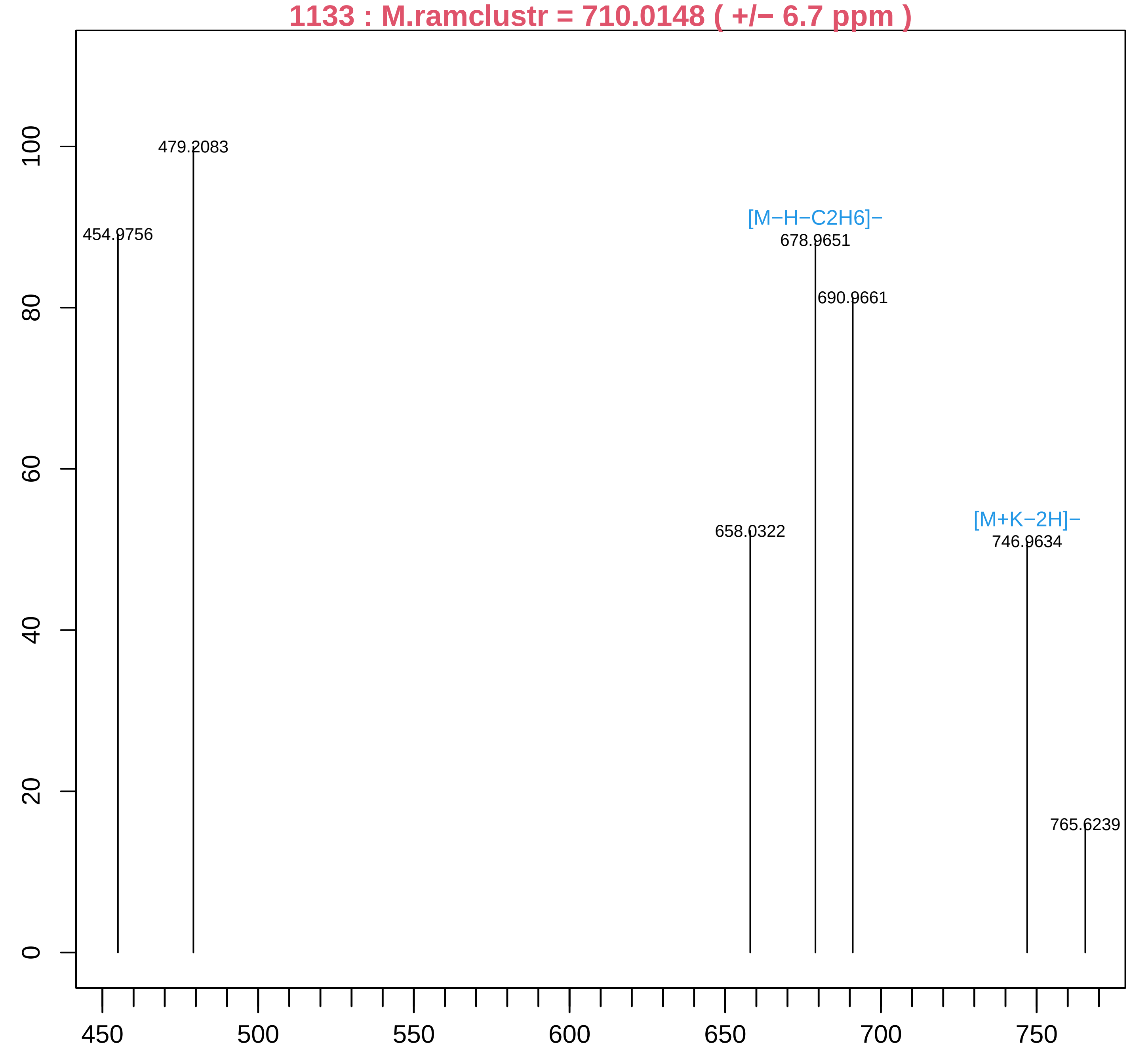
1133 : M.ramclustr = 710.0148 (+/− 6.7 ppm)
479.2083
454.9756
[M−H−C2H6]−
678.9651
690.9661
658.0322
[M+K−2H]−
746.9634
765.6239
0
20
40
60
80
100
450
500
550
600
650
700
750

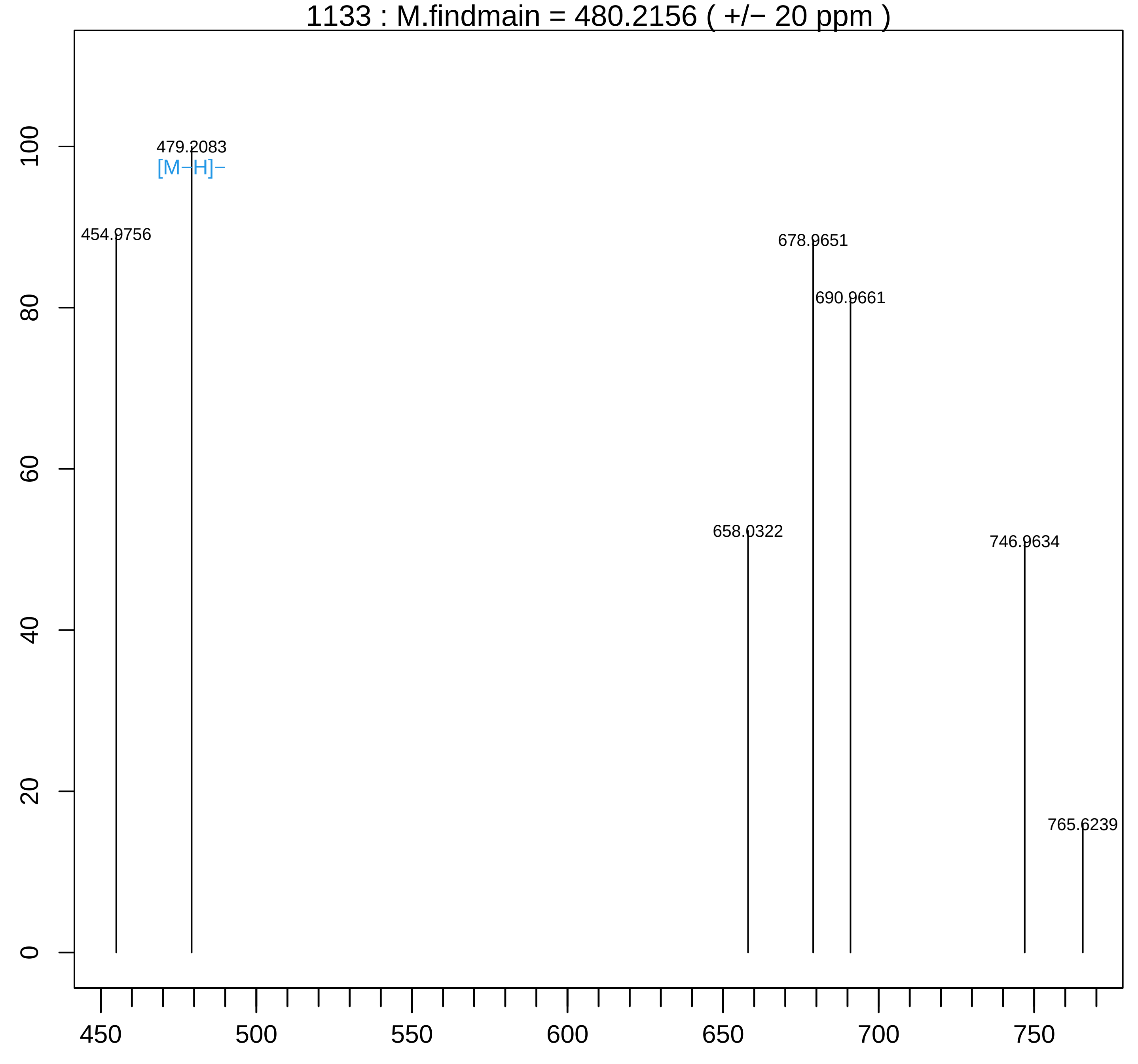
1133 : M.findmain = 480.2156 (+/− 20 ppm)
479.2083
[M−H]−
454.9756
678.9651
690.9661
658.0322
746.9634
765.6239
0
20
40
60
80
100
450
500
550
600
650
700
750

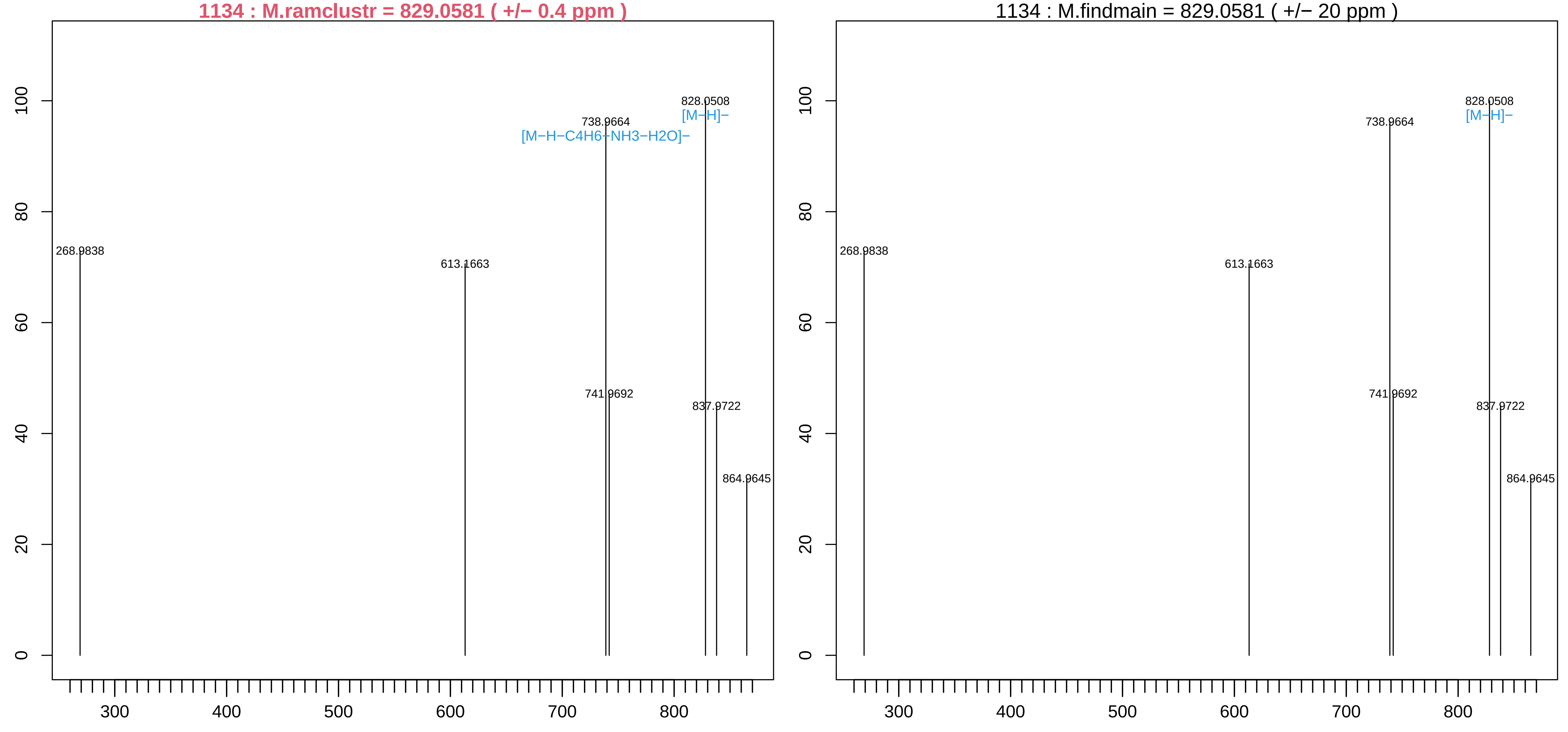
1134 : M.ramclustr = 829.0581 (+/− 0.4 ppm)
268.9838
613.1663
738.9664
[M−H−C4H6−NH3−H2O]−
741.9692
828.0508
[M−H]−
837.9722
864.9645
1134 : M.findmain = 829.0581 (+/− 20 ppm)
268.9838
613.1663
738.9664
741.9692
828.0508
[M−H]−
837.9722
864.9645
0
20
40
60
80
100
300
400
500
600
700
800

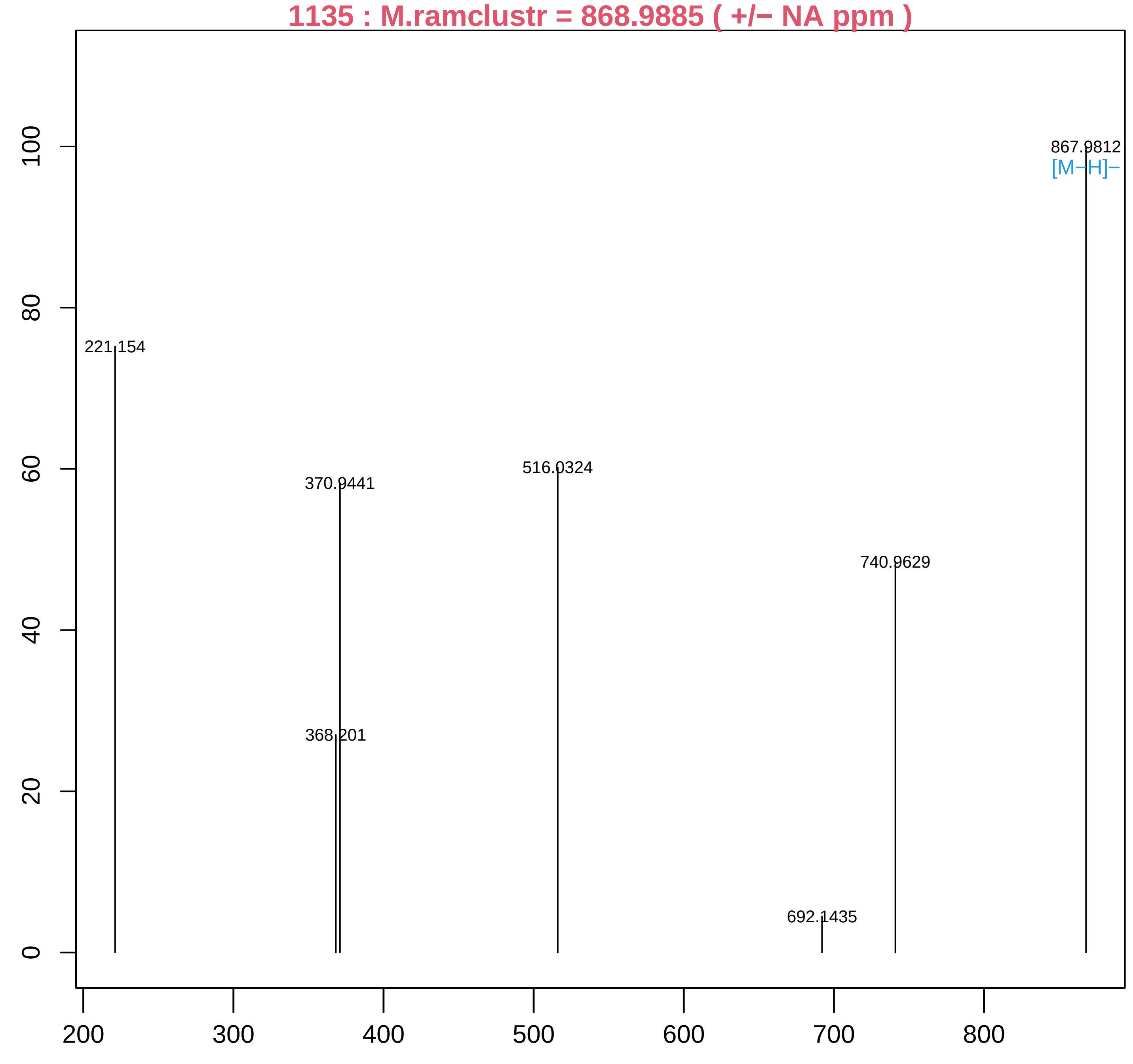

1135 : M.ramclustr = 868.9885 (+/− NA ppm)
867.9812
[M−H]−
221.154
370.9441
516.0324
740.9629
368.201
692.1435
0
20
40
60
80
100
200
300
400
500
600
700
800

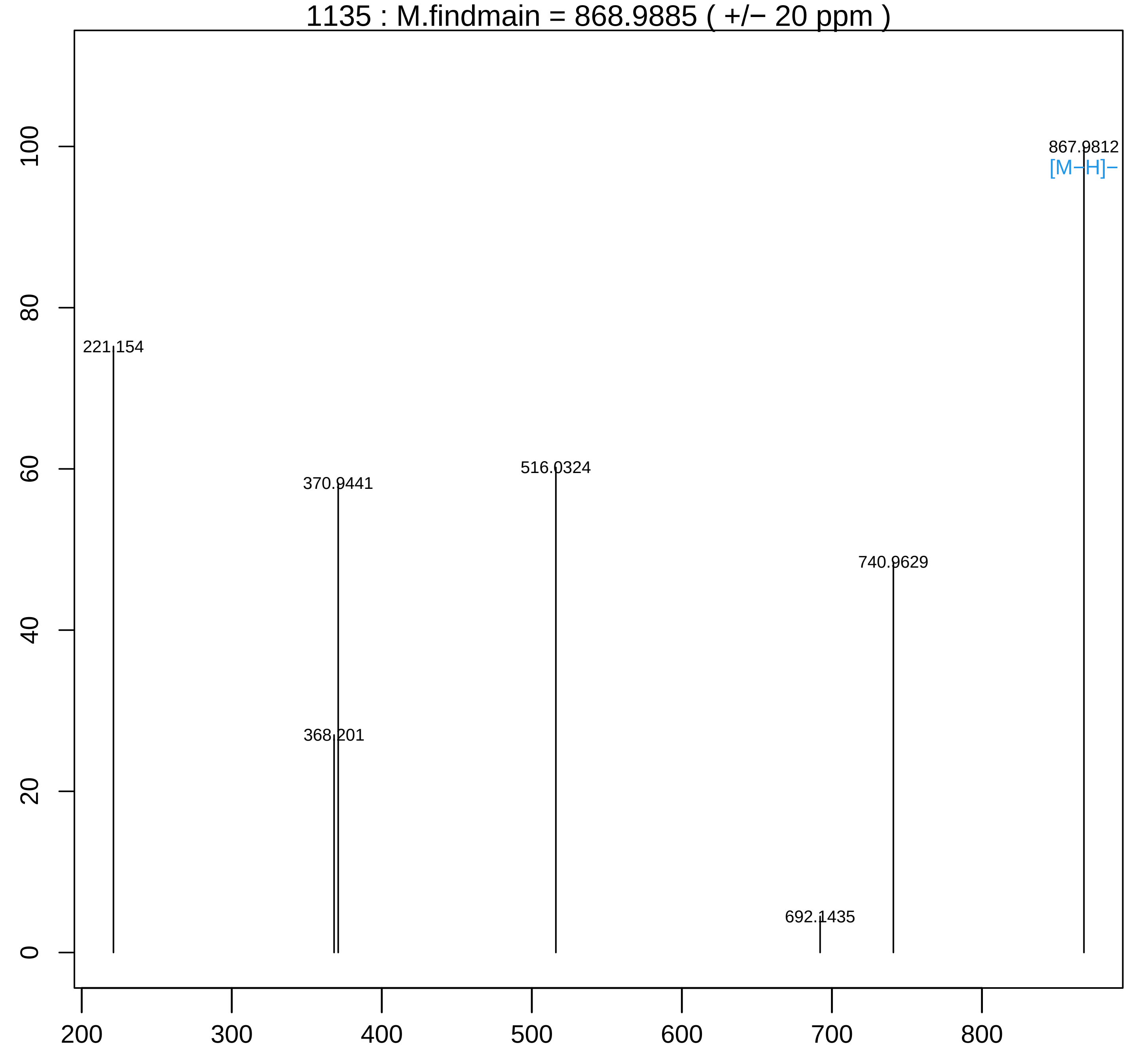

1135 : M.findmain = 868.9885 (+/− 20 ppm)
867.9812
[M−H]−
221.154
370.9441
516.0324
740.9629
368.201
692.1435
0
20
40
60
80
100
200
300
400
500
600
700
800

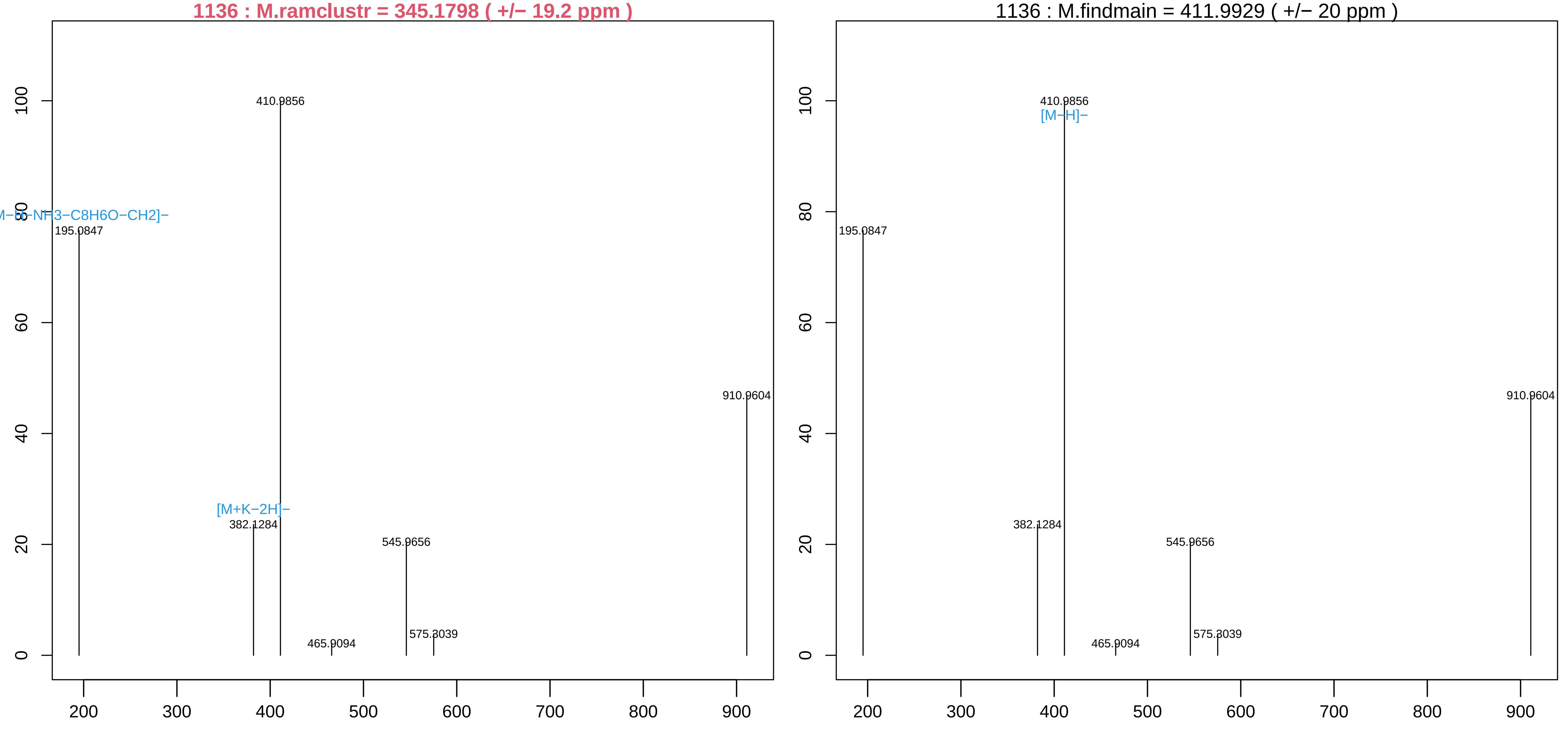

1136 : M.ramclustr = 345.1798 (+/− 19.2 ppm)
[M−H−NH3−C8H6O−CH2]−
195.0847
410.9856
[M+K−2H]−
382.1284
465.9094
545.9656
575.3039
910.9604
1136 : M.findmain = 411.9929 (+/− 20 ppm)
195.0847
410.9856
[M−H]−
382.1284
465.9094
545.9656
575.3039
910.9604

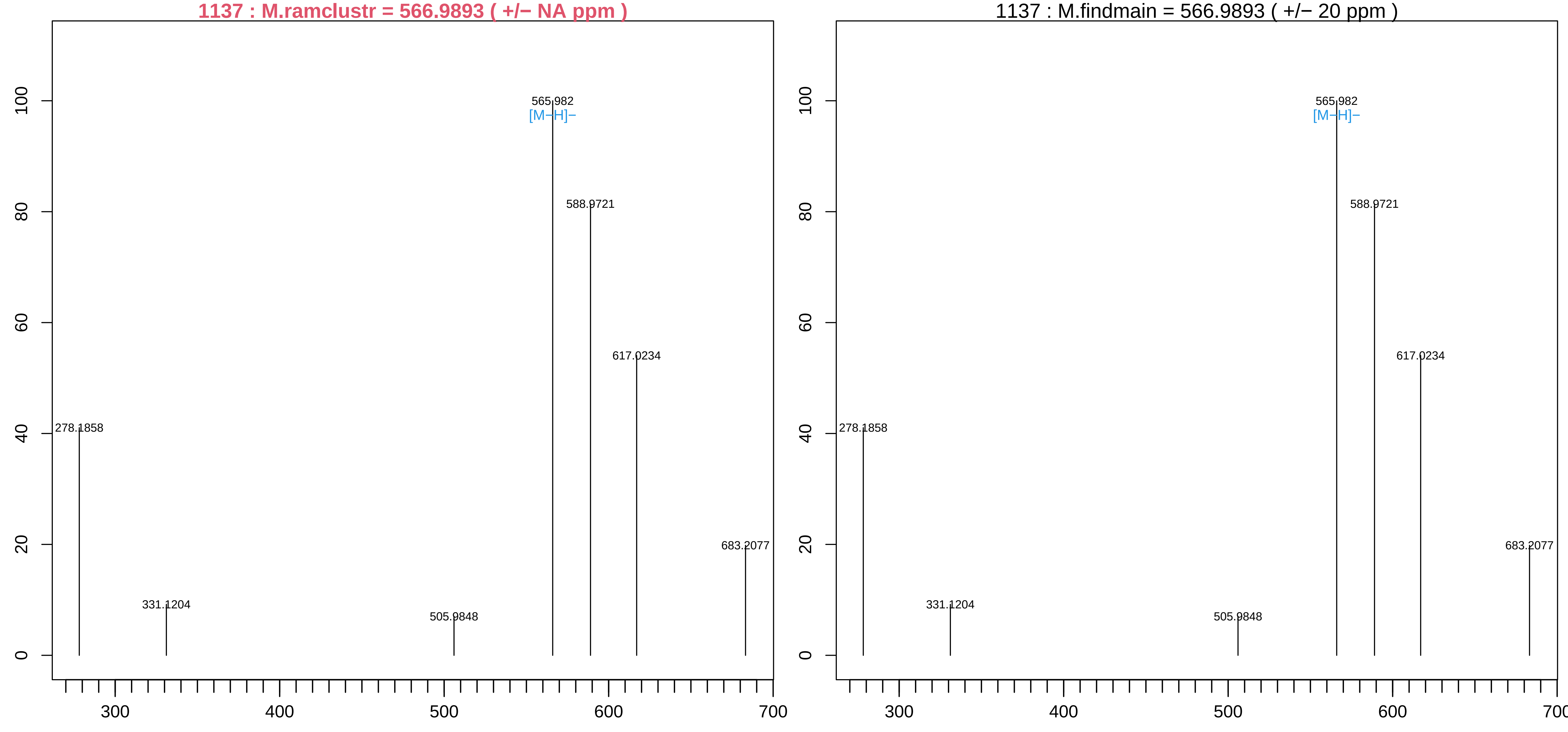
1137 : M.ramclustr = 566.9893 (+/− NA ppm)
1137 : M.findmain = 566.9893 (+/− 20 ppm)
565.982
[M−H]−
588.9721
617.0234
278.1858
683.2077
331.1204
505.9848
0
20
40
60
80
100
300
400
500
600
700

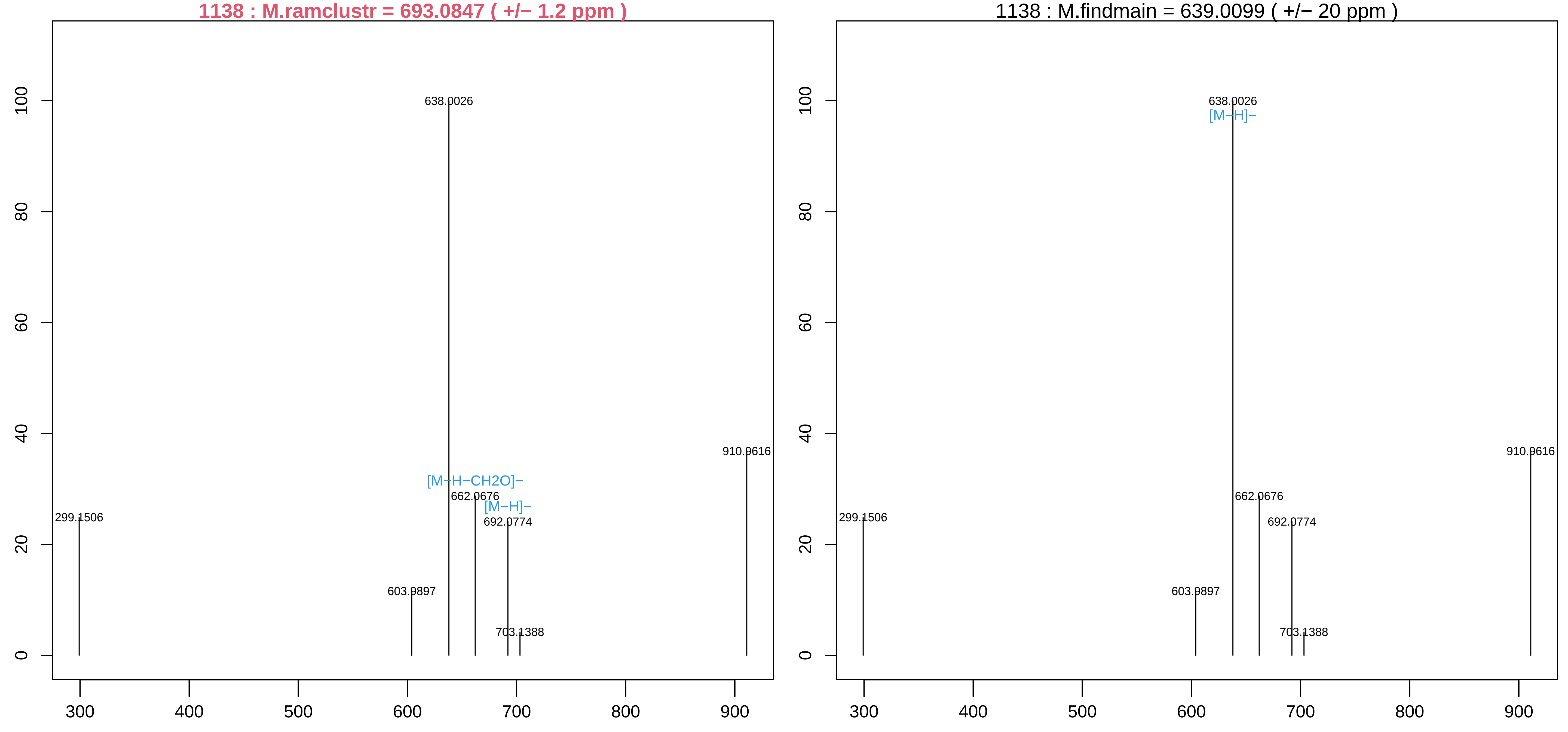
1138 : M.ramclustr = 693.0847 (+/− 1.2 ppm)
638.0026
[M−H−CH2O]−
662.0676
[M−H]−
692.0774
299.1506
603.9897
703.1388
910.9616
1138 : M.findmain = 639.0099 (+/− 20 ppm)
638.0026
[M−H]−
662.0676
692.0774
299.1506
603.9897
703.1388
910.9616
0
20
40
60
80
100
300
400
500
600
700
800
900

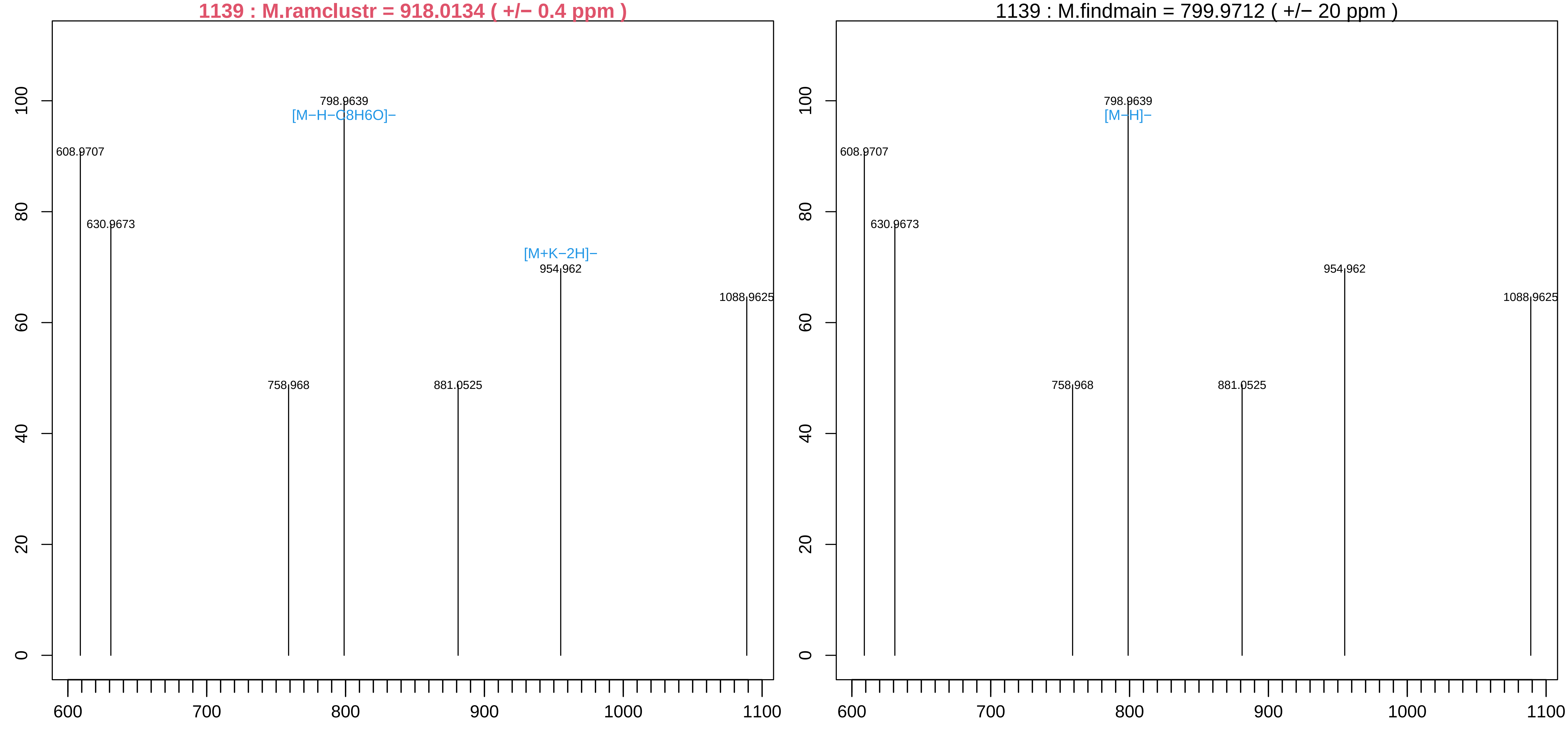

1139 : M.ramclustr = 918.0134 (+/− 0.4 ppm)
608.9707
630.9673
758.968
798.9639
[M−H−C8H6O]−
881.0525
[M+K−2H]−
954.962
1088.9625
1139 : M.findmain = 799.9712 (+/− 20 ppm)
608.9707
630.9673
758.968
798.9639
[M−H]−
881.0525
954.962
1088.9625
0
20
40
60
80
100
600
700
800
900
1000
1100

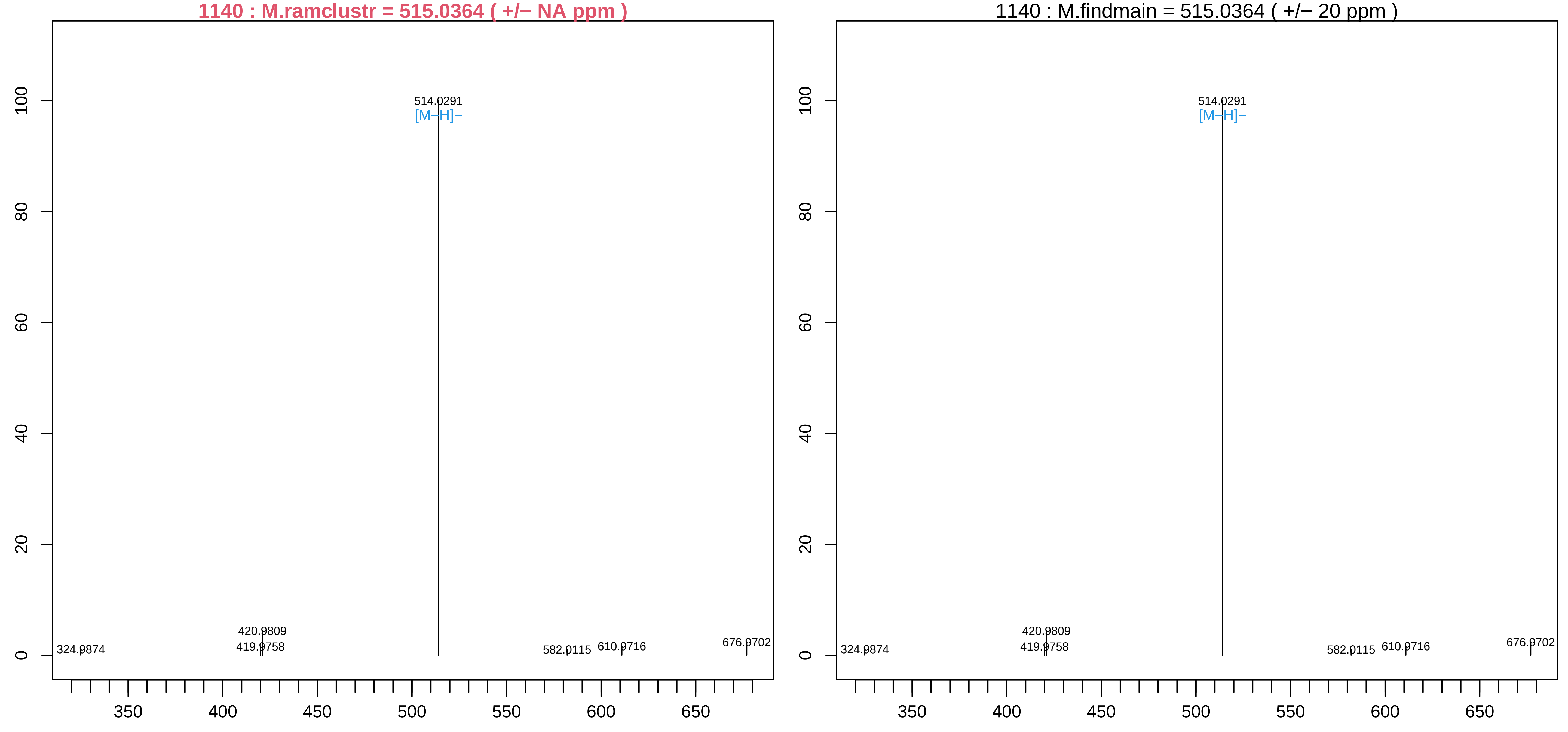
1140 : M.ramclustr = 515.0364 (+/− NA ppm)
514.0291
[M−H]−
420.9809
419.9758
324.9874
582.0115
610.9716
676.9702
1140 : M.findmain = 515.0364 (+/− 20 ppm)
514.0291
[M−H]−
420.9809
419.9758
324.9874
582.0115
610.9716
676.9702

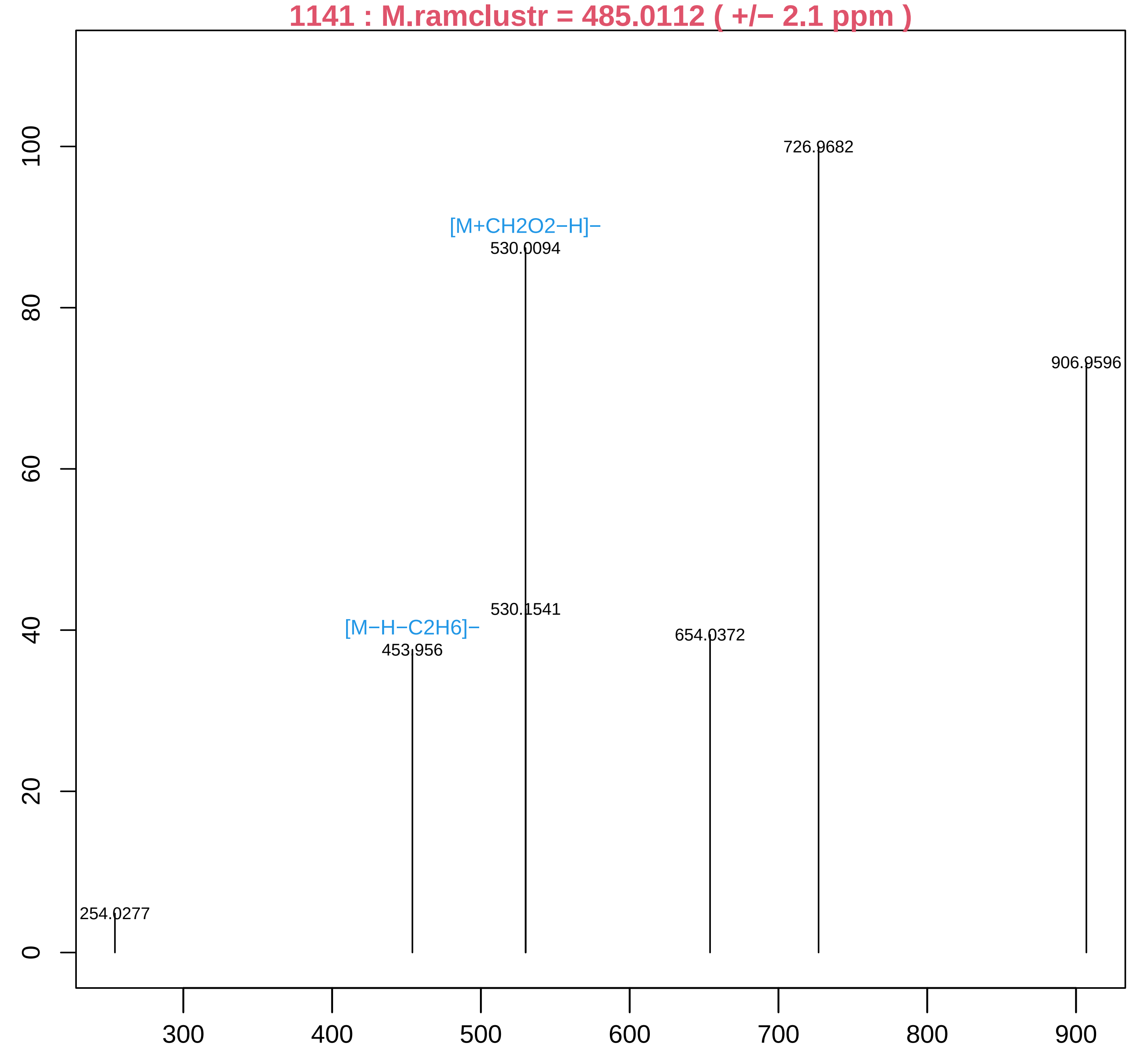
1141 : M.ramclustr = 485.0112 (+/− 2.1 ppm)
726.9682
[M+CH2O2−H]−
530.0094
906.9596
[M−H−C2H6]−
453.956
530.1541
654.0372
254.0277
0
20
40
60
80
100
300
400
500
600
700
800
900

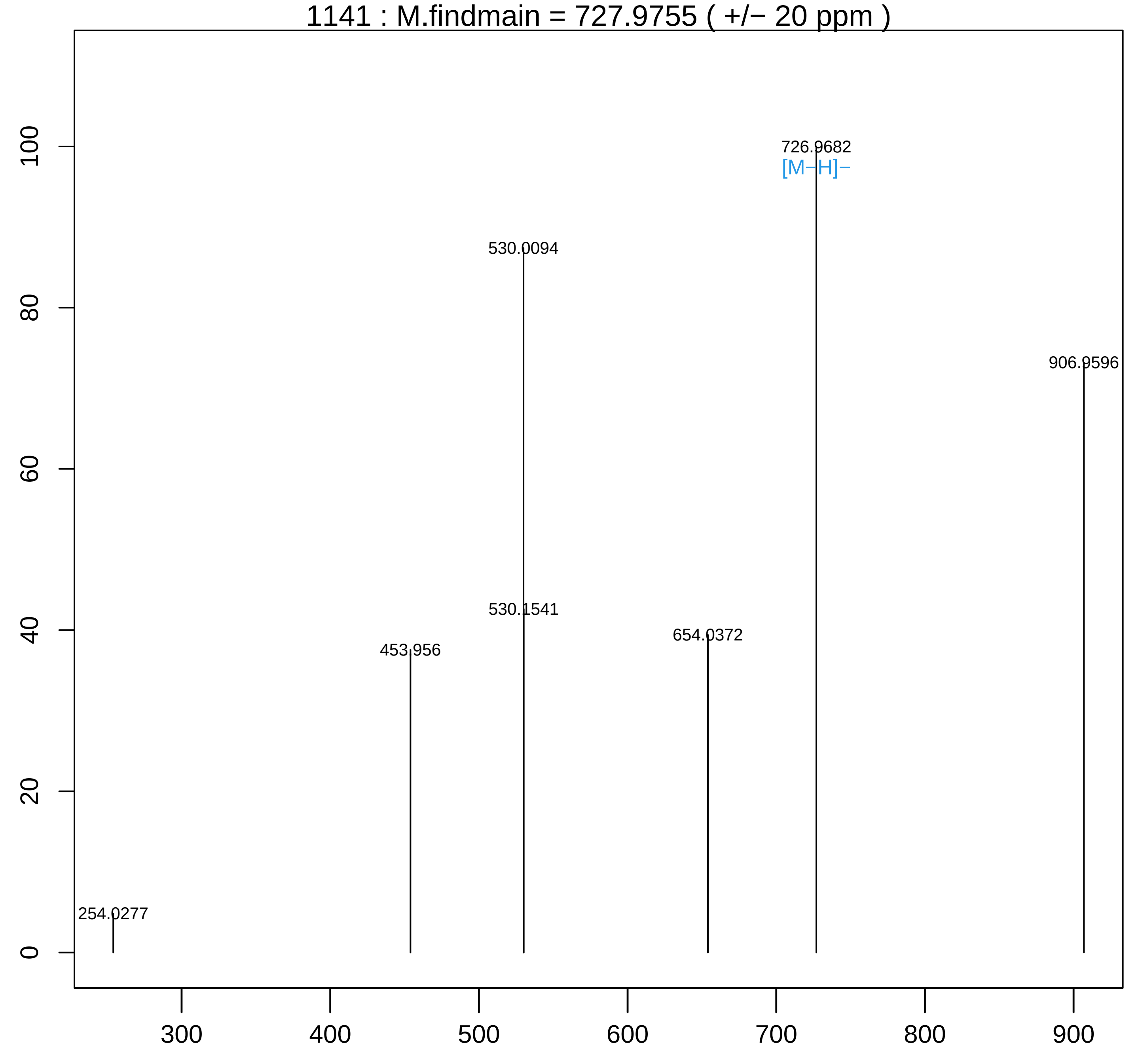
1141 : M.findmain = 727.9755 (+/− 20 ppm)
726.9682
[M−H]−
530.0094
906.9596
453.956
530.1541
654.0372
254.0277
0
20
40
60
80
100
300
400
500
600
700
800
900

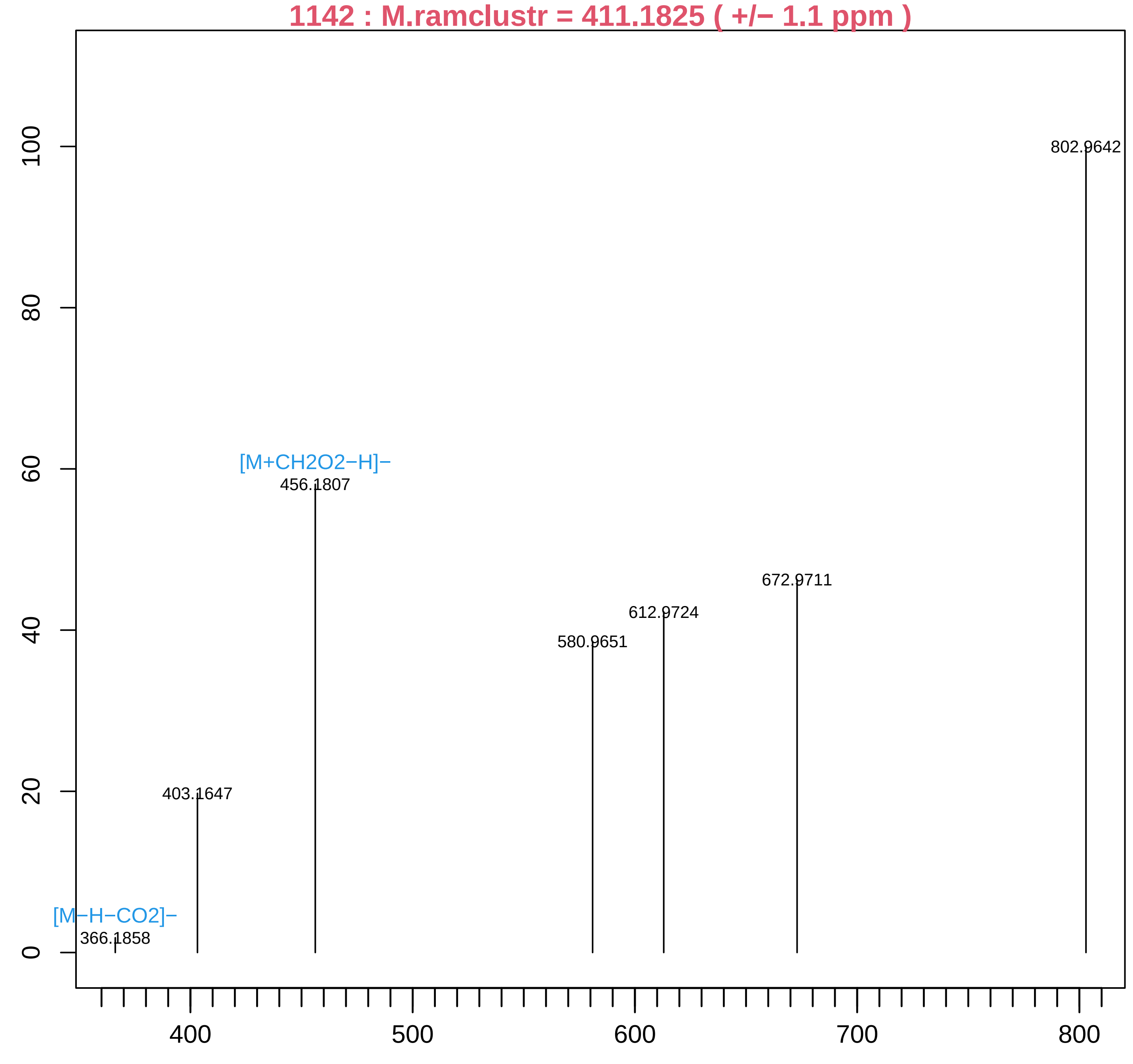
1142 : M.ramclustr = 411.1825 (+/− 1.1 ppm)
0
20
40
60
80
100
400
500
600
700
800
[M−H−CO2]−
366.1858
403.1647
[M+CH2O2−H]−
456.1807
580.9651
612.9724
672.9711
802.9642

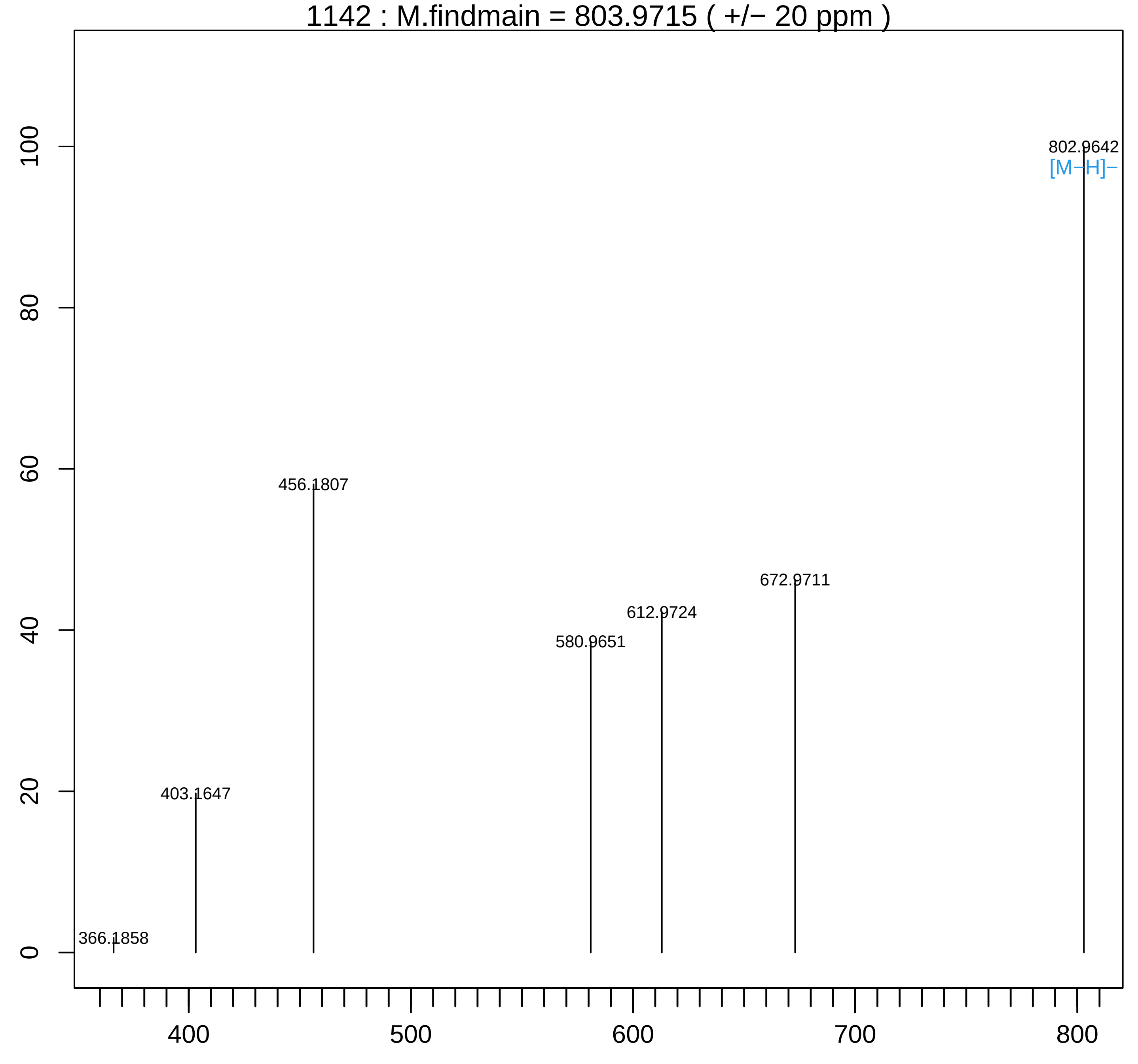
1142 : M.findmain = 803.9715 (+/− 20 ppm)
0
20
40
60
80
100
400
500
600
700
800
366.1858
403.1647
456.1807
580.9651
612.9724
672.9711
802.9642
[M−H]−

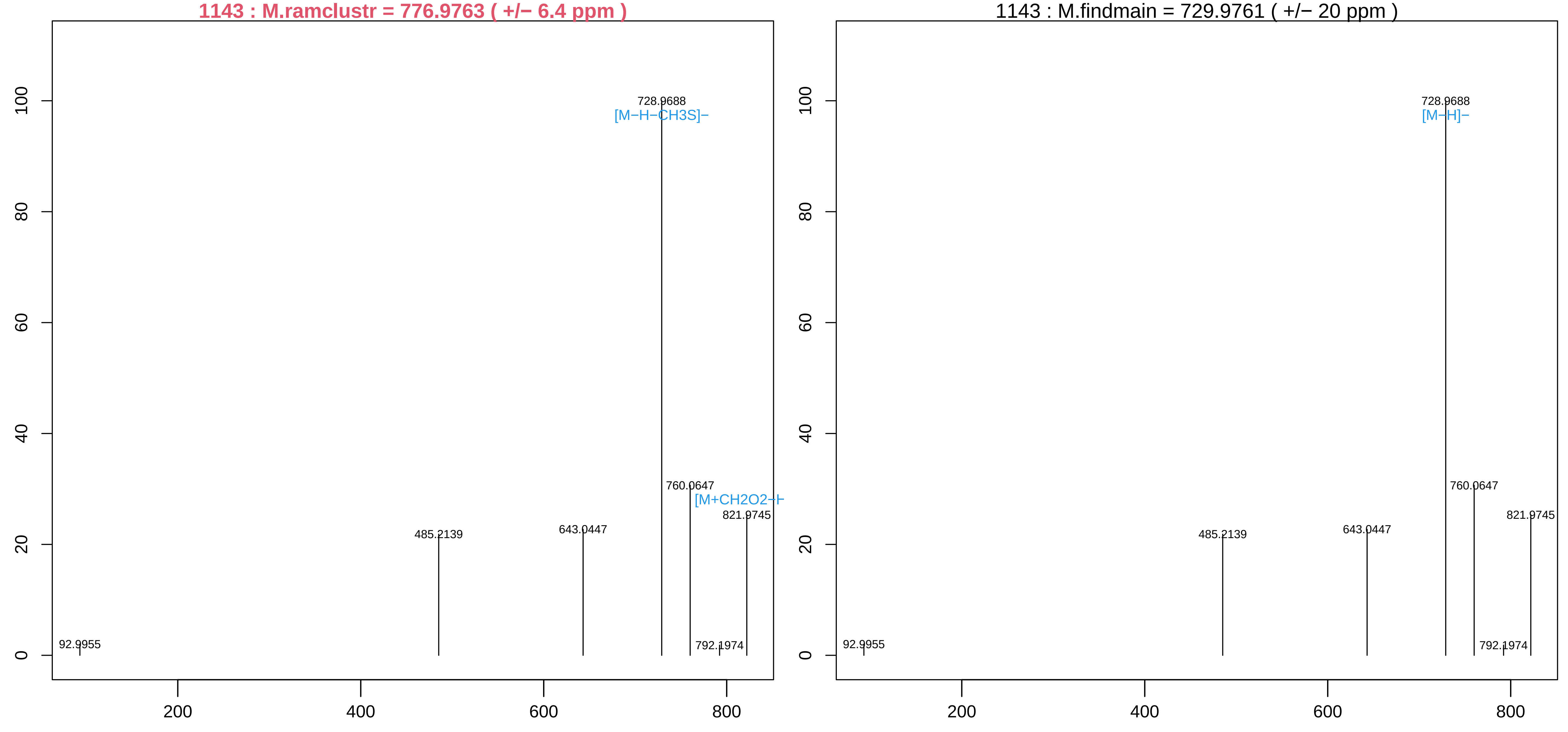
1143 : M.ramclustr = 776.9763 (+/− 6.4 ppm)
728.9688
[M−H−CH3S]−
760.0647
[M+CH2O2−H
821.9745
643.0447
485.2139
92.9955
792.1974
1143 : M.findmain = 729.9761 (+/− 20 ppm)
728.9688
[M−H]−
760.0647
821.9745
643.0447
485.2139
92.9955
792.1974
0
20
40
60
80
100
200
400
600
800

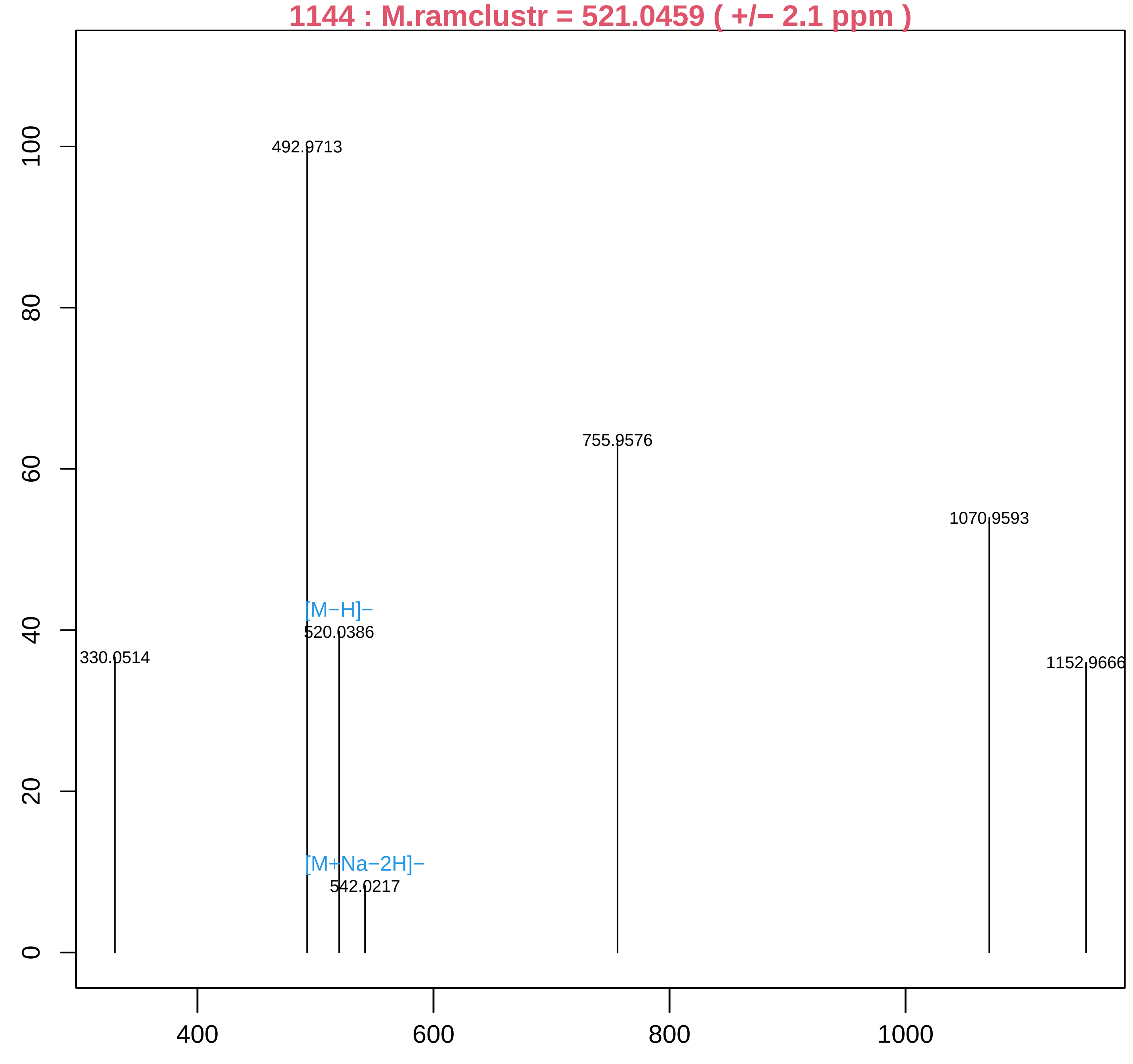

1144 : M.ramclustr = 521.0459 (+/− 2.1 ppm)
492.9713
755.9576
1070.9593
[M−H]−
520.0386
330.0514
1152.9666
[M+Na−2H]−
542.0217
0
20
40
60
80
100
400
600
800
1000

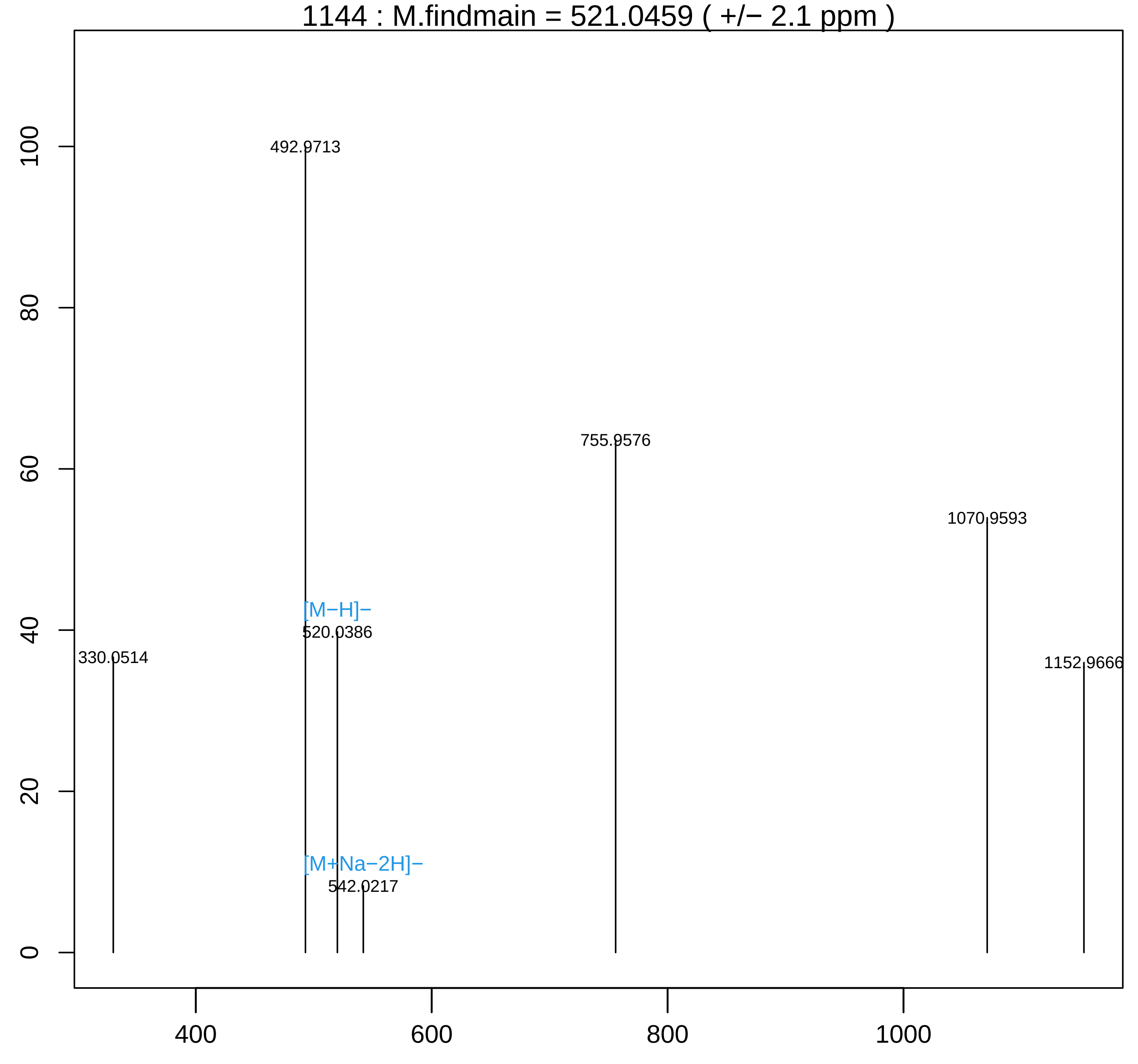

1144 : M.findmain = 521.0459 (+/− 2.1 ppm)
492.9713
755.9576
1070.9593
[M−H]−
520.0386
330.0514
1152.9666
[M+Na−2H]−
542.0217
0
20
40
60
80
100
400
600
800
1000

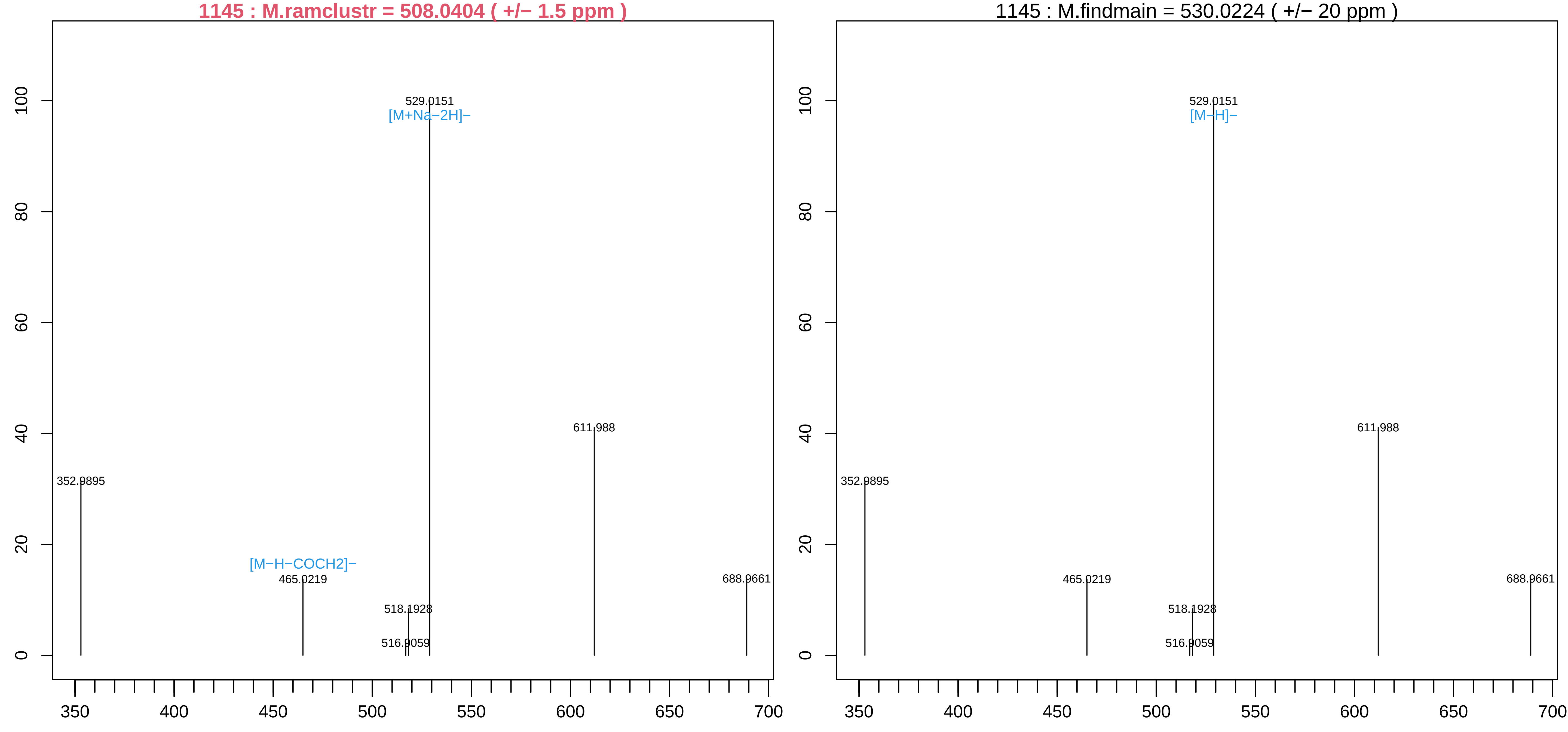

1145 : M.ramclustr = 508.0404 (+/− 1.5 ppm)
529.0151
[M+Na−2H]−
611.988
352.9895
[M−H−COCH2]−
465.0219
688.9661
518.1928
516.9059
1145 : M.findmain = 530.0224 (+/− 20 ppm)
529.0151
[M−H]−
611.988
352.9895
465.0219
688.9661
518.1928
516.9059
0
20
40
60
80
100
350
400
450
500
550
600
650
700

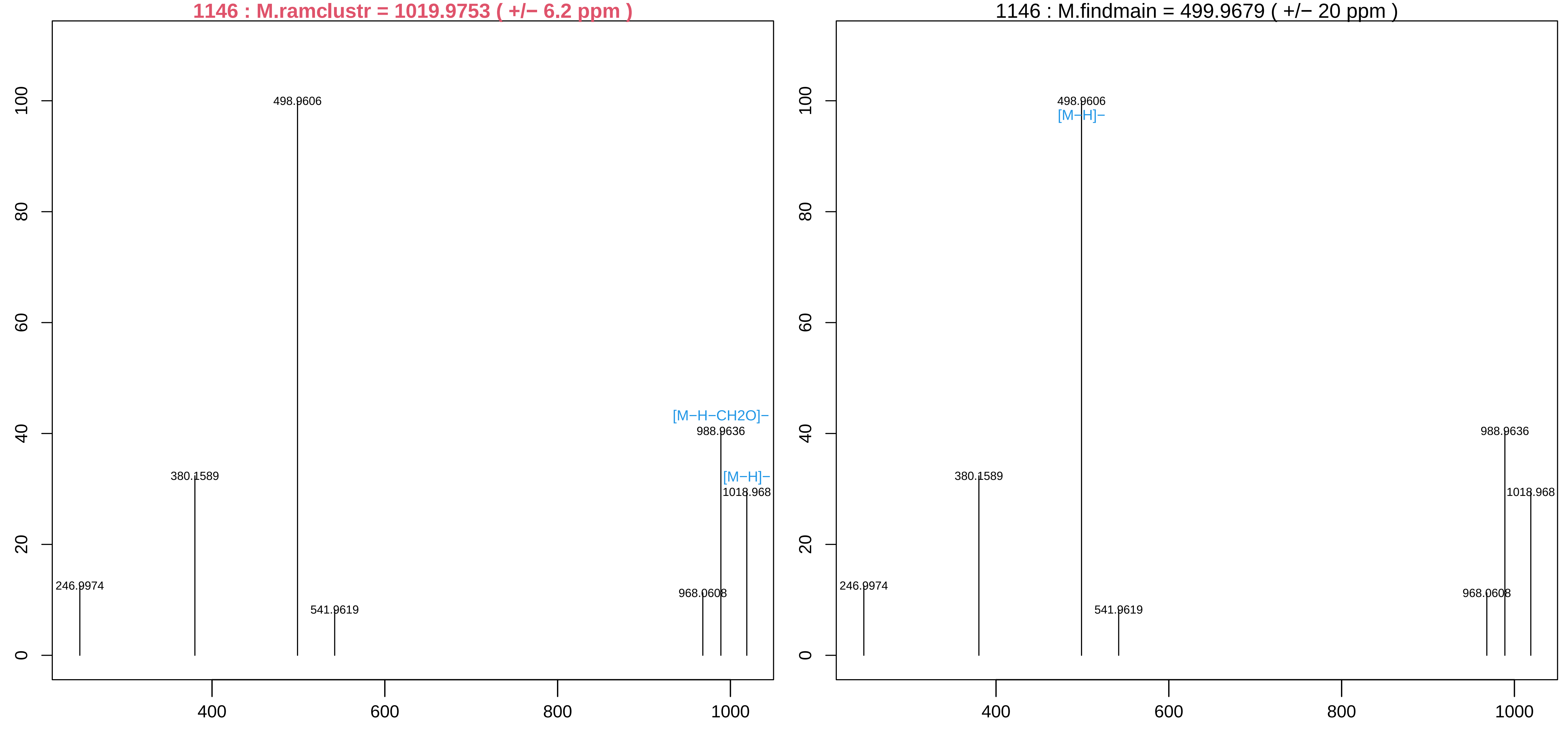
1146 : M.ramclustr = 1019.9753 (+/− 6.2 ppm)
1146 : M.findmain = 499.9679 (+/− 20 ppm)
498.9606
[M−H]−
380.1589
246.9974
541.9619
968.0608
988.9636
1018.968
[M−H−CH2O]−
0
20
40
60
80
100
400
600
800
1000

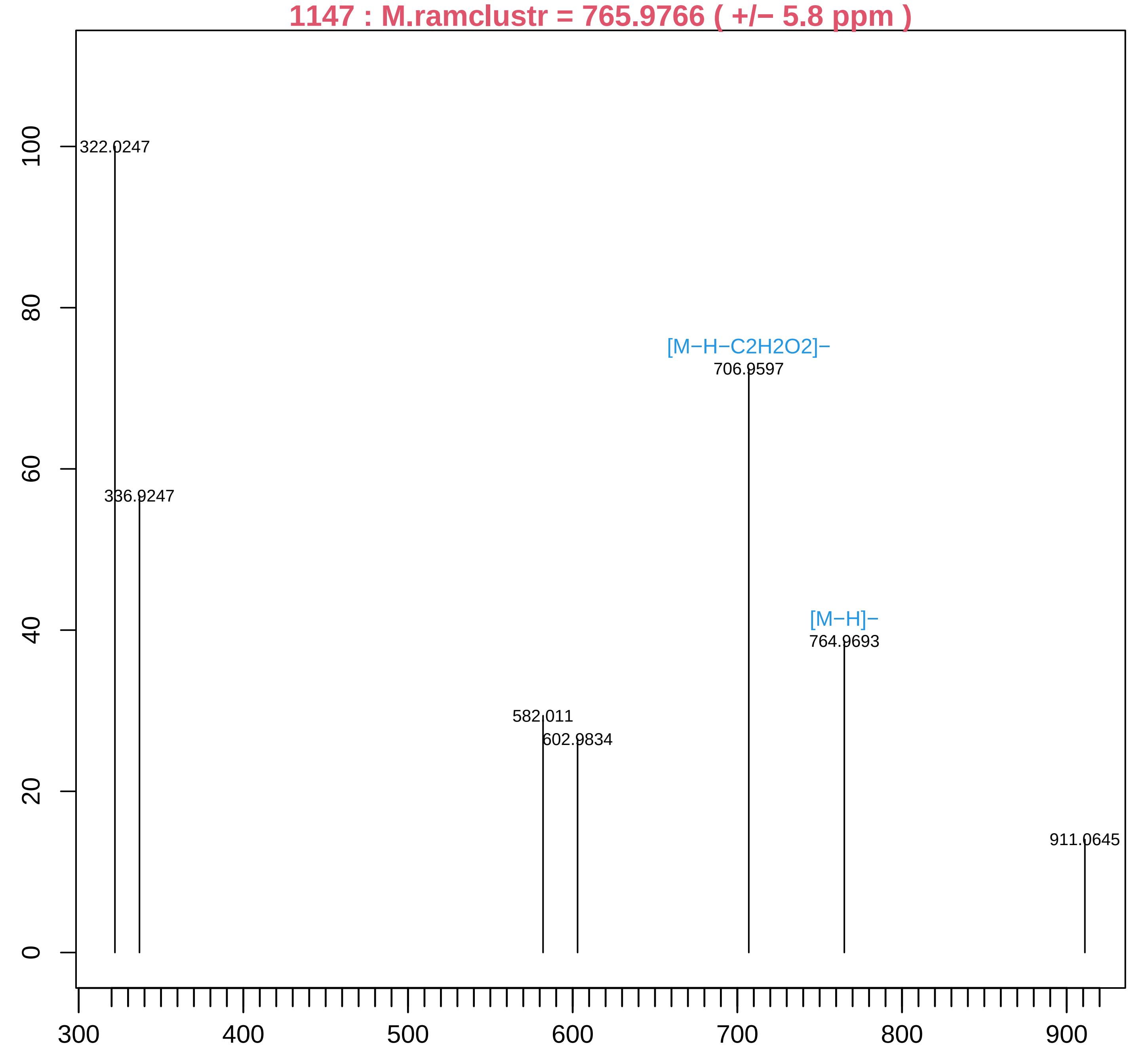
1147 : M.ramclustr = 765.9766 (+/− 5.8 ppm)
322.0247
336.9247
[M−H−C2H2O2]−
706.9597
[M−H]−
764.9693
582.011
602.9834
911.0645
0
20
40
60
80
100
300
400
500
600
700
800
900

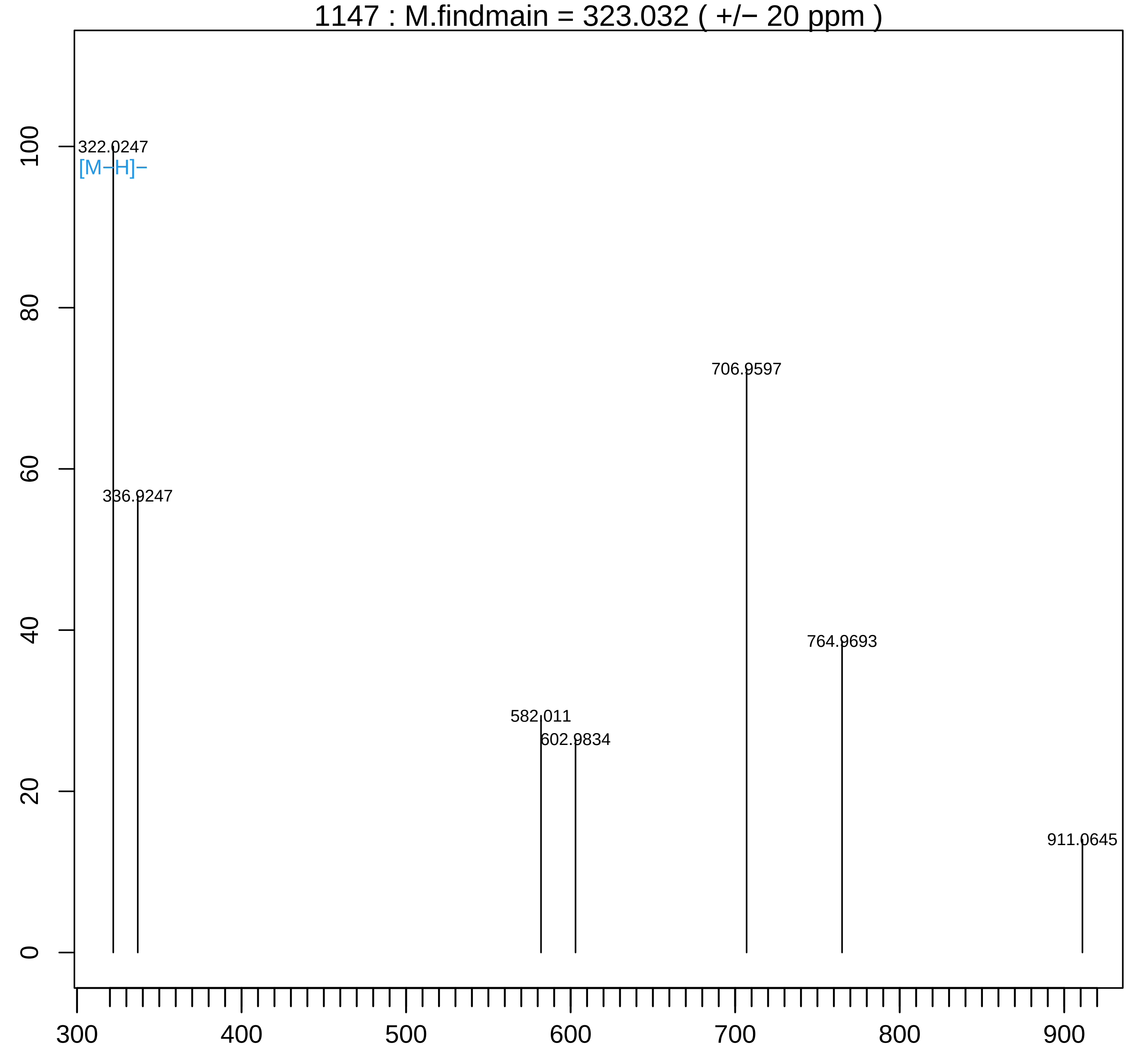
1147 : M.findmain = 323.032 (+/− 20 ppm)
322.0247
[M−H]−
336.9247
706.9597
764.9693
582.011
602.9834
911.0645
0
20
40
60
80
100
300
400
500
600
700
800
900

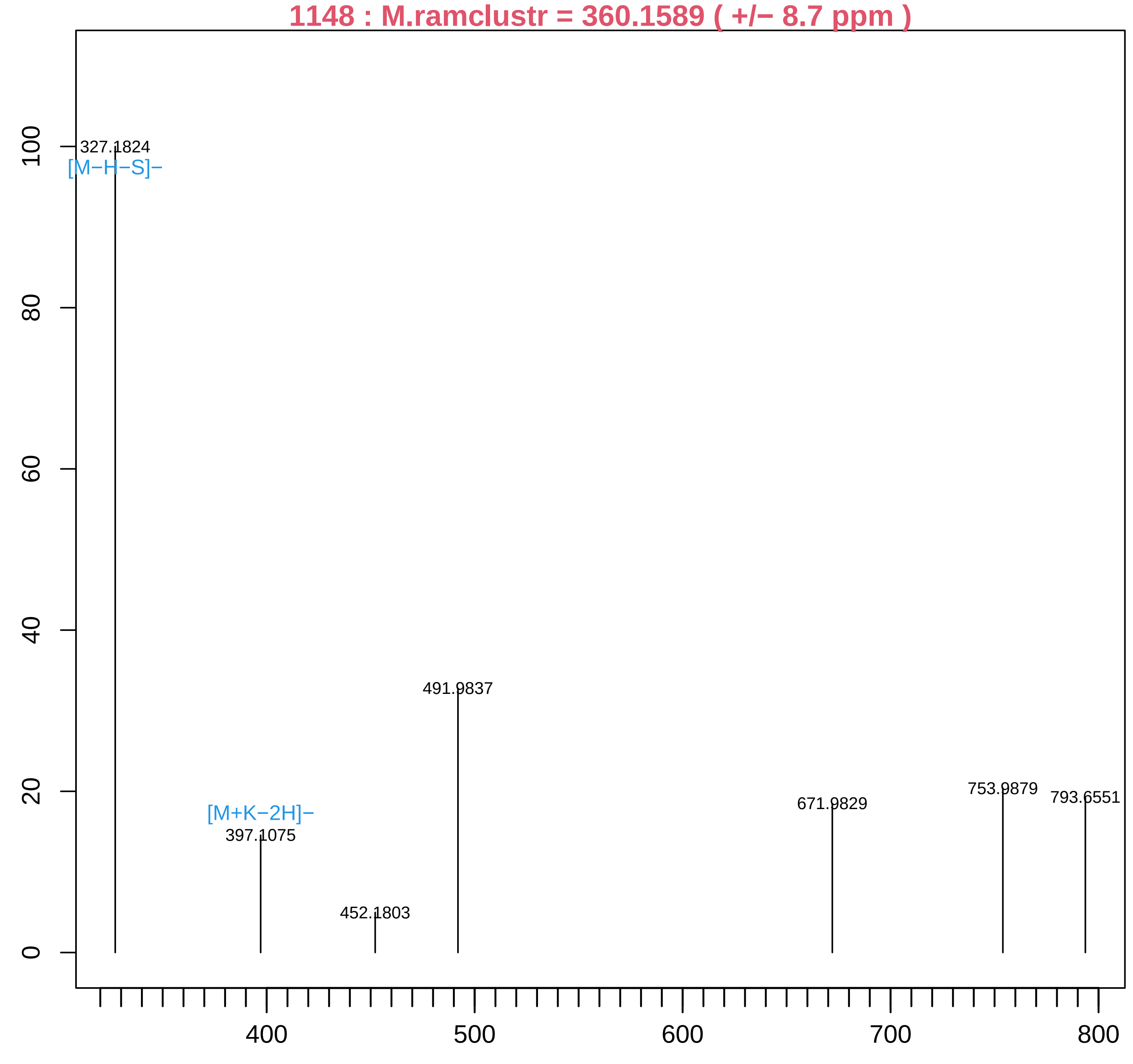
1148 : M.ramclustr = 360.1589 (+/− 8.7 ppm)
327.1824
[M−H−S]−
[M+K−2H]−
397.1075
452.1803
491.9837
671.9829
753.9879
793.6551
0
20
40
60
80
100
400
500
600
700
800

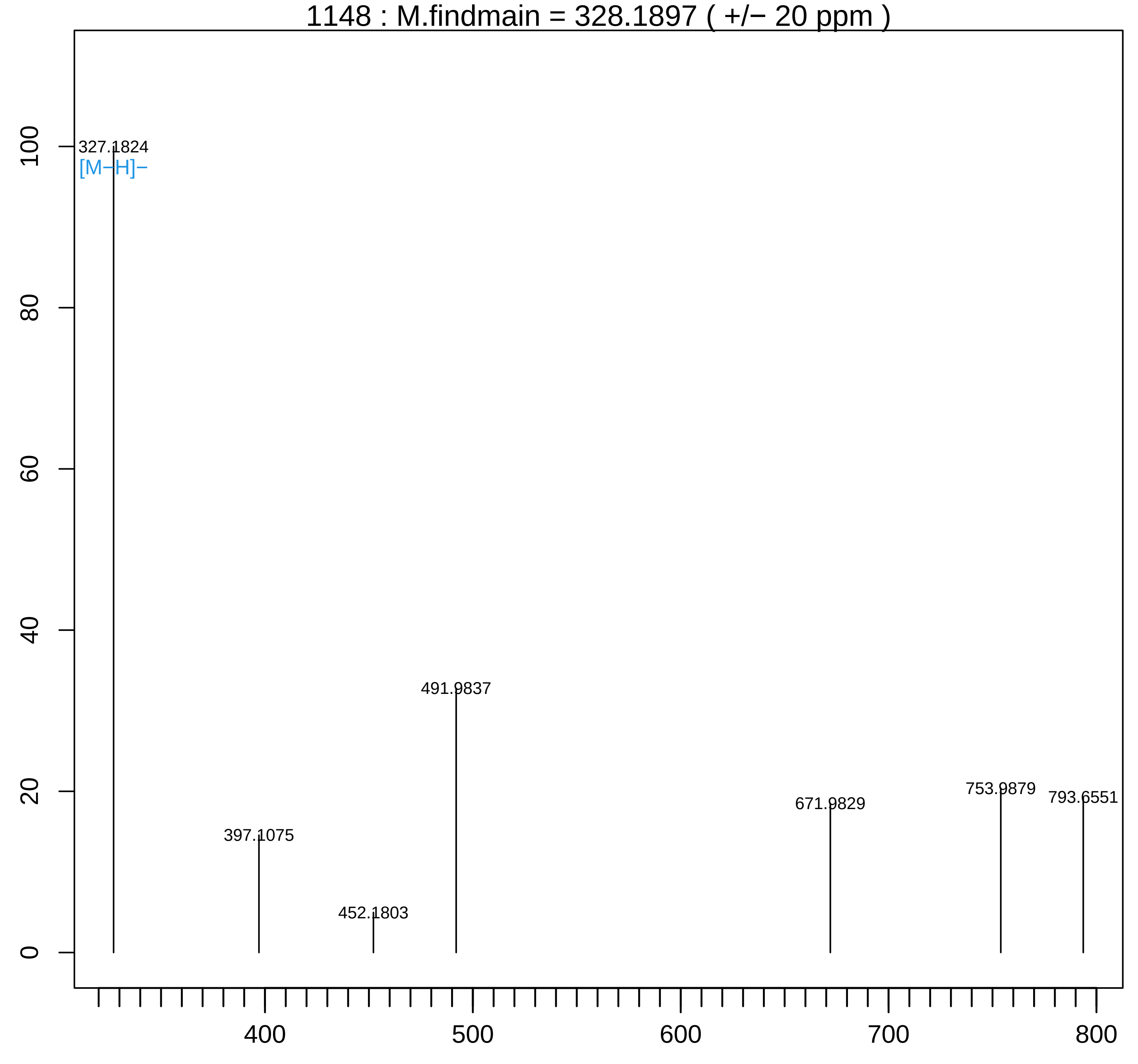
1148 : M.findmain = 328.1897 (+/− 20 ppm)
327.1824
[M−H]−
397.1075
452.1803
491.9837
671.9829
753.9879
793.6551
0
20
40
60
80
100
400
500
600
700
800

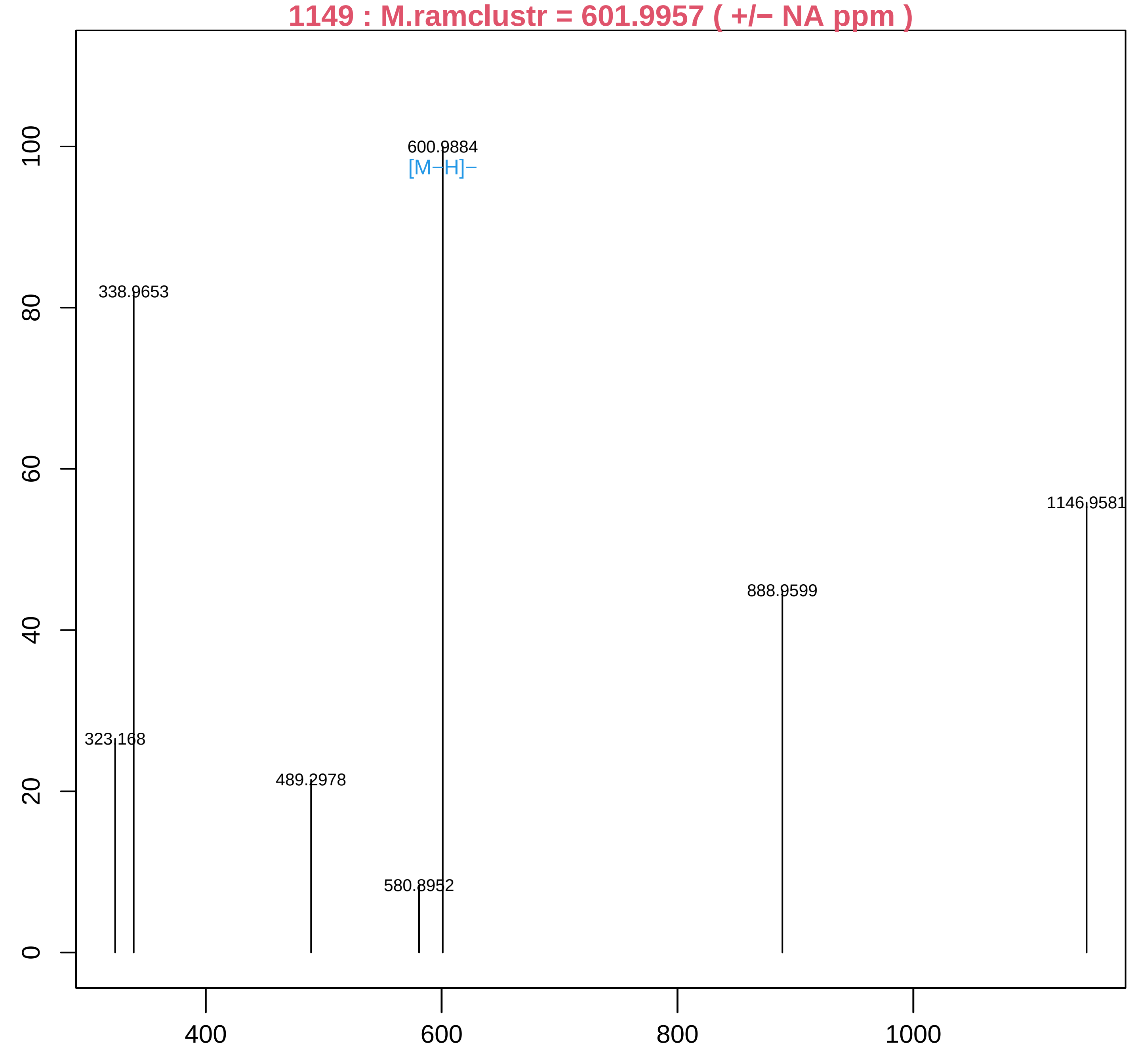
1149 : M.ramclustr = 601.9957 (+/− NA ppm)
600.9884
[M−H]−
338.9653
1146.9581
888.9599
323.168
489.2978
580.8952
0
20
40
60
80
100
400
600
800
1000

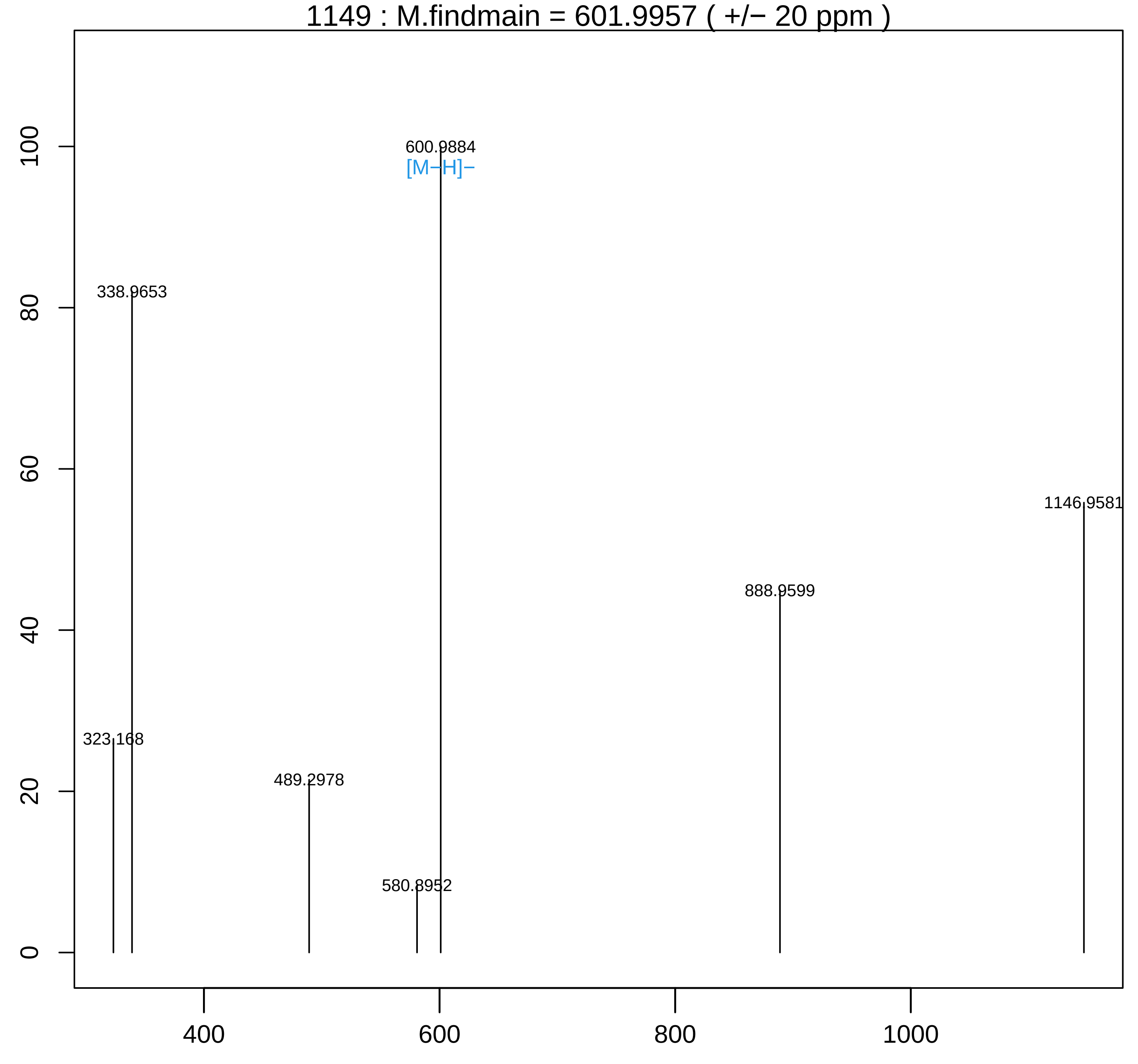
1149 : M.findmain = 601.9957 (+/− 20 ppm)
600.9884
[M−H]−
338.9653
1146.9581
888.9599
323.168
489.2978
580.8952
0
20
40
60
80
100
400
600
800
1000

1150 : M.ramclustr = 507.9786 (+/− 9.4 ppm)

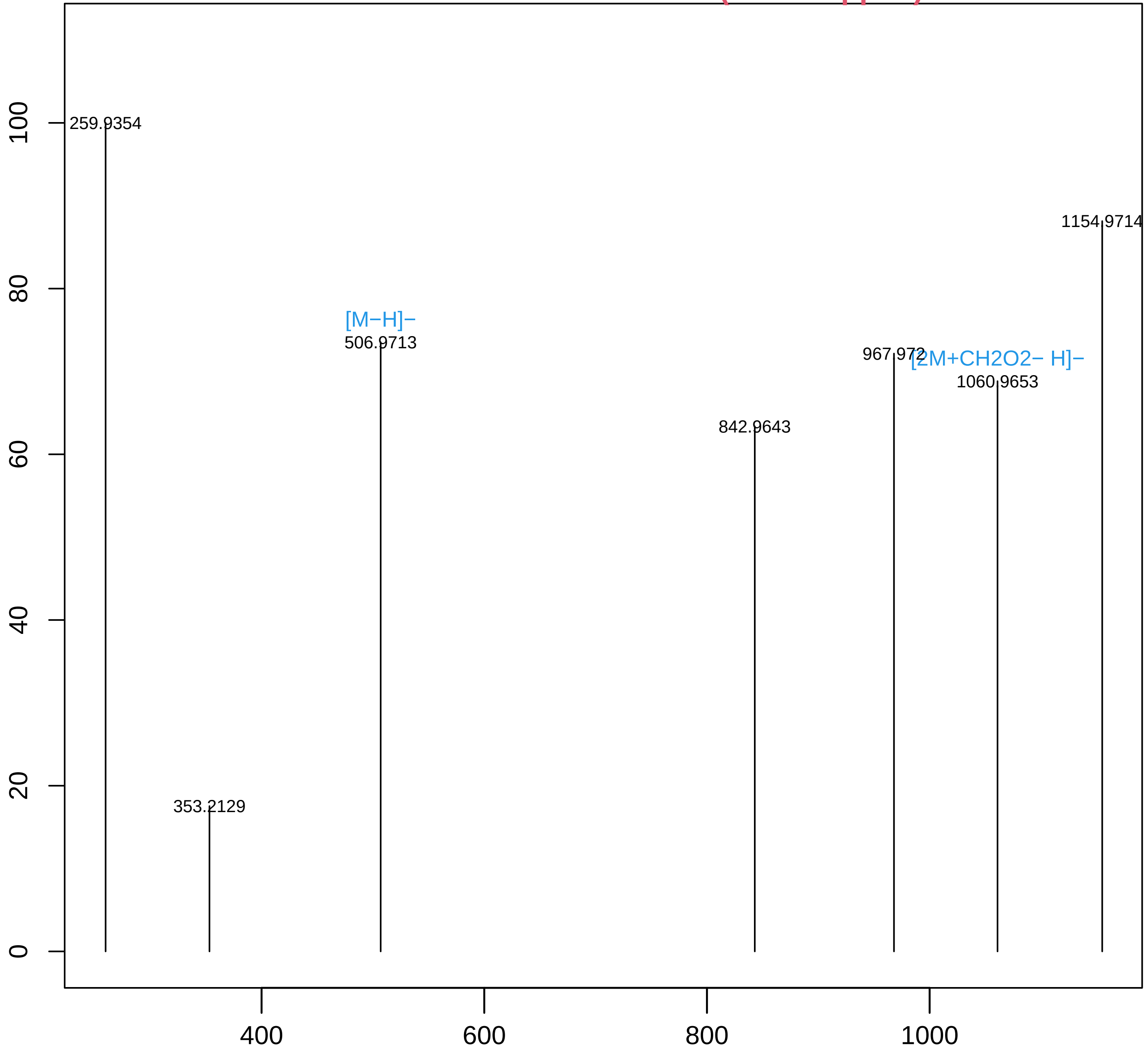

1150 : M.findmain = 507.9786 (+/− 9.4 ppm)

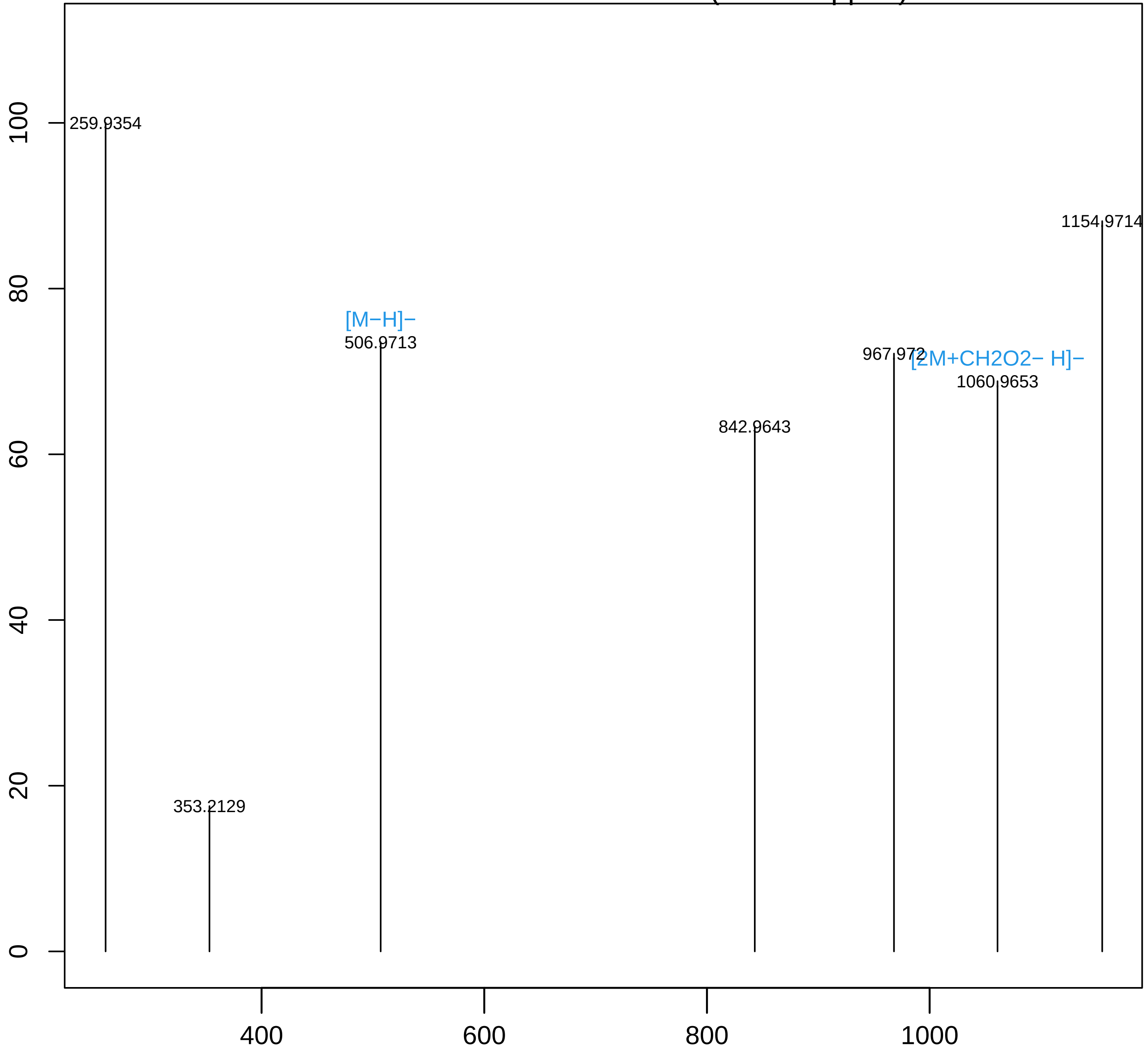

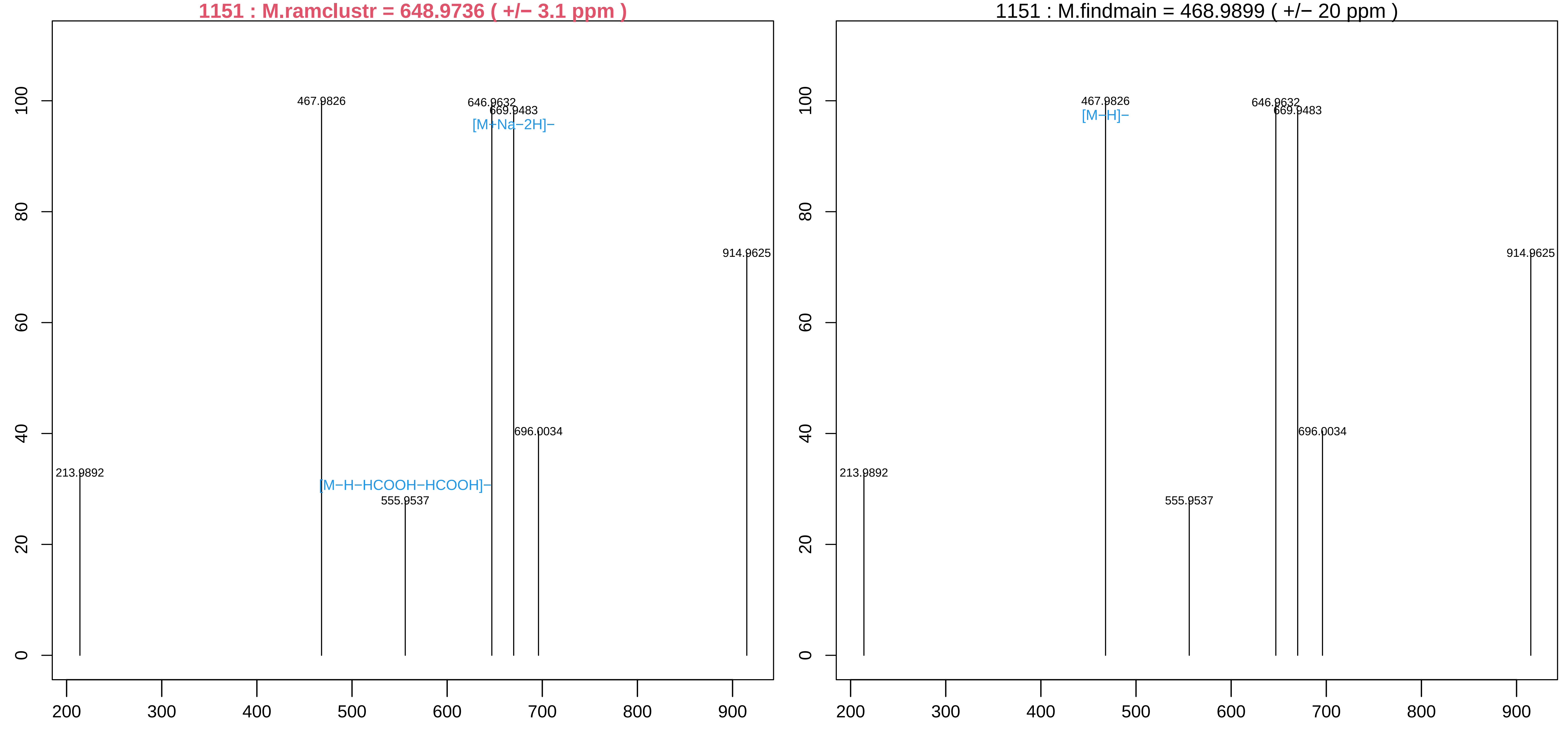

1151 : M.ramclustr = 648.9736 (+/− 3.1 ppm)
467.9826
646.9632
669.9483
[M+Na−2H]−
914.9625
696.0034
213.9892
[M−H−HCOOH−HCOOH]−
555.9537
0
20
40
60
80
100
200
300
400
500
600
700
800
900
1151 : M.findmain = 468.9899 (+/− 20 ppm)
467.9826
[M−H]−
646.9632
669.9483
914.9625
696.0034
213.9892
555.9537
0
20
40
60
80
100
200
300
400
500
600
700
800
900

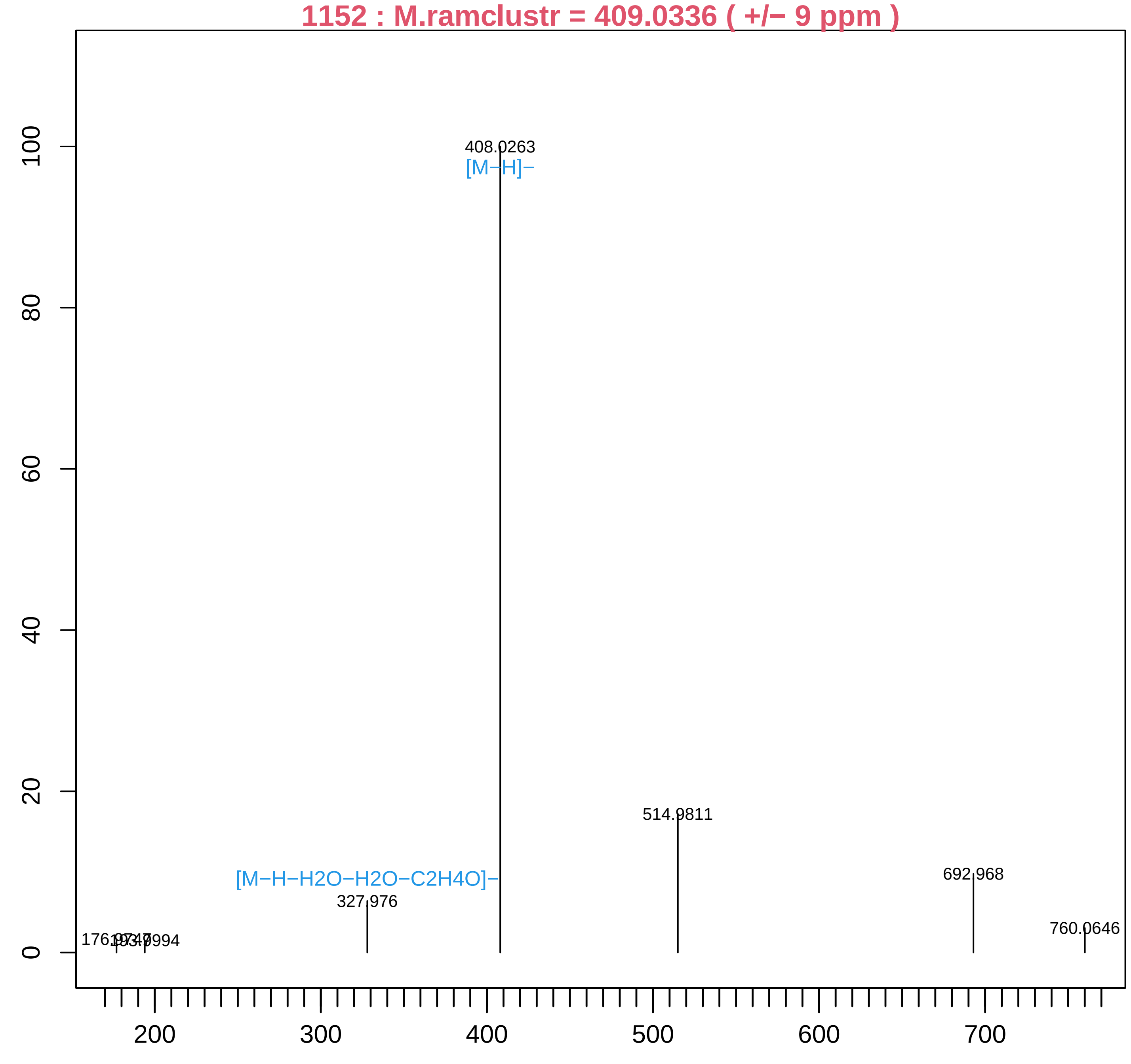
1152 : M.ramclustr = 409.0336 (+/− 9 ppm)
408.0263
[M−H]−
[M−H−H2O−H2O−C2H4O]−
327.976
514.9811
692.968
760.0646
0
20
40
60
80
100
200
300
400
500
600
700
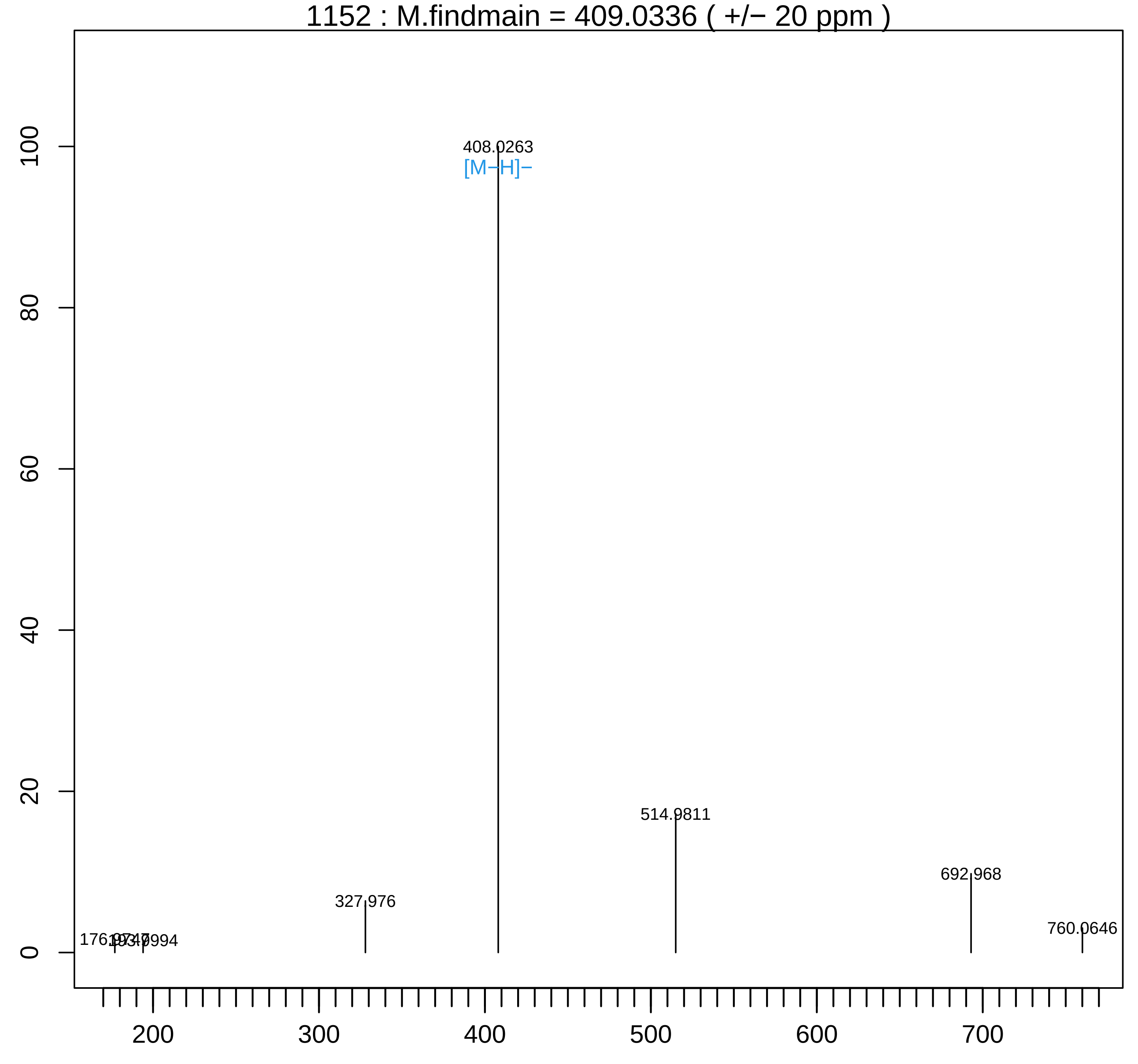
1152 : M.findmain = 409.0336 (+/− 20 ppm)
408.0263
[M−H]−
327.976
514.9811
692.968
760.0646
0
20
40
60
80
100
200
300
400
500
600
700

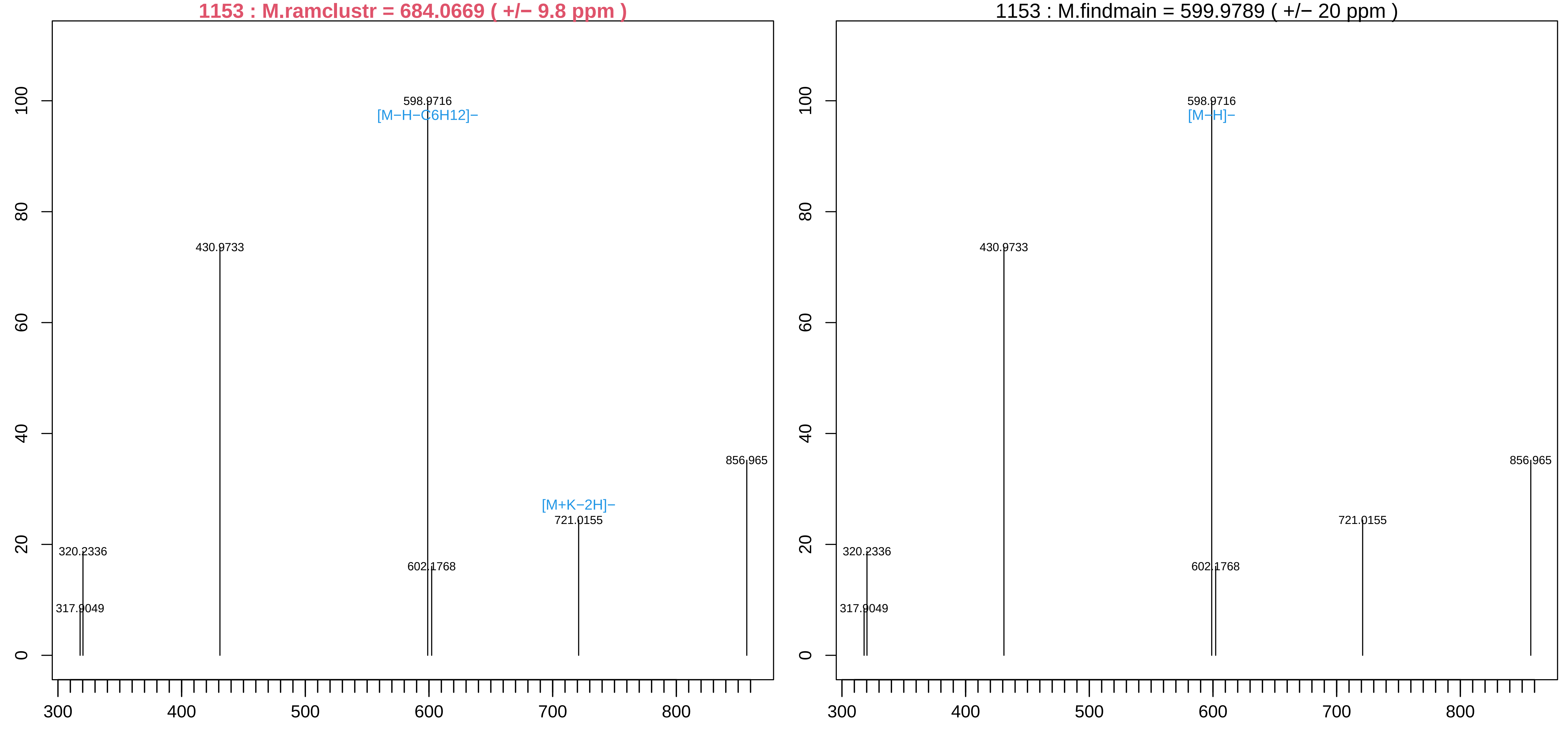
1153 : M.ramclustr = 684.0669 (+/− 9.8 ppm)
598.9716
[M−H−C6H12]−
430.9733
856.965
[M+K−2H]−
721.0155
320.2336
602.1768
317.9049
1153 : M.findmain = 599.9789 (+/− 20 ppm)
598.9716
[M−H]−
430.9733
856.965
721.0155
320.2336
602.1768
317.9049
0
20
40
60
80
100
300
400
500
600
700
800

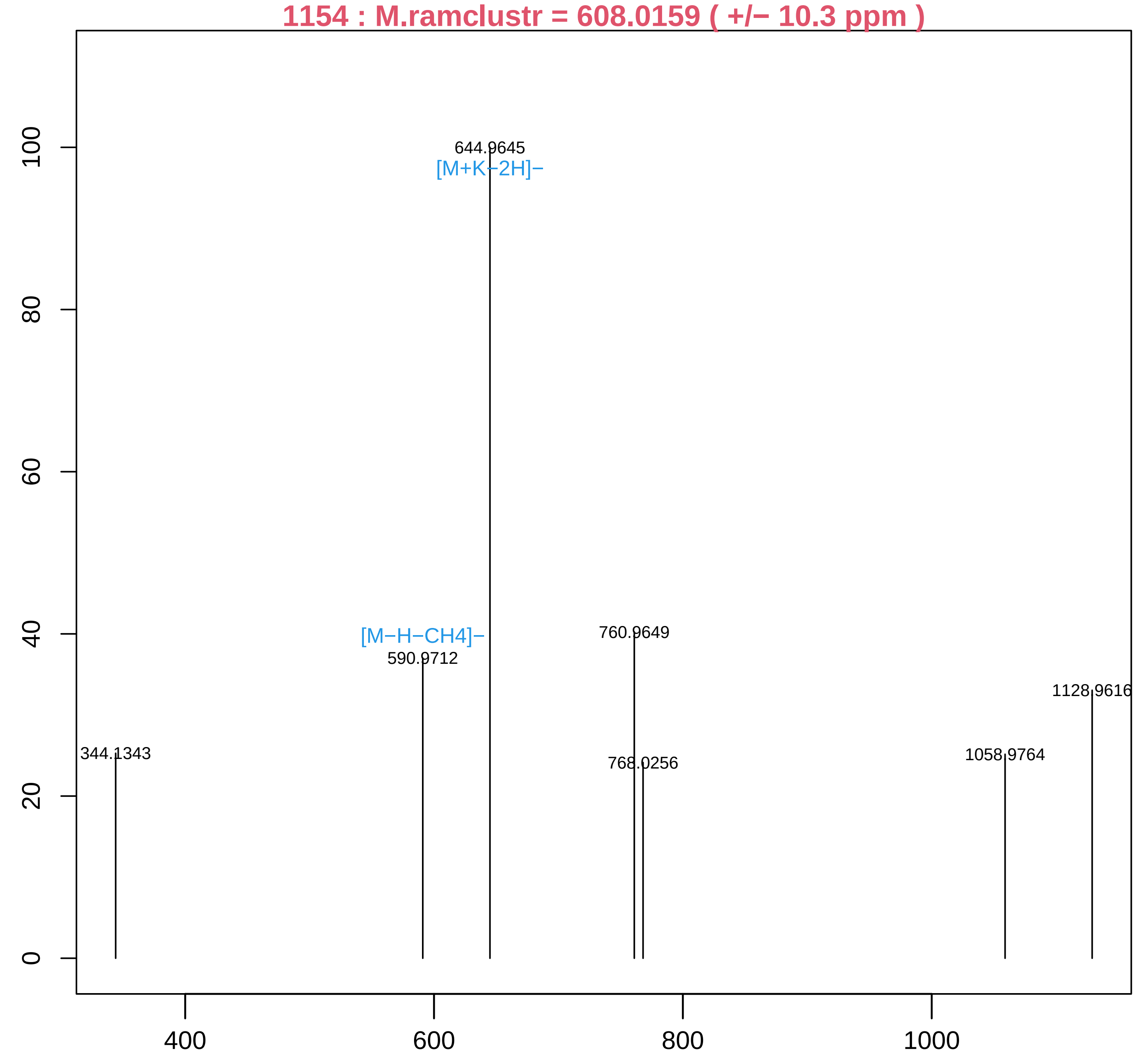
1154 : M.ramclustr = 608.0159 (+/− 10.3 ppm)
644.9645
[M+K−2H]−
[M−H−CH4]−
590.9712
760.9649
1128.9616
344.1343
768.0256
1058.9764
0
20
40
60
80
100
400
600
800
1000

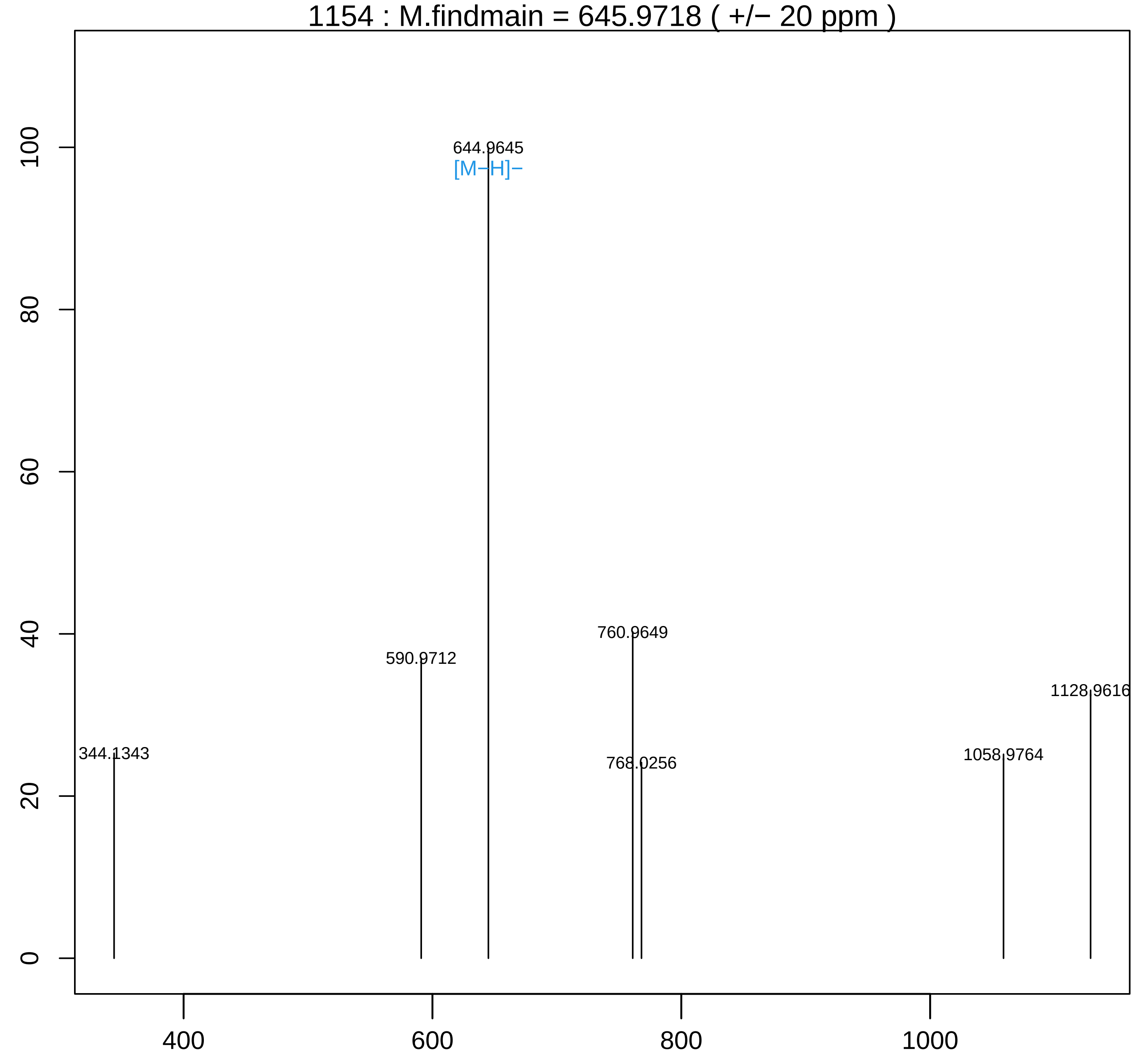
1154 : M.findmain = 645.9718 (+/− 20 ppm)
644.9645
[M−H]−
590.9712
760.9649
1128.9616
344.1343
768.0256
1058.9764
0
20
40
60
80
100
400
600
800
1000

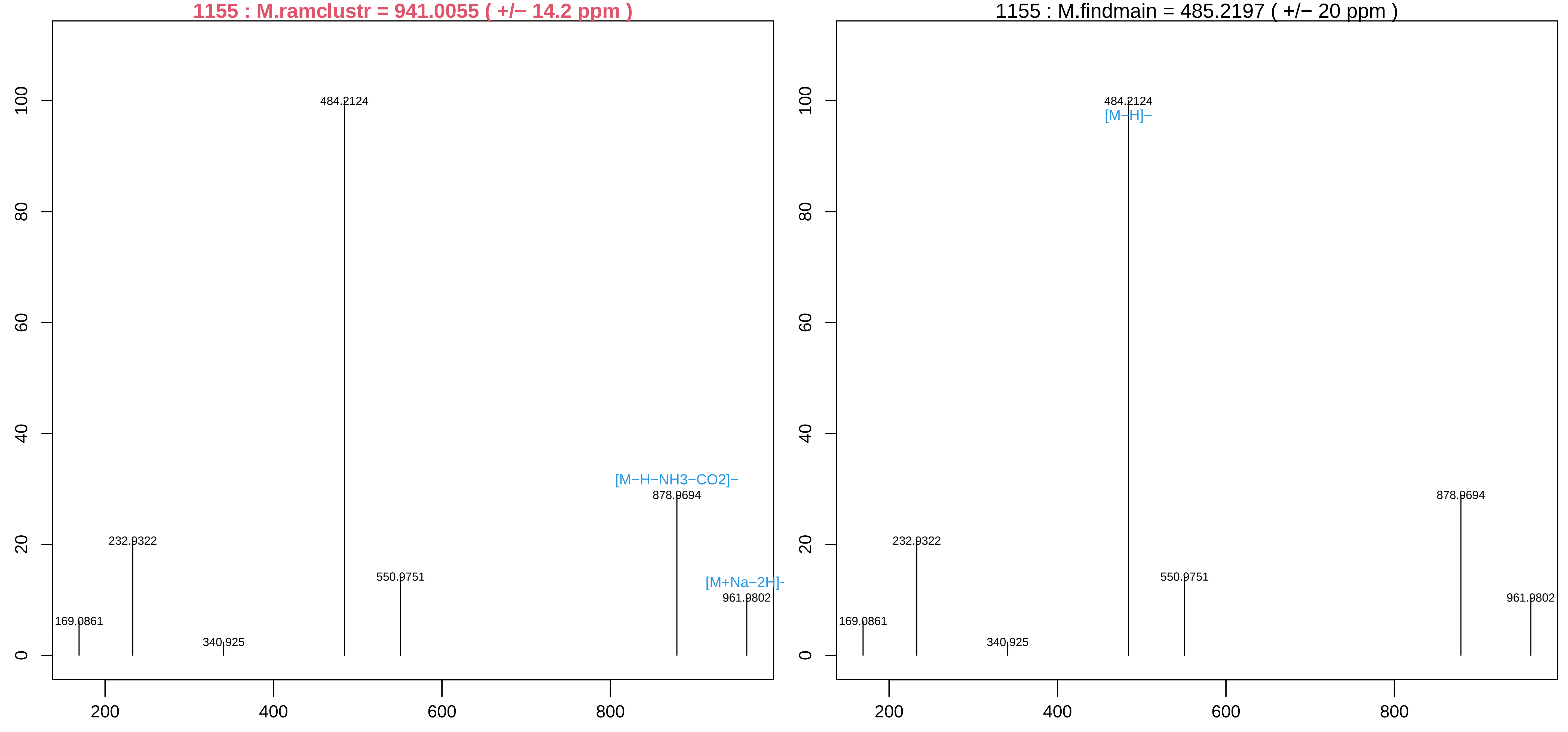
1155 : M.ramclustr = 941.0055 (+/− 14.2 ppm)
484.2124
[M−H−NH3−CO2]−
878.9694
232.9322
550.9751
[M+Na−2H]-
961.9802
169.0861
340.925
0
20
40
60
80
100
200
400
600
800
1155 : M.findmain = 485.2197 (+/− 20 ppm)
484.2124
[M−H]−
878.9694
232.9322
550.9751
961.9802
169.0861
340.925

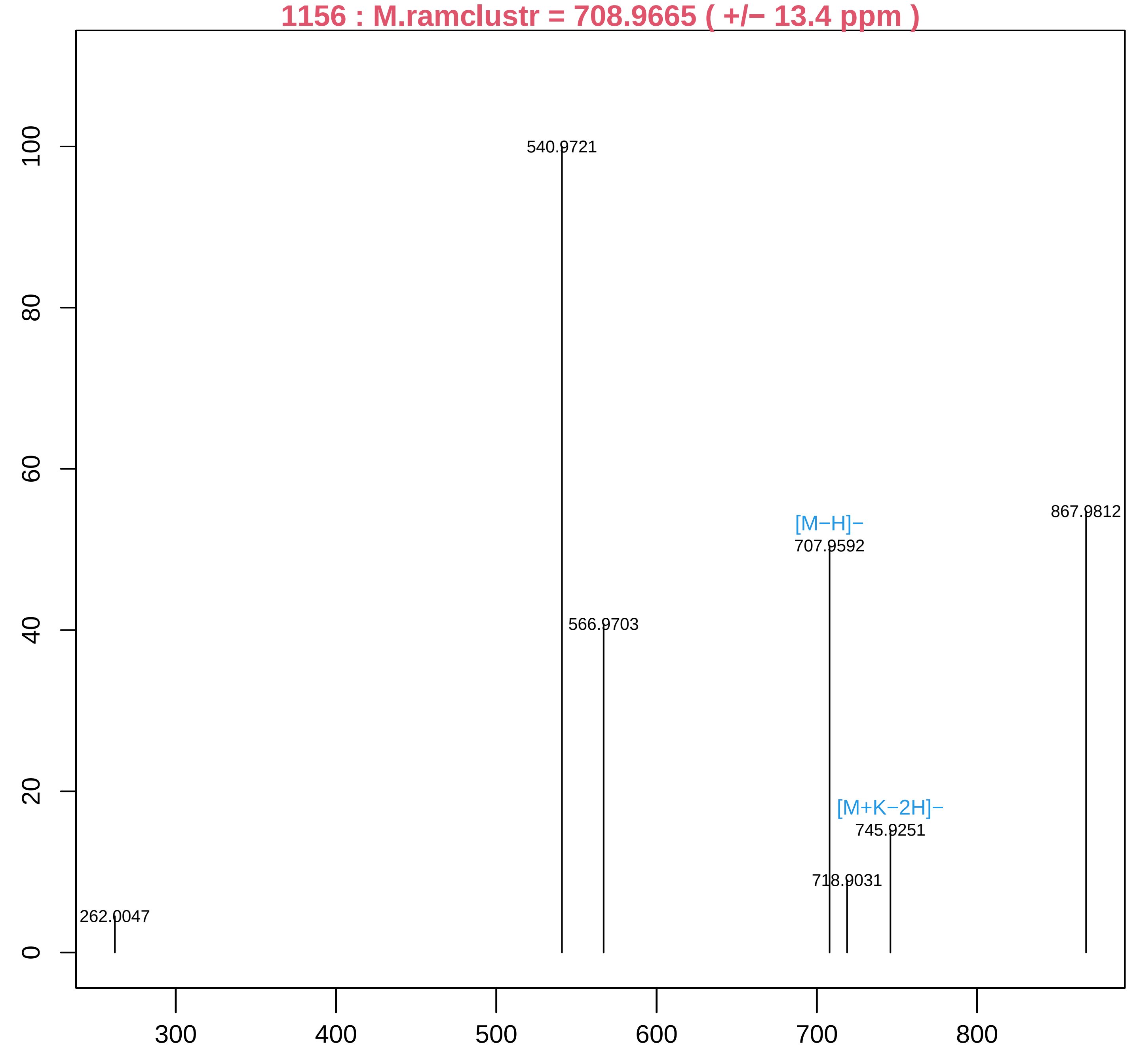
1156 : M.ramclustr = 708.9665 (+/− 13.4 ppm)
540.9721
566.9703
[M−H]−
707.9592
[M+K−2H]−
745.9251
718.9031
867.9812
262.0047
0
20
40
60
80
100
300
400
500
600
700
800

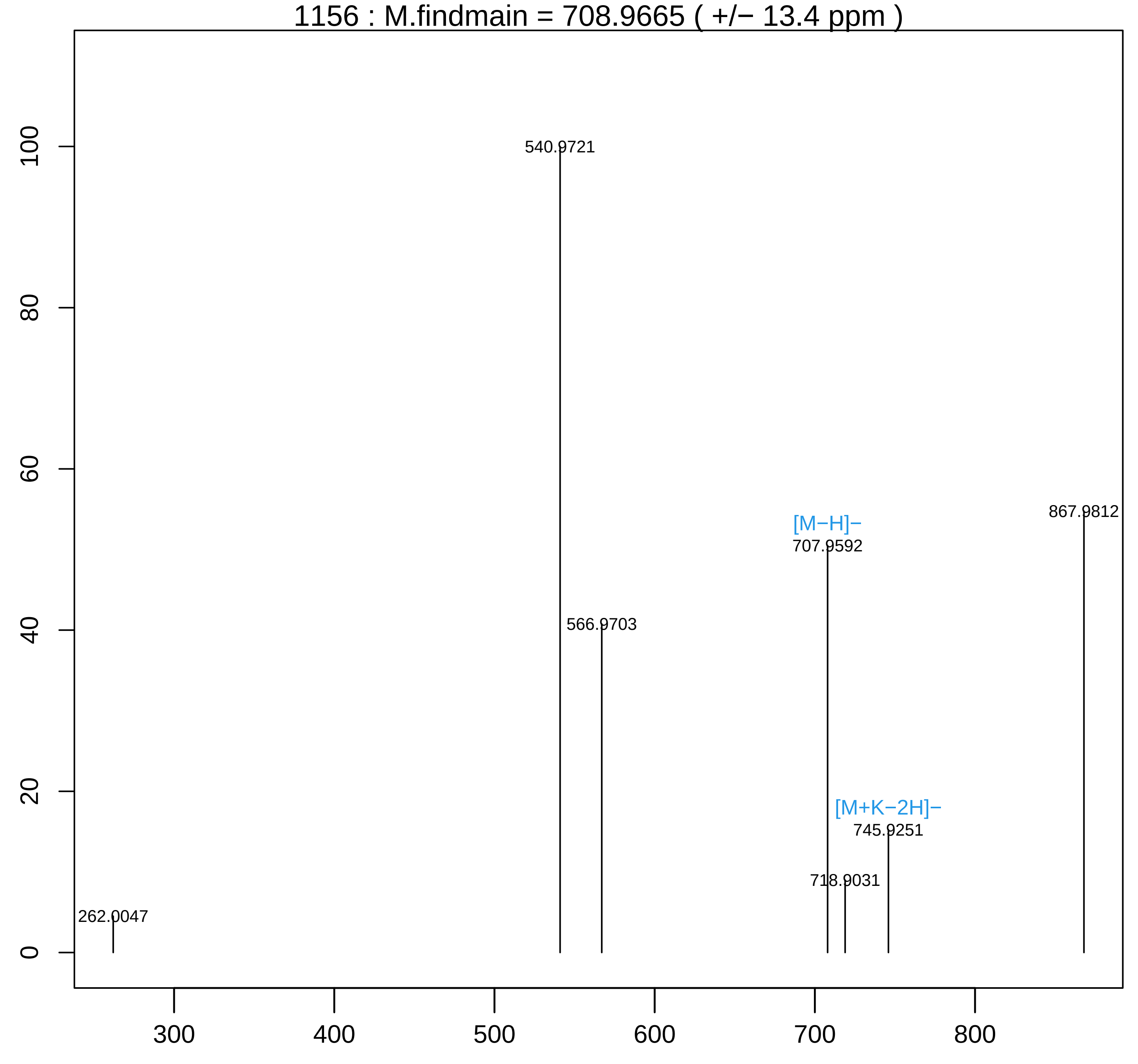
1156 : M.findmain = 708.9665 (+/− 13.4 ppm)
540.9721
566.9703
[M−H]−
707.9592
[M+K−2H]−
745.9251
718.9031
867.9812
262.0047
0
20
40
60
80
100
300
400
500
600
700
800

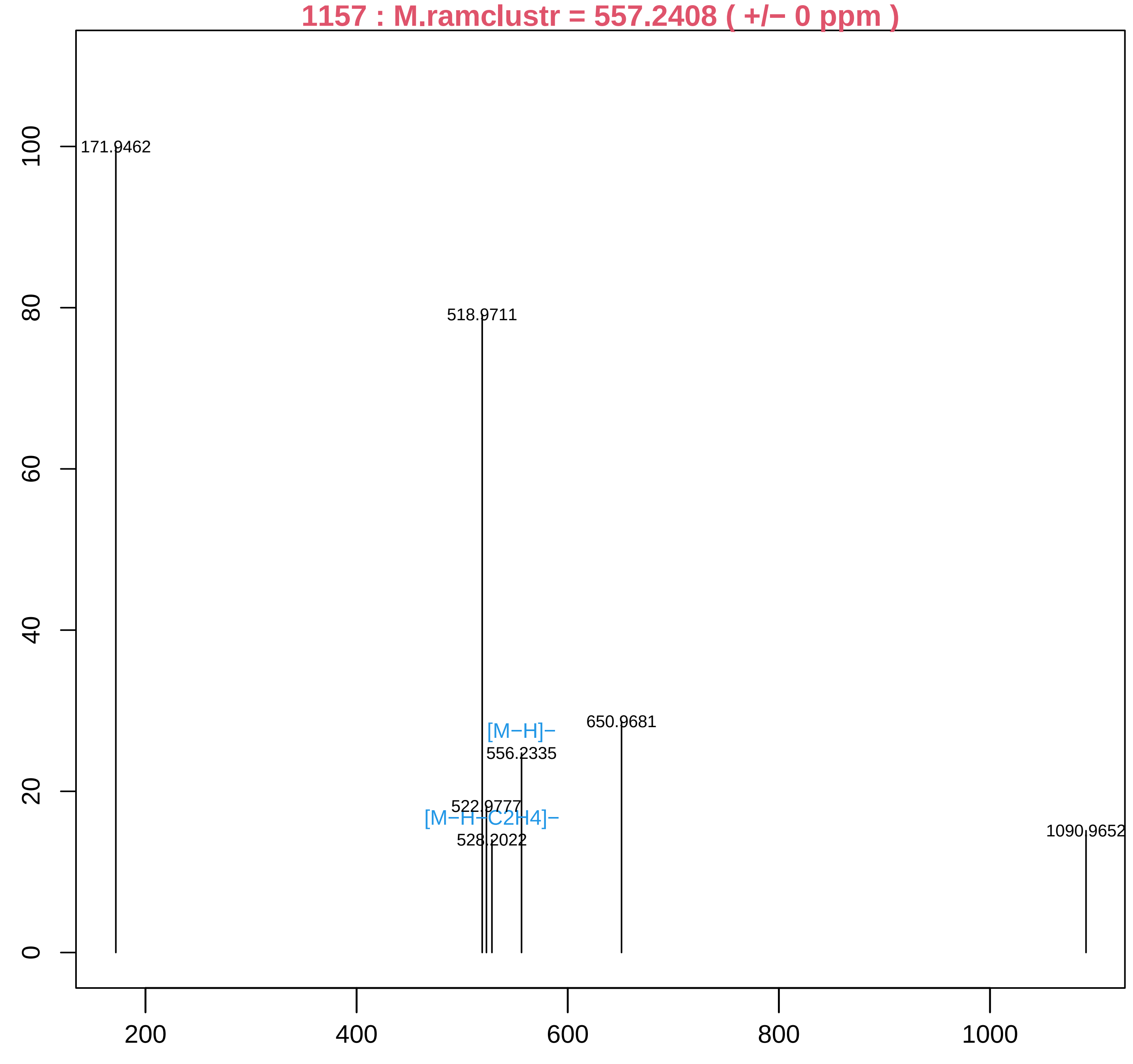
1157 : M.ramclustr = 557.2408 (+/− 0 ppm)
171.9462
518.9711
650.9681
[M−H]−
556.2335
522.9777
[M−H−C2H4]−
528.2022
1090.9652
0
20
40
60
80
100
200
400
600
800
1000

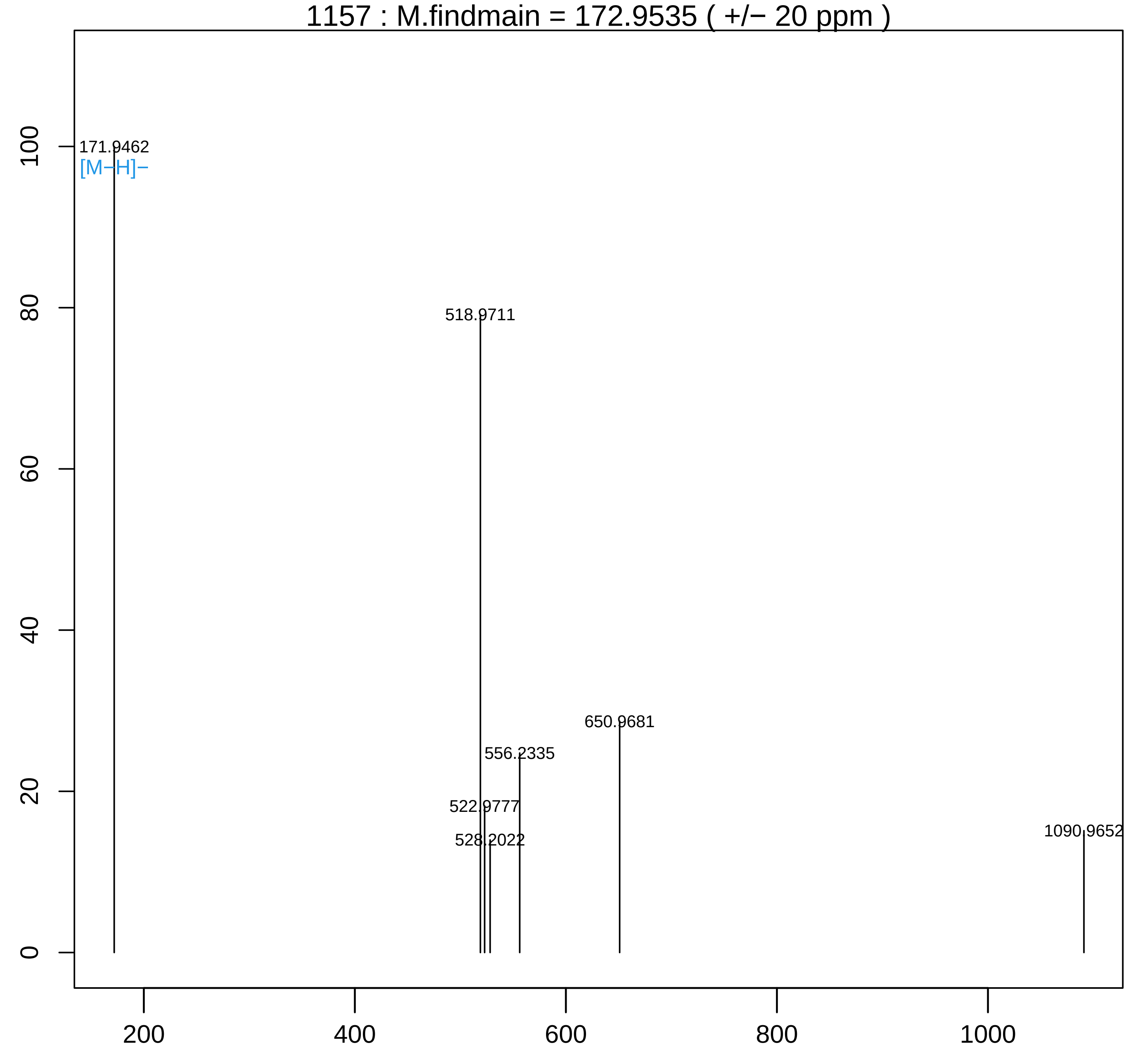
1157 : M.findmain = 172.9535 (+/− 20 ppm)
171.9462
[M−H]−
518.9711
650.9681
556.2335
522.9777
528.2022
1090.9652
0
20
40
60
80
100
200
400
600
800
1000

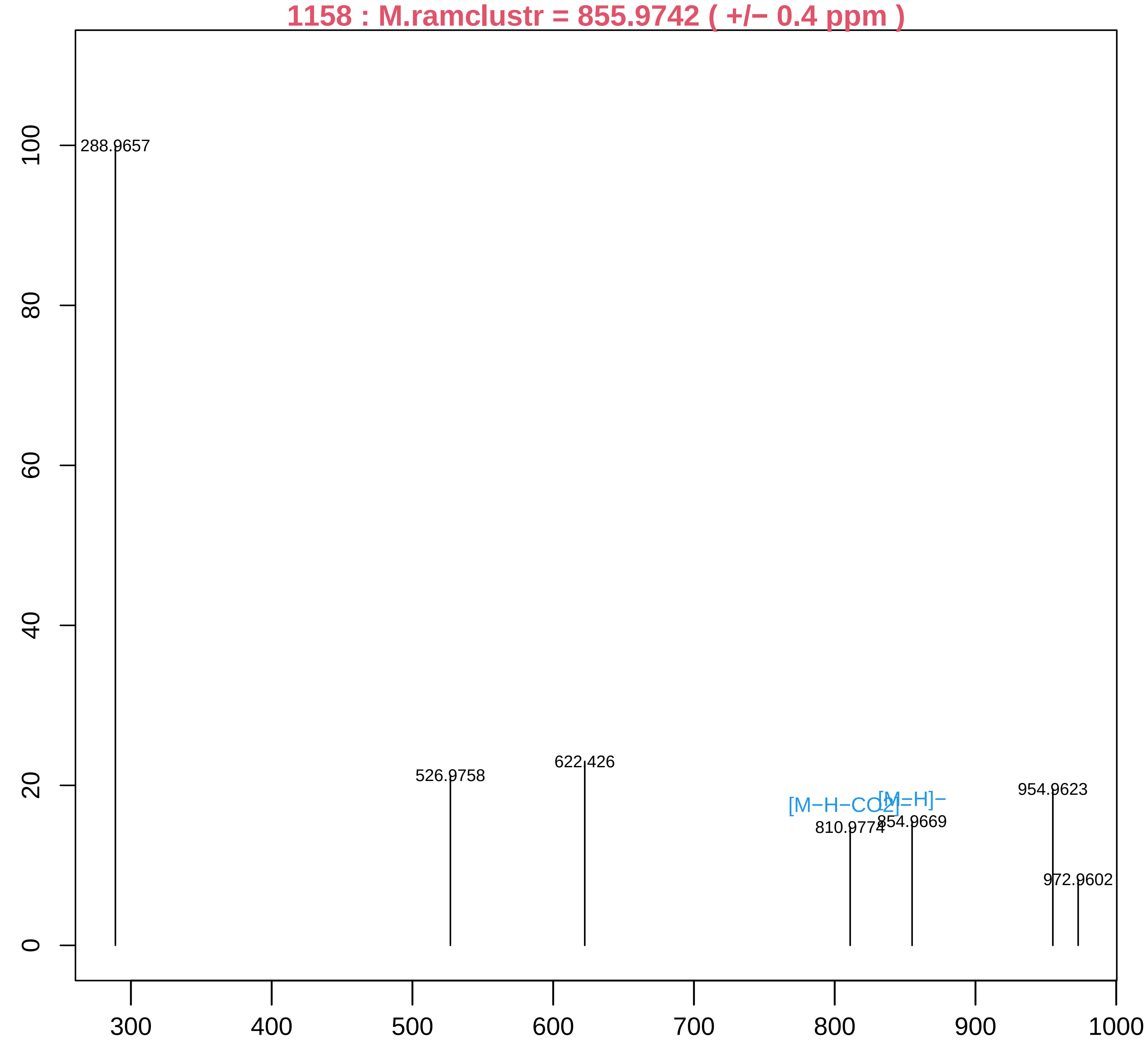
1158 : M.ramclustr = 855.9742 (+/− 0.4 ppm)
288.9657
526.9758
622.426
[M−H−CO2]−
[M−H]−
810.9774
854.9669
954.9623
972.9602
0
20
40
60
80
100
300
400
500
600
700
800
900
1000

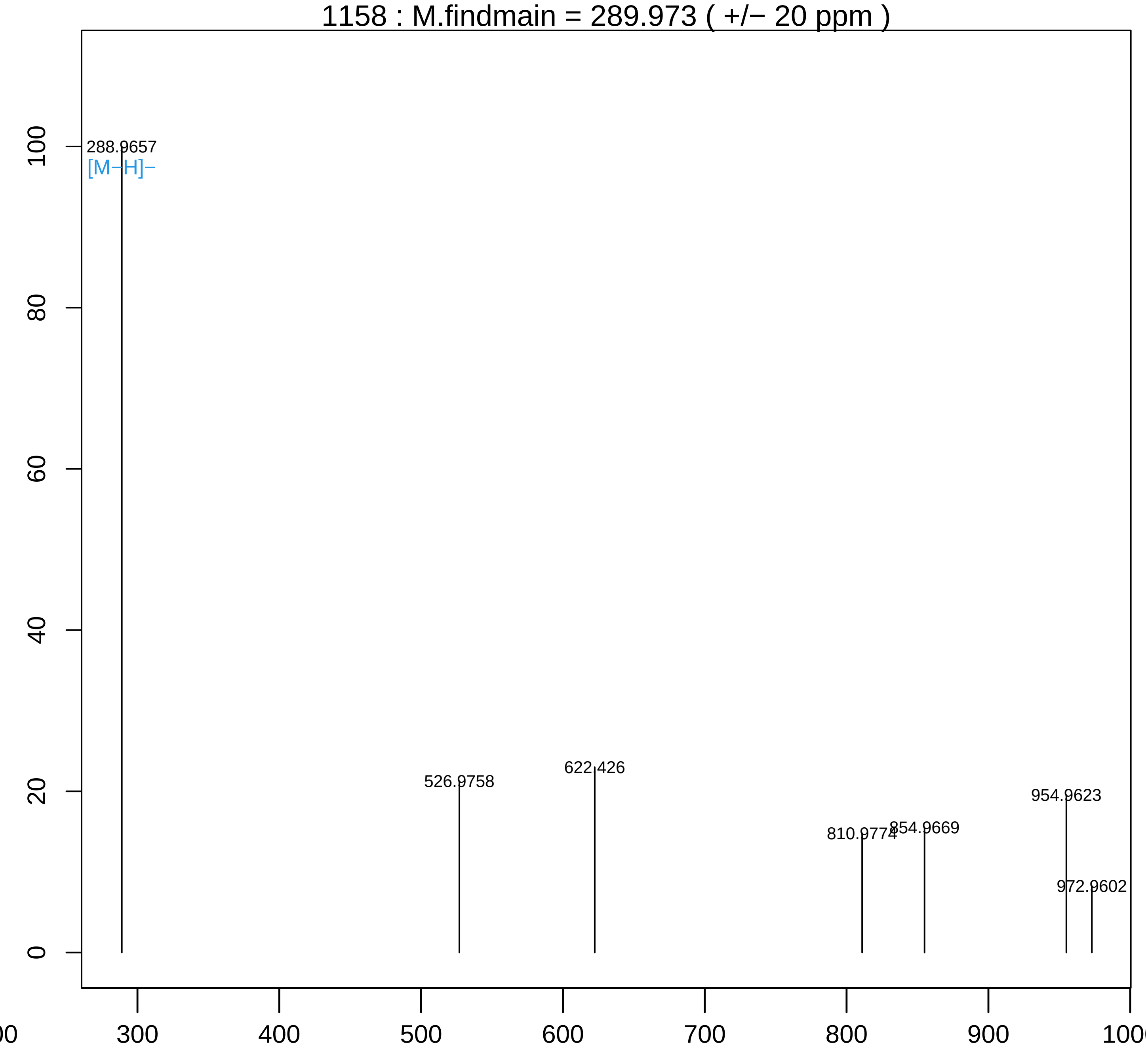
1158 : M.findmain = 289.973 (+/− 20 ppm)
288.9657
[M−H]−
526.9758
622.426
810.9774
854.9669
954.9623
972.9602
0
20
40
60
80
100
300
400
500
600
700
800
900

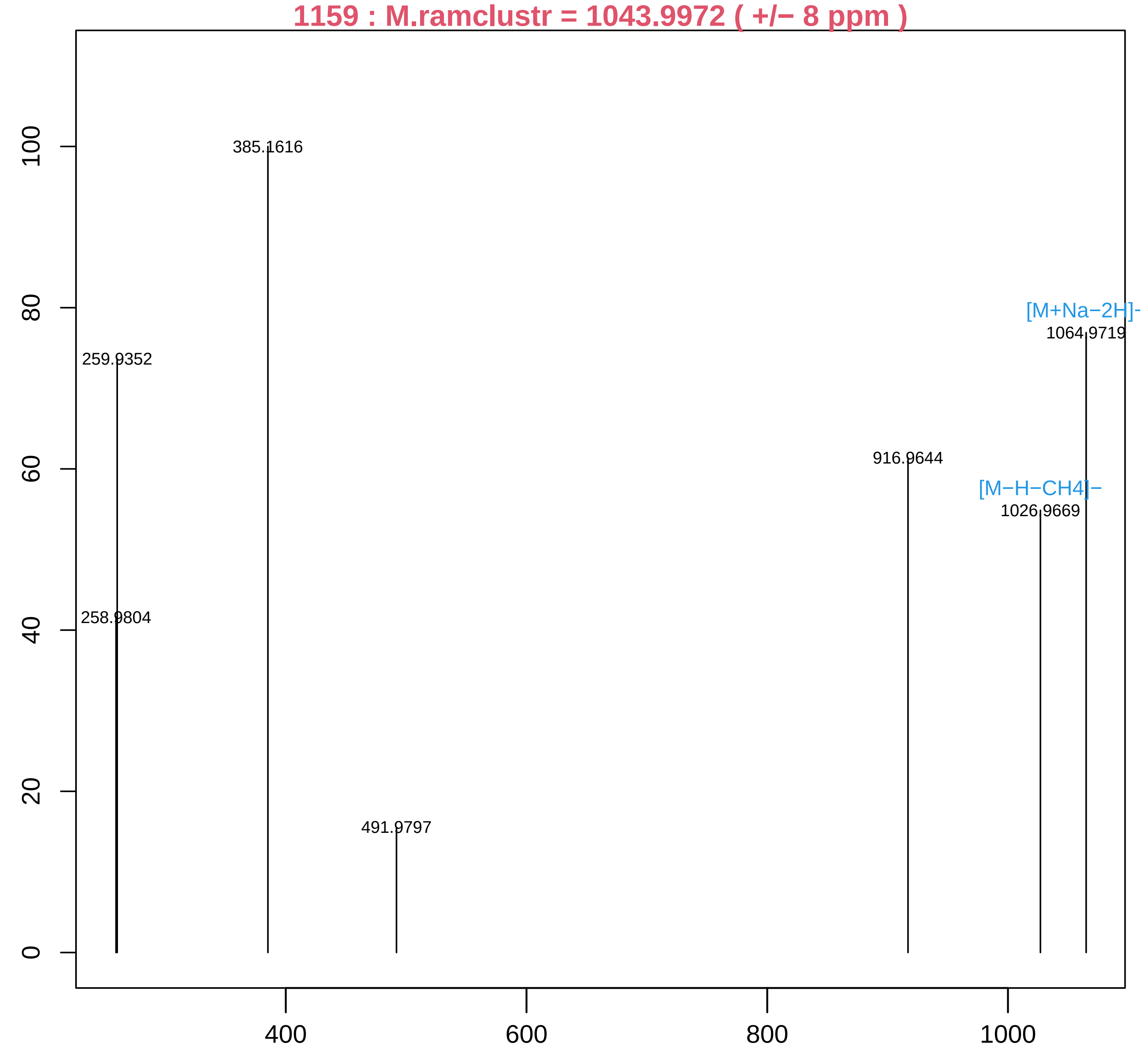
1159 : M.ramclustr = 1043.9972 (+/− 8 ppm)
385.1616
259.9352
258.9804
491.9797
916.9644
[M−H−CH4]−
1026.9669
[M+Na−2H]-
1064.9719

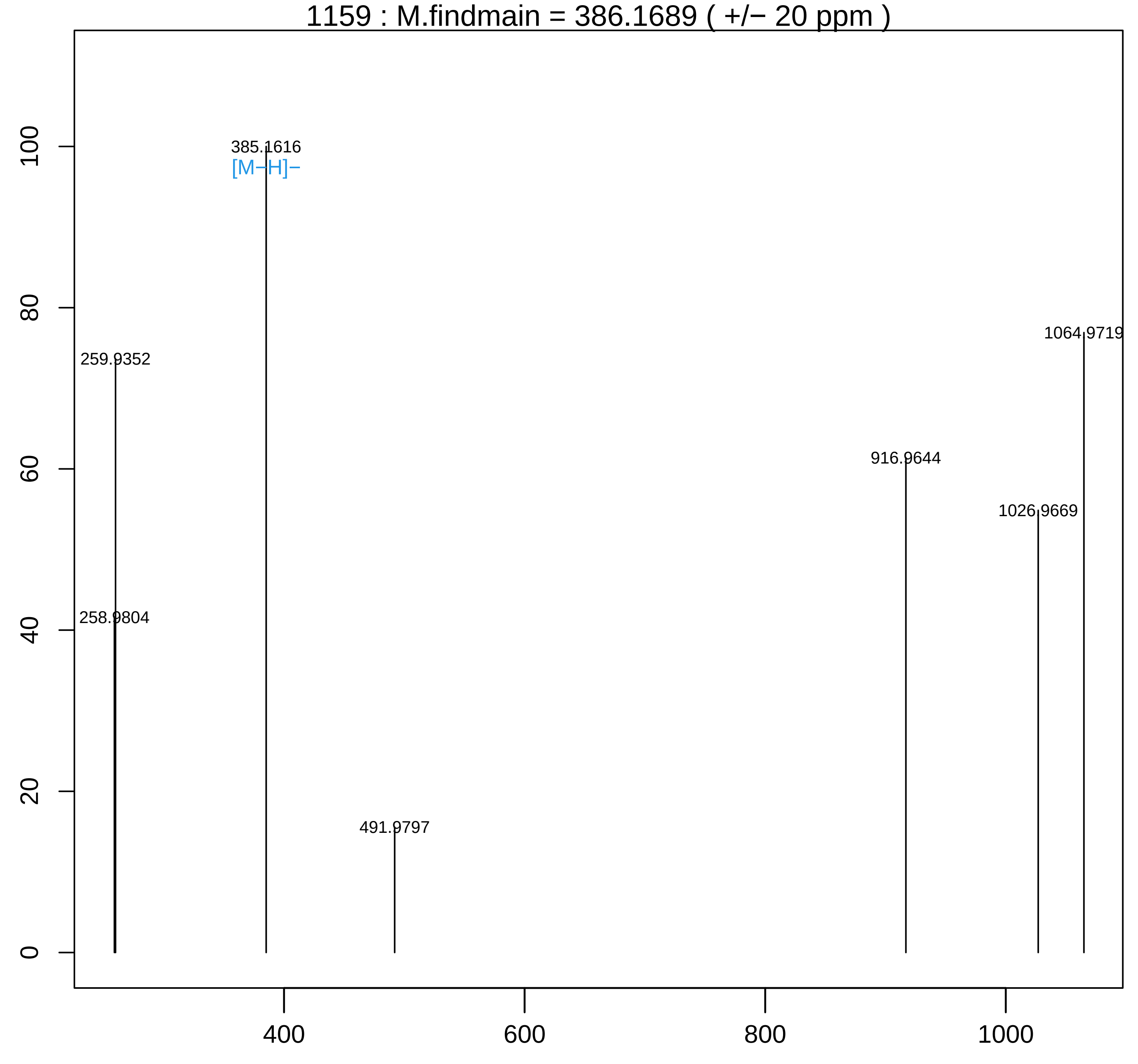
1159 : M.findmain = 386.1689 (+/− 20 ppm)
385.1616
[M−H]−
259.9352
258.9804
491.9797
916.9644
1026.9669
1064.9719

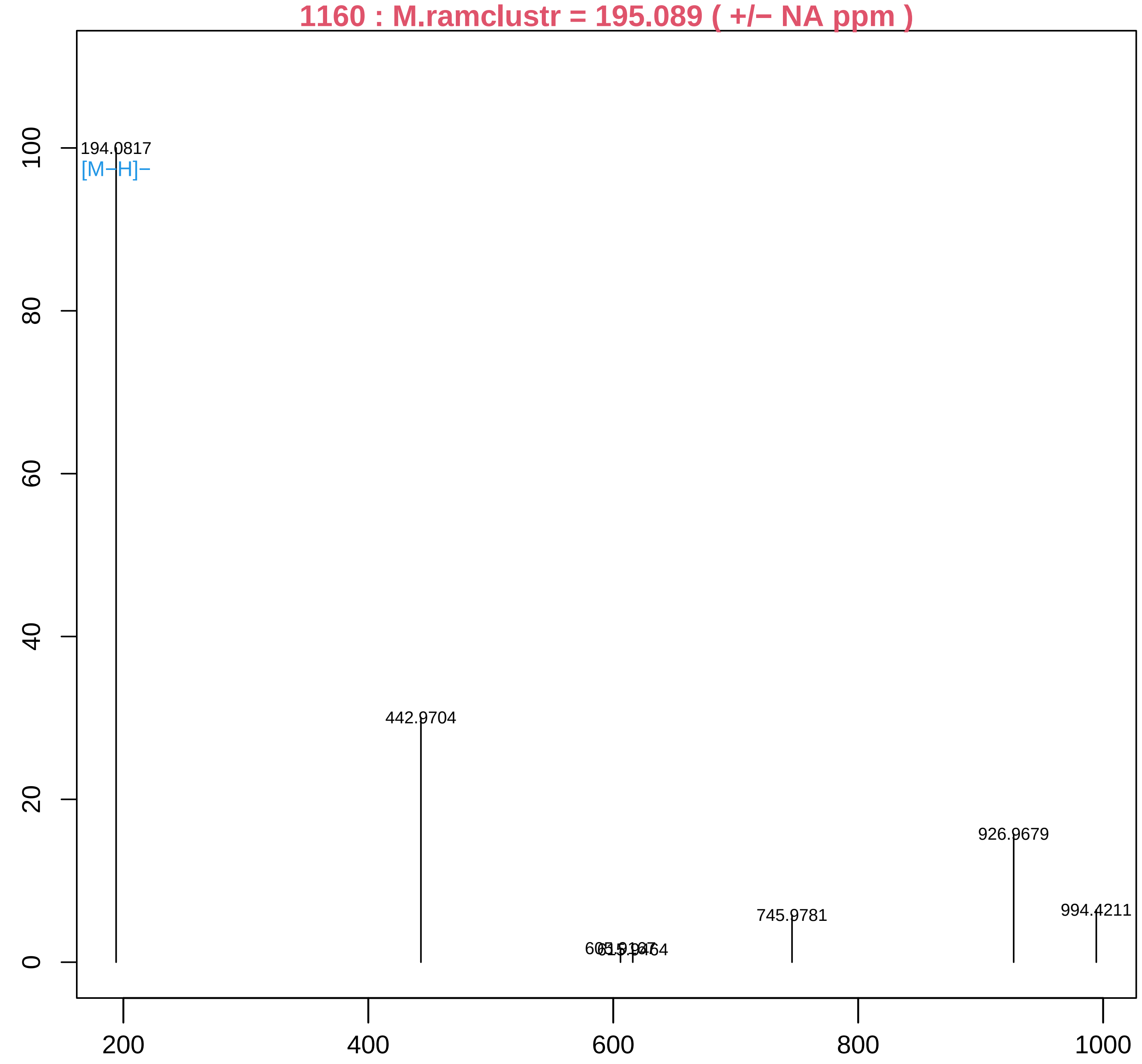
1160 : M.ramclustr = 195.089 (+/− NA ppm)
194.0817
[M−H]−
442.9704
745.9781
926.9679
994.4211
0
20
40
60
80
100
200
400
600
800
1000

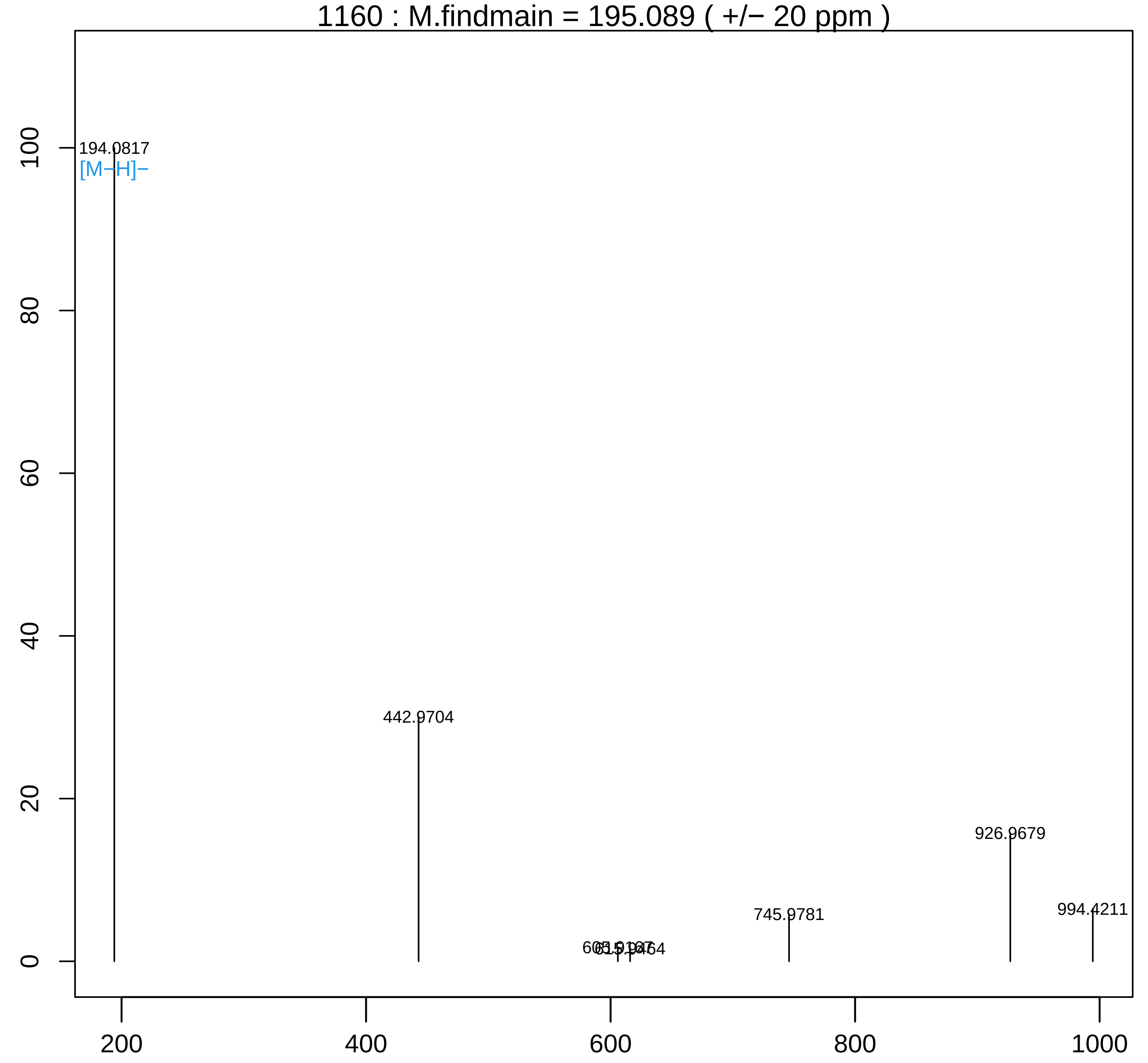
1160 : M.findmain = 195.089 (+/− 20 ppm)
194.0817
[M−H]−
442.9704
745.9781
926.9679
994.4211
0
20
40
60
80
100
200
400
600
800
1000

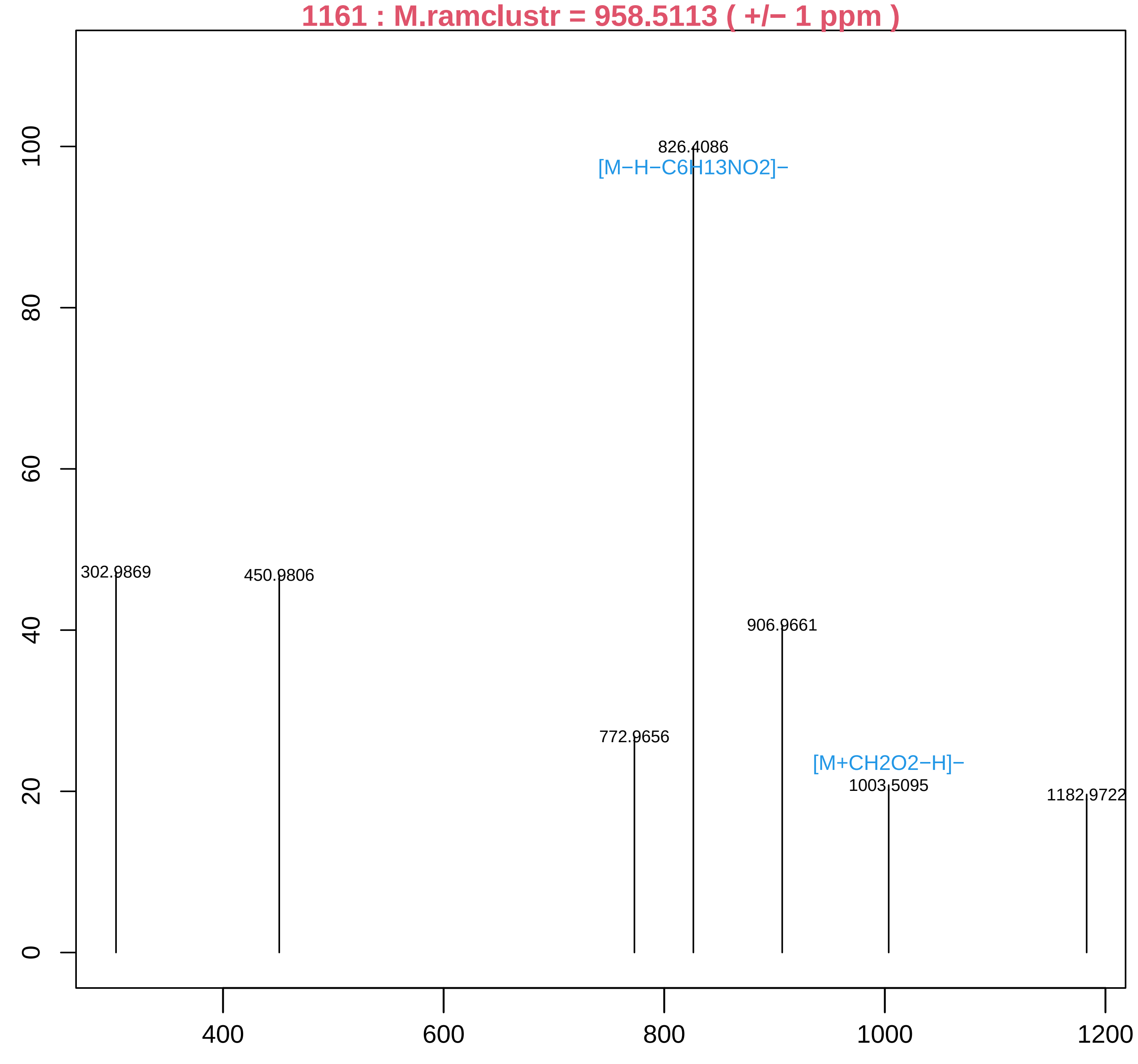
1161 : M.ramclustr = 958.5113 (+/− 1 ppm)
826.4086
[M−H−C6H13NO2]−
302.9869
450.9806
906.9661
772.9656
[M+CH2O2−H]−
1003.5095
1182.9722
0
20
40
60
80
100
400
600
800
1000
1200

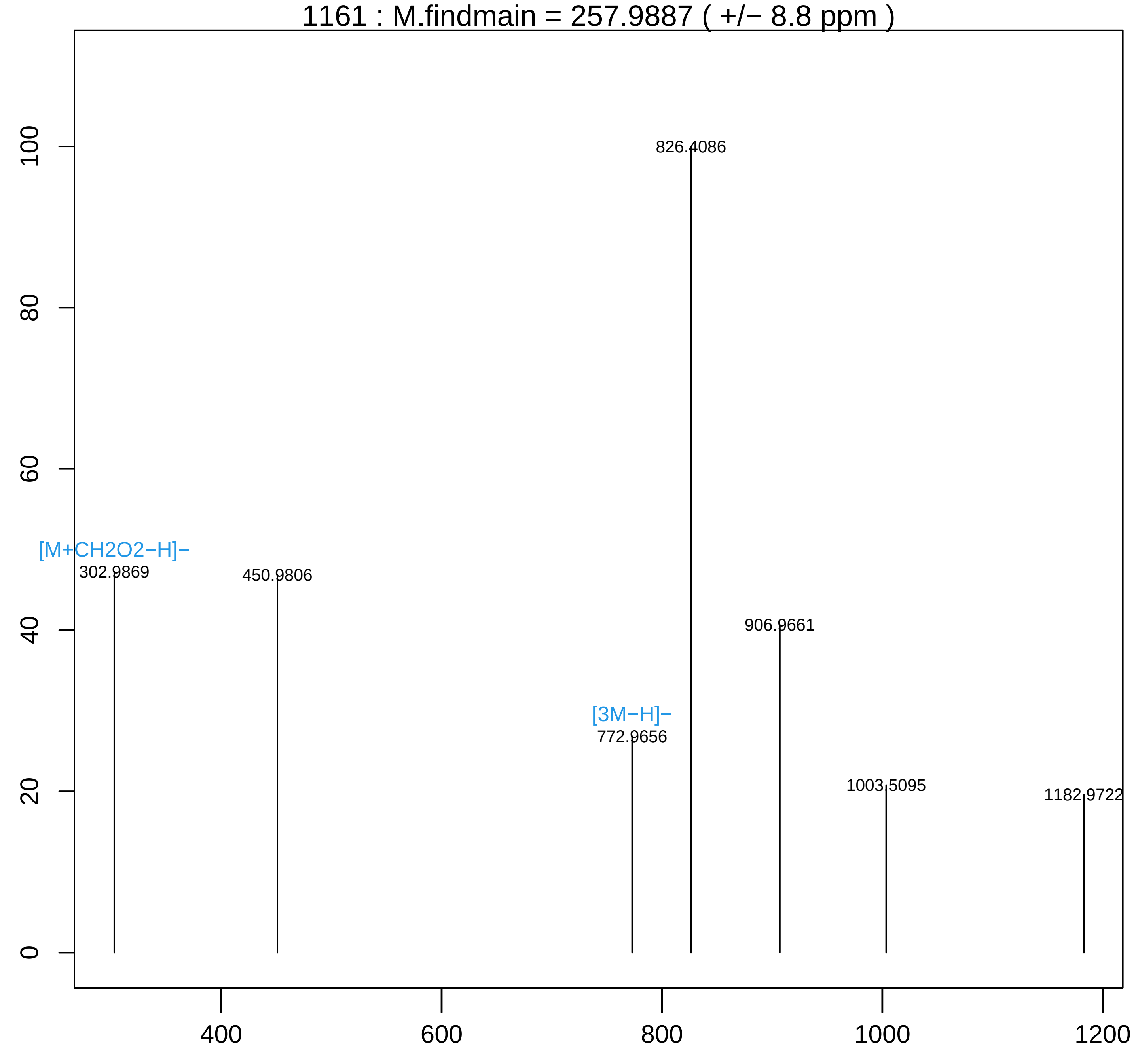
1161 : M.findmain = 257.9887 (+/− 8.8 ppm)
826.4086
[M+CH2O2−H]−
302.9869
450.9806
906.9661
[3M−H]−
772.9656
1003.5095
1182.9722
0
20
40
60
80
100
400
600
800
1000
1200

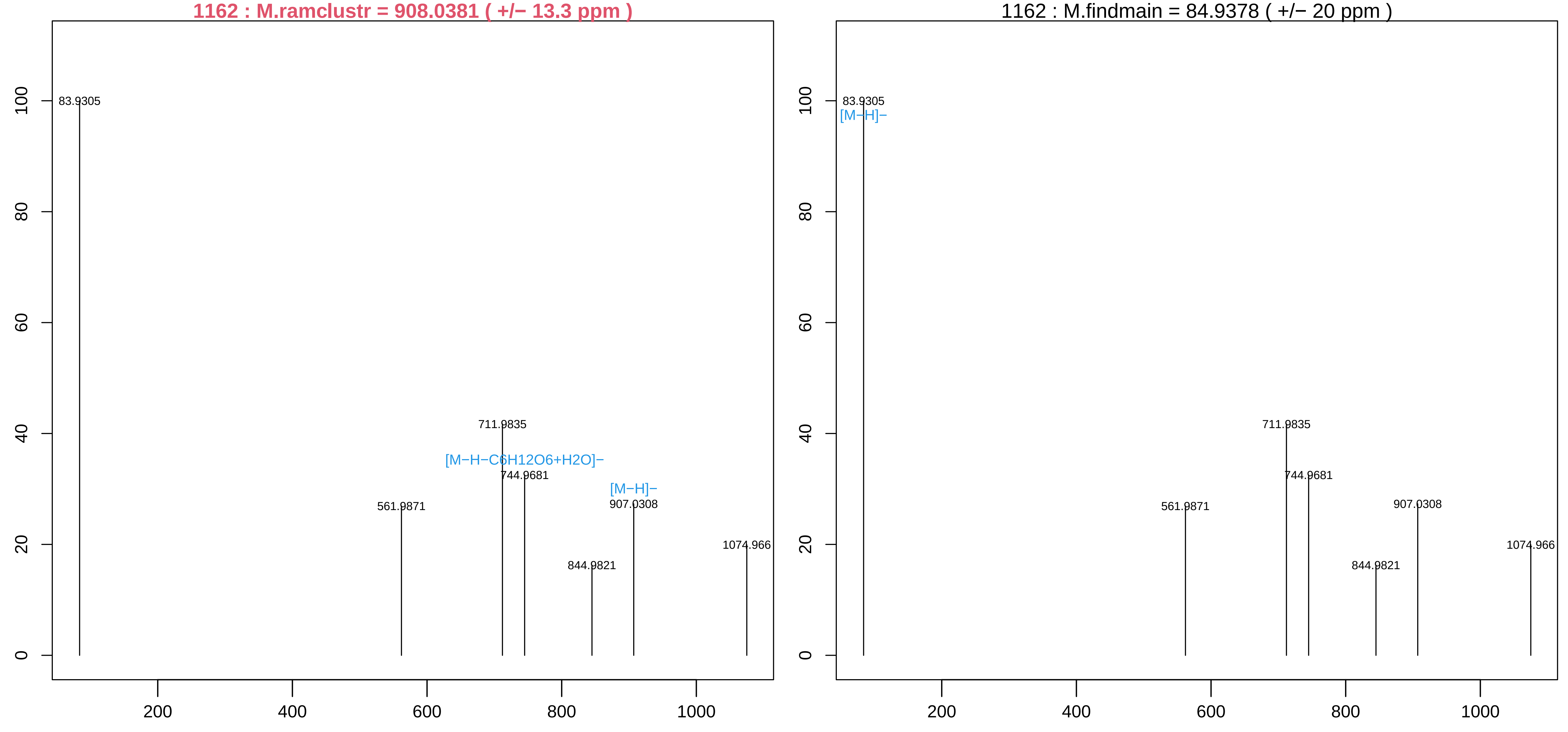
1162 : M.ramclustr = 908.0381 (+/− 13.3 ppm)
83.9305
711.9835
[M−H−C6H12O6+H2O]−
744.9681
[M−H]−
907.0308
561.9871
1074.966
844.9821
0
20
40
60
80
100
200
400
600
800
1000
1162 : M.findmain = 84.9378 (+/− 20 ppm)
83.9305
[M−H]−
711.9835
744.9681
561.9871
907.0308
1074.966
844.9821

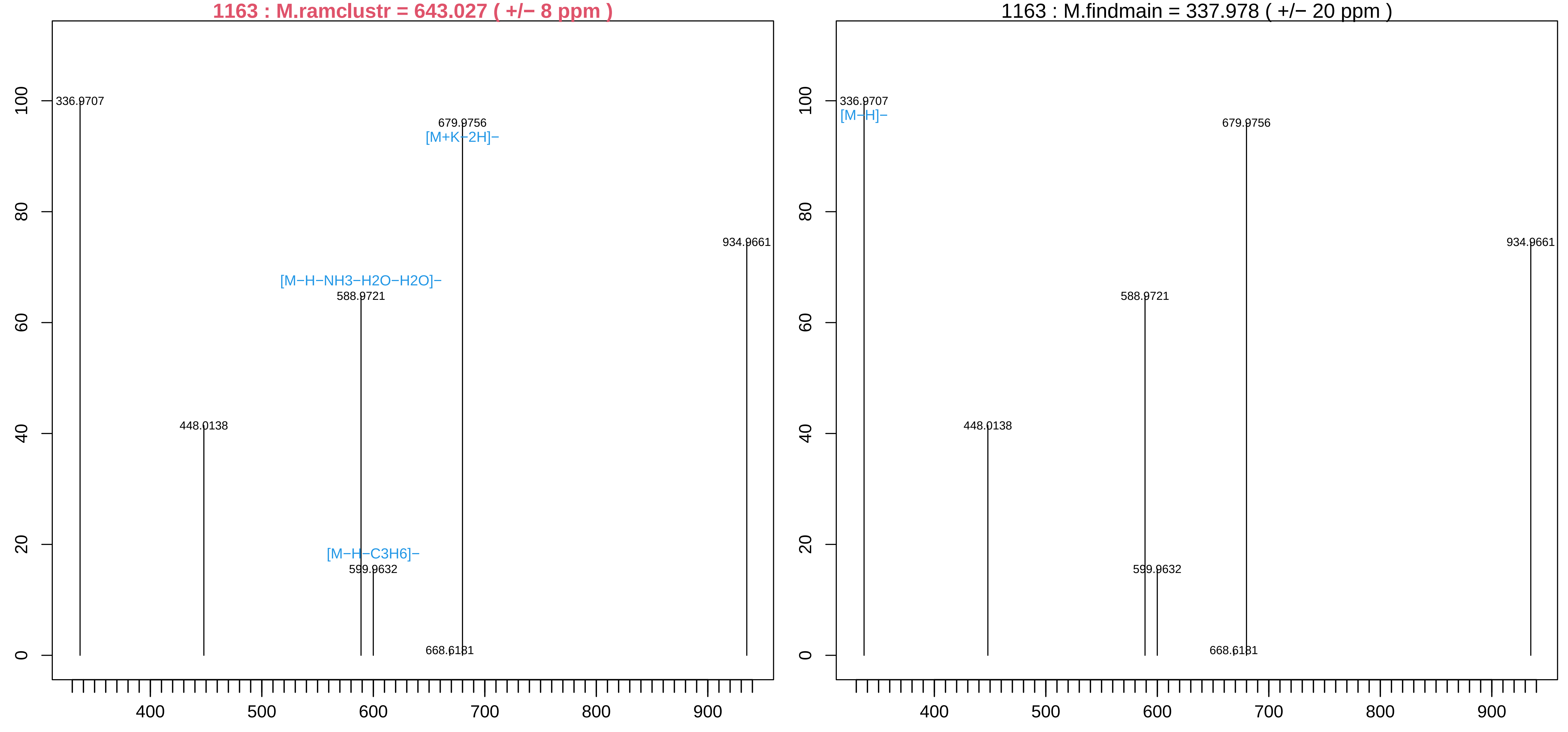
1163 : M.ramclustr = 643.027 (+/− 8 ppm)
336.9707
679.9756
[M+K−2H]−
934.9661
[M−H−NH3−H2O−H2O]−
588.9721
448.0138
[M−H−C3H6]−
599.9632
668.6131
1163 : M.findmain = 337.978 (+/− 20 ppm)
336.9707
[M−H]−
679.9756
934.9661
588.9721
448.0138
599.9632
668.6131
0
20
40
60
80
100
400
500
600
700
800
900

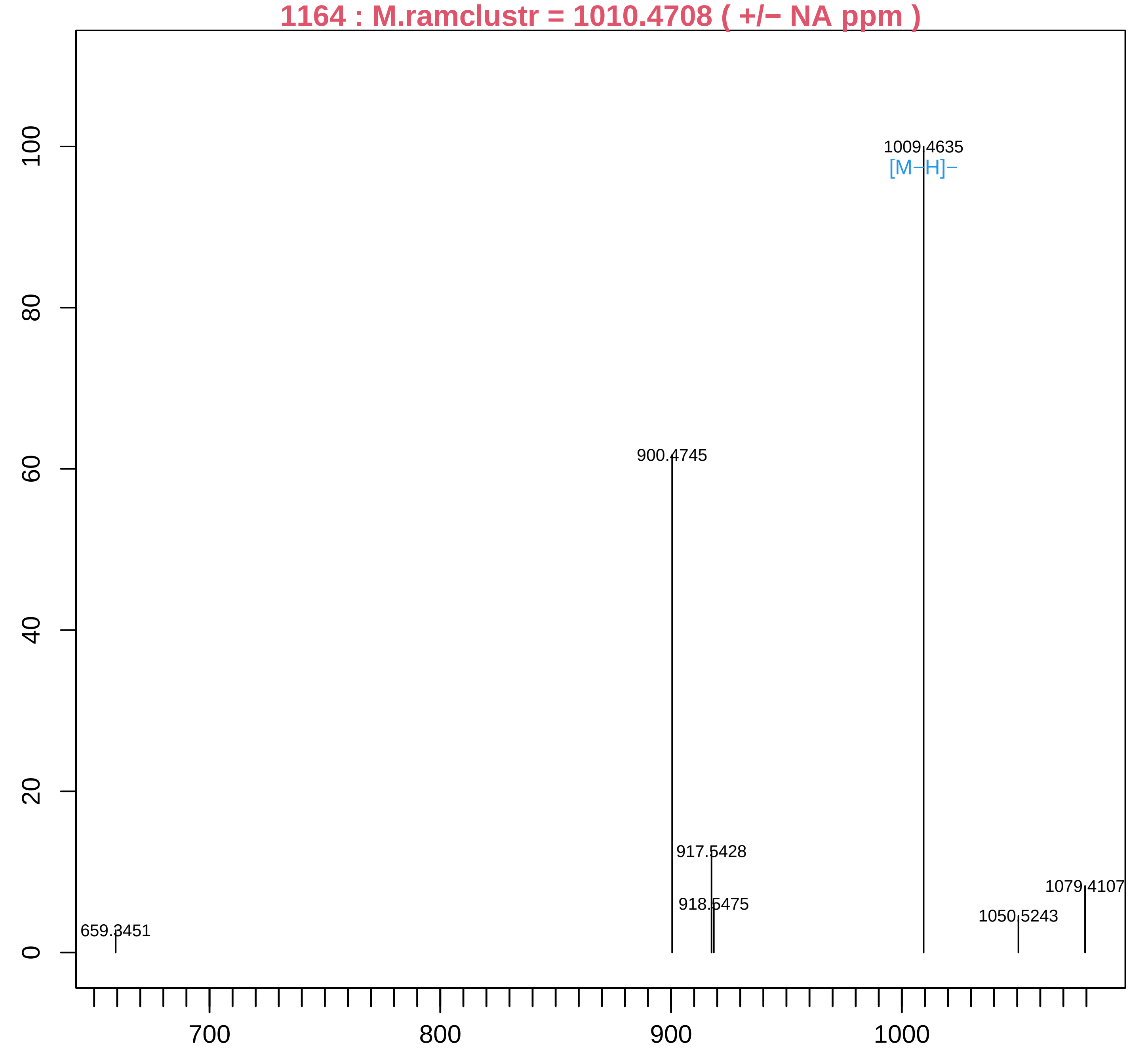
1164 : M.ramclustr = 1010.4708 (+/− NA ppm)
1009.4635
[M−H]−
900.4745
917.5428
918.5475
659.3451
1050.5243
1079.4107
0
20
40
60
80
100
700
800
900
1000

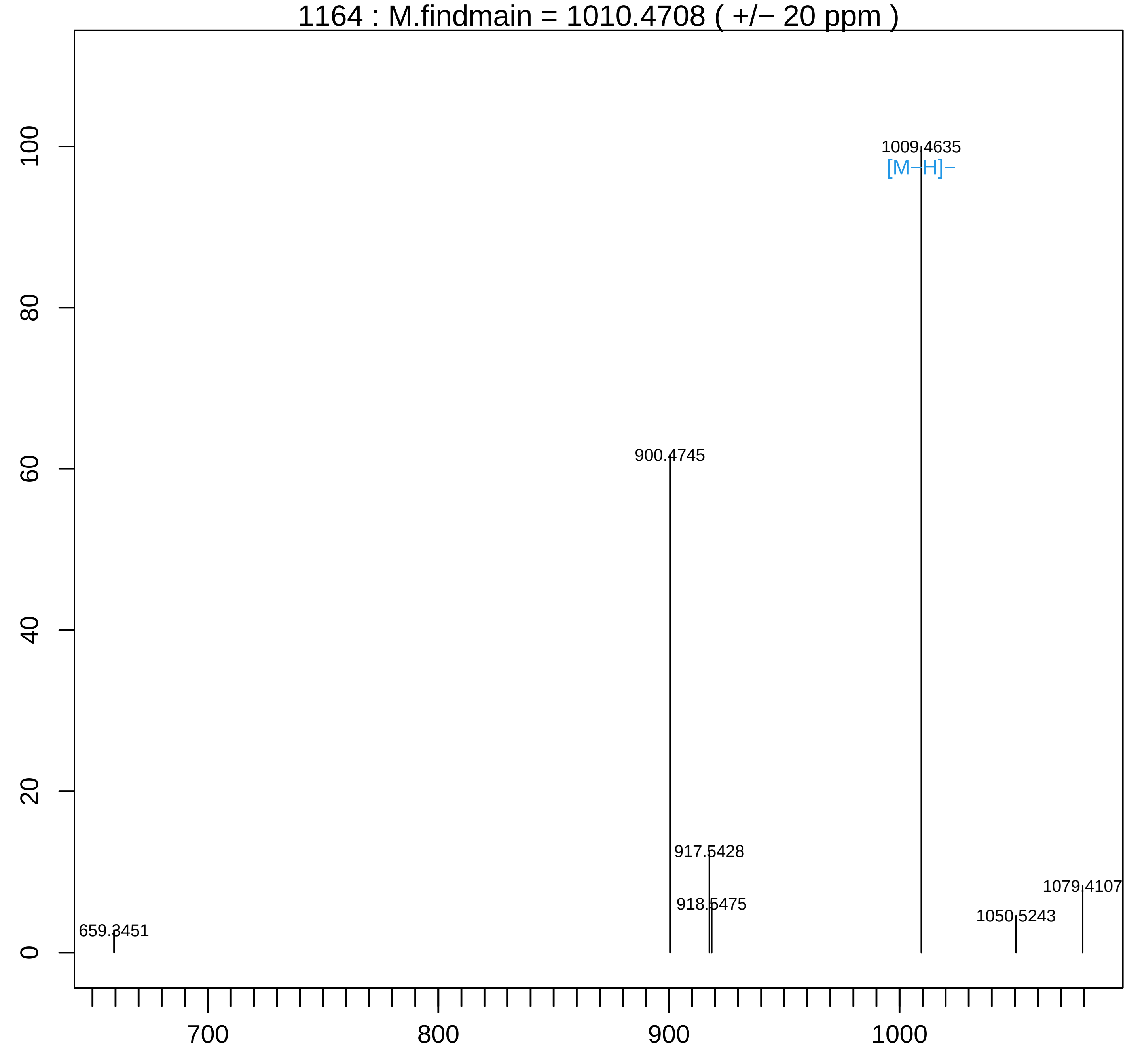
1164 : M.findmain = 1010.4708 (+/− 20 ppm)
1009.4635
[M−H]−
900.4745
917.5428
918.5475
659.3451
1050.5243
1079.4107
0
20
40
60
80
100
700
800
900
1000

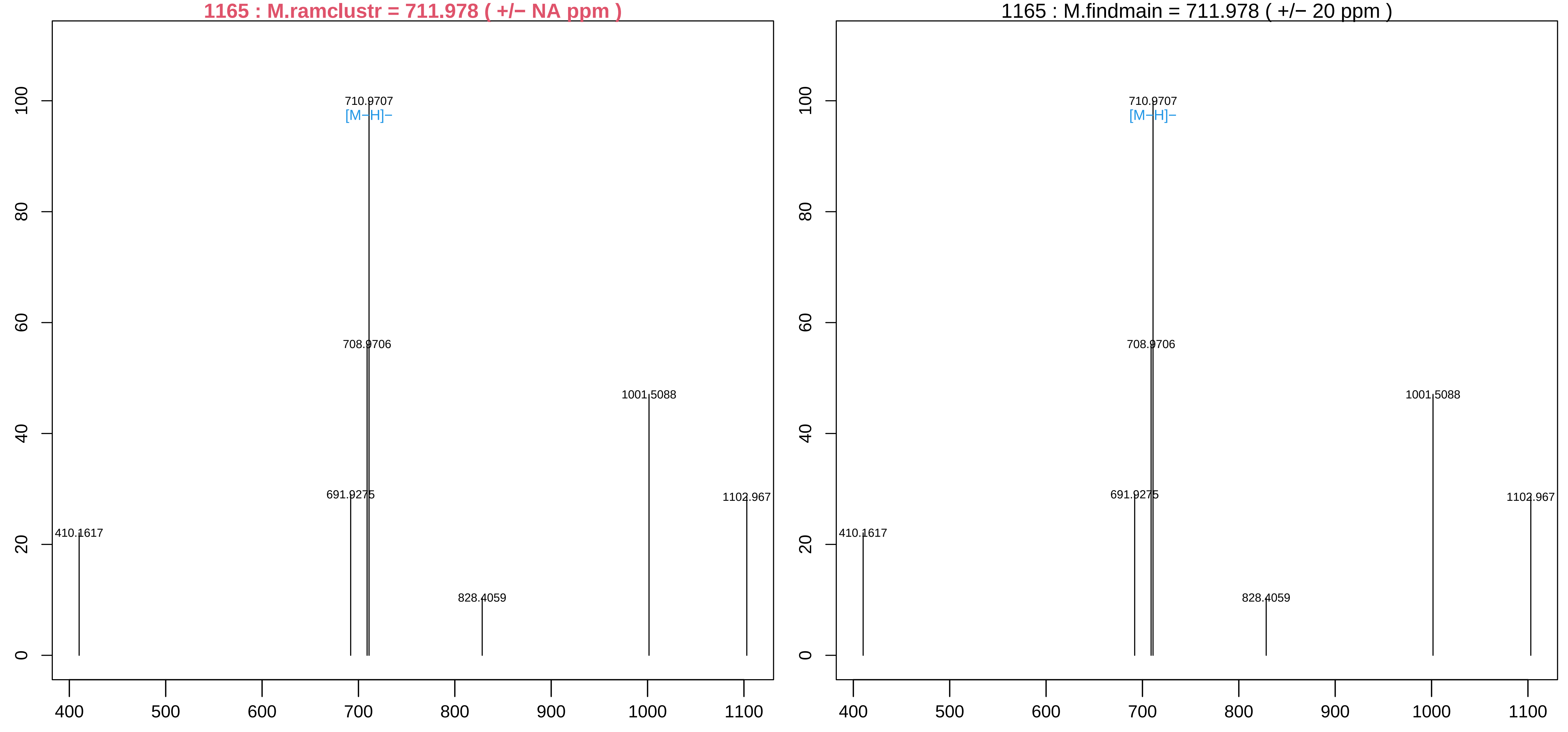
1165 : M.ramclustr = 711.978 (+/− NA ppm)
710.9707
[M−H]−
708.9706
1001.5088
691.9275
1102.967
410.1617
828.4059
0
20
40
60
80
100
400
500
600
700
800
900
1000
1100
1165 : M.findmain = 711.978 (+/− 20 ppm)
710.9707
[M−H]−
708.9706
1001.5088
691.9275
1102.967
410.1617
828.4059
0
20
40
60
80
100
400
500
600
700
800
900
1000
1100

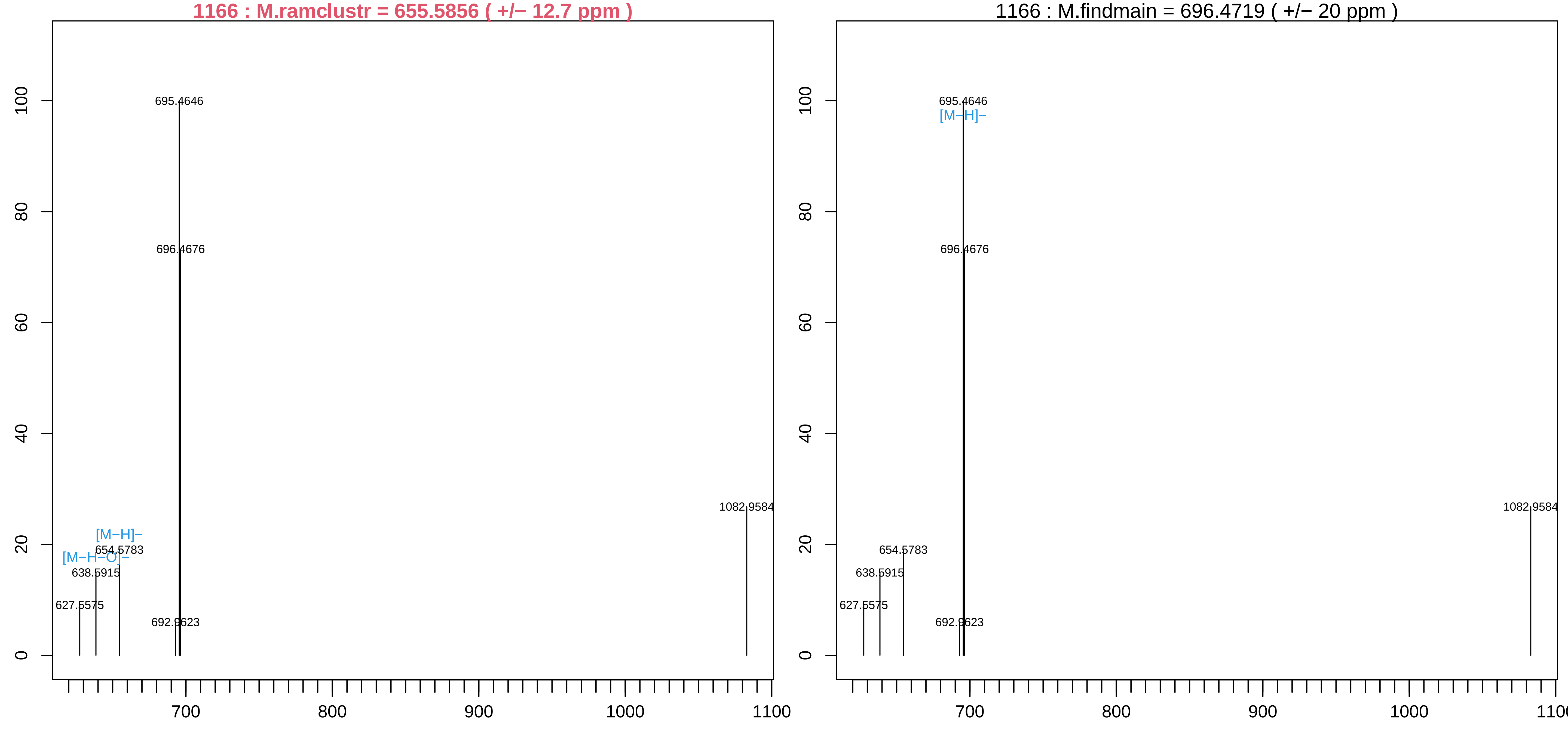

1166 : M.ramclustr = 655.5856 (+/− 12.7 ppm)
695.4646
696.4676
[M−H]−
[M−H−O]−
654.5783
638.5915
627.5575
692.9623
1082.9584
1166 : M.findmain = 696.4719 (+/− 20 ppm)
695.4646
[M−H]−
696.4676
654.5783
638.5915
627.5575
692.9623
1082.9584
0
20
40
60
80
100
700
800
900
1000
1100

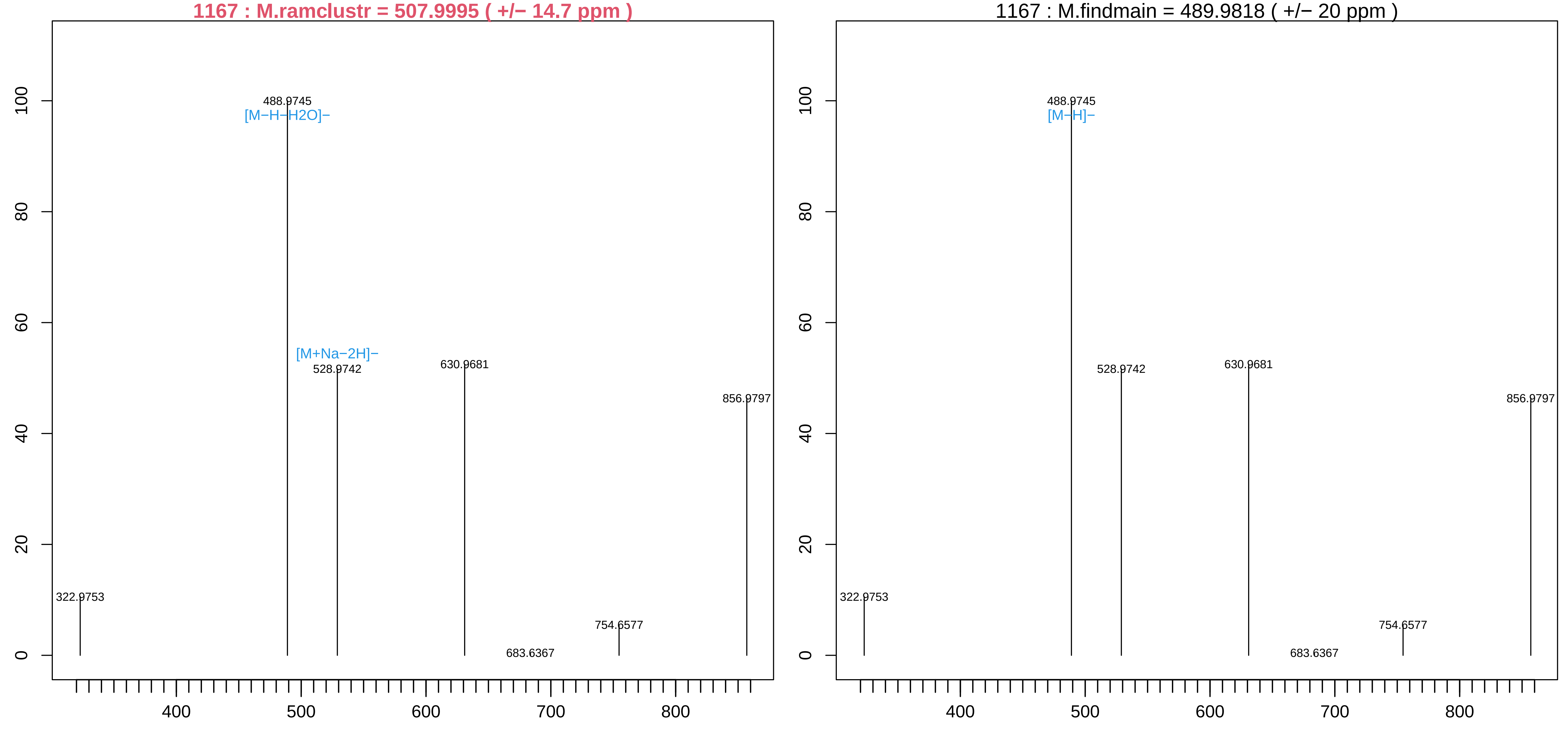
1167 : M.ramclustr = 507.9995 (+/− 14.7 ppm)
488.9745
[M−H−H2O]−
[M+Na−2H]−
528.9742
630.9681
856.9797
322.9753
754.6577
683.6367
1167 : M.findmain = 489.9818 (+/− 20 ppm)
488.9745
[M−H]−
528.9742
630.9681
856.9797
322.9753
754.6577
683.6367

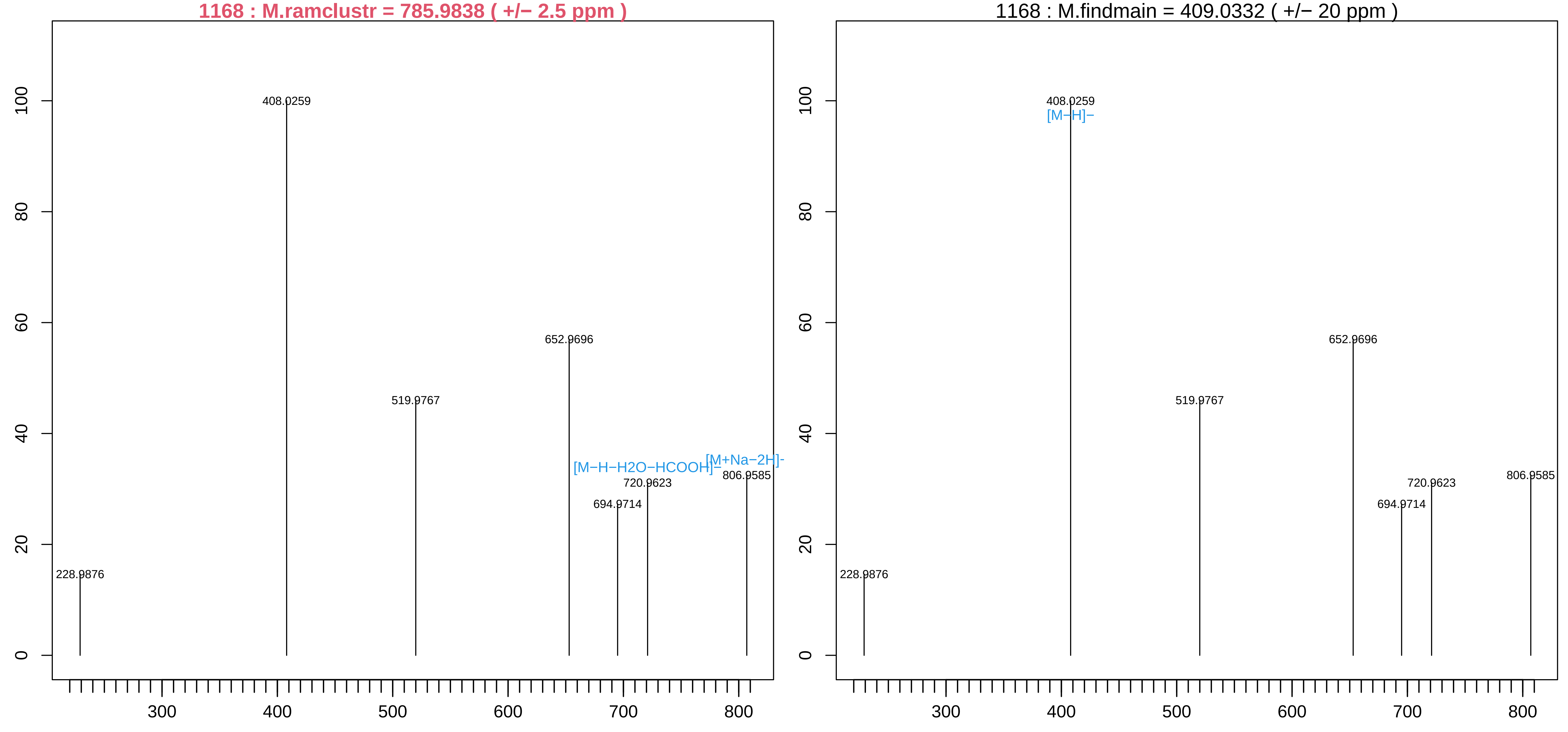

1168 : M.ramclustr = 785.9838 (+/− 2.5 ppm)
408.0259
519.9767
652.9696
[M−H−H2O−HCOOH]−
720.9623
694.9714
[M+Na−2H]-
806.9585
228.9876
1168 : M.findmain = 409.0332 (+/− 20 ppm)
408.0259
[M−H]−
519.9767
652.9696
720.9623
694.9714
806.9585
228.9876

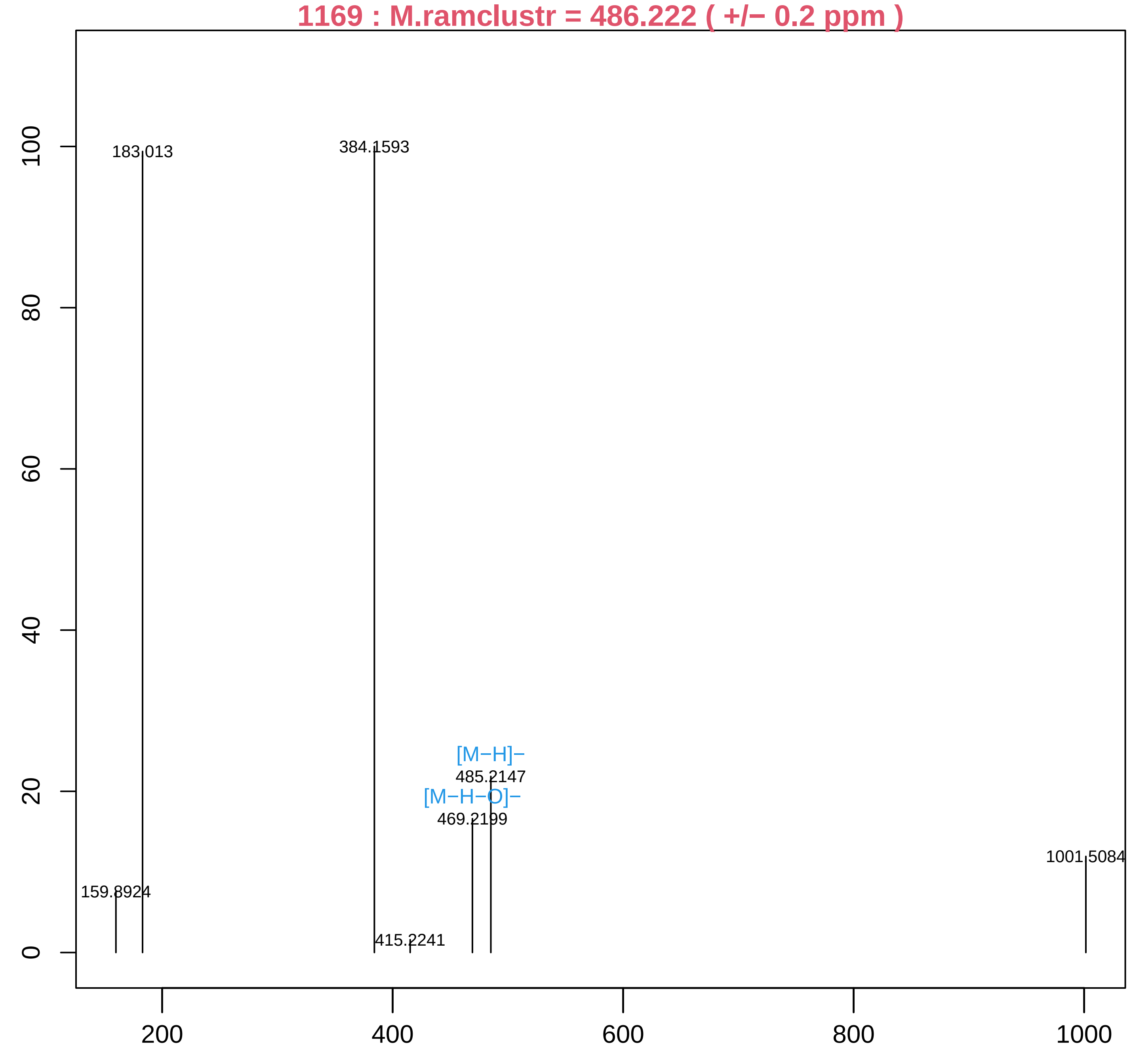
1169 : M.ramclustr = 486.222 (+/− 0.2 ppm)
183.013
384.1593
[M−H]−
485.2147
[M−H−O]−
469.2199
1001.5084
159.8924
415.2241
0
20
40
60
80
100
200
400
600
800
1000

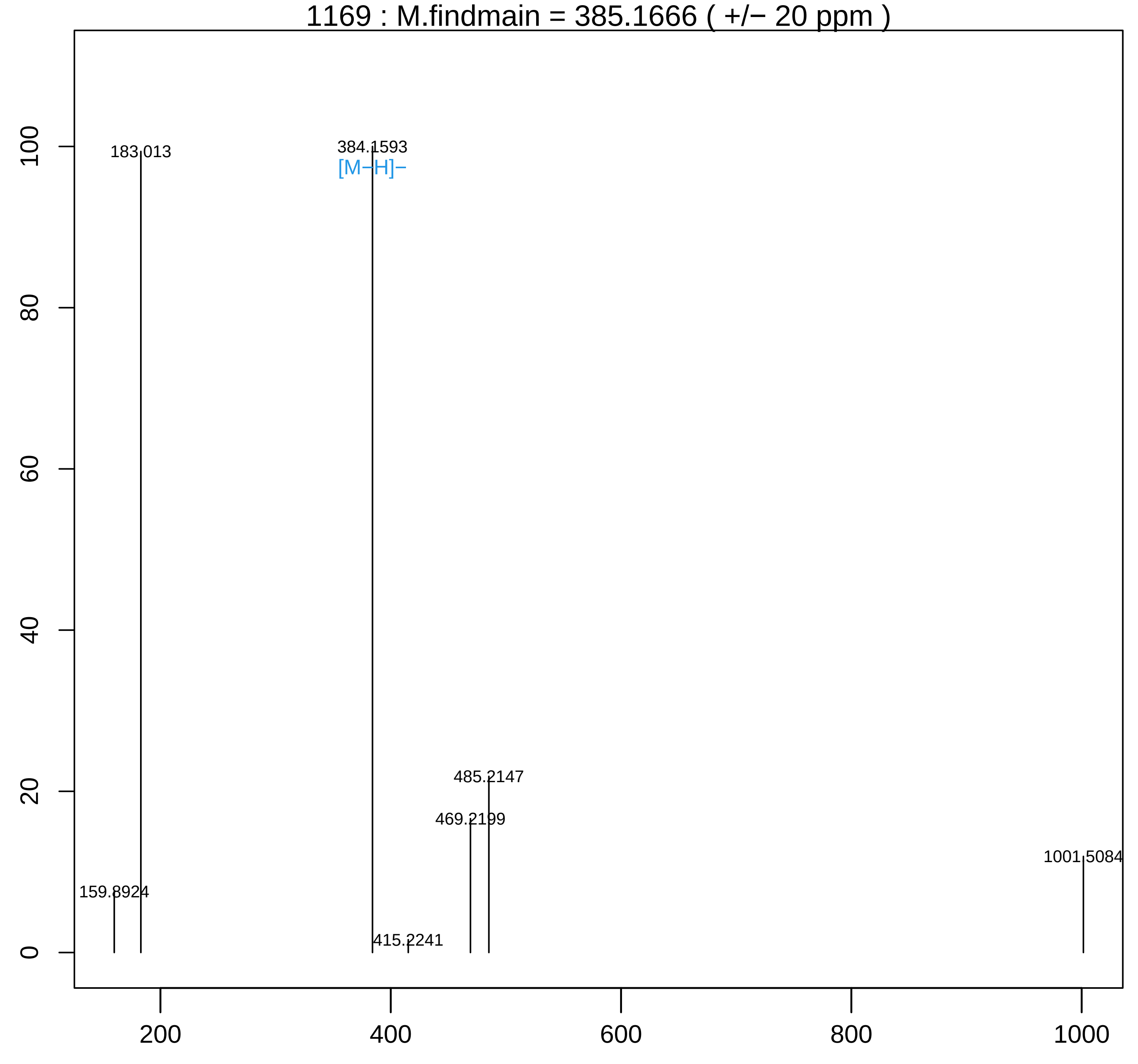
1169 : M.findmain = 385.1666 (+/− 20 ppm)
183.013
384.1593
[M−H]−
485.2147
469.2199
1001.5084
159.8924
415.2241
0
20
40
60
80
100
200
400
600
800
1000

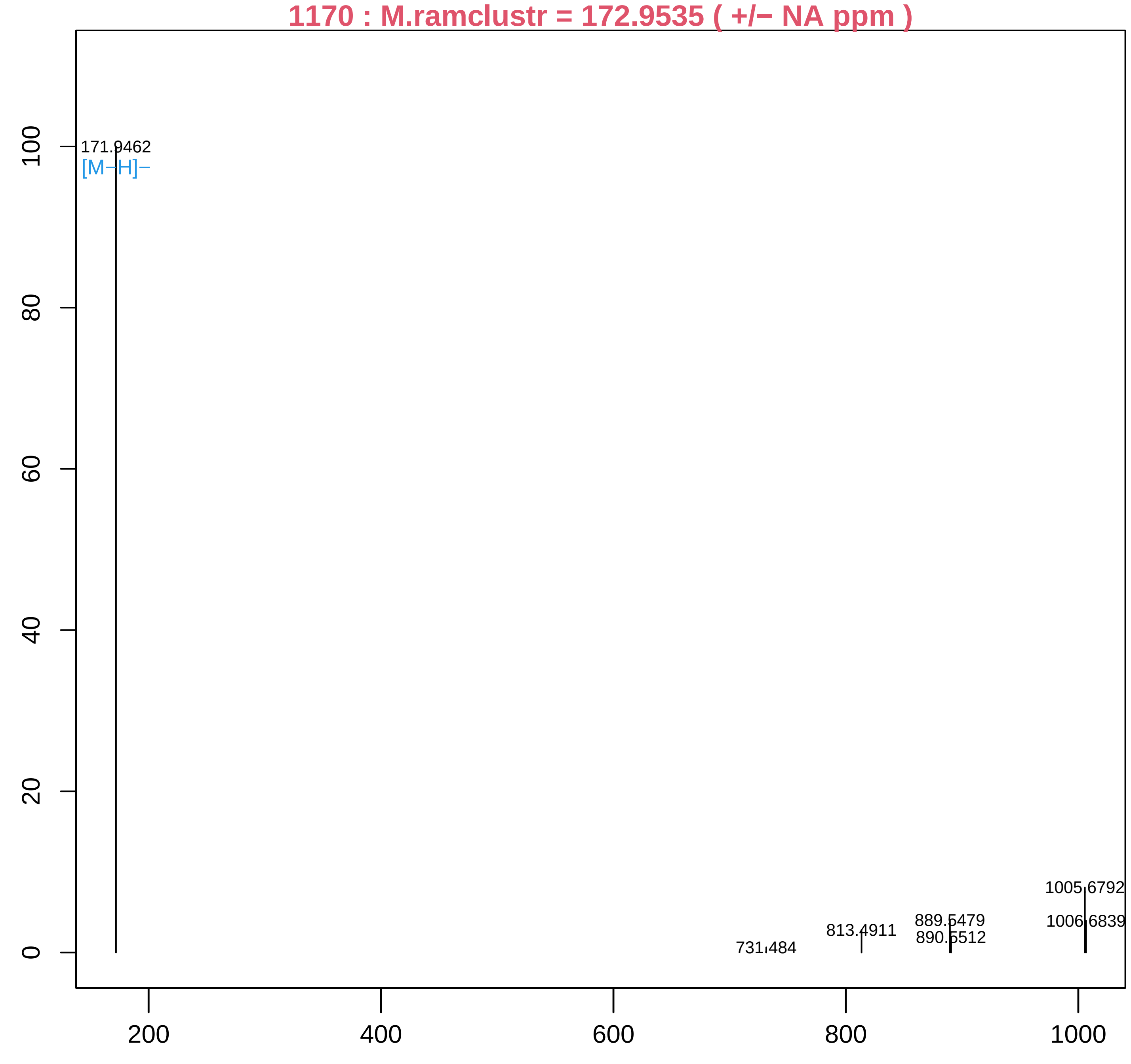
1170 : M.ramclustr = 172.9535 (+/− NA ppm)
171.9462
[M−H]−
731.484
813.4911
889.5479
890.5512
1005.6792
1006.6839
0
20
40
60
80
100
200
400
600
800
1000

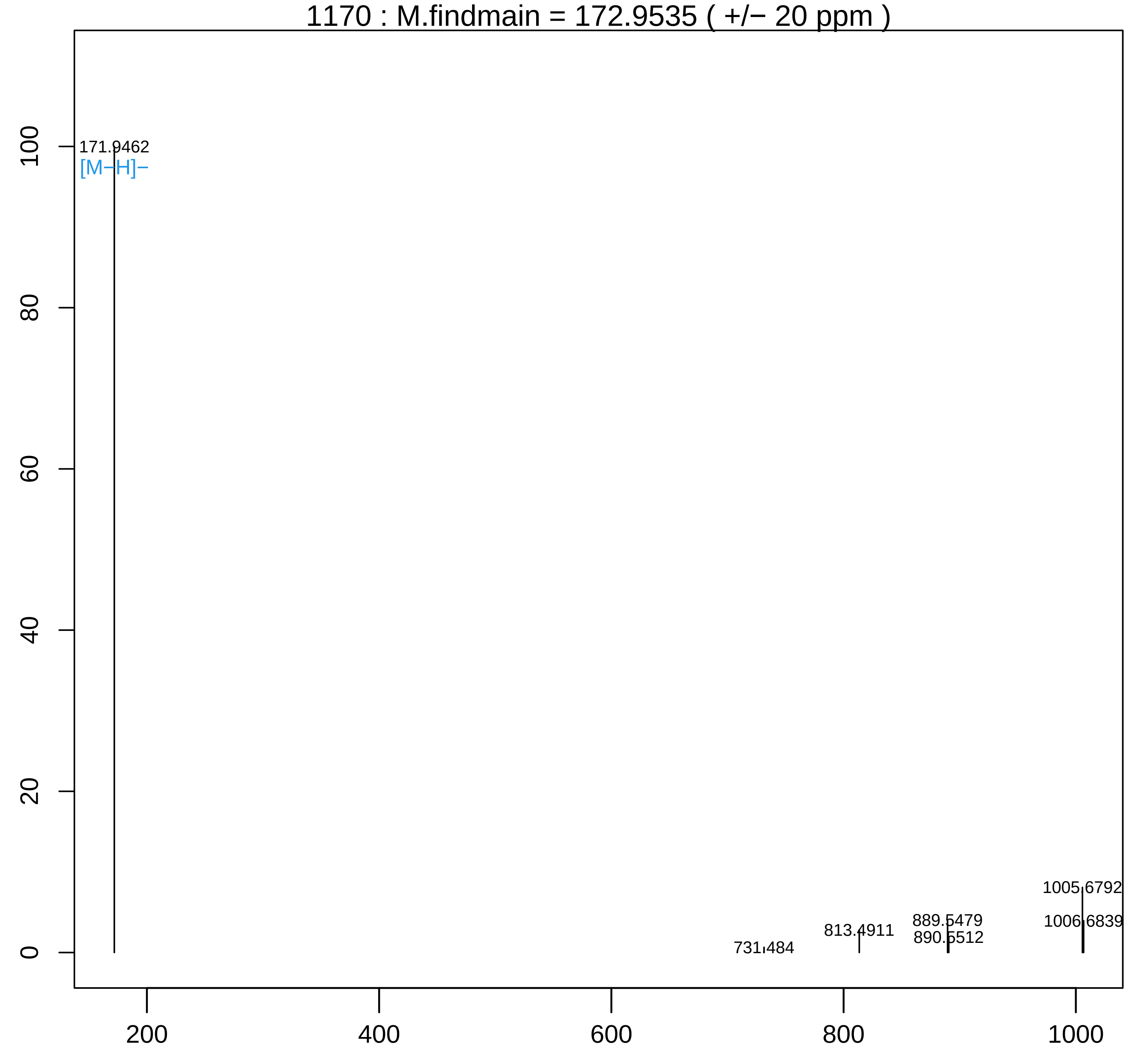
1170 : M.findmain = 172.9535 (+/− 20 ppm)
171.9462
[M−H]−
731.484
813.4911
889.5479
890.5512
1005.6792
1006.6839
0
20
40
60
80
100
200
400
600
800
1000

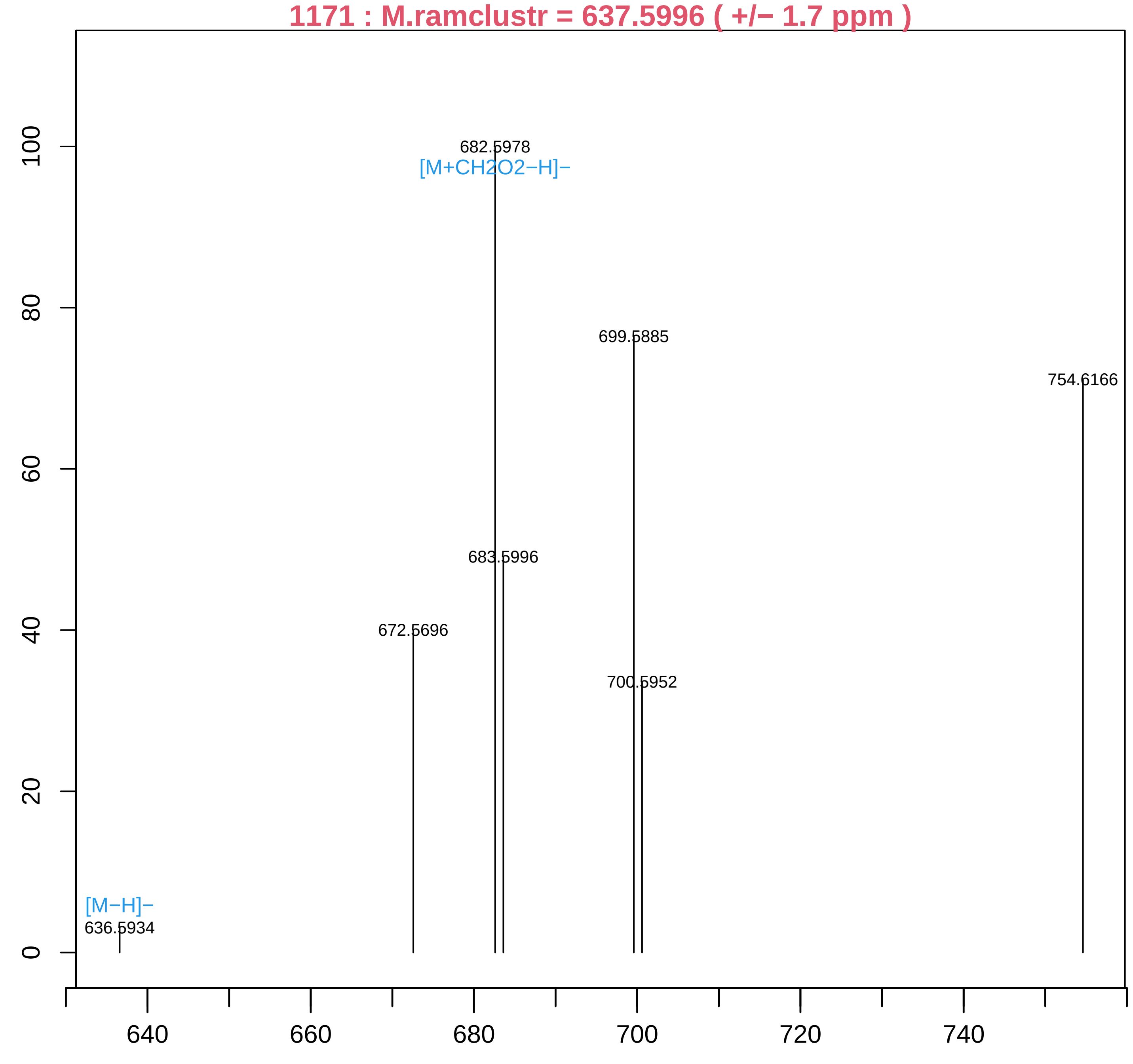
1171 : M.ramclustr = 637.5996 (+/− 1.7 ppm)
682.5978
[M+CH2O2−H]−
699.5885
754.6166
683.5996
672.5696
700.5952
[M−H]−
636.5934
0
20
40
60
80
100
640
660
680
700
720
740

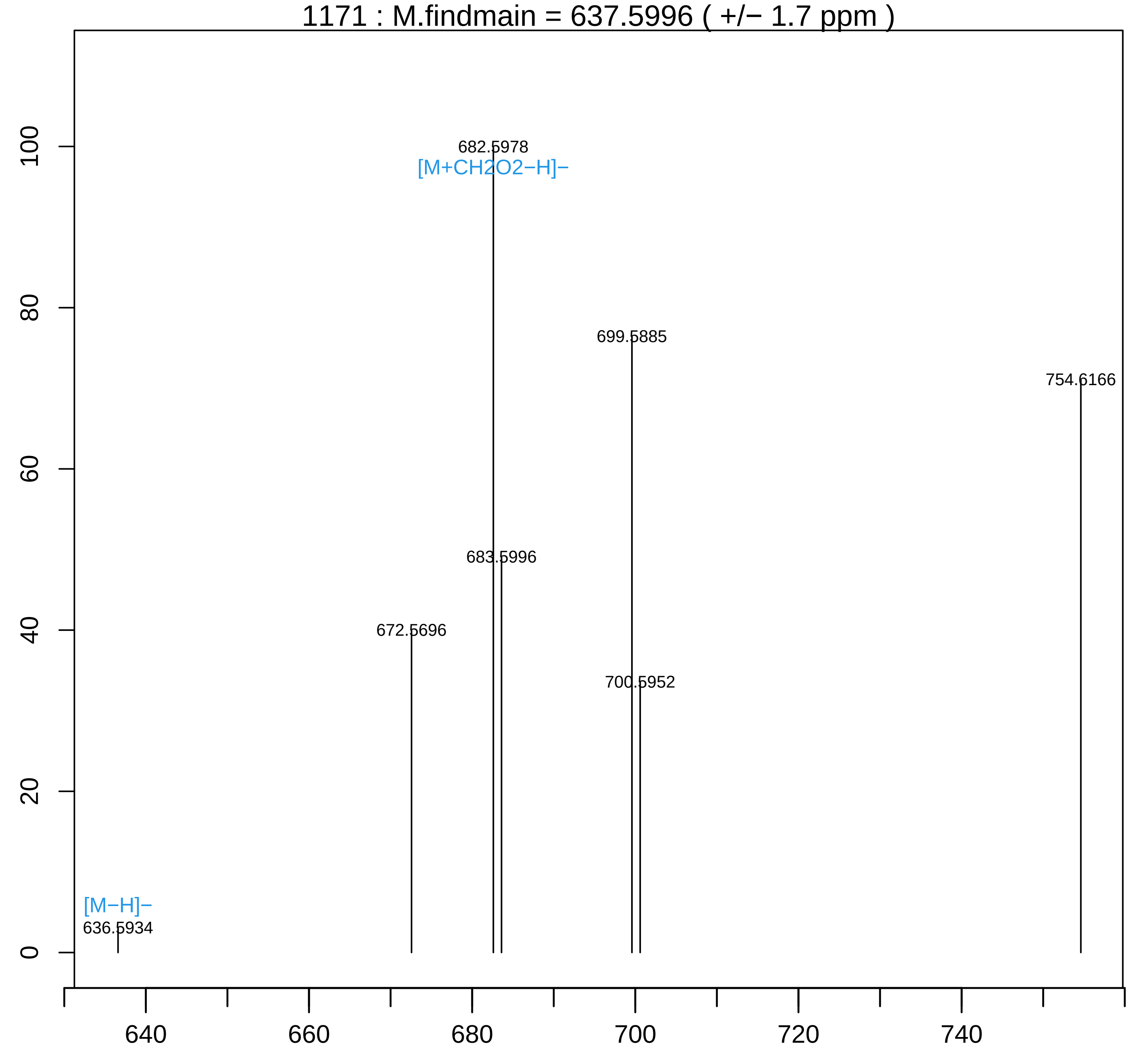
1171 : M.findmain = 637.5996 (+/− 1.7 ppm)
682.5978
[M+CH2O2−H]−
699.5885
754.6166
683.5996
672.5696
700.5952
[M−H]−
636.5934
0
20
40
60
80
100
640
660
680
700
720
740

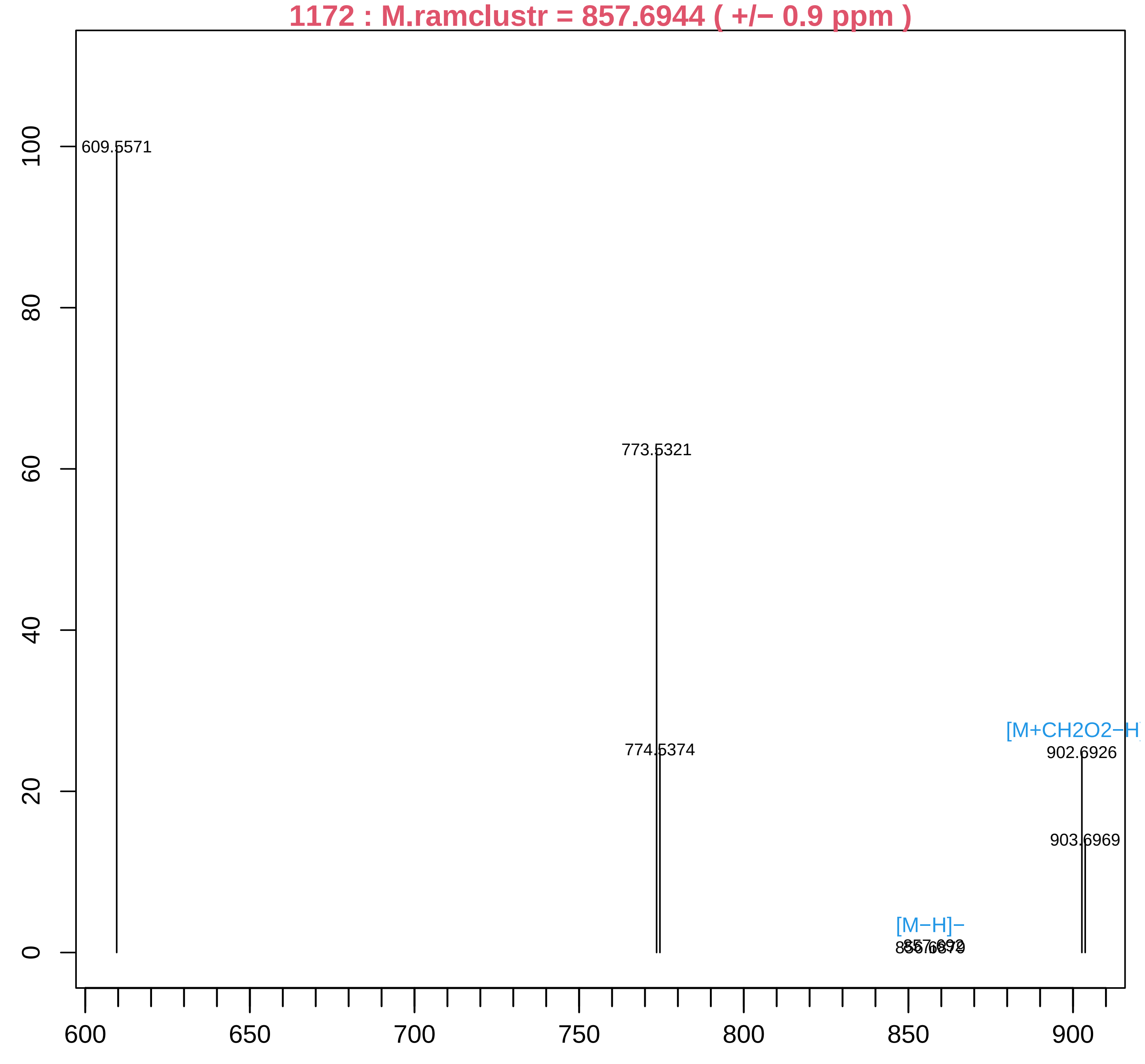
1172 : M.ramclustr = 857.6944 (+/− 0.9 ppm)
609.5571
773.5321
774.5374
[M+CH2O2−H
902.6926
903.6969
[M−H]−
0
20
40
60
80
100
600
650
700
750
800
850
900

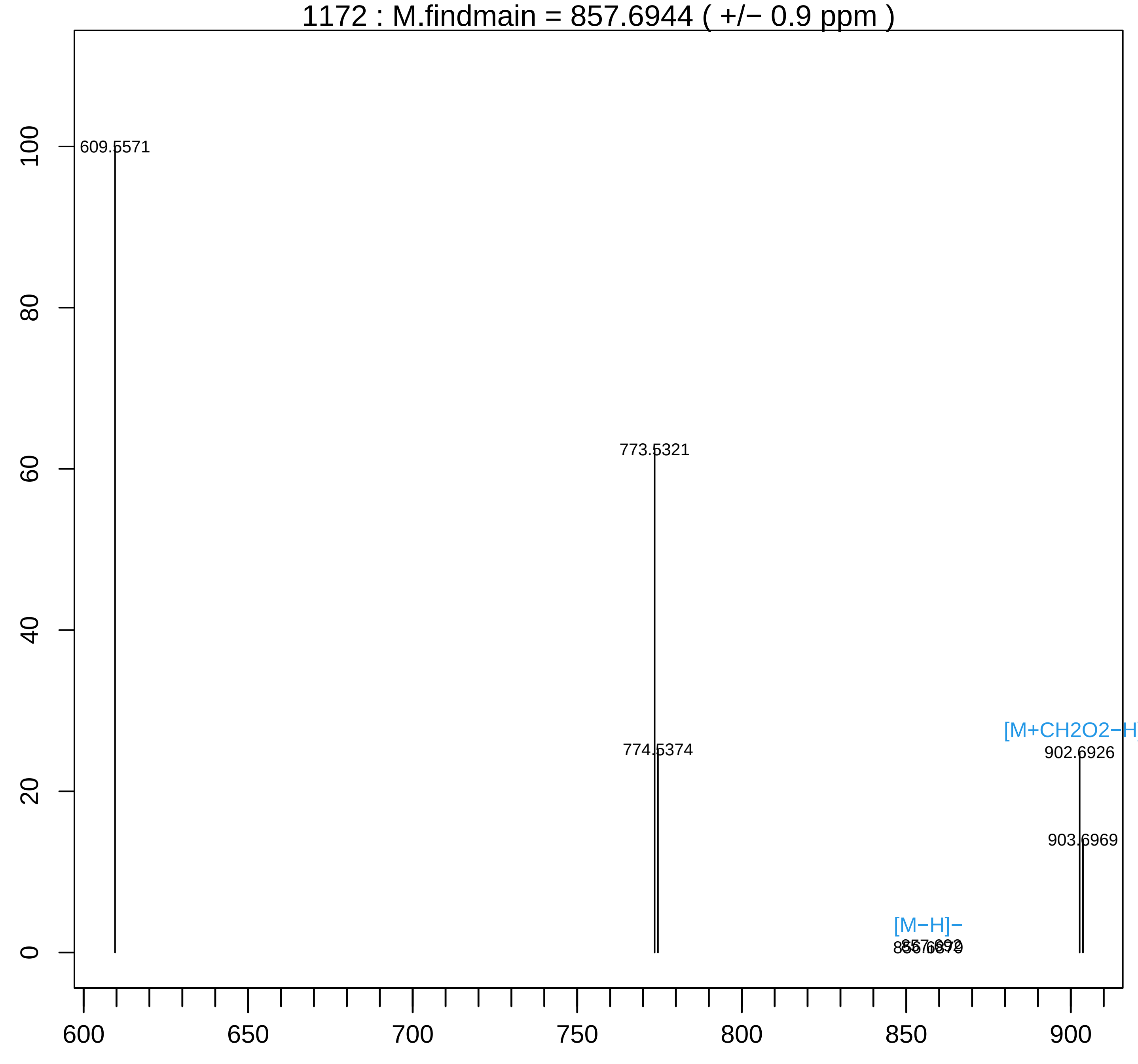
1172 : M.findmain = 857.6944 (+/− 0.9 ppm)
609.5571
773.5321
774.5374
[M+CH2O2−H
902.6926
903.6969
[M−H]−
0
20
40
60
80
100
600
650
700
750
800
850
900

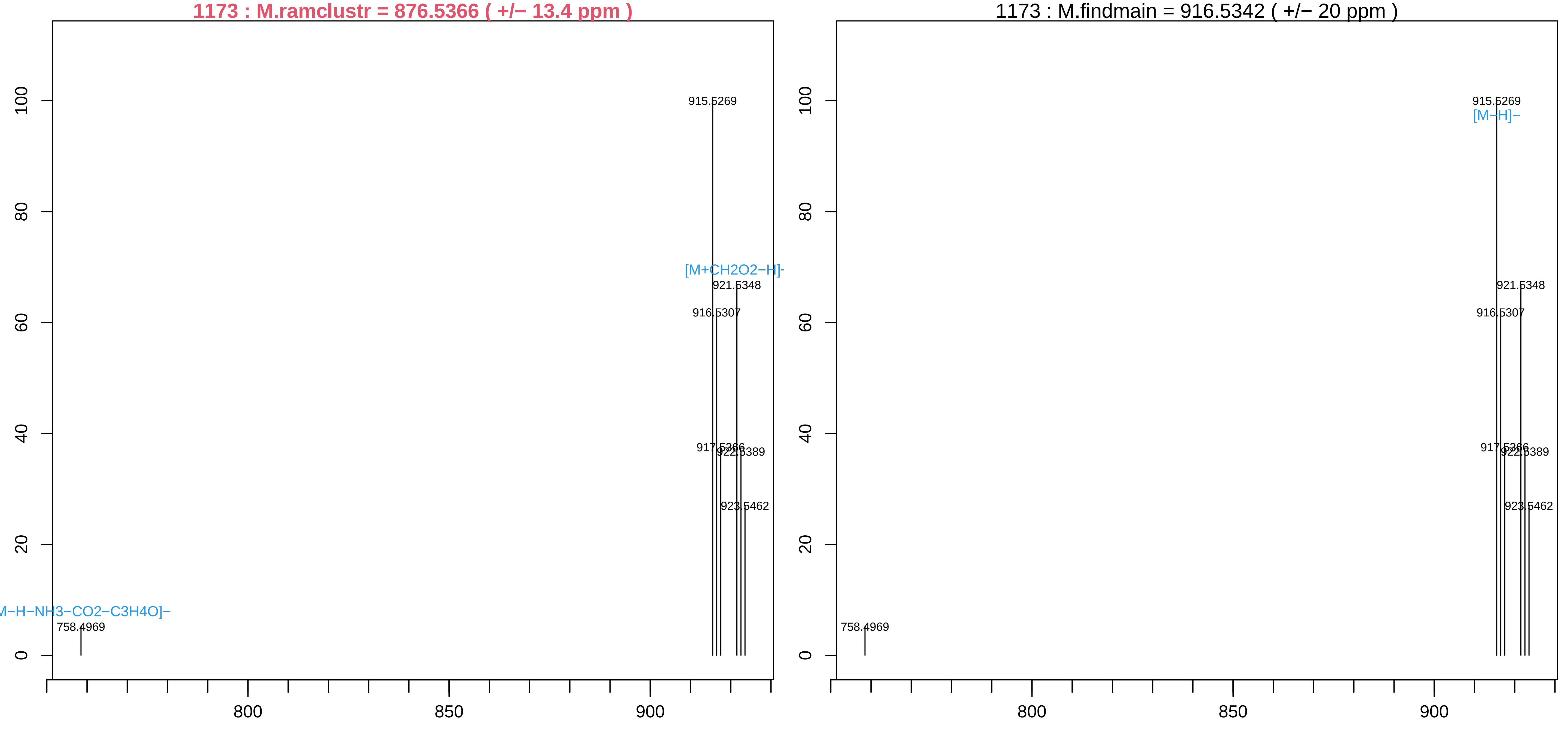
1173 : M.ramclustr = 876.5366 (+/− 13.4 ppm)
915.5269
[M+CH2O2−H]-
921.5348
916.5307
917.5366
922.5389
923.5462
M−H−NH3−CO2−C3H4O]−
758.4969
0
20
40
60
80
100
800
850
900
1173 : M.findmain = 916.5342 (+/− 20 ppm)
915.5269
[M−H]−
921.5348
916.5307
917.5366
922.5389
923.5462
758.4969
0
20
40
60
80
100
800
850
900

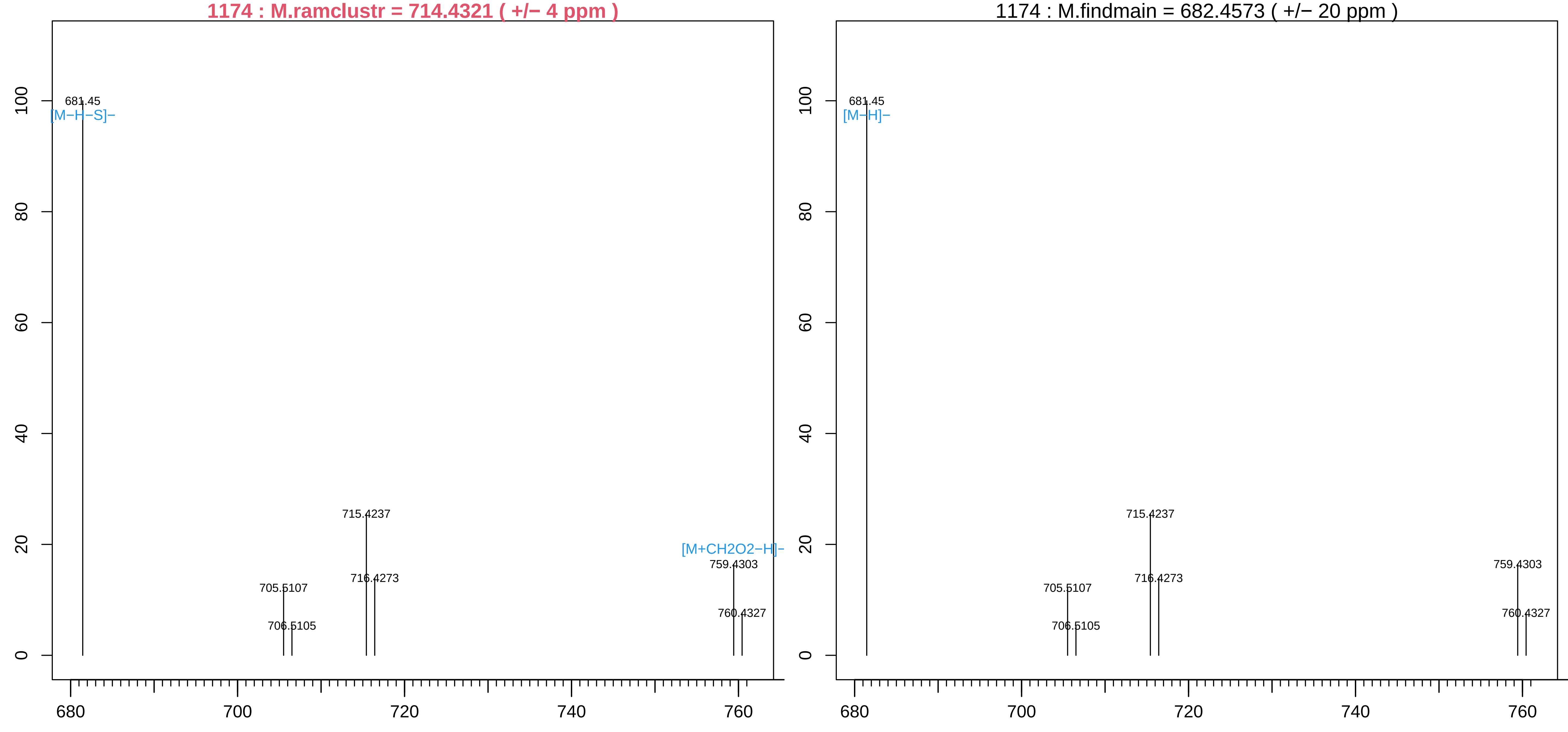
1174 : M.ramclustr = 714.4321 (+/− 4 ppm)
681.45
[M−H−S]−
705.5107
706.5105
715.4237
716.4273
[M+CH2O2−H]−
759.4303
760.4327
1174 : M.findmain = 682.4573 (+/− 20 ppm)
681.45
[M−H]−
705.5107
706.5105
715.4237
716.4273
759.4303
760.4327

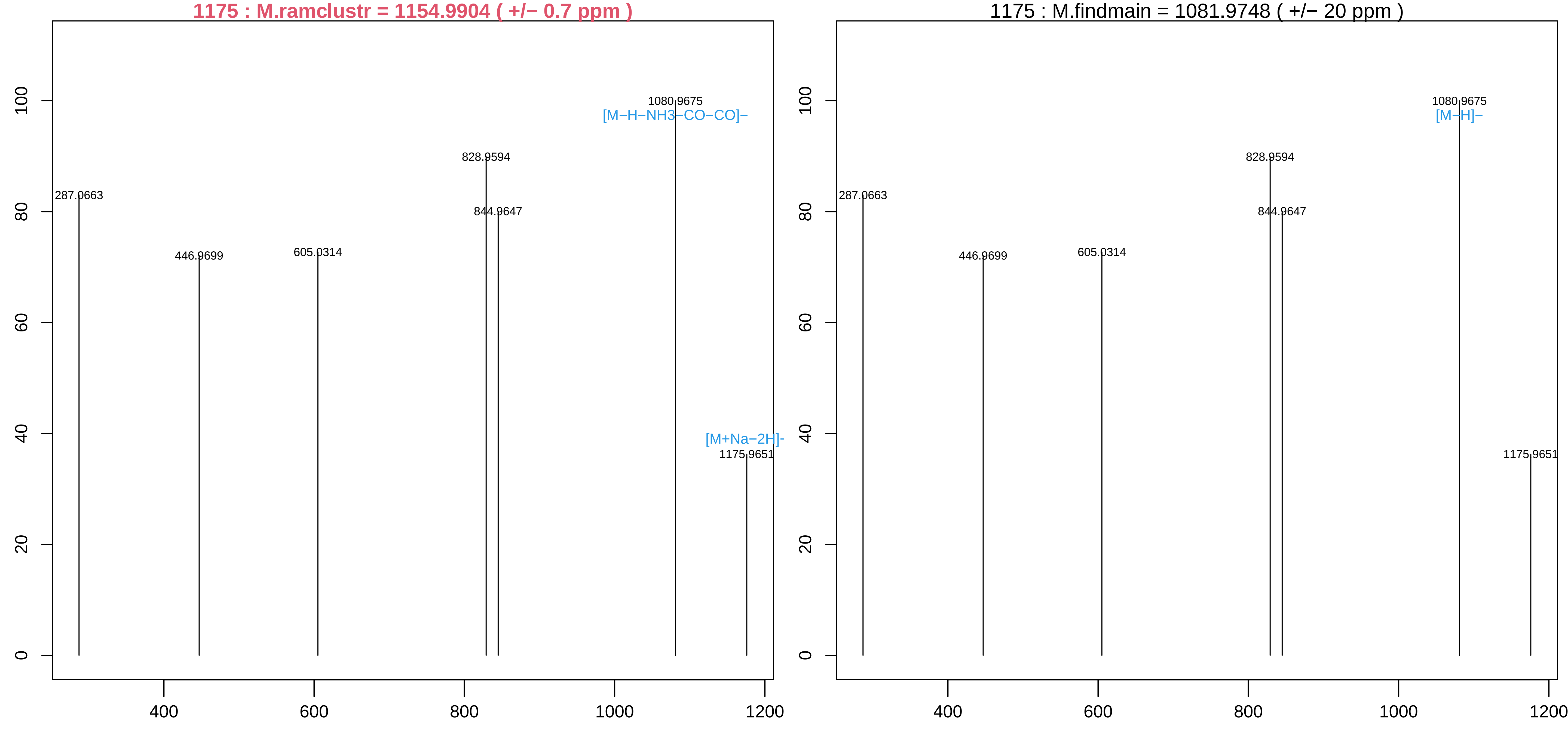
1175 : M.ramclustr = 1154.9904 (+/− 0.7 ppm)
1080.9675
[M−H−NH3−CO−CO]−
828.9594
287.0663
844.9647
446.9699
605.0314
[M+Na−2H]-
1175.9651
1175 : M.findmain = 1081.9748 (+/− 20 ppm)
1080.9675
[M−H]−
828.9594
287.0663
844.9647
446.9699
605.0314
1175.9651
0
20
40
60
80
100
400
600
800
1000
1200

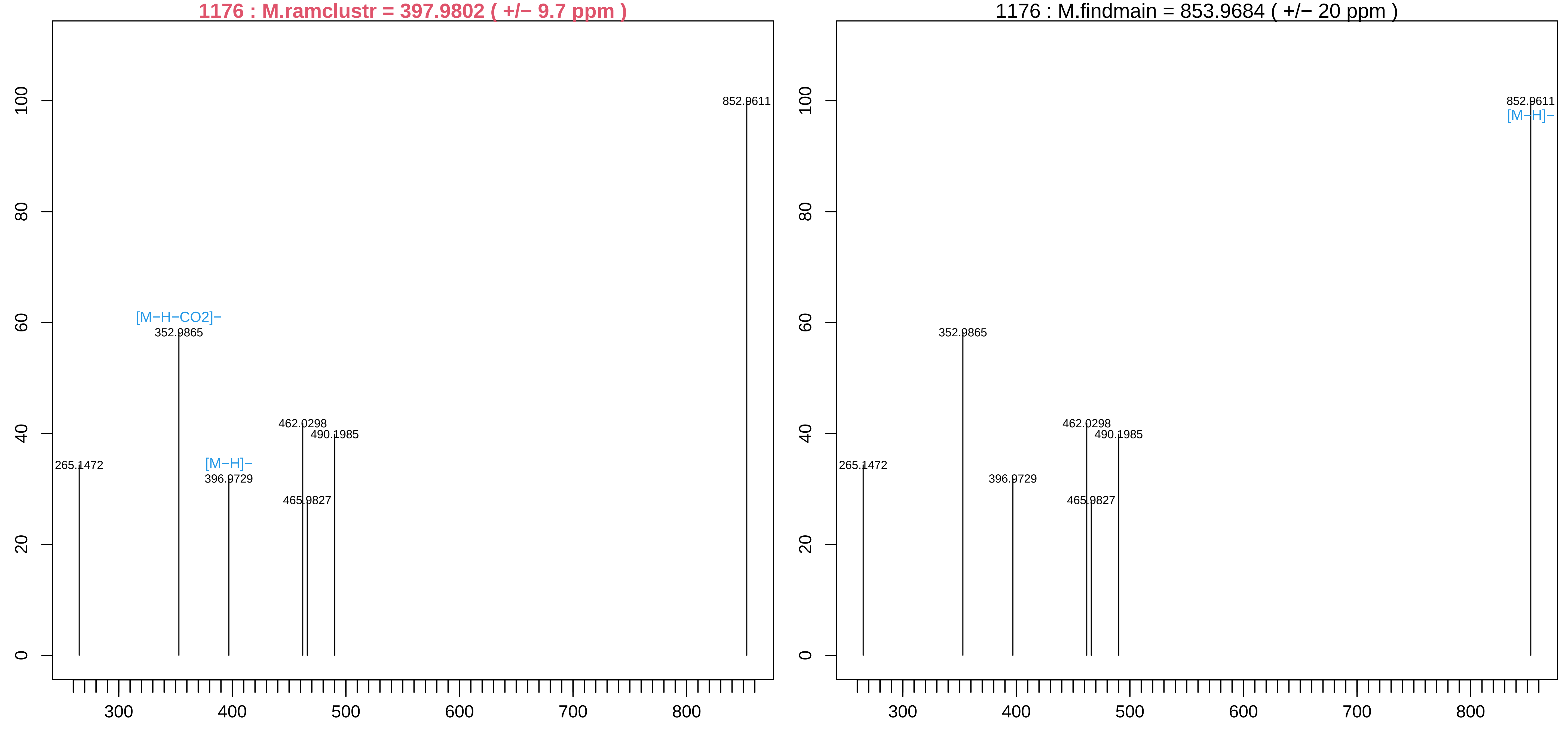
1176 : M.ramclustr = 397.9802 (+/− 9.7 ppm)
[M−H−CO2]−
352.9865
[M−H]−
396.9729
265.1472
462.0298
490.1985
465.9827
852.9611
1176 : M.findmain = 853.9684 (+/− 20 ppm)
852.9611
[M−H]−
352.9865
396.9729
265.1472
462.0298
490.1985
465.9827
0
20
40
60
80
100
300
400
500
600
700
800

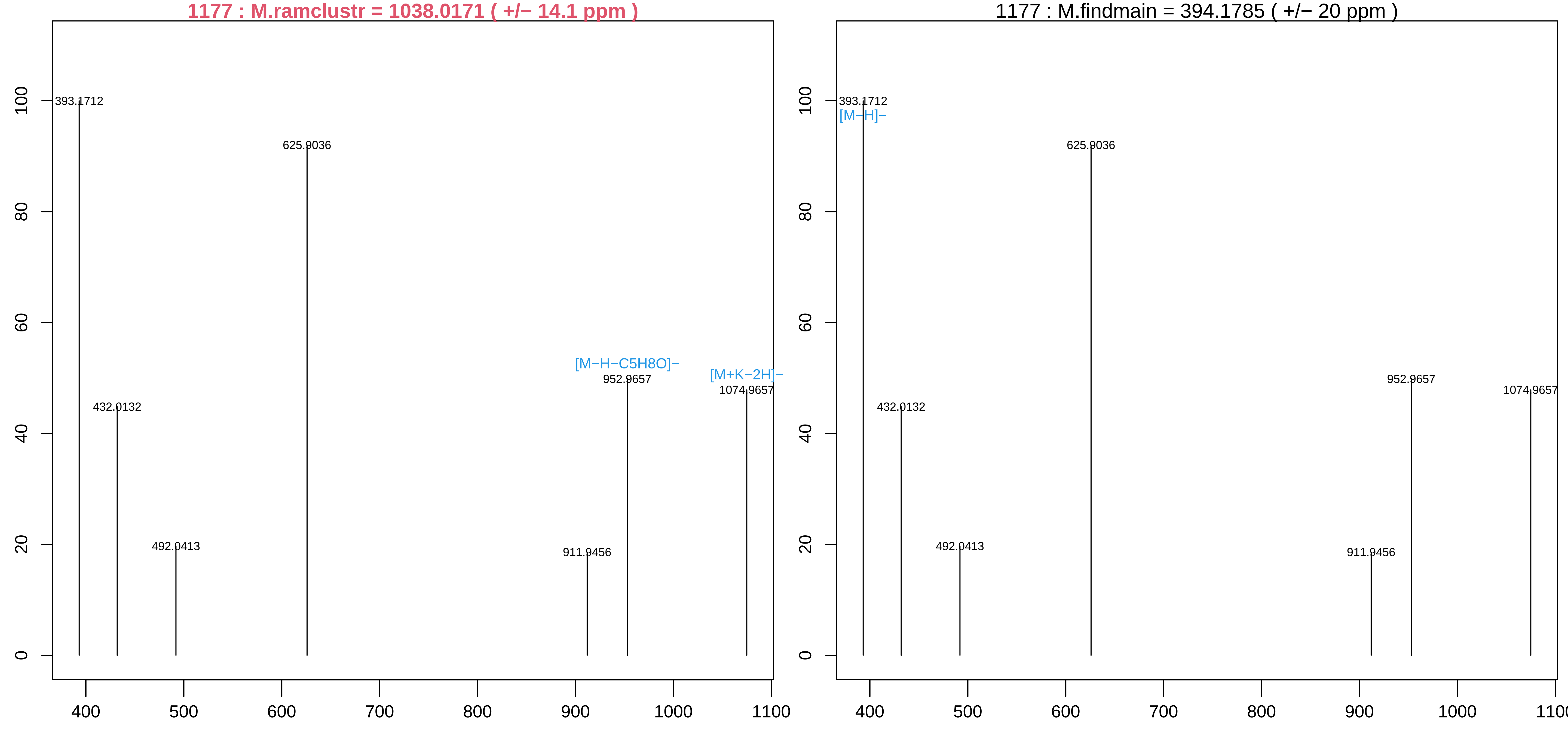

1177 : M.ramclustr = 1038.0171 (+/− 14.1 ppm)
393.1712
625.9036
432.0132
492.0413
911.9456
952.9657
[M−H−C5H8O]−
1074.9657
[M+K−2H]−
1177 : M.findmain = 394.1785 (+/− 20 ppm)
393.1712
[M−H]−
625.9036
432.0132
492.0413
911.9456
952.9657
1074.9657
0
20
40
60
80
100
400
500
600
700
800
900
1000
1100

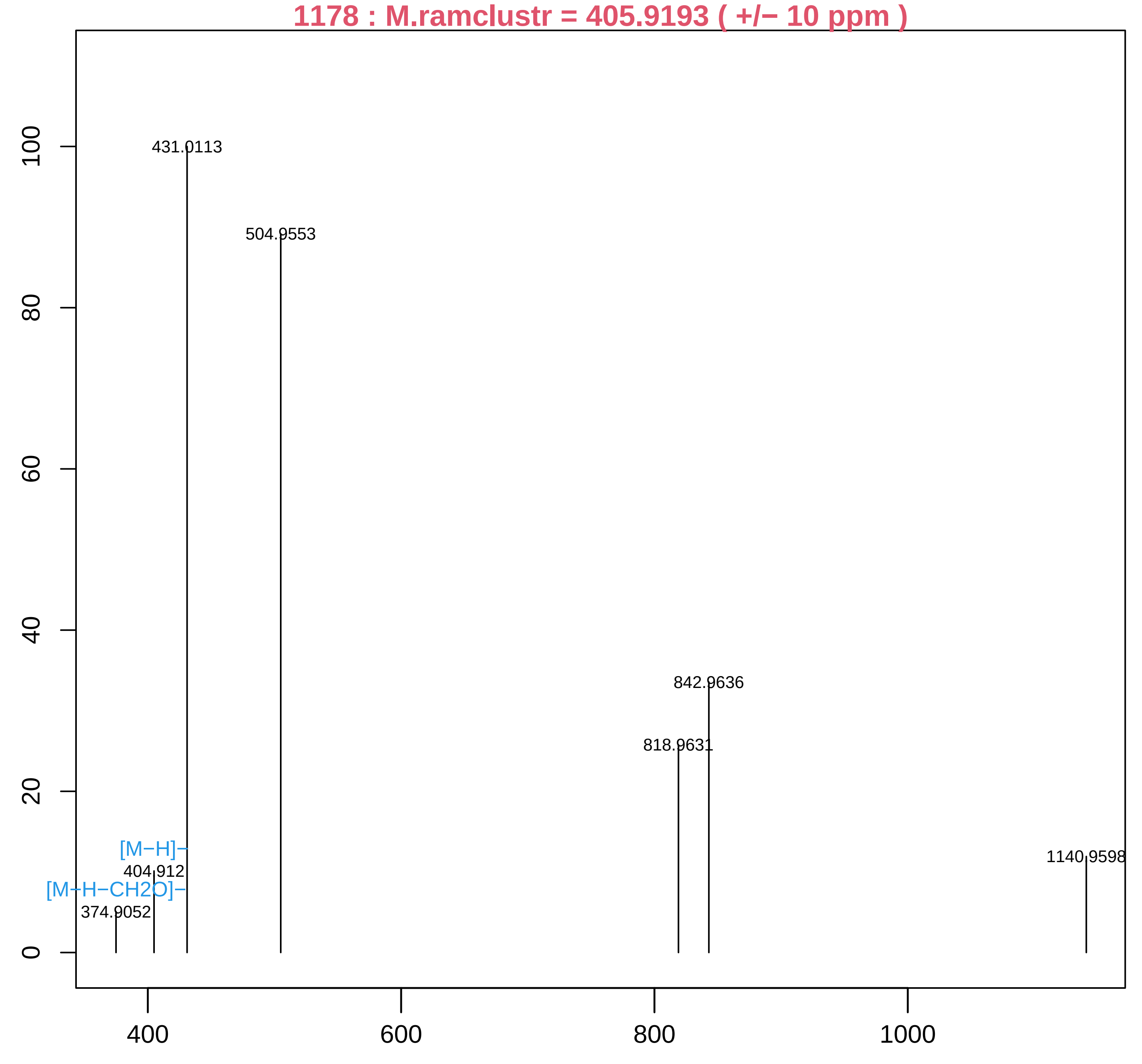

1178 : M.ramclustr = 405.9193 (+/− 10 ppm)
431.0113
504.9553
842.9636
818.9631
1140.9598
[M−H]−
404.912
[M−H−CH2O]−
374.9052
0
20
40
60
80
100
400
600
800
1000

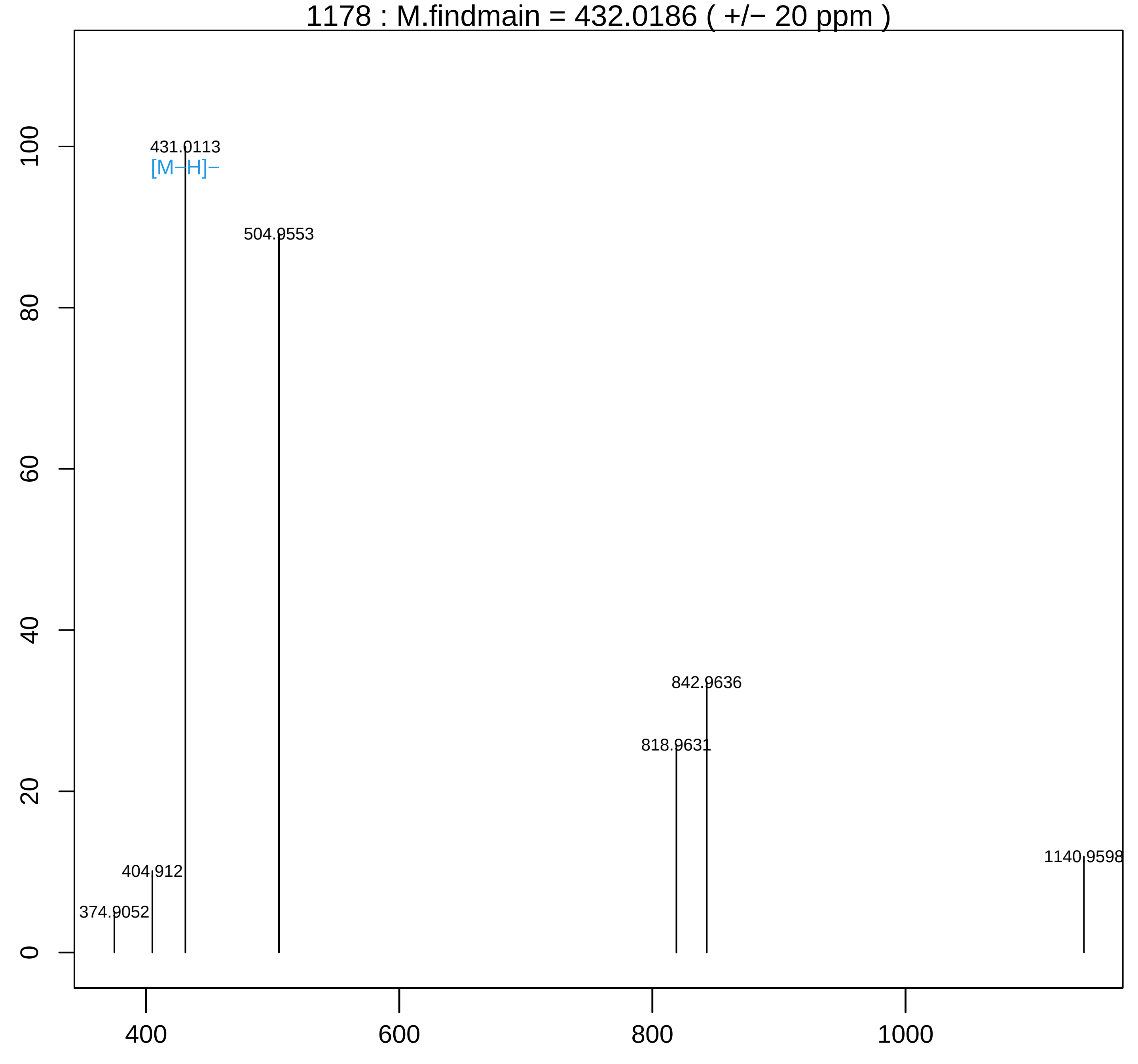

1178 : M.findmain = 432.0186 (+/− 20 ppm)
431.0113
[M−H]−
504.9553
842.9636
818.9631
1140.9598
404.912
374.9052
0
20
40
60
80
100
400
600
800
1000

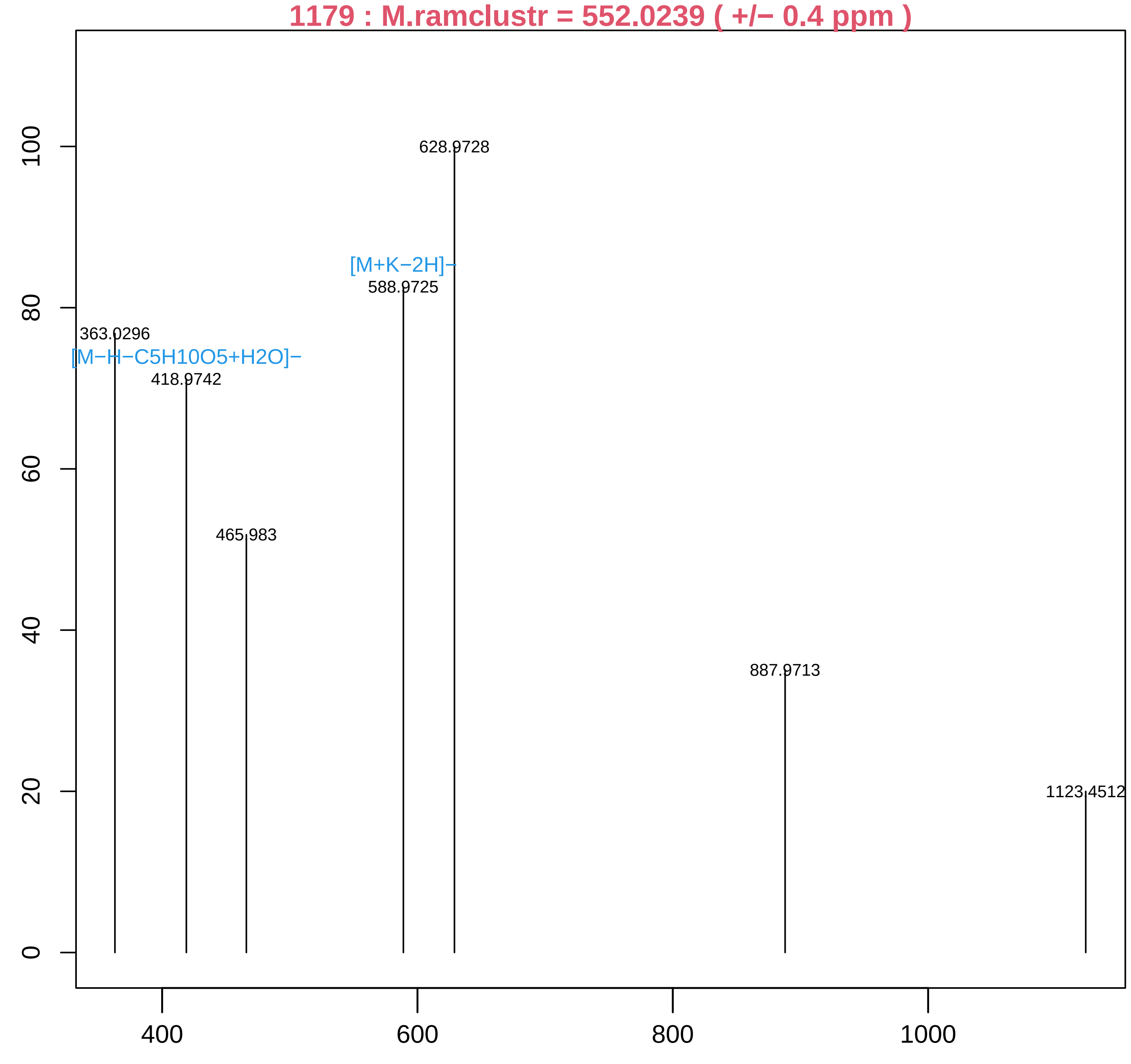
1179 : M.ramclustr = 552.0239 (+/− 0.4 ppm)
628.9728
[M+K−2H]−
588.9725
363.0296
[M−H−C5H10O5+H2O]−
418.9742
465.983
887.9713
1123.4512
0
20
40
60
80
100
400
600
800
1000

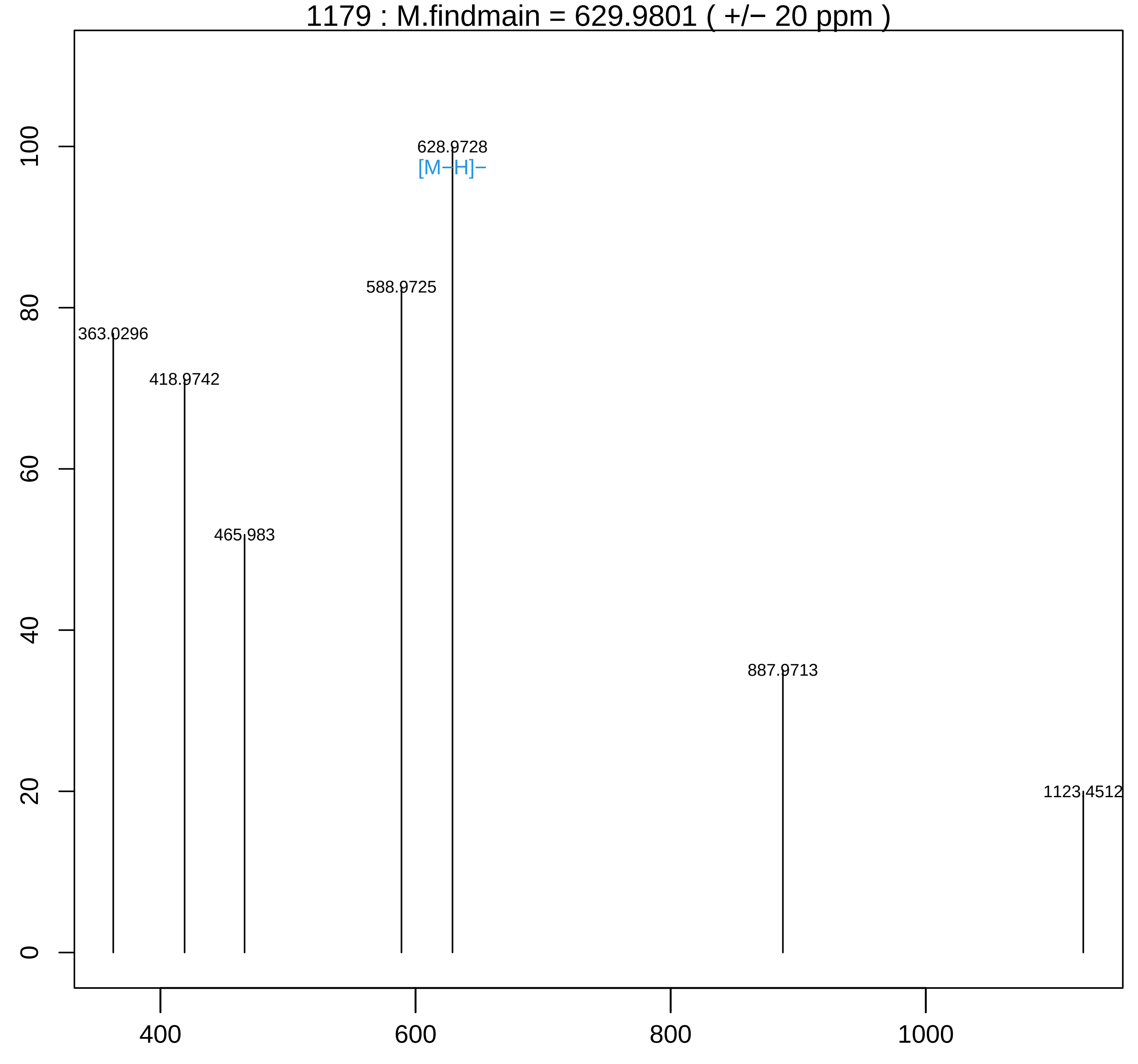
1179 : M.findmain = 629.9801 (+/− 20 ppm)
628.9728
[M−H]−
588.9725
363.0296
418.9742
465.983
887.9713
1123.4512
0
20
40
60
80
100
400
600
800
1000

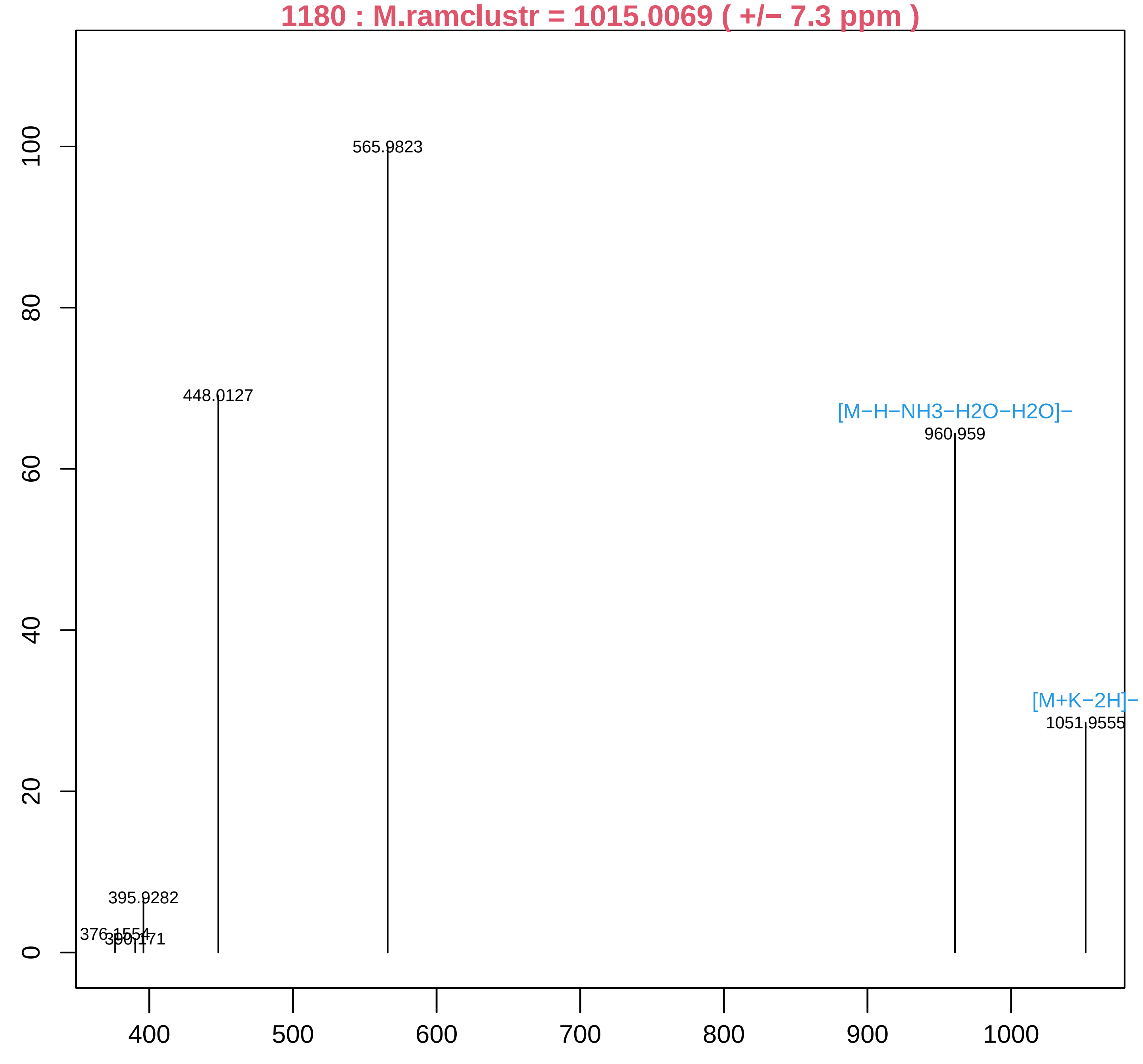

1180 : M.ramclustr = 1015.0069 (+/− 7.3 ppm)
565.9823
448.0127
[M−H−NH3−H2O−H2O]−
960.959
[M+K−2H]−
1051.9555
395.9282
376.1554
0
20
40
60
80
100
400
500
600
700
800
900
1000

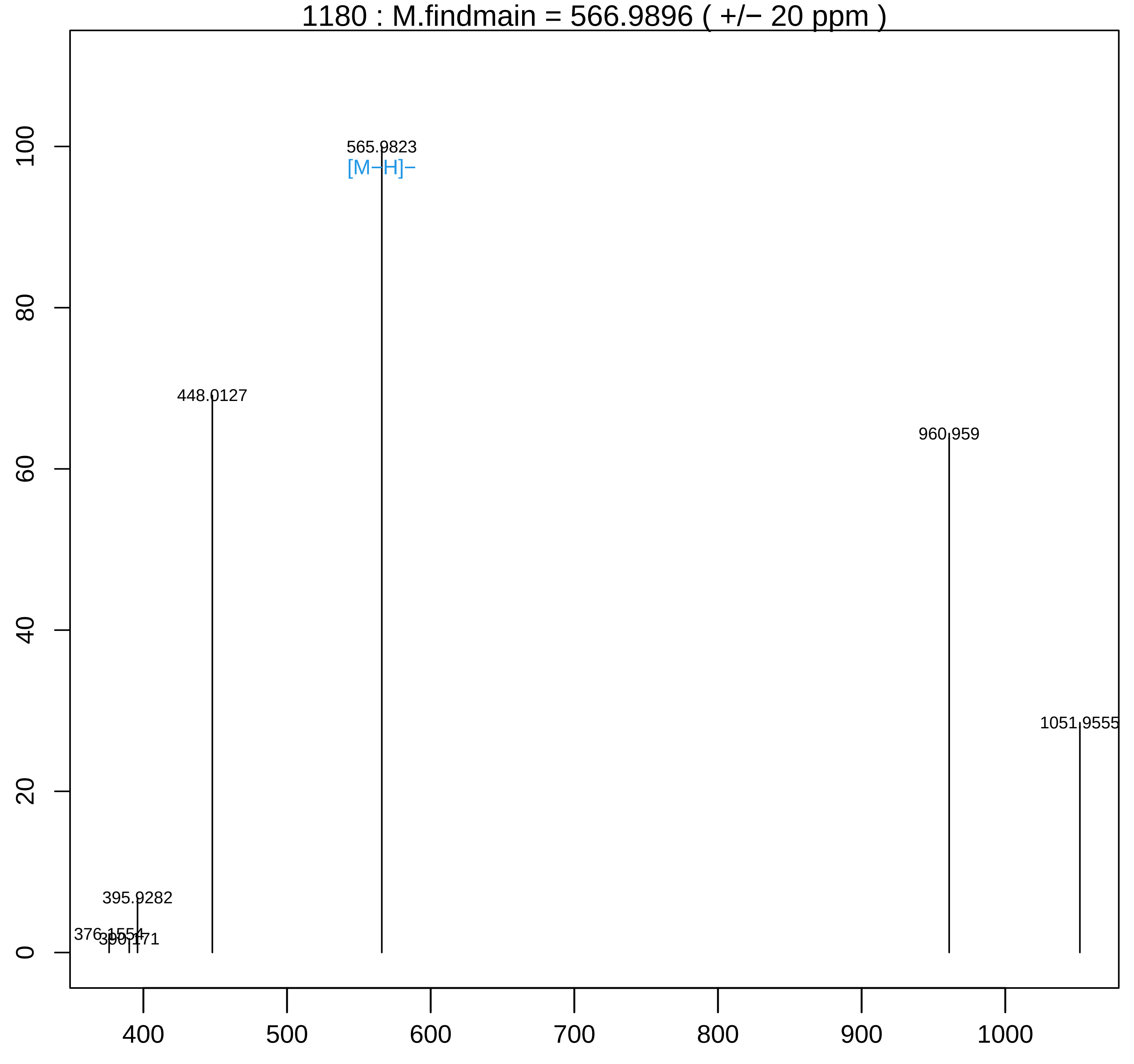

1180 : M.findmain = 566.9896 (+/− 20 ppm)
565.9823
[M−H]−
448.0127
960.959
1051.9555
395.9282
376.1554
0
20
40
60
80
100
400
500
600
700
800
900
1000

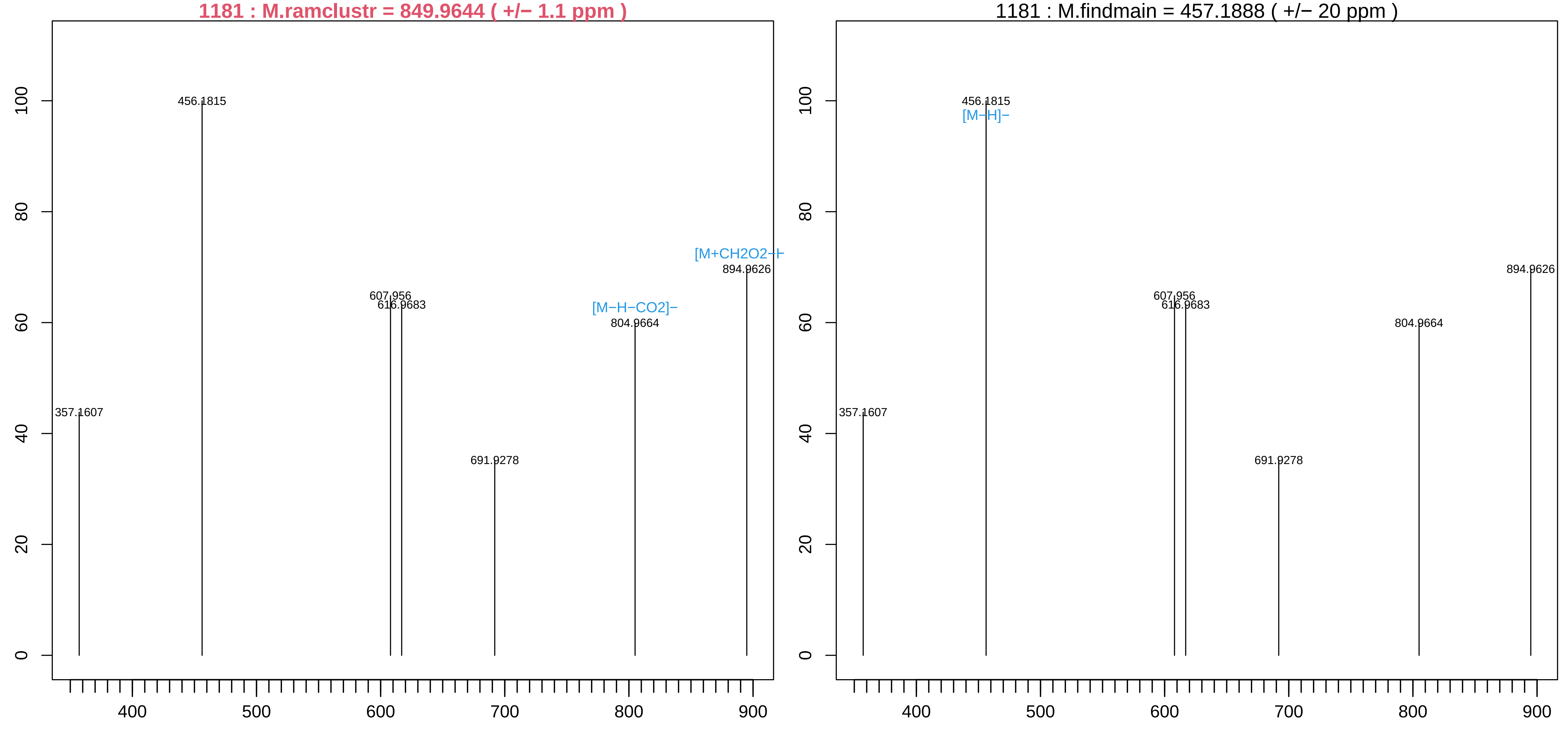
1181 : M.ramclustr = 849.9644 (+/− 1.1 ppm)
456.1815
357.1607
607.956
616.9683
691.9278
[M−H−CO2]−
804.9664
[M+CH2O2−H
894.9626
1181 : M.findmain = 457.1888 (+/− 20 ppm)
456.1815
[M−H]−
357.1607
607.956
616.9683
691.9278
804.9664
894.9626
0
20
40
60
80
100
400
500
600
700
800
900

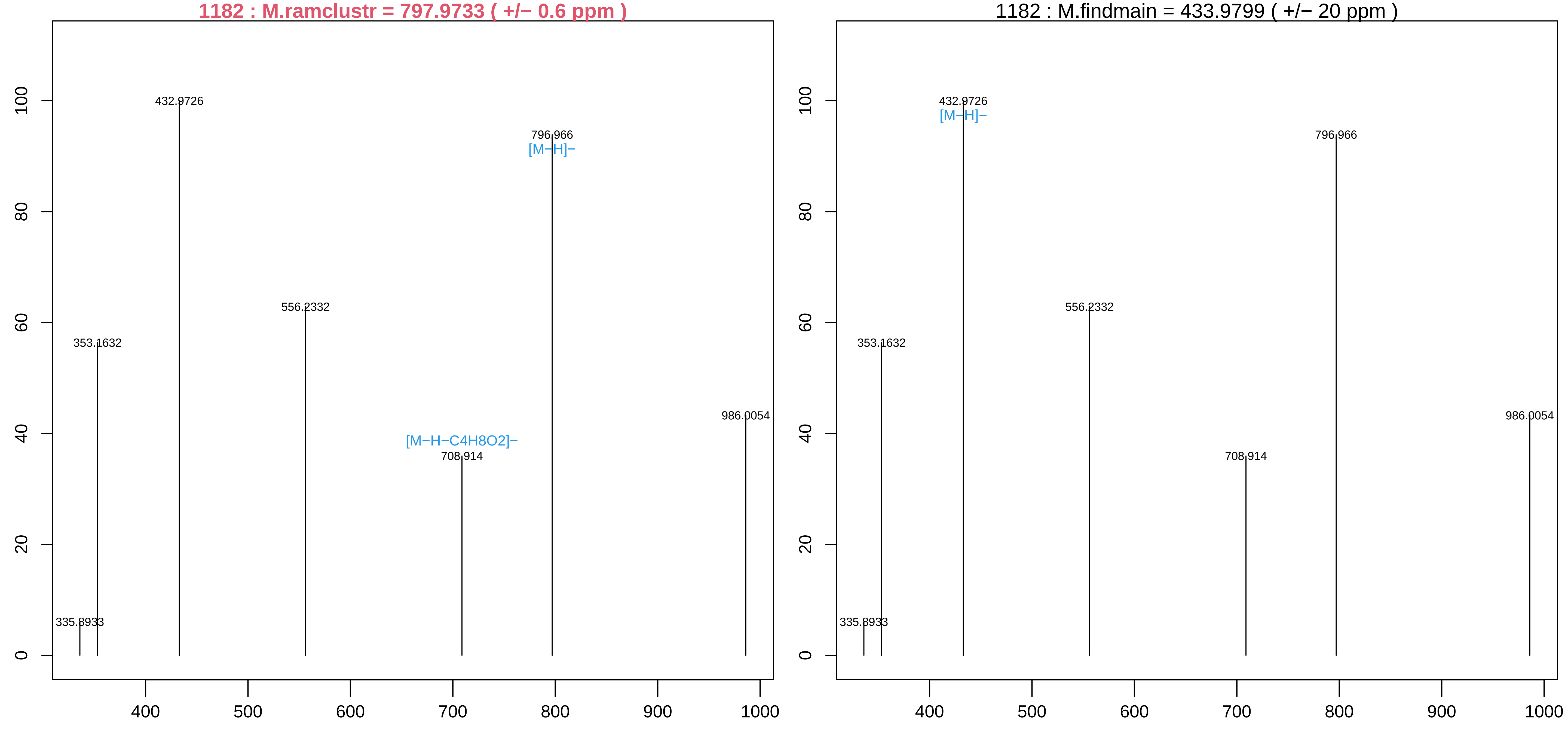

1182 : M.ramclustr = 797.9733 (+/− 0.6 ppm)
432.9726
796.966
[M−H]−
556.2332
353.1632
986.0054
[M−H−C4H8O2]−
708.914
335.8933
0
20
40
60
80
100
400
500
600
700
800
900
1000
1182 : M.findmain = 433.9799 (+/− 20 ppm)
432.9726
[M−H]−
796.966
556.2332
353.1632
986.0054
708.914
335.8933
0
20
40
60
80
100
400
500
600
700
800
900
1000

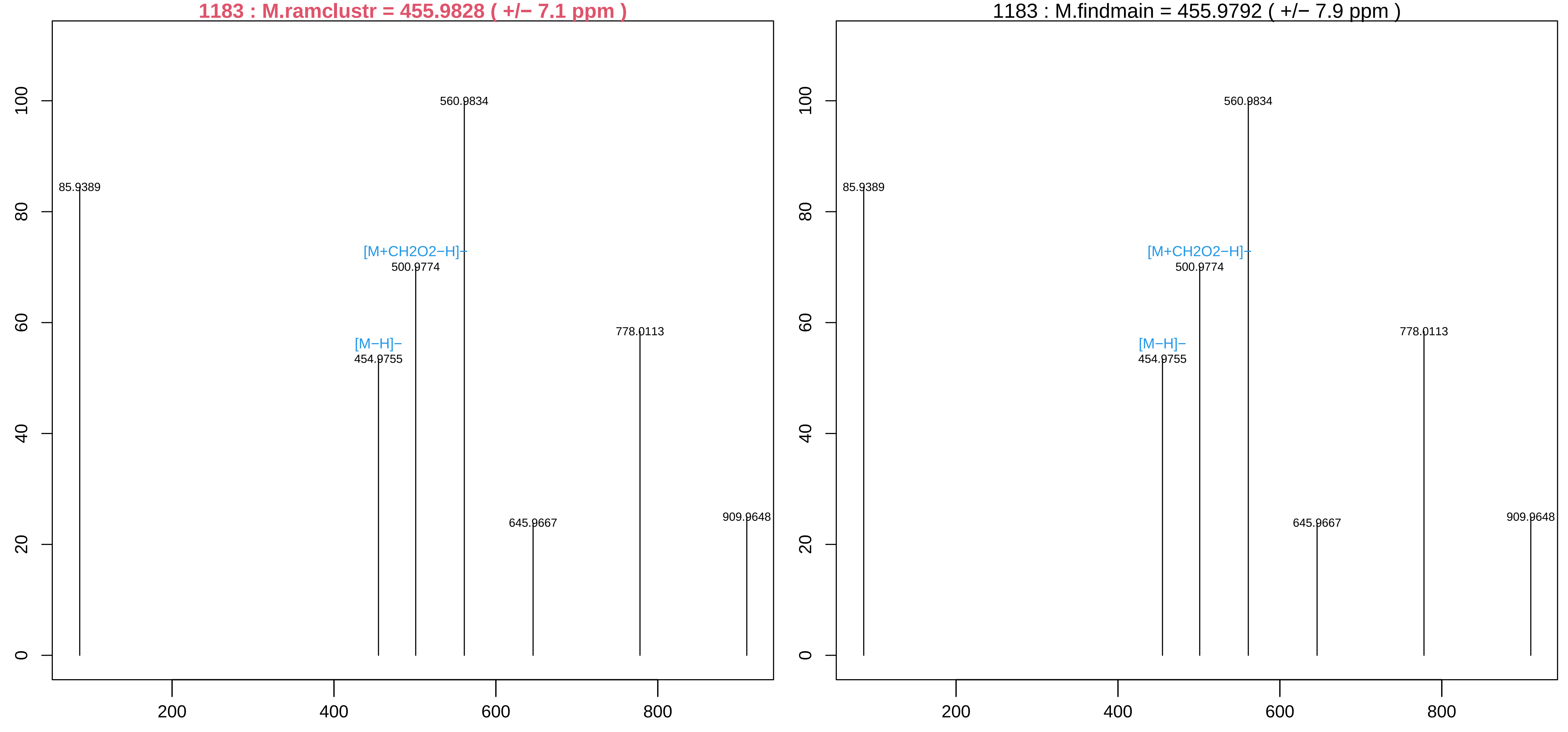
1183 : M.ramclustr = 455.9828 (+/− 7.1 ppm)
85.9389
560.9834
[M+CH2O2−H]−
500.9774
[M−H]−
454.9755
778.0113
645.9667
909.9648
0
20
40
60
80
100
200
400
600
800
1183 : M.findmain = 455.9792 (+/− 7.9 ppm)
85.9389
560.9834
[M+CH2O2−H]−
500.9774
[M−H]−
454.9755
778.0113
645.9667
909.9648
0
20
40
60
80
100
200
400
600
800

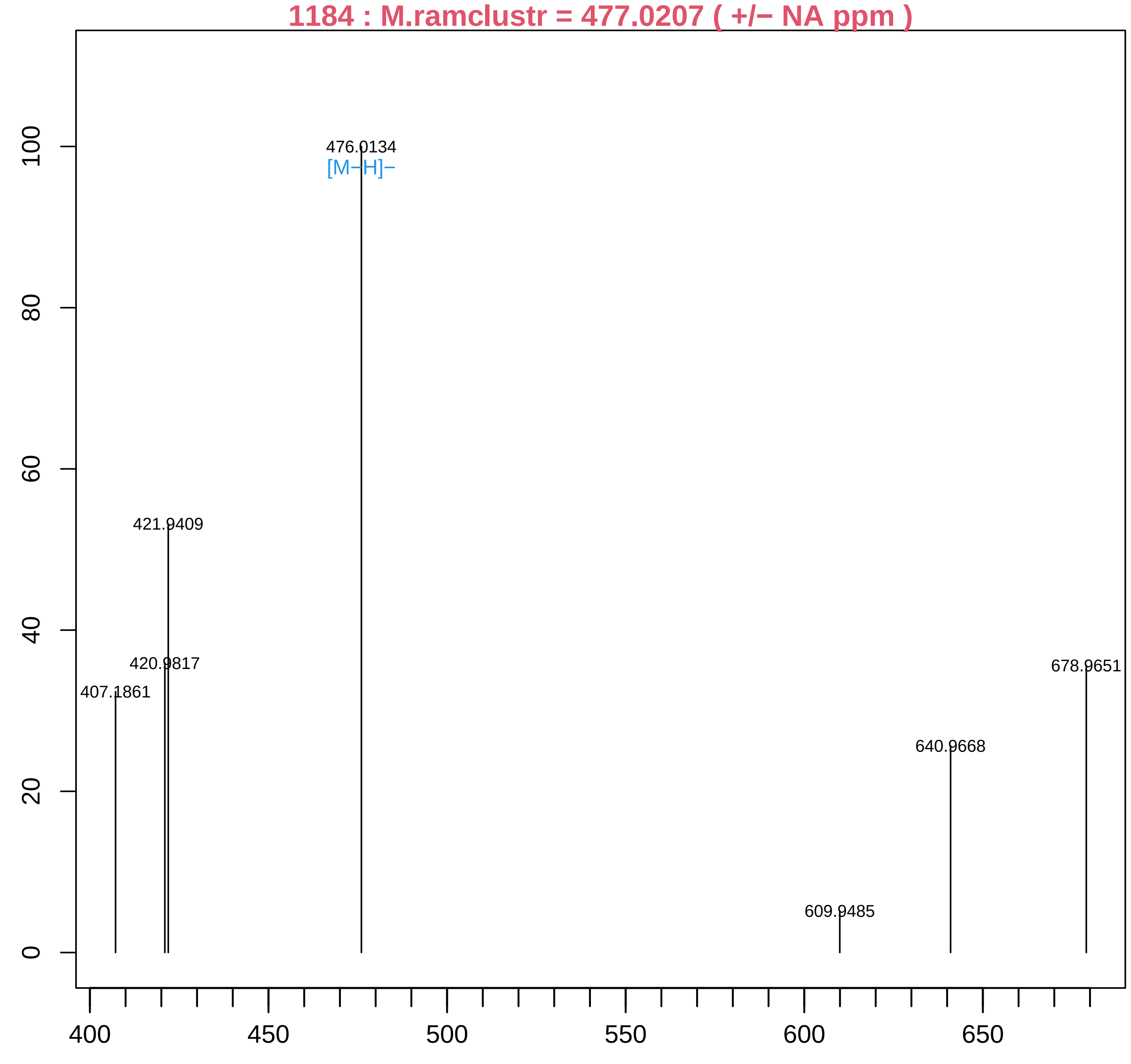
1184 : M.ramclustr = 477.0207 (+/− NA ppm)
476.0134
[M−H]−
421.9409
420.9817
407.1861
678.9651
640.9668
609.9485
0
20
40
60
80
100
400
450
500
550
600
650

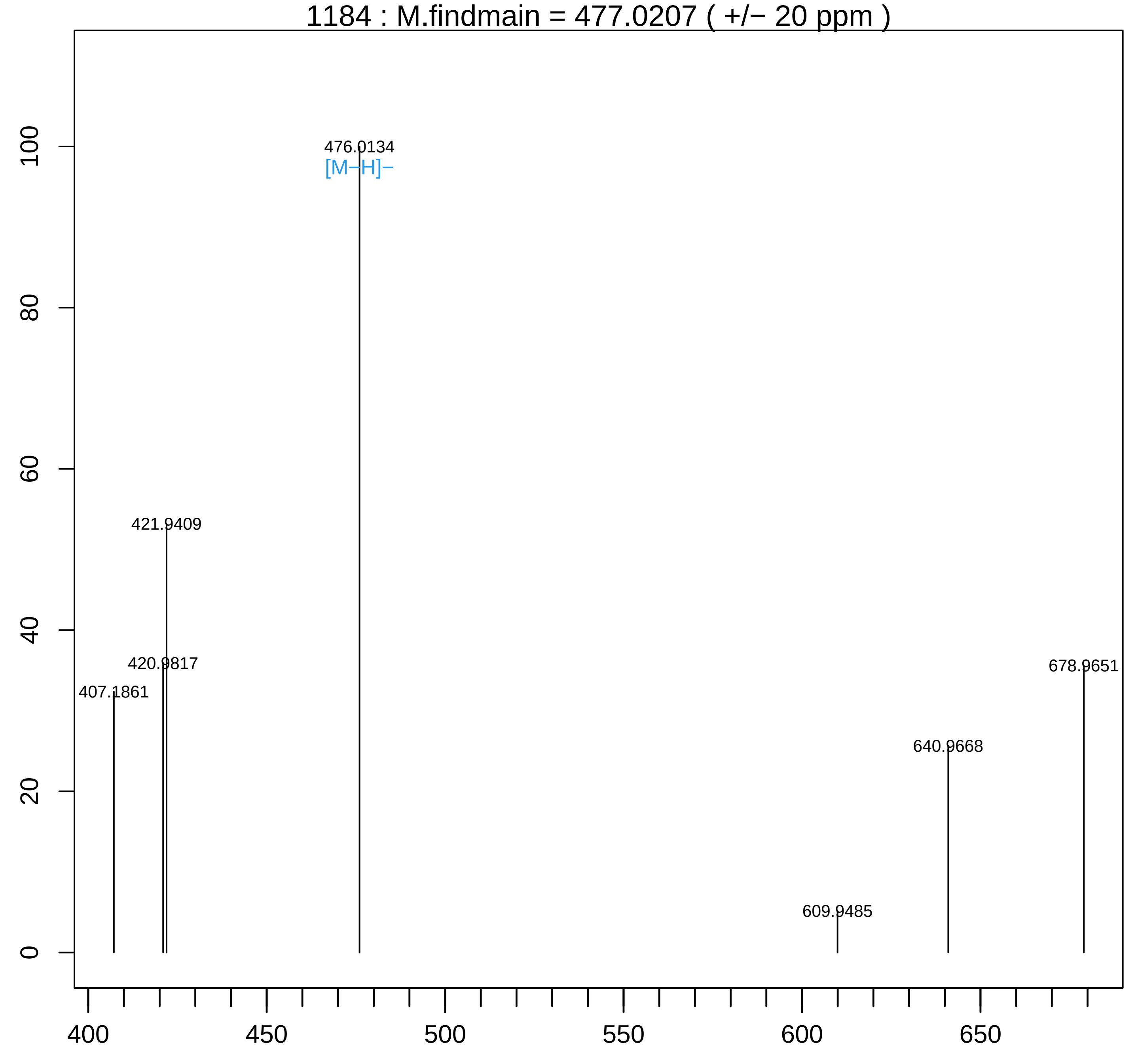
1184 : M.findmain = 477.0207 (+/− 20 ppm)
476.0134
[M−H]−
421.9409
420.9817
407.1861
678.9651
640.9668
609.9485
0
20
40
60
80
100
400
450
500
550
600
650

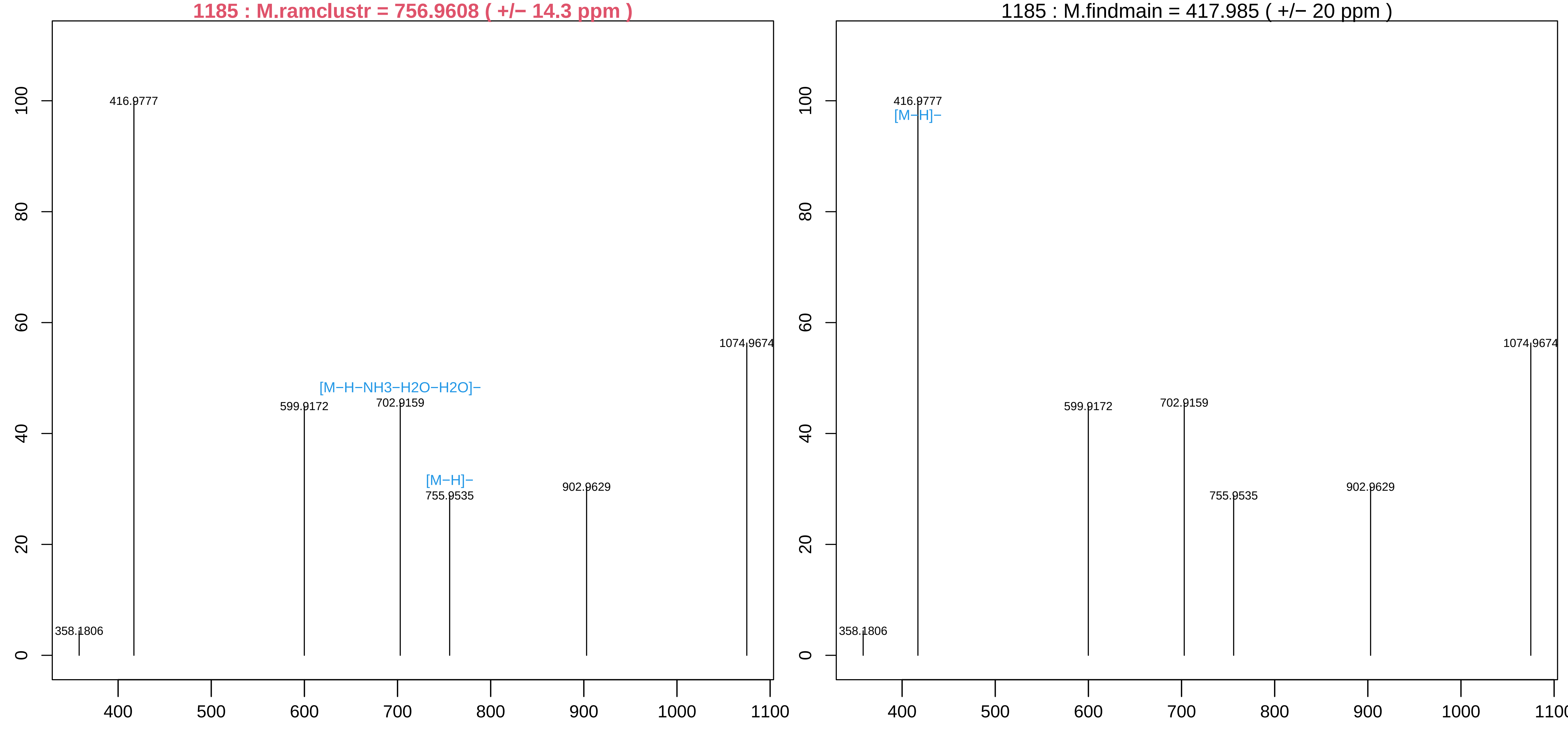
1185 : M.ramclustr = 756.9608 (+/− 14.3 ppm)
416.9777
358.1806
599.9172
702.9159
[M−H−NH3−H2O−H2O]−
[M−H]−
755.9535
902.9629
1074.9674
1185 : M.findmain = 417.985 (+/− 20 ppm)
416.9777
[M−H]−
358.1806
599.9172
702.9159
755.9535
902.9629
1074.9674
0
20
40
60
80
100
400
500
600
700
800
900
1000
1100

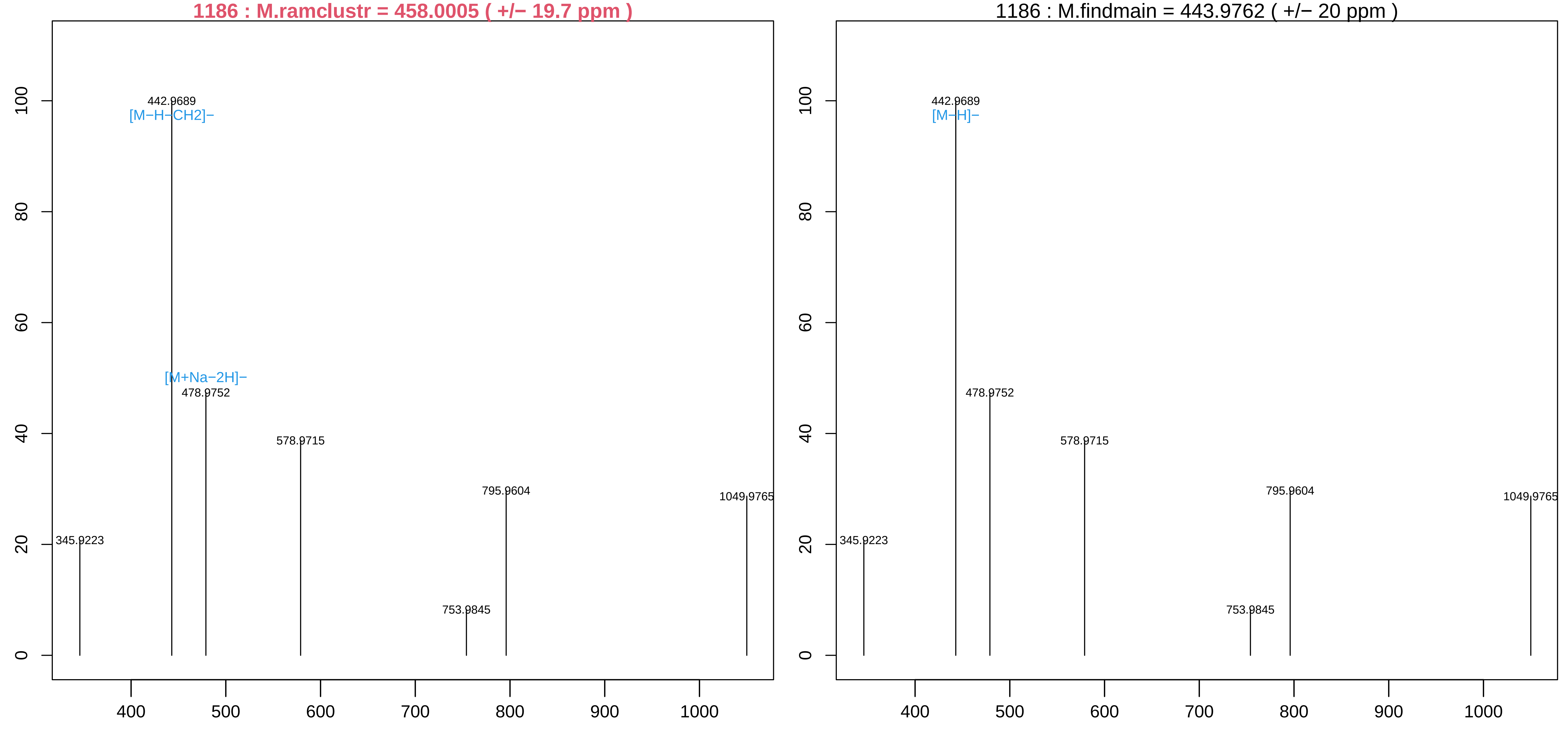

1186 : M.ramclustr = 458.0005 (+/− 19.7 ppm)
442.9689
[M−H−CH2]−
[M+Na−2H]−
478.9752
578.9715
795.9604
1049.9765
345.9223
753.9845
1186 : M.findmain = 443.9762 (+/− 20 ppm)
442.9689
[M−H]−
478.9752
578.9715
795.9604
1049.9765
345.9223
753.9845
0
20
40
60
80
100
400
500
600
700
800
900
1000

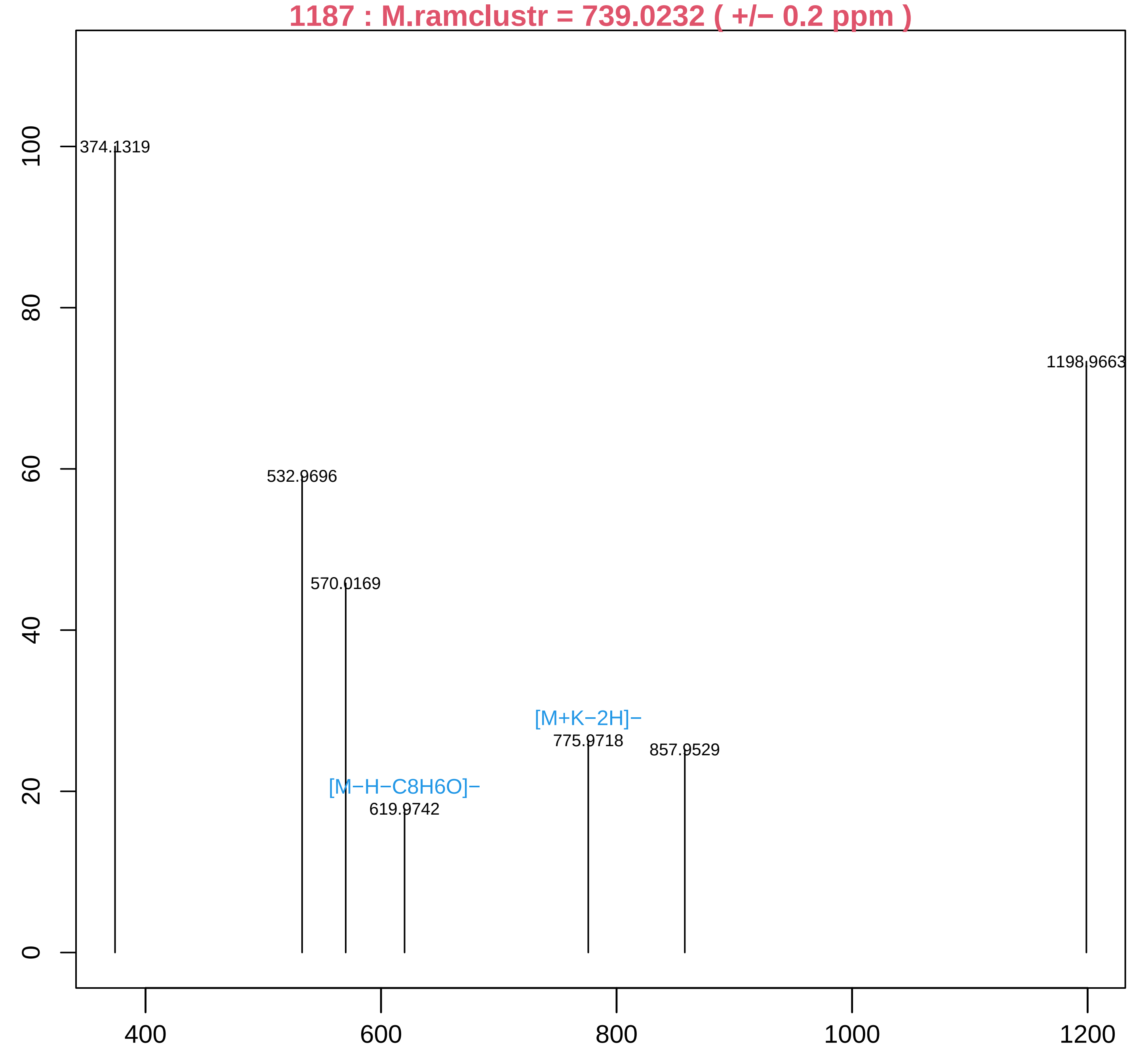
1187 : M.ramclustr = 739.0232 (+/− 0.2 ppm)
374.1319
532.9696
570.0169
[M−H−C8H6O]−
619.9742
[M+K−2H]−
775.9718
857.9529
1198.9663
0
20
40
60
80
100
400
600
800
1000
1200

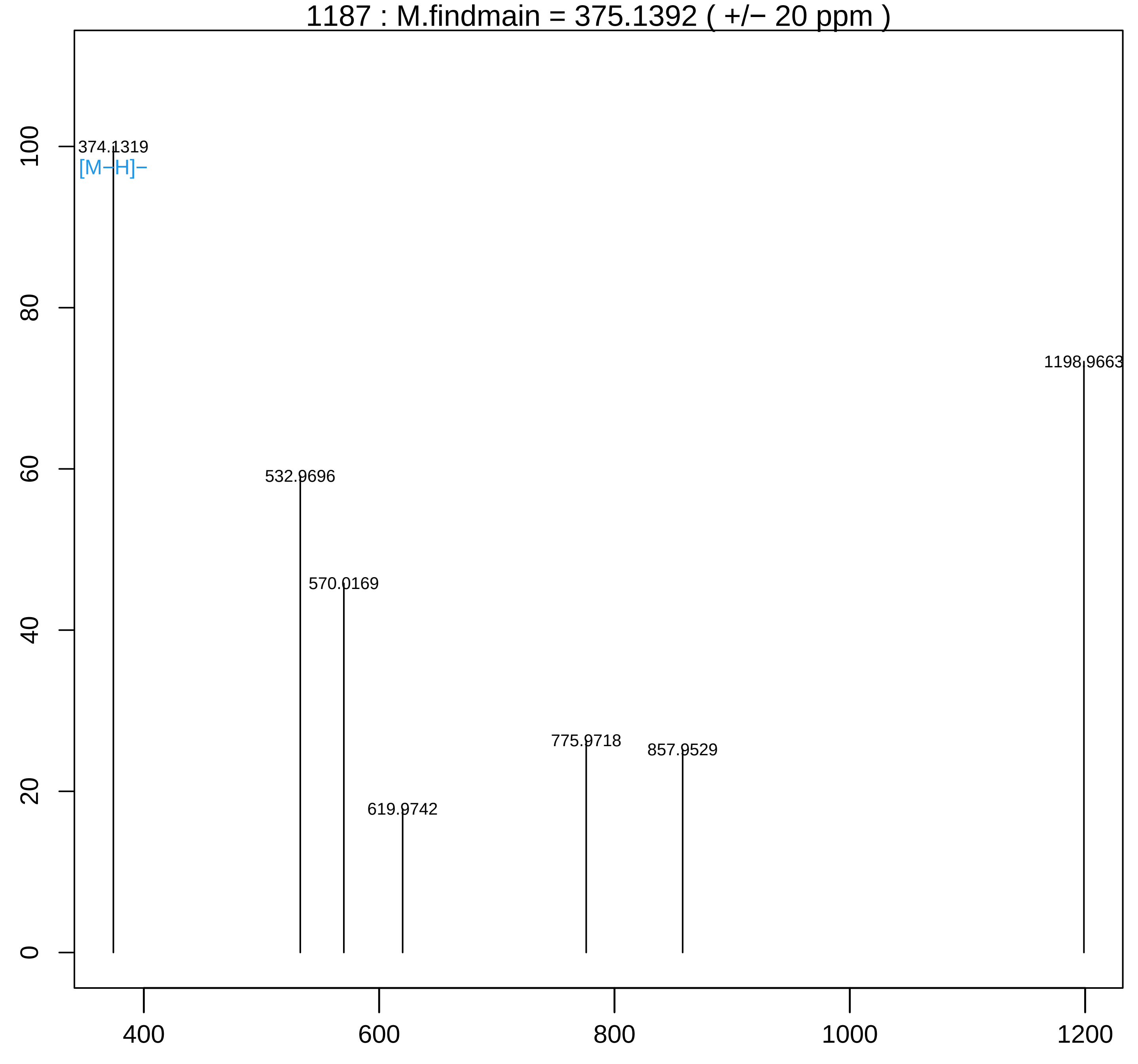
1187 : M.findmain = 375.1392 (+/− 20 ppm)
374.1319
[M−H]−
532.9696
570.0169
619.9742
775.9718
857.9529
1198.9663
0
20
40
60
80
100
400
600
800
1000
1200

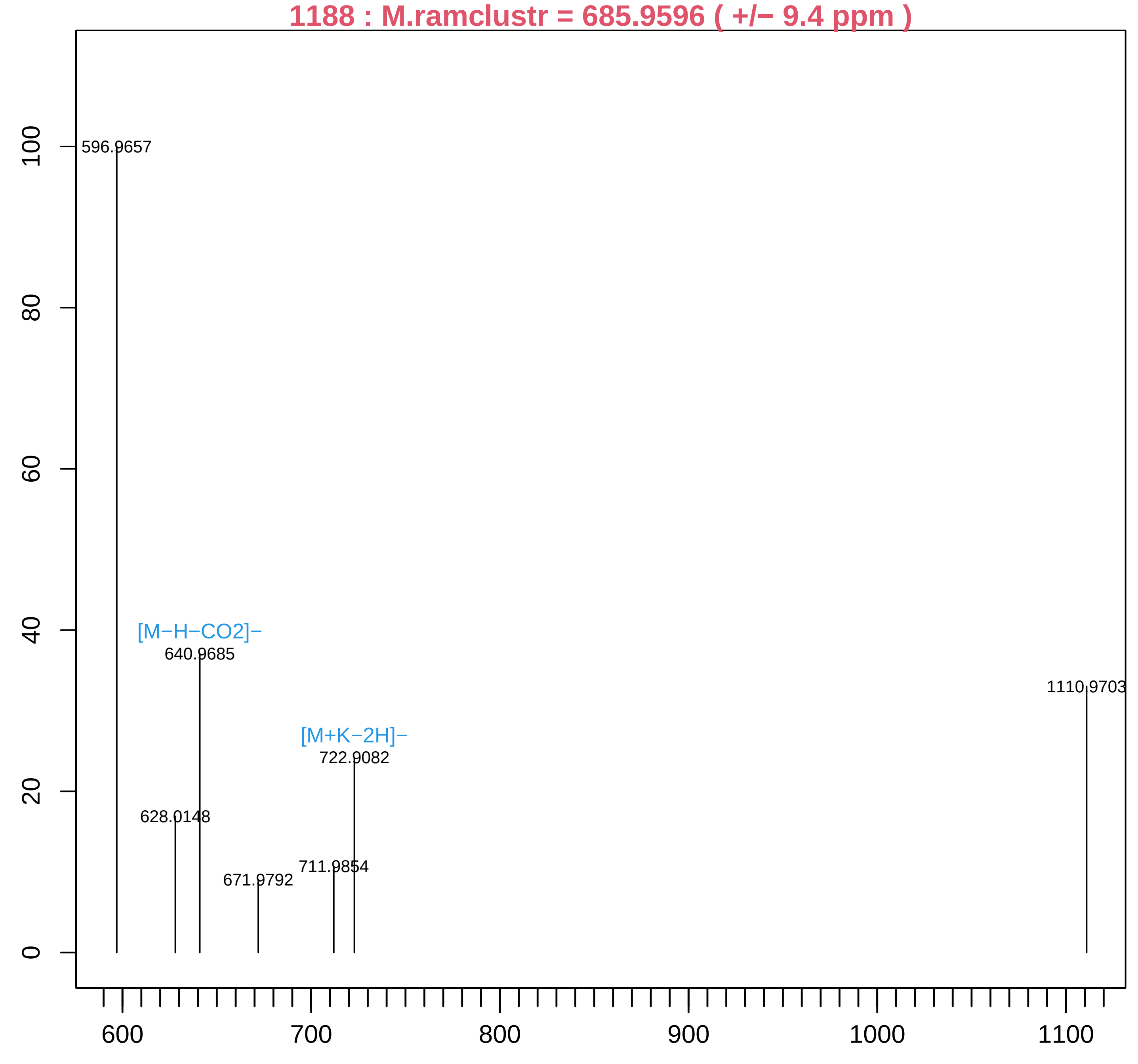
1188 : M.ramclustr = 685.9596 (+/− 9.4 ppm)
596.9657
[M−H−CO2]−
640.9685
1110.9703
[M+K−2H]−
722.9082
628.0148
671.9792
711.9854
0
20
40
60
80
100
600
700
800
900
1000
1100

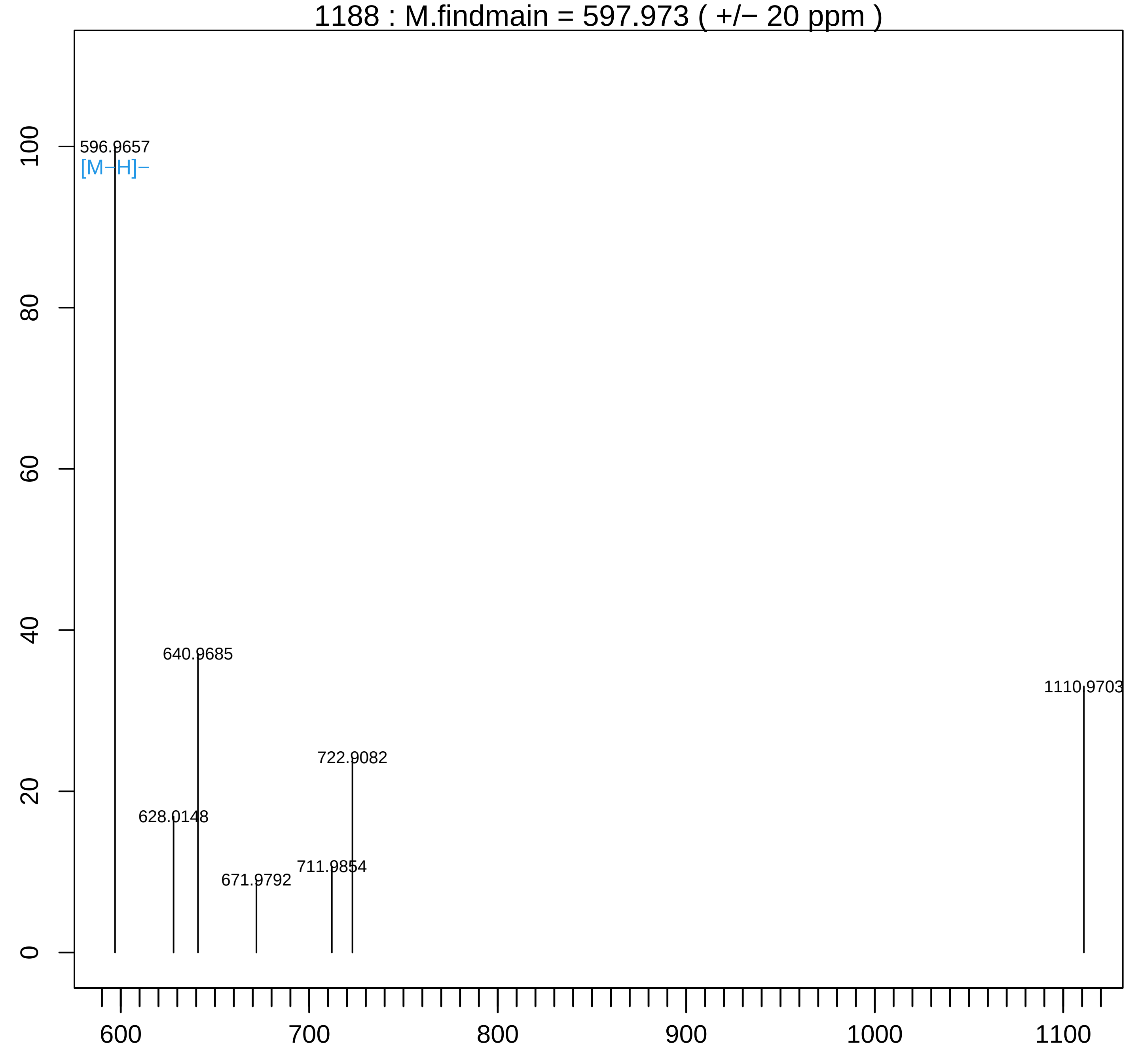
1188 : M.findmain = 597.973 (+/− 20 ppm)
596.9657
[M−H]−
640.9685
1110.9703
722.9082
628.0148
671.9792
711.9854
0
20
40
60
80
100
600
700
800
900
1000
1100

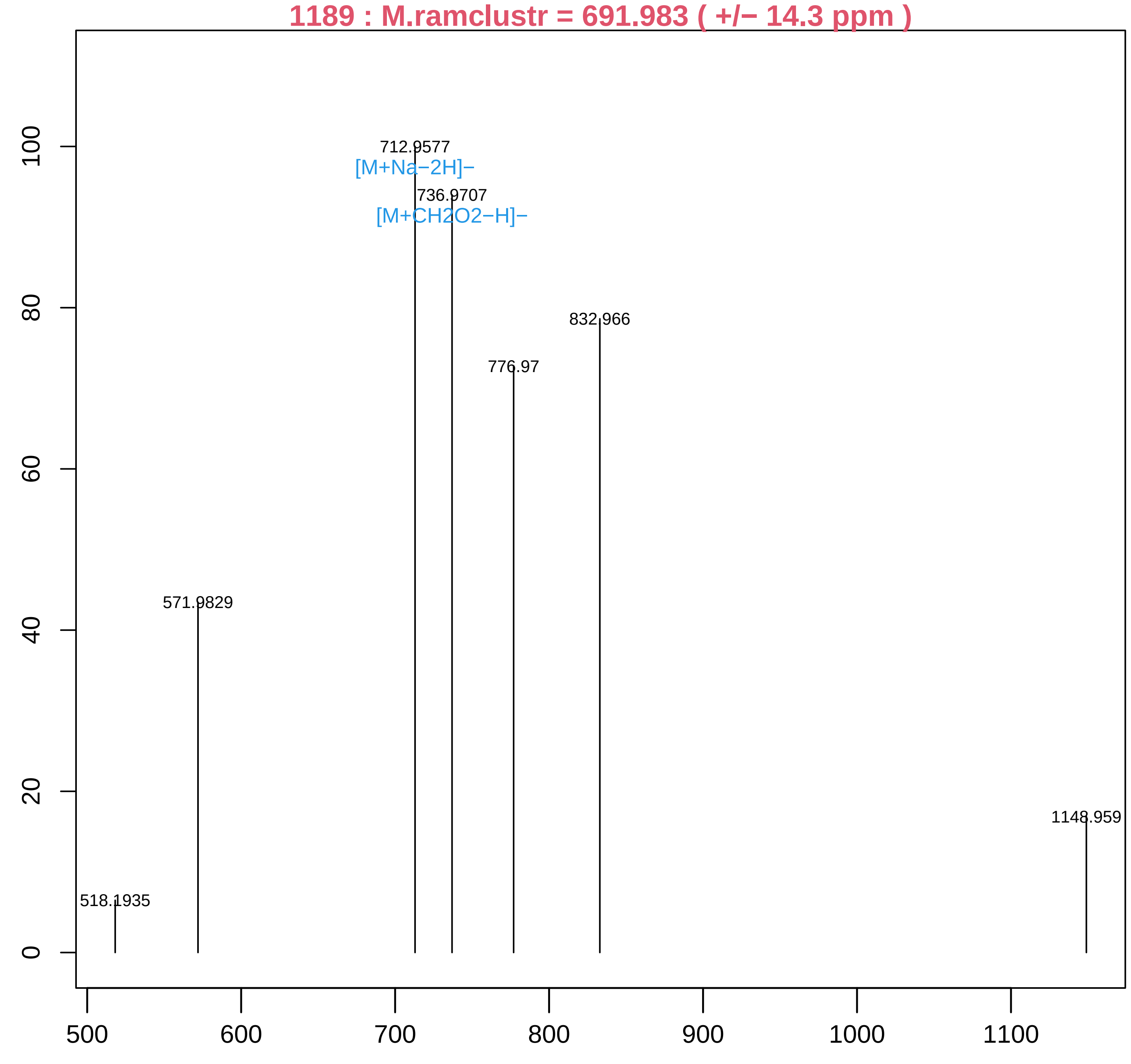
1189 : M.ramclustr = 691.983 (+/− 14.3 ppm)
712.9577
[M+Na−2H]−
736.9707
[M+CH2O2−H]−
832.966
776.97
571.9829
1148.959
518.1935
0
20
40
60
80
100
500
600
700
800
900
1000
1100

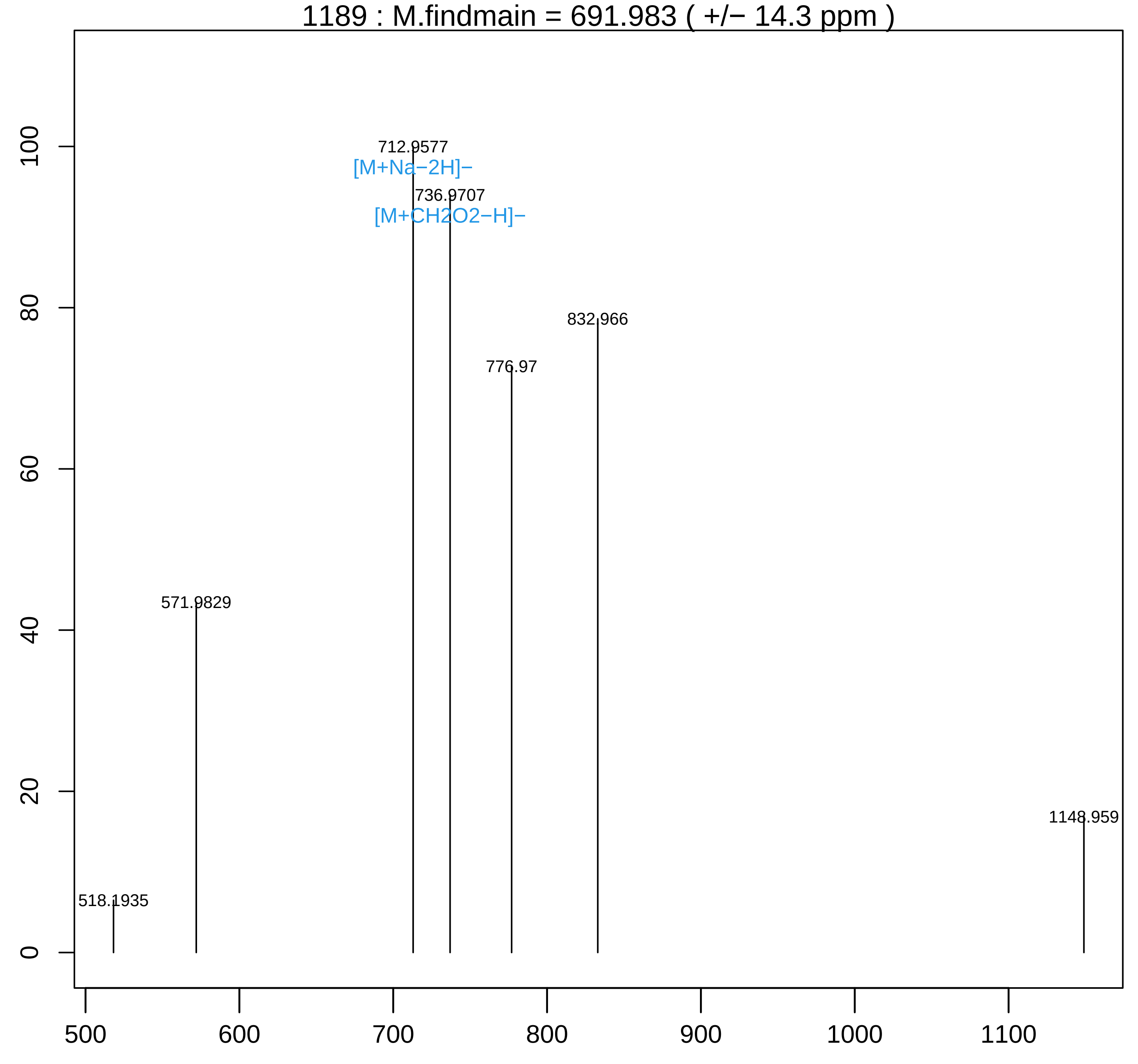
1189 : M.findmain = 691.983 (+/− 14.3 ppm)
712.9577
[M+Na−2H]−
736.9707
[M+CH2O2−H]−
832.966
776.97
571.9829
1148.959
518.1935
0
20
40
60
80
100
500
600
700
800
900
1000
1100

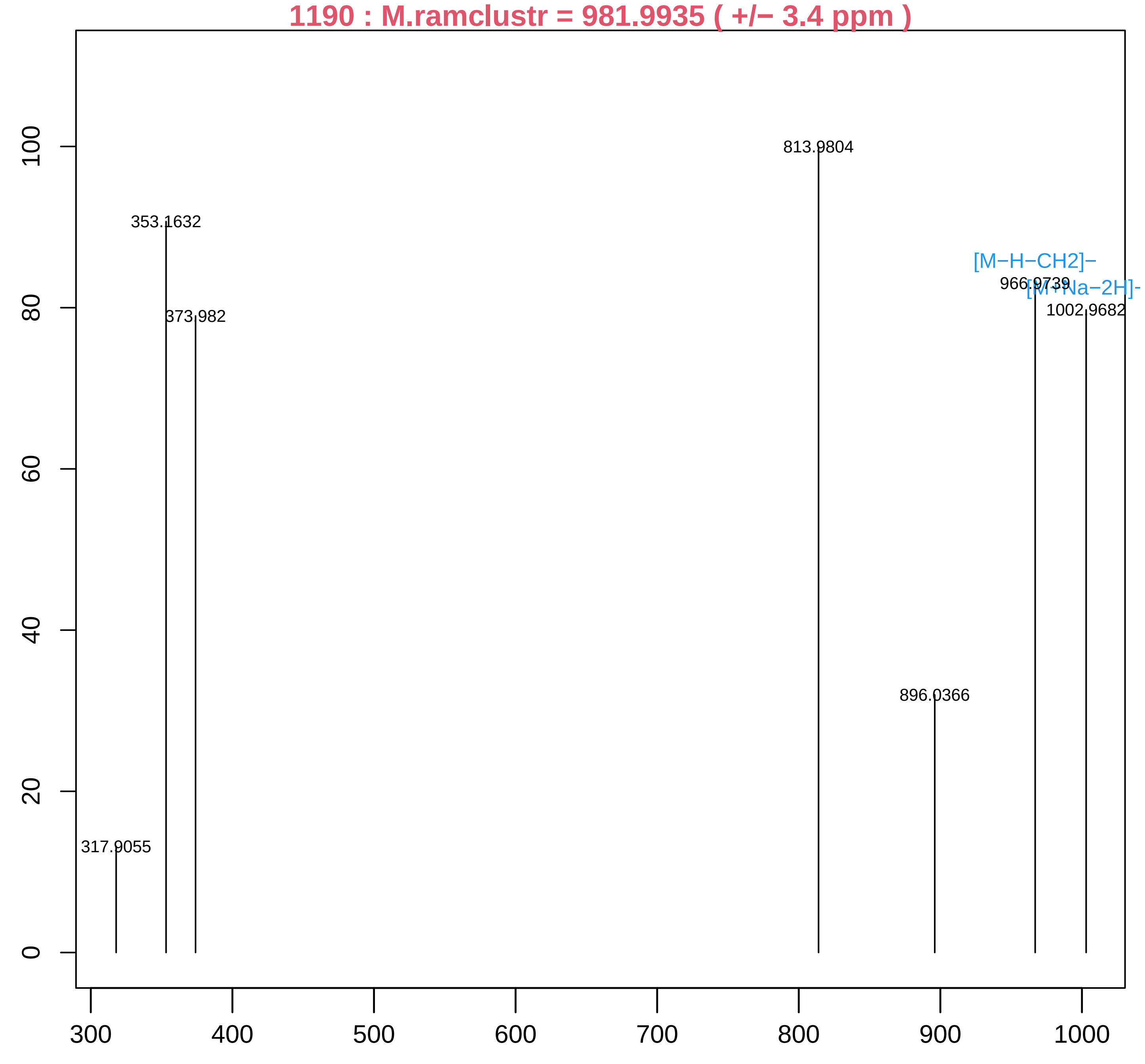
1190 : M.ramclustr = 981.9935 (+/− 3.4 ppm)
813.9804
353.1632
[M−H−CH2]−
966.9739
[M+Na−2H]-
1002.9682
373.982
896.0366
317.9055
0
20
40
60
80
100
300
400
500
600
700
800
900
1000

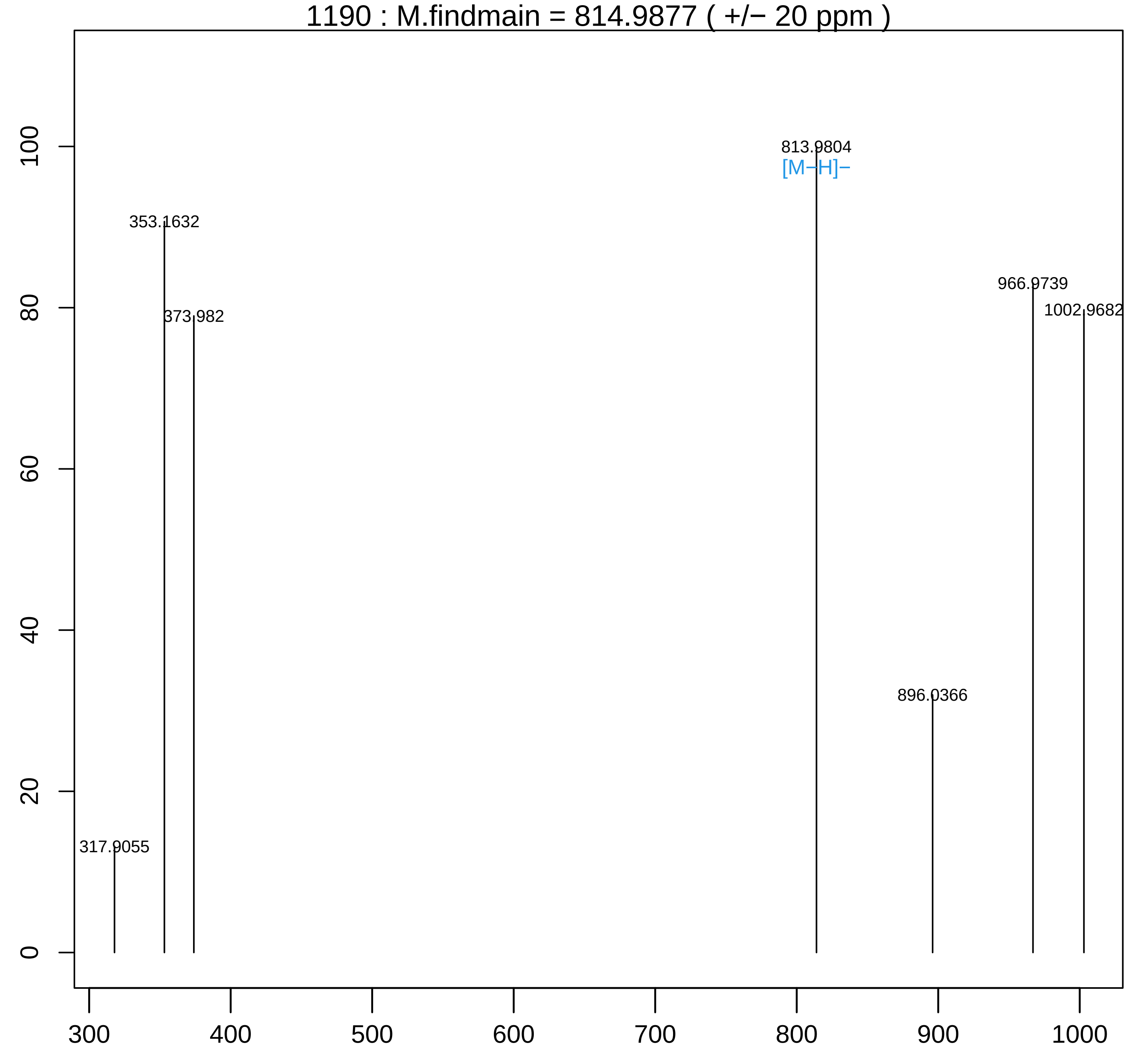
1190 : M.findmain = 814.9877 (+/− 20 ppm)
813.9804
[M−H]−
353.1632
966.9739
1002.9682
373.982
896.0366
317.9055
0
20
40
60
80
100
300
400
500
600
700
800
900
1000

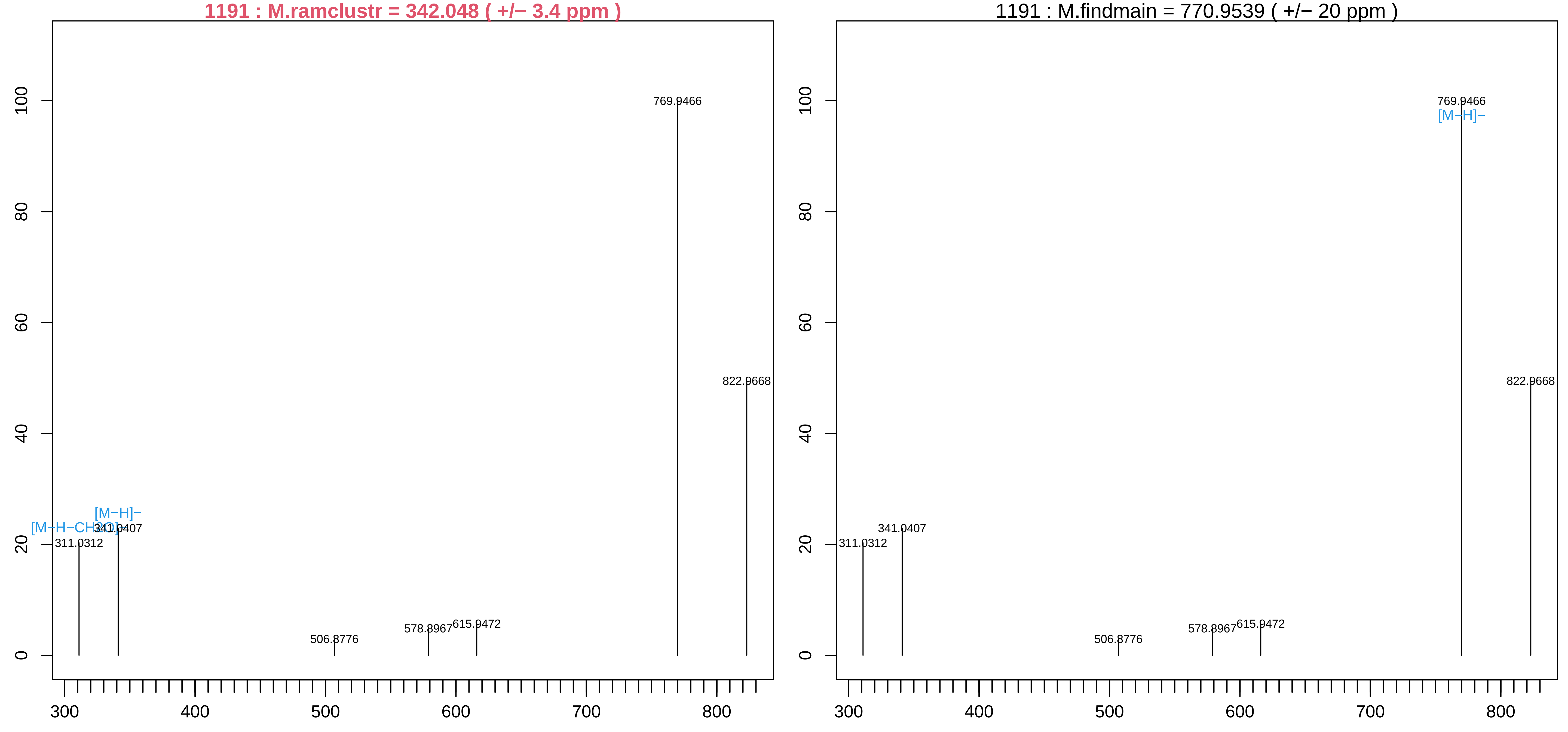
1191 : M.ramclustr = 342.048 (+/− 3.4 ppm)
[M−H]−
[M−H−CH2O]−
311.0312
341.0407
506.8776
578.8967
615.9472
769.9466
822.9668
1191 : M.findmain = 770.9539 (+/− 20 ppm)
311.0312
341.0407
506.8776
578.8967
615.9472
769.9466
[M−H]−
822.9668
0
20
40
60
80
100
300
400
500
600
700
800

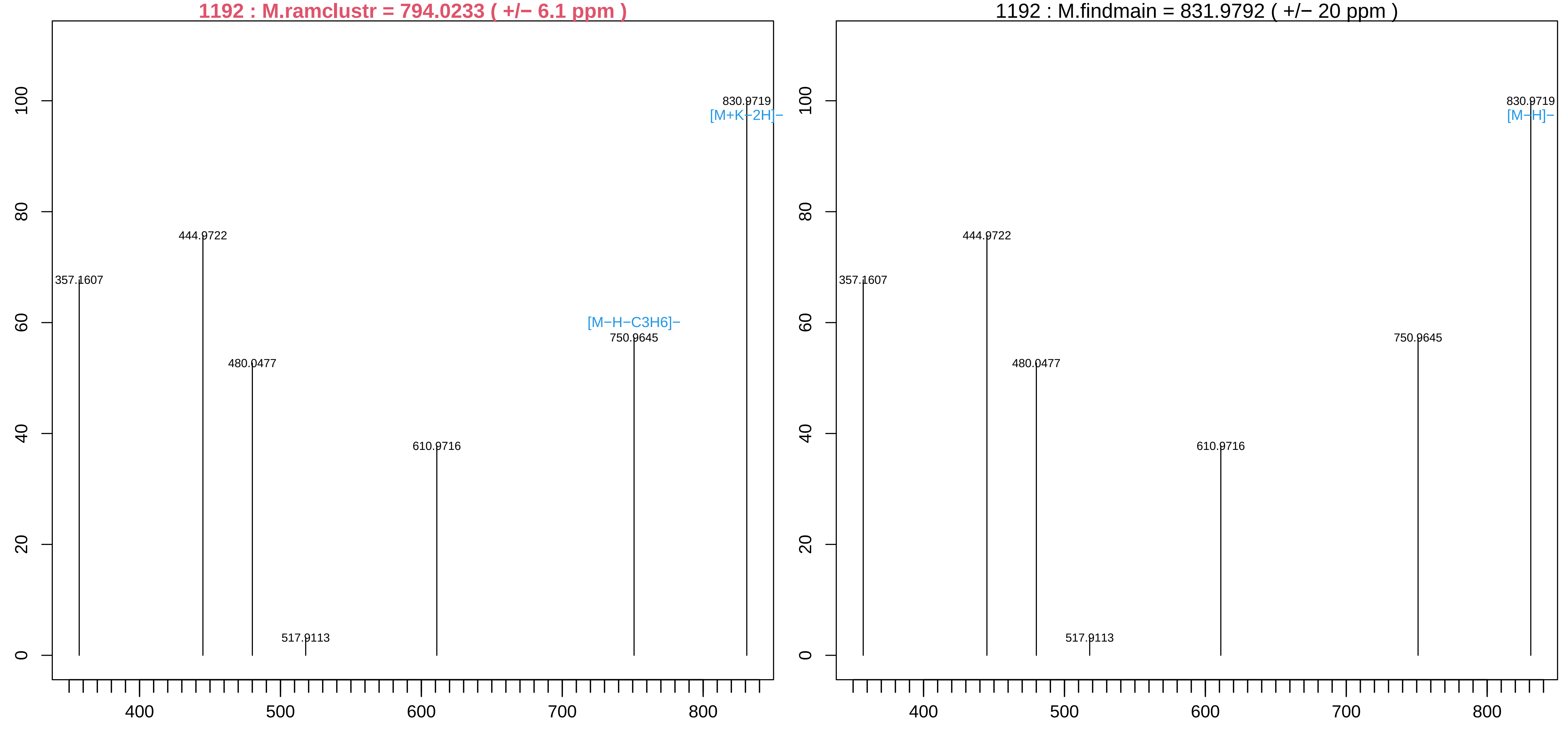
1192 : M.ramclustr = 794.0233 (+/− 6.1 ppm)
830.9719
[M+K−2H]−
444.9722
357.1607
480.0477
[M−H−C3H6]−
750.9645
610.9716
517.9113
0
20
40
60
80
100
400
500
600
700
800
1192 : M.findmain = 831.9792 (+/− 20 ppm)
830.9719
[M−H]−
444.9722
357.1607
480.0477
750.9645
610.9716
517.9113
0
20
40
60
80
100
400
500
600
700
800

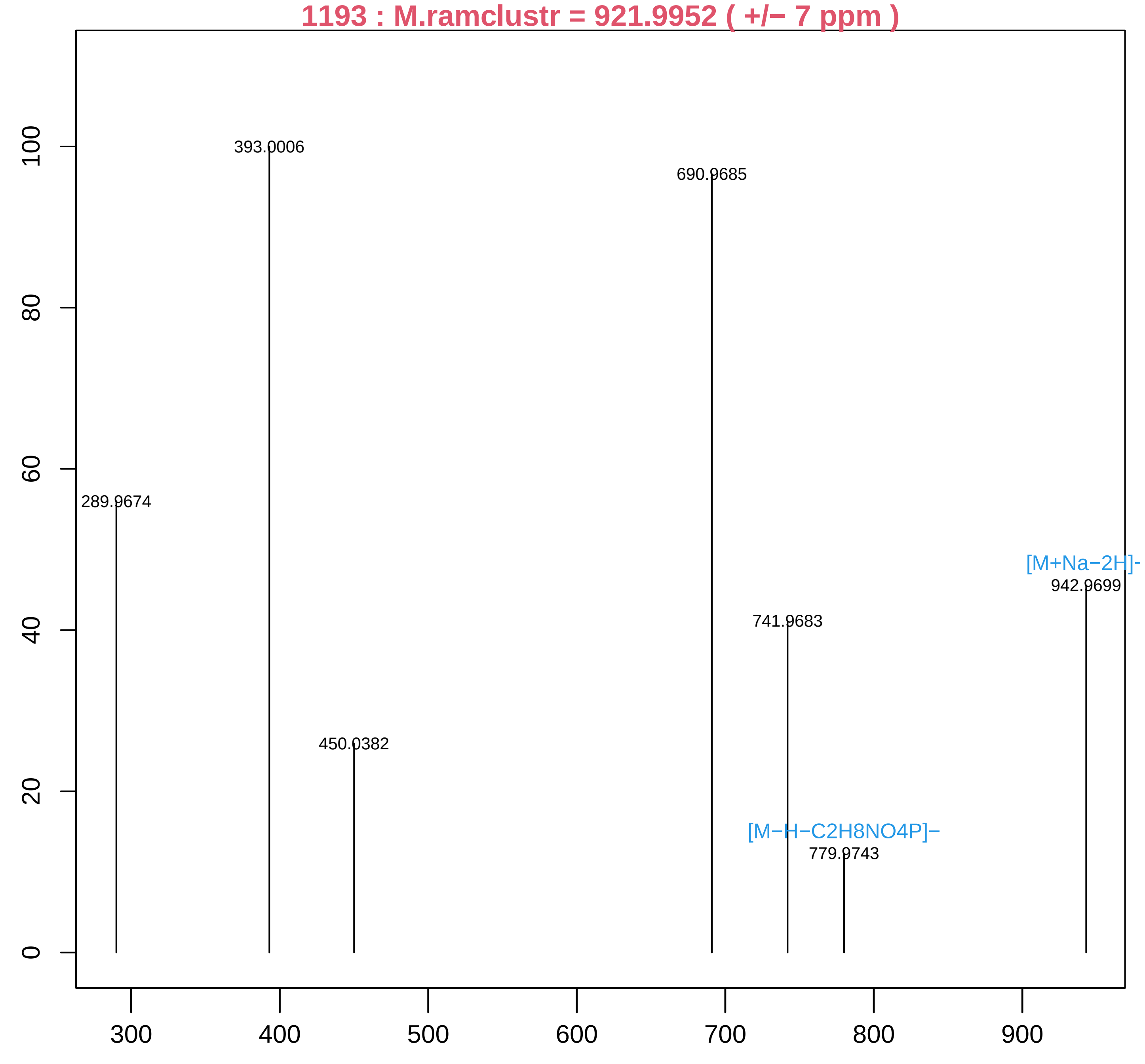

1193 : M.ramclustr = 921.9952 (+/− 7 ppm)
393.0006
690.9685
289.9674
[M+Na−2H]-
942.9699
741.9683
450.0382
[M−H−C2H8NO4P]−
779.9743
0
20
40
60
80
100
300
400
500
600
700
800
900

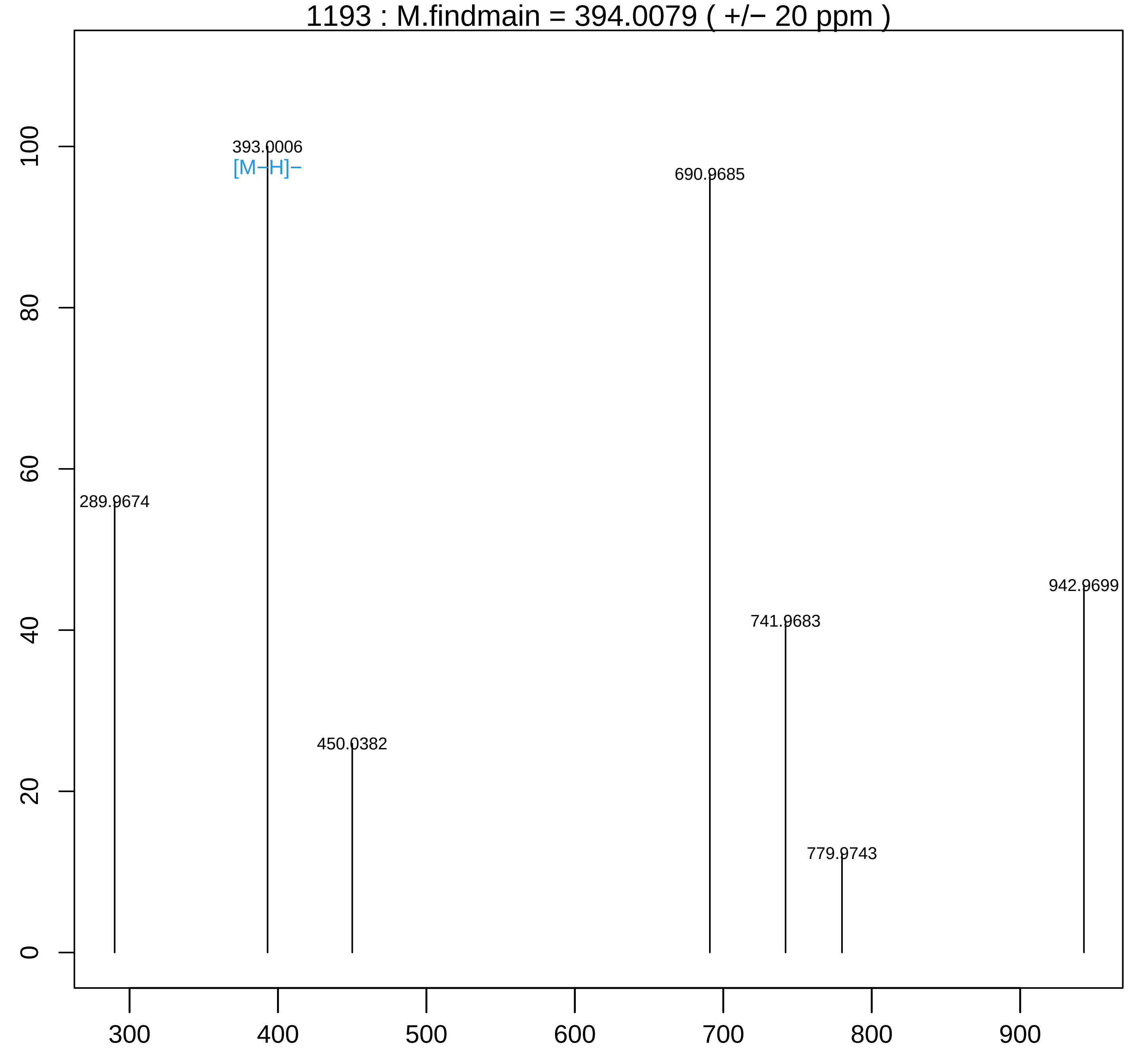

1193 : M.findmain = 394.0079 (+/− 20 ppm)
393.0006
[M−H]−
690.9685
289.9674
942.9699
741.9683
450.0382
779.9743
0
20
40
60
80
100
300
400
500
600
700
800
900

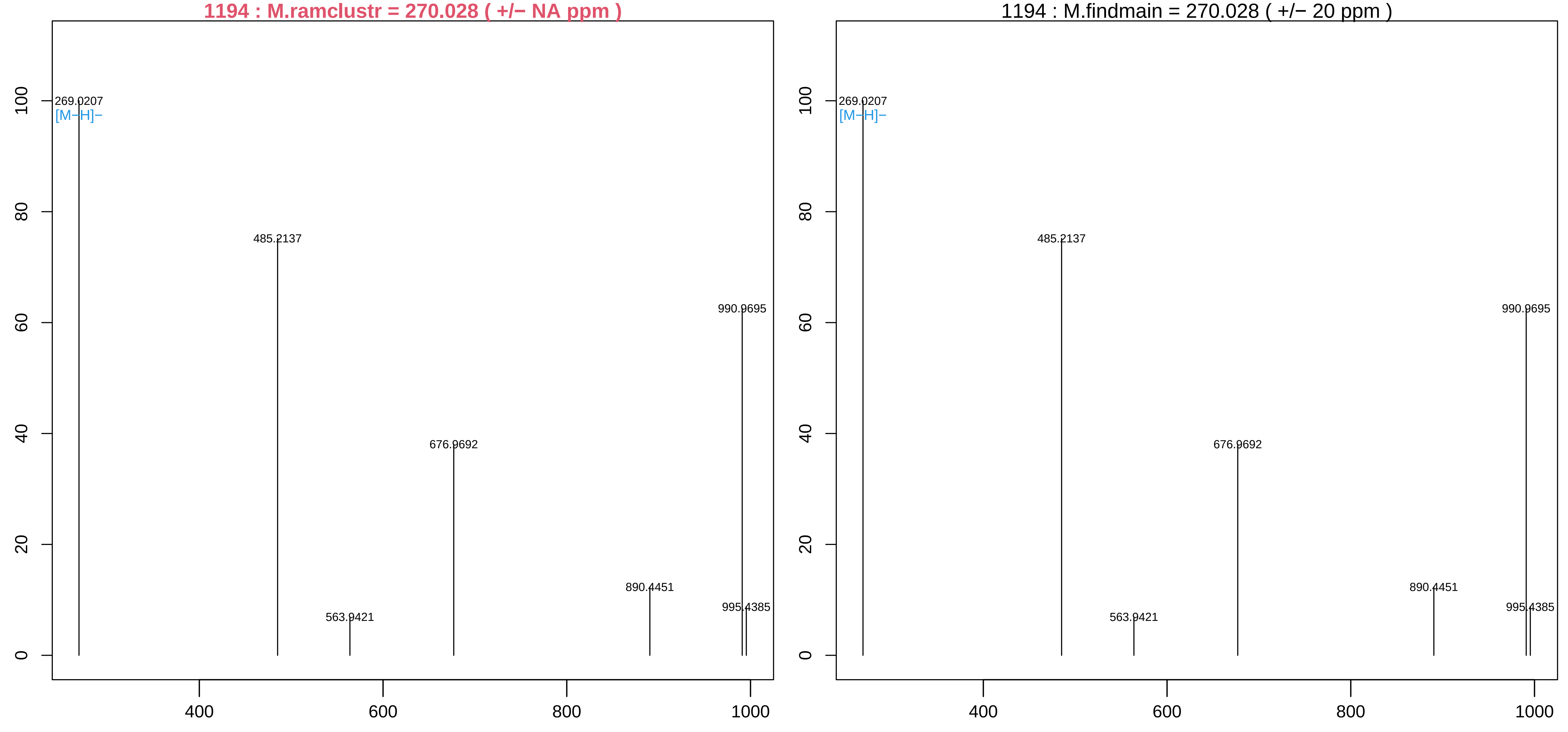
1194 : M.ramclustr = 270.028 (+/− NA ppm)
269.0207
[M−H]−
485.2137
563.9421
676.9692
890.4451
990.9695
995.4385
0
20
40
60
80
100
400
600
800
1000
1194 : M.findmain = 270.028 (+/− 20 ppm)
269.0207
[M−H]−
485.2137
563.9421
676.9692
890.4451
990.9695
995.4385
0
20
40
60
80
100
400
600
800
1000

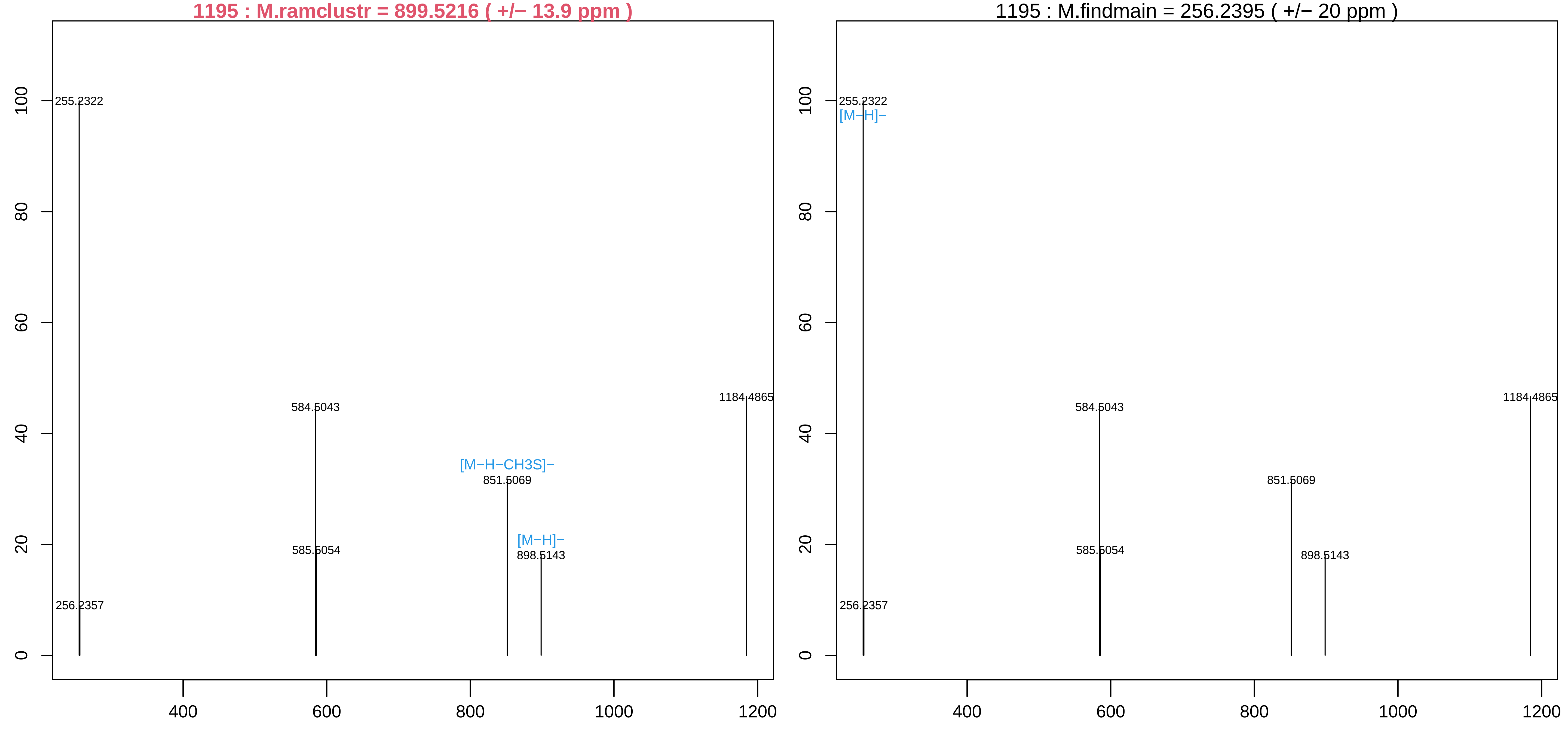

1195 : M.ramclustr = 899.5216 (+/− 13.9 ppm)
255.2322
256.2357
584.5043
585.5054
[M−H−CH3S]−
851.5069
[M−H]−
898.5143
1184.4865
1195 : M.findmain = 256.2395 (+/− 20 ppm)
255.2322
[M−H]−
256.2357
584.5043
585.5054
851.5069
898.5143
1184.4865
0
20
40
60
80
100
400
600
800
1000
1200

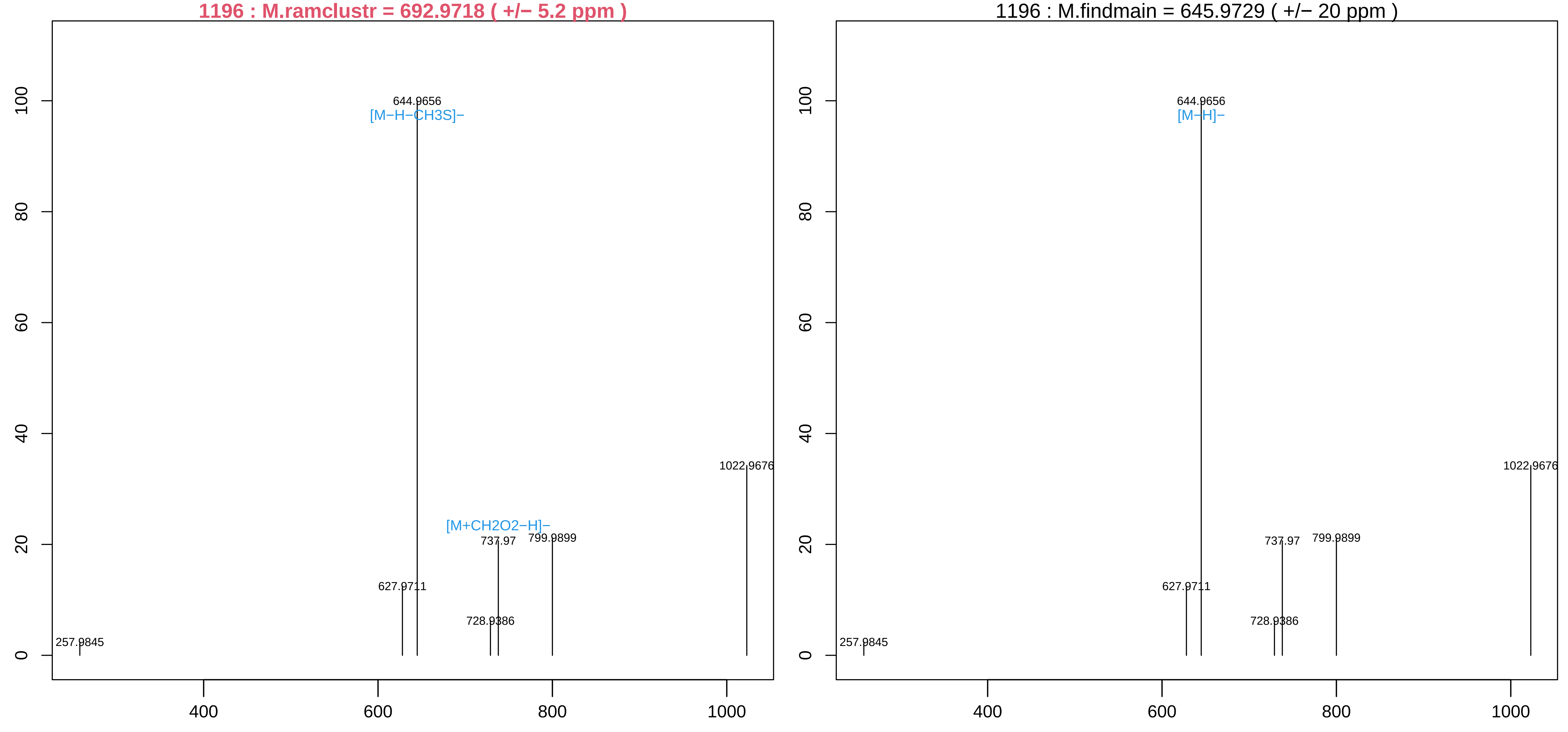

1196 : M.ramclustr = 692.9718 (+/− 5.2 ppm)
644.9656
[M−H−CH3S]−
[M+CH2O2−H]−
737.97
799.9899
1022.9676
627.9711
728.9386
257.9845
1196 : M.findmain = 645.9729 (+/− 20 ppm)
644.9656
[M−H]−
737.97
799.9899
1022.9676
627.9711
728.9386
257.9845

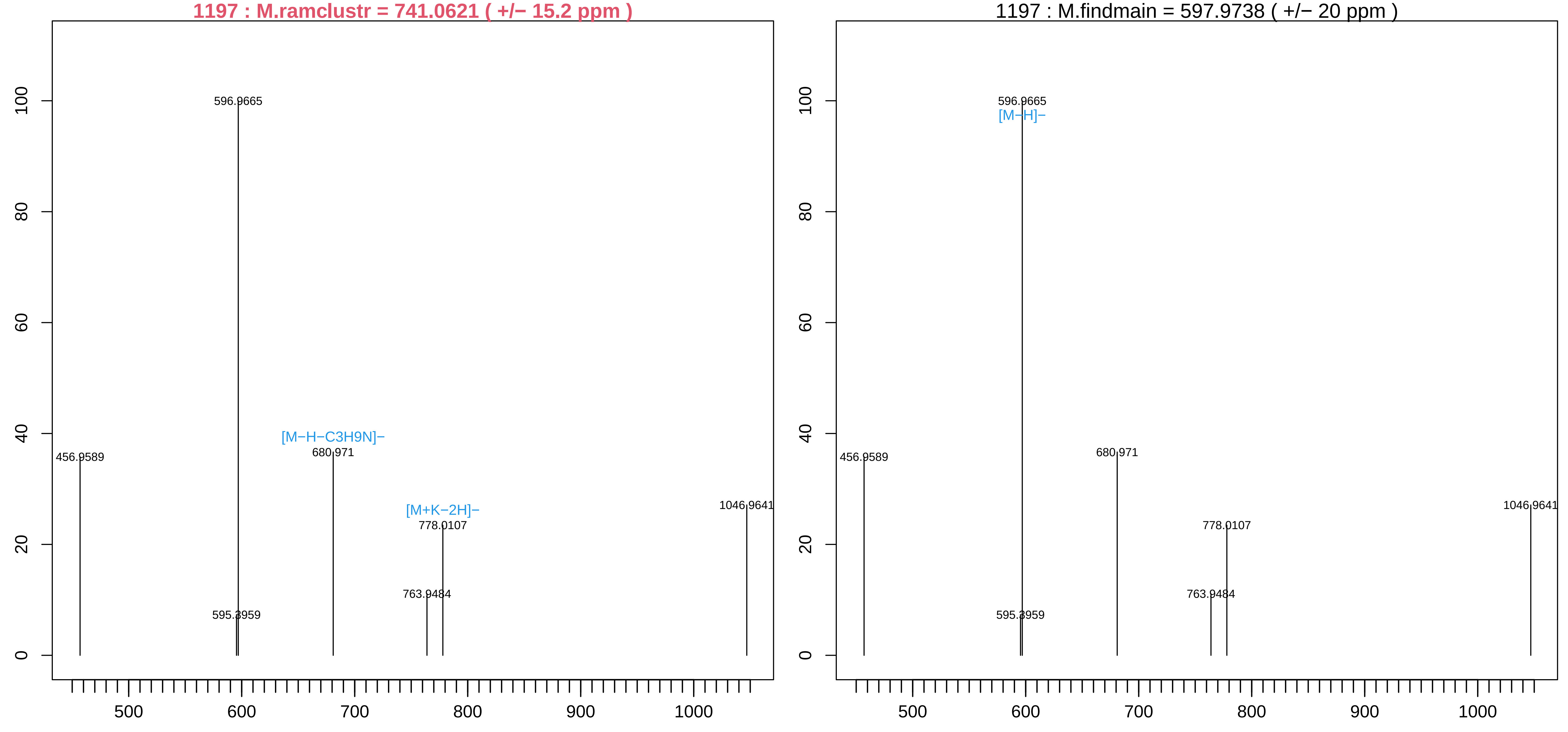
1197 : M.ramclustr = 741.0621 (+/− 15.2 ppm)
596.9665
456.9589
[M−H−C3H9N]−
680.971
[M+K−2H]−
778.0107
1046.9641
763.9484
595.3959
1197 : M.findmain = 597.9738 (+/− 20 ppm)
596.9665
[M−H]−
456.9589
680.971
778.0107
1046.9641
763.9484
595.3959

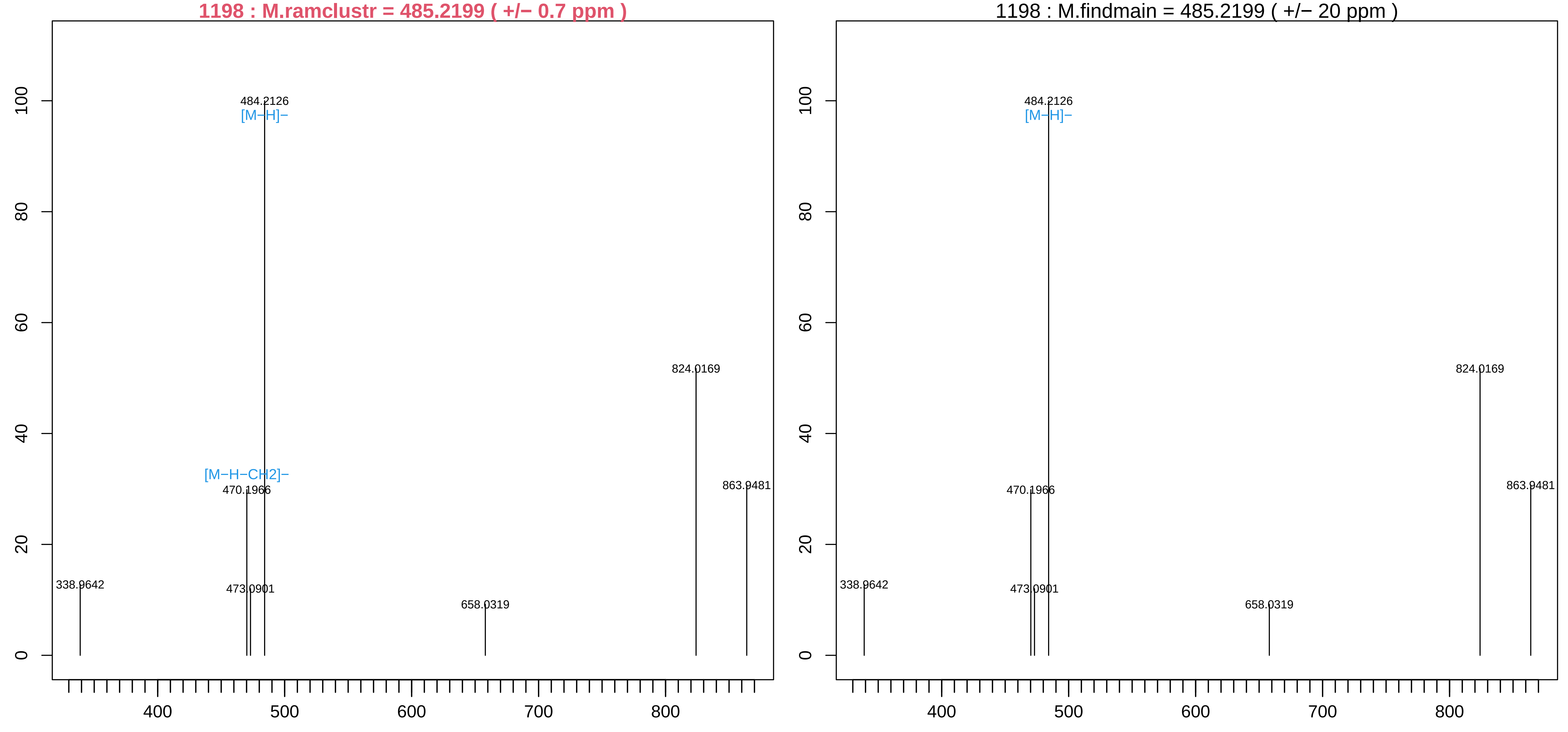
1198 : M.ramclustr = 485.2199 (+/− 0.7 ppm)
484.2126
[M−H]−
[M−H−CH2]−
470.1966
824.0169
863.9481
338.9642
473.0901
658.0319
1198 : M.findmain = 485.2199 (+/− 20 ppm)
484.2126
[M−H]−
470.1966
824.0169
863.9481
338.9642
473.0901
658.0319
0
20
40
60
80
100
400
500
600
700
800

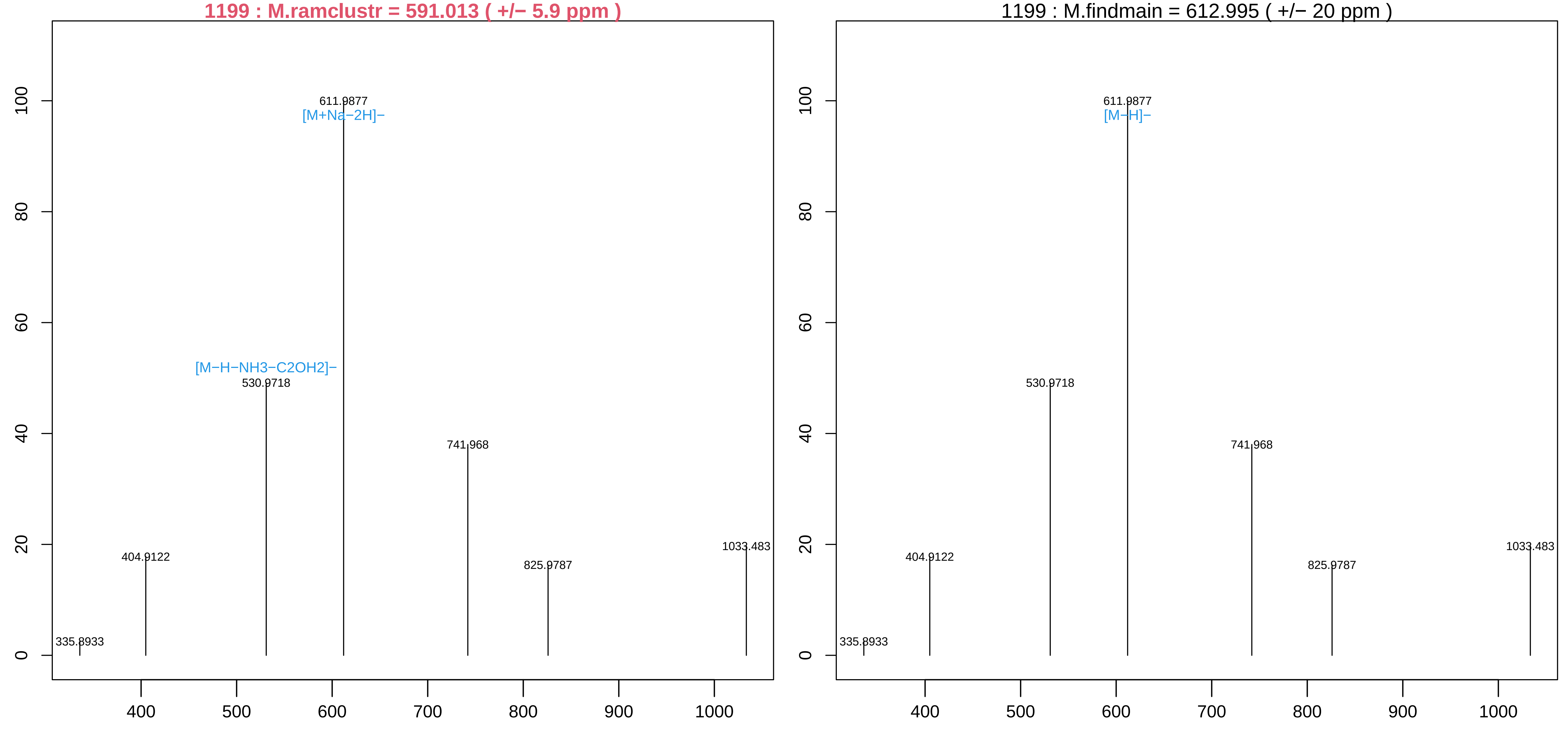
1199 : M.ramclustr = 591.013 (+/− 5.9 ppm)
611.9877
[M+Na−2H]−
[M−H−NH3−C2OH2]−
530.9718
741.968
404.9122
825.9787
1033.483
335.8933
1199 : M.findmain = 612.995 (+/− 20 ppm)
611.9877
[M−H]−
530.9718
741.968
404.9122
825.9787
1033.483
335.8933
0
20
40
60
80
100
400
500
600
700
800
900
1000

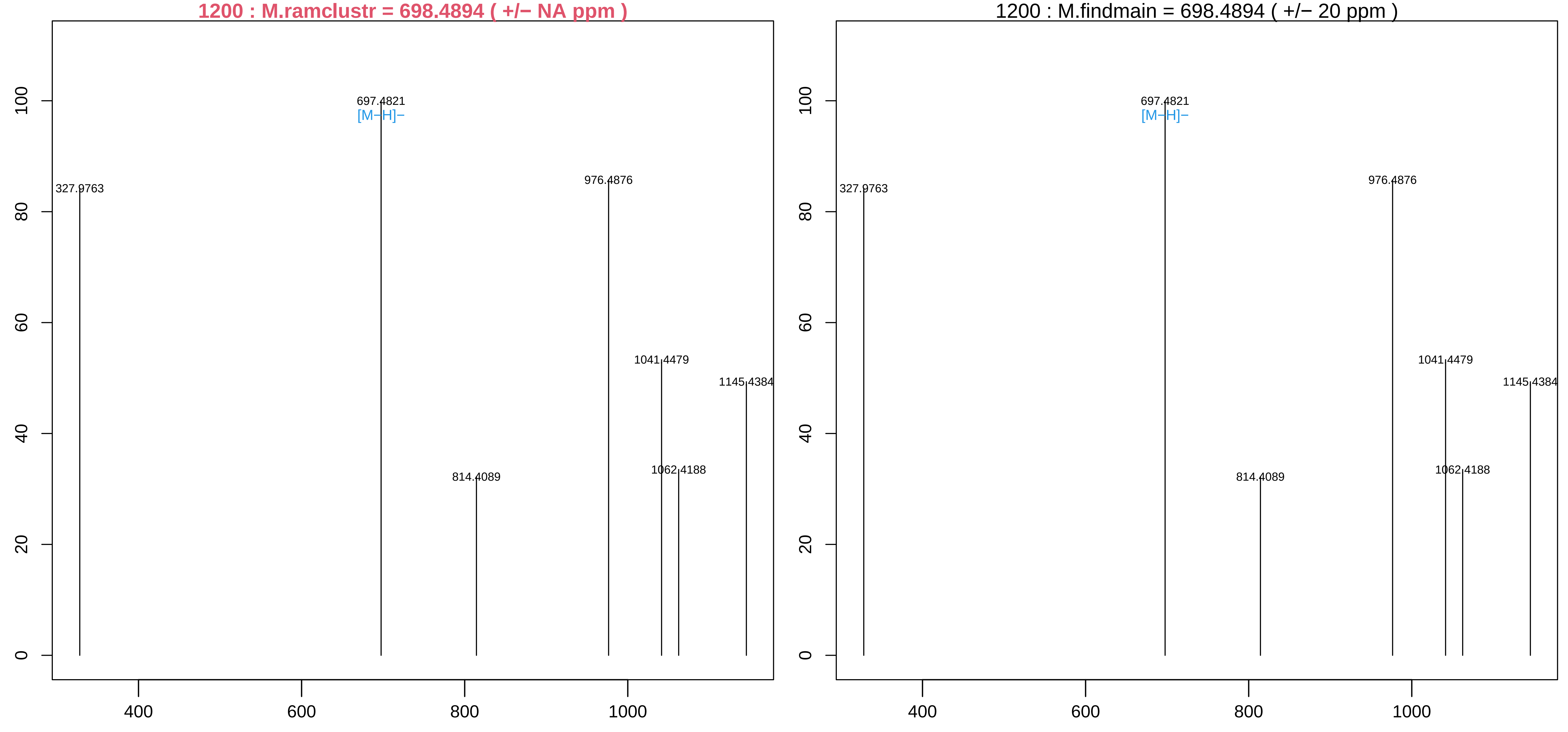
1200 : M.ramclustr = 698.4894 (+/− NA ppm)
1200 : M.findmain = 698.4894 (+/− 20 ppm)
327.9763
697.4821
[M−H]−
814.4089
976.4876
1041.4479
1062.4188
1145.4384
0
20
40
60
80
100
400
600
800
1000

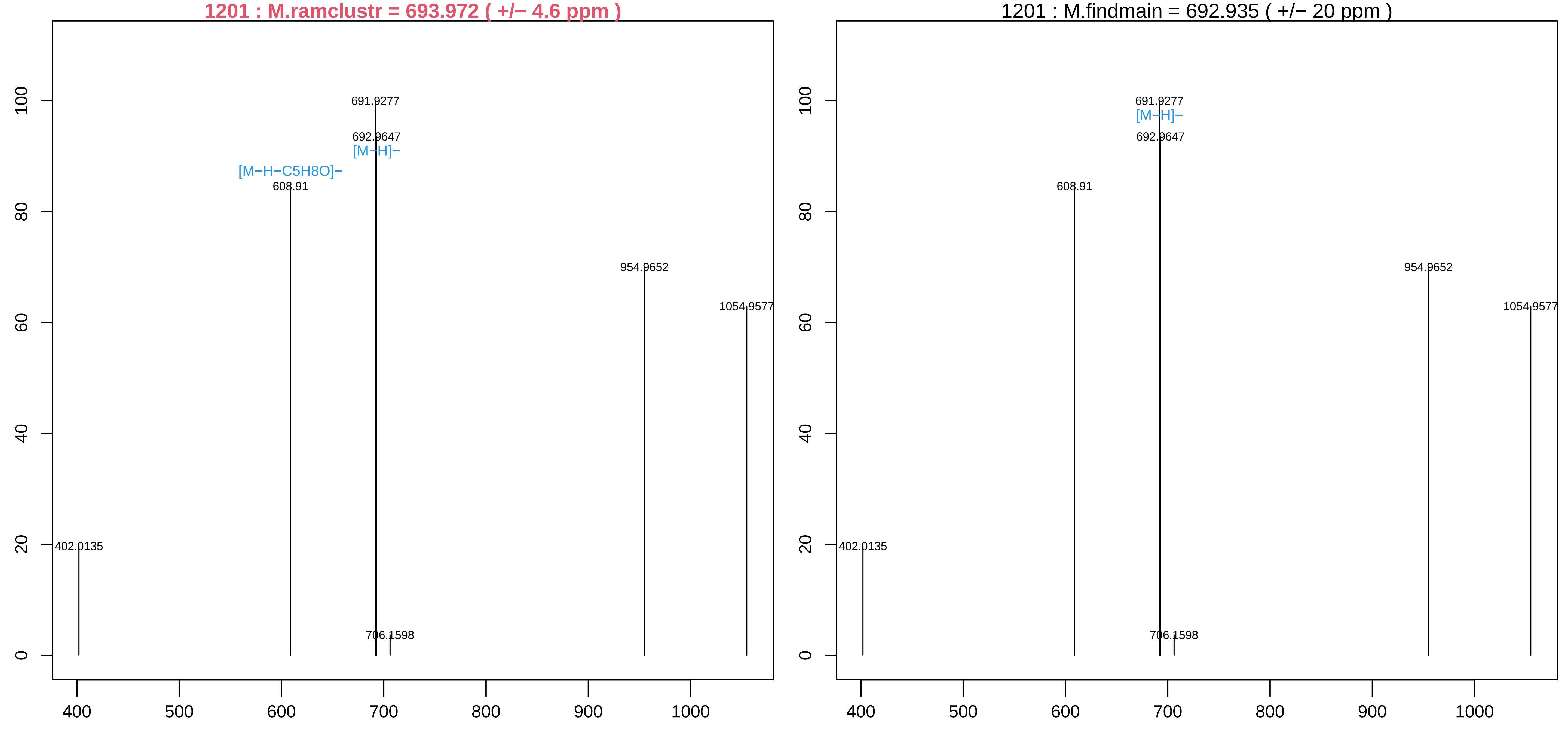
1201 : M.ramclustr = 693.972 (+/− 4.6 ppm)
691.9277
692.9647
[M−H]−
[M−H−C5H8O]−
608.91
954.9652
1054.9577
402.0135
706.1598
0
20
40
60
80
100
400
500
600
700
800
900
1000
1201 : M.findmain = 692.935 (+/− 20 ppm)
691.9277
[M−H]−
692.9647
608.91
954.9652
1054.9577
402.0135
706.1598

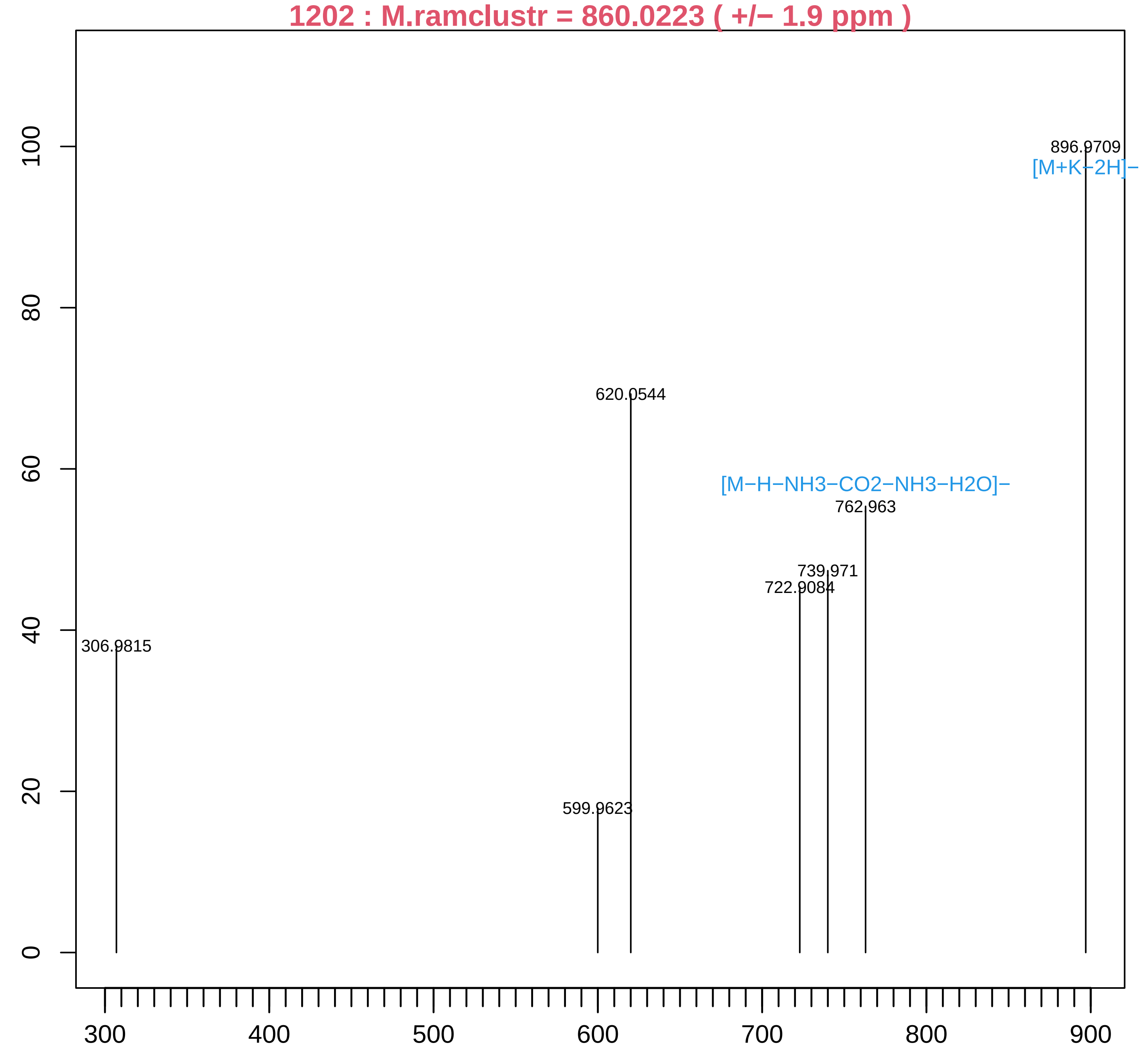
1202 : M.ramclustr = 860.0223 (+/− 1.9 ppm)
896.9709
[M+K−2H]−
620.0544
[M−H−NH3−CO2−NH3−H2O]−
762.963
739.971
722.9084
306.9815
599.9623
0
20
40
60
80
100
300
400
500
600
700
800
900

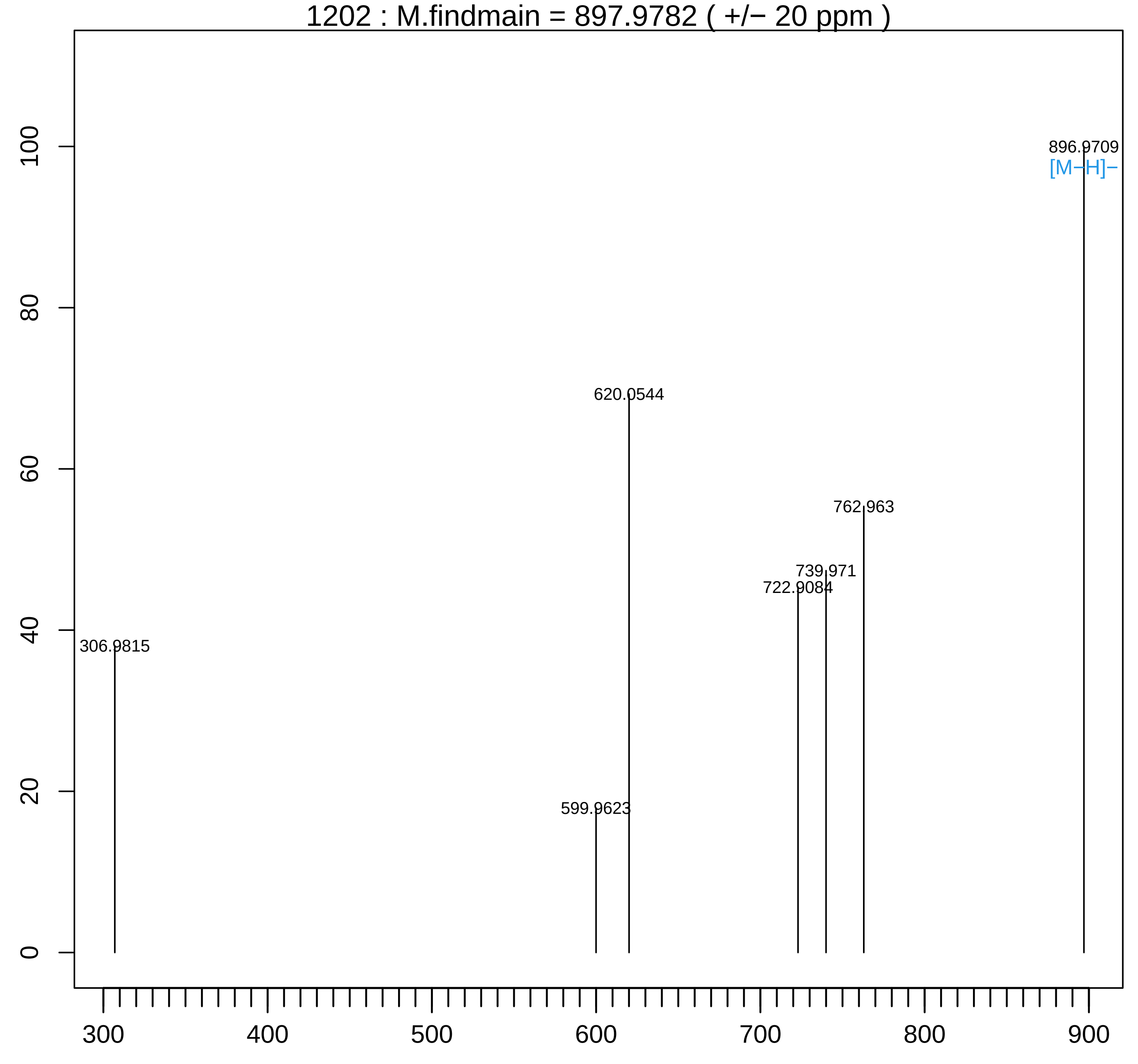
1202 : M.findmain = 897.9782 (+/− 20 ppm)
896.9709
[M−H]−
620.0544
762.963
739.971
722.9084
306.9815
599.9623
0
20
40
60
80
100
300
400
500
600
700
800
900

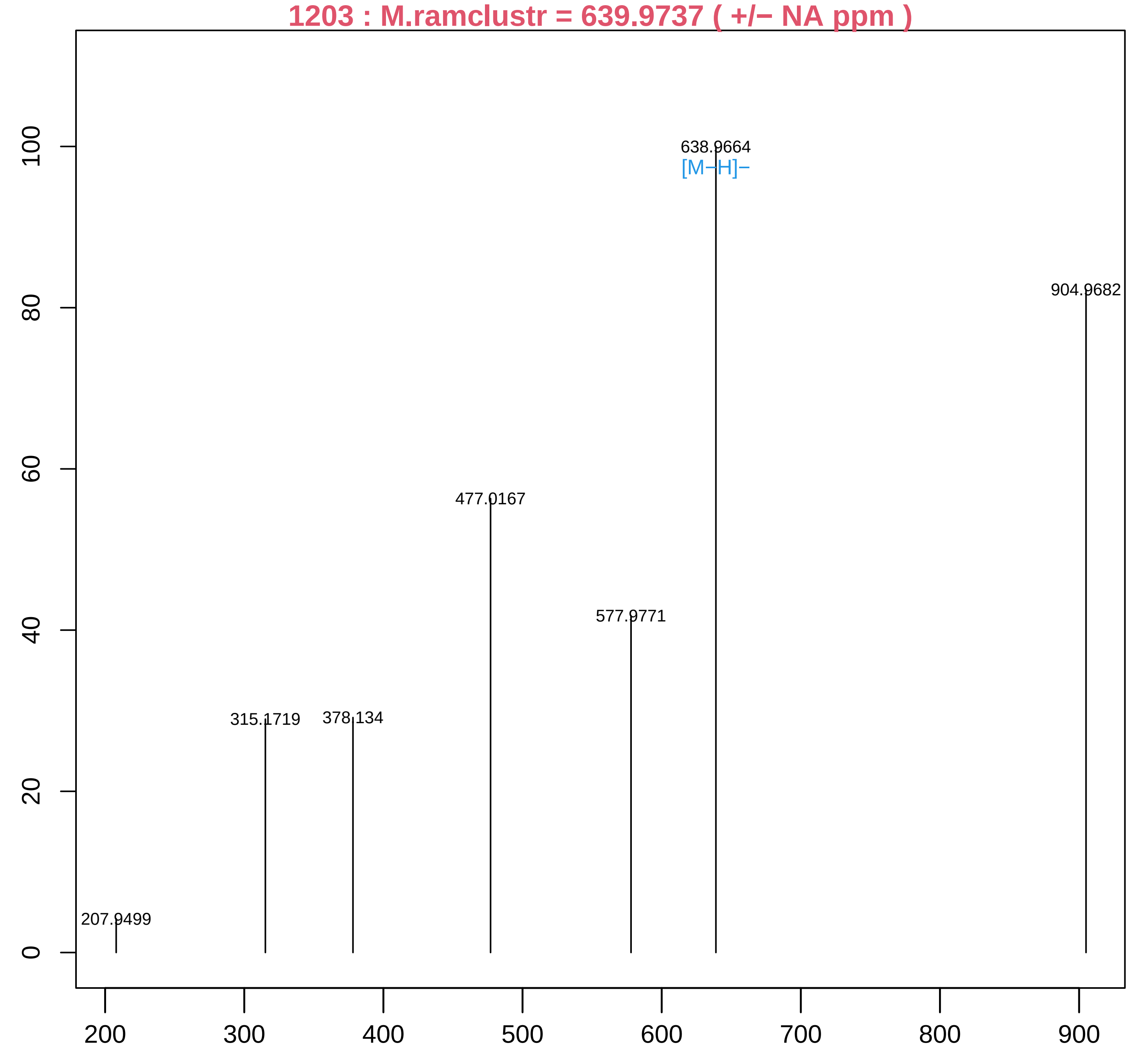
1203 : M.ramclustr = 639.9737 (+/− NA ppm)
638.9664
[M−H]−
904.9682
477.0167
577.9771
315.1719
378.134
207.9499
0
20
40
60
80
100
200
300
400
500
600
700
800
900

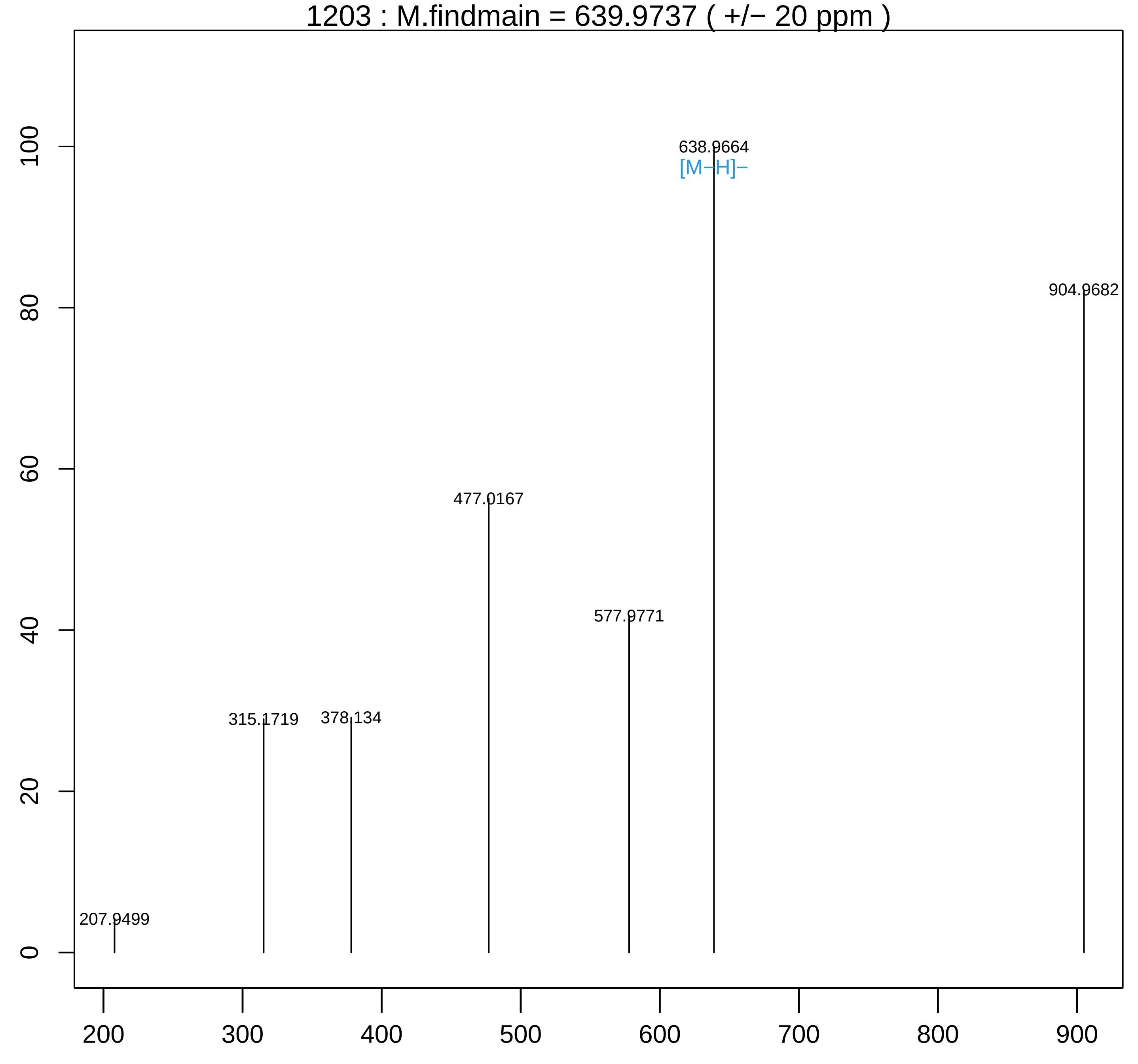
1203 : M.findmain = 639.9737 (+/− 20 ppm)
638.9664
[M−H]−
904.9682
477.0167
577.9771
315.1719
378.134
207.9499
0
20
40
60
80
100
200
300
400
500
600
700
800
900

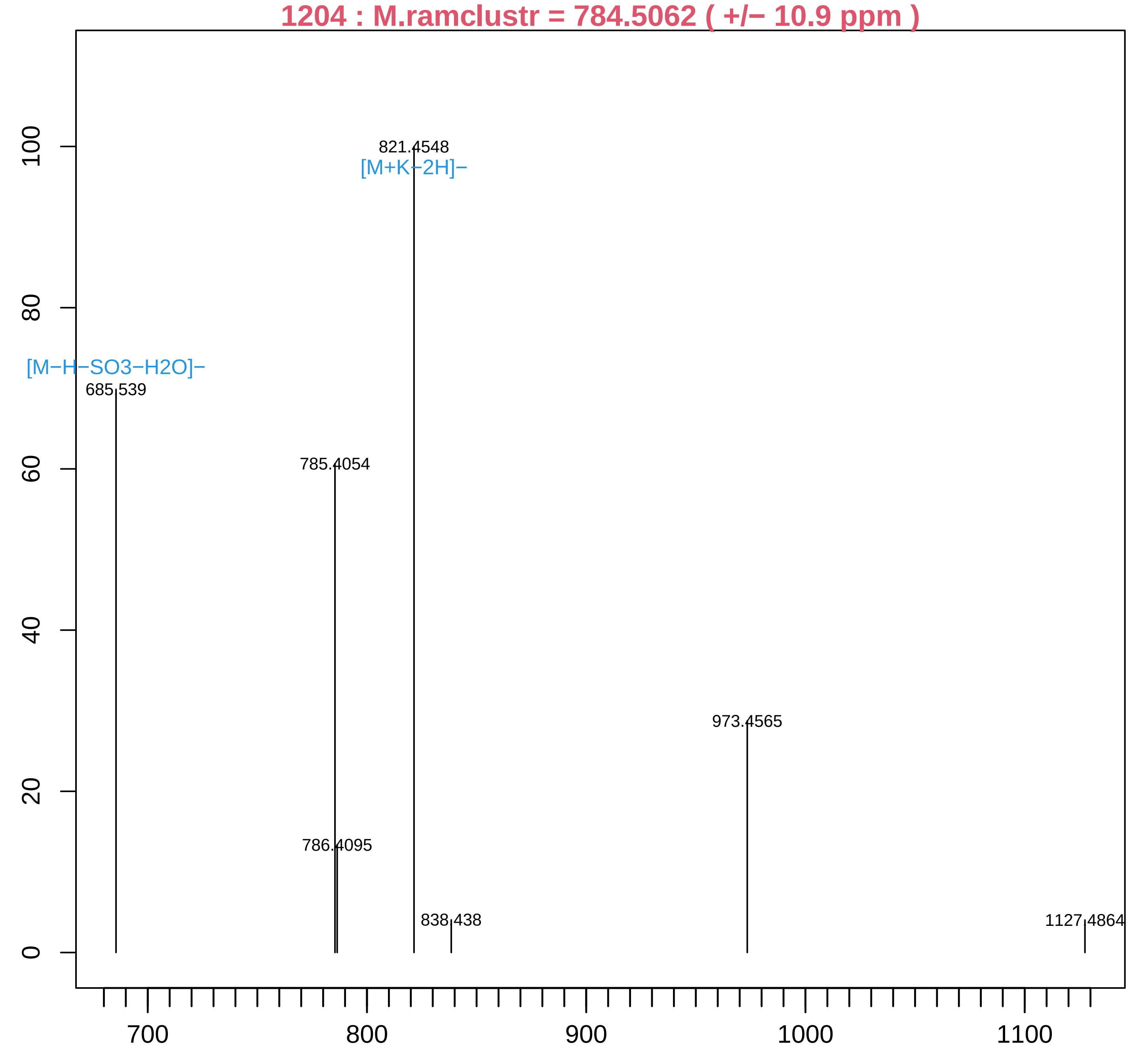

1204 : M.ramclustr = 784.5062 (+/− 10.9 ppm)
821.4548
[M+K−2H]−
[M−H−SO3−H2O]−
685.539
785.4054
973.4565
786.4095
838.438
1127.4864
0
20
40
60
80
100
700
800
900
1000
1100

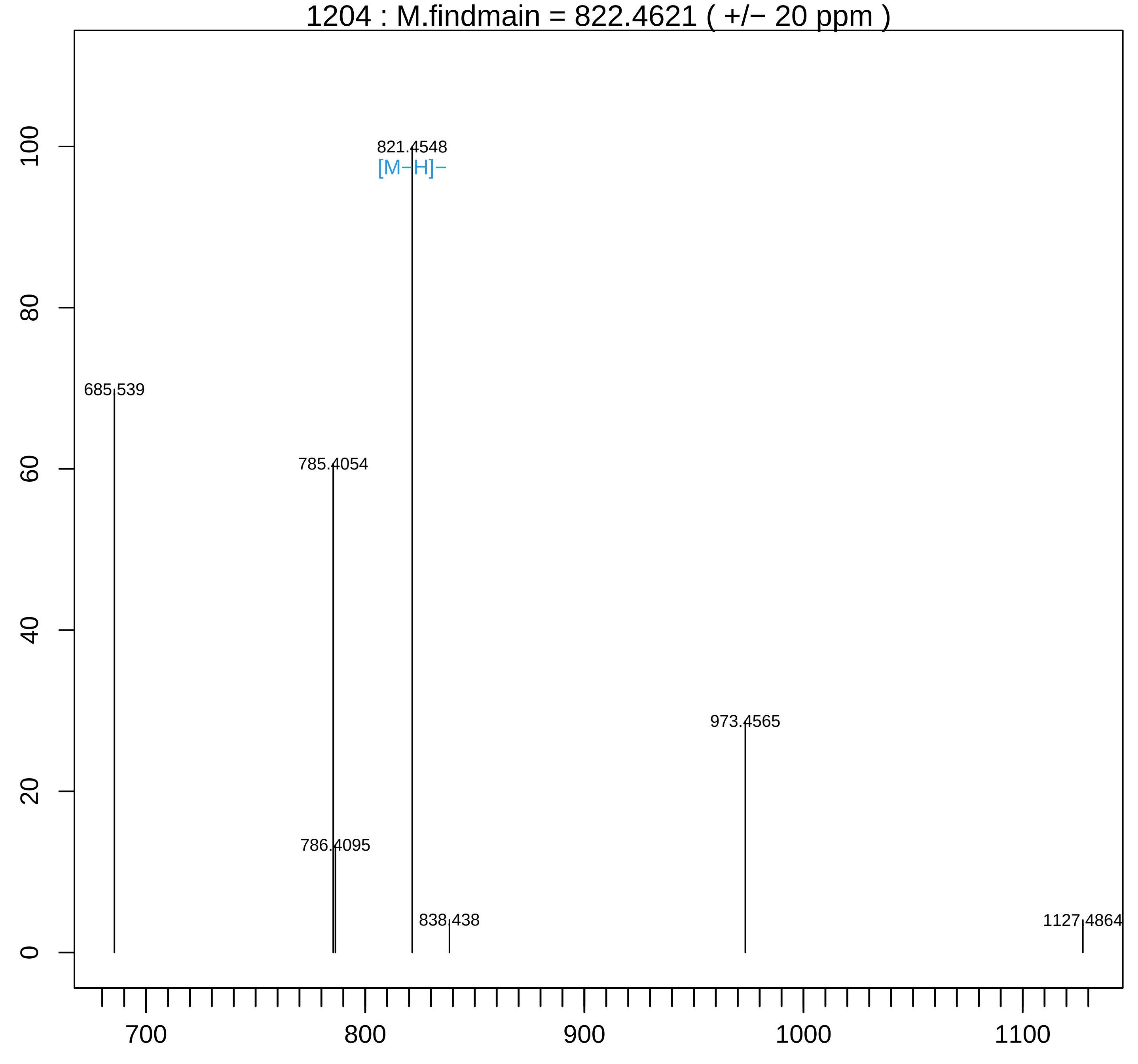

1204 : M.findmain = 822.4621 (+/− 20 ppm)
821.4548
[M−H]−
685.539
785.4054
973.4565
786.4095
838.438
1127.4864
0
20
40
60
80
100
700
800
900
1000
1100

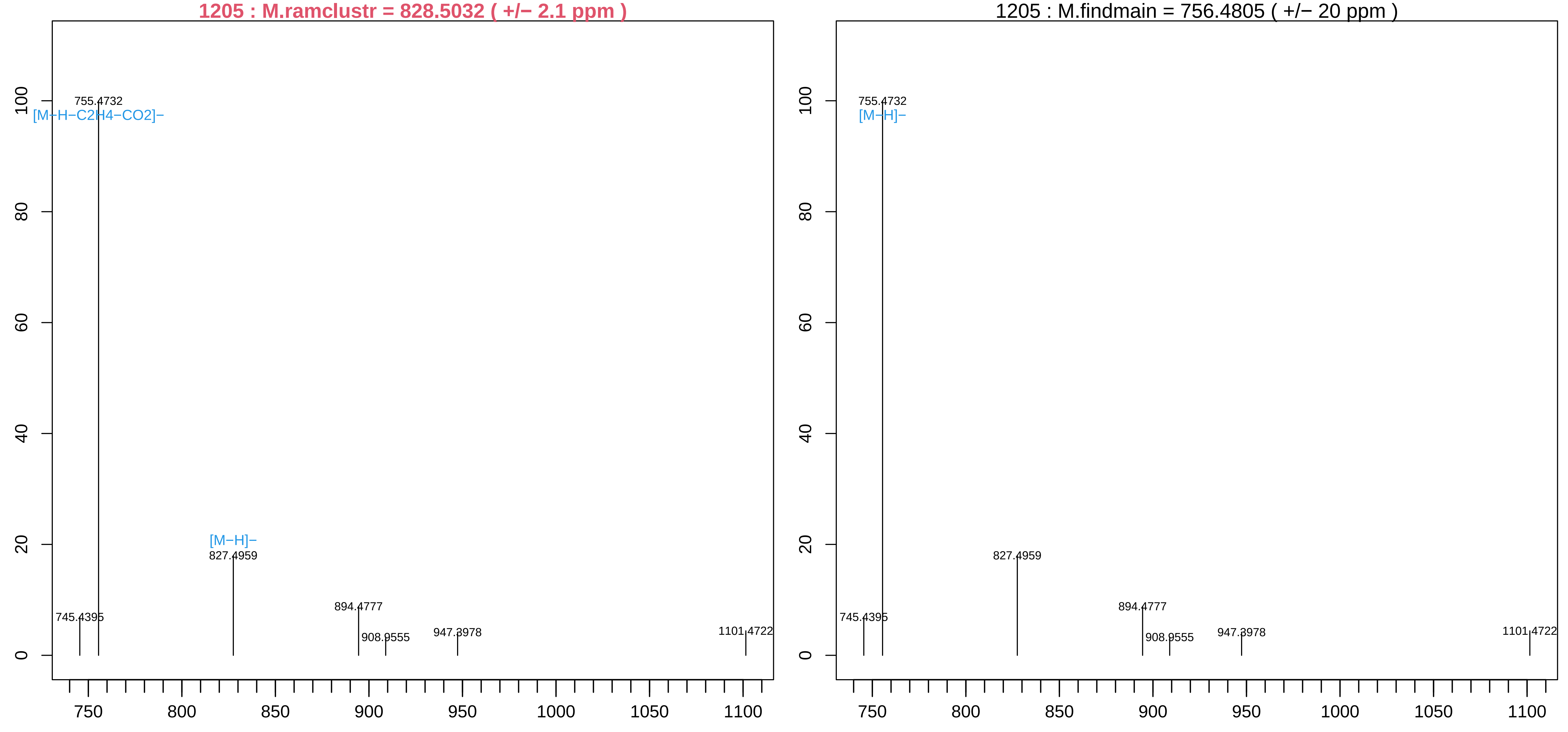

1205 : M.ramclustr = 828.5032 (+/− 2.1 ppm)
755.4732
[M−H−C2H4−CO2]−
[M−H]−
827.4959
745.4395
894.4777
908.9555
947.3978
1101.4722
1205 : M.findmain = 756.4805 (+/− 20 ppm)
755.4732
[M−H]−
827.4959
745.4395
894.4777
908.9555
947.3978
1101.4722

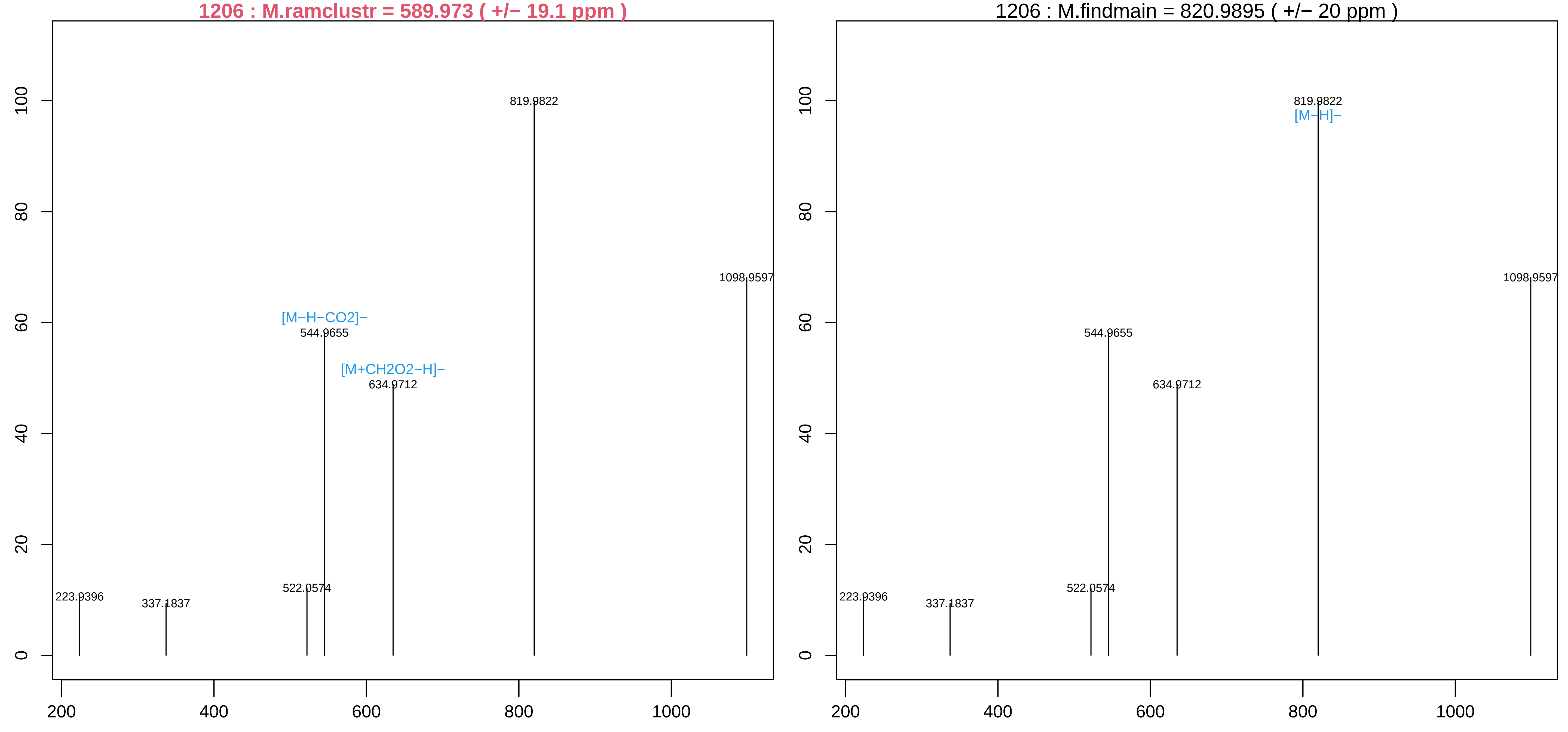
1206 : M.ramclustr = 589.973 (+/− 19.1 ppm)
819.9822
1098.9597
[M−H−CO2]−
544.9655
[M+CH2O2−H]−
634.9712
223.9396
337.1837
522.0574
1206 : M.findmain = 820.9895 (+/− 20 ppm)
819.9822
[M−H]−
1098.9597
544.9655
634.9712
223.9396
337.1837
522.0574
0
20
40
60
80
100
200
400
600
800
1000

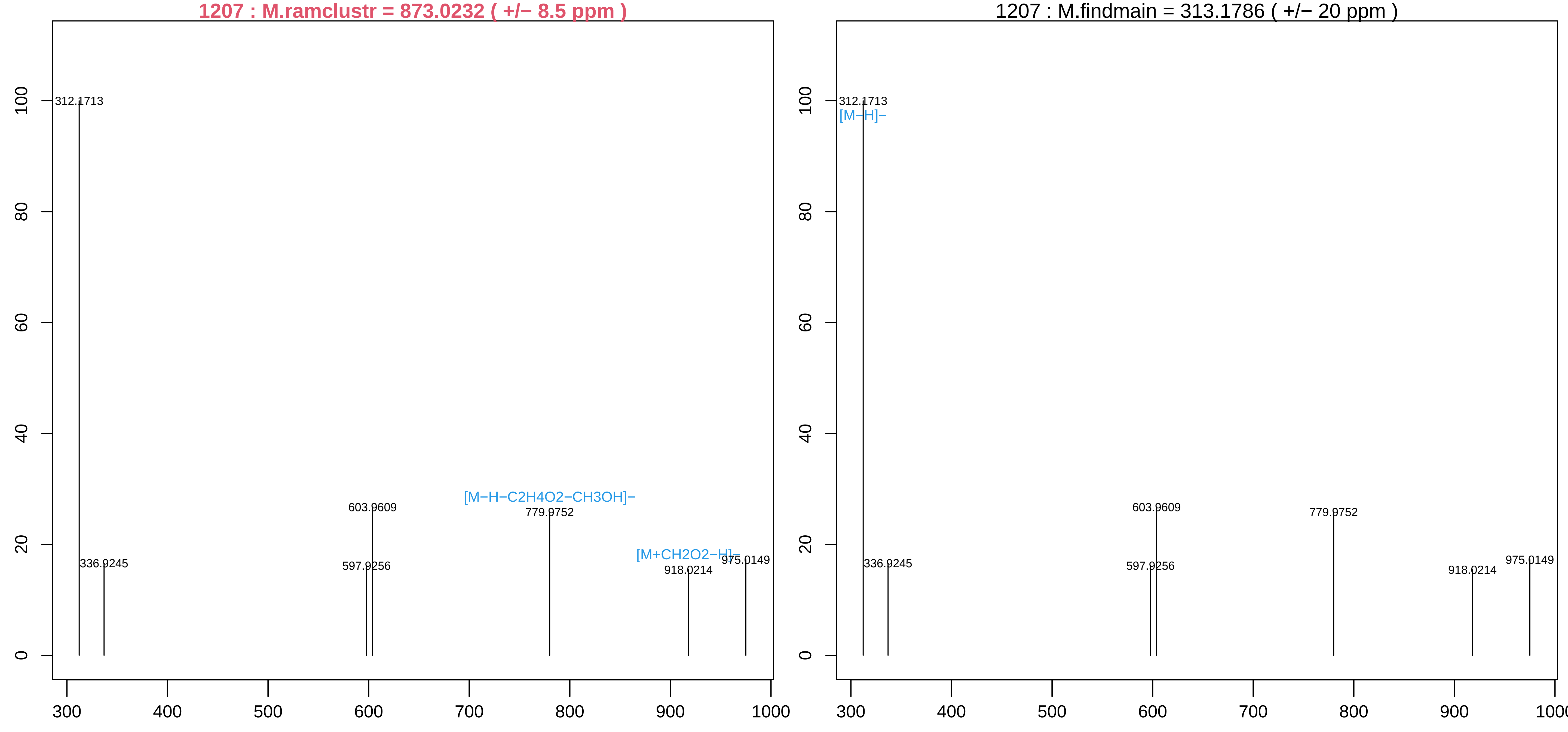

1207 : M.ramclustr = 873.0232 (+/− 8.5 ppm)
312.1713
336.9245
597.9256
603.9609
[M−H−C2H4O2−CH3OH]−
779.9752
[M+CH2O2−H]−
918.0214
975.0149
1207 : M.findmain = 313.1786 (+/− 20 ppm)
312.1713
[M−H]−
336.9245
597.9256
603.9609
779.9752
918.0214
975.0149
0
20
40
60
80
100
300
400
500
600
700
800
900
1000

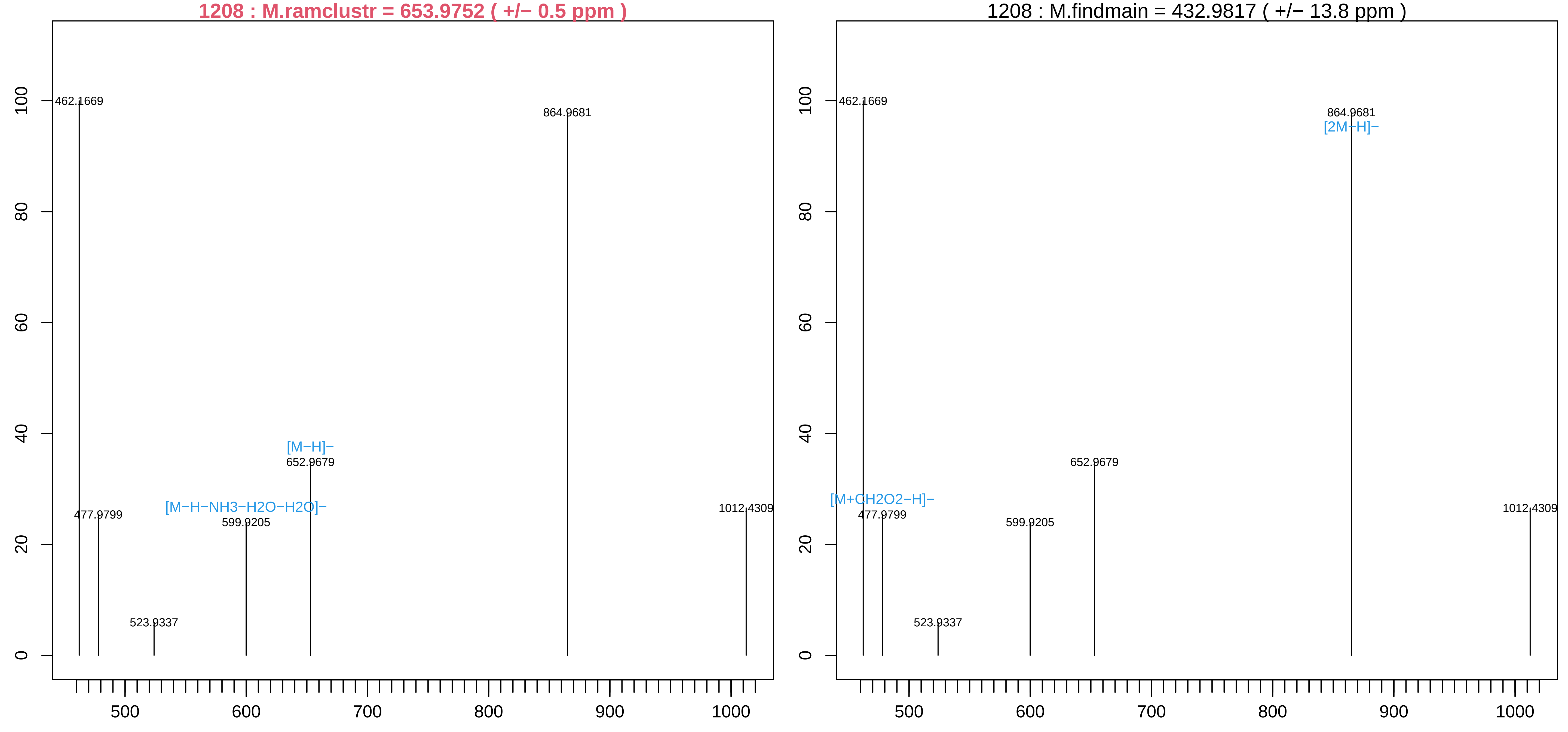
1208 : M.ramclustr = 653.9752 (+/− 0.5 ppm)
462.1669
864.9681
[M−H]−
652.9679
[M−H−NH3−H2O−H2O]−
599.9205
477.9799
1012.4309
523.9337
1208 : M.findmain = 432.9817 (+/− 13.8 ppm)
462.1669
864.9681
[2M−H]−
652.9679
[M+CH2O2−H]−
477.9799
599.9205
1012.4309
523.9337
0
20
40
60
80
100
500
600
700
800
900
1000

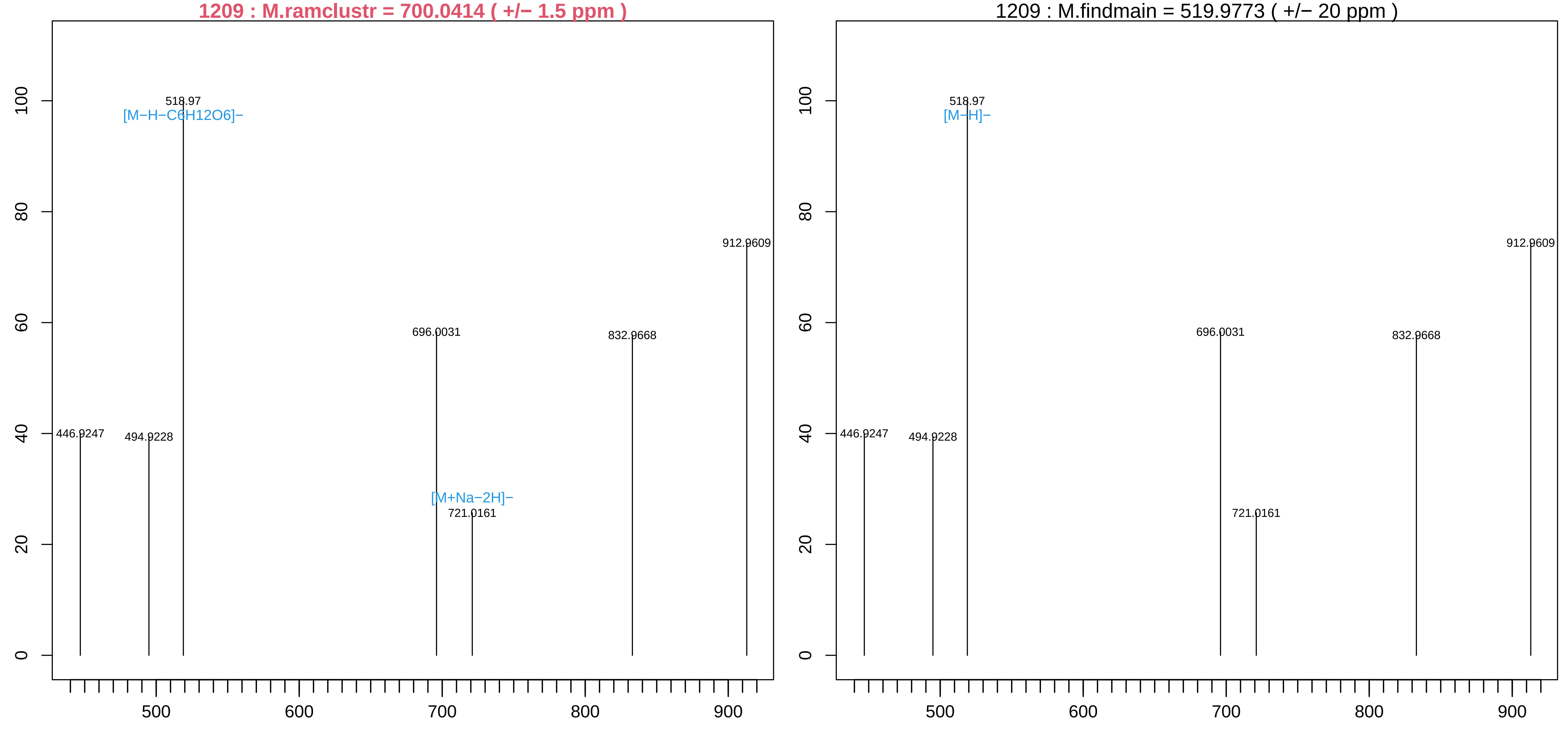
1209 : M.ramclustr = 700.0414 (+/− 1.5 ppm)
518.97
[M−H−C6H12O6]−
446.9247
494.9228
696.0031
[M+Na−2H]−
721.0161
832.9668
912.9609
1209 : M.findmain = 519.9773 (+/− 20 ppm)
518.97
[M−H]−
446.9247
494.9228
696.0031
721.0161
832.9668
912.9609

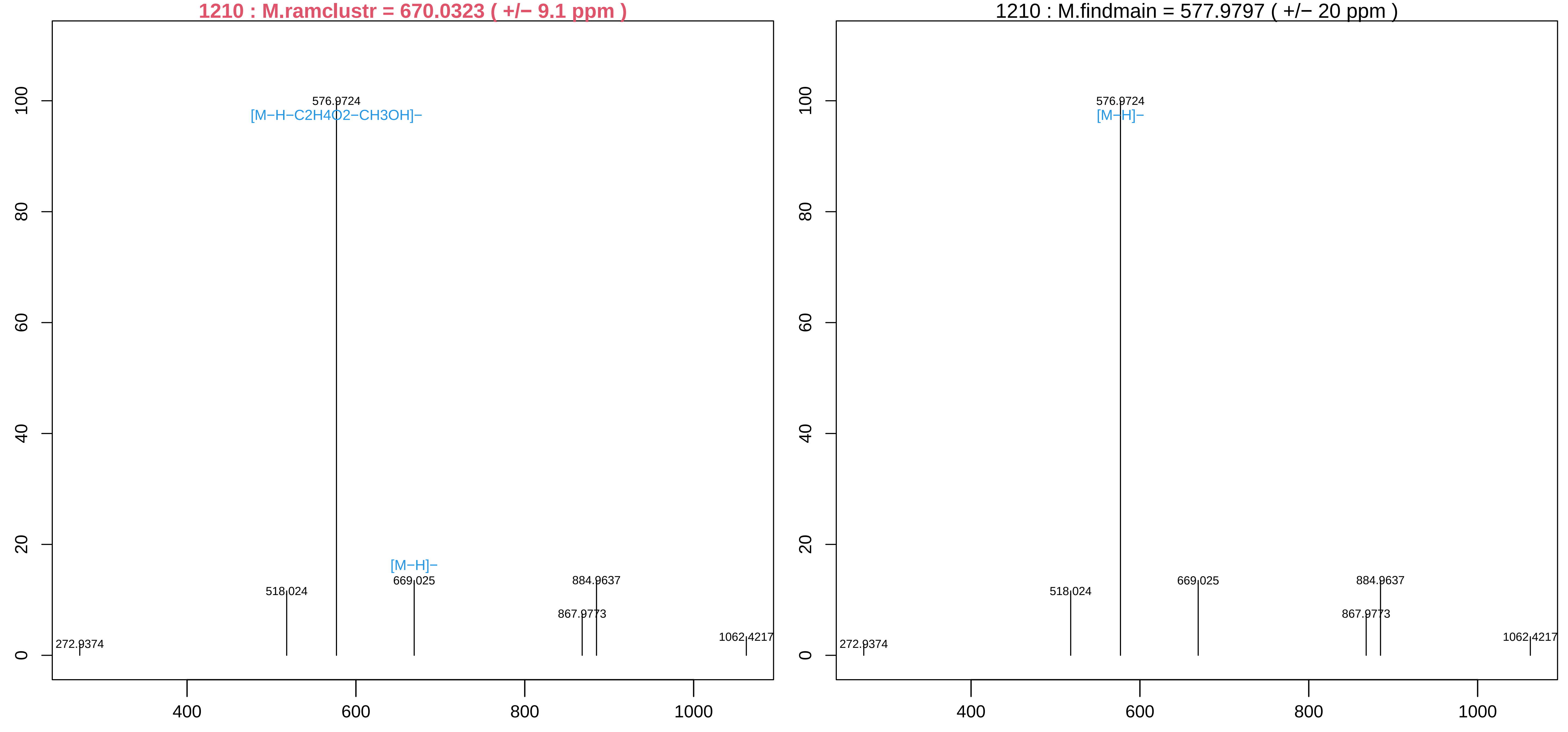

1210 : M.ramclustr = 670.0323 (+/− 9.1 ppm)
576.9724
[M−H−C2H4O2−CH3OH]−
[M−H]−
669.025
518.024
884.9637
867.9773
272.9374
1062.4217
1210 : M.findmain = 577.9797 (+/− 20 ppm)
576.9724
[M−H]−
669.025
518.024
884.9637
867.9773
272.9374
1062.4217
0
20
40
60
80
100
400
600
800
1000

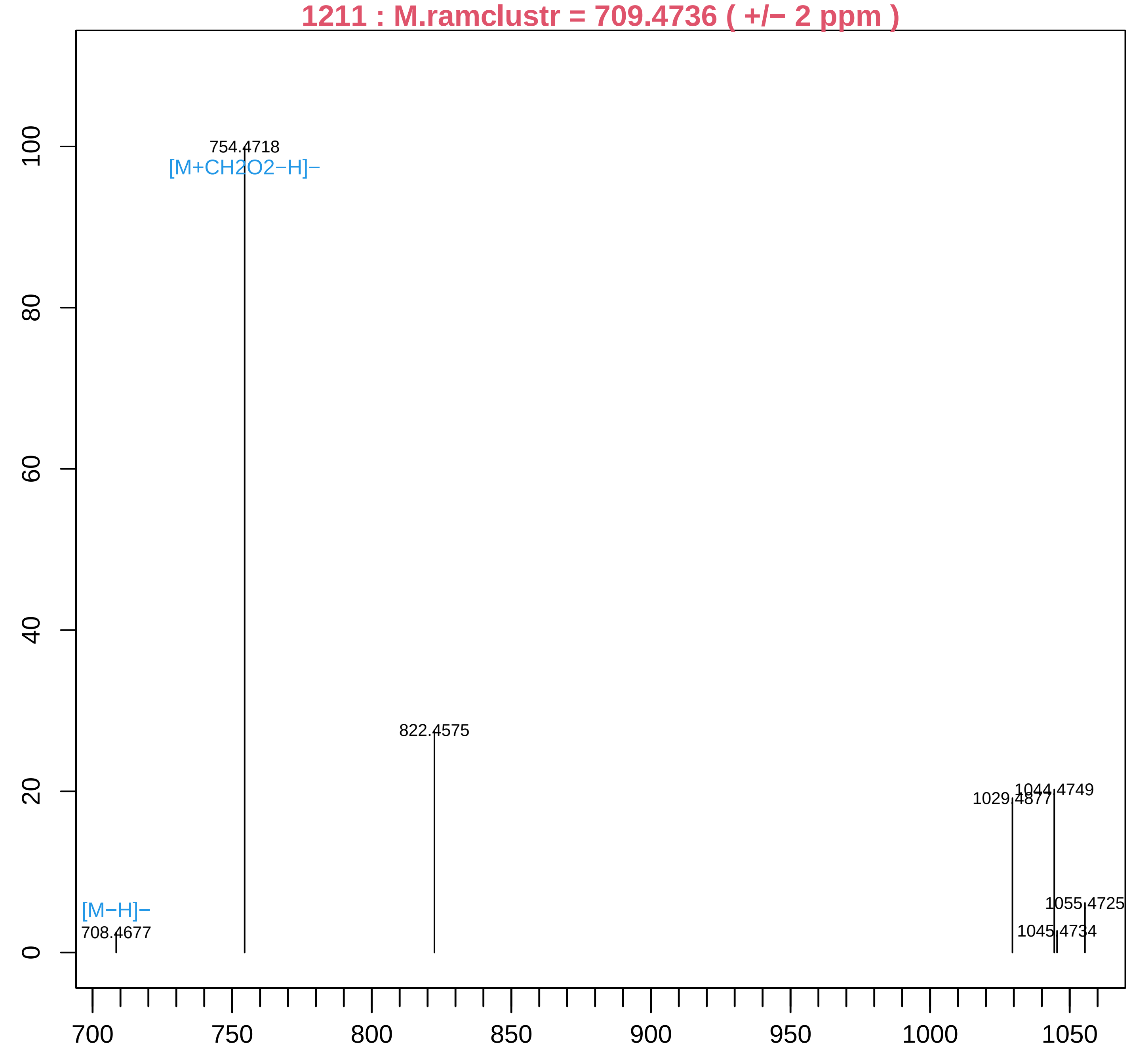
1211 : M.ramclustr = 709.4736 (+/− 2 ppm)
754.4718
[M+CH2O2−H]−
822.4575
1029.4877
1044.4749
1055.4725
1045.4734
[M−H]−
708.4677
0
20
40
60
80
100
700
750
800
850
900
950
1000
1050

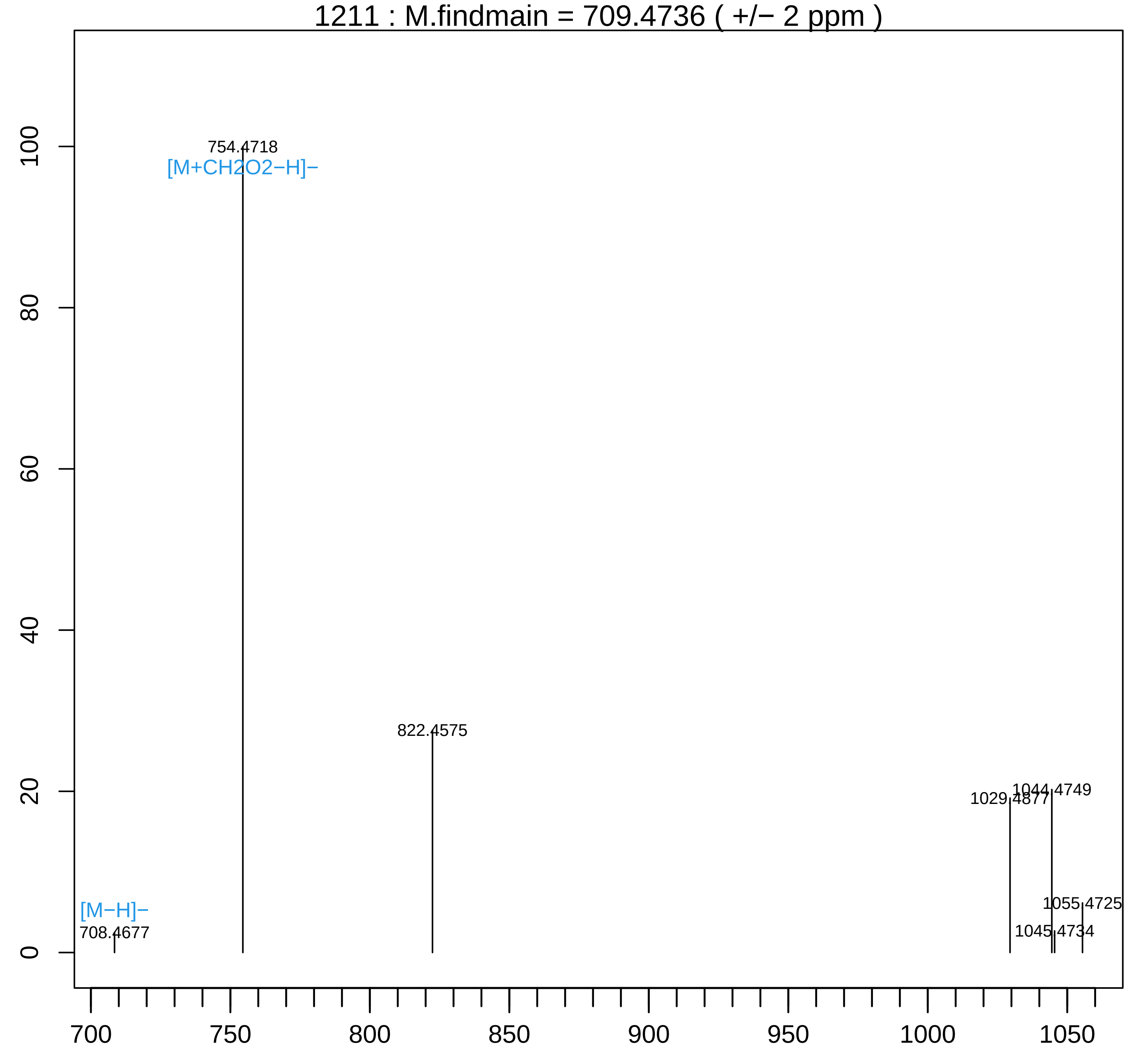
1211 : M.findmain = 709.4736 (+/− 2 ppm)
754.4718
[M+CH2O2−H]−
822.4575
1029.4877
1044.4749
1055.4725
1045.4734
[M−H]−
708.4677
0
20
40
60
80
100
700
750
800
850
900
950
1000
1050

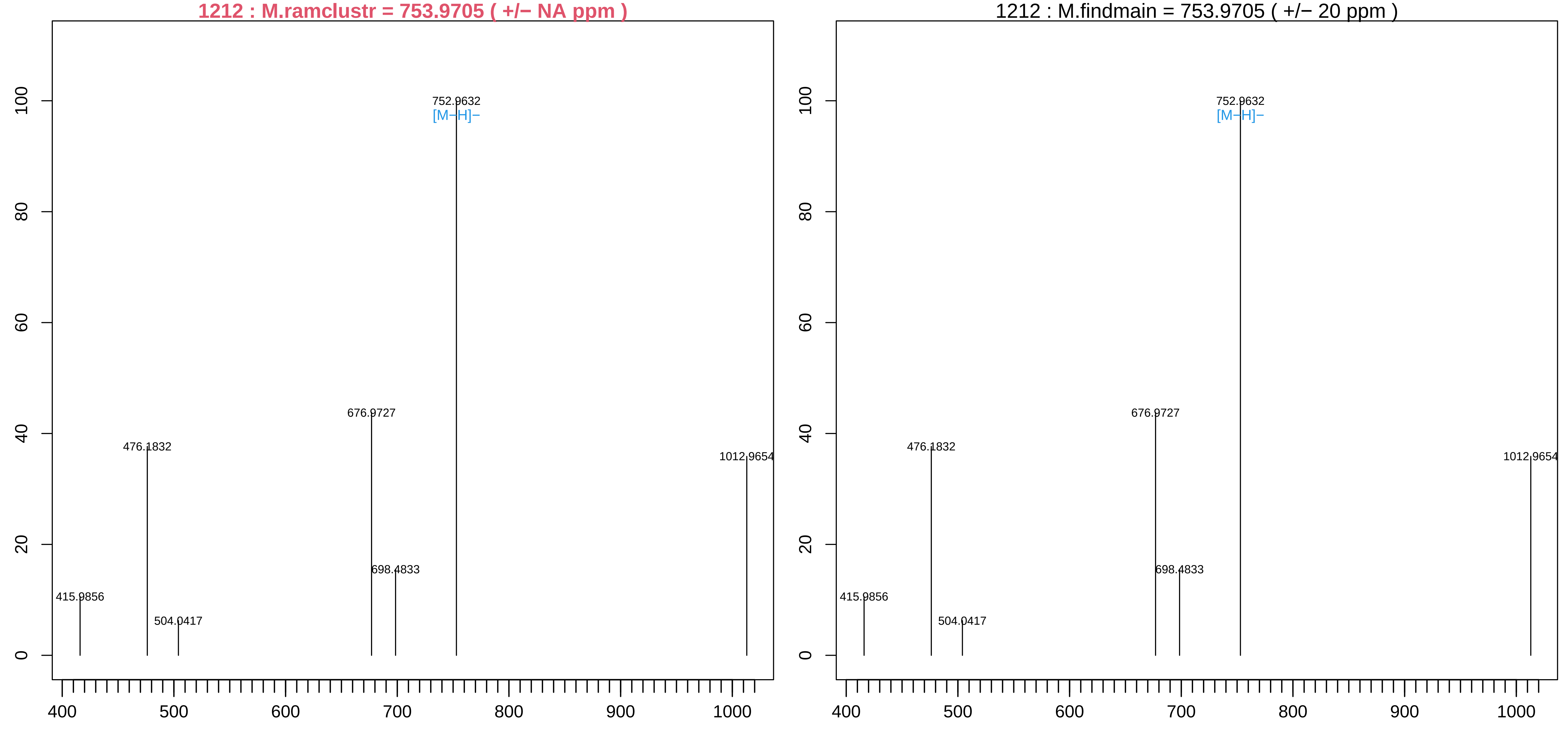
1212 : M.ramclustr = 753.9705 (+/− NA ppm)
752.9632
[M−H]−
676.9727
476.1832
1012.9654
698.4833
415.9856
504.0417
1212 : M.findmain = 753.9705 (+/− 20 ppm)
752.9632
[M−H]−
676.9727
476.1832
1012.9654
698.4833
415.9856
504.0417

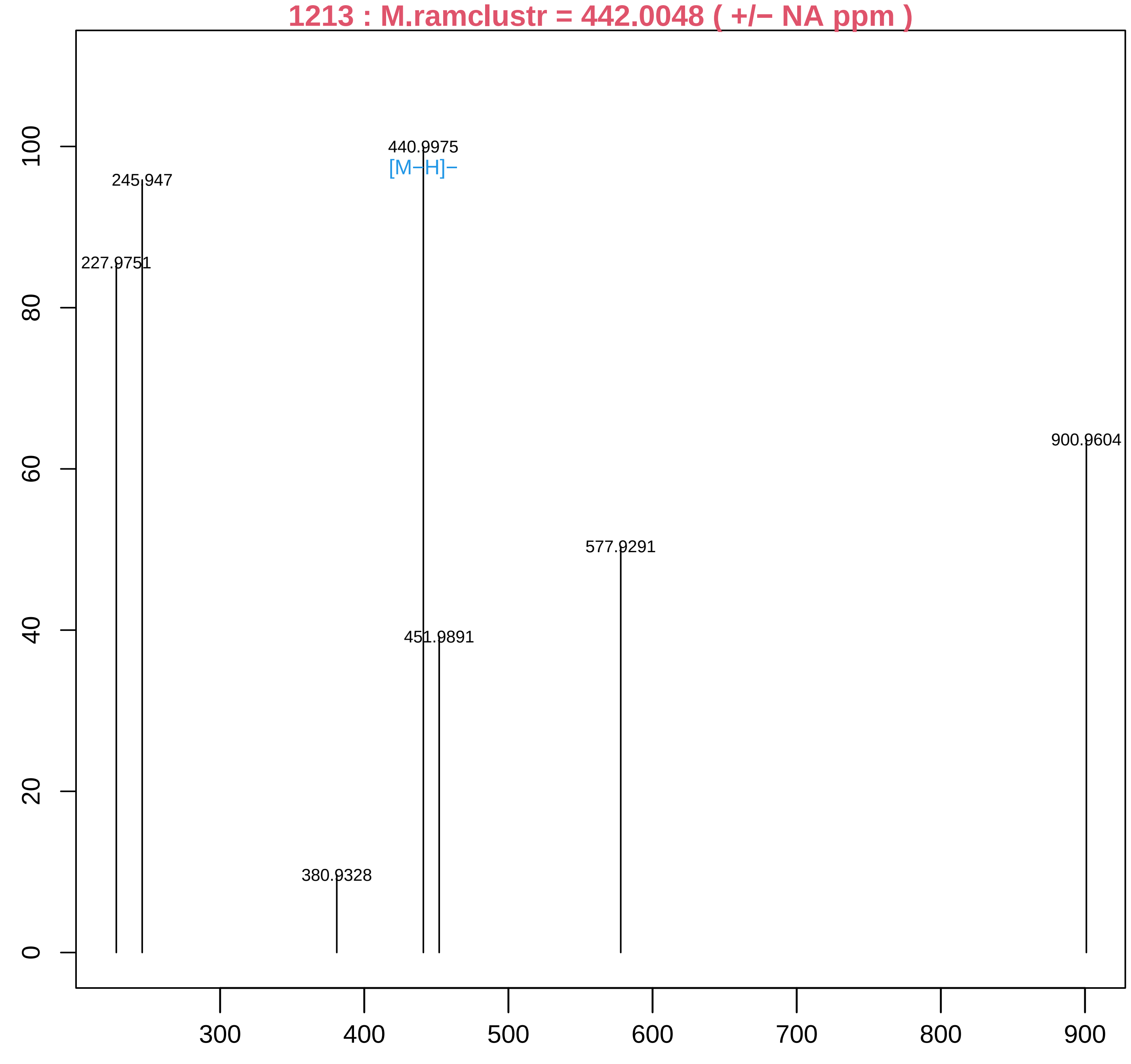
1213 : M.ramclustr = 442.0048 (+/− NA ppm)
440.9975
[M−H]−
245.947
227.9751
900.9604
577.9291
451.9891
380.9328
0
20
40
60
80
100
300
400
500
600
700
800
900

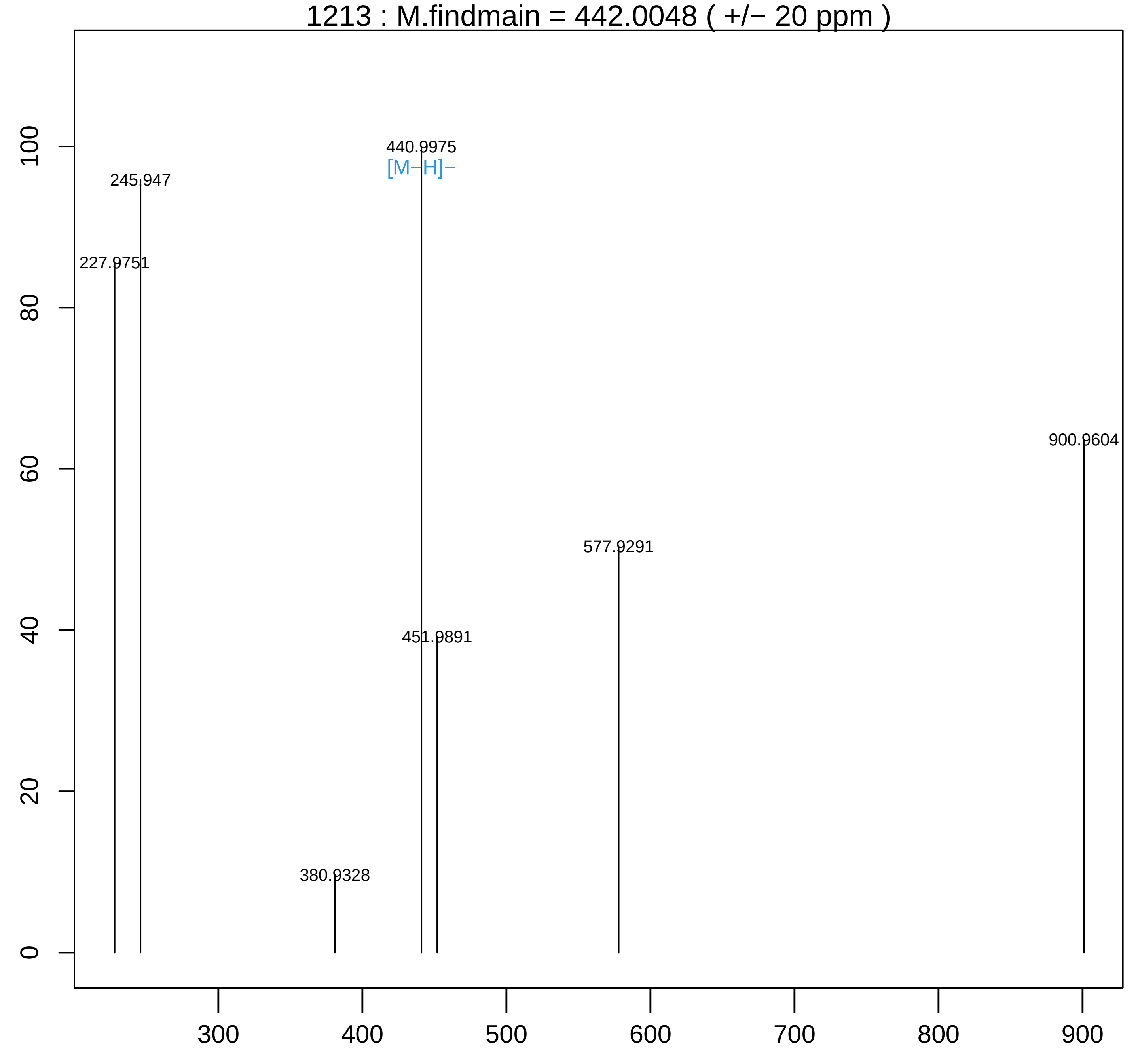
1213 : M.findmain = 442.0048 (+/− 20 ppm)
440.9975
[M−H]−
245.947
227.9751
900.9604
577.9291
451.9891
380.9328
0
20
40
60
80
100
300
400
500
600
700
800
900

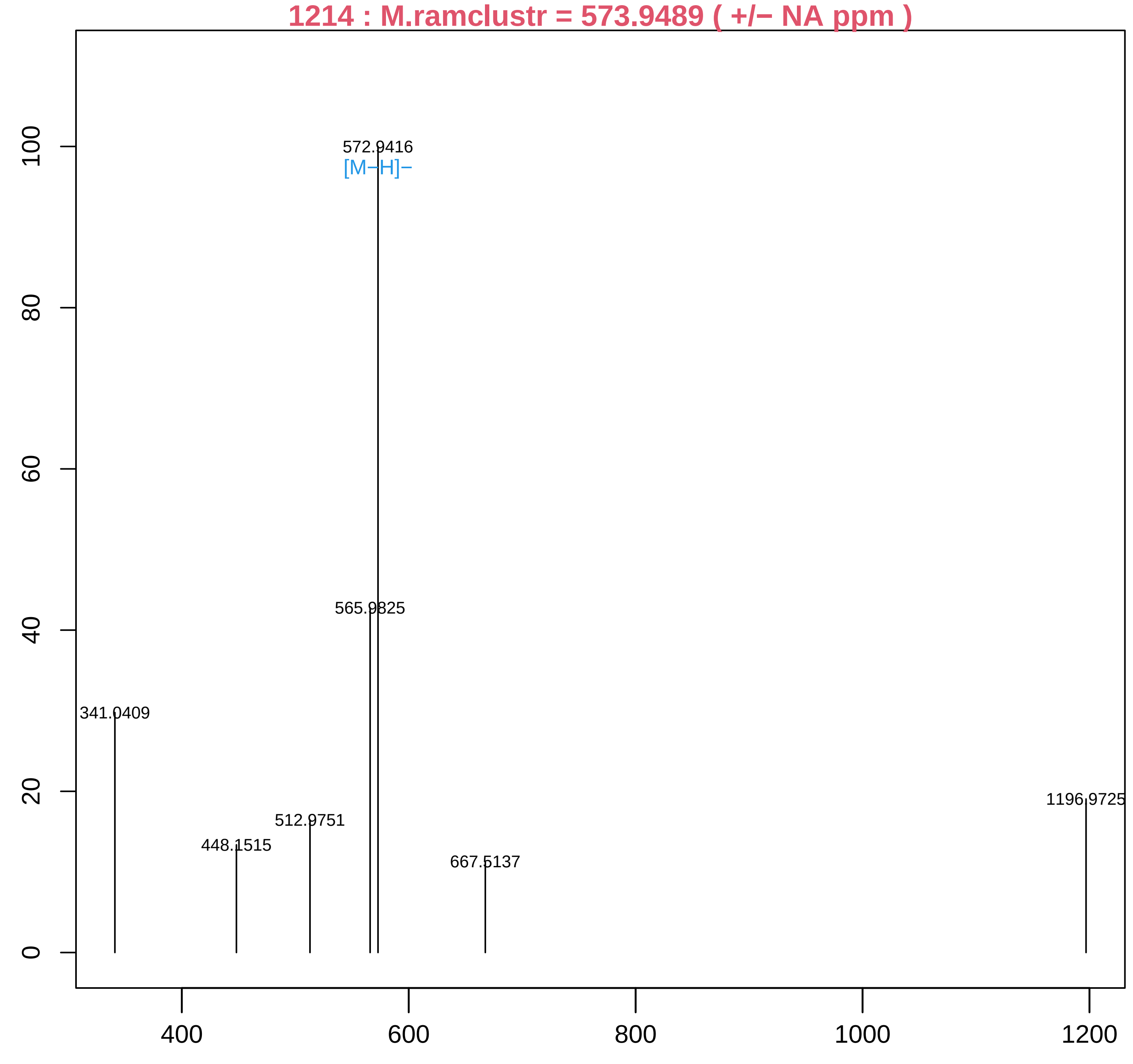
1214 : M.ramclustr = 573.9489 (+/− NA ppm)
572.9416
[M−H]−
565.9825
341.0409
512.9751
448.1515
667.5137
1196.9725
0
20
40
60
80
100
400
600
800
1000
1200

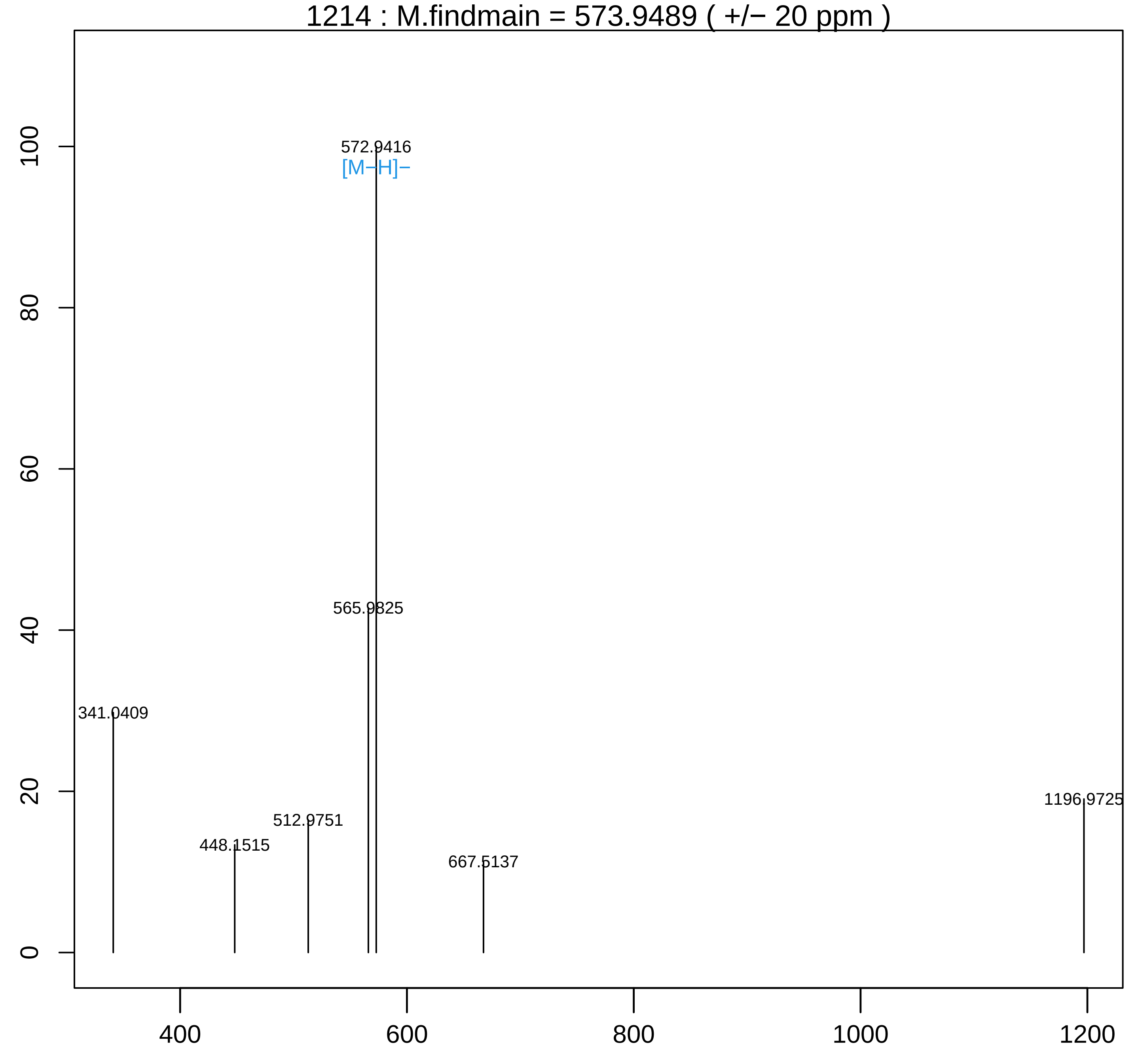
1214 : M.findmain = 573.9489 (+/− 20 ppm)
572.9416
[M−H]−
565.9825
341.0409
512.9751
448.1515
667.5137
1196.9725
0
20
40
60
80
100
400
600
800
1000
1200

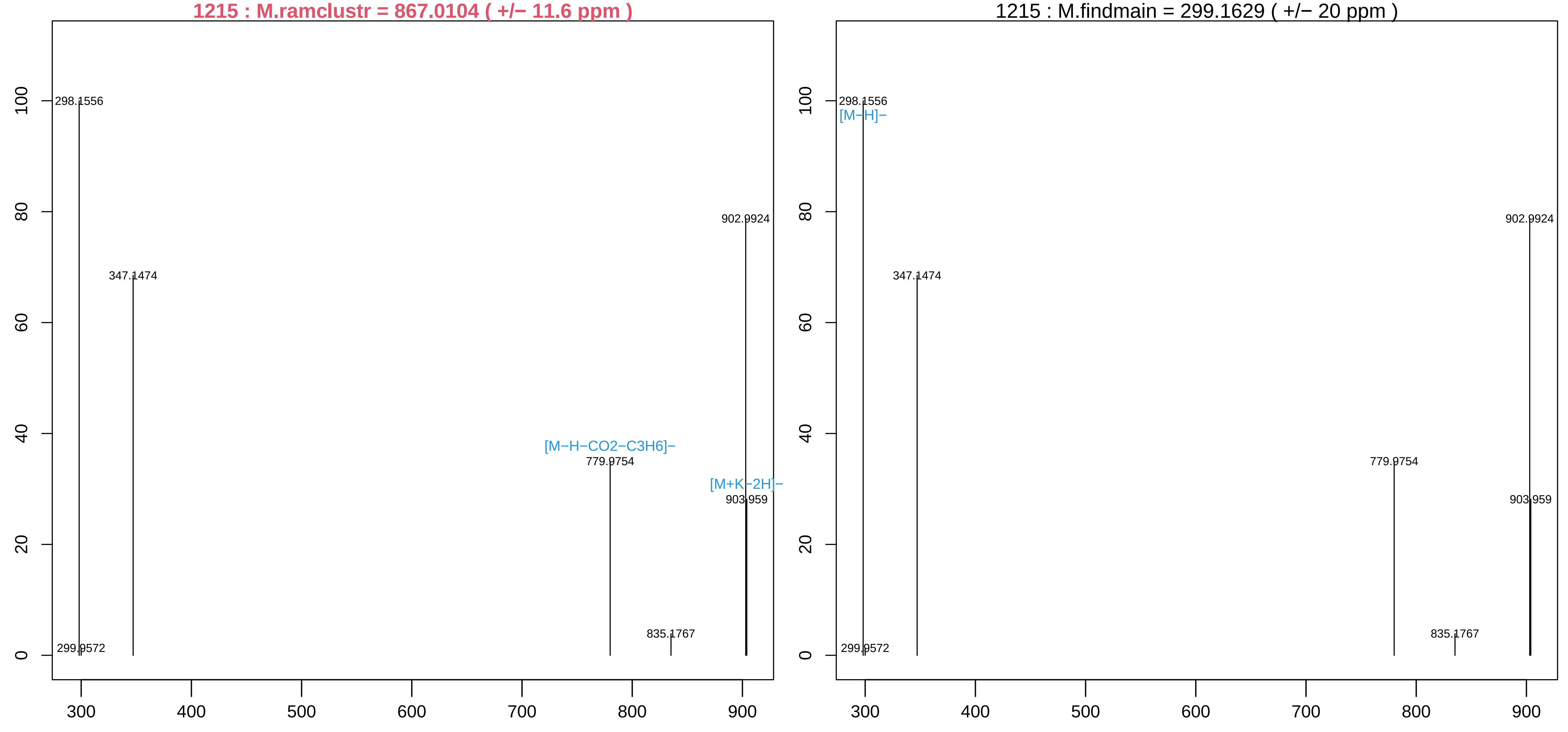

1215 : M.ramclustr = 867.0104 (+/− 11.6 ppm)
298.1556
347.1474
902.9924
[M−H−CO2−C3H6]−
779.9754
[M+K−2H]−
903.959
835.1767
299.9572
0
20
40
60
80
100
300
400
500
600
700
800
900
1215 : M.findmain = 299.1629 (+/− 20 ppm)
298.1556
[M−H]−
347.1474
902.9924
779.9754
903.959
835.1767
299.9572

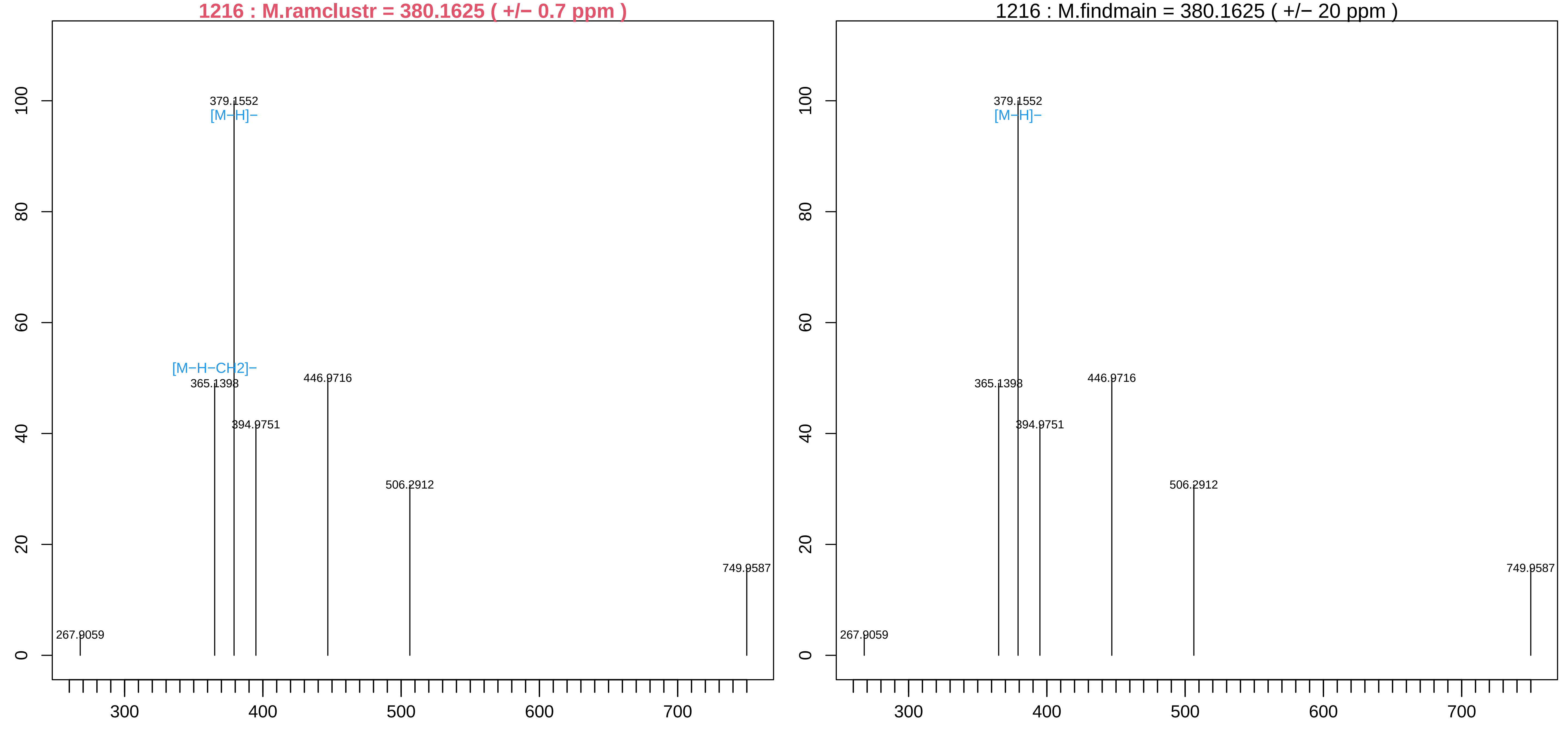
1216 : M.ramclustr = 380.1625 (+/− 0.7 ppm)
379.1552
[M−H]−
[M−H−CH2]−
365.1398
446.9716
394.9751
506.2912
749.9587
267.9059
0
20
40
60
80
100
300
400
500
600
700
1216 : M.findmain = 380.1625 (+/− 20 ppm)
379.1552
[M−H]−
365.1398
446.9716
394.9751
506.2912
749.9587
267.9059
0
20
40
60
80
100
300
400
500
600
700

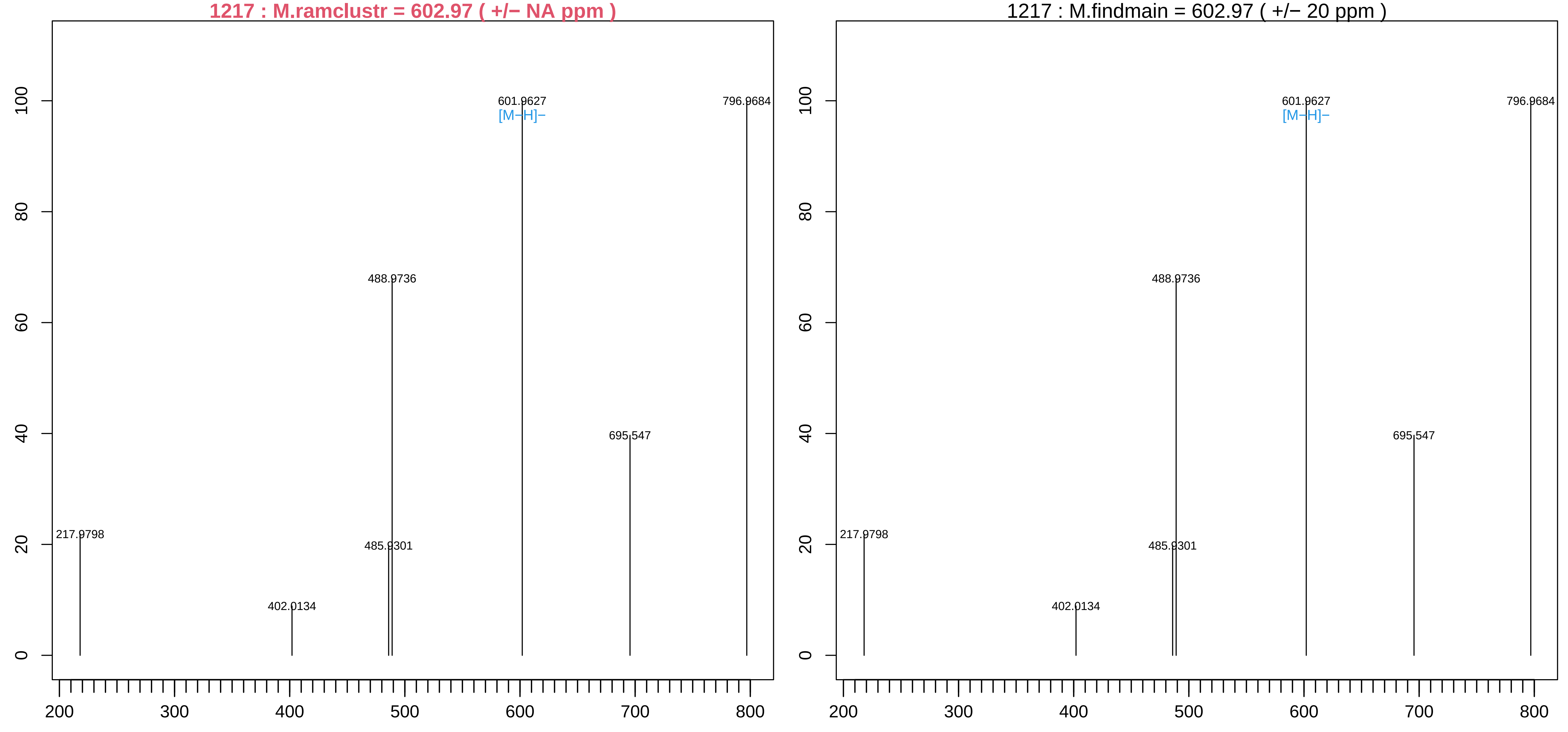
1217 : M.ramclustr = 602.97 (+/− NA ppm)
1217 : M.findmain = 602.97 (+/− 20 ppm)
601.9627
[M−H]−
796.9684
488.9736
695.547
217.9798
485.9301
402.0134
0
20
40
60
80
100
200
300
400
500
600
700
800

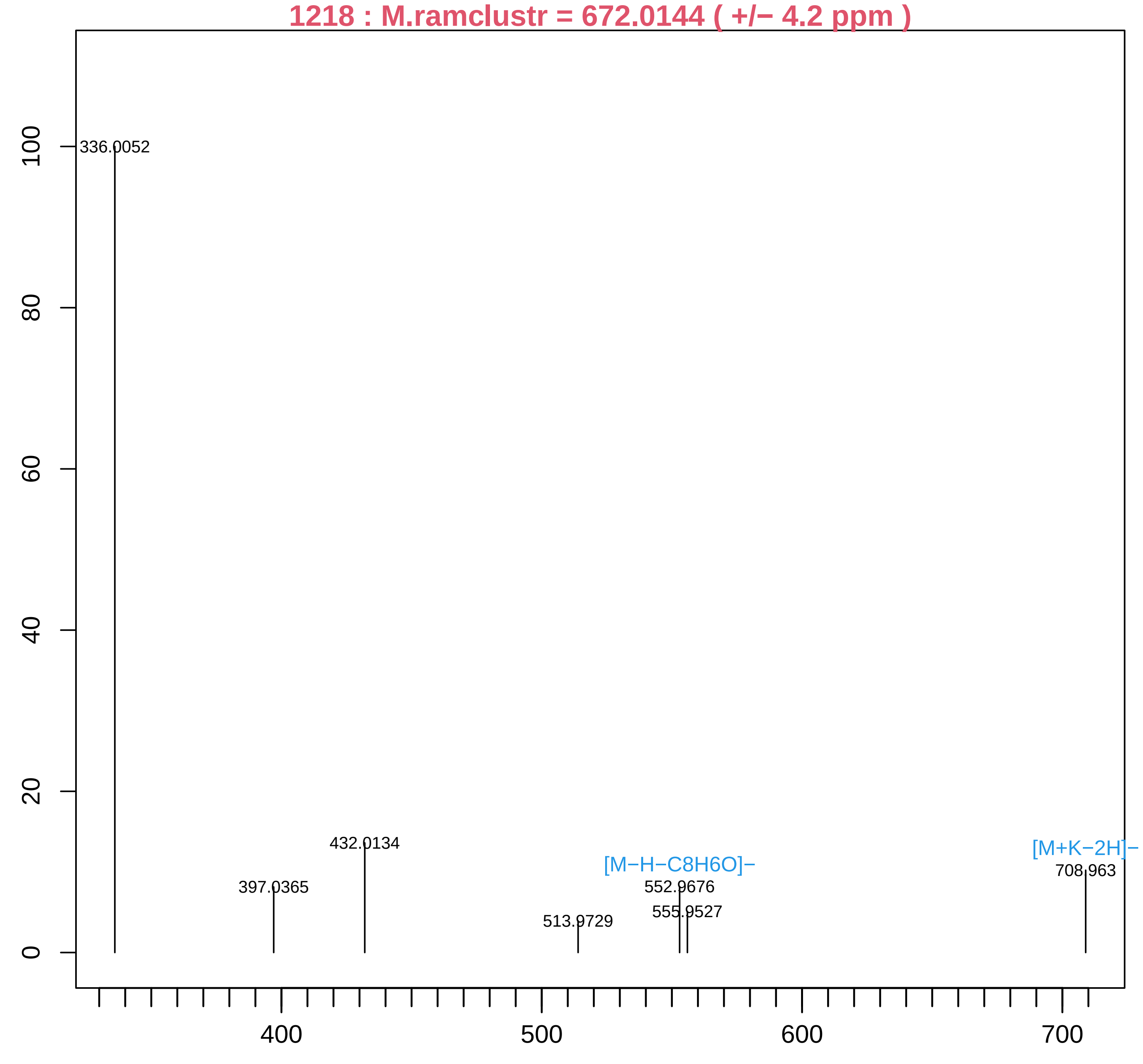

1218 : M.ramclustr = 672.0144 (+/− 4.2 ppm)
336.0052
397.0365
432.0134
513.9729
[M−H−C8H6O]−
552.9676
555.9527
[M+K−2H]−
708.963
0
20
40
60
80
100
400
500
600
700

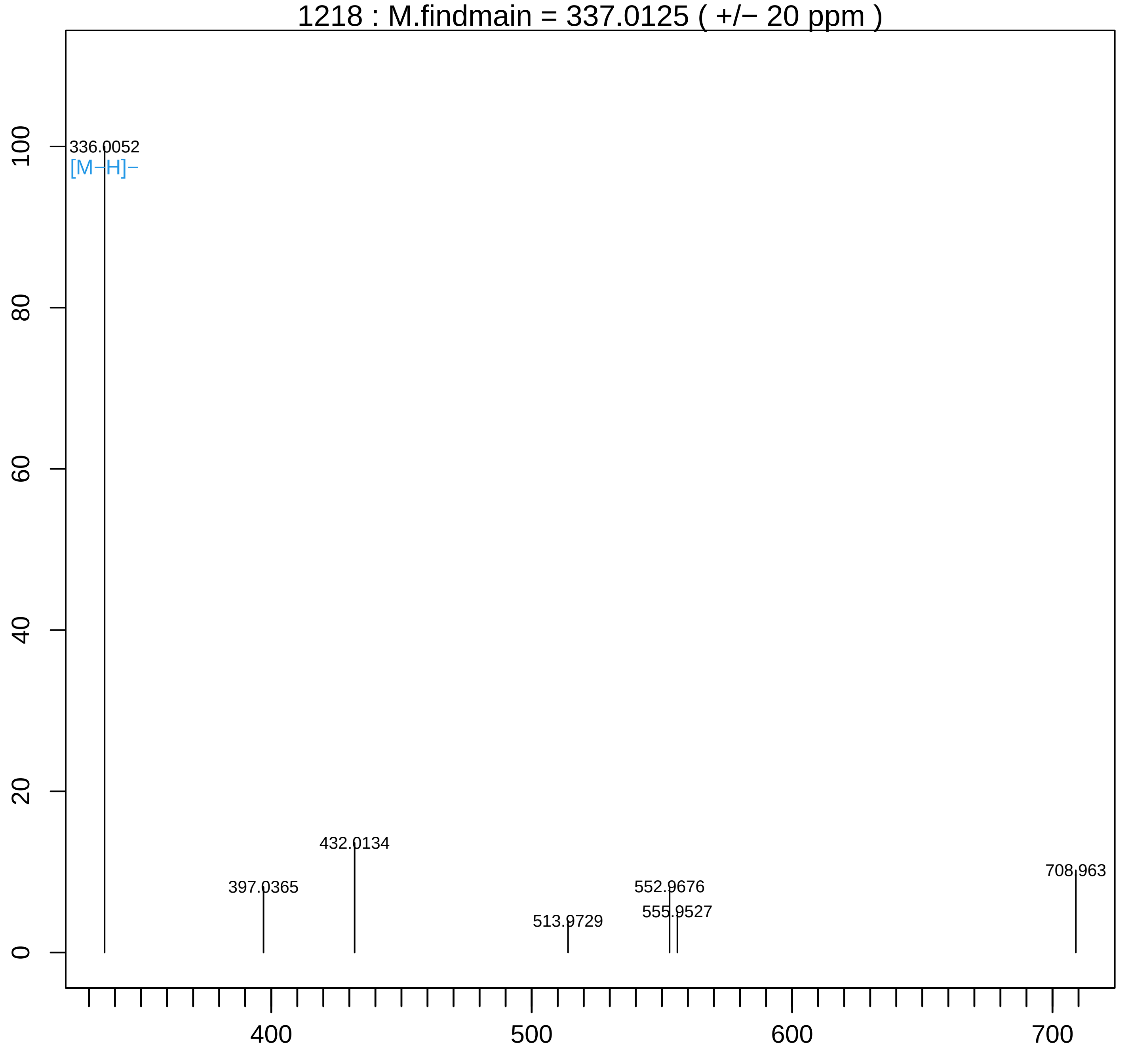

1218 : M.findmain = 337.0125 (+/− 20 ppm)
336.0052
[M−H]−
397.0365
432.0134
513.9729
552.9676
555.9527
708.963
0
20
40
60
80
100
400
500
600
700

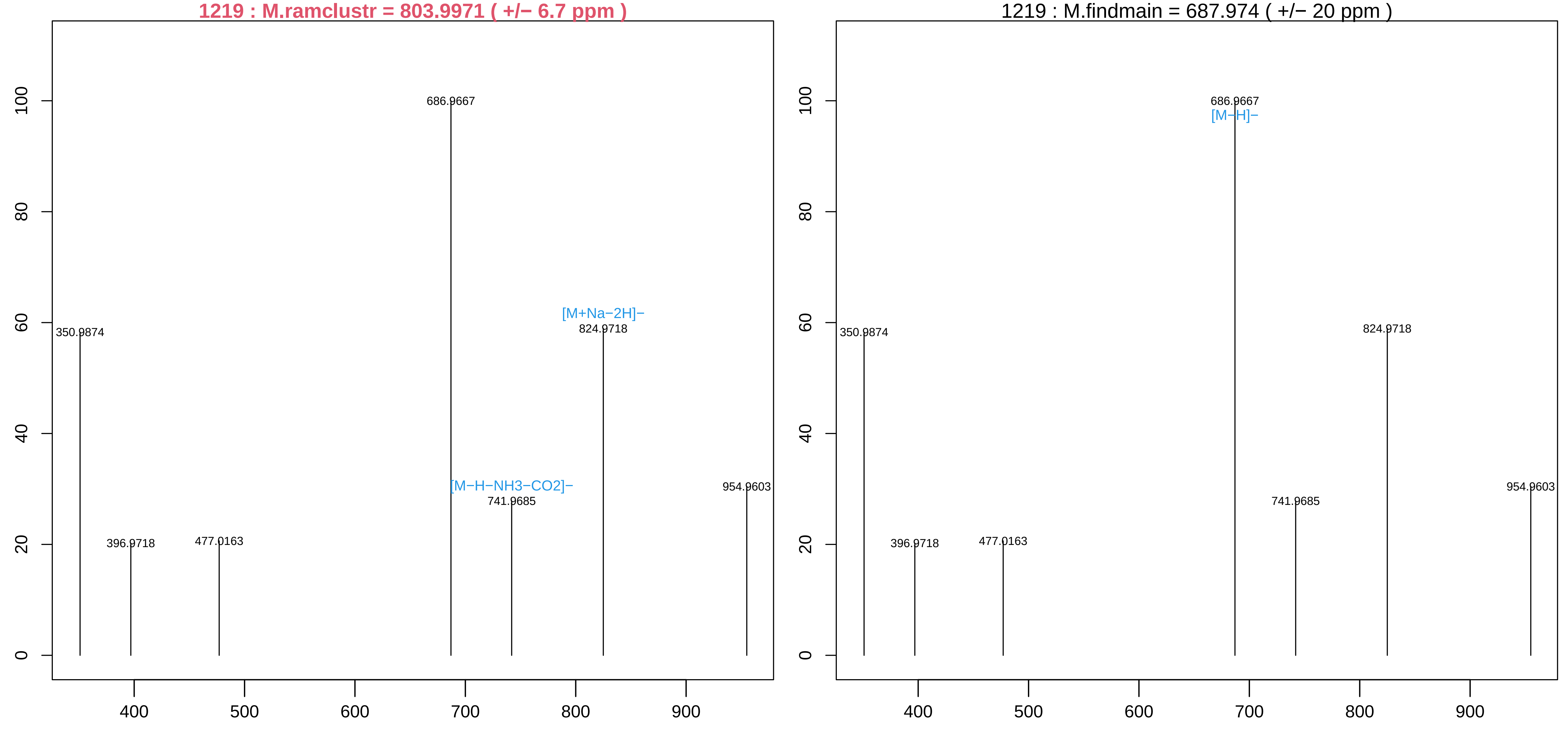

1219 : M.ramclustr = 803.9971 (+/− 6.7 ppm)
686.9667
350.9874
[M+Na−2H]−
824.9718
[M−H−NH3−CO2]−
741.9685
954.9603
396.9718
477.0163
1219 : M.findmain = 687.974 (+/− 20 ppm)
686.9667
[M−H]−
350.9874
824.9718
741.9685
954.9603
396.9718
477.0163
0
20
40
60
80
100
400
500
600
700
800
900

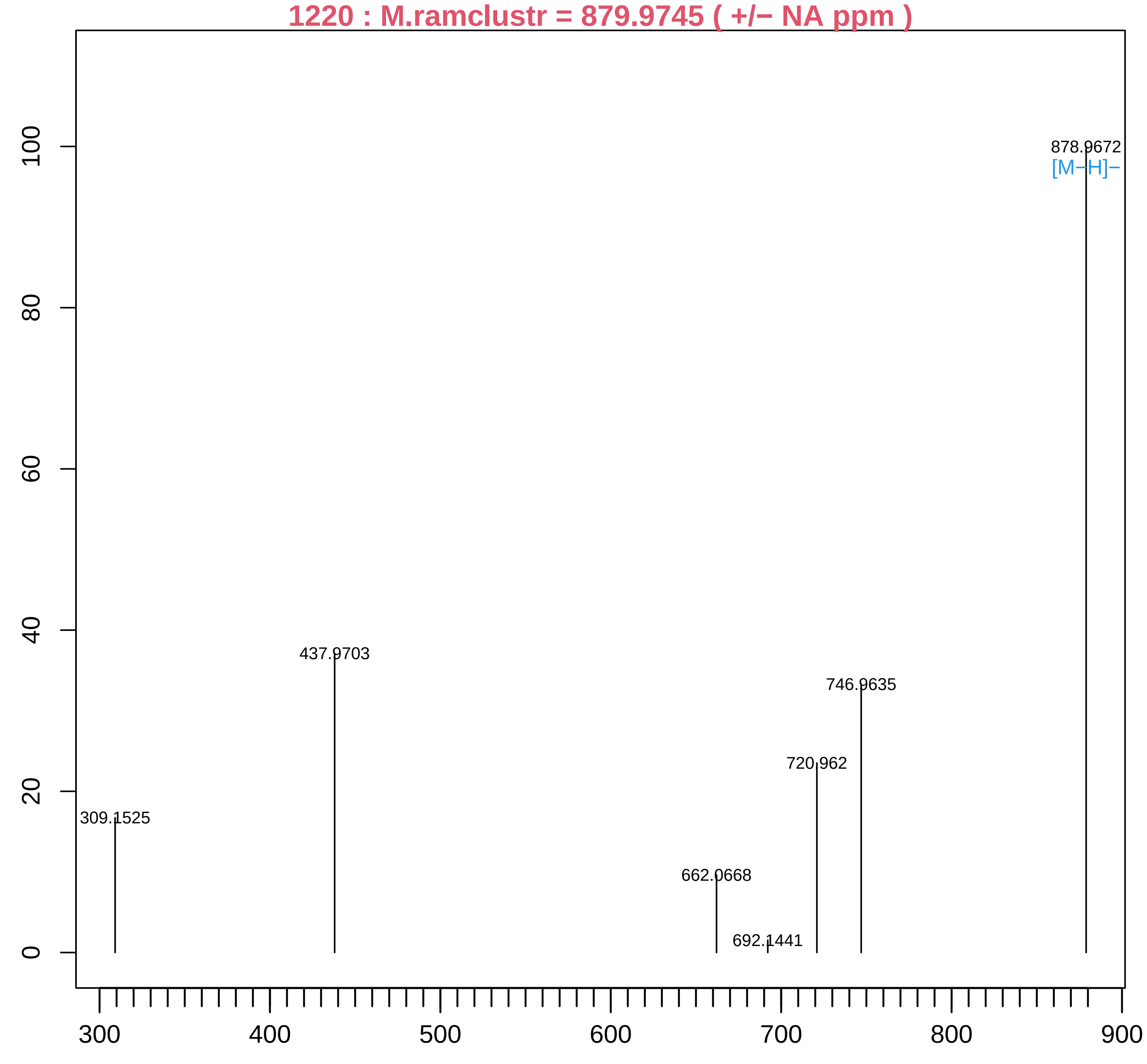
1220 : M.ramclustr = 879.9745 (+/− NA ppm)
878.9672
[M−H]−
437.9703
746.9635
720.962
309.1525
662.0668
692.1441
0
20
40
60
80
100
300
400
500
600
700
800
900

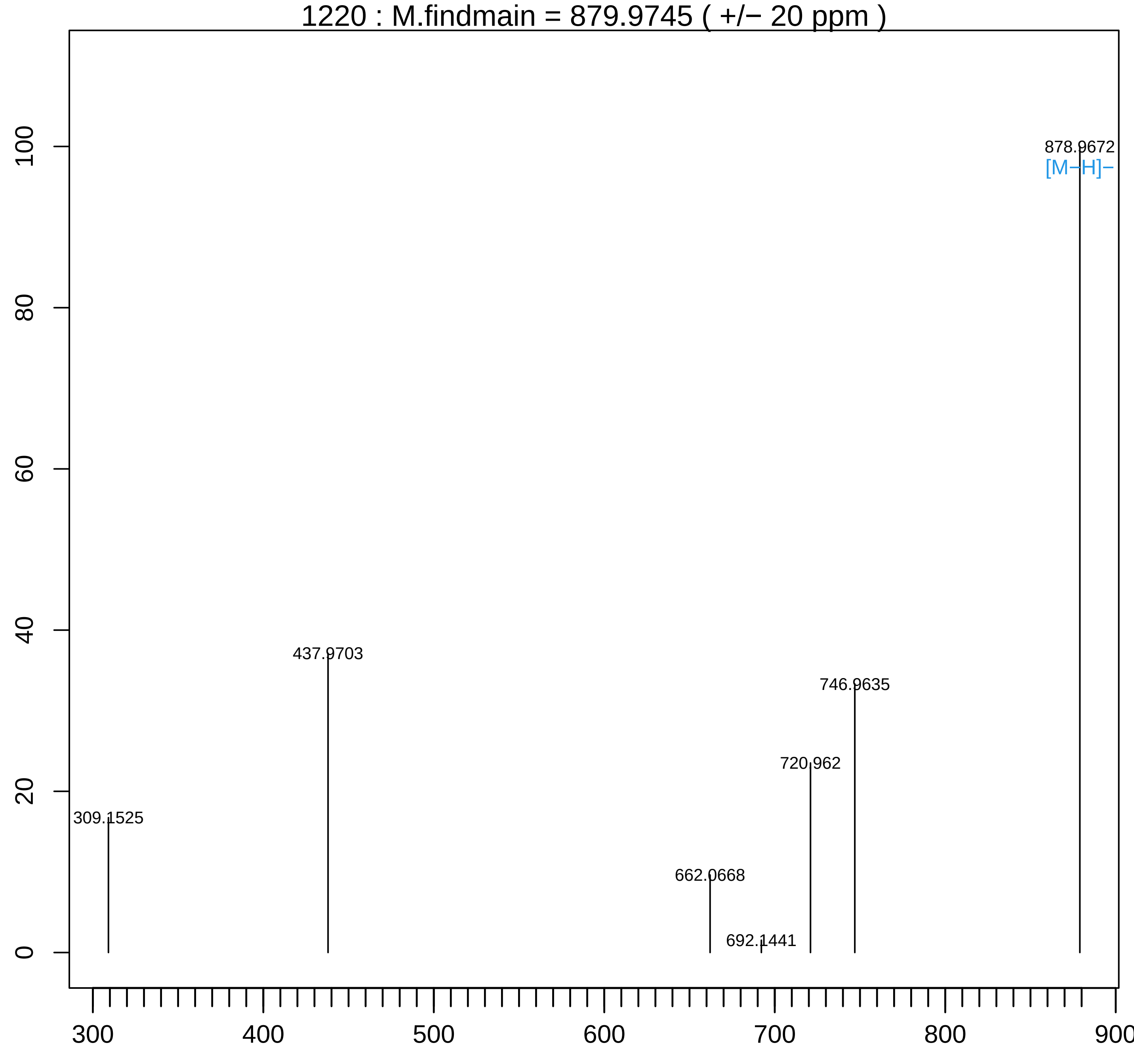
1220 : M.findmain = 879.9745 (+/− 20 ppm)
878.9672
[M−H]−
437.9703
746.9635
720.962
309.1525
662.0668
692.1441
0
20
40
60
80
100
300
400
500
600
700
800
900

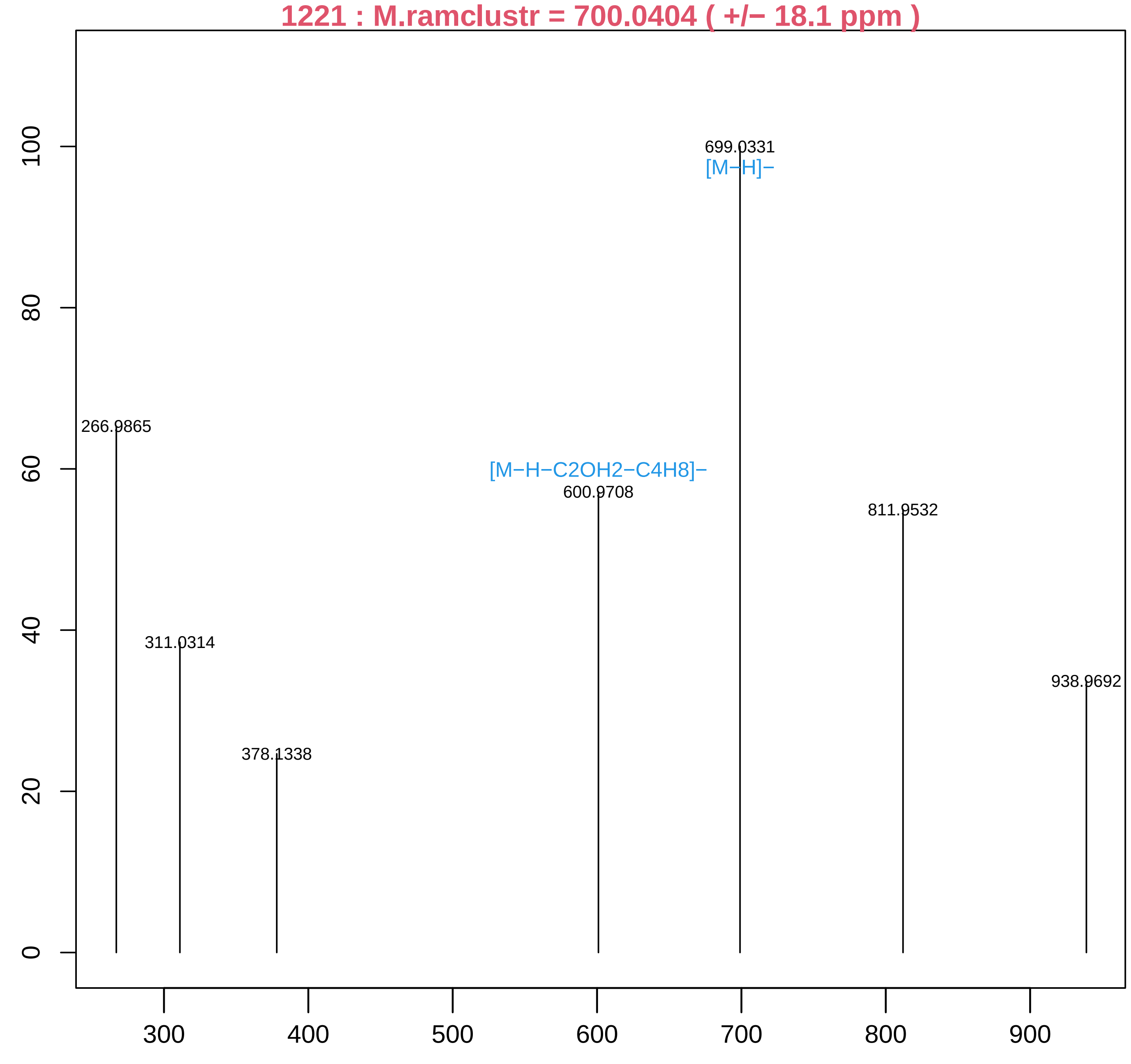
1221 : M.ramclustr = 700.0404 (+/− 18.1 ppm)
699.0331
[M−H]−
266.9865
[M−H−C2OH2−C4H8]−
600.9708
811.9532
311.0314
938.9692
378.1338
0
20
40
60
80
100
300
400
500
600
700
800
900

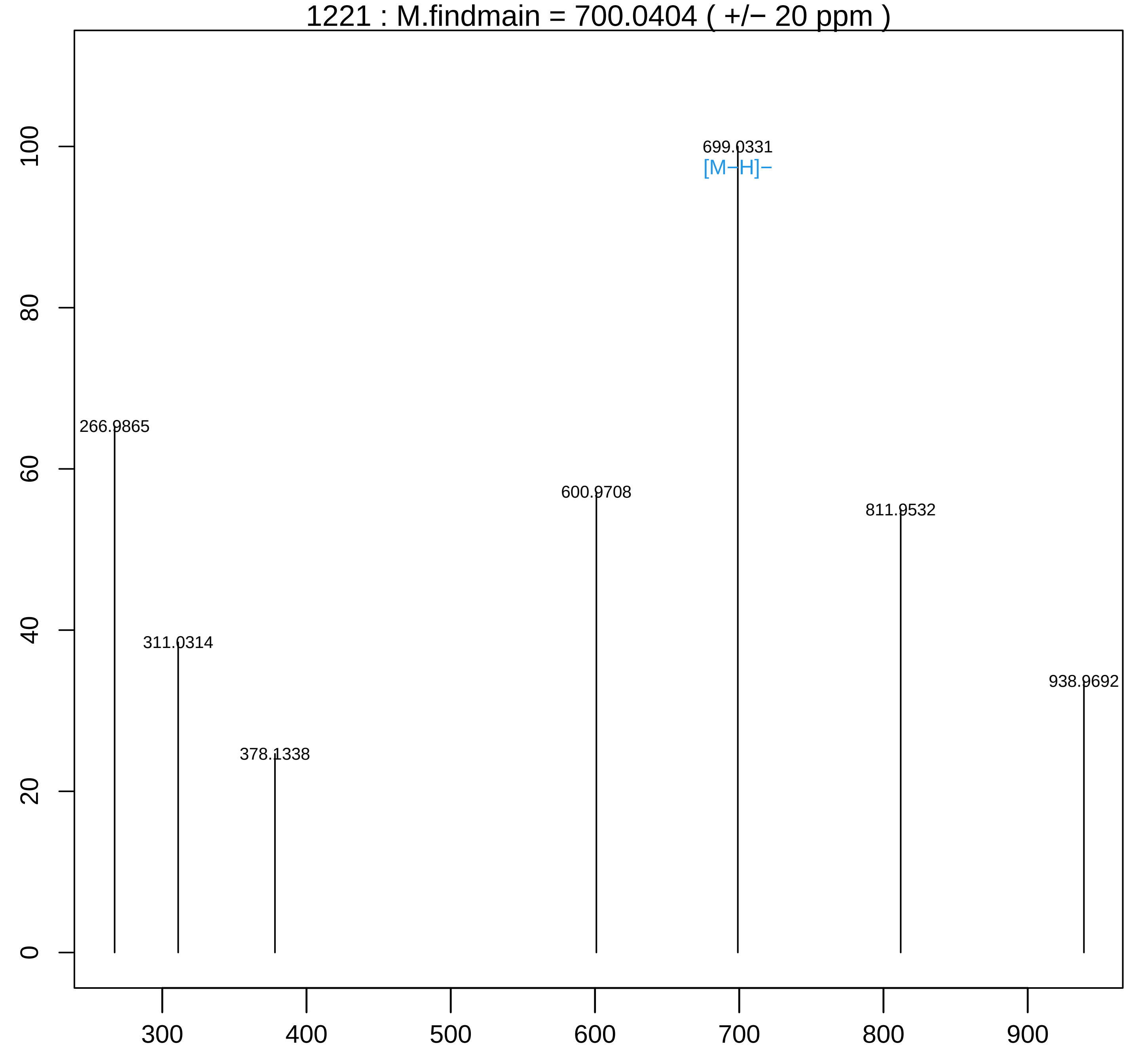
1221 : M.findmain = 700.0404 (+/− 20 ppm)
699.0331
[M−H]−
266.9865
600.9708
811.9532
311.0314
938.9692
378.1338
0
20
40
60
80
100
300
400
500
600
700
800
900

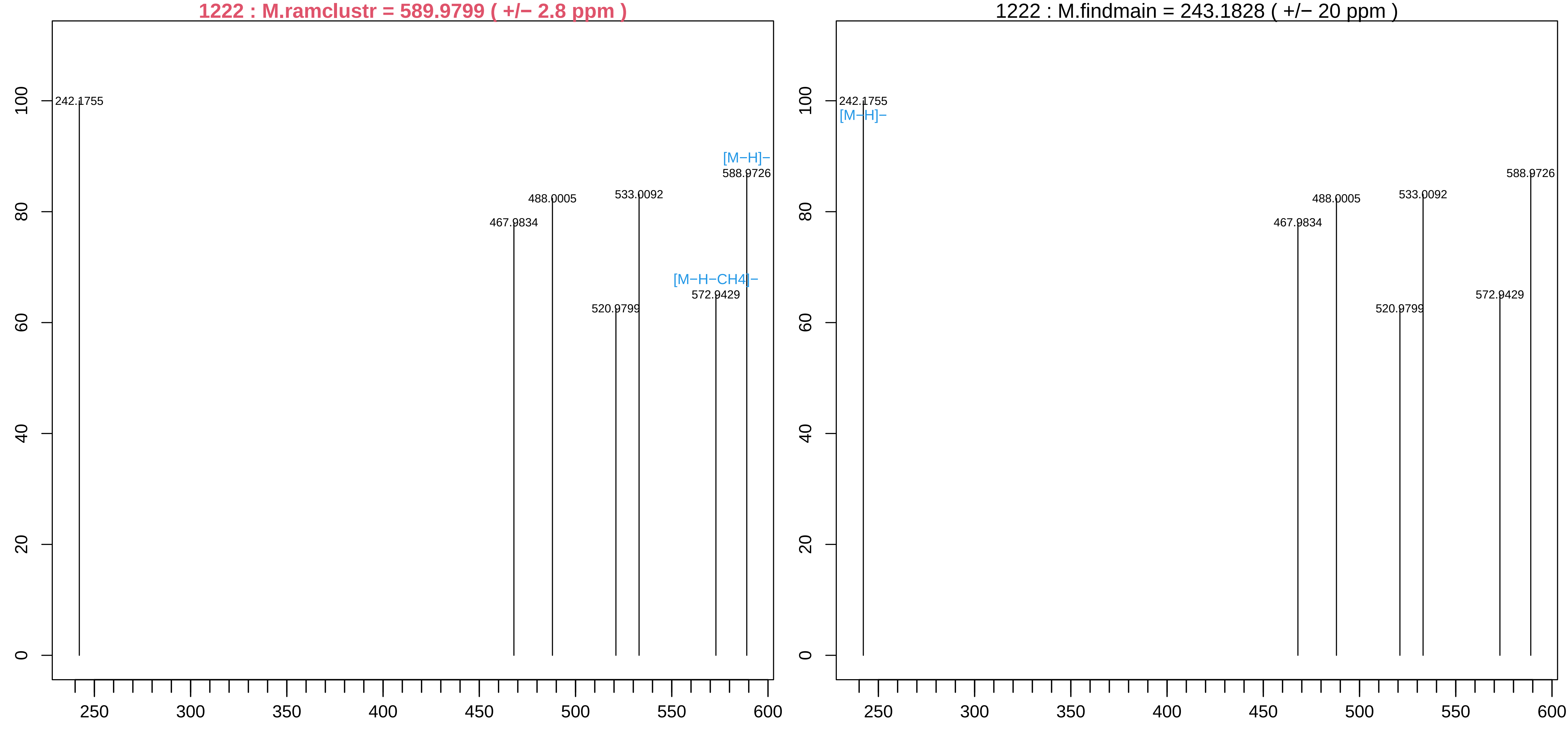
1222 : M.ramclustr = 589.9799 (+/− 2.8 ppm)
242.1755
467.9834
488.0005
520.9799
533.0092
[M−H−CH4]−
572.9429
[M−H]−
588.9726
1222 : M.findmain = 243.1828 (+/− 20 ppm)
242.1755
[M−H]−
467.9834
488.0005
520.9799
533.0092
572.9429
588.9726

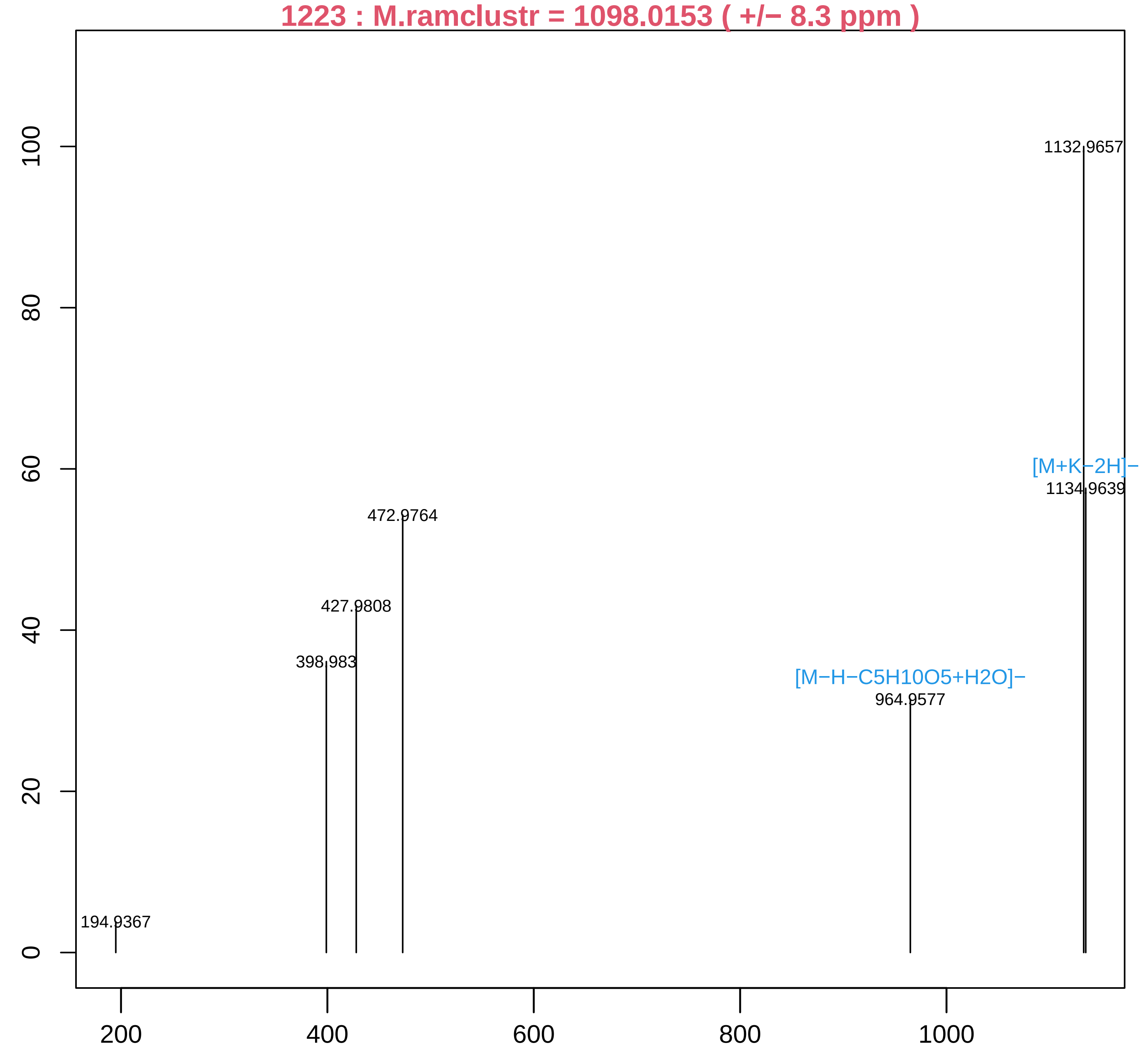
1223 : M.ramclustr = 1098.0153 (+/− 8.3 ppm)
1132.9657
[M+K−2H]−
1134.9639
472.9764
427.9808
398.983
[M−H−C5H10O5+H2O]−
964.9577
194.9367
0
20
40
60
80
100
200
400
600
800
1000

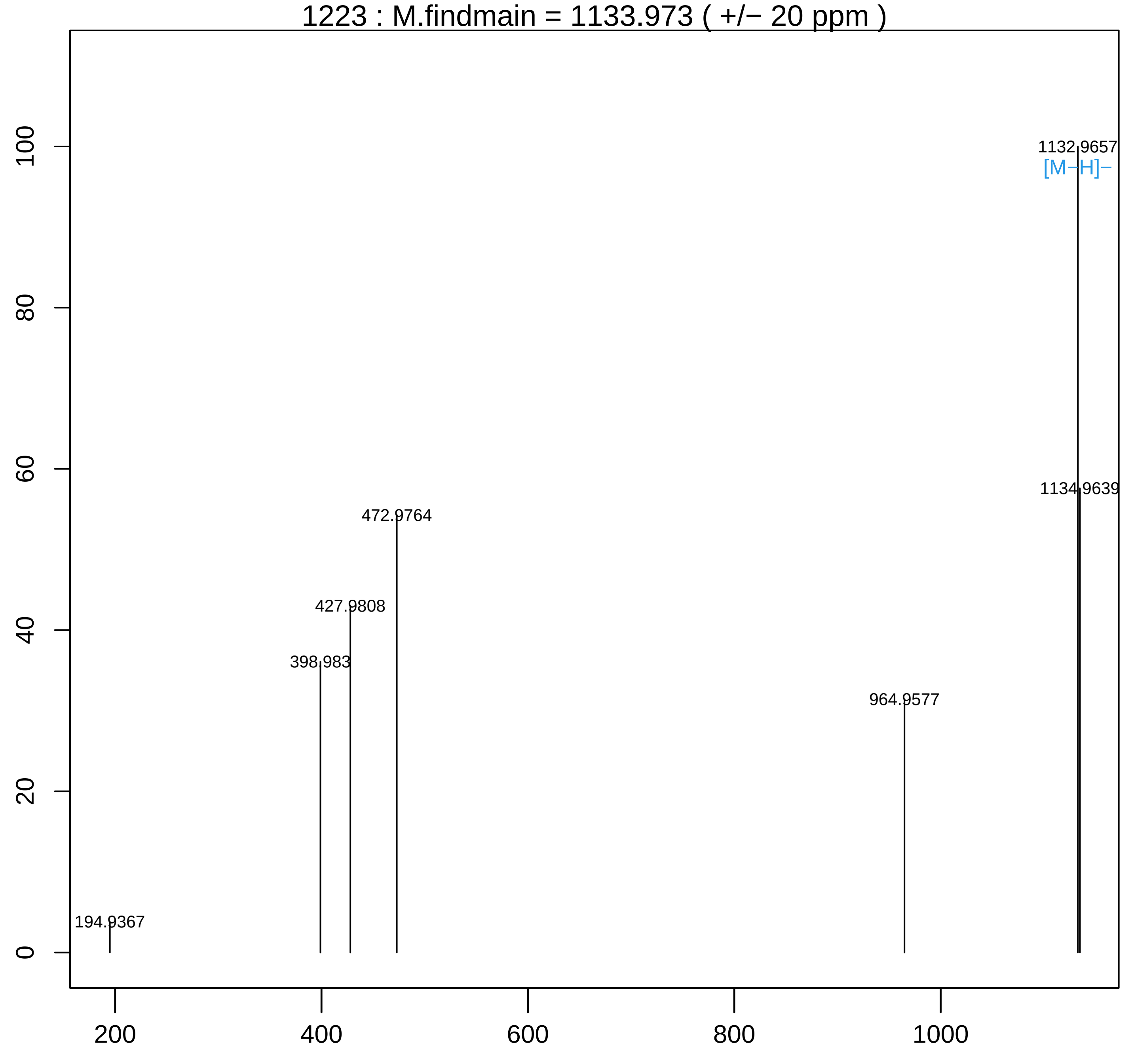
1223 : M.findmain = 1133.973 (+/− 20 ppm)
1132.9657
[M−H]−
1134.9639
472.9764
427.9808
398.983
964.9577
194.9367
0
20
40
60
80
100
200
400
600
800
1000

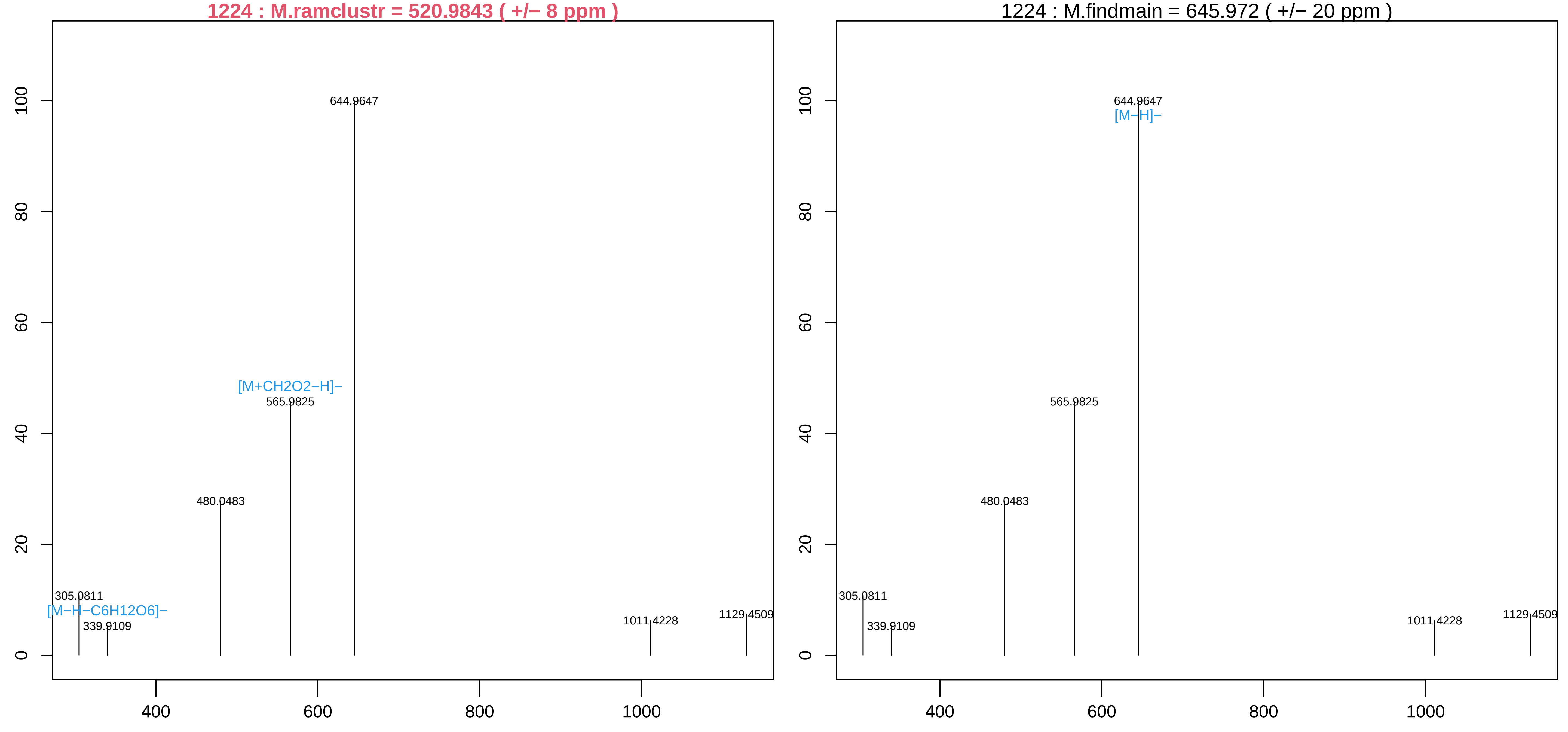
1224 : M.ramclustr = 520.9843 (+/− 8 ppm)
644.9647
[M+CH2O2−H]−
565.9825
480.0483
305.0811
[M−H−C6H12O6]−
339.9109
1011.4228
1129.4509
0
20
40
60
80
100
400
600
800
1000
1224 : M.findmain = 645.972 (+/− 20 ppm)
644.9647
[M−H]−
565.9825
480.0483
305.0811
339.9109
1011.4228
1129.4509

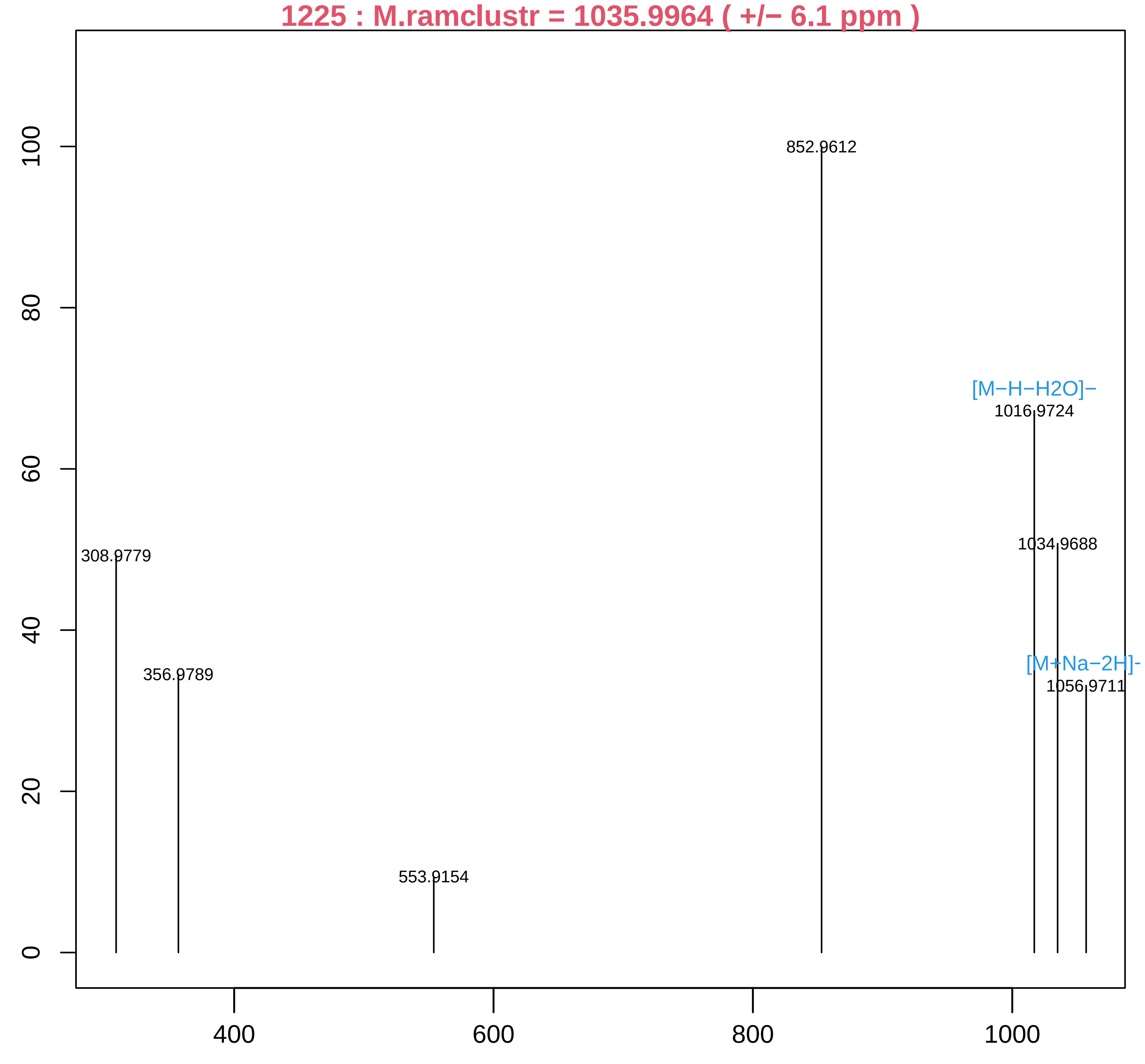

1225 : M.ramclustr = 1035.9964 (+/− 6.1 ppm)
852.9612
[M−H−H2O]−
1016.9724
1034.9688
308.9779
[M+Na−2H]-
356.9789
1056.9711
553.9154
0
20
40
60
80
100
400
600
800
1000

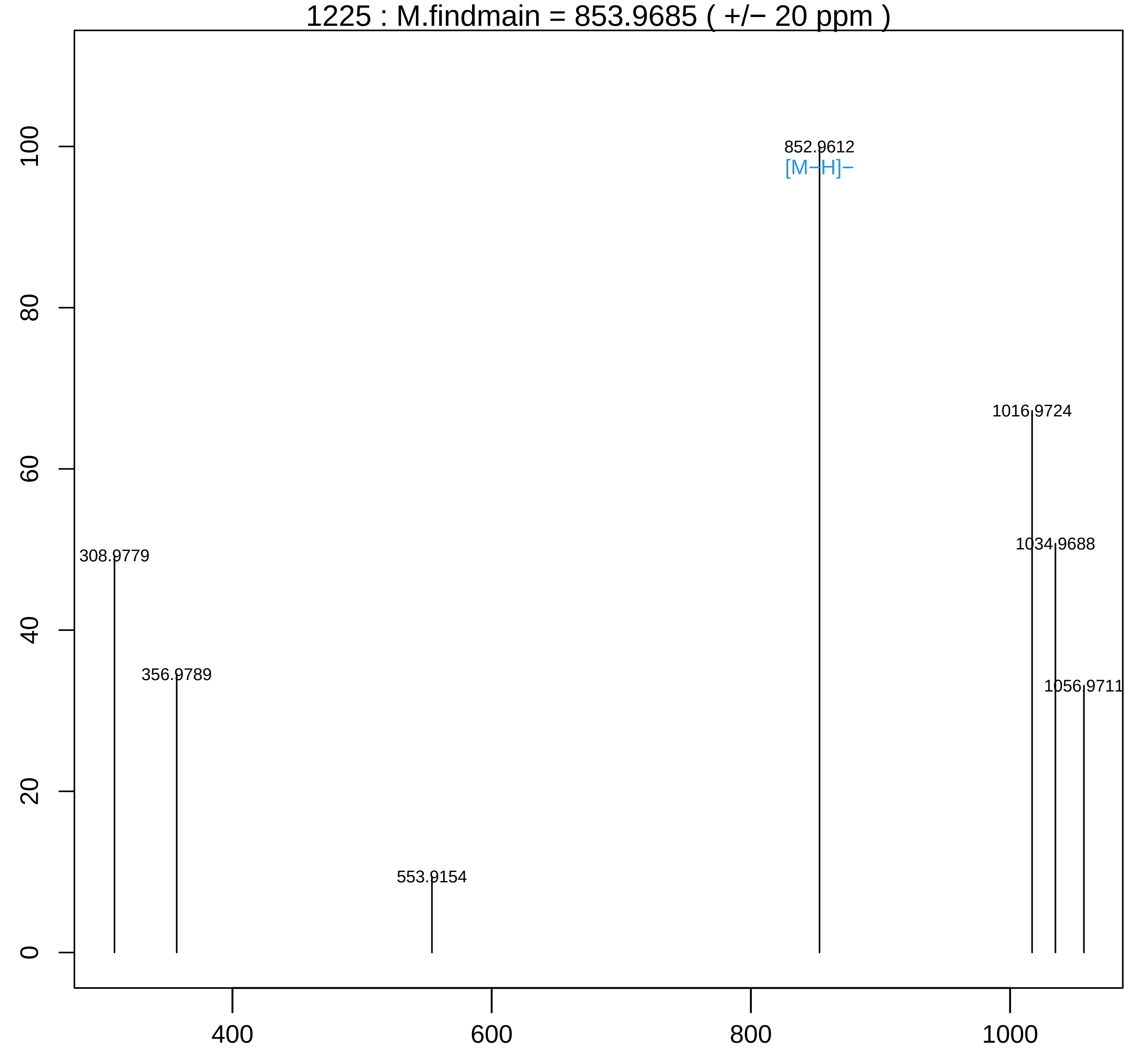

1225 : M.findmain = 853.9685 (+/− 20 ppm)
852.9612
[M−H]−
1016.9724
1034.9688
308.9779
356.9789
1056.9711
553.9154
0
20
40
60
80
100
400
600
800
1000

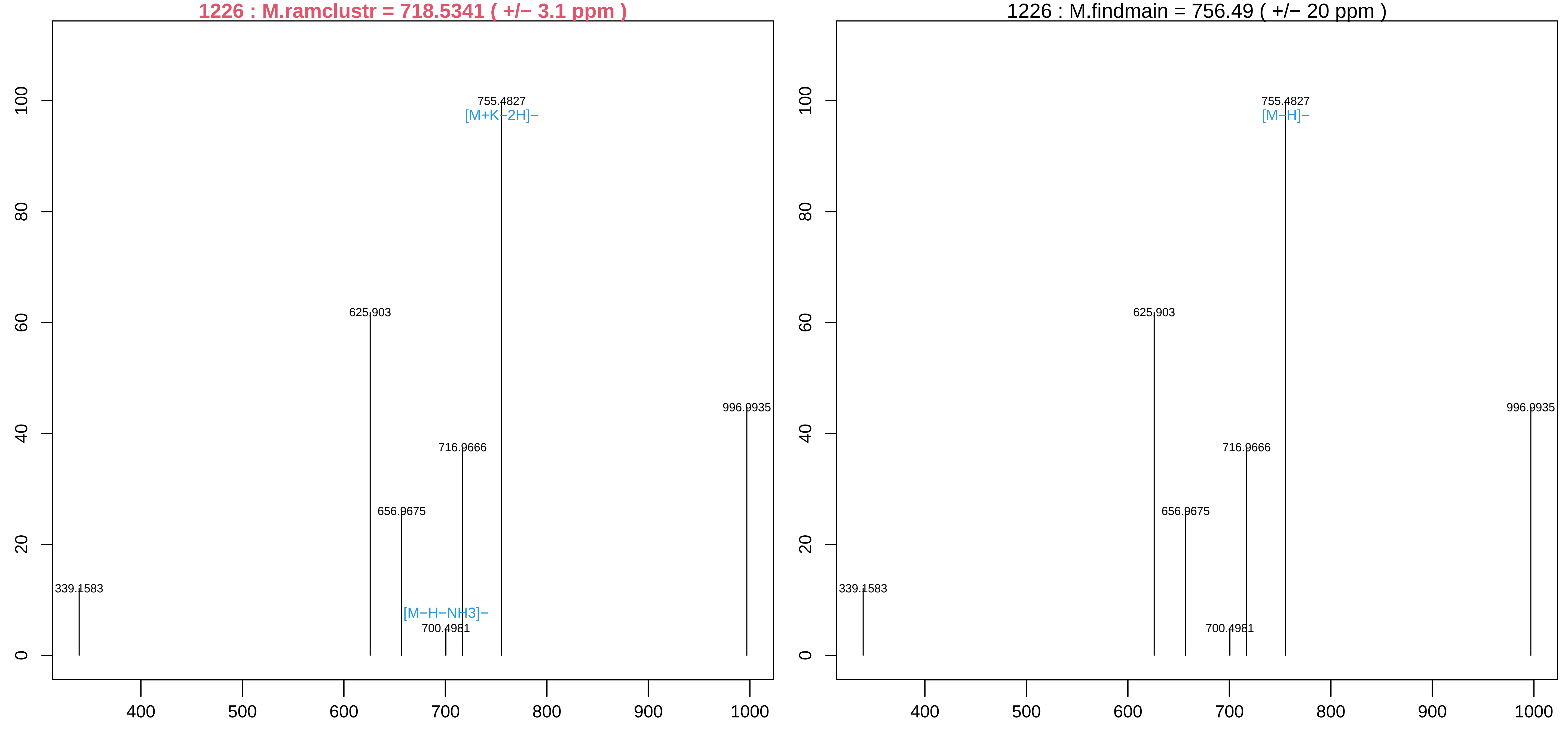

1226 : M.ramclustr = 718.5341 (+/− 3.1 ppm)
755.4827
[M+K−2H]−
625.903
996.9935
716.9666
656.9675
339.1583
[M−H−NH3]−
700.4981
1226 : M.findmain = 756.49 (+/− 20 ppm)
755.4827
[M−H]−
625.903
996.9935
716.9666
656.9675
339.1583
700.4981
0
20
40
60
80
100
400
500
600
700
800
900
1000

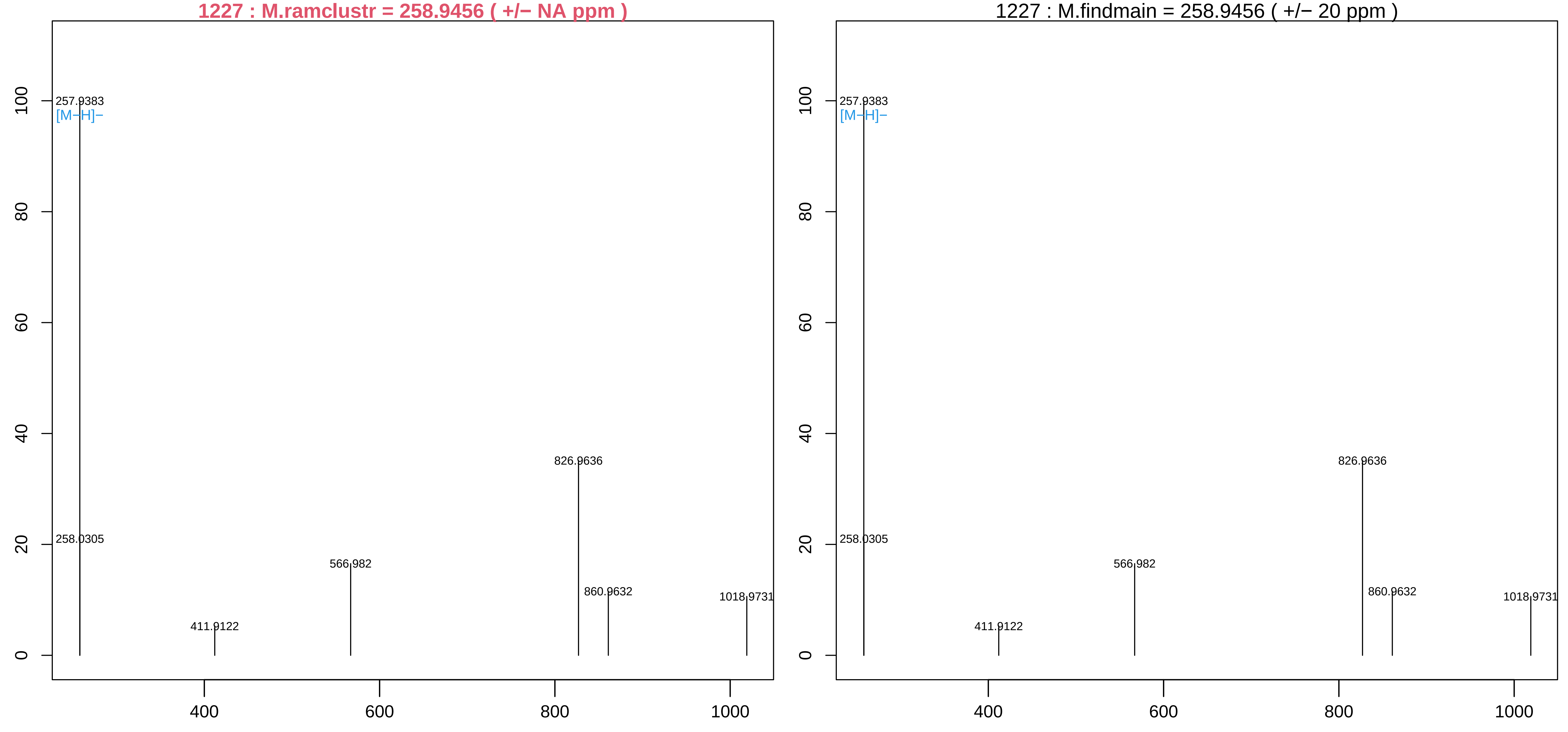
1227 : M.ramclustr = 258.9456 (+/− NA ppm)
257.9383
[M−H]−
258.0305
411.9122
566.982
826.9636
860.9632
1018.9731
1227 : M.findmain = 258.9456 (+/− 20 ppm)
257.9383
[M−H]−
258.0305
411.9122
566.982
826.9636
860.9632
1018.9731
0
20
40
60
80
100
400
600
800
1000

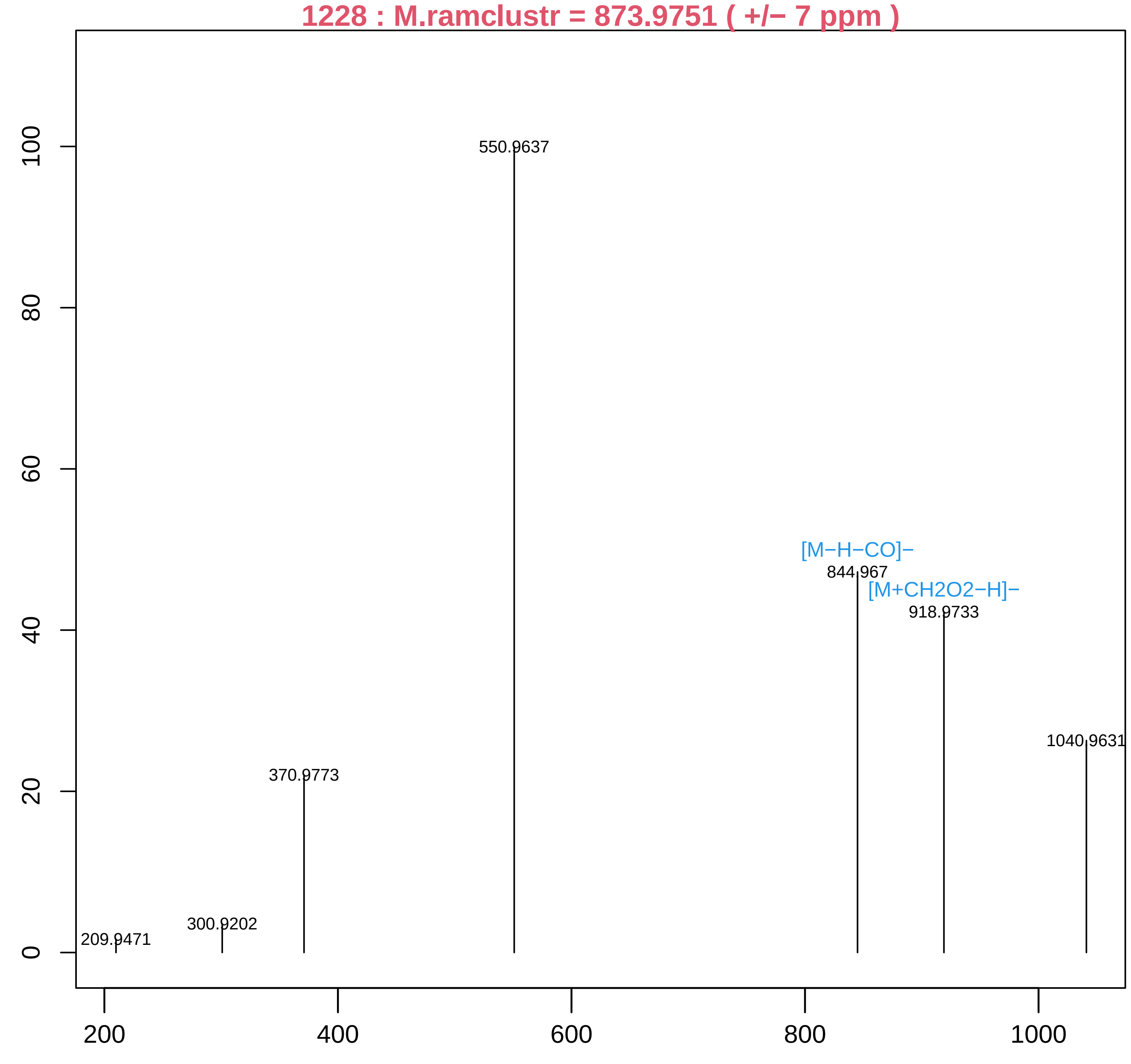
1228 : M.ramclustr = 873.9751 (+/− 7 ppm)
550.9637
[M−H−CO]−
844.967
[M+CH2O2−H]−
918.9733
1040.9631
370.9773
300.9202
209.9471
0
20
40
60
80
100
200
400
600
800
1000

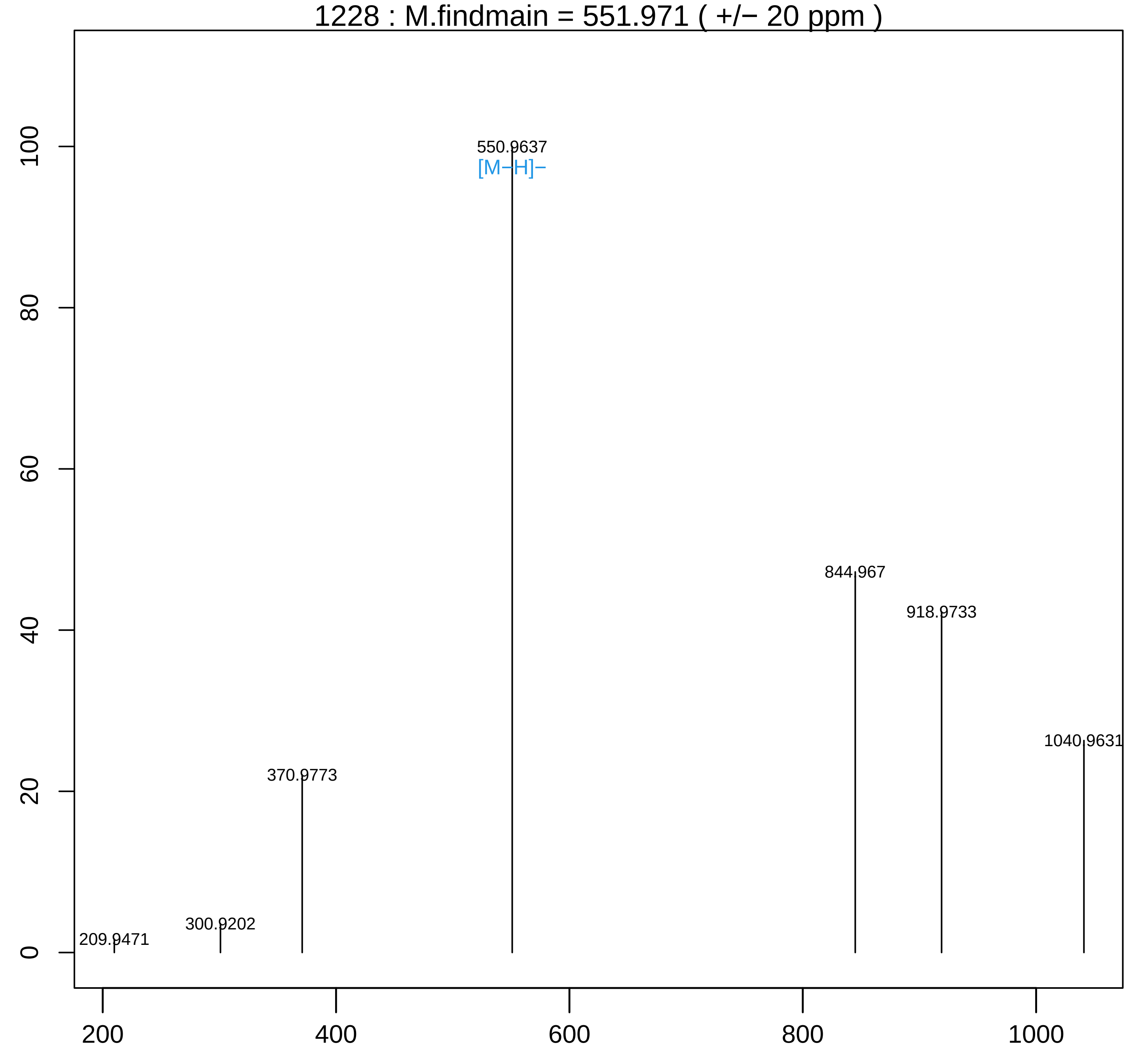
1228 : M.findmain = 551.971 (+/− 20 ppm)
550.9637
[M−H]−
844.967
918.9733
1040.9631
370.9773
300.9202
209.9471
0
20
40
60
80
100
200
400
600
800
1000

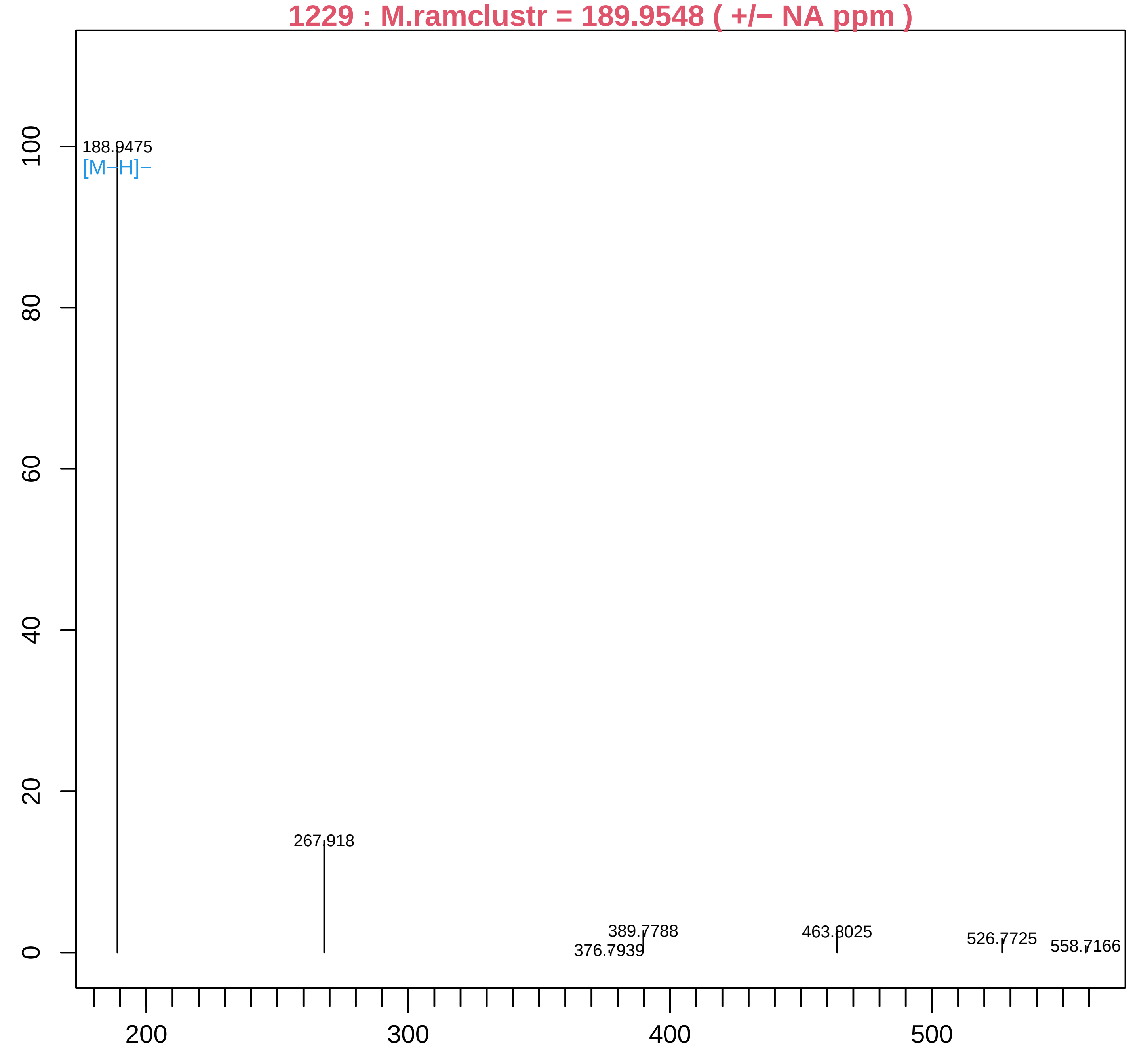

1229 : M.ramclustr = 189.9548 (+/− NA ppm)
188.9475
[M−H]−
267.918
389.7788
376.7939
463.8025
526.7725
558.7166
0
20
40
60
80
100
200
300
400
500

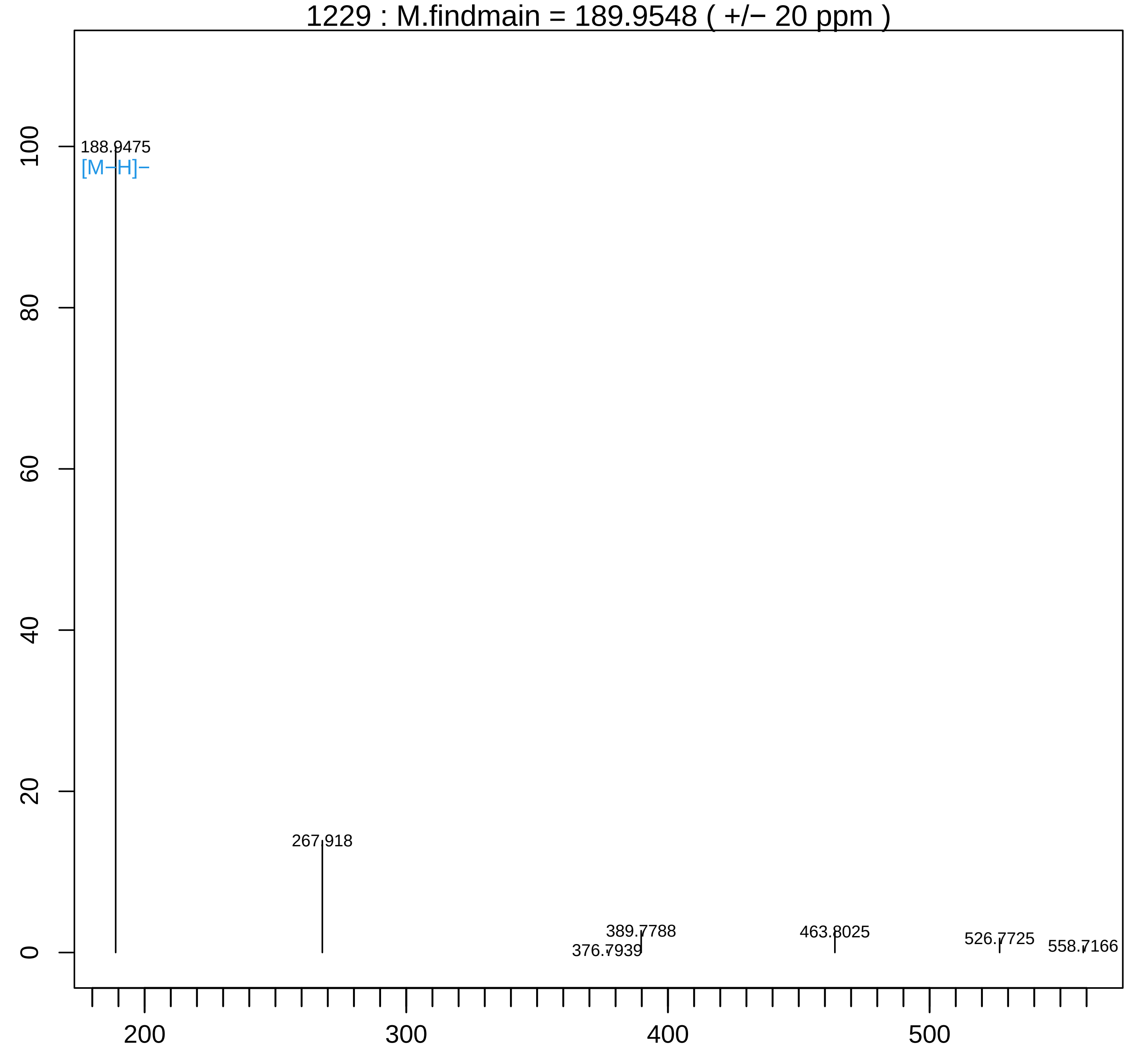

1229 : M.findmain = 189.9548 (+/− 20 ppm)
188.9475
[M−H]−
267.918
389.7788
376.7939
463.8025
526.7725
558.7166
0
20
40
60
80
100
200
300
400
500

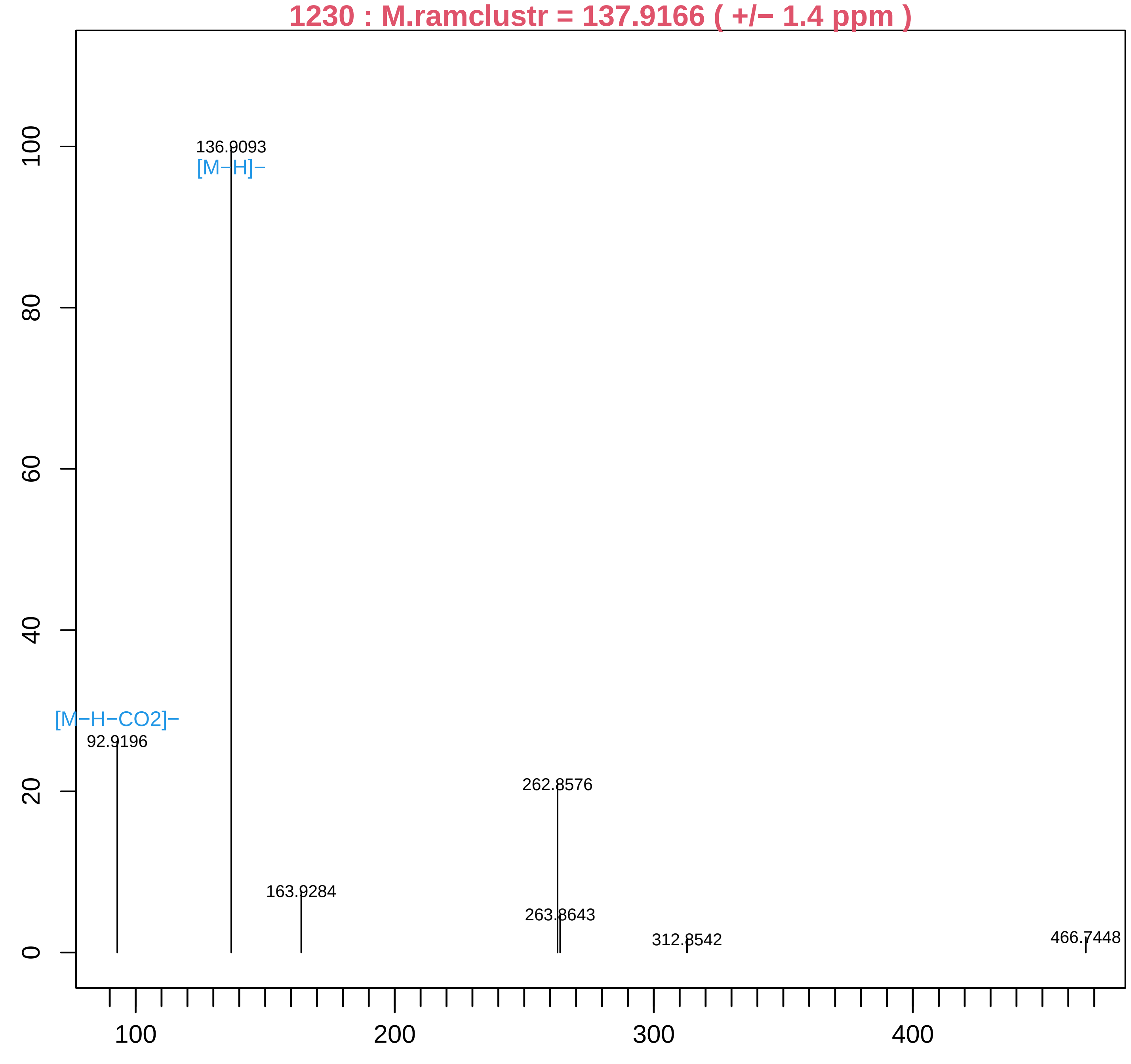
1230 : M.ramclustr = 137.9166 (+/− 1.4 ppm)
136.9093
[M−H]−
[M−H−CO2]−
92.9196
163.9284
262.8576
263.8643
312.8542
466.7448
0
20
40
60
80
100
100
200
300
400

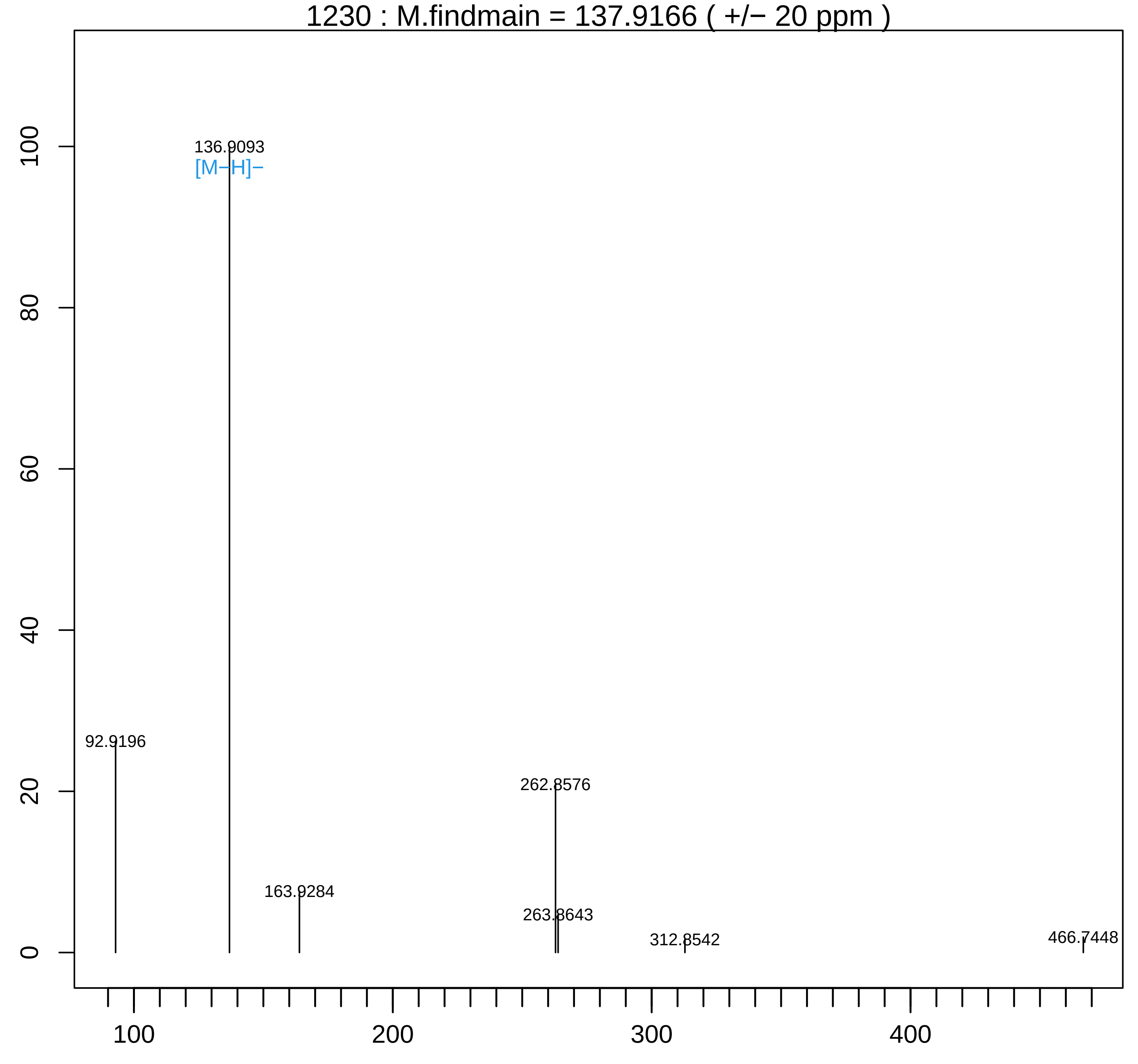
1230 : M.findmain = 137.9166 (+/− 20 ppm)
136.9093
[M−H]−
92.9196
163.9284
262.8576
263.8643
312.8542
466.7448
0
20
40
60
80
100
100
200
300
400

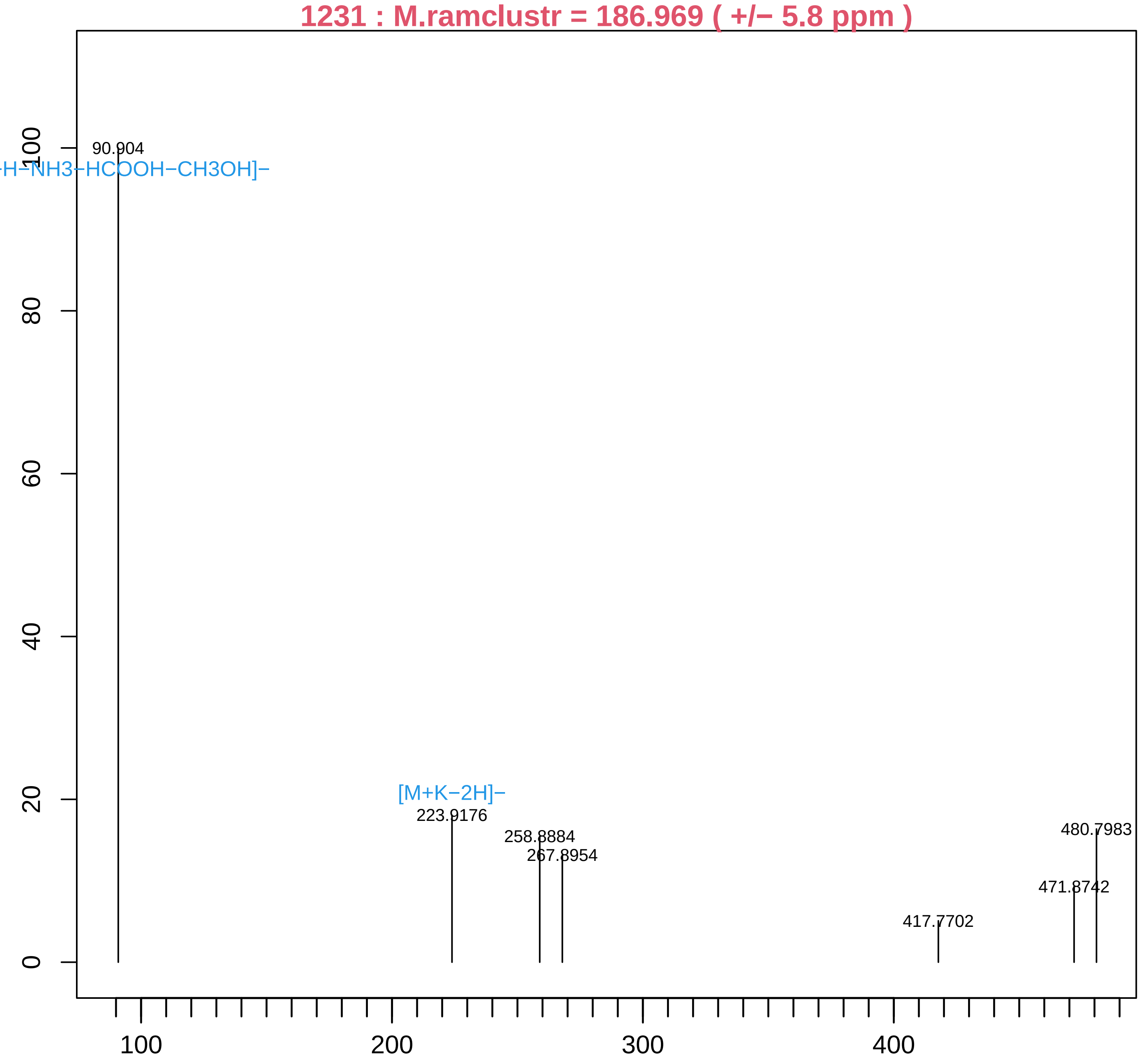
1231 : M.ramclustr = 186.969 (+/− 5.8 ppm)
90.904
H−NH3−HCOOH−CH3OH]−
[M+K−2H]−
223.9176
258.8884
267.8954
417.7702
471.8742
480.7983
0
20
40
60
80
100
100
200
300
400

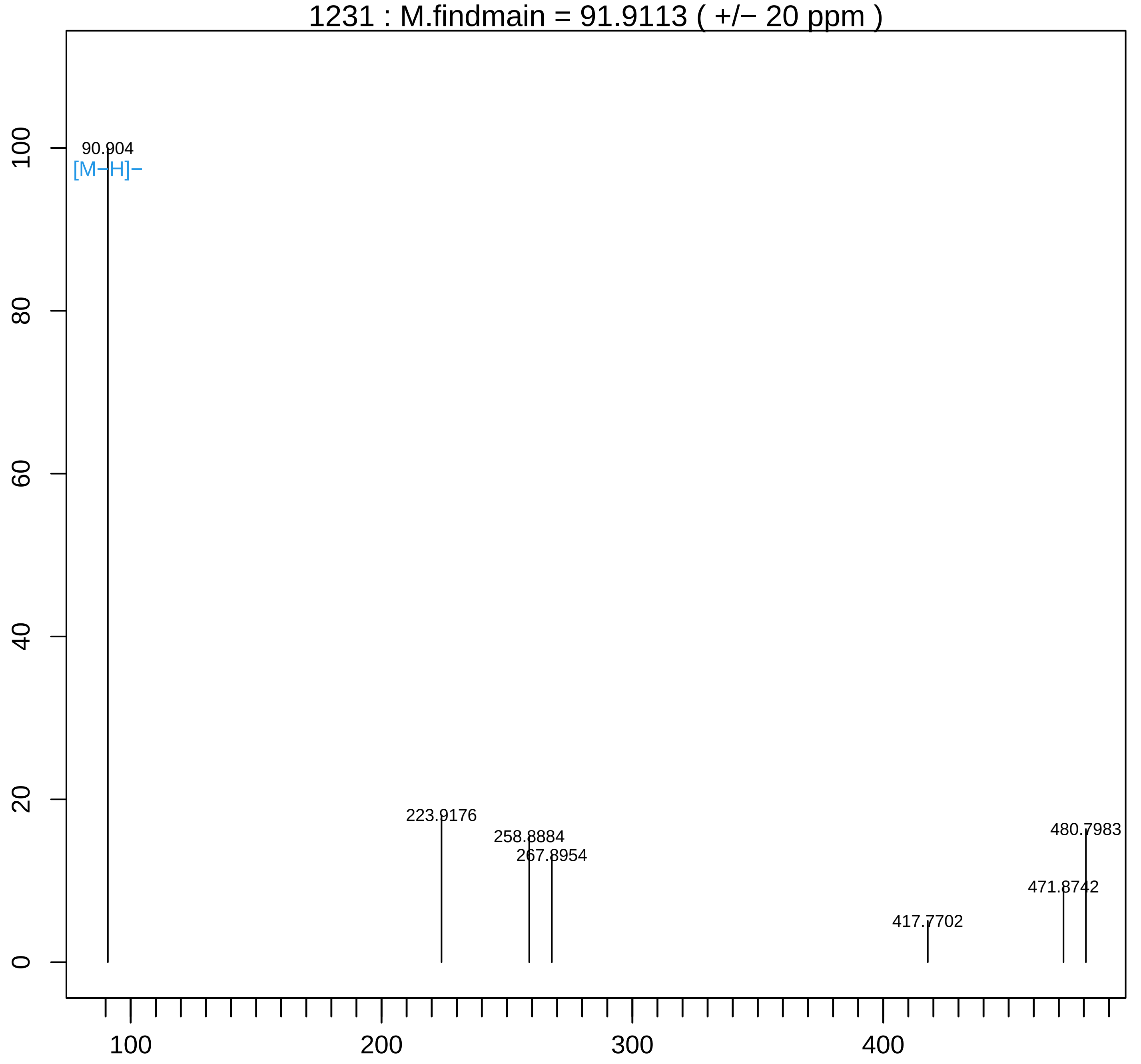
1231 : M.findmain = 91.9113 (+/− 20 ppm)
90.904
[M−H]−
223.9176
258.8884
267.8954
417.7702
471.8742
480.7983
0
20
40
60
80
100
100
200
300
400

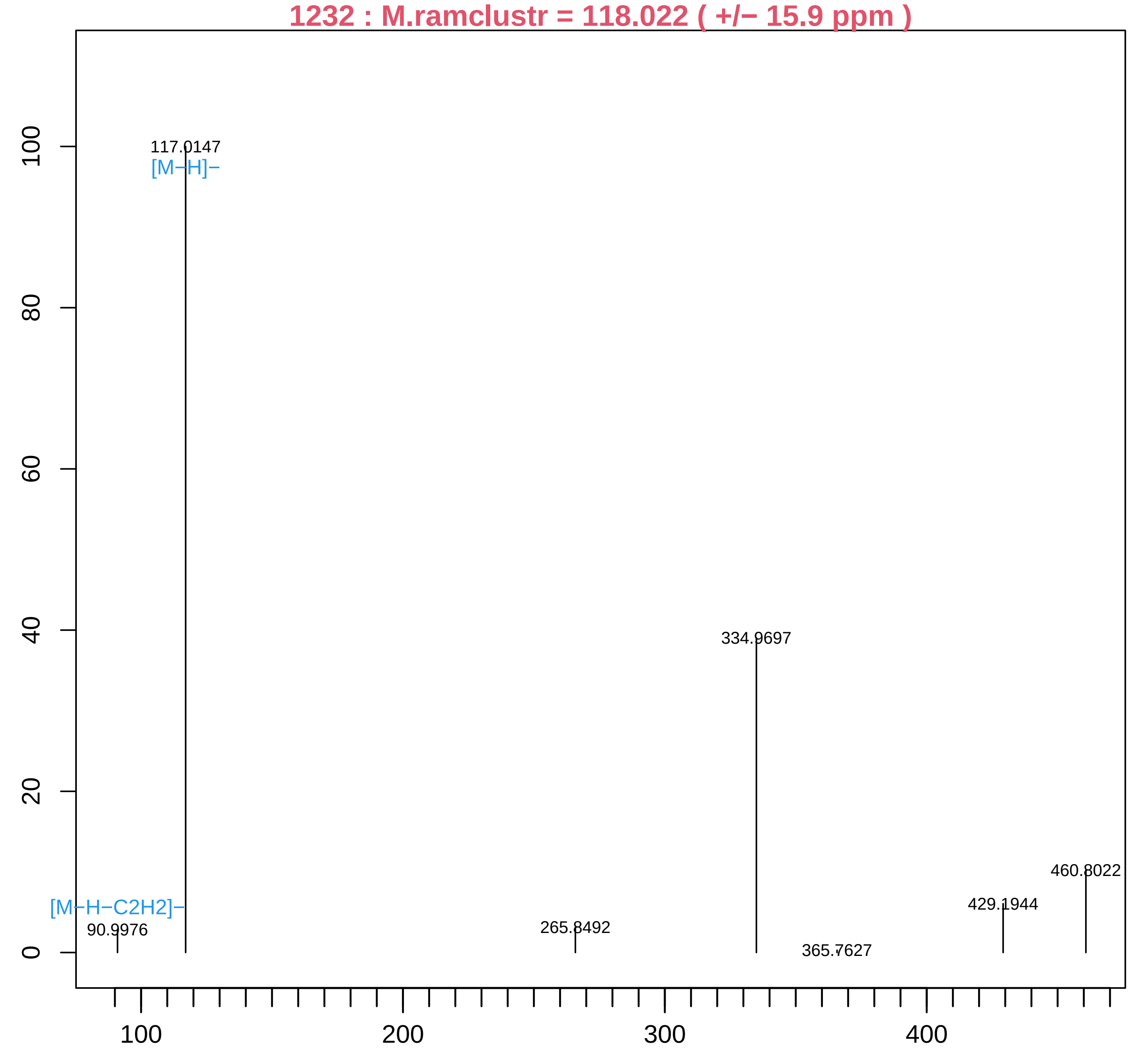
1232 : M.ramclustr = 118.022 (+/− 15.9 ppm)
117.0147
[M−H]−
334.9697
460.8022
[M−H−C2H2]−
429.1944
90.9976
265.8492
365.7627
0
20
40
60
80
100
100
200
300
400

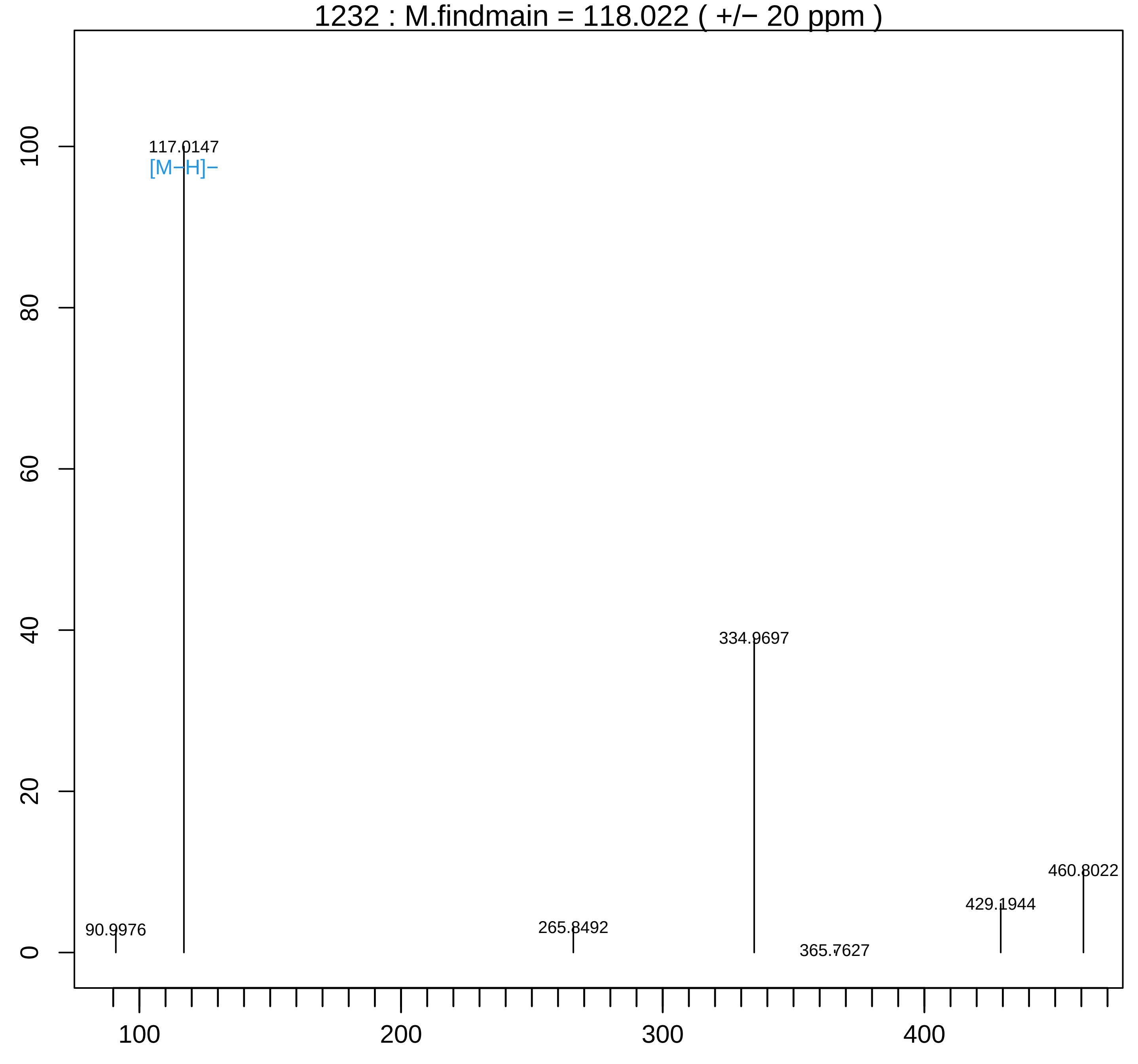
1232 : M.findmain = 118.022 (+/− 20 ppm)
117.0147
[M−H]−
334.9697
460.8022
429.1944
90.9976
265.8492
365.7627
0
20
40
60
80
100
100
200
300
400

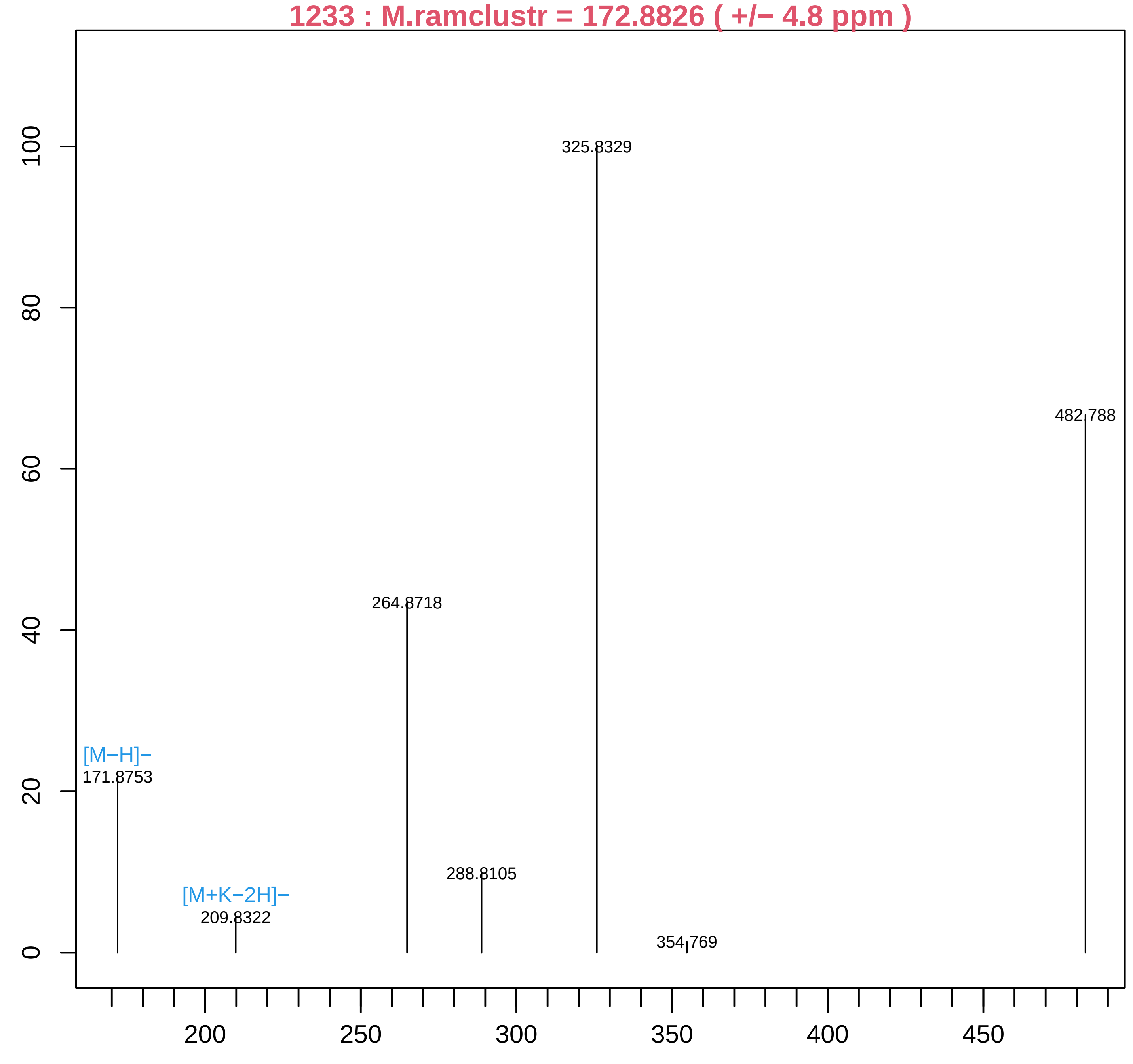
1233 : M.ramclustr = 172.8826 (+/− 4.8 ppm)
[M−H]−
171.8753
[M+K−2H]−
209.8322
264.8718
288.8105
325.8329
354.769
482.788
0
20
40
60
80
100
200
250
300
350
400
450

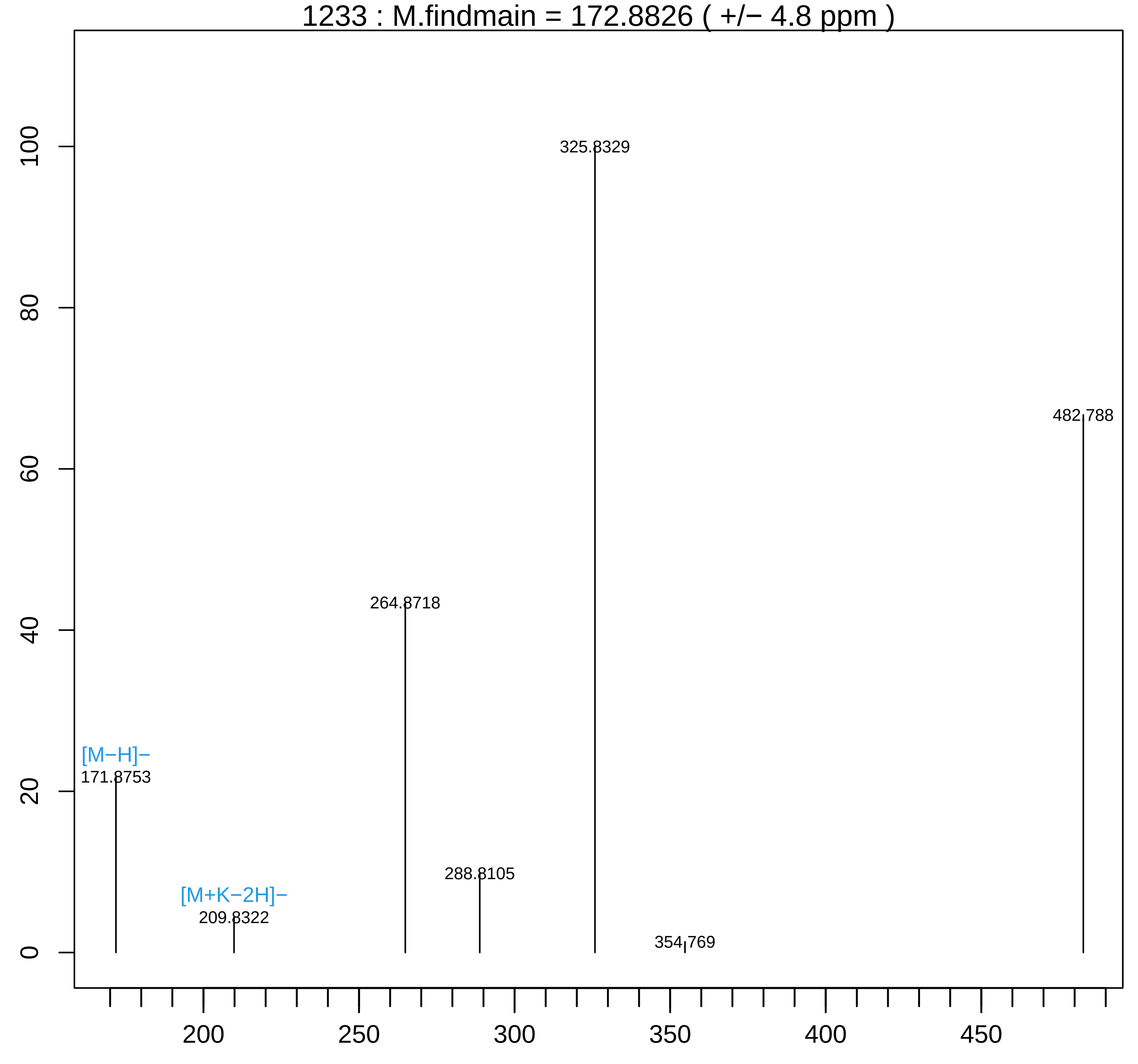
1233 : M.findmain = 172.8826 (+/− 4.8 ppm)
[M−H]−
171.8753
[M+K−2H]−
209.8322
264.8718
288.8105
325.8329
354.769
482.788
0
20
40
60
80
100
200
250
300
350
400
450

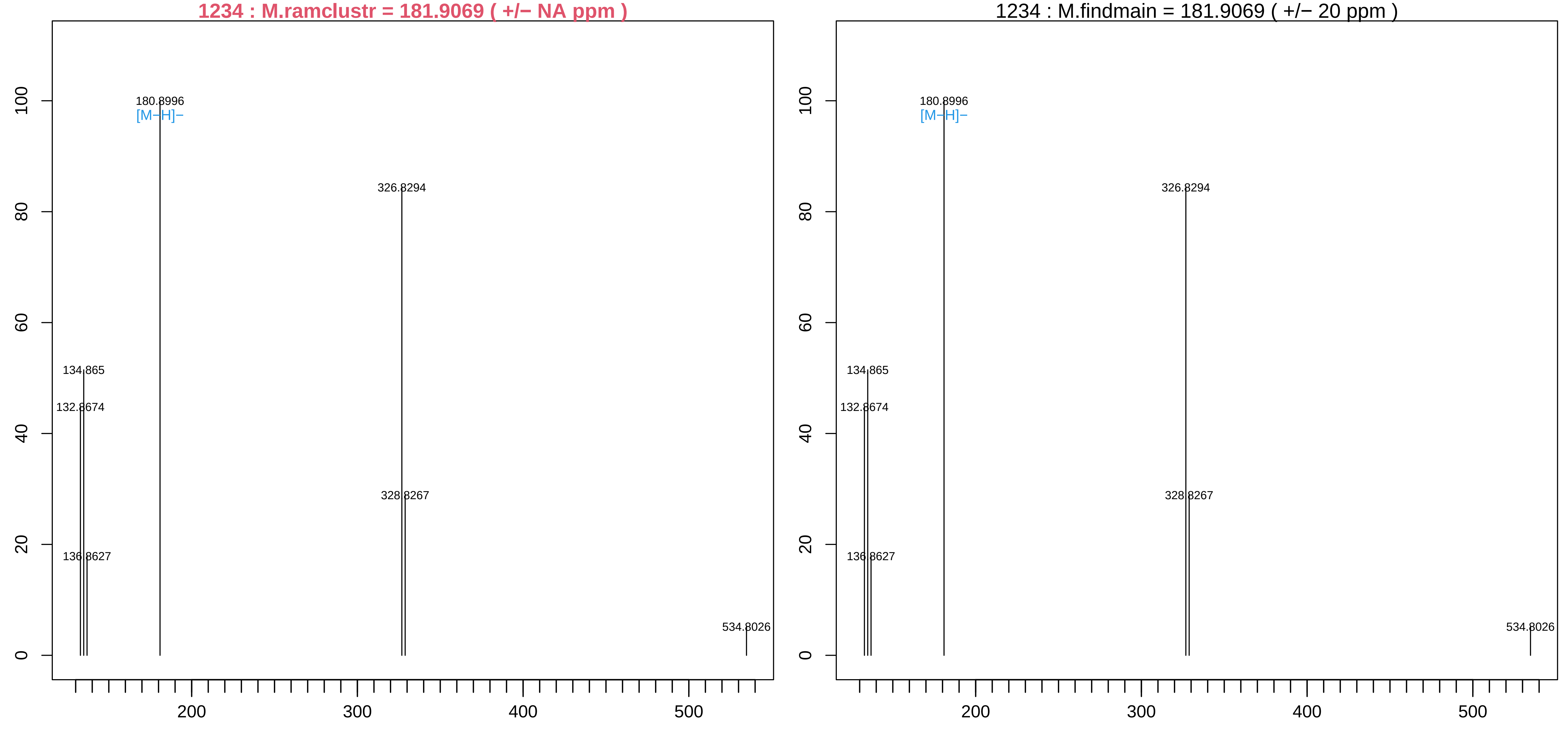
1234 : M.ramclustr = 181.9069 (+/− NA ppm)
180.8996
[M−H]−
326.8294
134.865
132.8674
328.8267
136.8627
534.8026
0
20
40
60
80
100
200
300
400
500
1234 : M.findmain = 181.9069 (+/− 20 ppm)
180.8996
[M−H]−
326.8294
134.865
132.8674
328.8267
136.8627
534.8026
0
20
40
60
80
100
200
300
400
500

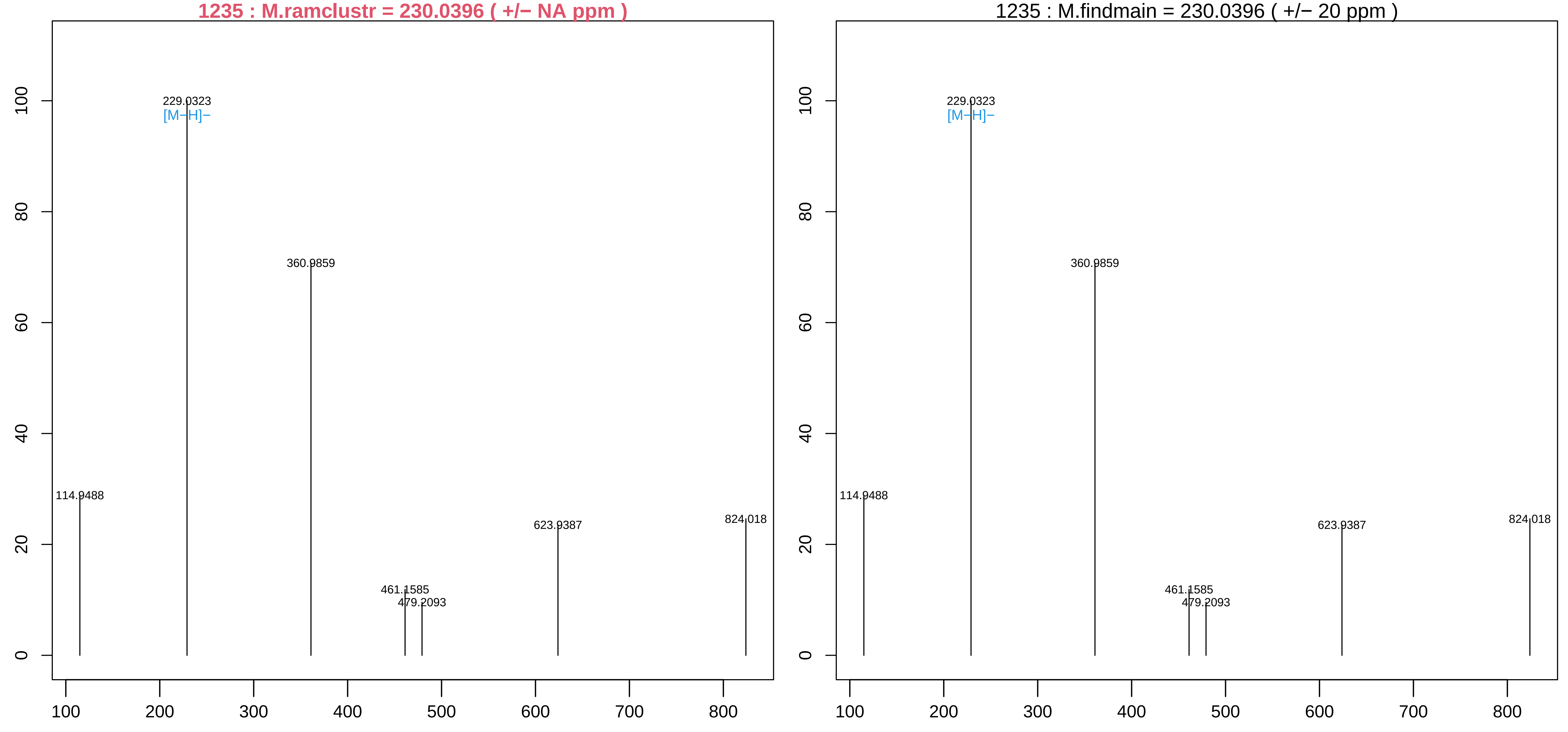
1235 : M.ramclustr = 230.0396 (+/− NA ppm)
1235 : M.findmain = 230.0396 (+/− 20 ppm)
229.0323
[M−H]−
360.9859
114.9488
461.1585
479.2093
623.9387
824.018
0
20
40
60
80
100
200
300
400
500
600
700
800

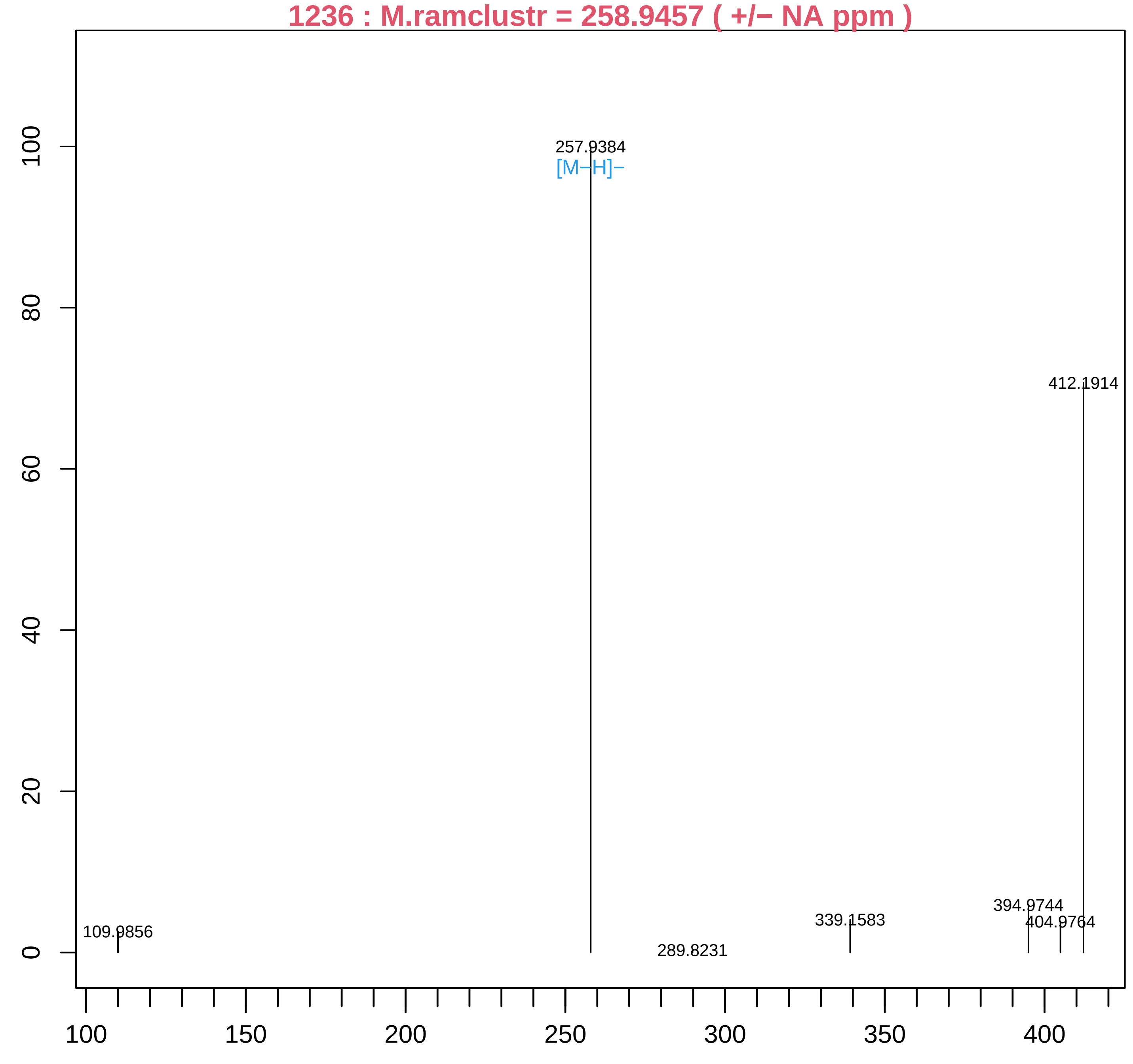
1236 : M.ramclustr = 258.9457 (+/− NA ppm)
257.9384
[M−H]−
412.1914
109.9856
289.8231
339.1583
394.9744
404.9764
0
20
40
60
80
100
100
150
200
250
300
350
400

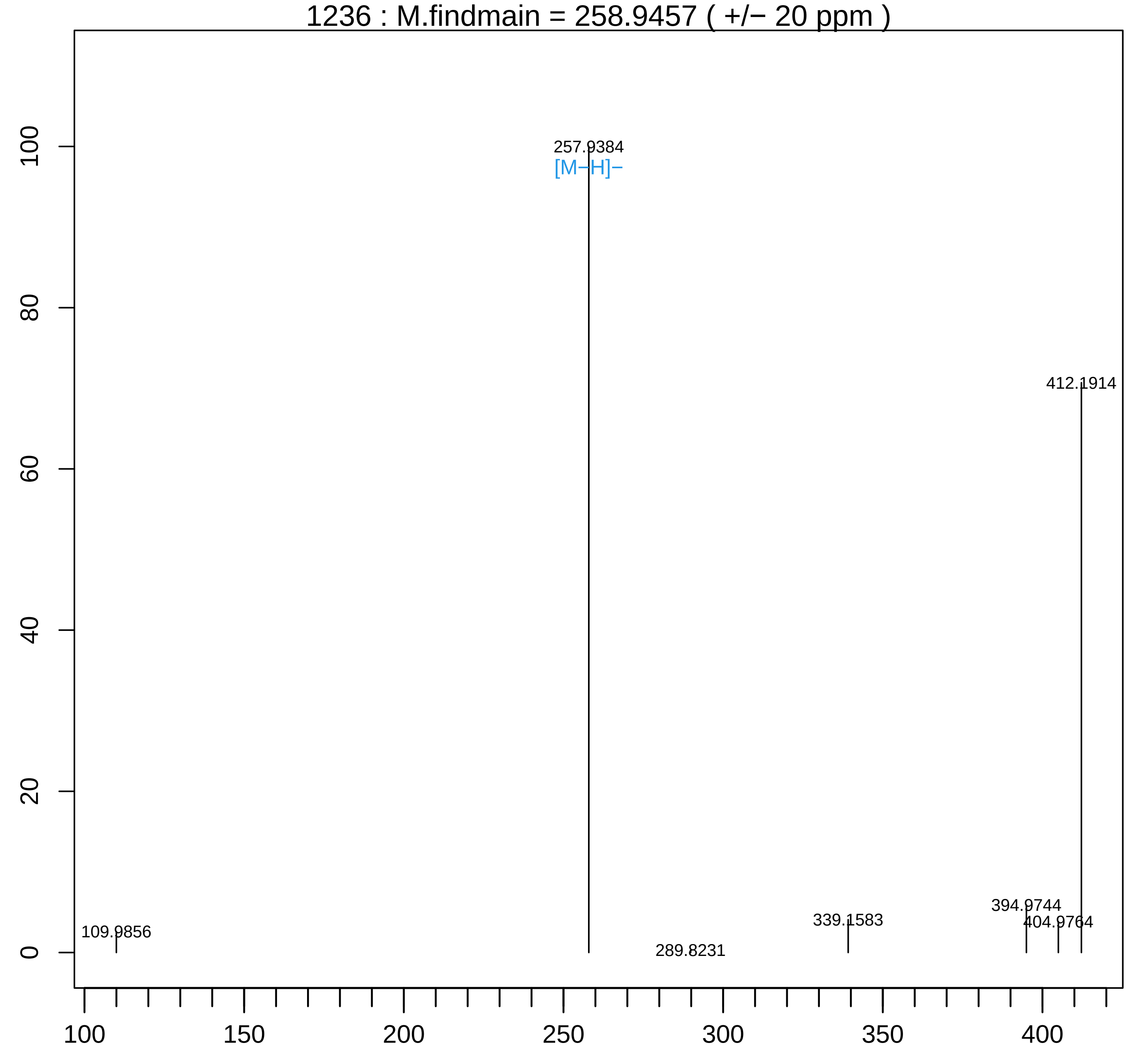
1236 : M.findmain = 258.9457 (+/− 20 ppm)
257.9384
[M−H]−
412.1914
109.9856
289.8231
339.1583
394.9744
404.9764
0
20
40
60
80
100
100
150
200
250
300
350
400

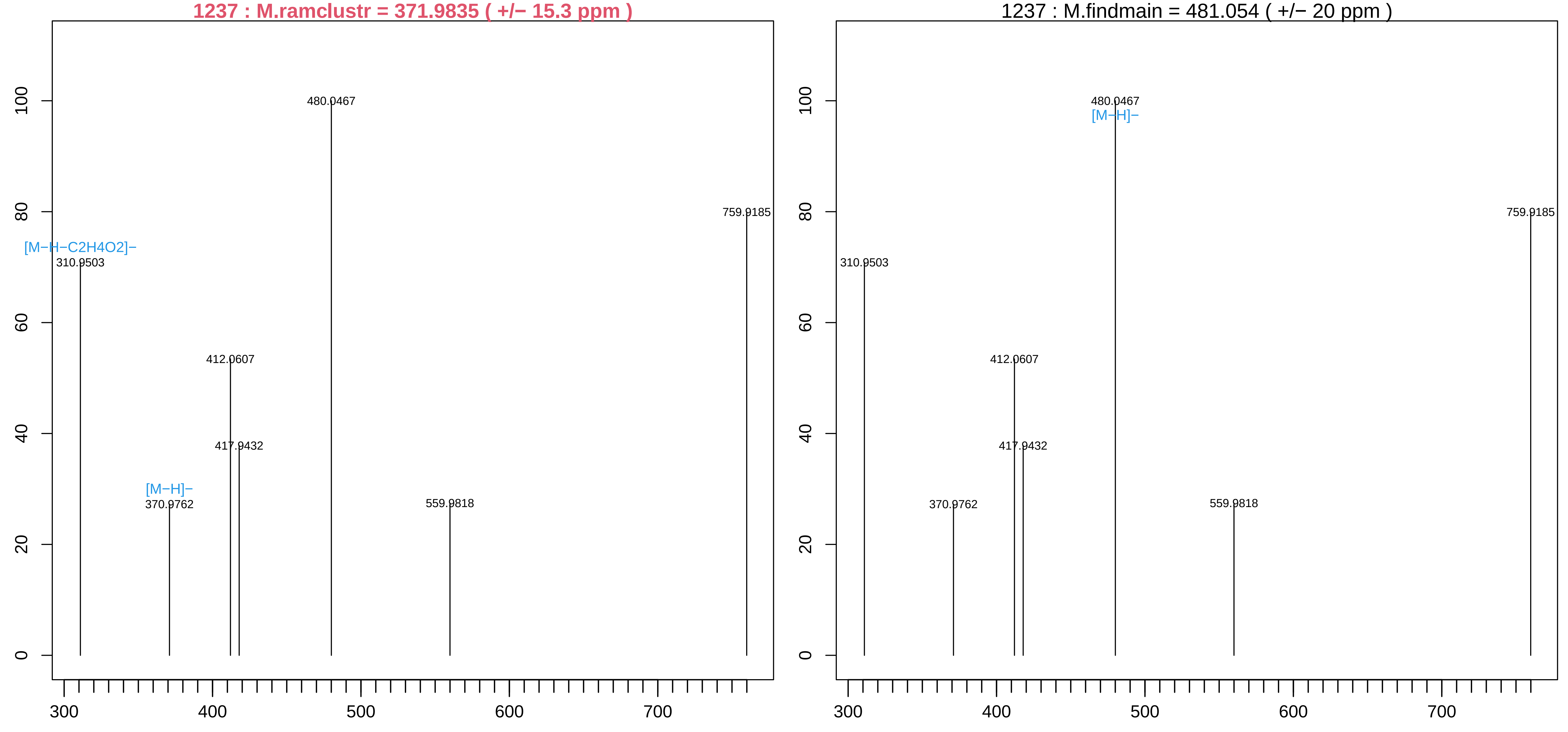
1237 : M.ramclustr = 371.9835 (+/− 15.3 ppm)
480.0467
759.9185
[M−H−C2H4O2]−
310.9503
412.0607
417.9432
[M−H]−
370.9762
559.9818
1237 : M.findmain = 481.054 (+/− 20 ppm)
480.0467
[M−H]−
759.9185
310.9503
412.0607
417.9432
370.9762
559.9818
0
20
40
60
80
100
300
400
500
600
700

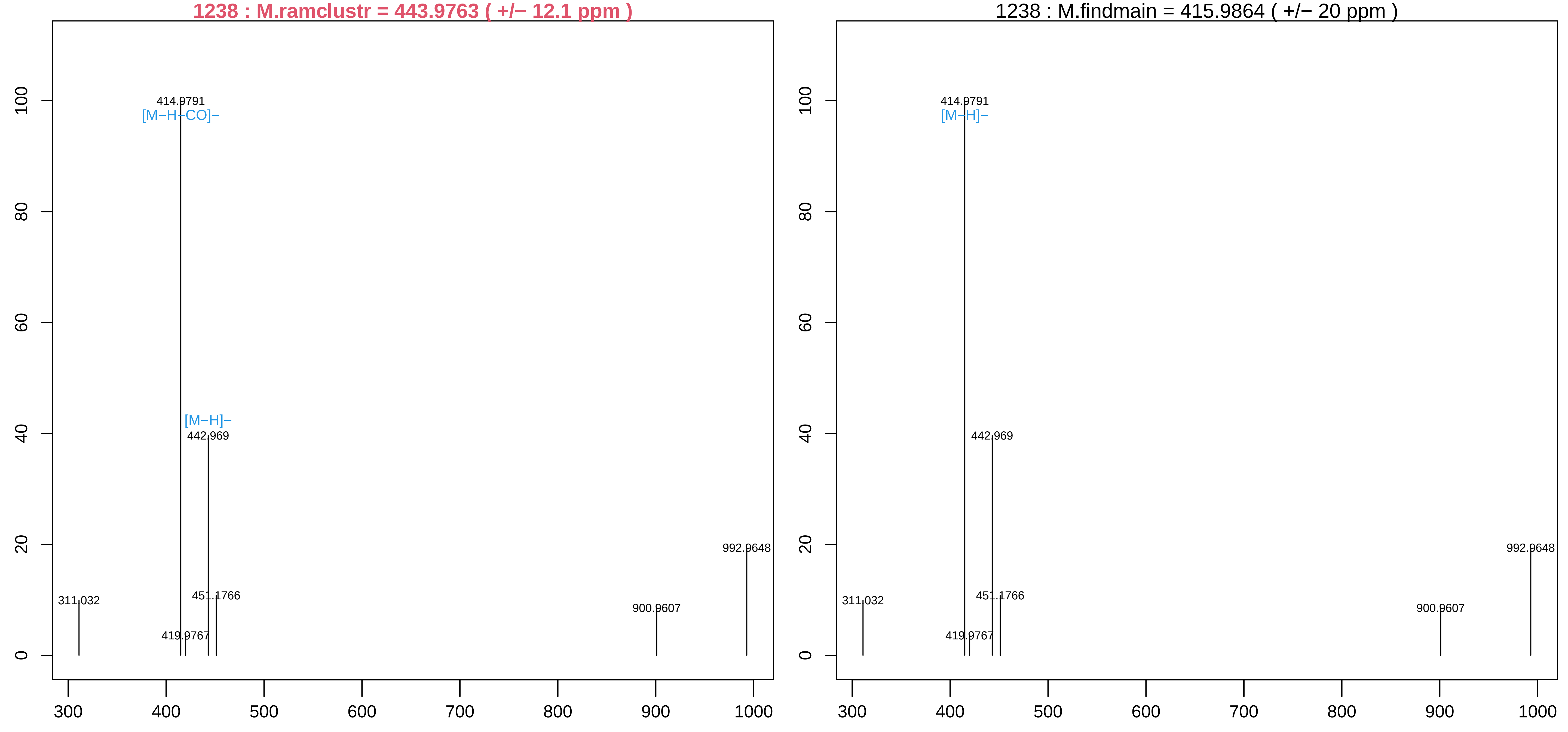
1238 : M.ramclustr = 443.9763 (+/− 12.1 ppm)
414.9791
[M−H−CO]−
[M−H]−
442.969
311.032
419.9767
451.1766
900.9607
992.9648
1238 : M.findmain = 415.9864 (+/− 20 ppm)
414.9791
[M−H]−
442.969
311.032
419.9767
451.1766
900.9607
992.9648

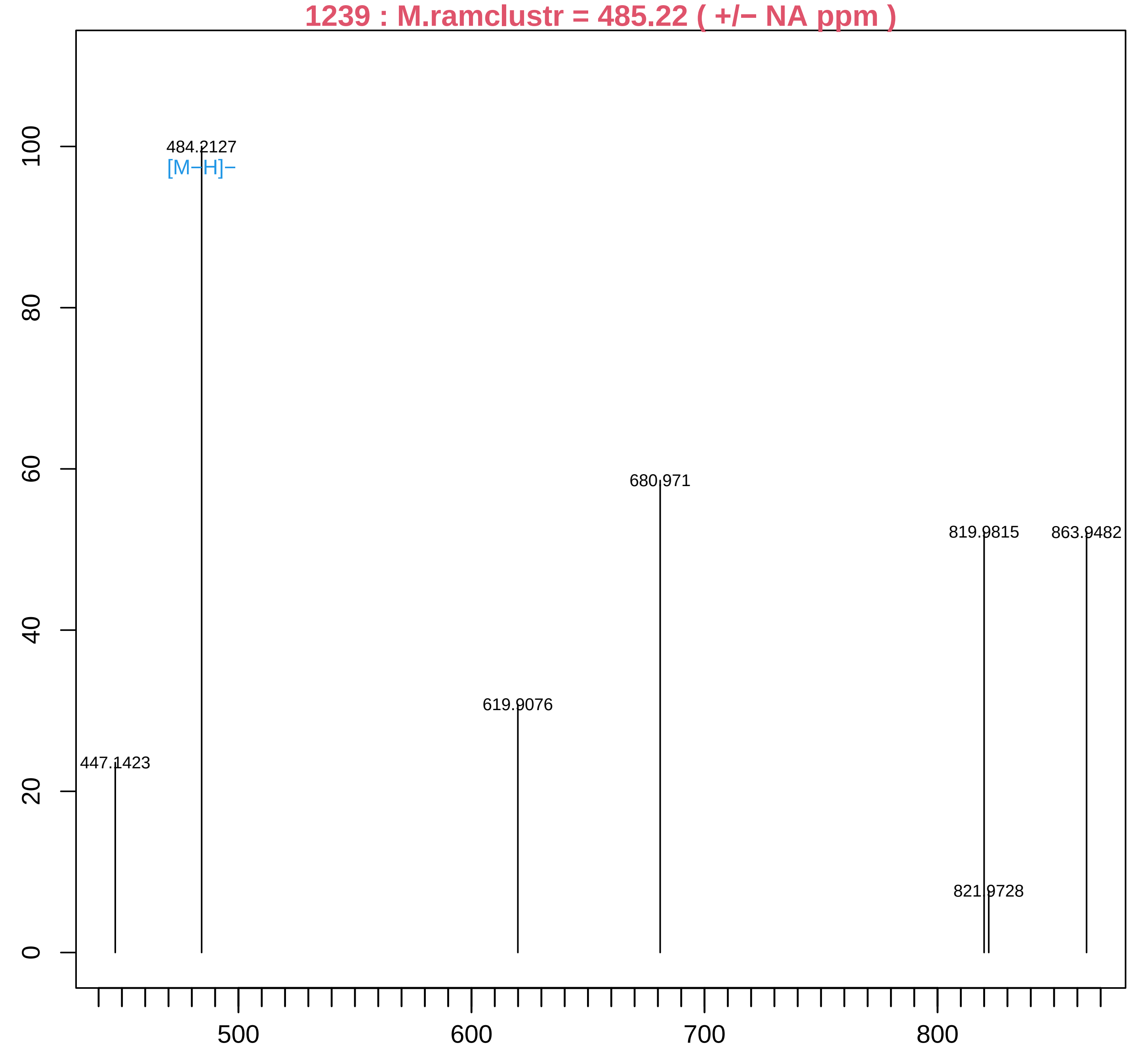
1239 : M.ramclustr = 485.22 (+/− NA ppm)
484.2127
[M−H]−
680.971
819.9815
863.9482
619.9076
447.1423
821.9728
0
20
40
60
80
100
500
600
700
800

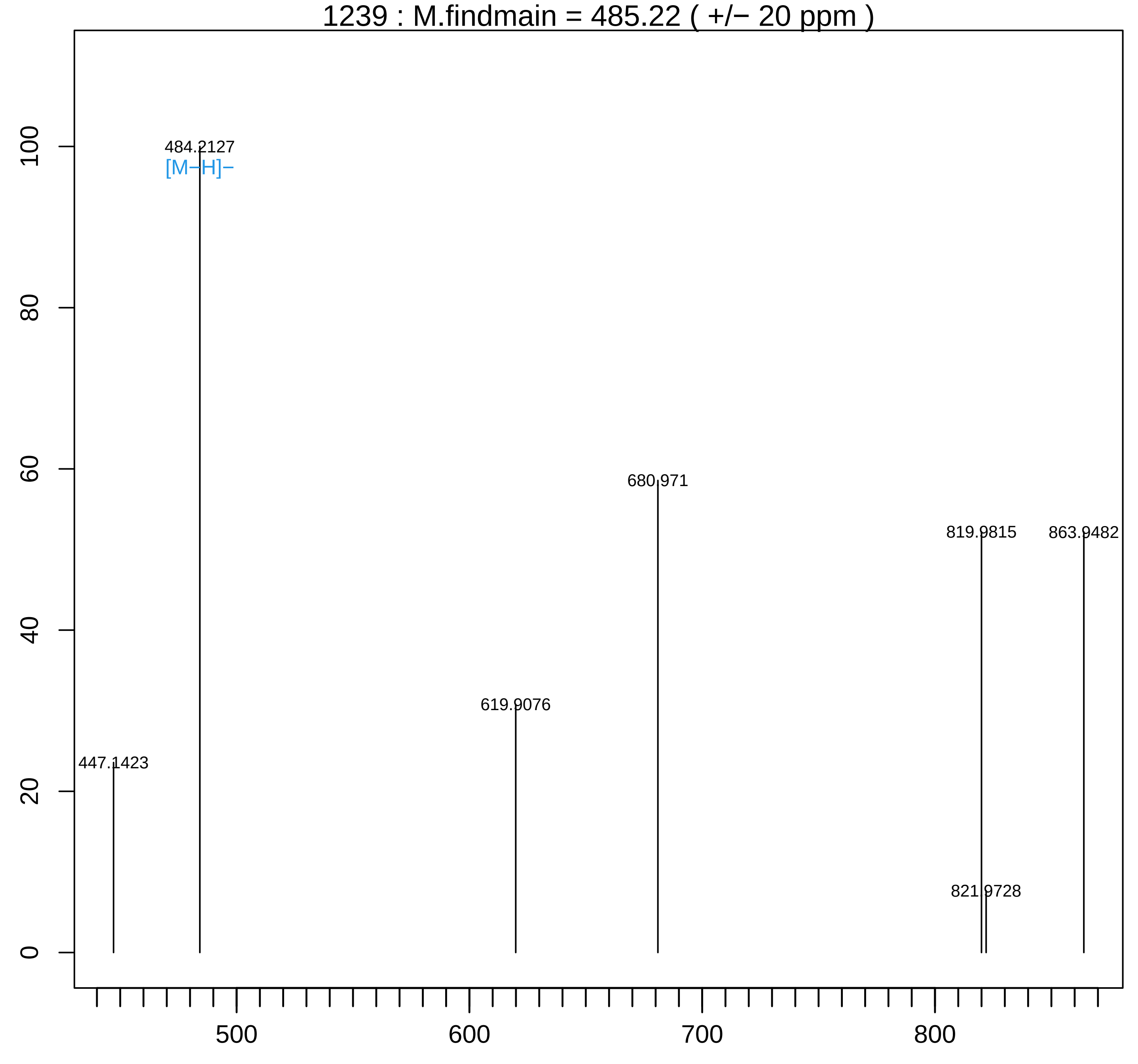
1239 : M.findmain = 485.22 (+/− 20 ppm)
484.2127
[M−H]−
680.971
819.9815
863.9482
619.9076
447.1423
821.9728
0
20
40
60
80
100
500
600
700
800

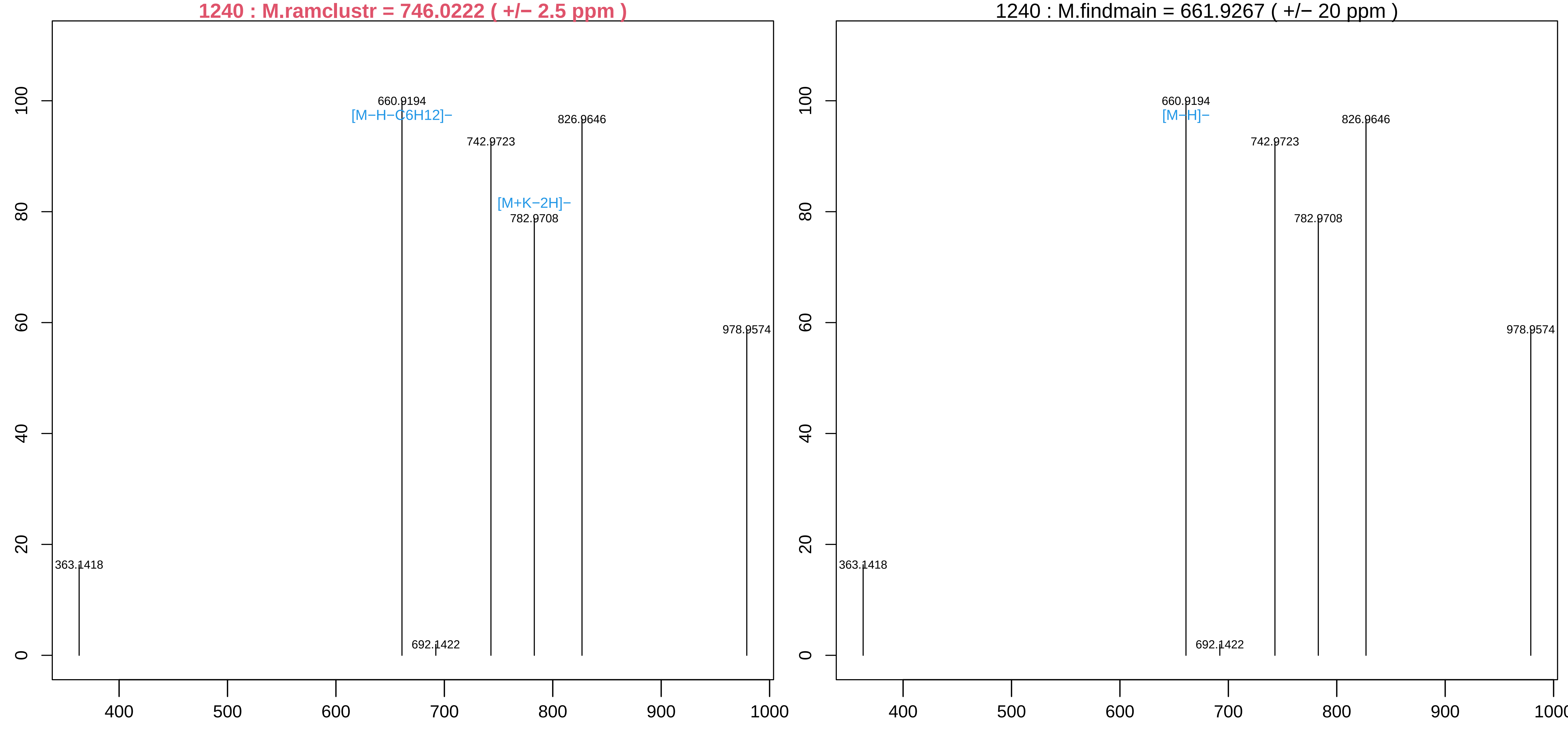
1240 : M.ramclustr = 746.0222 (+/− 2.5 ppm)
660.9194
[M−H−C6H12]−
742.9723
826.9646
[M+K−2H]−
782.9708
978.9574
363.1418
692.1422
1240 : M.findmain = 661.9267 (+/− 20 ppm)
660.9194
[M−H]−
742.9723
826.9646
782.9708
978.9574
363.1418
692.1422

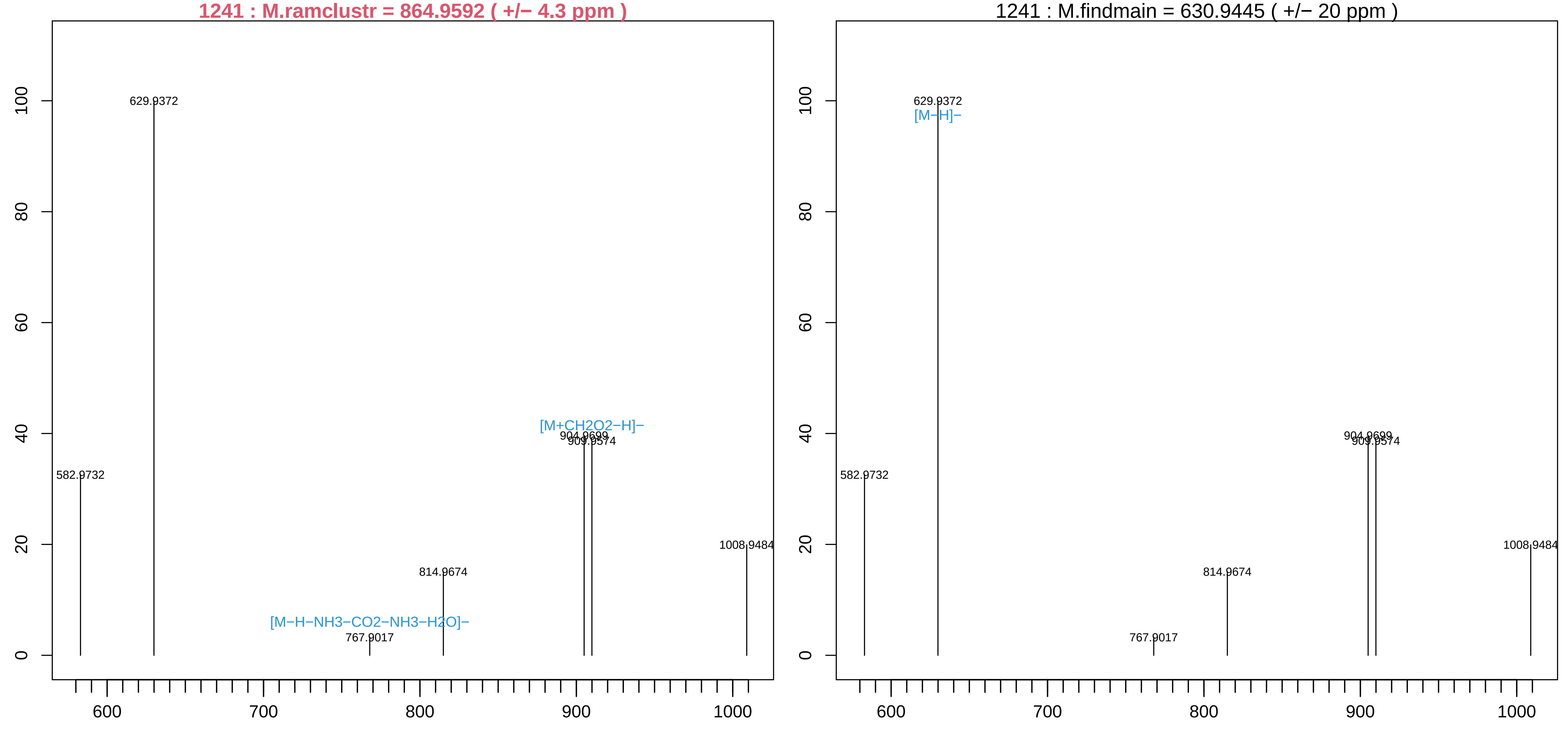
1241 : M.ramclustr = 864.9592 (+/− 4.3 ppm)
629.9372
582.9732
[M+CH2O2−H]−
904.9699
909.9574
814.9674
[M−H−NH3−CO2−NH3−H2O]−
767.9017
1008.9484
1241 : M.findmain = 630.9445 (+/− 20 ppm)
629.9372
[M−H]−
582.9732
904.9699
909.9574
814.9674
767.9017
1008.9484
0
20
40
60
80
100
600
700
800
900
1000

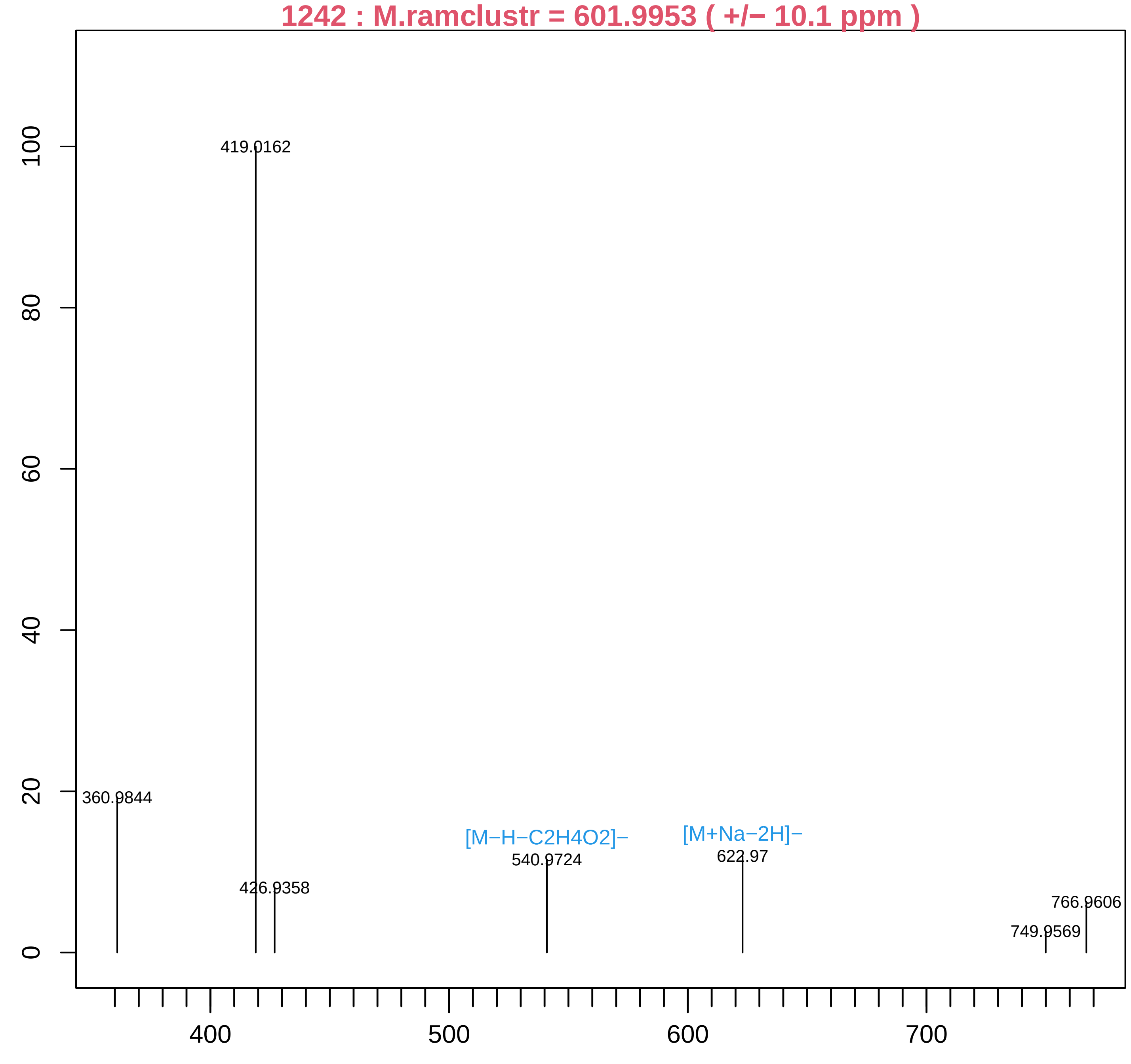
1242 : M.ramclustr = 601.9953 (+/− 10.1 ppm)
419.0162
360.9844
426.9358
[M−H−C2H4O2]−
540.9724
[M+Na−2H]−
622.97
766.9606
749.9569
0
20
40
60
80
100
400
500
600
700

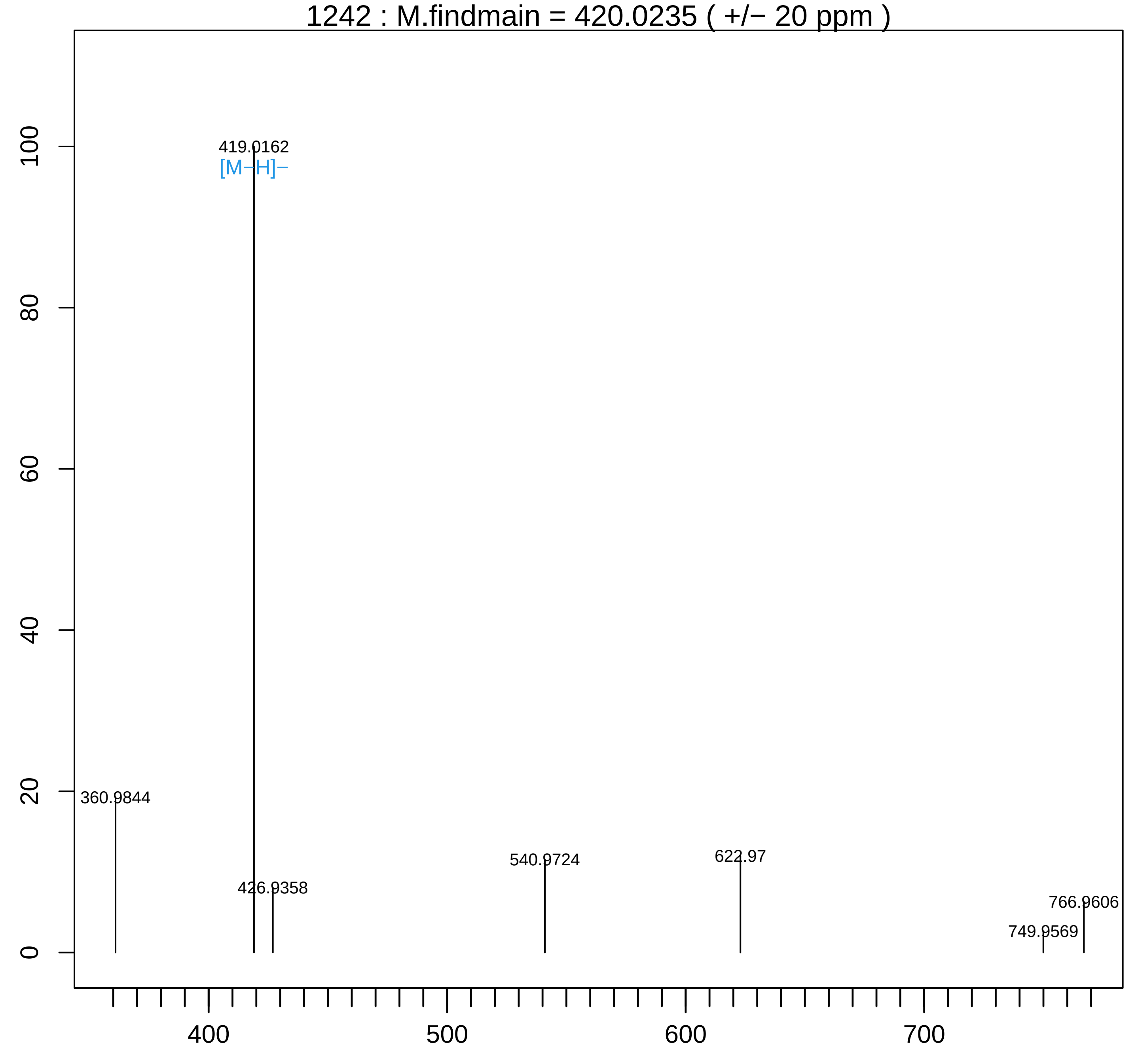
1242 : M.findmain = 420.0235 (+/− 20 ppm)
419.0162
[M−H]−
360.9844
426.9358
540.9724
622.97
766.9606
749.9569
0
20
40
60
80
100
400
500
600
700

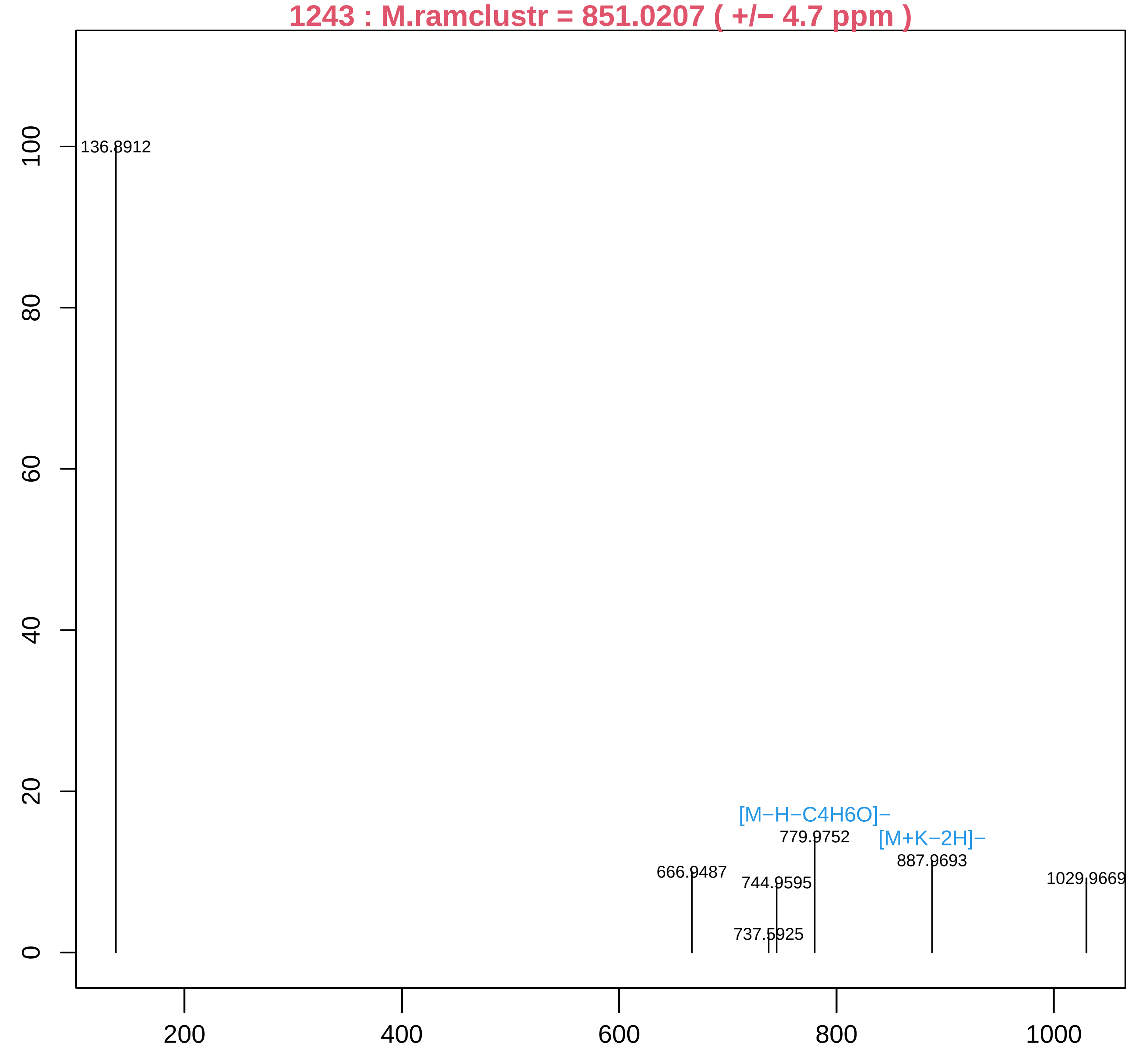
1243 : M.ramclustr = 851.0207 (+/− 4.7 ppm)
136.8912
[M−H−C4H6O]−
779.9752
[M+K−2H]−
887.9693
666.9487
744.9595
737.5925
1029.9669
0
20
40
60
80
100
200
400
600
800
1000

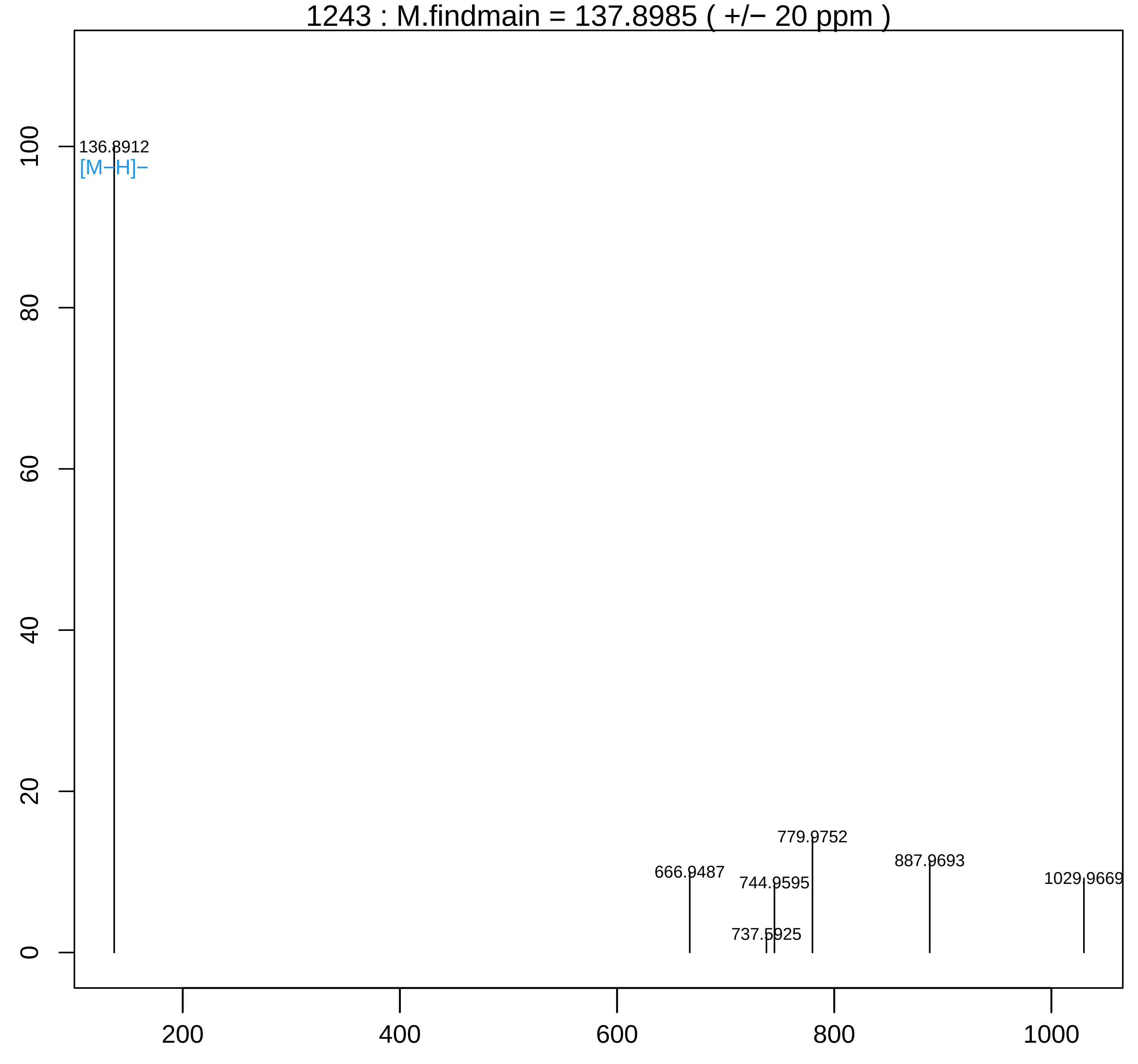
1243 : M.findmain = 137.8985 (+/− 20 ppm)
136.8912
[M−H]−
779.9752
887.9693
666.9487
744.9595
737.5925
1029.9669
0
20
40
60
80
100
200
400
600
800
1000

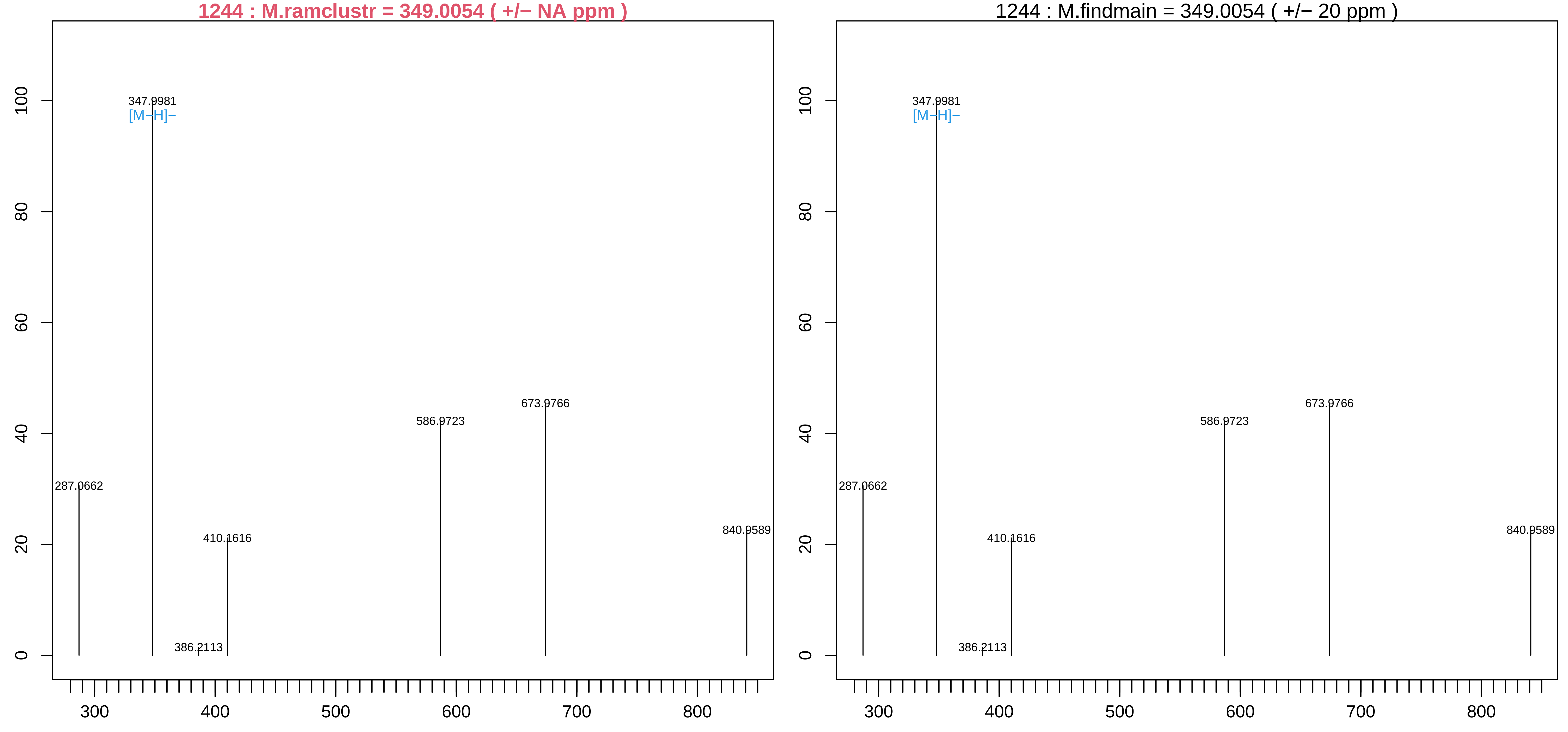
1244 : M.ramclustr = 349.0054 (+/− NA ppm)
347.9981
[M−H]−
287.0662
410.1616
386.2113
586.9723
673.9766
840.9589
0
20
40
60
80
100
300
400
500
600
700
800
1244 : M.findmain = 349.0054 (+/− 20 ppm)
347.9981
[M−H]−
287.0662
410.1616
386.2113
586.9723
673.9766
840.9589
0
20
40
60
80
100
300
400
500
600
700
800

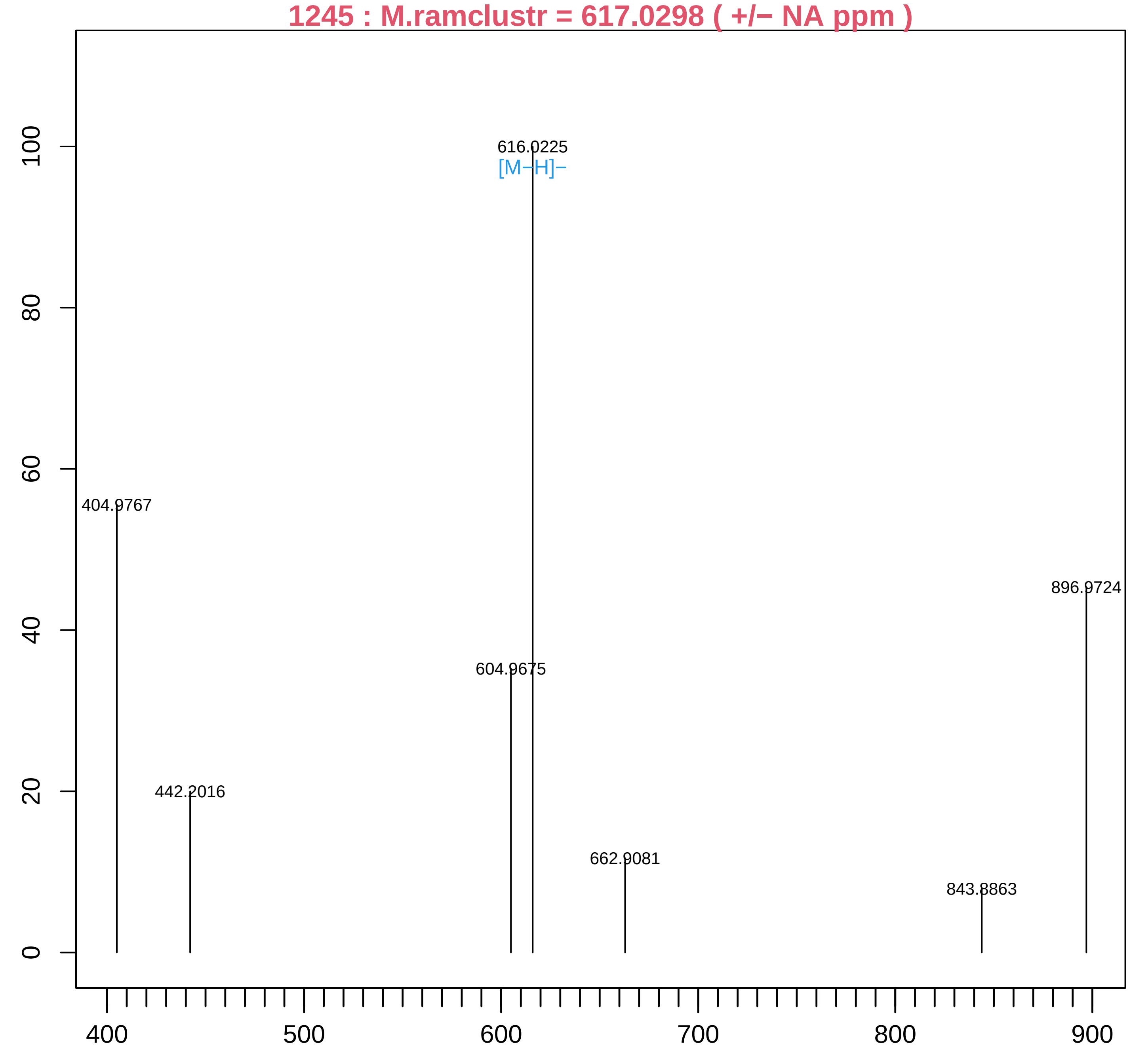
1245 : M.ramclustr = 617.0298 (+/− NA ppm)
616.0225
[M−H]−
404.9767
896.9724
604.9675
442.2016
662.9081
843.8863
0
20
40
60
80
100
400
500
600
700
800
900

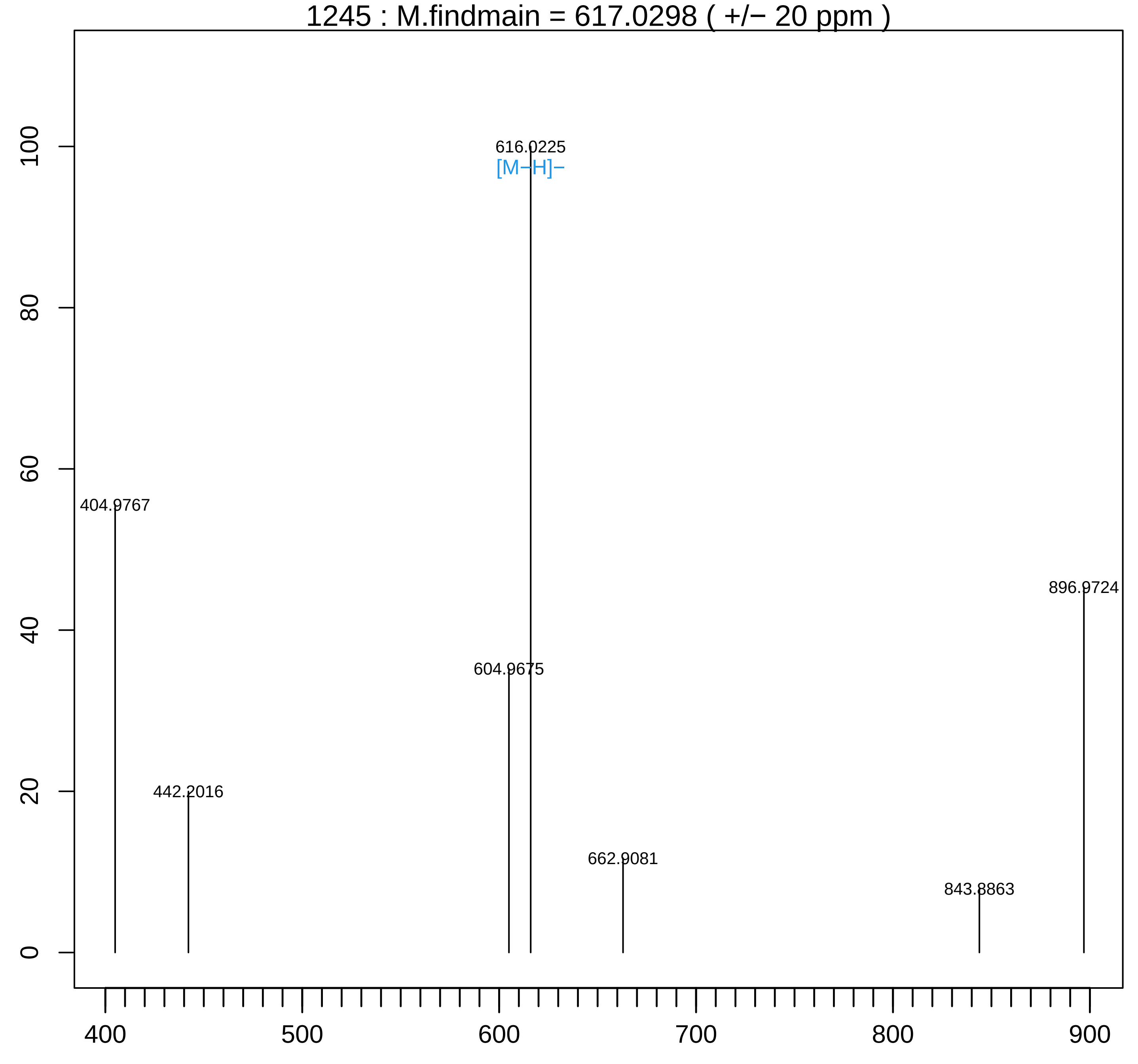
1245 : M.findmain = 617.0298 (+/− 20 ppm)
616.0225
[M−H]−
404.9767
896.9724
604.9675
442.2016
662.9081
843.8863
0
20
40
60
80
100
400
500
600
700
800
900

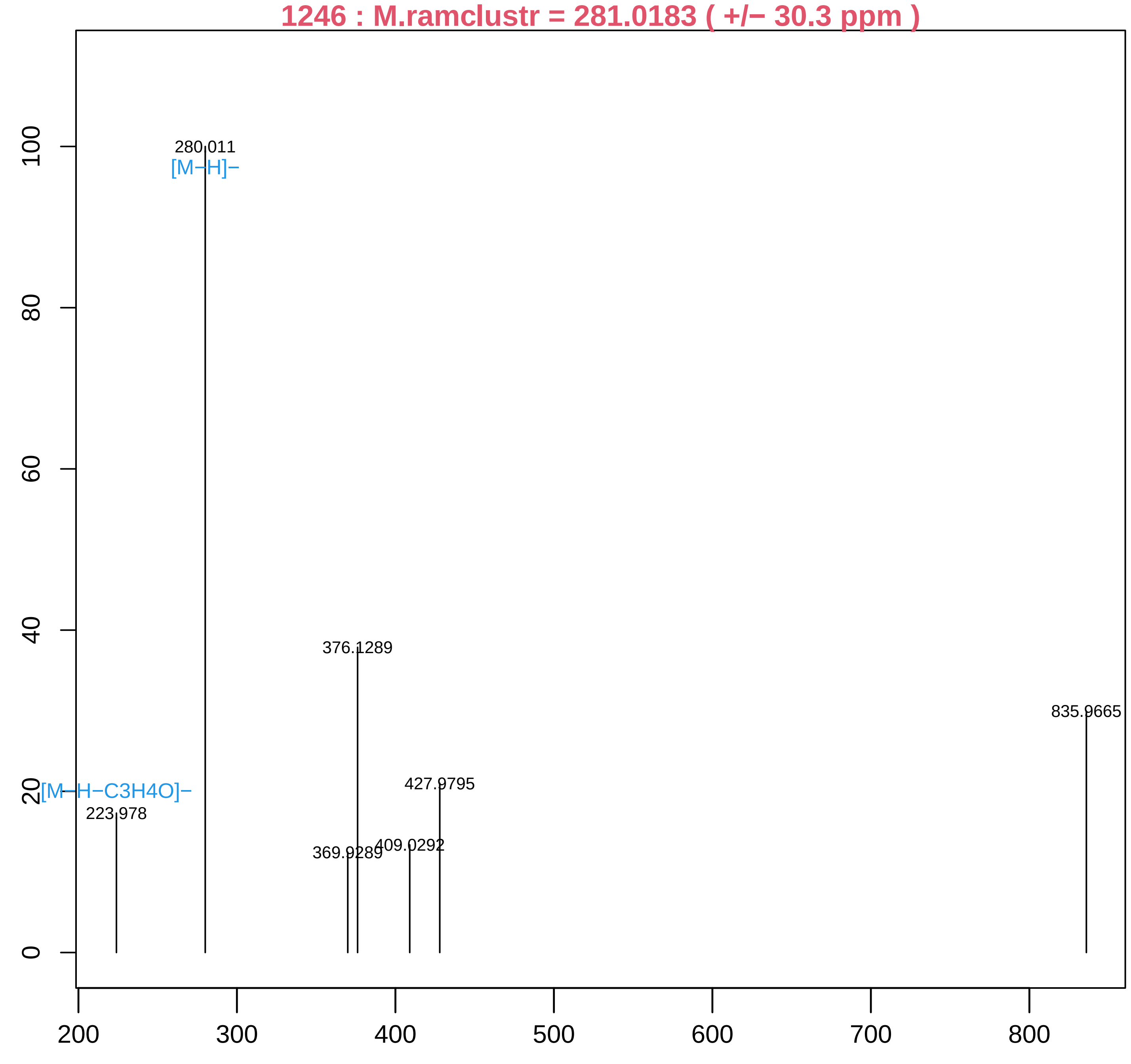
1246 : M.ramclustr = 281.0183 (+/− 30.3 ppm)
280.011
[M−H]−
376.1289
835.9665
427.9795
[M−H−C3H4O]−
223.978
369.9289
409.0292
0
20
40
60
80
100
200
300
400
500
600
700
800

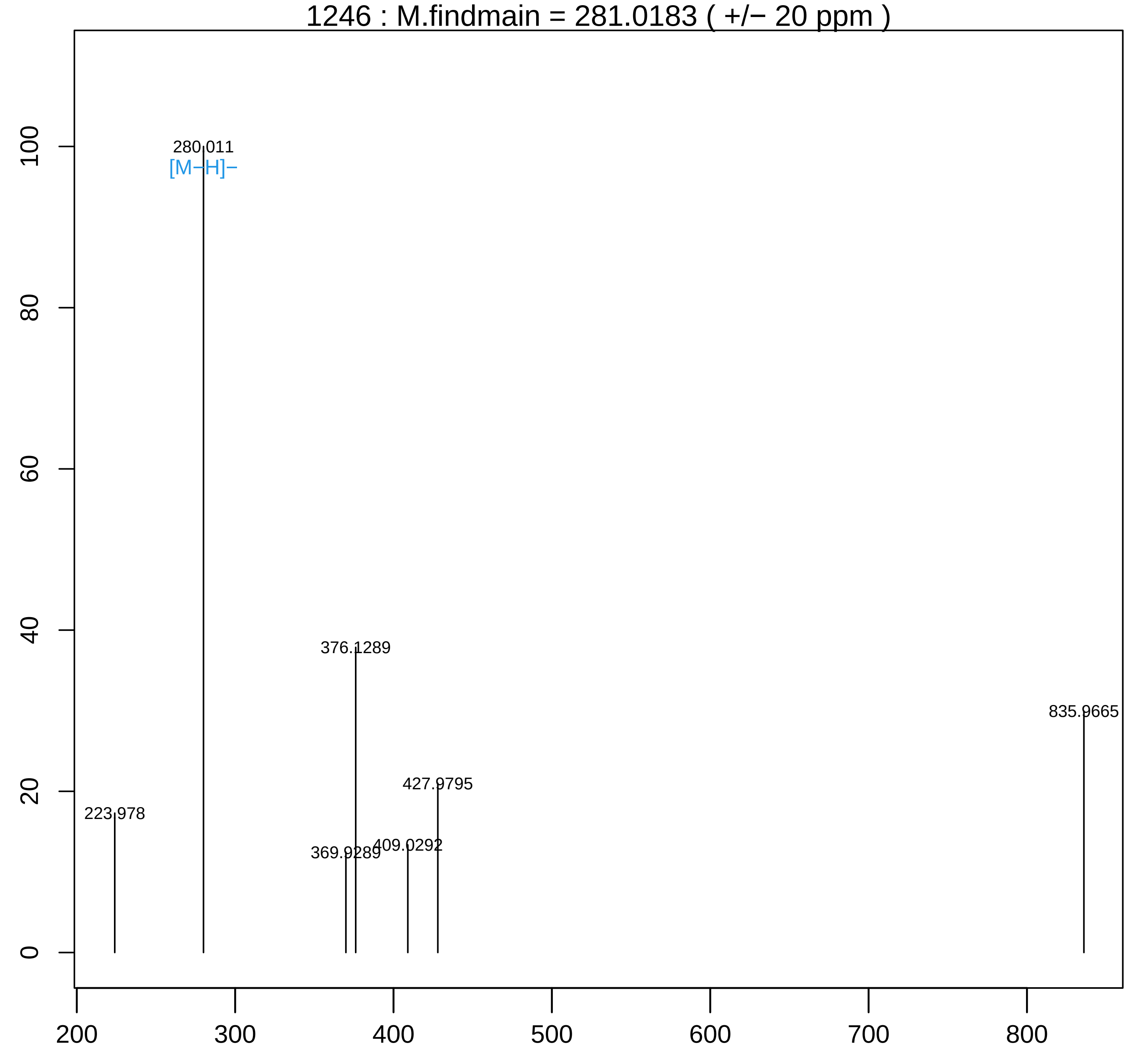
1246 : M.findmain = 281.0183 (+/− 20 ppm)
280.011
[M−H]−
376.1289
835.9665
427.9795
223.978
369.9289
409.0292
0
20
40
60
80
100
200
300
400
500
600
700
800

1247 : M.ramclustr = 420.0234 (+/− NA ppm)

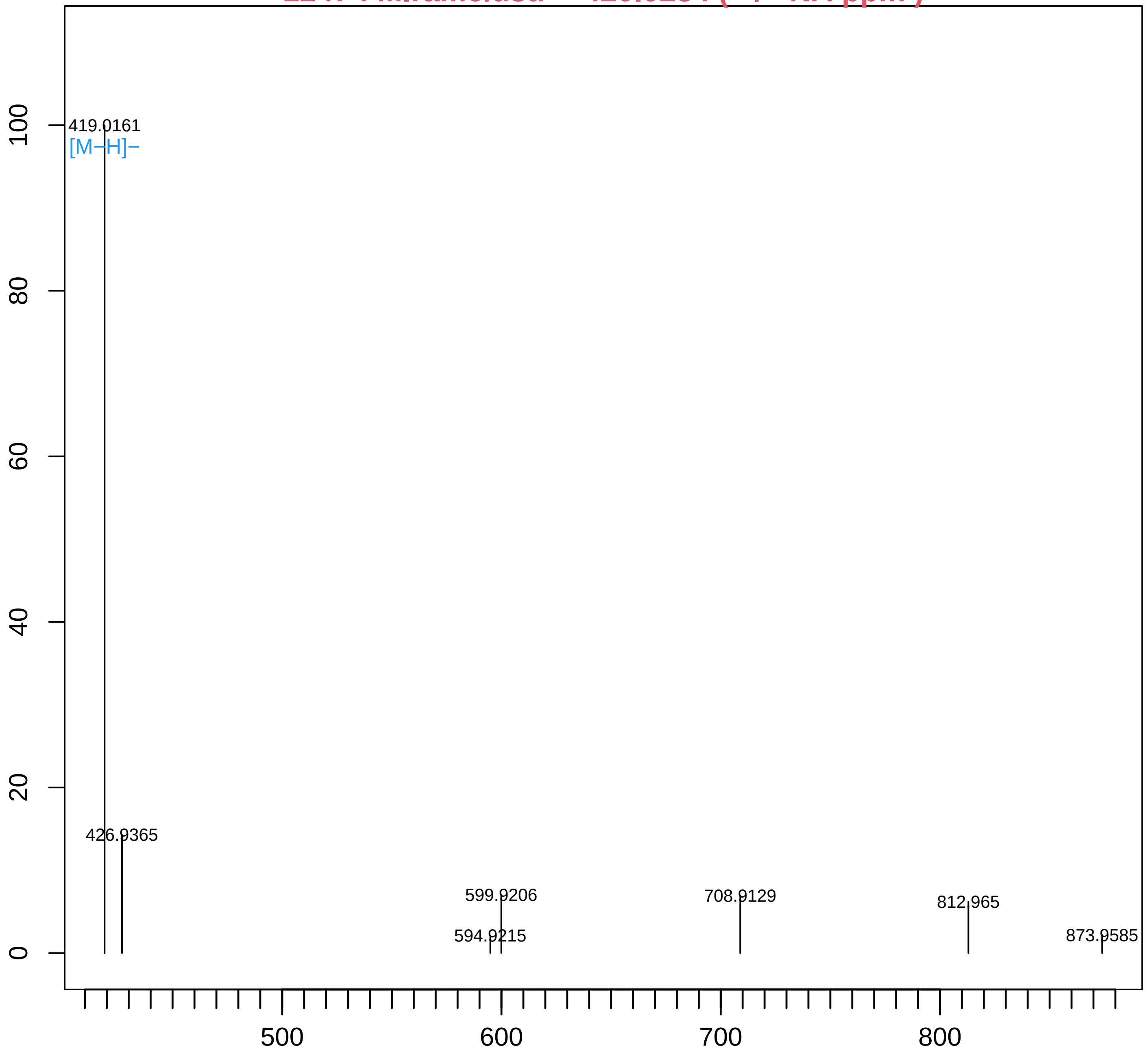

1247 : M.findmain = 420.0234 (+/− 20 ppm)

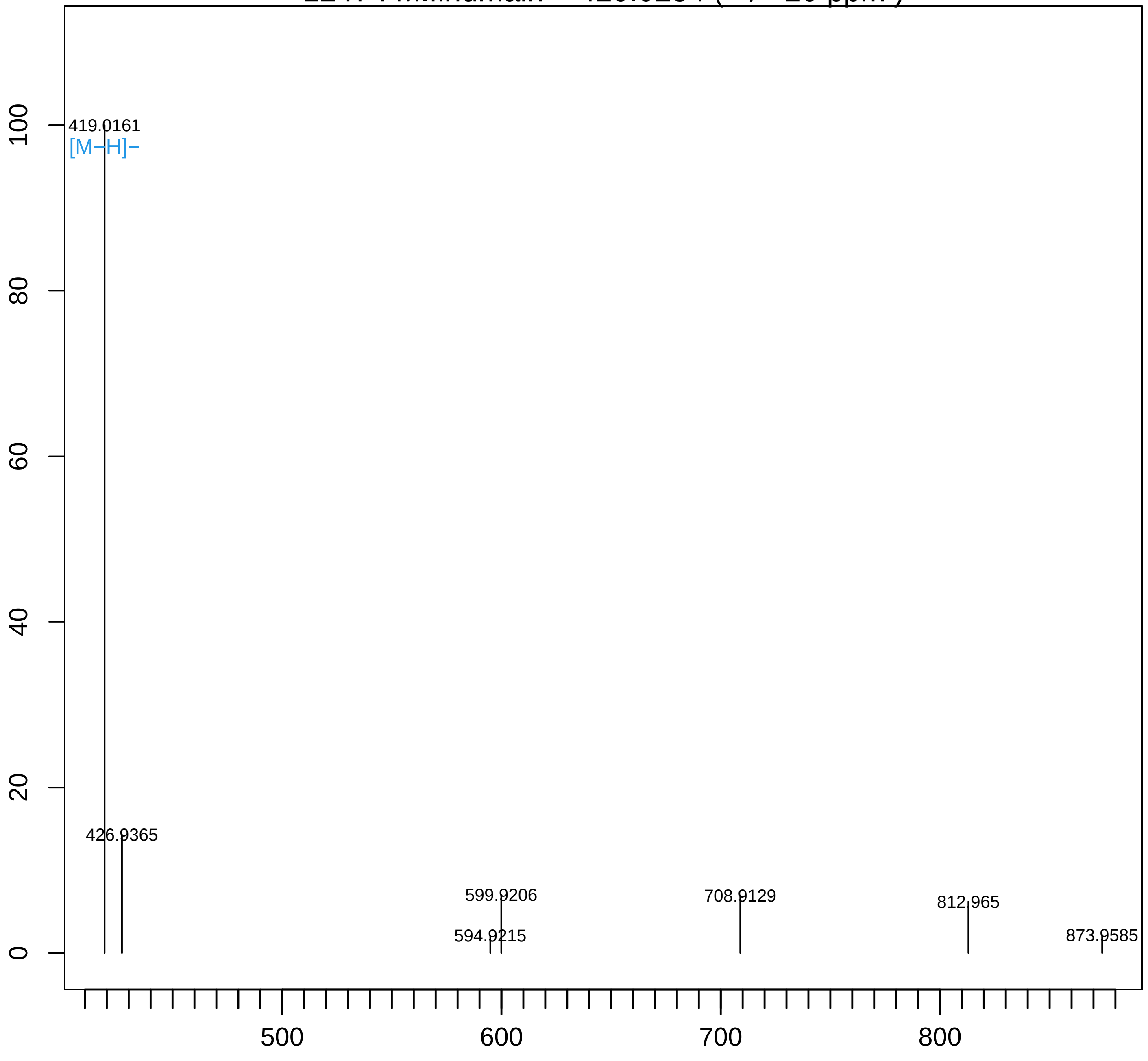

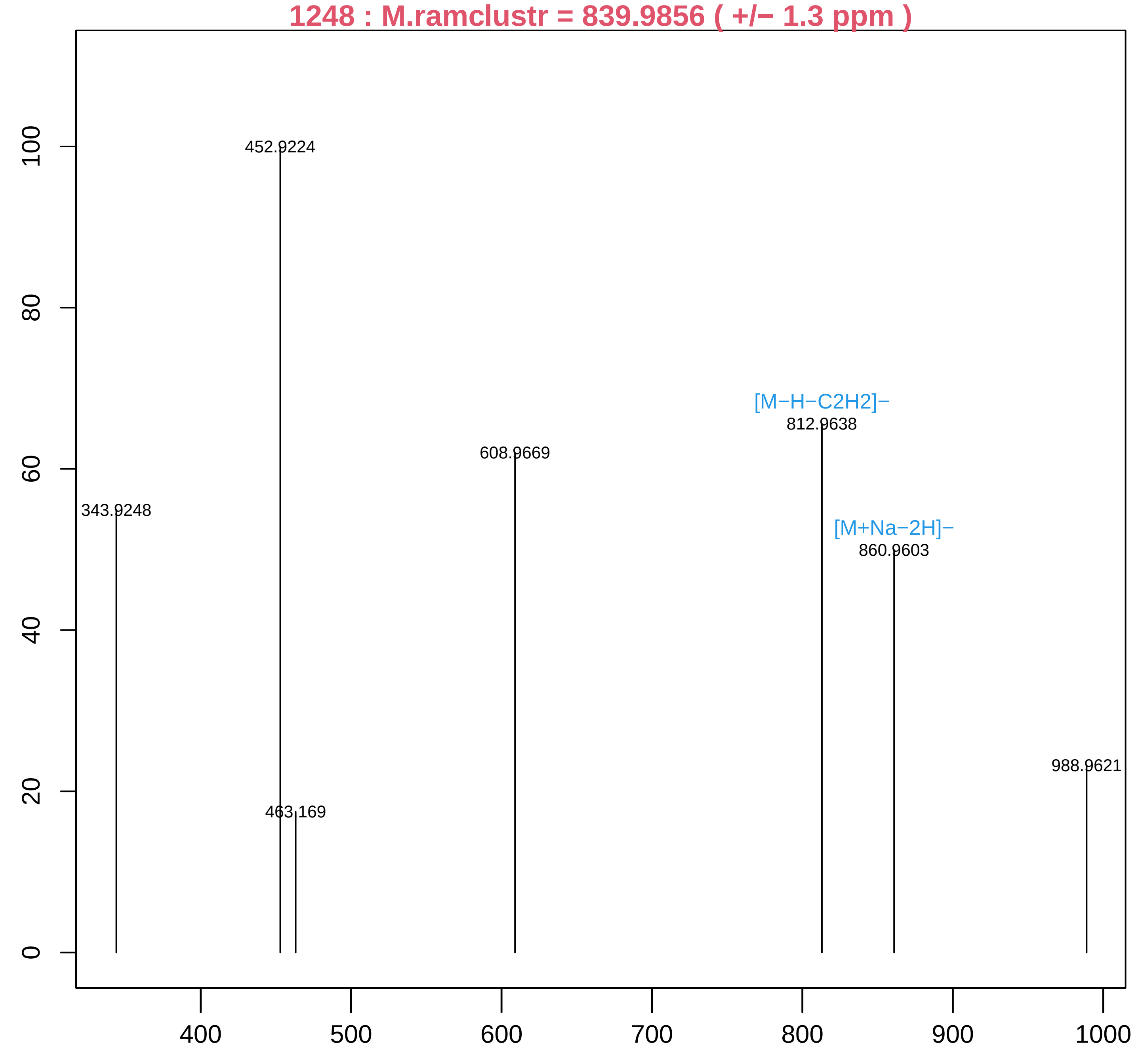
1248 : M.ramclustr = 839.9856 (+/− 1.3 ppm)
452.9224
343.9248
463.169
608.9669
[M−H−C2H2]−
812.9638
[M+Na−2H]−
860.9603
988.9621
0
20
40
60
80
100
400
500
600
700
800
900
1000

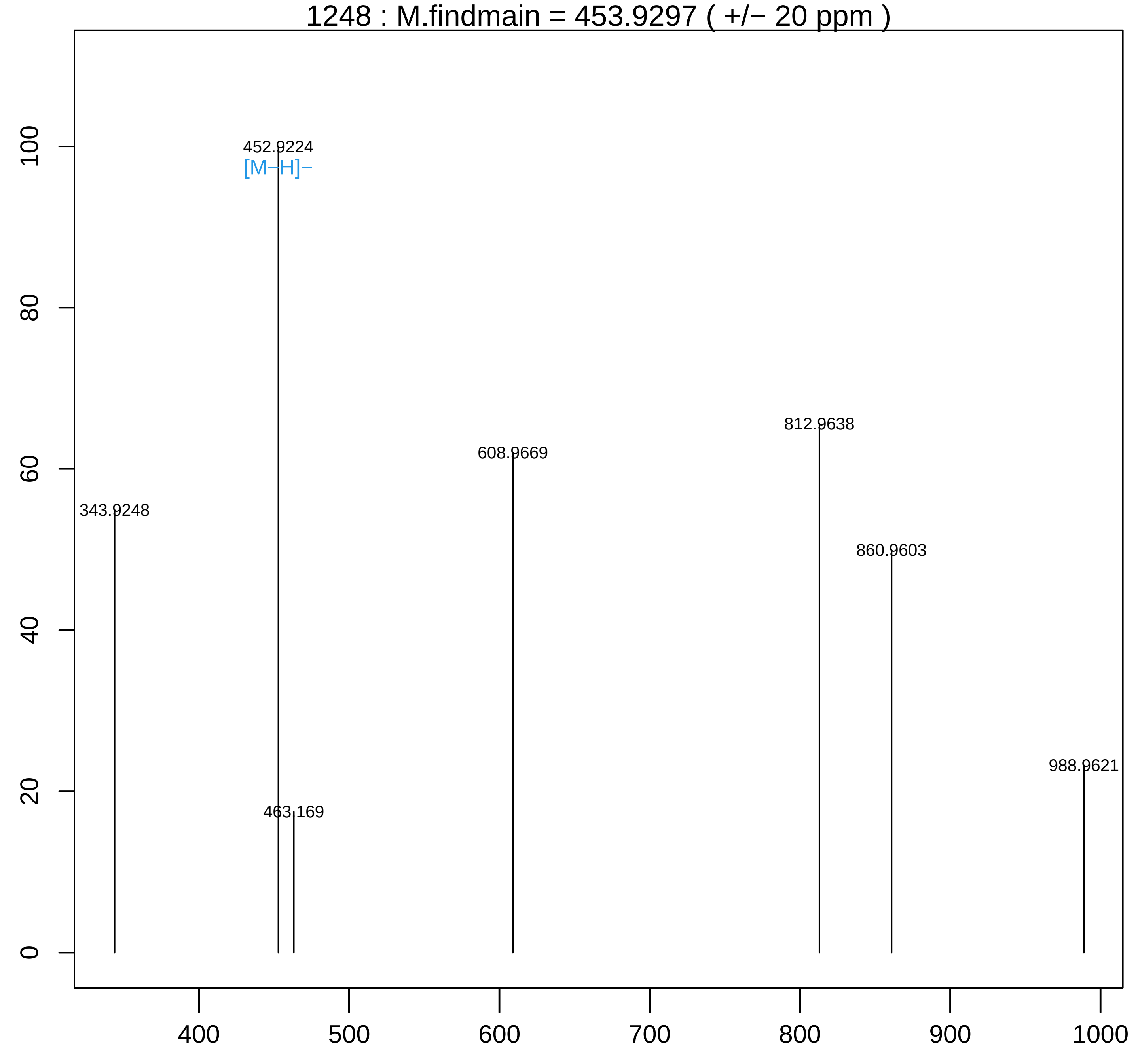
1248 : M.findmain = 453.9297 (+/− 20 ppm)
452.9224
[M−H]−
343.9248
463.169
608.9669
812.9638
860.9603
988.9621
0
20
40
60
80
100
400
500
600
700
800
900
1000

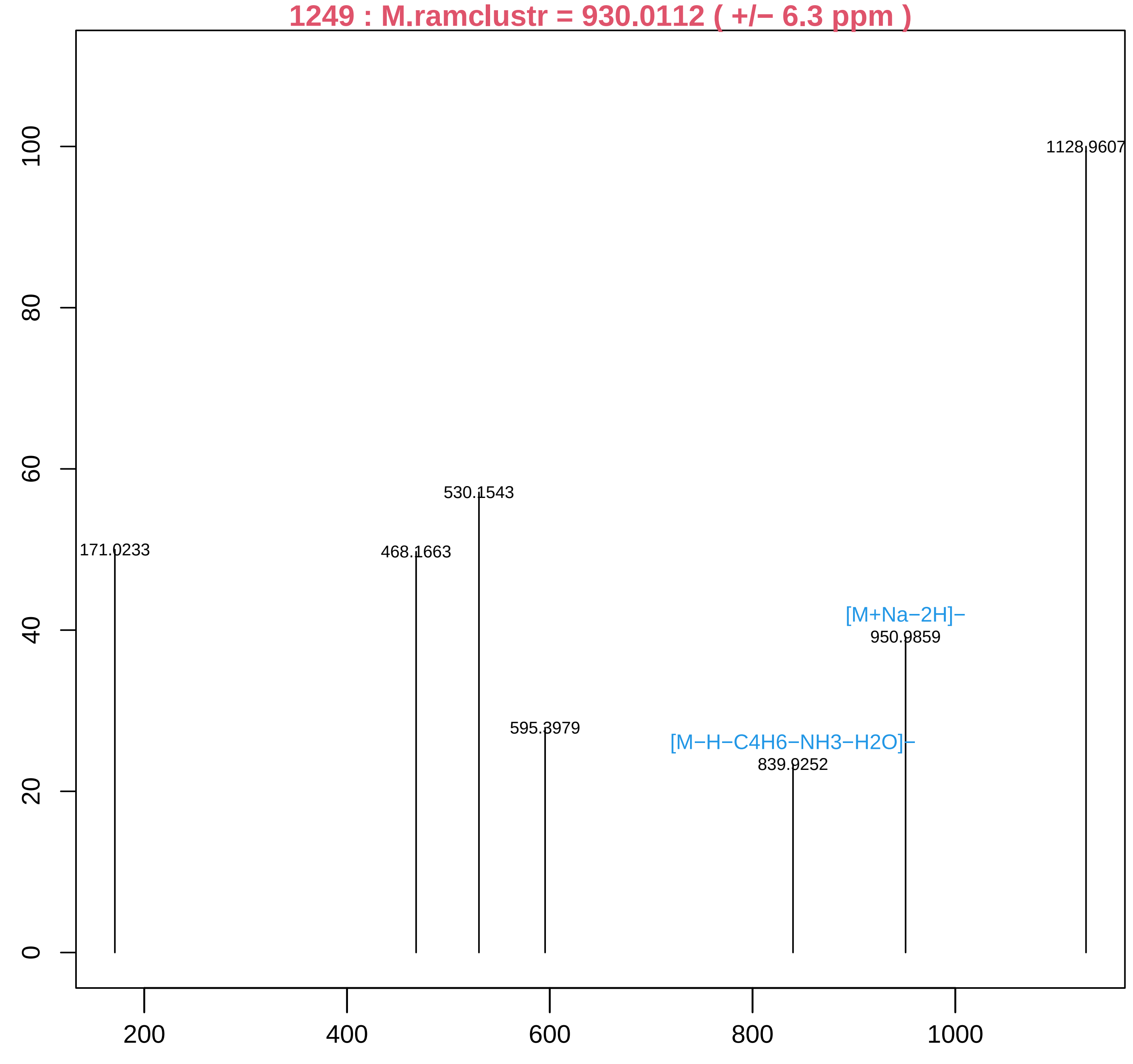
1249 : M.ramclustr = 930.0112 (+/− 6.3 ppm)
1128.9607
530.1543
171.0233
468.1663
[M+Na−2H]−
950.9859
595.3979
[M−H−C4H6−NH3−H2O]−
839.9252
0
20
40
60
80
100
200
400
600
800
1000

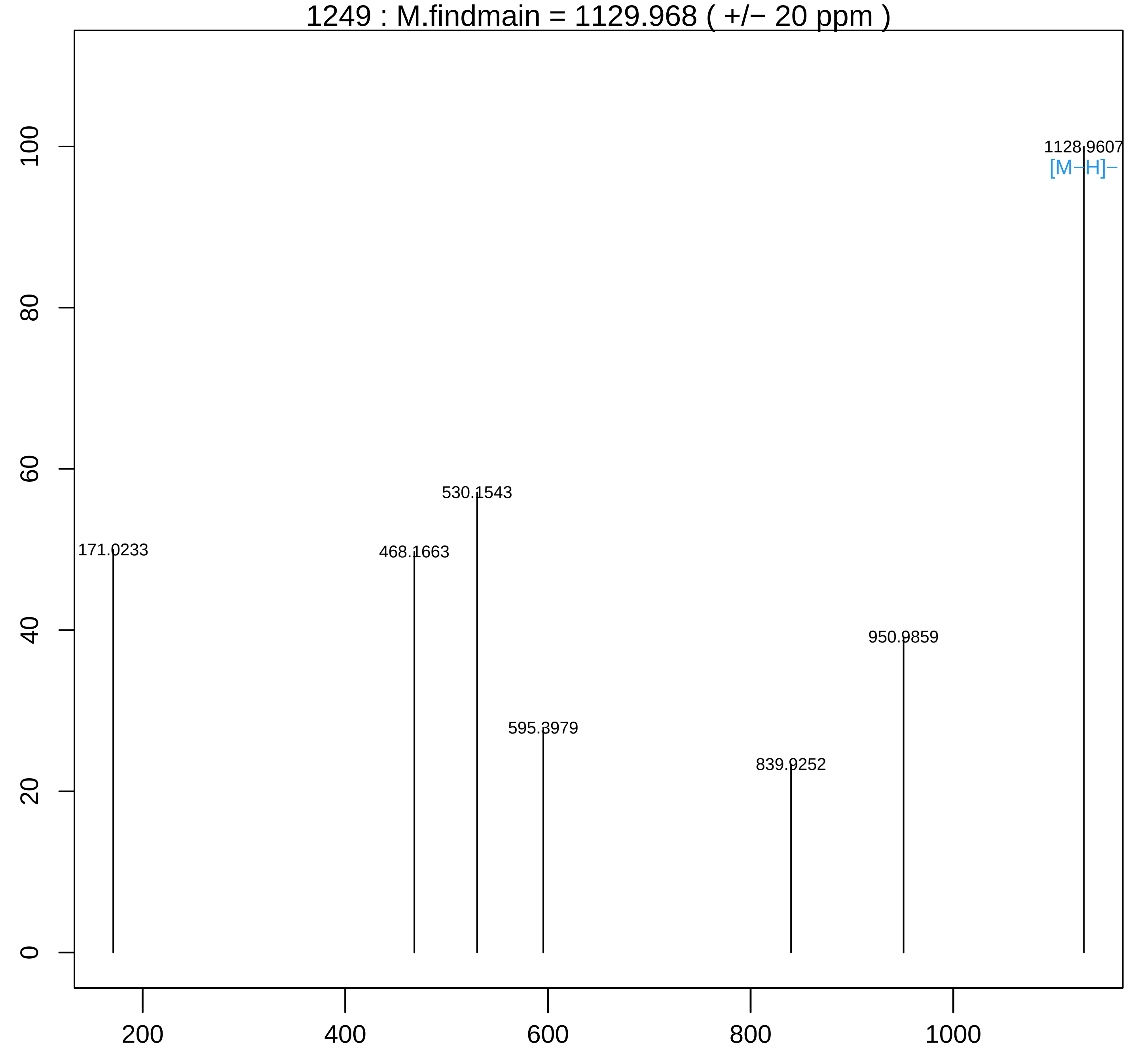
1249 : M.findmain = 1129.968 (+/− 20 ppm)
1128.9607
[M−H]−
530.1543
171.0233
468.1663
950.9859
595.3979
839.9252
0
20
40
60
80
100
200
400
600
800
1000

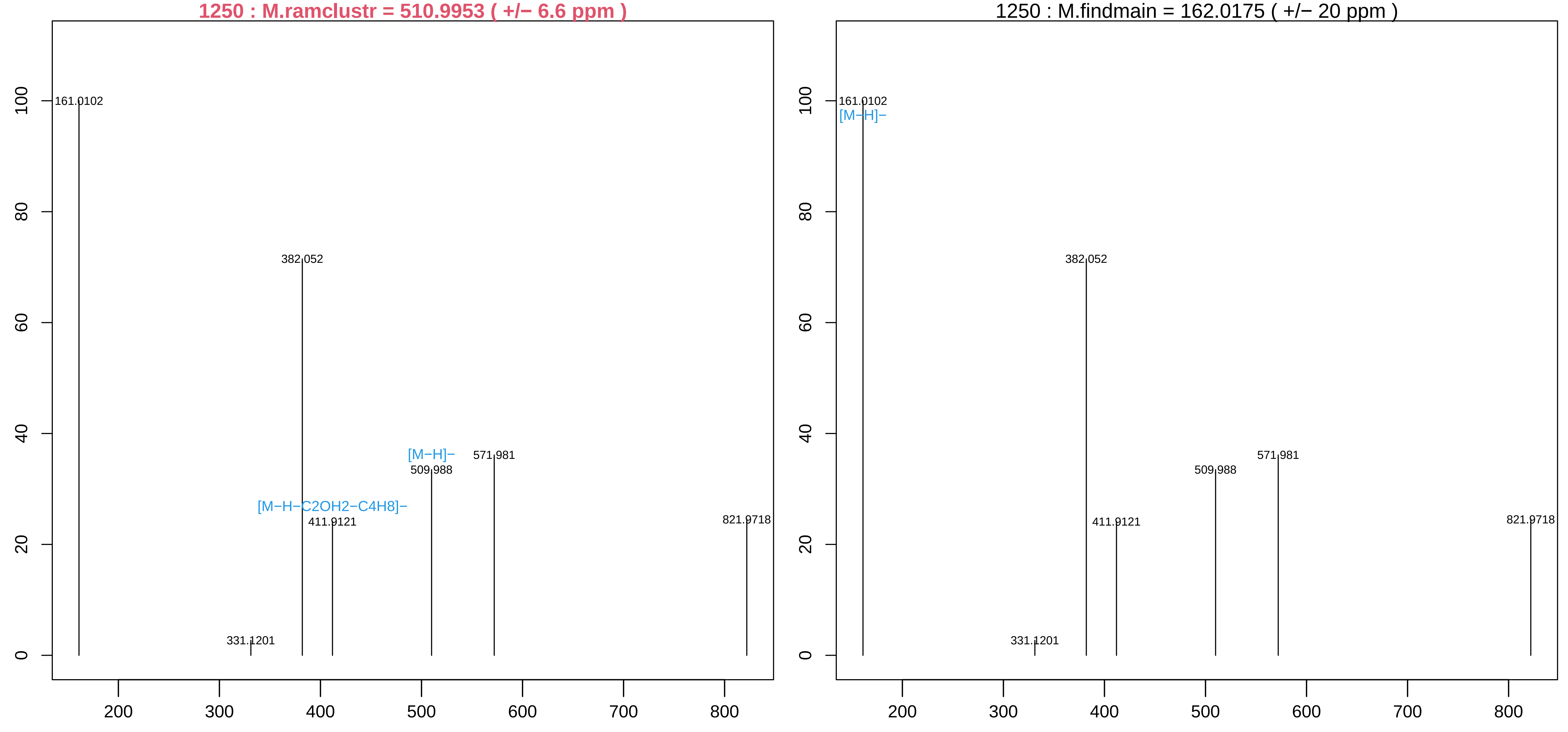
1250 : M.ramclustr = 510.9953 (+/− 6.6 ppm)
161.0102
382.052
[M−H]−
509.988
571.981
[M−H−C2OH2−C4H8]−
411.9121
821.9718
331.1201
0
20
40
60
80
100
200
300
400
500
600
700
800
1250 : M.findmain = 162.0175 (+/− 20 ppm)
161.0102
[M−H]−
382.052
509.988
571.981
411.9121
821.9718
331.1201
0
20
40
60
80
100
200
300
400
500
600
700
800

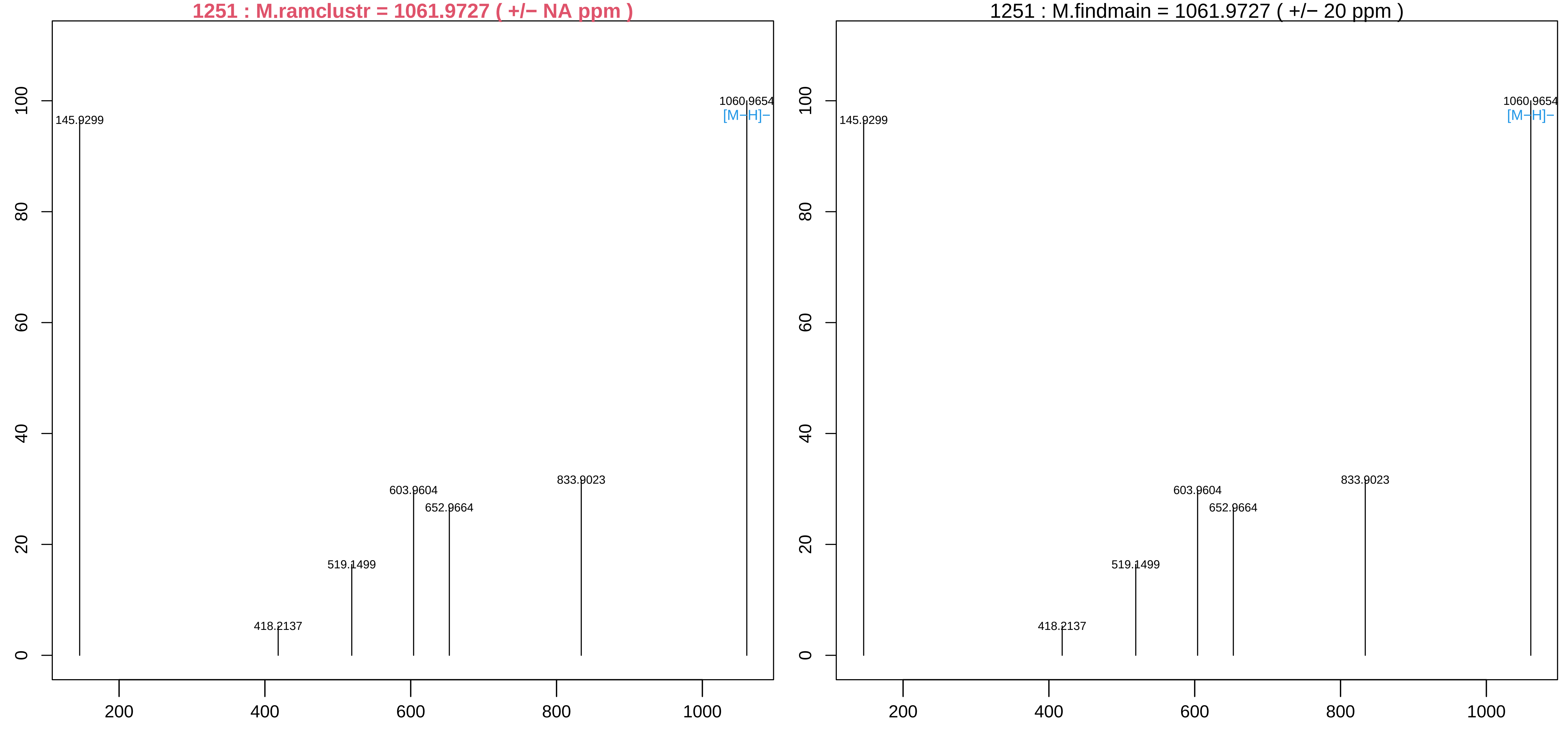

1251 : M.ramclustr = 1061.9727 (+/− NA ppm)
145.9299
418.2137
519.1499
603.9604
652.9664
833.9023
1060.9654
[M−H]−
1251 : M.findmain = 1061.9727 (+/− 20 ppm)
145.9299
418.2137
519.1499
603.9604
652.9664
833.9023
1060.9654
[M−H]−
0
20
40
60
80
100
200
400
600
800
1000

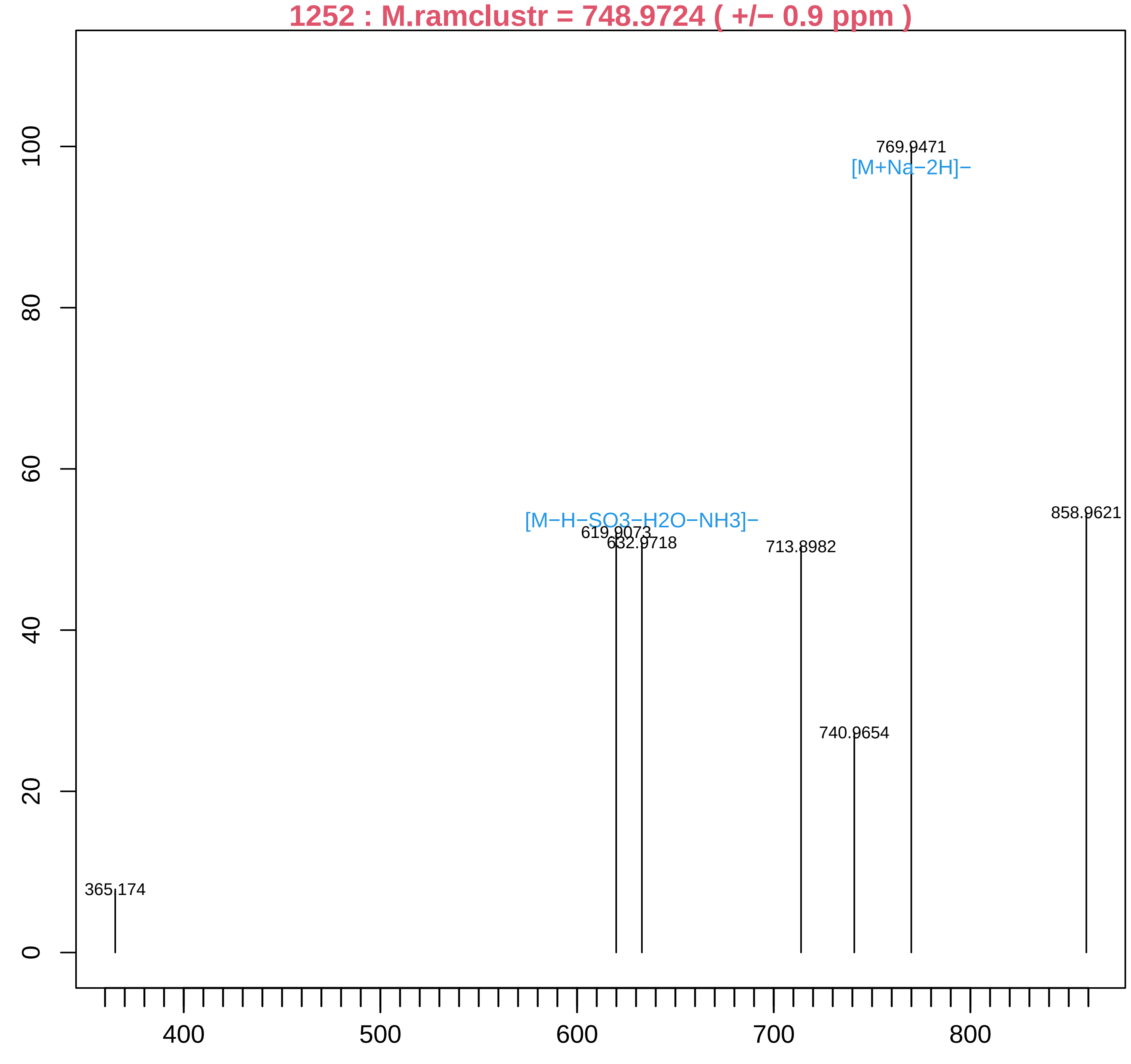
1252 : M.ramclustr = 748.9724 (+/− 0.9 ppm)
769.9471
[M+Na−2H]−
[M−H−SO3−H2O−NH3]−
619.9073
632.9718
713.8982
740.9654
858.9621
365.174
0
20
40
60
80
100
400
500
600
700
800

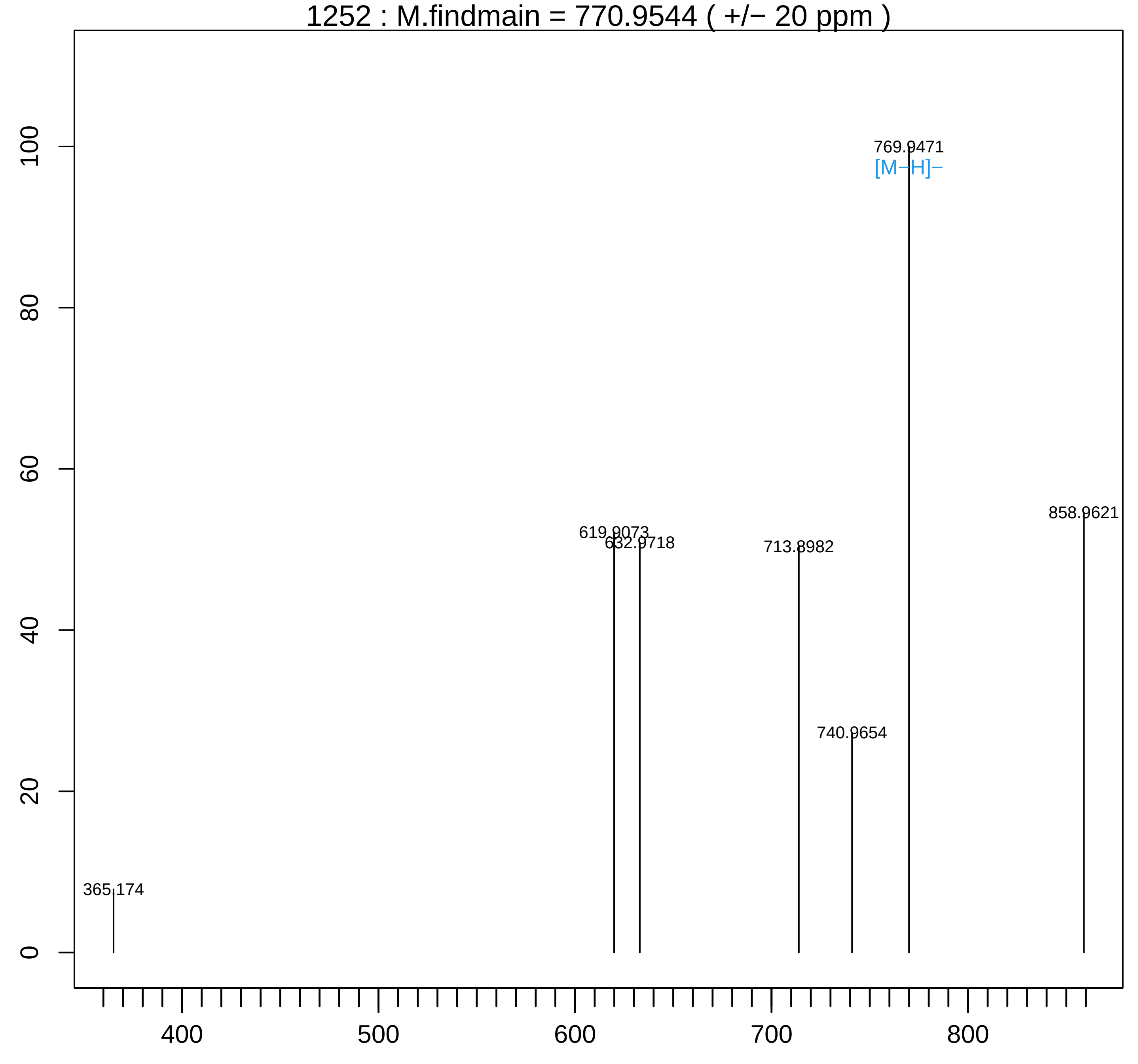
1252 : M.findmain = 770.9544 (+/− 20 ppm)
769.9471
[M−H]−
619.9073
632.9718
713.8982
740.9654
858.9621
365.174
0
20
40
60
80
100
400
500
600
700
800

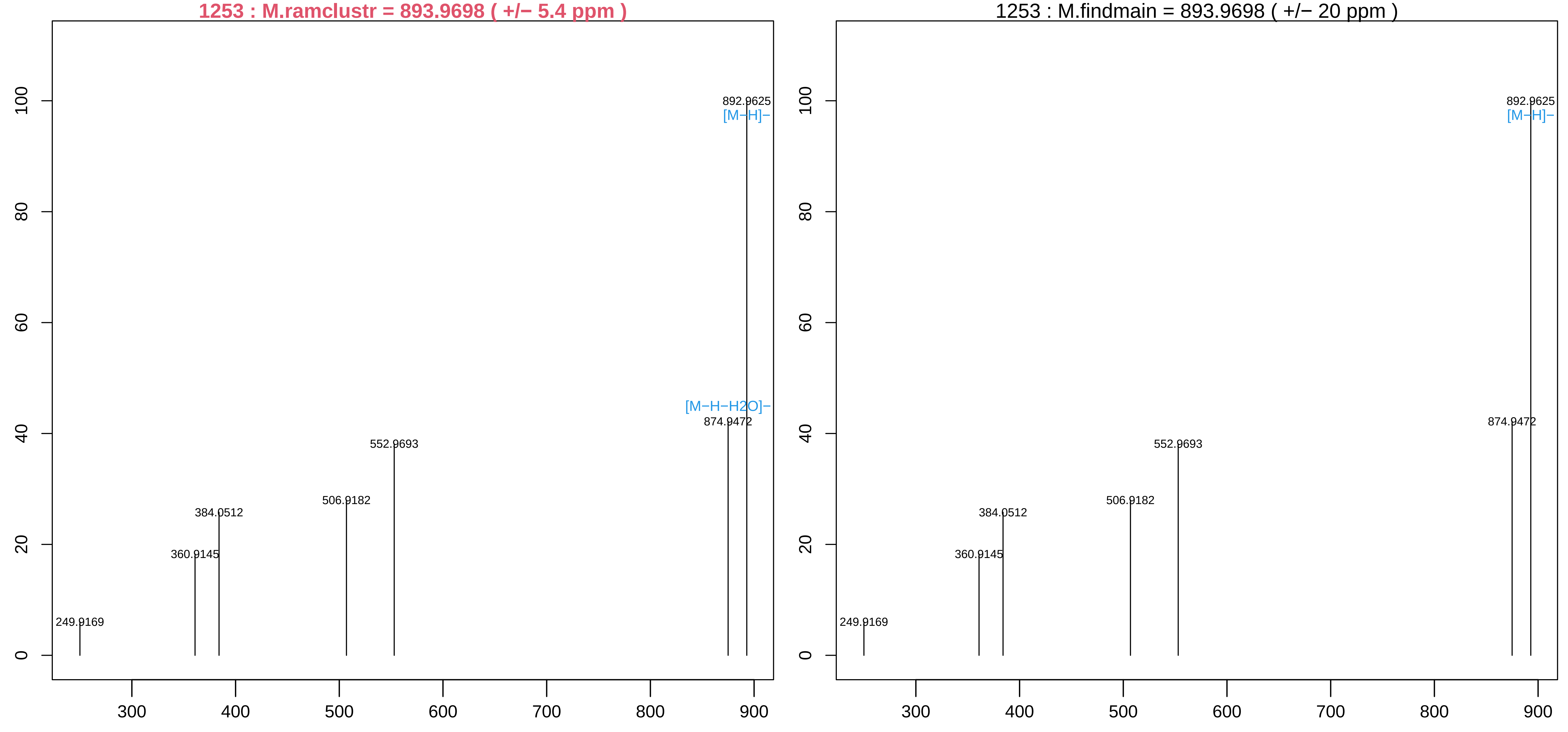
1253 : M.ramclustr = 893.9698 (+/− 5.4 ppm)
892.9625
[M−H]−
[M−H−H2O]−
874.9472
552.9693
506.9182
384.0512
360.9145
249.9169
1253 : M.findmain = 893.9698 (+/− 20 ppm)
892.9625
[M−H]−
874.9472
552.9693
506.9182
384.0512
360.9145
249.9169

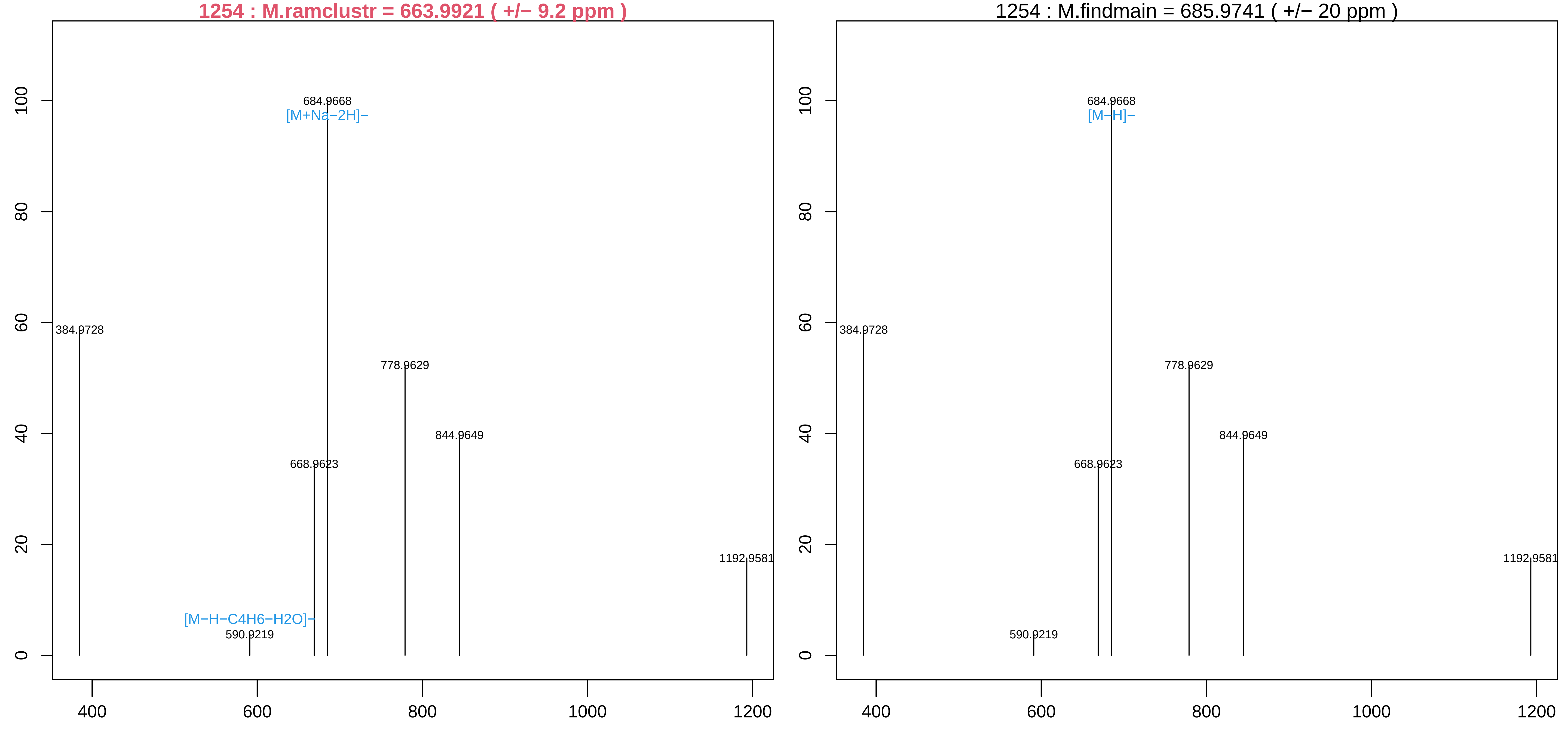
1254 : M.ramclustr = 663.9921 (+/− 9.2 ppm)
684.9668
[M+Na−2H]−
384.9728
778.9629
844.9649
668.9623
1192.9581
[M−H−C4H6−H2O]−
590.9219
1254 : M.findmain = 685.9741 (+/− 20 ppm)
684.9668
[M−H]−
384.9728
778.9629
844.9649
668.9623
1192.9581
590.9219
0
20
40
60
80
100
400
600
800
1000
1200

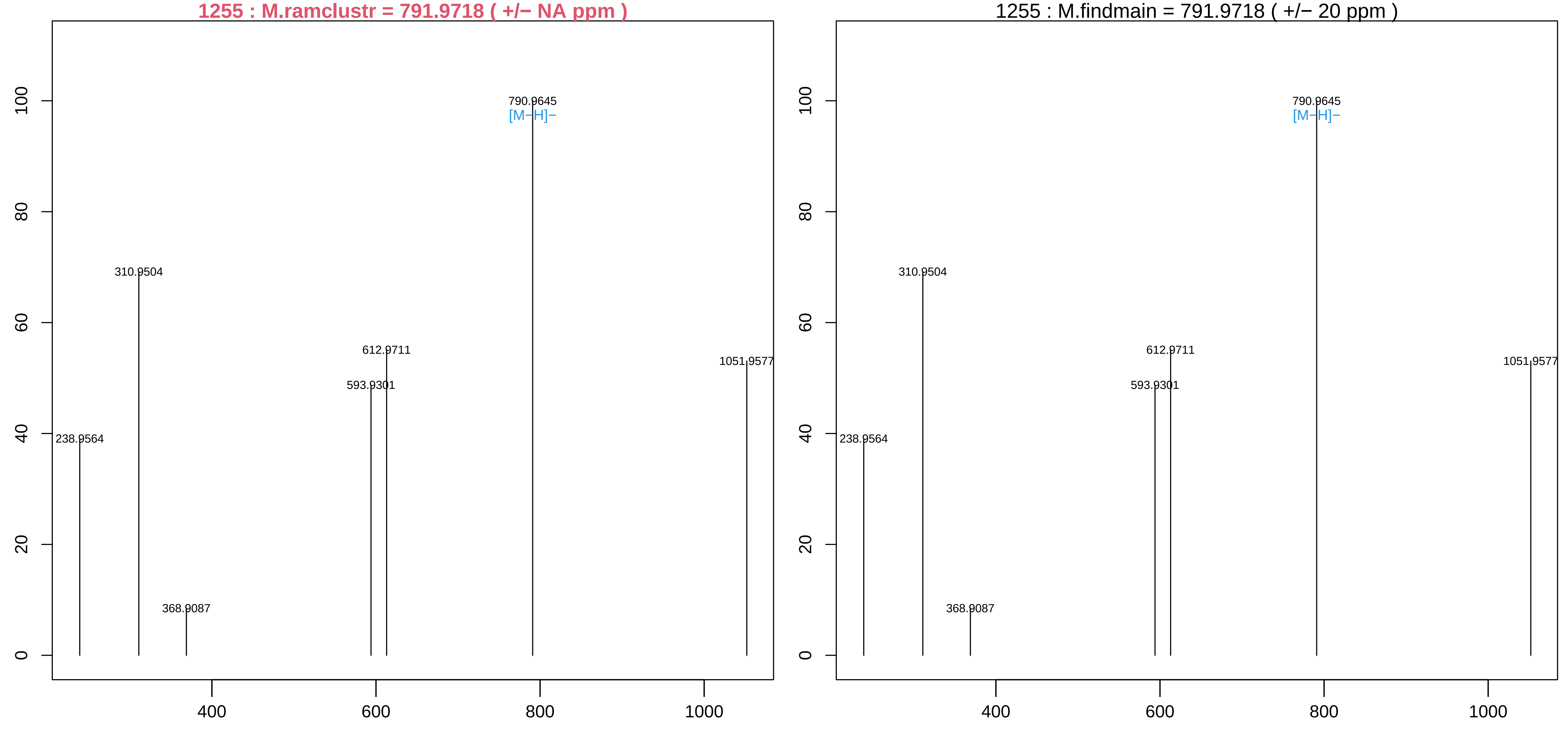
1255 : M.ramclustr = 791.9718 (+/− NA ppm)
1255 : M.findmain = 791.9718 (+/− 20 ppm)
790.9645
[M−H]−
310.9504
612.9711
1051.9577
593.9301
238.9564
368.9087
0
20
40
60
80
100
400
600
800
1000

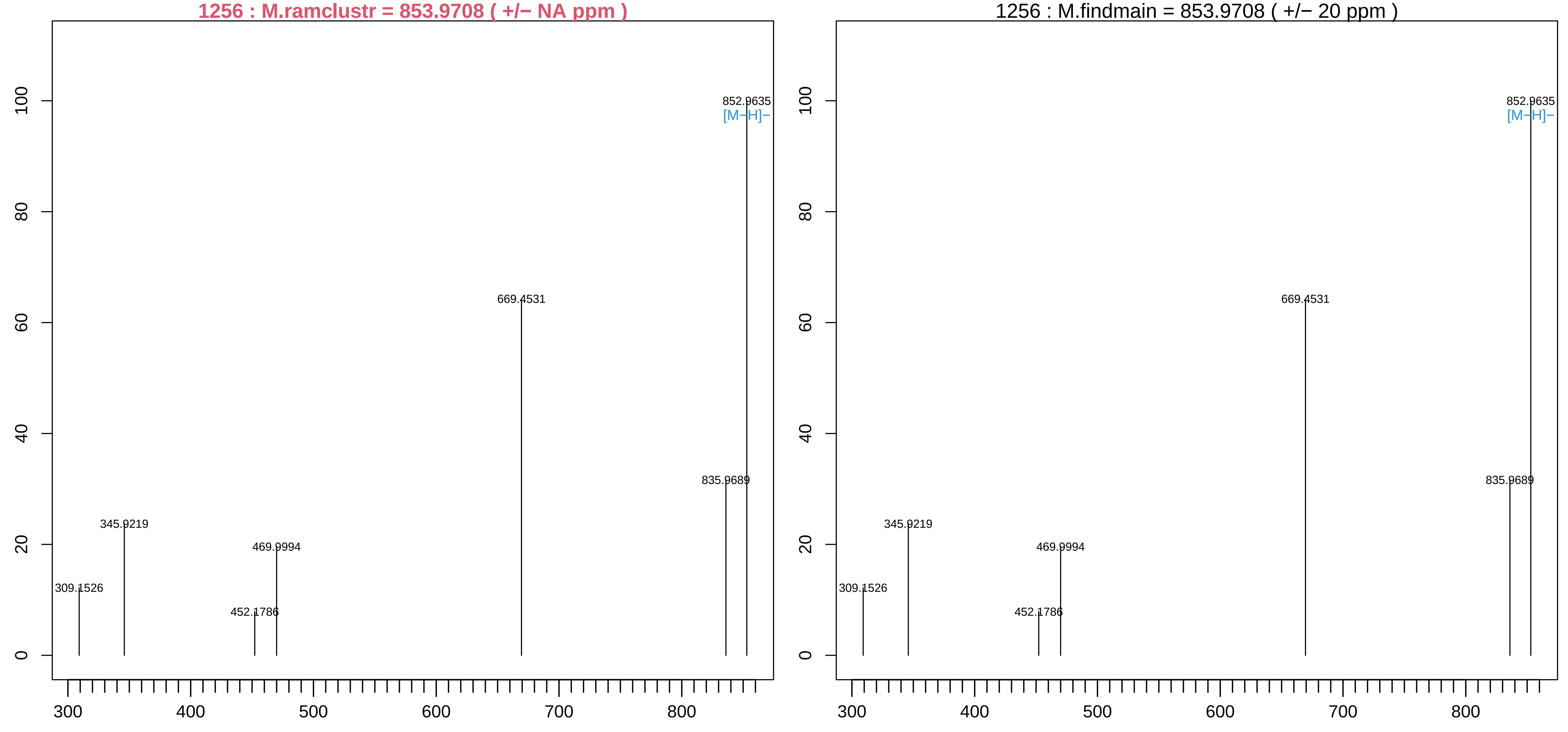

1256 : M.ramclustr = 853.9708 (+/− NA ppm)
852.9635
[M−H]−
669.4531
835.9689
345.9219
469.9994
309.1526
452.1786
1256 : M.findmain = 853.9708 (+/− 20 ppm)
852.9635
[M−H]−
669.4531
835.9689
345.9219
469.9994
309.1526
452.1786

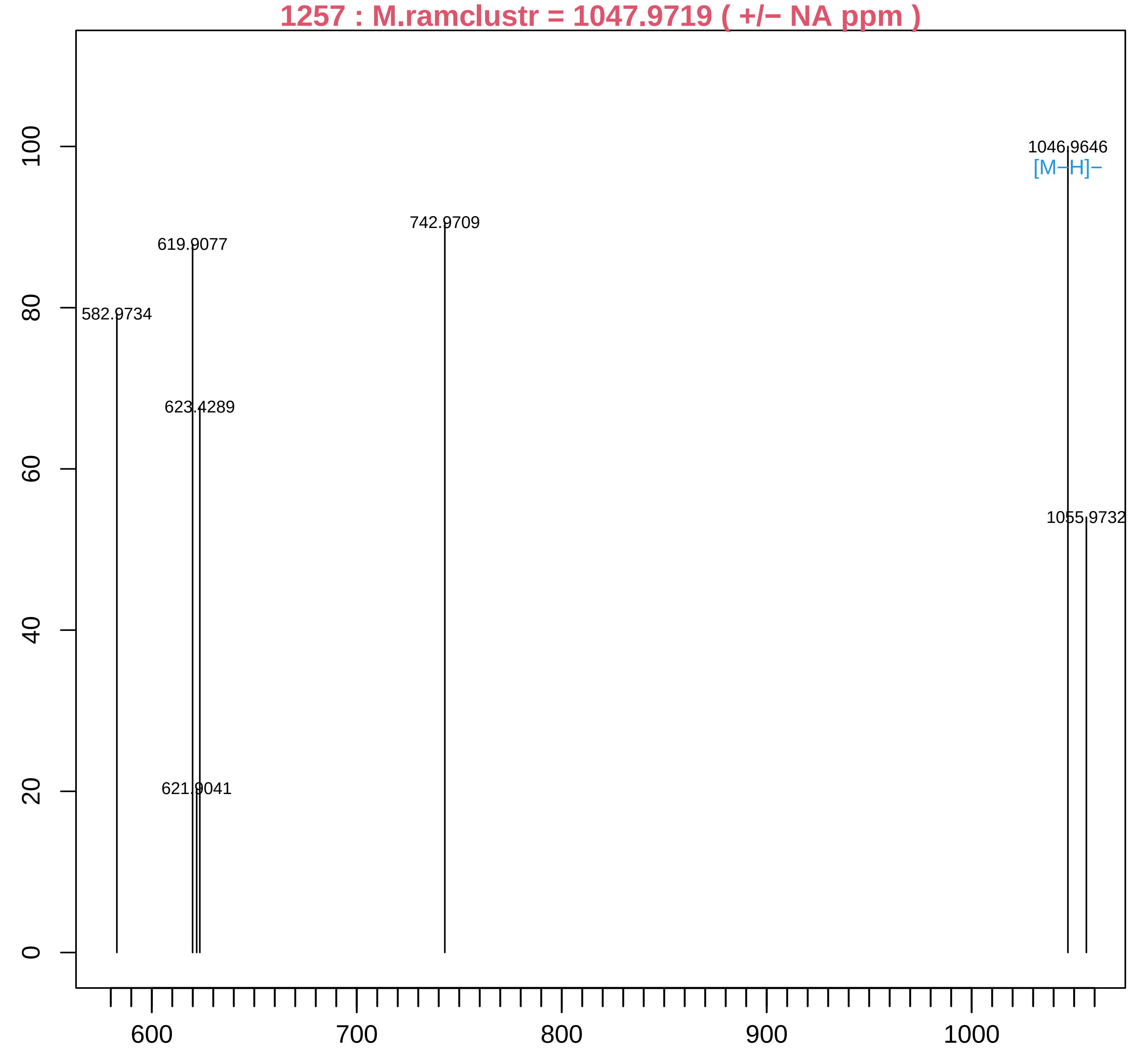
1257 : M.ramclustr = 1047.9719 (+/− NA ppm)
1046.9646
[M−H]−
742.9709
619.9077
582.9734
623.4289
1055.9732
621.9041
0
20
40
60
80
100
600
700
800
900
1000

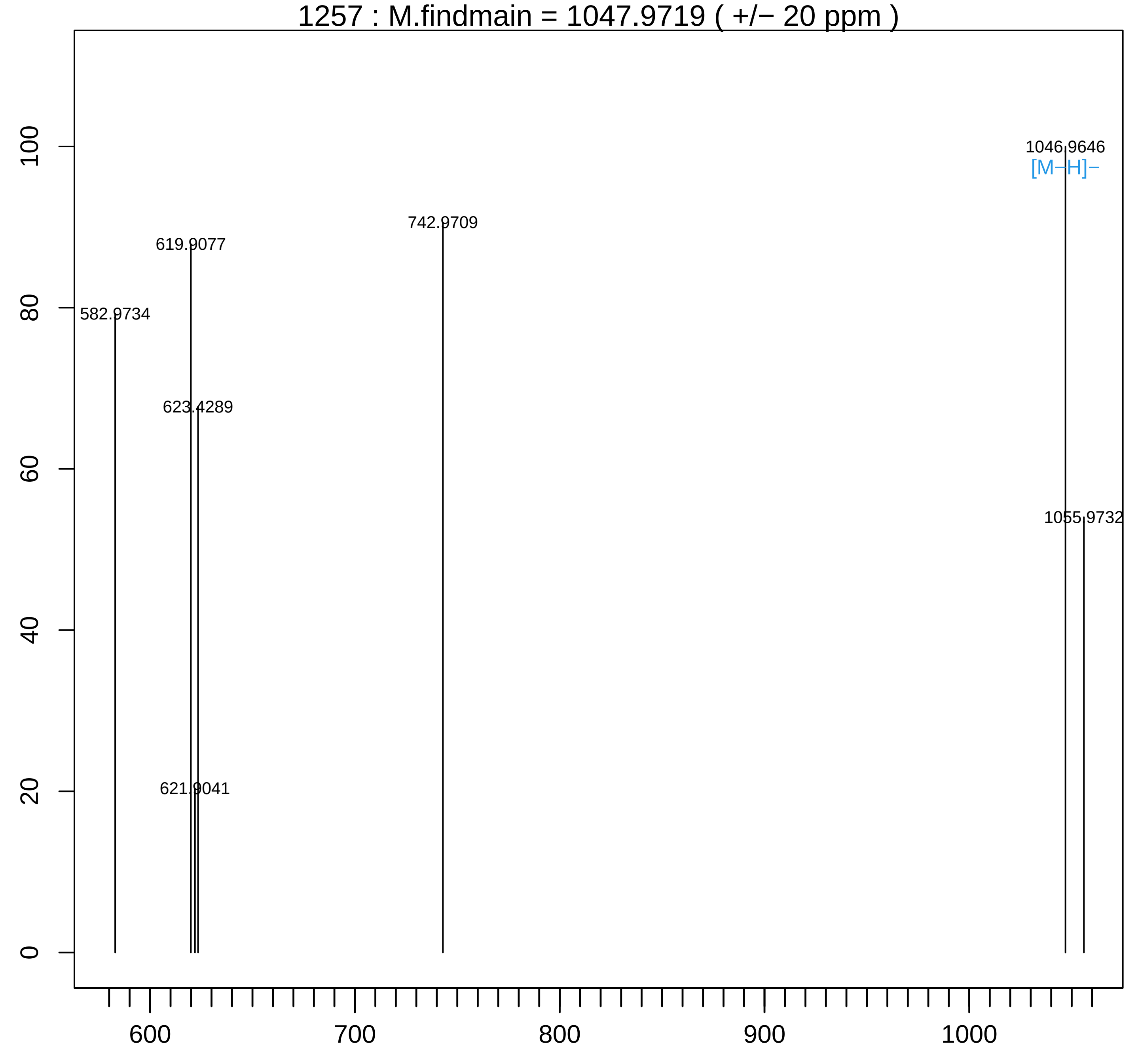
1257 : M.findmain = 1047.9719 (+/− 20 ppm)
1046.9646
[M−H]−
742.9709
619.9077
582.9734
623.4289
1055.9732
621.9041
0
20
40
60
80
100
600
700
800
900
1000

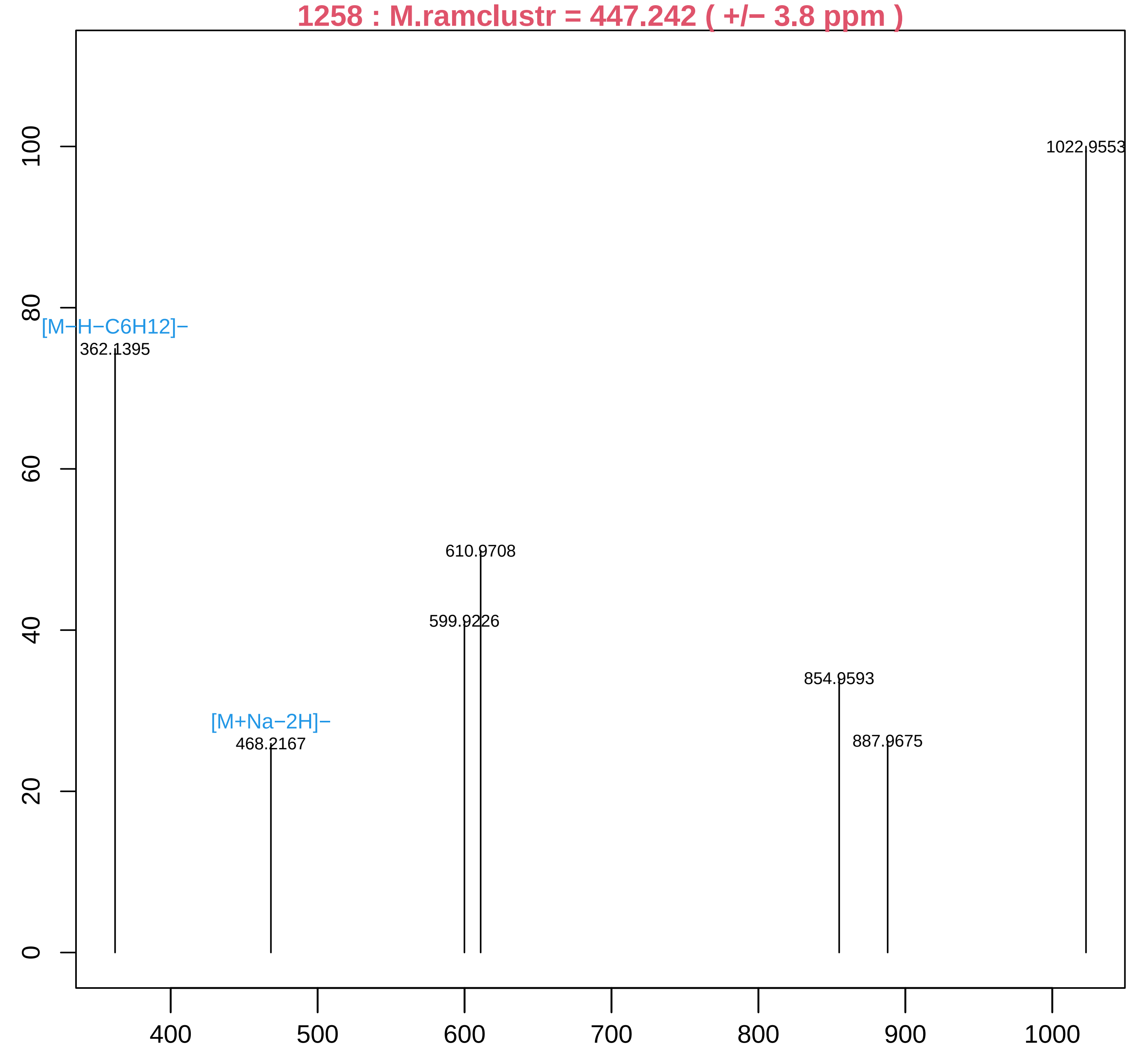
1258 : M.ramclustr = 447.242 (+/− 3.8 ppm)
[M−H−C6H12]−
362.1395
[M+Na−2H]−
468.2167
599.9226
610.9708
854.9593
887.9675
1022.9553
0
20
40
60
80
100
400
500
600
700
800
900
1000

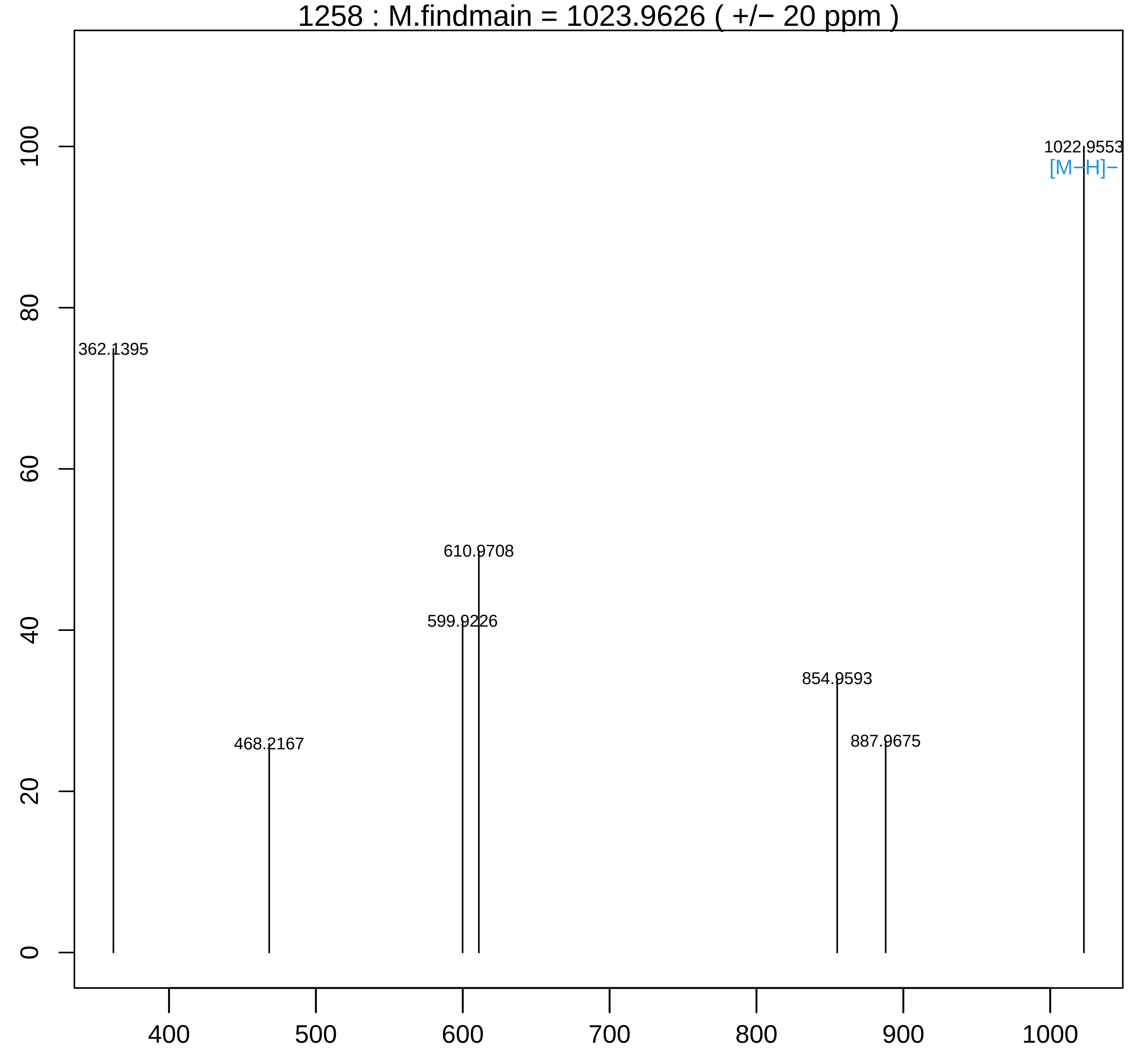
1258 : M.findmain = 1023.9626 (+/− 20 ppm)
362.1395
468.2167
599.9226
610.9708
854.9593
887.9675
1022.9553
[M−H]−
0
20
40
60
80
100
400
500
600
700
800
900
1000

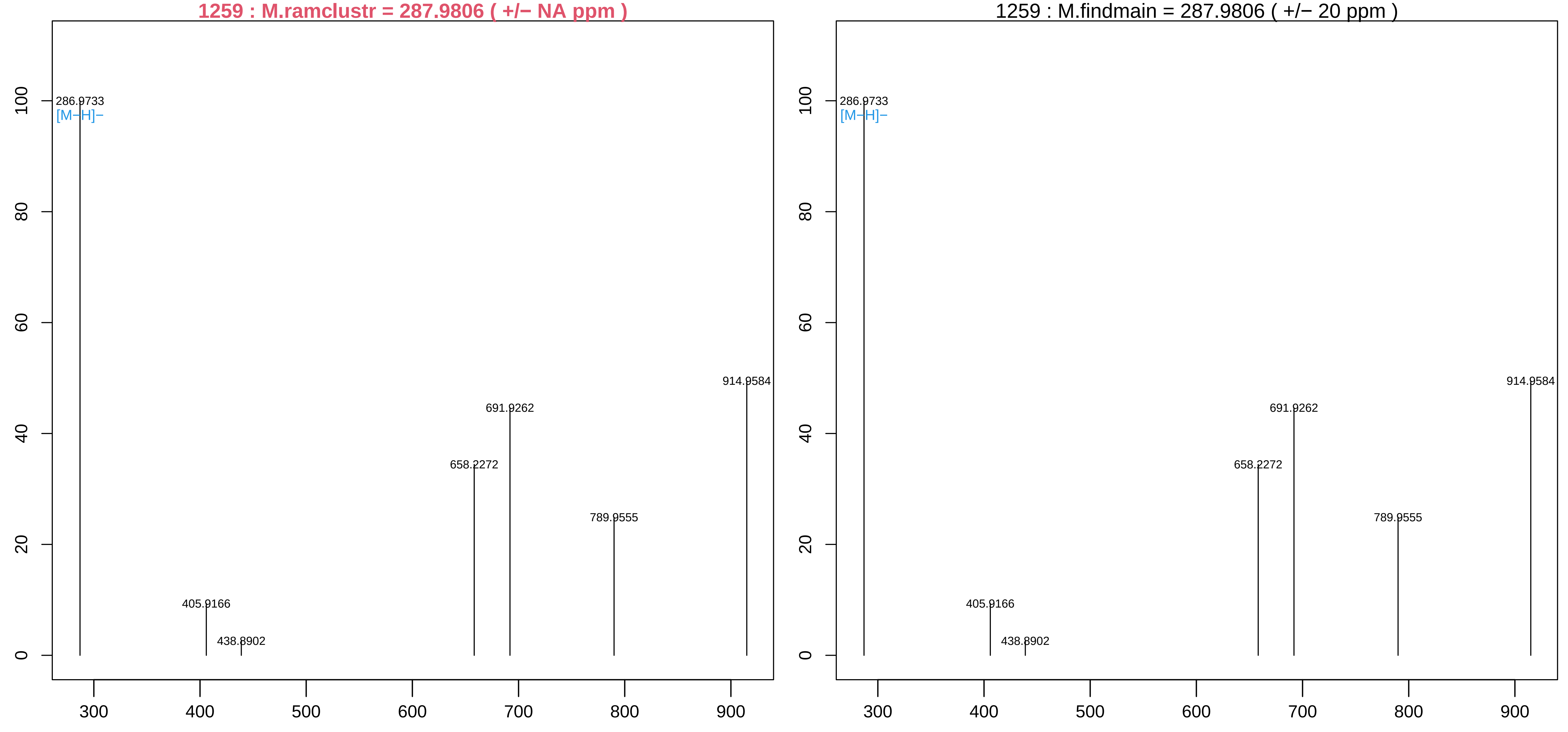
1259 : M.ramclustr = 287.9806 (+/− NA ppm)
286.9733
[M−H]−
405.9166
438.8902
658.2272
691.9262
789.9555
914.9584
0
20
40
60
80
100
300
400
500
600
700
800
900
1259 : M.findmain = 287.9806 (+/− 20 ppm)
286.9733
[M−H]−
405.9166
438.8902
658.2272
691.9262
789.9555
914.9584
0
20
40
60
80
100
300
400
500
600
700
800
900

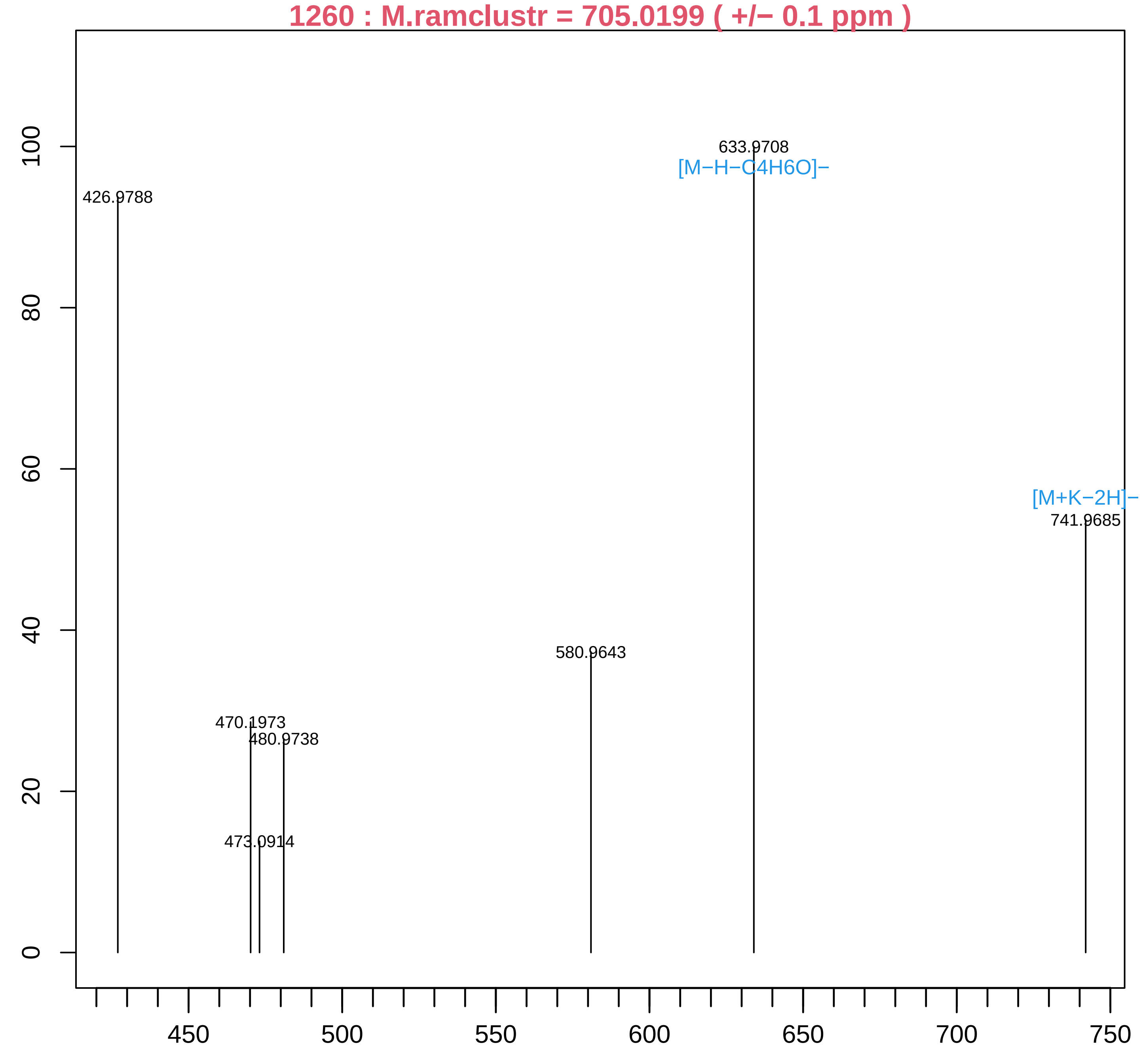
1260 : M.ramclustr = 705.0199 (+/− 0.1 ppm)
633.9708
[M−H−C4H6O]−
426.9788
[M+K−2H]−
741.9685
580.9643
470.1973
480.9738
473.0914
0
20
40
60
80
100
450
500
550
600
650
700
750
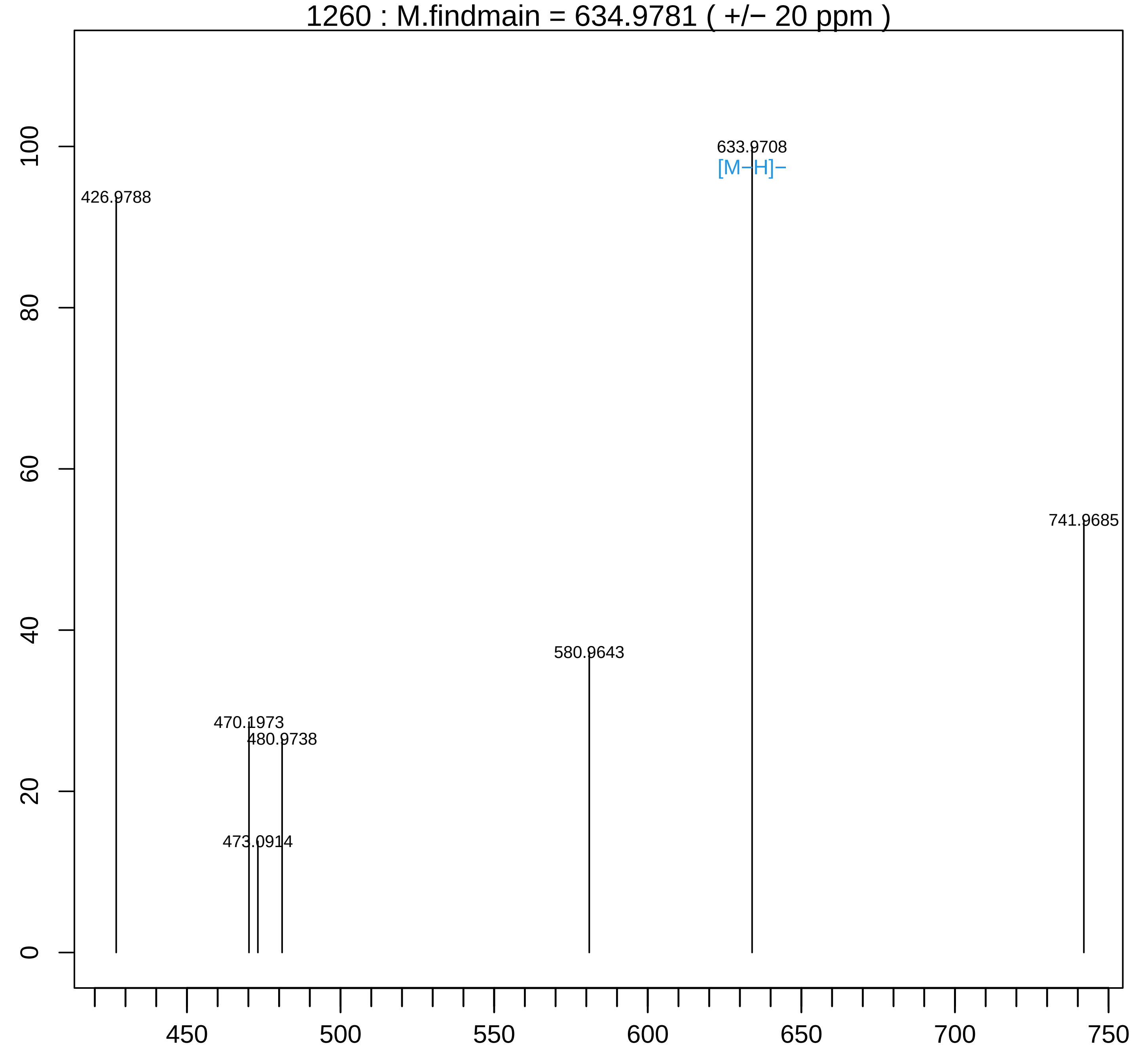
1260 : M.findmain = 634.9781 (+/− 20 ppm)
633.9708
[M−H]−
426.9788
741.9685
580.9643
470.1973
480.9738
473.0914
0
20
40
60
80
100
450
500
550
600
650
700
750

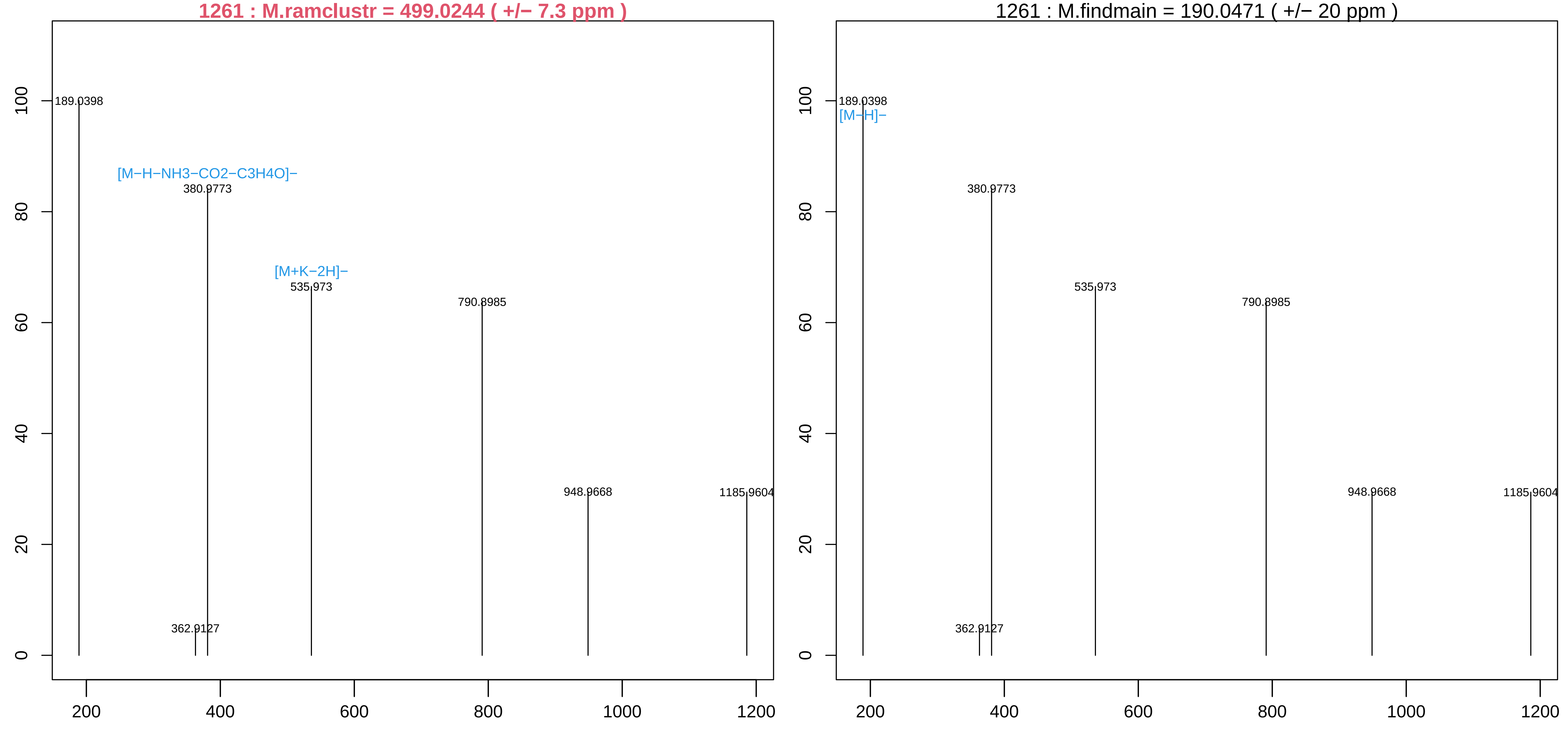
1261 : M.ramclustr = 499.0244 (+/− 7.3 ppm)
189.0398
[M−H−NH3−CO2−C3H4O]−
380.9773
[M+K−2H]−
535.973
790.8985
948.9668
1185.9604
362.9127
100
80
60
40
20
0
200
400
600
800
1000
1200
1261 : M.findmain = 190.0471 (+/− 20 ppm)
189.0398
[M−H]−
380.9773
535.973
790.8985
948.9668
1185.9604
362.9127

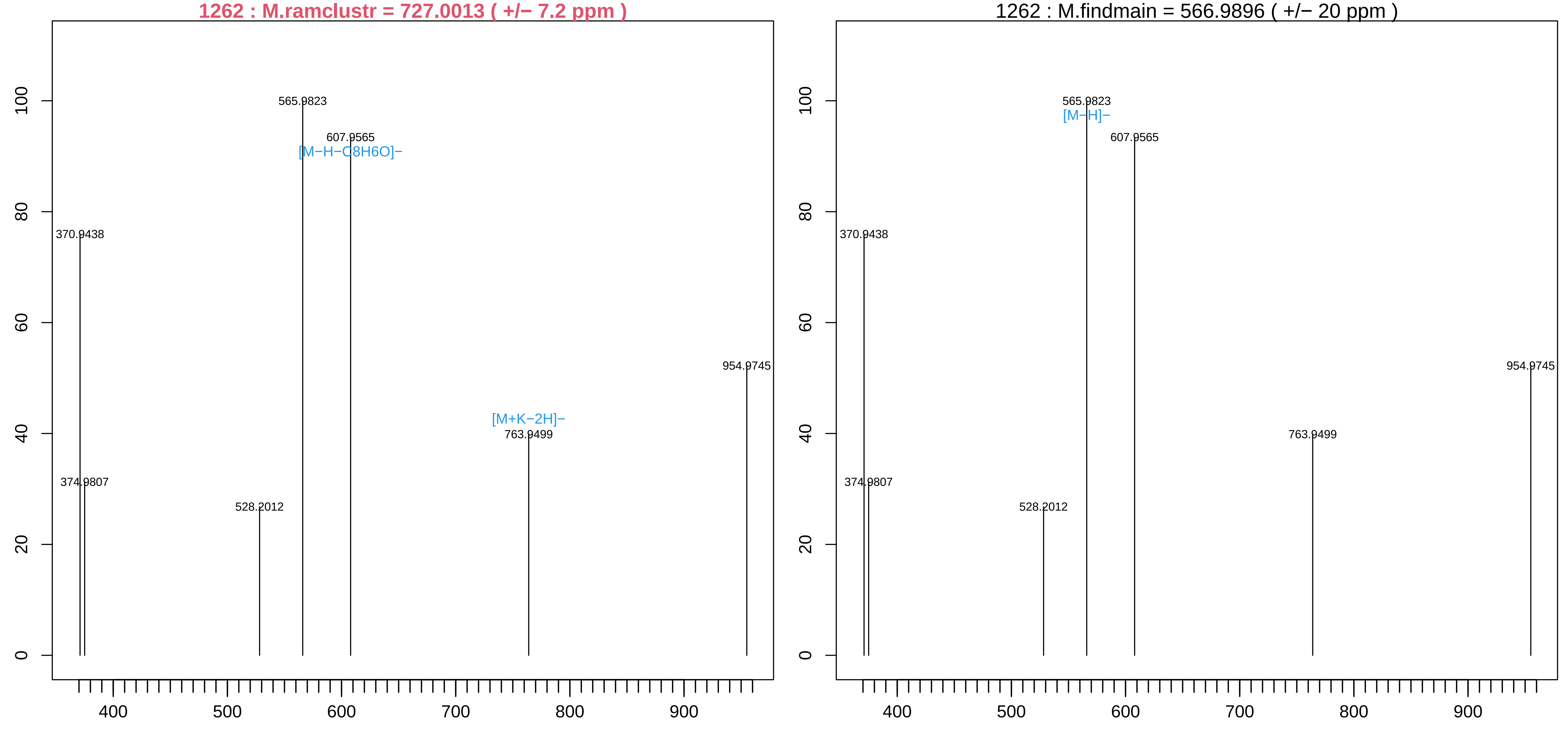
1262 : M.ramclustr = 727.0013 (+/− 7.2 ppm)
565.9823
607.9565
[M−H−C8H6O]−
370.9438
374.9807
528.2012
[M+K−2H]−
763.9499
954.9745
1262 : M.findmain = 566.9896 (+/− 20 ppm)
565.9823
[M−H]−
607.9565
370.9438
374.9807
528.2012
763.9499
954.9745
0
20
40
60
80
100
400
500
600
700
800
900

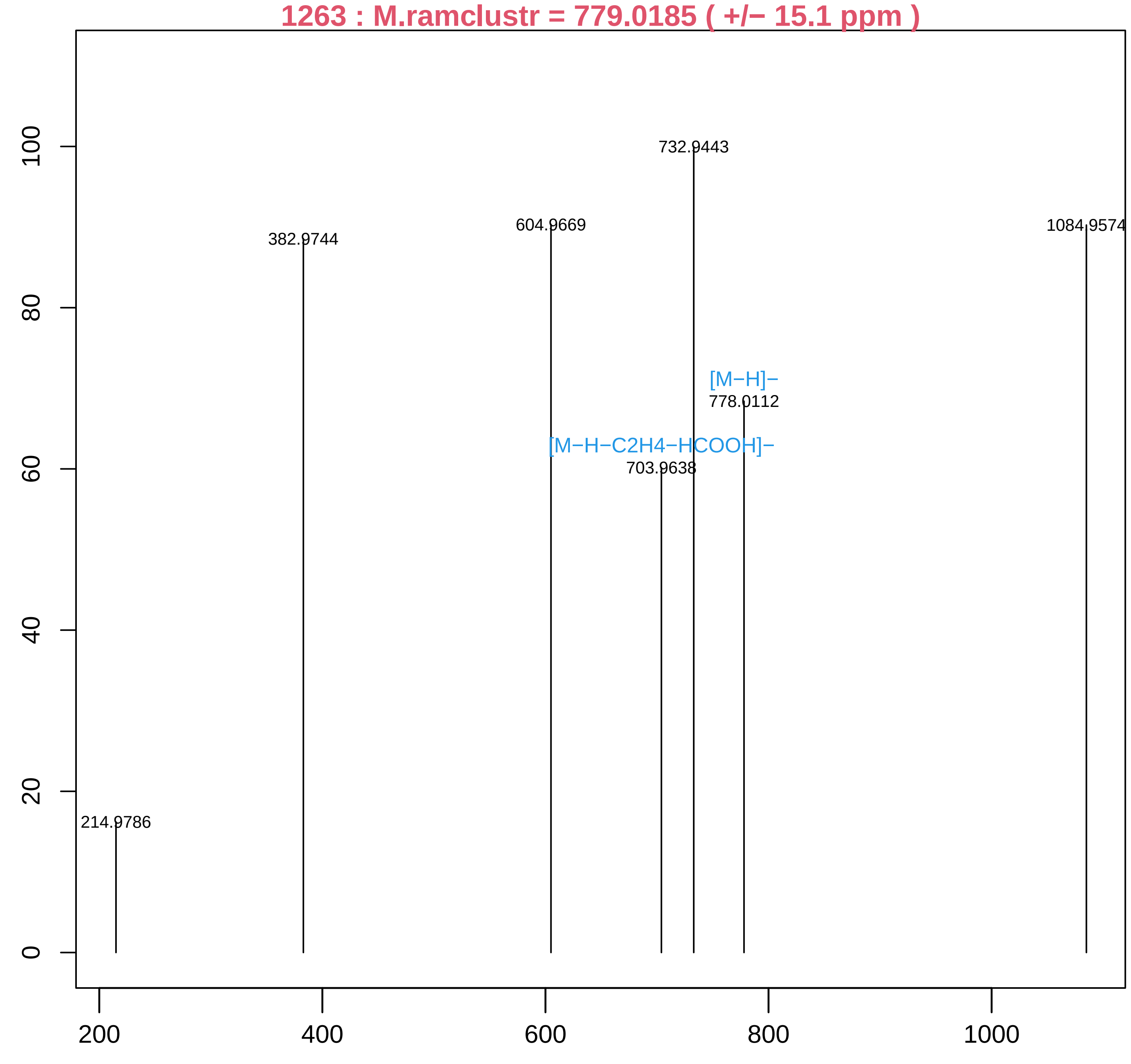
1263 : M.ramclustr = 779.0185 (+/− 15.1 ppm)
732.9443
382.9744
604.9669
1084.9574
[M−H]−
778.0112
[M−H−C2H4−HCOOH]−
703.9638
214.9786
0
20
40
60
80
100
200
400
600
800
1000

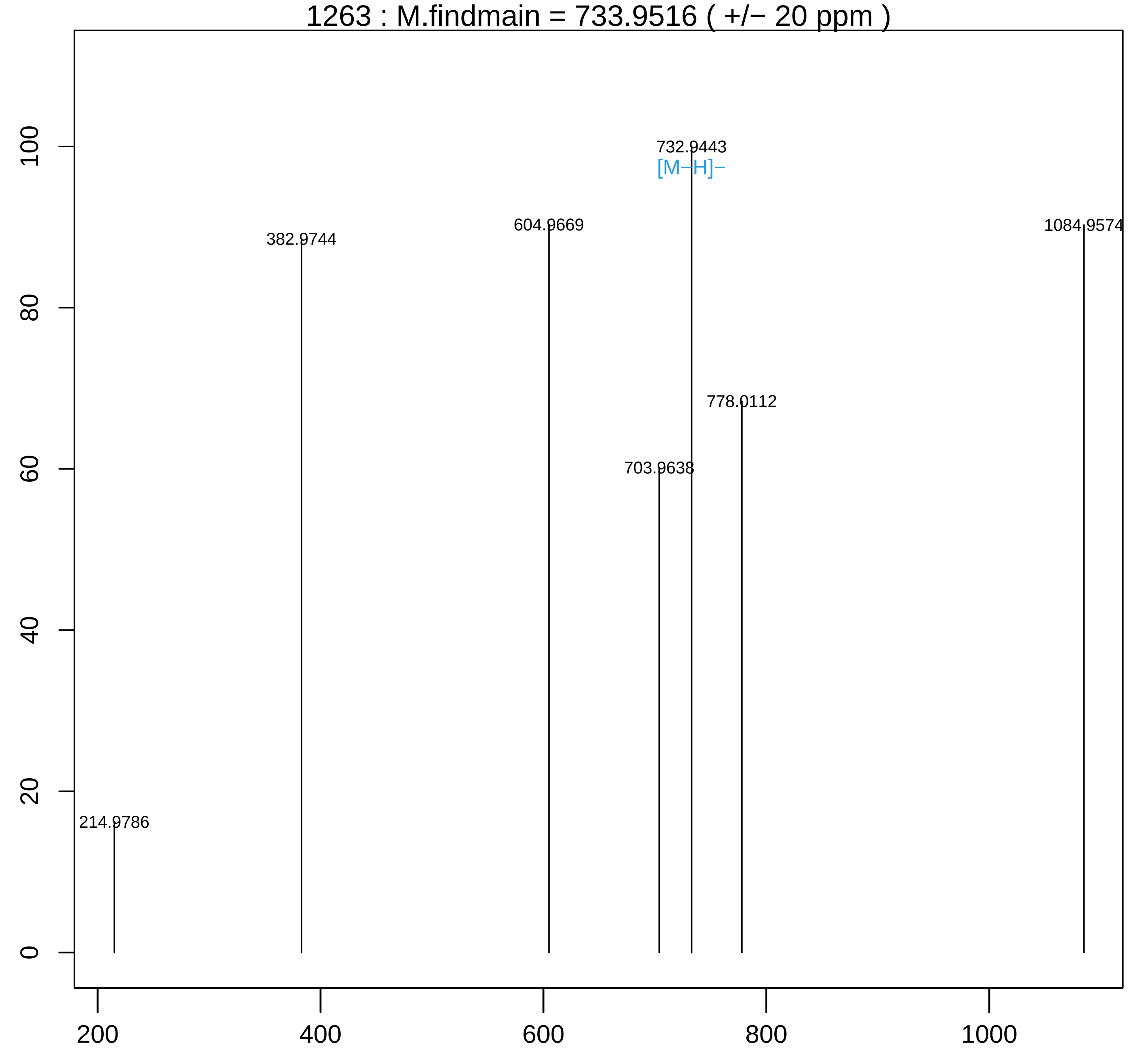
1263 : M.findmain = 733.9516 (+/− 20 ppm)
732.9443
[M−H]−
382.9744
604.9669
1084.9574
778.0112
703.9638
214.9786
0
20
40
60
80
100
200
400
600
800
1000

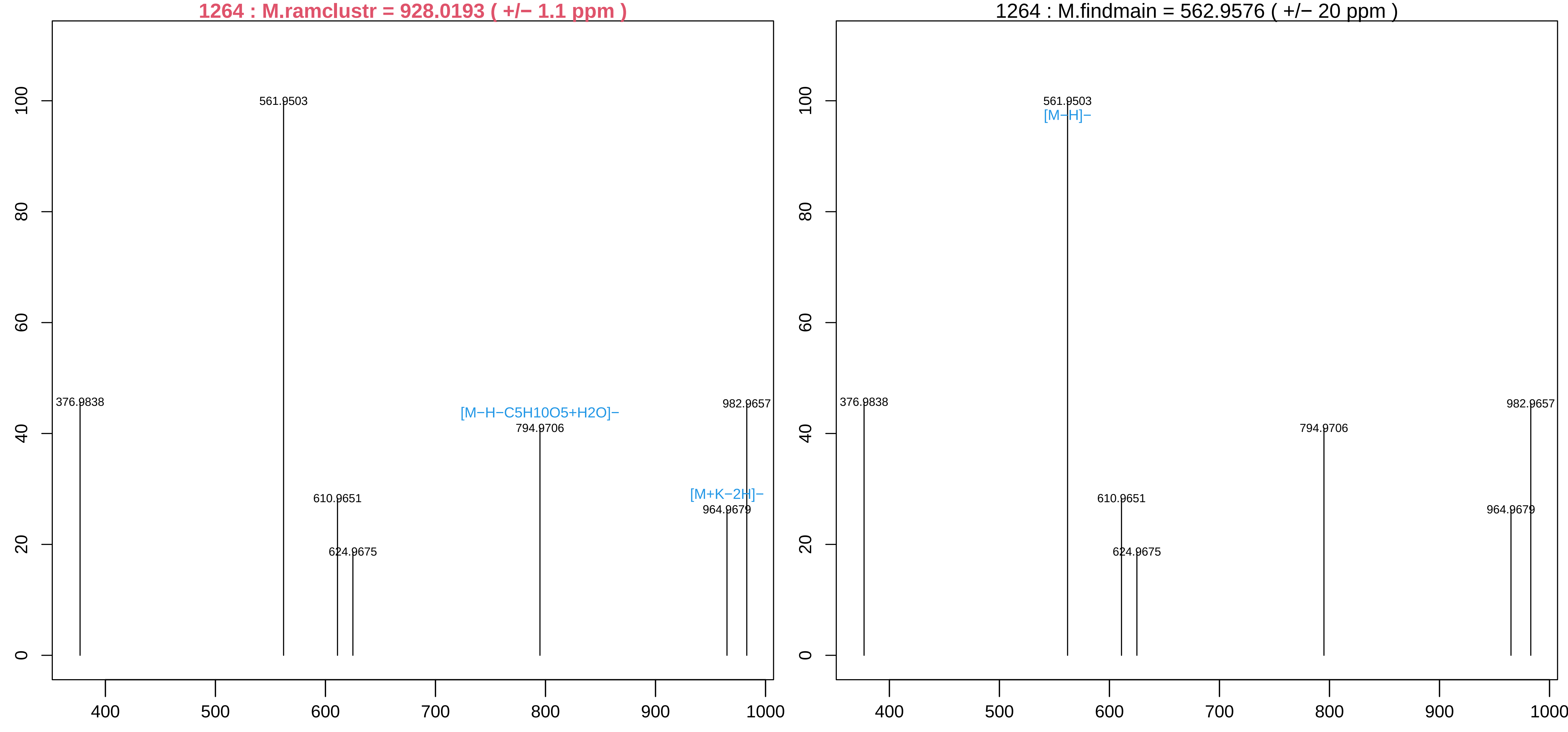
1264 : M.ramclustr = 928.0193 (+/− 1.1 ppm)
561.9503
376.9838
982.9657
[M−H−C5H10O5+H2O]−
794.9706
[M+K−2H]−
964.9679
610.9651
624.9675
1264 : M.findmain = 562.9576 (+/− 20 ppm)
561.9503
[M−H]−
376.9838
982.9657
794.9706
610.9651
964.9679
624.9675

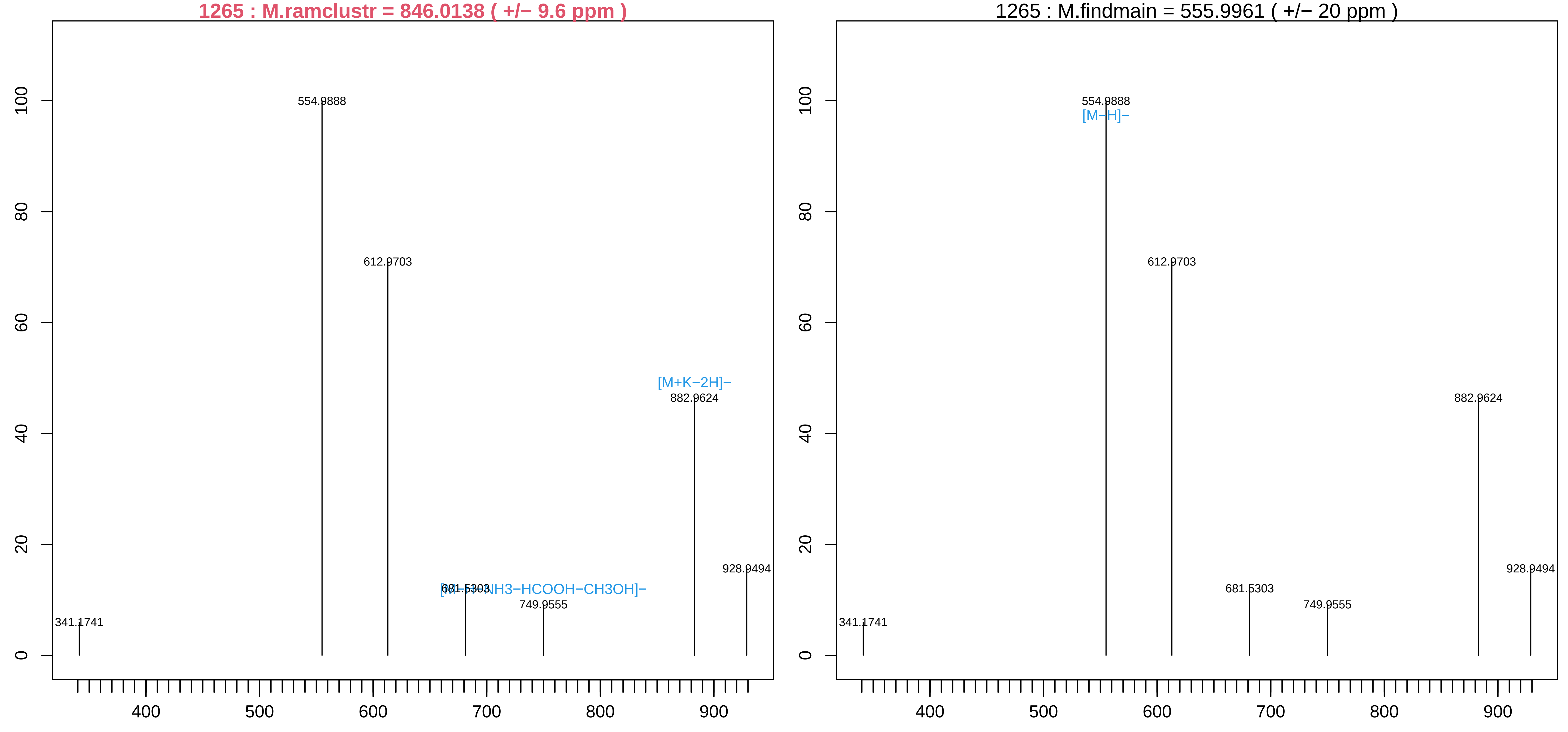

1265 : M.ramclustr = 846.0138 (+/− 9.6 ppm)
554.9888
612.9703
[M+K−2H]−
882.9624
928.9494
681.5303
NH3−HCOOH−CH3OH]−
749.9555
341.1741
0
20
40
60
80
100
400
500
600
700
800
900
1265 : M.findmain = 555.9961 (+/− 20 ppm)
554.9888
[M−H]−
612.9703
882.9624
928.9494
681.5303
749.9555
341.1741
0
20
40
60
80
100
400
500
600
700
800
900

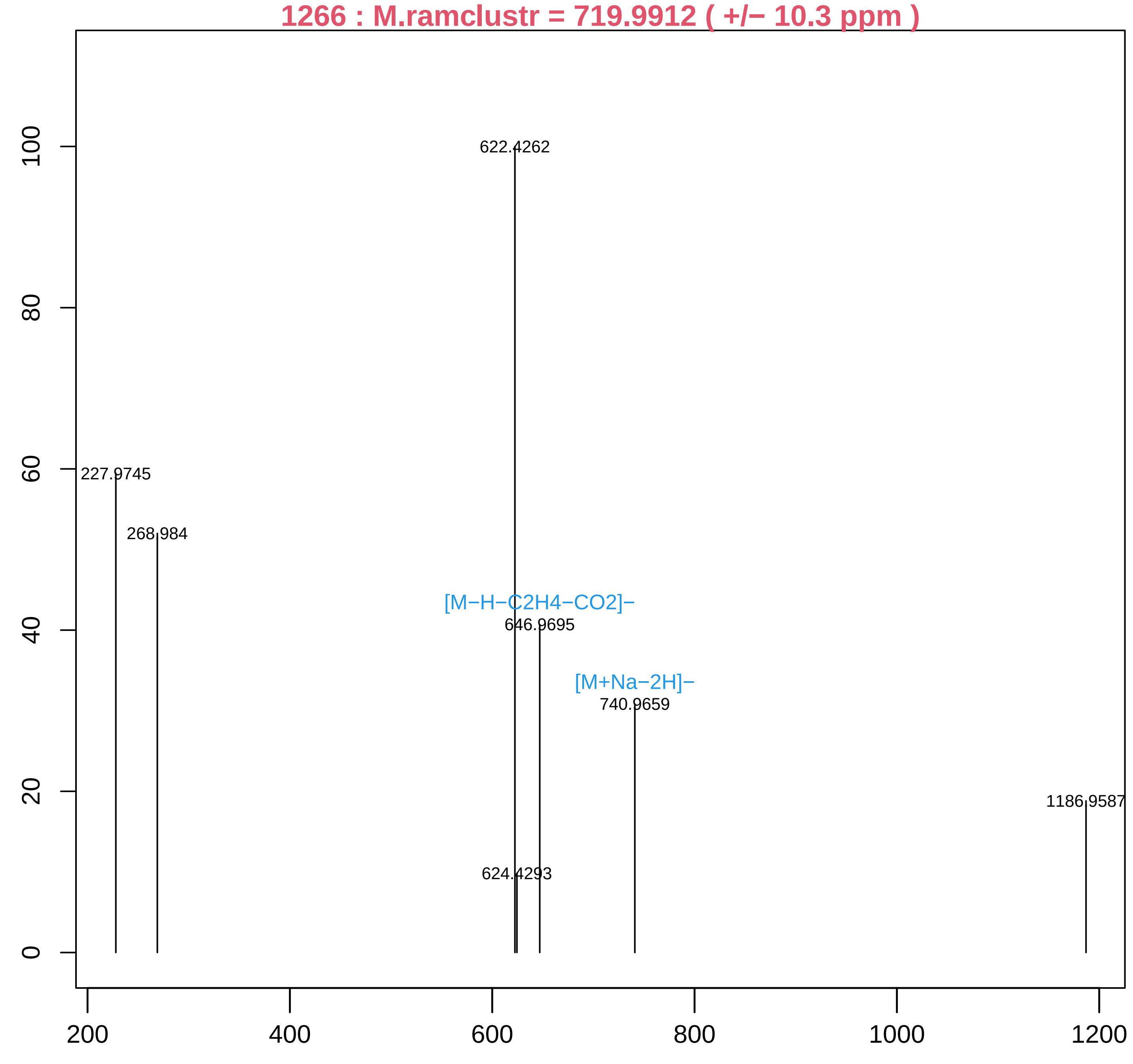

1266 : M.ramclustr = 719.9912 (+/− 10.3 ppm)
622.4262
227.9745
268.984
[M−H−C2H4−CO2]−
646.9695
[M+Na−2H]−
740.9659
1186.9587
624.4293
0
20
40
60
80
100
200
400
600
800
1000
1200

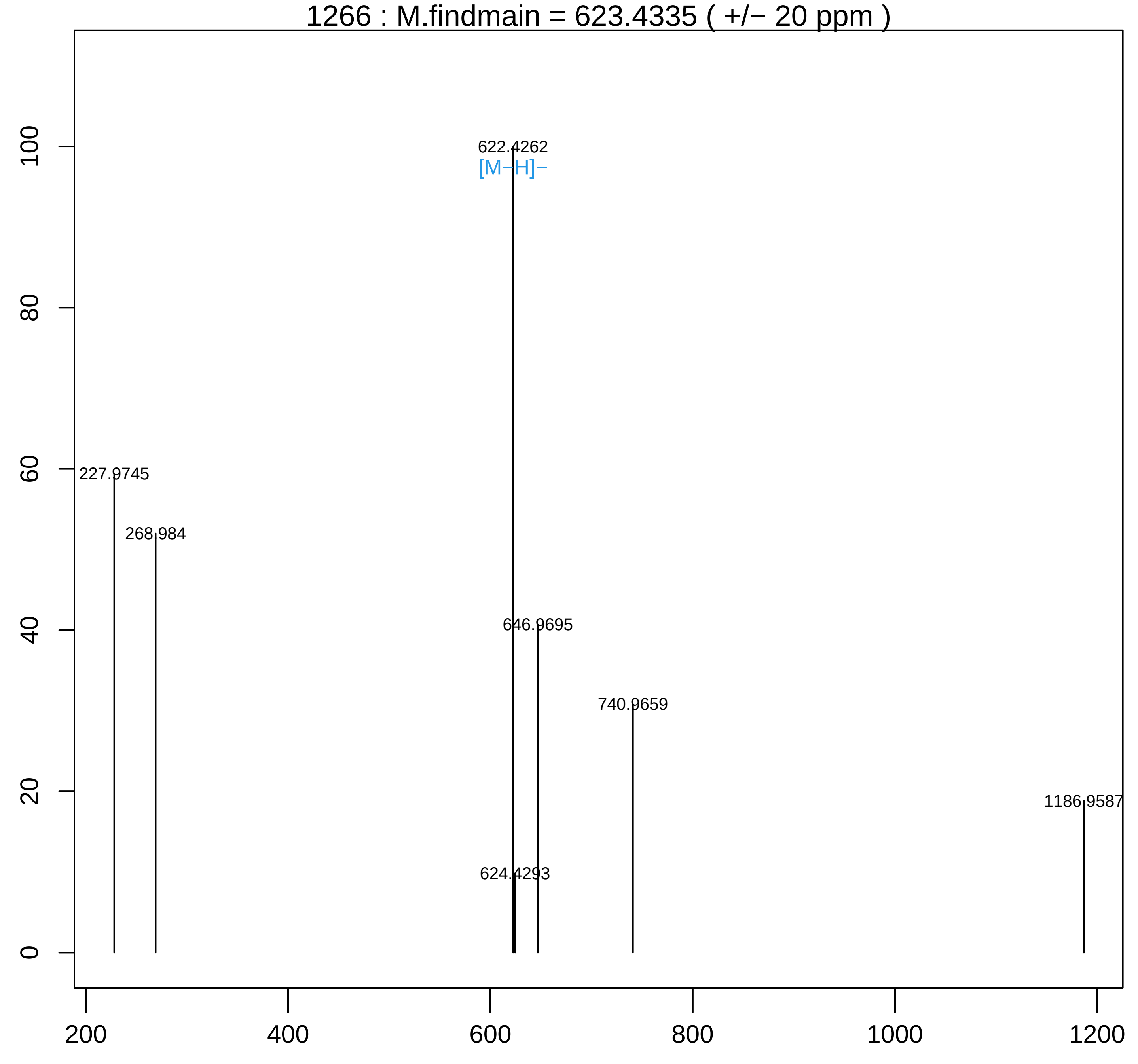

1266 : M.findmain = 623.4335 (+/− 20 ppm)
622.4262
[M−H]−
227.9745
268.984
646.9695
740.9659
1186.9587
624.4293
0
20
40
60
80
100
200
400
600
800
1000
1200

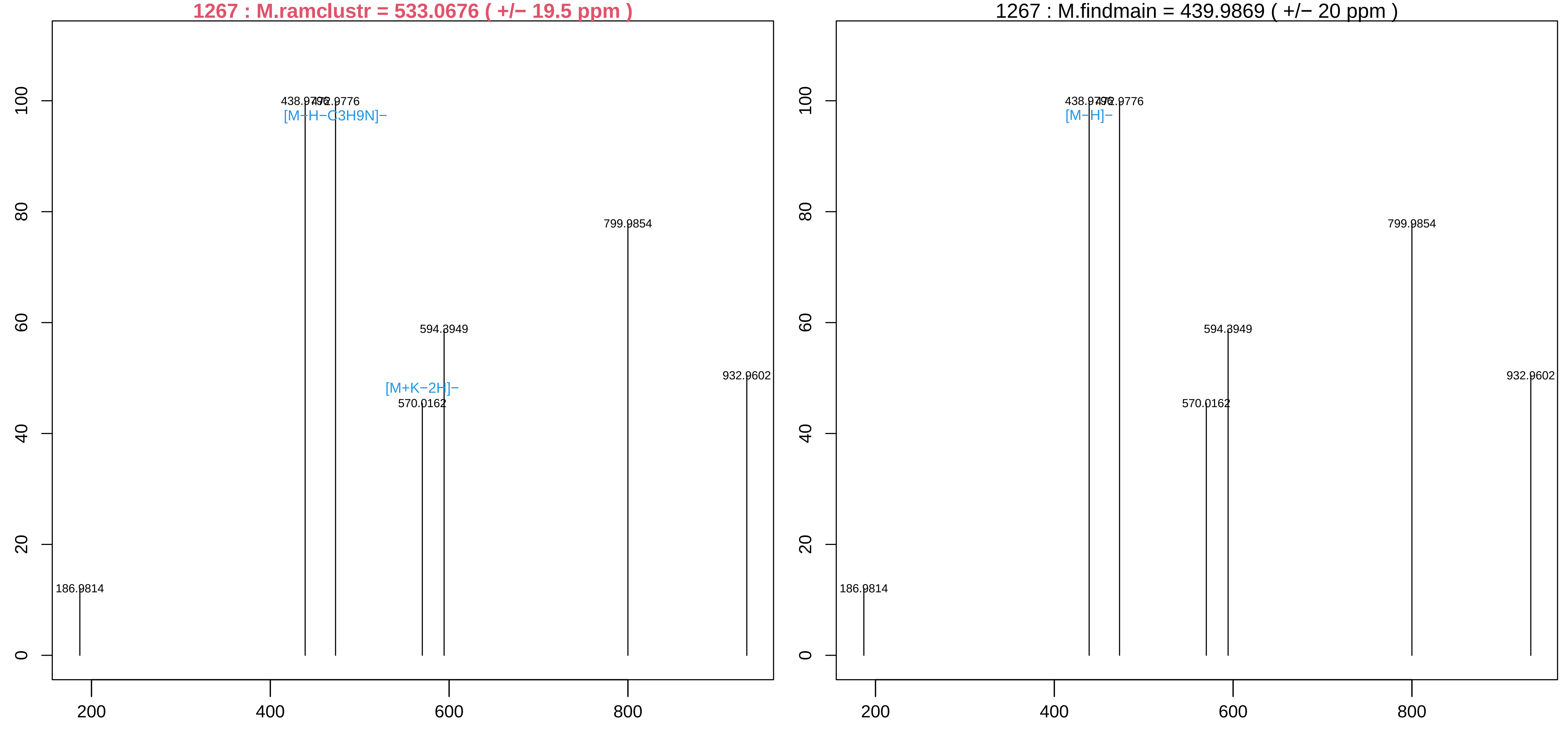

1267 : M.ramclustr = 533.0676 (+/− 19.5 ppm)
438.9796
472.9776
[M−H−C3H9N]−
799.9854
594.3949
[M+K−2H]−
570.0162
932.9602
186.9814
0
20
40
60
80
100
200
400
600
800
1267 : M.findmain = 439.9869 (+/− 20 ppm)
438.9796
472.9776
[M−H]−
799.9854
594.3949
570.0162
932.9602
186.9814

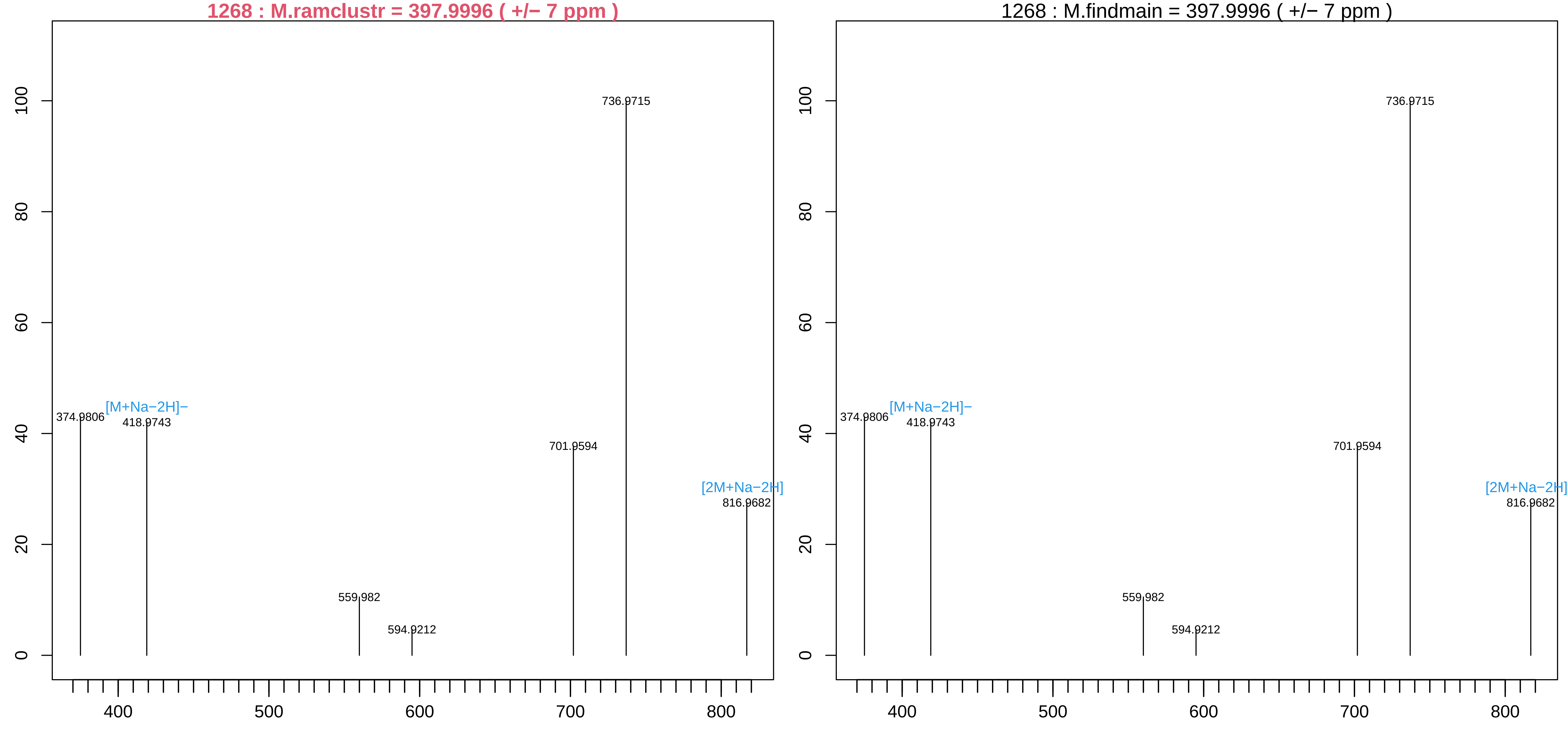
1268 : M.ramclustr = 397.9996 (+/− 7 ppm)
1268 : M.findmain = 397.9996 (+/− 7 ppm)
736.9715
[M+Na−2H]−
374.9806
418.9743
701.9594
[2M+Na−2H]
816.9682
559.982
594.9212
0
20
40
60
80
100
400
500
600
700
800

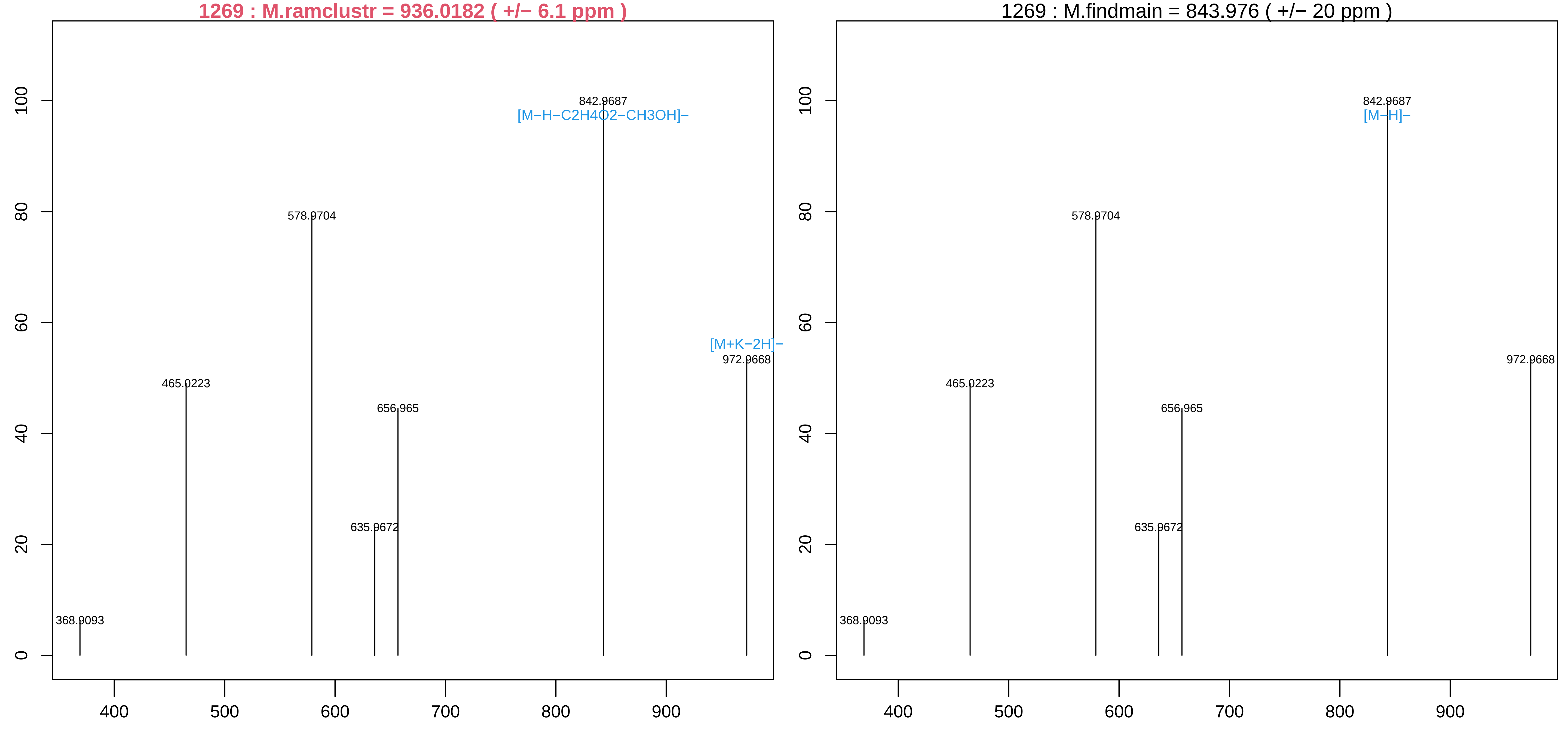
1269 : M.ramclustr = 936.0182 (+/− 6.1 ppm)
1269 : M.findmain = 843.976 (+/− 20 ppm)
842.9687
[M−H−C2H4O2−CH3OH]−
[M−H]−
578.9704
[M+K−2H]−
972.9668
465.0223
656.965
635.9672
368.9093
0
20
40
60
80
100
400
500
600
700
800
900

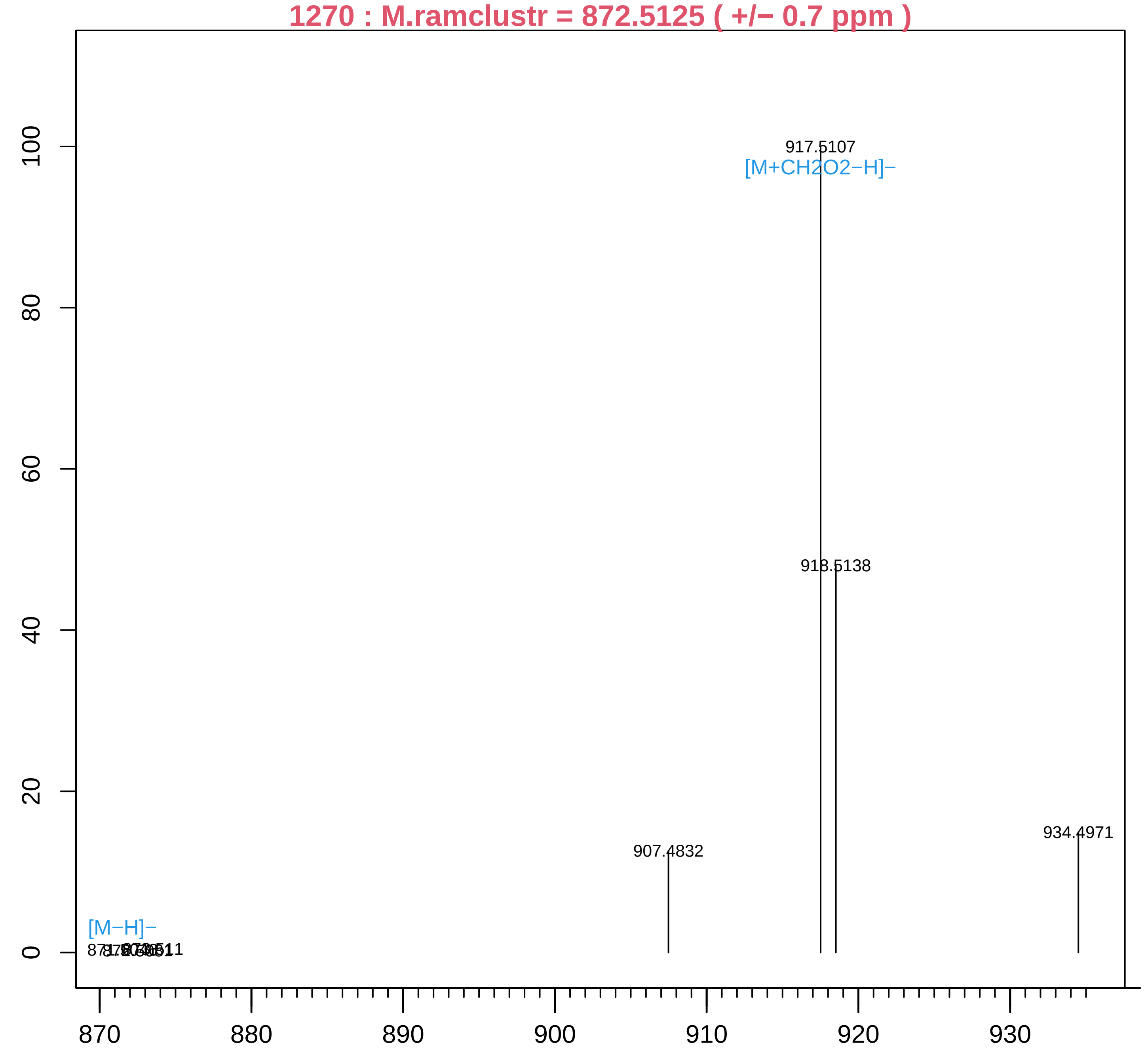
1270 : M.ramclustr = 872.5125 (+/− 0.7 ppm)
917.5107
[M+CH2O2−H]−
918.5138
907.4832
934.4971
[M−H]−
0
20
40
60
80
100
870
880
890
900
910
920
930

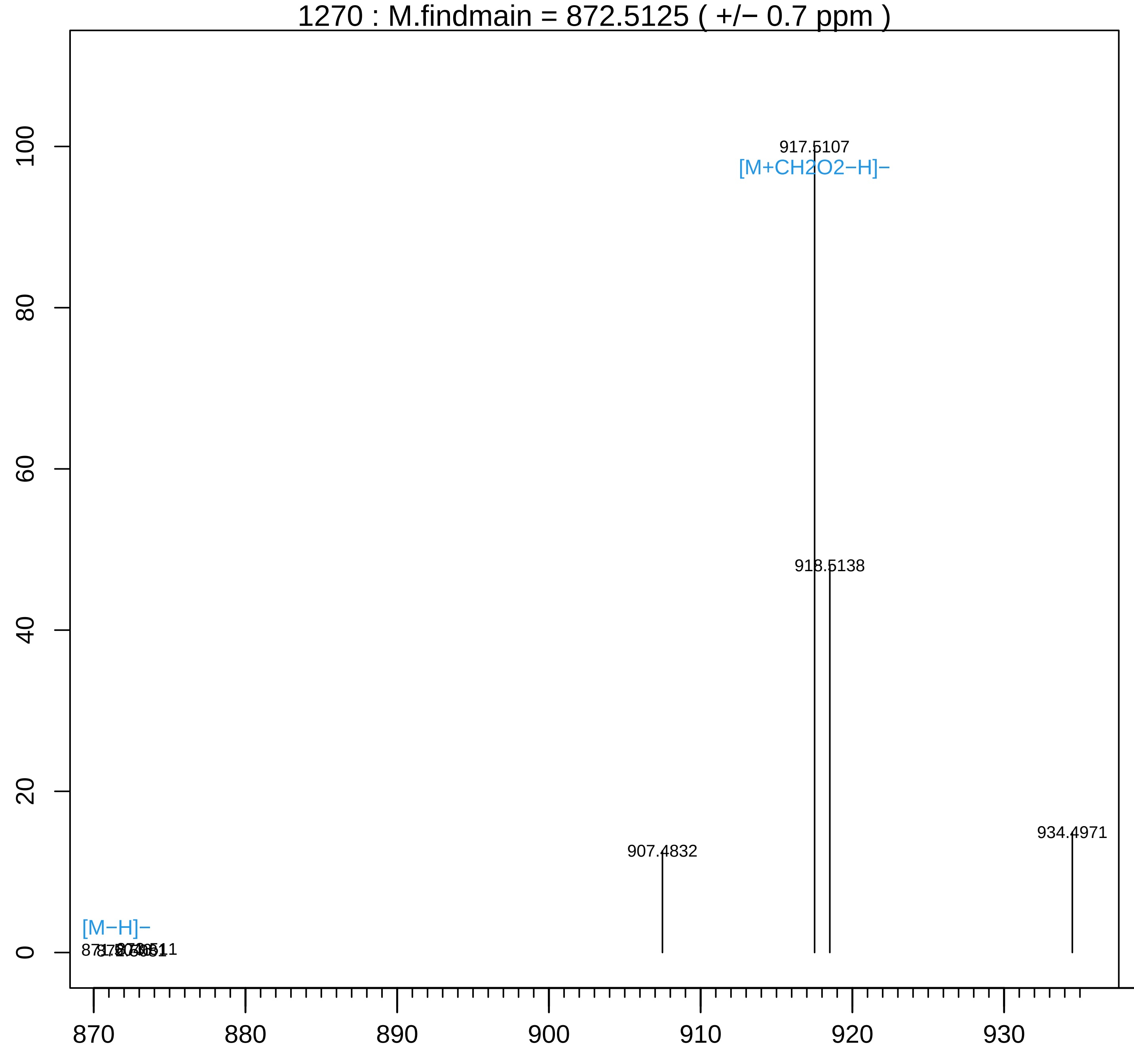
1270 : M.findmain = 872.5125 (+/− 0.7 ppm)
917.5107
[M+CH2O2−H]−
918.5138
907.4832
934.4971
[M−H]−
0
20
40
60
80
100
870
880
890
900
910
920
930

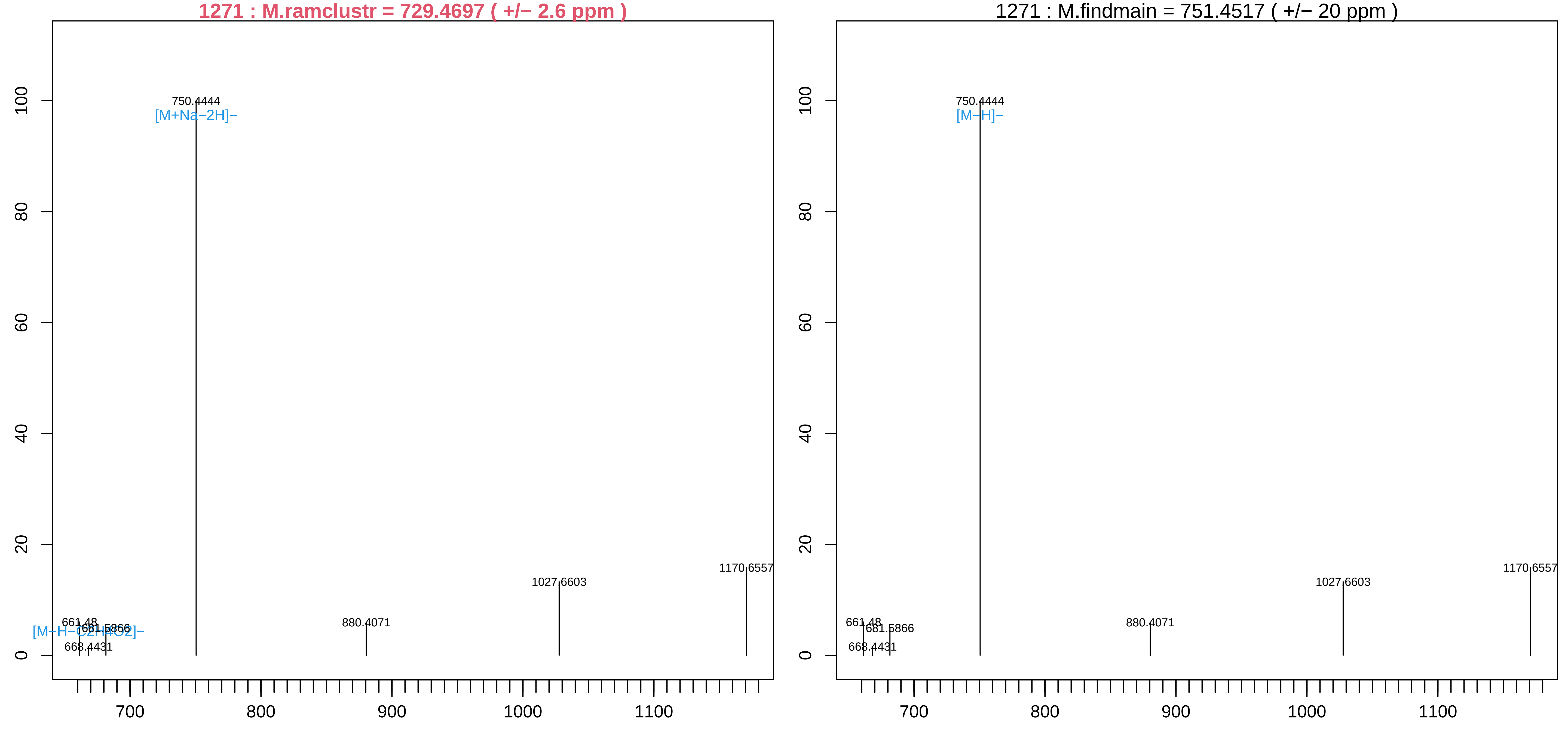

1271 : M.ramclustr = 729.4697 (+/− 2.6 ppm)
750.4444
[M+Na−2H]−
661.48
681.5866
[M−H−C2H4O2]−
668.4431
880.4071
1027.6603
1170.6557
1271 : M.findmain = 751.4517 (+/− 20 ppm)
750.4444
[M−H]−
661.48
681.5866
668.4431
880.4071
1027.6603
1170.6557
0
20
40
60
80
100
700
800
900
1000
1100

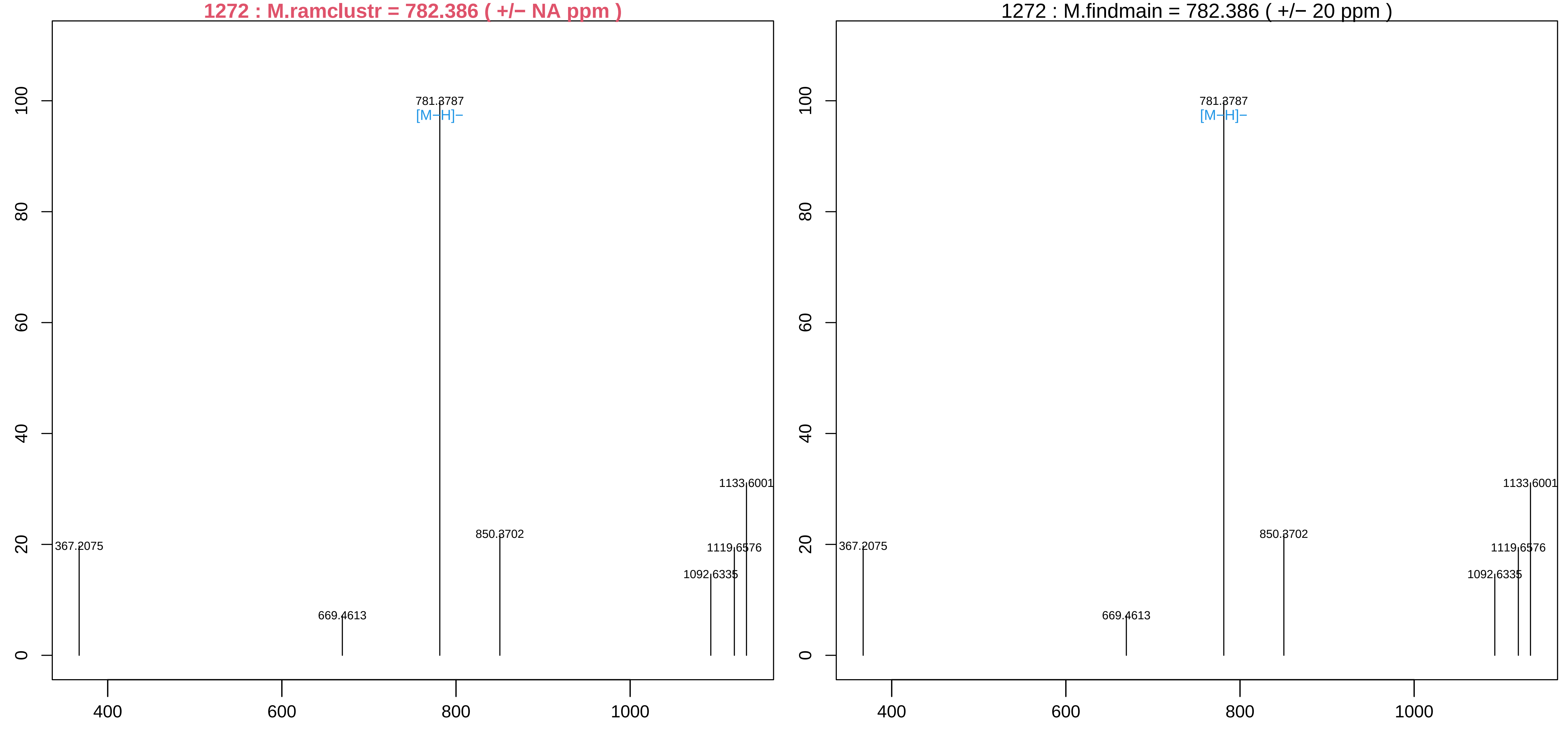

1272 : M.ramclustr = 782.386 (+/− NA ppm)
1272 : M.findmain = 782.386 (+/− 20 ppm)
781.3787
[M−H]−
367.2075
669.4613
850.3702
1092.6335
1119.6576
1133.6001
0
20
40
60
80
100
400
600
800
1000

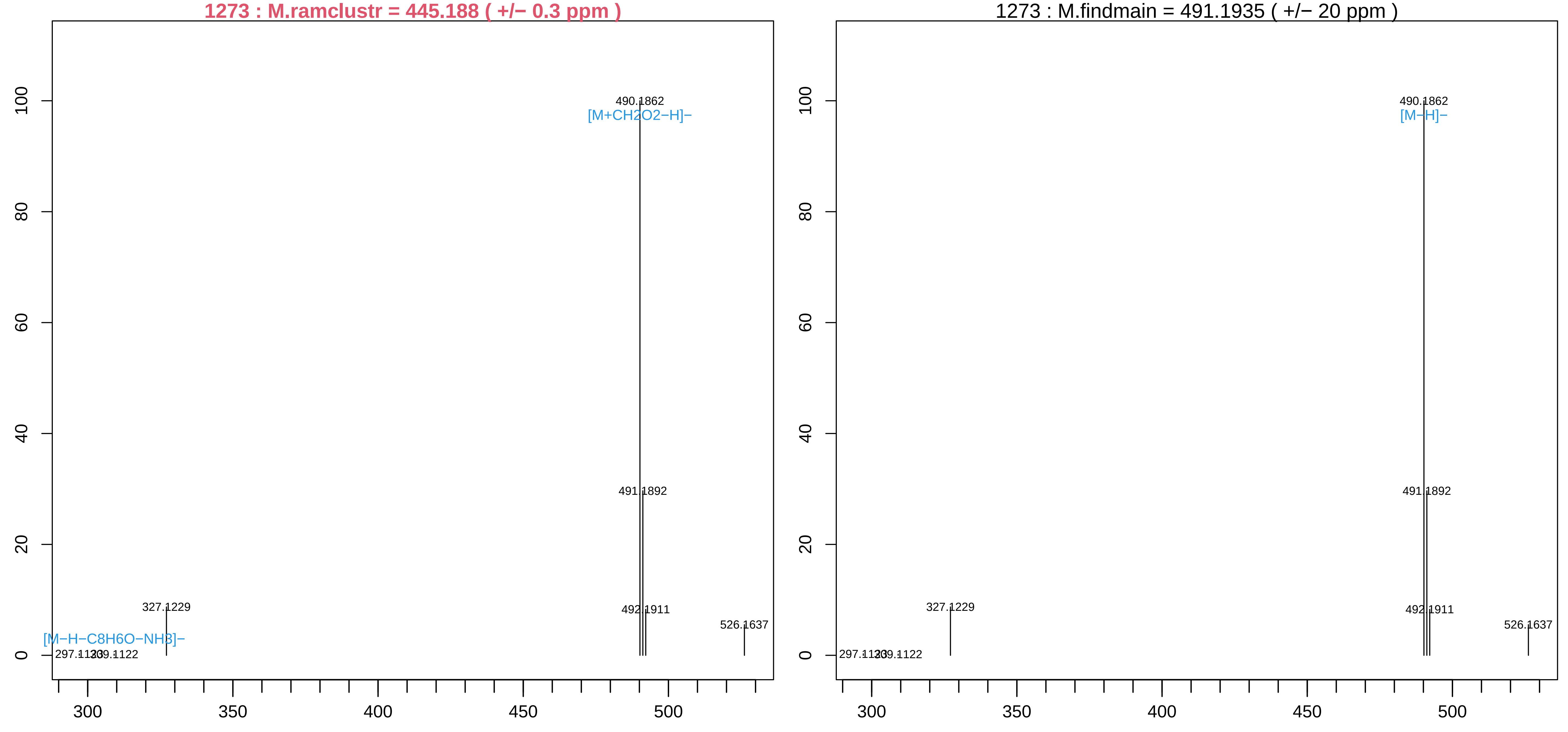

1273 : M.ramclustr = 445.188 (+/− 0.3 ppm)
490.1862
[M+CH2O2−H]−
491.1892
492.1911
526.1637
327.1229
[M−H−C8H6O−NH3]−
297.1123
299.1122
1273 : M.findmain = 491.1935 (+/− 20 ppm)
490.1862
[M−H]−
491.1892
492.1911
526.1637
327.1229
297.1123
299.1122

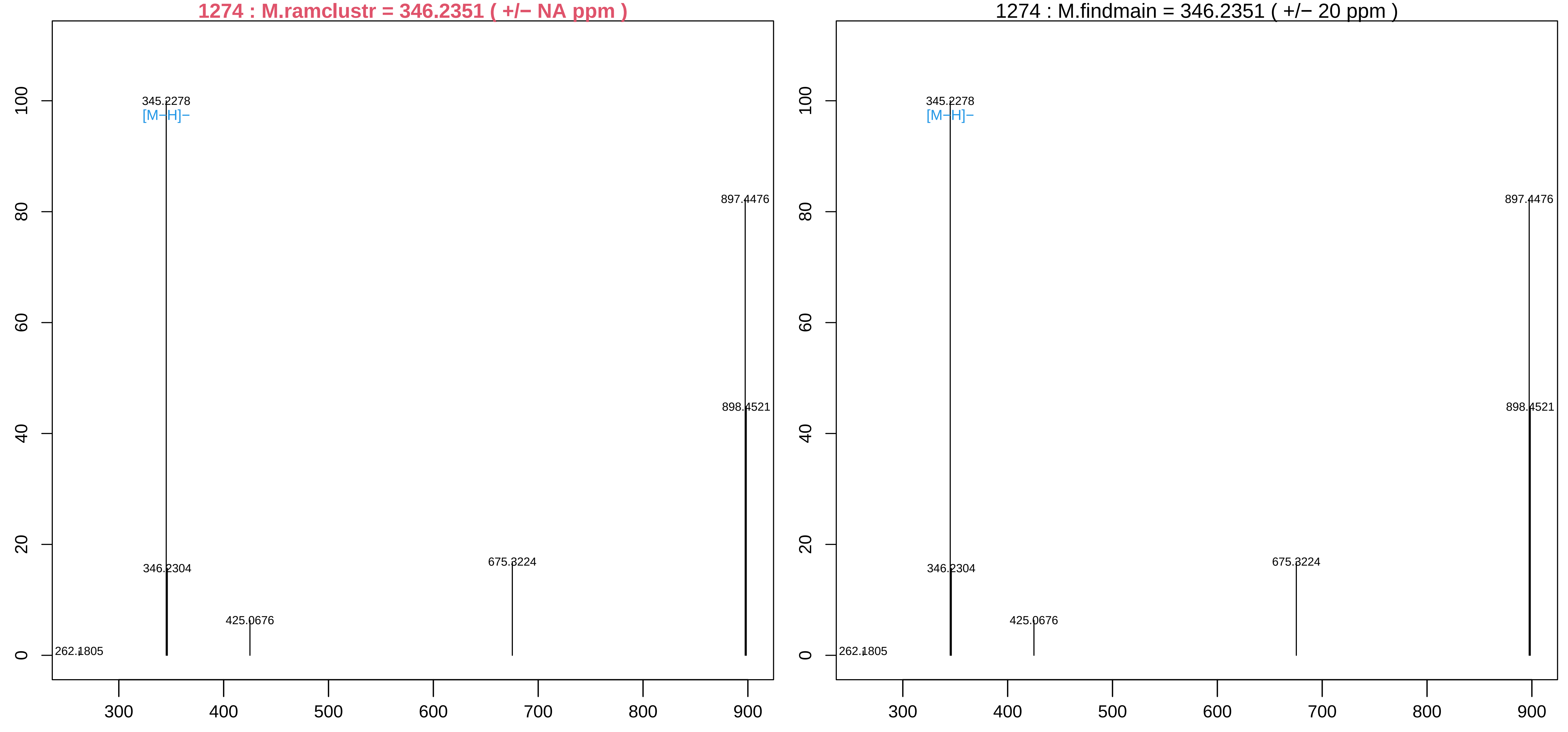

1274 : M.ramclustr = 346.2351 (+/− NA ppm)
345.2278
[M−H]−
897.4476
898.4521
675.3224
346.2304
425.0676
262.1805
0
20
40
60
80
100
300
400
500
600
700
800
900
1274 : M.findmain = 346.2351 (+/− 20 ppm)
345.2278
[M−H]−
897.4476
898.4521
675.3224
346.2304
425.0676
262.1805
0
20
40
60
80
100
300
400
500
600
700
800
900

1275 : M.ramclustr = 216.1354 (+/− NA ppm)

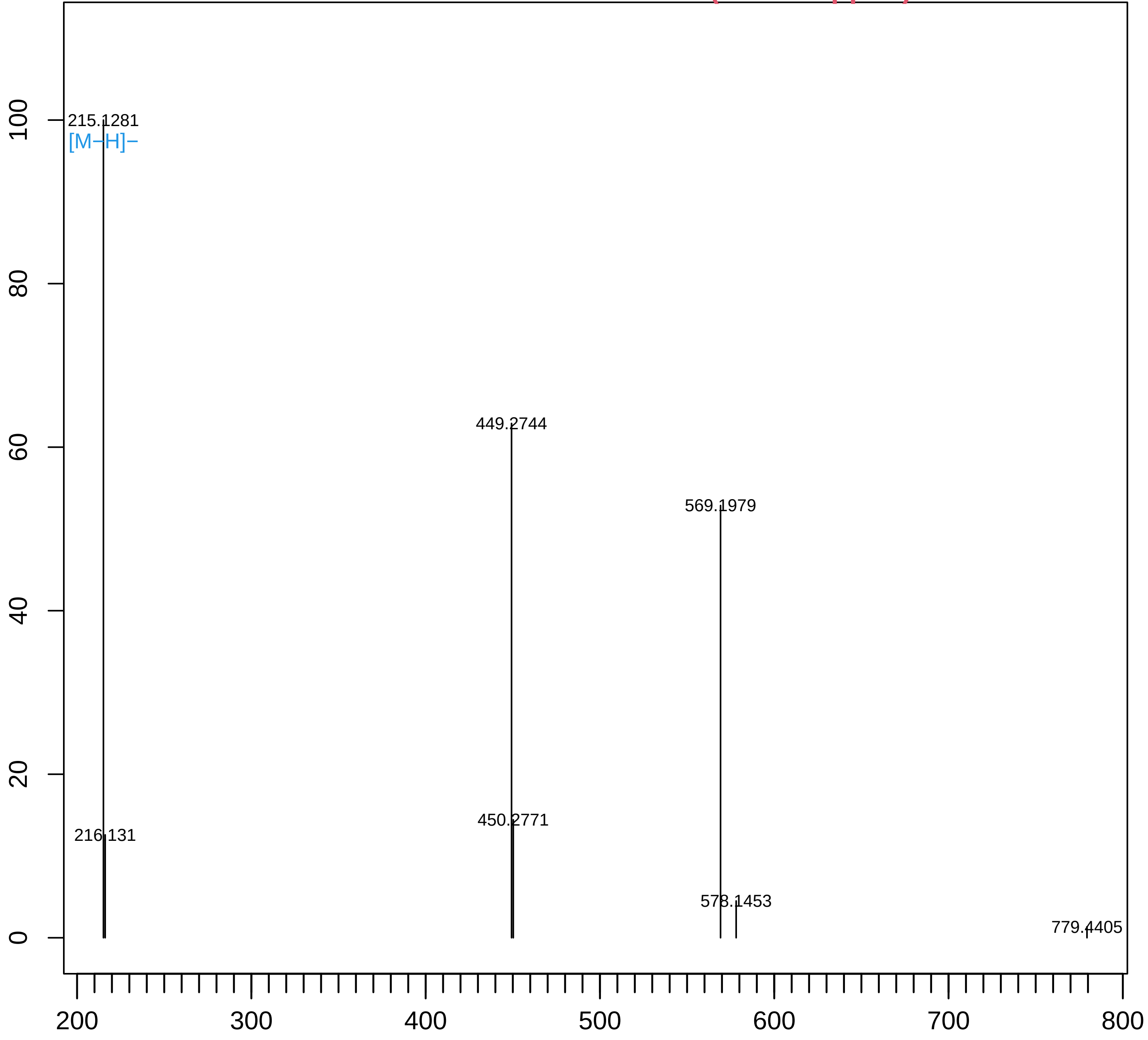

1275 : M.findmain = 216.1354 (+/− 20 ppm)

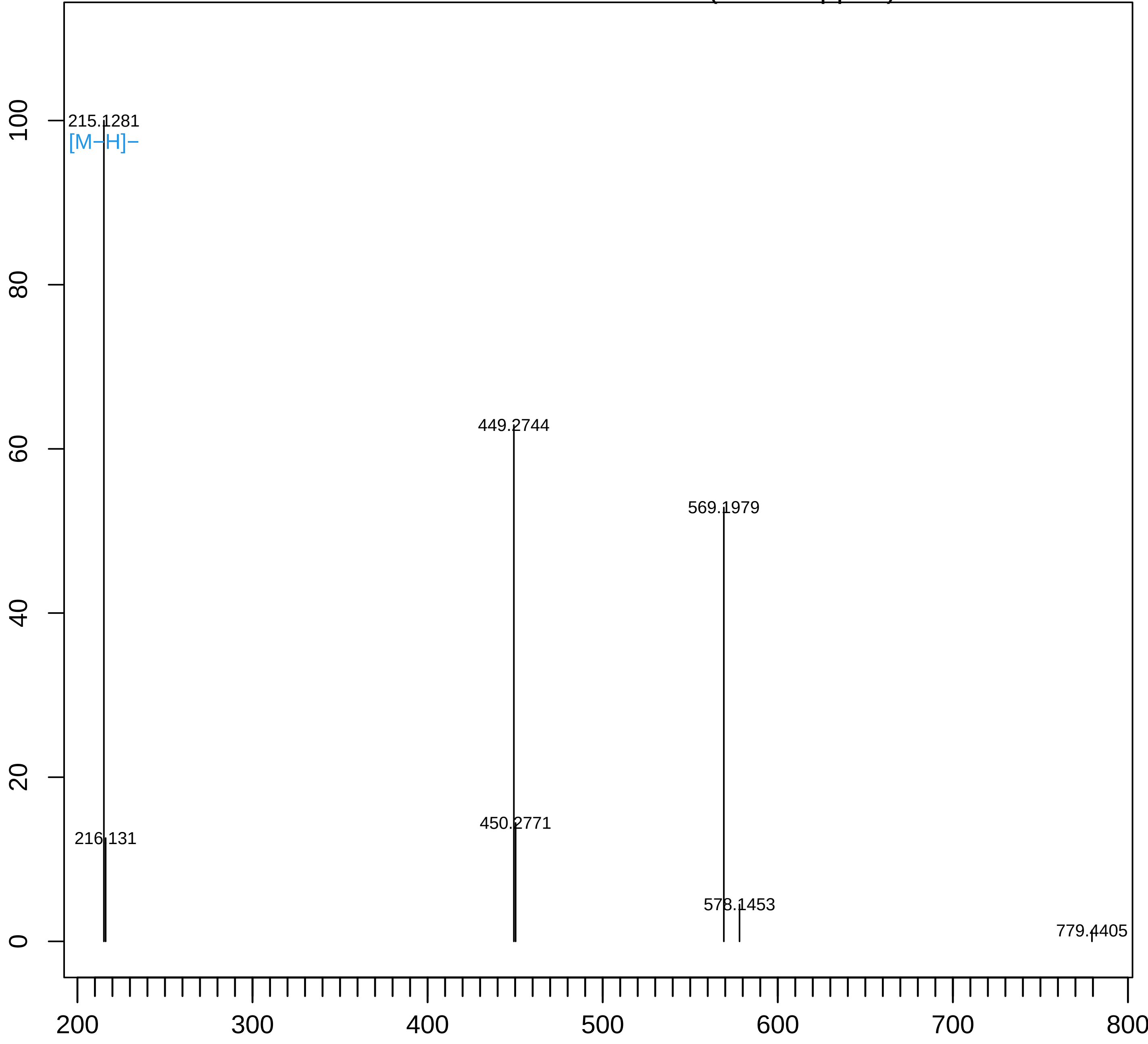

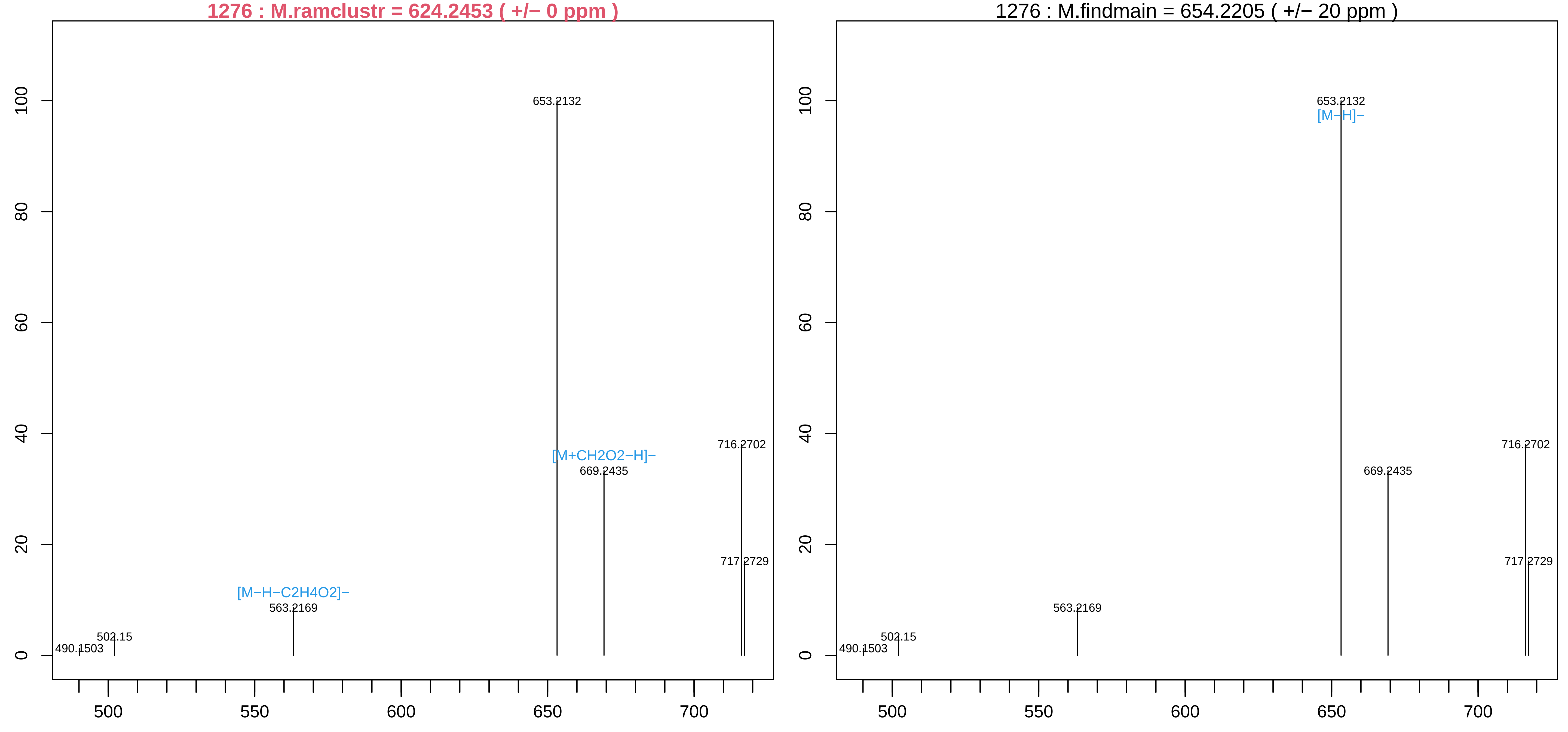

1276 : M.ramclustr = 624.2453 (+/− 0 ppm)
653.2132
[M+CH2O2−H]−
669.2435
716.2702
717.2729
[M−H−C2H4O2]−
563.2169
502.15
490.1503
1276 : M.findmain = 654.2205 (+/− 20 ppm)
653.2132
[M−H]−
669.2435
716.2702
717.2729
563.2169
502.15
490.1503
0
20
40
60
80
100
500
550
600
650
700

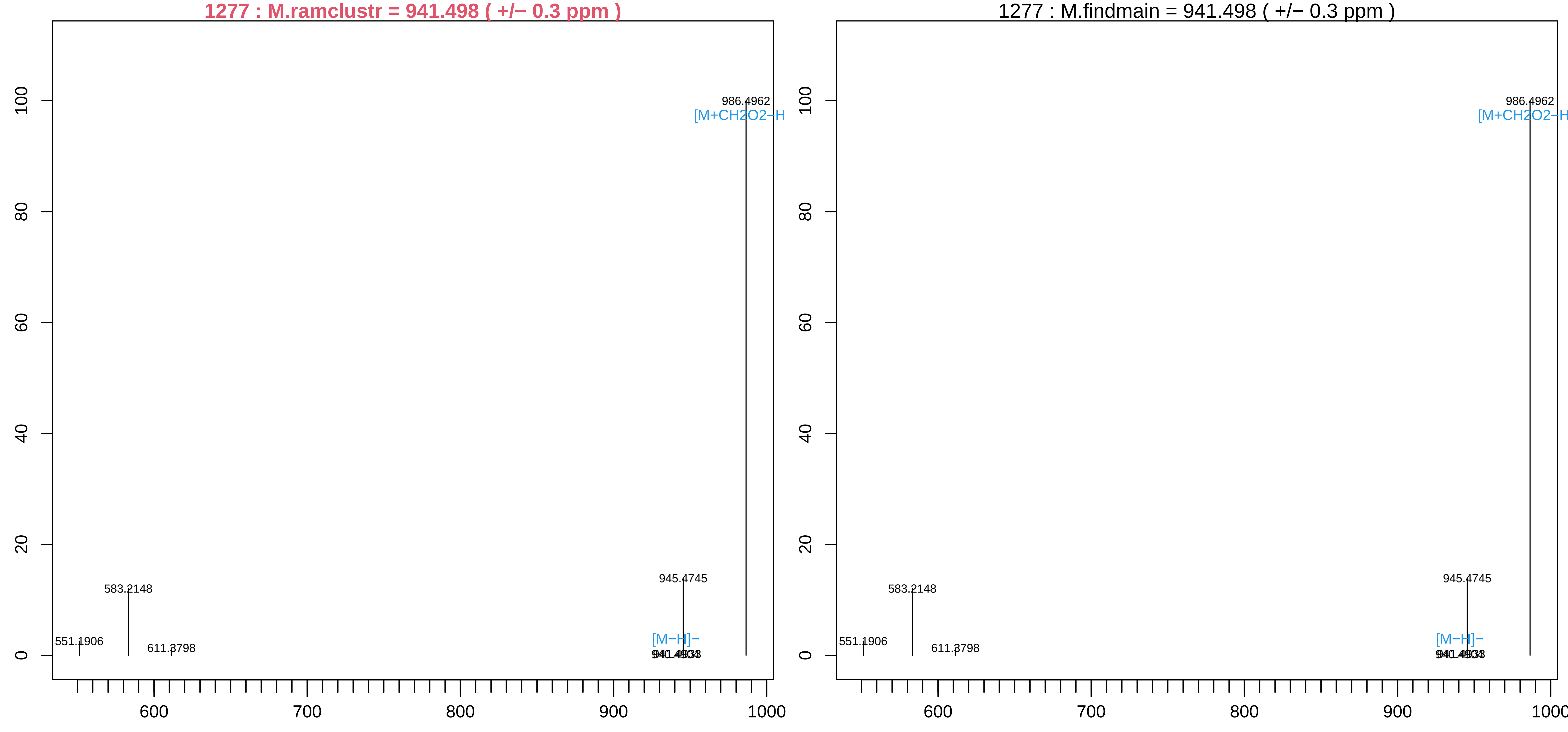
1277 : M.ramclustr = 941.498 (+/− 0.3 ppm)
986.4962
[M+CH2O2−H
945.4745
583.2148
551.1906
611.3798
[M−H]−
940.4933
1277 : M.findmain = 941.498 (+/− 0.3 ppm)
986.4962
[M+CH2O2−H
945.4745
583.2148
551.1906
611.3798
[M−H]−
940.4933
0
20
40
60
80
100
600
700
800
900
1000

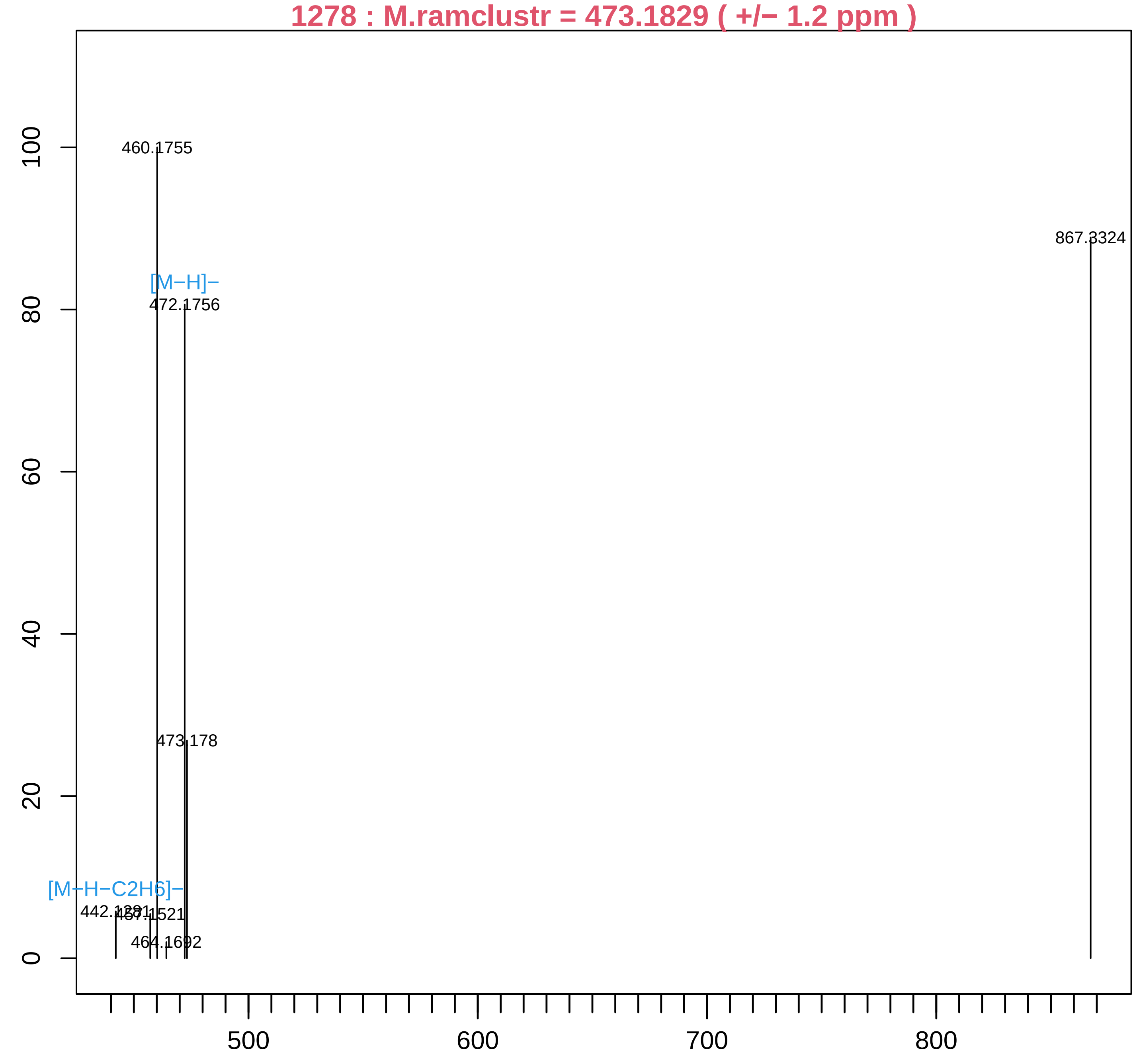
1278 : M.ramclustr = 473.1829 (+/− 1.2 ppm)
460.1755
867.3324
[M−H]−
472.1756
473.178
[M−H−C2H6]−
442.1291
457.1521
464.1692
0
20
40
60
80
100
500
600
700
800

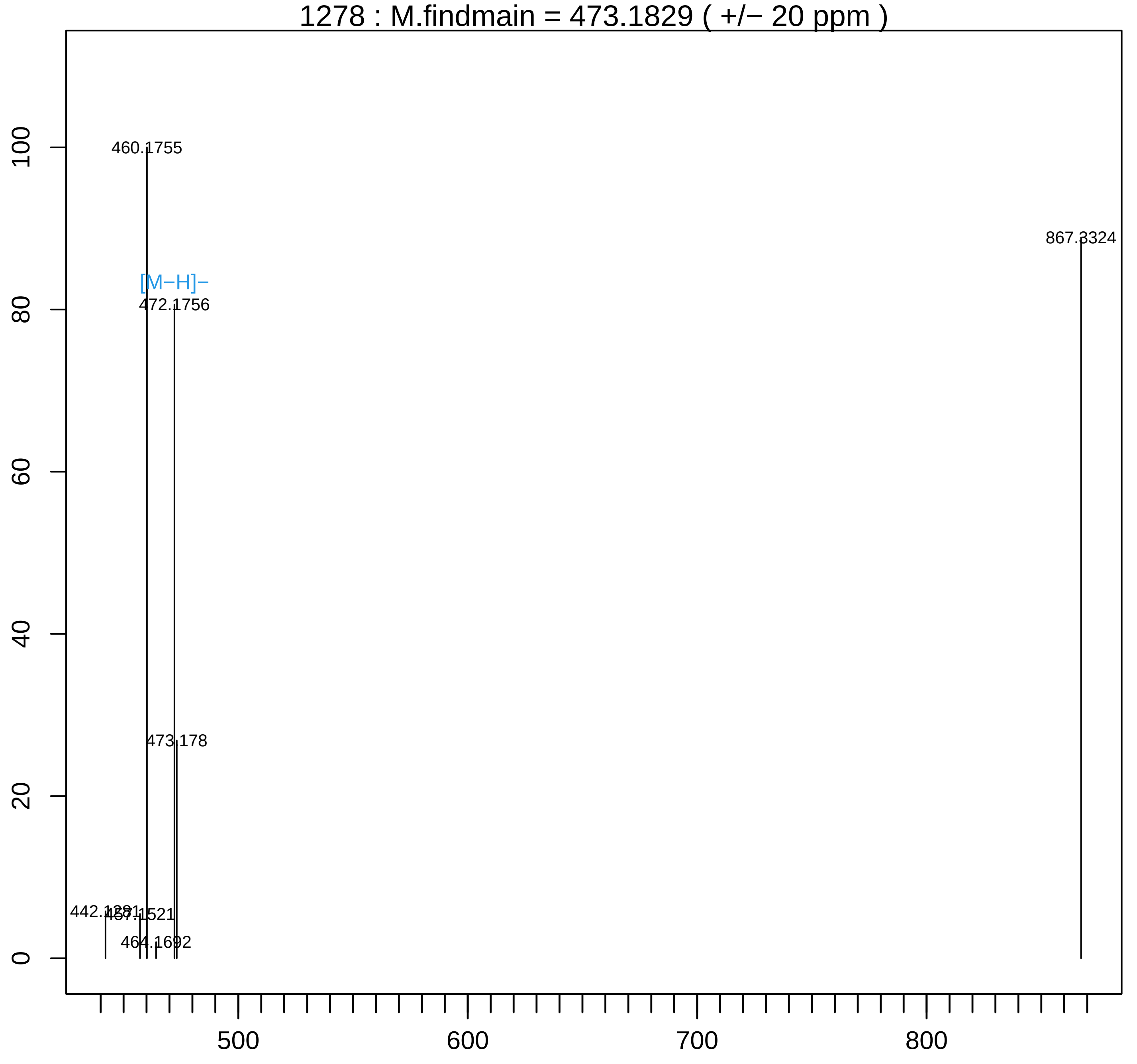
1278 : M.findmain = 473.1829 (+/− 20 ppm)
460.1755
867.3324
[M−H]−
472.1756
473.178
442.1291
457.1521
464.1692
0
20
40
60
80
100
500
600
700
800

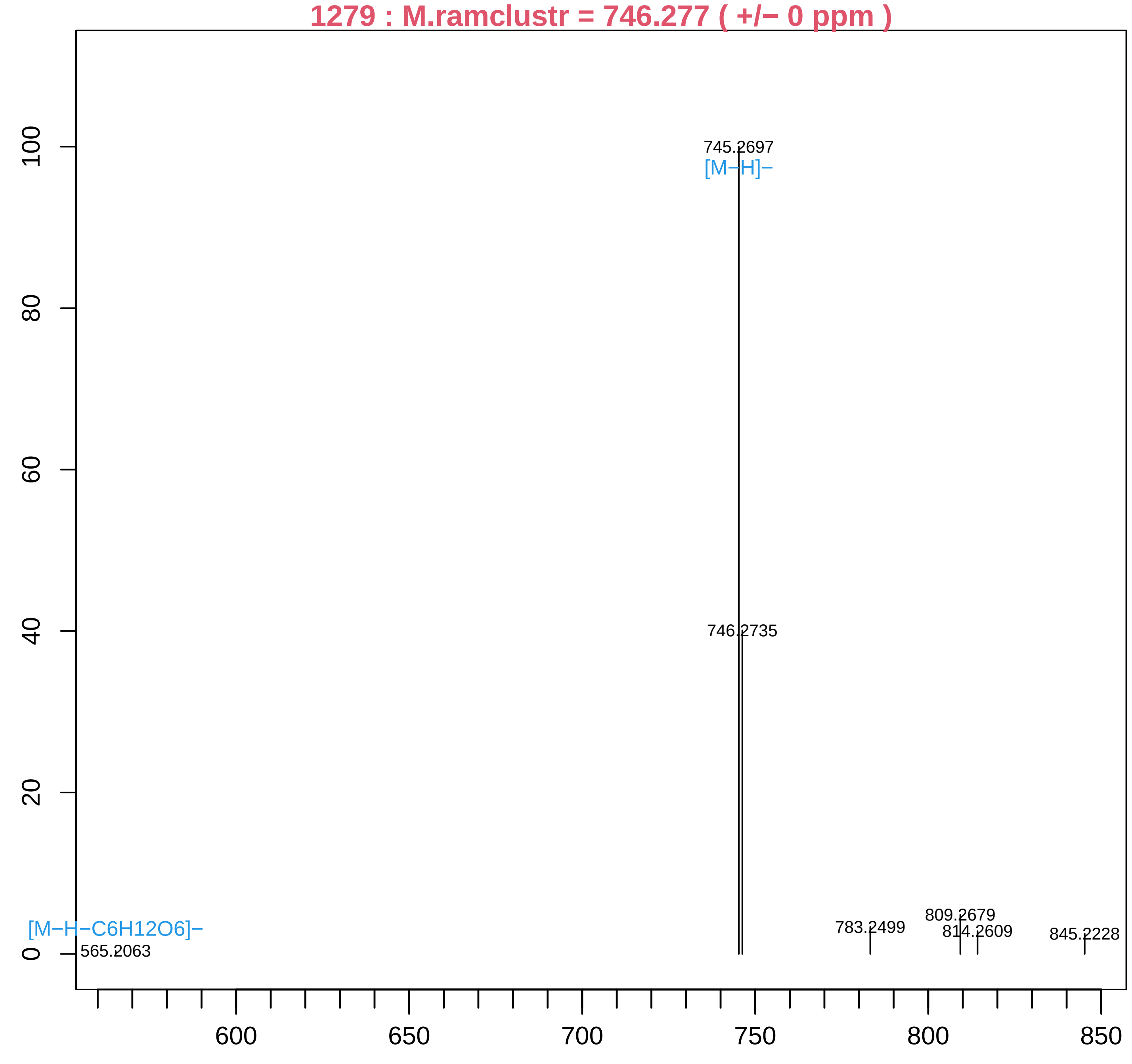
1279 : M.ramclustr = 746.277 (+/− 0 ppm)
745.2697
[M−H]−
746.2735
[M−H−C6H12O6]−
565.2063
783.2499
809.2679
814.2609
845.2228
0
20
40
60
80
100
600
650
700
750
800
850

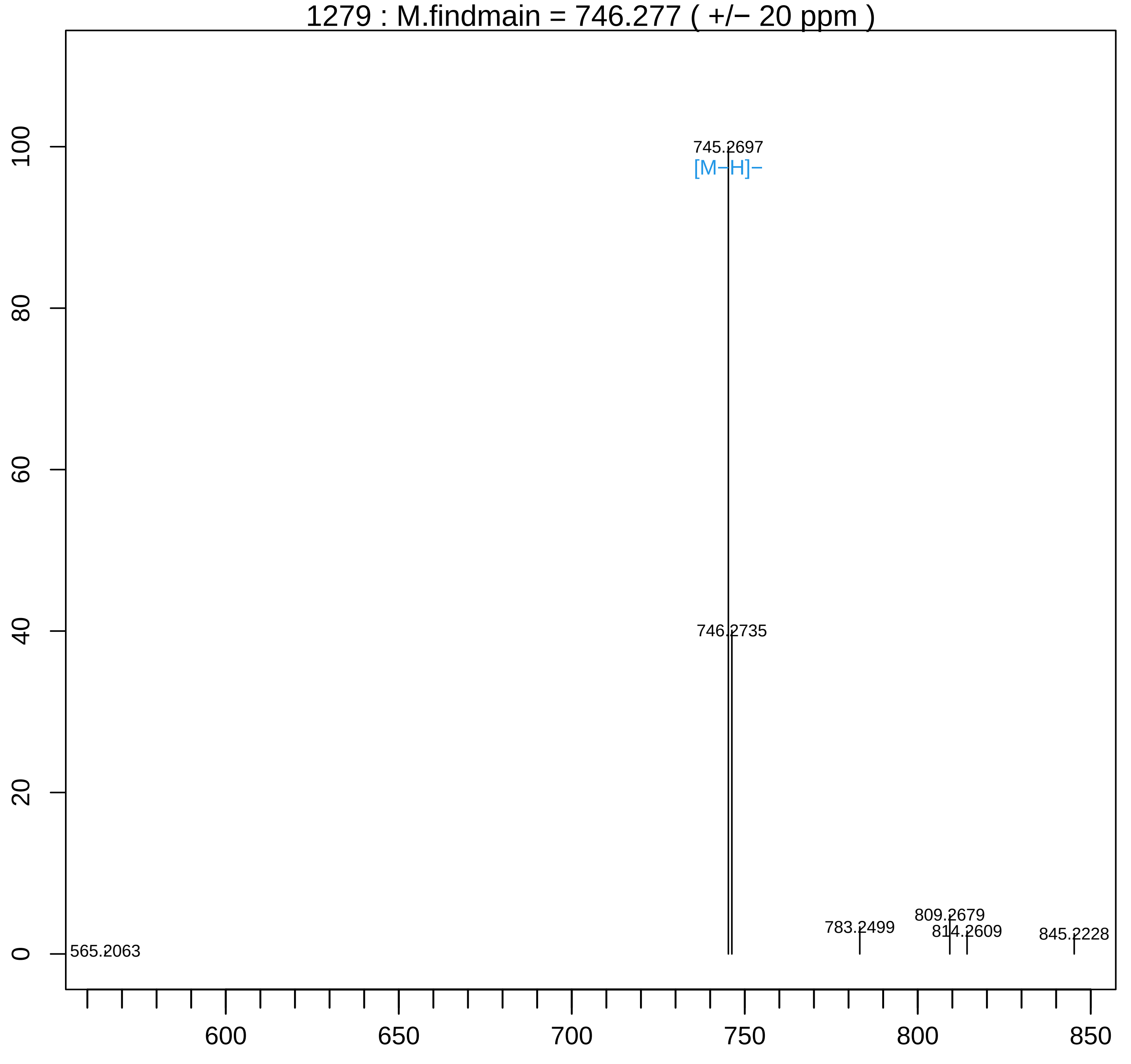
1279 : M.findmain = 746.277 (+/− 20 ppm)
745.2697
[M−H]−
746.2735
565.2063
783.2499
809.2679
814.2609
845.2228
0
20
40
60
80
100
600
650
700
750
800
850

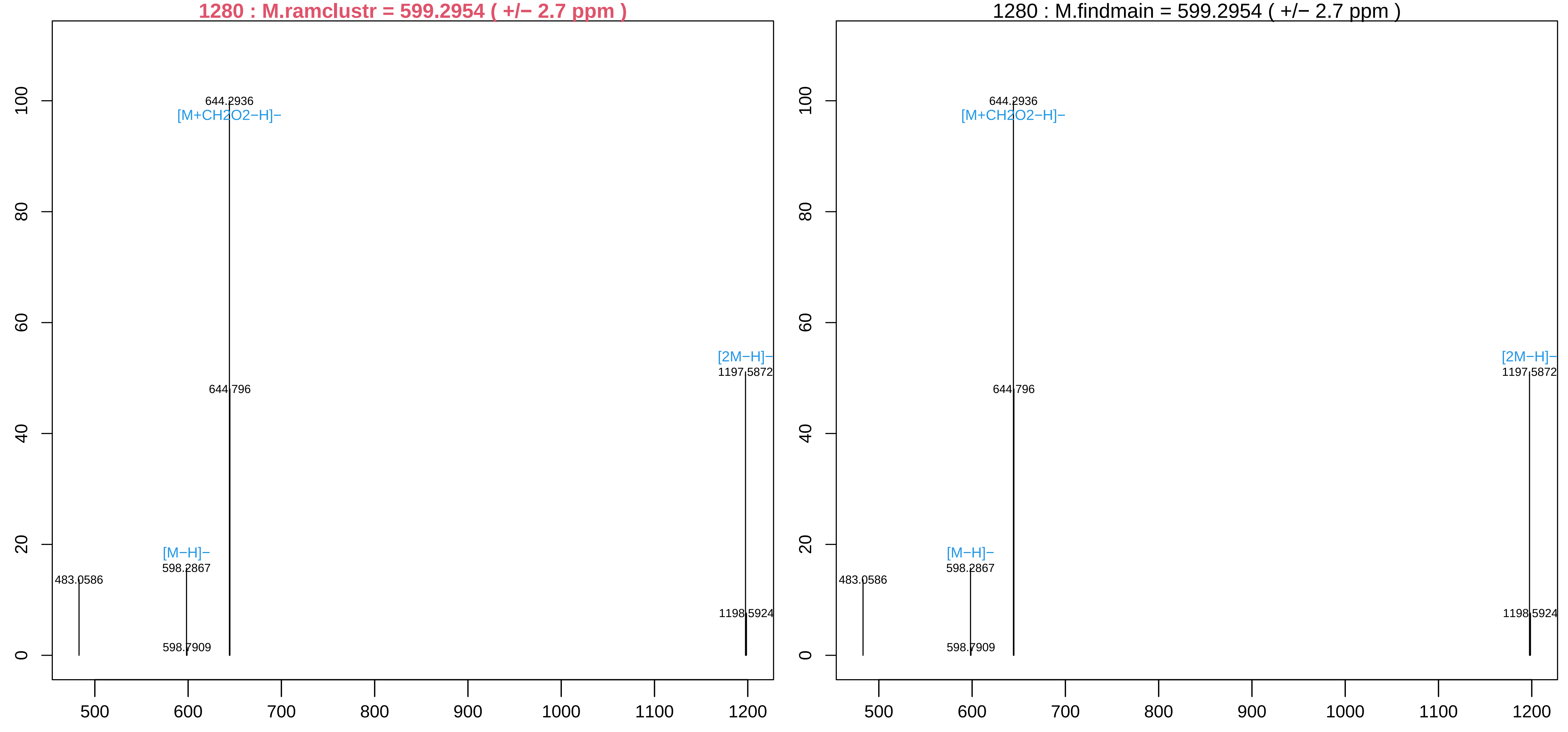

1280 : M.ramclustr = 599.2954 (+/− 2.7 ppm)
644.2936
[M+CH2O2−H]−
644.796
[2M−H]−
1197.5872
1198.5924
[M−H]−
598.2867
483.0586
598.7909
1280 : M.findmain = 599.2954 (+/− 2.7 ppm)
644.2936
[M+CH2O2−H]−
644.796
[2M−H]−
1197.5872
1198.5924
[M−H]−
598.2867
483.0586
598.7909

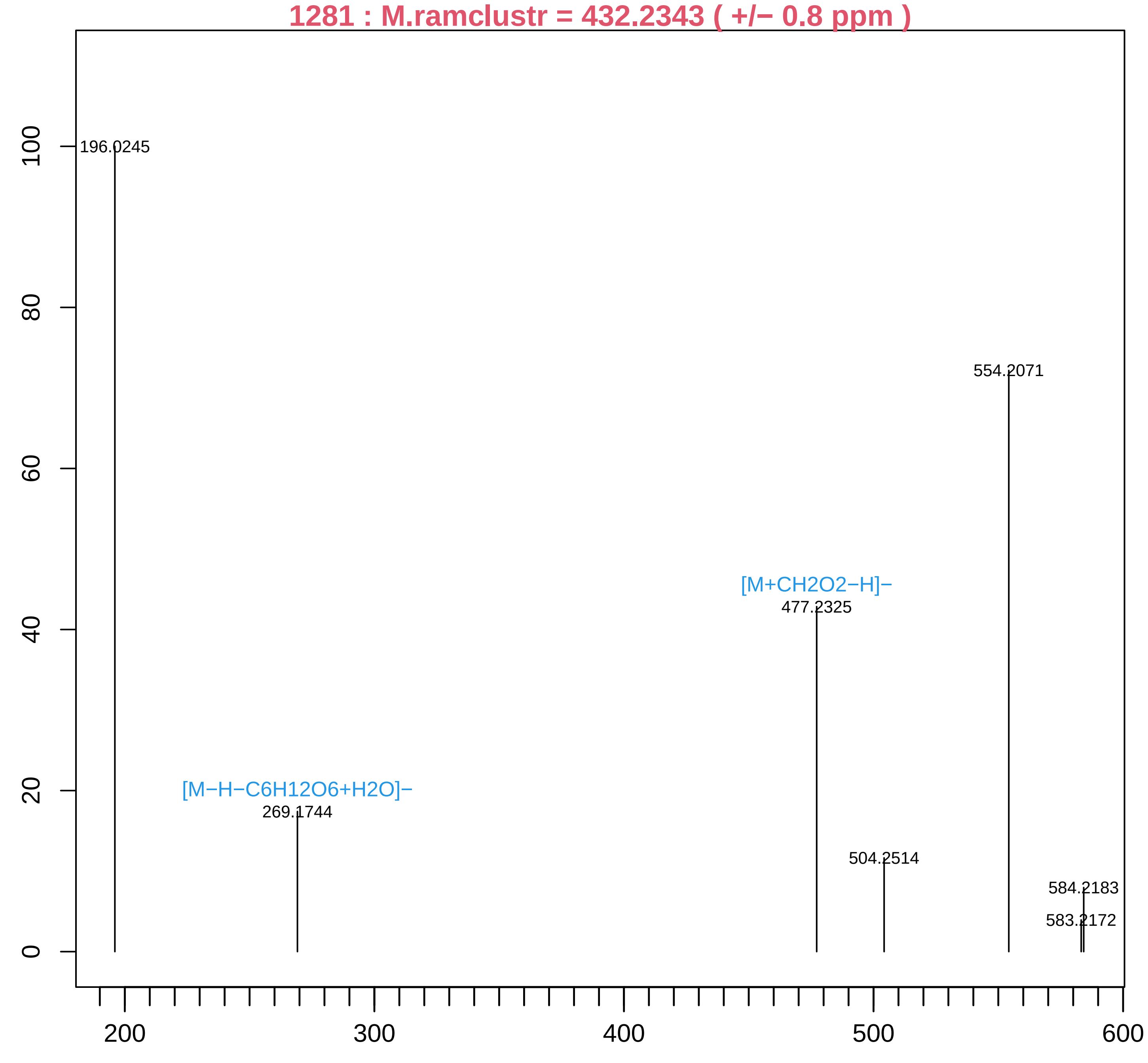
1281 : M.ramclustr = 432.2343 (+/− 0.8 ppm)
196.0245
[M−H−C6H12O6+H2O]−
269.1744
[M+CH2O2−H]−
477.2325
504.2514
554.2071
584.2183
583.2172
0
20
40
60
80
100
200
300
400
500
600

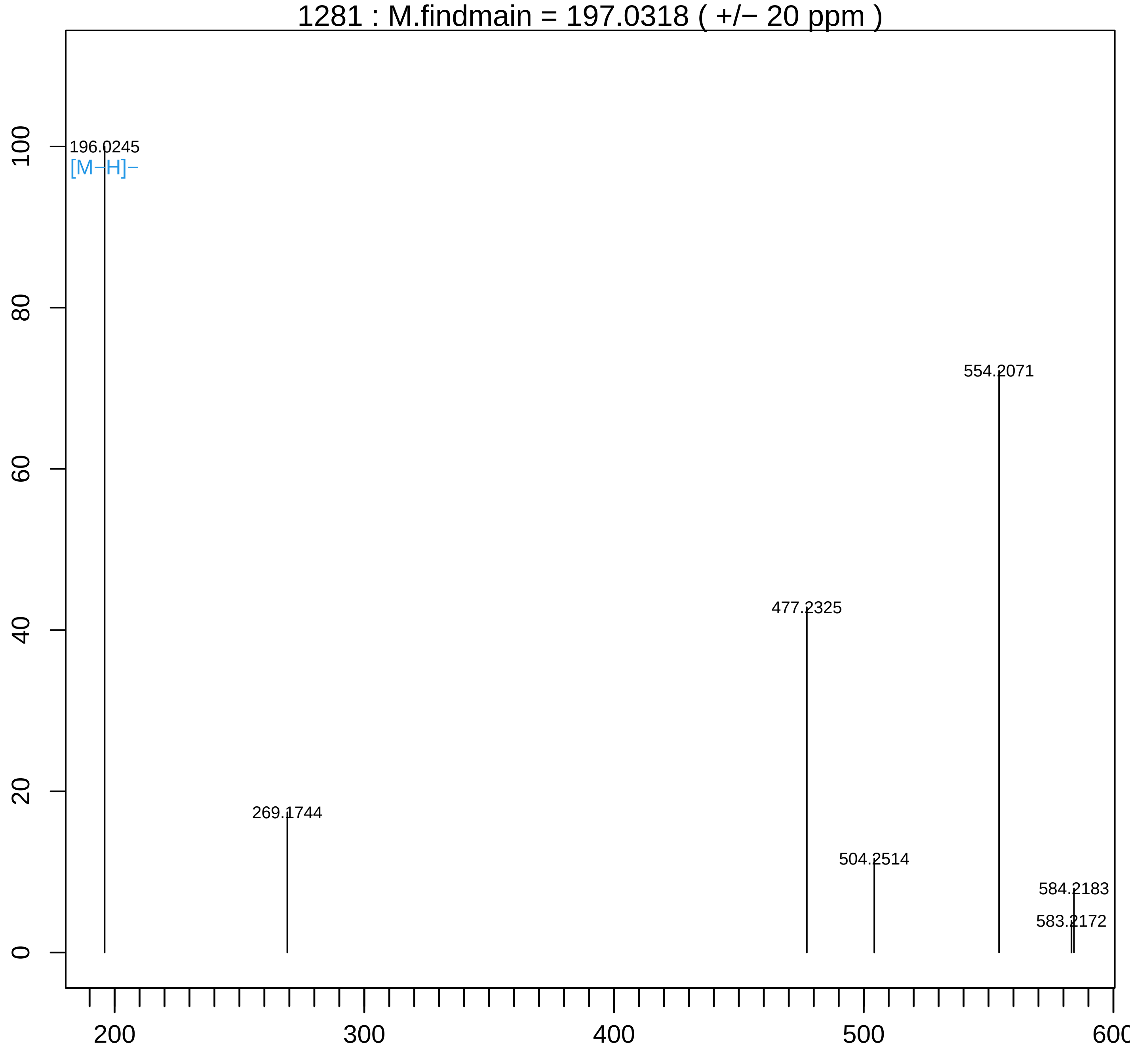
1281 : M.findmain = 197.0318 (+/− 20 ppm)
196.0245
[M−H]−
269.1744
477.2325
504.2514
554.2071
584.2183
583.2172
0
20
40
60
80
100
200
300
400
500
600

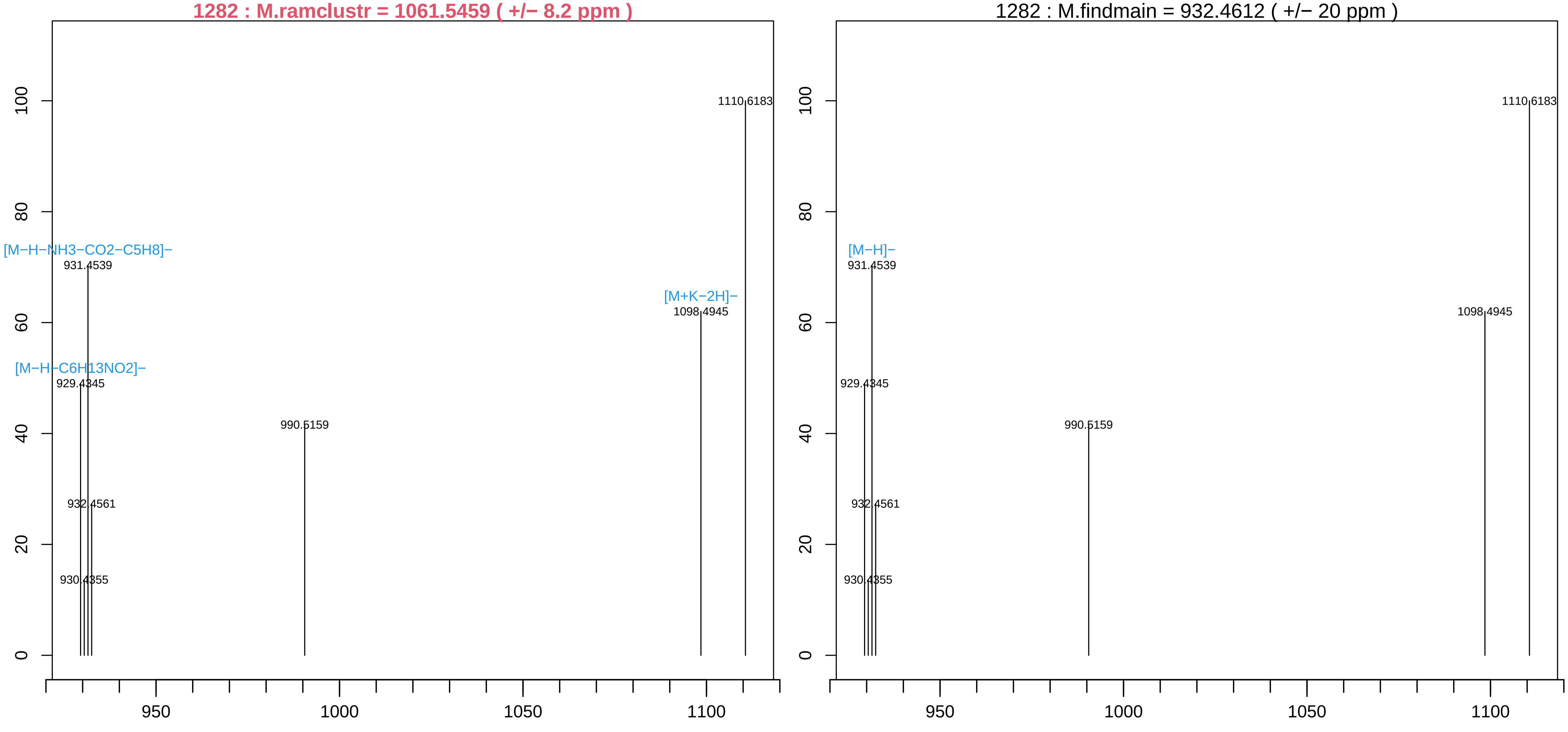
1282 : M.ramclustr = 1061.5459 (+/− 8.2 ppm)
[M−H−NH3−CO2−C5H8]−
931.4539
[M−H−C6H13NO2]−
929.4345
932.4561
930.4355
990.5159
[M+K−2H]−
1098.4945
1110.6183
1282 : M.findmain = 932.4612 (+/− 20 ppm)
[M−H]−
931.4539
929.4345
932.4561
930.4355
990.5159
1098.4945
1110.6183
0
20
40
60
80
100
950
1000
1050
1100

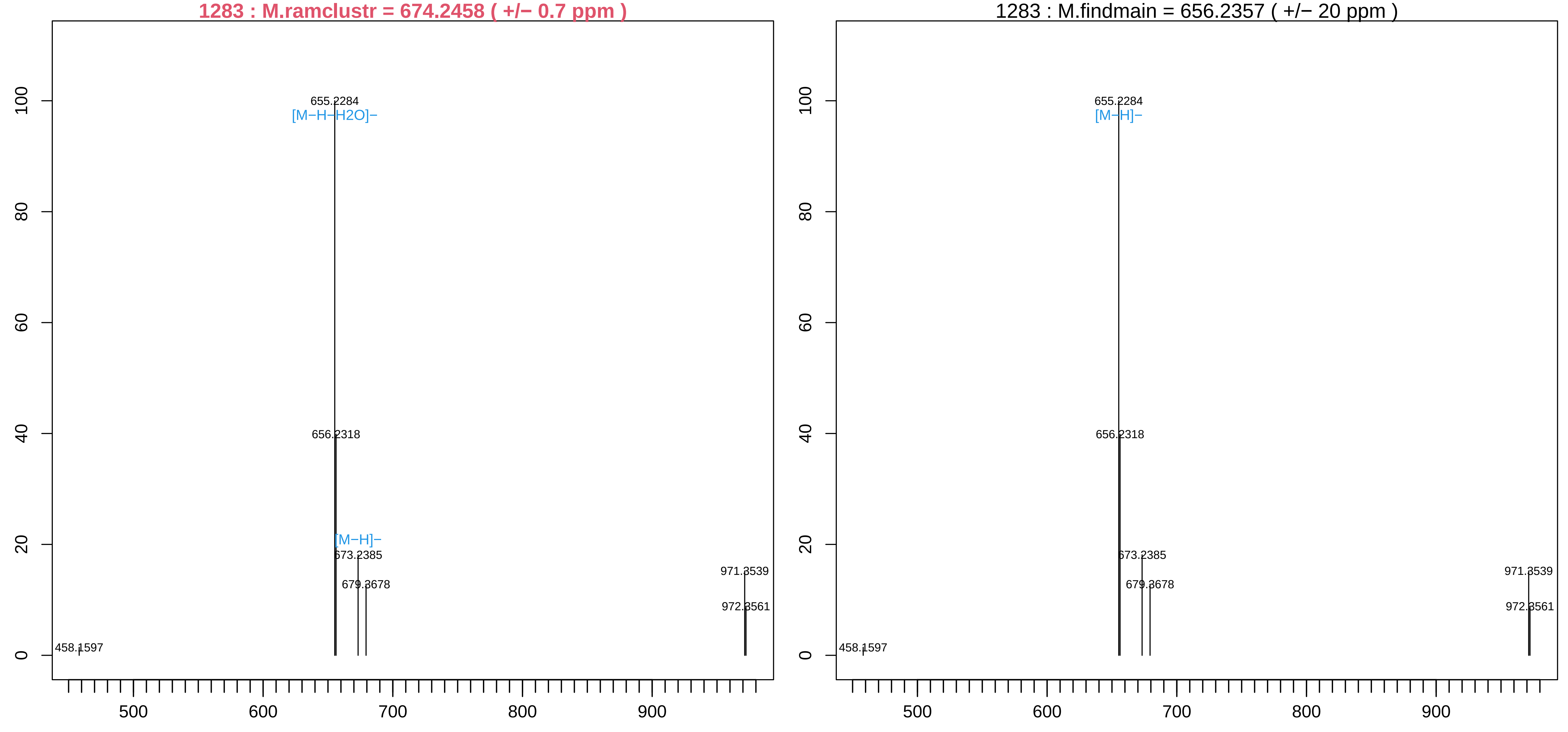

1283 : M.ramclustr = 674.2458 (+/− 0.7 ppm)
655.2284
[M−H−H2O]−
656.2318
[M−H]−
673.2385
679.3678
971.3539
972.3561
458.1597
0
20
40
60
80
100
500
600
700
800
900
1283 : M.findmain = 656.2357 (+/− 20 ppm)
655.2284
[M−H]−
656.2318
673.2385
679.3678
971.3539
972.3561
458.1597
0
20
40
60
80
100
500
600
700
800
900

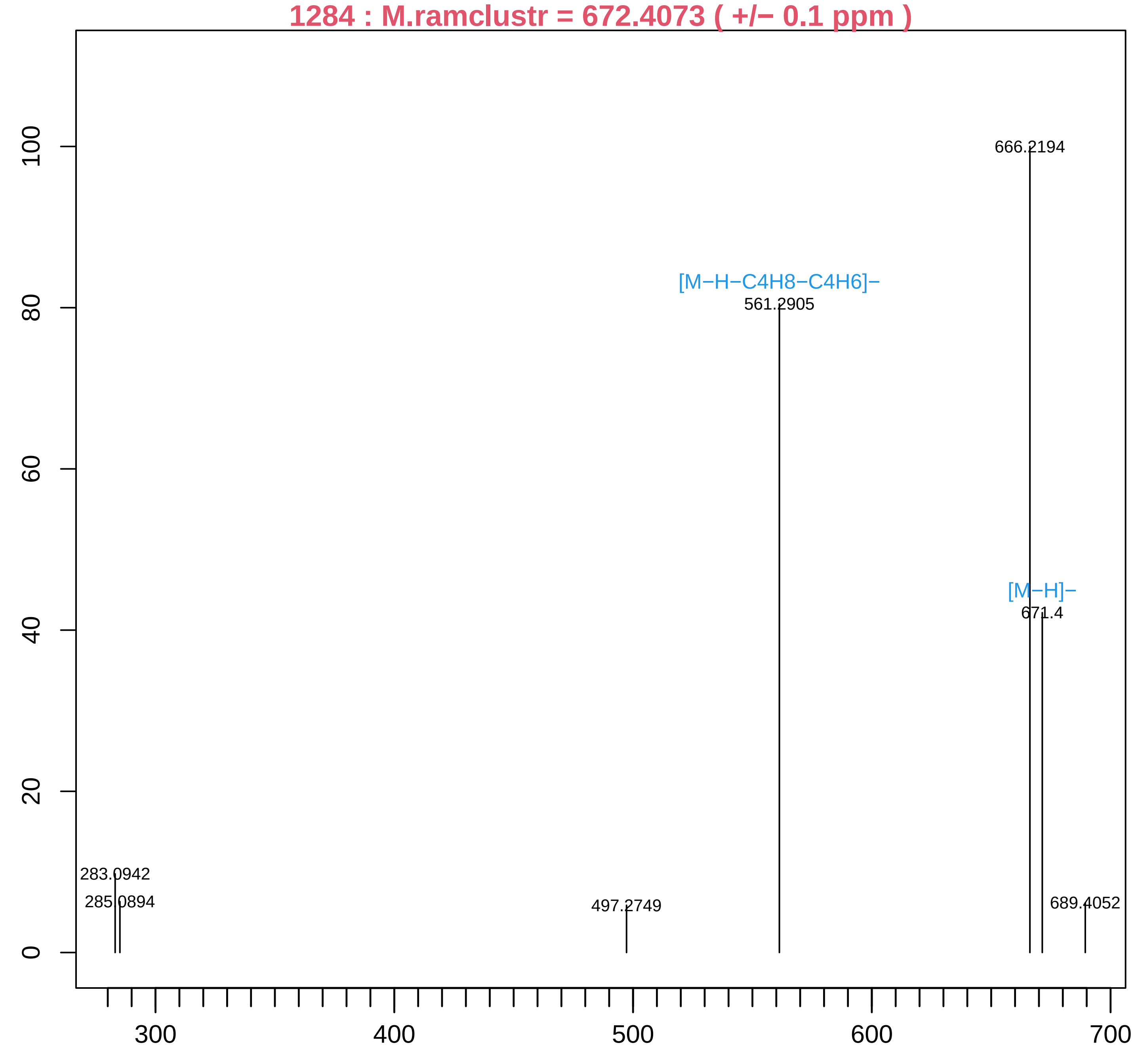

1284 : M.ramclustr = 672.4073 (+/− 0.1 ppm)
666.2194
[M−H−C4H8−C4H6]−
561.2905
[M−H]−
671.4
283.0942
285.0894
497.2749
689.4052
0
20
40
60
80
100
300
400
500
600
700

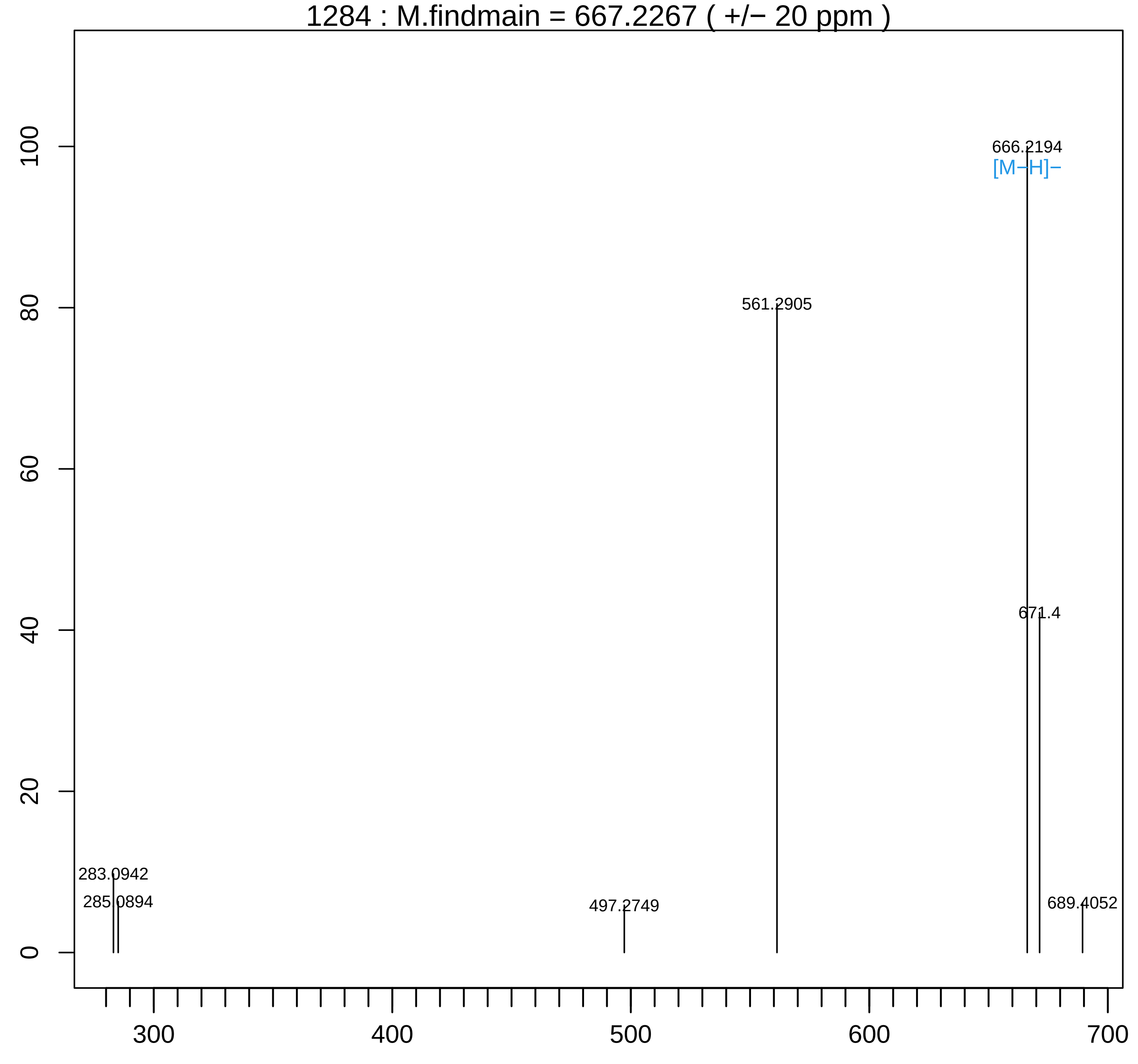

1284 : M.findmain = 667.2267 (+/− 20 ppm)
666.2194
[M−H]−
561.2905
671.4
283.0942
285.0894
497.2749
689.4052
0
20
40
60
80
100
300
400
500
600
700

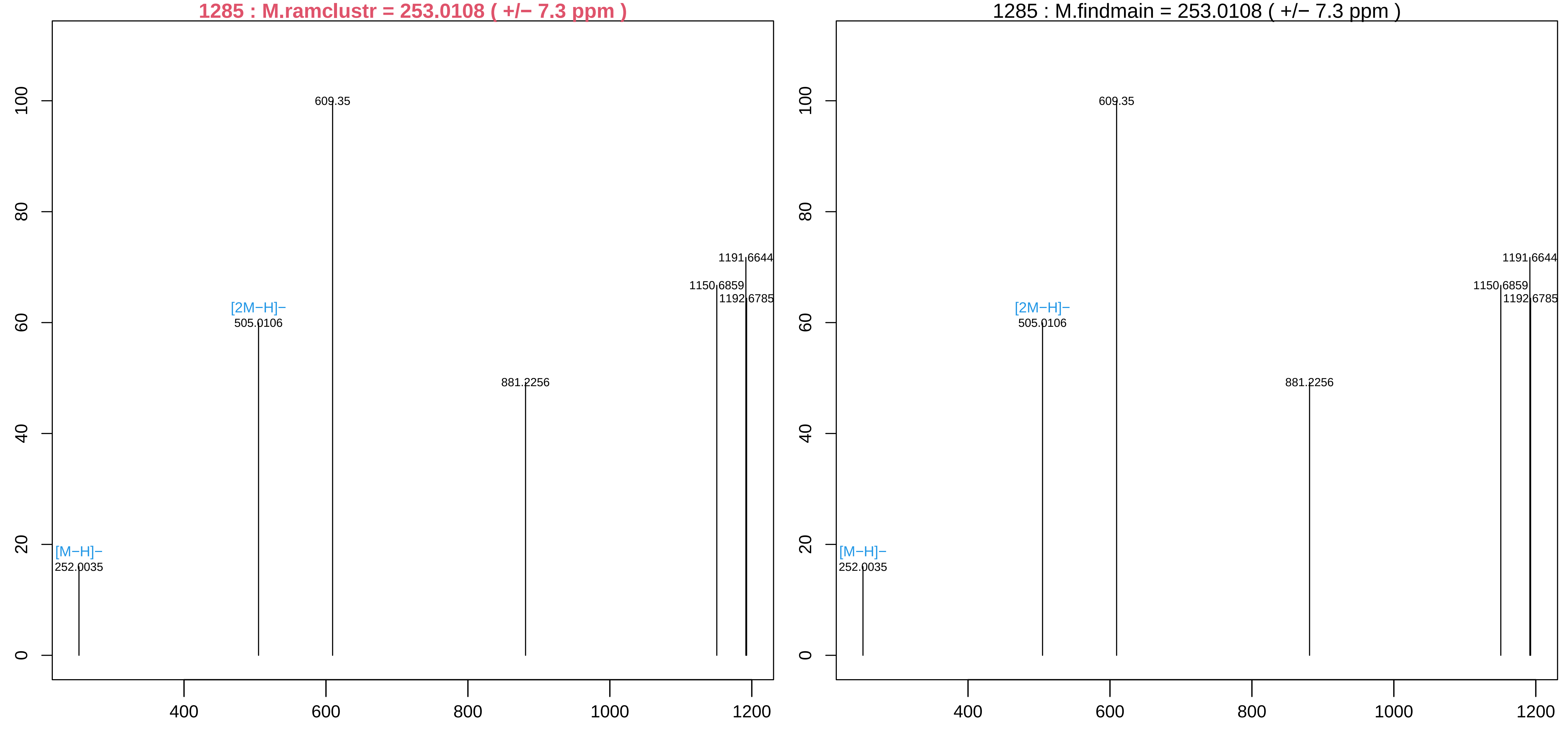
1285 : M.ramclustr = 253.0108 (+/− 7.3 ppm)
1285 : M.findmain = 253.0108 (+/− 7.3 ppm)
[M−H]−
252.0035
[2M−H]−
505.0106
609.35
881.2256
1150.6859
1191.6644
1192.6785
0
20
40
60
80
100
400
600
800
1000
1200

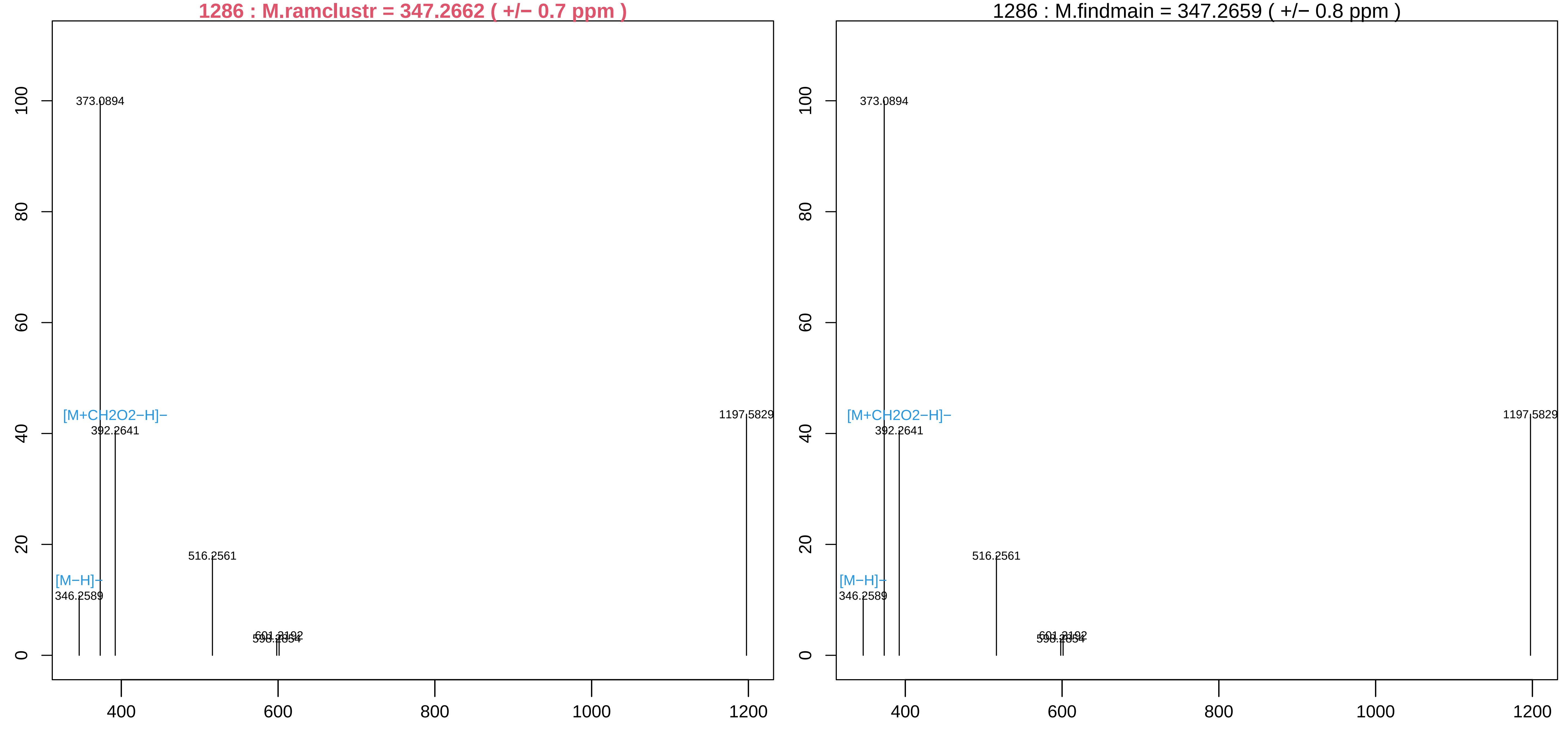
1286 : M.ramclustr = 347.2662 (+/− 0.7 ppm)
1286 : M.findmain = 347.2659 (+/− 0.8 ppm)
373.0894
[M+CH2O2−H]−
392.2641
1197.5829
516.2561
[M−H]−
346.2589
601.2192
598.2654
0
20
40
60
80
100
400
600
800
1000
1200

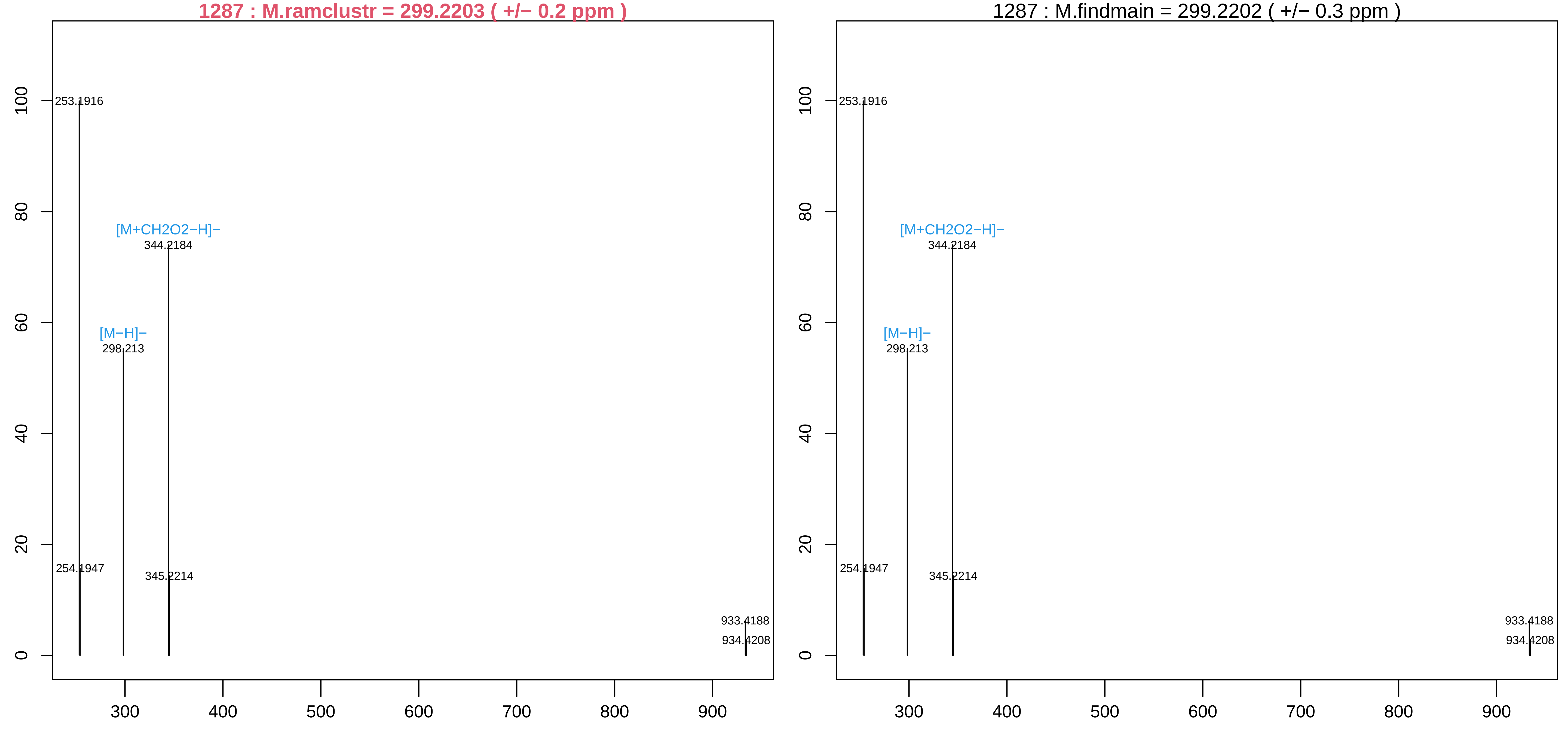
1287 : M.ramclustr = 299.2203 (+/− 0.2 ppm)
253.1916
[M+CH2O2−H]−
344.2184
[M−H]−
298.213
254.1947
345.2214
933.4188
934.4208
0
20
40
60
80
100
300
400
500
600
700
800
900
1287 : M.findmain = 299.2202 (+/− 0.3 ppm)
253.1916
[M+CH2O2−H]−
344.2184
[M−H]−
298.213
254.1947
345.2214
933.4188
934.4208

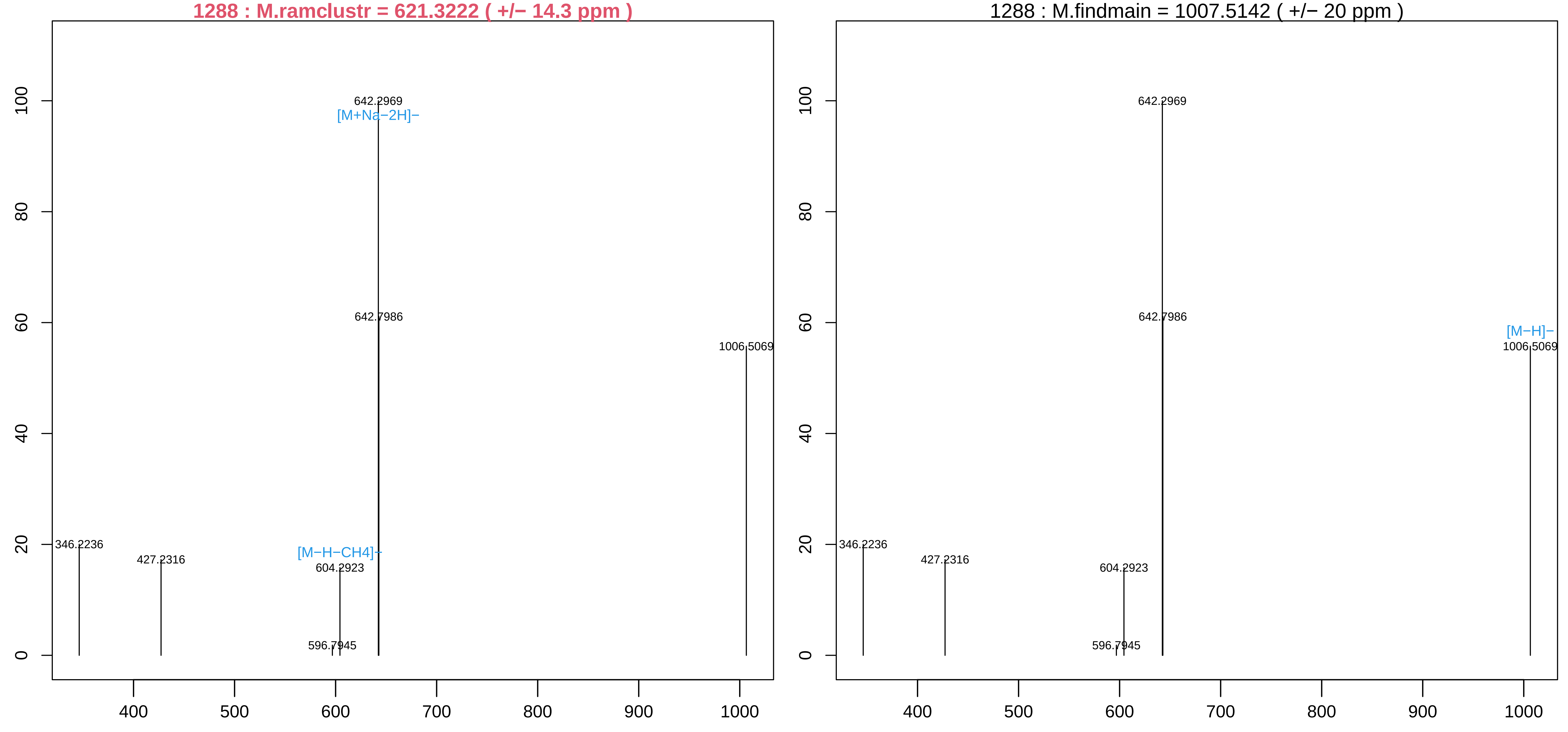
1288 : M.ramclustr = 621.3222 (+/− 14.3 ppm)
642.2969
[M+Na−2H]−
642.7986
1006.5069
346.2236
427.2316
[M−H−CH4]−
604.2923
596.7945
1288 : M.findmain = 1007.5142 (+/− 20 ppm)
642.2969
642.7986
[M−H]−
1006.5069
346.2236
427.2316
604.2923
596.7945

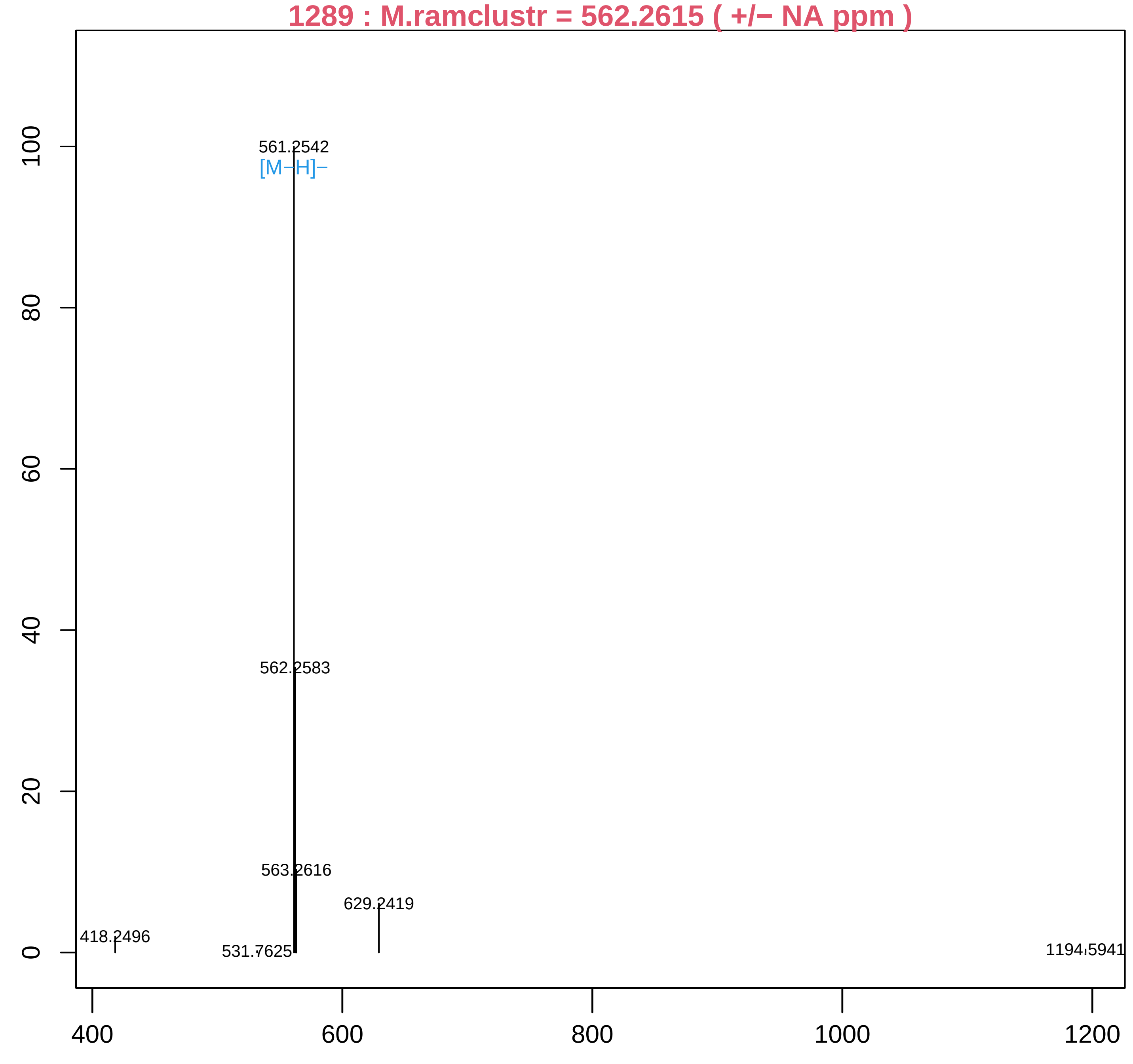
1289 : M.ramclustr = 562.2615 (+/− NA ppm)
561.2542
[M−H]−
562.2583
563.2616
629.2419
418.2496
531.7625
1194.5941
0
20
40
60
80
100
400
600
800
1000
1200

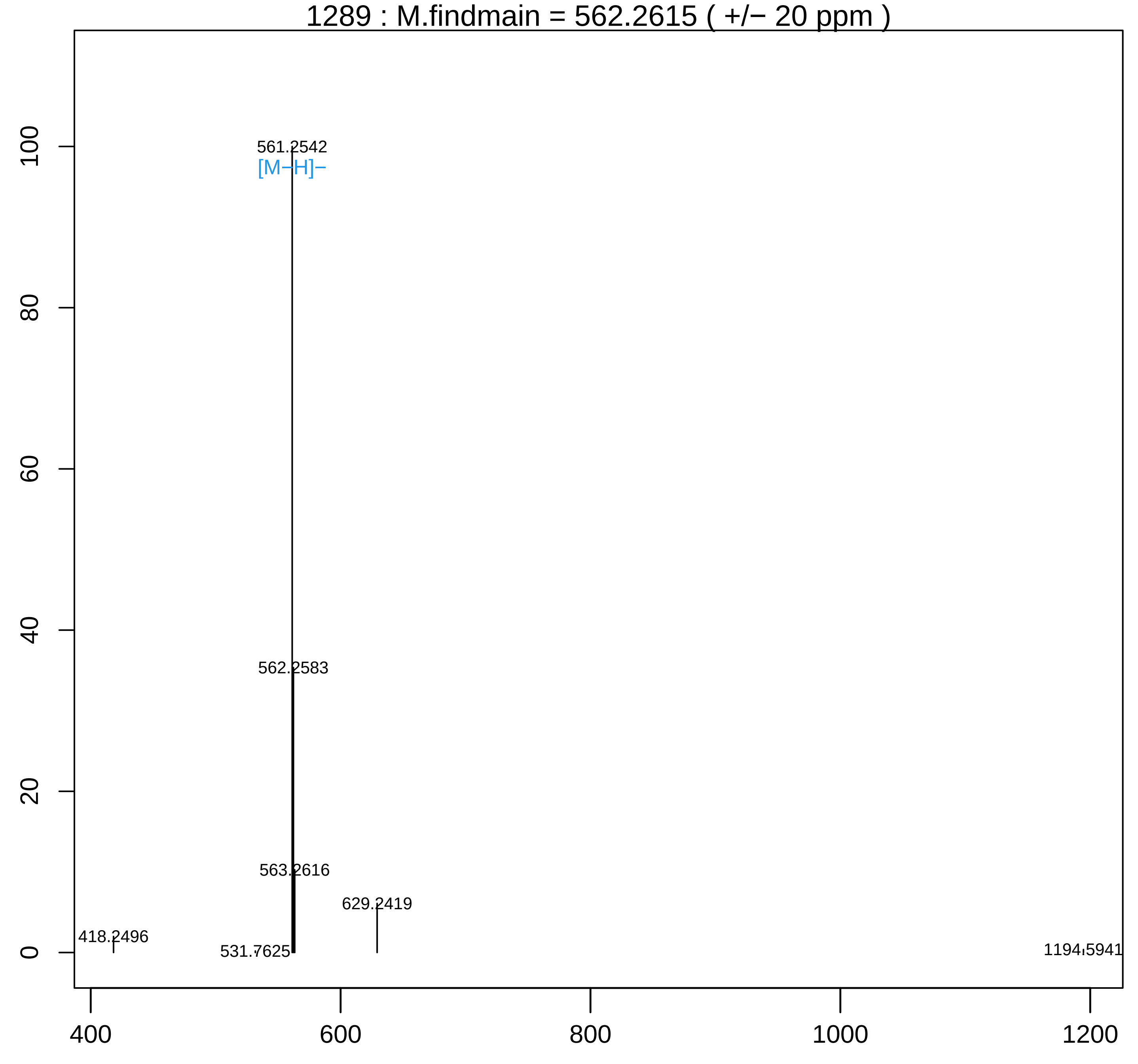
1289 : M.findmain = 562.2615 (+/− 20 ppm)
561.2542
[M−H]−
562.2583
563.2616
629.2419
418.2496
531.7625
1194.5941
0
20
40
60
80
100
400
600
800
1000
1200

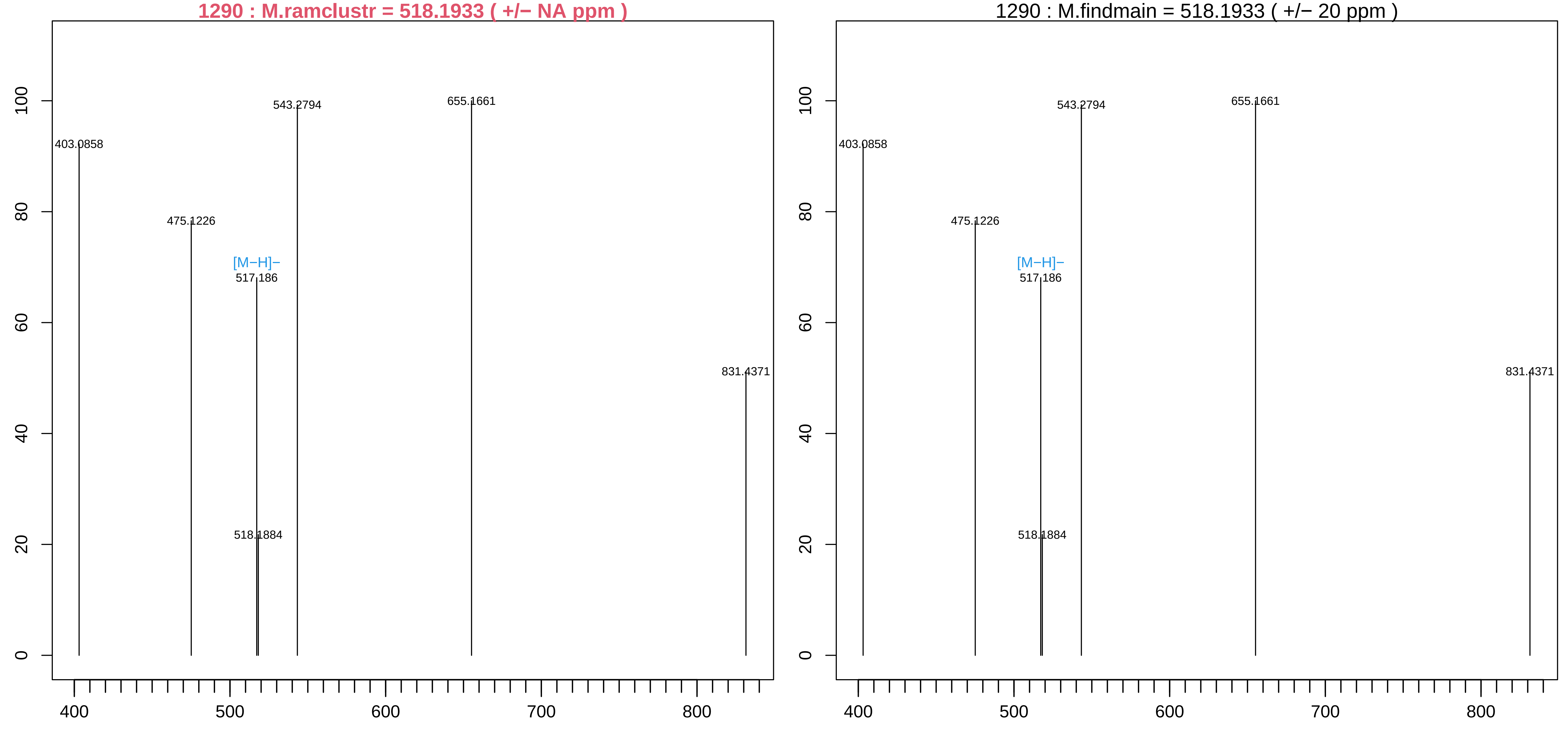

1290 : M.ramclustr = 518.1933 (+/− NA ppm)
403.0858
475.1226
[M−H]−
517.186
518.1884
543.2794
655.1661
831.4371
0
20
40
60
80
100
400
500
600
700
800
1290 : M.findmain = 518.1933 (+/− 20 ppm)
403.0858
475.1226
[M−H]−
517.186
518.1884
543.2794
655.1661
831.4371
0
20
40
60
80
100
400
500
600
700
800

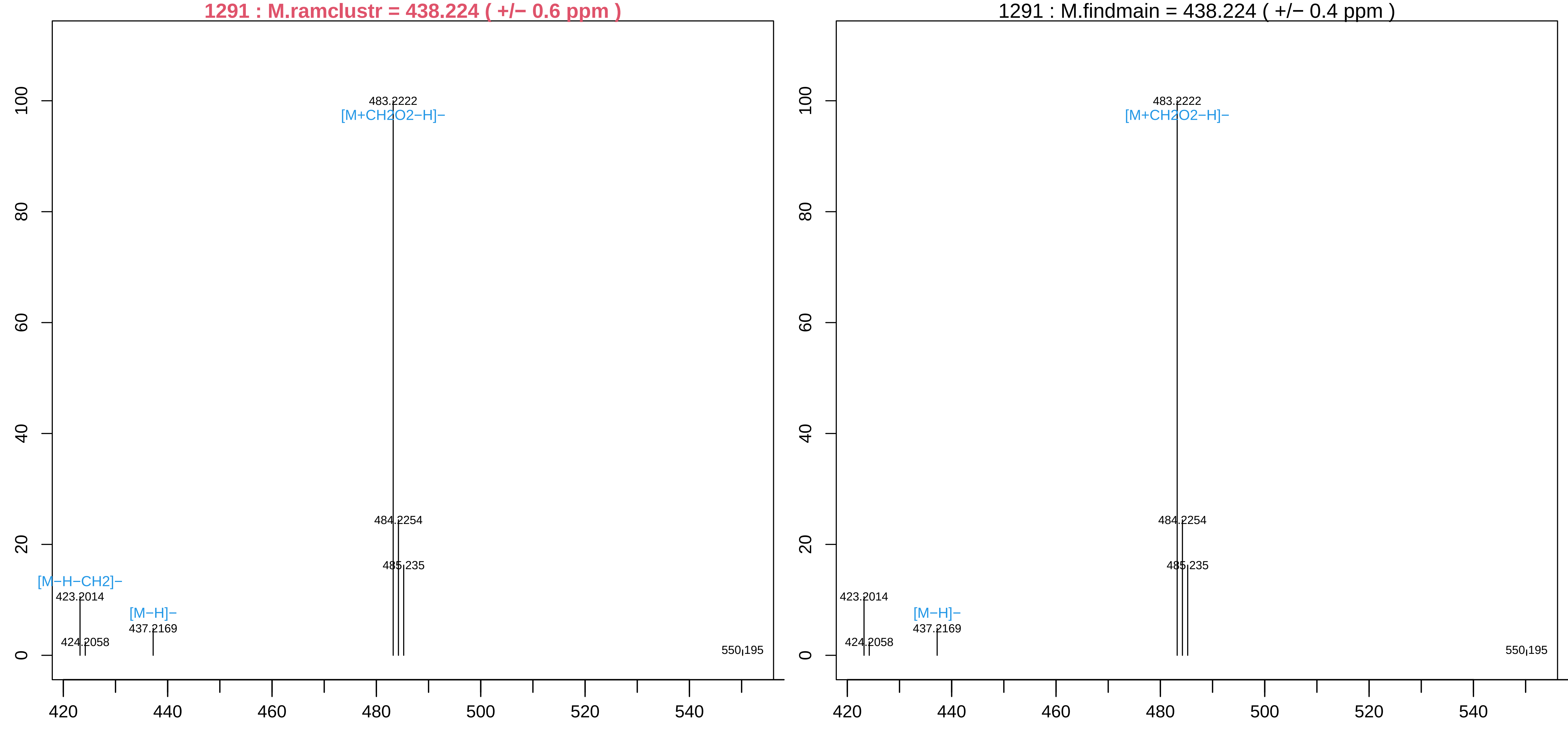

1291 : M.ramclustr = 438.224 (+/− 0.6 ppm)
483.2222
[M+CH2O2−H]−
484.2254
485.235
[M−H−CH2]−
423.2014
[M−H]−
437.2169
424.2058
550.195
1291 : M.findmain = 438.224 (+/− 0.4 ppm)
483.2222
[M+CH2O2−H]−
484.2254
485.235
423.2014
[M−H]−
437.2169
424.2058
550.195

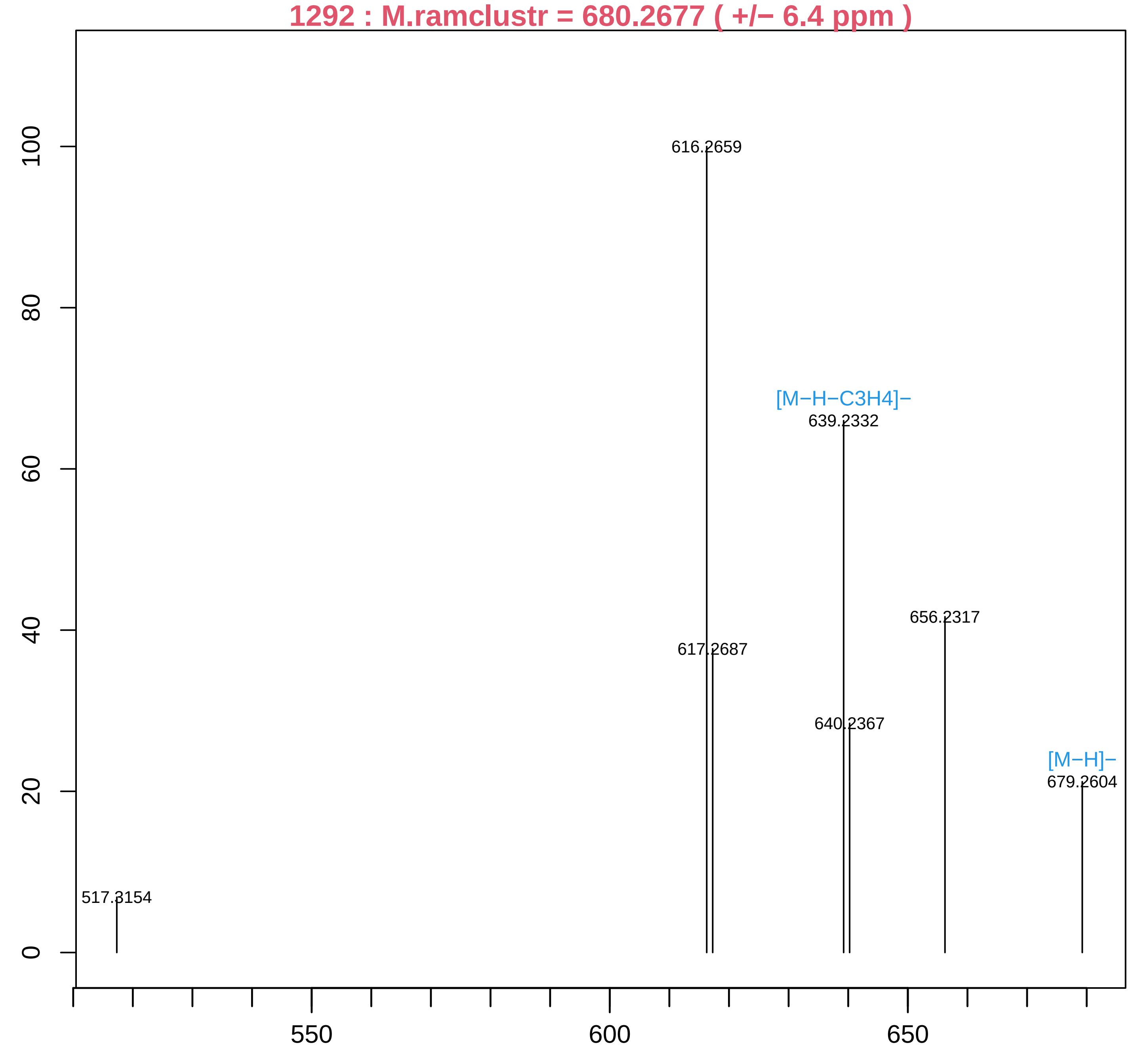
1292 : M.ramclustr = 680.2677 (+/− 6.4 ppm)
616.2659
[M−H−C3H4]−
639.2332
656.2317
617.2687
640.2367
[M−H]−
679.2604
517.3154
0
20
40
60
80
100
550
600
650

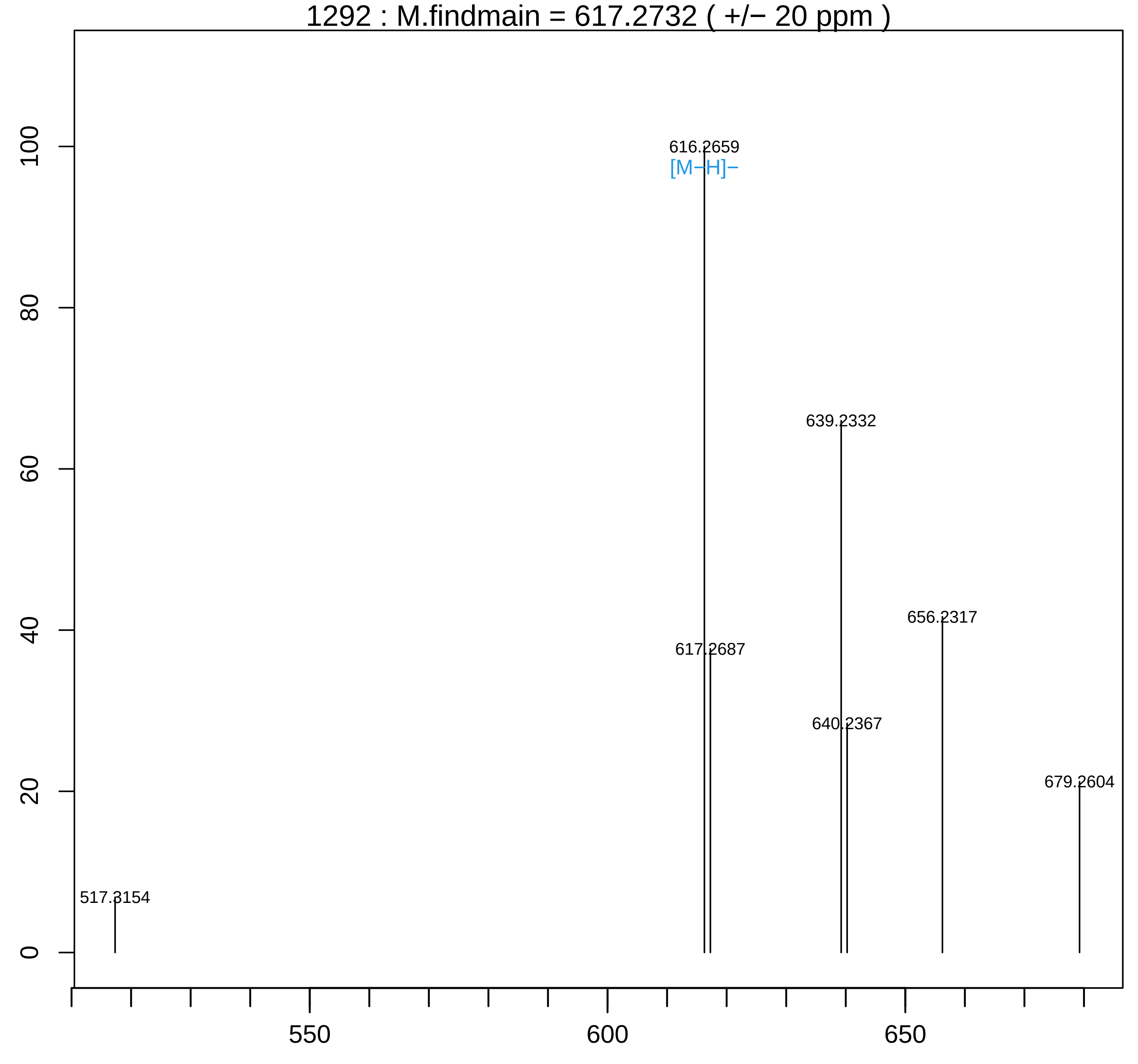
1292 : M.findmain = 617.2732 (+/− 20 ppm)
616.2659
[M−H]−
639.2332
656.2317
617.2687
640.2367
679.2604
517.3154
0
20
40
60
80
100
550
600
650

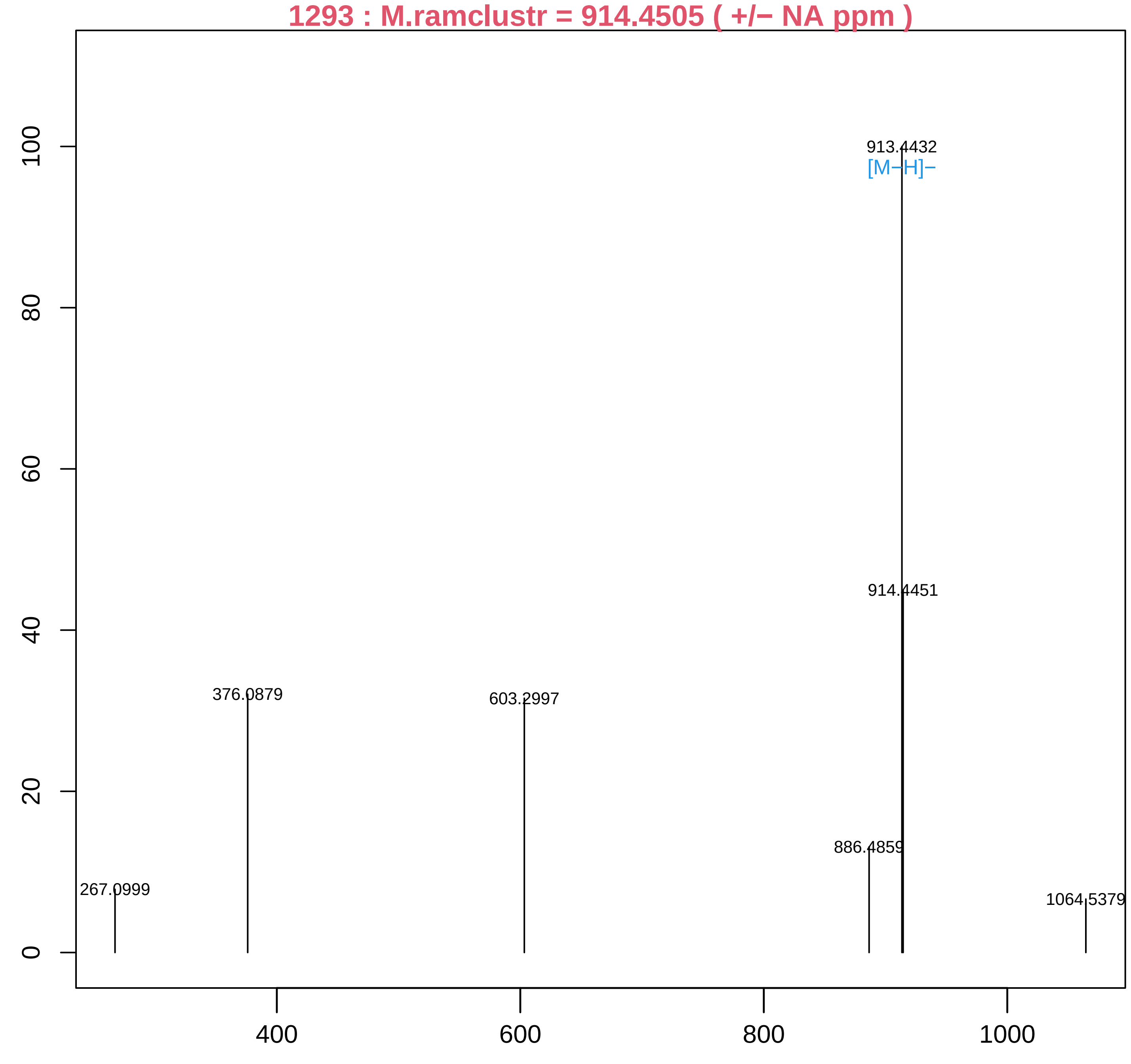
1293 : M.ramclustr = 914.4505 (+/− NA ppm)
913.4432
[M−H]−
914.4451
376.0879
603.2997
886.4859
267.0999
1064.5379
0
20
40
60
80
100
400
600
800
1000

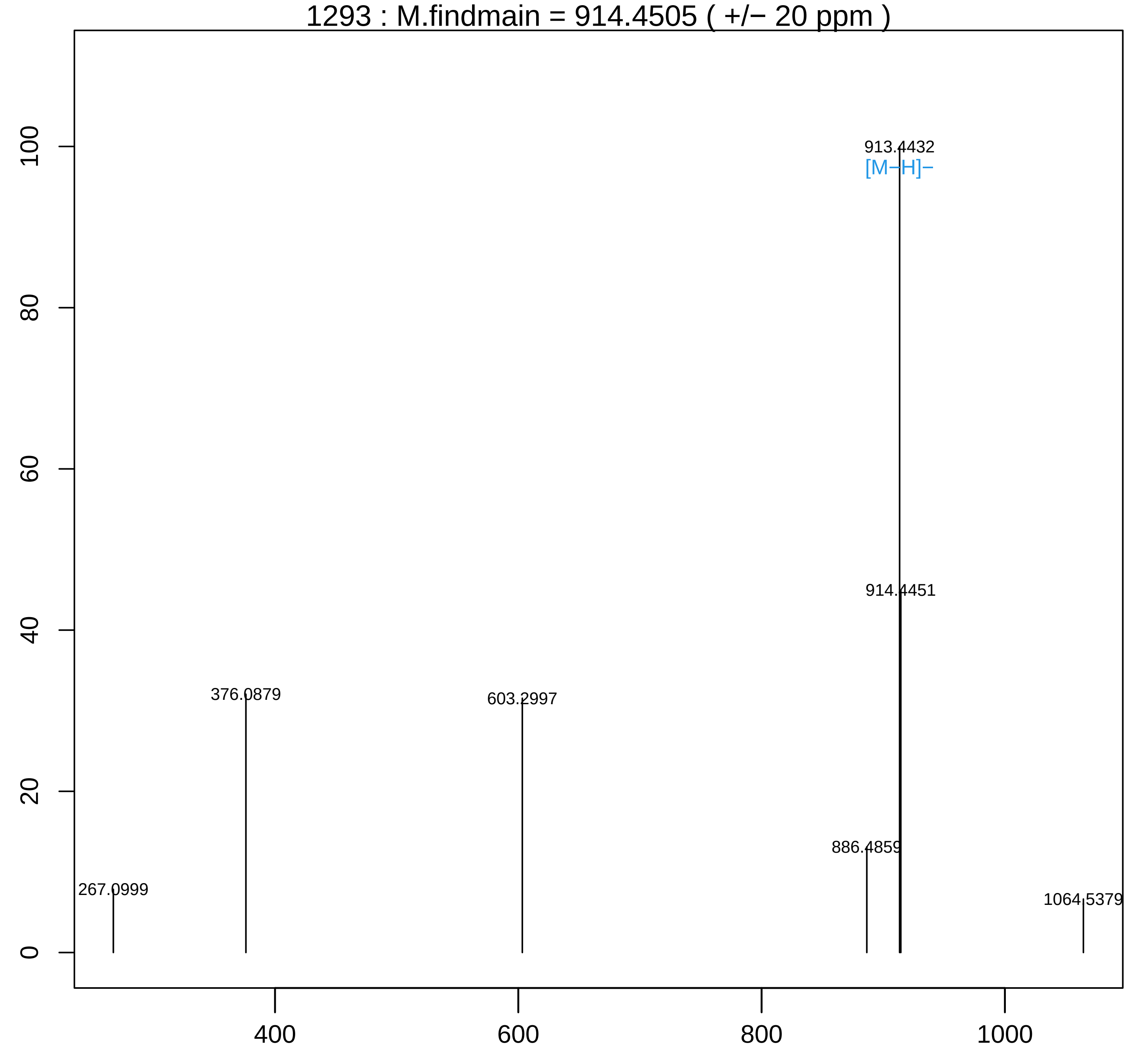
1293 : M.findmain = 914.4505 (+/− 20 ppm)
913.4432
[M−H]−
914.4451
376.0879
603.2997
886.4859
267.0999
1064.5379
0
20
40
60
80
100
400
600
800
1000

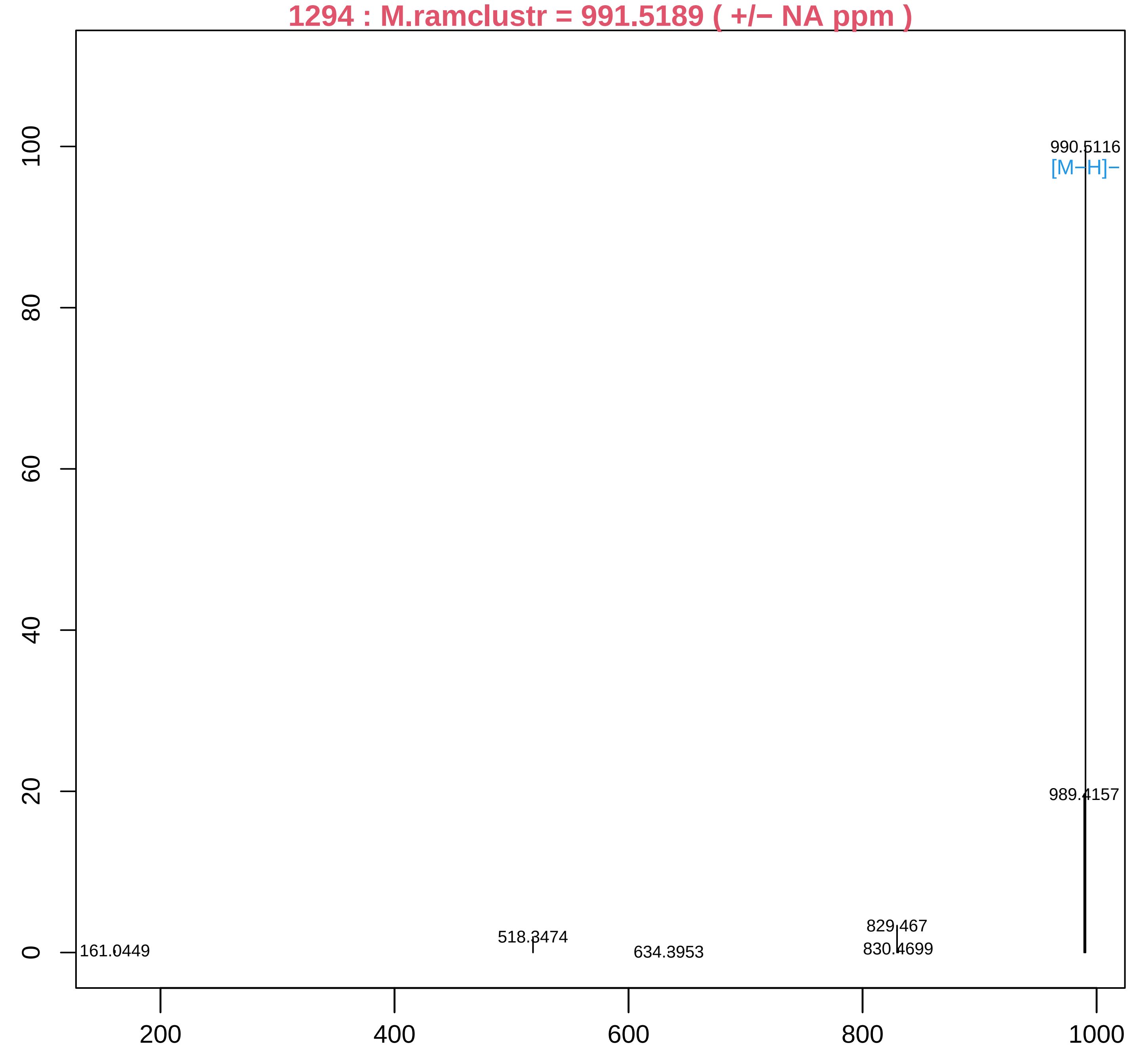

1294 : M.ramclustr = 991.5189 (+/− NA ppm)
990.5116
[M−H]−
989.4157
161.0449
518.3474
634.3953
829.467
830.4699
0
20
40
60
80
100
200
400
600
800
1000

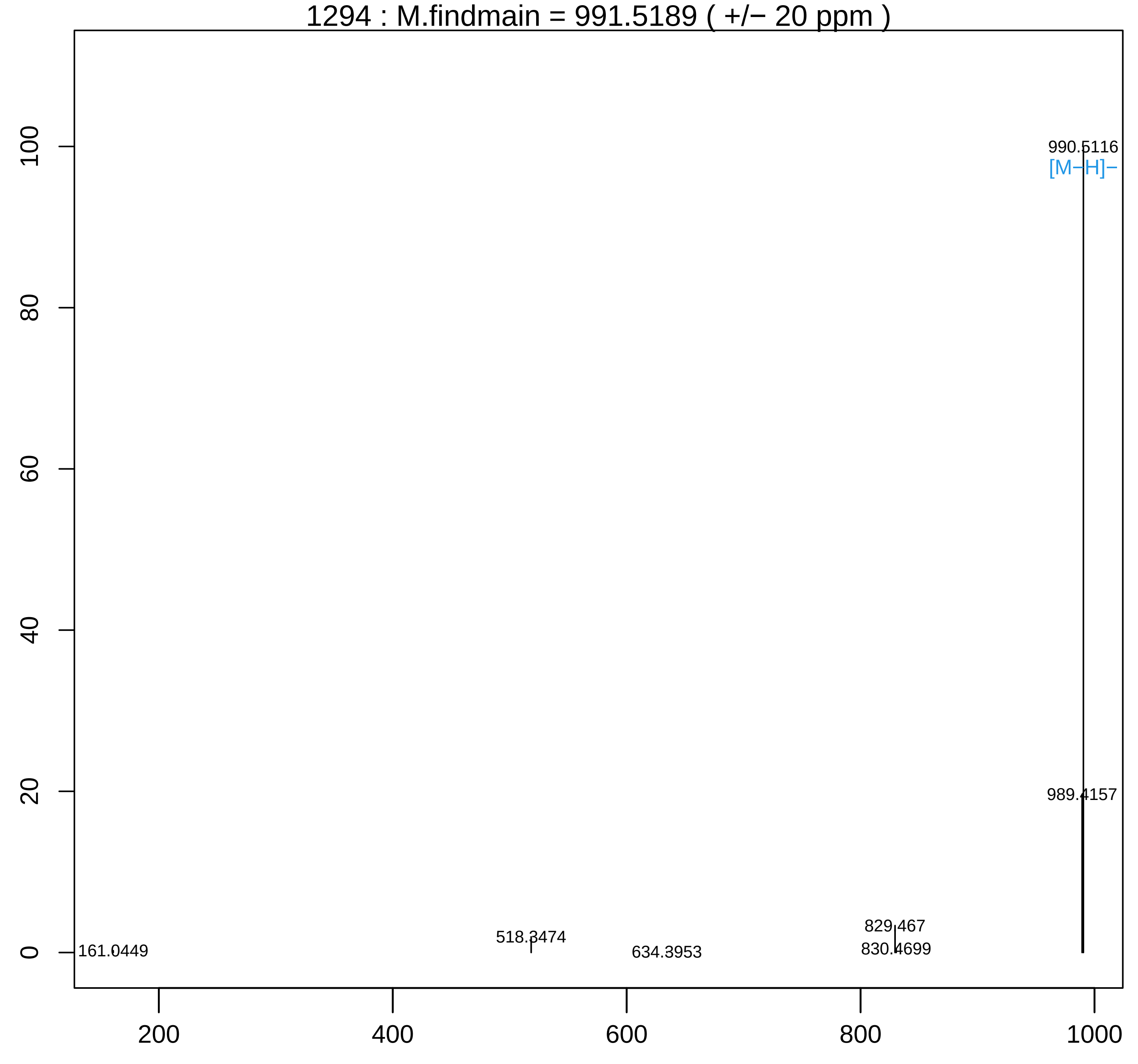

1294 : M.findmain = 991.5189 (+/− 20 ppm)
990.5116
[M−H]−
989.4157
161.0449
518.3474
634.3953
829.467
830.4699
0
20
40
60
80
100
200
400
600
800
1000

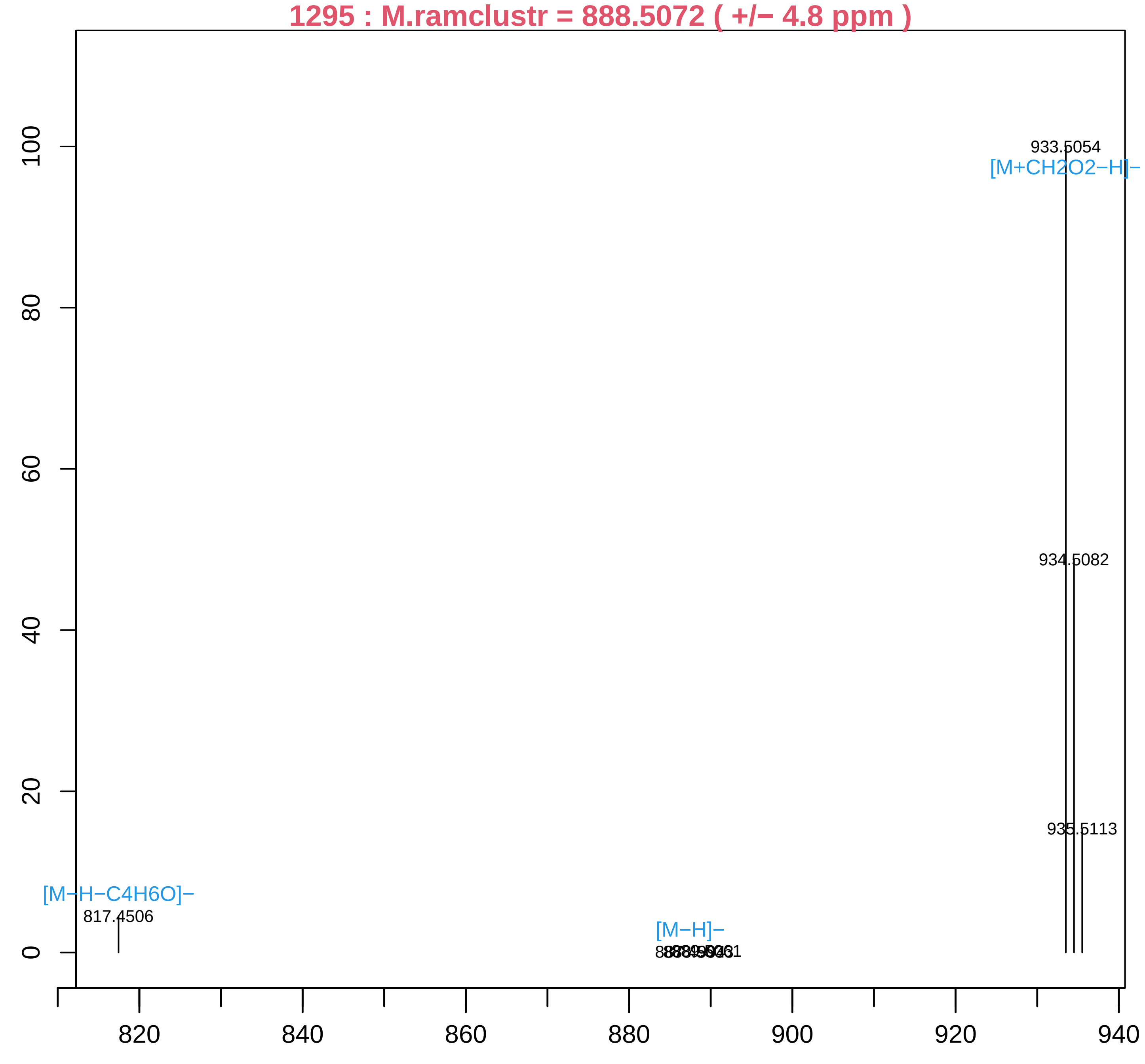
1295 : M.ramclustr = 888.5072 (+/− 4.8 ppm)
933.5054
[M+CH2O2−H]−
934.5082
935.5113
[M−H−C4H6O]−
817.4506
[M−H]−
0
20
40
60
80
100
820
840
860
880
900
920
940

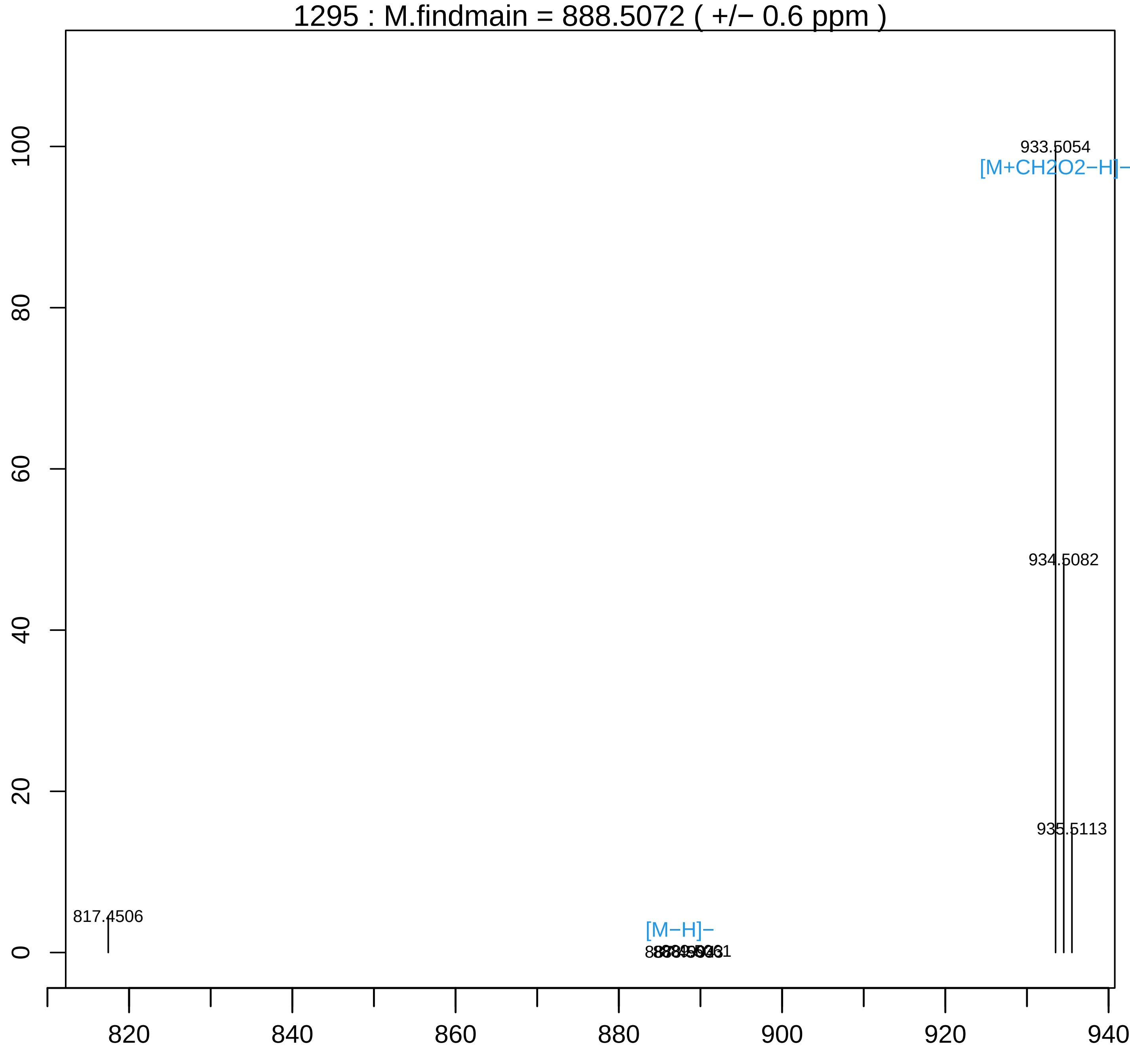
1295 : M.findmain = 888.5072 (+/− 0.6 ppm)
933.5054
[M+CH2O2−H]−
934.5082
935.5113
817.4506
[M−H]−
0
20
40
60
80
100
820
840
860
880
900
920
940

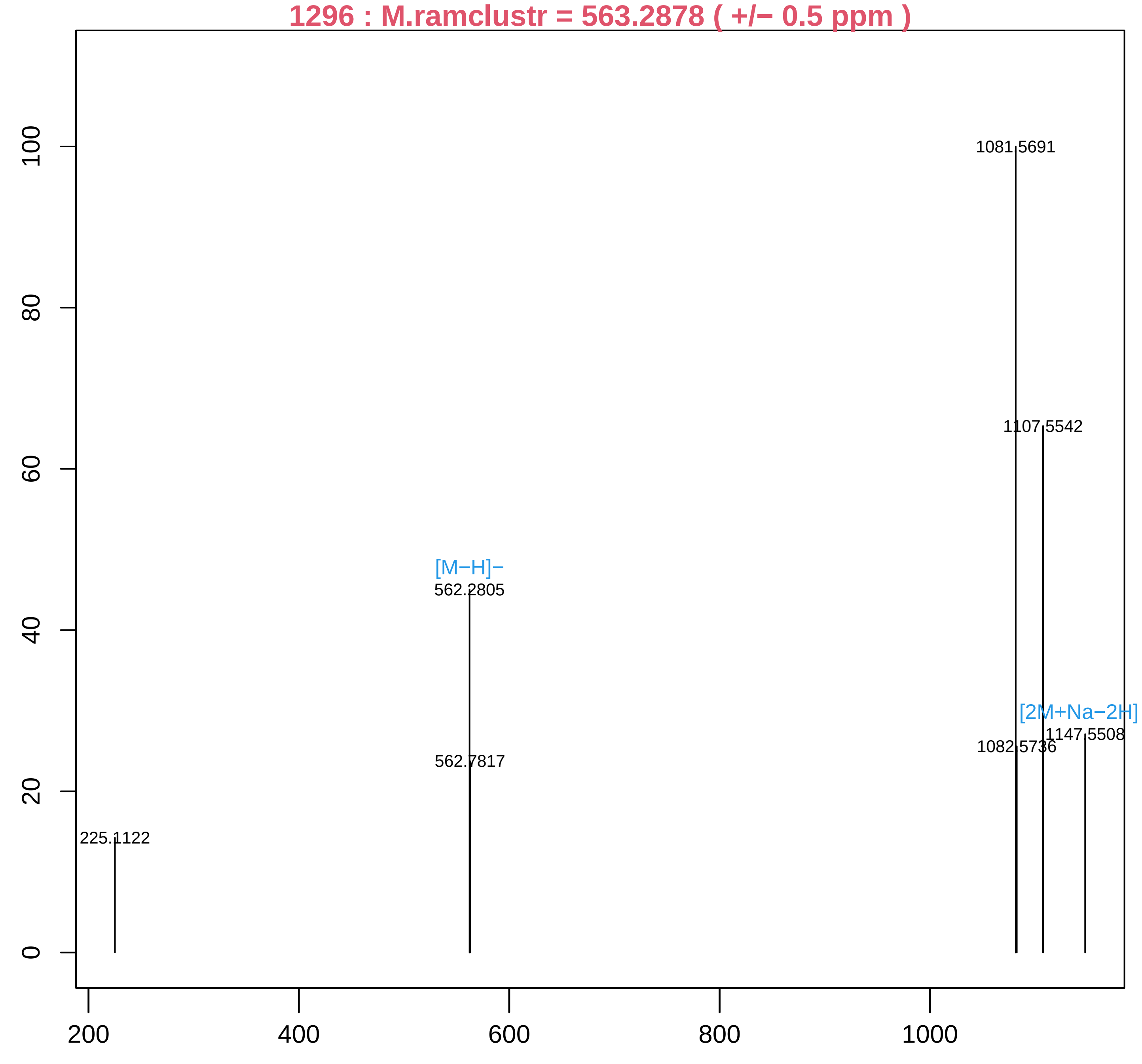
1296 : M.ramclustr = 563.2878 (+/− 0.5 ppm)
1081.5691
1107.5542
[M−H]−
562.2805
[2M+Na−2H]
1147.5508
1082.5736
562.7817
225.1122
0
20
40
60
80
100
200
400
600
800
1000

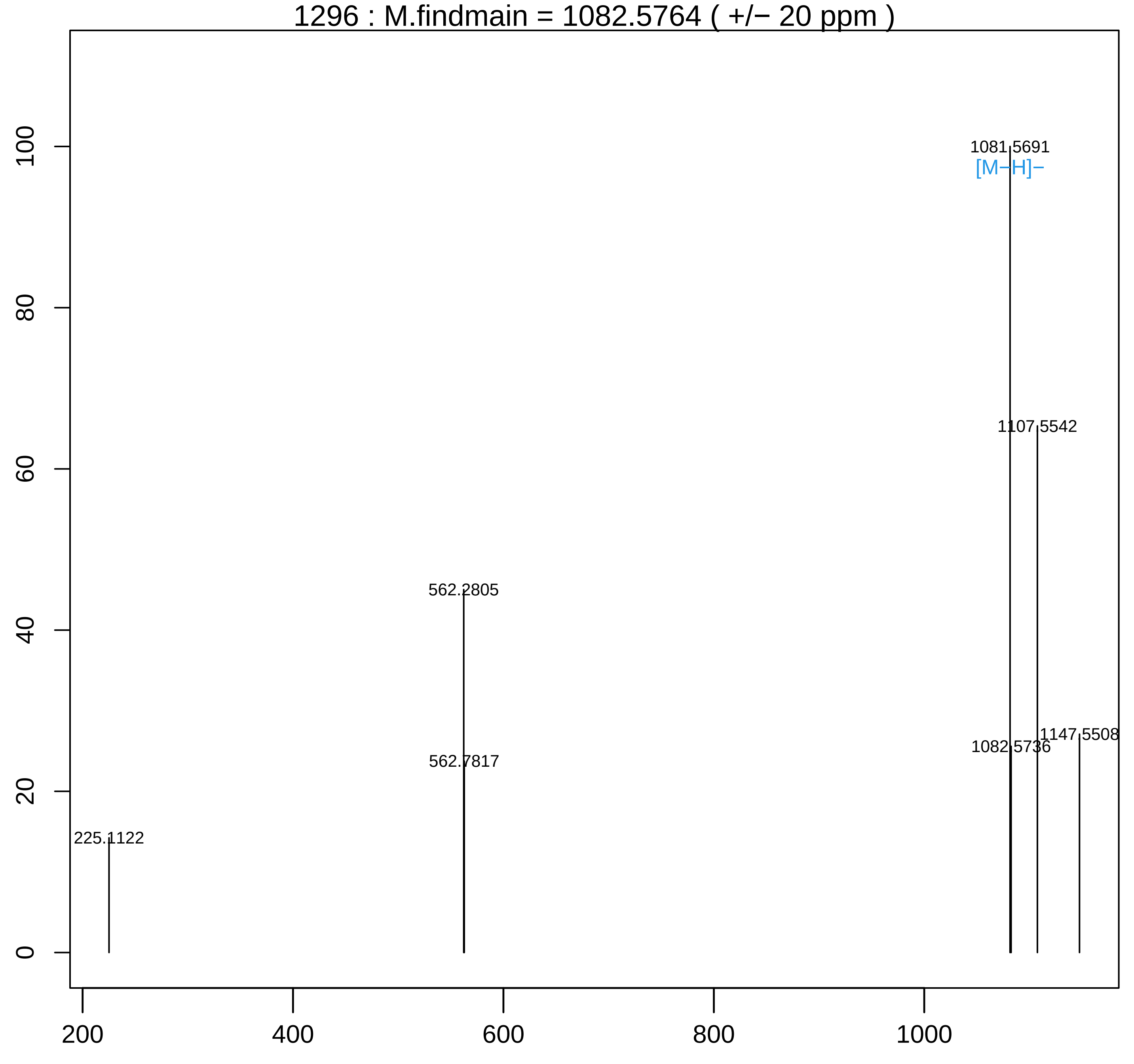
1296 : M.findmain = 1082.5764 (+/− 20 ppm)
1081.5691
[M−H]−
1107.5542
562.2805
1147.5508
1082.5736
562.7817
225.1122
0
20
40
60
80
100
200
400
600
800
1000

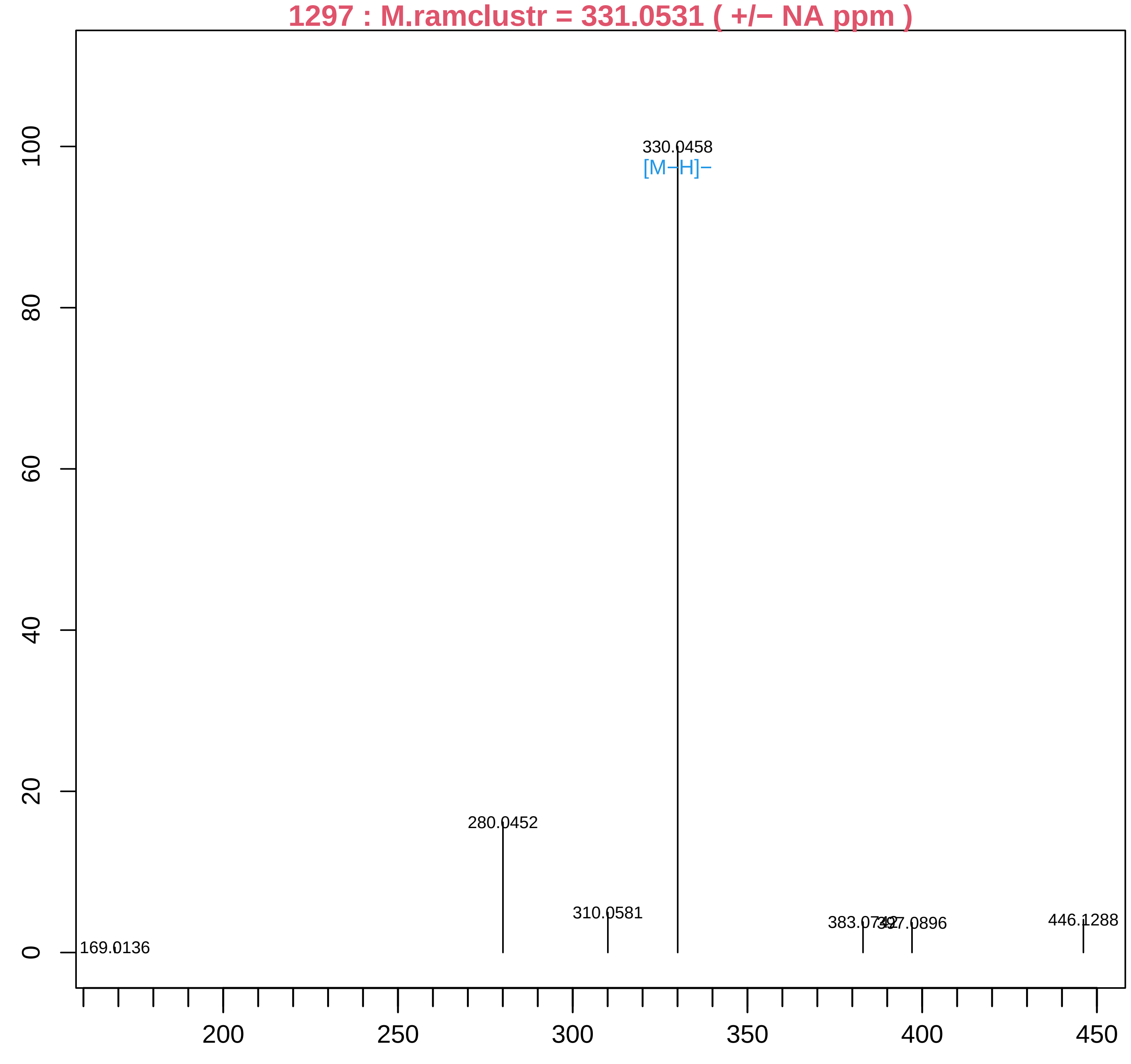
1297 : M.ramclustr = 331.0531 (+/− NA ppm)
330.0458
[M−H]−
280.0452
310.0581
383.0742
397.0896
446.1288
169.0136
0
20
40
60
80
100
200
250
300
350
400
450

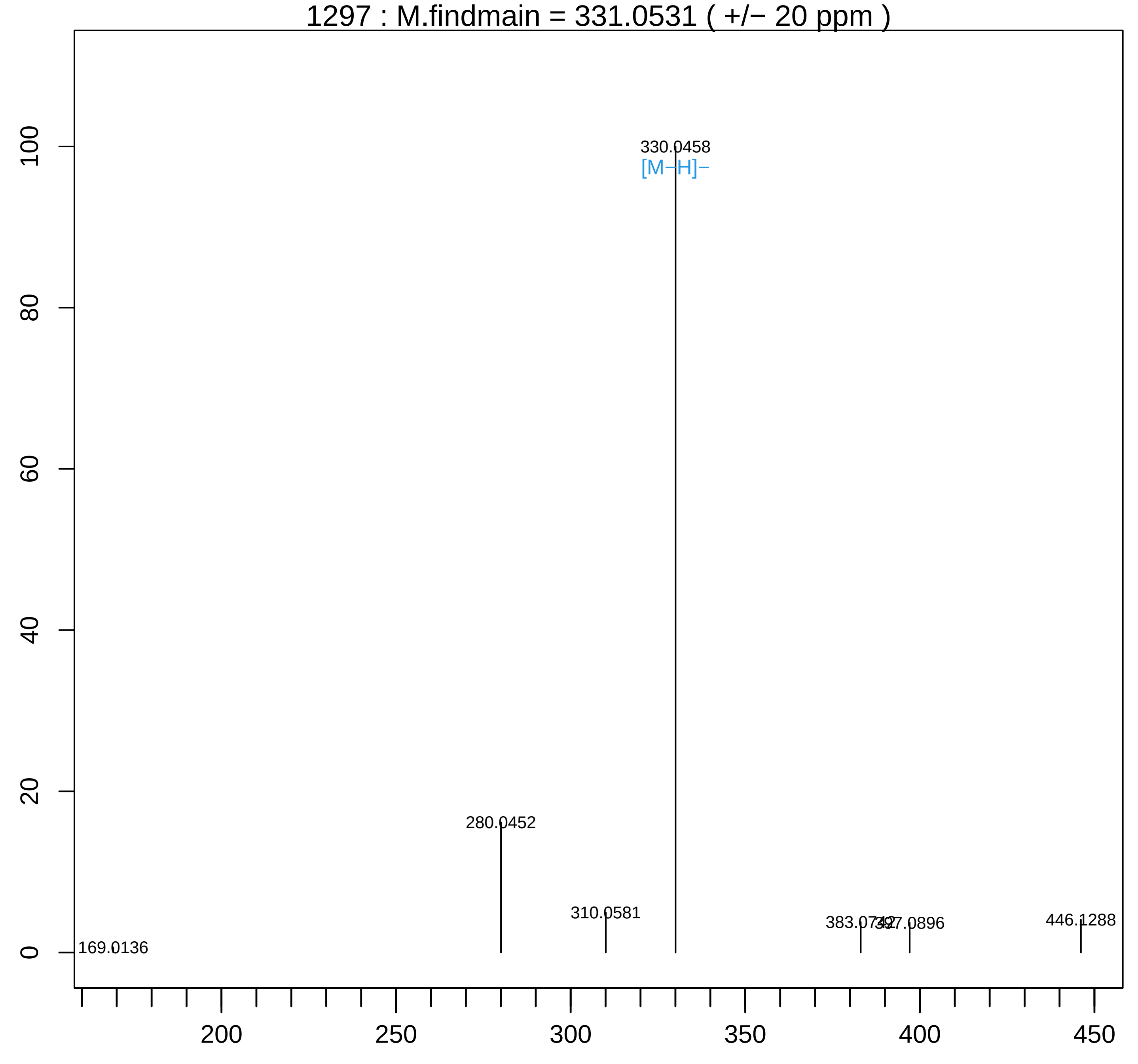
1297 : M.findmain = 331.0531 (+/− 20 ppm)
330.0458
[M−H]−
280.0452
310.0581
383.0742
397.0896
446.1288
169.0136
0
20
40
60
80
100
200
250
300
350
400
450

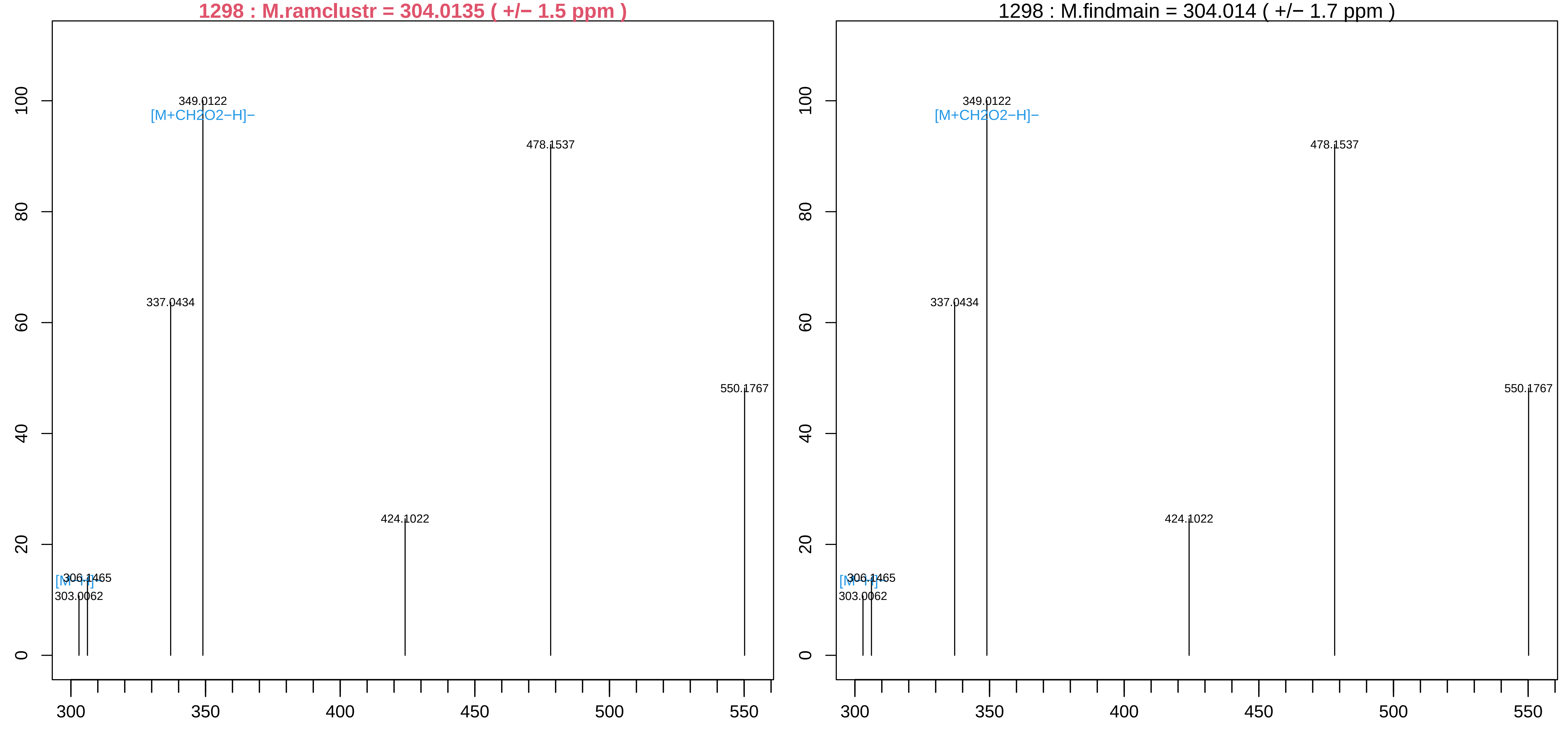
1298 : M.ramclustr = 304.0135 (+/− 1.5 ppm)
349.0122
[M+CH2O2−H]−
478.1537
337.0434
550.1767
424.1022
306.1465
[M−H]−
303.0062
0
20
40
60
80
100
300
350
400
450
500
550
1298 : M.findmain = 304.014 (+/− 1.7 ppm)
349.0122
[M+CH2O2−H]−
478.1537
337.0434
550.1767
424.1022
306.1465
[M−H]−
303.0062
0
20
40
60
80
100
300
350
400
450
500
550

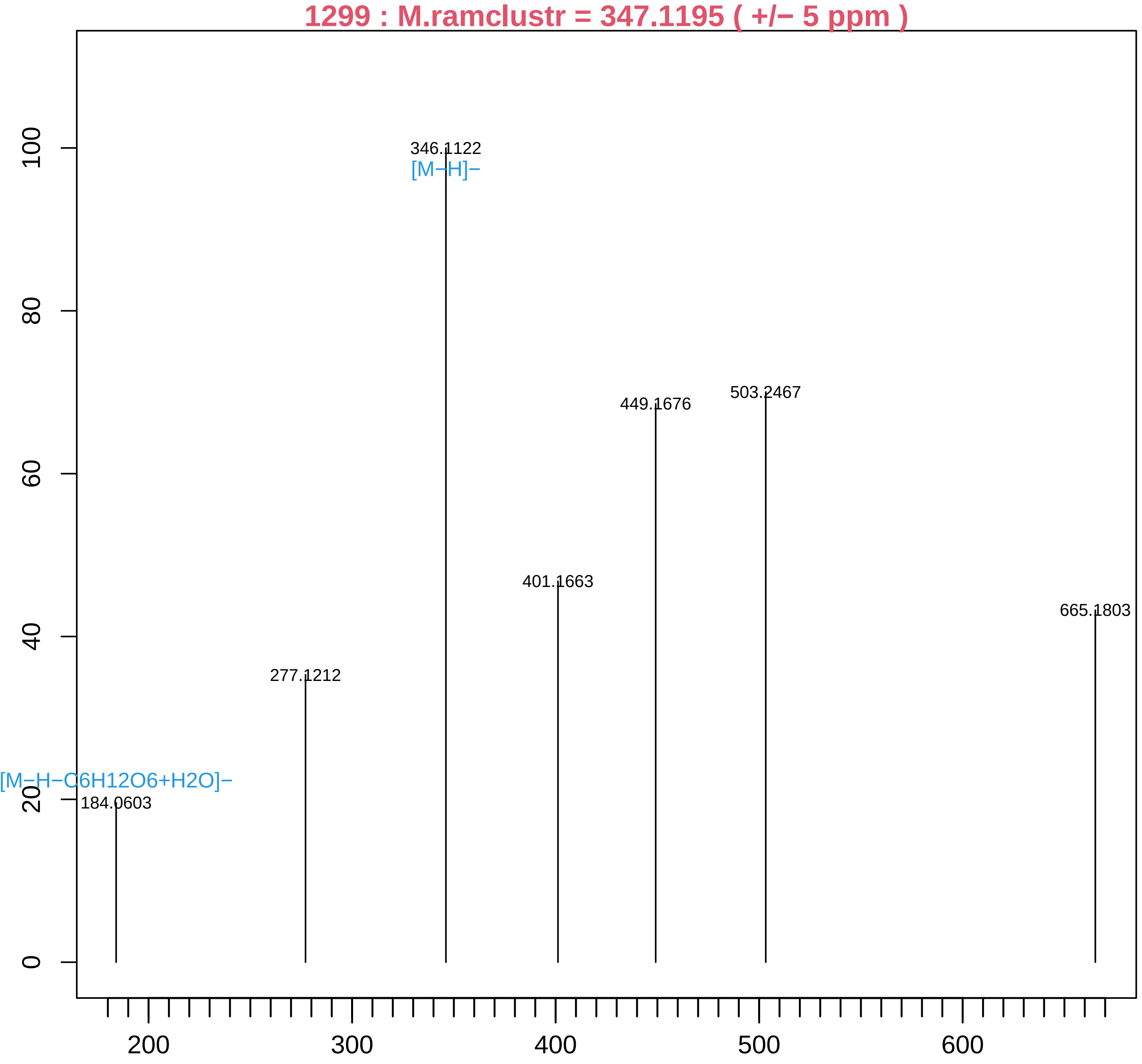
1299 : M.ramclustr = 347.1195 (+/− 5 ppm)
346.1122
[M−H]−
449.1676
503.2467
401.1663
665.1803
277.1212
[M−H−C6H12O6+H2O]−
184.0603
0
20
40
60
80
100
200
300
400
500
600

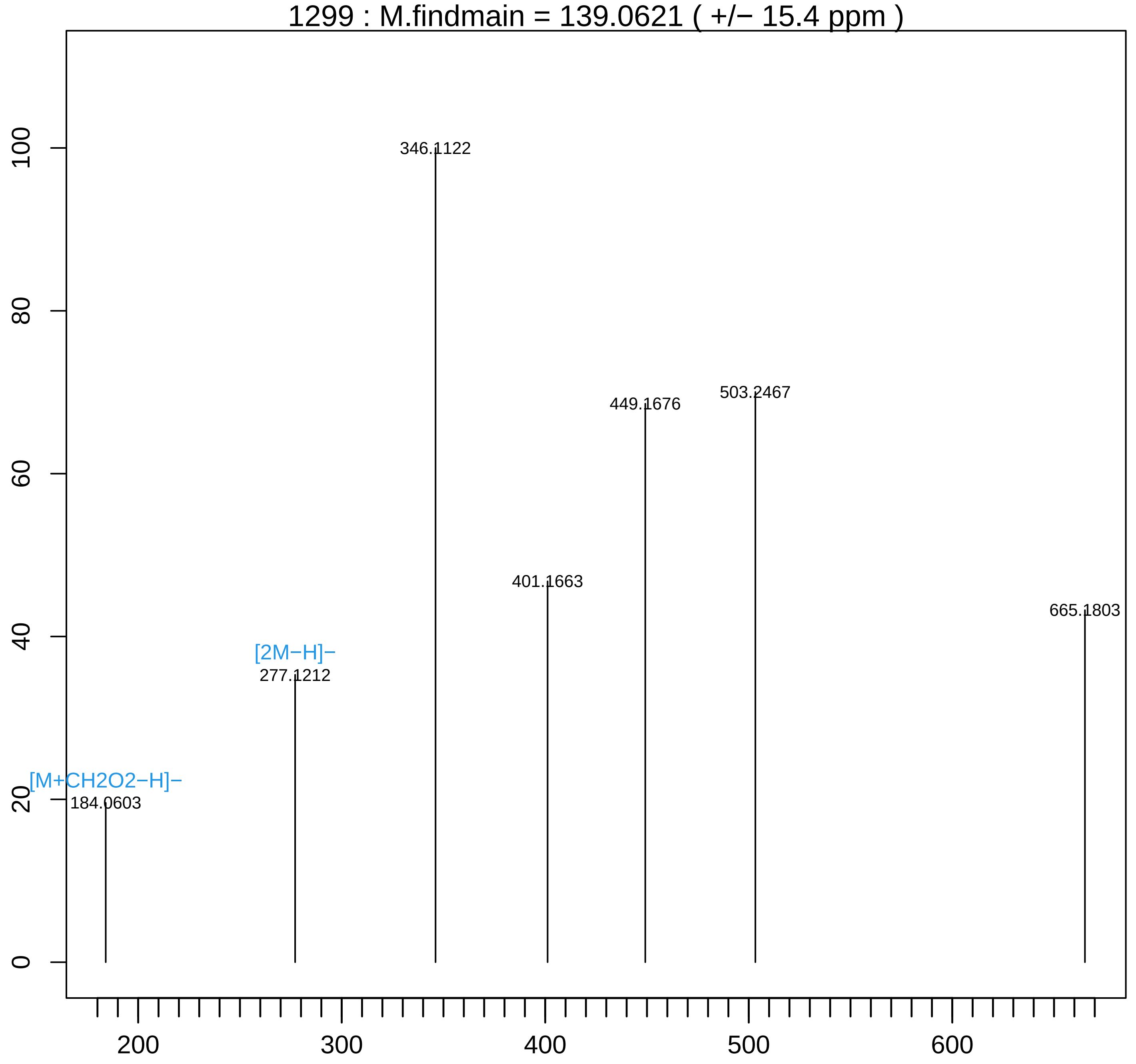
1299 : M.findmain = 139.0621 (+/− 15.4 ppm)
346.1122
449.1676
503.2467
401.1663
665.1803
[2M−H]−
277.1212
[M+CH2O2−H]−
184.0603
0
20
40
60
80
100
200
300
400
500
600

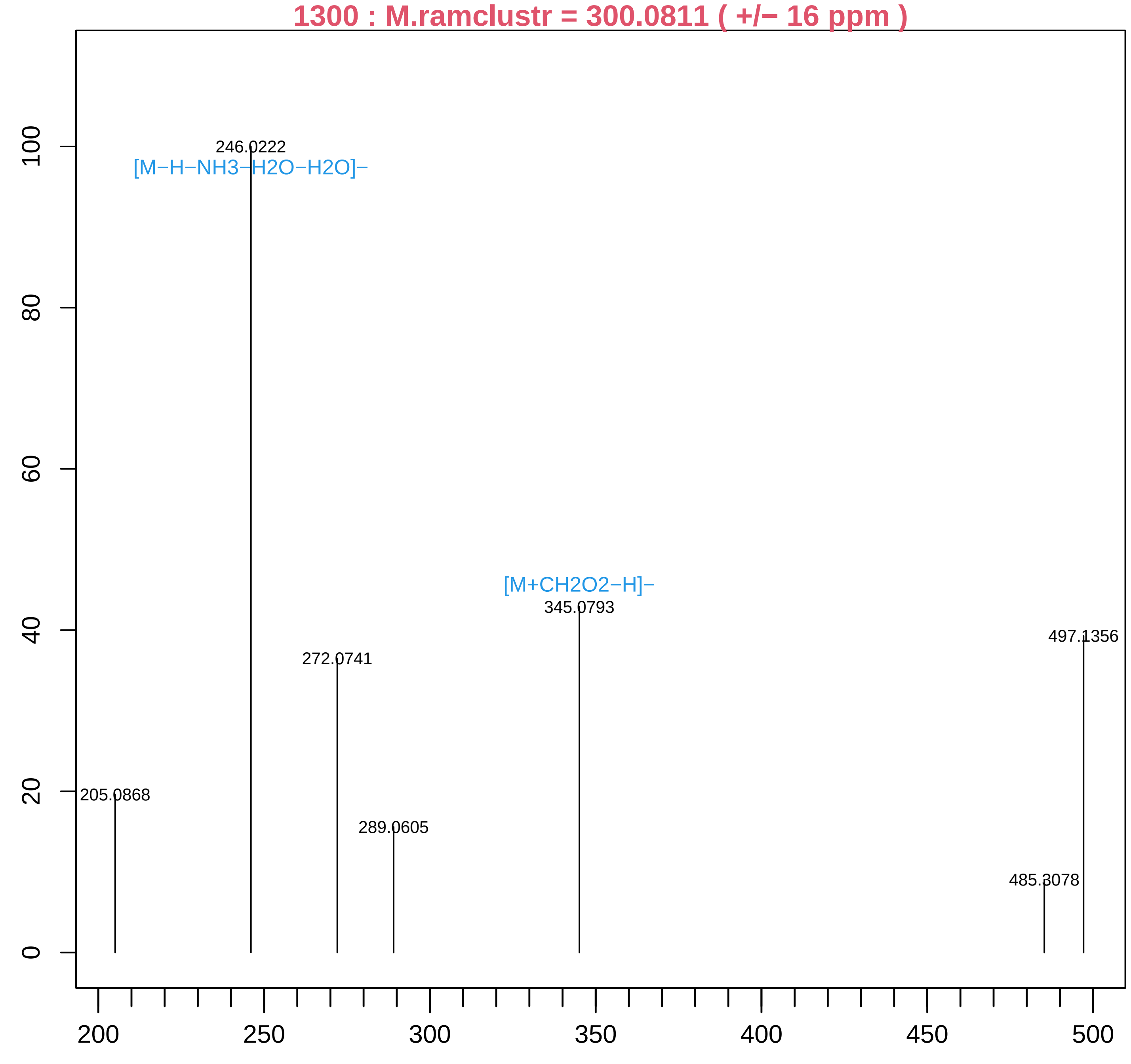

1300 : M.ramclustr = 300.0811 (+/− 16 ppm)
246.0222
[M−H−NH3−H2O−H2O]−
[M+CH2O2−H]−
345.0793
497.1356
272.0741
205.0868
289.0605
485.3078
0
20
40
60
80
100
200
250
300
350
400
450
500

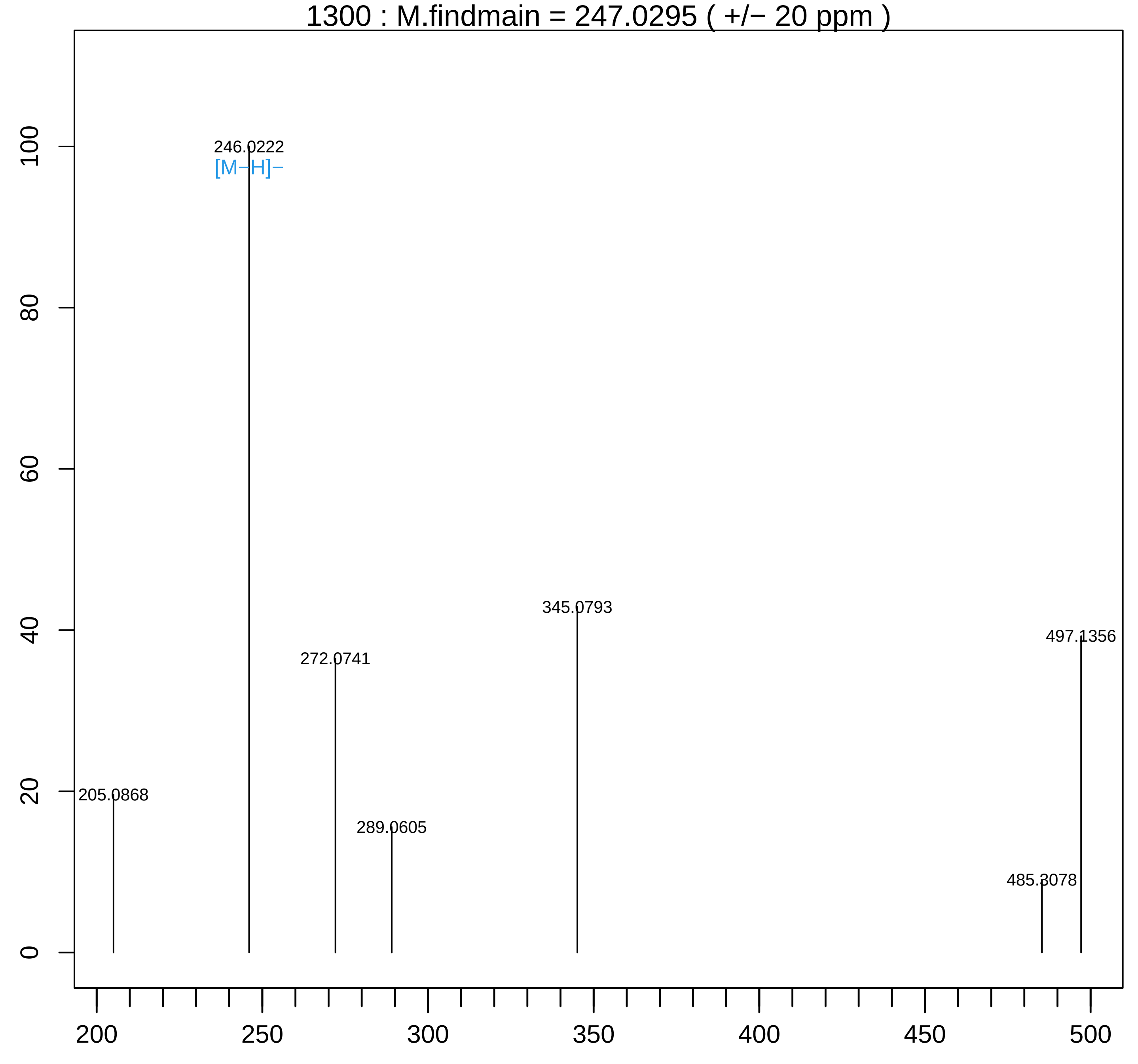

1300 : M.findmain = 247.0295 (+/− 20 ppm)
246.0222
[M−H]−
345.0793
497.1356
272.0741
205.0868
289.0605
485.3078
0
20
40
60
80
100
200
250
300
350
400
450
500

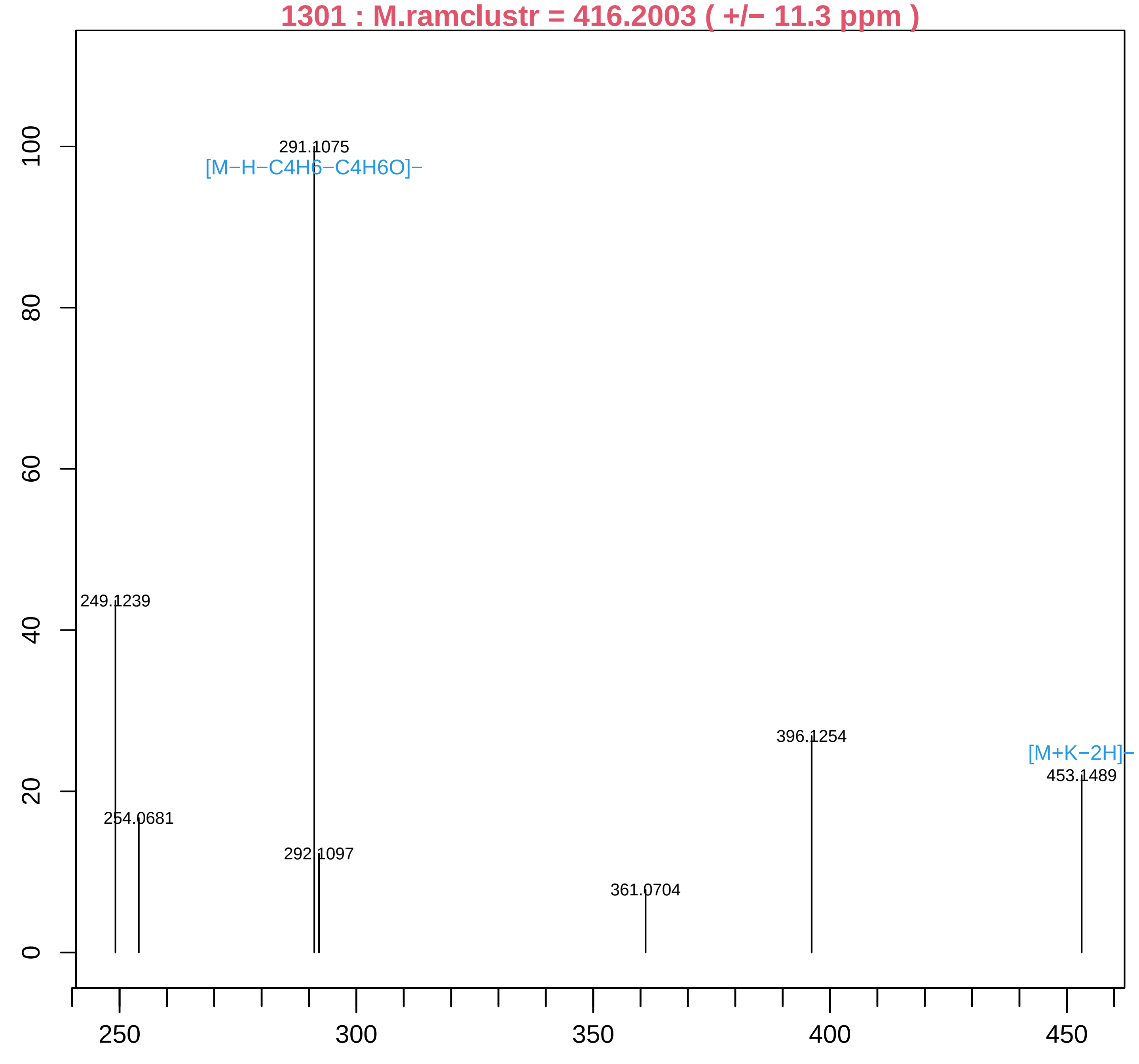
1301 : M.ramclustr = 416.2003 (+/− 11.3 ppm)
291.1075
[M−H−C4H6−C4H6O]−
249.1239
254.0681
292.1097
361.0704
396.1254
[M+K−2H]−
453.1489
0
20
40
60
80
100
250
300
350
400
450

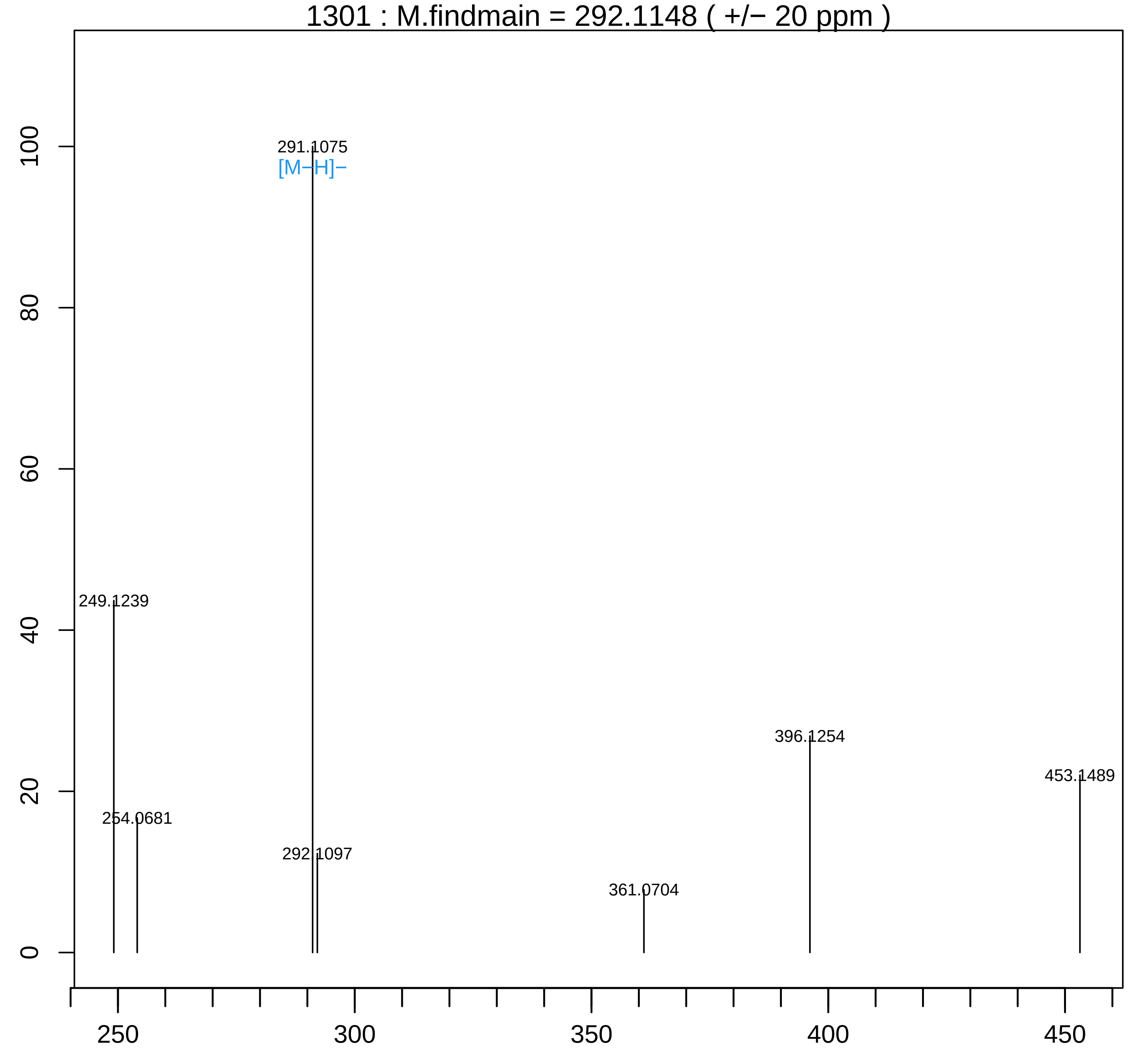
1301 : M.findmain = 292.1148 (+/− 20 ppm)
291.1075
[M−H]−
249.1239
254.0681
292.1097
361.0704
396.1254
453.1489
0
20
40
60
80
100
250
300
350
400
450

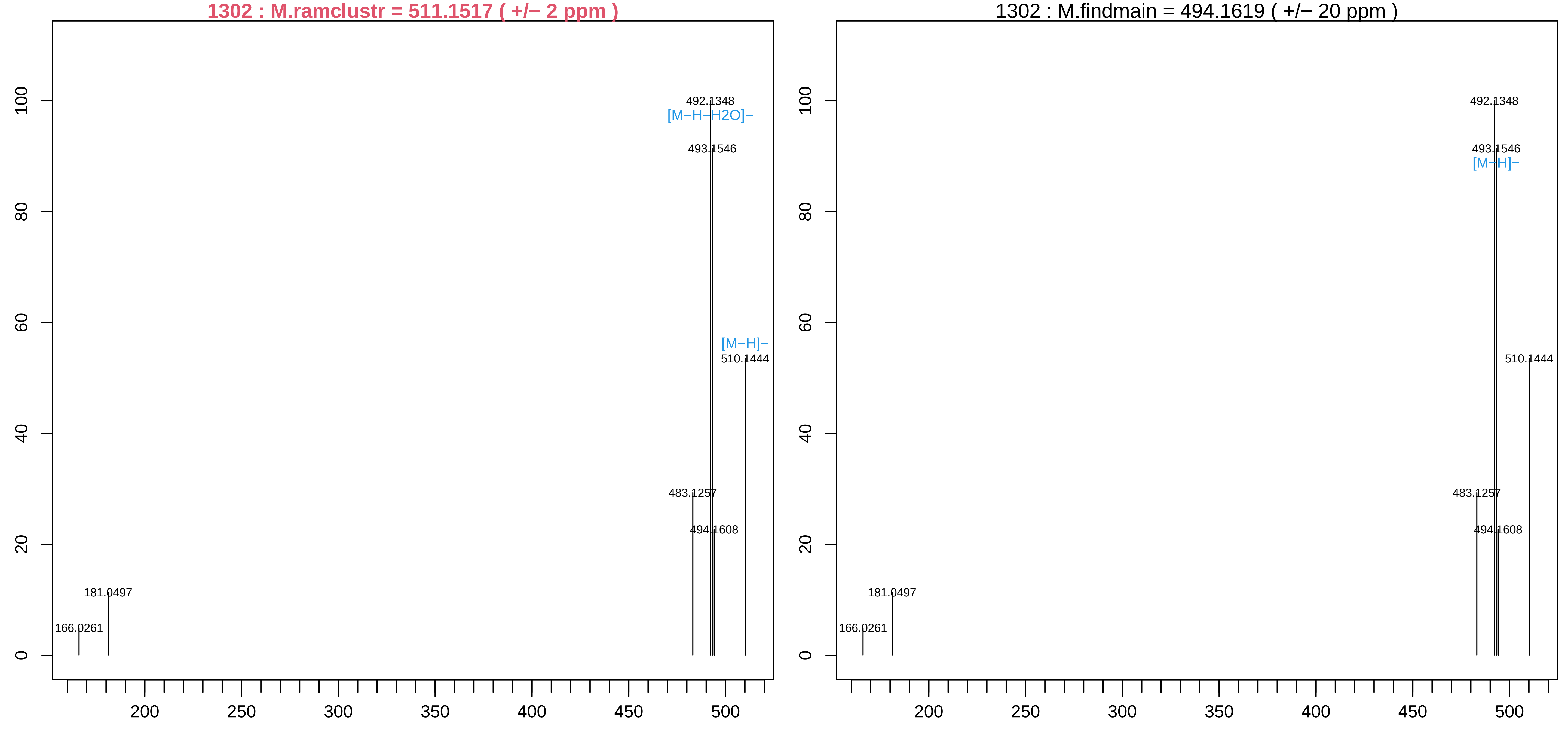
1302 : M.ramclustr = 511.1517 (+/− 2 ppm)
492.1348
[M−H−H2O]−
493.1546
[M−H]−
510.1444
483.1257
494.1608
181.0497
166.0261
1302 : M.findmain = 494.1619 (+/− 20 ppm)
492.1348
493.1546
[M−H]−
510.1444
483.1257
494.1608
181.0497
166.0261

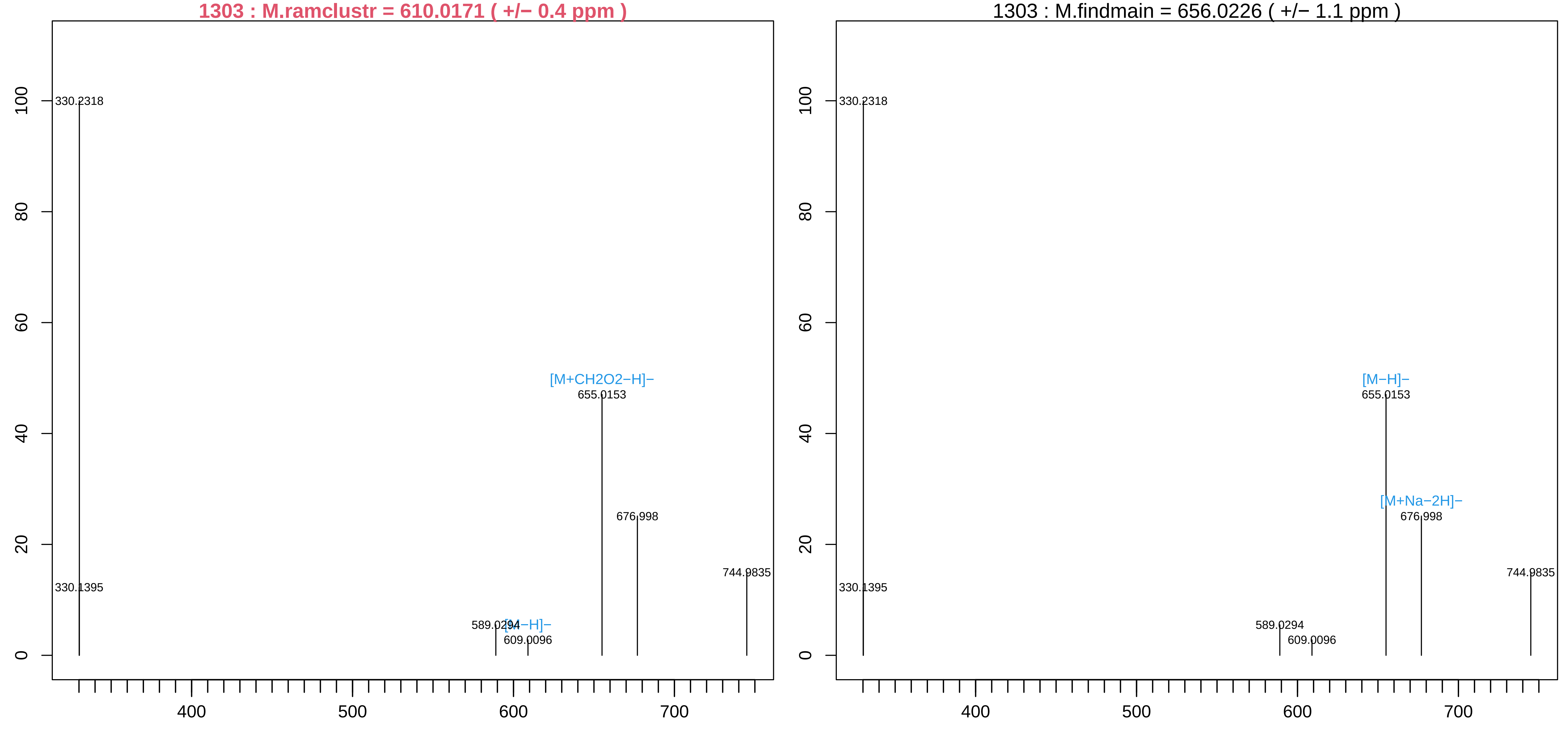

1303 : M.ramclustr = 610.0171 (+/− 0.4 ppm)
330.2318
330.1395
589.0294
[M−H]−
609.0096
[M+CH2O2−H]−
655.0153
676.998
744.9835
1303 : M.findmain = 656.0226 (+/− 1.1 ppm)
330.2318
330.1395
589.0294
609.0096
[M−H]−
655.0153
[M+Na−2H]−
676.998
744.9835
0
20
40
60
80
100
400
500
600
700

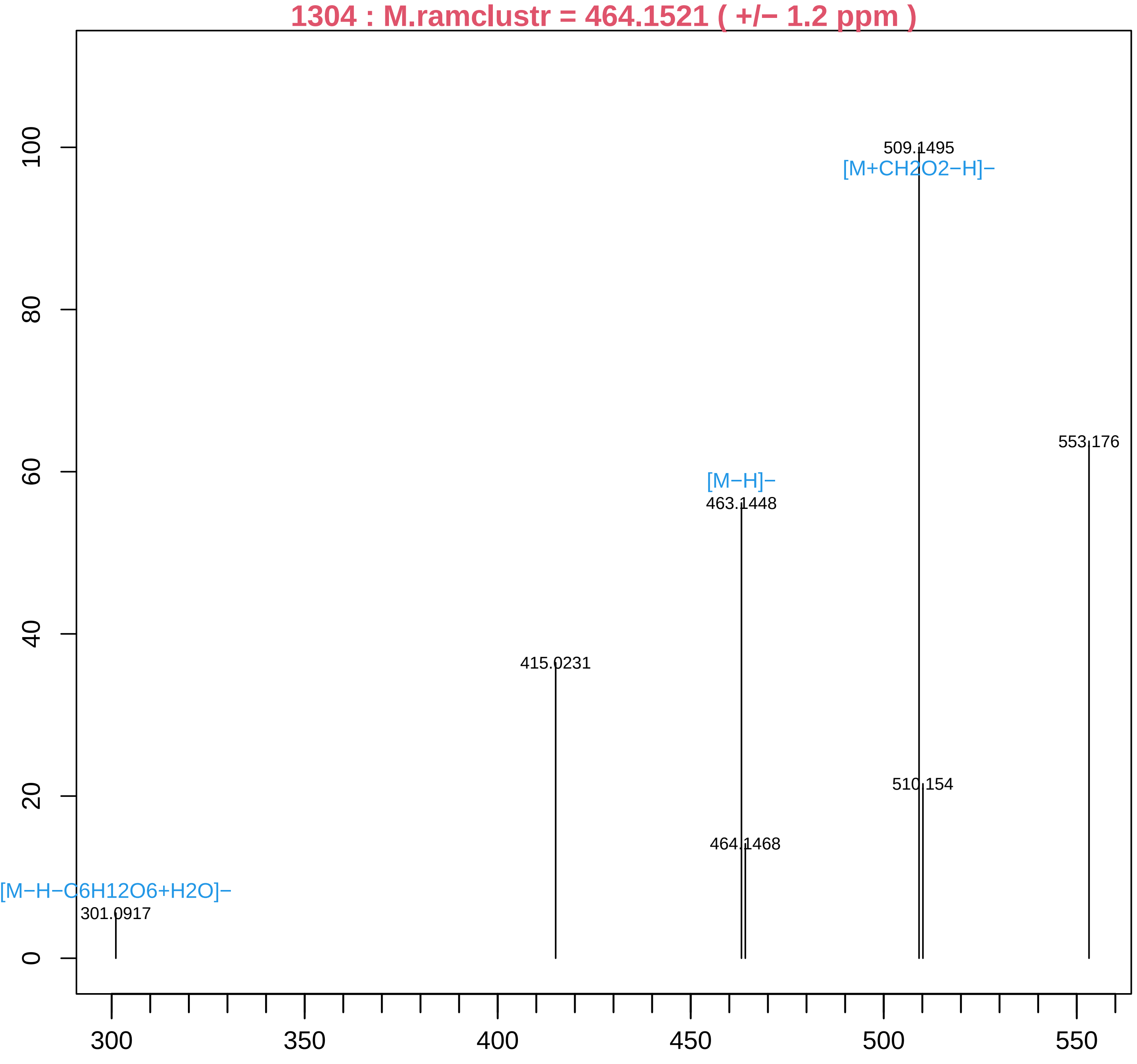
1304 : M.ramclustr = 464.1521 (+/− 1.2 ppm)
509.1495
[M+CH2O2−H]−
553.176
[M−H]−
463.1448
415.0231
510.154
464.1468
[M−H−C6H12O6+H2O]−
301.0917
0
20
40
60
80
100
300
350
400
450
500
550
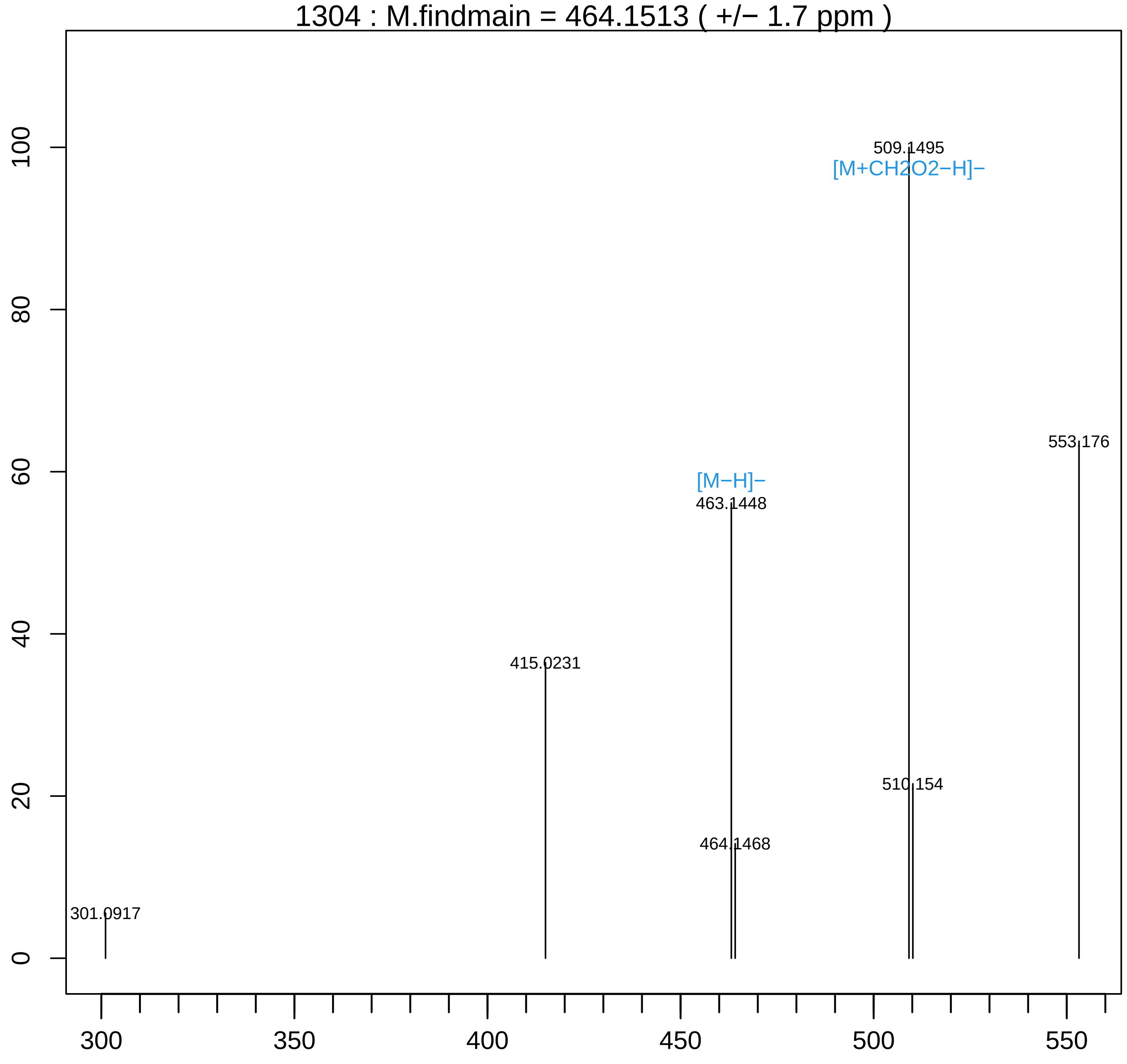
1304 : M.findmain = 464.1513 (+/− 1.7 ppm)
509.1495
[M+CH2O2−H]−
553.176
[M−H]−
463.1448
415.0231
510.154
464.1468
301.0917
0
20
40
60
80
100
300
350
400
450
500
550

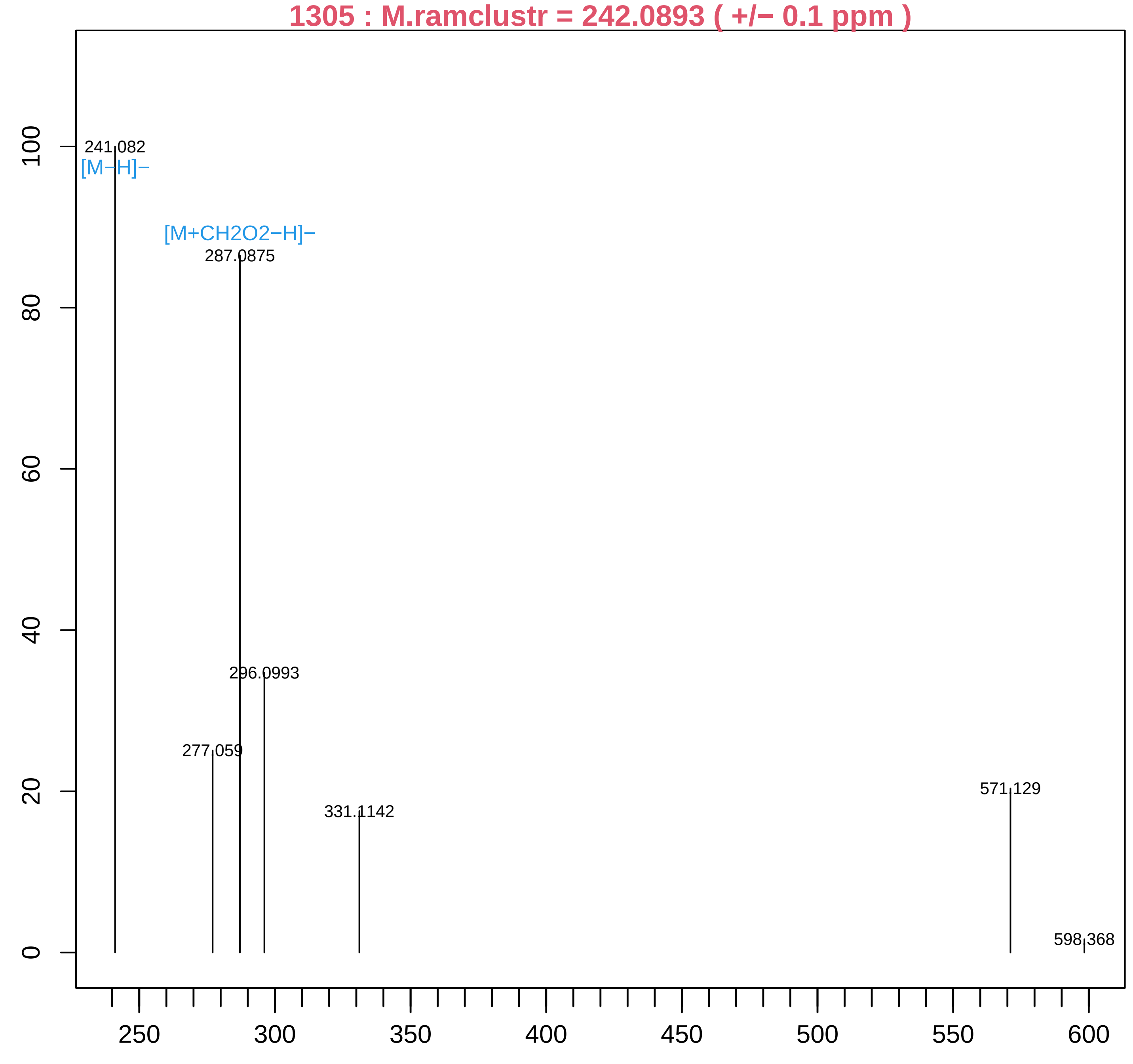

1305 : M.ramclustr = 242.0893 (+/− 0.1 ppm)
241.082
[M−H]−
[M+CH2O2−H]−
287.0875
296.0993
277.059
331.1142
571.129
598.368
0
20
40
60
80
100
250
300
350
400
450
500
550
600

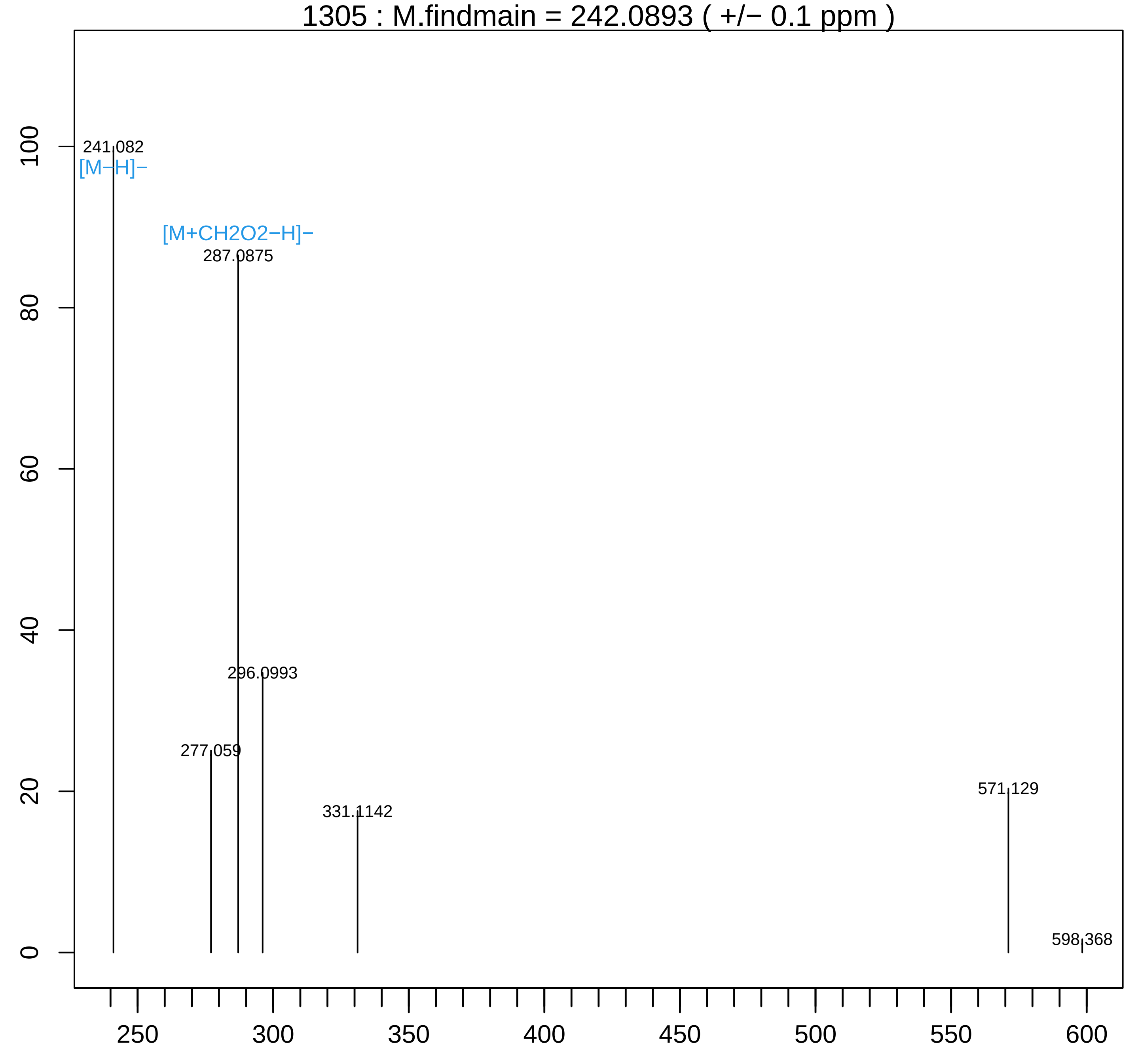

1305 : M.findmain = 242.0893 (+/− 0.1 ppm)
241.082
[M−H]−
[M+CH2O2−H]−
287.0875
296.0993
277.059
331.1142
571.129
598.368
0
20
40
60
80
100
250
300
350
400
450
500
550
600

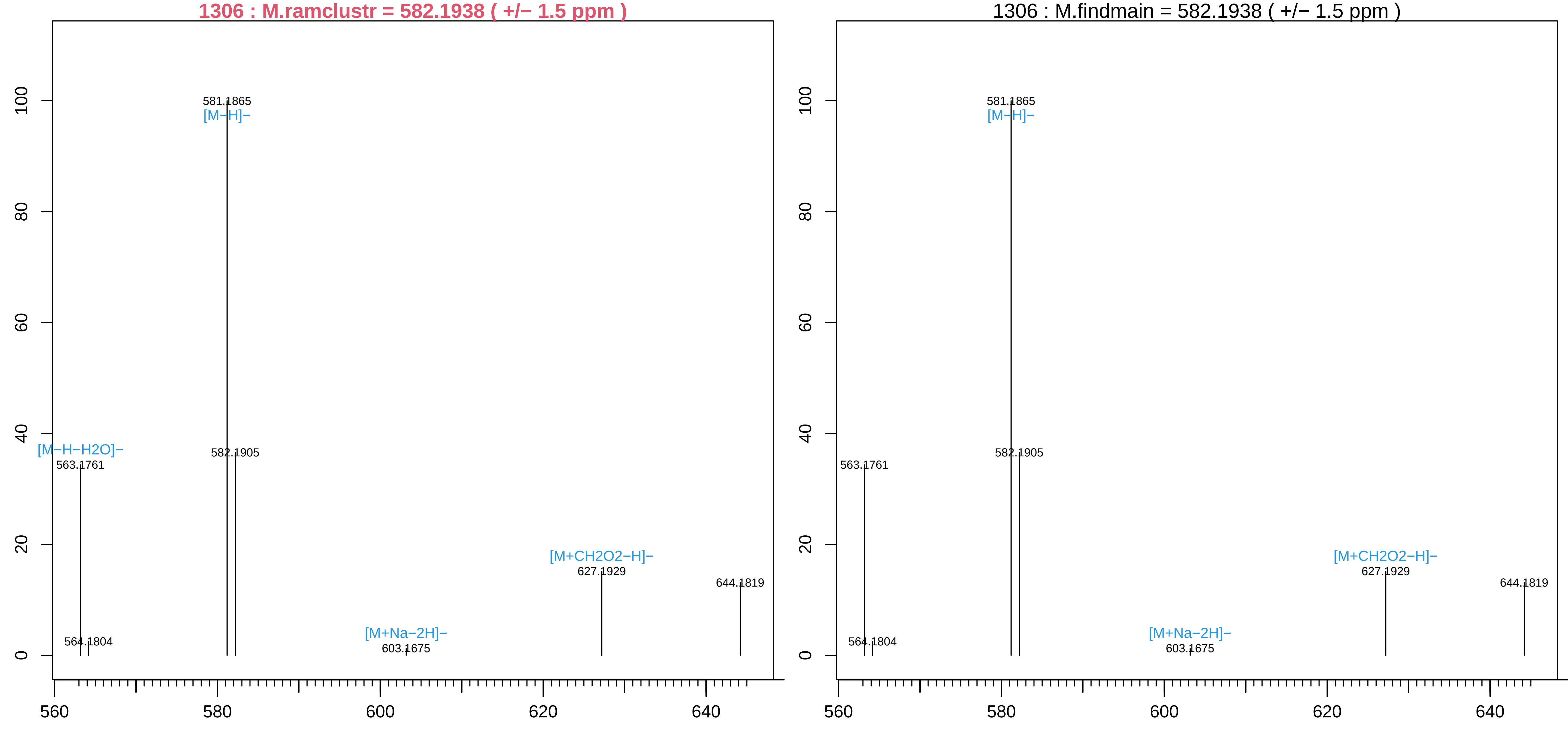
1306 : M.ramclustr = 582.1938 (+/− 1.5 ppm)
581.1865
[M−H]−
[M−H−H2O]−
563.1761
582.1905
564.1804
[M+Na−2H]−
603.1675
[M+CH2O2−H]−
627.1929
644.1819
1306 : M.findmain = 582.1938 (+/− 1.5 ppm)
581.1865
[M−H]−
563.1761
582.1905
564.1804
[M+Na−2H]−
603.1675
[M+CH2O2−H]−
627.1929
644.1819
0
20
40
60
80
100
560
580
600
620
640

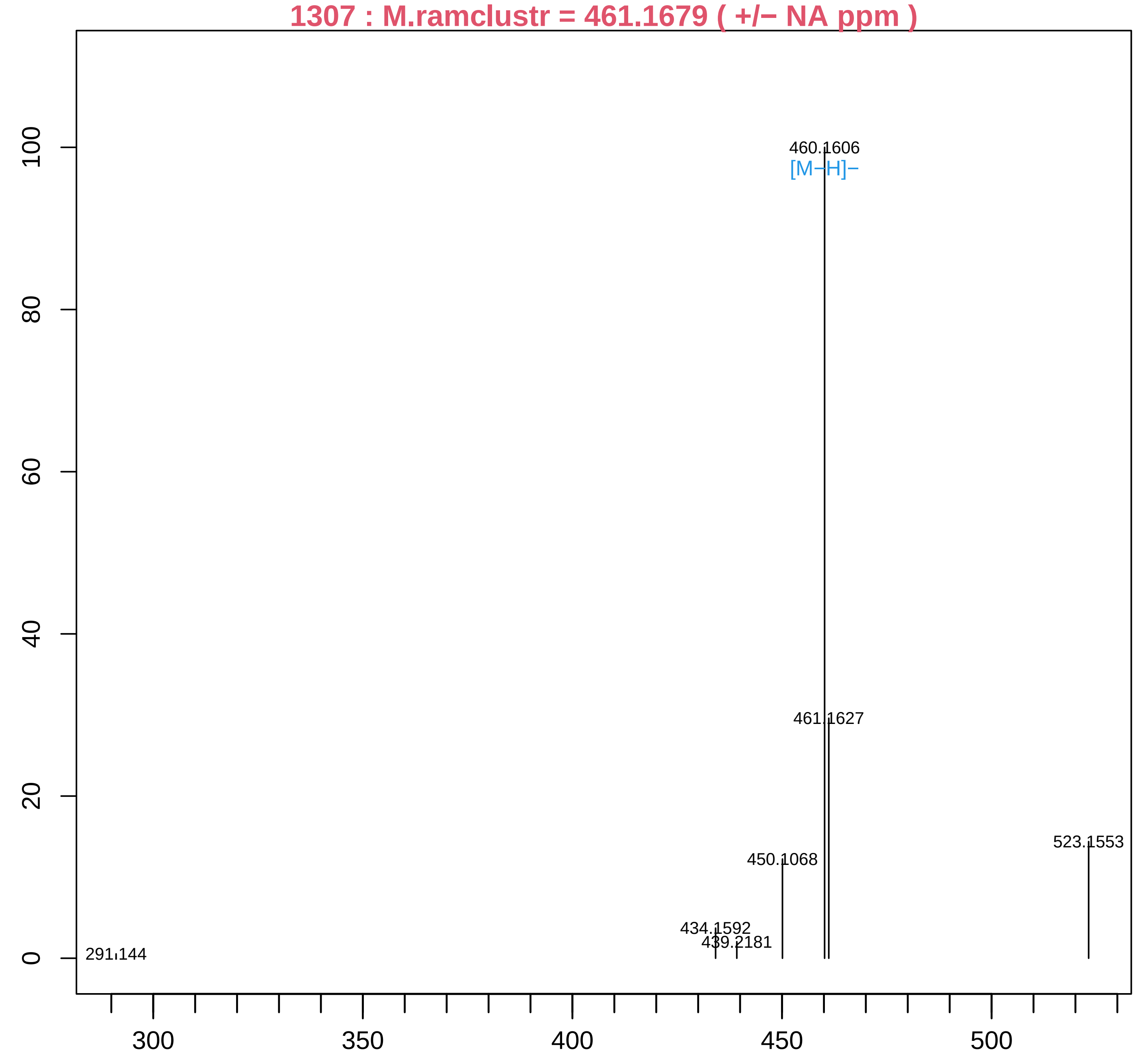

1307 : M.ramclustr = 461.1679 (+/− NA ppm)
460.1606
[M−H]−
461.1627
450.1068
523.1553
434.1592
439.2181
291.144
0
20
40
60
80
100
300
350
400
450
500

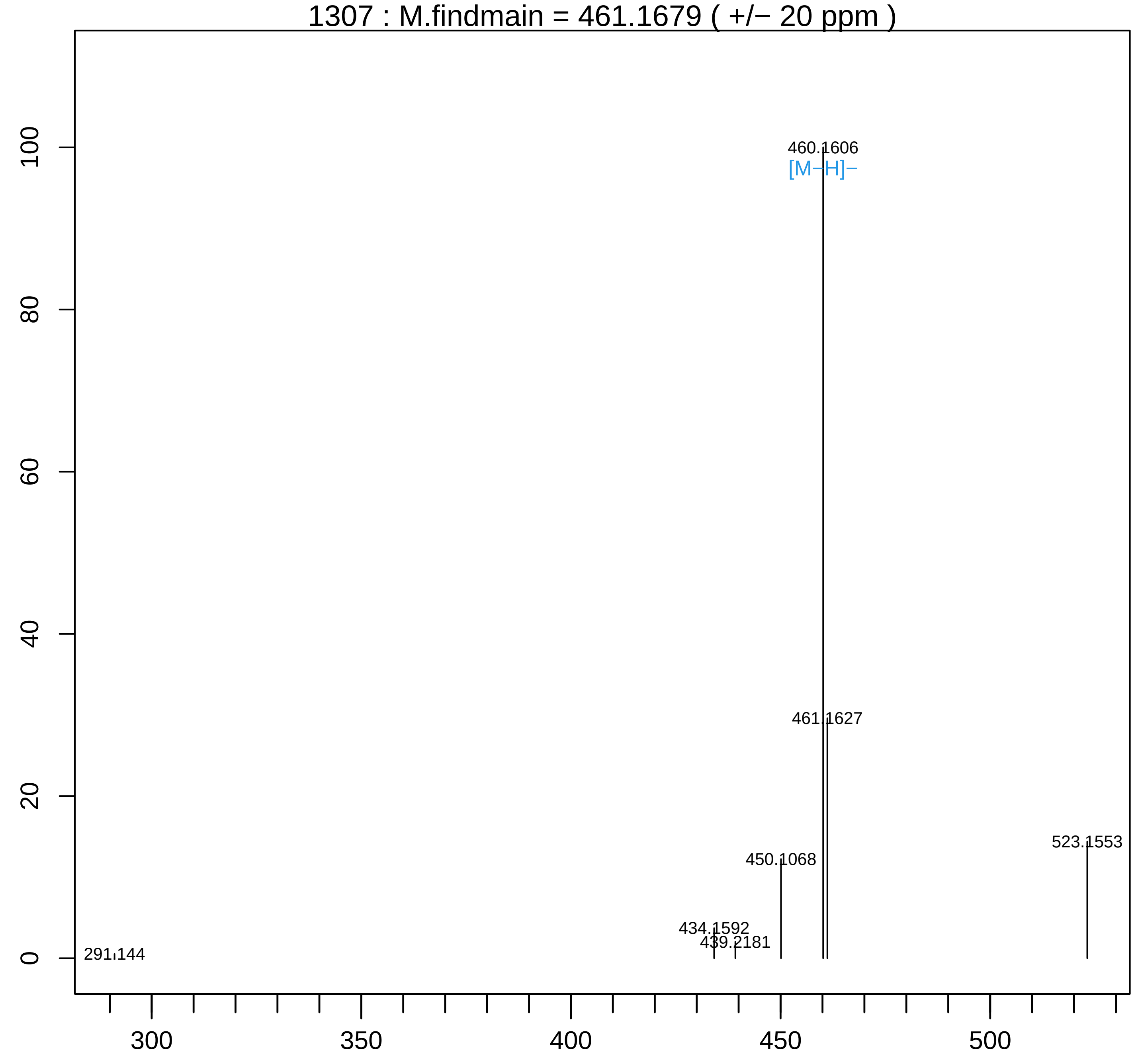

1307 : M.findmain = 461.1679 (+/− 20 ppm)
460.1606
[M−H]−
461.1627
450.1068
523.1553
434.1592
439.2181
291.144
0
20
40
60
80
100
300
350
400
450
500

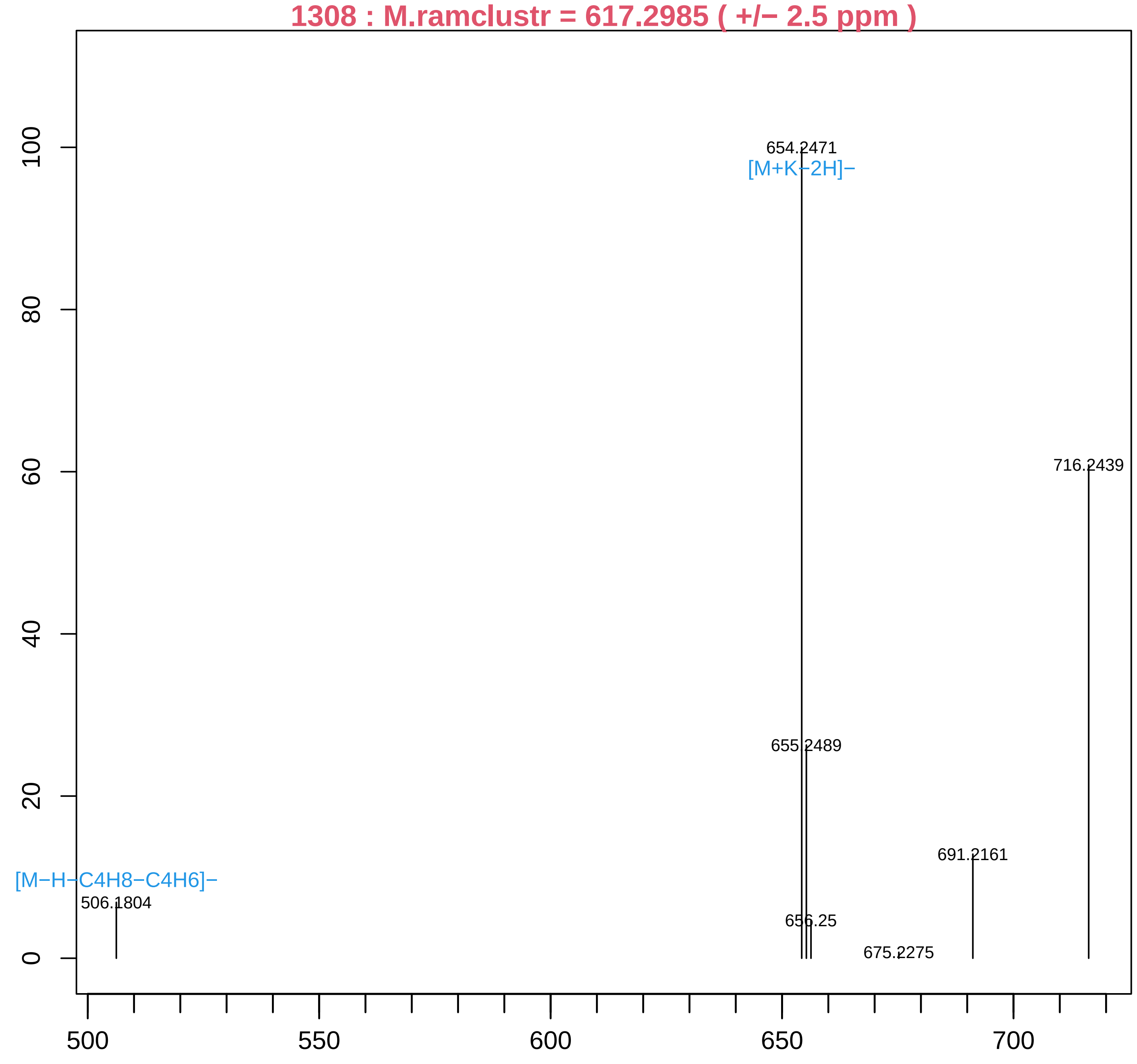

1308 : M.ramclustr = 617.2985 (+/− 2.5 ppm)
654.2471
[M+K−2H]−
716.2439
655.2489
691.2161
[M−H−C4H8−C4H6]−
506.1804
656.25
675.2275
0
20
40
60
80
100
500
550
600
650
700

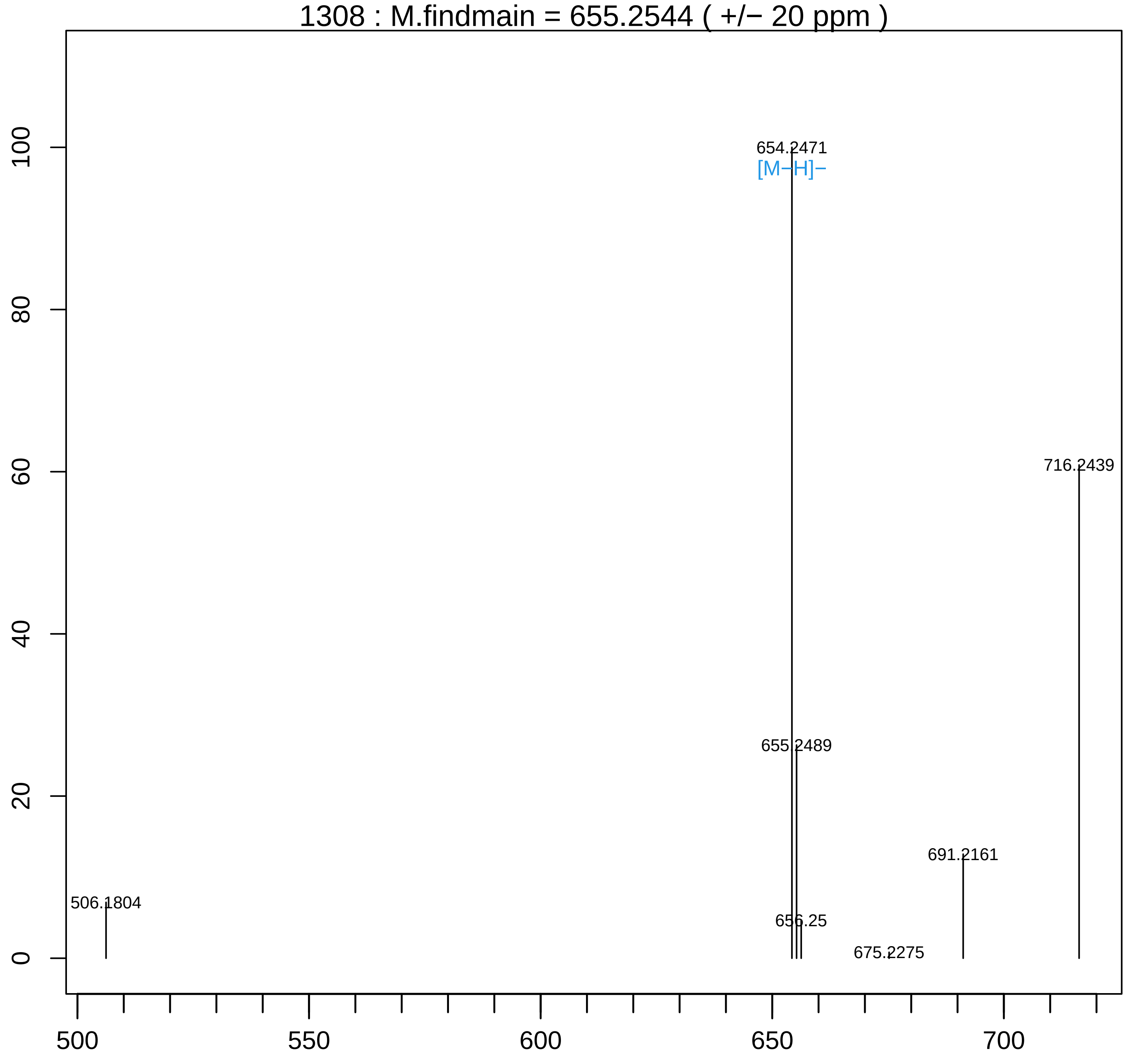

1308 : M.findmain = 655.2544 (+/− 20 ppm)
654.2471
[M−H]−
716.2439
655.2489
691.2161
506.1804
656.25
675.2275
0
20
40
60
80
100
500
550
600
650
700

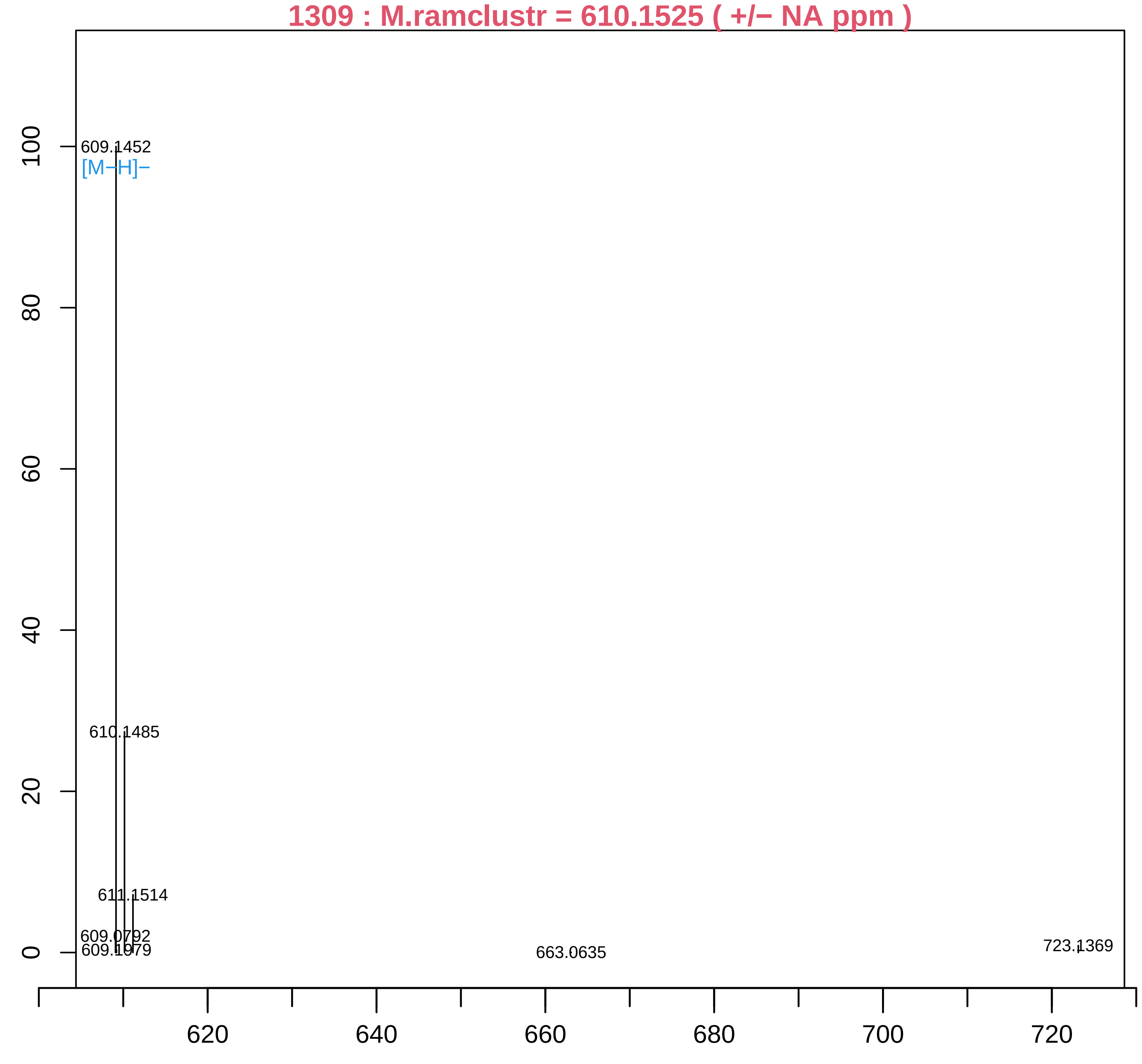
1309 : M.ramclustr = 610.1525 (+/− NA ppm)
609.1452
[M−H]−
610.1485
611.1514
609.0792
609.1979
663.0635
723.1369
0
20
40
60
80
100
620
640
660
680
700
720

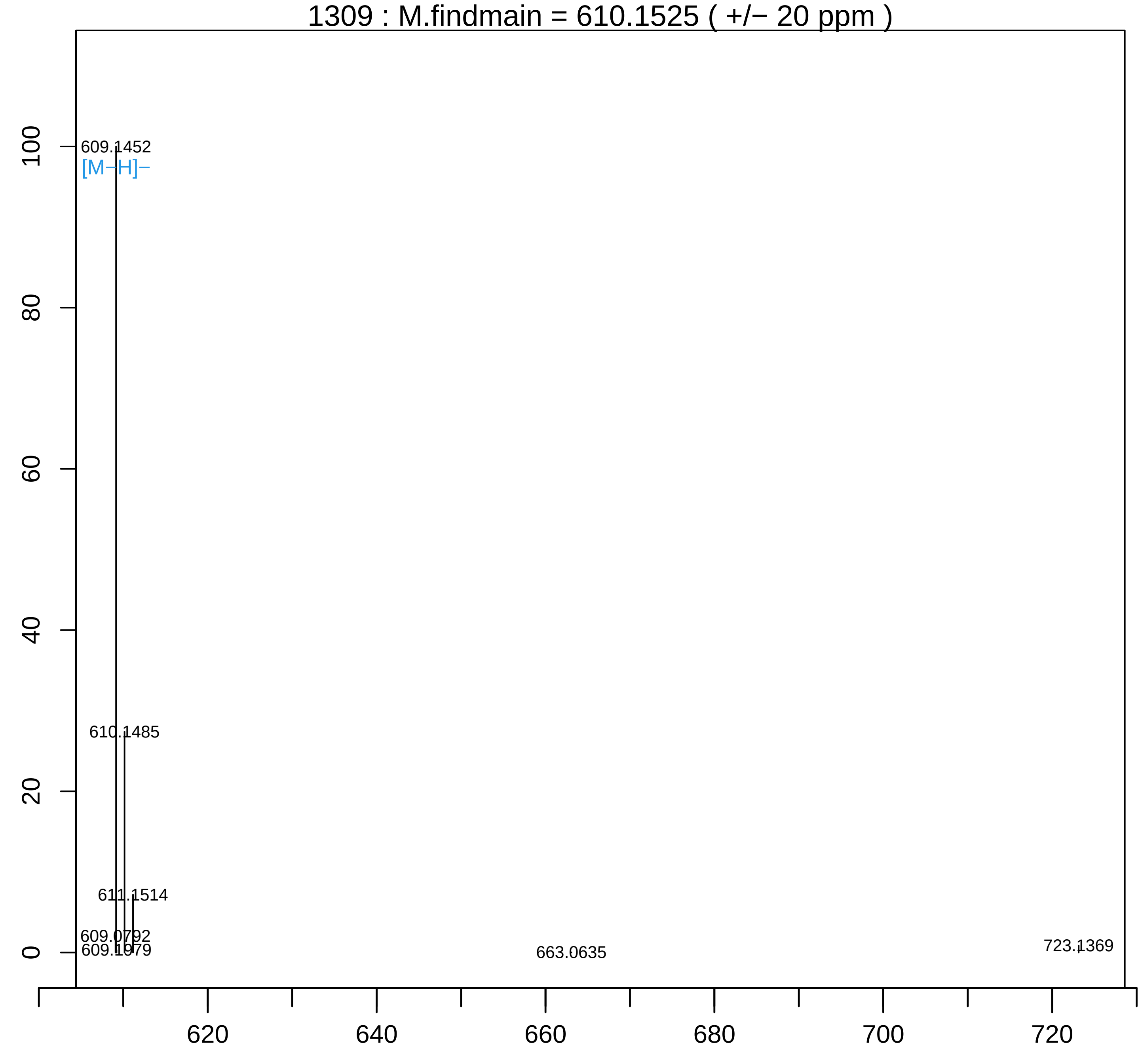
1309 : M.findmain = 610.1525 (+/− 20 ppm)
609.1452
[M−H]−
610.1485
611.1514
609.0792
609.1979
663.0635
723.1369
0
20
40
60
80
100
620
640
660
680
700
720

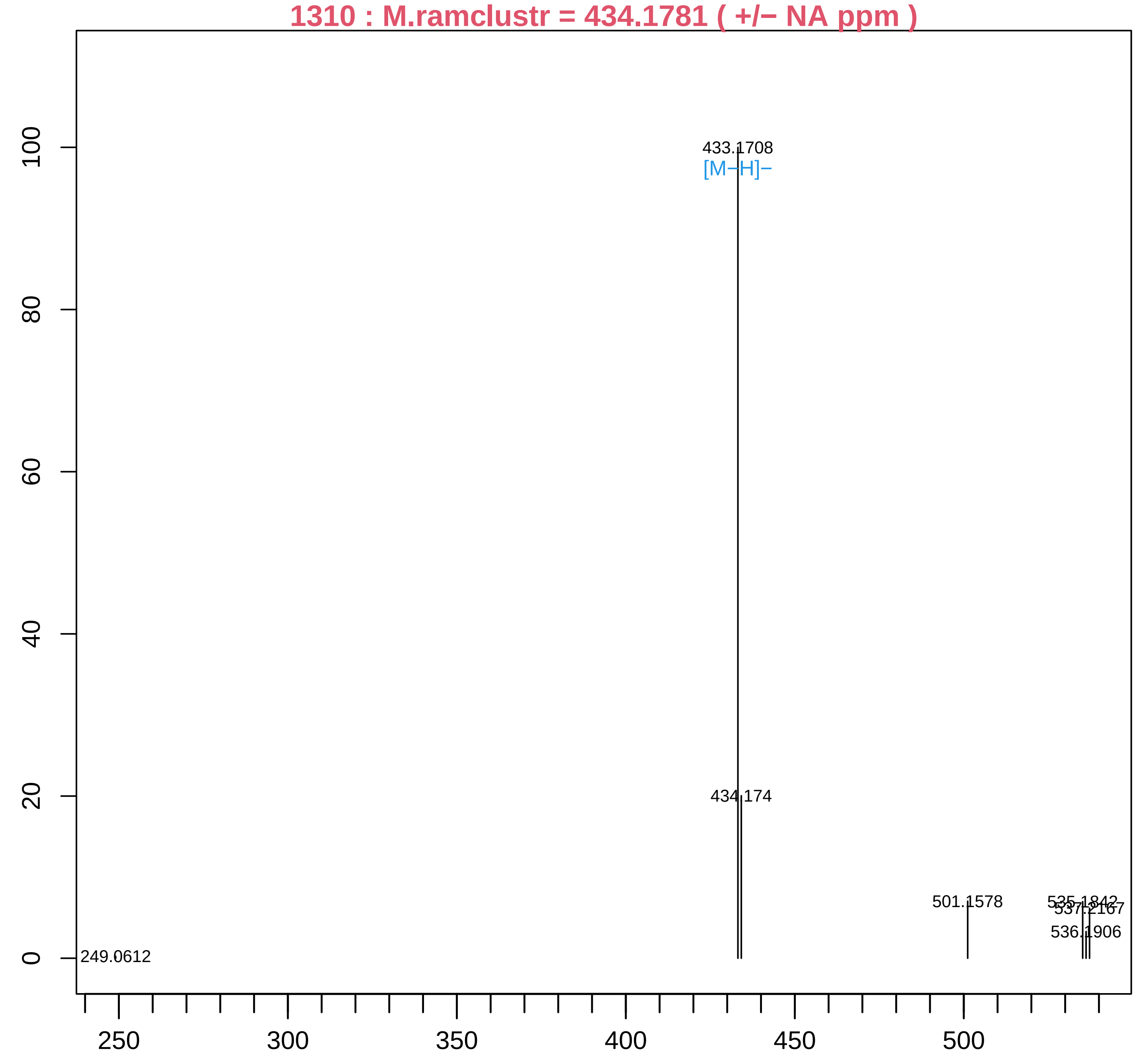

1310 : M.ramclustr = 434.1781 (+/− NA ppm)
433.1708
[M−H]−
434.174
501.1578
535.1842
537.2167
536.1906
249.0612
0
20
40
60
80
100
250
300
350
400
450
500

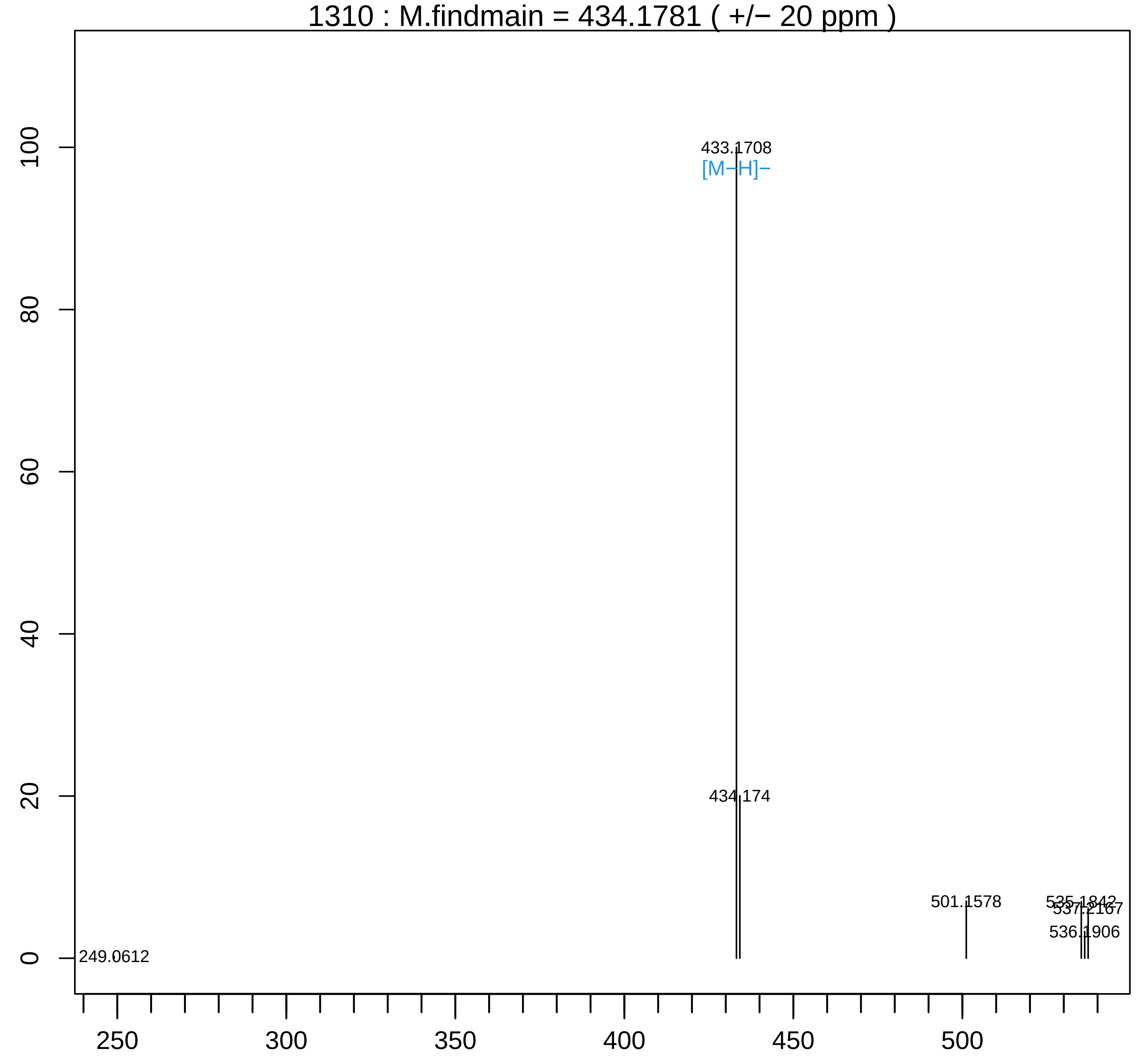

1310 : M.findmain = 434.1781 (+/− 20 ppm)
433.1708
[M−H]−
434.174
501.1578
535.1842
537.2167
536.1906
249.0612
0
20
40
60
80
100
250
300
350
400
450
500

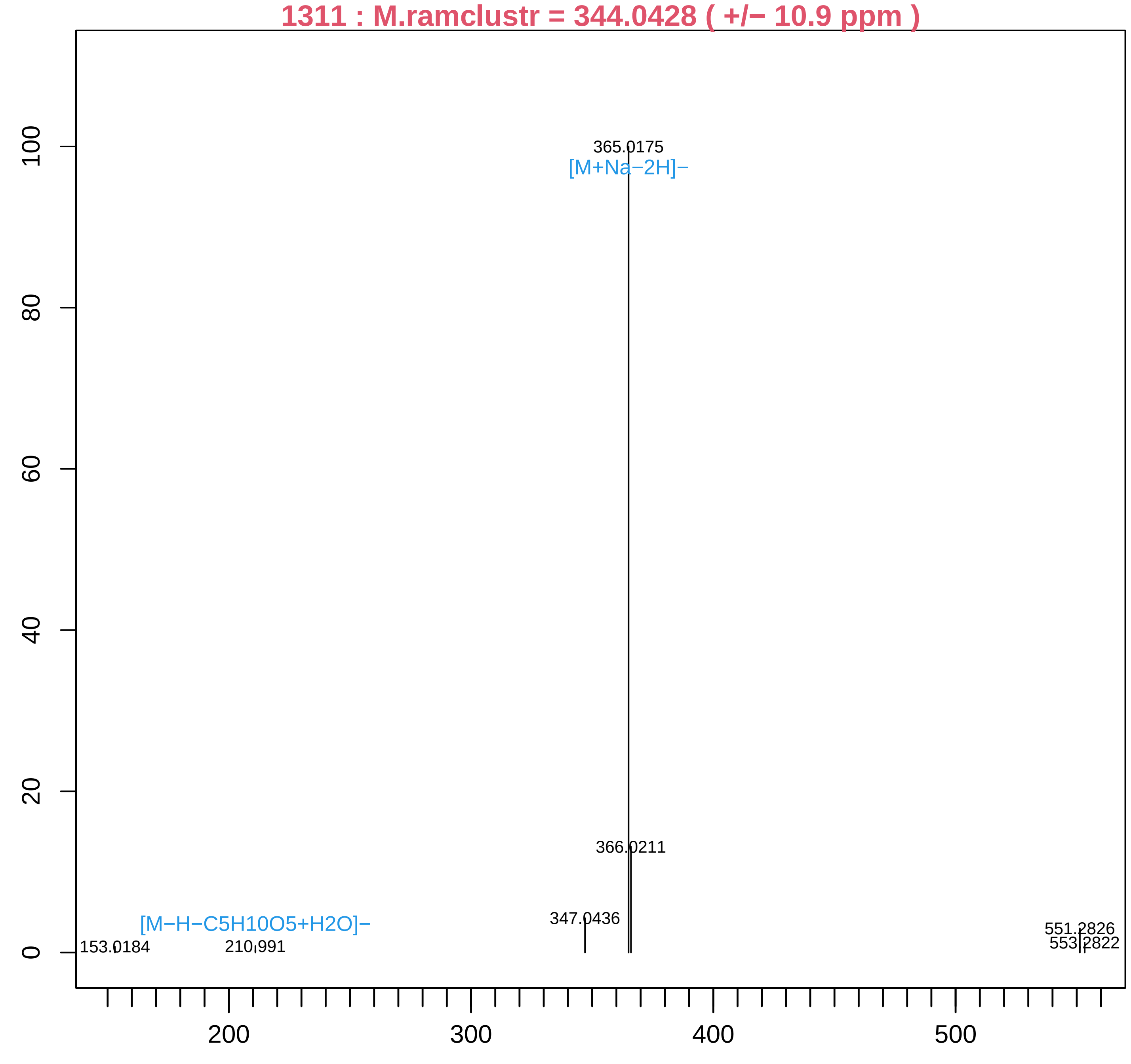

1311 : M.ramclustr = 344.0428 (+/− 10.9 ppm)
365.0175
[M+Na−2H]−
366.0211
347.0436
[M−H−C5H10O5+H2O]−
153.0184
210.991
551.2826
553.2822
0
20
40
60
80
100
200
300
400
500

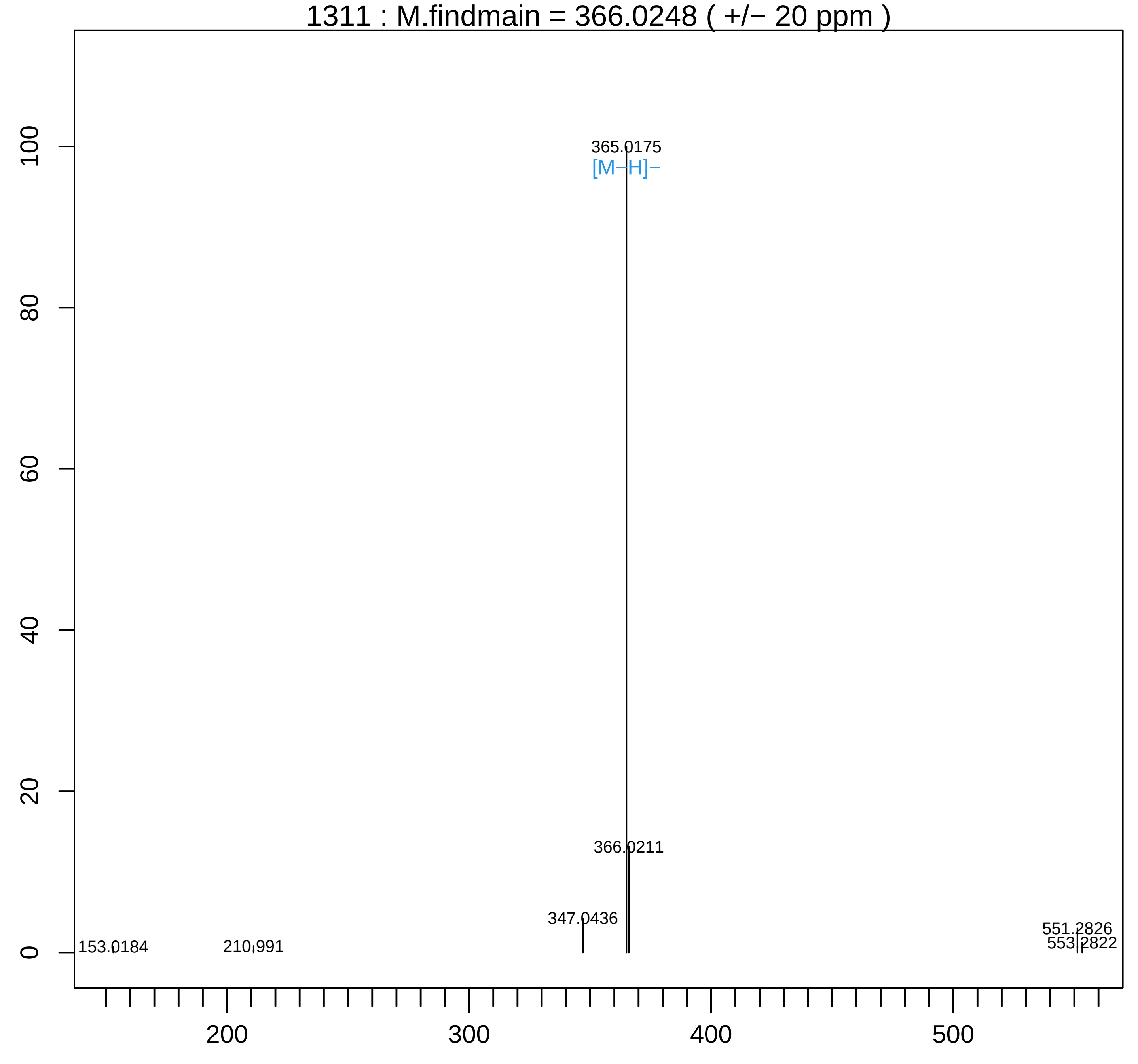

1311 : M.findmain = 366.0248 (+/− 20 ppm)
365.0175
[M−H]−
366.0211
347.0436
153.0184
210.991
551.2826
553.2822
0
20
40
60
80
100
200
300
400
500

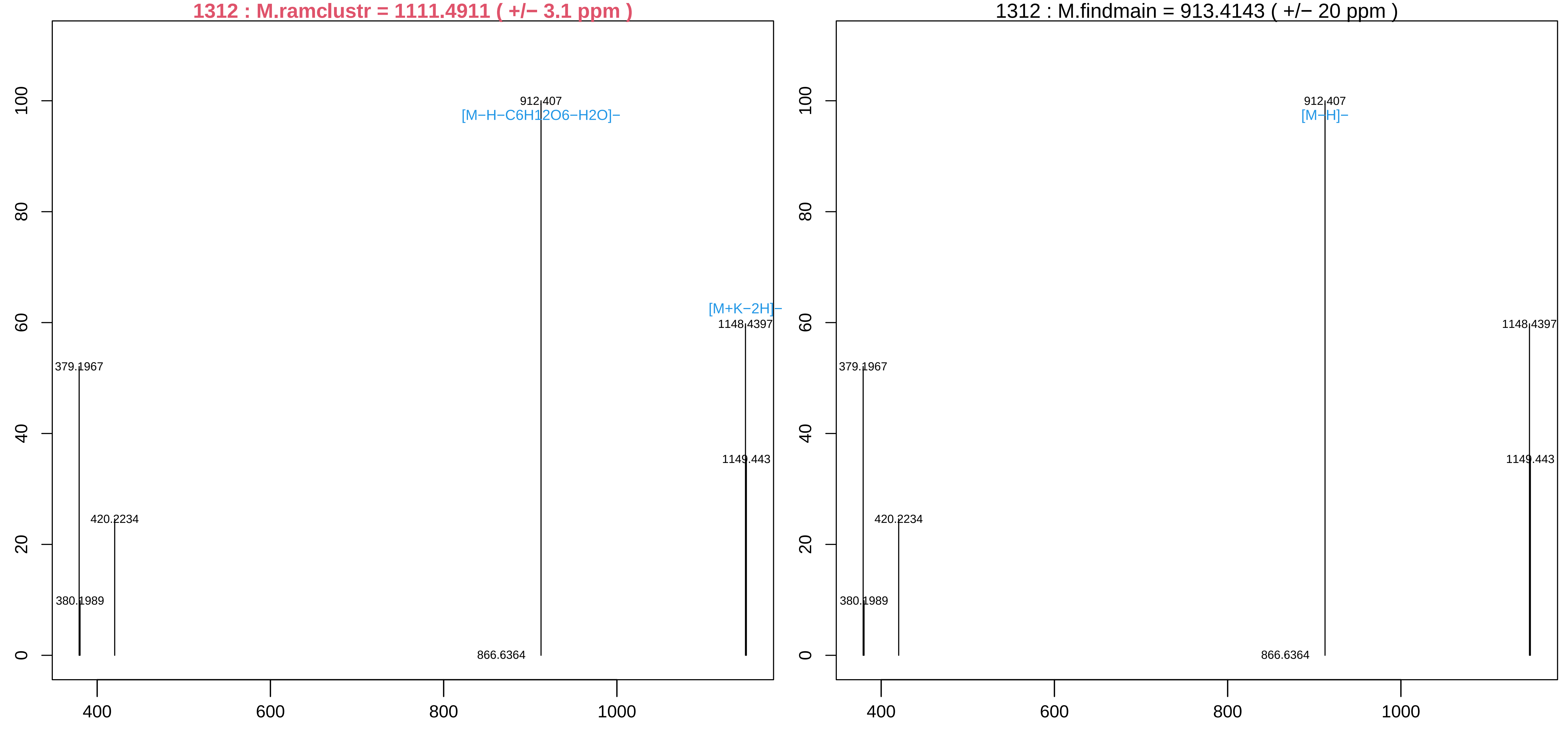

1312 : M.ramclustr = 1111.4911 (+/− 3.1 ppm)
912.407
[M−H−C6H12O6−H2O]−
[M+K−2H]−
1148.4397
379.1967
1149.443
420.2234
380.1989
866.6364
1312 : M.findmain = 913.4143 (+/− 20 ppm)
912.407
[M−H]−
1148.4397
379.1967
1149.443
420.2234
380.1989
866.6364

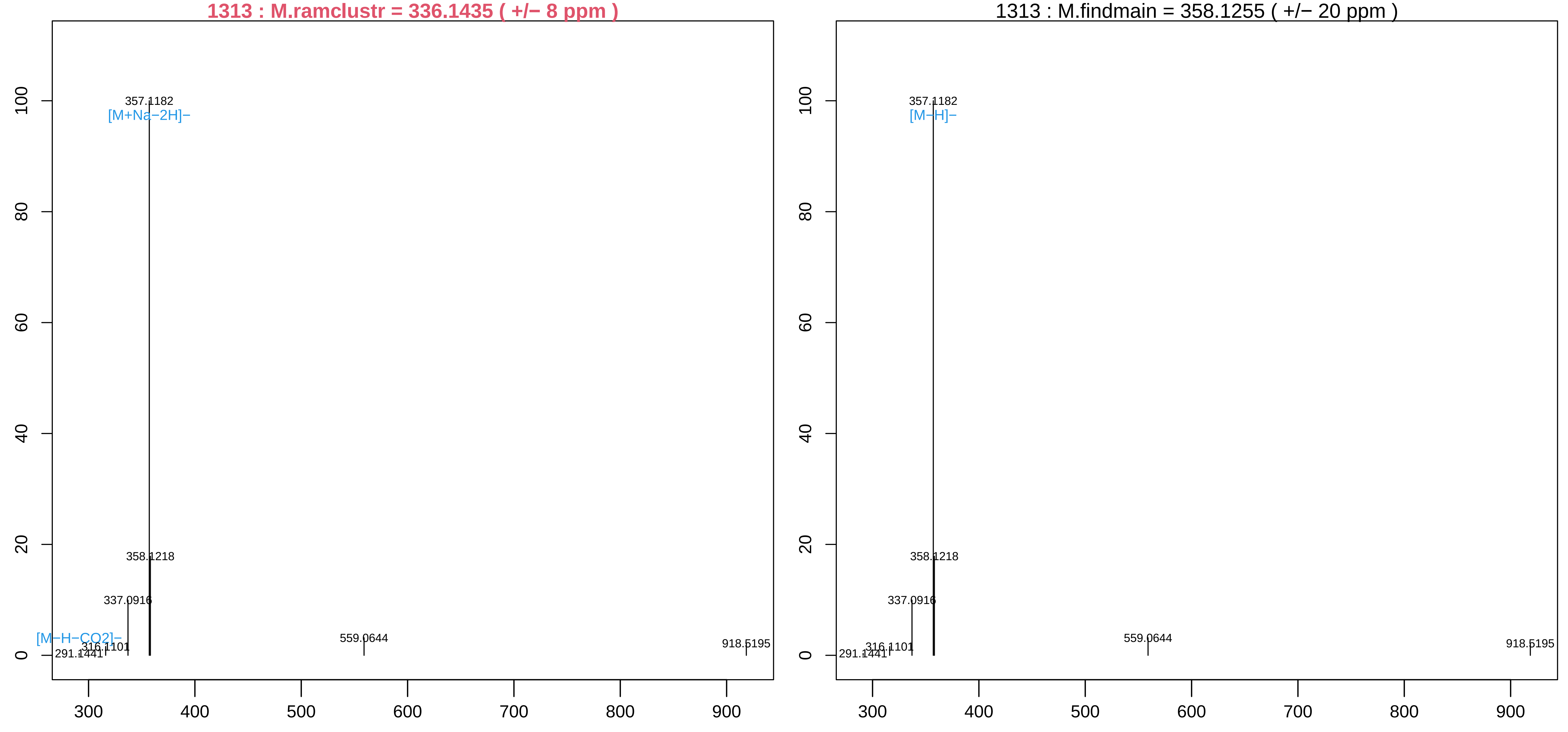

1313 : M.ramclustr = 336.1435 (+/− 8 ppm)
357.1182
[M+Na−2H]−
358.1218
337.0916
[M−H−CO2]−
316.1101
291.1441
559.0644
918.5195
1313 : M.findmain = 358.1255 (+/− 20 ppm)
357.1182
[M−H]−
358.1218
337.0916
316.1101
291.1441
559.0644
918.5195

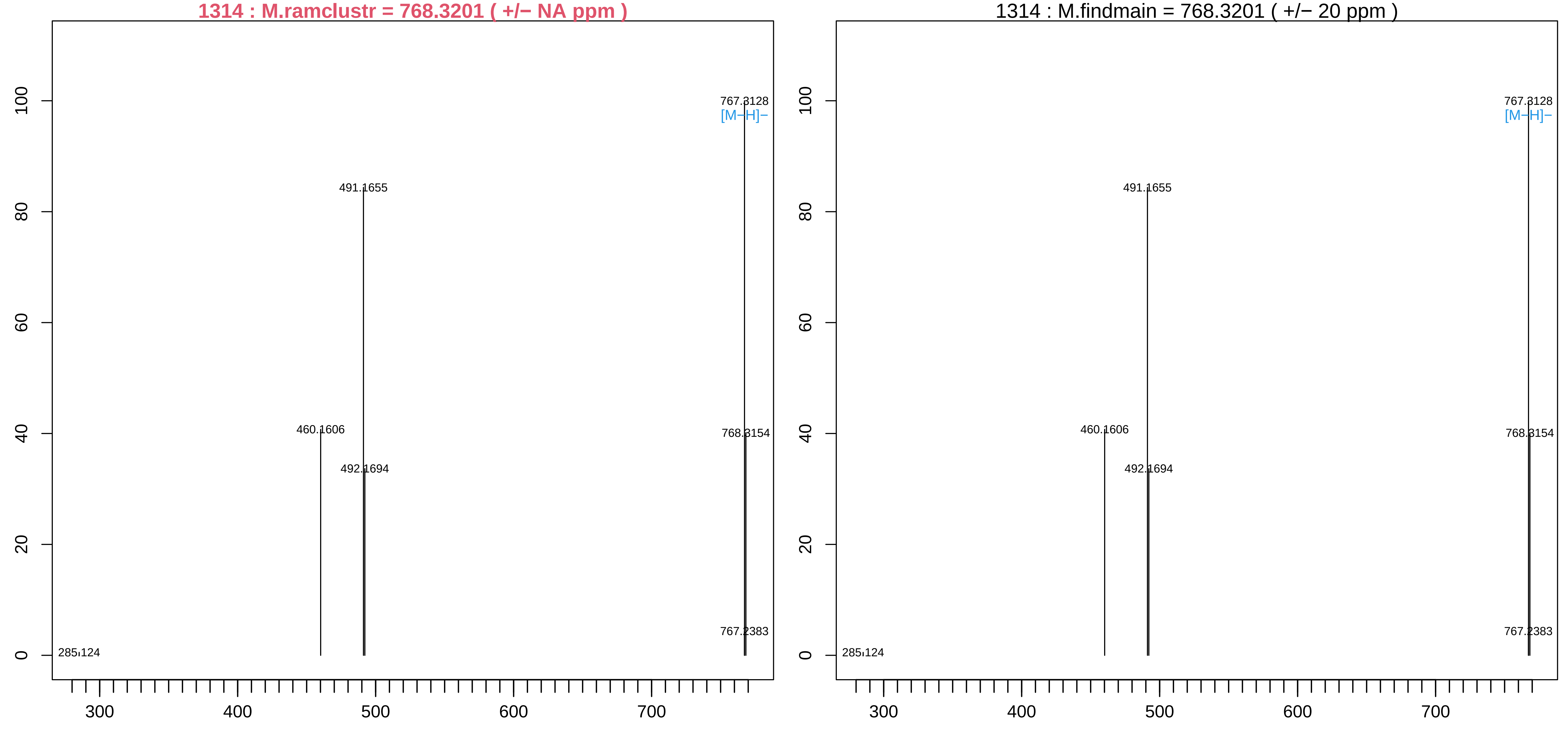
1314 : M.ramclustr = 768.3201 (+/− NA ppm)
767.3128
[M−H]−
491.1655
460.1606
492.1694
768.3154
767.2383
285.124
1314 : M.findmain = 768.3201 (+/− 20 ppm)
767.3128
[M−H]−
491.1655
460.1606
492.1694
768.3154
767.2383
285.124
0
20
40
60
80
100
300
400
500
600
700

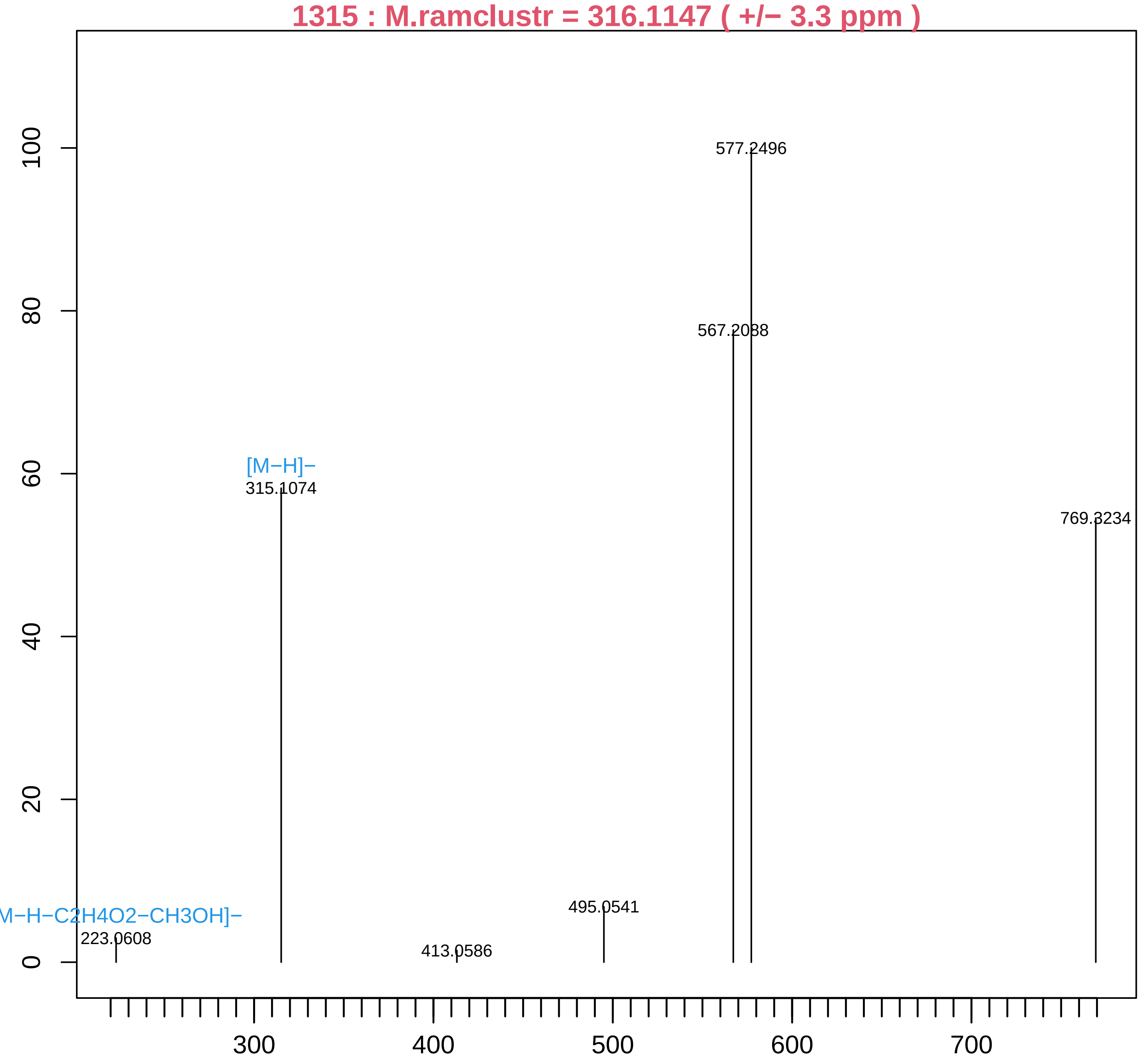
1315 : M.ramclustr = 316.1147 (+/− 3.3 ppm)
577.2496
567.2088
[M−H]−
315.1074
769.3234
495.0541
M−H−C2H4O2−CH3OH]−
223.0608
413.0586
0
20
40
60
80
100
300
400
500
600
700

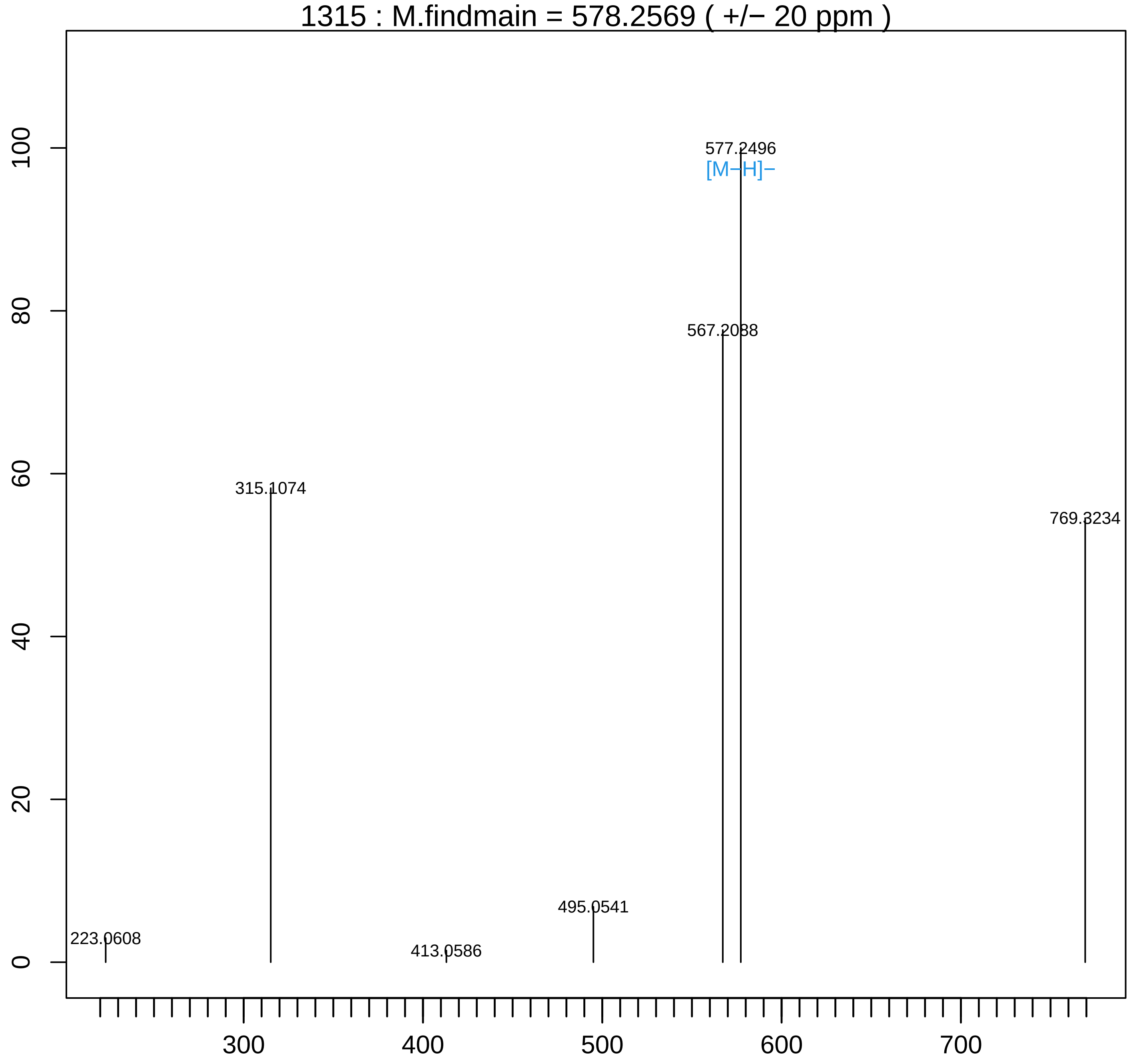
1315 : M.findmain = 578.2569 (+/− 20 ppm)
577.2496
[M−H]−
567.2088
315.1074
769.3234
495.0541
223.0608
413.0586
0
20
40
60
80
100
300
400
500
600
700

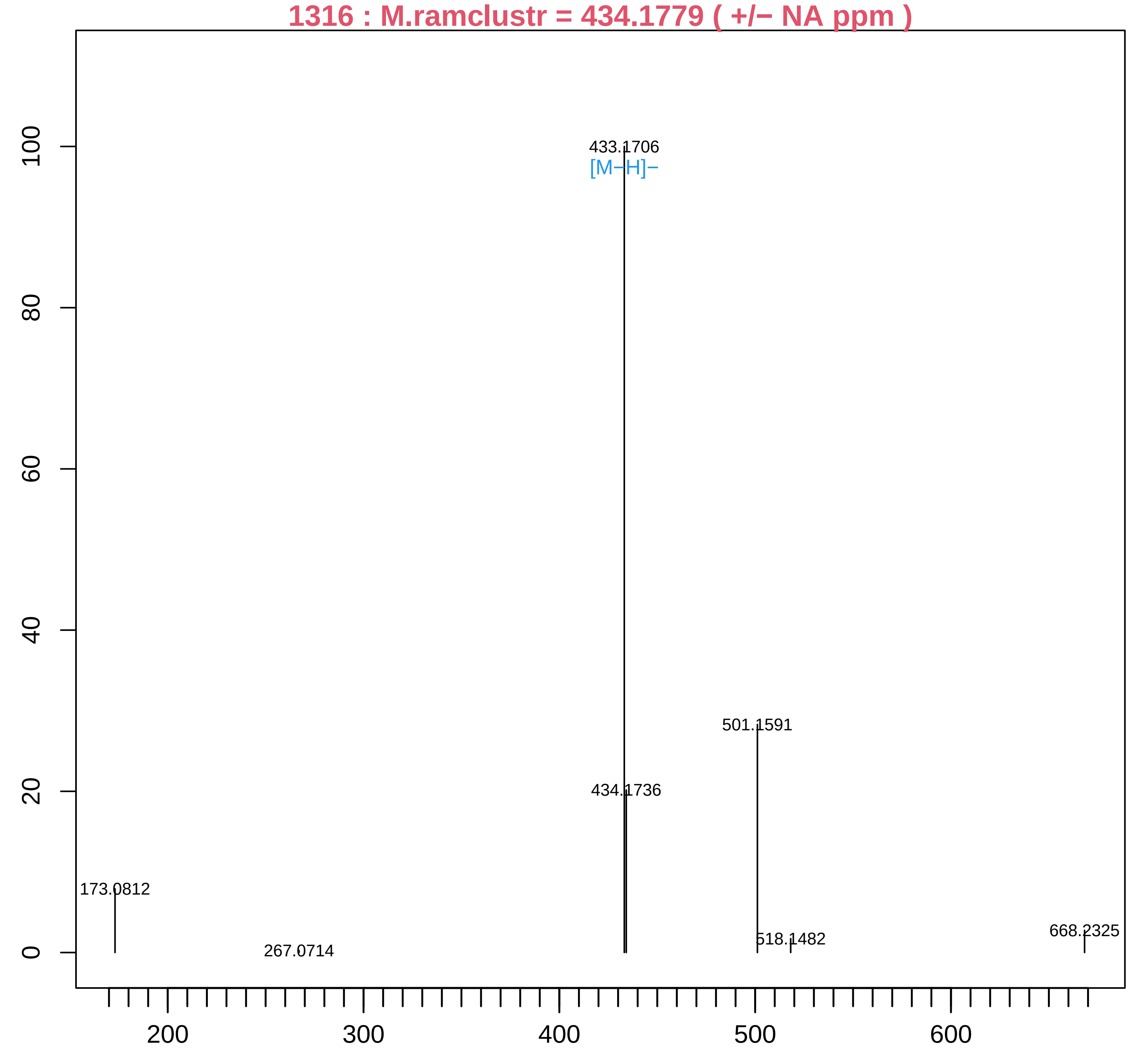
1316 : M.ramclustr = 434.1779 (+/− NA ppm)
433.1706
[M−H]−
501.1591
434.1736
173.0812
518.1482
668.2325
267.0714
0
20
40
60
80
100
200
300
400
500
600

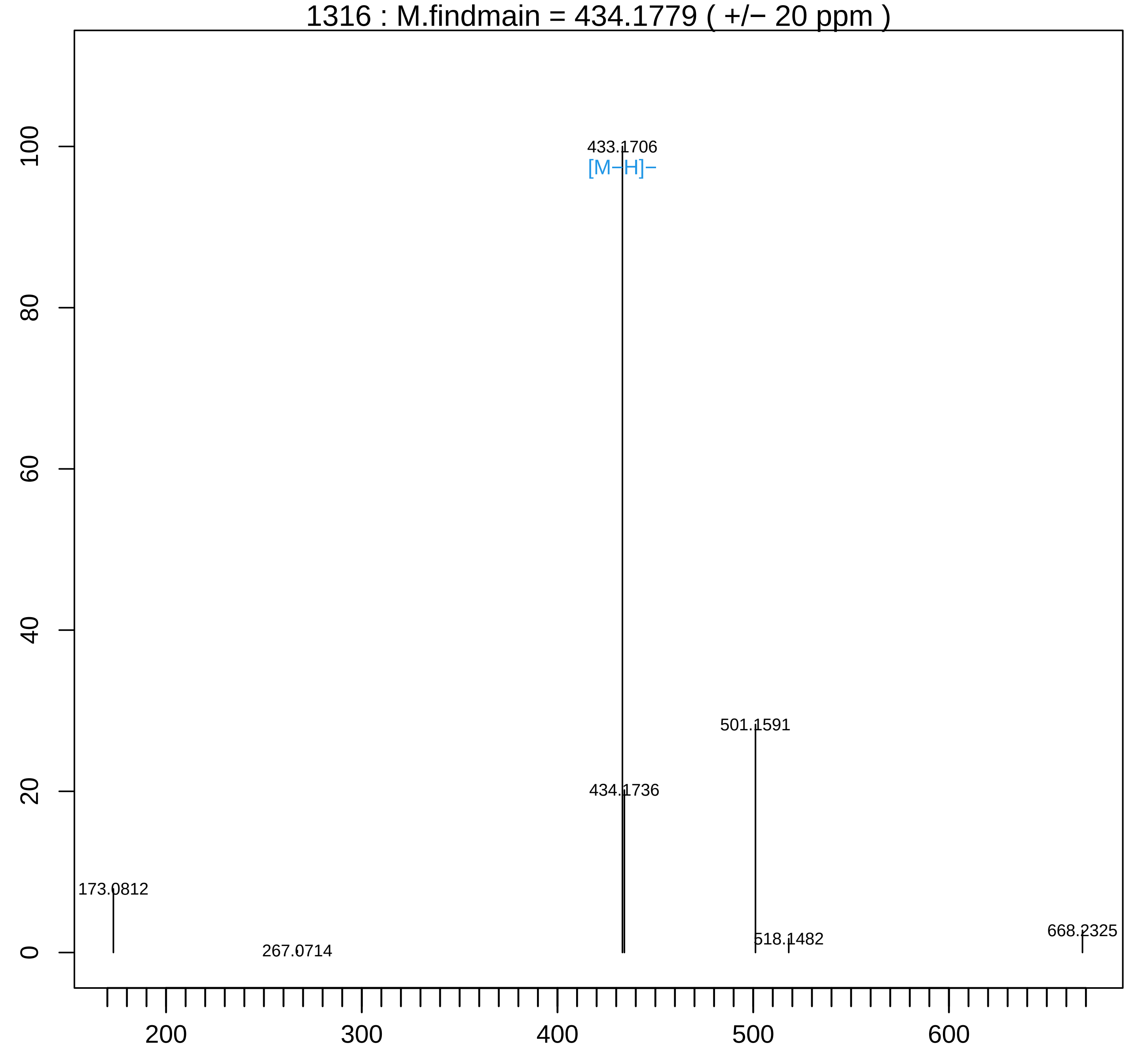
1316 : M.findmain = 434.1779 (+/− 20 ppm)
433.1706
[M−H]−
501.1591
434.1736
173.0812
518.1482
668.2325
267.0714
0
20
40
60
80
100
200
300
400
500
600

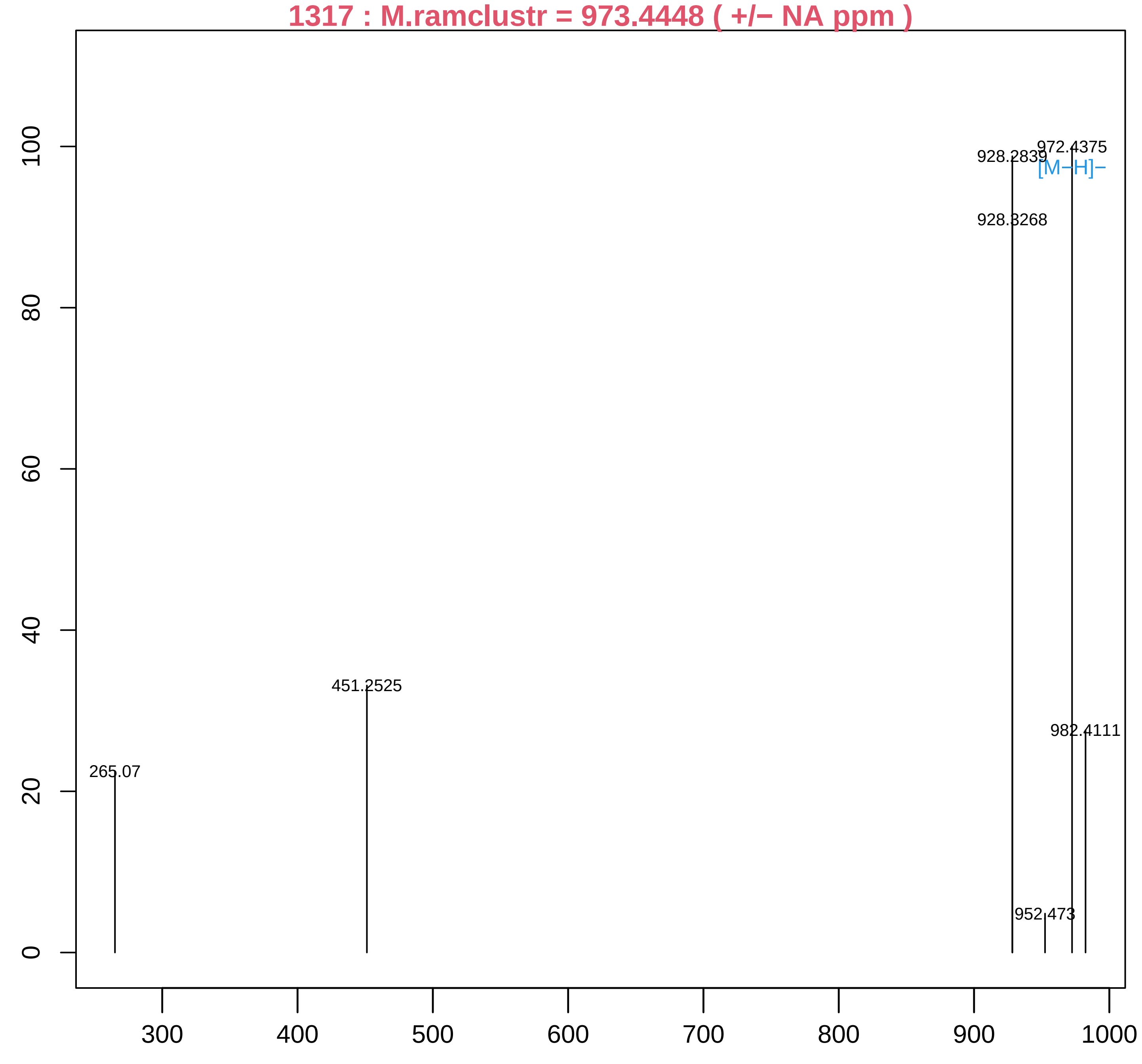
1317 : M.ramclustr = 973.4448 (+/− NA ppm)
0
20
40
60
80
100
300
400
500
600
700
800
900
1000
265.07
451.2525
928.2839
928.3268
952.473
972.4375
[M−H]−
982.4111

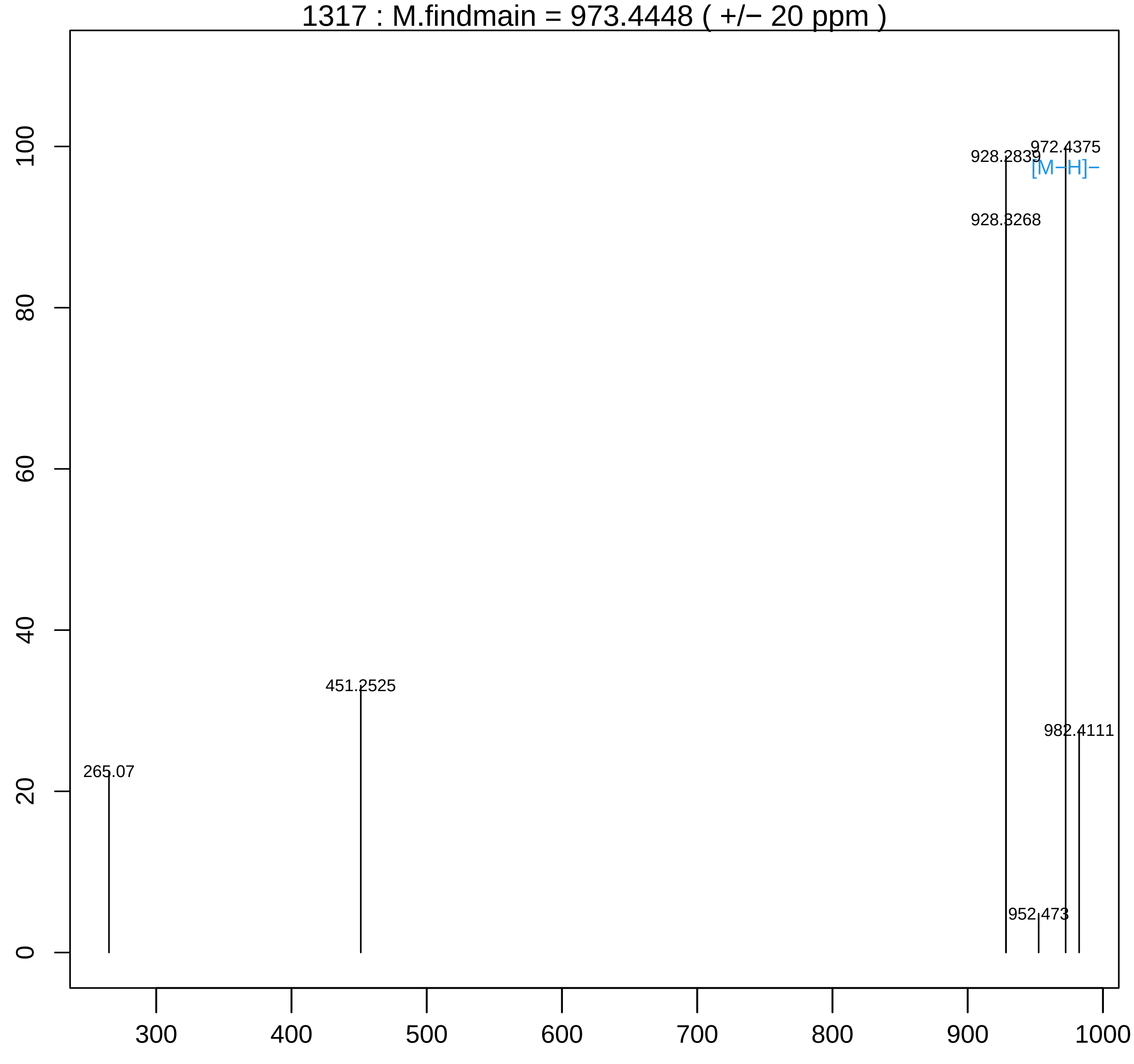
1317 : M.findmain = 973.4448 (+/− 20 ppm)
0
20
40
60
80
100
300
400
500
600
700
800
900
1000
265.07
451.2525
928.2839
928.3268
952.473
972.4375
[M−H]−
982.4111

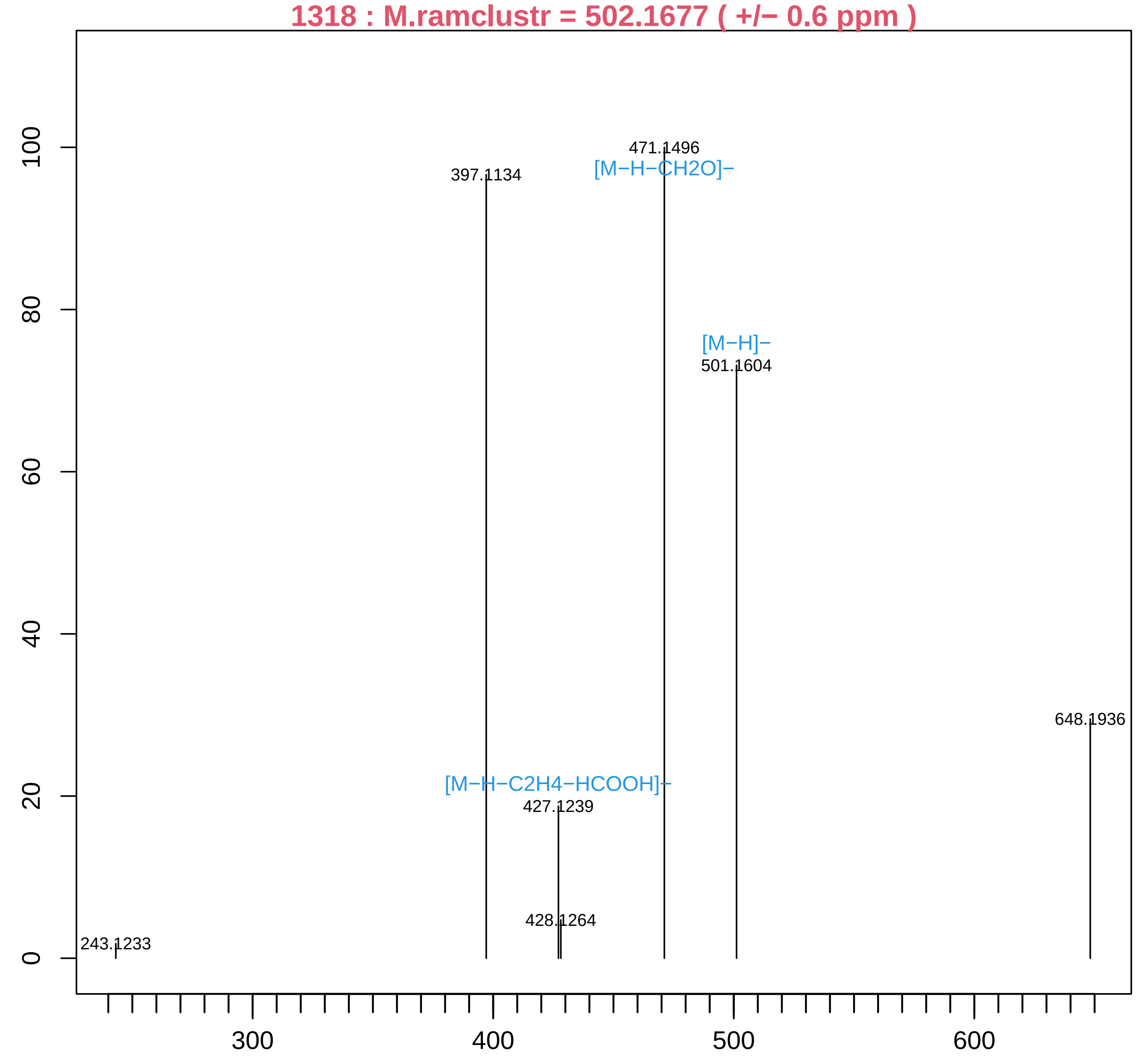
1318 : M.ramclustr = 502.1677 (+/− 0.6 ppm)
471.1496
[M−H−CH2O]−
397.1134
[M−H]−
501.1604
648.1936
[M−H−C2H4−HCOOH]−
427.1239
428.1264
243.1233
0
20
40
60
80
100
300
400
500
600

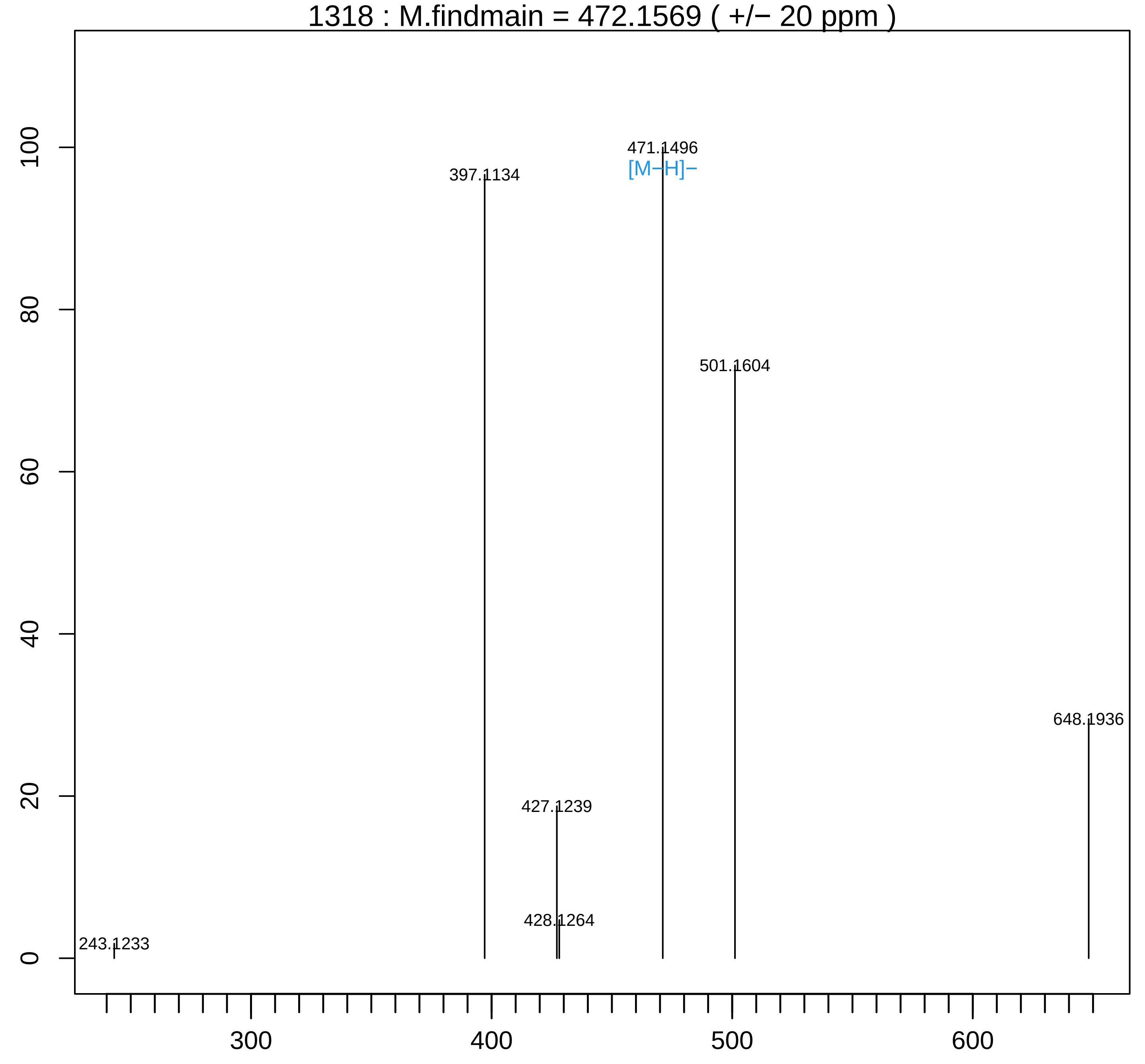
1318 : M.findmain = 472.1569 (+/− 20 ppm)
471.1496
[M−H]−
397.1134
501.1604
648.1936
427.1239
428.1264
243.1233
0
20
40
60
80
100
300
400
500
600

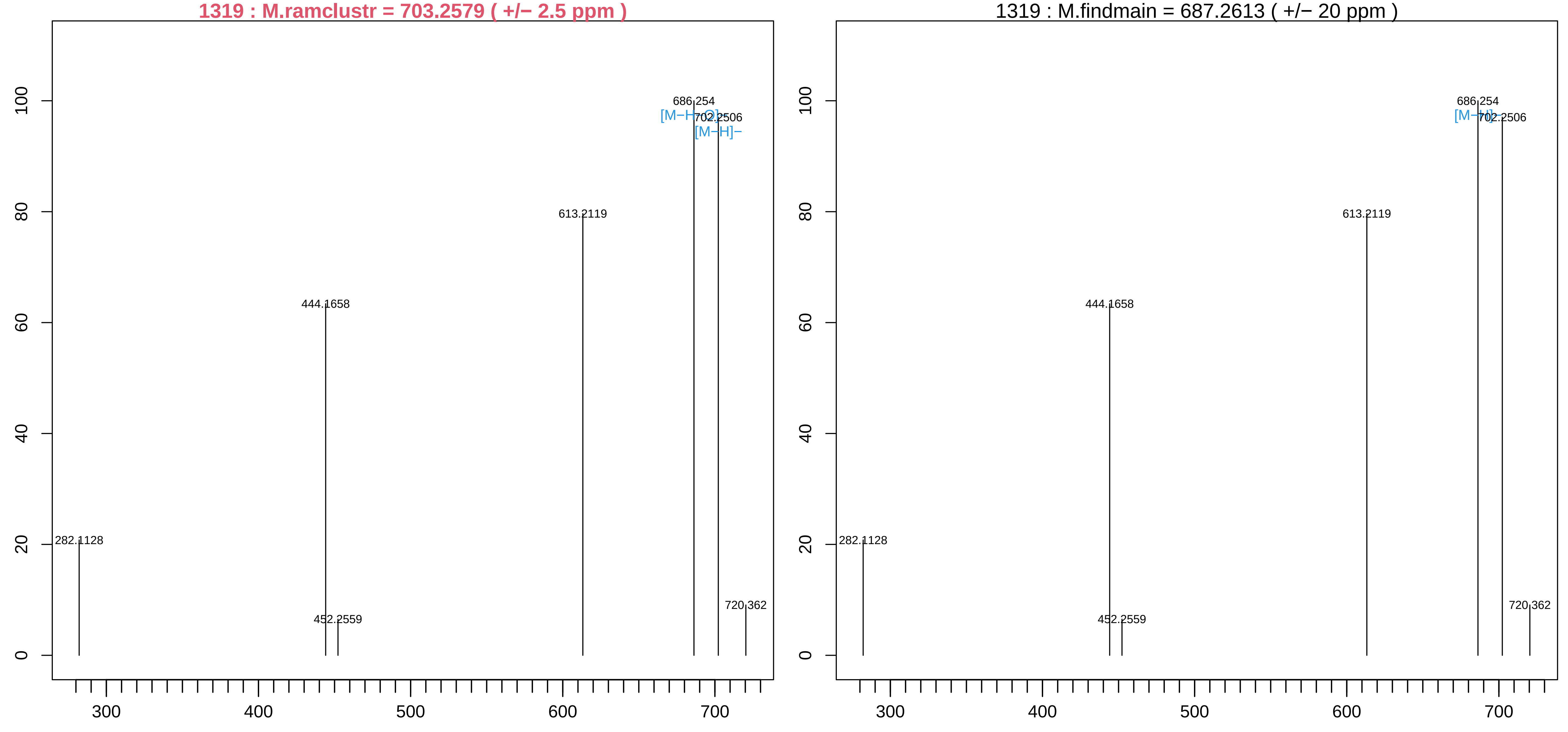
1319 : M.ramclustr = 703.2579 (+/− 2.5 ppm)
1319 : M.findmain = 687.2613 (+/− 20 ppm)
686.254
[M−H−O]−
702.2506
[M−H]−
613.2119
444.1658
452.2559
282.1128
720.362
0
20
40
60
80
100
300
400
500
600
700

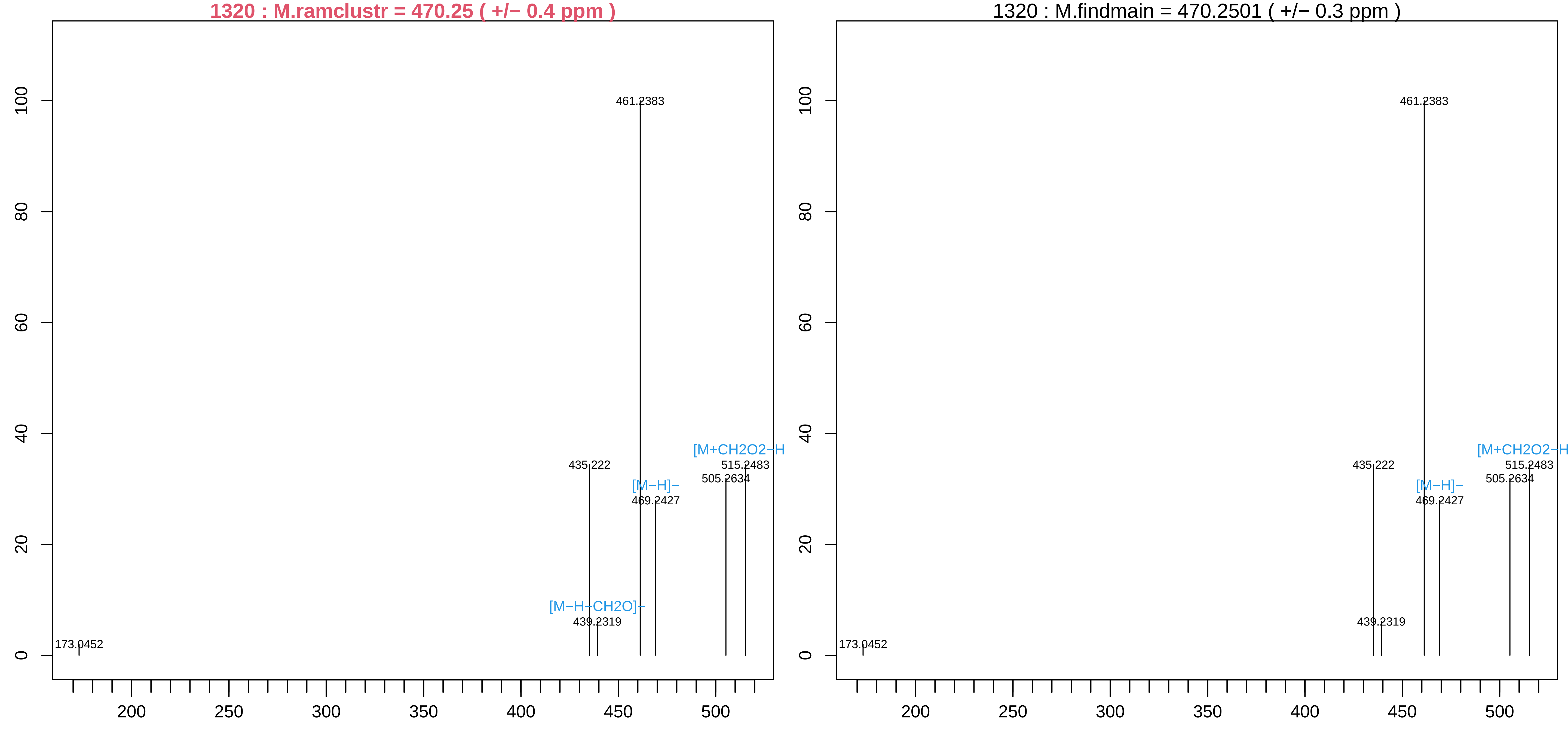
1320 : M.ramclustr = 470.25 (+/− 0.4 ppm)
1320 : M.findmain = 470.2501 (+/− 0.3 ppm)
461.2383
435.222
515.2483
505.2634
469.2427
439.2319
173.0452
[M−H]−
[M−H−CH2O]−
[M+CH2O2−H
0
20
40
60
80
100
200
250
300
350
400
450
500

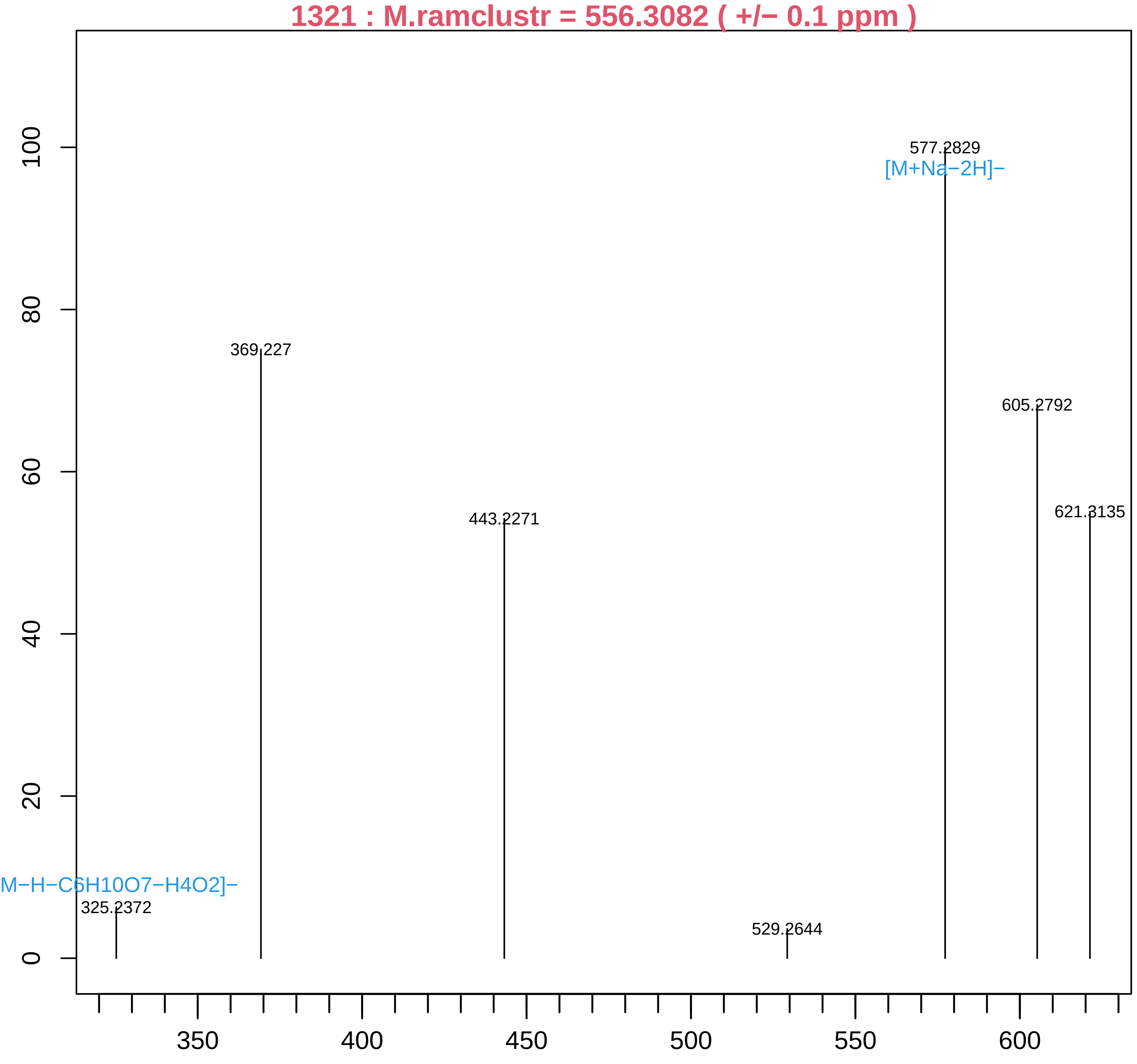

1321 : M.ramclustr = 556.3082 (+/− 0.1 ppm)
577.2829
[M+Na−2H]−
369.227
605.2792
621.3135
443.2271
M−H−C6H10O7−H4O2]−
325.2372
529.2644
0
20
40
60
80
100
350
400
450
500
550
600

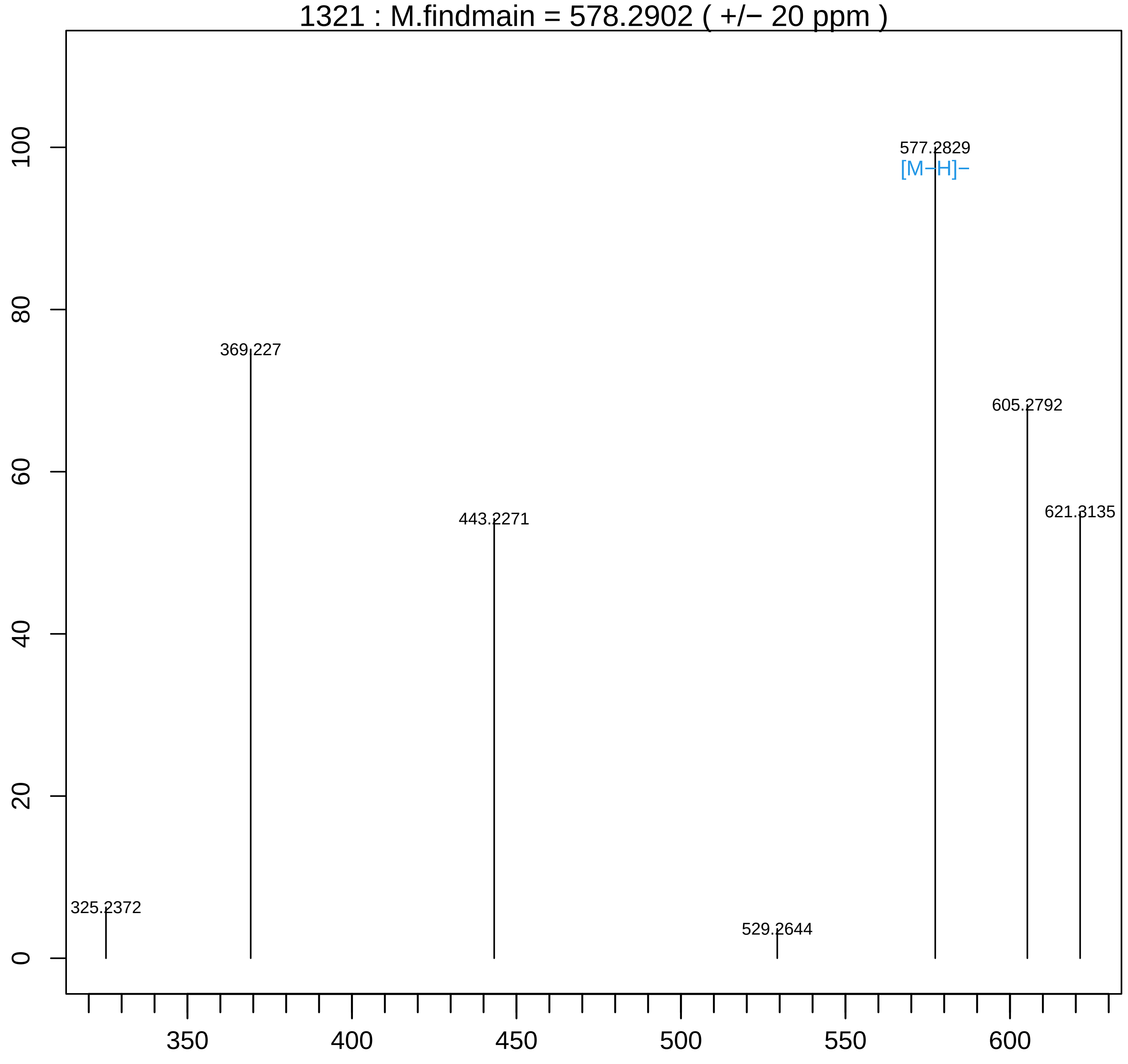

1321 : M.findmain = 578.2902 (+/− 20 ppm)
577.2829
[M−H]−
369.227
605.2792
621.3135
443.2271
325.2372
529.2644
0
20
40
60
80
100
350
400
450
500
550
600

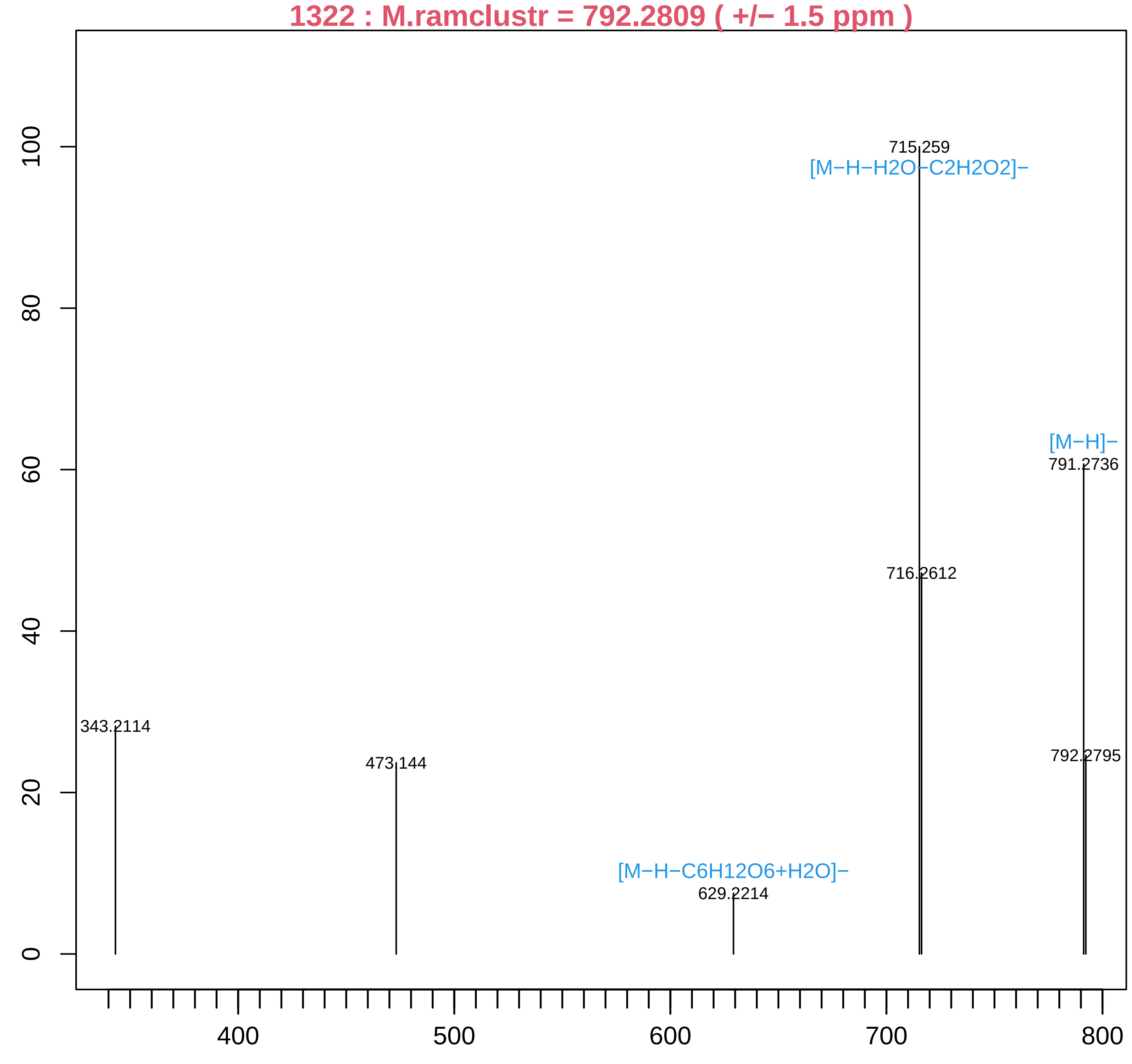

1322 : M.ramclustr = 792.2809 (+/− 1.5 ppm)
715.259
[M−H−H2O−C2H2O2]−
[M−H]−
791.2736
716.2612
343.2114
473.144
792.2795
[M−H−C6H12O6+H2O]−
629.2214
0
20
40
60
80
100
400
500
600
700
800

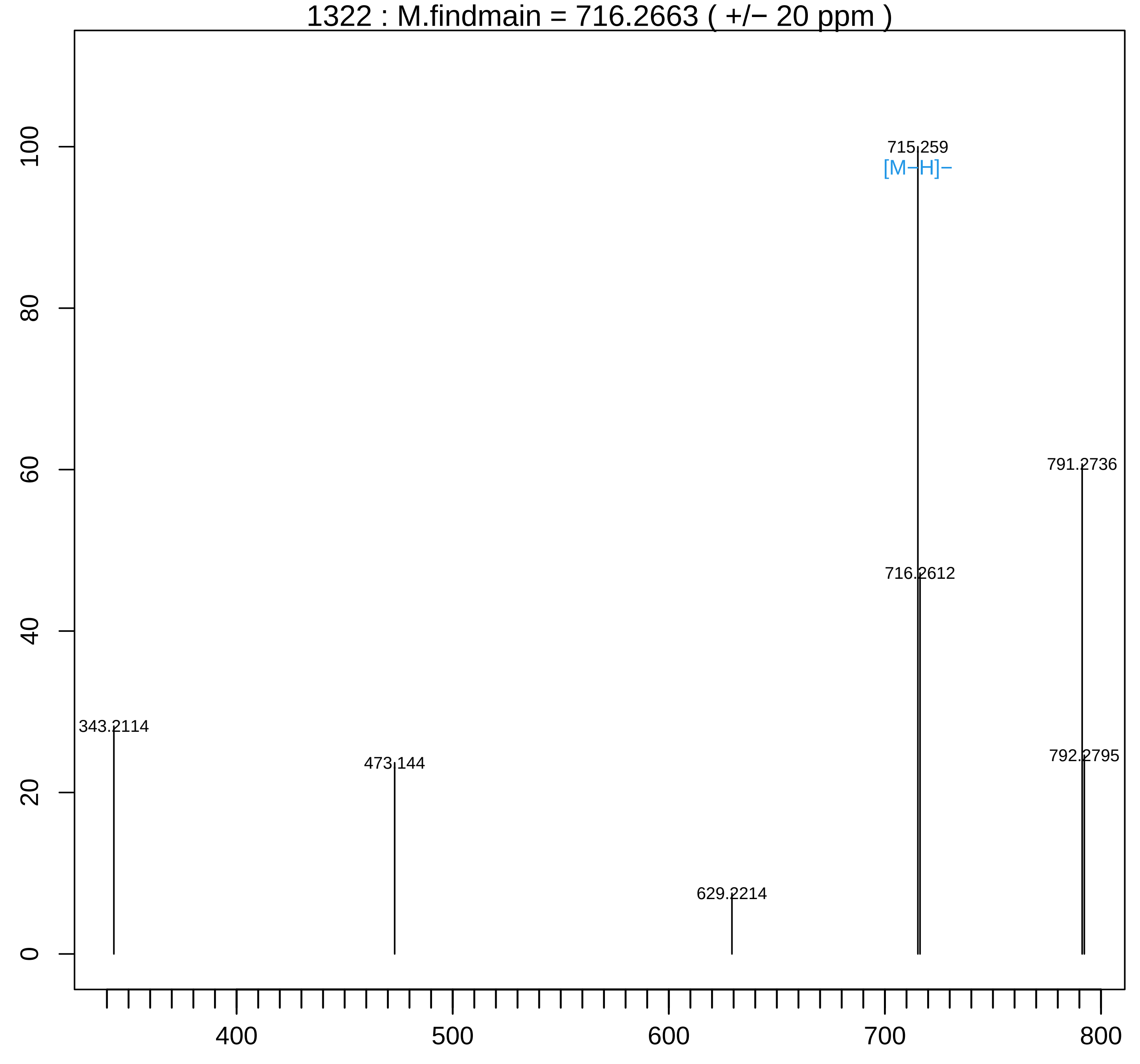

1322 : M.findmain = 716.2663 (+/− 20 ppm)
715.259
[M−H]−
791.2736
716.2612
343.2114
473.144
792.2795
629.2214
0
20
40
60
80
100
400
500
600
700
800

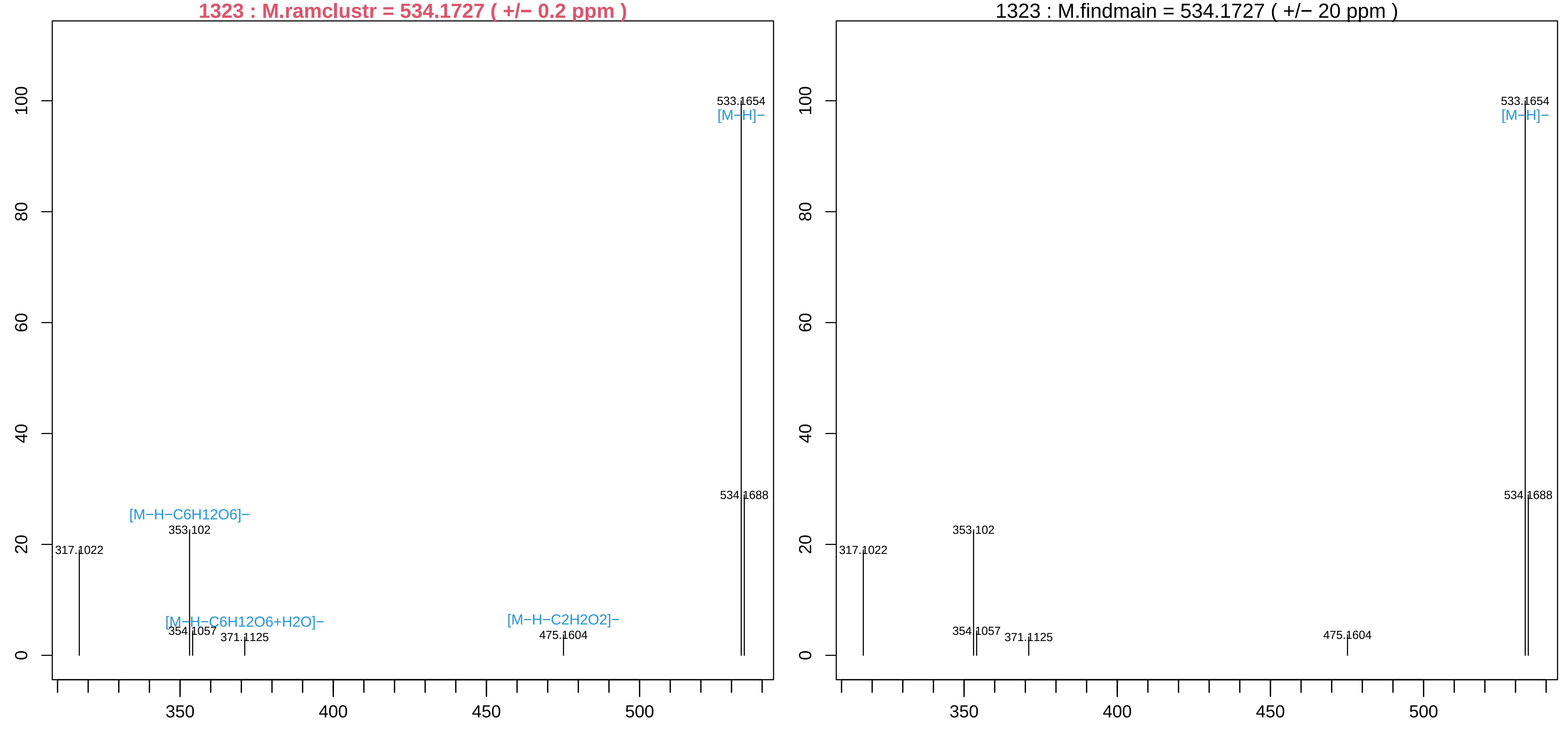
1323 : M.ramclustr = 534.1727 (+/− 0.2 ppm)
533.1654
[M−H]−
534.1688
[M−H−C6H12O6]−
353.102
317.1022
[M−H−C6H12O6+H2O]−
354.1057
371.1125
[M−H−C2H2O2]−
475.1604
0
20
40
60
80
100
350
400
450
500
1323 : M.findmain = 534.1727 (+/− 20 ppm)
533.1654
[M−H]−
534.1688
353.102
317.1022
354.1057
371.1125
475.1604
0
20
40
60
80
100
350
400
450
500

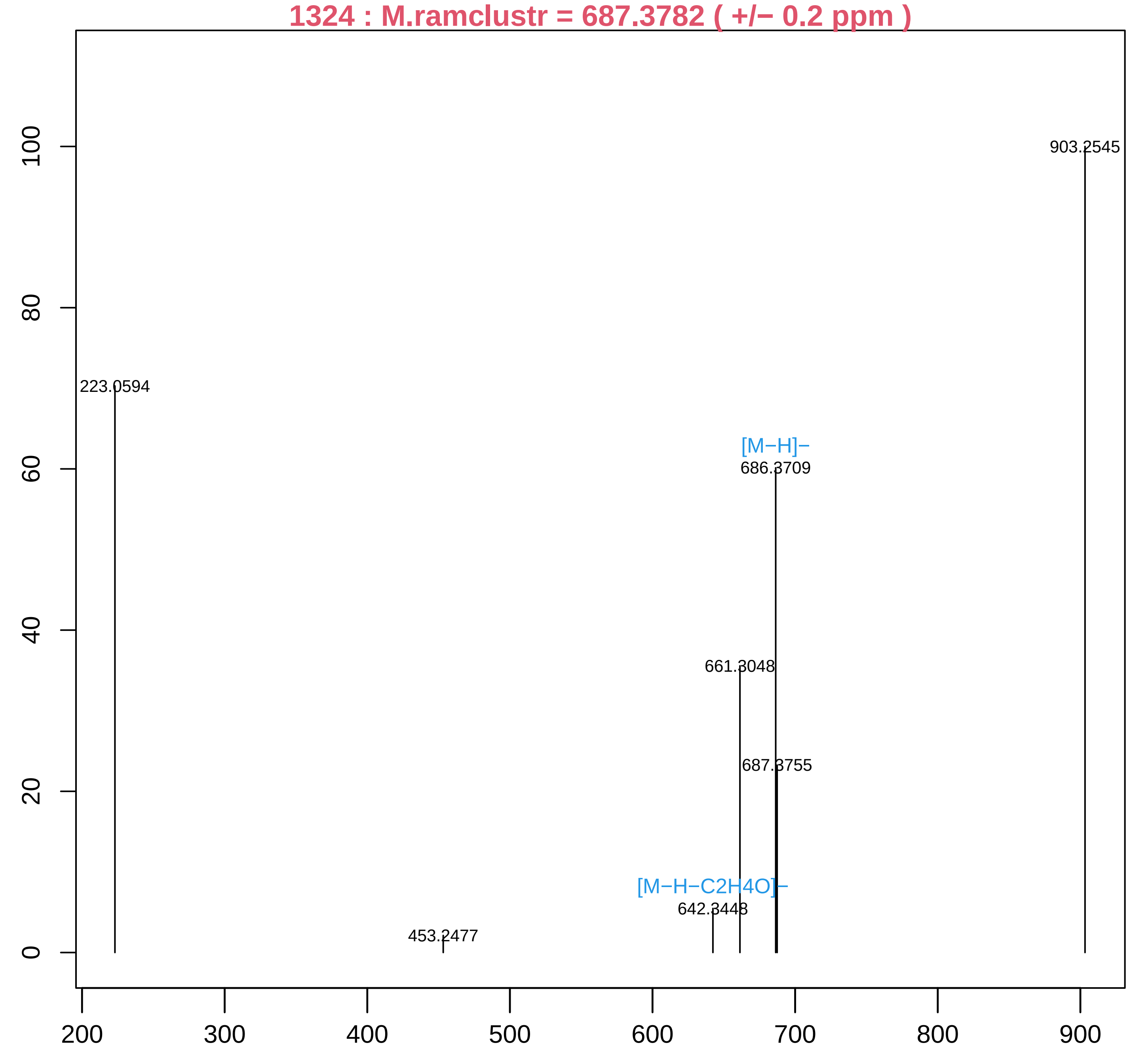
1324 : M.ramclustr = 687.3782 (+/− 0.2 ppm)
903.2545
223.0594
[M−H]−
686.3709
661.3048
687.3755
[M−H−C2H4O]−
642.3448
453.2477
0
20
40
60
80
100
200
300
400
500
600
700
800
900

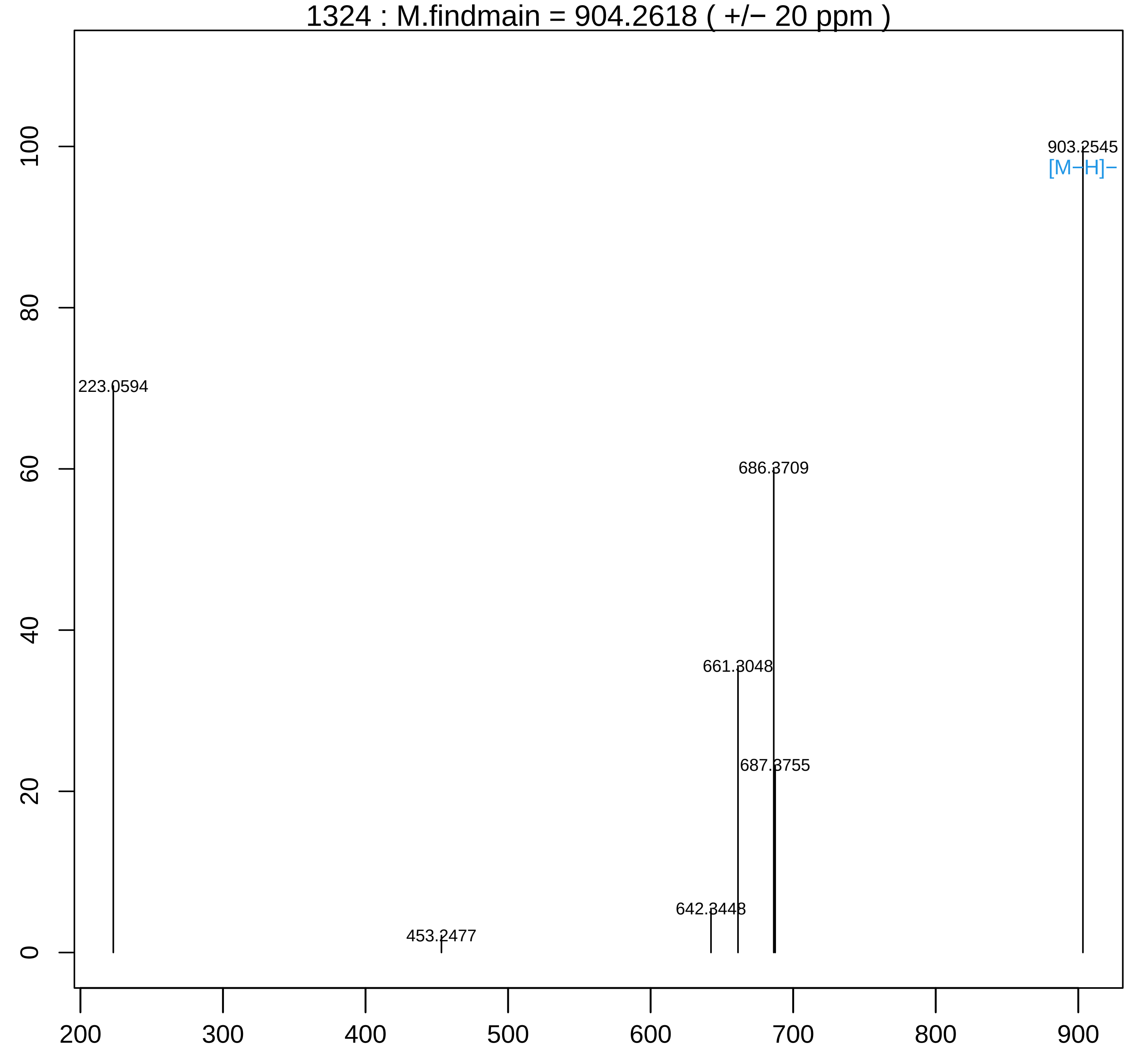
1324 : M.findmain = 904.2618 (+/− 20 ppm)
903.2545
[M−H]−
223.0594
686.3709
661.3048
687.3755
642.3448
453.2477
0
20
40
60
80
100
200
300
400
500
600
700
800
900

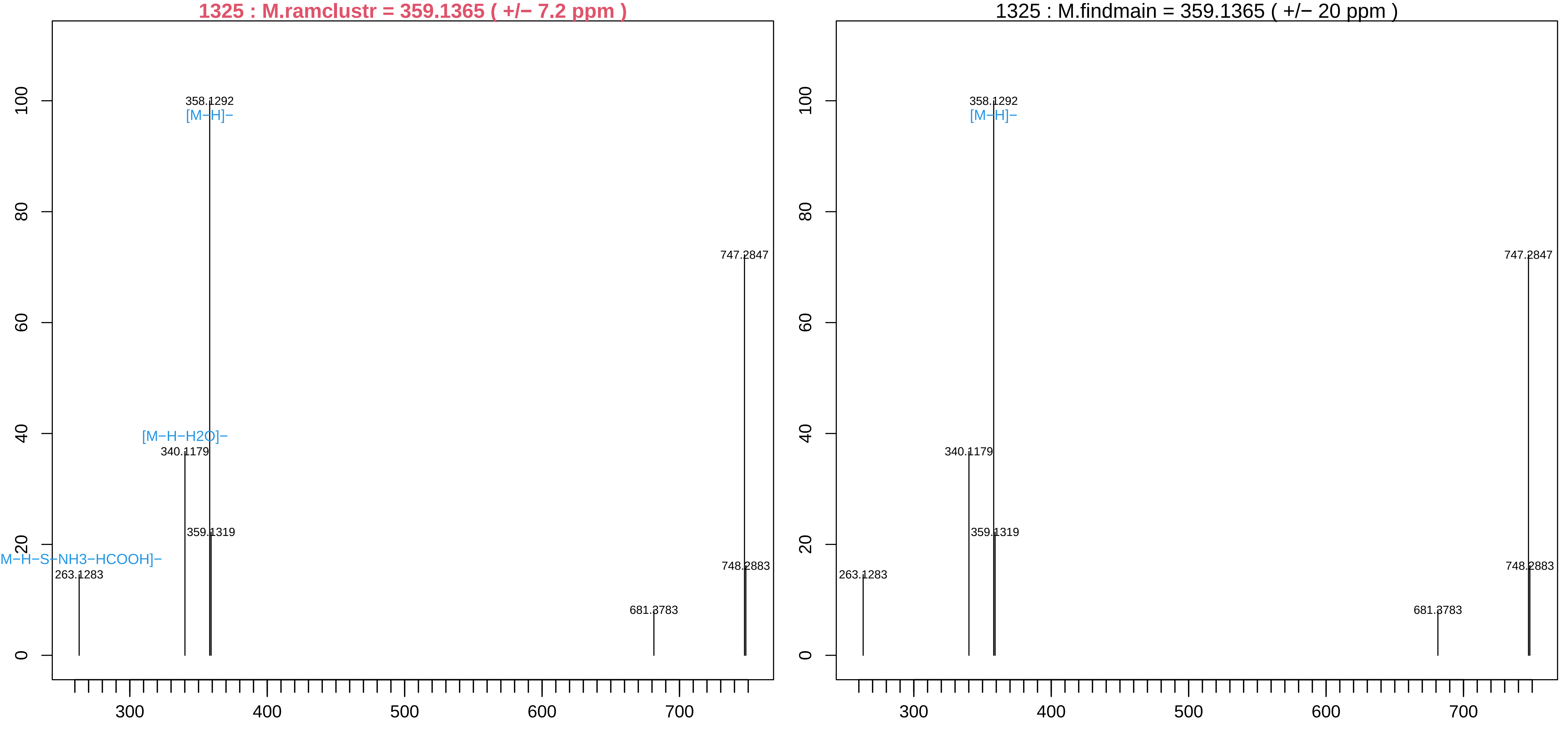
1325 : M.ramclustr = 359.1365 (+/− 7.2 ppm)
358.1292
[M−H]−
[M−H−H2O]−
340.1179
359.1319
M−H−S−NH3−HCOOH]−
263.1283
747.2847
748.2883
681.3783
0
20
40
60
80
100
300
400
500
600
700
1325 : M.findmain = 359.1365 (+/− 20 ppm)
358.1292
[M−H]−
340.1179
359.1319
263.1283
747.2847
748.2883
681.3783
0
20
40
60
80
100
300
400
500
600
700

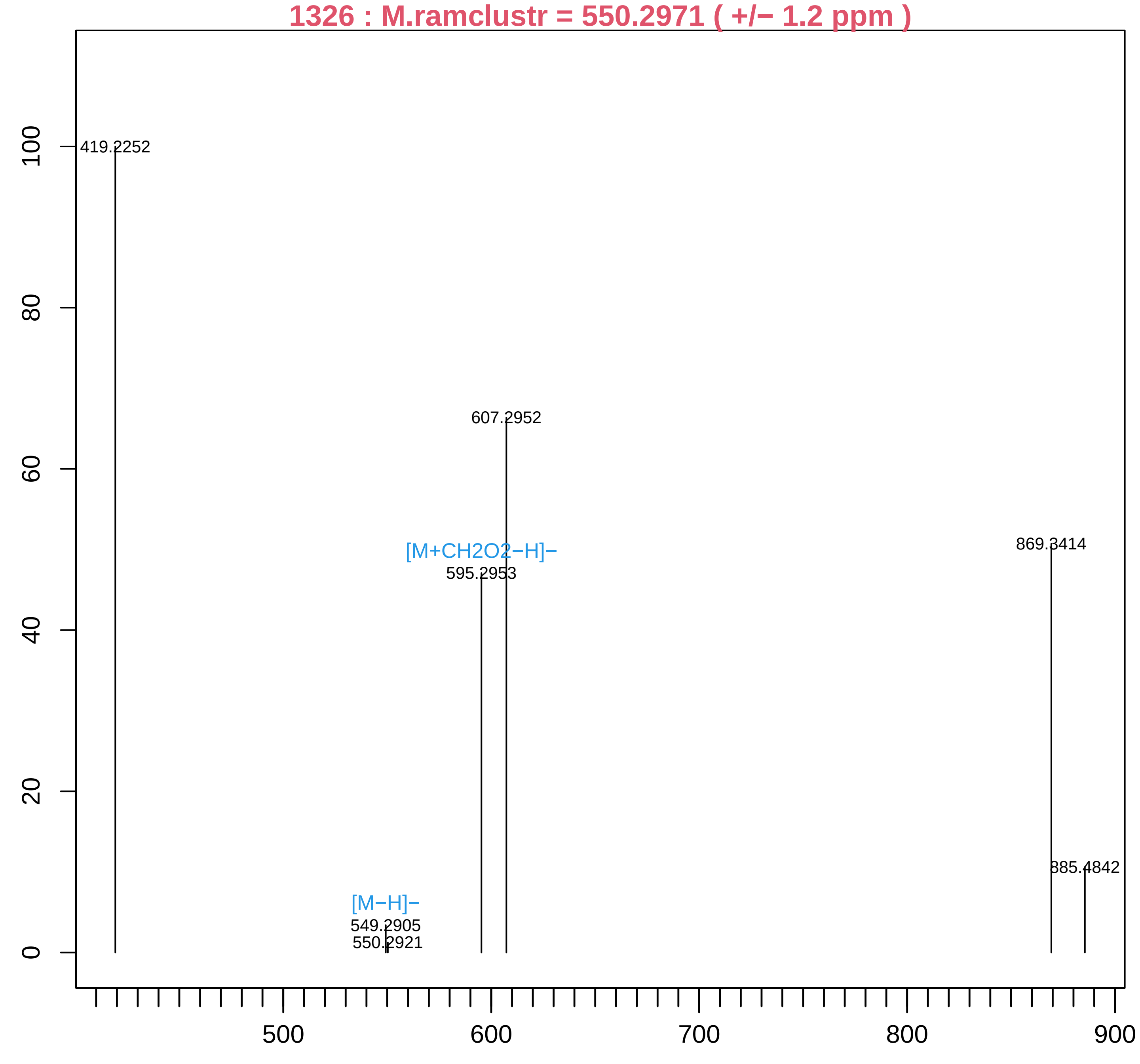
1326 : M.ramclustr = 550.2971 (+/− 1.2 ppm)
419.2252
607.2952
[M+CH2O2−H]−
595.2953
869.3414
885.4842
[M−H]−
549.2905
550.2921
0
20
40
60
80
100
500
600
700
800
900

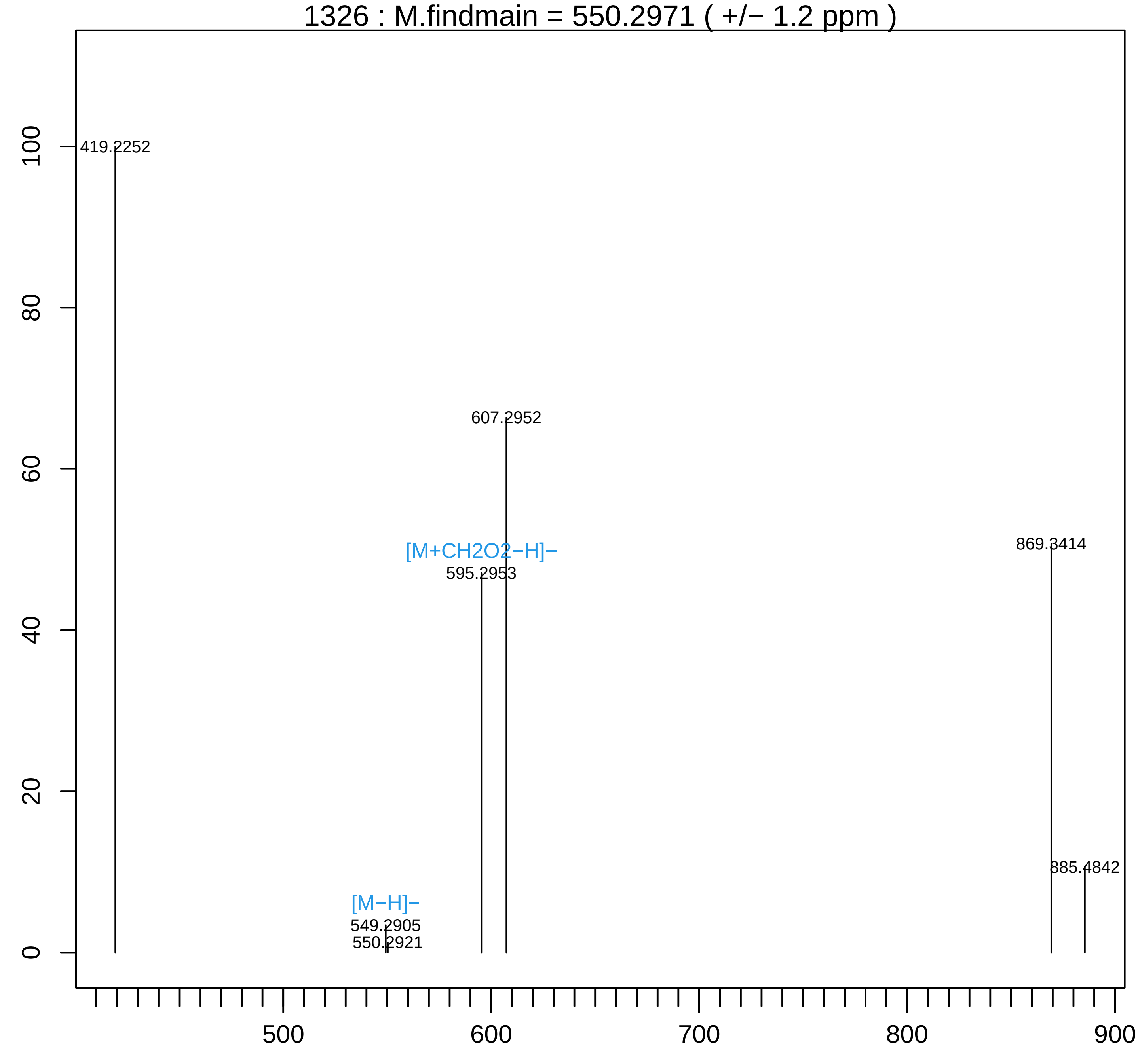
1326 : M.findmain = 550.2971 (+/− 1.2 ppm)
419.2252
607.2952
[M+CH2O2−H]−
595.2953
869.3414
885.4842
[M−H]−
549.2905
550.2921
0
20
40
60
80
100
500
600
700
800
900

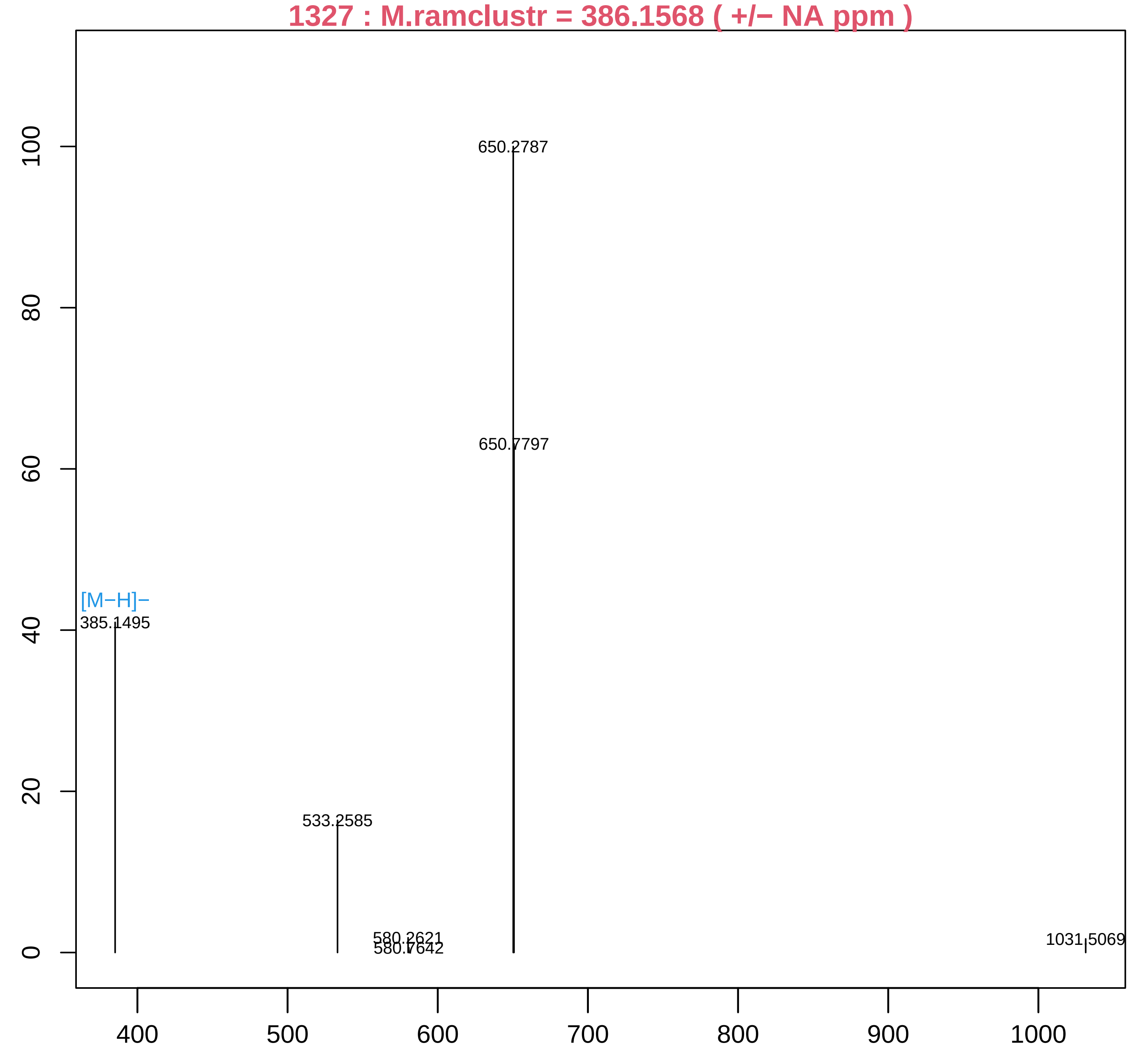

1327 : M.ramclustr = 386.1568 (+/− NA ppm)
650.2787
650.7797
[M−H]−
385.1495
533.2585
580.2621
580.7642
1031.5069
0
20
40
60
80
100
400
500
600
700
800
900
1000

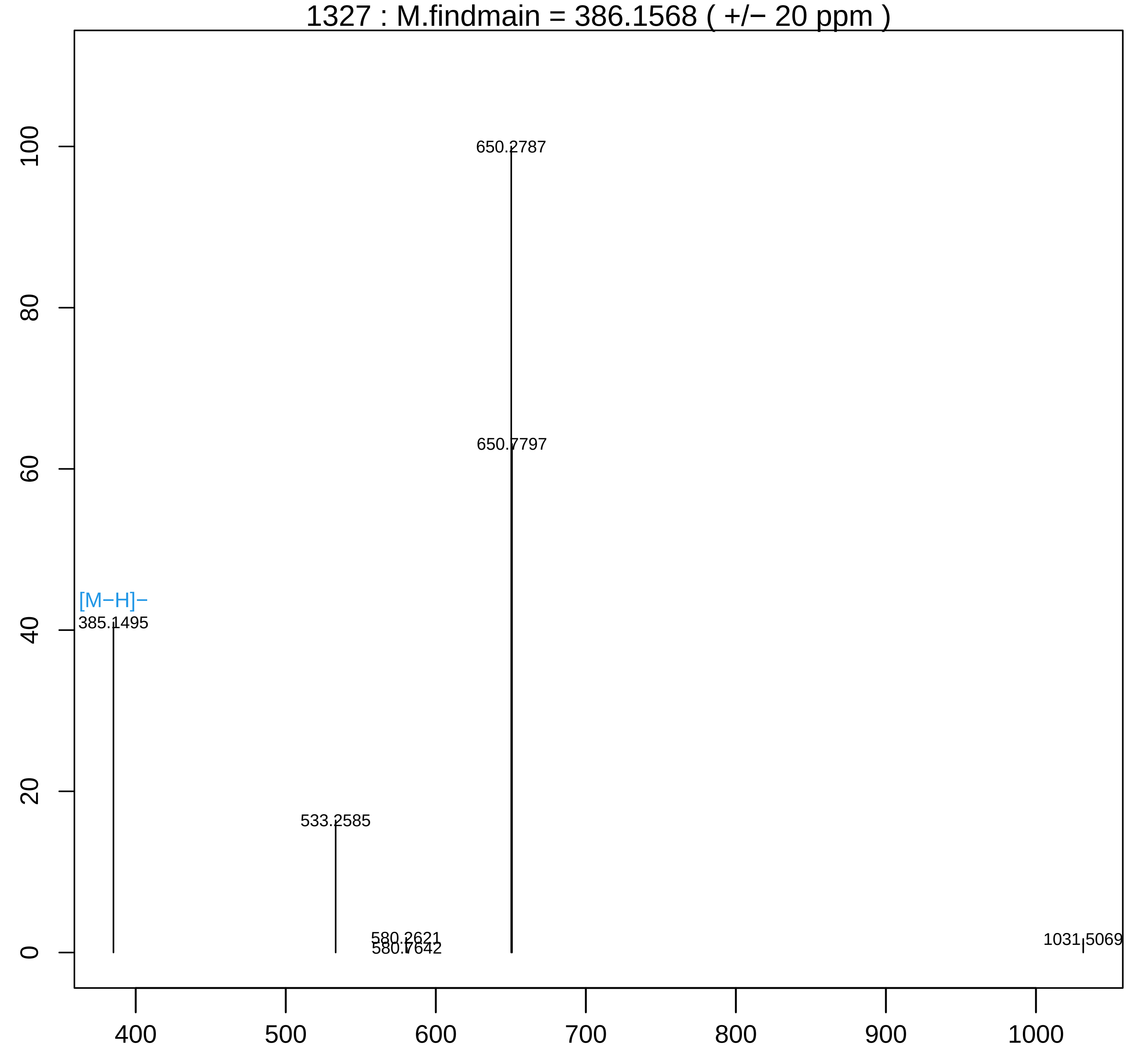

1327 : M.findmain = 386.1568 (+/− 20 ppm)
650.2787
650.7797
[M−H]−
385.1495
533.2585
580.2621
580.7642
1031.5069
0
20
40
60
80
100
400
500
600
700
800
900
1000

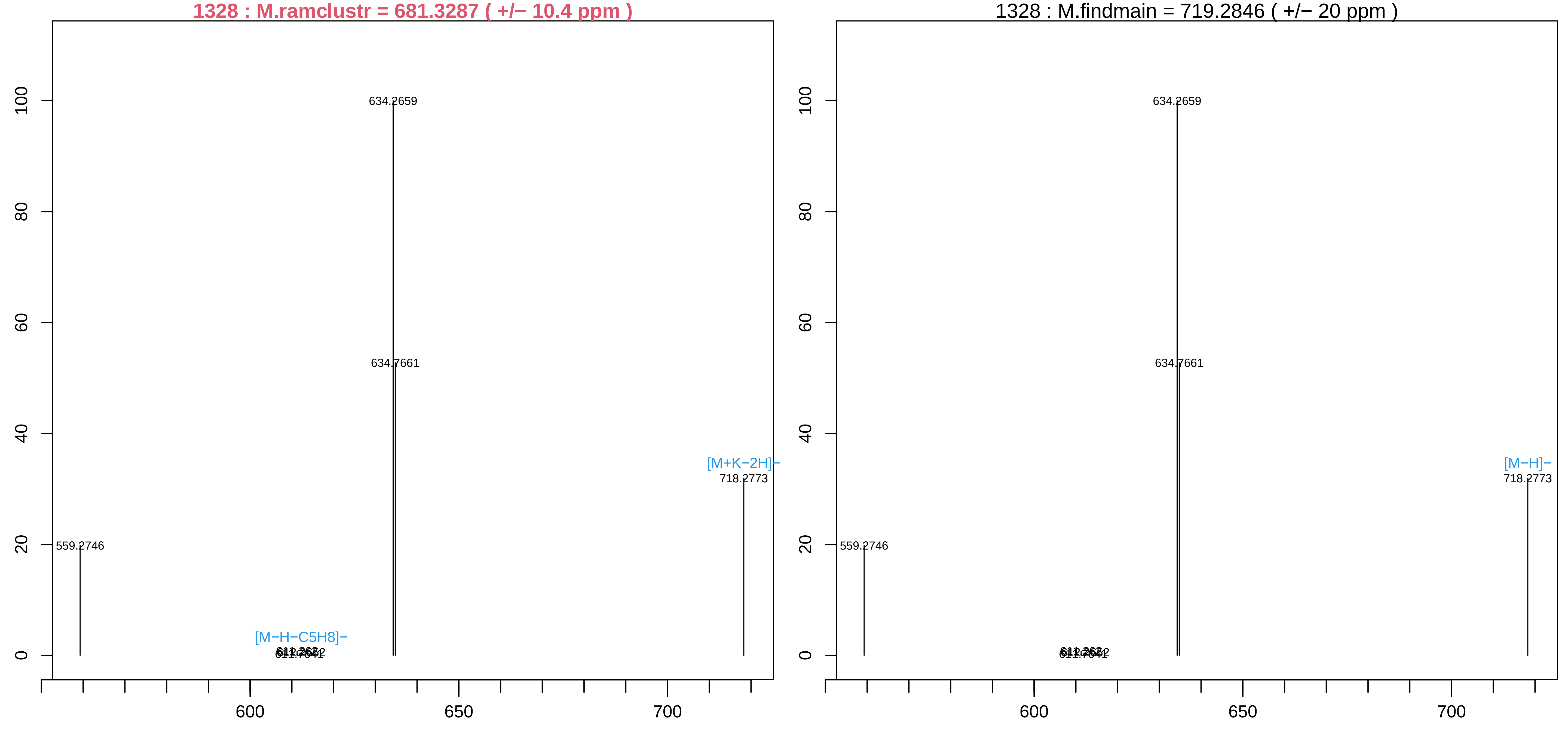
1328 : M.ramclustr = 681.3287 (+/− 10.4 ppm)
634.2659
634.7661
[M+K−2H]−
718.2773
559.2746
[M−H−C5H8]−
0
20
40
60
80
100
600
650
700
1328 : M.findmain = 719.2846 (+/− 20 ppm)
634.2659
634.7661
[M−H]−
718.2773
559.2746
0
20
40
60
80
100
600
650
700

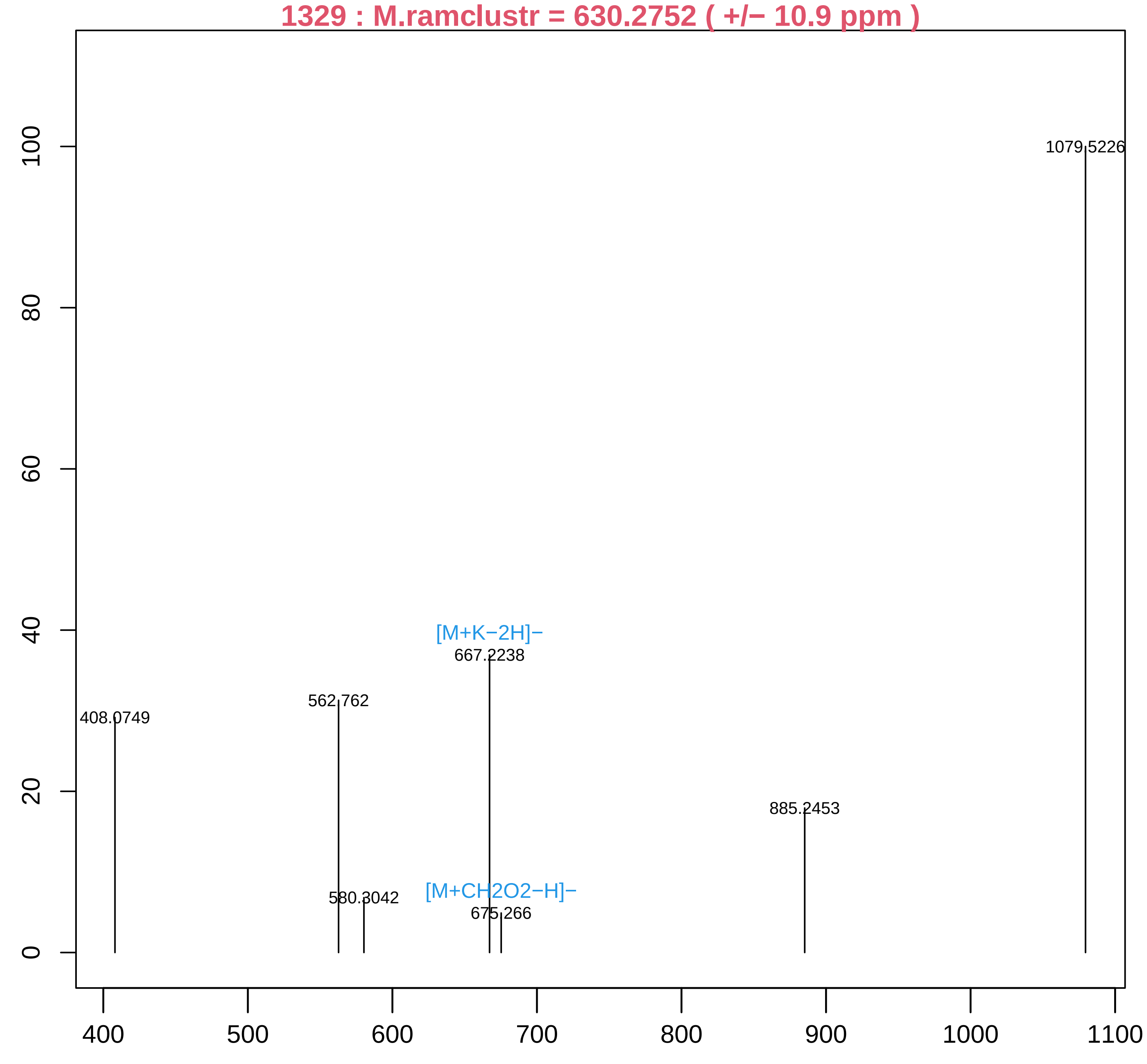

1329 : M.ramclustr = 630.2752 (+/− 10.9 ppm)
1079.5226
[M+K−2H]−
667.2238
562.762
408.0749
885.2453
580.3042
[M+CH2O2−H]−
675.266

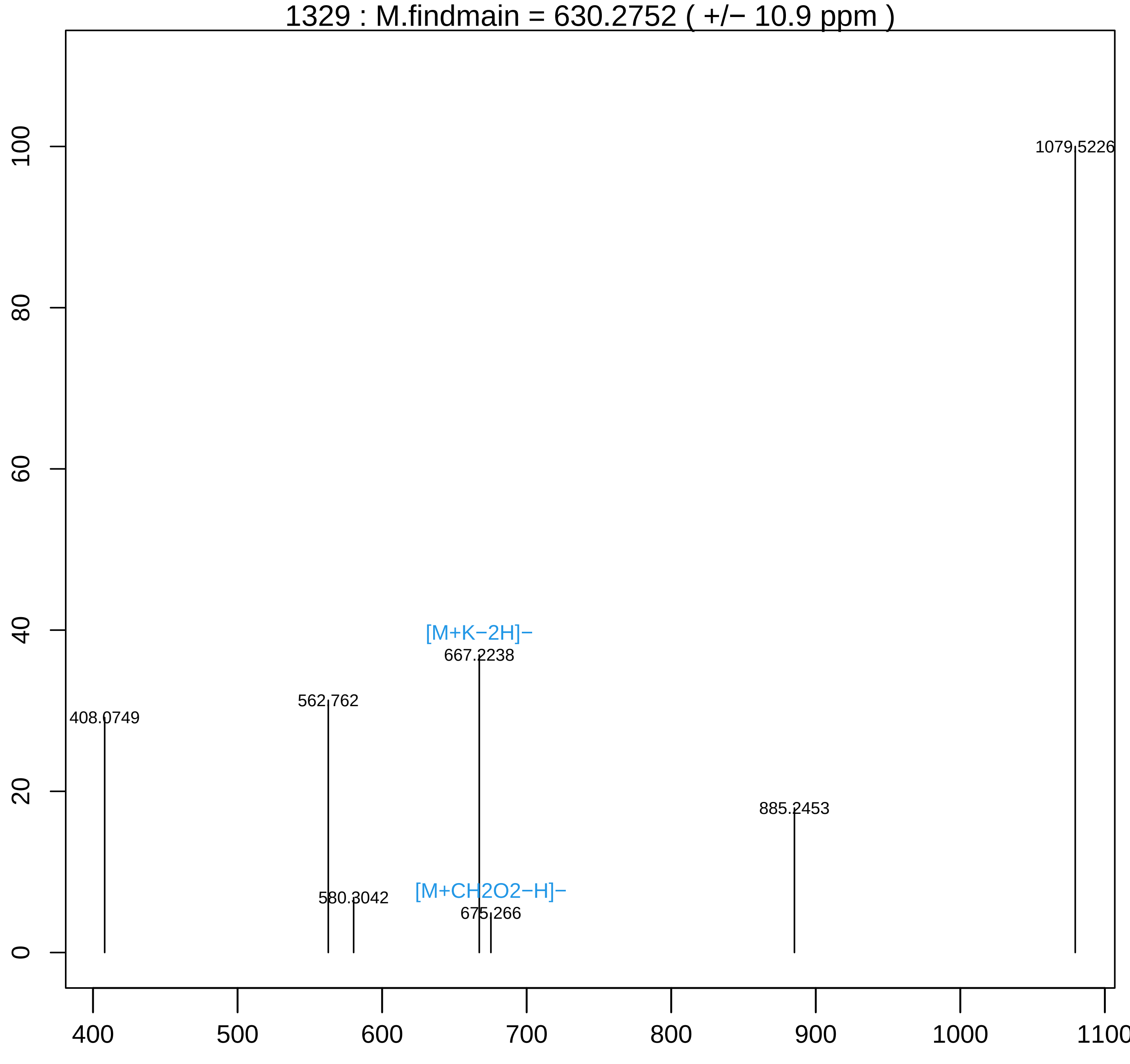

1329 : M.findmain = 630.2752 (+/− 10.9 ppm)
1079.5226
[M+K−2H]−
667.2238
562.762
408.0749
885.2453
580.3042
[M+CH2O2−H]−
675.266

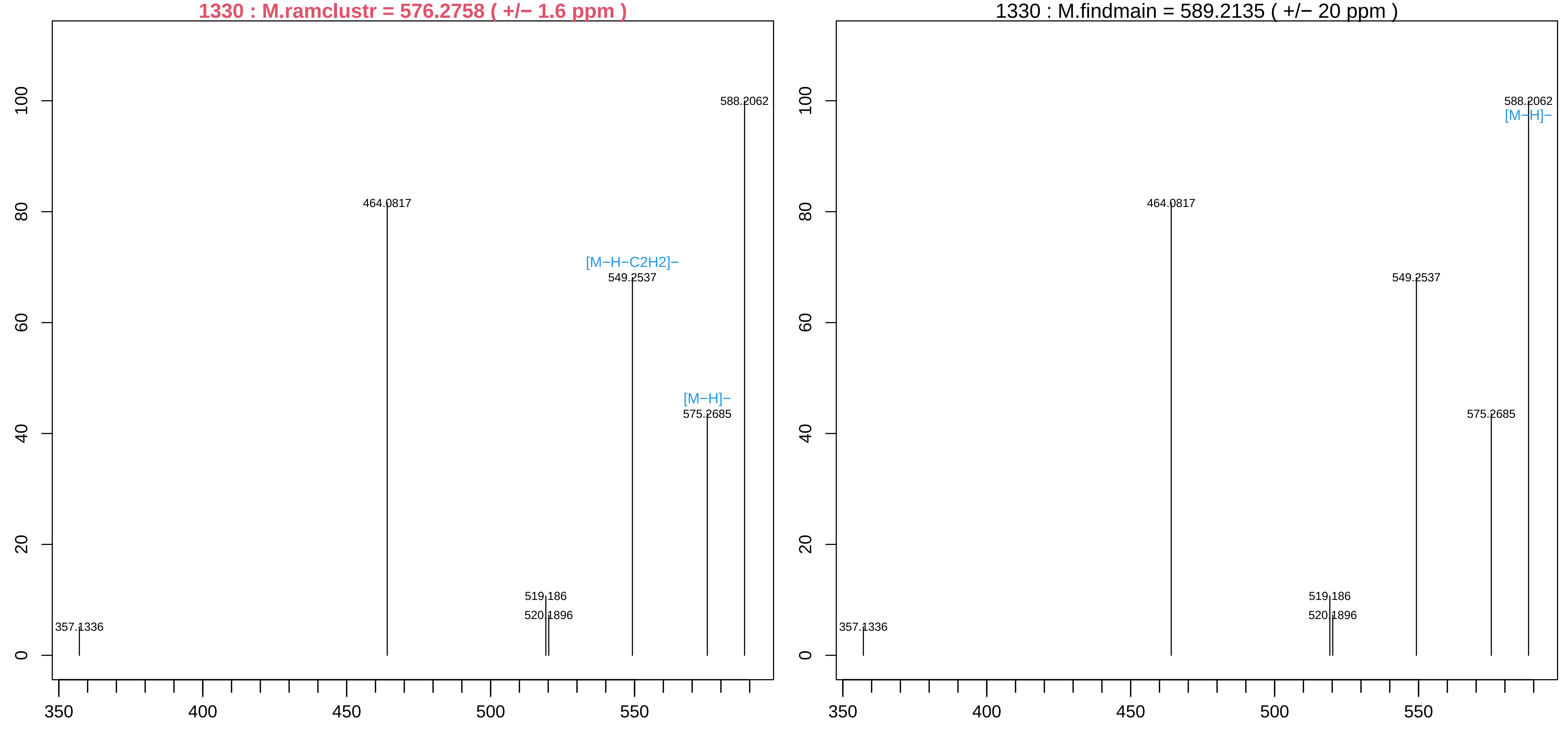
1330 : M.ramclustr = 576.2758 (+/− 1.6 ppm)
1330 : M.findmain = 589.2135 (+/− 20 ppm)
588.2062
464.0817
[M−H−C2H2]−
549.2537
[M−H]−
575.2685
519.186
520.1896
357.1336
[M−H]−
0
20
40
60
80
100
350
400
450
500
550

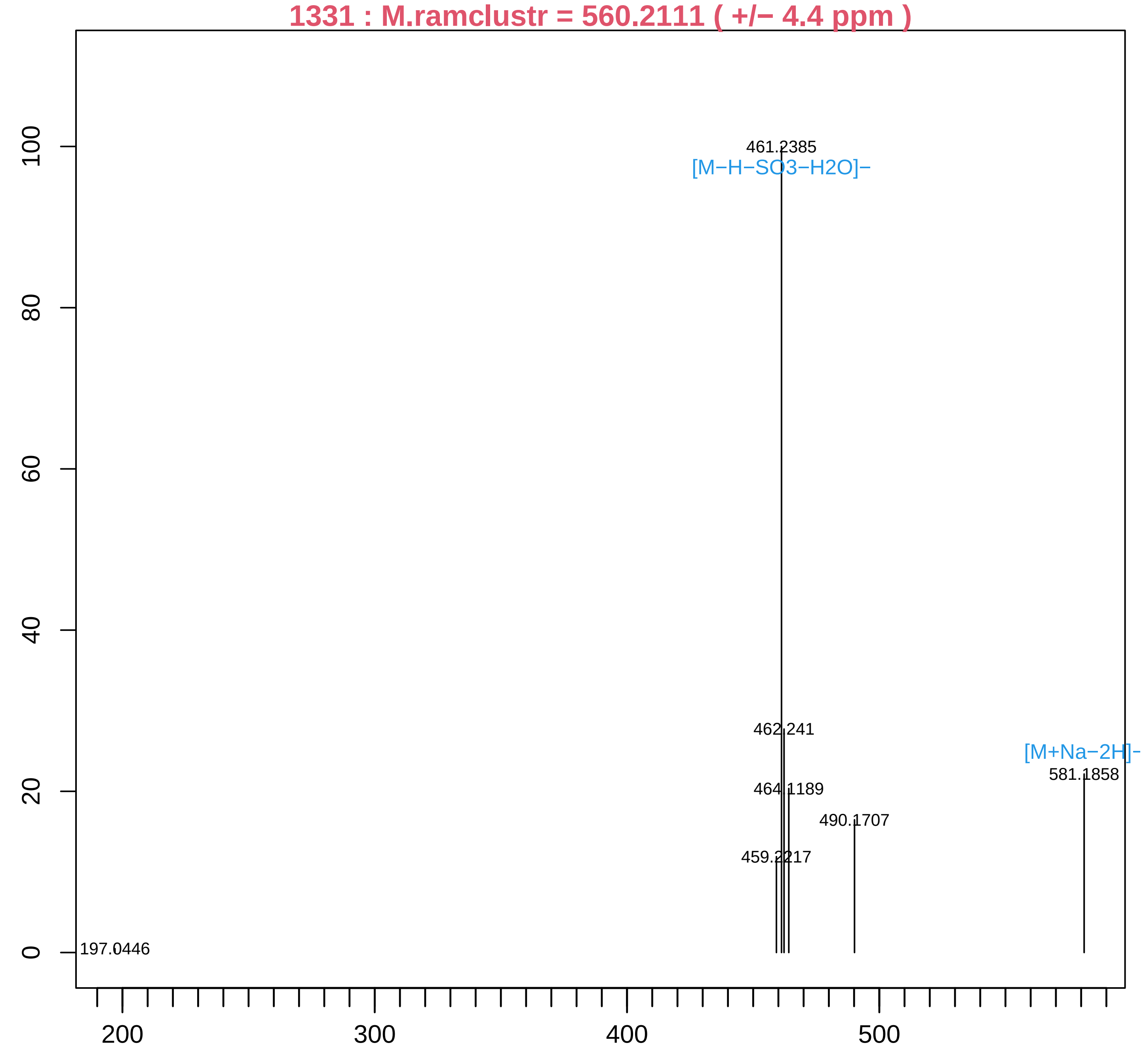
1331 : M.ramclustr = 560.2111 (+/− 4.4 ppm)
461.2385
[M−H−SO3−H2O]−
462.241
464.1189
490.1707
459.2217
[M+Na−2H]−
581.1858
197.0446

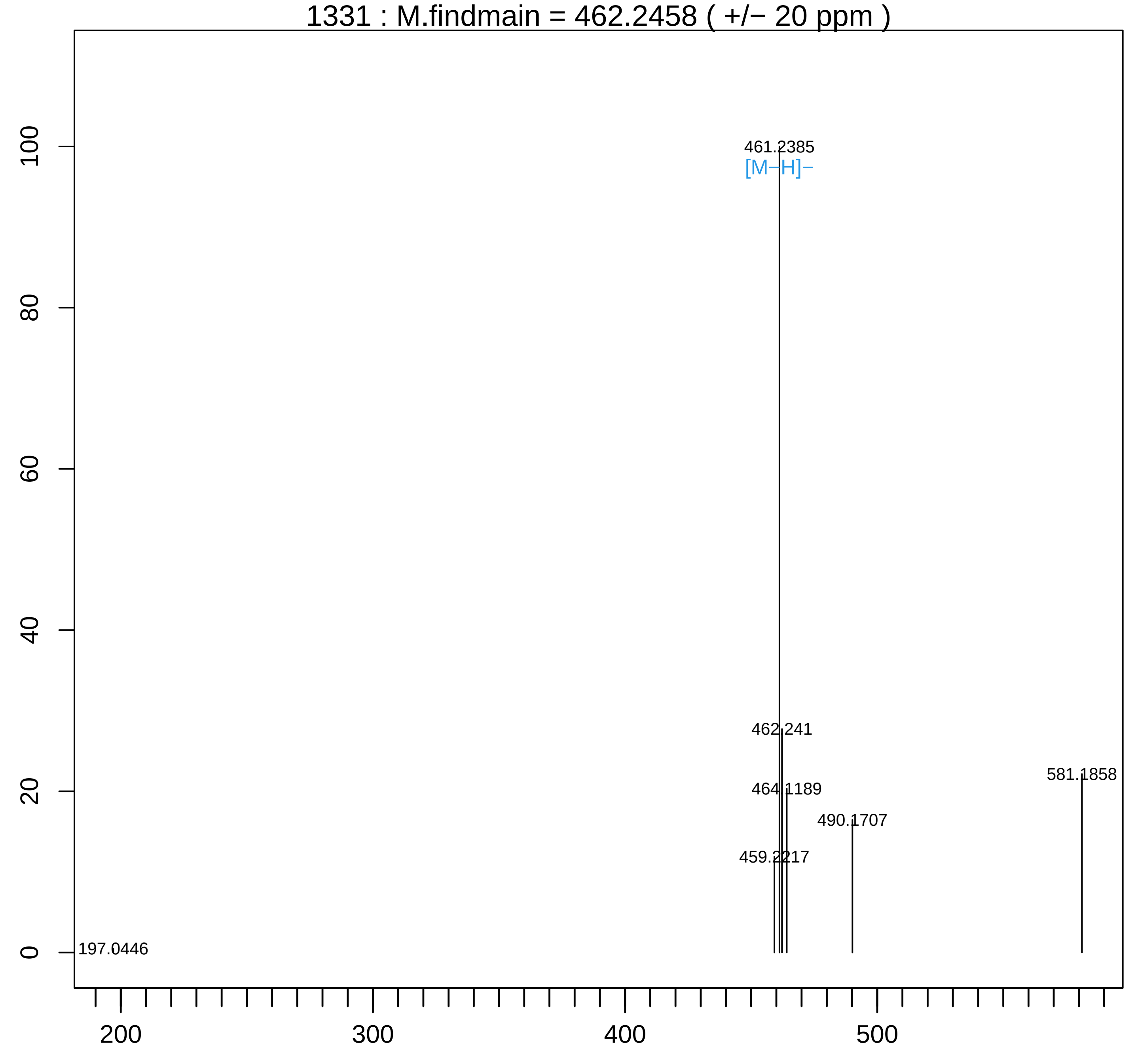
1331 : M.findmain = 462.2458 (+/− 20 ppm)
461.2385
[M−H]−
462.241
464.1189
490.1707
459.2217
581.1858
197.0446

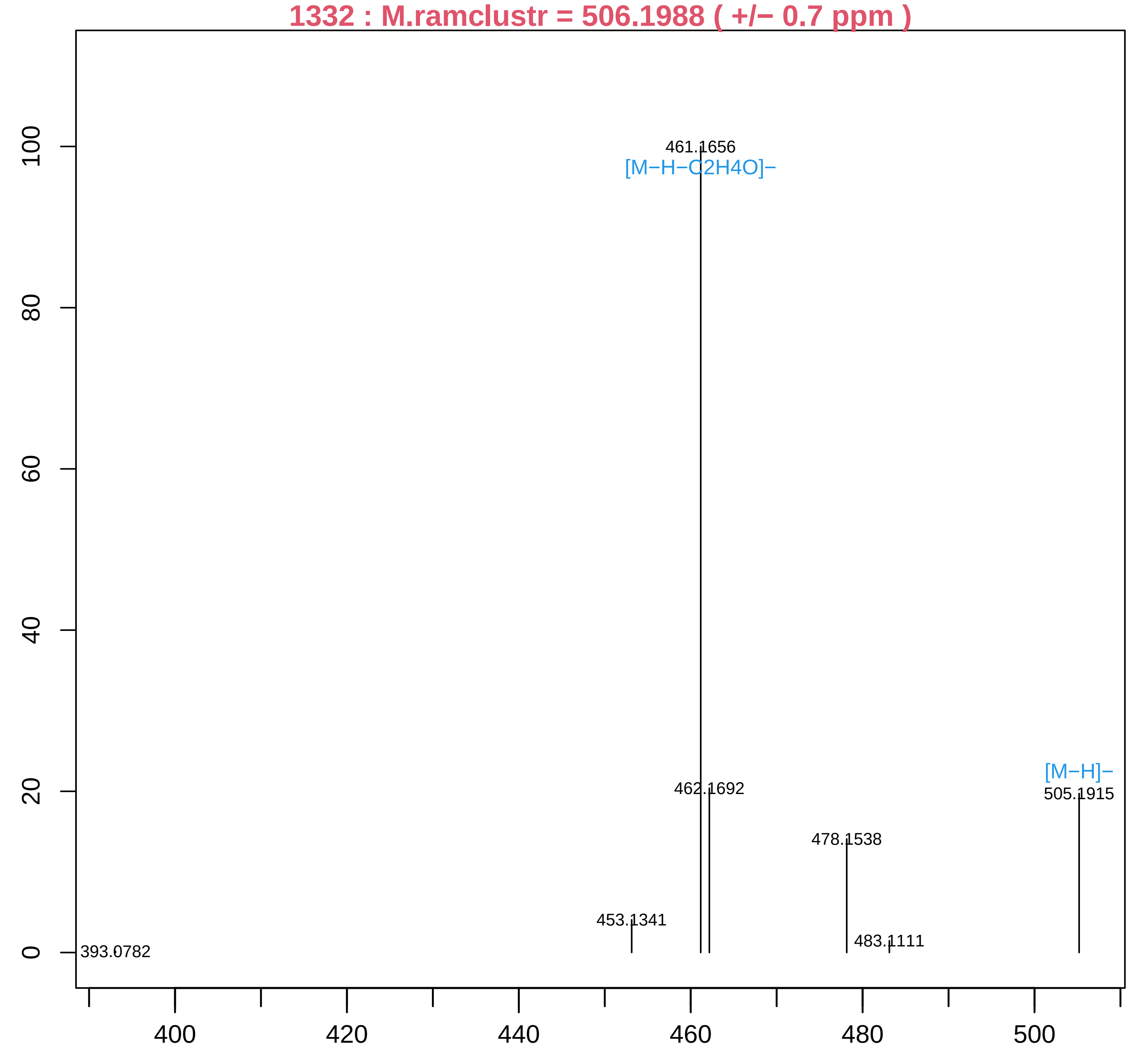

1332 : M.ramclustr = 506.1988 (+/− 0.7 ppm)
461.1656
[M−H−C2H4O]−
[M−H]−
505.1915
462.1692
478.1538
453.1341
483.1111
393.0782
0
20
40
60
80
100
400
420
440
460
480
500

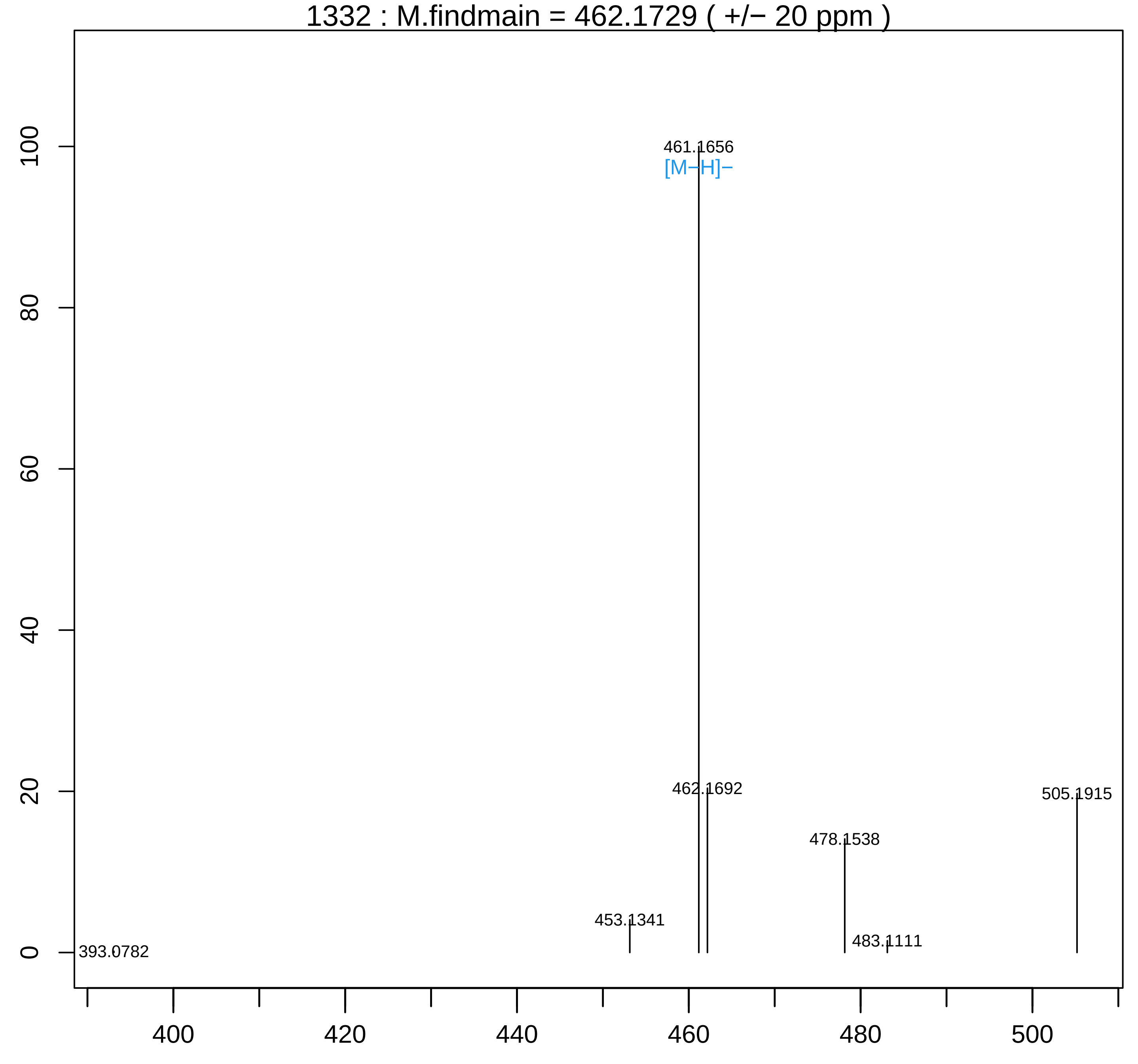

1332 : M.findmain = 462.1729 (+/− 20 ppm)
461.1656
[M−H]−
462.1692
505.1915
478.1538
453.1341
483.1111
393.0782
0
20
40
60
80
100
400
420
440
460
480
500

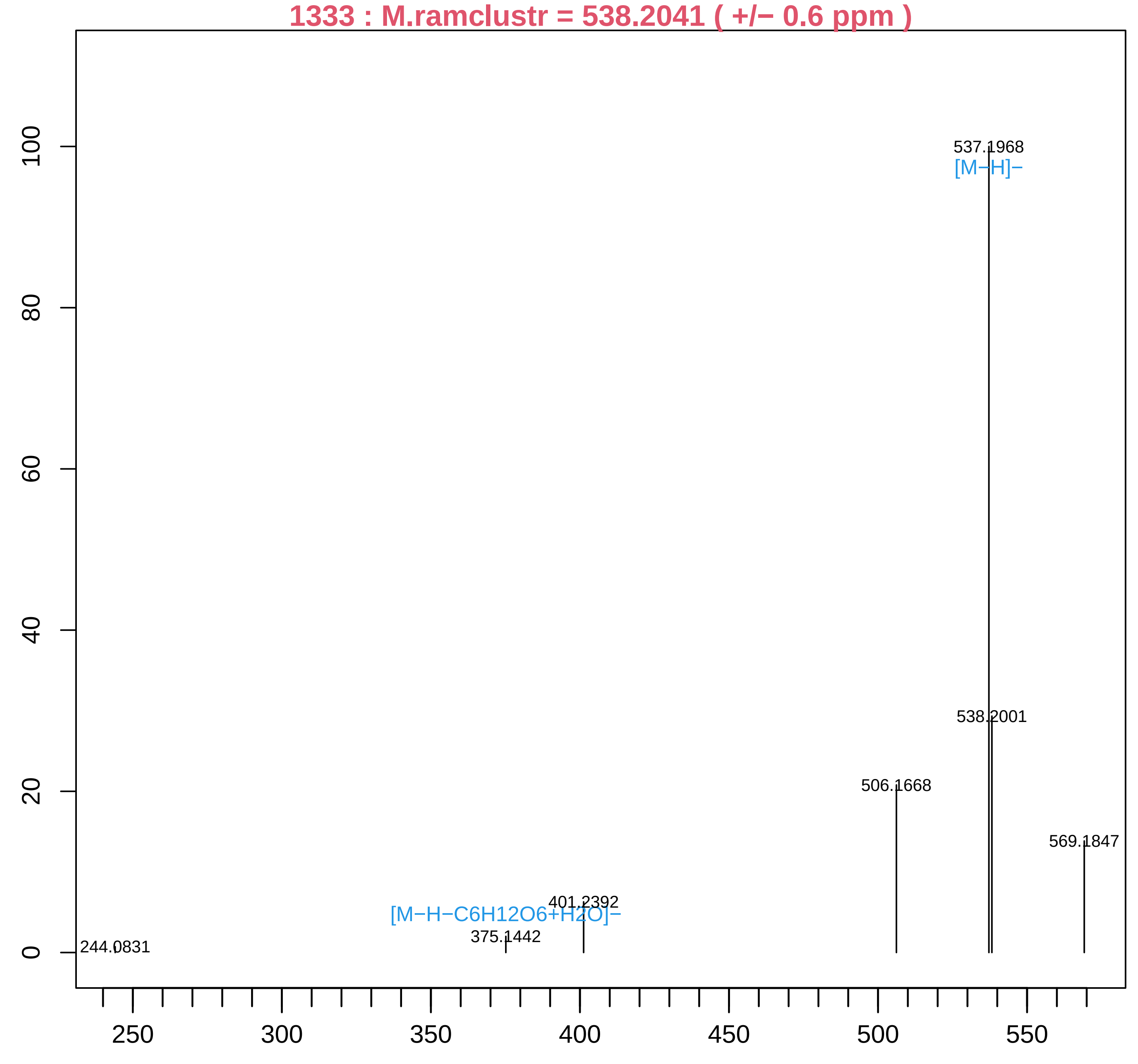
1333 : M.ramclustr = 538.2041 (+/− 0.6 ppm)
537.1968
[M−H]−
538.2001
506.1668
569.1847
401.2392
[M−H−C6H12O6+H2O]−
375.1442
244.0831
0
20
40
60
80
100
250
300
350
400
450
500
550

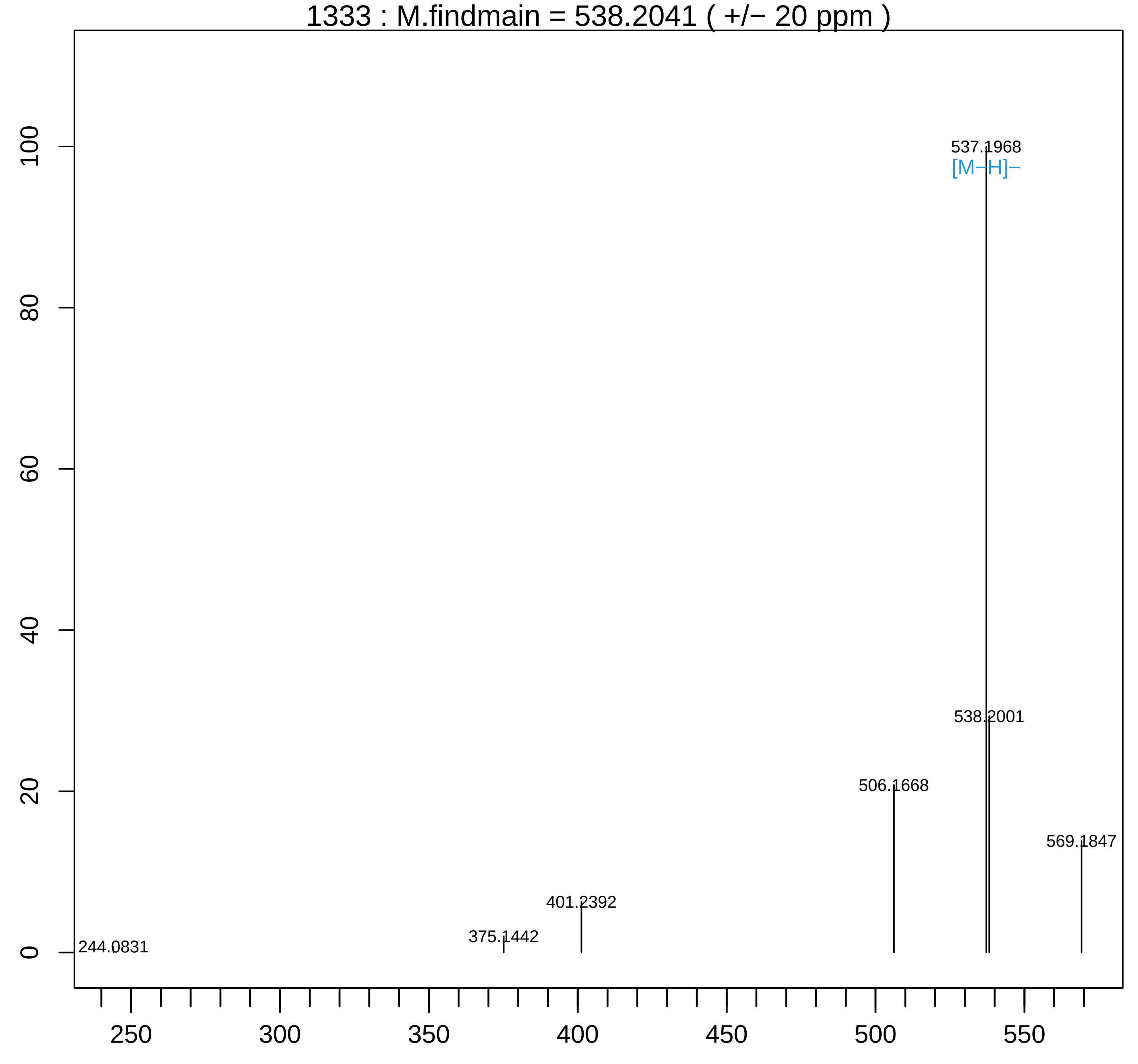
1333 : M.findmain = 538.2041 (+/− 20 ppm)
537.1968
[M−H]−
538.2001
506.1668
569.1847
401.2392
375.1442
244.0831
0
20
40
60
80
100
250
300
350
400
450
500
550

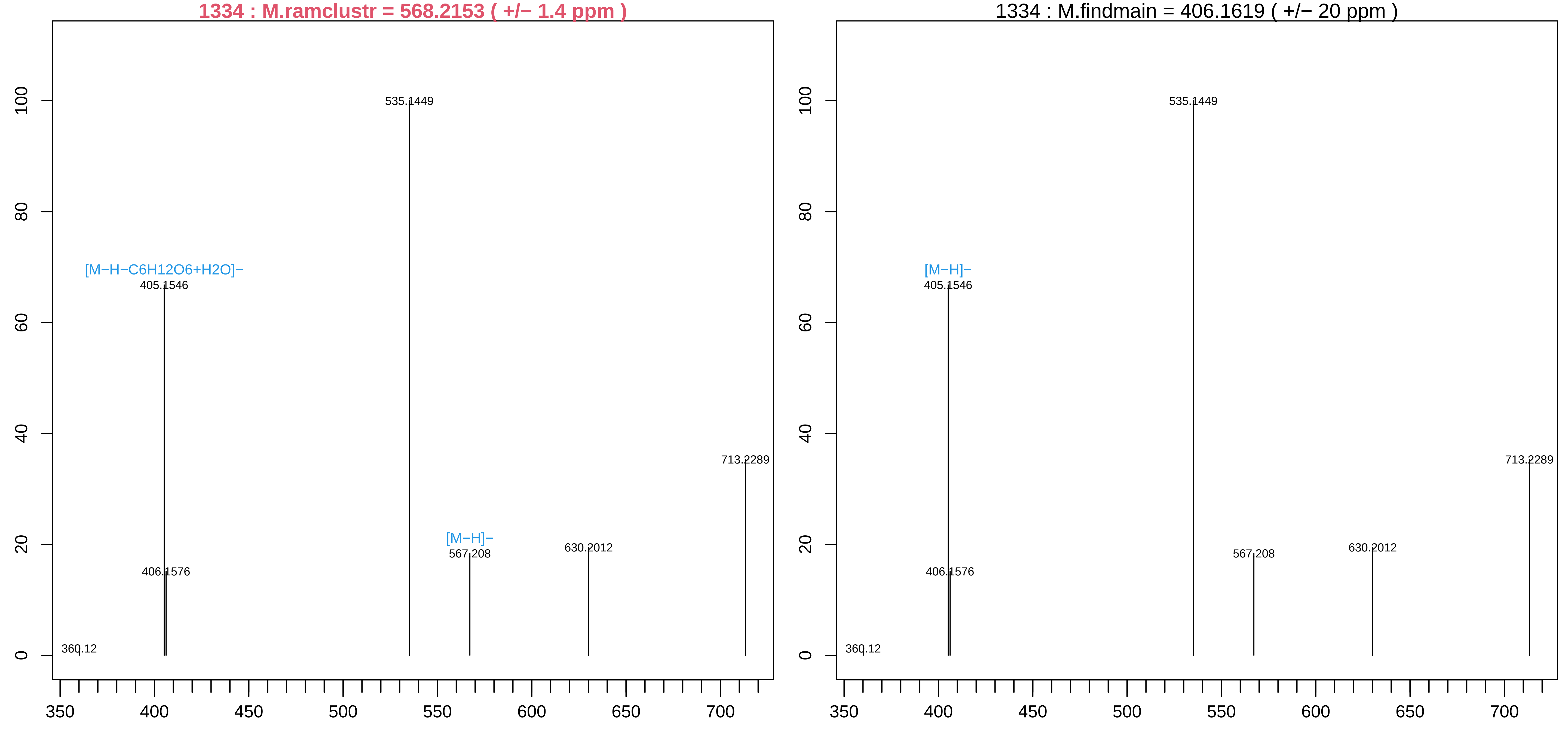
1334 : M.ramclustr = 568.2153 (+/− 1.4 ppm)
535.1449
[M−H−C6H12O6+H2O]−
405.1546
406.1576
360.12
[M−H]−
567.208
630.2012
713.2289
1334 : M.findmain = 406.1619 (+/− 20 ppm)
535.1449
[M−H]−
405.1546
406.1576
360.12
567.208
630.2012
713.2289
0
20
40
60
80
100
350
400
450
500
550
600
650
700

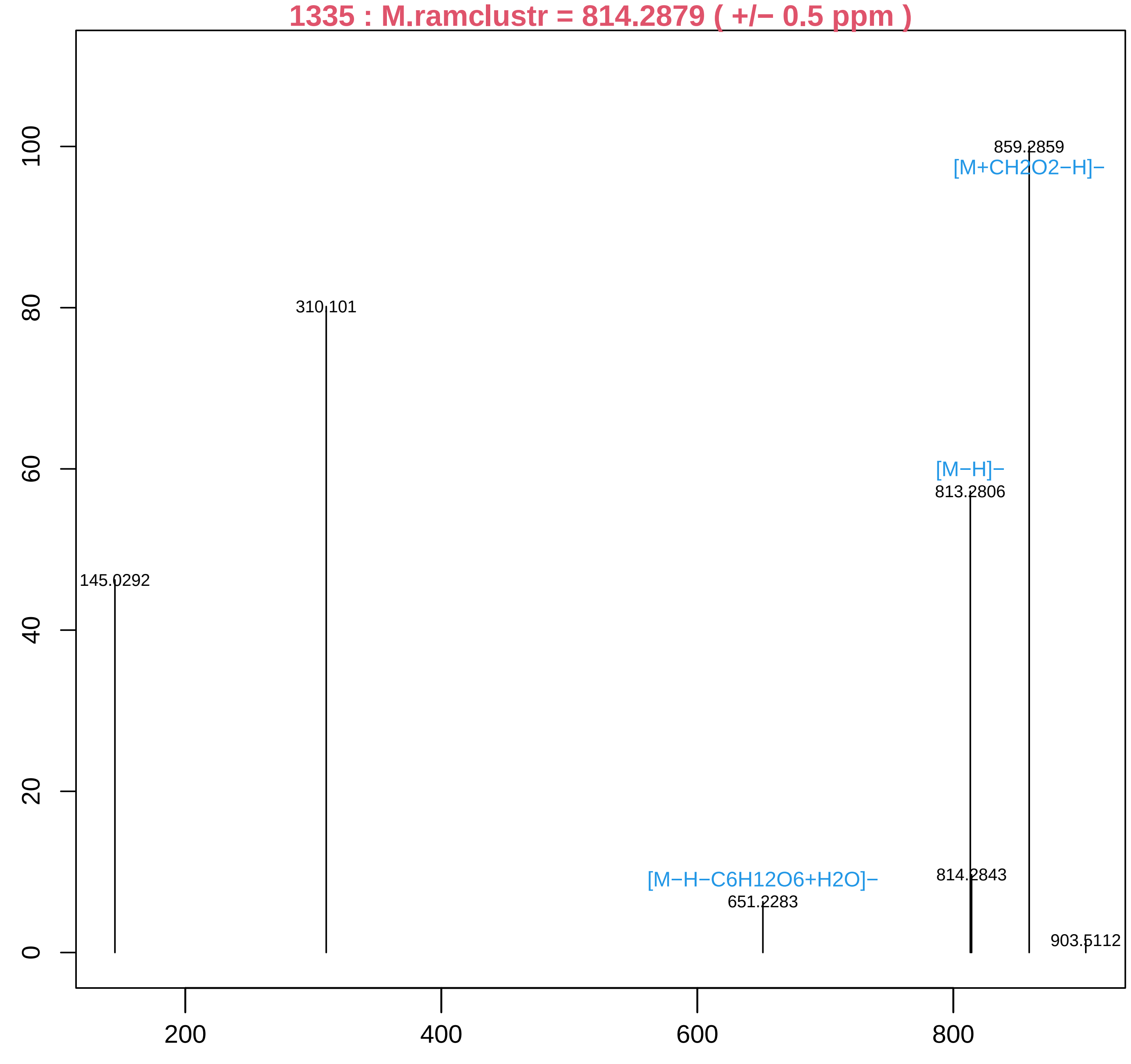
1335 : M.ramclustr = 814.2879 (+/− 0.5 ppm)
859.2859
[M+CH2O2−H]−
310.101
[M−H]−
813.2806
145.0292
[M−H−C6H12O6+H2O]−
814.2843
651.2283
903.5112
0
20
40
60
80
100
200
400
600
800
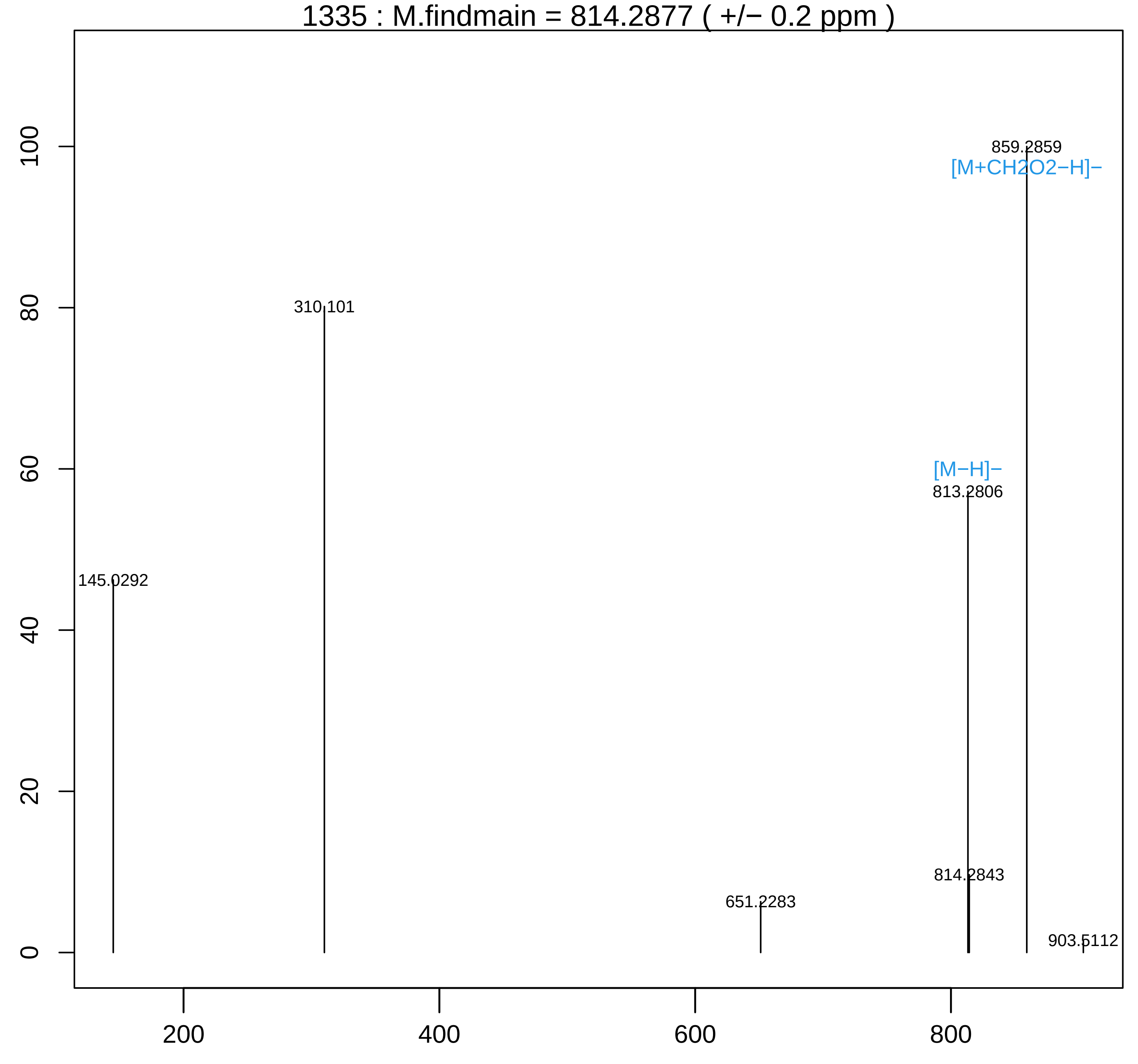
1335 : M.findmain = 814.2877 (+/− 0.2 ppm)
859.2859
[M+CH2O2−H]−
310.101
[M−H]−
813.2806
145.0292
814.2843
651.2283
903.5112
0
20
40
60
80
100
200
400
600
800

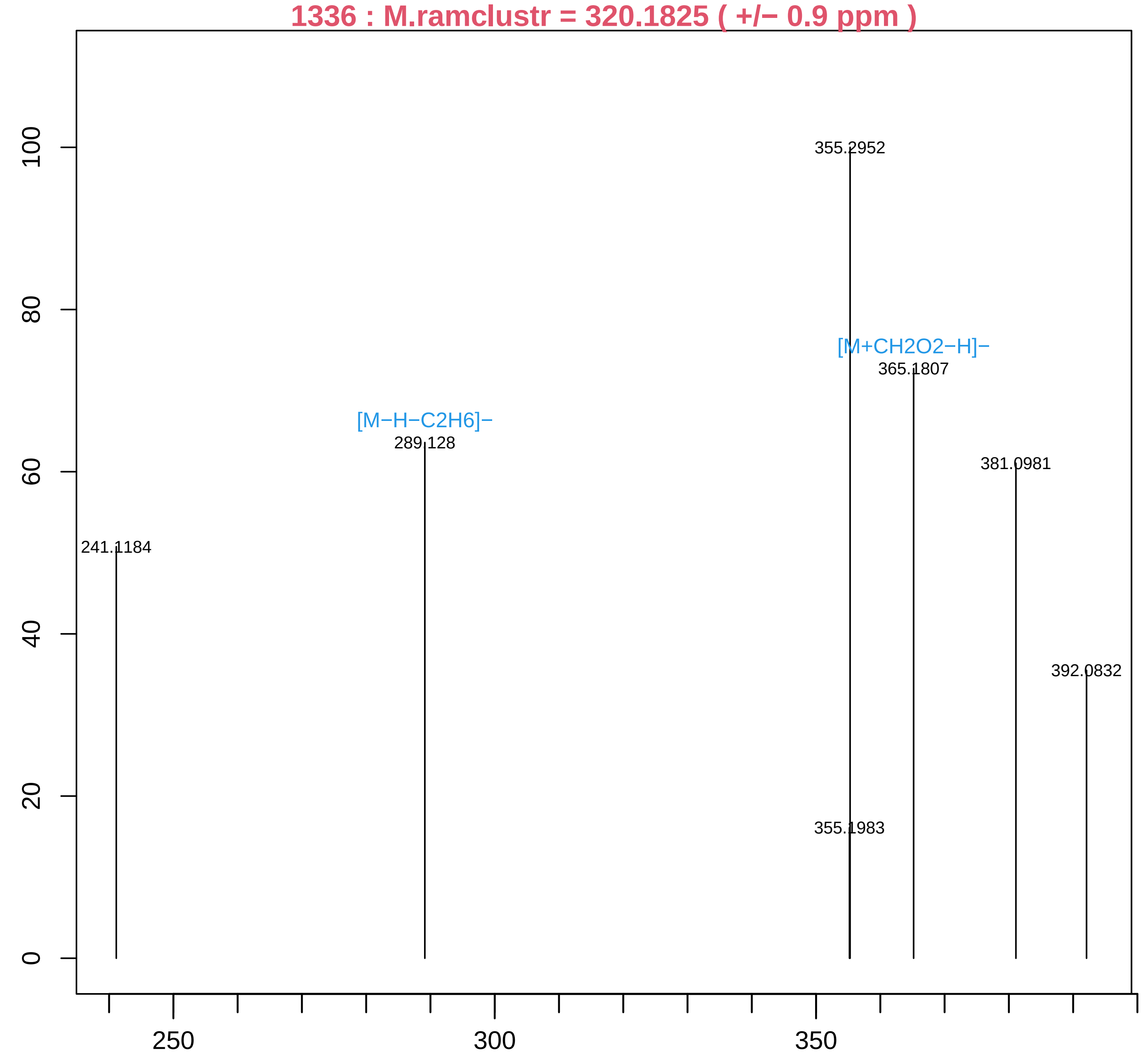

1336 : M.ramclustr = 320.1825 (+/− 0.9 ppm)
355.2952
[M+CH2O2−H]−
365.1807
[M−H−C2H6]−
289.128
381.0981
241.1184
392.0832
355.1983
0
20
40
60
80
100
250
300
350

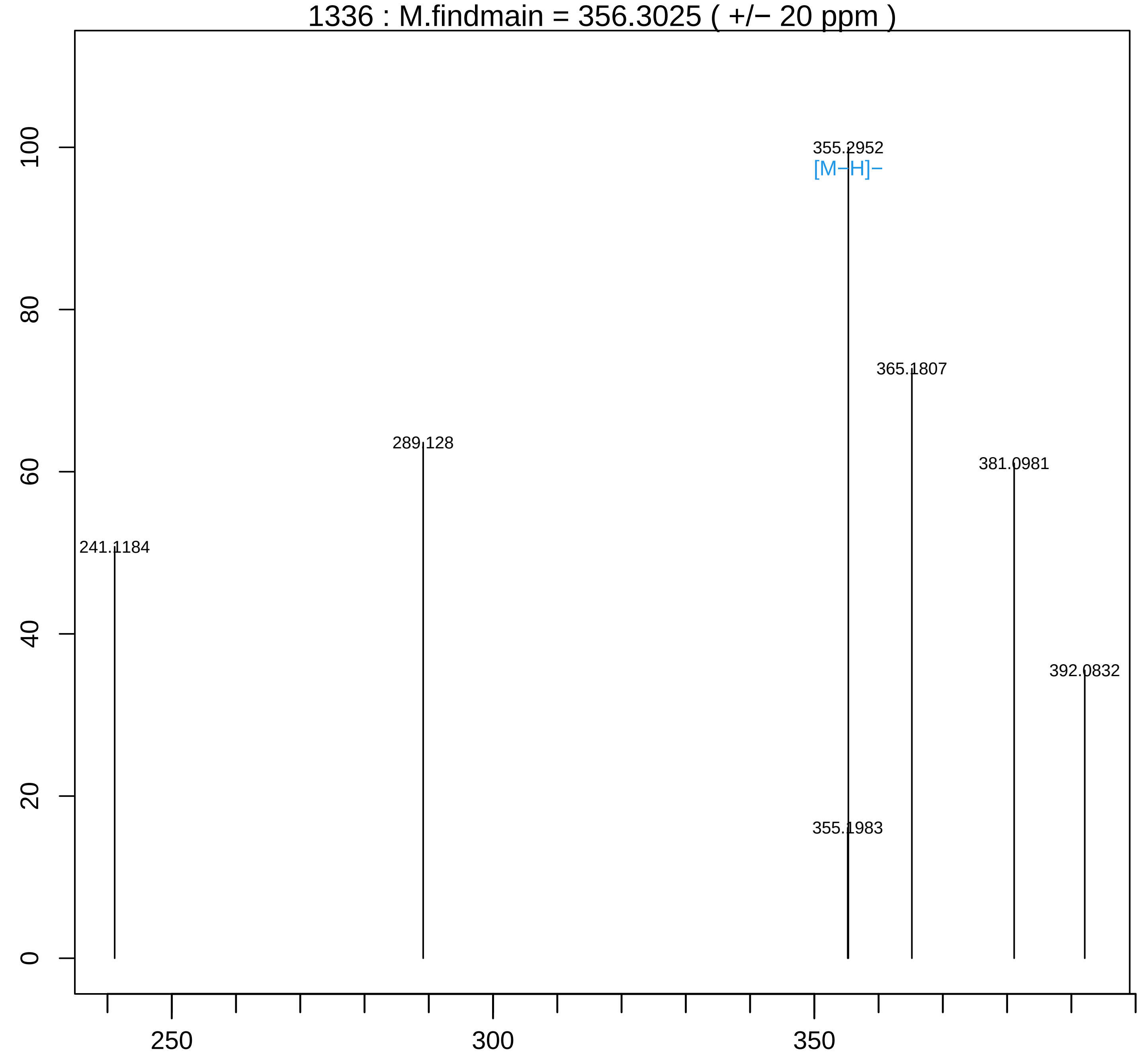

1336 : M.findmain = 356.3025 (+/− 20 ppm)
355.2952
[M−H]−
365.1807
289.128
381.0981
241.1184
392.0832
355.1983
0
20
40
60
80
100
250
300
350

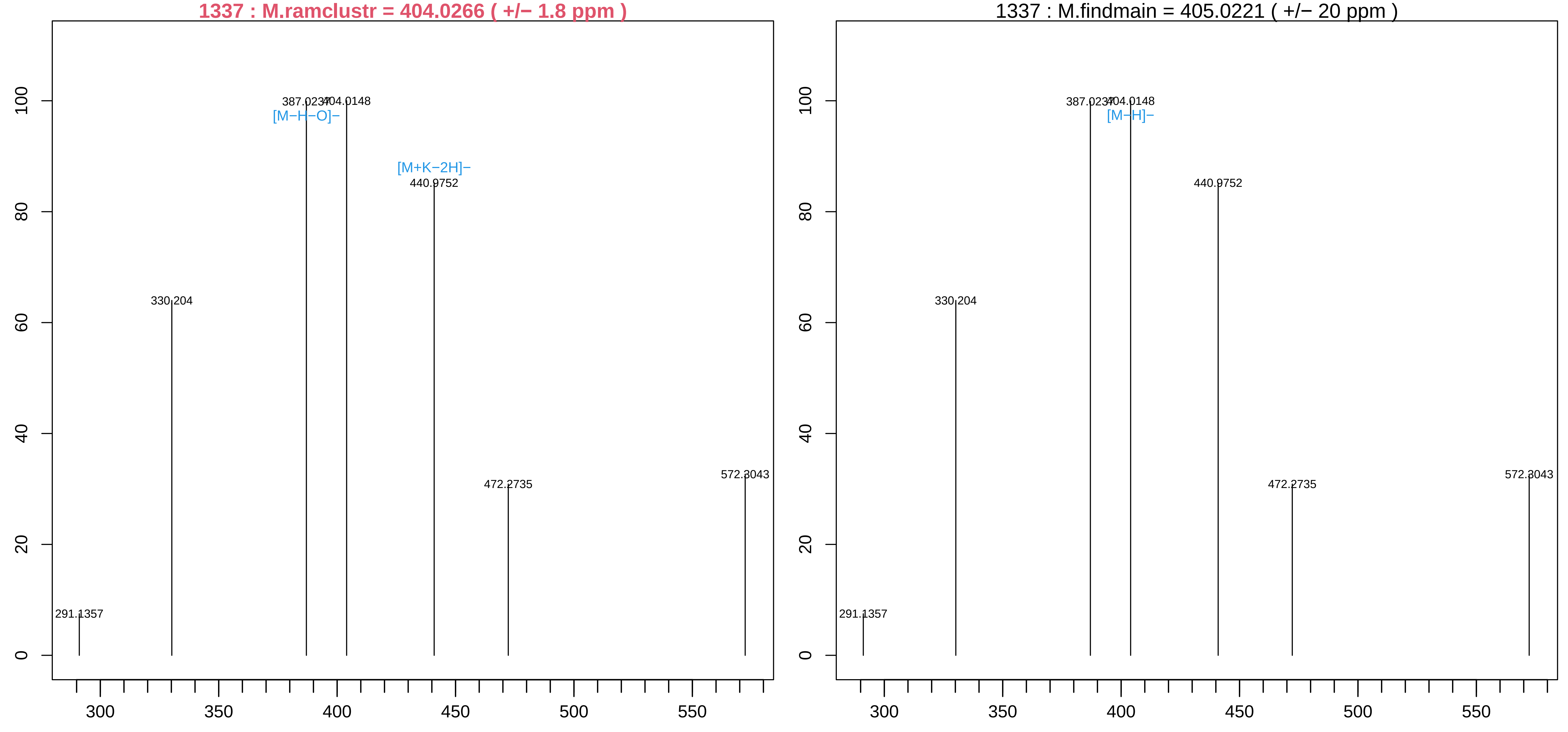
1337 : M.ramclustr = 404.0266 (+/− 1.8 ppm)
387.0234
404.0148
[M−H−O]−
[M+K−2H]−
440.9752
330.204
472.2735
572.3043
291.1357
1337 : M.findmain = 405.0221 (+/− 20 ppm)
[M−H]−
0
20
40
60
80
100
300
350
400
450
500
550

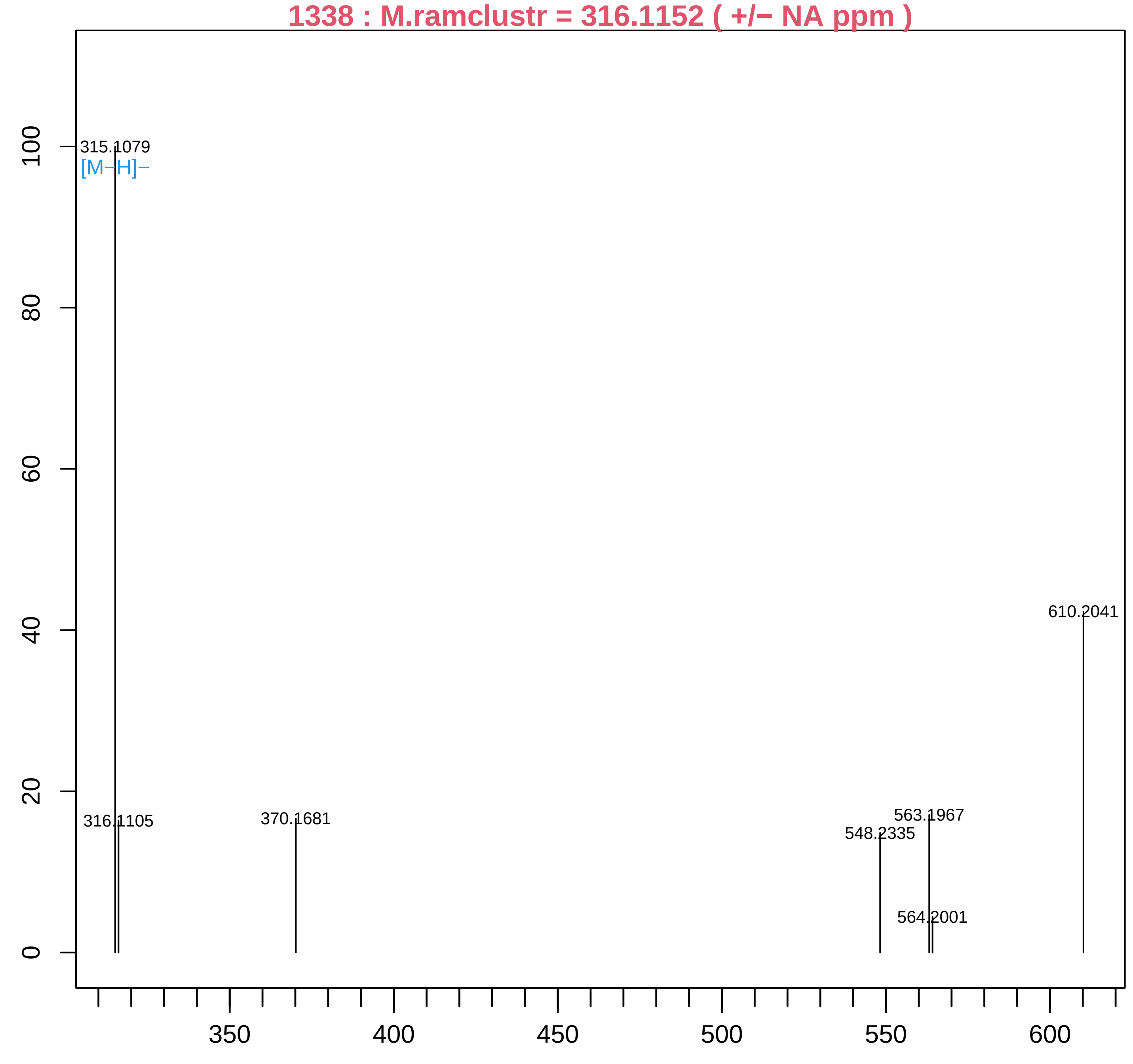
1338 : M.ramclustr = 316.1152 (+/− NA ppm)
315.1079
[M−H]−
316.1105
370.1681
548.2335
563.1967
564.2001
610.2041
0
20
40
60
80
100
350
400
450
500
550
600

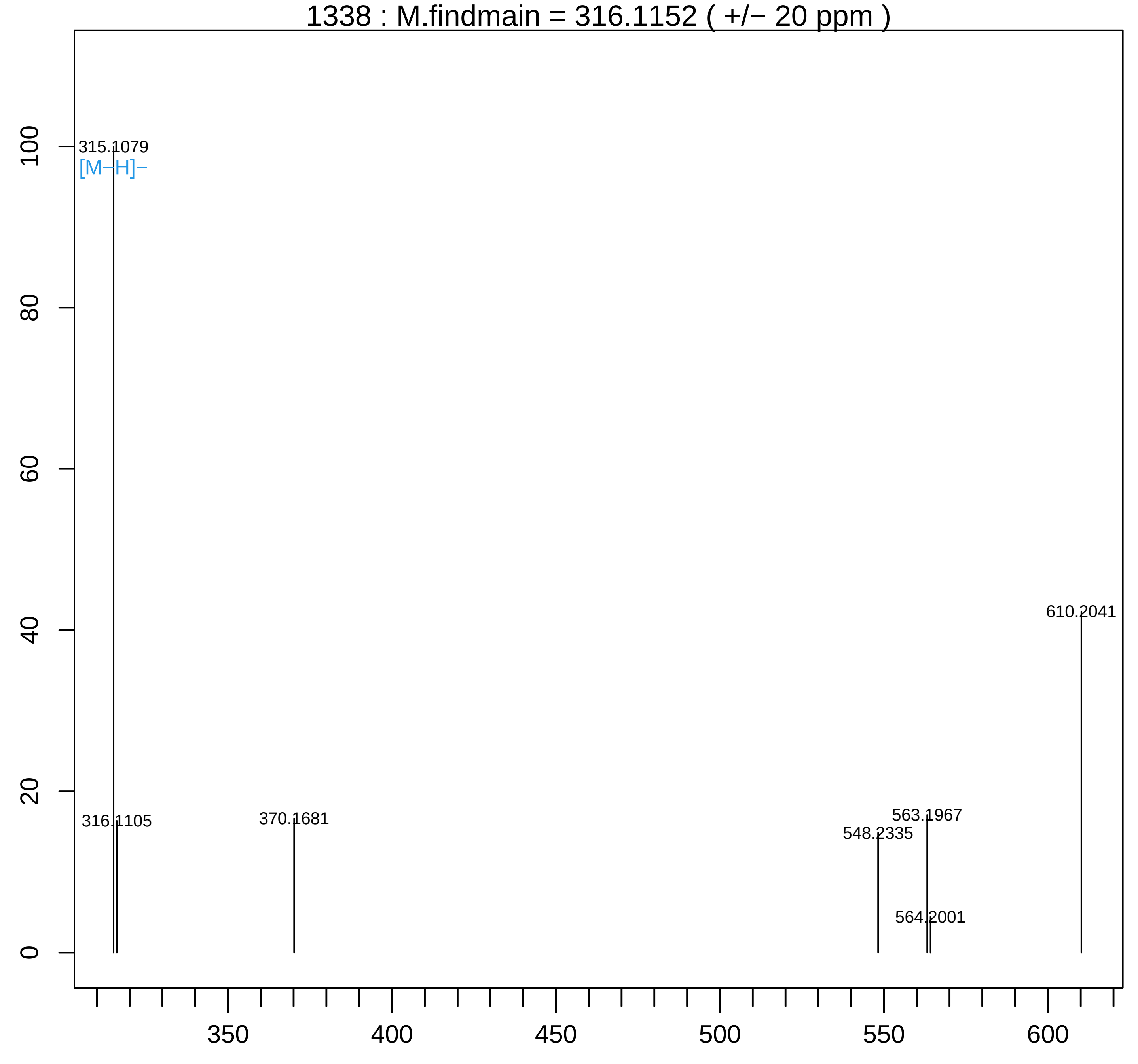
1338 : M.findmain = 316.1152 (+/− 20 ppm)
315.1079
[M−H]−
316.1105
370.1681
548.2335
563.1967
564.2001
610.2041
0
20
40
60
80
100
350
400
450
500
550
600

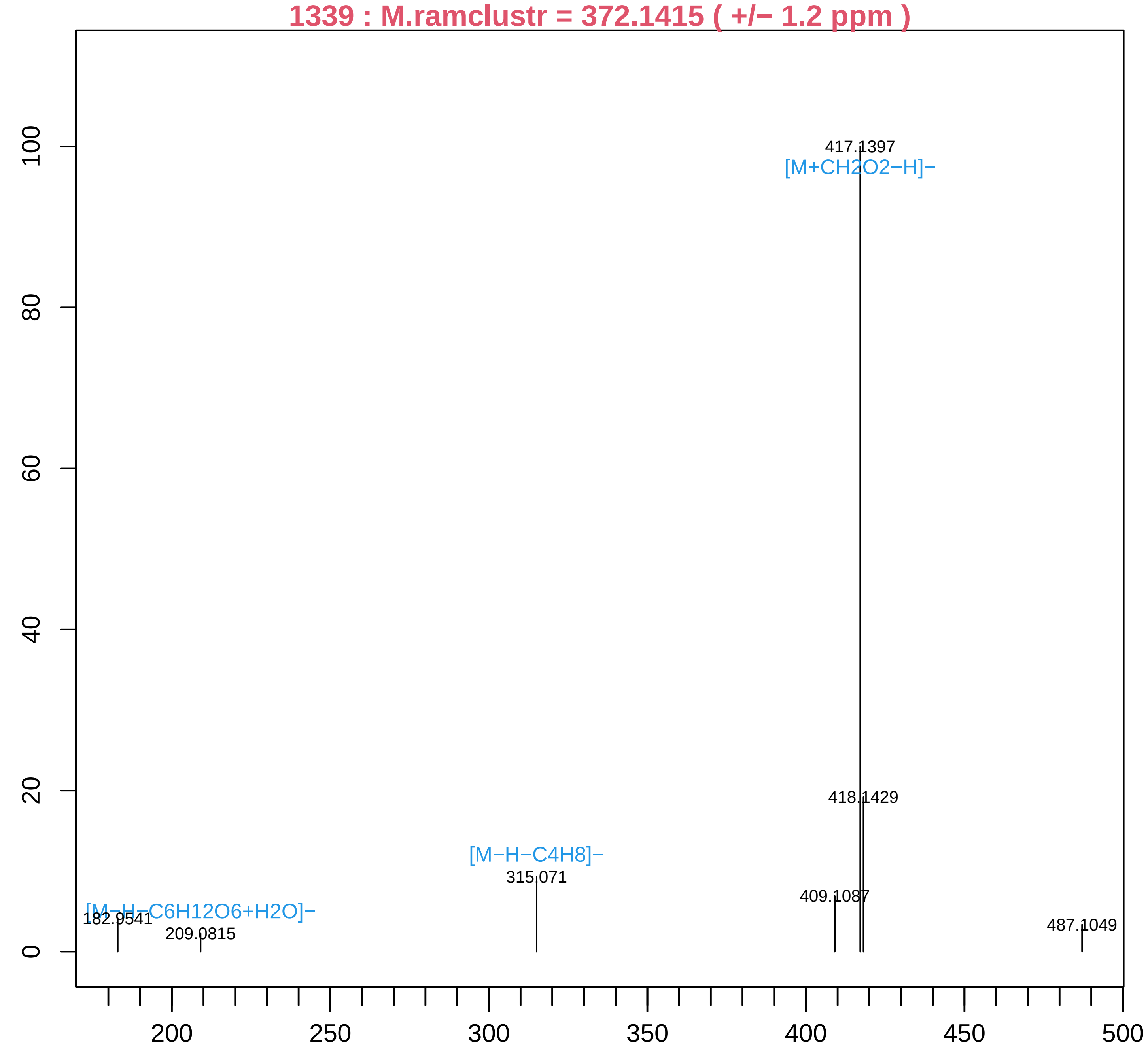
1339 : M.ramclustr = 372.1415 (+/− 1.2 ppm)
417.1397
[M+CH2O2−H]−
418.1429
[M−H−C4H8]−
315.071
409.1087
[M−H−C6H12O6+H2O]−
182.9541
209.0815
487.1049
0
20
40
60
80
100
200
250
300
350
400
450
500

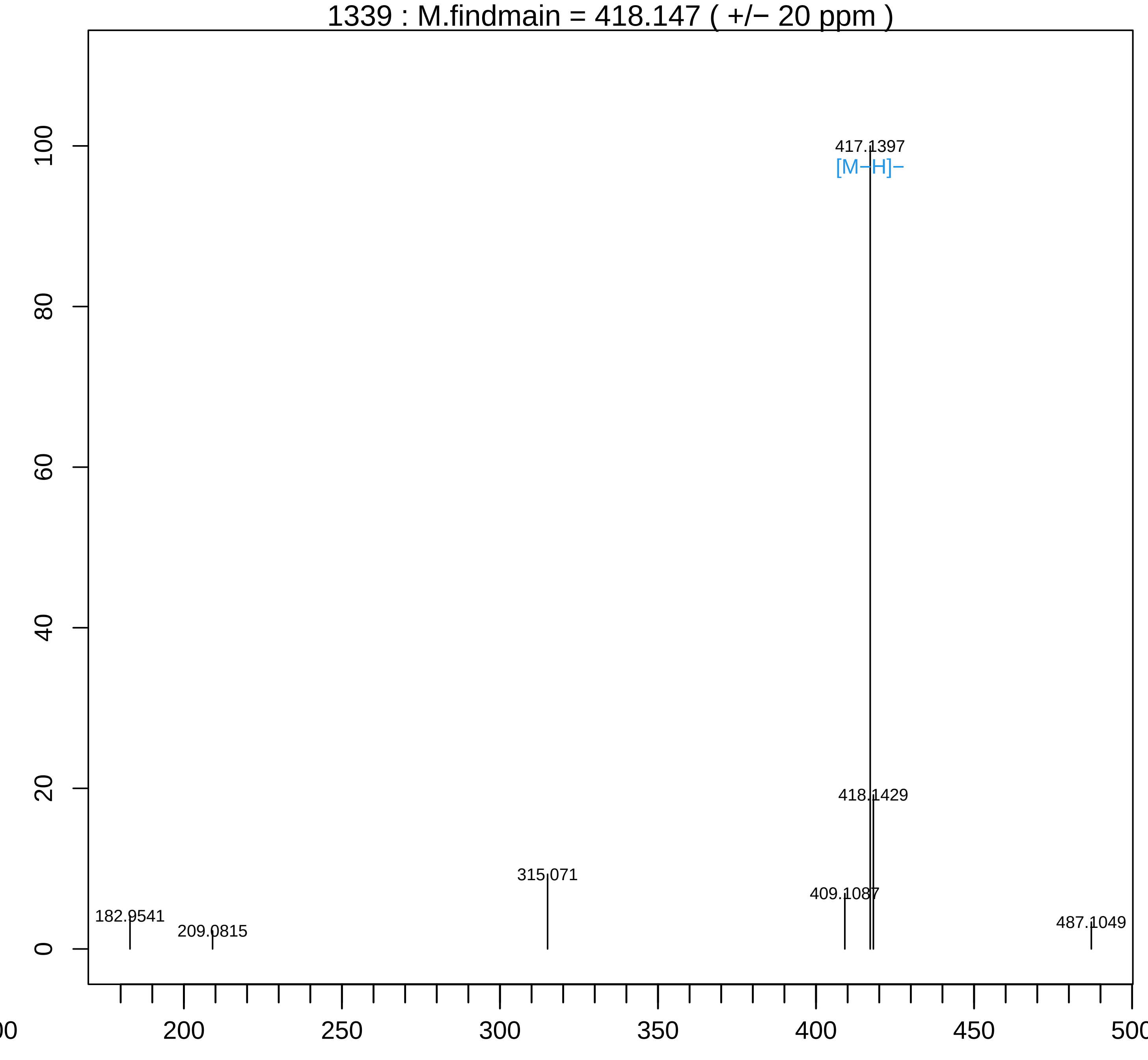
1339 : M.findmain = 418.147 (+/− 20 ppm)
417.1397
[M−H]−
418.1429
315.071
409.1087
182.9541
209.0815
487.1049
0
20
40
60
80
100
200
250
300
350
400
450
500

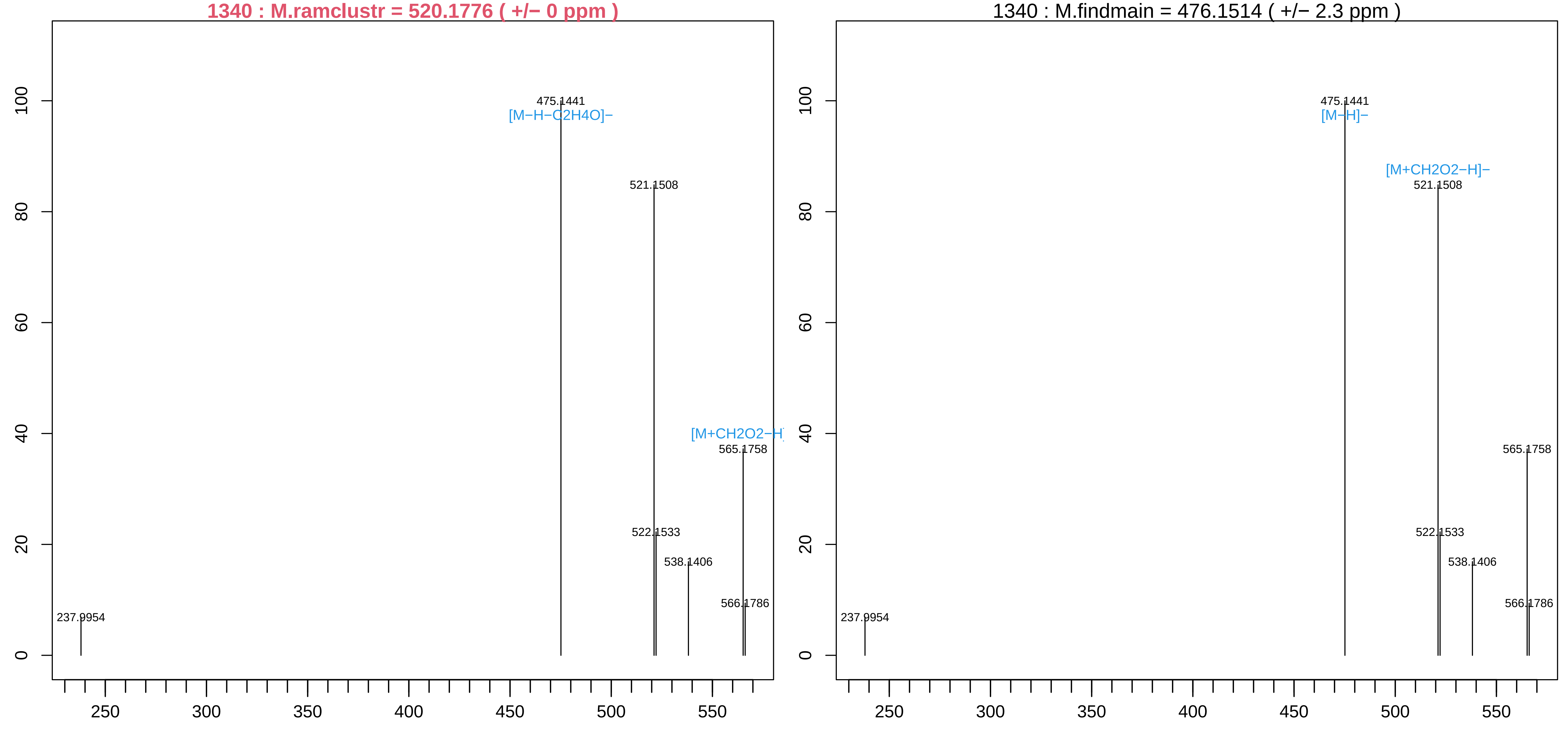
1340 : M.ramclustr = 520.1776 (+/− 0 ppm)
475.1441
[M−H−C2H4O]−
521.1508
[M+CH2O2−H]
565.1758
522.1533
538.1406
566.1786
237.9954
1340 : M.findmain = 476.1514 (+/− 2.3 ppm)
475.1441
[M−H]−
[M+CH2O2−H]−
521.1508
565.1758
522.1533
538.1406
566.1786
237.9954
0
20
40
60
80
100
250
300
350
400
450
500
550

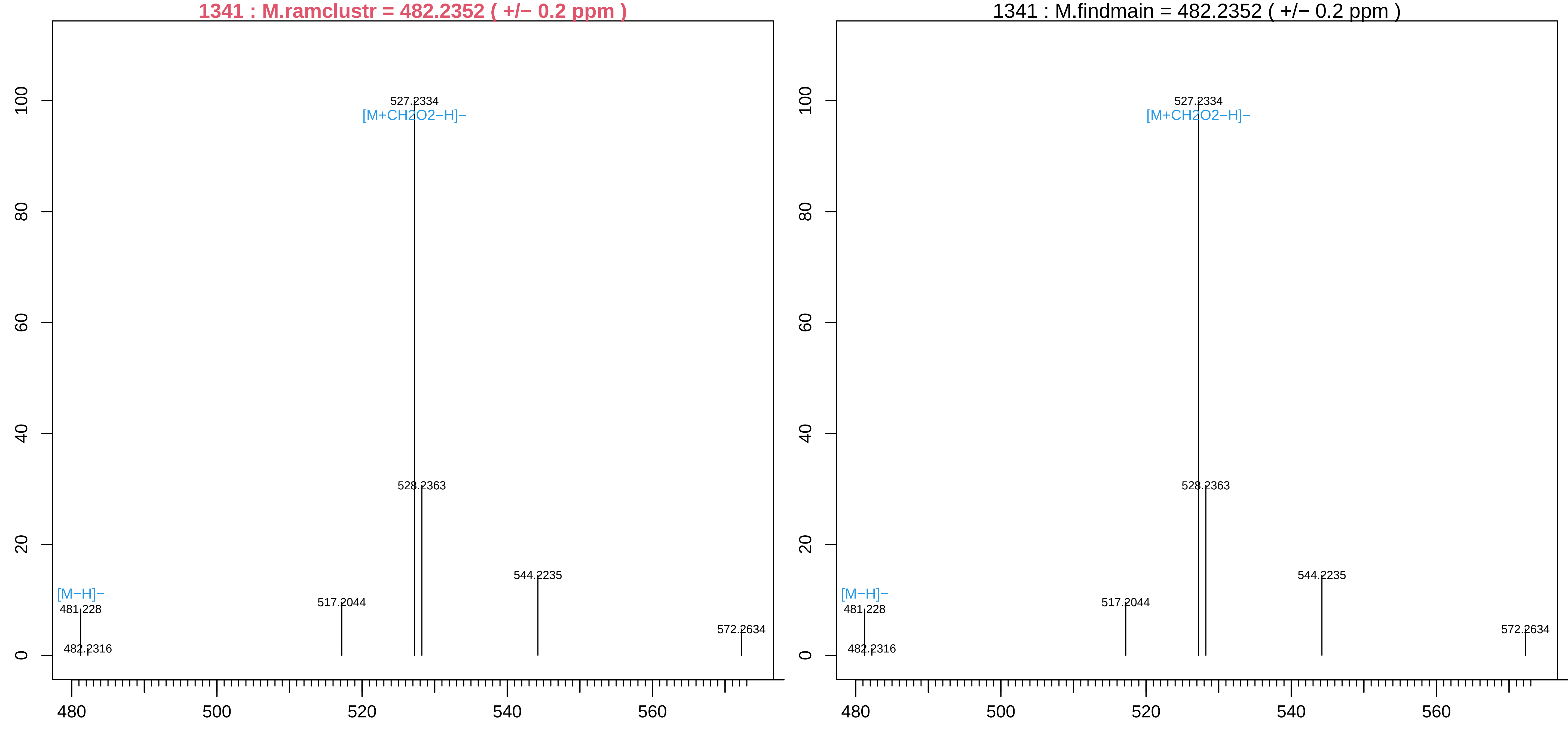
1341 : M.ramclustr = 482.2352 (+/− 0.2 ppm)
527.2334
[M+CH2O2−H]−
528.2363
544.2235
[M−H]−
481.228
517.2044
572.2634
482.2316
1341 : M.findmain = 482.2352 (+/− 0.2 ppm)
527.2334
[M+CH2O2−H]−
528.2363
544.2235
[M−H]−
481.228
517.2044
572.2634
482.2316
0
20
40
60
80
100
480
500
520
540
560

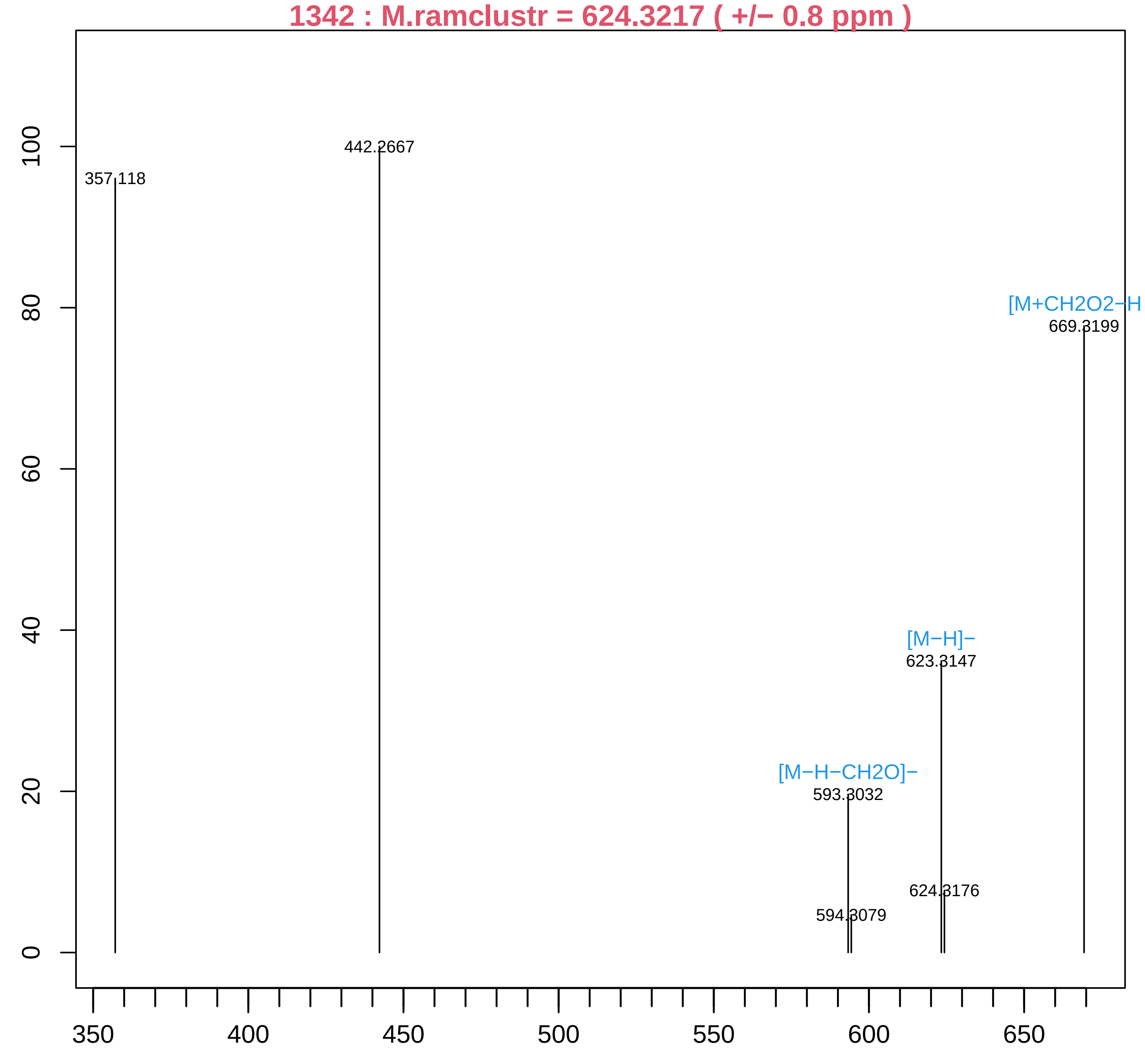
1342 : M.ramclustr = 624.3217 (+/− 0.8 ppm)
357.118
442.2667
[M+CH2O2−H
669.3199
[M−H]−
623.3147
[M−H−CH2O]−
593.3032
624.3176
594.3079
0
20
40
60
80
100
350
400
450
500
550
600
650

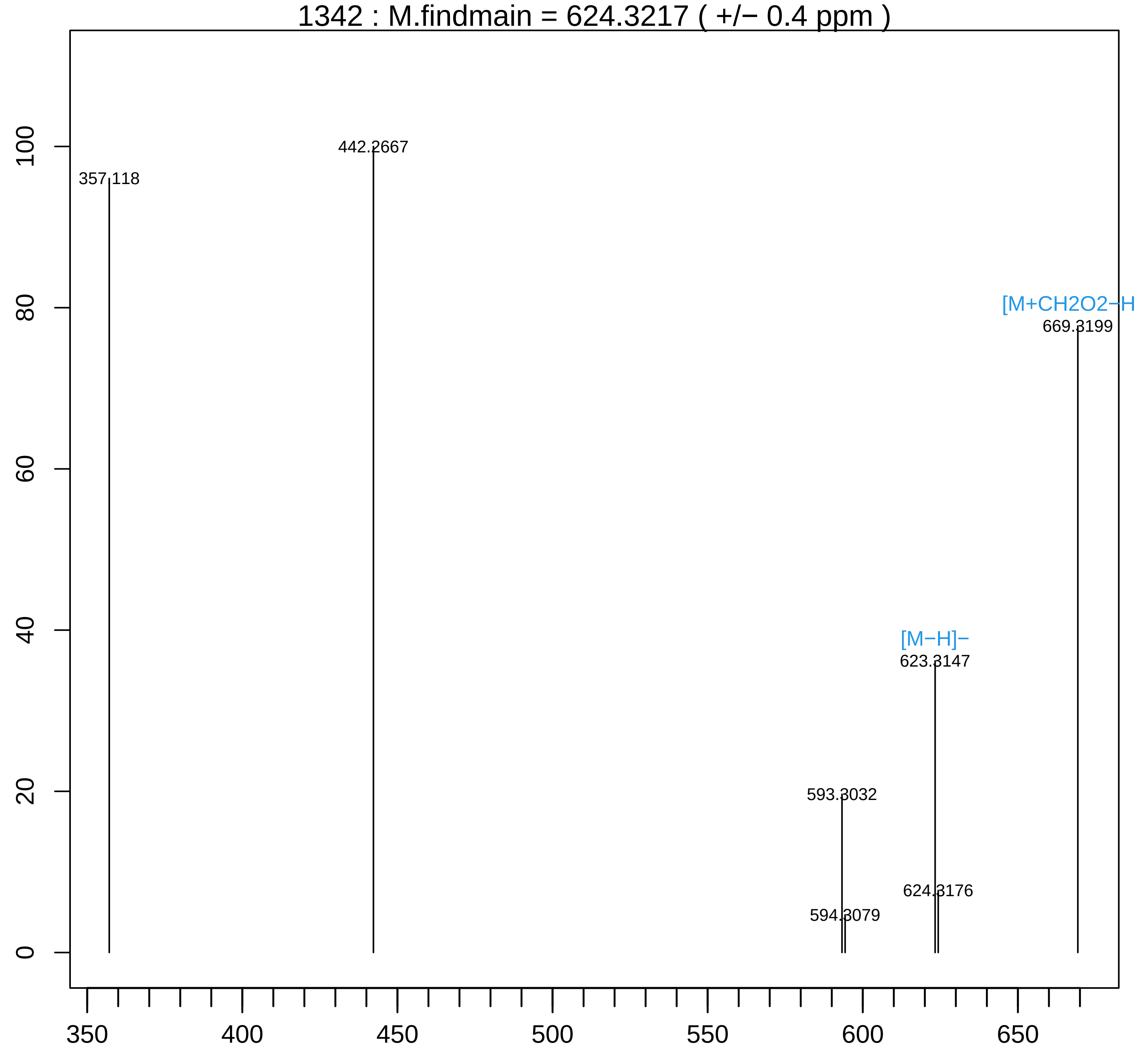
1342 : M.findmain = 624.3217 (+/− 0.4 ppm)
357.118
442.2667
[M+CH2O2−H
669.3199
[M−H]−
623.3147
593.3032
624.3176
594.3079
0
20
40
60
80
100
350
400
450
500
550
600
650

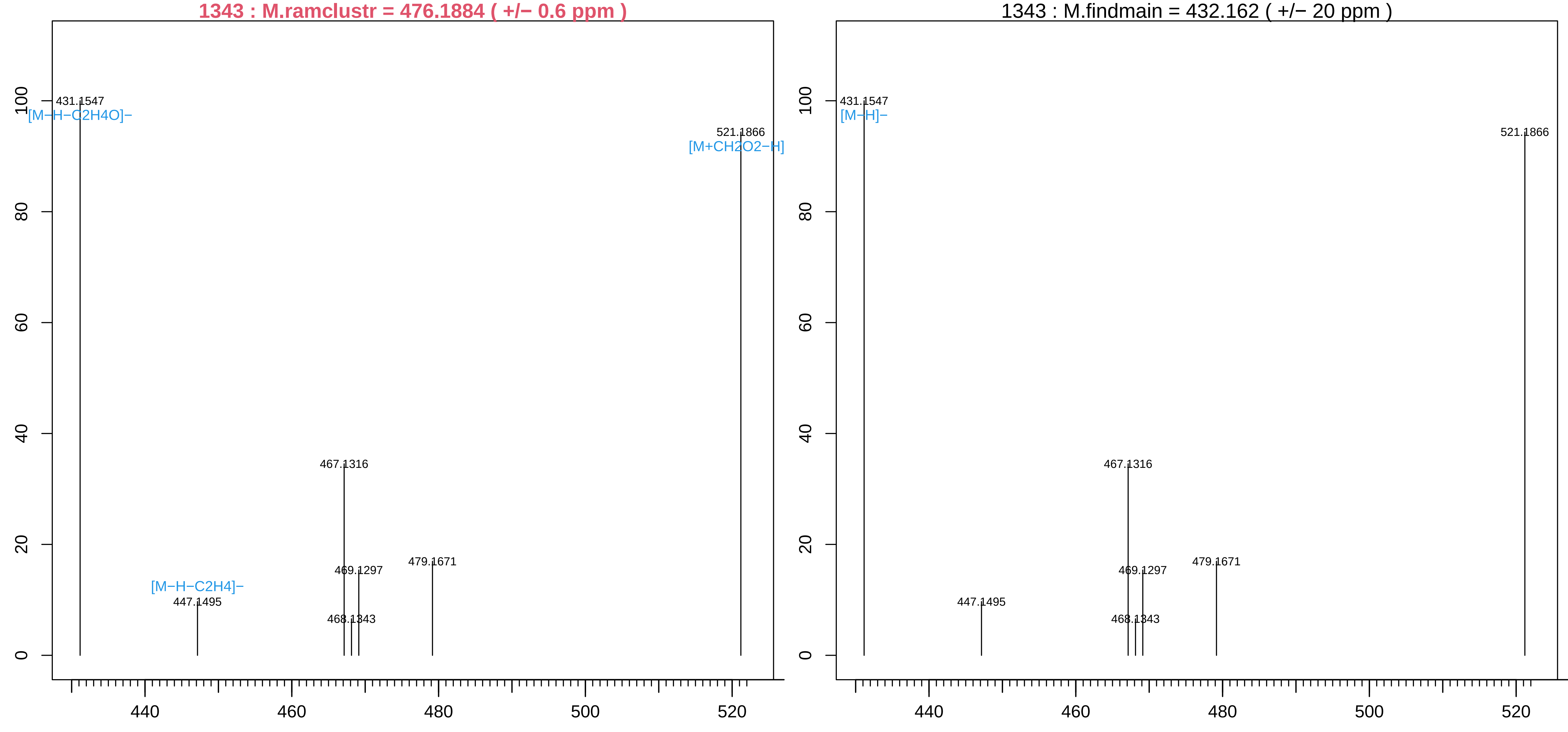
1343 : M.ramclustr = 476.1884 (+/− 0.6 ppm)
431.1547
[M−H−C2H4O]−
521.1866
[M+CH2O2−H]
467.1316
469.1297
479.1671
[M−H−C2H4]−
447.1495
468.1343
1343 : M.findmain = 432.162 (+/− 20 ppm)
431.1547
[M−H]−
521.1866
467.1316
469.1297
479.1671
447.1495
468.1343

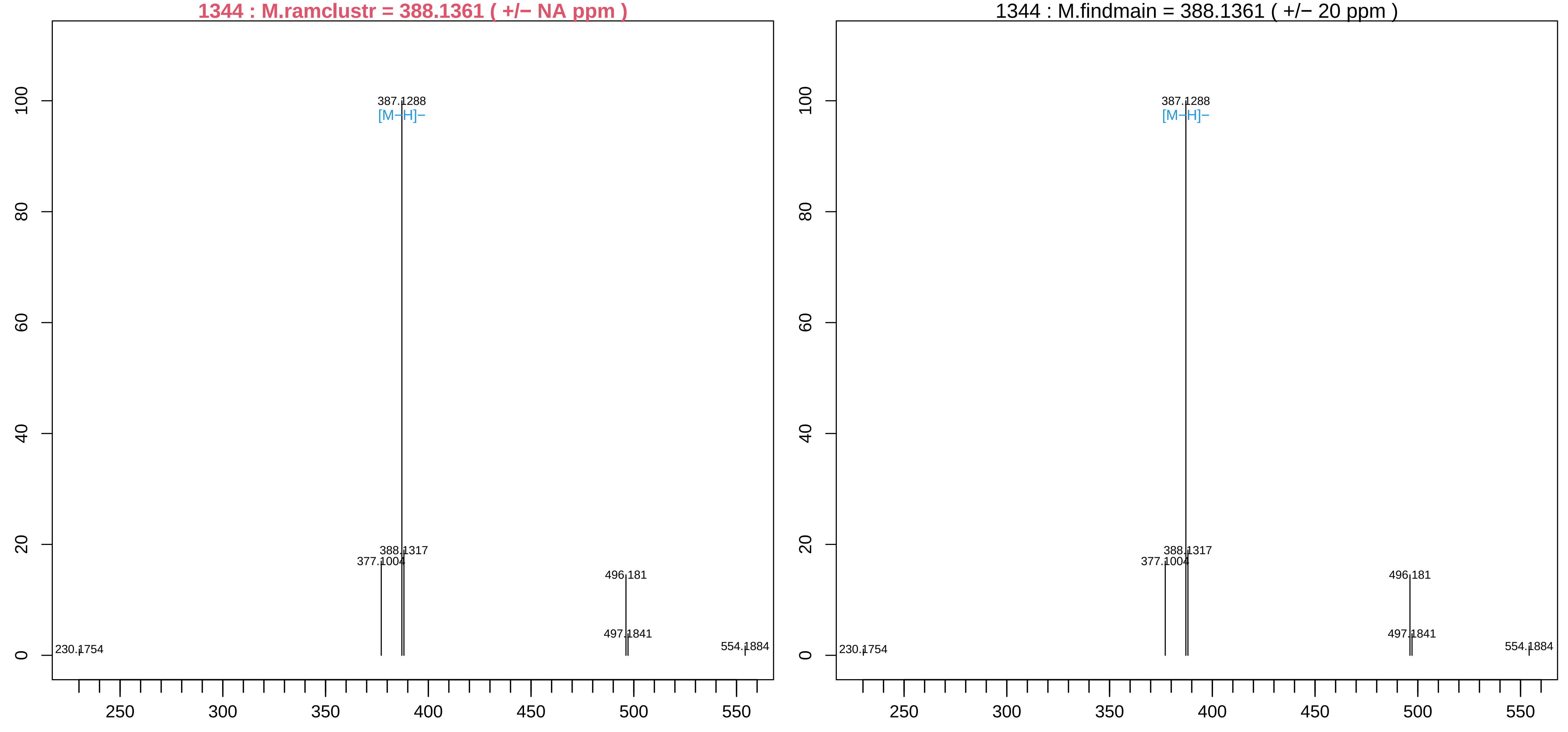
1344 : M.ramclustr = 388.1361 (+/− NA ppm)
1344 : M.findmain = 388.1361 (+/− 20 ppm)
387.1288
[M−H]−
388.1317
377.1004
496.181
497.1841
554.1884
230.1754
0
20
40
60
80
100
250
300
350
400
450
500
550

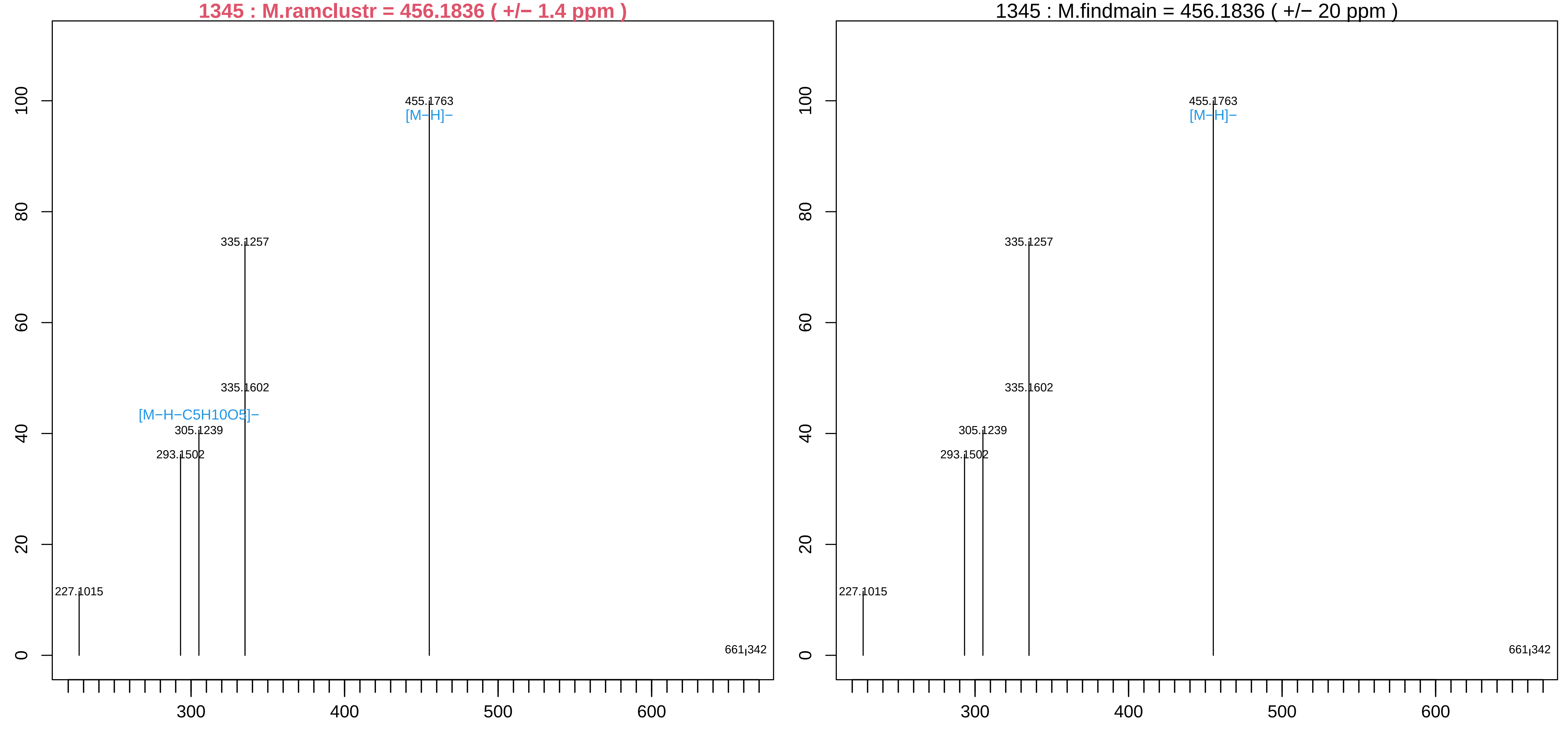

1345 : M.ramclustr = 456.1836 (+/− 1.4 ppm)
455.1763
[M−H]−
335.1257
335.1602
[M−H−C5H10O5]−
305.1239
293.1502
227.1015
661.342
1345 : M.findmain = 456.1836 (+/− 20 ppm)
455.1763
[M−H]−
335.1257
335.1602
305.1239
293.1502
227.1015
661.342

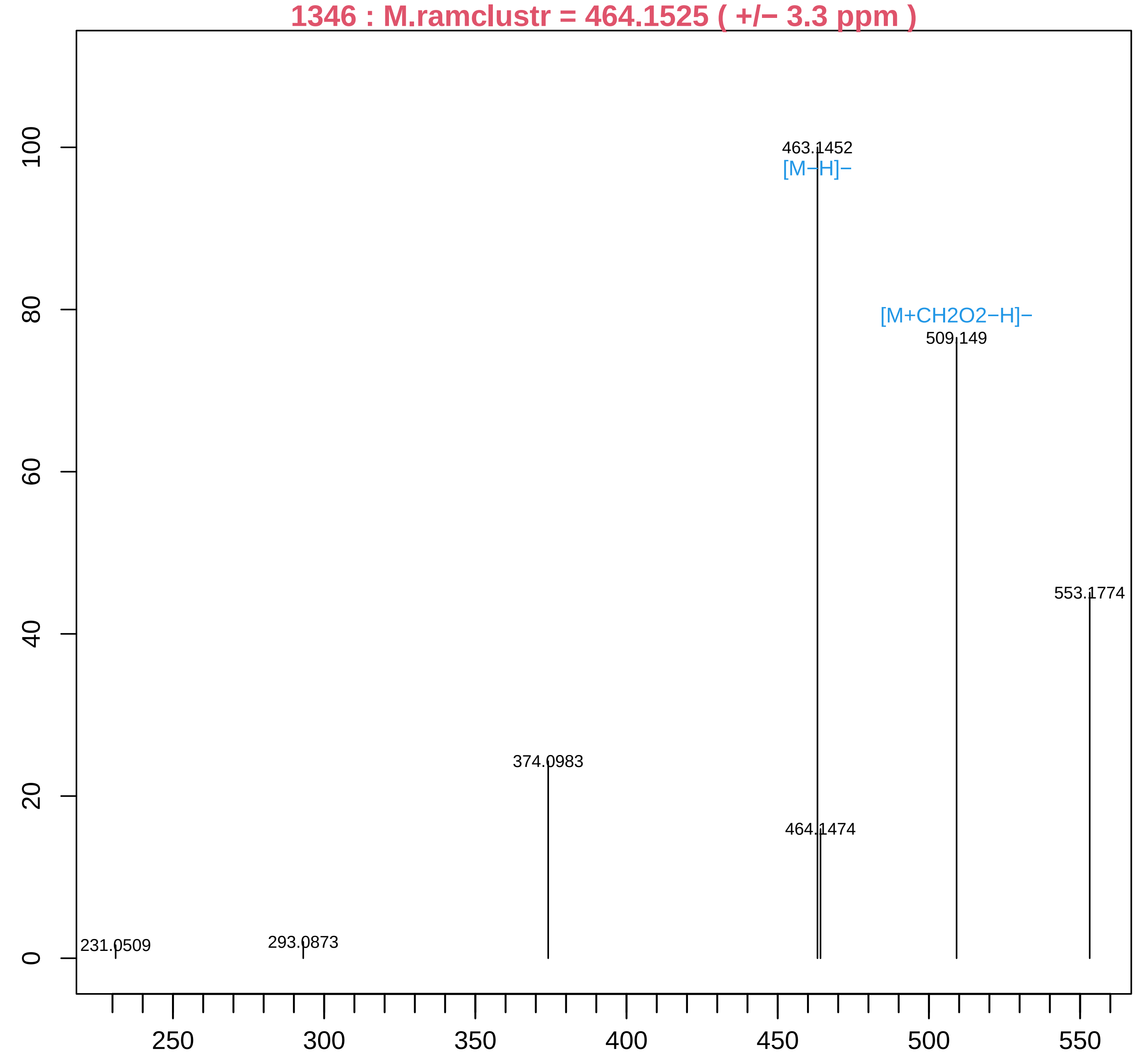
1346 : M.ramclustr = 464.1525 (+/− 3.3 ppm)
463.1452
[M−H]−
[M+CH2O2−H]−
509.149
553.1774
374.0983
464.1474
231.0509
293.0873
0
20
40
60
80
100
250
300
350
400
450
500
550
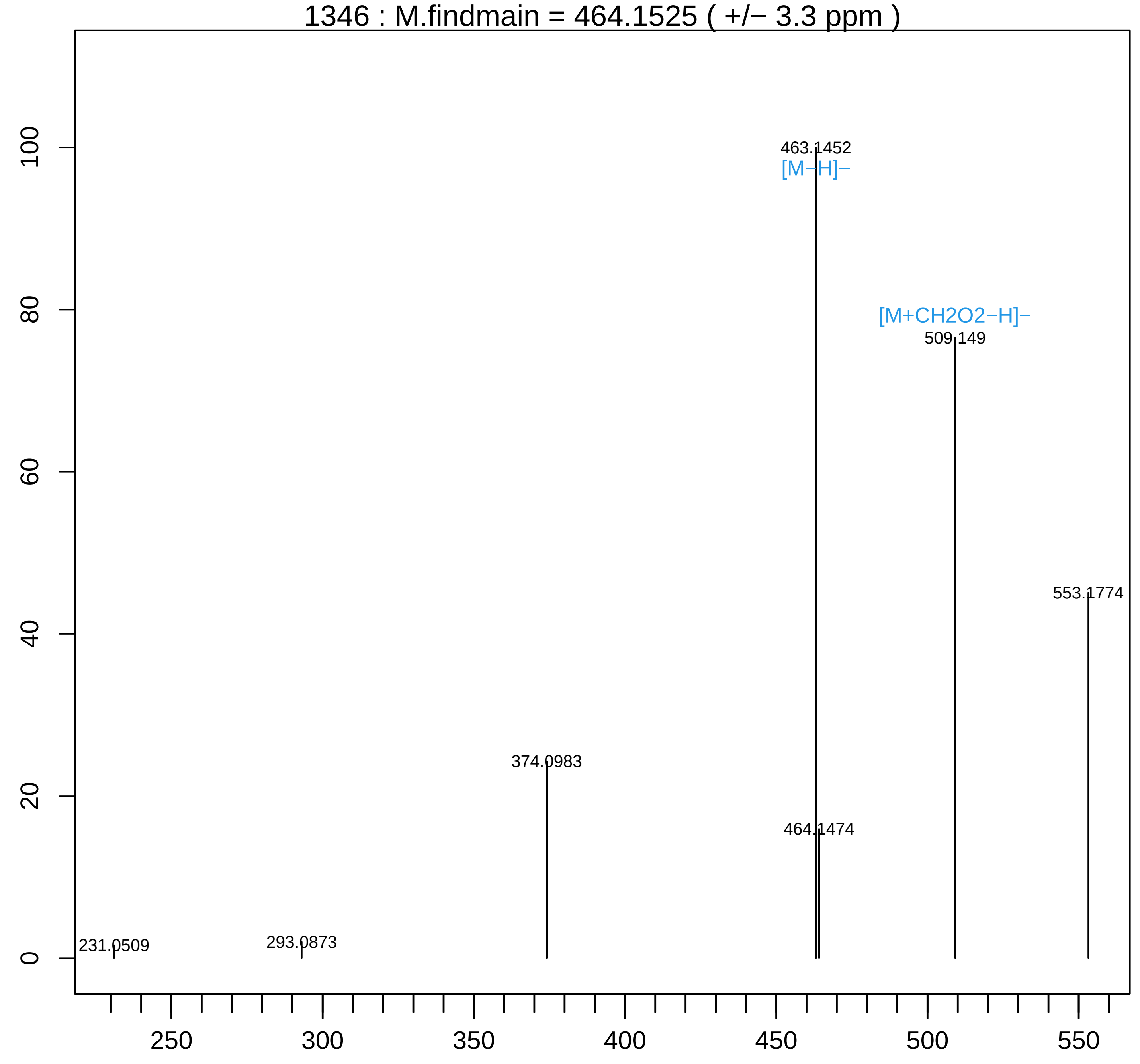
1346 : M.findmain = 464.1525 (+/− 3.3 ppm)
463.1452
[M−H]−
[M+CH2O2−H]−
509.149
553.1774
374.0983
464.1474
231.0509
293.0873
0
20
40
60
80
100
250
300
350
400
450
500
550

1347 : M.ramclustr = 588.2898 (+/− 2 ppm)

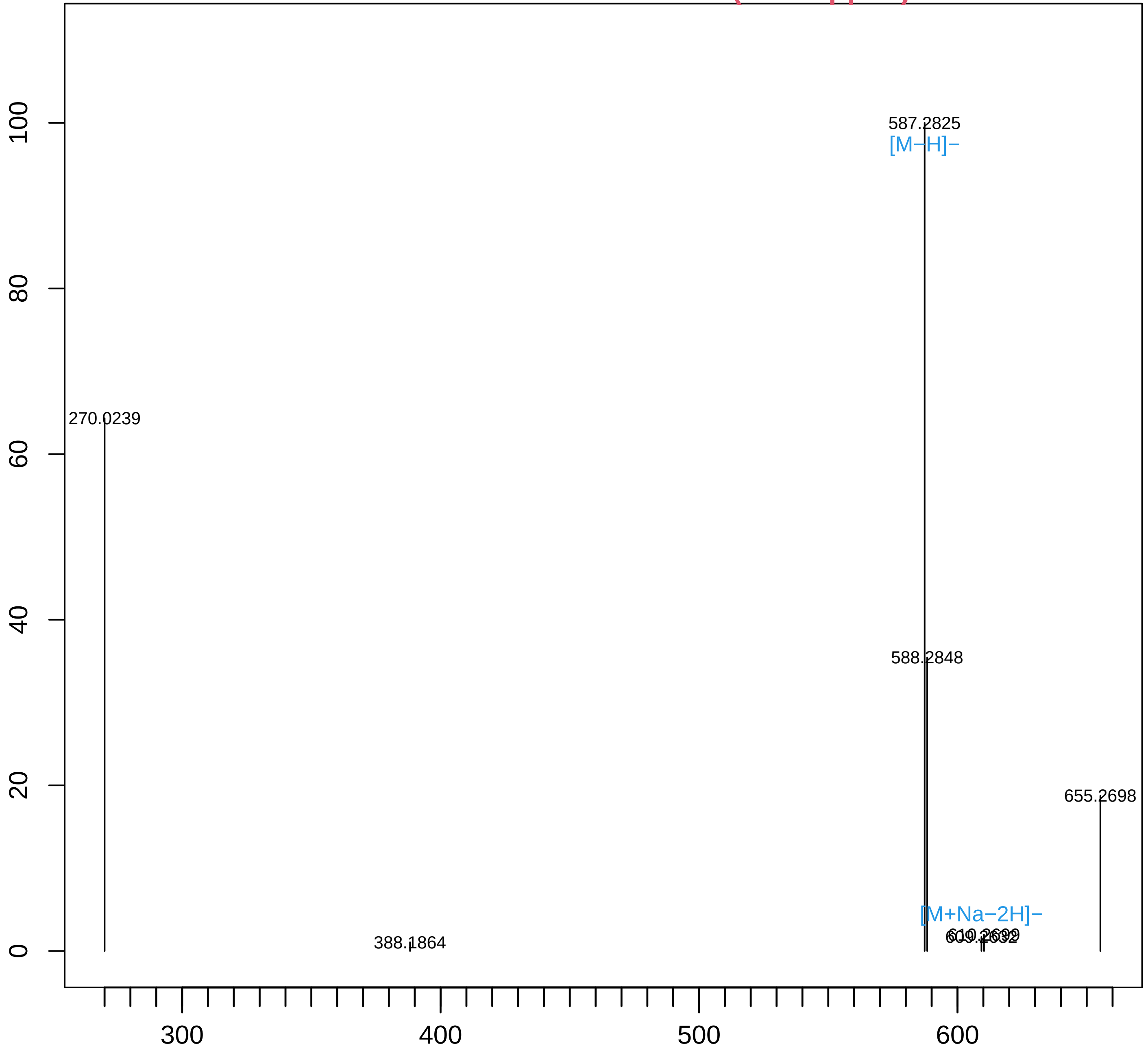

1347 : M.findmain = 588.2898 (+/− 2 ppm)

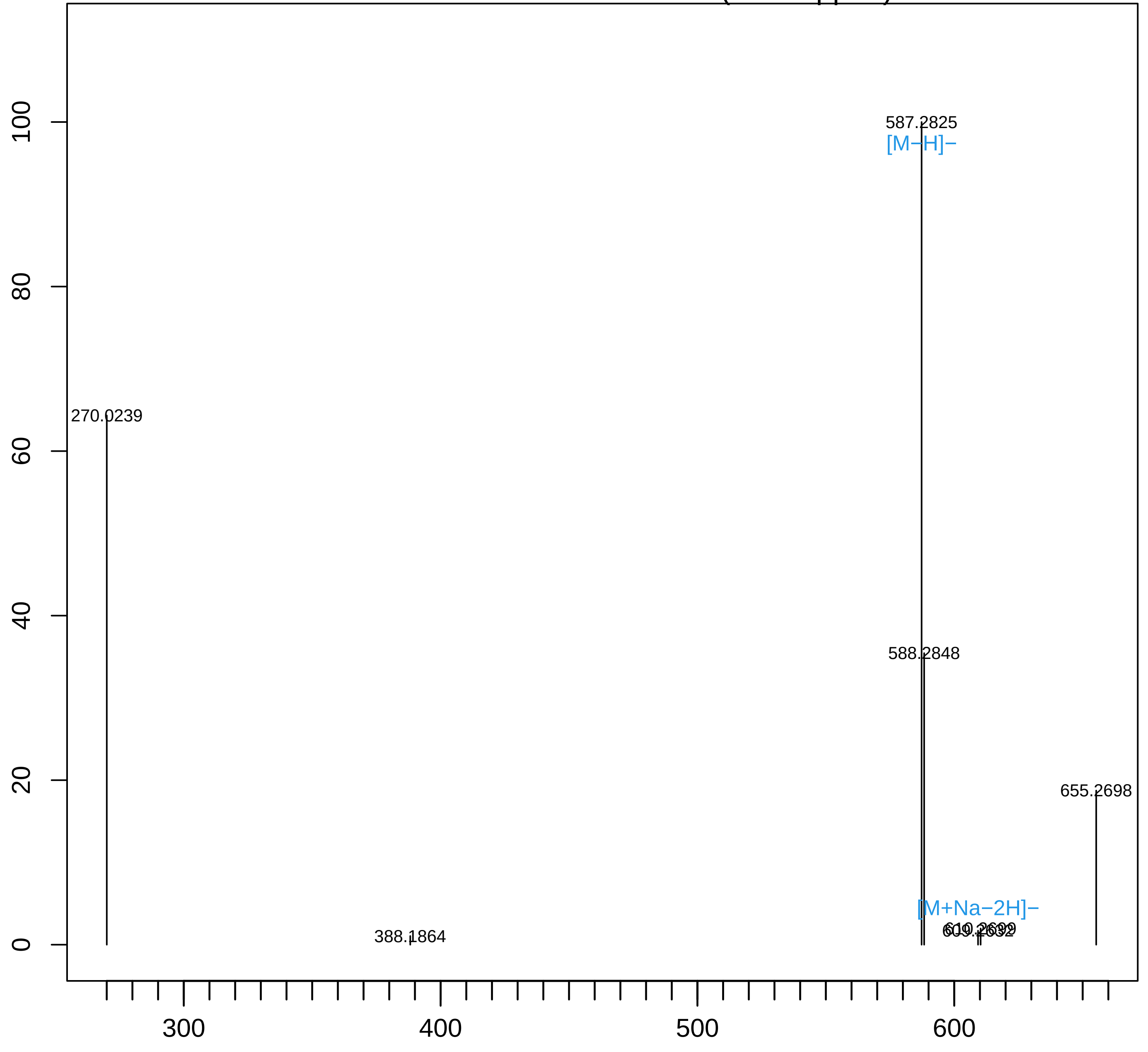

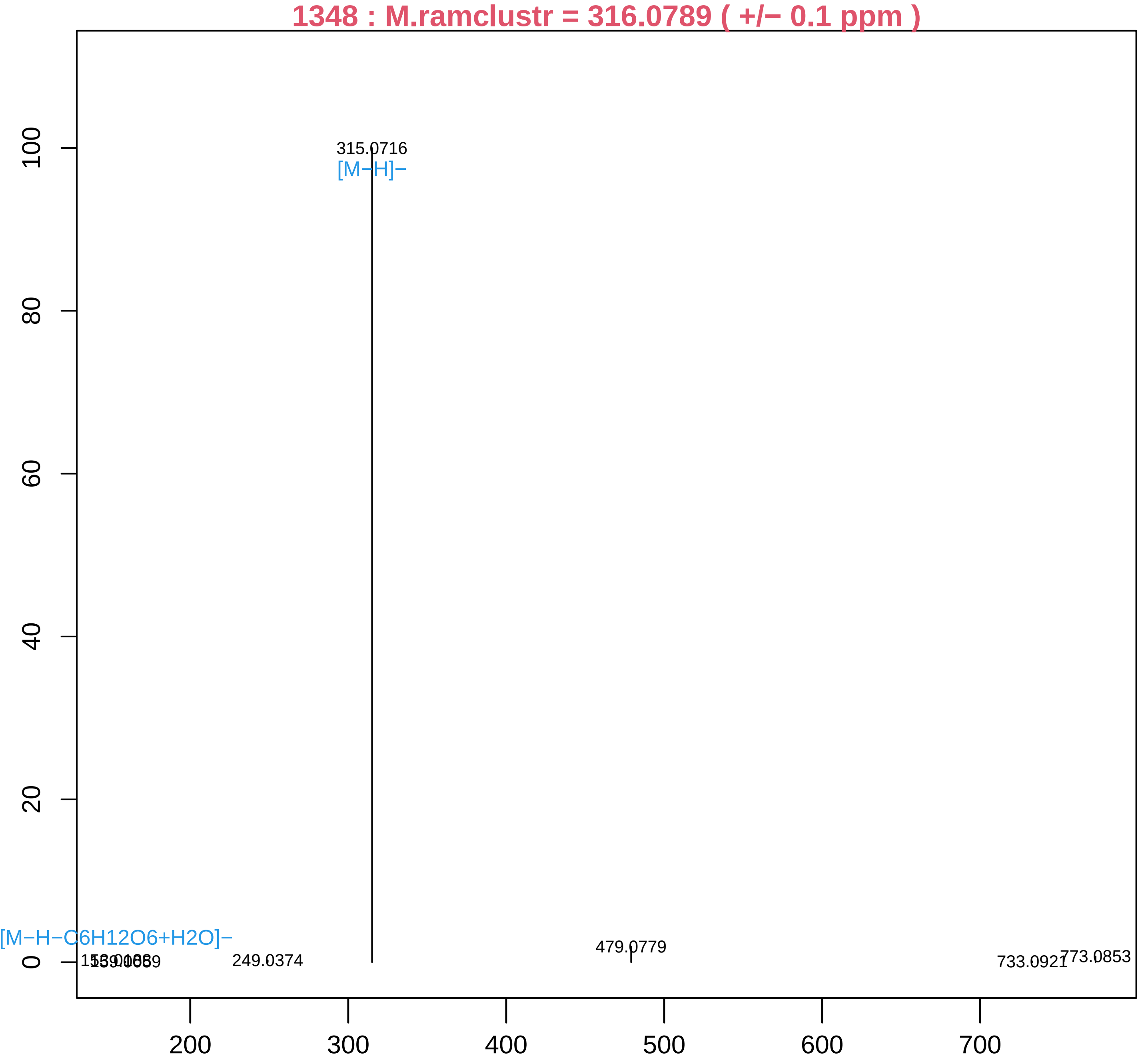
1348 : M.ramclustr = 316.0789 (+/− 0.1 ppm)
315.0716
[M−H]−
[M−H−C6H12O6+H2O]−
249.0374
479.0779
733.0921
773.0853
0
20
40
60
80
100
200
300
400
500
600
700

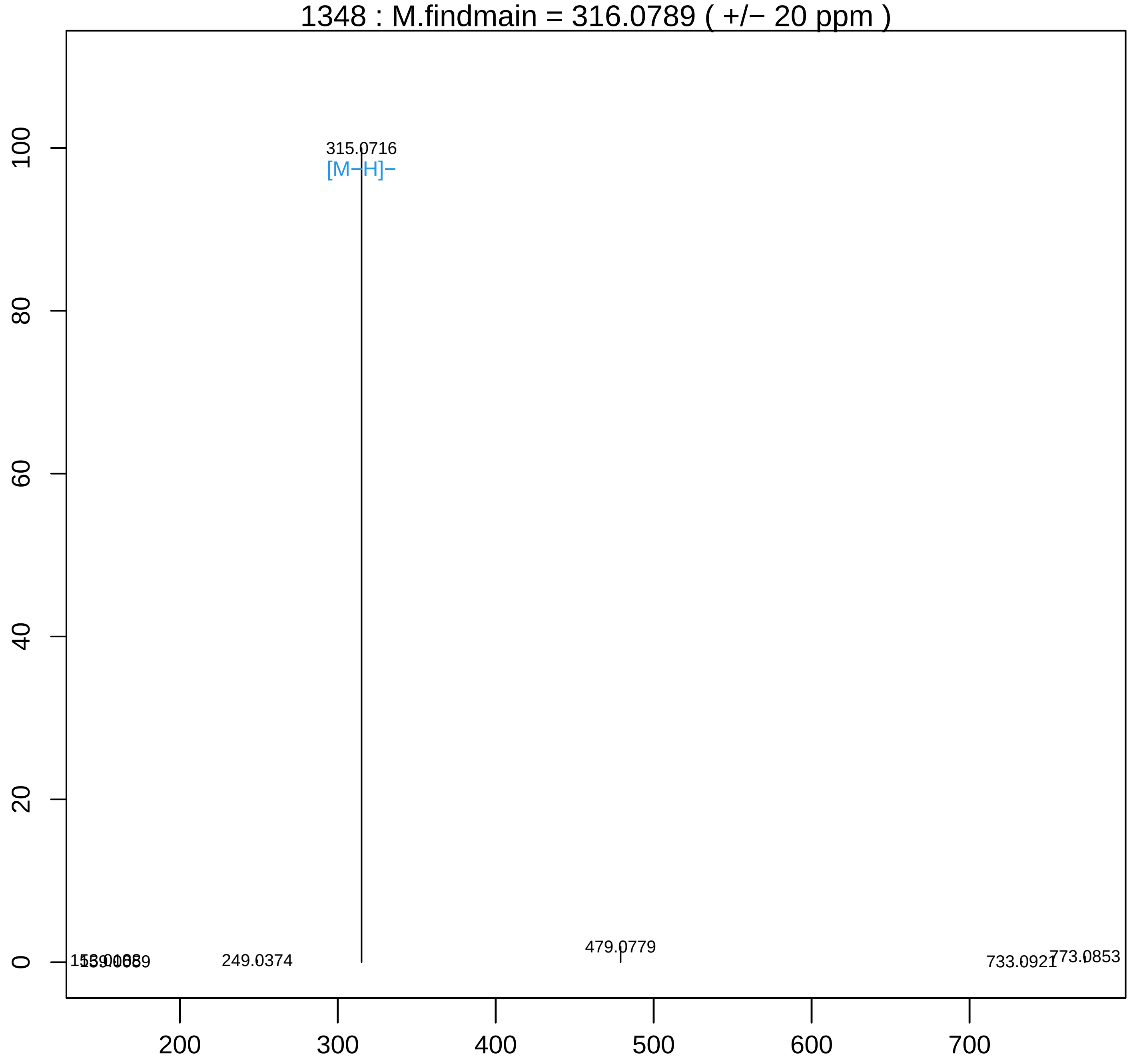
1348 : M.findmain = 316.0789 (+/− 20 ppm)
315.0716
[M−H]−
249.0374
479.0779
733.0921
773.0853
0
20
40
60
80
100
200
300
400
500
600
700

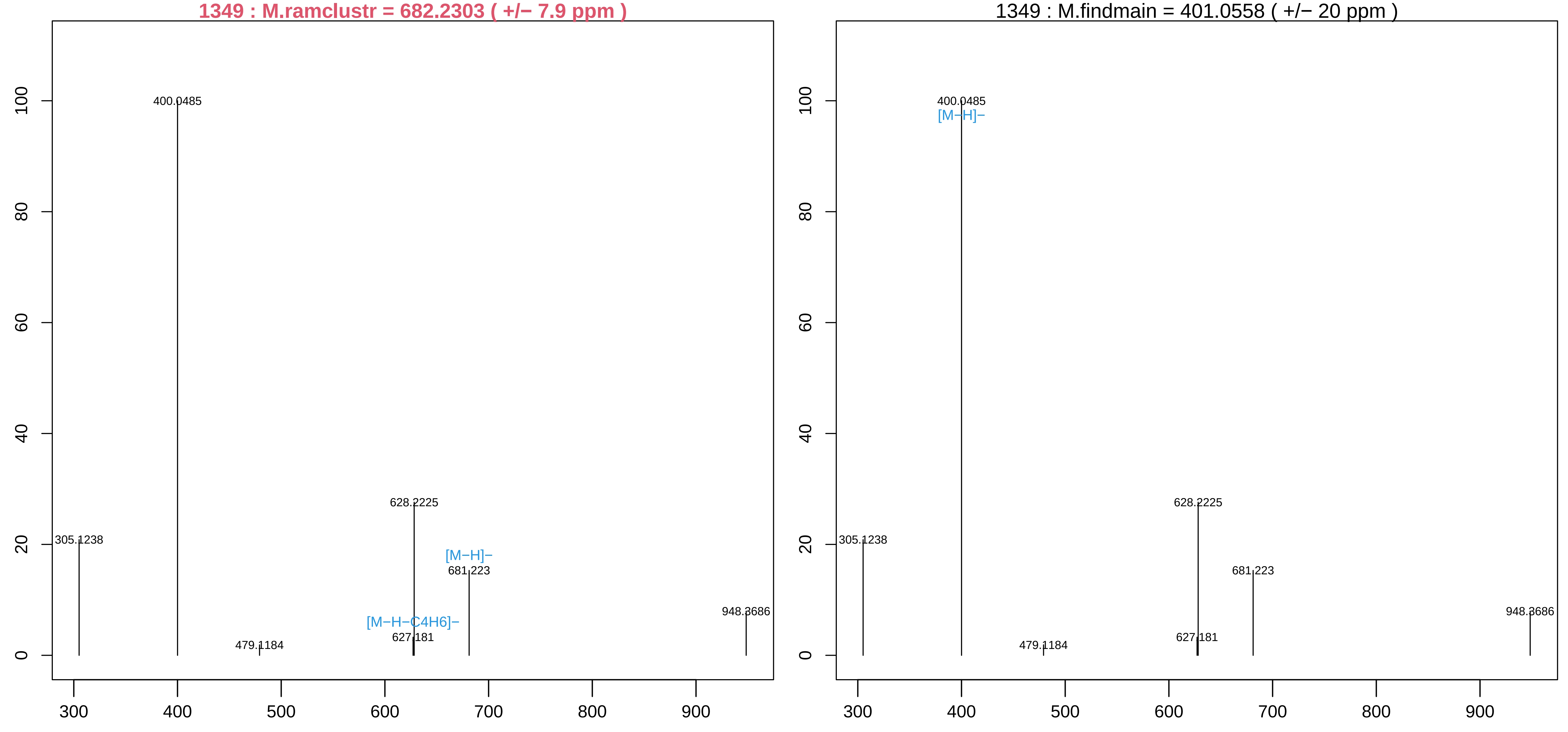
1349 : M.ramclustr = 682.2303 (+/− 7.9 ppm)
400.0485
305.1238
479.1184
628.2225
[M−H]−
681.223
[M−H−C4H6]−
627.181
948.3686
1349 : M.findmain = 401.0558 (+/− 20 ppm)
400.0485
[M−H]−
305.1238
479.1184
628.2225
681.223
627.181
948.3686
0
20
40
60
80
100
300
400
500
600
700
800
900

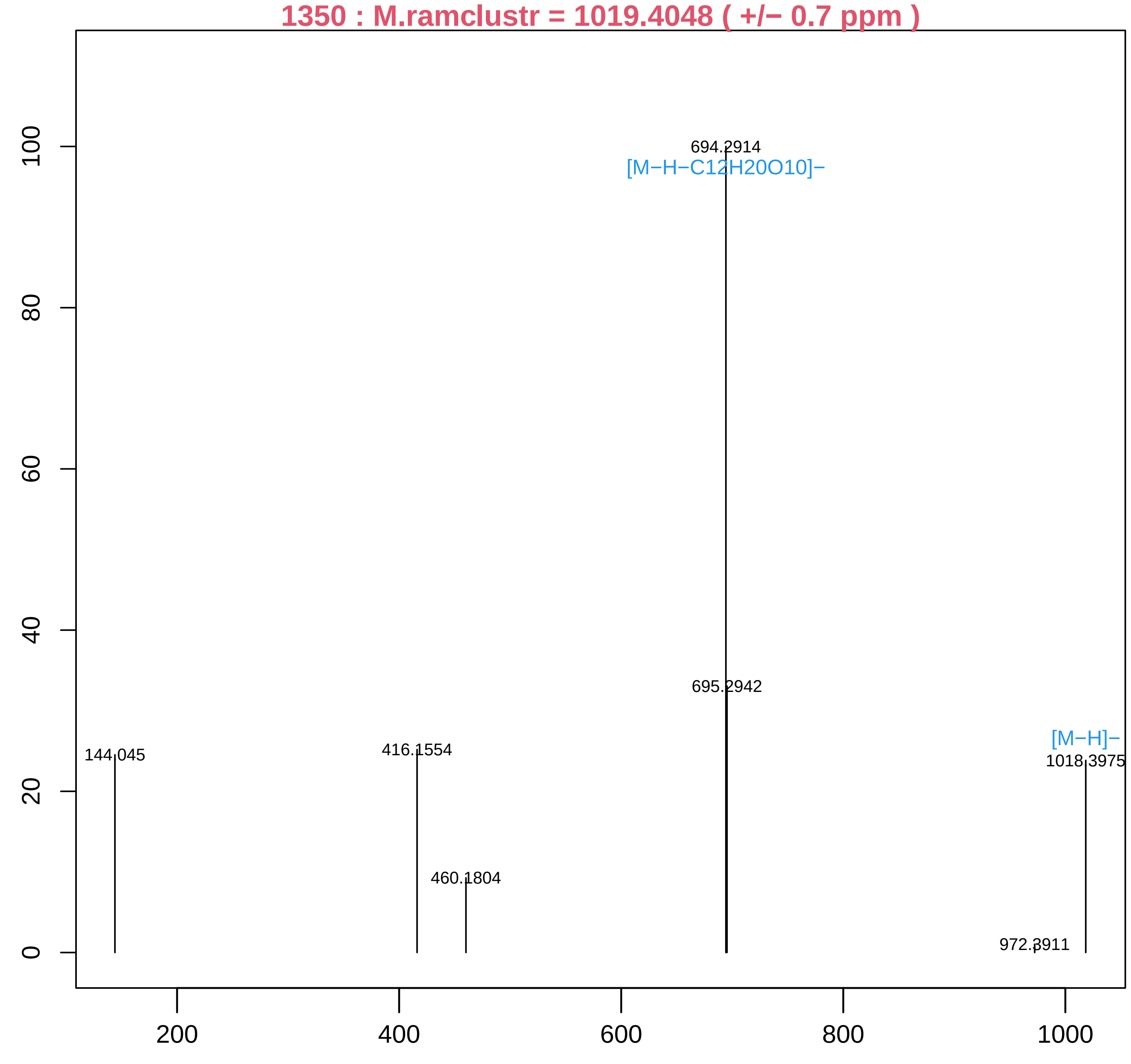

1350 : M.ramclustr = 1019.4048 (+/− 0.7 ppm)
694.2914
[M−H−C12H20O10]−
695.2942
144.045
416.1554
460.1804
[M−H]−
1018.3975
972.3911
0
20
40
60
80
100
200
400
600
800
1000

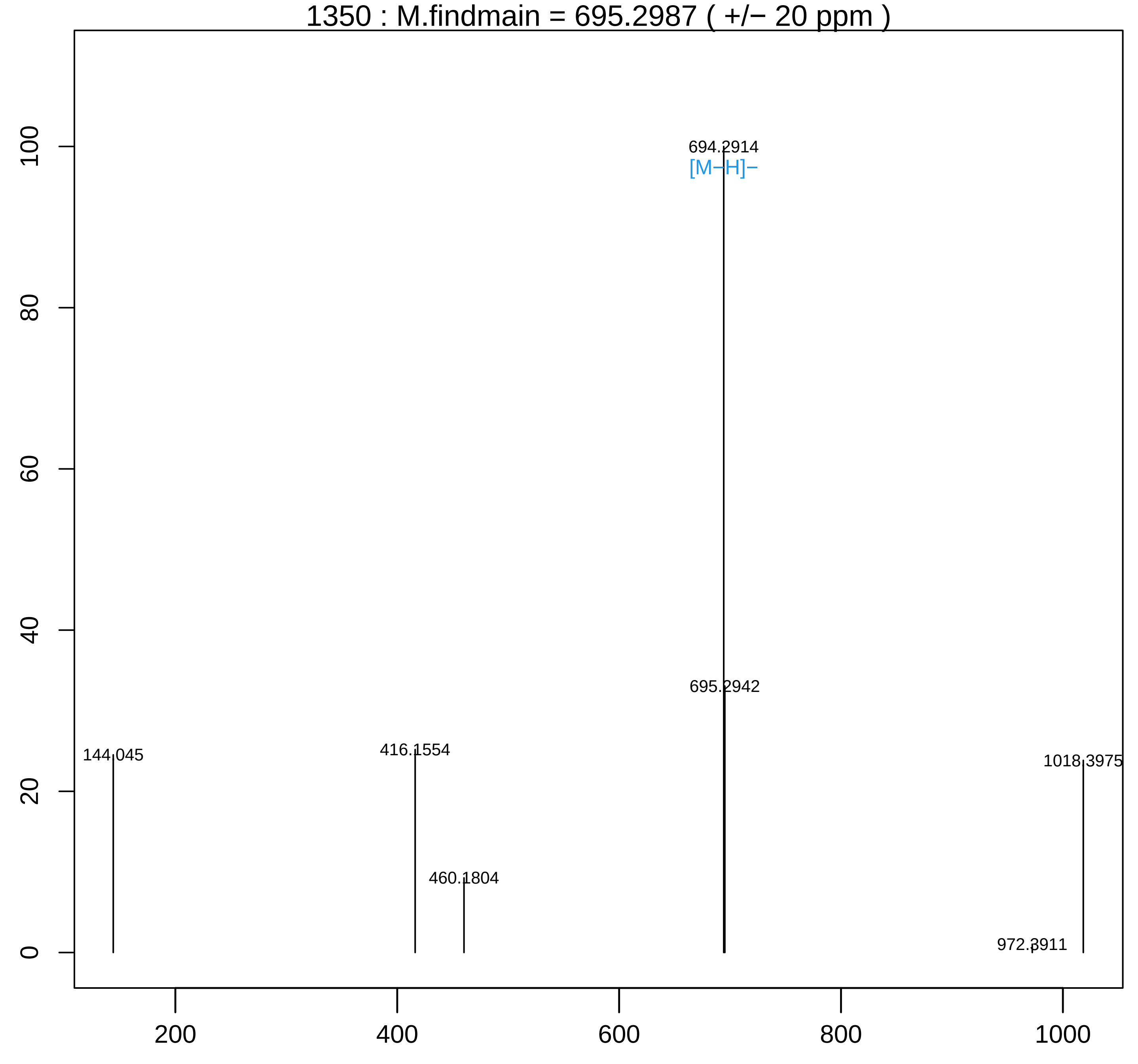

1350 : M.findmain = 695.2987 (+/− 20 ppm)
694.2914
[M−H]−
695.2942
144.045
416.1554
460.1804
1018.3975
972.3911
0
20
40
60
80
100
200
400
600
800
1000

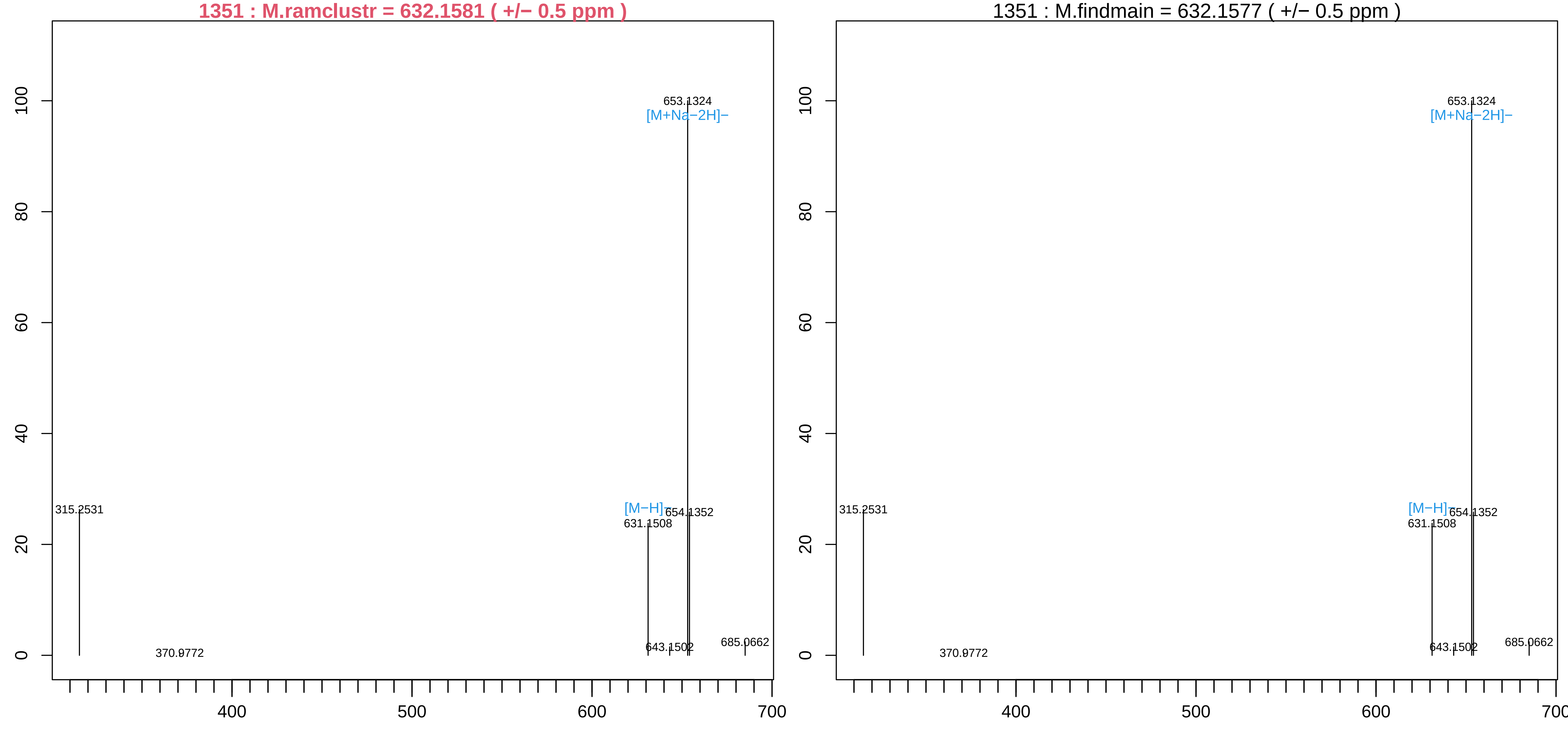
1351 : M.ramclustr = 632.1581 (+/− 0.5 ppm)
653.1324
[M+Na−2H]−
315.2531
[M−H]−
631.1508
654.1352
370.9772
643.1502
685.0662
0
20
40
60
80
100
400
500
600
700
1351 : M.findmain = 632.1577 (+/− 0.5 ppm)
653.1324
[M+Na−2H]−
315.2531
[M−H]−
631.1508
654.1352
370.9772
643.1502
685.0662
0
20
40
60
80
100
400
500
600
700

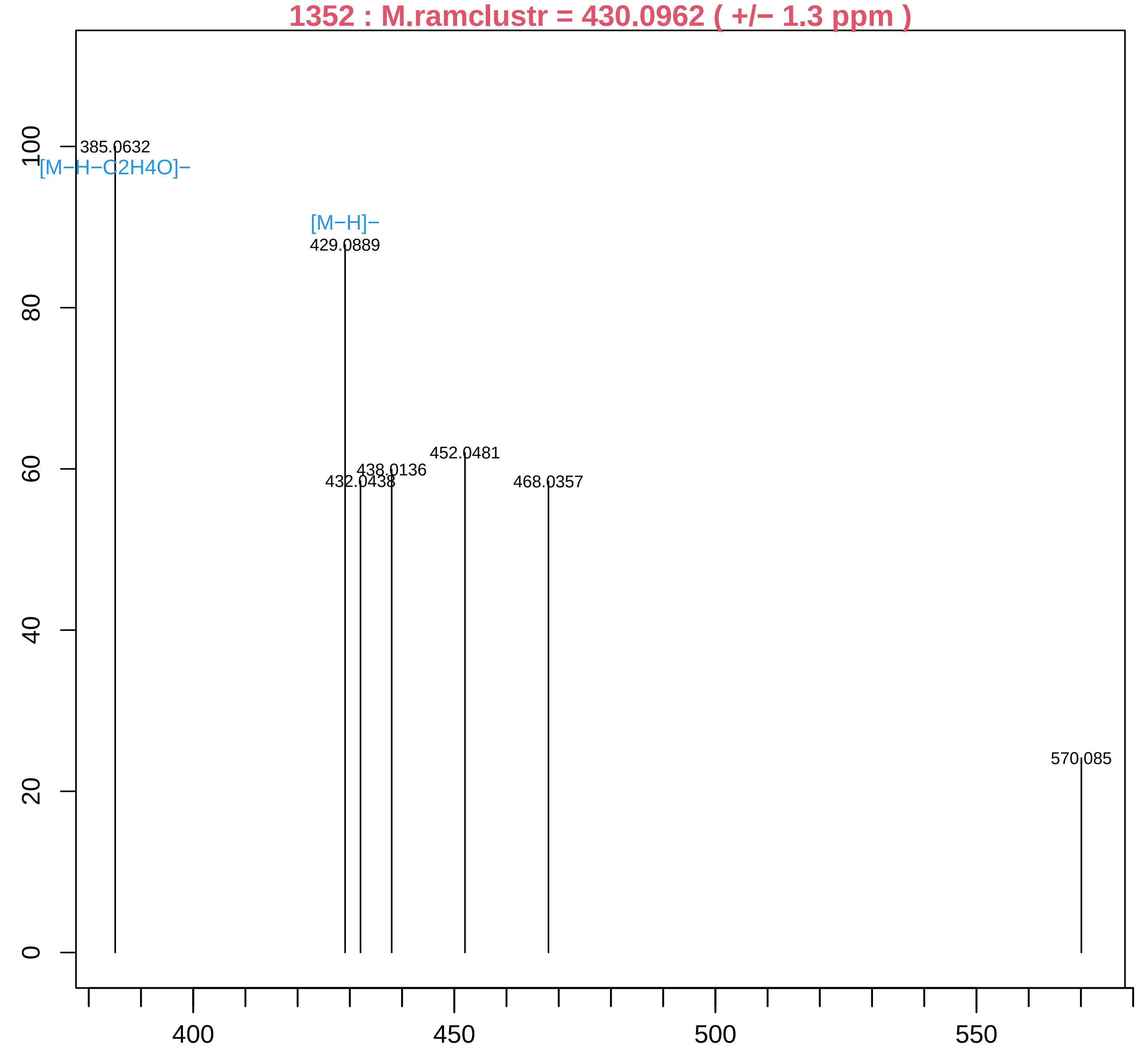
1352 : M.ramclustr = 430.0962 (+/− 1.3 ppm)
385.0632
[M−H−C2H4O]−
[M−H]−
429.0889
432.0438
438.0136
452.0481
468.0357
570.085
0
20
40
60
80
100
400
450
500
550

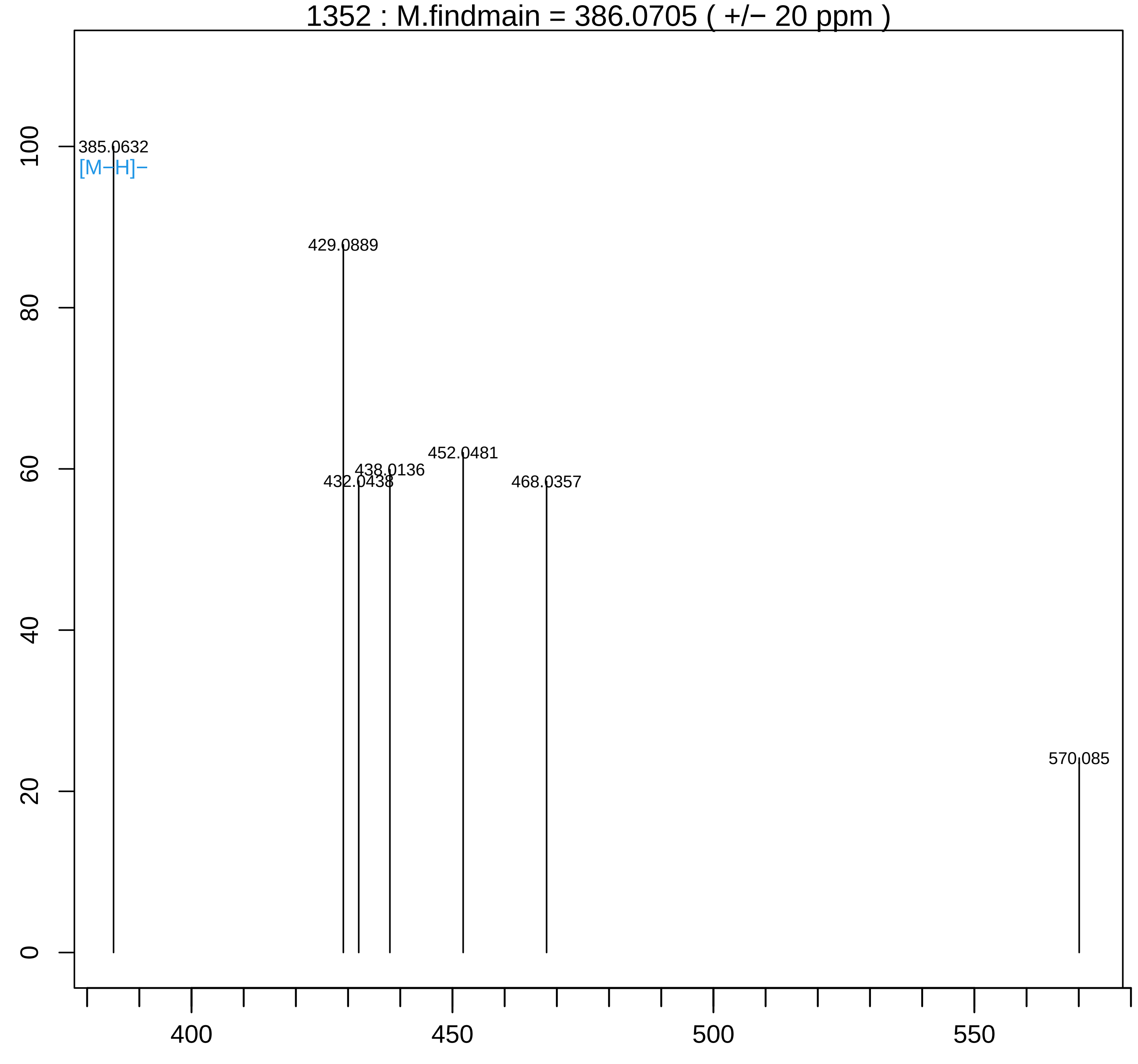
1352 : M.findmain = 386.0705 (+/− 20 ppm)
385.0632
[M−H]−
429.0889
432.0438
438.0136
452.0481
468.0357
570.085
0
20
40
60
80
100
400
450
500
550

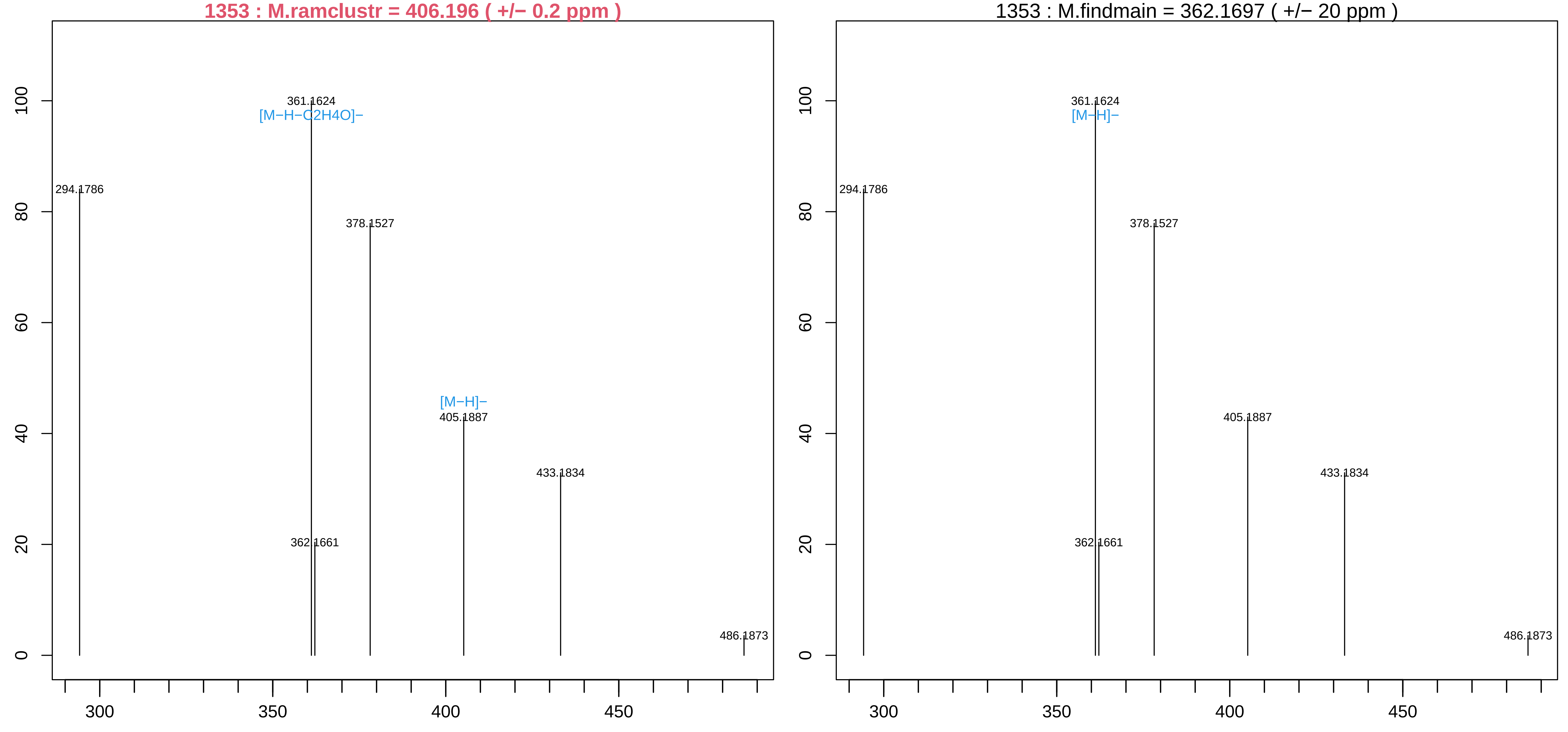
1353 : M.ramclustr = 406.196 (+/− 0.2 ppm)
294.1786
361.1624
[M−H−C2H4O]−
362.1661
378.1527
[M−H]−
405.1887
433.1834
486.1873
1353 : M.findmain = 362.1697 (+/− 20 ppm)
294.1786
361.1624
[M−H]−
362.1661
378.1527
405.1887
433.1834
486.1873
0
20
40
60
80
100
300
350
400
450

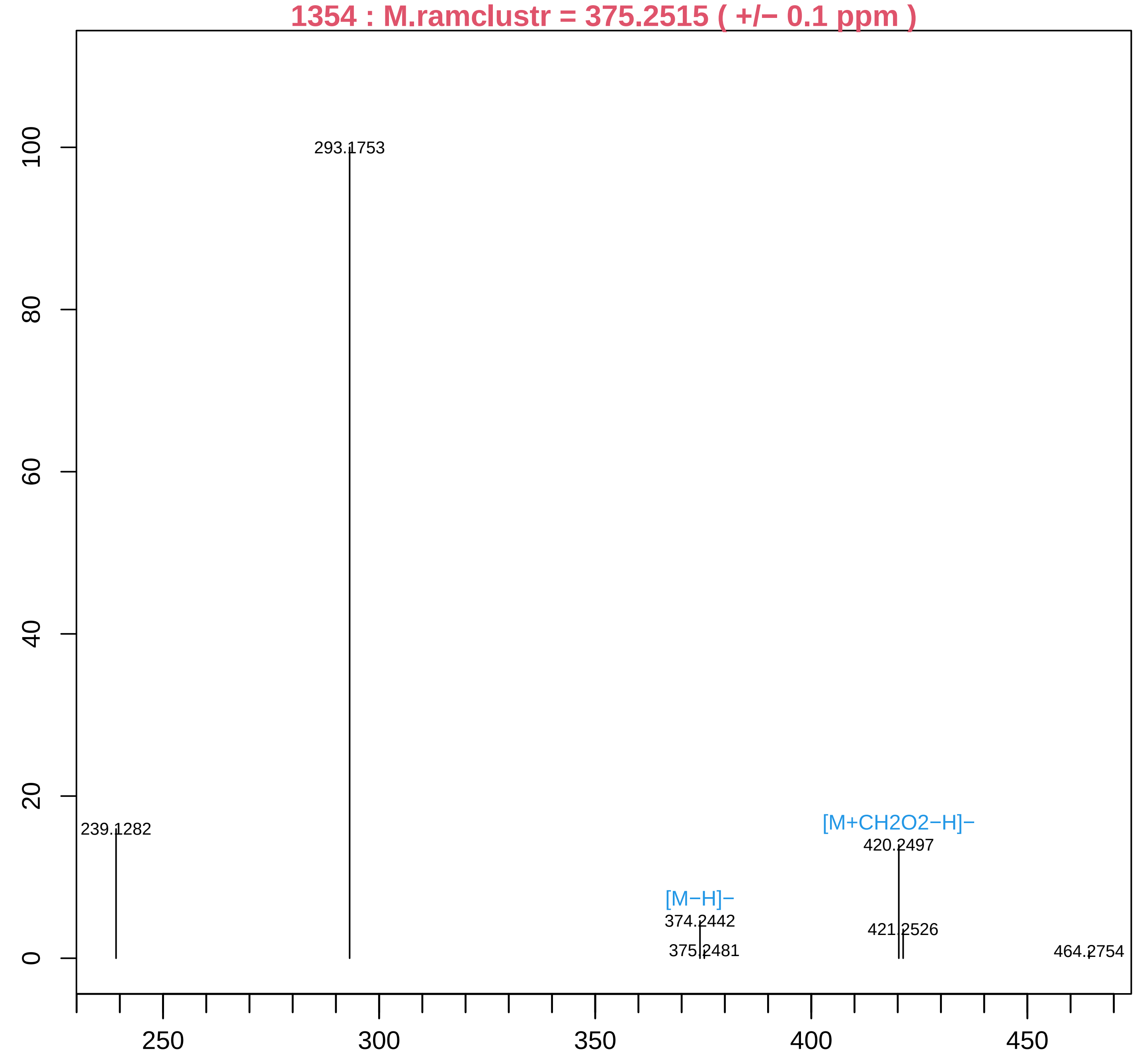
1354 : M.ramclustr = 375.2515 (+/− 0.1 ppm)
293.1753
239.1282
[M+CH2O2−H]−
420.2497
[M−H]−
374.2442
375.2481
421.2526
464.2754
0
20
40
60
80
100
250
300
350
400
450

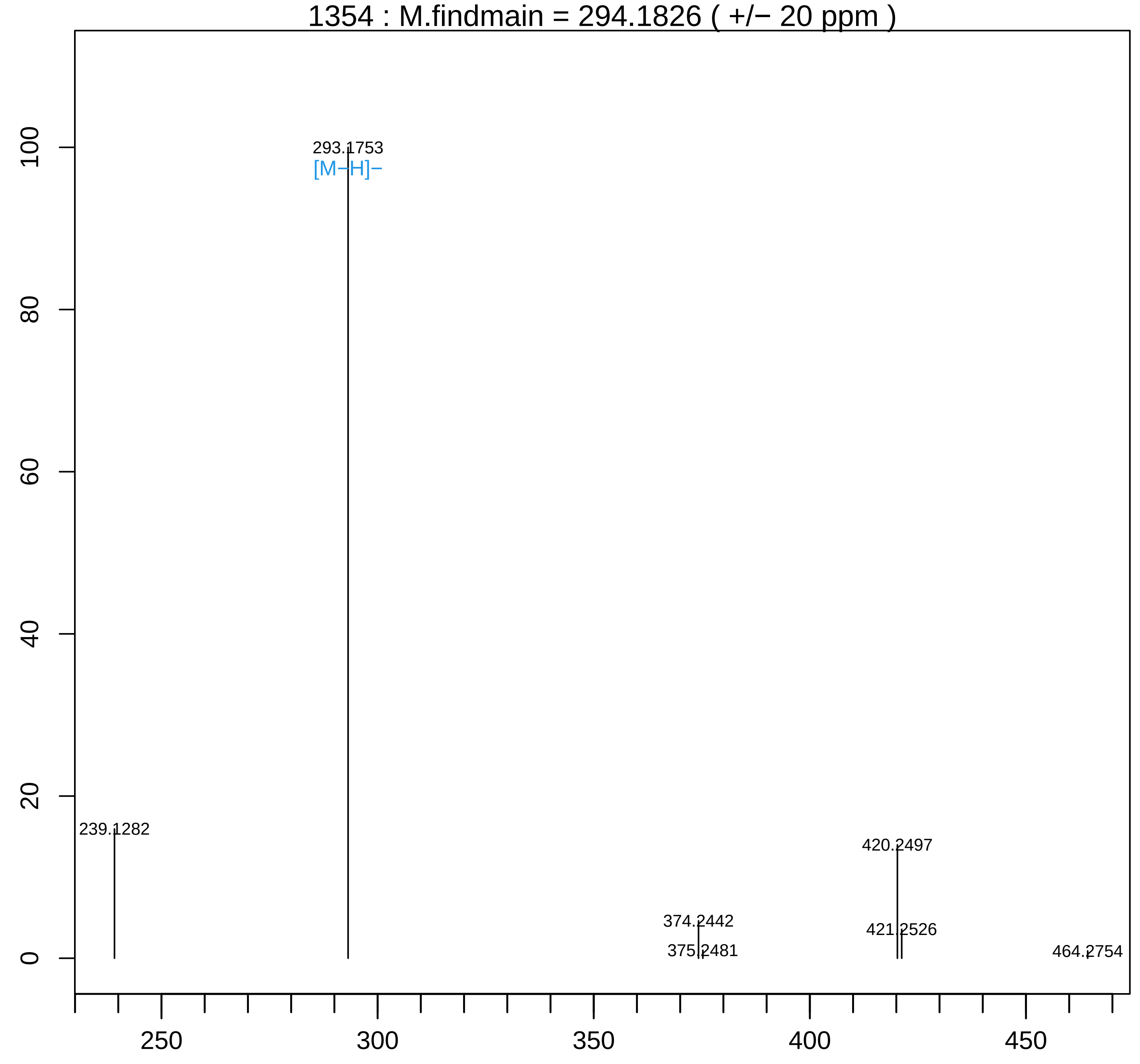
1354 : M.findmain = 294.1826 (+/− 20 ppm)
293.1753
[M−H]−
239.1282
420.2497
374.2442
375.2481
421.2526
464.2754
0
20
40
60
80
100
250
300
350
400
450

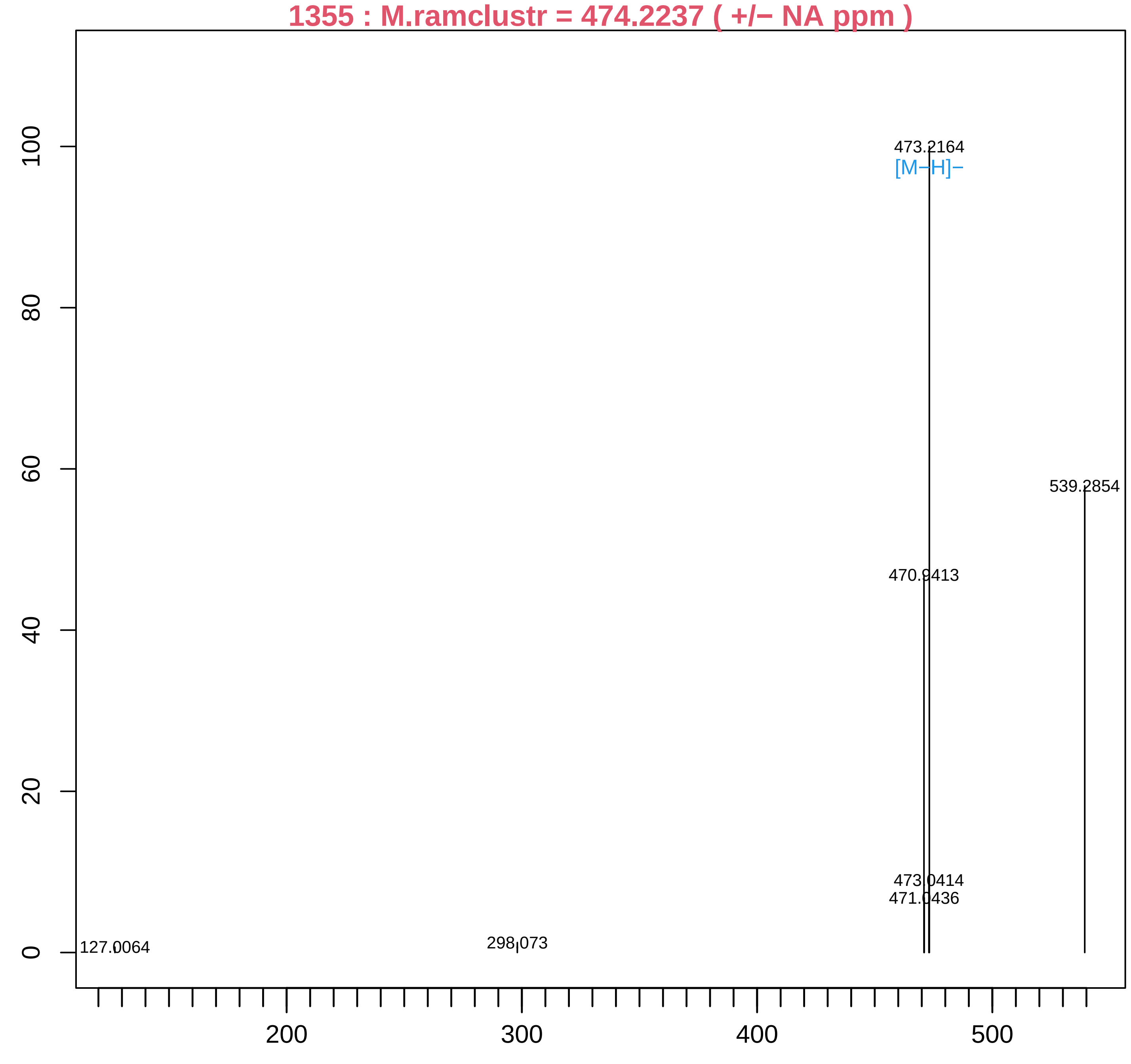
1355 : M.ramclustr = 474.2237 (+/− NA ppm)
473.2164
[M−H]−
539.2854
470.9413
473.0414
471.0436
127.0064
298.073
0
20
40
60
80
100
200
300
400
500

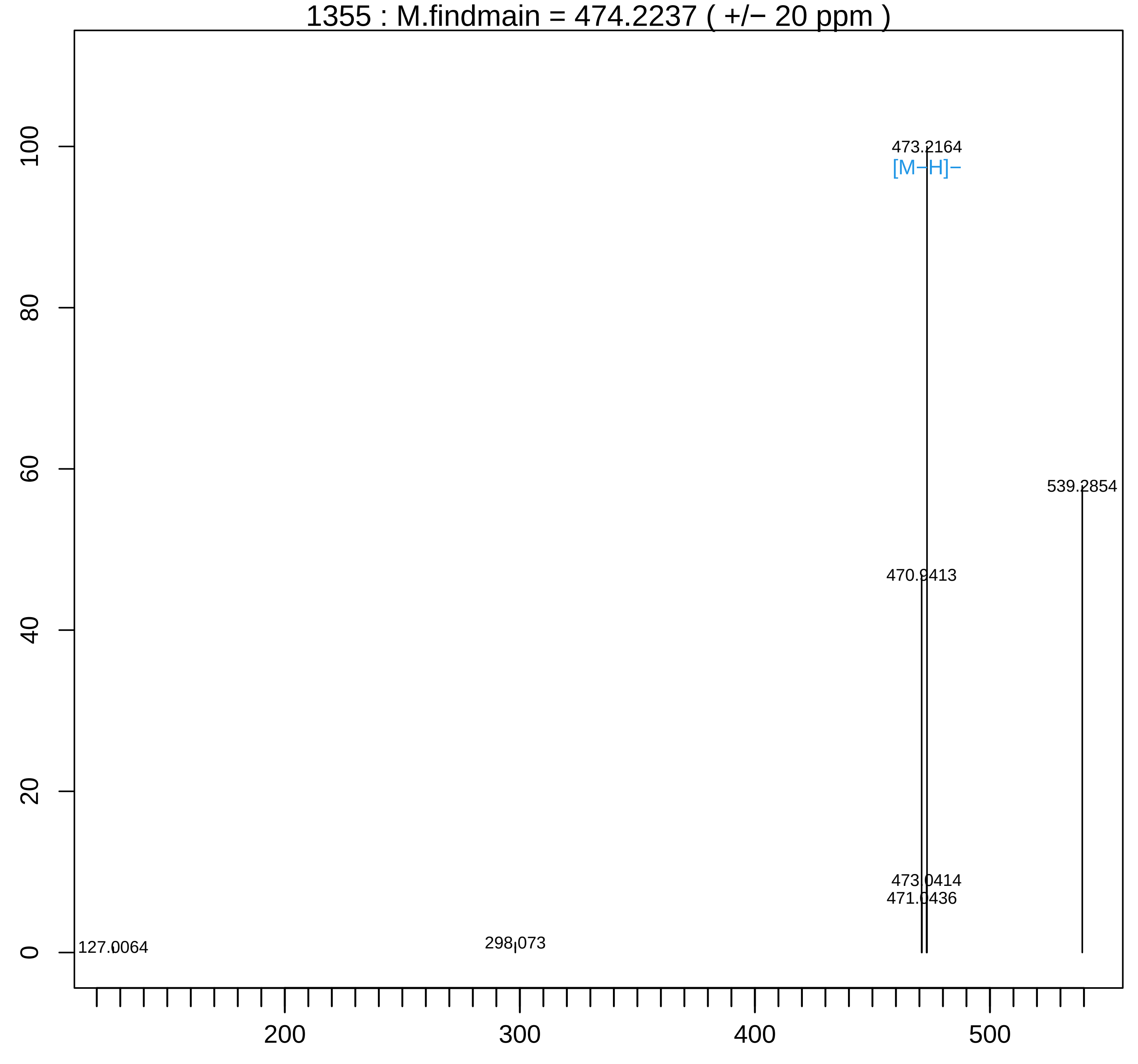
1355 : M.findmain = 474.2237 (+/− 20 ppm)
473.2164
[M−H]−
539.2854
470.9413
473.0414
471.0436
127.0064
298.073
0
20
40
60
80
100
200
300
400
500

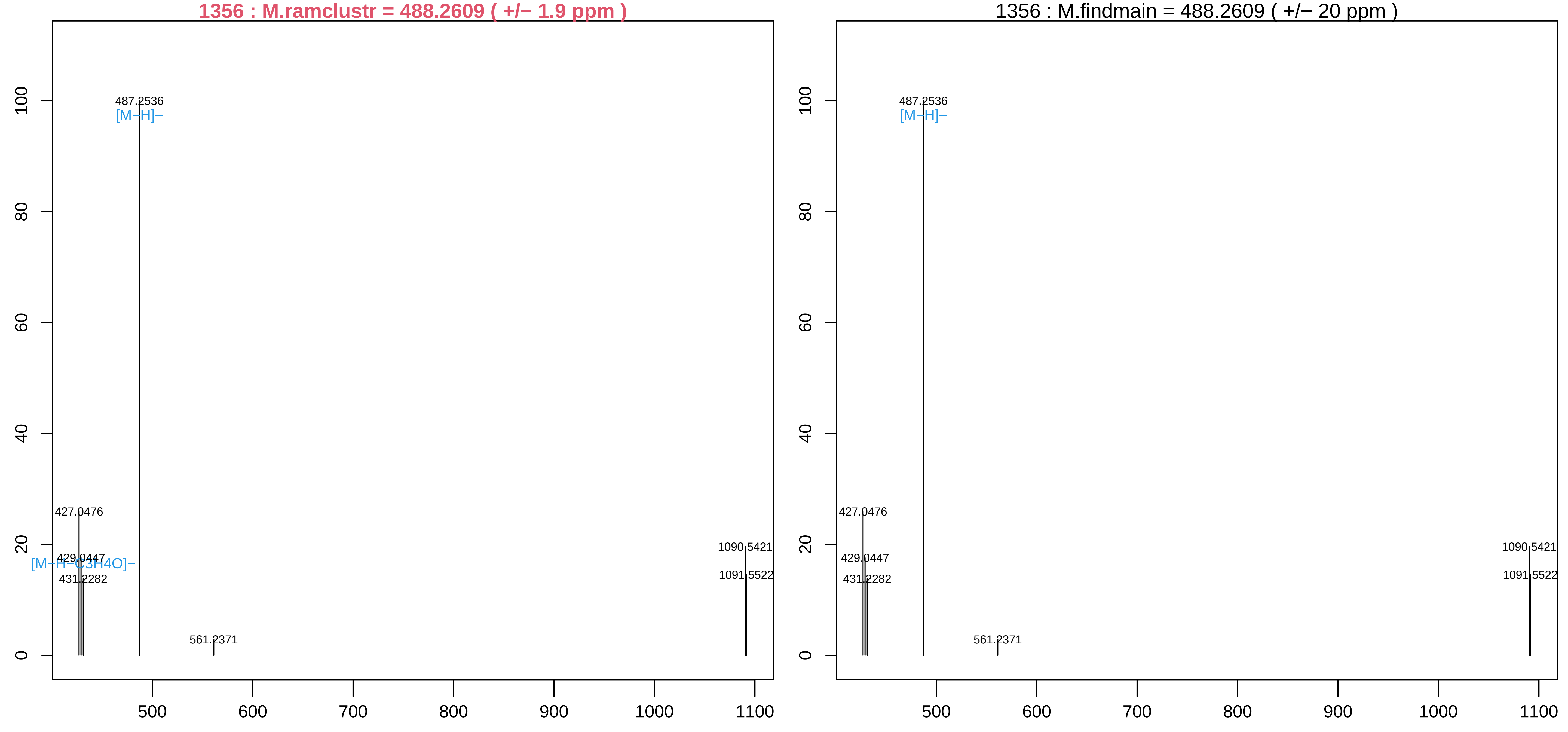
1356 : M.ramclustr = 488.2609 (+/− 1.9 ppm)
1356 : M.findmain = 488.2609 (+/− 20 ppm)
487.2536
[M−H]−
427.0476
429.0447
[M−H−C3H4O]−
431.2282
561.2371
1090.5421
1091.5522
487.2536
[M−H]−
427.0476
429.0447
431.2282
561.2371
1090.5421
1091.5522
0
20
40
60
80
100
500
600
700
800
900
1000
1100

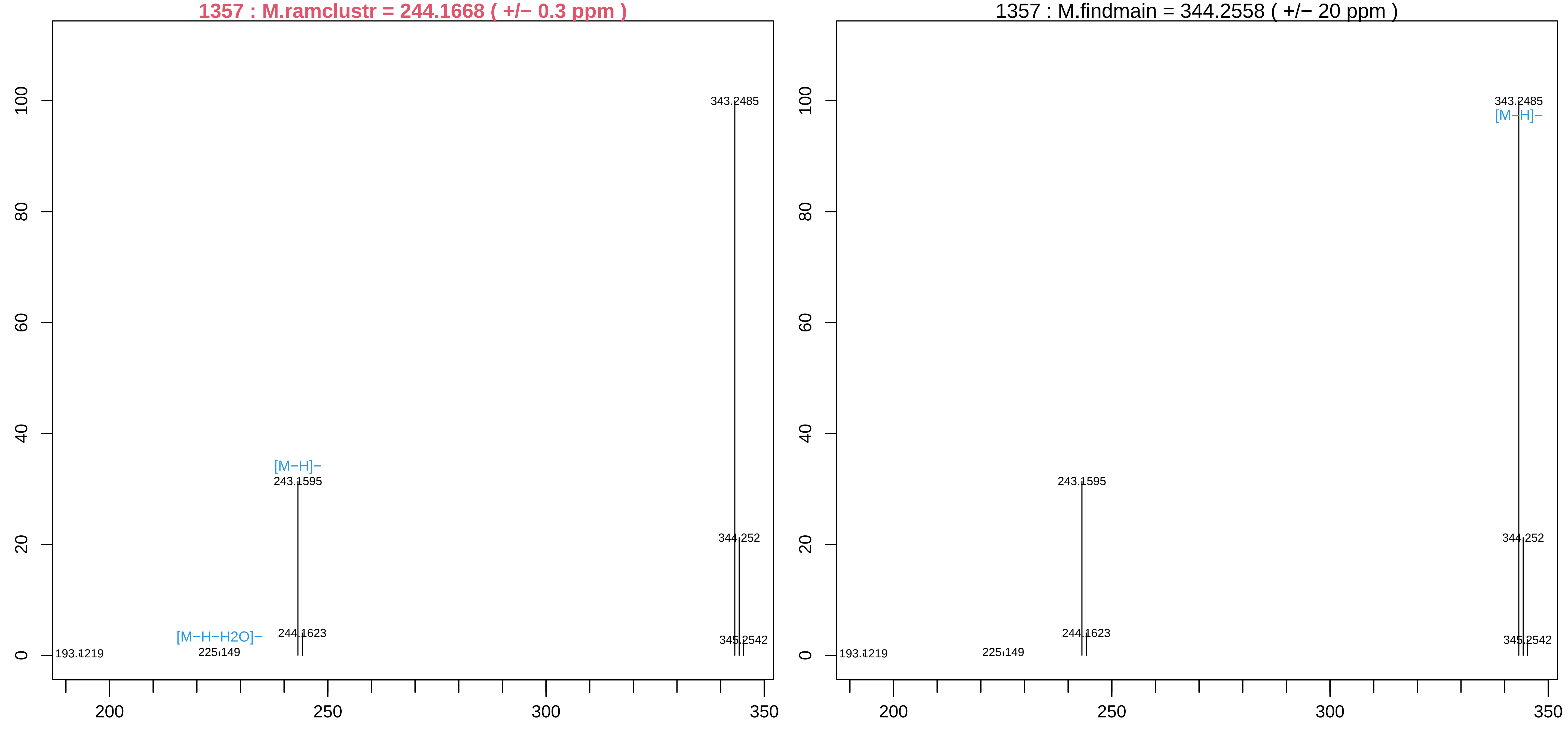

1357 : M.ramclustr = 244.1668 (+/− 0.3 ppm)
[M−H]−
243.1595
[M−H−H2O]−
225.149
193.1219
244.1623
343.2485
344.252
345.2542
1357 : M.findmain = 344.2558 (+/− 20 ppm)
343.2485
[M−H]−
243.1595
244.1623
225.149
193.1219
344.252
345.2542

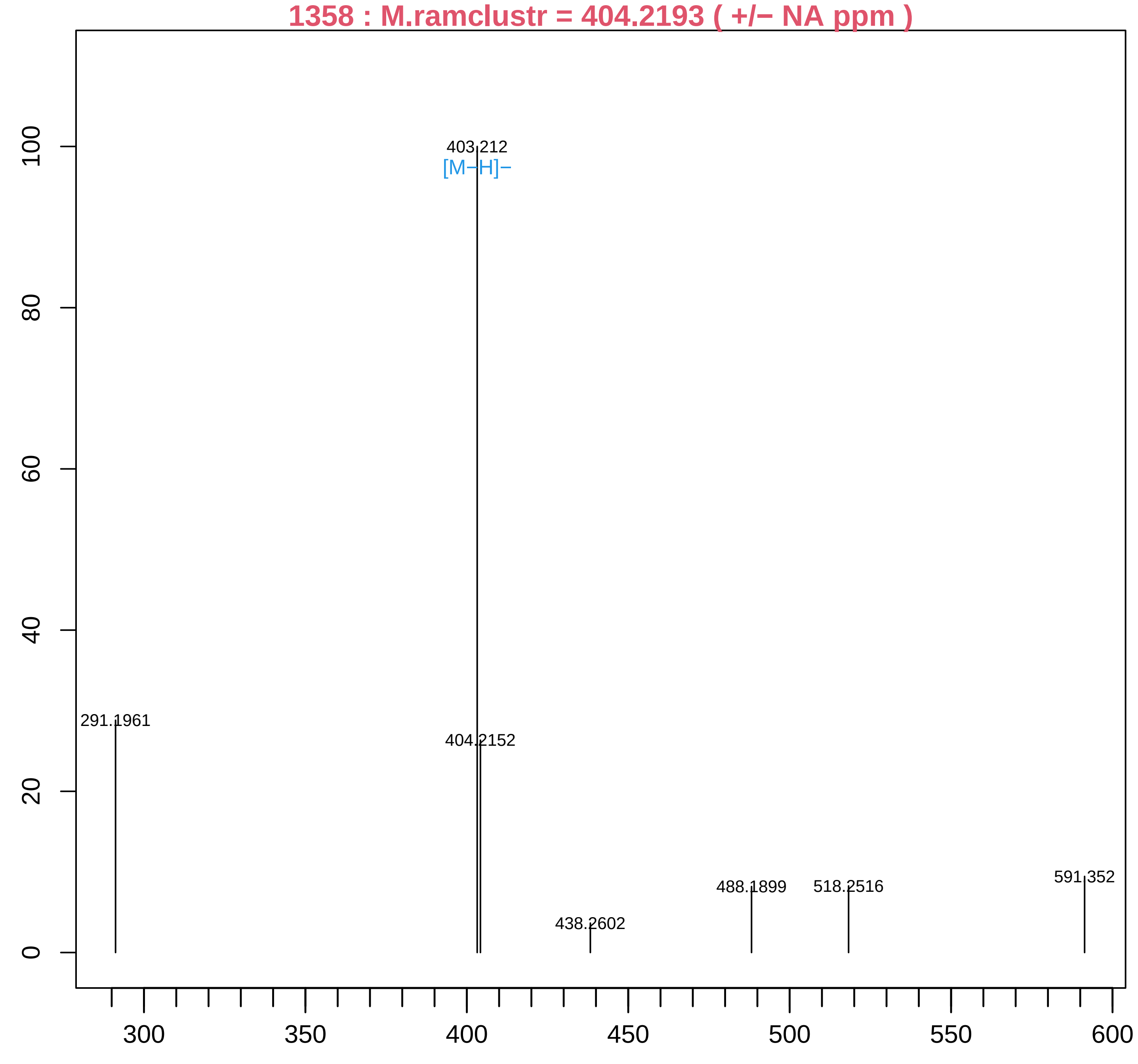

1358 : M.ramclustr = 404.2193 (+/− NA ppm)
403.212
[M−H]−
291.1961
404.2152
438.2602
488.1899
518.2516
591.352
0
20
40
60
80
100
300
350
400
450
500
550
600

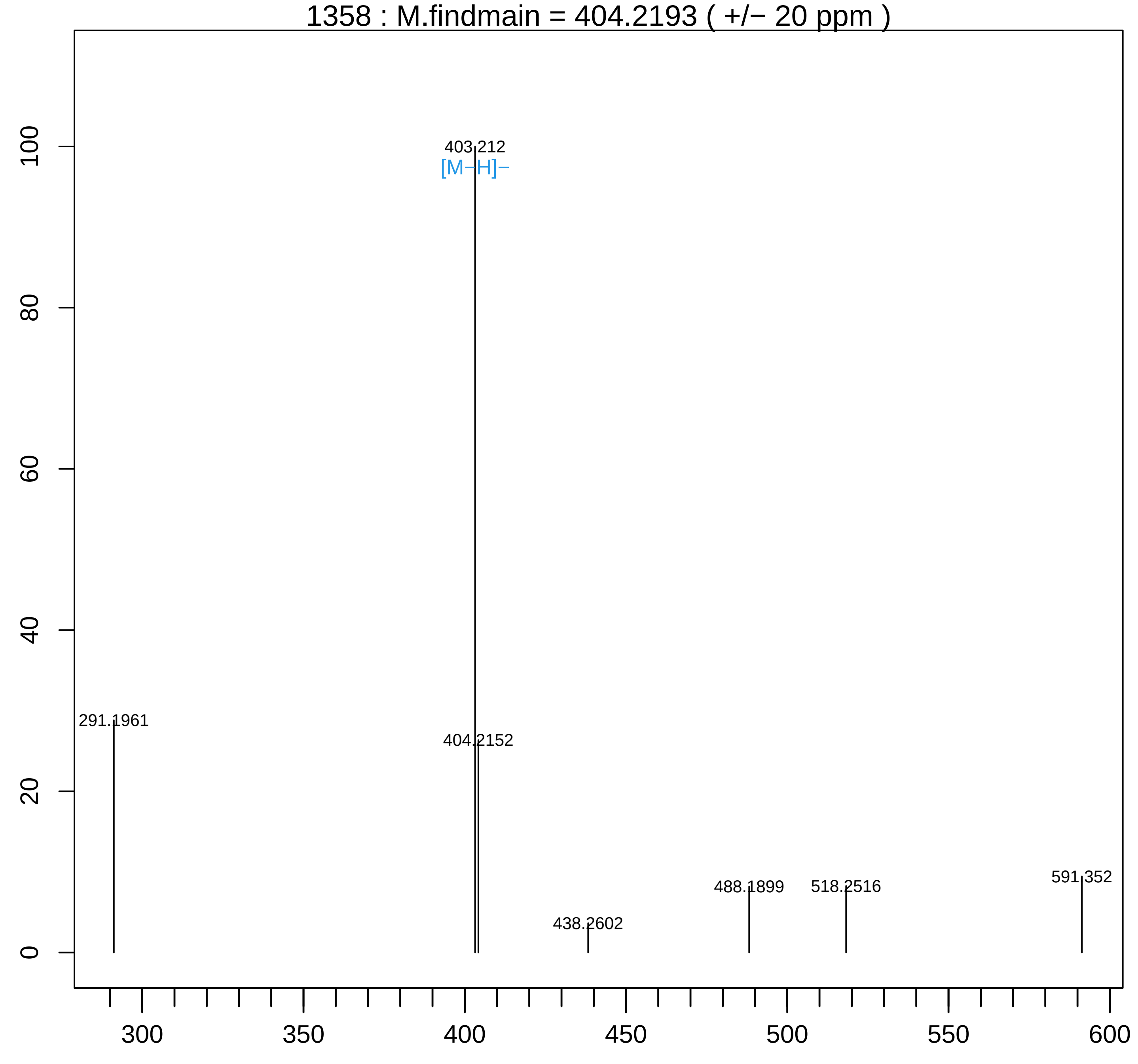

1358 : M.findmain = 404.2193 (+/− 20 ppm)
403.212
[M−H]−
291.1961
404.2152
438.2602
488.1899
518.2516
591.352
0
20
40
60
80
100
300
350
400
450
500
550
600

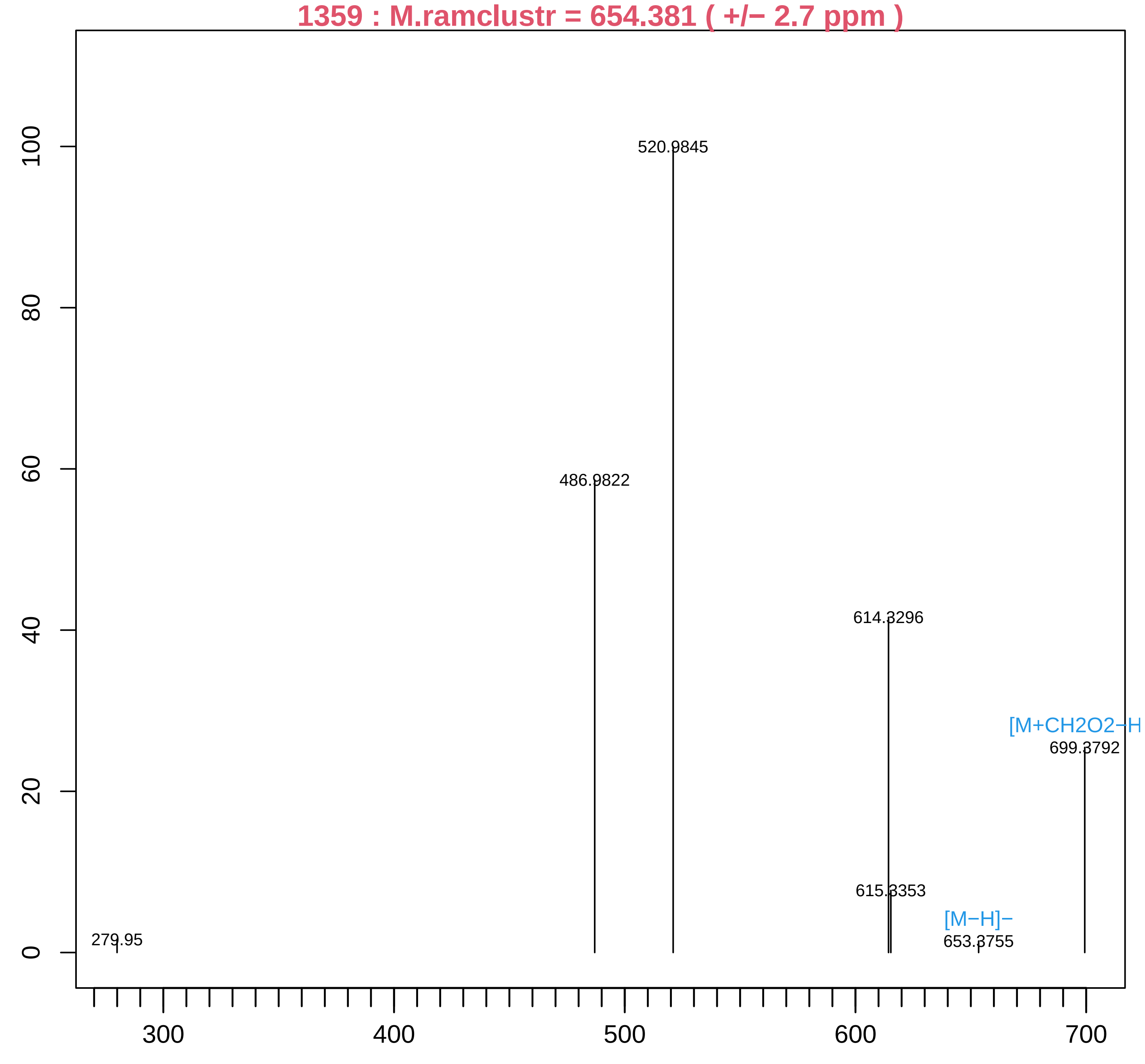
1359 : M.ramclustr = 654.381 (+/− 2.7 ppm)
520.9845
486.9822
614.3296
615.3353
[M+CH2O2−H
699.3792
[M−H]−
653.3755
279.95
0
20
40
60
80
100
300
400
500
600
700

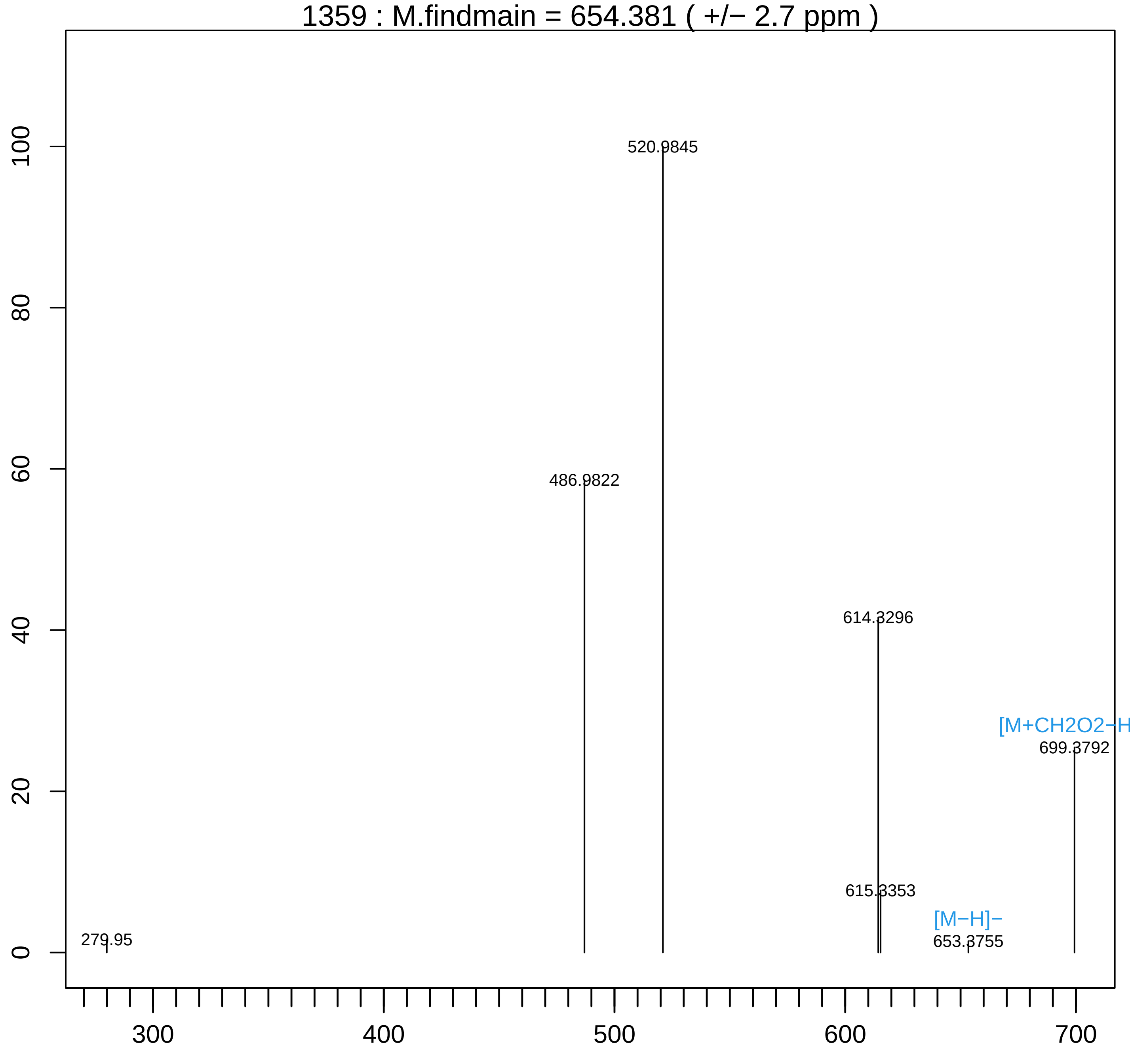
1359 : M.findmain = 654.381 (+/− 2.7 ppm)
520.9845
486.9822
614.3296
615.3353
[M+CH2O2−H
699.3792
[M−H]−
653.3755
279.95
0
20
40
60
80
100
300
400
500
600
700

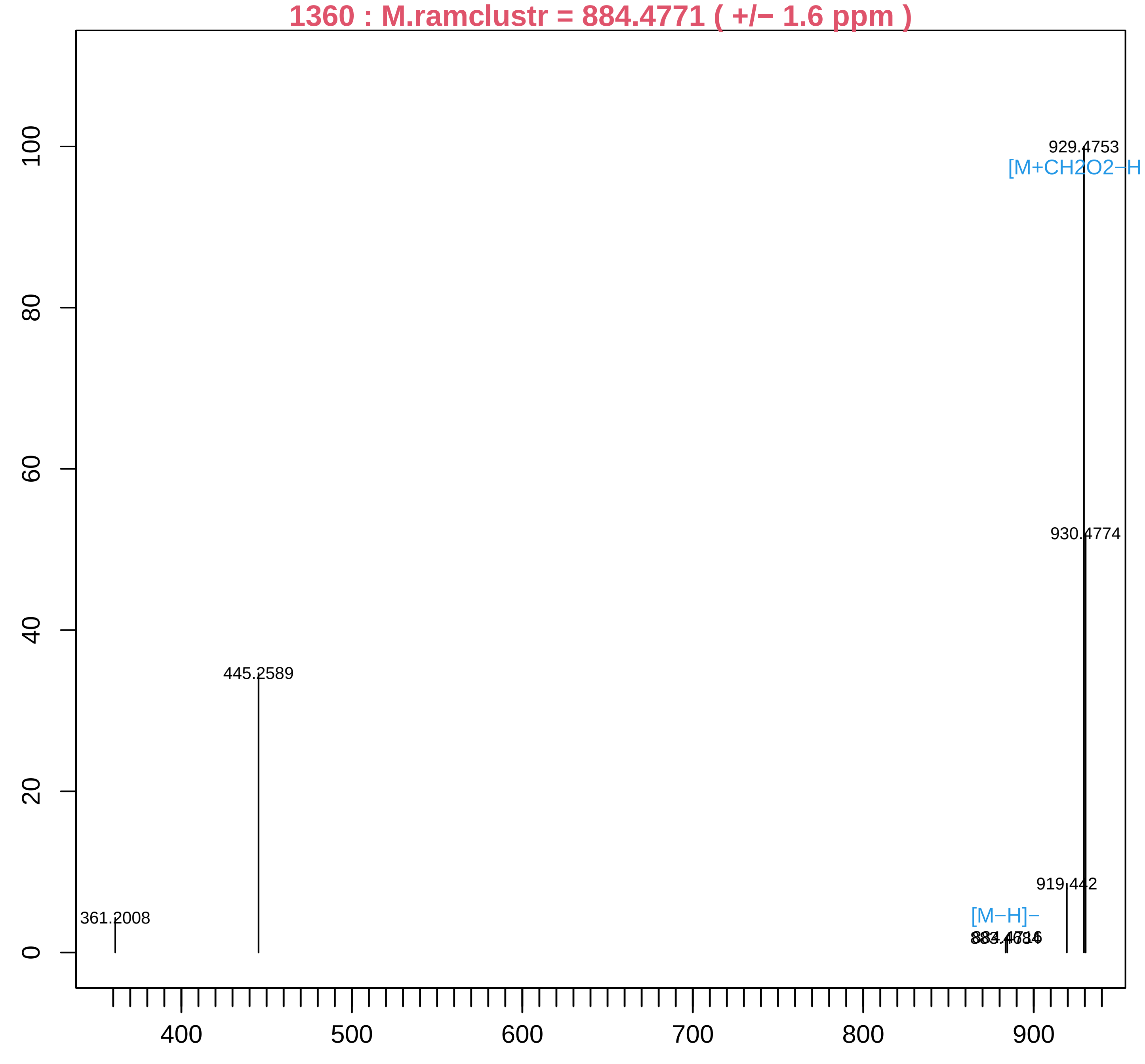
1360 : M.ramclustr = 884.4771 (+/− 1.6 ppm)
929.4753
[M+CH2O2−H
930.4774
445.2589
919.442
[M−H]−
361.2008
0
20
40
60
80
100
400
500
600
700
800
900

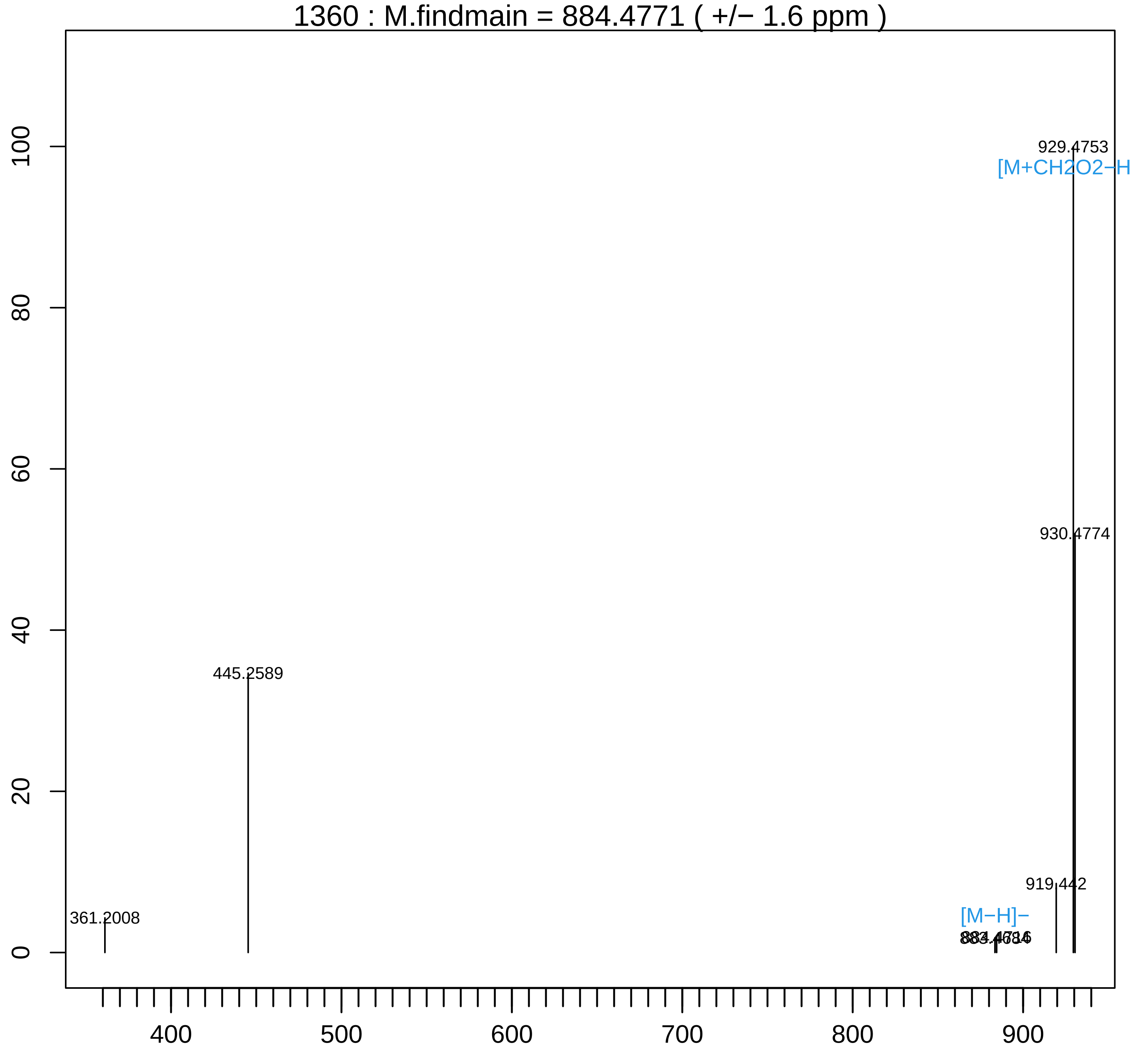
1360 : M.findmain = 884.4771 (+/− 1.6 ppm)
929.4753
[M+CH2O2−H
930.4774
445.2589
919.442
[M−H]−
361.2008
0
20
40
60
80
100
400
500
600
700
800
900

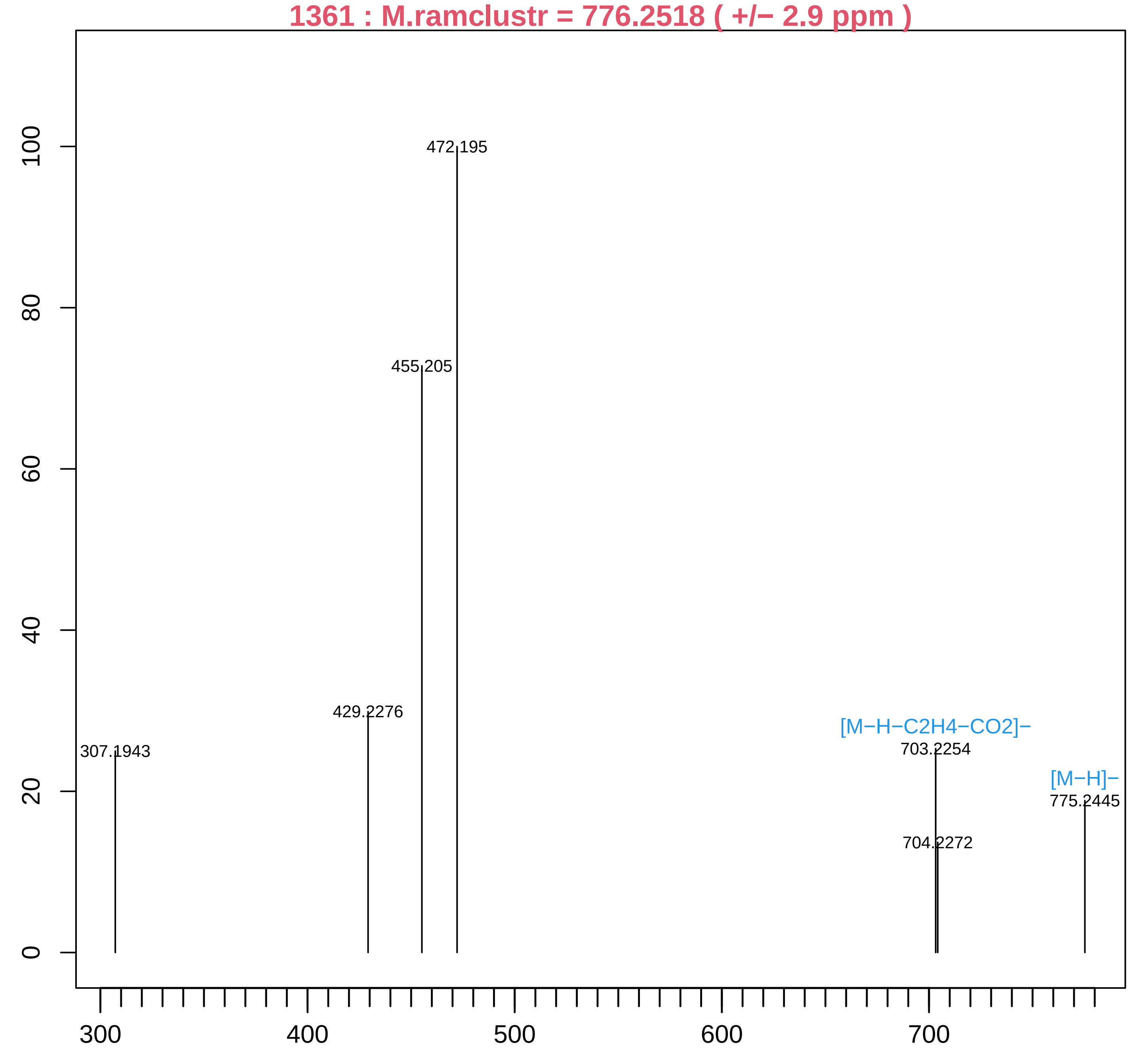
1361 : M.ramclustr = 776.2518 (+/− 2.9 ppm)
472.195
455.205
429.2276
307.1943
[M−H−C2H4−CO2]−
703.2254
704.2272
[M−H]−
775.2445
0
20
40
60
80
100
300
400
500
600
700

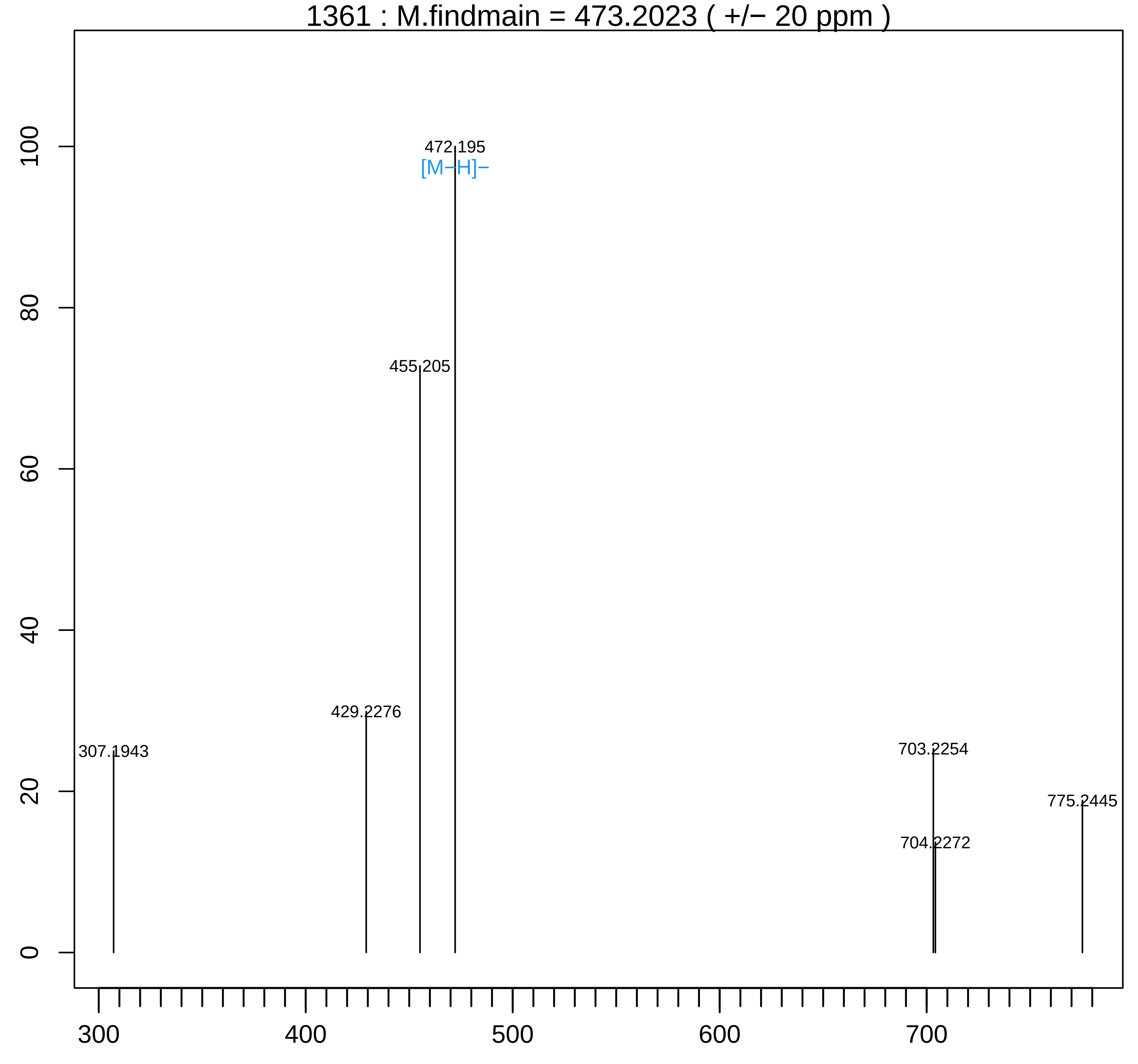
1361 : M.findmain = 473.2023 (+/− 20 ppm)
472.195
[M−H]−
455.205
429.2276
307.1943
703.2254
704.2272
775.2445
0
20
40
60
80
100
300
400
500
600
700

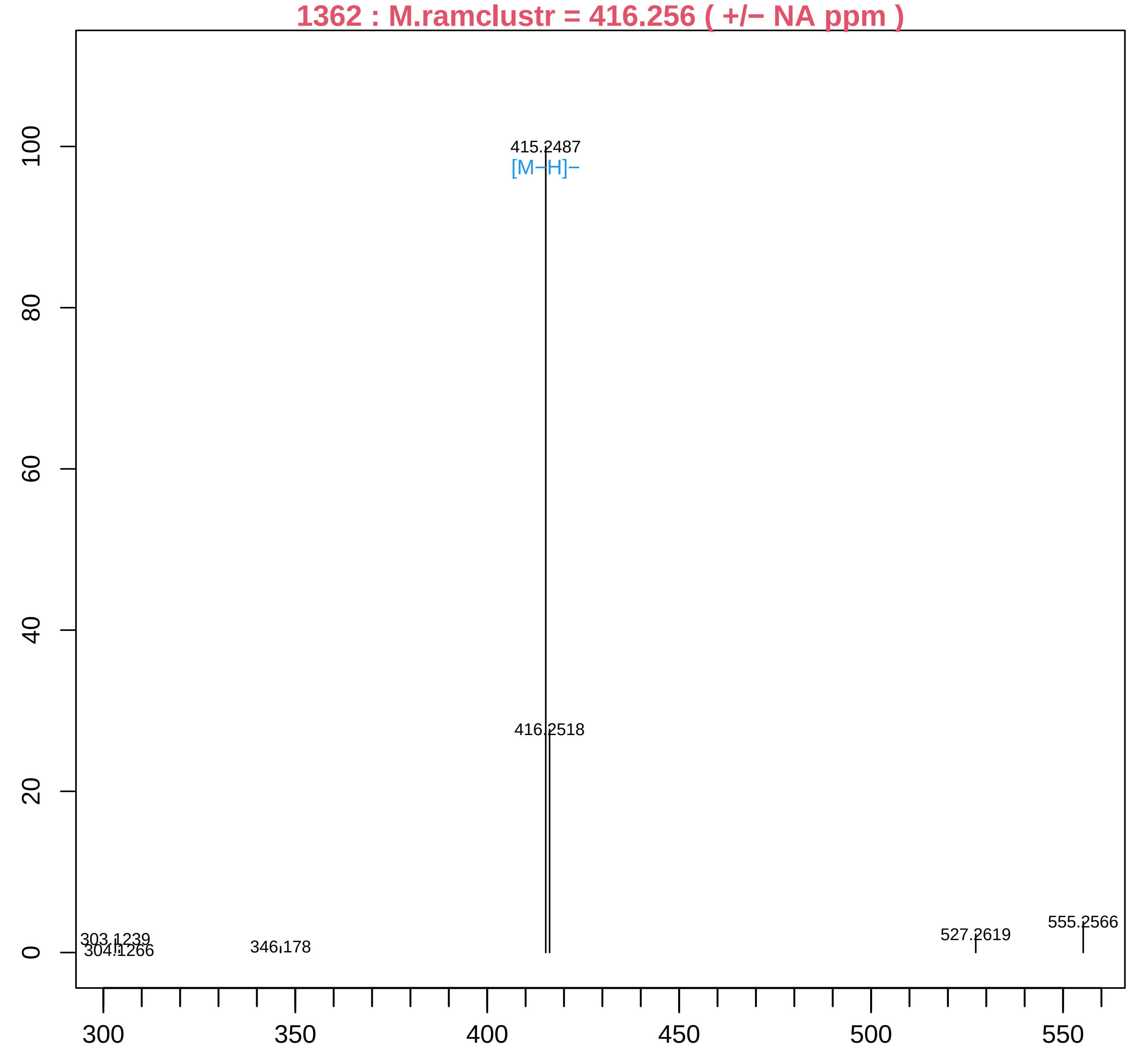
1362 : M.ramclustr = 416.256 (+/− NA ppm)
415.2487
[M−H]−
416.2518
303.1239
304.1266
346.178
527.2619
555.2566
0
20
40
60
80
100
300
350
400
450
500
550

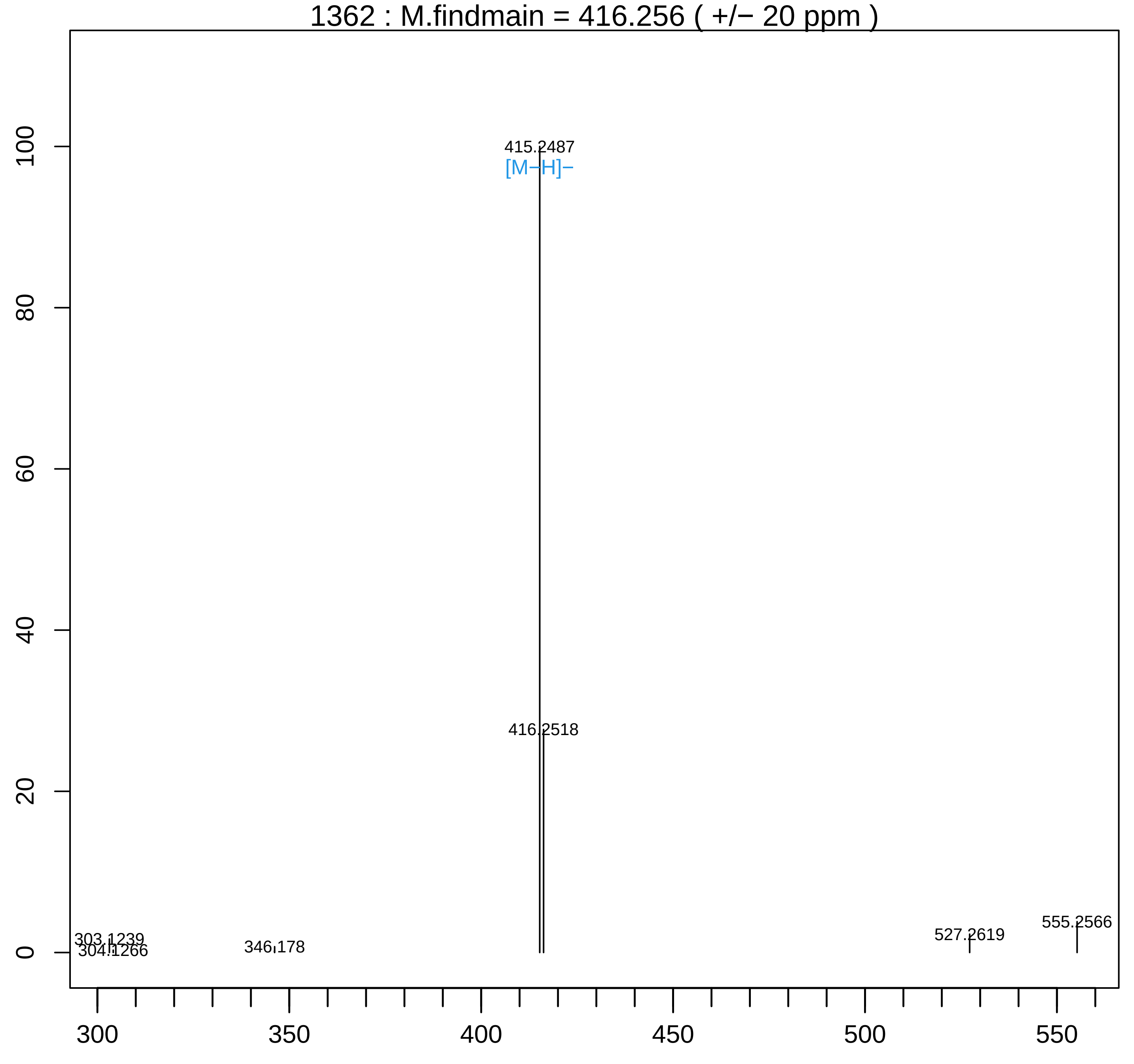
1362 : M.findmain = 416.256 (+/− 20 ppm)
415.2487
[M−H]−
416.2518
303.1239
304.1266
346.178
527.2619
555.2566
0
20
40
60
80
100
300
350
400
450
500
550

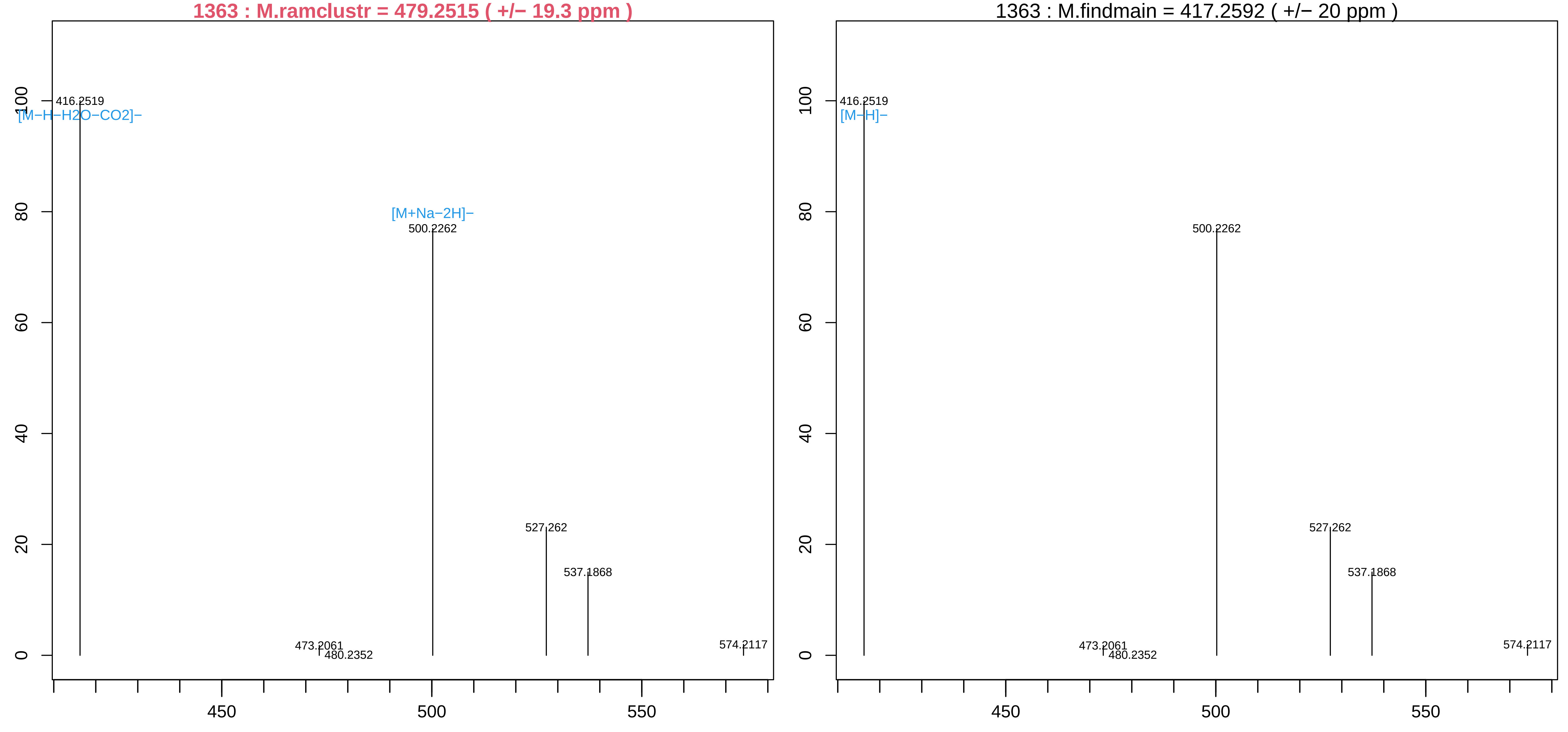
1363 : M.ramclustr = 479.2515 (+/− 19.3 ppm)
416.2519
[M−H−H2O−CO2]−
[M+Na−2H]−
500.2262
527.262
537.1868
473.2061
480.2352
574.2117
1363 : M.findmain = 417.2592 (+/− 20 ppm)
416.2519
[M−H]−
500.2262
527.262
537.1868
473.2061
480.2352
574.2117
0
20
40
60
80
100
450
500
550

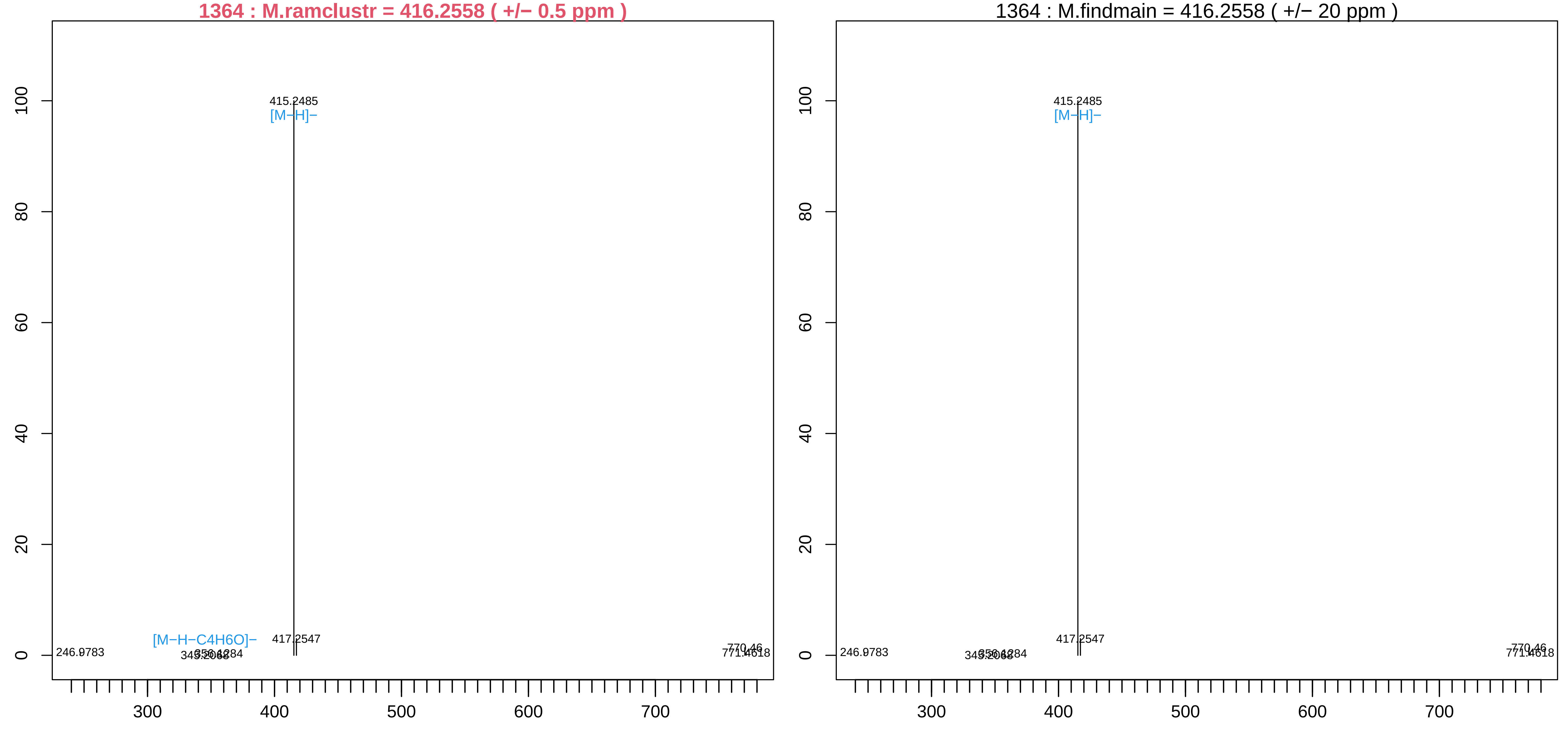

1364 : M.ramclustr = 416.2558 (+/− 0.5 ppm)
415.2485
[M−H]−
[M−H−C4H6O]−
417.2547
246.9783
345.2068
356.1284
770.46
771.4618
1364 : M.findmain = 416.2558 (+/− 20 ppm)
415.2485
[M−H]−
417.2547
246.9783
345.2068
356.1284
770.46
771.4618

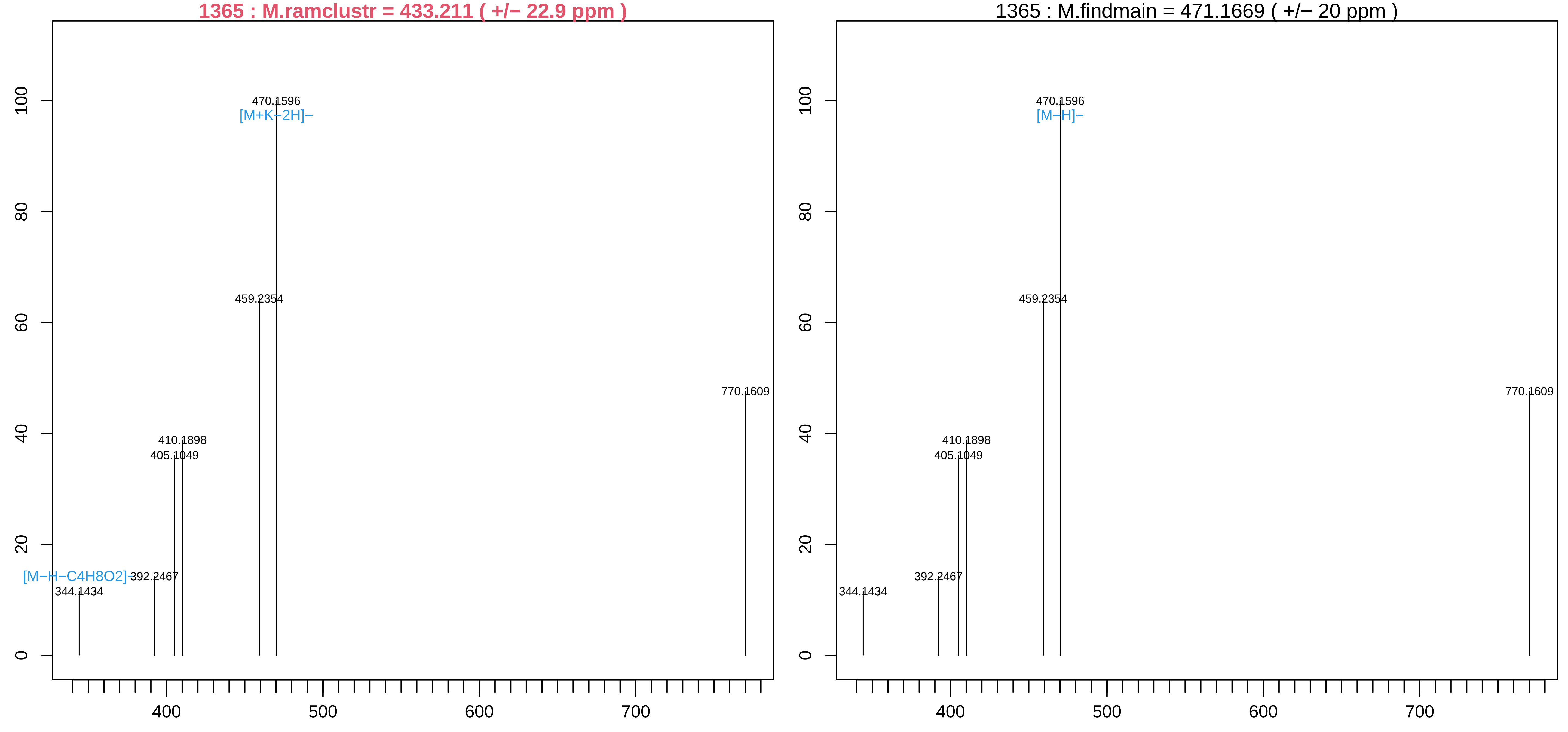

1365 : M.ramclustr = 433.211 (+/− 22.9 ppm)
470.1596
[M+K−2H]−
459.2354
770.1609
410.1898
405.1049
[M−H−C4H8O2]−
392.2467
344.1434
0
20
40
60
80
100
400
500
600
700
1365 : M.findmain = 471.1669 (+/− 20 ppm)
470.1596
[M−H]−
459.2354
770.1609
410.1898
405.1049
392.2467
344.1434
0
20
40
60
80
100
400
500
600
700

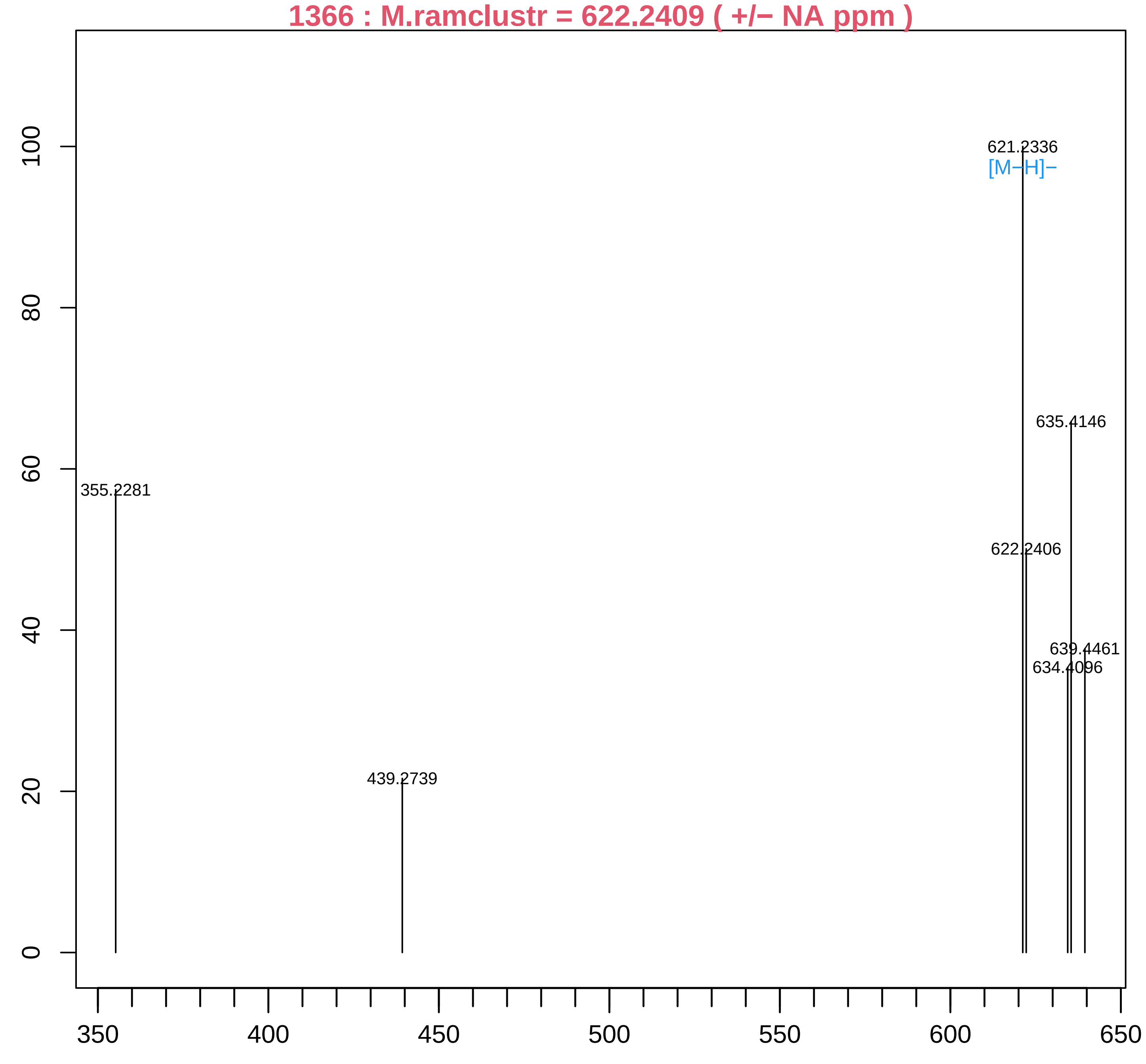
1366 : M.ramclustr = 622.2409 (+/− NA ppm)
621.2336
[M−H]−
635.4146
355.2281
622.2406
639.4461
634.4096
439.2739
0
20
40
60
80
100
350
400
450
500
550
600
650

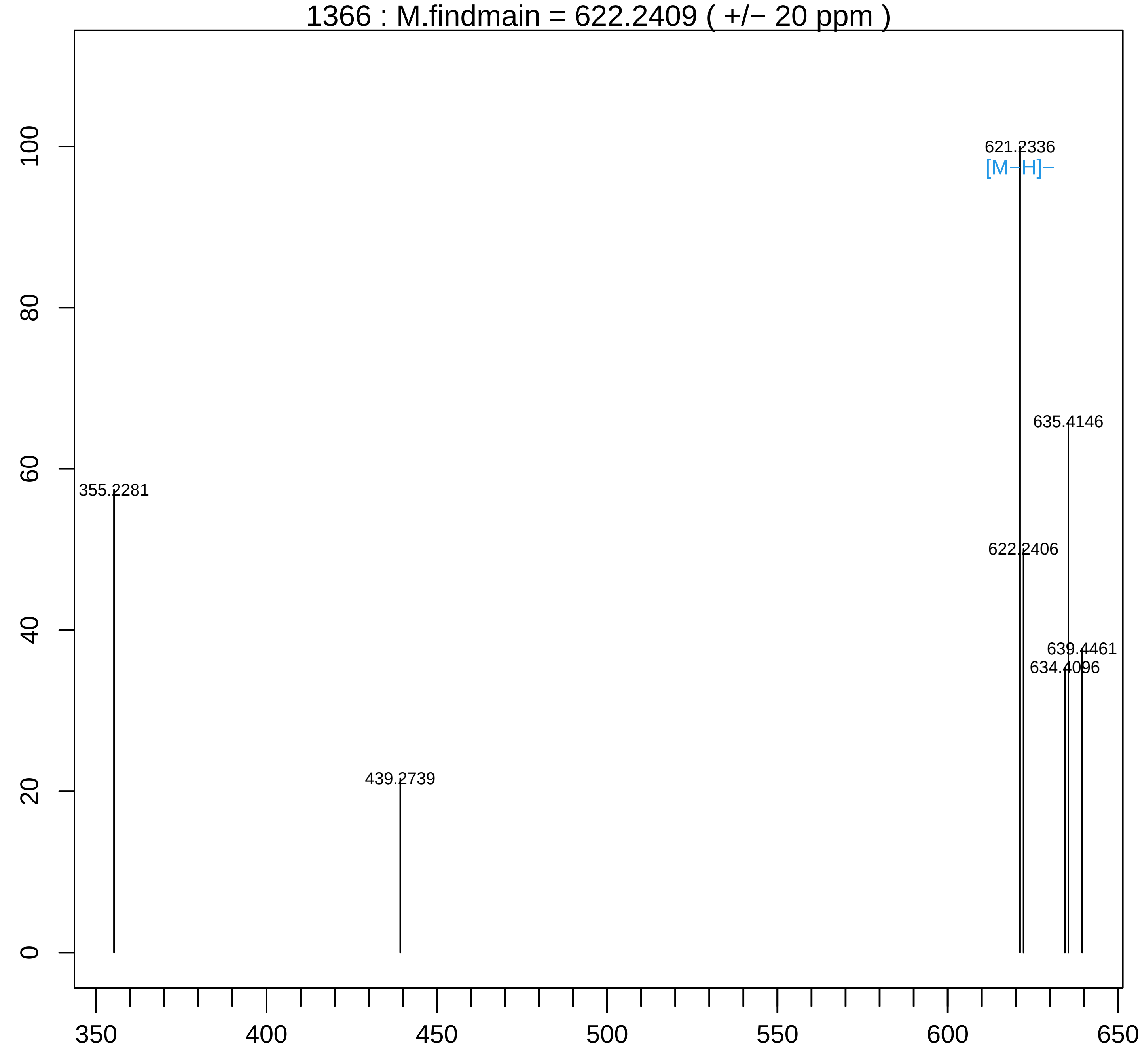
1366 : M.findmain = 622.2409 (+/− 20 ppm)
621.2336
[M−H]−
635.4146
355.2281
622.2406
639.4461
634.4096
439.2739
0
20
40
60
80
100
350
400
450
500
550
600
650

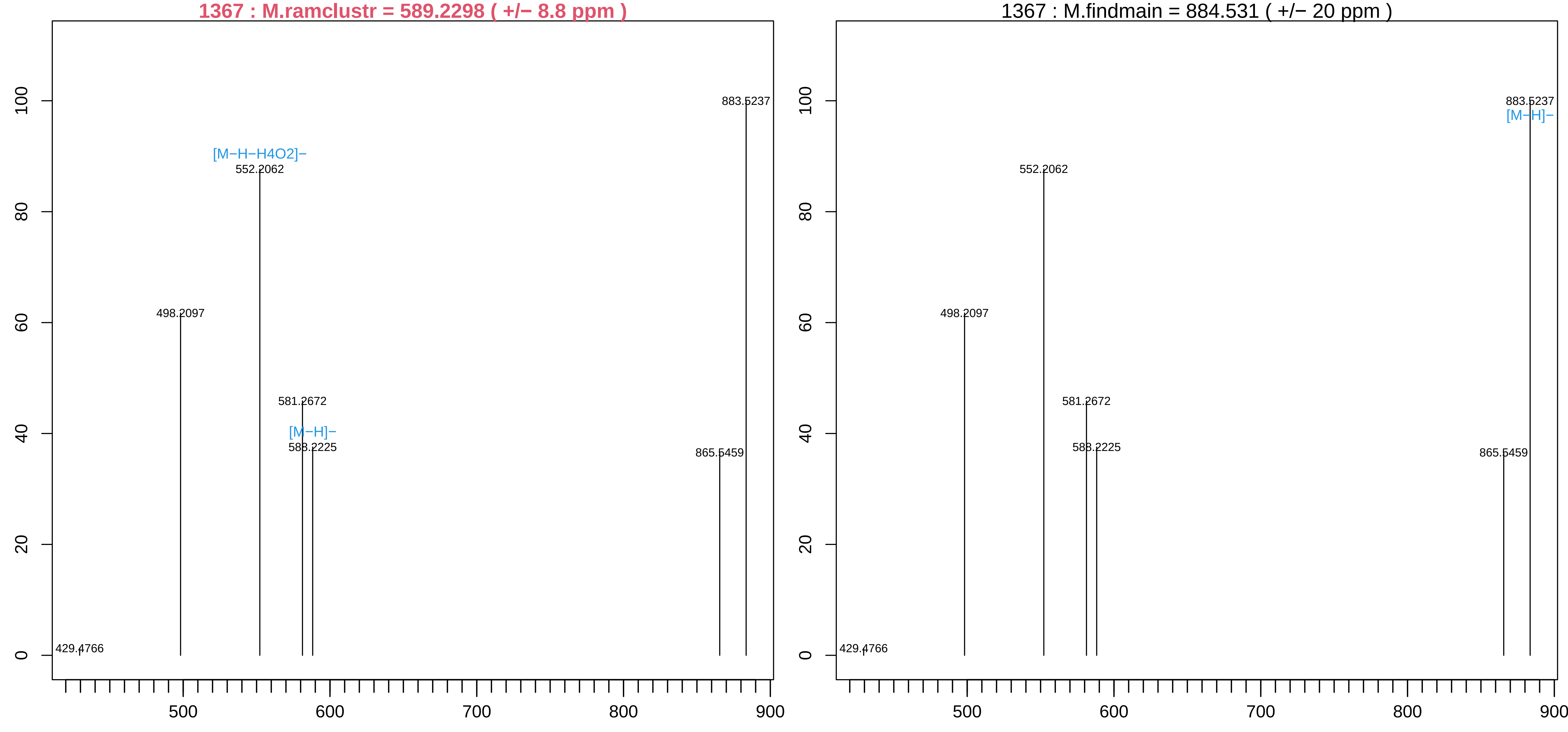
1367 : M.ramclustr = 589.2298 (+/− 8.8 ppm)
[M−H−H4O2]−
552.2062
498.2097
581.2672
[M−H]−
588.2225
865.5459
883.5237
429.4766
1367 : M.findmain = 884.531 (+/− 20 ppm)
883.5237
[M−H]−
552.2062
498.2097
581.2672
588.2225
865.5459
429.4766
0
20
40
60
80
100
500
600
700
800
900

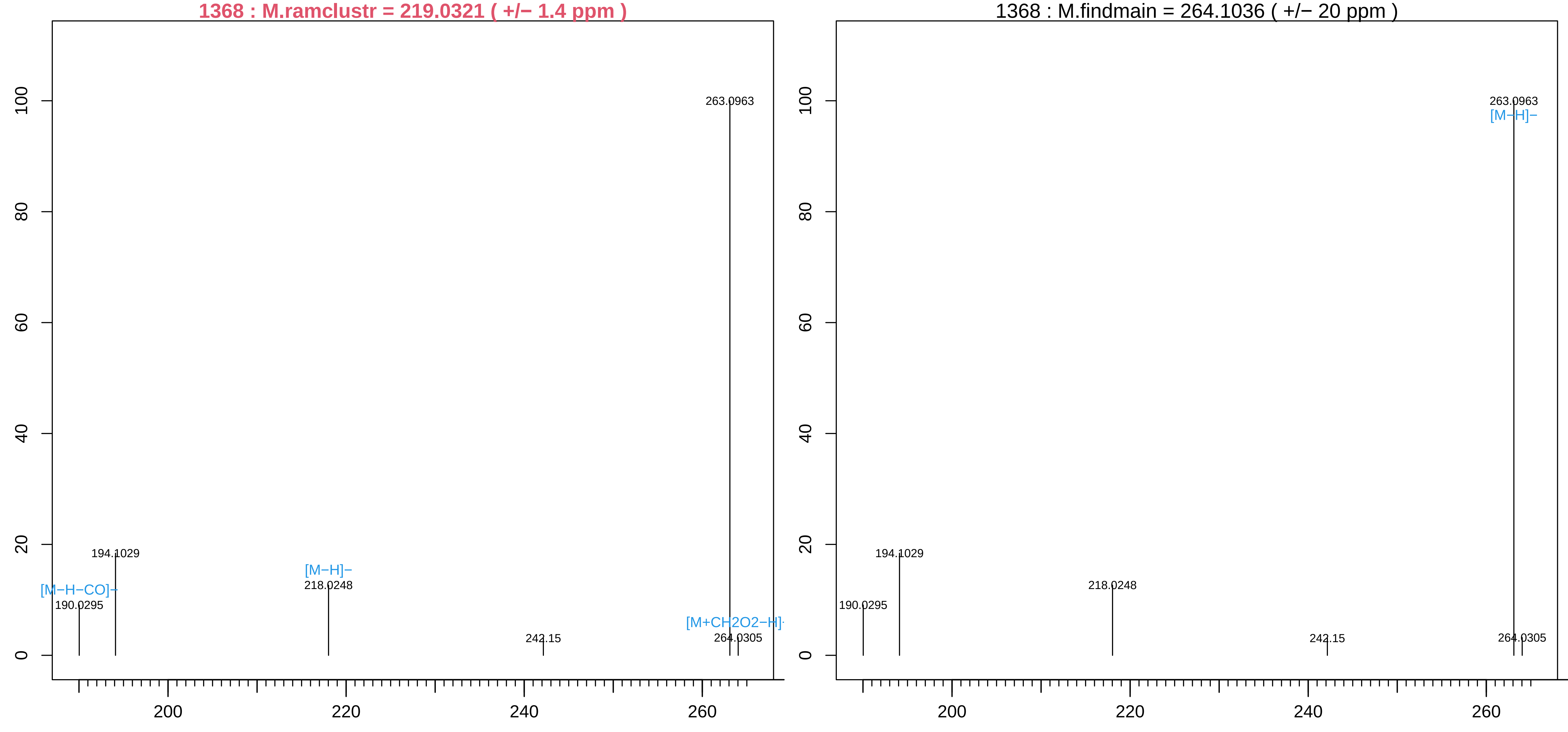
1368 : M.ramclustr = 219.0321 (+/− 1.4 ppm)
263.0963
194.1029
[M−H]−
218.0248
[M−H−CO]−
190.0295
242.15
[M+CH2O2−H]−
264.0305
1368 : M.findmain = 264.1036 (+/− 20 ppm)
263.0963
[M−H]−
194.1029
218.0248
190.0295
242.15
264.0305
0
20
40
60
80
100
200
220
240
260

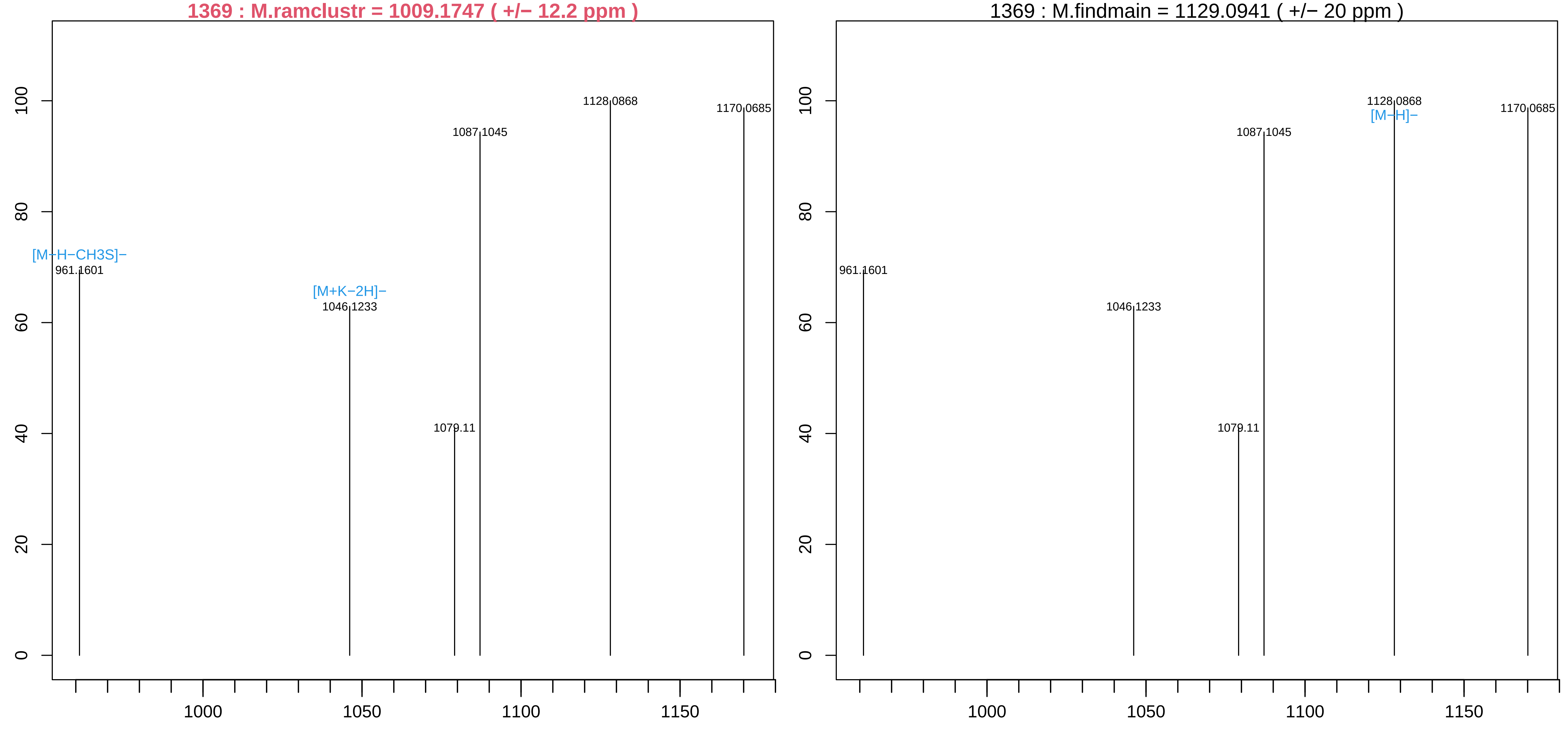

1369 : M.ramclustr = 1009.1747 (+/− 12.2 ppm)
[M−H−CH3S]−
961.1601
[M+K−2H]−
1046.1233
1079.11
1087.1045
1128.0868
1170.0685
1369 : M.findmain = 1129.0941 (+/− 20 ppm)
961.1601
1046.1233
1079.11
1087.1045
1128.0868
[M−H]−
1170.0685
0
20
40
60
80
100
1000
1050
1100
1150

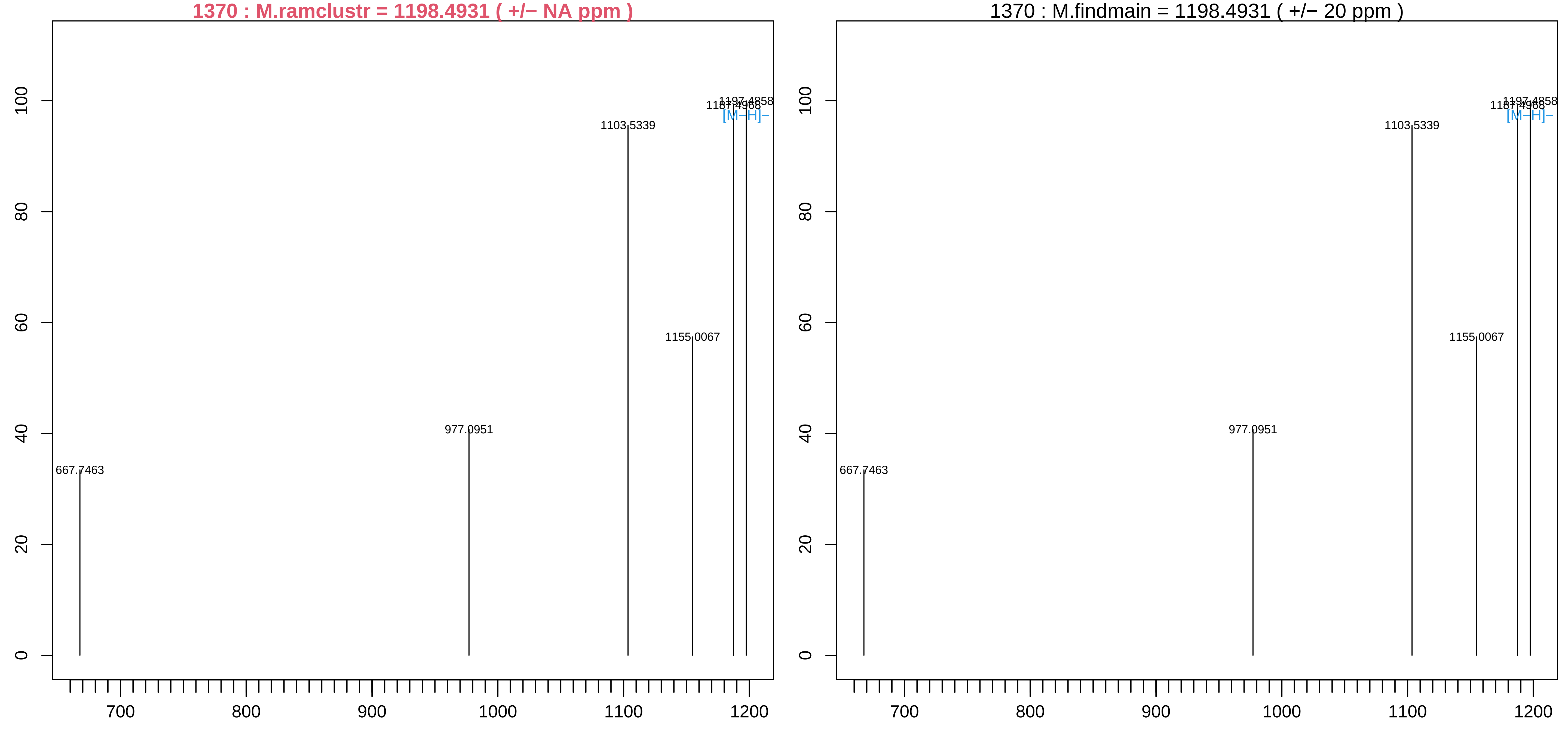
1370 : M.ramclustr = 1198.4931 (+/− NA ppm)
667.7463
977.0951
1103.5339
1155.0067
1187.4968
1197.4858
[M−H]−
1370 : M.findmain = 1198.4931 (+/− 20 ppm)
667.7463
977.0951
1103.5339
1155.0067
1187.4968
1197.4858
[M−H]−

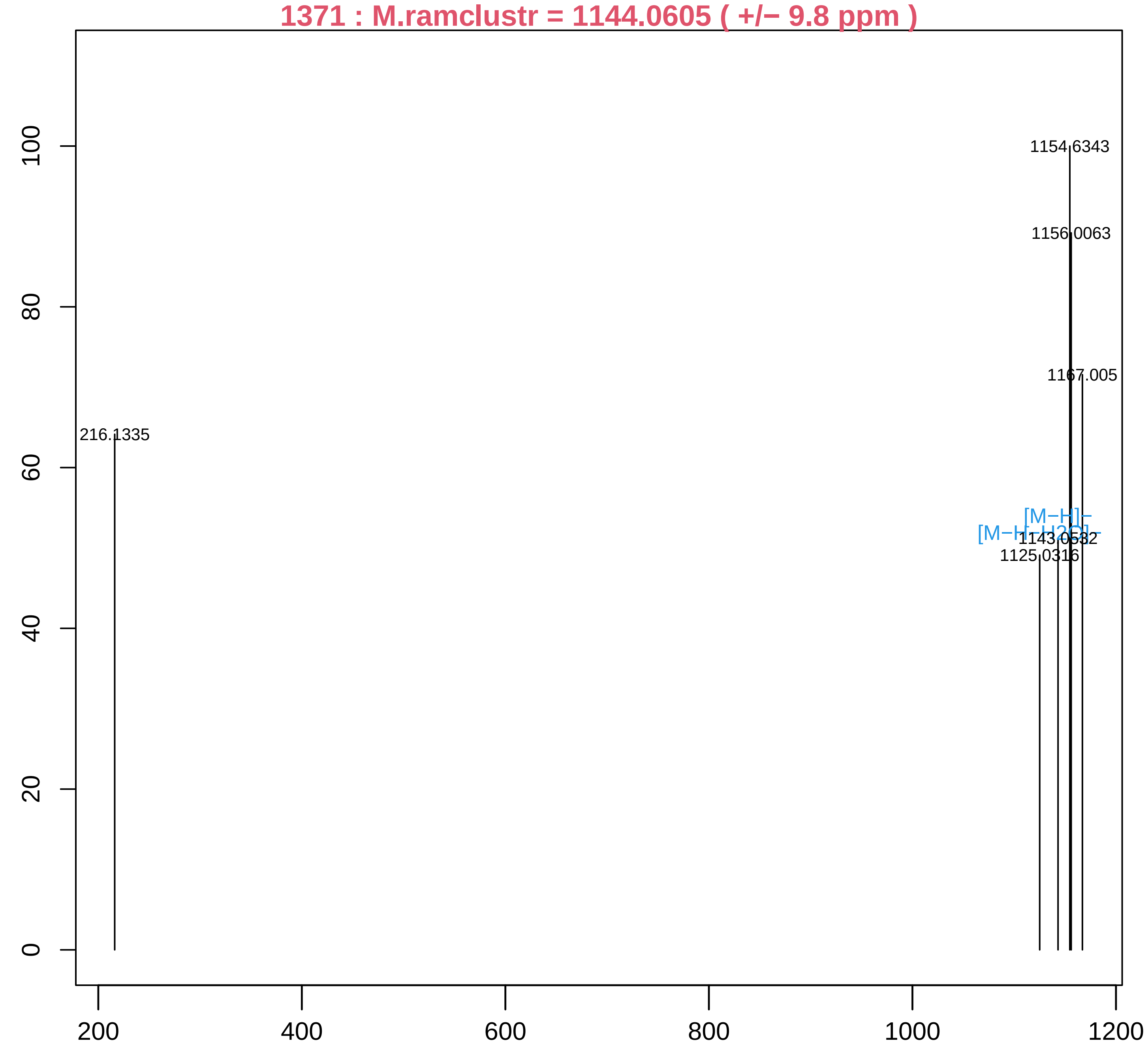
1371 : M.ramclustr = 1144.0605 (+/− 9.8 ppm)
1154.6343
1156.0063
1167.005
216.1335
[M−H]−
1143.0532
1125.0316
0
20
40
60
80
100
200
400
600
800
1000
1200

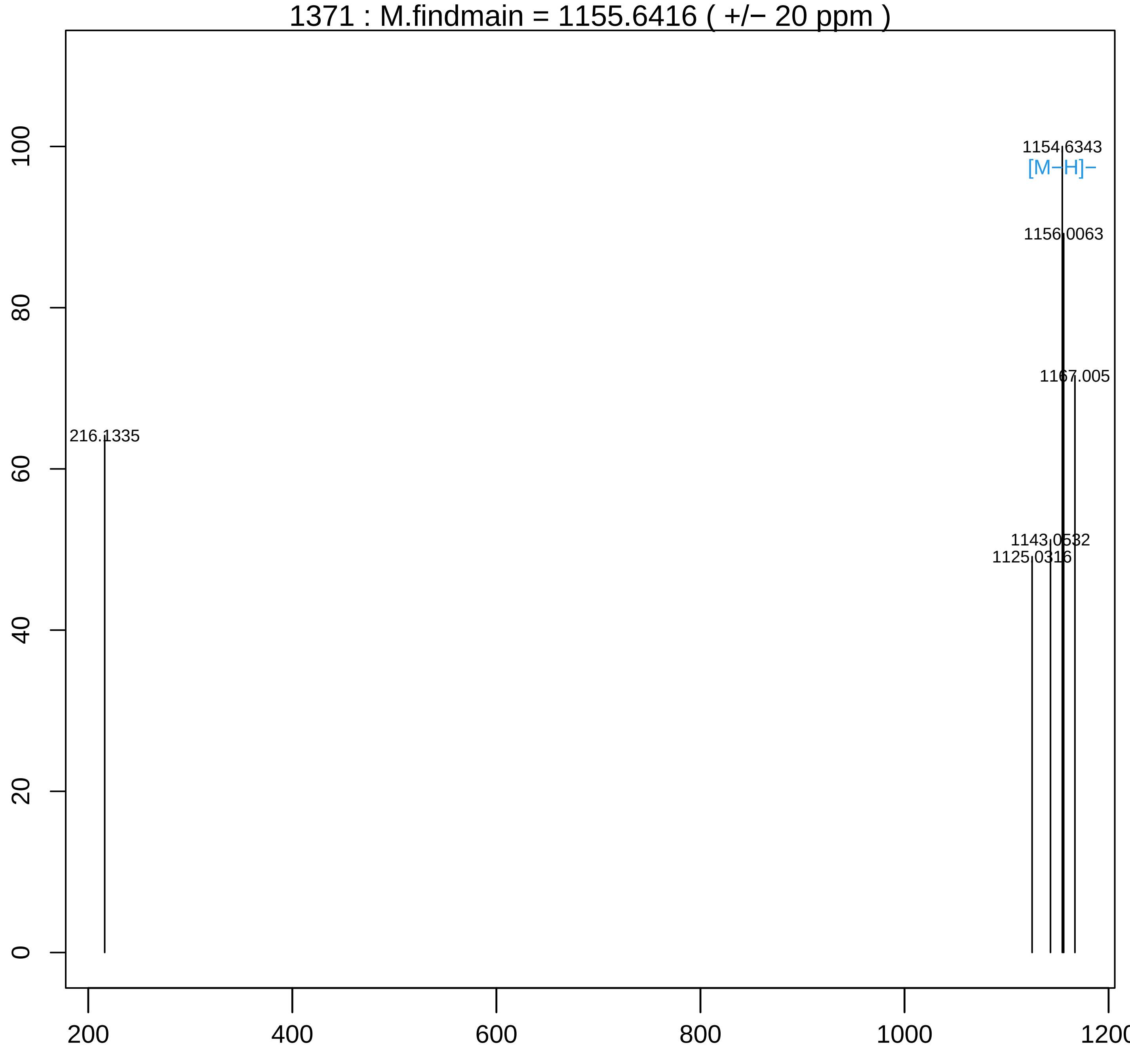
1371 : M.findmain = 1155.6416 (+/− 20 ppm)
1154.6343
[M−H]−
1156.0063
1167.005
216.1335
1143.0532
1125.0316
0
20
40
60
80
100
200
400
600
800
1000
1200

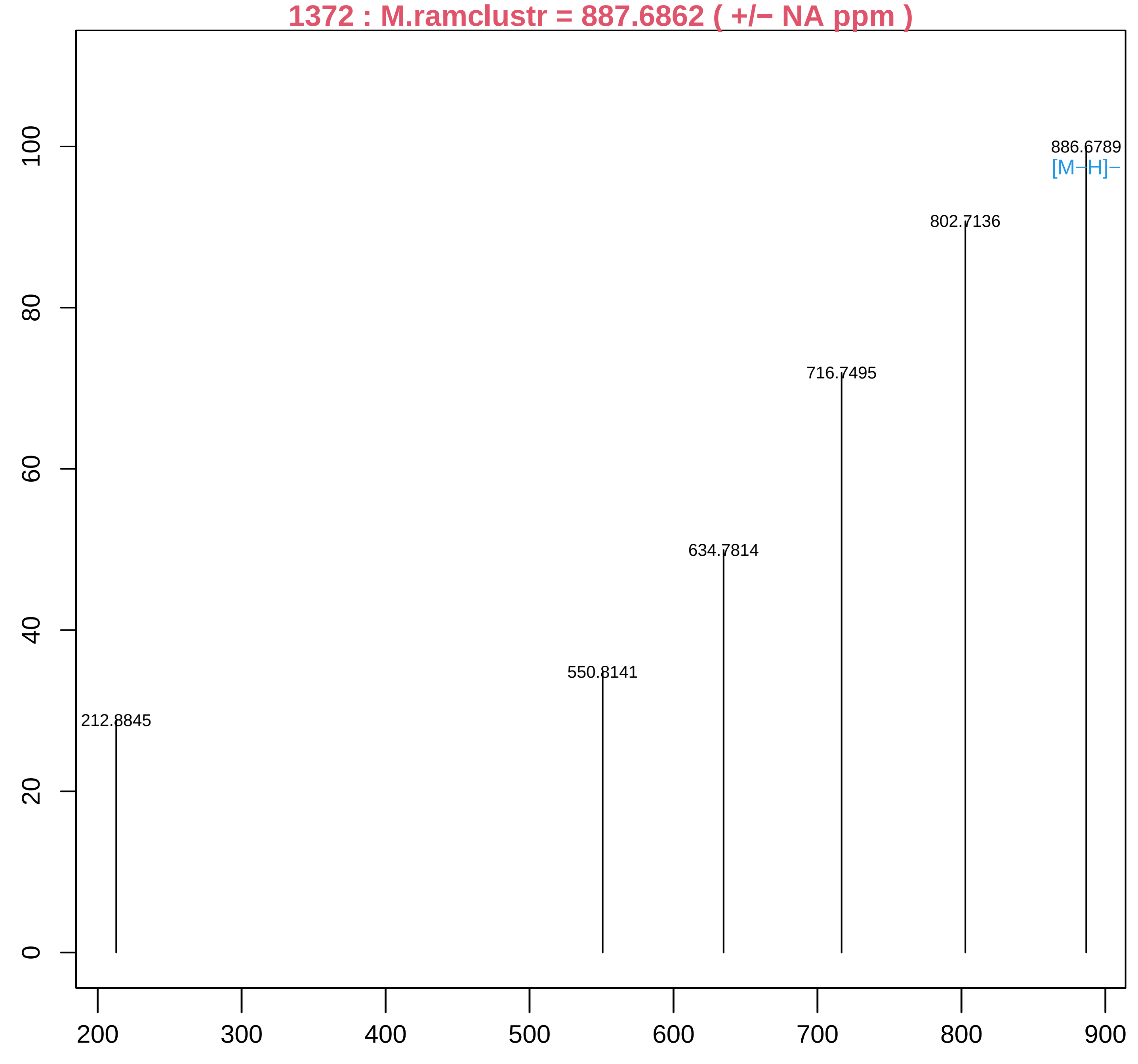
1372 : M.ramclustr = 887.6862 (+/− NA ppm)
212.8845
550.8141
634.7814
716.7495
802.7136
886.6789
[M−H]−
0
20
40
60
80
100
200
300
400
500
600
700
800
900

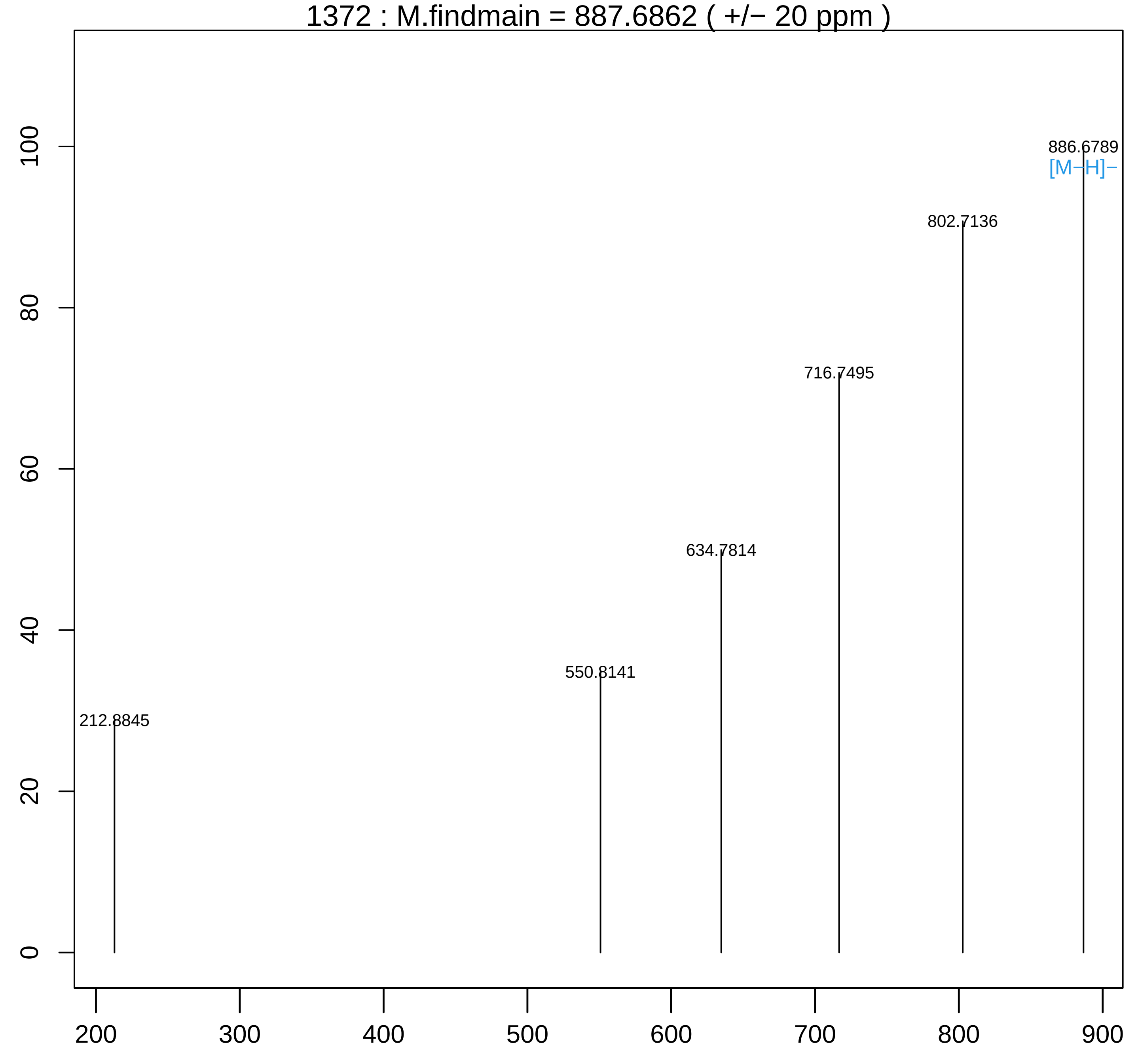
1372 : M.findmain = 887.6862 (+/− 20 ppm)
212.8845
550.8141
634.7814
716.7495
802.7136
886.6789
[M−H]−
0
20
40
60
80
100
200
300
400
500
600
700
800
900

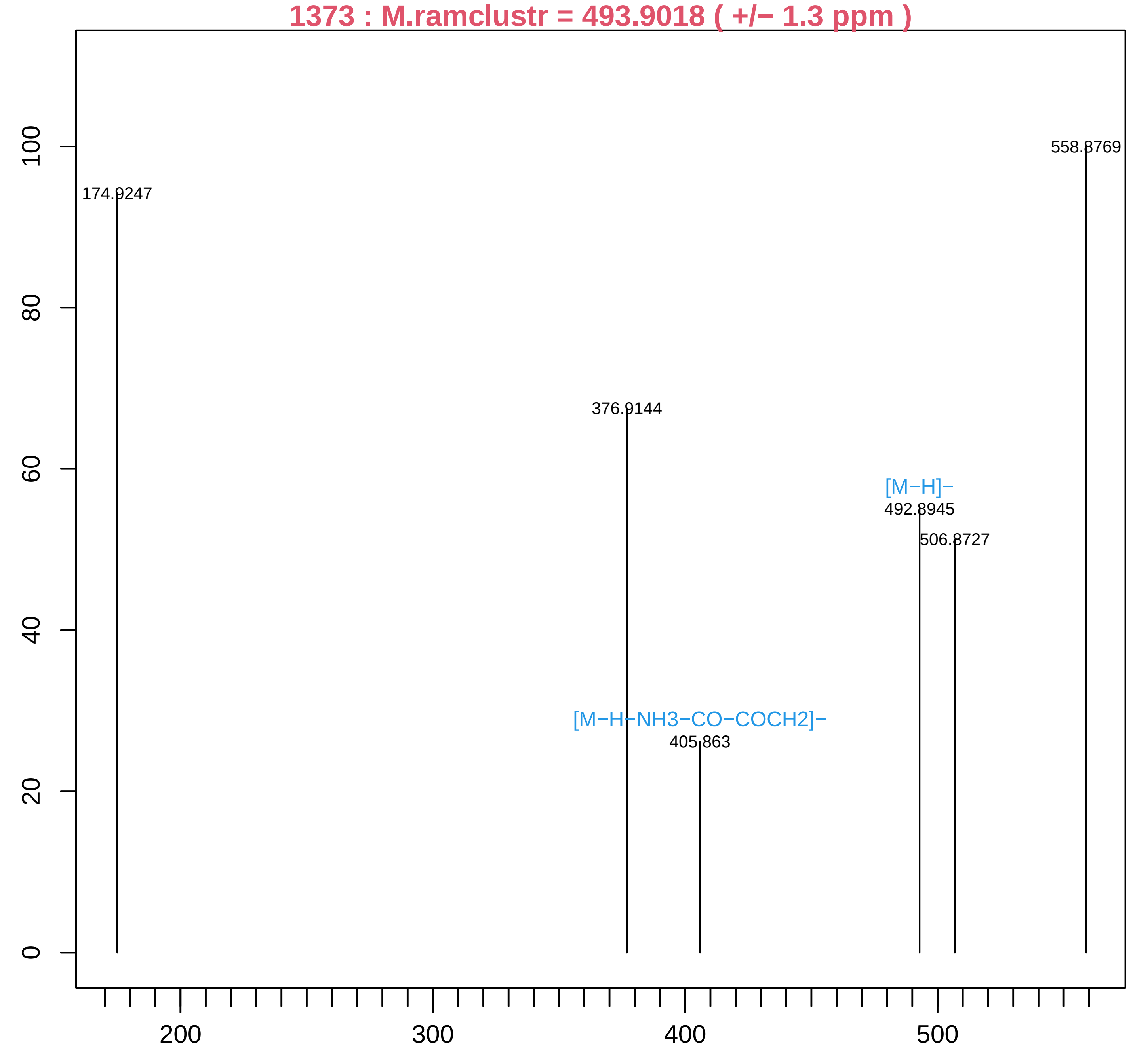
1373 : M.ramclustr = 493.9018 (+/− 1.3 ppm)
174.9247
376.9144
[M−H−NH3−CO−COCH2]−
405.863
[M−H]−
492.8945
506.8727
558.8769
0
20
40
60
80
100
200
300
400
500

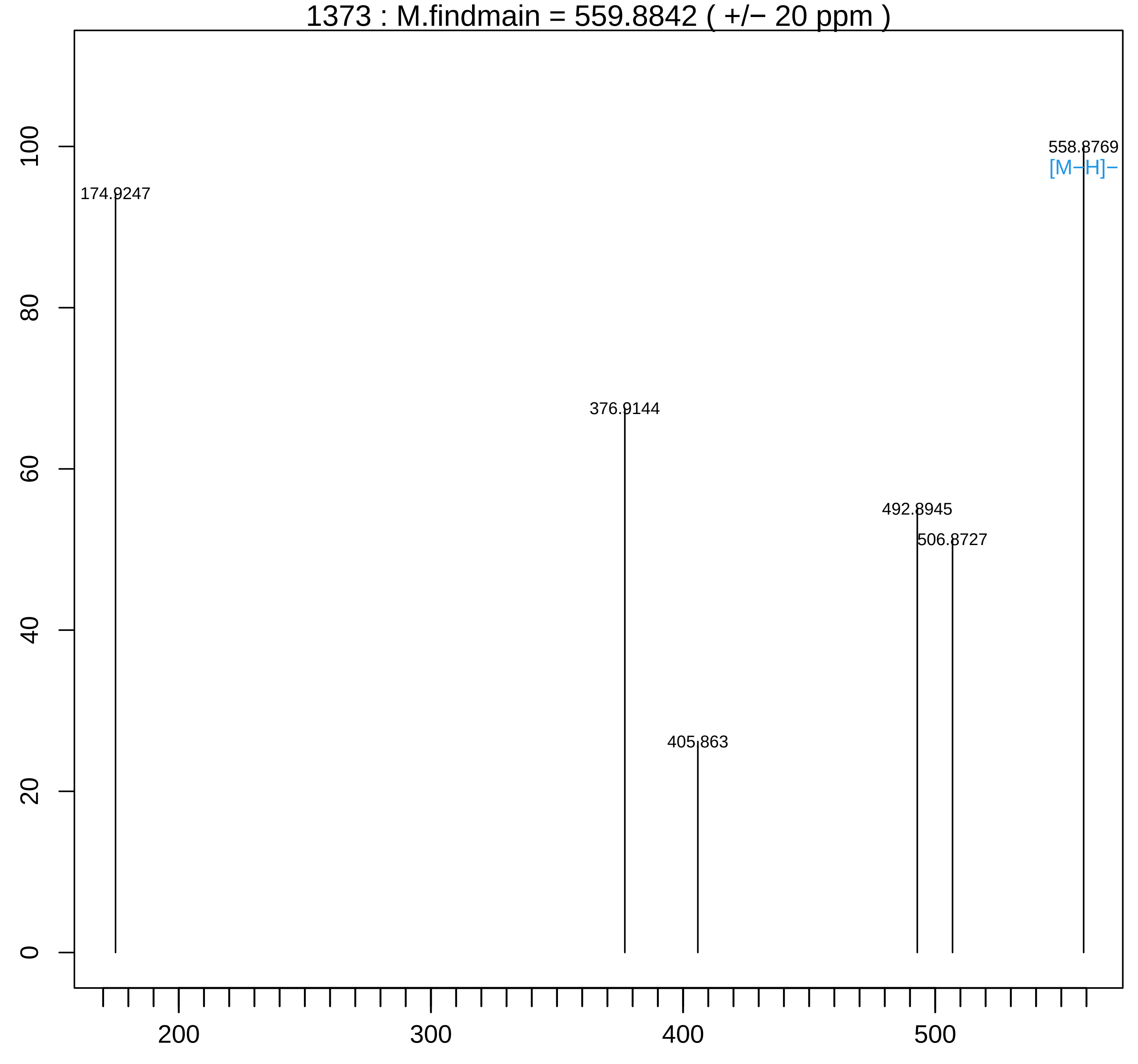
1373 : M.findmain = 559.8842 (+/− 20 ppm)
174.9247
376.9144
405.863
492.8945
506.8727
558.8769
[M−H]−
0
20
40
60
80
100
200
300
400
500

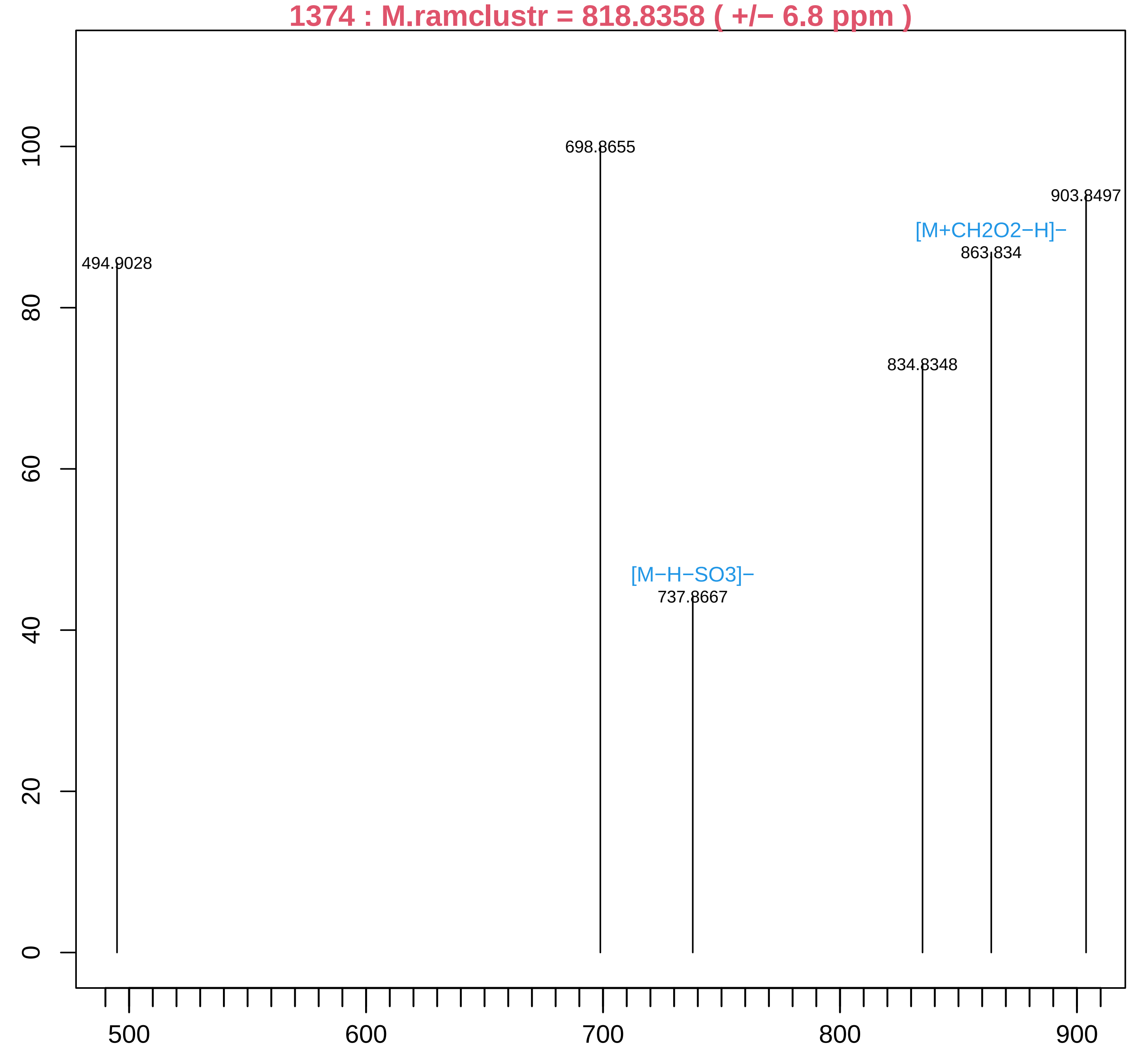
1374 : M.ramclustr = 818.8358 (+/− 6.8 ppm)
494.9028
698.8655
[M−H−SO3]−
737.8667
834.8348
[M+CH2O2−H]−
863.834
903.8497
0
20
40
60
80
100
500
600
700
800
900

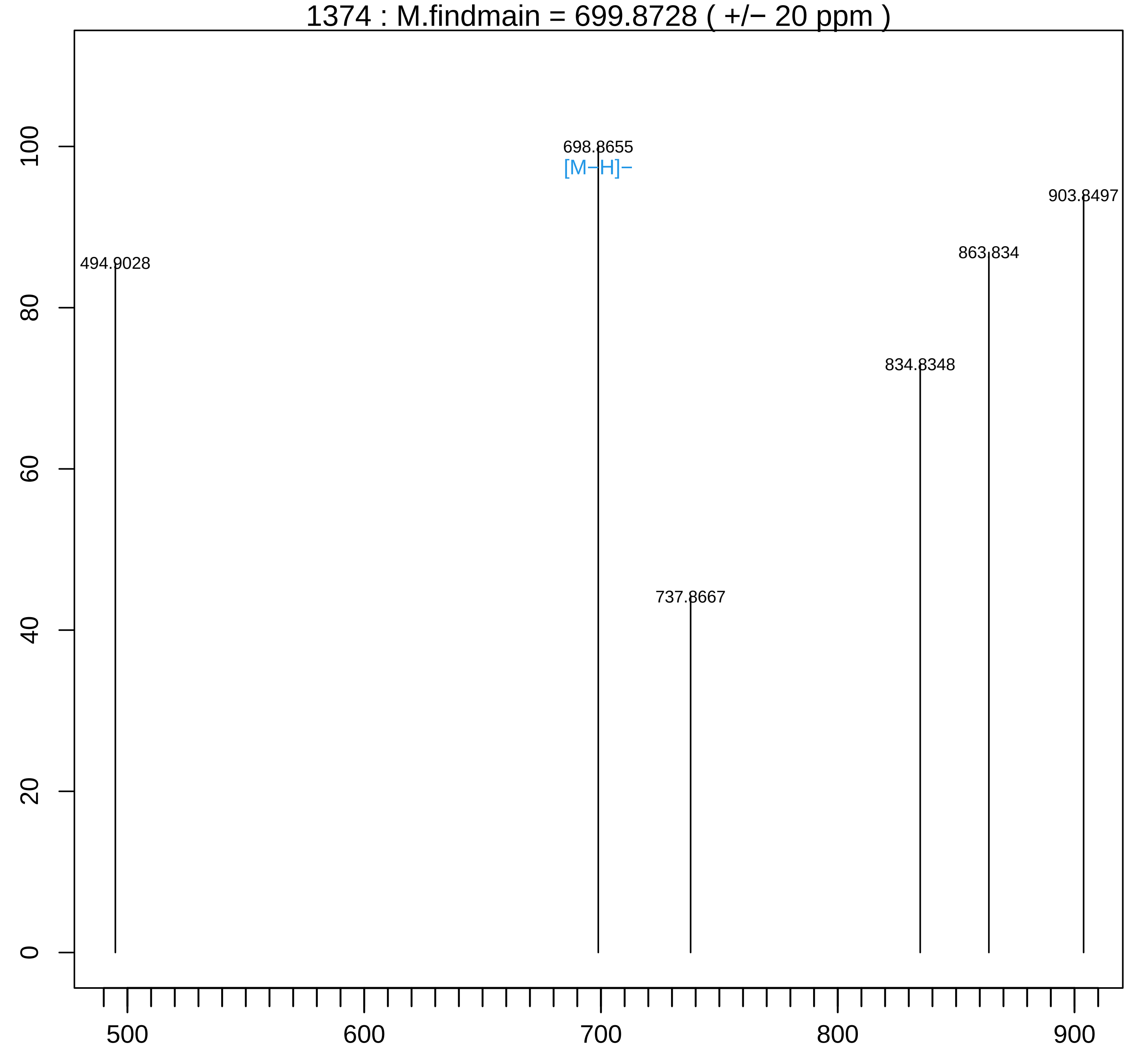
1374 : M.findmain = 699.8728 (+/− 20 ppm)
494.9028
698.8655
[M−H]−
737.8667
834.8348
863.834
903.8497
0
20
40
60
80
100
500
600
700
800
900

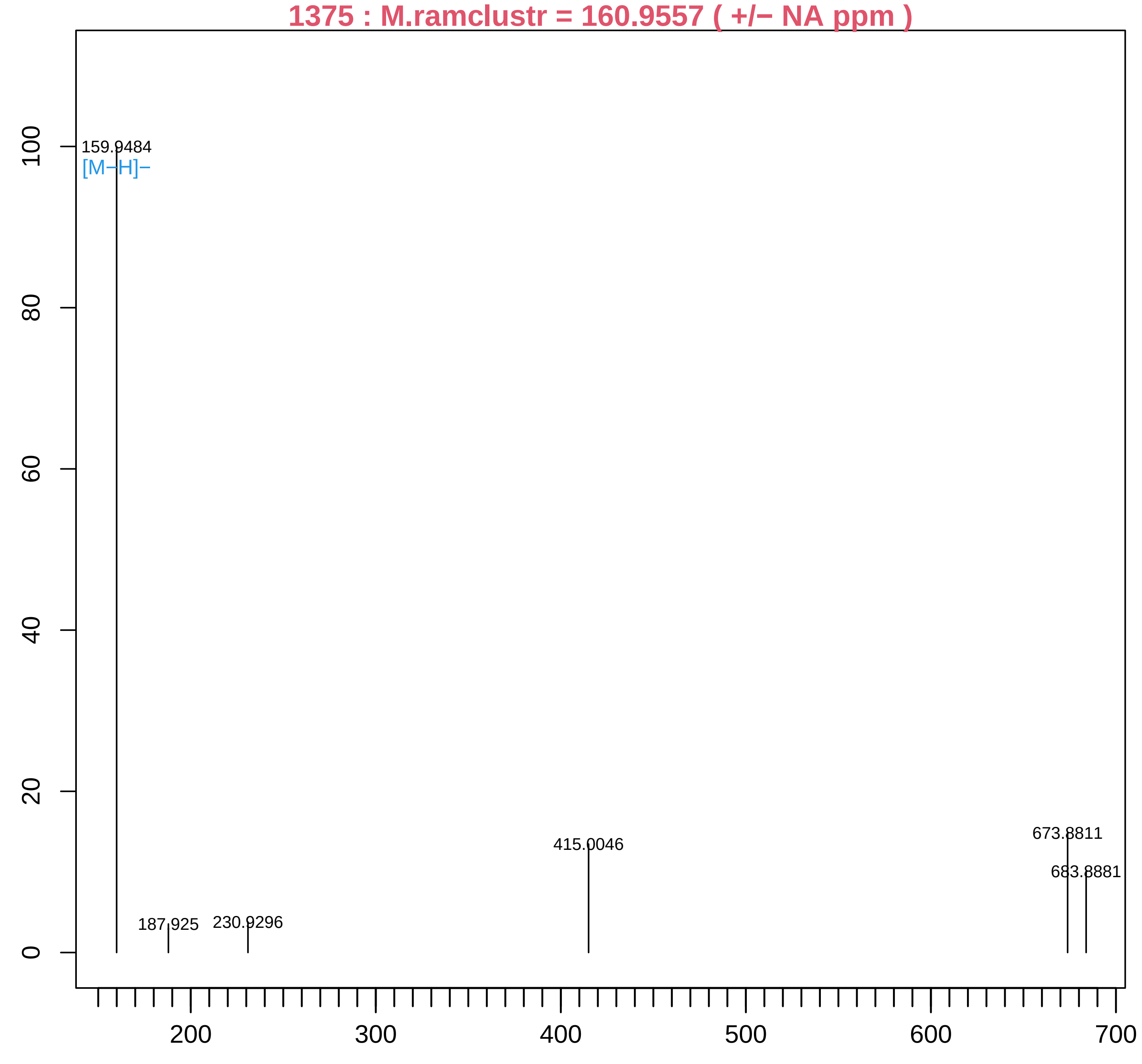
1375 : M.ramclustr = 160.9557 (+/− NA ppm)
159.9484
[M−H]−
187.925
230.9296
415.0046
673.8811
683.8881
0
20
40
60
80
100
200
300
400
500
600
700

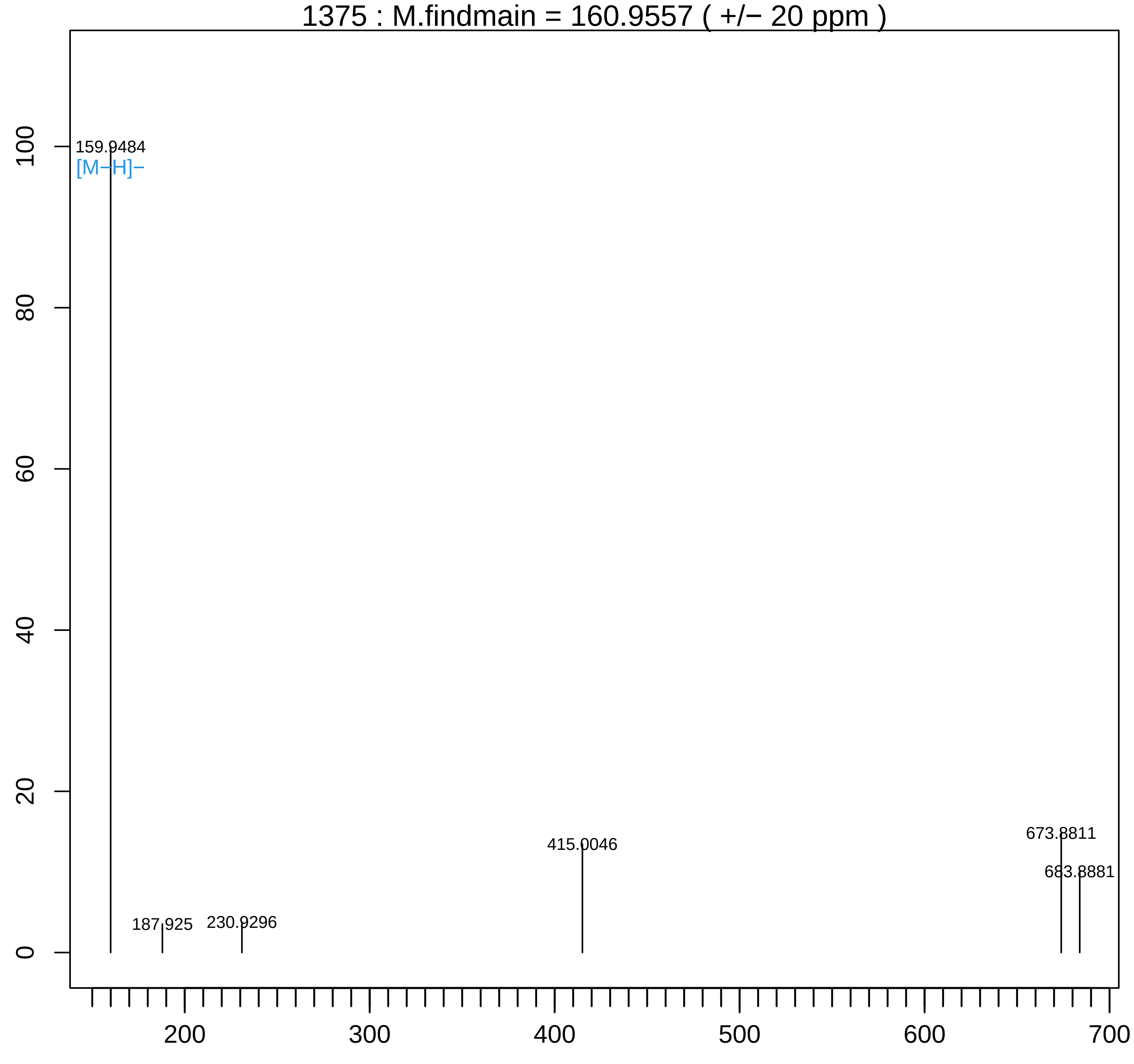
1375 : M.findmain = 160.9557 (+/− 20 ppm)
159.9484
[M−H]−
187.925
230.9296
415.0046
673.8811
683.8881
0
20
40
60
80
100
200
300
400
500
600
700

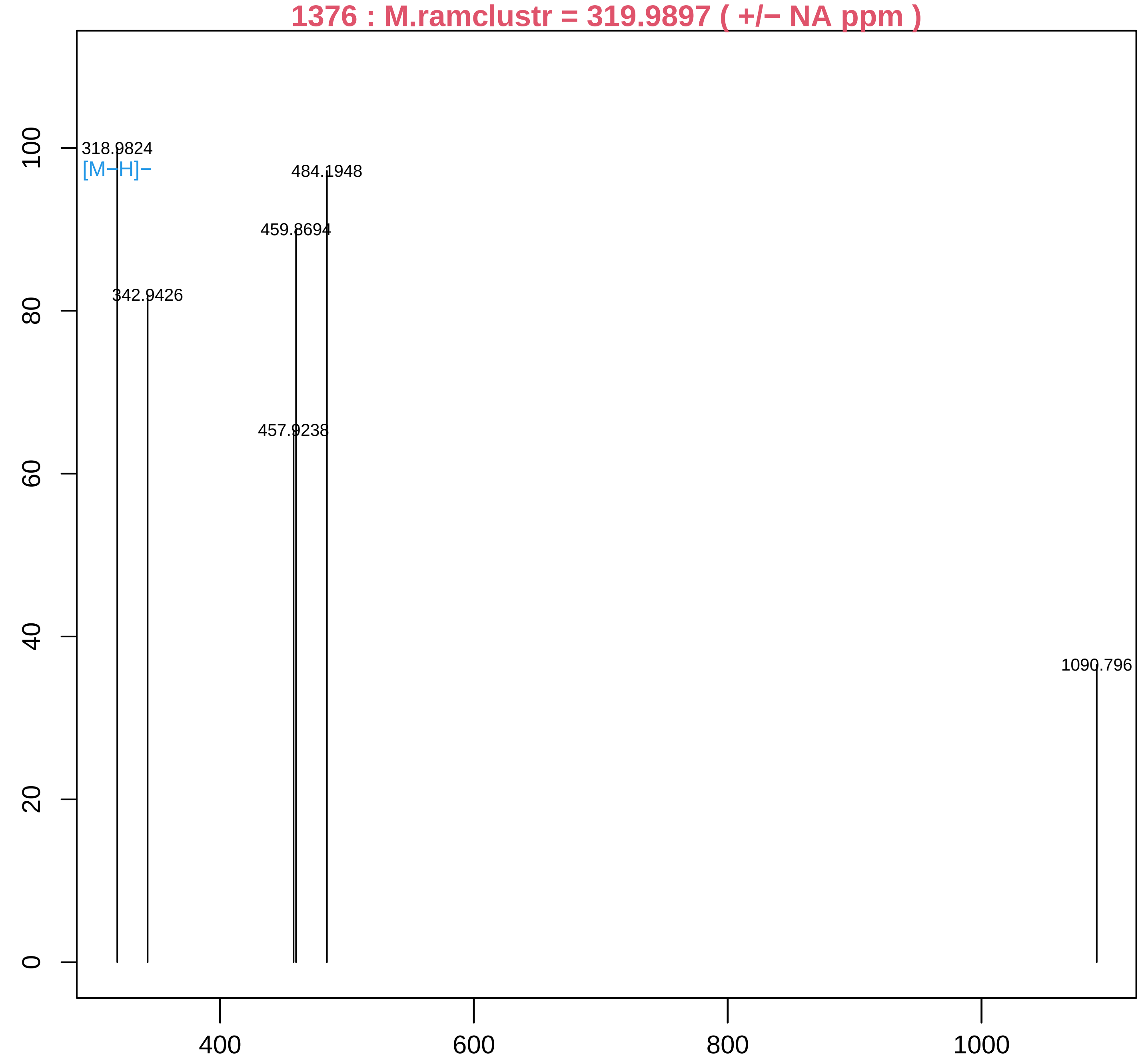
1376 : M.ramclustr = 319.9897 (+/− NA ppm)
318.9824
[M−H]−
484.1948
459.8694
342.9426
457.9238
1090.796
0
20
40
60
80
100
400
600
800
1000

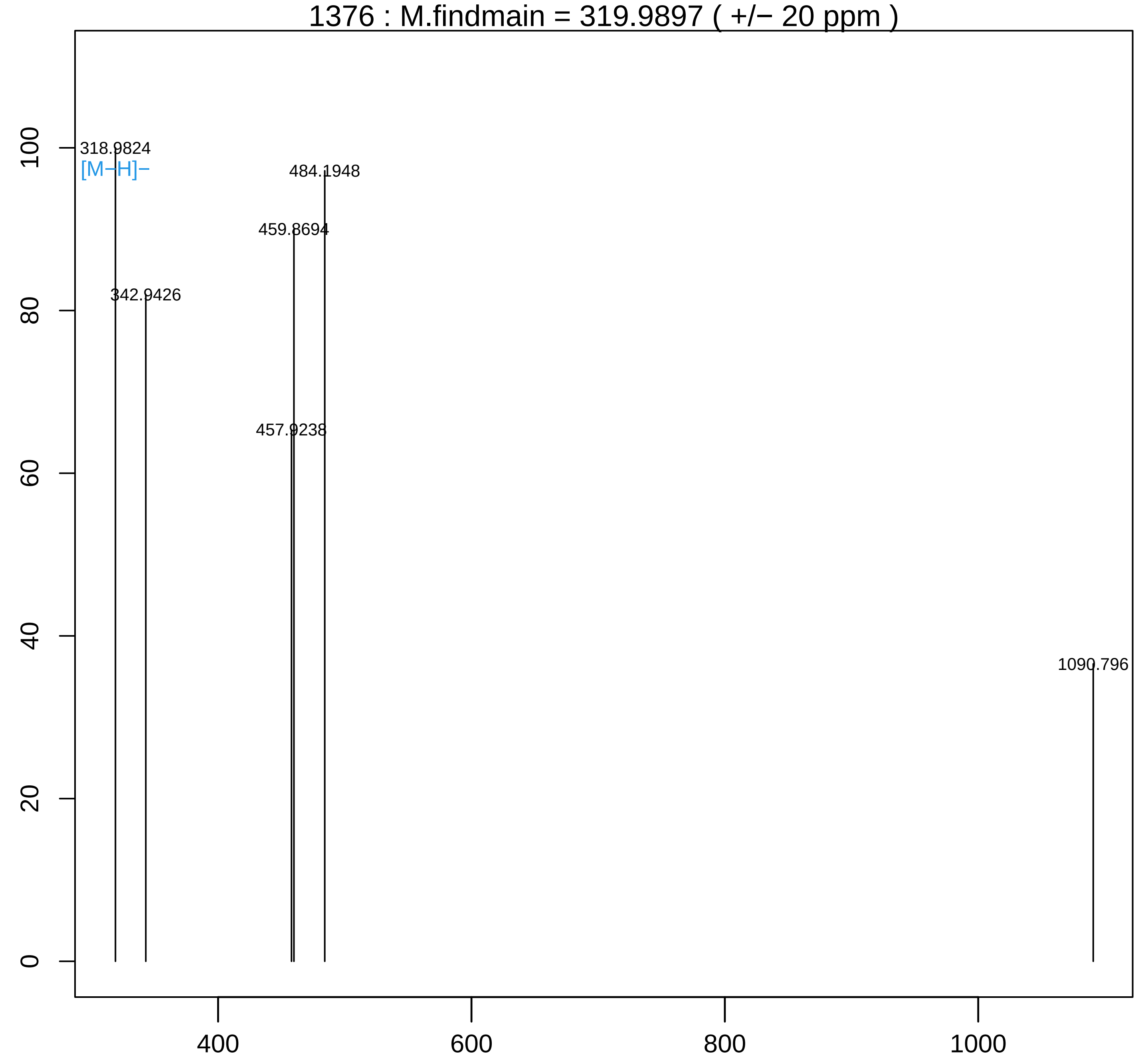
1376 : M.findmain = 319.9897 (+/− 20 ppm)
318.9824
[M−H]−
484.1948
459.8694
342.9426
457.9238
1090.796
0
20
40
60
80
100
400
600
800
1000

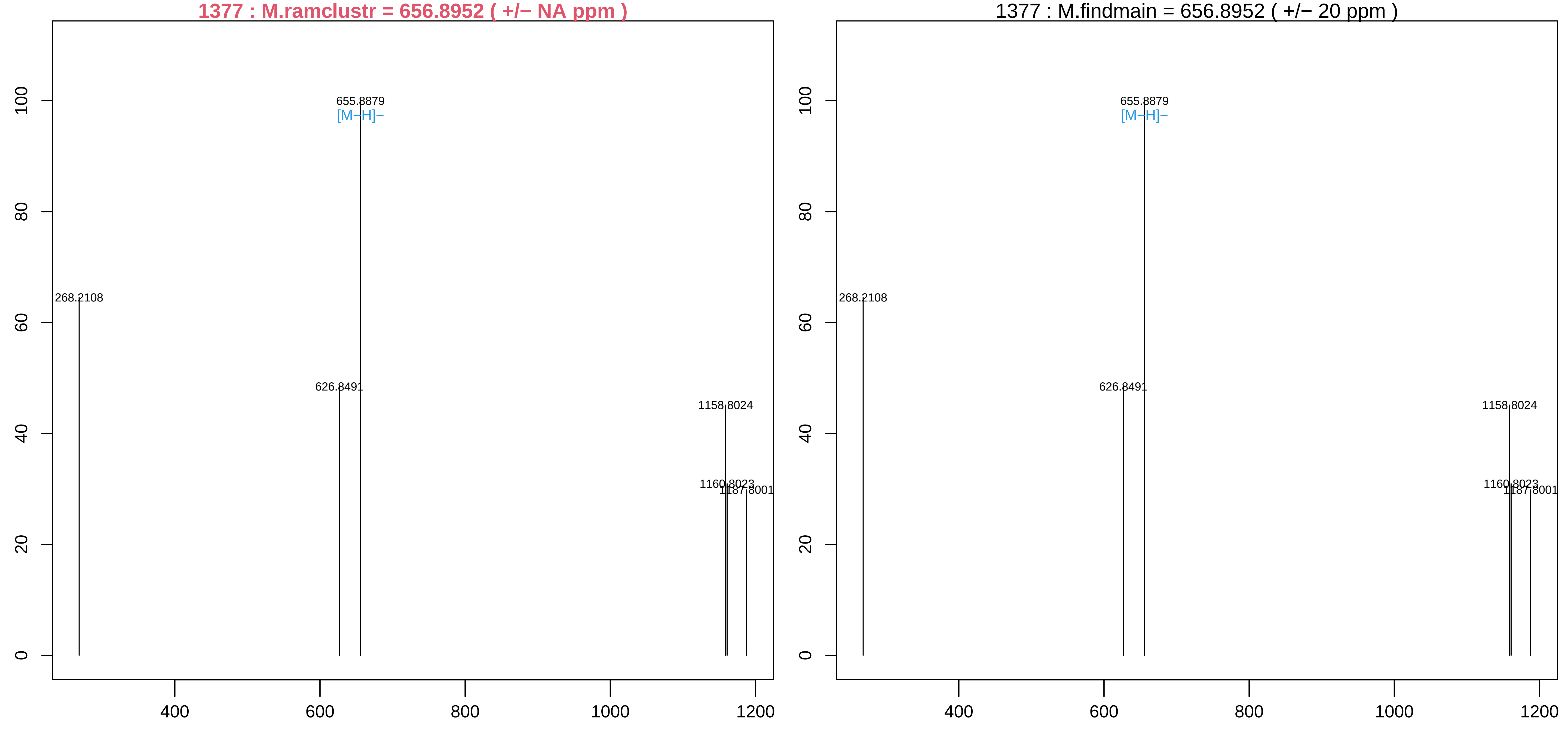

1377 : M.ramclustr = 656.8952 (+/− NA ppm)
268.2108
626.8491
655.8879
[M−H]−
1158.8024
1160.8023
1187.8001
0
20
40
60
80
100
400
600
800
1000
1200
1377 : M.findmain = 656.8952 (+/− 20 ppm)
268.2108
626.8491
655.8879
[M−H]−
1158.8024
1160.8023
1187.8001
0
20
40
60
80
100
400
600
800
1000
1200

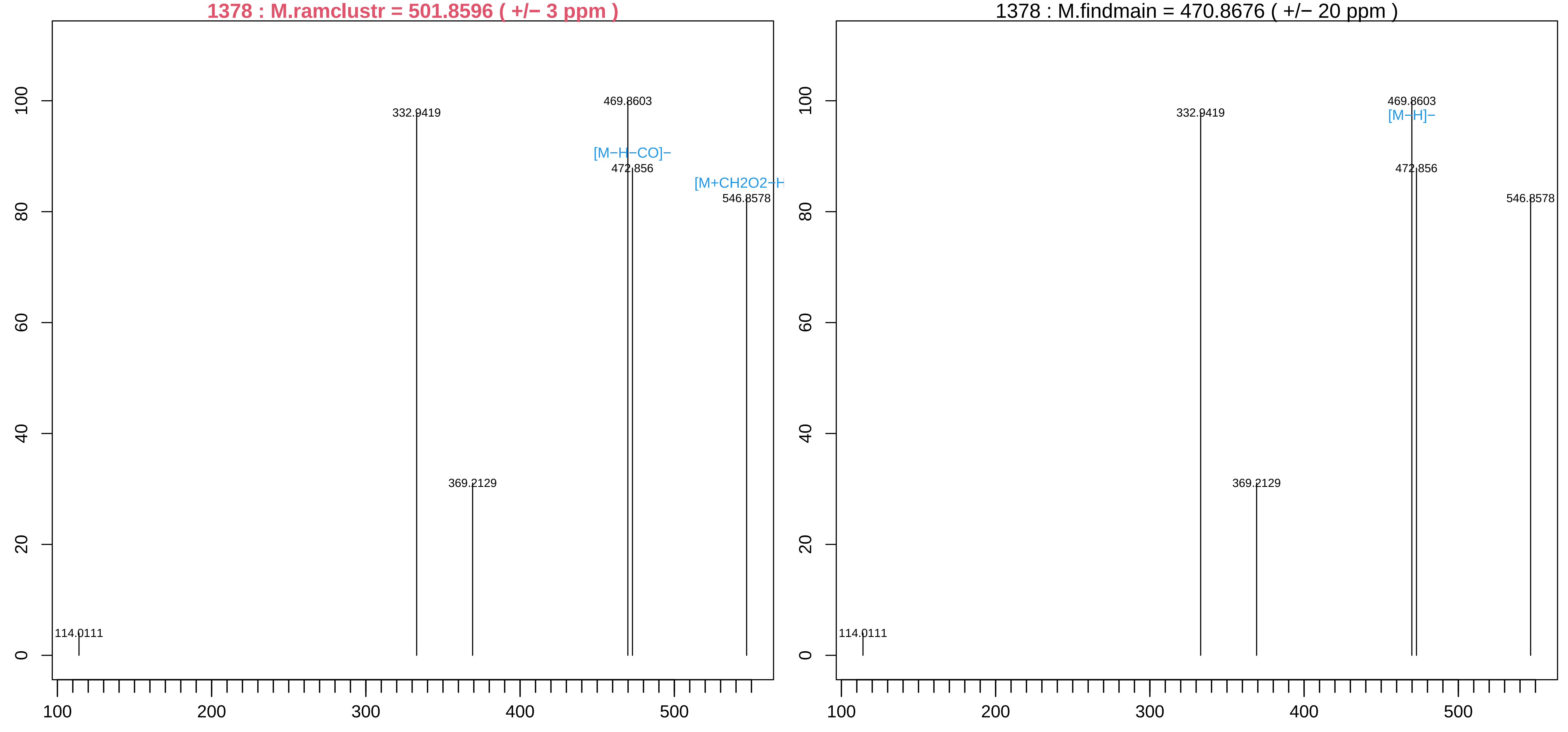
1378 : M.ramclustr = 501.8596 (+/− 3 ppm)
332.9419
369.2129
469.8603
[M−H−CO]−
472.856
[M+CH2O2−H
546.8578
114.0111
1378 : M.findmain = 470.8676 (+/− 20 ppm)
332.9419
369.2129
469.8603
[M−H]−
472.856
546.8578
114.0111

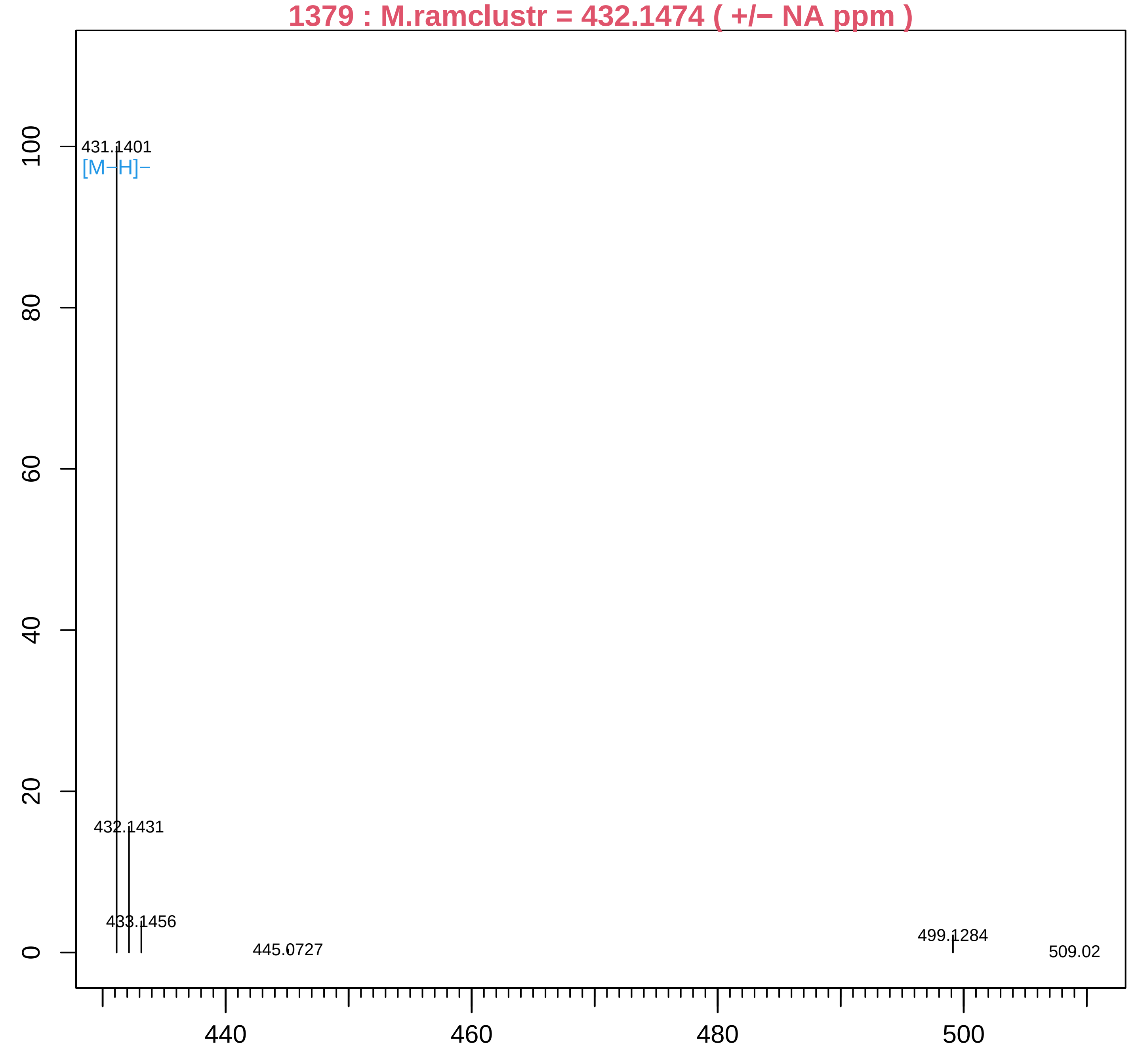
1379 : M.ramclustr = 432.1474 (+/− NA ppm)
431.1401
[M−H]−
432.1431
433.1456
445.0727
499.1284
509.02
0
20
40
60
80
100
440
460
480
500

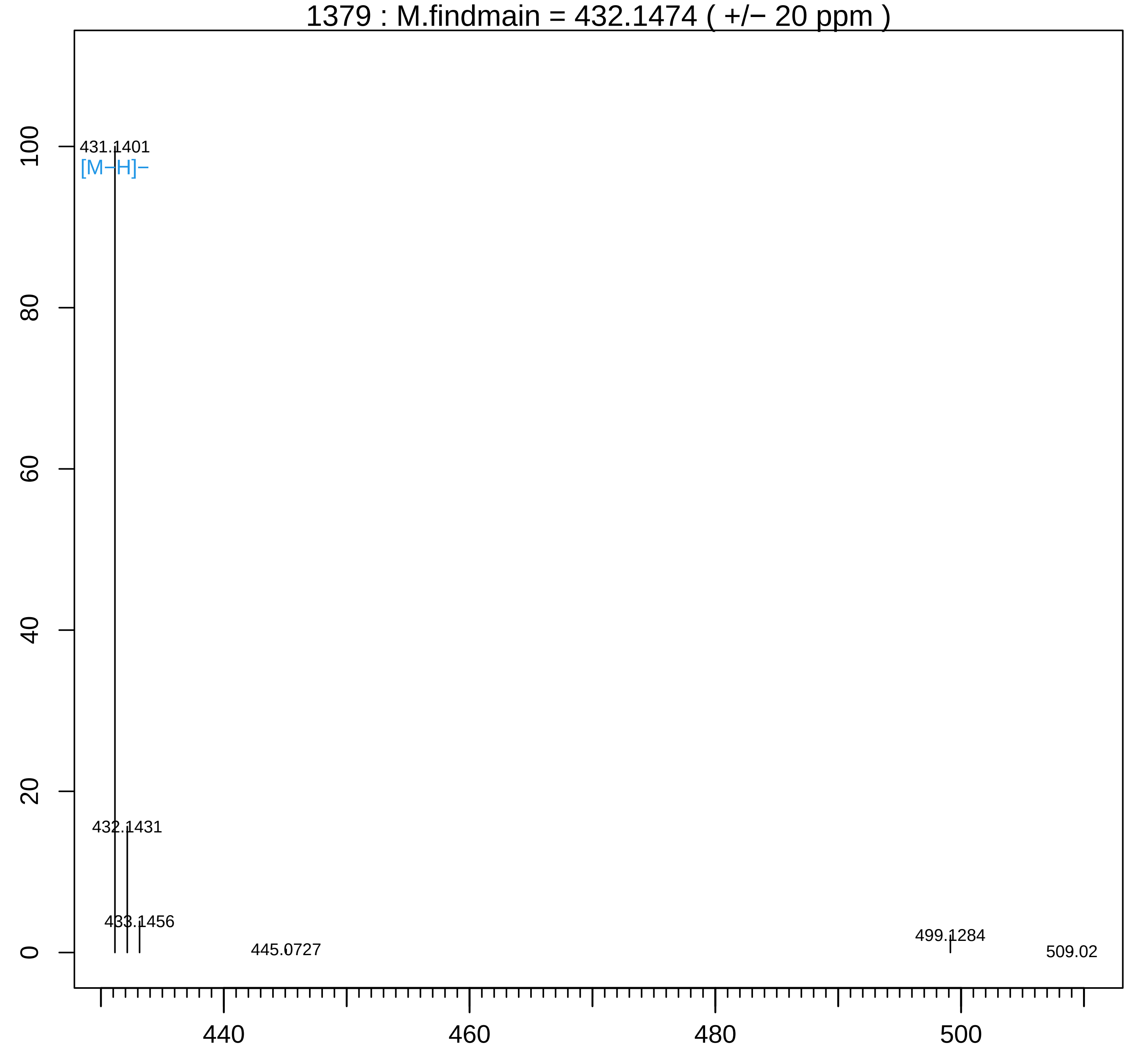
1379 : M.findmain = 432.1474 (+/− 20 ppm)
431.1401
[M−H]−
432.1431
433.1456
445.0727
499.1284
509.02
0
20
40
60
80
100
440
460
480
500

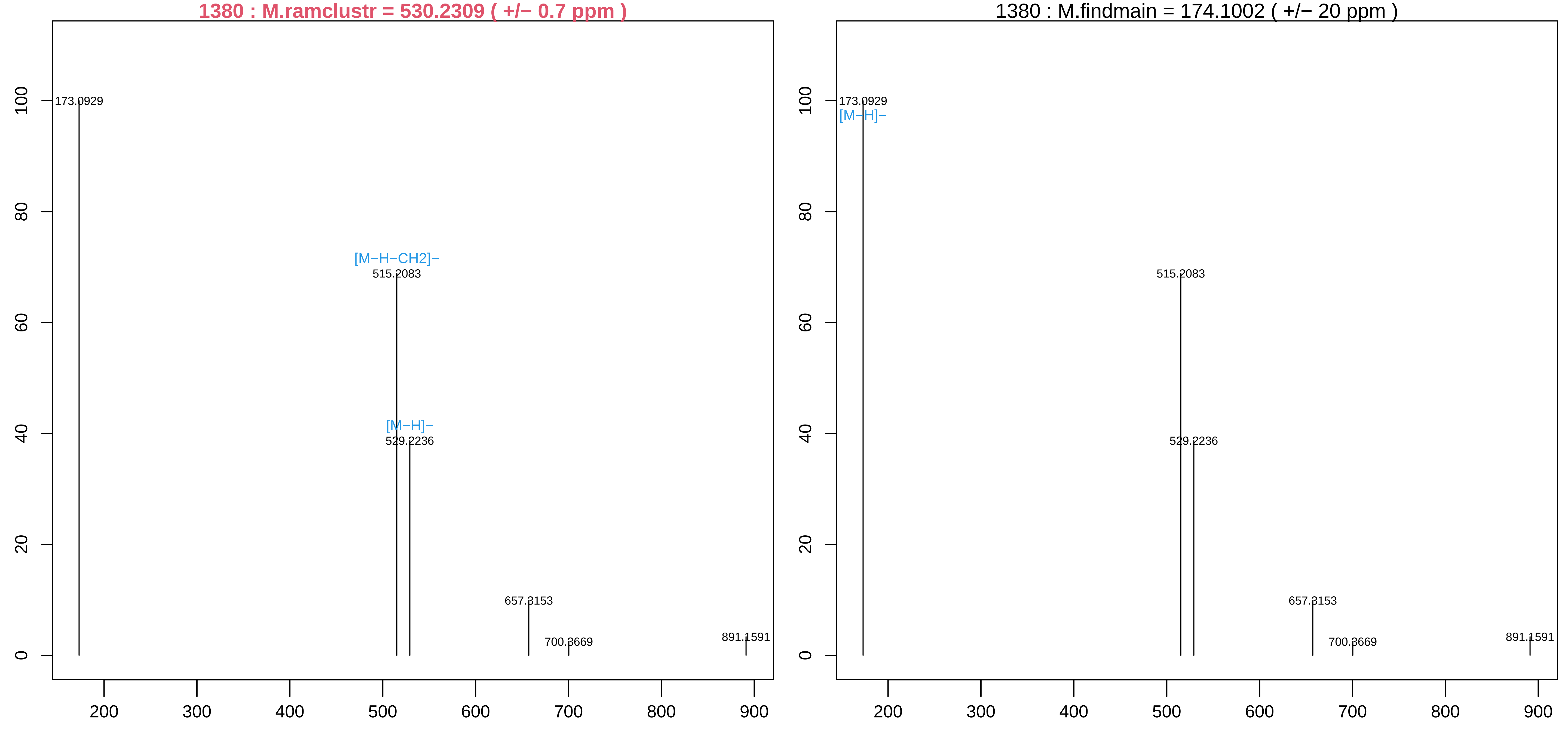
1380 : M.ramclustr = 530.2309 (+/− 0.7 ppm)
173.0929
[M−H−CH2]−
515.2083
[M−H]−
529.2236
657.3153
700.3669
891.1591
1380 : M.findmain = 174.1002 (+/− 20 ppm)
173.0929
[M−H]−
515.2083
529.2236
657.3153
700.3669
891.1591

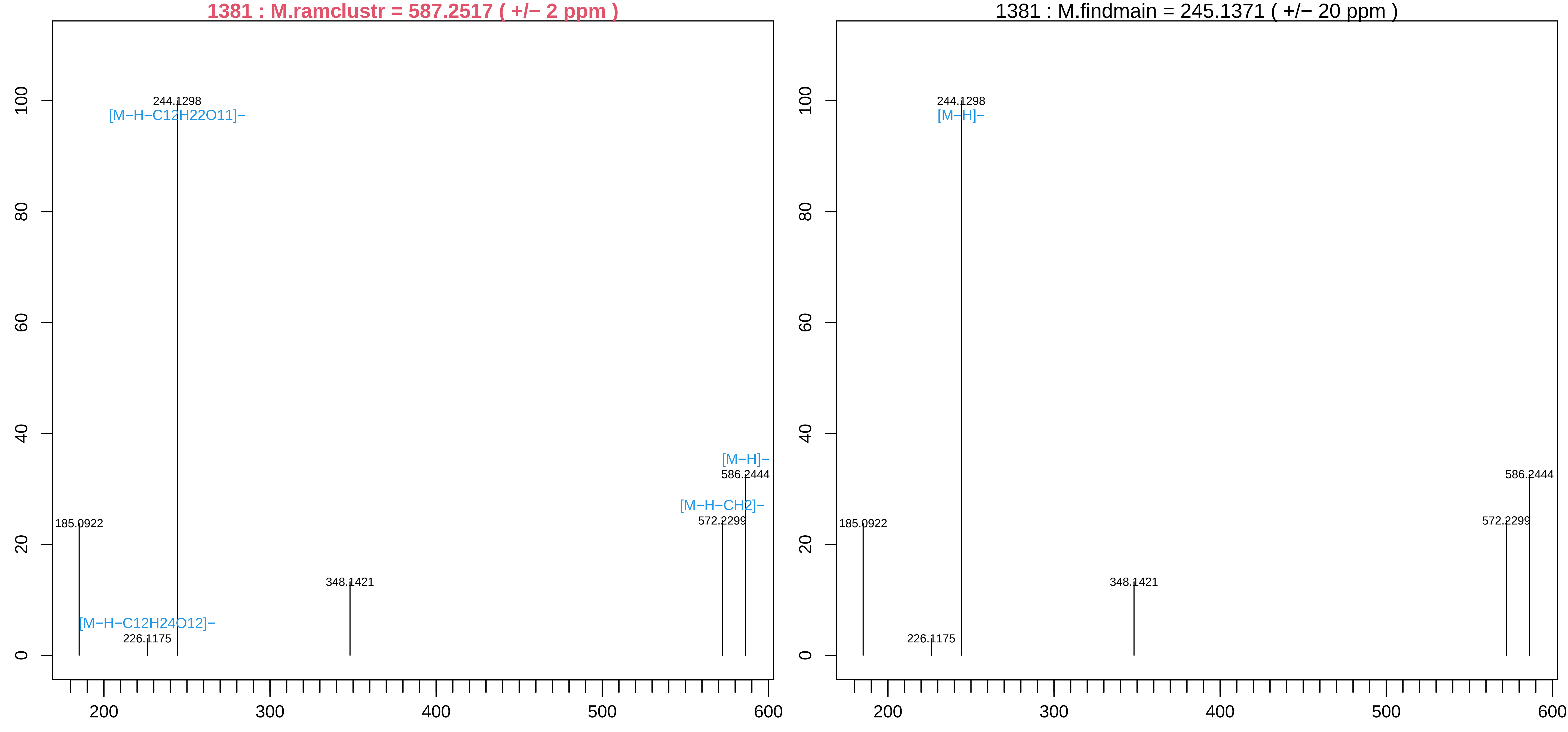
1381 : M.ramclustr = 587.2517 (+/− 2 ppm)
244.1298
[M−H−C12H22O11]−
185.0922
[M−H−C12H24O12]−
226.1175
348.1421
[M−H]−
586.2444
[M−H−CH2]−
572.2299
1381 : M.findmain = 245.1371 (+/− 20 ppm)
244.1298
[M−H]−
185.0922
226.1175
348.1421
572.2299
586.2444
0
20
40
60
80
100
200
300
400
500
600

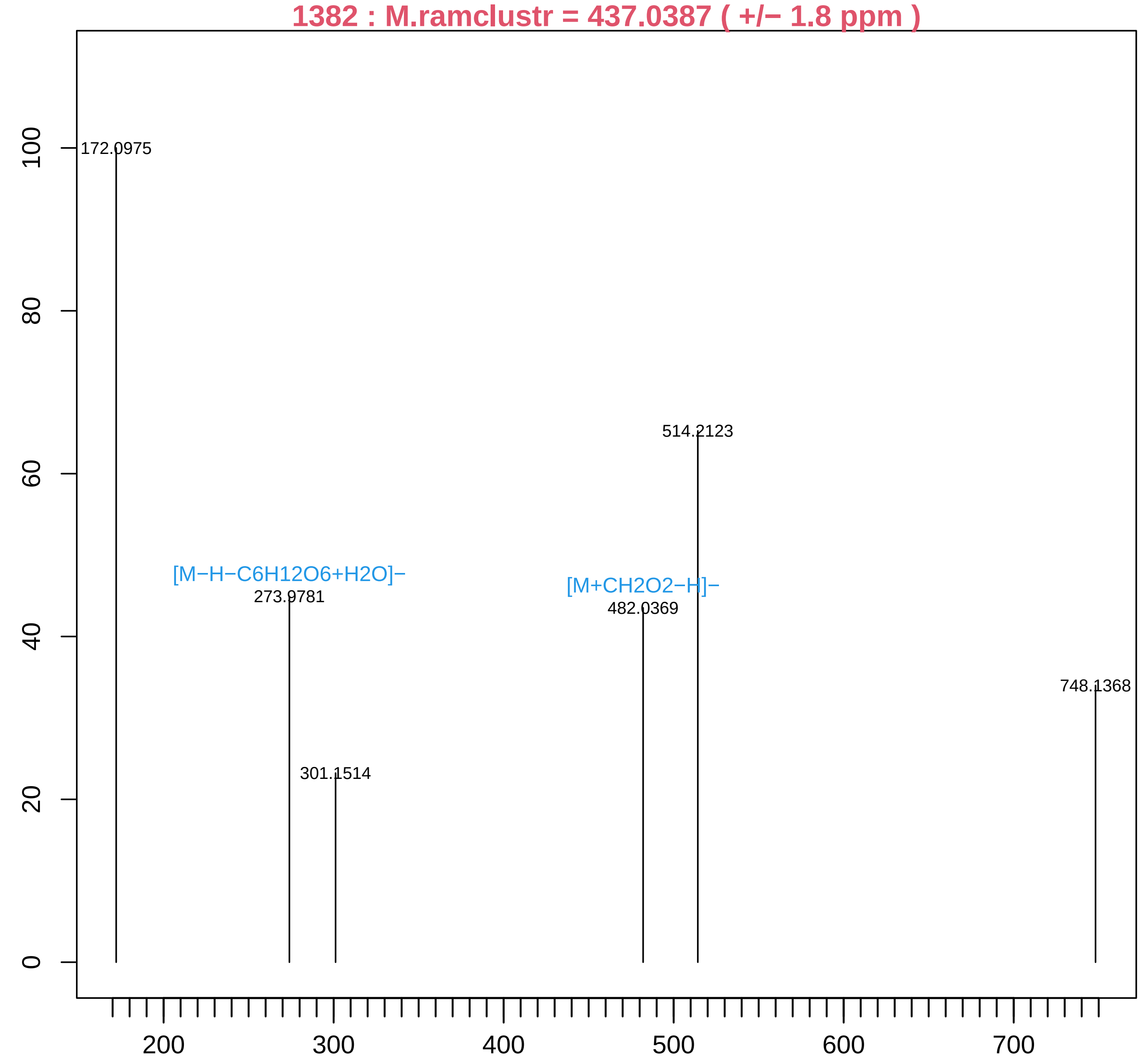
1382 : M.ramclustr = 437.0387 (+/− 1.8 ppm)
172.0975
[M−H−C6H12O6+H2O]−
273.9781
301.1514
[M+CH2O2−H]−
482.0369
514.2123
748.1368
0
20
40
60
80
100
200
300
400
500
600
700

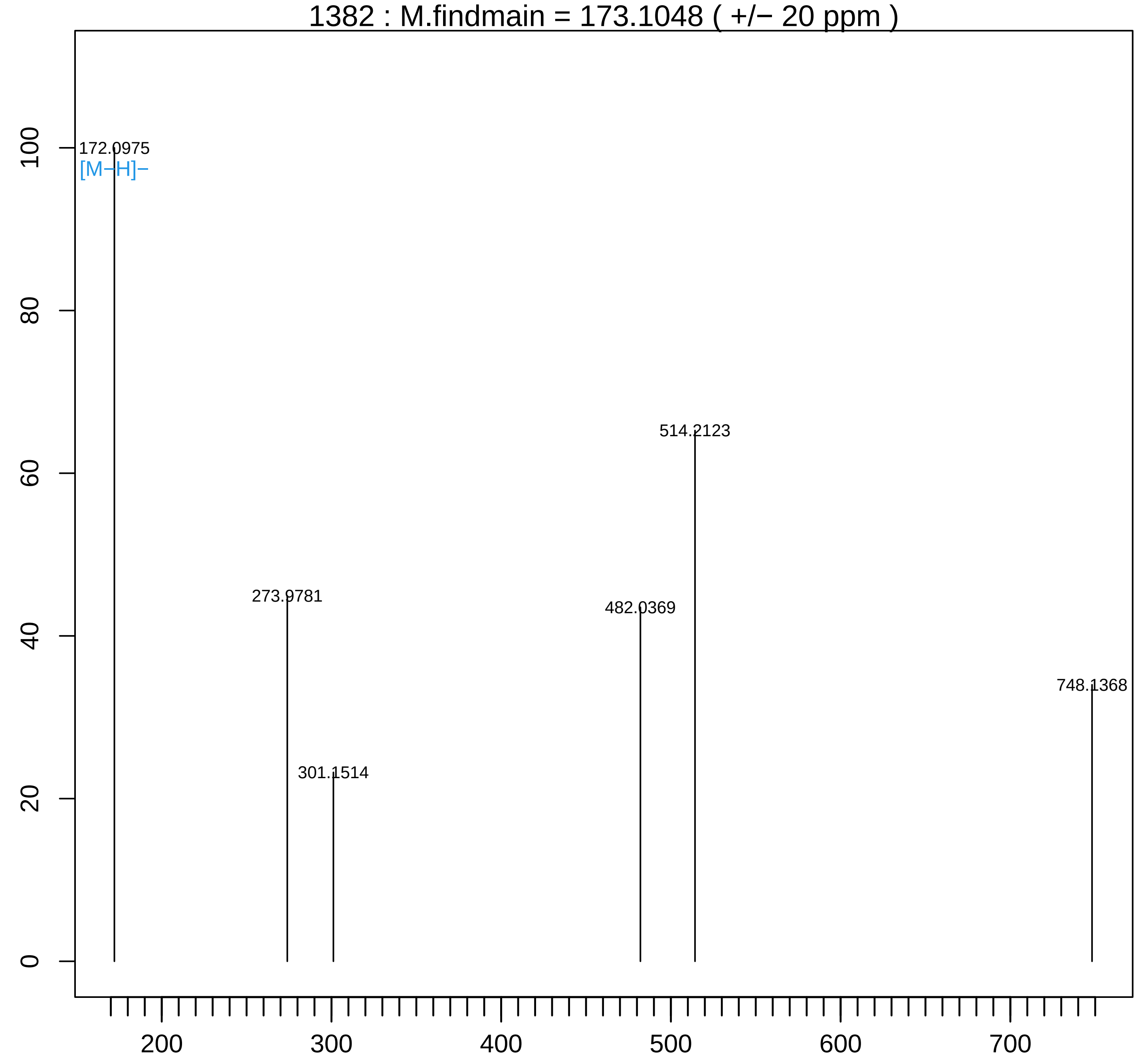
1382 : M.findmain = 173.1048 (+/− 20 ppm)
172.0975
[M−H]−
273.9781
301.1514
482.0369
514.2123
748.1368
0
20
40
60
80
100
200
300
400
500
600
700

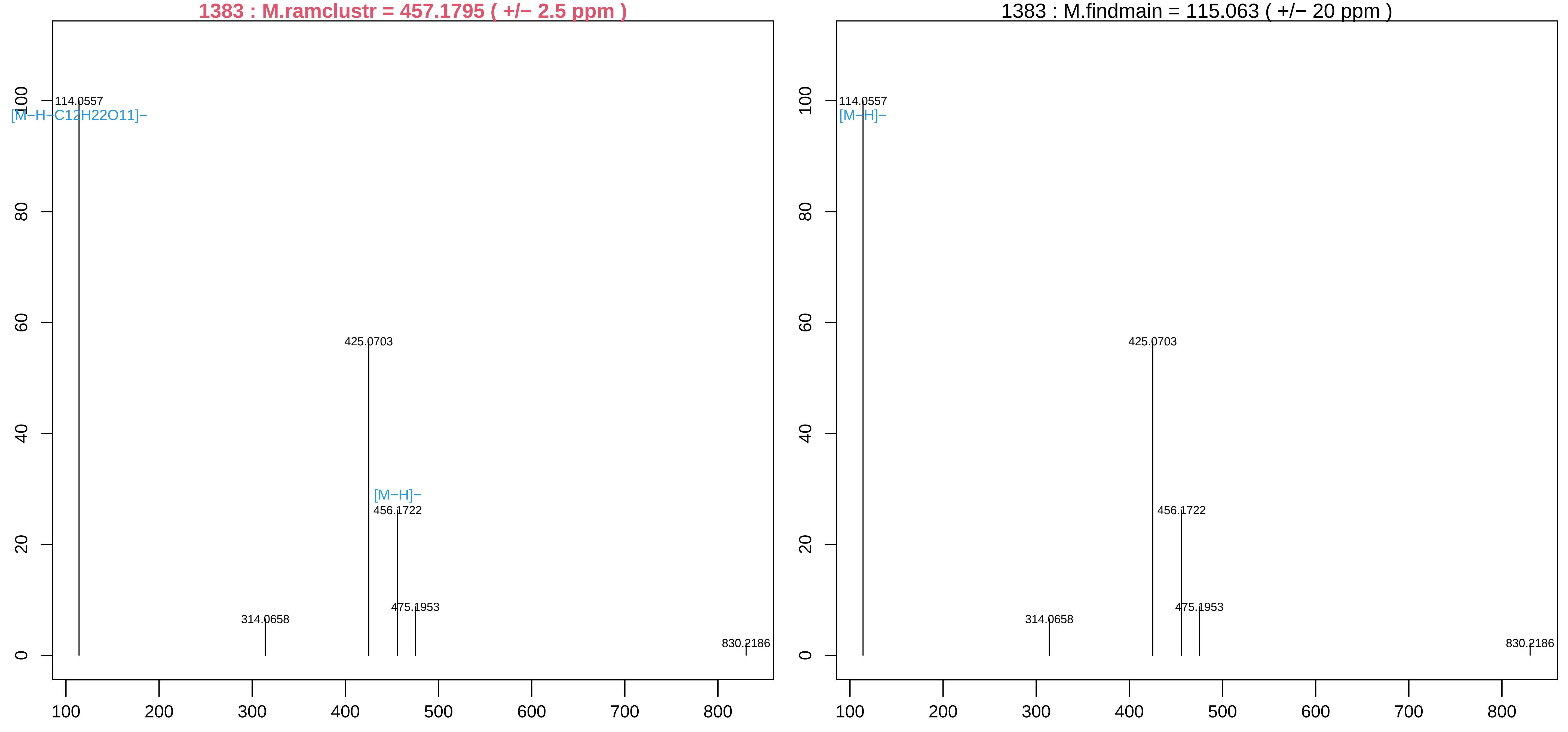

1383 : M.ramclustr = 457.1795 (+/− 2.5 ppm)
114.0557
[M−H−C12H22O11]−
425.0703
[M−H]−
456.1722
314.0658
475.1953
830.2186
1383 : M.findmain = 115.063 (+/− 20 ppm)
114.0557
[M−H]−
425.0703
456.1722
314.0658
475.1953
830.2186
0
20
40
60
80
100
200
300
400
500
600
700
800

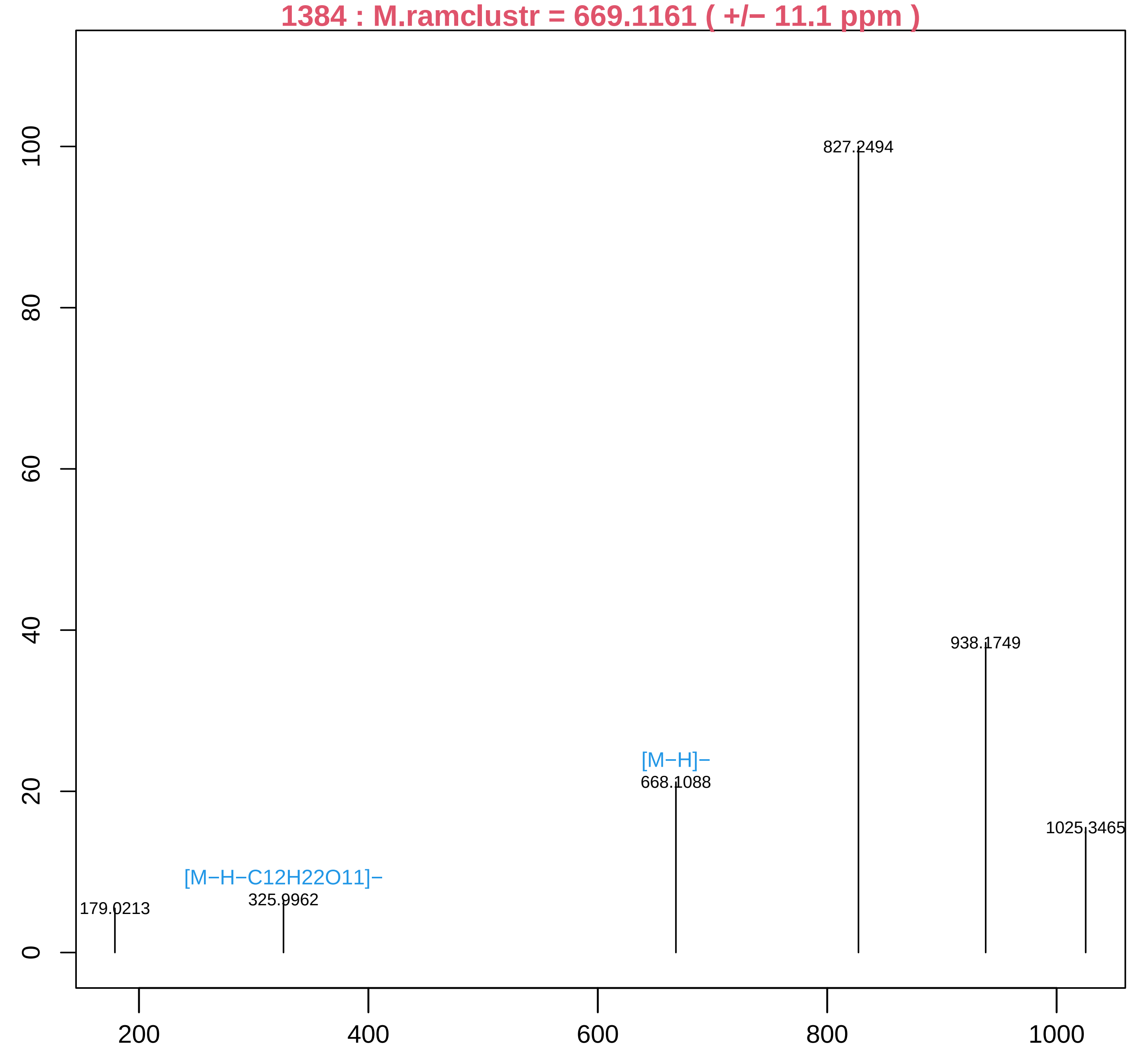
1384 : M.ramclustr = 669.1161 (+/− 11.1 ppm)
827.2494
938.1749
1025.3465
[M−H]−
668.1088
[M−H−C12H22O11]−
325.9962
179.0213
0
20
40
60
80
100
200
400
600
800
1000

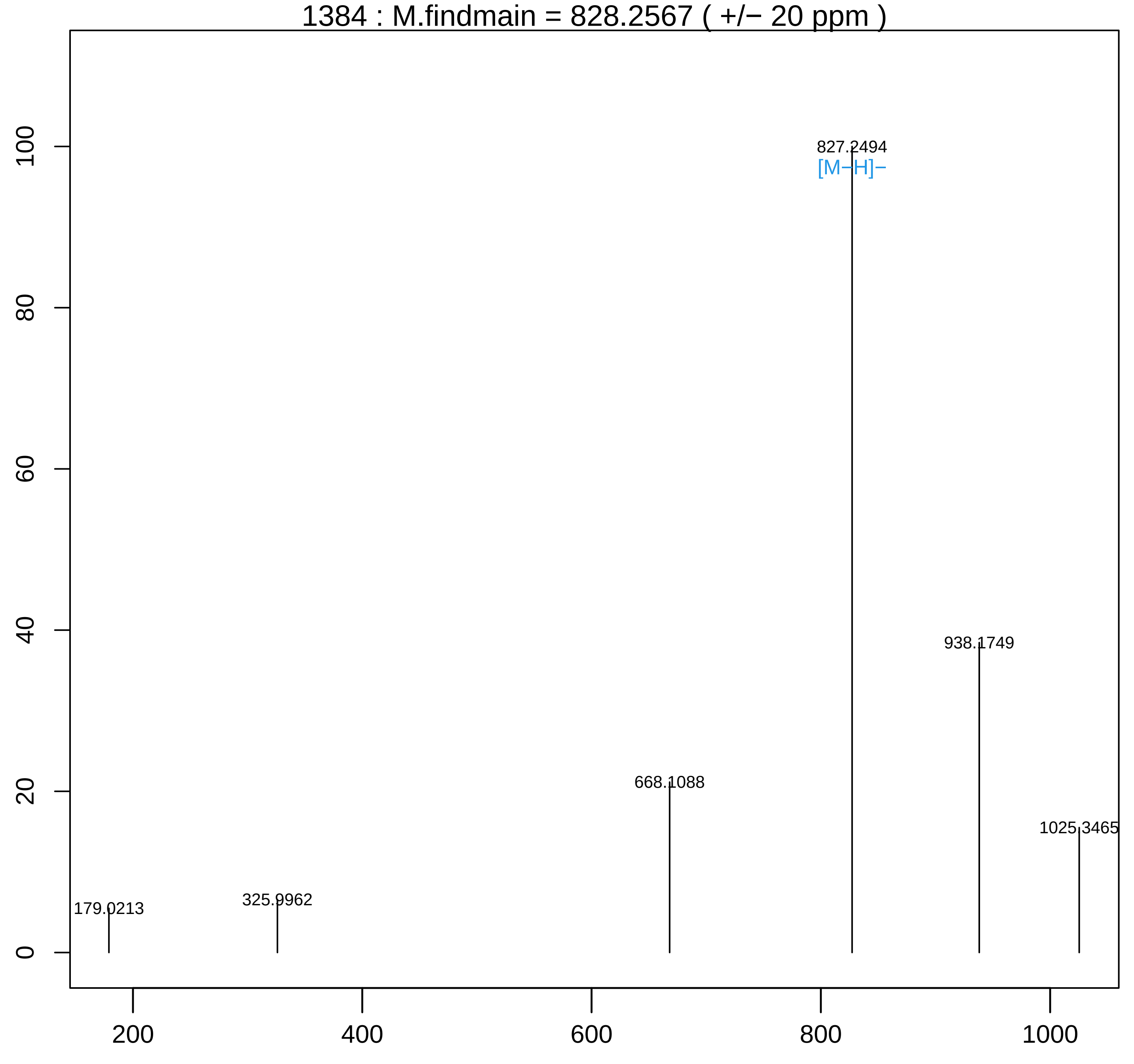
1384 : M.findmain = 828.2567 (+/− 20 ppm)
827.2494
[M−H]−
938.1749
1025.3465
668.1088
325.9962
179.0213
0
20
40
60
80
100
200
400
600
800
1000

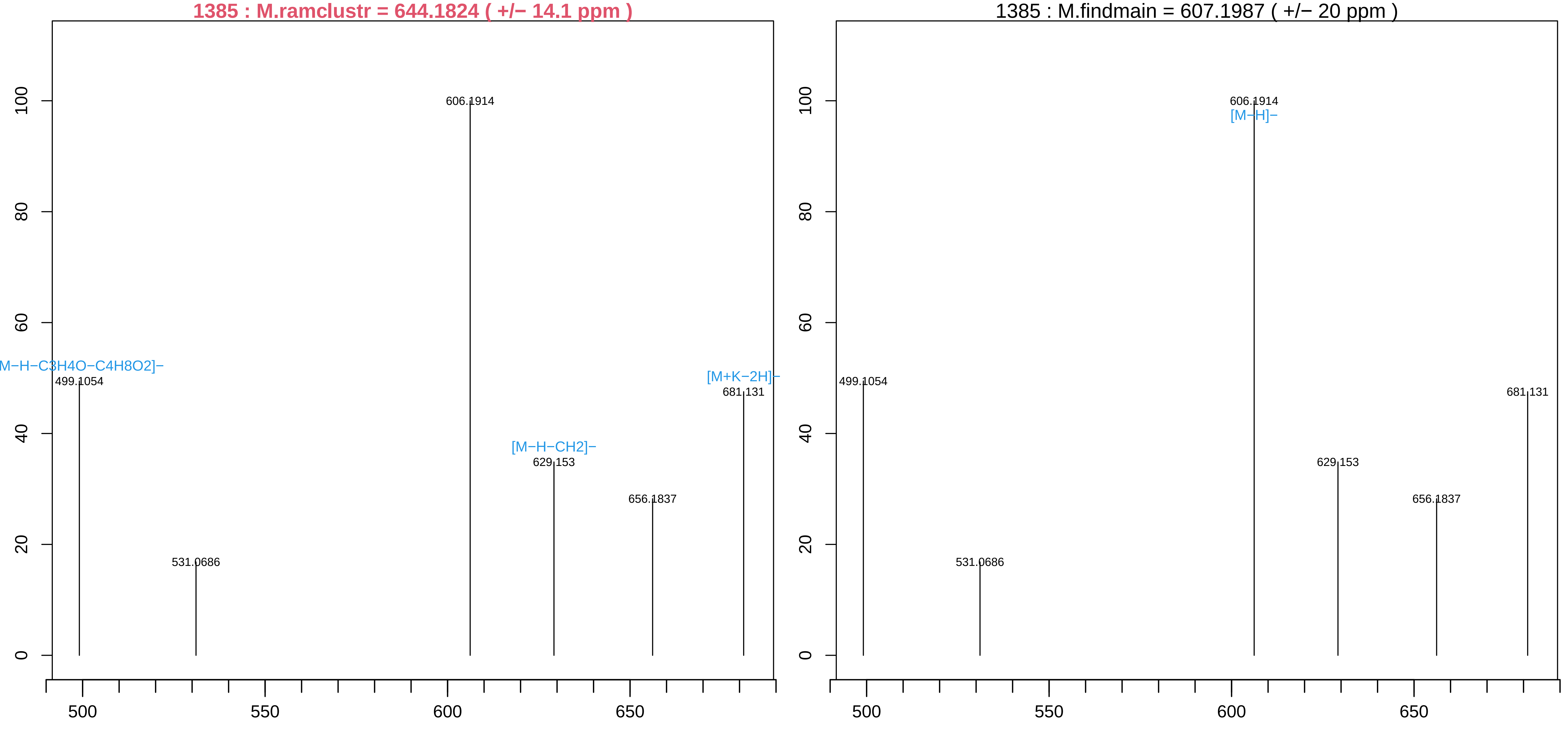

1385 : M.ramclustr = 644.1824 (+/− 14.1 ppm)
1385 : M.findmain = 607.1987 (+/− 20 ppm)
606.1914
[M−H]−
[M−H−C3H4O−C4H8O2]−
499.1054
[M+K−2H]−
681.131
[M−H−CH2]−
629.153
656.1837
531.0686
0
20
40
60
80
100
500
550
600
650

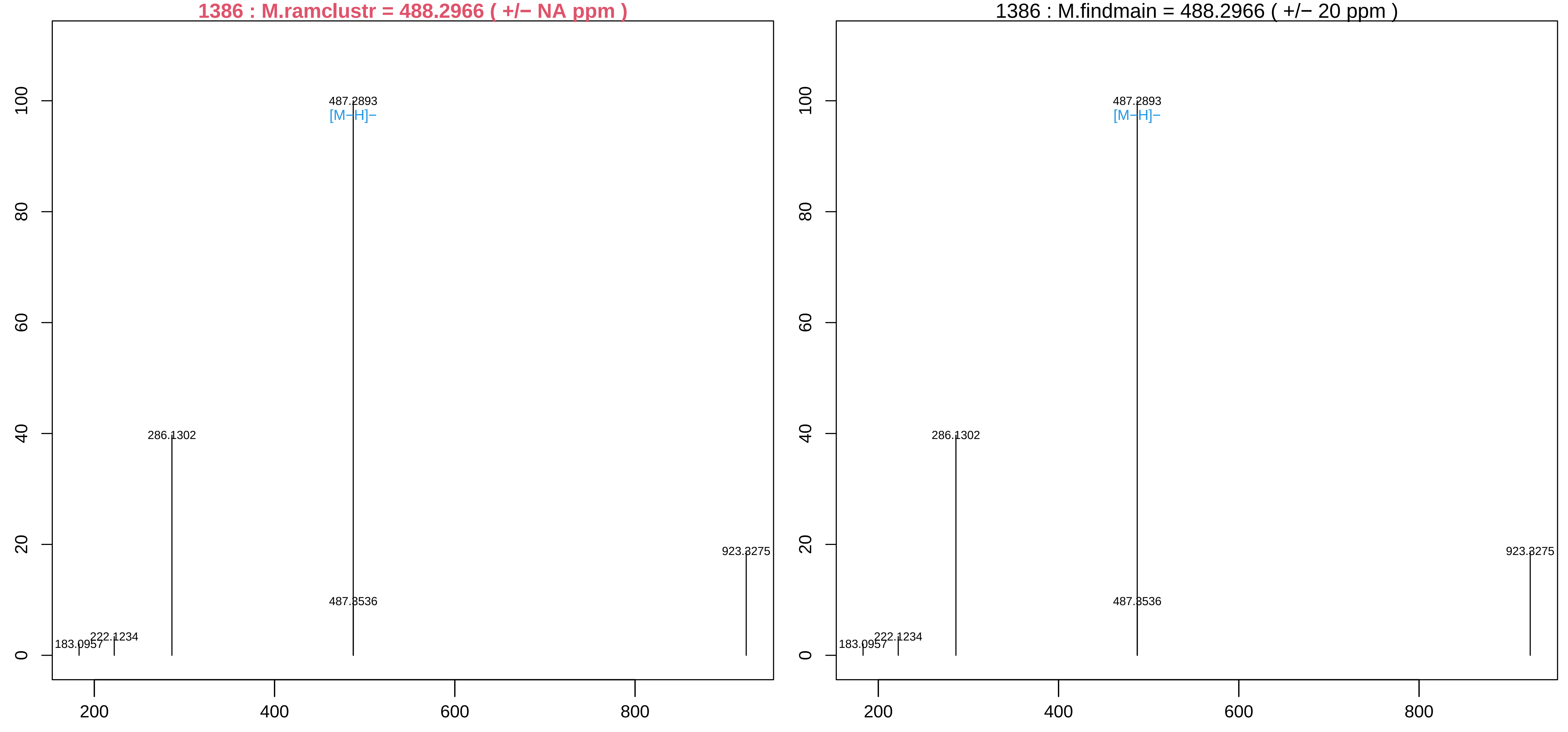
1386 : M.ramclustr = 488.2966 (+/− NA ppm)
1386 : M.findmain = 488.2966 (+/− 20 ppm)
0
20
40
60
80
100
200
400
600
800
183.0957
222.1234
286.1302
487.2893
[M−H]−
487.3536
923.3275

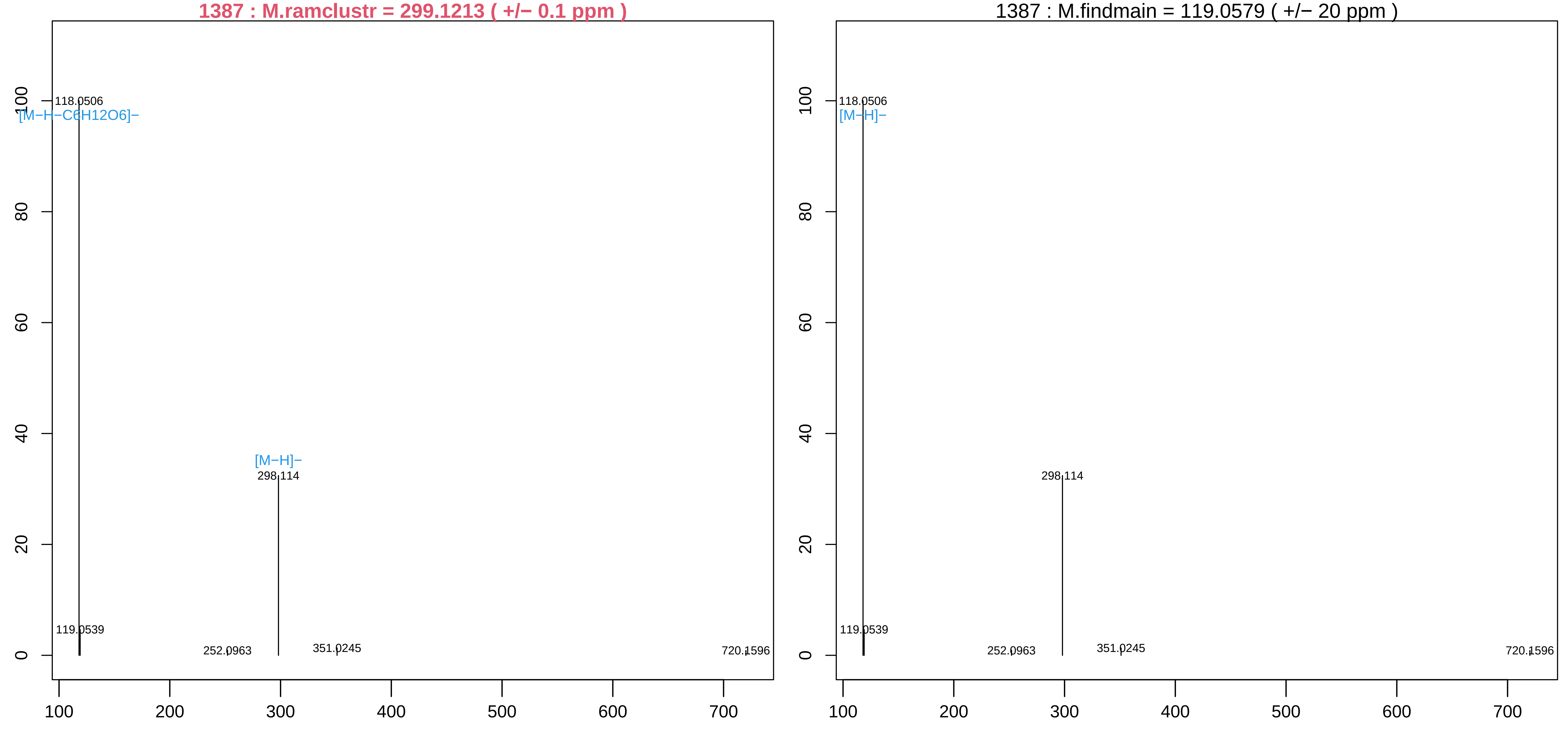
1387 : M.ramclustr = 299.1213 (+/− 0.1 ppm)
118.0506
[M−H−C6H12O6]−
[M−H]−
298.114
119.0539
252.0963
351.0245
720.1596
0
20
40
60
80
100
100
200
300
400
500
600
700
1387 : M.findmain = 119.0579 (+/− 20 ppm)
118.0506
[M−H]−
298.114
119.0539
252.0963
351.0245
720.1596
0
20
40
60
80
100
100
200
300
400
500
600
700

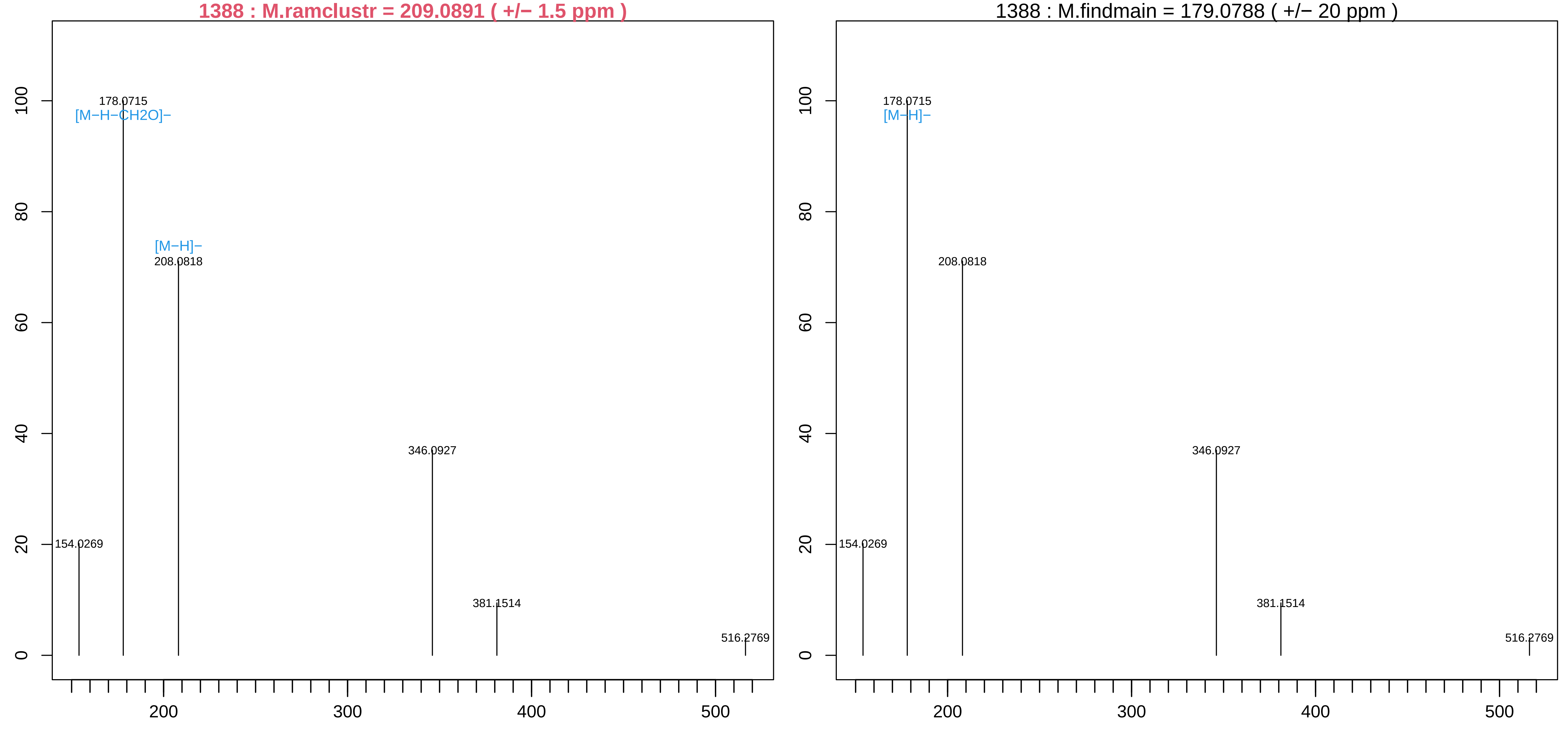
1388 : M.ramclustr = 209.0891 (+/− 1.5 ppm)
178.0715
[M−H−CH2O]−
[M−H]−
208.0818
154.0269
346.0927
381.1514
516.2769
1388 : M.findmain = 179.0788 (+/− 20 ppm)
178.0715
[M−H]−
208.0818
154.0269
346.0927
381.1514
516.2769

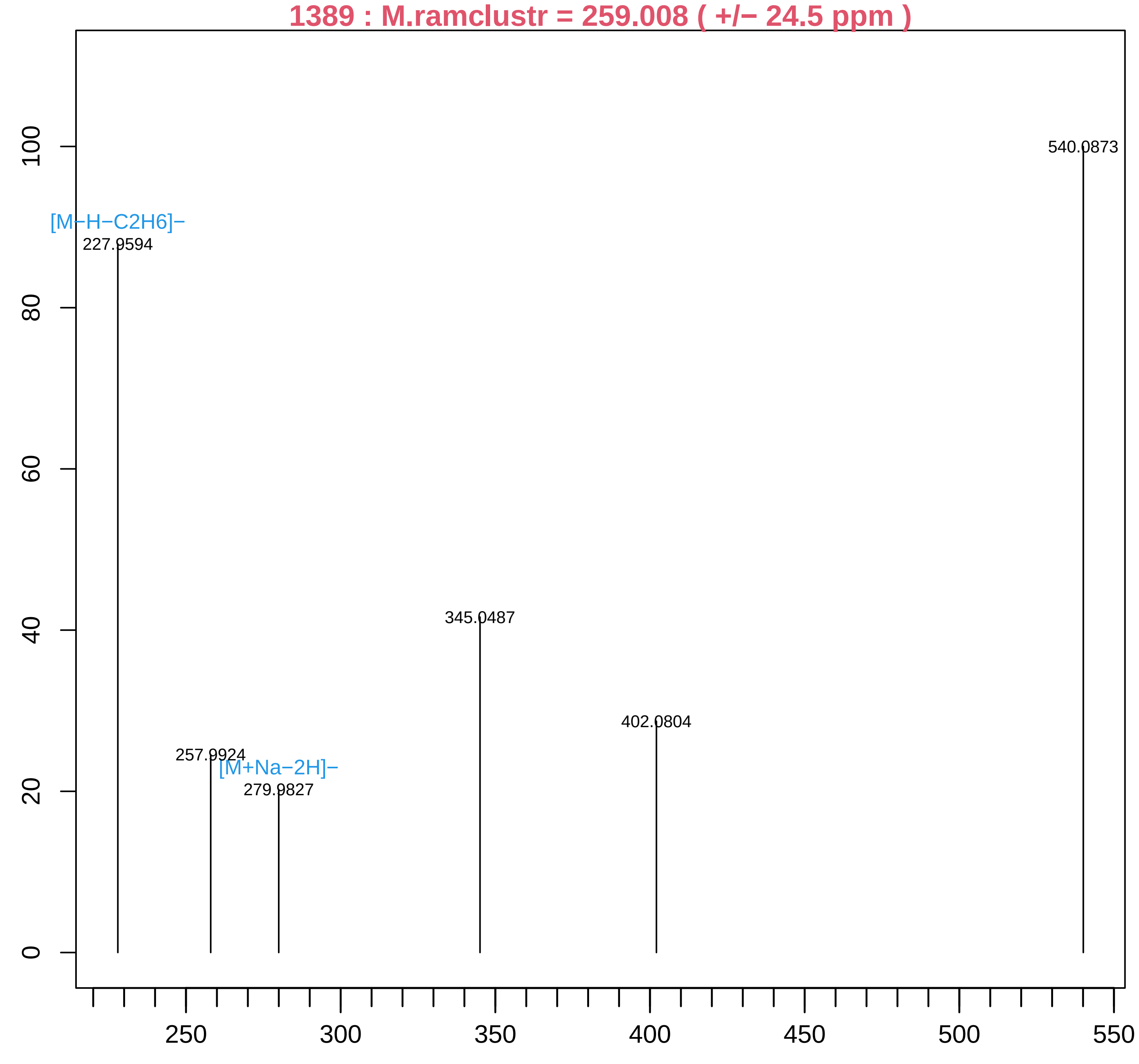
1389 : M.ramclustr = 259.008 (+/− 24.5 ppm)
[M−H−C2H6]−
227.9594
257.9924
[M+Na−2H]−
279.9827
345.0487
402.0804
540.0873
0
20
40
60
80
100
250
300
350
400
450
500
550

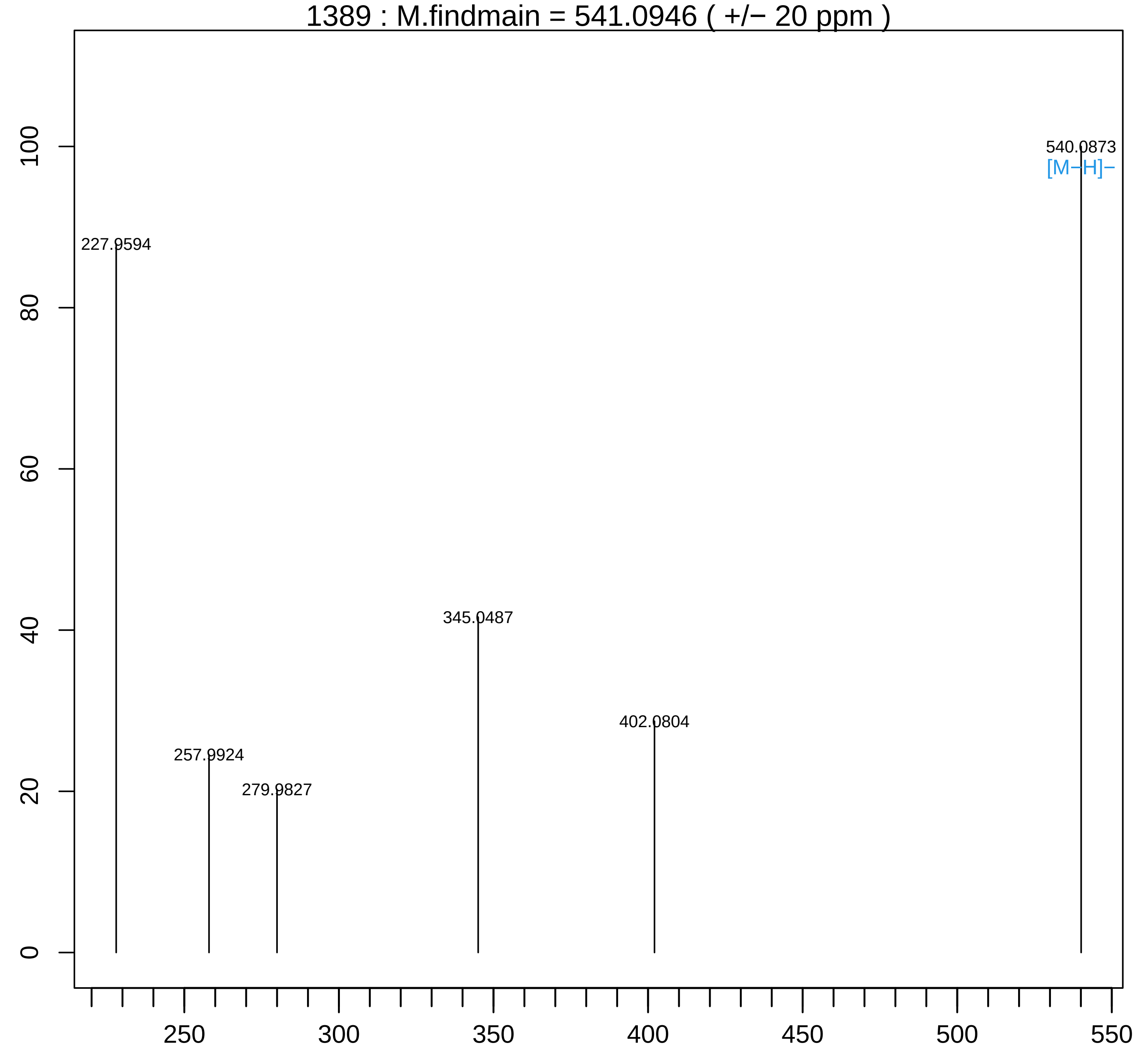
1389 : M.findmain = 541.0946 (+/− 20 ppm)
227.9594
257.9924
279.9827
345.0487
402.0804
540.0873
[M−H]−
0
20
40
60
80
100
250
300
350
400
450
500
550

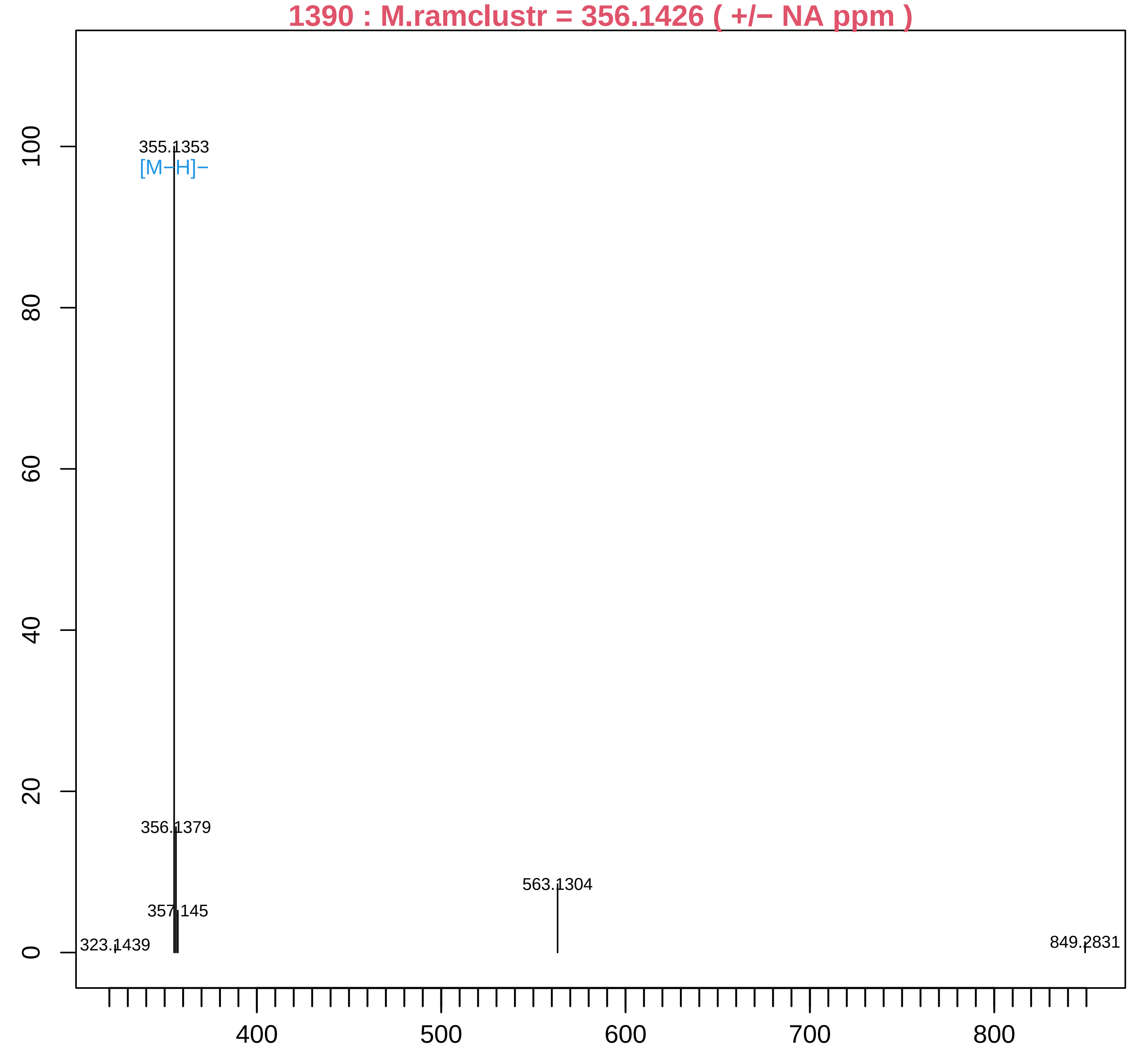
1390 : M.ramclustr = 356.1426 (+/− NA ppm)
355.1353
[M−H]−
356.1379
357.145
323.1439
563.1304
849.2831
0
20
40
60
80
100
400
500
600
700
800

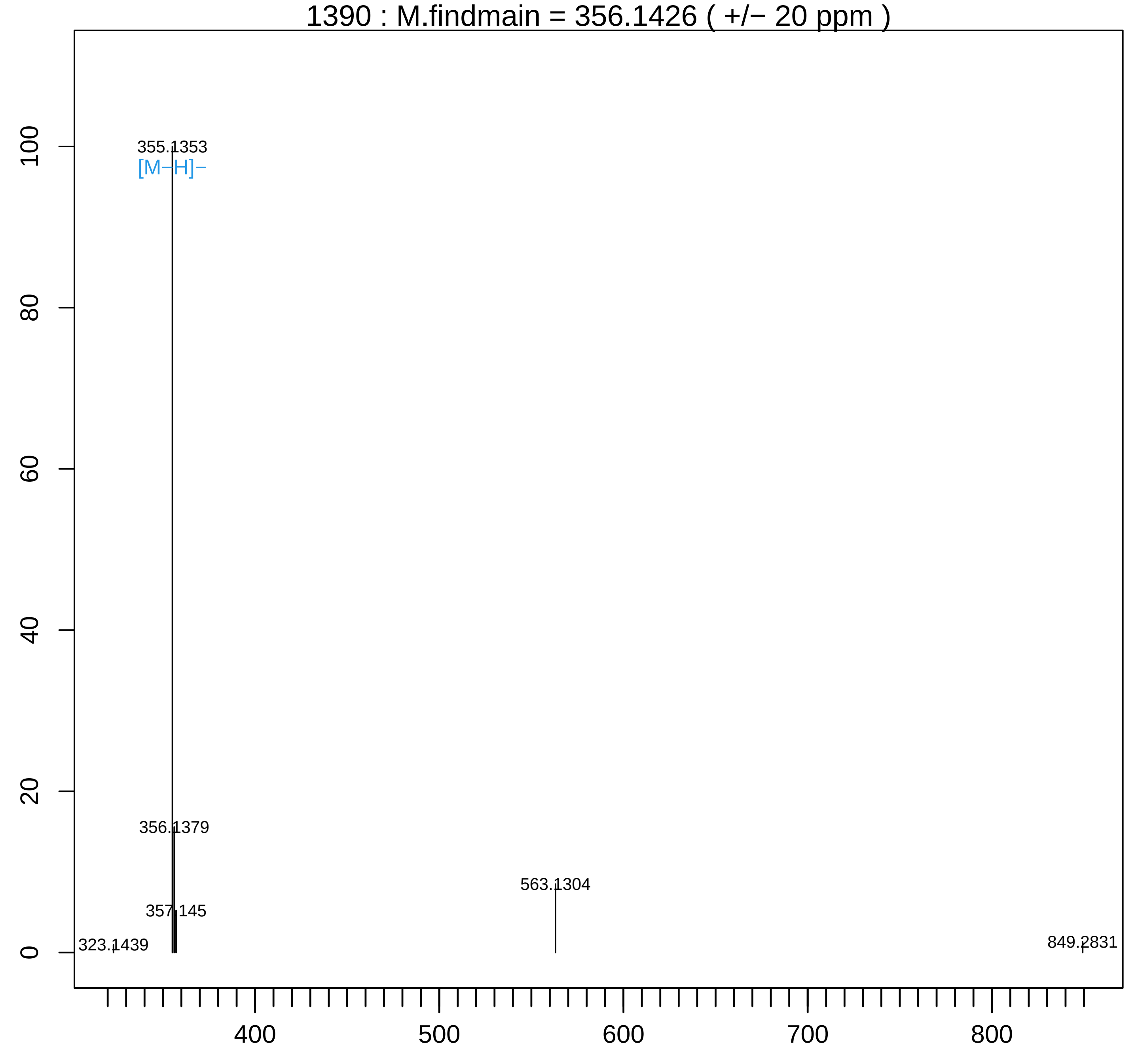
1390 : M.findmain = 356.1426 (+/− 20 ppm)
355.1353
[M−H]−
356.1379
357.145
323.1439
563.1304
849.2831
0
20
40
60
80
100
400
500
600
700
800

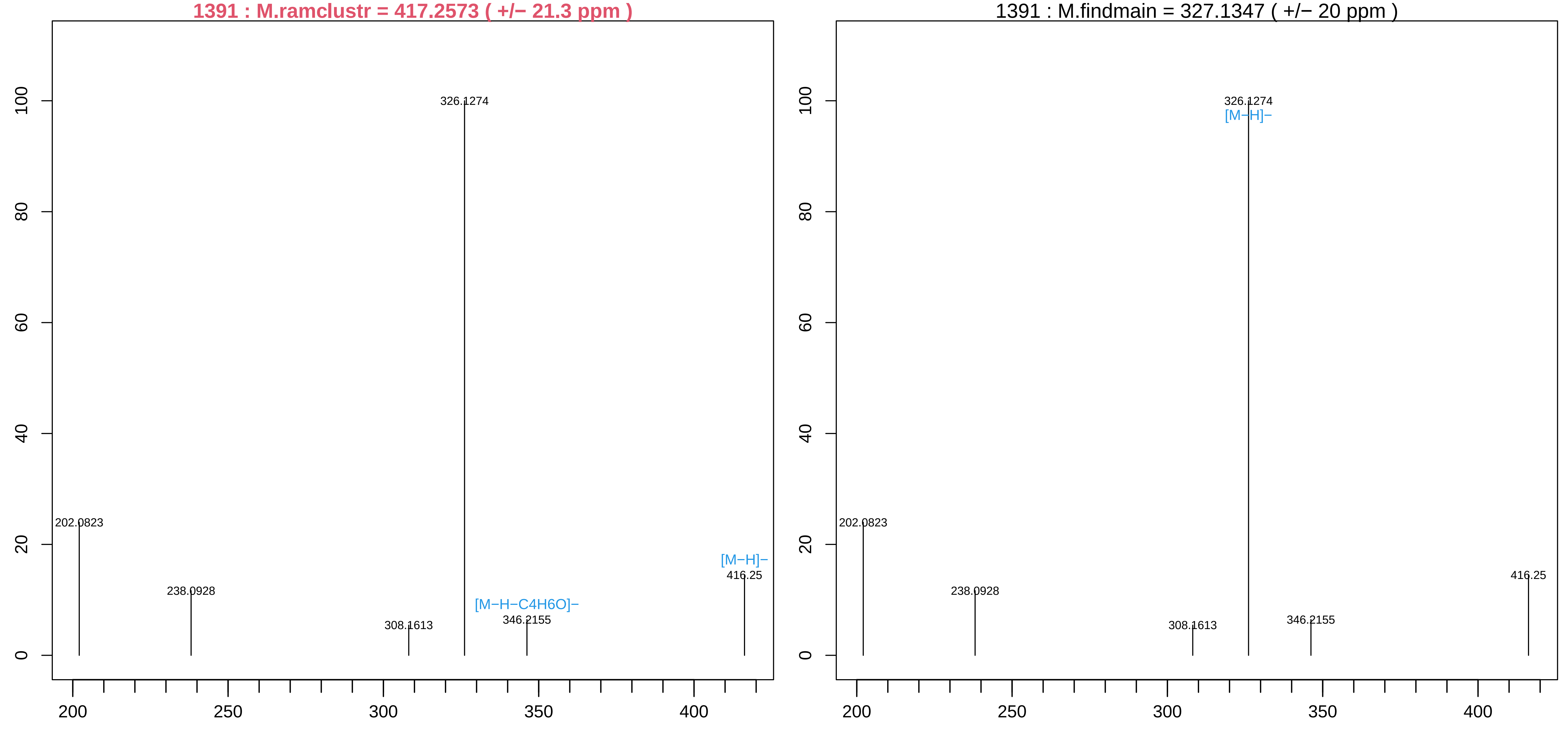

1391 : M.ramclustr = 417.2573 (+/− 21.3 ppm)
326.1274
202.0823
238.0928
308.1613
[M−H−C4H6O]−
346.2155
[M−H]−
416.25
1391 : M.findmain = 327.1347 (+/− 20 ppm)
326.1274
[M−H]−
202.0823
238.0928
308.1613
346.2155
416.25
0
20
40
60
80
100
200
250
300
350
400

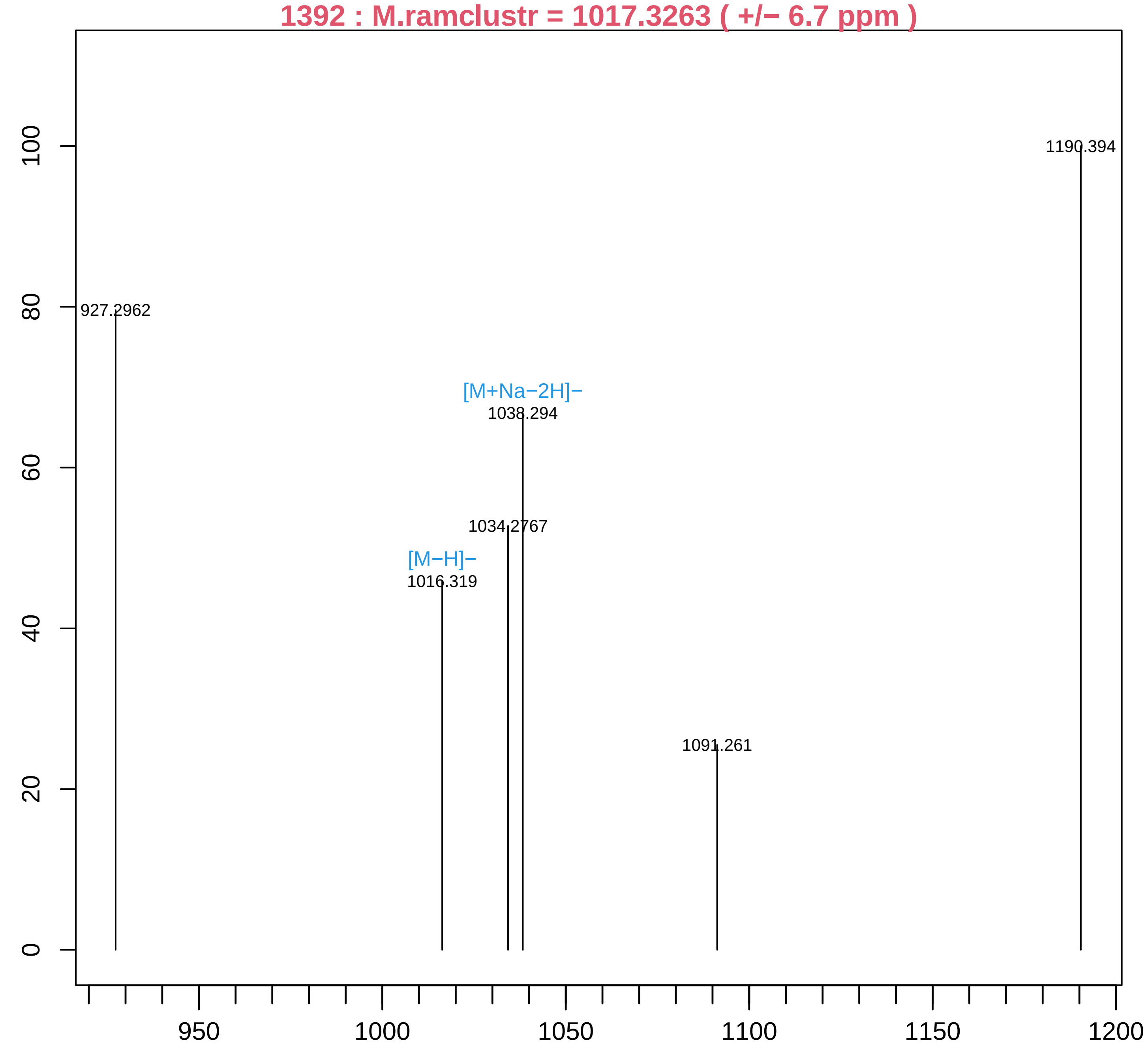
1392 : M.ramclustr = 1017.3263 (+/− 6.7 ppm)
1190.394
927.2962
[M+Na−2H]−
1038.294
1034.2767
[M−H]−
1016.319
1091.261
0
20
40
60
80
100
950
1000
1050
1100
1150
1200

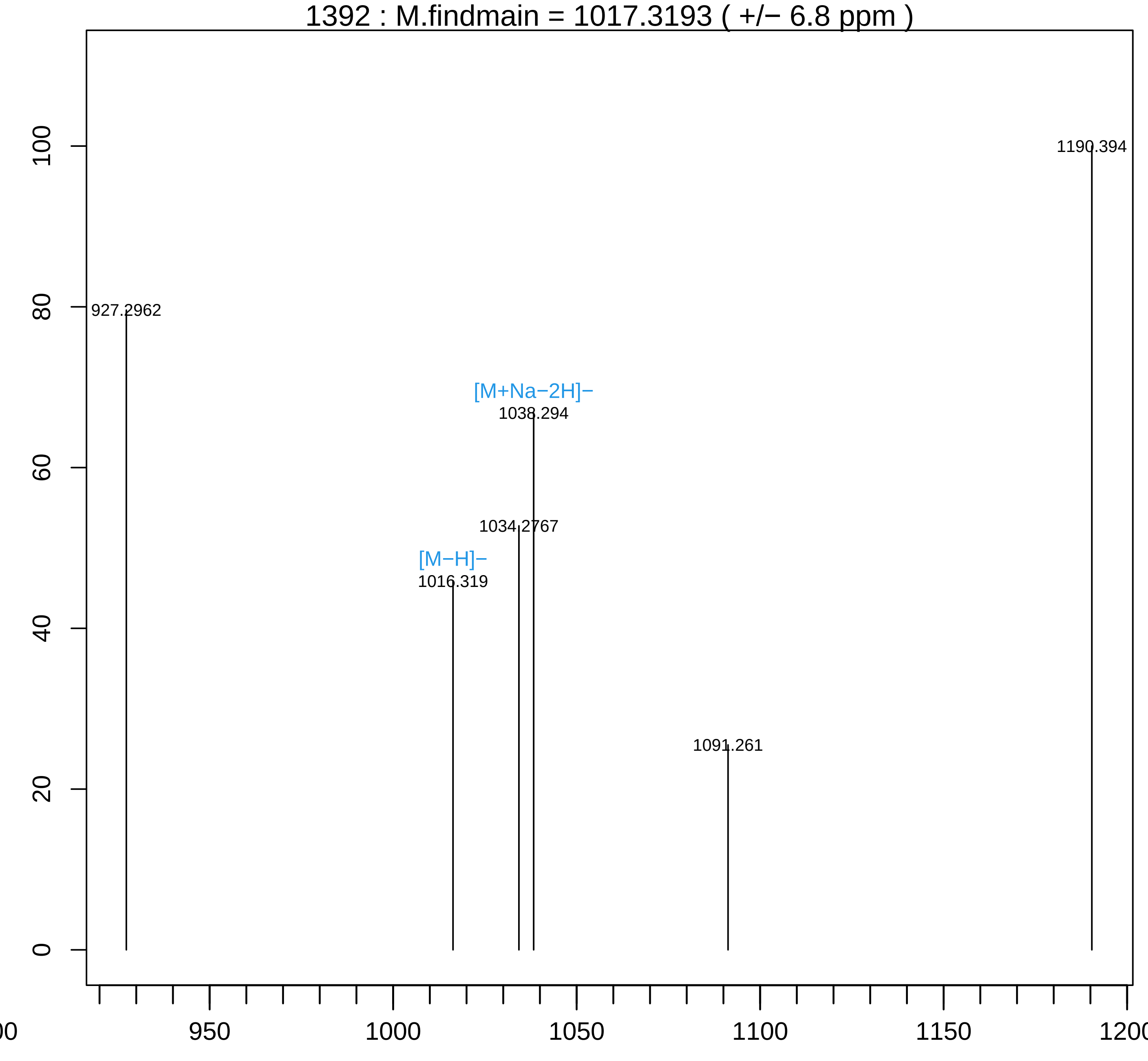
1392 : M.findmain = 1017.3193 (+/− 6.8 ppm)
1190.394
927.2962
[M+Na−2H]−
1038.294
1034.2767
[M−H]−
1016.319
1091.261
0
20
40
60
80
100
950
1000
1050
1100
1150
1200

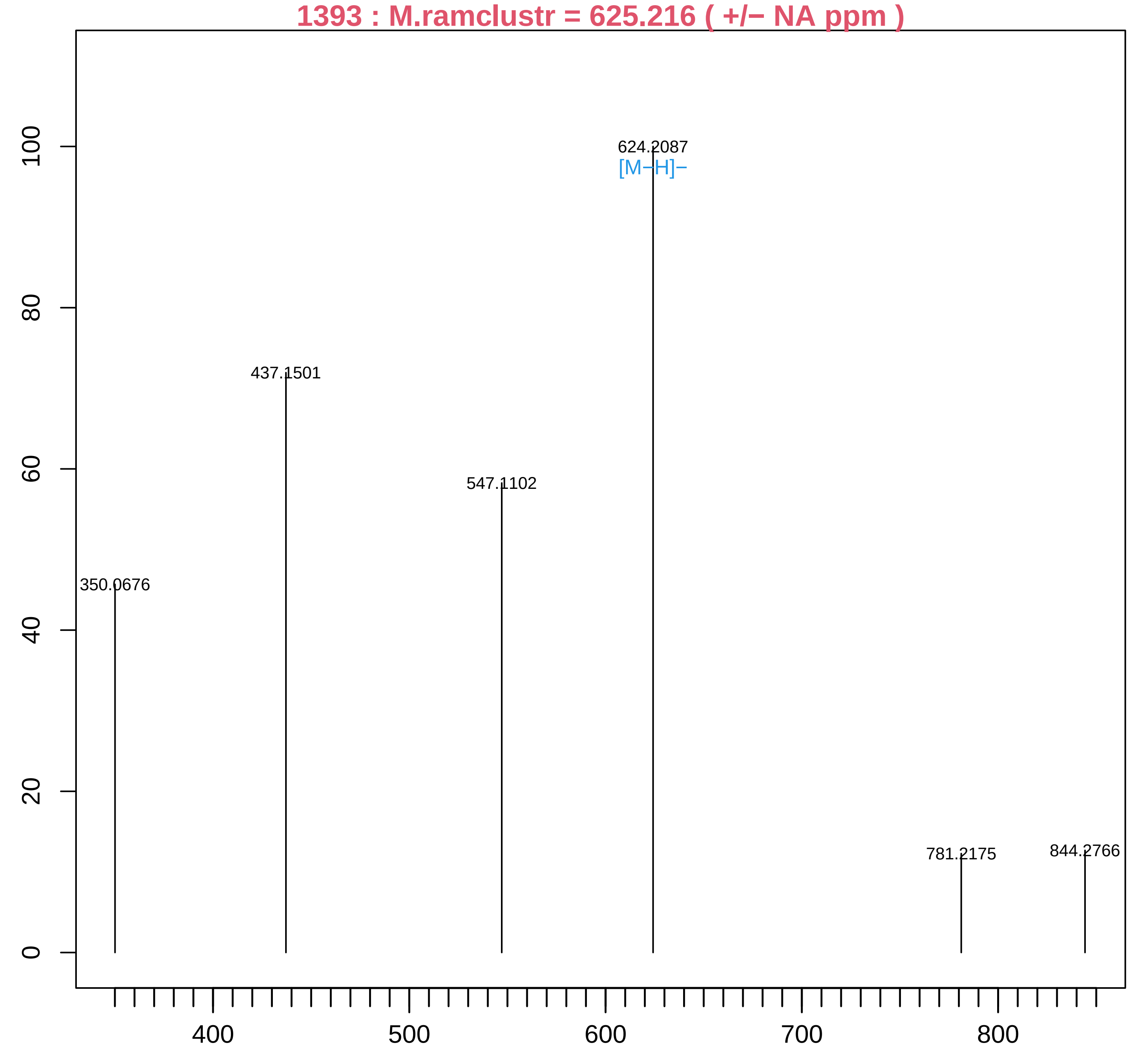

1393 : M.ramclustr = 625.216 (+/− NA ppm)
100
80
60
40
20
0
400
500
600
700
800
624.2087
[M−H]−
437.1501
547.1102
350.0676
781.2175
844.2766

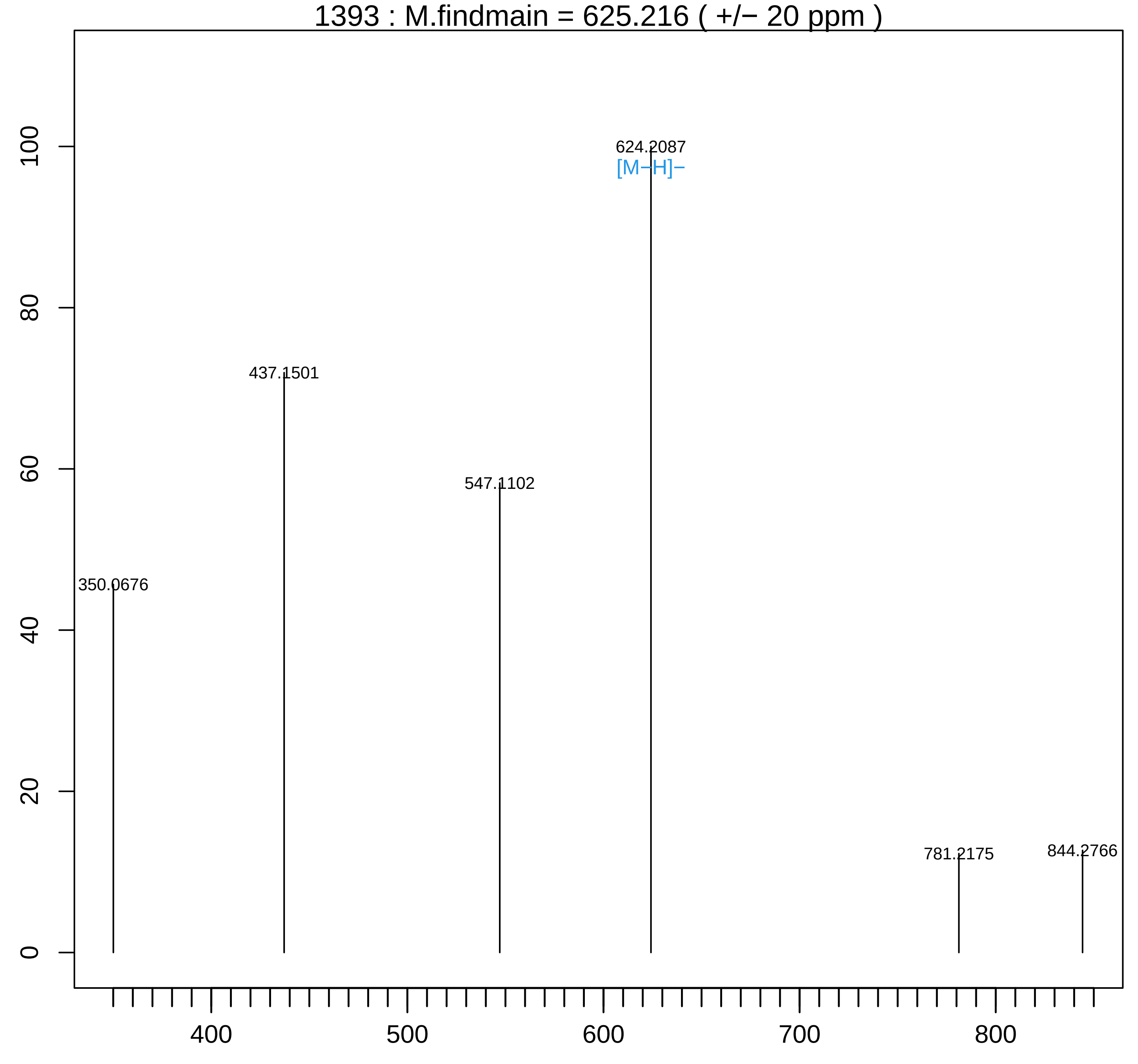

1393 : M.findmain = 625.216 (+/− 20 ppm)
100
80
60
40
20
0
400
500
600
700
800
624.2087
[M−H]−
437.1501
547.1102
350.0676
781.2175
844.2766

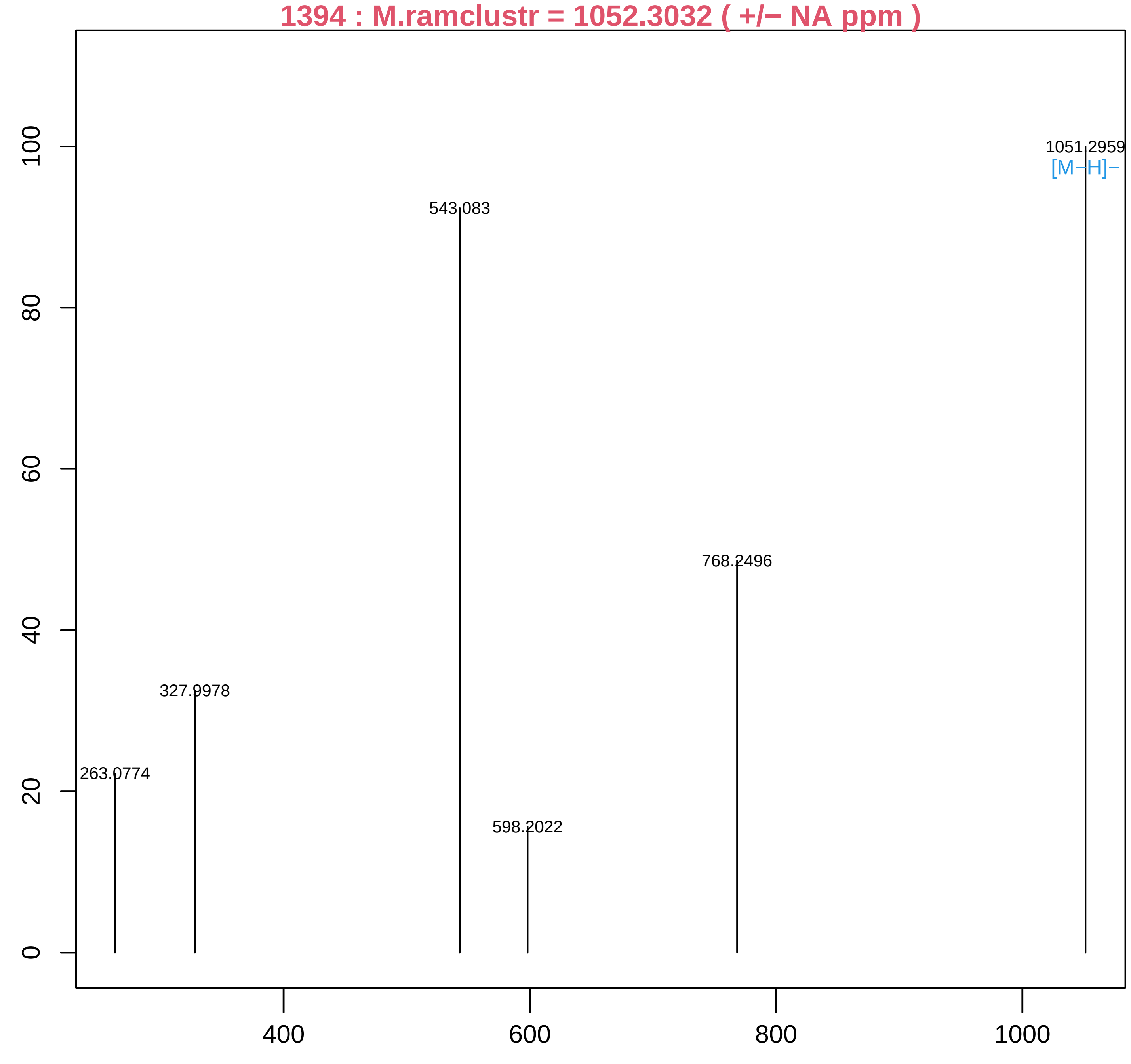
1394 : M.ramclustr = 1052.3032 (+/− NA ppm)
1051.2959
[M−H]−
543.083
768.2496
327.9978
263.0774
598.2022
0
20
40
60
80
100
400
600
800
1000

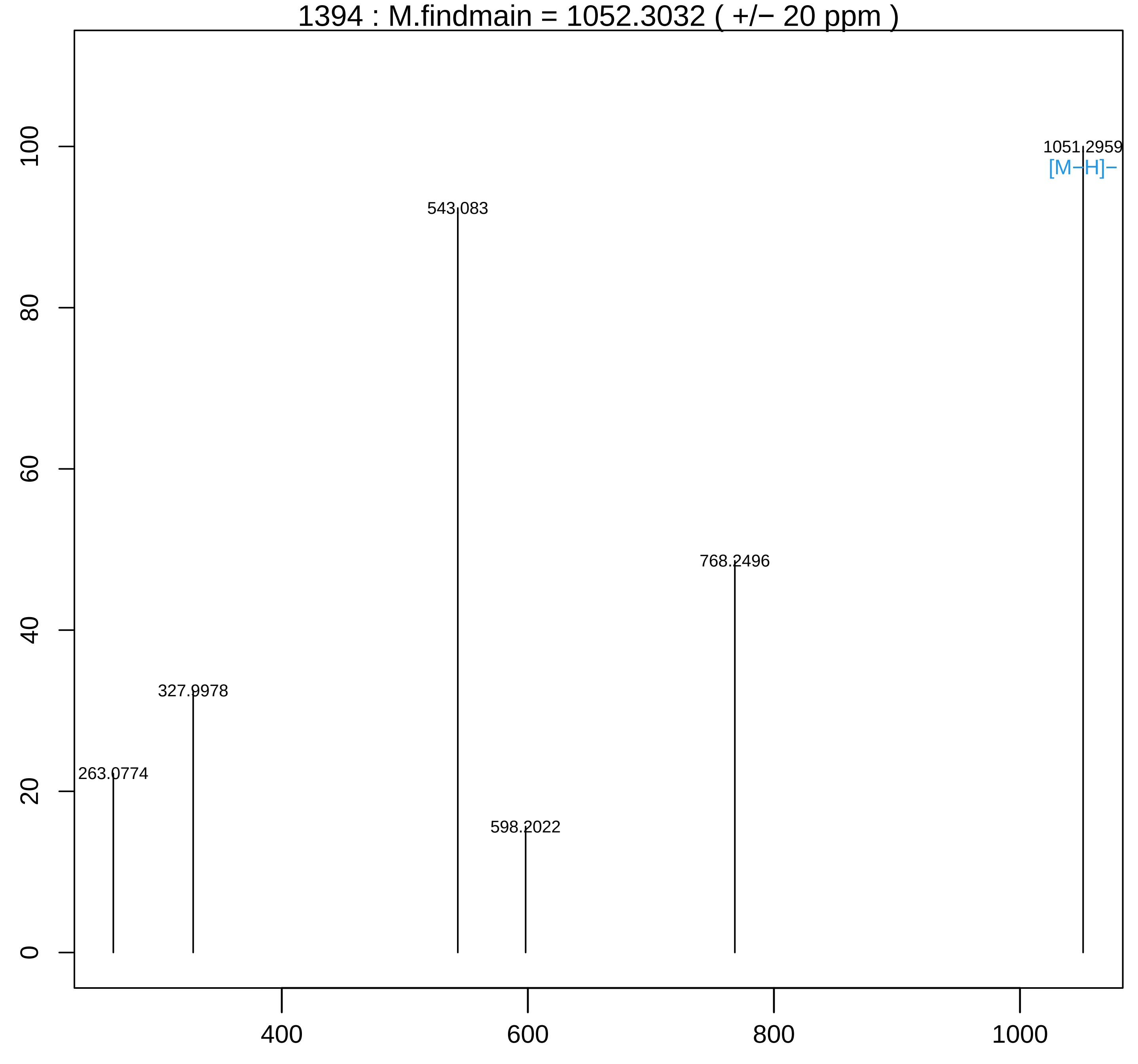
1394 : M.findmain = 1052.3032 (+/− 20 ppm)
1051.2959
[M−H]−
543.083
768.2496
327.9978
263.0774
598.2022
0
20
40
60
80
100
400
600
800
1000

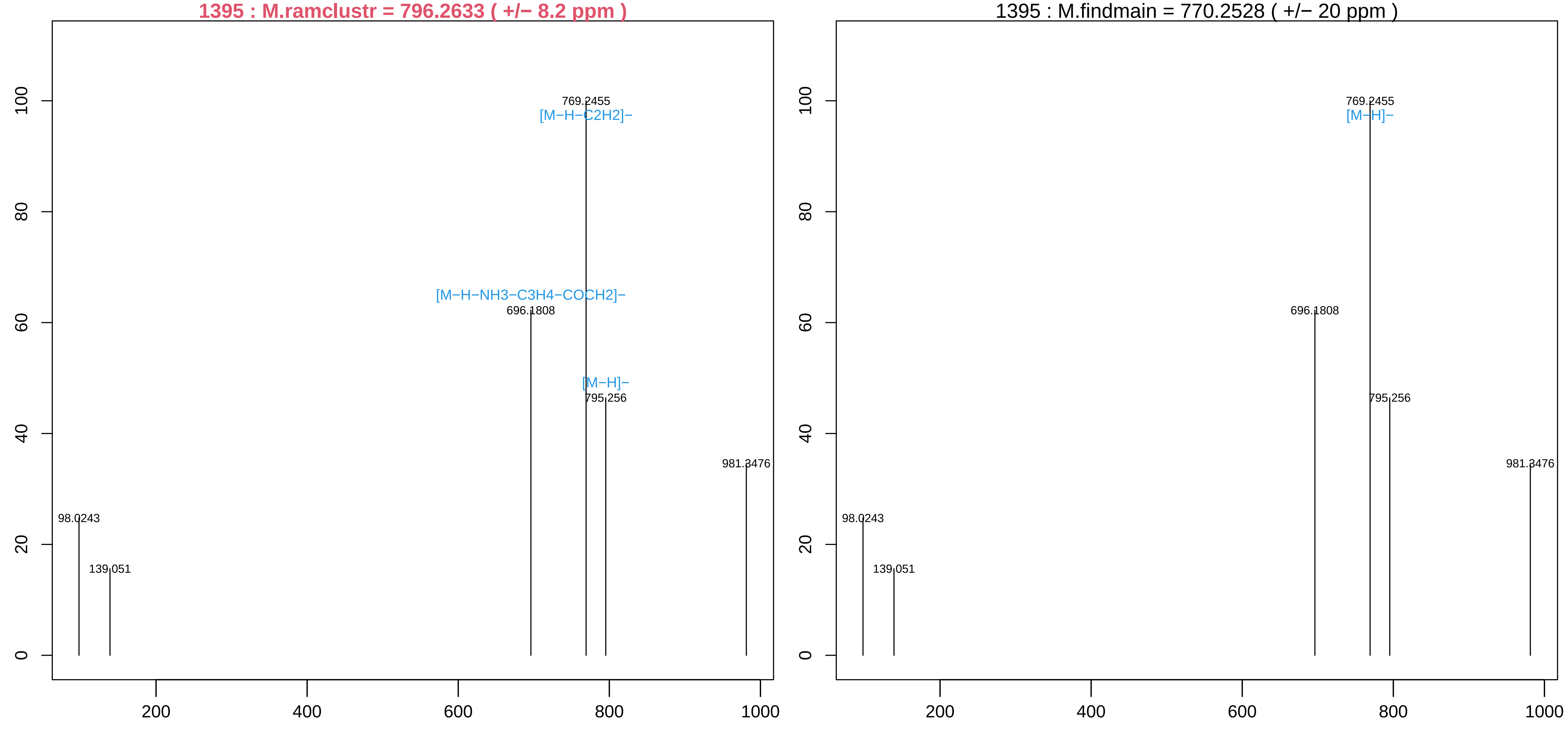

1395 : M.ramclustr = 796.2633 (+/− 8.2 ppm)
769.2455
[M−H−C2H2]−
[M−H−NH3−C3H4−COCH2]−
696.1808
[M−H]−
795.256
981.3476
98.0243
139.051
1395 : M.findmain = 770.2528 (+/− 20 ppm)
769.2455
[M−H]−
696.1808
795.256
981.3476
98.0243
139.051
0
20
40
60
80
100
200
400
600
800
1000

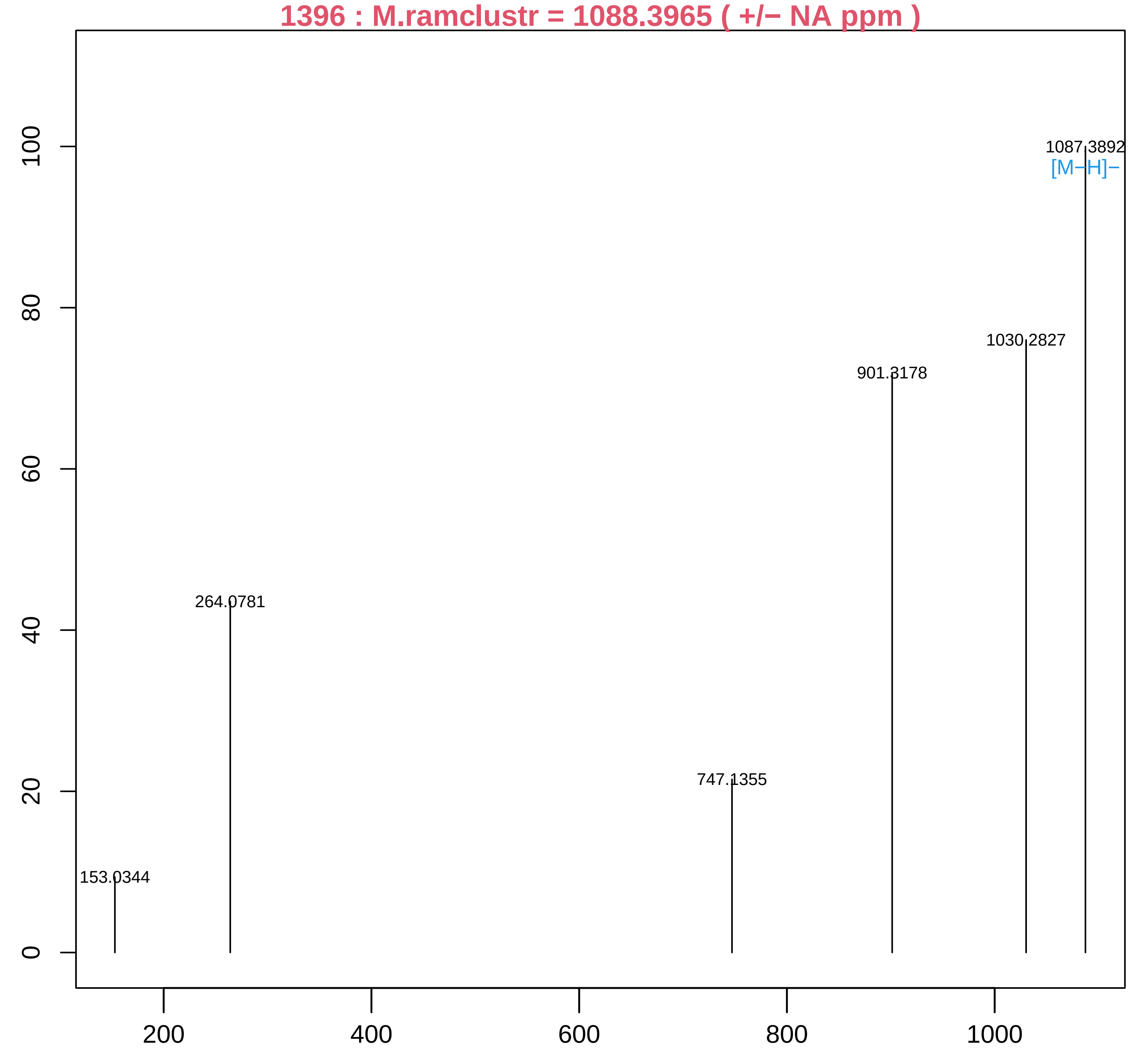
1396 : M.ramclustr = 1088.3965 (+/− NA ppm)
1087.3892
[M−H]−
1030.2827
901.3178
747.1355
264.0781
153.0344
0
20
40
60
80
100
200
400
600
800
1000

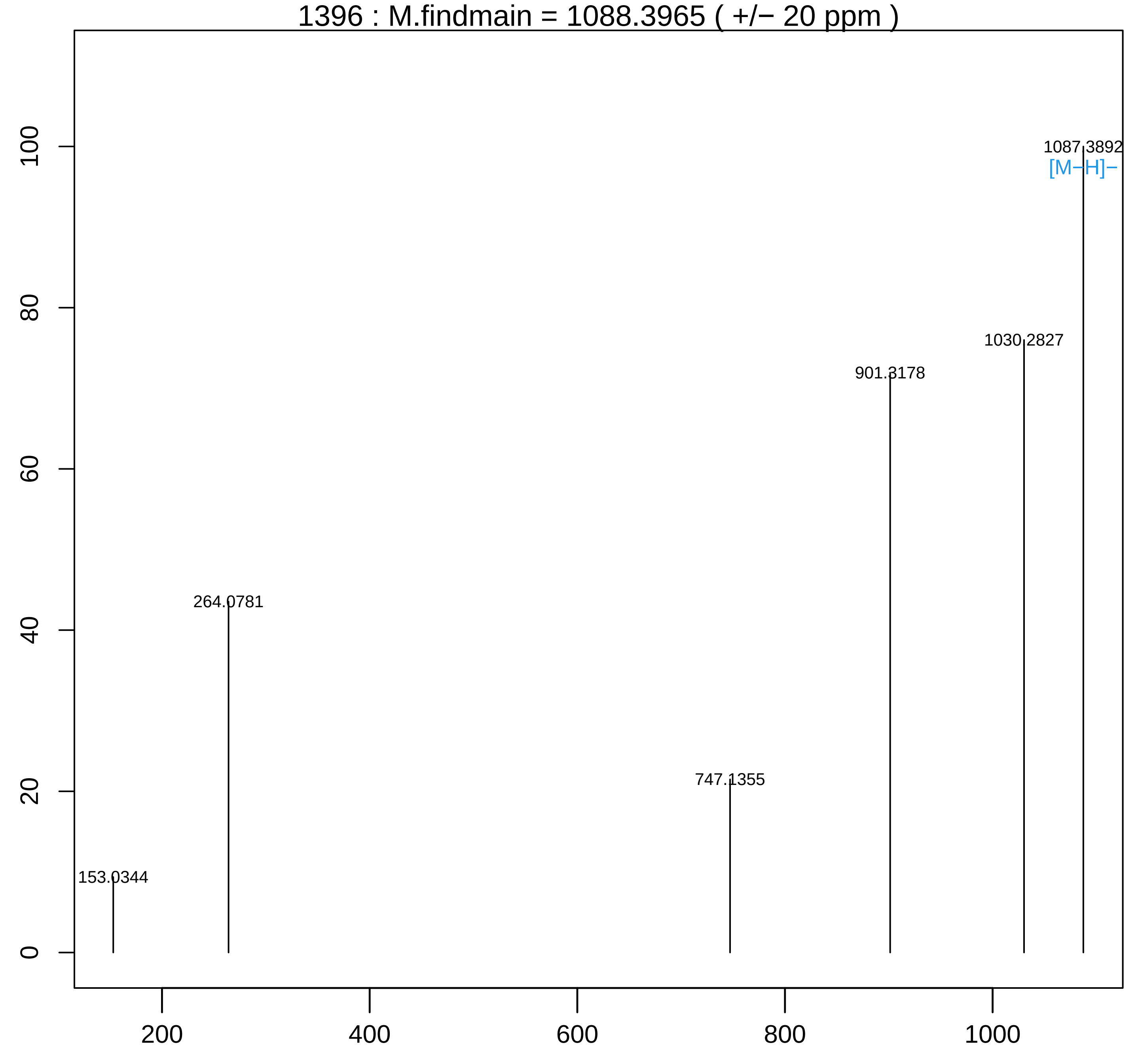
1396 : M.findmain = 1088.3965 (+/− 20 ppm)
1087.3892
[M−H]−
1030.2827
901.3178
747.1355
264.0781
153.0344
0
20
40
60
80
100
200
400
600
800
1000

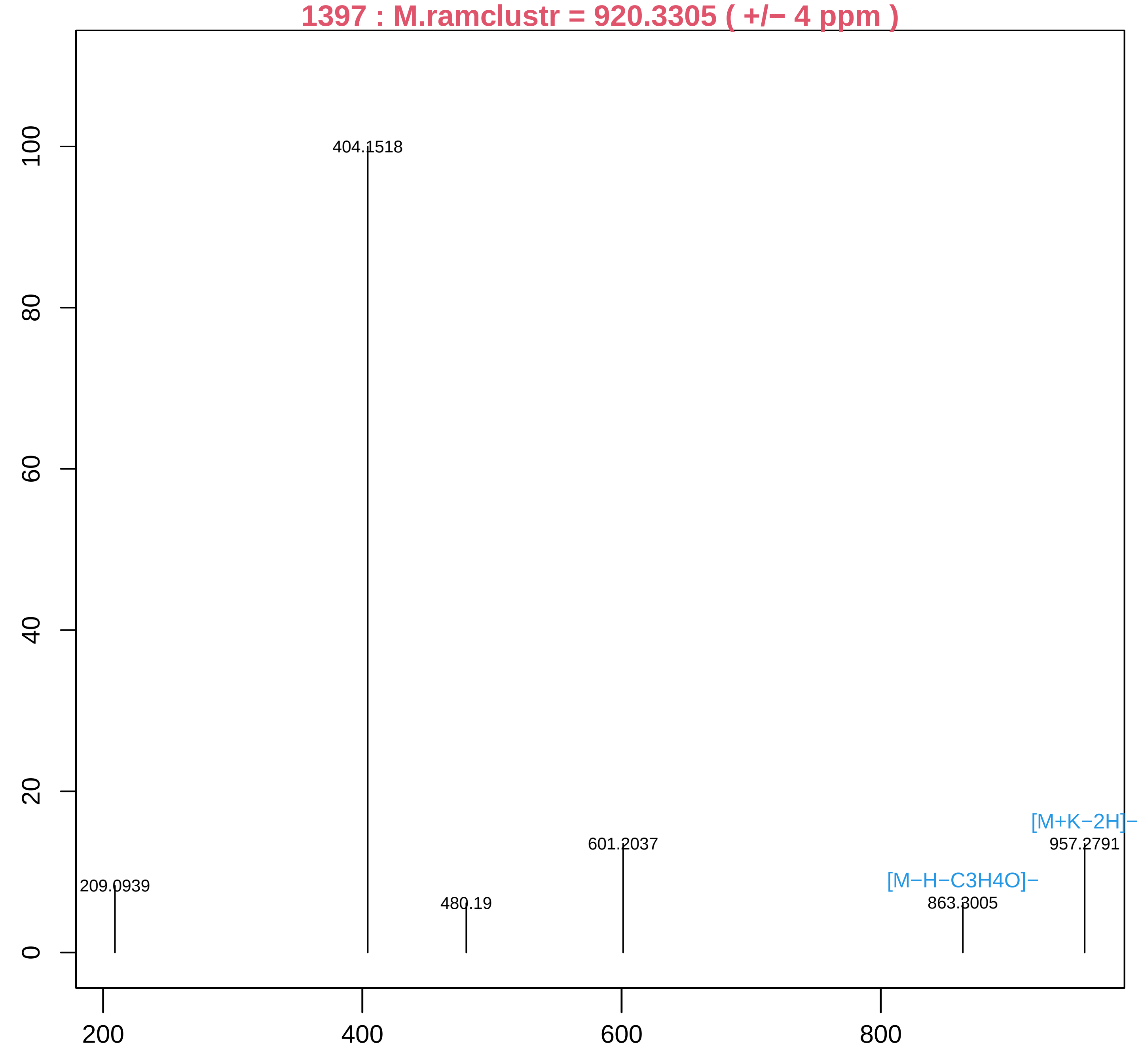
1397 : M.ramclustr = 920.3305 (+/− 4 ppm)
404.1518
601.2037
[M+K−2H]−
957.2791
209.0939
480.19
[M−H−C3H4O]−
863.3005
0
20
40
60
80
100
200
400
600
800

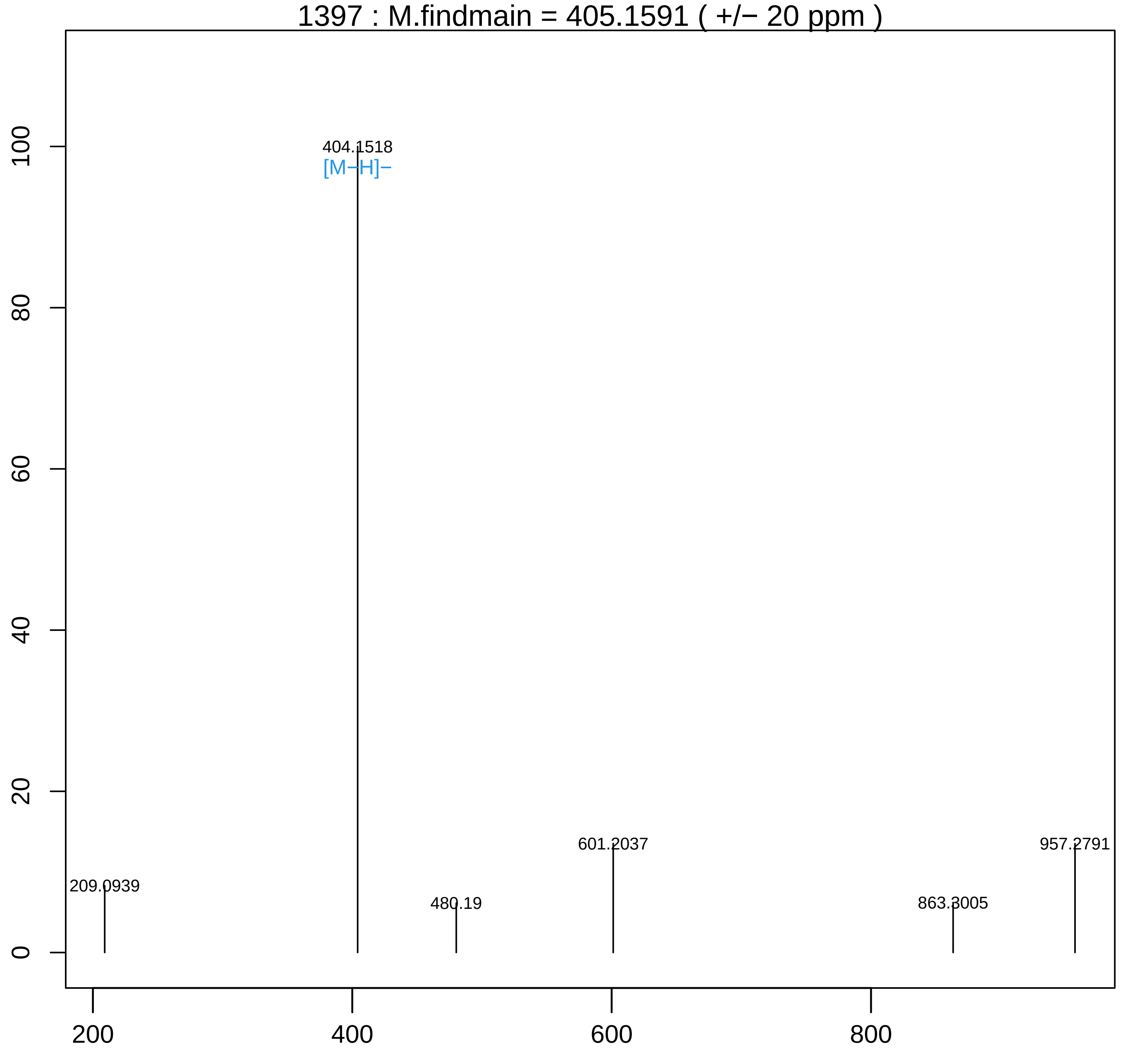
1397 : M.findmain = 405.1591 (+/− 20 ppm)
404.1518
[M−H]−
601.2037
957.2791
209.0939
480.19
863.3005
0
20
40
60
80
100
200
400
600
800

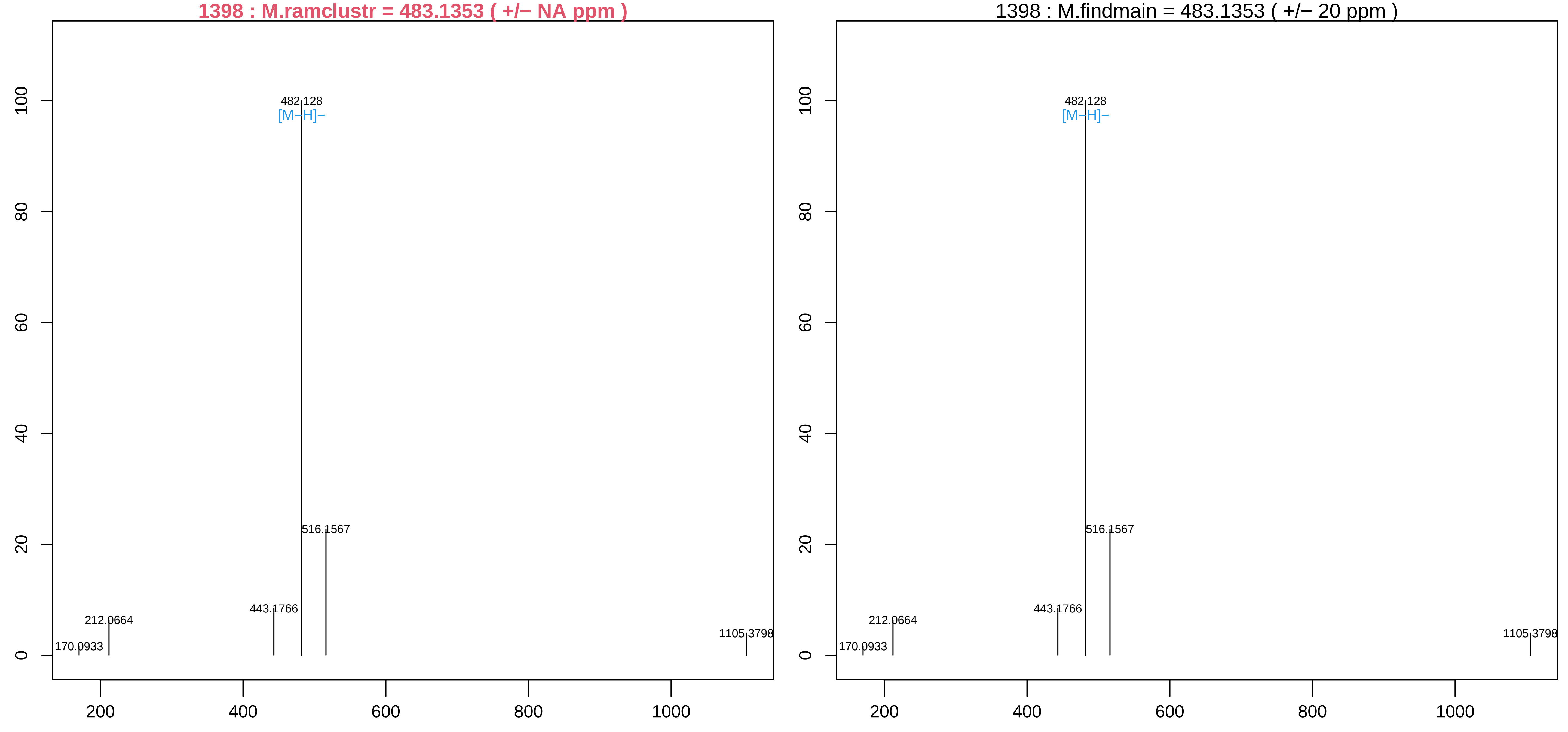
1398 : M.ramclustr = 483.1353 (+/− NA ppm)
482.128
[M−H]−
516.1567
443.1766
212.0664
170.0933
1105.3798
0
20
40
60
80
100
200
400
600
800
1000
1398 : M.findmain = 483.1353 (+/− 20 ppm)
482.128
[M−H]−
516.1567
443.1766
212.0664
170.0933
1105.3798
0
20
40
60
80
100
200
400
600
800
1000

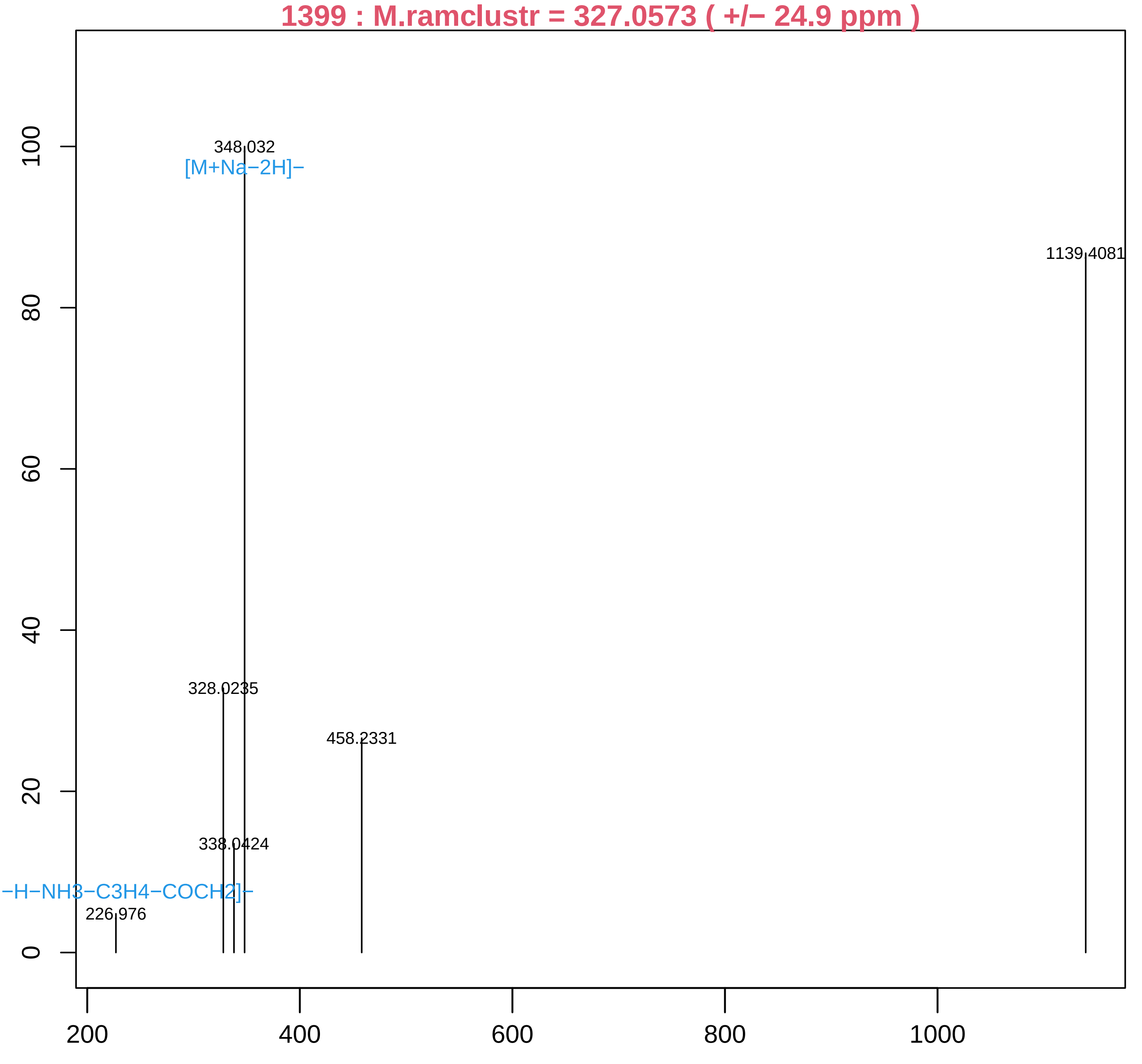
1399 : M.ramclustr = 327.0573 (+/− 24.9 ppm)
348.032
[M+Na−2H]−
1139.4081
328.0235
458.2331
338.0424
−H−NH3−C3H4−COCH2]−
226.976
0
20
40
60
80
100
200
400
600
800
1000

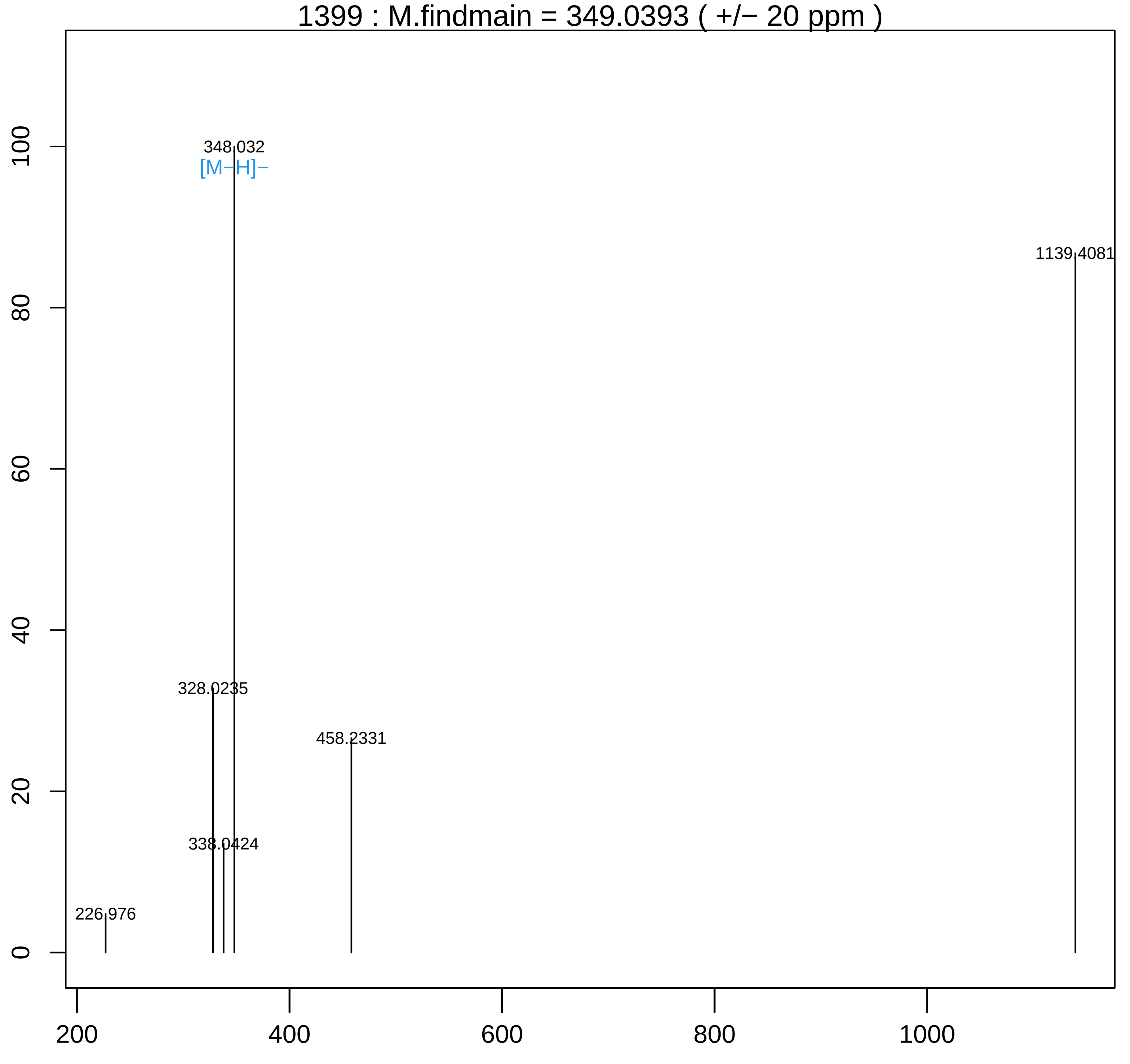
1399 : M.findmain = 349.0393 (+/− 20 ppm)
348.032
[M−H]−
1139.4081
328.0235
458.2331
338.0424
226.976
0
20
40
60
80
100
200
400
600
800
1000

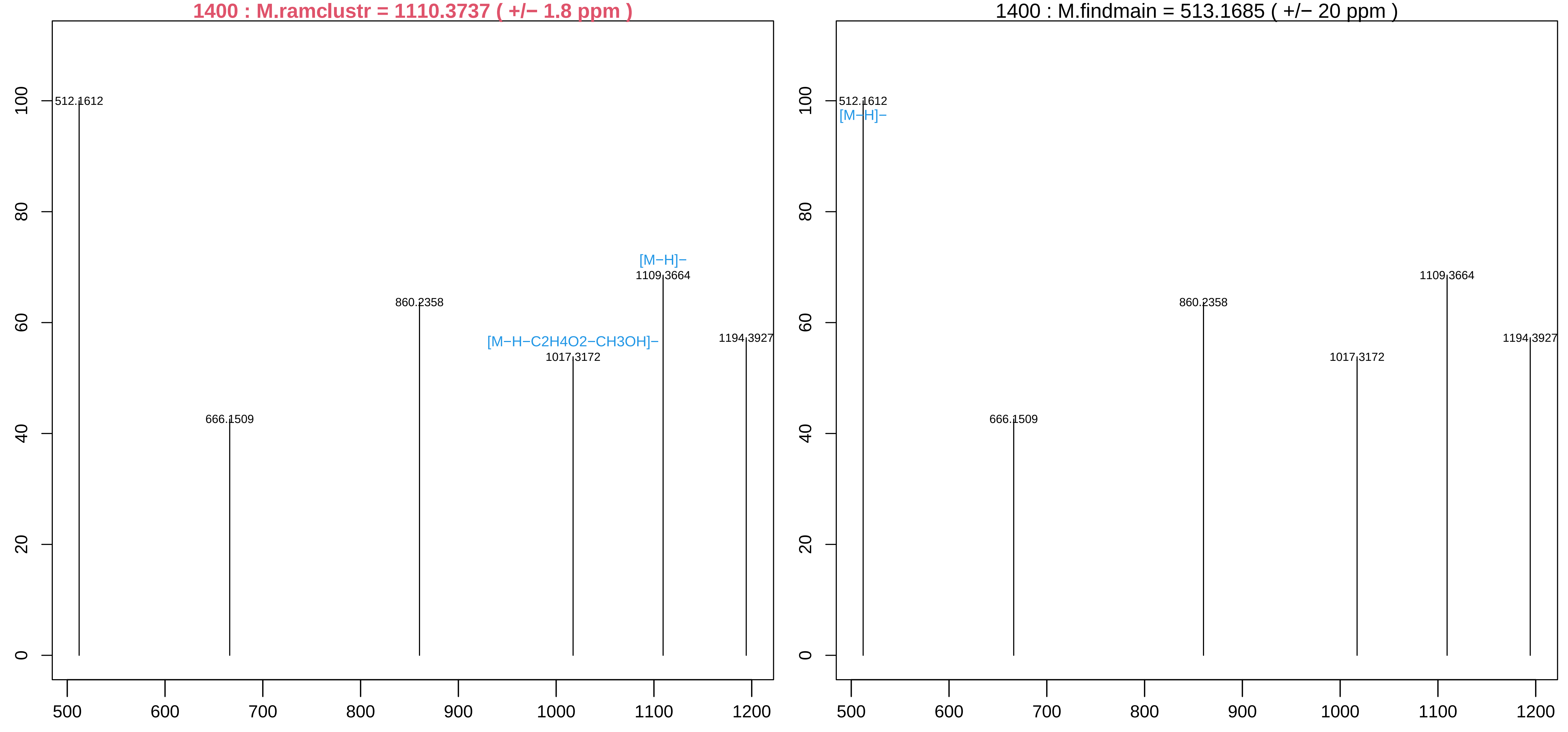

1400 : M.ramclustr = 1110.3737 (+/− 1.8 ppm)
512.1612
666.1509
860.2358
[M−H−C2H4O2−CH3OH]−
1017.3172
[M−H]−
1109.3664
1194.3927
1400 : M.findmain = 513.1685 (+/− 20 ppm)
512.1612
[M−H]−
666.1509
860.2358
1017.3172
1109.3664
1194.3927
0
20
40
60
80
100
500
600
700
800
900
1000
1100
1200

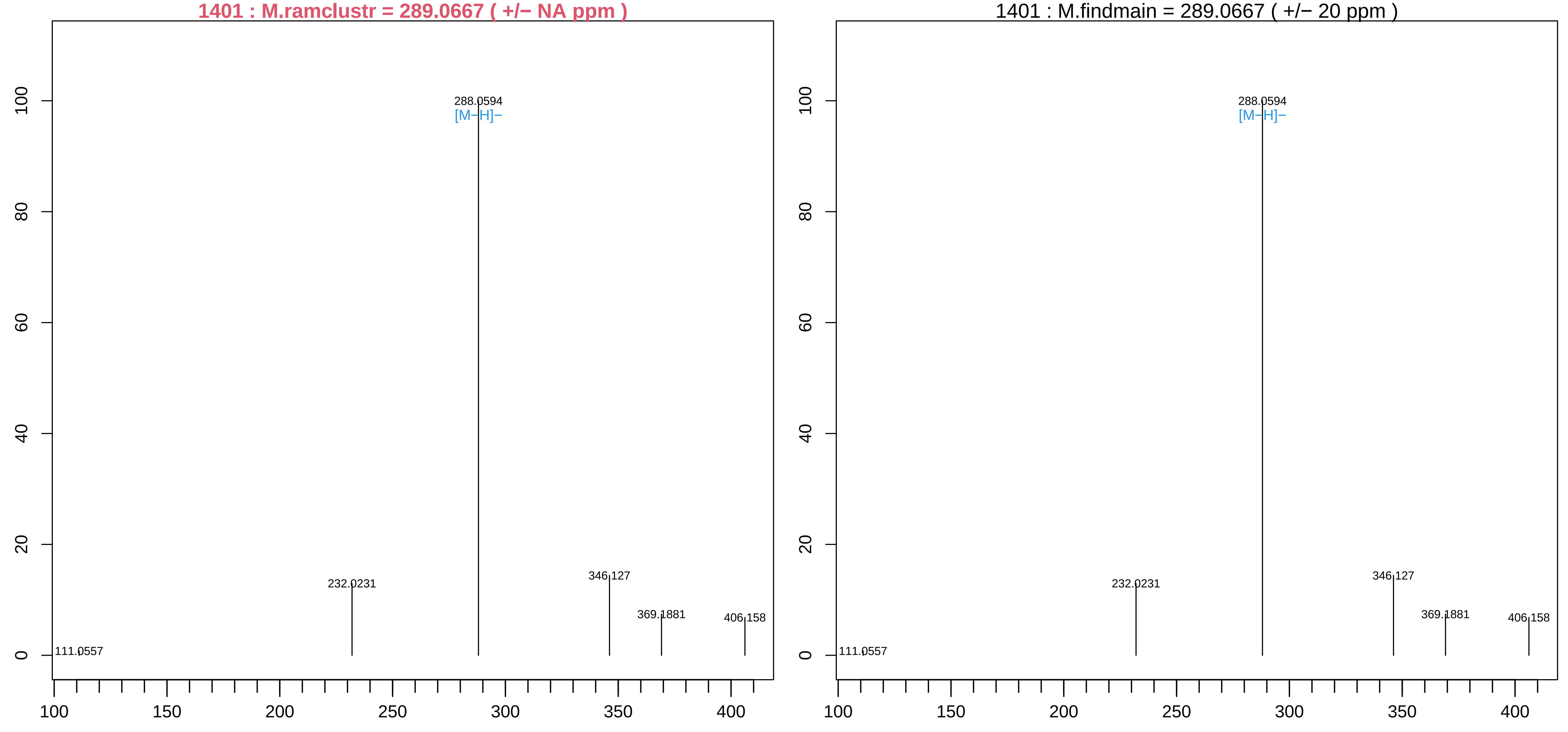
1401 : M.ramclustr = 289.0667 (+/− NA ppm)
288.0594
[M−H]−
232.0231
346.127
369.1881
406.158
111.0557
1401 : M.findmain = 289.0667 (+/− 20 ppm)
288.0594
[M−H]−
232.0231
346.127
369.1881
406.158
111.0557

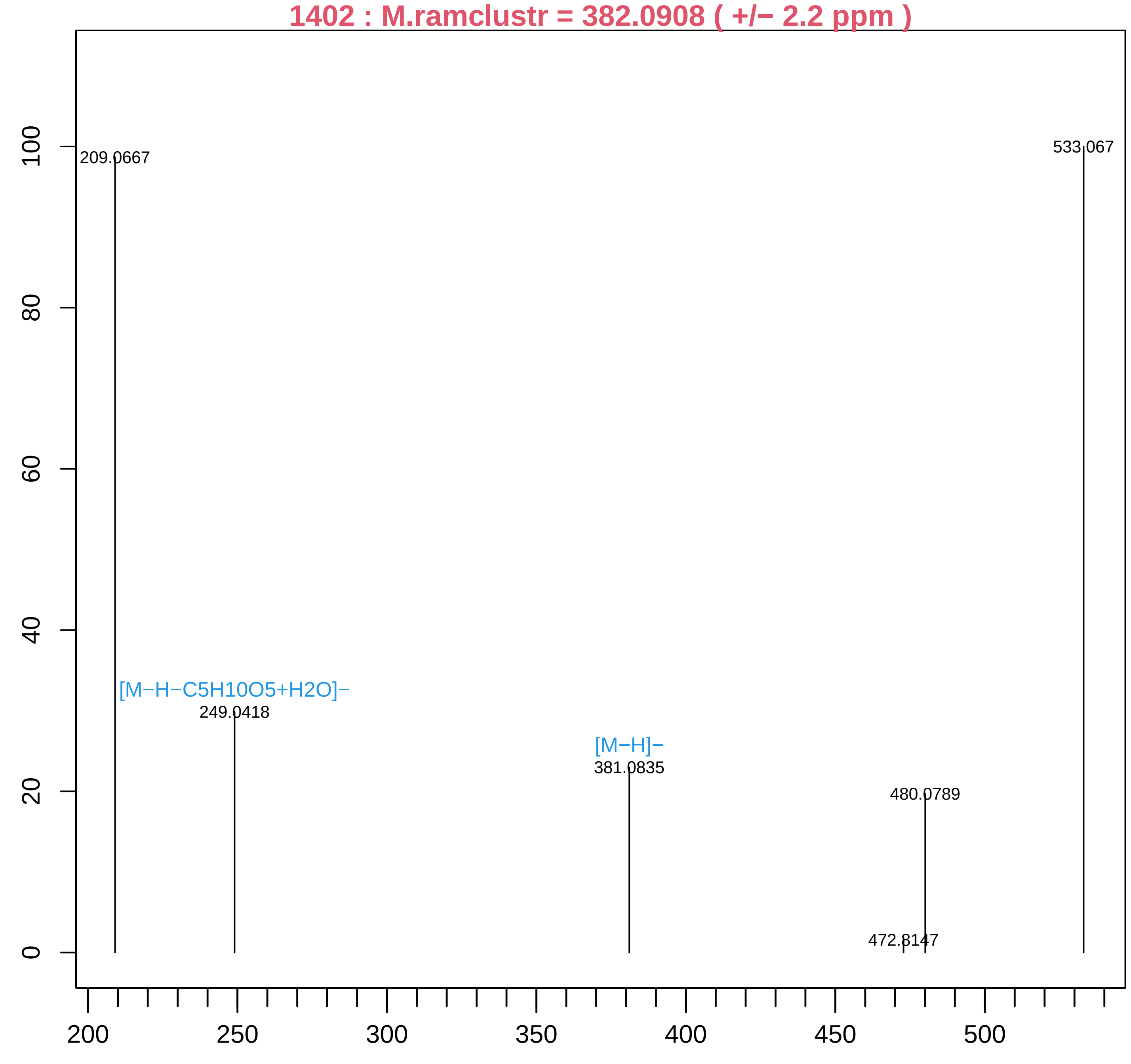
1402 : M.ramclustr = 382.0908 (+/− 2.2 ppm)
209.0667
533.067
[M−H−C5H10O5+H2O]−
249.0418
[M−H]−
381.0835
480.0789
472.8147
0
20
40
60
80
100
200
250
300
350
400
450
500

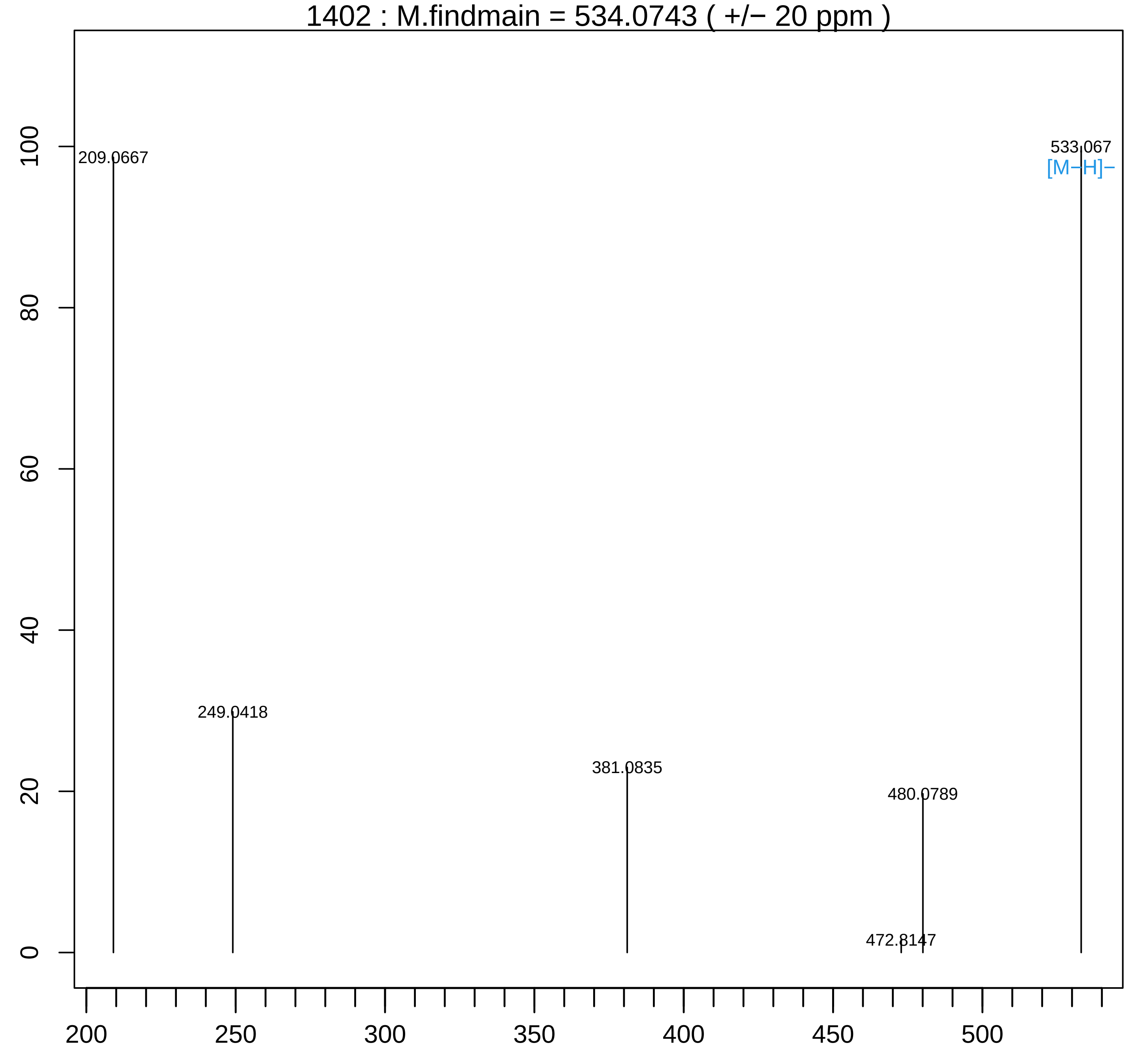
1402 : M.findmain = 534.0743 (+/− 20 ppm)
209.0667
533.067
[M−H]−
249.0418
381.0835
480.0789
472.8147
0
20
40
60
80
100
200
250
300
350
400
450
500

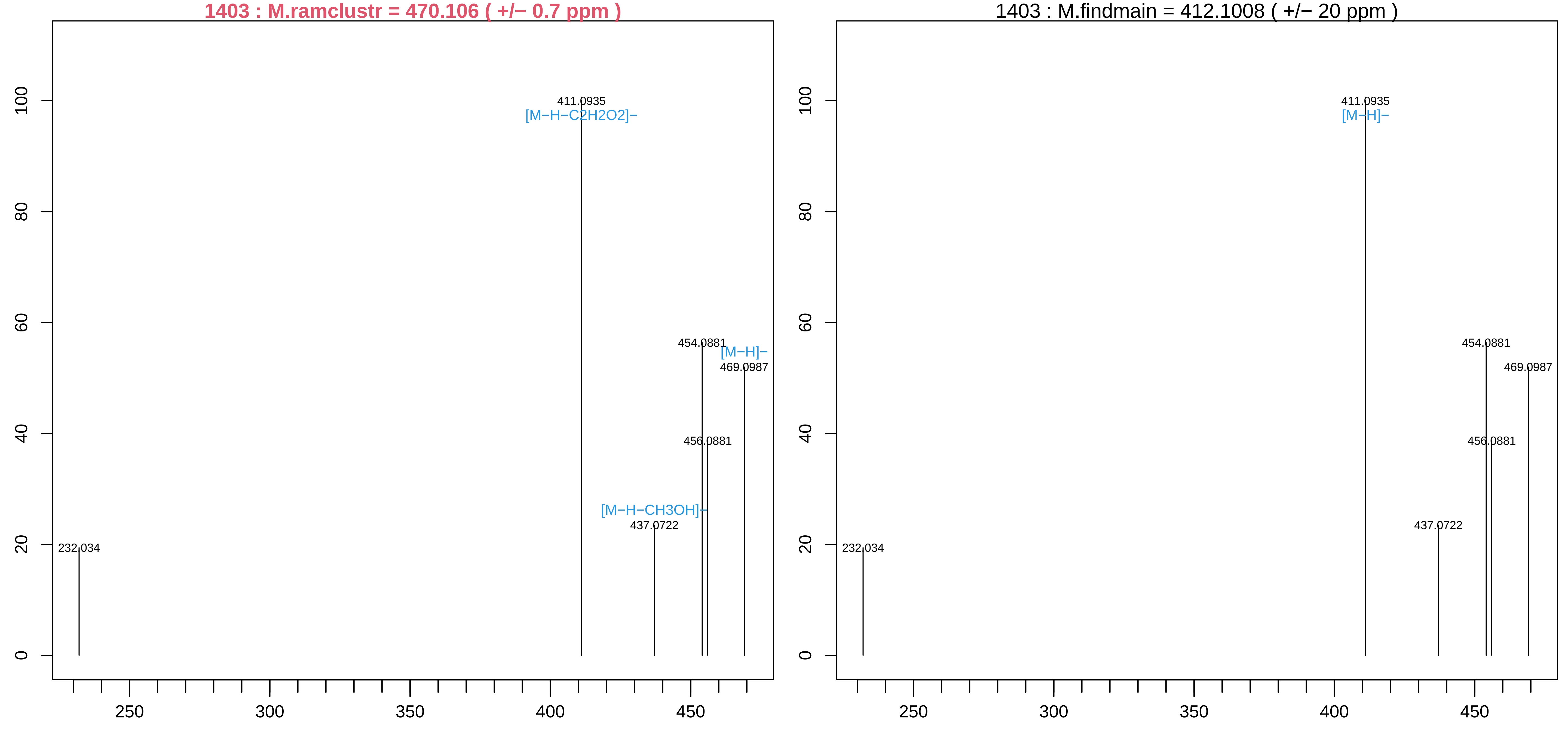
1403 : M.ramclustr = 470.106 (+/− 0.7 ppm)
411.0935
[M−H−C2H2O2]−
454.0881
[M−H]−
469.0987
456.0881
[M−H−CH3OH]−
437.0722
232.034
0
20
40
60
80
100
250
300
350
400
450
1403 : M.findmain = 412.1008 (+/− 20 ppm)
411.0935
[M−H]−
454.0881
469.0987
456.0881
437.0722
232.034
0
20
40
60
80
100
250
300
350
400
450

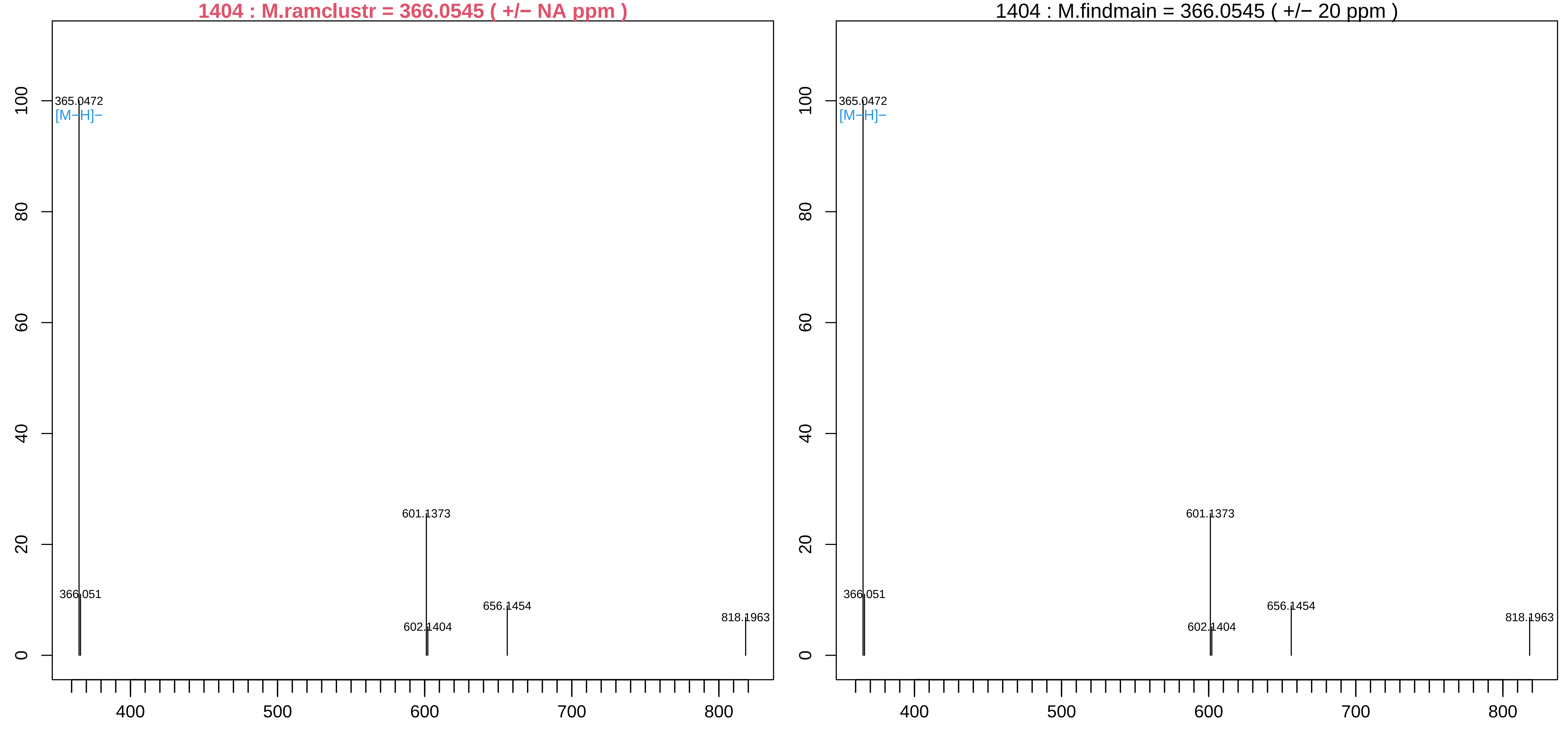
1404 : M.ramclustr = 366.0545 (+/− NA ppm)
365.0472
[M−H]−
366.051
601.1373
602.1404
656.1454
818.1963
1404 : M.findmain = 366.0545 (+/− 20 ppm)
365.0472
[M−H]−
366.051
601.1373
602.1404
656.1454
818.1963
0
20
40
60
80
100
400
500
600
700
800

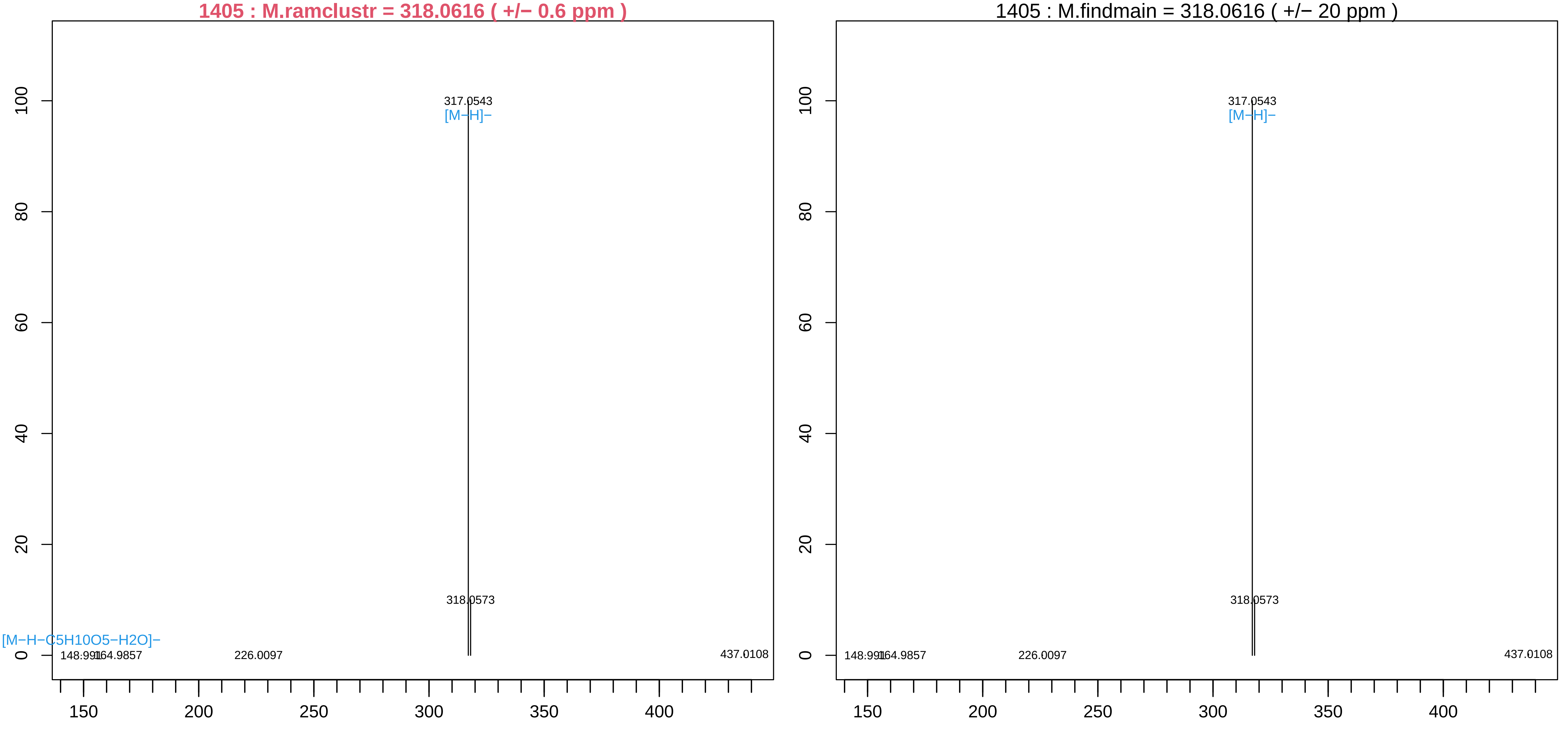
1405 : M.ramclustr = 318.0616 (+/− 0.6 ppm)
317.0543
[M−H]−
318.0573
[M−H−C5H10O5−H2O]−
148.991
164.9857
226.0097
437.0108
1405 : M.findmain = 318.0616 (+/− 20 ppm)
317.0543
[M−H]−
318.0573
148.991
164.9857
226.0097
437.0108
0
20
40
60
80
100
150
200
250
300
350
400

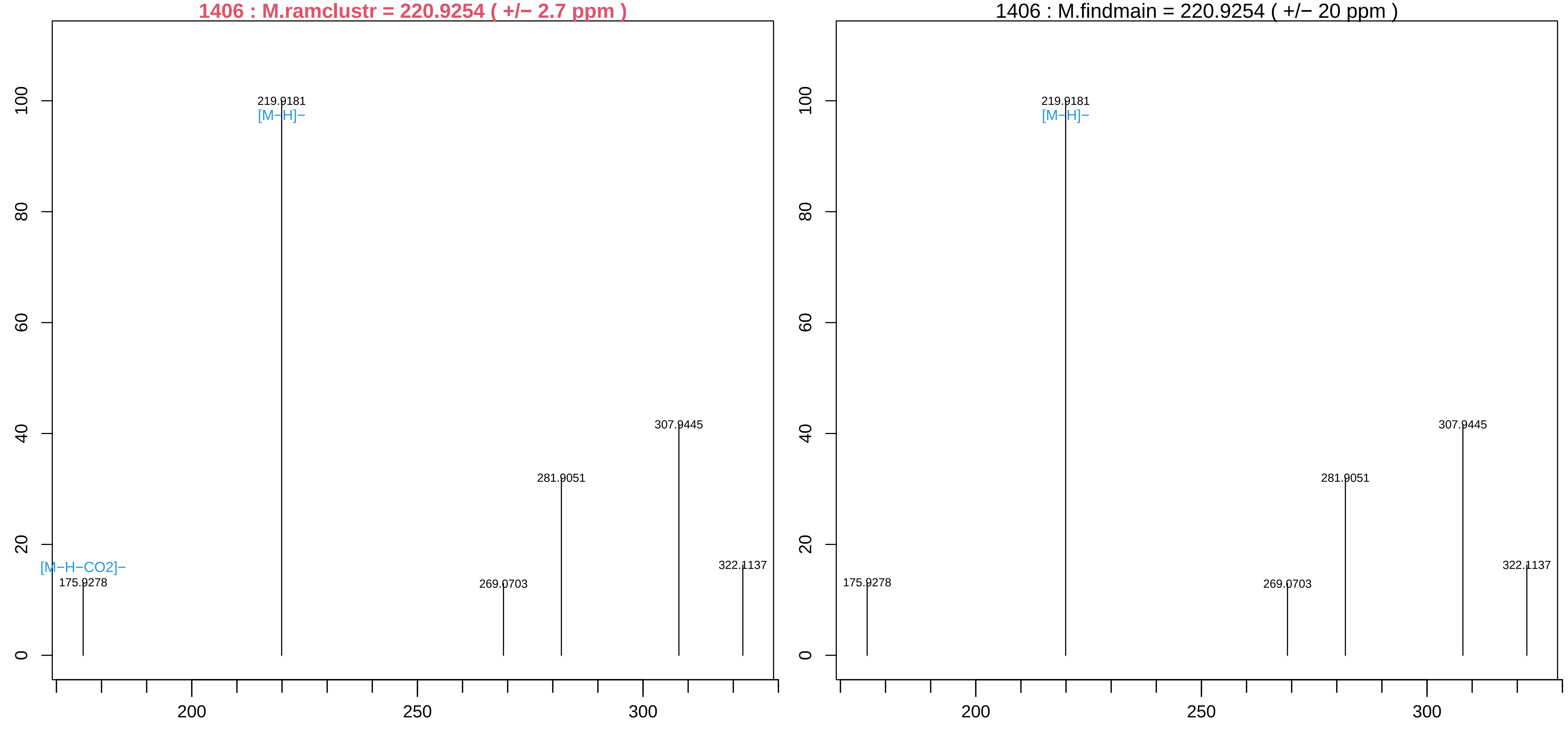

1406 : M.ramclustr = 220.9254 (+/− 2.7 ppm)
219.9181
[M−H]−
[M−H−CO2]−
175.9278
269.0703
281.9051
307.9445
322.1137
0
20
40
60
80
100
200
250
300
1406 : M.findmain = 220.9254 (+/− 20 ppm)
219.9181
[M−H]−
175.9278
269.0703
281.9051
307.9445
322.1137
0
20
40
60
80
100
200
250
300

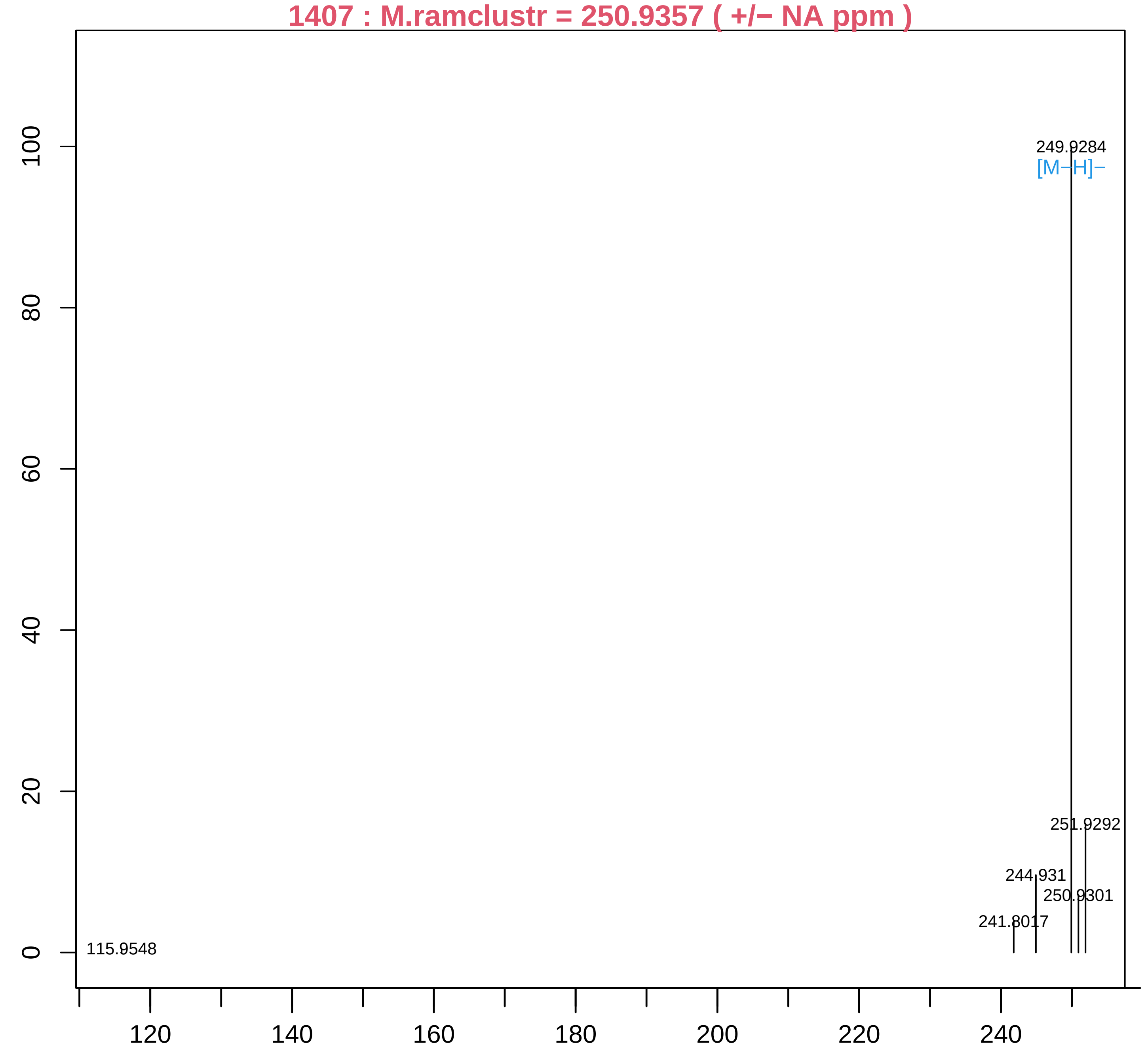
1407 : M.ramclustr = 250.9357 (+/− NA ppm)
249.9284
[M−H]−
251.9292
244.931
250.9301
241.8017
115.9548
0
20
40
60
80
100
120
140
160
180
200
220
240

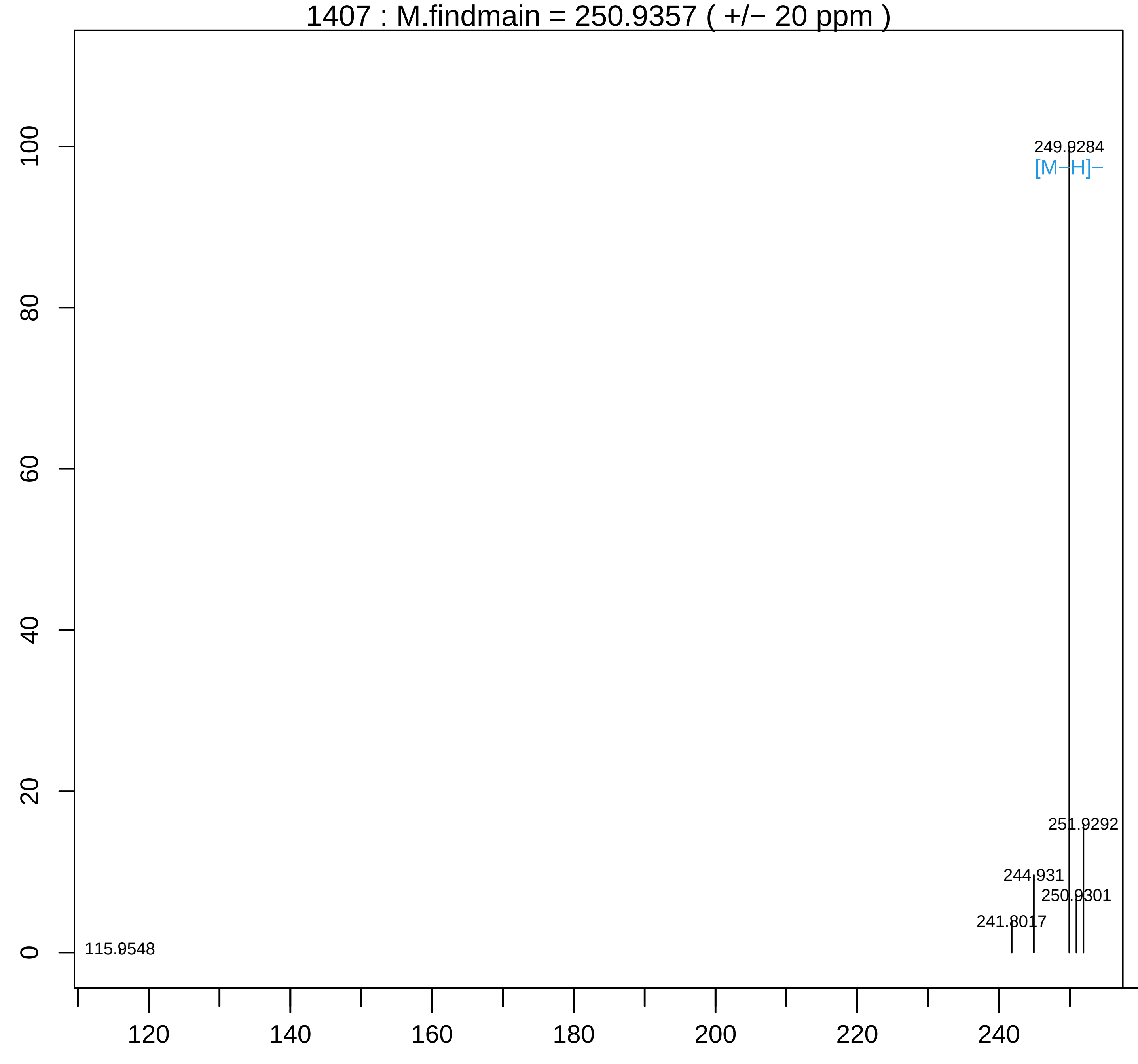
1407 : M.findmain = 250.9357 (+/− 20 ppm)
249.9284
[M−H]−
251.9292
244.931
250.9301
241.8017
115.9548
0
20
40
60
80
100
120
140
160
180
200
220
240

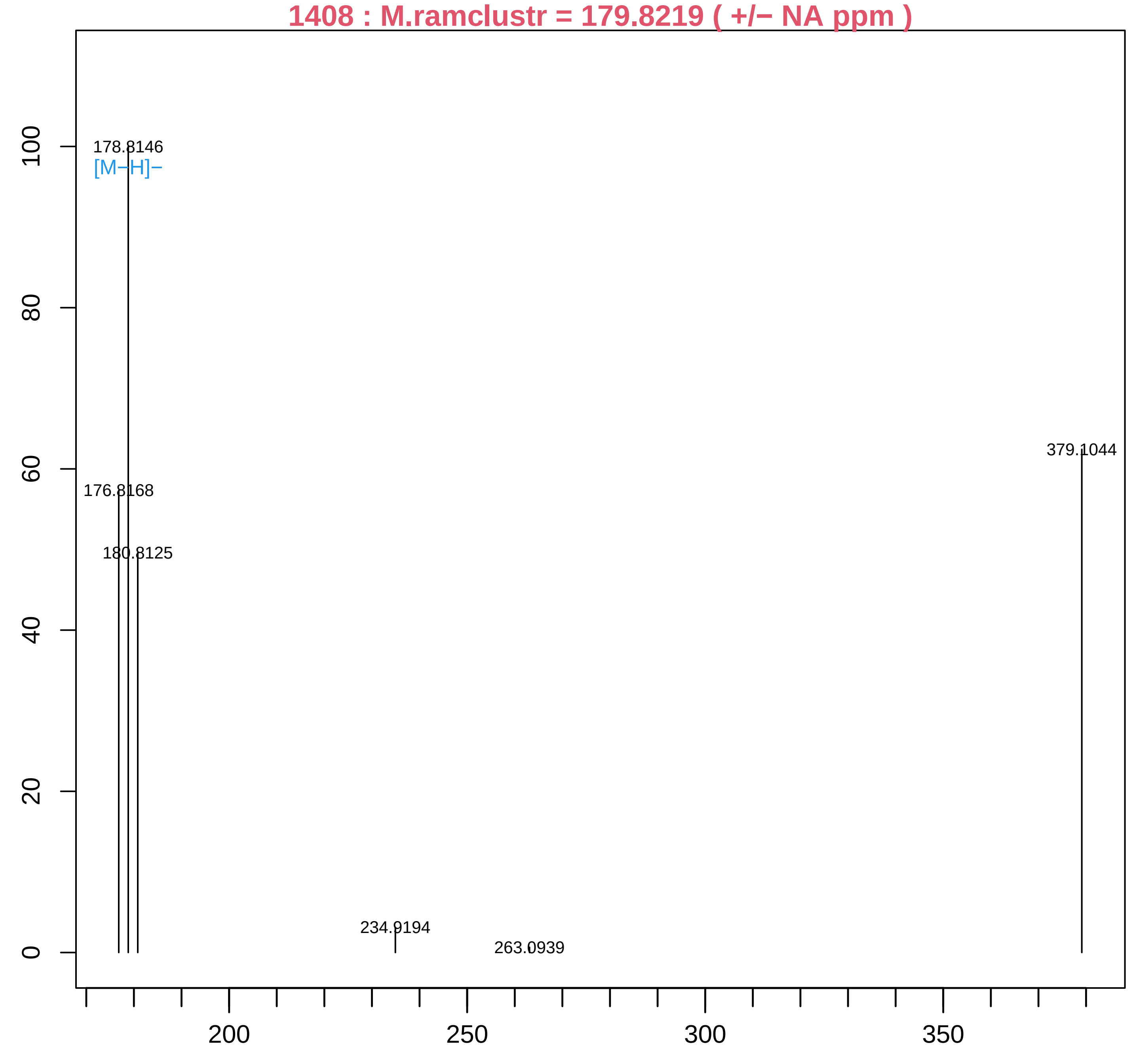
1408 : M.ramclustr = 179.8219 (+/− NA ppm)
178.8146
[M−H]−
176.8168
180.8125
379.1044
234.9194
263.0939
0
20
40
60
80
100
200
250
300
350

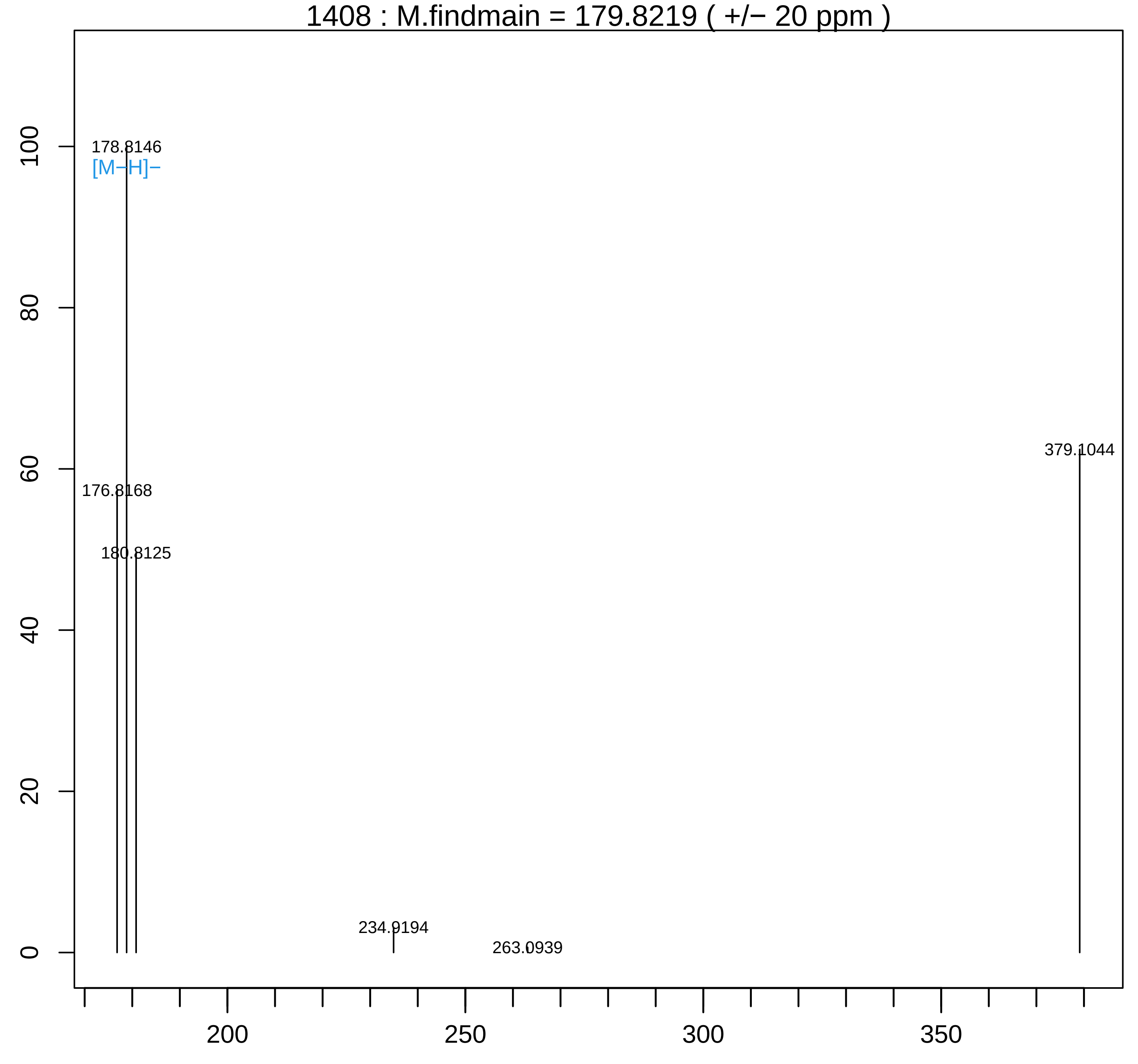
1408 : M.findmain = 179.8219 (+/− 20 ppm)
178.8146
[M−H]−
176.8168
180.8125
379.1044
234.9194
263.0939
0
20
40
60
80
100
200
250
300
350

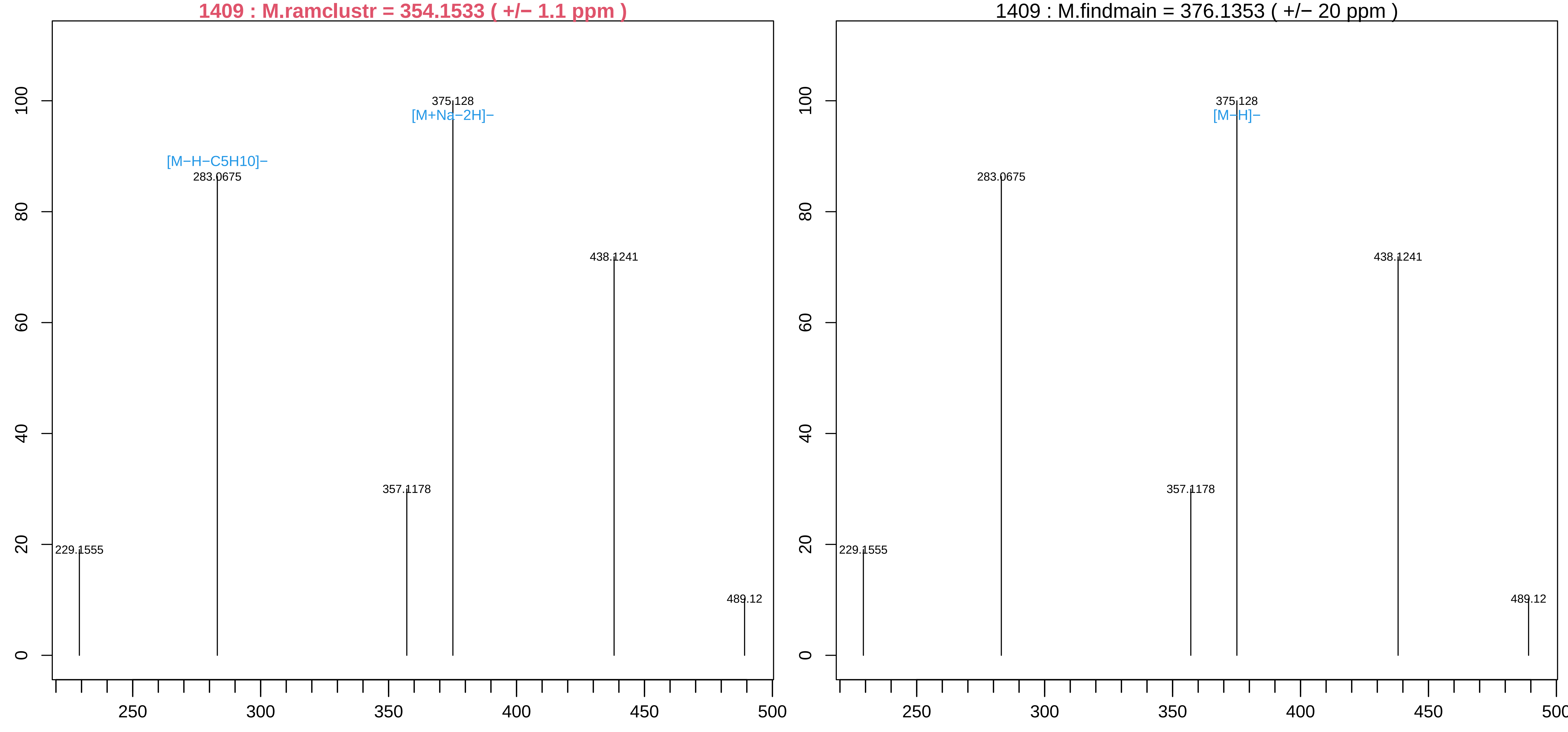
1409 : M.ramclustr = 354.1533 (+/− 1.1 ppm)
[M+Na−2H]−
375.128
[M−H−C5H10]−
283.0675
438.1241
357.1178
229.1555
489.12
1409 : M.findmain = 376.1353 (+/− 20 ppm)
375.128
[M−H]−
283.0675
438.1241
357.1178
229.1555
489.12
0
20
40
60
80
100
250
300
350
400
450
500

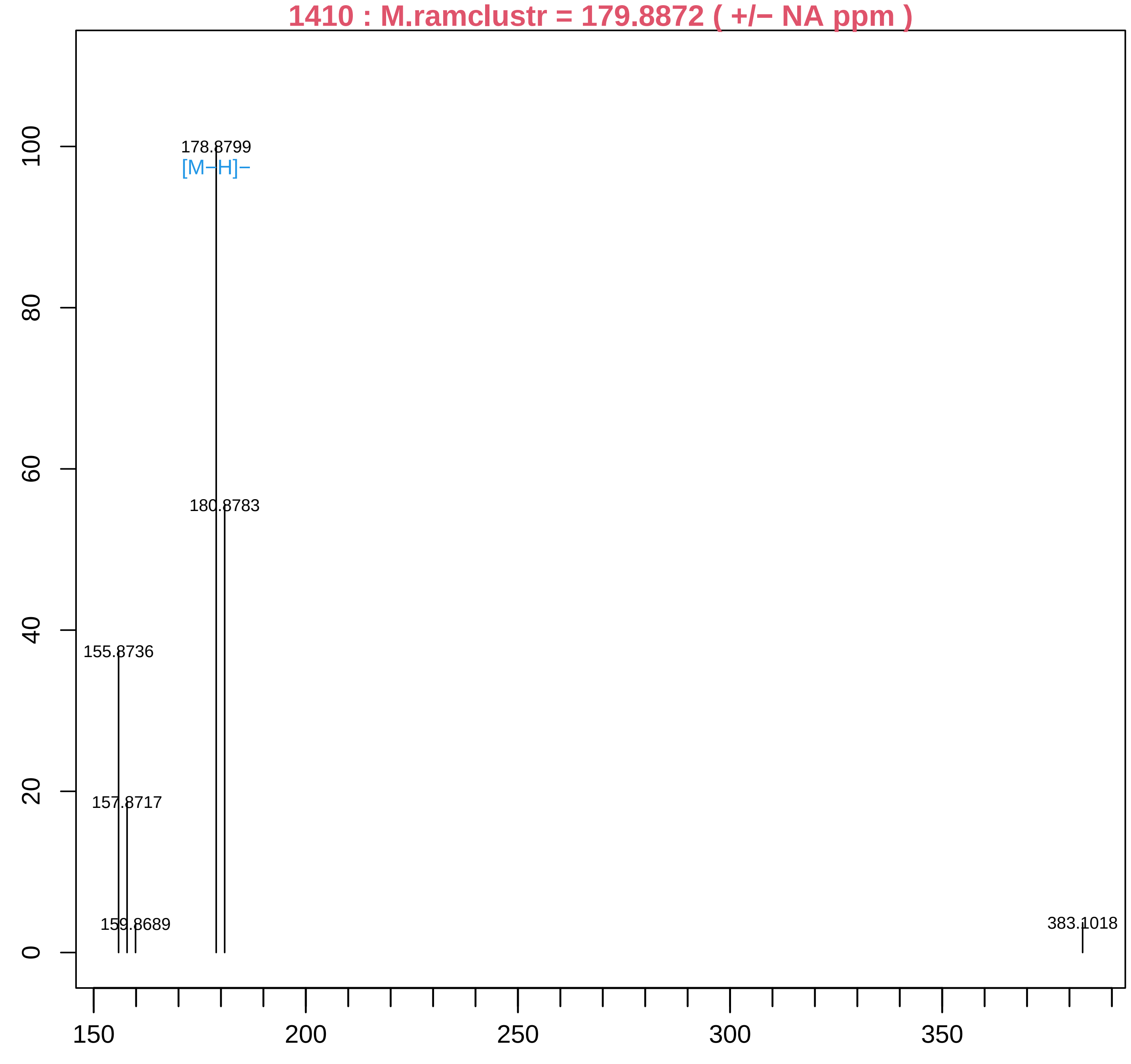

1410 : M.ramclustr = 179.8872 (+/− NA ppm)
178.8799
[M−H]−
180.8783
155.8736
157.8717
159.8689
383.1018
0
20
40
60
80
100
150
200
250
300
350

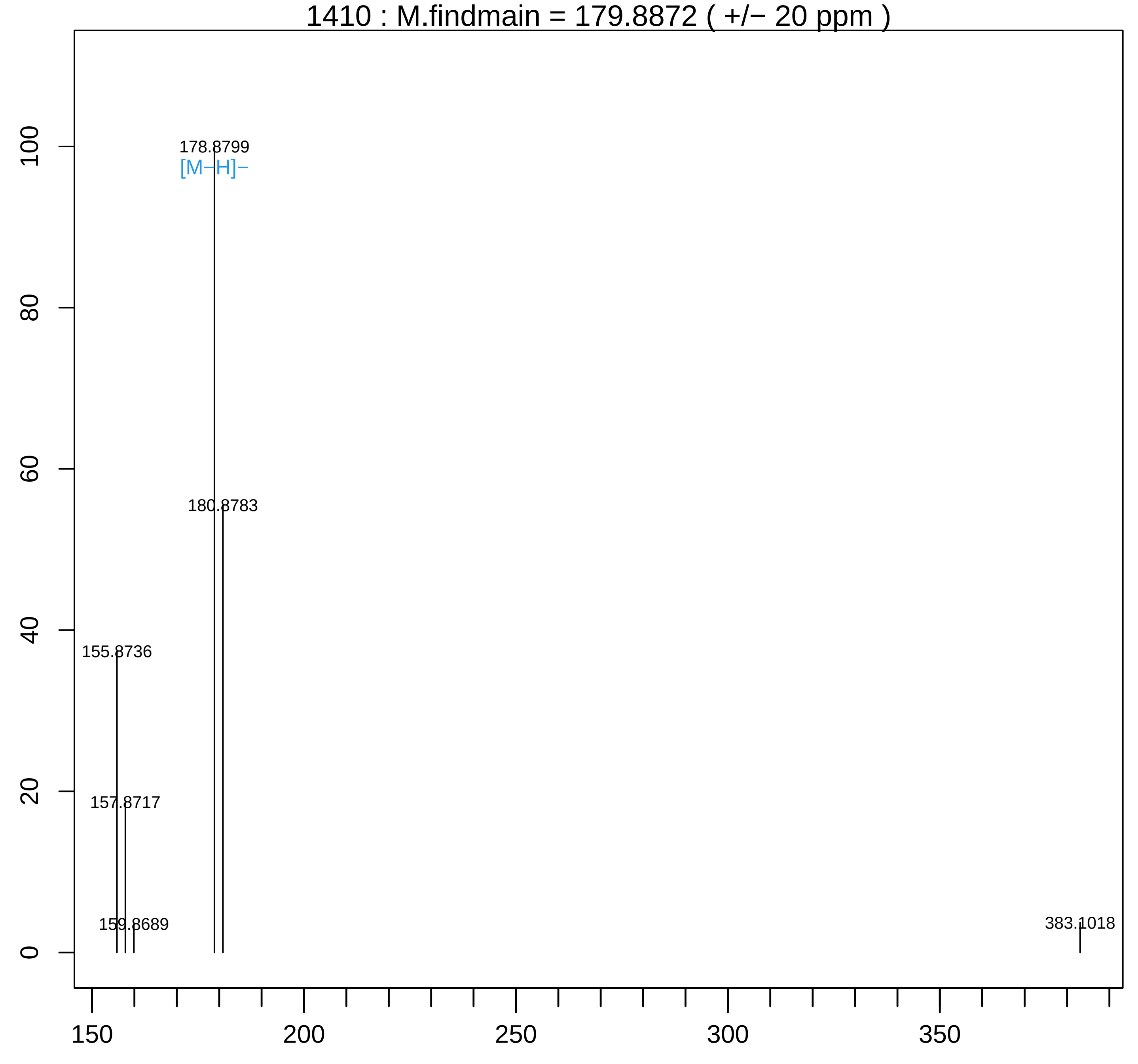

1410 : M.findmain = 179.8872 (+/− 20 ppm)
178.8799
[M−H]−
180.8783
155.8736
157.8717
159.8689
383.1018
0
20
40
60
80
100
150
200
250
300
350

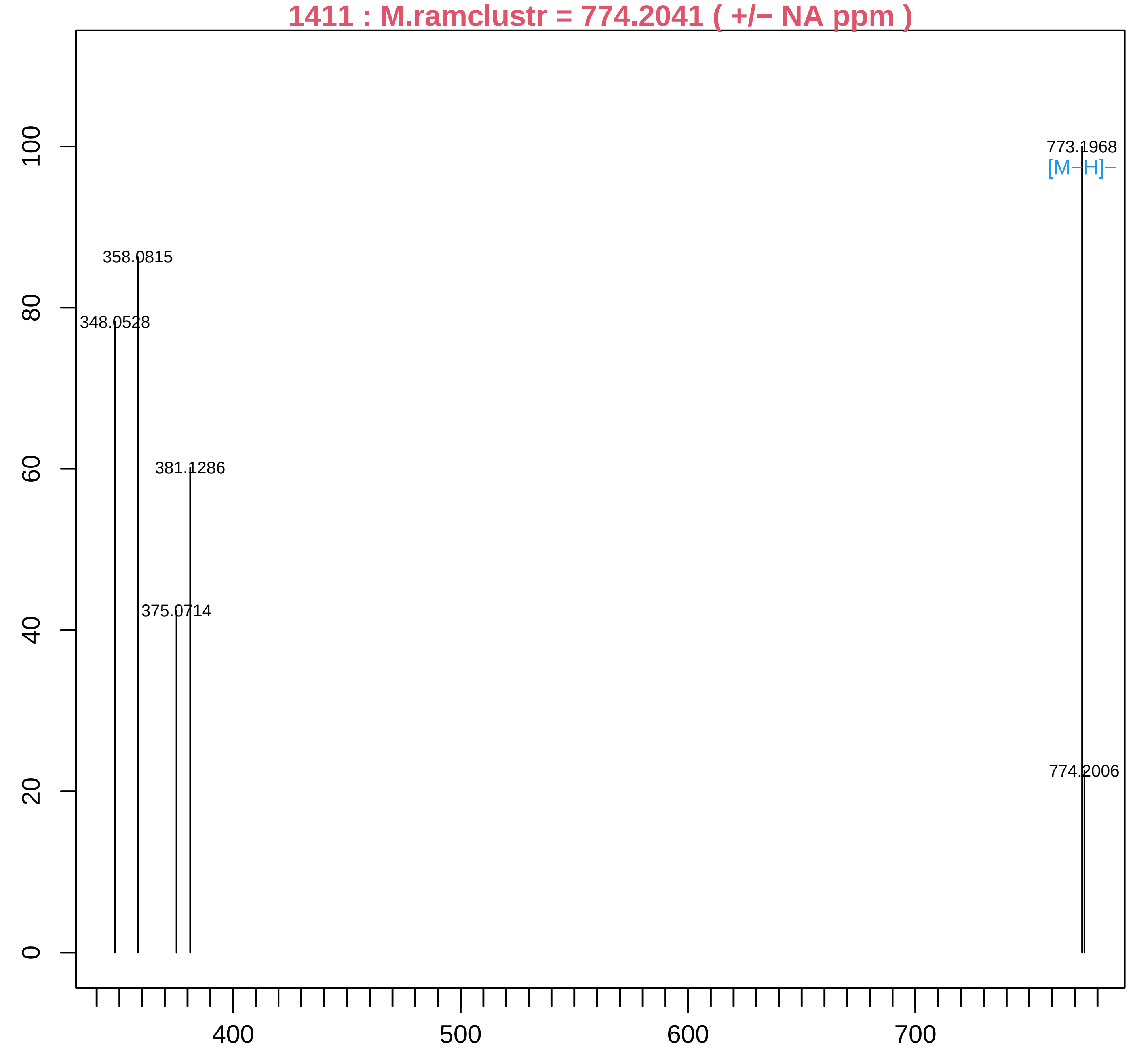
1411 : M.ramclustr = 774.2041 (+/− NA ppm)
773.1968
[M−H]−
358.0815
348.0528
381.1286
375.0714
774.2006
0
20
40
60
80
100
400
500
600
700

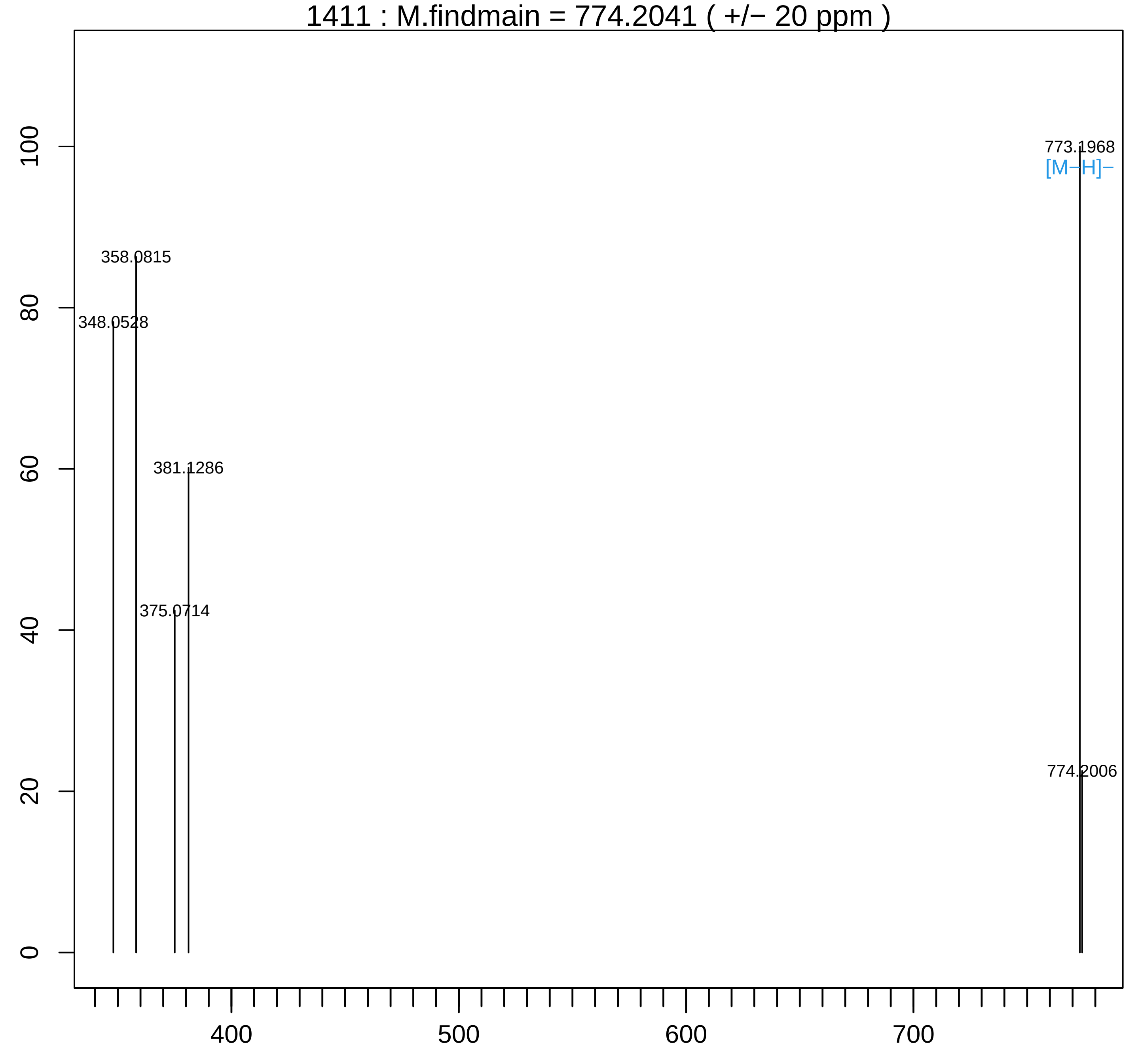
1411 : M.findmain = 774.2041 (+/− 20 ppm)
773.1968
[M−H]−
358.0815
348.0528
381.1286
375.0714
774.2006
0
20
40
60
80
100
400
500
600
700

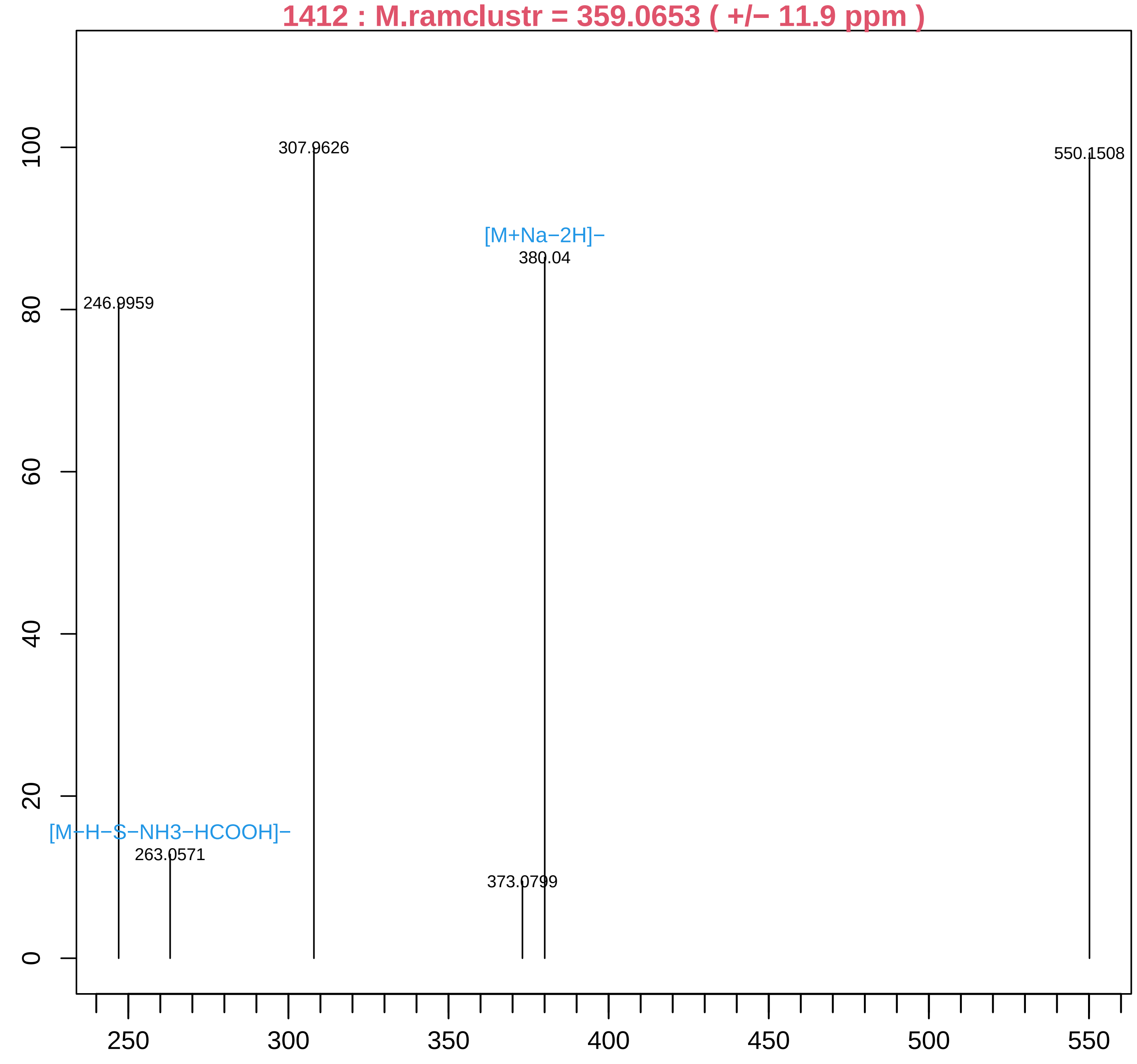
1412 : M.ramclustr = 359.0653 (+/− 11.9 ppm)
307.9626
550.1508
[M+Na−2H]−
380.04
246.9959
[M−H−S−NH3−HCOOH]−
263.0571
373.0799
0
20
40
60
80
100
250
300
350
400
450
500
550
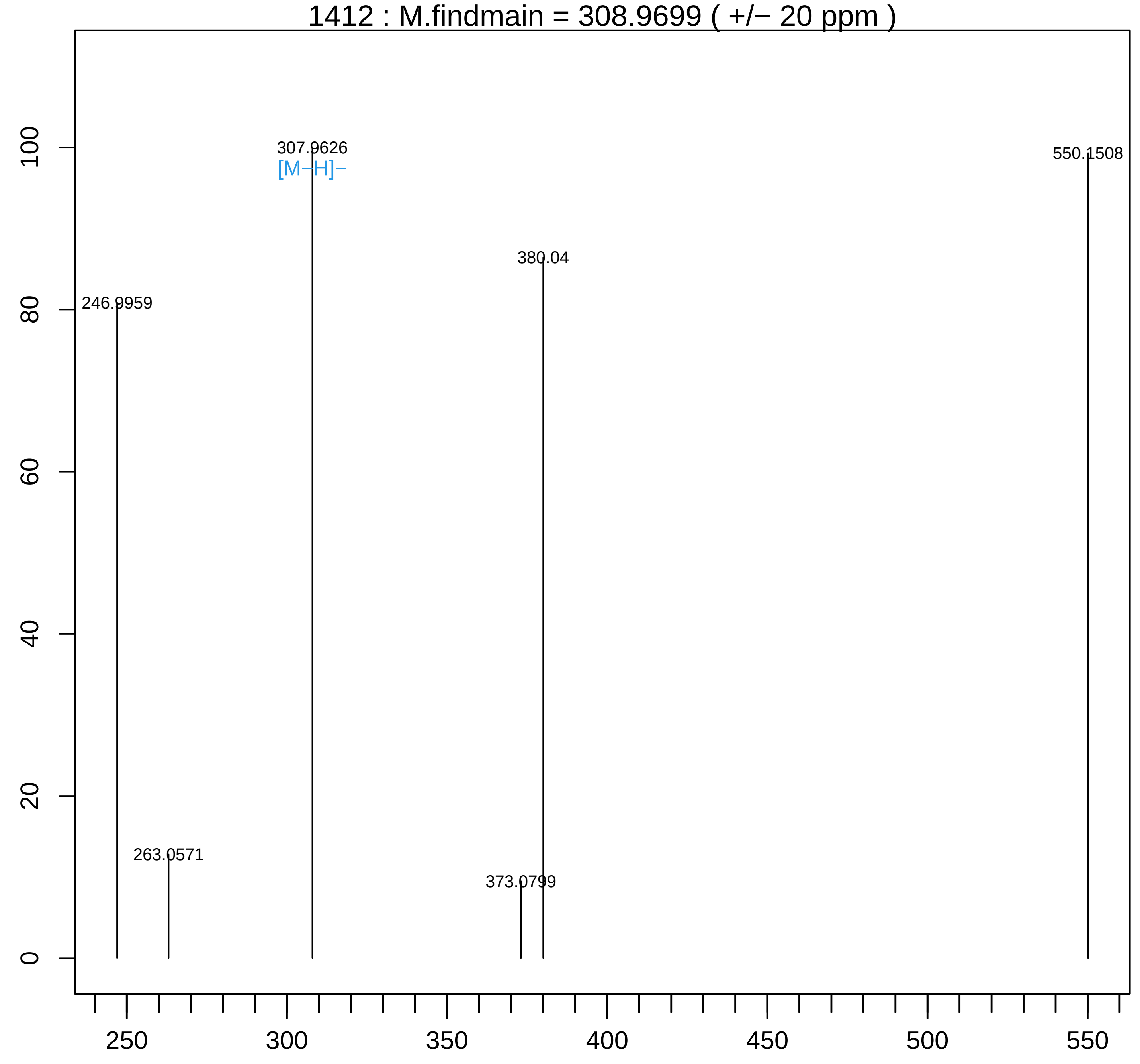
1412 : M.findmain = 308.9699 (+/− 20 ppm)
307.9626
[M−H]−
550.1508
380.04
246.9959
263.0571
373.0799
0
20
40
60
80
100
250
300
350
400
450
500
550

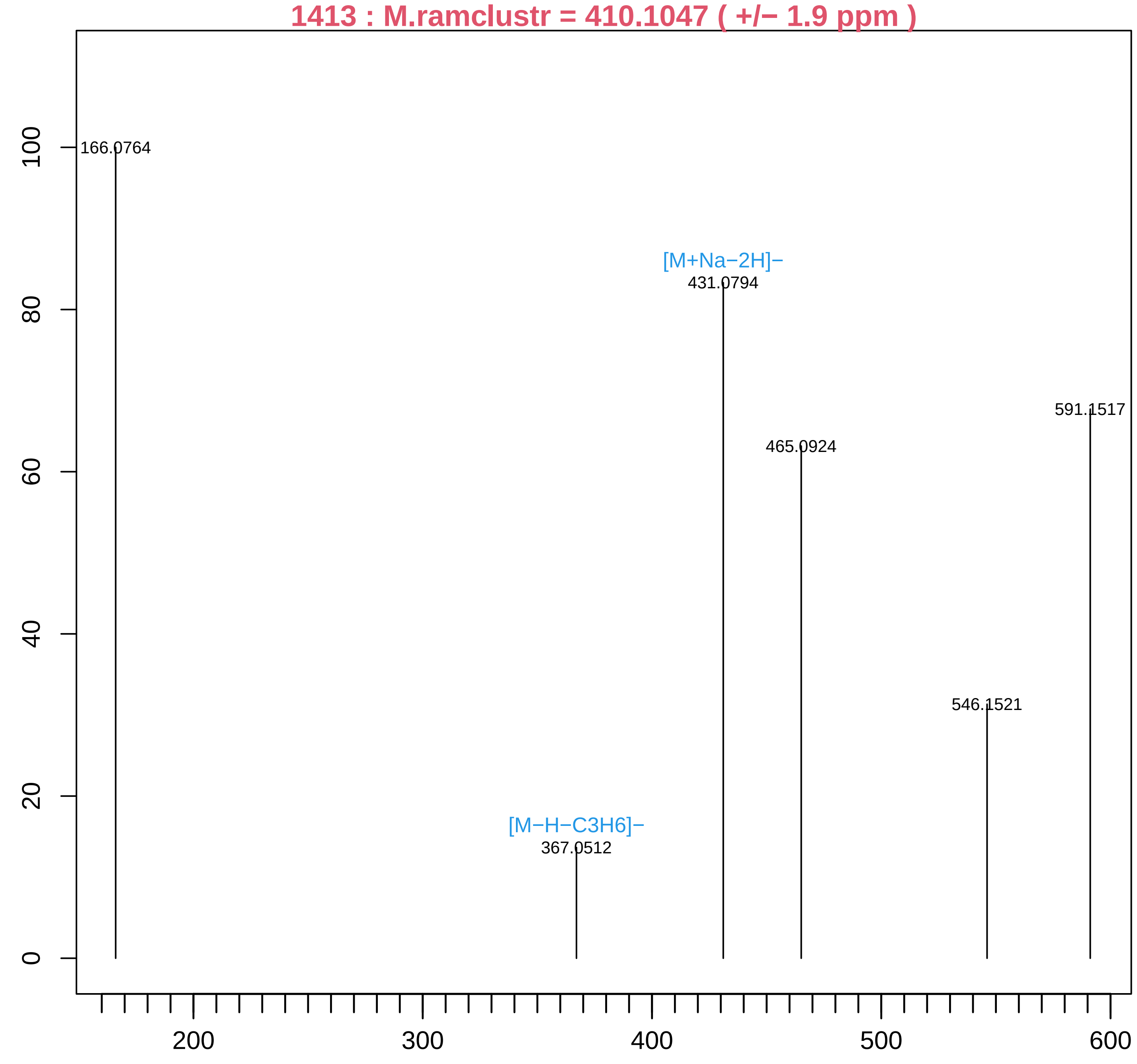
1413 : M.ramclustr = 410.1047 (+/− 1.9 ppm)
166.0764
[M+Na−2H]−
431.0794
465.0924
591.1517
546.1521
[M−H−C3H6]−
367.0512
0
20
40
60
80
100
200
300
400
500
600
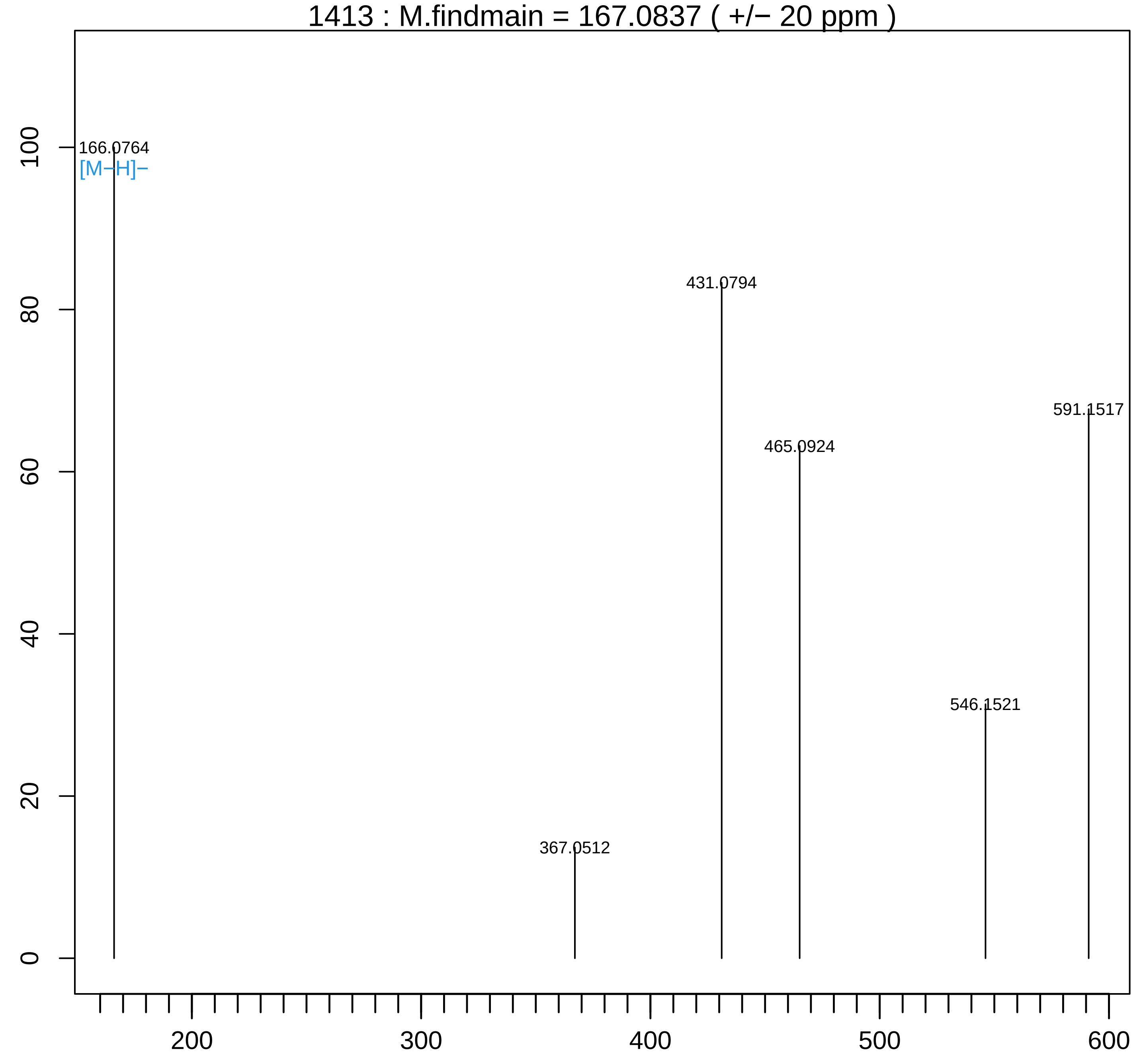
1413 : M.findmain = 167.0837 (+/− 20 ppm)
166.0764
[M−H]−
431.0794
465.0924
591.1517
546.1521
367.0512
0
20
40
60
80
100
200
300
400
500
600

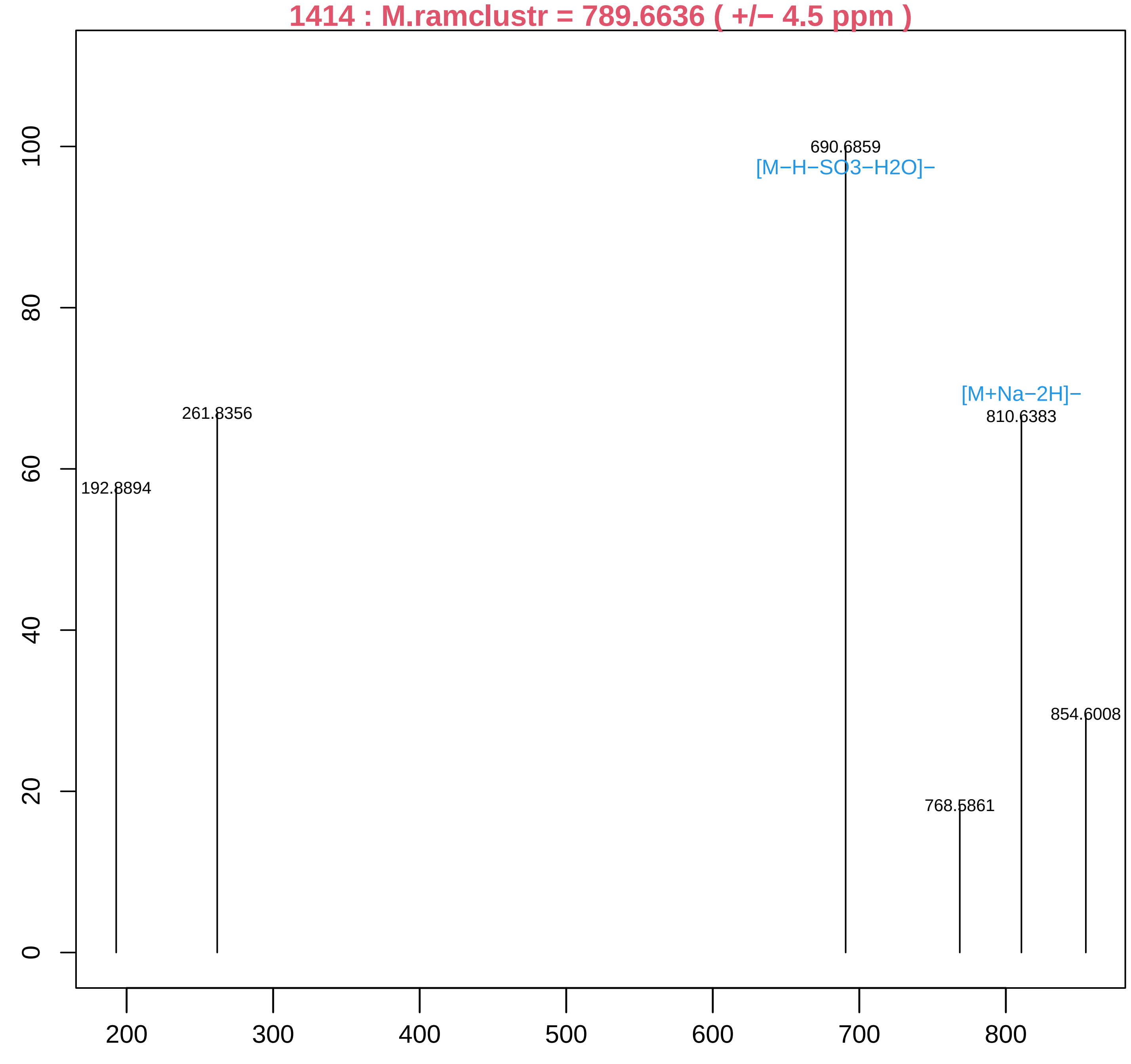
1414 : M.ramclustr = 789.6636 (+/− 4.5 ppm)
690.6859
[M−H−SO3−H2O]−
[M+Na−2H]−
810.6383
261.8356
192.8894
854.6008
768.5861
0
20
40
60
80
100
200
300
400
500
600
700
800
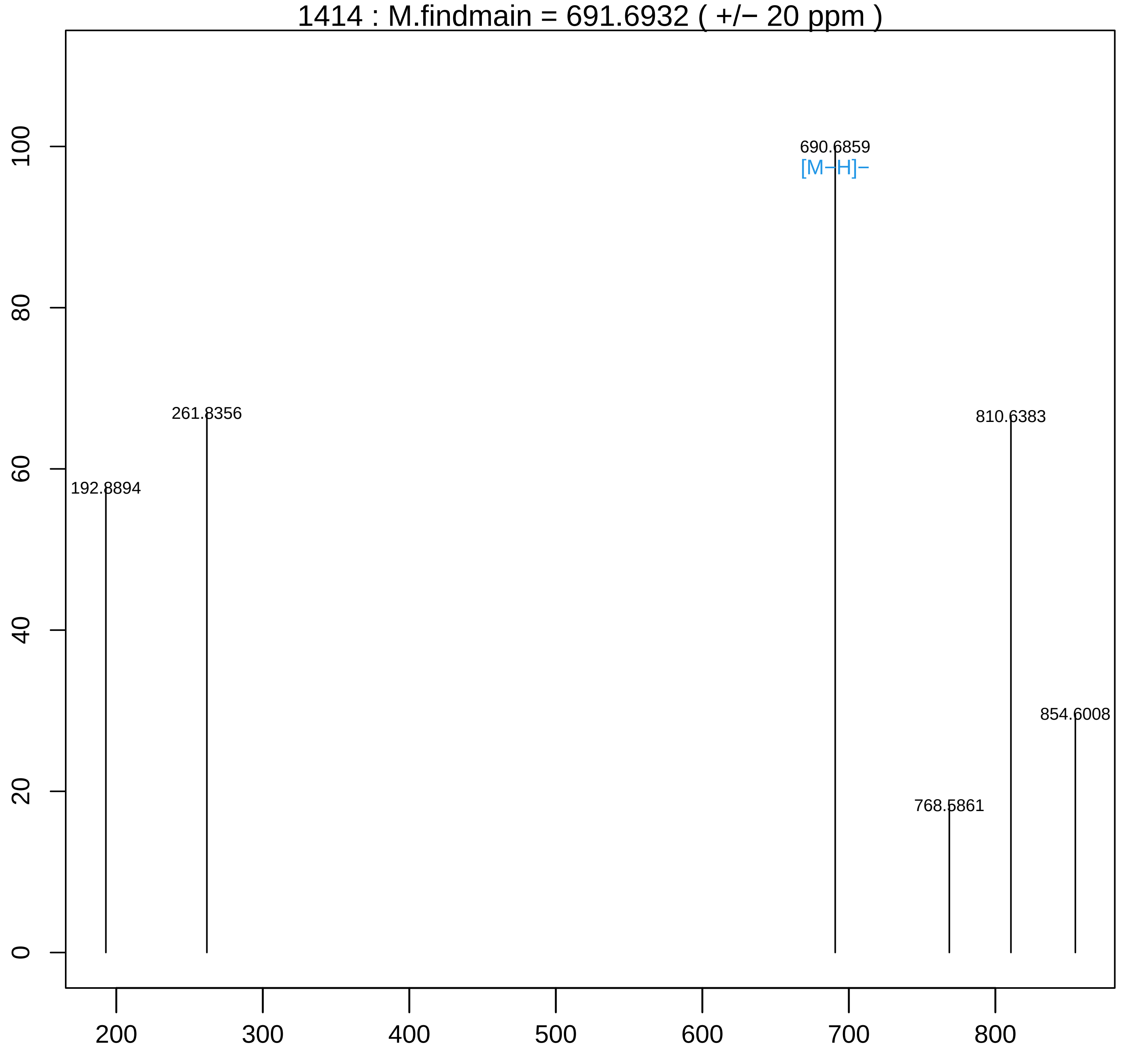
1414 : M.findmain = 691.6932 (+/− 20 ppm)
690.6859
[M−H]−
261.8356
810.6383
192.8894
854.6008
768.5861
0
20
40
60
80
100
200
300
400
500
600
700
800

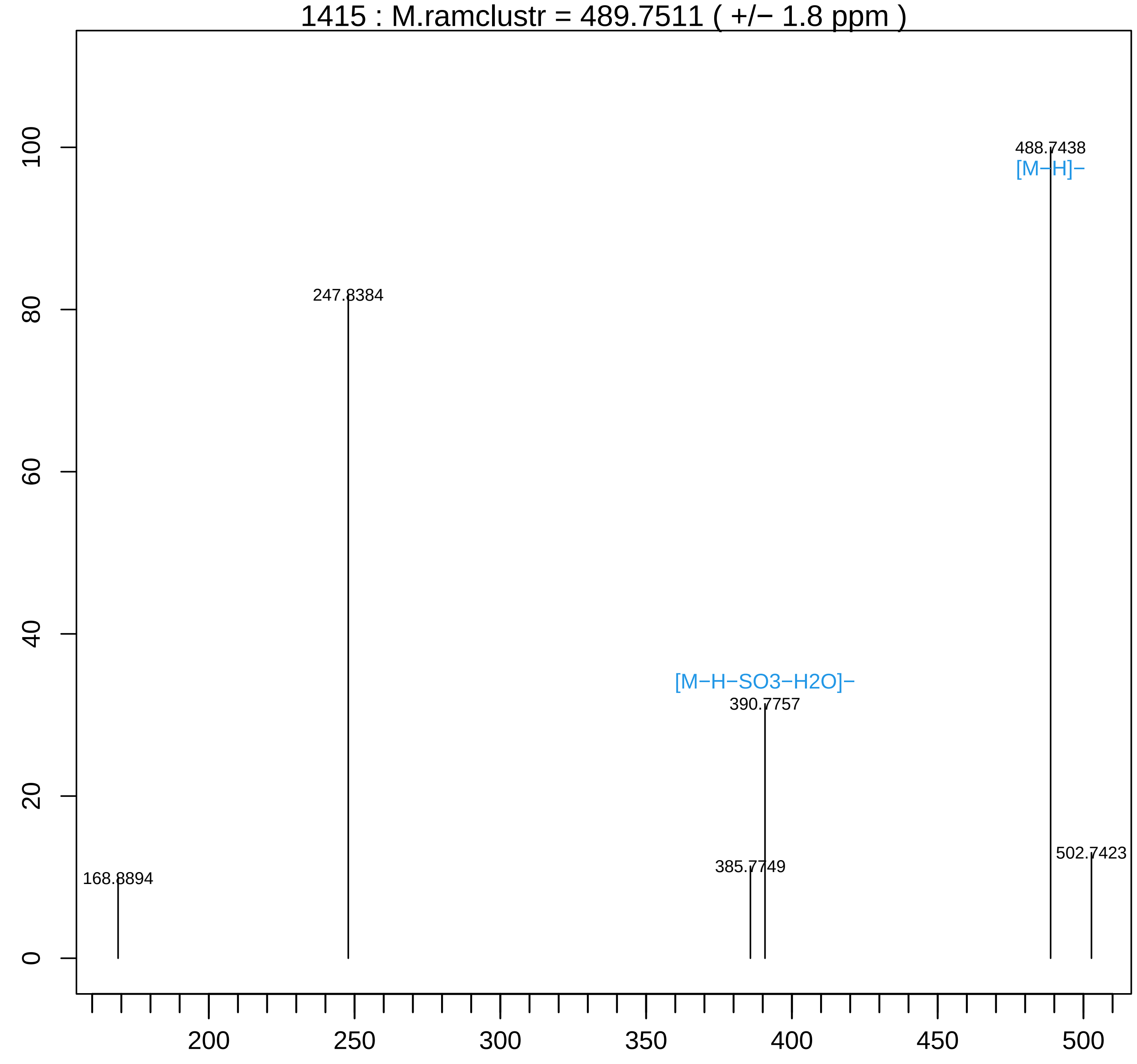
1415 : M.ramclustr = 489.7511 (+/− 1.8 ppm)
488.7438
[M−H]−
247.8384
[M−H−SO3−H2O]−
390.7757
385.7749
502.7423
168.8894
0
20
40
60
80
100
200
250
300
350
400
450
500

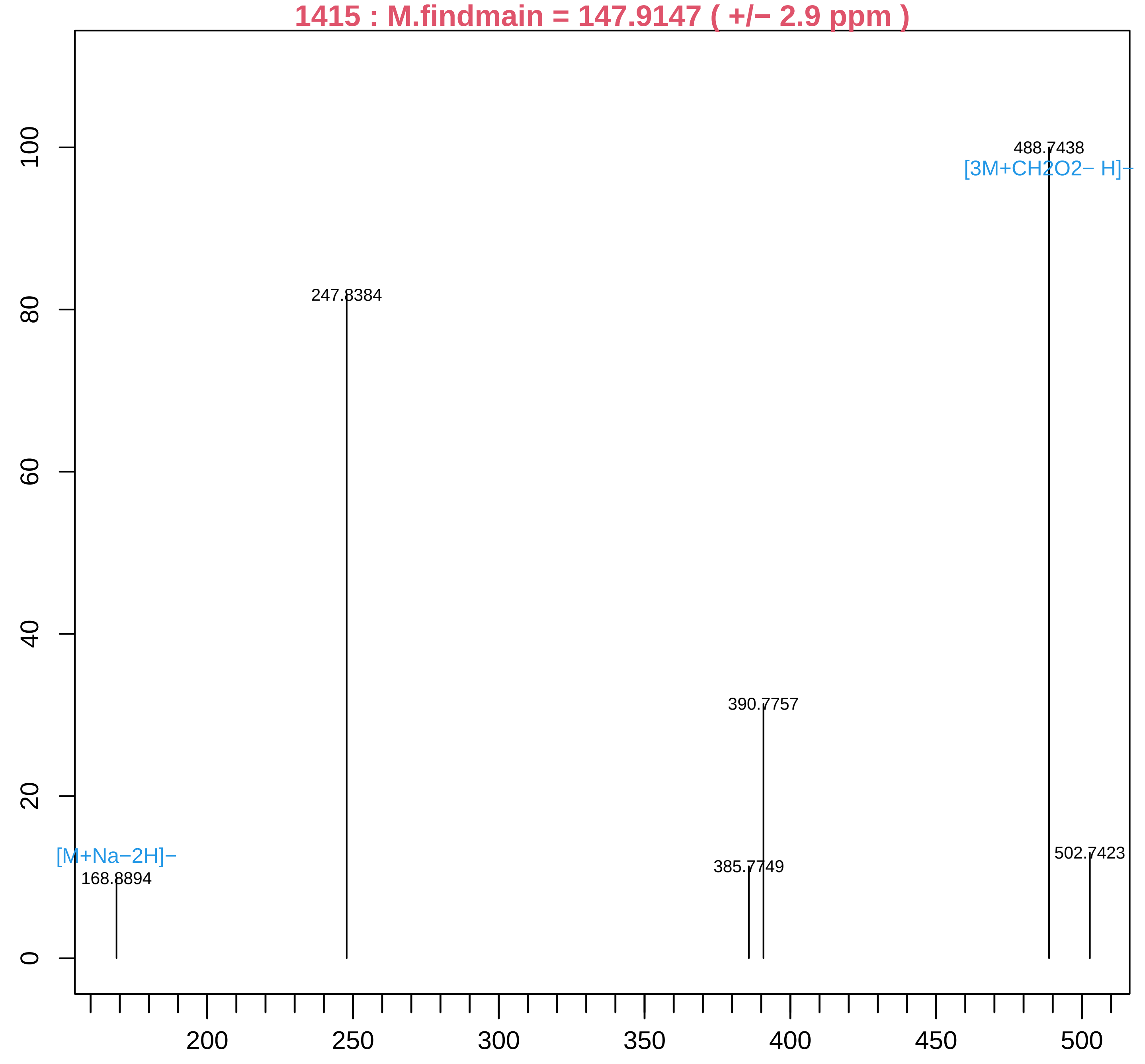
1415 : M.findmain = 147.9147 (+/− 2.9 ppm)
488.7438
[3M+CH2O2− H]−
247.8384
390.7757
[M+Na−2H]−
168.8894
385.7749
502.7423
0
20
40
60
80
100
200
250
300
350
400
450
500

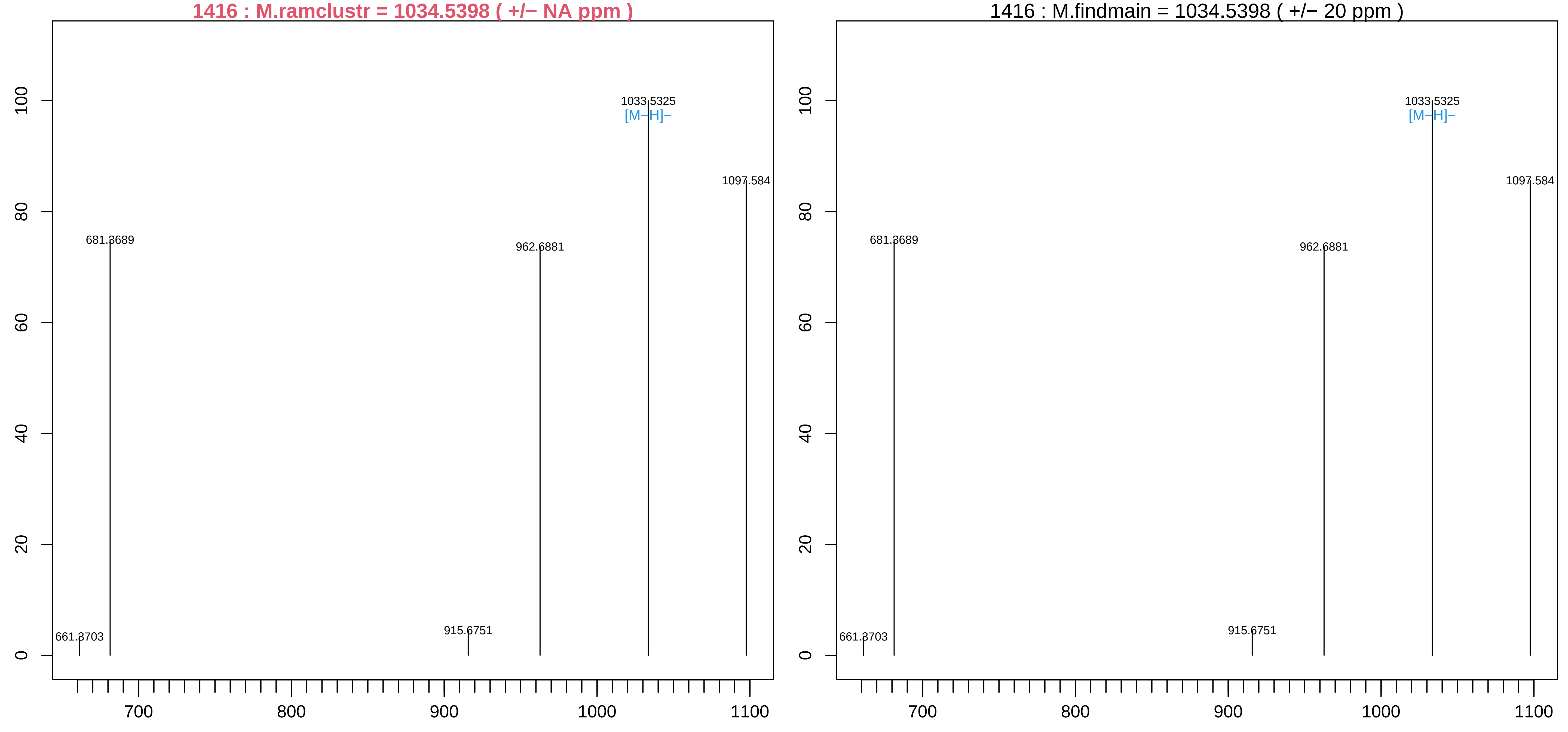

1416 : M.ramclustr = 1034.5398 (+/− NA ppm)
1416 : M.findmain = 1034.5398 (+/− 20 ppm)
1033.5325
[M−H]−
1097.584
681.3689
962.6881
661.3703
915.6751
0
20
40
60
80
100
700
800
900
1000
1100

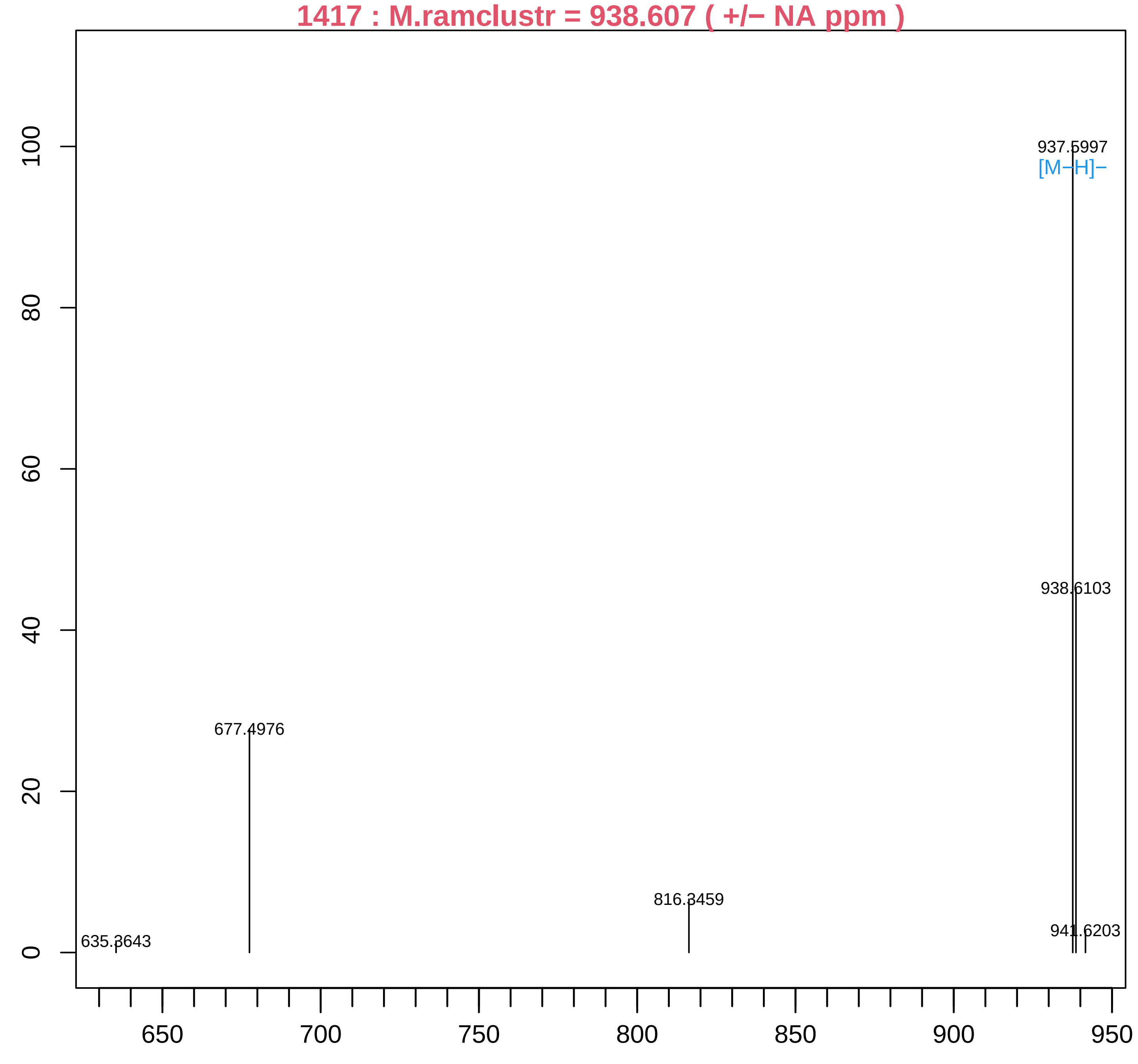
1417 : M.ramclustr = 938.607 (+/− NA ppm)
937.5997
[M−H]−
938.6103
677.4976
816.3459
941.6203
635.3643
0
20
40
60
80
100
650
700
750
800
850
900
950

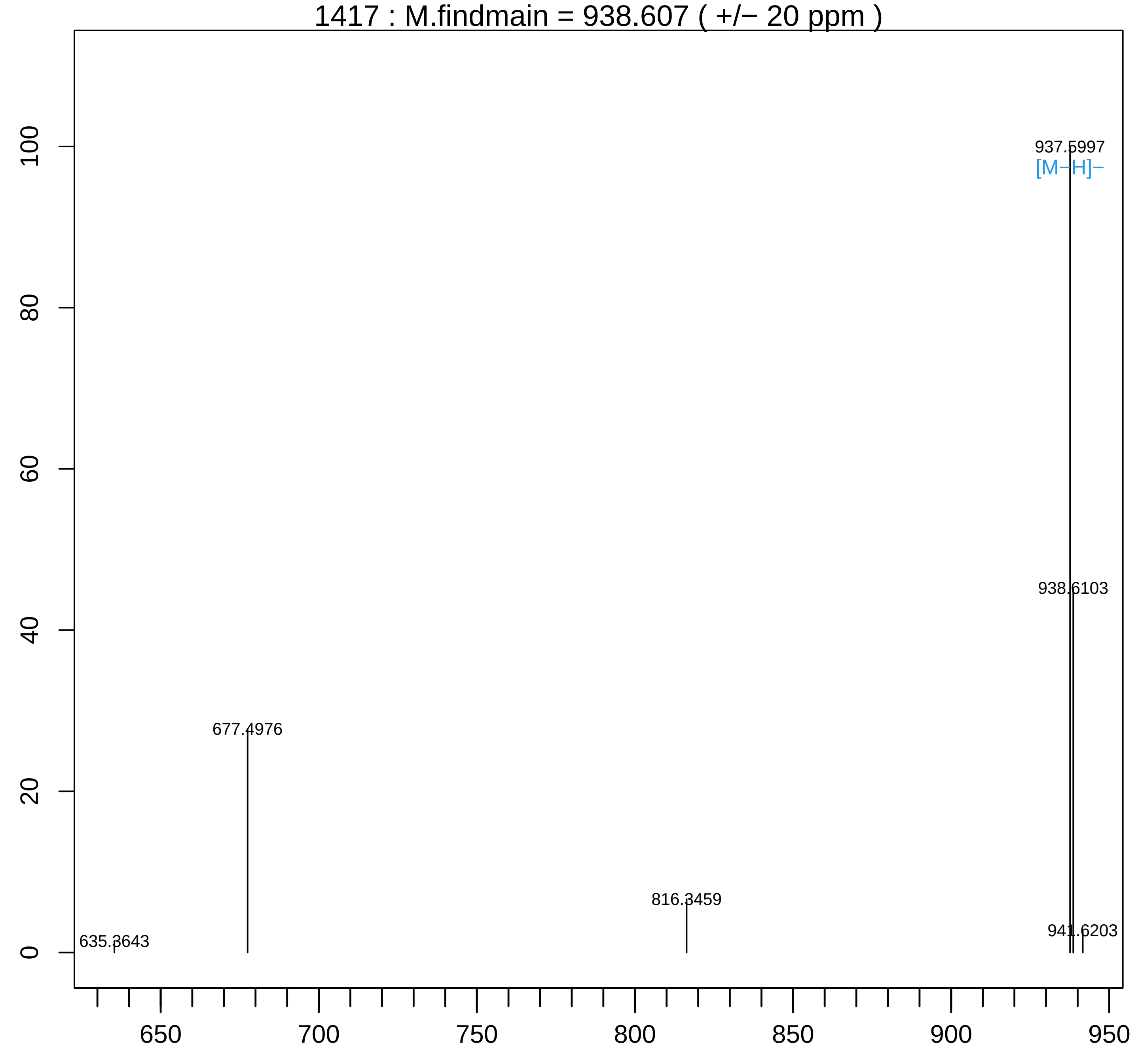
1417 : M.findmain = 938.607 (+/− 20 ppm)
937.5997
[M−H]−
938.6103
677.4976
816.3459
941.6203
635.3643
0
20
40
60
80
100
650
700
750
800
850
900
950

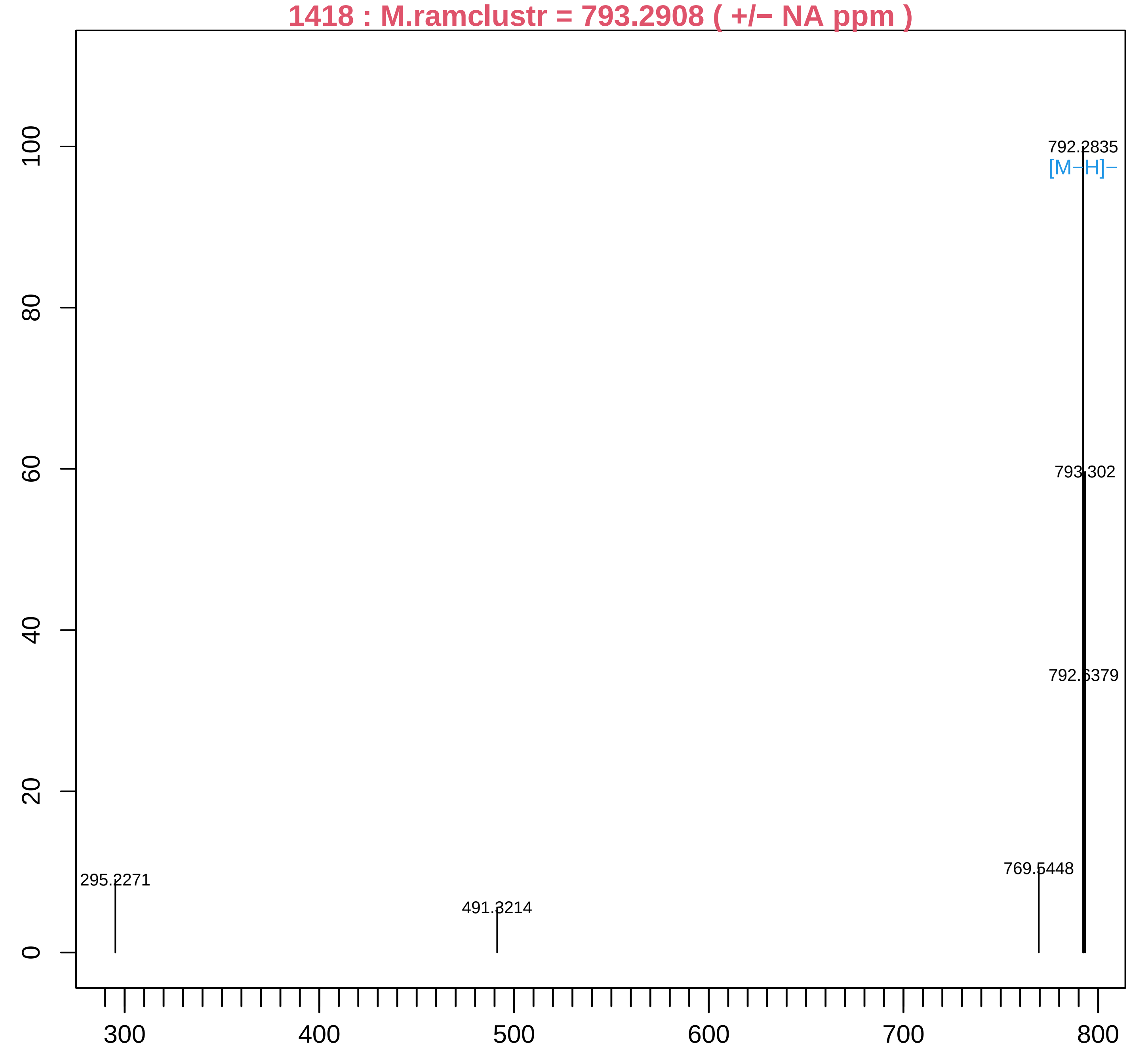
1418 : M.ramclustr = 793.2908 (+/− NA ppm)
792.2835
[M−H]−
793.302
792.6379
769.5448
295.2271
491.3214

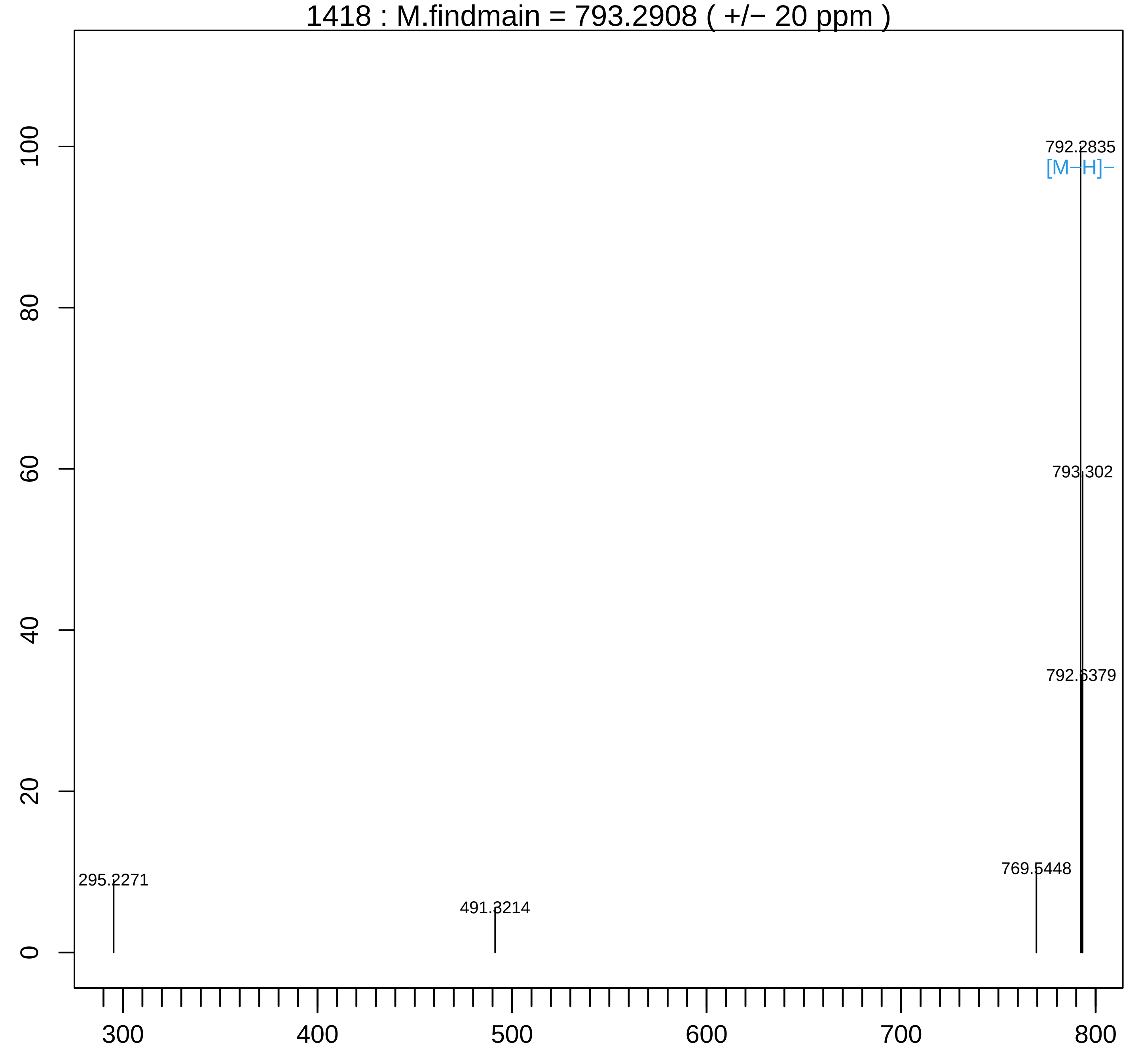
1418 : M.findmain = 793.2908 (+/− 20 ppm)
792.2835
[M−H]−
793.302
792.6379
769.5448
295.2271
491.3214

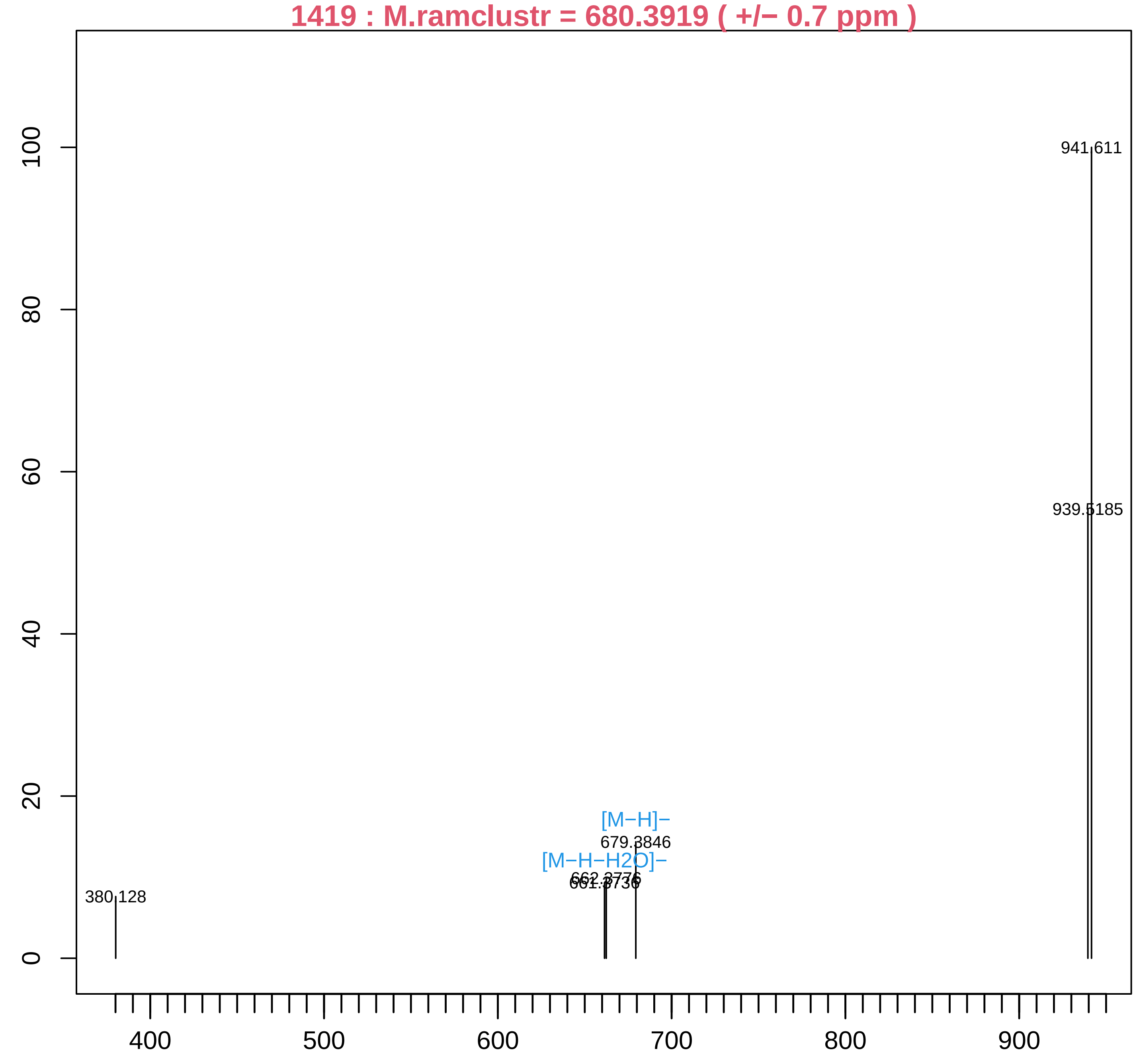
1419 : M.ramclustr = 680.3919 (+/− 0.7 ppm)
941.611
939.5185
[M−H]−
679.3846
[M−H−H2O]−
662.3776
661.3736
380.128
0
20
40
60
80
100
400
500
600
700
800
900
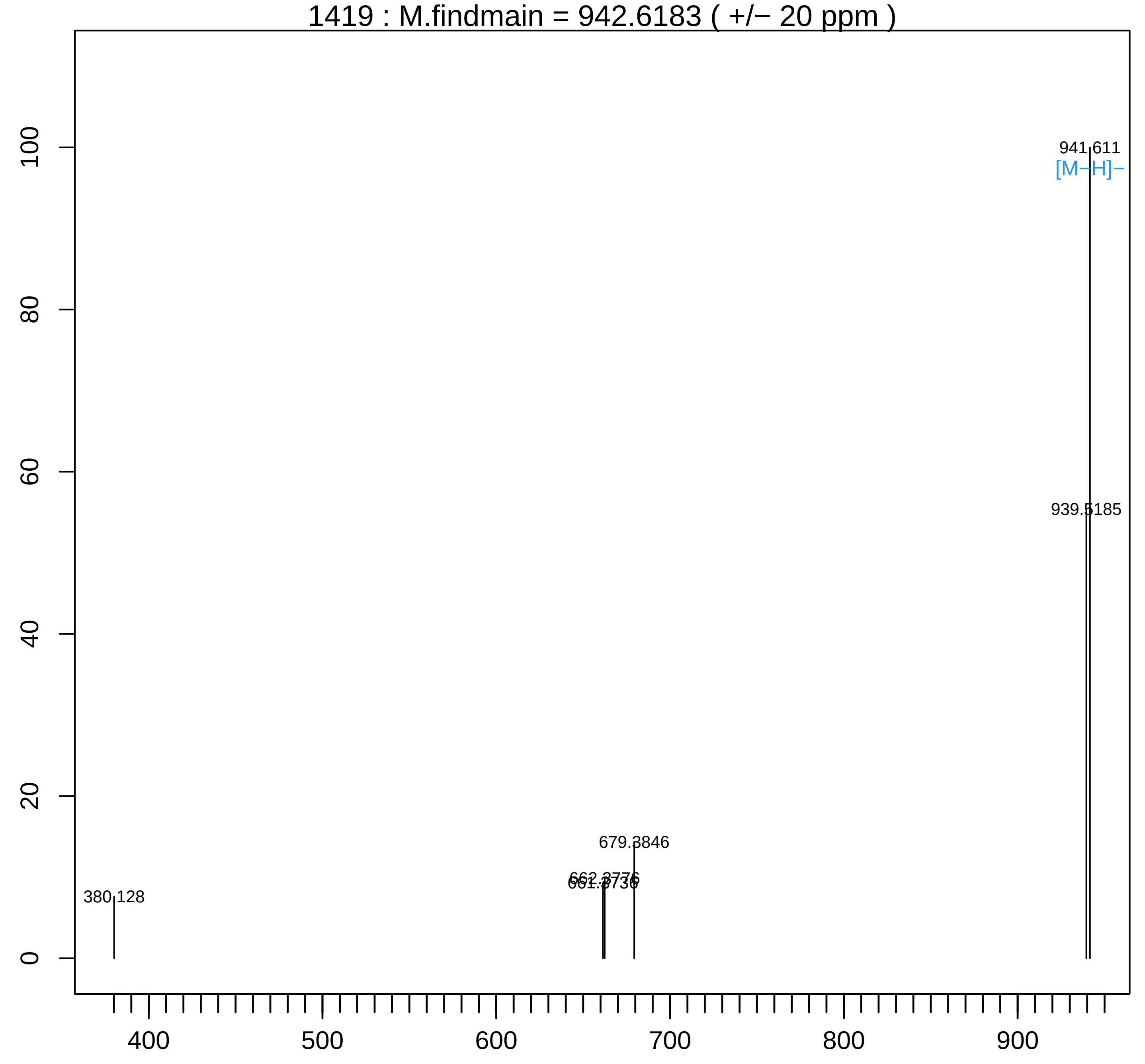
1419 : M.findmain = 942.6183 (+/− 20 ppm)
941.611
[M−H]−
939.5185
679.3846
662.3776
661.3736
380.128
0
20
40
60
80
100
400
500
600
700
800
900

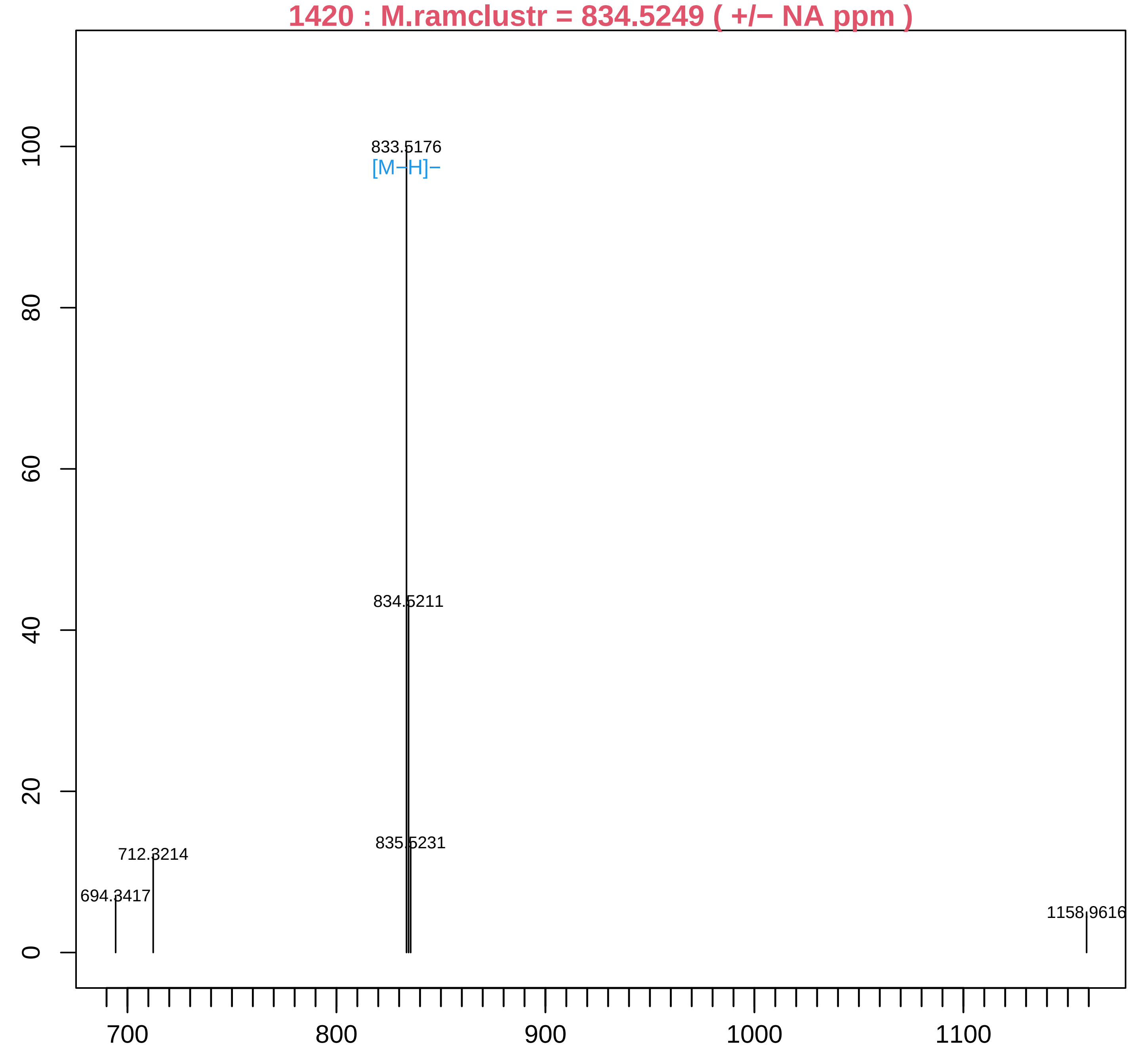
1420 : M.ramclustr = 834.5249 (+/− NA ppm)
833.5176
[M−H]−
834.5211
835.5231
712.3214
694.3417
1158.9616
0
20
40
60
80
100
700
800
900
1000
1100

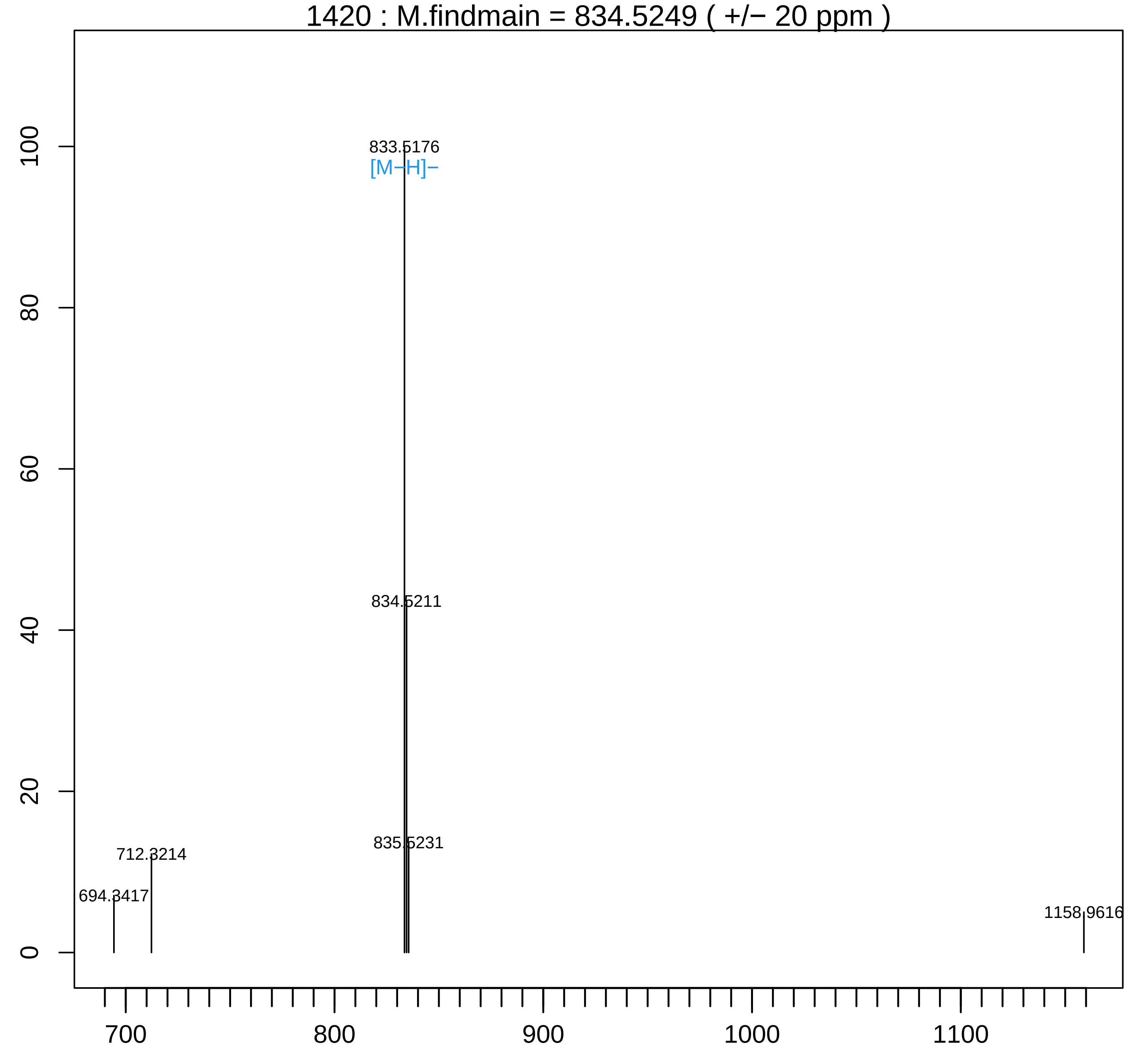
1420 : M.findmain = 834.5249 (+/− 20 ppm)
833.5176
[M−H]−
834.5211
835.5231
712.3214
694.3417
1158.9616
0
20
40
60
80
100
700
800
900
1000
1100

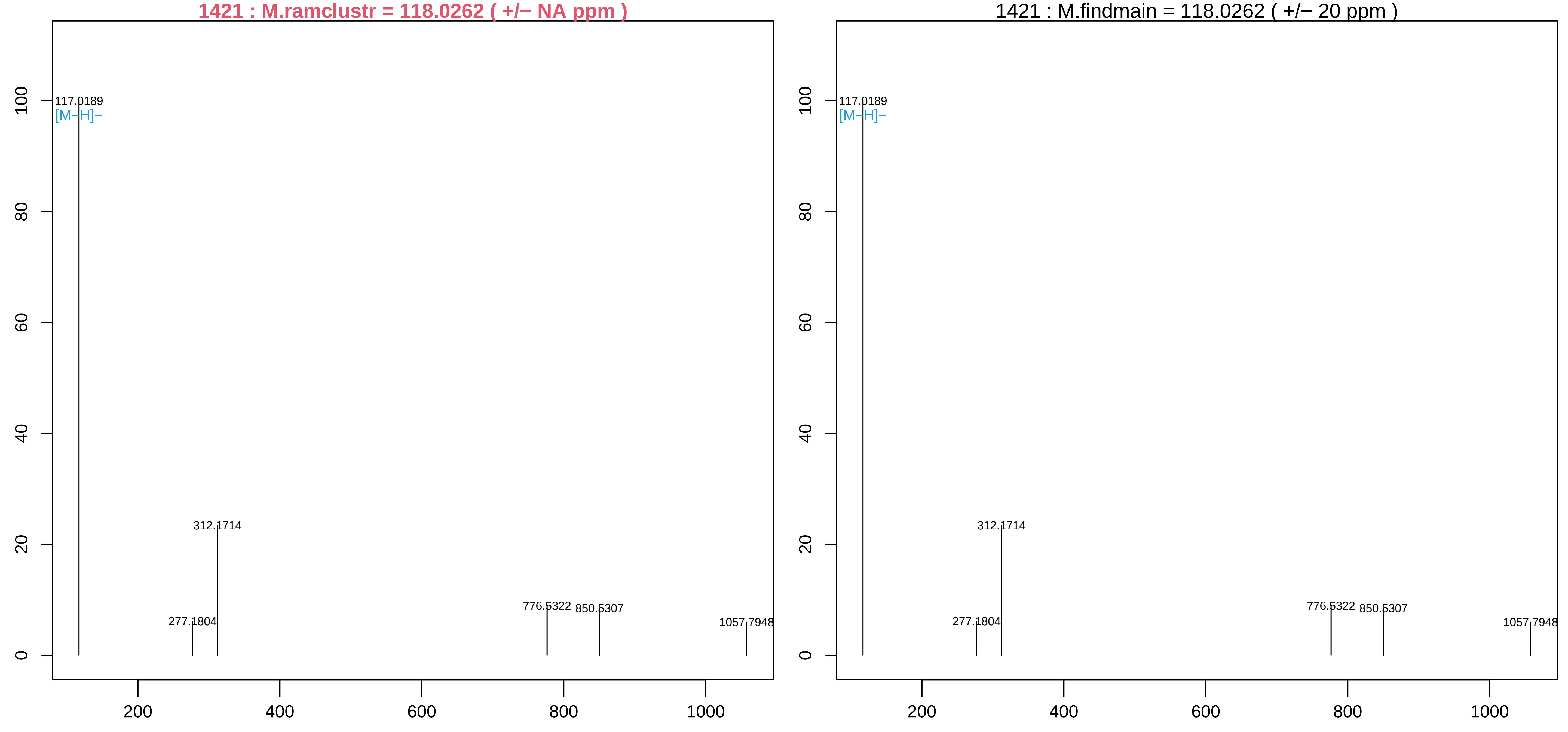
1421 : M.ramclustr = 118.0262 (+/− NA ppm)
117.0189
[M−H]−
312.1714
277.1804
776.5322
850.5307
1057.7948
1421 : M.findmain = 118.0262 (+/− 20 ppm)
117.0189
[M−H]−
312.1714
277.1804
776.5322
850.5307
1057.7948
0
20
40
60
80
100
200
400
600
800
1000

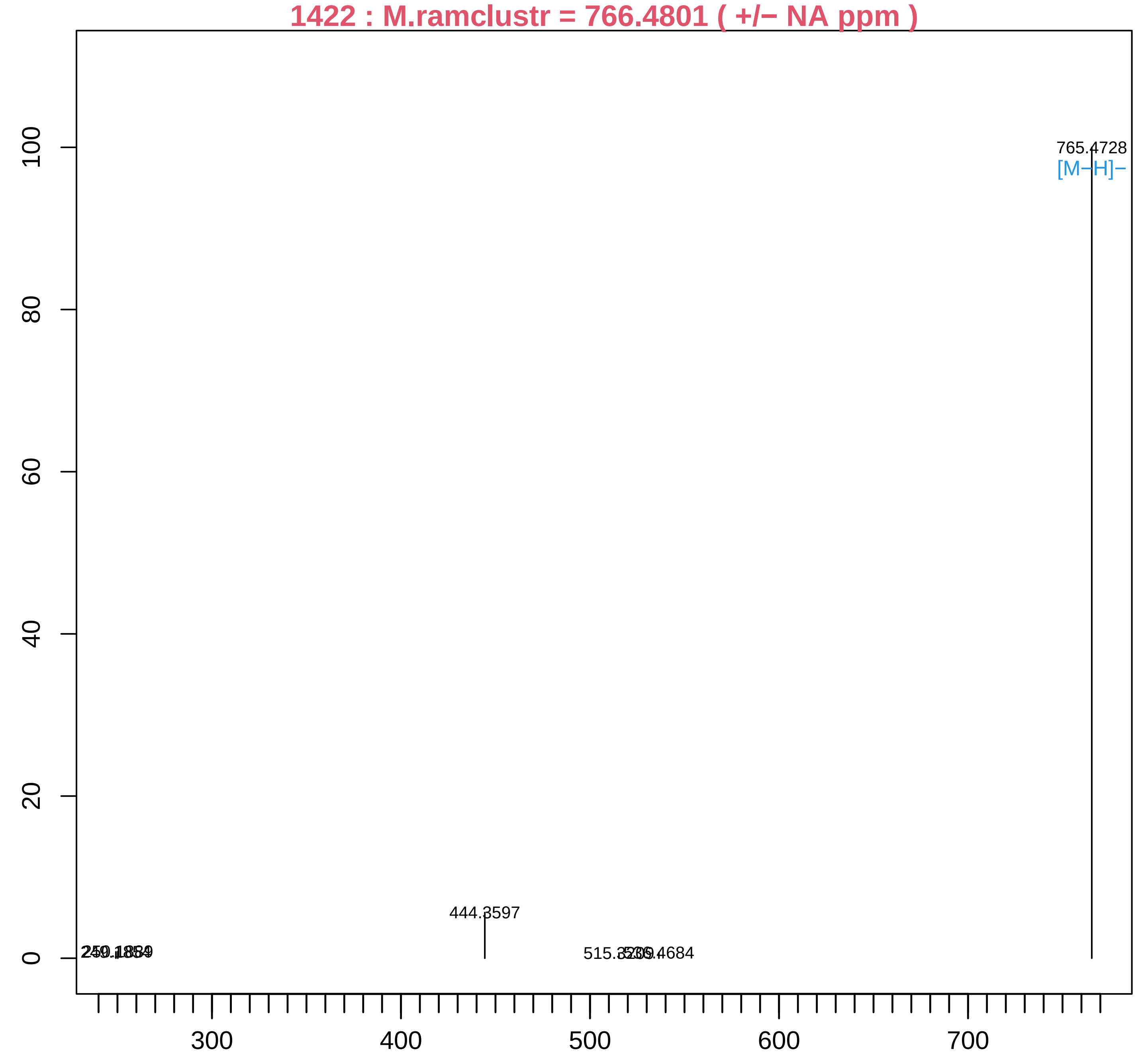
1422 : M.ramclustr = 766.4801 (+/− NA ppm)
765.4728
[M−H]−
444.3597
0
20
40
60
80
100
300
400
500
600
700

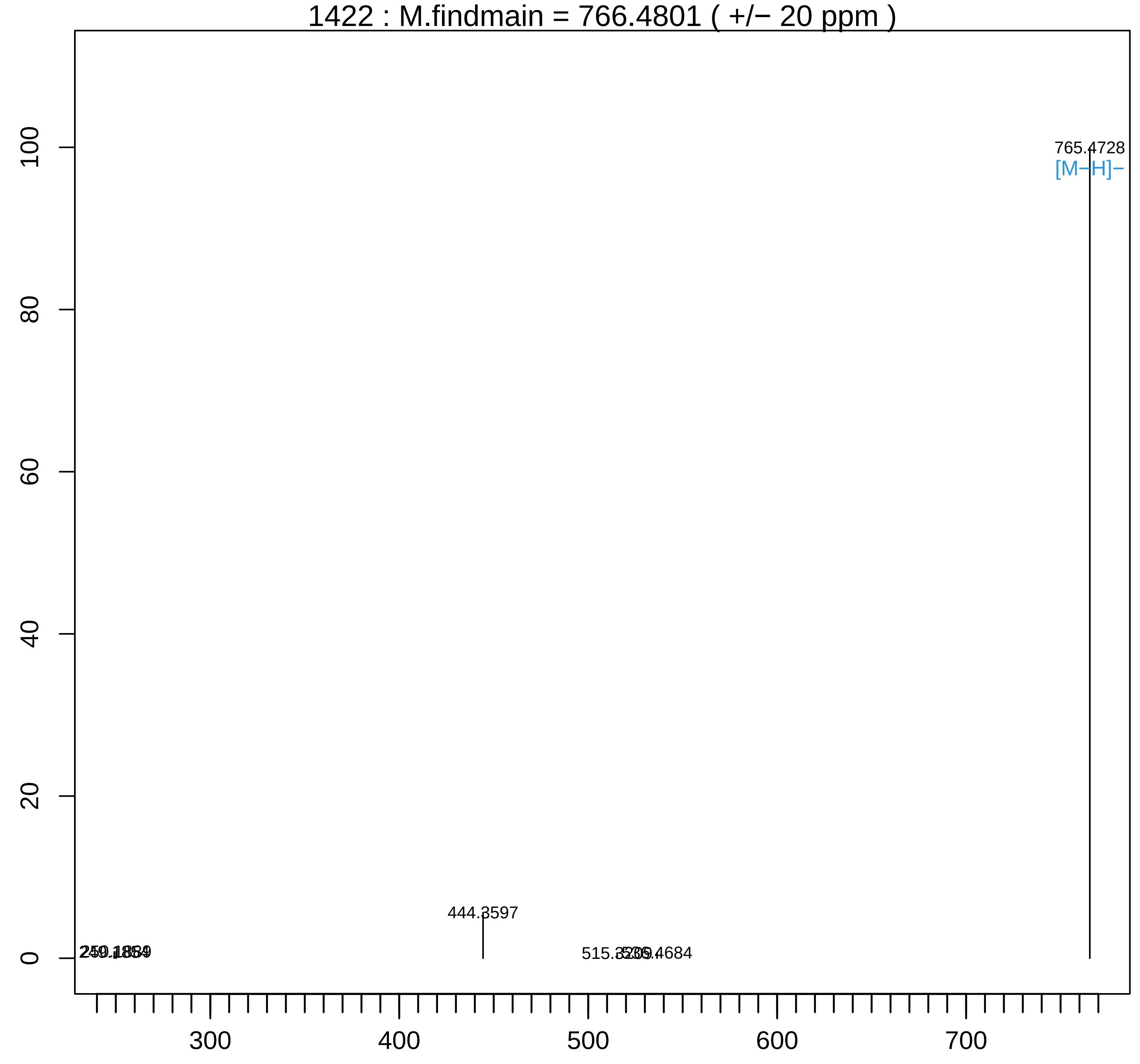
1422 : M.findmain = 766.4801 (+/− 20 ppm)
765.4728
[M−H]−
444.3597
0
20
40
60
80
100
300
400
500
600
700

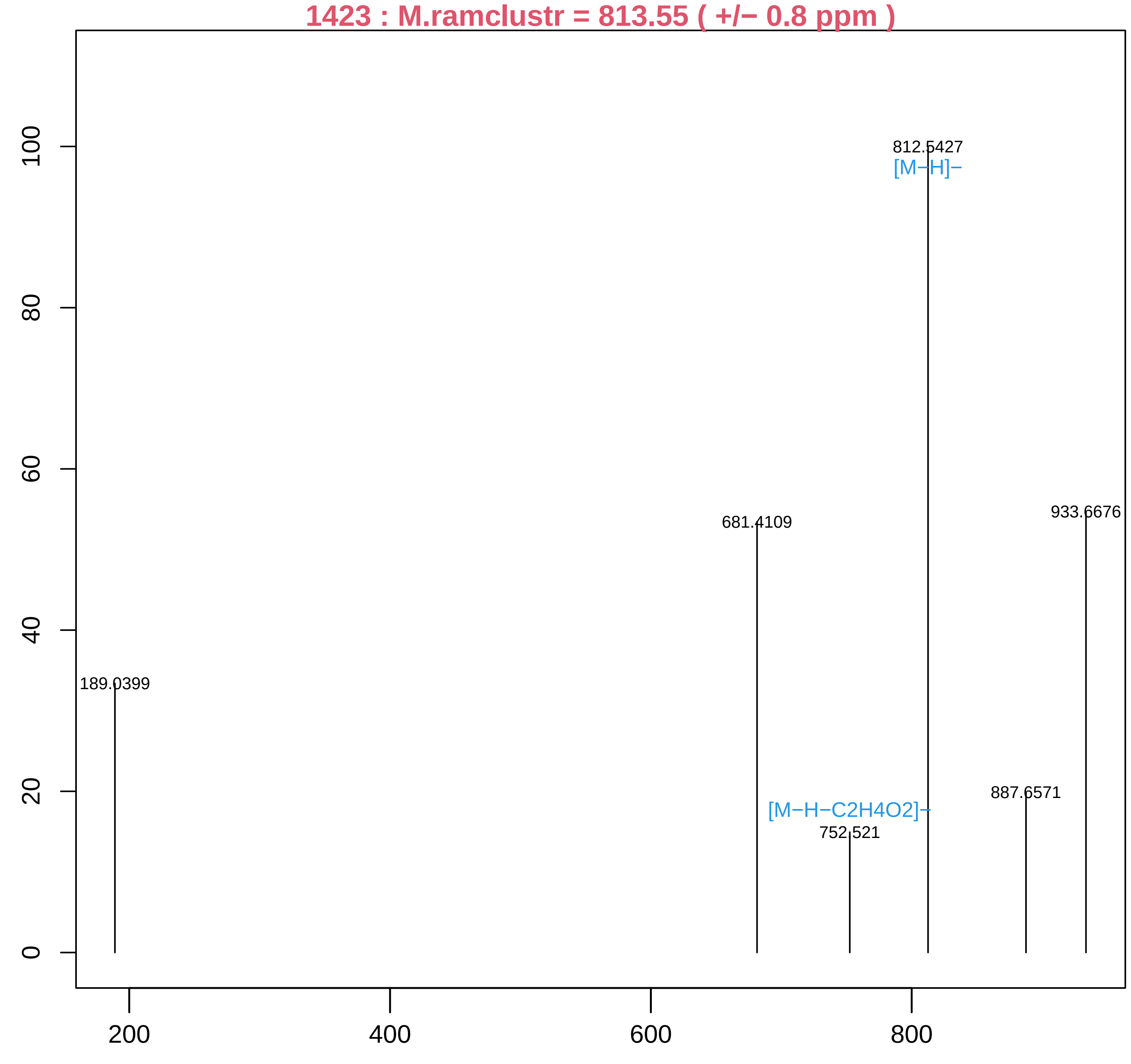

1423 : M.ramclustr = 813.55 (+/− 0.8 ppm)
812.5427
[M−H]−
681.4109
933.6676
189.0399
887.6571
[M−H−C2H4O2]−
752.521
0
20
40
60
80
100
200
400
600
800

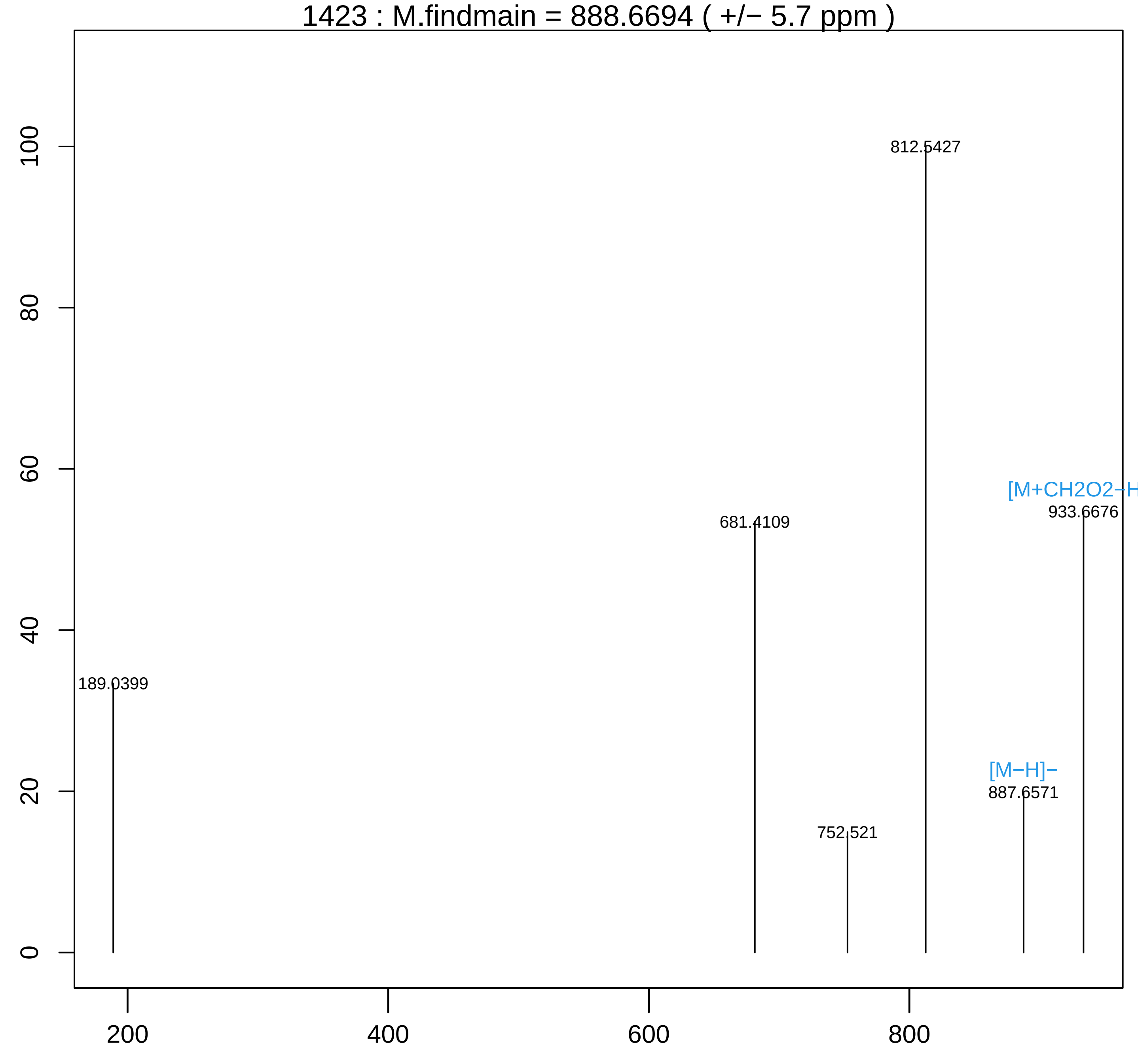

1423 : M.findmain = 888.6694 (+/− 5.7 ppm)
812.5427
681.4109
[M+CH2O2−H
933.6676
189.0399
[M−H]−
887.6571
752.521
0
20
40
60
80
100
200
400
600
800

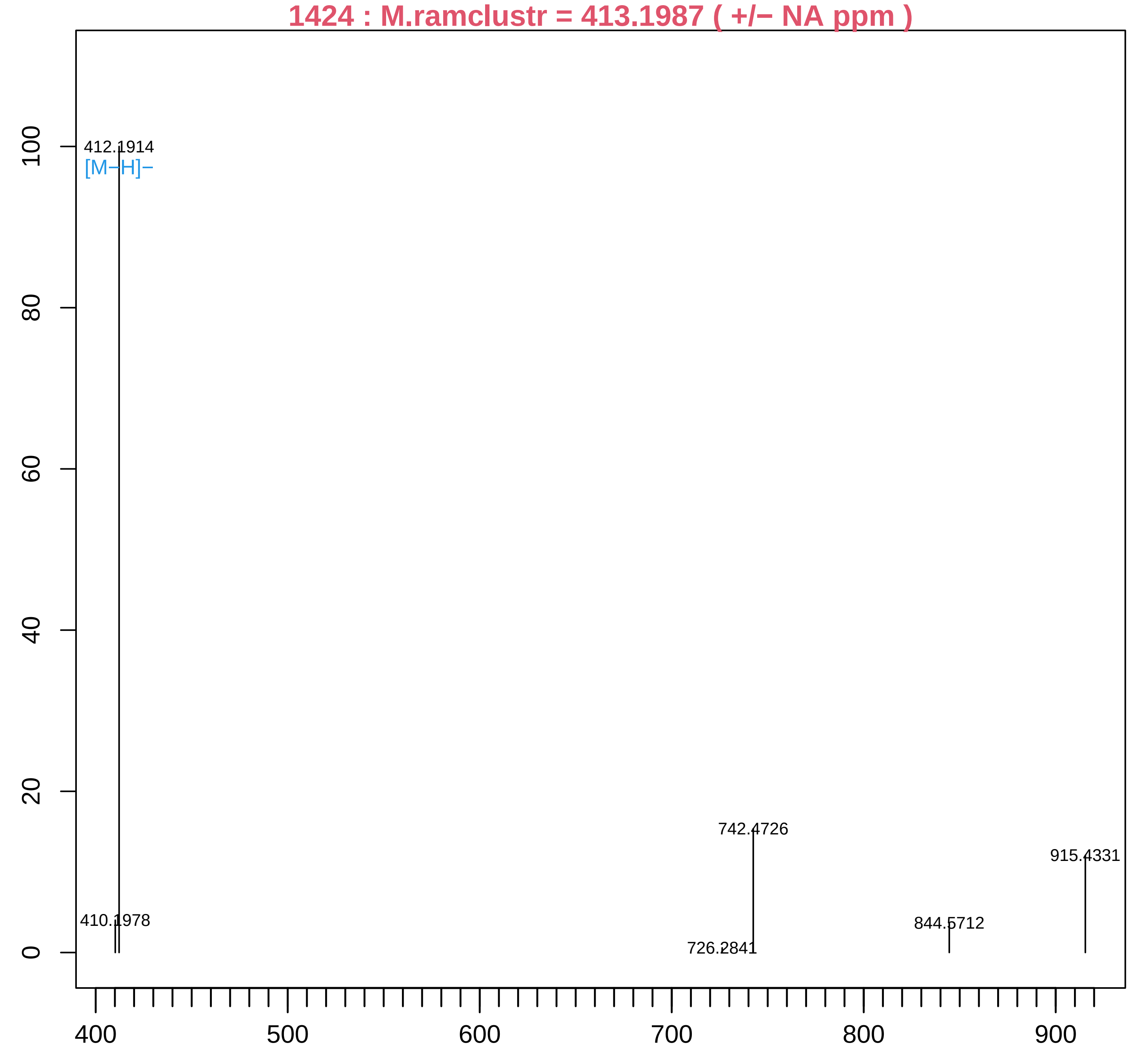
1424 : M.ramclustr = 413.1987 (+/− NA ppm)
412.1914
[M−H]−
410.1978
742.4726
726.2841
844.5712
915.4331
0
20
40
60
80
100
400
500
600
700
800
900

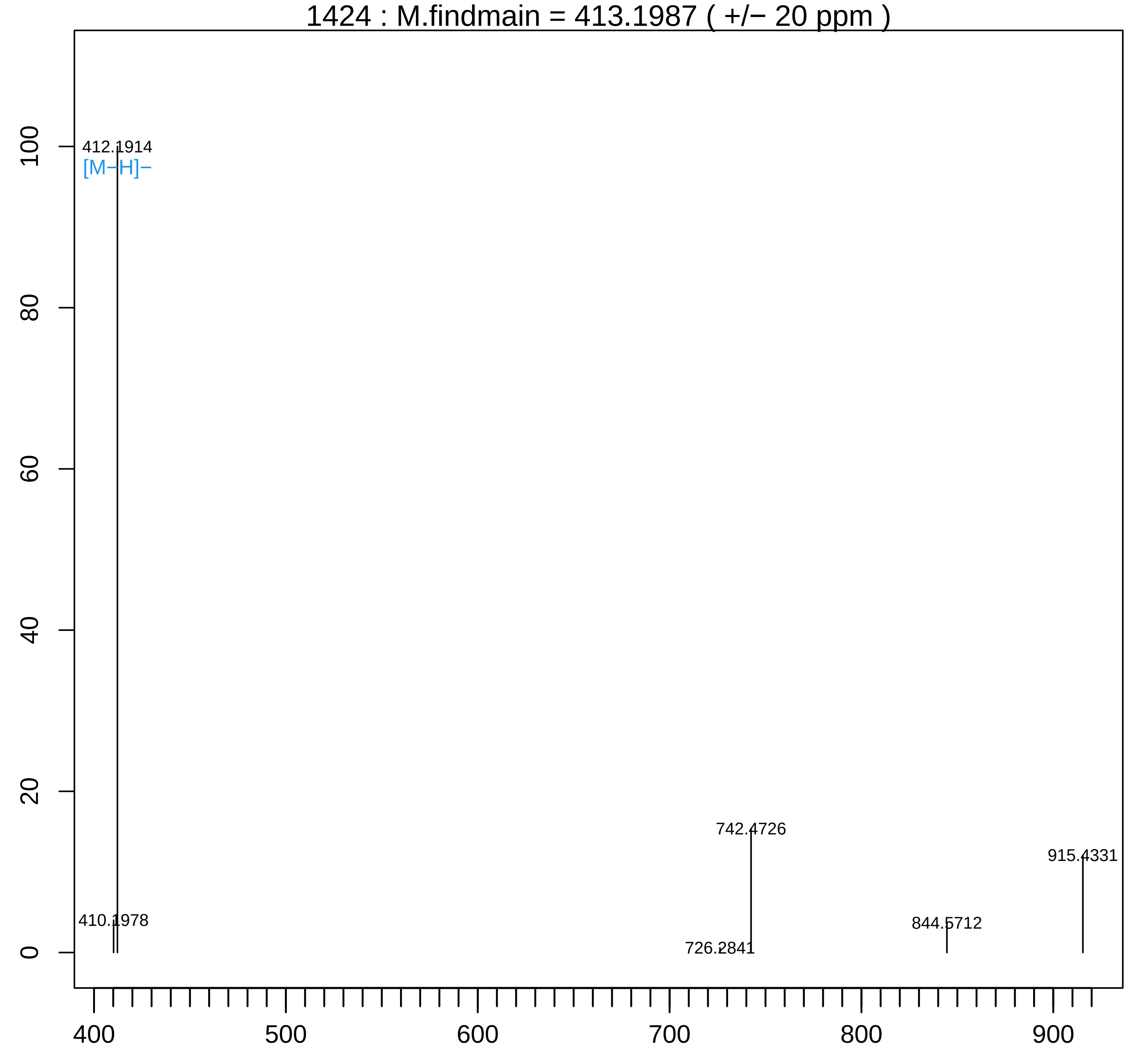
1424 : M.findmain = 413.1987 (+/− 20 ppm)
412.1914
[M−H]−
410.1978
742.4726
726.2841
844.5712
915.4331
0
20
40
60
80
100
400
500
600
700
800
900

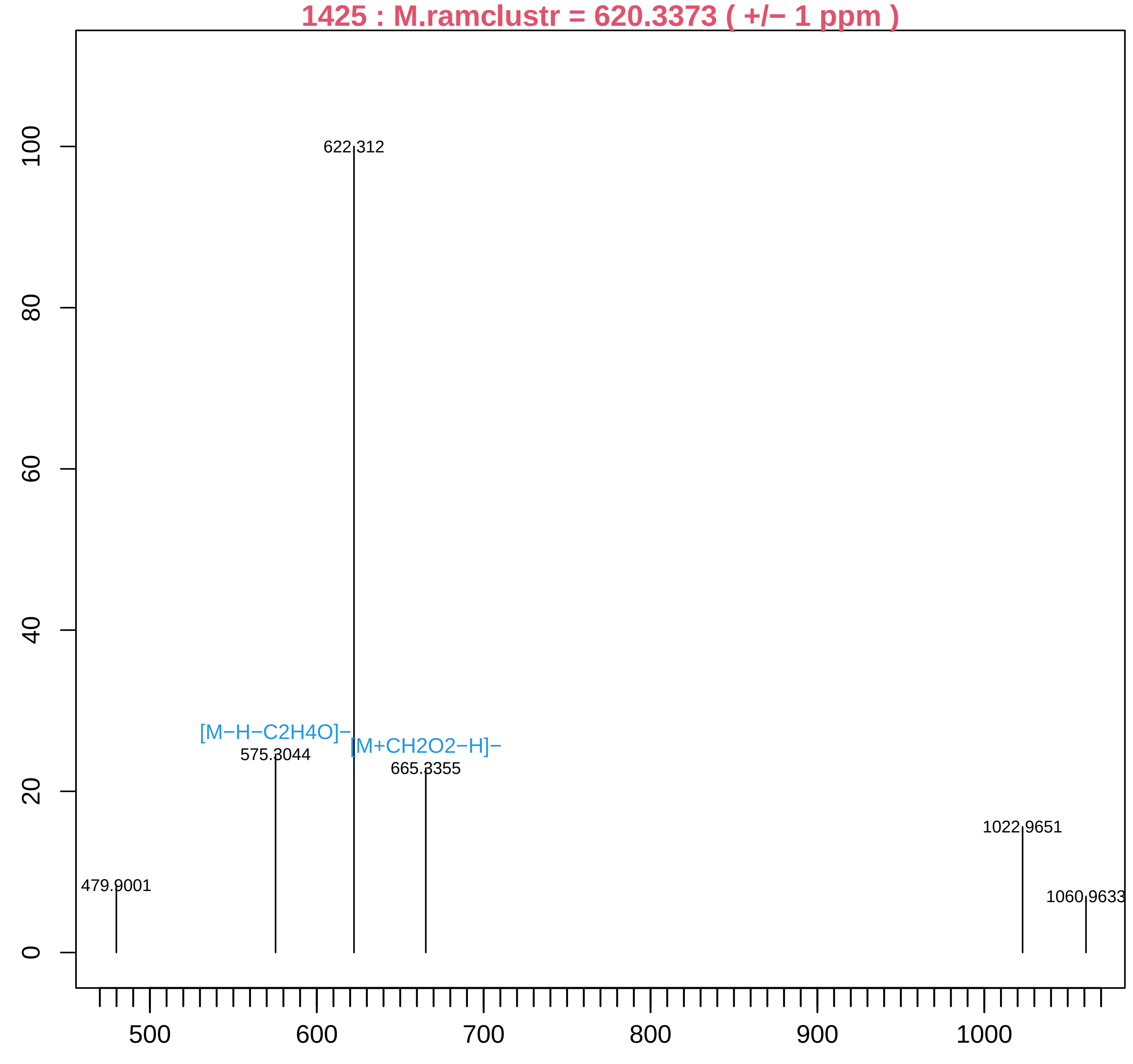
1425 : M.ramclustr = 620.3373 (+/− 1 ppm)
622.312
[M−H−C2H4O]−
575.3044
[M+CH2O2−H]−
665.3355
1022.9651
479.9001
1060.9633
0
20
40
60
80
100
500
600
700
800
900
1000
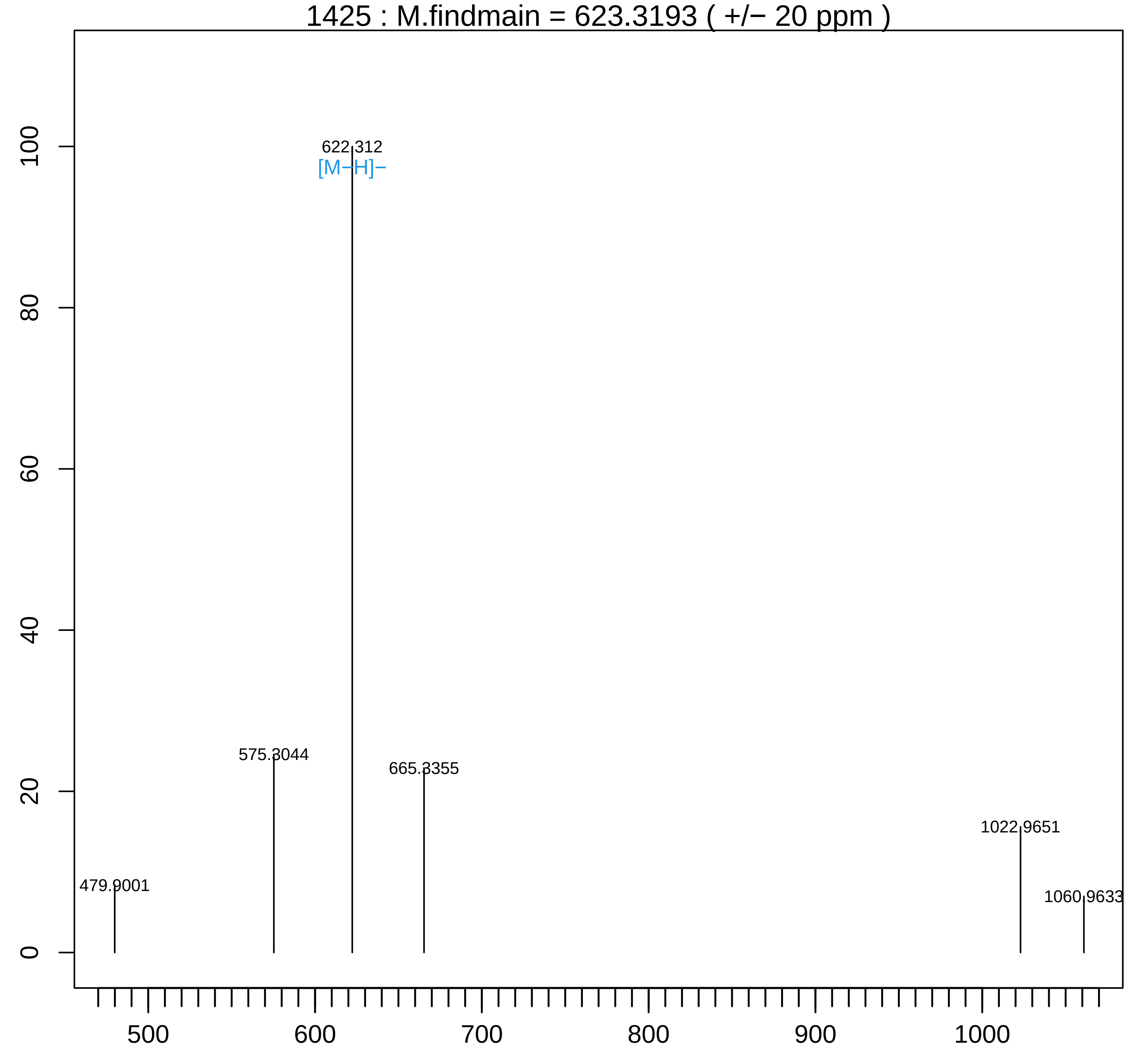
1425 : M.findmain = 623.3193 (+/− 20 ppm)
622.312
[M−H]−
575.3044
665.3355
1022.9651
479.9001
1060.9633
0
20
40
60
80
100
500
600
700
800
900
1000

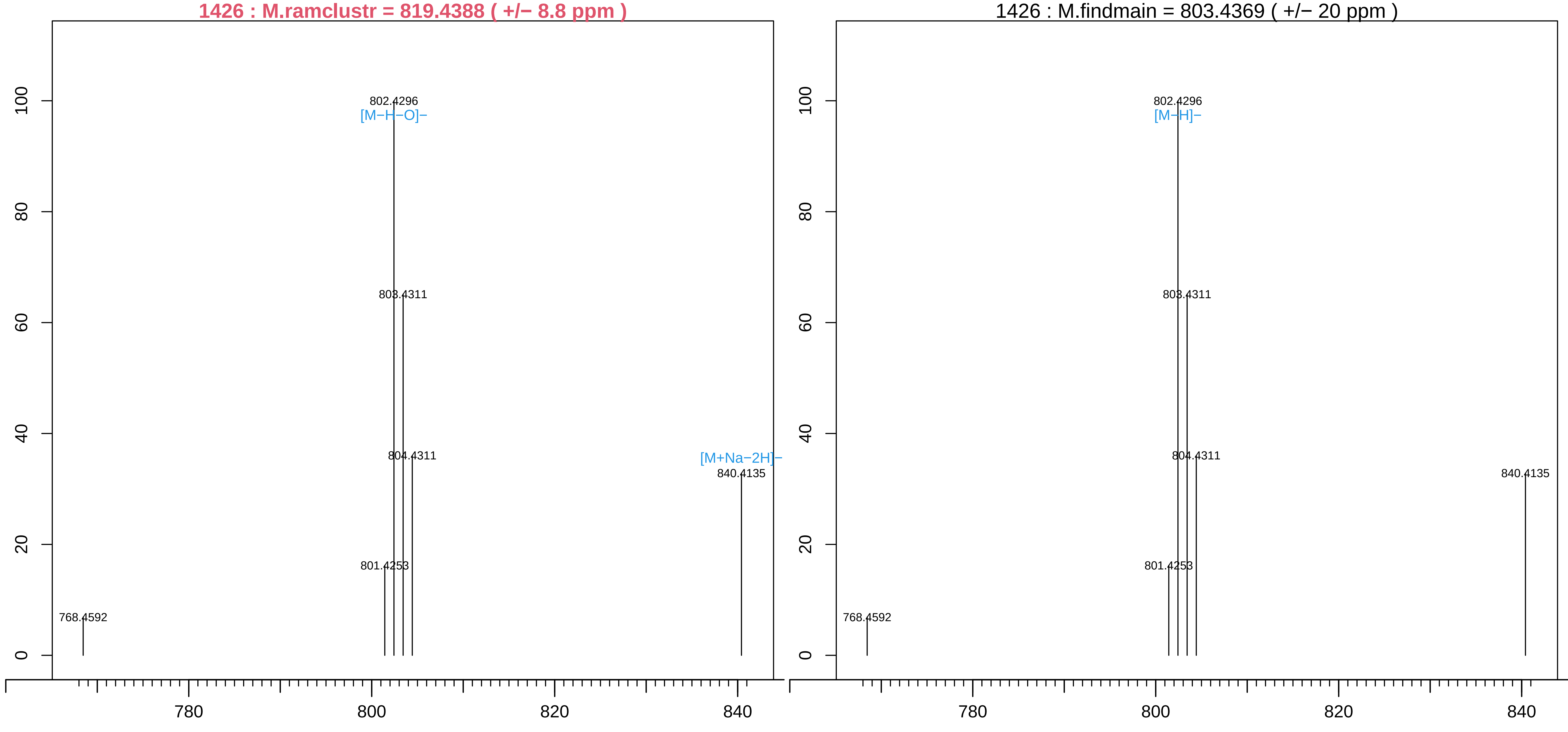

1426 : M.ramclustr = 819.4388 (+/− 8.8 ppm)
802.4296
[M−H−O]−
803.4311
804.4311
[M+Na−2H]−
840.4135
801.4253
768.4592
1426 : M.findmain = 803.4369 (+/− 20 ppm)
802.4296
[M−H]−
803.4311
804.4311
840.4135
801.4253
768.4592
0
20
40
60
80
100
780
800
820
840

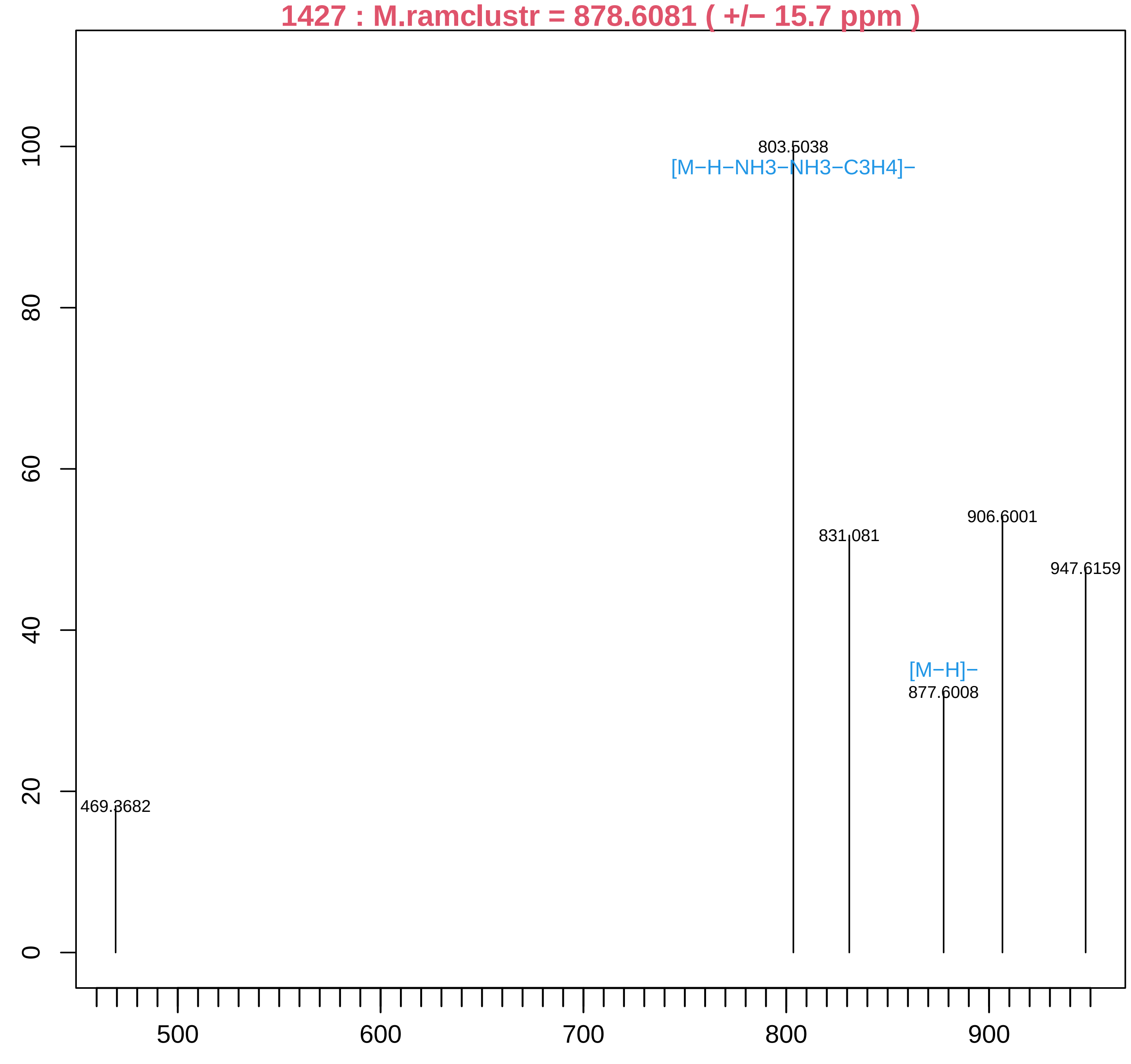
1427 : M.ramclustr = 878.6081 (+/− 15.7 ppm)
803.5038
[M−H−NH3−NH3−C3H4]−
831.081
906.6001
947.6159
[M−H]−
877.6008
469.3682
0
20
40
60
80
100
500
600
700
800
900

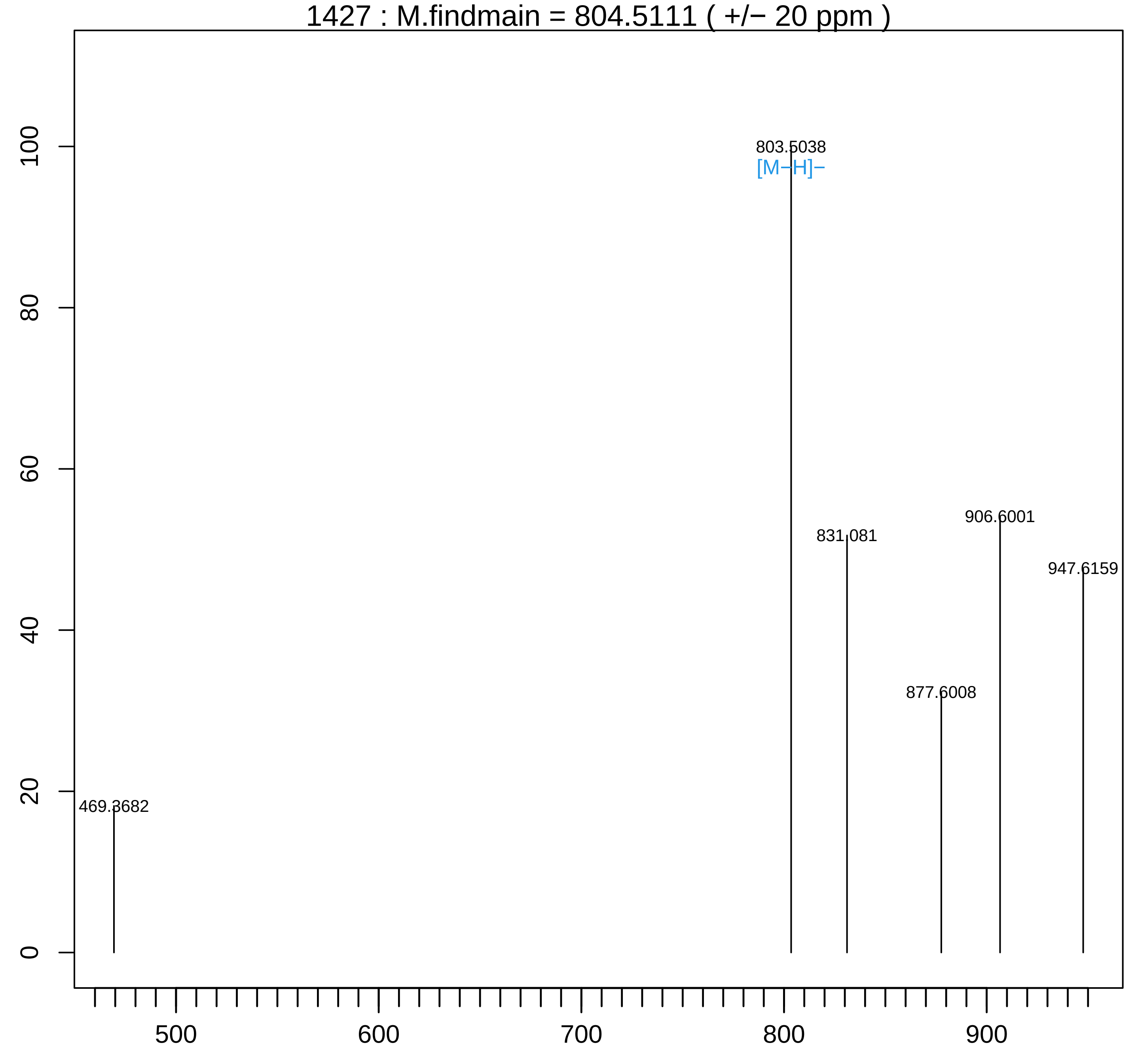
1427 : M.findmain = 804.5111 (+/− 20 ppm)
803.5038
[M−H]−
831.081
906.6001
947.6159
877.6008
469.3682
0
20
40
60
80
100
500
600
700
800
900

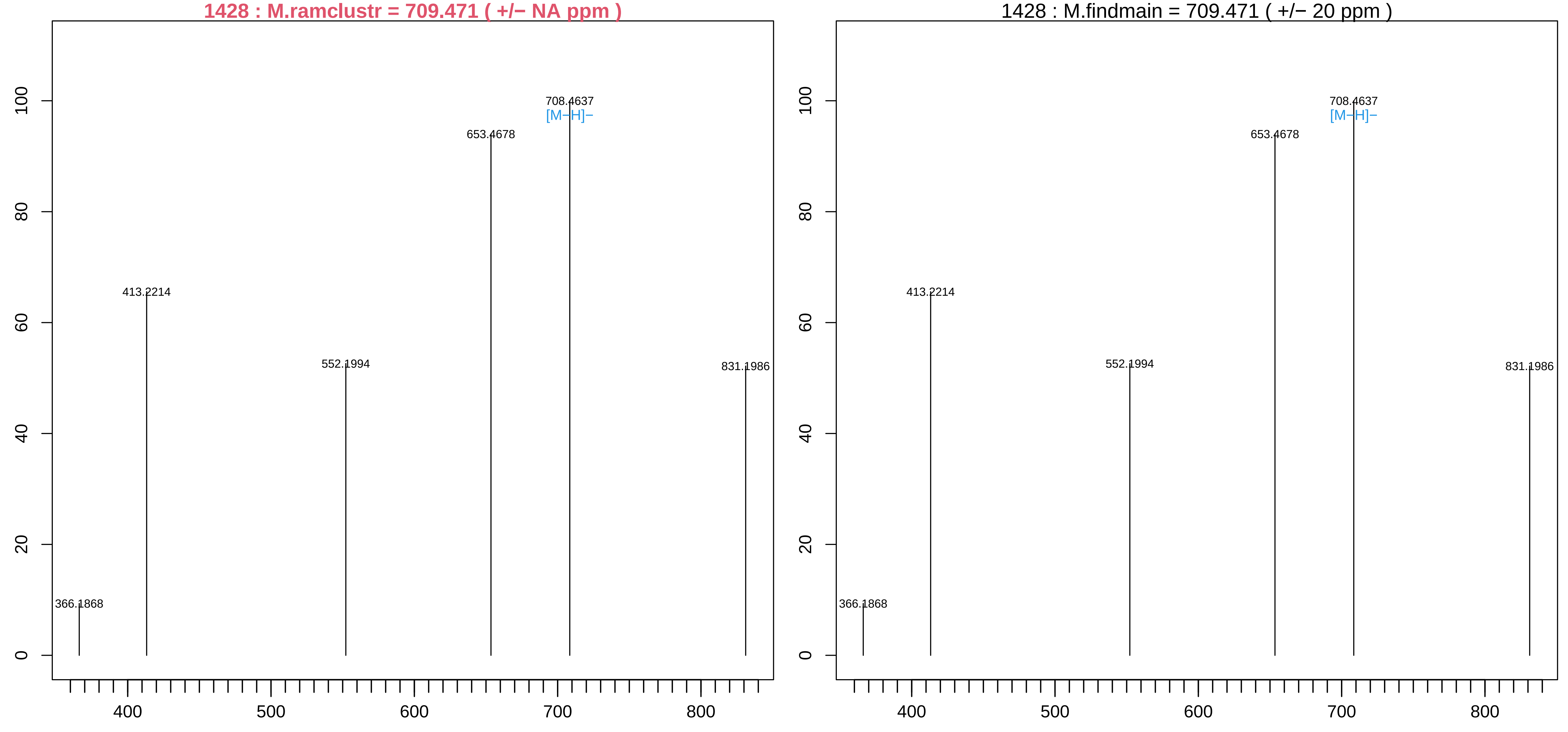
1428 : M.ramclustr = 709.471 (+/− NA ppm)
1428 : M.findmain = 709.471 (+/− 20 ppm)
708.4637
[M−H]−
653.4678
413.2214
552.1994
831.1986
366.1868
0
20
40
60
80
100
400
500
600
700
800

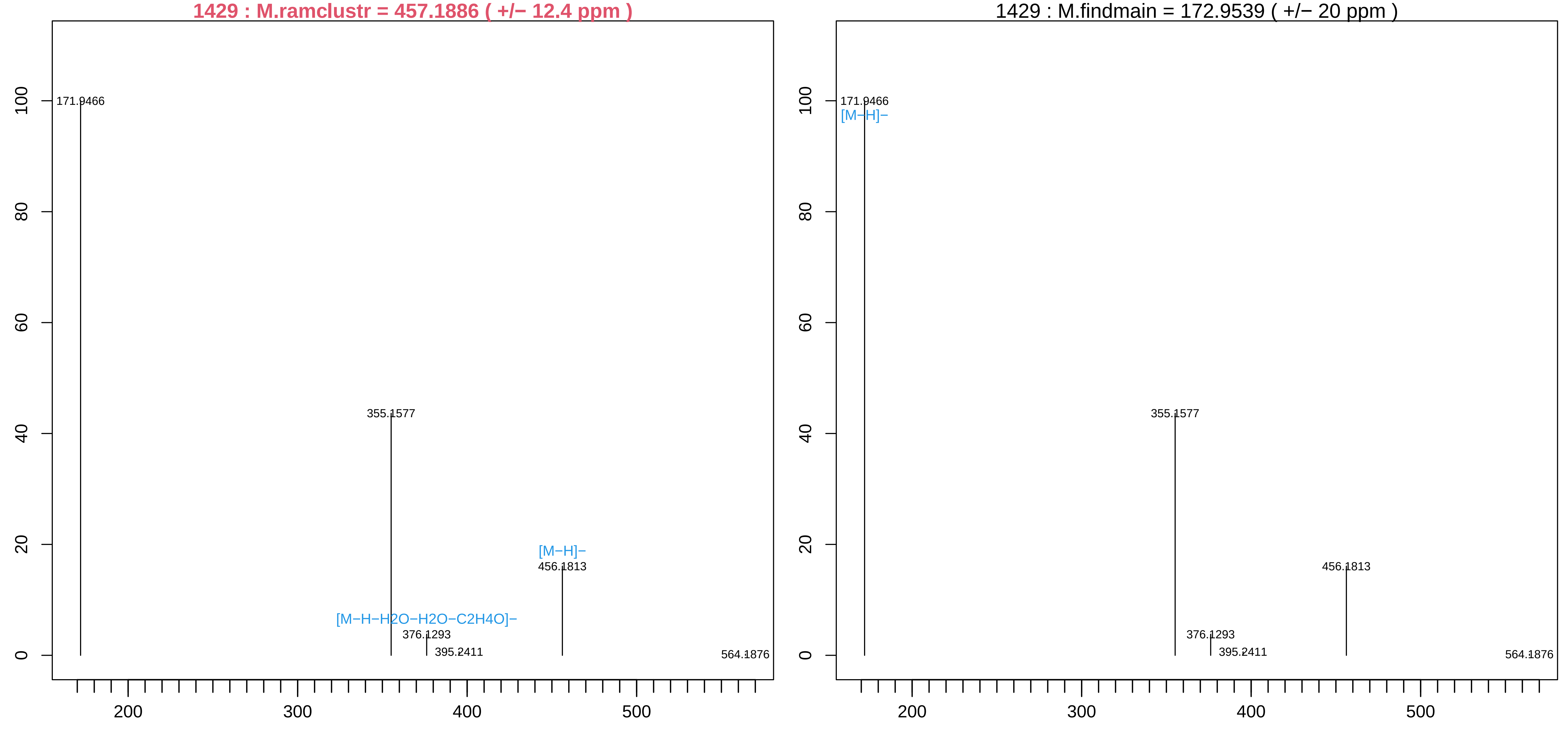

1429 : M.ramclustr = 457.1886 (+/− 12.4 ppm)
171.9466
355.1577
[M−H−H2O−H2O−C2H4O]−
376.1293
395.2411
[M−H]−
456.1813
564.1876
0
20
40
60
80
100
200
300
400
500
1429 : M.findmain = 172.9539 (+/− 20 ppm)
171.9466
[M−H]−
355.1577
376.1293
395.2411
456.1813
564.1876
0
20
40
60
80
100
200
300
400
500

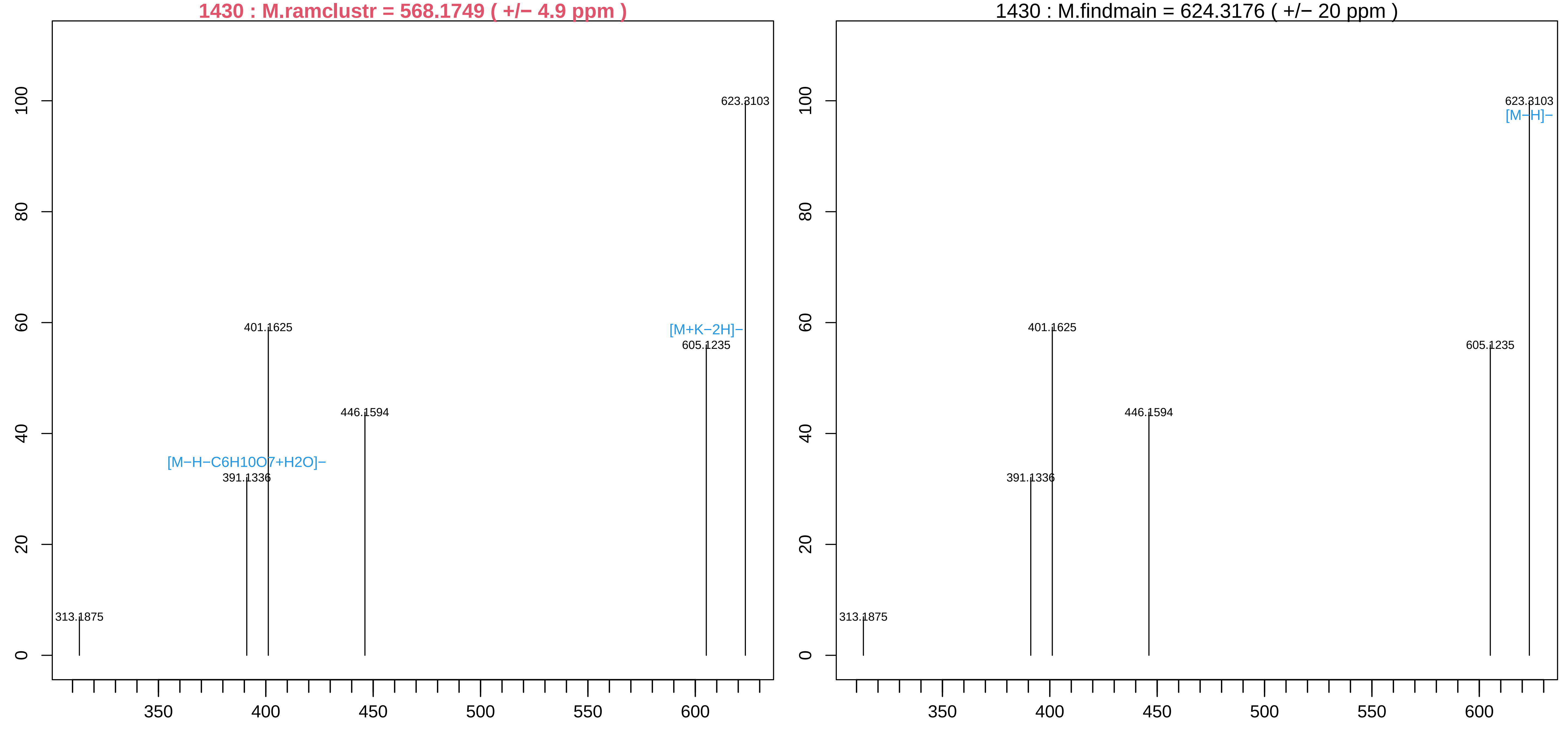
1430 : M.ramclustr = 568.1749 (+/− 4.9 ppm)
623.3103
401.1625
[M+K−2H]−
605.1235
446.1594
[M−H−C6H10O7+H2O]−
391.1336
313.1875
1430 : M.findmain = 624.3176 (+/− 20 ppm)
623.3103
[M−H]−
401.1625
605.1235
446.1594
391.1336
313.1875

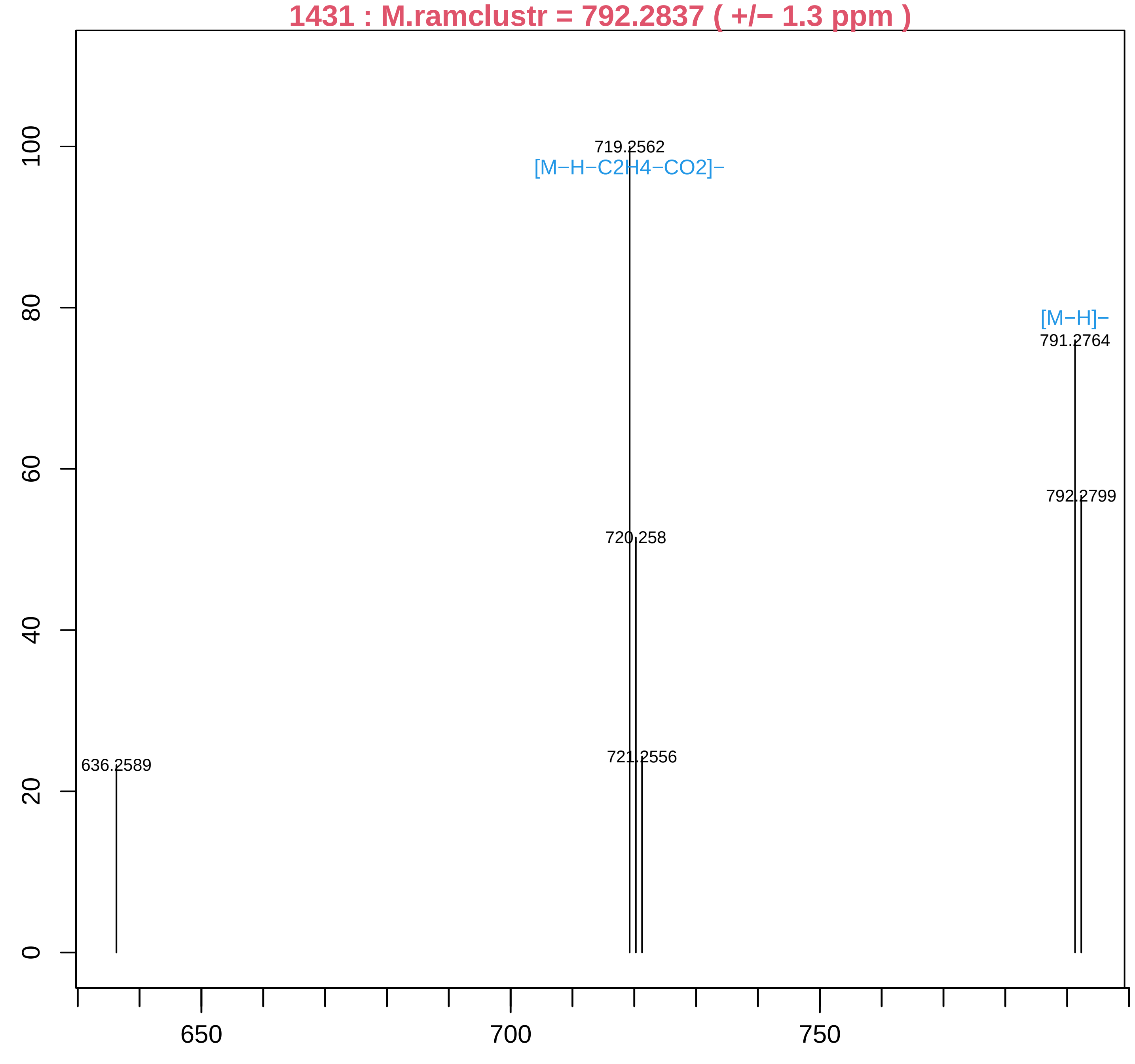

1431 : M.ramclustr = 792.2837 (+/− 1.3 ppm)
719.2562
[M−H−C2H4−CO2]−
[M−H]−
791.2764
792.2799
720.258
721.2556
636.2589
0
20
40
60
80
100
650
700
750

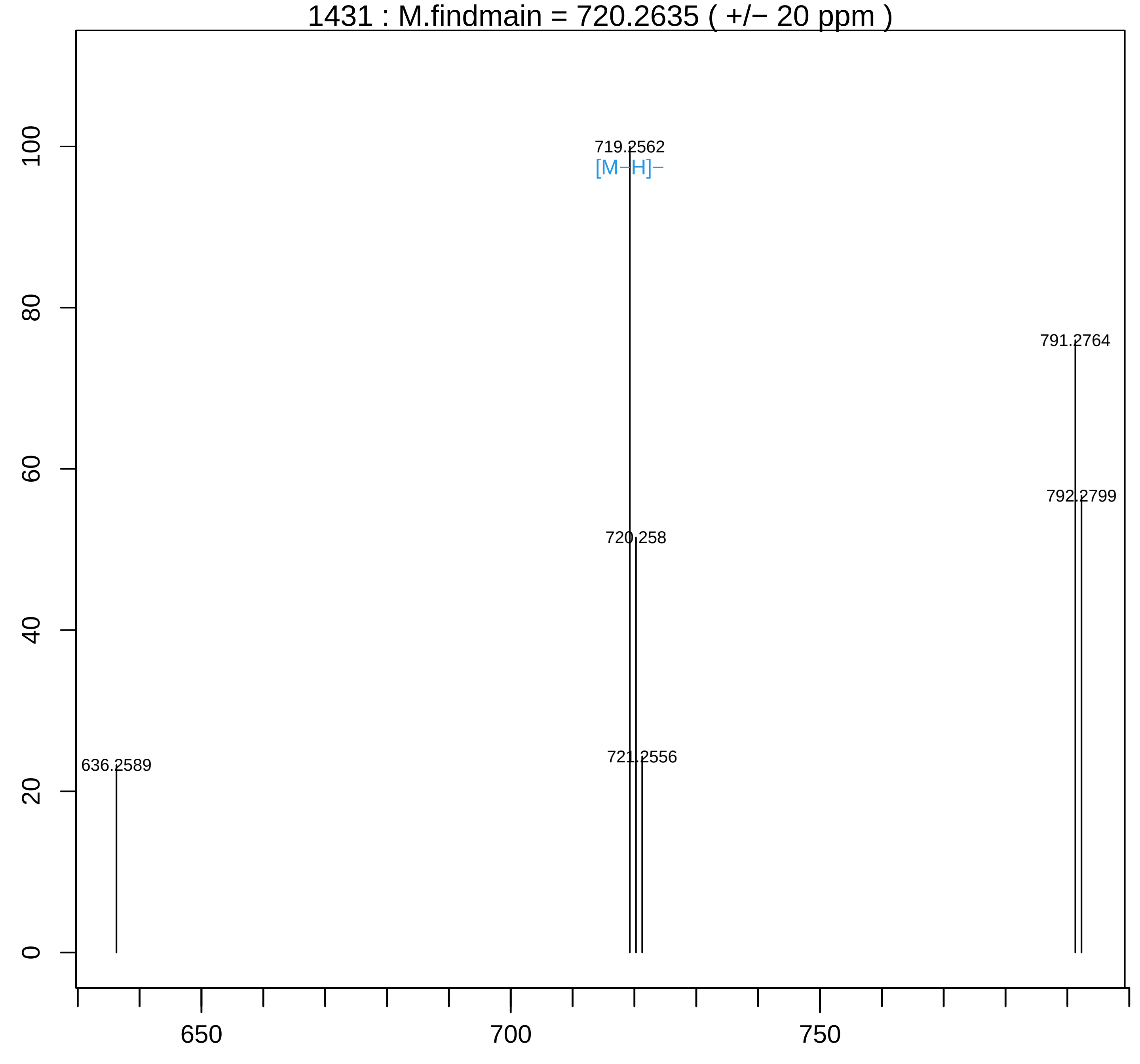

1431 : M.findmain = 720.2635 (+/− 20 ppm)
719.2562
[M−H]−
791.2764
792.2799
720.258
721.2556
636.2589
0
20
40
60
80
100
650
700
750

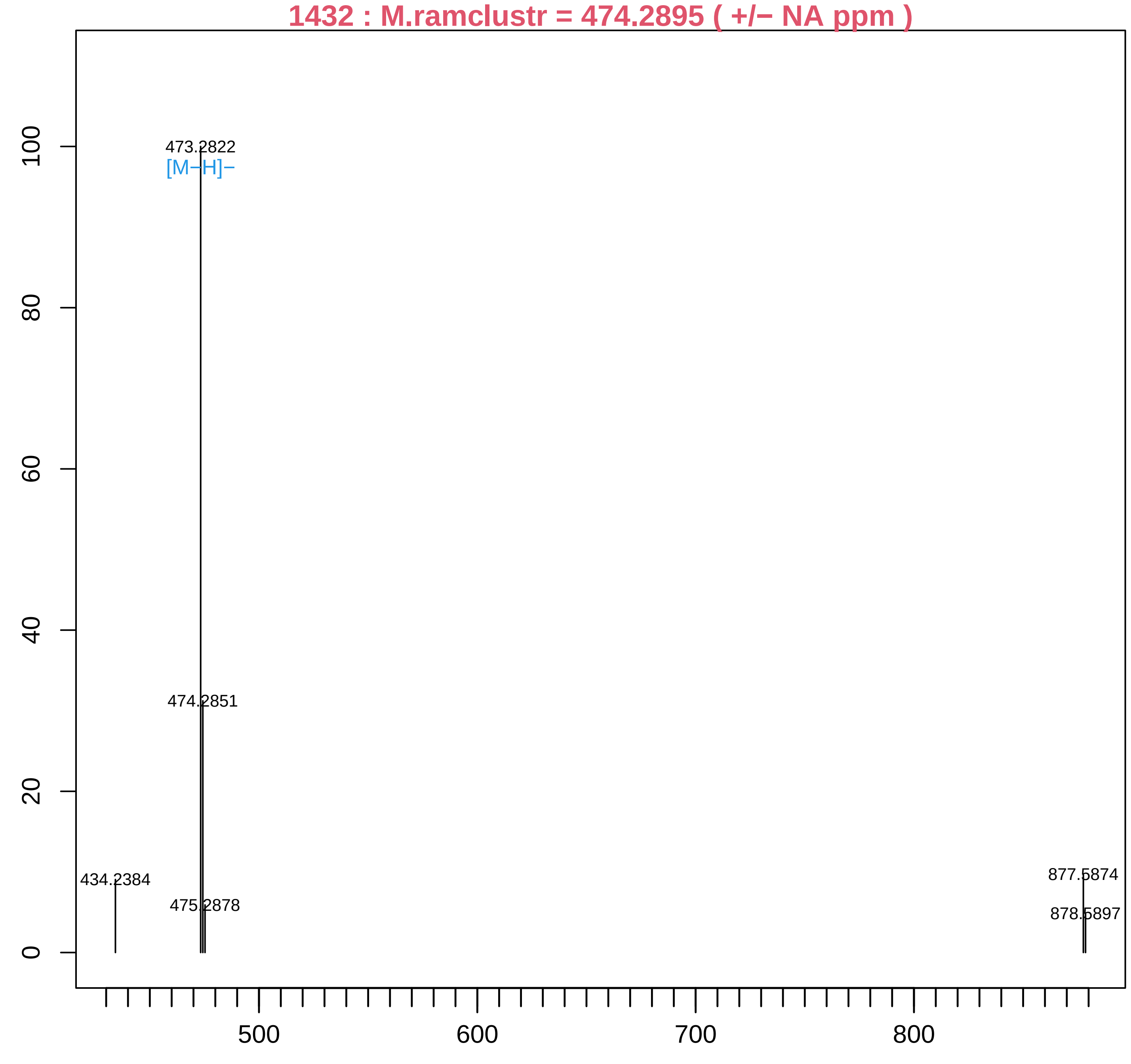
1432 : M.ramclustr = 474.2895 (+/− NA ppm)
473.2822
[M−H]−
474.2851
434.2384
475.2878
877.5874
878.5897
0
20
40
60
80
100
500
600
700
800

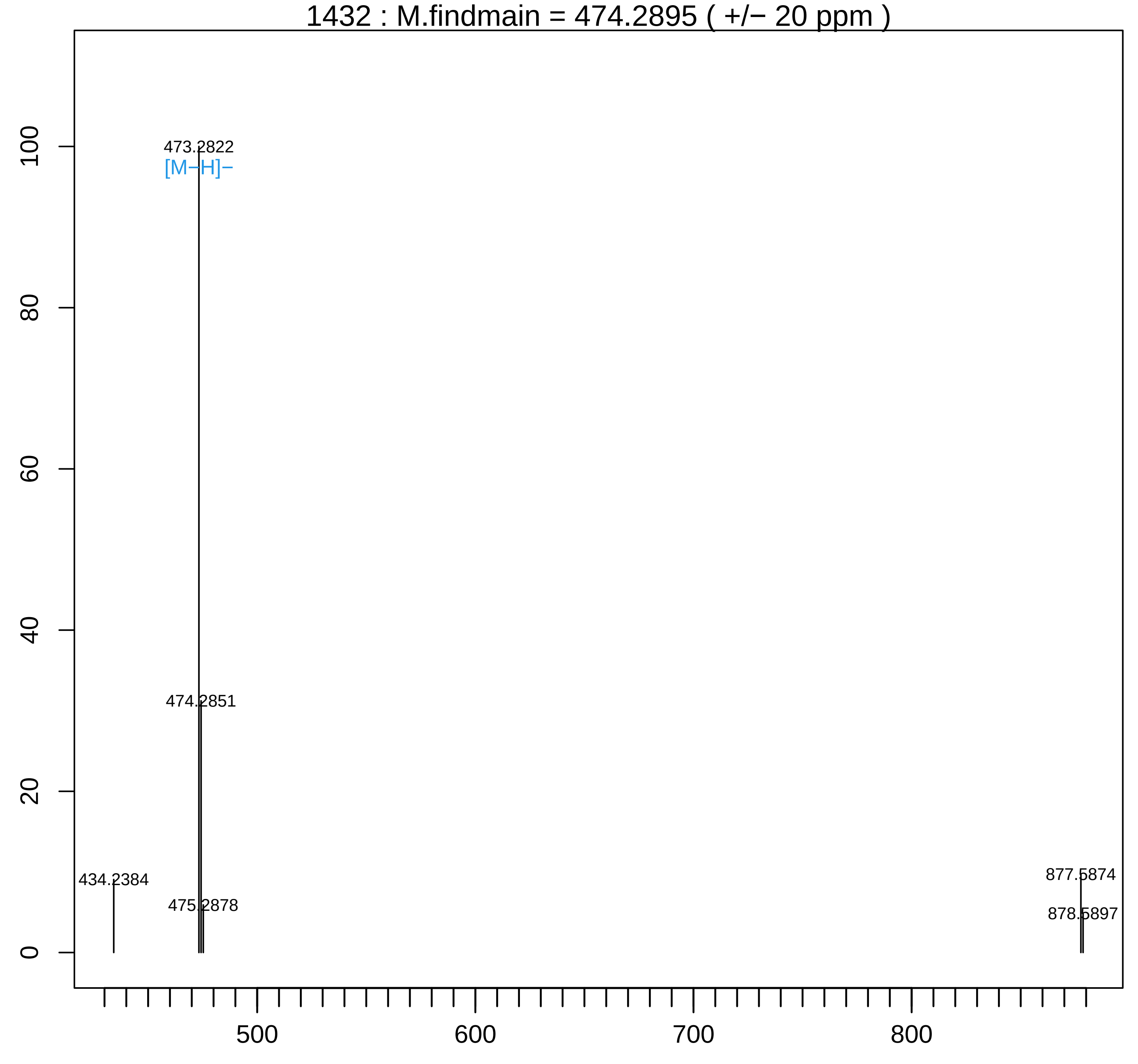
1432 : M.findmain = 474.2895 (+/− 20 ppm)
473.2822
[M−H]−
474.2851
434.2384
475.2878
877.5874
878.5897
0
20
40
60
80
100
500
600
700
800

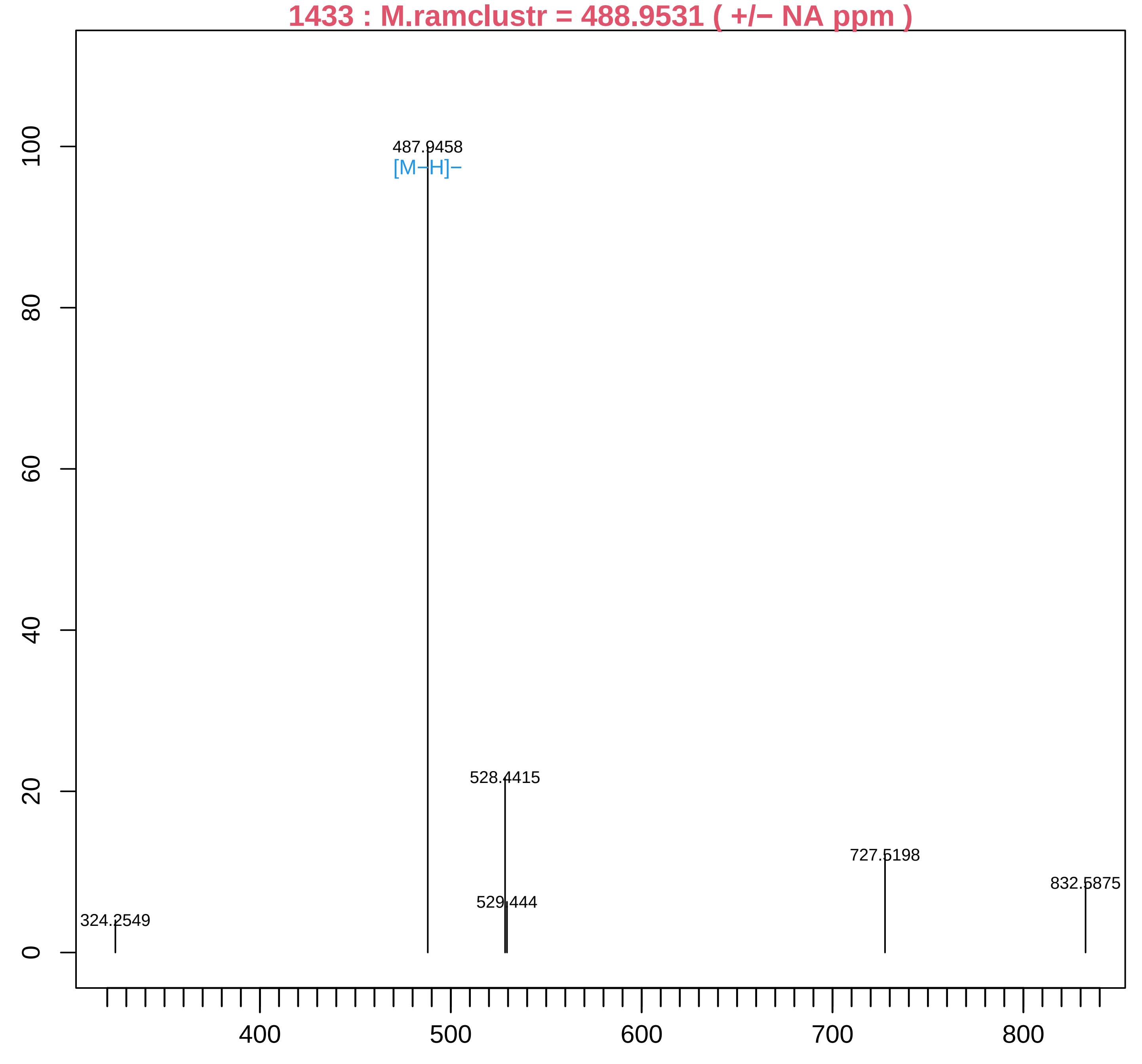
1433 : M.ramclustr = 488.9531 (+/− NA ppm)
487.9458
[M−H]−
528.4415
529.444
727.5198
832.5875
324.2549
0
20
40
60
80
100
400
500
600
700
800

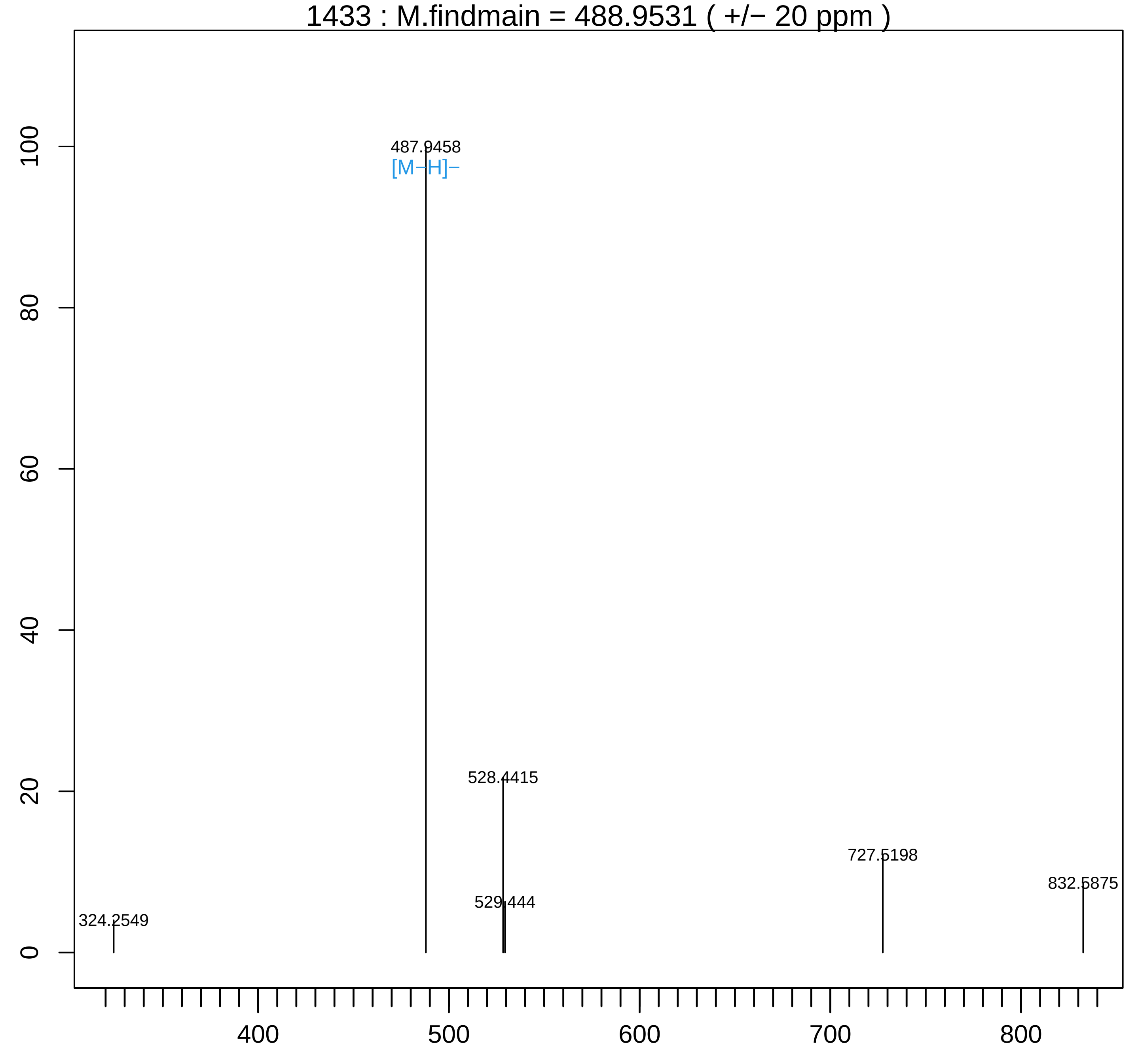
1433 : M.findmain = 488.9531 (+/− 20 ppm)
487.9458
[M−H]−
528.4415
529.444
727.5198
832.5875
324.2549
0
20
40
60
80
100
400
500
600
700
800

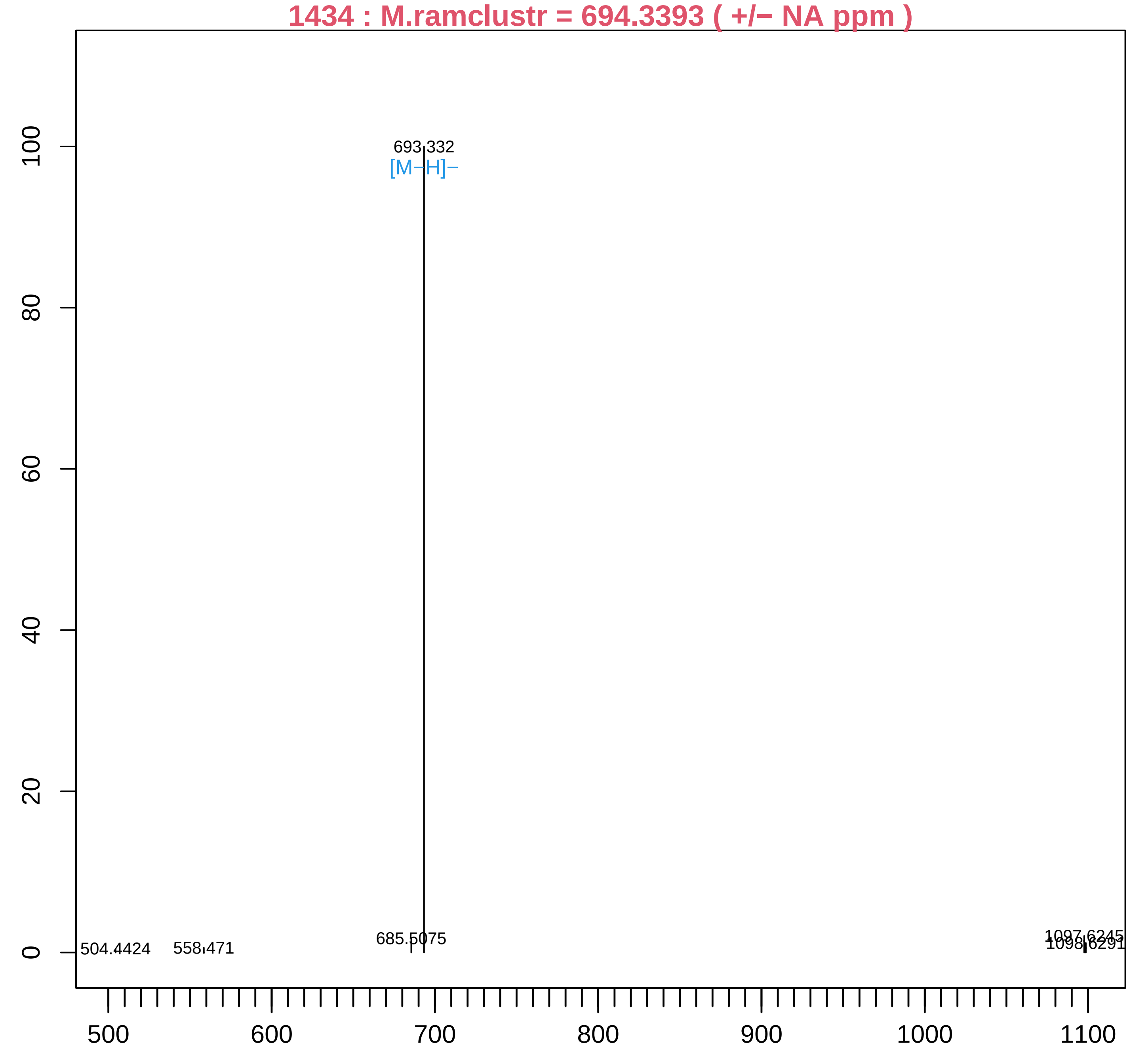

1434 : M.ramclustr = 694.3393 (+/− NA ppm)
693.332
[M−H]−
504.4424
558.471
685.5075
1097.6245
1098.6291
0
20
40
60
80
100
500
600
700
800
900
1000
1100

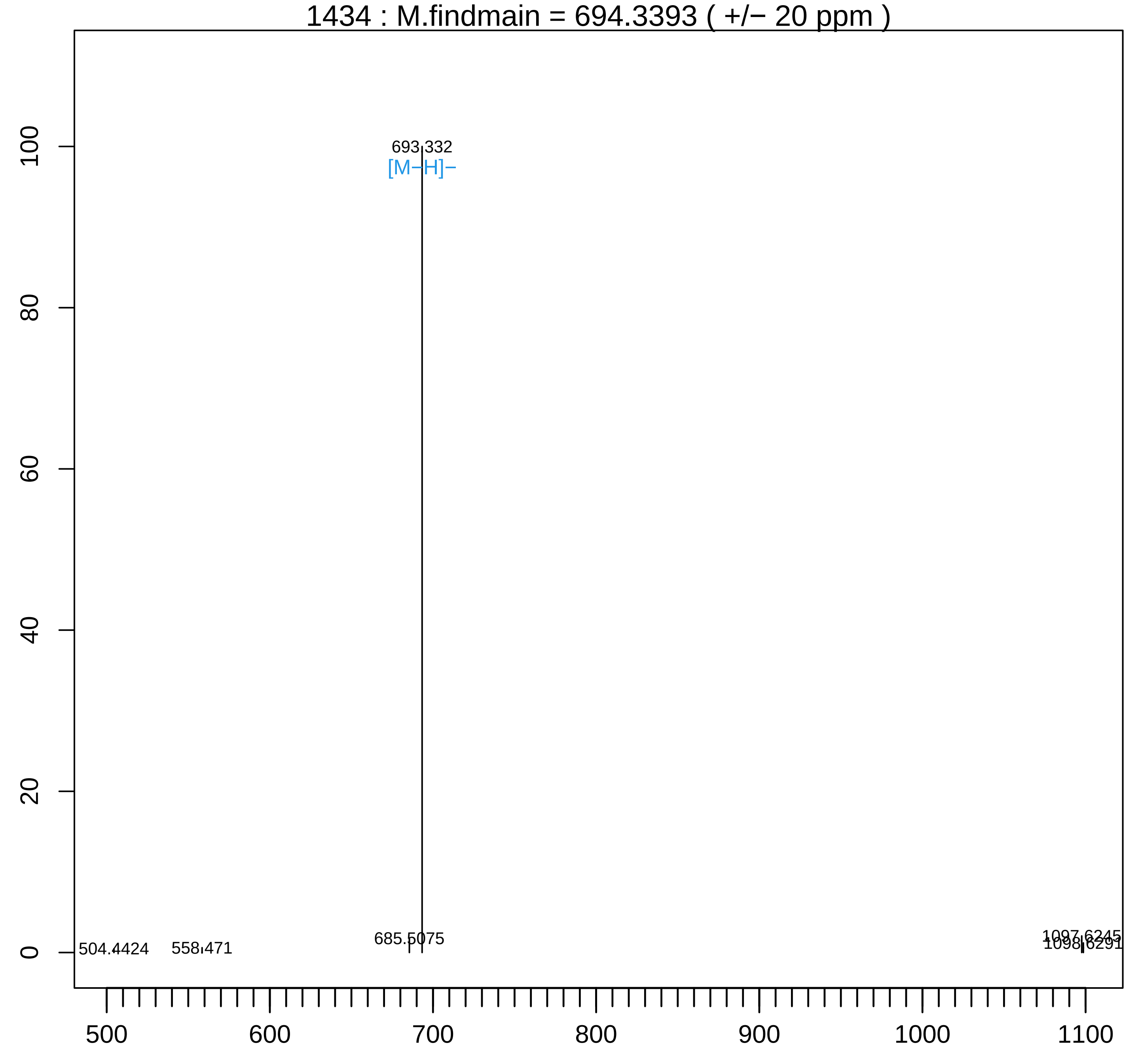

1434 : M.findmain = 694.3393 (+/− 20 ppm)
693.332
[M−H]−
504.4424
558.471
685.5075
1097.6245
1098.6291
0
20
40
60
80
100
500
600
700
800
900
1000
1100

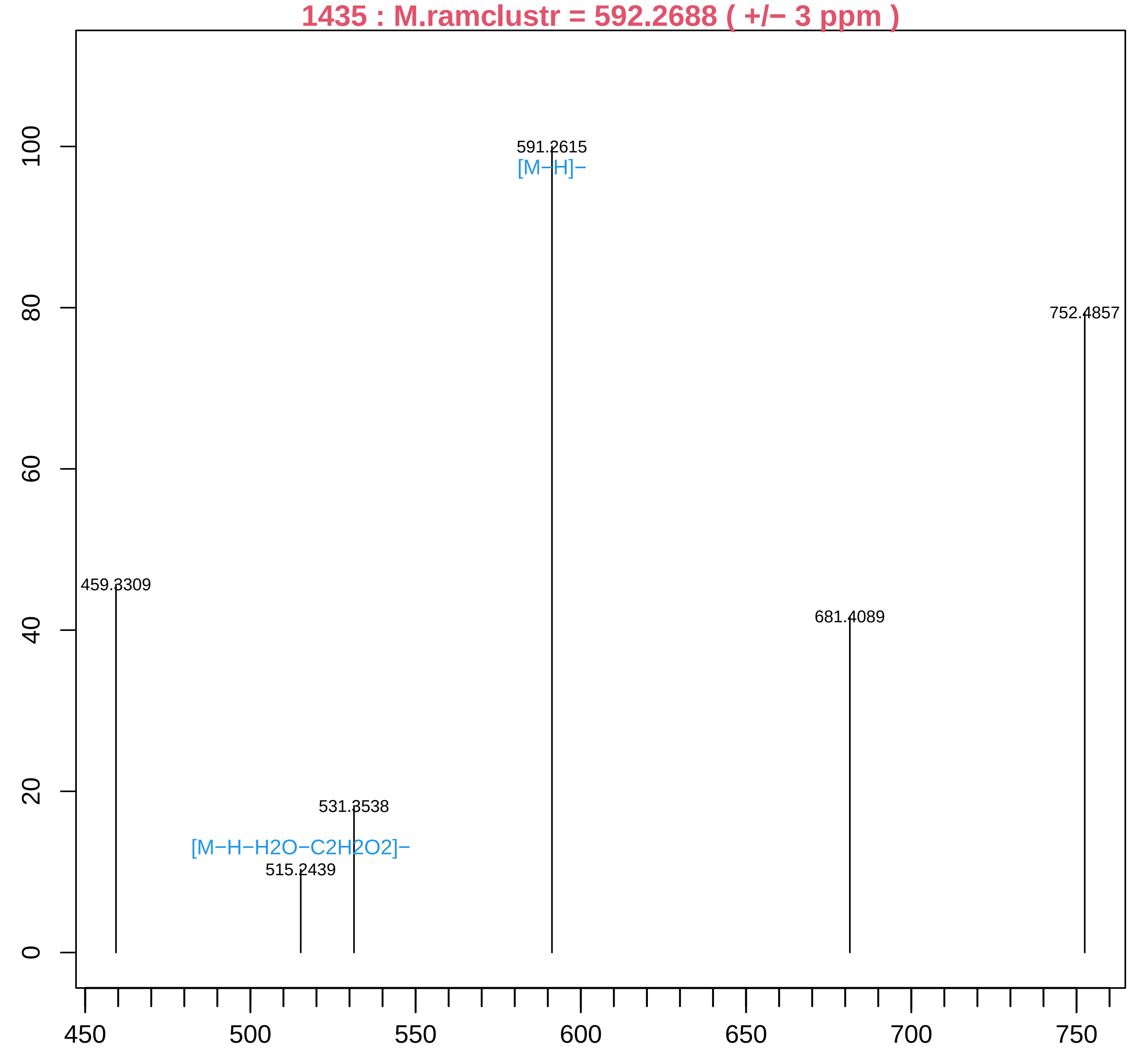

1435 : M.ramclustr = 592.2688 (+/− 3 ppm)
591.2615
[M−H]−
752.4857
459.3309
681.4089
531.3538
[M−H−H2O−C2H2O2]−
515.2439
0
20
40
60
80
100
450
500
550
600
650
700
750

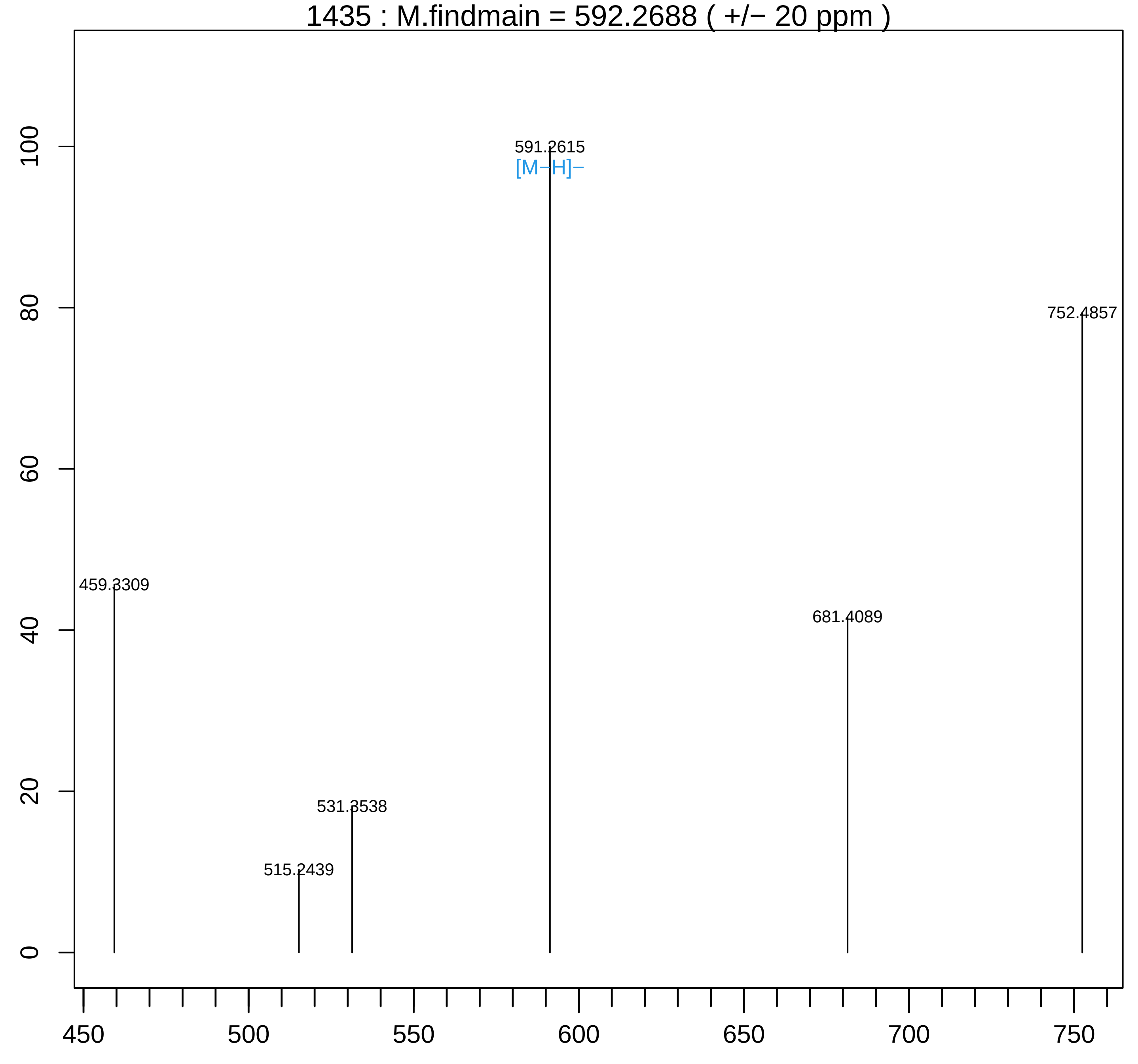

1435 : M.findmain = 592.2688 (+/− 20 ppm)
591.2615
[M−H]−
752.4857
459.3309
681.4089
531.3538
515.2439
0
20
40
60
80
100
450
500
550
600
650
700
750

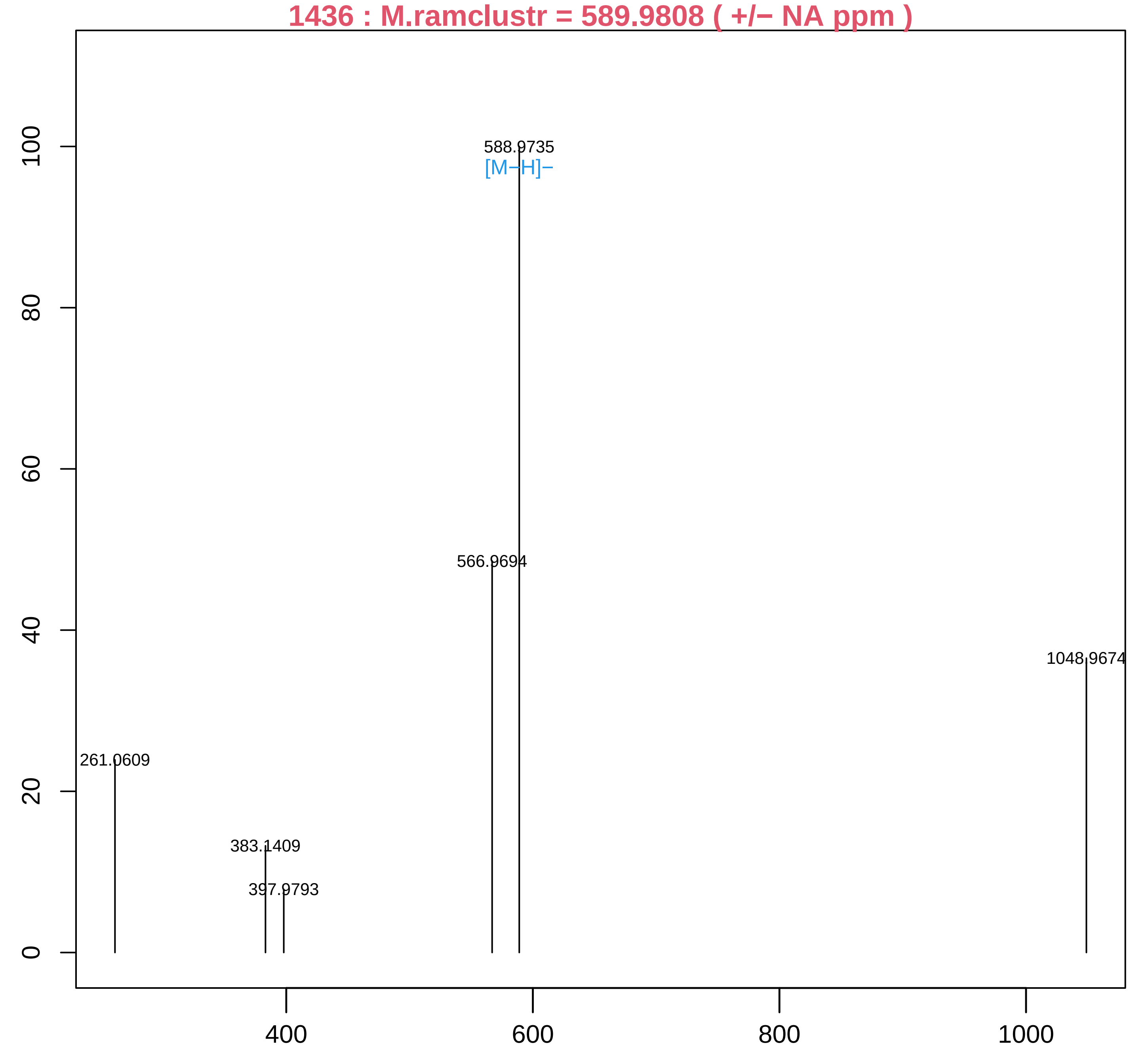
1436 : M.ramclustr = 589.9808 (+/− NA ppm)
588.9735
[M−H]−
566.9694
1048.9674
261.0609
383.1409
397.9793
0
20
40
60
80
100
400
600
800
1000

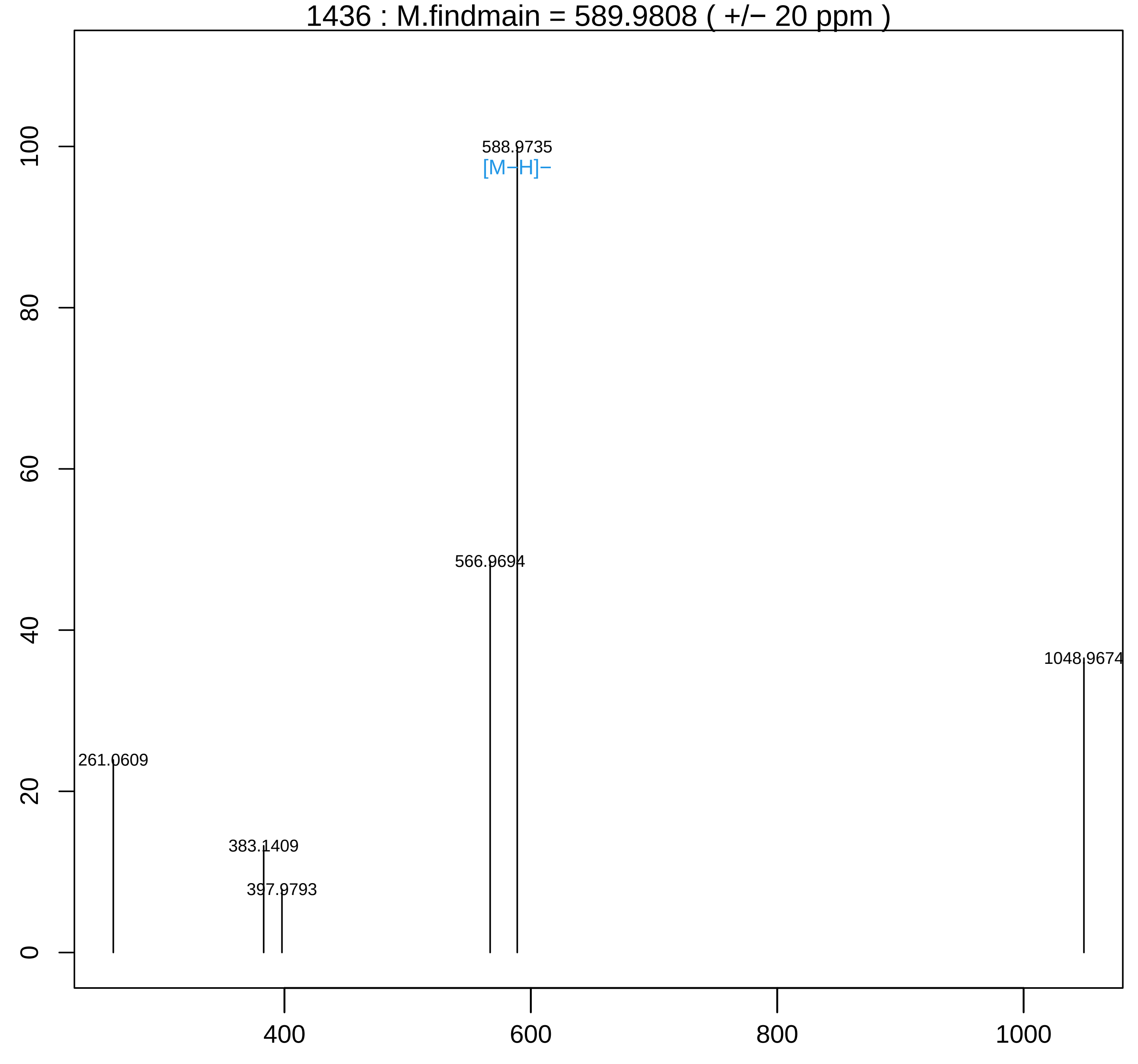
1436 : M.findmain = 589.9808 (+/− 20 ppm)
588.9735
[M−H]−
566.9694
1048.9674
261.0609
383.1409
397.9793
0
20
40
60
80
100
400
600
800
1000

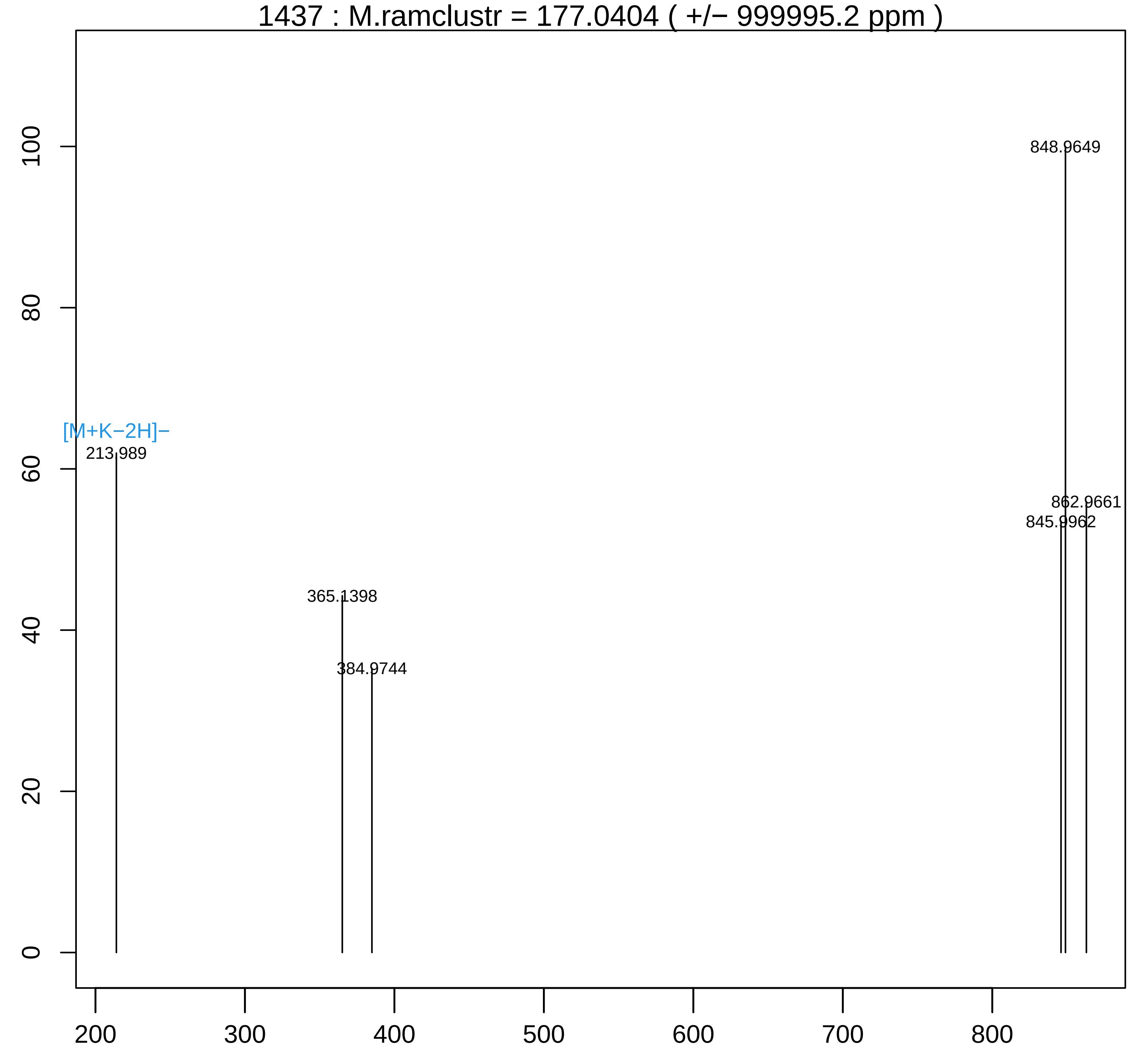
1437 : M.ramclustr = 177.0404 (+/− 999995.2 ppm)
[M+K−2H]−
213.989
365.1398
384.9744
848.9649
862.9661
845.9962
0
20
40
60
80
100
200
300
400
500
600
700
800

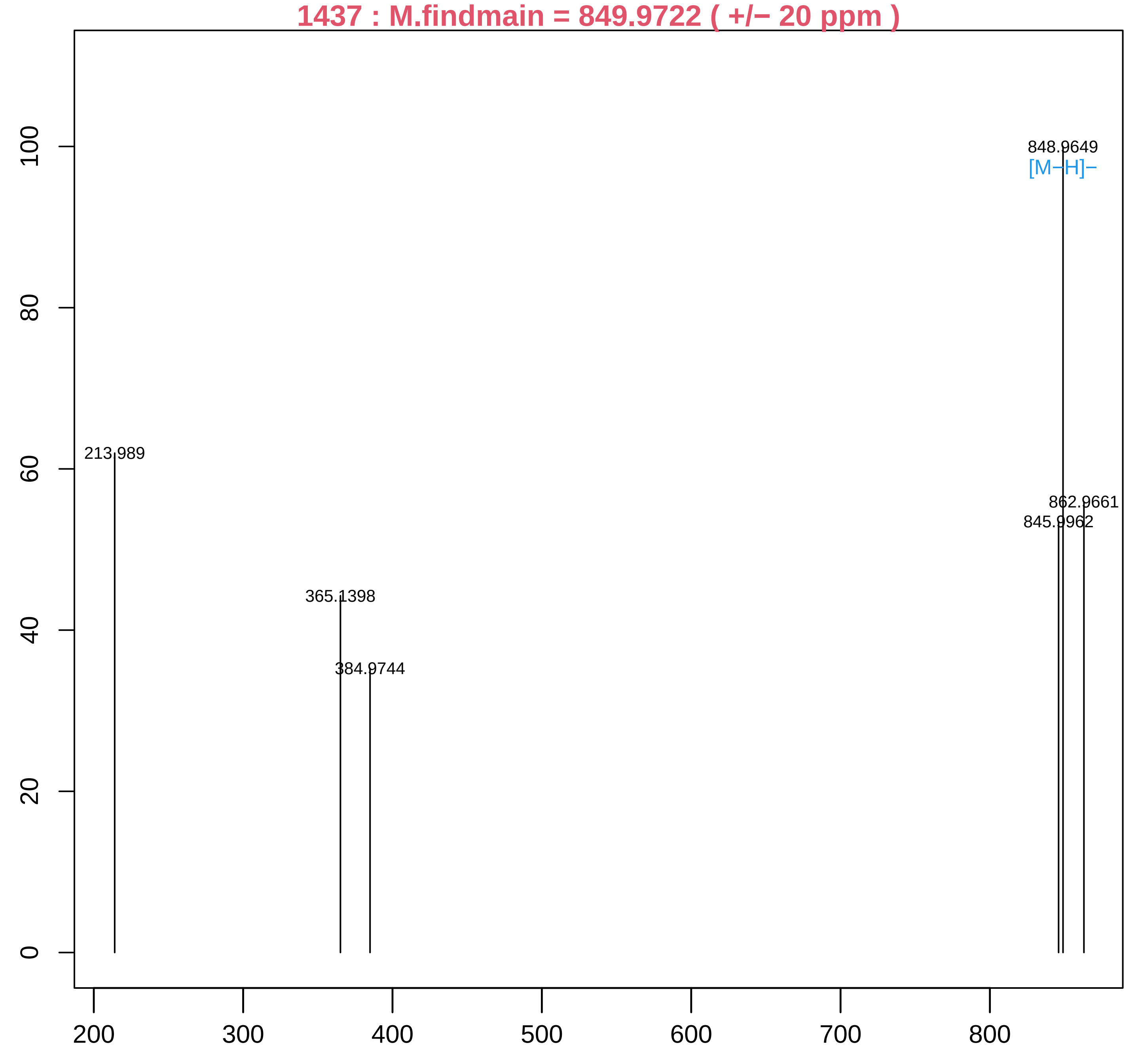
1437 : M.findmain = 849.9722 (+/− 20 ppm)
848.9649
[M−H]−
213.989
365.1398
384.9744
862.9661
845.9962
0
20
40
60
80
100
200
300
400
500
600
700
800

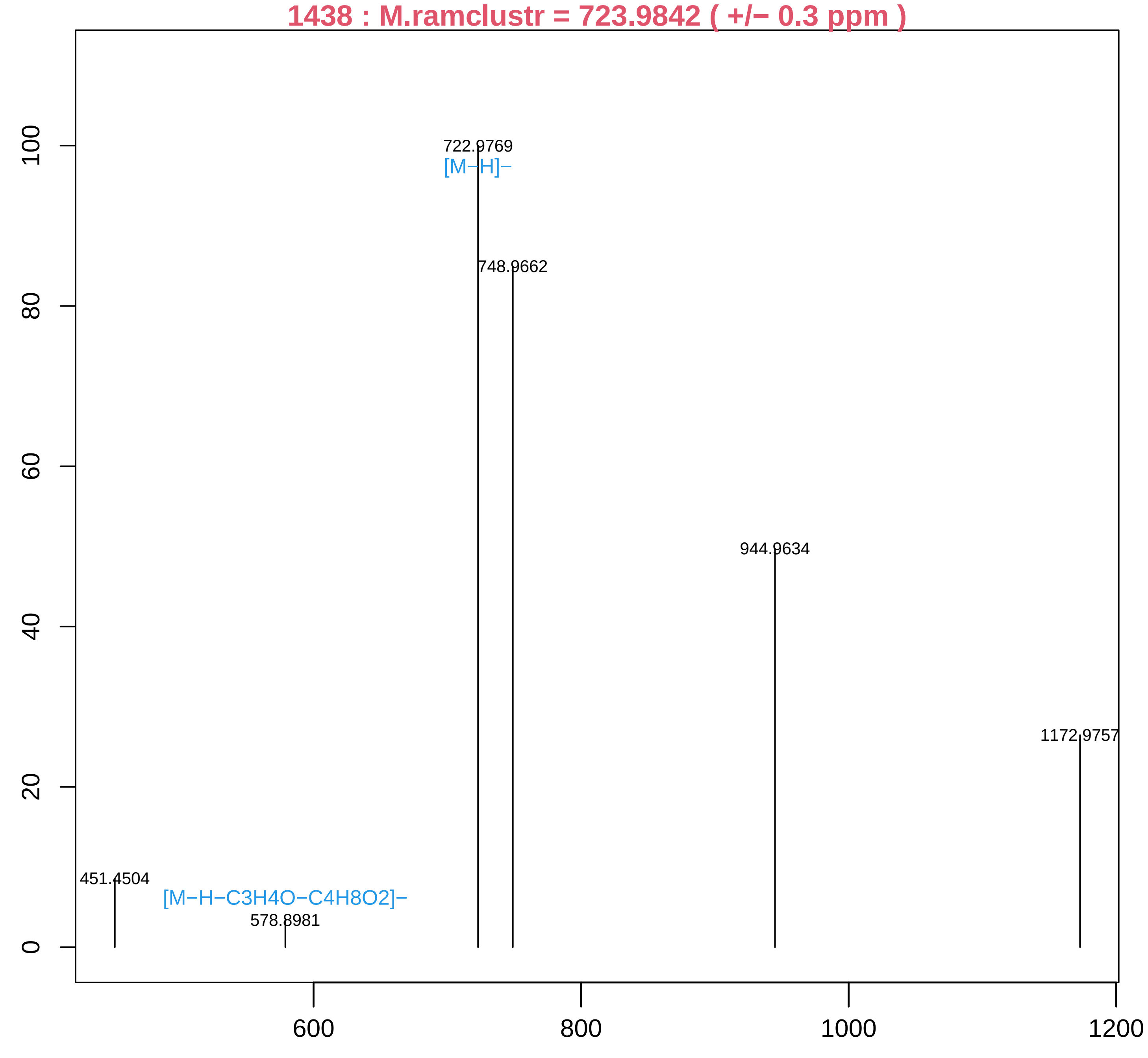
1438 : M.ramclustr = 723.9842 (+/− 0.3 ppm)
722.9769
[M−H]−
748.9662
944.9634
1172.9757
451.4504
[M−H−C3H4O−C4H8O2]−
578.8981
0
20
40
60
80
100
600
800
1000
1200

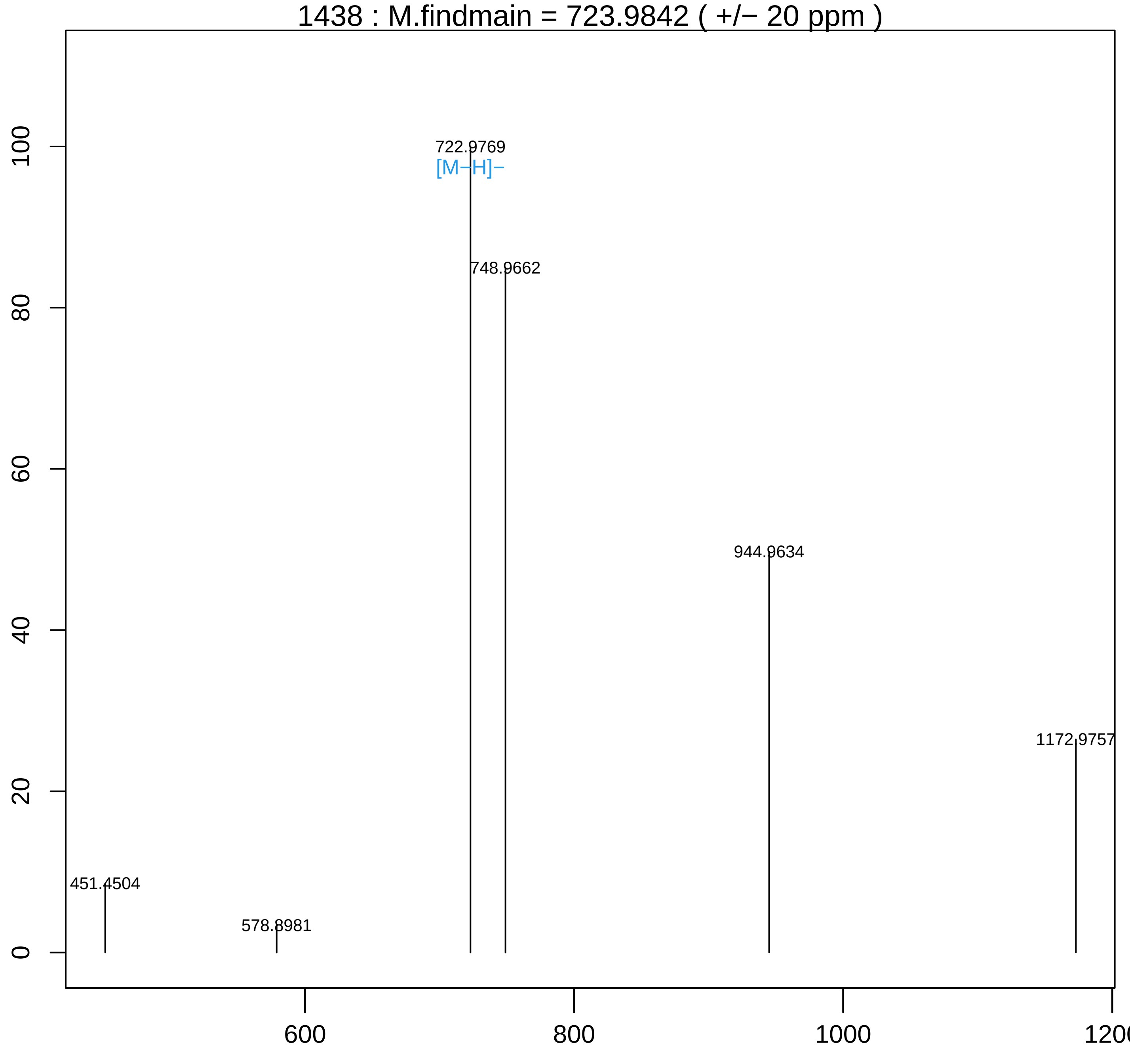
1438 : M.findmain = 723.9842 (+/− 20 ppm)
722.9769
[M−H]−
748.9662
944.9634
1172.9757
451.4504
578.8981
0
20
40
60
80
100
600
800
1000

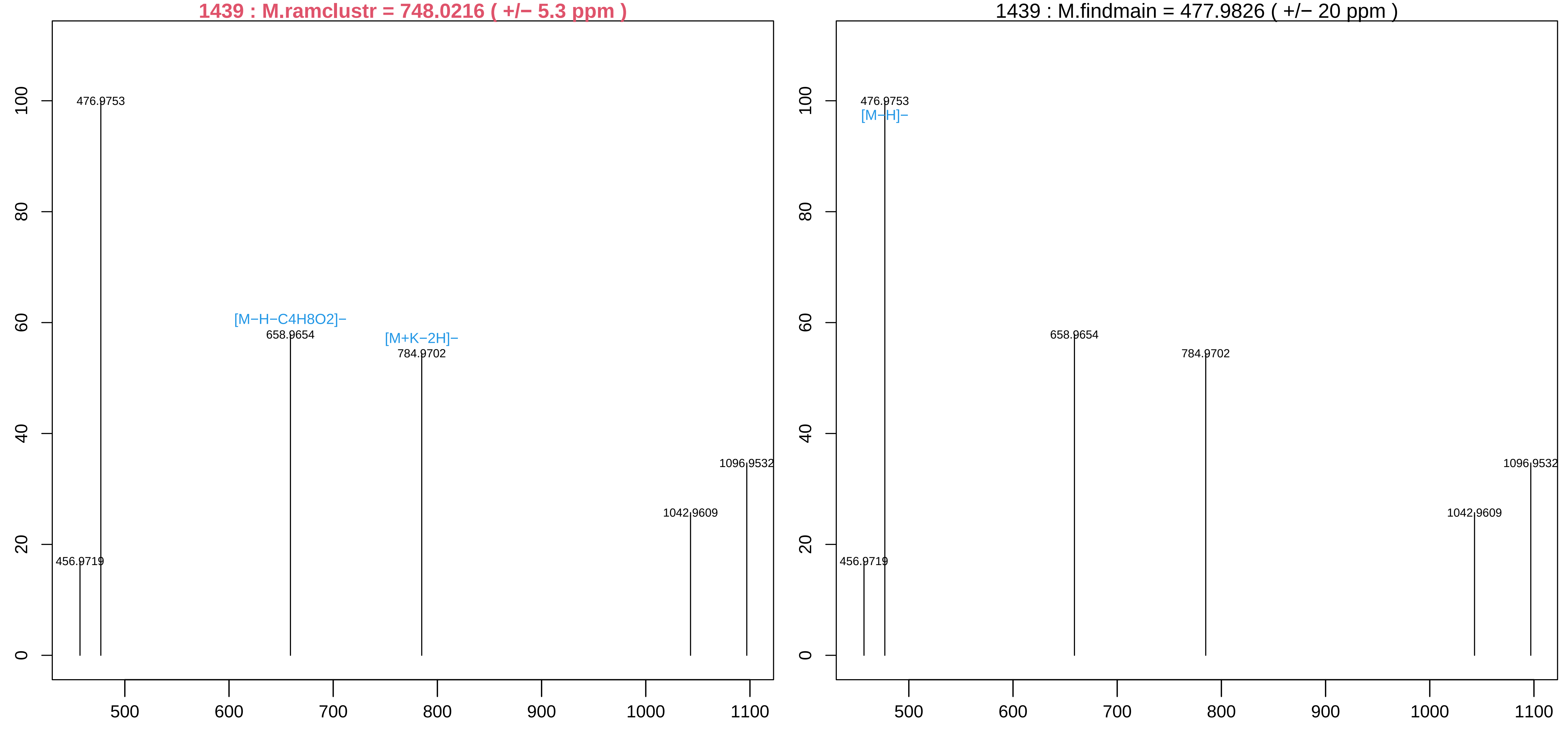
1439 : M.ramclustr = 748.0216 (+/− 5.3 ppm)
476.9753
456.9719
[M−H−C4H8O2]−
658.9654
[M+K−2H]−
784.9702
1042.9609
1096.9532
1439 : M.findmain = 477.9826 (+/− 20 ppm)
476.9753
[M−H]−
456.9719
658.9654
784.9702
1042.9609
1096.9532

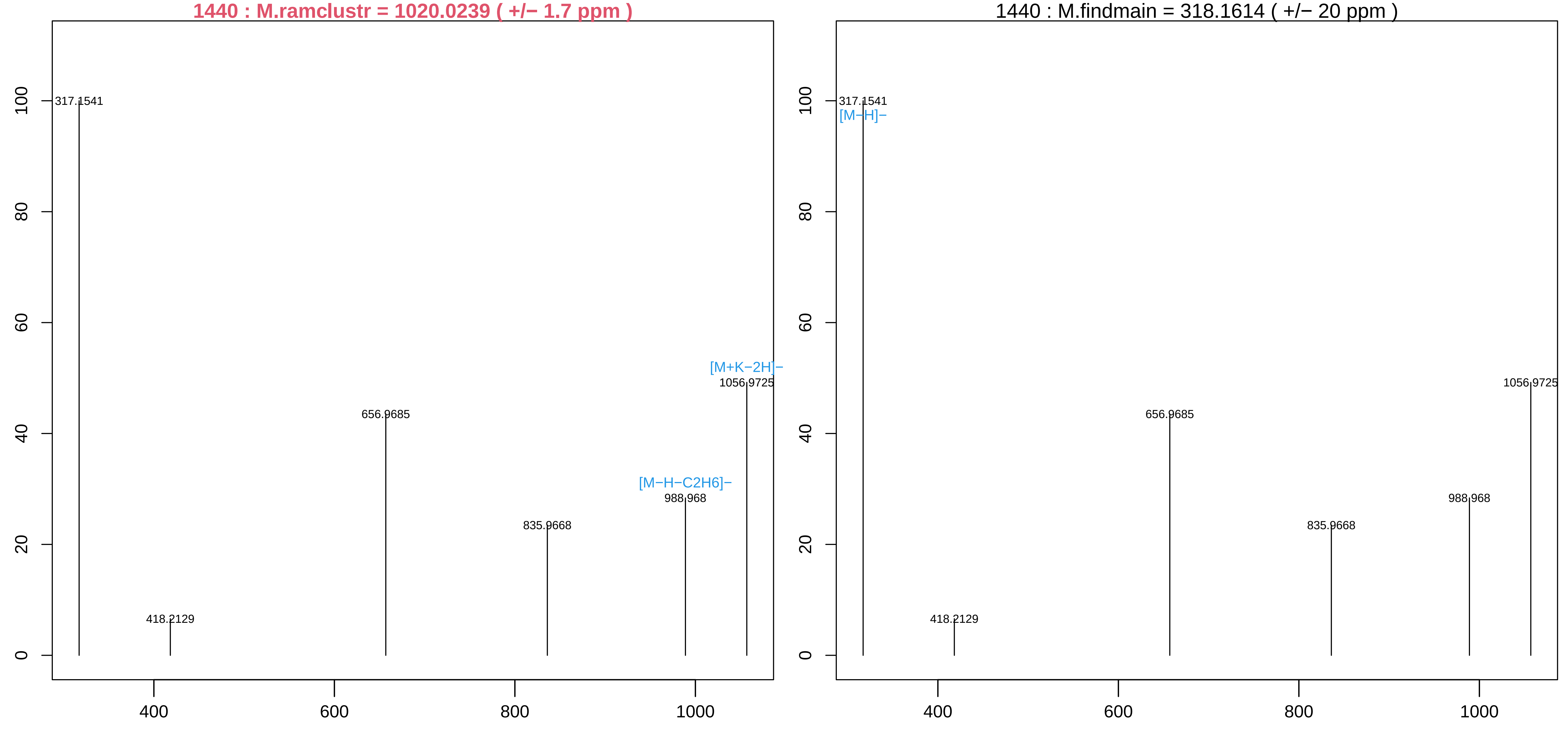
1440 : M.ramclustr = 1020.0239 (+/− 1.7 ppm)
317.1541
418.2129
656.9685
835.9668
[M−H−C2H6]−
988.968
[M+K−2H]−
1056.9725
1440 : M.findmain = 318.1614 (+/− 20 ppm)
317.1541
[M−H]−
418.2129
656.9685
835.9668
988.968
1056.9725
0
20
40
60
80
100
400
600
800
1000

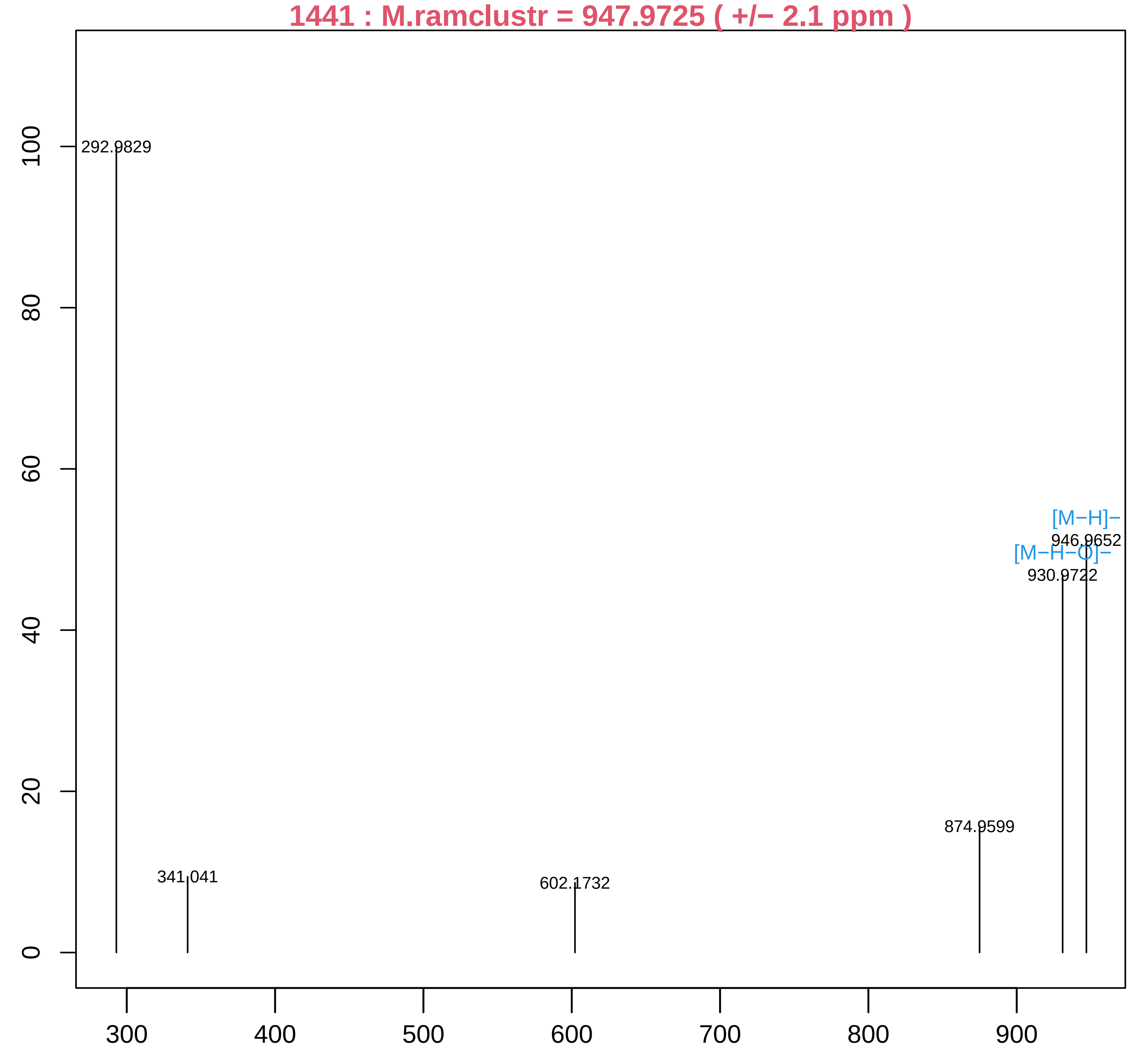
1441 : M.ramclustr = 947.9725 (+/− 2.1 ppm)
292.9829
341.041
602.1732
874.9599
930.9722
946.9652
[M−H]−
[M−H−O]−
0
20
40
60
80
100
300
400
500
600
700
800
900

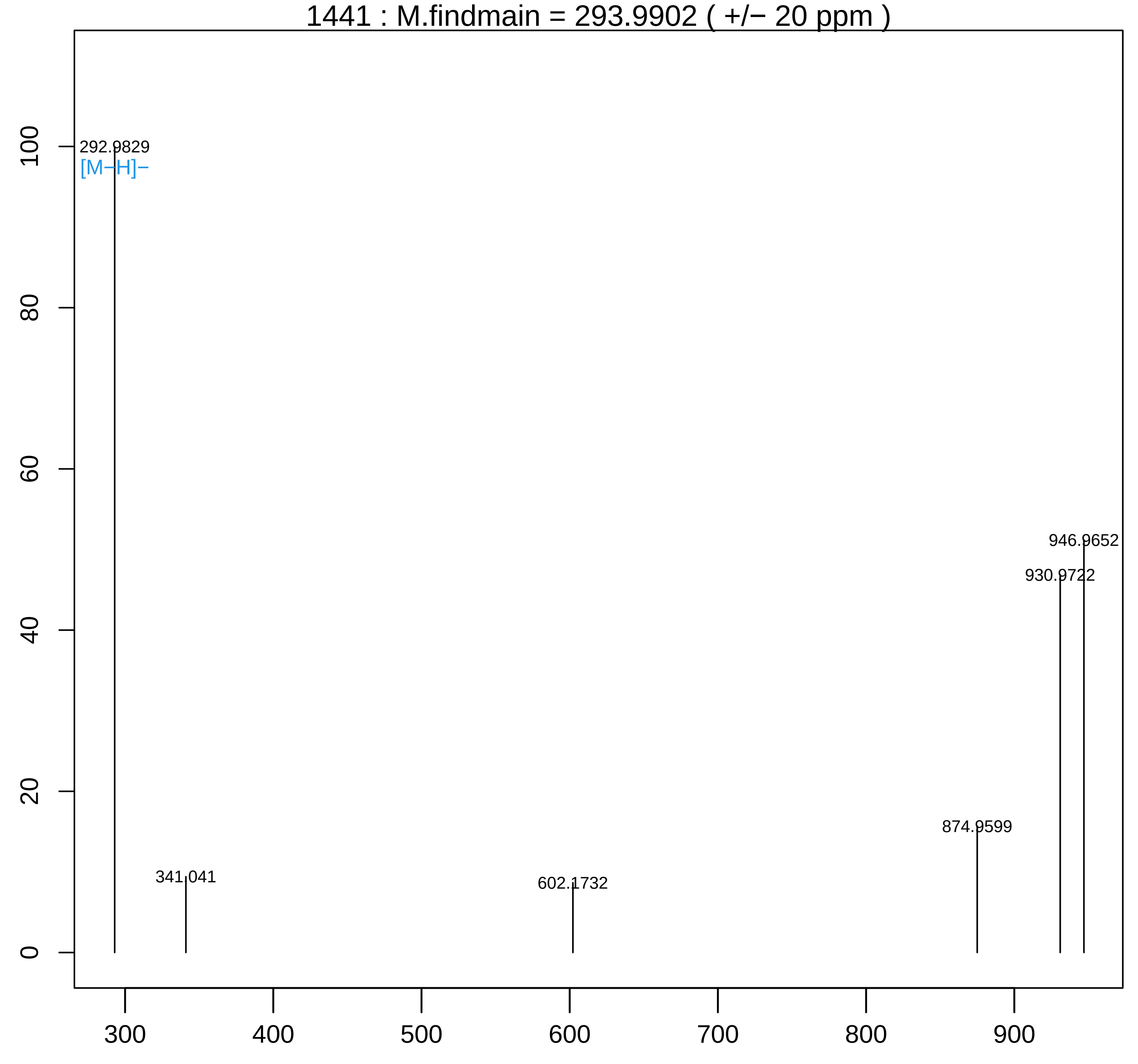
1441 : M.findmain = 293.9902 (+/− 20 ppm)
292.9829
[M−H]−
341.041
602.1732
874.9599
930.9722
946.9652
0
20
40
60
80
100
300
400
500
600
700
800
900

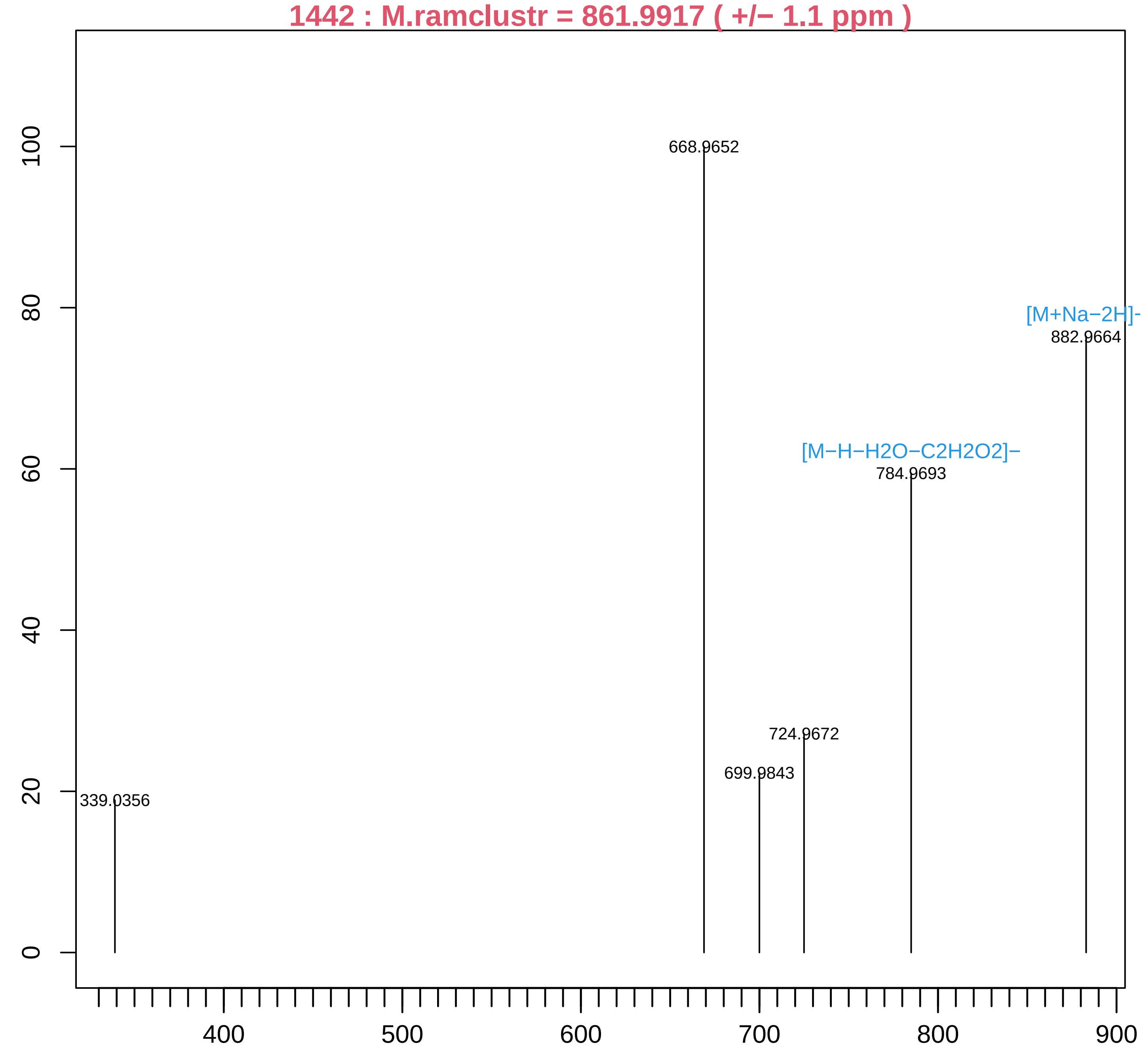
1442 : M.ramclustr = 861.9917 (+/− 1.1 ppm)
668.9652
[M+Na−2H]-
882.9664
[M−H−H2O−C2H2O2]−
784.9693
724.9672
699.9843
339.0356
0
20
40
60
80
100
400
500
600
700
800
900

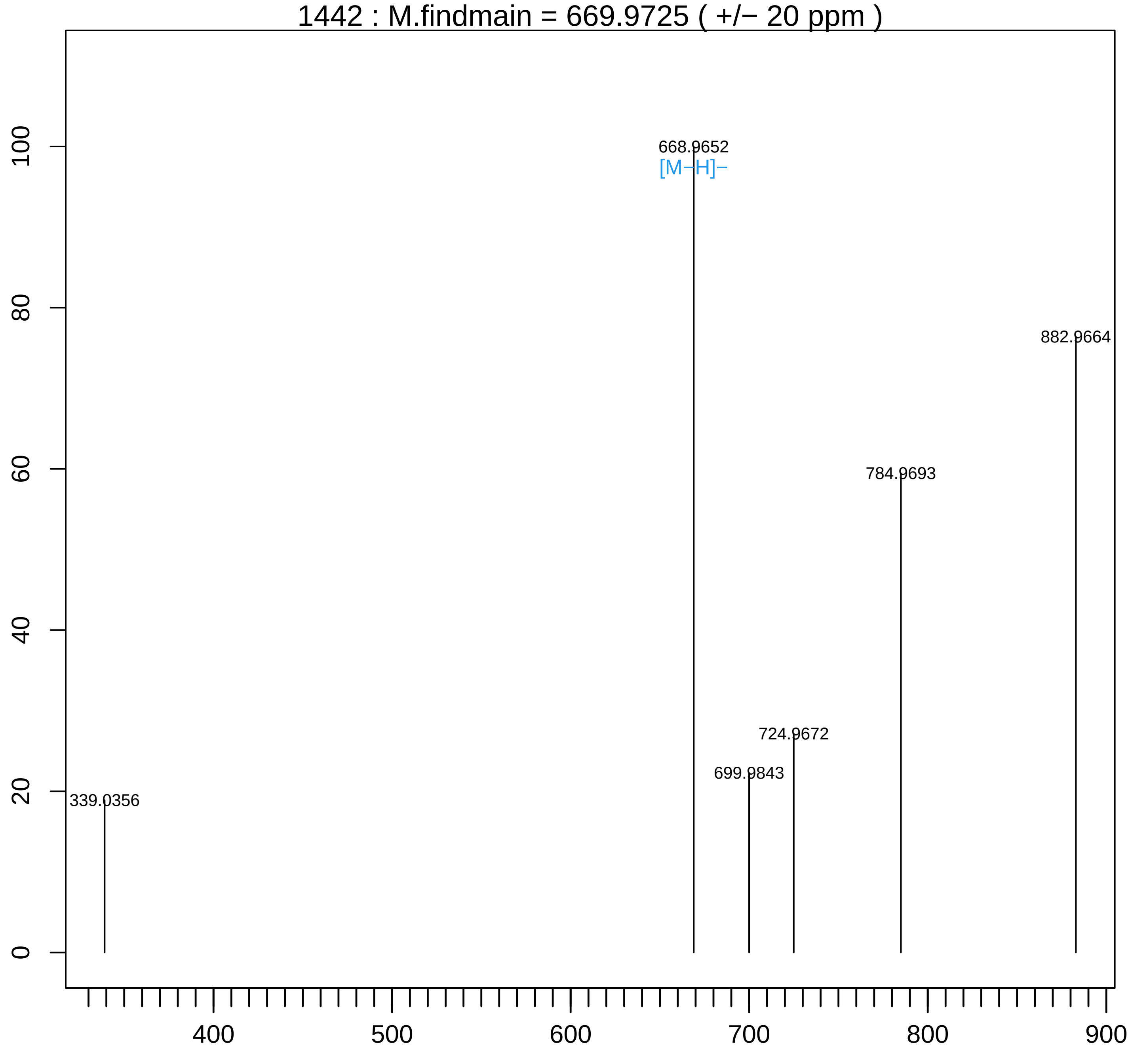
1442 : M.findmain = 669.9725 (+/− 20 ppm)
668.9652
[M−H]−
882.9664
784.9693
724.9672
699.9843
339.0356
0
20
40
60
80
100
400
500
600
700
800
900

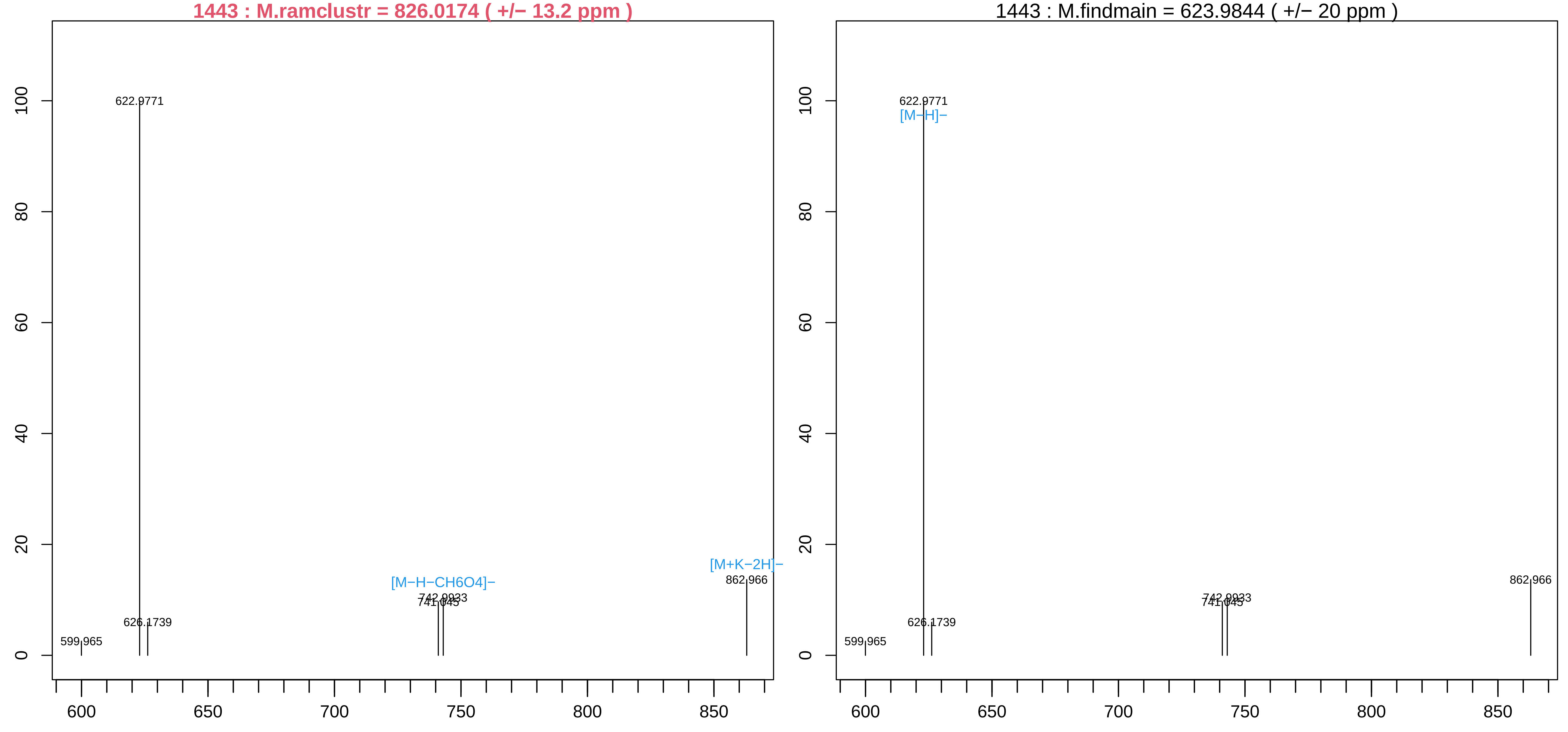
1443 : M.ramclustr = 826.0174 (+/− 13.2 ppm)
622.9771
626.1739
599.965
[M−H−CH6O4]−
742.0933
741.045
[M+K−2H]−
862.966
1443 : M.findmain = 623.9844 (+/− 20 ppm)
622.9771
[M−H]−
626.1739
599.965
742.0933
741.045
862.966
0
20
40
60
80
100
600
650
700
750
800
850

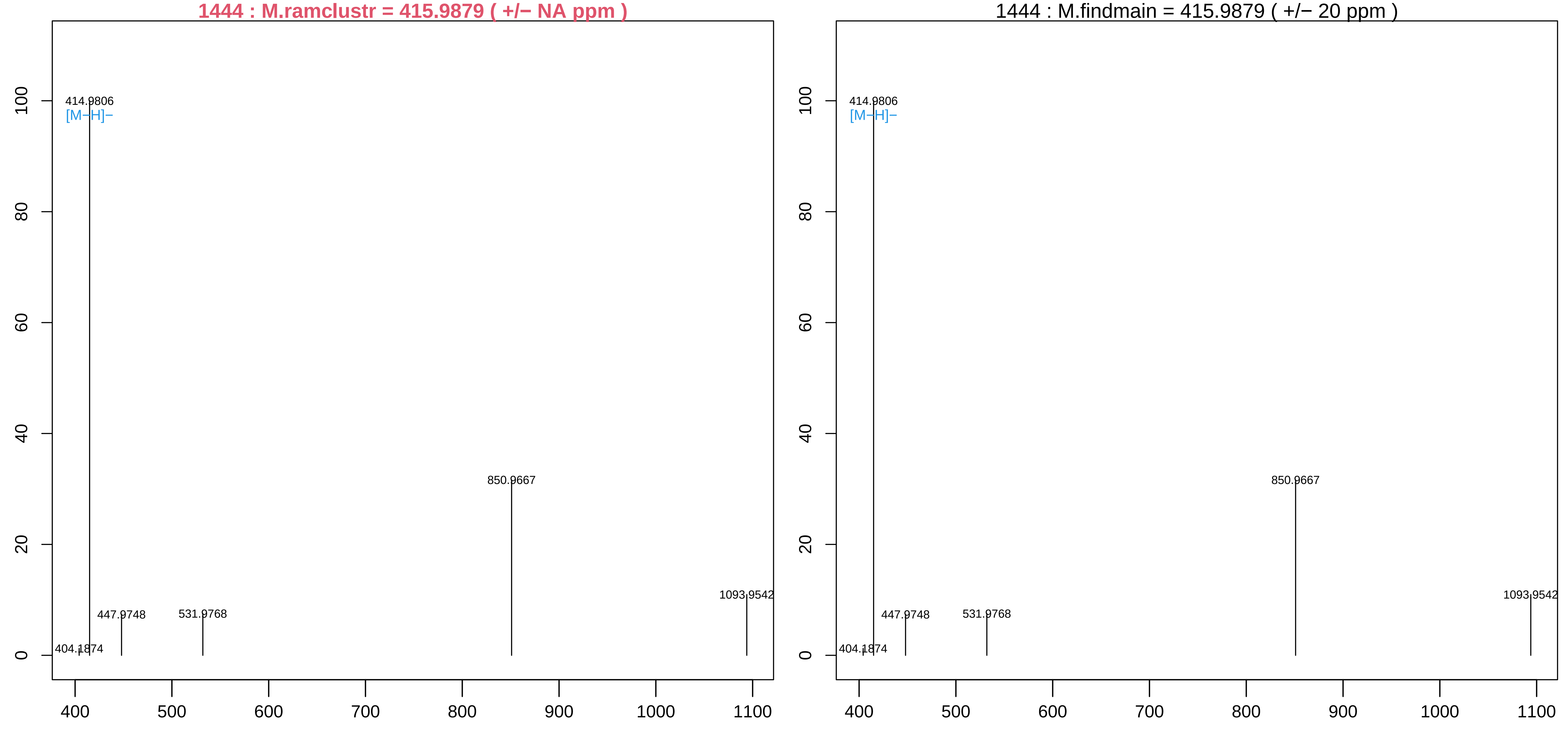

1444 : M.ramclustr = 415.9879 (+/− NA ppm)
414.9806
[M−H]−
850.9667
1093.9542
447.9748
531.9768
404.1874
1444 : M.findmain = 415.9879 (+/− 20 ppm)
414.9806
[M−H]−
850.9667
1093.9542
447.9748
531.9768
404.1874

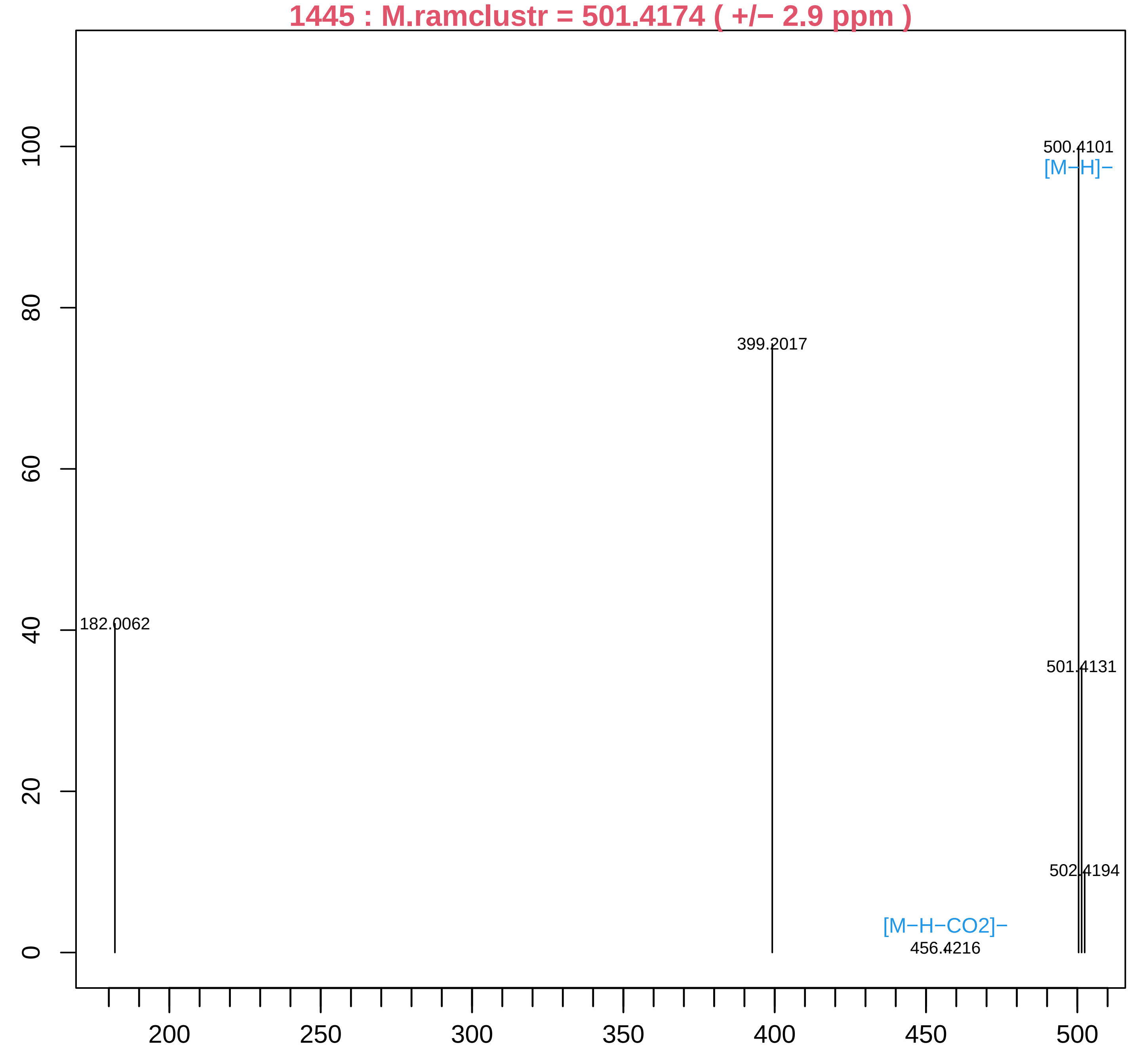
1445 : M.ramclustr = 501.4174 (+/− 2.9 ppm)
500.4101
[M−H]−
399.2017
182.0062
501.4131
502.4194
[M−H−CO2]−
456.4216
0
20
40
60
80
100
200
250
300
350
400
450
500

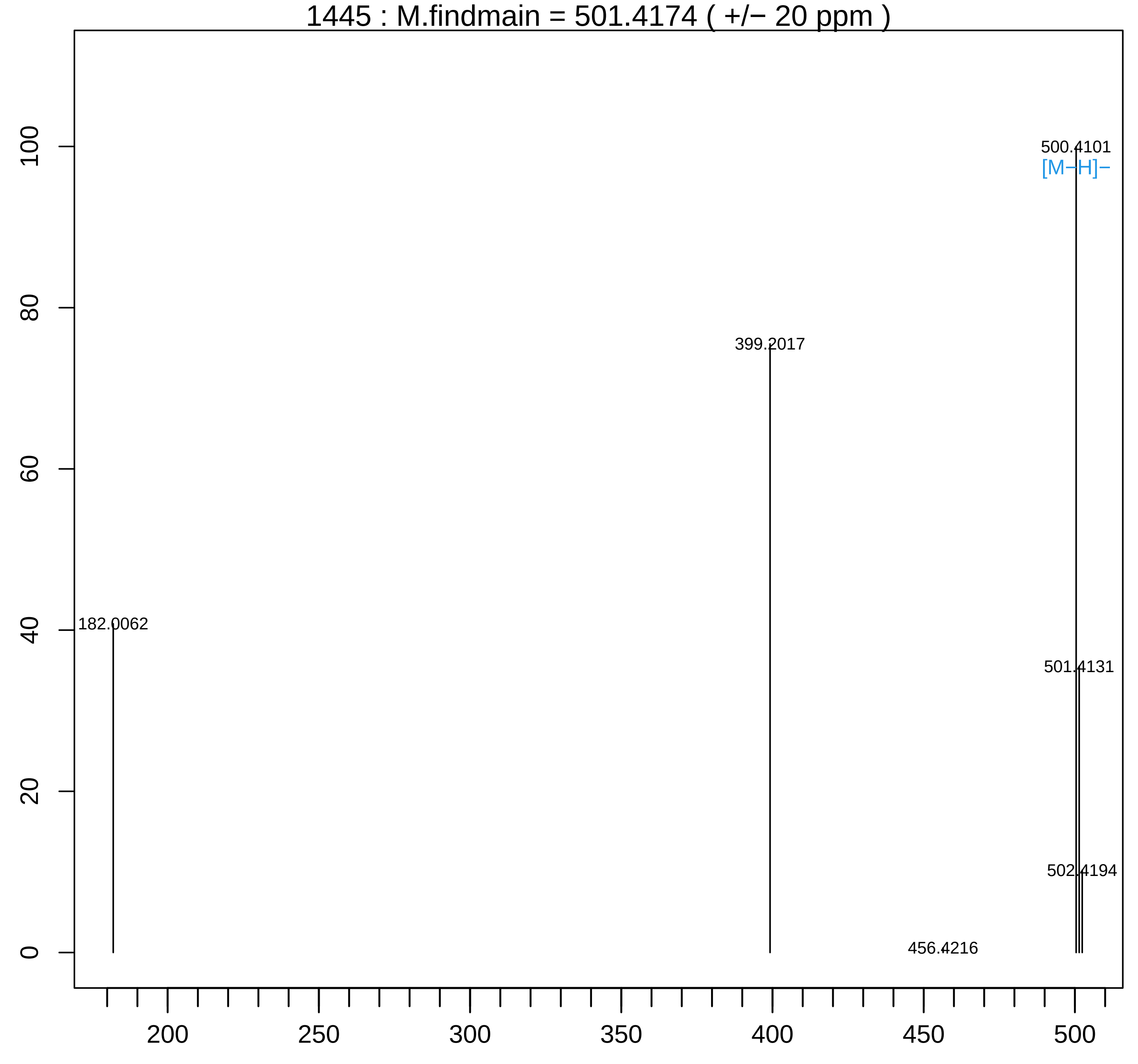
1445 : M.findmain = 501.4174 (+/− 20 ppm)
500.4101
[M−H]−
399.2017
182.0062
501.4131
502.4194
456.4216
0
20
40
60
80
100
200
250
300
350
400
450
500

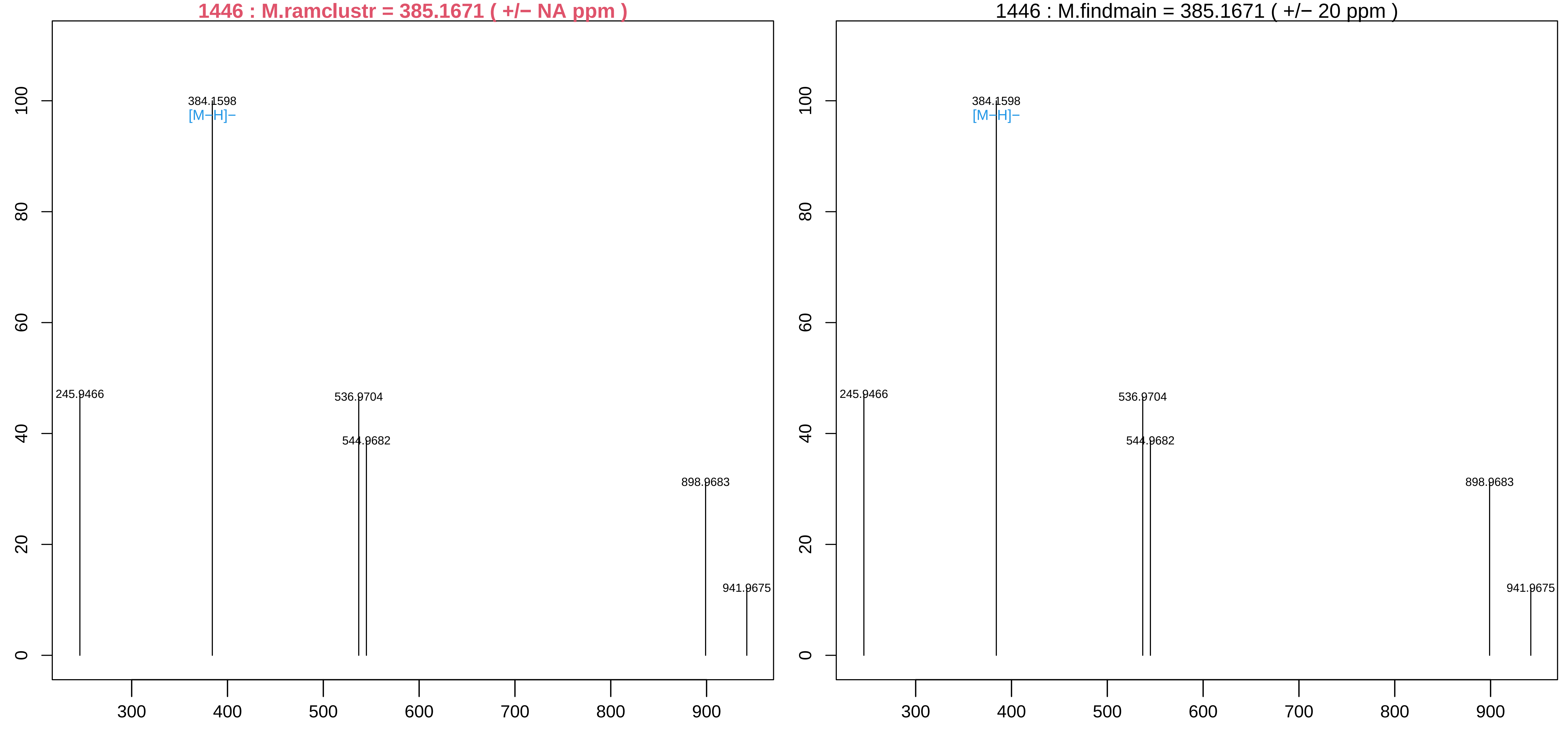
1446 : M.ramclustr = 385.1671 (+/− NA ppm)
1446 : M.findmain = 385.1671 (+/− 20 ppm)
384.1598
[M−H]−
245.9466
536.9704
544.9682
898.9683
941.9675
0
20
40
60
80
100
300
400
500
600
700
800
900

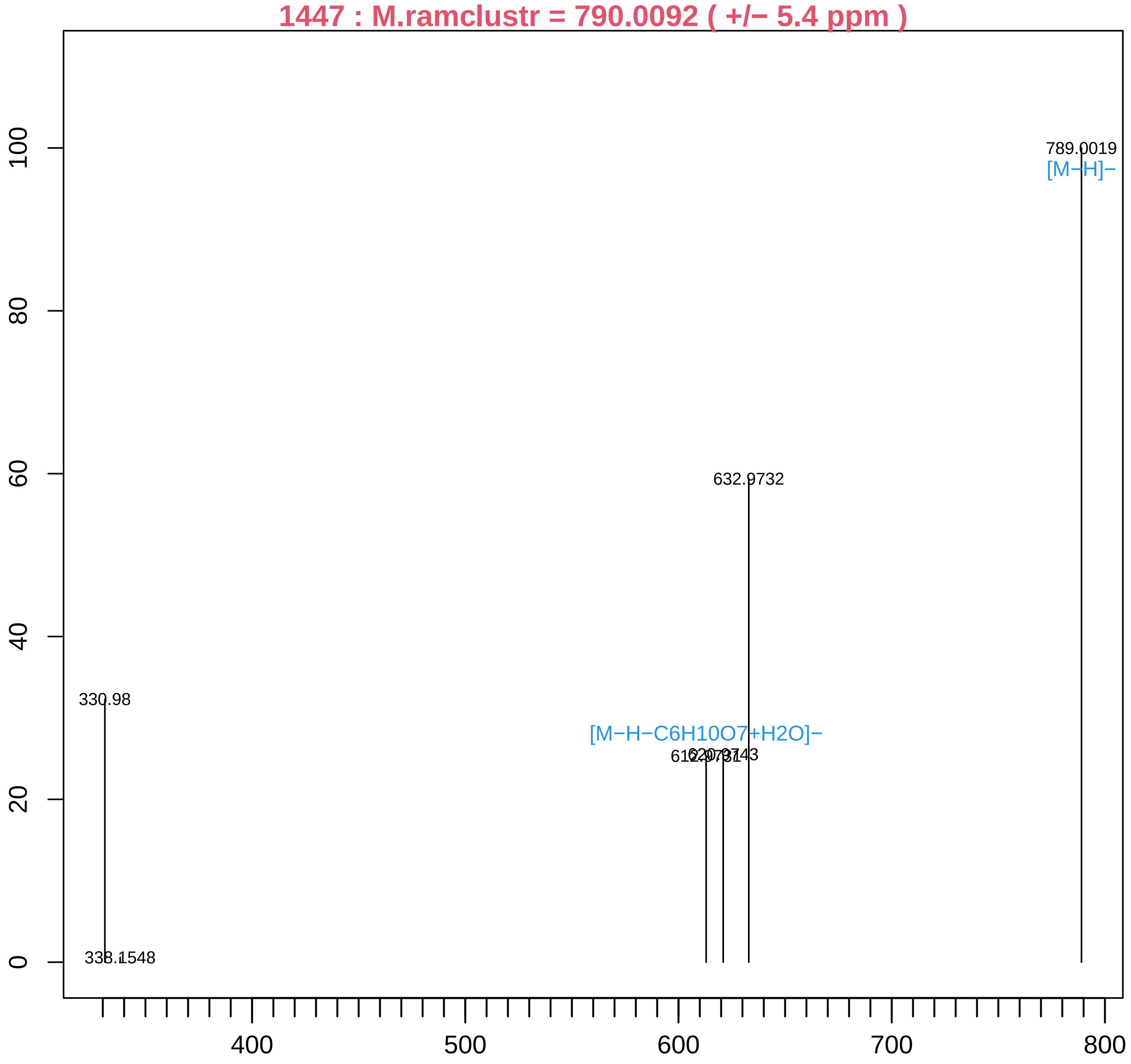
1447 : M.ramclustr = 790.0092 (+/− 5.4 ppm)
789.0019
[M−H]−
632.9732
[M−H−C6H10O7+H2O]−
330.98
338.1548
0
20
40
60
80
100
400
500
600
700
800

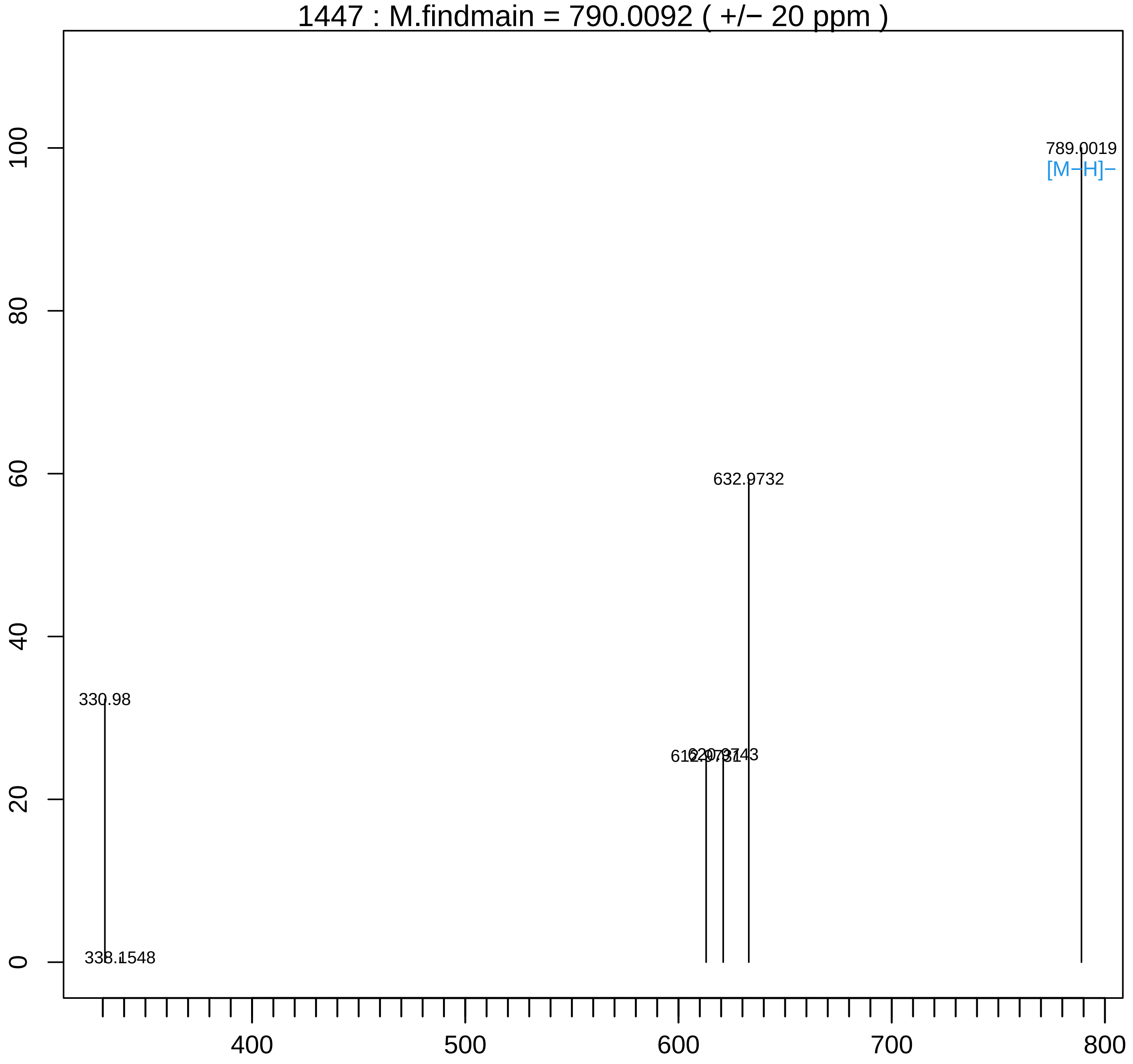
1447 : M.findmain = 790.0092 (+/− 20 ppm)
789.0019
[M−H]−
632.9732
330.98
338.1548
0
20
40
60
80
100
400
500
600
700
800

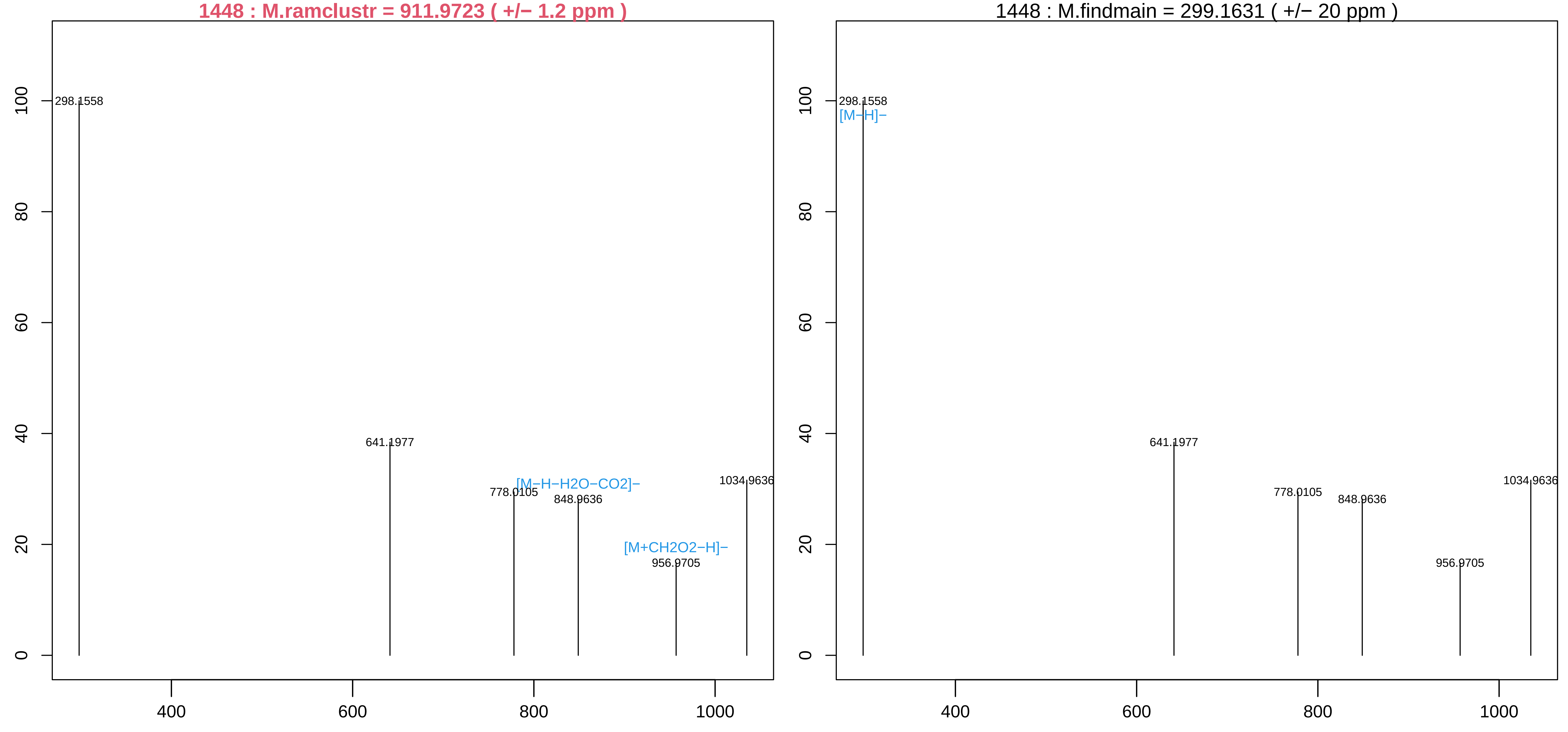
1448 : M.ramclustr = 911.9723 (+/− 1.2 ppm)
298.1558
641.1977
778.0105
[M−H−H2O−CO2]−
848.9636
[M+CH2O2−H]−
956.9705
1034.9636
1448 : M.findmain = 299.1631 (+/− 20 ppm)
298.1558
[M−H]−
641.1977
778.0105
848.9636
956.9705
1034.9636
0
20
40
60
80
100
400
600
800
1000

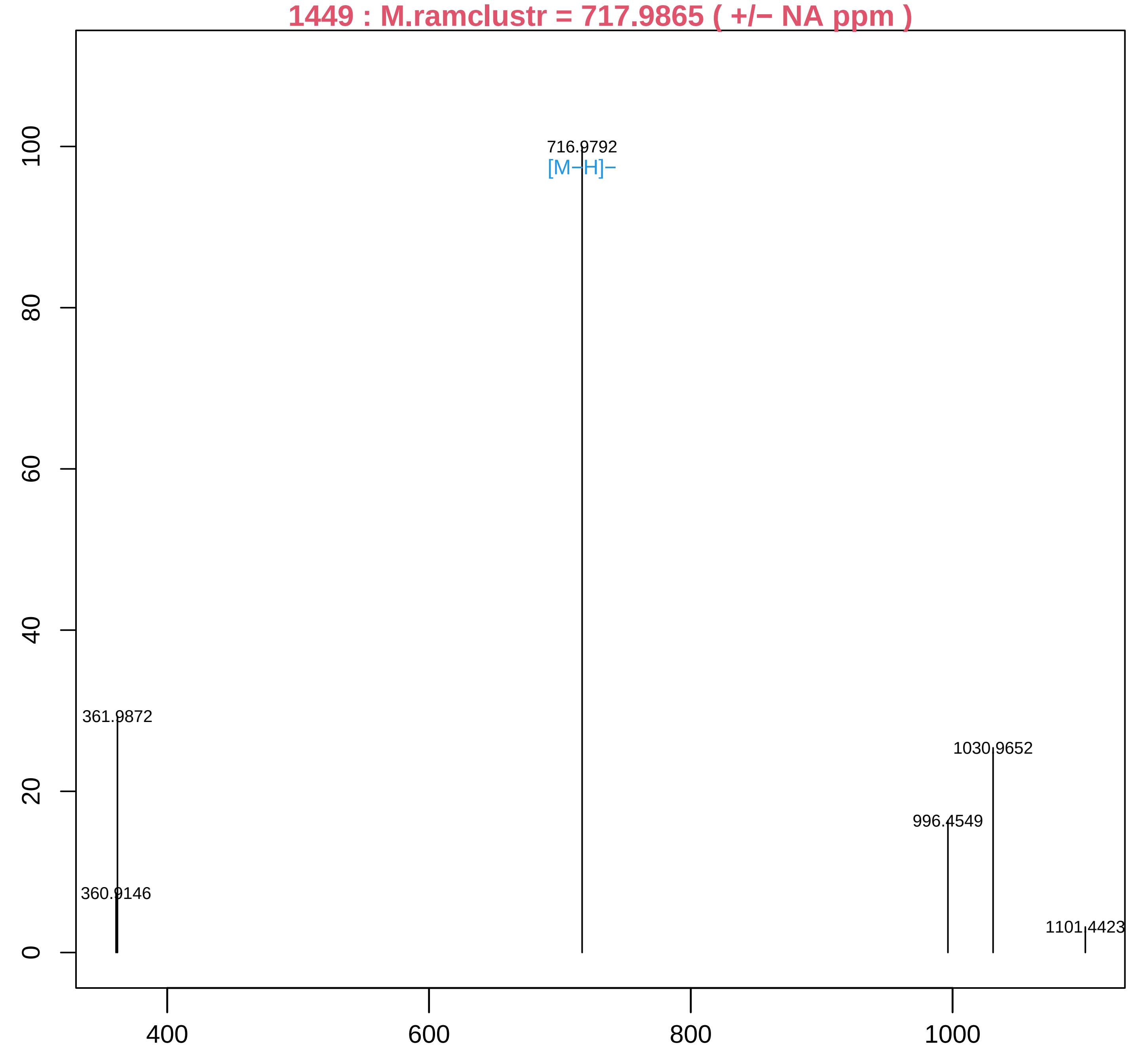
1449 : M.ramclustr = 717.9865 (+/− NA ppm)
716.9792
[M−H]−
361.9872
360.9146
996.4549
1030.9652
1101.4423
0
20
40
60
80
100
400
600
800
1000

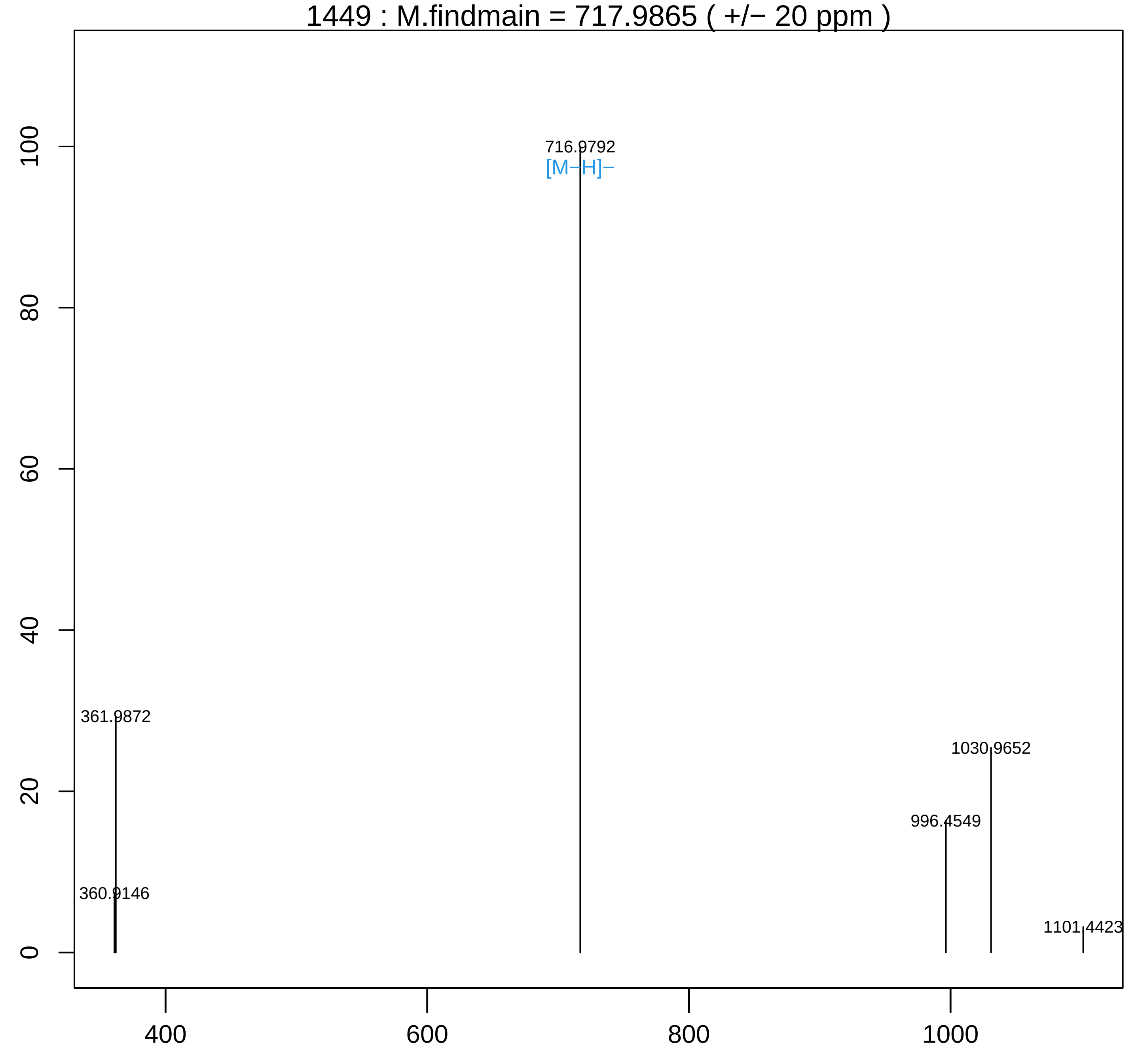
1449 : M.findmain = 717.9865 (+/− 20 ppm)
716.9792
[M−H]−
361.9872
360.9146
996.4549
1030.9652
1101.4423
0
20
40
60
80
100
400
600
800
1000

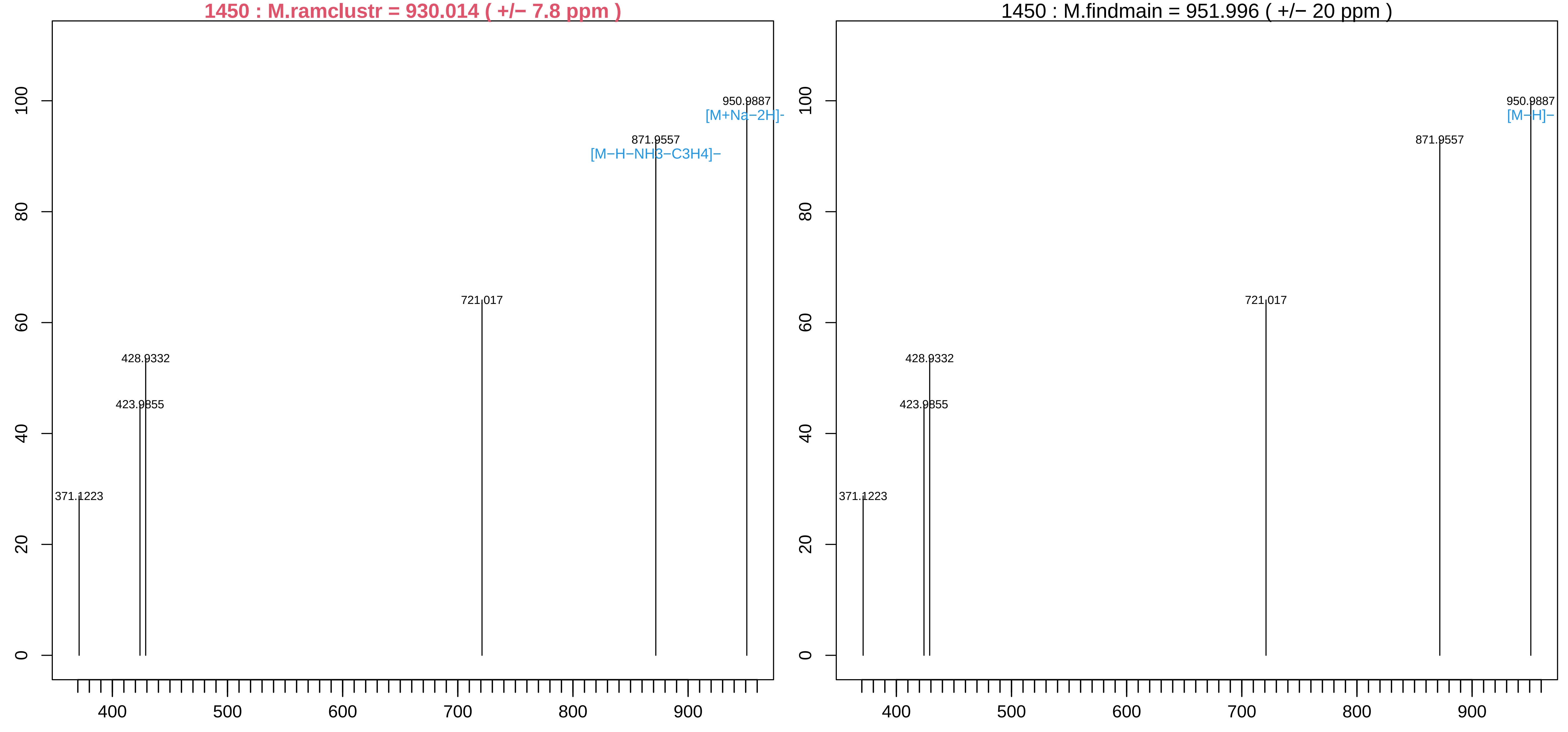
1450 : M.ramclustr = 930.014 (+/− 7.8 ppm)
950.9887
[M+Na−2H]-
871.9557
[M−H−NH3−C3H4]−
721.017
428.9332
423.9855
371.1223
1450 : M.findmain = 951.996 (+/− 20 ppm)
950.9887
[M−H]−
871.9557
721.017
428.9332
423.9855
371.1223
0
20
40
60
80
100
400
500
600
700
800
900

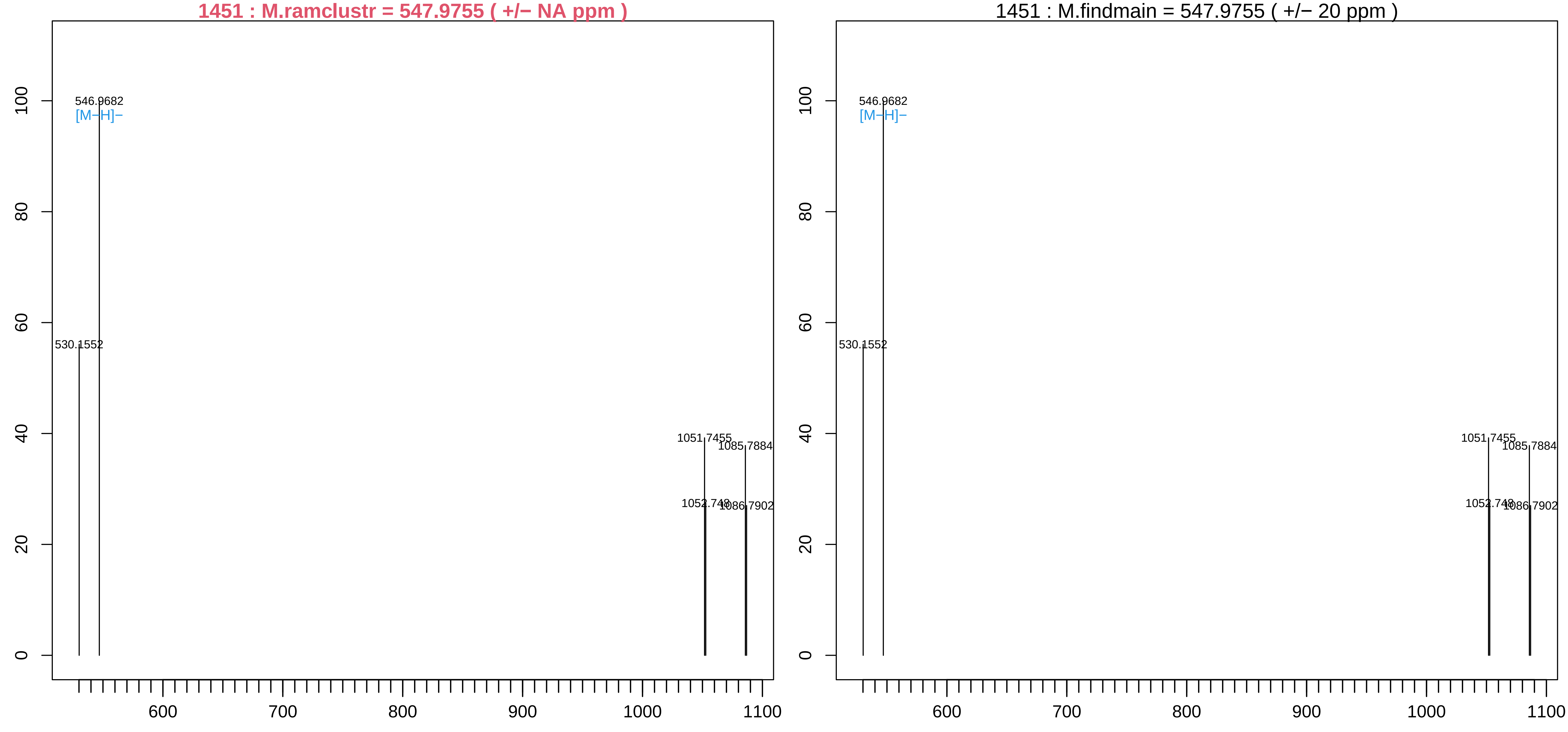
1451 : M.ramclustr = 547.9755 (+/− NA ppm)
546.9682
[M−H]−
530.1552
1051.7455
1085.7884
1052.748
1086.7902
1451 : M.findmain = 547.9755 (+/− 20 ppm)
546.9682
[M−H]−
530.1552
1051.7455
1085.7884
1052.748
1086.7902

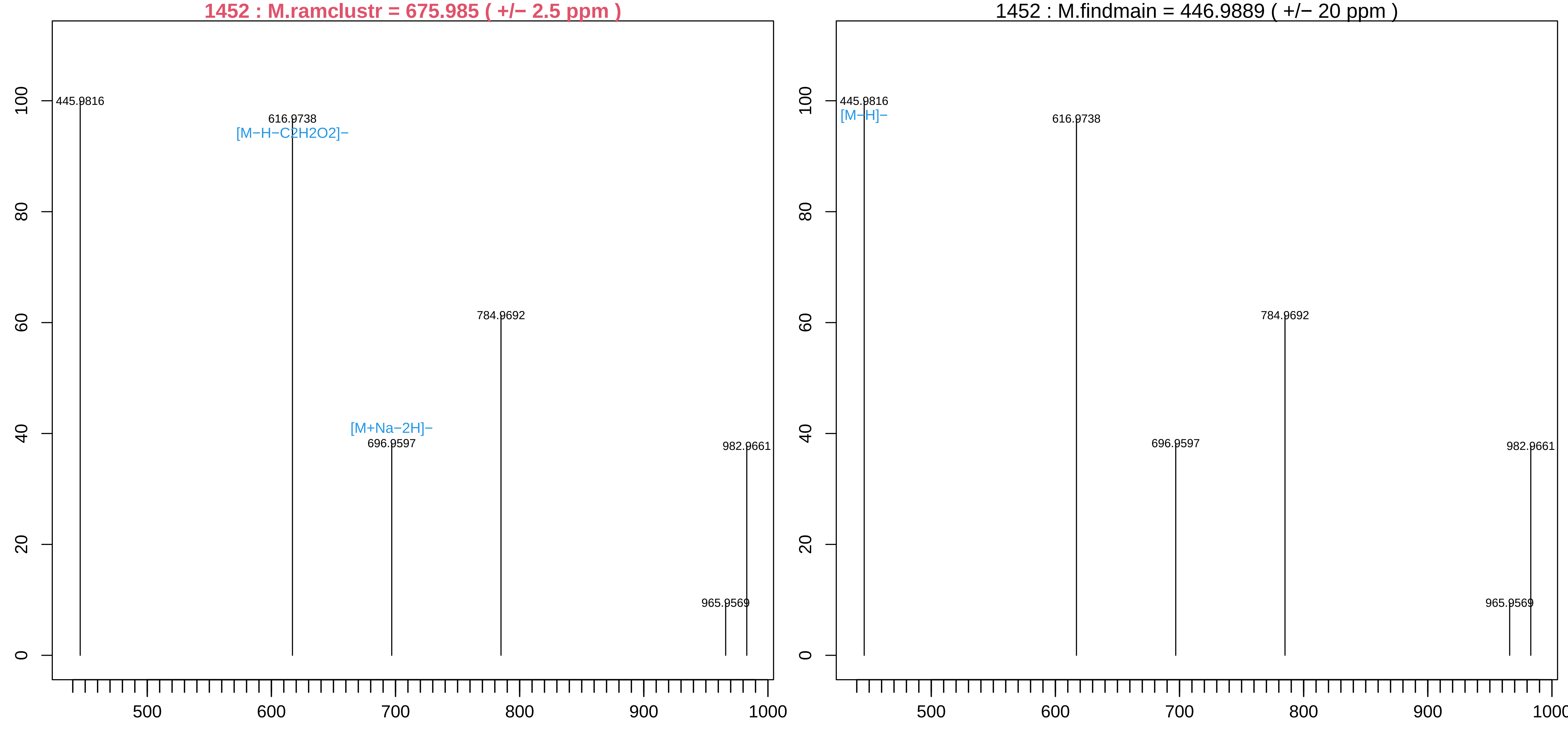

1452 : M.ramclustr = 675.985 (+/− 2.5 ppm)
445.9816
616.9738
[M−H−C2H2O2]−
[M+Na−2H]−
696.9597
784.9692
982.9661
965.9569
1452 : M.findmain = 446.9889 (+/− 20 ppm)
445.9816
[M−H]−
616.9738
696.9597
784.9692
982.9661
965.9569
0
20
40
60
80
100
500
600
700
800
900
1000

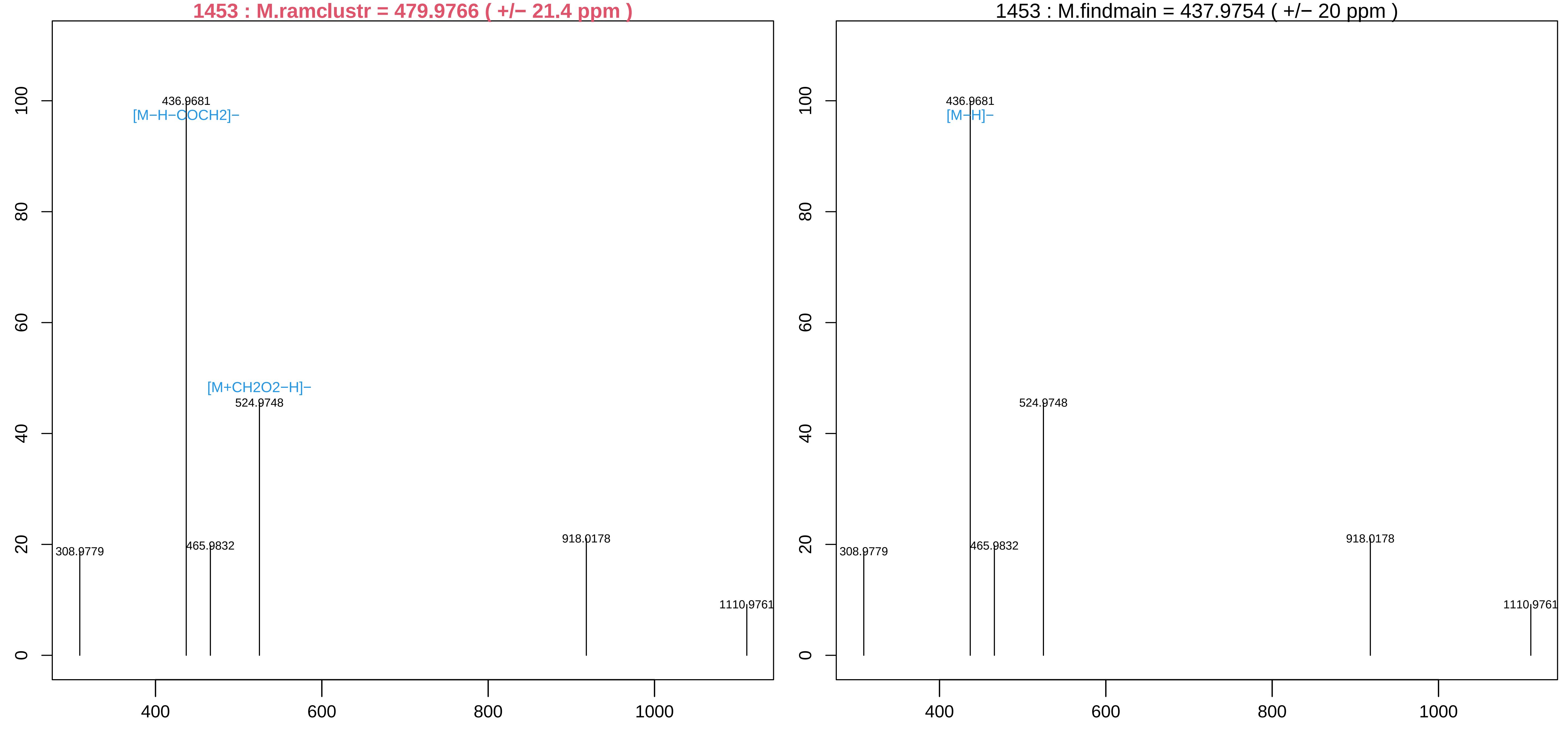
1453 : M.ramclustr = 479.9766 (+/− 21.4 ppm)
436.9681
[M−H−COCH2]−
[M+CH2O2−H]−
524.9748
308.9779
465.9832
918.0178
1110.9761
0
20
40
60
80
100
400
600
800
1000
1453 : M.findmain = 437.9754 (+/− 20 ppm)
436.9681
[M−H]−
524.9748
308.9779
465.9832
918.0178
1110.9761

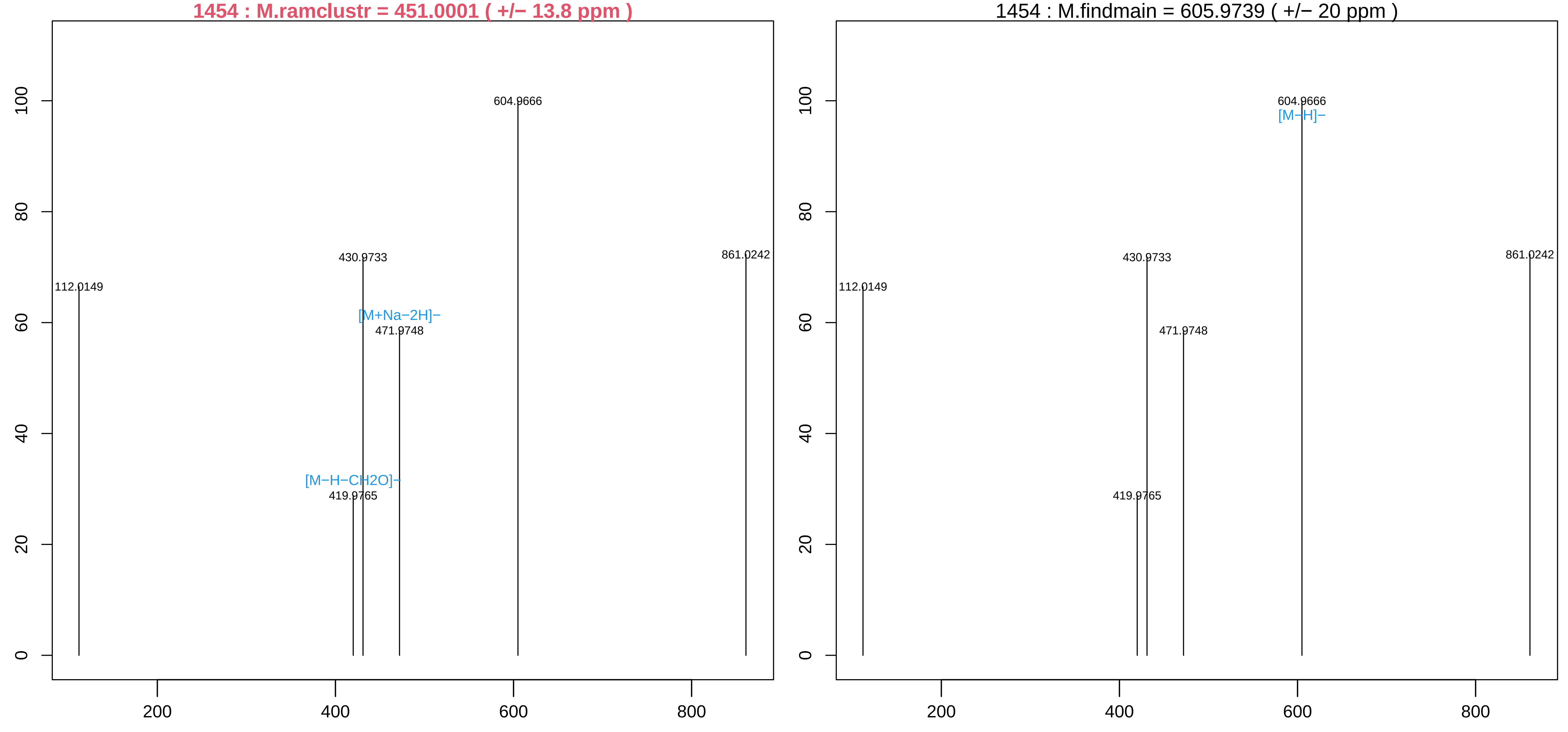

1454 : M.ramclustr = 451.0001 (+/− 13.8 ppm)
604.9666
430.9733
861.0242
112.0149
[M+Na−2H]−
471.9748
[M−H−CH2O]−
419.9765
0
20
40
60
80
100
200
400
600
800
1454 : M.findmain = 605.9739 (+/− 20 ppm)
604.9666
[M−H]−
430.9733
861.0242
112.0149
471.9748
419.9765
0
20
40
60
80
100
200
400
600
800

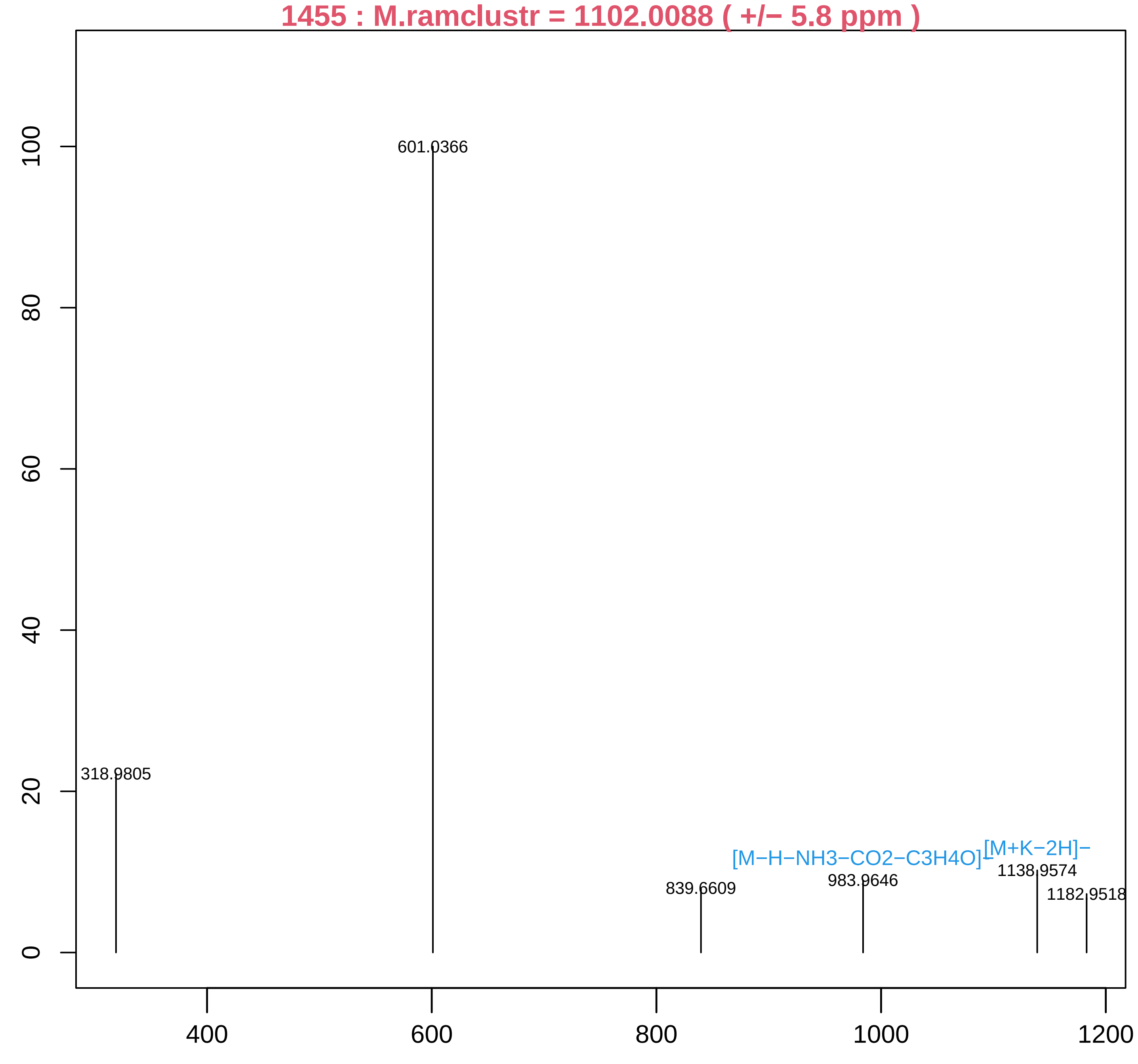
1455 : M.ramclustr = 1102.0088 (+/− 5.8 ppm)
601.0366
318.9805
839.6609
983.9646
1138.9574
1182.9518
[M−H−NH3−CO2−C3H4O]−
[M+K−2H]−
0
20
40
60
80
100
400
600
800
1000
1200

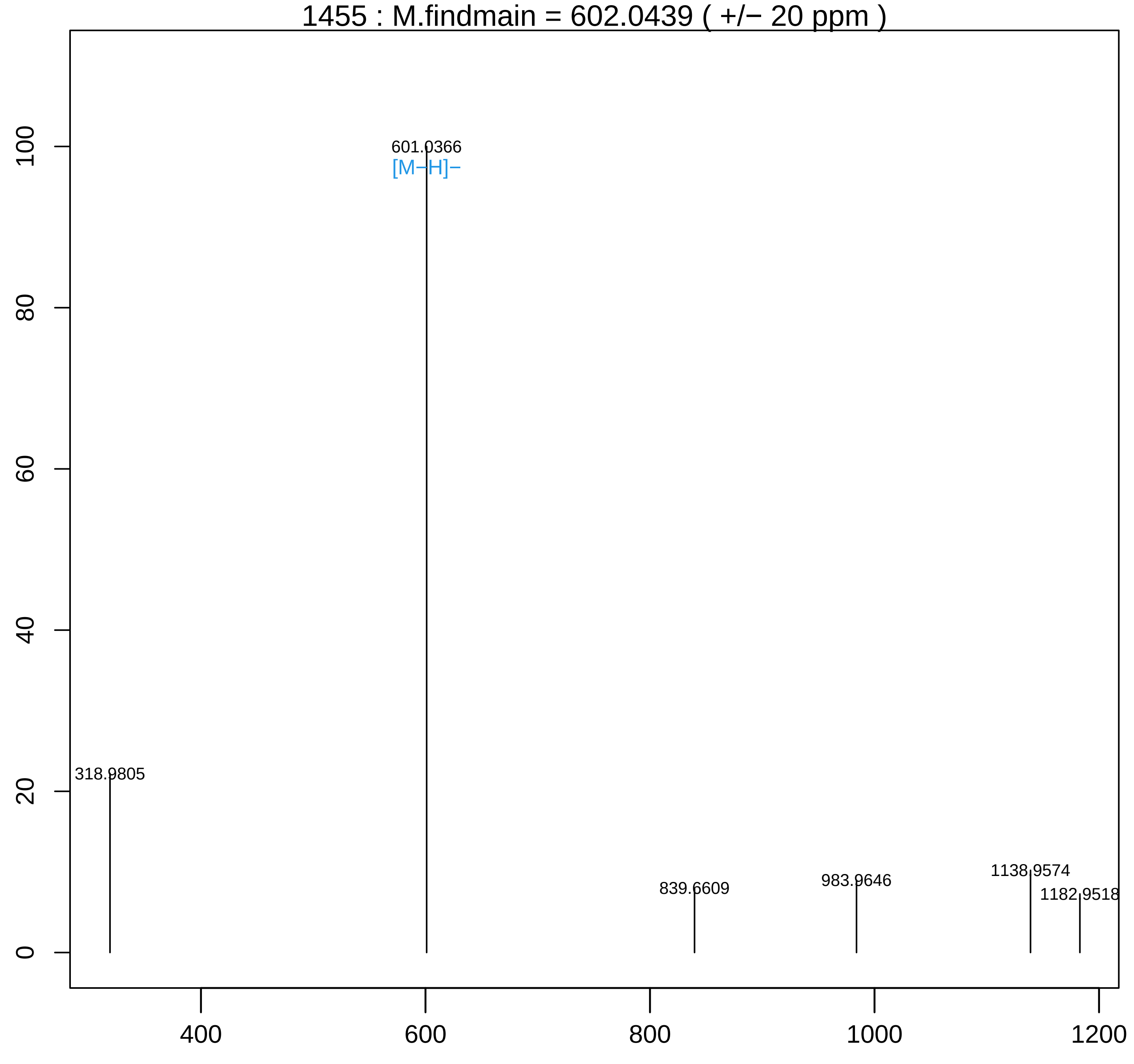
1455 : M.findmain = 602.0439 (+/− 20 ppm)
601.0366
[M−H]−
318.9805
839.6609
983.9646
1138.9574
1182.9518
0
20
40
60
80
100
400
600
800
1000
1200

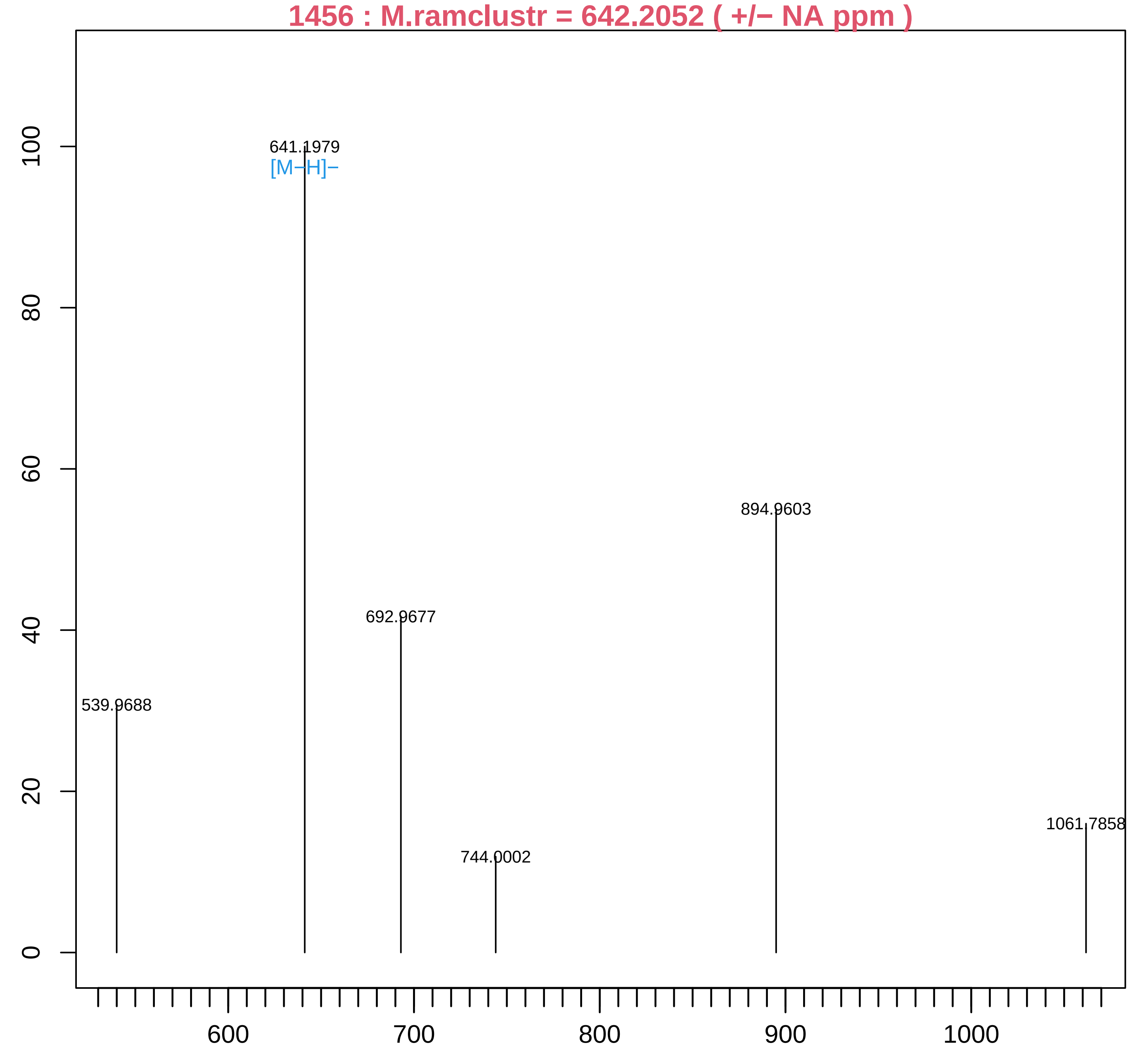
1456 : M.ramclustr = 642.2052 (+/− NA ppm)
641.1979
[M−H]−
539.9688
692.9677
744.0002
894.9603
1061.7858
0
20
40
60
80
100
600
700
800
900
1000

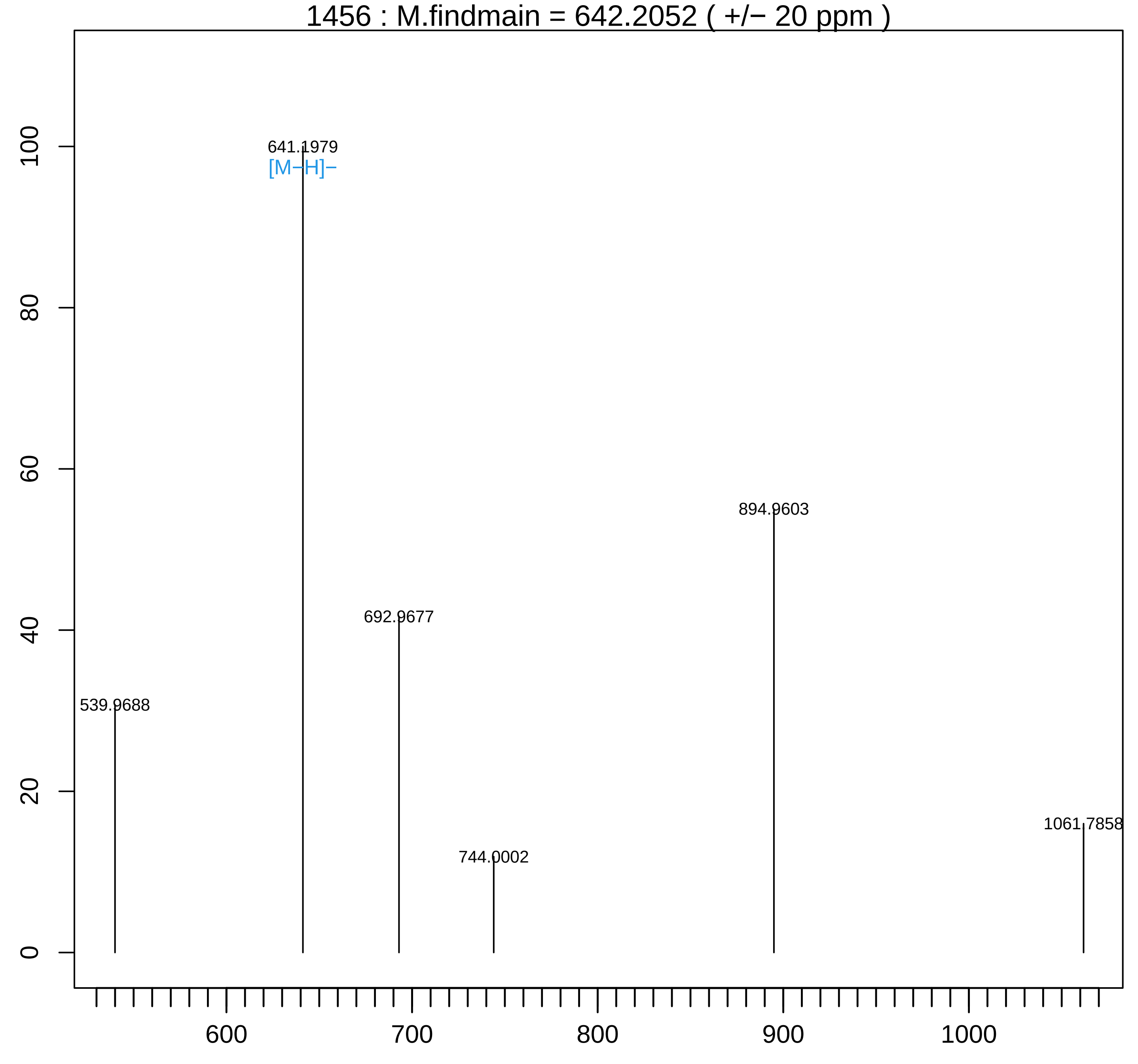
1456 : M.findmain = 642.2052 (+/− 20 ppm)
641.1979
[M−H]−
539.9688
692.9677
744.0002
894.9603
1061.7858
0
20
40
60
80
100
600
700
800
900
1000

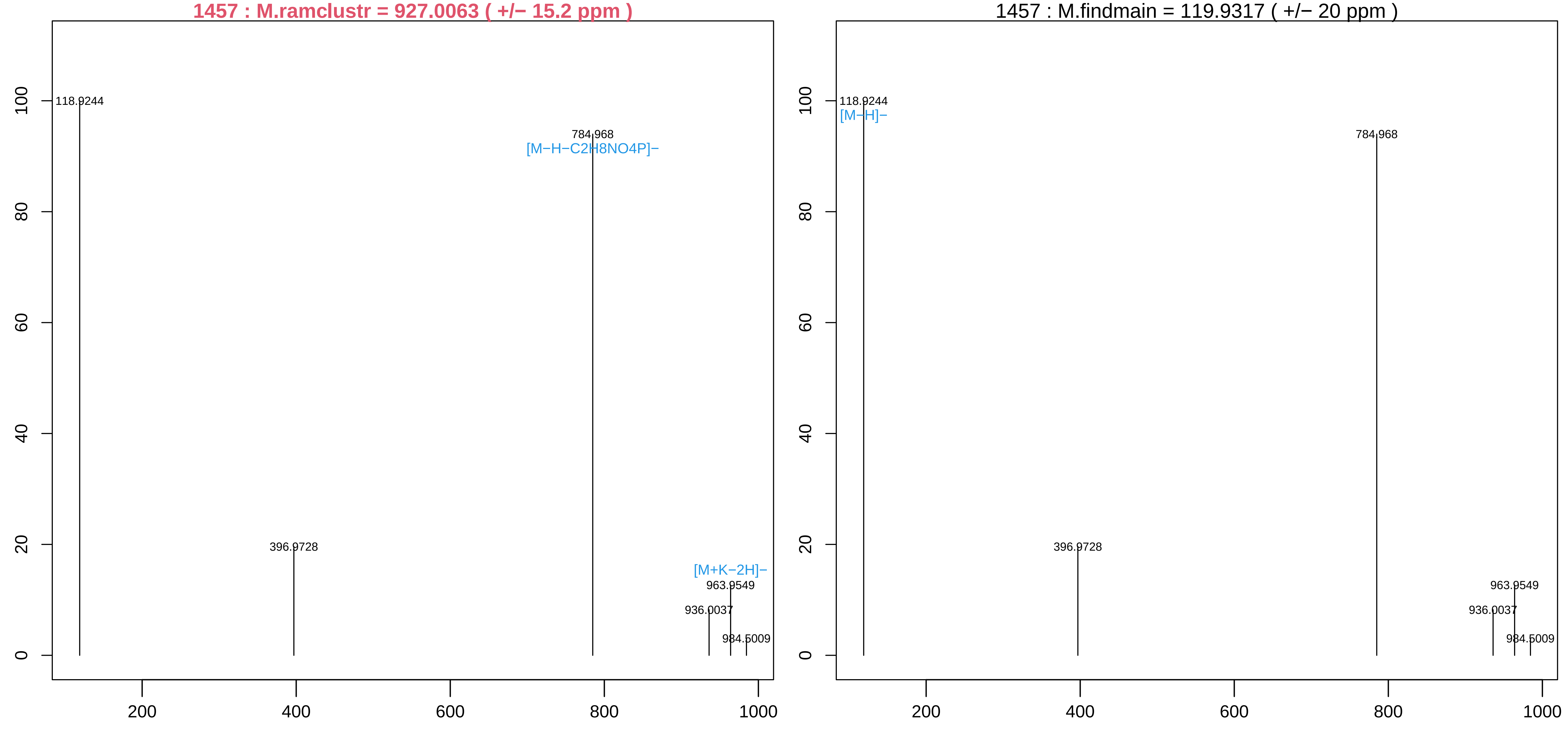
1457 : M.ramclustr = 927.0063 (+/− 15.2 ppm)
118.9244
784.968
[M−H−C2H8NO4P]−
396.9728
[M+K−2H]−
963.9549
936.0037
984.5009
1457 : M.findmain = 119.9317 (+/− 20 ppm)
118.9244
[M−H]−
784.968
396.9728
963.9549
936.0037
984.5009

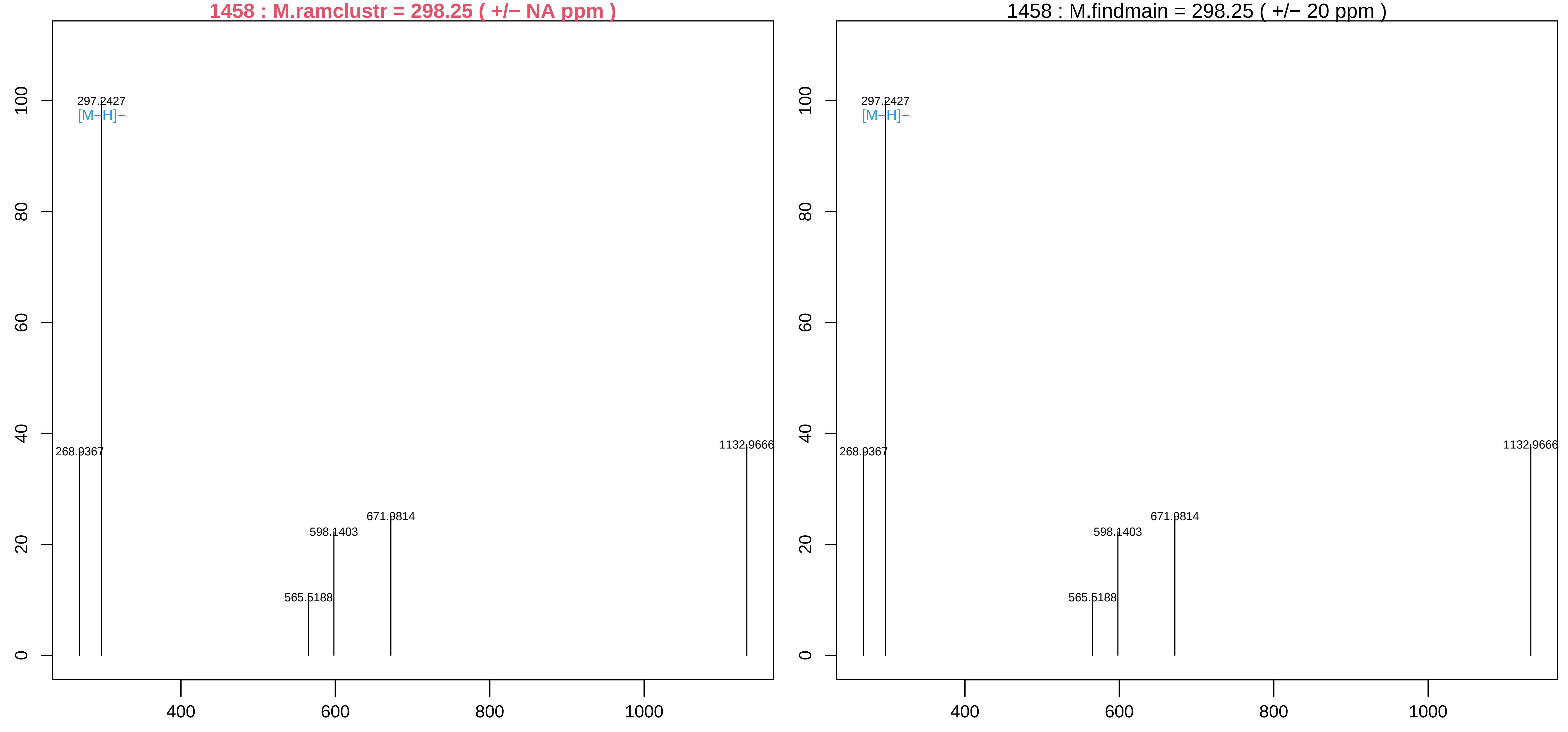
1458 : M.ramclustr = 298.25 (+/− NA ppm)
297.2427
[M−H]−
268.9367
565.5188
598.1403
671.9814
1132.9666
0
20
40
60
80
100
400
600
800
1000
1458 : M.findmain = 298.25 (+/− 20 ppm)
297.2427
[M−H]−
268.9367
565.5188
598.1403
671.9814
1132.9666
0
20
40
60
80
100
400
600
800
1000

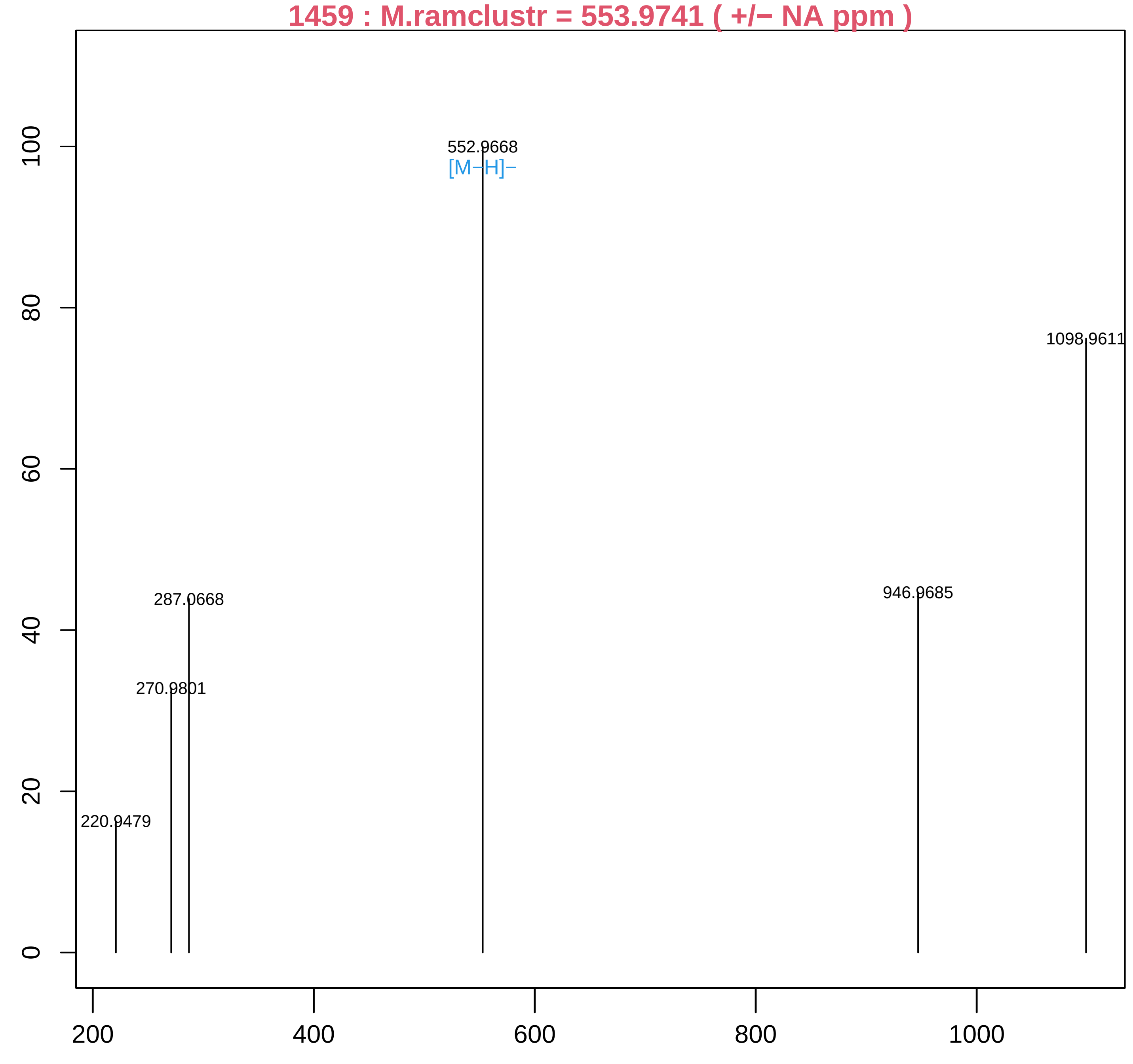
1459 : M.ramclustr = 553.9741 (+/− NA ppm)
552.9668
[M−H]−
1098.9611
287.0668
270.9801
946.9685
220.9479
0
20
40
60
80
100
200
400
600
800
1000

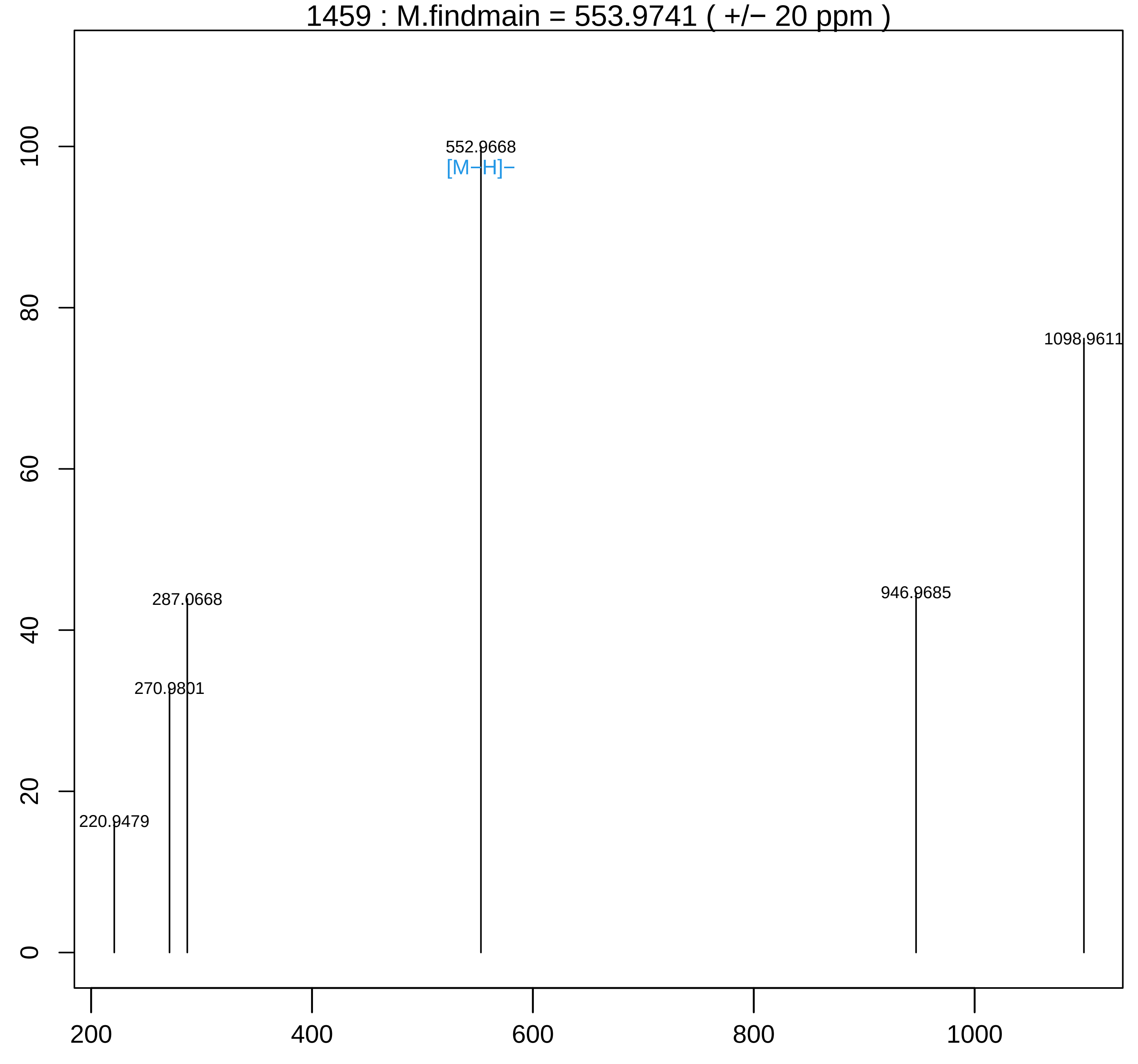
1459 : M.findmain = 553.9741 (+/− 20 ppm)
552.9668
[M−H]−
1098.9611
287.0668
270.9801
946.9685
220.9479
0
20
40
60
80
100
200
400
600
800
1000

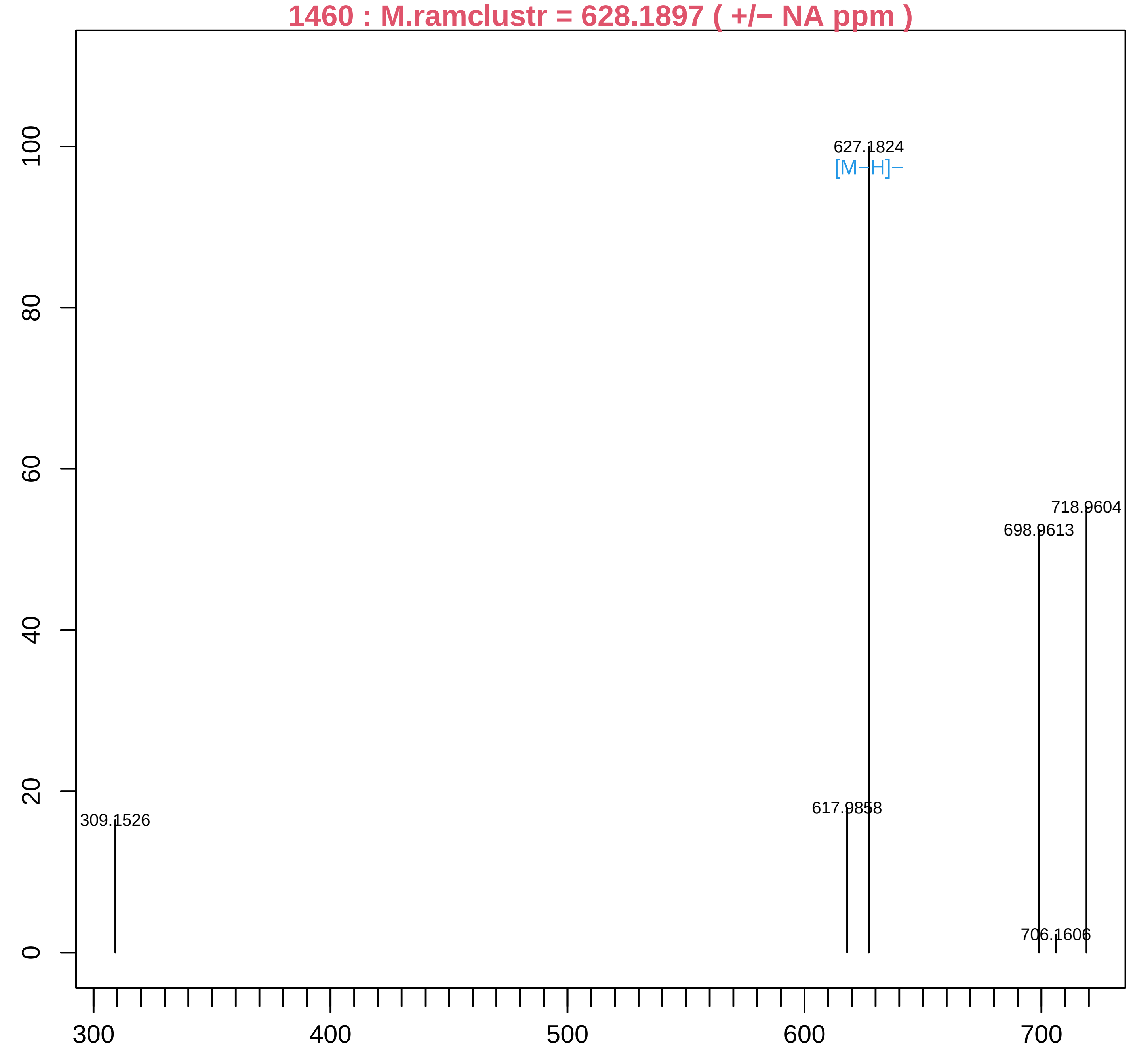

1460 : M.ramclustr = 628.1897 (+/− NA ppm)
627.1824
[M−H]−
718.9604
698.9613
617.9858
309.1526
706.1606
0
20
40
60
80
100
300
400
500
600
700

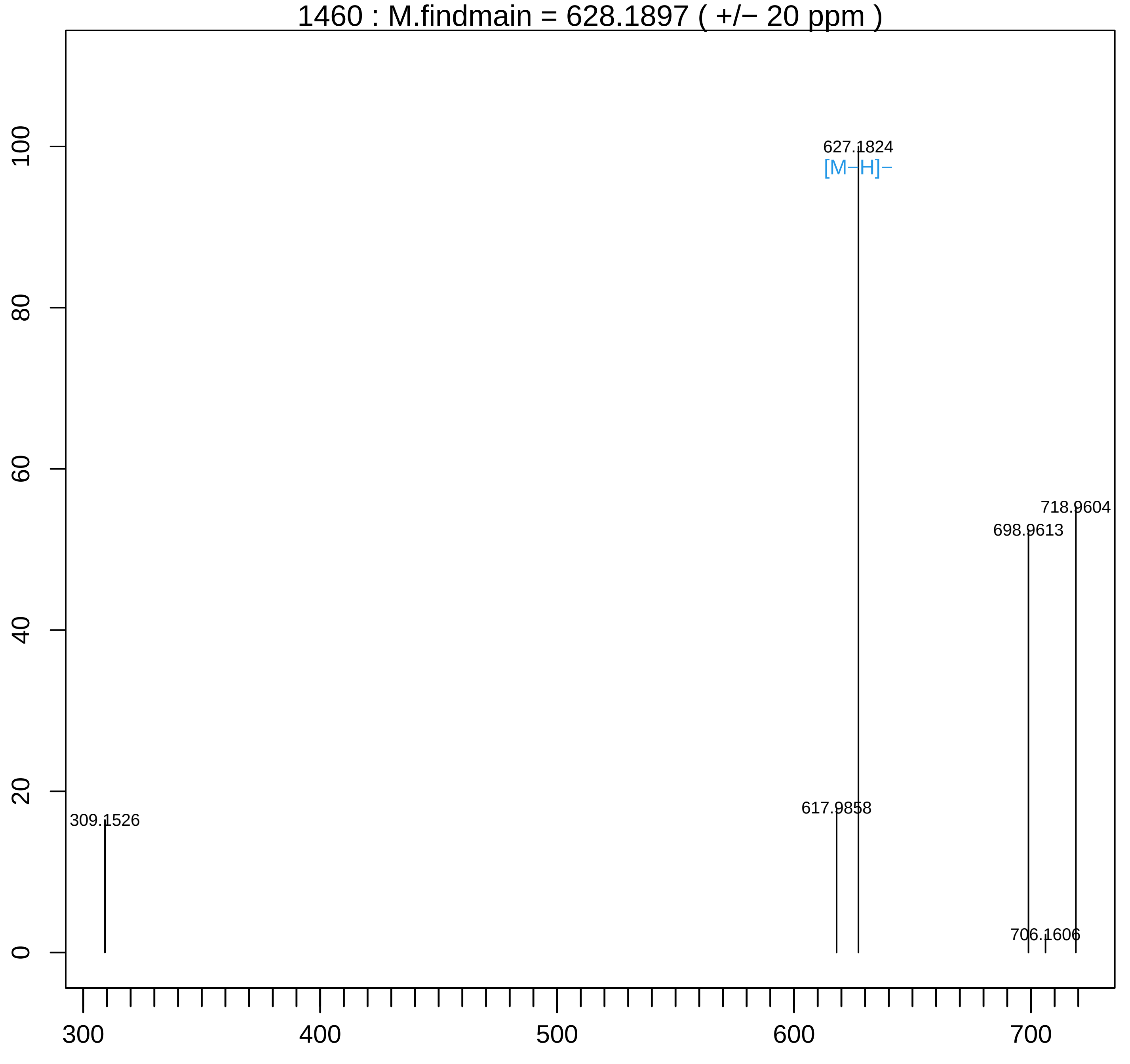

1460 : M.findmain = 628.1897 (+/− 20 ppm)
627.1824
[M−H]−
718.9604
698.9613
617.9858
309.1526
706.1606
0
20
40
60
80
100
300
400
500
600
700

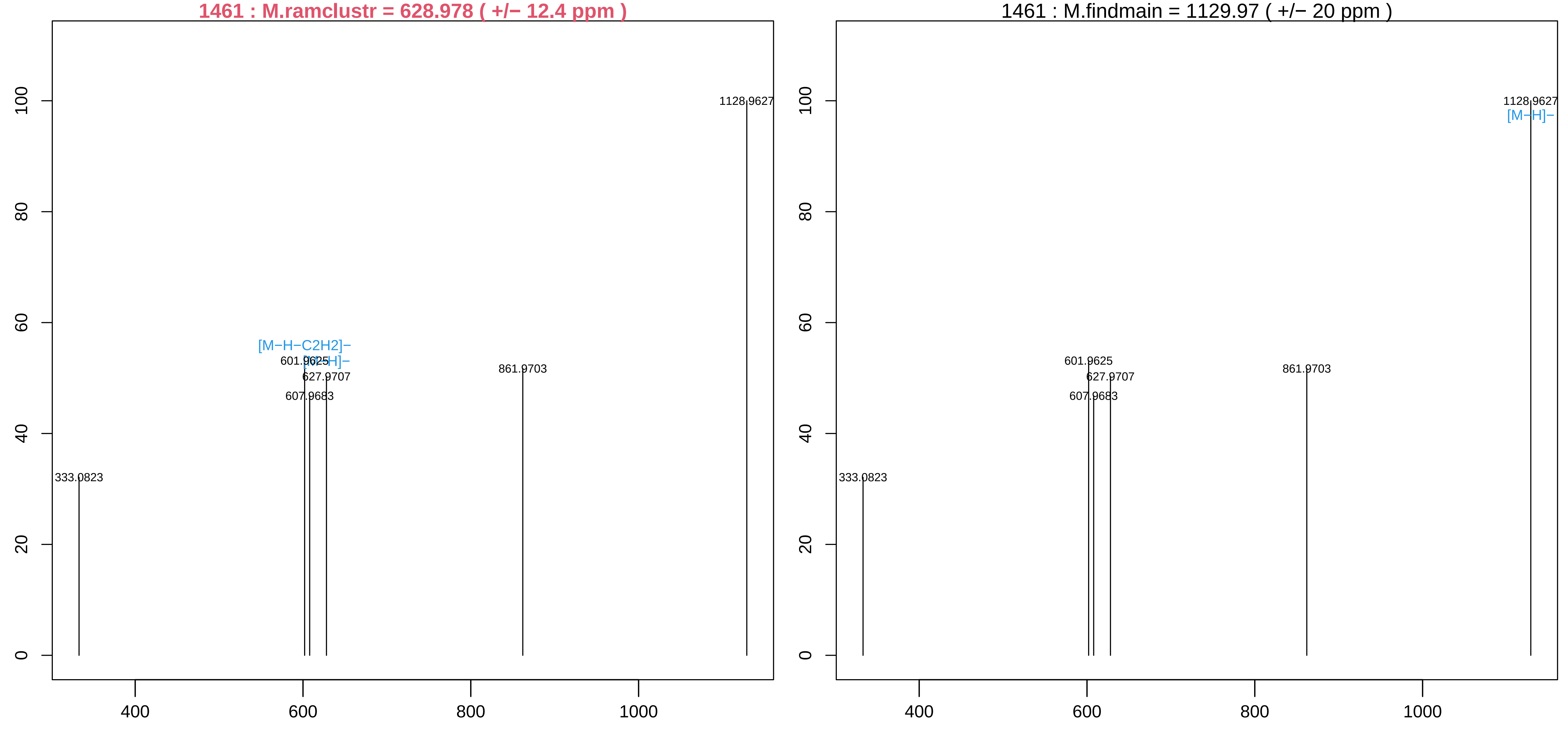
1461 : M.ramclustr = 628.978 (+/− 12.4 ppm)
1128.9627
[M−H−C2H2]−
601.9625
[M−H]−
627.9707
607.9683
861.9703
333.0823
1461 : M.findmain = 1129.97 (+/− 20 ppm)
1128.9627
[M−H]−
601.9625
627.9707
607.9683
861.9703
333.0823

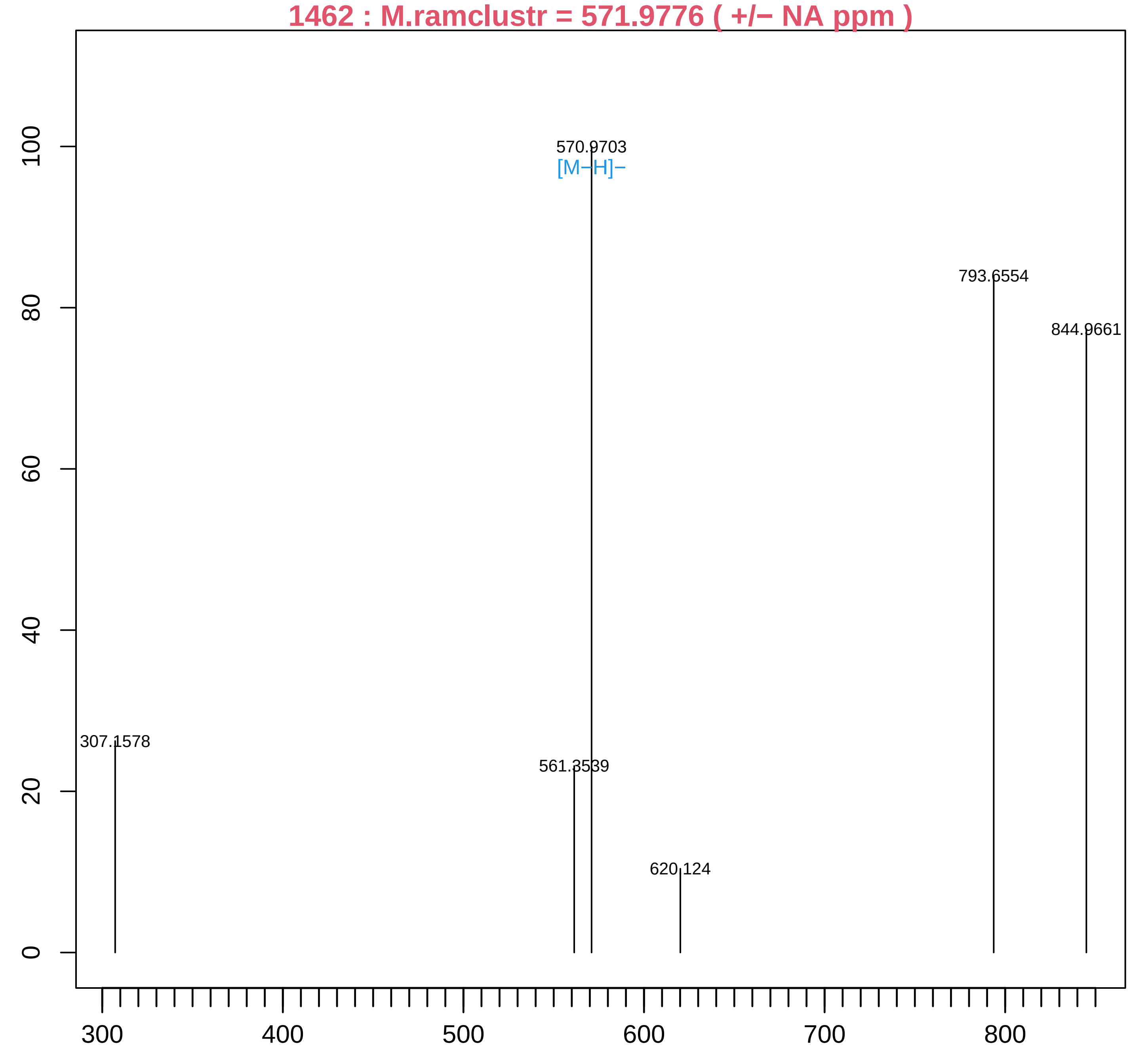
1462 : M.ramclustr = 571.9776 (+/− NA ppm)
570.9703
[M−H]−
793.6554
844.9661
307.1578
561.3539
620.124
0
20
40
60
80
100
300
400
500
600
700
800

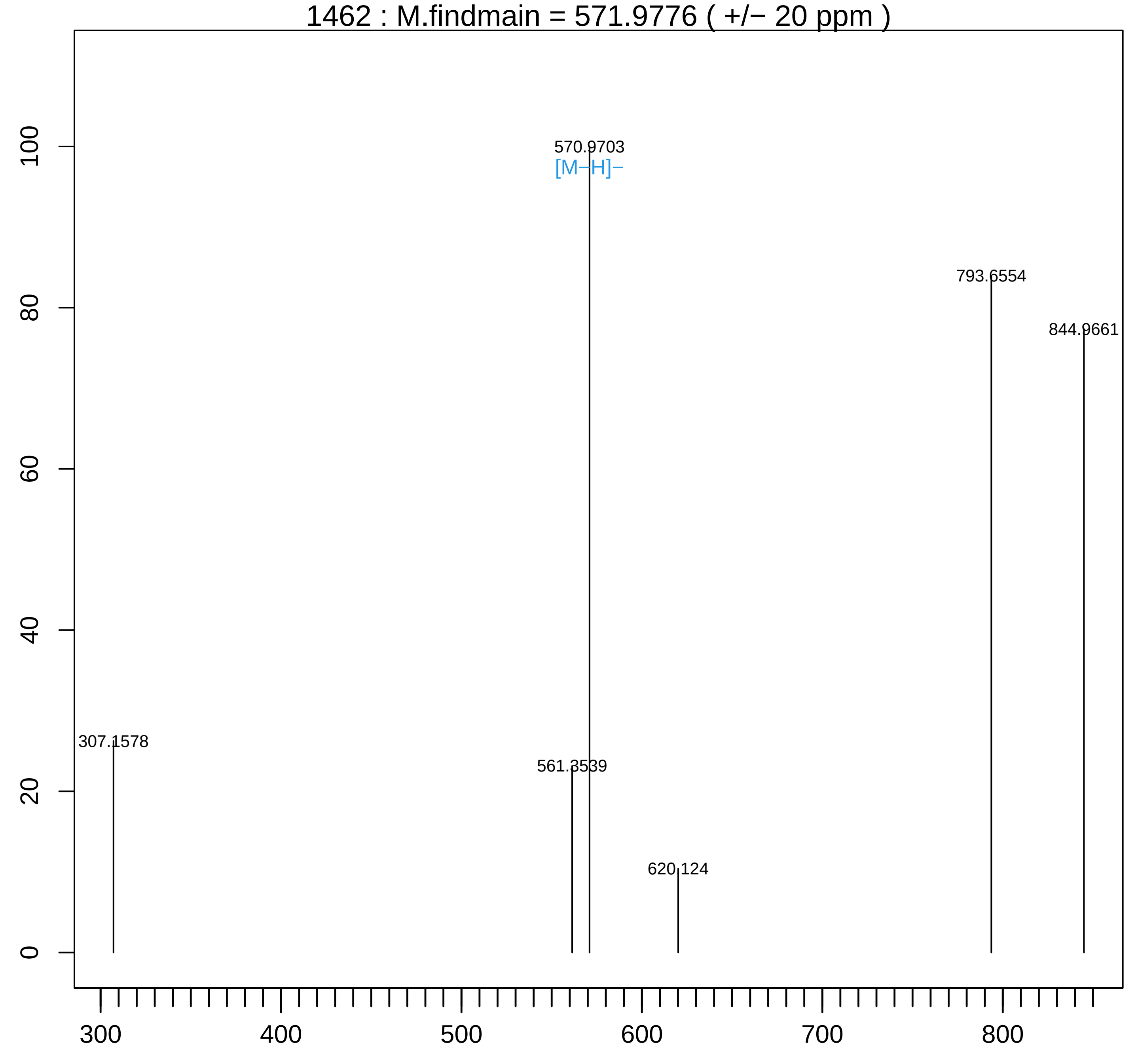
1462 : M.findmain = 571.9776 (+/− 20 ppm)
570.9703
[M−H]−
793.6554
844.9661
307.1578
561.3539
620.124
0
20
40
60
80
100
300
400
500
600
700
800

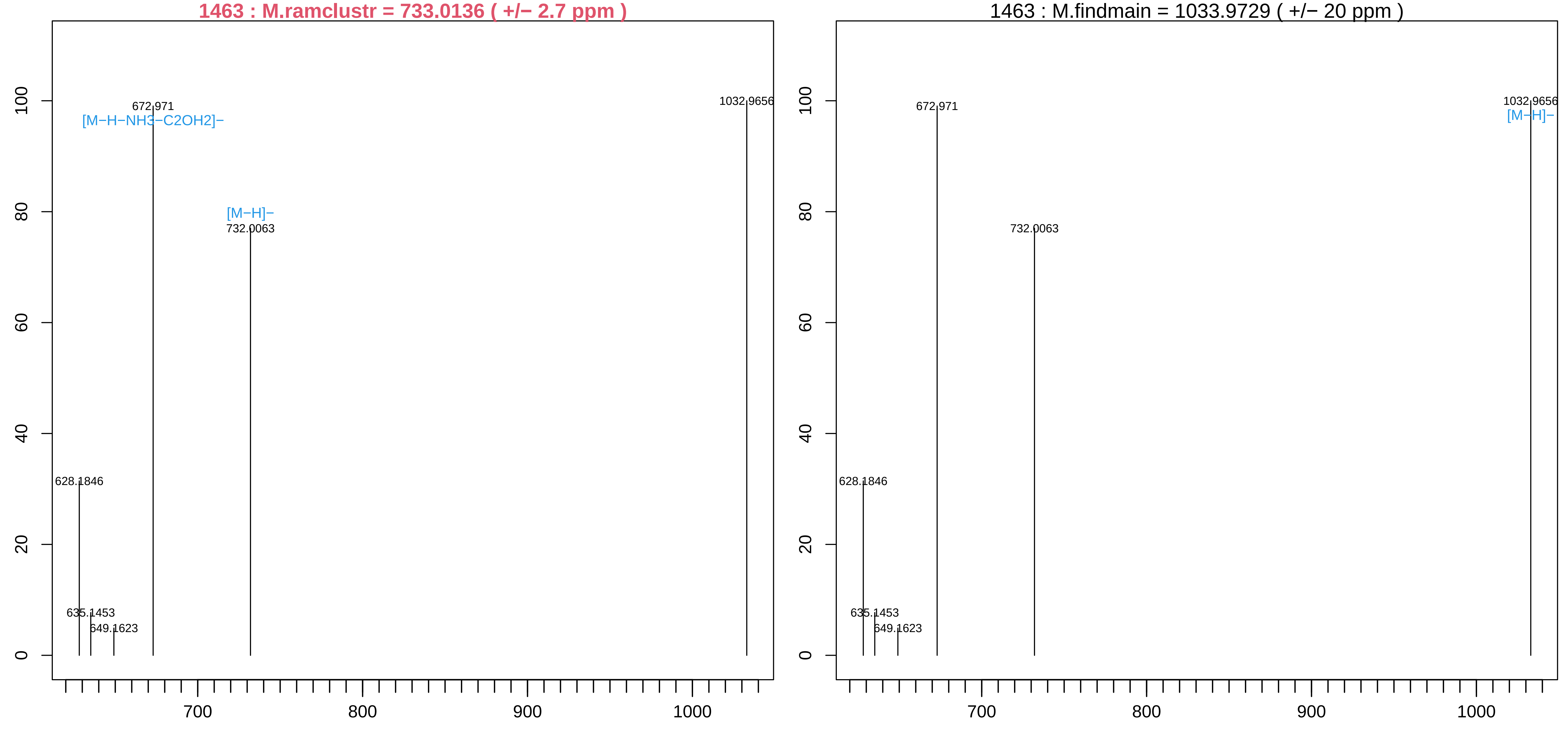

1463 : M.ramclustr = 733.0136 (+/− 2.7 ppm)
1463 : M.findmain = 1033.9729 (+/− 20 ppm)
[M−H−NH3−C2OH2]−
[M−H]−
628.1846
635.1453
649.1623
672.971
732.0063
1032.9656
0
20
40
60
80
100
700
800
900
1000

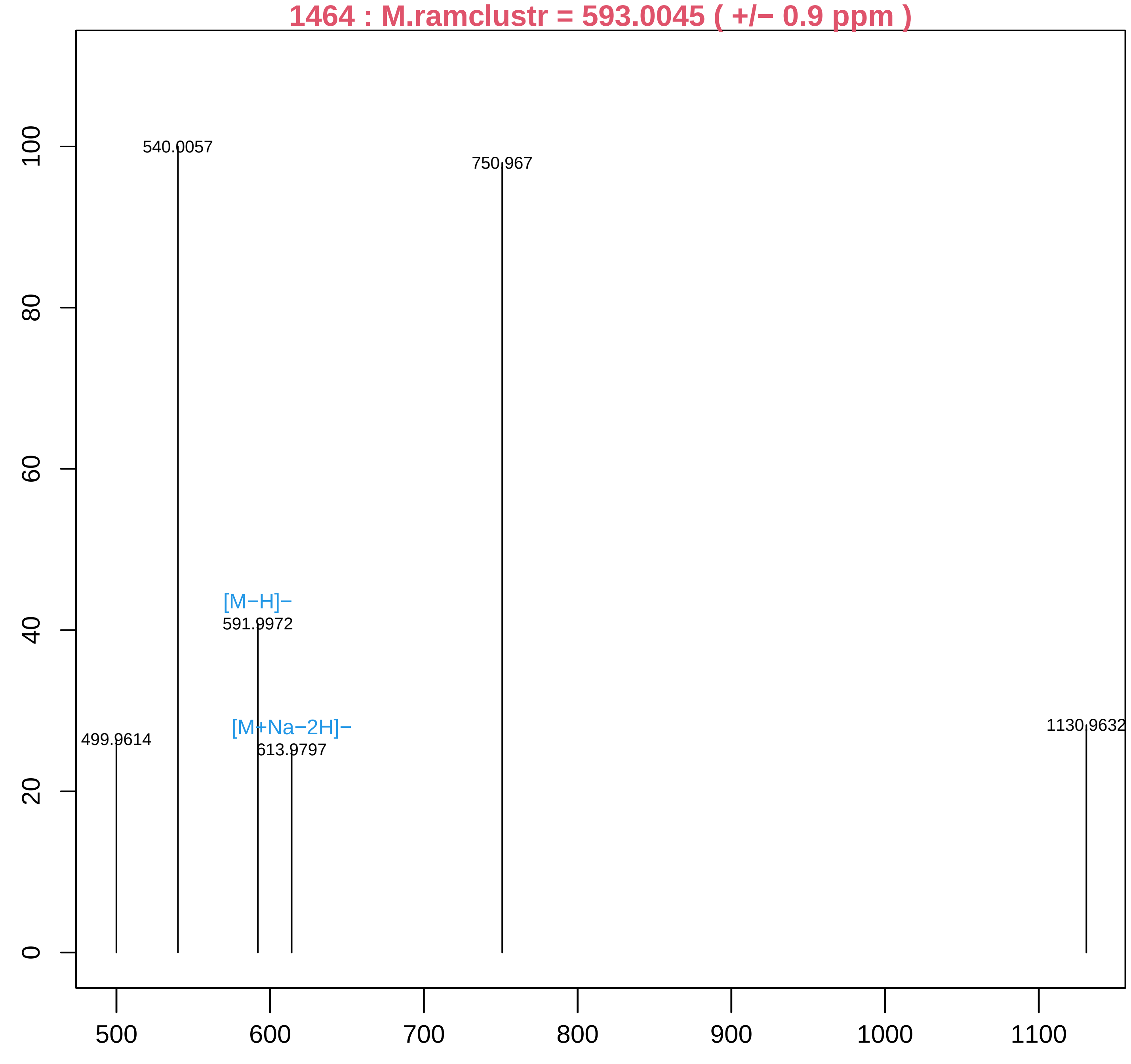
1464 : M.ramclustr = 593.0045 (+/− 0.9 ppm)
540.0057
750.967
[M−H]−
591.9972
[M+Na−2H]−
613.9797
499.9614
1130.9632
0
20
40
60
80
100
500
600
700
800
900
1000
1100

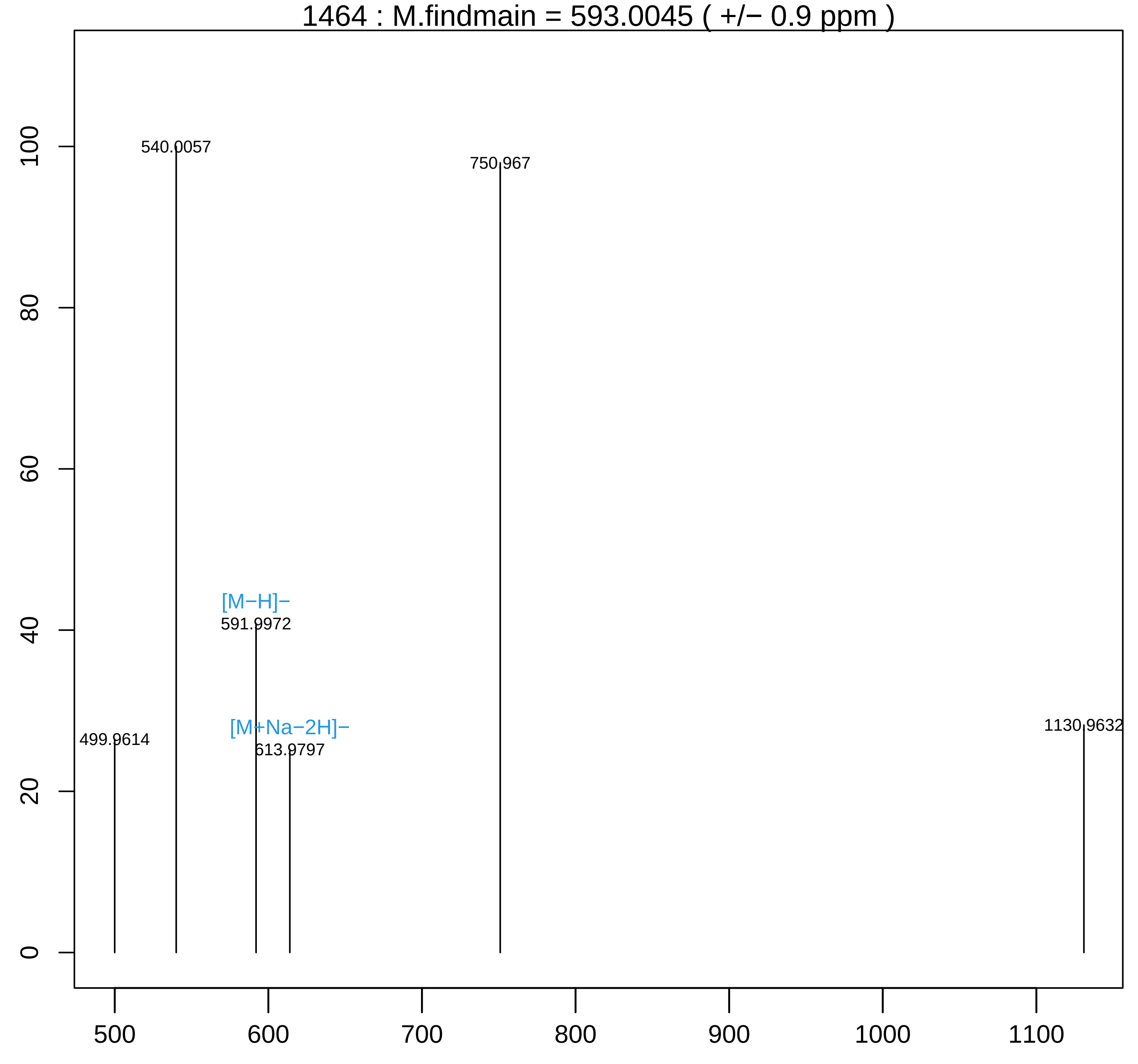
1464 : M.findmain = 593.0045 (+/− 0.9 ppm)
540.0057
750.967
[M−H]−
591.9972
[M+Na−2H]−
613.9797
499.9614
1130.9632
0
20
40
60
80
100
500
600
700
800
900
1000
1100

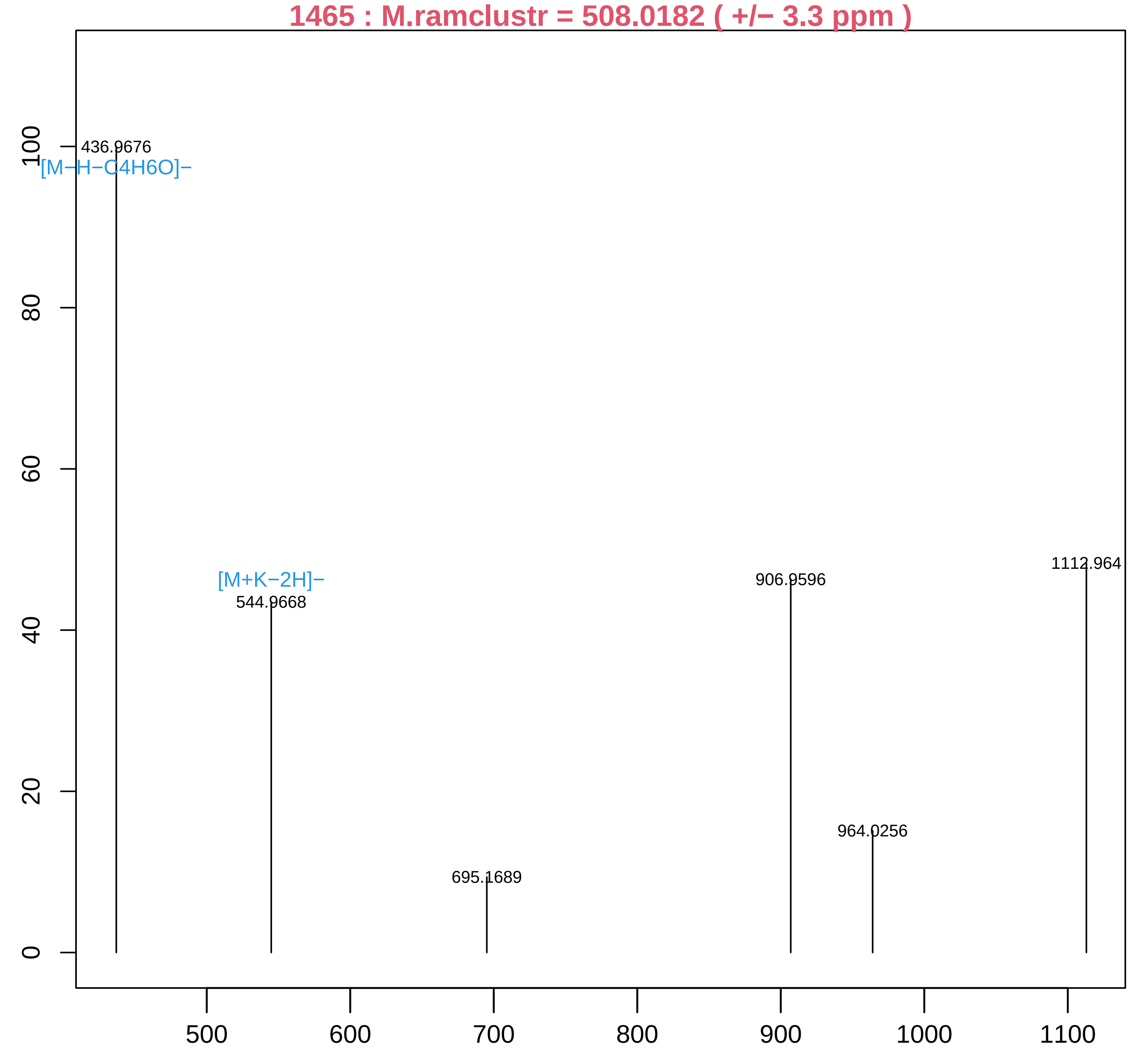

1465 : M.ramclustr = 508.0182 (+/− 3.3 ppm)
436.9676
[M−H−C4H6O]−
[M+K−2H]−
544.9668
695.1689
906.9596
964.0256
1112.964
0
20
40
60
80
100
500
600
700
800
900
1000
1100

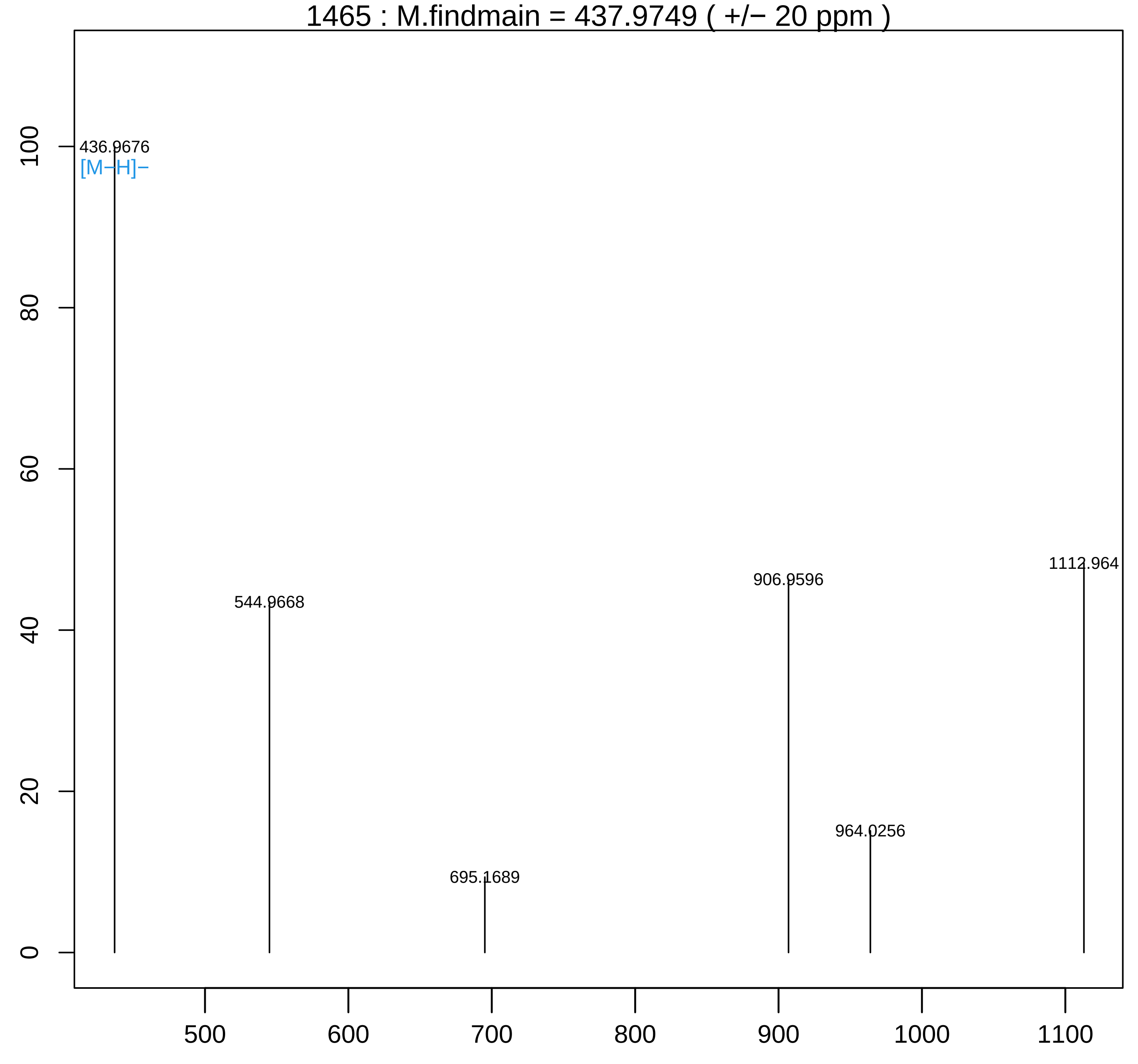

1465 : M.findmain = 437.9749 (+/− 20 ppm)
436.9676
[M−H]−
544.9668
695.1689
906.9596
964.0256
1112.964
0
20
40
60
80
100
500
600
700
800
900
1000
1100

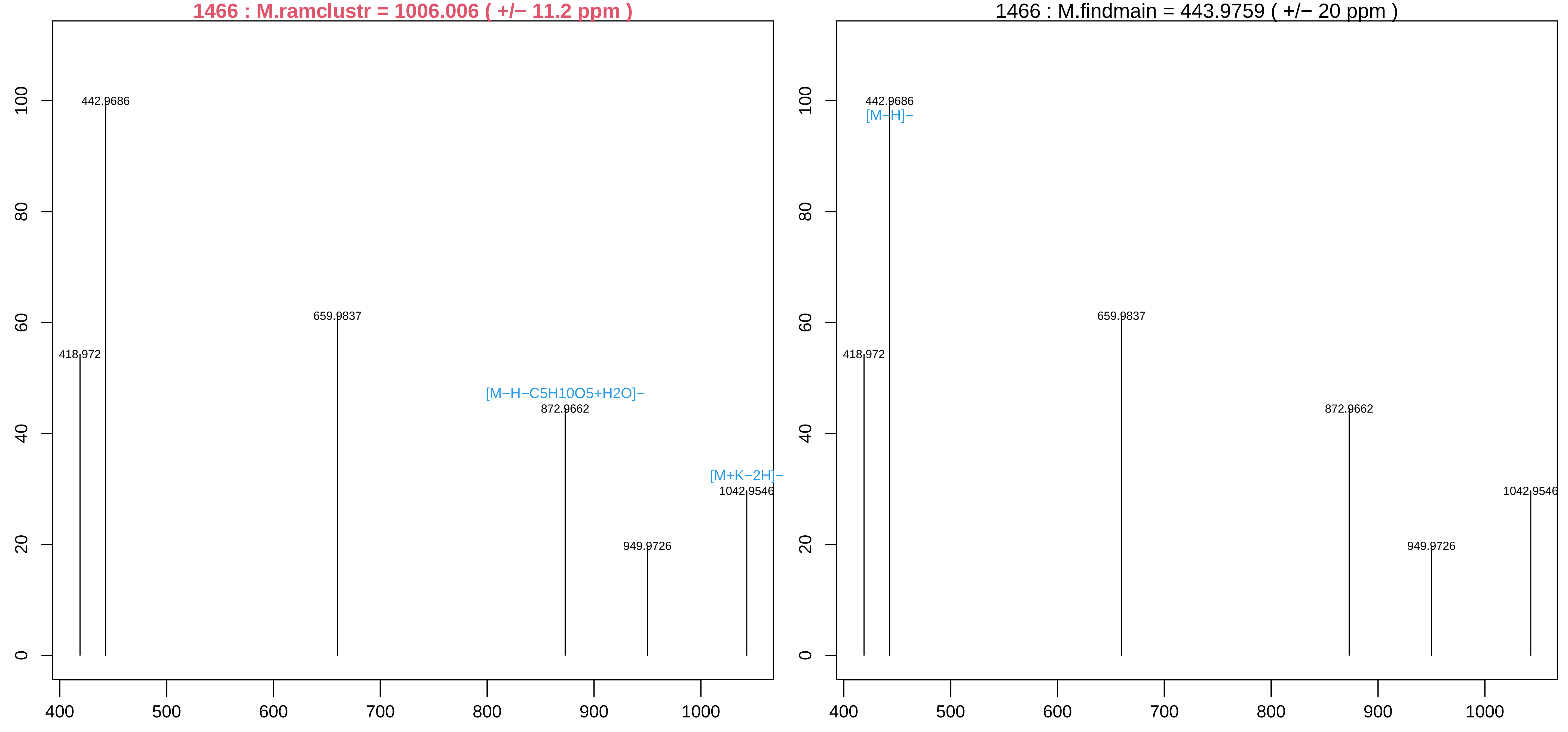
1466 : M.ramclustr = 1006.006 (+/− 11.2 ppm)
442.9686
418.972
659.9837
[M−H−C5H10O5+H2O]−
872.9662
[M+K−2H]−
1042.9546
949.9726
1466 : M.findmain = 443.9759 (+/− 20 ppm)
442.9686
[M−H]−
418.972
659.9837
872.9662
1042.9546
949.9726
0
20
40
60
80
100
400
500
600
700
800
900
1000

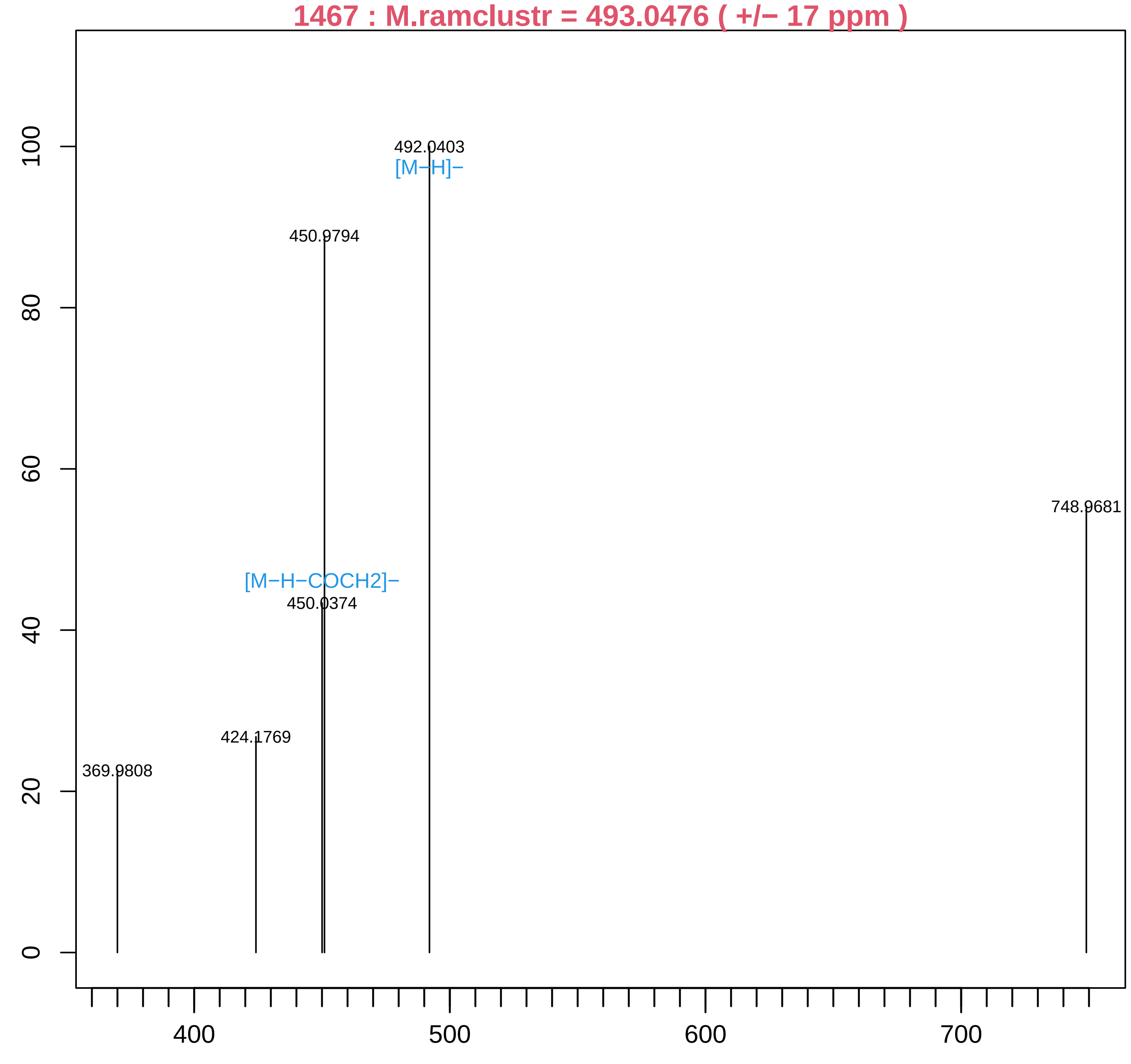
1467 : M.ramclustr = 493.0476 (+/− 17 ppm)
492.0403
[M−H]−
450.9794
748.9681
[M−H−COCH2]−
450.0374
424.1769
369.9808
0
20
40
60
80
100
400
500
600
700

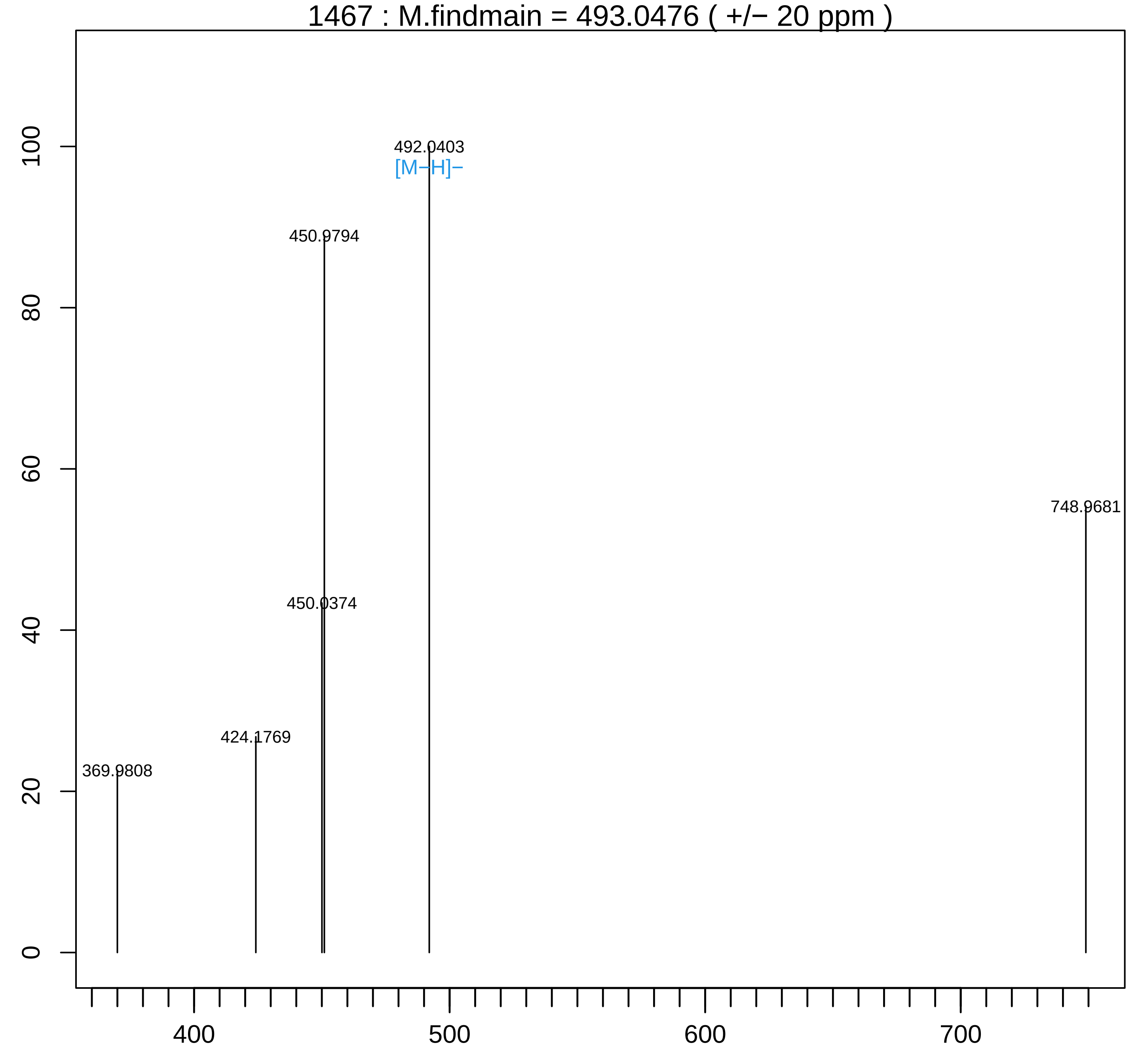
1467 : M.findmain = 493.0476 (+/− 20 ppm)
492.0403
[M−H]−
450.9794
748.9681
450.0374
424.1769
369.9808
0
20
40
60
80
100
400
500
600
700

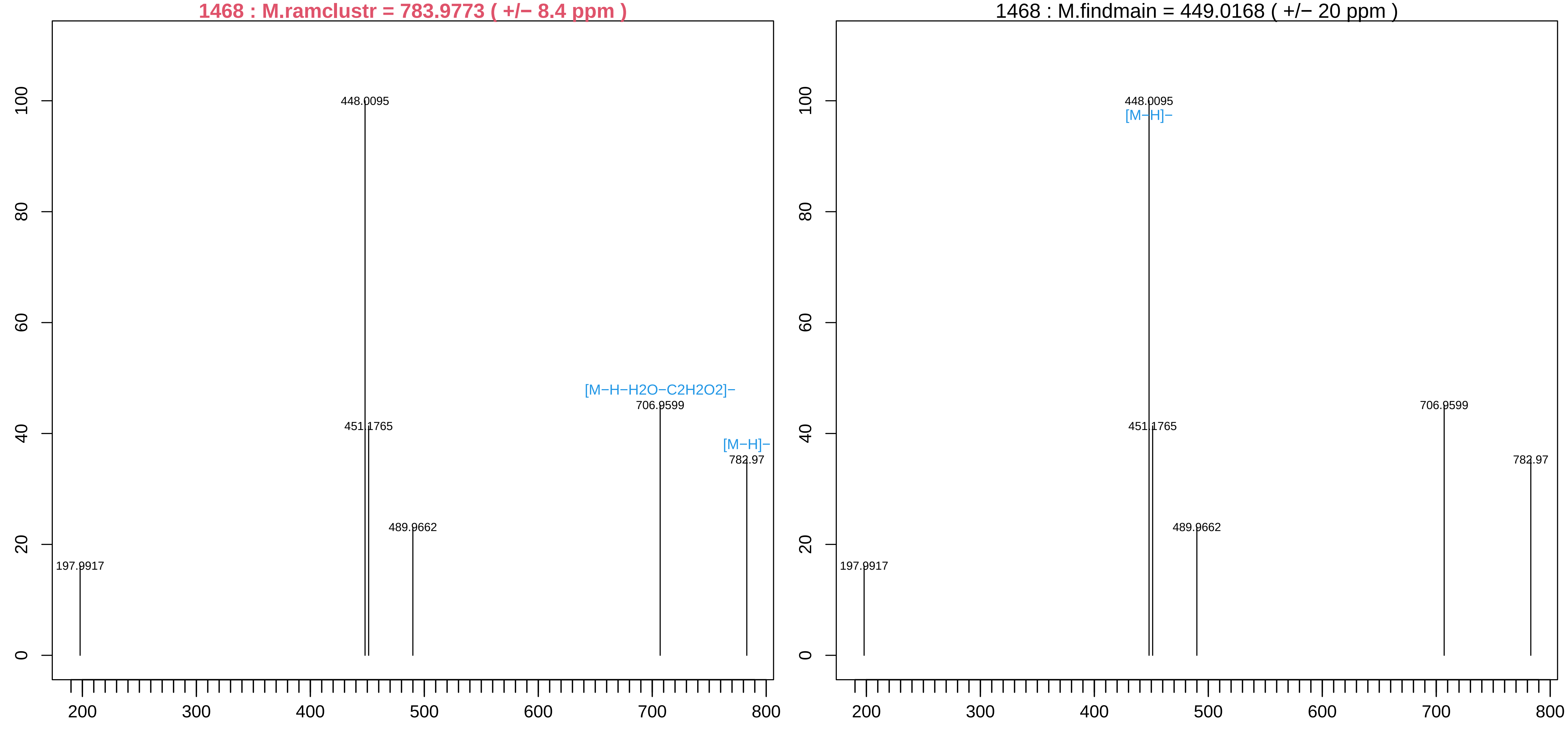
1468 : M.ramclustr = 783.9773 (+/− 8.4 ppm)
448.0095
451.1765
489.9662
197.9917
[M−H−H2O−C2H2O2]−
706.9599
[M−H]−
782.97
1468 : M.findmain = 449.0168 (+/− 20 ppm)
448.0095
[M−H]−
451.1765
489.9662
197.9917
706.9599
782.97
0
20
40
60
80
100
200
300
400
500
600
700
800

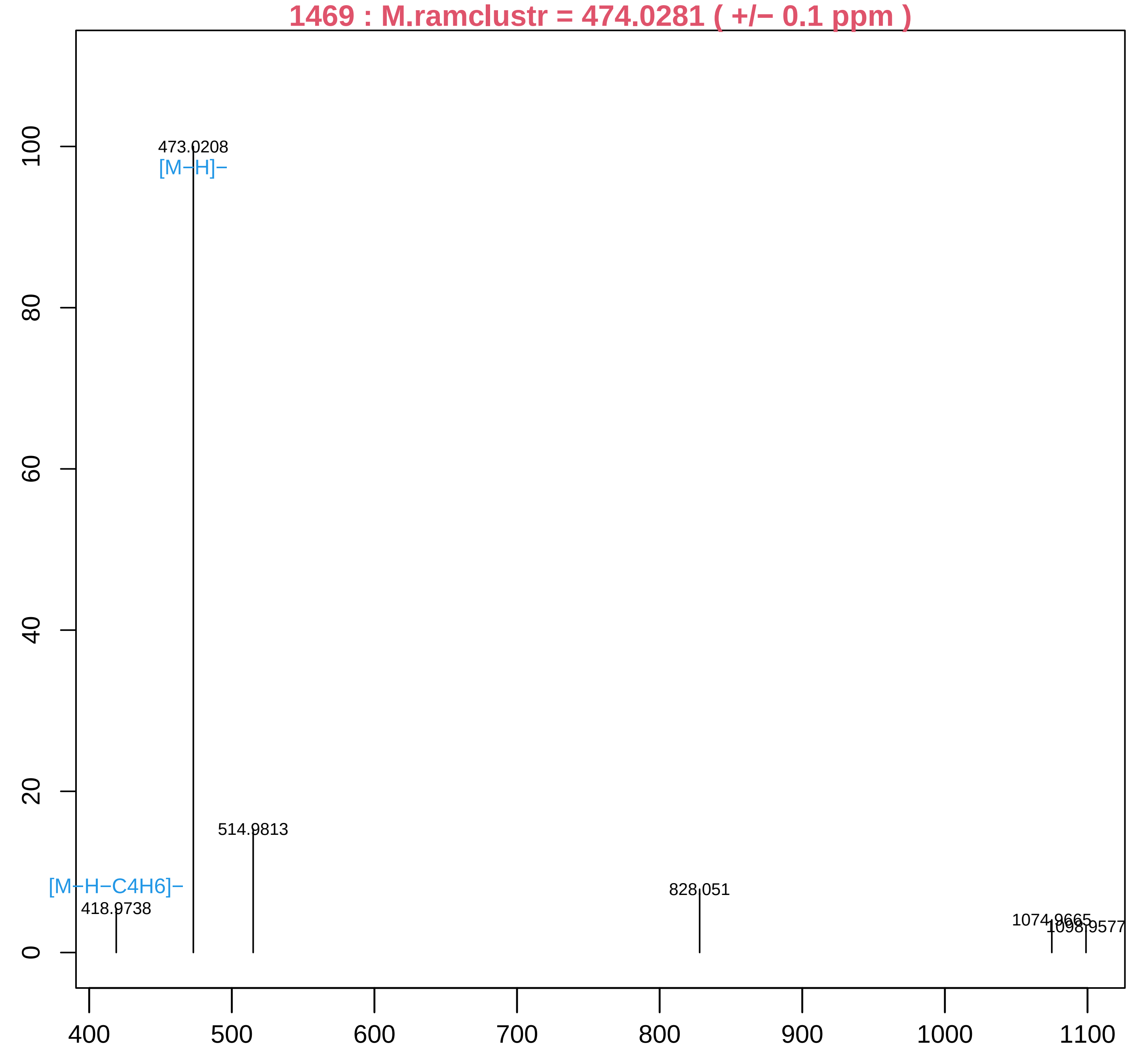
1469 : M.ramclustr = 474.0281 (+/− 0.1 ppm)
473.0208
[M−H]−
514.9813
[M−H−C4H6]−
418.9738
828.051
1074.9665
1098.9577
0
20
40
60
80
100
400
500
600
700
800
900
1000
1100

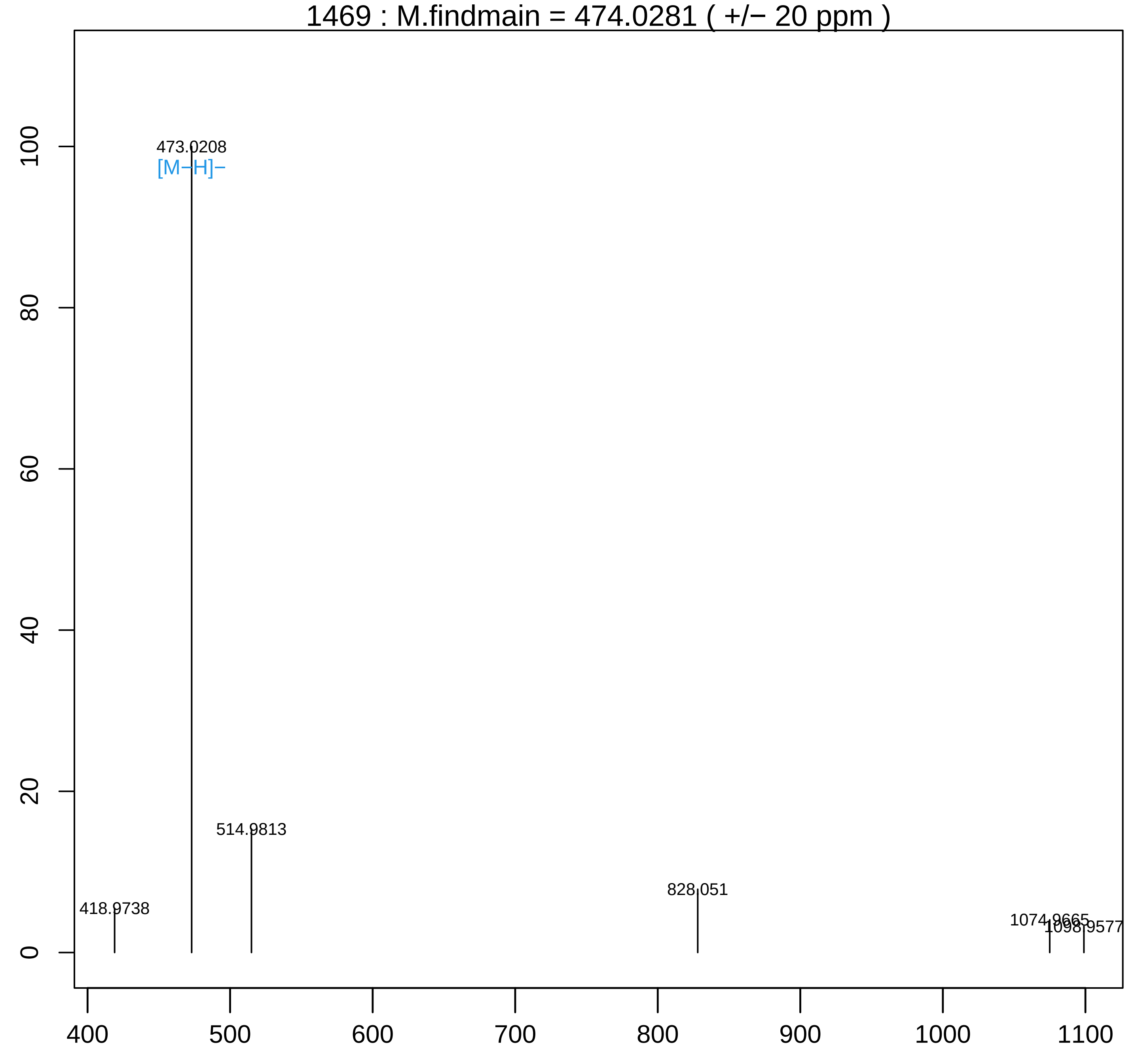
1469 : M.findmain = 474.0281 (+/− 20 ppm)
473.0208
[M−H]−
514.9813
418.9738
828.051
1074.9665
1098.9577
0
20
40
60
80
100
400
500
600
700
800
900
1000
1100

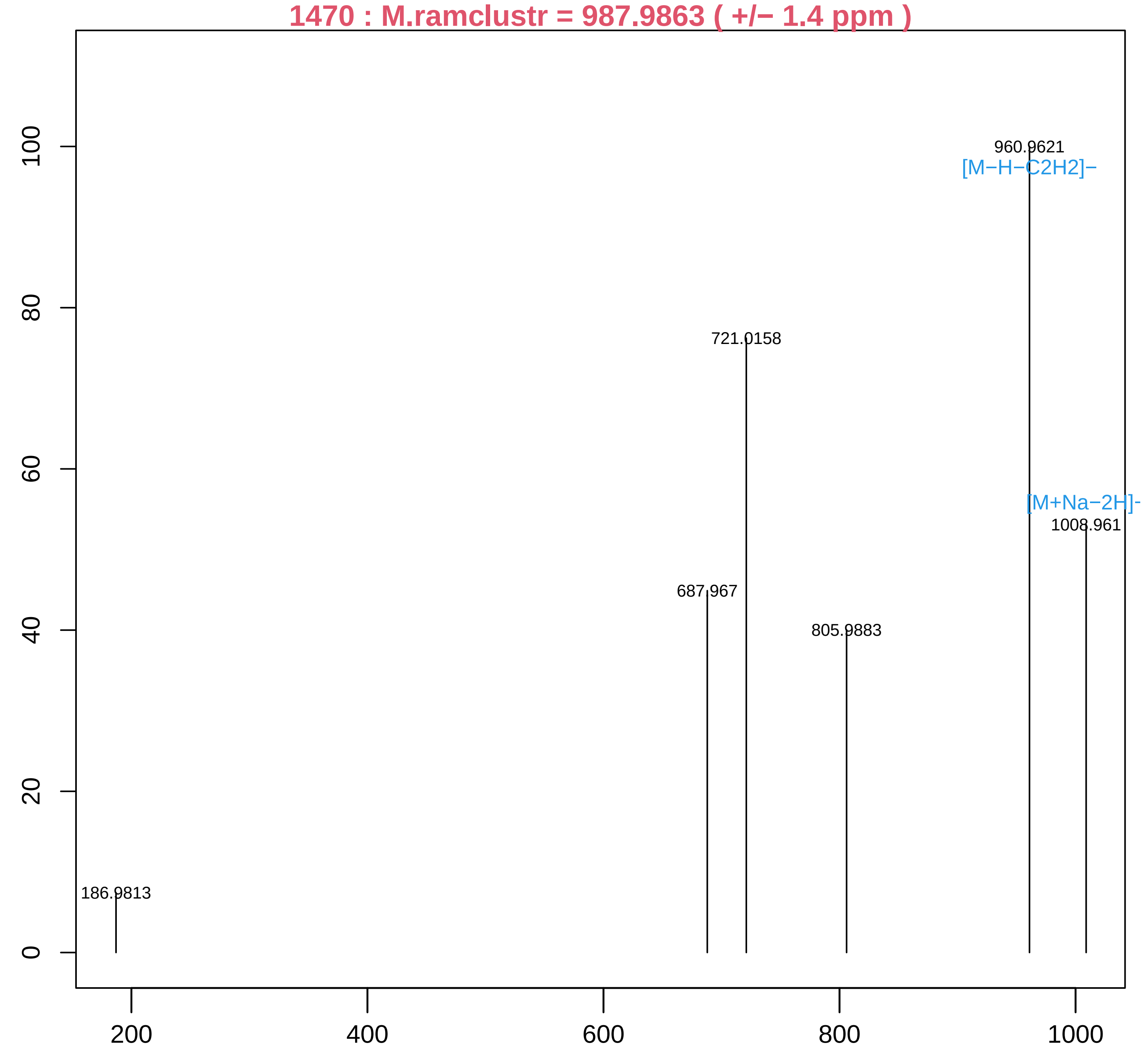

1470 : M.ramclustr = 987.9863 (+/− 1.4 ppm)
960.9621
[M−H−C2H2]−
721.0158
[M+Na−2H]-
1008.961
687.967
805.9883
186.9813
0
20
40
60
80
100
200
400
600
800
1000

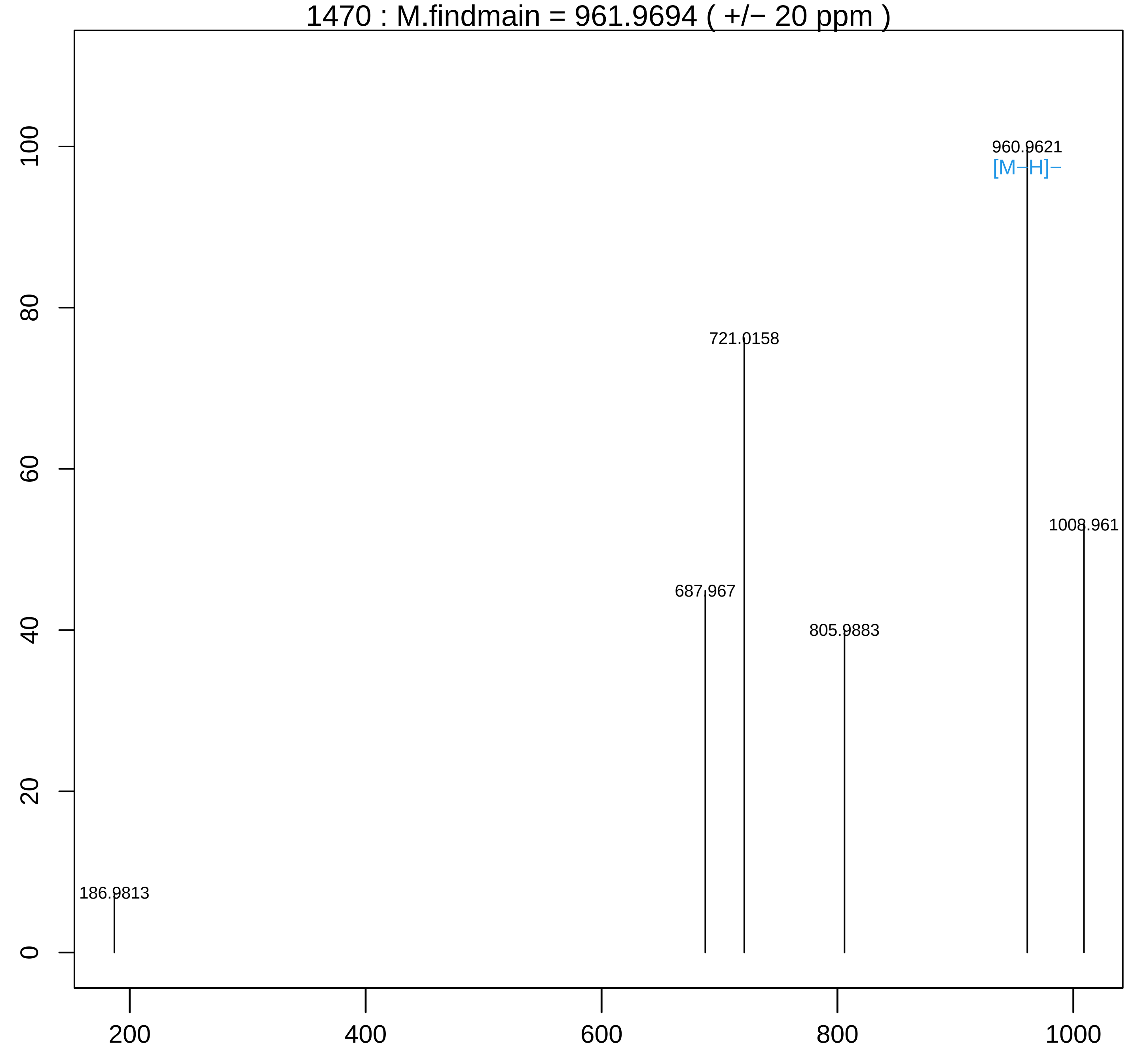

1470 : M.findmain = 961.9694 (+/− 20 ppm)
960.9621
[M−H]−
721.0158
1008.961
687.967
805.9883
186.9813
0
20
40
60
80
100
200
400
600
800
1000

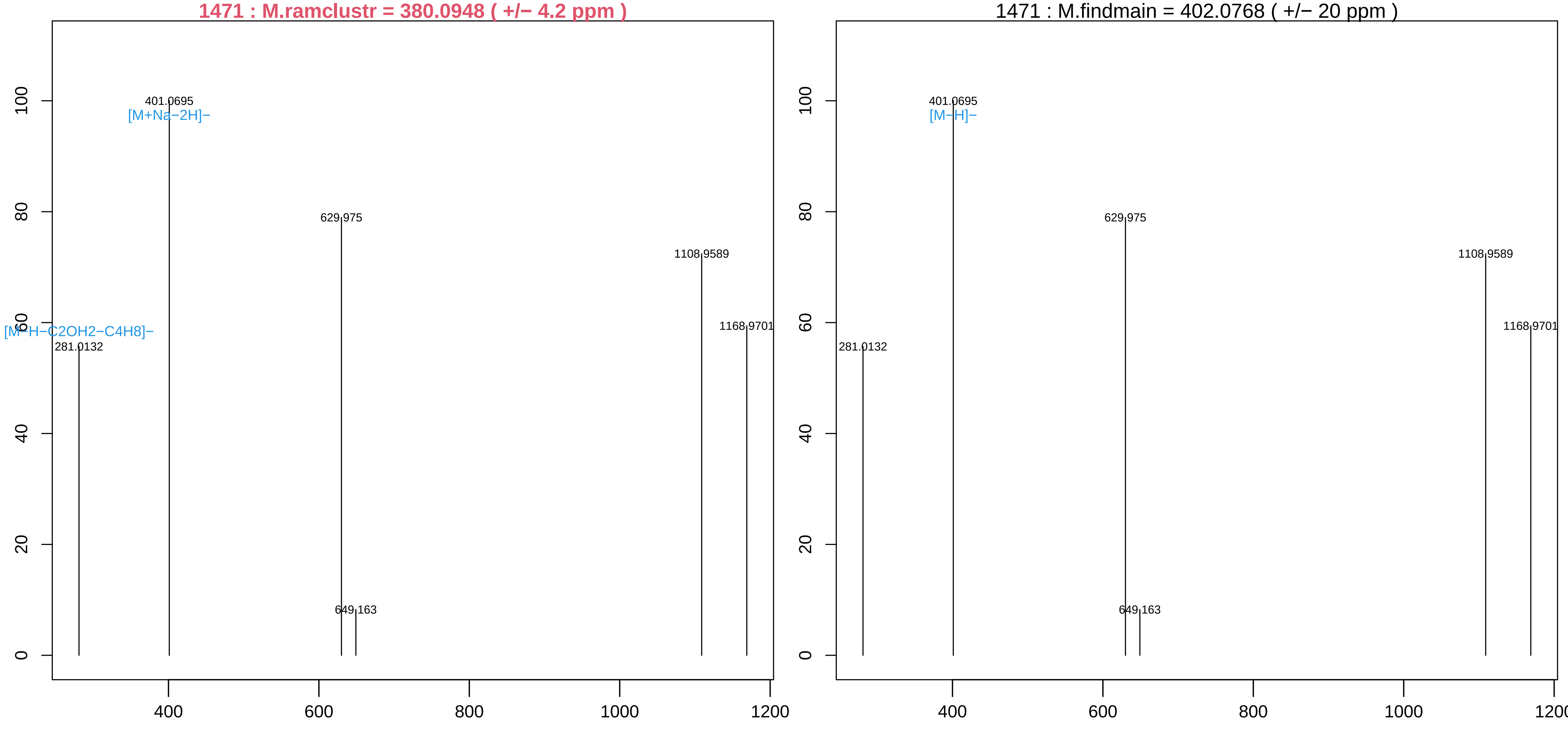
1471 : M.ramclustr = 380.0948 (+/− 4.2 ppm)
401.0695
[M+Na−2H]−
629.975
1108.9589
1168.9701
[M−H−C2OH2−C4H8]−
281.0132
649.163
1471 : M.findmain = 402.0768 (+/− 20 ppm)
401.0695
[M−H]−
629.975
1108.9589
1168.9701
281.0132
649.163
0
20
40
60
80
100
400
600
800
1000
1200

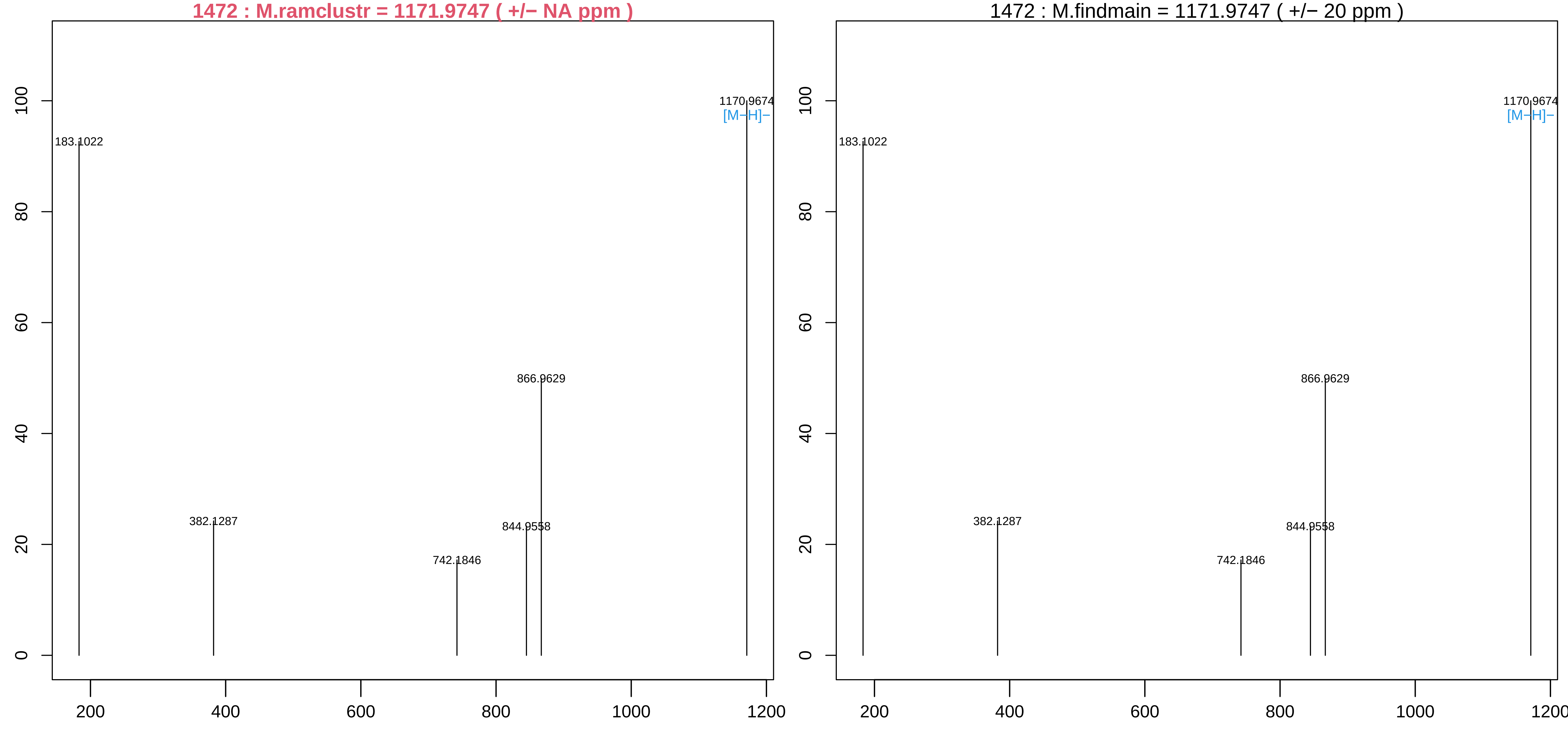
1472 : M.ramclustr = 1171.9747 (+/− NA ppm)
1170.9674
[M−H]−
183.1022
866.9629
382.1287
844.9558
742.1846
1472 : M.findmain = 1171.9747 (+/− 20 ppm)
1170.9674
[M−H]−
183.1022
866.9629
382.1287
844.9558
742.1846
0
20
40
60
80
100
200
400
600
800
1000
1200

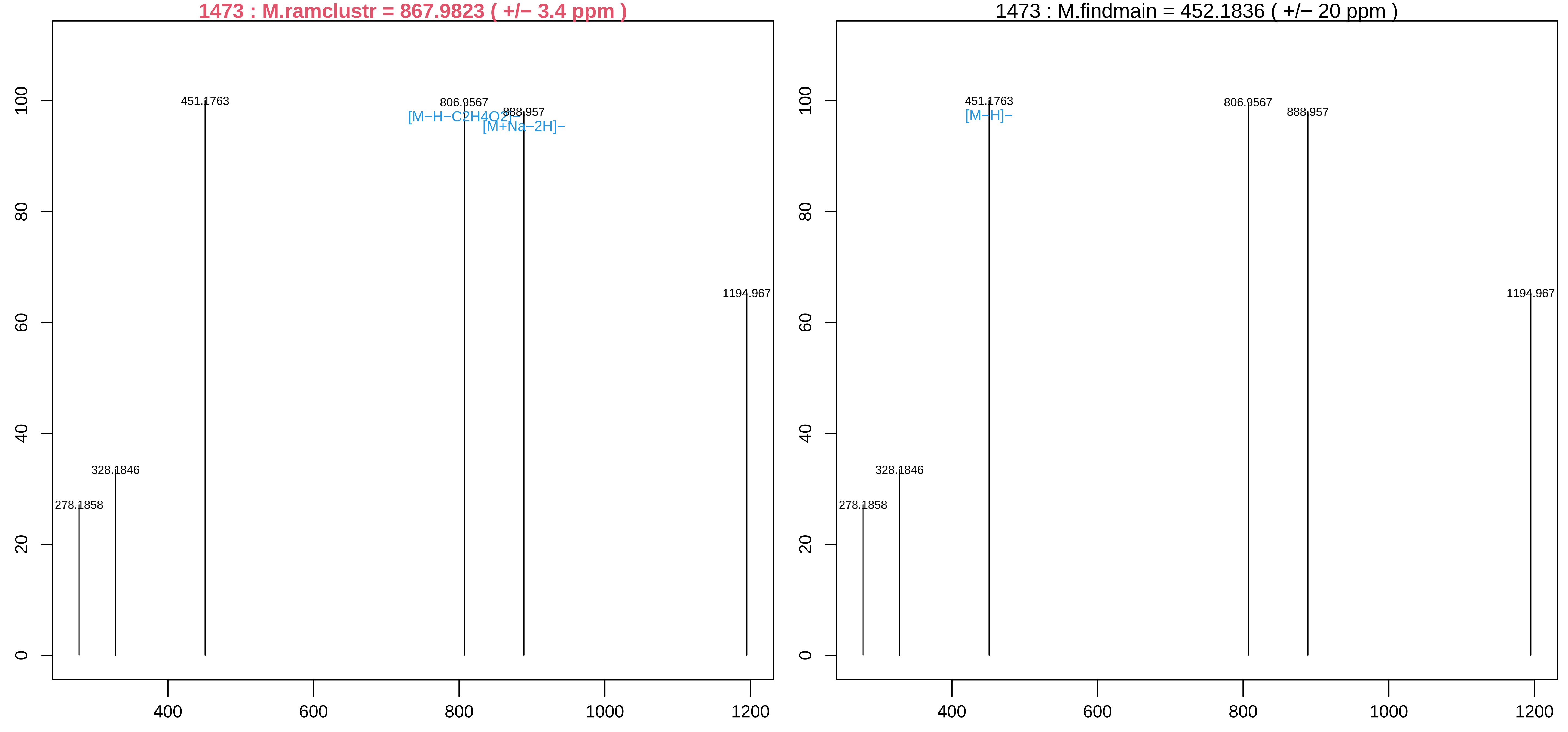
1473 : M.ramclustr = 867.9823 (+/− 3.4 ppm)
451.1763
806.9567
[M−H−C2H4O2]−
888.957
[M+Na−2H]−
1194.967
328.1846
278.1858
0
20
40
60
80
100
400
600
800
1000
1200
1473 : M.findmain = 452.1836 (+/− 20 ppm)
451.1763
[M−H]−
806.9567
888.957
1194.967
328.1846
278.1858

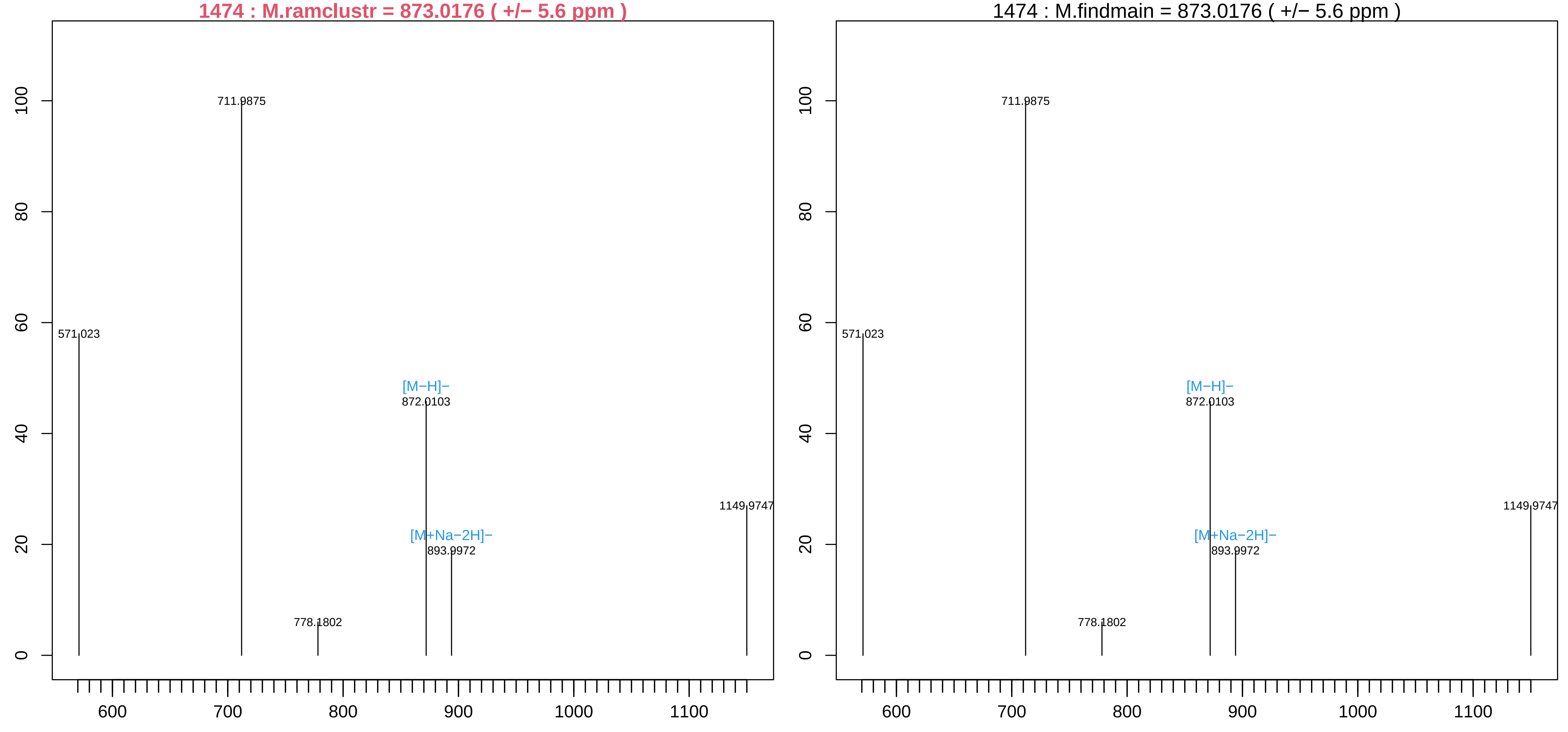
1474 : M.ramclustr = 873.0176 (+/− 5.6 ppm)
711.9875
571.023
[M−H]−
872.0103
1149.9747
[M+Na−2H]−
893.9972
778.1802
1474 : M.findmain = 873.0176 (+/− 5.6 ppm)
711.9875
571.023
[M−H]−
872.0103
1149.9747
[M+Na−2H]−
893.9972
778.1802

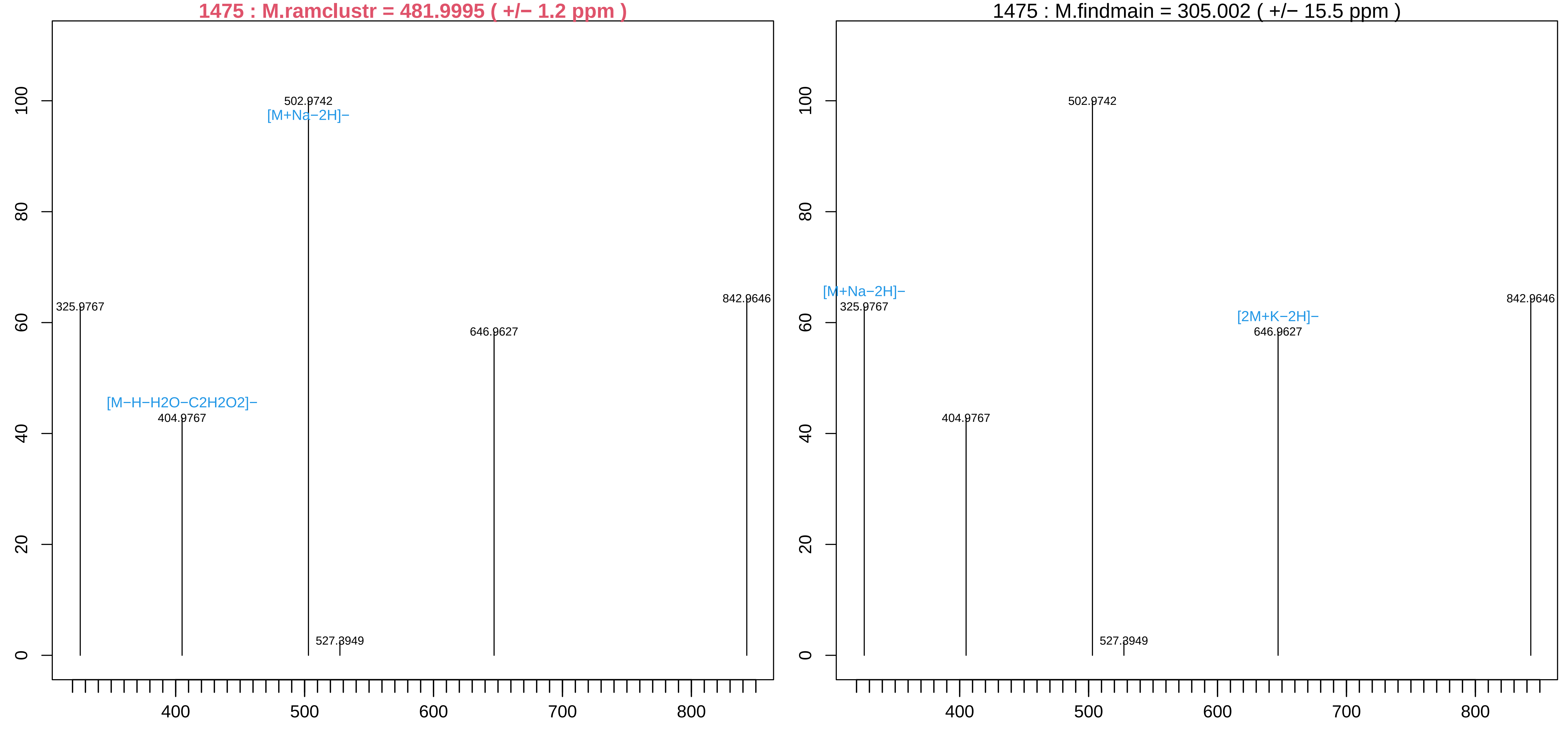

1475 : M.ramclustr = 481.9995 (+/− 1.2 ppm)
502.9742
[M+Na−2H]−
325.9767
[M−H−H2O−C2H2O2]−
404.9767
527.3949
646.9627
842.9646
1475 : M.findmain = 305.002 (+/− 15.5 ppm)
502.9742
[M+Na−2H]−
325.9767
404.9767
527.3949
[2M+K−2H]−
646.9627
842.9646
0
20
40
60
80
100
400
500
600
700
800

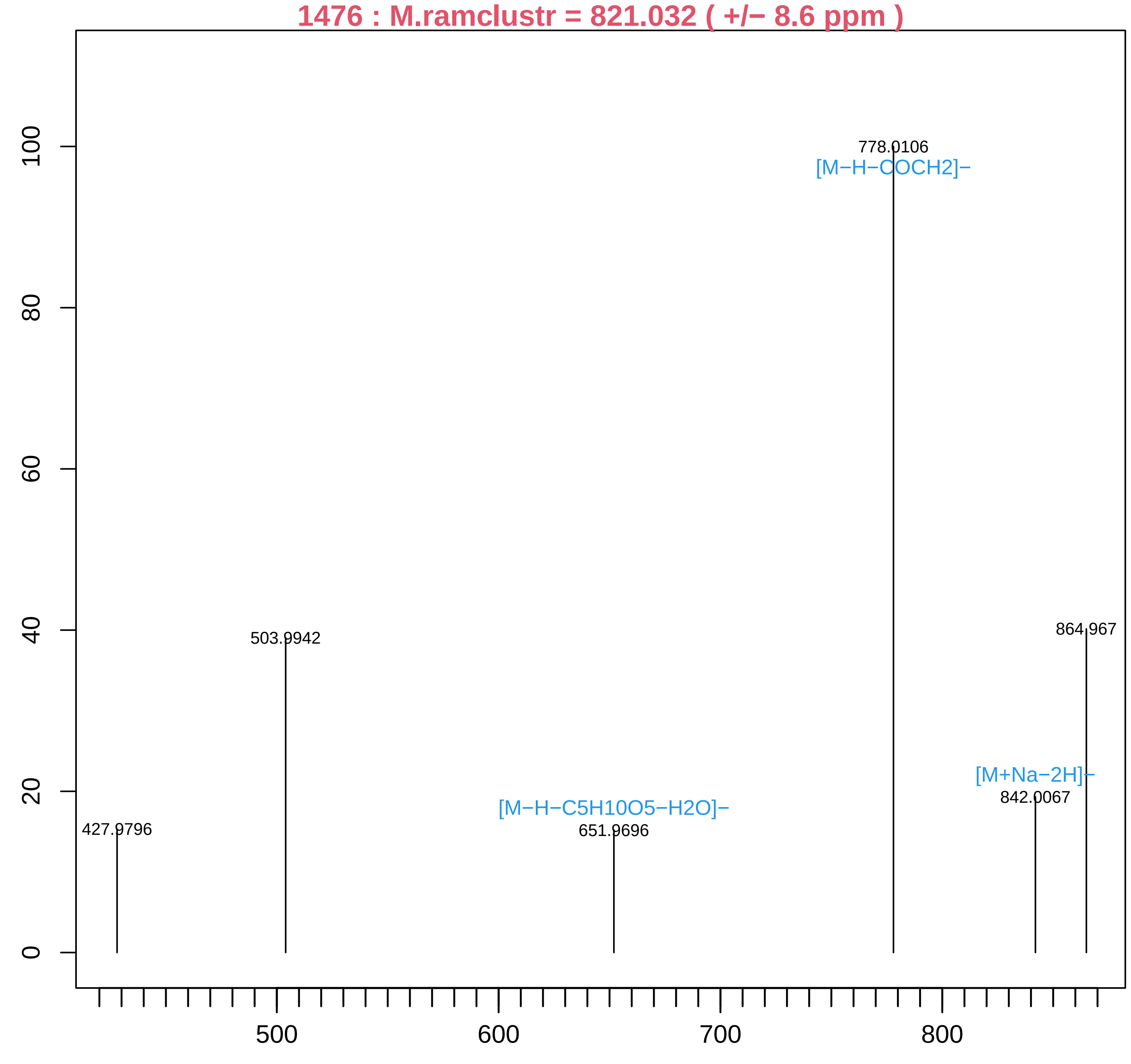
1476 : M.ramclustr = 821.032 (+/− 8.6 ppm)
778.0106
[M−H−COCH2]−
503.9942
864.967
[M+Na−2H]−
842.0067
[M−H−C5H10O5−H2O]−
651.9696
427.9796
0
20
40
60
80
100
500
600
700
800

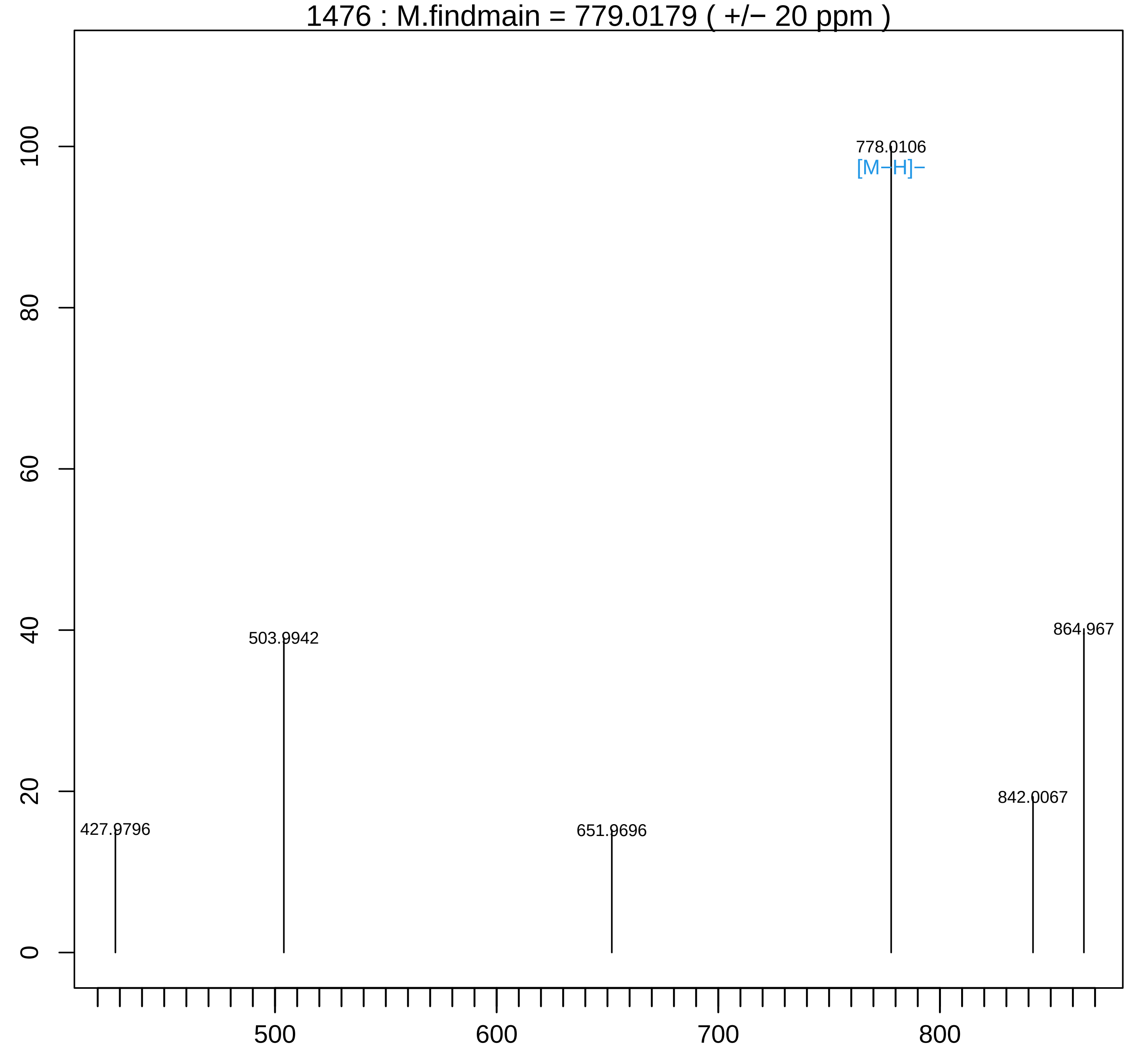
1476 : M.findmain = 779.0179 (+/− 20 ppm)
778.0106
[M−H]−
503.9942
864.967
842.0067
651.9696
427.9796
0
20
40
60
80
100
500
600
700
800

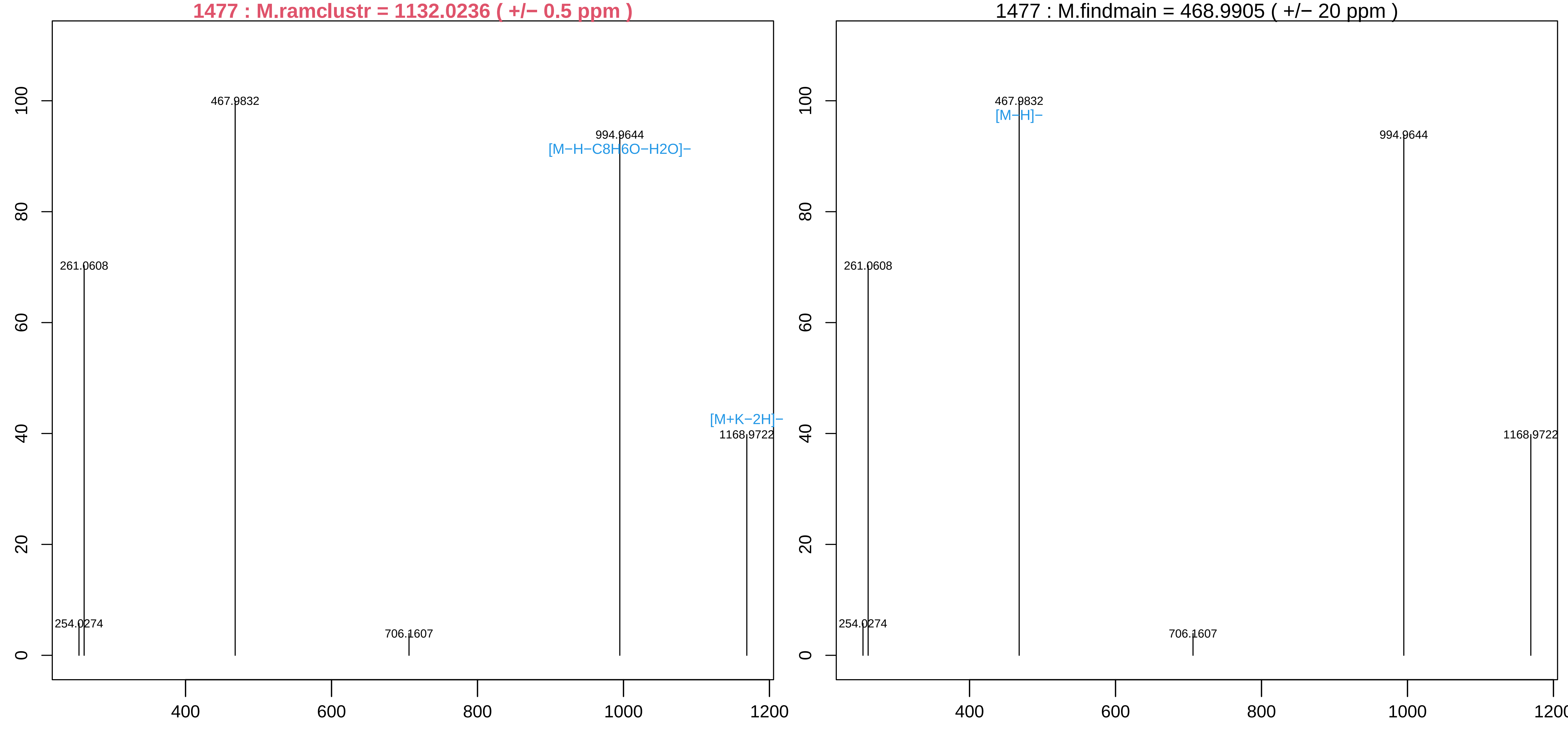
1477 : M.ramclustr = 1132.0236 (+/− 0.5 ppm)
467.9832
994.9644
[M−H−C8H6O−H2O]−
261.0608
[M+K−2H]−
1168.9722
254.0274
706.1607
0
20
40
60
80
100
400
600
800
1000
1200
1477 : M.findmain = 468.9905 (+/− 20 ppm)
467.9832
[M−H]−
994.9644
261.0608
1168.9722
254.0274
706.1607

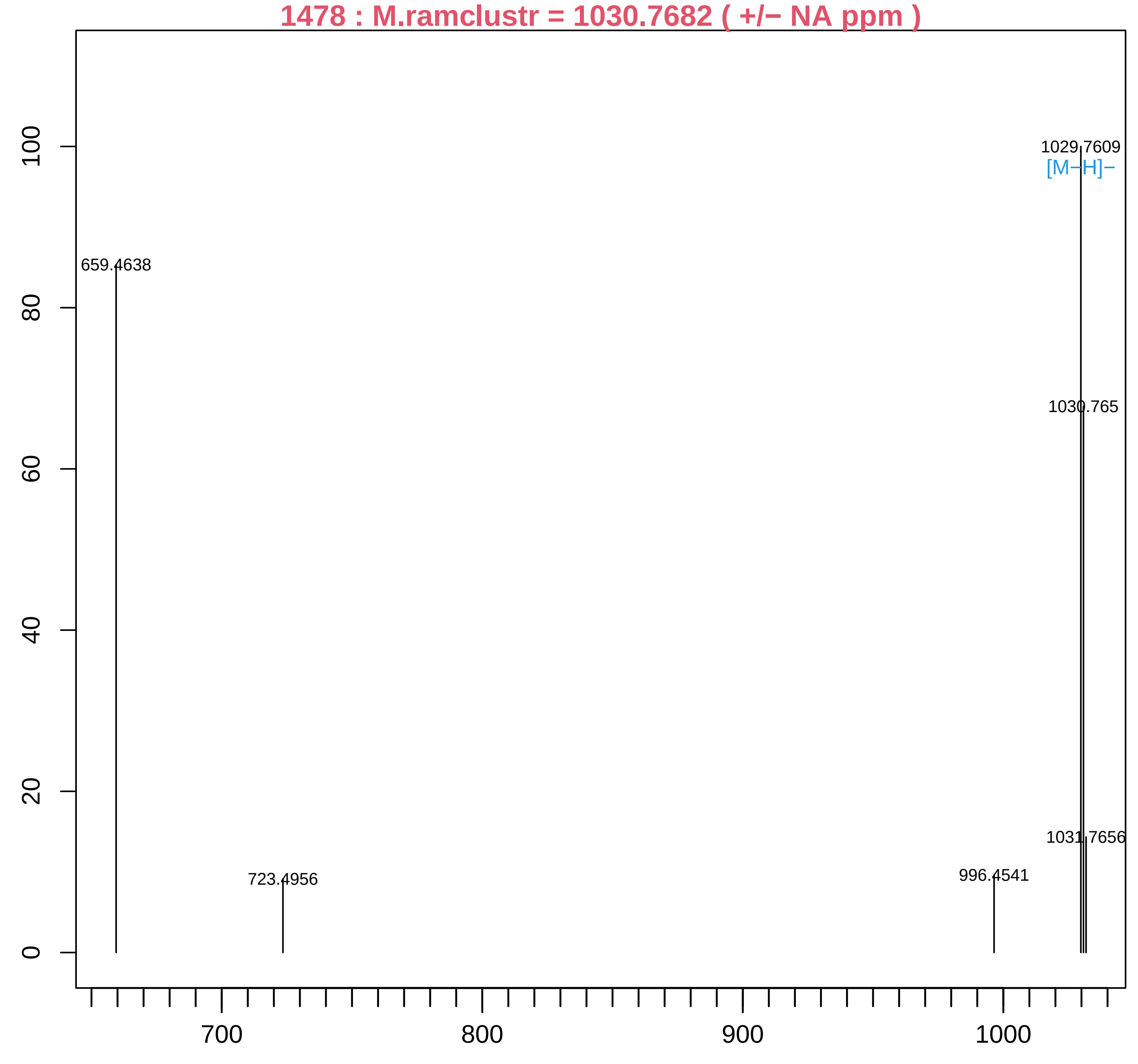
1478 : M.ramclustr = 1030.7682 (+/− NA ppm)
659.4638
723.4956
996.4541
1029.7609
[M−H]−
1030.765
1031.7656
0
20
40
60
80
100
700
800
900
1000

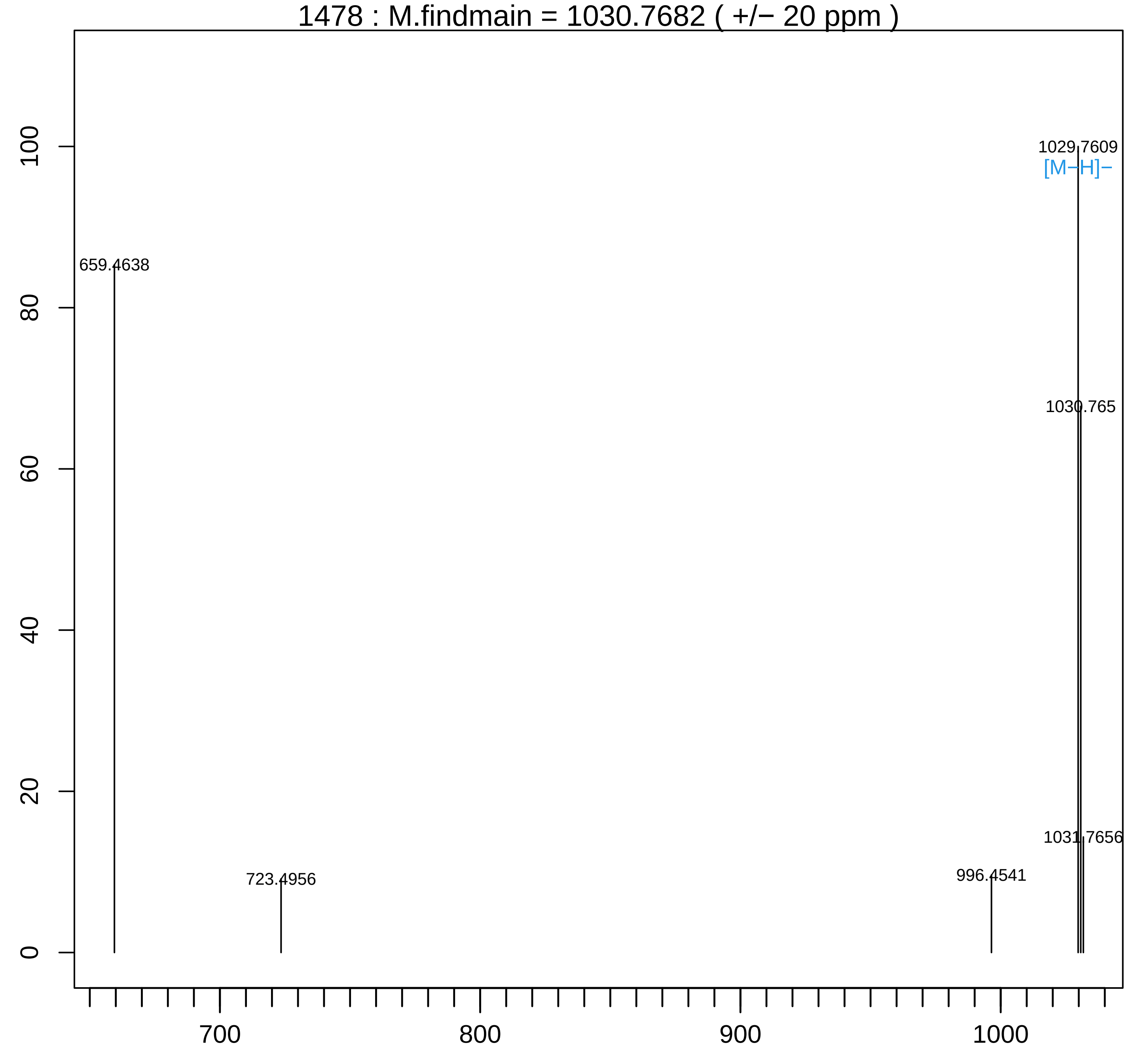
1478 : M.findmain = 1030.7682 (+/− 20 ppm)
659.4638
723.4956
996.4541
1029.7609
[M−H]−
1030.765
1031.7656
0
20
40
60
80
100
700
800
900
1000

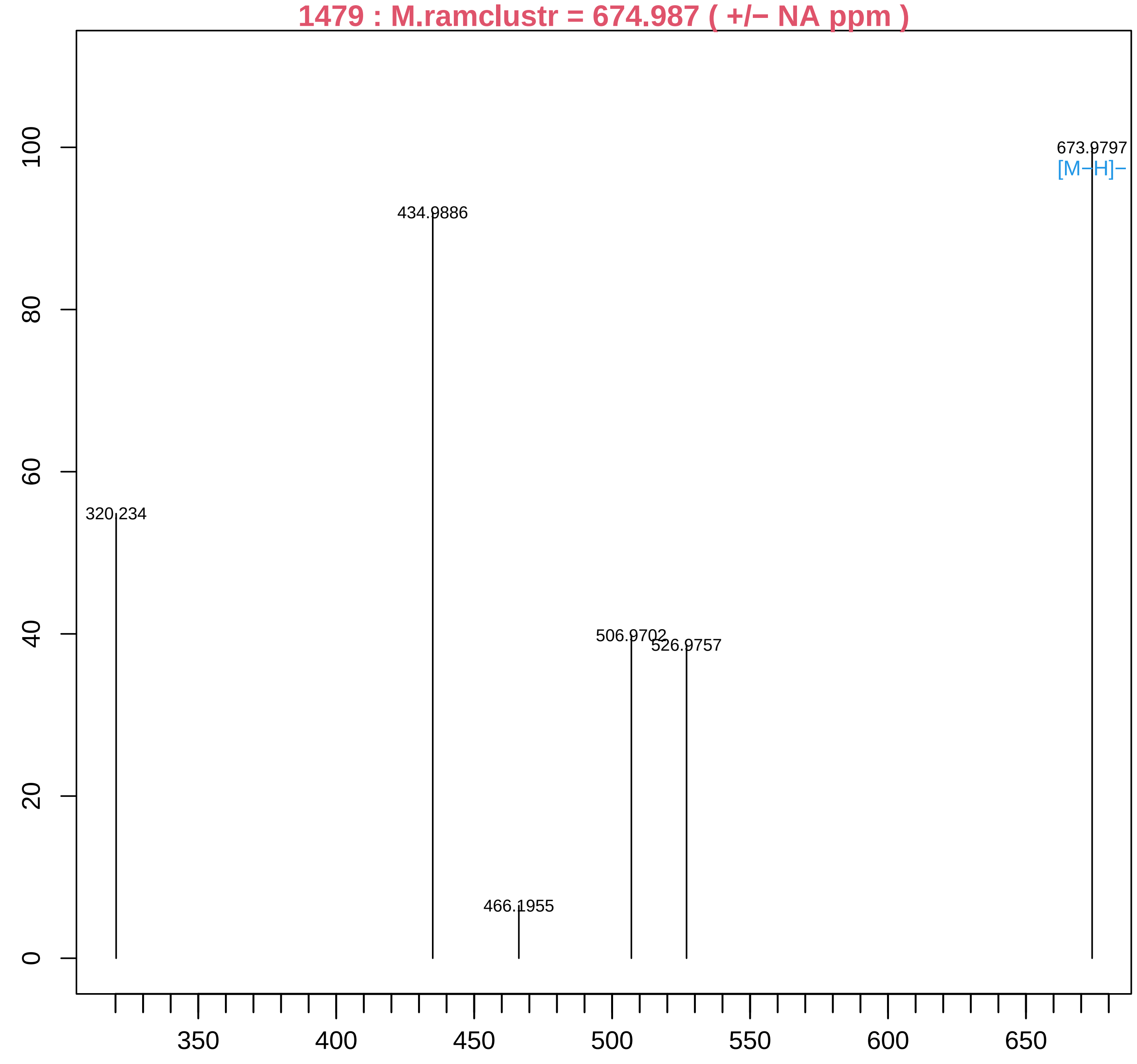
1479 : M.ramclustr = 674.987 (+/− NA ppm)
673.9797
[M−H]−
434.9886
320.234
506.9702
526.9757
466.1955
0
20
40
60
80
100
350
400
450
500
550
600
650

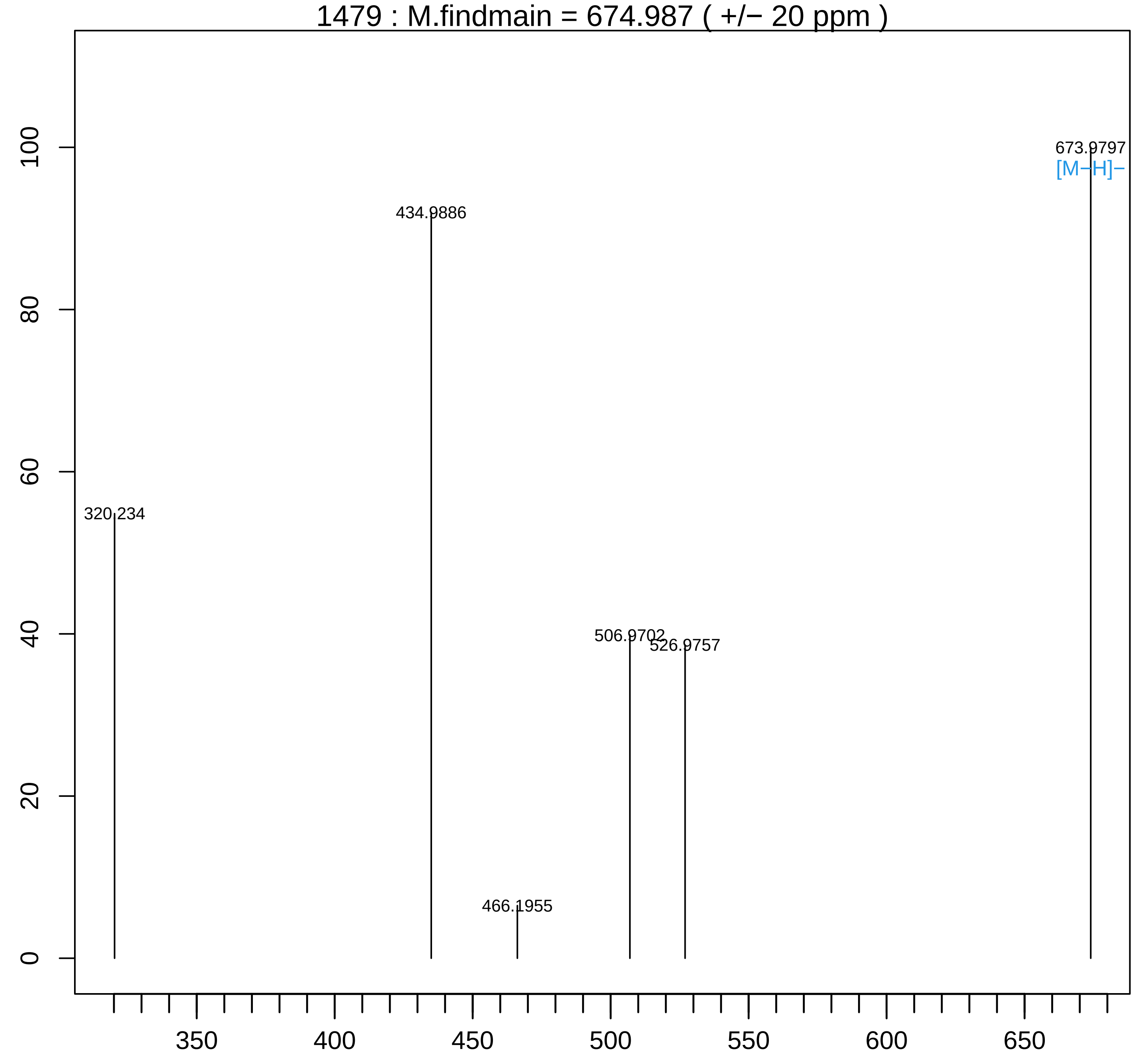
1479 : M.findmain = 674.987 (+/− 20 ppm)
673.9797
[M−H]−
434.9886
320.234
506.9702
526.9757
466.1955
0
20
40
60
80
100
350
400
450
500
550
600
650

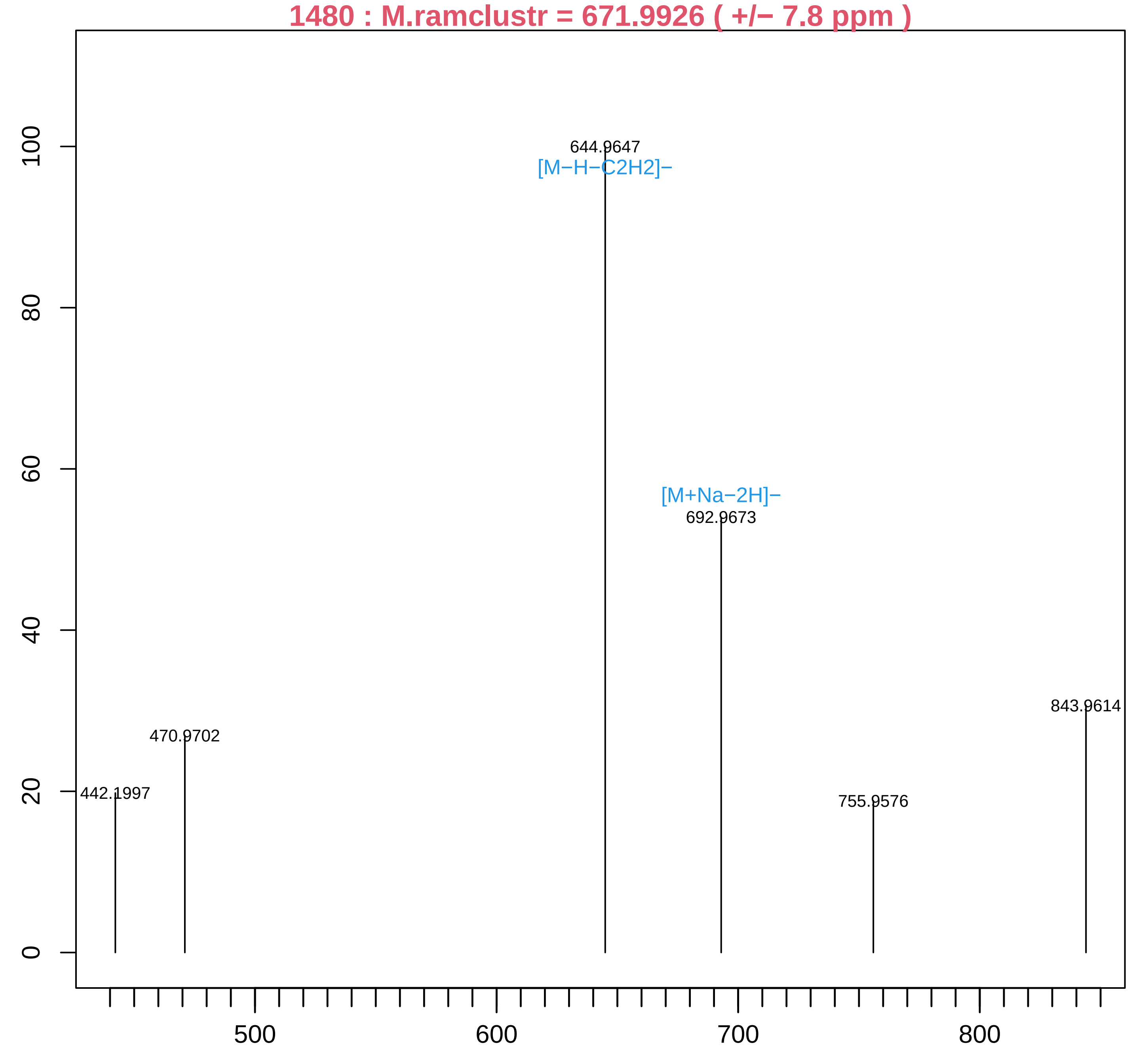

1480 : M.ramclustr = 671.9926 (+/− 7.8 ppm)
644.9647
[M−H−C2H2]−
[M+Na−2H]−
692.9673
843.9614
470.9702
442.1997
755.9576
0
20
40
60
80
100
500
600
700
800

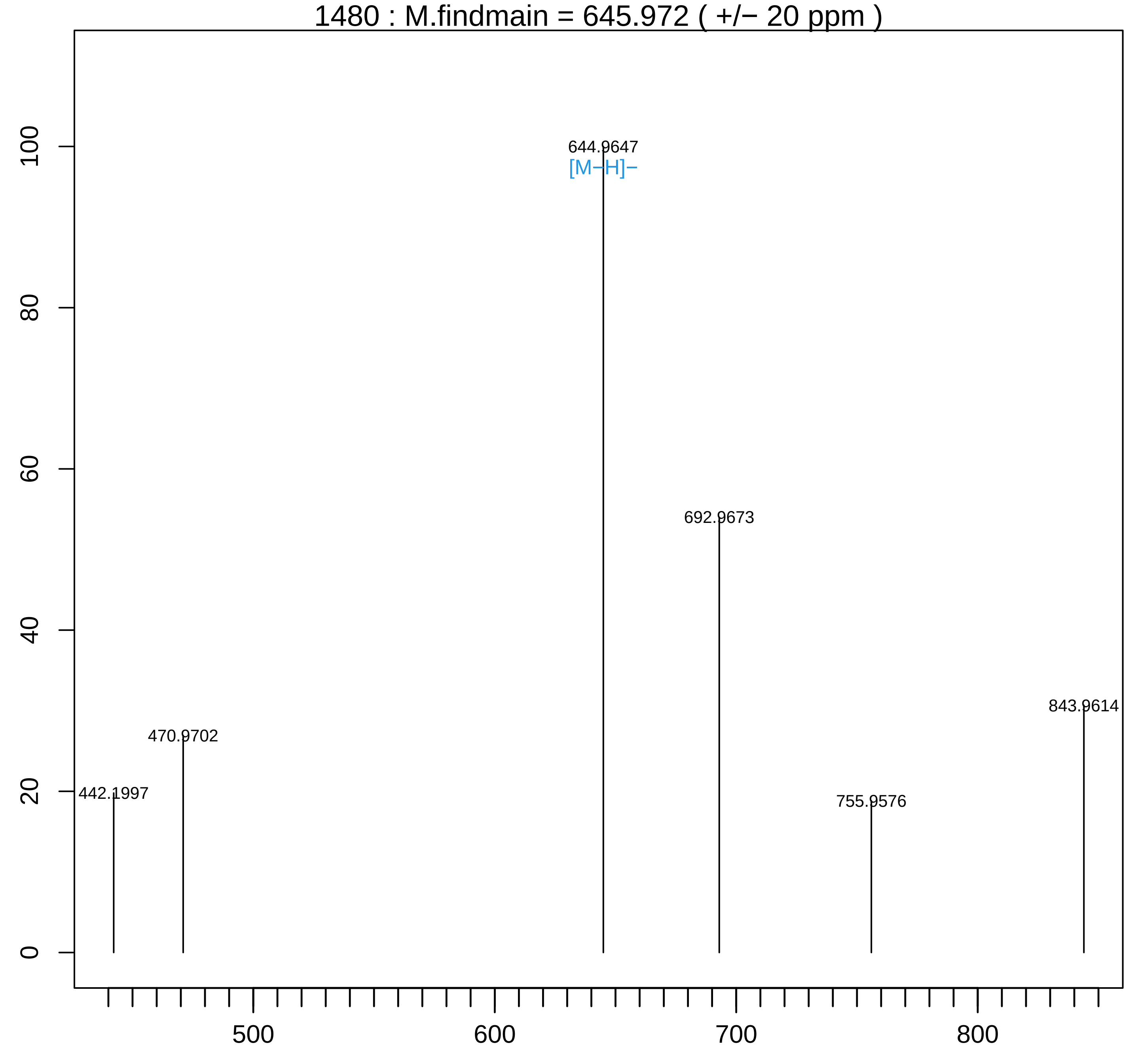

1480 : M.findmain = 645.972 (+/− 20 ppm)
644.9647
[M−H]−
692.9673
843.9614
470.9702
442.1997
755.9576
0
20
40
60
80
100
500
600
700
800

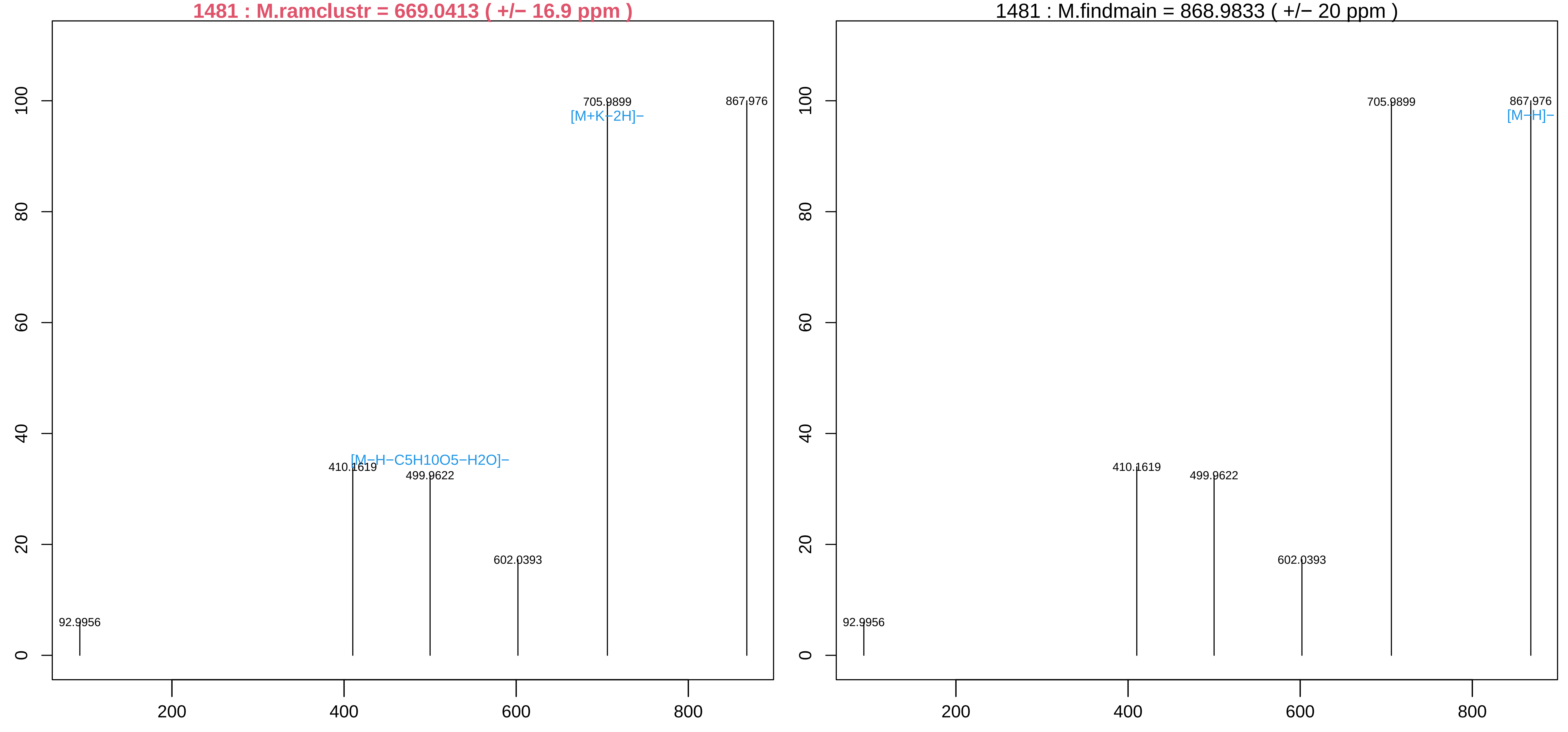

1481 : M.ramclustr = 669.0413 (+/− 16.9 ppm)
1481 : M.findmain = 868.9833 (+/− 20 ppm)
0
20
40
60
80
100
200
400
600
800
92.9956
410.1619
499.9622
602.0393
705.9899
867.976
[M+K−2H]−
[M−H−C5H10O5−H2O]−
[M−H]−

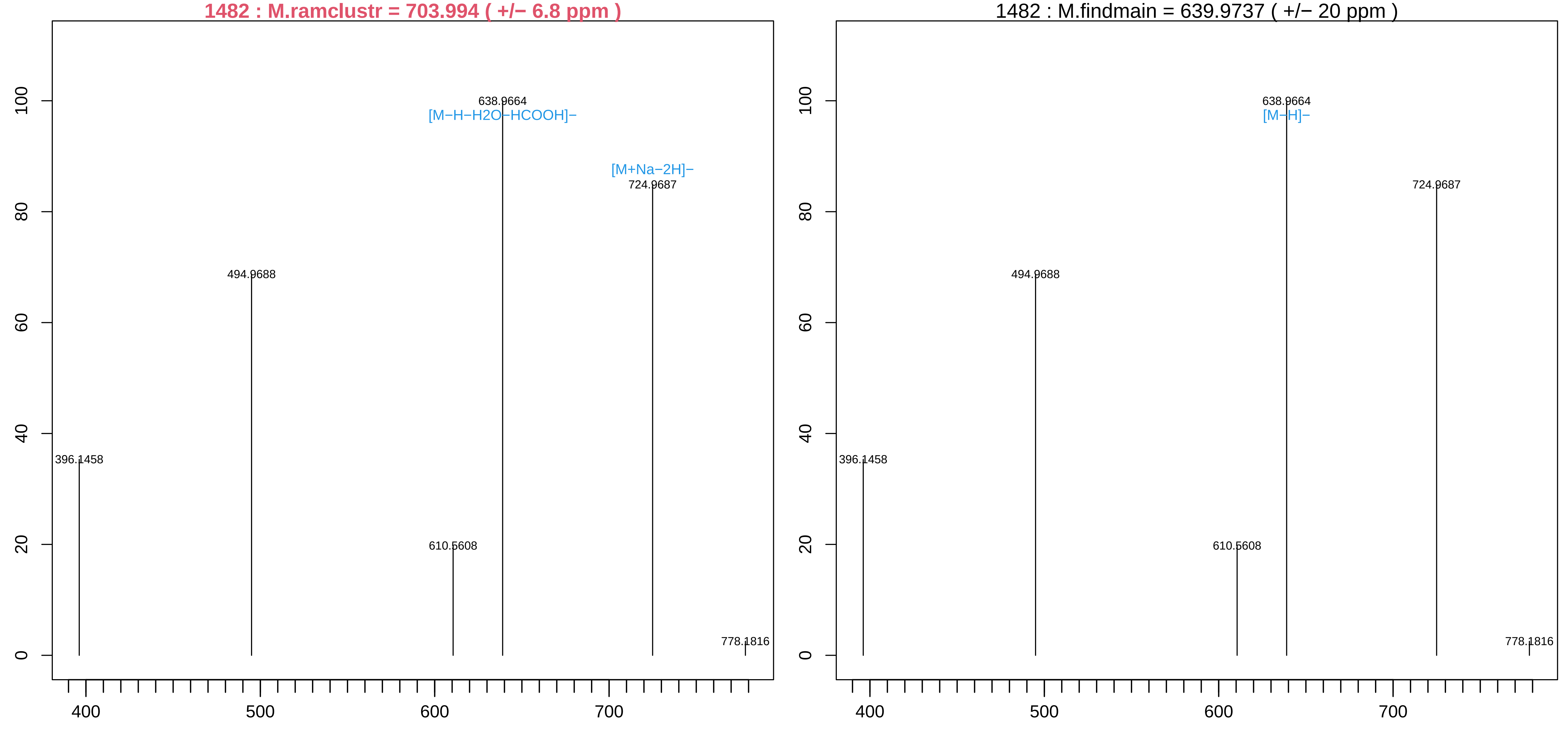
1482 : M.ramclustr = 703.994 (+/− 6.8 ppm)
638.9664
[M−H−H2O−HCOOH]−
[M+Na−2H]−
724.9687
494.9688
396.1458
610.5608
778.1816
1482 : M.findmain = 639.9737 (+/− 20 ppm)
638.9664
[M−H]−
724.9687
494.9688
396.1458
610.5608
778.1816

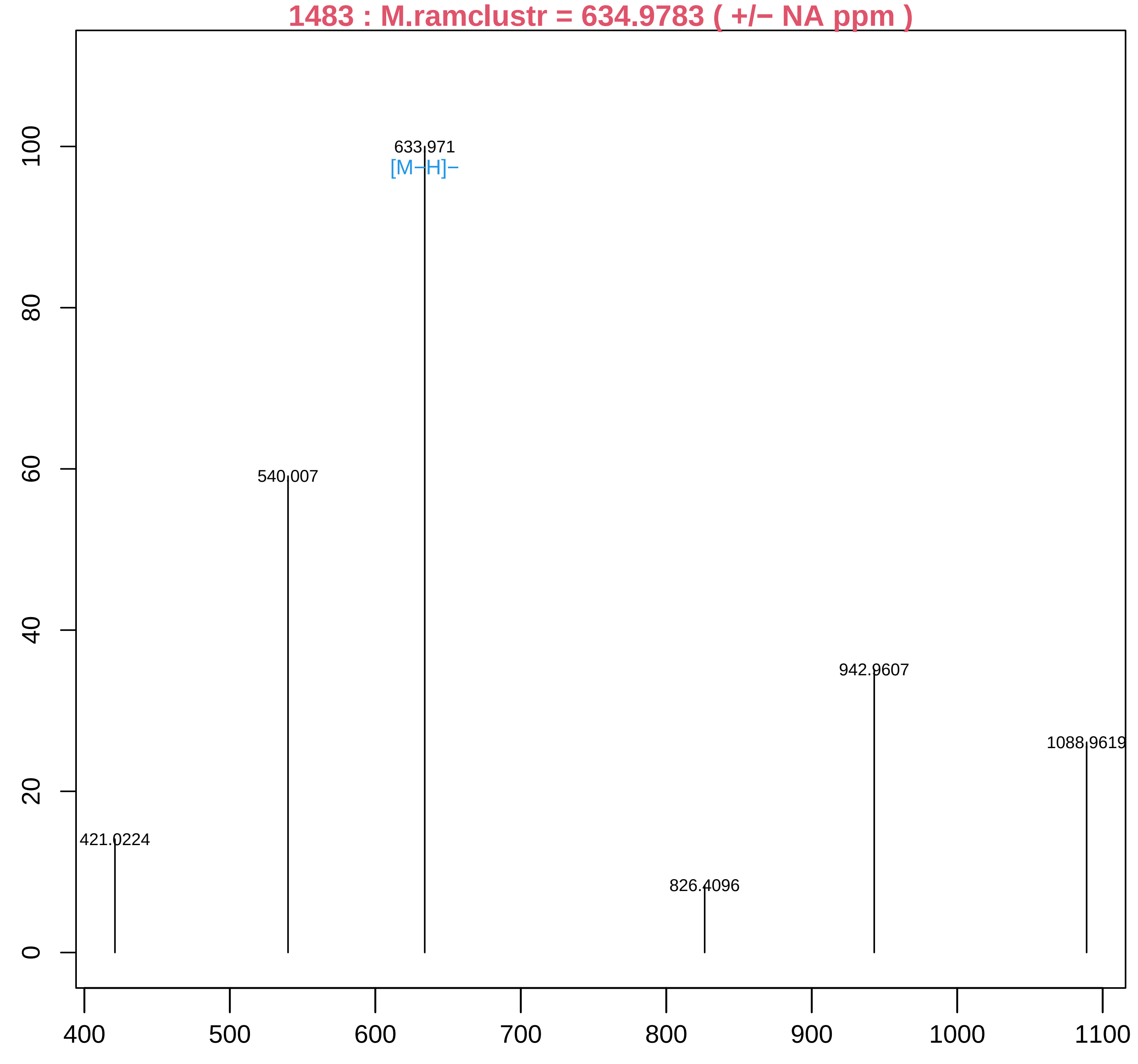
1483 : M.ramclustr = 634.9783 (+/− NA ppm)
633.971
[M−H]−
540.007
942.9607
1088.9619
421.0224
826.4096
0
20
40
60
80
100
400
500
600
700
800
900
1000
1100

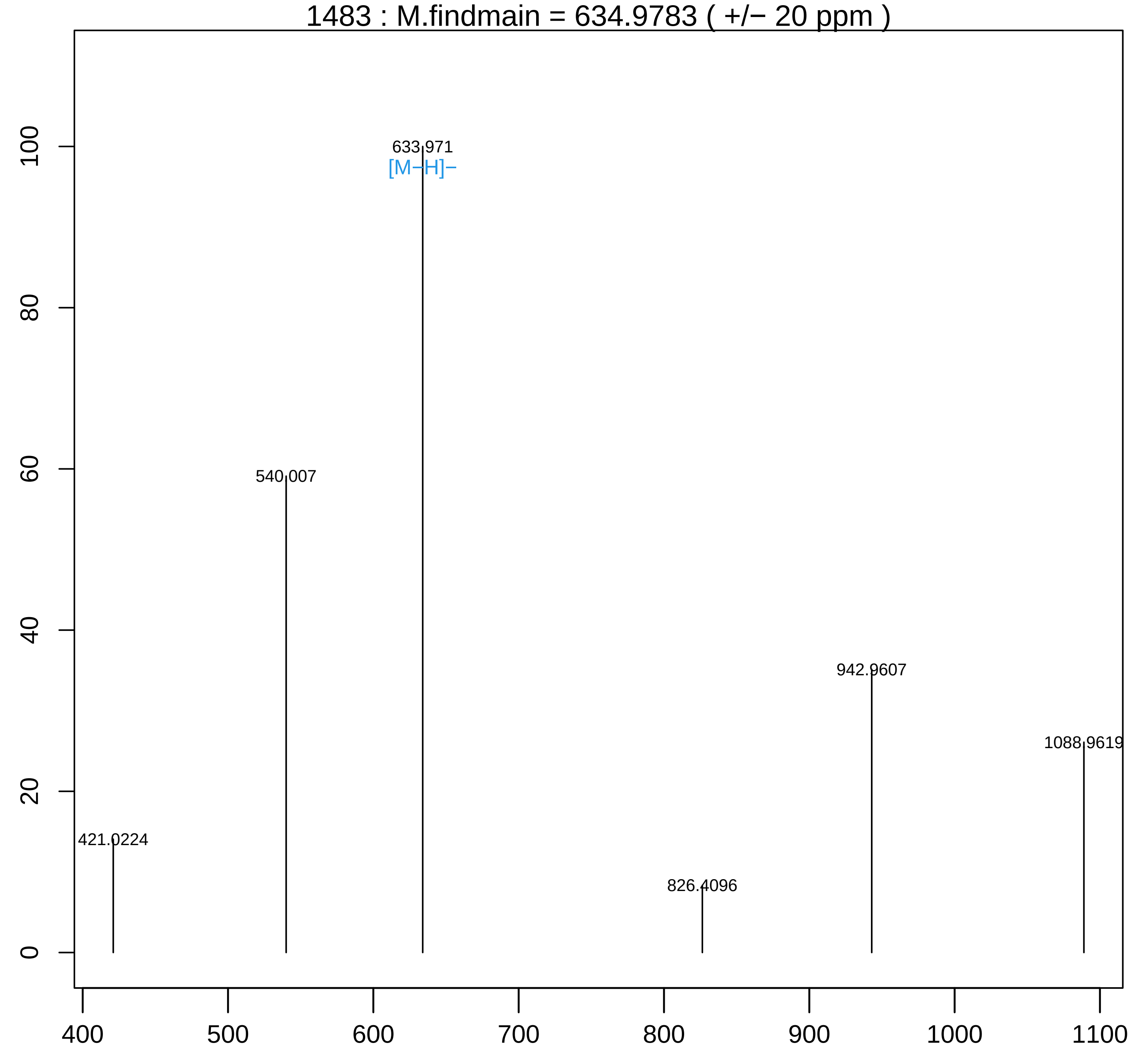
1483 : M.findmain = 634.9783 (+/− 20 ppm)
633.971
[M−H]−
540.007
942.9607
1088.9619
421.0224
826.4096
0
20
40
60
80
100
400
500
600
700
800
900
1000
1100

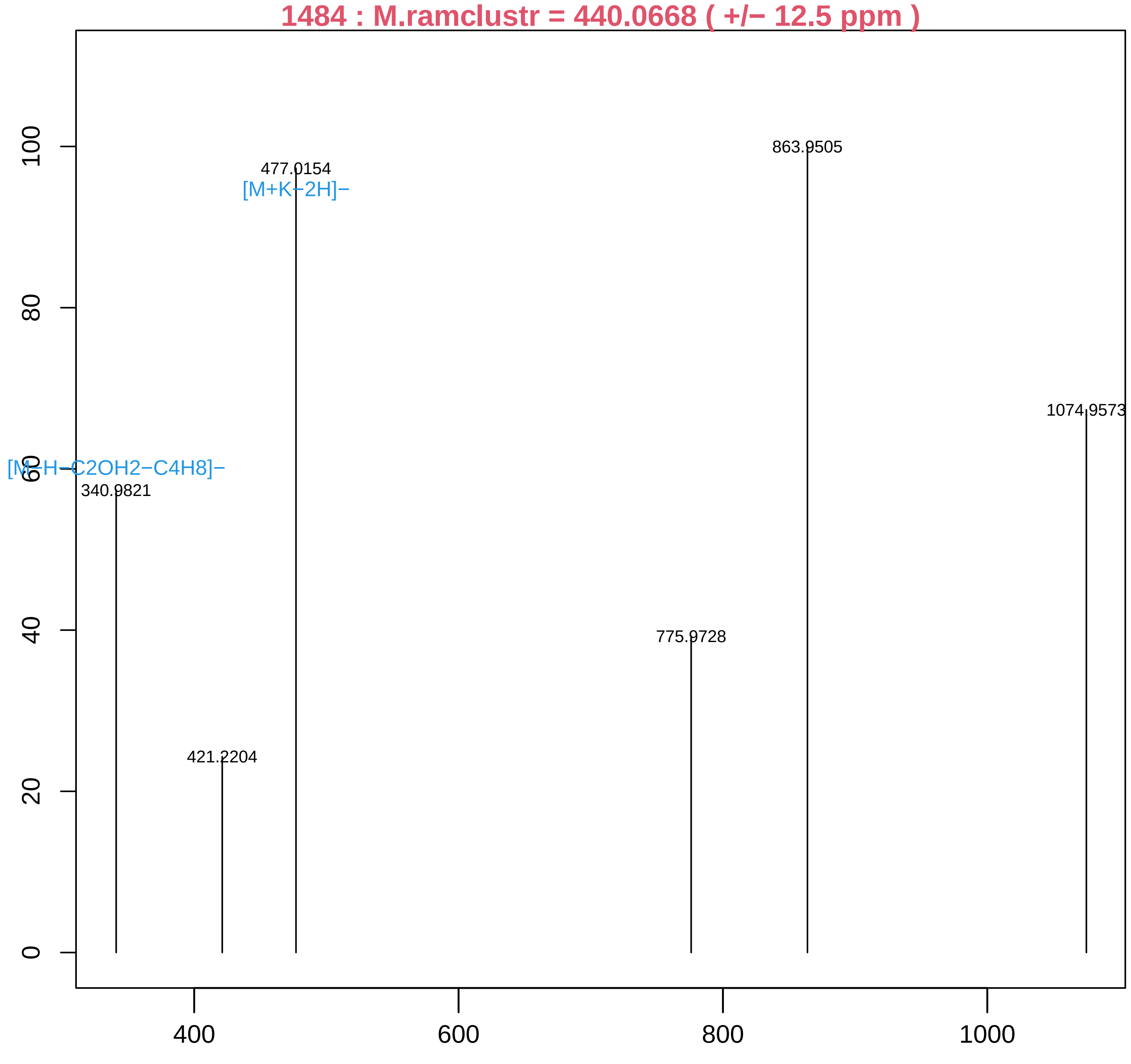
1484 : M.ramclustr = 440.0668 (+/− 12.5 ppm)
[M-H−C2OH2−C4H8]−
340.9821
421.2204
477.0154
[M+K-2H]−
775.9728
863.9505
1074.9573
0
20
40
60
80
100
400
600
800
1000

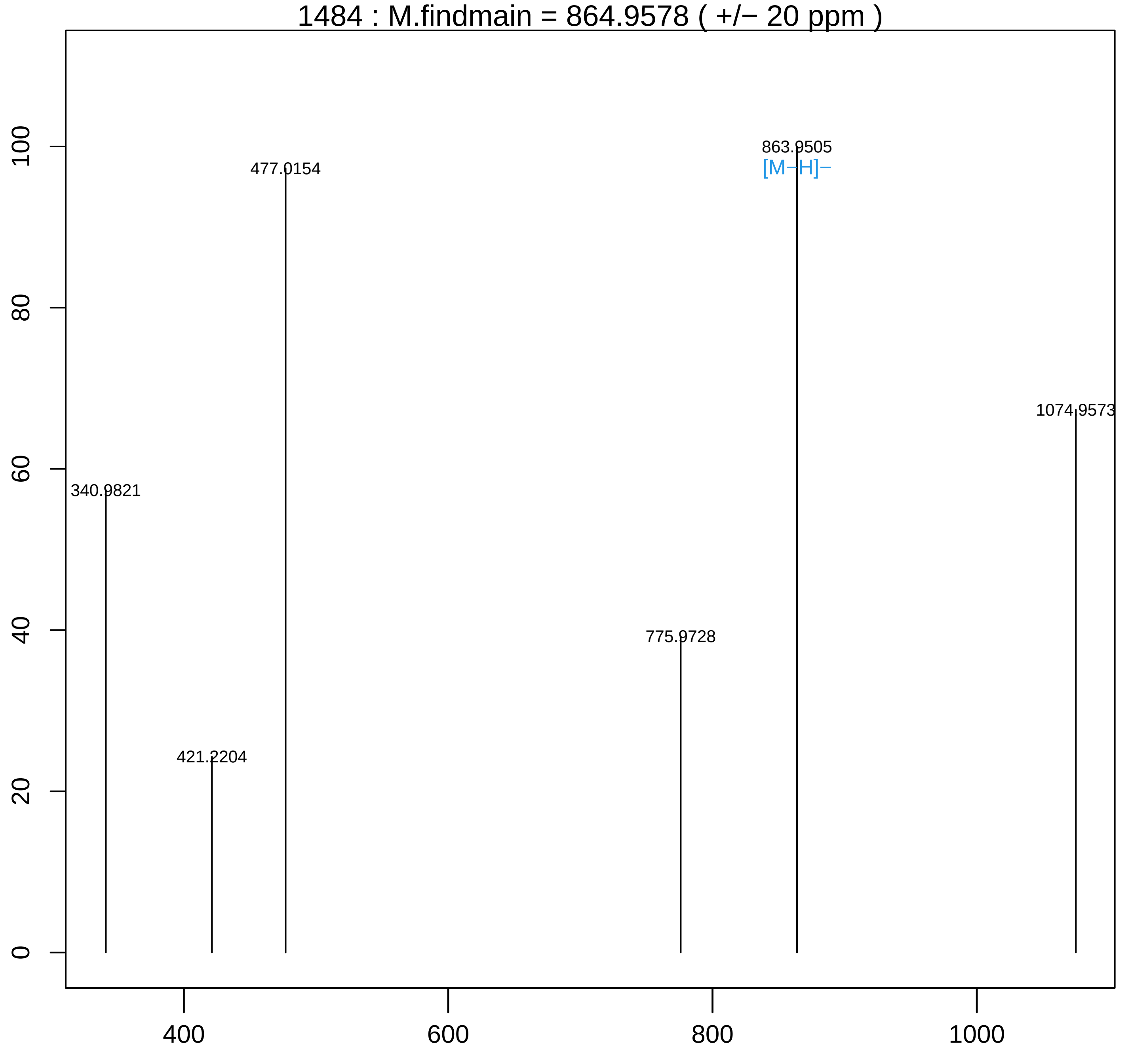
1484 : M.findmain = 864.9578 (+/− 20 ppm)
340.9821
421.2204
477.0154
775.9728
863.9505
[M-H]−
1074.9573
0
20
40
60
80
100
400
600
800
1000

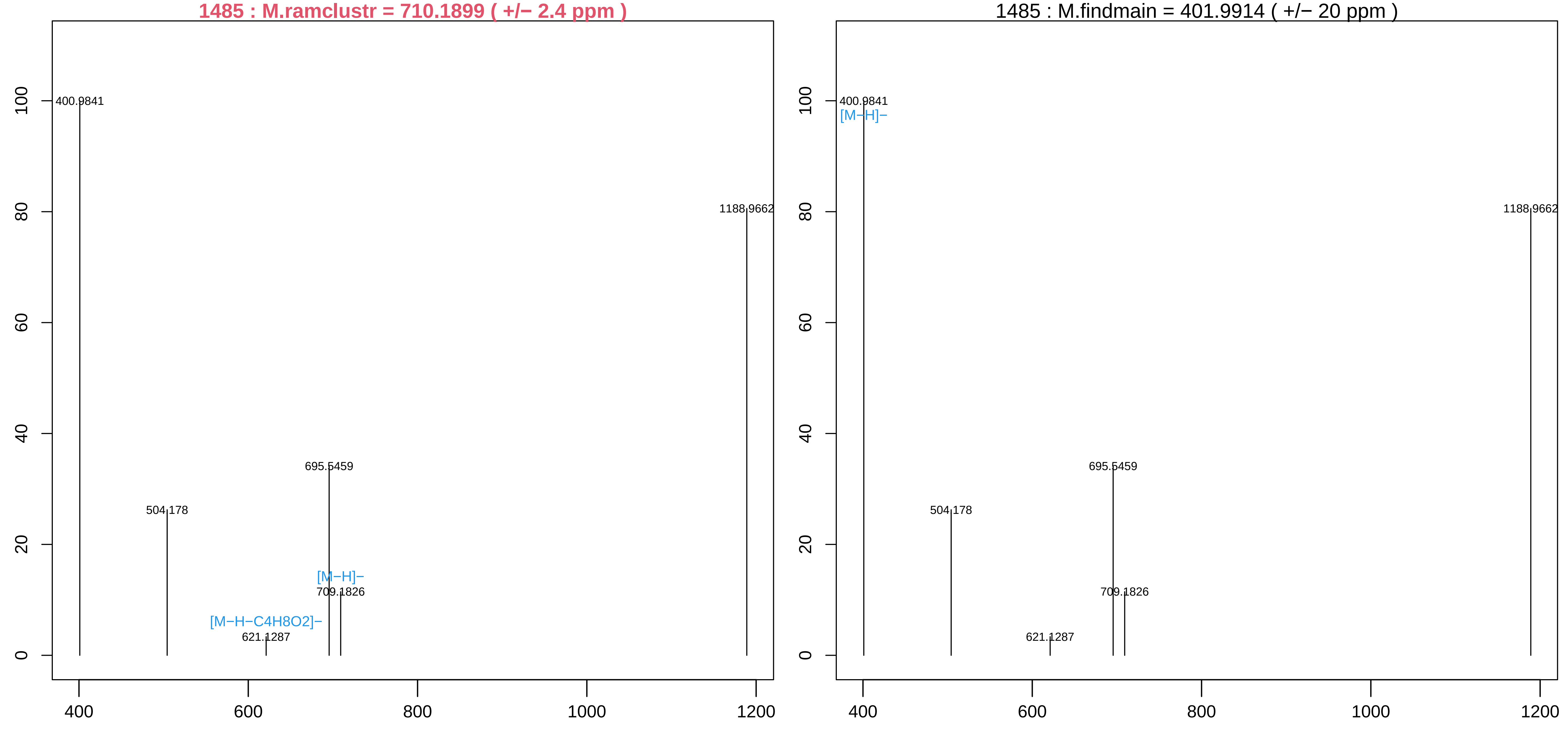
1485 : M.ramclustr = 710.1899 (+/− 2.4 ppm)
400.9841
1188.9662
695.5459
504.178
[M−H]−
709.1826
[M−H−C4H8O2]−
621.1287
0
20
40
60
80
100
400
600
800
1000
1200
1485 : M.findmain = 401.9914 (+/− 20 ppm)
400.9841
[M−H]−
1188.9662
695.5459
504.178
709.1826
621.1287

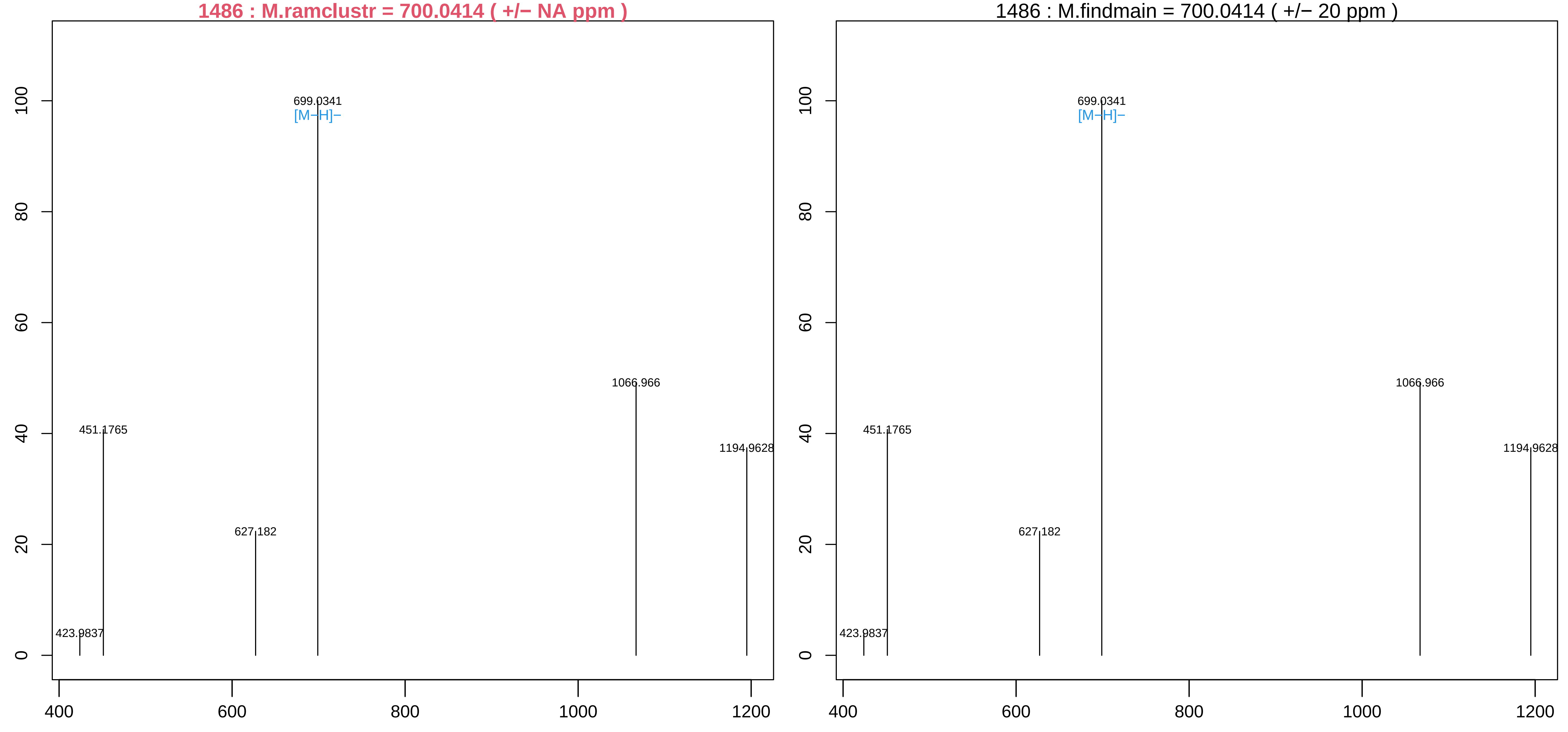
1486 : M.ramclustr = 700.0414 (+/− NA ppm)
1486 : M.findmain = 700.0414 (+/− 20 ppm)
699.0341
[M−H]−
1066.966
451.1765
1194.9628
627.182
423.9837
699.0341
[M−H]−
1066.966
451.1765
1194.9628
627.182
423.9837

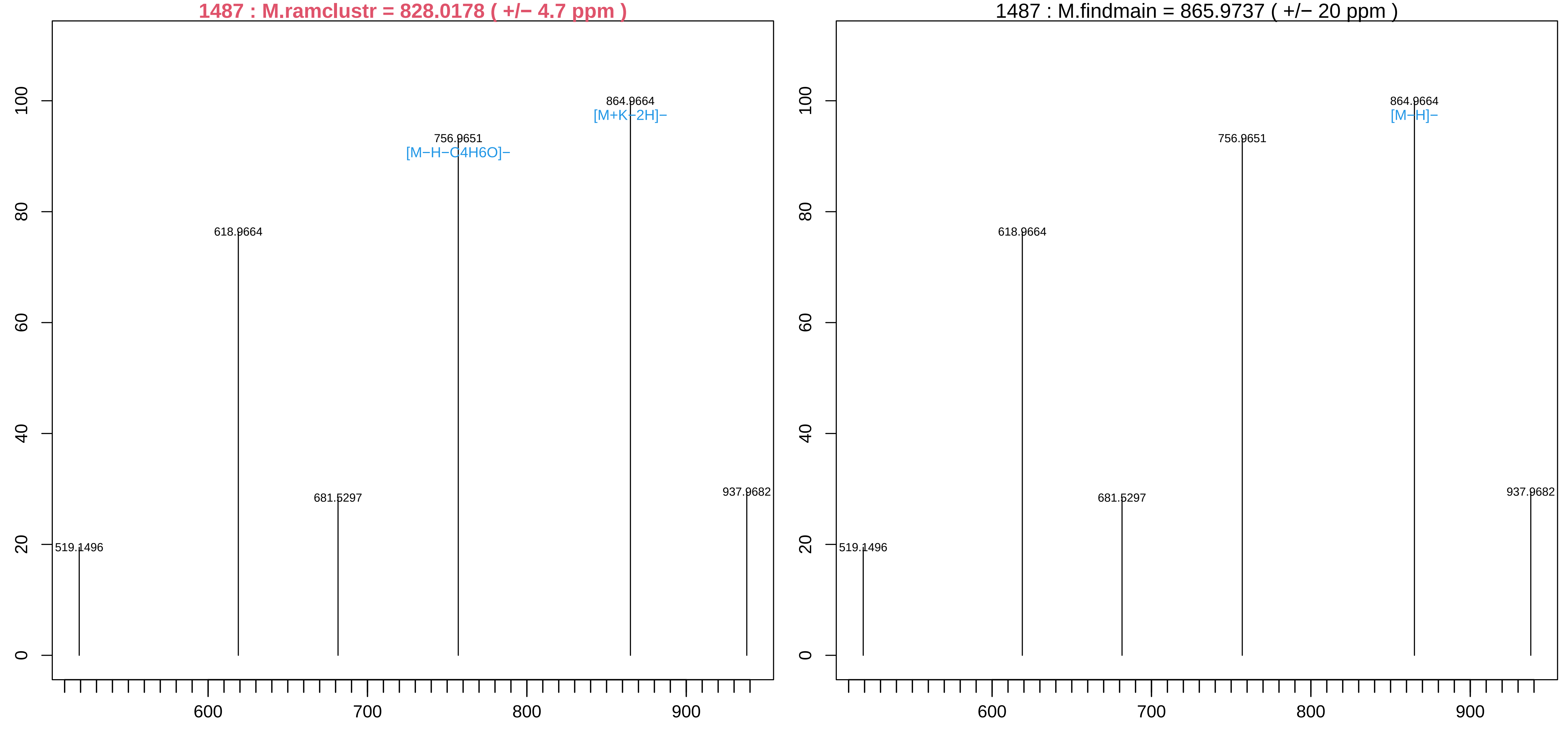
1487 : M.ramclustr = 828.0178 (+/− 4.7 ppm)
864.9664
[M+K−2H]−
756.9651
[M−H−C4H6O]−
618.9664
681.5297
937.9682
519.1496
1487 : M.findmain = 865.9737 (+/− 20 ppm)
864.9664
[M−H]−
756.9651
618.9664
681.5297
937.9682
519.1496
0
20
40
60
80
100
600
700
800
900

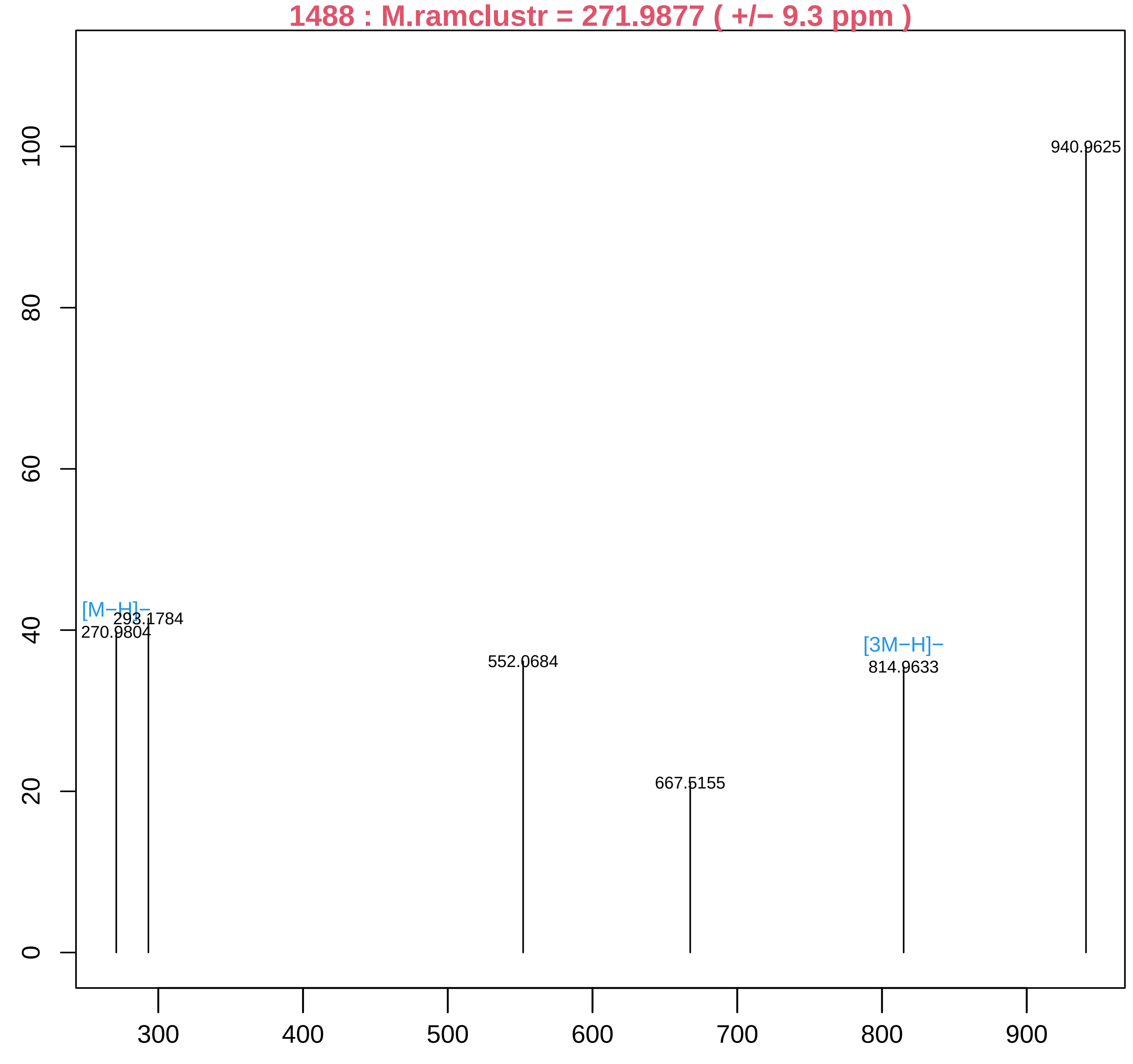

1488 : M.ramclustr = 271.9877 (+/− 9.3 ppm)
940.9625
[M−H]−
293.1784
270.9804
552.0684
667.5155
[3M−H]−
814.9633
0
20
40
60
80
100
300
400
500
600
700
800
900

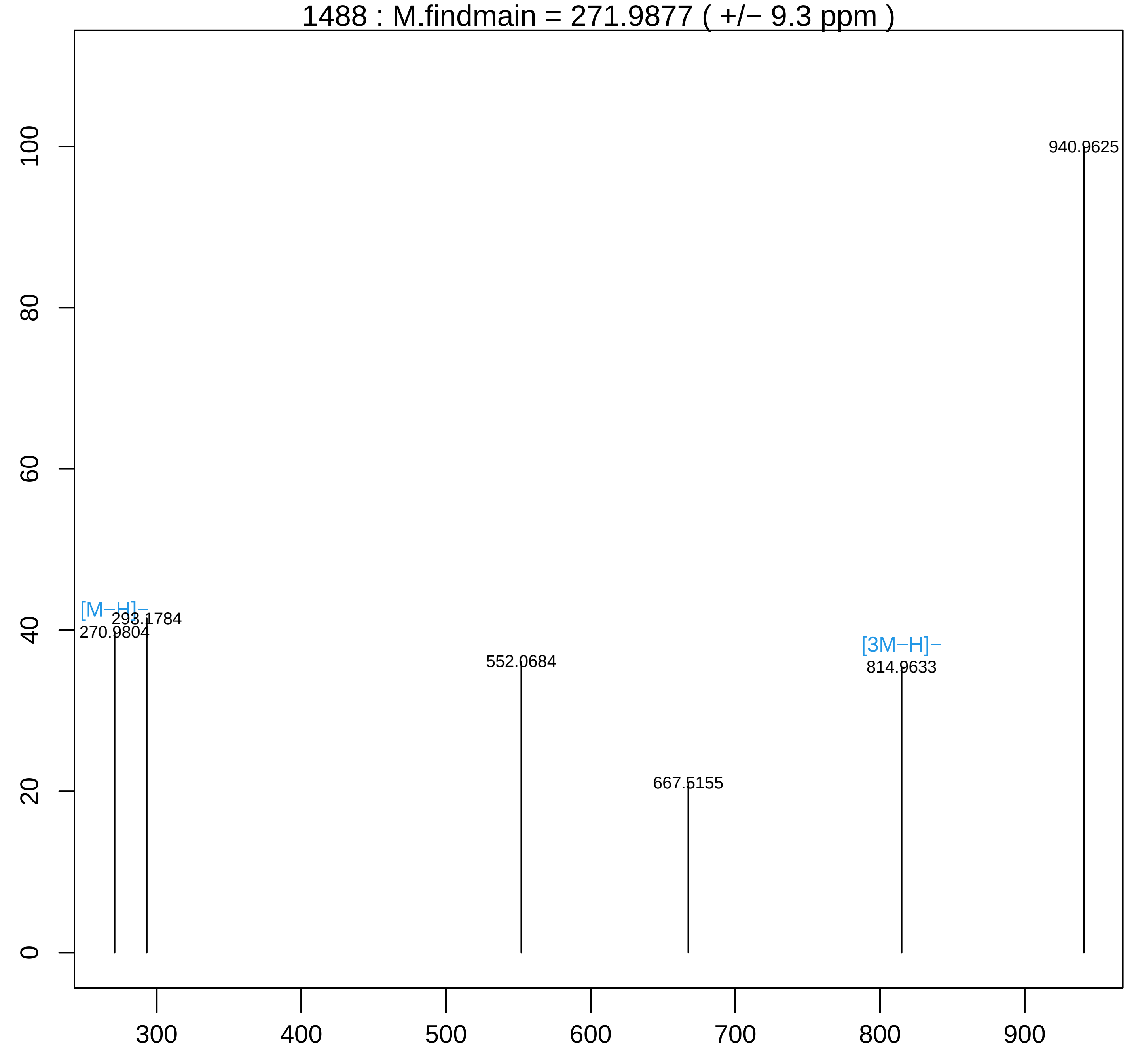

1488 : M.findmain = 271.9877 (+/− 9.3 ppm)
940.9625
[M−H]−
293.1784
270.9804
552.0684
667.5155
[3M−H]−
814.9633
0
20
40
60
80
100
300
400
500
600
700
800
900

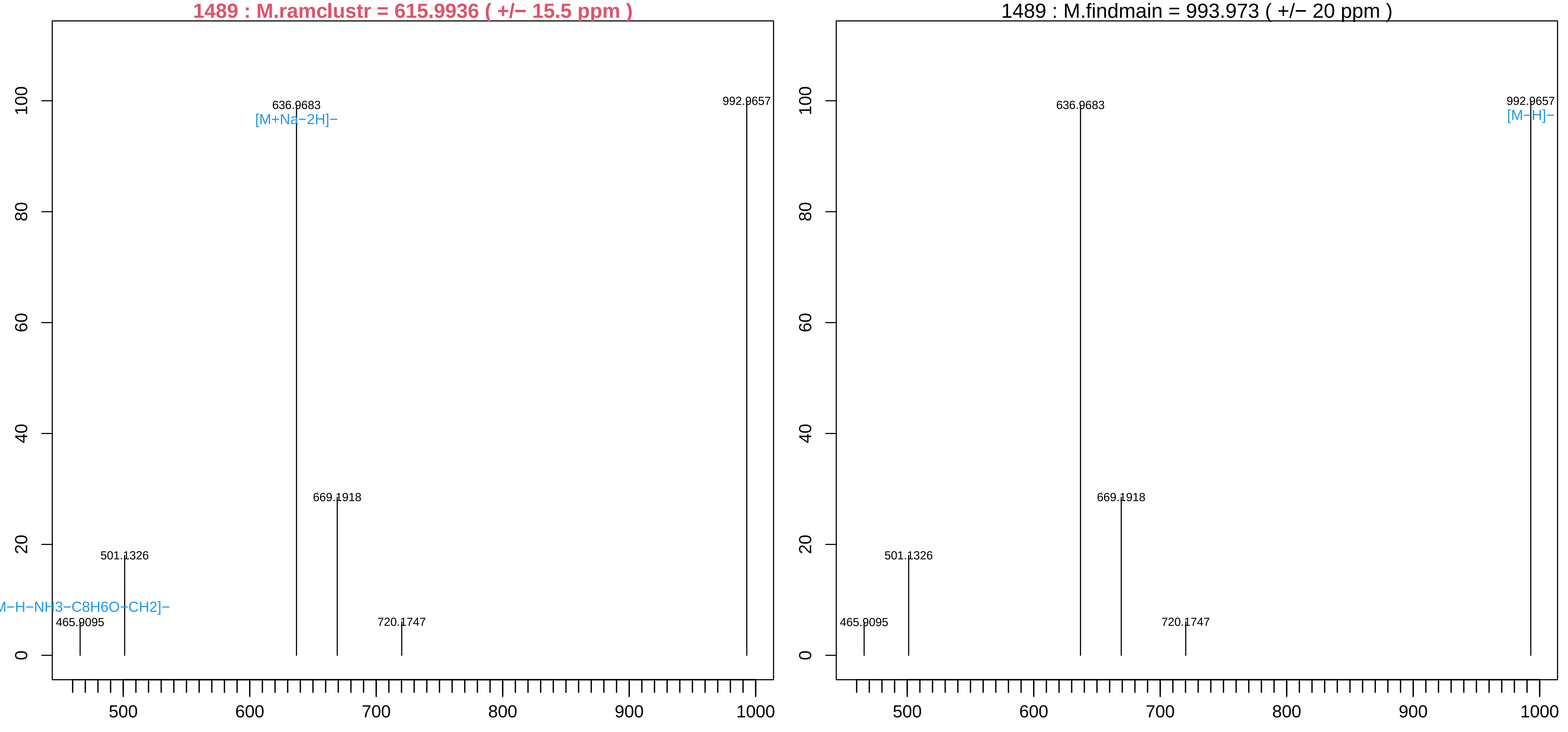
1489 : M.ramclustr = 615.9936 (+/− 15.5 ppm)
636.9683
[M+Na−2H]−
992.9657
669.1918
501.1326
M−H−NH3−C8H6O−CH2]−
465.9095
720.1747
0
20
40
60
80
100
500
600
700
800
900
1000
1489 : M.findmain = 993.973 (+/− 20 ppm)
636.9683
992.9657
[M−H]−
669.1918
501.1326
465.9095
720.1747
0
20
40
60
80
100
500
600
700
800
900
1000

1490 : M.ramclustr = 569.9963 (+/− 7.6 ppm)

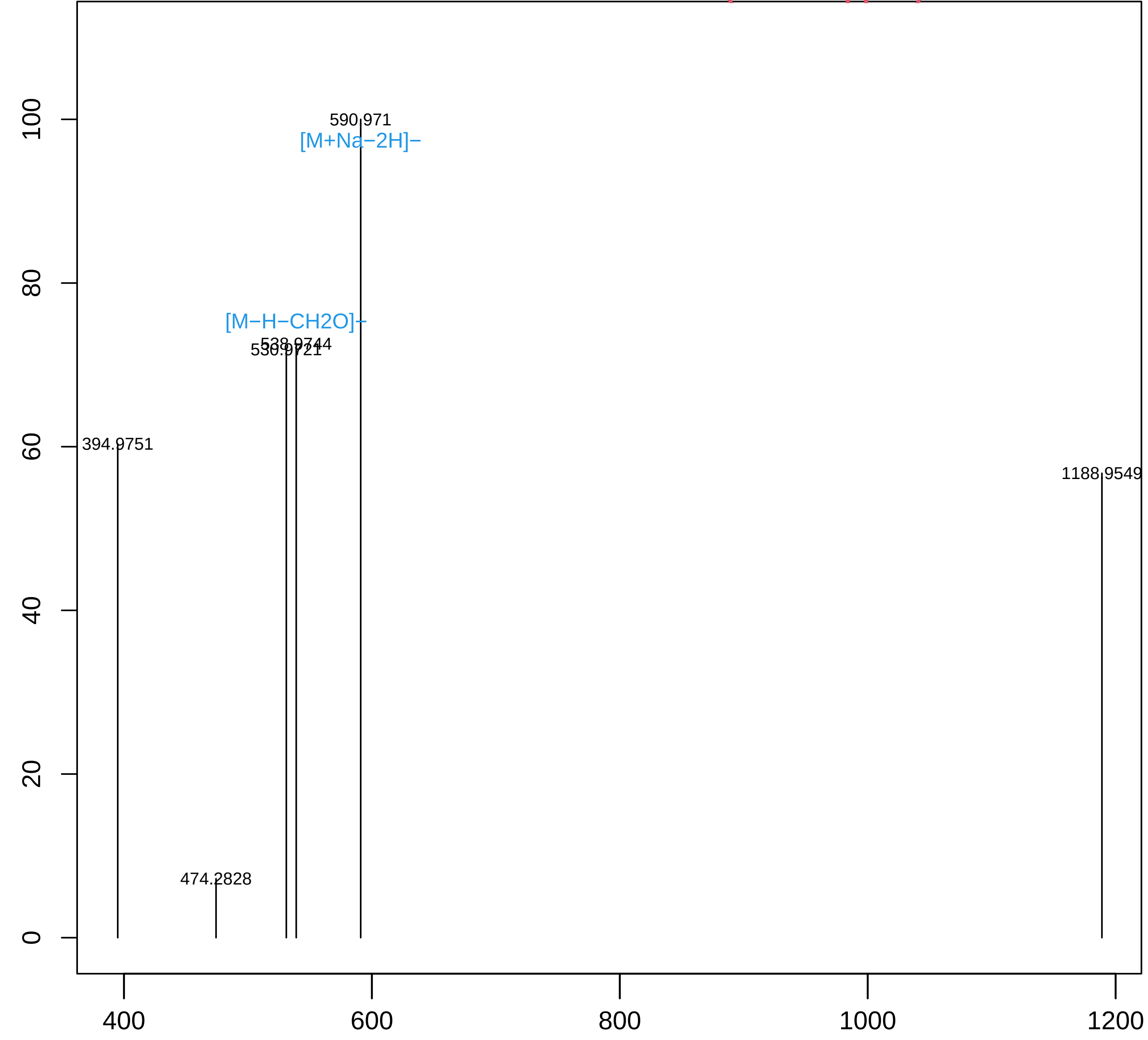

1490 : M.findmain = 591.9783 (+/− 20 ppm)

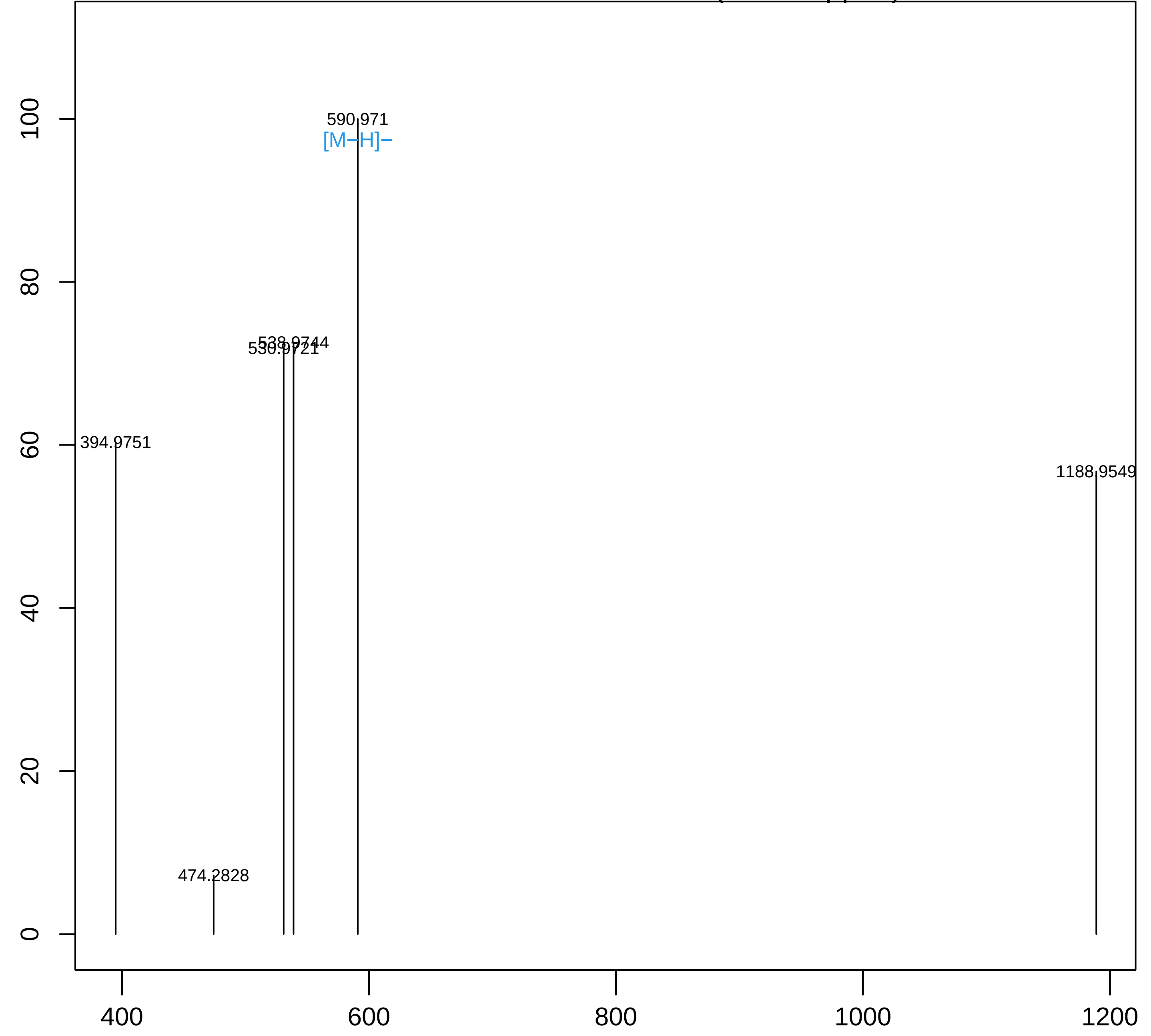

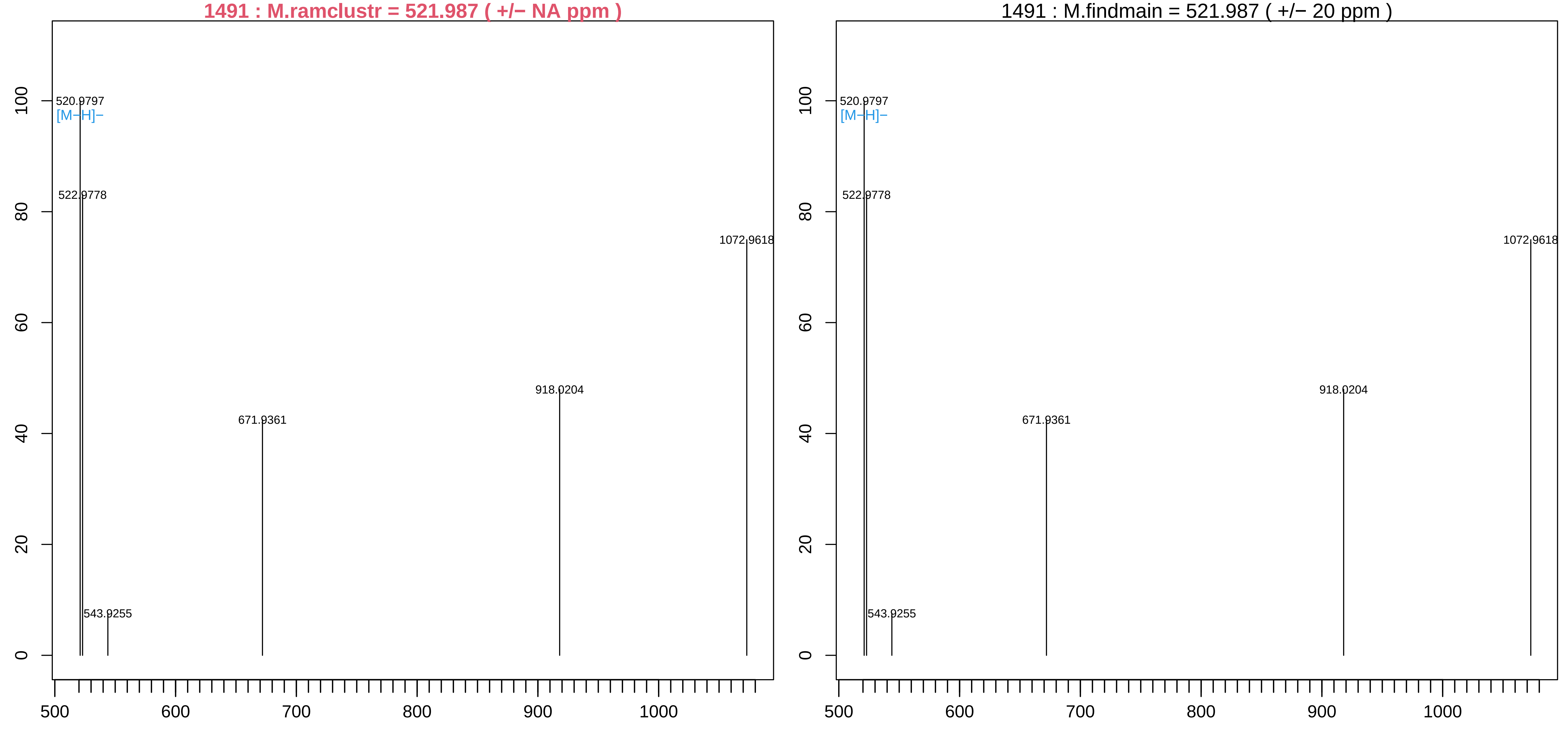
1491 : M.ramclustr = 521.987 (+/− NA ppm)
1491 : M.findmain = 521.987 (+/− 20 ppm)
520.9797
[M−H]−
522.9778
543.9255
671.9361
918.0204
1072.9618
0
20
40
60
80
100
500
600
700
800
900
1000

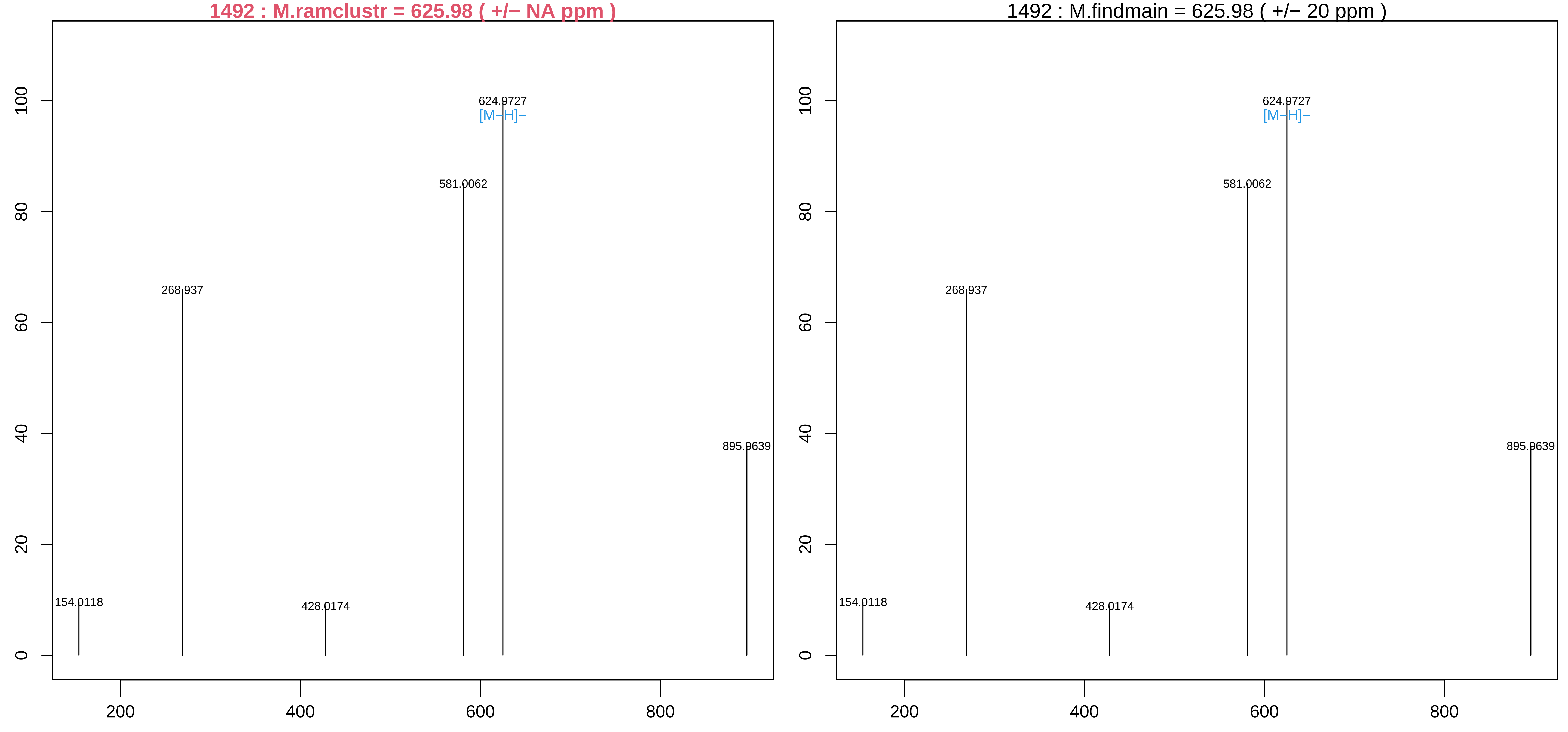

1492 : M.ramclustr = 625.98 (+/− NA ppm)
624.9727
[M−H]−
581.0062
268.937
895.9639
154.0118
428.0174
0
20
40
60
80
100
200
400
600
800
1492 : M.findmain = 625.98 (+/− 20 ppm)
624.9727
[M−H]−
581.0062
268.937
895.9639
154.0118
428.0174
0
20
40
60
80
100
200
400
600
800

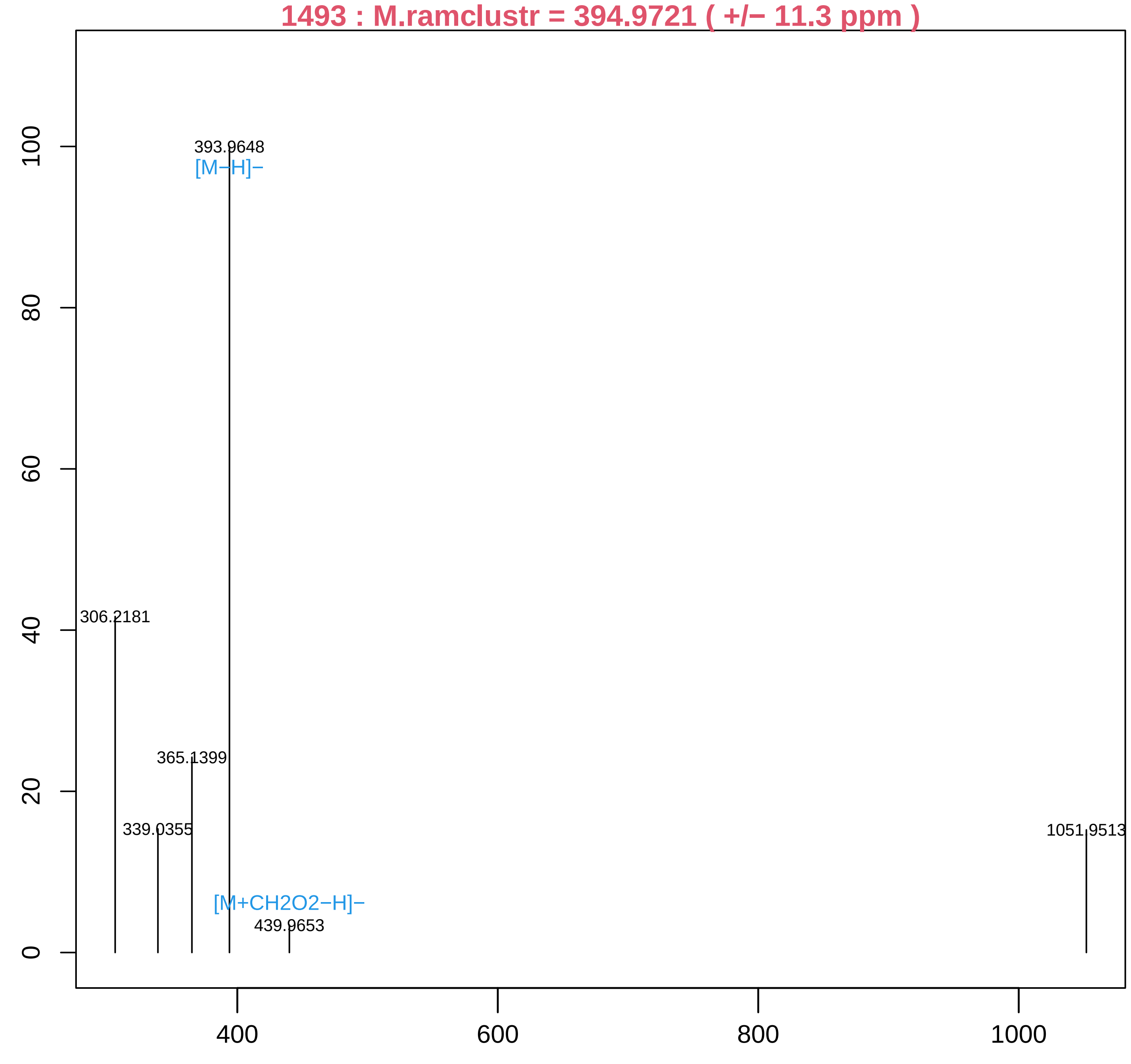
1493 : M.ramclustr = 394.9721 (+/− 11.3 ppm)
393.9648
[M−H]−
306.2181
365.1399
339.0355
1051.9513
[M+CH2O2−H]−
439.9653
0
20
40
60
80
100
400
600
800
1000

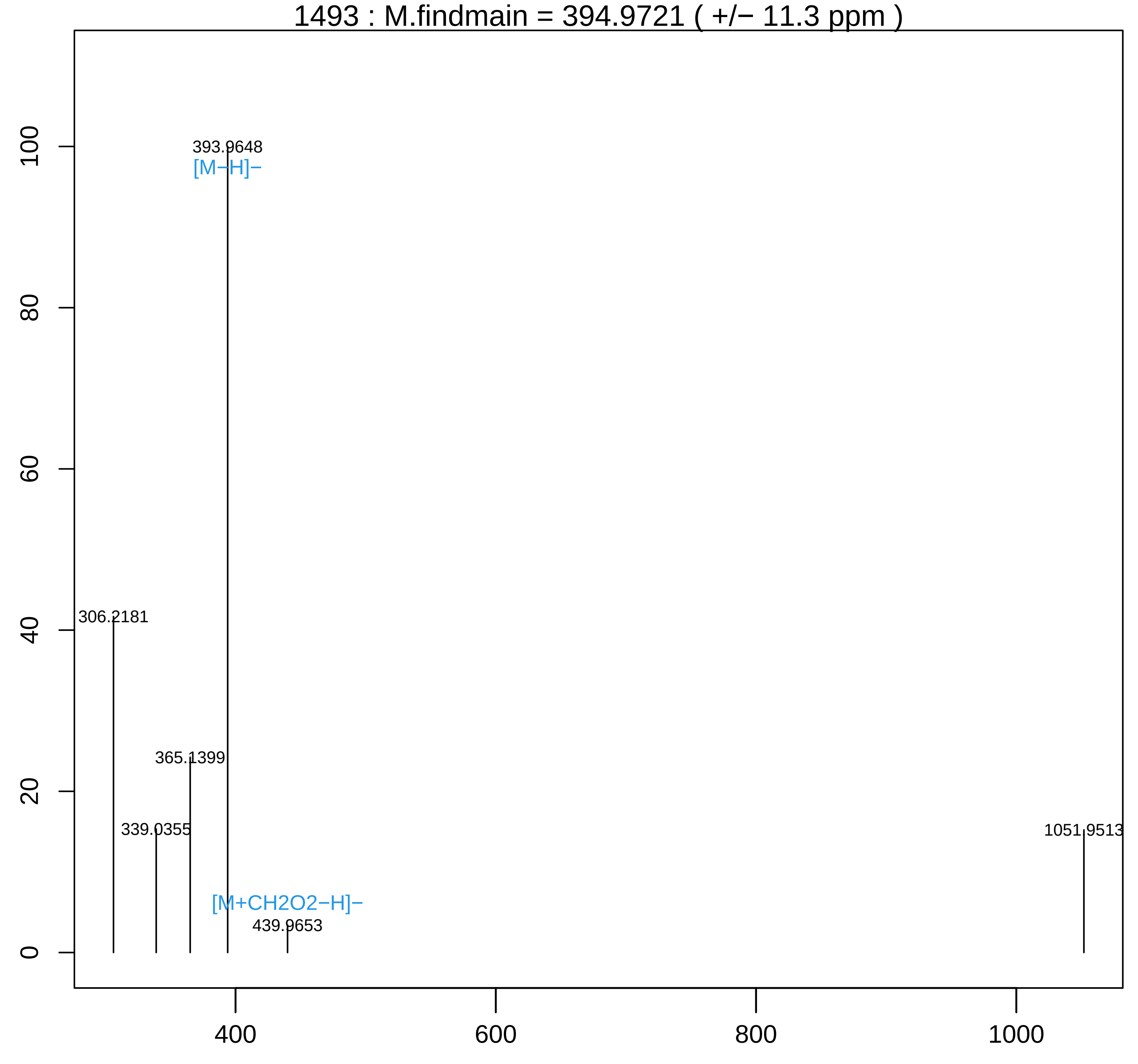
1493 : M.findmain = 394.9721 (+/− 11.3 ppm)
393.9648
[M−H]−
306.2181
365.1399
339.0355
1051.9513
[M+CH2O2−H]−
439.9653
0
20
40
60
80
100
400
600
800
1000

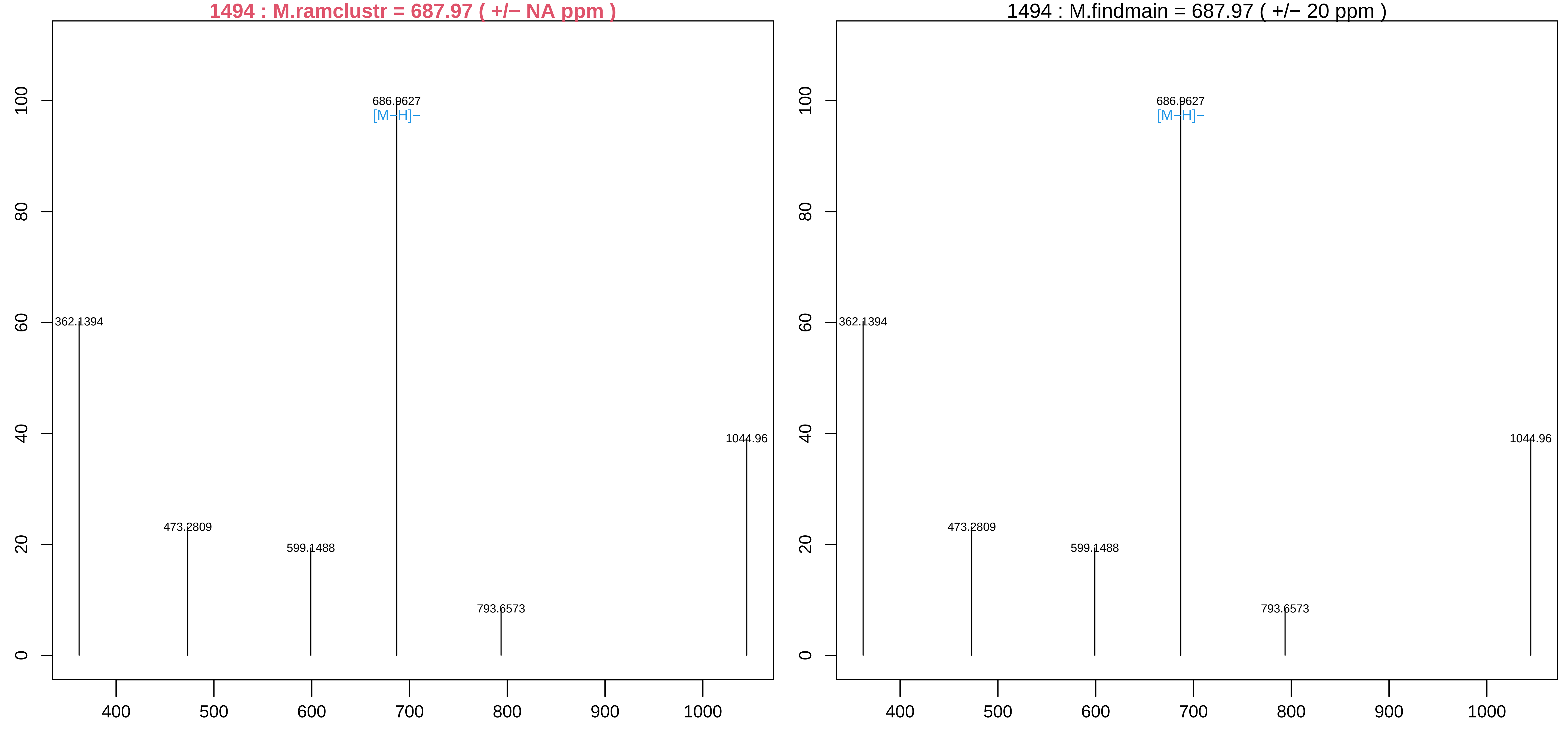

1494 : M.ramclustr = 687.97 (+/− NA ppm)
686.9627
[M−H]−
362.1394
473.2809
599.1488
793.6573
1044.96
0
20
40
60
80
100
400
500
600
700
800
900
1000
1494 : M.findmain = 687.97 (+/− 20 ppm)
686.9627
[M−H]−
362.1394
473.2809
599.1488
793.6573
1044.96
0
20
40
60
80
100
400
500
600
700
800
900
1000

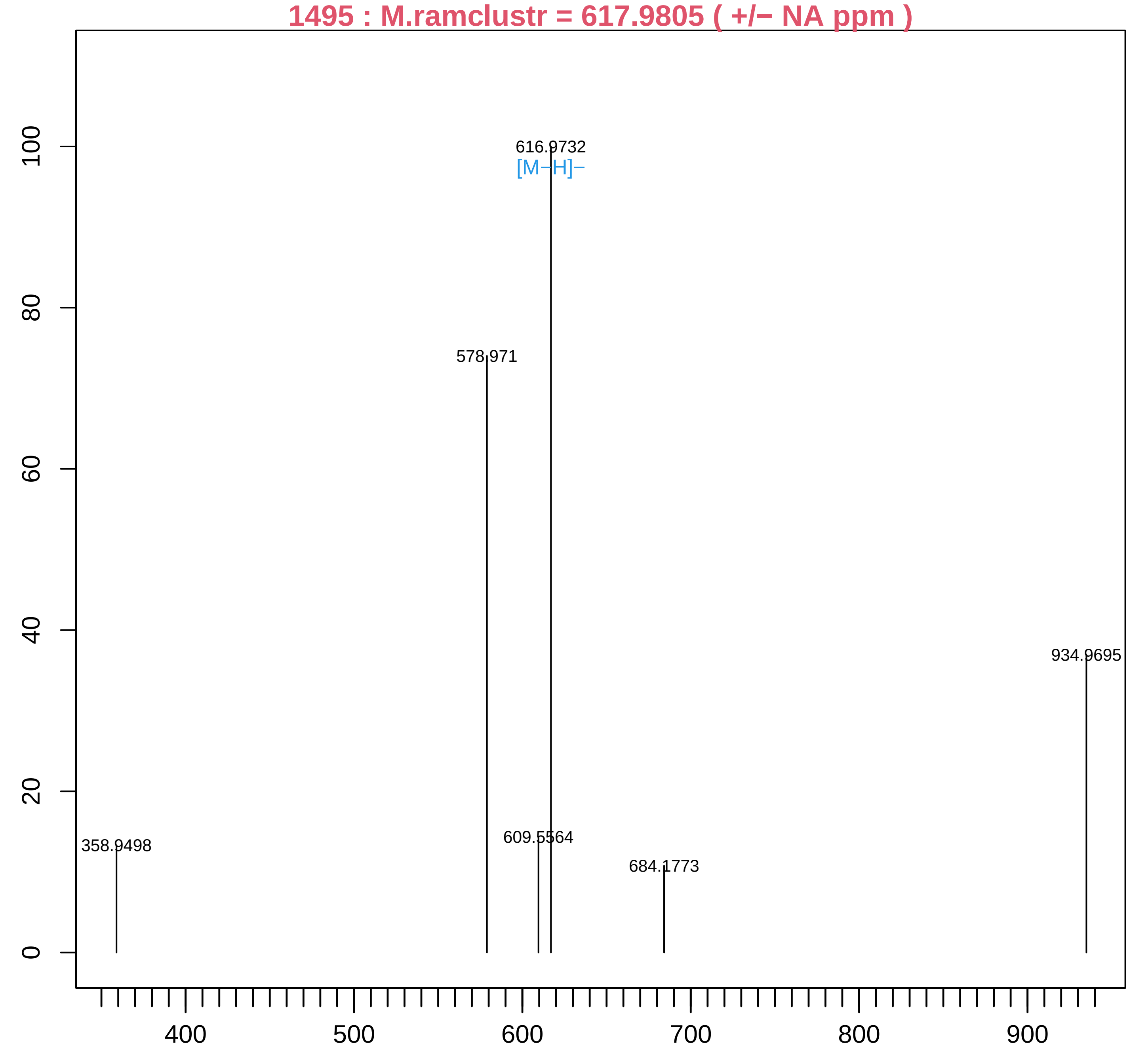
1495 : M.ramclustr = 617.9805 (+/− NA ppm)
616.9732
[M−H]−
578.971
934.9695
358.9498
609.5564
684.1773
0
20
40
60
80
100
400
500
600
700
800
900

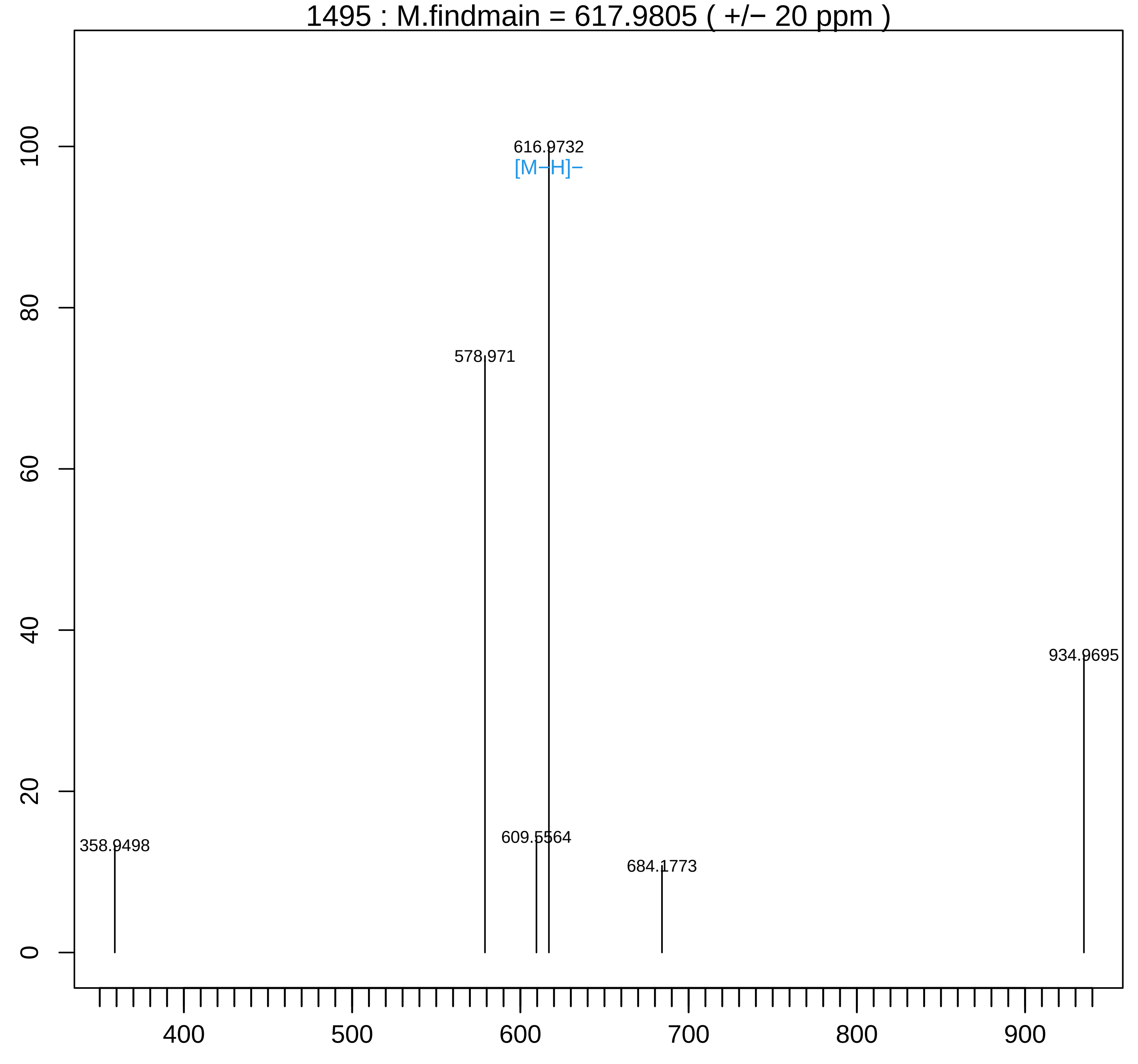
1495 : M.findmain = 617.9805 (+/− 20 ppm)
616.9732
[M−H]−
578.971
934.9695
358.9498
609.5564
684.1773
0
20
40
60
80
100
400
500
600
700
800
900

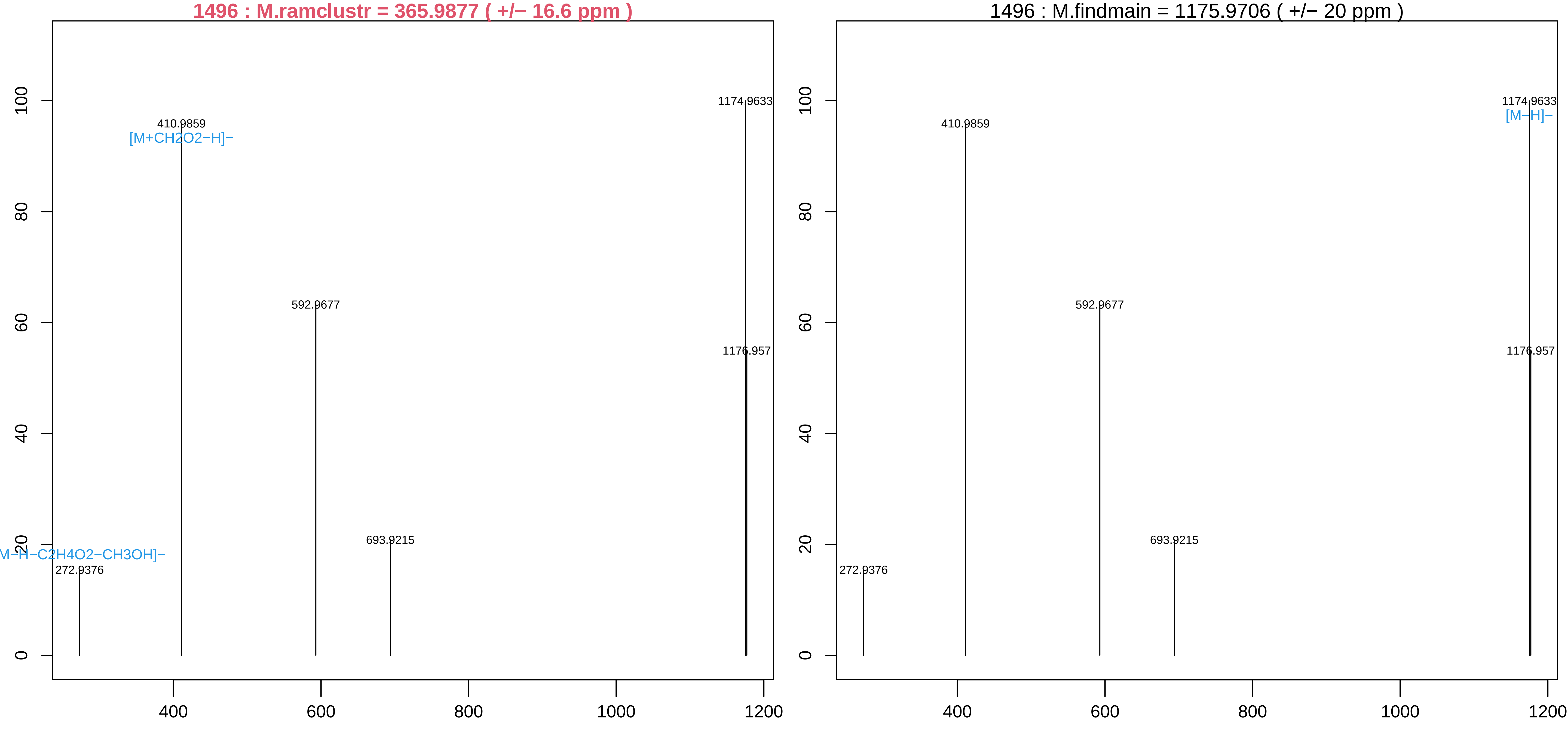
1496 : M.ramclustr = 365.9877 (+/− 16.6 ppm)
410.9859
[M+CH2O2−H]−
272.9376
M−H−C2H4O2−CH3OH]−
592.9677
693.9215
1174.9633
1176.957
1496 : M.findmain = 1175.9706 (+/− 20 ppm)
410.9859
272.9376
592.9677
693.9215
1174.9633
[M−H]−
1176.957
0
20
40
60
80
100
400
600
800
1000
1200

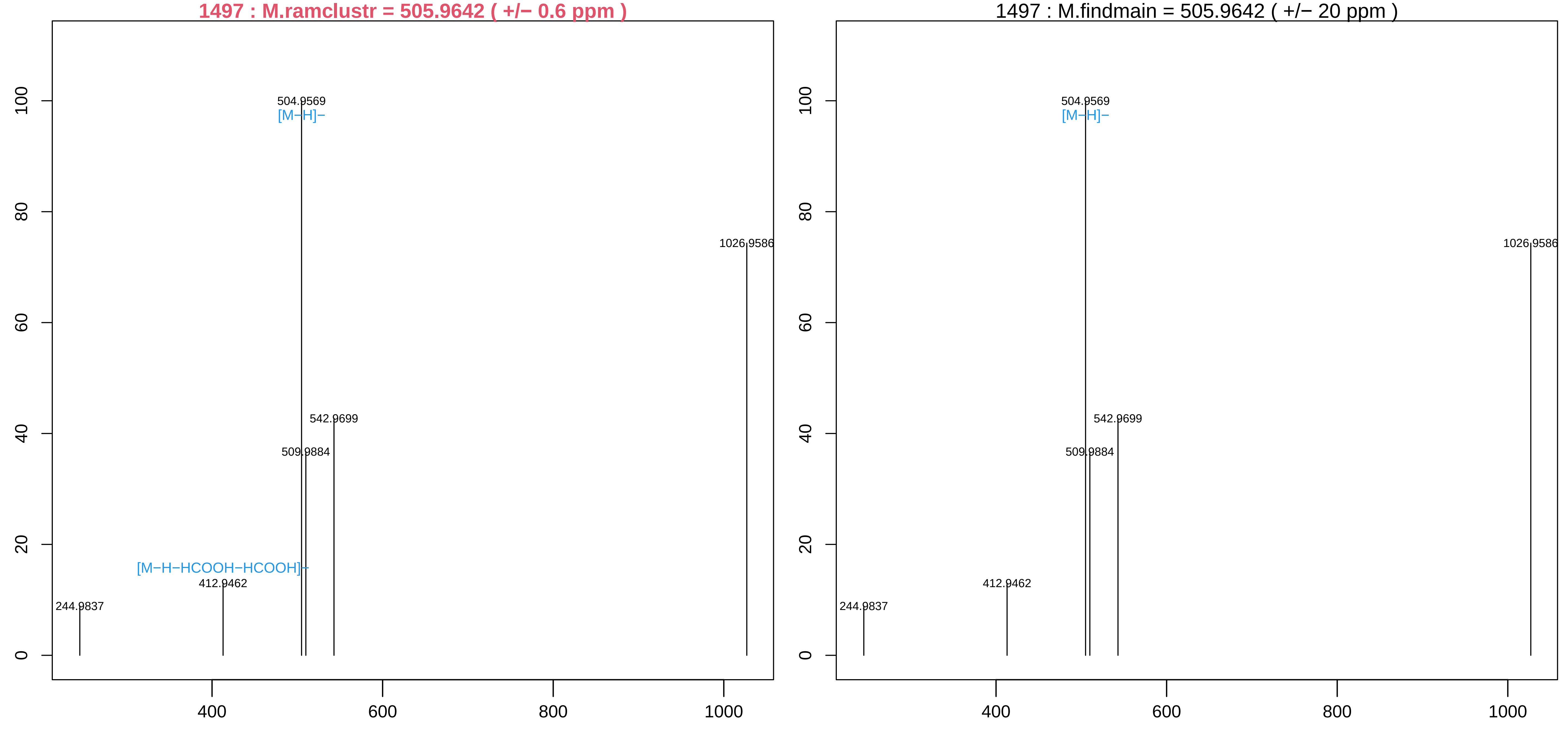

1497 : M.ramclustr = 505.9642 (+/− 0.6 ppm)
504.9569
[M−H]−
1026.9586
542.9699
509.9884
[M−H−HCOOH−HCOOH]−
412.9462
244.9837
0
20
40
60
80
100
400
600
800
1000
1497 : M.findmain = 505.9642 (+/− 20 ppm)
504.9569
[M−H]−
1026.9586
542.9699
509.9884
412.9462
244.9837
0
20
40
60
80
100
400
600
800
1000

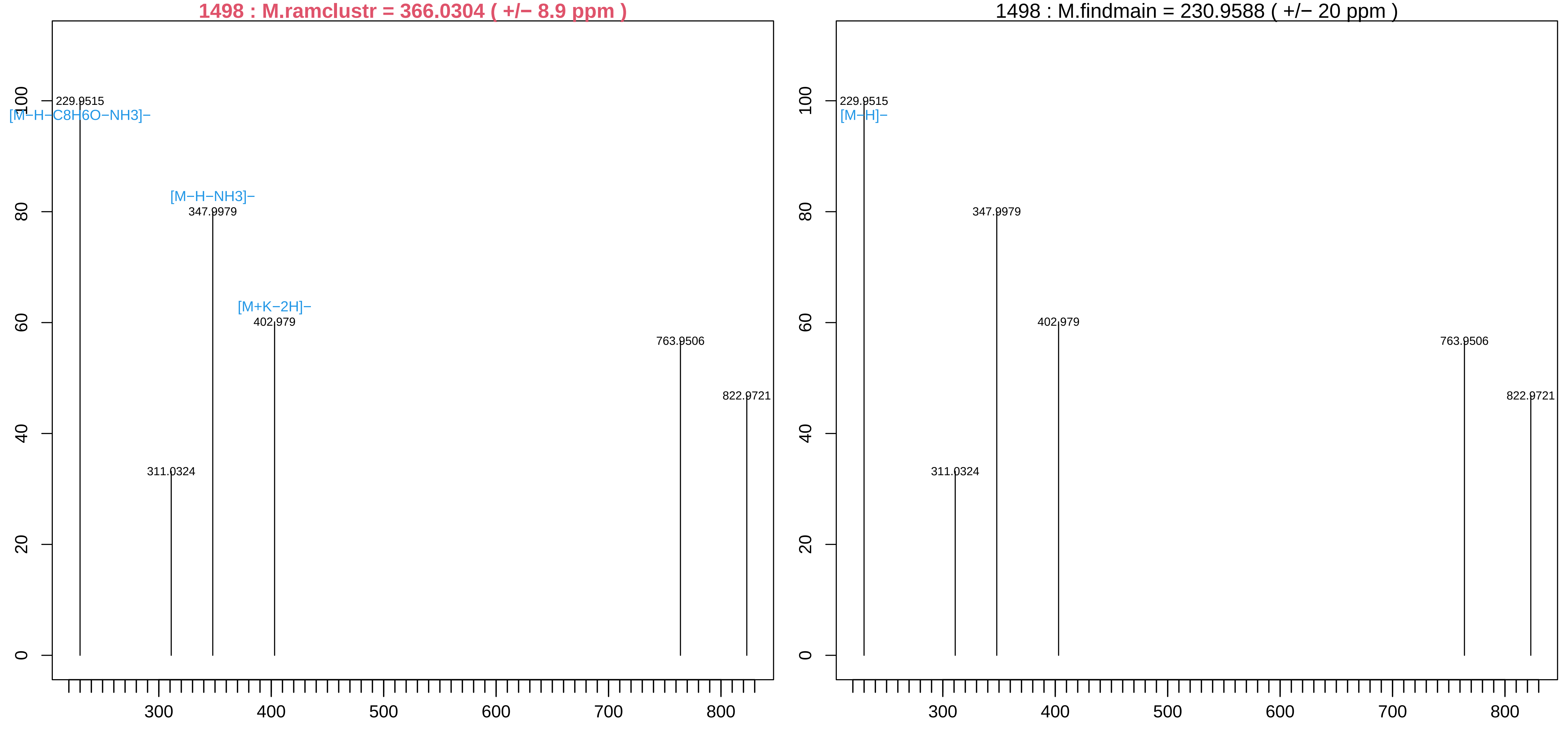
1498 : M.ramclustr = 366.0304 (+/− 8.9 ppm)
229.9515
[M−H−C8H6O−NH3]−
[M−H−NH3]−
347.9979
[M+K−2H]−
402.979
311.0324
763.9506
822.9721
1498 : M.findmain = 230.9588 (+/− 20 ppm)
229.9515
[M−H]−
347.9979
402.979
311.0324
763.9506
822.9721
0
20
40
60
80
100
300
400
500
600
700
800

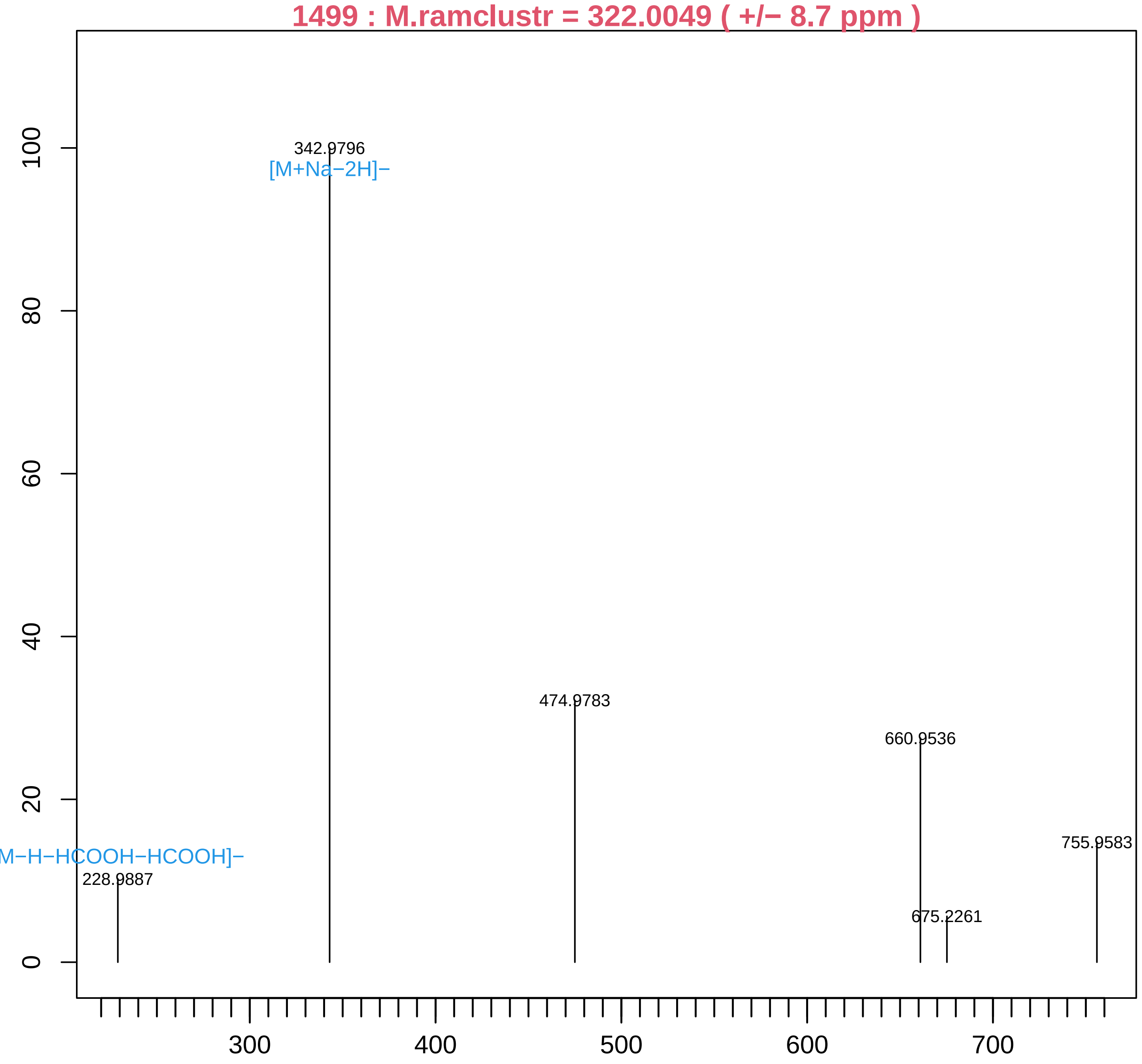
1499 : M.ramclustr = 322.0049 (+/− 8.7 ppm)
342.9796
[M+Na−2H]−
474.9783
660.9536
755.9583
M−H−HCOOH−HCOOH]−
228.9887
675.2261
0
20
40
60
80
100
300
400
500
600
700

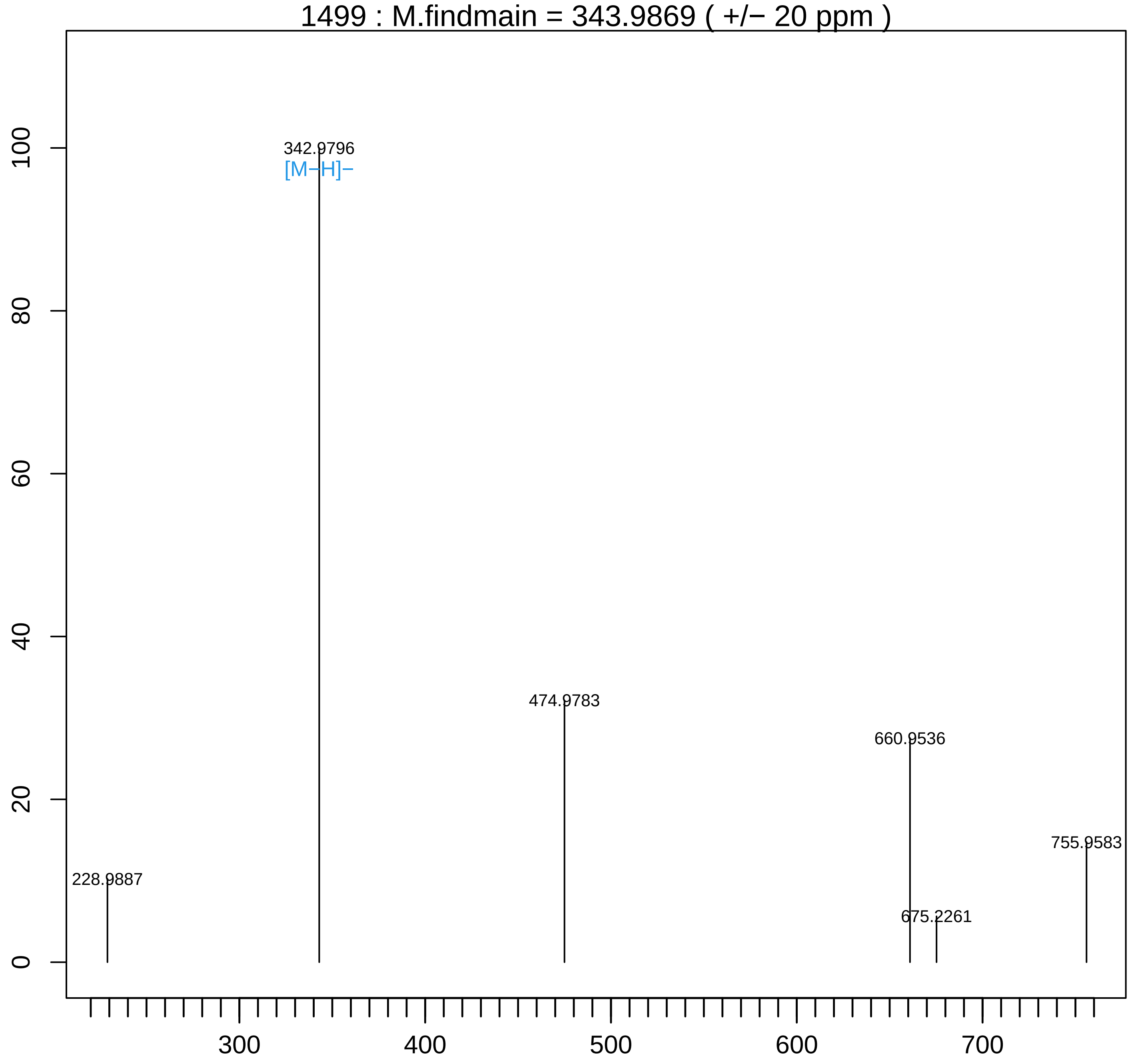
1499 : M.findmain = 343.9869 (+/− 20 ppm)
342.9796
[M−H]−
474.9783
660.9536
755.9583
228.9887
675.2261
0
20
40
60
80
100
300
400
500
600
700

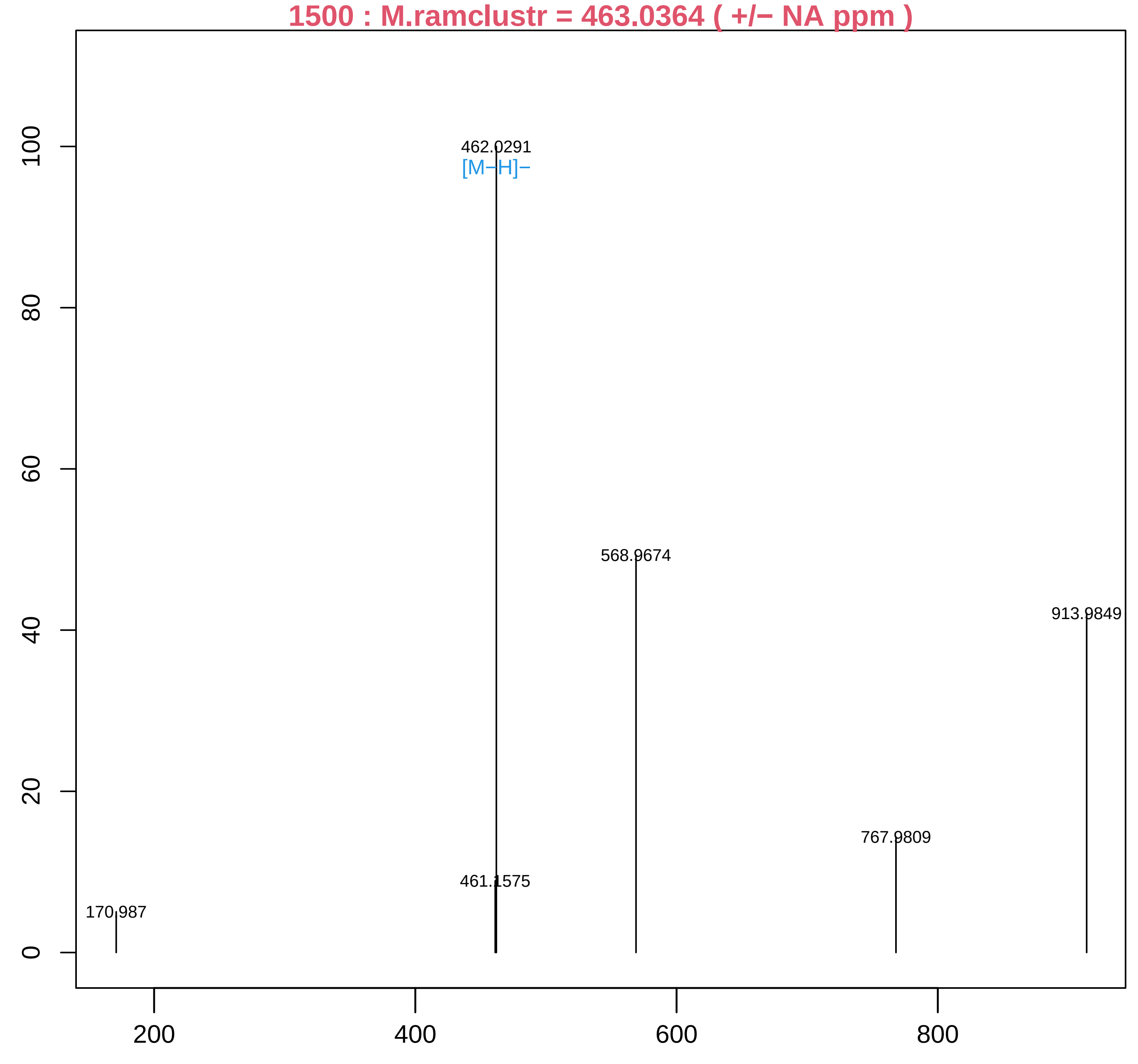
1500 : M.ramclustr = 463.0364 (+/− NA ppm)
462.0291
[M−H]−
568.9674
913.9849
767.9809
461.1575
170.987
0
20
40
60
80
100
200
400
600
800

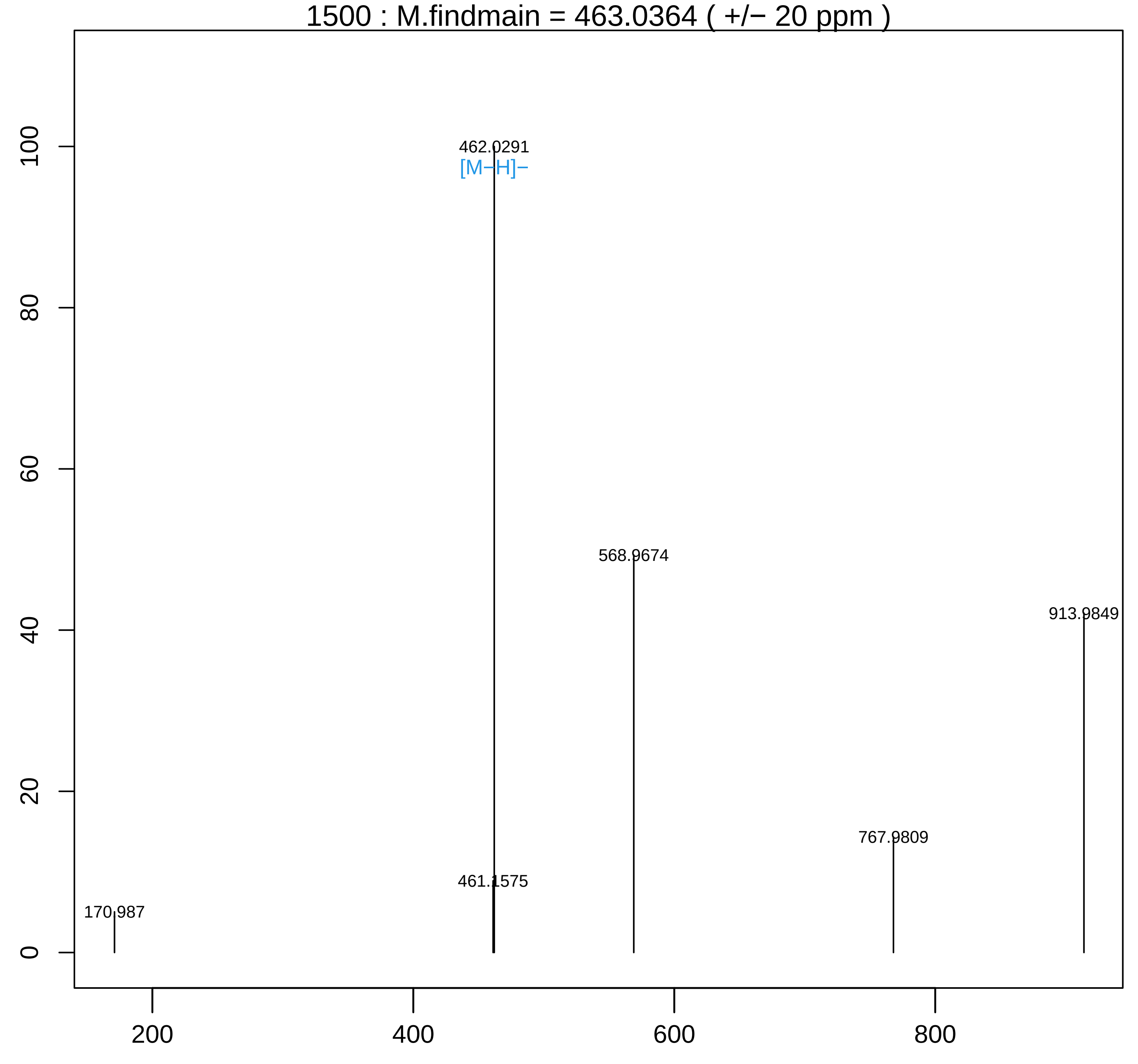
1500 : M.findmain = 463.0364 (+/− 20 ppm)
462.0291
[M−H]−
568.9674
913.9849
767.9809
461.1575
170.987
0
20
40
60
80
100
200
400
600
800

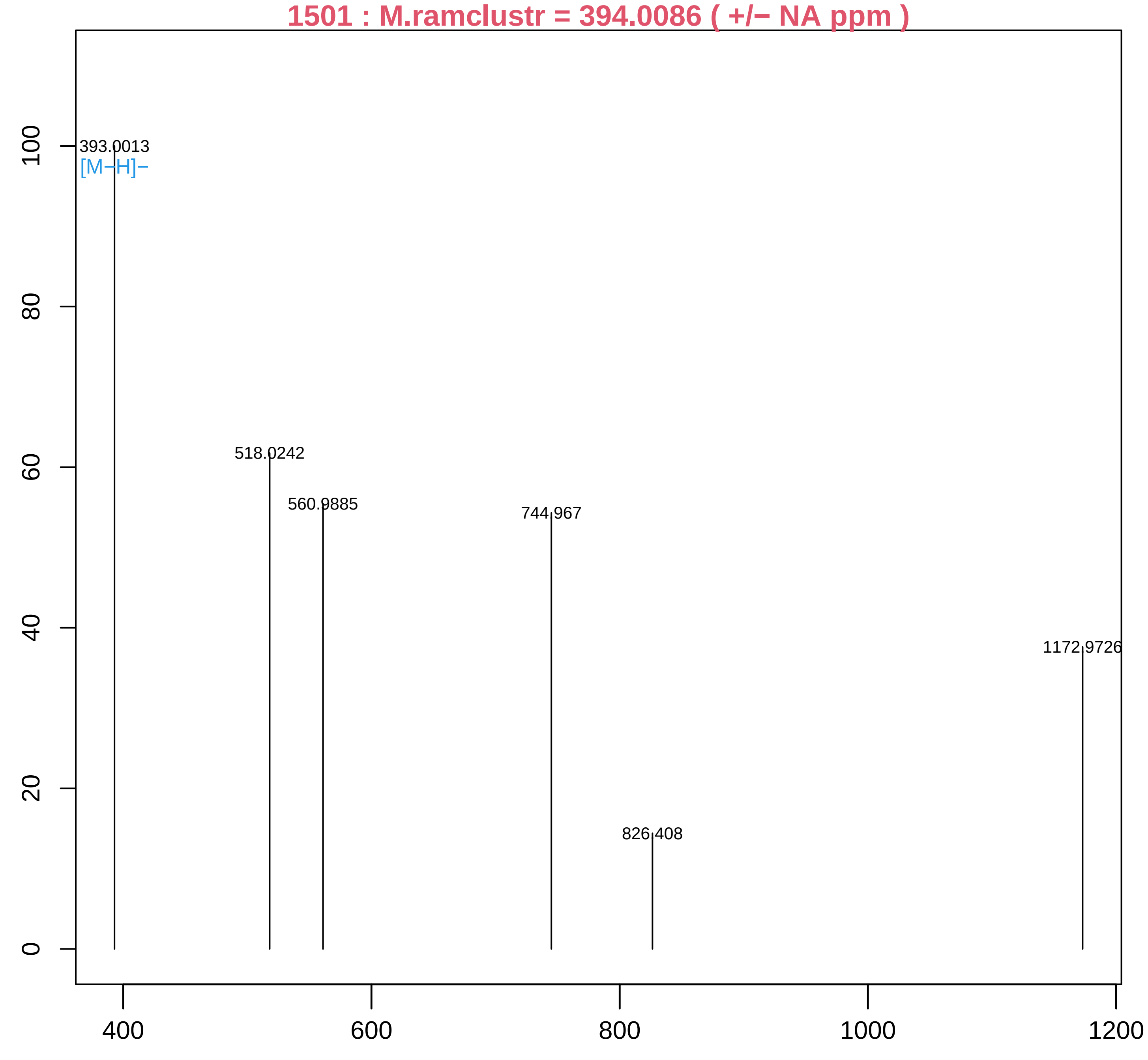

1501 : M.ramclustr = 394.0086 (+/− NA ppm)
393.0013
[M−H]−
518.0242
560.9885
744.967
826.408
1172.9726
0
20
40
60
80
100
400
600
800
1000
1200

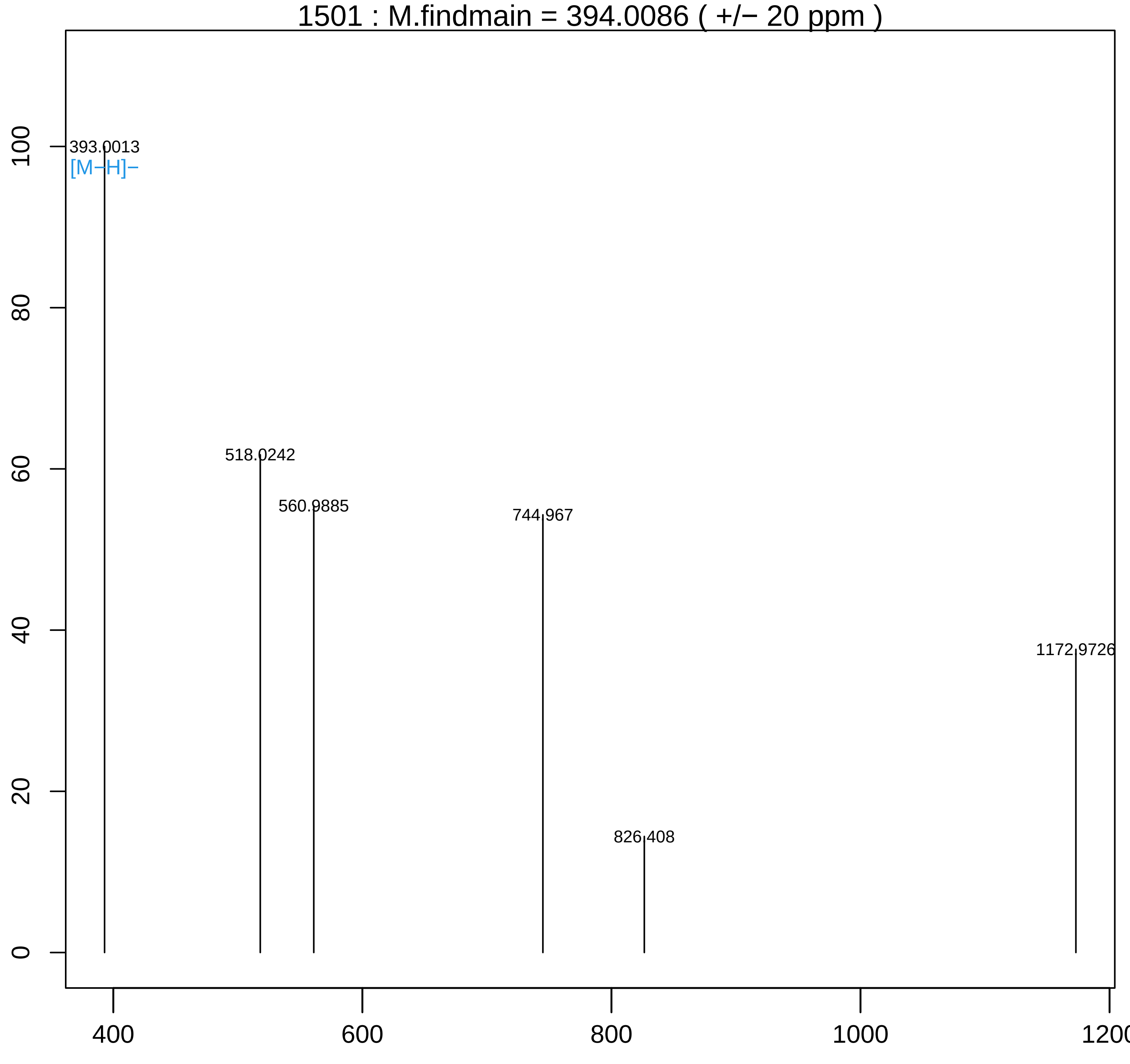

1501 : M.findmain = 394.0086 (+/− 20 ppm)
393.0013
[M−H]−
518.0242
560.9885
744.967
826.408
1172.9726
0
20
40
60
80
100
400
600
800
1000
1200

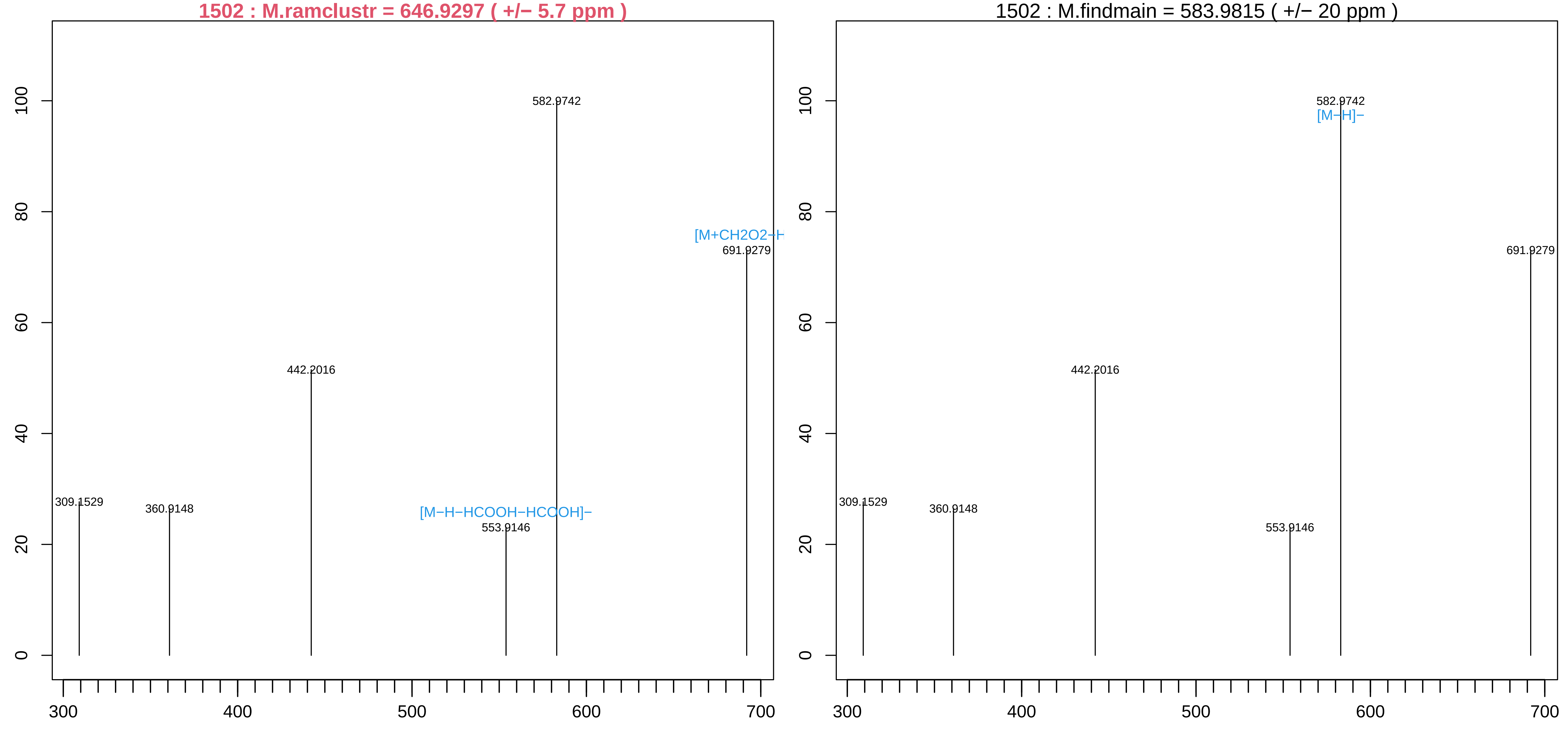

1502 : M.ramclustr = 646.9297 (+/− 5.7 ppm)
582.9742
[M+CH2O2−H
691.9279
442.2016
309.1529
360.9148
[M−H−HCOOH−HCOOH]−
553.9146
1502 : M.findmain = 583.9815 (+/− 20 ppm)
582.9742
[M−H]−
691.9279
442.2016
309.1529
360.9148
553.9146
0
20
40
60
80
100
300
400
500
600
700

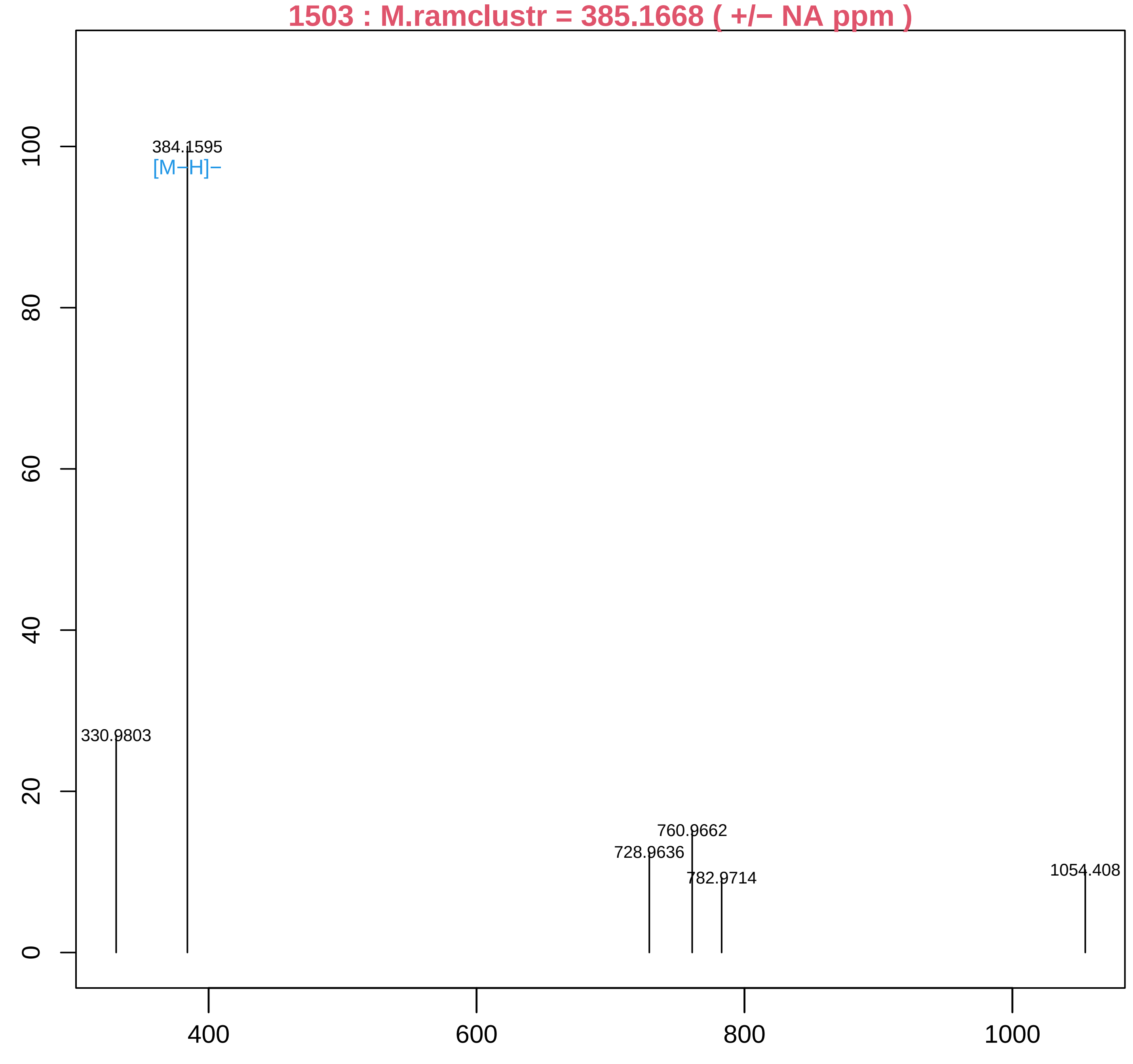
1503 : M.ramclustr = 385.1668 (+/− NA ppm)
384.1595
[M−H]−
330.9803
760.9662
728.9636
782.9714
1054.408
0
20
40
60
80
100
400
600
800
1000

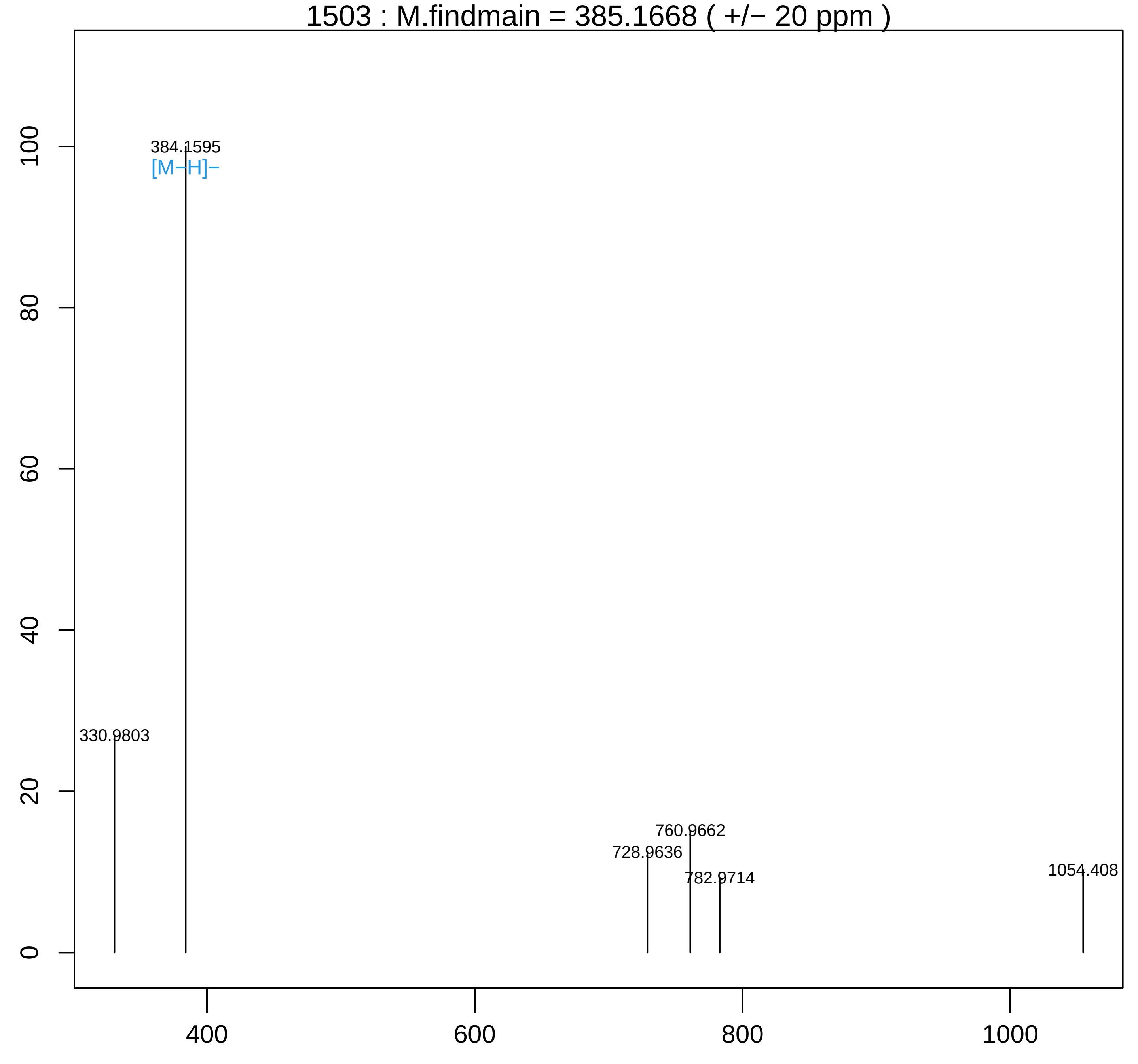
1503 : M.findmain = 385.1668 (+/− 20 ppm)
384.1595
[M−H]−
330.9803
760.9662
728.9636
782.9714
1054.408
0
20
40
60
80
100
400
600
800
1000

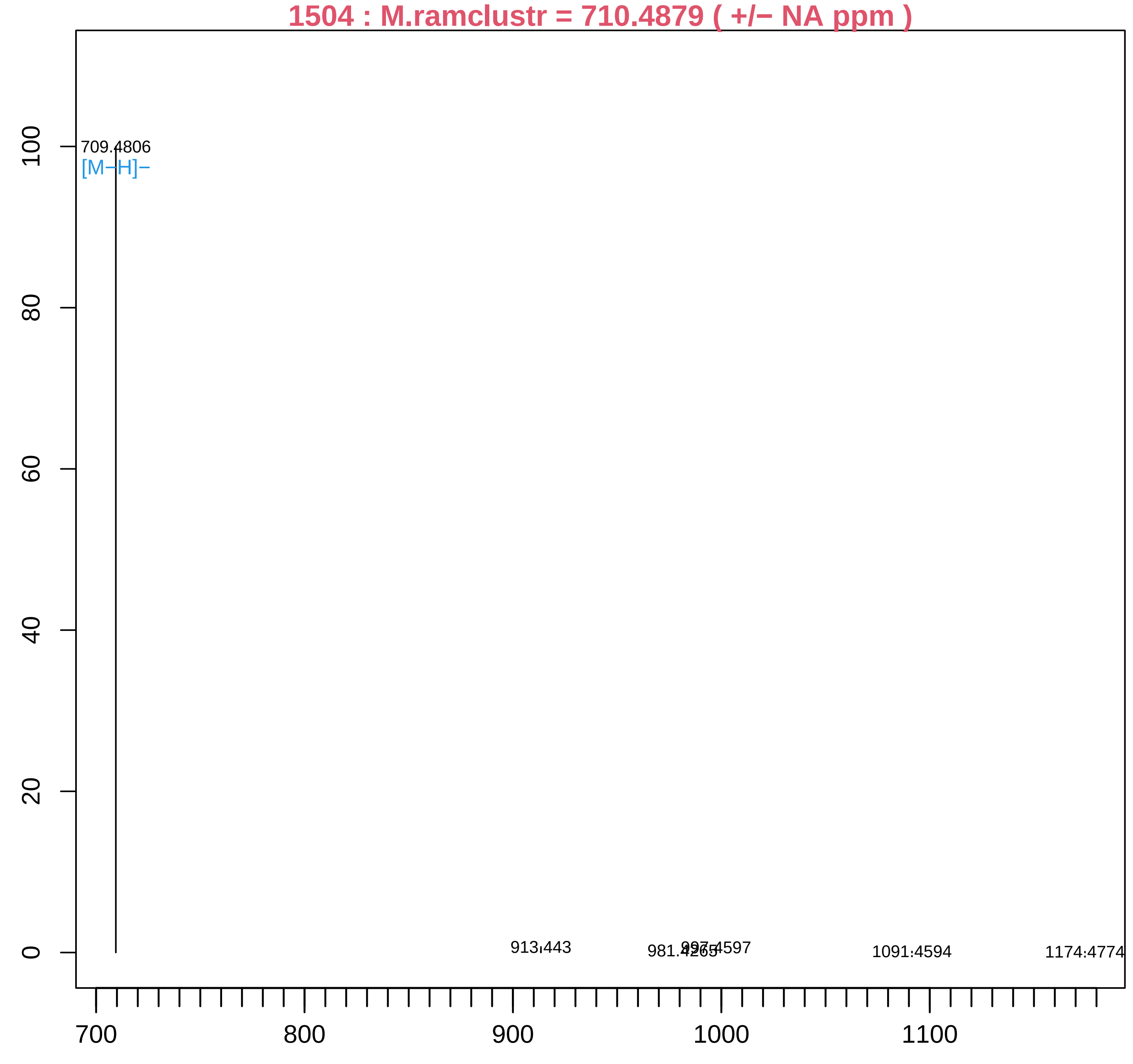

1504 : M.ramclustr = 710.4879 (+/− NA ppm)
709.4806
[M−H]−
913.443
981.4265
997.4597
1091.4594
1174.4774
0
20
40
60
80
100
700
800
900
1000
1100

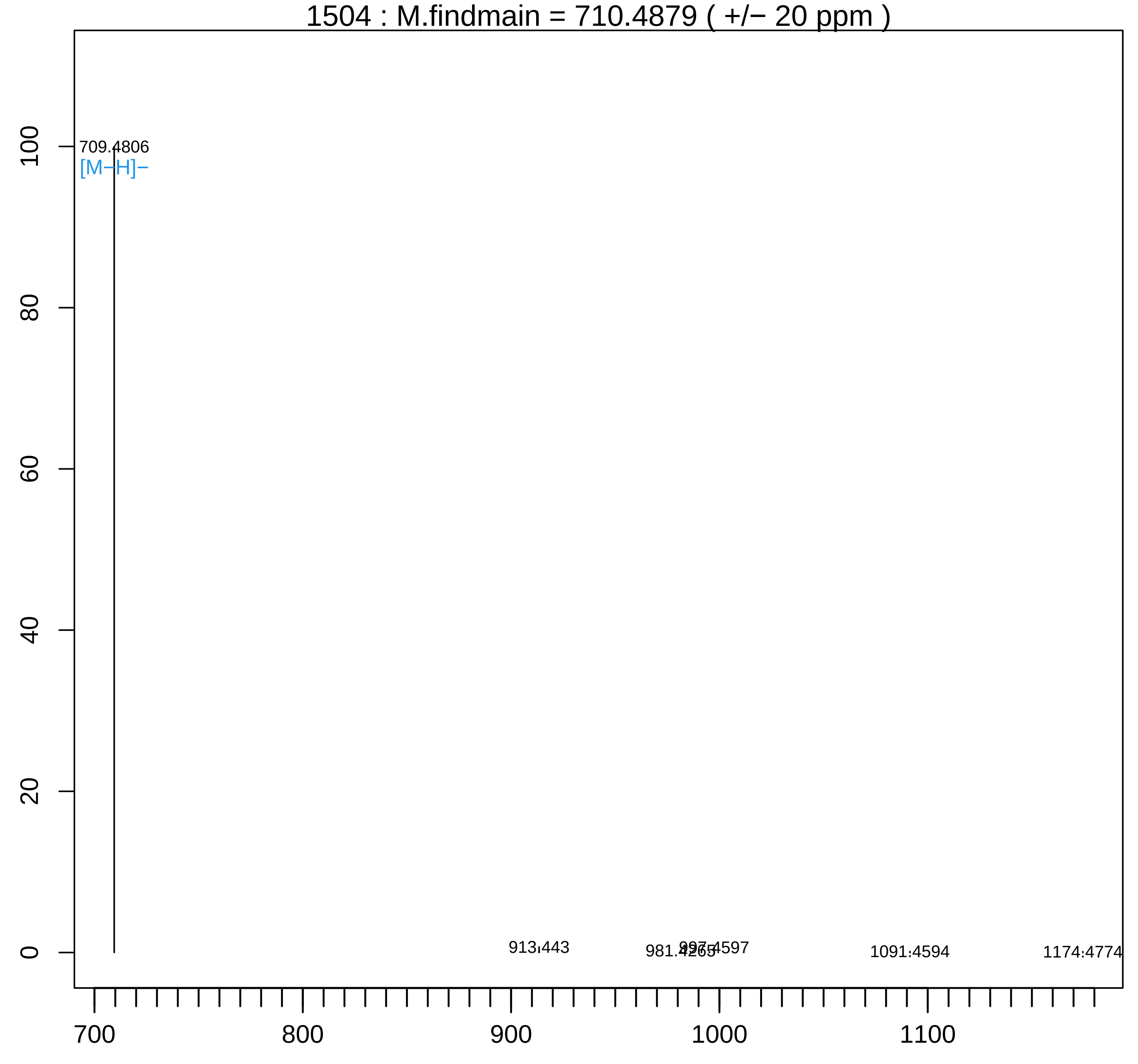

1504 : M.findmain = 710.4879 (+/− 20 ppm)
709.4806
[M−H]−
913.443
981.4265
997.4597
1091.4594
1174.4774
0
20
40
60
80
100
700
800
900
1000
1100

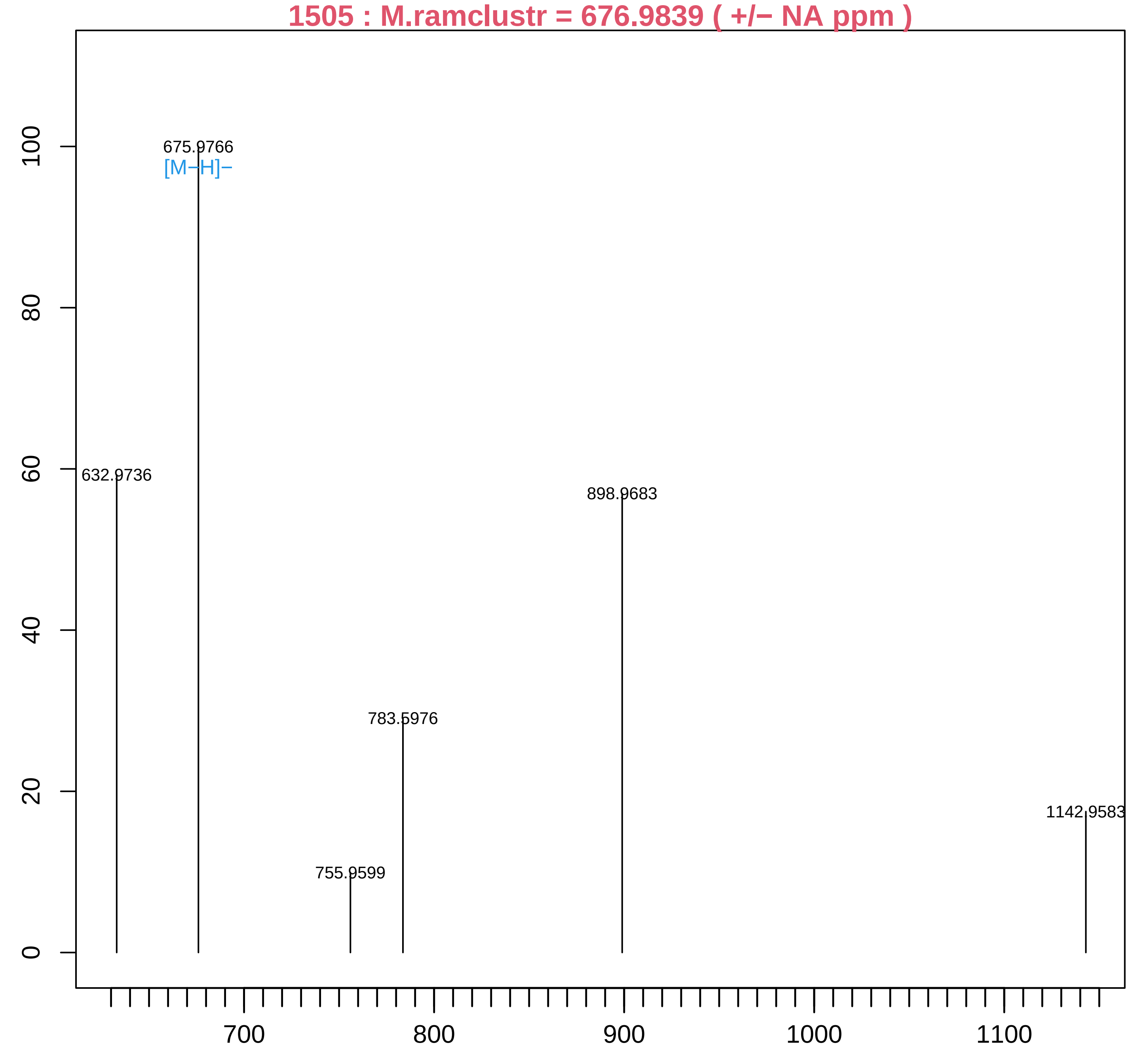
1505 : M.ramclustr = 676.9839 (+/− NA ppm)
675.9766
[M−H]−
632.9736
898.9683
783.5976
755.9599
1142.9583
0
20
40
60
80
100
700
800
900
1000
1100

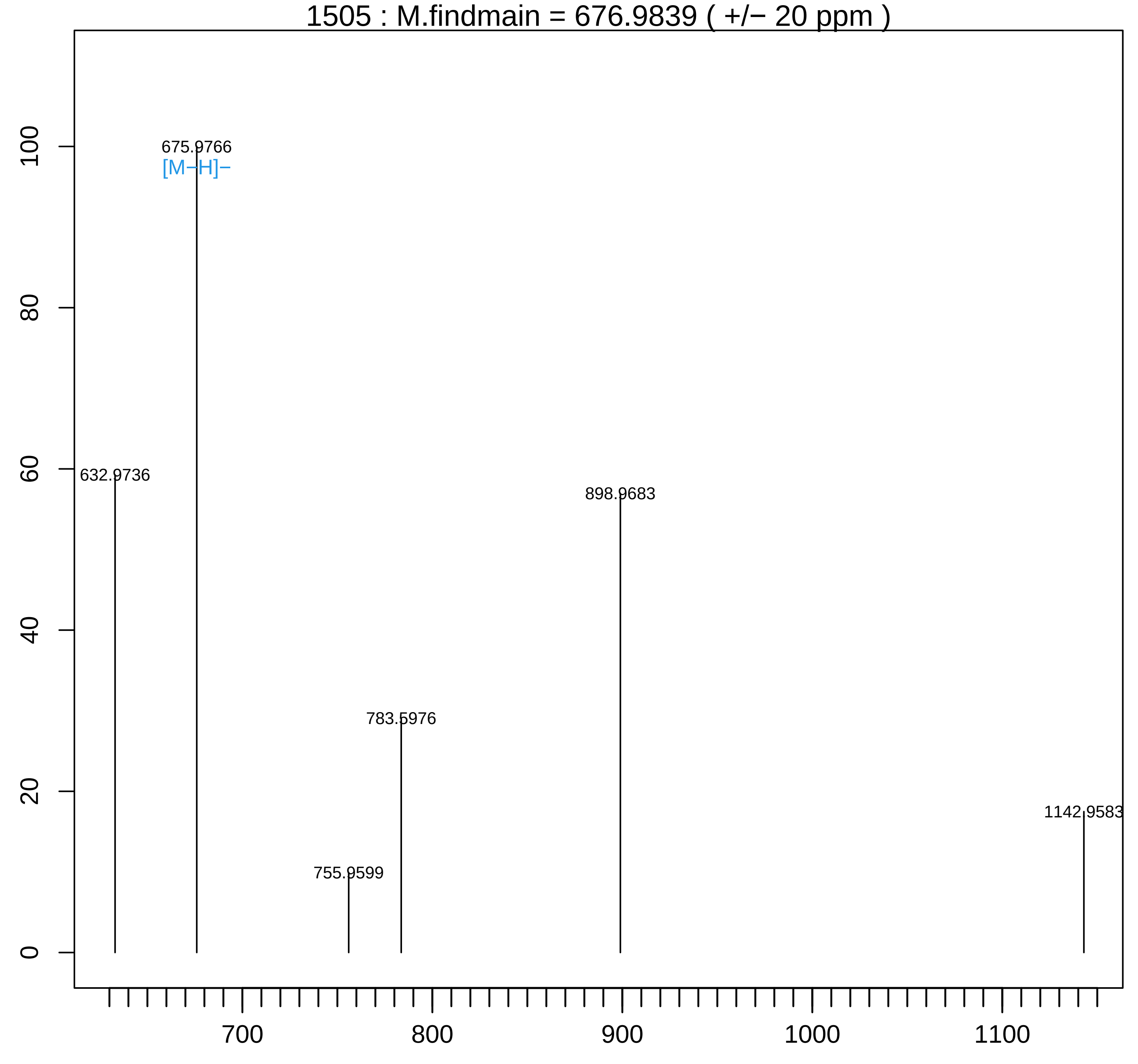
1505 : M.findmain = 676.9839 (+/− 20 ppm)
675.9766
[M−H]−
632.9736
898.9683
783.5976
755.9599
1142.9583
0
20
40
60
80
100
700
800
900
1000
1100

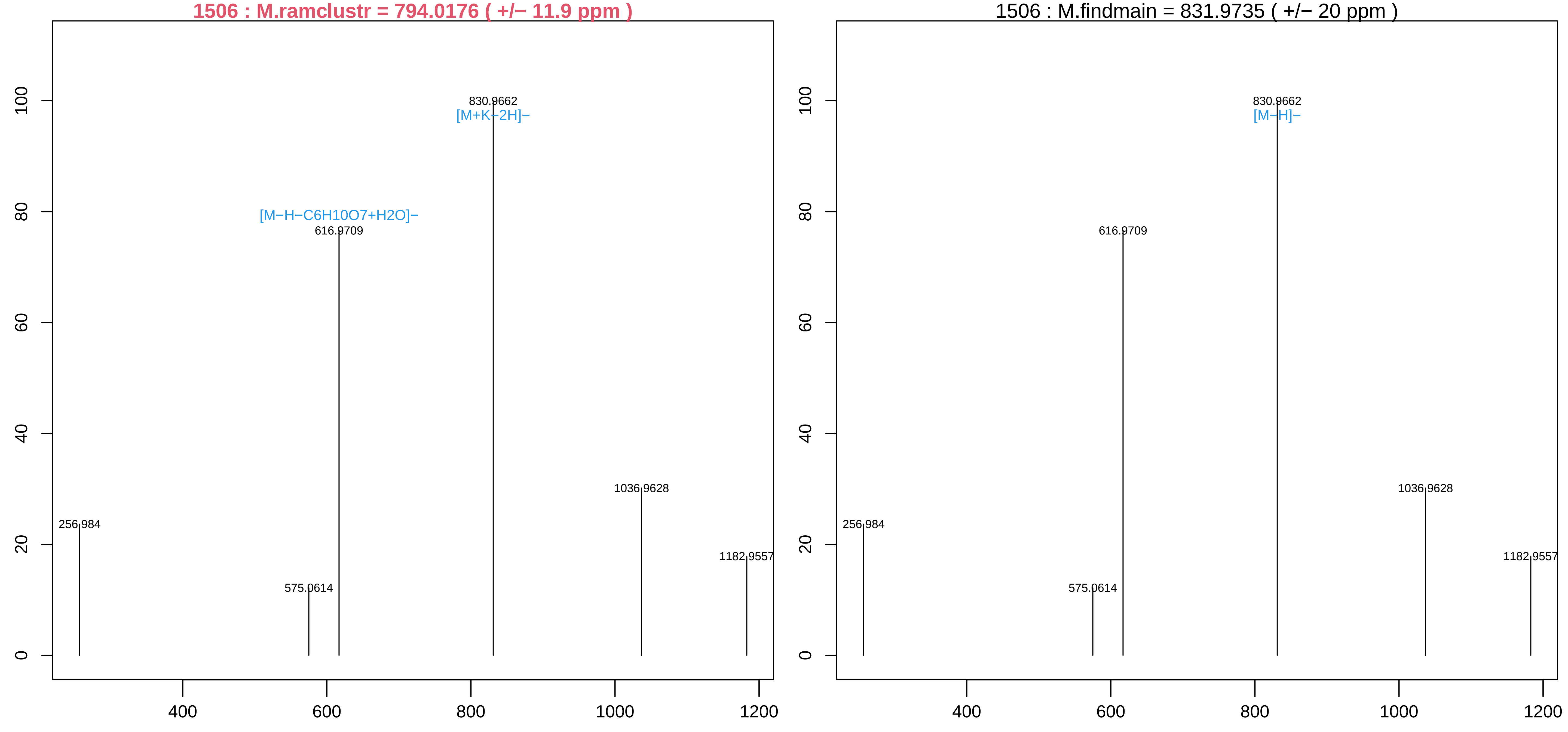
1506 : M.ramclustr = 794.0176 (+/− 11.9 ppm)
830.9662
[M+K−2H]−
[M−H−C6H10O7+H2O]−
616.9709
1036.9628
256.984
1182.9557
575.0614
1506 : M.findmain = 831.9735 (+/− 20 ppm)
830.9662
[M−H]−
616.9709
1036.9628
256.984
1182.9557
575.0614
0
20
40
60
80
100
400
600
800
1000
1200

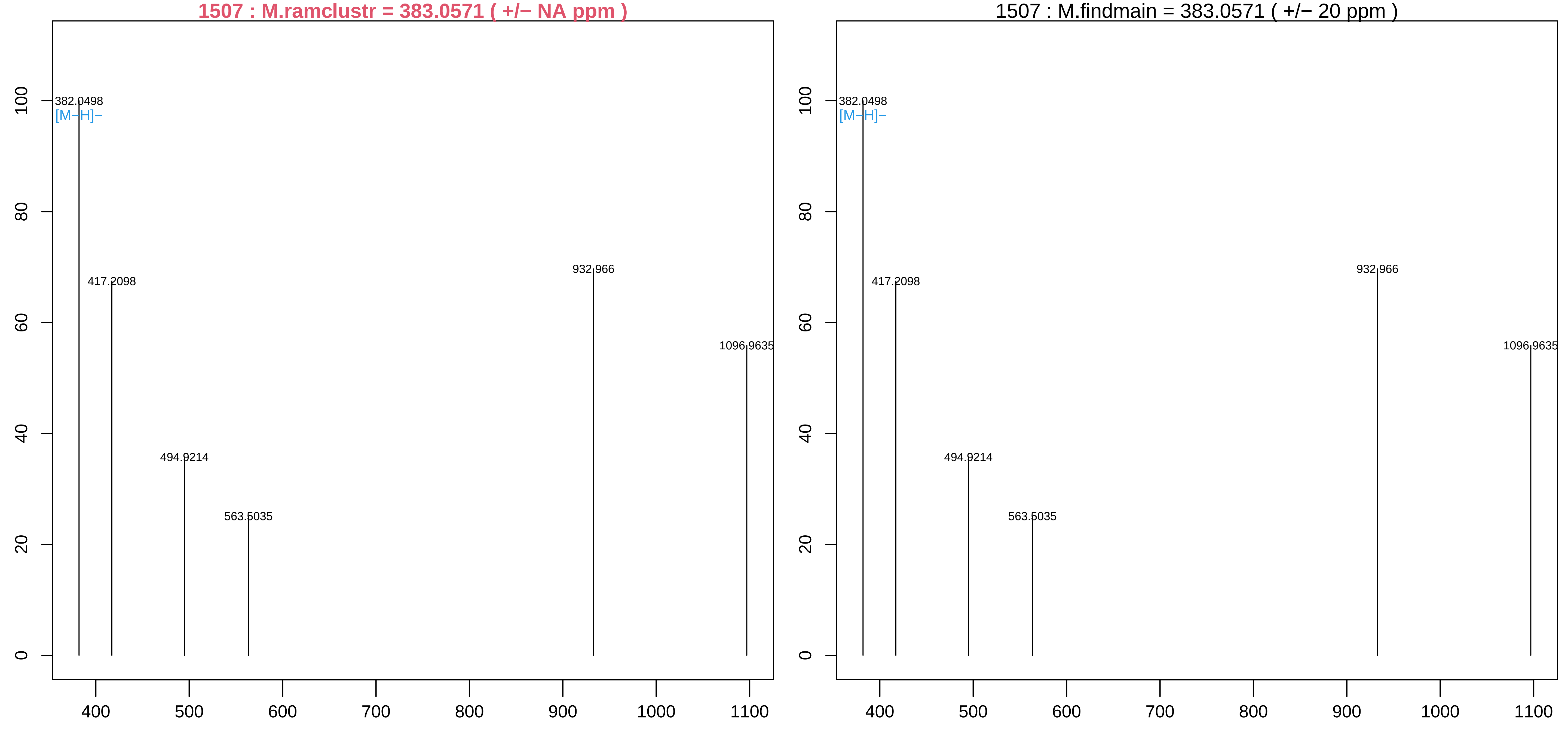

1507 : M.ramclustr = 383.0571 (+/− NA ppm)
382.0498
[M−H]−
417.2098
494.9214
563.5035
932.966
1096.9635
1507 : M.findmain = 383.0571 (+/− 20 ppm)
382.0498
[M−H]−
417.2098
494.9214
563.5035
932.966
1096.9635
0
20
40
60
80
100
400
500
600
700
800
900
1000
1100

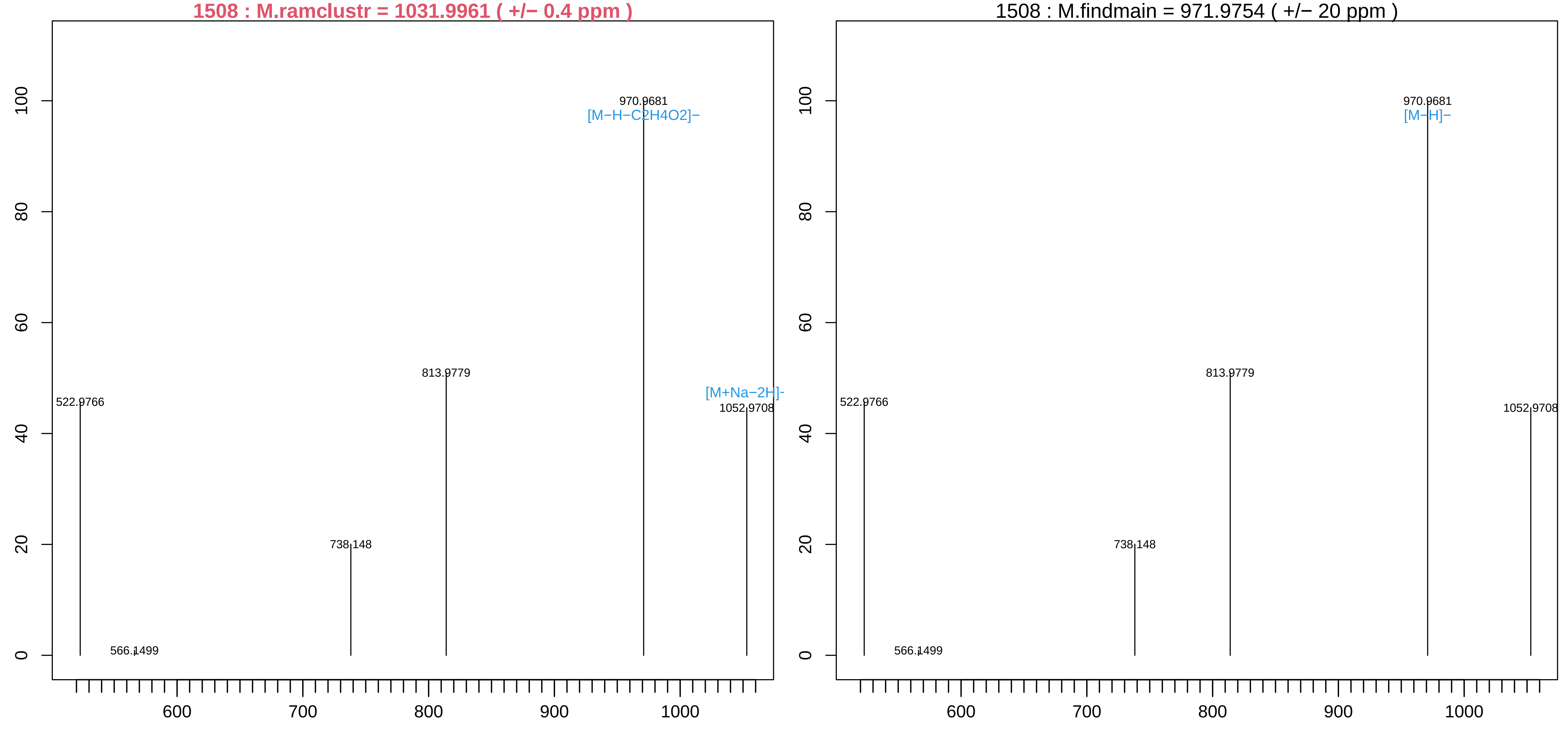
1508 : M.ramclustr = 1031.9961 (+/− 0.4 ppm)
970.9681
[M−H−C2H4O2]−
813.9779
[M+Na−2H]-
522.9766
1052.9708
738.148
566.1499
1508 : M.findmain = 971.9754 (+/− 20 ppm)
970.9681
[M−H]−
813.9779
522.9766
1052.9708
738.148
566.1499
0
20
40
60
80
100
600
700
800
900
1000

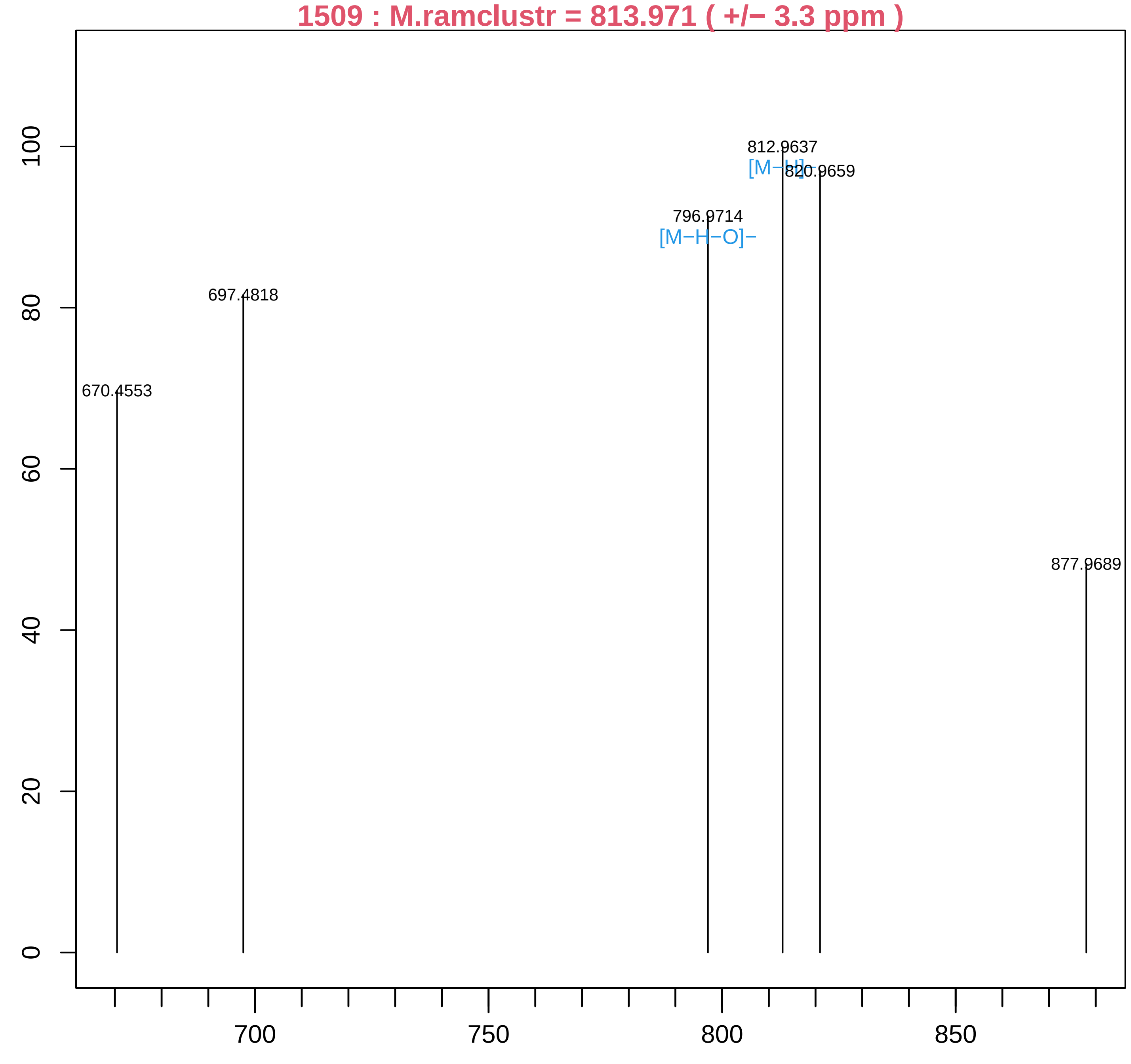
1509 : M.ramclustr = 813.971 (+/− 3.3 ppm)
670.4553
697.4818
796.9714
[M−H−O]−
812.9637
[M−H]−
820.9659
877.9689
0
20
40
60
80
100
700
750
800
850

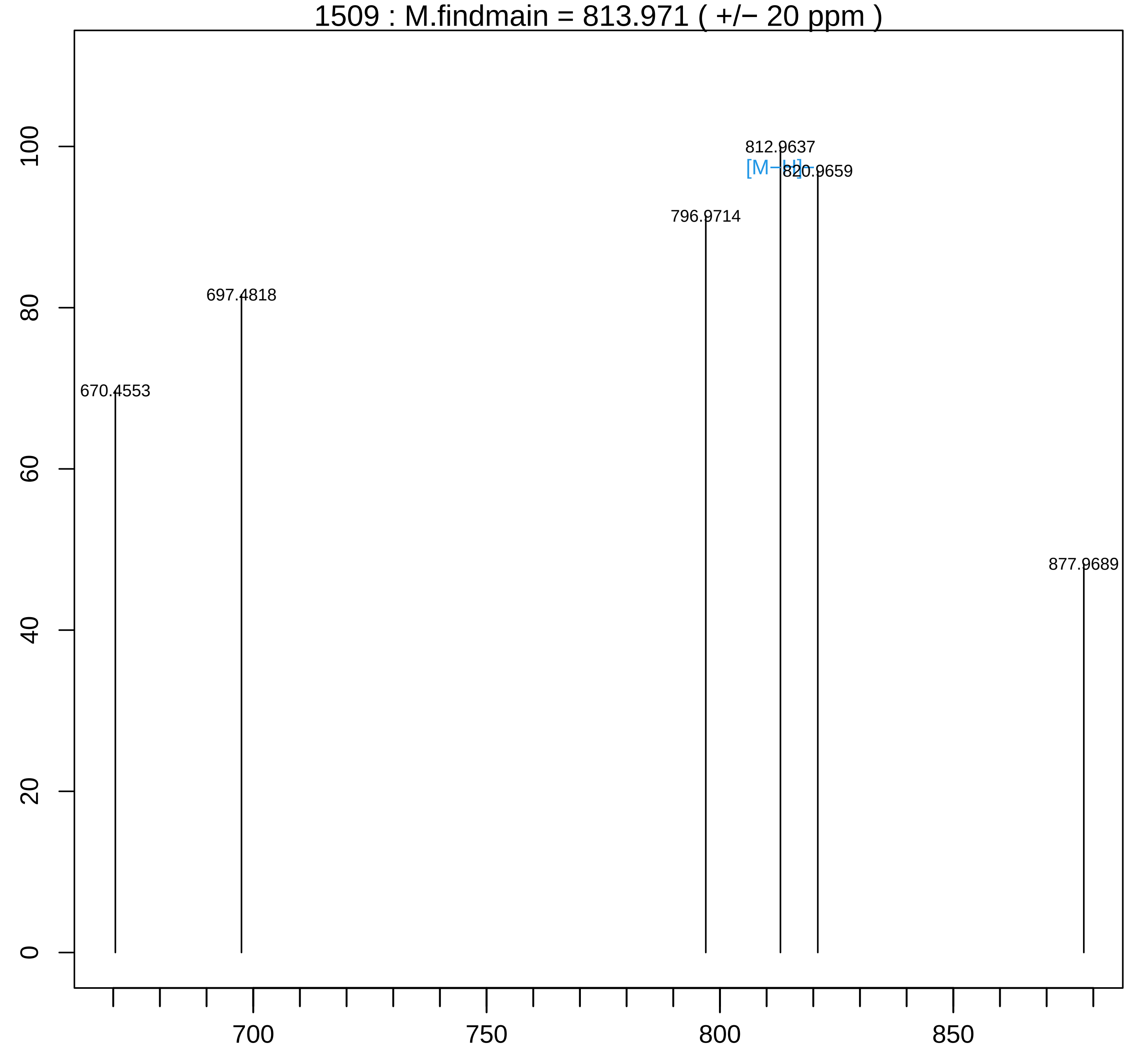
1509 : M.findmain = 813.971 (+/− 20 ppm)
670.4553
697.4818
796.9714
812.9637
[M−H]−
820.9659
877.9689
0
20
40
60
80
100
700
750
800
850

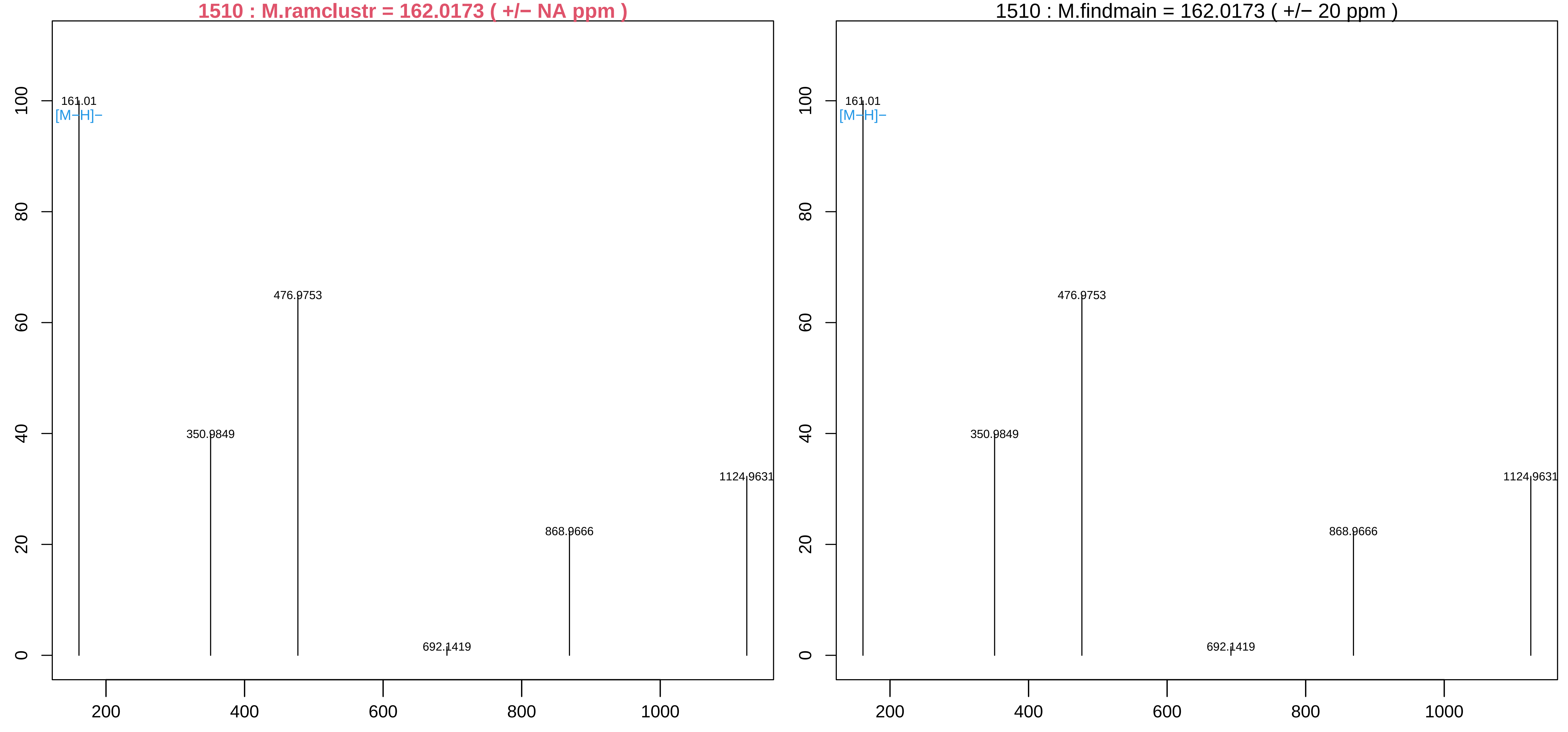
1510 : M.ramclustr = 162.0173 (+/− NA ppm)
1510 : M.findmain = 162.0173 (+/− 20 ppm)
161.01
[M−H]−
350.9849
476.9753
692.1419
868.9666
1124.9631
0
20
40
60
80
100
200
400
600
800
1000

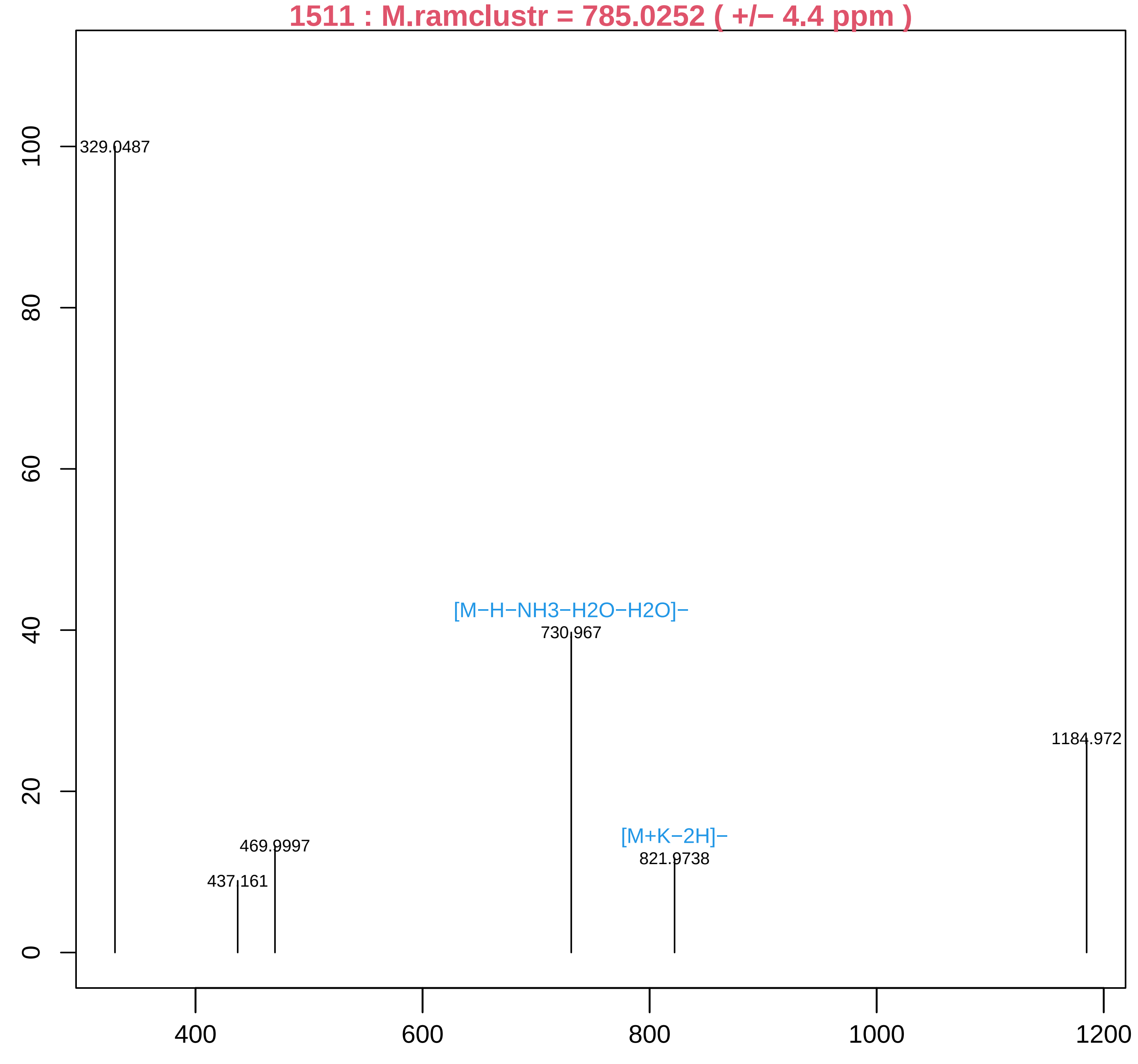
1511 : M.ramclustr = 785.0252 (+/− 4.4 ppm)
329.0487
437.161
469.9997
[M−H−NH3−H2O−H2O]−
730.967
[M+K−2H]−
821.9738
1184.972
0
20
40
60
80
100
400
600
800
1000
1200

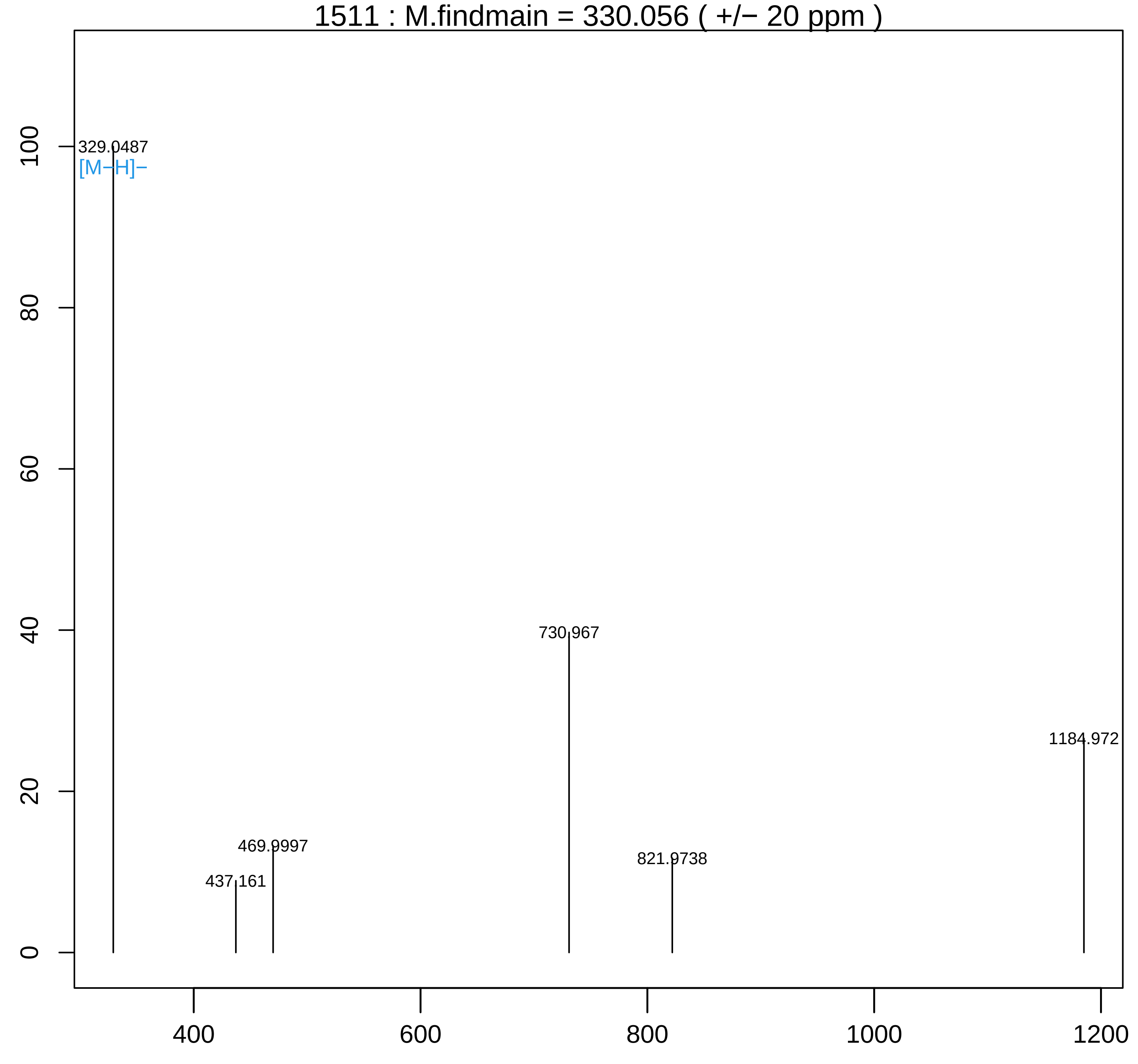
1511 : M.findmain = 330.056 (+/− 20 ppm)
329.0487
[M−H]−
437.161
469.9997
730.967
821.9738
1184.972
0
20
40
60
80
100
400
600
800
1000
1200

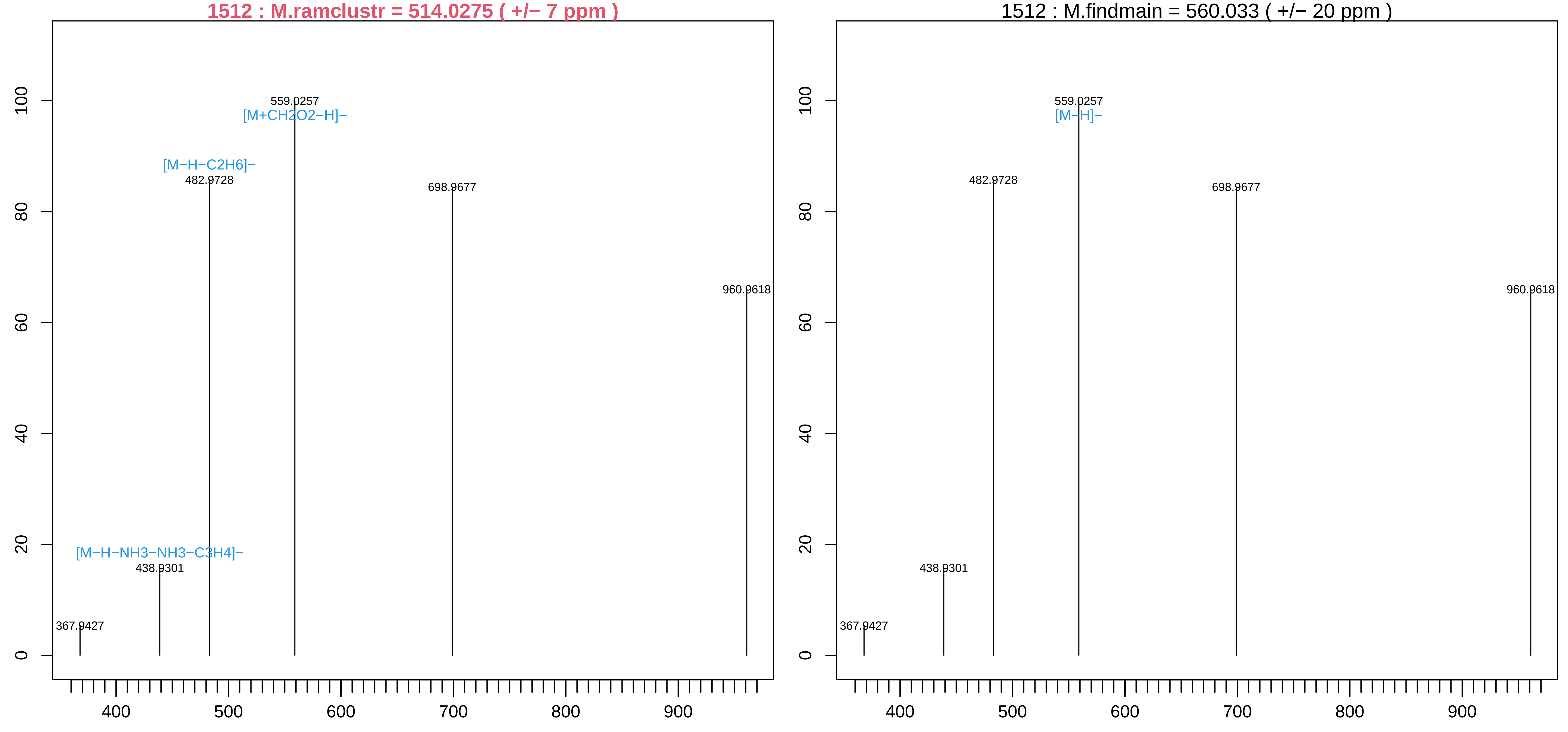

1512 : M.ramclustr = 514.0275 (+/− 7 ppm)
559.0257
[M+CH2O2−H]−
[M−H−C2H6]−
482.9728
698.9677
960.9618
[M−H−NH3−NH3−C3H4]−
438.9301
367.9427
0
20
40
60
80
100
400
500
600
700
800
900
1512 : M.findmain = 560.033 (+/− 20 ppm)
559.0257
[M−H]−
482.9728
698.9677
960.9618
438.9301
367.9427

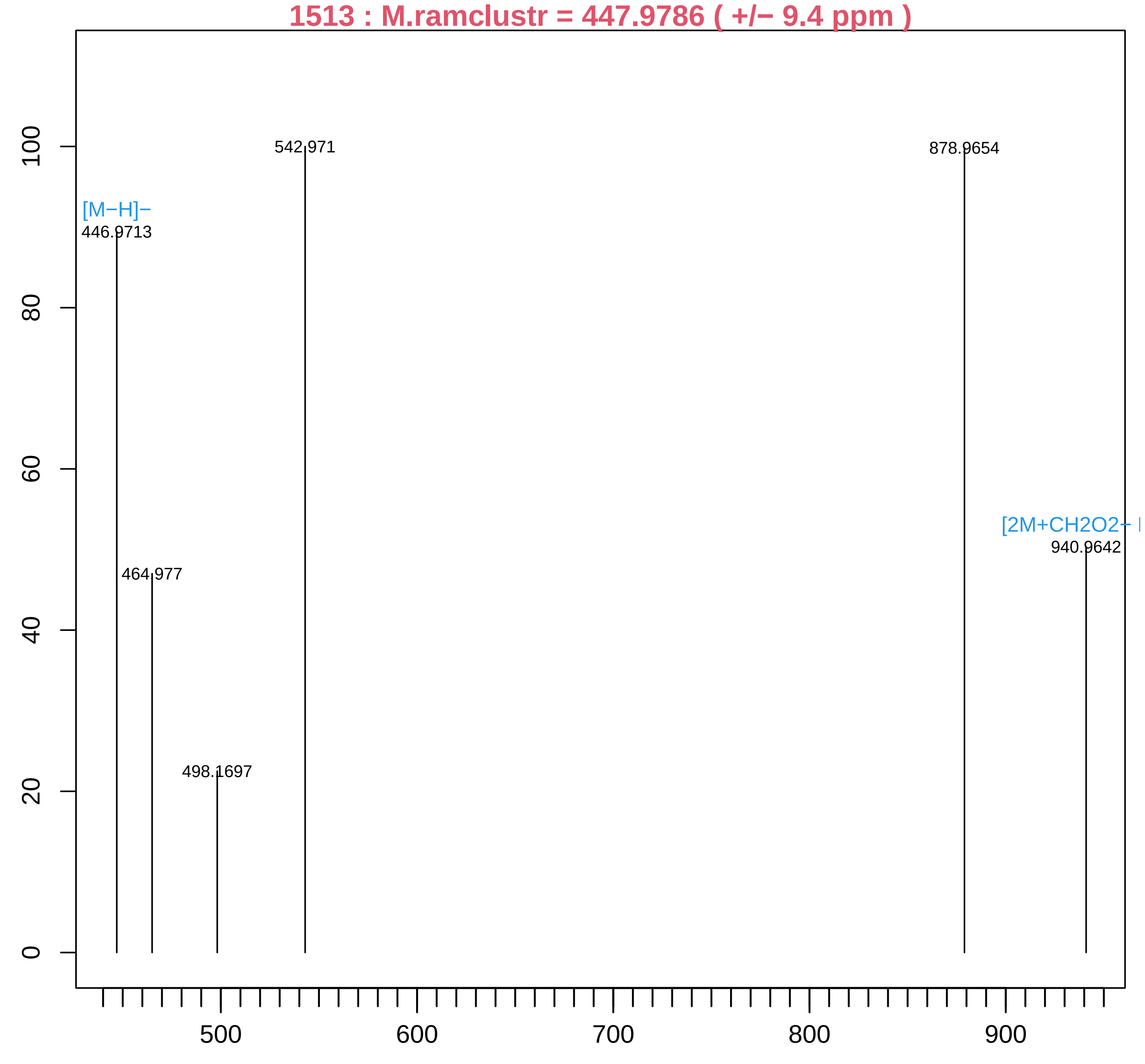
1513 : M.ramclustr = 447.9786 (+/− 9.4 ppm)
[M−H]−
446.9713
464.977
498.1697
542.971
878.9654
[2M+CH2O2−
940.9642
0
20
40
60
80
100
500
600
700
800
900

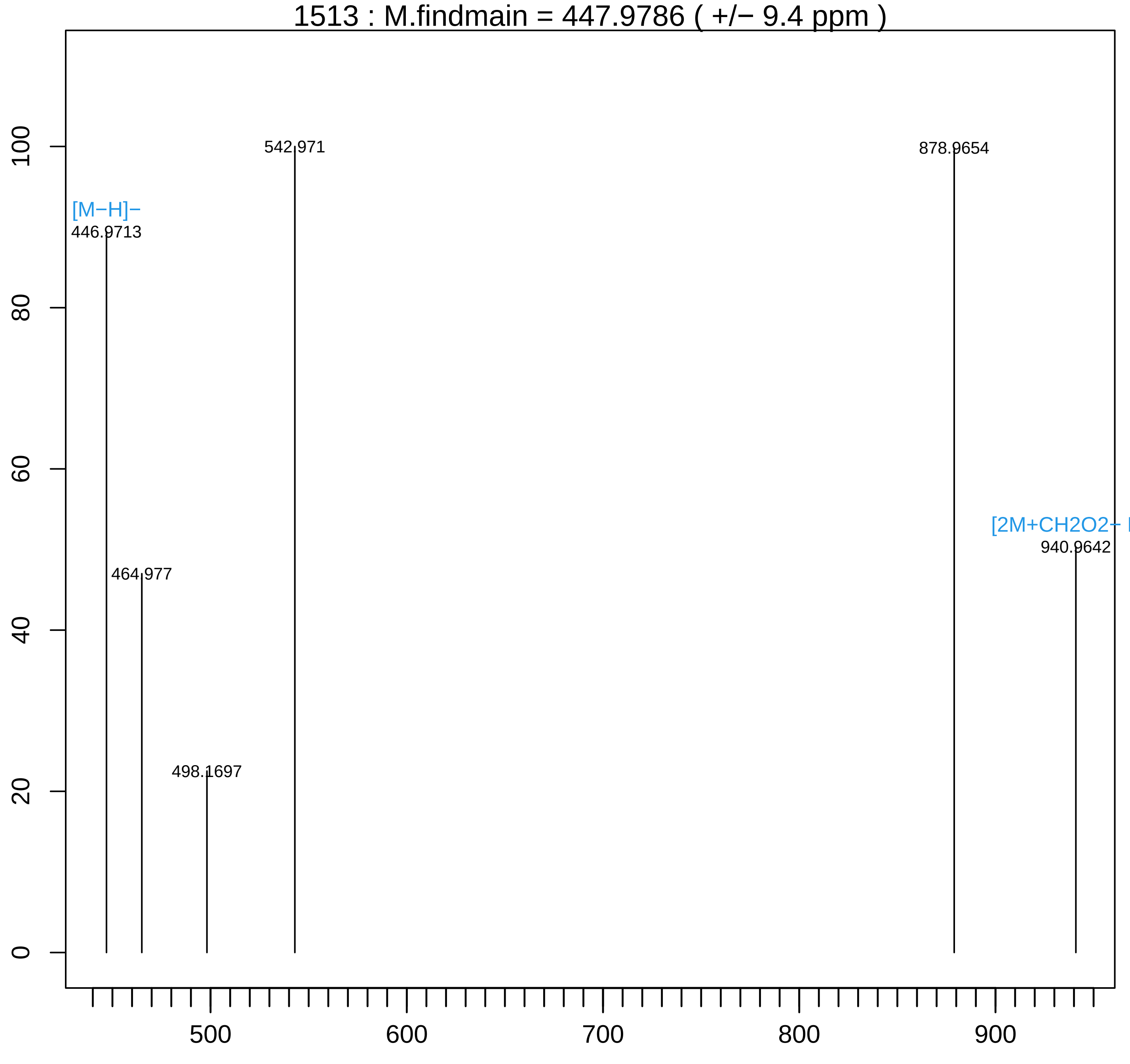
1513 : M.findmain = 447.9786 (+/− 9.4 ppm)
[M−H]−
446.9713
464.977
498.1697
542.971
878.9654
[2M+CH2O2−
940.9642
0
20
40
60
80
100
500
600
700
800
900

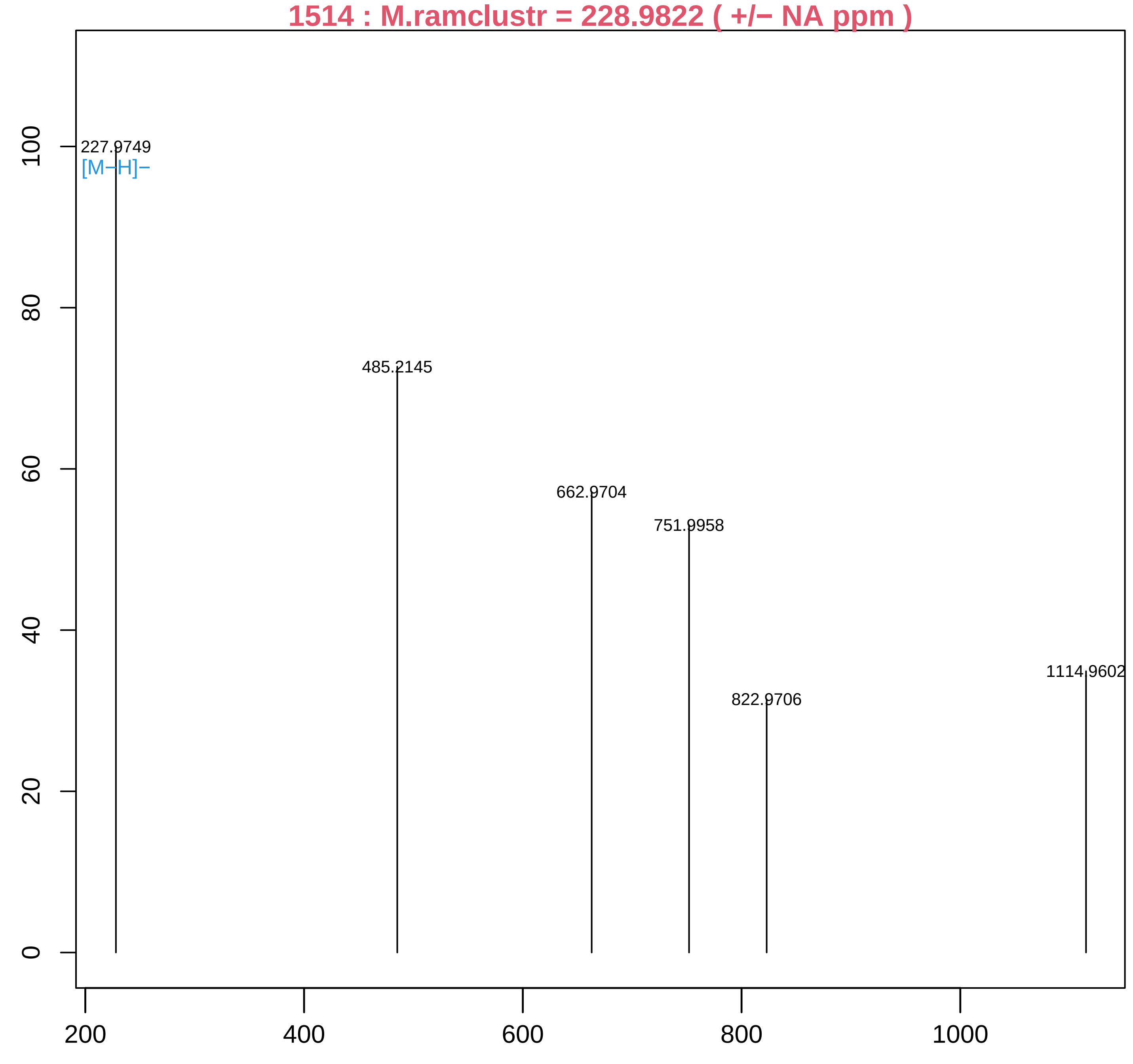
1514 : M.ramclustr = 228.9822 (+/− NA ppm)
227.9749
[M−H]−
485.2145
662.9704
751.9958
822.9706
1114.9602
0
20
40
60
80
100
200
400
600
800
1000

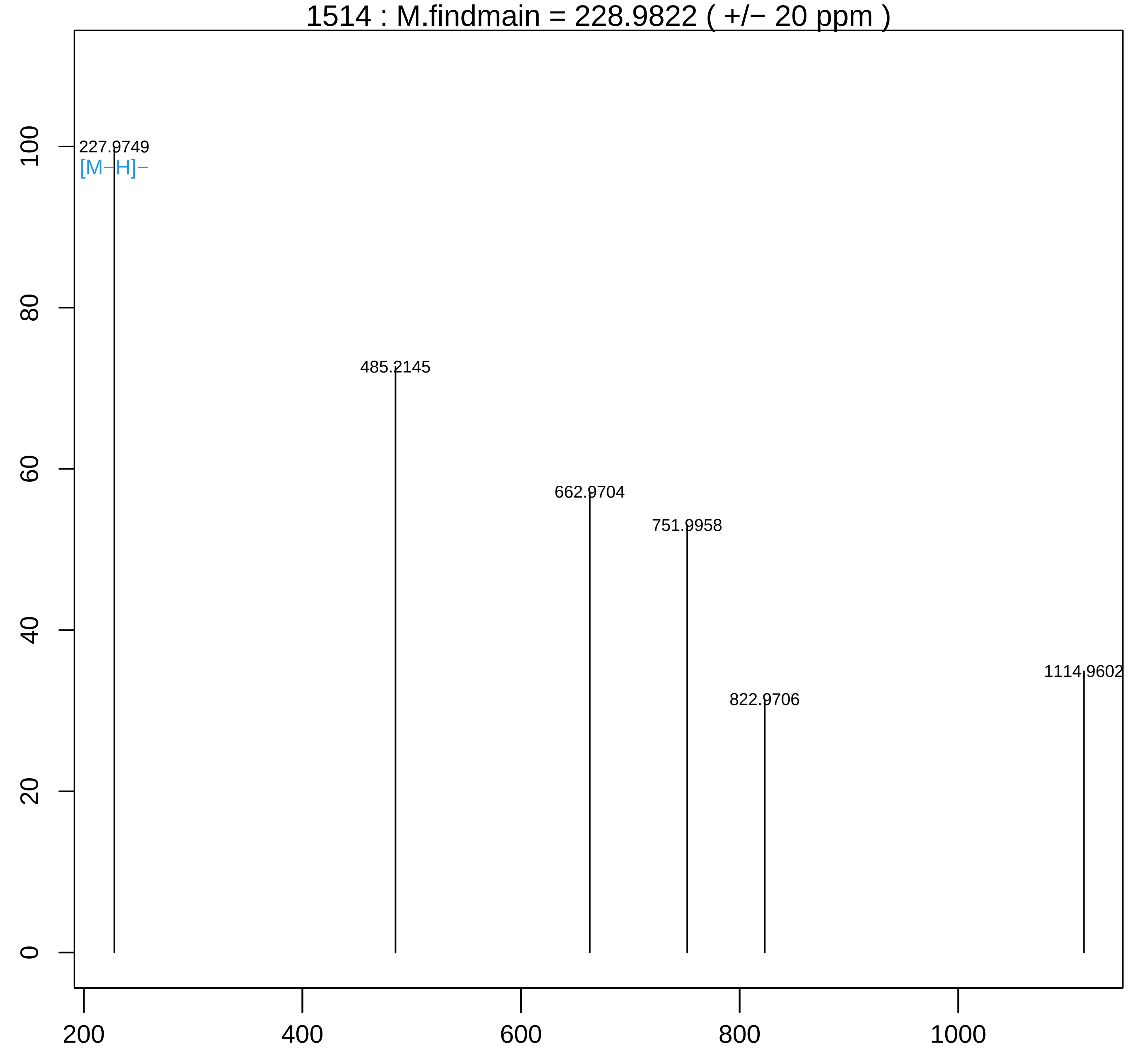
1514 : M.findmain = 228.9822 (+/− 20 ppm)
227.9749
[M−H]−
485.2145
662.9704
751.9958
822.9706
1114.9602
0
20
40
60
80
100
200
400
600
800
1000

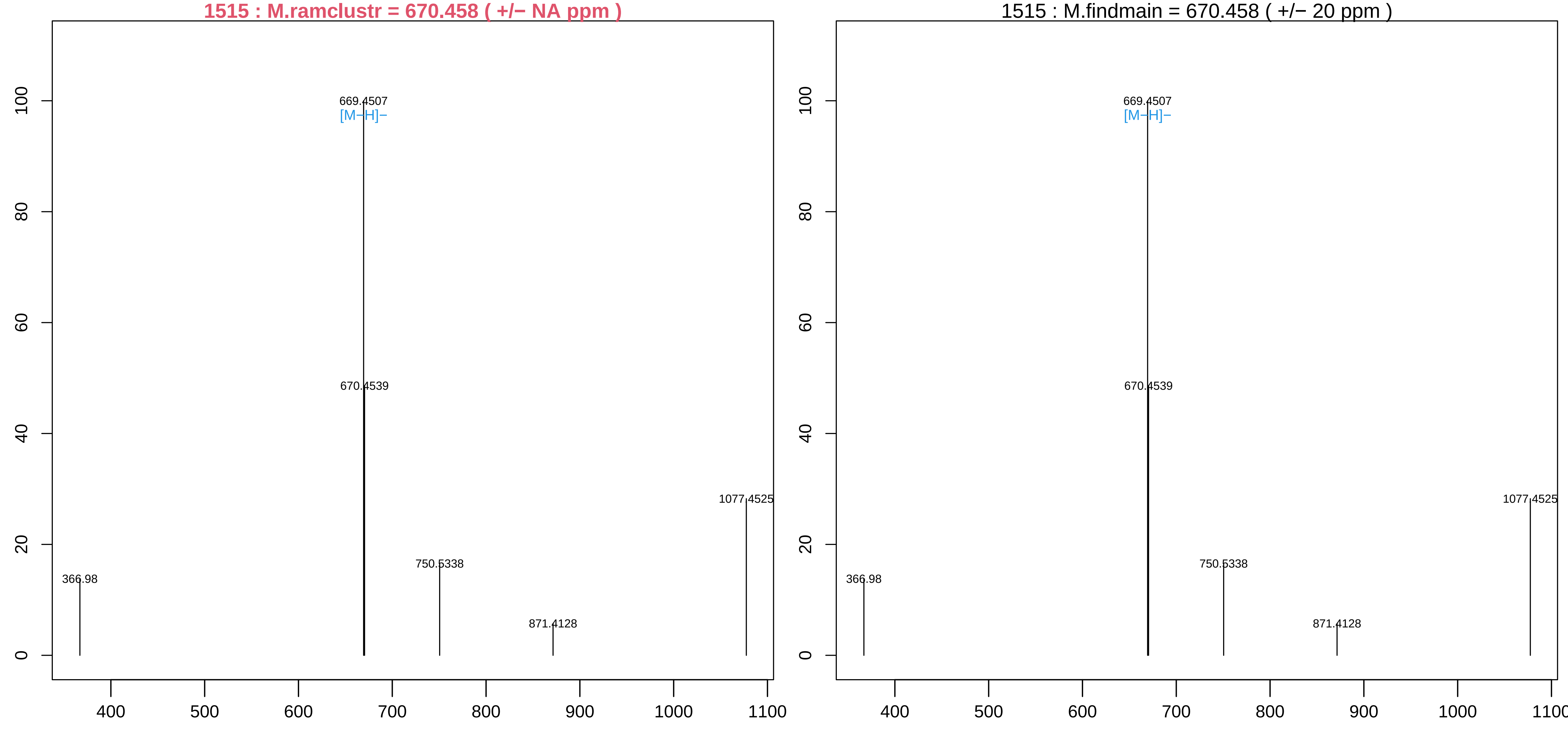
1515 : M.ramclustr = 670.458 (+/− NA ppm)
669.4507
[M−H]−
670.4539
366.98
750.5338
871.4128
1077.4525
1515 : M.findmain = 670.458 (+/− 20 ppm)
669.4507
[M−H]−
670.4539
366.98
750.5338
871.4128
1077.4525

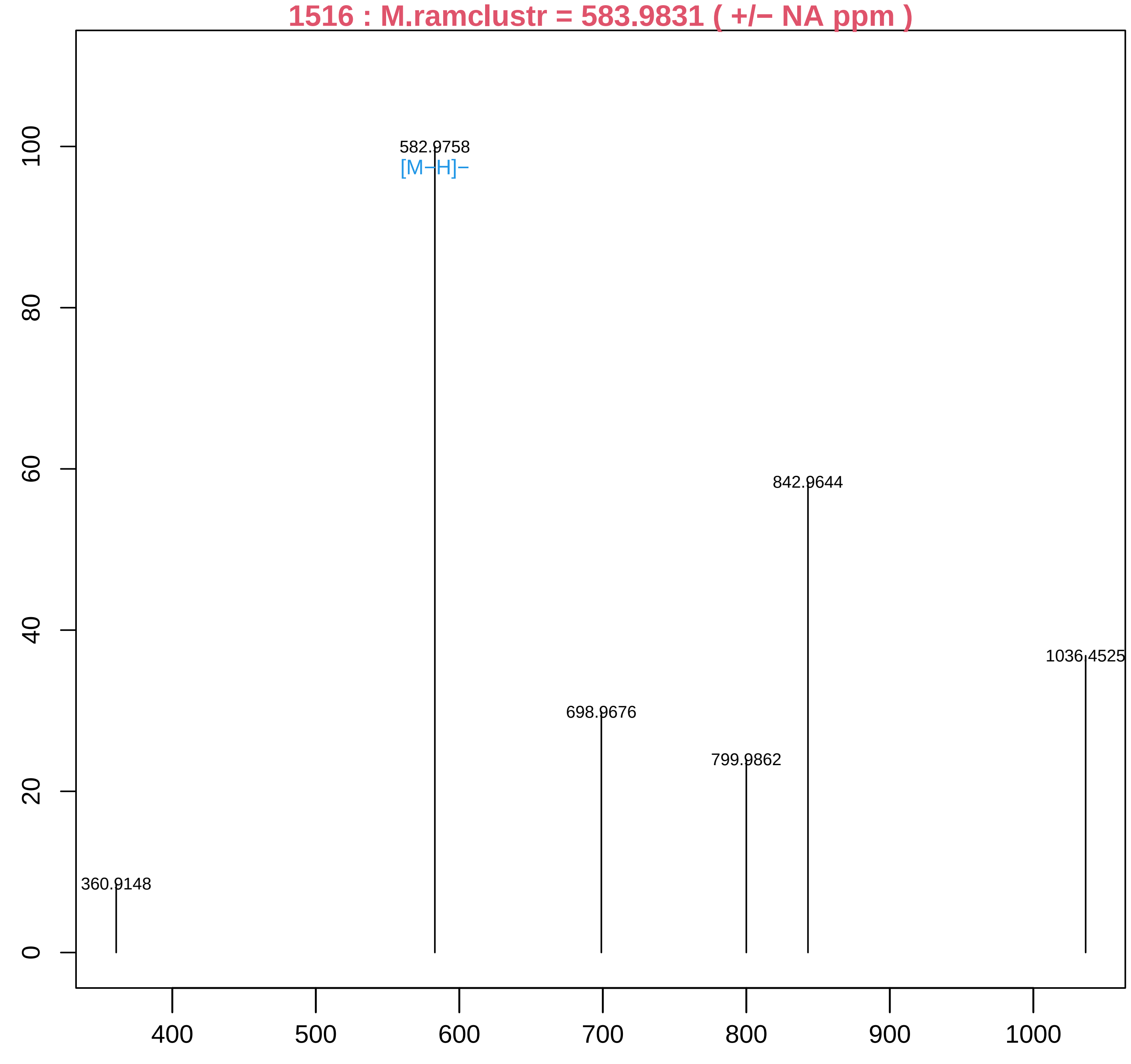
1516 : M.ramclustr = 583.9831 (+/− NA ppm)
582.9758
[M−H]−
842.9644
1036.4525
698.9676
799.9862
360.9148
0
20
40
60
80
100
400
500
600
700
800
900
1000

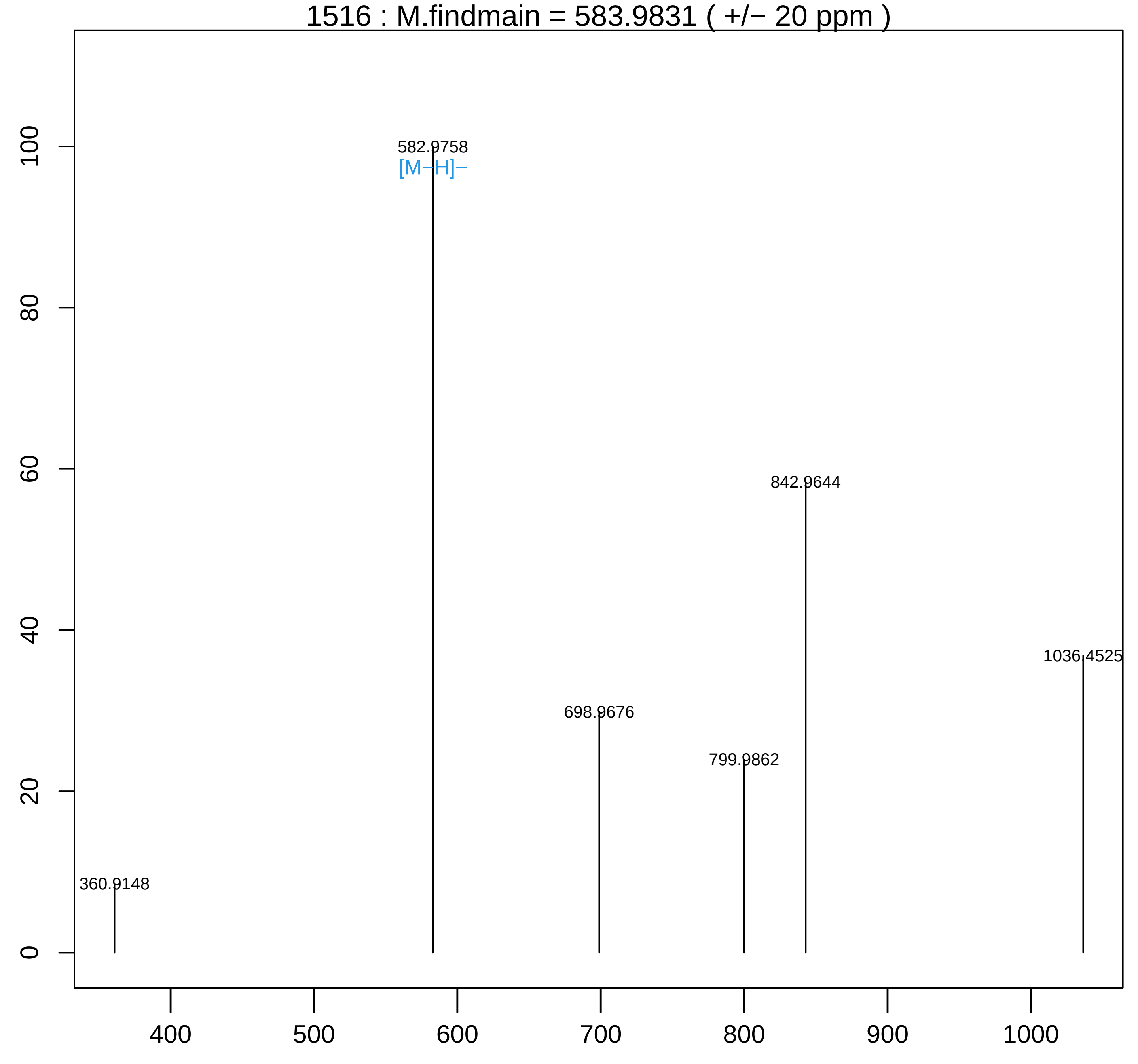
1516 : M.findmain = 583.9831 (+/− 20 ppm)
582.9758
[M−H]−
842.9644
1036.4525
698.9676
799.9862
360.9148
0
20
40
60
80
100
400
500
600
700
800
900
1000

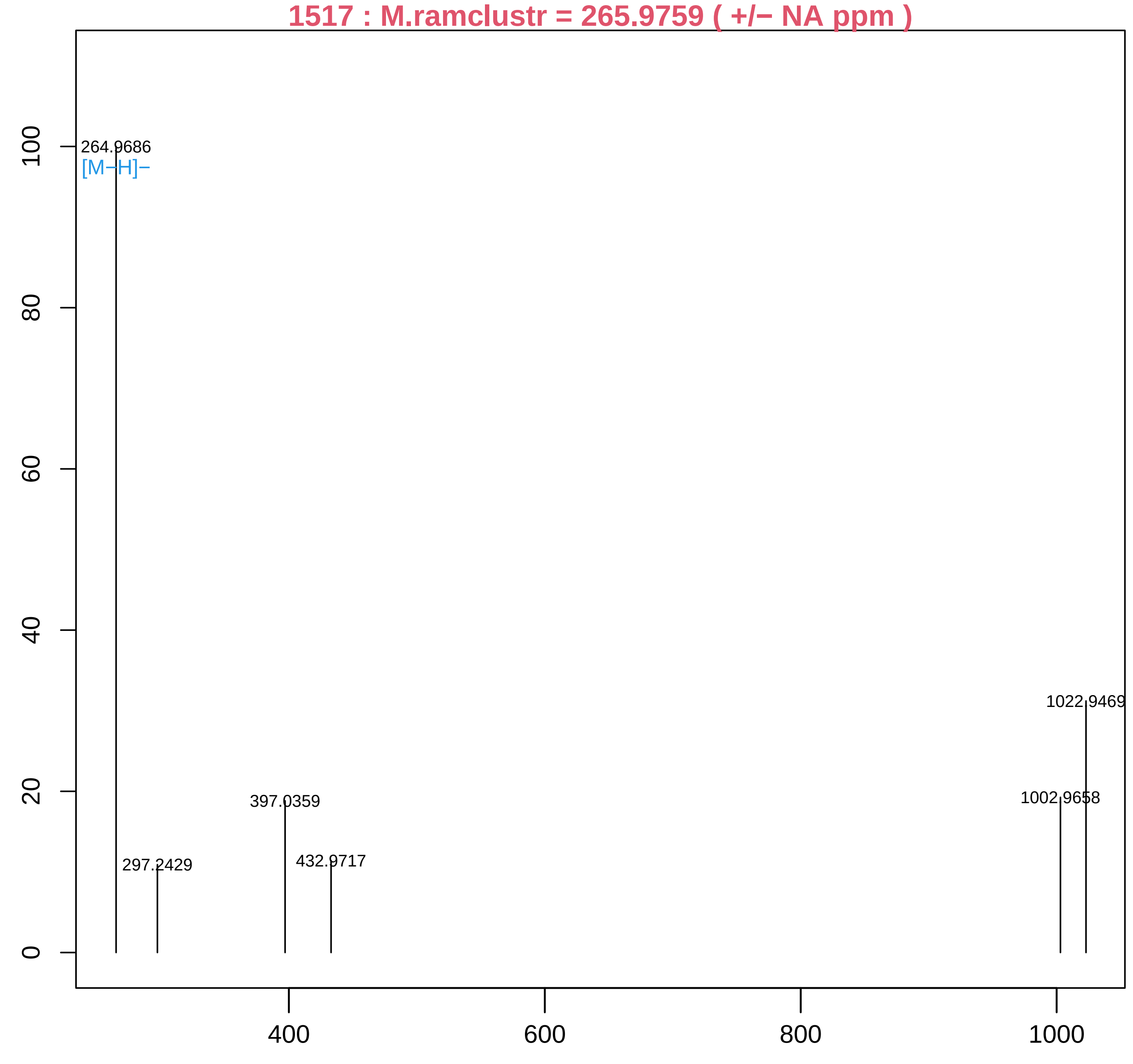

1517 : M.ramclustr = 265.9759 (+/− NA ppm)
264.9686
[M−H]−
297.2429
397.0359
432.9717
1002.9658
1022.9469
0
20
40
60
80
100
400
600
800
1000

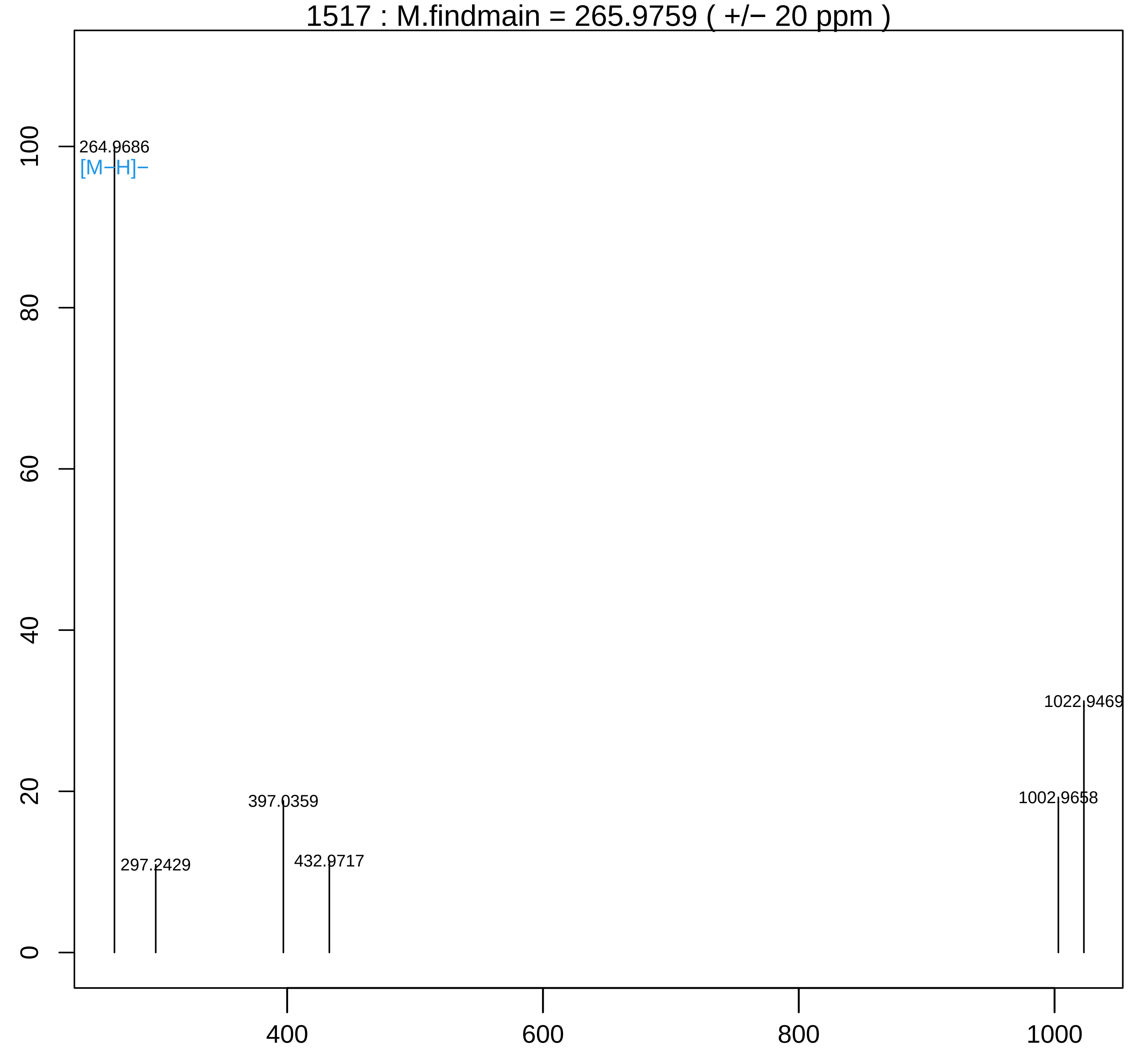

1517 : M.findmain = 265.9759 (+/− 20 ppm)
264.9686
[M−H]−
297.2429
397.0359
432.9717
1002.9658
1022.9469
0
20
40
60
80
100
400
600
800
1000

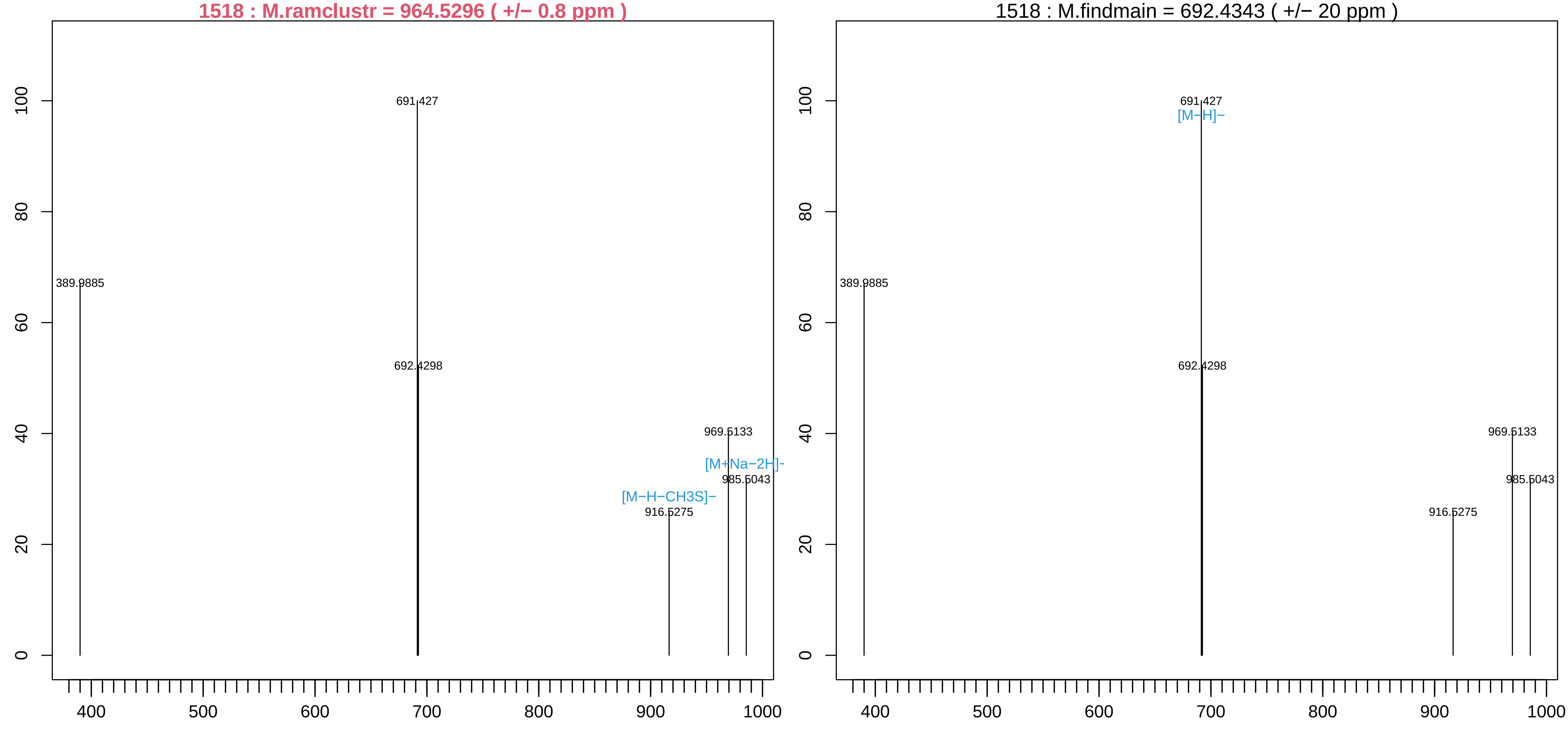
1518 : M.ramclustr = 964.5296 (+/− 0.8 ppm)
691.427
389.9885
692.4298
969.5133
[M+Na−2H]-
985.5043
[M−H−CH3S]−
916.5275
1518 : M.findmain = 692.4343 (+/− 20 ppm)
691.427
[M−H]−
389.9885
692.4298
969.5133
985.5043
916.5275
0
20
40
60
80
100
400
500
600
700
800
900
1000

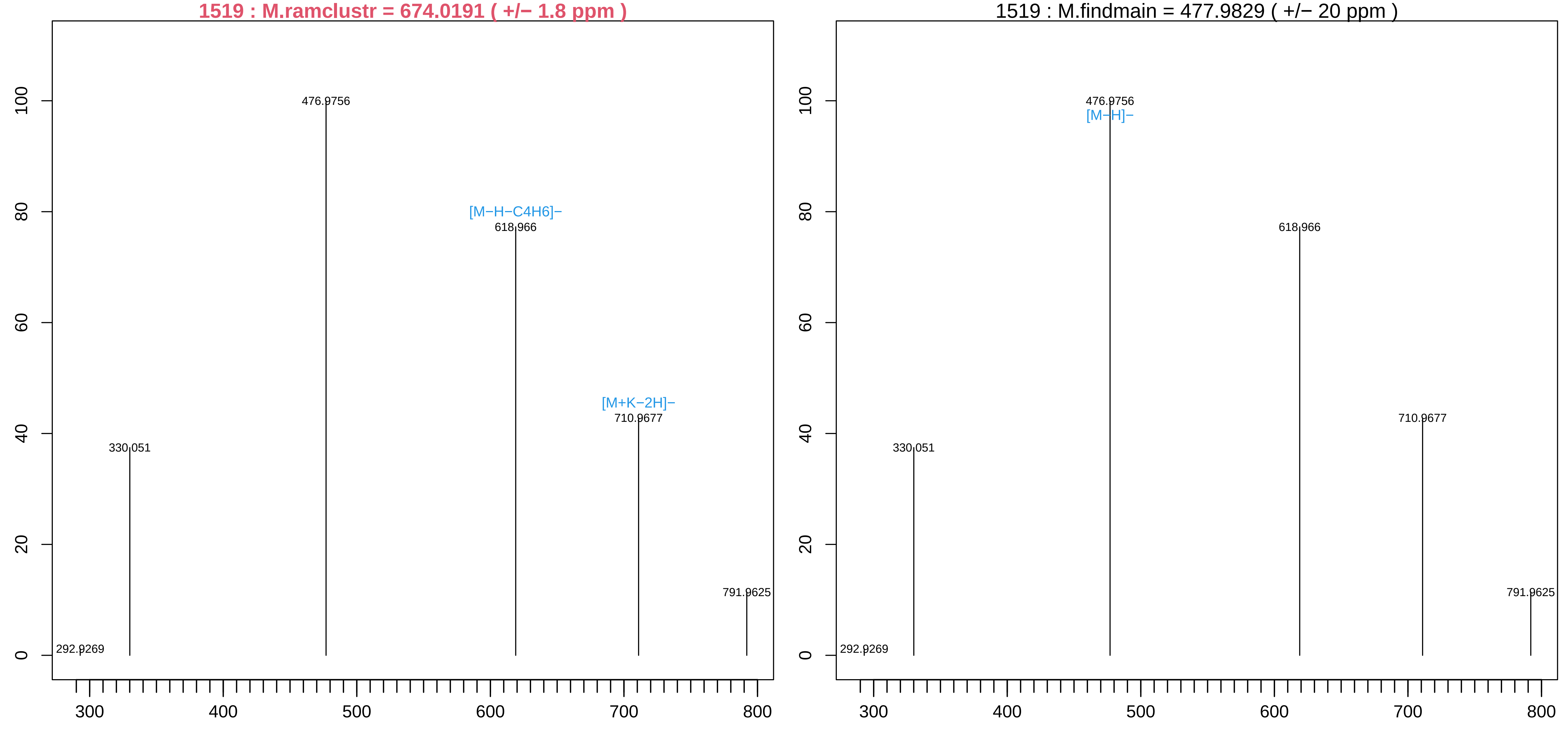

1519 : M.ramclustr = 674.0191 (+/− 1.8 ppm)
476.9756
[M−H−C4H6]−
618.966
[M+K−2H]−
710.9677
330.051
791.9625
292.9269
1519 : M.findmain = 477.9829 (+/− 20 ppm)
476.9756
[M−H]−
618.966
710.9677
330.051
791.9625
292.9269
0
20
40
60
80
100
300
400
500
600
700
800

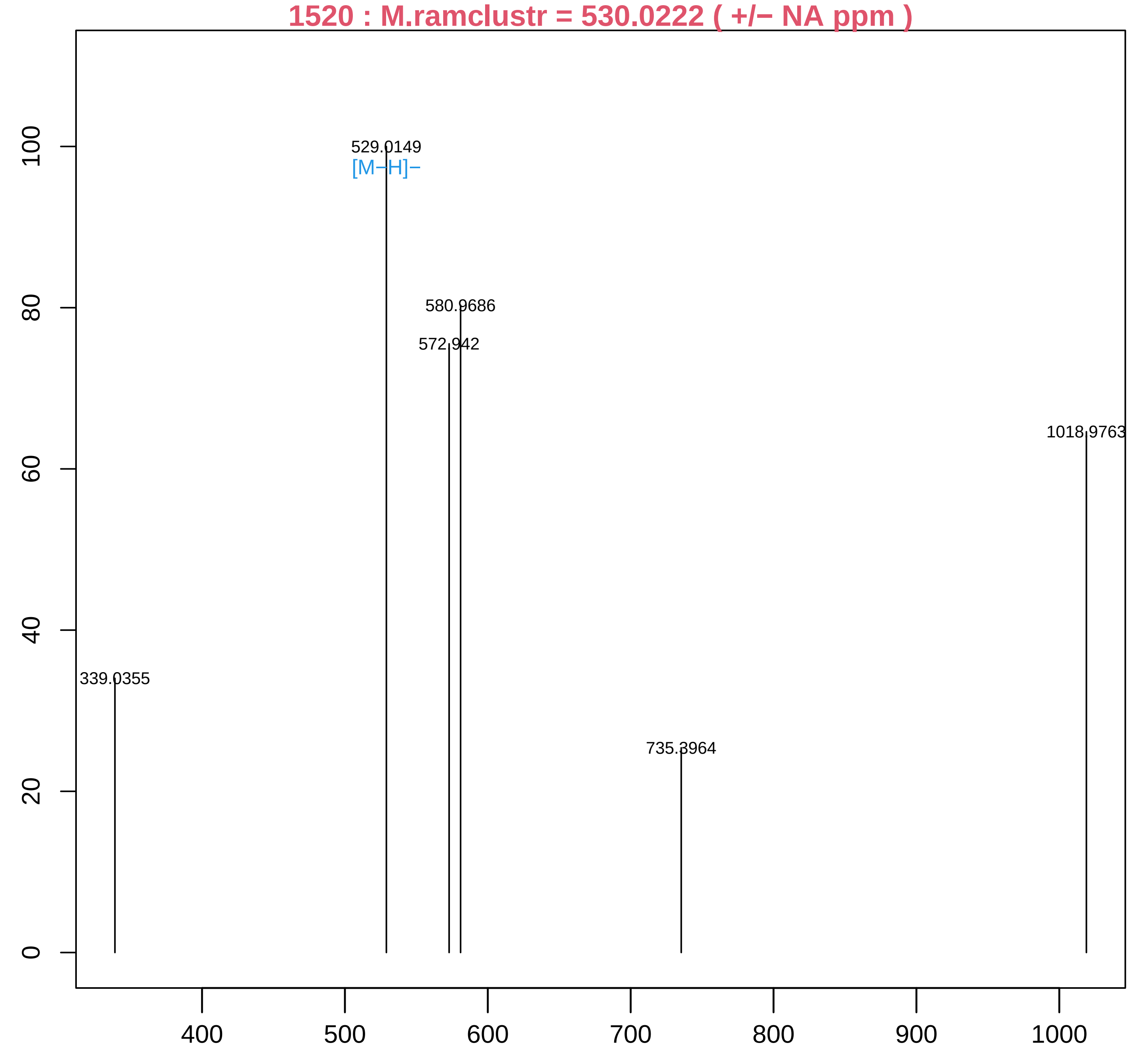
1520 : M.ramclustr = 530.0222 (+/− NA ppm)
529.0149
[M−H]−
580.9686
572.942
1018.9763
339.0355
735.3964
0
20
40
60
80
100
400
500
600
700
800
900
1000

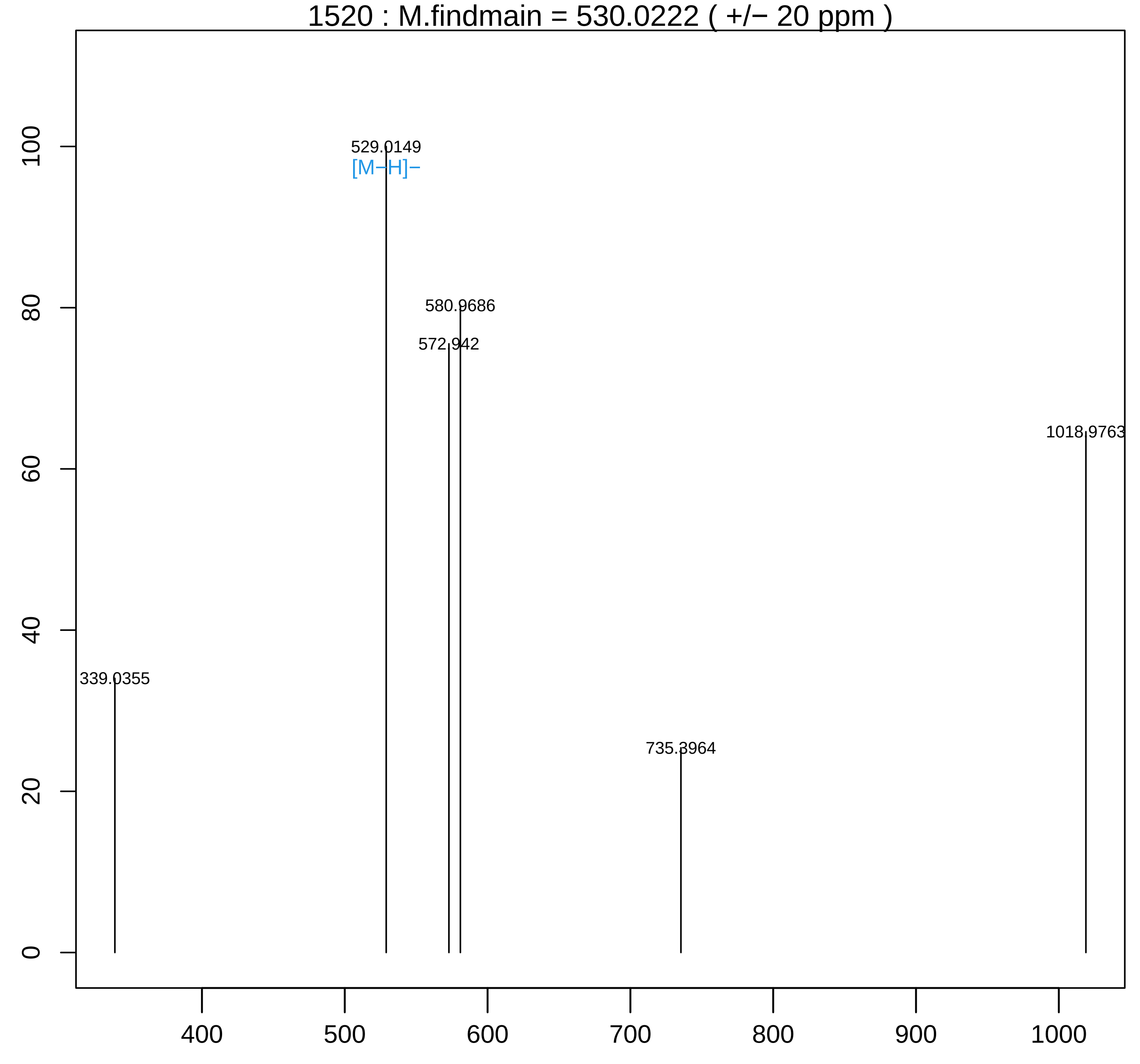
1520 : M.findmain = 530.0222 (+/− 20 ppm)
529.0149
[M−H]−
580.9686
572.942
1018.9763
339.0355
735.3964
0
20
40
60
80
100
400
500
600
700
800
900
1000

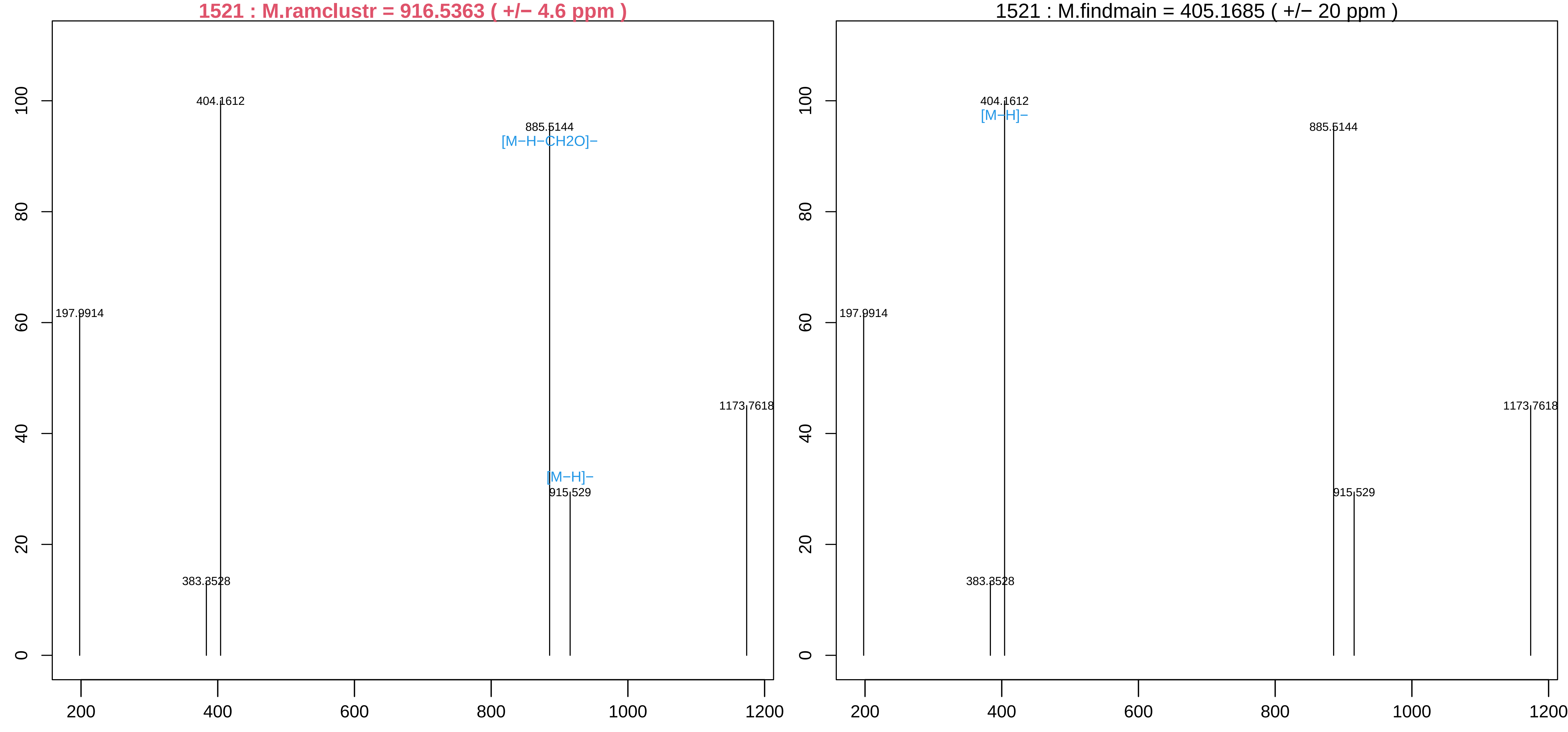
1521 : M.ramclustr = 916.5363 (+/− 4.6 ppm)
404.1612
885.5144
[M−H−CH2O]−
197.9914
1173.7618
[M−H]−
915.529
383.3528
0
20
40
60
80
100
200
400
600
800
1000
1200
1521 : M.findmain = 405.1685 (+/− 20 ppm)
404.1612
[M−H]−
885.5144
197.9914
1173.7618
915.529
383.3528

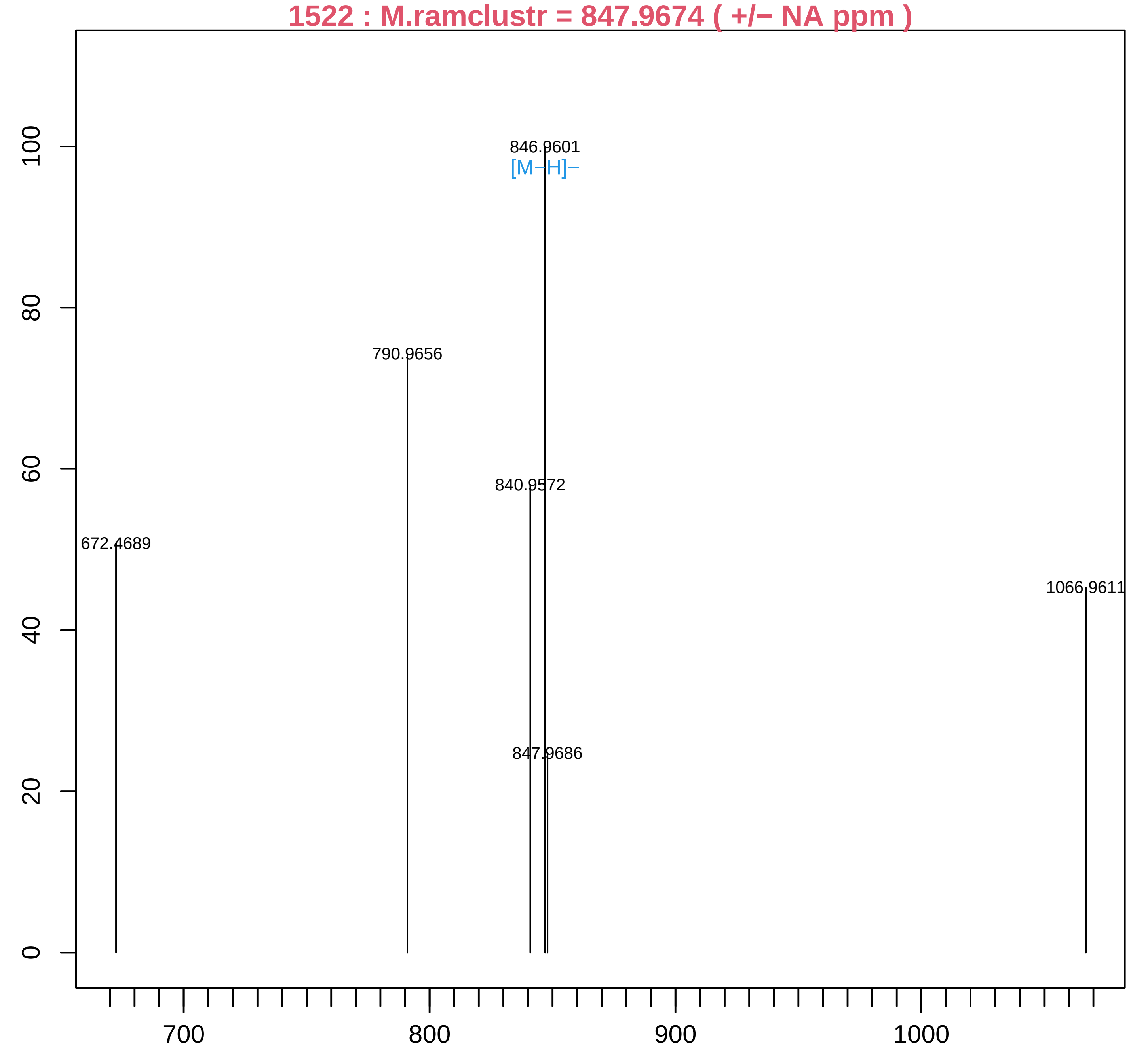
1522 : M.ramclustr = 847.9674 (+/− NA ppm)
846.9601
[M−H]−
790.9656
840.9572
672.4689
1066.9611
847.9686
0
20
40
60
80
100
700
800
900
1000

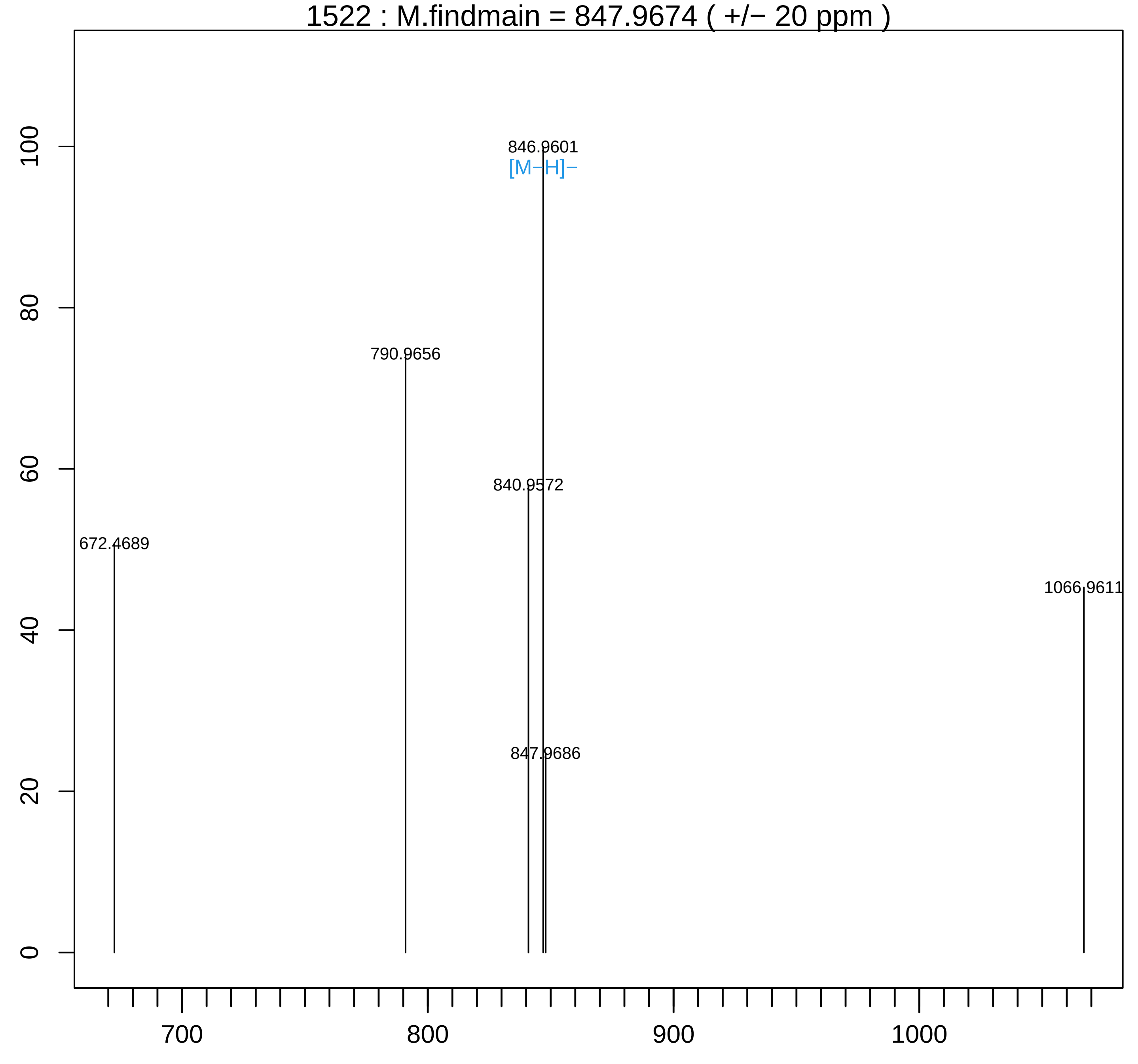
1522 : M.findmain = 847.9674 (+/− 20 ppm)
846.9601
[M−H]−
790.9656
840.9572
672.4689
1066.9611
847.9686
0
20
40
60
80
100
700
800
900
1000

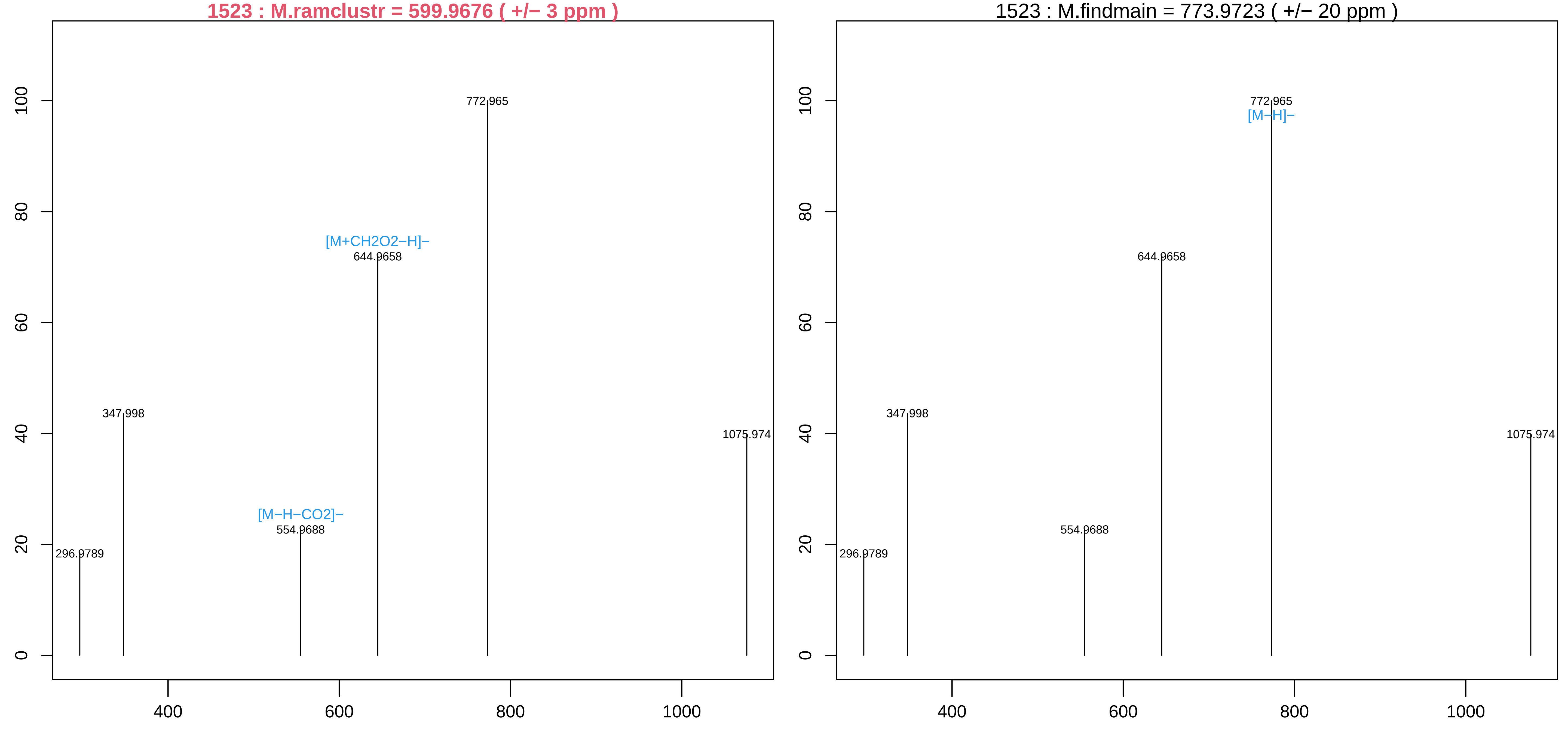

1523 : M.ramclustr = 599.9676 (+/− 3 ppm)
1523 : M.findmain = 773.9723 (+/− 20 ppm)
[M+CH2O2−H]−
[M−H−CO2]−
[M−H]−
296.9789
347.998
554.9688
644.9658
772.965
1075.974
0
20
40
60
80
100
400
600
800
1000

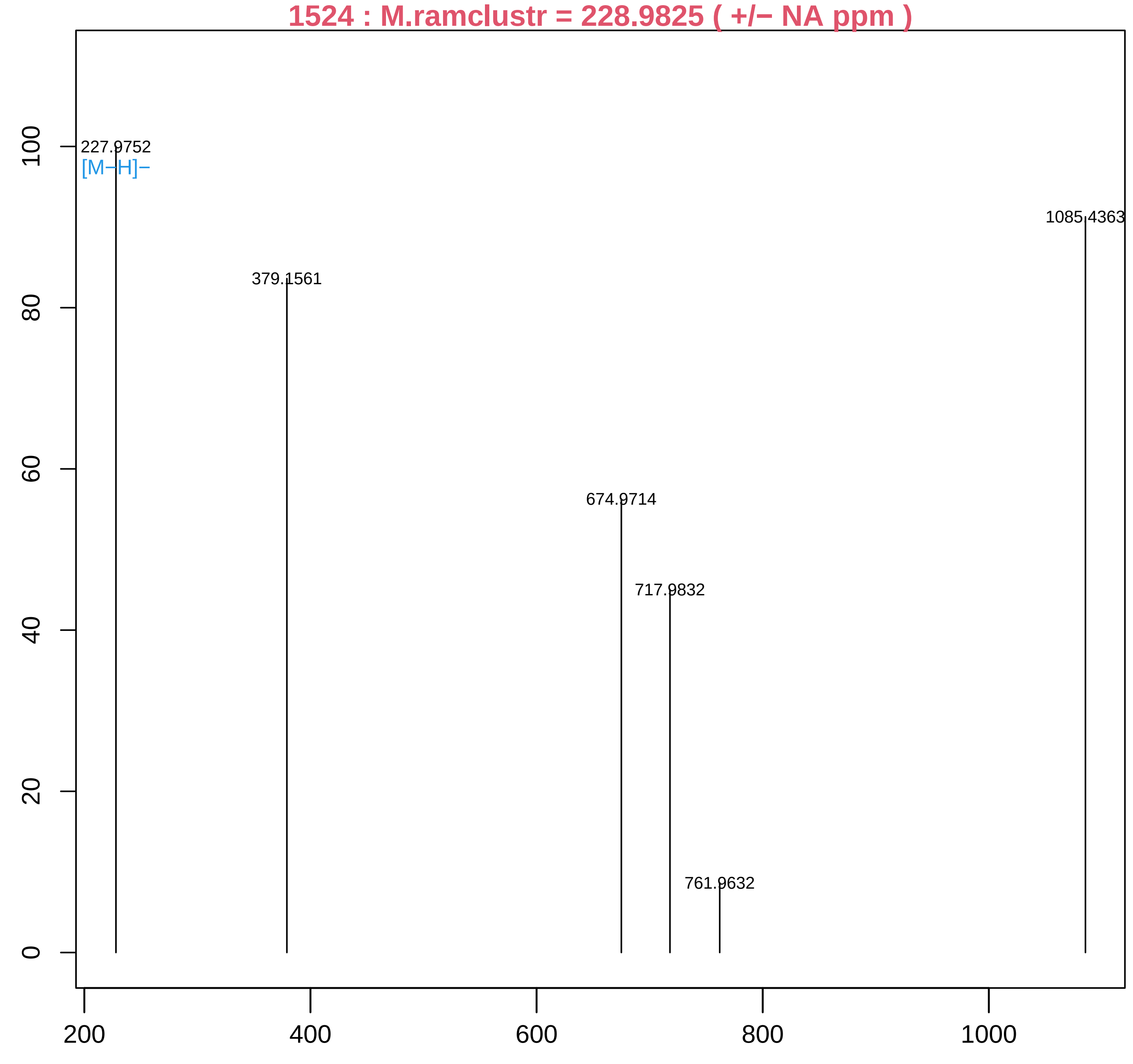
1524 : M.ramclustr = 228.9825 (+/− NA ppm)
227.9752
[M−H]−
379.1561
674.9714
717.9832
761.9632
1085.4363

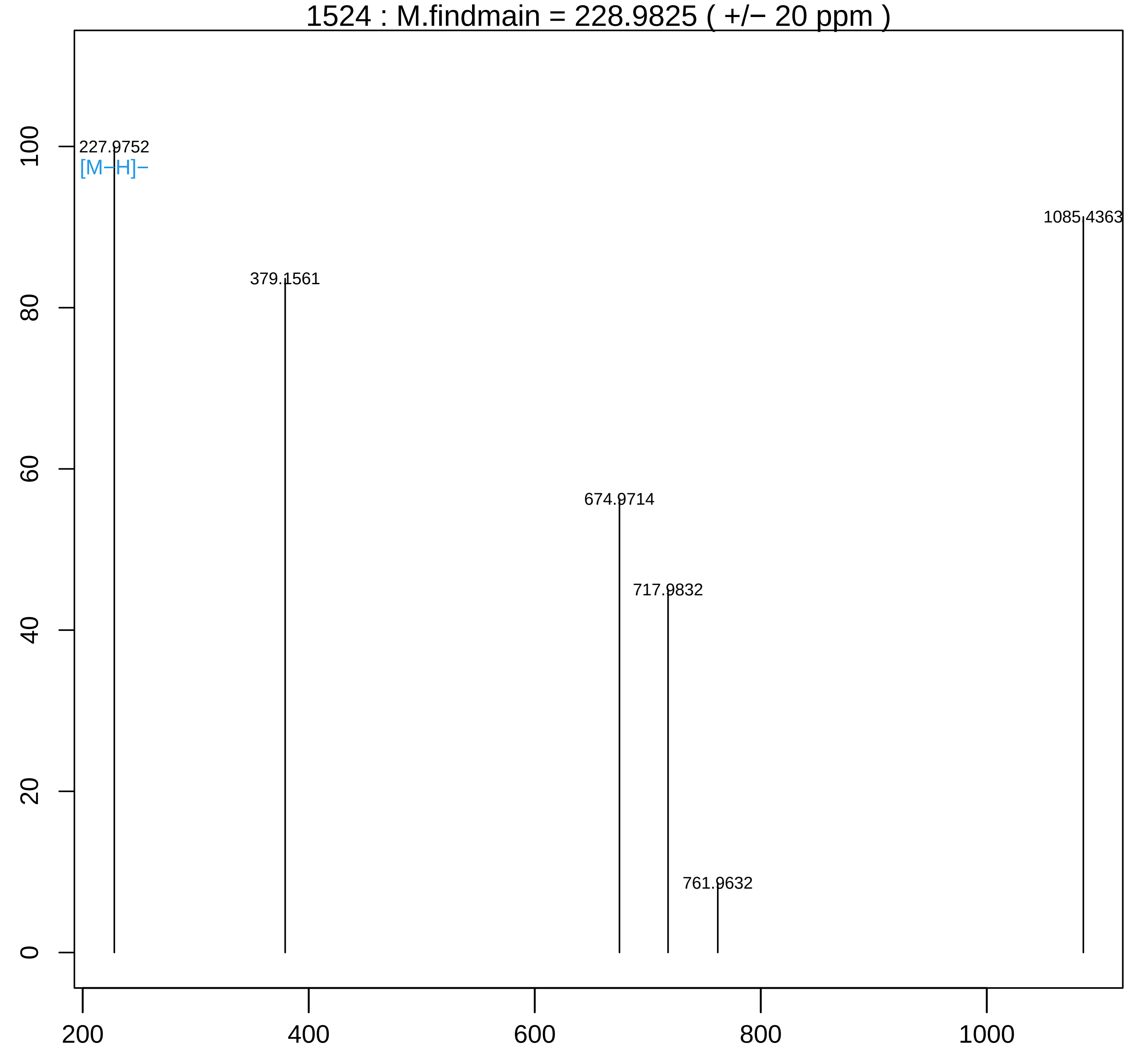
1524 : M.findmain = 228.9825 (+/− 20 ppm)
227.9752
[M−H]−
379.1561
674.9714
717.9832
761.9632
1085.4363

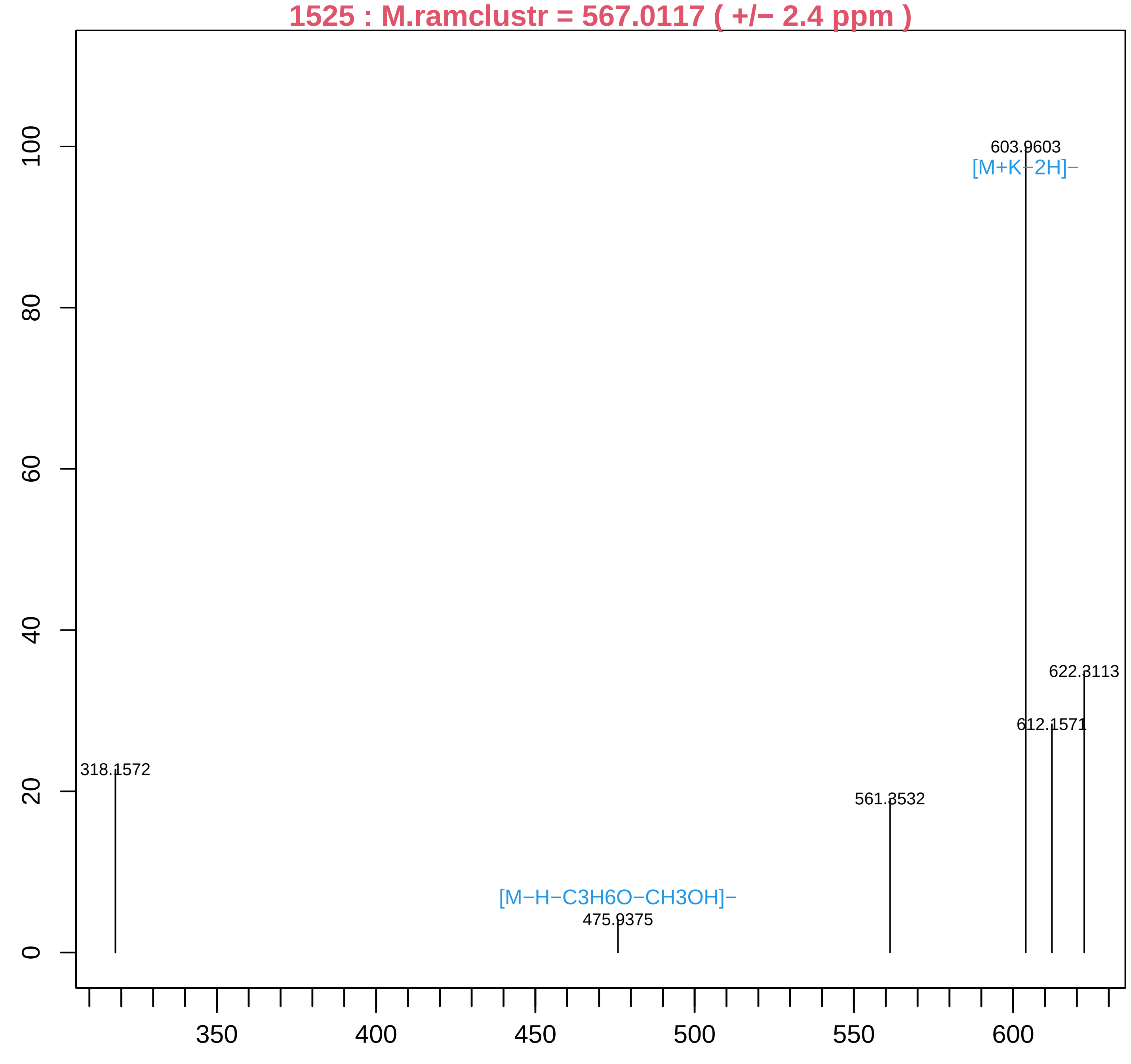

1525 : M.ramclustr = 567.0117 (+/− 2.4 ppm)
603.9603
[M+K−2H]−
622.3113
612.1571
318.1572
561.3532
[M−H−C3H6O−CH3OH]−
475.9375
0
20
40
60
80
100
350
400
450
500
550
600

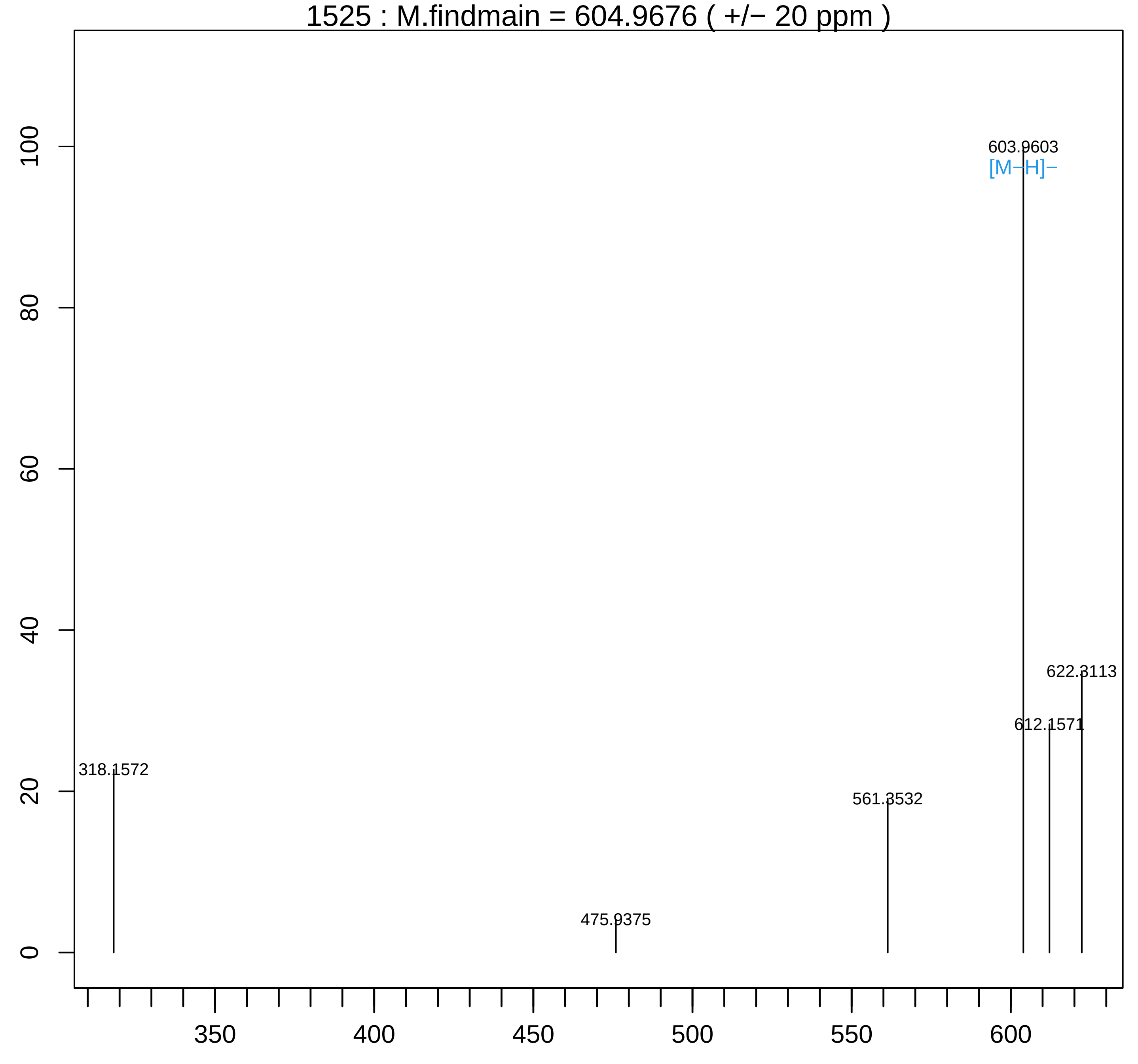

1525 : M.findmain = 604.9676 (+/− 20 ppm)
603.9603
[M−H]−
622.3113
612.1571
318.1572
561.3532
475.9375
0
20
40
60
80
100
350
400
450
500
550
600

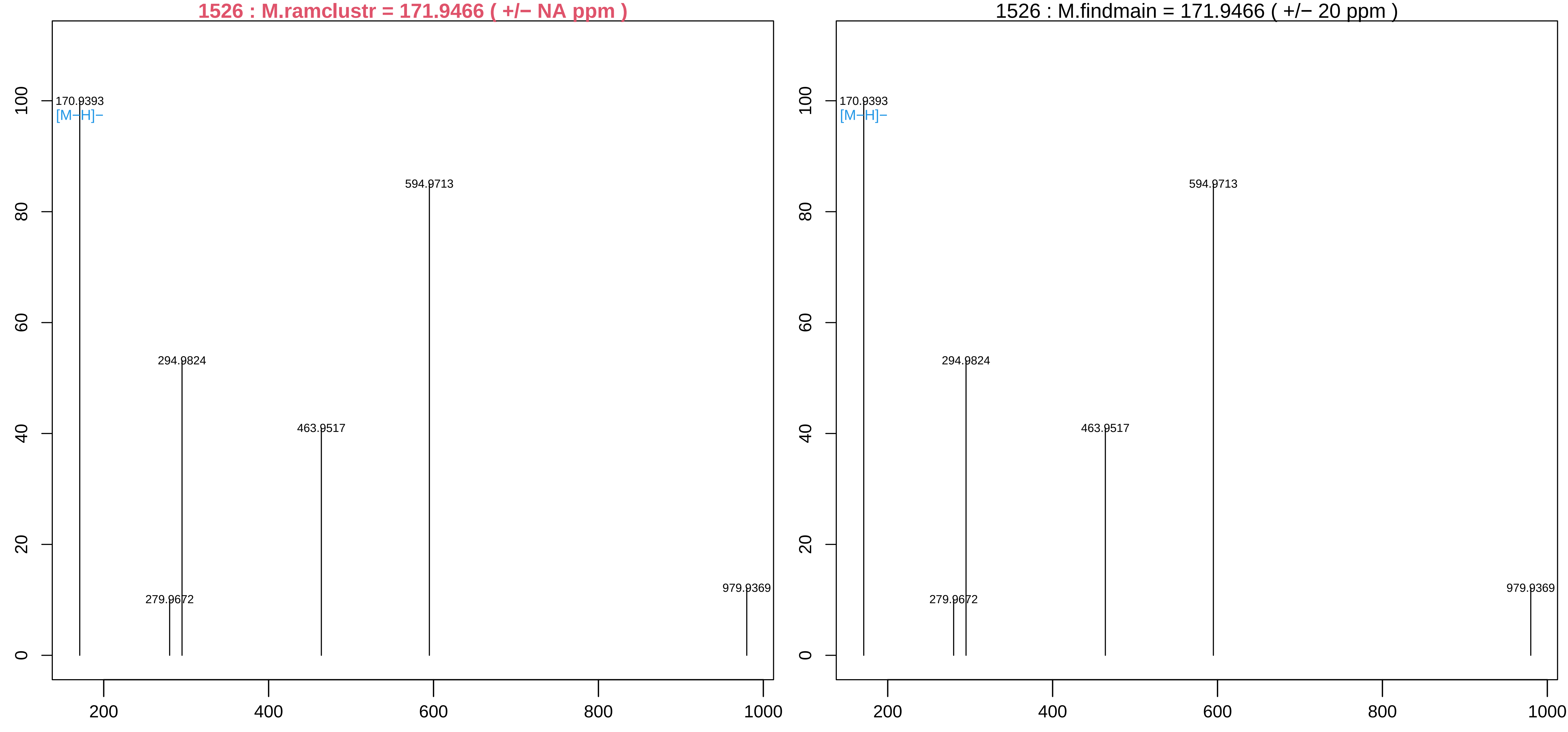
1526 : M.ramclustr = 171.9466 (+/− NA ppm)
1526 : M.findmain = 171.9466 (+/− 20 ppm)
170.9393
[M−H]−
294.9824
279.9672
463.9517
594.9713
979.9369
0
20
40
60
80
100
200
400
600
800
1000

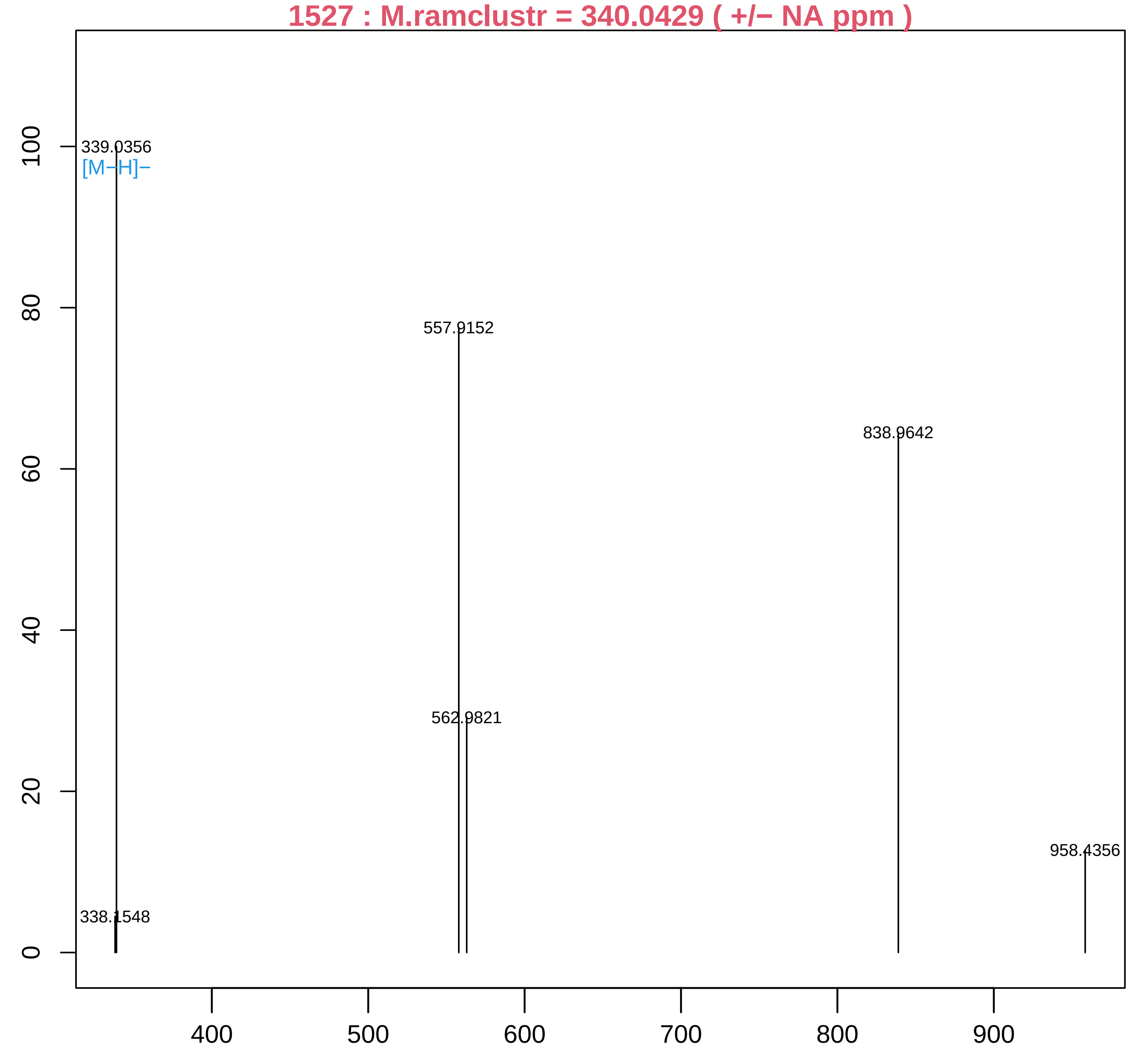
1527 : M.ramclustr = 340.0429 (+/− NA ppm)
339.0356
[M−H]−
557.9152
838.9642
562.9821
958.4356
338.1548
0
20
40
60
80
100
400
500
600
700
800
900

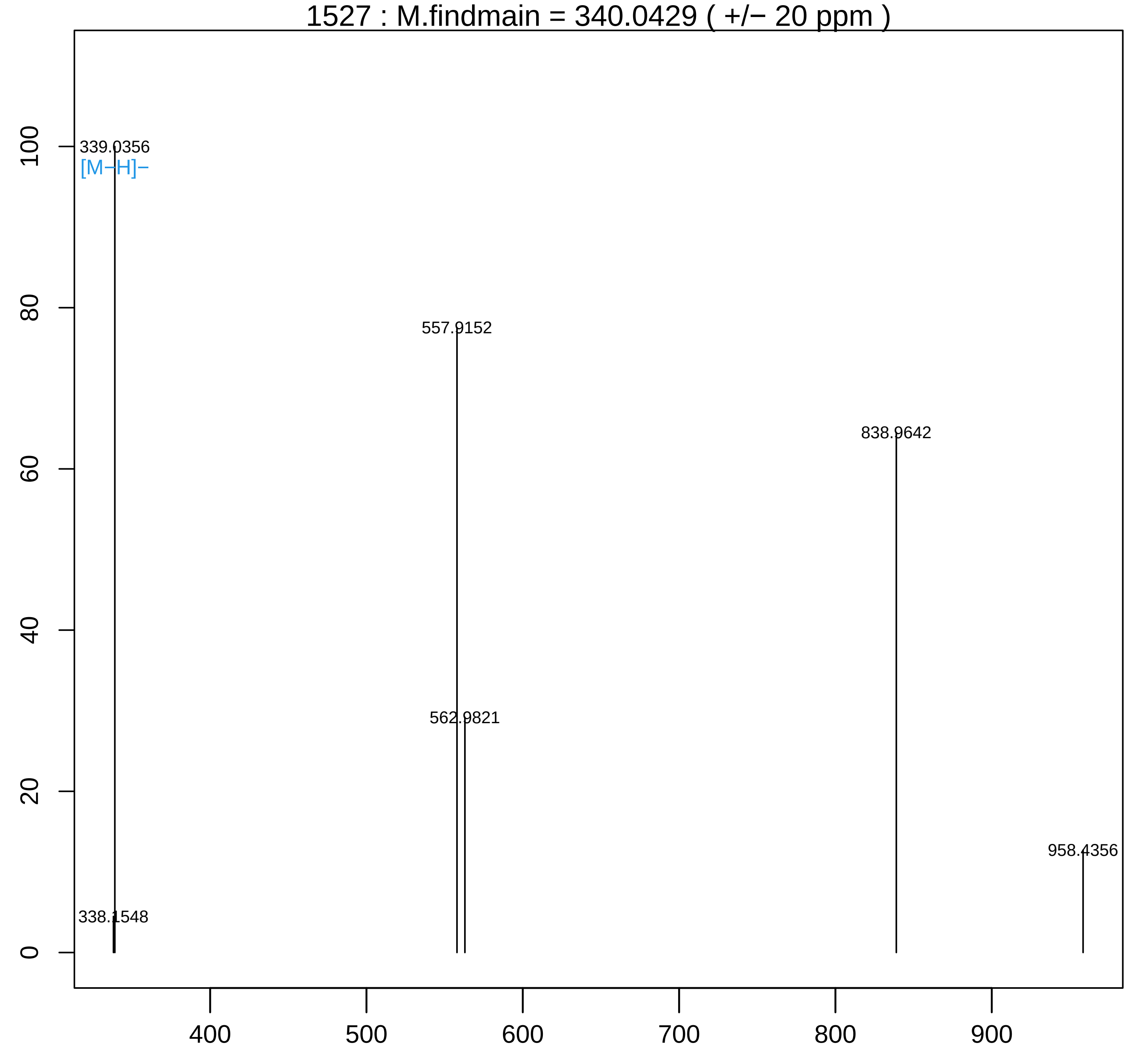
1527 : M.findmain = 340.0429 (+/− 20 ppm)
339.0356
[M−H]−
557.9152
838.9642
562.9821
958.4356
338.1548
0
20
40
60
80
100
400
500
600
700
800
900

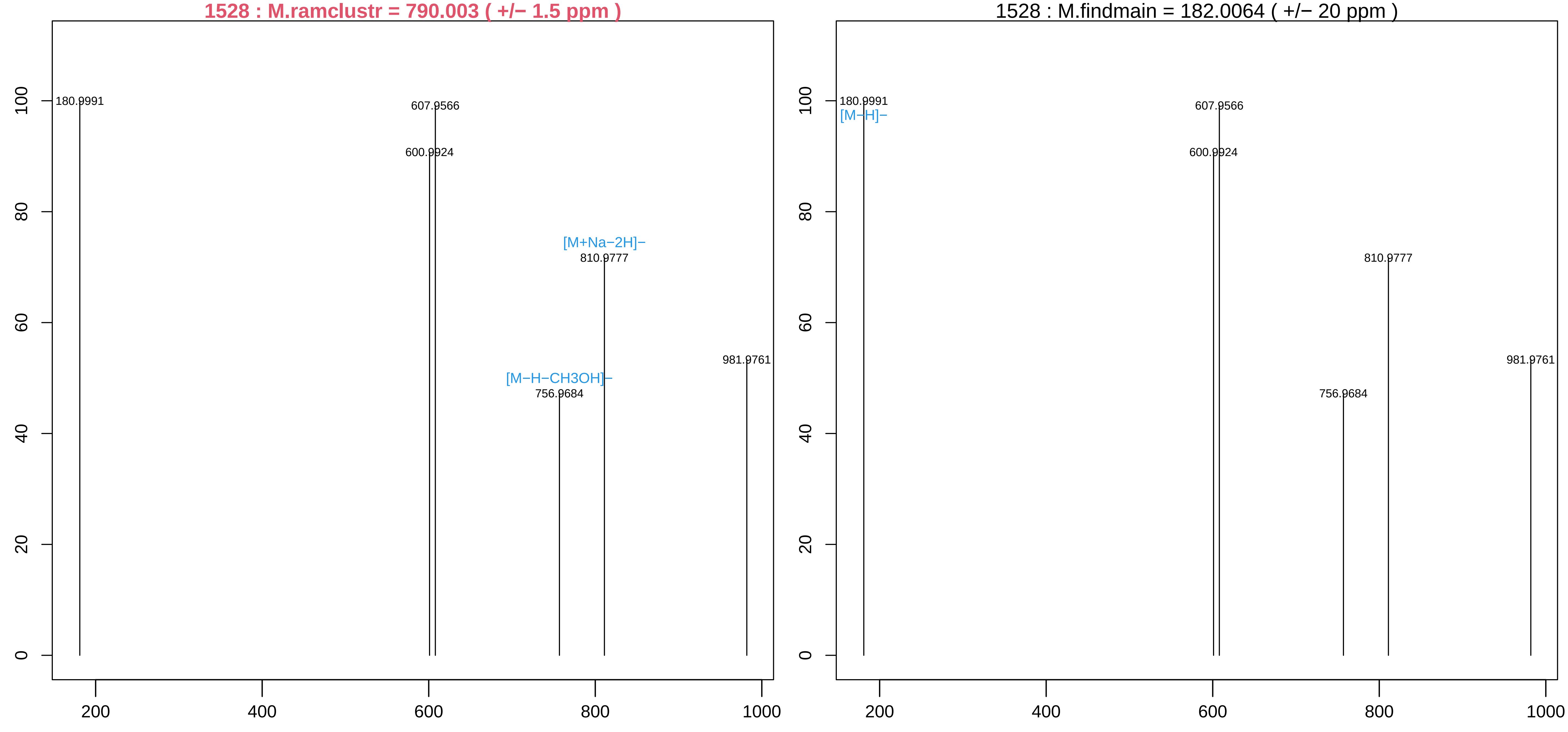

1528 : M.ramclustr = 790.003 (+/− 1.5 ppm)
180.9991
607.9566
600.9924
[M+Na−2H]−
810.9777
981.9761
[M−H−CH3OH]−
756.9684
1528 : M.findmain = 182.0064 (+/− 20 ppm)
180.9991
[M−H]−
607.9566
600.9924
810.9777
981.9761
756.9684

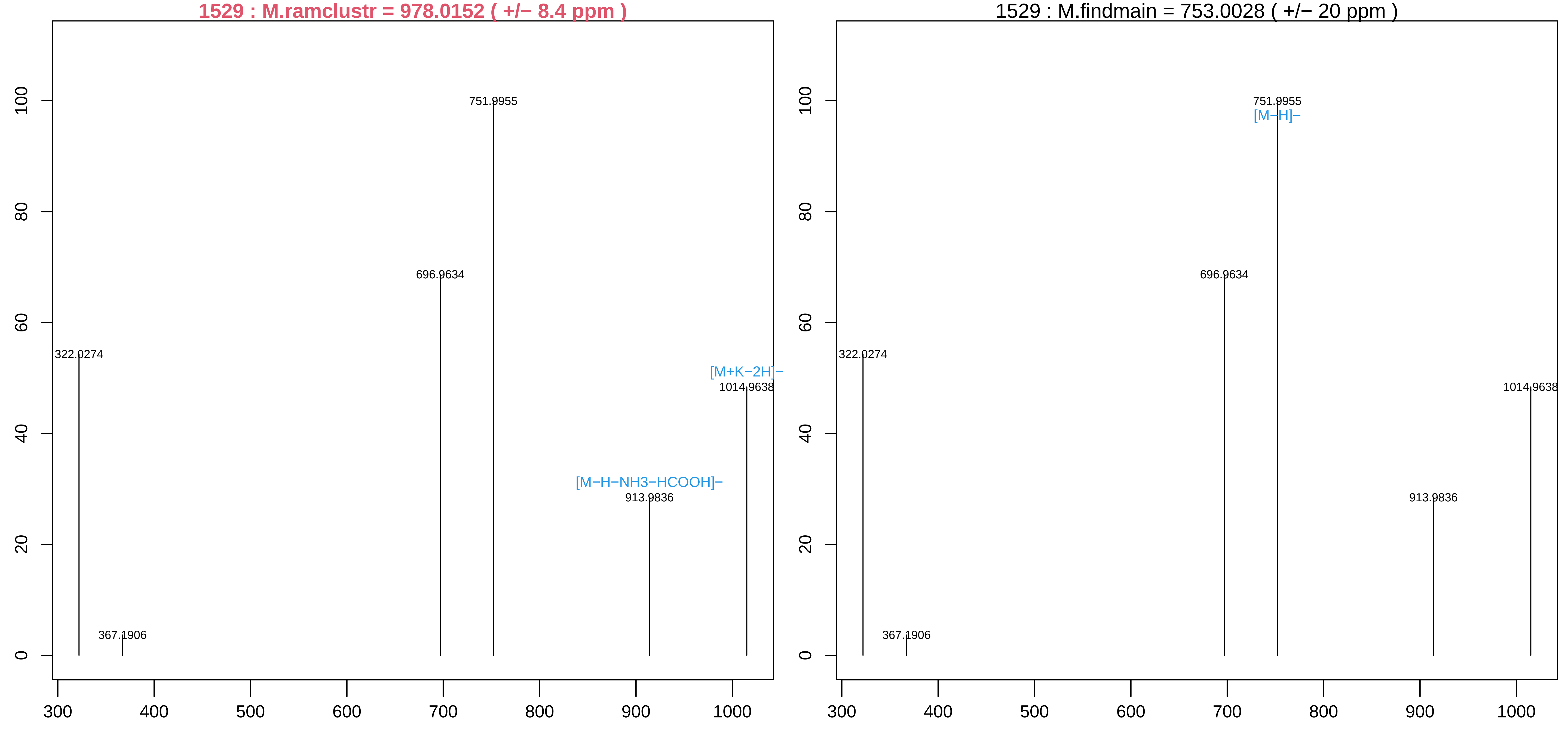
1529 : M.ramclustr = 978.0152 (+/− 8.4 ppm)
751.9955
696.9634
322.0274
[M+K−2H]−
1014.9638
[M−H−NH3−HCOOH]−
913.9836
367.1906
1529 : M.findmain = 753.0028 (+/− 20 ppm)
751.9955
[M−H]−
696.9634
322.0274
1014.9638
913.9836
367.1906
0
20
40
60
80
100
300
400
500
600
700
800
900
1000

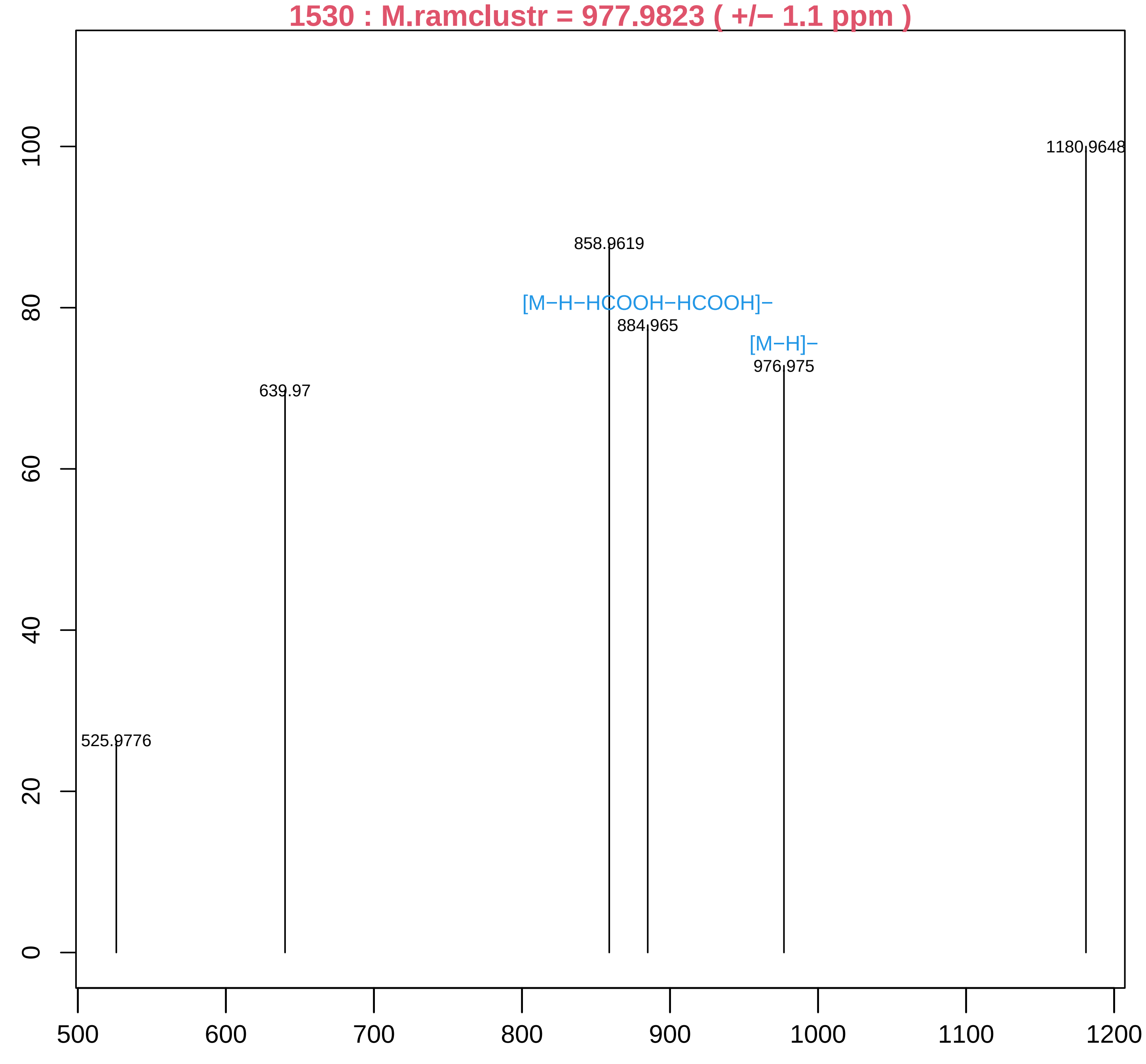
1530 : M.ramclustr = 977.9823 (+/− 1.1 ppm)
1180.9648
858.9619
[M−H−HCOOH−HCOOH]−
884.965
[M−H]−
976.975
639.97
525.9776
0
20
40
60
80
100
500
600
700
800
900
1000
1100
1200

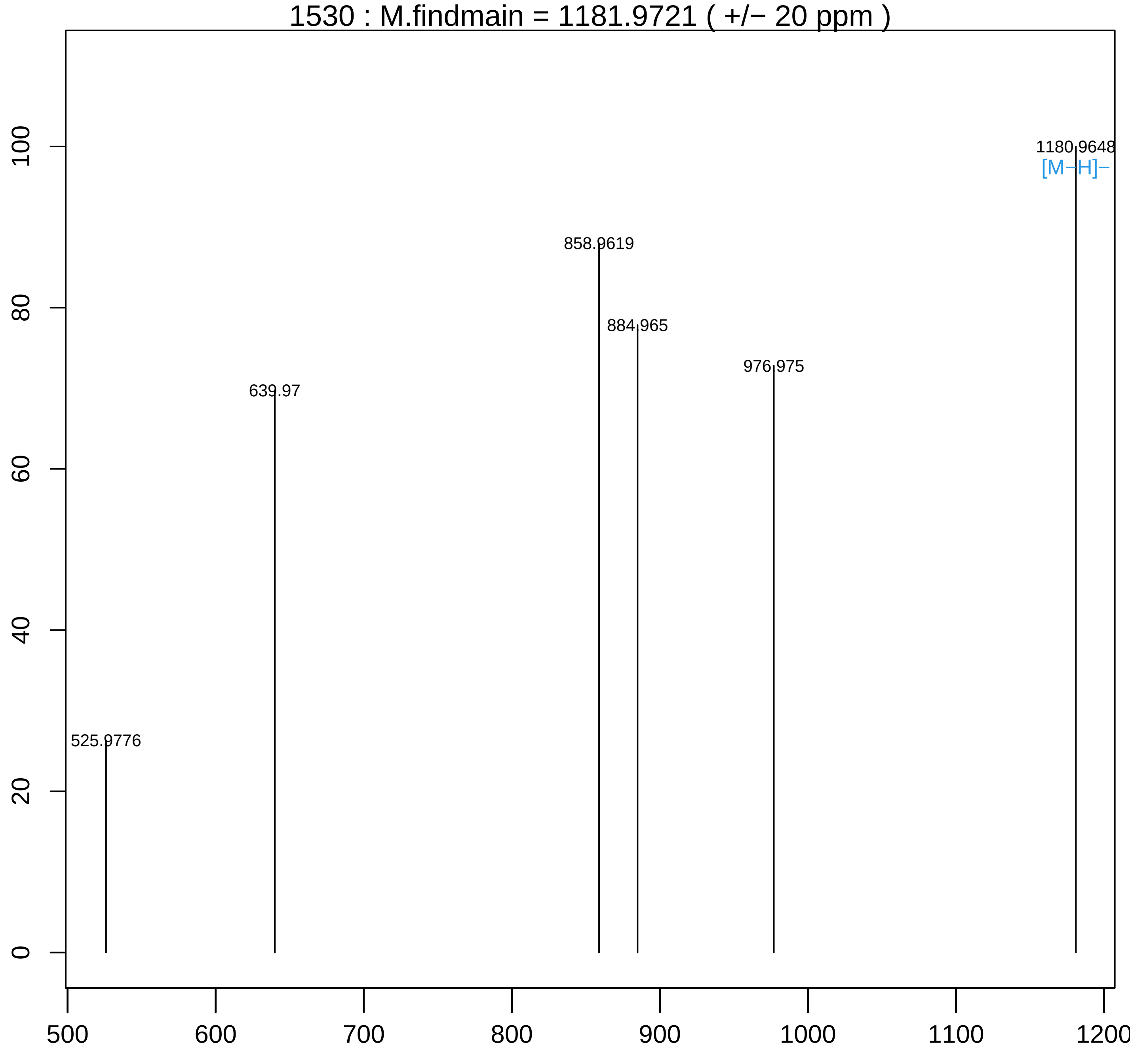
1530 : M.findmain = 1181.9721 (+/− 20 ppm)
1180.9648
[M−H]−
858.9619
884.965
976.975
639.97
525.9776
0
20
40
60
80
100
500
600
700
800
900
1000
1100
1200

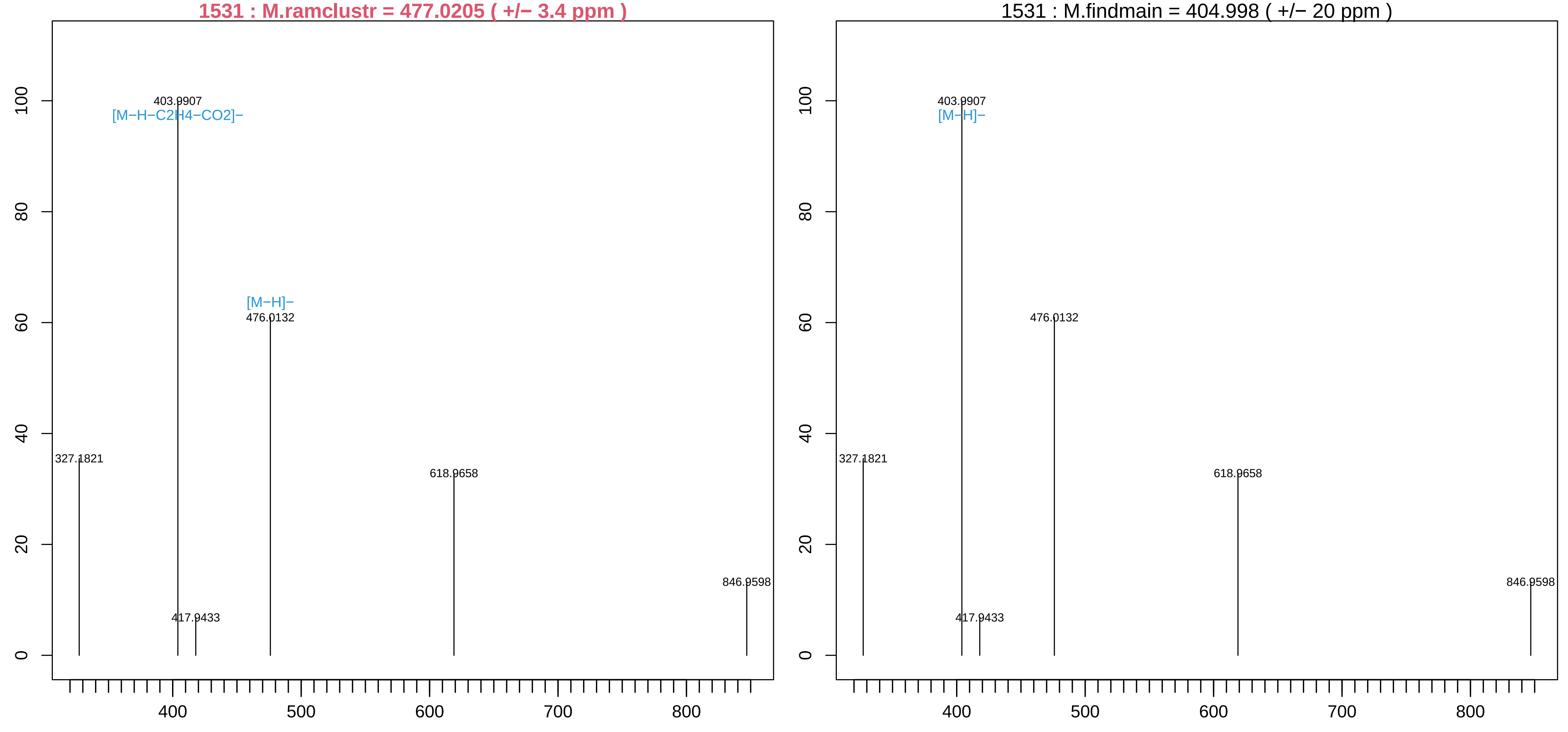
1531 : M.ramclustr = 477.0205 (+/− 3.4 ppm)
403.9907
[M−H−C2H4−CO2]−
[M−H]−
476.0132
327.1821
618.9658
846.9598
417.9433
1531 : M.findmain = 404.998 (+/− 20 ppm)
403.9907
[M−H]−
476.0132
327.1821
618.9658
846.9598
417.9433
0
20
40
60
80
100
400
500
600
700
800

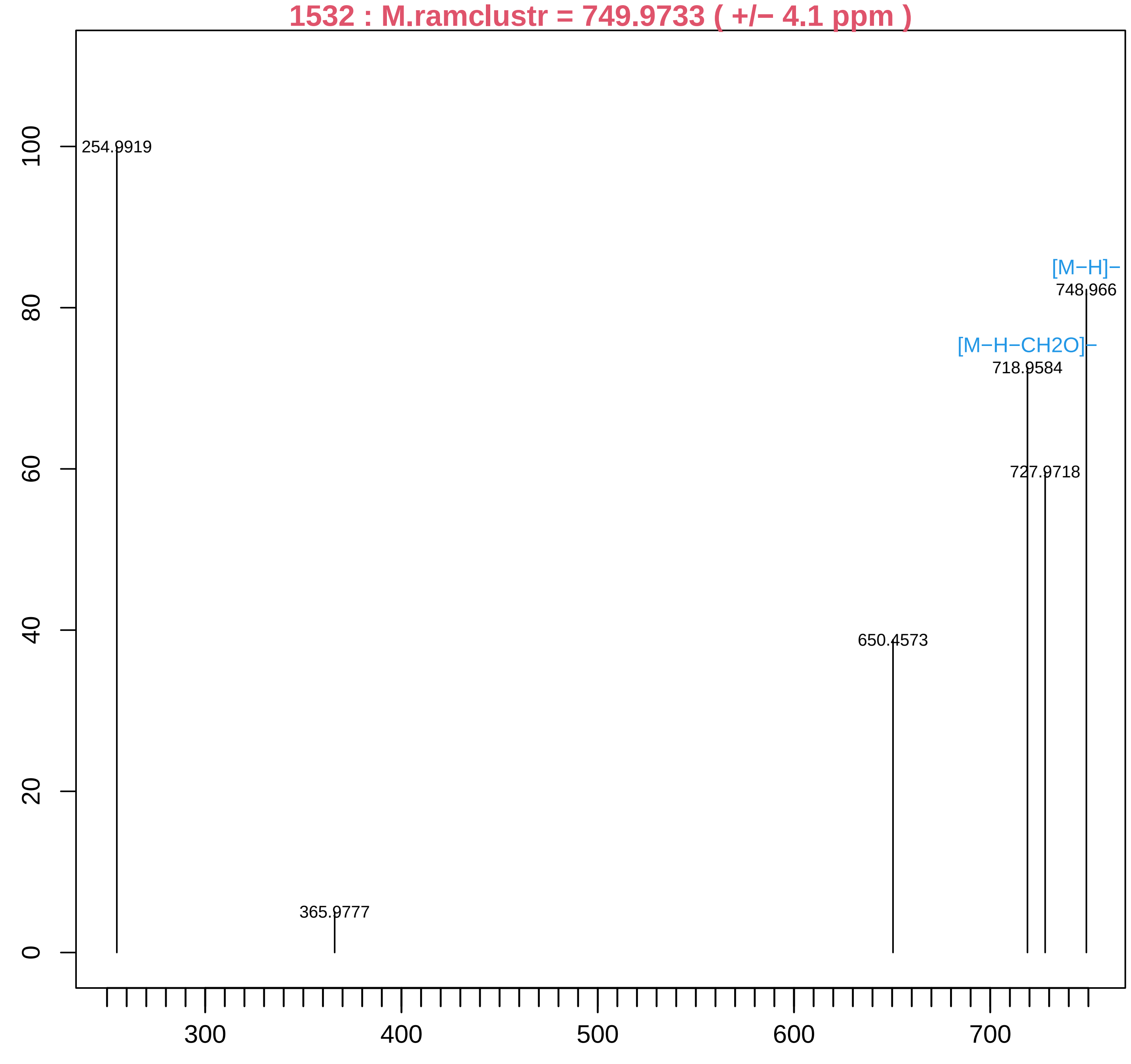
1532 : M.ramclustr = 749.9733 (+/− 4.1 ppm)
254.9919
365.9777
650.4573
[M−H−CH2O]−
718.9584
727.9718
[M−H]−
748.966
0
20
40
60
80
100
300
400
500
600
700

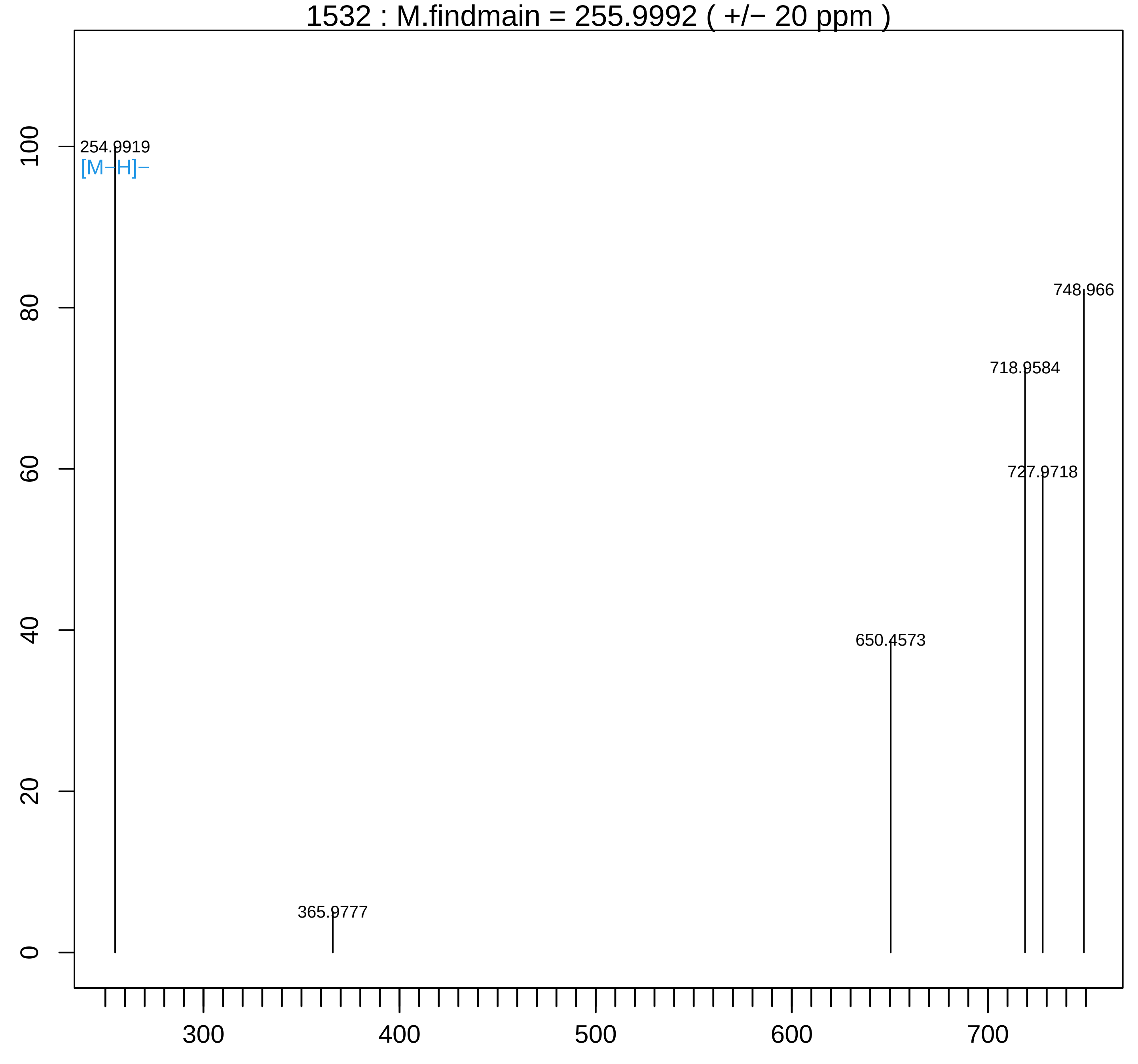
1532 : M.findmain = 255.9992 (+/− 20 ppm)
254.9919
[M−H]−
365.9777
650.4573
718.9584
727.9718
748.966
0
20
40
60
80
100
300
400
500
600
700

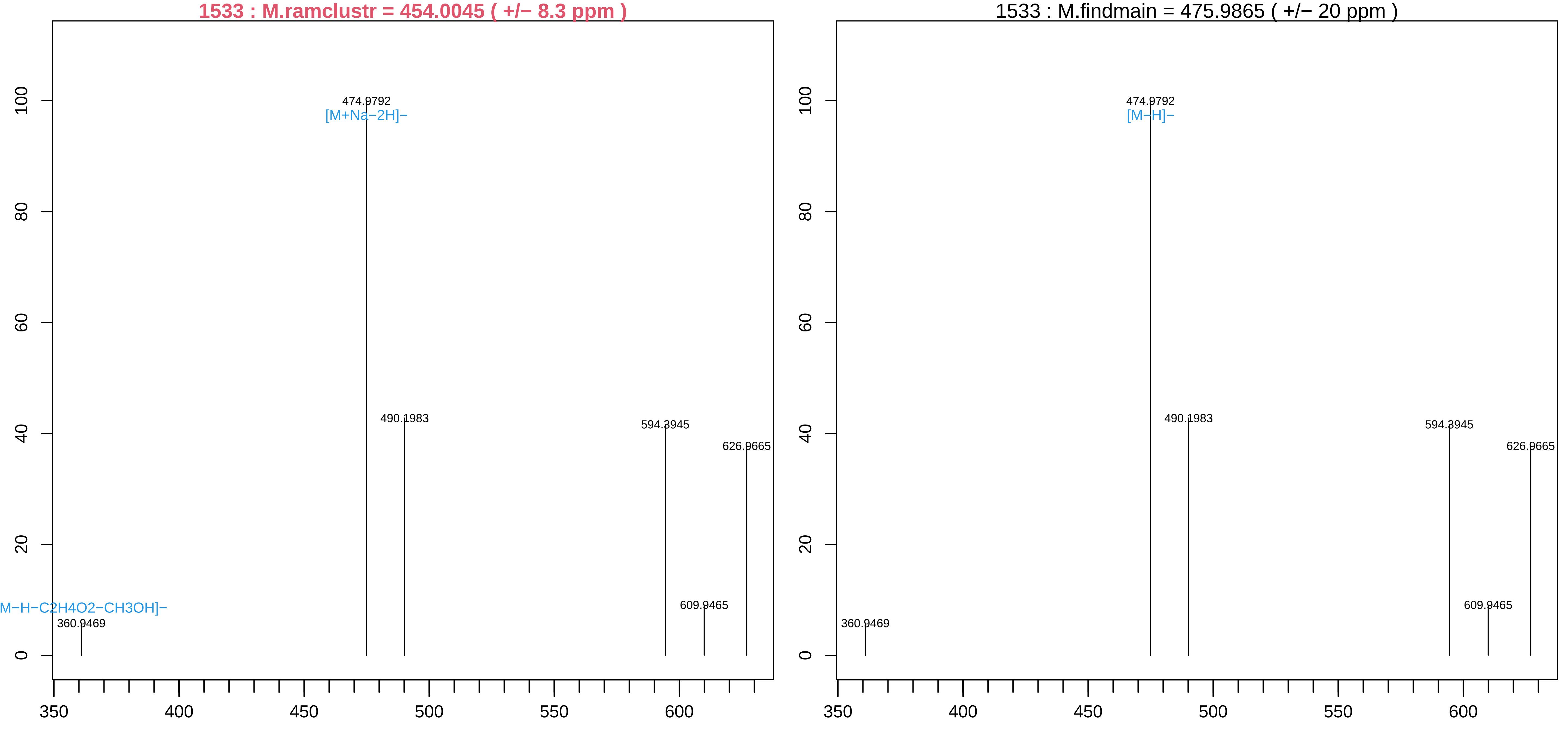

1533 : M.ramclustr = 454.0045 (+/− 8.3 ppm)
474.9792
[M+Na−2H]−
490.1983
594.3945
626.9665
609.9465
[M−H−C2H4O2−CH3OH]−
360.9469
1533 : M.findmain = 475.9865 (+/− 20 ppm)
474.9792
[M−H]−
490.1983
594.3945
626.9665
609.9465
360.9469

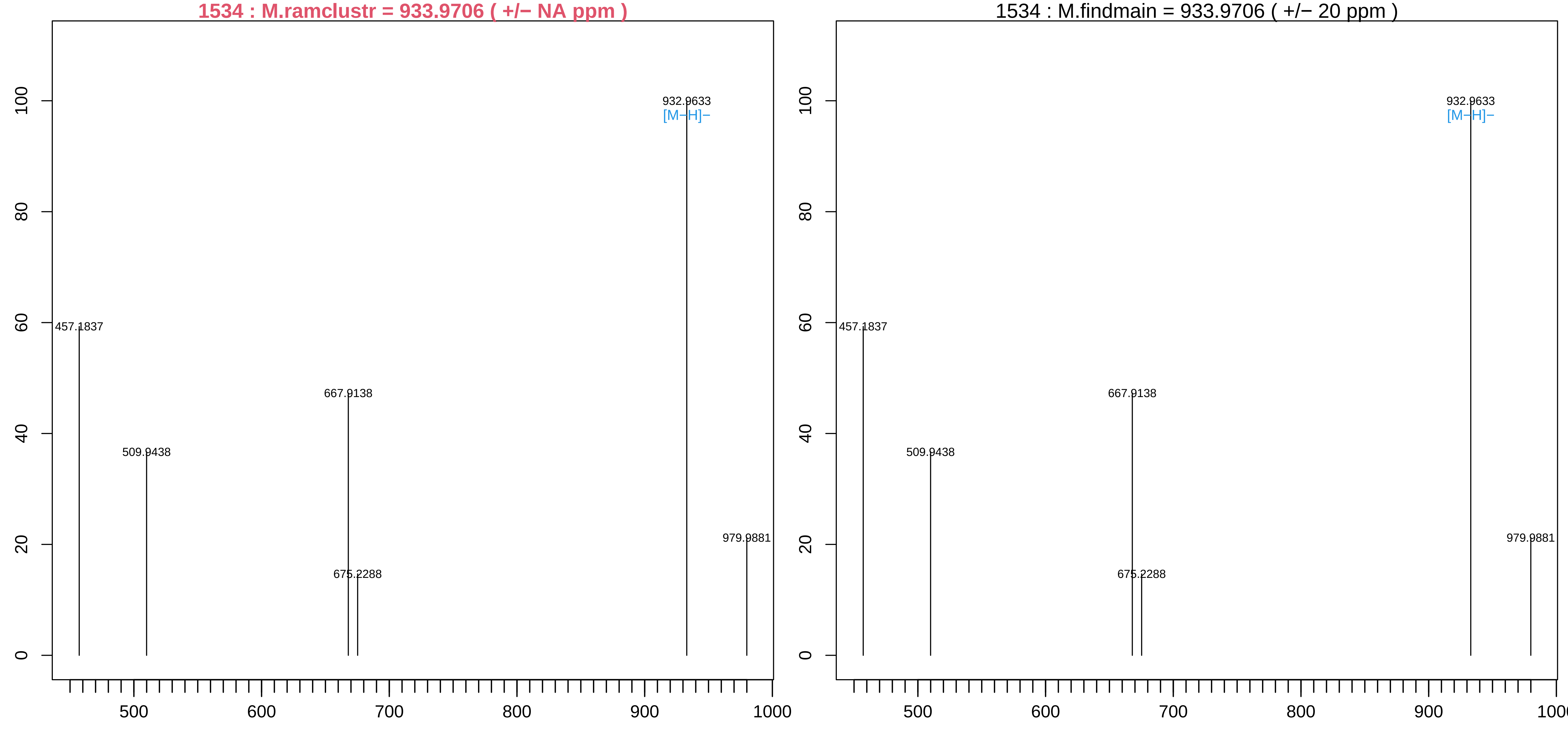

1534 : M.ramclustr = 933.9706 (+/− NA ppm)
1534 : M.findmain = 933.9706 (+/− 20 ppm)
932.9633
[M−H]−
457.1837
667.9138
509.9438
979.9881
675.2288
0
20
40
60
80
100
500
600
700
800
900
1000

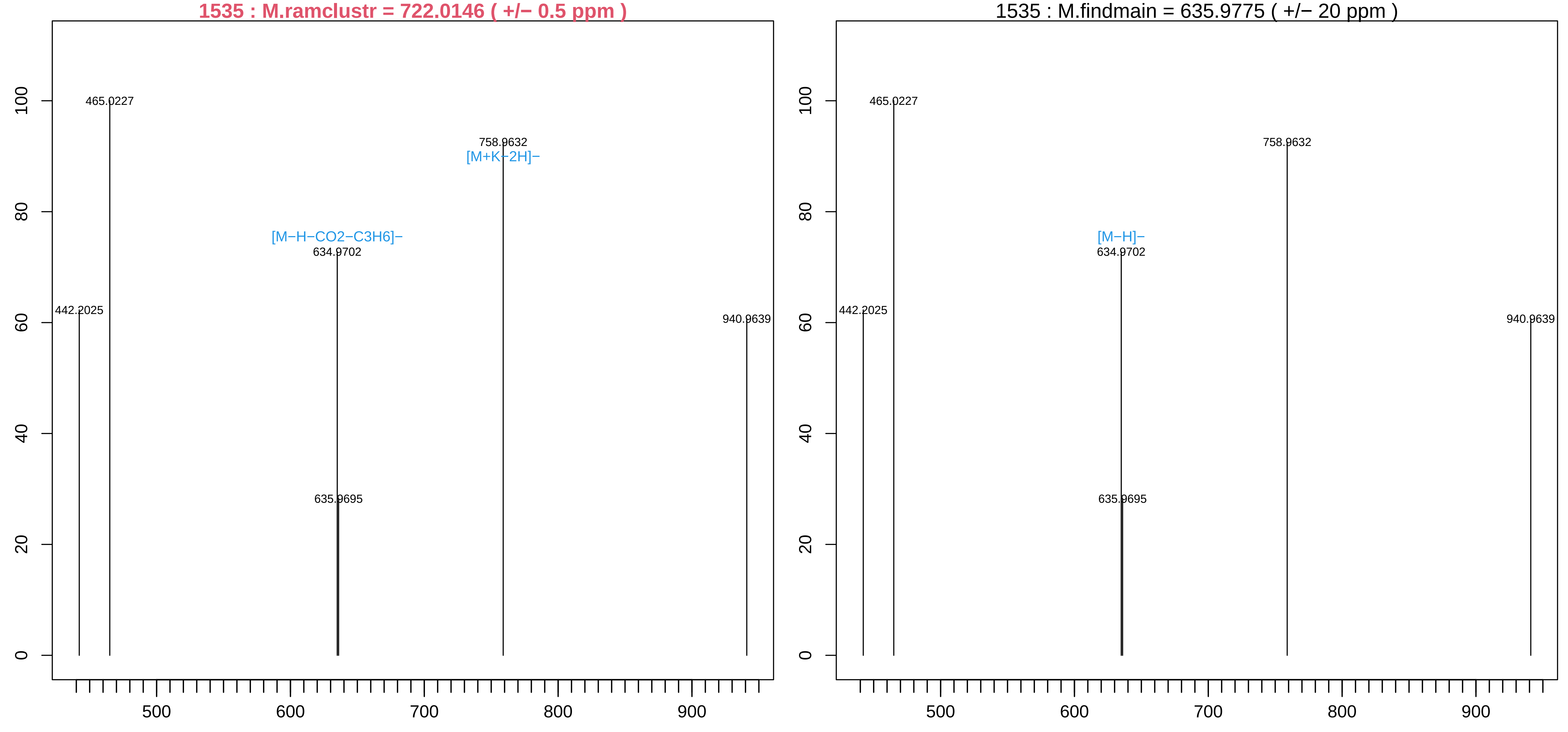

1535 : M.ramclustr = 722.0146 (+/− 0.5 ppm)
465.0227
758.9632
[M+K−2H]−
[M−H−CO2−C3H6]−
634.9702
442.2025
940.9639
635.9695
0
20
40
60
80
100
500
600
700
800
900
1535 : M.findmain = 635.9775 (+/− 20 ppm)
465.0227
758.9632
[M−H]−
634.9702
442.2025
940.9639
635.9695

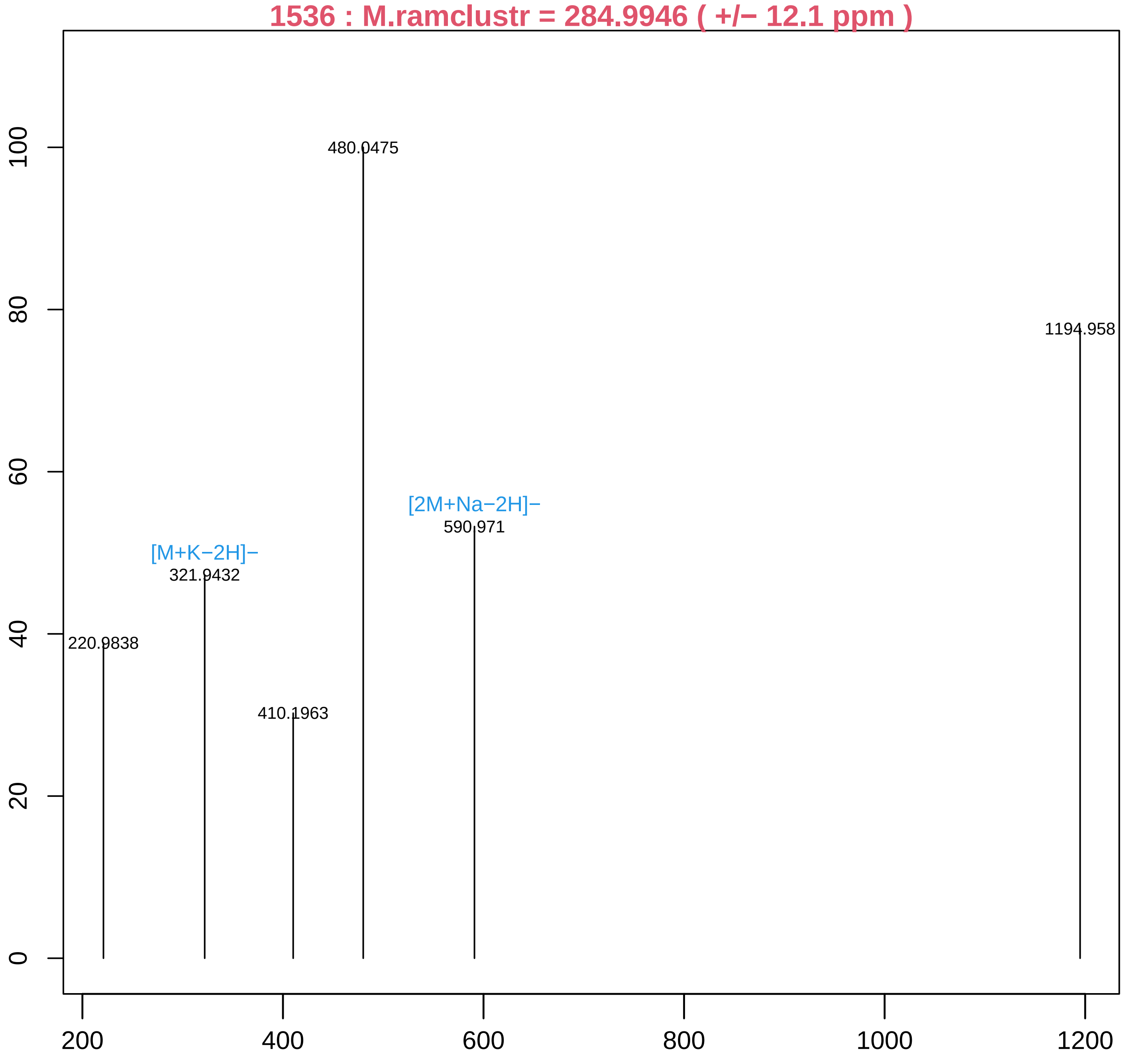
1536 : M.ramclustr = 284.9946 (+/− 12.1 ppm)
480.0475
1194.958
[2M+Na−2H]−
590.971
[M+K−2H]−
321.9432
220.9838
410.1963
0
20
40
60
80
100
200
400
600
800
1000
1200

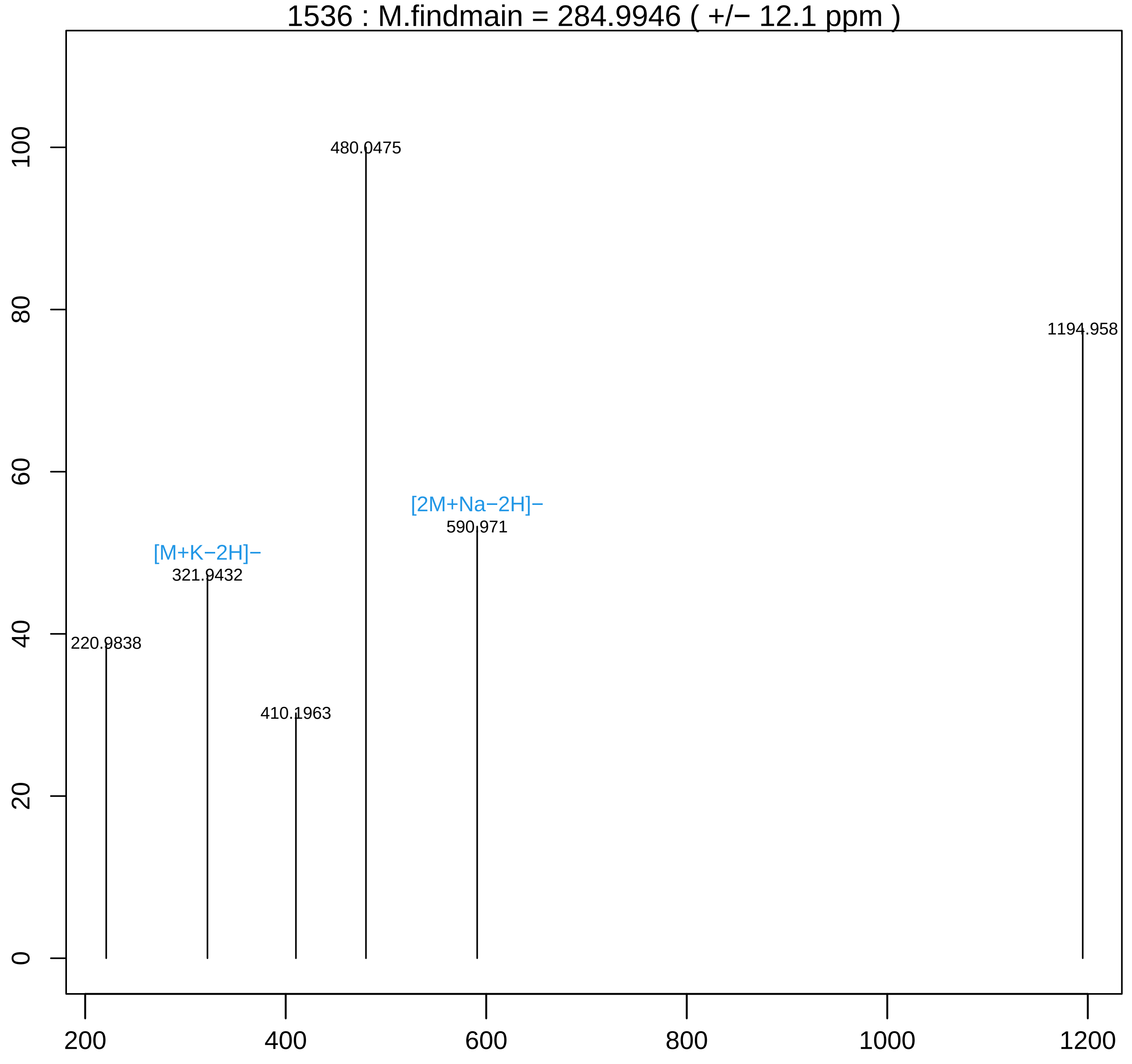
1536 : M.findmain = 284.9946 (+/− 12.1 ppm)
480.0475
1194.958
[2M+Na−2H]−
590.971
[M+K−2H]−
321.9432
220.9838
410.1963
0
20
40
60
80
100
200
400
600
800
1000
1200

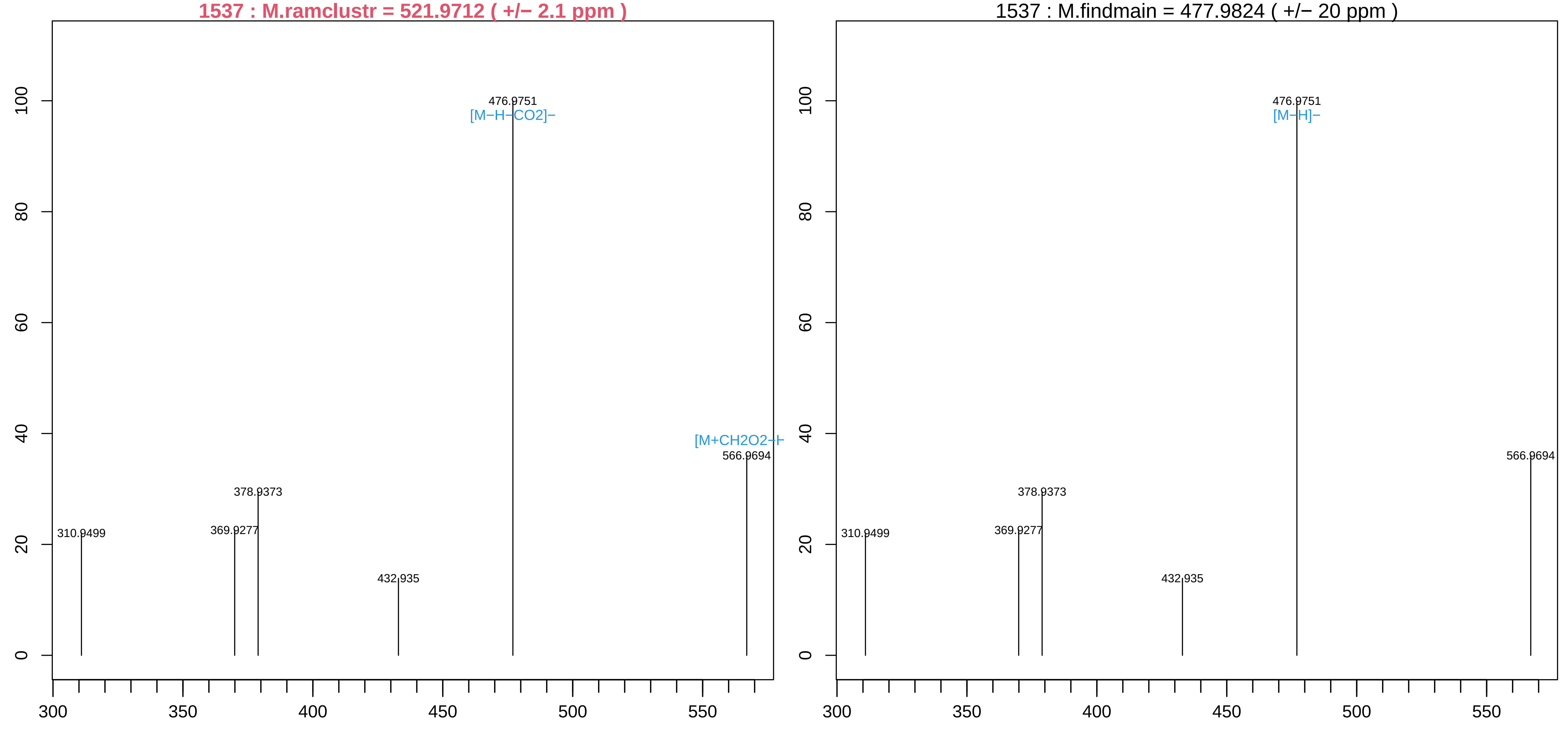
1537 : M.ramclustr = 521.9712 (+/− 2.1 ppm)
476.9751
[M−H−CO2]−
[M+CH2O2−H
566.9694
378.9373
369.9277
310.9499
432.935
1537 : M.findmain = 477.9824 (+/− 20 ppm)
476.9751
[M−H]−
566.9694
378.9373
369.9277
310.9499
432.935
0
20
40
60
80
100
300
350
400
450
500
550

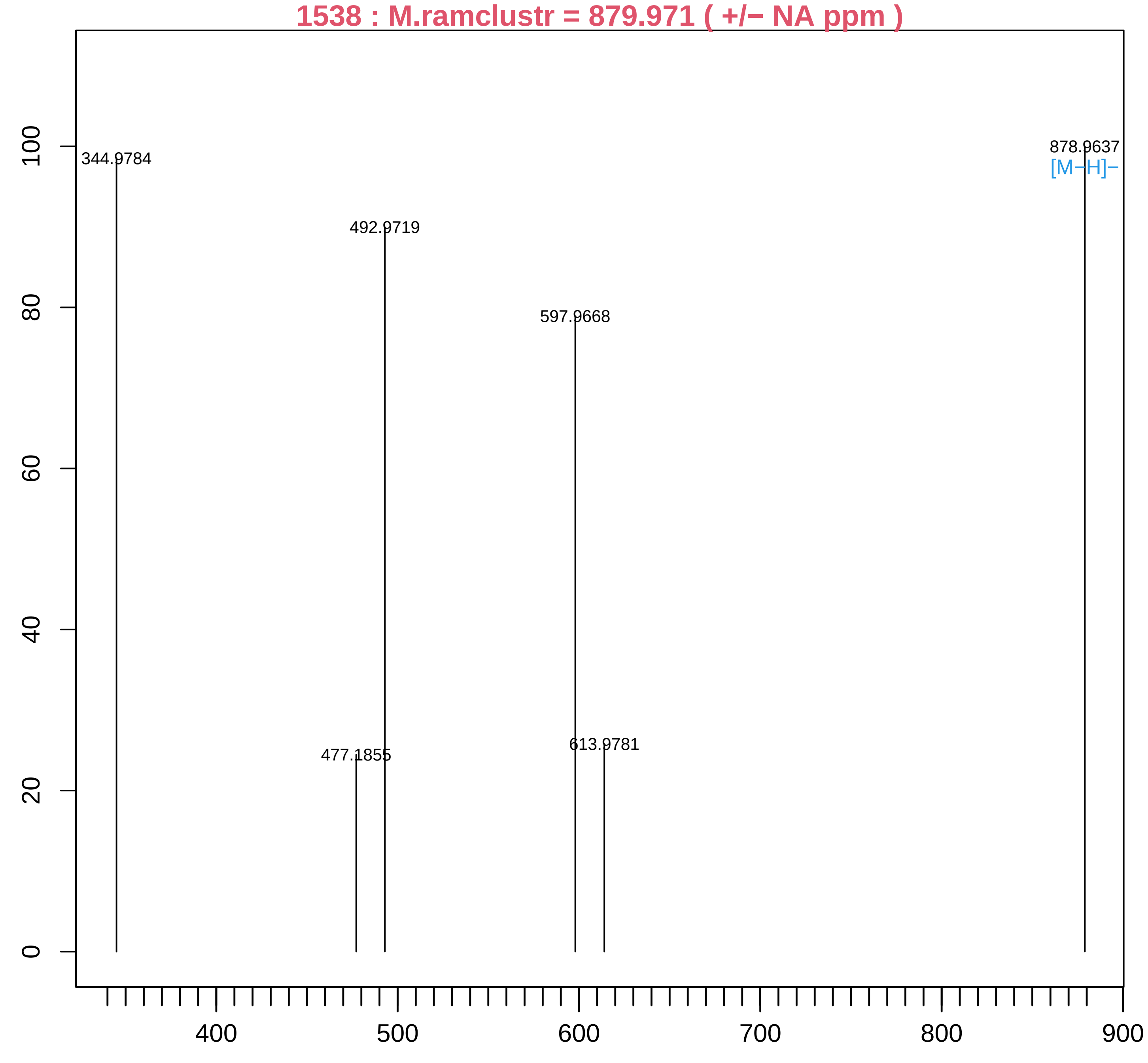

1538 : M.ramclustr = 879.971 (+/− NA ppm)
344.9784
492.9719
597.9668
477.1855
613.9781
878.9637
[M−H]−
0
20
40
60
80
100
400
500
600
700
800
900

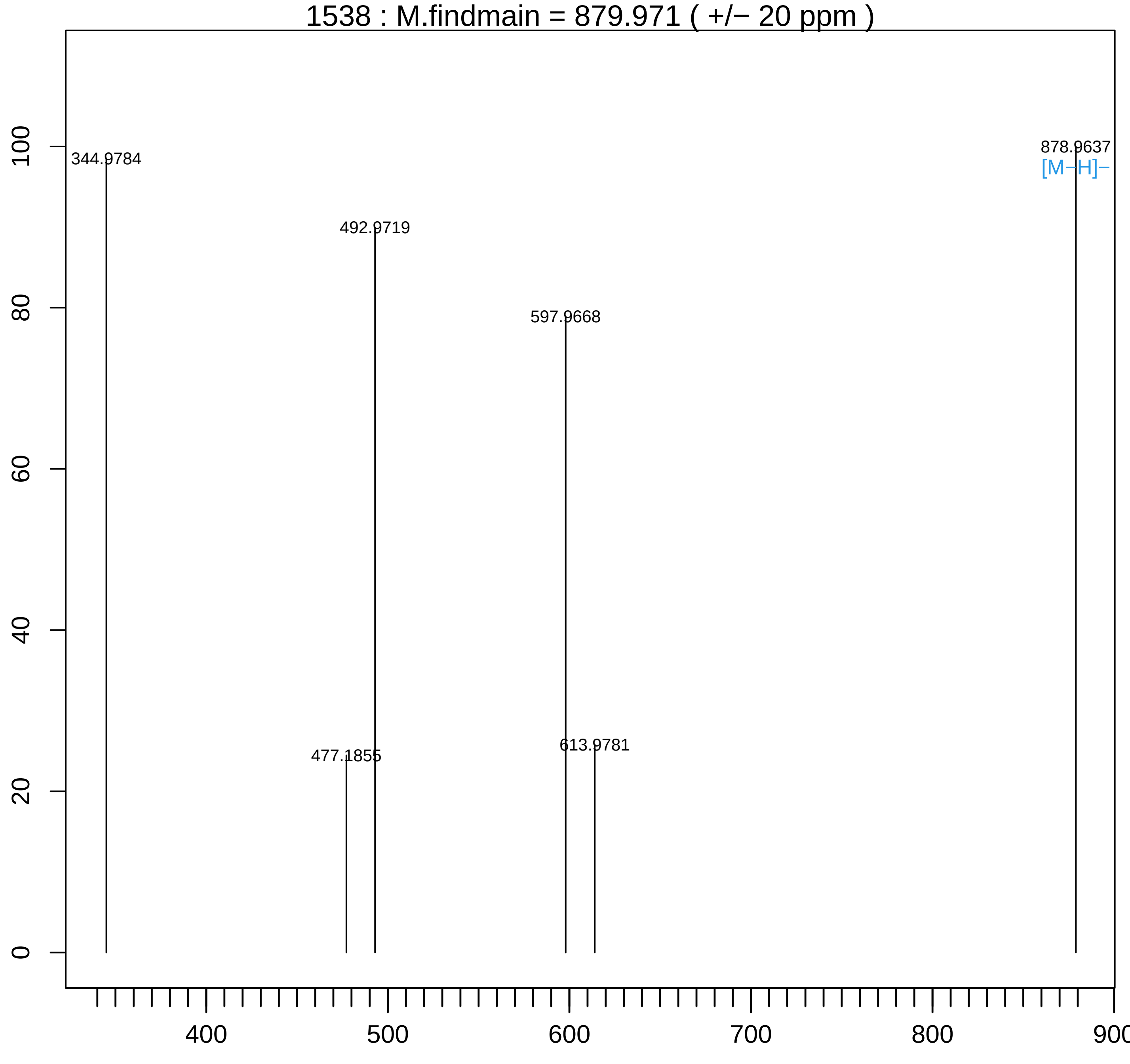

1538 : M.findmain = 879.971 (+/− 20 ppm)
344.9784
492.9719
597.9668
477.1855
613.9781
878.9637
[M−H]−
0
20
40
60
80
100
400
500
600
700
800
900

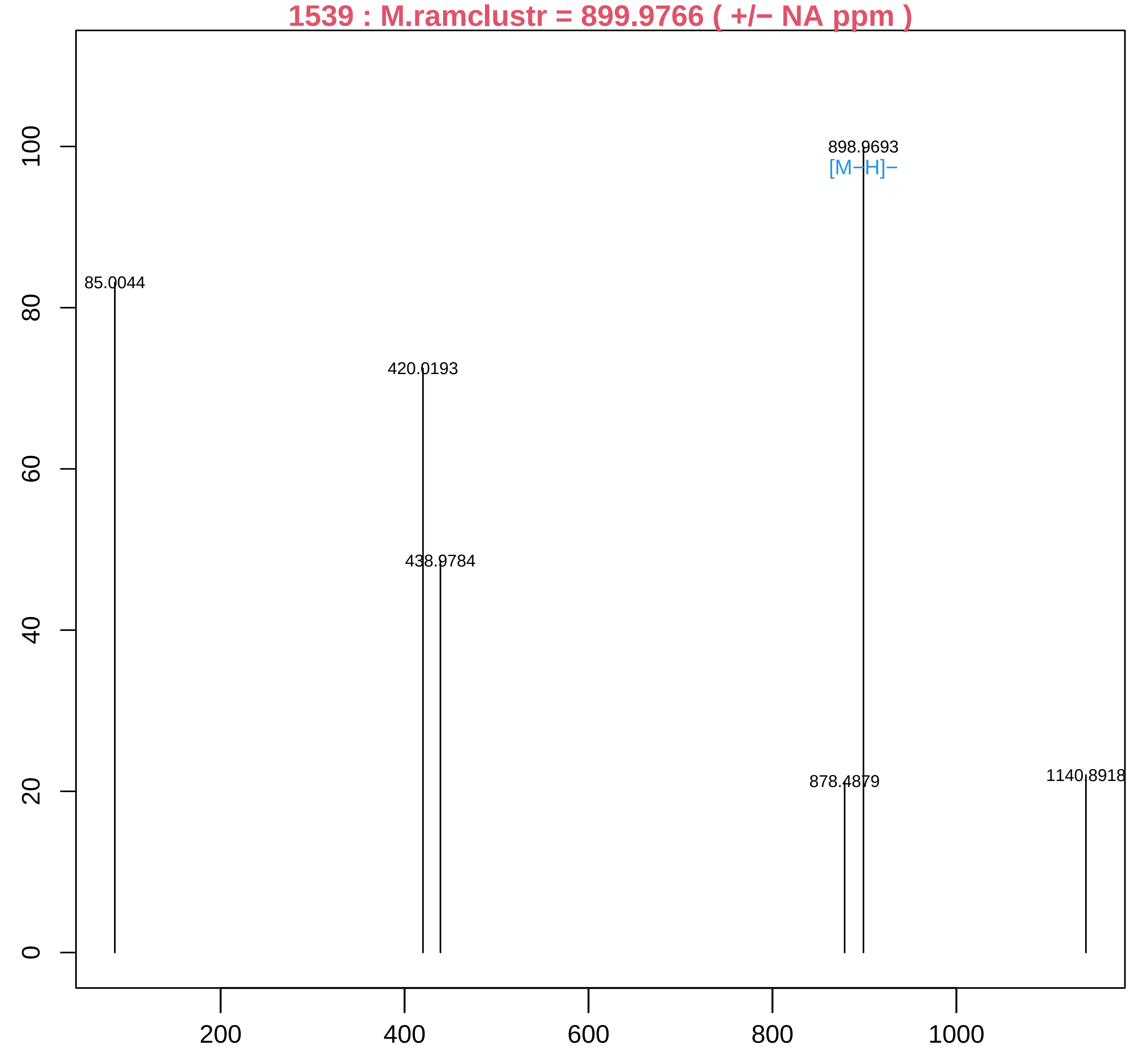
1539 : M.ramclustr = 899.9766 (+/− NA ppm)
898.9693
[M−H]−
85.0044
420.0193
438.9784
878.4879
1140.8918
0
20
40
60
80
100
200
400
600
800
1000

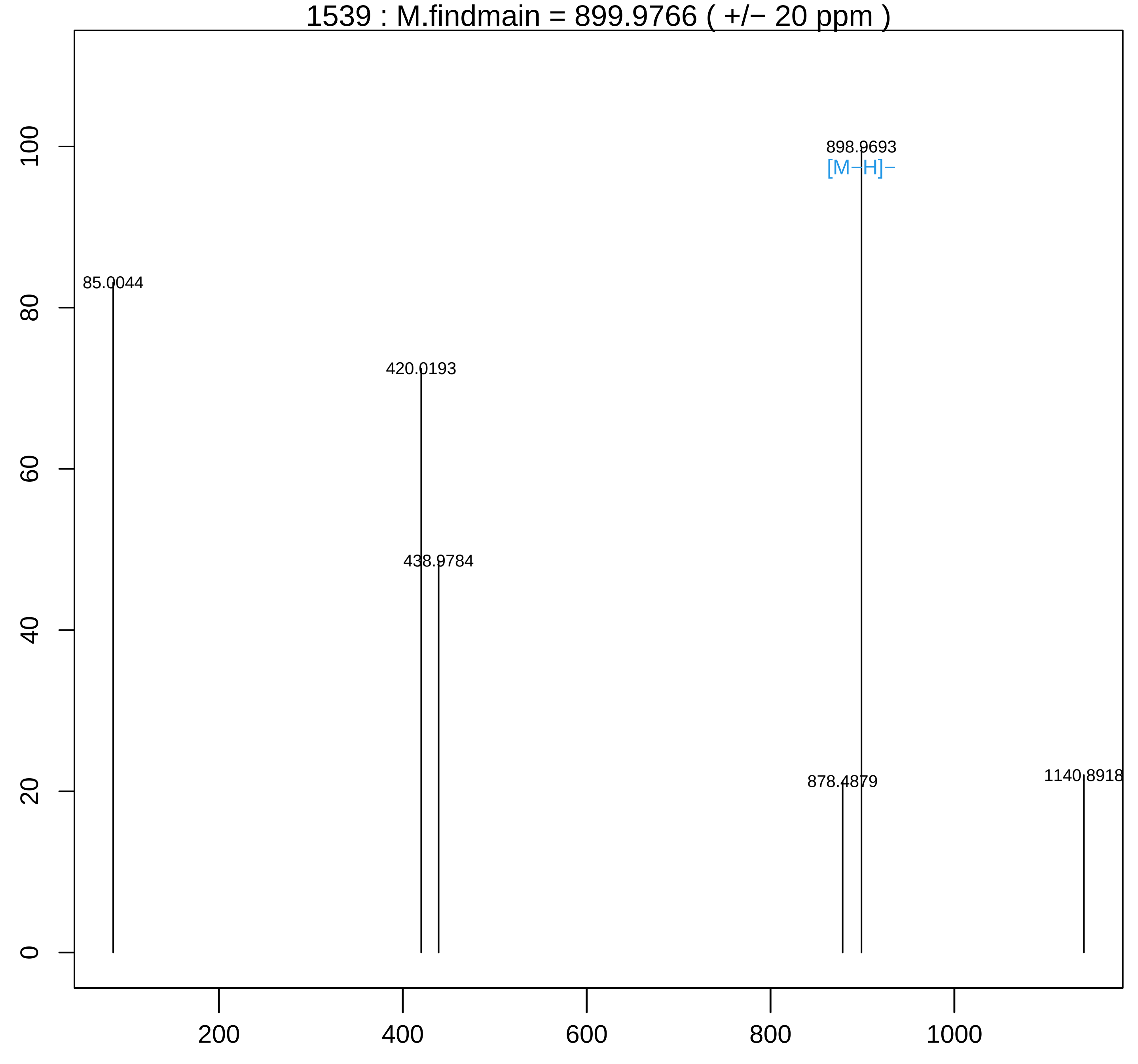
1539 : M.findmain = 899.9766 (+/− 20 ppm)
898.9693
[M−H]−
85.0044
420.0193
438.9784
878.4879
1140.8918
0
20
40
60
80
100
200
400
600
800
1000

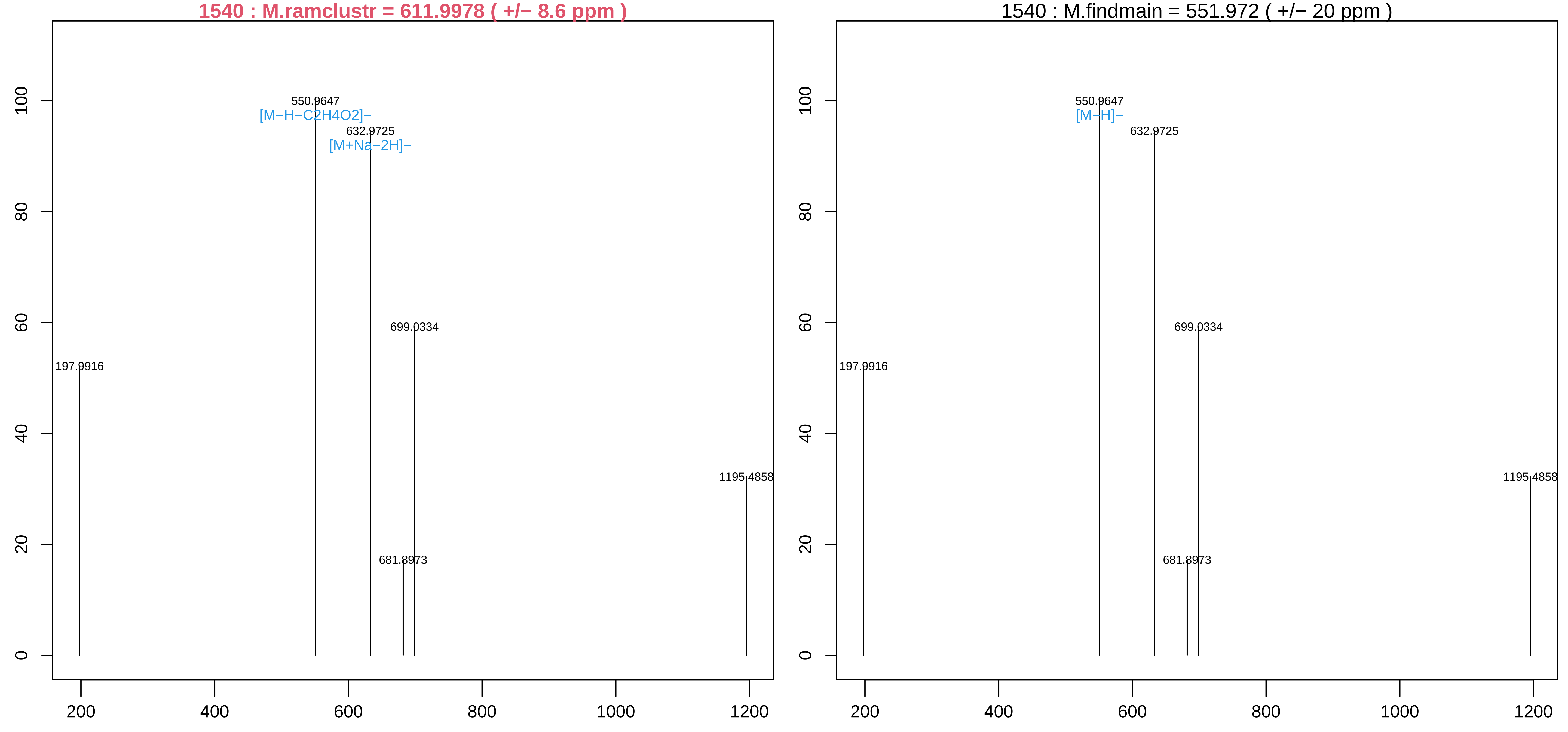
1540 : M.ramclustr = 611.9978 (+/− 8.6 ppm)
550.9647
[M−H−C2H4O2]−
632.9725
[M+Na−2H]−
699.0334
197.9916
681.8973
1195.4858
1540 : M.findmain = 551.972 (+/− 20 ppm)
550.9647
[M−H]−
632.9725
699.0334
197.9916
681.8973
1195.4858

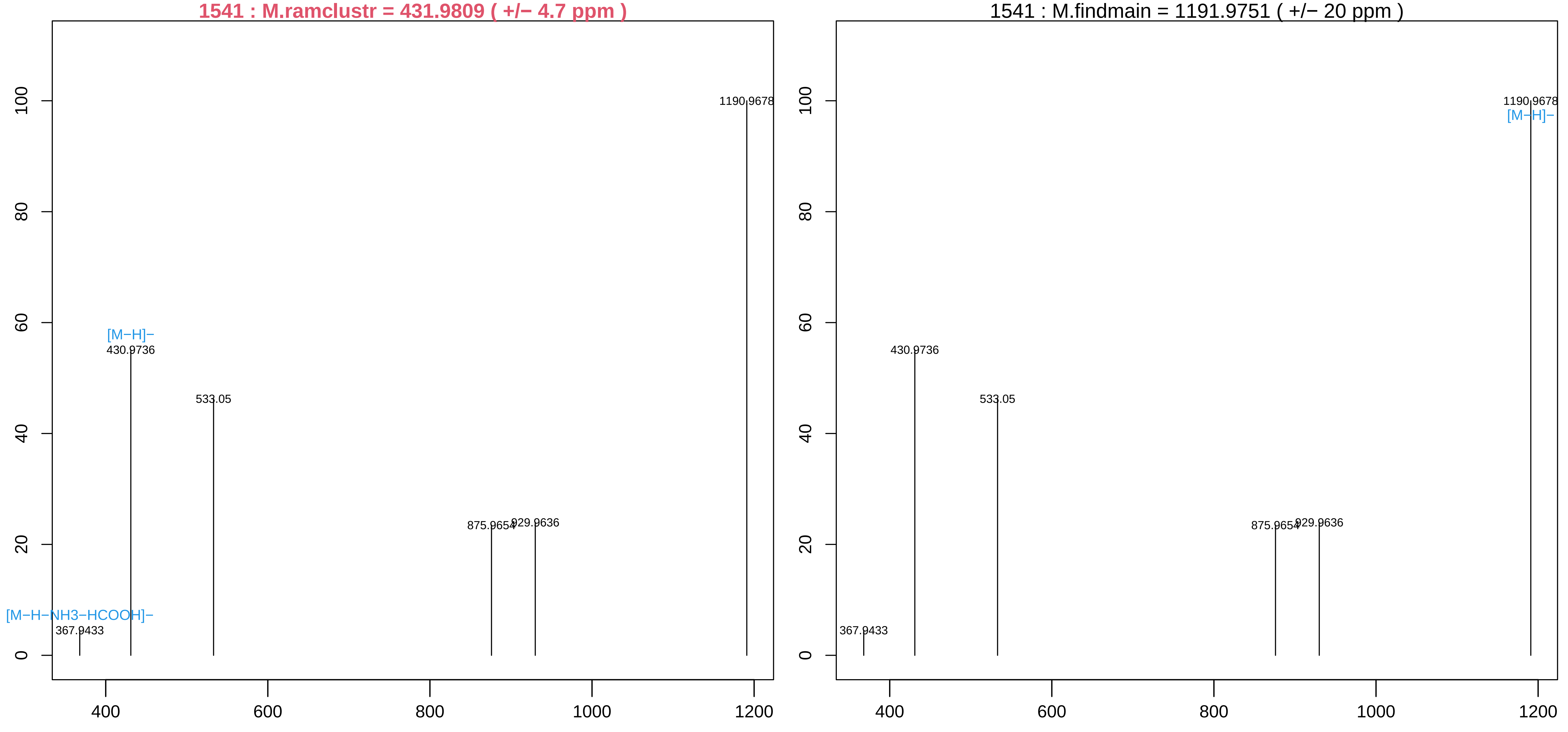

1541 : M.ramclustr = 431.9809 (+/− 4.7 ppm)
1190.9678
[M−H]−
430.9736
533.05
875.9654
929.9636
[M−H−NH3−HCOOH]−
367.9433
0
20
40
60
80
100
400
600
800
1000
1200
1541 : M.findmain = 1191.9751 (+/− 20 ppm)
1190.9678
[M−H]−
430.9736
533.05
875.9654
929.9636
367.9433

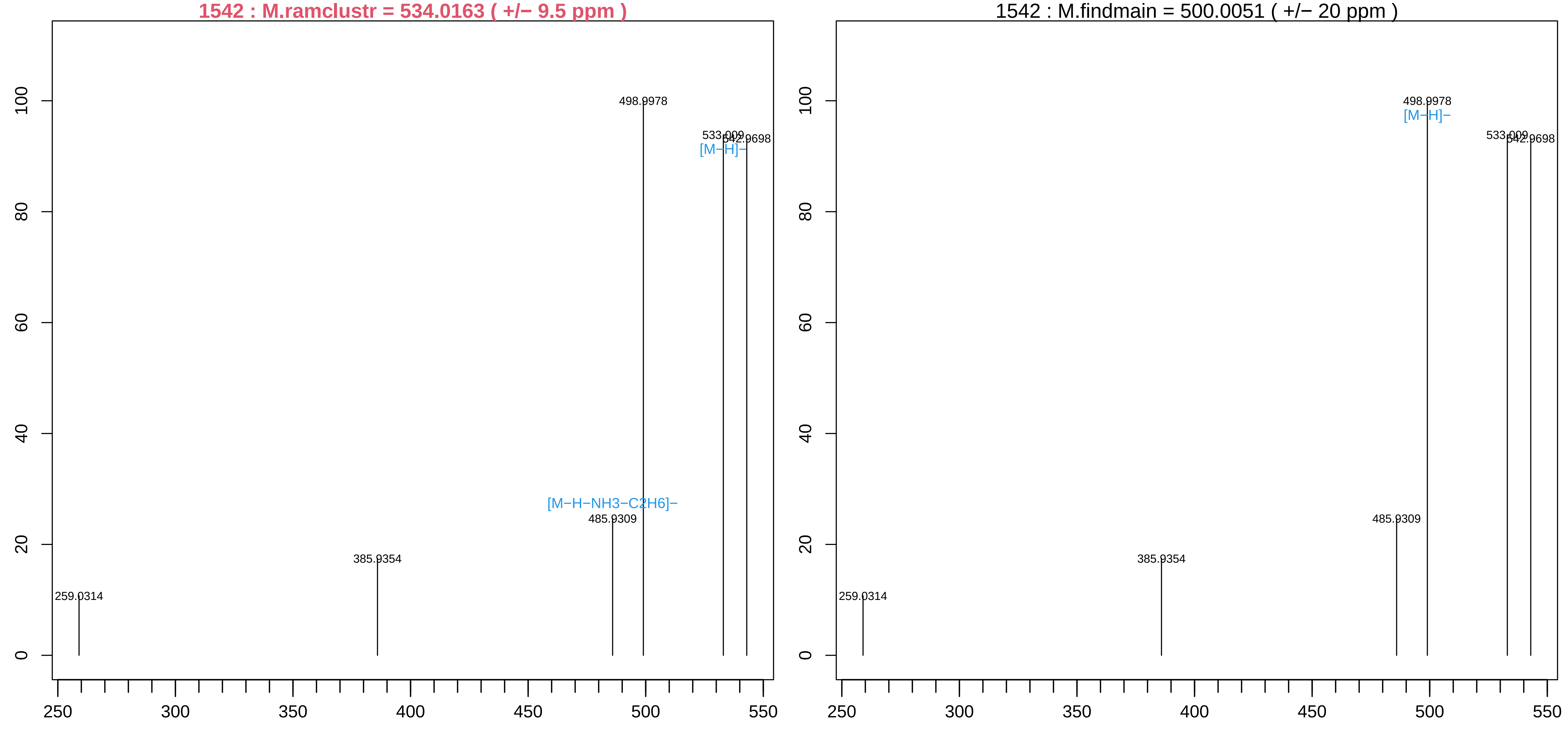

1542 : M.ramclustr = 534.0163 (+/− 9.5 ppm)
498.9978
533.009
542.9698
[M−H]−
[M−H−NH3−C2H6]−
485.9309
385.9354
259.0314
1542 : M.findmain = 500.0051 (+/− 20 ppm)
498.9978
[M−H]−
533.009
542.9698
485.9309
385.9354
259.0314

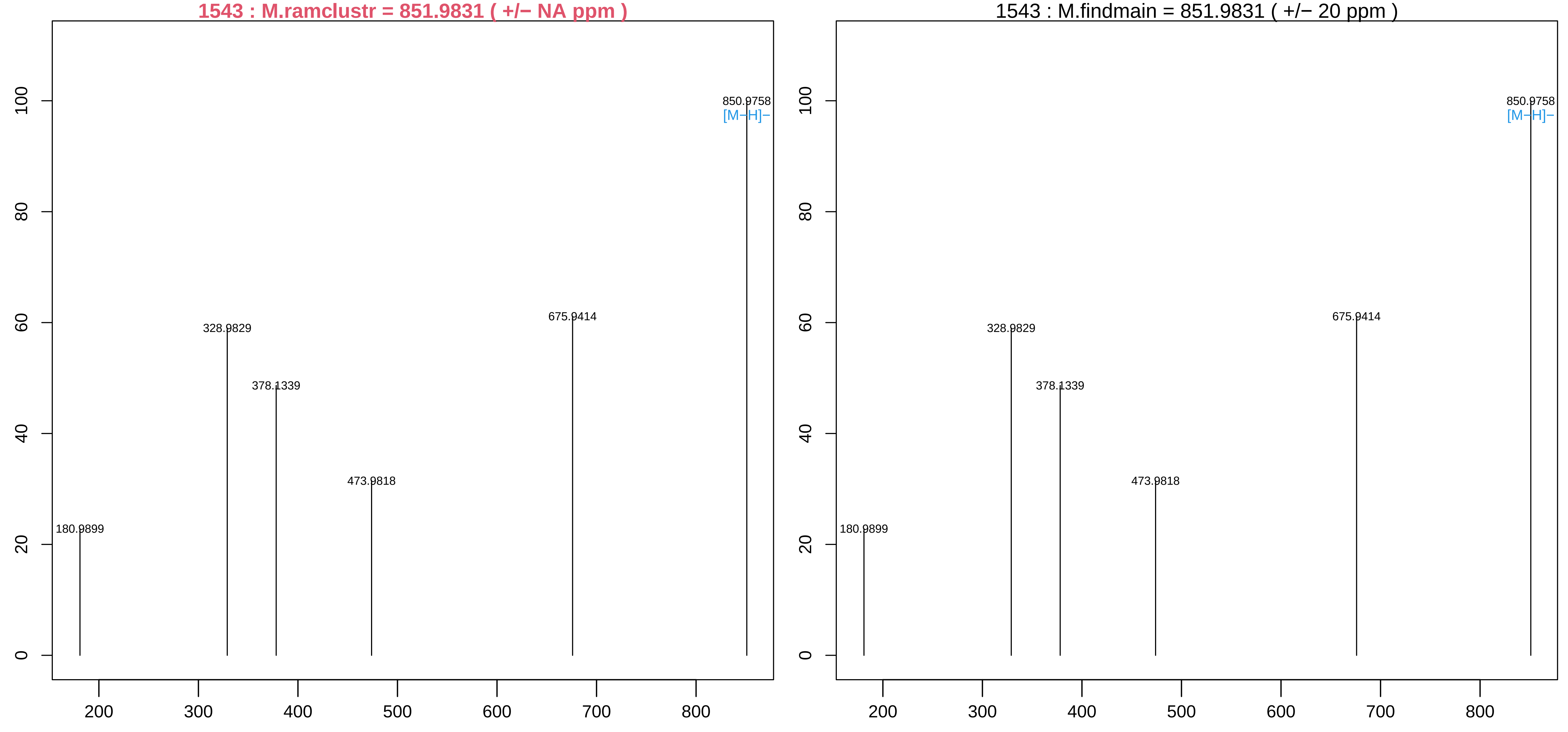
1543 : M.ramclustr = 851.9831 (+/− NA ppm)
1543 : M.findmain = 851.9831 (+/− 20 ppm)
850.9758
[M−H]−
675.9414
328.9829
378.1339
473.9818
180.9899
0
20
40
60
80
100
200
300
400
500
600
700
800

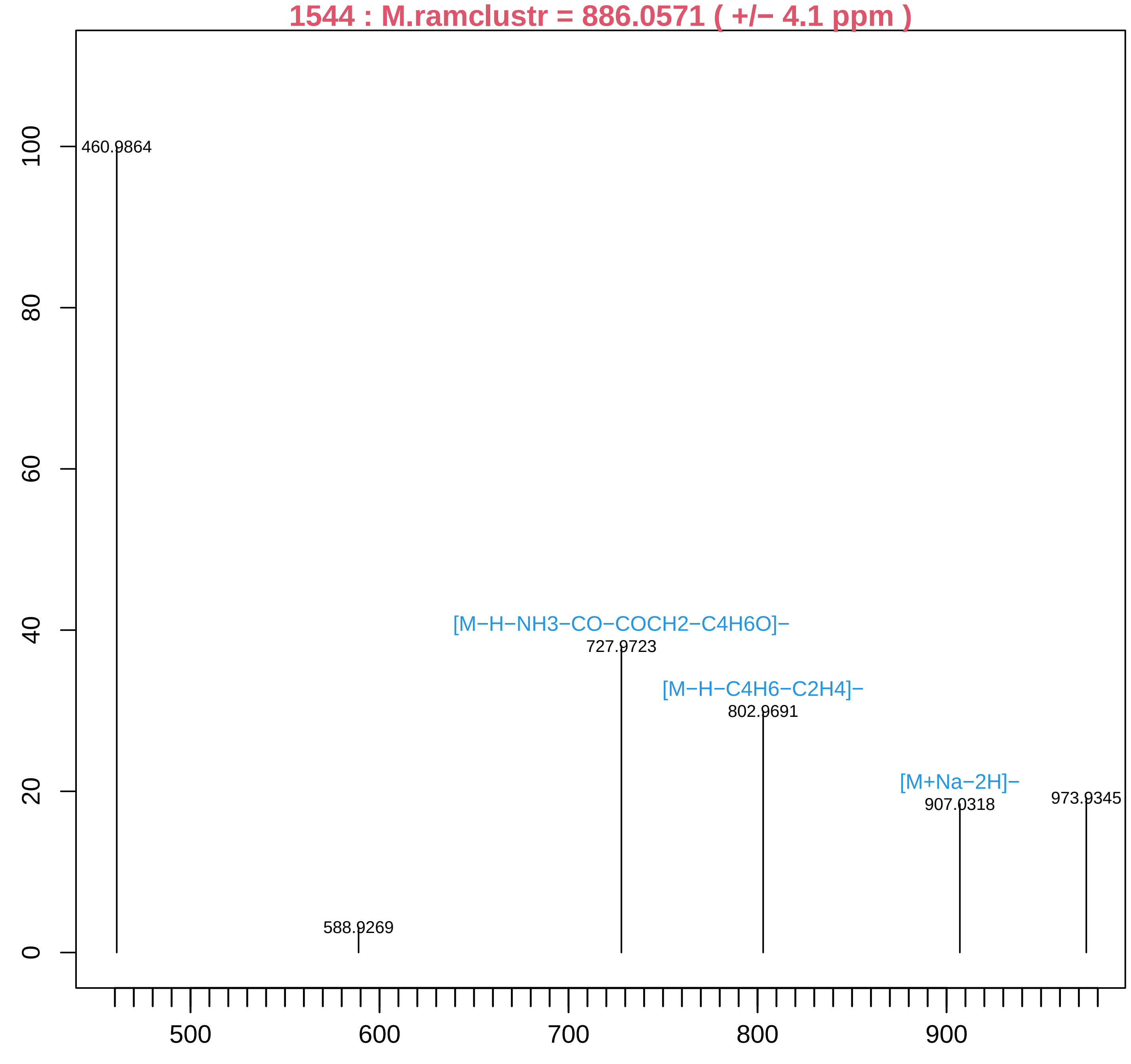

1544 : M.ramclustr = 886.0571 (+/− 4.1 ppm)
460.9864
588.9269
[M−H−NH3−CO−COCH2−C4H6O]−
727.9723
[M−H−C4H6−C2H4]−
802.9691
[M+Na−2H]−
907.0318
973.9345
0
20
40
60
80
100
500
600
700
800
900

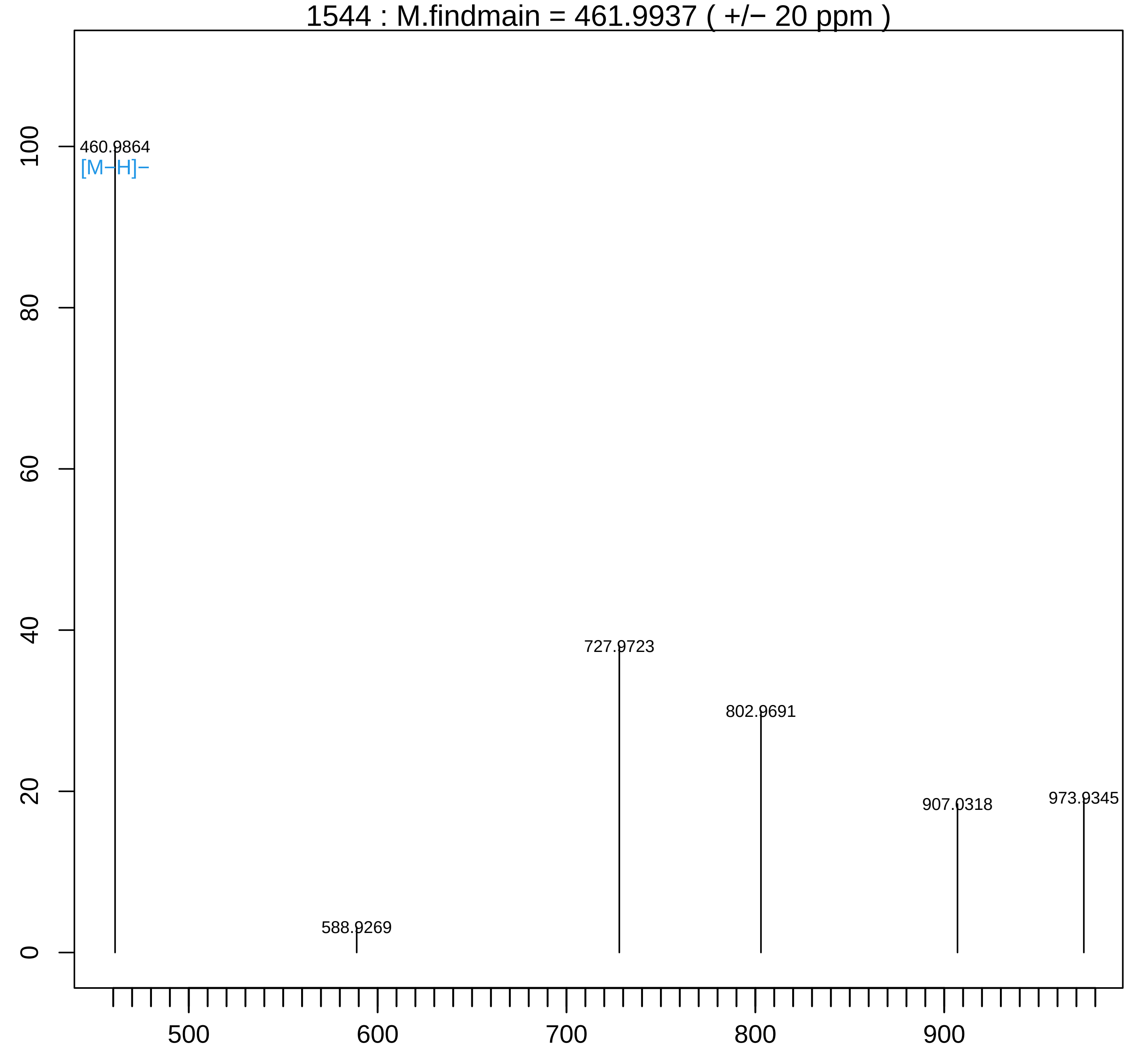

1544 : M.findmain = 461.9937 (+/− 20 ppm)
460.9864
[M−H]−
588.9269
727.9723
802.9691
907.0318
973.9345
0
20
40
60
80
100
500
600
700
800
900

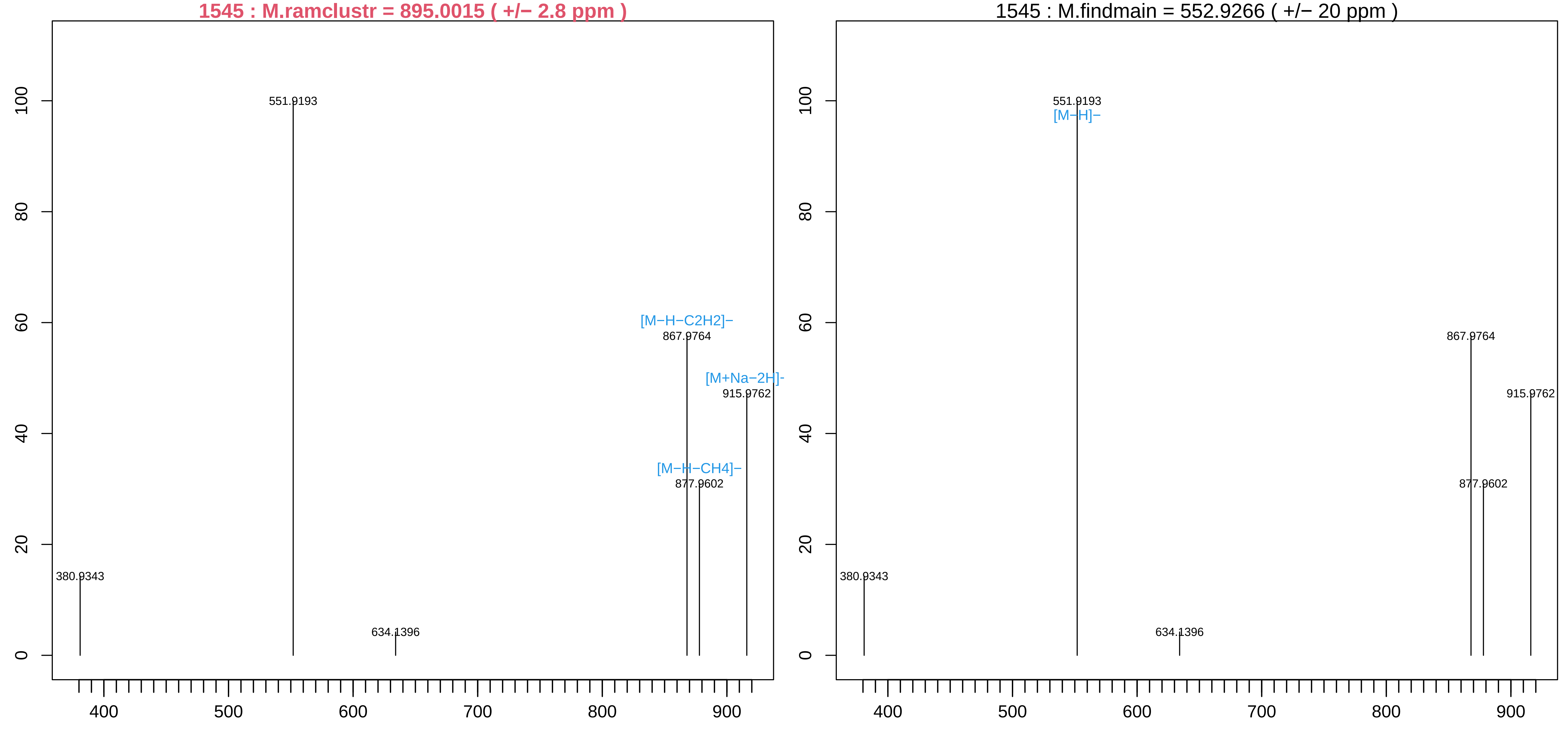

1545 : M.ramclustr = 895.0015 (+/− 2.8 ppm)
551.9193
[M−H−C2H2]−
867.9764
[M+Na−2H]-
915.9762
[M−H−CH4]−
877.9602
380.9343
634.1396
1545 : M.findmain = 552.9266 (+/− 20 ppm)
551.9193
[M−H]−
867.9764
915.9762
877.9602
380.9343
634.1396
0
20
40
60
80
100
400
500
600
700
800
900

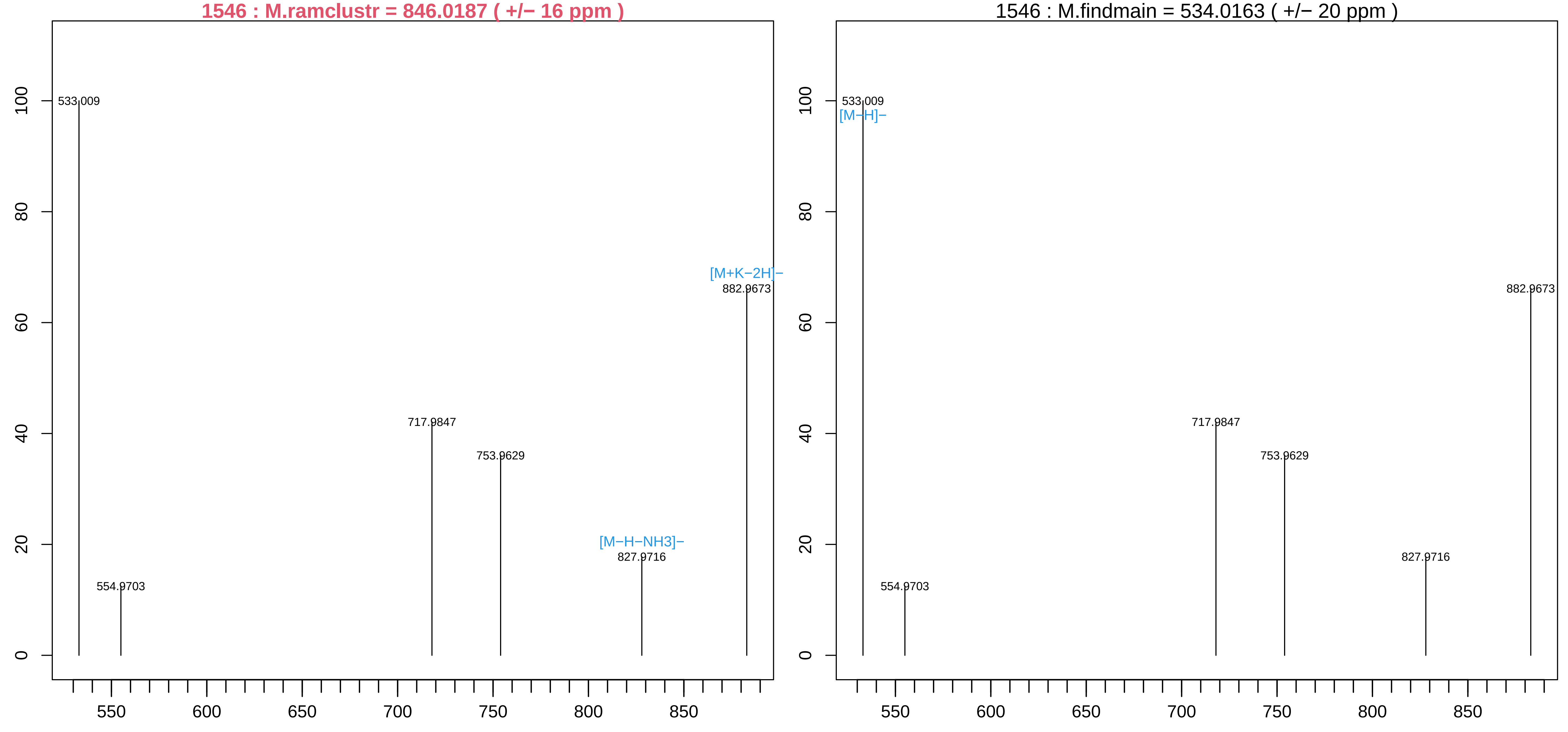

1546 : M.ramclustr = 846.0187 (+/− 16 ppm)
533.009
554.9703
717.9847
753.9629
[M−H−NH3]−
827.9716
[M+K−2H]−
882.9673
0
20
40
60
80
100
550
600
650
700
750
800
850
1546 : M.findmain = 534.0163 (+/− 20 ppm)
533.009
[M−H]−
554.9703
717.9847
753.9629
827.9716
882.9673
0
20
40
60
80
100
550
600
650
700
750
800
850

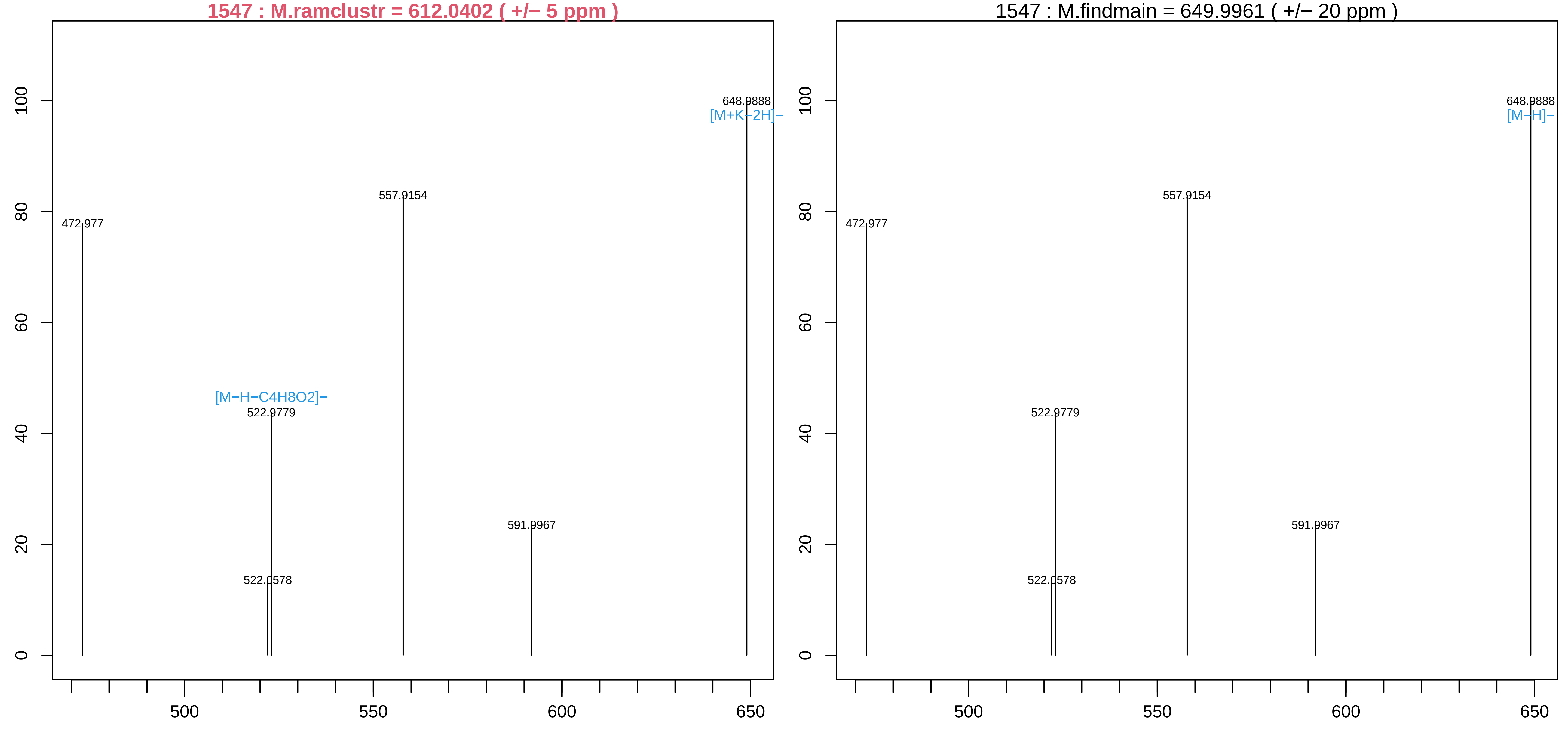
1547 : M.ramclustr = 612.0402 (+/− 5 ppm)
648.9888
[M+K−2H]−
557.9154
472.977
[M−H−C4H8O2]−
522.9779
591.9967
522.0578
0
20
40
60
80
100
500
550
600
650
1547 : M.findmain = 649.9961 (+/− 20 ppm)
648.9888
[M−H]−
557.9154
472.977
522.9779
591.9967
522.0578
0
20
40
60
80
100
500
550
600
650

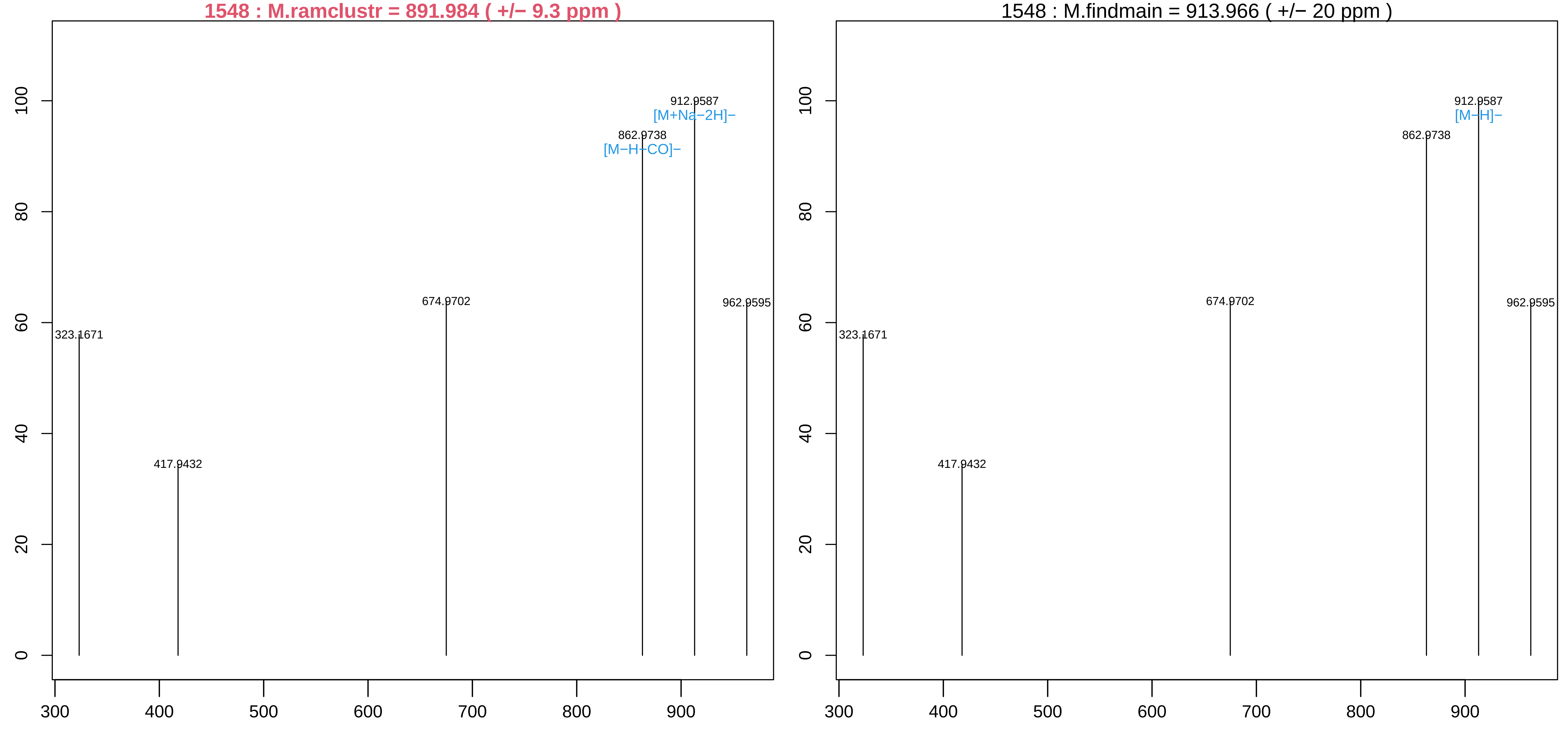
1548 : M.ramclustr = 891.984 (+/− 9.3 ppm)
912.9587
[M+Na−2H]−
862.9738
[M−H−CO]−
674.9702
962.9595
323.1671
417.9432
0
20
40
60
80
100
300
400
500
600
700
800
900
1548 : M.findmain = 913.966 (+/− 20 ppm)
912.9587
[M−H]−
862.9738
674.9702
962.9595
323.1671
417.9432
0
20
40
60
80
100
300
400
500
600
700
800
900

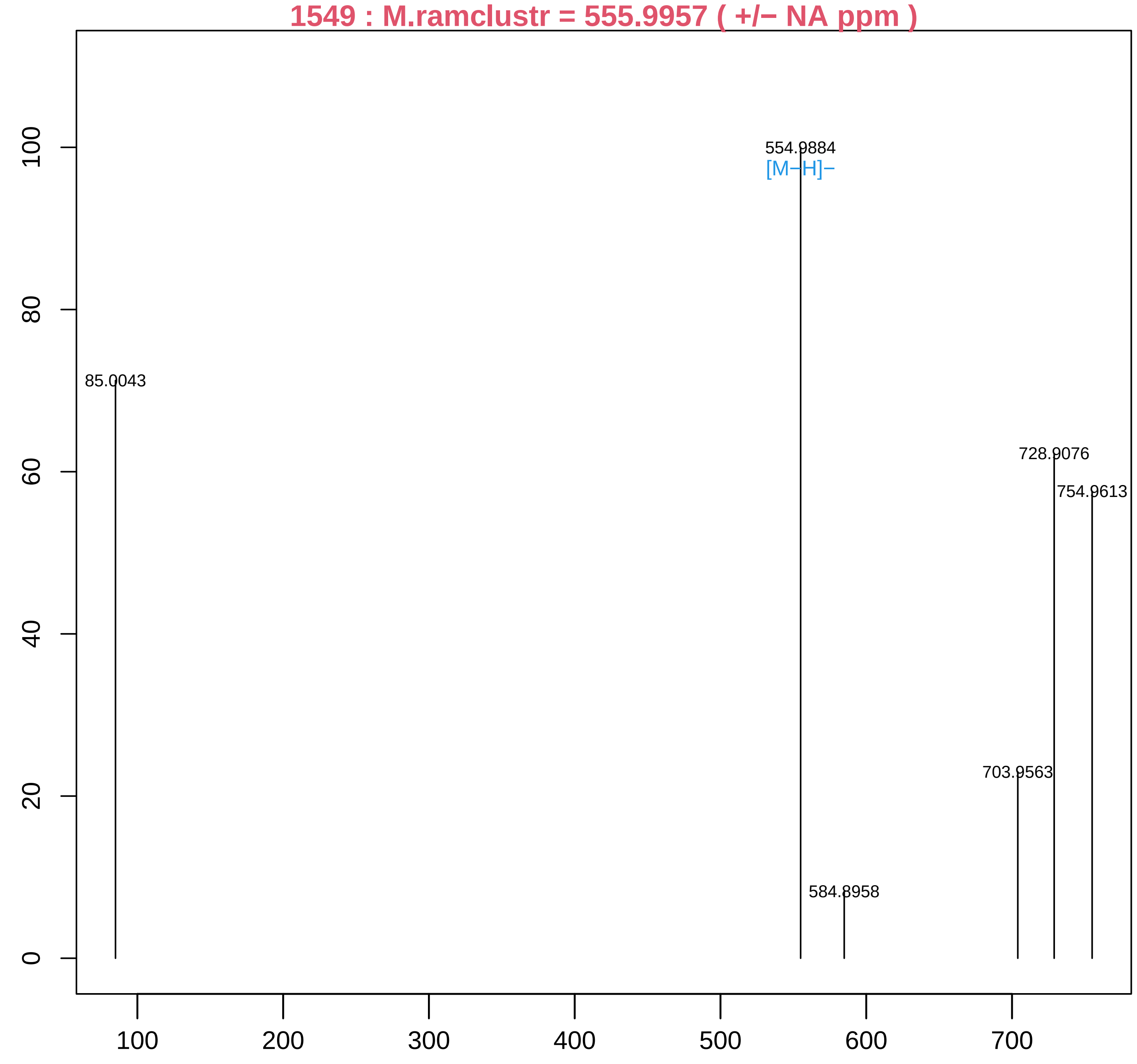
1549 : M.ramclustr = 555.9957 (+/− NA ppm)
554.9884
[M−H]−
85.0043
728.9076
754.9613
703.9563
584.8958
0
20
40
60
80
100
100
200
300
400
500
600
700

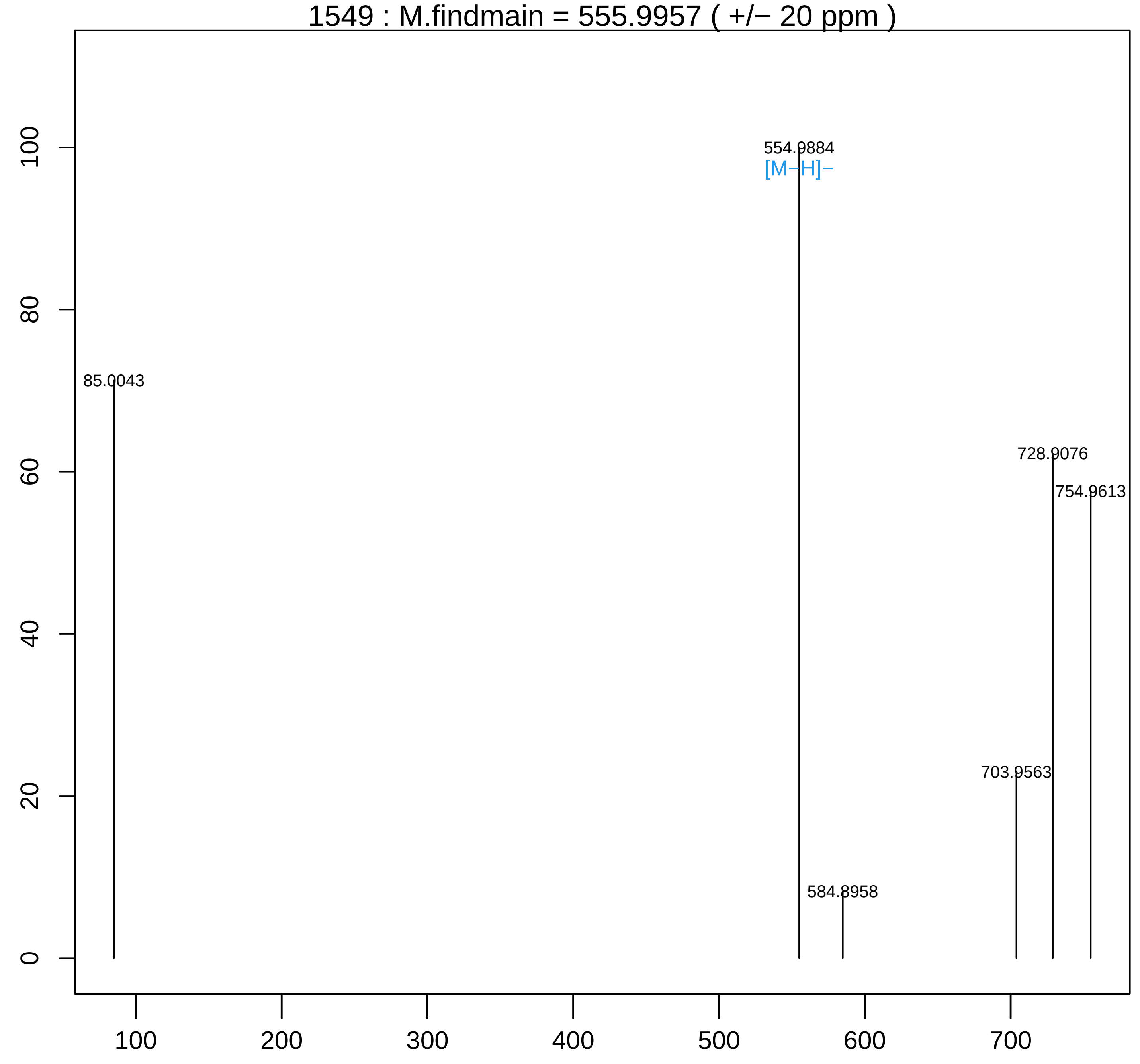
1549 : M.findmain = 555.9957 (+/− 20 ppm)
554.9884
[M−H]−
85.0043
728.9076
754.9613
703.9563
584.8958
0
20
40
60
80
100
100
200
300
400
500
600
700

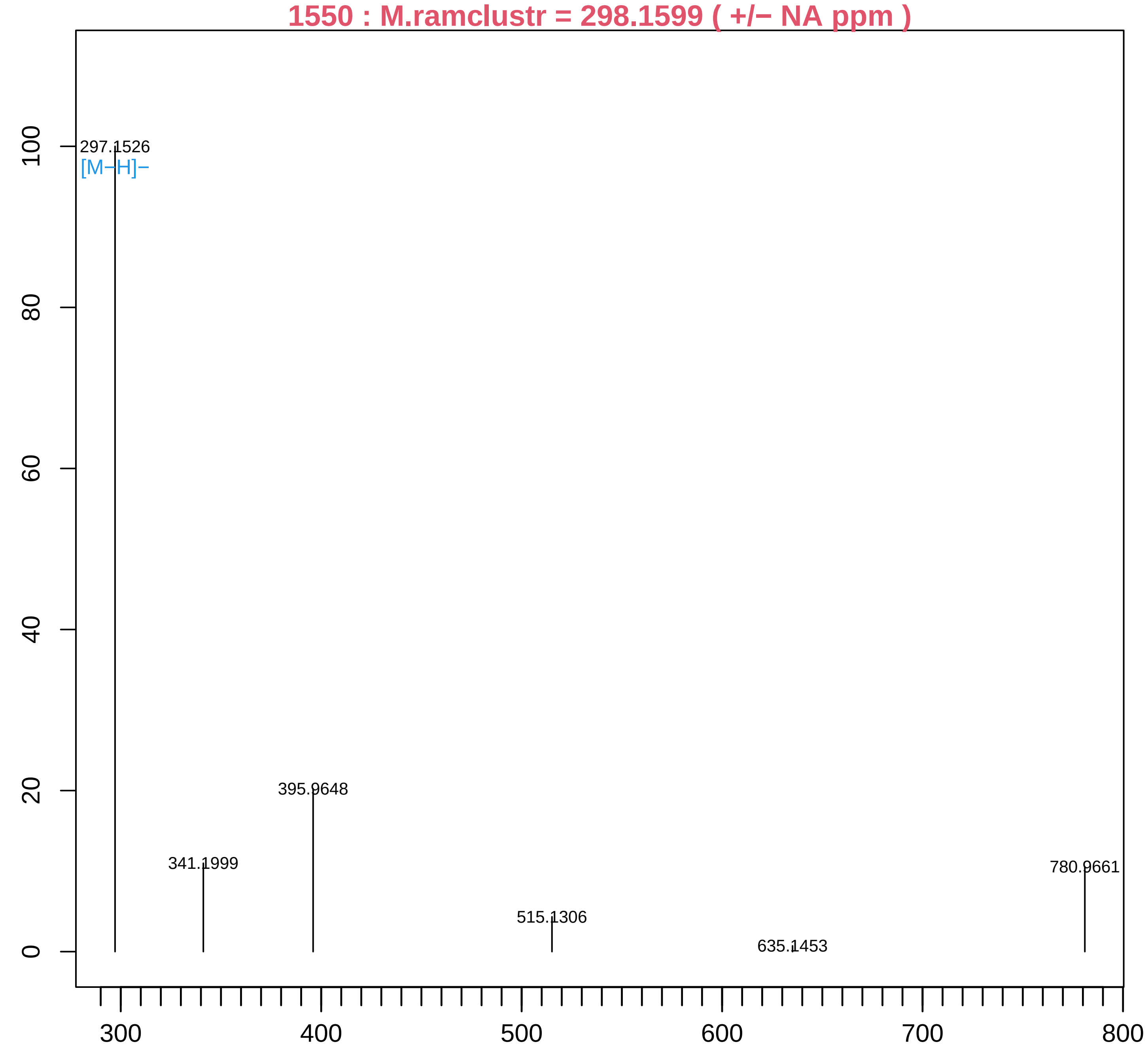
1550 : M.ramclustr = 298.1599 (+/− NA ppm)
297.1526
[M−H]−
341.1999
395.9648
515.1306
635.1453
780.9661
0
20
40
60
80
100
300
400
500
600
700
800

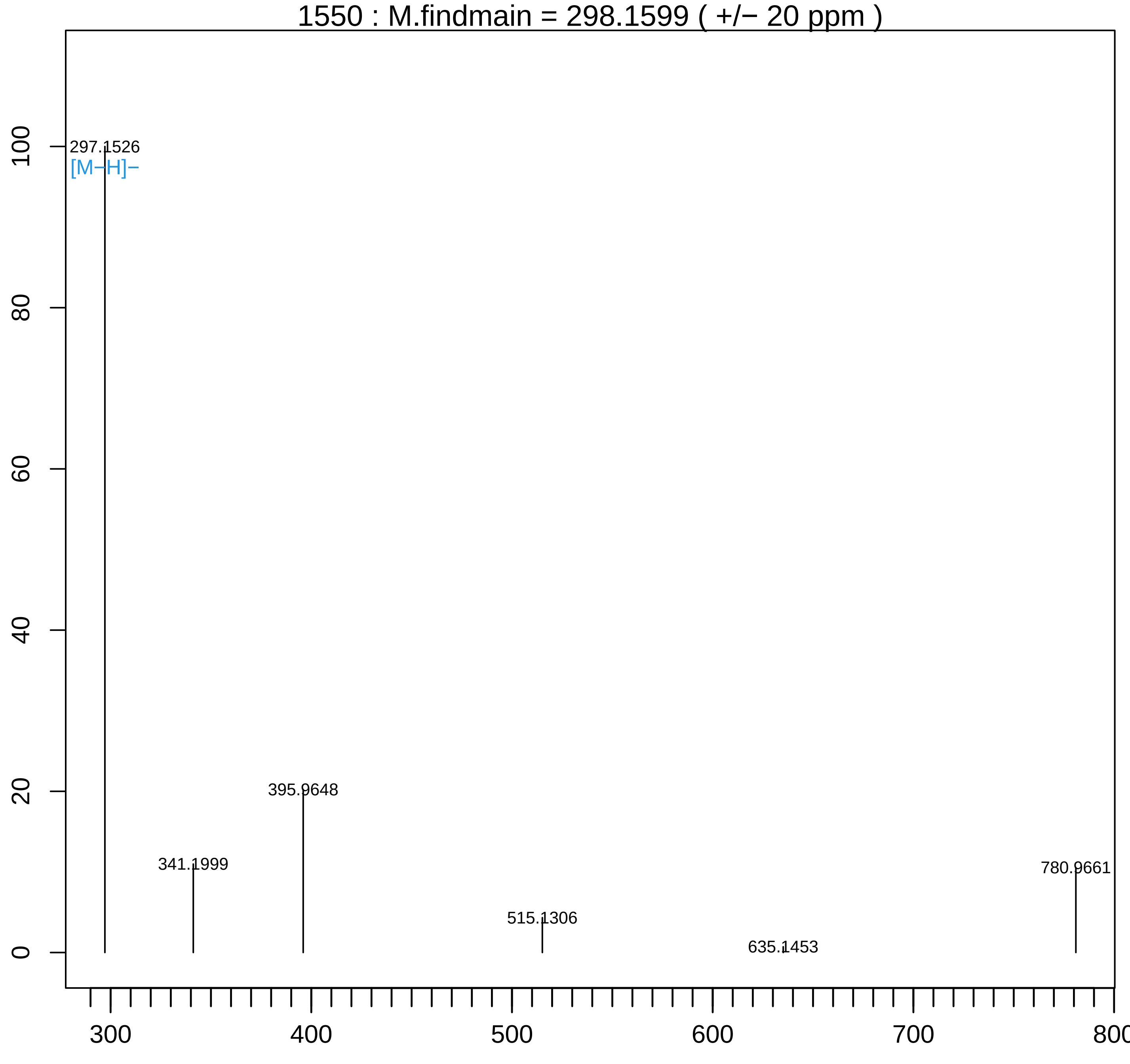
1550 : M.findmain = 298.1599 (+/− 20 ppm)
297.1526
[M−H]−
341.1999
395.9648
515.1306
635.1453
780.9661
0
20
40
60
80
100
300
400
500
600
700
800

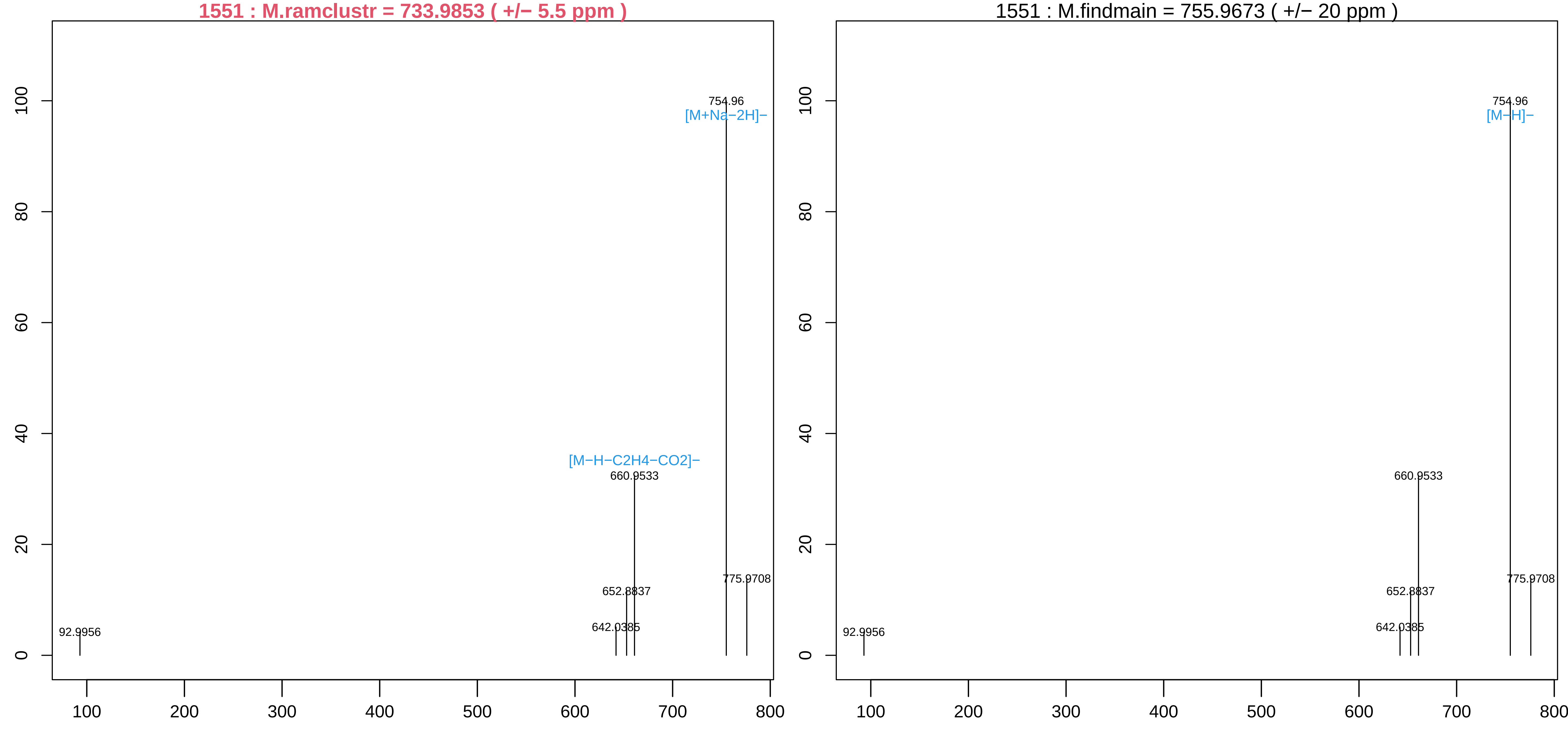

1551 : M.ramclustr = 733.9853 (+/− 5.5 ppm)
754.96
[M+Na−2H]−
[M−H−C2H4−CO2]−
660.9533
775.9708
652.8837
642.0385
92.9956
0
20
40
60
80
100
100
200
300
400
500
600
700
800
1551 : M.findmain = 755.9673 (+/− 20 ppm)
754.96
[M−H]−
660.9533
775.9708
652.8837
642.0385
92.9956

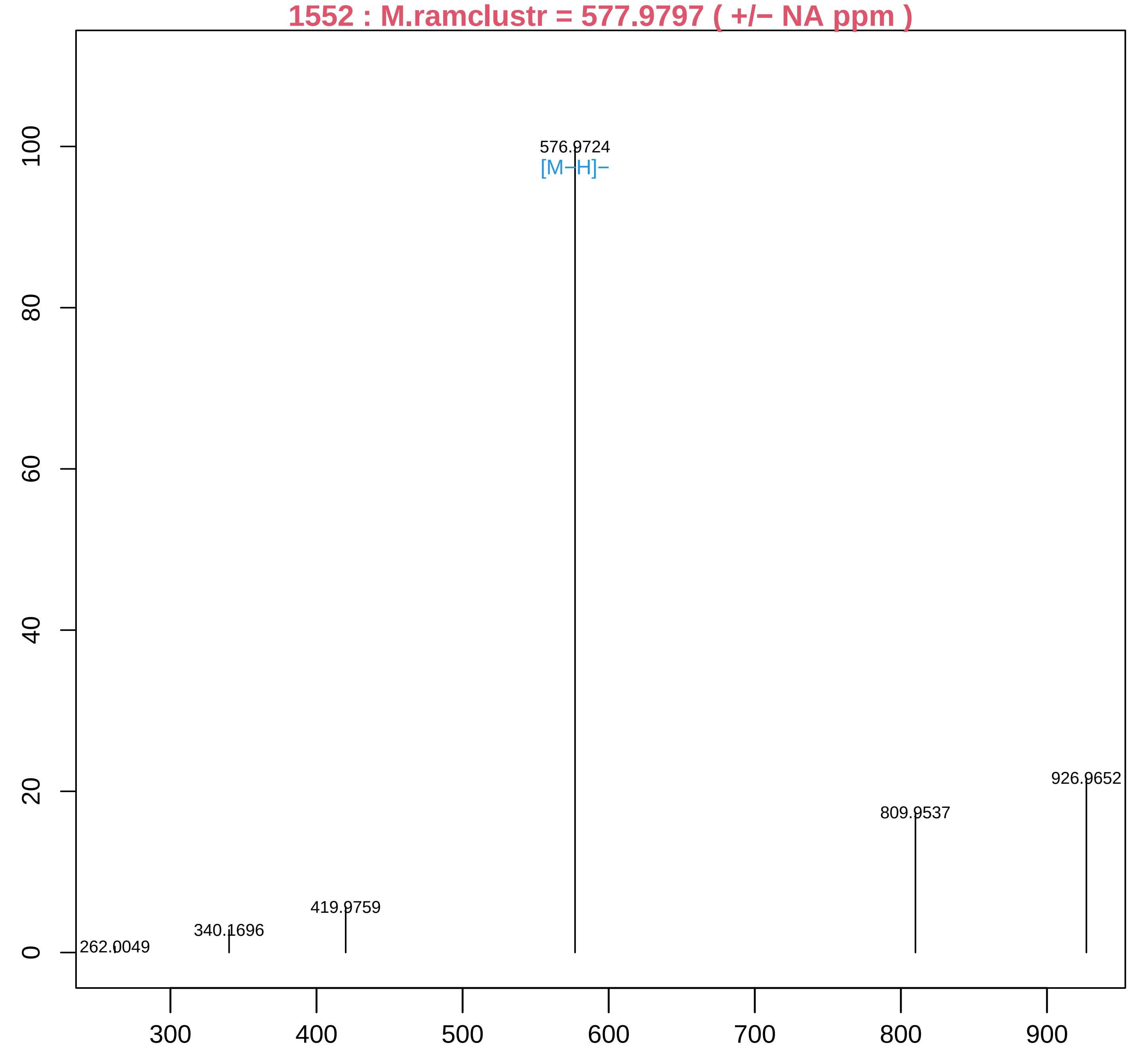
1552 : M.ramclustr = 577.9797 (+/− NA ppm)
576.9724
[M−H]−
926.9652
809.9537
419.9759
340.1696
262.0049
0
20
40
60
80
100
300
400
500
600
700
800
900

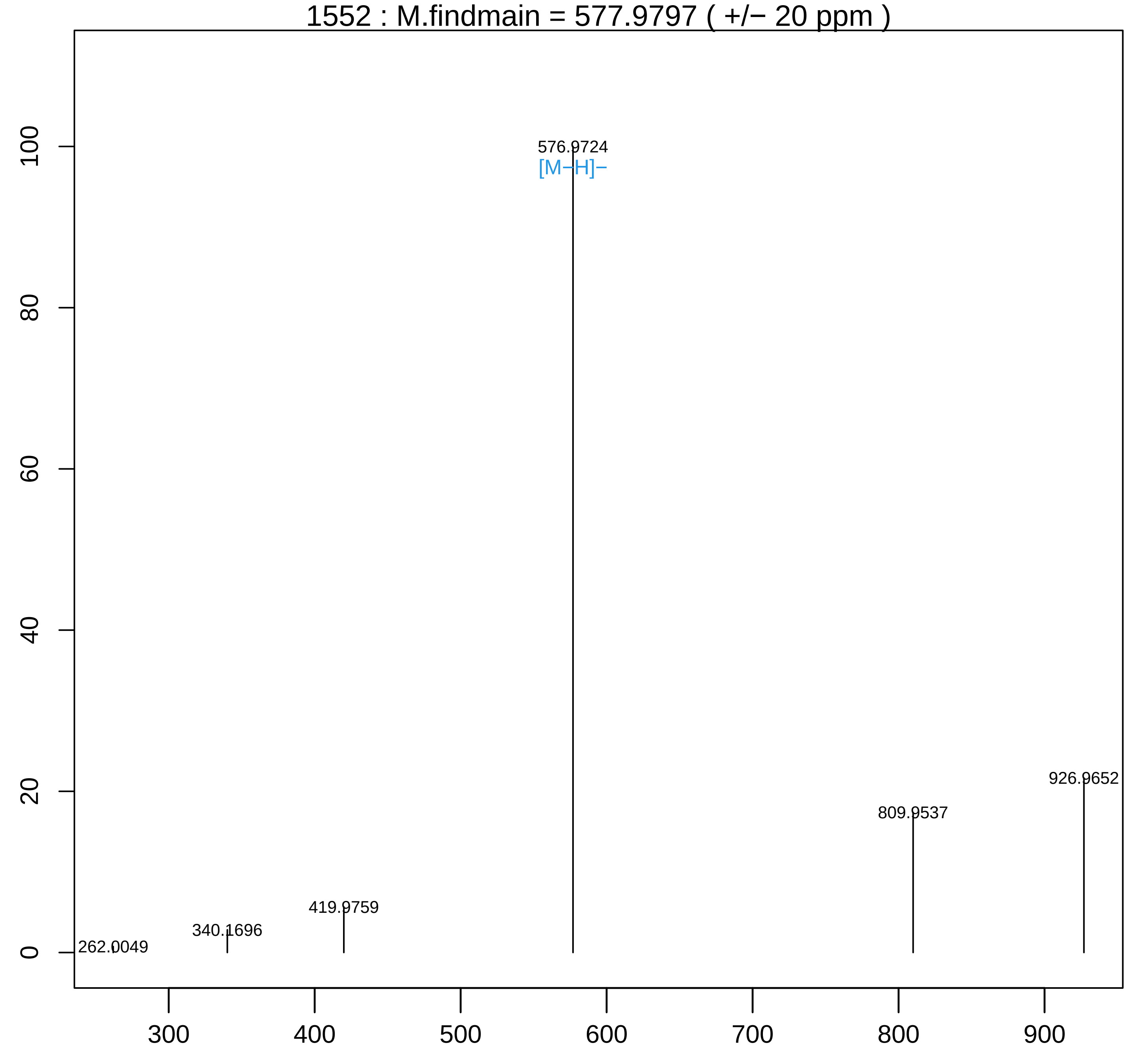
1552 : M.findmain = 577.9797 (+/− 20 ppm)
576.9724
[M−H]−
926.9652
809.9537
419.9759
340.1696
262.0049
0
20
40
60
80
100
300
400
500
600
700
800
900

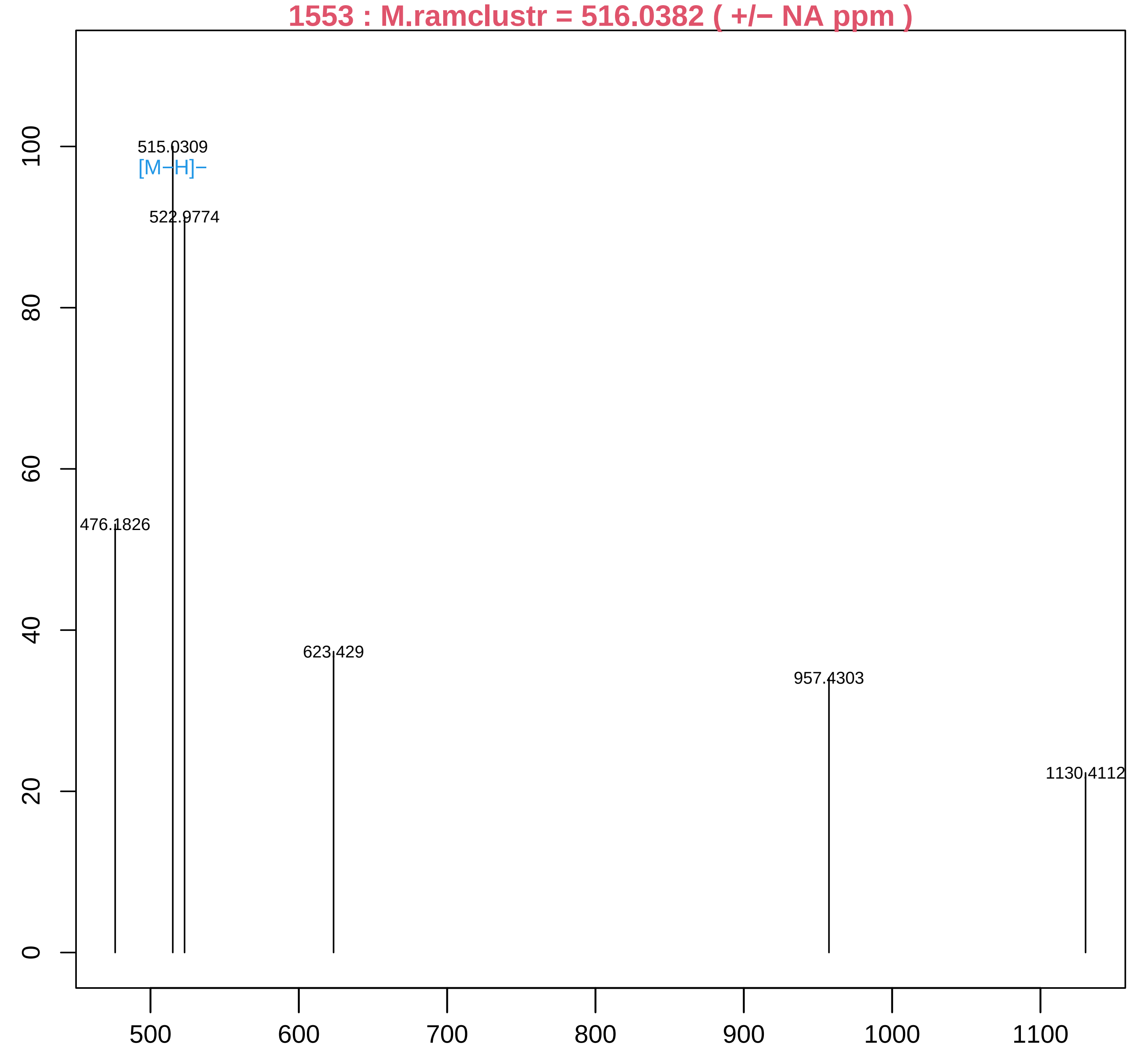
1553 : M.ramclustr = 516.0382 (+/− NA ppm)
515.0309
[M−H]−
522.9774
476.1826
623.429
957.4303
1130.4112
0
20
40
60
80
100
500
600
700
800
900
1000
1100

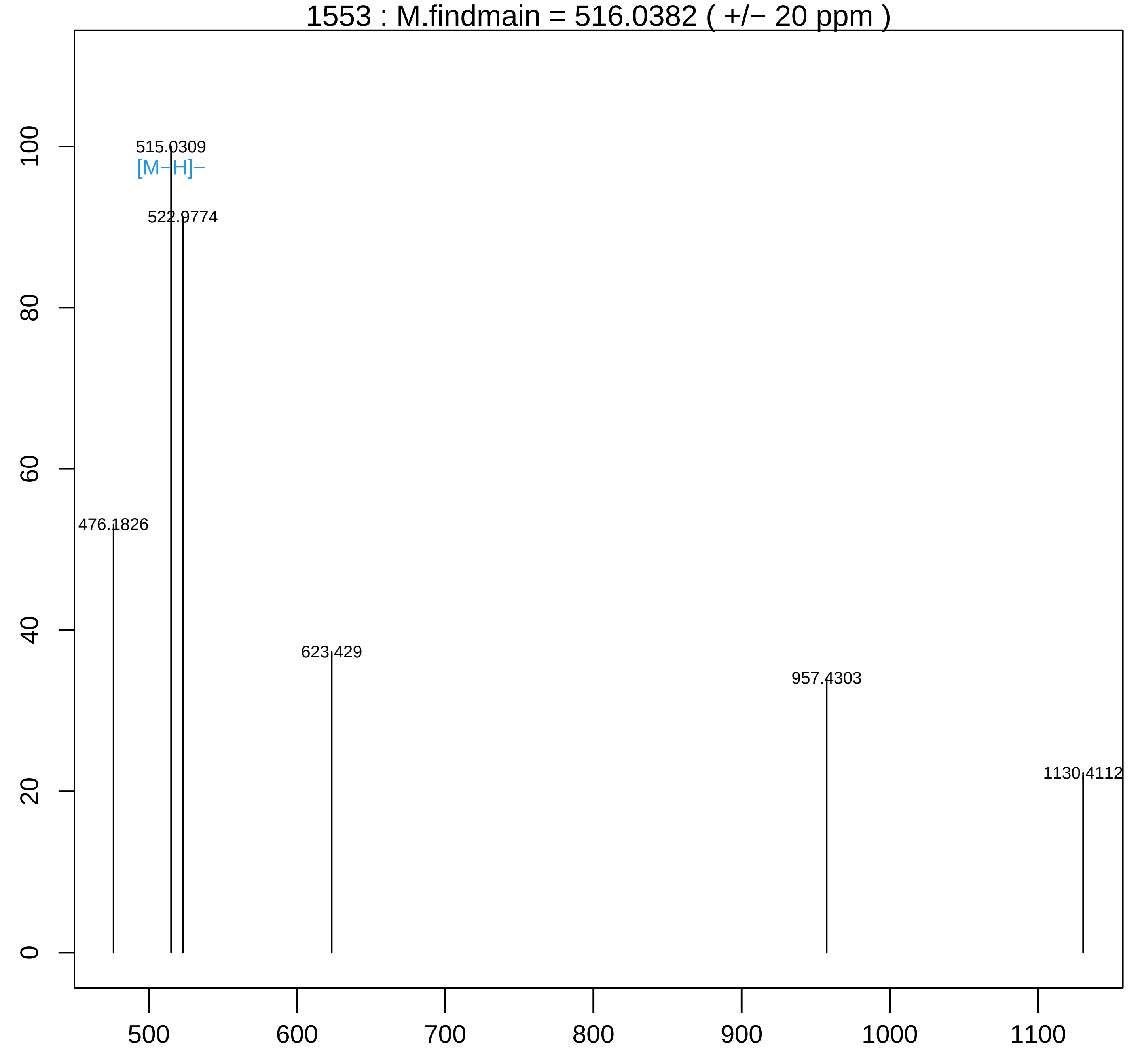
1553 : M.findmain = 516.0382 (+/− 20 ppm)
515.0309
[M−H]−
522.9774
476.1826
623.429
957.4303
1130.4112
0
20
40
60
80
100
500
600
700
800
900
1000
1100

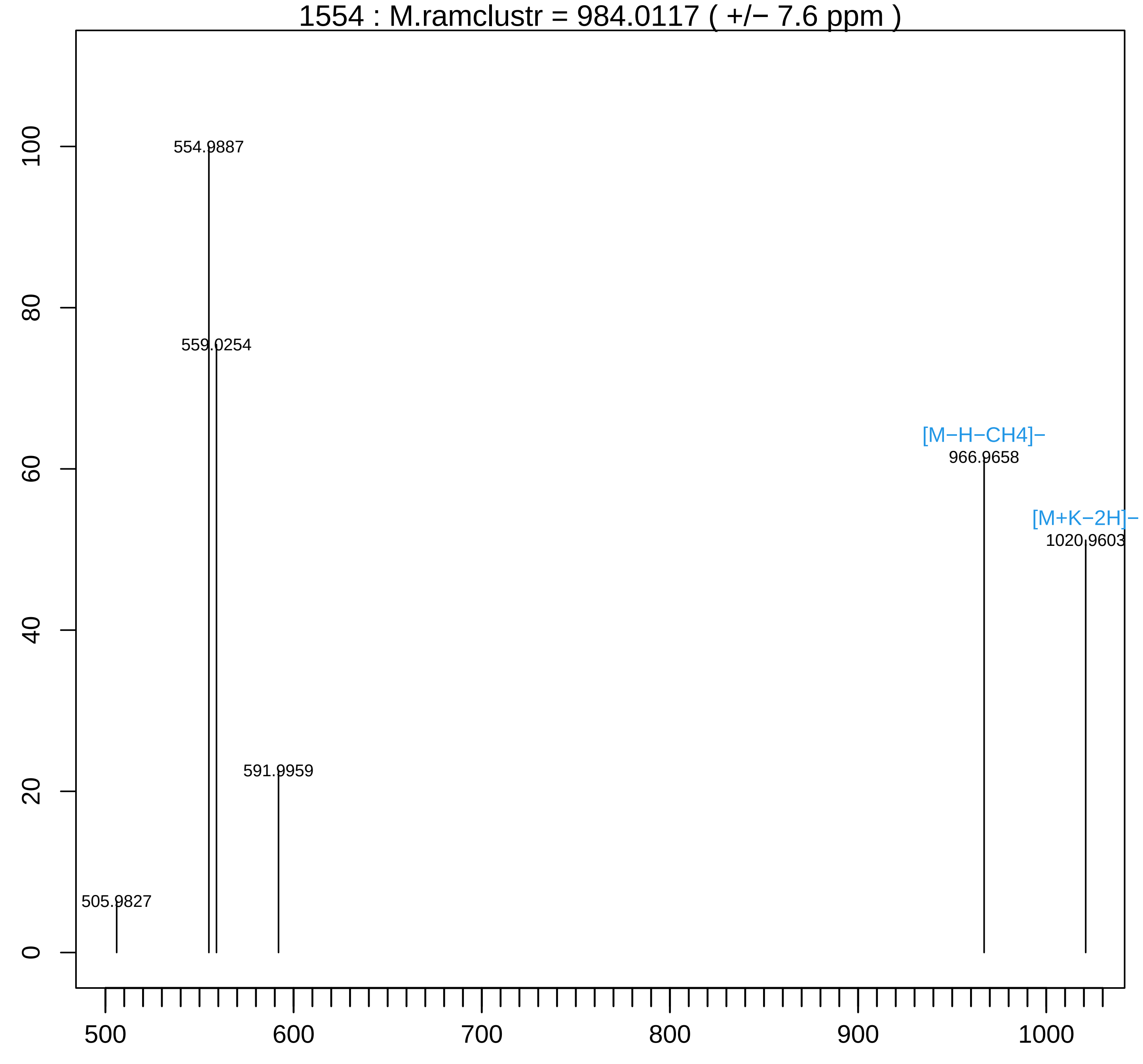
1554 : M.ramclustr = 984.0117 (+/− 7.6 ppm)
554.9887
559.0254
591.9959
505.9827
[M−H−CH4]−
966.9658
[M+K−2H]−
1020.9603
0
20
40
60
80
100
500
600
700
800
900
1000

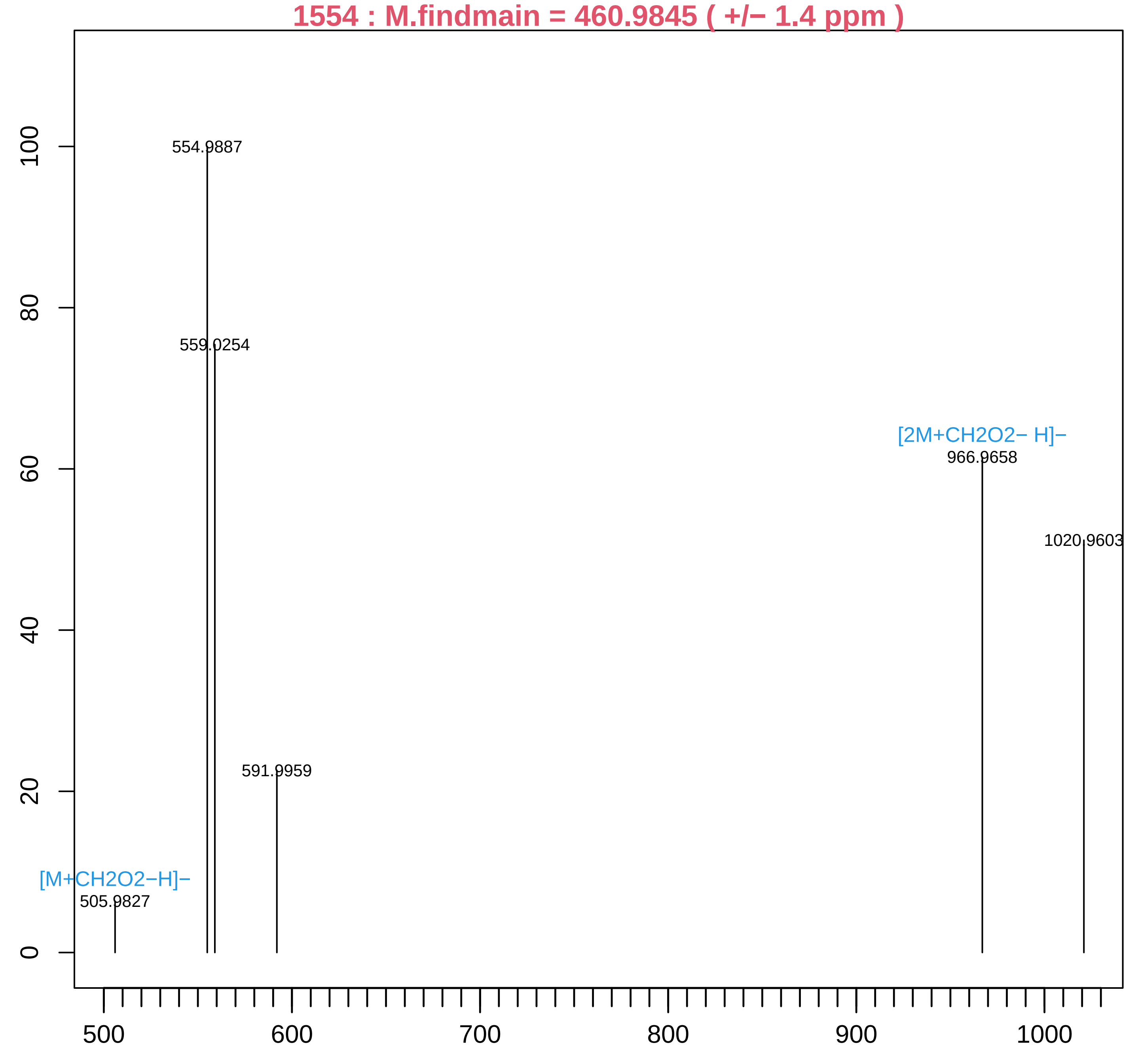
1554 : M.findmain = 460.9845 (+/− 1.4 ppm)
554.9887
559.0254
591.9959
[M+CH2O2−H]−
505.9827
[2M+CH2O2− H]−
966.9658
1020.9603
0
20
40
60
80
100
500
600
700
800
900
1000

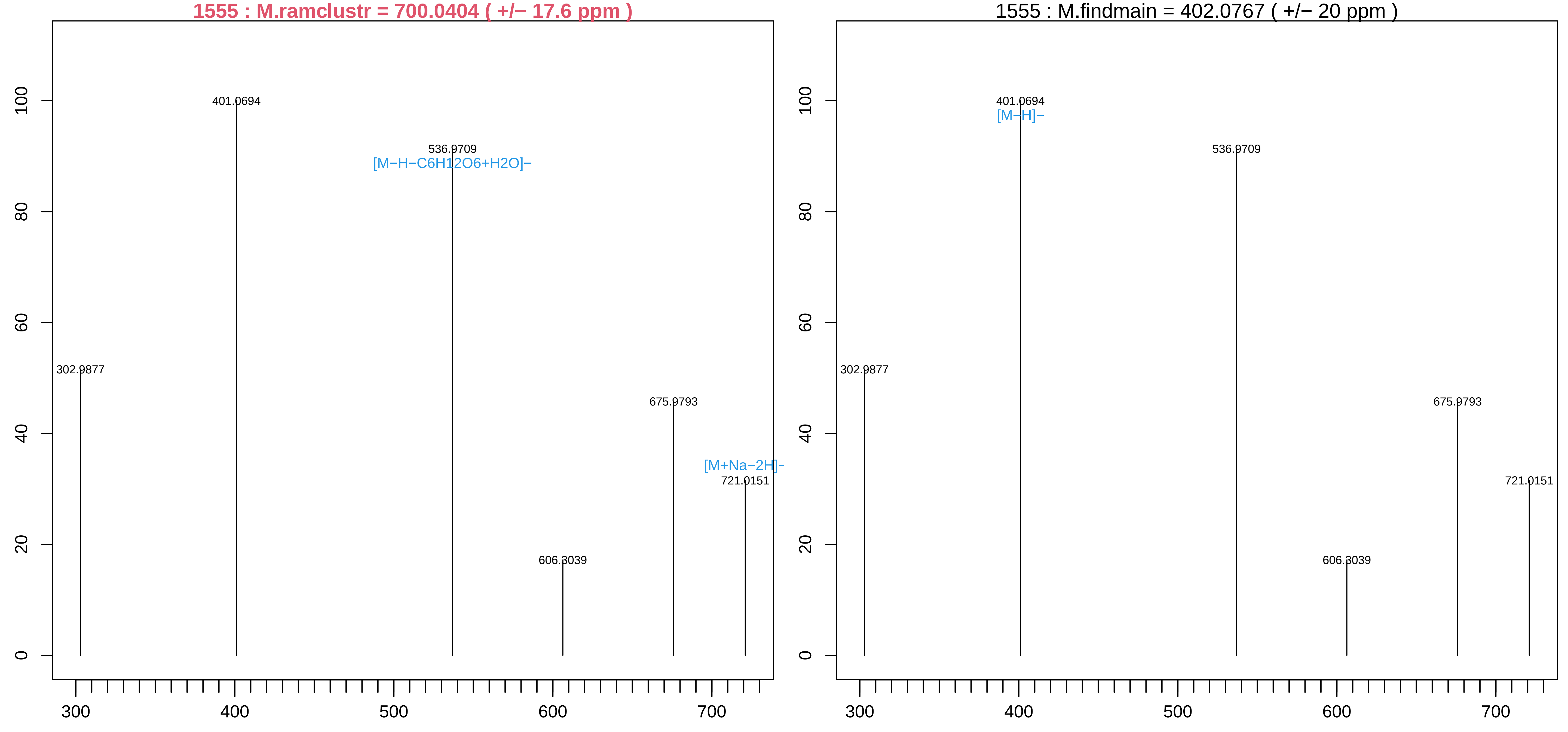

1555 : M.ramclustr = 700.0404 (+/− 17.6 ppm)
401.0694
536.9709
[M−H−C6H12O6+H2O]−
302.9877
675.9793
[M+Na−2H]−
721.0151
606.3039
0
20
40
60
80
100
300
400
500
600
700
1555 : M.findmain = 402.0767 (+/− 20 ppm)
401.0694
[M−H]−
536.9709
302.9877
675.9793
721.0151
606.3039
0
20
40
60
80
100
300
400
500
600
700

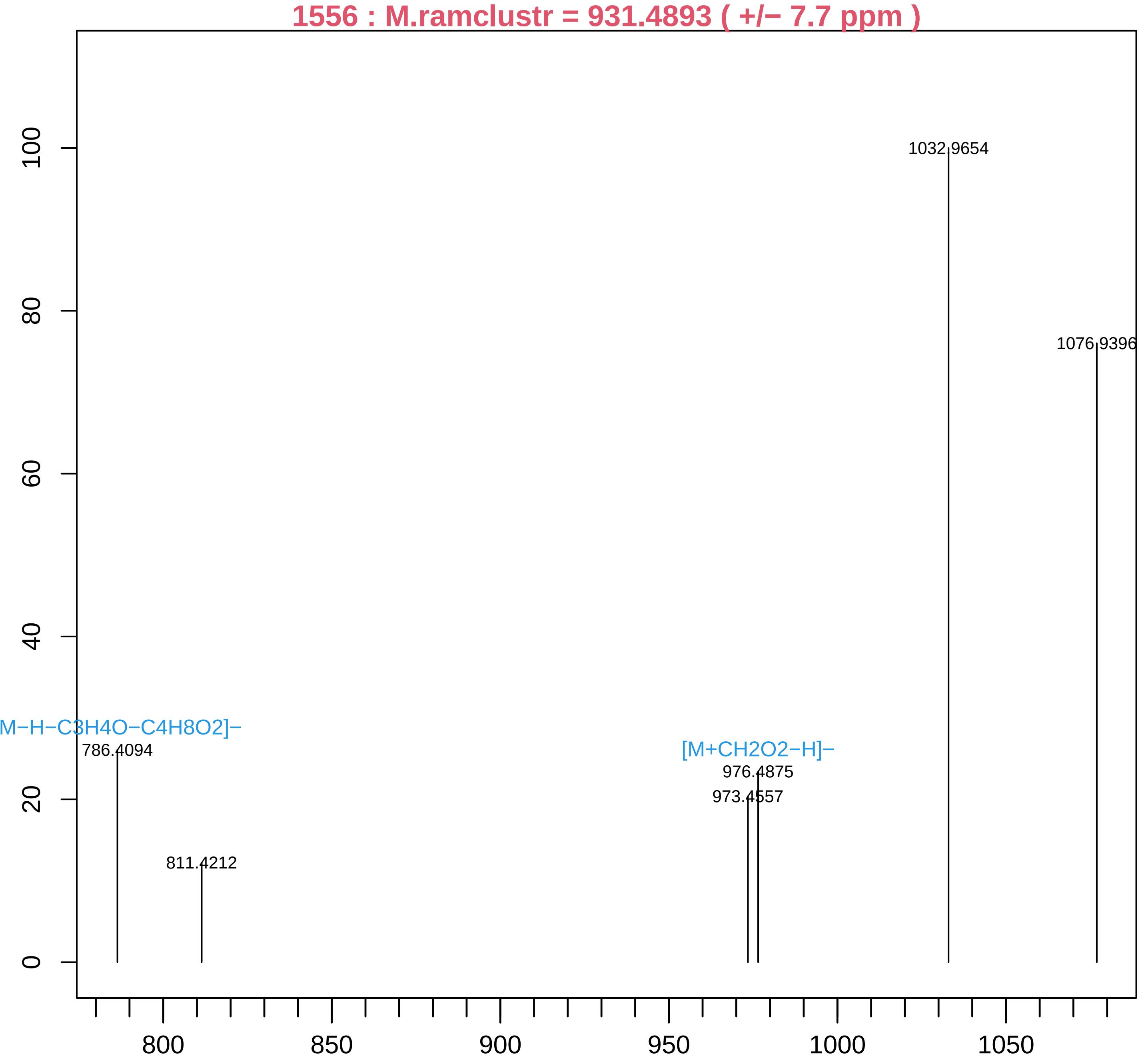

1556 : M.ramclustr = 931.4893 (+/− 7.7 ppm)
1032.9654
1076.9396
[M−H−C3H4O−C4H8O2]−
786.4094
811.4212
[M+CH2O2−H]−
976.4875
973.4557
0
20
40
60
80
100
800
850
900
950
1000
1050

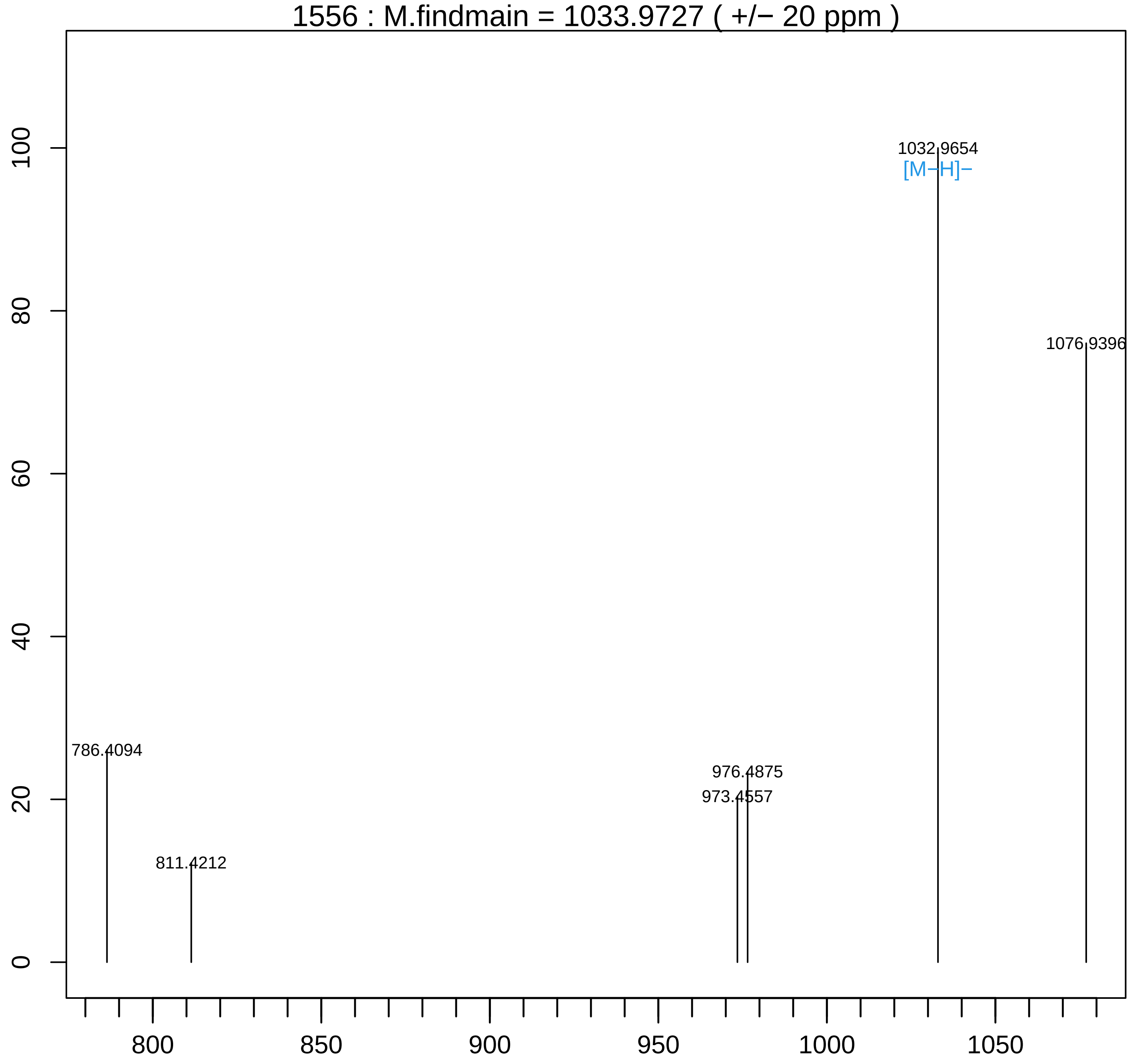

1556 : M.findmain = 1033.9727 (+/− 20 ppm)
1032.9654
[M−H]−
1076.9396
786.4094
811.4212
976.4875
973.4557
0
20
40
60
80
100
800
850
900
950
1000
1050

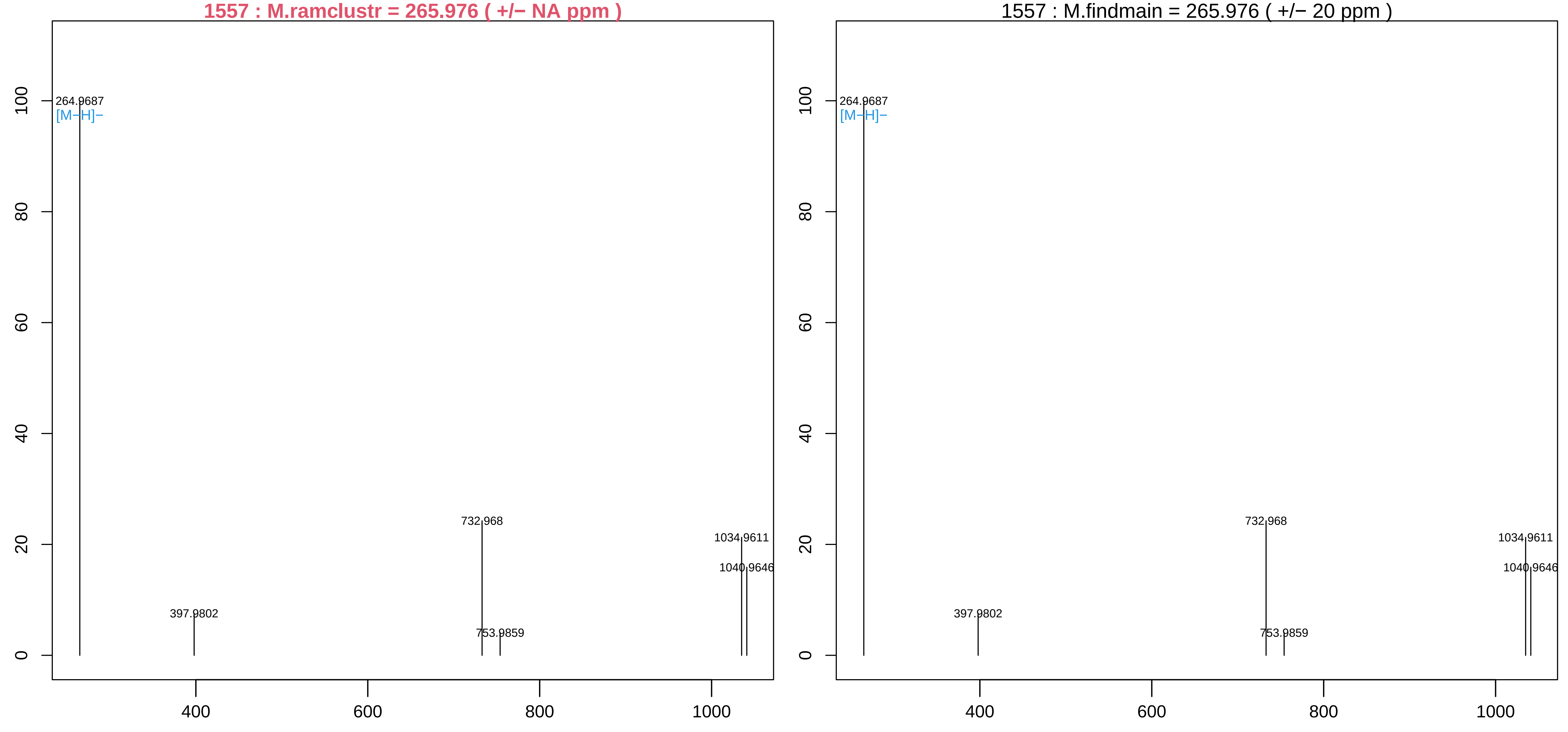
1557 : M.ramclustr = 265.976 (+/− NA ppm)
264.9687
[M−H]−
397.9802
732.968
753.9859
1034.9611
1040.9646
0
20
40
60
80
100
400
600
800
1000
1557 : M.findmain = 265.976 (+/− 20 ppm)
264.9687
[M−H]−
397.9802
732.968
753.9859
1034.9611
1040.9646
0
20
40
60
80
100
400
600
800
1000

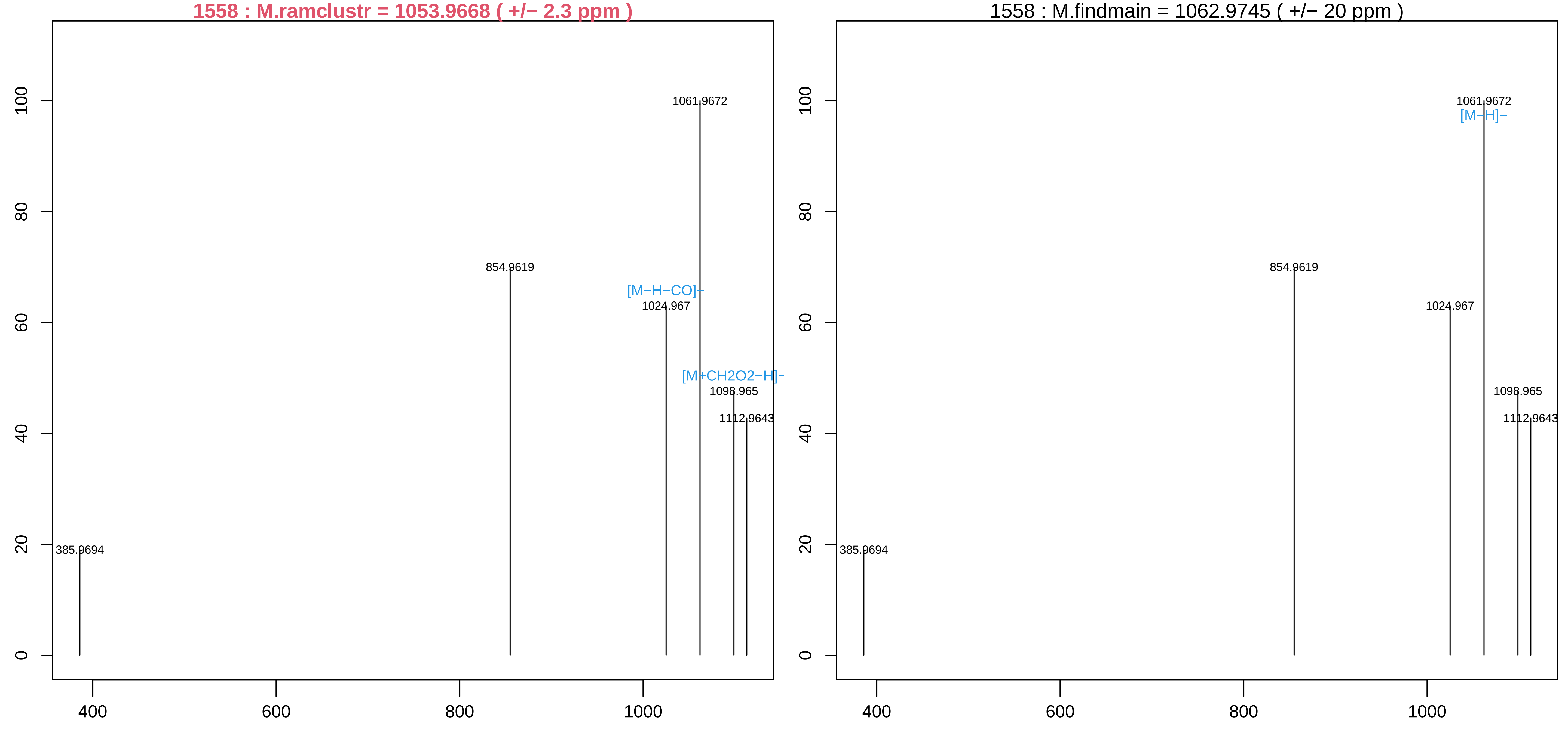

1558 : M.ramclustr = 1053.9668 (+/− 2.3 ppm)
1061.9672
854.9619
[M−H−CO]−
1024.967
[M+CH2O2−H]−
1098.965
1112.9643
385.9694
1558 : M.findmain = 1062.9745 (+/− 20 ppm)
1061.9672
[M−H]−
854.9619
1024.967
1098.965
1112.9643
385.9694
0
20
40
60
80
100
400
600
800
1000

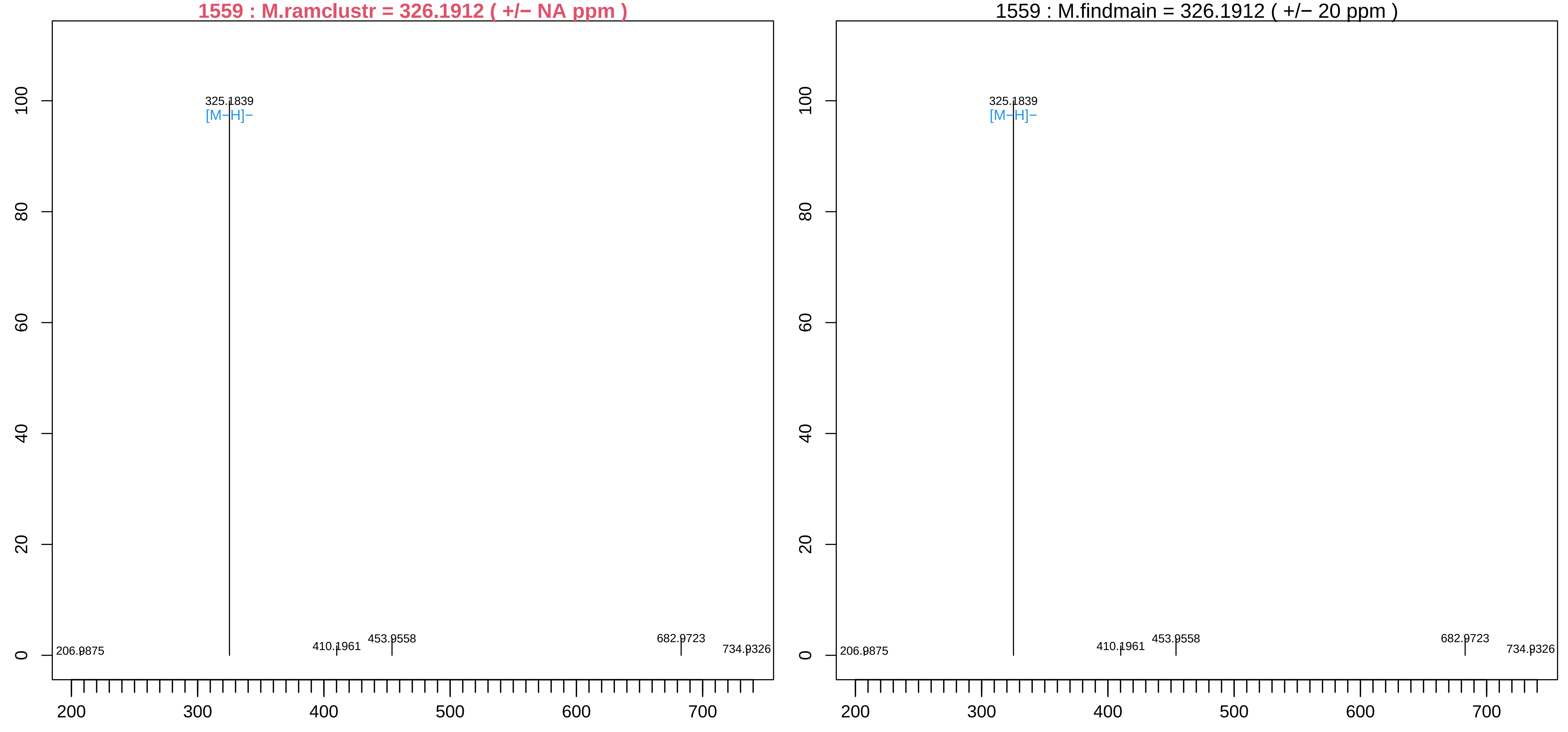
1559 : M.ramclustr = 326.1912 (+/− NA ppm)
325.1839
[M−H]−
206.9875
410.1961
453.9558
682.9723
734.9326
1559 : M.findmain = 326.1912 (+/− 20 ppm)
325.1839
[M−H]−
206.9875
410.1961
453.9558
682.9723
734.9326

1560 : M.ramclustr = 158.9379 (+/− NA ppm)

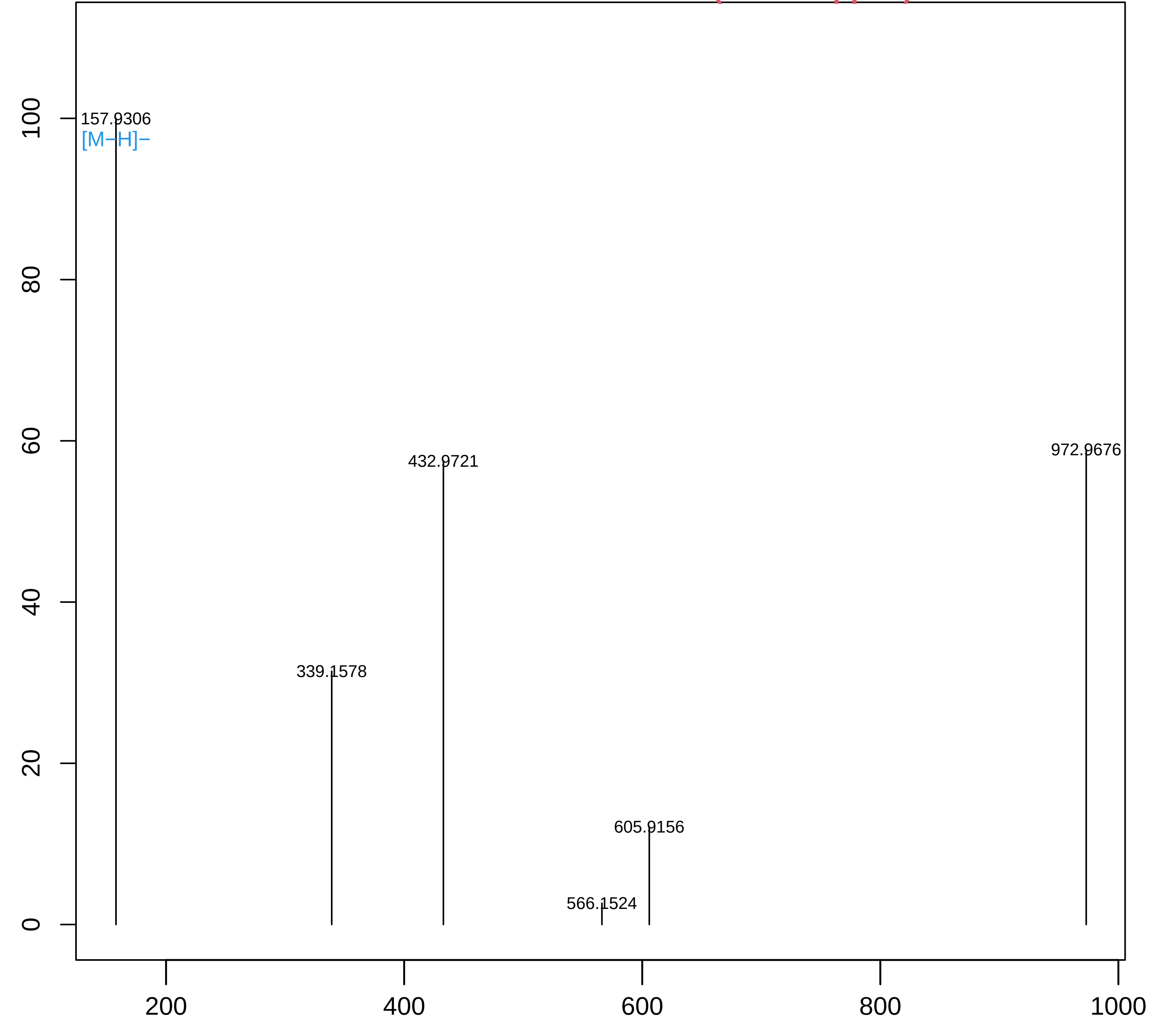

1560 : M.findmain = 158.9379 (+/− 20 ppm)

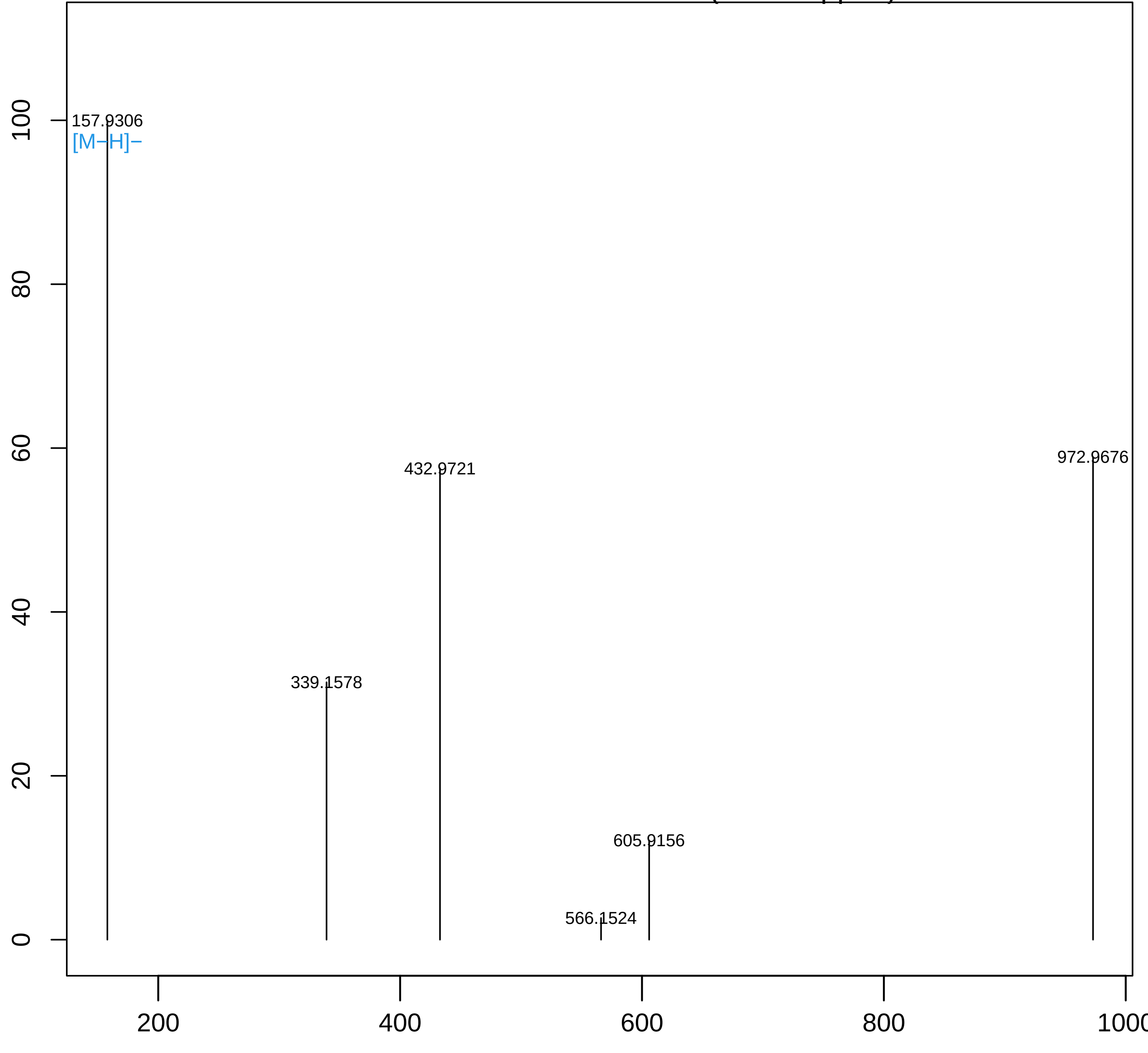

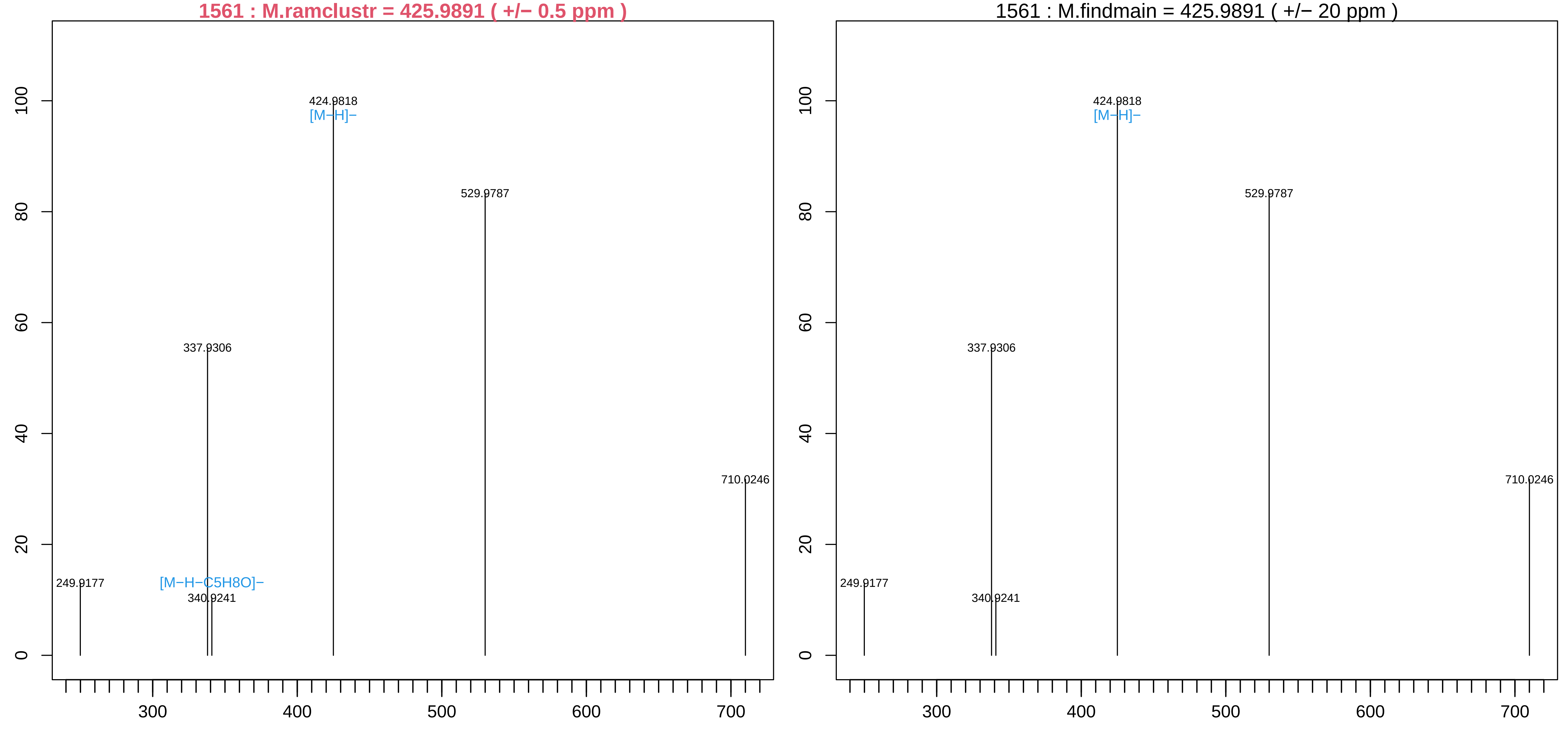

1561 : M.ramclustr = 425.9891 (+/− 0.5 ppm)
424.9818
[M−H]−
529.9787
337.9306
710.0246
249.9177
[M−H−C5H8O]−
340.9241
1561 : M.findmain = 425.9891 (+/− 20 ppm)
424.9818
[M−H]−
529.9787
337.9306
710.0246
249.9177
340.9241

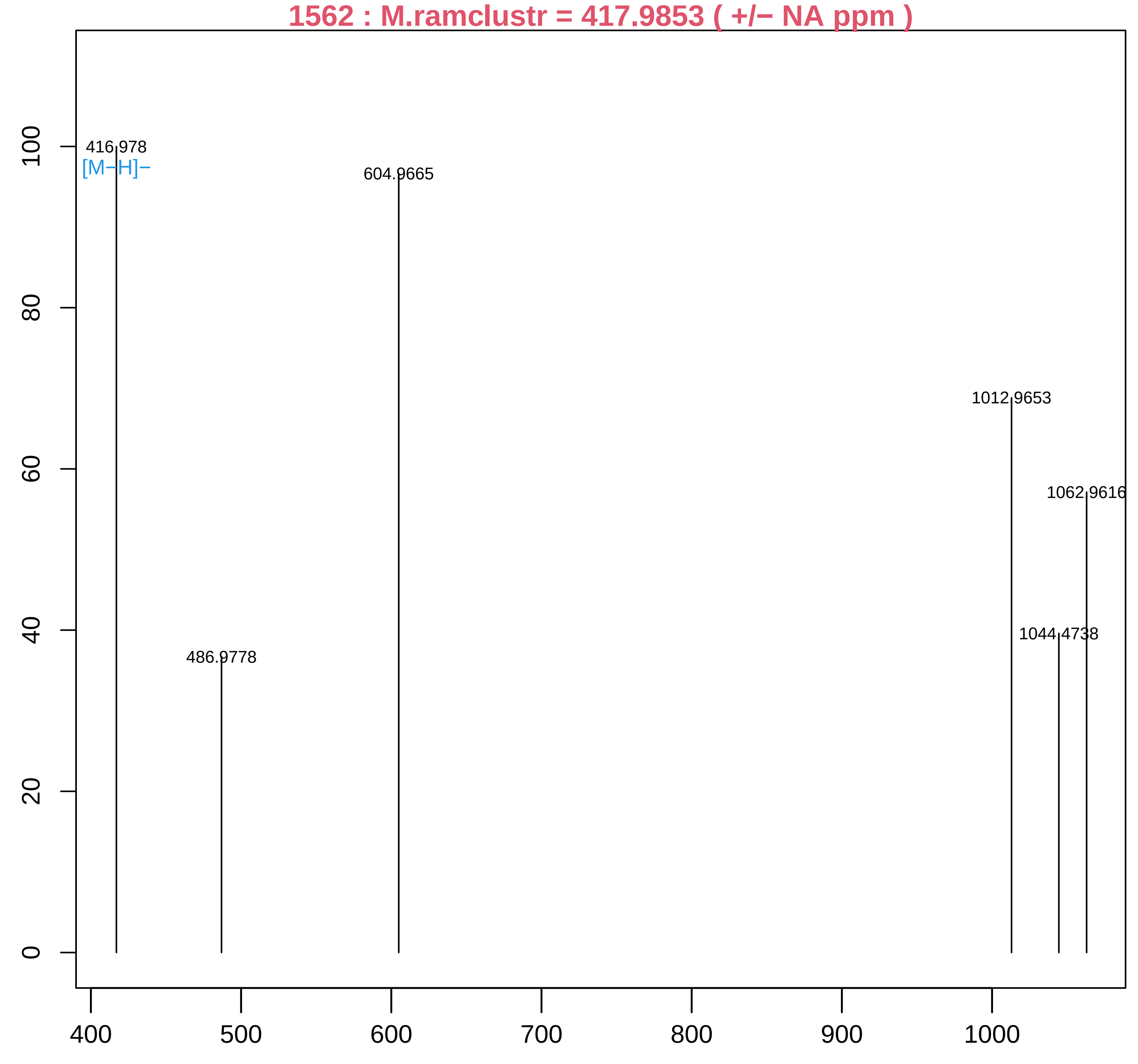

1562 : M.ramclustr = 417.9853 (+/− NA ppm)
416.978
[M−H]−
604.9665
486.9778
1012.9653
1062.9616
1044.4738
0
20
40
60
80
100
400
500
600
700
800
900
1000

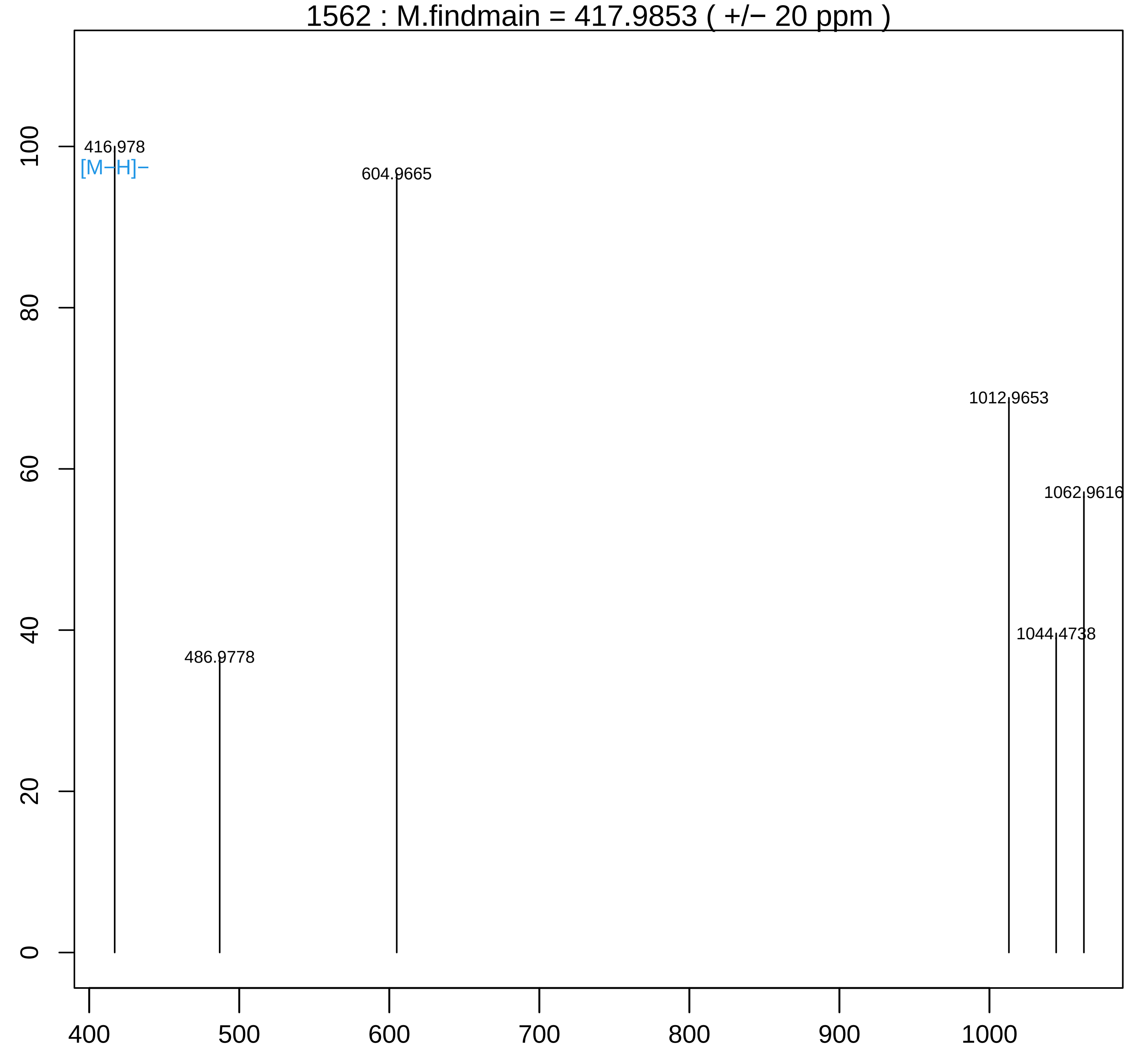

1562 : M.findmain = 417.9853 (+/− 20 ppm)
416.978
[M−H]−
604.9665
486.9778
1012.9653
1062.9616
1044.4738
0
20
40
60
80
100
400
500
600
700
800
900
1000

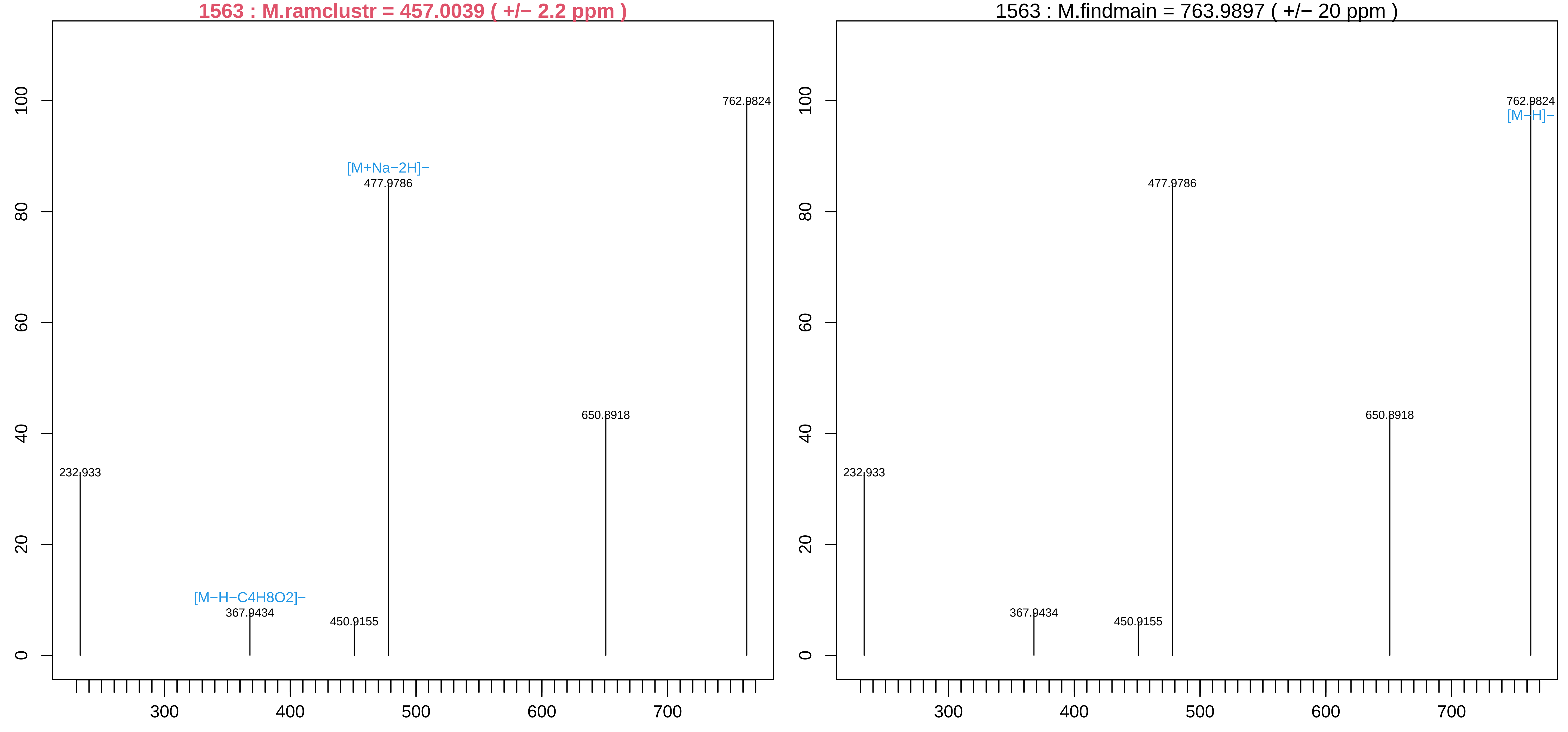
1563 : M.ramclustr = 457.0039 (+/− 2.2 ppm)
762.9824
[M+Na−2H]−
477.9786
650.8918
232.933
[M−H−C4H8O2]−
367.9434
450.9155
1563 : M.findmain = 763.9897 (+/− 20 ppm)
762.9824
[M−H]−
477.9786
650.8918
232.933
367.9434
450.9155
0
20
40
60
80
100
300
400
500
600
700

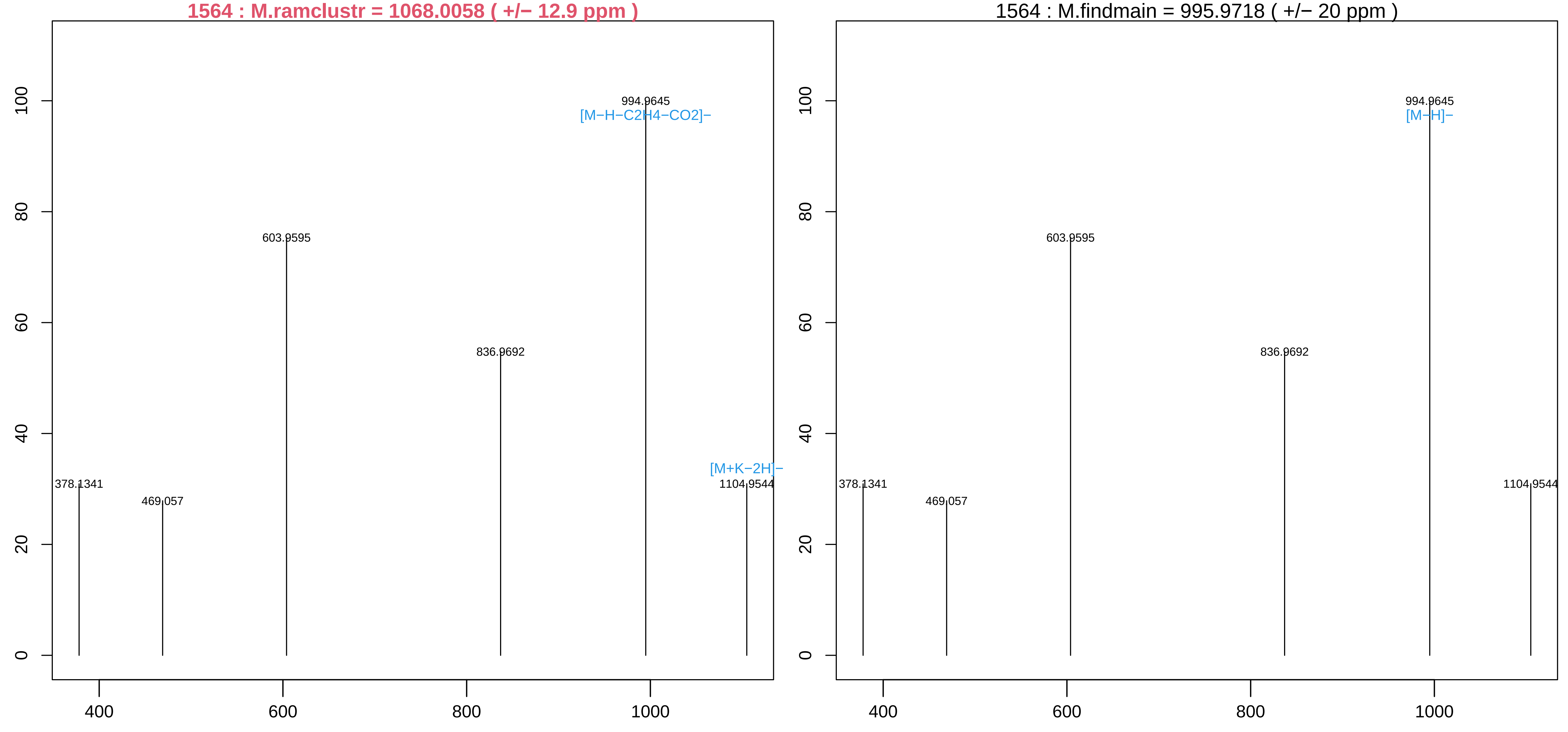
1564 : M.ramclustr = 1068.0058 (+/− 12.9 ppm)
994.9645
[M−H−C2H4−CO2]−
603.9595
836.9692
[M+K−2H]−
378.1341
469.057
1104.9544
1564 : M.findmain = 995.9718 (+/− 20 ppm)
994.9645
[M−H]−
603.9595
836.9692
378.1341
469.057
1104.9544

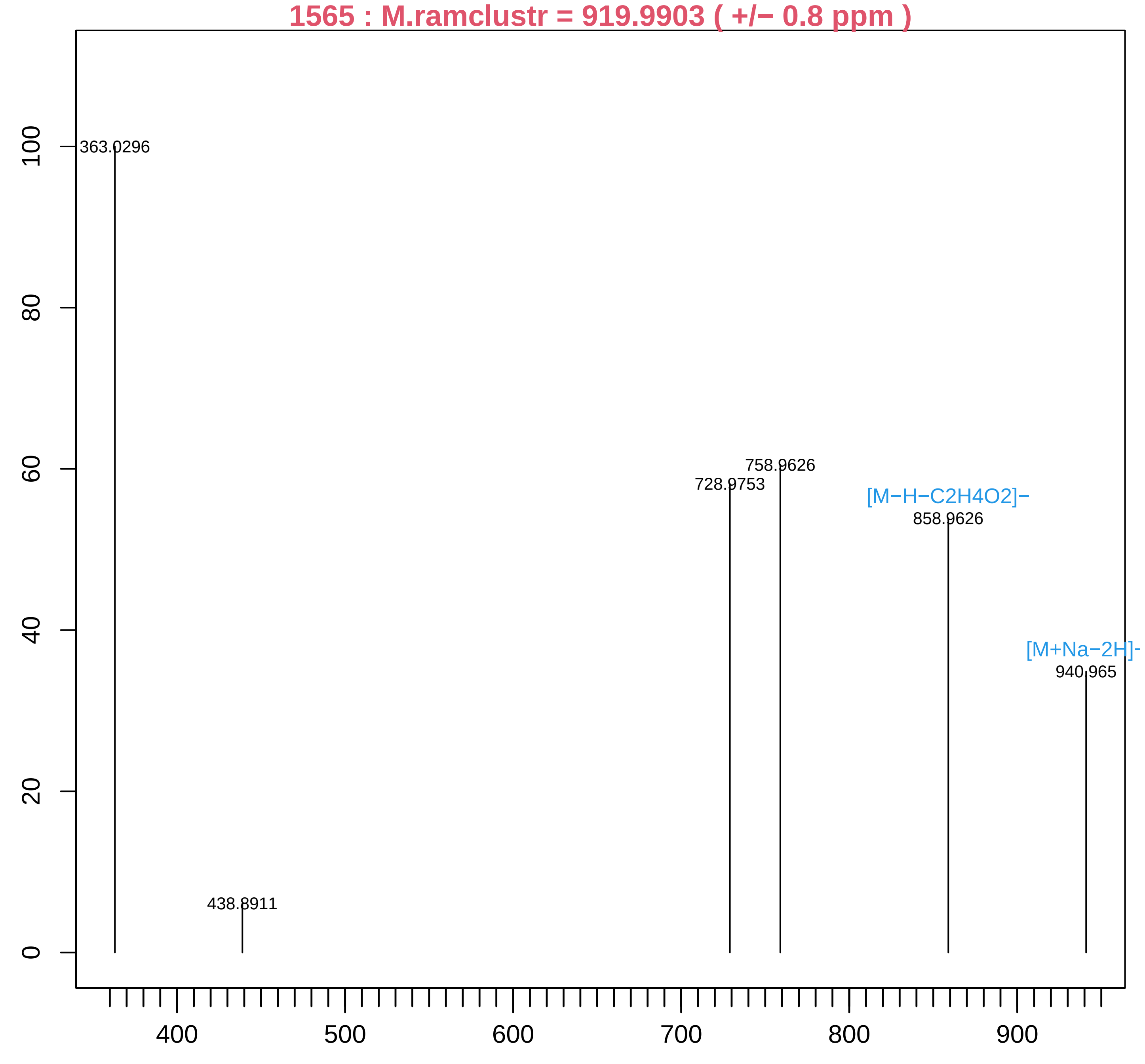

1565 : M.ramclustr = 919.9903 (+/− 0.8 ppm)
363.0296
438.8911
728.9753
758.9626
[M−H−C2H4O2]−
858.9626
[M+Na−2H]-
940.965
0
20
40
60
80
100
400
500
600
700
800
900

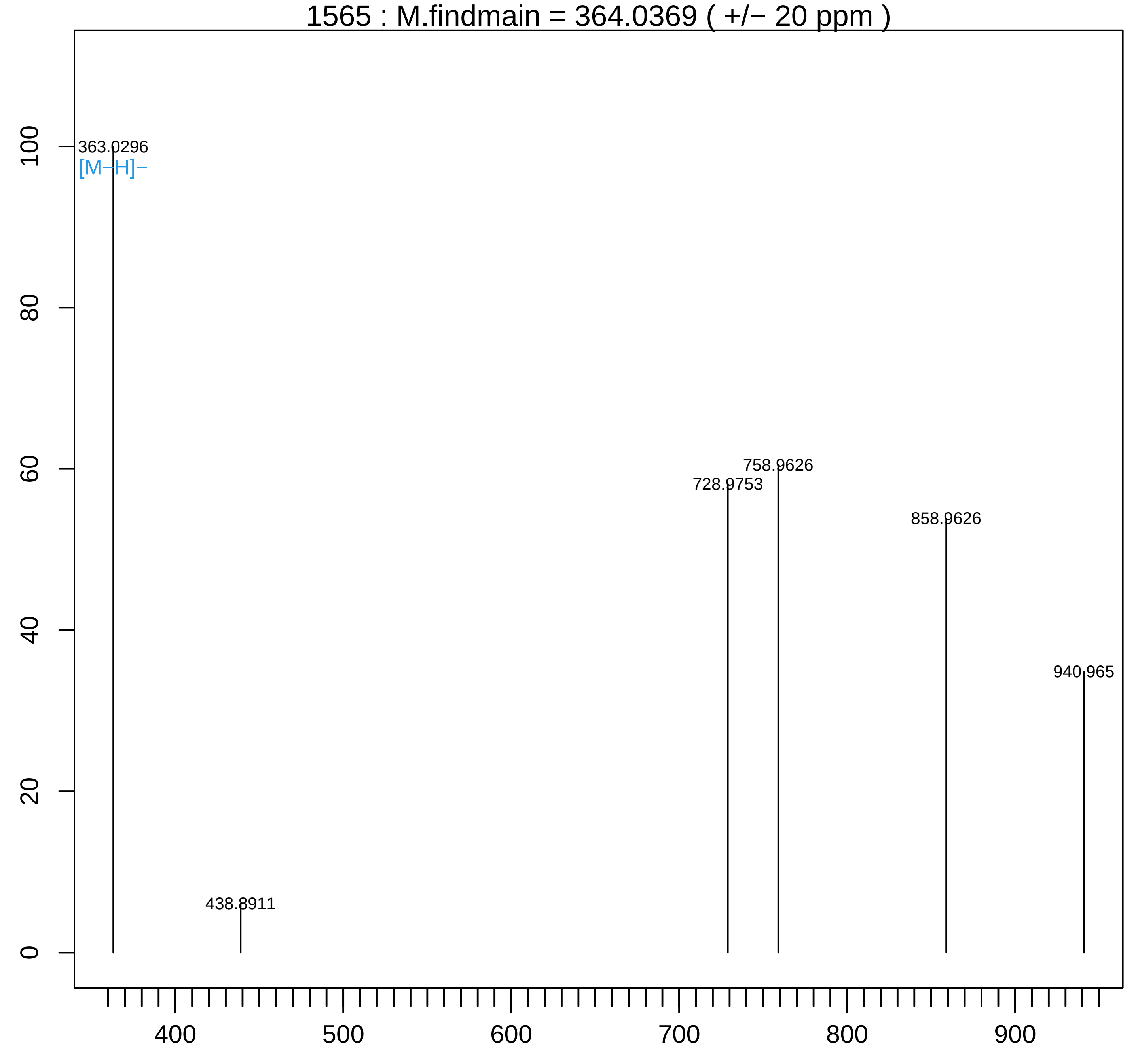

1565 : M.findmain = 364.0369 (+/− 20 ppm)
363.0296
[M−H]−
438.8911
728.9753
758.9626
858.9626
940.965
0
20
40
60
80
100
400
500
600
700
800
900

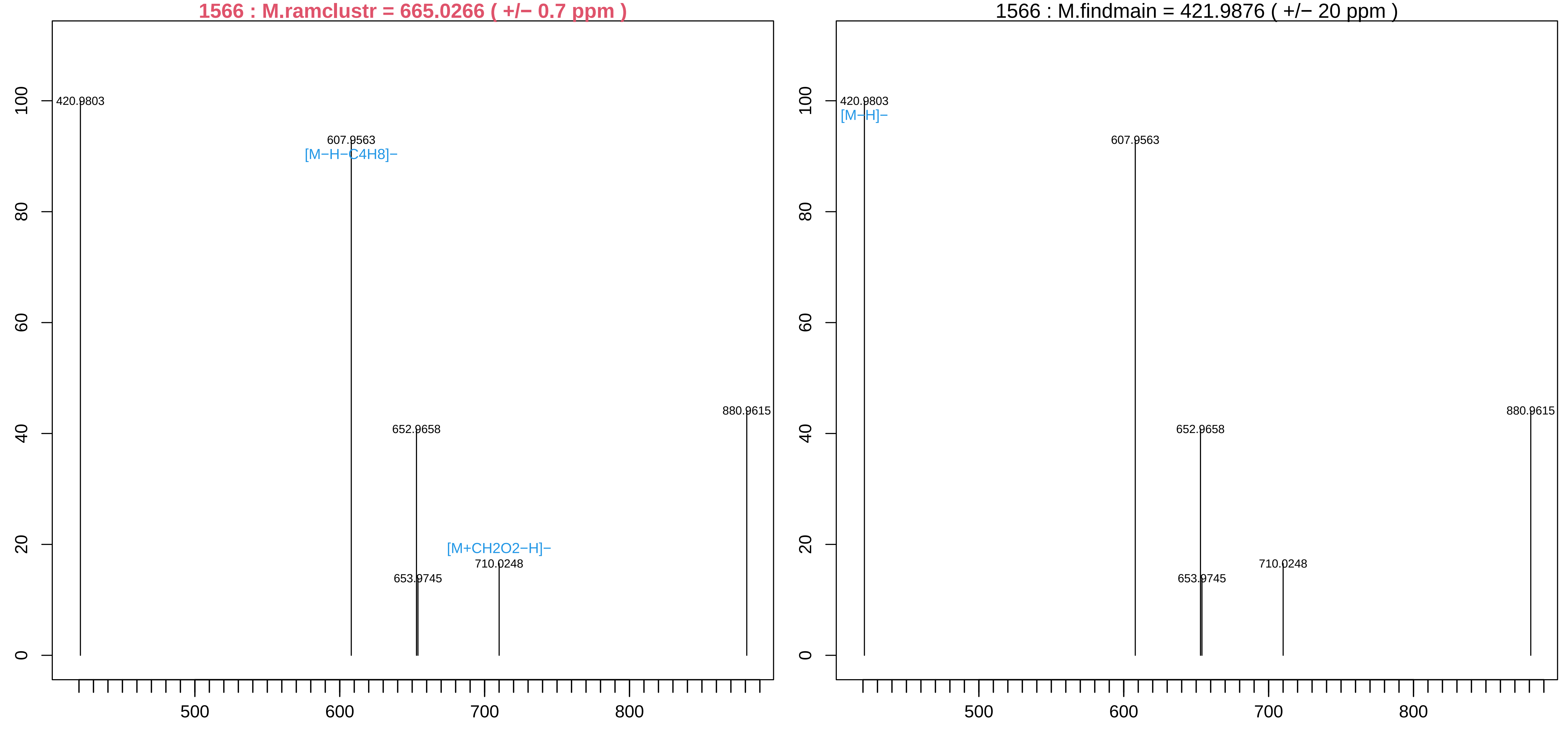

1566 : M.ramclustr = 665.0266 (+/− 0.7 ppm)
420.9803
607.9563
[M−H−C4H8]−
652.9658
653.9745
[M+CH2O2−H]−
710.0248
880.9615
1566 : M.findmain = 421.9876 (+/− 20 ppm)
420.9803
[M−H]−
607.9563
652.9658
653.9745
710.0248
880.9615
0
20
40
60
80
100
500
600
700
800

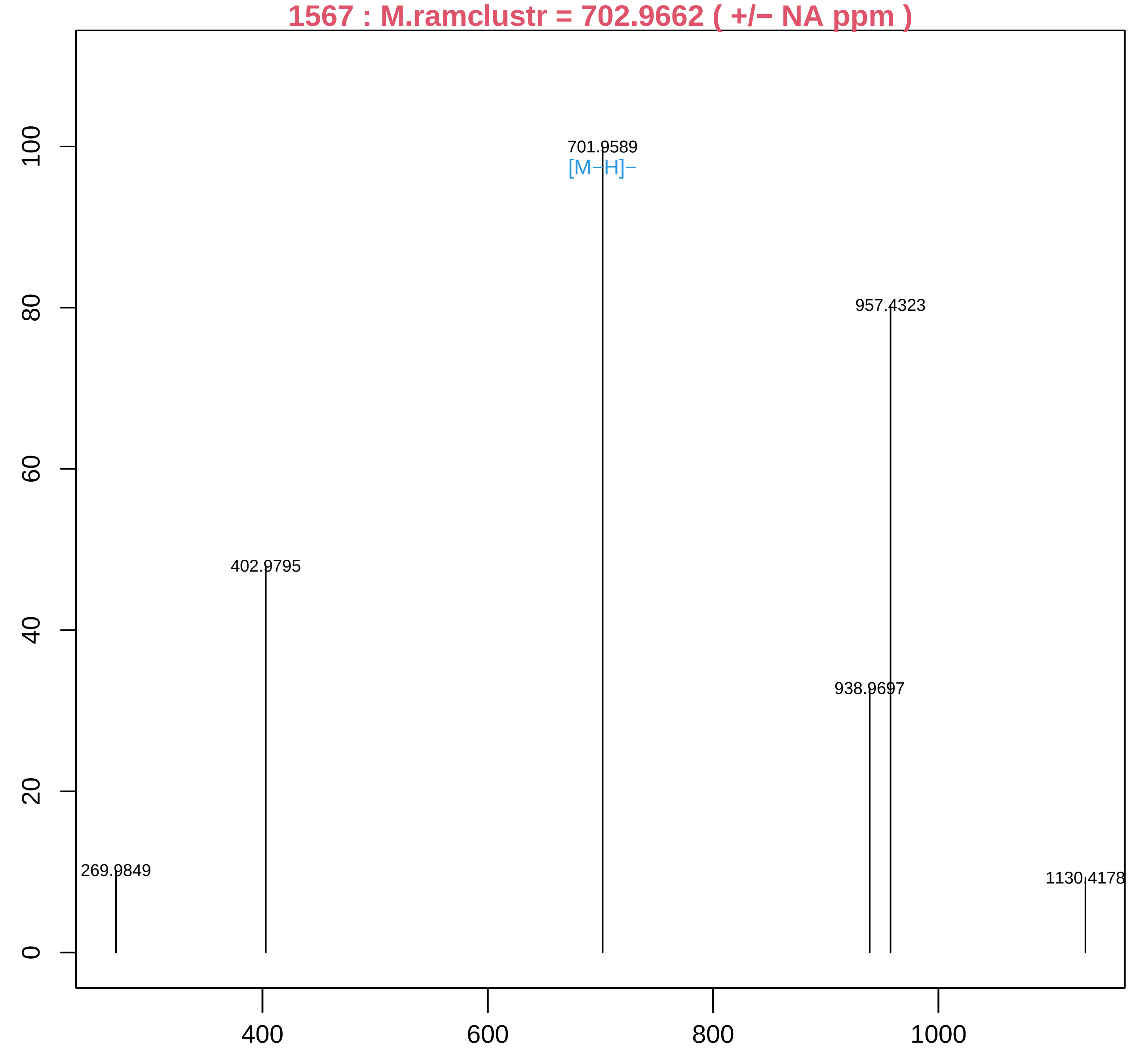
1567 : M.ramclustr = 702.9662 (+/− NA ppm)
701.9589
[M−H]−
957.4323
402.9795
938.9697
269.9849
1130.4178
0
20
40
60
80
100
400
600
800
1000

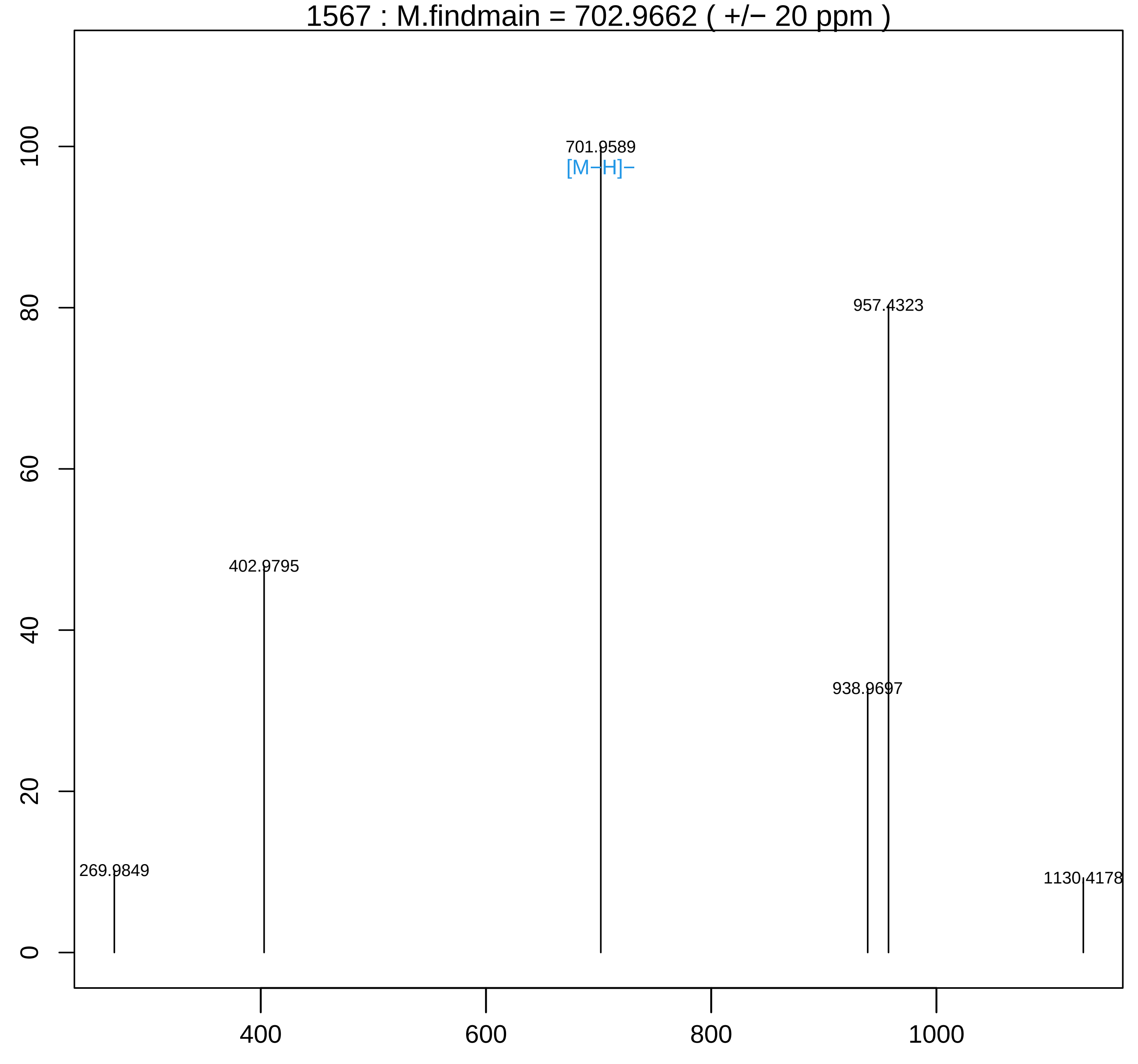
1567 : M.findmain = 702.9662 (+/− 20 ppm)
701.9589
[M−H]−
957.4323
402.9795
938.9697
269.9849
1130.4178
0
20
40
60
80
100
400
600
800
1000

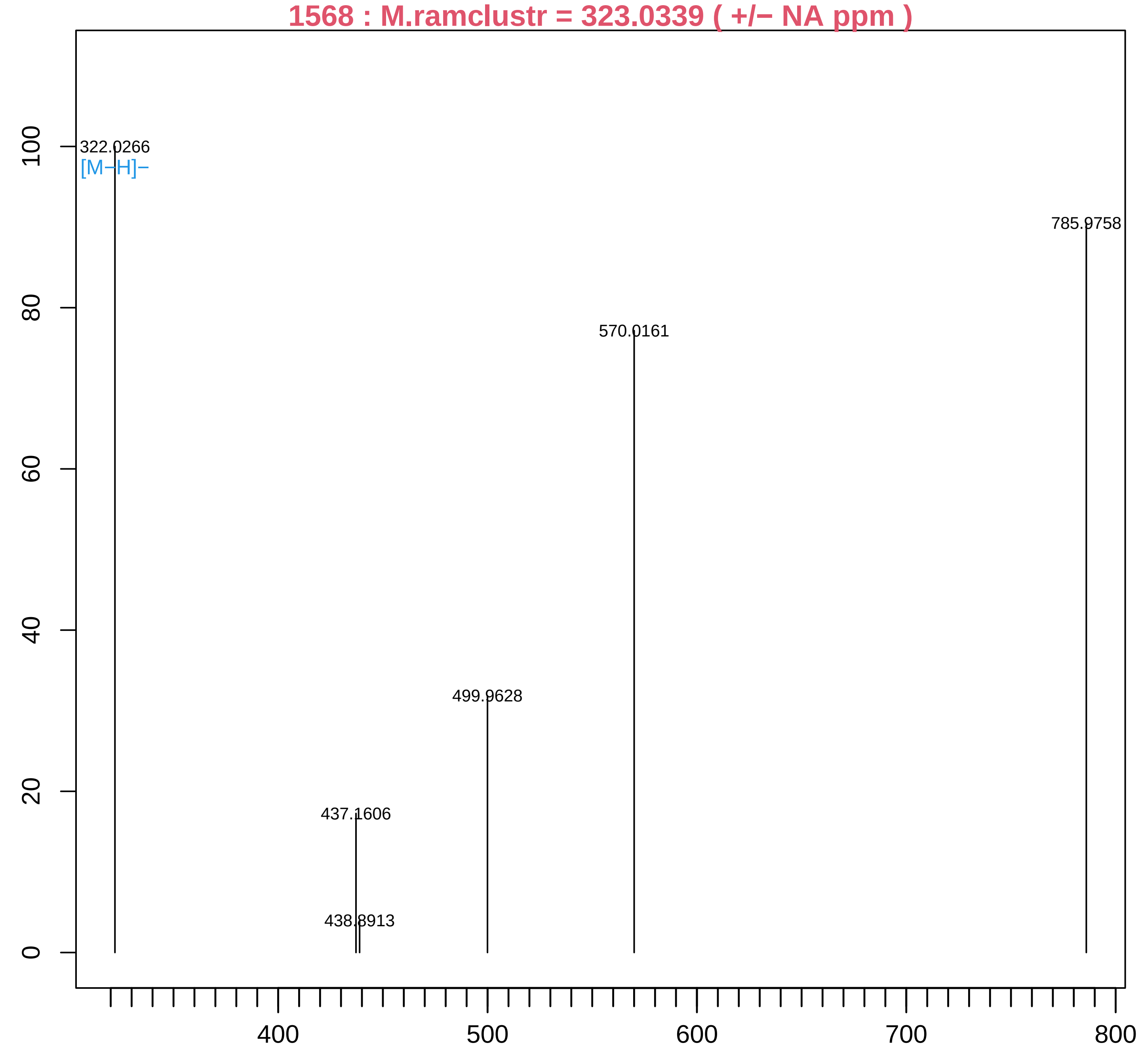
1568 : M.ramclustr = 323.0339 (+/− NA ppm)
322.0266
[M−H]−
785.9758
570.0161
499.9628
437.1606
438.8913
0
20
40
60
80
100
400
500
600
700
800

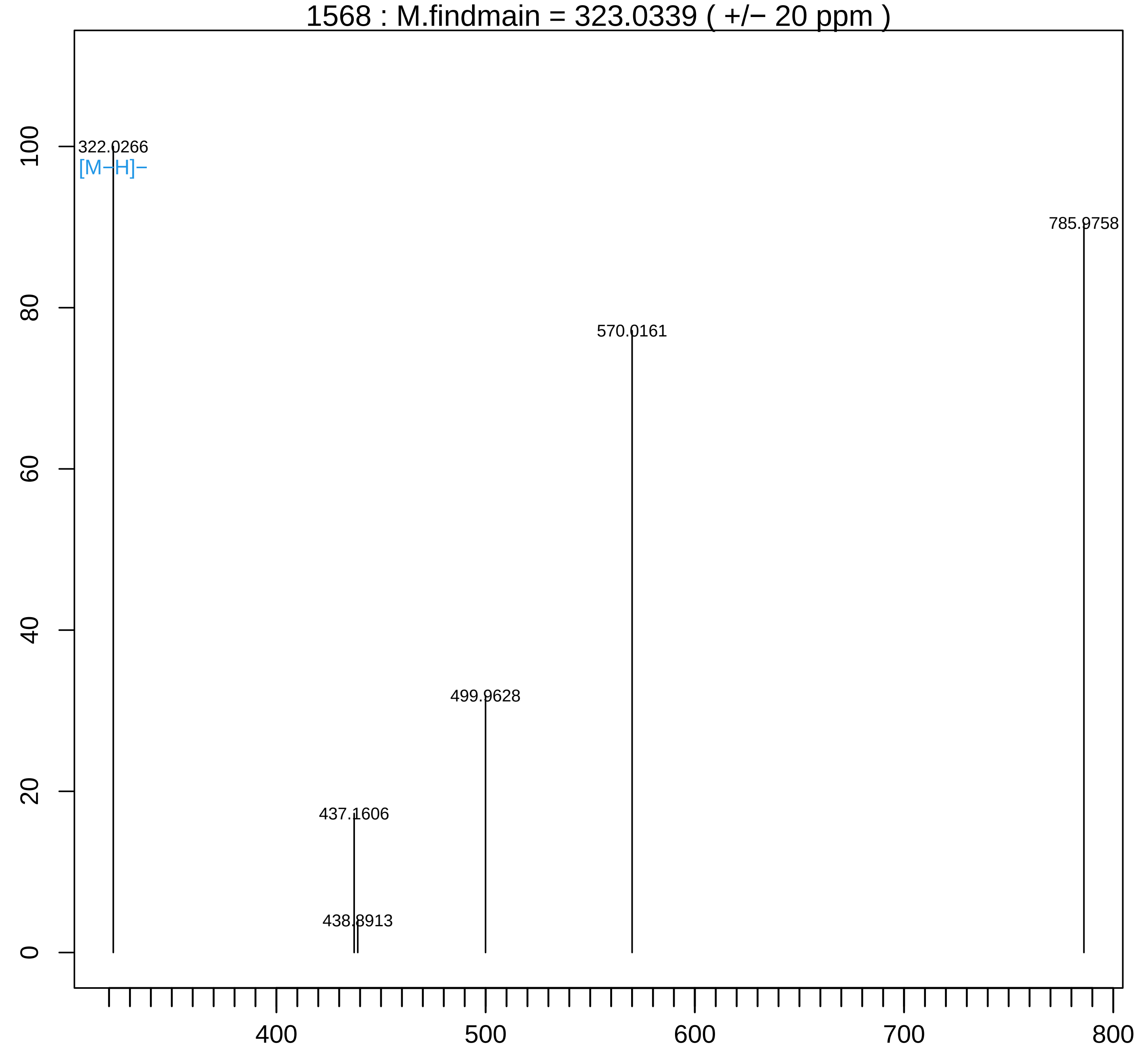
1568 : M.findmain = 323.0339 (+/− 20 ppm)
322.0266
[M−H]−
785.9758
570.0161
499.9628
437.1606
438.8913
0
20
40
60
80
100
400
500
600
700
800

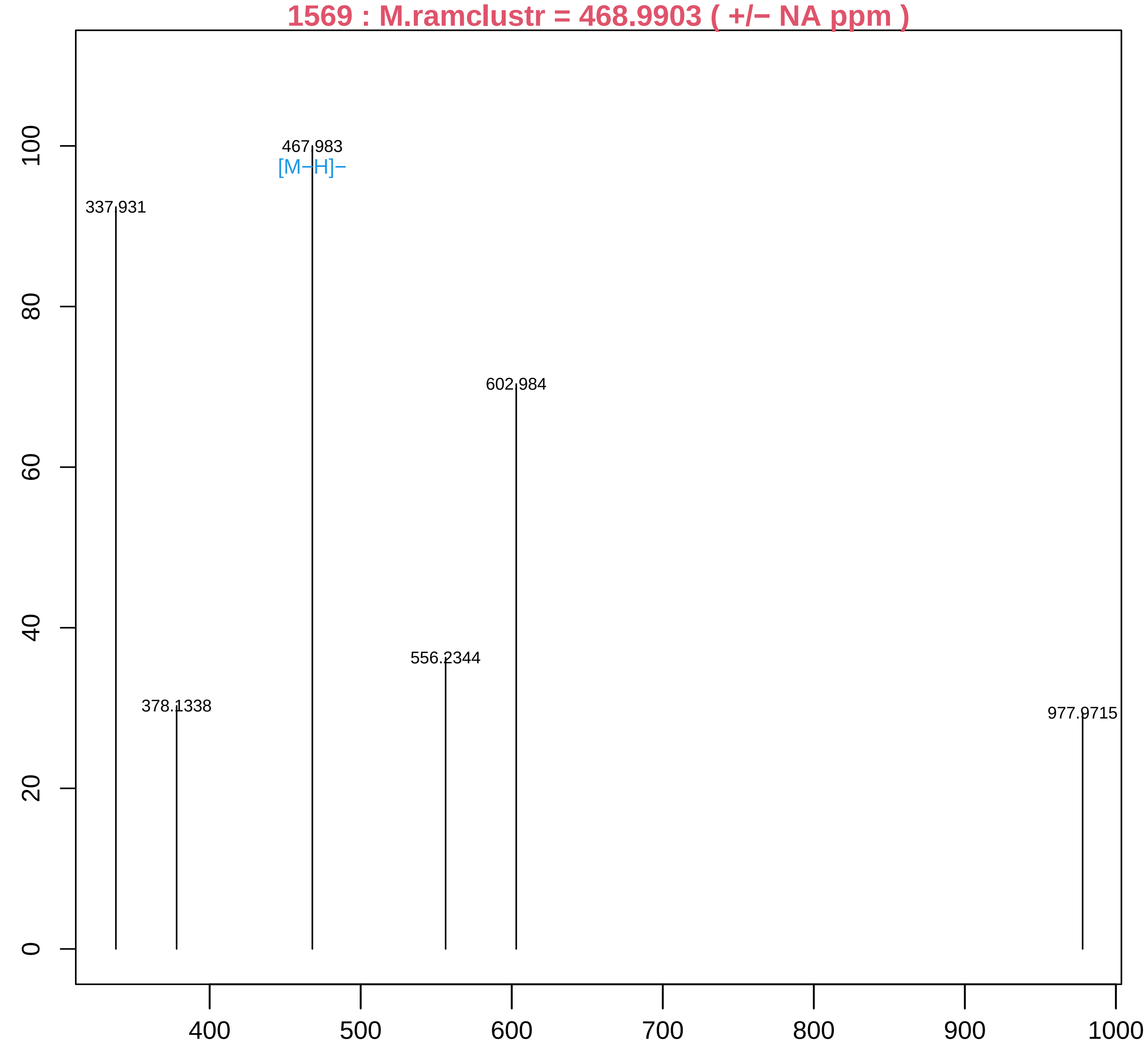
1569 : M.ramclustr = 468.9903 (+/− NA ppm)
467.983
[M−H]−
337.931
602.984
556.2344
378.1338
977.9715
0
20
40
60
80
100
400
500
600
700
800
900
1000

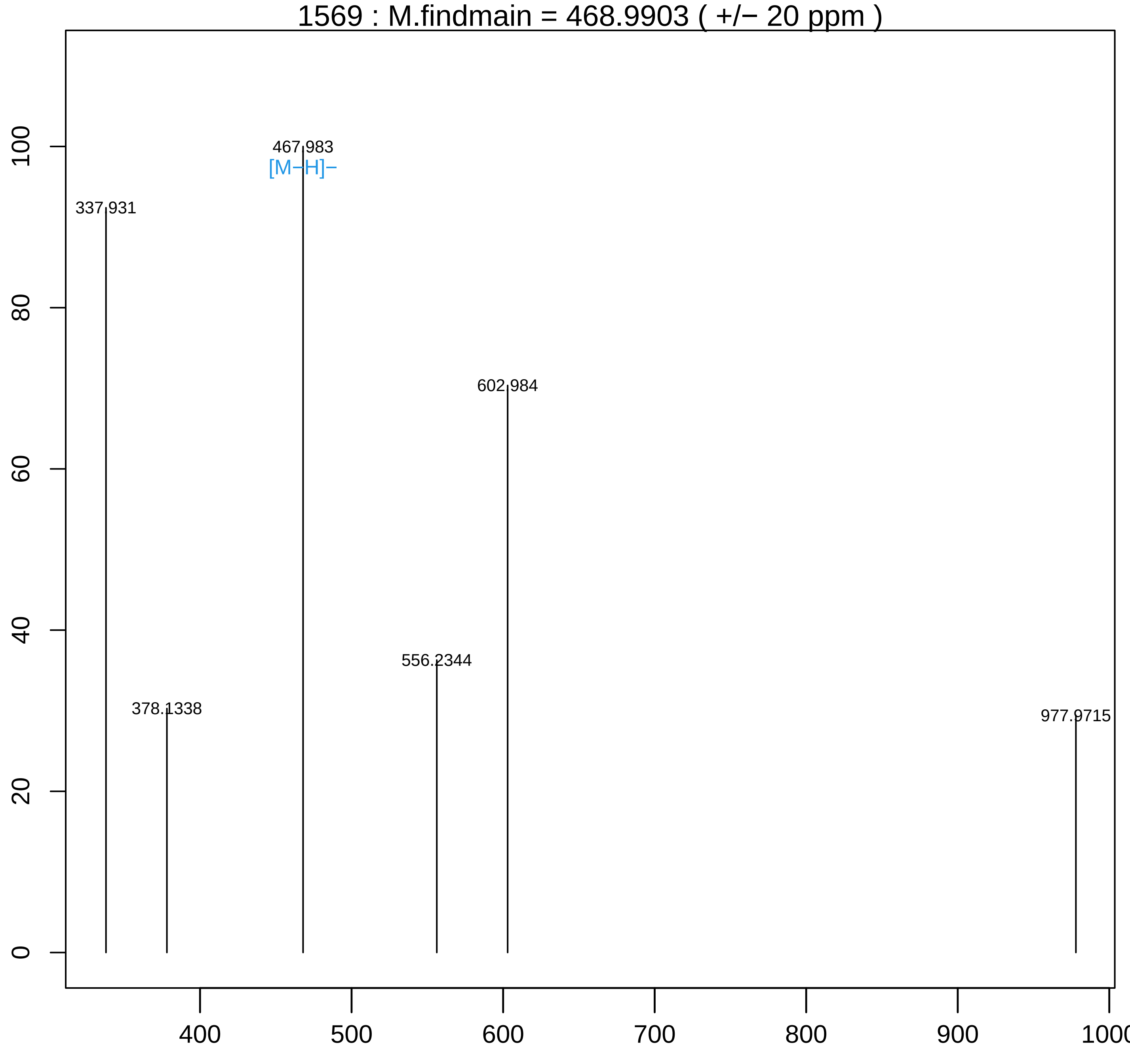
1569 : M.findmain = 468.9903 (+/− 20 ppm)
467.983
[M−H]−
337.931
602.984
556.2344
378.1338
977.9715
0
20
40
60
80
100
400
500
600
700
800
900
1000

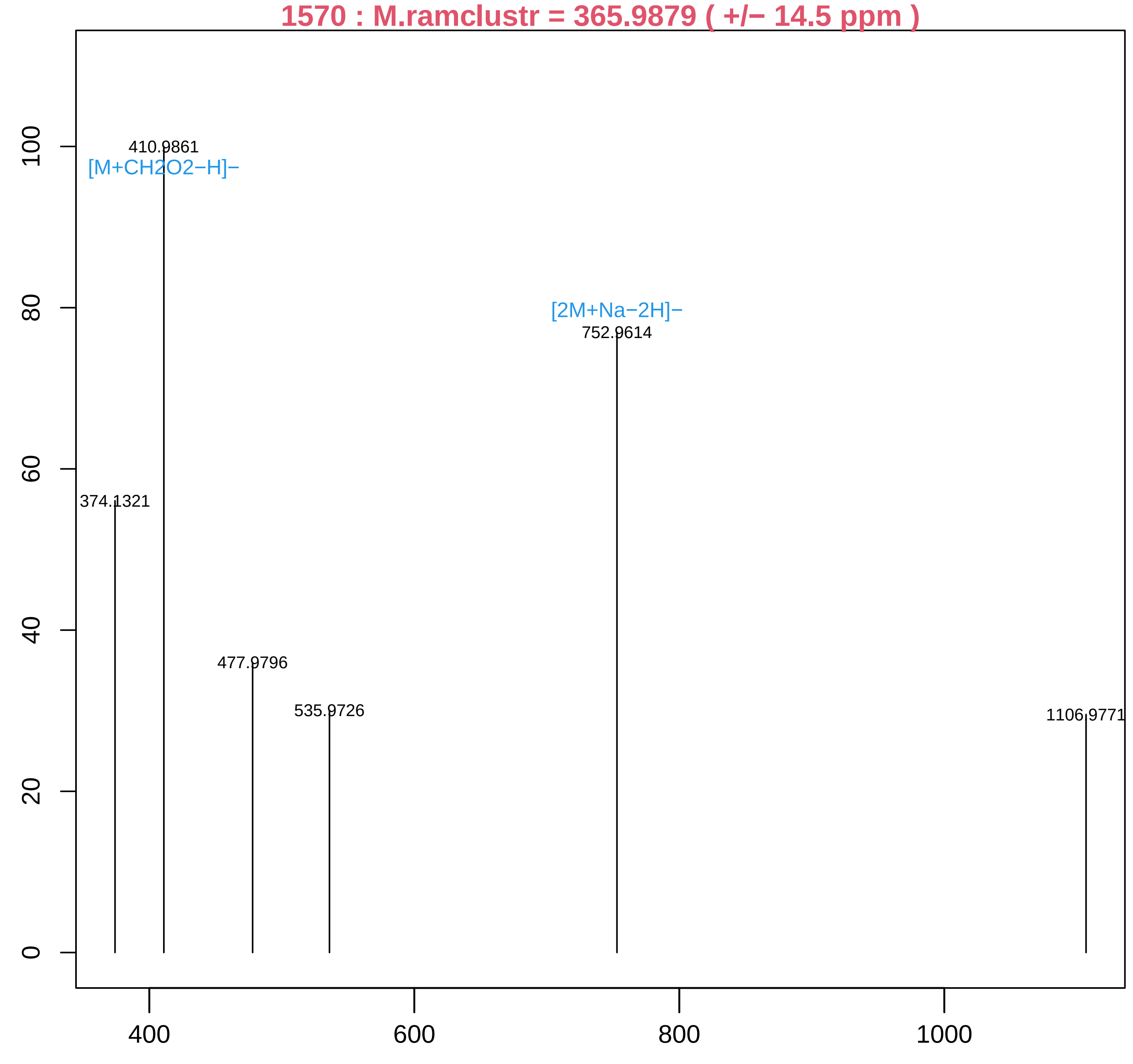

1570 : M.ramclustr = 365.9879 (+/− 14.5 ppm)
410.9861
[M+CH2O2−H]−
[2M+Na−2H]−
752.9614
374.1321
477.9796
535.9726
1106.9771
0
20
40
60
80
100
400
600
800
1000

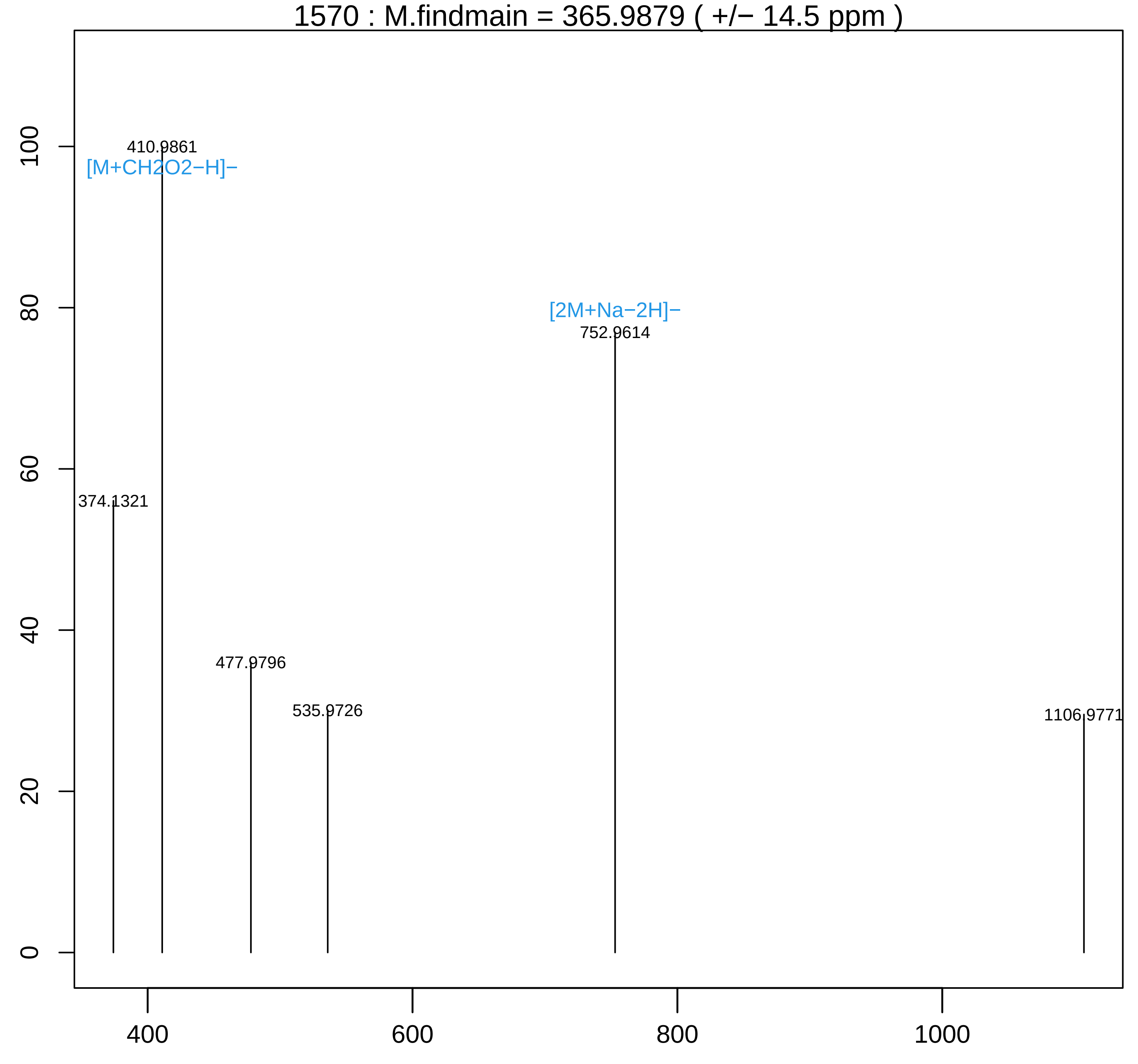

1570 : M.findmain = 365.9879 (+/− 14.5 ppm)
410.9861
[M+CH2O2−H]−
[2M+Na−2H]−
752.9614
374.1321
477.9796
535.9726
1106.9771
0
20
40
60
80
100
400
600
800
1000

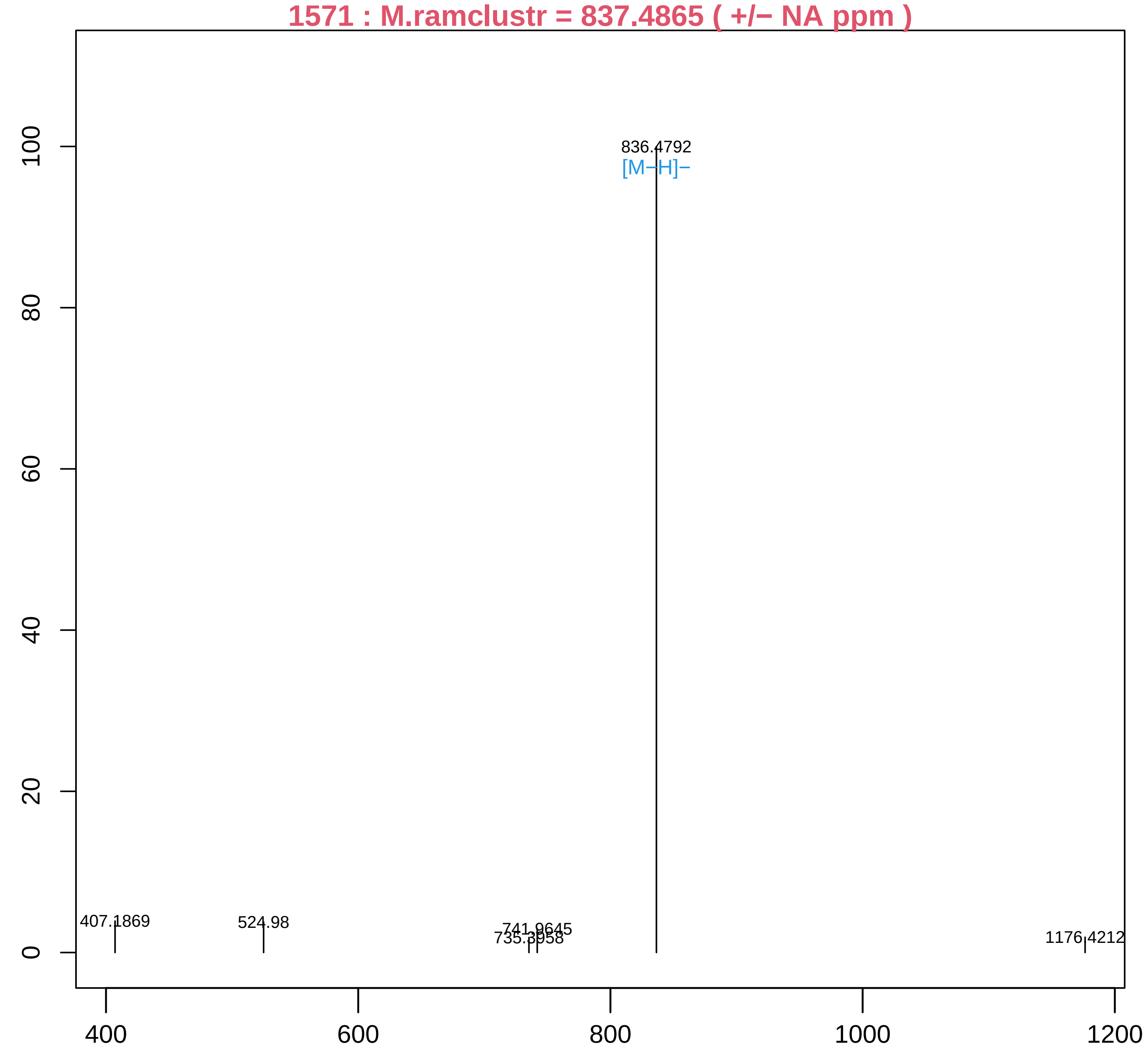
1571 : M.ramclustr = 837.4865 (+/− NA ppm)
836.4792
[M−H]−
407.1869
524.98
741.9645
735.3958
1176.4212
0
20
40
60
80
100
400
600
800
1000
1200

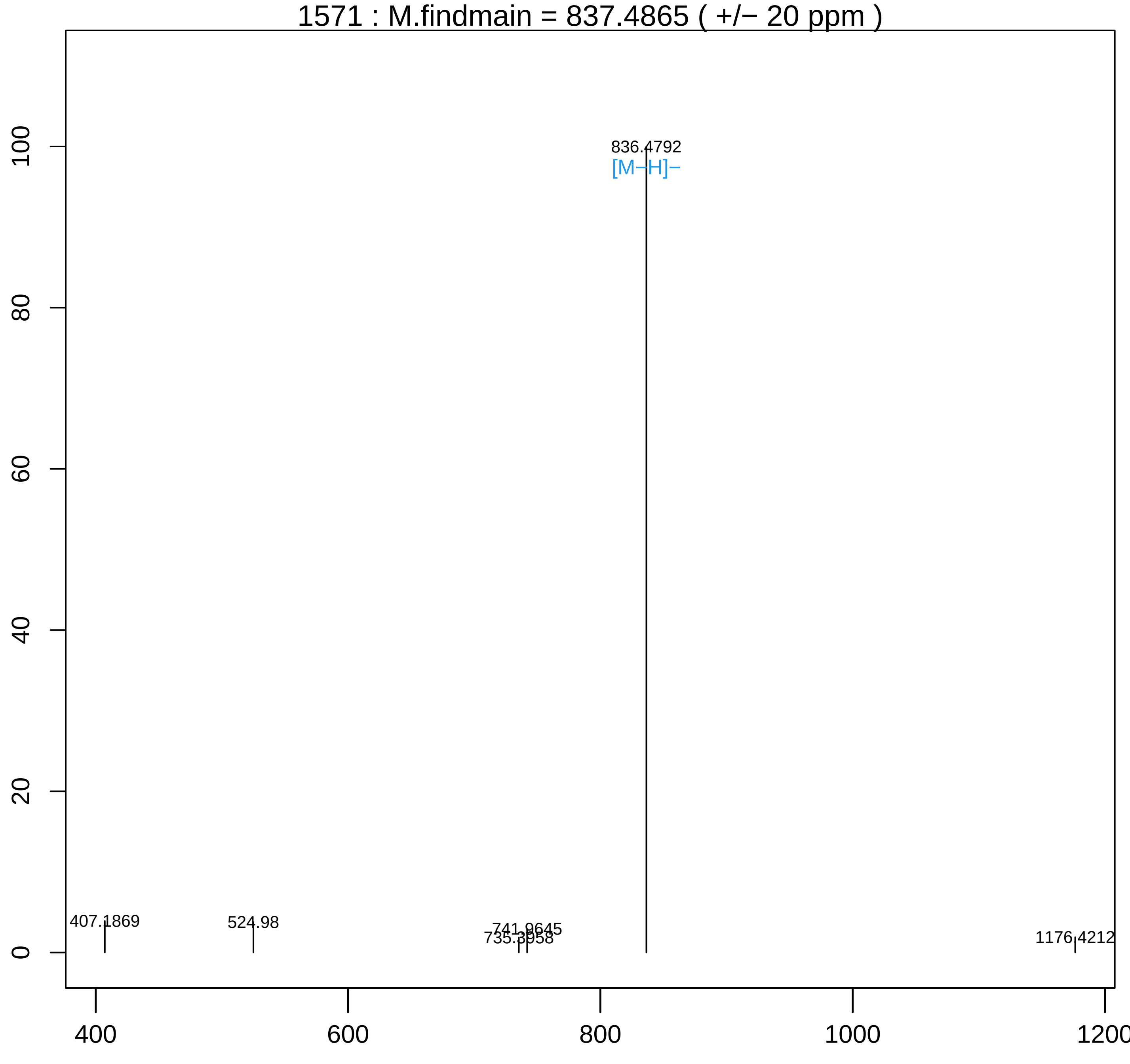
1571 : M.findmain = 837.4865 (+/− 20 ppm)
836.4792
[M−H]−
407.1869
524.98
741.9645
735.3958
1176.4212
0
20
40
60
80
100
400
600
800
1000
1200

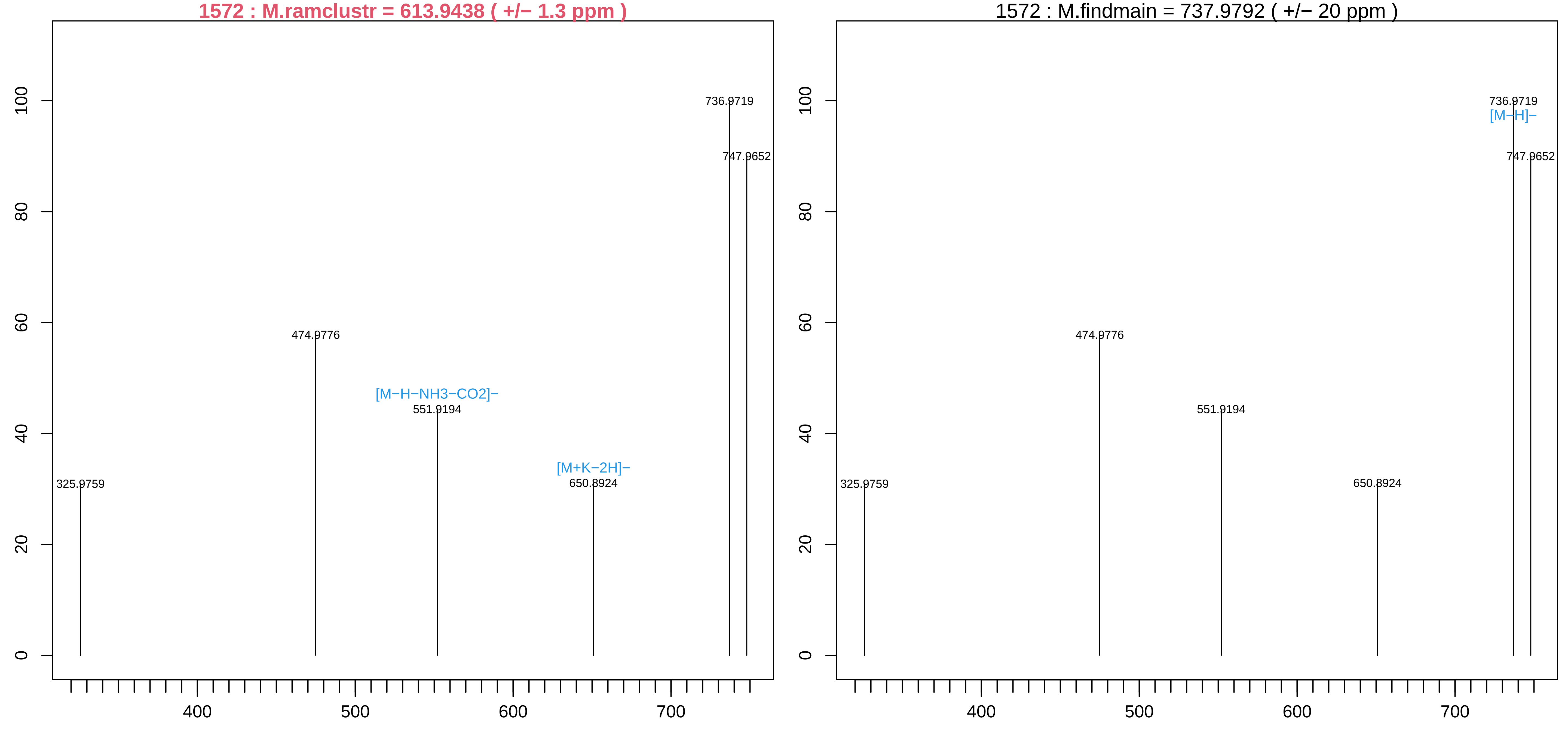
1572 : M.ramclustr = 613.9438 (+/− 1.3 ppm)
736.9719
747.9652
474.9776
[M−H−NH3−CO2]−
551.9194
[M+K−2H]−
650.8924
325.9759
1572 : M.findmain = 737.9792 (+/− 20 ppm)
736.9719
[M−H]−
747.9652
474.9776
551.9194
650.8924
325.9759
0
20
40
60
80
100
400
500
600
700

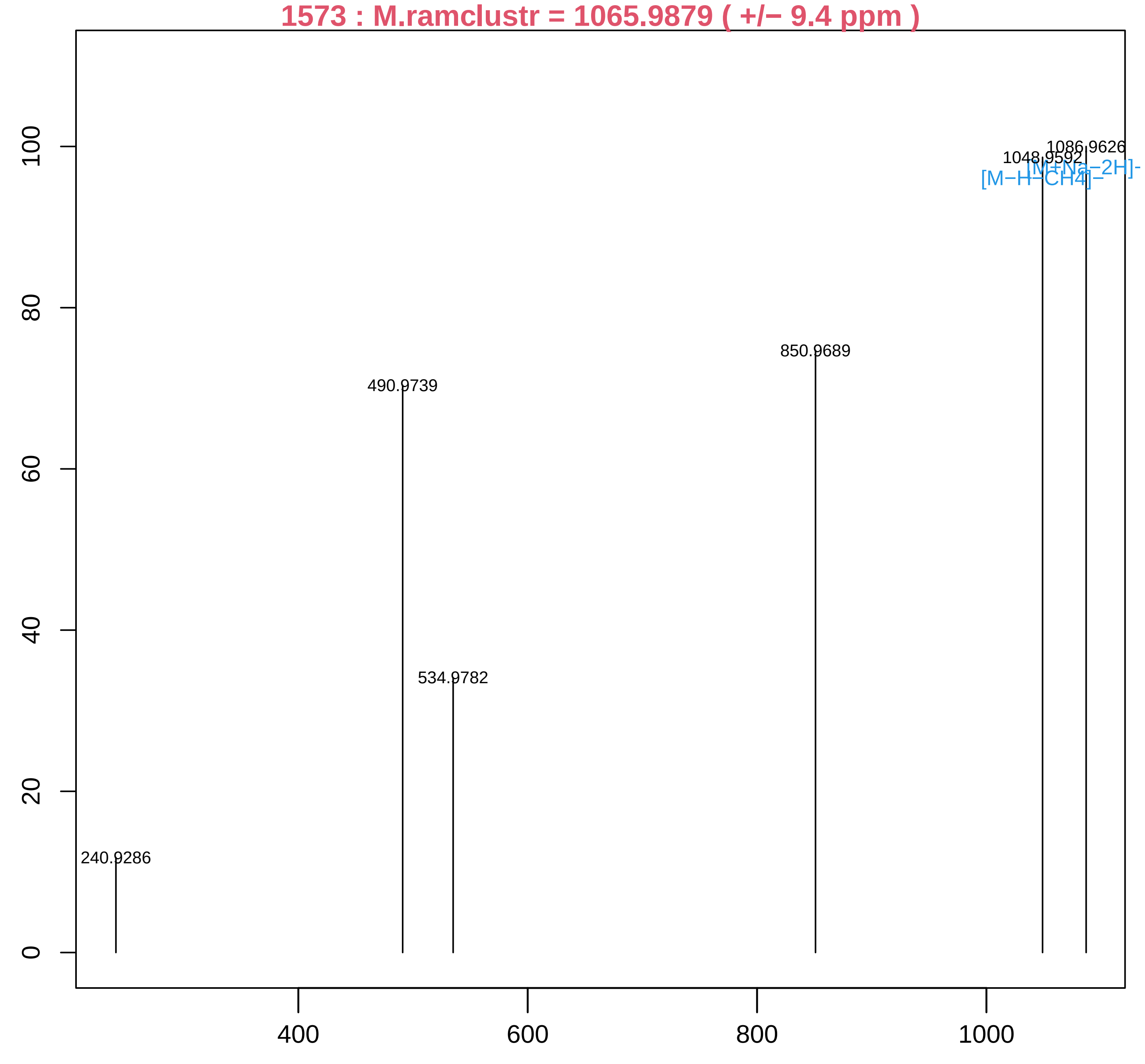
1573 : M.ramclustr = 1065.9879 (+/− 9.4 ppm)
240.9286
490.9739
534.9782
850.9689
1048.9592
1086.9626
[M+Na−2H]-
[M−H−CH4]−
0
20
40
60
80
100
400
600
800
1000

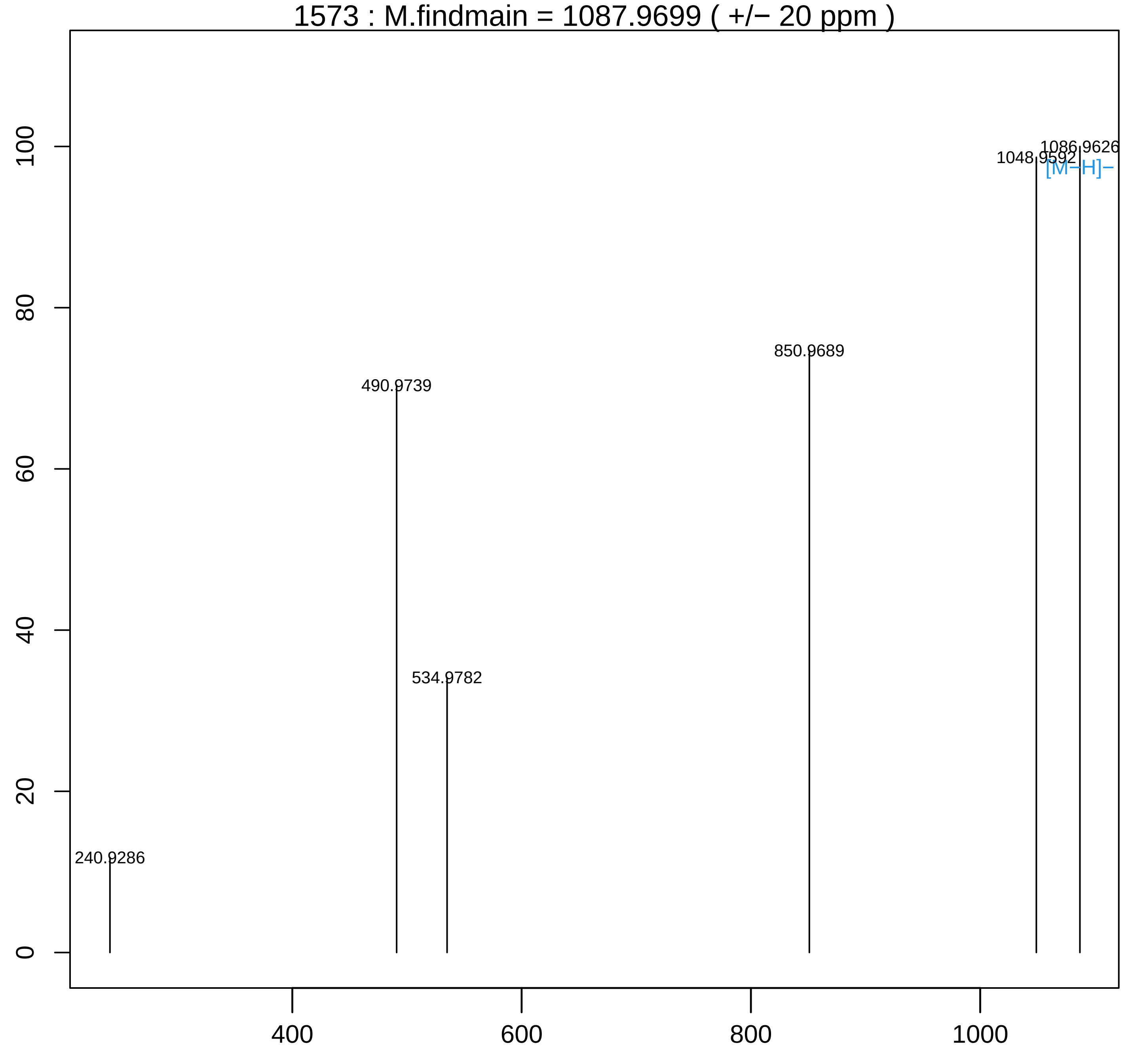
1573 : M.findmain = 1087.9699 (+/− 20 ppm)
240.9286
490.9739
534.9782
850.9689
1048.9592
1086.9626
[M−H]−
0
20
40
60
80
100
400
600
800
1000

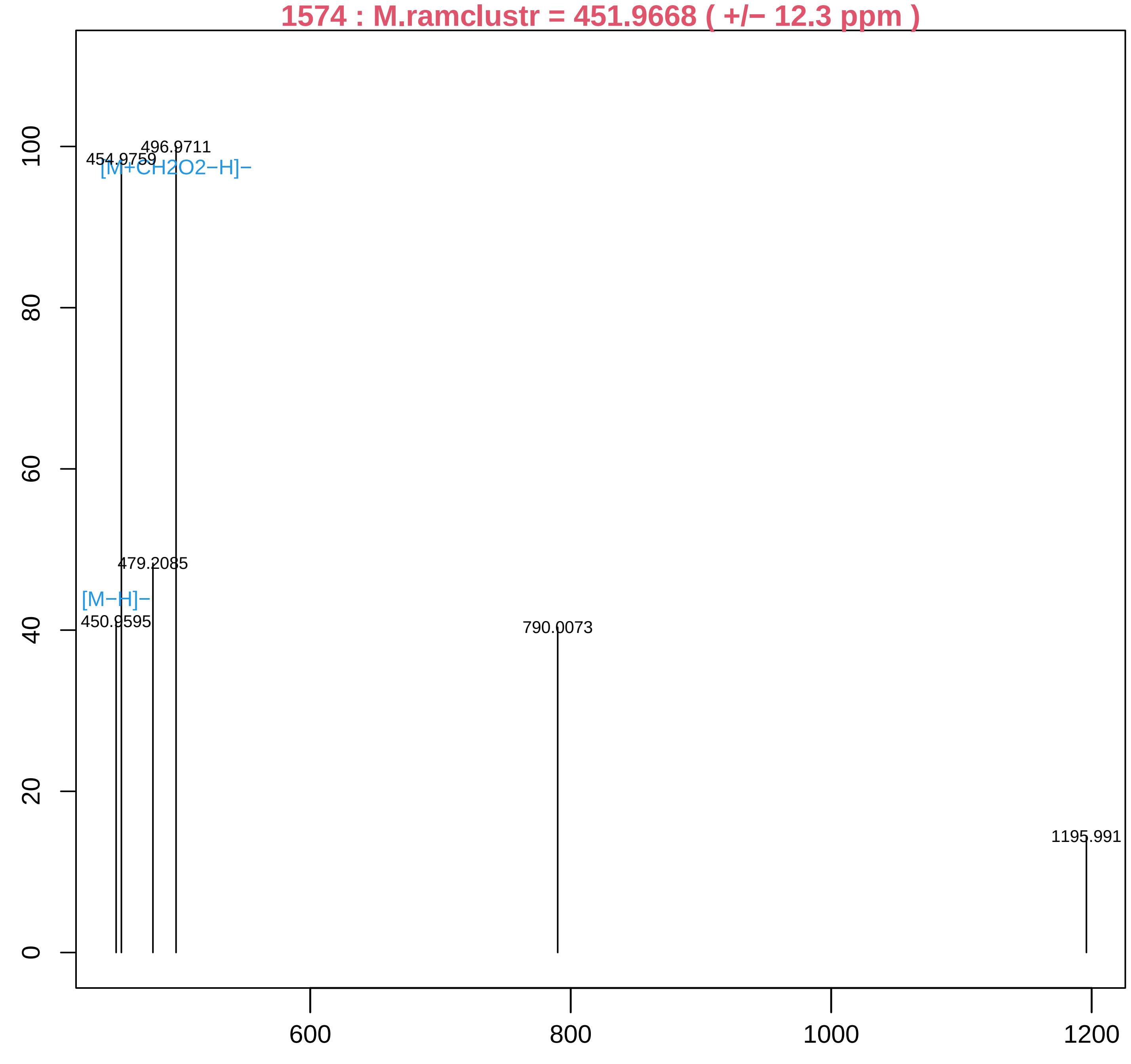

1574 : M.ramclustr = 451.9668 (+/− 12.3 ppm)
496.9711
454.9759
[M+CH2O2−H]−
479.2085
[M−H]−
450.9595
790.0073
1195.991
0
20
40
60
80
100
600
800
1000
1200

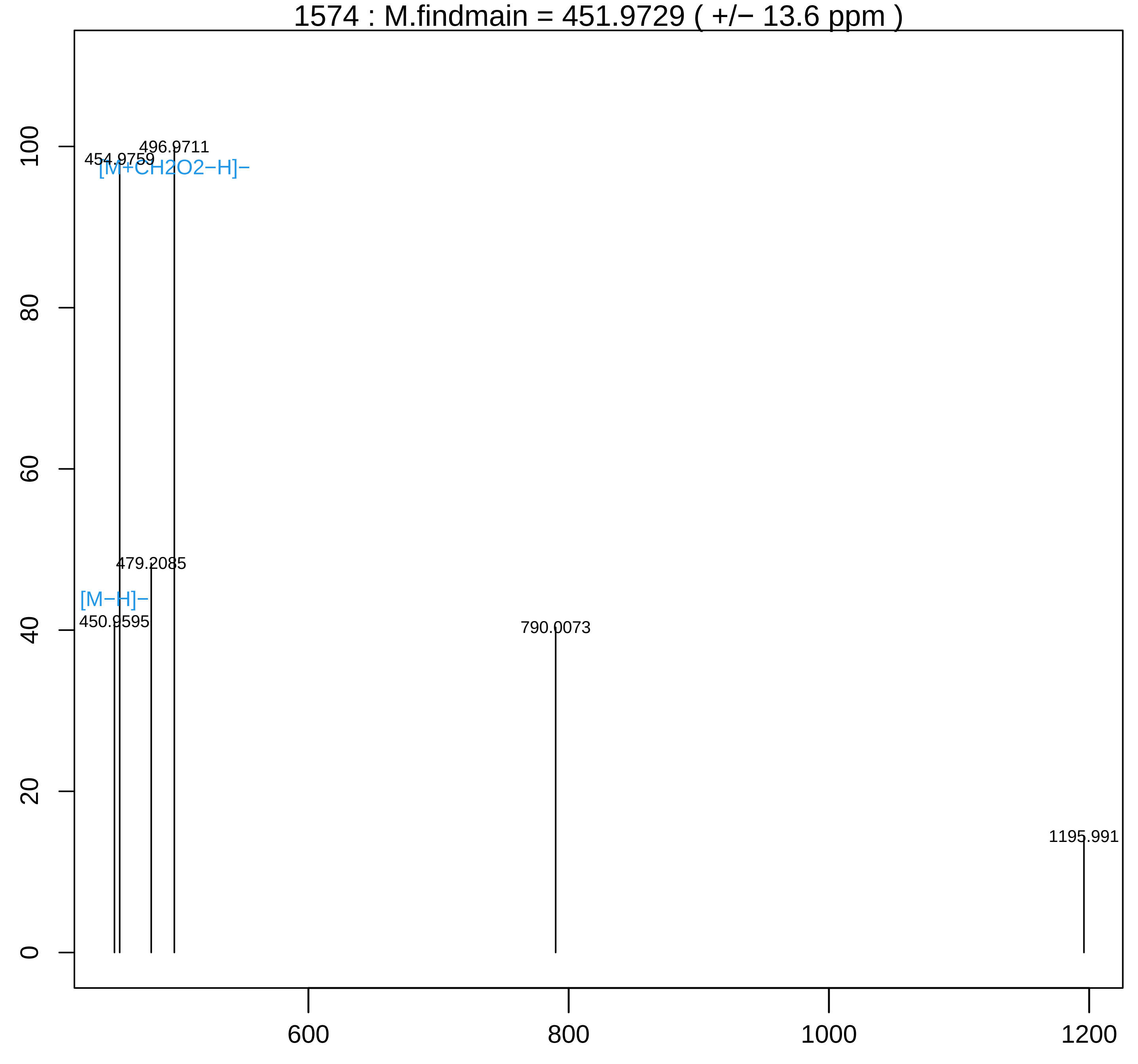

1574 : M.findmain = 451.9729 (+/− 13.6 ppm)
496.9711
454.9759
[M+CH2O2−H]−
479.2085
[M−H]−
450.9595
790.0073
1195.991
0
20
40
60
80
100
600
800
1000
1200

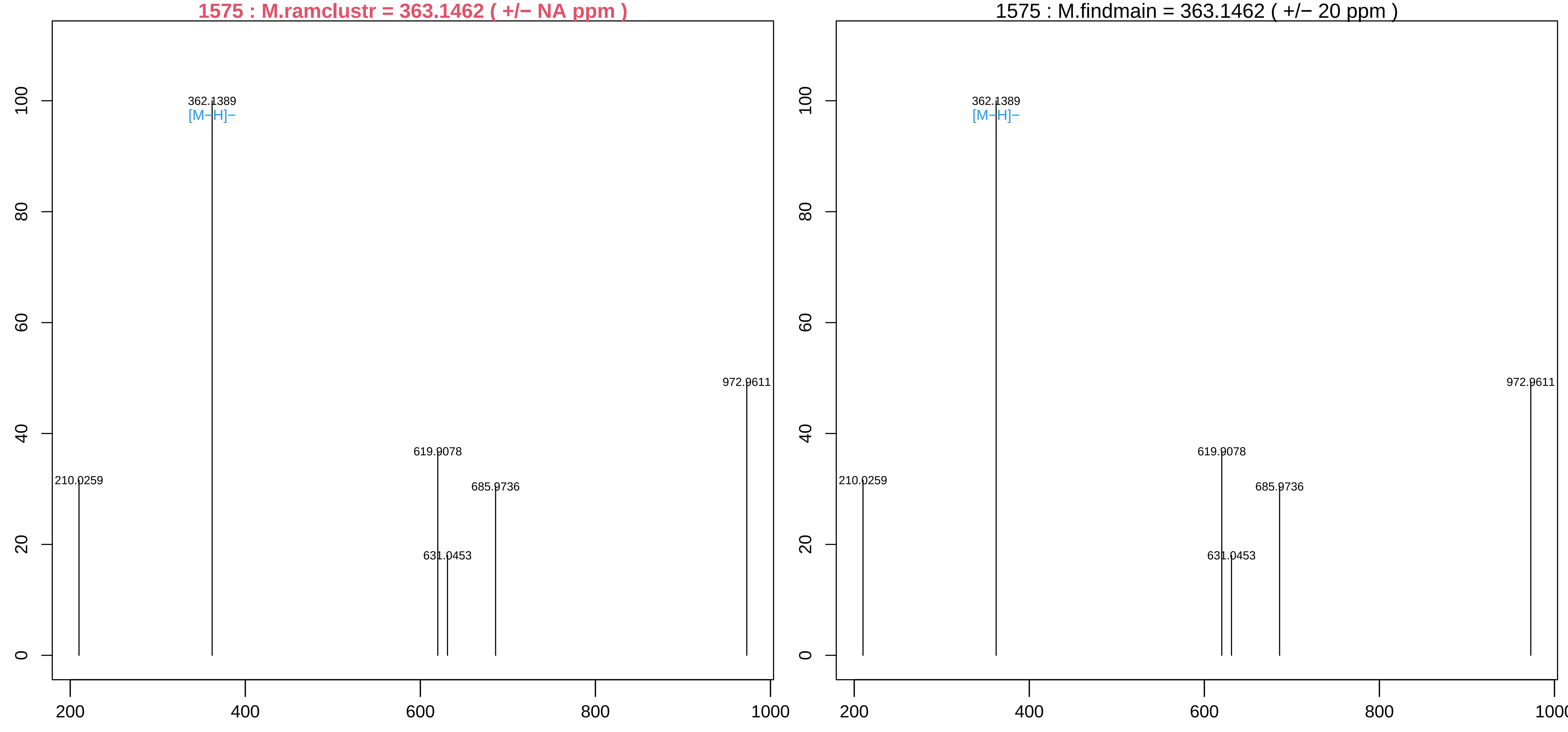

1575 : M.ramclustr = 363.1462 (+/− NA ppm)
362.1389
[M−H]−
972.9611
619.9078
210.0259
685.9736
631.0453
0
20
40
60
80
100
200
400
600
800
1000
1575 : M.findmain = 363.1462 (+/− 20 ppm)
362.1389
[M−H]−
972.9611
619.9078
210.0259
685.9736
631.0453
0
20
40
60
80
100
200
400
600
800
1000

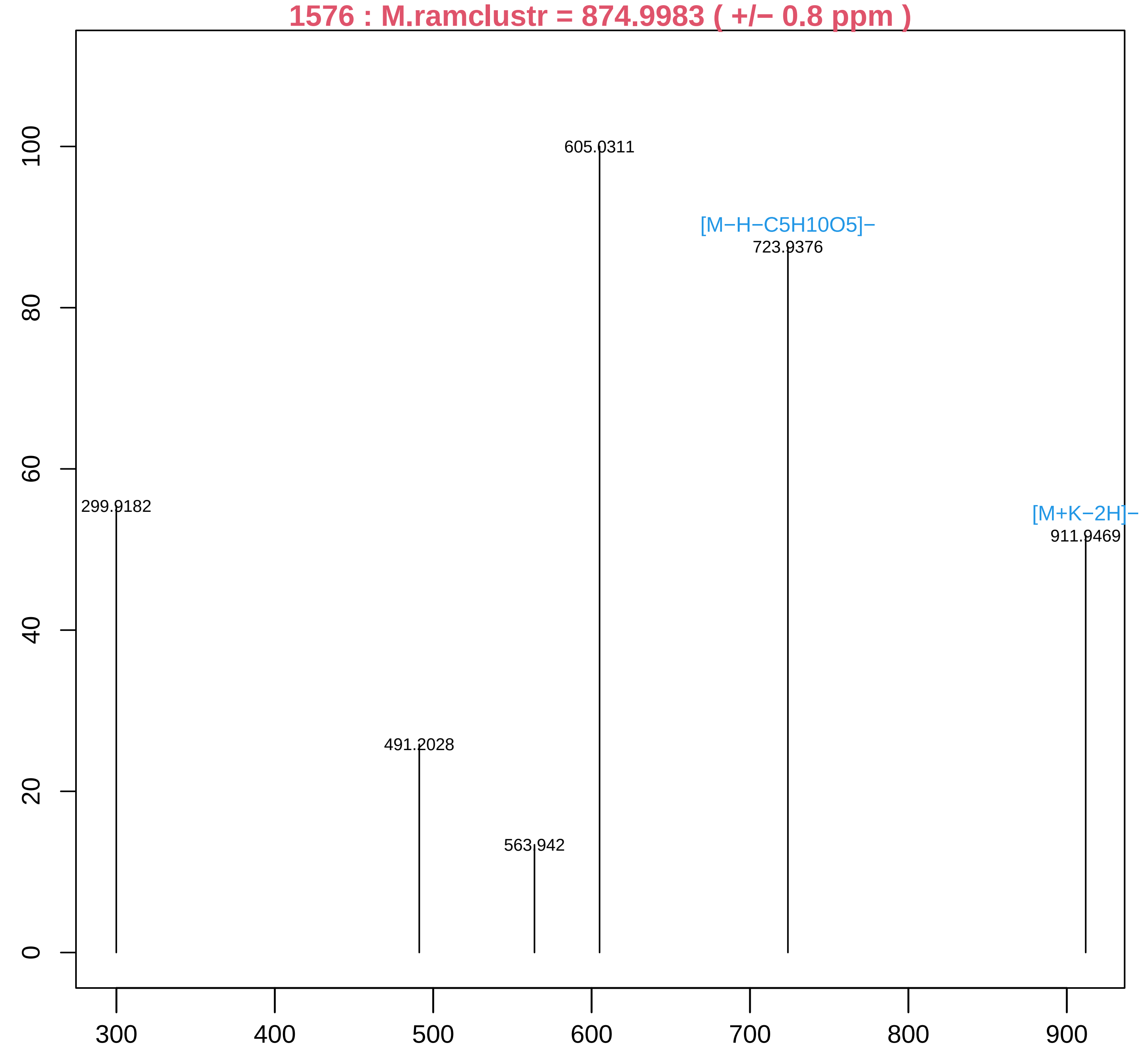

1576 : M.ramclustr = 874.9983 (+/− 0.8 ppm)
605.0311
[M−H−C5H10O5]−
723.9376
299.9182
[M+K−2H]−
911.9469
491.2028
563.942
0
20
40
60
80
100
300
400
500
600
700
800
900

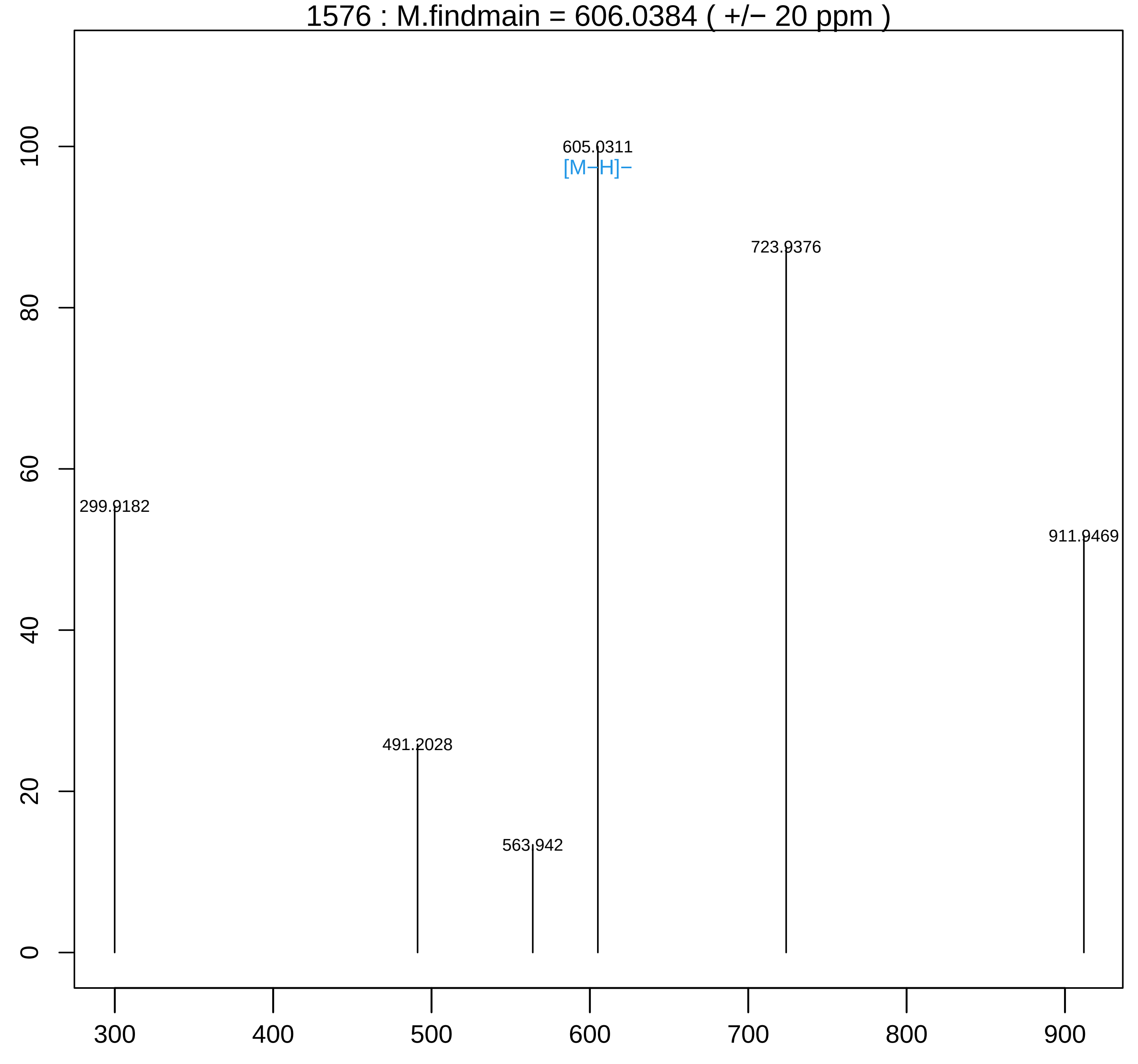

1576 : M.findmain = 606.0384 (+/− 20 ppm)
605.0311
[M−H]−
723.9376
299.9182
911.9469
491.2028
563.942
0
20
40
60
80
100
300
400
500
600
700
800
900

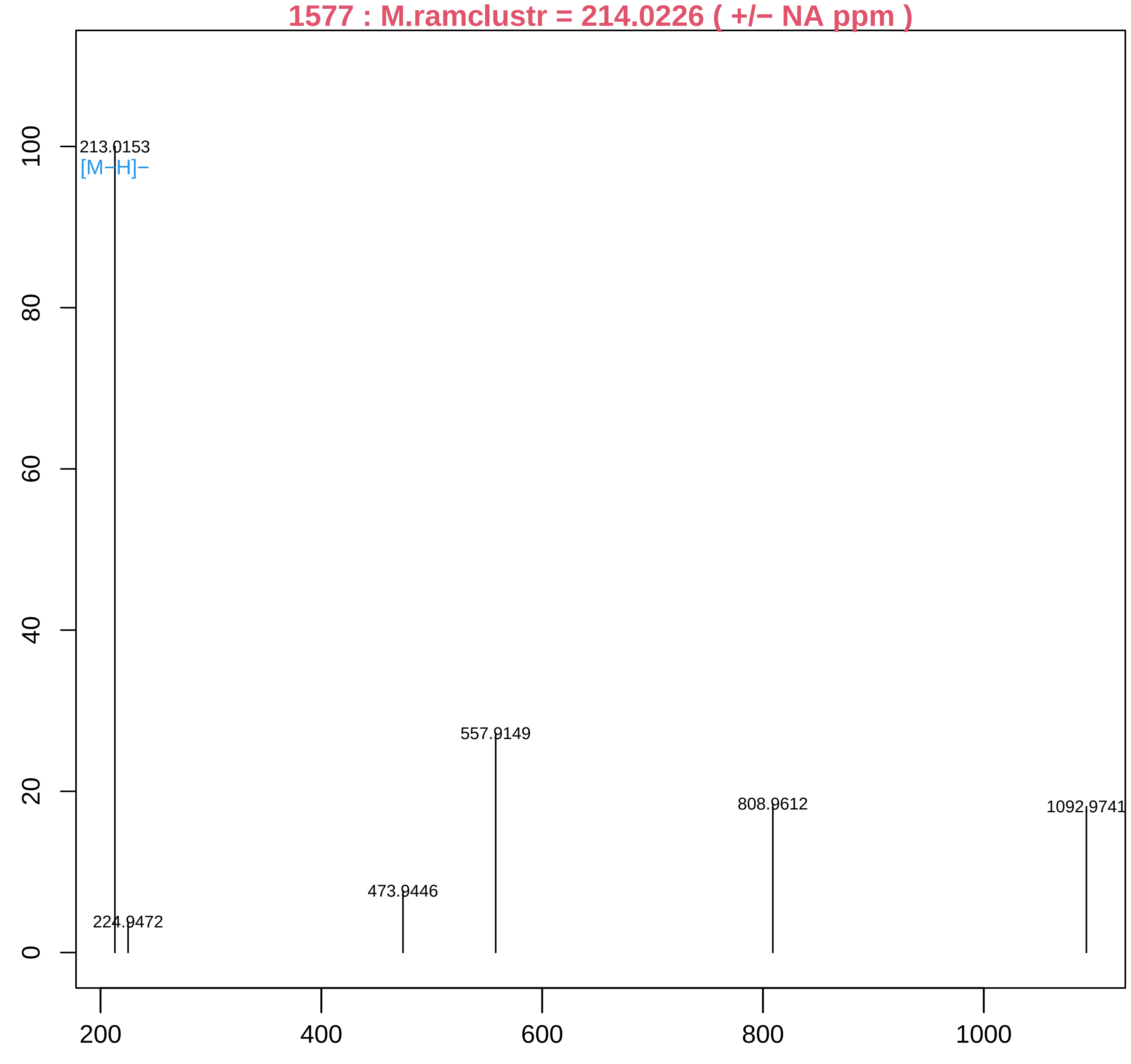
1577 : M.ramclustr = 214.0226 (+/− NA ppm)
213.0153
[M−H]−
224.9472
473.9446
557.9149
808.9612
1092.9741
0
20
40
60
80
100
200
400
600
800
1000

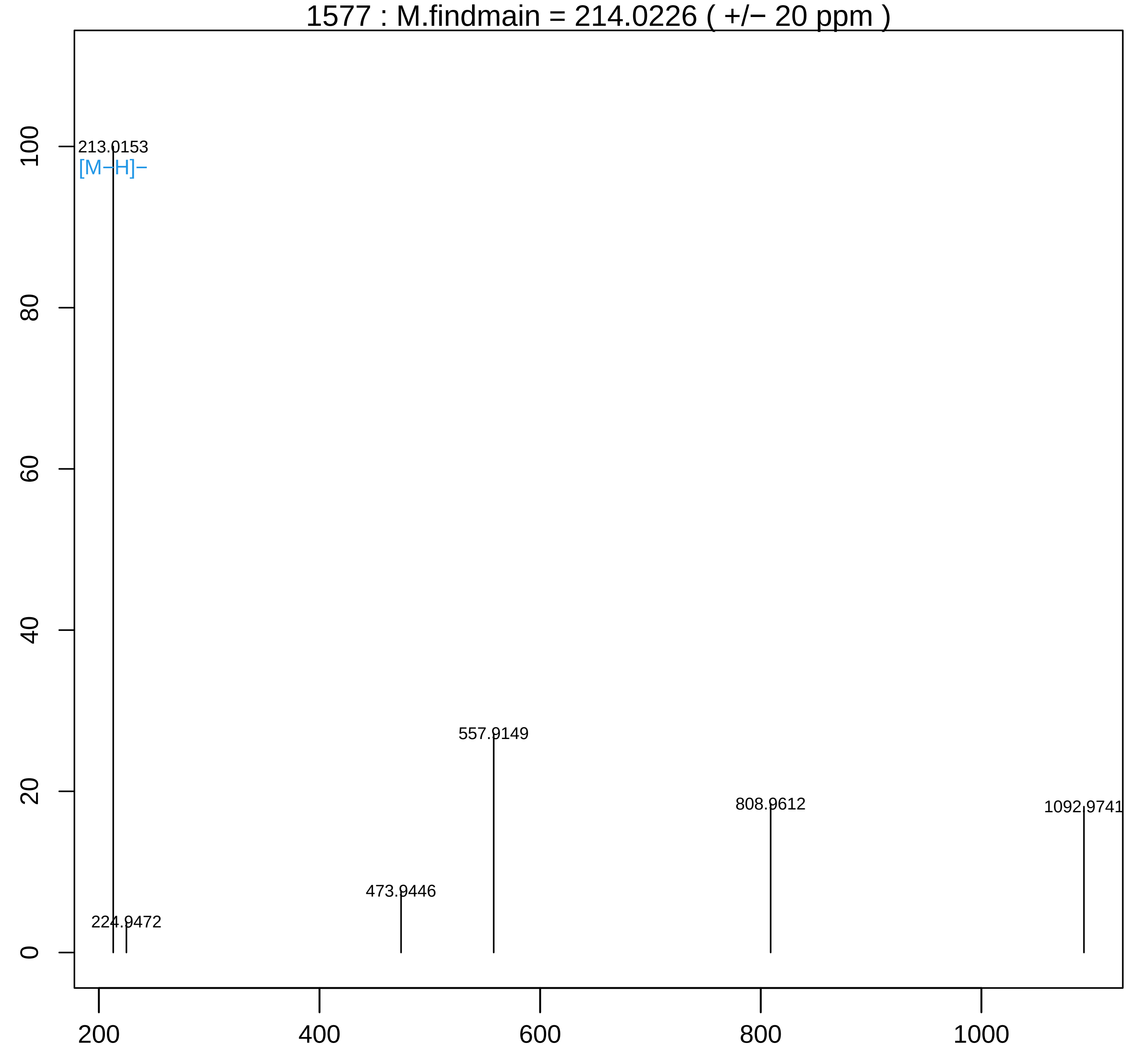
1577 : M.findmain = 214.0226 (+/− 20 ppm)
213.0153
[M−H]−
224.9472
473.9446
557.9149
808.9612
1092.9741
0
20
40
60
80
100
200
400
600
800
1000

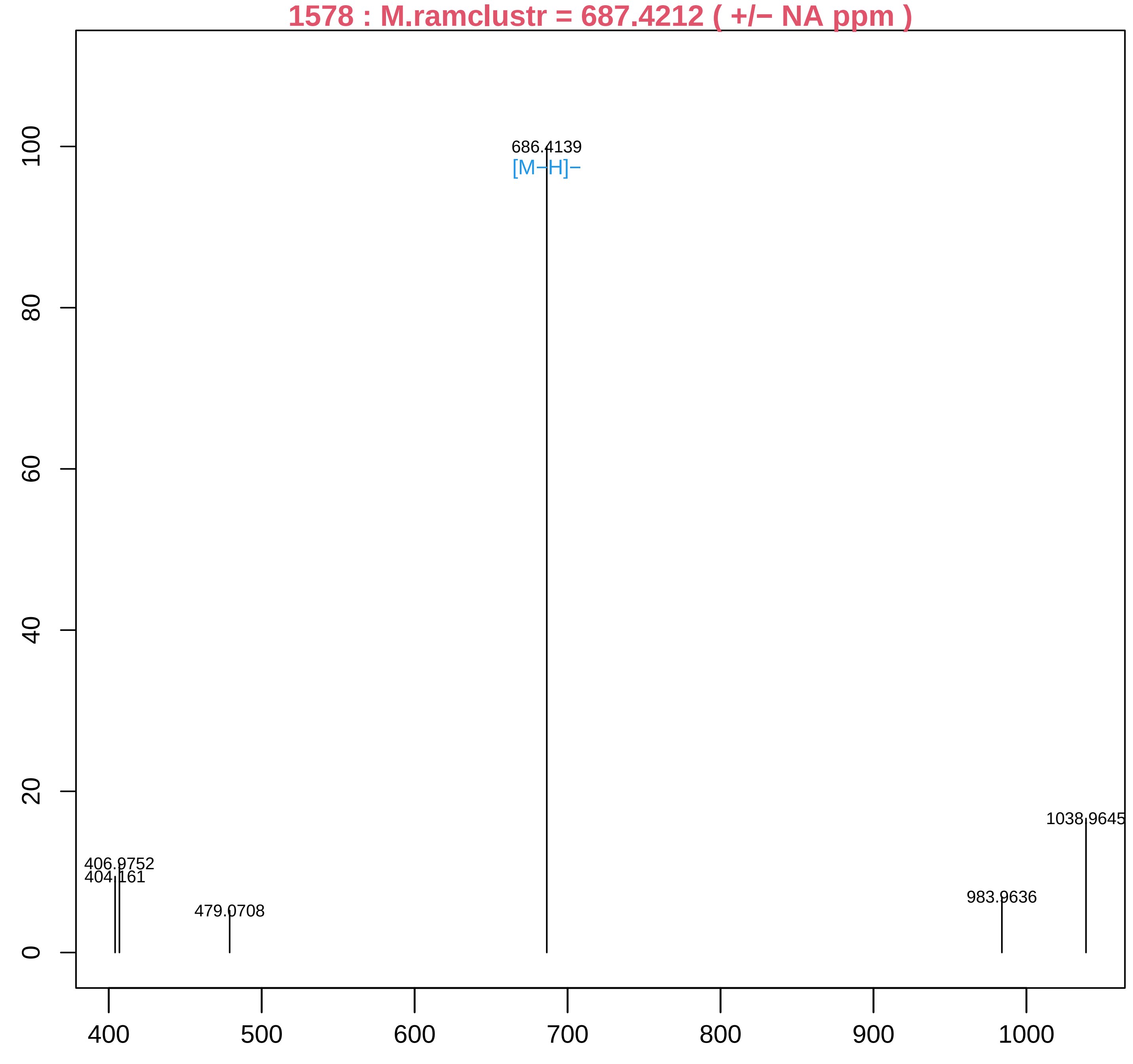

1578 : M.ramclustr = 687.4212 (+/− NA ppm)
686.4139
[M−H]−
406.9752
404.161
479.0708
983.9636
1038.9645
0
20
40
60
80
100
400
500
600
700
800
900
1000

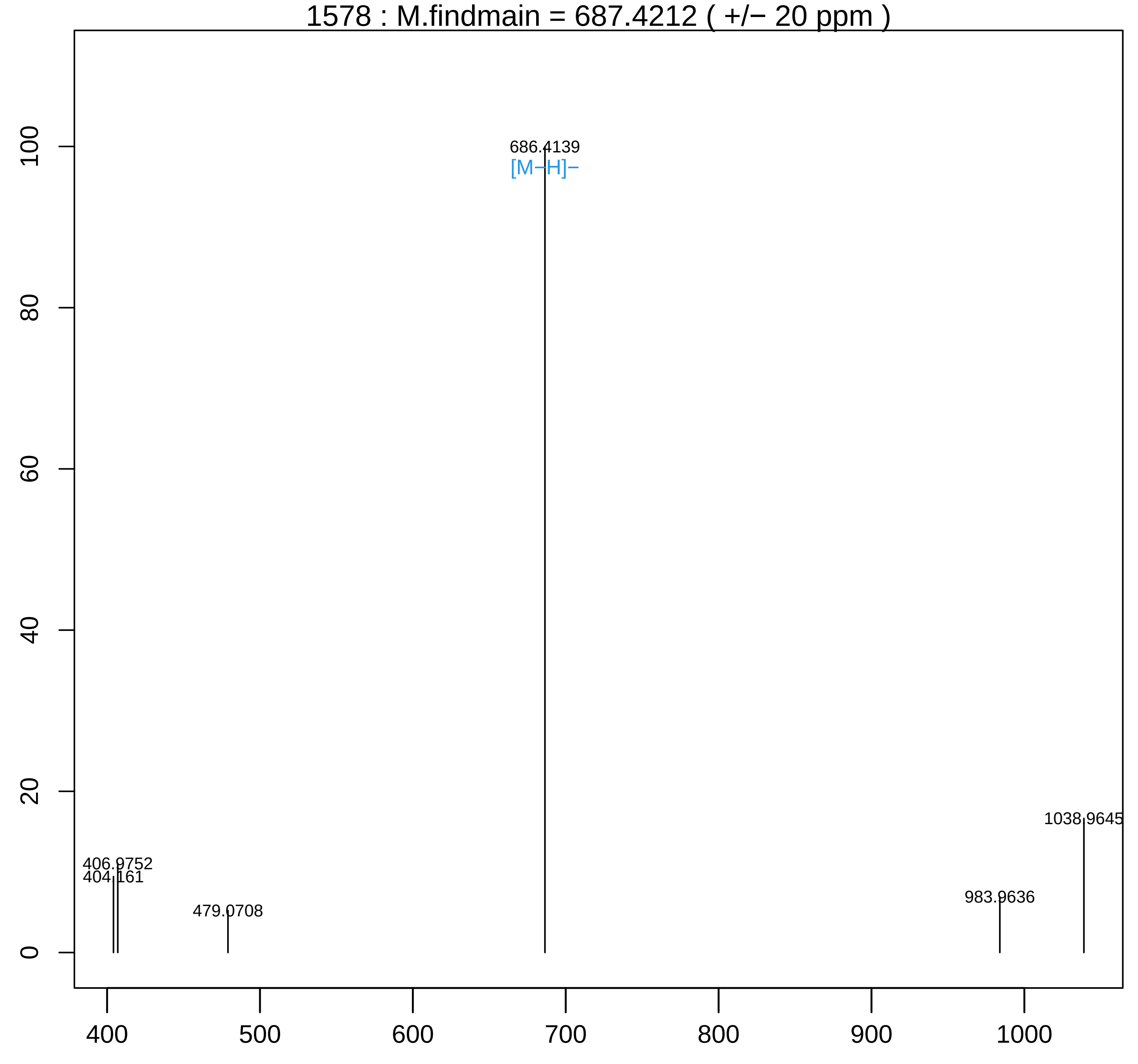

1578 : M.findmain = 687.4212 (+/− 20 ppm)
686.4139
[M−H]−
406.9752
404.161
479.0708
983.9636
1038.9645
0
20
40
60
80
100
400
500
600
700
800
900
1000

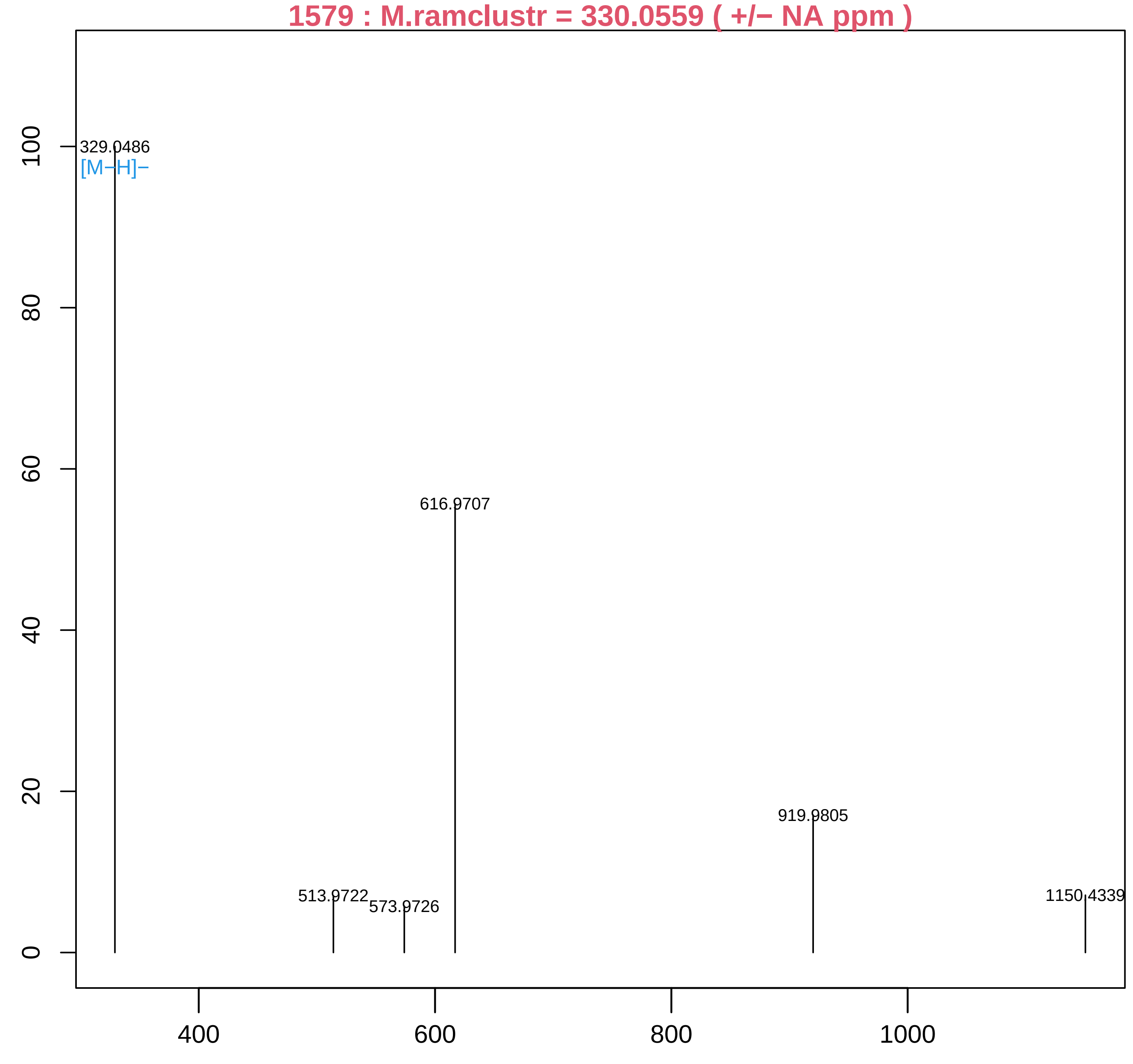
1579 : M.ramclustr = 330.0559 (+/− NA ppm)
329.0486
[M−H]−
616.9707
919.9805
513.9722
573.9726
1150.4339
0
20
40
60
80
100
400
600
800
1000

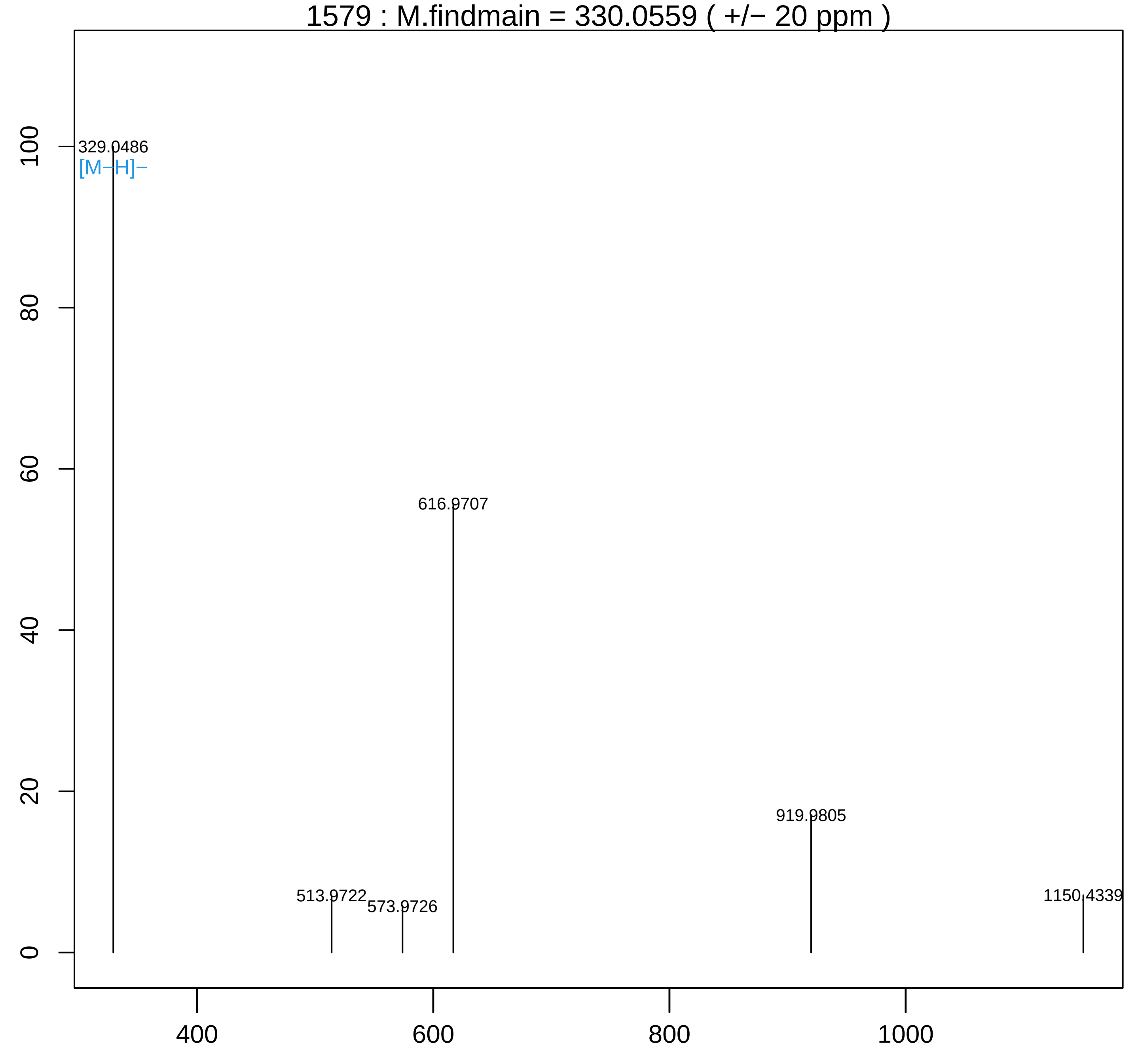
1579 : M.findmain = 330.0559 (+/− 20 ppm)
329.0486
[M−H]−
616.9707
919.9805
513.9722
573.9726
1150.4339
0
20
40
60
80
100
400
600
800
1000

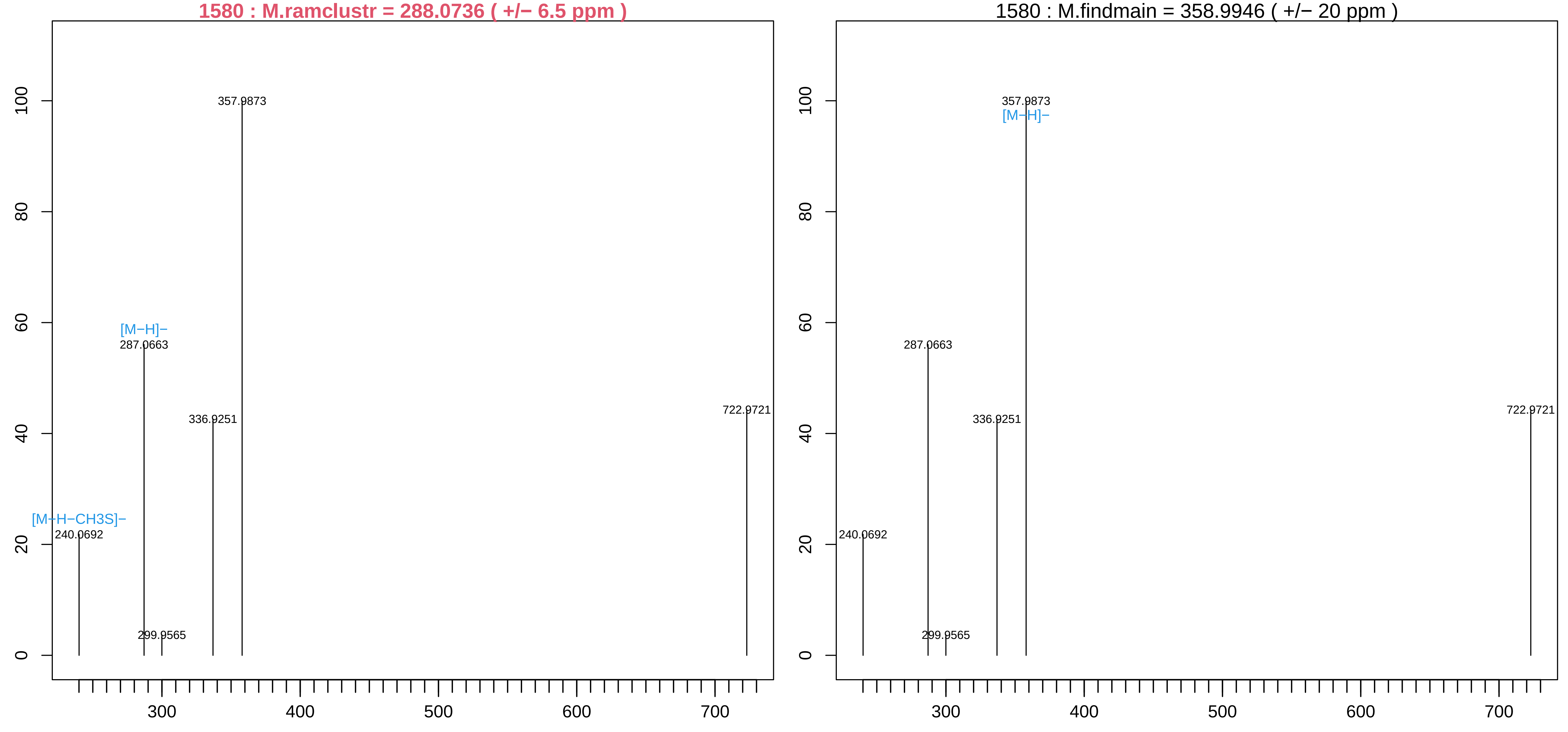
1580 : M.ramclustr = 288.0736 (+/− 6.5 ppm)
357.9873
[M−H]−
287.0663
336.9251
722.9721
[M−H−CH3S]−
240.0692
299.9565
1580 : M.findmain = 358.9946 (+/− 20 ppm)
357.9873
[M−H]−
287.0663
336.9251
722.9721
240.0692
299.9565
0
20
40
60
80
100
300
400
500
600
700

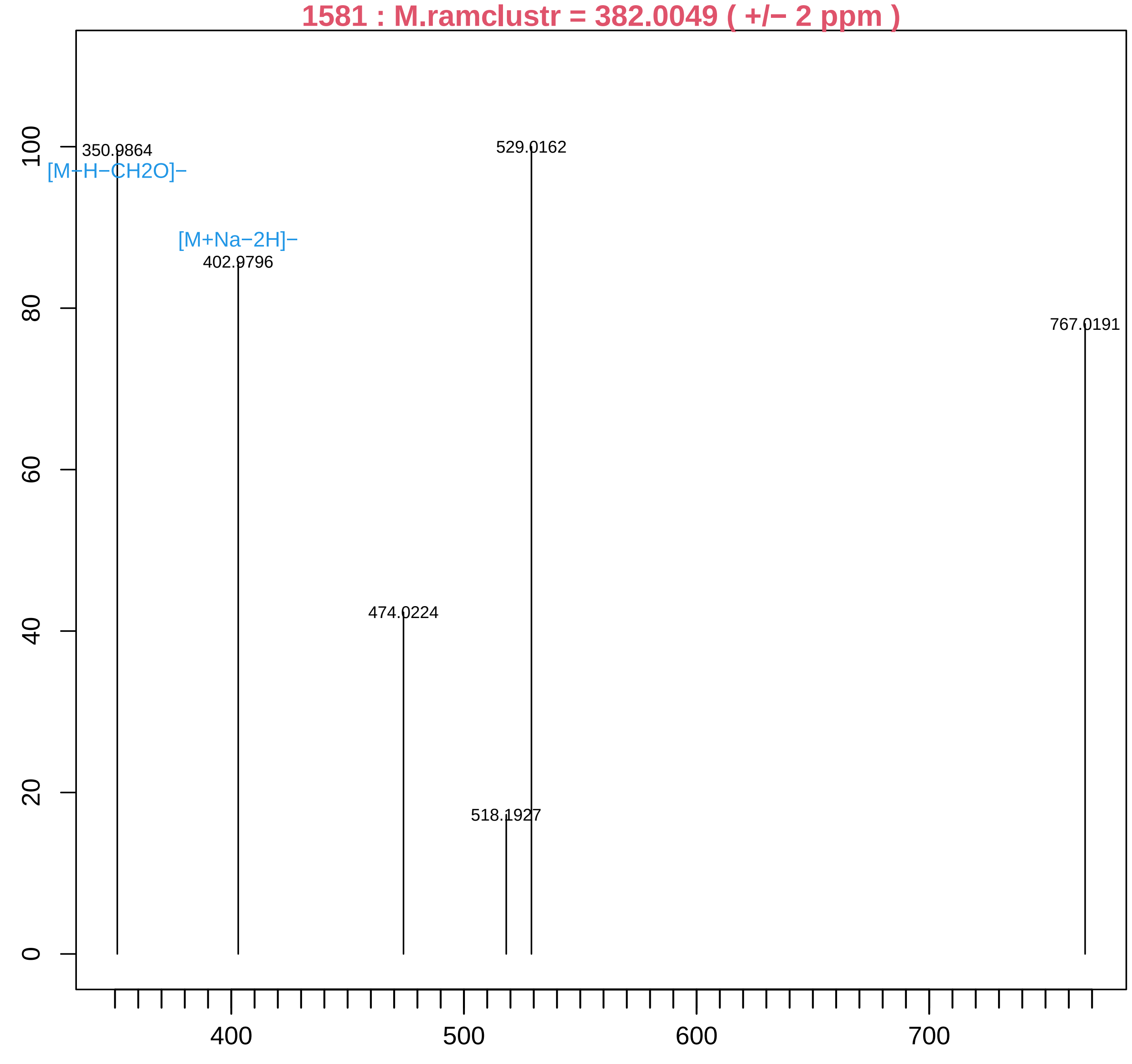
1581 : M.ramclustr = 382.0049 (+/− 2 ppm)
350.9864
[M−H−CH2O]−
[M+Na−2H]−
402.9796
474.0224
518.1927
529.0162
767.0191
0
20
40
60
80
100
400
500
600
700

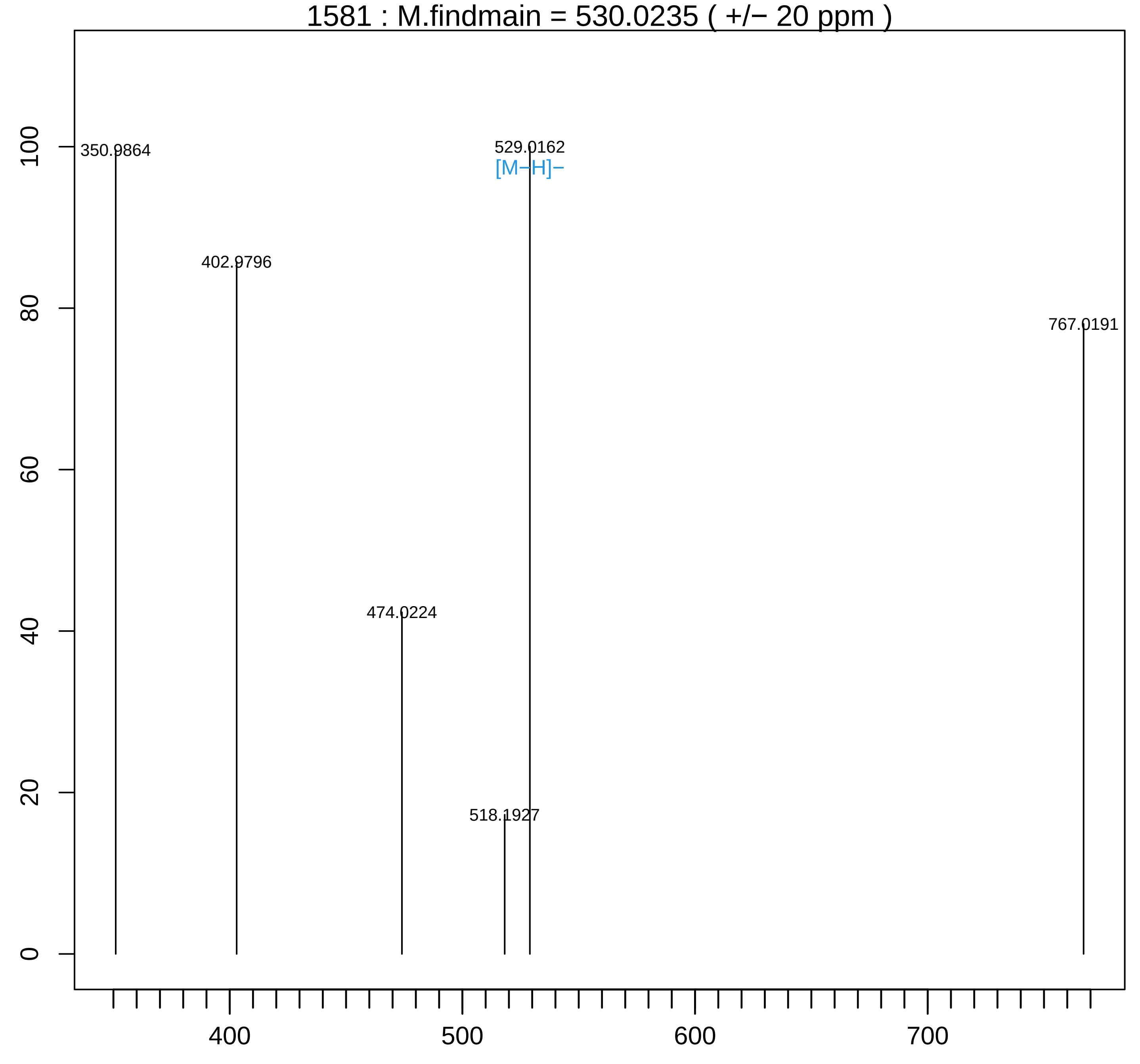
1581 : M.findmain = 530.0235 (+/− 20 ppm)
350.9864
402.9796
474.0224
518.1927
529.0162
[M−H]−
767.0191
0
20
40
60
80
100
400
500
600
700

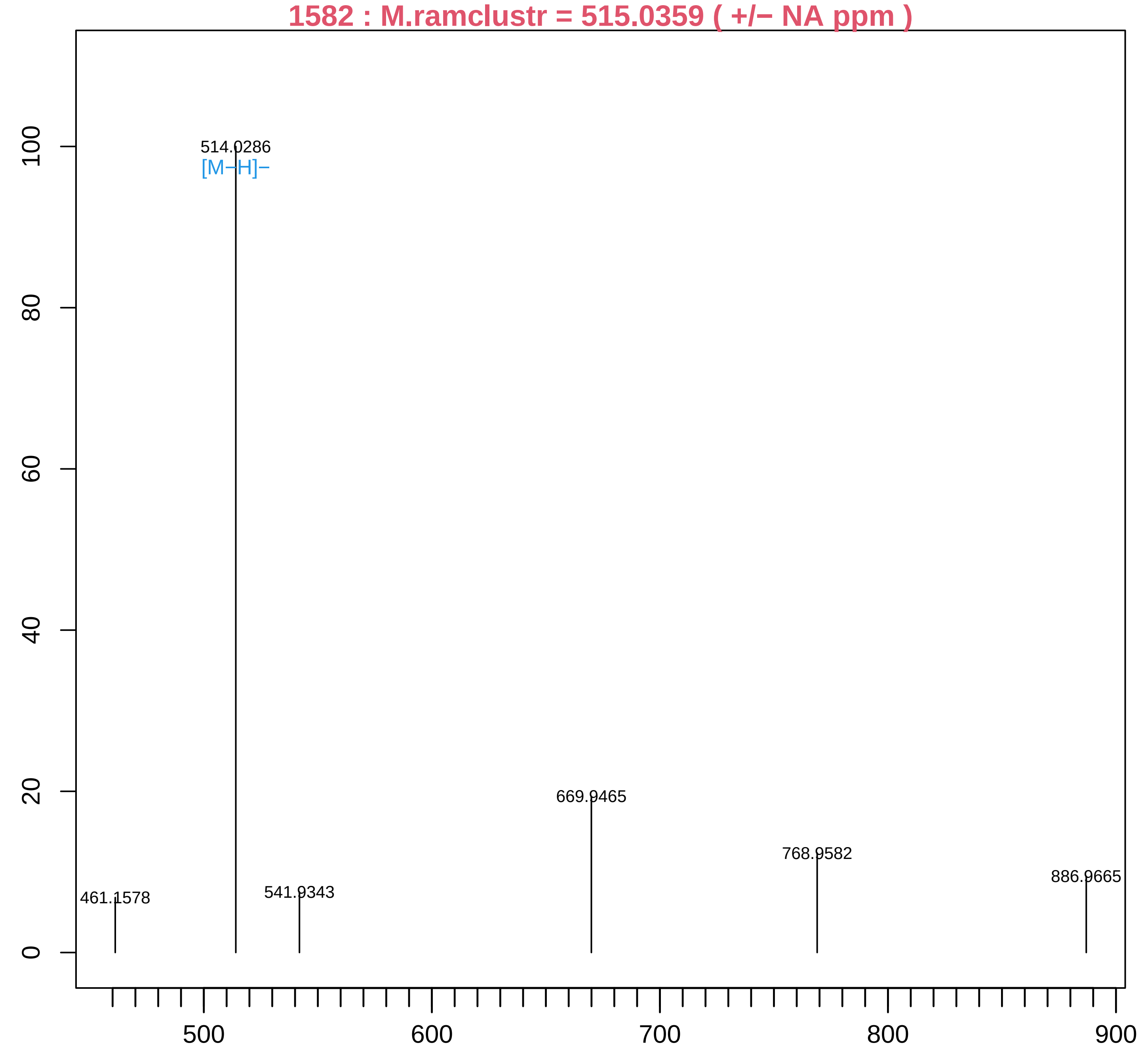
1582 : M.ramclustr = 515.0359 (+/− NA ppm)
514.0286
[M−H]−
461.1578
541.9343
669.9465
768.9582
886.9665
0
20
40
60
80
100
500
600
700
800
900

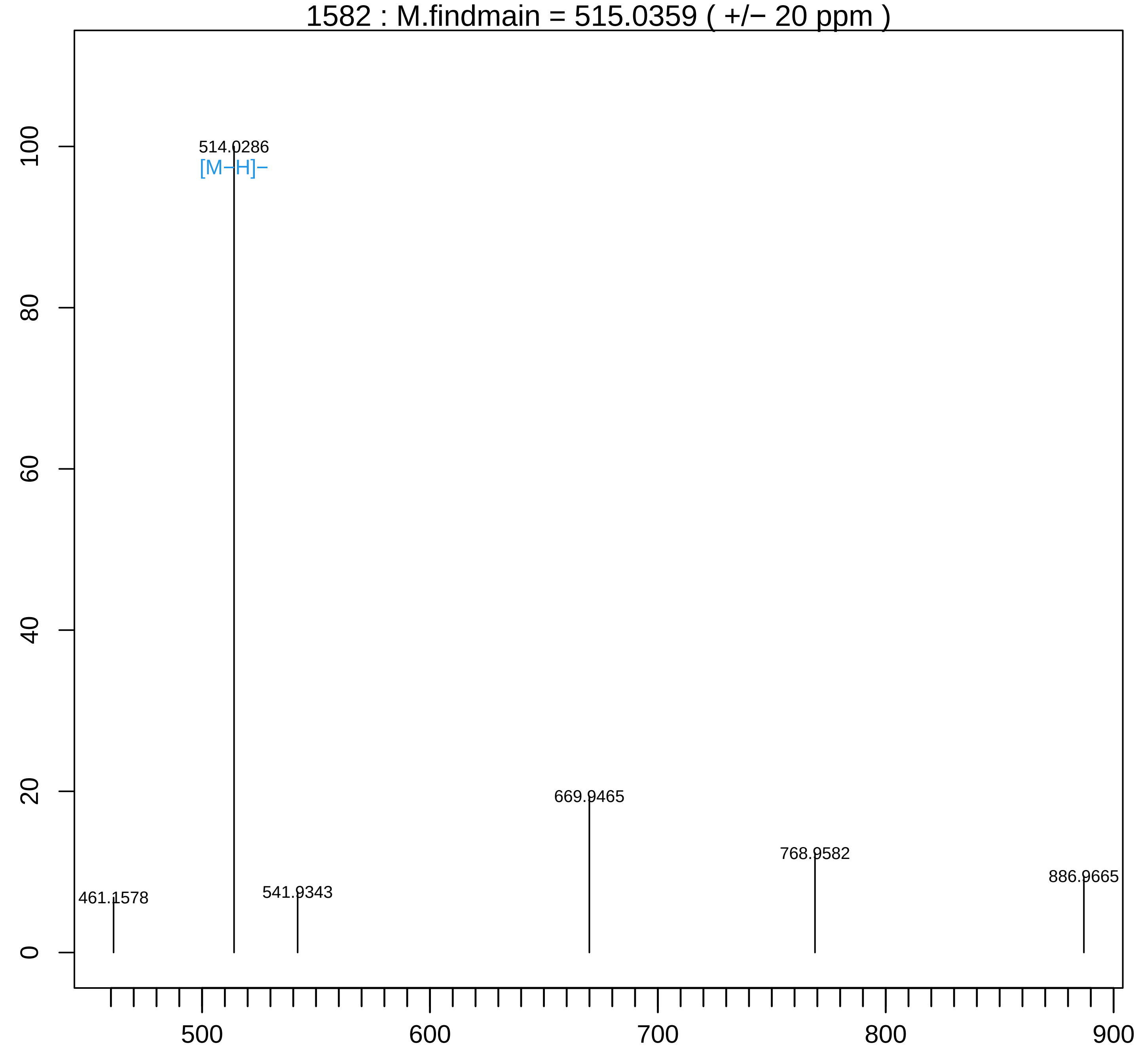
1582 : M.findmain = 515.0359 (+/− 20 ppm)
514.0286
[M−H]−
461.1578
541.9343
669.9465
768.9582
886.9665
0
20
40
60
80
100
500
600
700
800
900

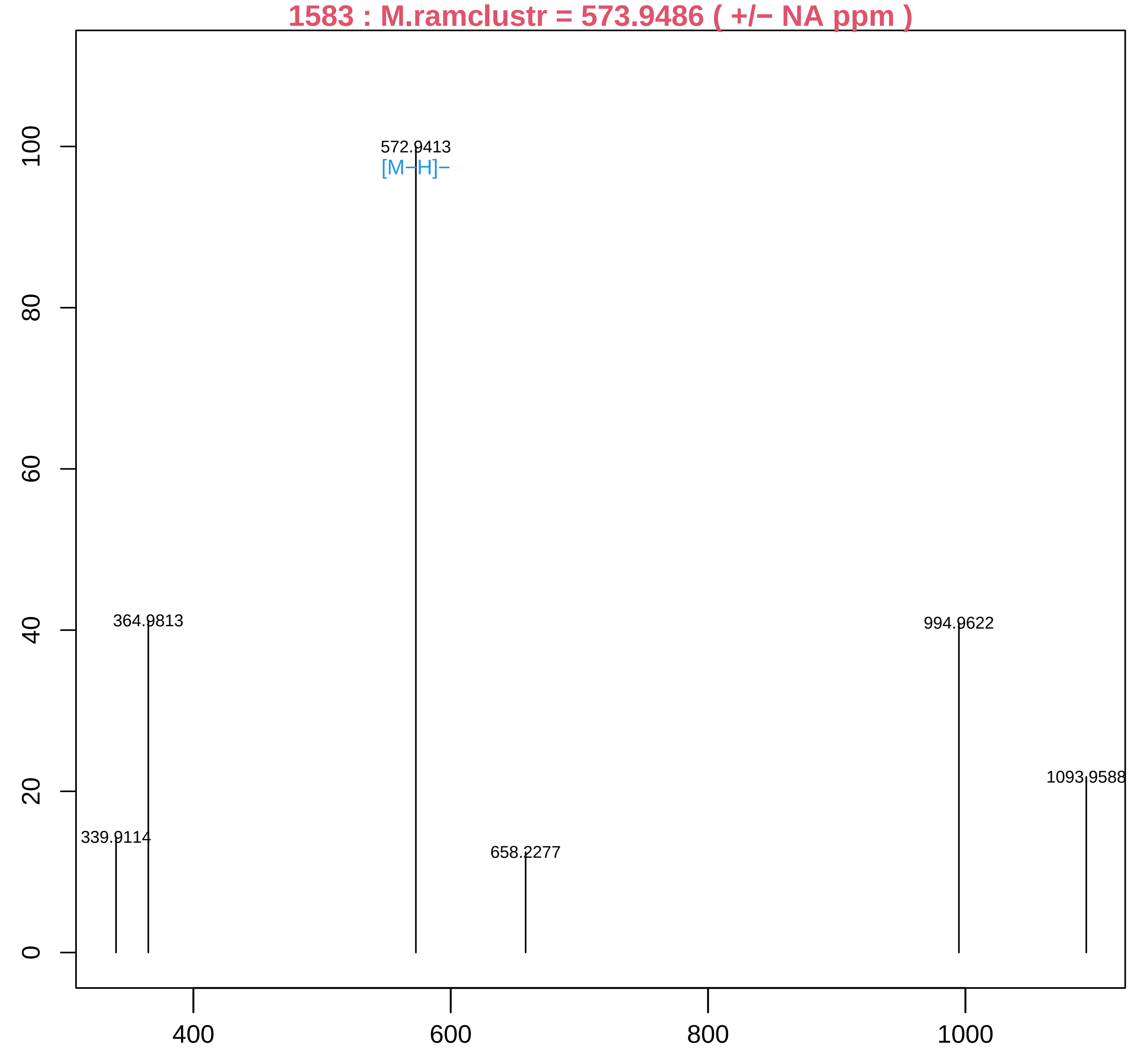

1583 : M.ramclustr = 573.9486 (+/− NA ppm)
572.9413
[M−H]−
364.9813
994.9622
1093.9588
339.9114
658.2277
0
20
40
60
80
100
400
600
800
1000

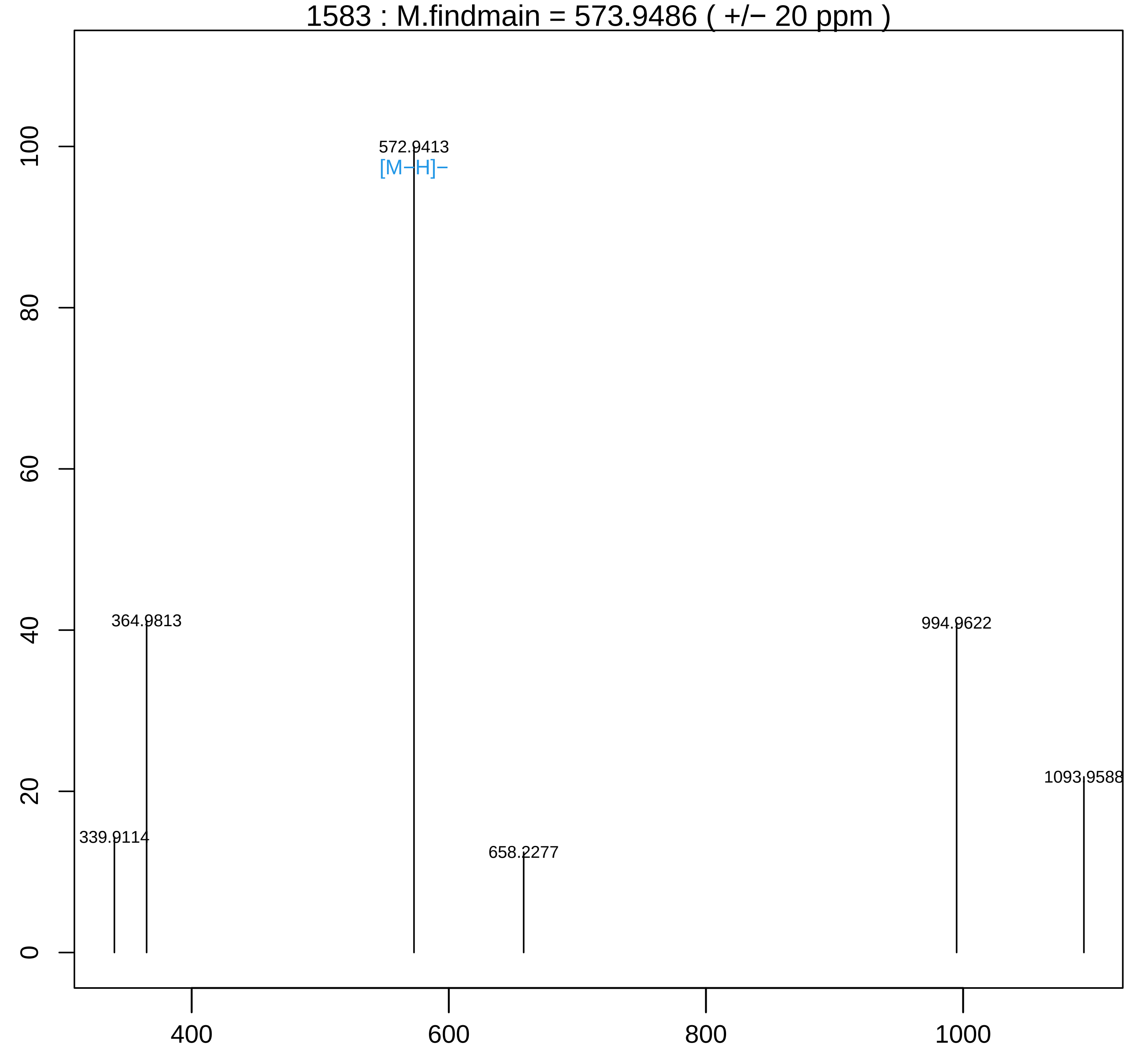

1583 : M.findmain = 573.9486 (+/− 20 ppm)
572.9413
[M−H]−
364.9813
994.9622
1093.9588
339.9114
658.2277
0
20
40
60
80
100
400
600
800
1000

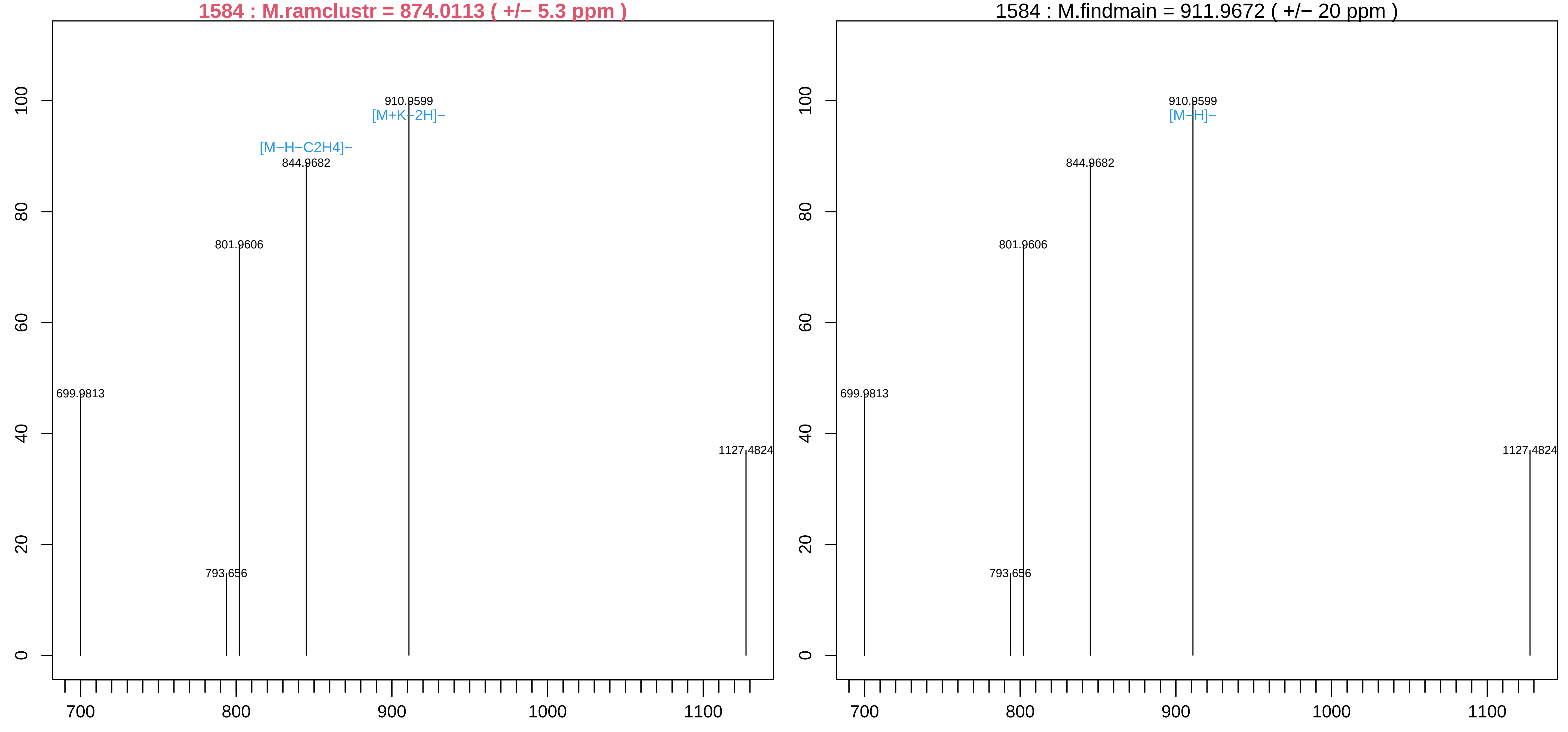
1584 : M.ramclustr = 874.0113 (+/− 5.3 ppm)
910.9599
[M+K−2H]−
[M−H−C2H4]−
844.9682
801.9606
699.9813
793.656
1127.4824
1584 : M.findmain = 911.9672 (+/− 20 ppm)
910.9599
[M−H]−
844.9682
801.9606
699.9813
793.656
1127.4824
0
20
40
60
80
100
700
800
900
1000
1100

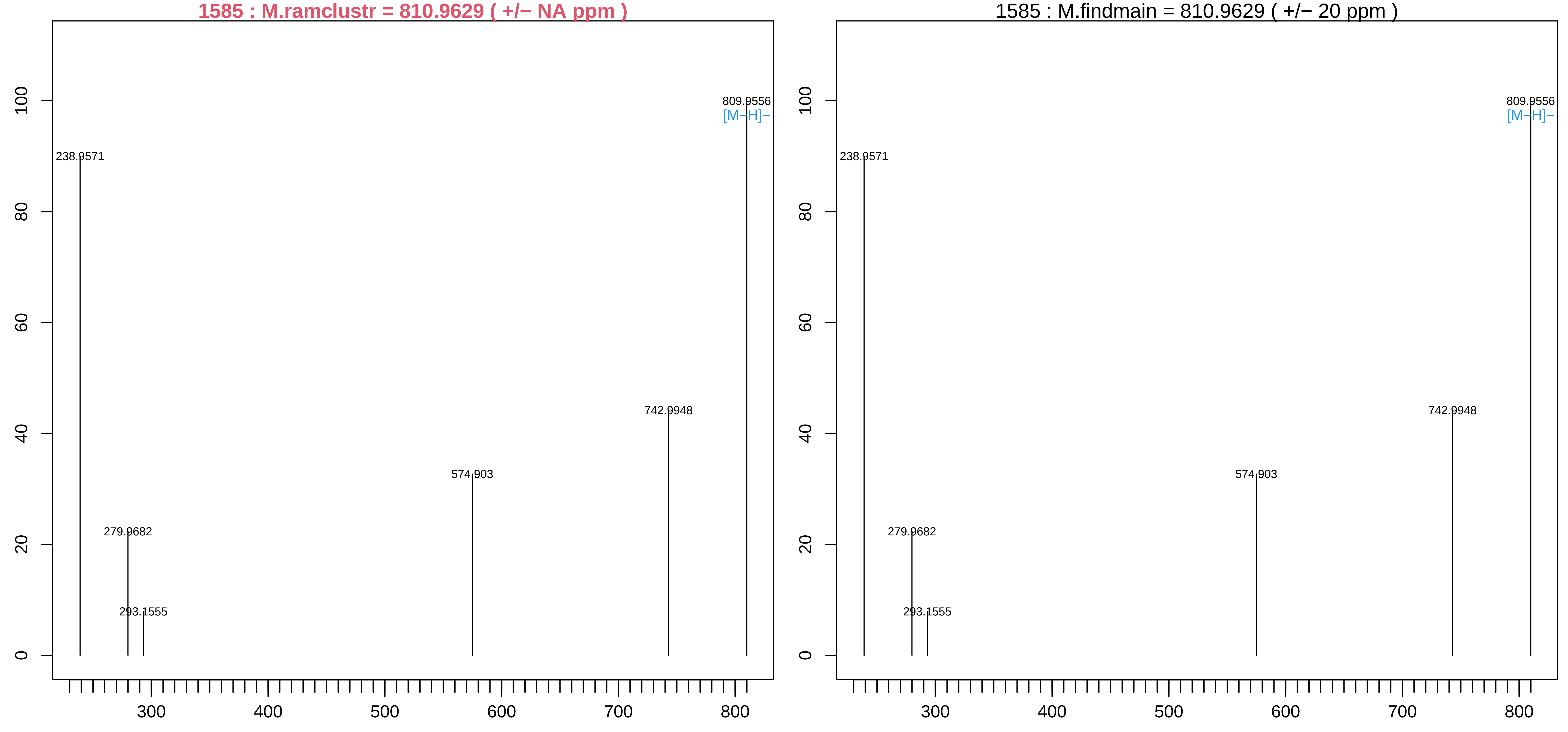

1585 : M.ramclustr = 810.9629 (+/− NA ppm)
809.9556
[M−H]−
238.9571
742.9948
574.903
279.9682
293.1555
1585 : M.findmain = 810.9629 (+/− 20 ppm)
809.9556
[M−H]−
238.9571
742.9948
574.903
279.9682
293.1555
0
20
40
60
80
100
300
400
500
600
700
800

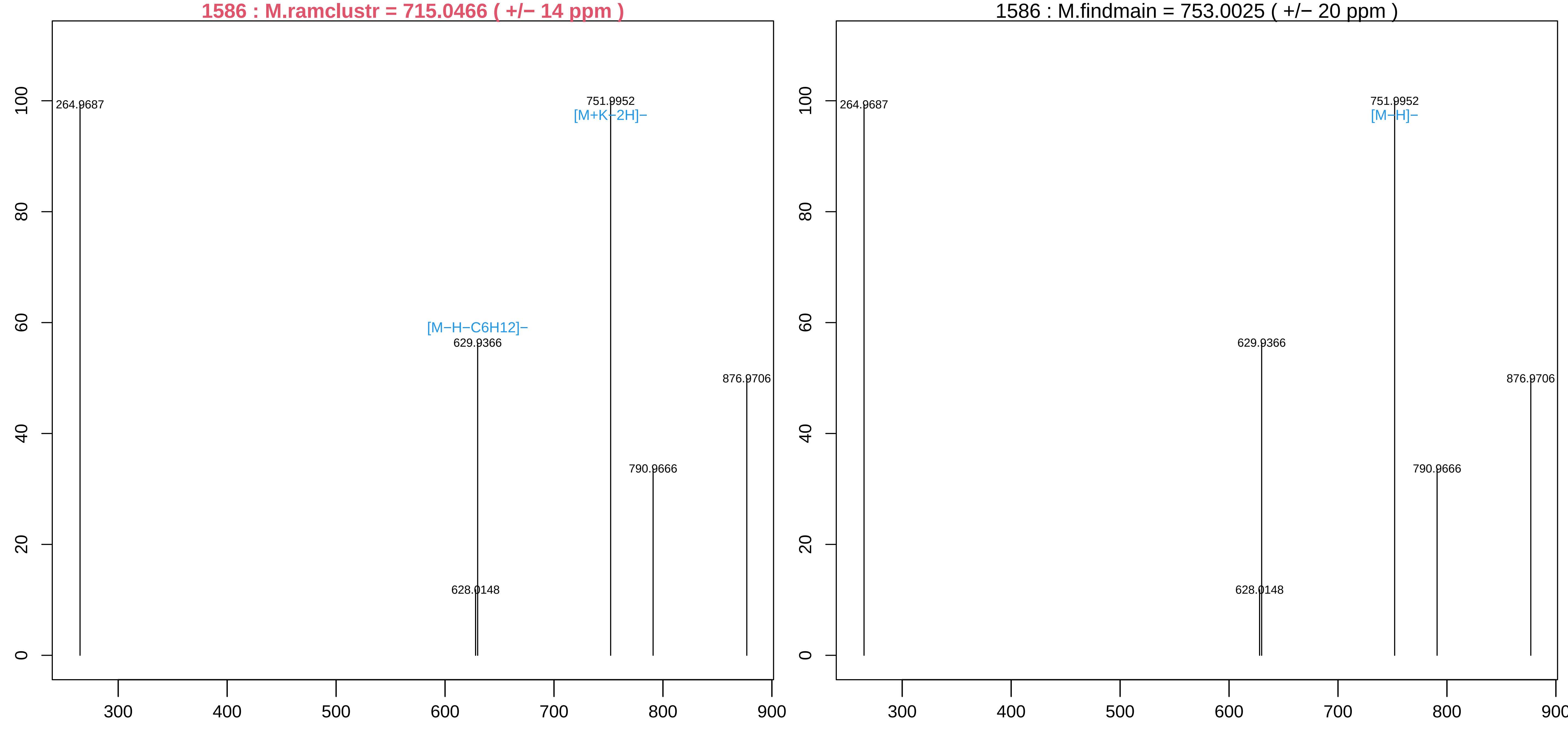

1586 : M.ramclustr = 715.0466 (+/− 14 ppm)
264.9687
751.9952
[M+K−2H]−
[M−H−C6H12]−
629.9366
876.9706
790.9666
628.0148
0
20
40
60
80
100
300
400
500
600
700
800
900
1586 : M.findmain = 753.0025 (+/− 20 ppm)
264.9687
751.9952
[M−H]−
629.9366
876.9706
790.9666
628.0148
0
20
40
60
80
100
300
400
500
600
700
800
900

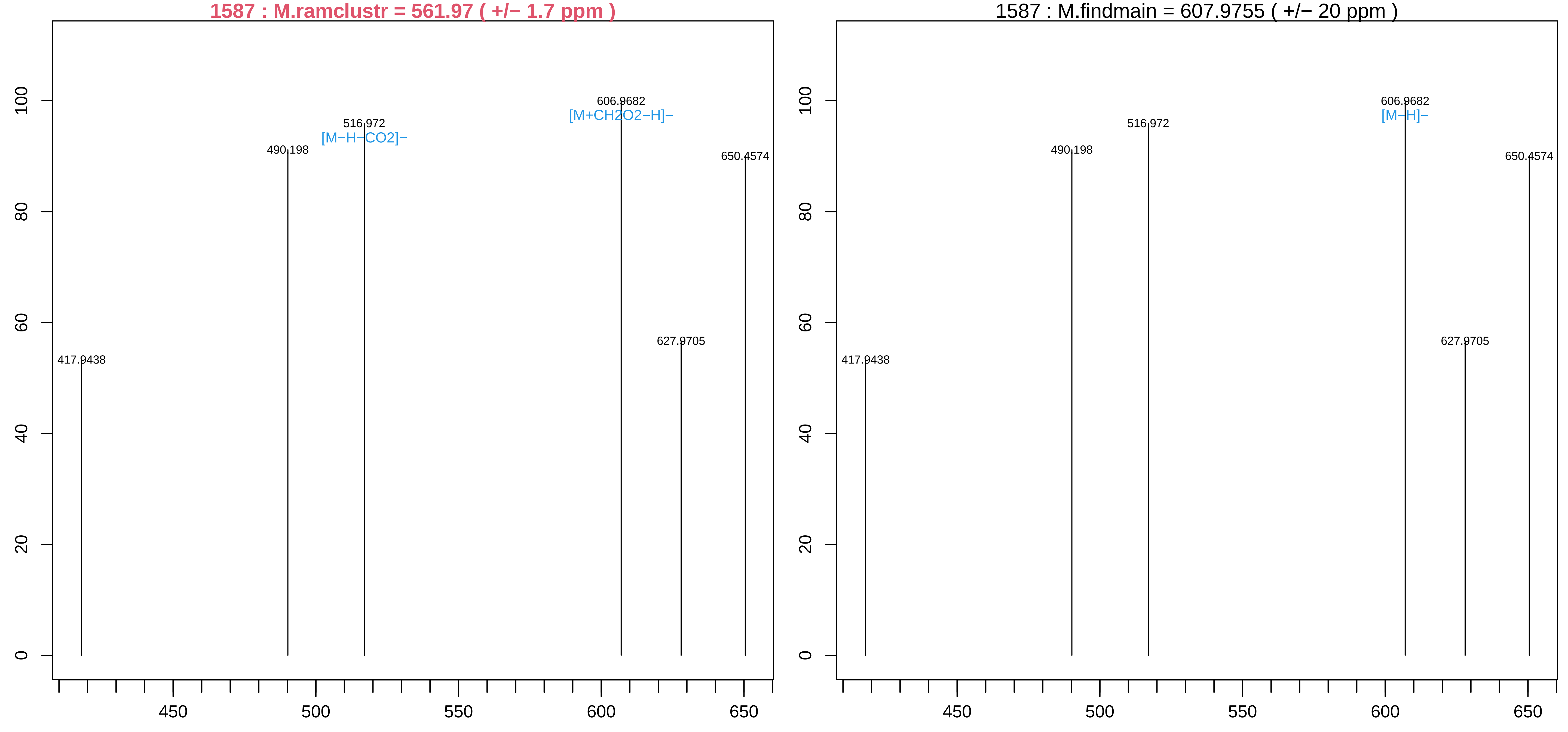

1587 : M.ramclustr = 561.97 (+/− 1.7 ppm)
417.9438
490.198
516.972
[M−H−CO2]−
606.9682
[M+CH2O2−H]−
627.9705
650.4574
1587 : M.findmain = 607.9755 (+/− 20 ppm)
417.9438
490.198
516.972
606.9682
[M−H]−
627.9705
650.4574
0
20
40
60
80
100
450
500
550
600
650

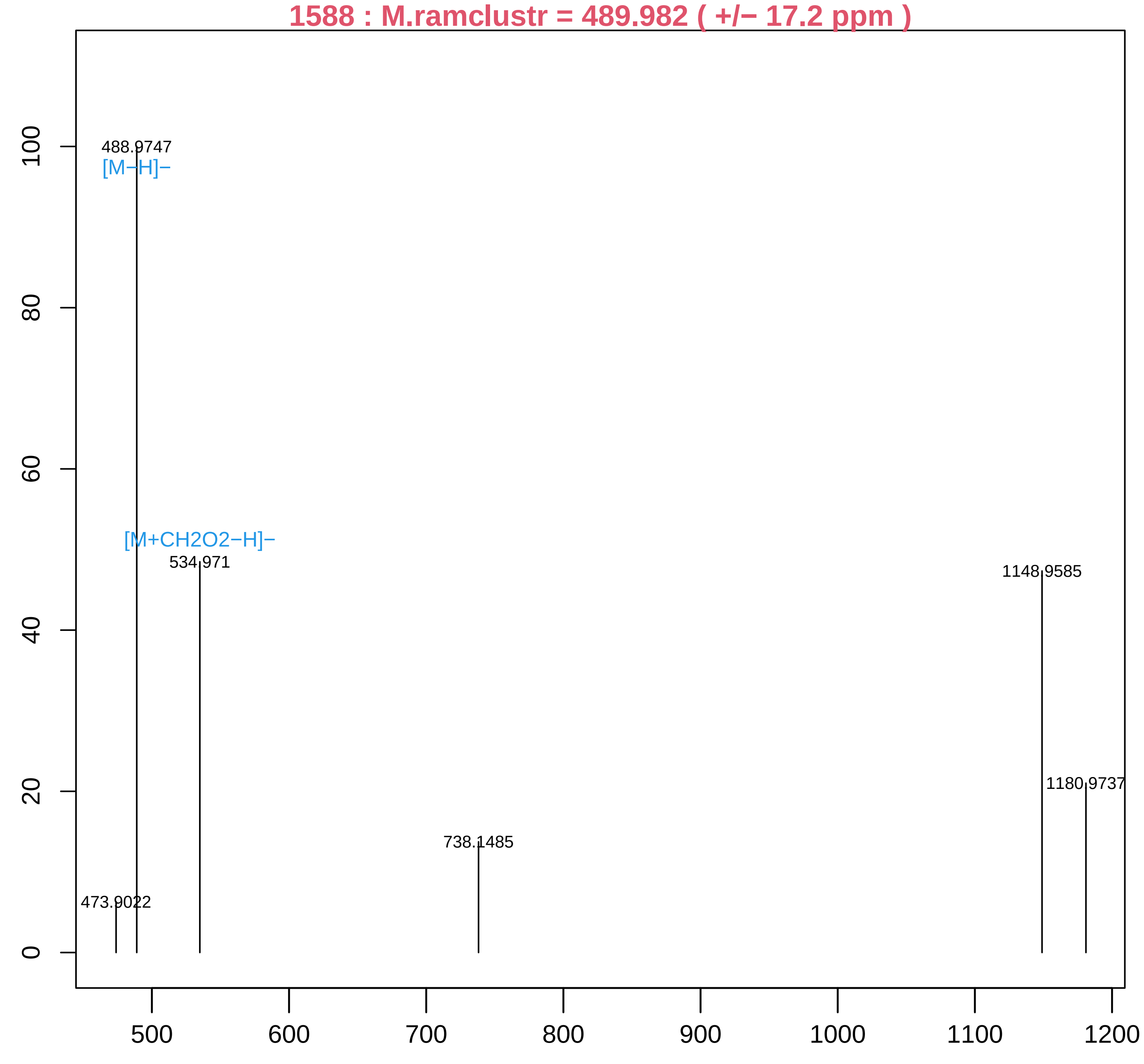
1588 : M.ramclustr = 489.982 (+/− 17.2 ppm)
488.9747
[M−H]−
[M+CH2O2−H]−
534.971
1148.9585
1180.9737
738.1485
473.9022
0
20
40
60
80
100
500
600
700
800
900
1000
1100
1200
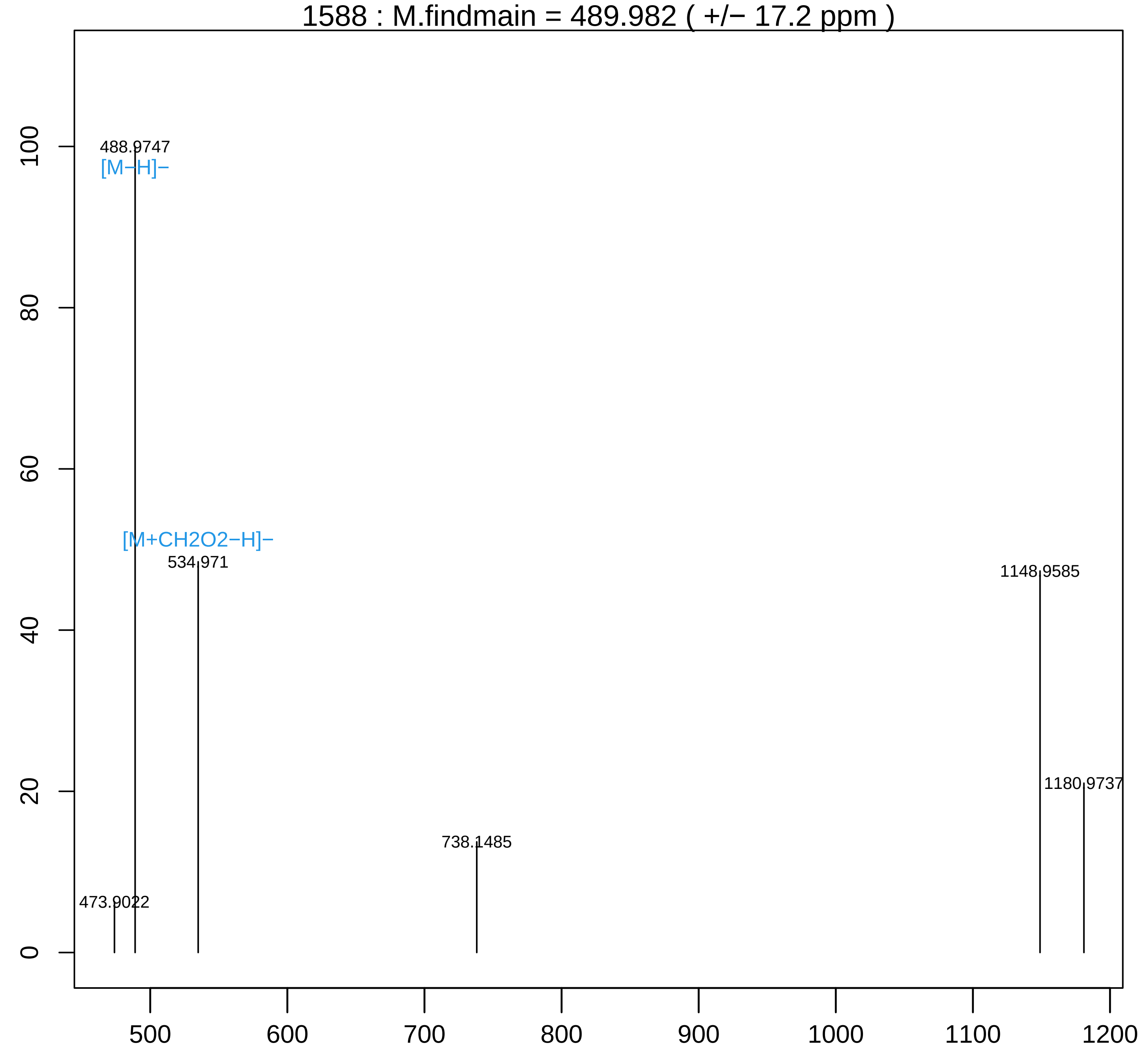
1588 : M.findmain = 489.982 (+/− 17.2 ppm)
488.9747
[M−H]−
[M+CH2O2−H]−
534.971
1148.9585
1180.9737
738.1485
473.9022
0
20
40
60
80
100
500
600
700
800
900
1000
1100
1200

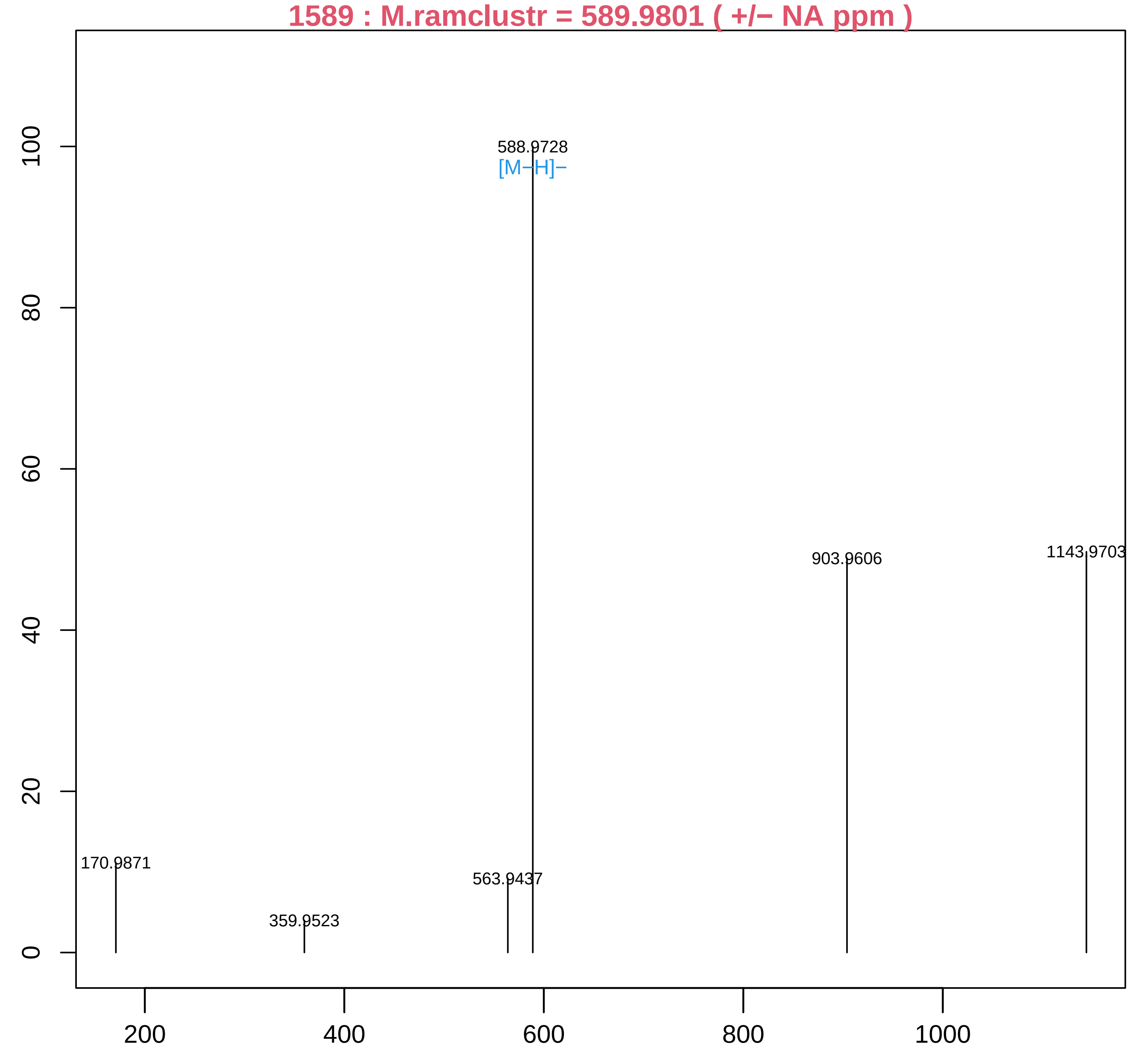
1589 : M.ramclustr = 589.9801 (+/− NA ppm)
588.9728
[M−H]−
903.9606
1143.9703
170.9871
359.9523
563.9437
0
20
40
60
80
100
200
400
600
800
1000

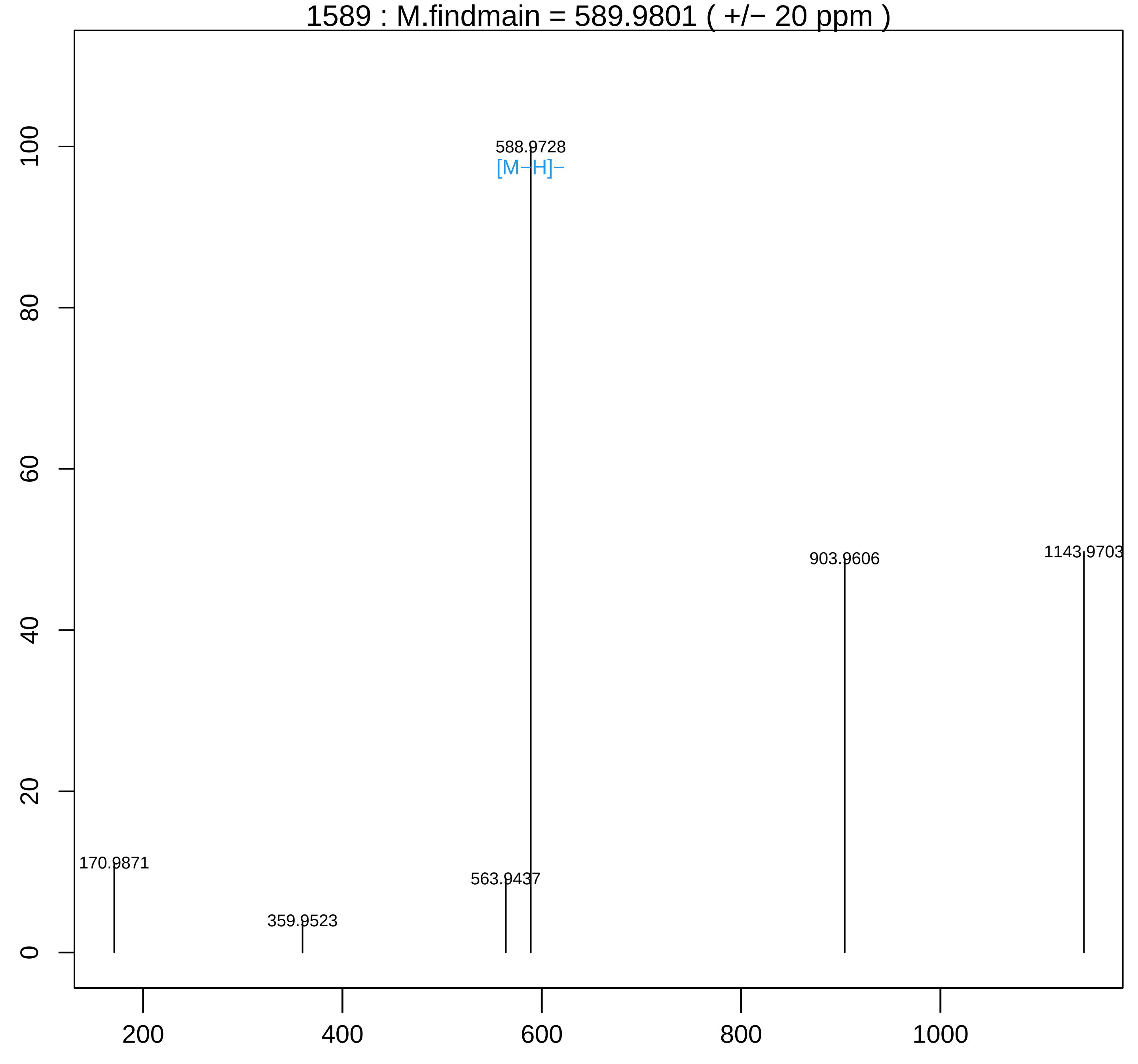
1589 : M.findmain = 589.9801 (+/− 20 ppm)
588.9728
[M−H]−
903.9606
1143.9703
170.9871
359.9523
563.9437
0
20
40
60
80
100
200
400
600
800
1000

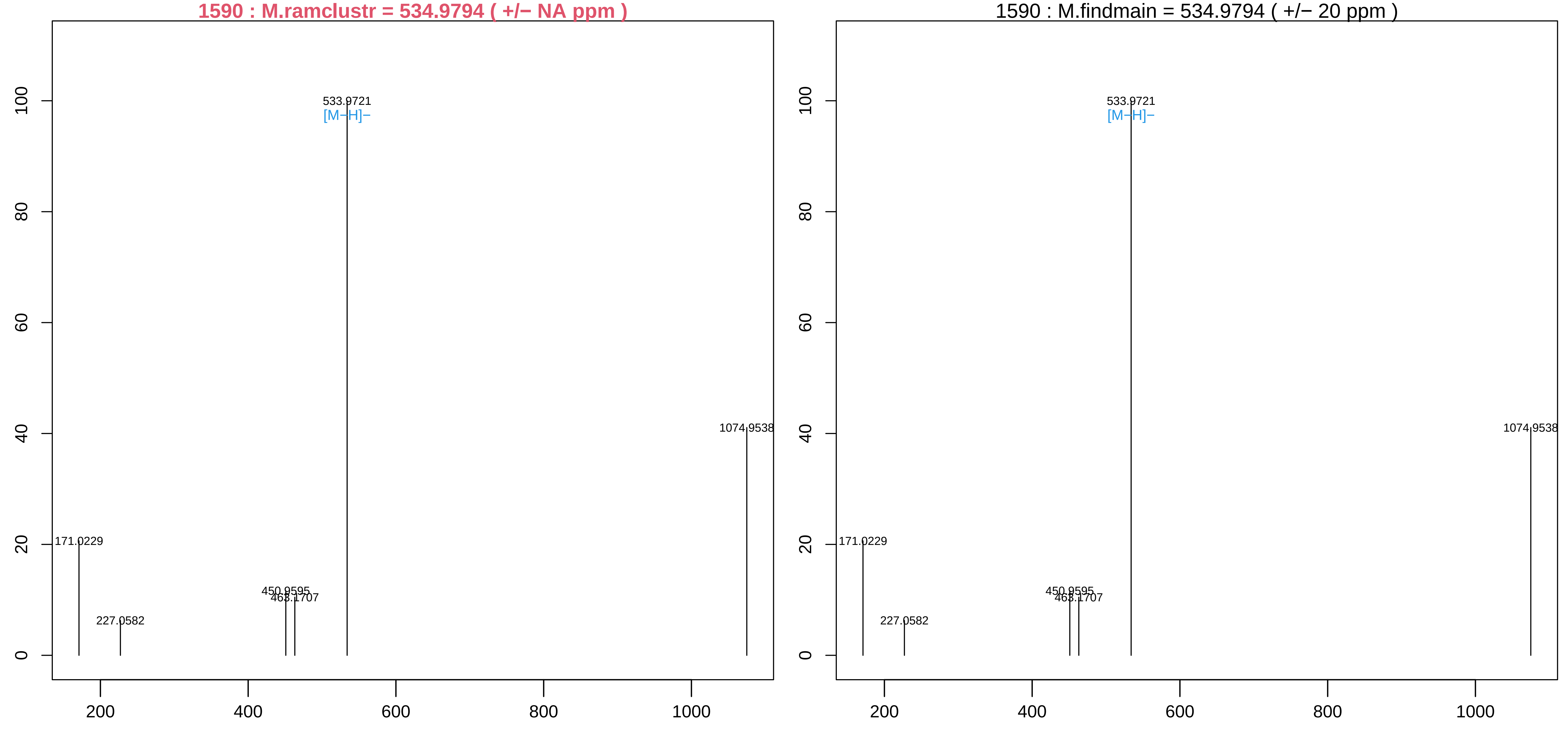
1590 : M.ramclustr = 534.9794 (+/− NA ppm)
1590 : M.findmain = 534.9794 (+/− 20 ppm)
533.9721
[M−H]−
1074.9538
171.0229
227.0582
450.9595
463.1707
0
20
40
60
80
100
200
400
600
800
1000

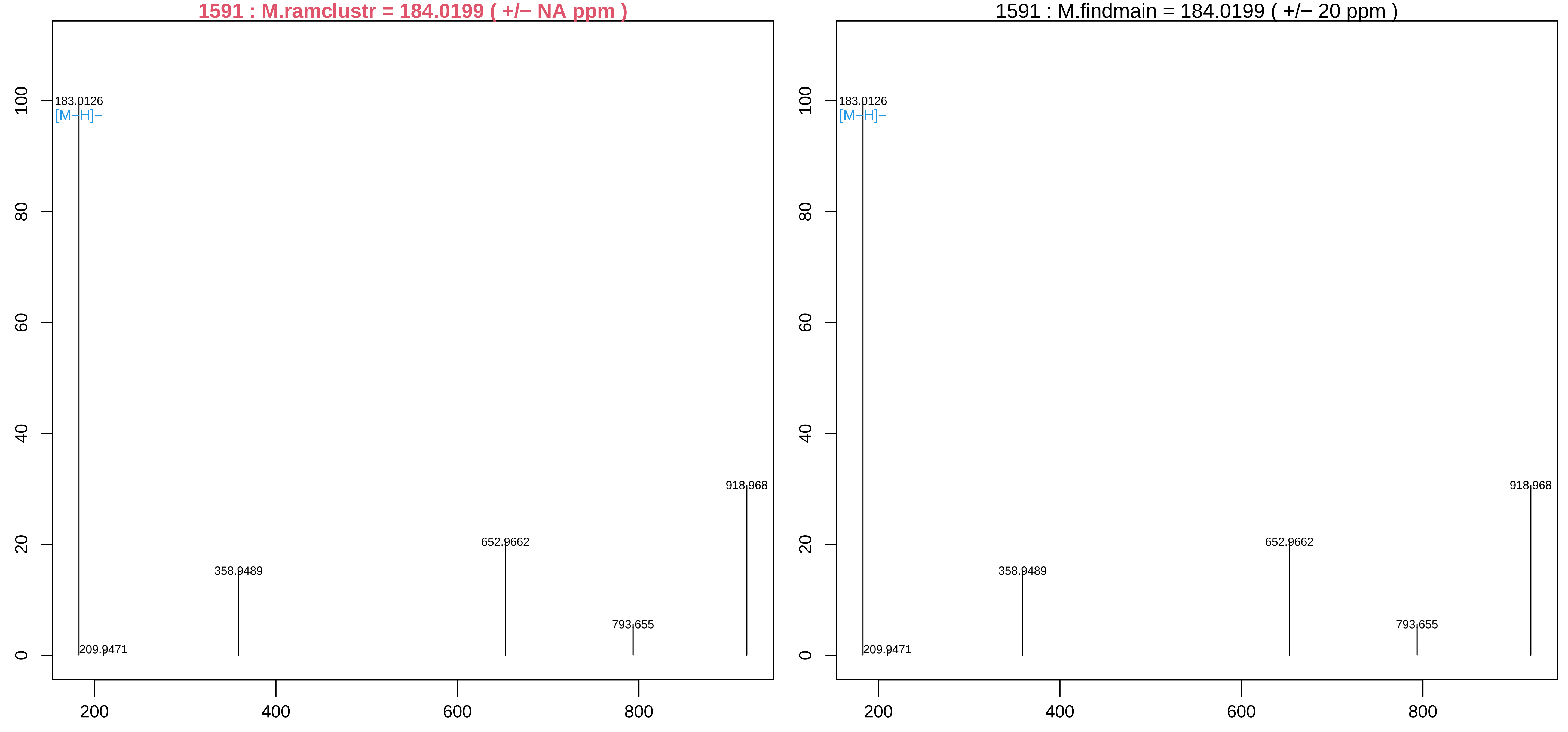
1591 : M.ramclustr = 184.0199 (+/− NA ppm)
183.0126
[M−H]−
209.9471
358.9489
652.9662
793.655
918.968
1591 : M.findmain = 184.0199 (+/− 20 ppm)
183.0126
[M−H]−
209.9471
358.9489
652.9662
793.655
918.968

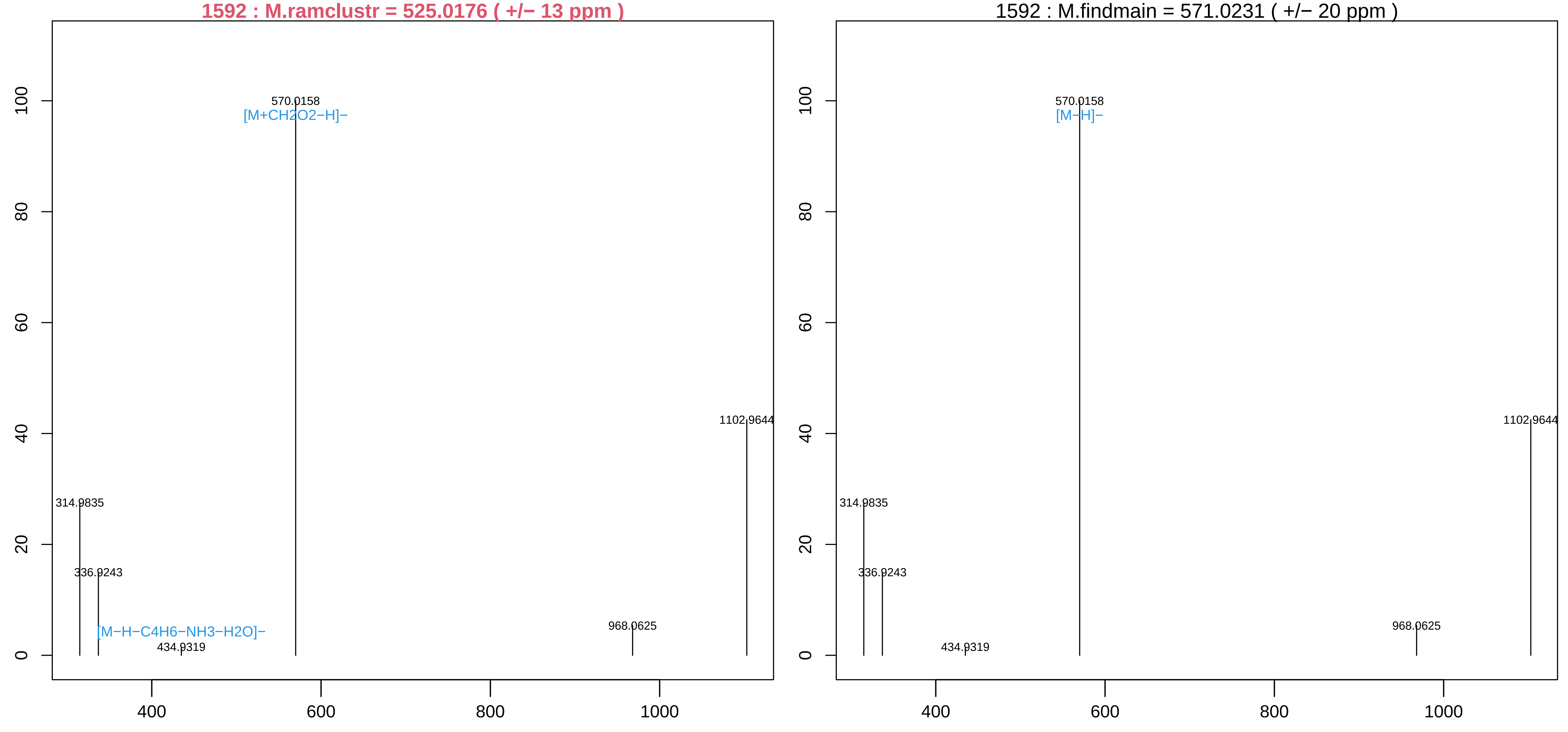
1592 : M.ramclustr = 525.0176 (+/− 13 ppm)
570.0158
[M+CH2O2−H]−
1102.9644
314.9835
336.9243
[M−H−C4H6−NH3−H2O]−
434.9319
968.0625
1592 : M.findmain = 571.0231 (+/− 20 ppm)
570.0158
[M−H]−
1102.9644
314.9835
336.9243
434.9319
968.0625
0
20
40
60
80
100
400
600
800
1000

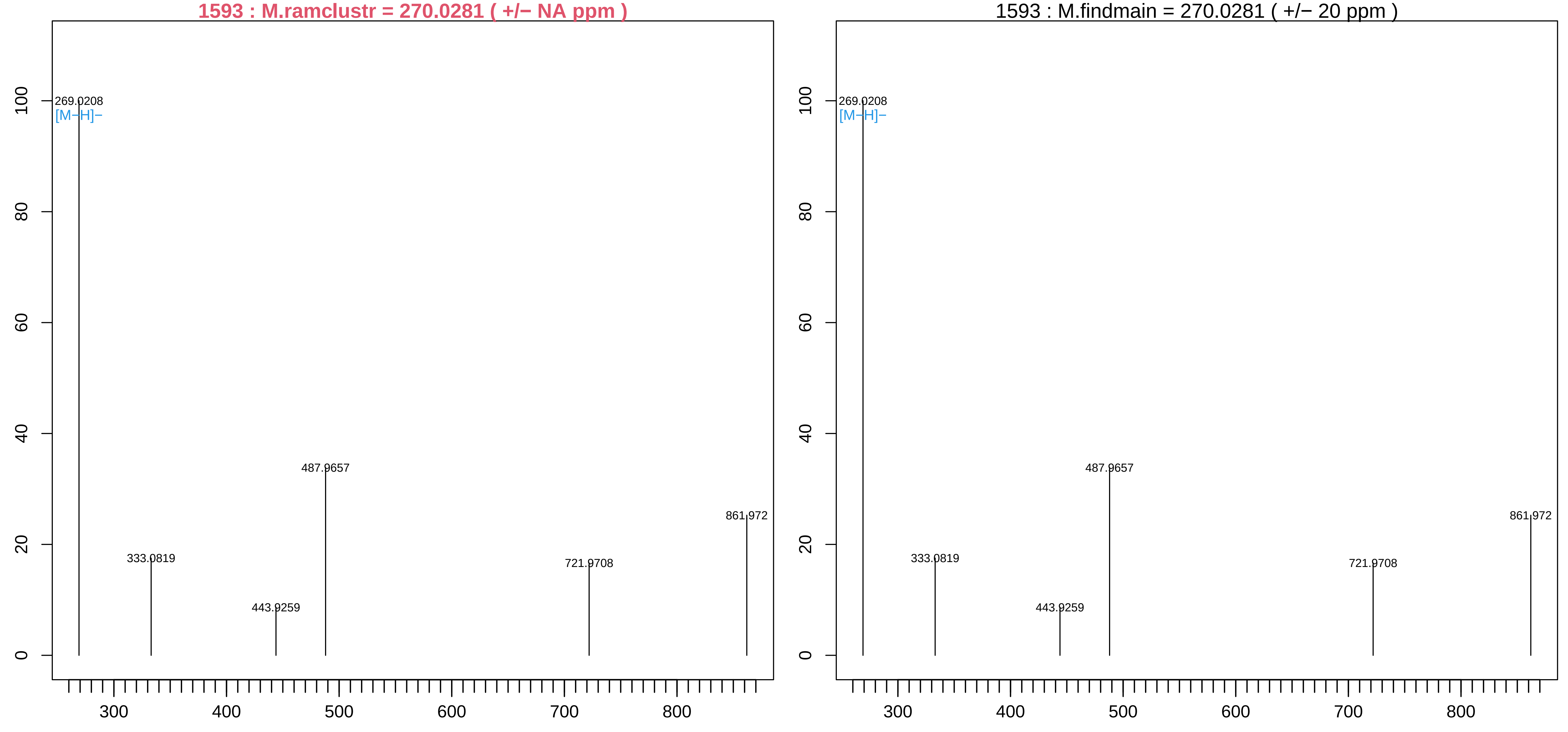

1593 : M.ramclustr = 270.0281 (+/− NA ppm)
269.0208
[M−H]−
333.0819
443.9259
487.9657
721.9708
861.972
1593 : M.findmain = 270.0281 (+/− 20 ppm)
269.0208
[M−H]−
333.0819
443.9259
487.9657
721.9708
861.972

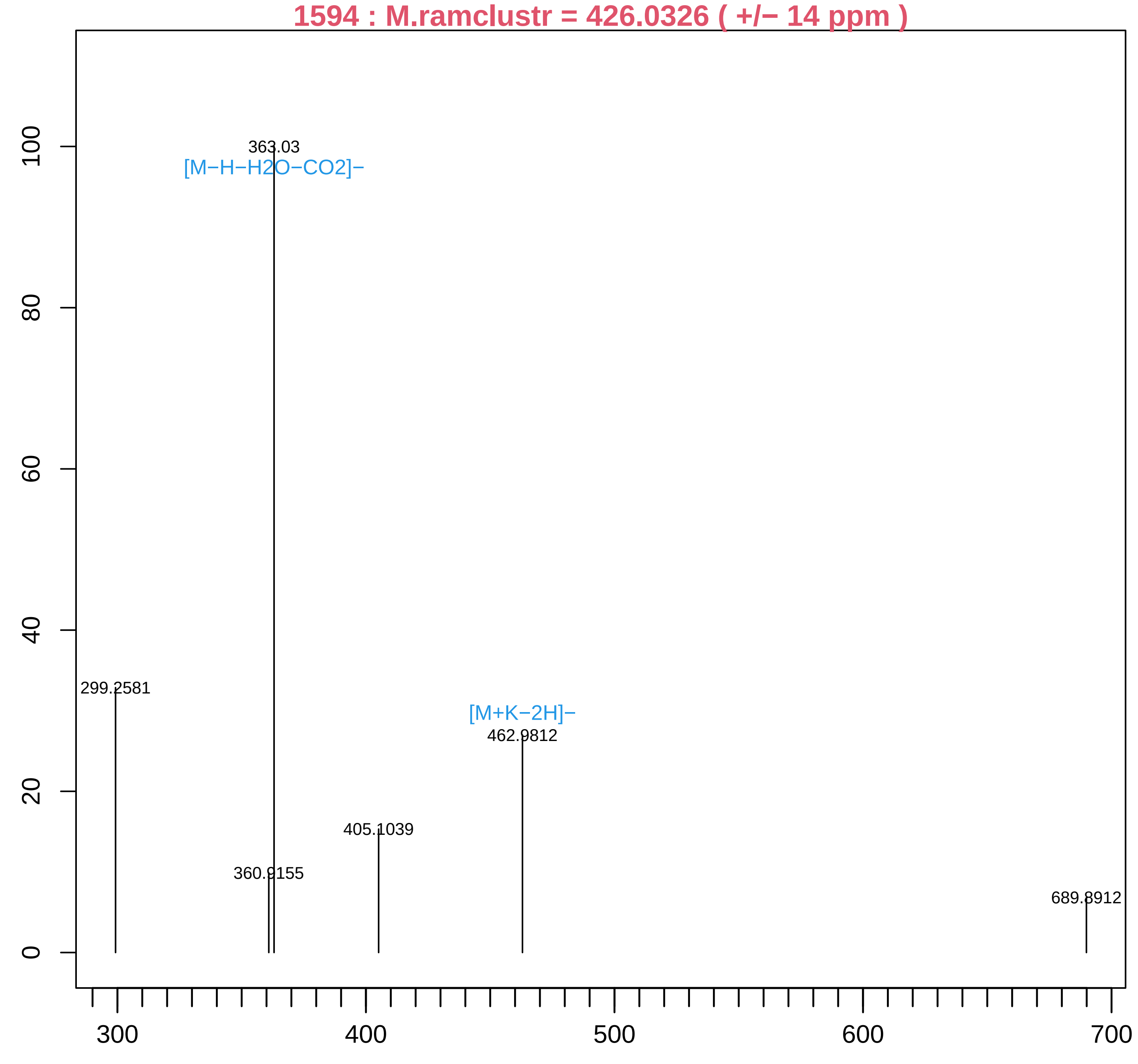

1594 : M.ramclustr = 426.0326 (+/− 14 ppm)
363.03
[M−H−H2O−CO2]−
299.2581
360.9155
405.1039
[M+K−2H]−
462.9812
689.8912
0
20
40
60
80
100
300
400
500
600
700

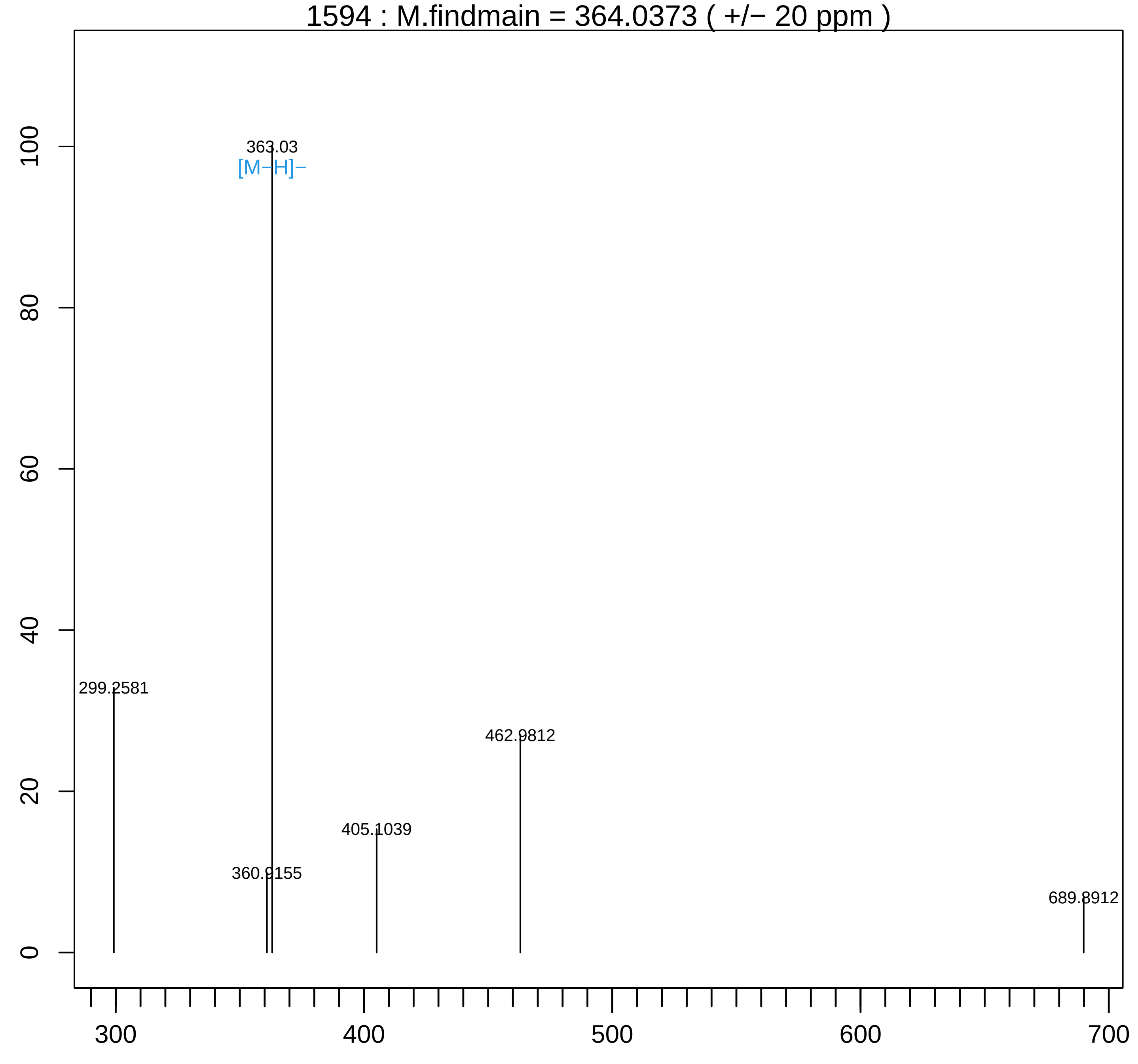

1594 : M.findmain = 364.0373 (+/− 20 ppm)
363.03
[M−H]−
299.2581
360.9155
405.1039
462.9812
689.8912
0
20
40
60
80
100
300
400
500
600
700

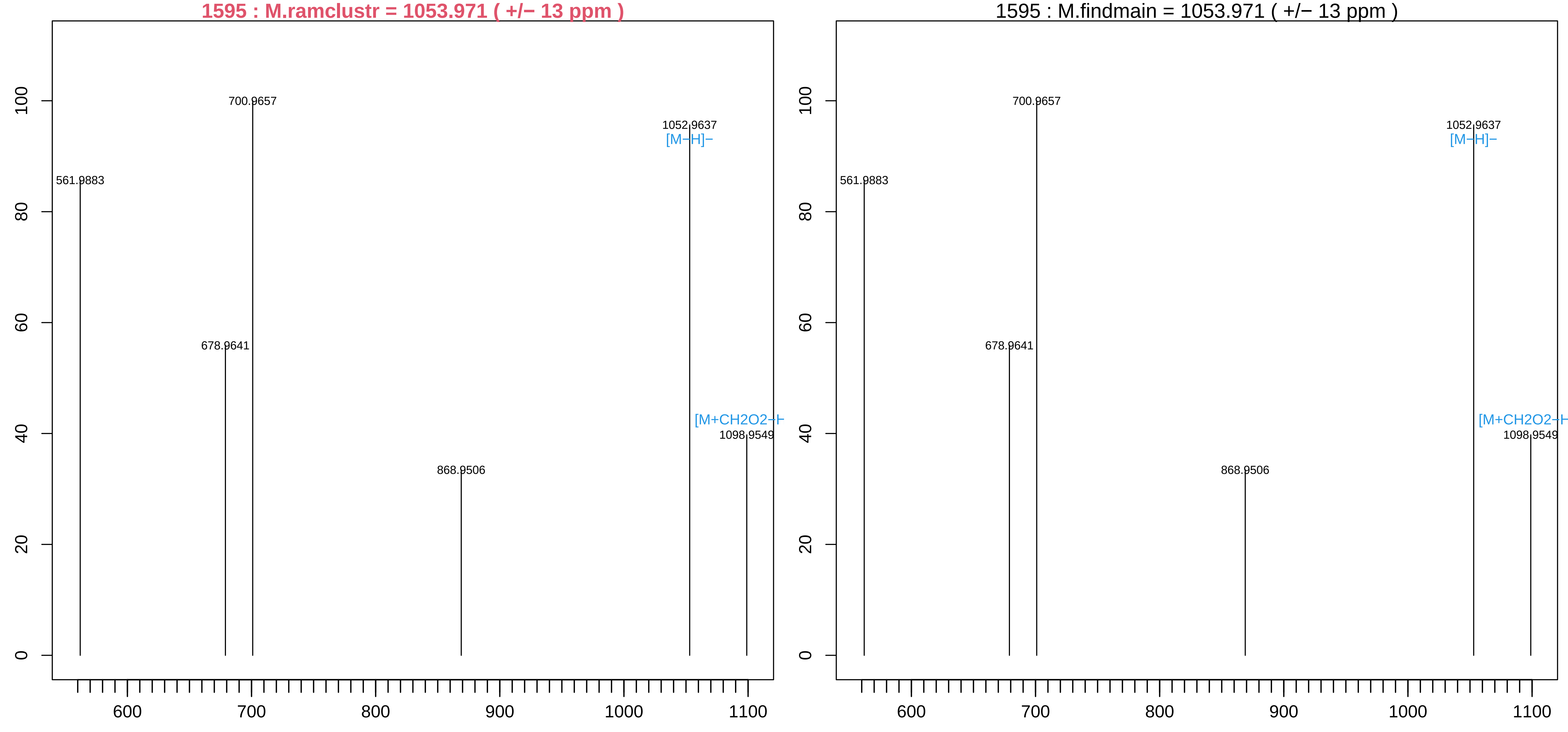
1595 : M.ramclustr = 1053.971 (+/− 13 ppm)
1595 : M.findmain = 1053.971 (+/− 13 ppm)
561.9883
678.9641
700.9657
868.9506
1052.9637
[M−H]−
[M+CH2O2−H
1098.9549
0
20
40
60
80
100
600
700
800
900
1000
1100

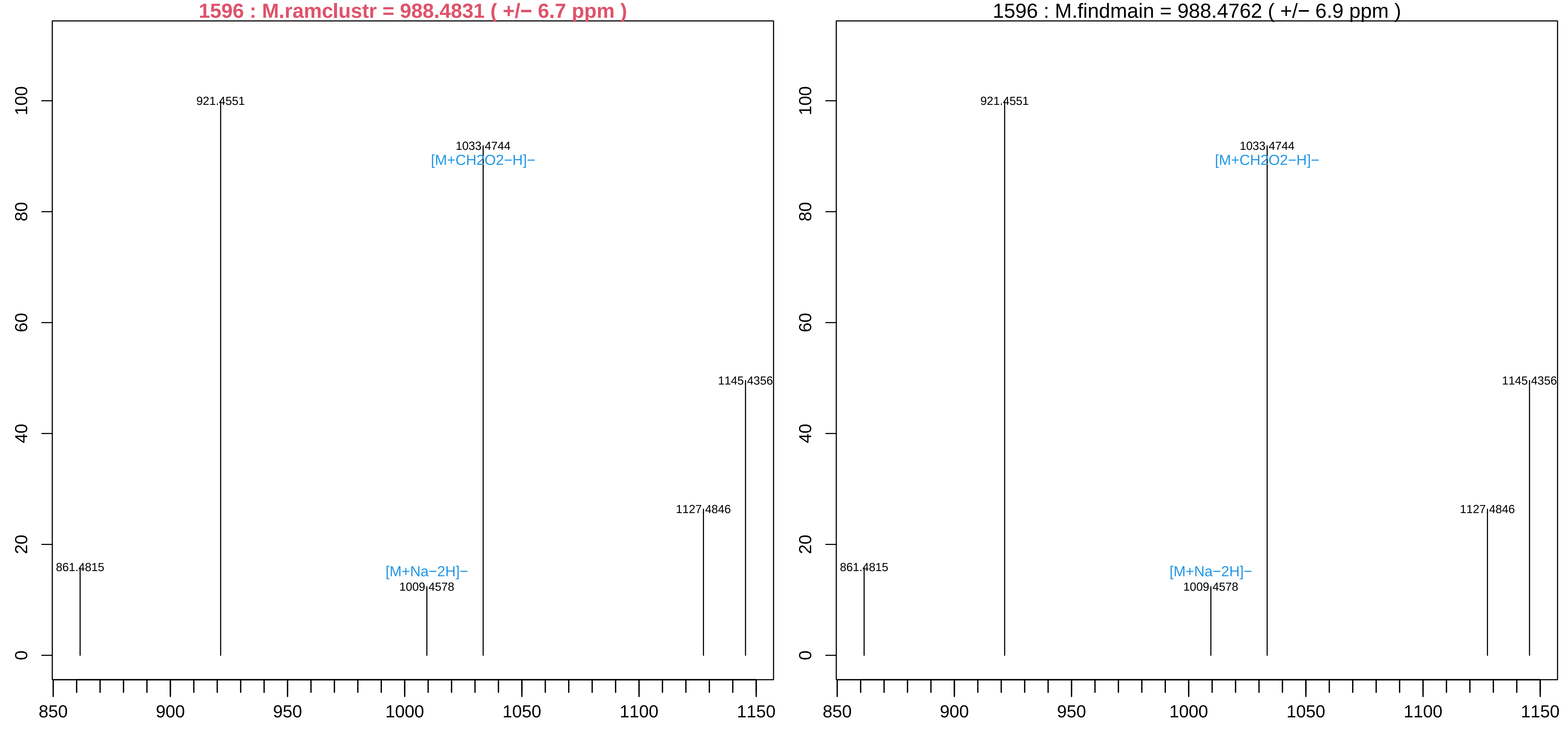
1596 : M.ramclustr = 988.4831 (+/− 6.7 ppm)
921.4551
1033.4744
[M+CH2O2−H]−
1145.4356
1127.4846
861.4815
[M+Na−2H]−
1009.4578
1596 : M.findmain = 988.4762 (+/− 6.9 ppm)
921.4551
1033.4744
[M+CH2O2−H]−
1145.4356
1127.4846
861.4815
[M+Na−2H]−
1009.4578
0
20
40
60
80
100
850
900
950
1000
1050
1100
1150

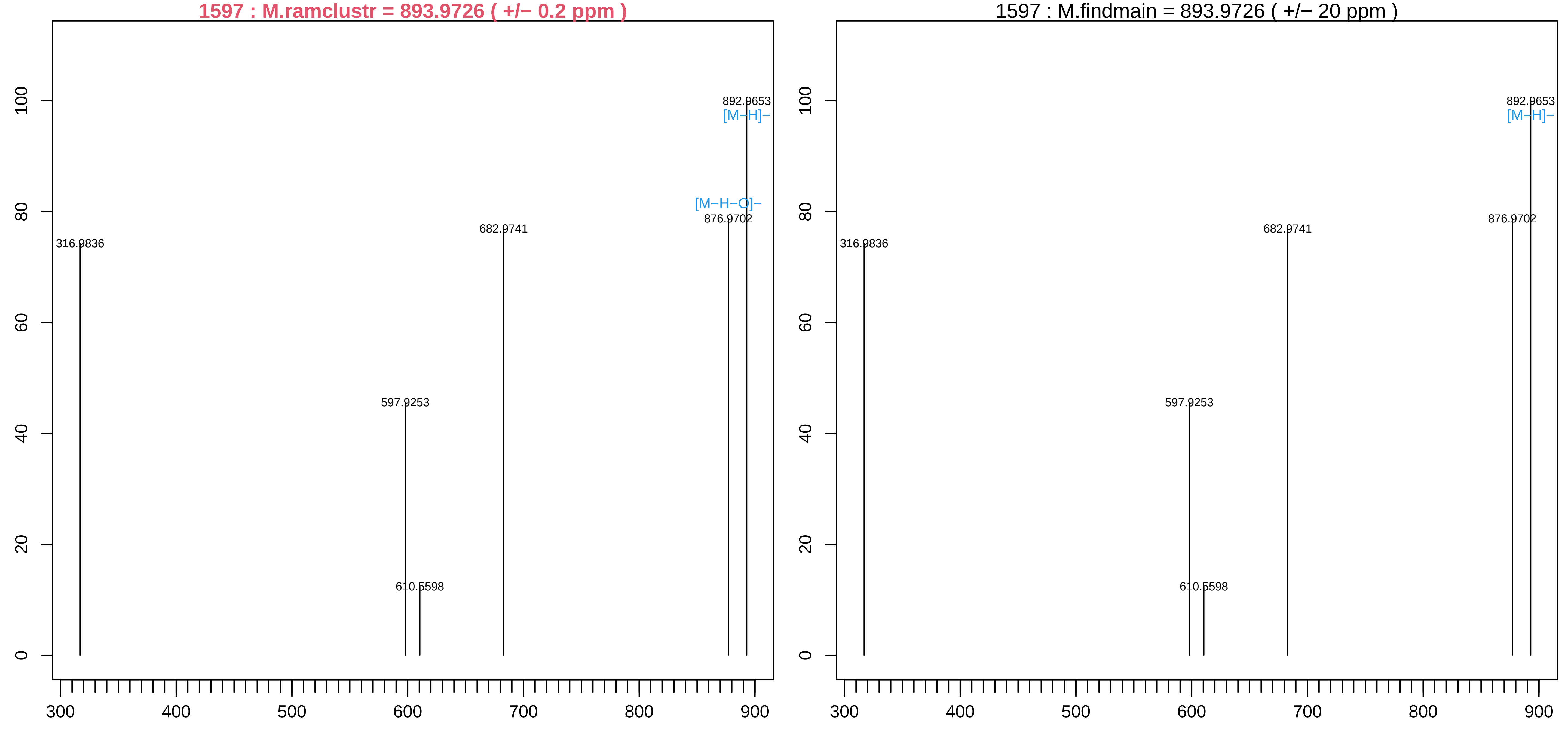
1597 : M.ramclustr = 893.9726 (+/− 0.2 ppm)
892.9653
[M−H]−
[M−H−O]−
876.9702
682.9741
316.9836
597.9253
610.5598
0
20
40
60
80
100
300
400
500
600
700
800
900
1597 : M.findmain = 893.9726 (+/− 20 ppm)
892.9653
[M−H]−
876.9702
682.9741
316.9836
597.9253
610.5598
0
20
40
60
80
100
300
400
500
600
700
800
900

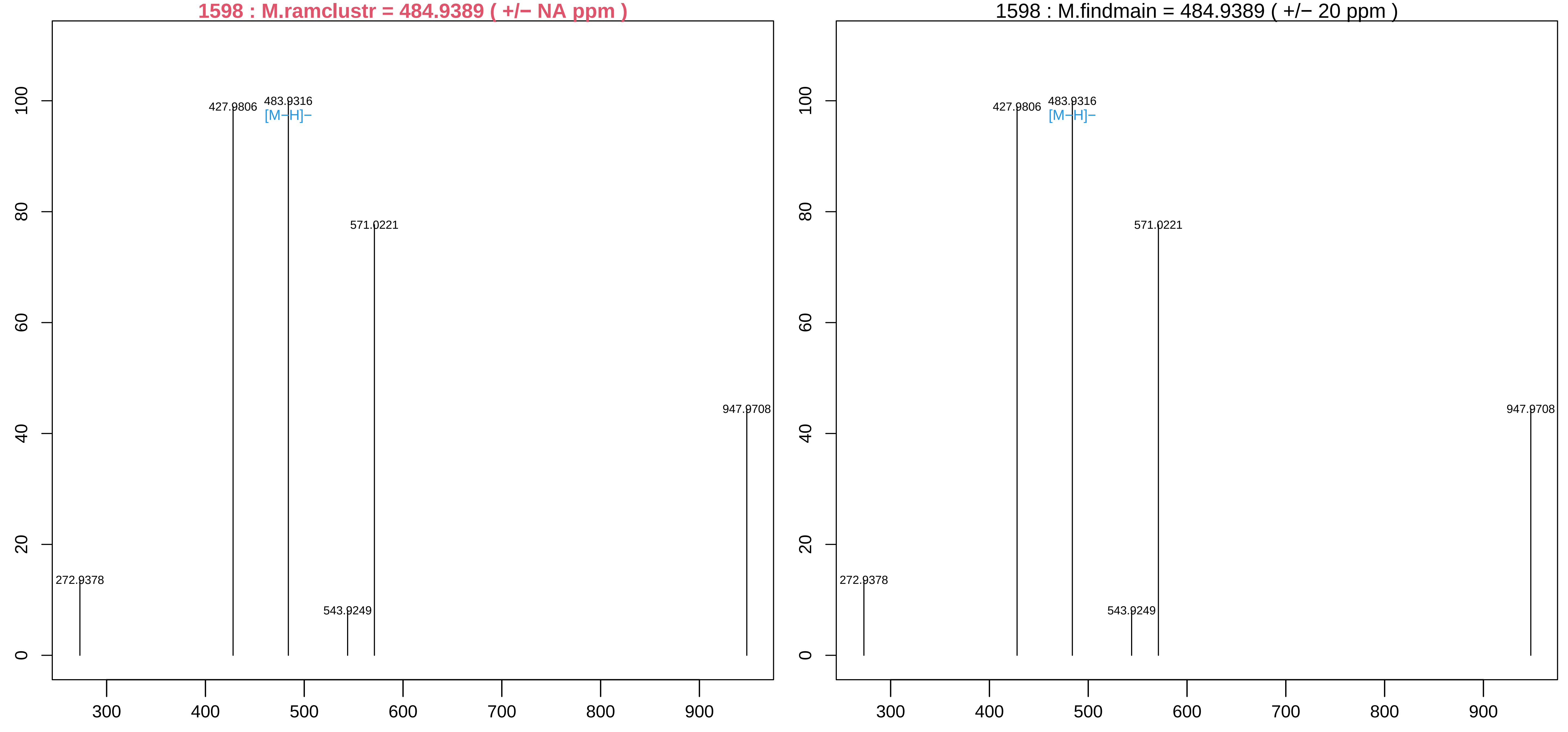

1598 : M.ramclustr = 484.9389 (+/− NA ppm)
427.9806
483.9316
[M−H]−
571.0221
947.9708
272.9378
543.9249
1598 : M.findmain = 484.9389 (+/− 20 ppm)
427.9806
483.9316
[M−H]−
571.0221
947.9708
272.9378
543.9249

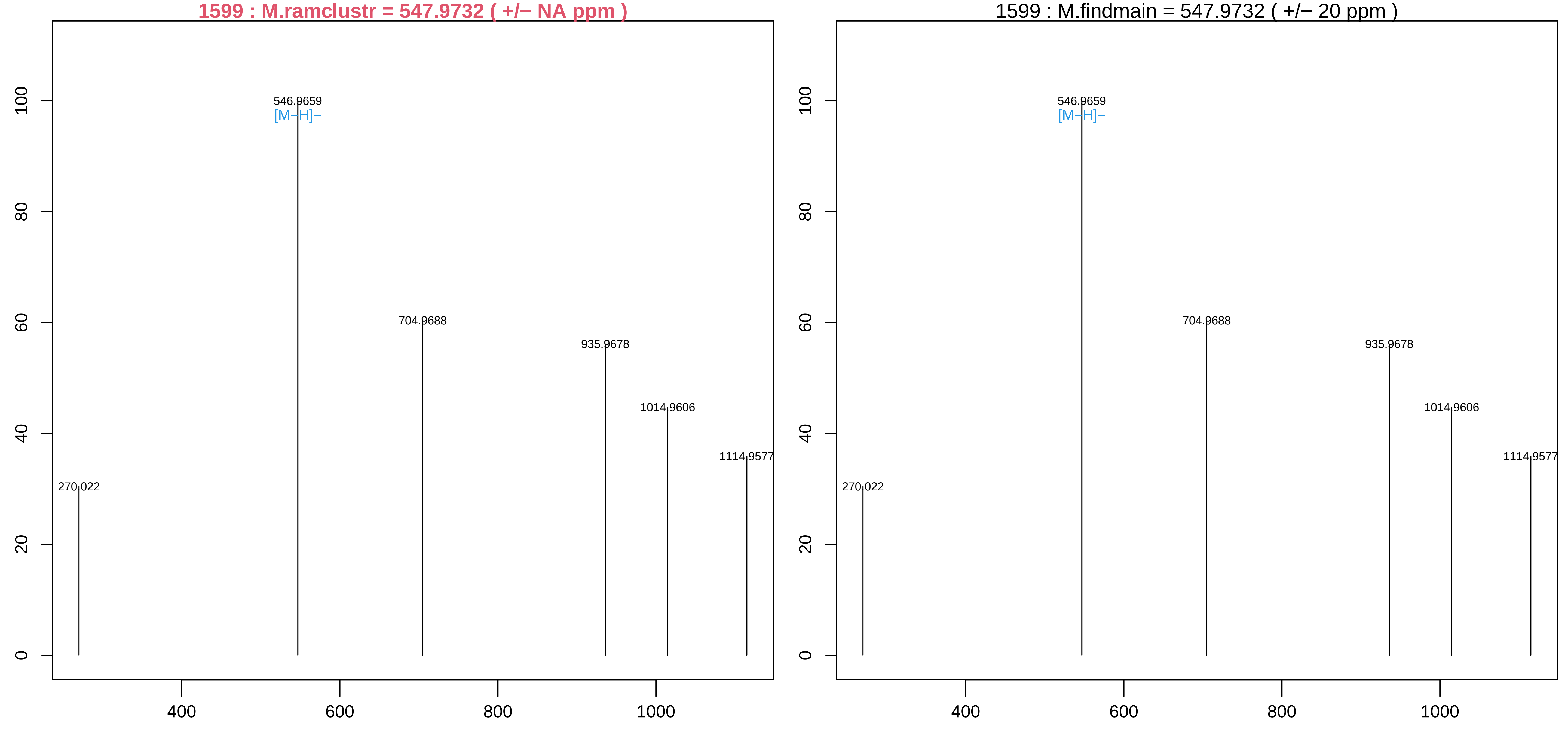
1599 : M.ramclustr = 547.9732 (+/− NA ppm)
1599 : M.findmain = 547.9732 (+/− 20 ppm)
546.9659
[M−H]−
704.9688
935.9678
1014.9606
1114.9577
270.022
0
20
40
60
80
100
400
600
800
1000

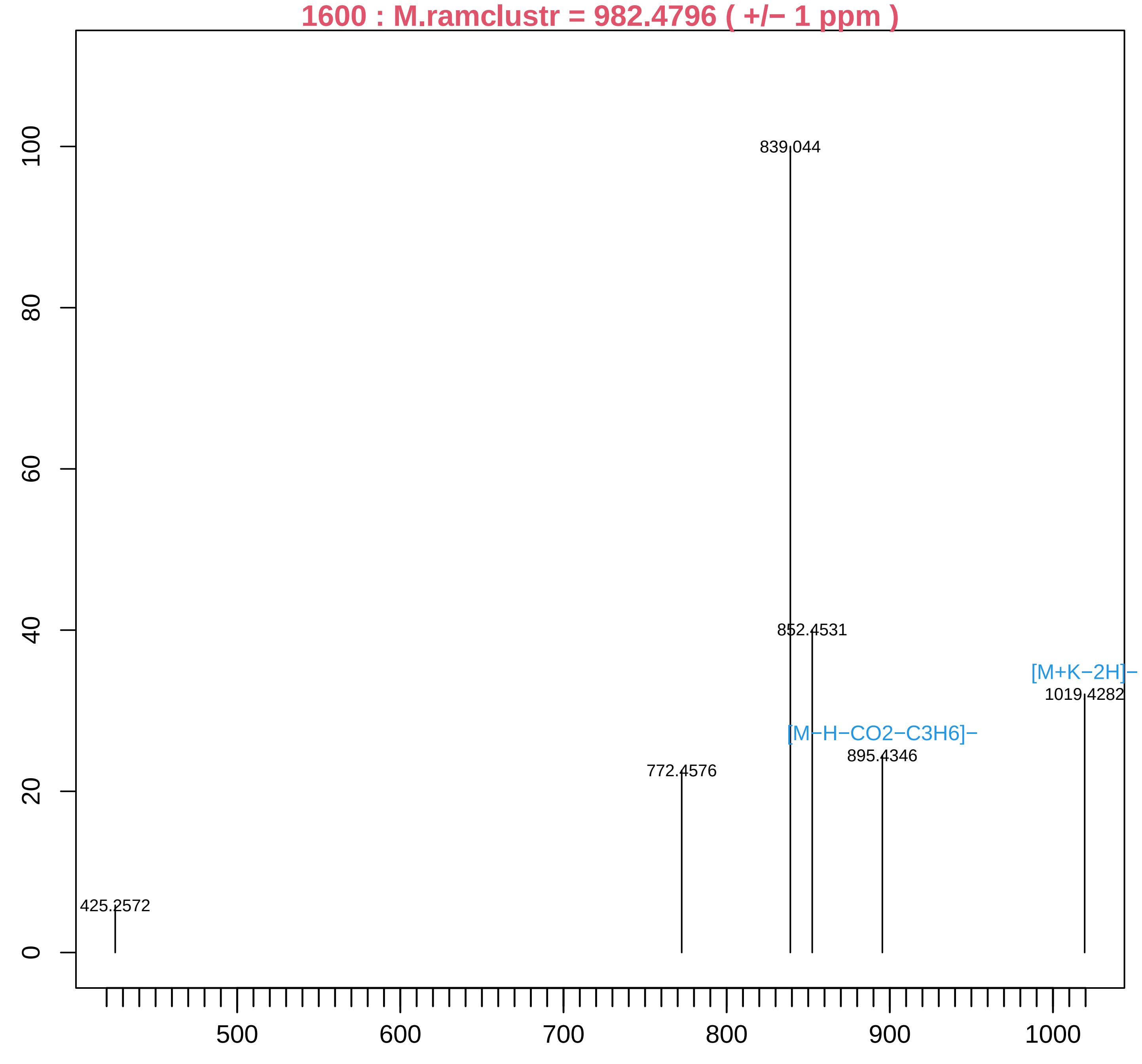
1600 : M.ramclustr = 982.4796 (+/− 1 ppm)
839.044
852.4531
[M+K−2H]−
1019.4282
[M−H−CO2−C3H6]−
895.4346
772.4576
425.2572
0
20
40
60
80
100
500
600
700
800
900
1000

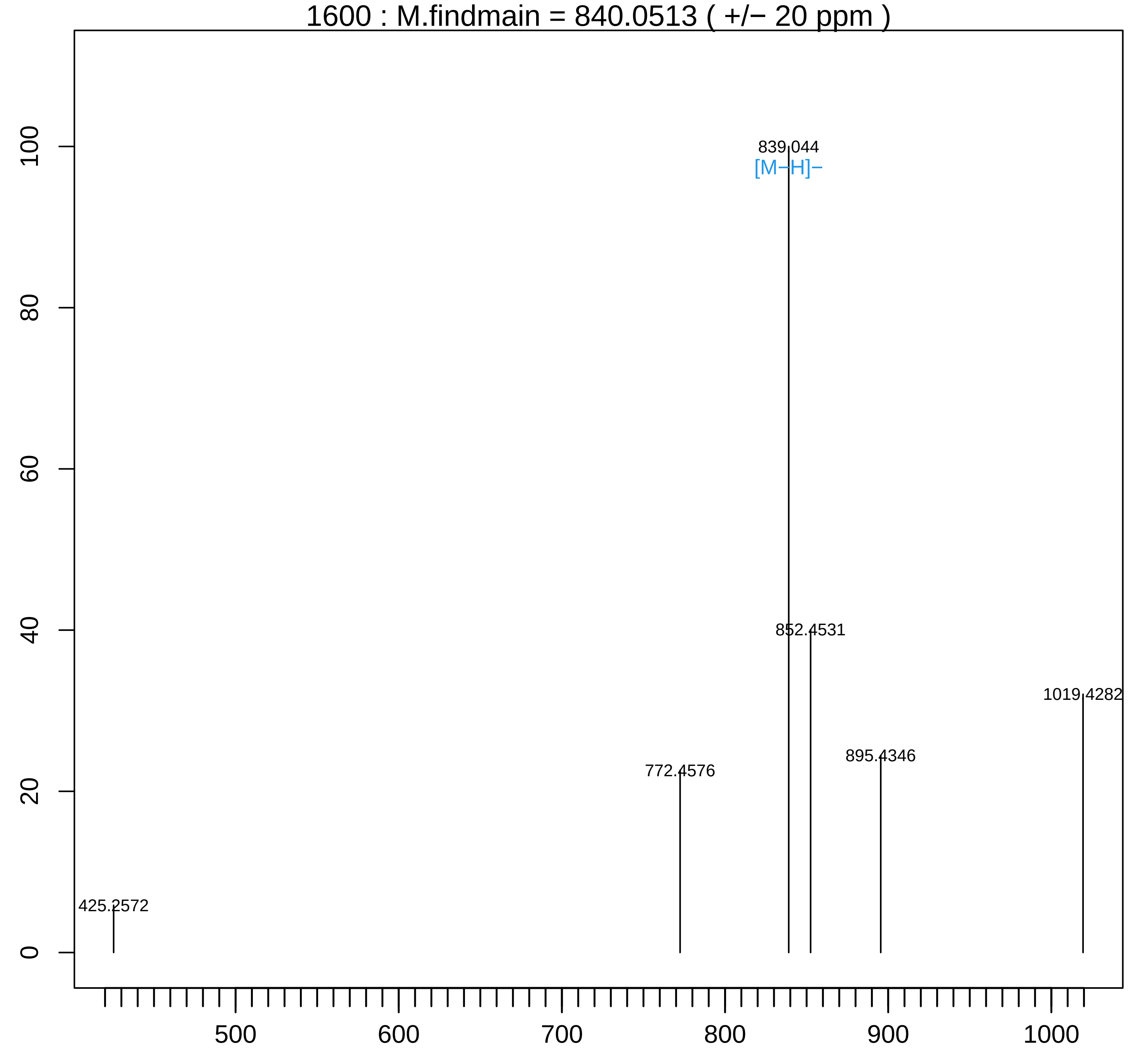
1600 : M.findmain = 840.0513 (+/− 20 ppm)
839.044
[M−H]−
852.4531
1019.4282
895.4346
772.4576
425.2572
0
20
40
60
80
100
500
600
700
800
900
1000

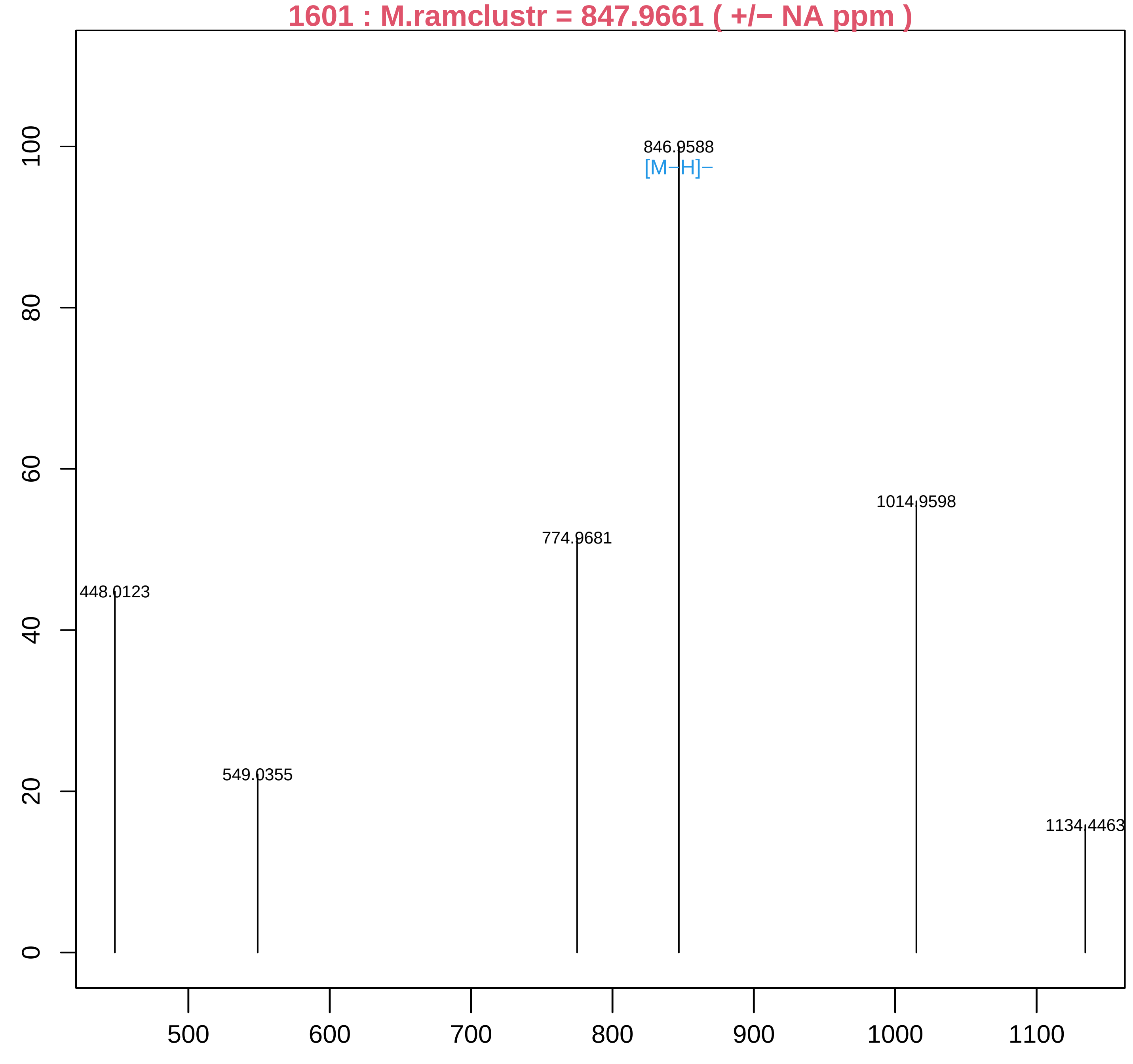

1601 : M.ramclustr = 847.9661 (+/− NA ppm)
846.9588
[M−H]−
1014.9598
774.9681
448.0123
549.0355
1134.4463
0
20
40
60
80
100
500
600
700
800
900
1000
1100

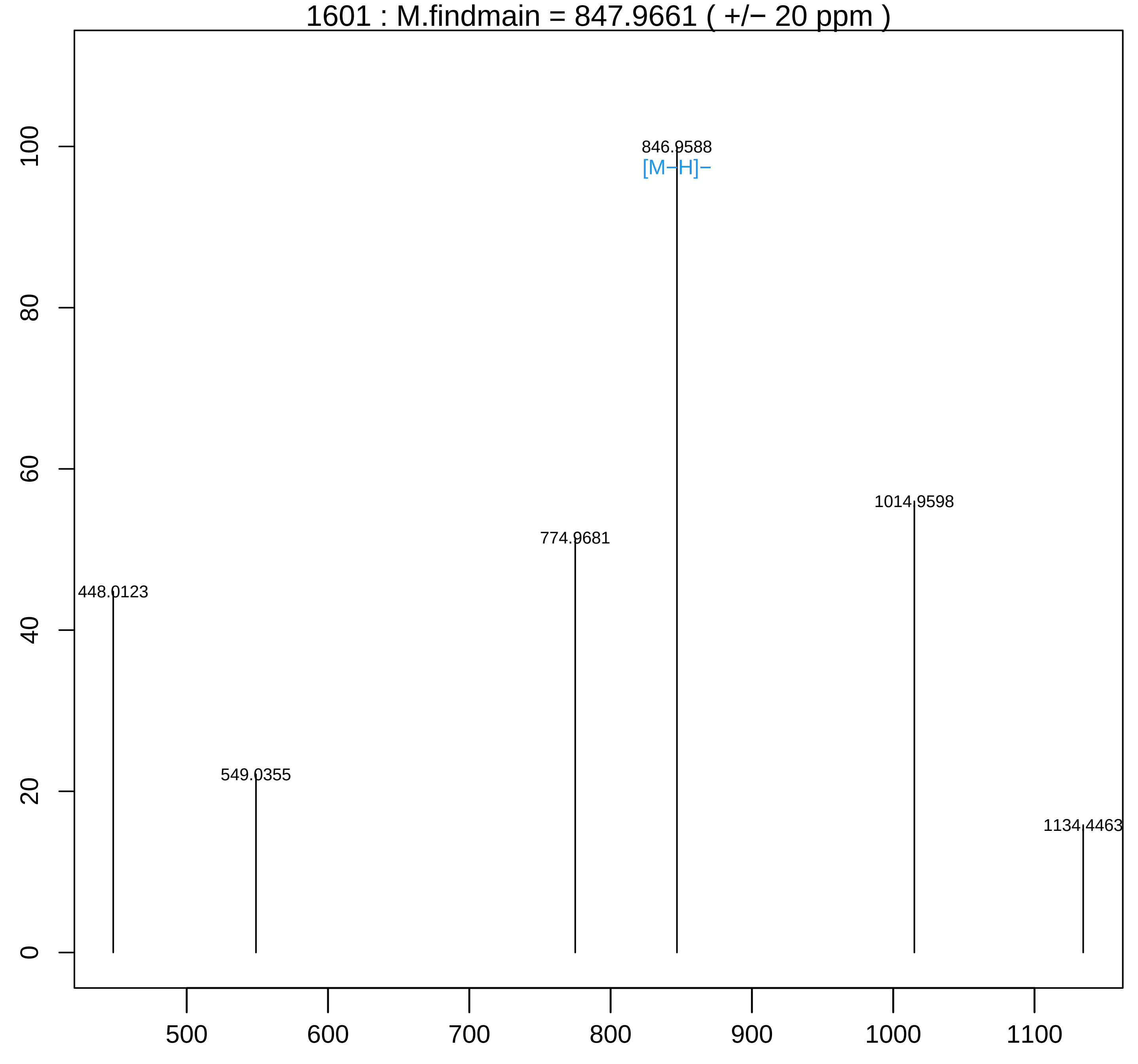

1601 : M.findmain = 847.9661 (+/− 20 ppm)
846.9588
[M−H]−
1014.9598
774.9681
448.0123
549.0355
1134.4463
0
20
40
60
80
100
500
600
700
800
900
1000
1100

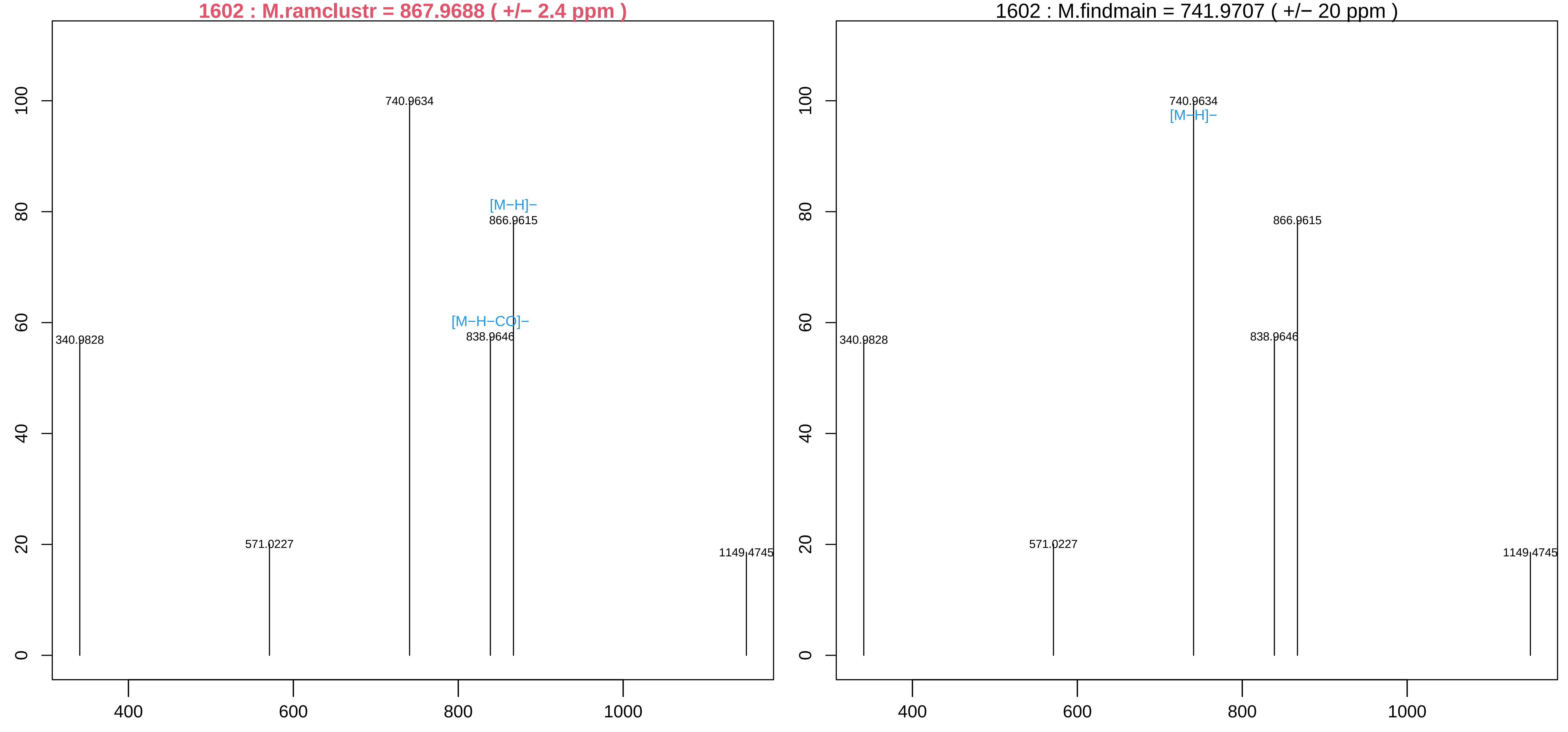

1602 : M.ramclustr = 867.9688 (+/− 2.4 ppm)
740.9634
[M−H]−
866.9615
[M−H−CO]−
838.9646
340.9828
571.0227
1149.4745
1602 : M.findmain = 741.9707 (+/− 20 ppm)
740.9634
[M−H]−
866.9615
838.9646
340.9828
571.0227
1149.4745

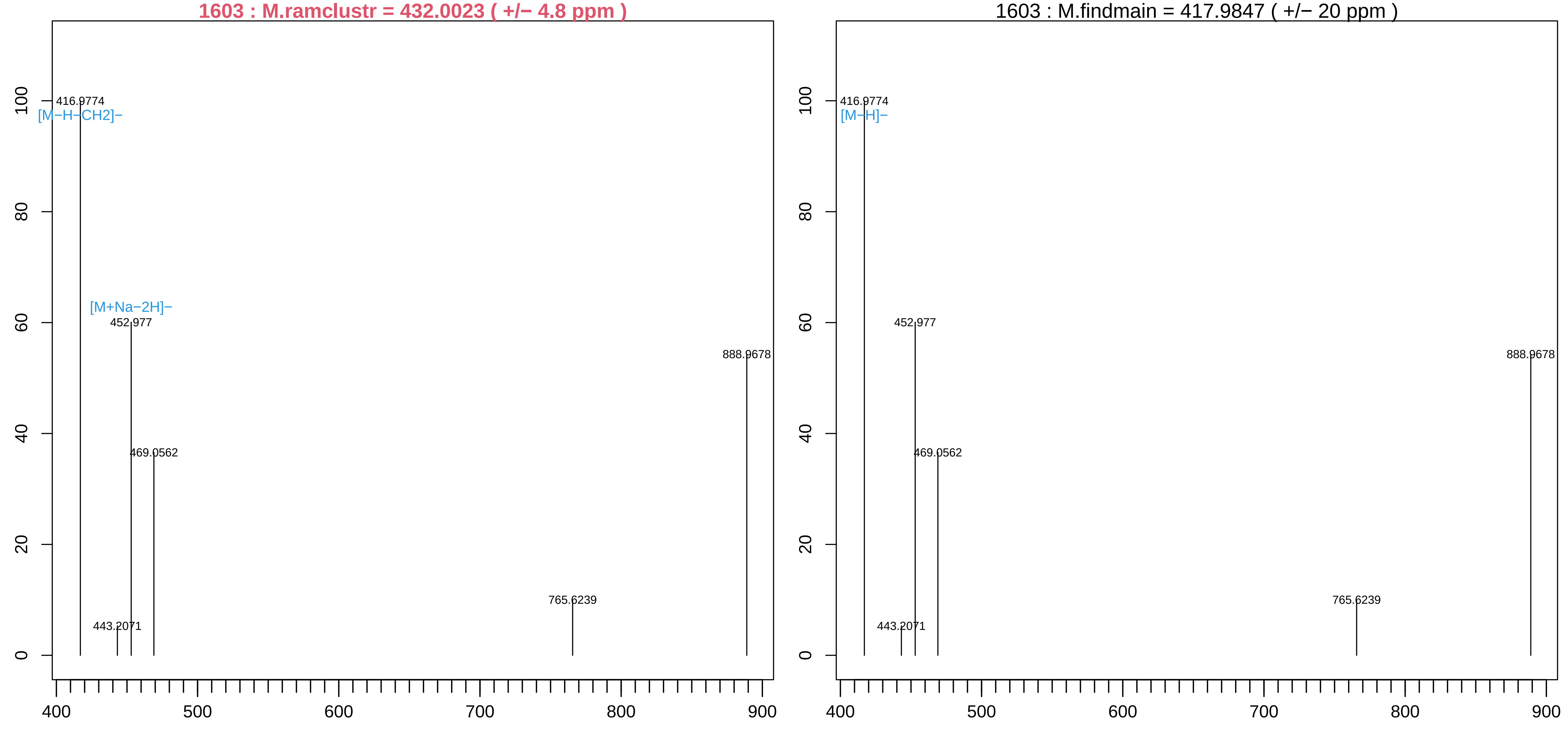
1603 : M.ramclustr = 432.0023 (+/− 4.8 ppm)
416.9774
[M−H−CH2]−
[M+Na−2H]−
452.977
469.0562
443.2071
765.6239
888.9678
1603 : M.findmain = 417.9847 (+/− 20 ppm)
416.9774
[M−H]−
452.977
469.0562
443.2071
765.6239
888.9678
0
20
40
60
80
100
400
500
600
700
800
900

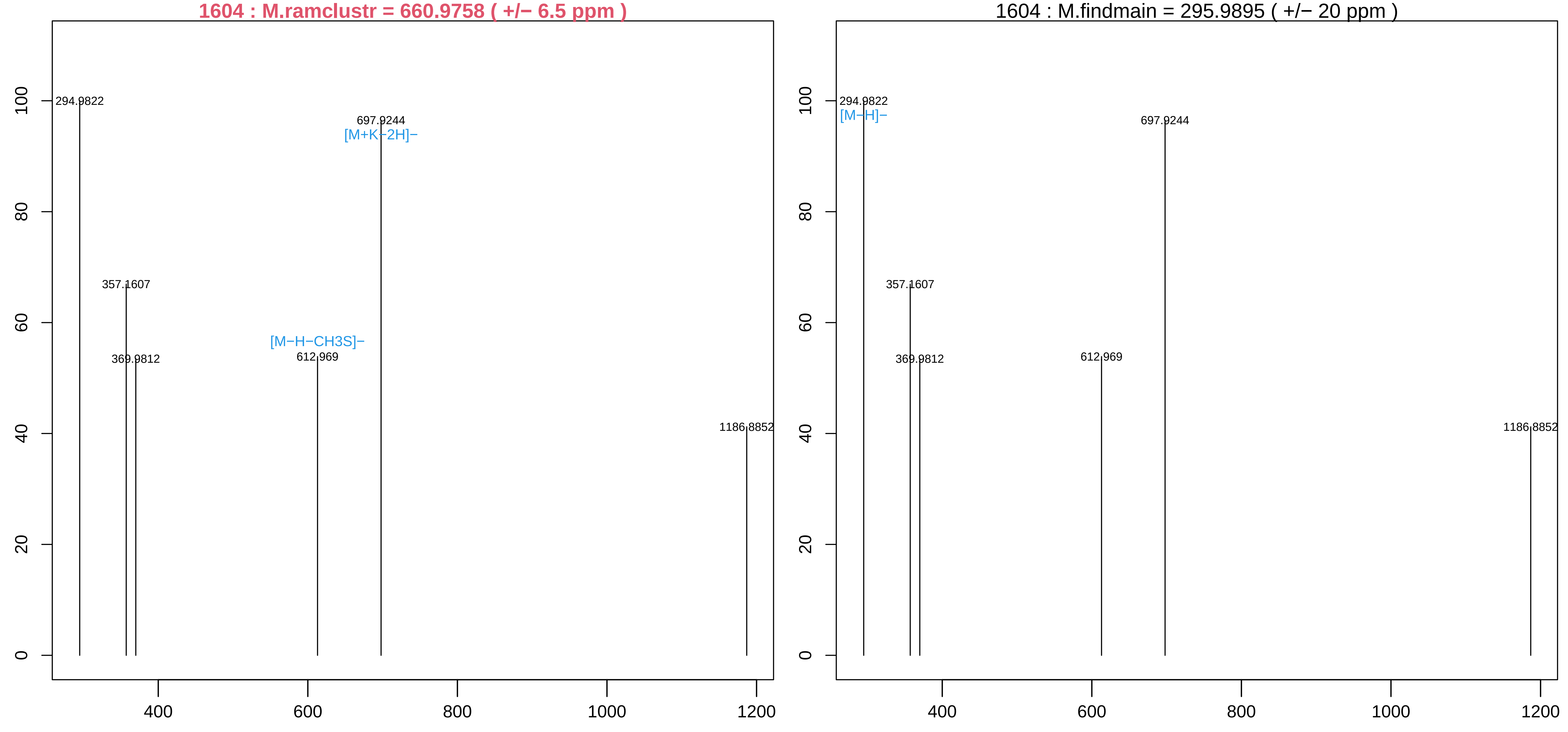
1604 : M.ramclustr = 660.9758 (+/− 6.5 ppm)
294.9822
357.1607
369.9812
[M−H−CH3S]−
612.969
697.9244
[M+K−2H]−
1186.8852
1604 : M.findmain = 295.9895 (+/− 20 ppm)
294.9822
[M−H]−
357.1607
369.9812
612.969
697.9244
1186.8852

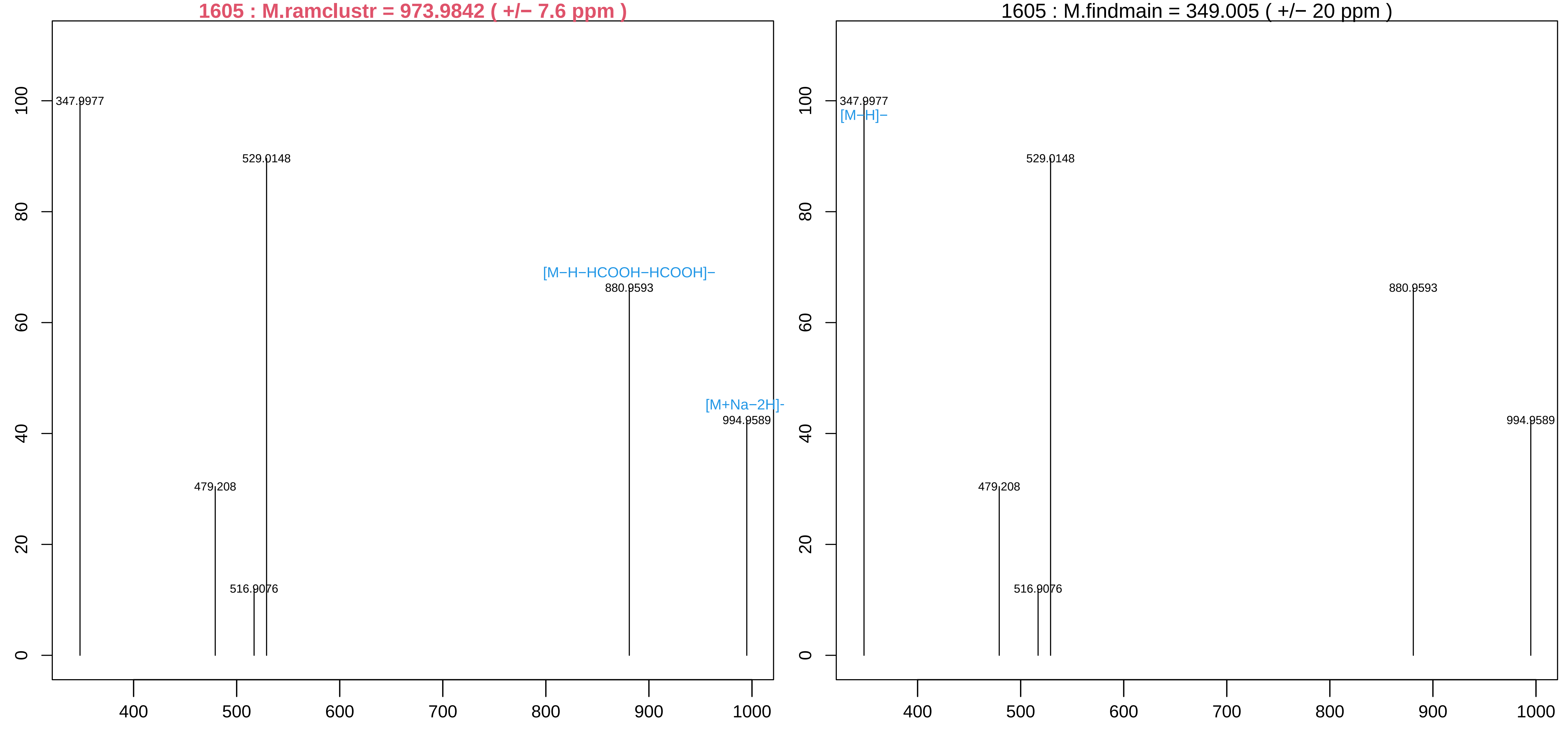

1605 : M.ramclustr = 973.9842 (+/− 7.6 ppm)
347.9977
529.0148
[M−H−HCOOH−HCOOH]−
880.9593
[M+Na−2H]-
994.9589
479.208
516.9076
0
20
40
60
80
100
400
500
600
700
800
900
1000
1605 : M.findmain = 349.005 (+/− 20 ppm)
347.9977
[M−H]−
529.0148
880.9593
994.9589
479.208
516.9076

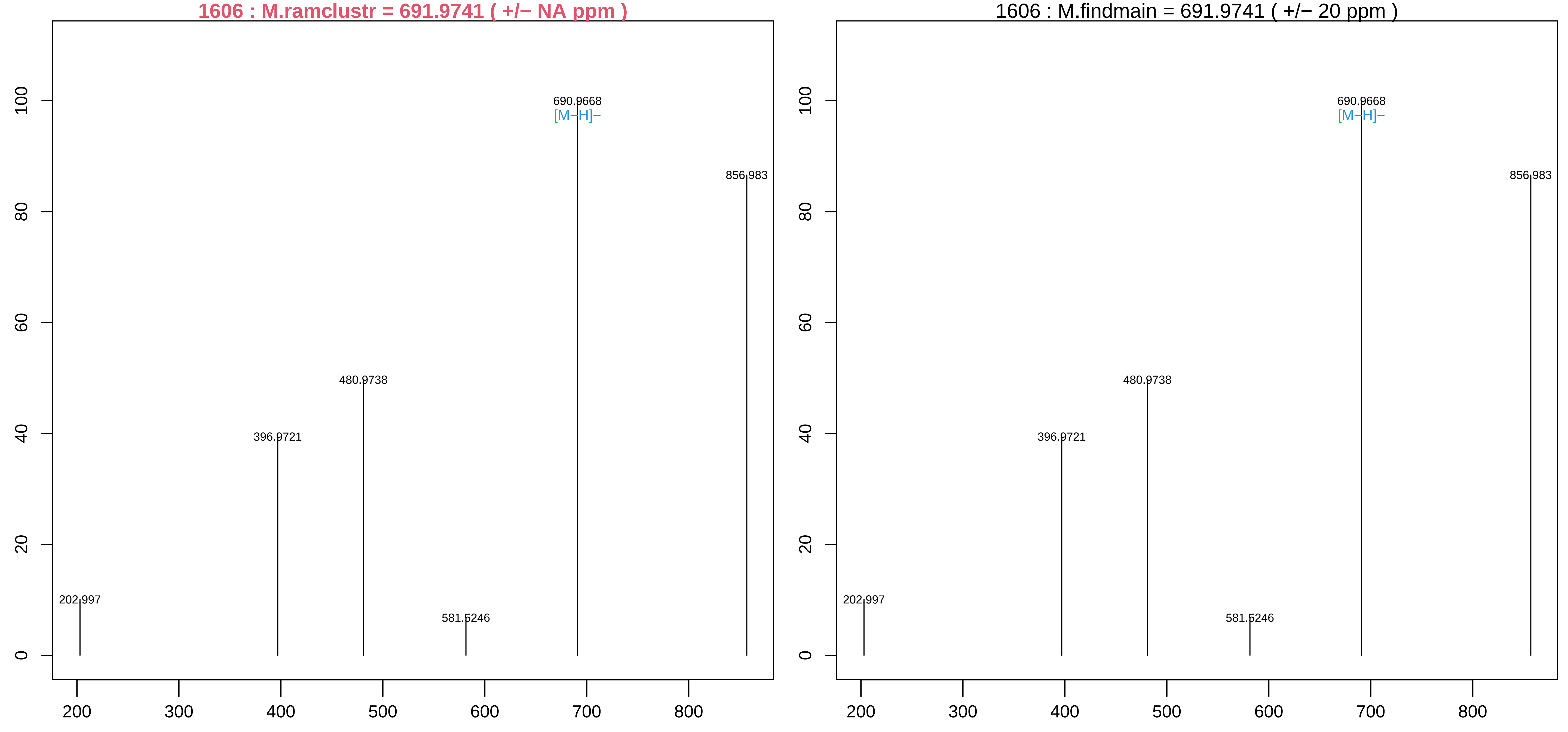
1606 : M.ramclustr = 691.9741 (+/− NA ppm)
1606 : M.findmain = 691.9741 (+/− 20 ppm)
690.9668
[M−H]−
856.983
480.9738
396.9721
202.997
581.5246
0
20
40
60
80
100
200
300
400
500
600
700
800

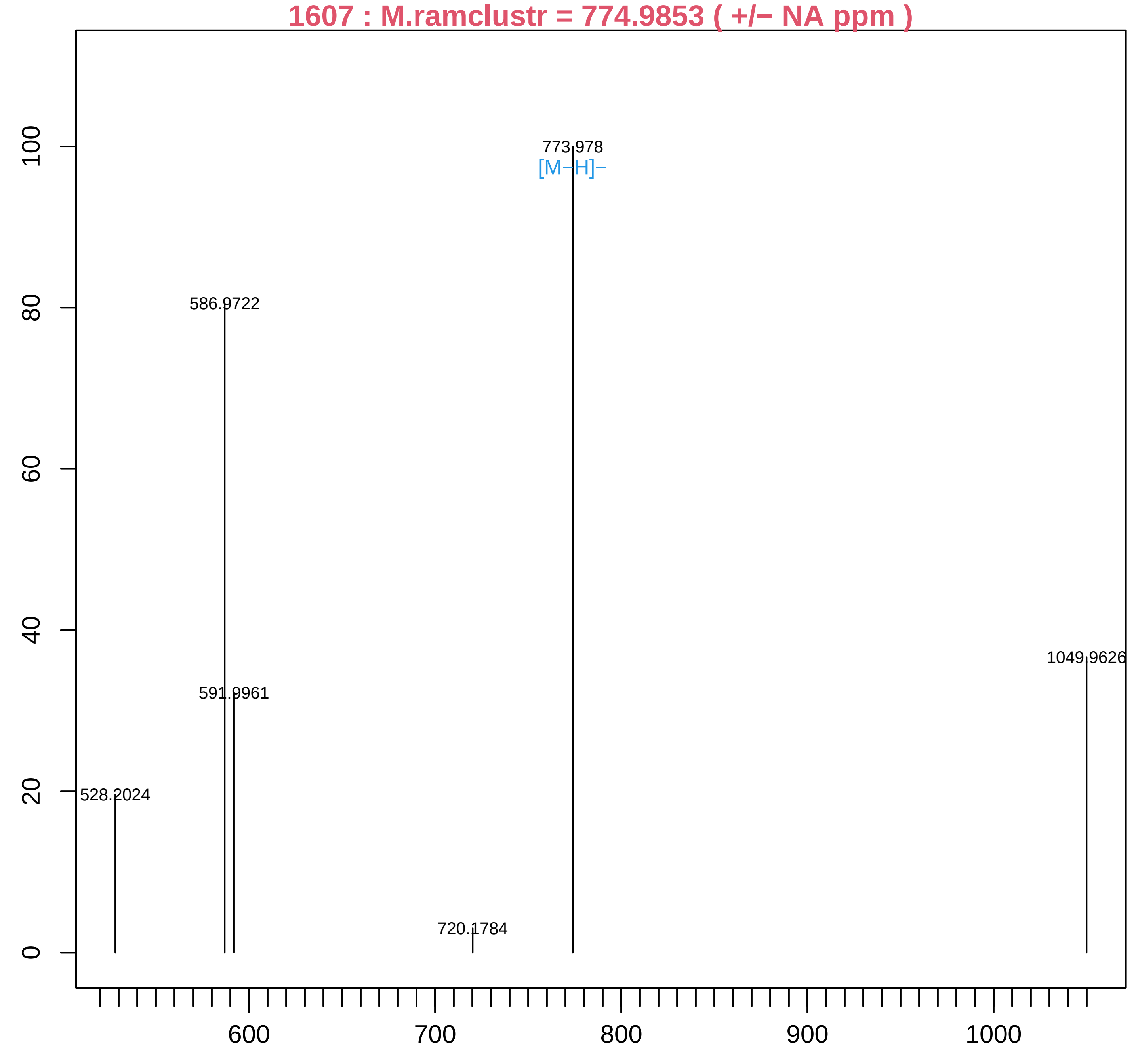
1607 : M.ramclustr = 774.9853 (+/− NA ppm)
773.978
[M−H]−
586.9722
591.9961
528.2024
720.1784
1049.9626
0
20
40
60
80
100
600
700
800
900
1000

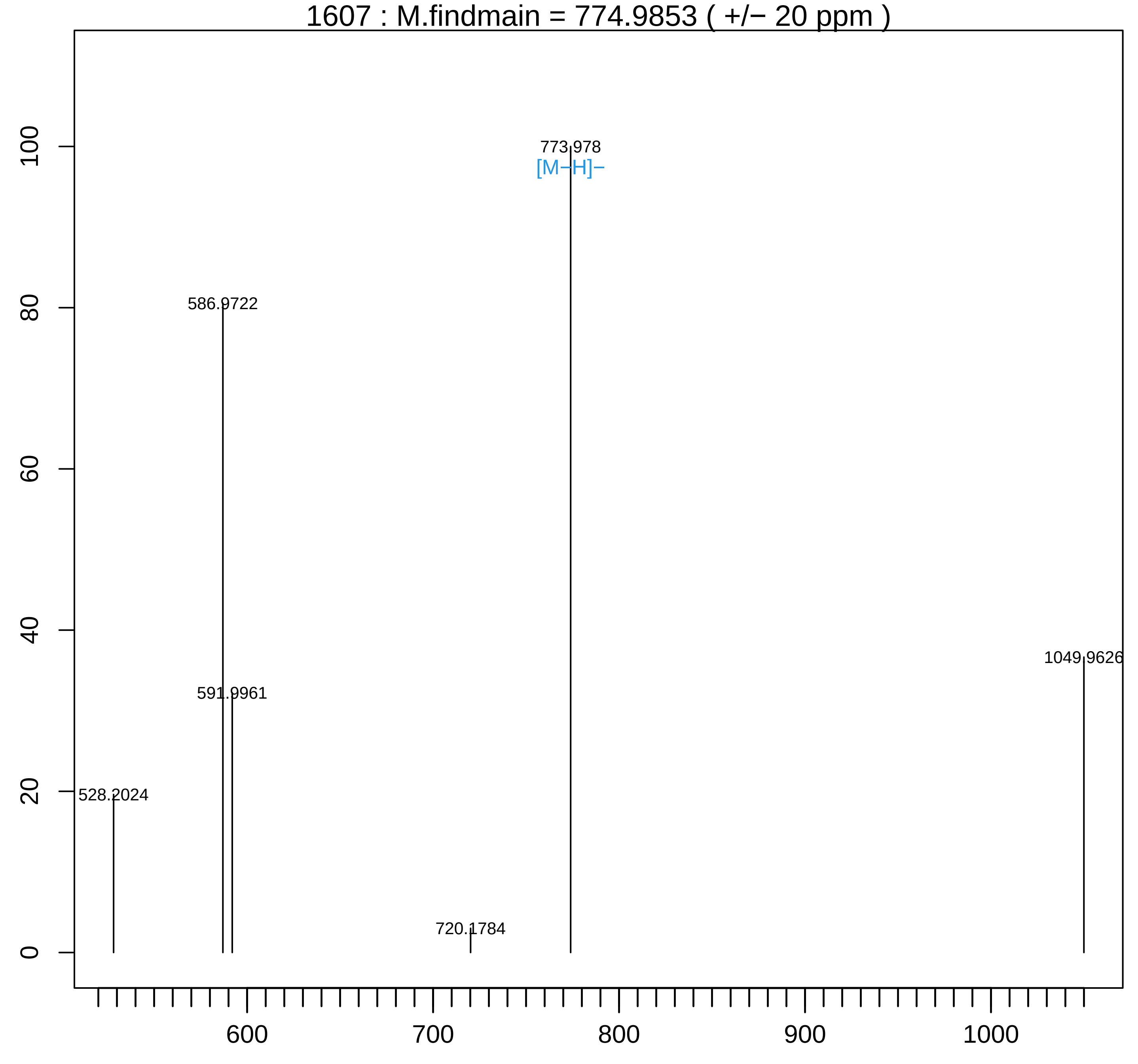
1607 : M.findmain = 774.9853 (+/− 20 ppm)
773.978
[M−H]−
586.9722
591.9961
528.2024
720.1784
1049.9626
0
20
40
60
80
100
600
700
800
900
1000

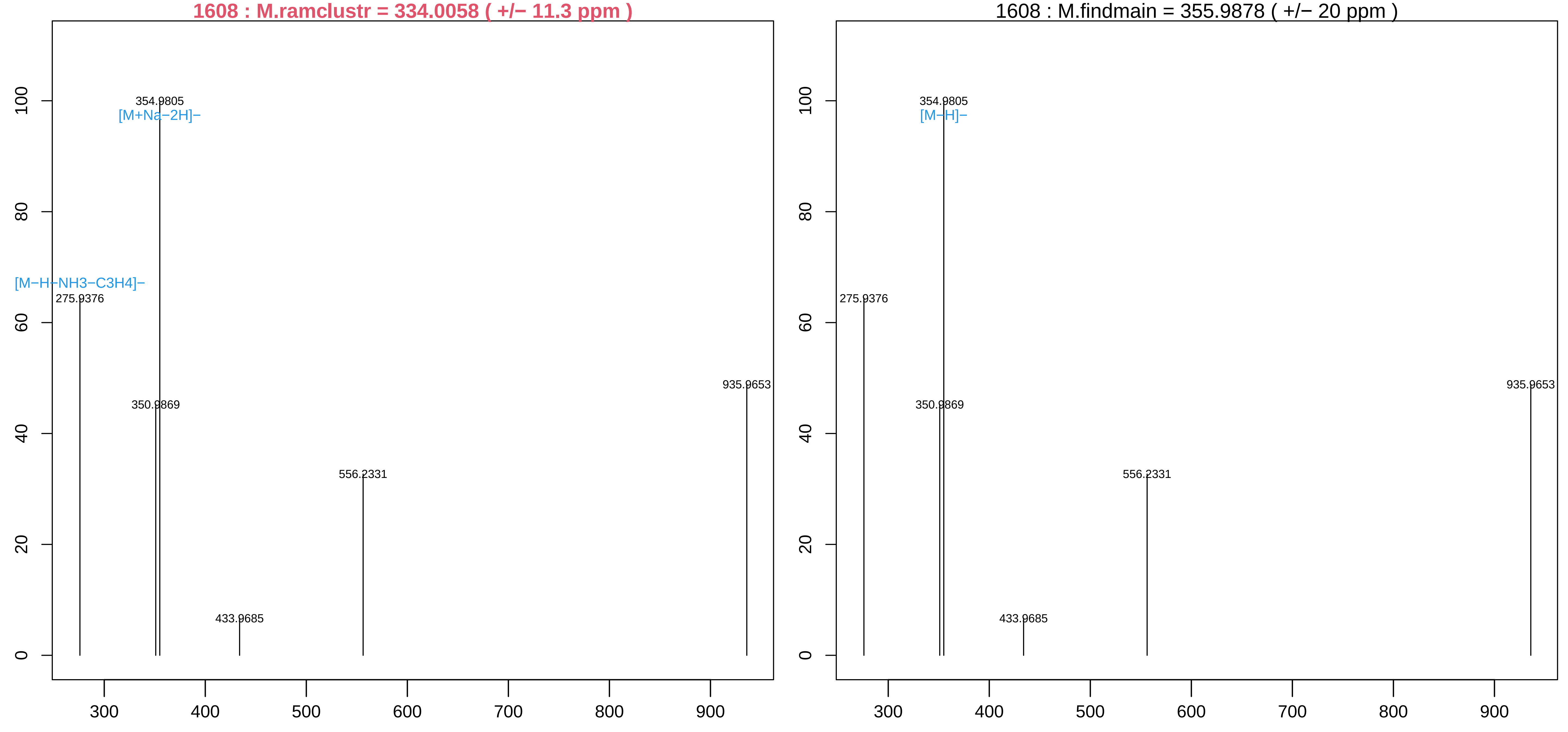
1608 : M.ramclustr = 334.0058 (+/− 11.3 ppm)
354.9805
[M+Na−2H]−
[M−H−NH3−C3H4]−
275.9376
350.9869
433.9685
556.2331
935.9653
1608 : M.findmain = 355.9878 (+/− 20 ppm)
354.9805
[M−H]−
275.9376
350.9869
433.9685
556.2331
935.9653

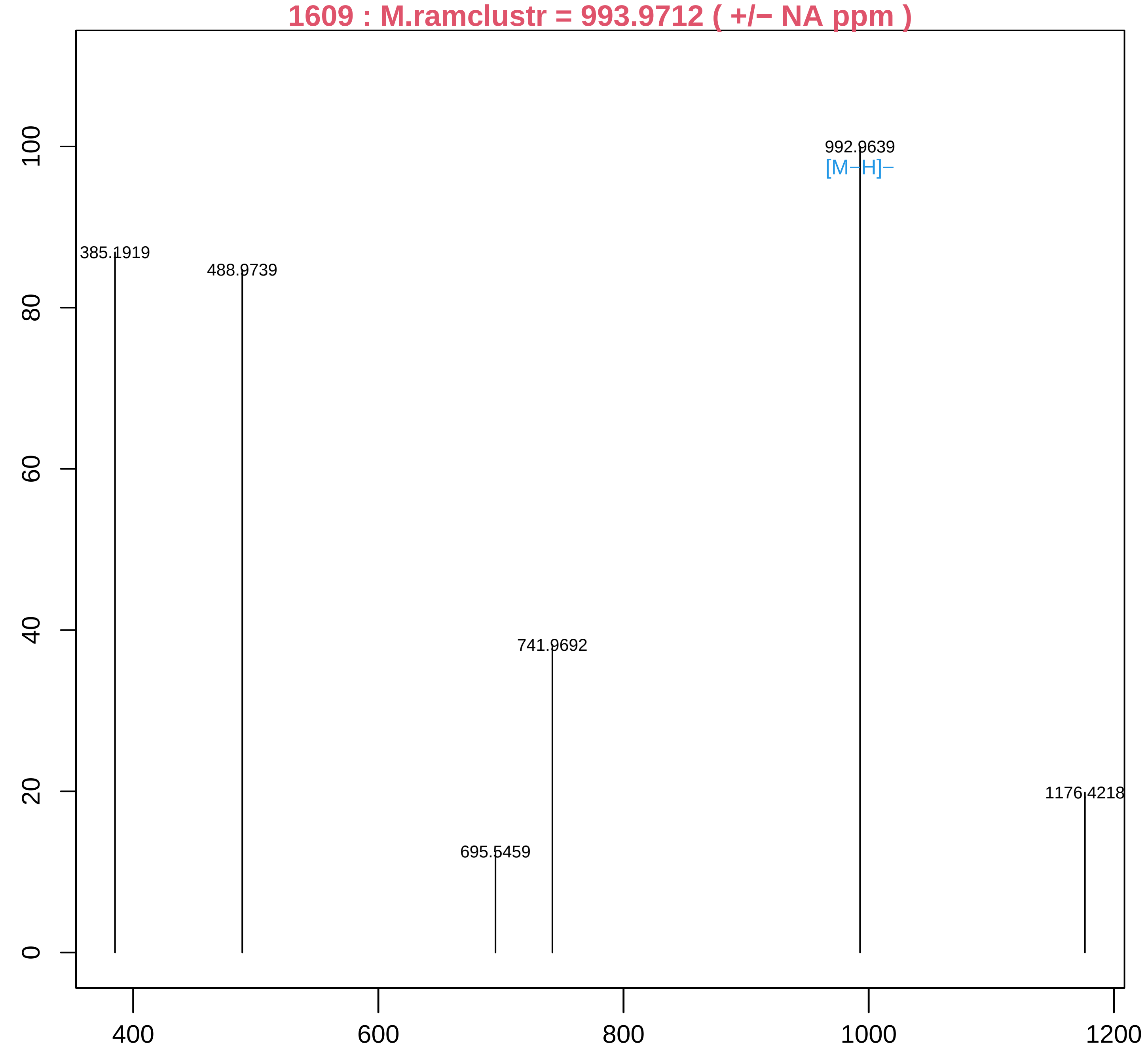
1609 : M.ramclustr = 993.9712 (+/− NA ppm)
992.9639
[M−H]−
385.1919
488.9739
741.9692
695.5459
1176.4218
0
20
40
60
80
100
400
600
800
1000
1200

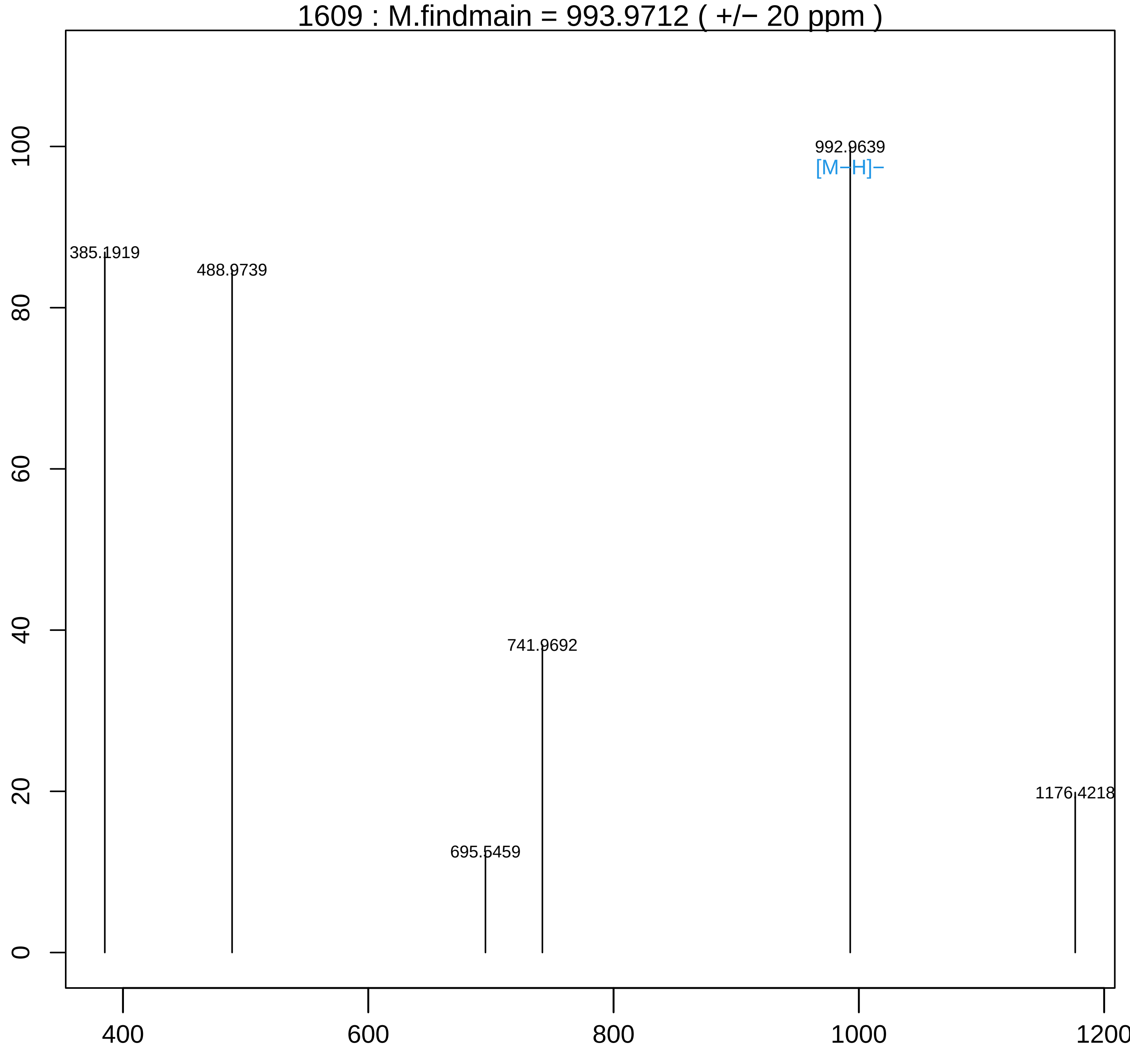
1609 : M.findmain = 993.9712 (+/− 20 ppm)
992.9639
[M−H]−
385.1919
488.9739
741.9692
695.5459
1176.4218
0
20
40
60
80
100
400
600
800
1000
1200

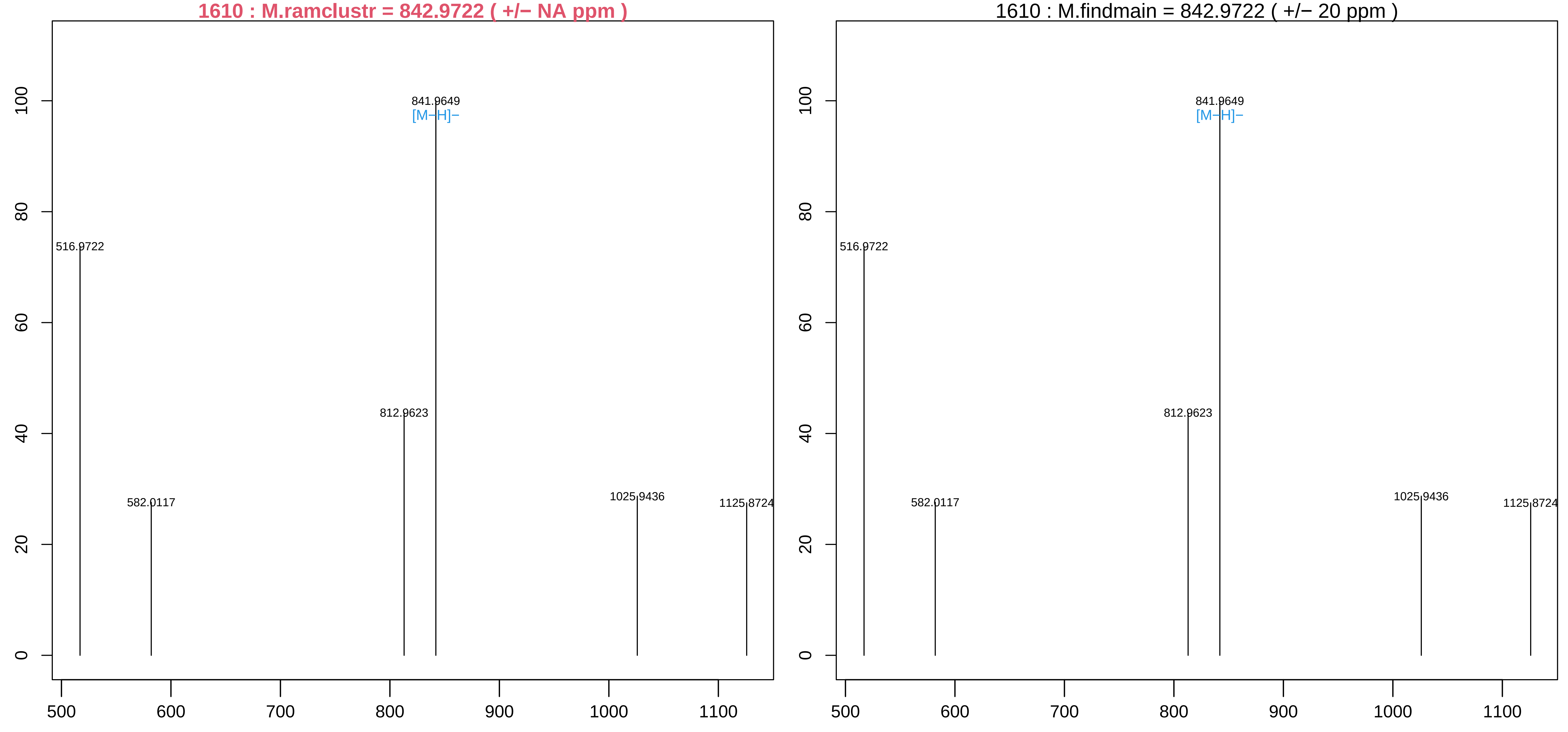
1610 : M.ramclustr = 842.9722 (+/− NA ppm)
1610 : M.findmain = 842.9722 (+/− 20 ppm)
841.9649
[M−H]−
516.9722
582.0117
812.9623
1025.9436
1125.8724
0
20
40
60
80
100
500
600
700
800
900
1000
1100

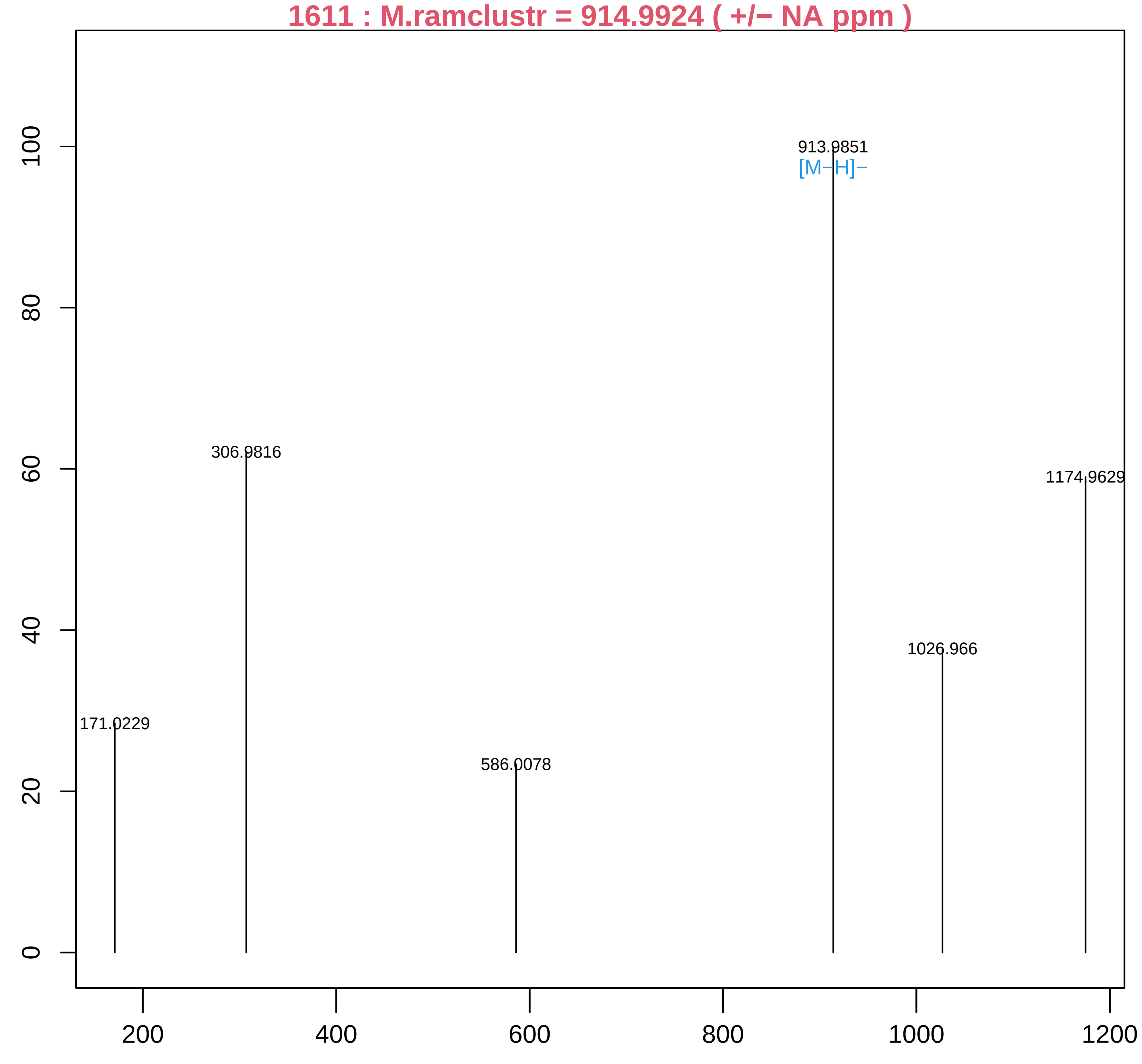
1611 : M.ramclustr = 914.9924 (+/− NA ppm)
913.9851
[M−H]−
306.9816
1174.9629
1026.966
171.0229
586.0078
0
20
40
60
80
100
200
400
600
800
1000
1200

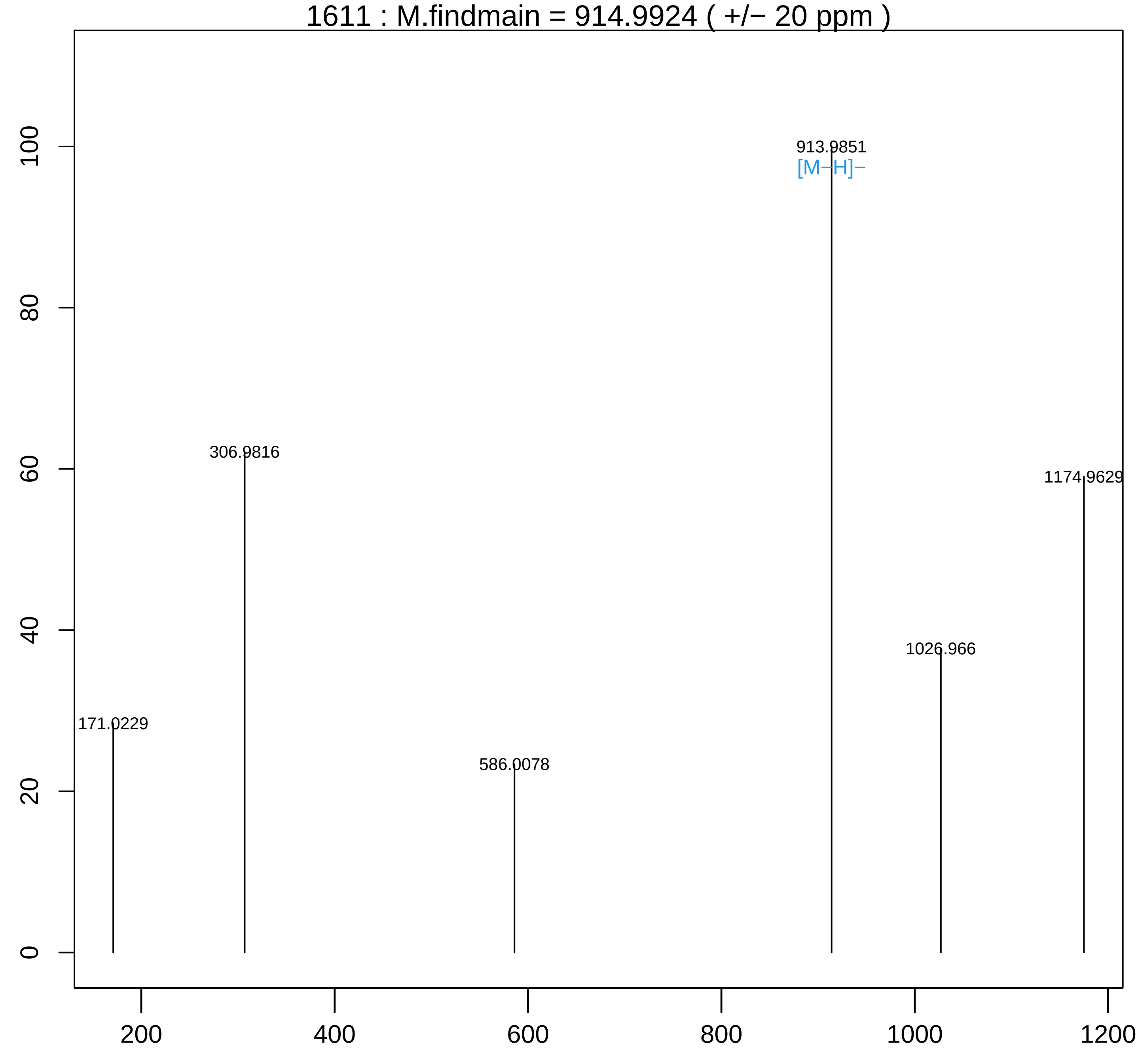
1611 : M.findmain = 914.9924 (+/− 20 ppm)
913.9851
[M−H]−
306.9816
1174.9629
1026.966
171.0229
586.0078
0
20
40
60
80
100
200
400
600
800
1000
1200

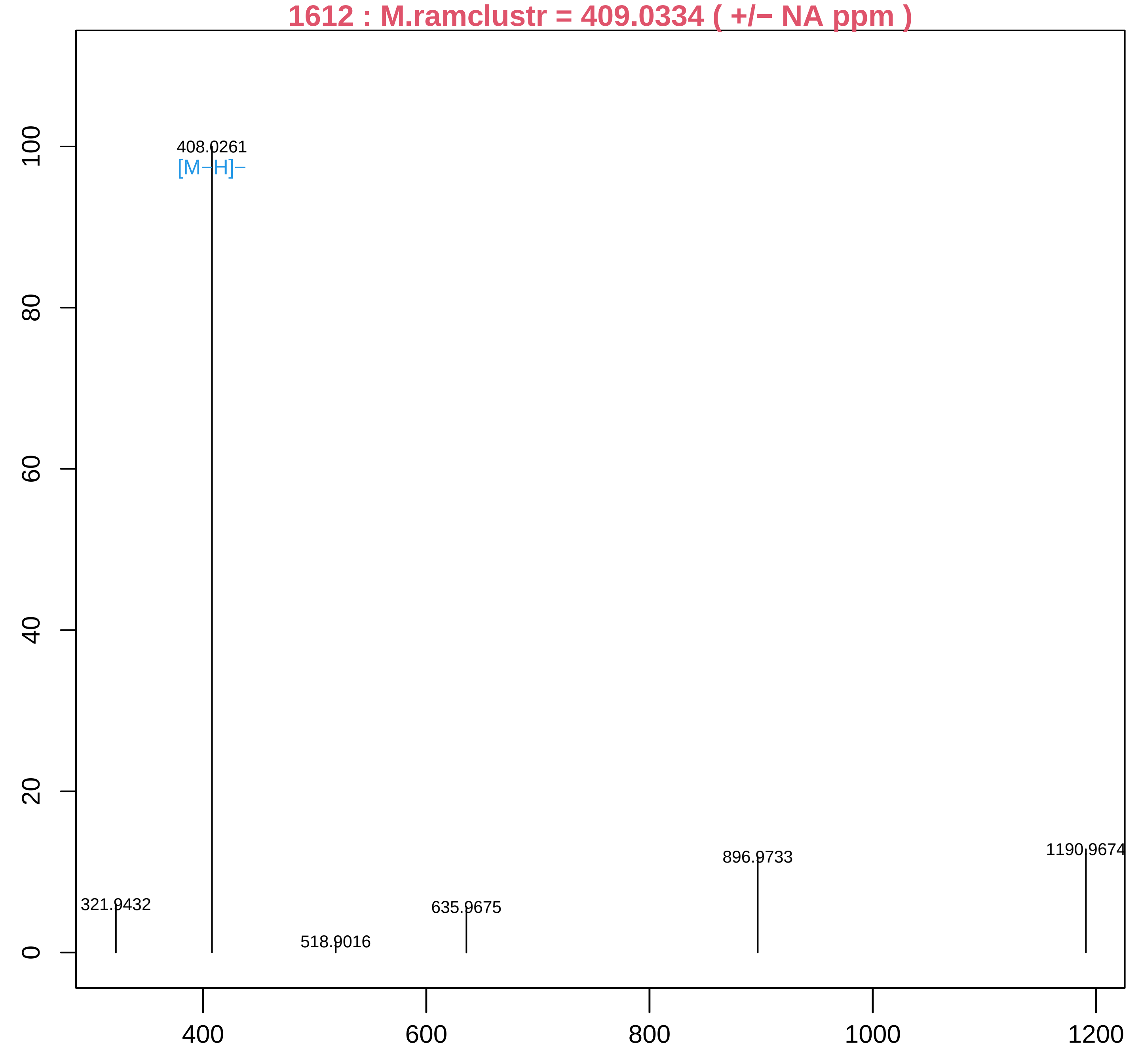
1612 : M.ramclustr = 409.0334 (+/− NA ppm)
408.0261
[M−H]−
321.9432
518.9016
635.9675
896.9733
1190.9674
0
20
40
60
80
100
400
600
800
1000
1200

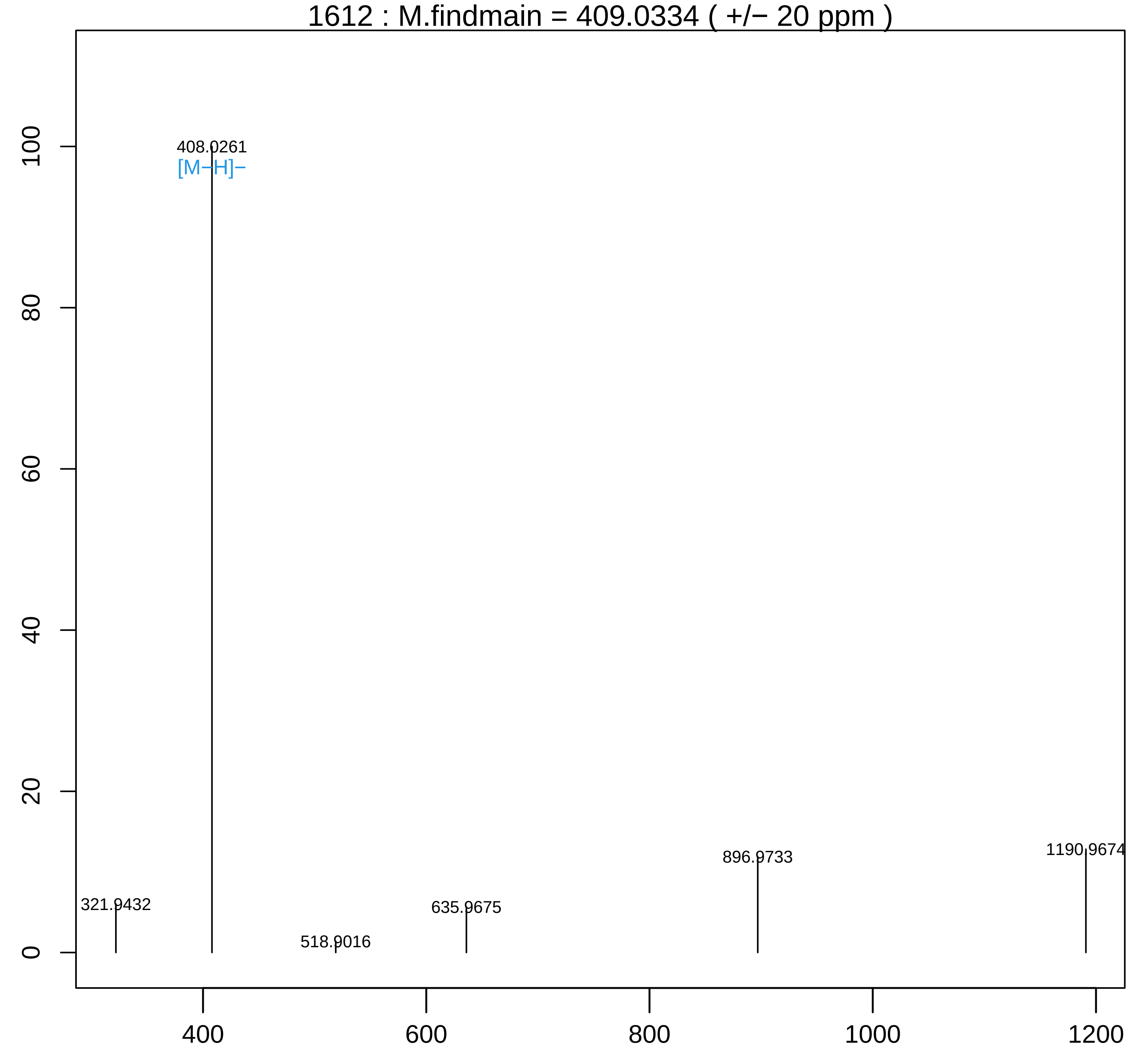
1612 : M.findmain = 409.0334 (+/− 20 ppm)
408.0261
[M−H]−
321.9432
518.9016
635.9675
896.9733
1190.9674
0
20
40
60
80
100
400
600
800
1000
1200

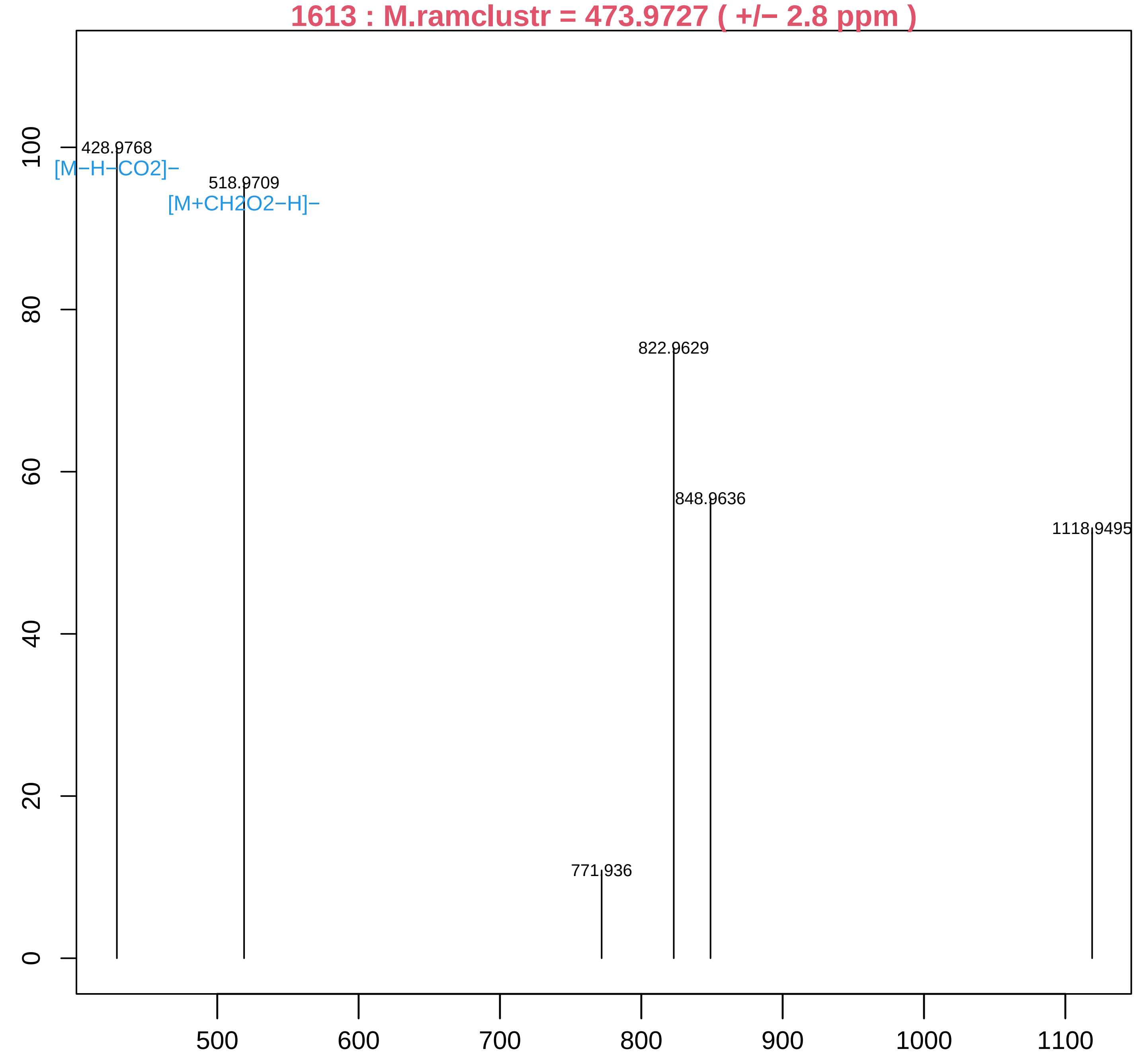
1613 : M.ramclustr = 473.9727 (+/− 2.8 ppm)
428.9768
[M−H−CO2]−
518.9709
[M+CH2O2−H]−
822.9629
848.9636
1118.9495
771.936
0
20
40
60
80
100
500
600
700
800
900
1000
1100

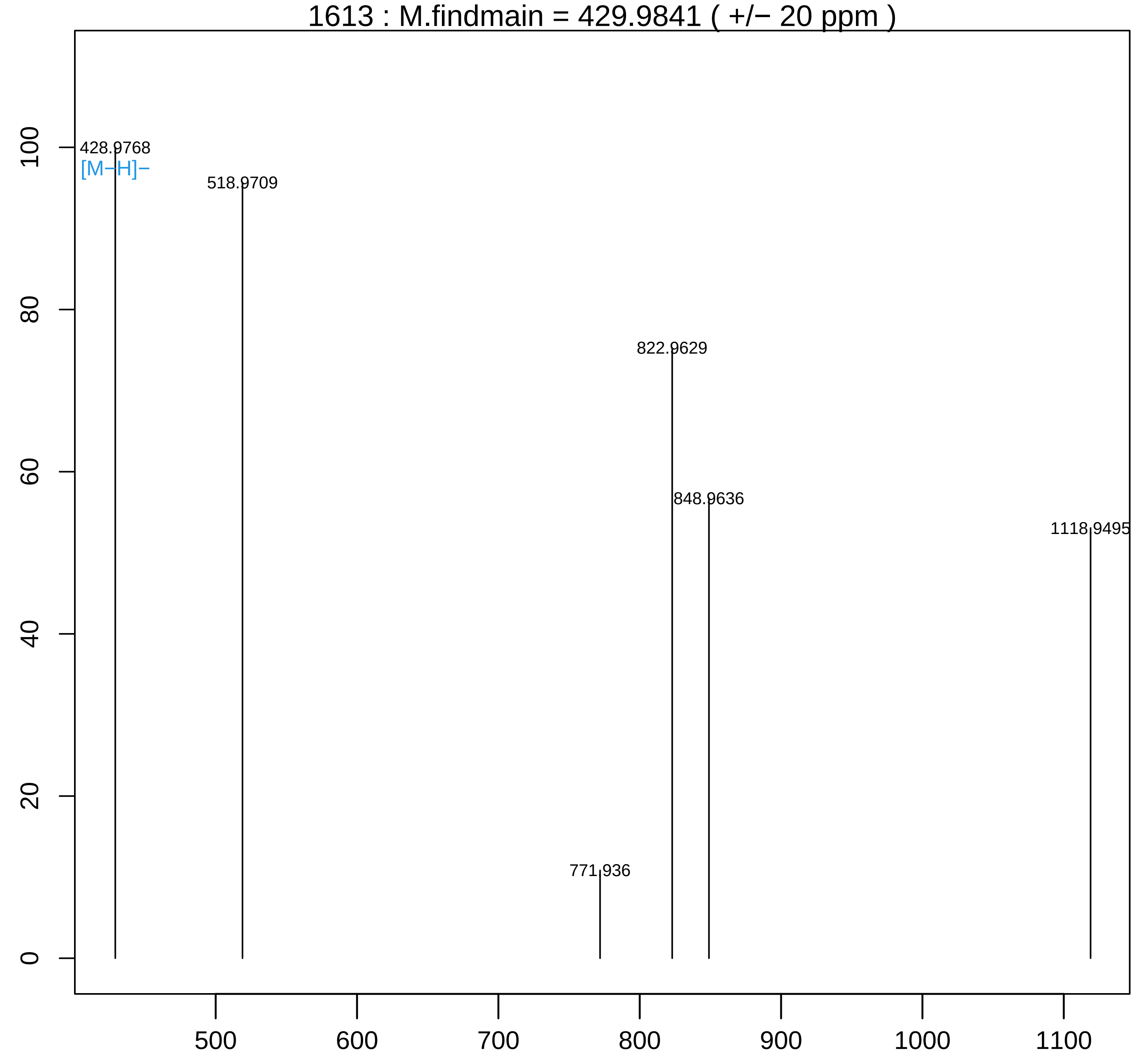
1613 : M.findmain = 429.9841 (+/− 20 ppm)
428.9768
[M−H]−
518.9709
822.9629
848.9636
1118.9495
771.936
0
20
40
60
80
100
500
600
700
800
900
1000
1100

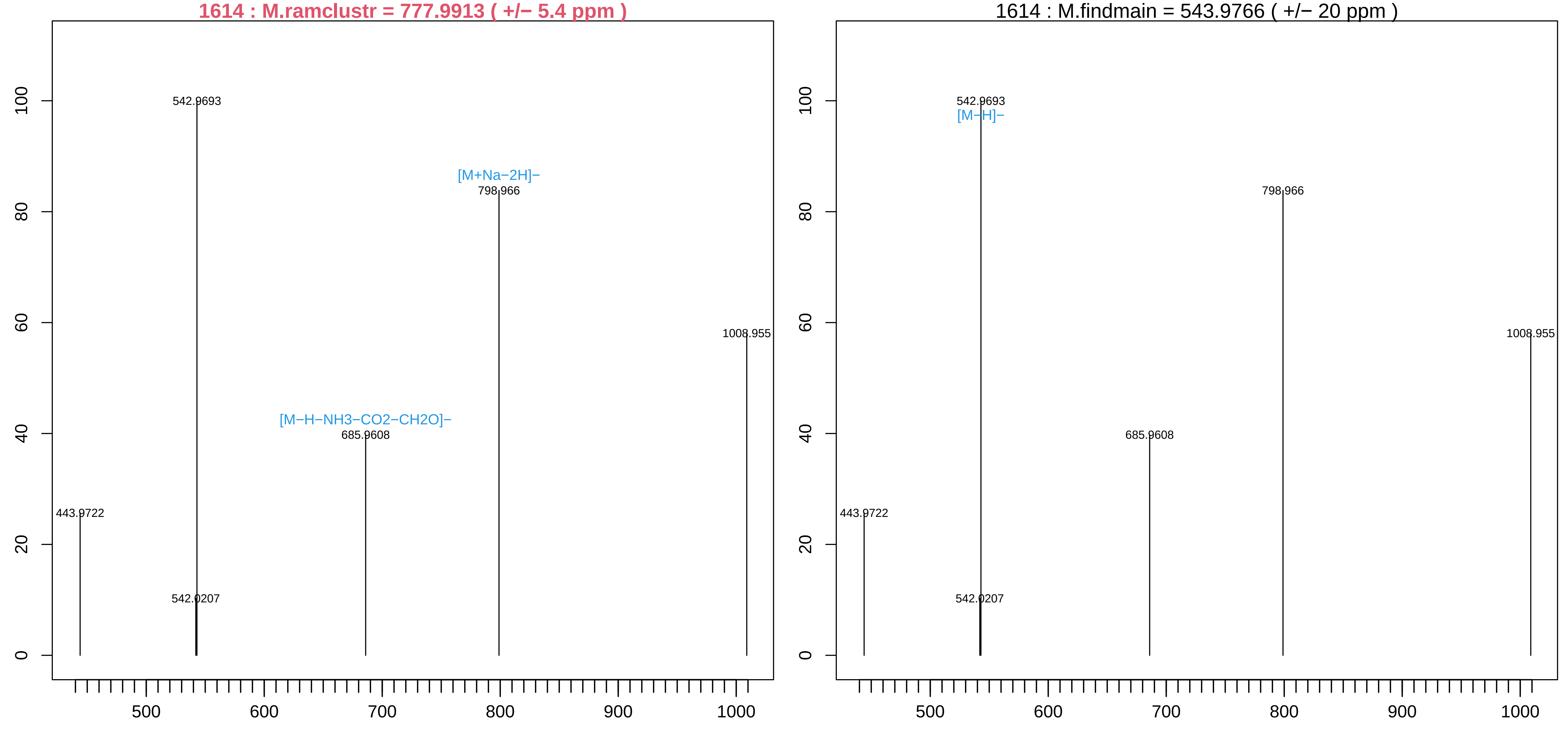
1614 : M.ramclustr = 777.9913 (+/− 5.4 ppm)
542.9693
[M+Na−2H]−
798.966
1008.955
[M−H−NH3−CO2−CH2O]−
685.9608
443.9722
542.0207
1614 : M.findmain = 543.9766 (+/− 20 ppm)
542.9693
[M−H]−
798.966
1008.955
685.9608
443.9722
542.0207
0
20
40
60
80
100
500
600
700
800
900
1000

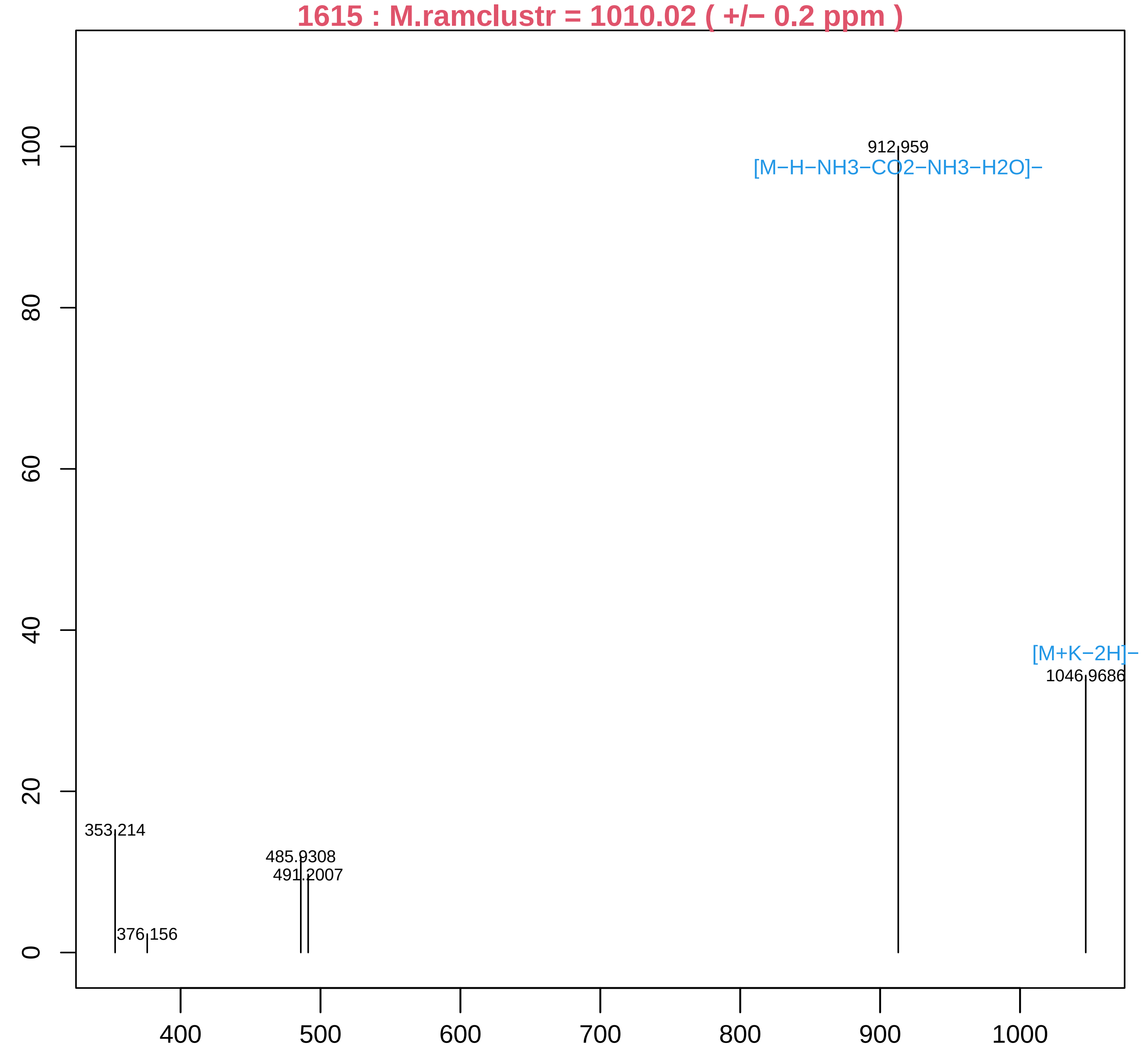

1615 : M.ramclustr = 1010.02 (+/− 0.2 ppm)
912.959
[M−H−NH3−CO2−NH3−H2O]−
[M+K−2H]−
1046.9686
353.214
485.9308
491.2007
376.156
0
20
40
60
80
100
400
500
600
700
800
900
1000

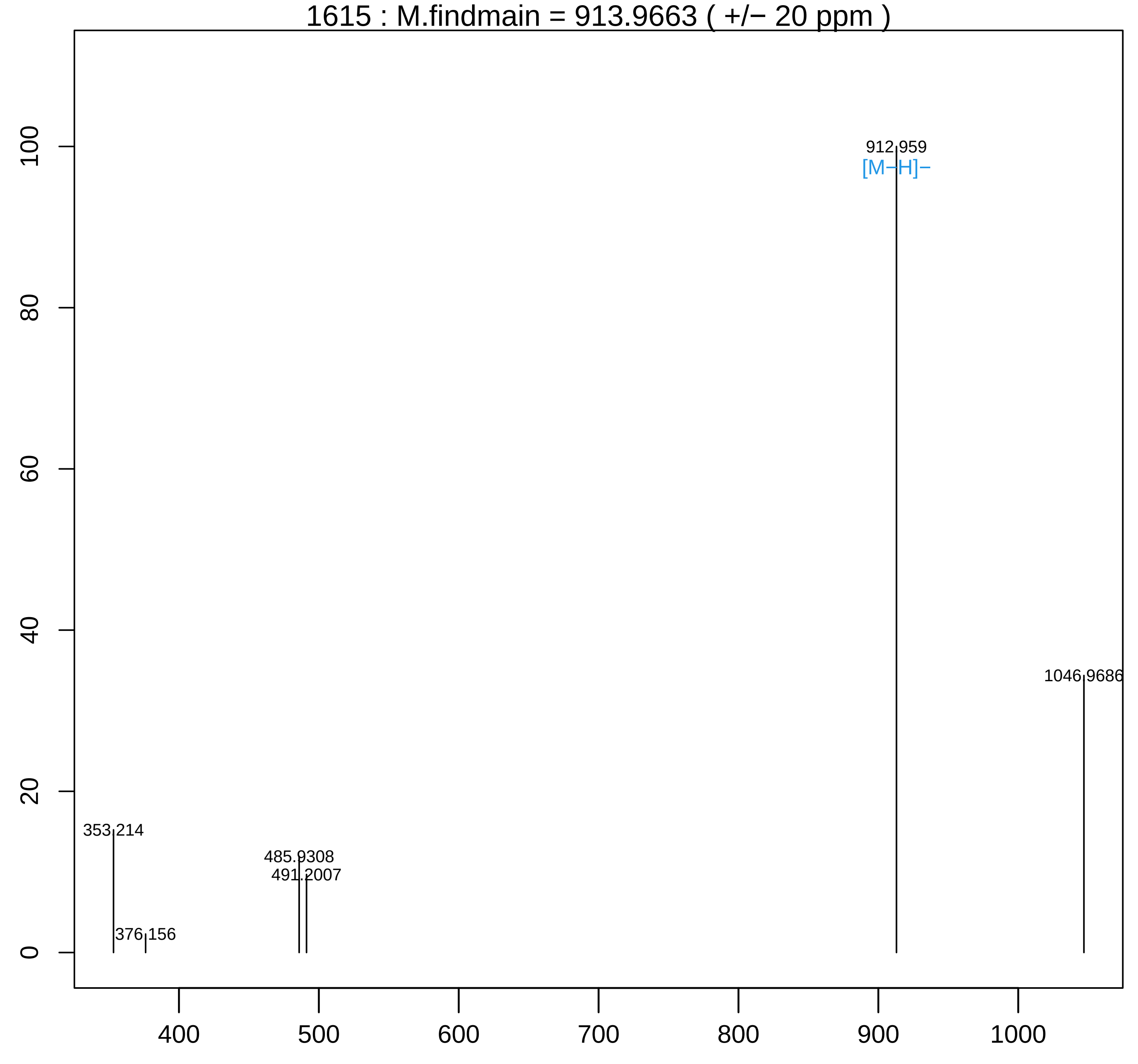

1615 : M.findmain = 913.9663 (+/− 20 ppm)
912.959
[M−H]−
1046.9686
353.214
485.9308
491.2007
376.156
0
20
40
60
80
100
400
500
600
700
800
900
1000

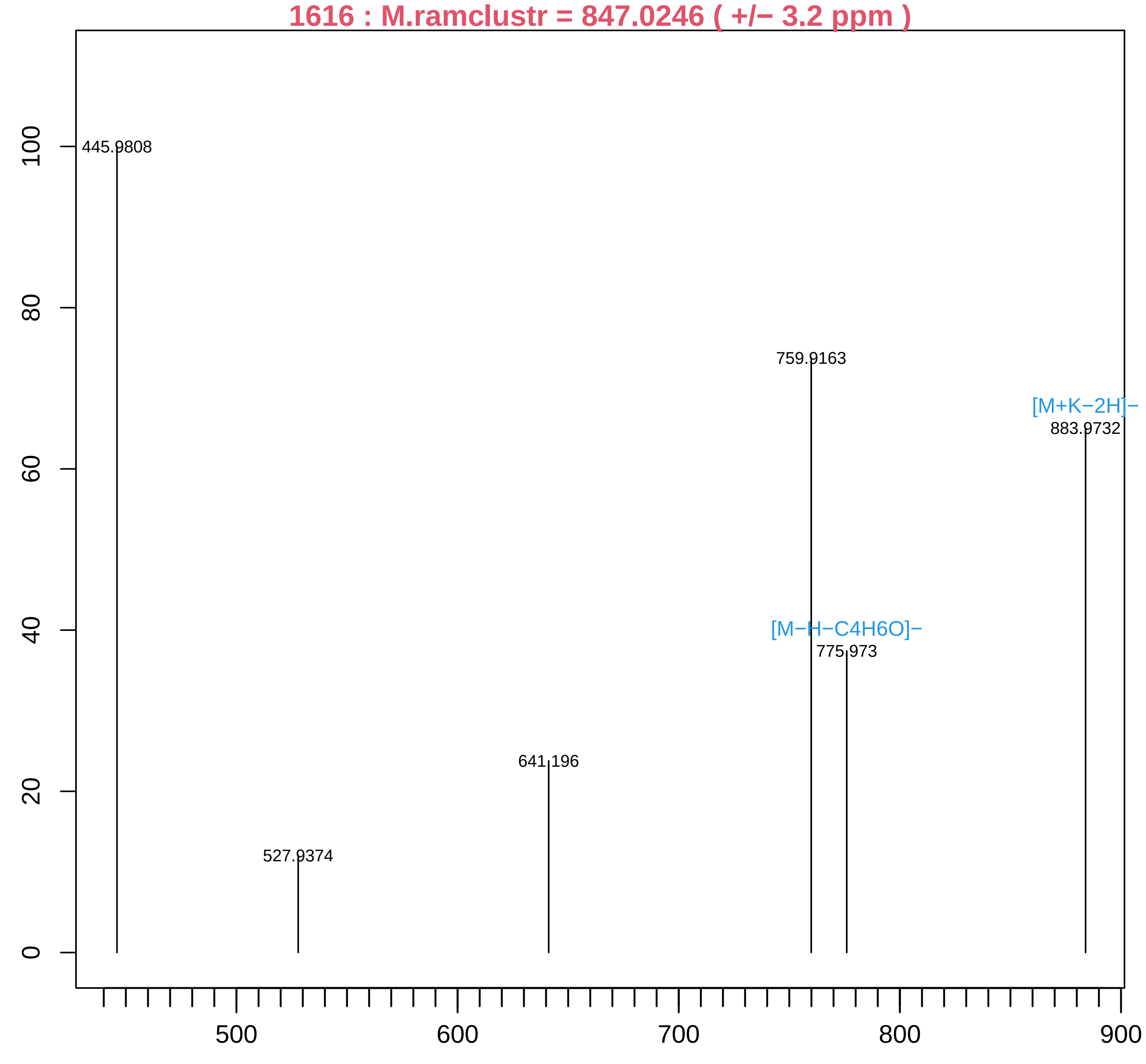

1616 : M.ramclustr = 847.0246 (+/− 3.2 ppm)
445.9808
527.9374
641.196
759.9163
[M−H−C4H6O]−
775.973
[M+K−2H]−
883.9732
0
20
40
60
80
100
500
600
700
800
900

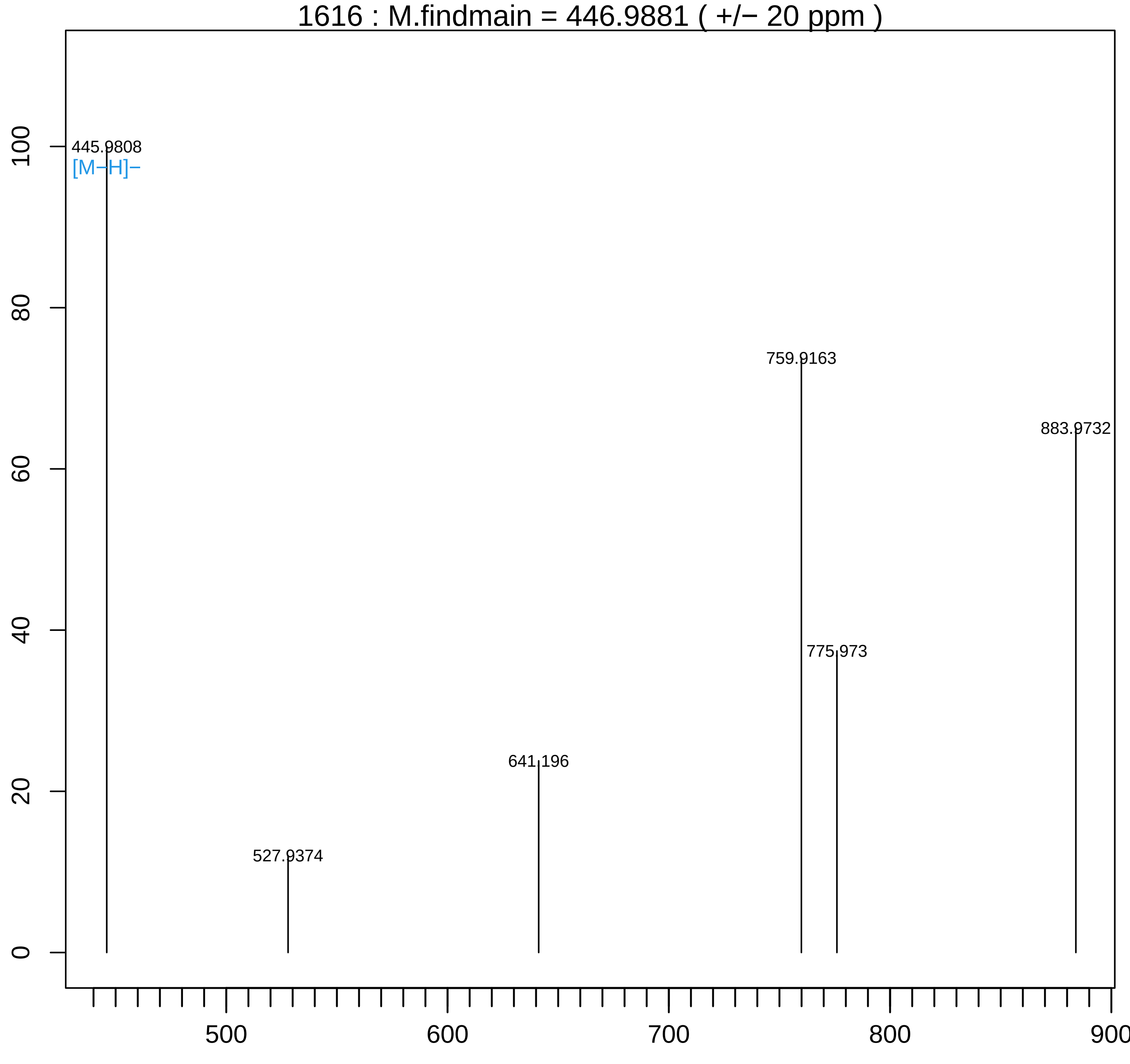

1616 : M.findmain = 446.9881 (+/− 20 ppm)
445.9808
[M−H]−
527.9374
641.196
759.9163
775.973
883.9732
0
20
40
60
80
100
500
600
700
800
900

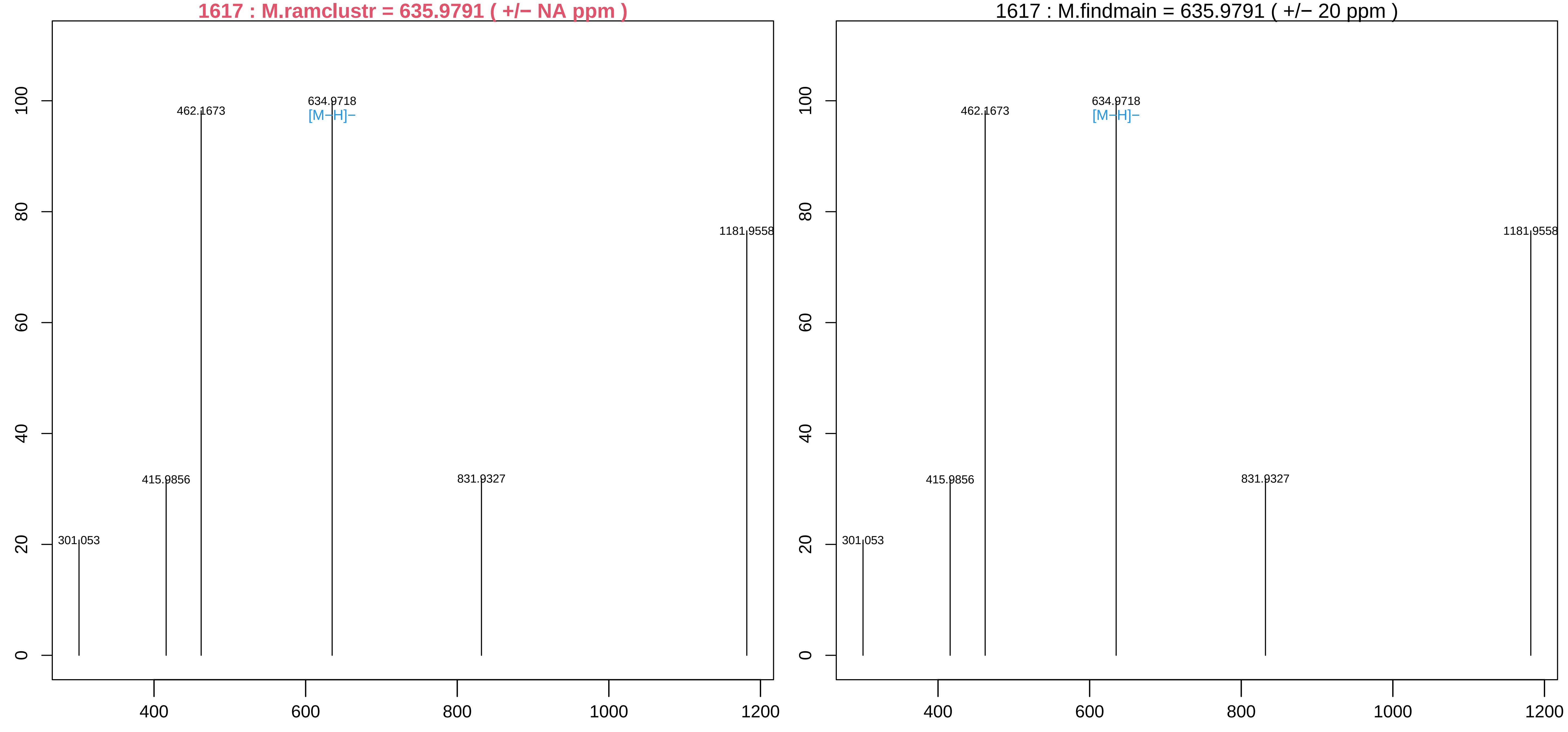
1617 : M.ramclustr = 635.9791 (+/− NA ppm)
1617 : M.findmain = 635.9791 (+/− 20 ppm)
301.053
415.9856
462.1673
634.9718
[M−H]−
831.9327
1181.9558
0
20
40
60
80
100
400
600
800
1000
1200

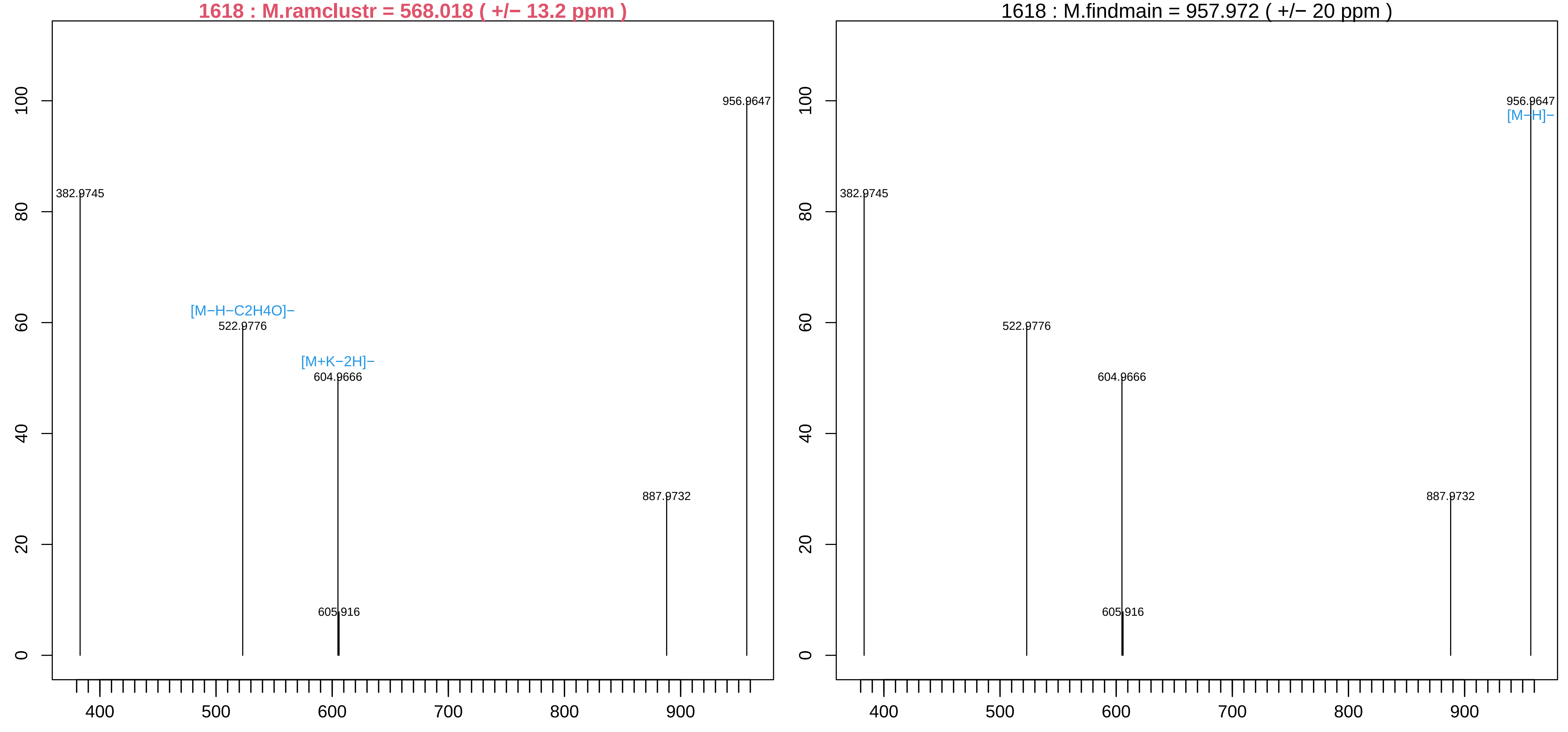
1618 : M.ramclustr = 568.018 (+/− 13.2 ppm)
956.9647
382.9745
[M−H−C2H4O]−
522.9776
[M+K−2H]−
604.9666
887.9732
605.916
0
20
40
60
80
100
400
500
600
700
800
900
1618 : M.findmain = 957.972 (+/− 20 ppm)
956.9647
[M−H]−
382.9745
522.9776
604.9666
887.9732
605.916
0
20
40
60
80
100
400
500
600
700
800
900

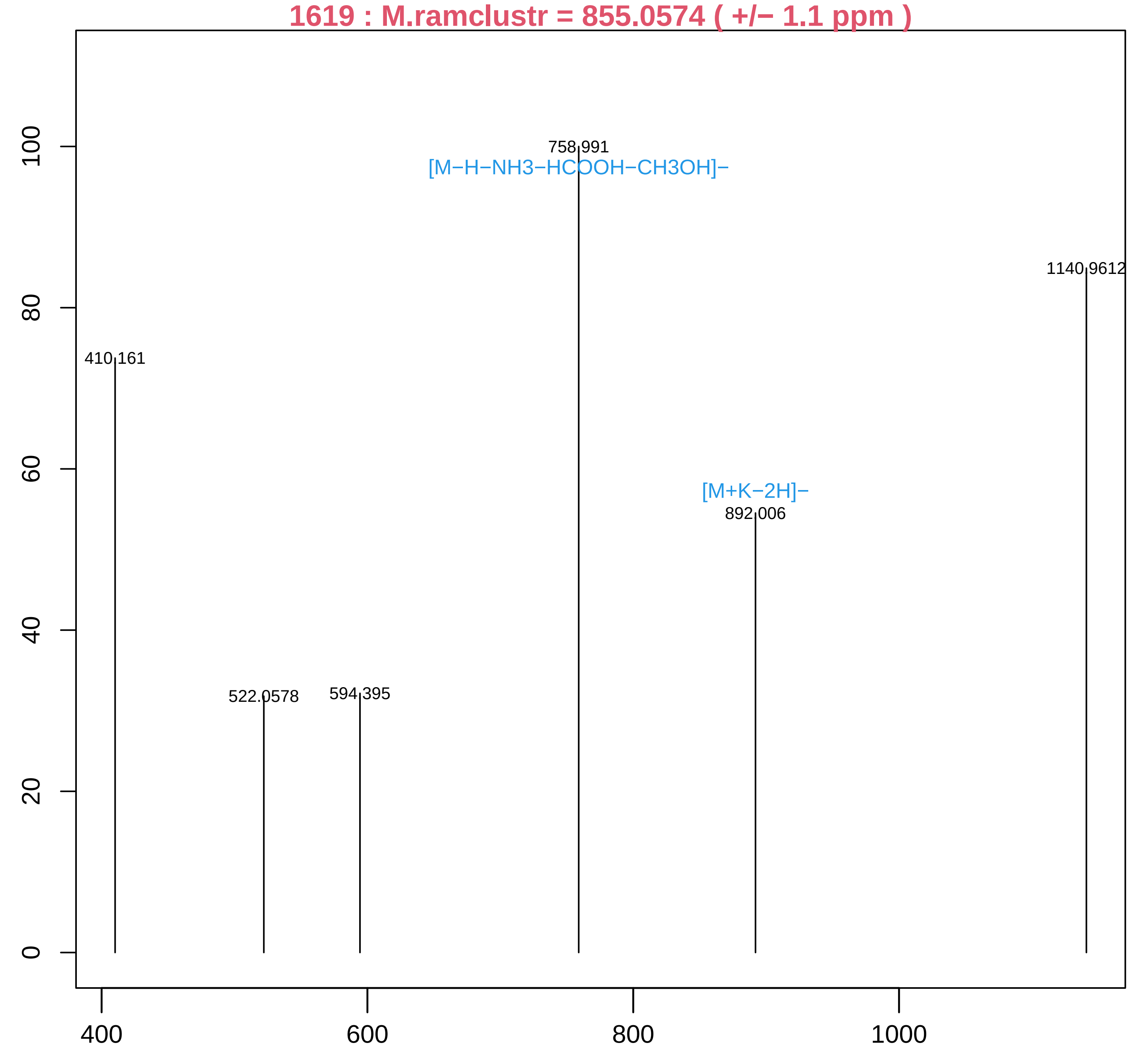
1619 : M.ramclustr = 855.0574 (+/− 1.1 ppm)
758.991
[M−H−NH3−HCOOH−CH3OH]−
1140.9612
410.161
[M+K−2H]−
892.006
522.0578
594.395
0
20
40
60
80
100
400
600
800
1000

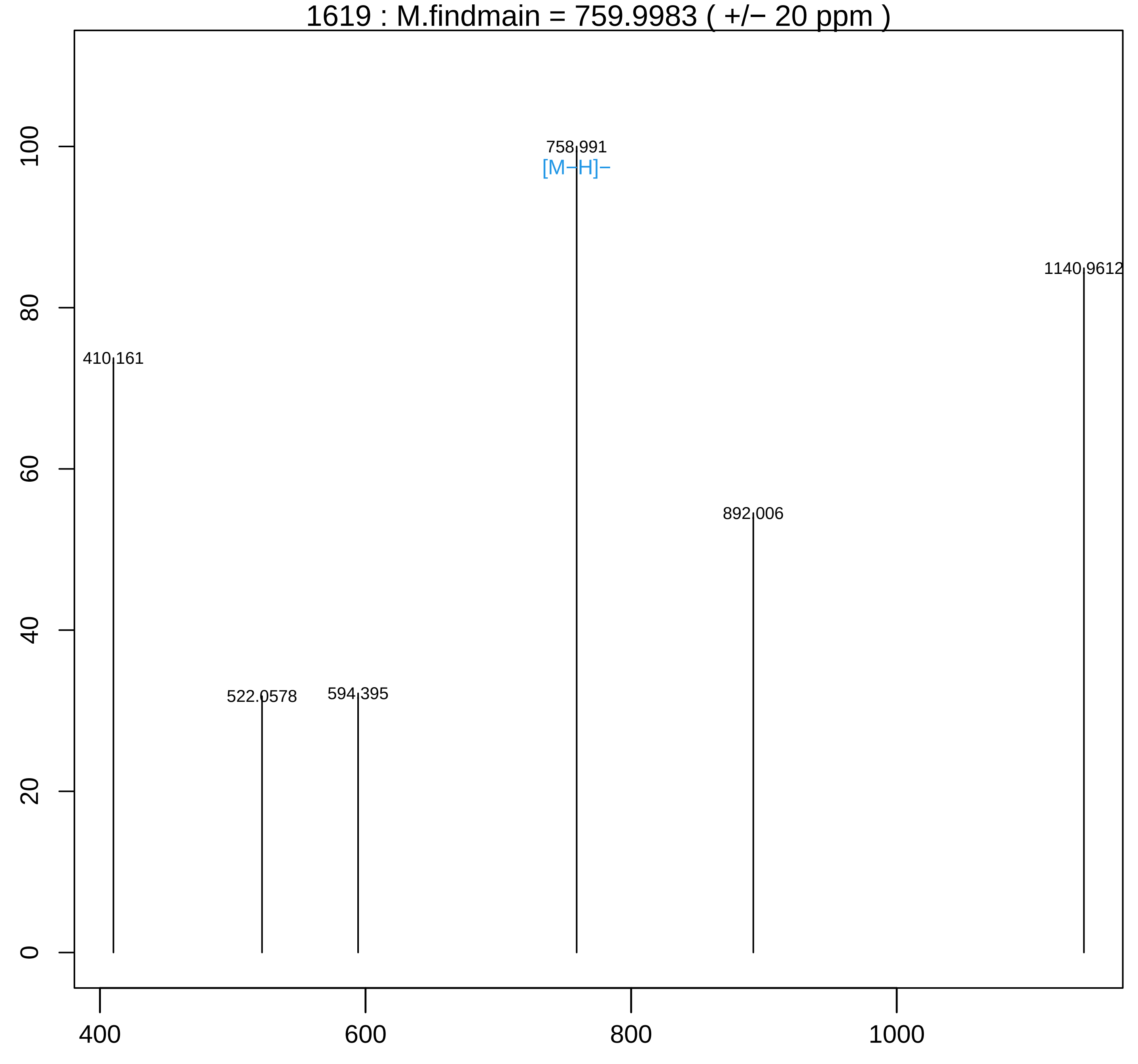
1619 : M.findmain = 759.9983 (+/− 20 ppm)
758.991
[M−H]−
1140.9612
410.161
892.006
522.0578
594.395
0
20
40
60
80
100
400
600
800
1000

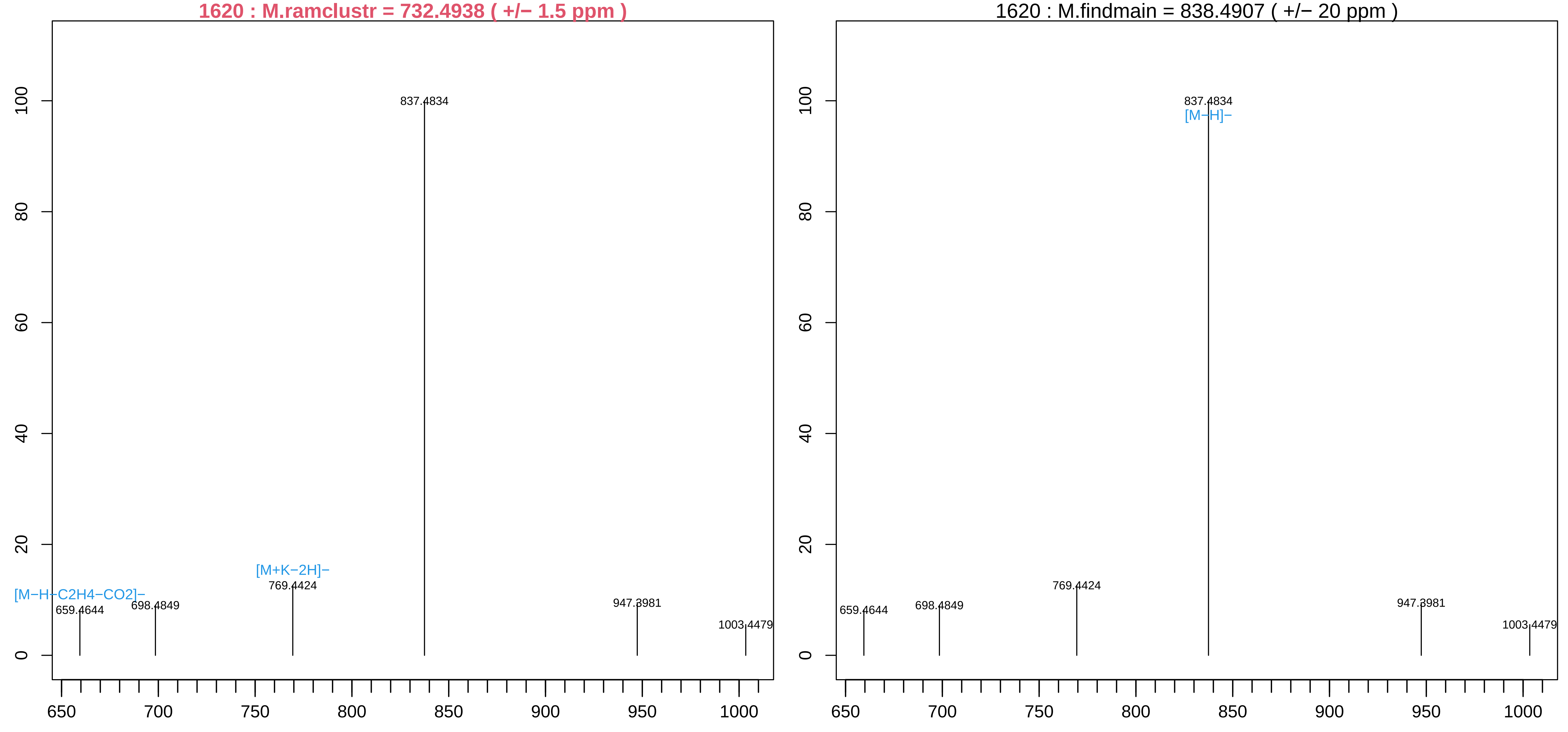
1620 : M.ramclustr = 732.4938 (+/− 1.5 ppm)
837.4834
[M+K−2H]−
769.4424
[M−H−C2H4−CO2]−
659.4644
698.4849
947.3981
1003.4479
0
20
40
60
80
100
650
700
750
800
850
900
950
1000
1620 : M.findmain = 838.4907 (+/− 20 ppm)
837.4834
[M−H]−
659.4644
698.4849
769.4424
947.3981
1003.4479

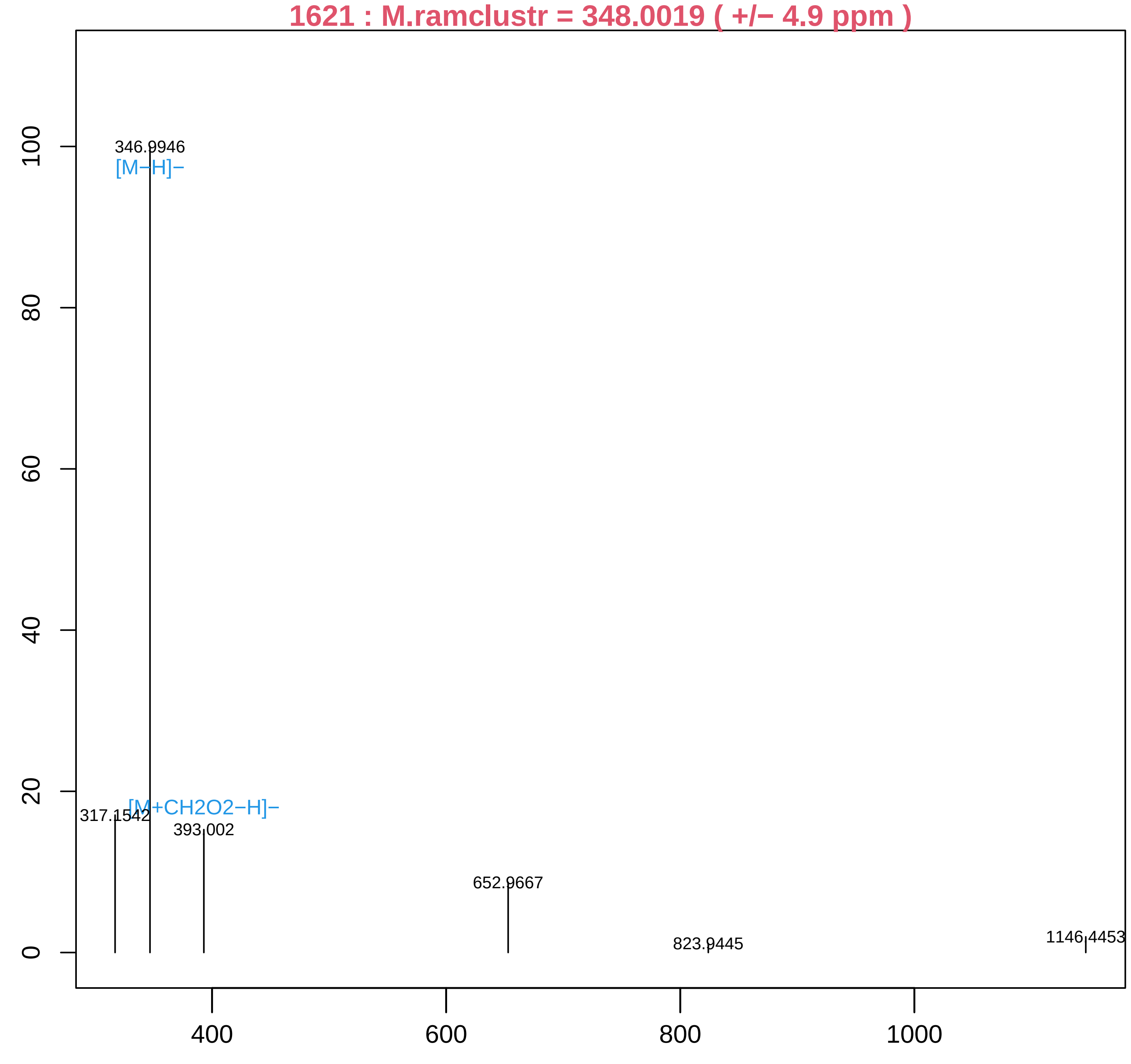
1621 : M.ramclustr = 348.0019 (+/− 4.9 ppm)
346.9946
[M−H]−
317.1542
[M+CH2O2−H]−
393.002
652.9667
823.9445
1146.4453
0
20
40
60
80
100
400
600
800
1000

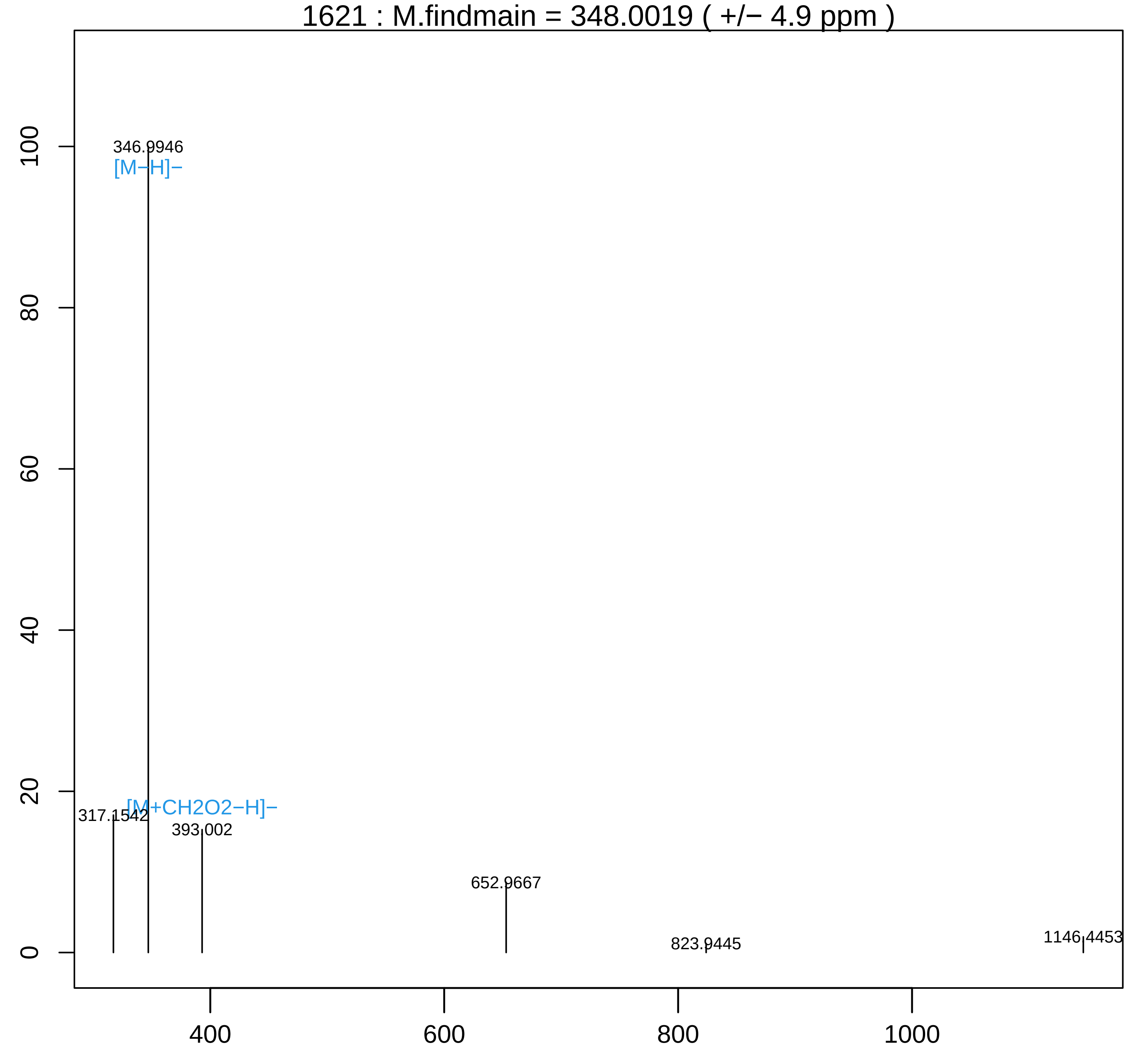
1621 : M.findmain = 348.0019 (+/− 4.9 ppm)
346.9946
[M−H]−
317.1542
[M+CH2O2−H]−
393.002
652.9667
823.9445
1146.4453
0
20
40
60
80
100
400
600
800
1000

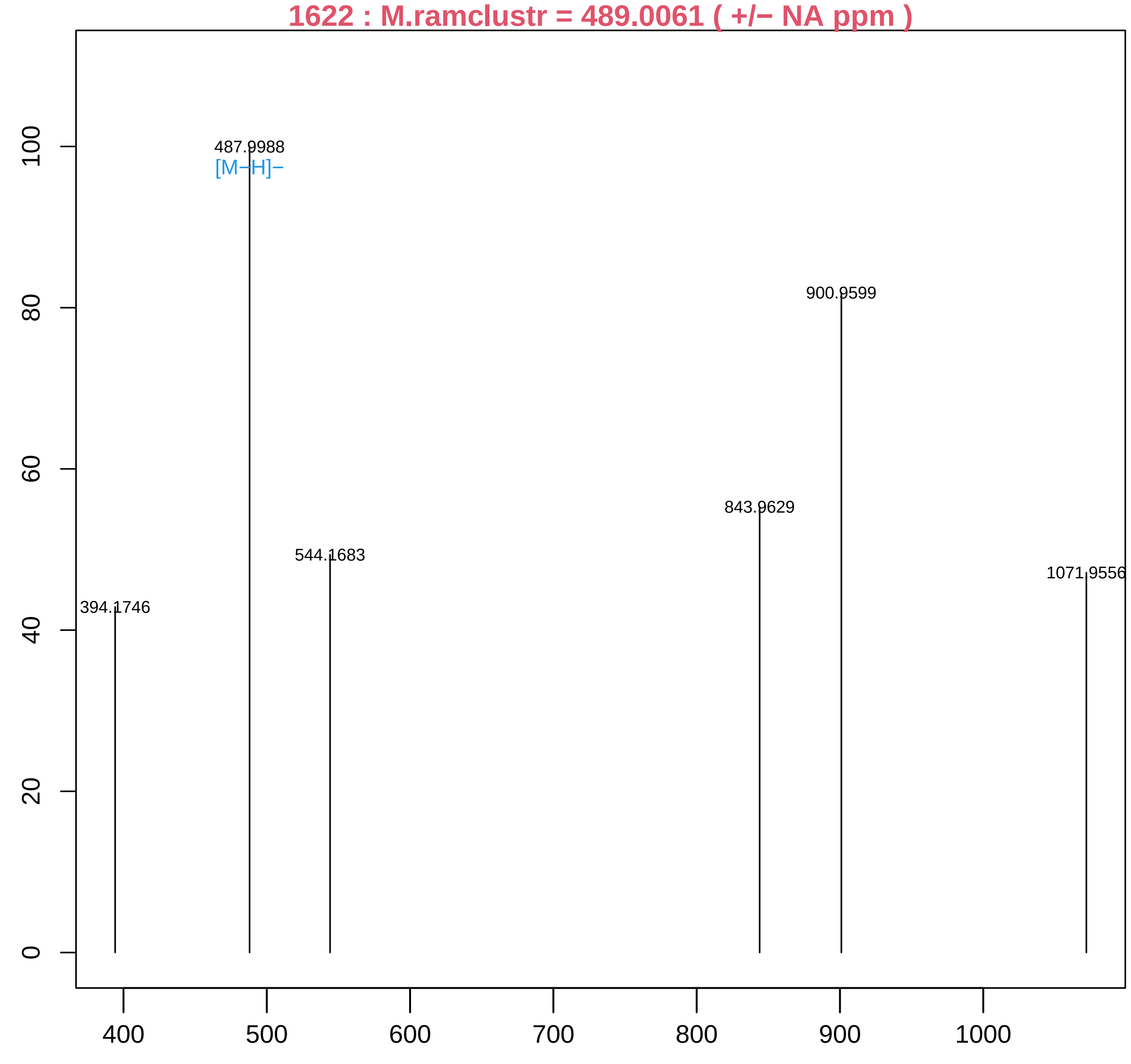

1622 : M.ramclustr = 489.0061 (+/− NA ppm)
487.9988
[M−H]−
900.9599
843.9629
544.1683
1071.9556
394.1746
0
20
40
60
80
100
400
500
600
700
800
900
1000

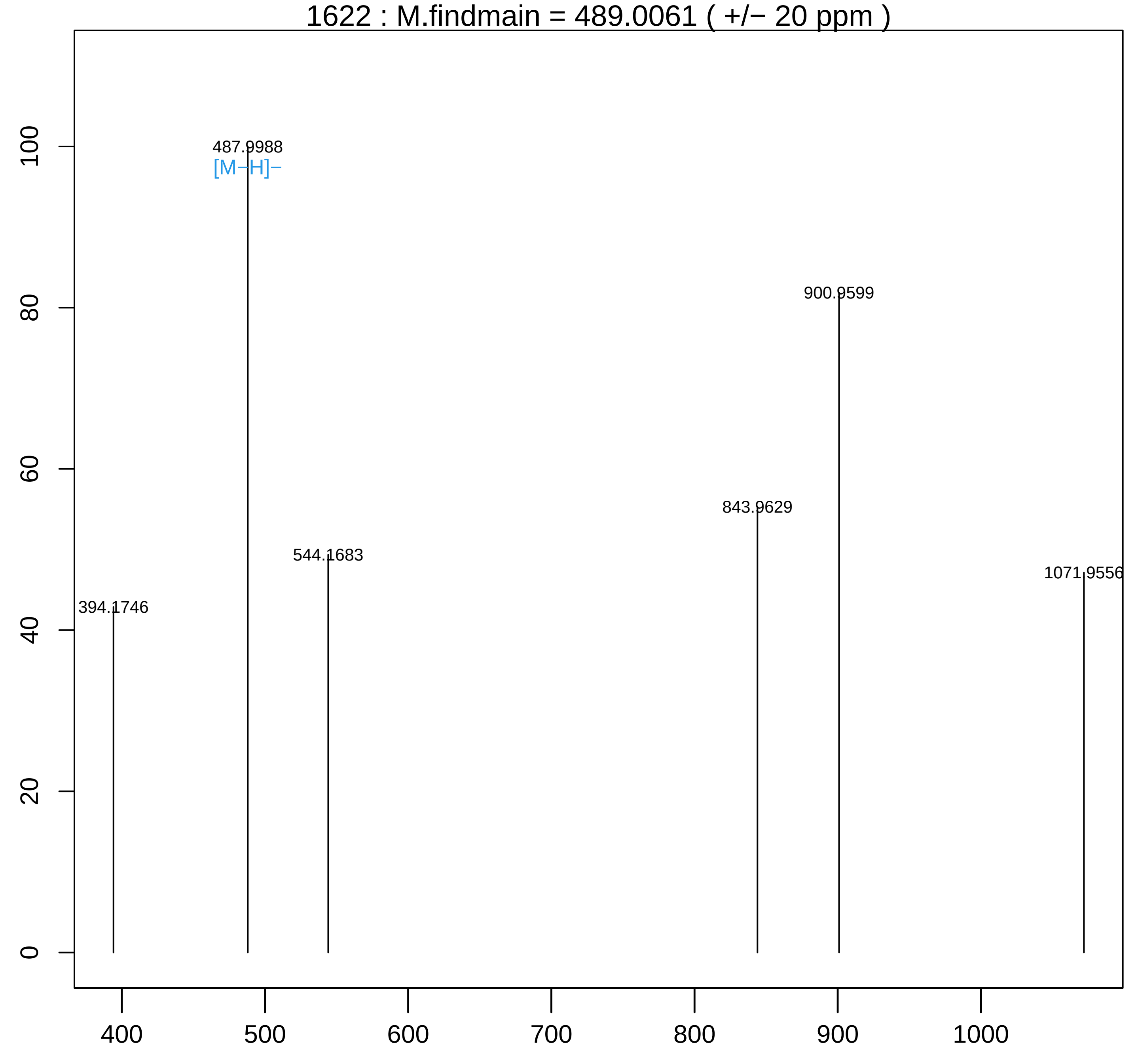

1622 : M.findmain = 489.0061 (+/− 20 ppm)
487.9988
[M−H]−
900.9599
843.9629
544.1683
1071.9556
394.1746
0
20
40
60
80
100
400
500
600
700
800
900
1000

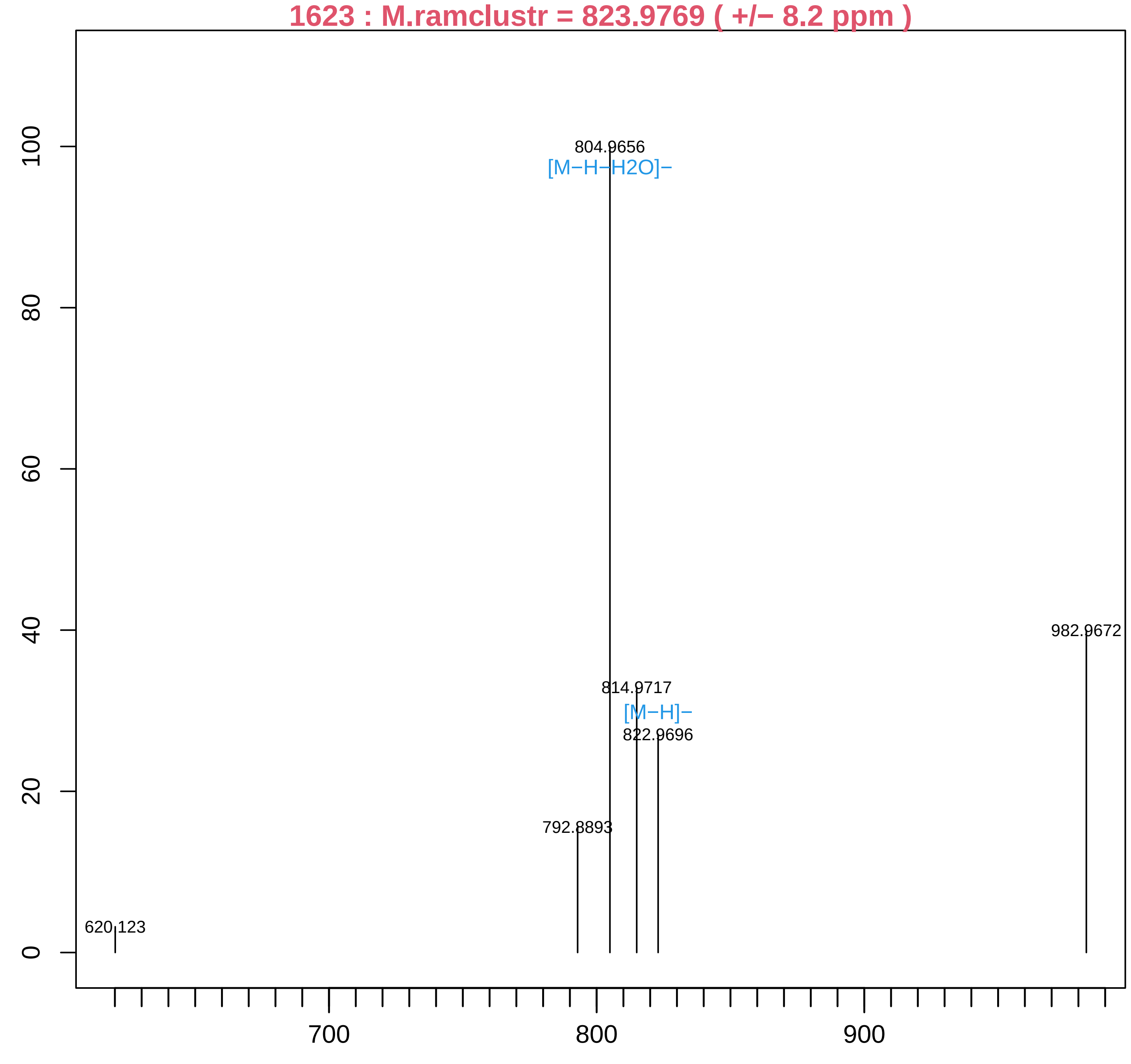

1623 : M.ramclustr = 823.9769 (+/− 8.2 ppm)
804.9656
[M−H−H2O]−
982.9672
814.9717
[M−H]−
822.9696
792.8893
620.123
0
20
40
60
80
100
700
800
900

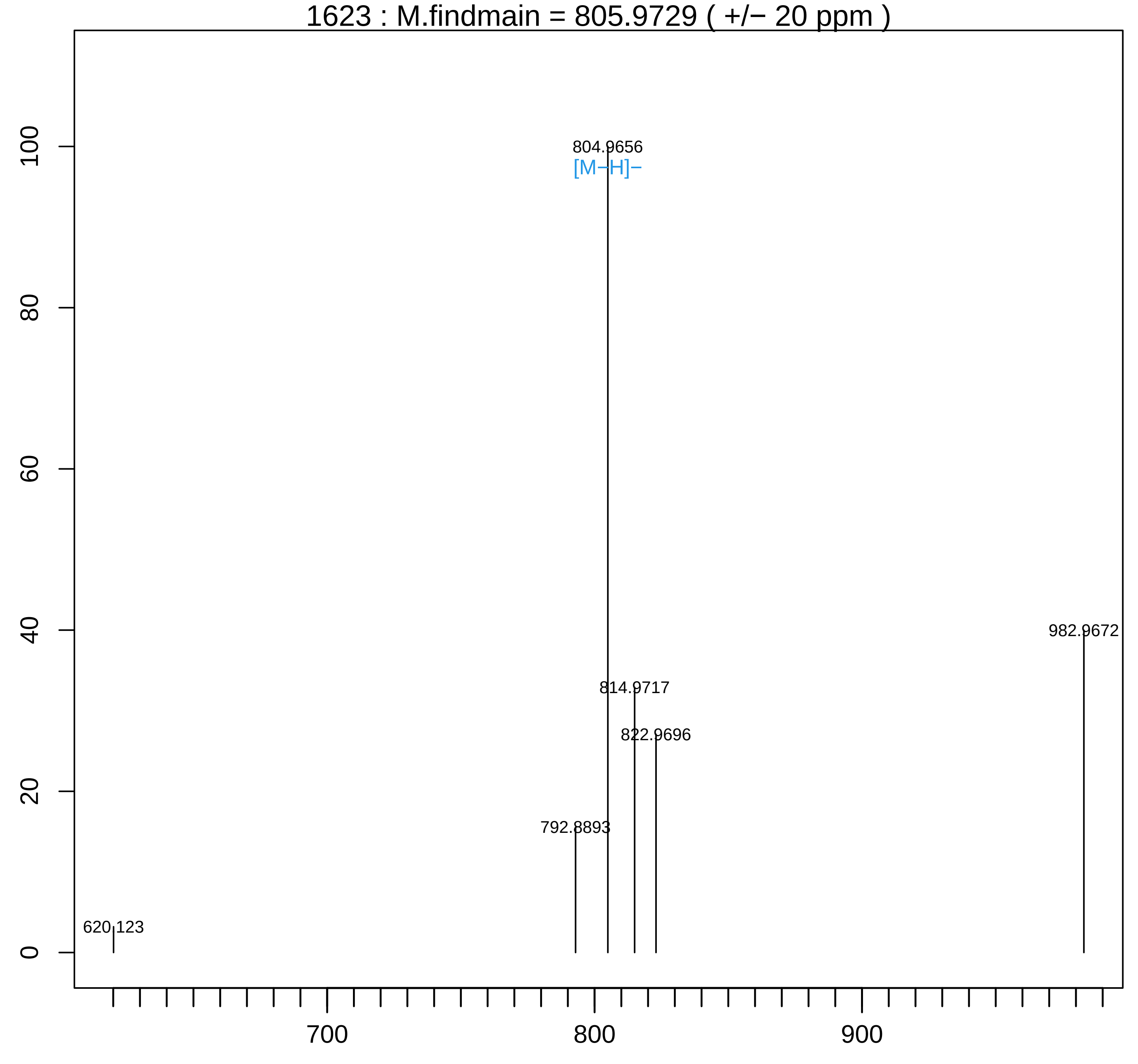

1623 : M.findmain = 805.9729 (+/− 20 ppm)
804.9656
[M−H]−
982.9672
814.9717
822.9696
792.8893
620.123
0
20
40
60
80
100
700
800
900

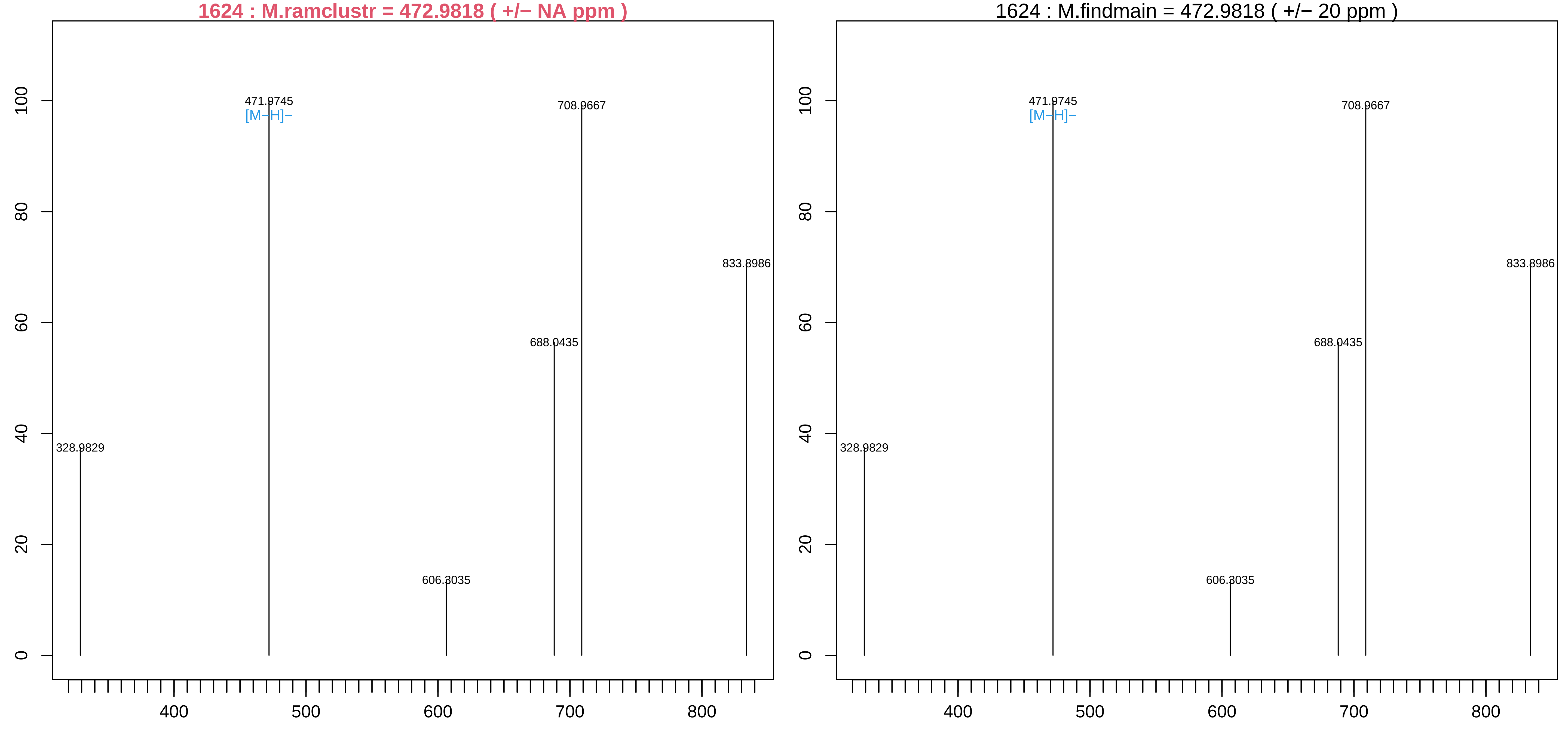
1624 : M.ramclustr = 472.9818 (+/− NA ppm)
471.9745
[M−H]−
708.9667
833.8986
688.0435
328.9829
606.3035
1624 : M.findmain = 472.9818 (+/− 20 ppm)
471.9745
[M−H]−
708.9667
833.8986
688.0435
328.9829
606.3035
0
20
40
60
80
100
400
500
600
700
800

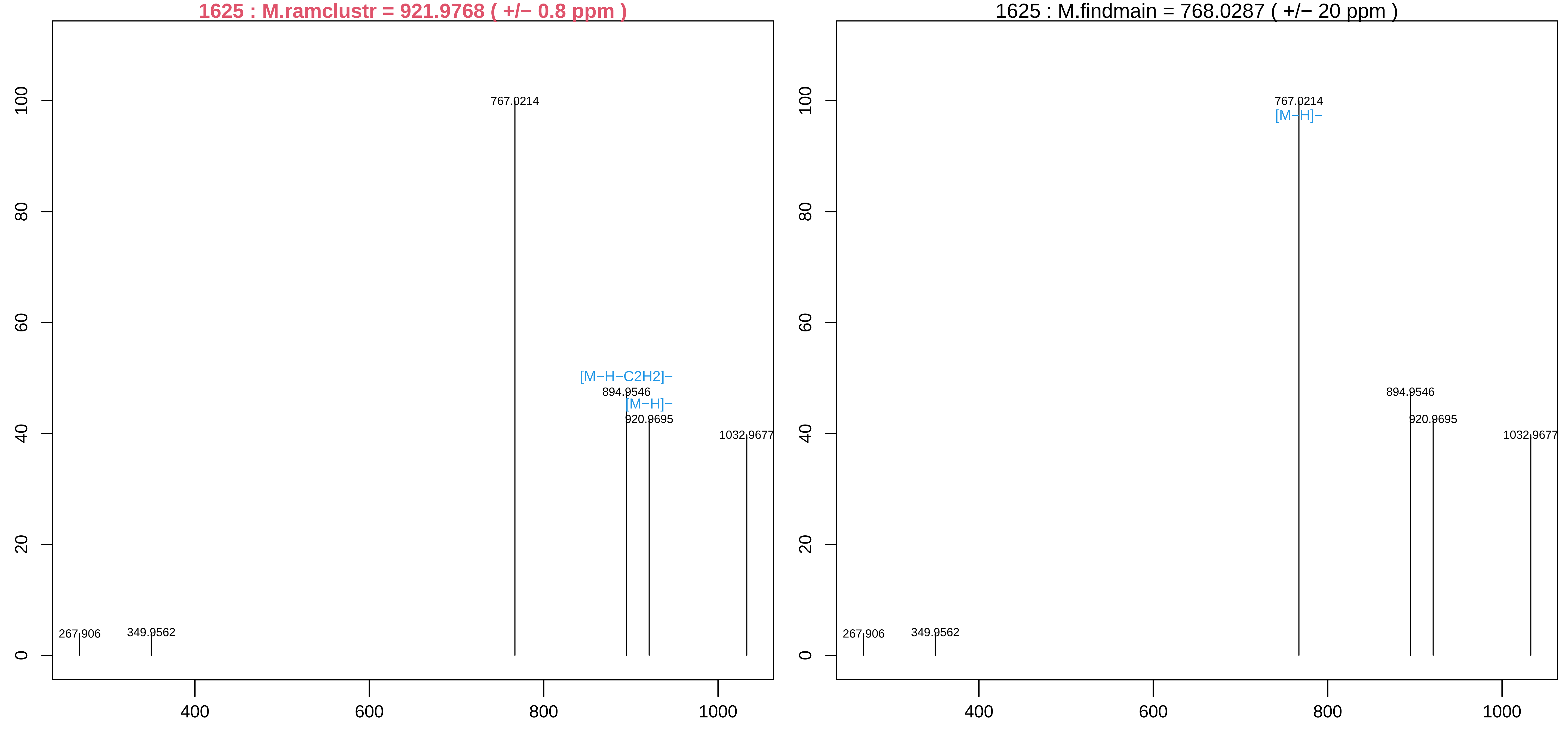

1625 : M.ramclustr = 921.9768 (+/− 0.8 ppm)
767.0214
[M−H−C2H2]−
894.9546
[M−H]−
920.9695
1032.9677
267.906
349.9562
1625 : M.findmain = 768.0287 (+/− 20 ppm)
767.0214
[M−H]−
894.9546
920.9695
1032.9677
267.906
349.9562

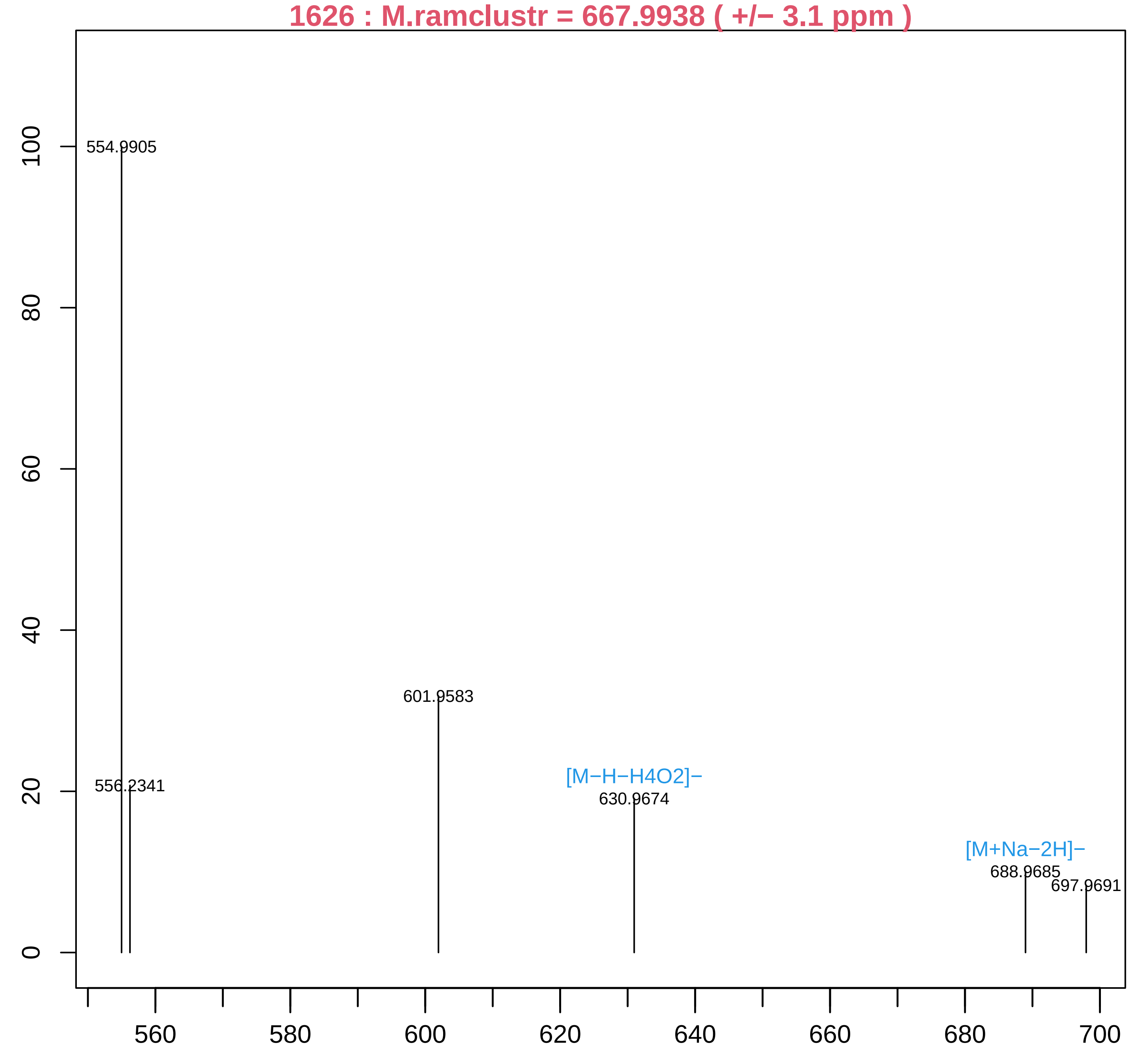
1626 : M.ramclustr = 667.9938 (+/− 3.1 ppm)
554.9905
556.2341
601.9583
[M−H−H4O2]−
630.9674
[M+Na−2H]−
688.9685
697.9691
0
20
40
60
80
100
560
580
600
620
640
660
680
700

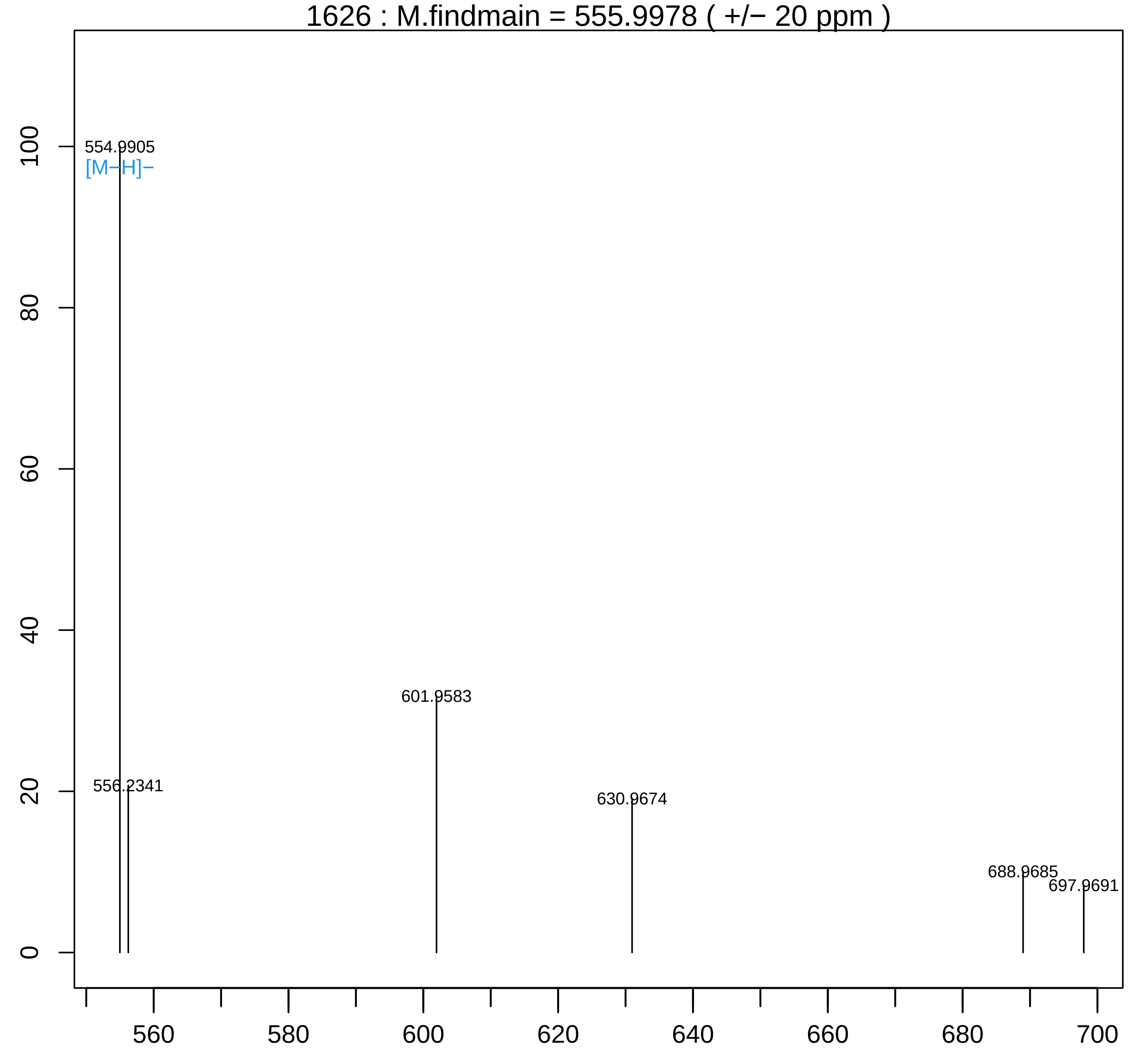
1626 : M.findmain = 555.9978 (+/− 20 ppm)
554.9905
[M−H]−
556.2341
601.9583
630.9674
688.9685
697.9691
0
20
40
60
80
100
560
580
600
620
640
660
680
700

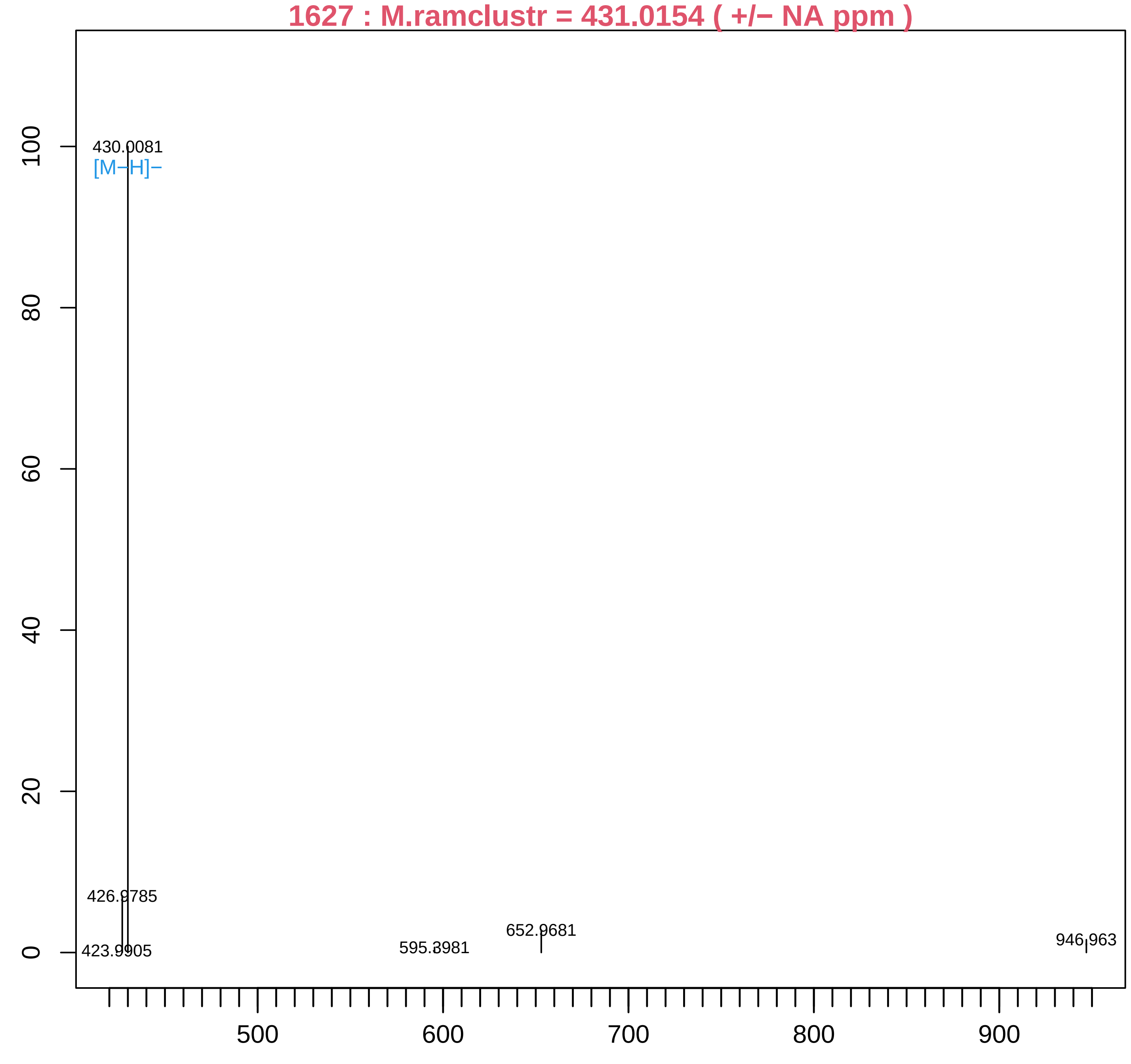
1627 : M.ramclustr = 431.0154 (+/− NA ppm)
430.0081
[M−H]−
426.9785
423.9905
595.8981
652.9681
946.963
0
20
40
60
80
100
500
600
700
800
900

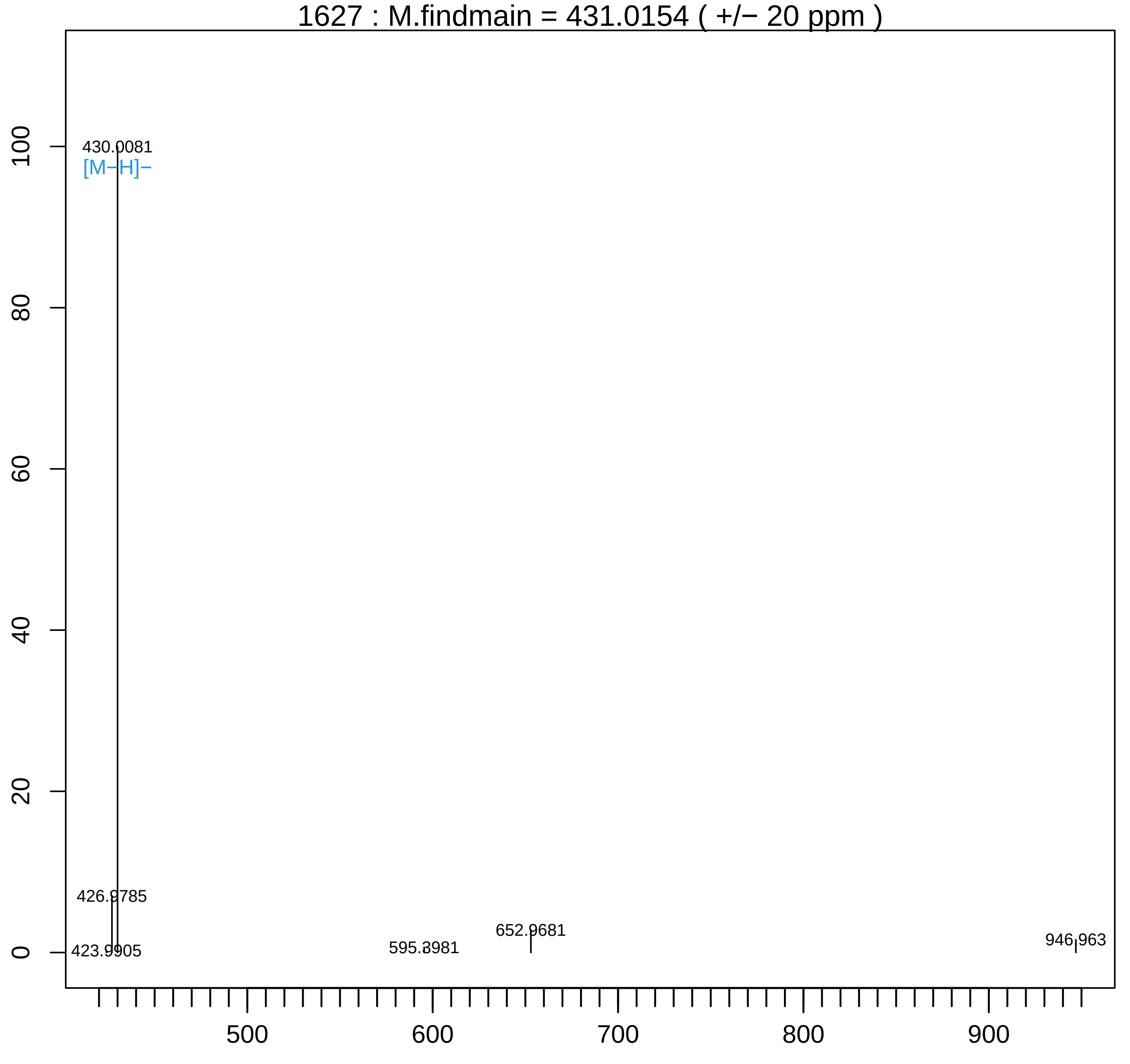
1627 : M.findmain = 431.0154 (+/− 20 ppm)
430.0081
[M−H]−
426.9785
423.9905
595.8981
652.9681
946.963
0
20
40
60
80
100
500
600
700
800
900

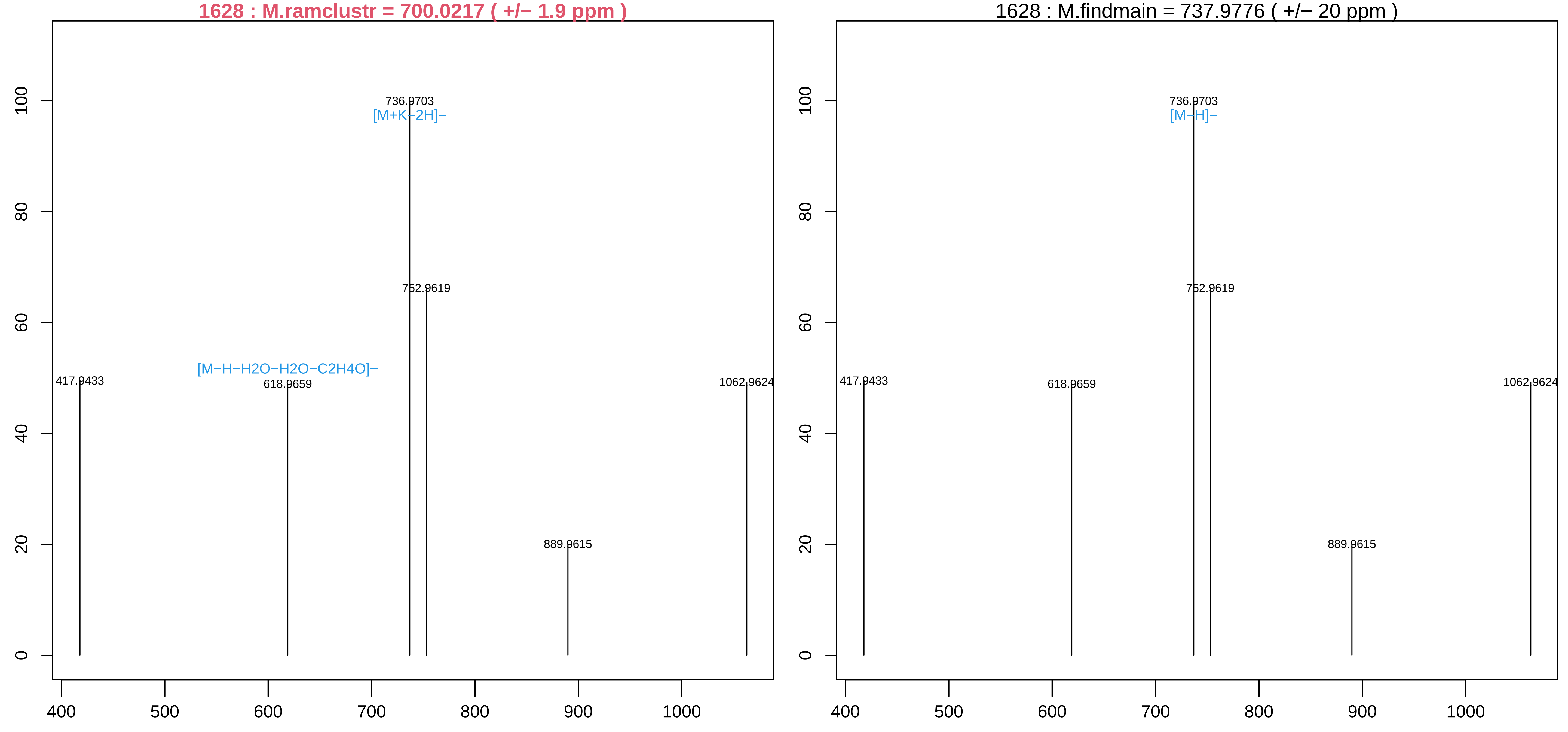
1628 : M.ramclustr = 700.0217 (+/− 1.9 ppm)
736.9703
[M+K−2H]−
752.9619
[M−H−H2O−H2O−C2H4O]−
417.9433
618.9659
1062.9624
889.9615
1628 : M.findmain = 737.9776 (+/− 20 ppm)
736.9703
[M−H]−
752.9619
417.9433
618.9659
1062.9624
889.9615
0
20
40
60
80
100
400
500
600
700
800
900
1000

1629 : M.ramclustr = 745.9759 (+/− NA ppm)

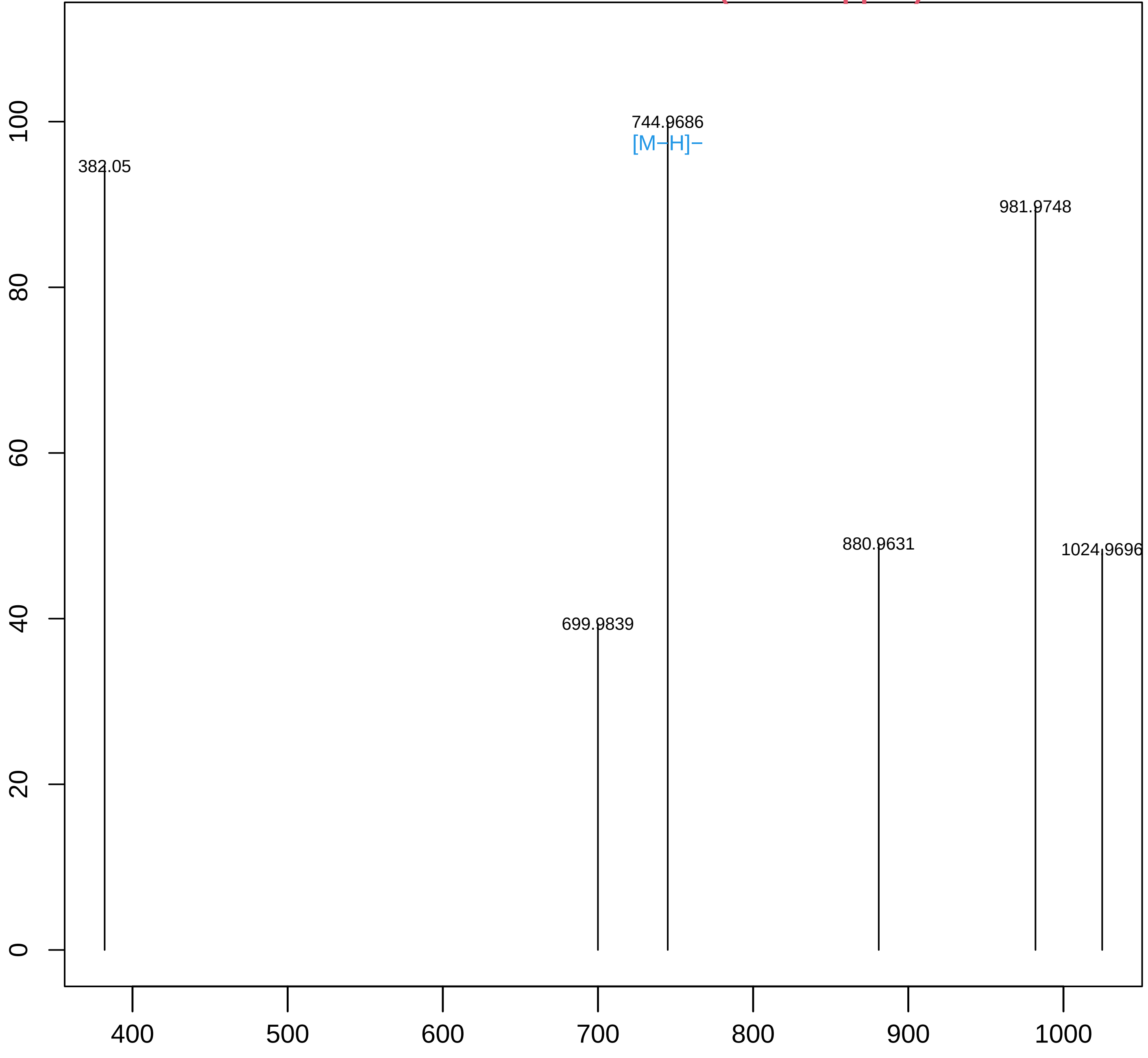

1629 : M.findmain = 745.9759 (+/− 20 ppm)

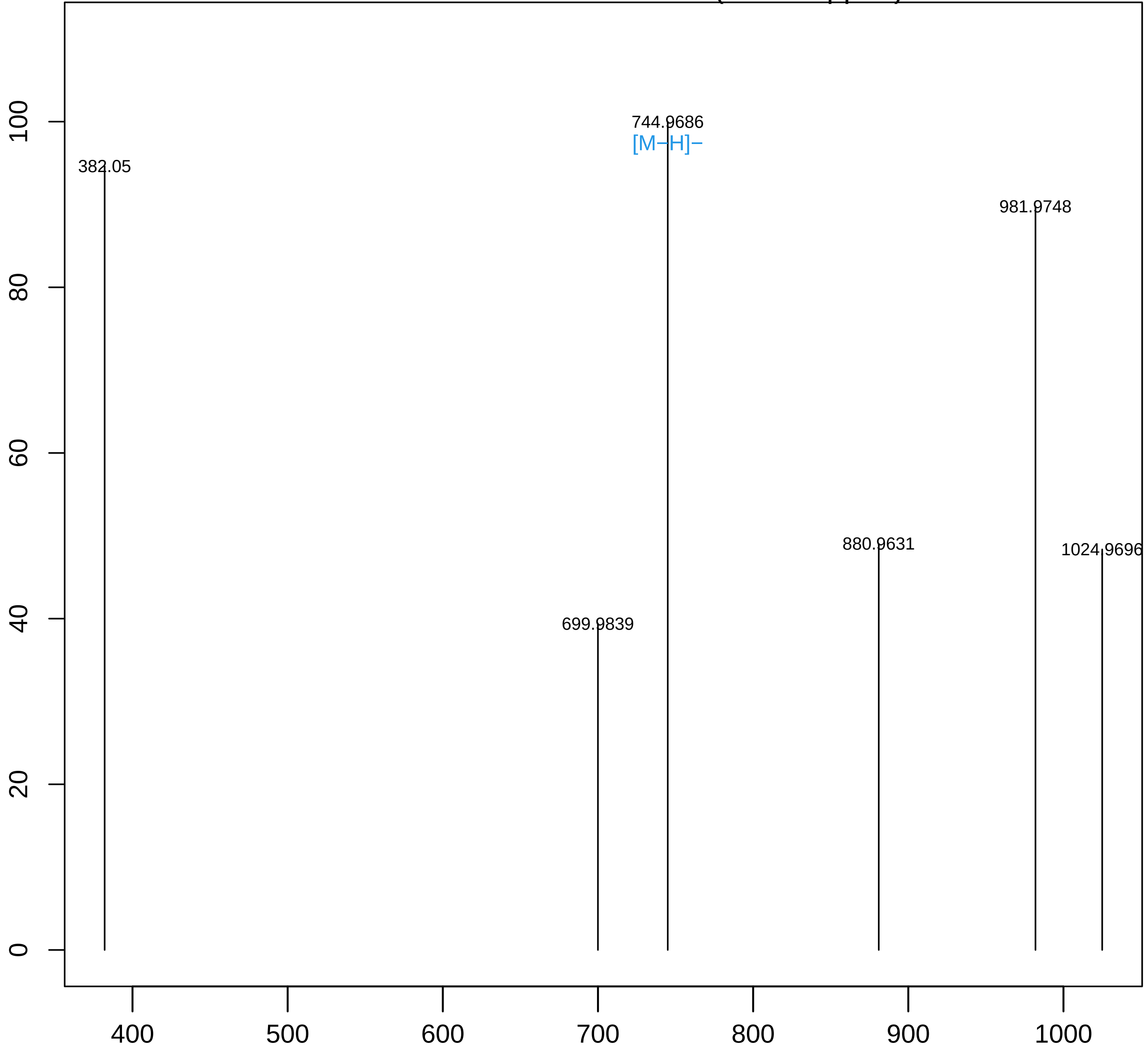

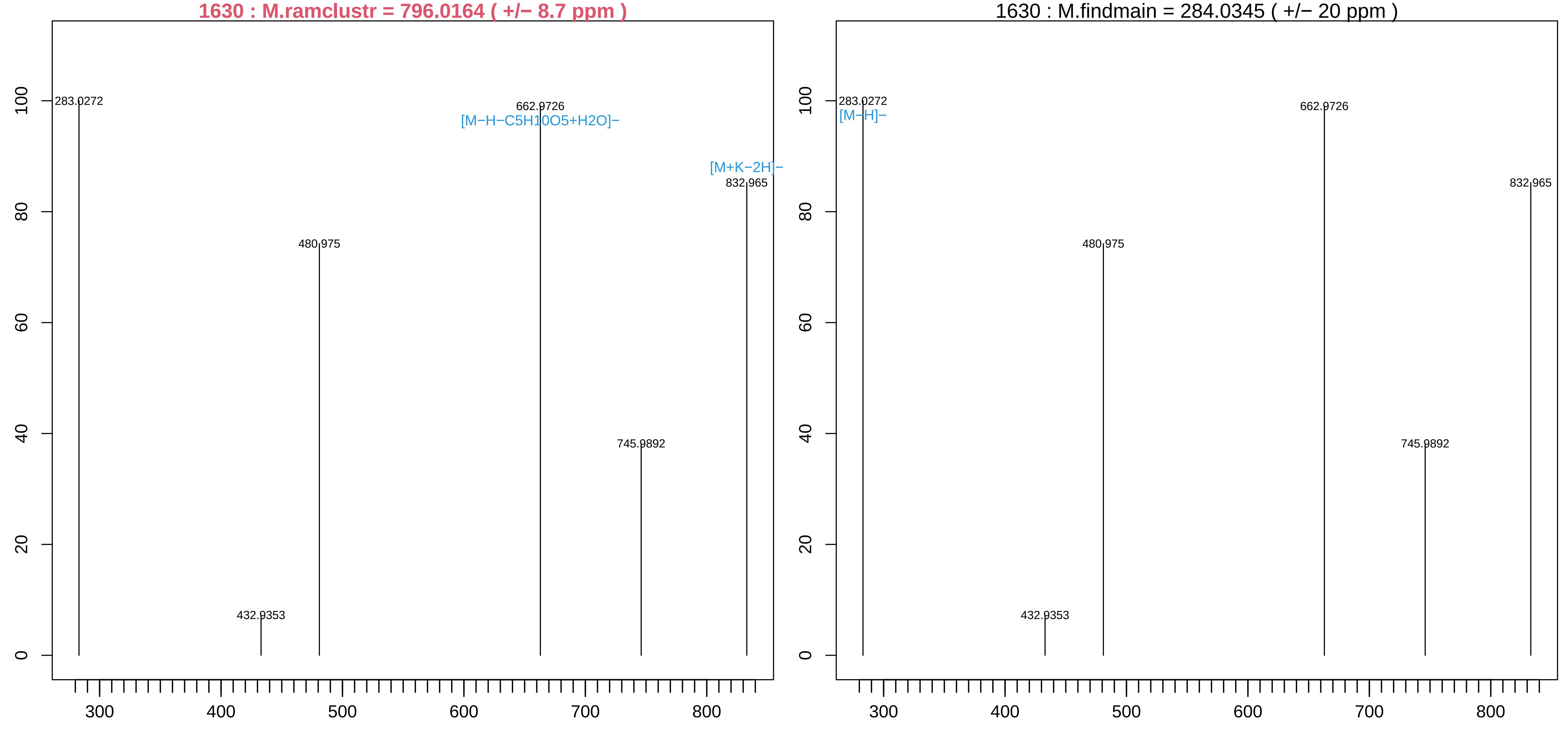
1630 : M.ramclustr = 796.0164 (+/− 8.7 ppm)
283.0272
432.9353
480.975
662.9726
[M−H−C5H10O5+H2O]−
745.9892
[M+K−2H]−
832.965
1630 : M.findmain = 284.0345 (+/− 20 ppm)
283.0272
[M−H]−
432.9353
480.975
662.9726
745.9892
832.965
0
20
40
60
80
100
300
400
500
600
700
800

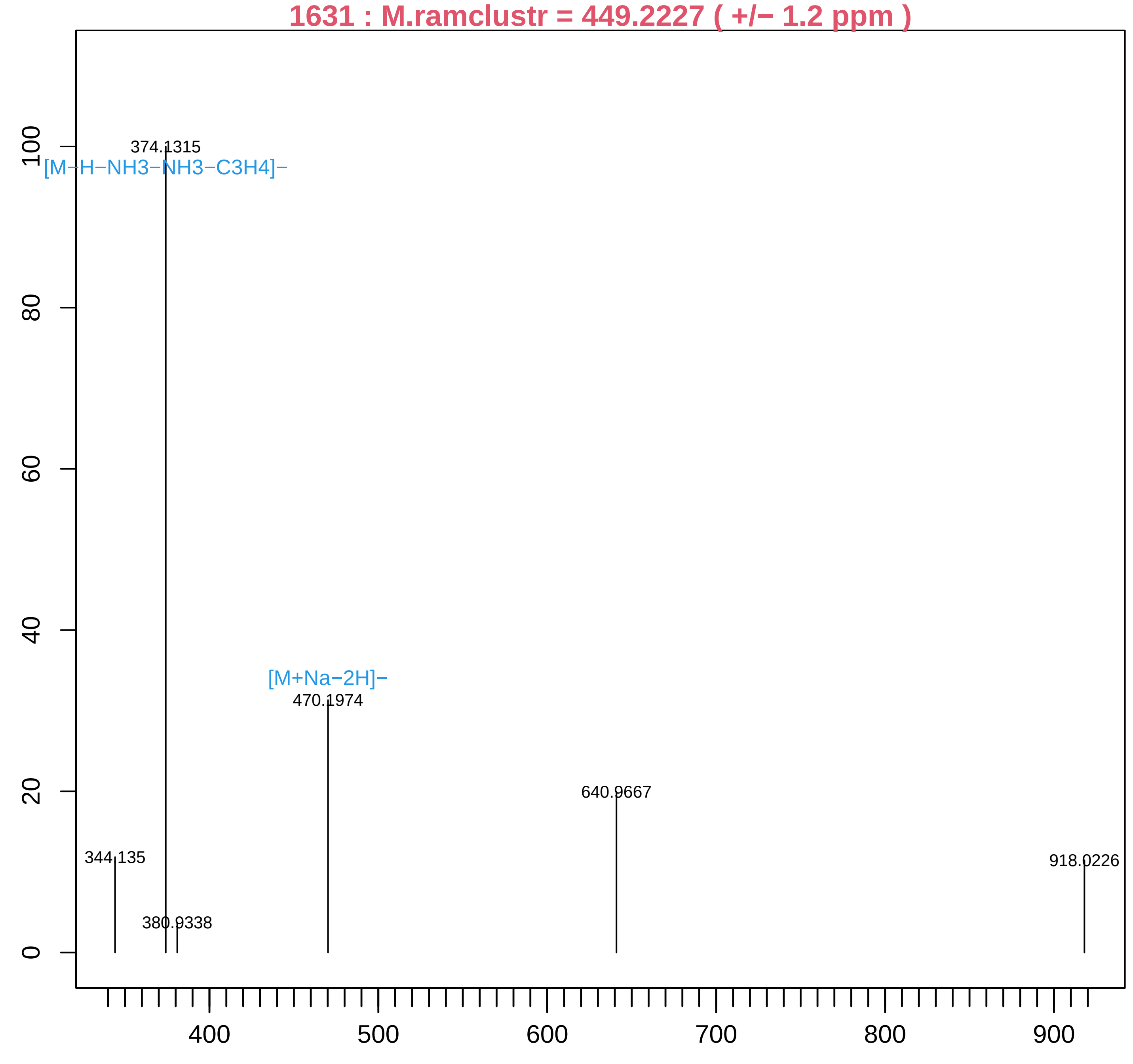
1631 : M.ramclustr = 449.2227 (+/− 1.2 ppm)
374.1315
[M−H−NH3−NH3−C3H4]−
[M+Na−2H]−
470.1974
640.9667
344.135
918.0226
380.9338
0
20
40
60
80
100
400
500
600
700
800
900

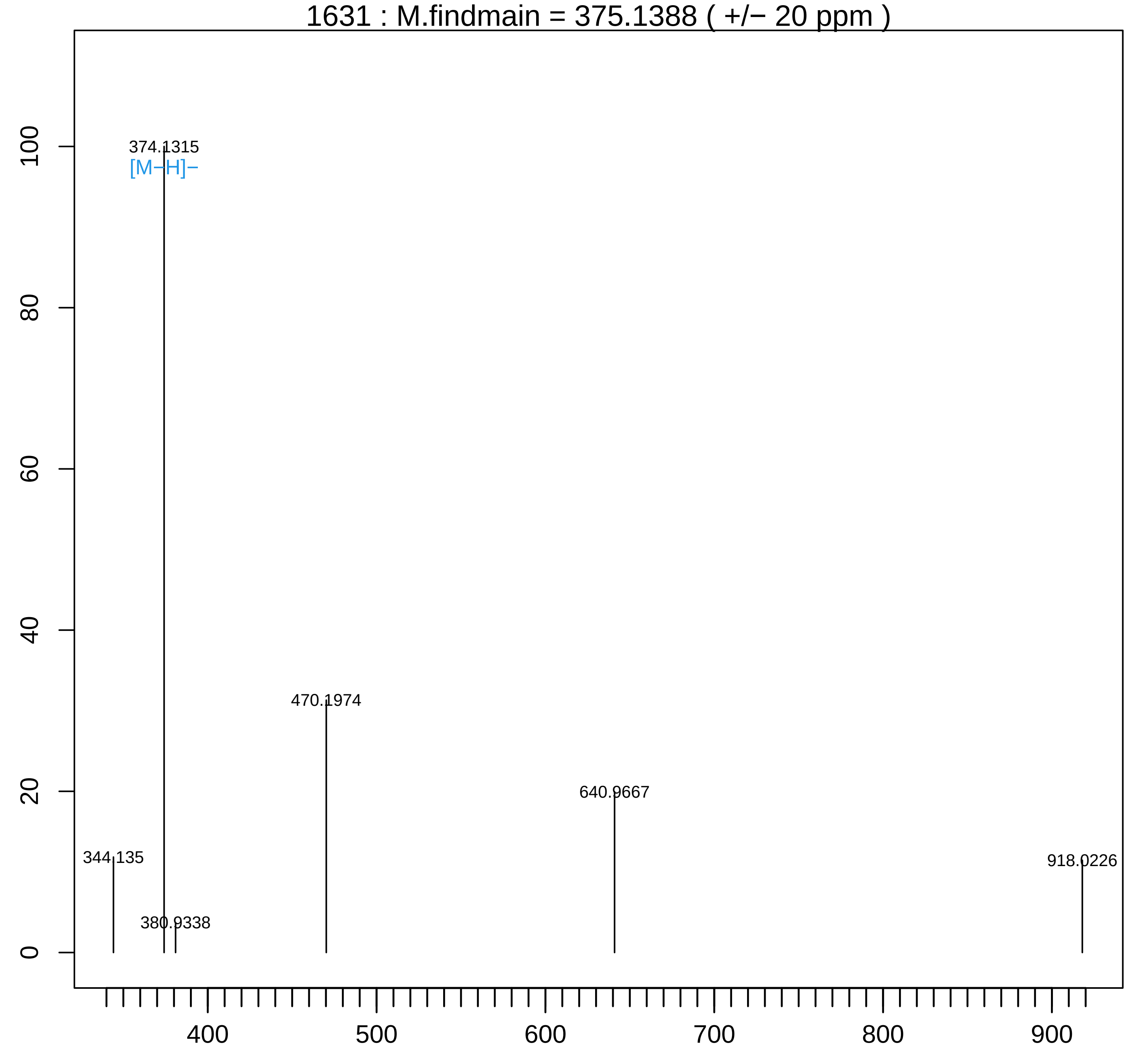
1631 : M.findmain = 375.1388 (+/− 20 ppm)
374.1315
[M−H]−
470.1974
640.9667
344.135
918.0226
380.9338
0
20
40
60
80
100
400
500
600
700
800
900

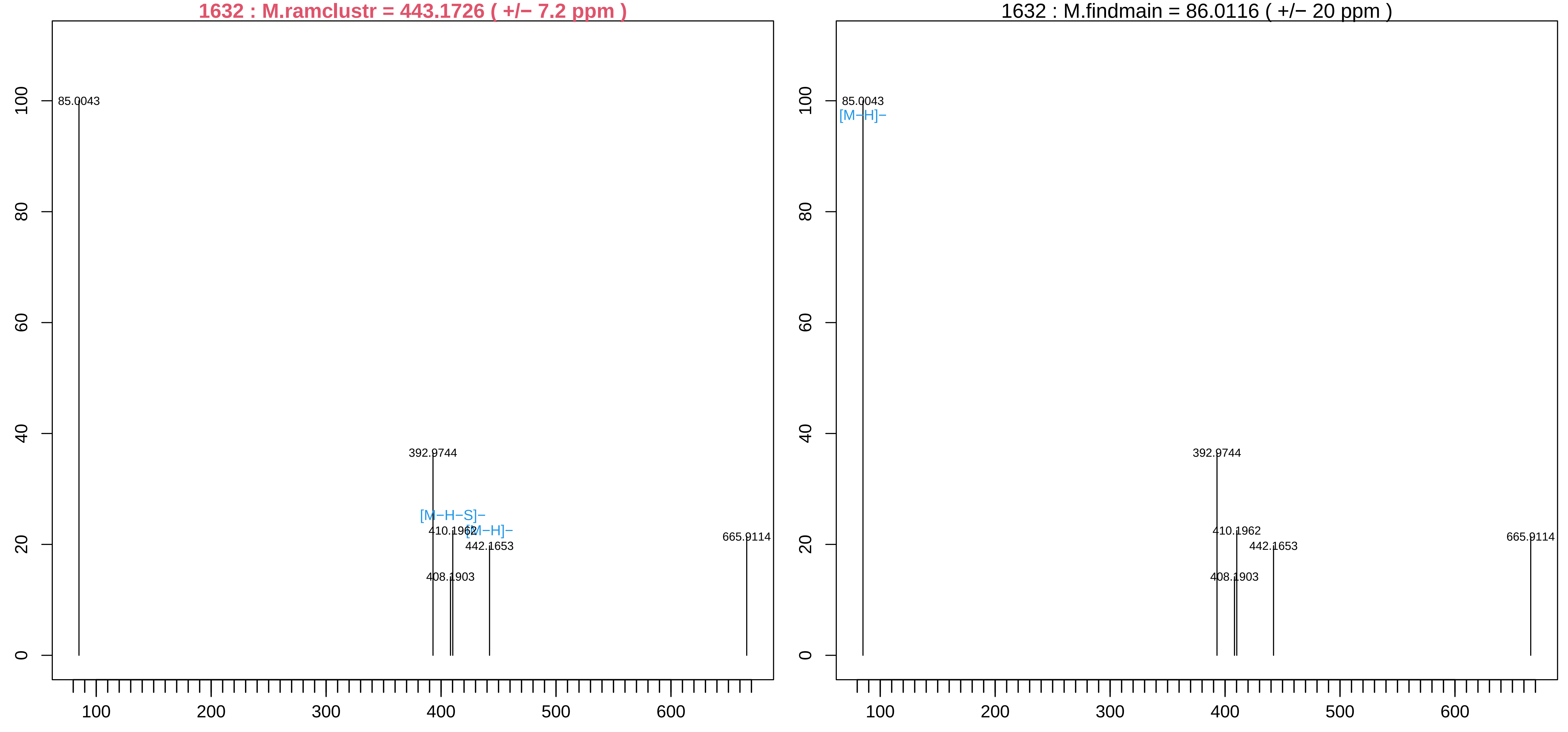
1632 : M.ramclustr = 443.1726 (+/− 7.2 ppm)
85.0043
392.9744
[M−H−S]−
410.1962
[M−H]−
442.1653
408.1903
665.9114
1632 : M.findmain = 86.0116 (+/− 20 ppm)
85.0043
[M−H]−
392.9744
410.1962
442.1653
408.1903
665.9114

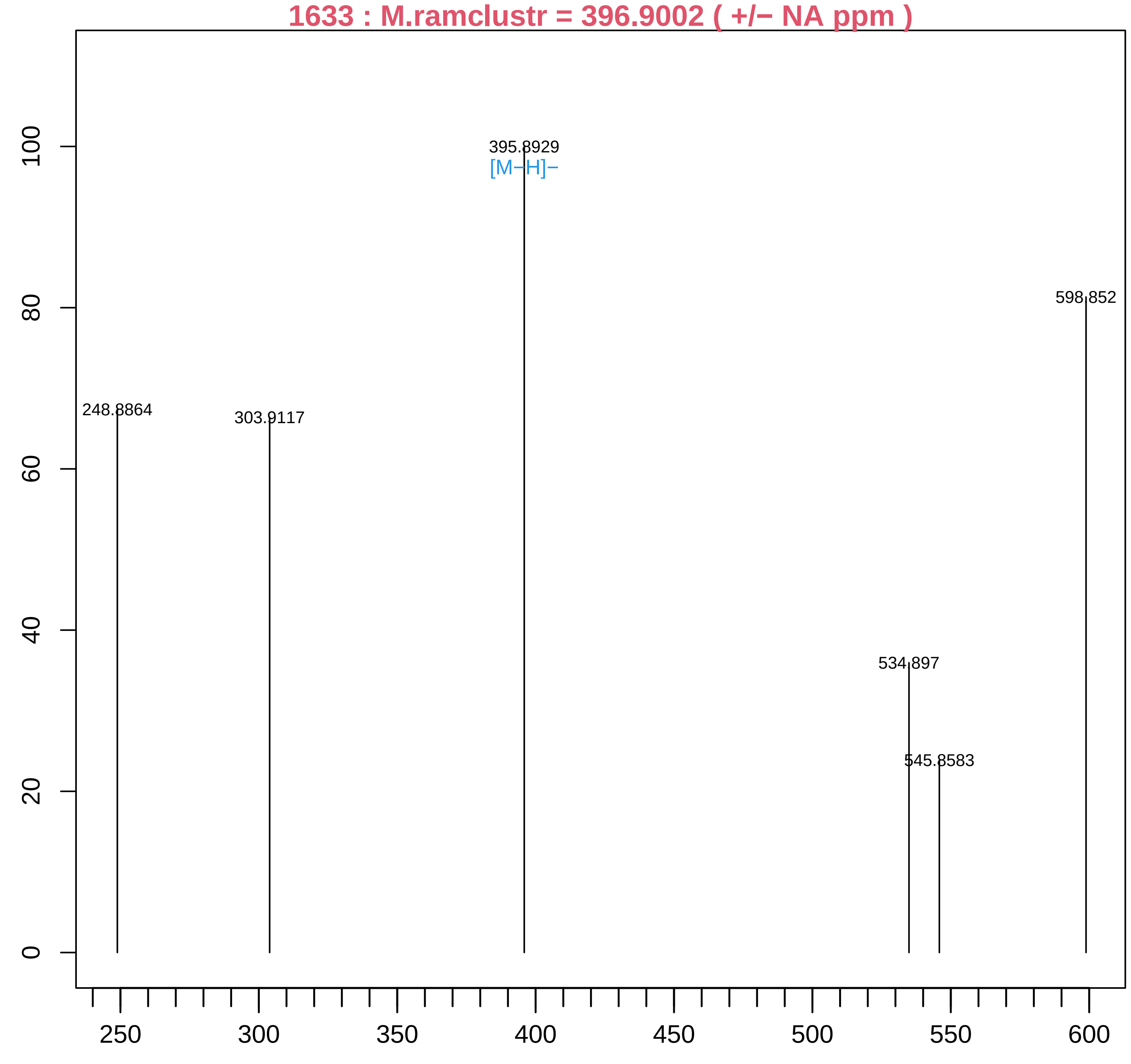
1633 : M.ramclustr = 396.9002 (+/− NA ppm)
395.8929
[M−H]−
598.852
248.8864
303.9117
534.897
545.8583
0
20
40
60
80
100
250
300
350
400
450
500
550
600

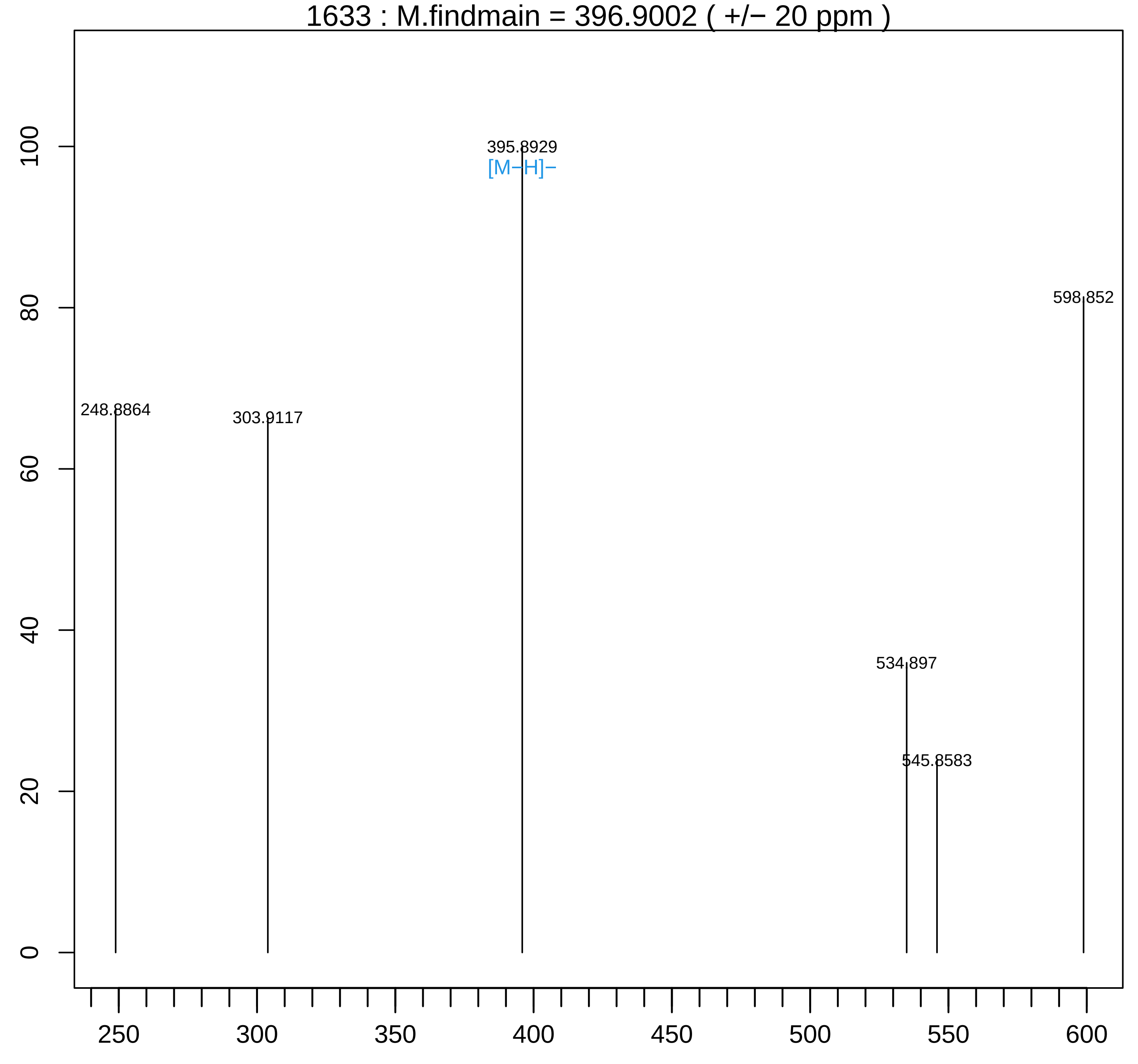
1633 : M.findmain = 396.9002 (+/− 20 ppm)
395.8929
[M−H]−
598.852
248.8864
303.9117
534.897
545.8583
0
20
40
60
80
100
250
300
350
400
450
500
550
600

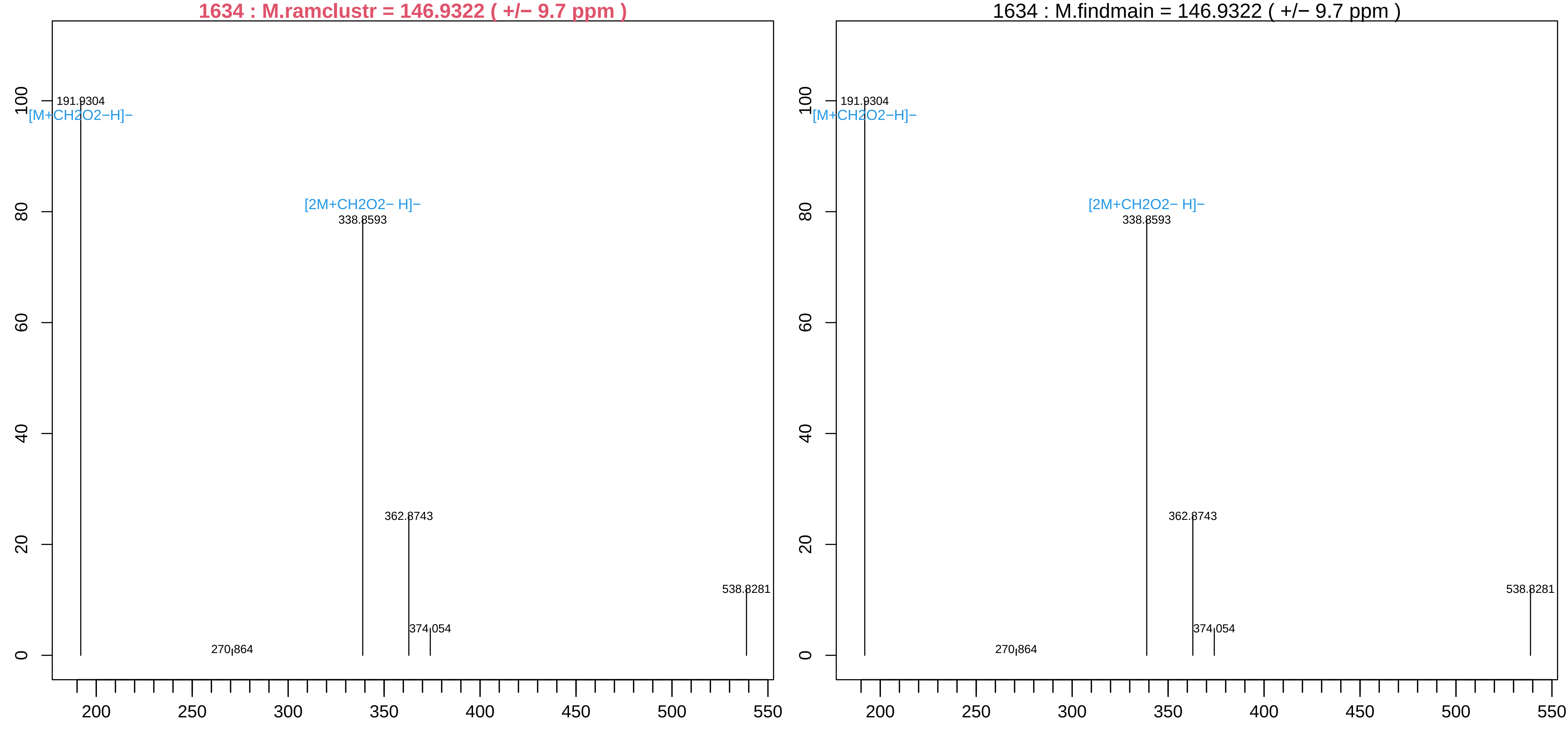
1634 : M.ramclustr = 146.9322 (+/− 9.7 ppm)
191.9304
[M+CH2O2−H]−
[2M+CH2O2− H]−
338.8593
362.8743
374.054
270.864
538.8281
1634 : M.findmain = 146.9322 (+/− 9.7 ppm)
191.9304
[M+CH2O2−H]−
[2M+CH2O2− H]−
338.8593
362.8743
374.054
270.864
538.8281

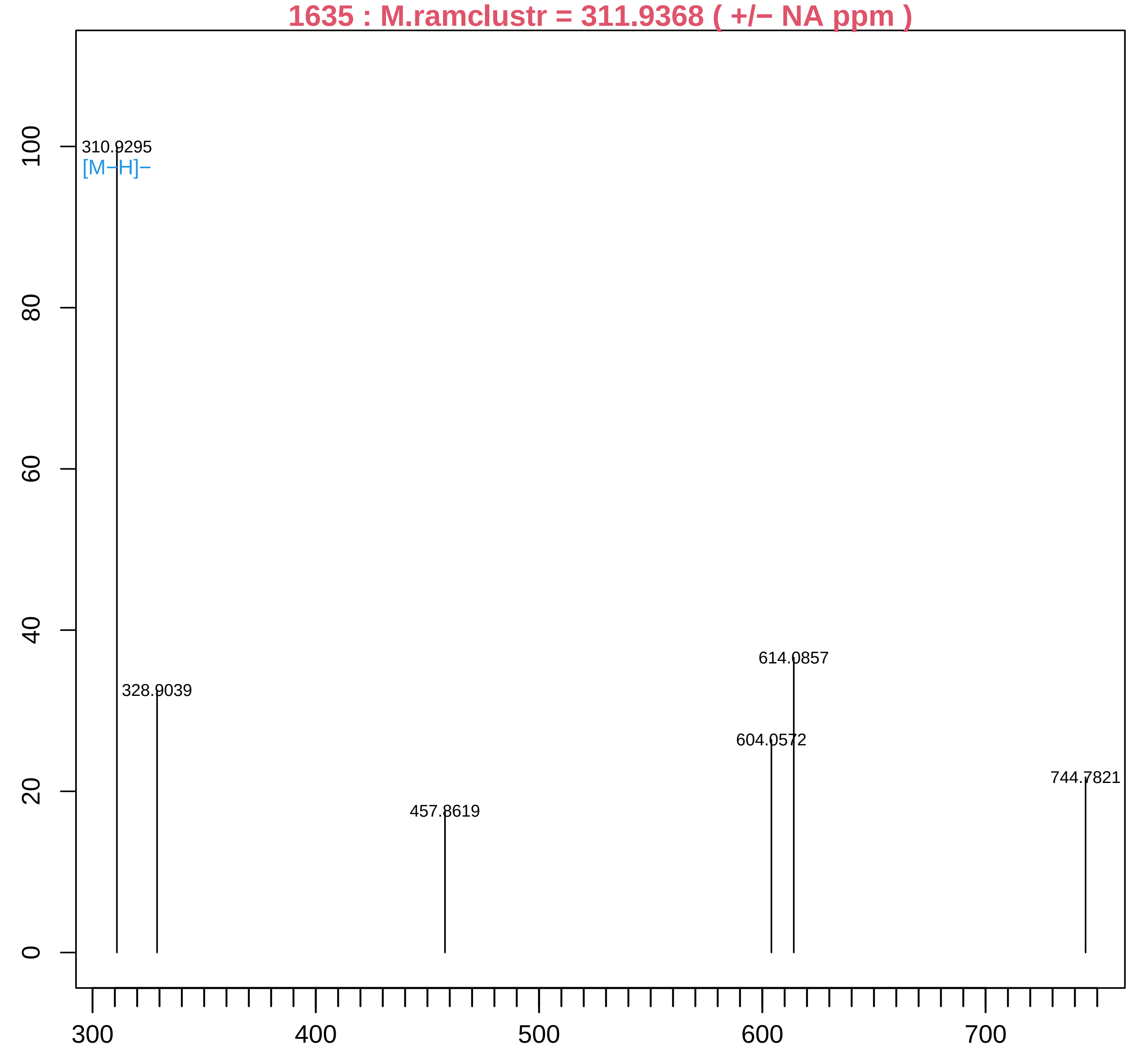
1635 : M.ramclustr = 311.9368 (+/− NA ppm)
310.9295
[M−H]−
328.9039
457.8619
604.0572
614.0857
744.7821
0
20
40
60
80
100
300
400
500
600
700

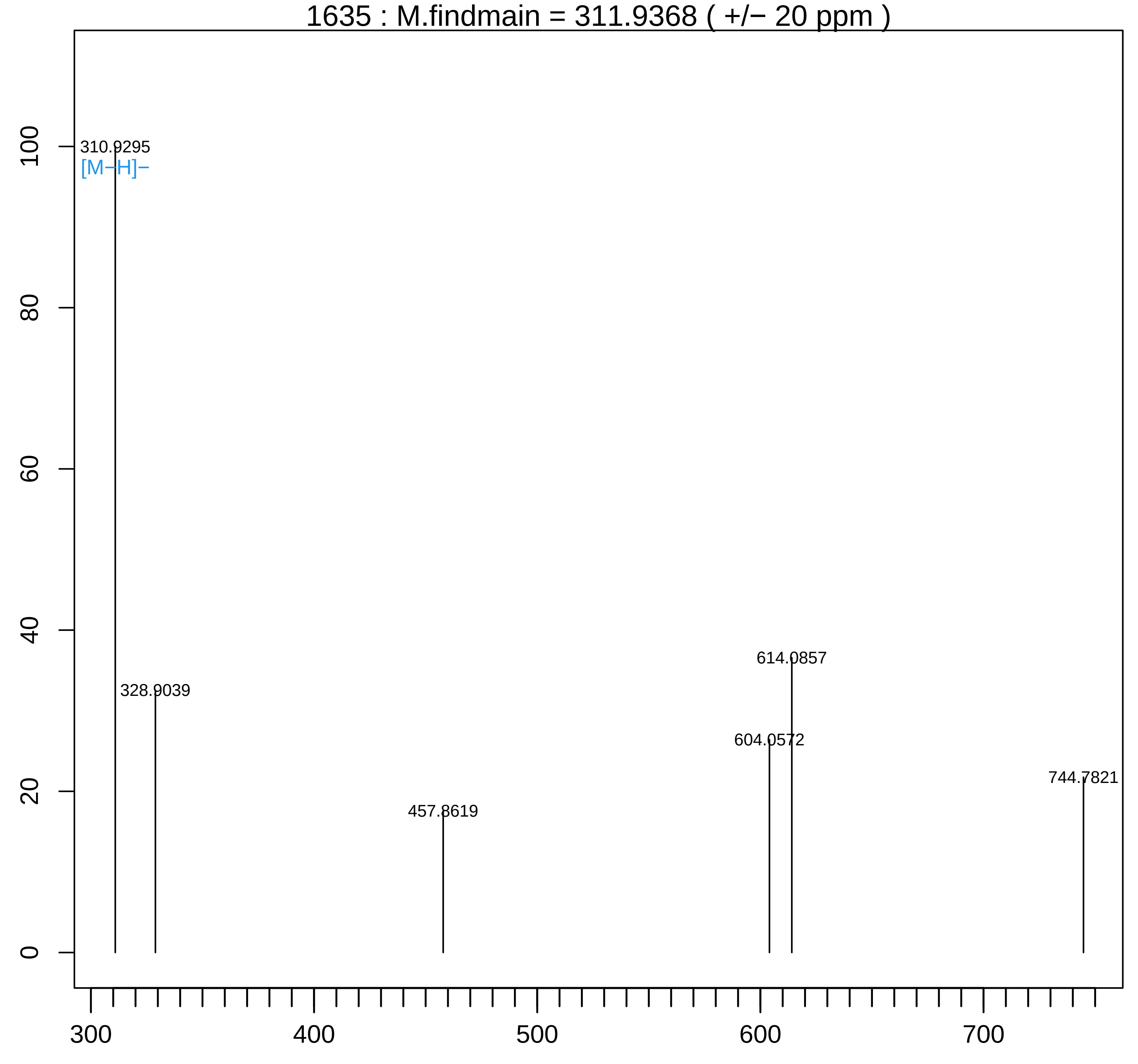
1635 : M.findmain = 311.9368 (+/− 20 ppm)
310.9295
[M−H]−
328.9039
457.8619
604.0572
614.0857
744.7821
0
20
40
60
80
100
300
400
500
600
700

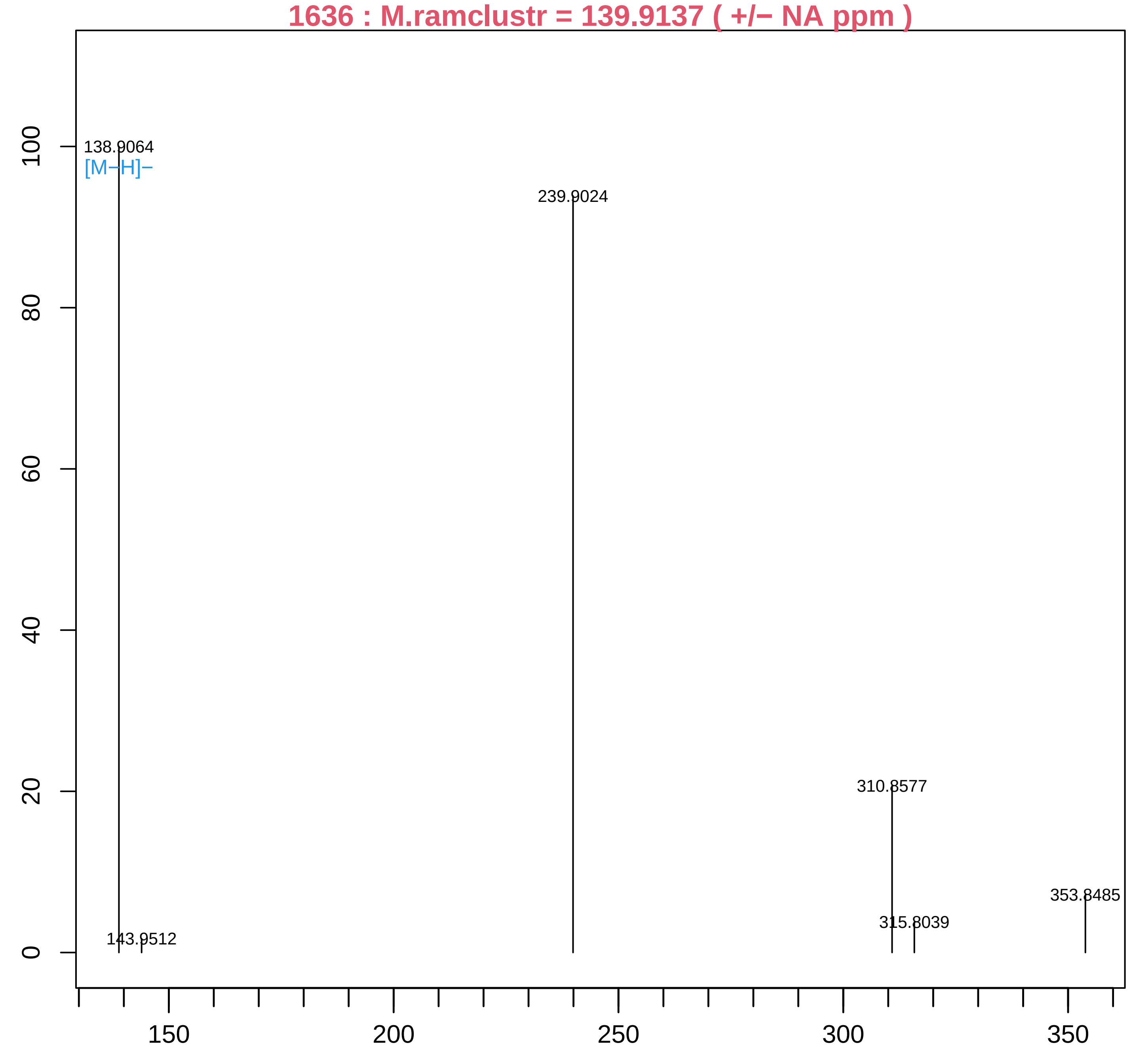

1636 : M.ramclustr = 139.9137 (+/− NA ppm)
138.9064
[M−H]−
239.9024
310.8577
353.8485
315.8039
143.9512
0
20
40
60
80
100
150
200
250
300
350

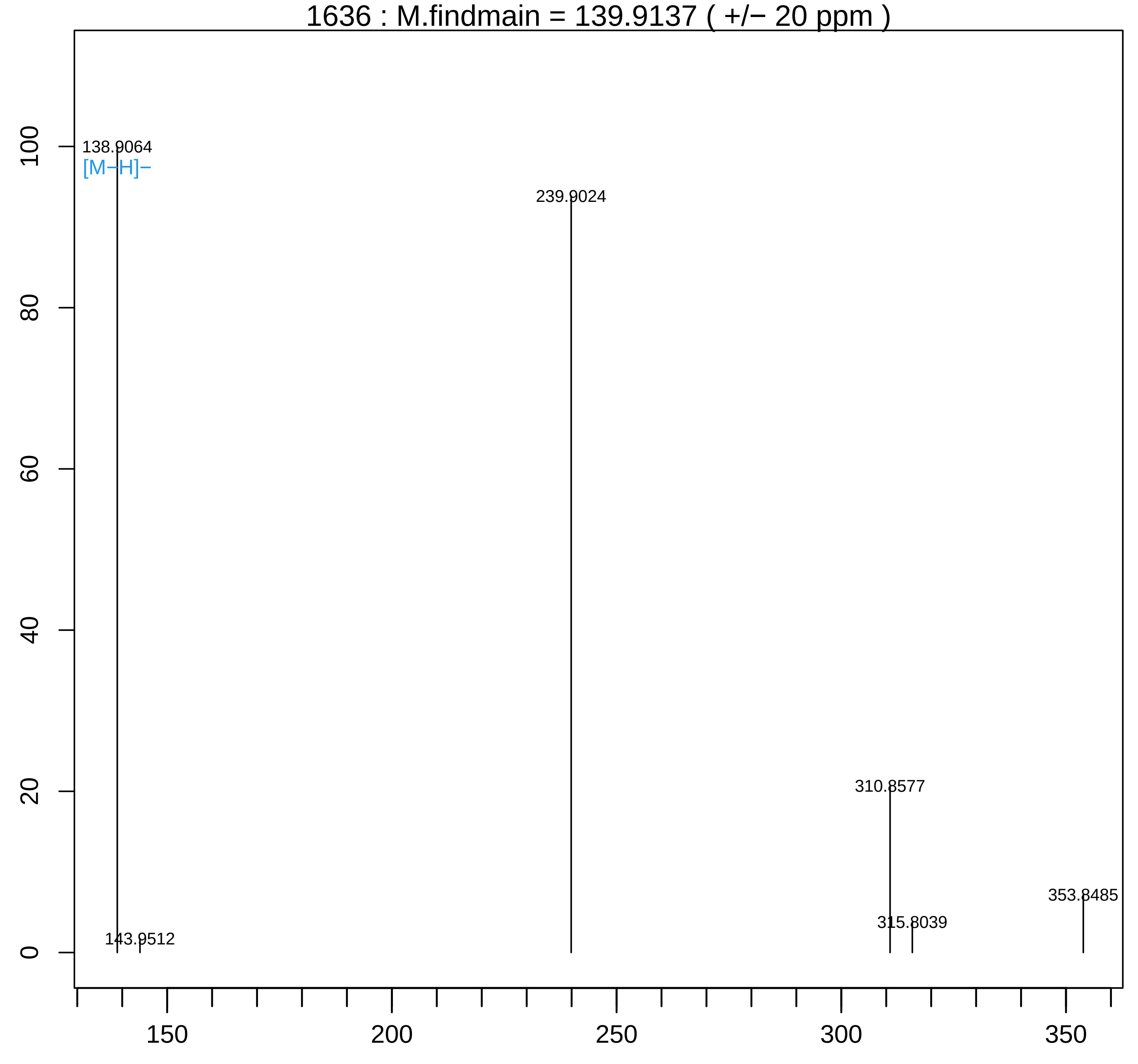

1636 : M.findmain = 139.9137 (+/− 20 ppm)
138.9064
[M−H]−
239.9024
310.8577
353.8485
315.8039
143.9512
0
20
40
60
80
100
150
200
250
300
350

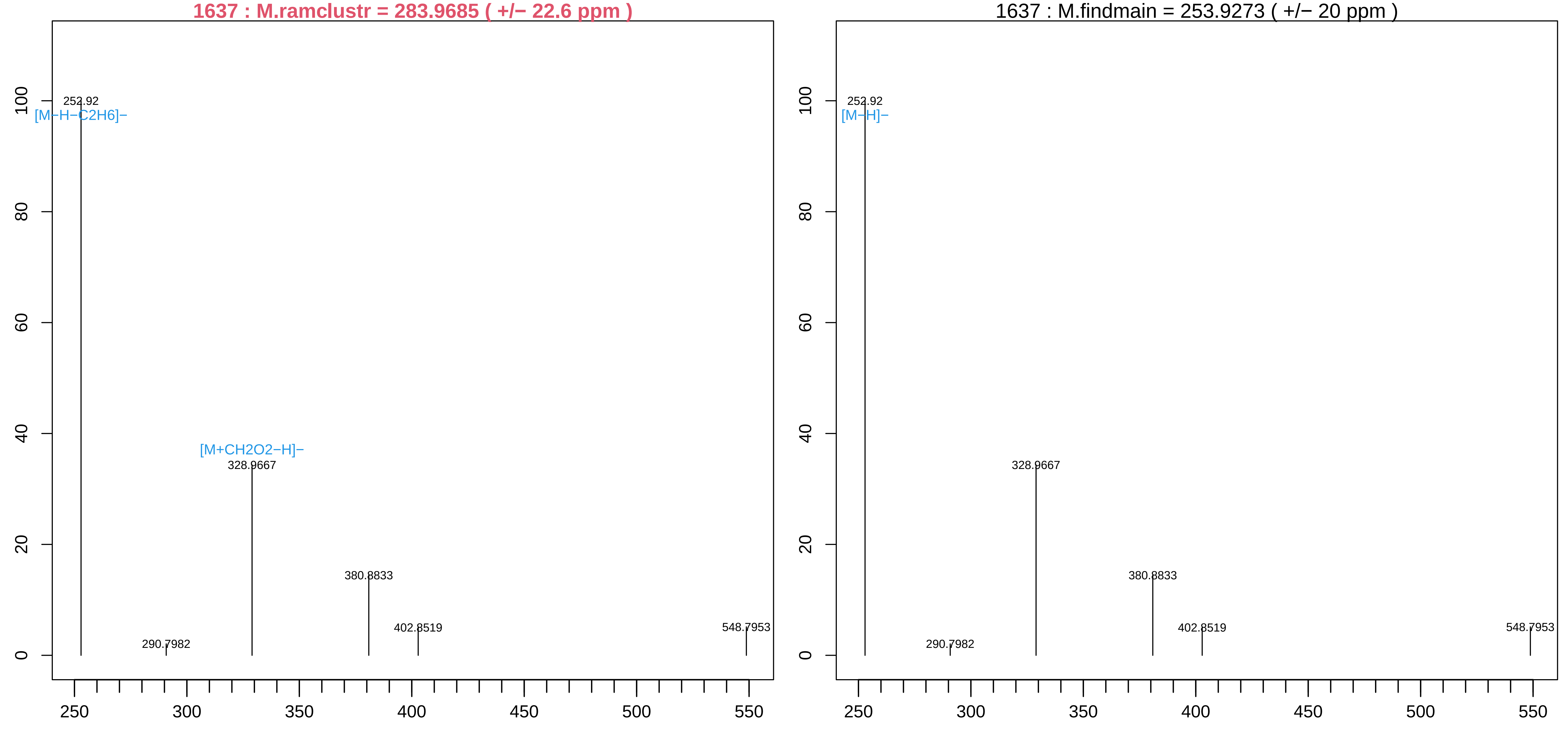

1637 : M.ramclustr = 283.9685 (+/− 22.6 ppm)
252.92
[M−H−C2H6]−
[M+CH2O2−H]−
328.9667
290.7982
380.8833
402.8519
548.7953
1637 : M.findmain = 253.9273 (+/− 20 ppm)
252.92
[M−H]−
328.9667
290.7982
380.8833
402.8519
548.7953

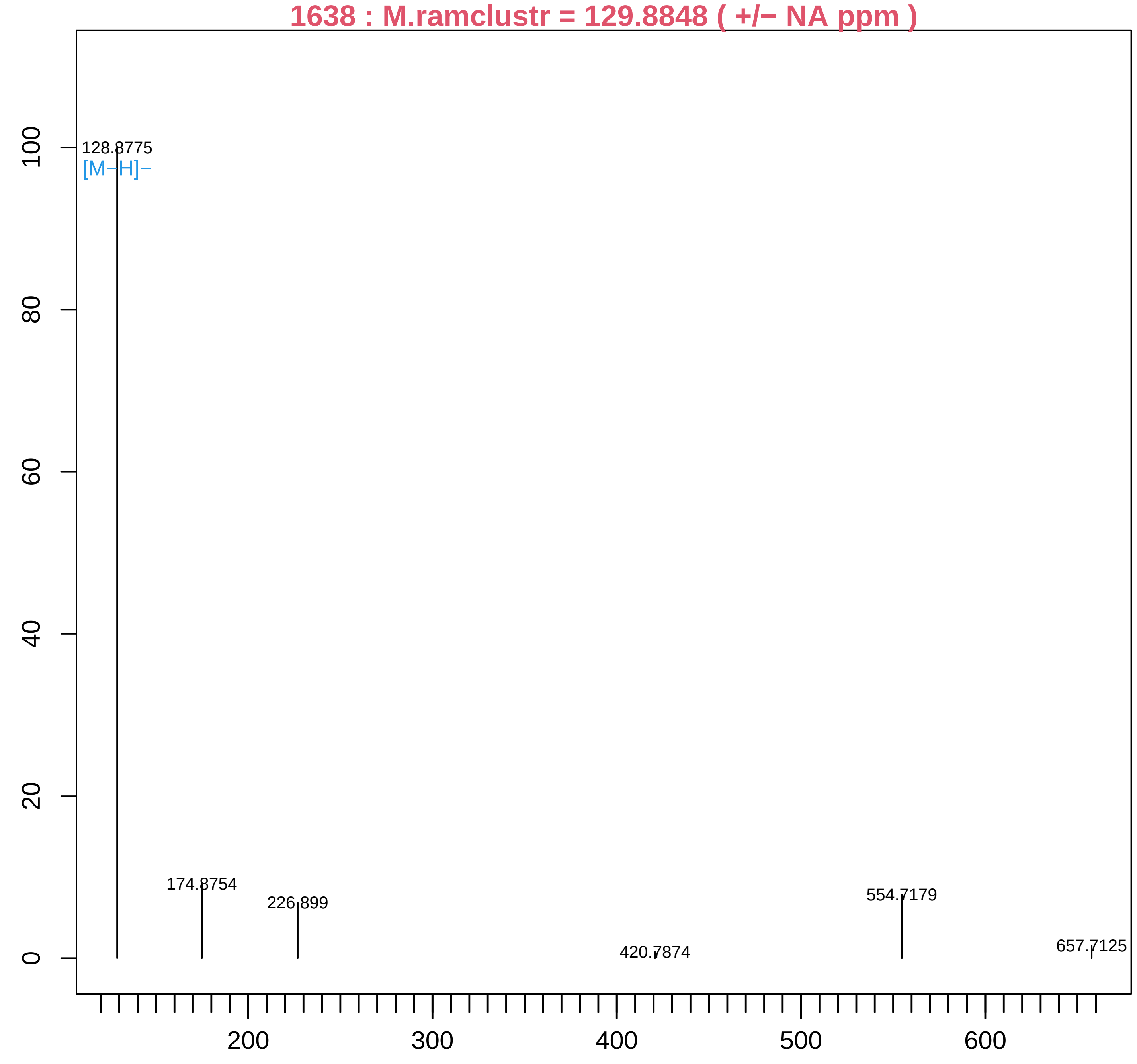
1638 : M.ramclustr = 129.8848 (+/− NA ppm)
128.8775
[M−H]−
174.8754
226.899
420.7874
554.7179
657.7125
0
20
40
60
80
100
200
300
400
500
600

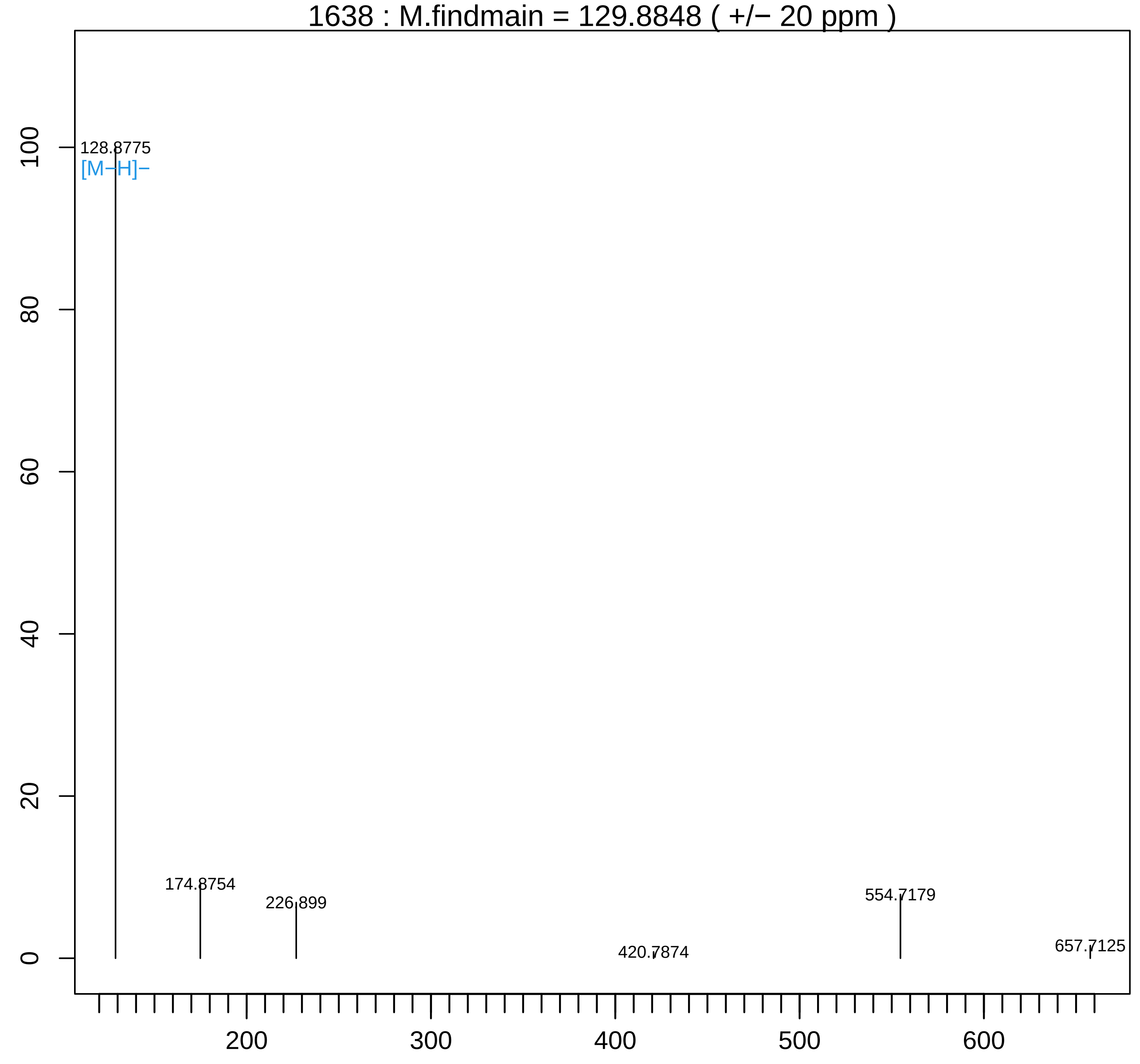
1638 : M.findmain = 129.8848 (+/− 20 ppm)
128.8775
[M−H]−
174.8754
226.899
420.7874
554.7179
657.7125
0
20
40
60
80
100
200
300
400
500
600

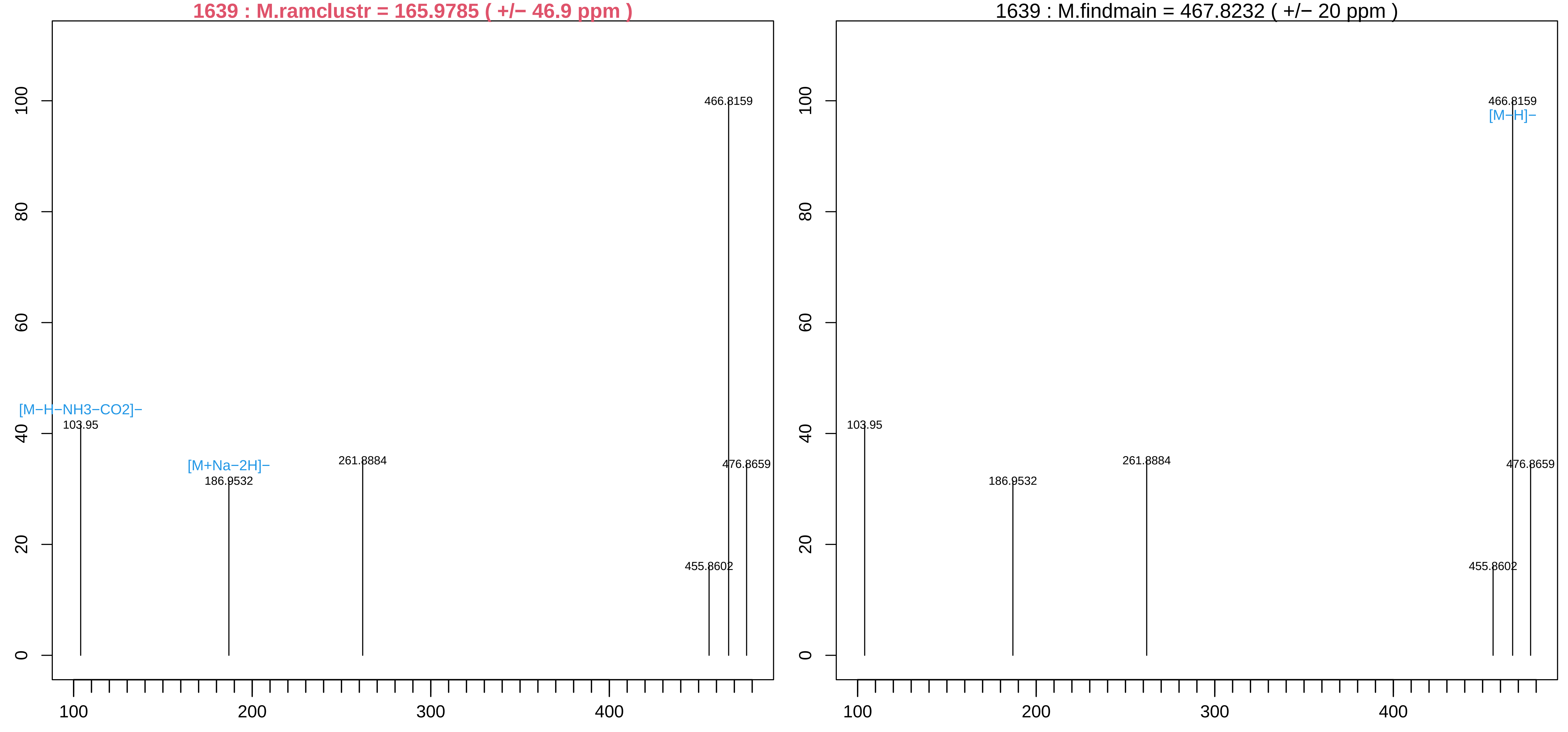
1639 : M.ramclustr = 165.9785 (+/− 46.9 ppm)
466.8159
[M−H−NH3−CO2]−
103.95
[M+Na−2H]−
186.9532
261.8884
476.8659
455.8602
1639 : M.findmain = 467.8232 (+/− 20 ppm)
466.8159
[M−H]−
103.95
186.9532
261.8884
476.8659
455.8602
0
20
40
60
80
100
100
200
300
400

1640 : M.ramclustr = 317.8079 (+/− NA ppm)

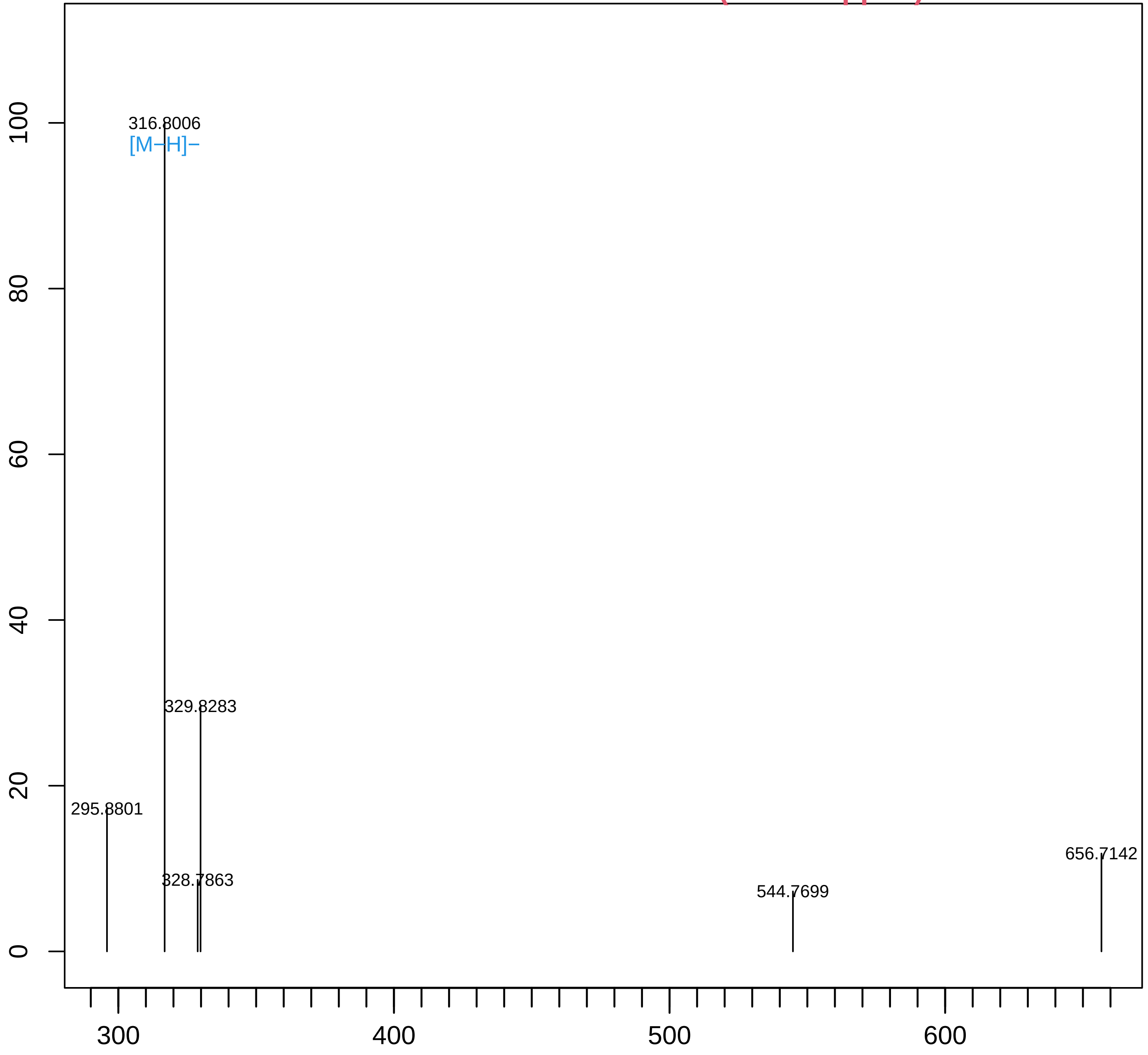

1640 : M.findmain = 317.8079 (+/− 20 ppm)

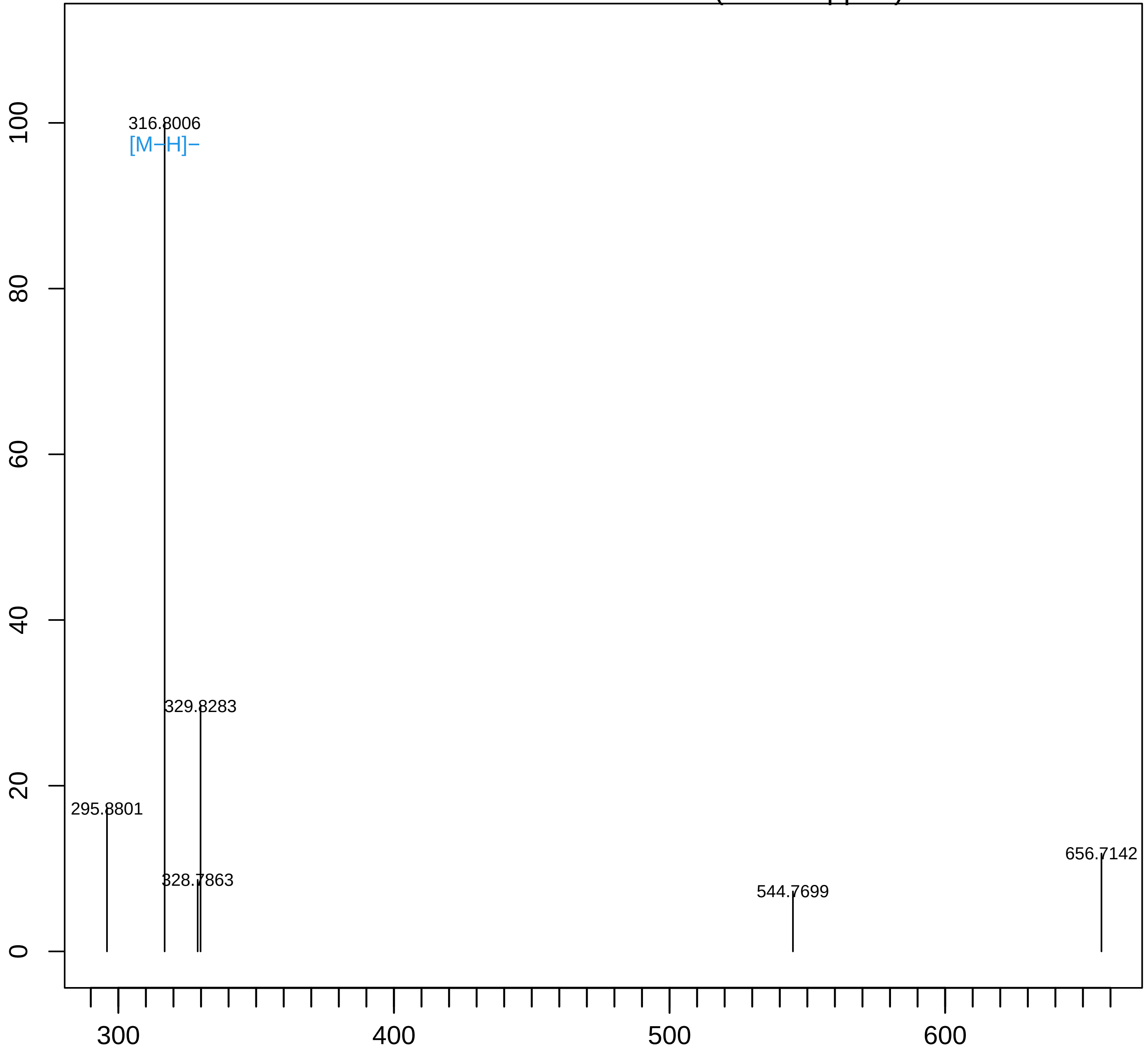

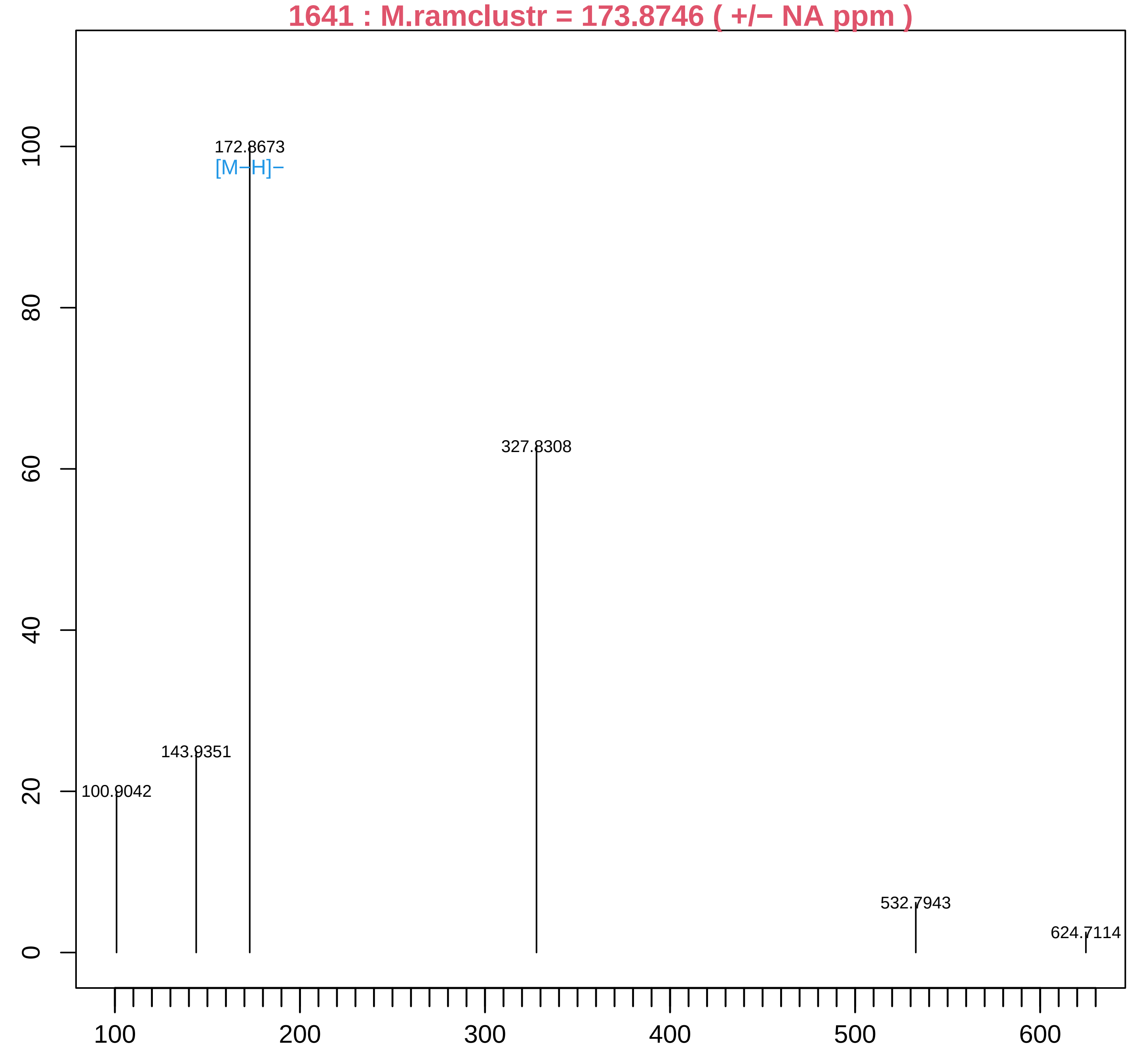
1641 : M.ramclustr = 173.8746 (+/− NA ppm)
172.8673
[M−H]−
327.8308
143.9351
100.9042
532.7943
624.7114
0
20
40
60
80
100
100
200
300
400
500
600

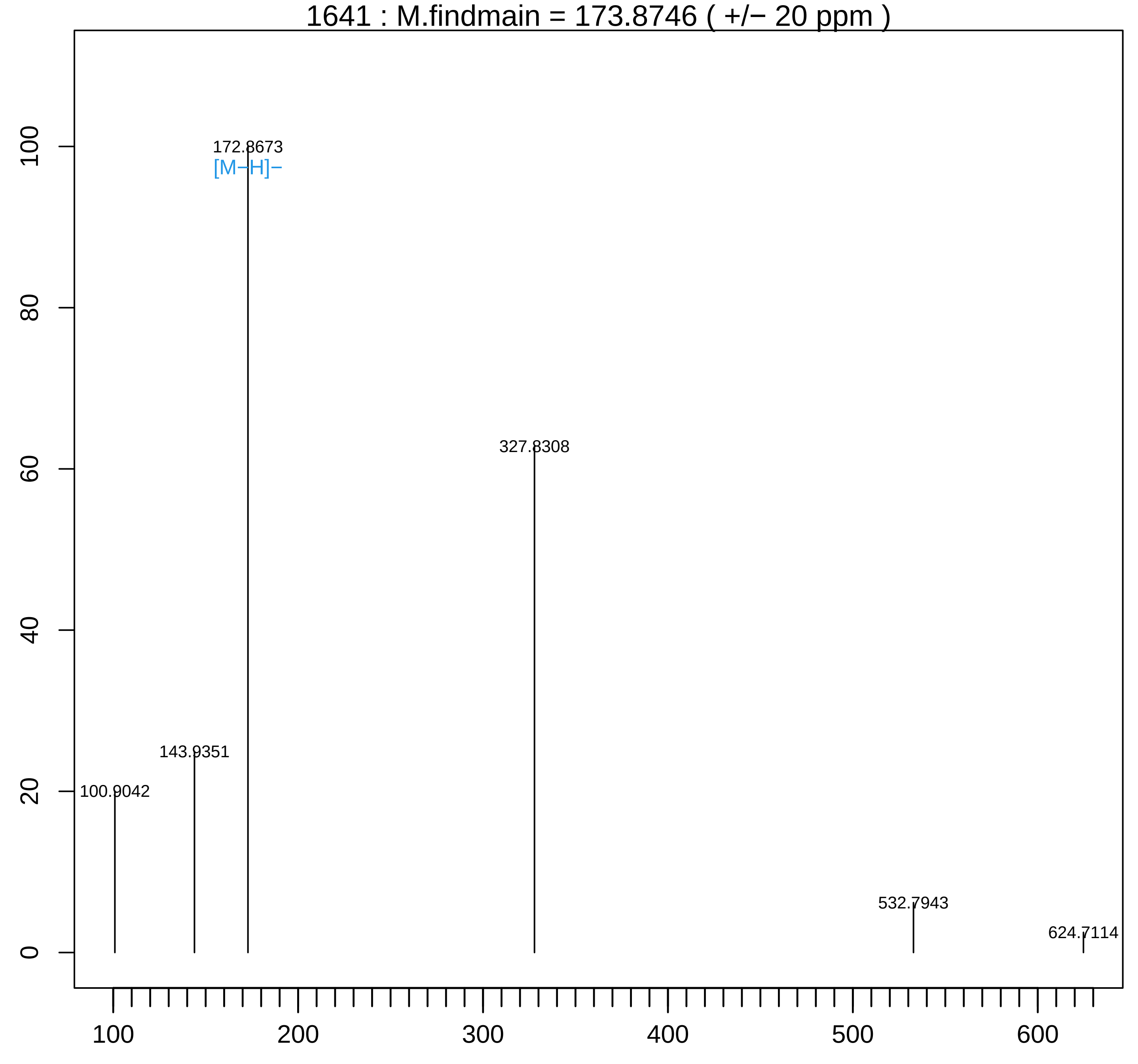
1641 : M.findmain = 173.8746 (+/− 20 ppm)
172.8673
[M−H]−
327.8308
143.9351
100.9042
532.7943
624.7114
0
20
40
60
80
100
100
200
300
400
500
600

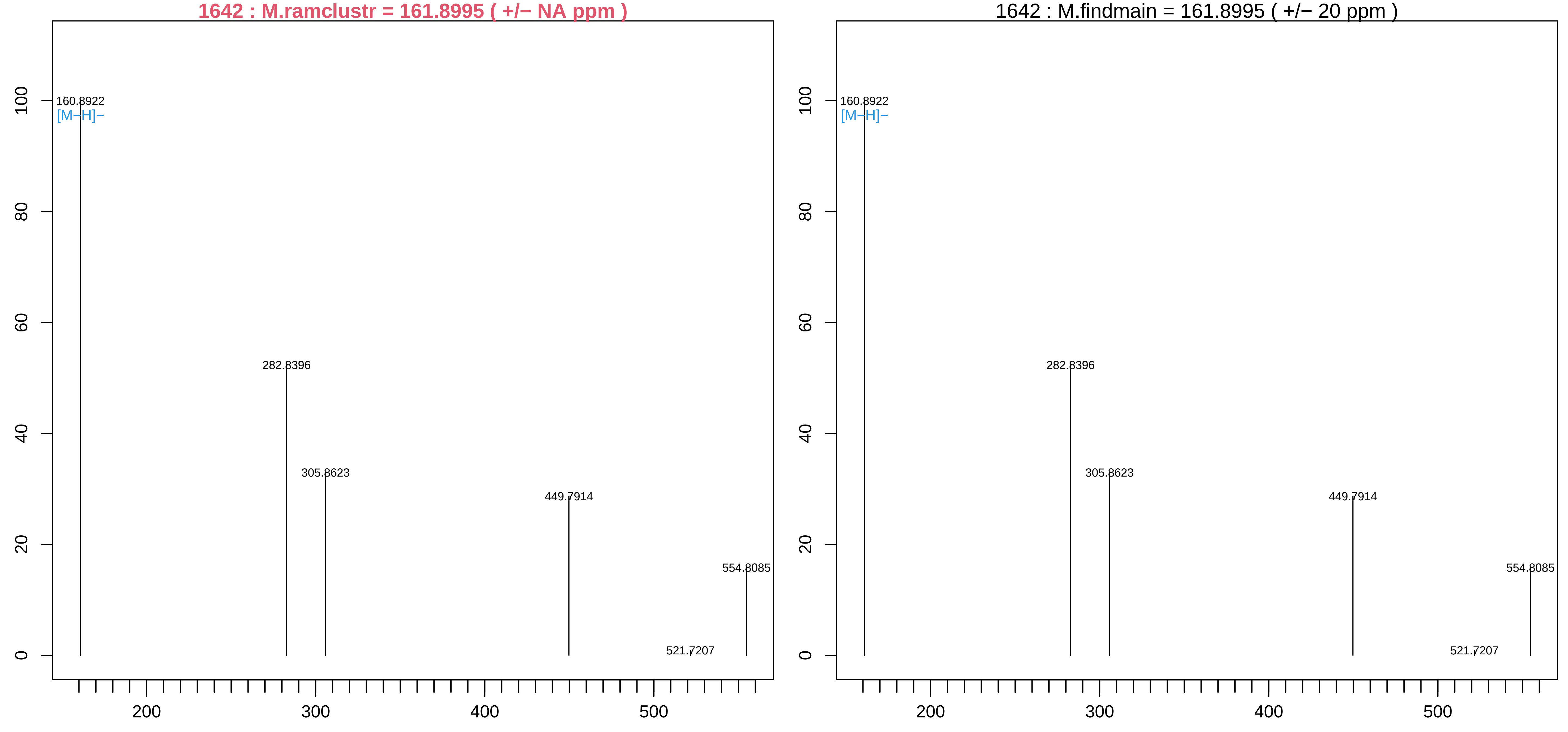
1642 : M.ramclustr = 161.8995 (+/− NA ppm)
160.8922
[M−H]−
282.8396
305.8623
449.7914
554.8085
521.7207
0
20
40
60
80
100
200
300
400
500
1642 : M.findmain = 161.8995 (+/− 20 ppm)
160.8922
[M−H]−
282.8396
305.8623
449.7914
554.8085
521.7207
0
20
40
60
80
100
200
300
400
500

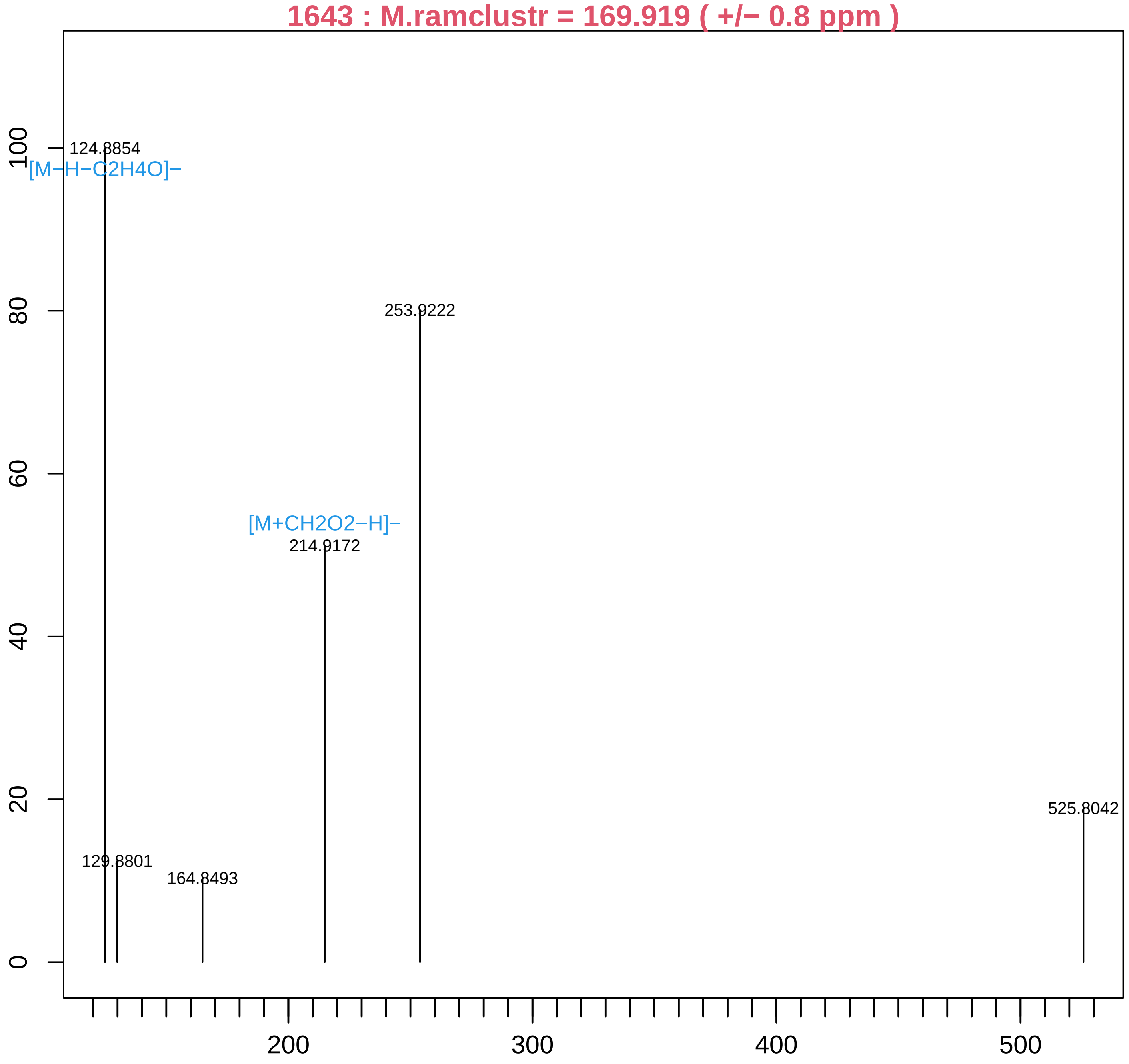

1643 : M.ramclustr = 169.919 (+/− 0.8 ppm)
124.8854
[M−H−C2H4O]−
253.9222
[M+CH2O2−H]−
214.9172
129.8801
164.8493
525.8042
0
20
40
60
80
100
200
300
400
500

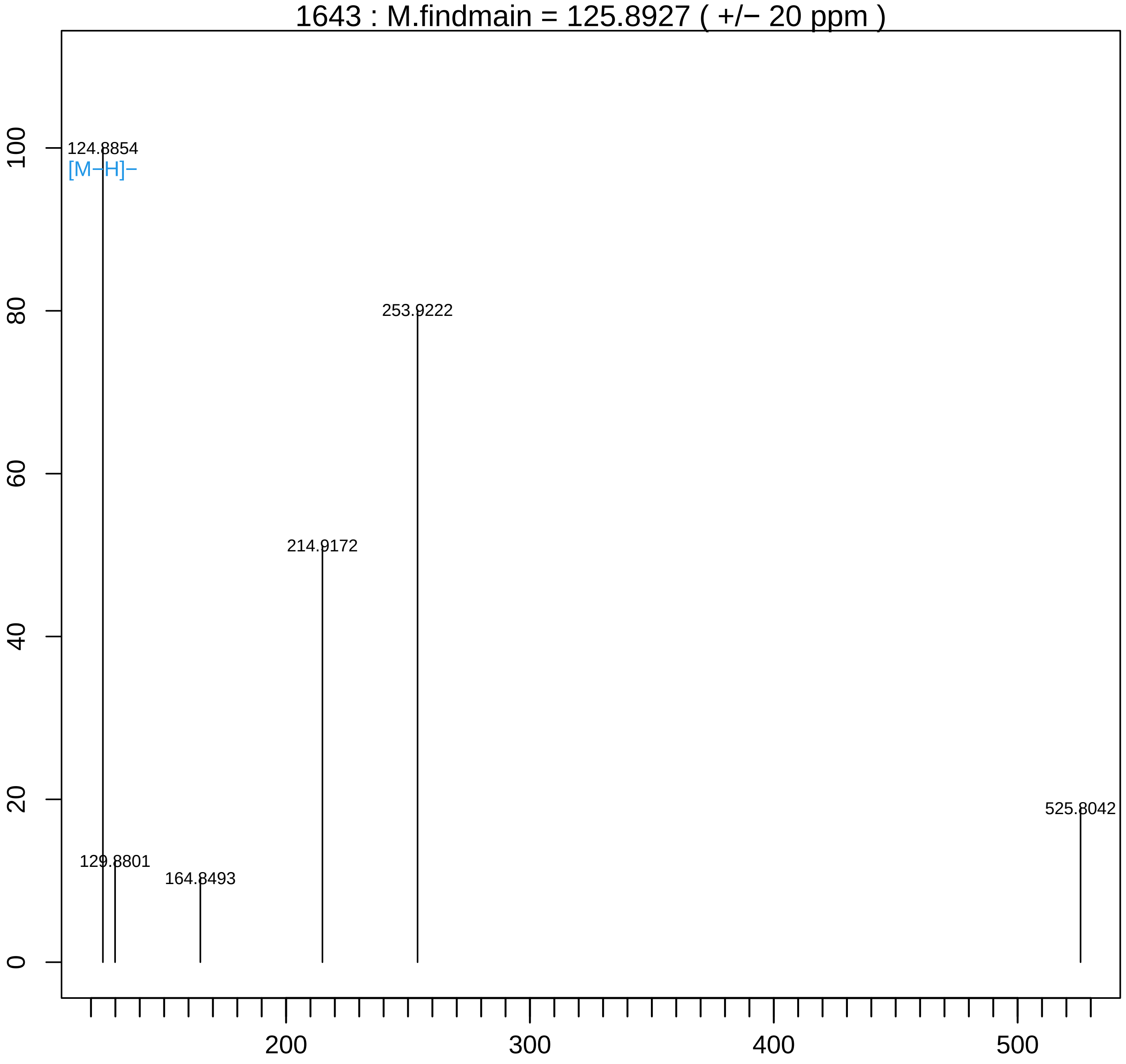

1643 : M.findmain = 125.8927 (+/− 20 ppm)
124.8854
[M−H]−
253.9222
214.9172
129.8801
164.8493
525.8042
0
20
40
60
80
100
200
300
400
500

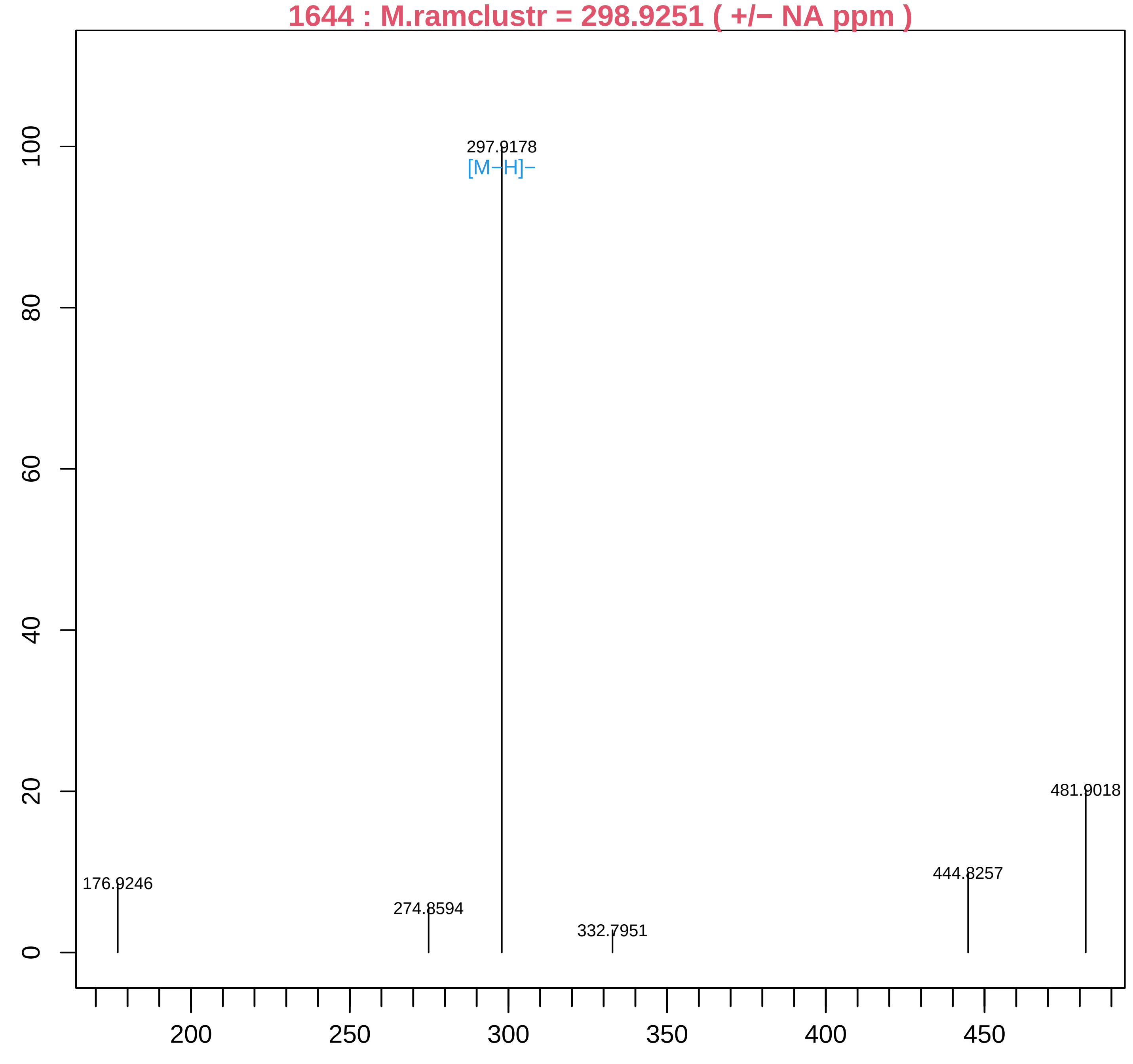

1644 : M.ramclustr = 298.9251 (+/− NA ppm)
297.9178
[M−H]−
176.9246
274.8594
332.7951
444.8257
481.9018
0
20
40
60
80
100
200
250
300
350
400
450

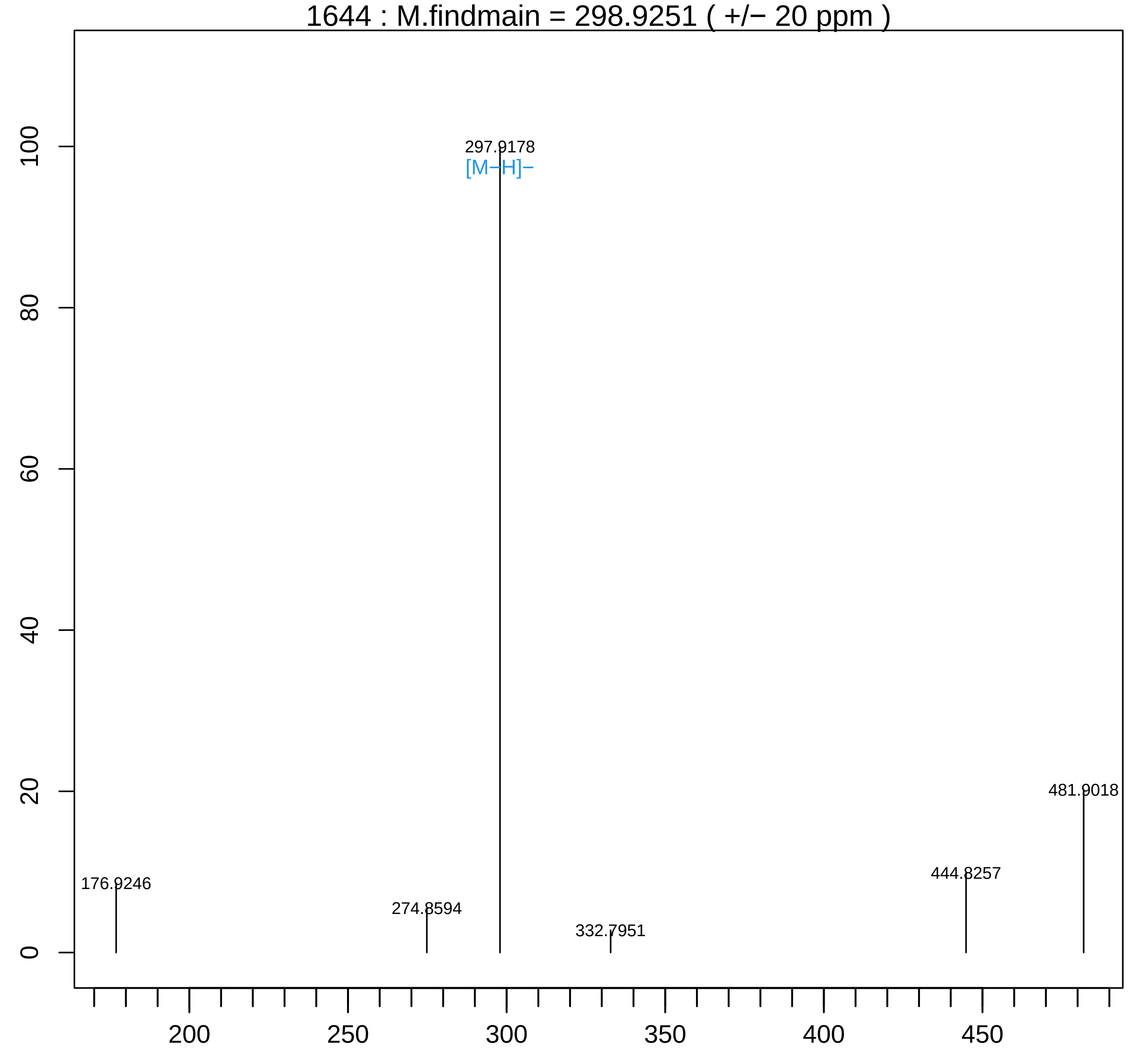

1644 : M.findmain = 298.9251 (+/− 20 ppm)
297.9178
[M−H]−
176.9246
274.8594
332.7951
444.8257
481.9018
0
20
40
60
80
100
200
250
300
350
400
450

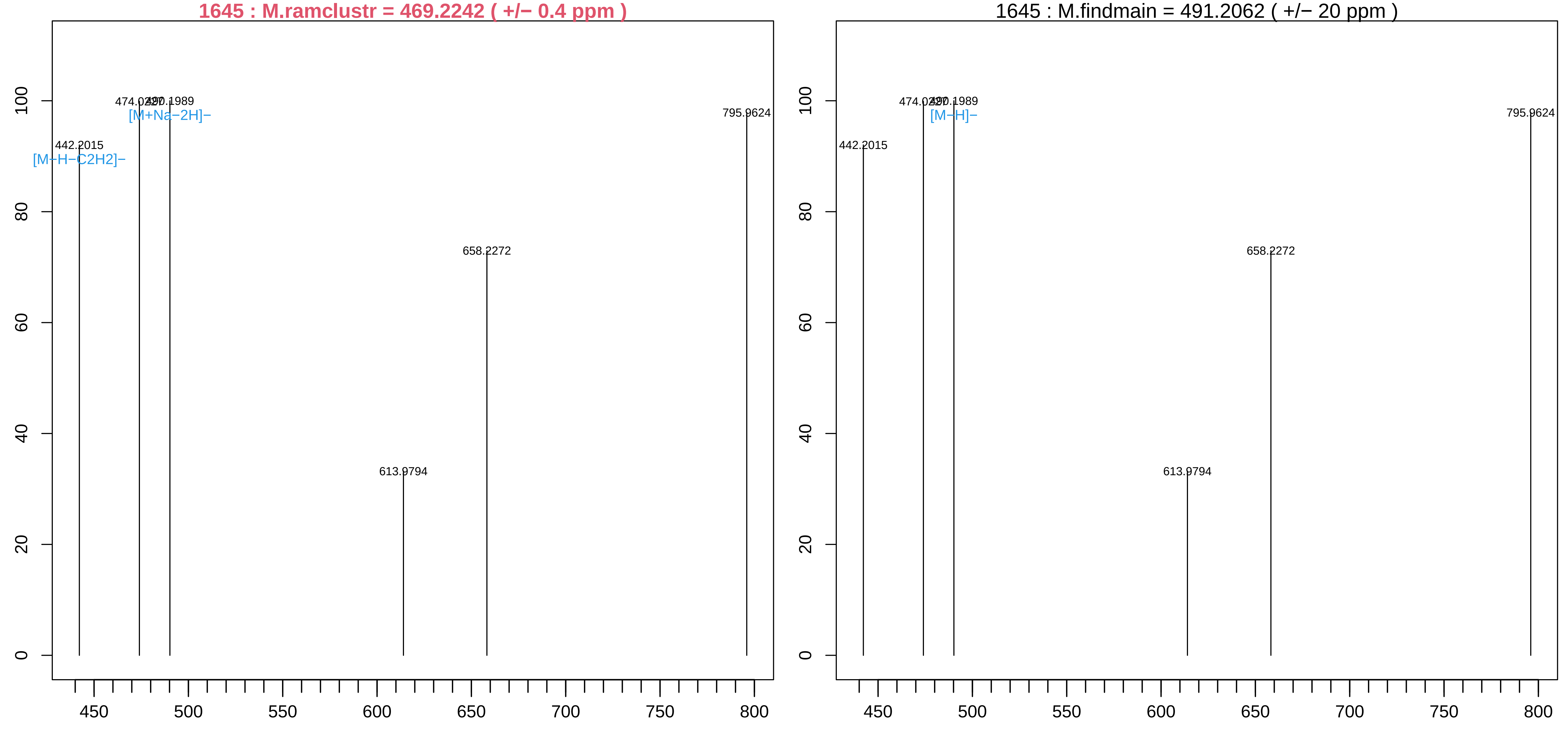

1645 : M.ramclustr = 469.2242 (+/− 0.4 ppm)
442.2015
[M−H−C2H2]−
474.0497
490.1989
[M+Na−2H]−
613.9794
658.2272
795.9624
1645 : M.findmain = 491.2062 (+/− 20 ppm)
442.2015
474.0497
490.1989
[M−H]−
613.9794
658.2272
795.9624
0
20
40
60
80
100
450
500
550
600
650
700
750
800

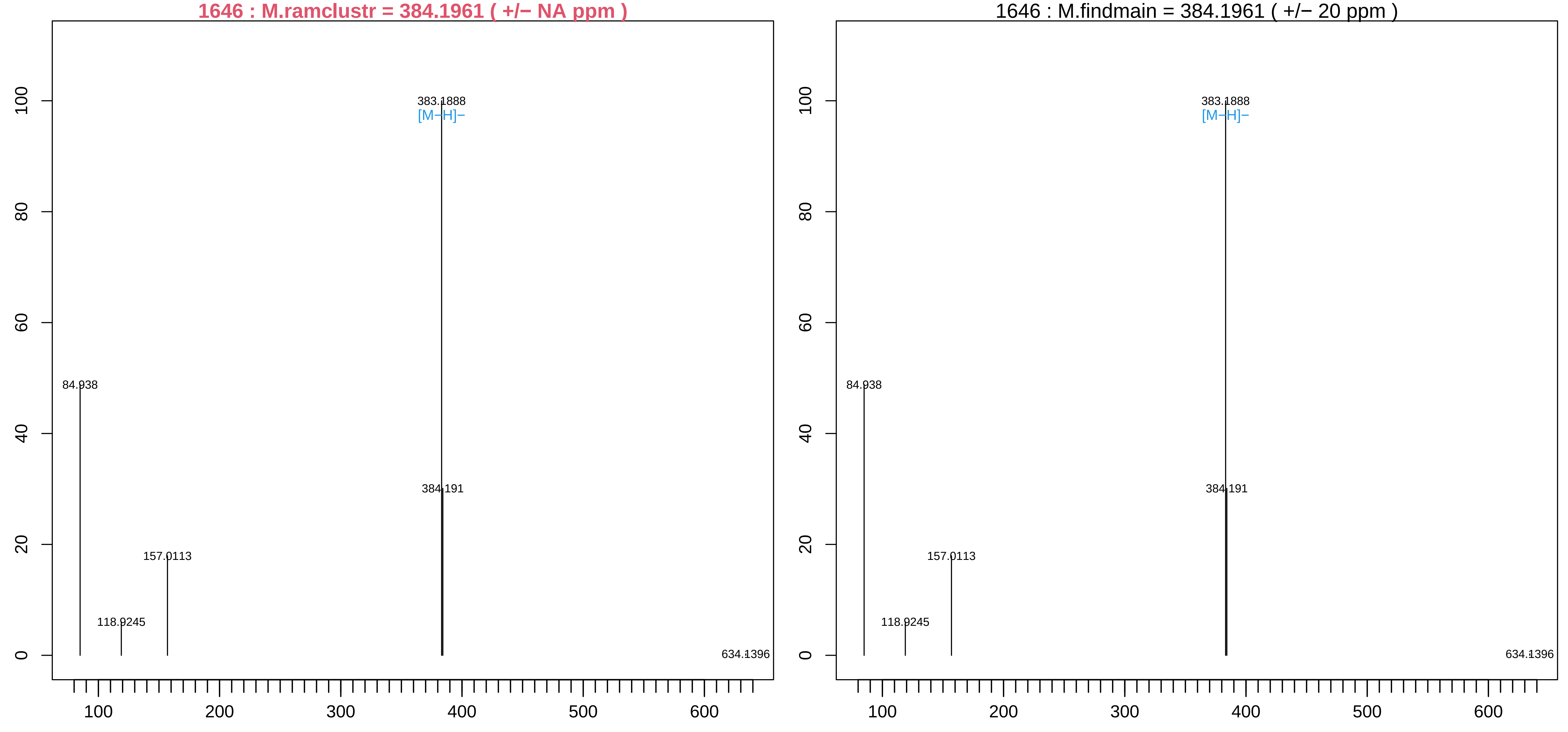
1646 : M.ramclustr = 384.1961 (+/− NA ppm)
383.1888
[M−H]−
84.938
384.191
157.0113
118.9245
634.1396
0
20
40
60
80
100
200
300
400
500
600
1646 : M.findmain = 384.1961 (+/− 20 ppm)
383.1888
[M−H]−
84.938
384.191
157.0113
118.9245
634.1396

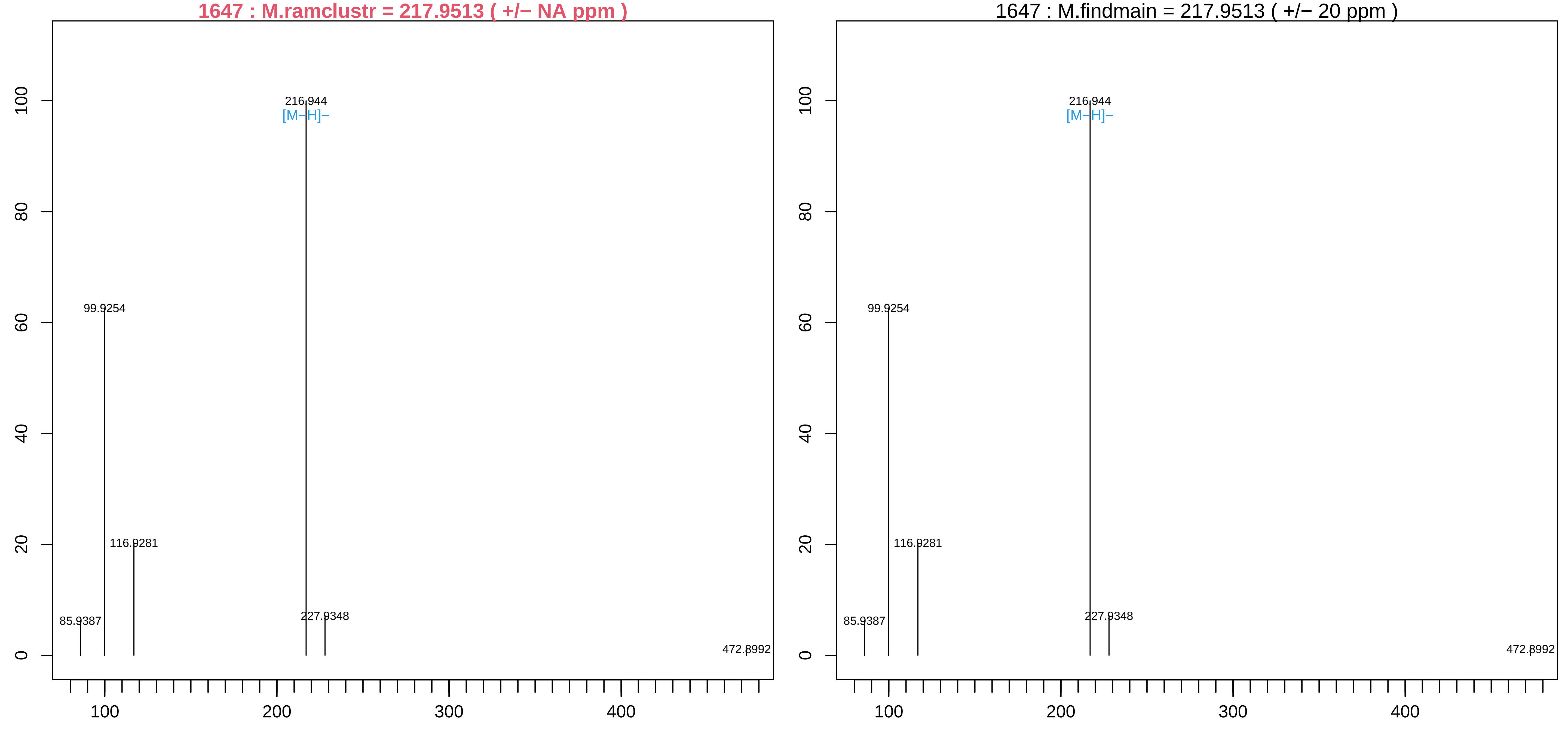
1647 : M.ramclustr = 217.9513 (+/− NA ppm)
1647 : M.findmain = 217.9513 (+/− 20 ppm)
216.944
[M−H]−
99.9254
116.9281
85.9387
227.9348
472.8992
0
20
40
60
80
100
200
300
400

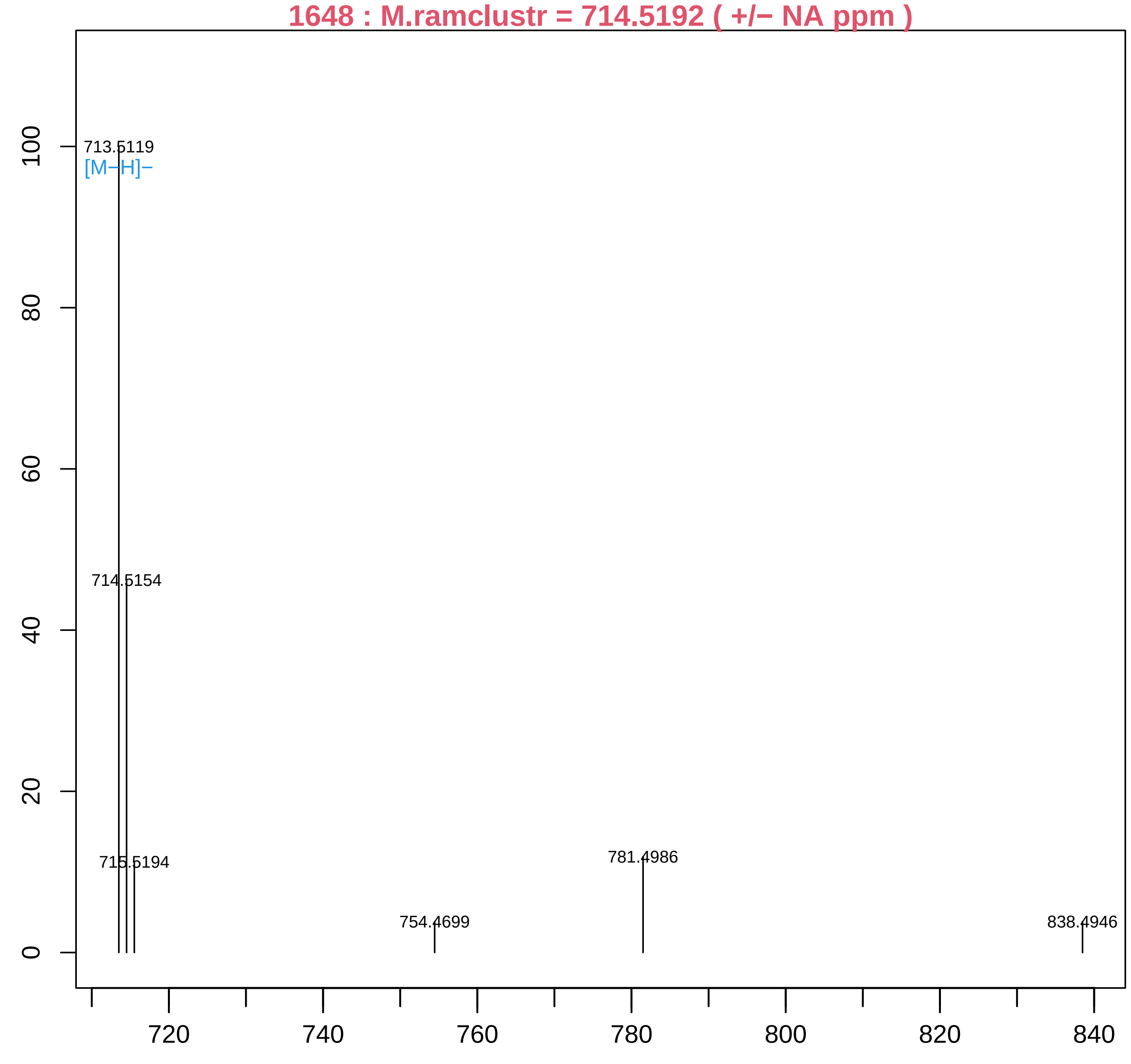
1648 : M.ramclustr = 714.5192 (+/− NA ppm)
713.5119
[M−H]−
714.5154
715.5194
754.4699
781.4986
838.4946
0
20
40
60
80
100
720
740
760
780
800
820
840

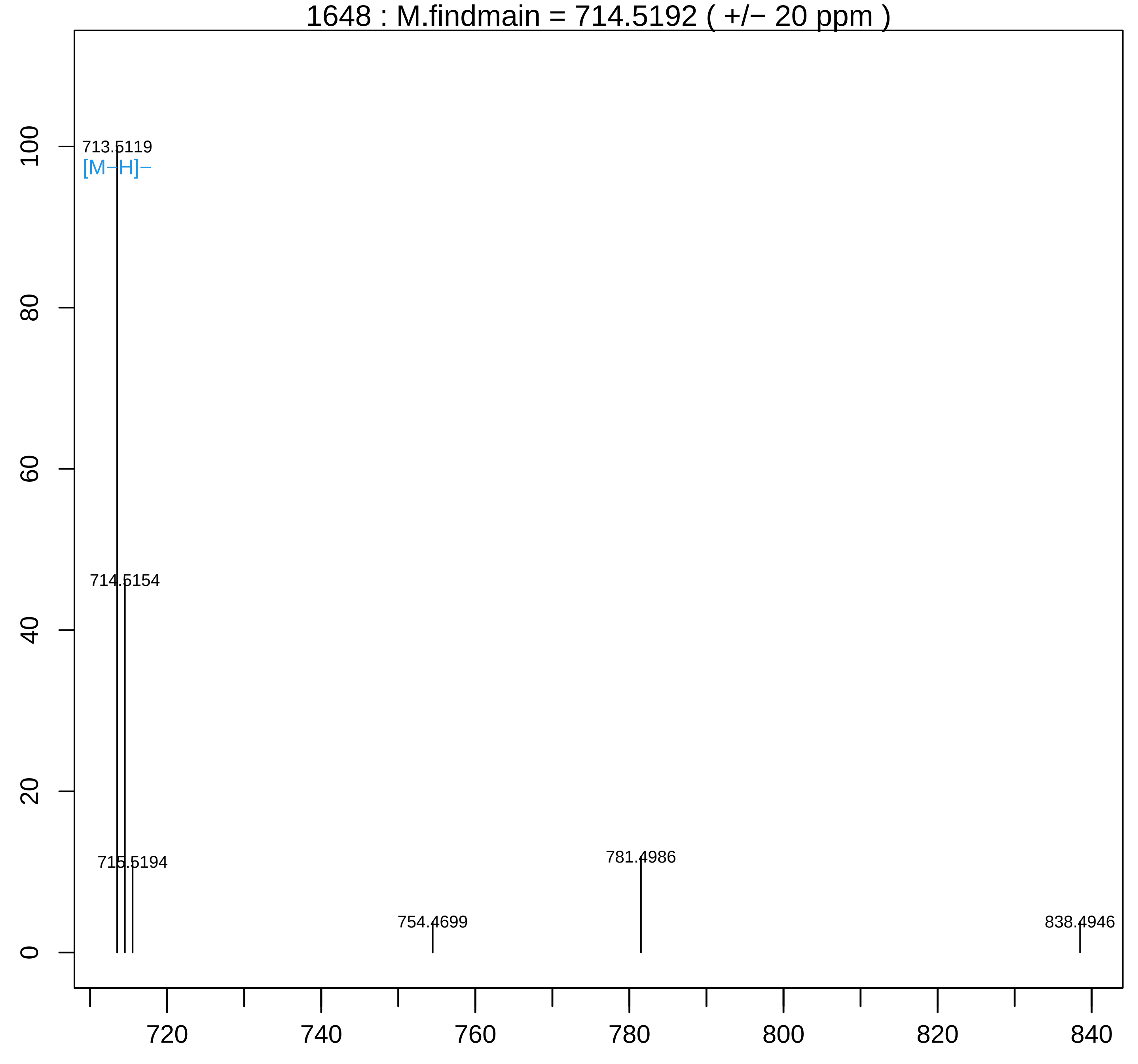
1648 : M.findmain = 714.5192 (+/− 20 ppm)
713.5119
[M−H]−
714.5154
715.5194
754.4699
781.4986
838.4946
0
20
40
60
80
100
720
740
760
780
800
820
840

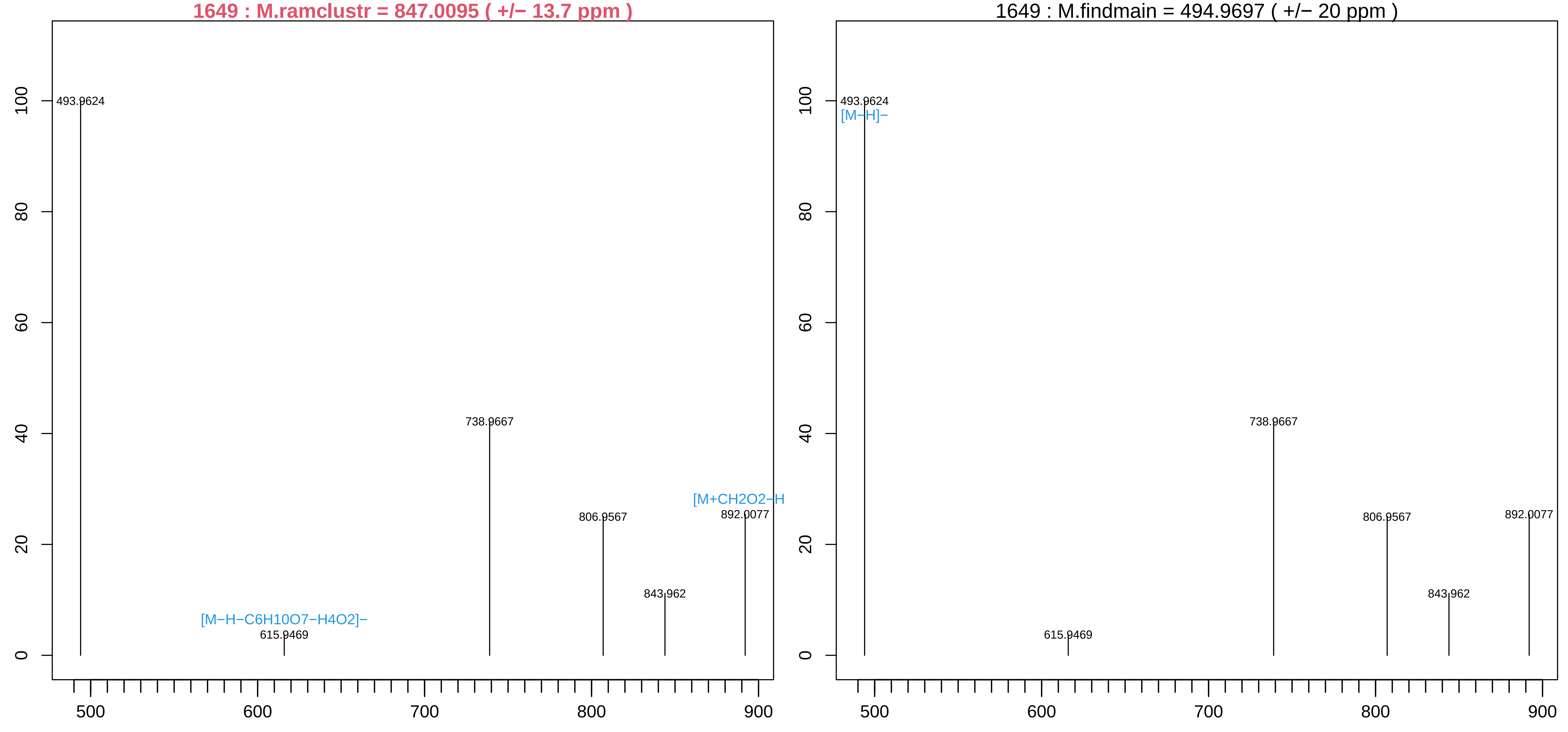

1649 : M.ramclustr = 847.0095 (+/− 13.7 ppm)
493.9624
[M−H−C6H10O7−H4O2]−
615.9469
738.9667
806.9567
843.962
[M+CH2O2−H
892.0077
1649 : M.findmain = 494.9697 (+/− 20 ppm)
493.9624
[M−H]−
615.9469
738.9667
806.9567
843.962
892.0077
0
20
40
60
80
100
500
600
700
800
900

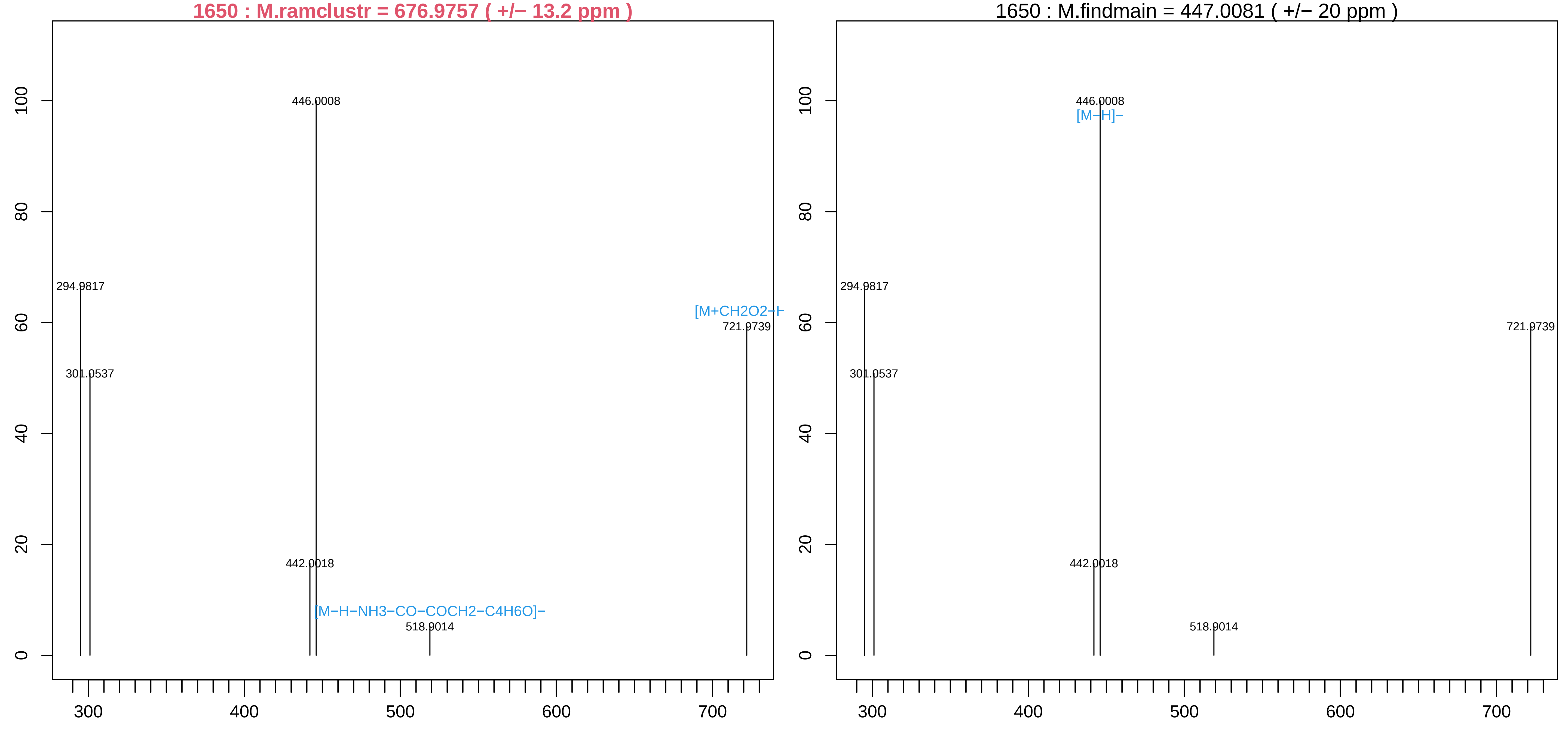
1650 : M.ramclustr = 676.9757 (+/− 13.2 ppm)
446.0008
294.9817
301.0537
[M+CH2O2−H
721.9739
442.0018
[M−H−NH3−CO−COCH2−C4H6O]−
518.9014
1650 : M.findmain = 447.0081 (+/− 20 ppm)
446.0008
[M−H]−
294.9817
301.0537
721.9739
442.0018
518.9014
0
20
40
60
80
100
300
400
500
600
700

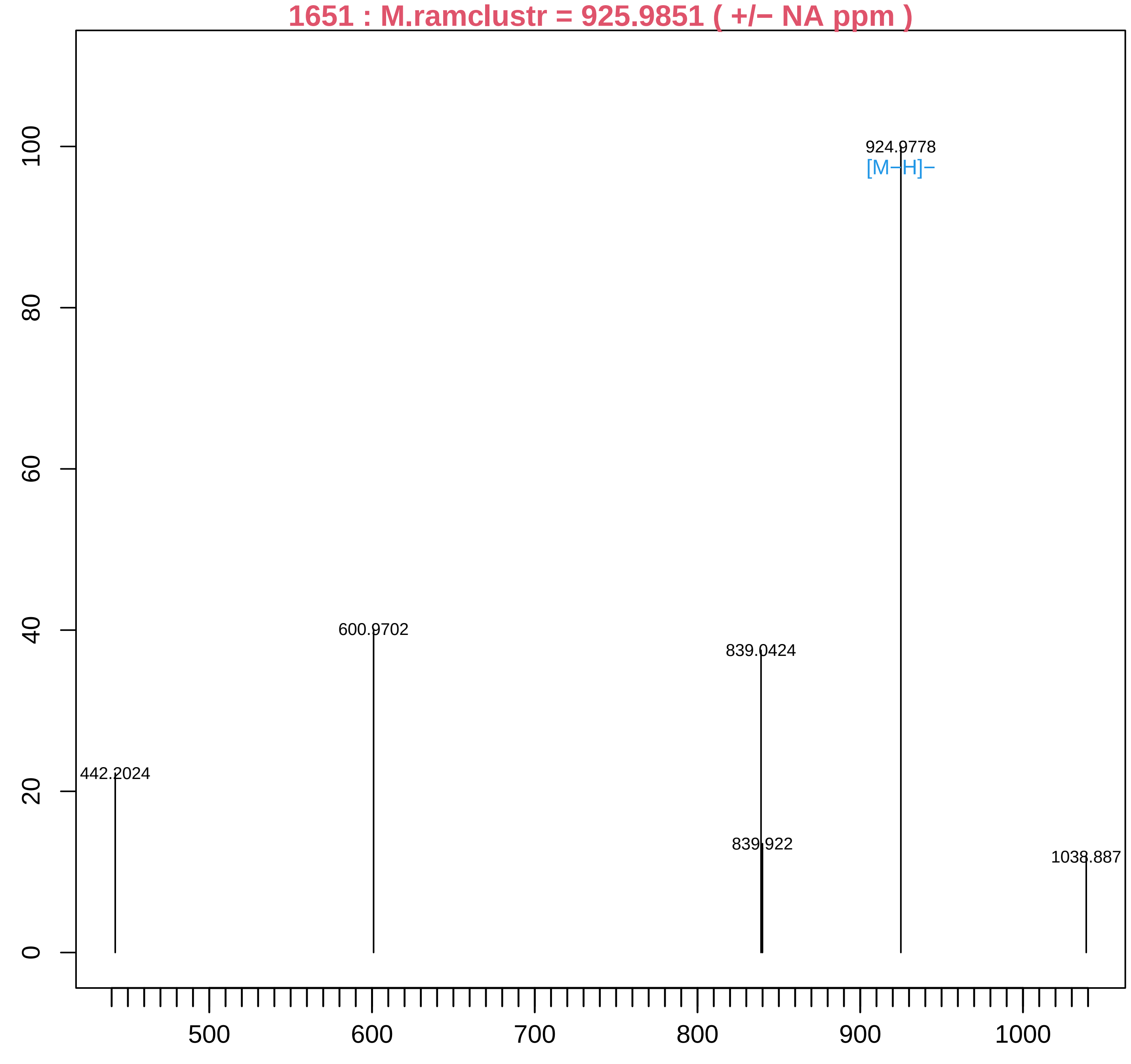
1651 : M.ramclustr = 925.9851 (+/− NA ppm)
924.9778
[M−H]−
600.9702
839.0424
442.2024
839.922
1038.887
0
20
40
60
80
100
500
600
700
800
900
1000

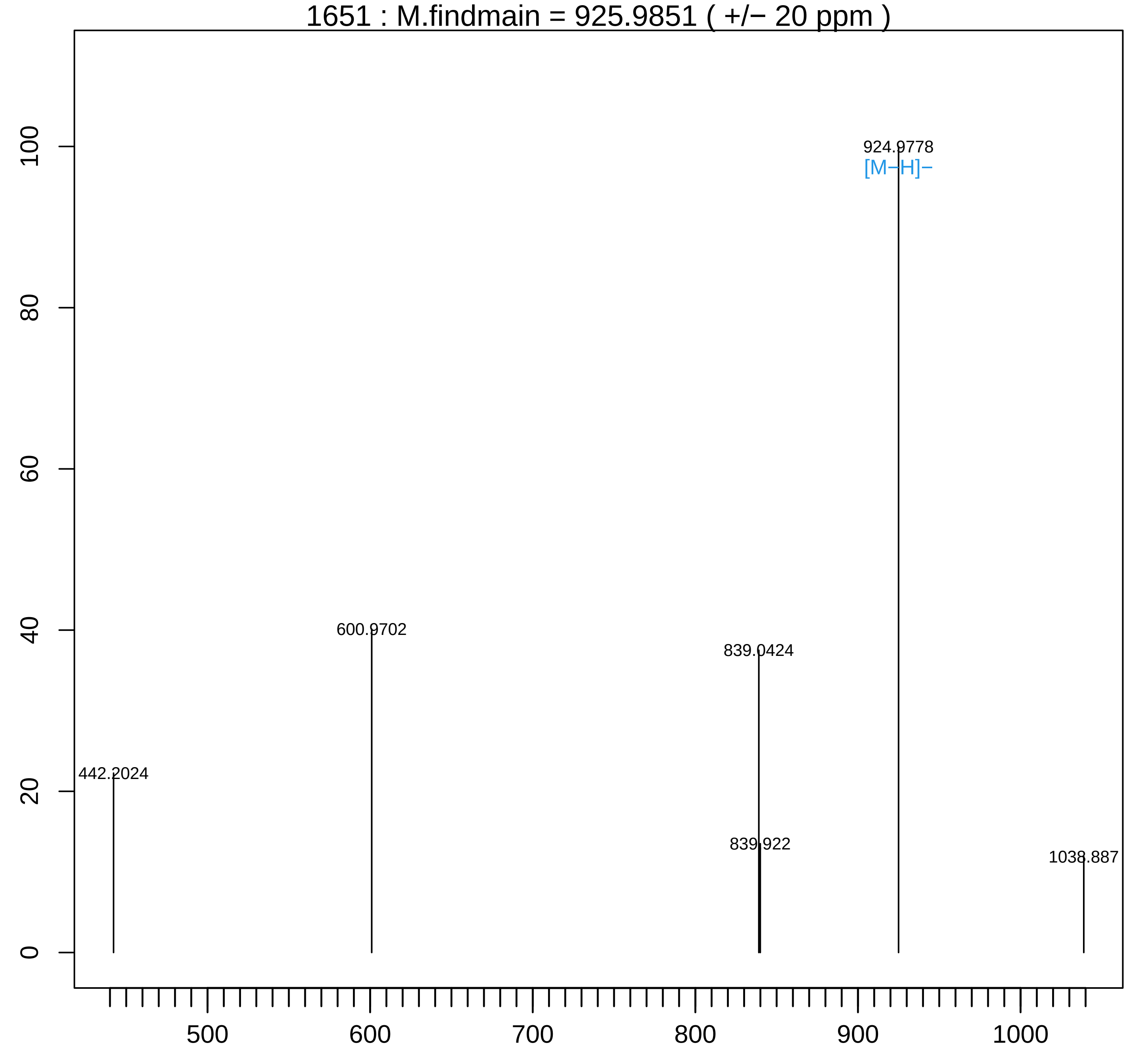
1651 : M.findmain = 925.9851 (+/− 20 ppm)
924.9778
[M−H]−
600.9702
839.0424
442.2024
839.922
1038.887
0
20
40
60
80
100
500
600
700
800
900
1000

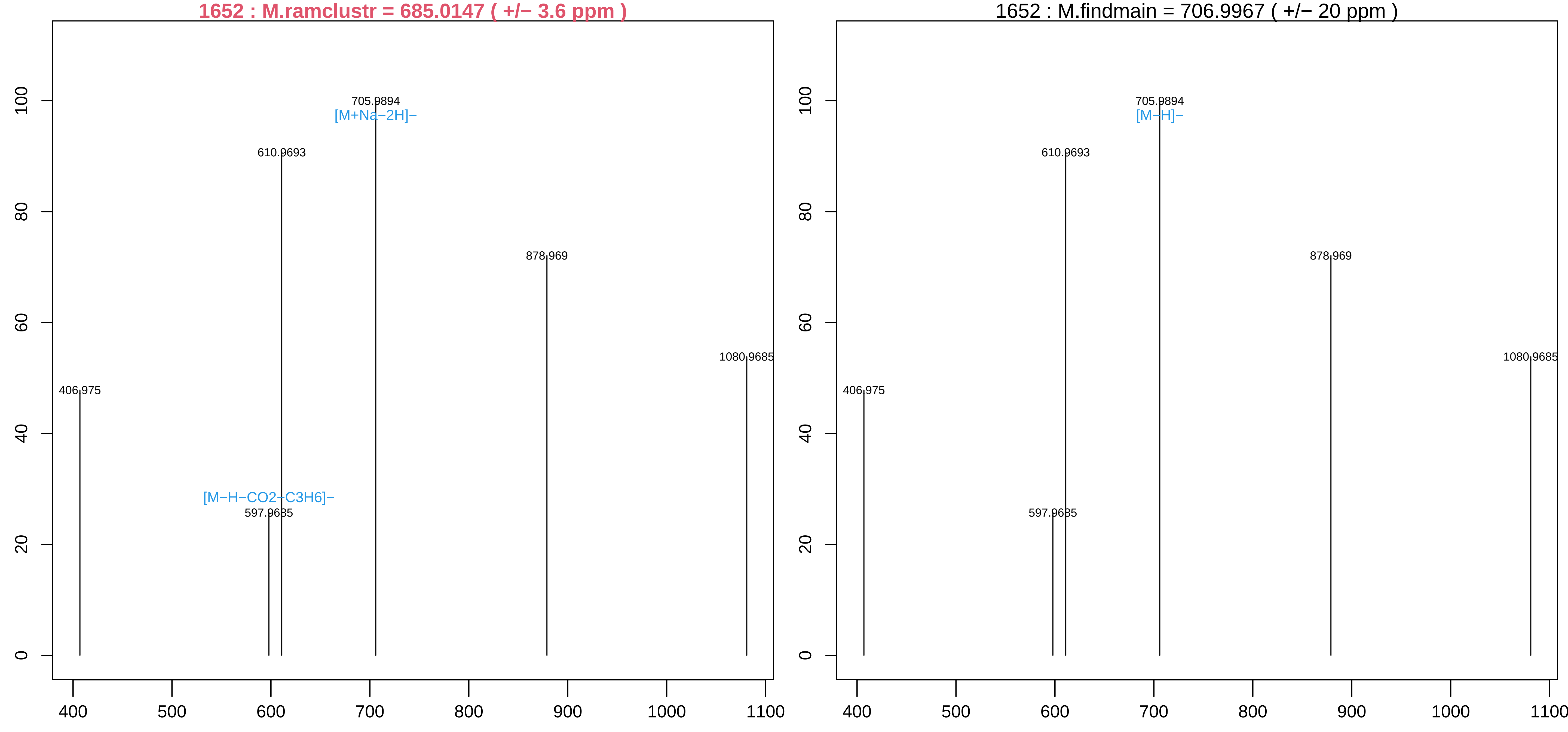

1652 : M.ramclustr = 685.0147 (+/− 3.6 ppm)
705.9894
[M+Na−2H]−
610.9693
878.969
1080.9685
406.975
[M−H−CO2−C3H6]−
597.9685
1652 : M.findmain = 706.9967 (+/− 20 ppm)
705.9894
[M−H]−
610.9693
878.969
1080.9685
406.975
597.9685

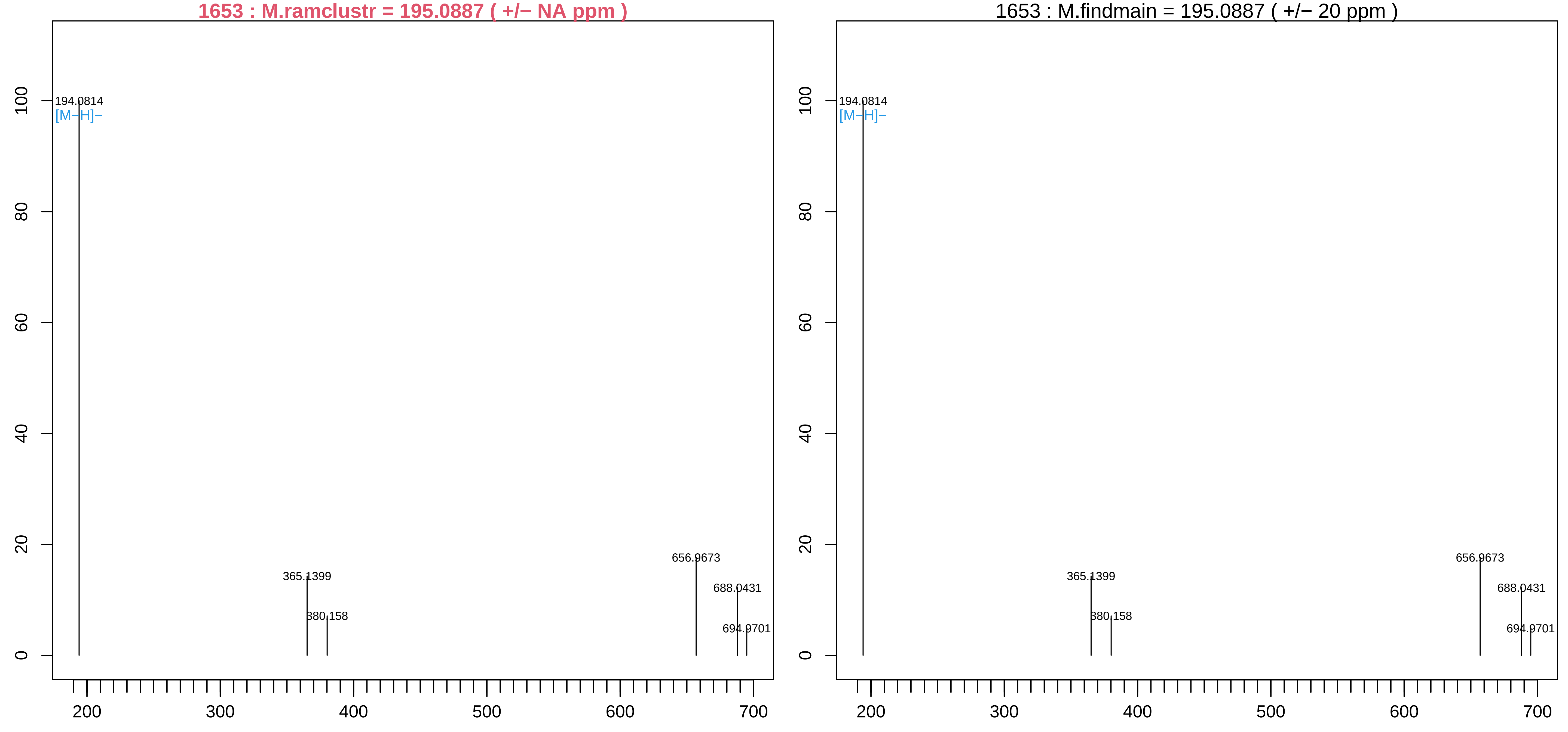
1653 : M.ramclustr = 195.0887 (+/− NA ppm)
194.0814
[M−H]−
365.1399
380.158
656.9673
688.0431
694.9701
0
20
40
60
80
100
200
300
400
500
600
700
1653 : M.findmain = 195.0887 (+/− 20 ppm)
194.0814
[M−H]−
365.1399
380.158
656.9673
688.0431
694.9701
0
20
40
60
80
100
200
300
400
500
600
700

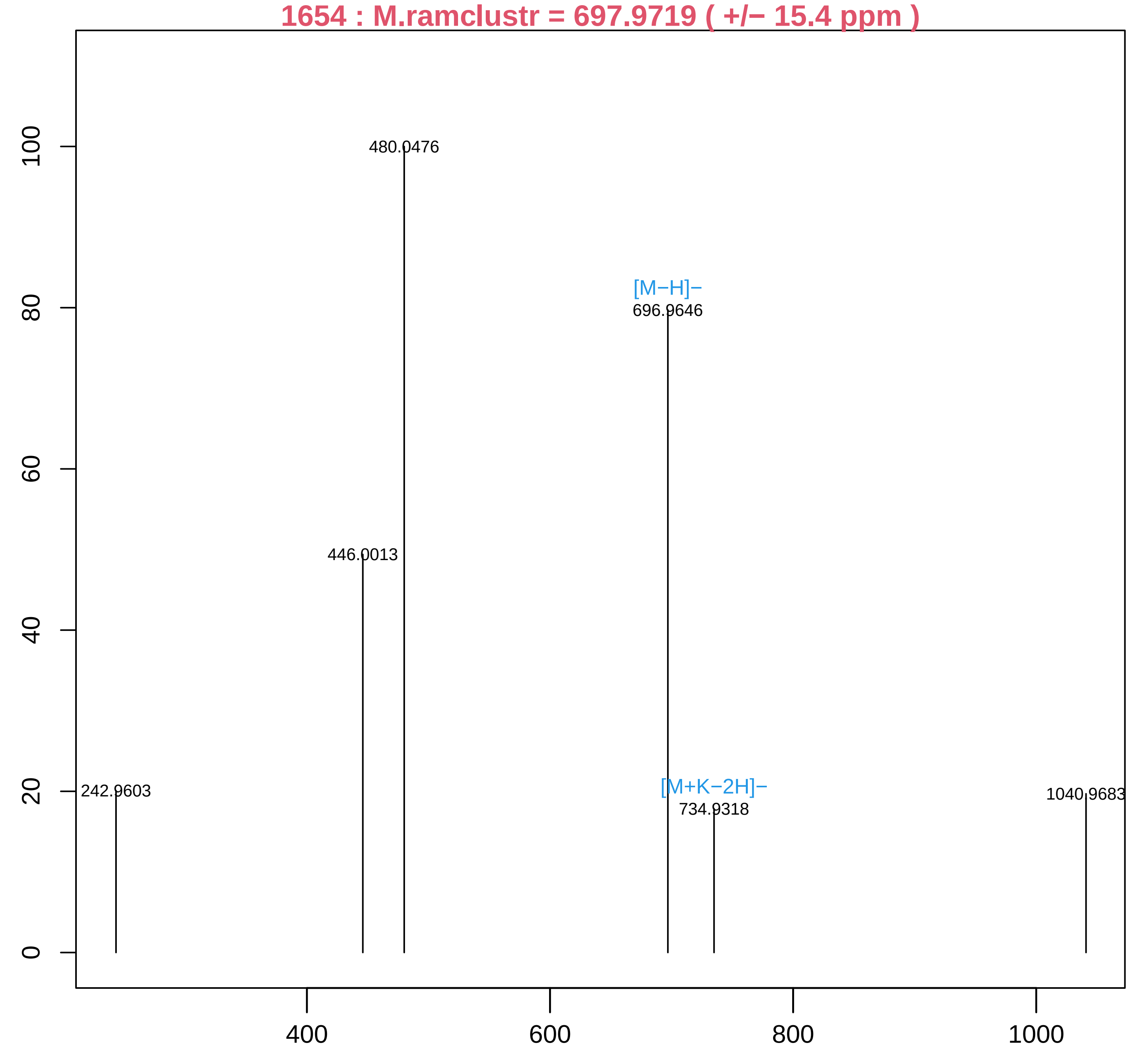
1654 : M.ramclustr = 697.9719 (+/− 15.4 ppm)
0
20
40
60
80
100
400
600
800
1000
480.0476
[M−H]−
696.9646
446.0013
242.9603
[M+K−2H]−
734.9318
1040.9683

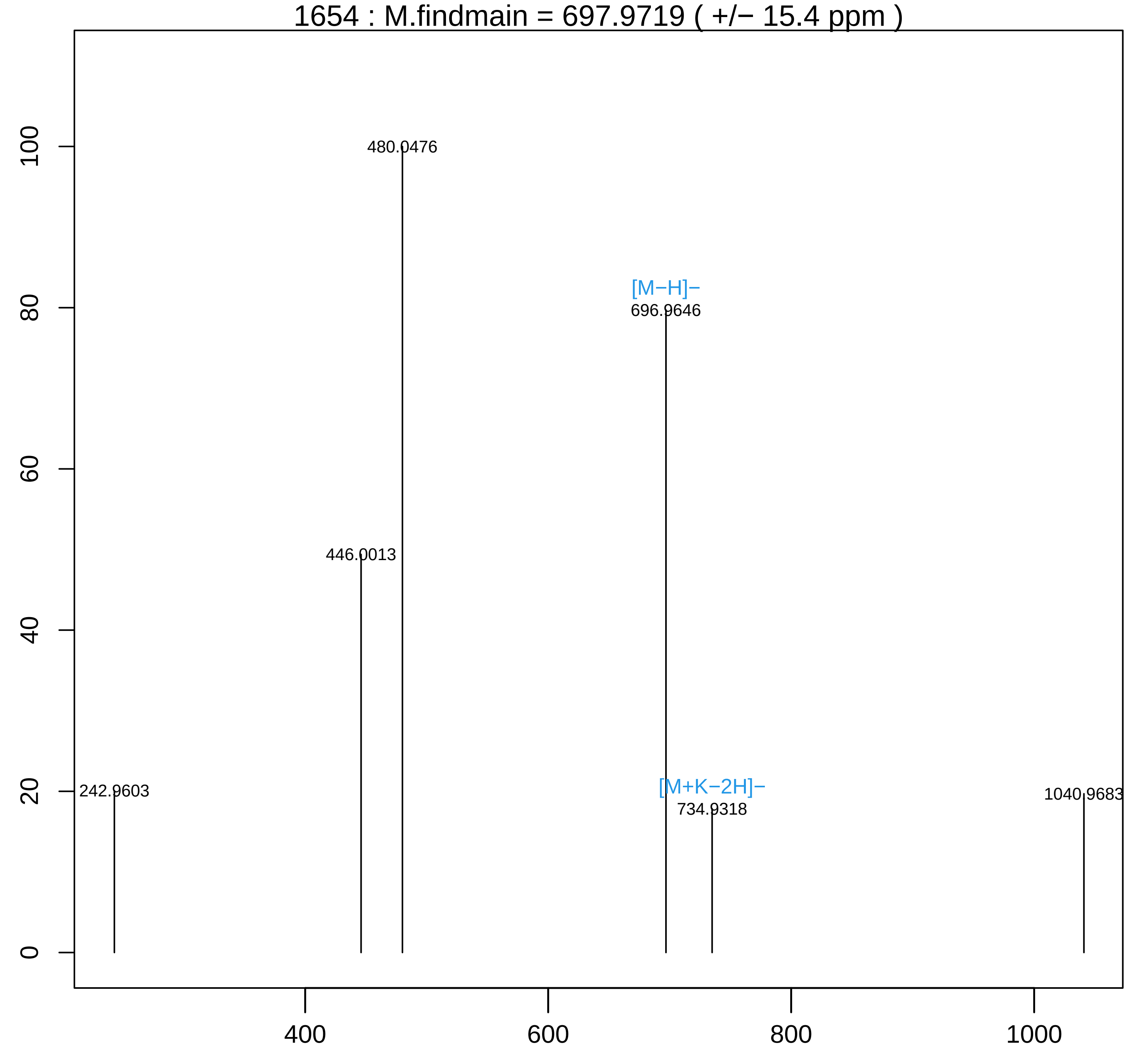
1654 : M.findmain = 697.9719 (+/− 15.4 ppm)
0
20
40
60
80
100
400
600
800
1000
480.0476
[M−H]−
696.9646
446.0013
242.9603
[M+K−2H]−
734.9318
1040.9683

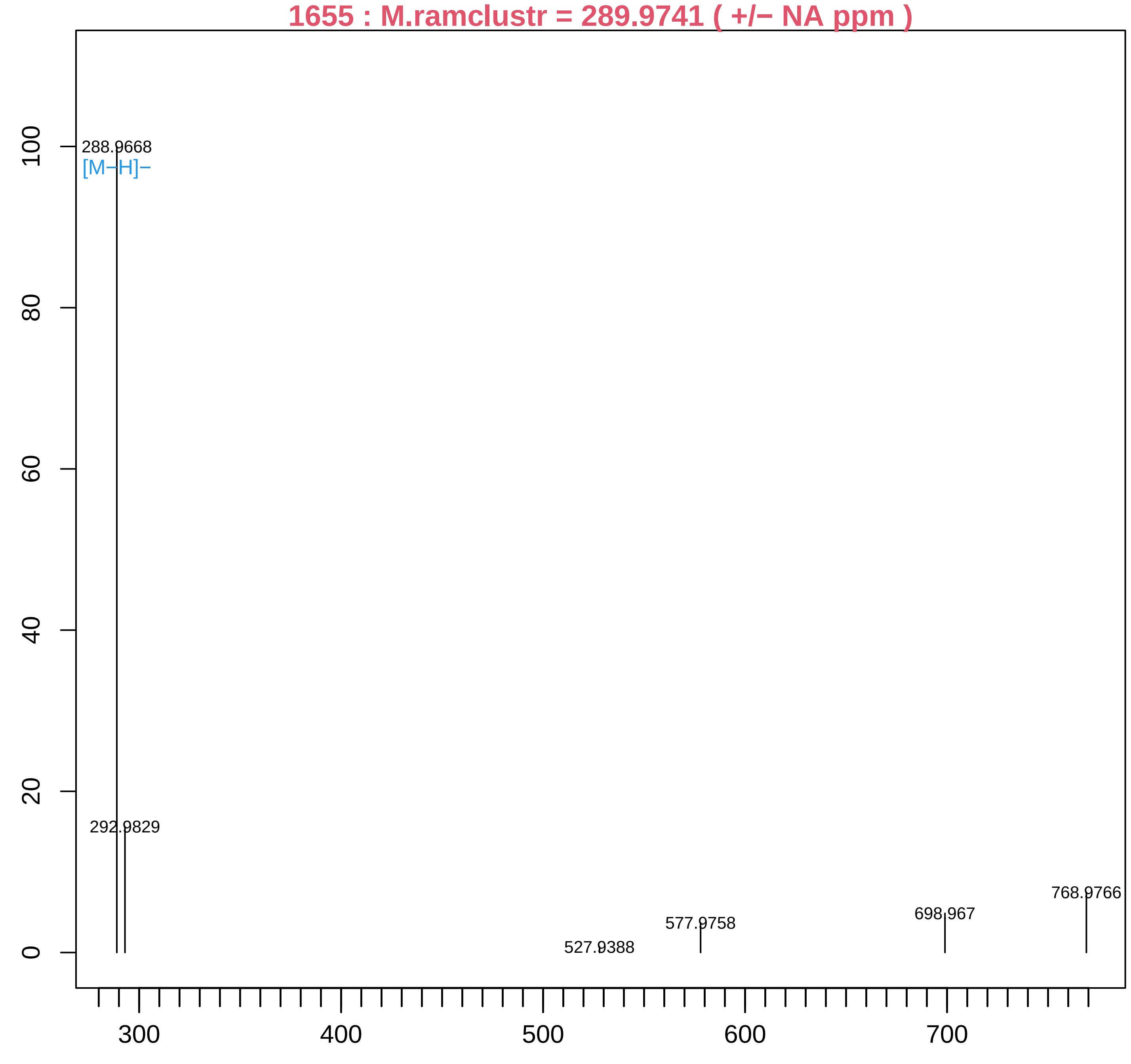
1655 : M.ramclustr = 289.9741 (+/− NA ppm)
288.9668
[M−H]−
292.9829
527.9388
577.9758
698.967
768.9766
0
20
40
60
80
100
300
400
500
600
700

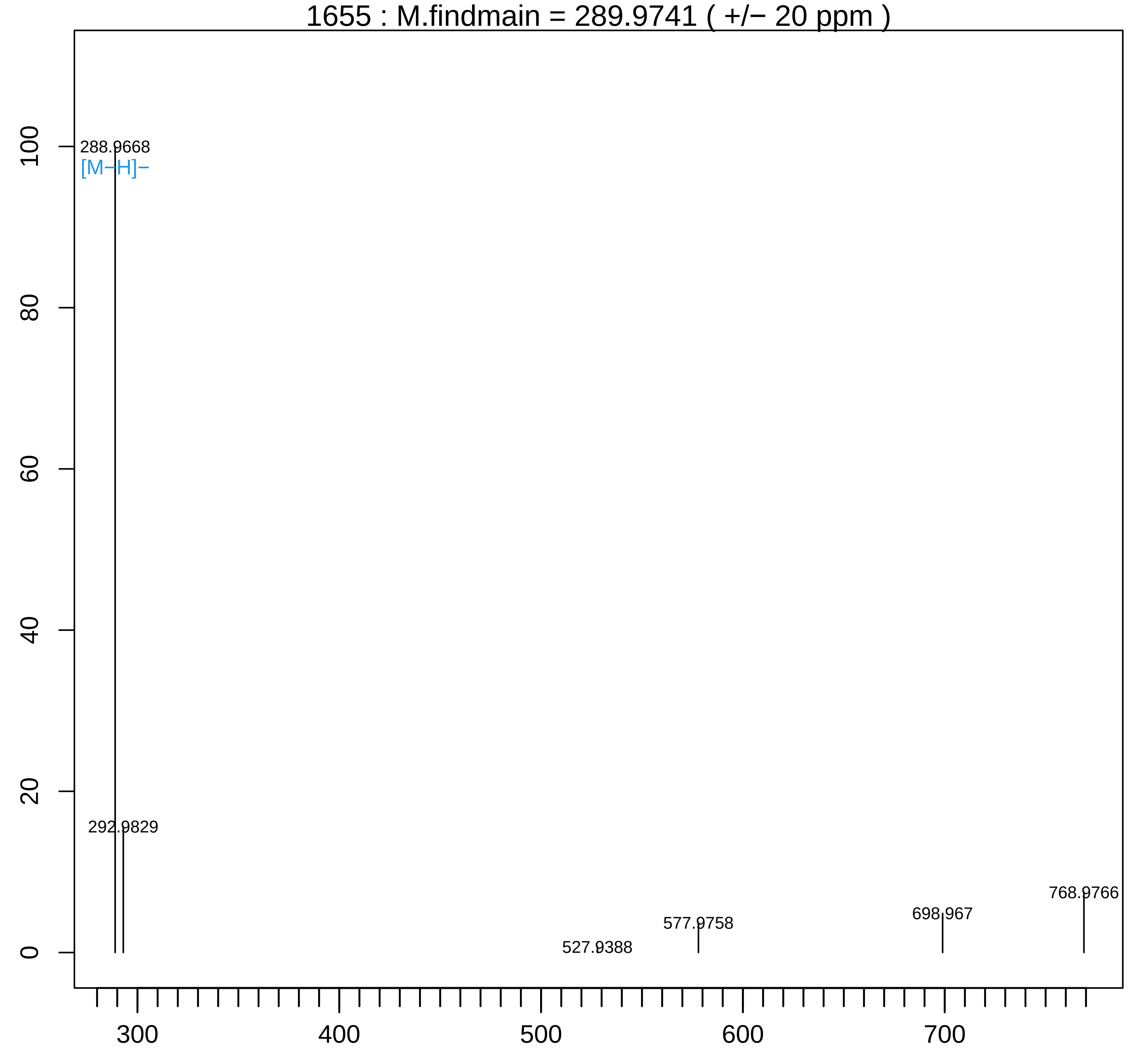
1655 : M.findmain = 289.9741 (+/− 20 ppm)
288.9668
[M−H]−
292.9829
527.9388
577.9758
698.967
768.9766
0
20
40
60
80
100
300
400
500
600
700

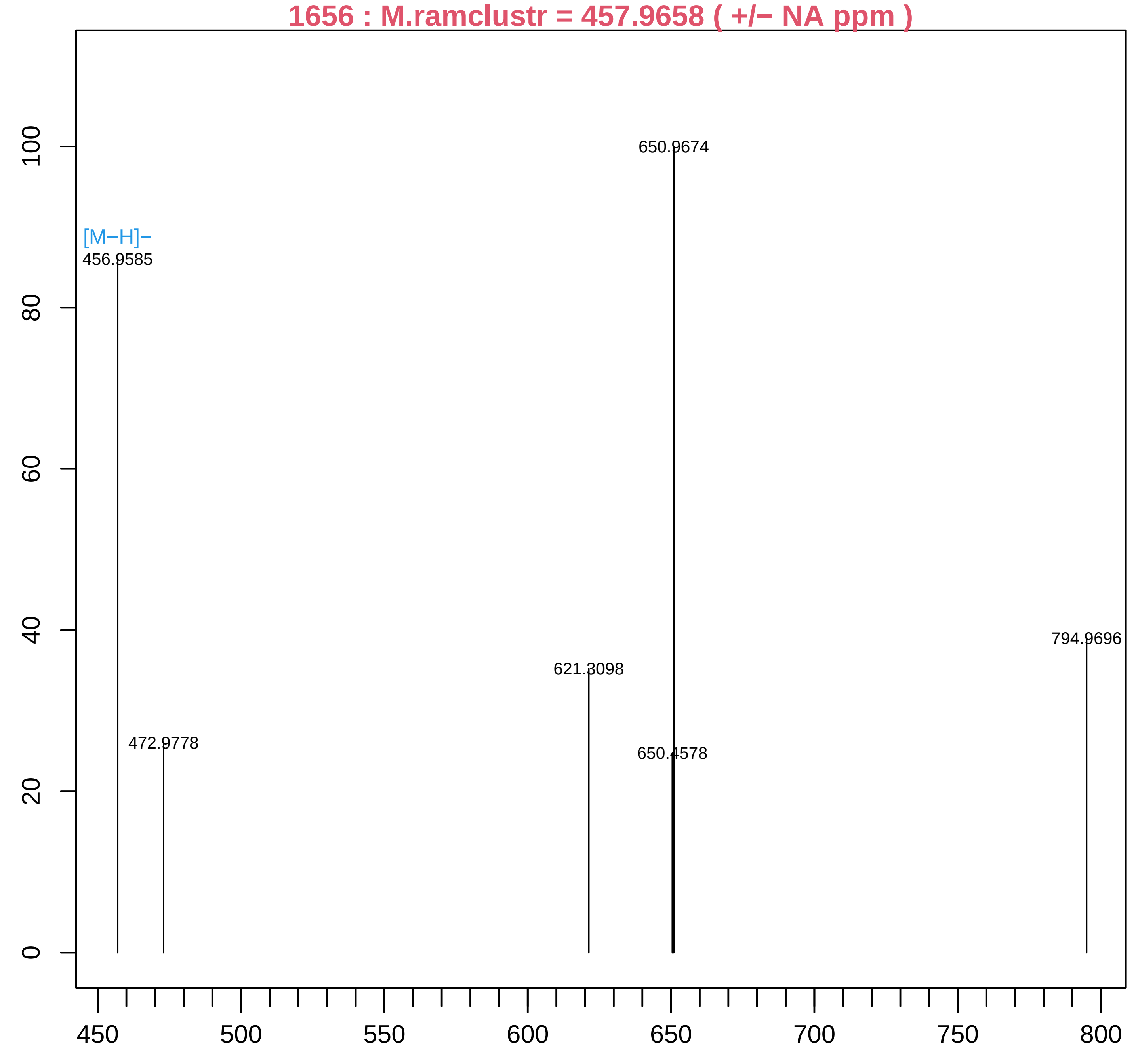
1656 : M.ramclustr = 457.9658 (+/− NA ppm)
[M−H]−
456.9585
472.9778
621.3098
650.9674
650.4578
794.9696
0
20
40
60
80
100
450
500
550
600
650
700
750
800

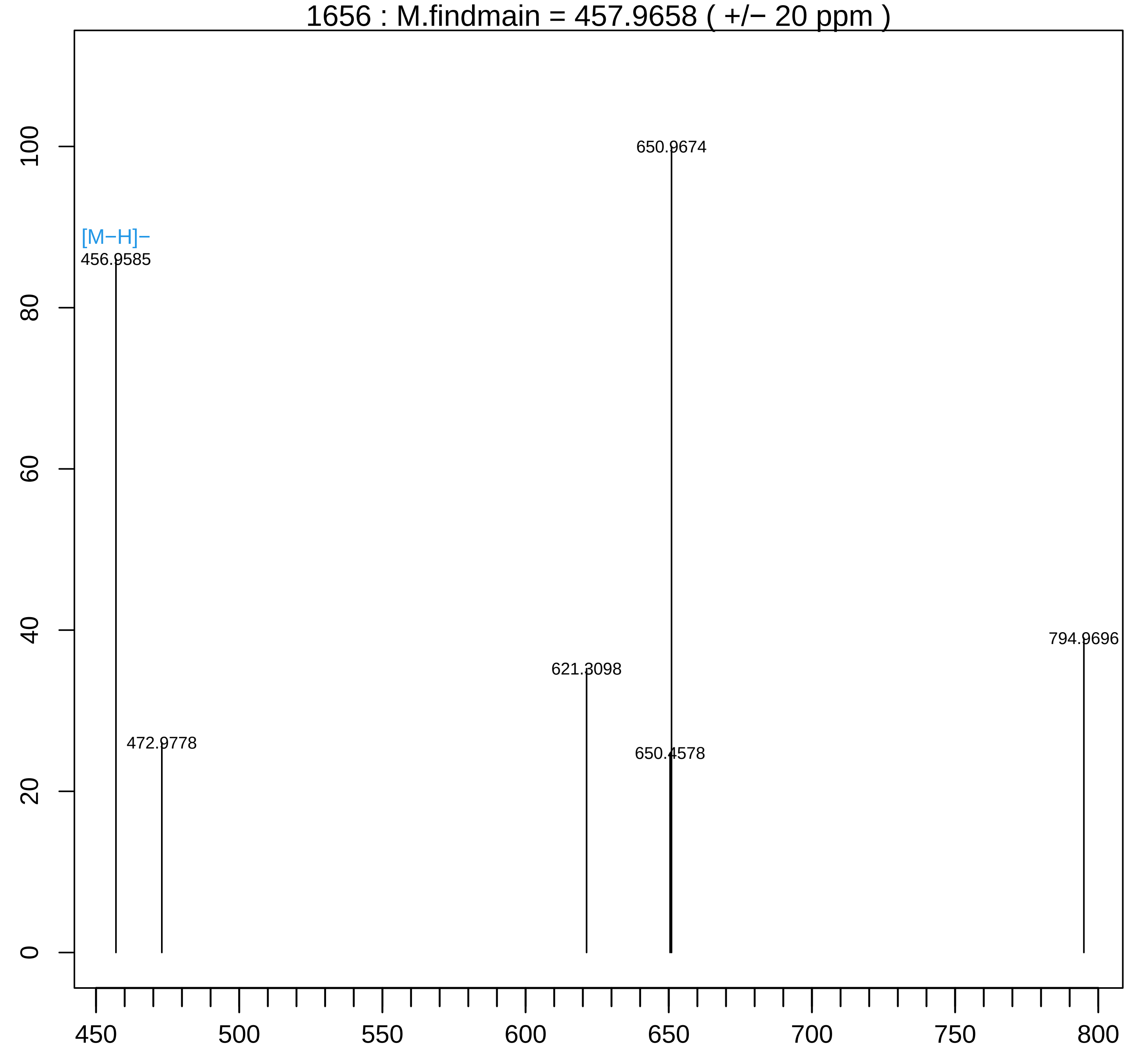
1656 : M.findmain = 457.9658 (+/− 20 ppm)
[M−H]−
456.9585
472.9778
621.3098
650.9674
650.4578
794.9696
0
20
40
60
80
100
450
500
550
600
650
700
750
800

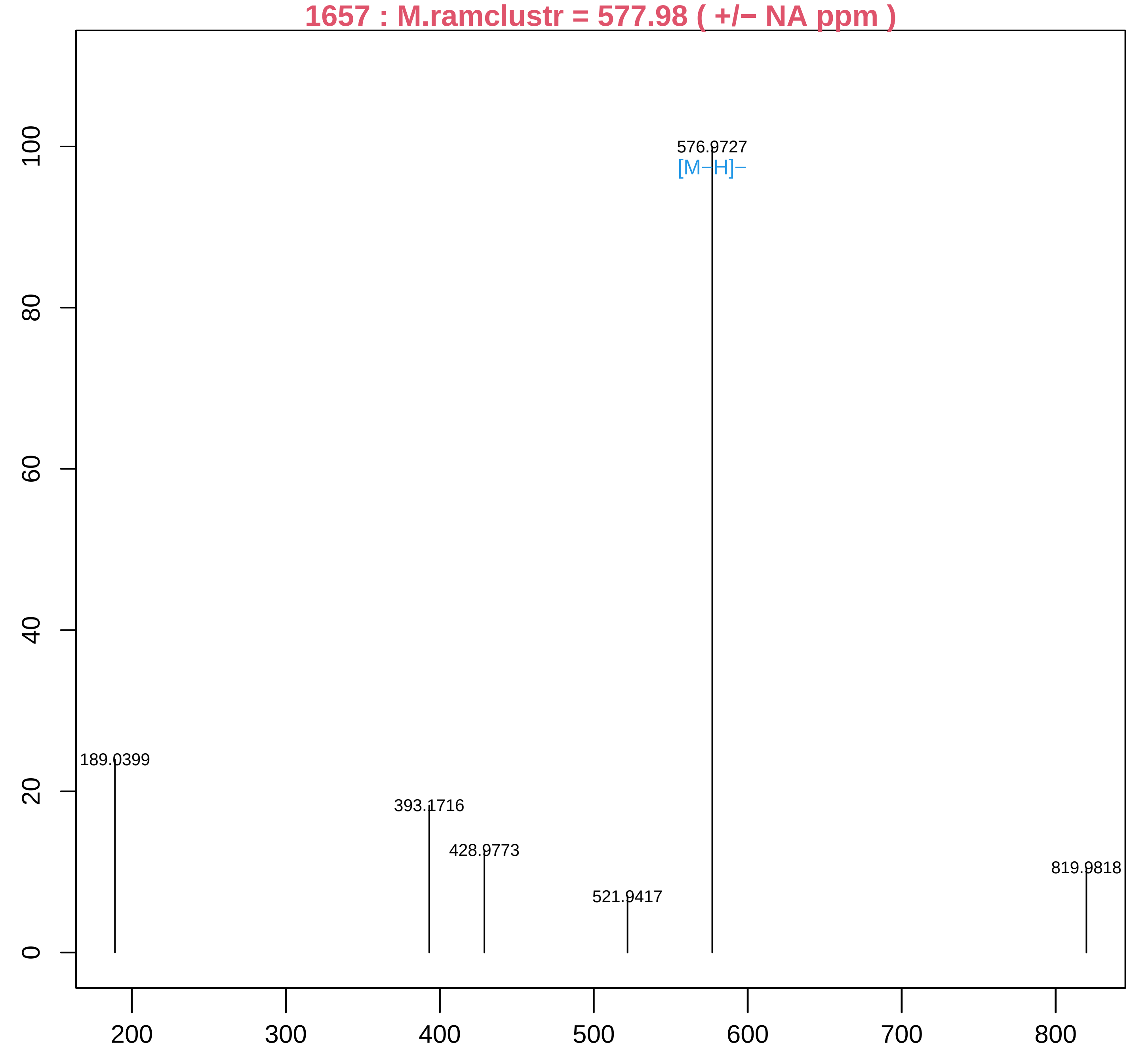
1657 : M.ramclustr = 577.98 (+/− NA ppm)
576.9727
[M−H]−
189.0399
393.1716
428.9773
521.9417
819.9818
0
20
40
60
80
100
200
300
400
500
600
700
800

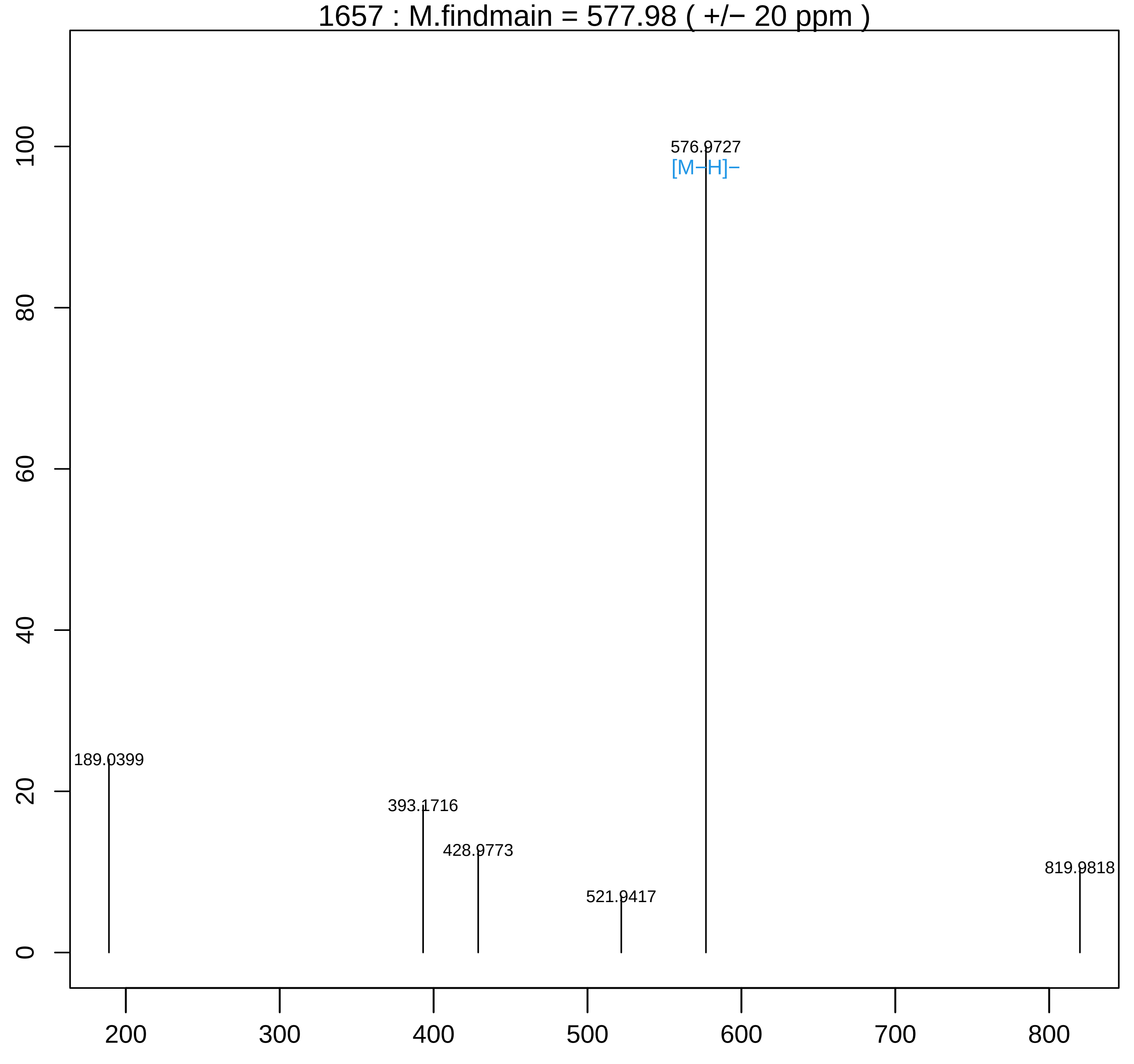
1657 : M.findmain = 577.98 (+/− 20 ppm)
576.9727
[M−H]−
189.0399
393.1716
428.9773
521.9417
819.9818
0
20
40
60
80
100
200
300
400
500
600
700
800

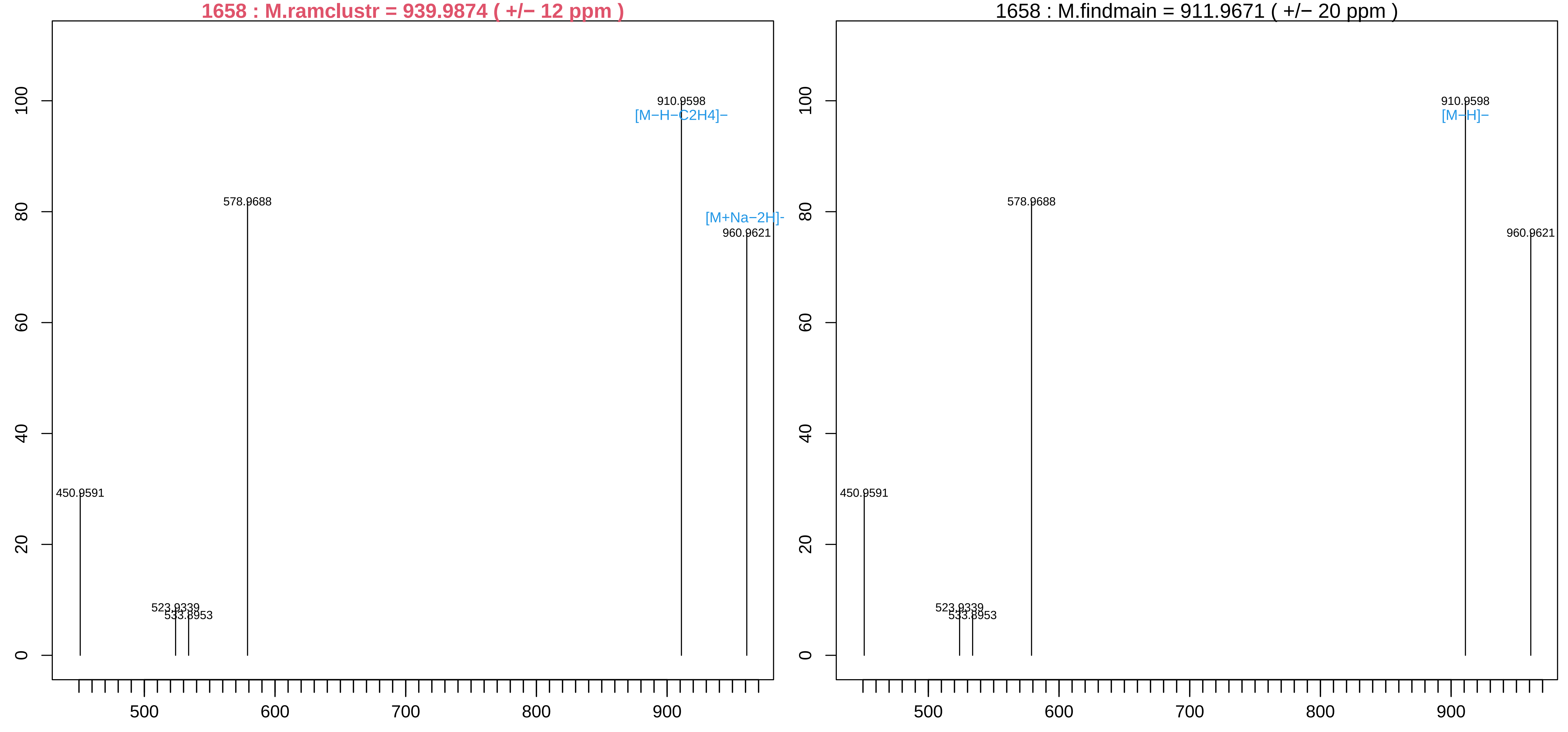

1658 : M.ramclustr = 939.9874 (+/− 12 ppm)
1658 : M.findmain = 911.9671 (+/− 20 ppm)
910.9598
[M−H−C2H4]−
[M−H]−
578.9688
[M+Na−2H]-
960.9621
450.9591
523.9339
533.8953
0
20
40
60
80
100
500
600
700
800
900

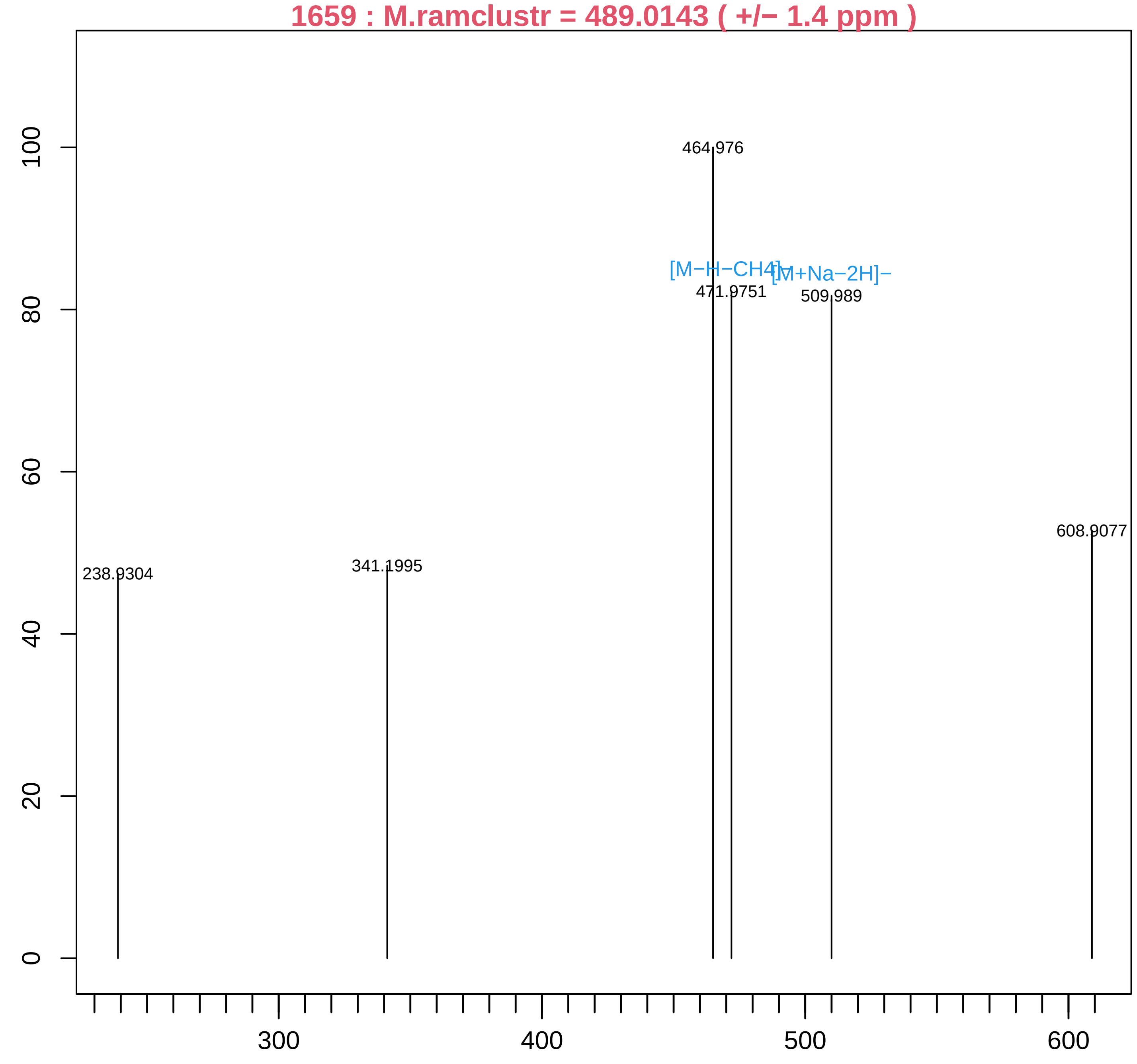
1659 : M.ramclustr = 489.0143 (+/− 1.4 ppm)
464.976
[M−H−CH4]−
471.9751
[M+Na−2H]−
509.989
608.9077
238.9304
341.1995
0
20
40
60
80
100
300
400
500
600

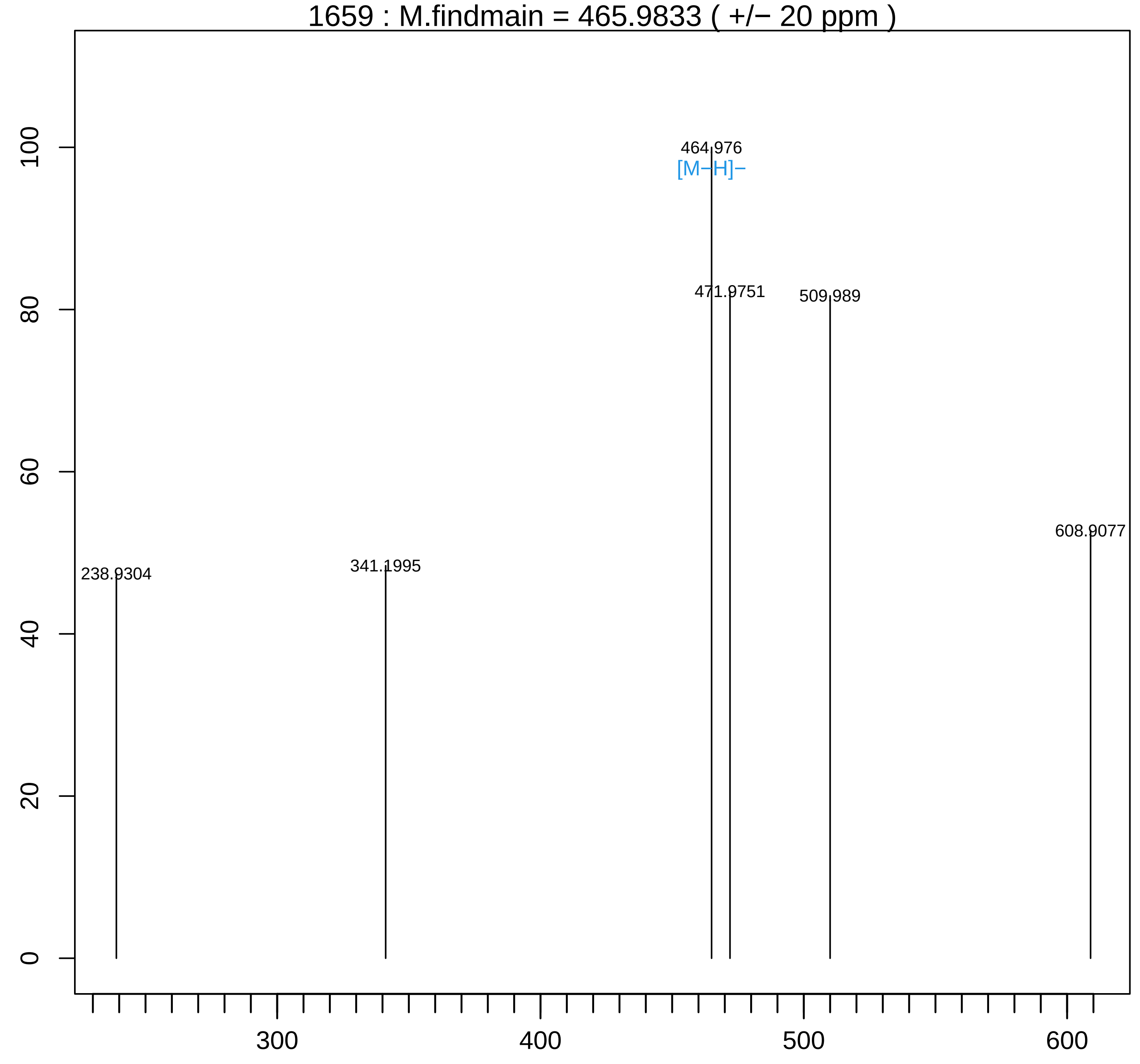
1659 : M.findmain = 465.9833 (+/− 20 ppm)
464.976
[M−H]−
471.9751
509.989
608.9077
238.9304
341.1995
0
20
40
60
80
100
300
400
500
600

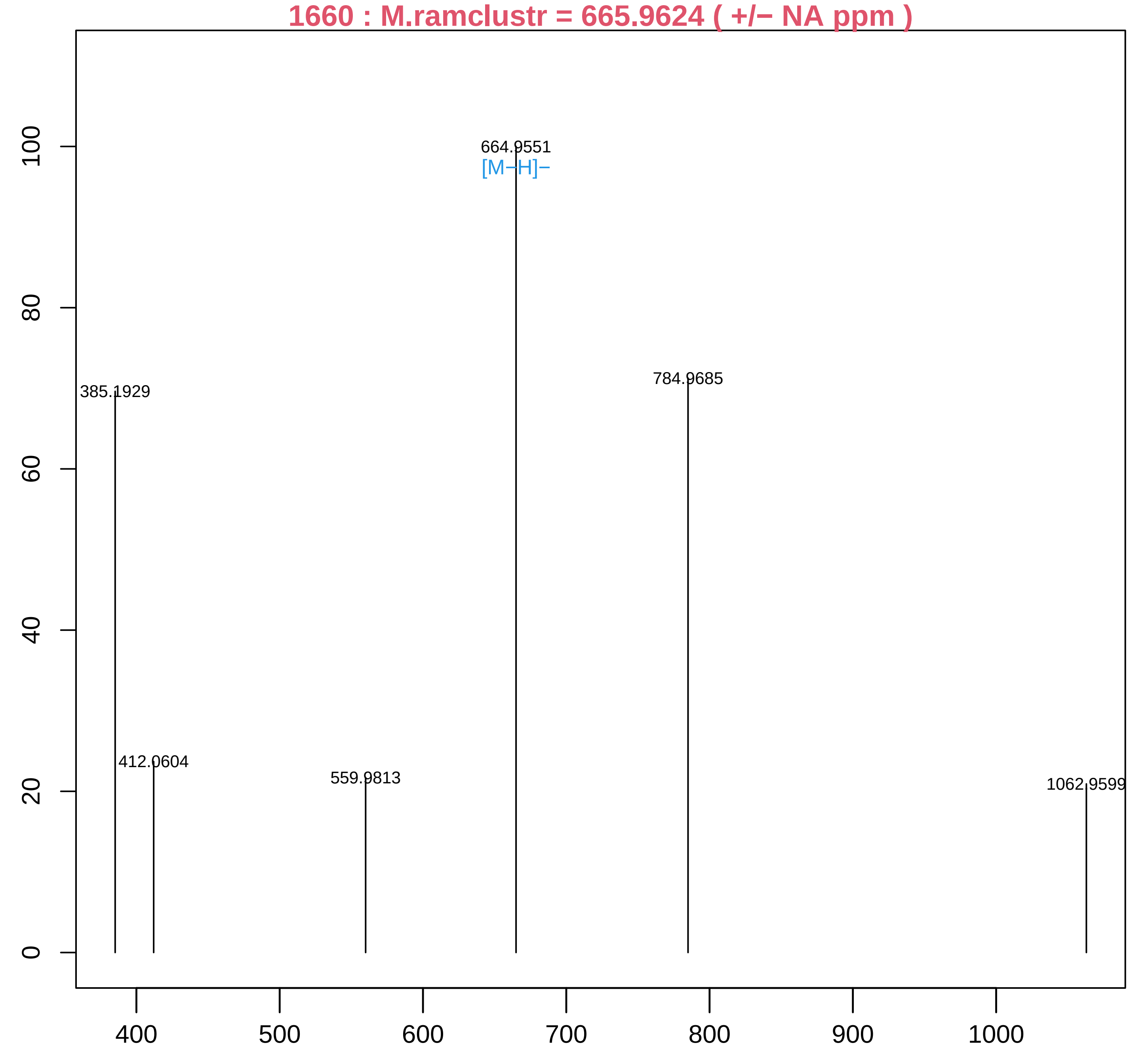

1660 : M.ramclustr = 665.9624 (+/− NA ppm)
664.9551
[M−H]−
784.9685
385.1929
412.0604
559.9813
1062.9599

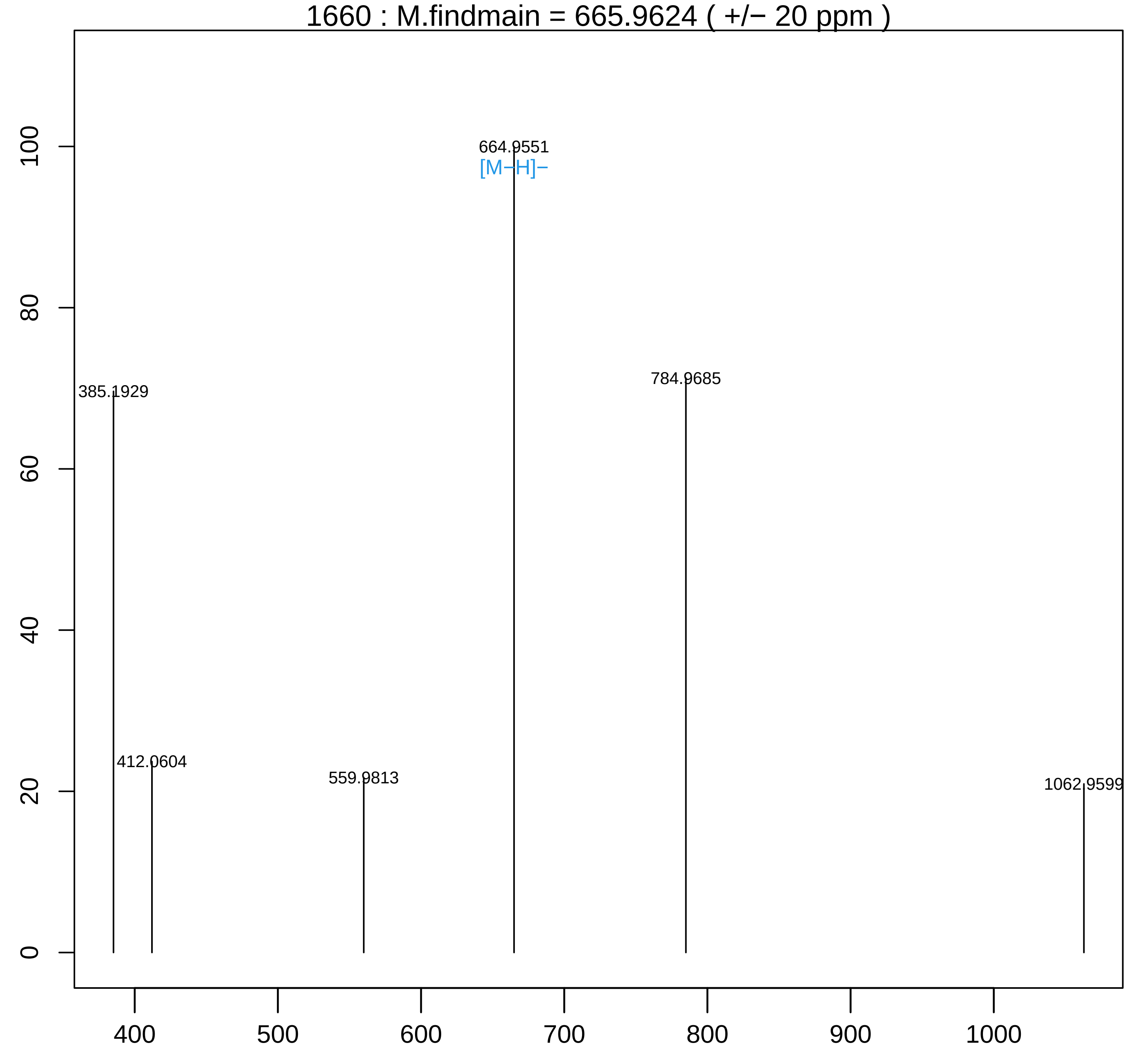

1660 : M.findmain = 665.9624 (+/− 20 ppm)
664.9551
[M−H]−
784.9685
385.1929
412.0604
559.9813
1062.9599

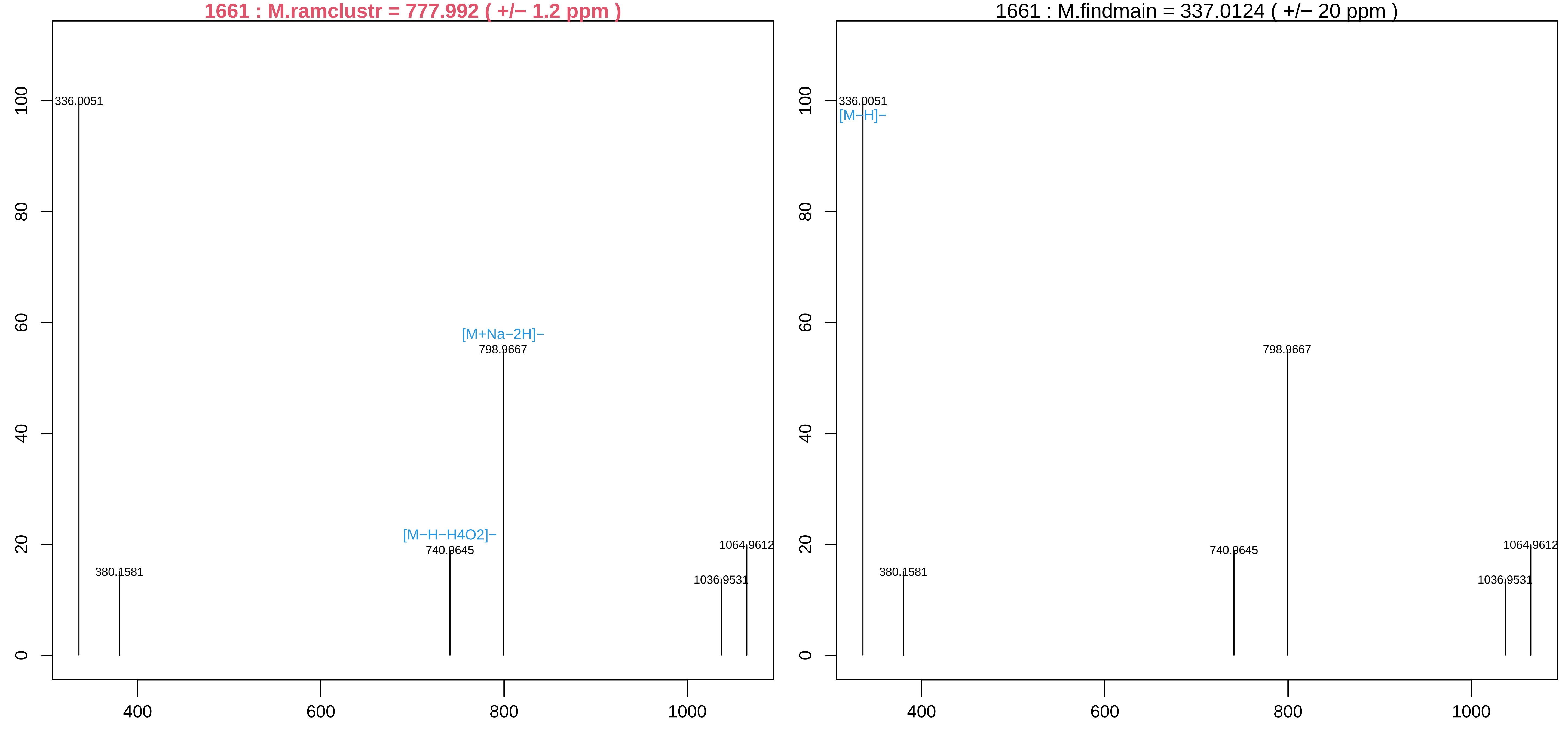
1661 : M.ramclustr = 777.992 (+/− 1.2 ppm)
336.0051
380.1581
[M−H−H4O2]−
740.9645
[M+Na−2H]−
798.9667
1036.9531
1064.9612
1661 : M.findmain = 337.0124 (+/− 20 ppm)
336.0051
[M−H]−
380.1581
740.9645
798.9667
1036.9531
1064.9612
0
20
40
60
80
100
400
600
800
1000

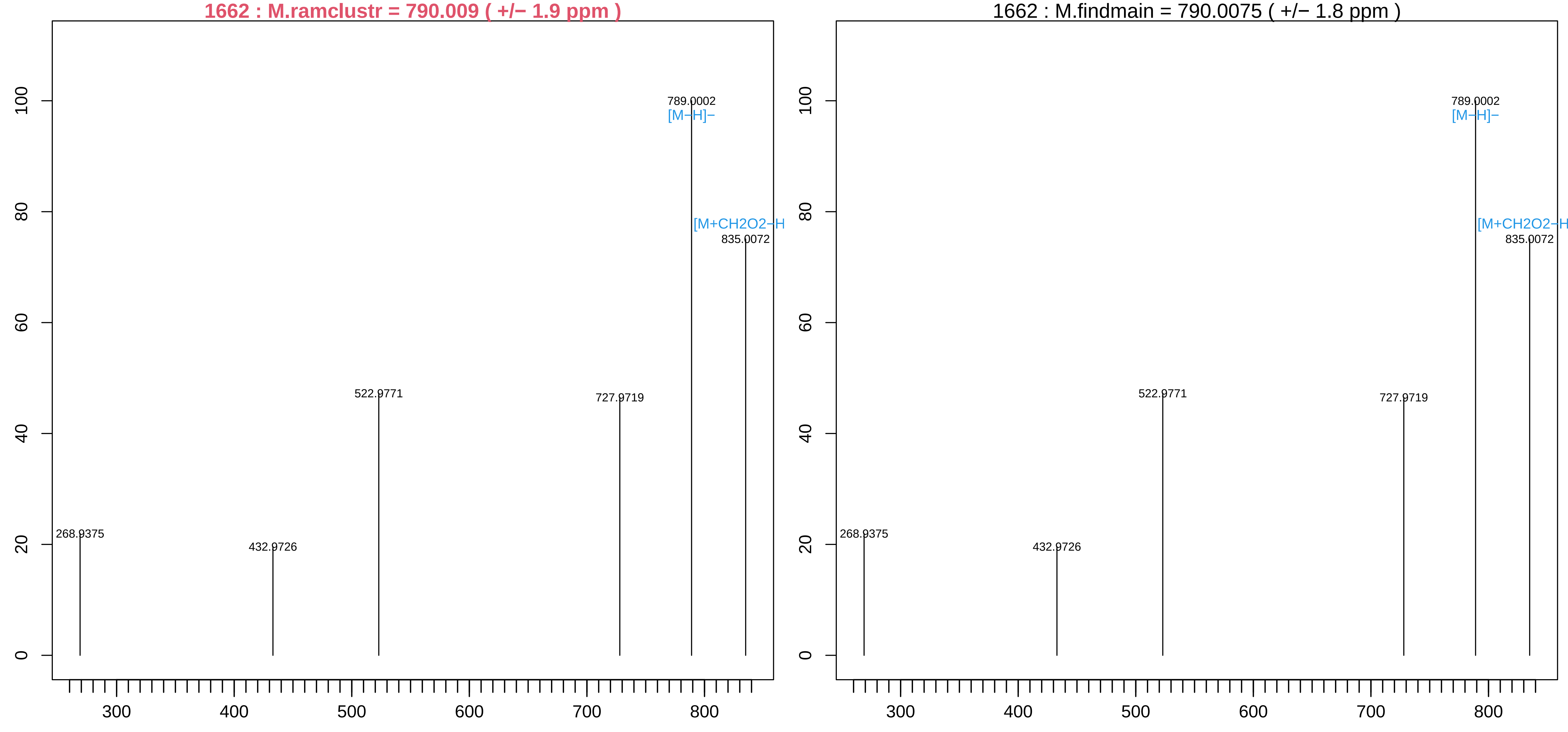
1662 : M.ramclustr = 790.009 (+/− 1.9 ppm)
789.0002
[M−H]−
[M+CH2O2−H
835.0072
522.9771
727.9719
268.9375
432.9726
0
20
40
60
80
100
300
400
500
600
700
800
1662 : M.findmain = 790.0075 (+/− 1.8 ppm)
789.0002
[M−H]−
[M+CH2O2−H
835.0072
522.9771
727.9719
268.9375
432.9726
0
20
40
60
80
100
300
400
500
600
700
800

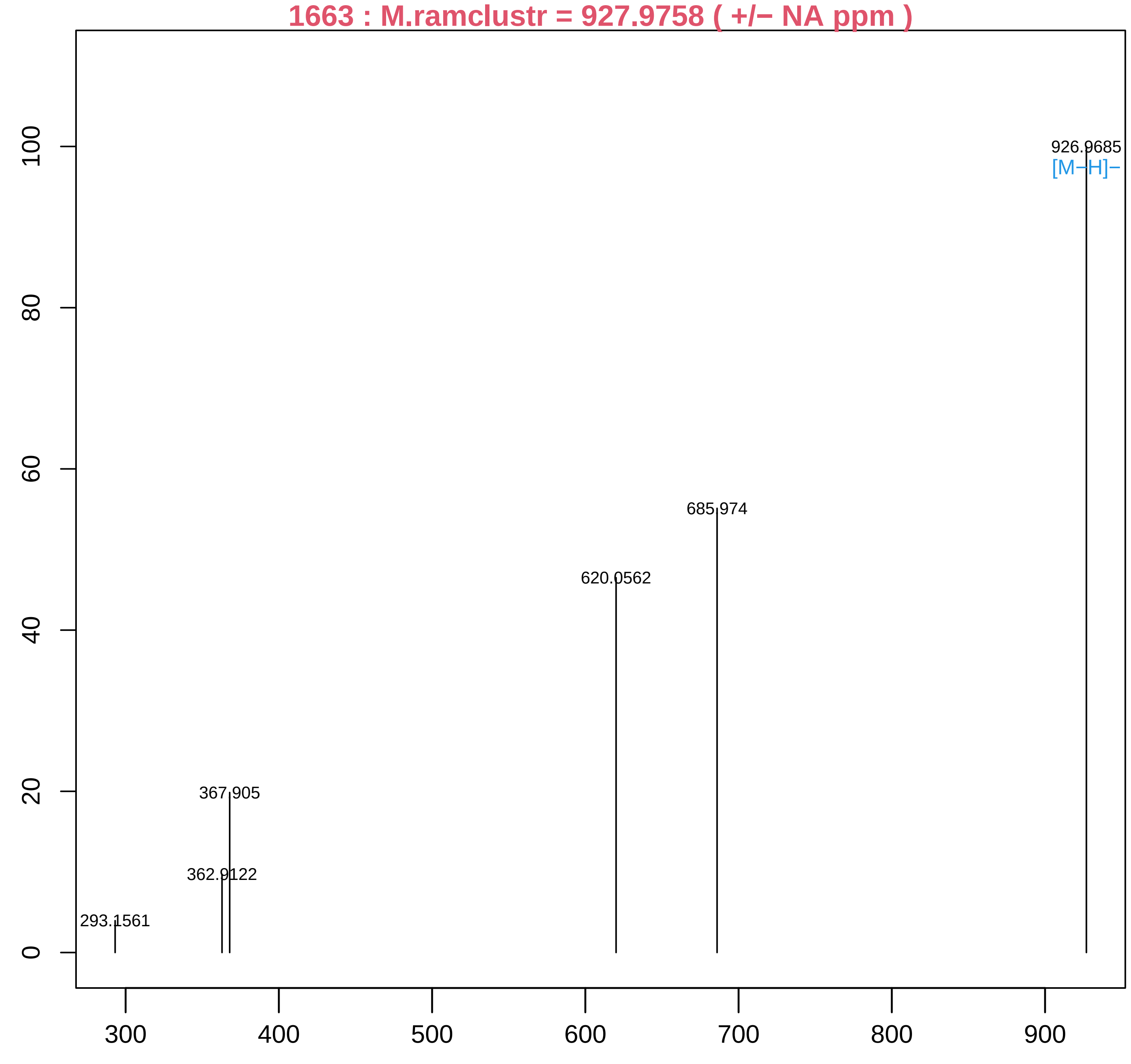
1663 : M.ramclustr = 927.9758 (+/− NA ppm)
926.9685
[M−H]−
685.974
620.0562
367.905
362.9122
293.1561
0
20
40
60
80
100
300
400
500
600
700
800
900

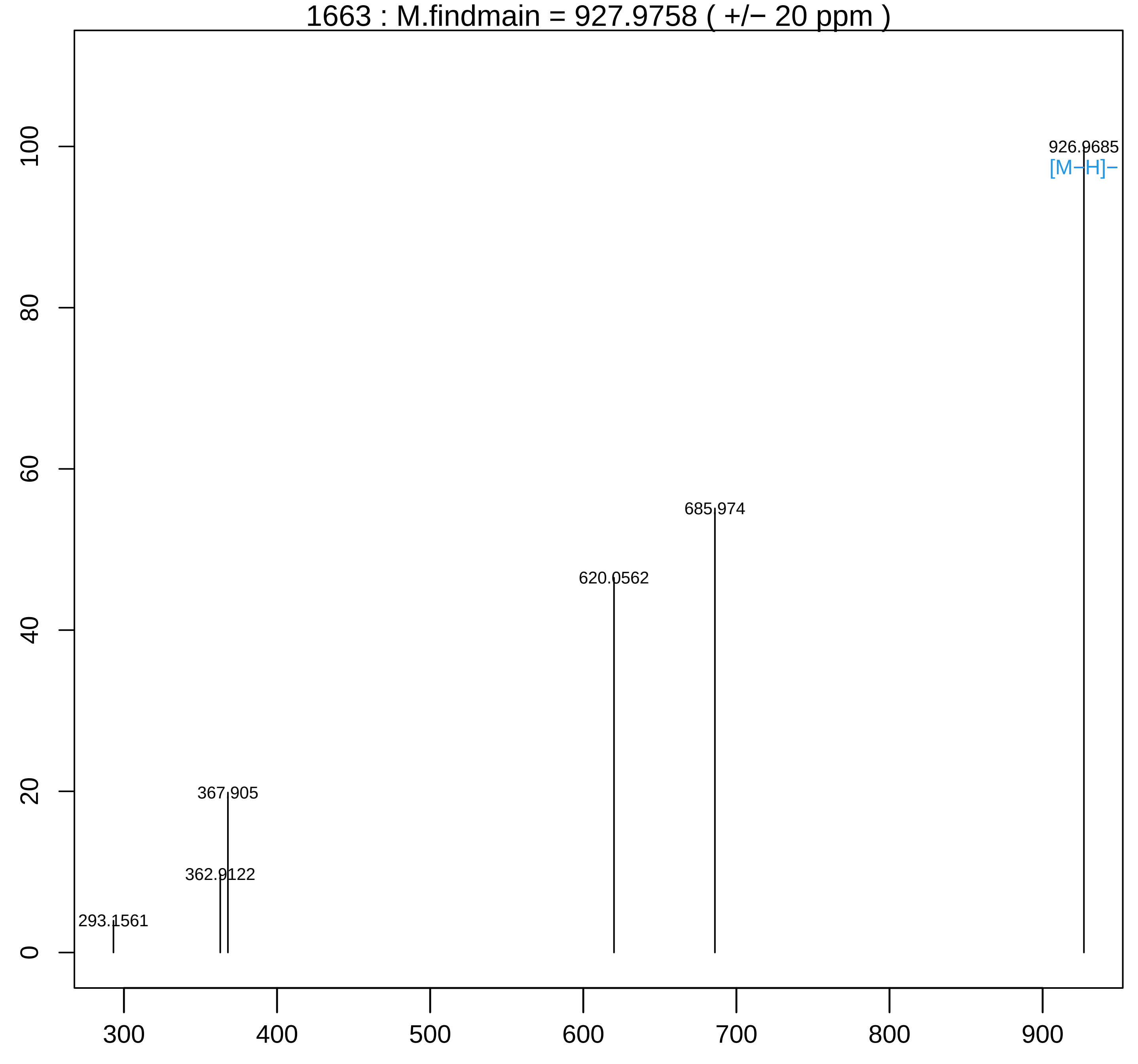
1663 : M.findmain = 927.9758 (+/− 20 ppm)
926.9685
[M−H]−
685.974
620.0562
367.905
362.9122
293.1561
0
20
40
60
80
100
300
400
500
600
700
800
900

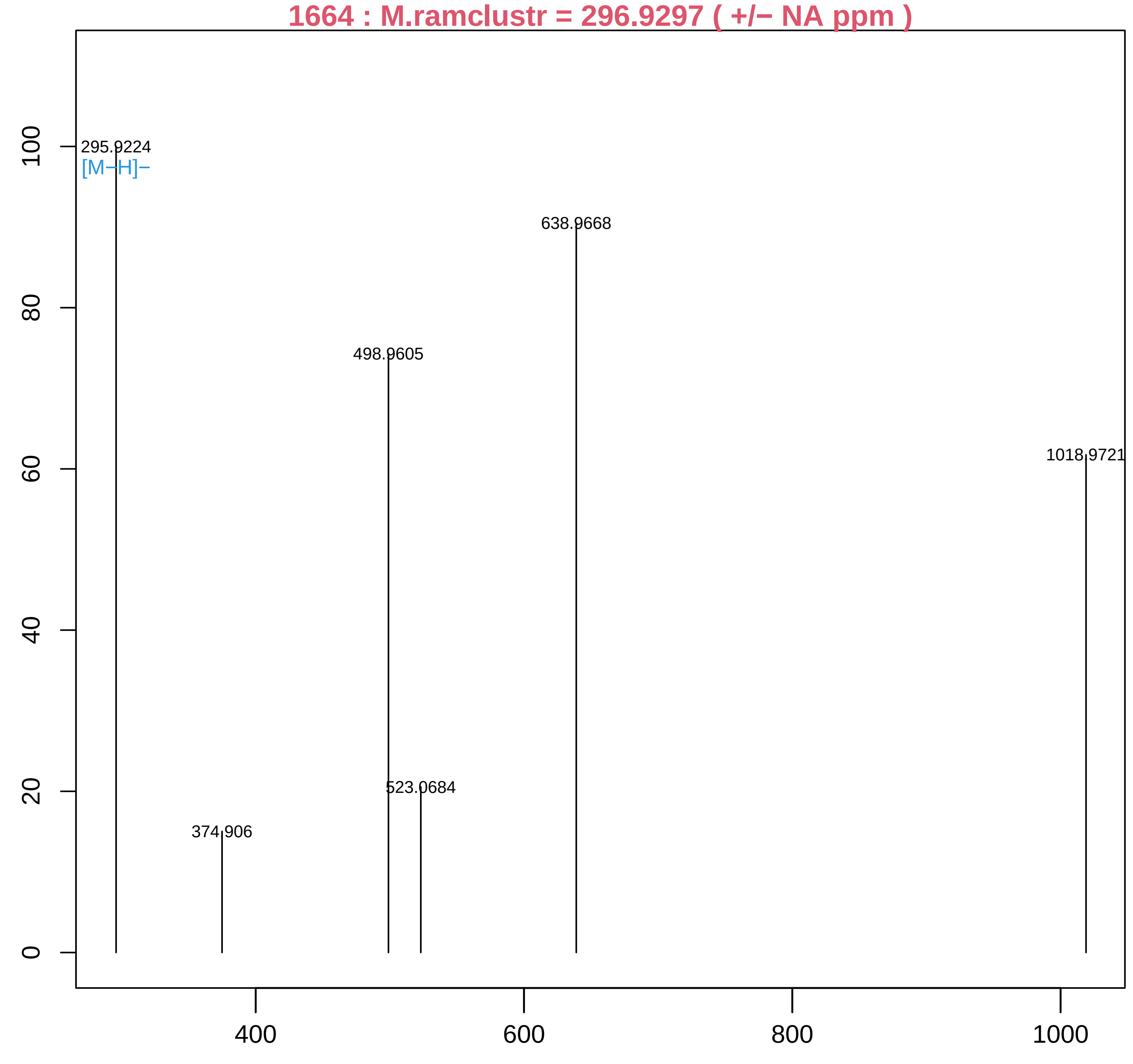

1664 : M.ramclustr = 296.9297 (+/− NA ppm)
295.9224
[M−H]−
638.9668
498.9605
1018.9721
523.0684
374.906
0
20
40
60
80
100
400
600
800
1000

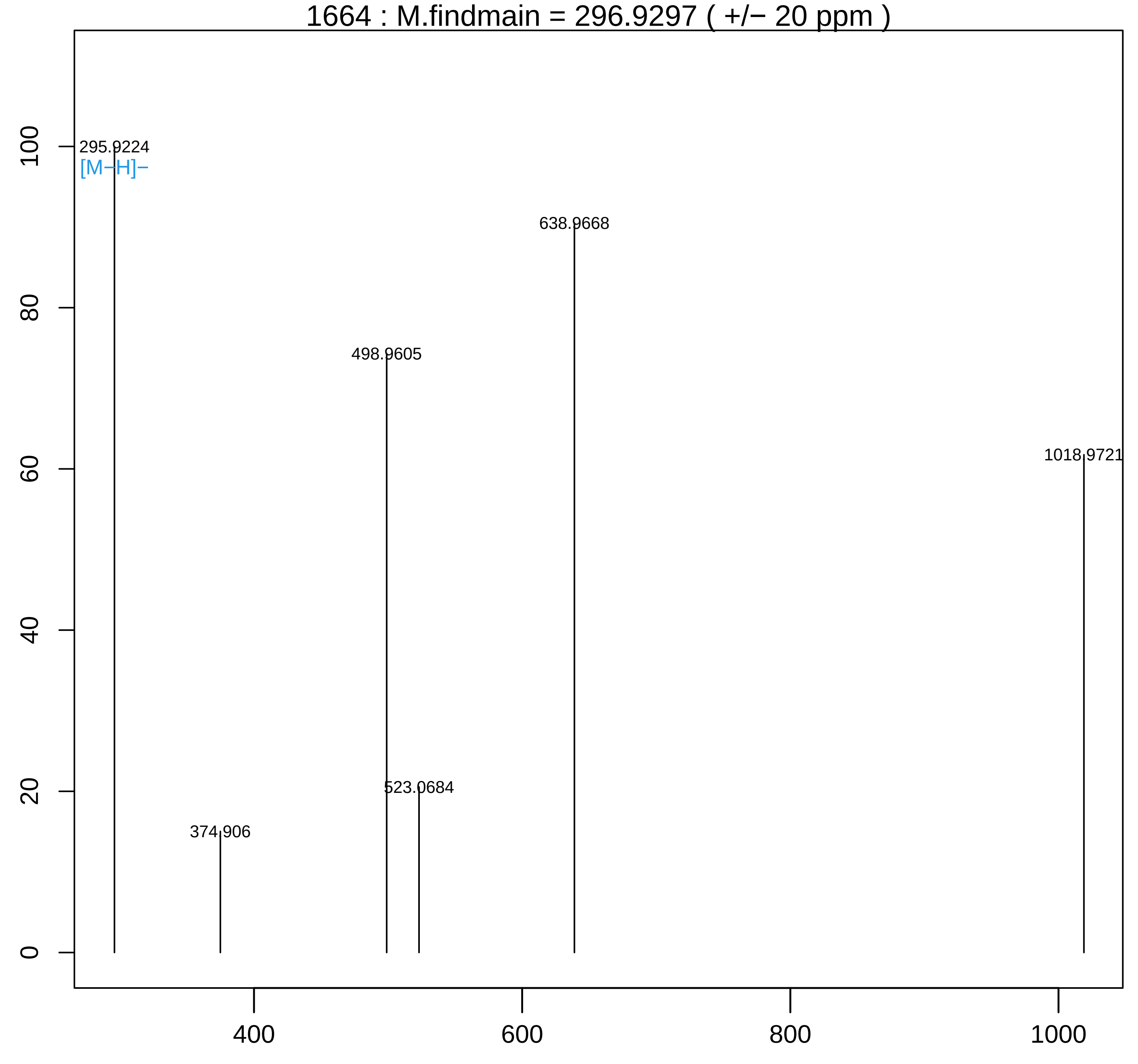

1664 : M.findmain = 296.9297 (+/− 20 ppm)
295.9224
[M−H]−
638.9668
498.9605
1018.9721
523.0684
374.906
0
20
40
60
80
100
400
600
800
1000

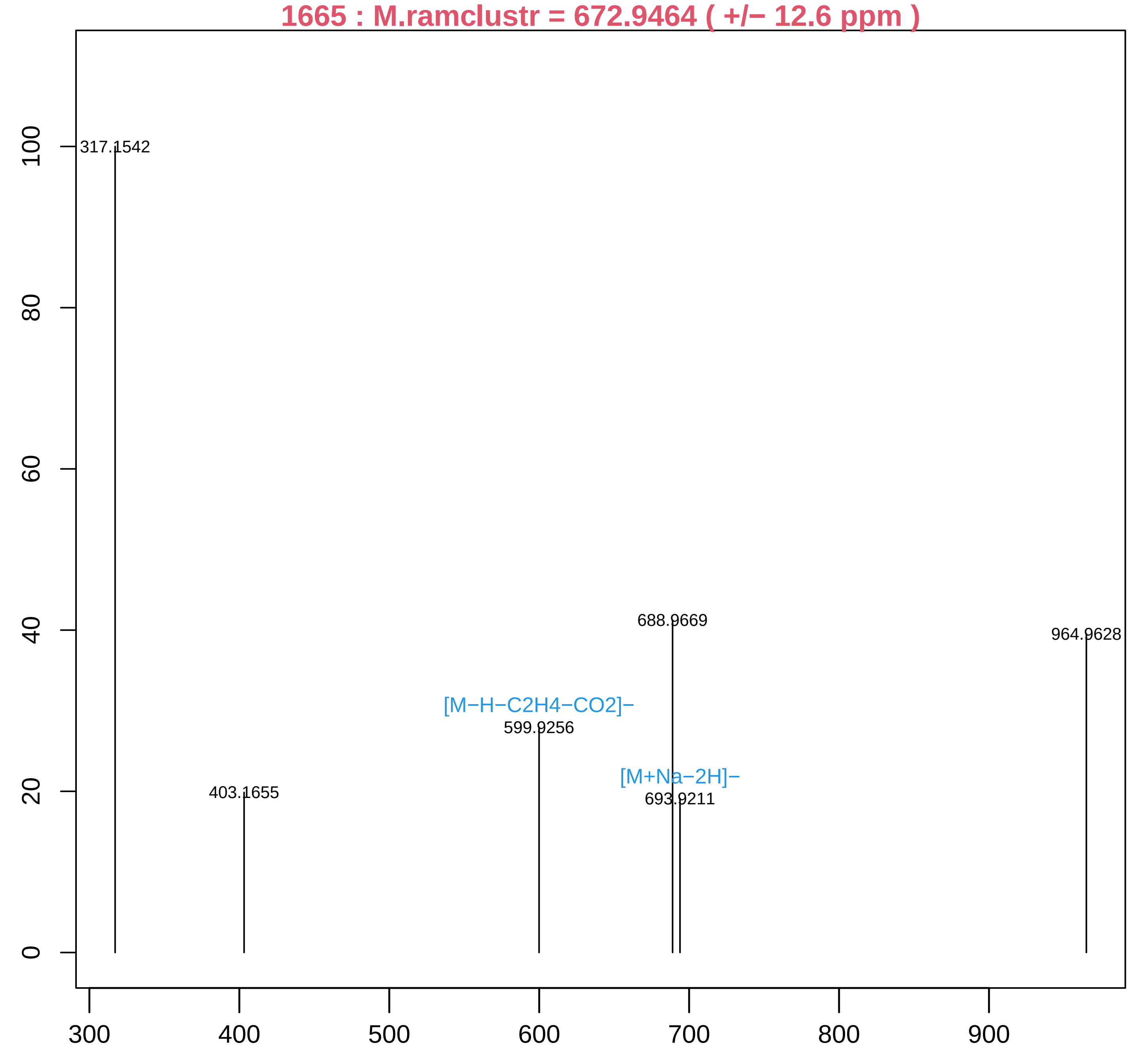
1665 : M.ramclustr = 672.9464 (+/− 12.6 ppm)
317.1542
403.1655
[M−H−C2H4−CO2]−
599.9256
688.9669
[M+Na−2H]−
693.9211
964.9628
0
20
40
60
80
100
300
400
500
600
700
800
900

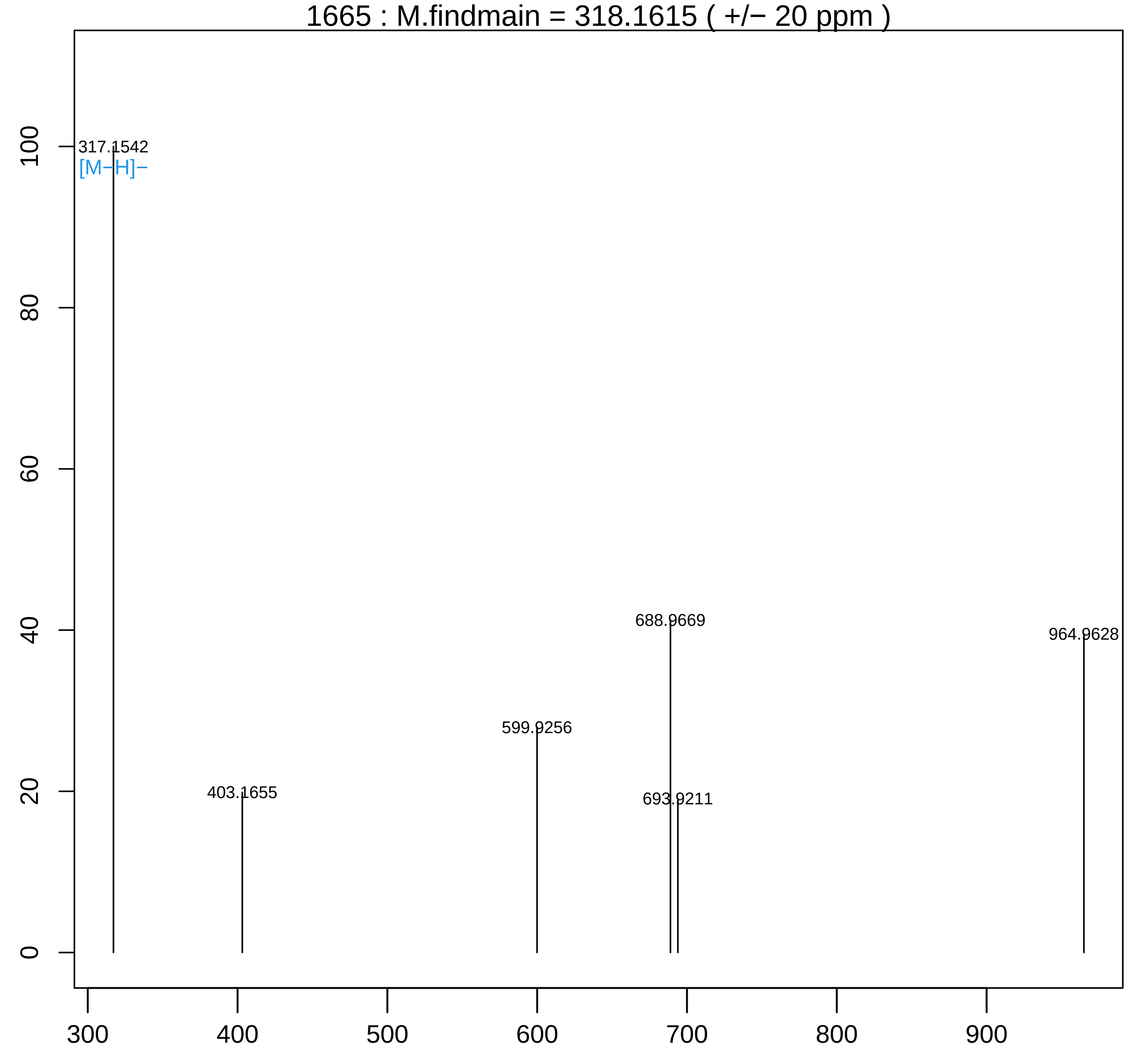
1665 : M.findmain = 318.1615 (+/− 20 ppm)
317.1542
[M−H]−
403.1655
599.9256
688.9669
693.9211
964.9628
0
20
40
60
80
100
300
400
500
600
700
800
900

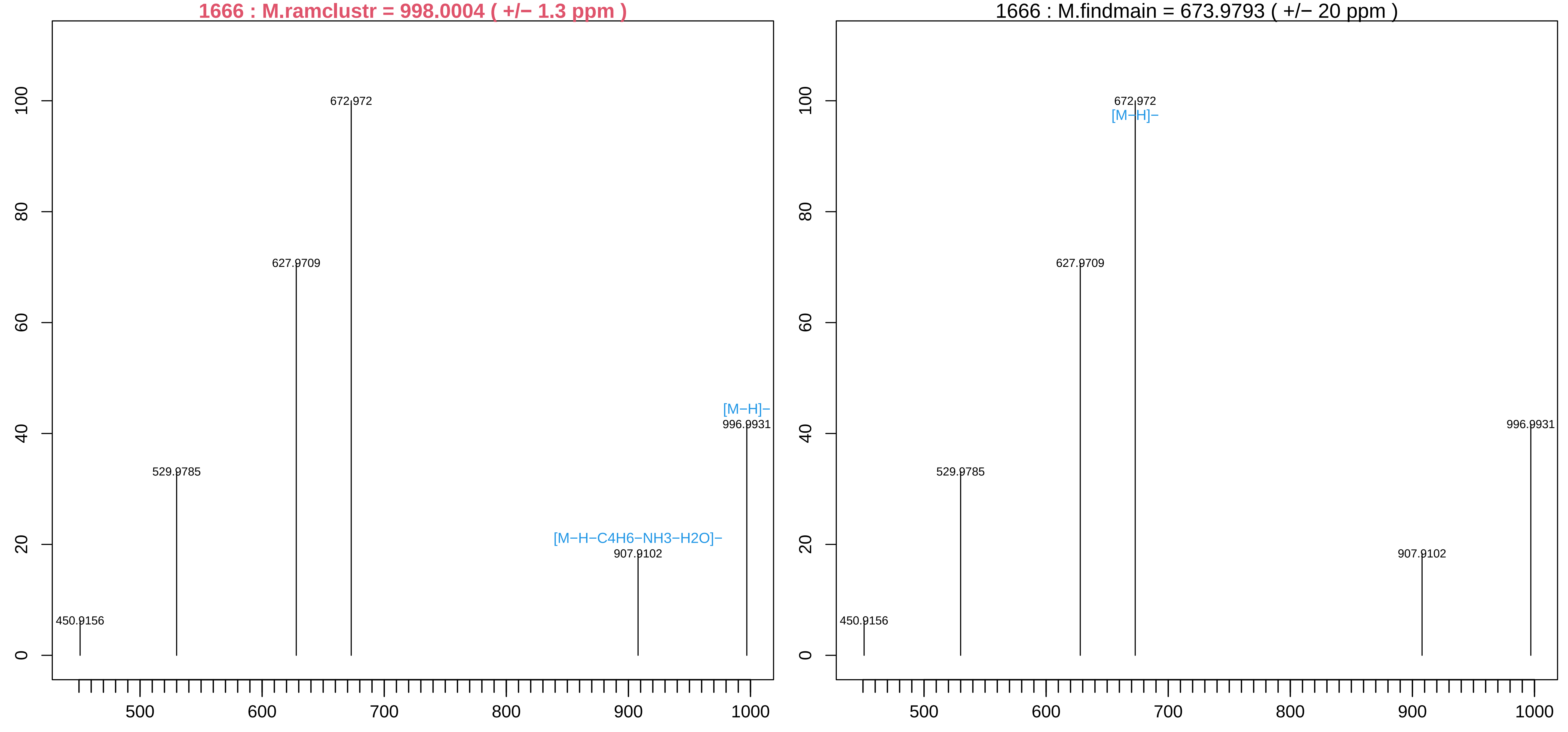

1666 : M.ramclustr = 998.0004 (+/− 1.3 ppm)
672.972
627.9709
529.9785
450.9156
[M−H]−
996.9931
[M−H−C4H6−NH3−H2O]−
907.9102
1666 : M.findmain = 673.9793 (+/− 20 ppm)
672.972
[M−H]−
627.9709
529.9785
450.9156
996.9931
907.9102

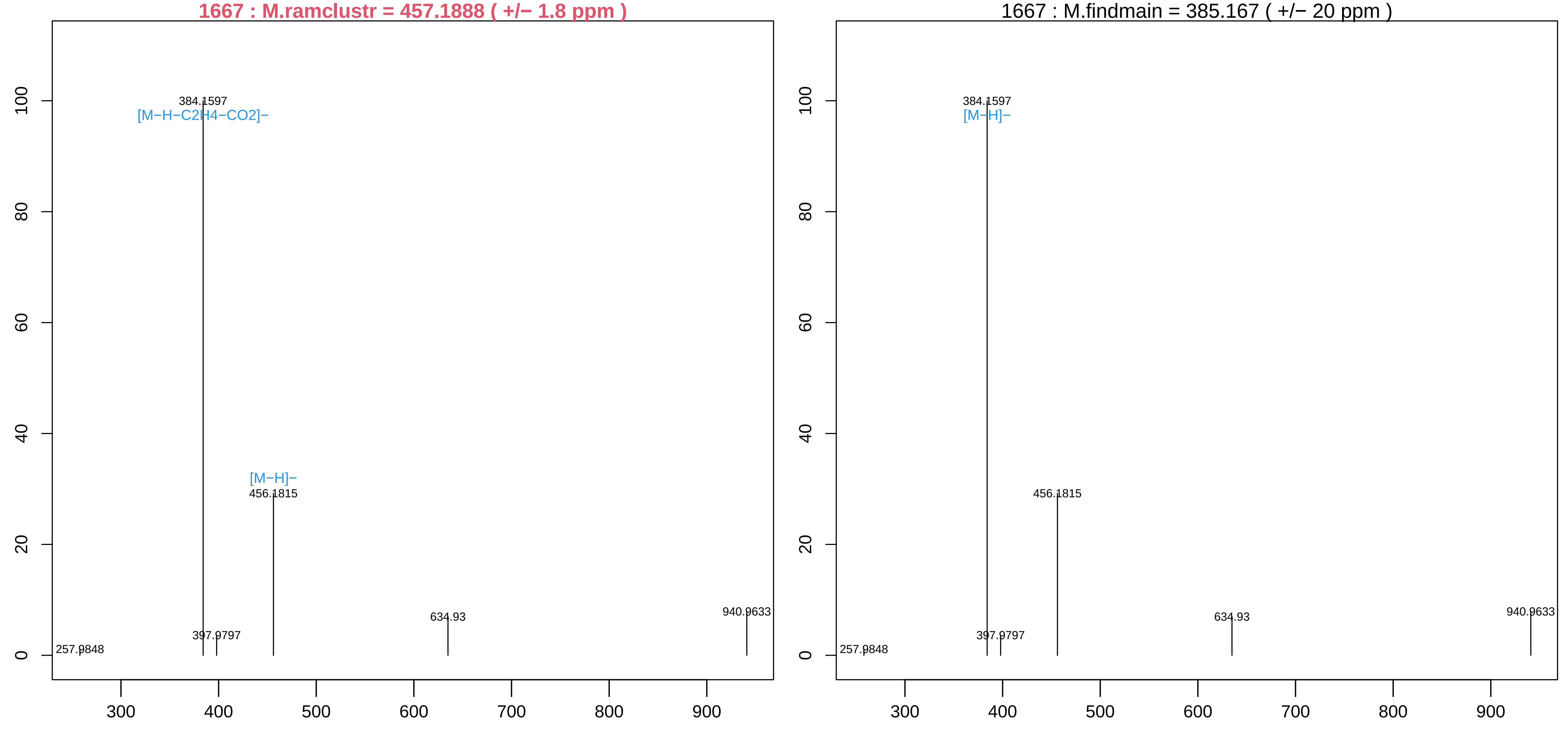

1667 : M.ramclustr = 457.1888 (+/− 1.8 ppm)
384.1597
[M−H−C2H4−CO2]−
[M−H]−
456.1815
257.9848
397.9797
634.93
940.9633
1667 : M.findmain = 385.167 (+/− 20 ppm)
384.1597
[M−H]−
456.1815
257.9848
397.9797
634.93
940.9633
0
20
40
60
80
100
300
400
500
600
700
800
900

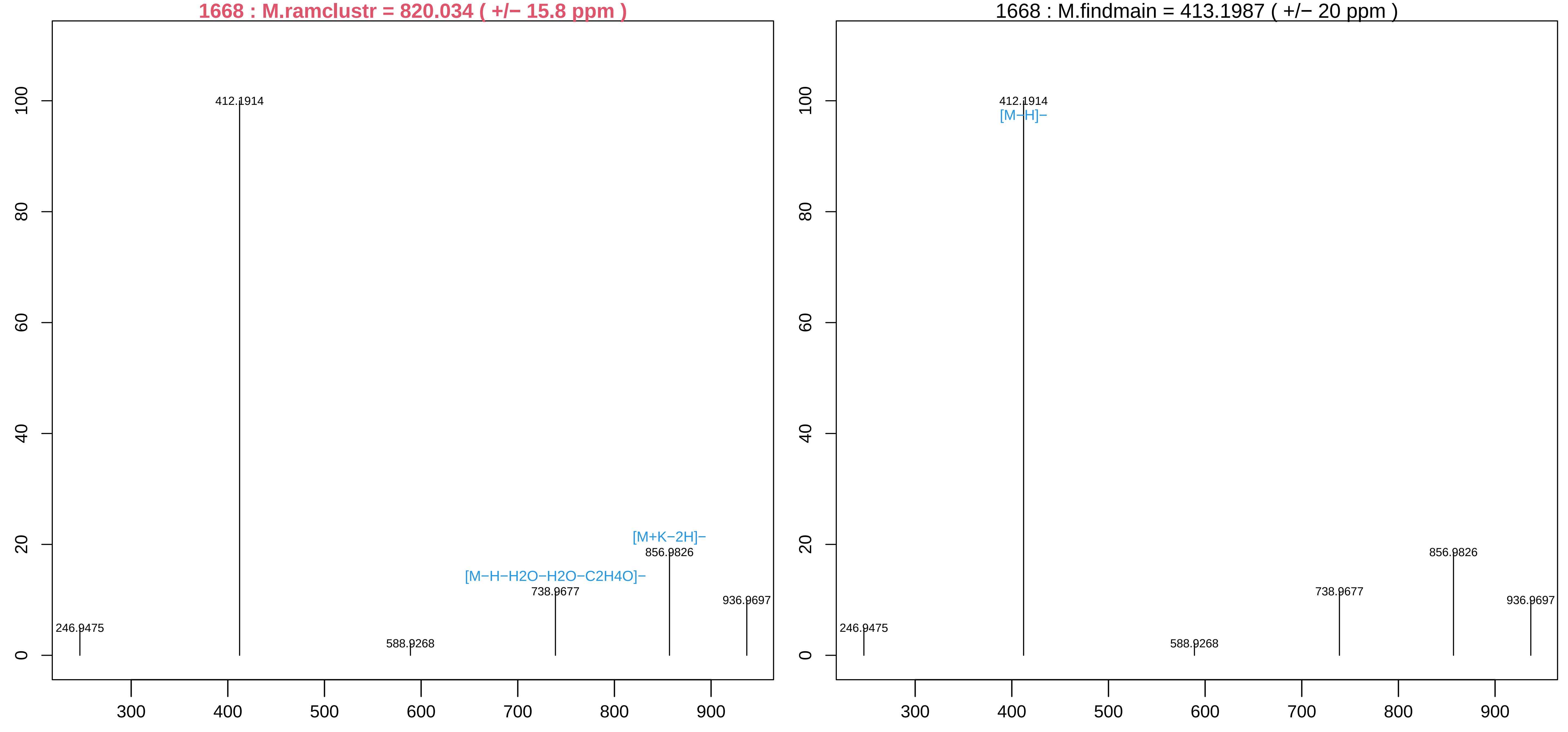
1668 : M.ramclustr = 820.034 (+/− 15.8 ppm)
412.1914
[M−H−H2O−H2O−C2H4O]−
738.9677
[M+K−2H]−
856.9826
936.9697
246.9475
588.9268
1668 : M.findmain = 413.1987 (+/− 20 ppm)
412.1914
[M−H]−
738.9677
856.9826
936.9697
246.9475
588.9268

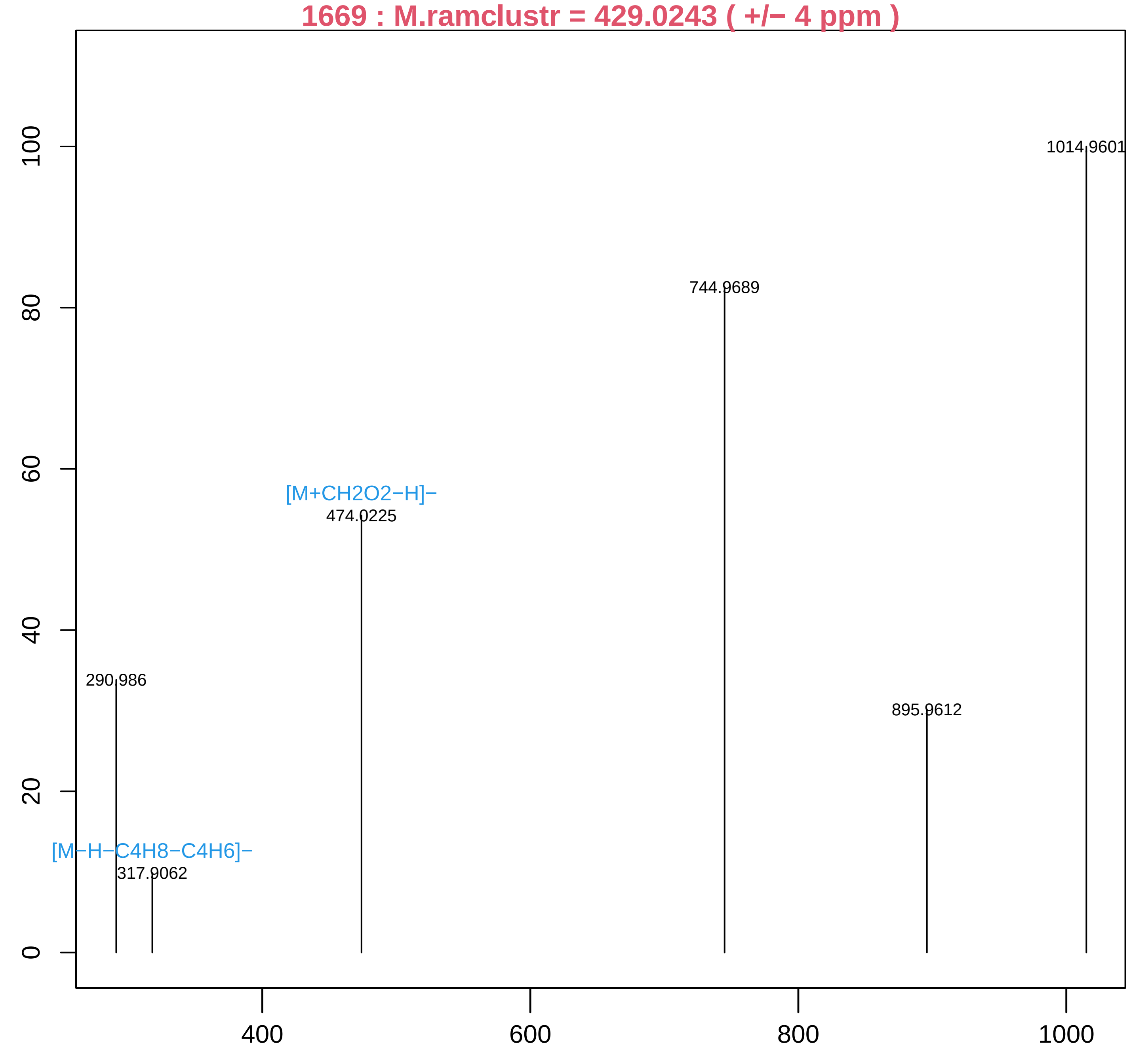
1669 : M.ramclustr = 429.0243 (+/− 4 ppm)
1014.9601
744.9689
[M+CH2O2−H]−
474.0225
290.986
895.9612
[M−H−C4H8−C4H6]−
317.9062
0
20
40
60
80
100
400
600
800
1000

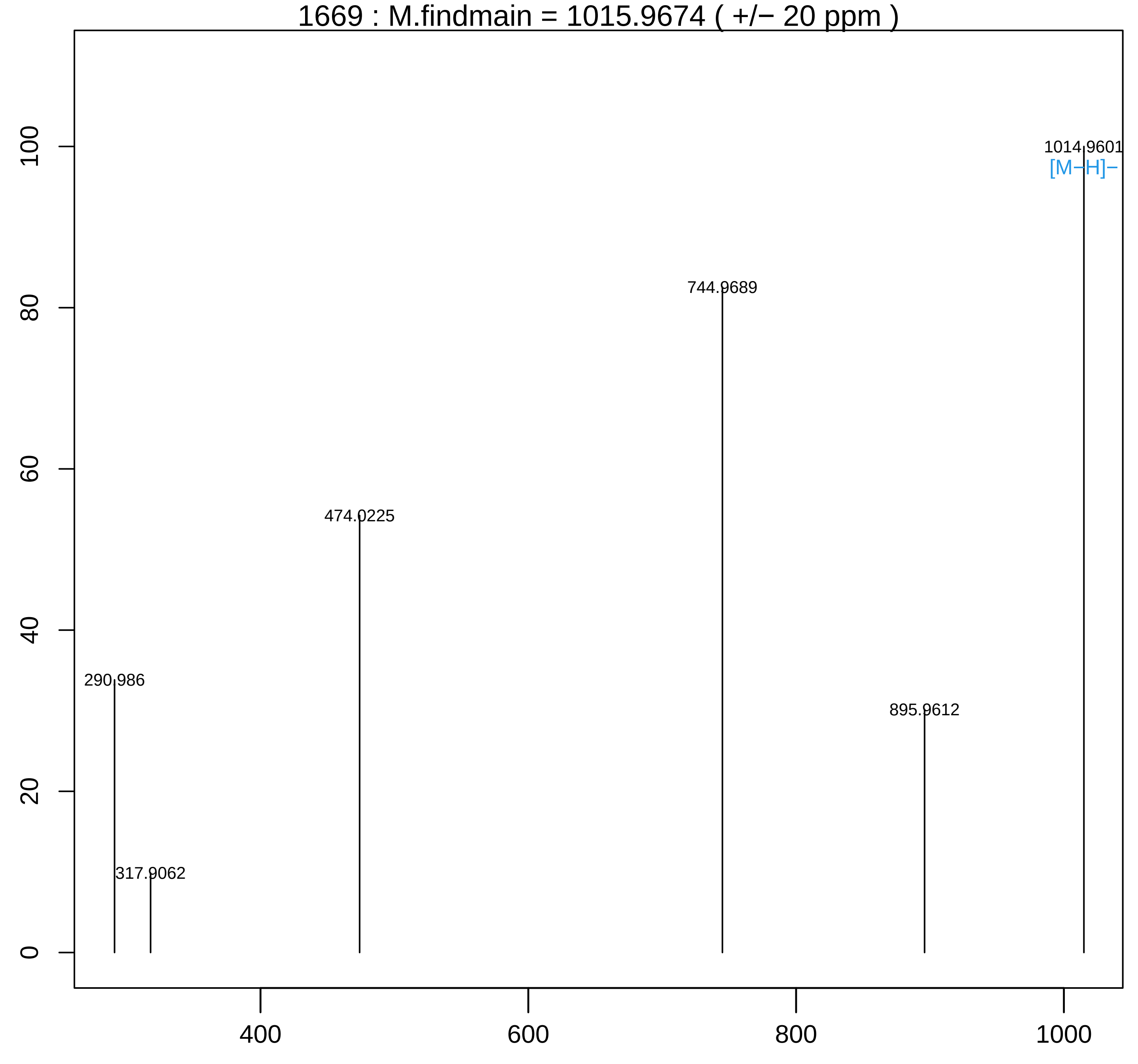
1669 : M.findmain = 1015.9674 (+/− 20 ppm)
1014.9601
[M−H]−
744.9689
474.0225
290.986
895.9612
317.9062
0
20
40
60
80
100
400
600
800
1000

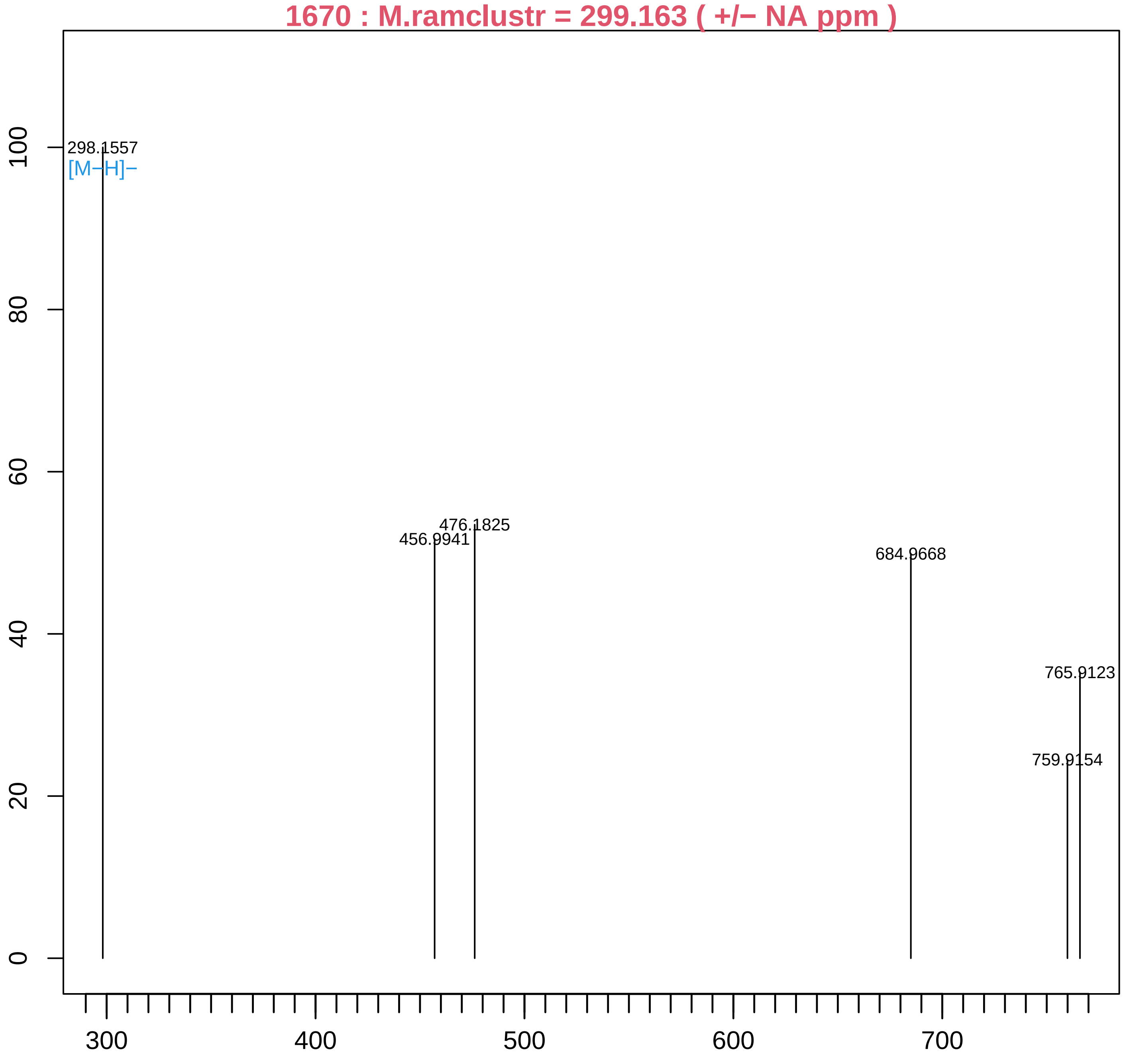
1670 : M.ramclustr = 299.163 (+/− NA ppm)
298.1557
[M−H]−
456.9941
476.1825
684.9668
765.9123
759.9154
0
20
40
60
80
100
300
400
500
600
700

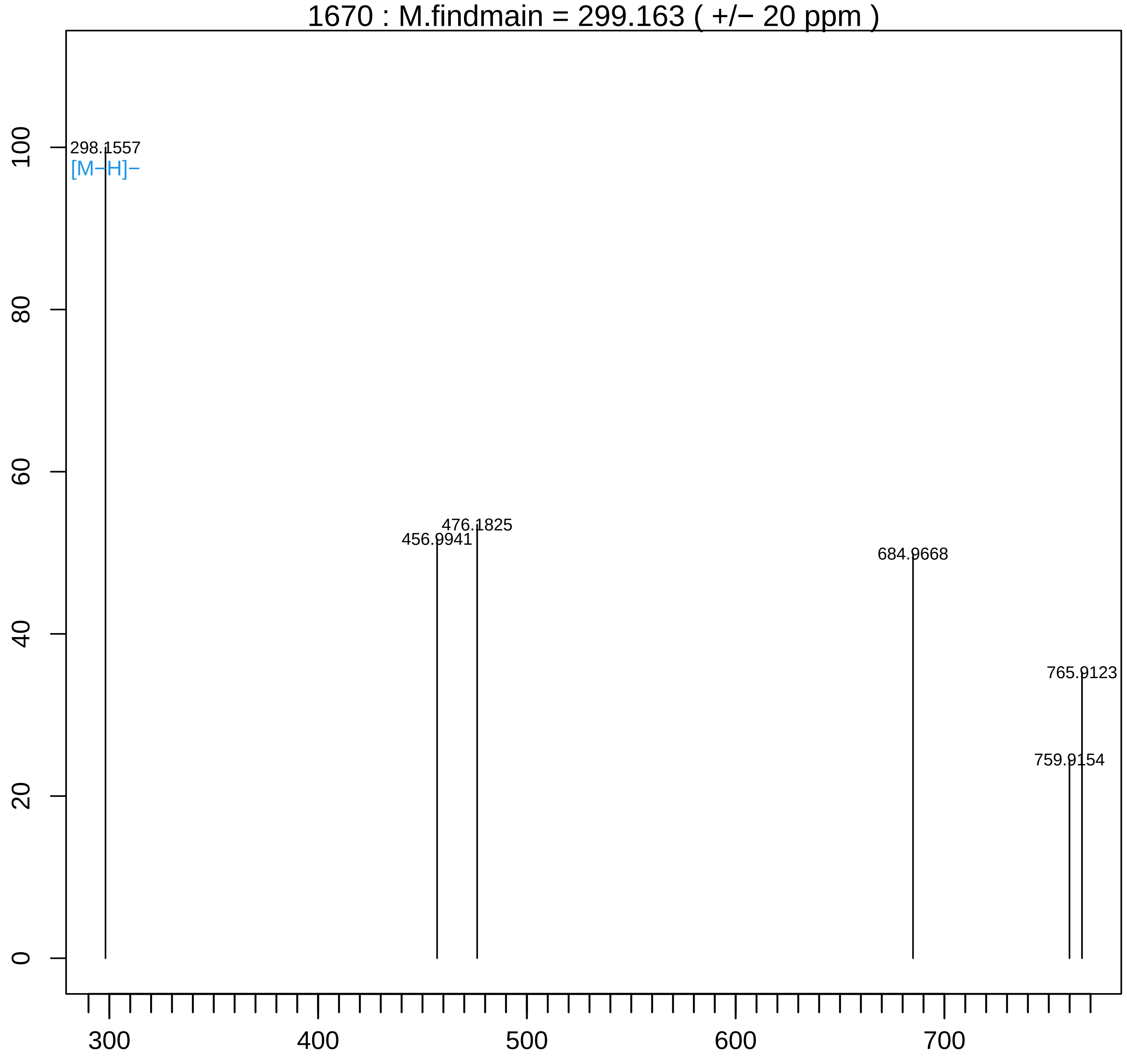
1670 : M.findmain = 299.163 (+/− 20 ppm)
298.1557
[M−H]−
456.9941
476.1825
684.9668
765.9123
759.9154
0
20
40
60
80
100
300
400
500
600
700

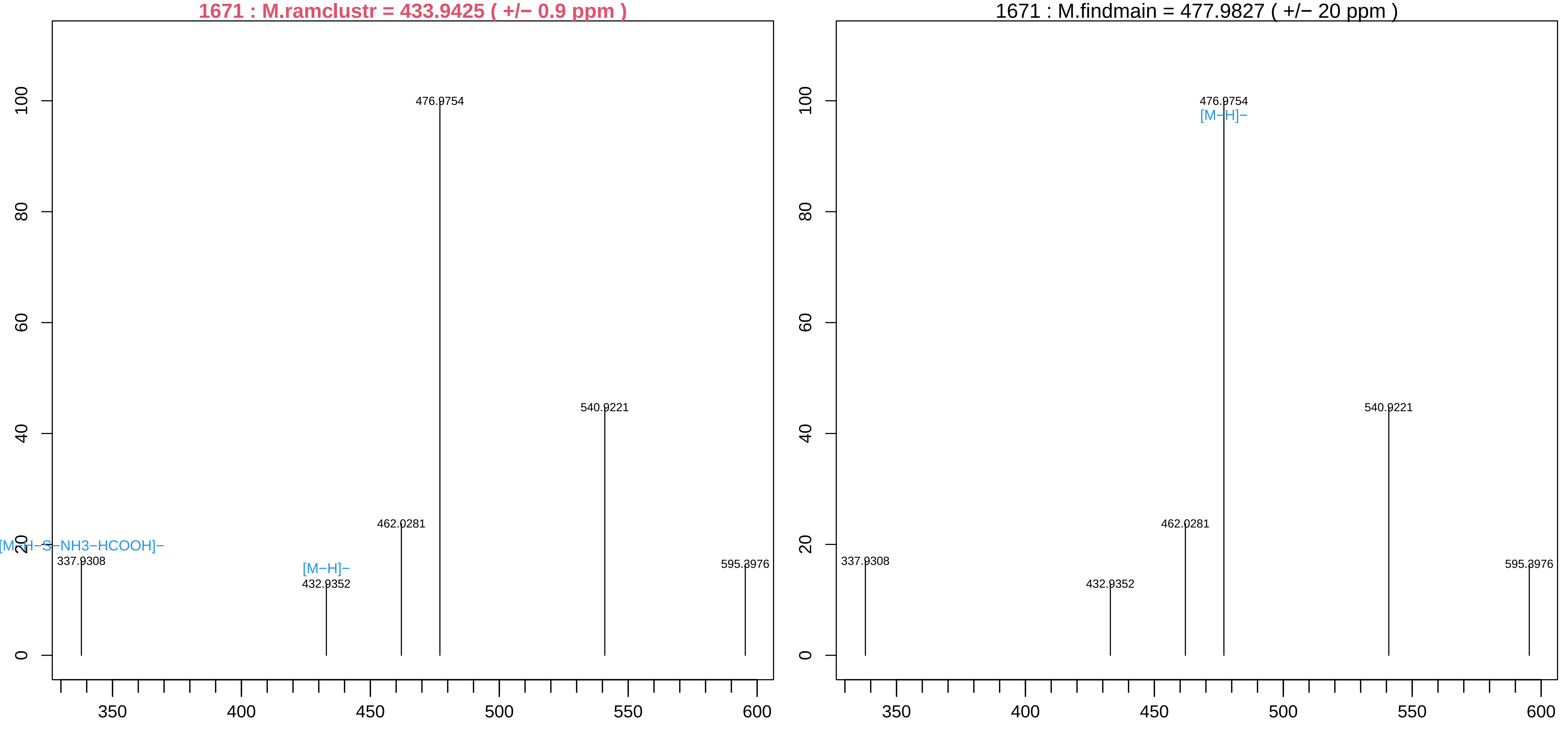

1671 : M.ramclustr = 433.9425 (+/− 0.9 ppm)
[M−H−S−NH3−HCOOH]−
337.9308
[M−H]−
432.9352
462.0281
476.9754
540.9221
595.3976
1671 : M.findmain = 477.9827 (+/− 20 ppm)
337.9308
432.9352
462.0281
476.9754
[M−H]−
540.9221
595.3976
0
20
40
60
80
100
350
400
450
500
550
600

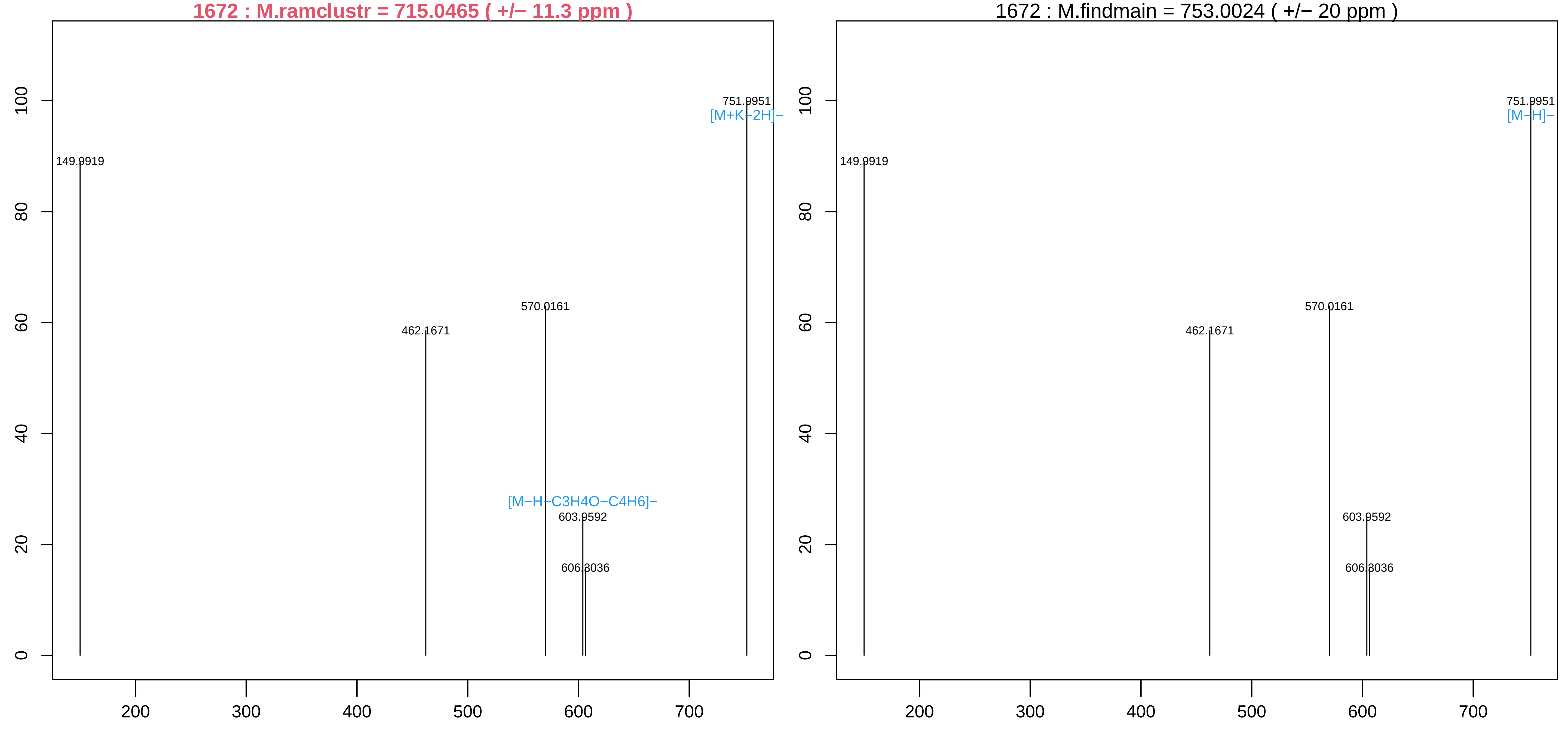
1672 : M.ramclustr = 715.0465 (+/− 11.3 ppm)
1672 : M.findmain = 753.0024 (+/− 20 ppm)
149.9919
462.1671
570.0161
603.9592
606.3036
751.9951
[M+K−2H]−
[M−H−C3H4O−C4H6]−
[M−H]−
0
20
40
60
80
100
200
300
400
500
600
700

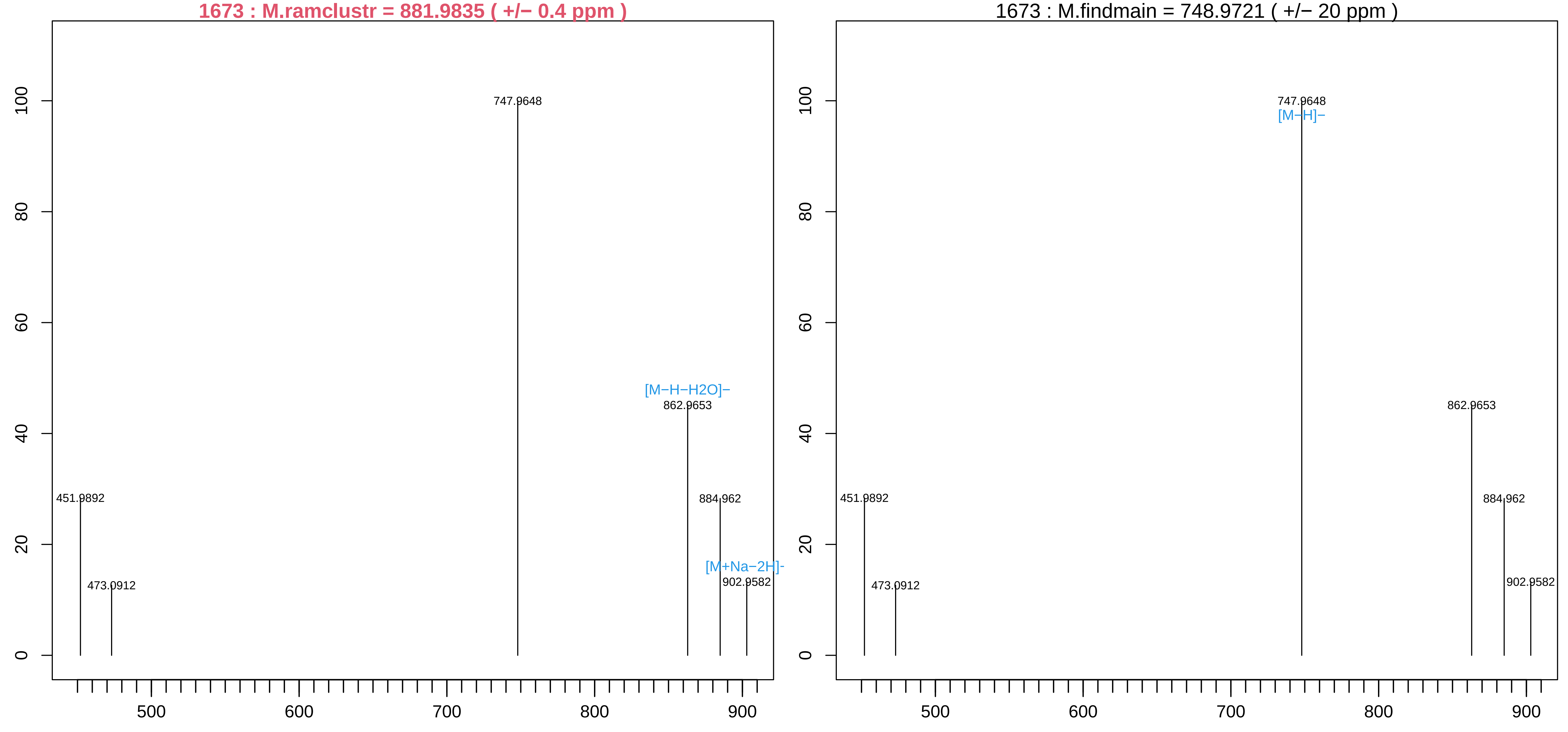

1673 : M.ramclustr = 881.9835 (+/− 0.4 ppm)
747.9648
[M−H−H2O]−
862.9653
451.9892
884.962
473.0912
[M+Na−2H]-
902.9582
0
20
40
60
80
100
500
600
700
800
900
1673 : M.findmain = 748.9721 (+/− 20 ppm)
747.9648
[M−H]−
862.9653
451.9892
884.962
473.0912
902.9582
0
20
40
60
80
100
500
600
700
800
900

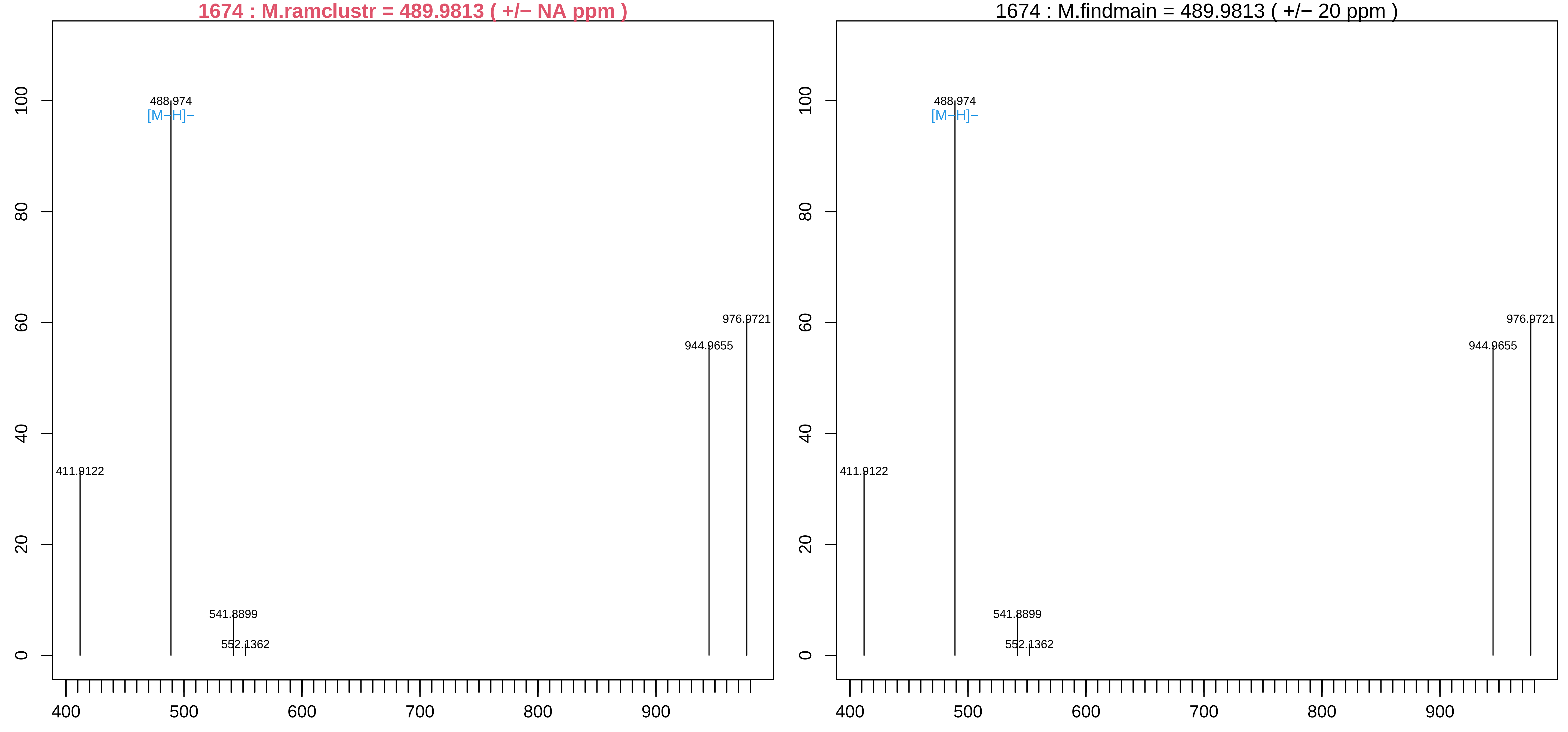
1674 : M.ramclustr = 489.9813 (+/− NA ppm)
1674 : M.findmain = 489.9813 (+/− 20 ppm)
488.974
[M−H]−
411.9122
541.8899
552.1362
944.9655
976.9721
0
20
40
60
80
100
400
500
600
700
800
900

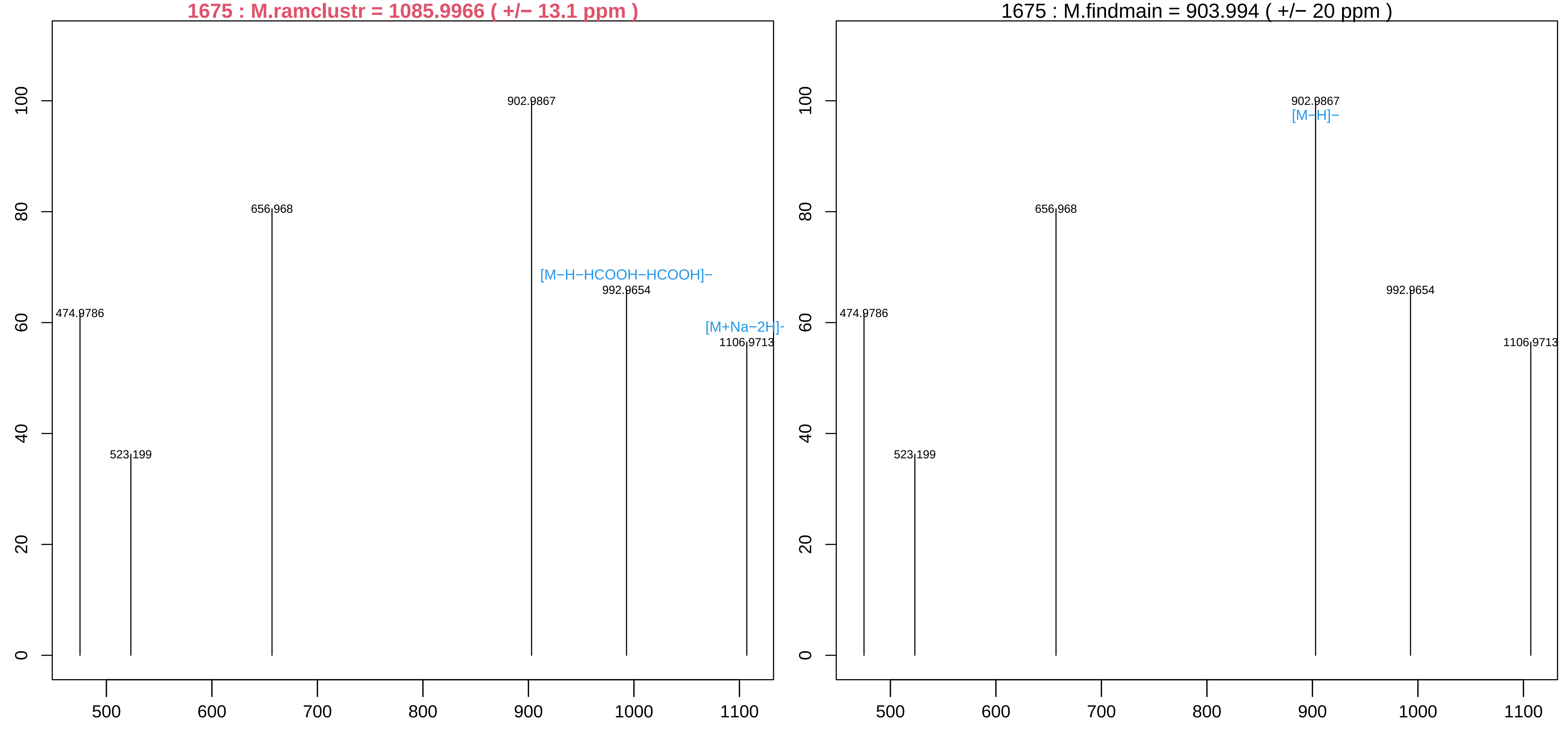
1675 : M.ramclustr = 1085.9966 (+/− 13.1 ppm)
1675 : M.findmain = 903.994 (+/− 20 ppm)
474.9786
523.199
656.968
902.9867
[M−H]−
[M−H−HCOOH−HCOOH]−
992.9654
[M+Na−2H]-
1106.9713
0
20
40
60
80
100
500
600
700
800
900
1000
1100

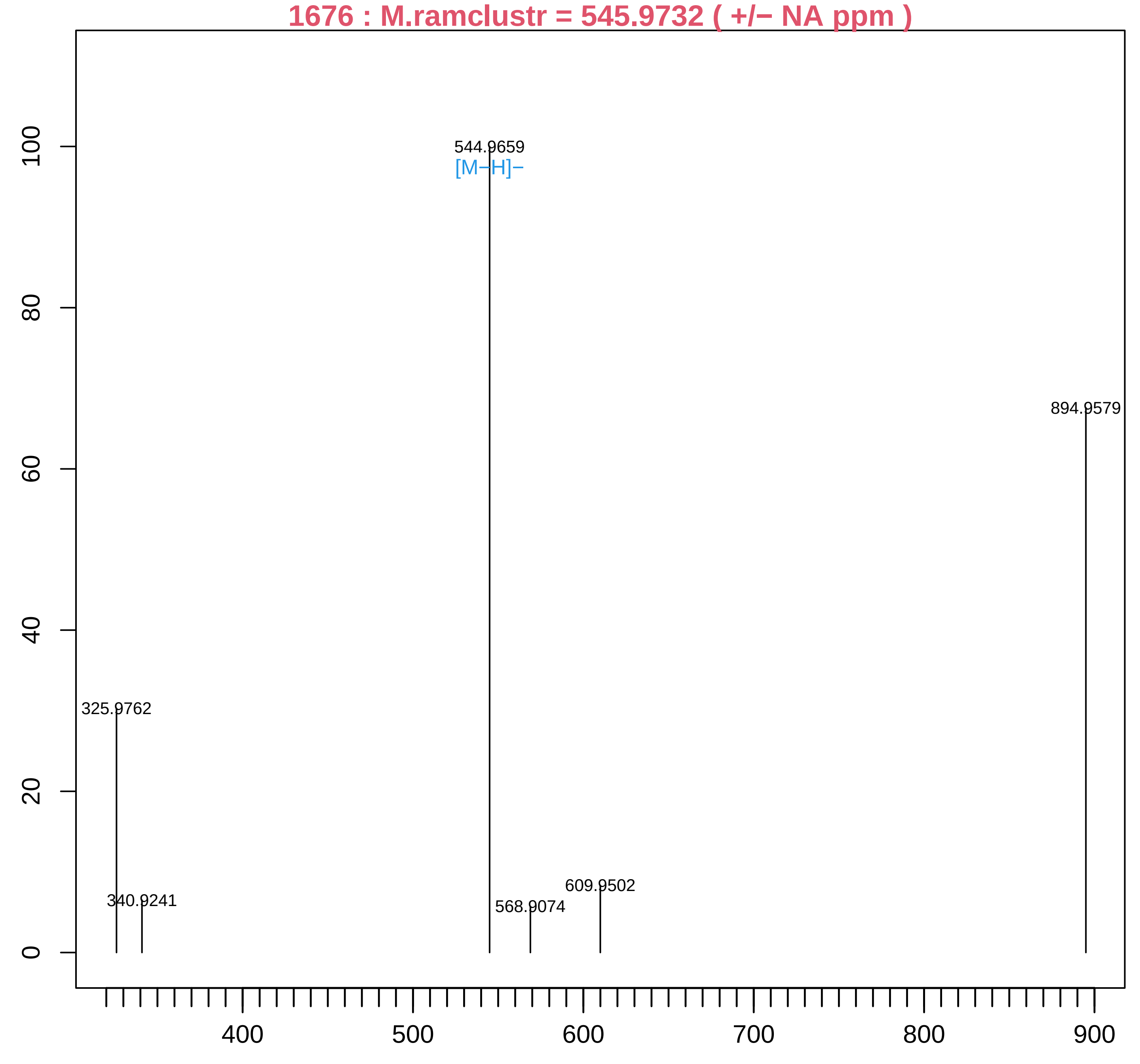
1676 : M.ramclustr = 545.9732 (+/− NA ppm)
544.9659
[M−H]−
894.9579
325.9762
340.9241
568.9074
609.9502
0
20
40
60
80
100
400
500
600
700
800
900

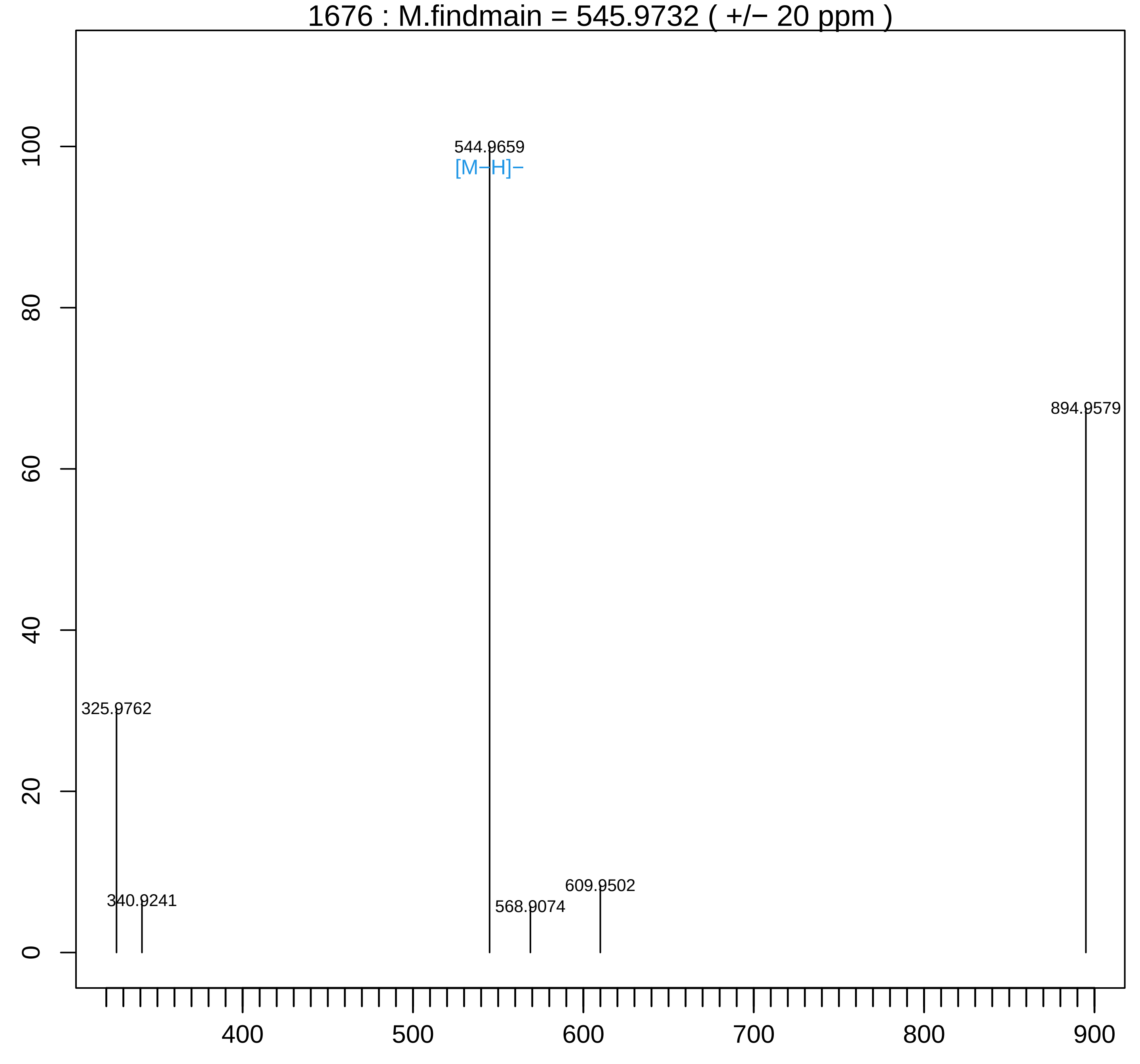
1676 : M.findmain = 545.9732 (+/− 20 ppm)
544.9659
[M−H]−
894.9579
325.9762
340.9241
568.9074
609.9502
0
20
40
60
80
100
400
500
600
700
800
900

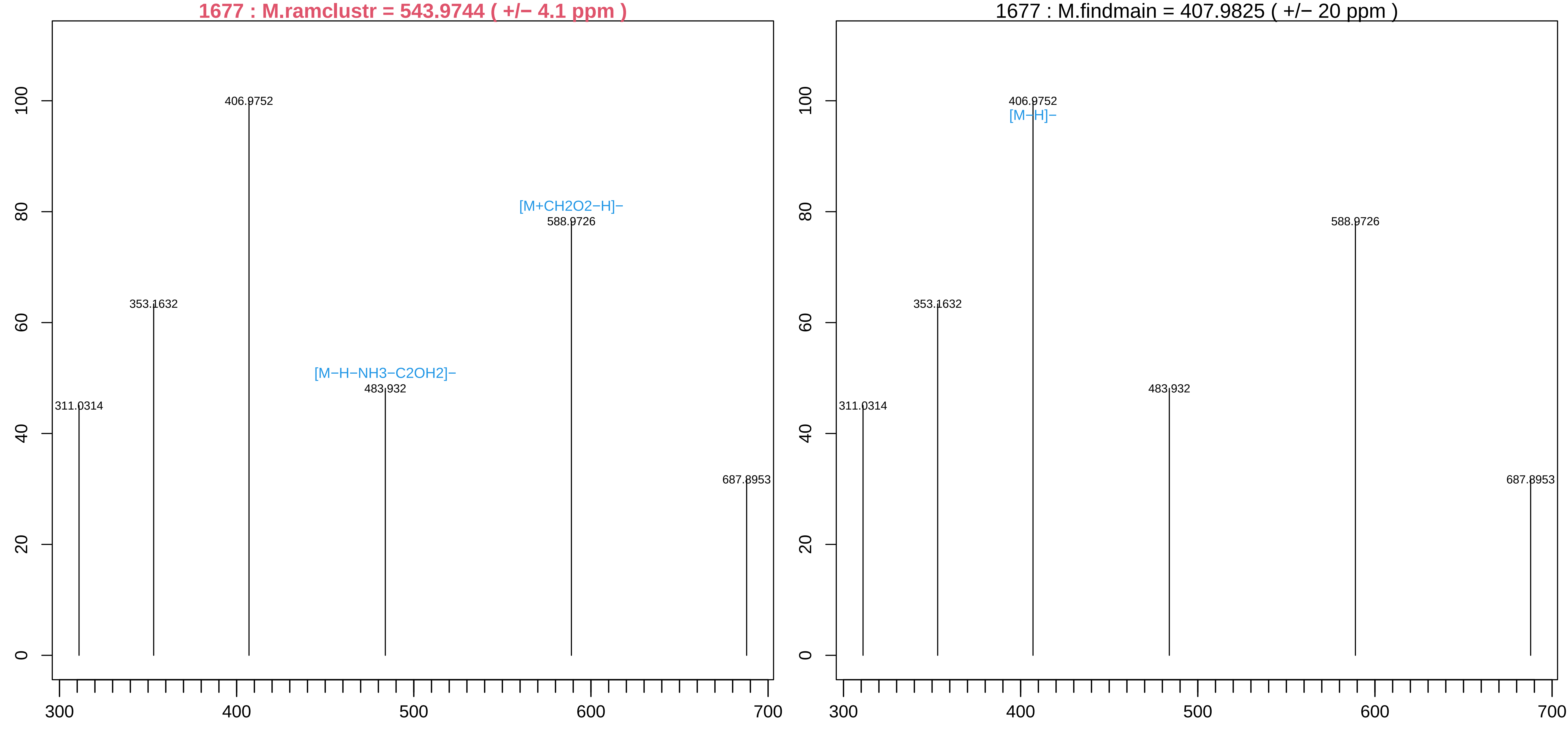
1677 : M.ramclustr = 543.9744 (+/− 4.1 ppm)
406.9752
353.1632
311.0314
[M−H−NH3−C2OH2]−
483.932
[M+CH2O2−H]−
588.9726
687.8953
1677 : M.findmain = 407.9825 (+/− 20 ppm)
406.9752
[M−H]−
353.1632
311.0314
483.932
588.9726
687.8953
0
20
40
60
80
100
300
400
500
600
700

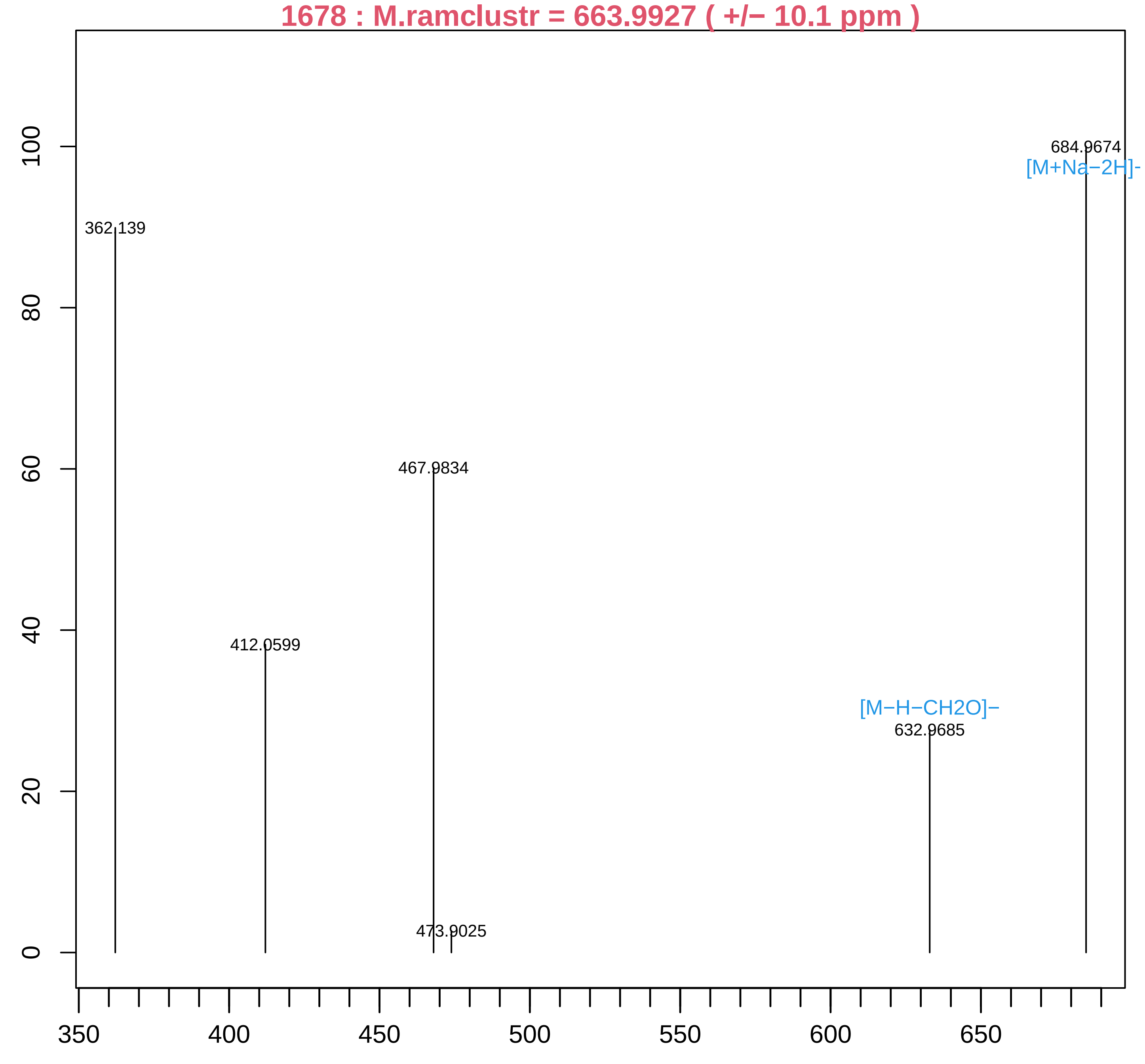
1678 : M.ramclustr = 663.9927 (+/− 10.1 ppm)
684.9674
[M+Na−2H]-
362.139
412.0599
467.9834
473.9025
[M−H−CH2O]−
632.9685
0
20
40
60
80
100
350
400
450
500
550
600
650

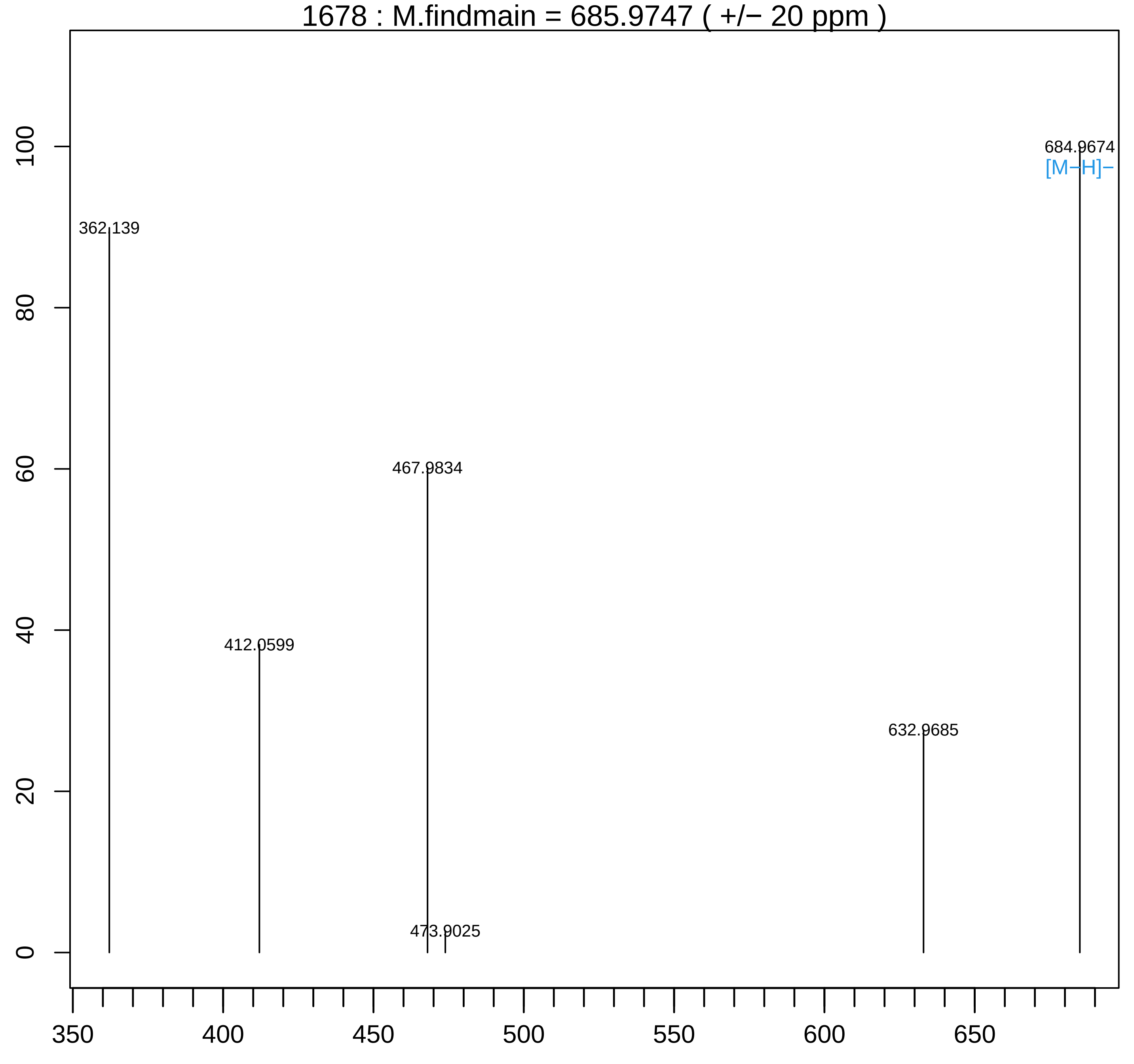
1678 : M.findmain = 685.9747 (+/− 20 ppm)
684.9674
[M−H]−
362.139
412.0599
467.9834
473.9025
632.9685
0
20
40
60
80
100
350
400
450
500
550
600
650

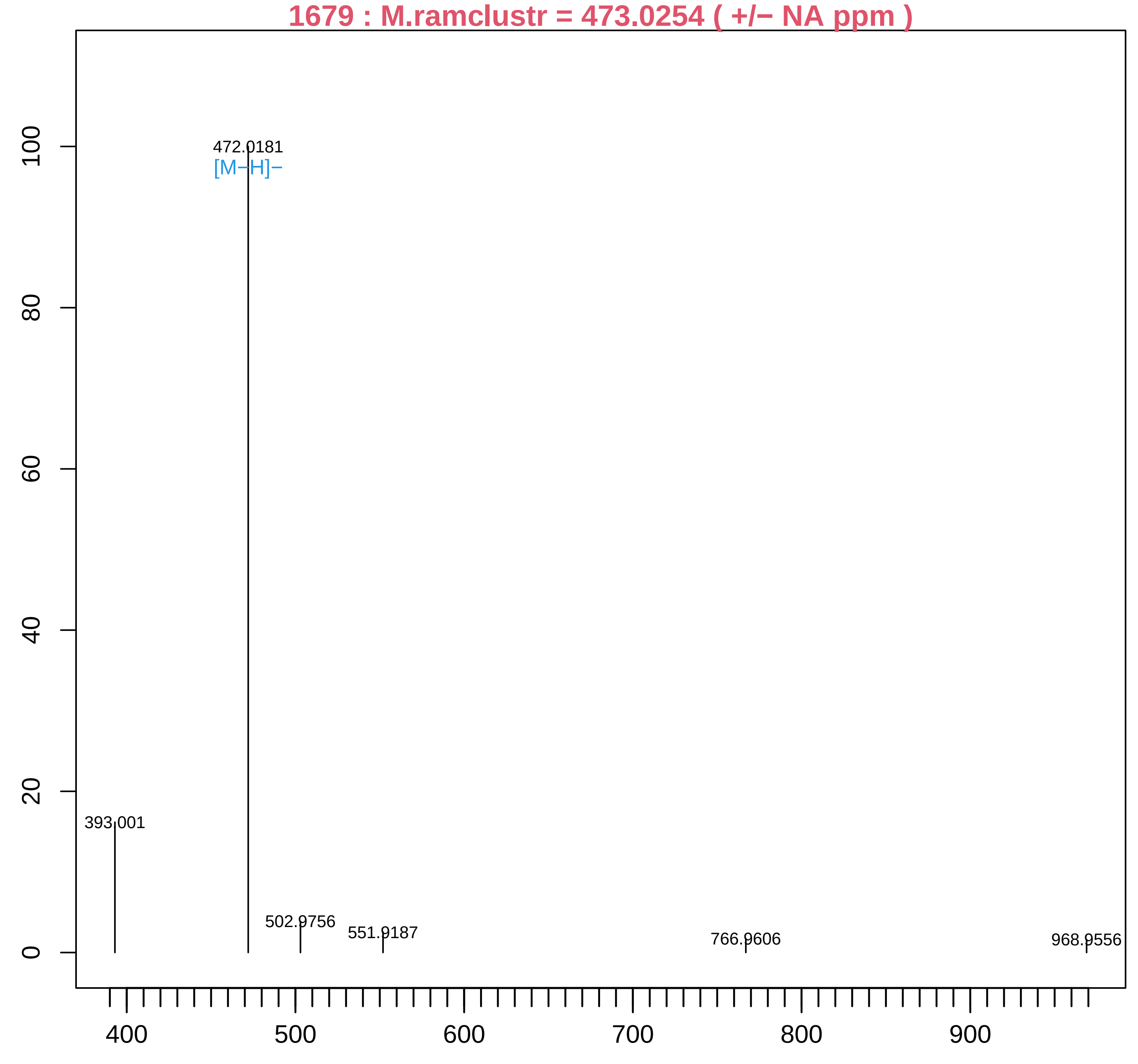
1679 : M.ramclustr = 473.0254 (+/− NA ppm)
472.0181
[M−H]−
393.001
502.9756
551.9187
766.9606
968.9556
0
20
40
60
80
100
400
500
600
700
800
900

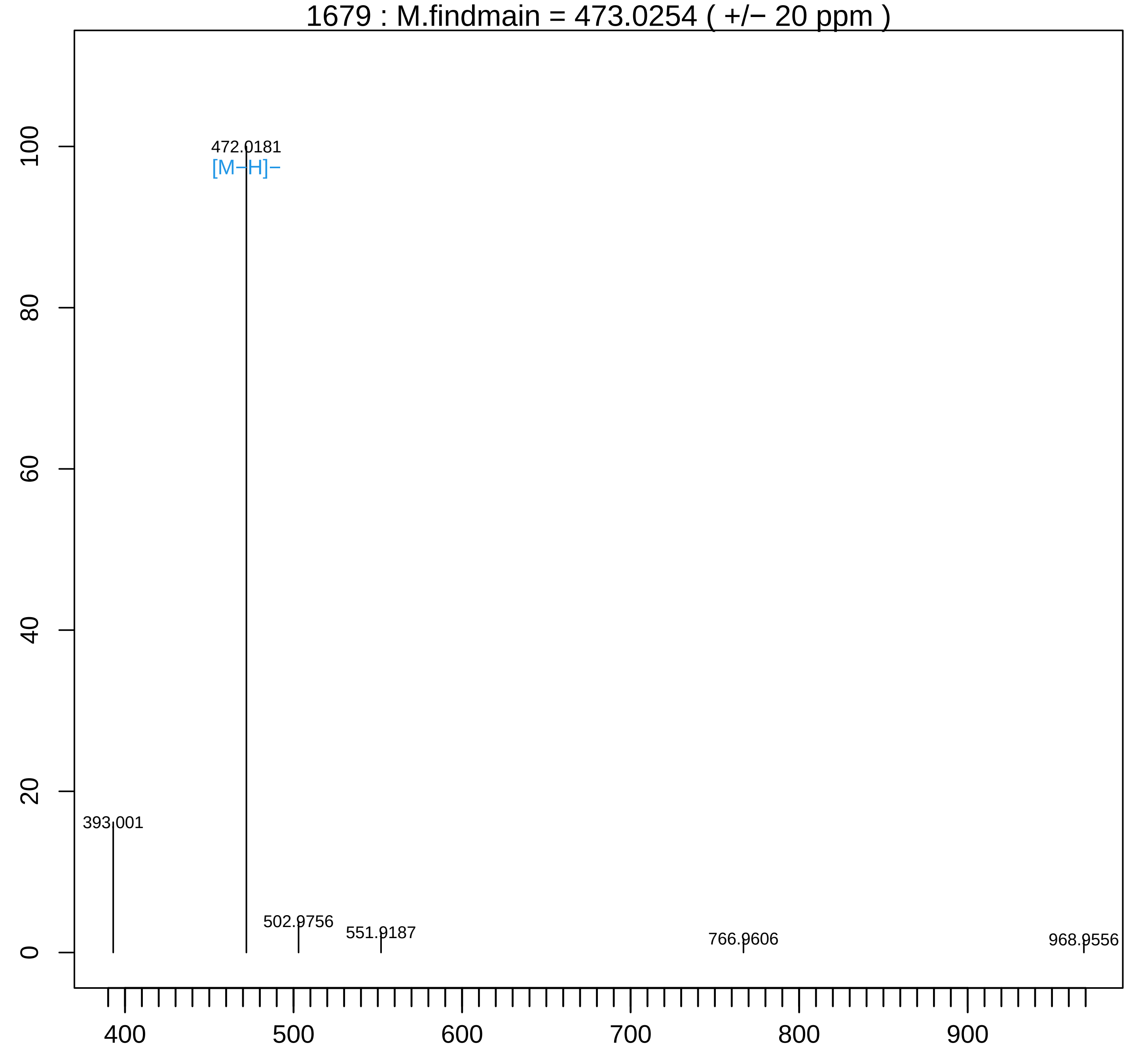
1679 : M.findmain = 473.0254 (+/− 20 ppm)
472.0181
[M−H]−
393.001
502.9756
551.9187
766.9606
968.9556
0
20
40
60
80
100
400
500
600
700
800
900

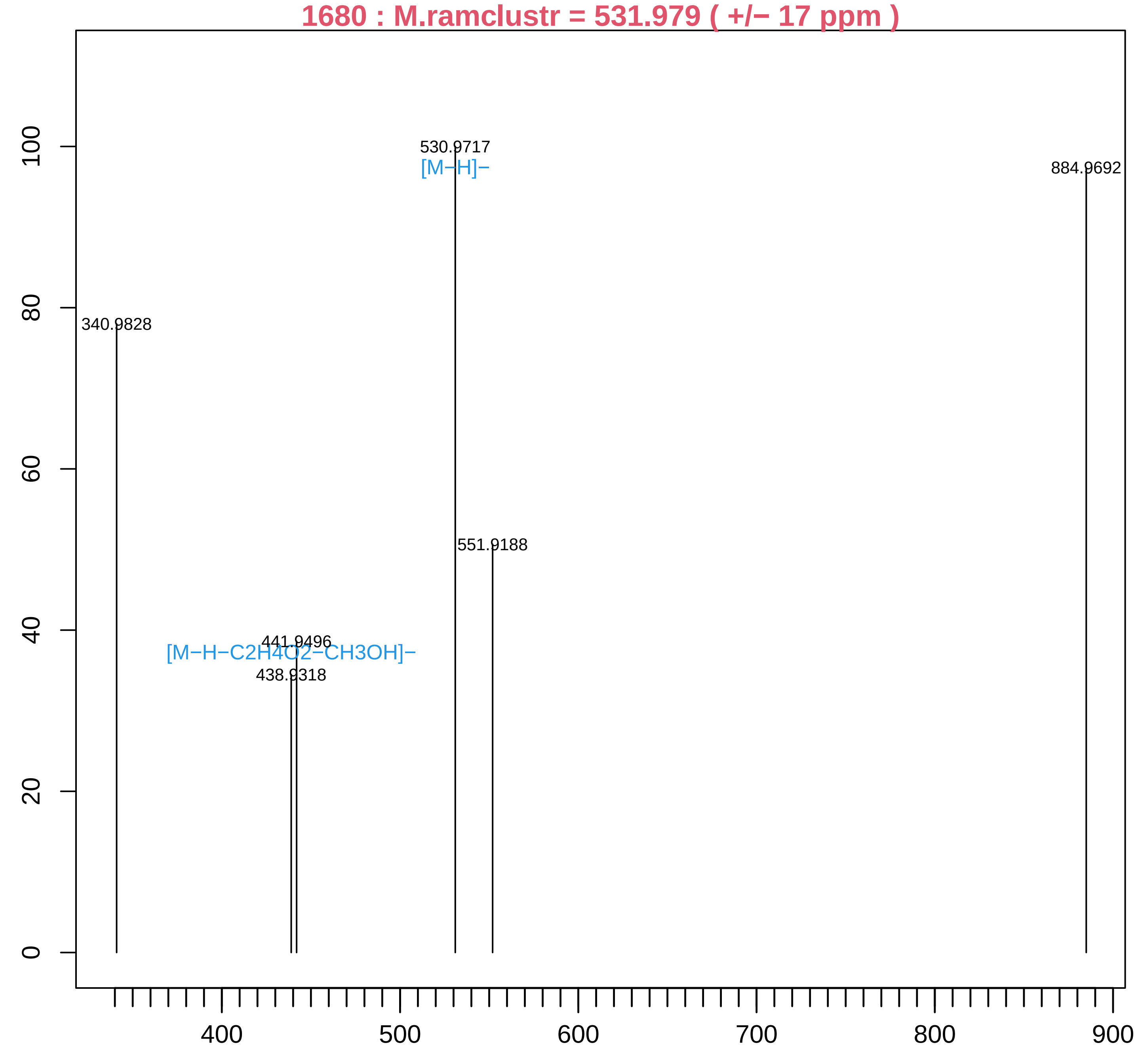
1680 : M.ramclustr = 531.979 (+/− 17 ppm)
340.9828
441.9496
[M−H−C2H4O2−CH3OH]−
438.9318
530.9717
[M−H]−
551.9188
884.9692
0
20
40
60
80
100
400
500
600
700
800
900

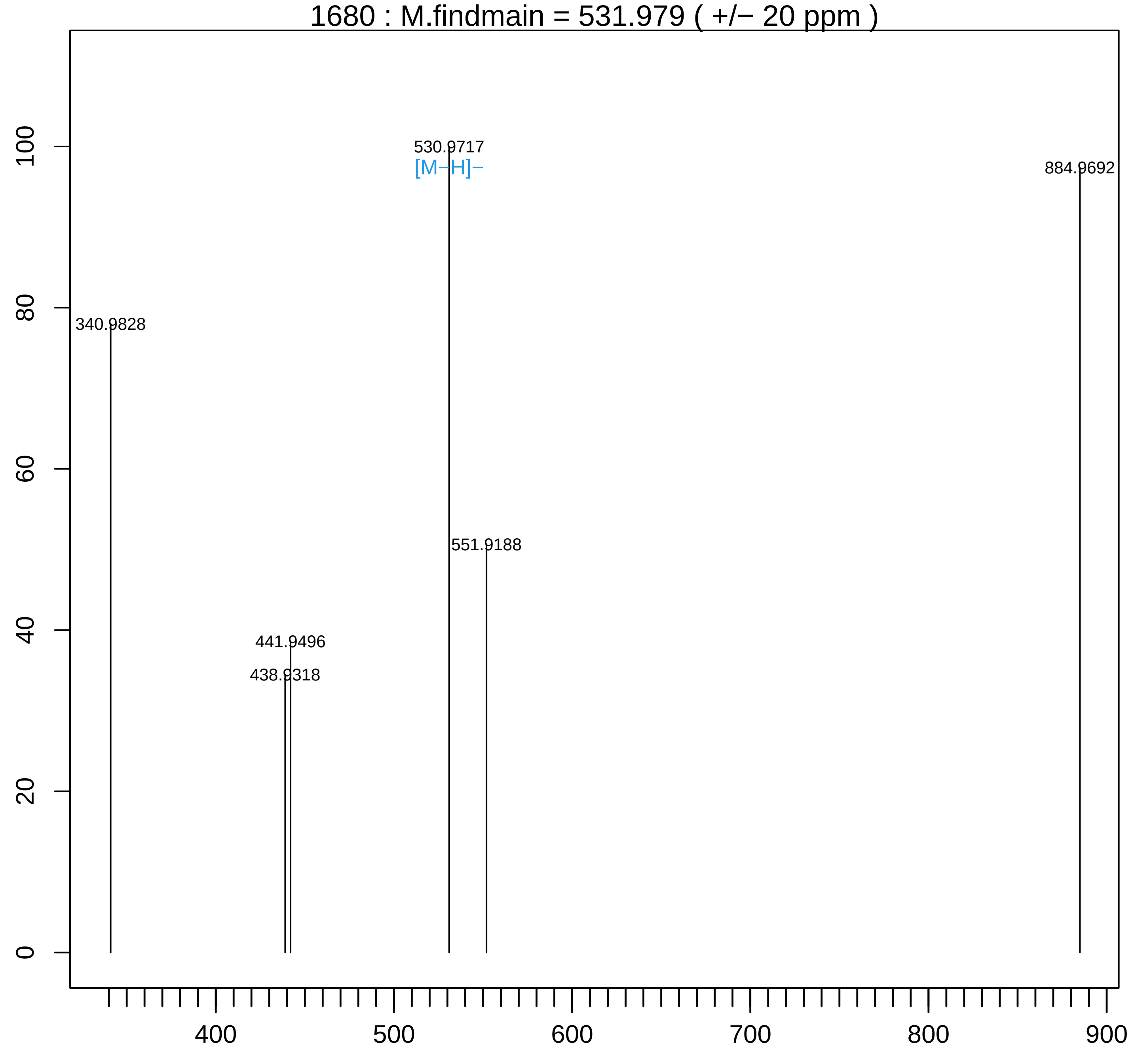
1680 : M.findmain = 531.979 (+/− 20 ppm)
340.9828
441.9496
438.9318
530.9717
[M−H]−
551.9188
884.9692
0
20
40
60
80
100
400
500
600
700
800
900

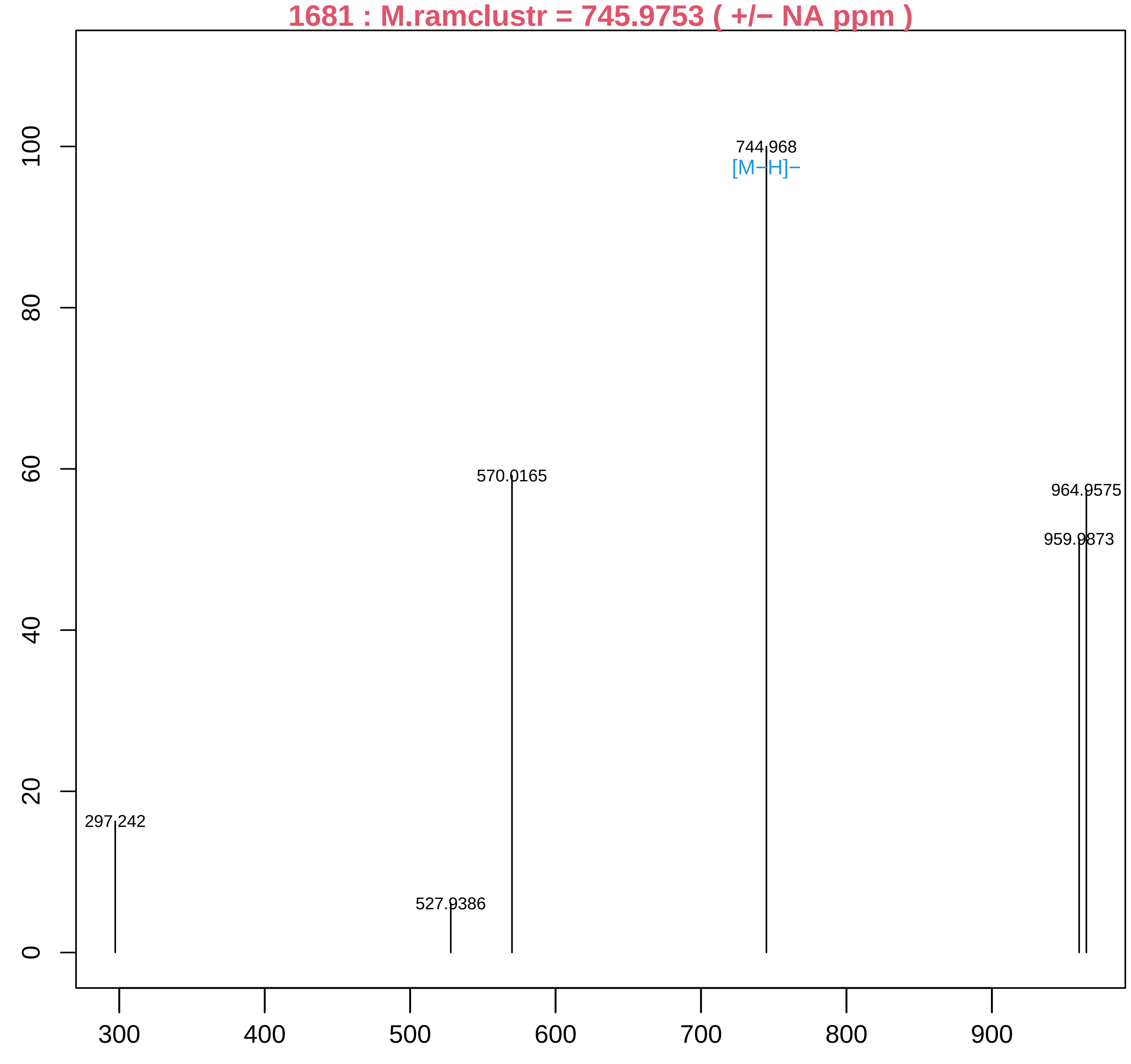
1681 : M.ramclustr = 745.9753 (+/− NA ppm)
744.968
[M−H]−
570.0165
964.9575
959.9873
297.242
527.9386
0
20
40
60
80
100
300
400
500
600
700
800
900

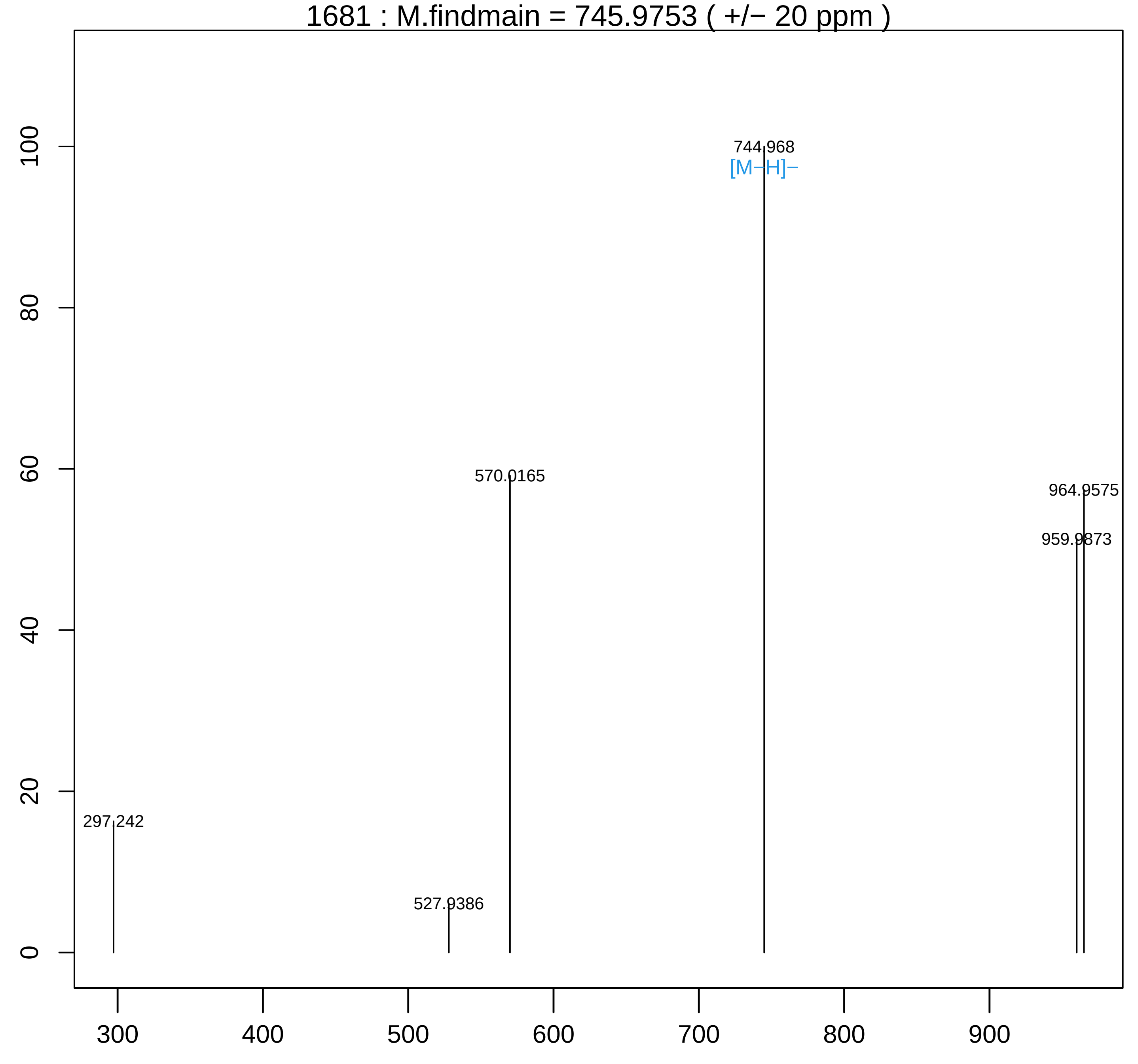
1681 : M.findmain = 745.9753 (+/− 20 ppm)
744.968
[M−H]−
570.0165
964.9575
959.9873
297.242
527.9386
0
20
40
60
80
100
300
400
500
600
700
800
900

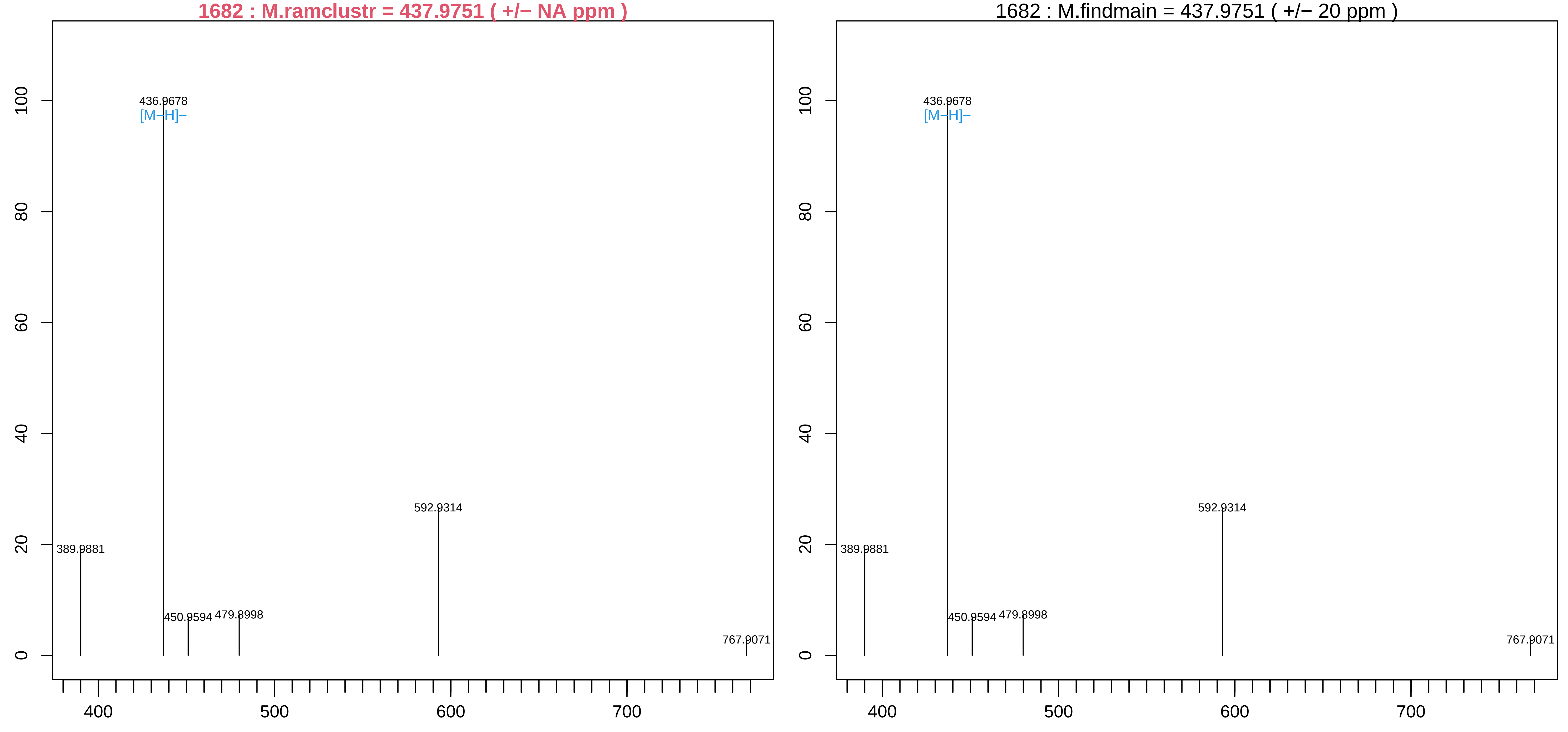
1682 : M.ramclustr = 437.9751 (+/− NA ppm)
436.9678
[M−H]−
389.9881
450.9594
479.8998
592.9314
767.9071
1682 : M.findmain = 437.9751 (+/− 20 ppm)
436.9678
[M−H]−
389.9881
450.9594
479.8998
592.9314
767.9071

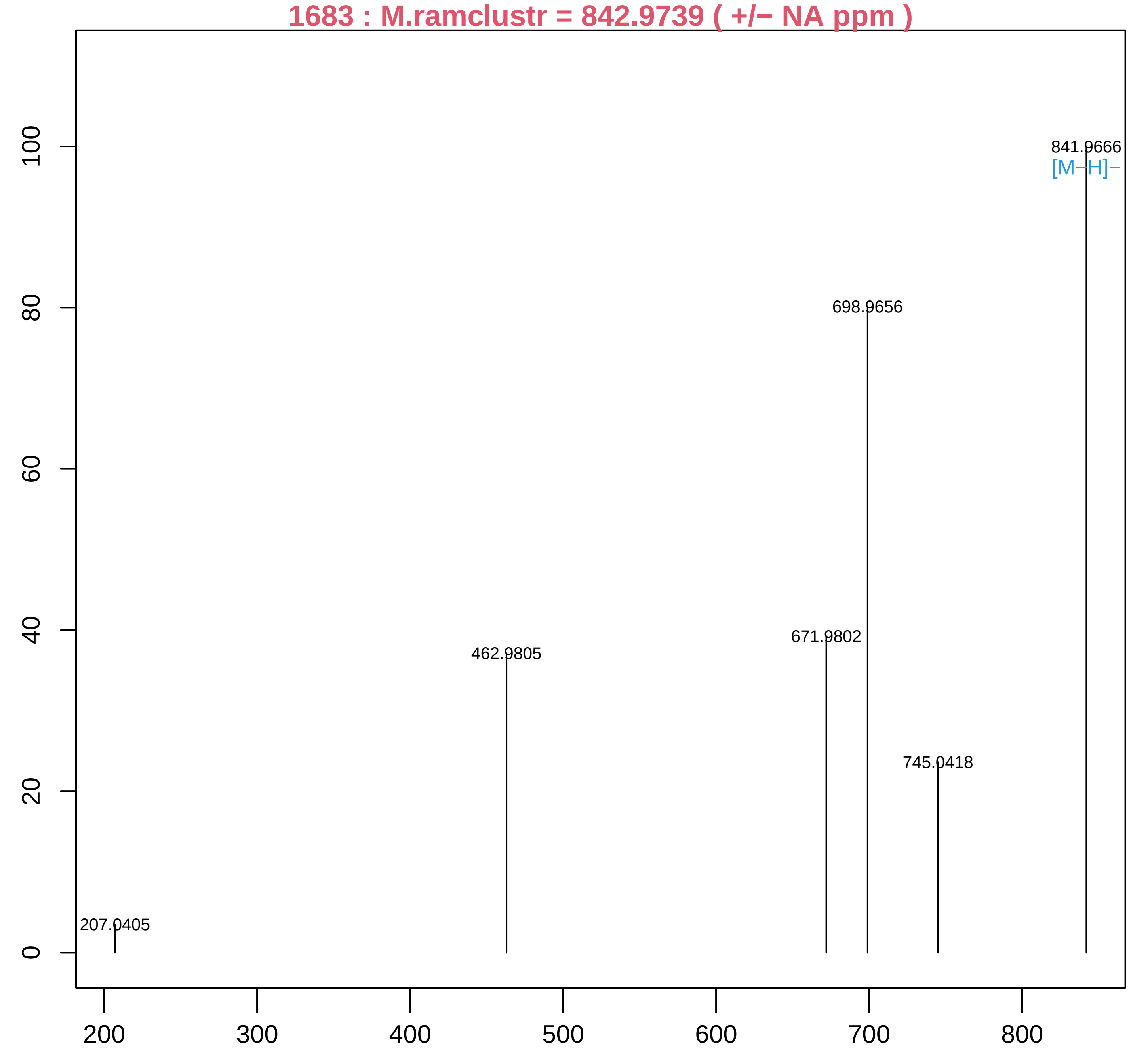
1683 : M.ramclustr = 842.9739 (+/− NA ppm)
841.9666
[M−H]−
698.9656
671.9802
462.9805
745.0418
207.0405
0
20
40
60
80
100
200
300
400
500
600
700
800

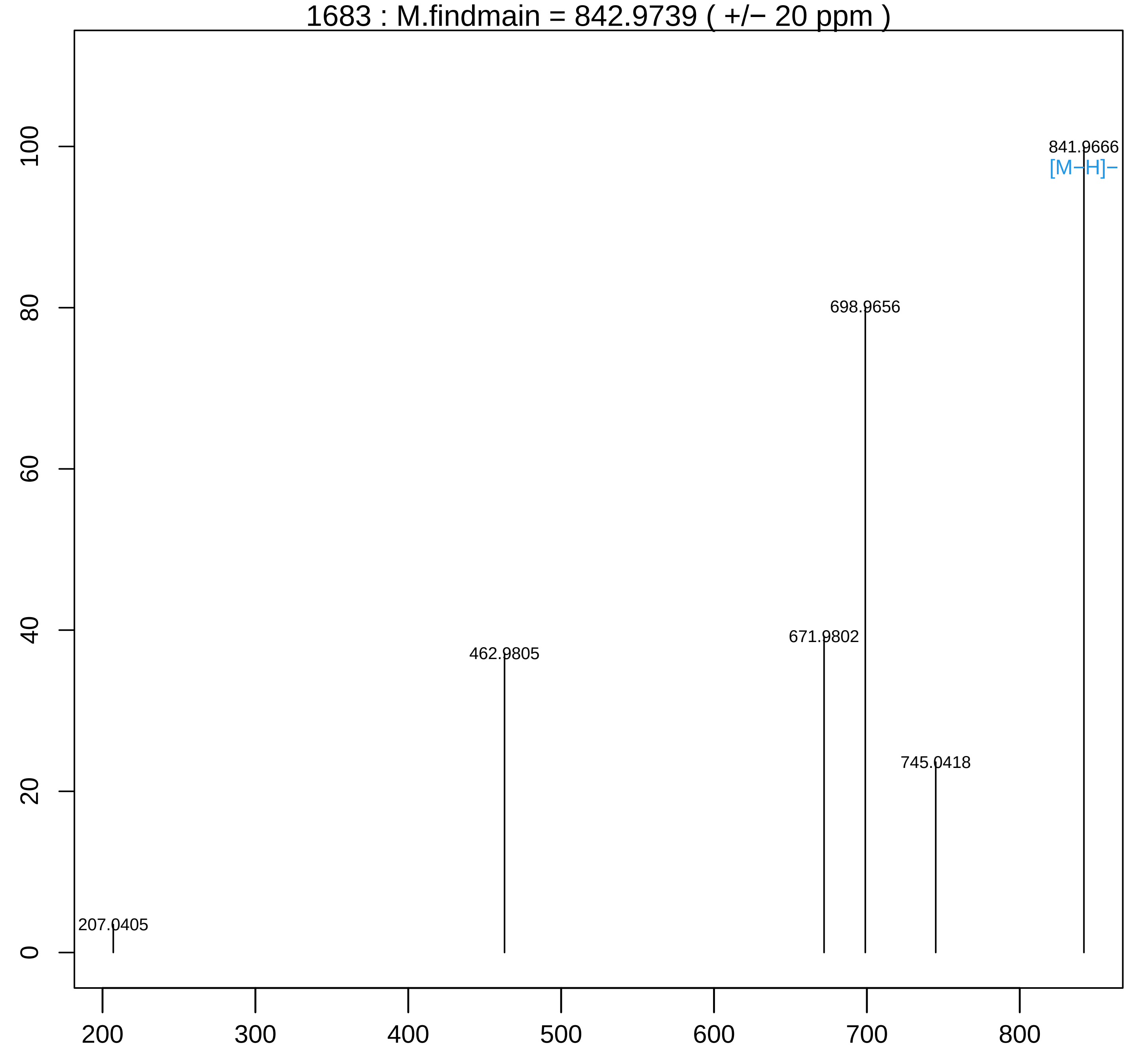
1683 : M.findmain = 842.9739 (+/− 20 ppm)
841.9666
[M−H]−
698.9656
671.9802
462.9805
745.0418
207.0405
0
20
40
60
80
100
200
300
400
500
600
700
800

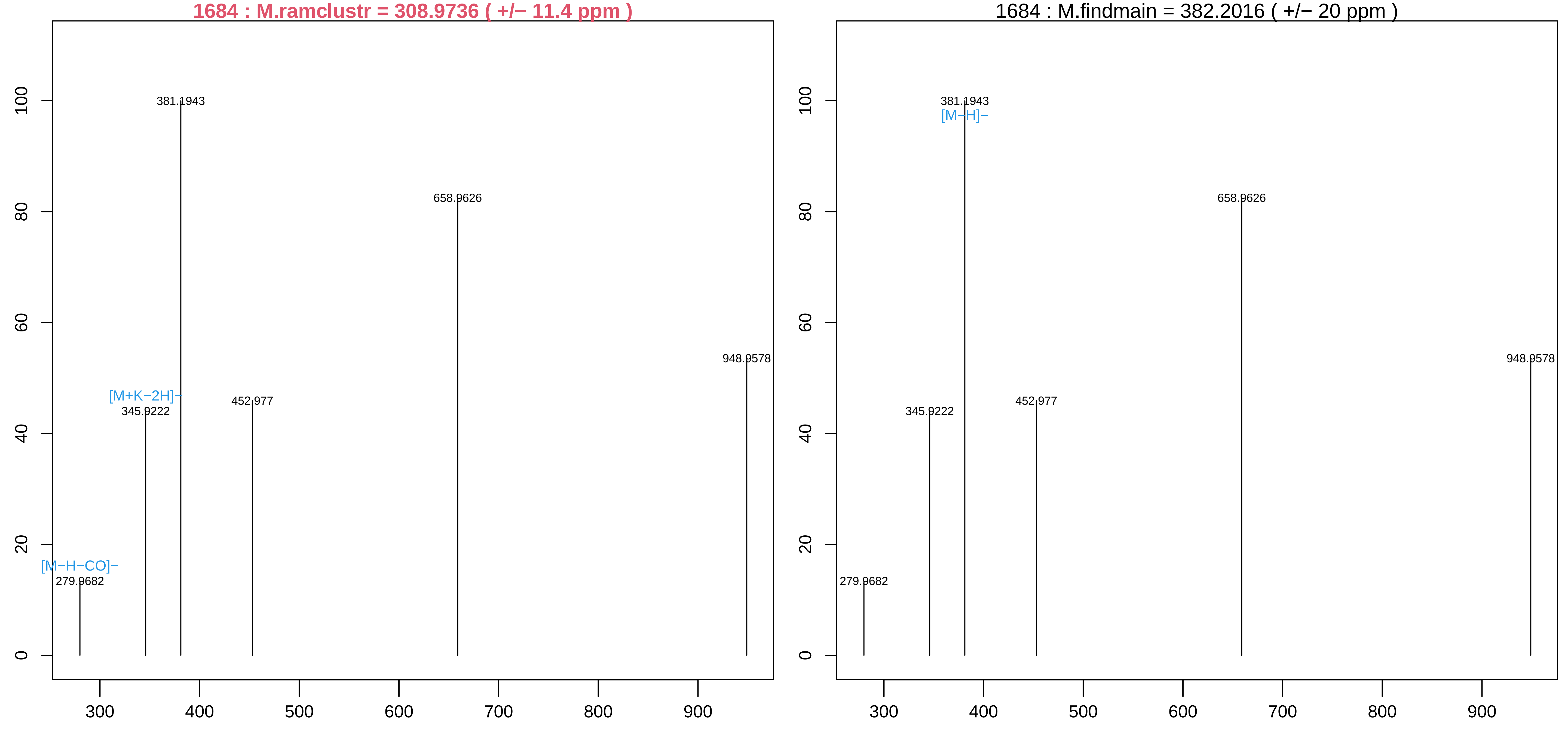

1684 : M.ramclustr = 308.9736 (+/− 11.4 ppm)
381.1943
658.9626
948.9578
[M+K−2H]−
345.9222
452.977
[M−H−CO]−
279.9682
0
20
40
60
80
100
300
400
500
600
700
800
900
1684 : M.findmain = 382.2016 (+/− 20 ppm)
381.1943
[M−H]−
658.9626
948.9578
345.9222
452.977
279.9682
0
20
40
60
80
100
300
400
500
600
700
800
900

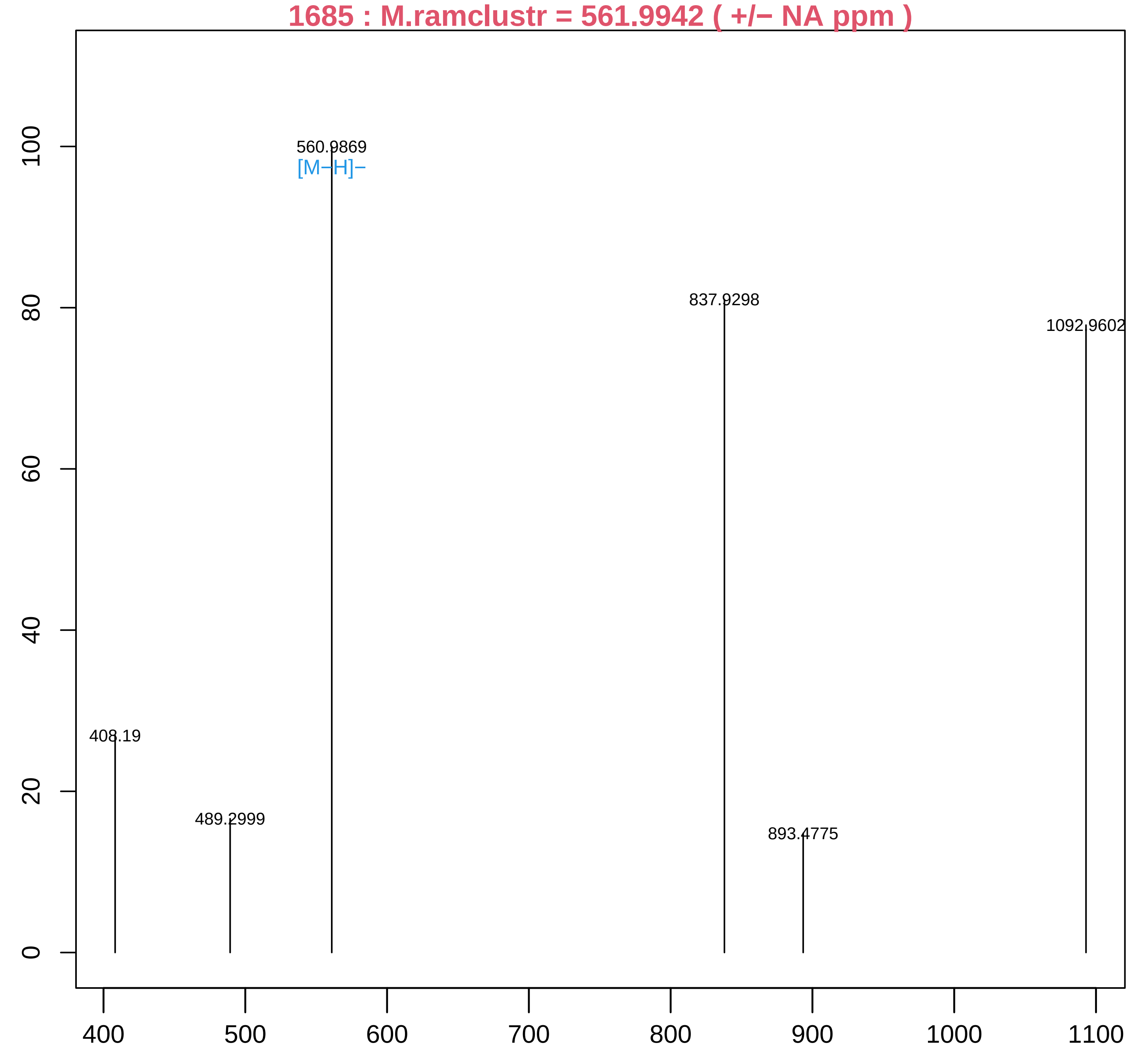

1685 : M.ramclustr = 561.9942 (+/− NA ppm)
560.9869
[M−H]−
837.9298
1092.9602
408.19
489.2999
893.4775
0
20
40
60
80
100
400
500
600
700
800
900
1000
1100

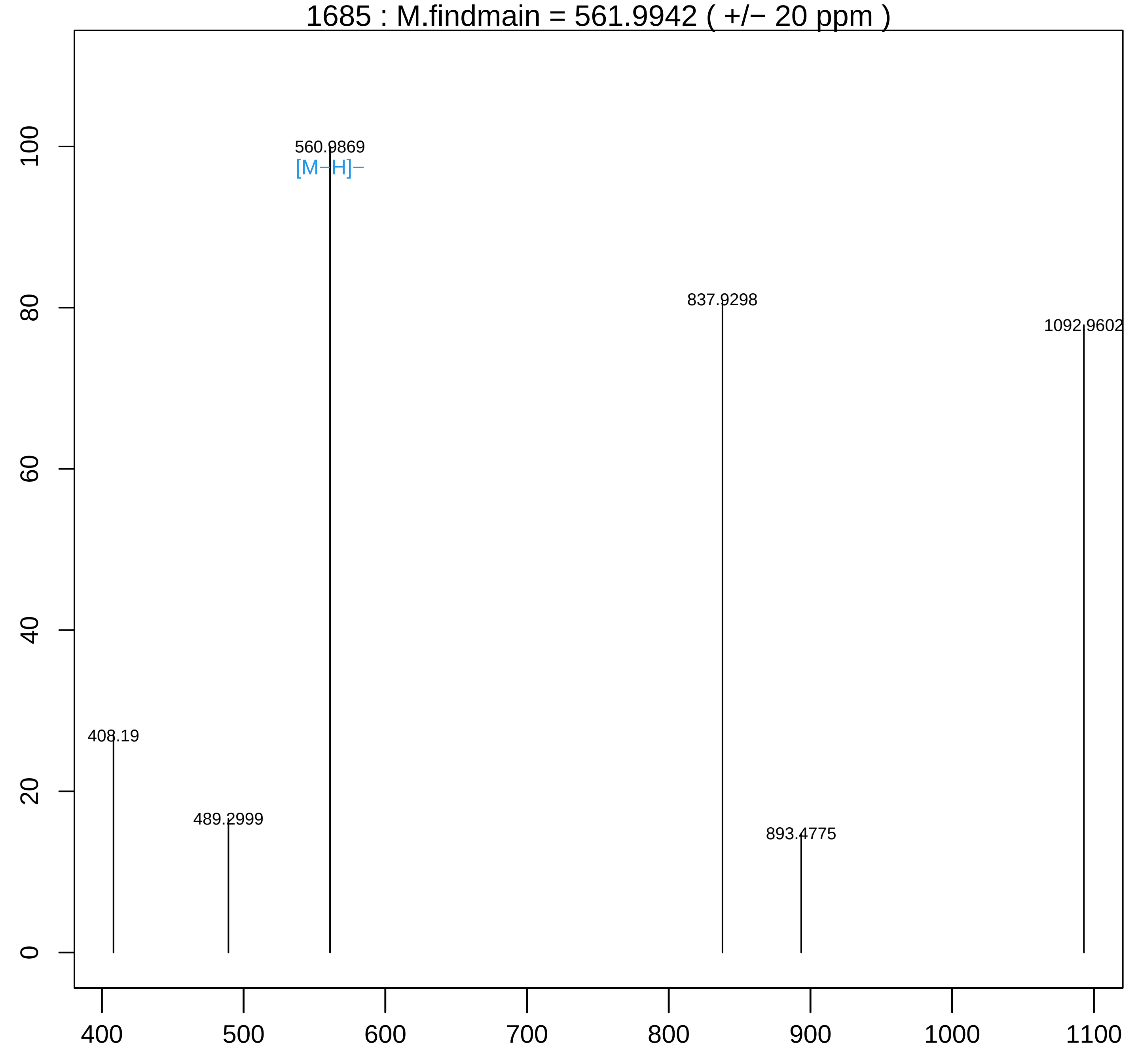

1685 : M.findmain = 561.9942 (+/− 20 ppm)
560.9869
[M−H]−
837.9298
1092.9602
408.19
489.2999
893.4775
0
20
40
60
80
100
400
500
600
700
800
900
1000
1100

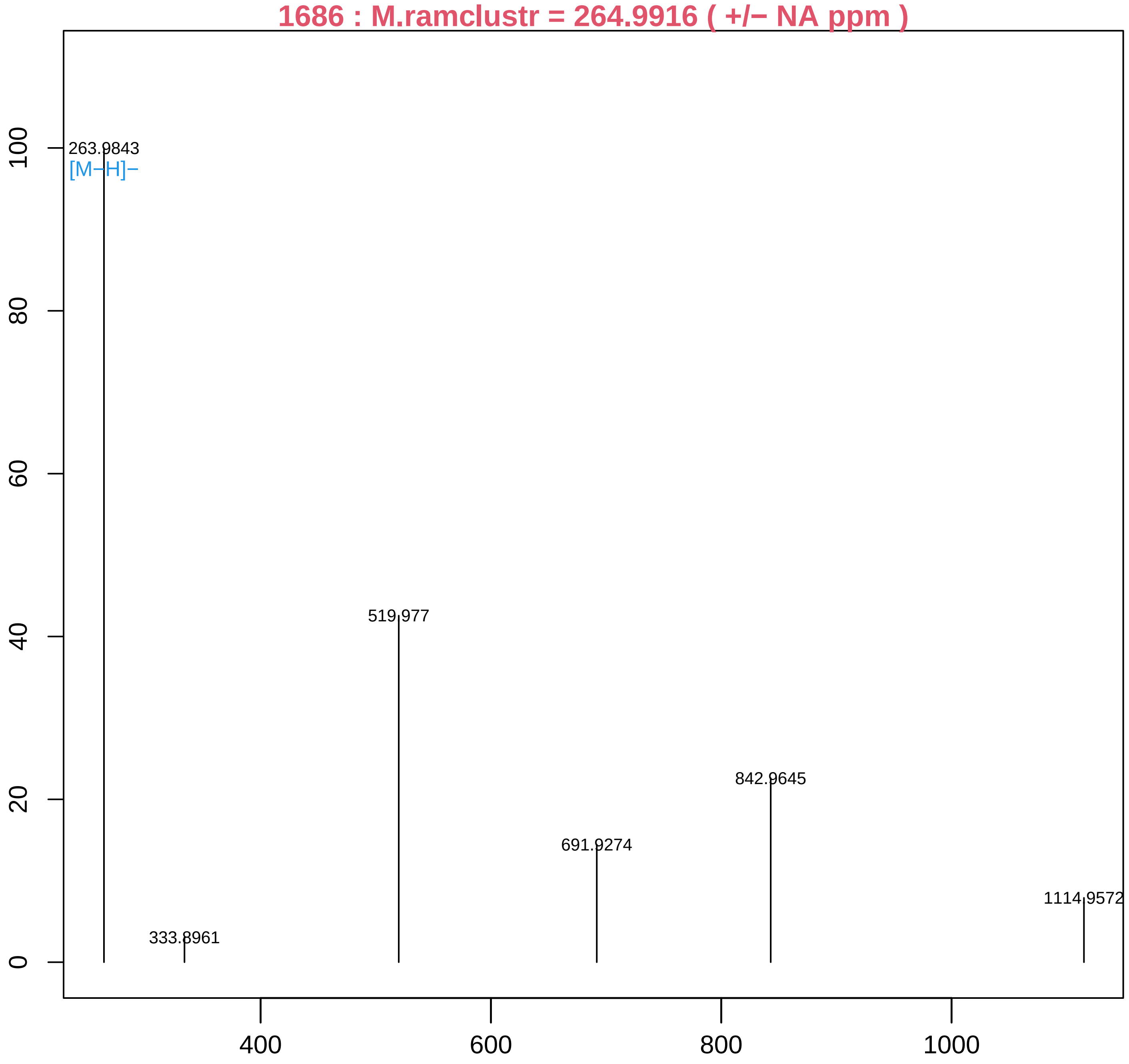
1686 : M.ramclustr = 264.9916 (+/− NA ppm)
263.9843
[M−H]−
333.8961
519.977
691.9274
842.9645
1114.9572
0
20
40
60
80
100
400
600
800
1000

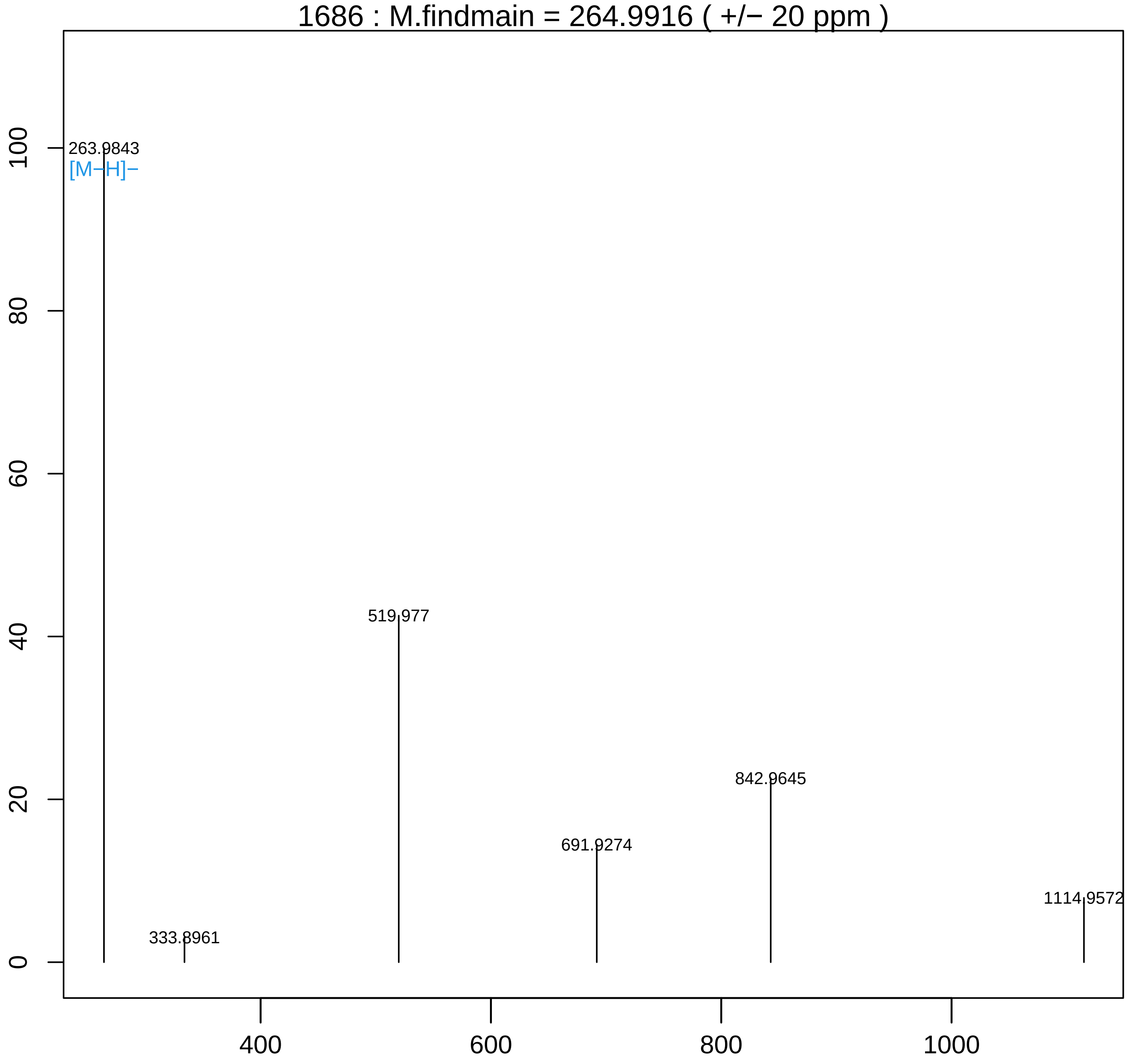
1686 : M.findmain = 264.9916 (+/− 20 ppm)
263.9843
[M−H]−
333.8961
519.977
691.9274
842.9645
1114.9572
0
20
40
60
80
100
400
600
800
1000

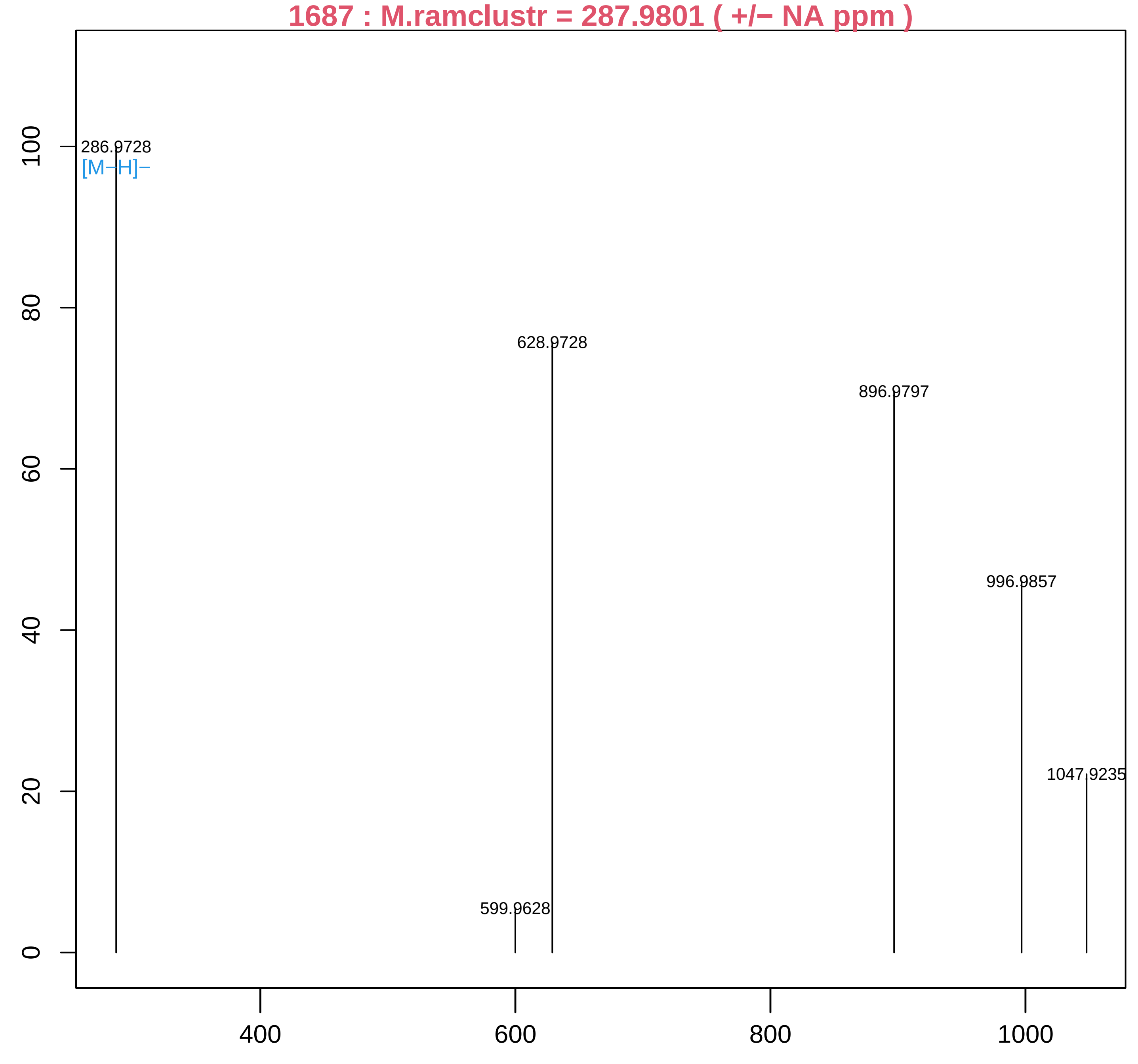
1687 : M.ramclustr = 287.9801 (+/− NA ppm)
286.9728
[M−H]−
628.9728
896.9797
996.9857
1047.9235
599.9628
0
20
40
60
80
100
400
600
800
1000

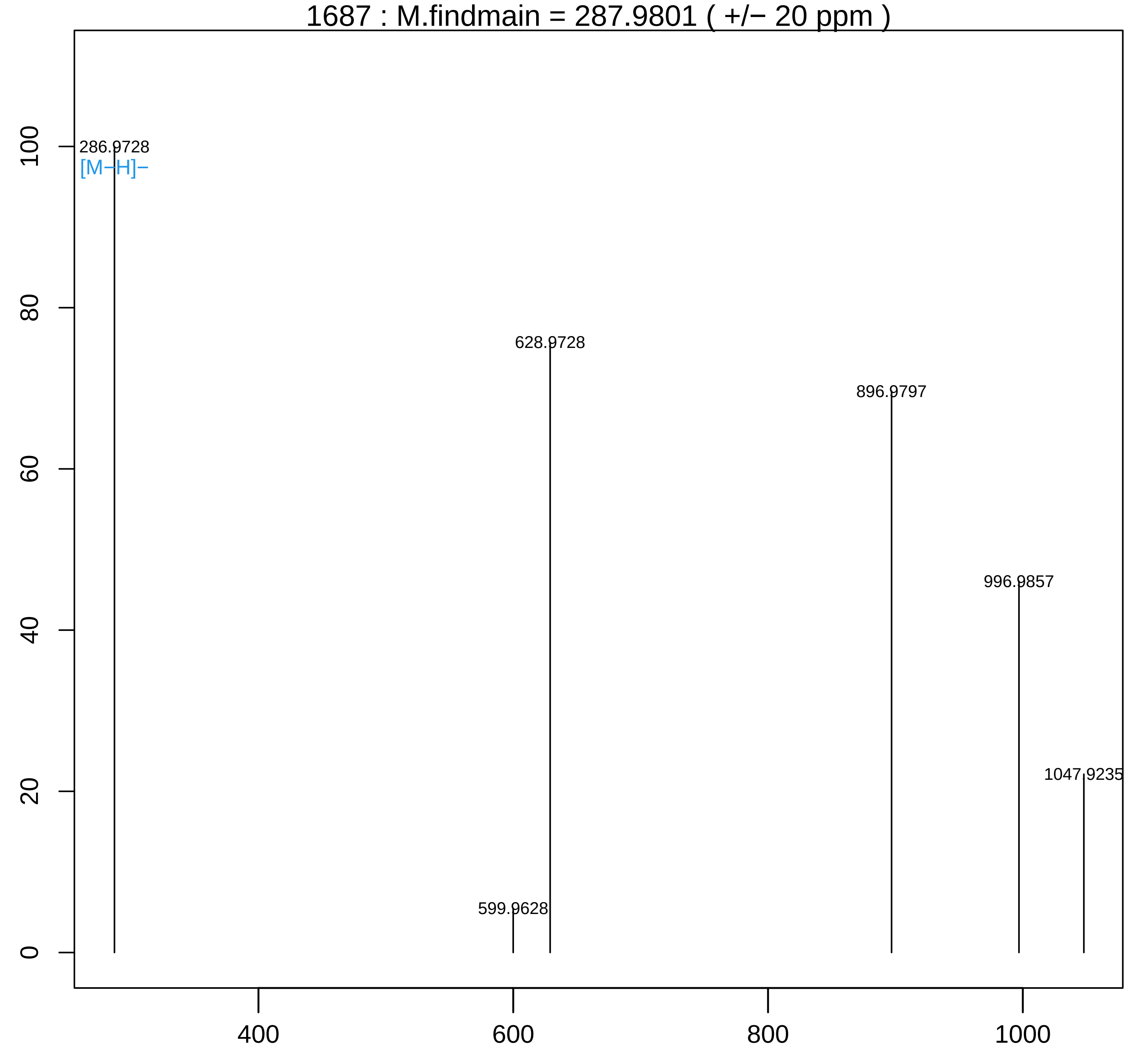
1687 : M.findmain = 287.9801 (+/− 20 ppm)
286.9728
[M−H]−
628.9728
896.9797
996.9857
1047.9235
599.9628
0
20
40
60
80
100
400
600
800
1000

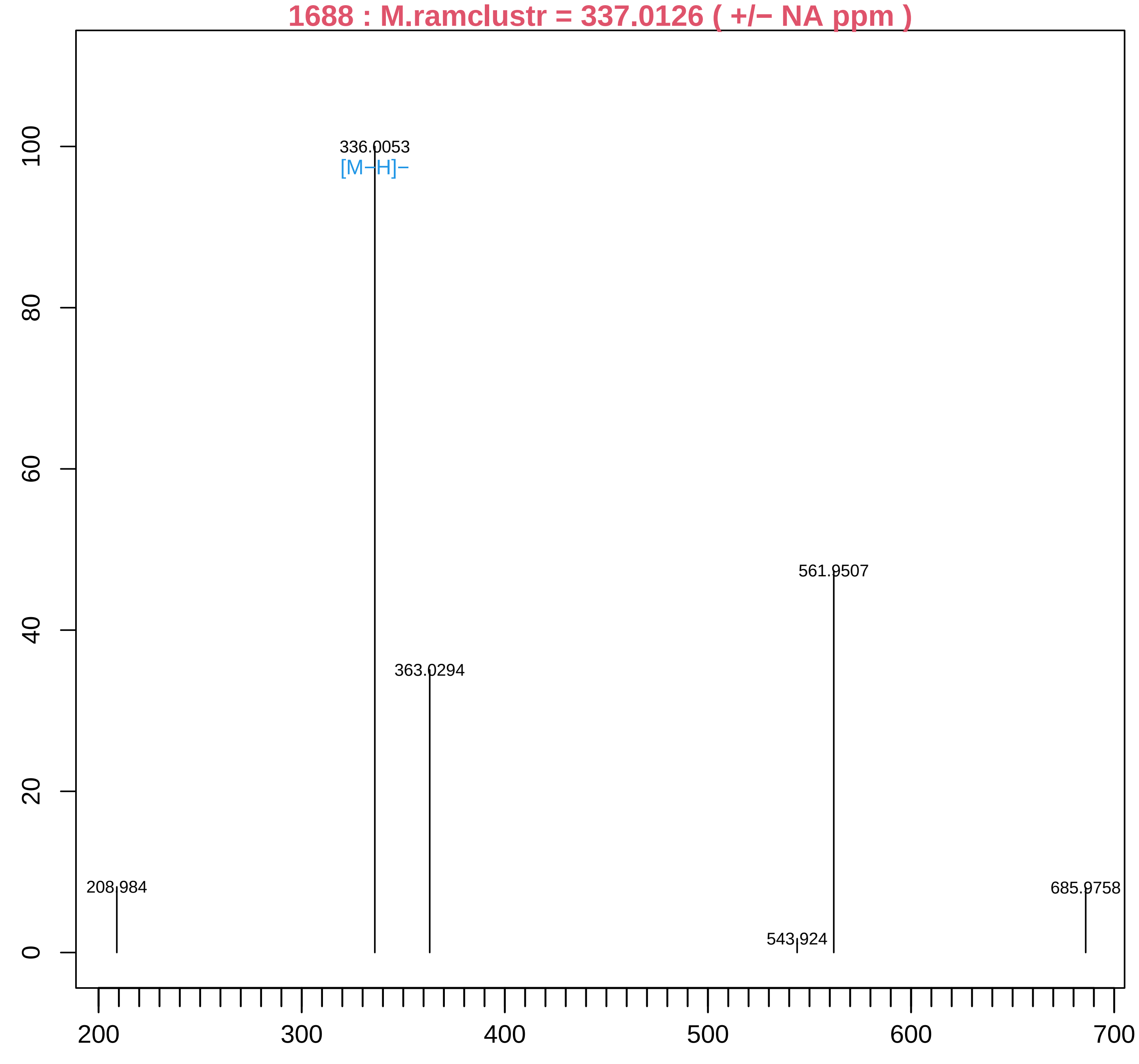

1688 : M.ramclustr = 337.0126 (+/− NA ppm)
336.0053
[M−H]−
363.0294
561.9507
543.924
208.984
685.9758
0
20
40
60
80
100
200
300
400
500
600
700

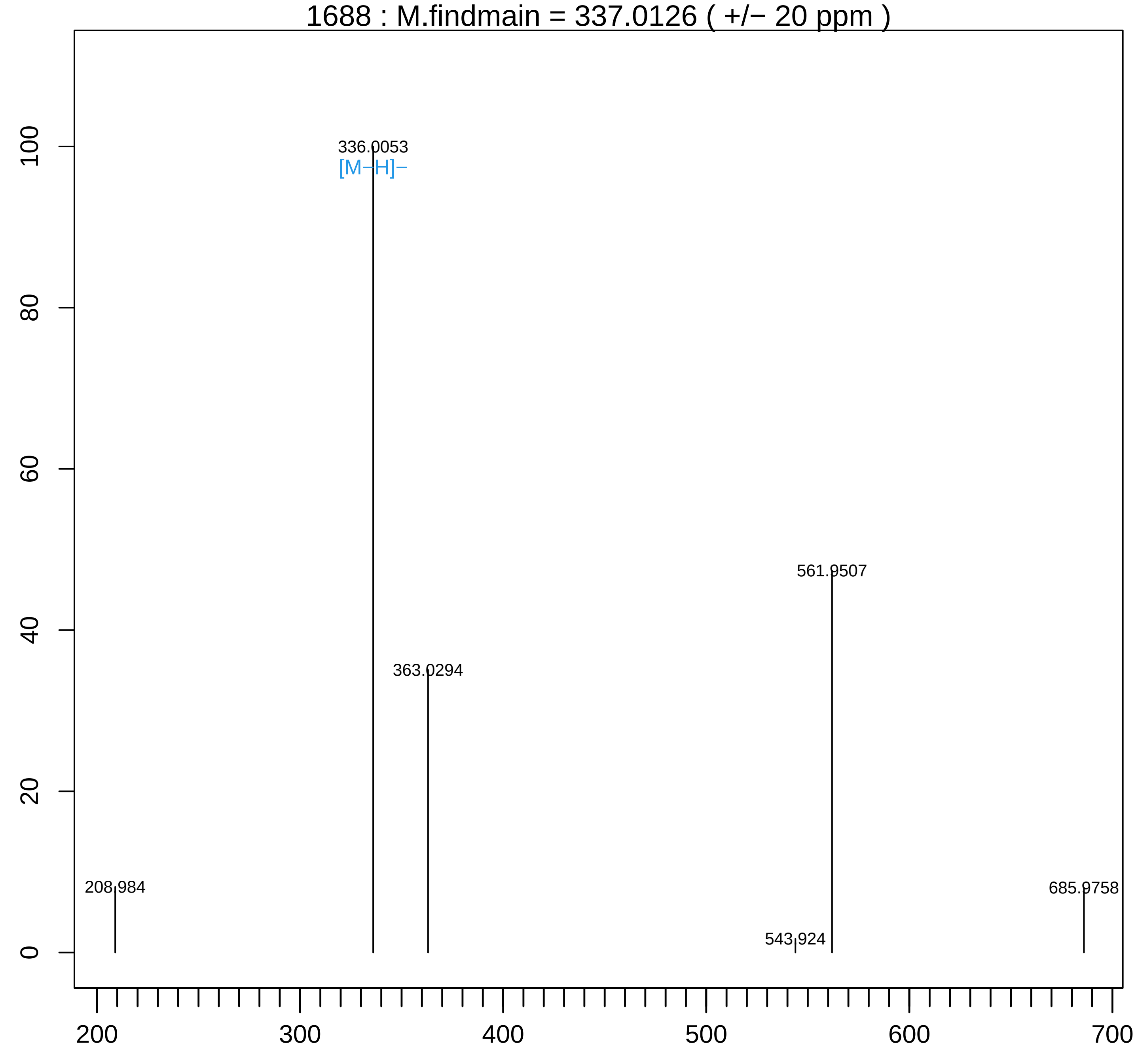

1688 : M.findmain = 337.0126 (+/− 20 ppm)
336.0053
[M−H]−
363.0294
561.9507
543.924
208.984
685.9758
0
20
40
60
80
100
200
300
400
500
600
700

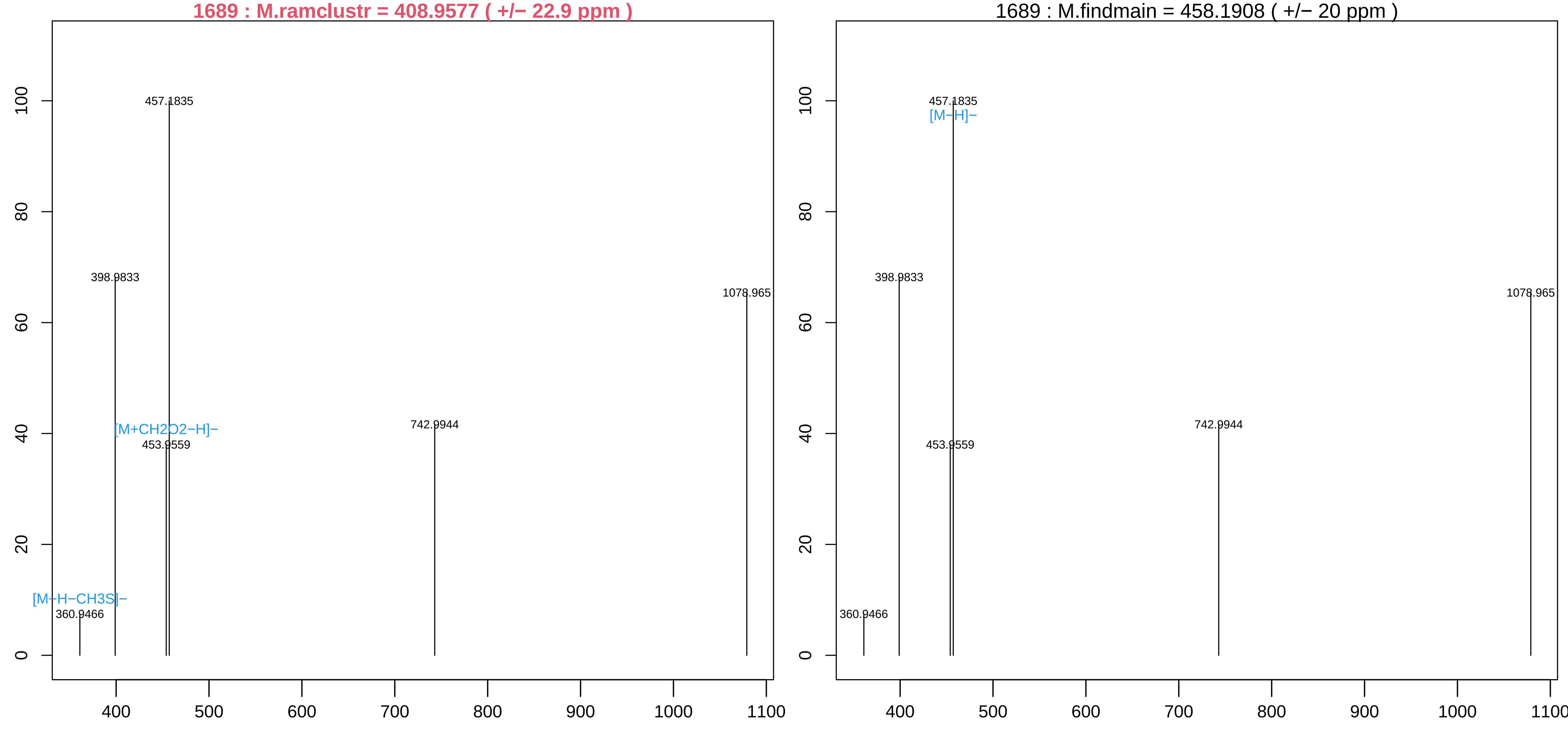

1689 : M.ramclustr = 408.9577 (+/− 22.9 ppm)
457.1835
398.9833
1078.965
742.9944
[M+CH2O2−H]−
453.9559
[M−H−CH3S]−
360.9466
1689 : M.findmain = 458.1908 (+/− 20 ppm)
457.1835
[M−H]−
398.9833
1078.965
742.9944
453.9559
360.9466
0
20
40
60
80
100
400
500
600
700
800
900
1000
1100

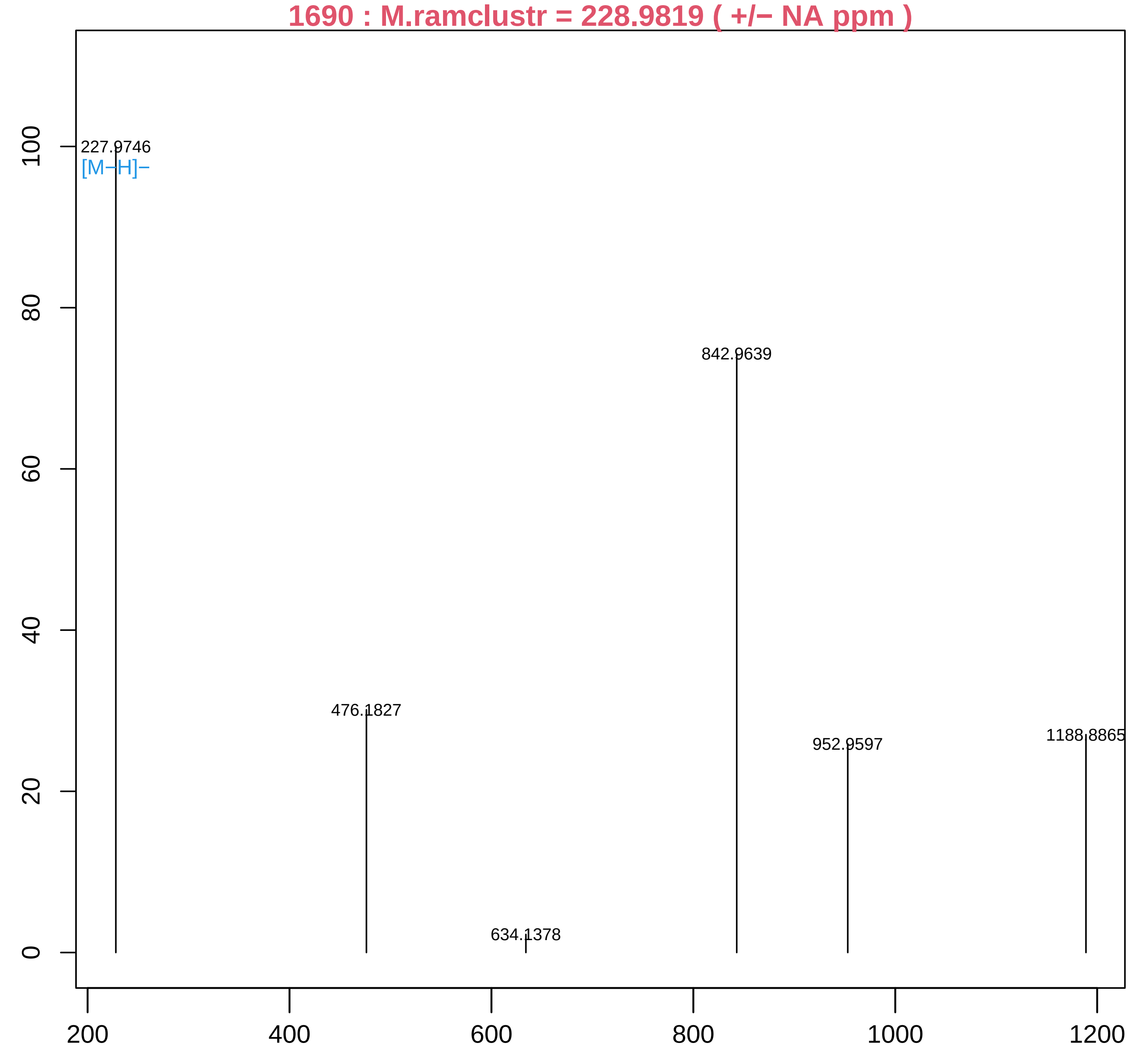
1690 : M.ramclustr = 228.9819 (+/− NA ppm)
227.9746
[M−H]−
476.1827
634.1378
842.9639
952.9597
1188.8865
0
20
40
60
80
100
200
400
600
800
1000
1200

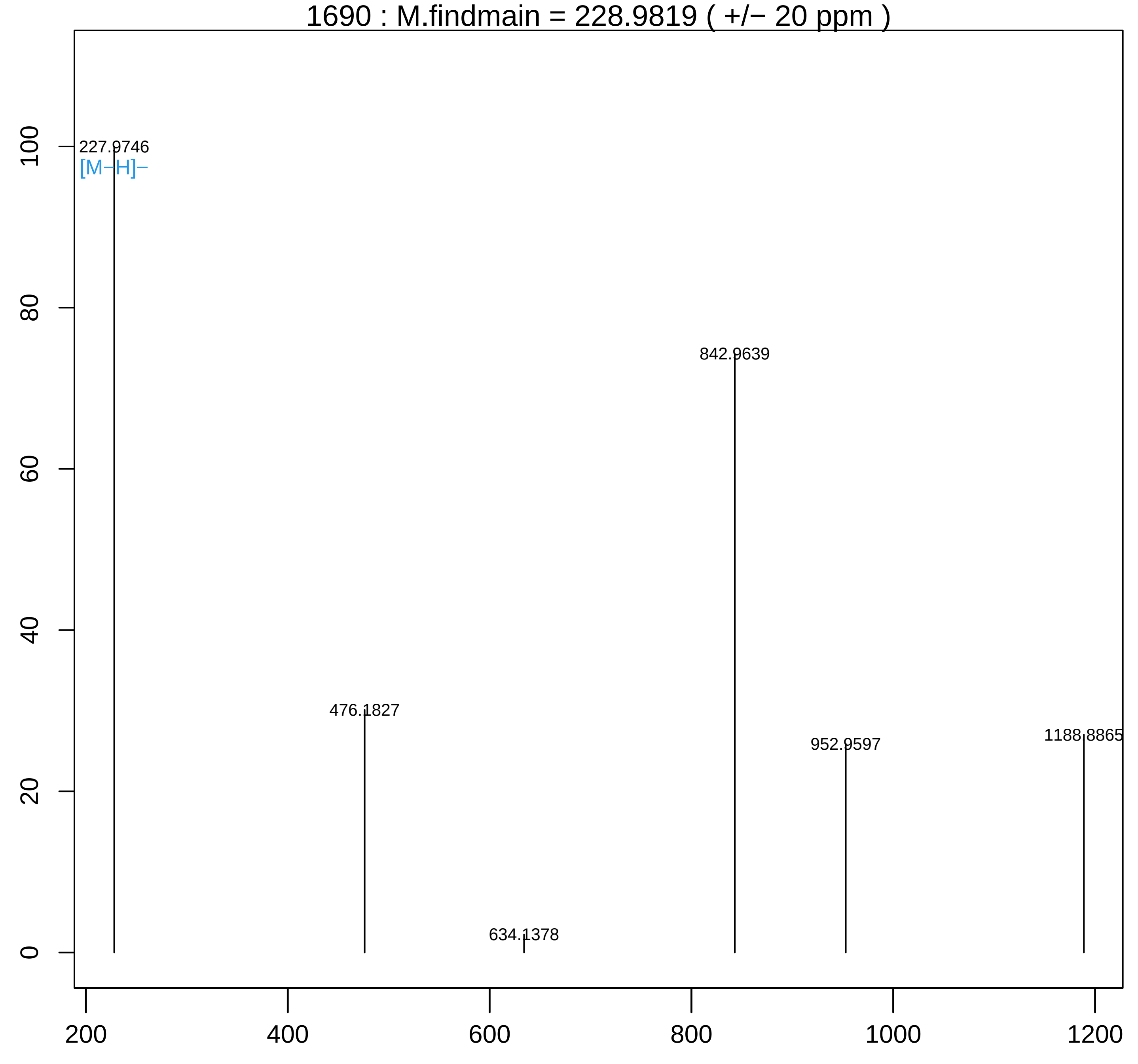
1690 : M.findmain = 228.9819 (+/− 20 ppm)
227.9746
[M−H]−
476.1827
634.1378
842.9639
952.9597
1188.8865
0
20
40
60
80
100
200
400
600
800
1000
1200

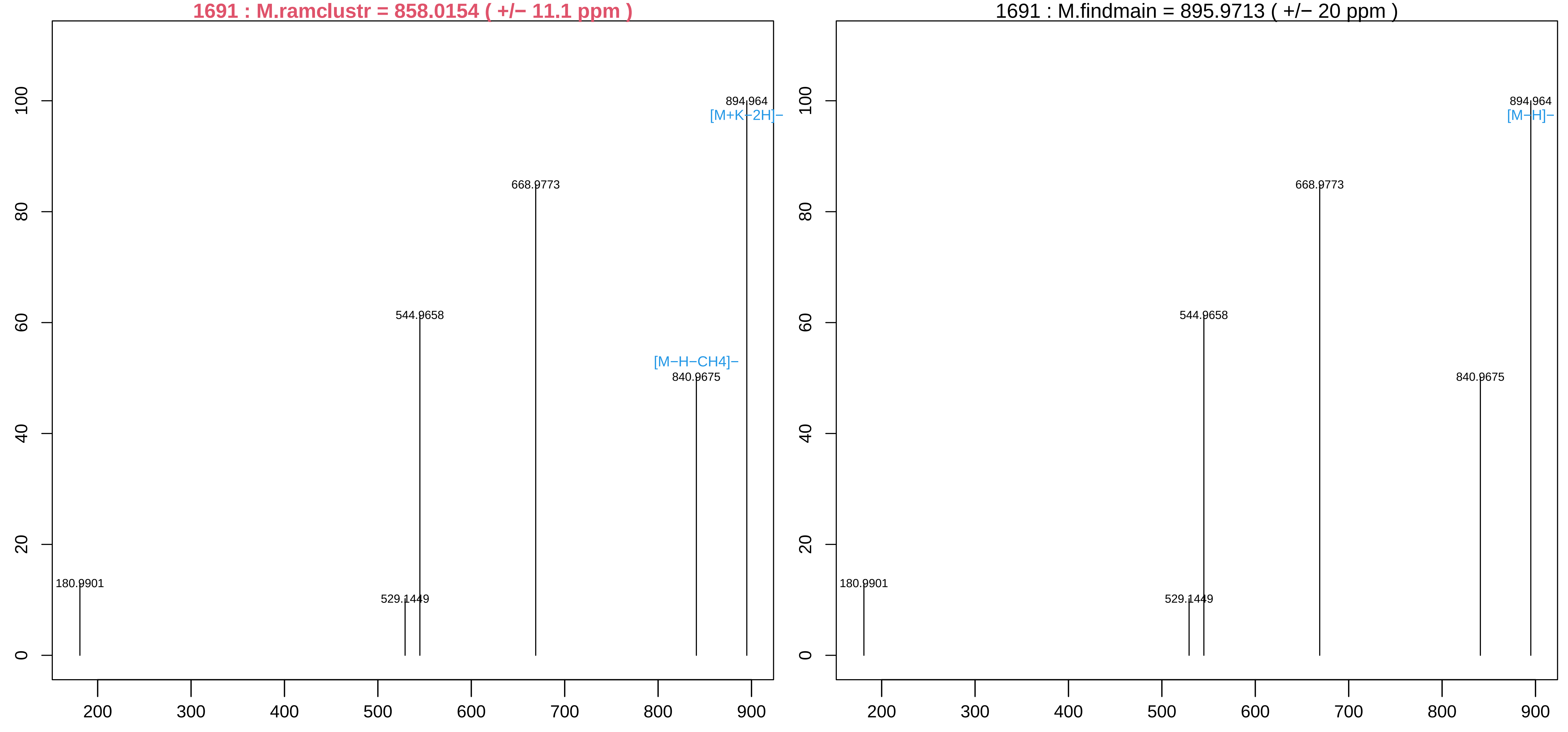
1691 : M.ramclustr = 858.0154 (+/− 11.1 ppm)
894.964
[M+K−2H]−
668.9773
544.9658
[M−H−CH4]−
840.9675
180.9901
529.1449
0
20
40
60
80
100
200
300
400
500
600
700
800
900
1691 : M.findmain = 895.9713 (+/− 20 ppm)
894.964
[M−H]−
668.9773
544.9658
840.9675
180.9901
529.1449
0
20
40
60
80
100
200
300
400
500
600
700
800
900

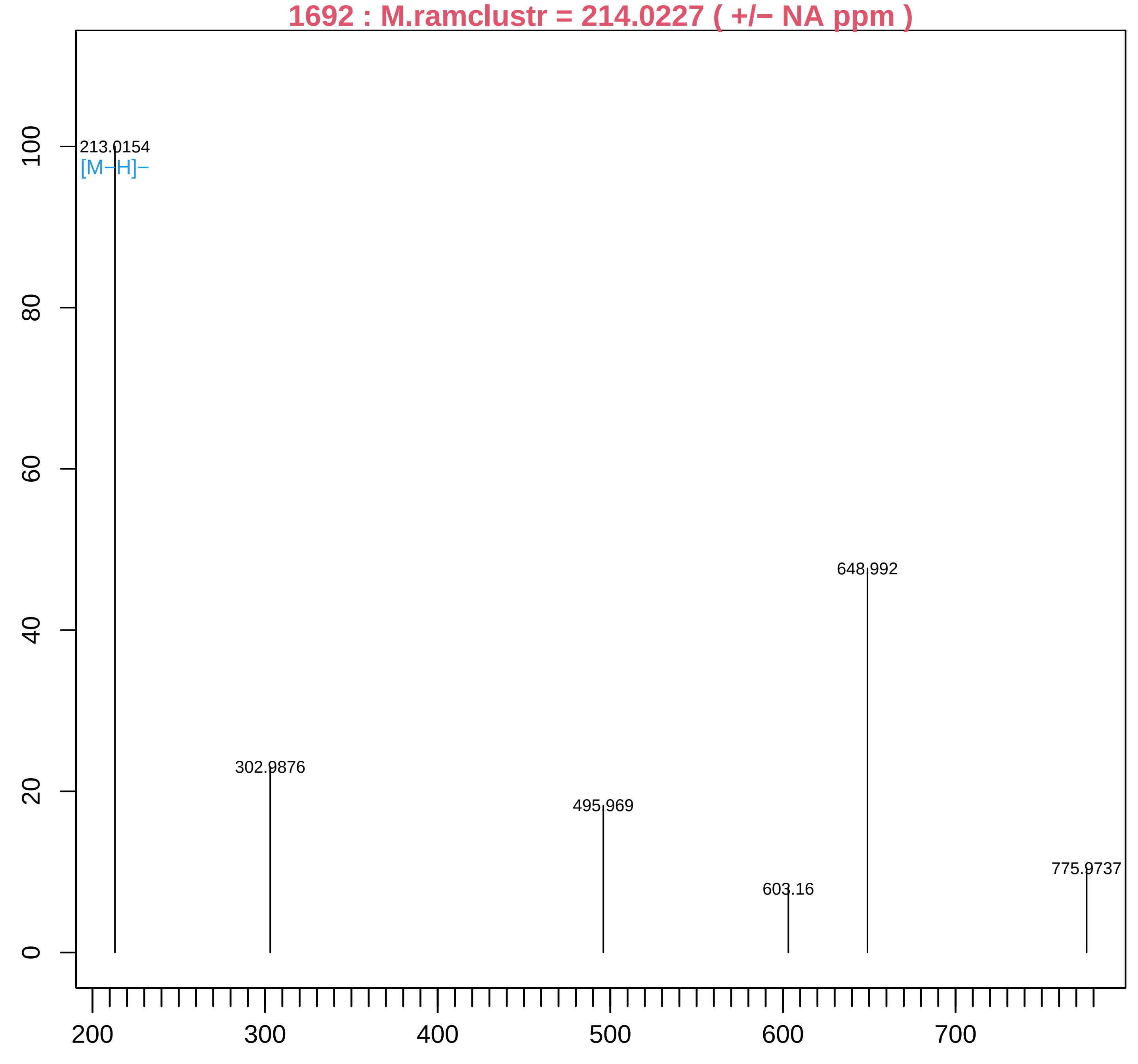
1692 : M.ramclustr = 214.0227 (+/− NA ppm)
213.0154
[M−H]−
302.9876
495.969
603.16
648.992
775.9737
0
20
40
60
80
100
200
300
400
500
600
700

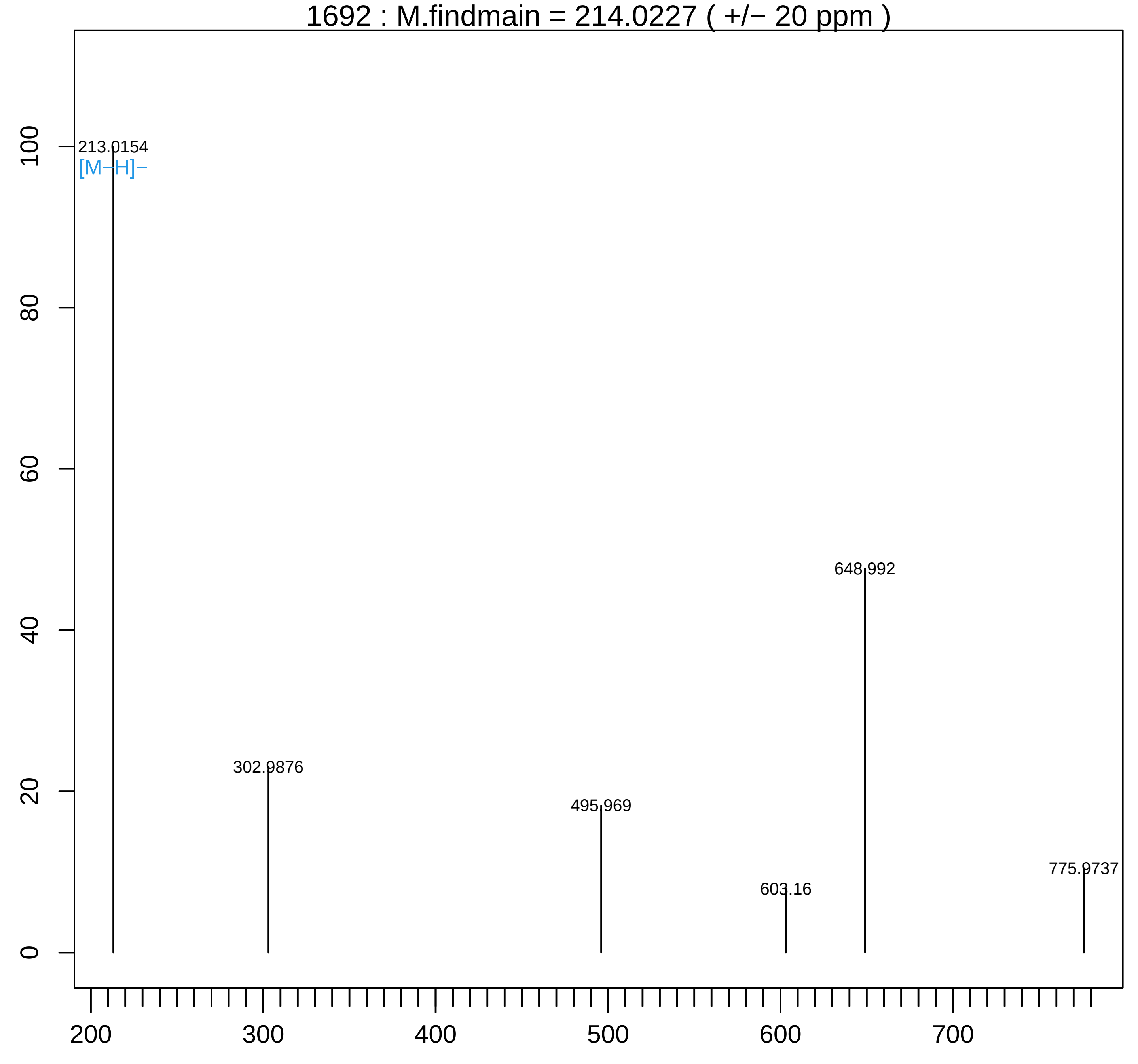
1692 : M.findmain = 214.0227 (+/− 20 ppm)
213.0154
[M−H]−
302.9876
495.969
603.16
648.992
775.9737
0
20
40
60
80
100
200
300
400
500
600
700

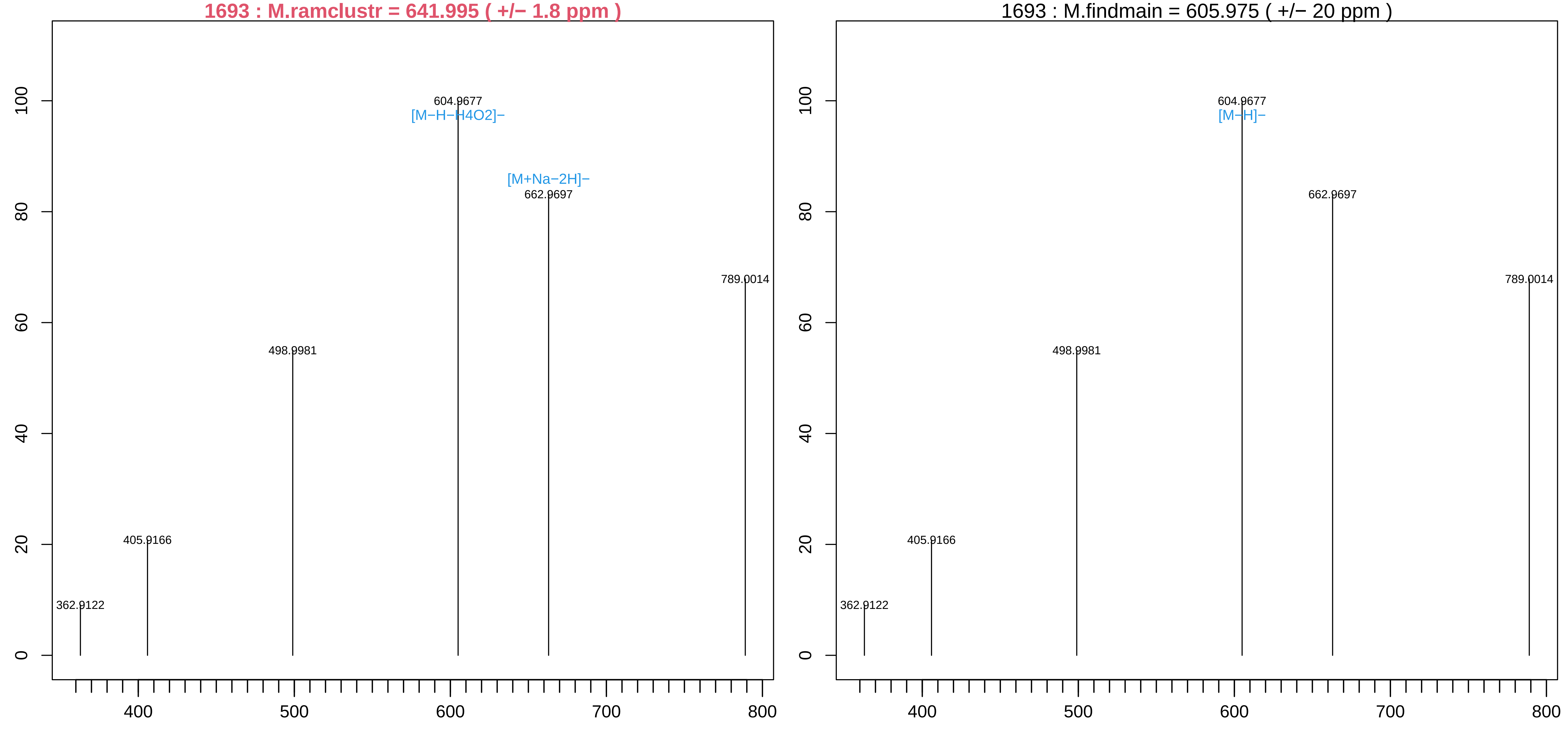

1693 : M.ramclustr = 641.995 (+/− 1.8 ppm)
604.9677
[M−H−H4O2]−
[M+Na−2H]−
662.9697
789.0014
498.9981
405.9166
362.9122
1693 : M.findmain = 605.975 (+/− 20 ppm)
604.9677
[M−H]−
662.9697
789.0014
498.9981
405.9166
362.9122

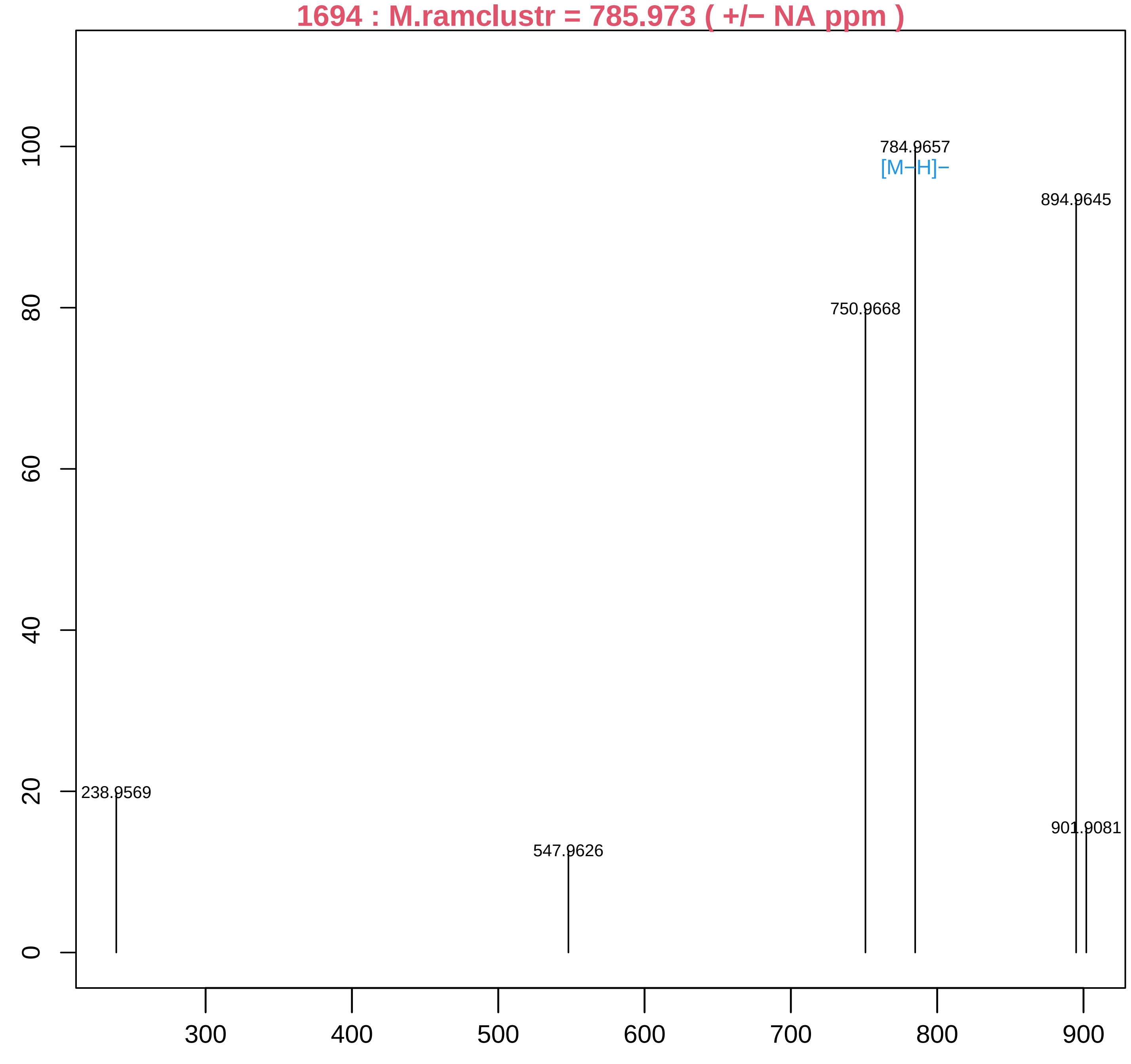
1694 : M.ramclustr = 785.973 (+/− NA ppm)
784.9657
[M−H]−
894.9645
750.9668
238.9569
547.9626
901.9081
0
20
40
60
80
100
300
400
500
600
700
800
900

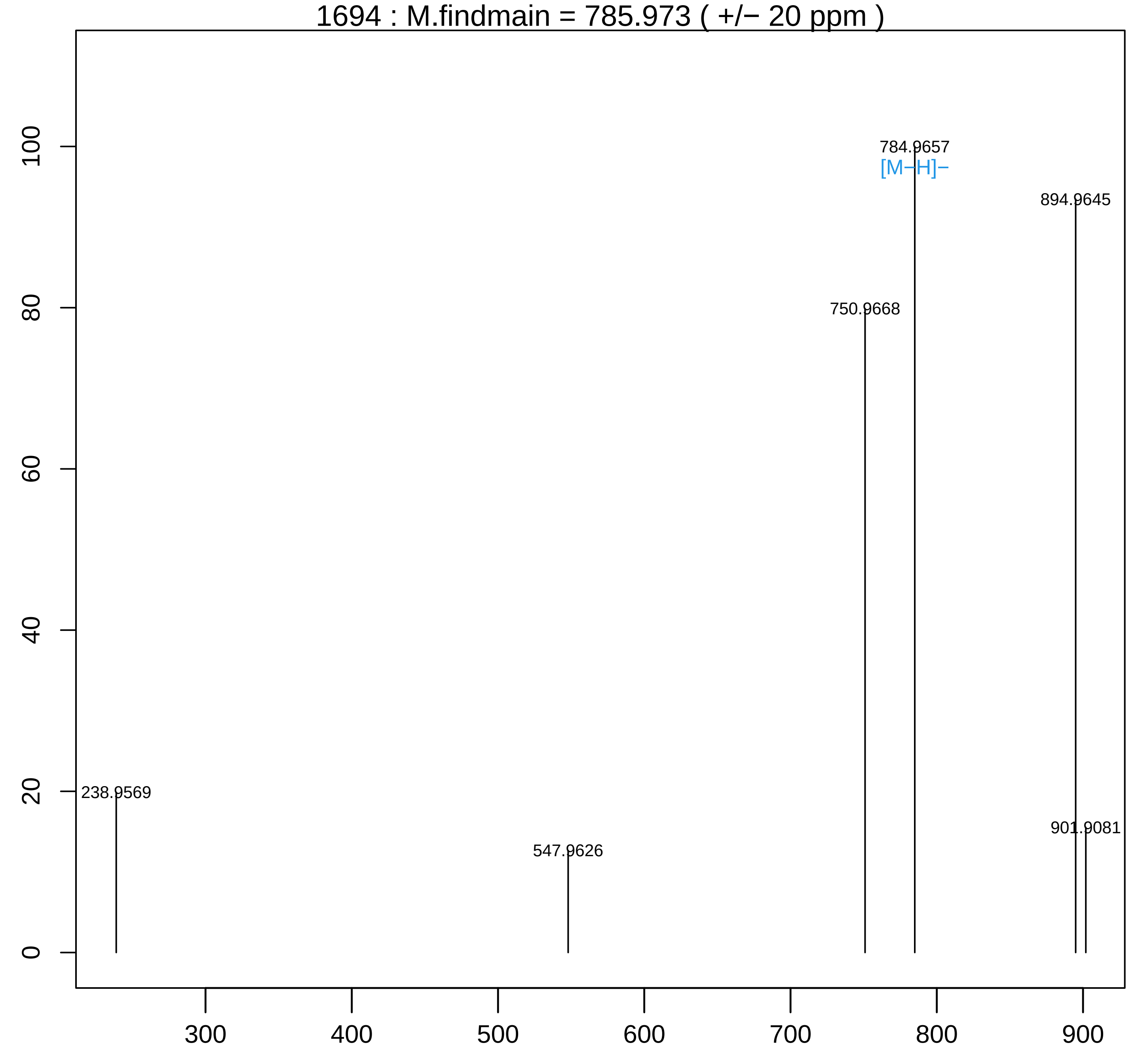
1694 : M.findmain = 785.973 (+/− 20 ppm)
784.9657
[M−H]−
894.9645
750.9668
238.9569
547.9626
901.9081
0
20
40
60
80
100
300
400
500
600
700
800
900

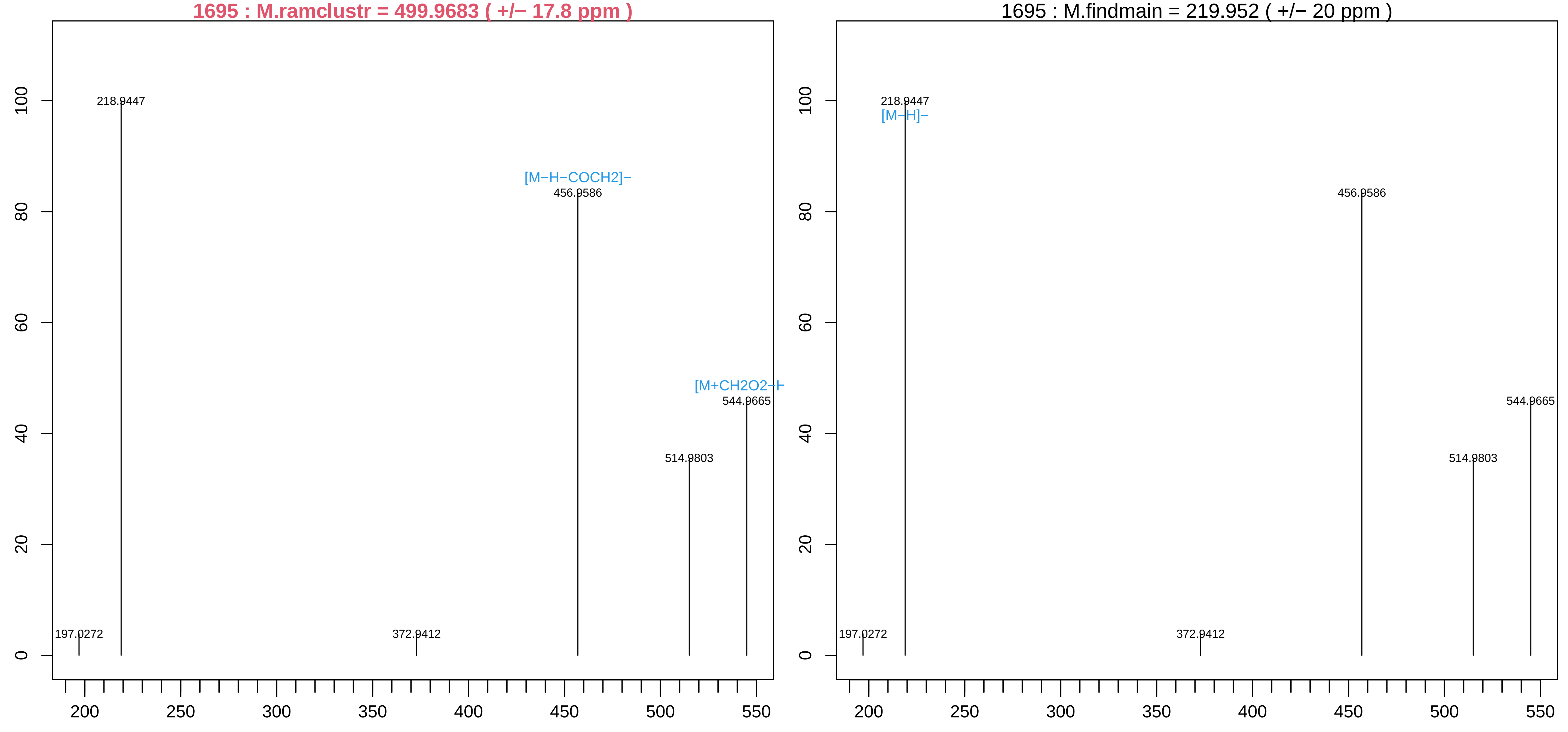

1695 : M.ramclustr = 499.9683 (+/− 17.8 ppm)
218.9447
[M−H−COCH2]−
456.9586
[M+CH2O2−H
544.9665
514.9803
197.0272
372.9412
1695 : M.findmain = 219.952 (+/− 20 ppm)
218.9447
[M−H]−
456.9586
544.9665
514.9803
197.0272
372.9412

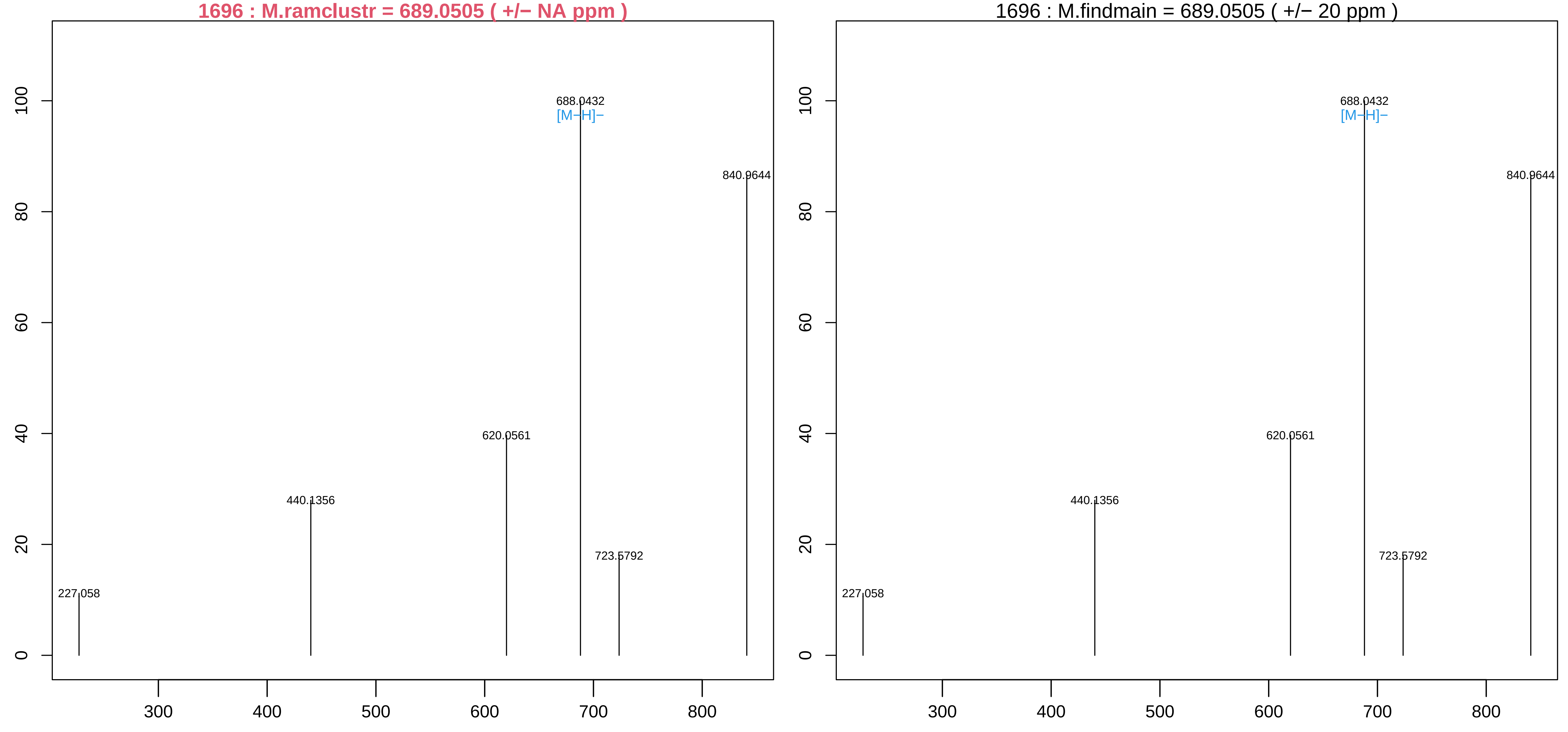
1696 : M.ramclustr = 689.0505 (+/− NA ppm)
1696 : M.findmain = 689.0505 (+/− 20 ppm)
688.0432
[M−H]−
840.9644
620.0561
440.1356
723.5792
227.058
0
20
40
60
80
100
300
400
500
600
700
800

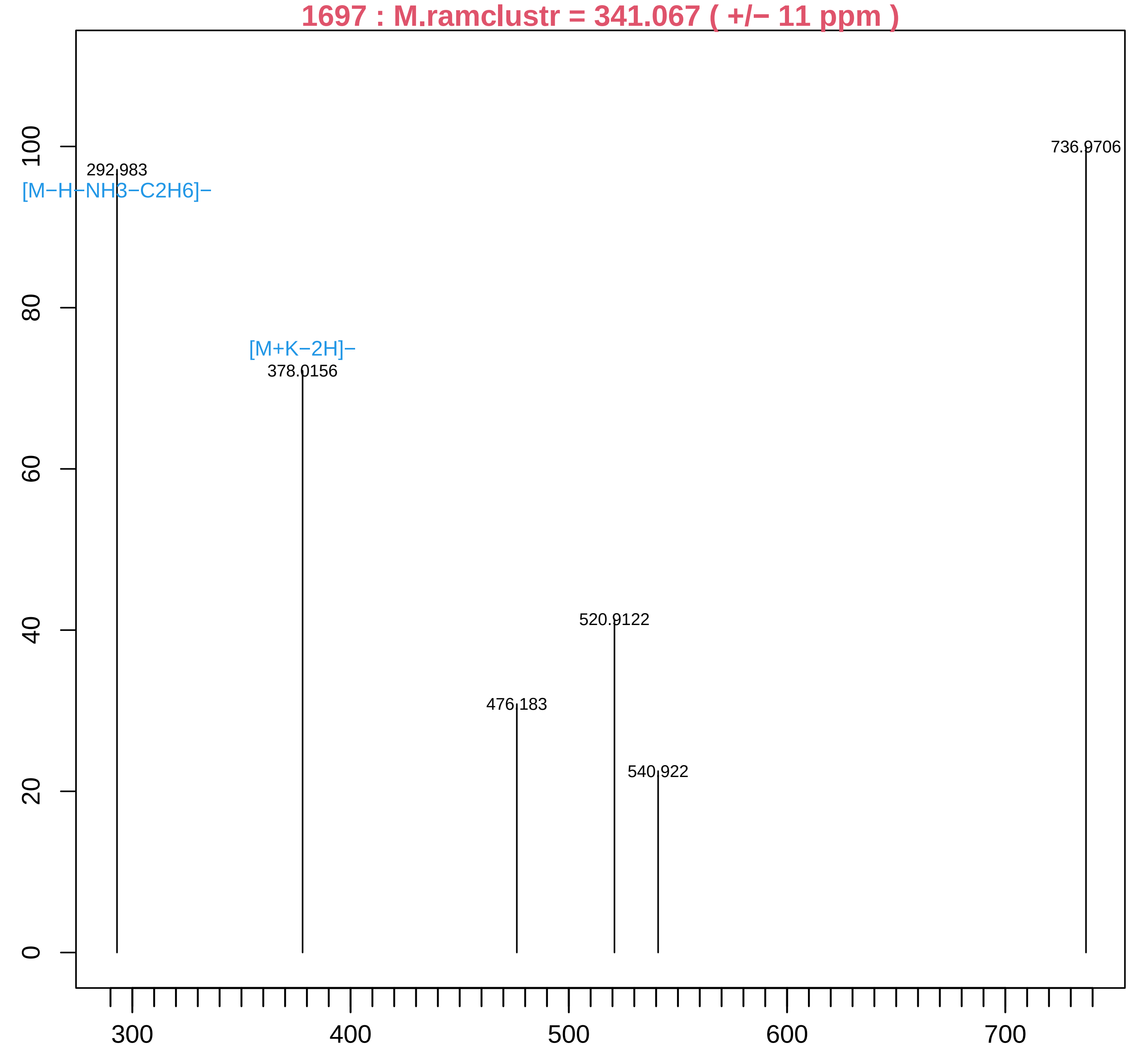
1697 : M.ramclustr = 341.067 (+/− 11 ppm)
292.983
[M−H−NH3−C2H6]−
[M+K−2H]−
378.0156
476.183
520.9122
540.922
736.9706
0
20
40
60
80
100
300
400
500
600
700
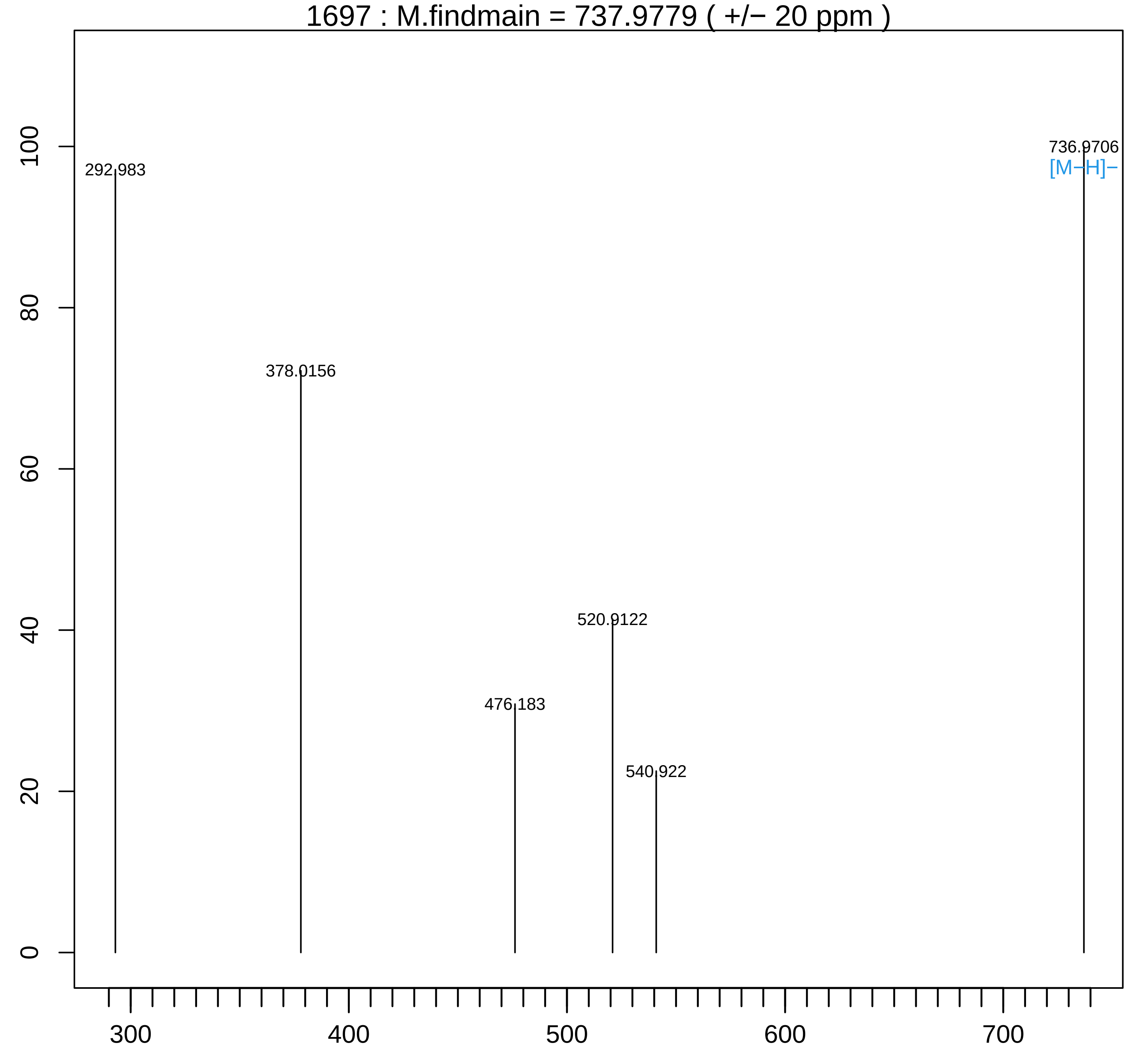
1697 : M.findmain = 737.9779 (+/− 20 ppm)
292.983
378.0156
476.183
520.9122
540.922
736.9706
[M−H]−
0
20
40
60
80
100
300
400
500
600
700

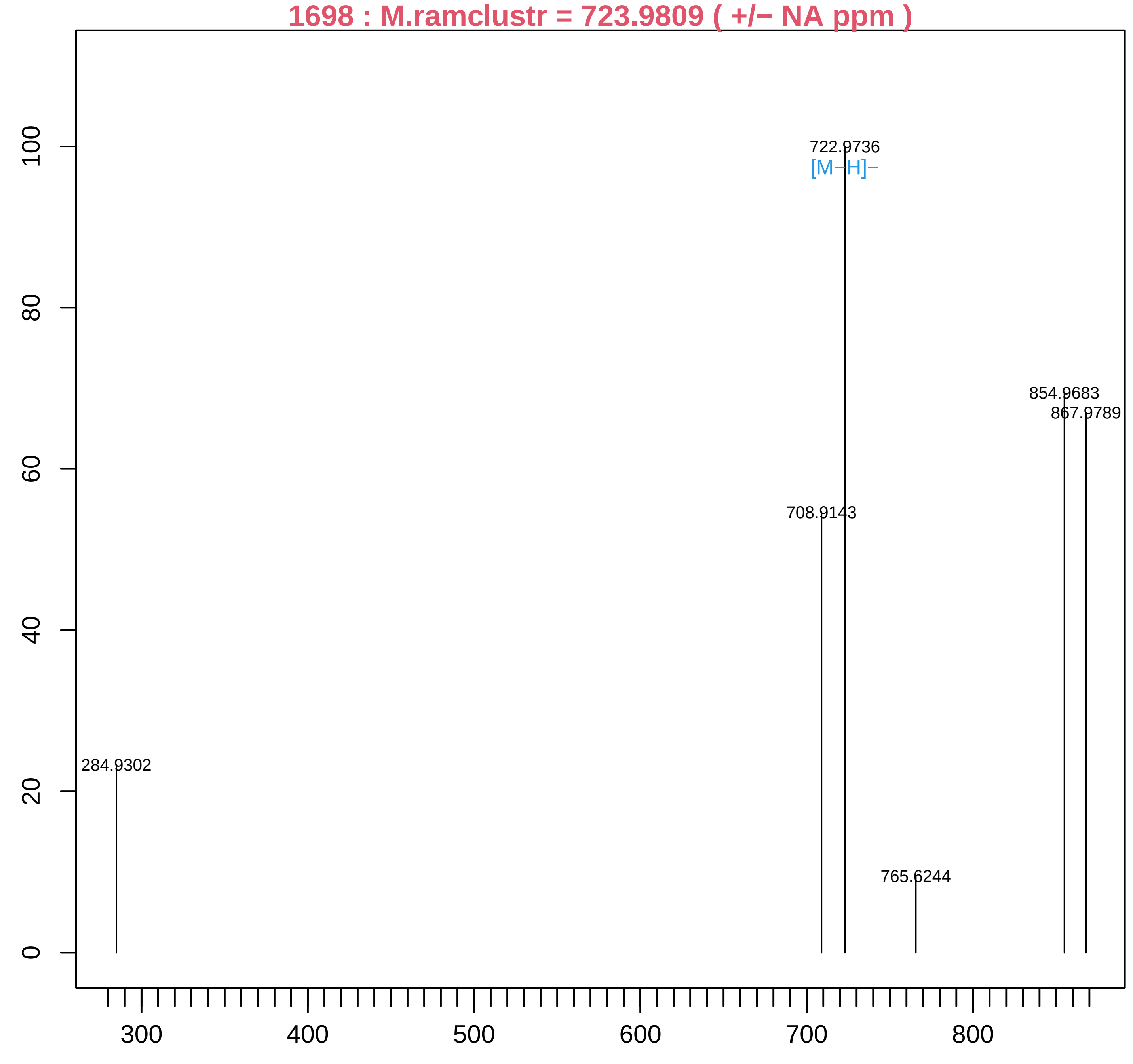
1698 : M.ramclustr = 723.9809 (+/− NA ppm)
722.9736
[M−H]−
854.9683
867.9789
708.9143
284.9302
765.6244
0
20
40
60
80
100
300
400
500
600
700
800

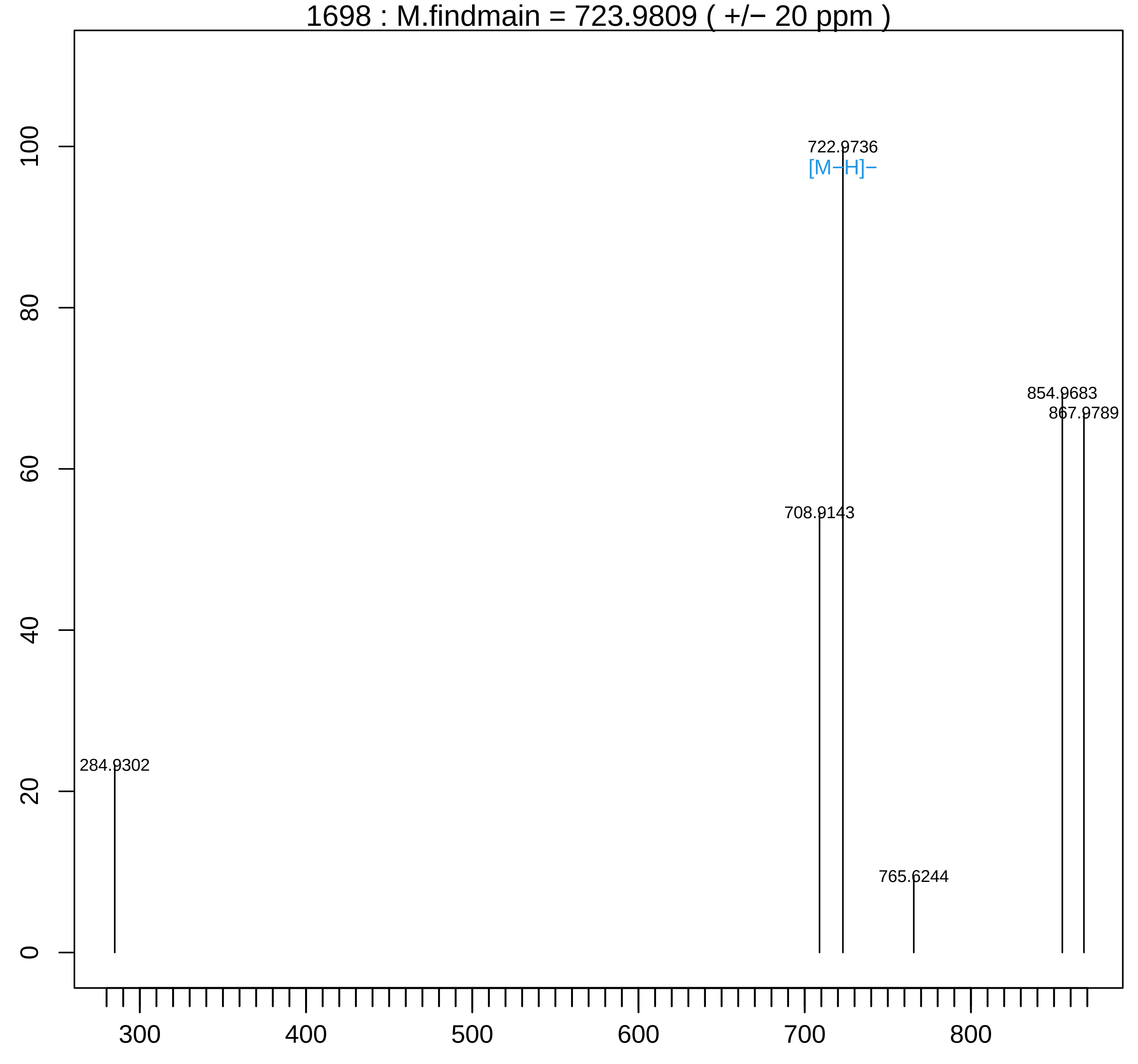
1698 : M.findmain = 723.9809 (+/− 20 ppm)
722.9736
[M−H]−
854.9683
867.9789
708.9143
284.9302
765.6244
0
20
40
60
80
100
300
400
500
600
700
800

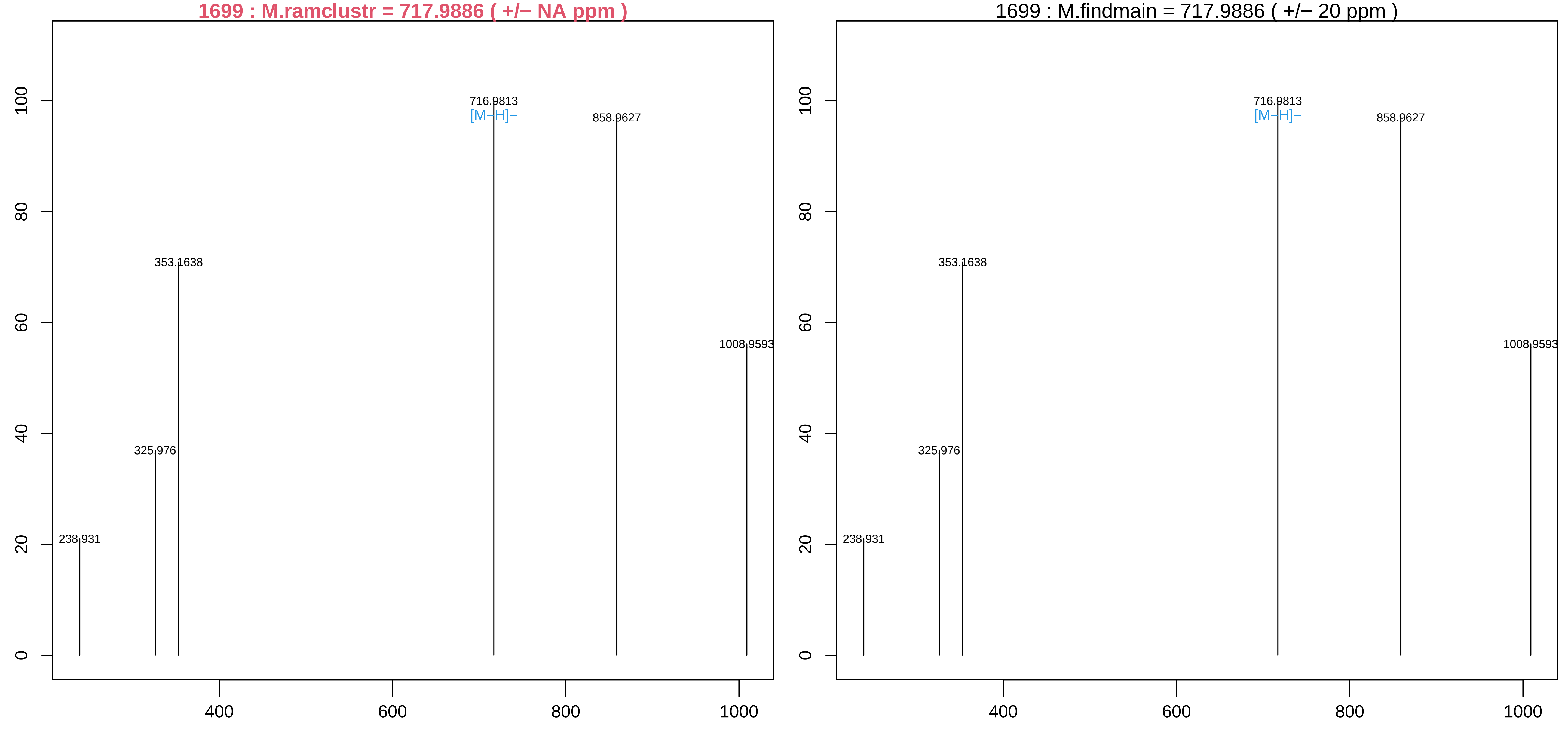
1699 : M.ramclustr = 717.9886 (+/− NA ppm)
1699 : M.findmain = 717.9886 (+/− 20 ppm)
716.9813
[M−H]−
858.9627
353.1638
1008.9593
325.976
238.931
0
20
40
60
80
100
400
600
800
1000

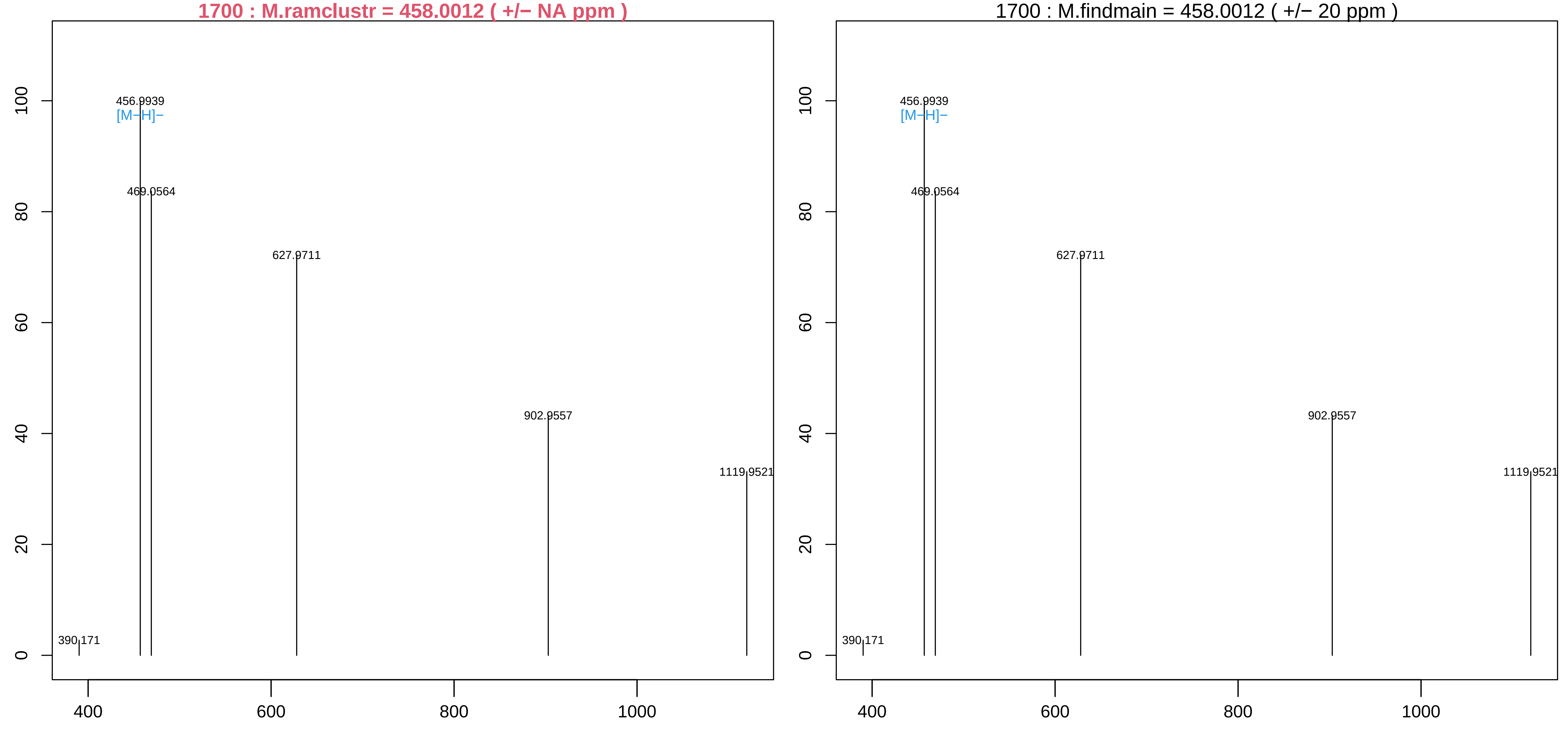
1700 : M.ramclustr = 458.0012 (+/− NA ppm)
456.9939
[M−H]−
469.0564
627.9711
902.9557
1119.9521
390.171
1700 : M.findmain = 458.0012 (+/− 20 ppm)
456.9939
[M−H]−
469.0564
627.9711
902.9557
1119.9521
390.171

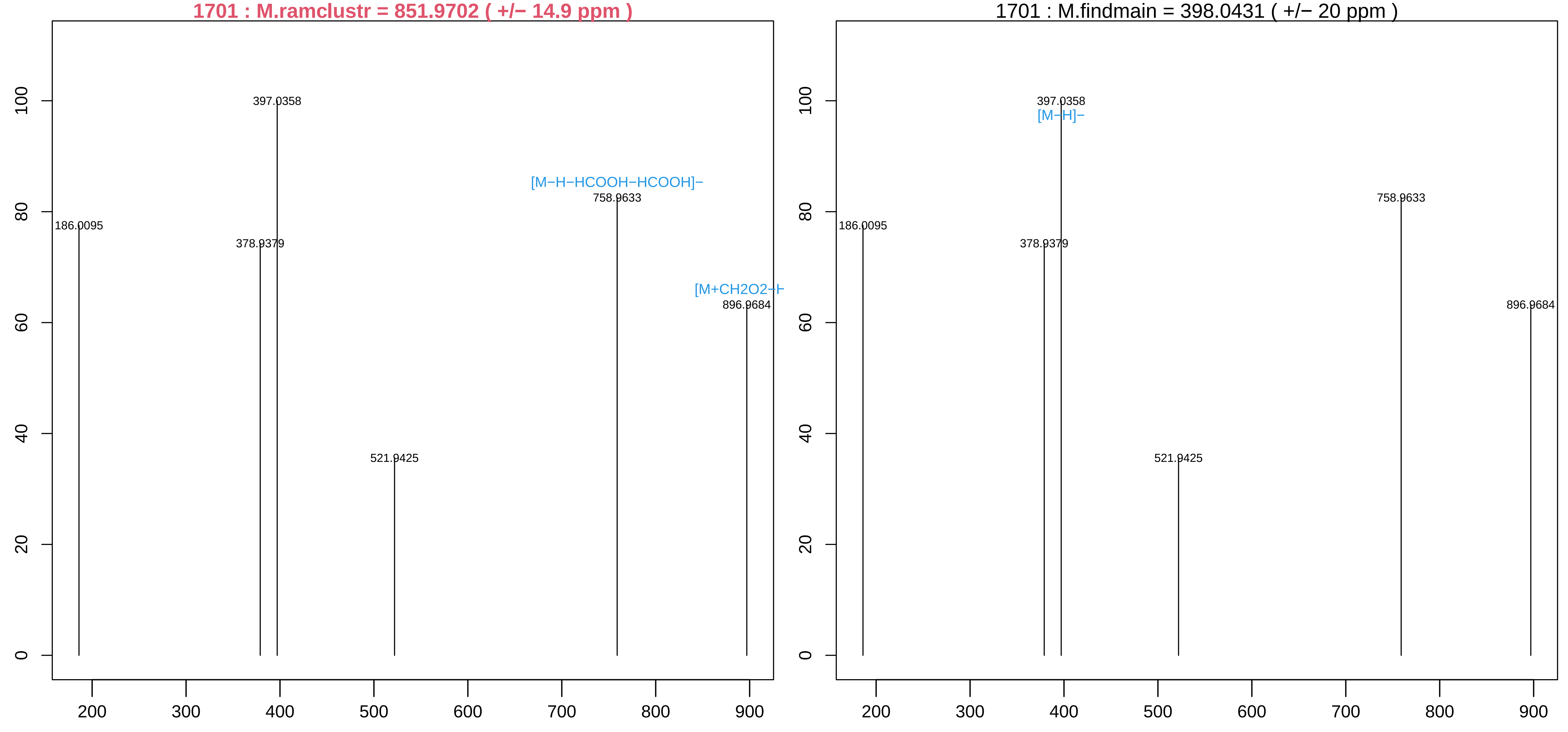

1701 : M.ramclustr = 851.9702 (+/− 14.9 ppm)
186.0095
378.9379
397.0358
521.9425
[M−H−HCOOH−HCOOH]−
758.9633
[M+CH2O2−H
896.9684
1701 : M.findmain = 398.0431 (+/− 20 ppm)
186.0095
378.9379
397.0358
[M−H]−
521.9425
758.9633
896.9684

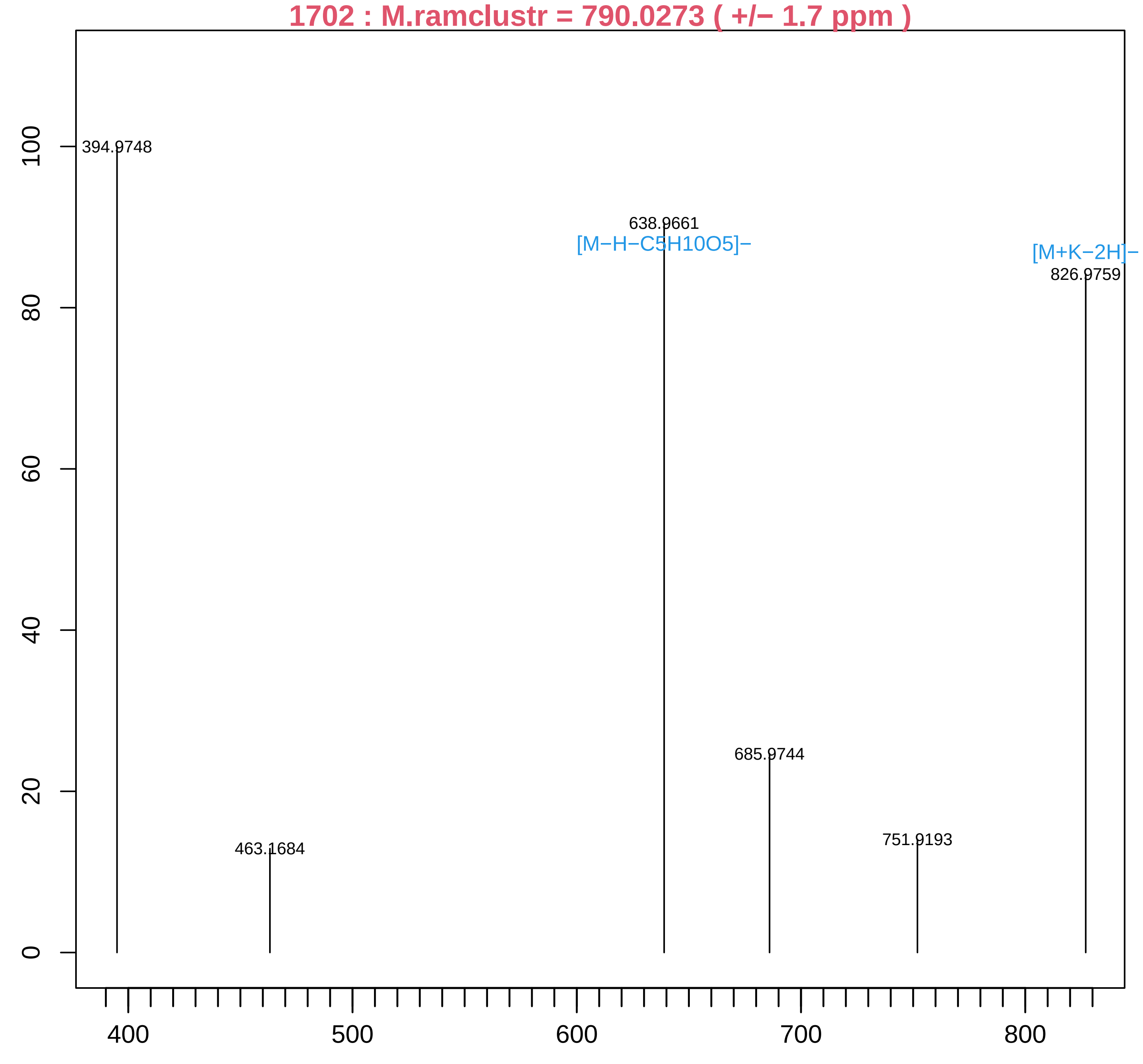

1702 : M.ramclustr = 790.0273 (+/− 1.7 ppm)
394.9748
463.1684
638.9661
[M−H−C5H10O5]−
685.9744
751.9193
[M+K−2H]−
826.9759
0
20
40
60
80
100
400
500
600
700
800

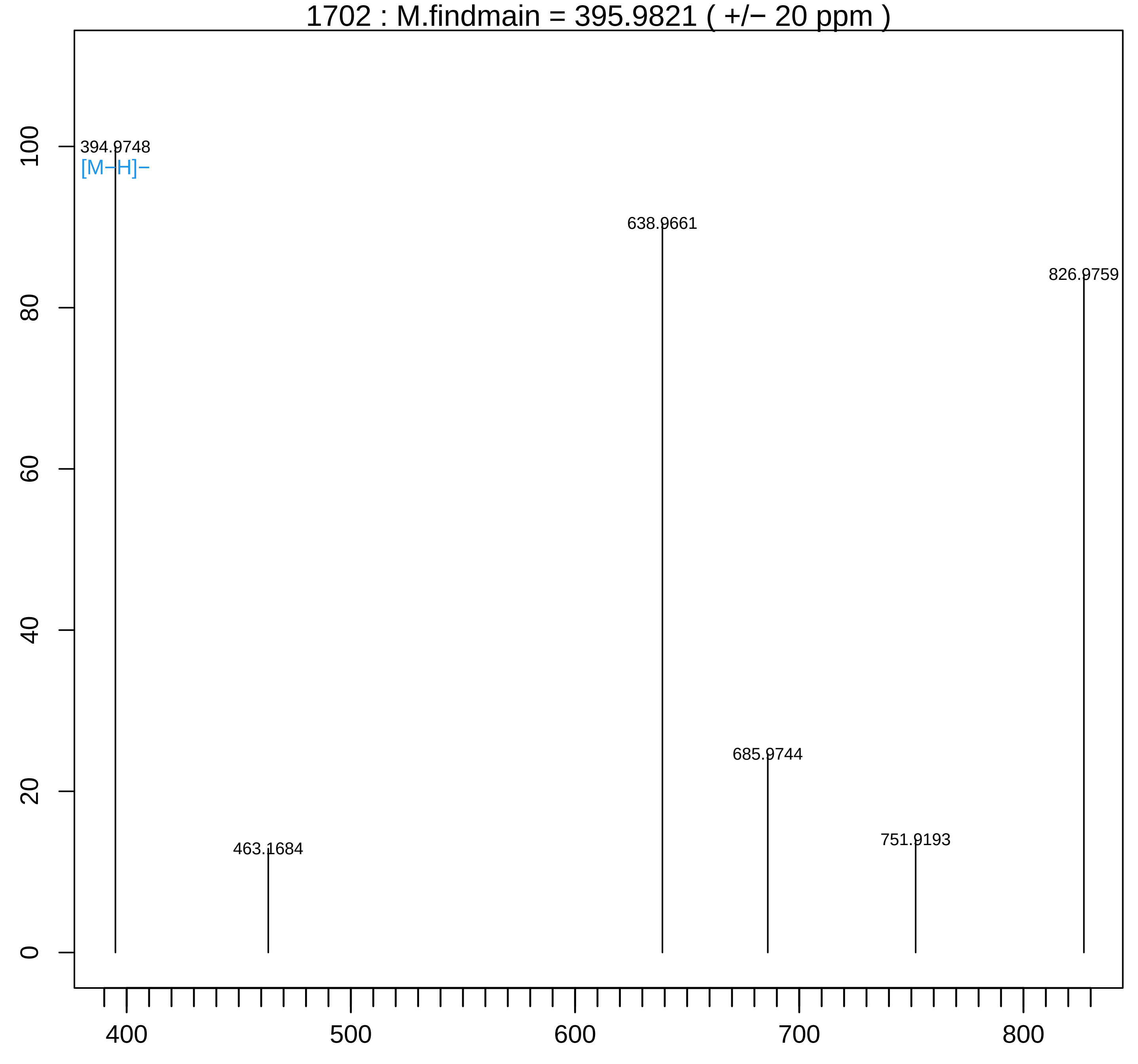

1702 : M.findmain = 395.9821 (+/− 20 ppm)
394.9748
[M−H]−
463.1684
638.9661
685.9744
751.9193
826.9759
0
20
40
60
80
100
400
500
600
700
800

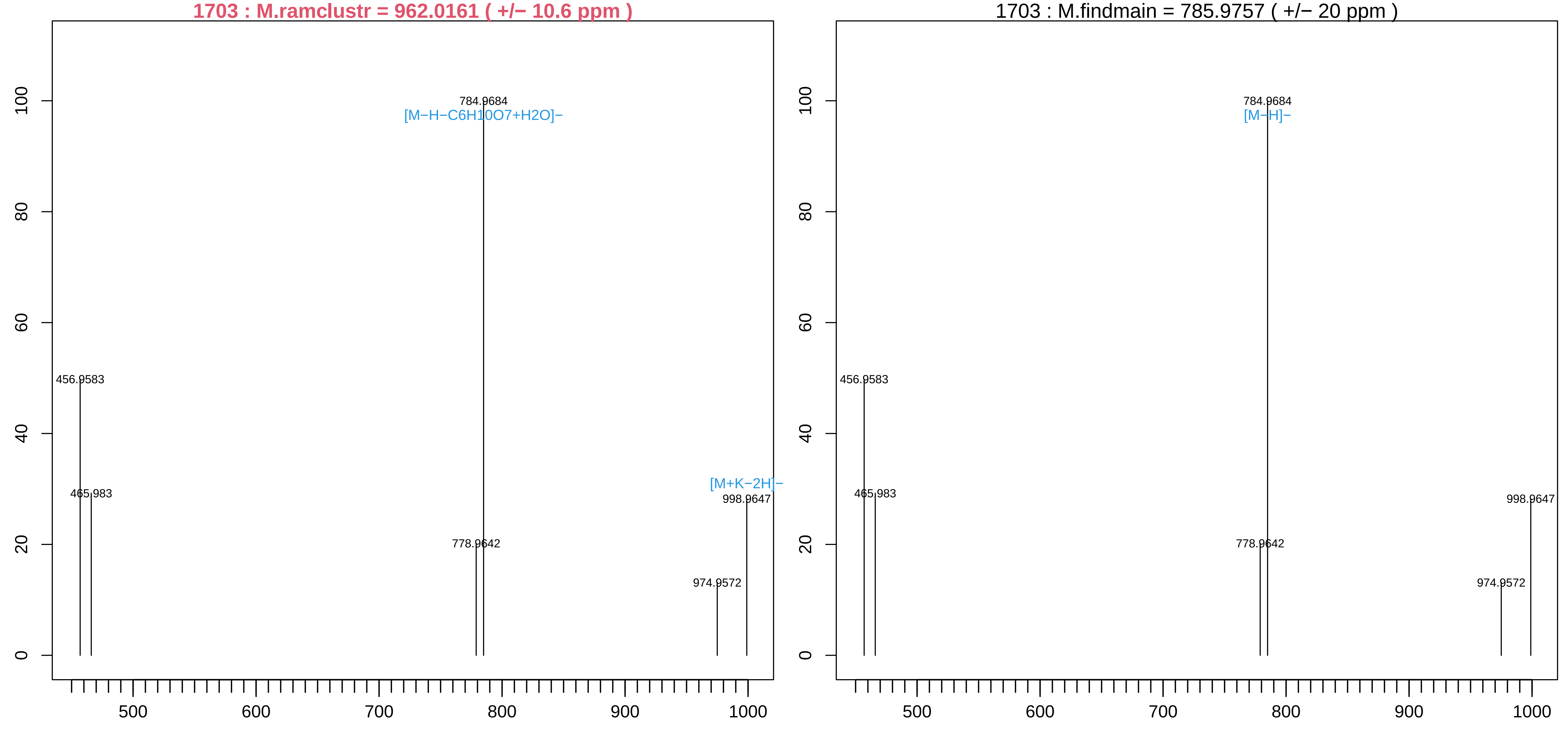

1703 : M.ramclustr = 962.0161 (+/− 10.6 ppm)
784.9684
[M−H−C6H10O7+H2O]−
456.9583
465.983
778.9642
974.9572
998.9647
[M+K−2H]−
1703 : M.findmain = 785.9757 (+/− 20 ppm)
784.9684
[M−H]−
456.9583
465.983
778.9642
974.9572
998.9647
0
20
40
60
80
100
500
600
700
800
900
1000

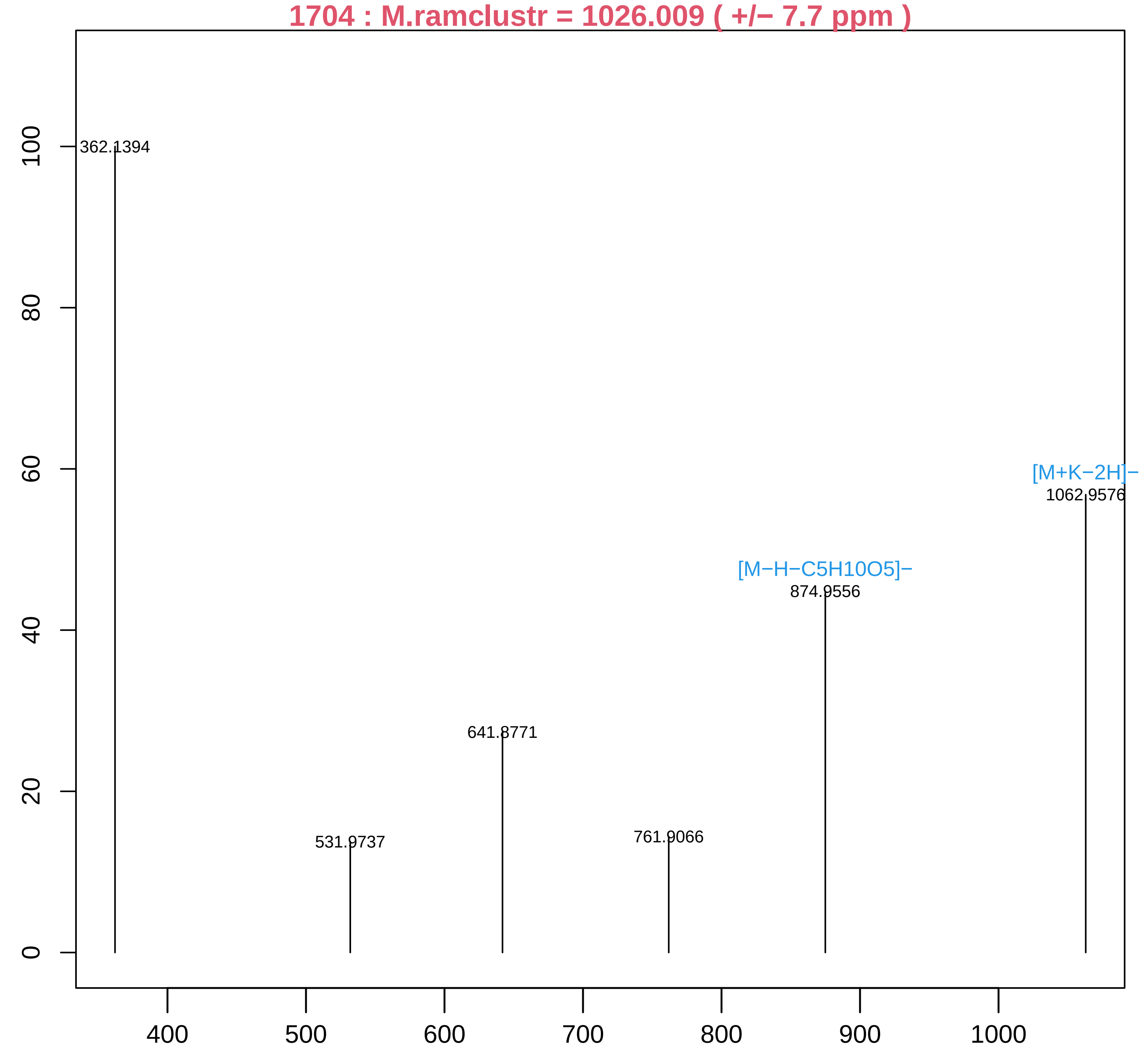

1704 : M.ramclustr = 1026.009 (+/− 7.7 ppm)
362.1394
[M+K−2H]−
1062.9576
[M−H−C5H10O5]−
874.9556
641.8771
531.9737
761.9066
0
20
40
60
80
100
400
500
600
700
800
900
1000

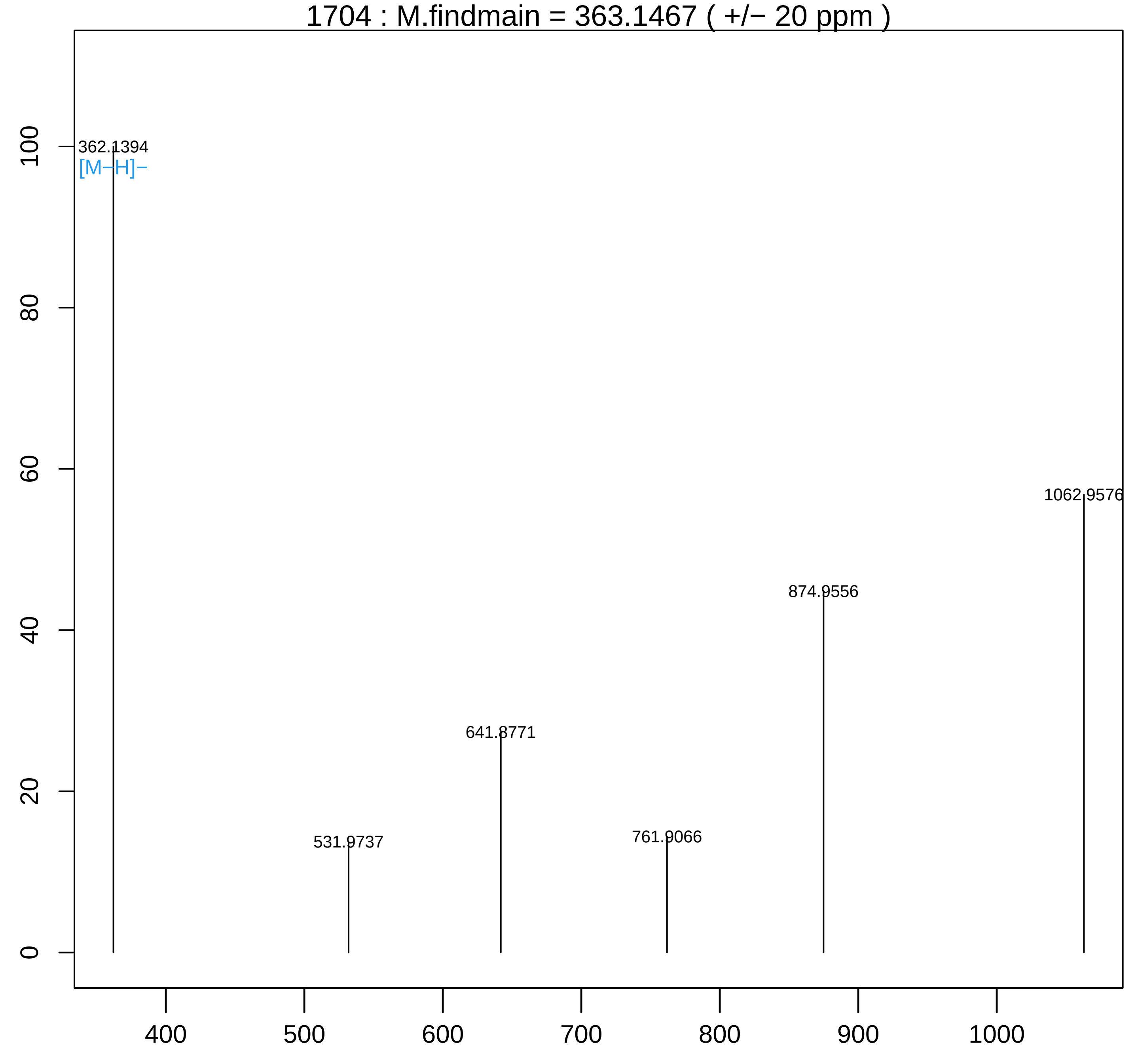

1704 : M.findmain = 363.1467 (+/− 20 ppm)
362.1394
[M−H]−
1062.9576
874.9556
641.8771
531.9737
761.9066
0
20
40
60
80
100
400
500
600
700
800
900
1000

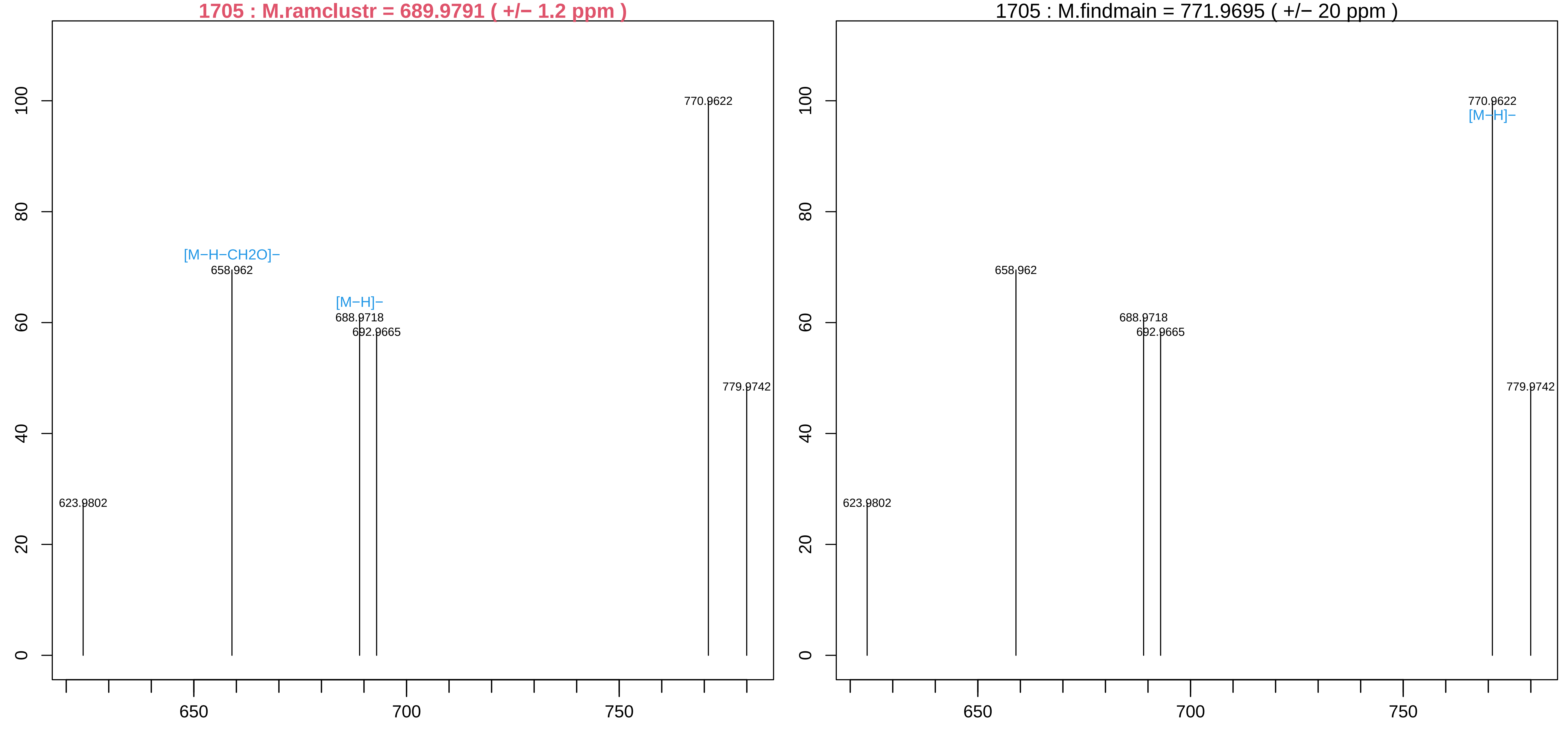

1705 : M.ramclustr = 689.9791 (+/− 1.2 ppm)
[M−H−CH2O]−
658.962
[M−H]−
688.9718
692.9665
770.9622
779.9742
623.9802
0
20
40
60
80
100
650
700
750
1705 : M.findmain = 771.9695 (+/− 20 ppm)
770.9622
[M−H]−
658.962
688.9718
692.9665
779.9742
623.9802
0
20
40
60
80
100
650
700
750

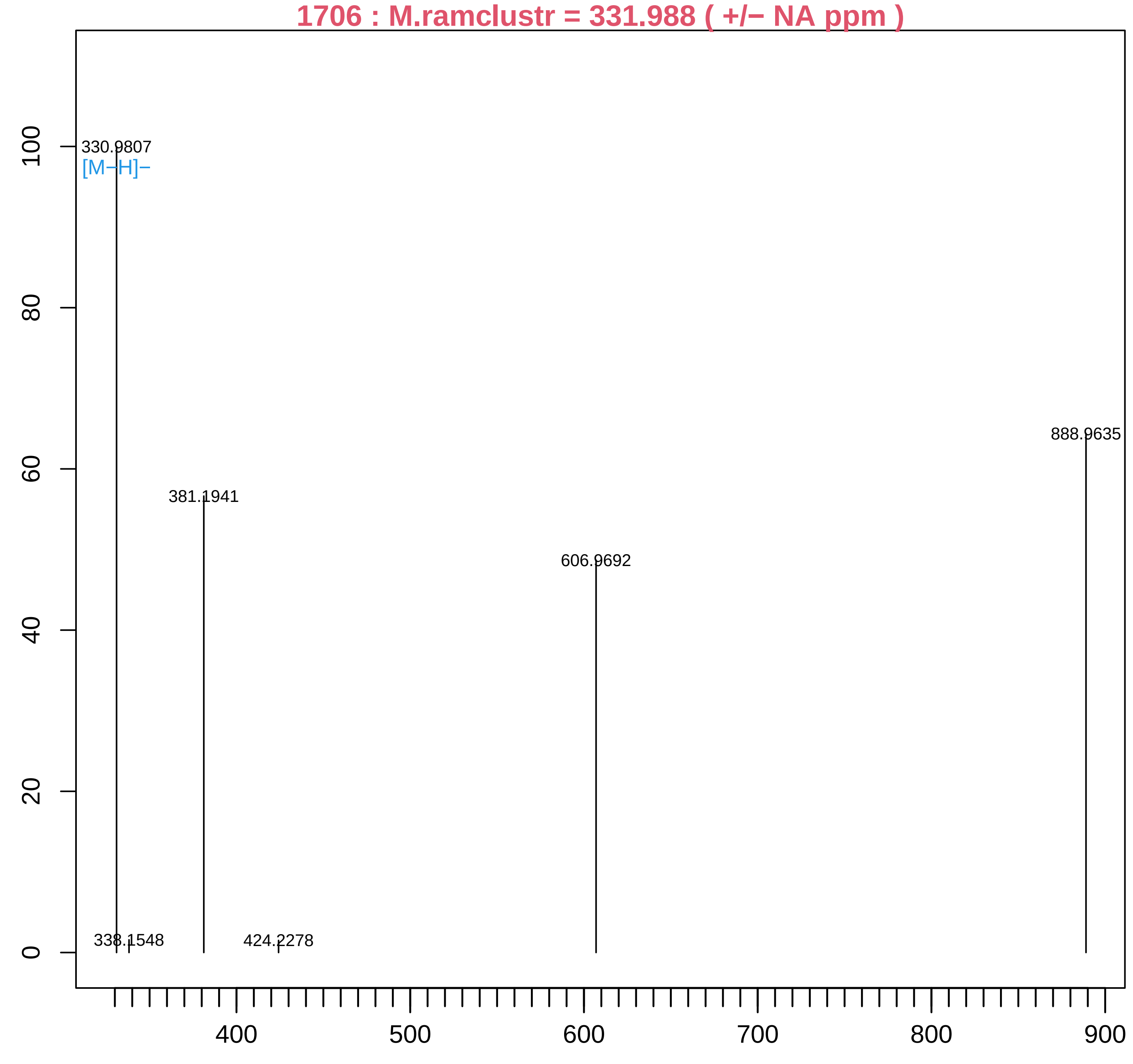

1706 : M.ramclustr = 331.988 (+/− NA ppm)
330.9807
[M−H]−
381.1941
606.9692
888.9635
338.1548
424.2278
0
20
40
60
80
100
400
500
600
700
800
900

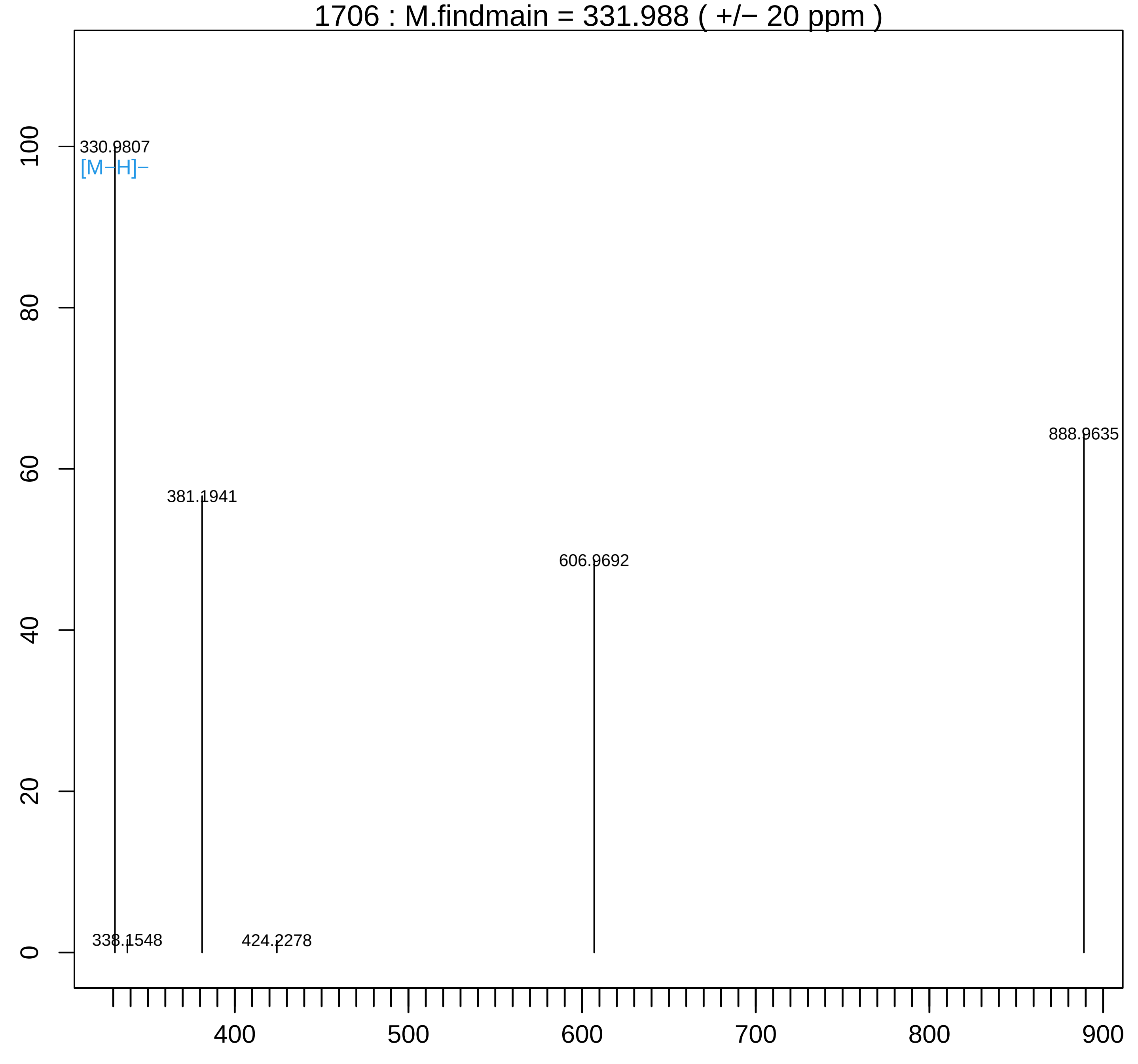

1706 : M.findmain = 331.988 (+/− 20 ppm)
330.9807
[M−H]−
381.1941
606.9692
888.9635
338.1548
424.2278
0
20
40
60
80
100
400
500
600
700
800
900

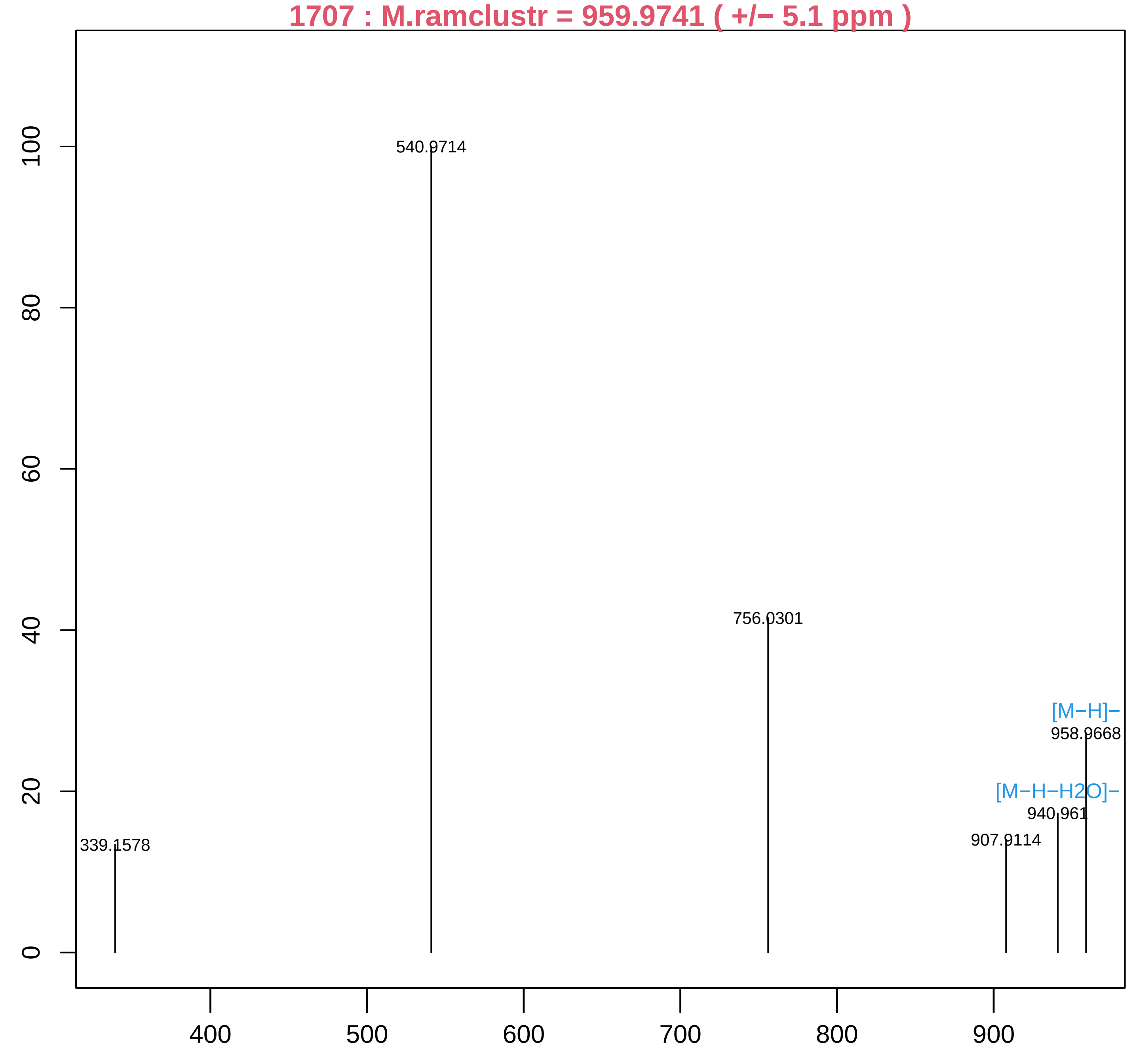
1707 : M.ramclustr = 959.9741 (+/− 5.1 ppm)
540.9714
756.0301
[M−H]−
958.9668
[M−H−H2O]−
940.961
907.9114
339.1578
0
20
40
60
80
100
400
500
600
700
800
900

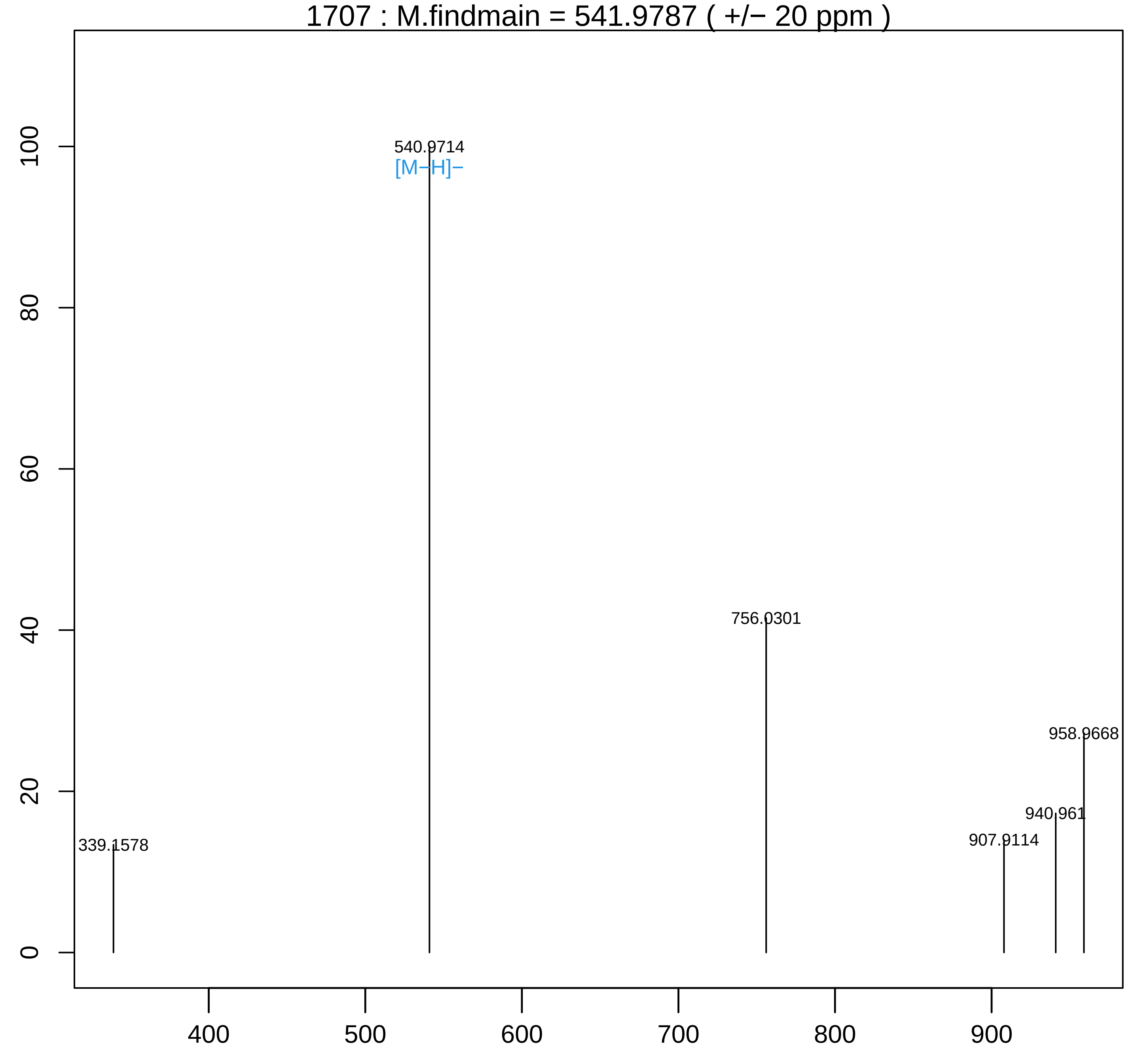
1707 : M.findmain = 541.9787 (+/− 20 ppm)
540.9714
[M−H]−
756.0301
958.9668
940.961
907.9114
339.1578
0
20
40
60
80
100
400
500
600
700
800
900

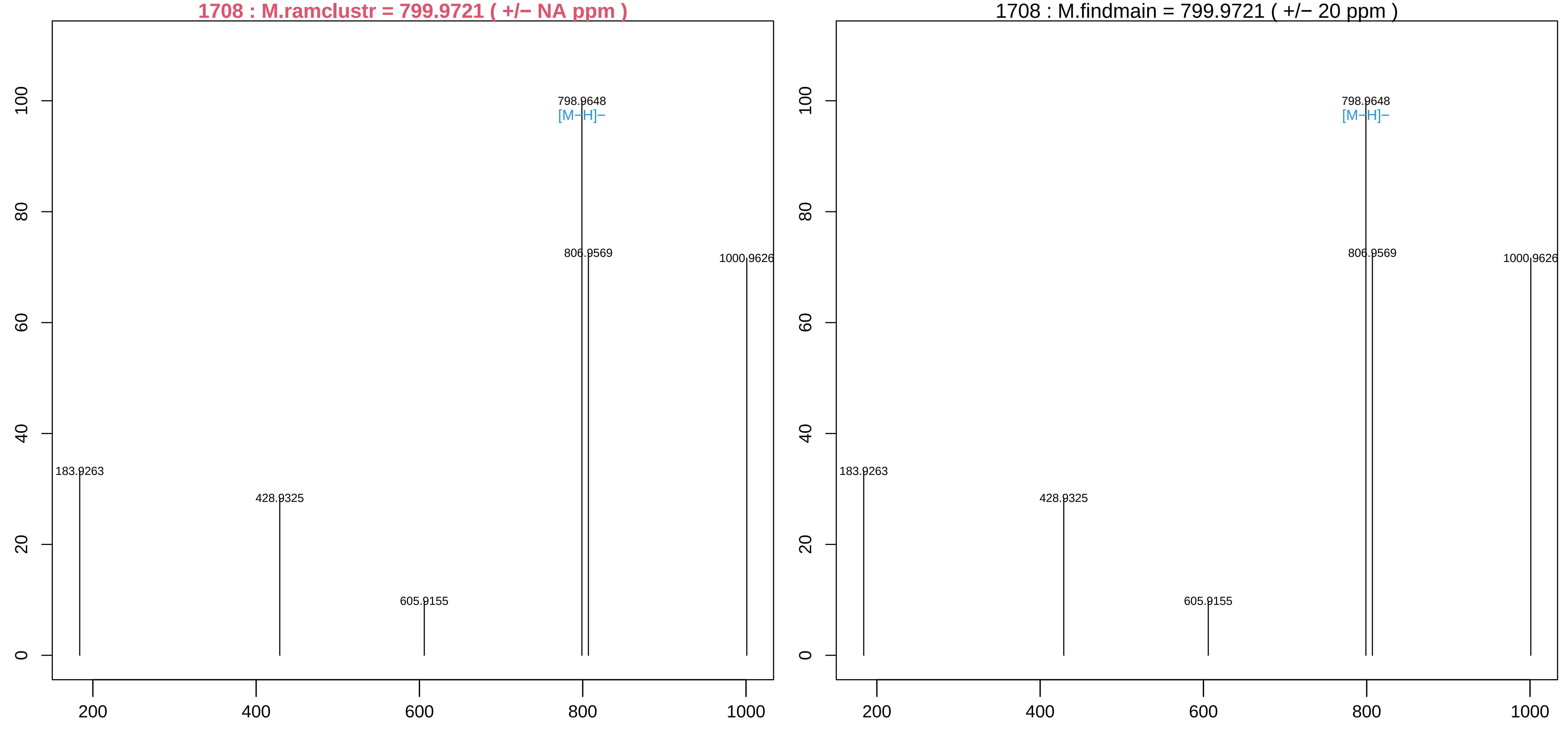
1708 : M.ramclustr = 799.9721 (+/− NA ppm)
1708 : M.findmain = 799.9721 (+/− 20 ppm)
798.9648
[M−H]−
806.9569
1000.9626
183.9263
428.9325
605.9155
0
20
40
60
80
100
200
400
600
800
1000

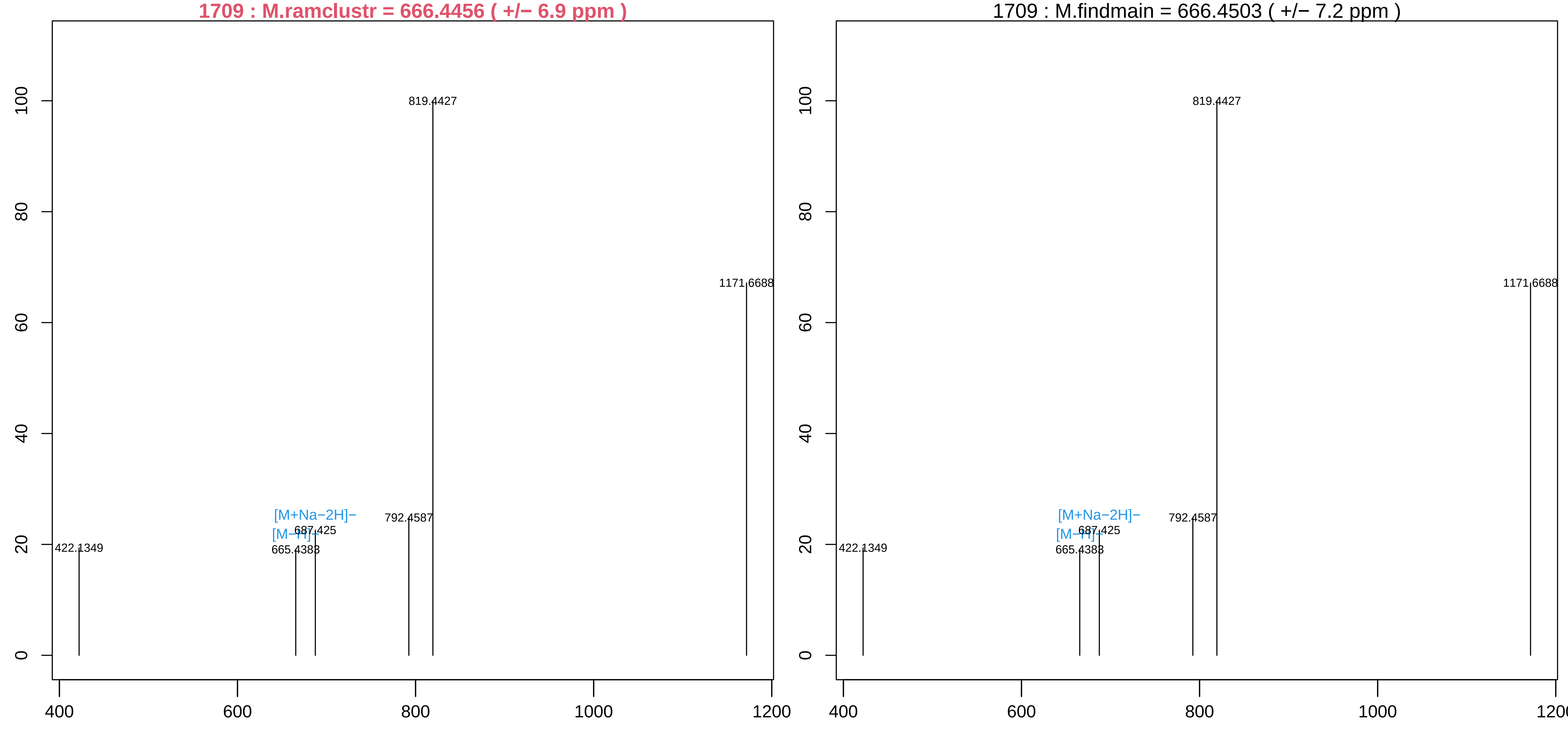
1709 : M.ramclustr = 666.4456 (+/− 6.9 ppm)
819.4427
1171.6688
792.4587
[M+Na−2H]−
687.425
[M−H]−
665.4383
422.1349
0
20
40
60
80
100
400
600
800
1000
1200
1709 : M.findmain = 666.4503 (+/− 7.2 ppm)
819.4427
1171.6688
792.4587
[M+Na−2H]−
687.425
[M−H]−
665.4383
422.1349
0
20
40
60
80
100
400
600
800
1000

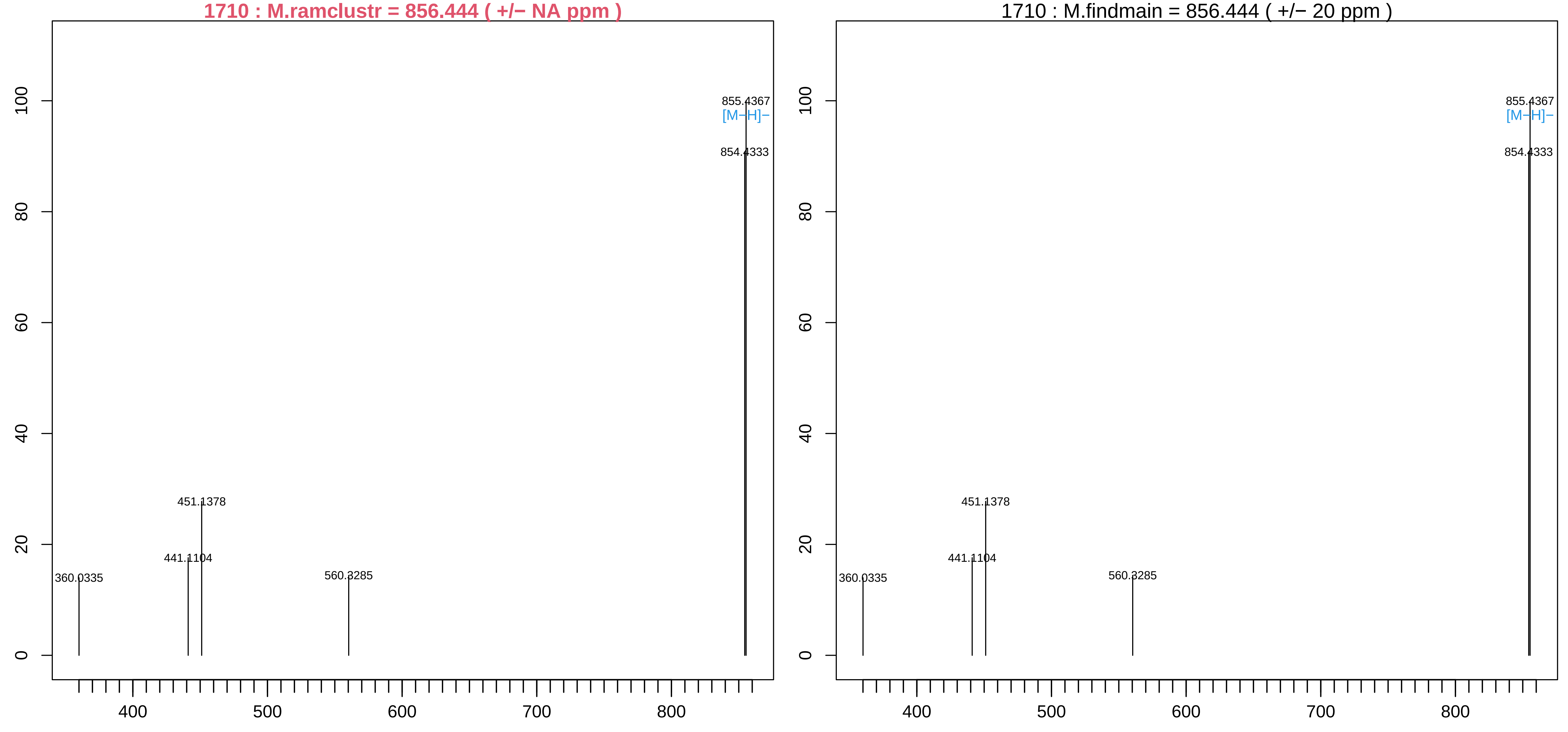
1710 : M.ramclustr = 856.444 (+/− NA ppm)
855.4367
[M−H]−
854.4333
451.1378
441.1104
360.0335
560.3285
0
20
40
60
80
100
400
500
600
700
800
1710 : M.findmain = 856.444 (+/− 20 ppm)
855.4367
[M−H]−
854.4333
451.1378
441.1104
360.0335
560.3285
0
20
40
60
80
100
400
500
600
700
800

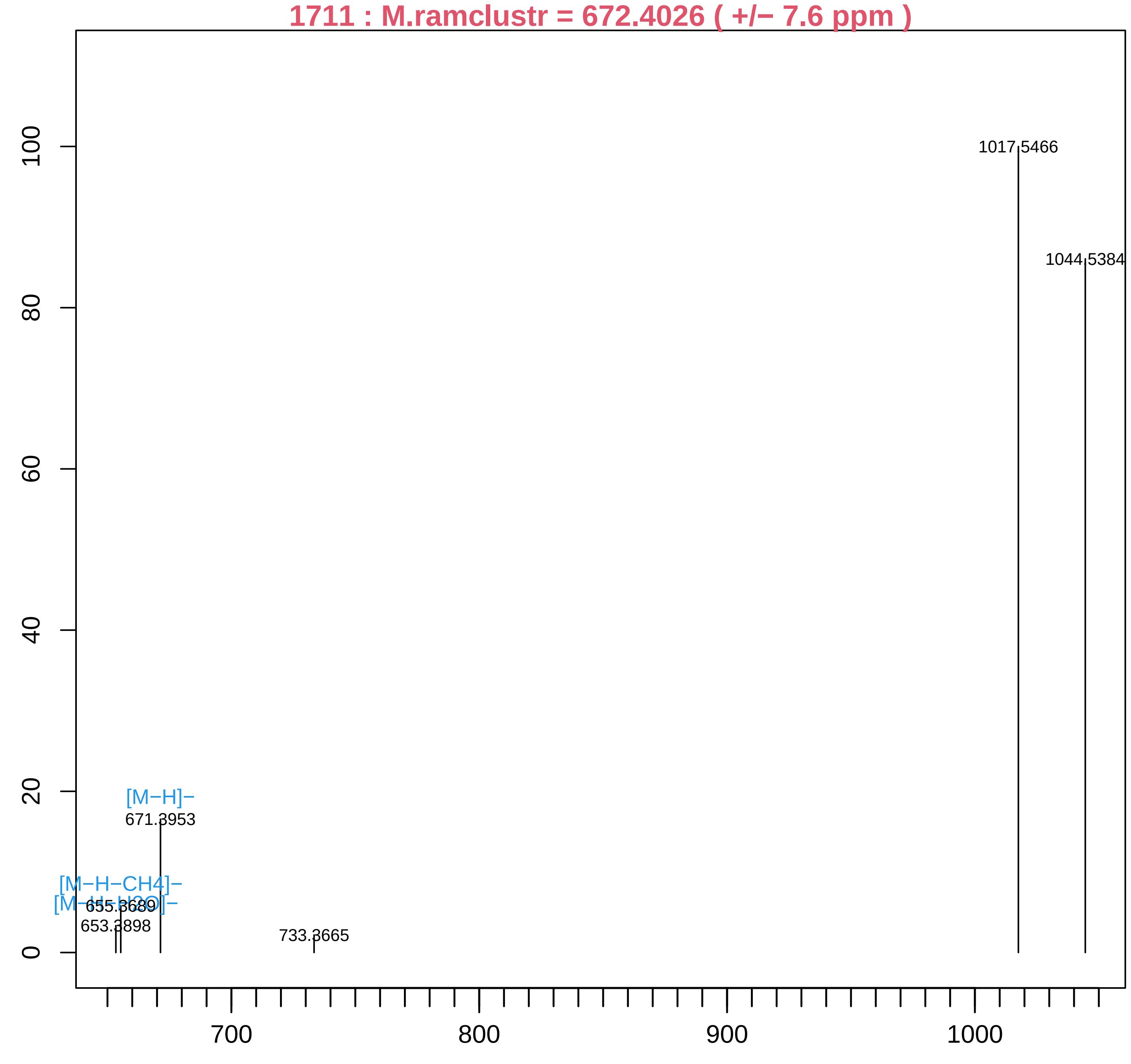
1711 : M.ramclustr = 672.4026 (+/− 7.6 ppm)
1017.5466
1044.5384
[M−H]−
671.3953
[M−H−CH4]−
[M−H−H2O]−
655.3689
653.3898
733.3665
0
20
40
60
80
100
700
800
900
1000

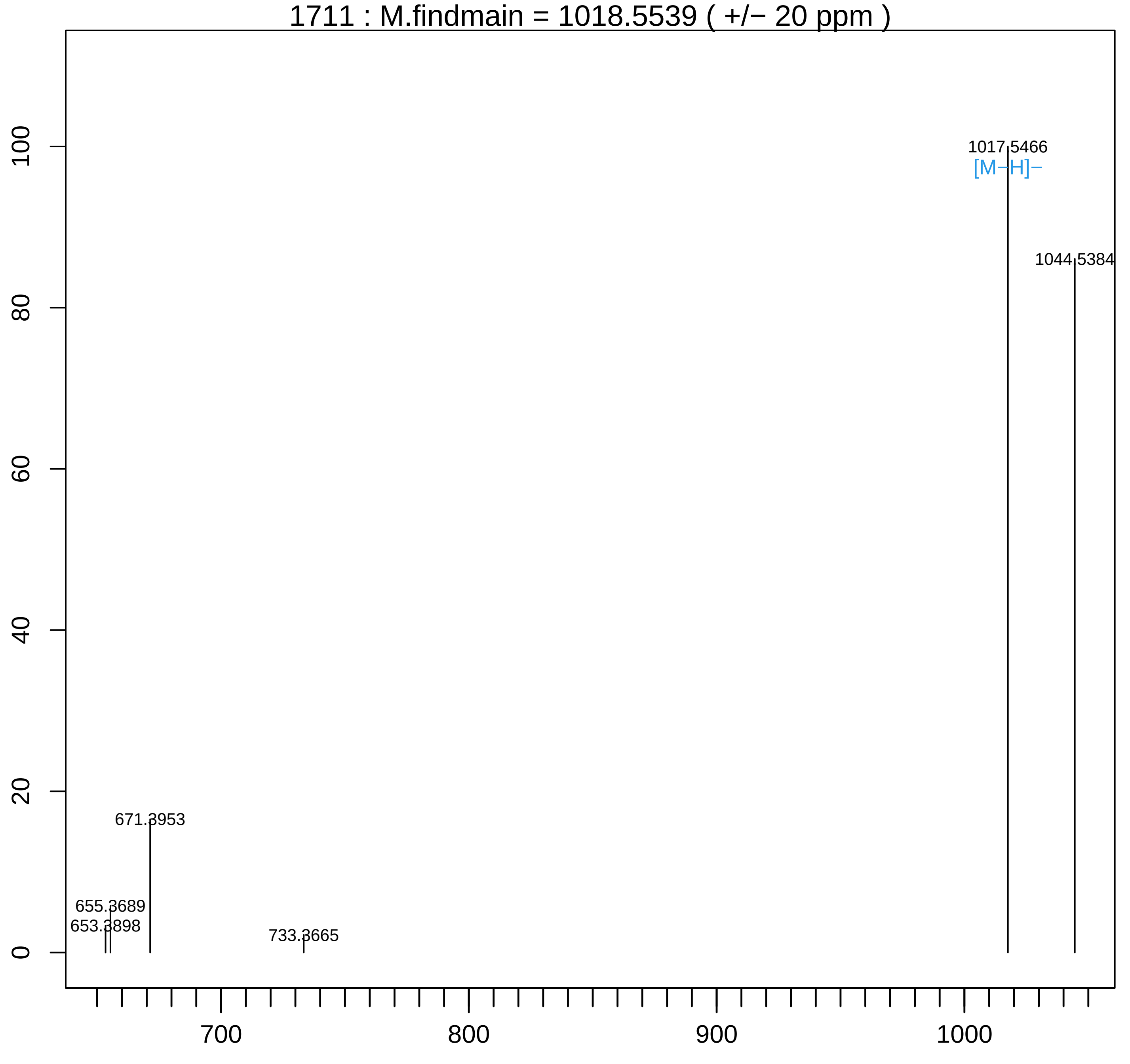
1711 : M.findmain = 1018.5539 (+/− 20 ppm)
1017.5466
[M−H]−
1044.5384
671.3953
655.3689
653.3898
733.3665
0
20
40
60
80
100
700
800
900
1000

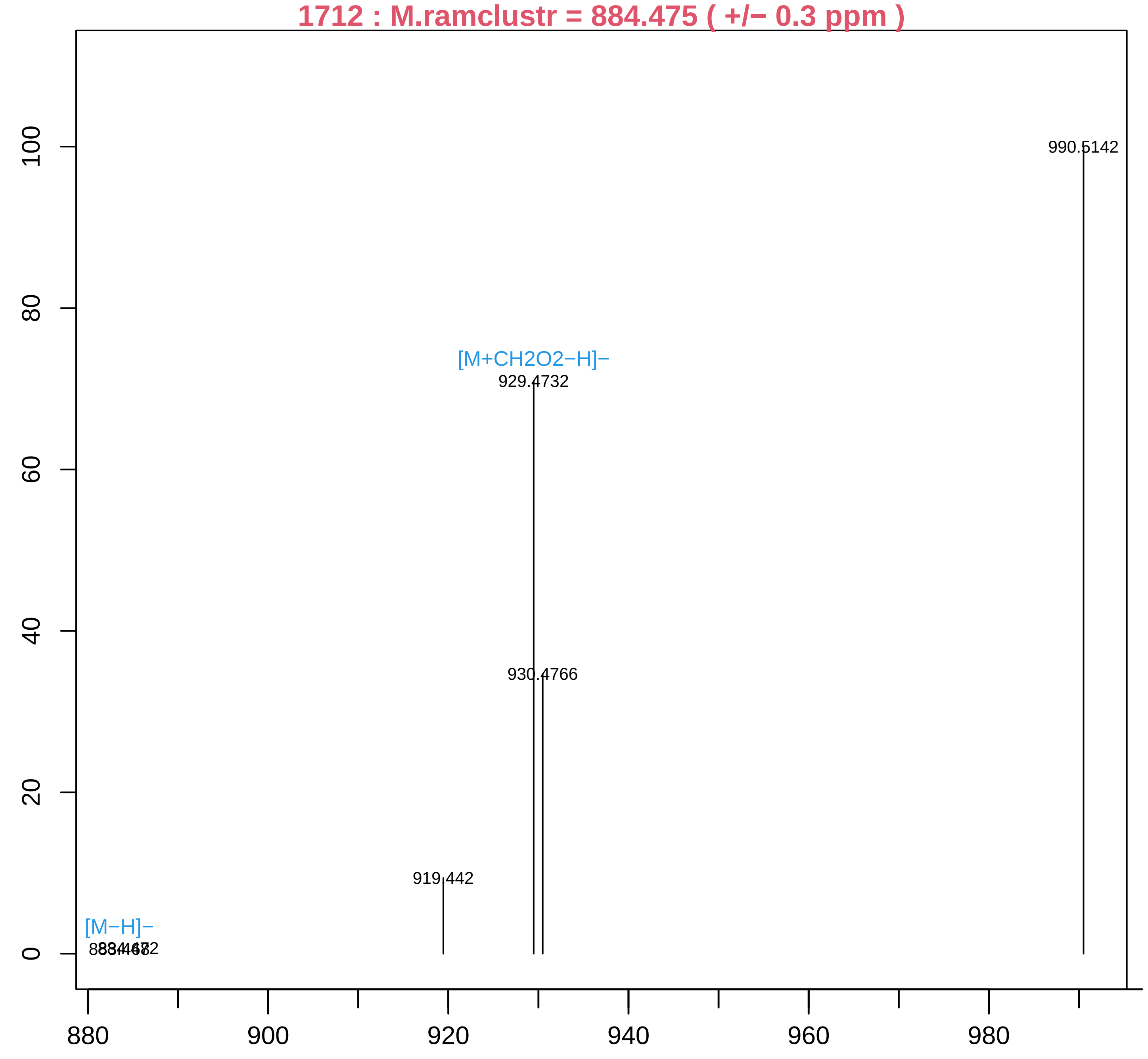
1712 : M.ramclustr = 884.475 (+/− 0.3 ppm)
990.5142
[M+CH2O2−H]−
929.4732
930.4766
919.442
[M−H]−
0
20
40
60
80
100
880
900
920
940
960
980

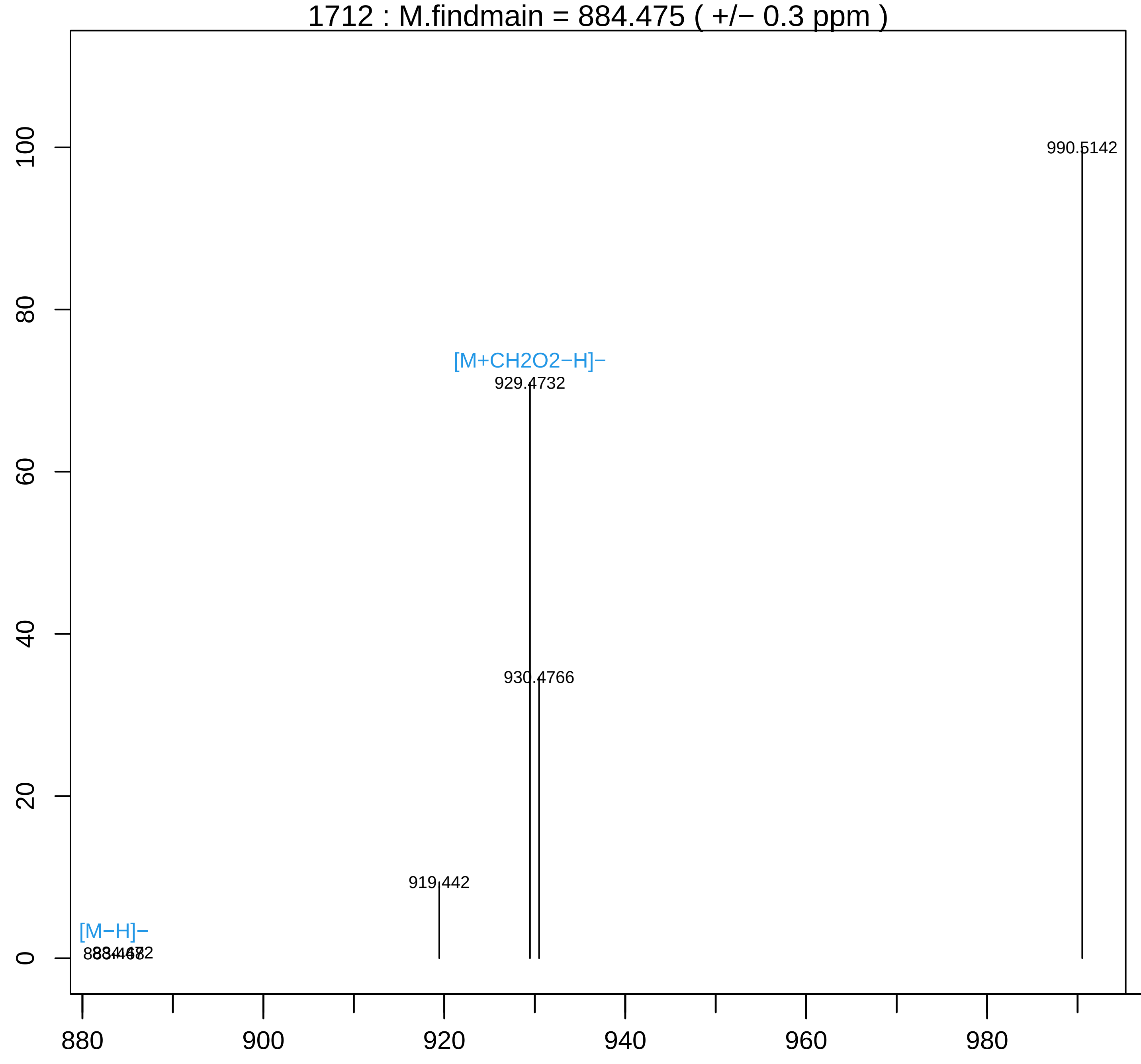
1712 : M.findmain = 884.475 (+/− 0.3 ppm)
990.5142
[M+CH2O2−H]−
929.4732
930.4766
919.442
[M−H]−
0
20
40
60
80
100
880
900
920
940
960
980

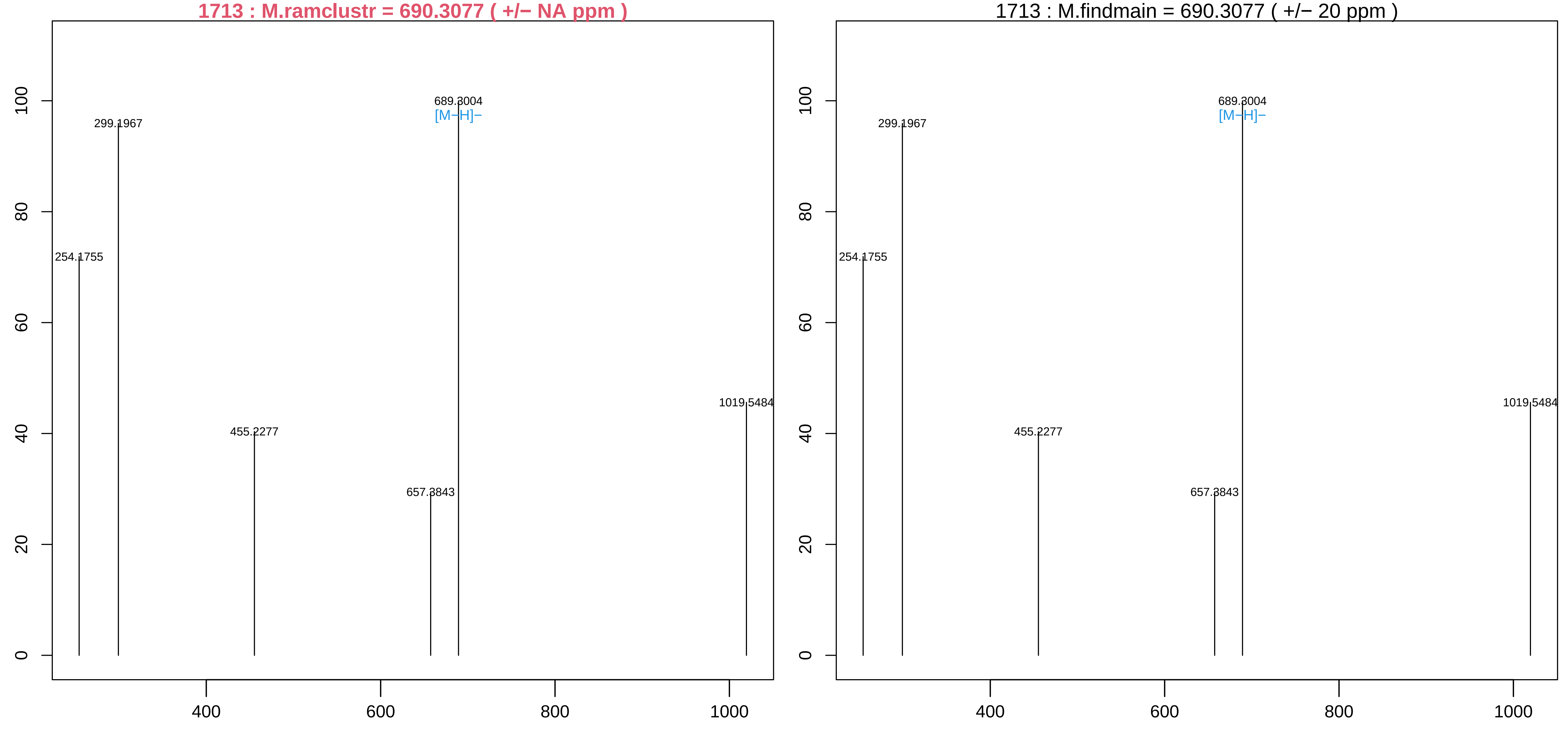

1713 : M.ramclustr = 690.3077 (+/− NA ppm)
254.1755
299.1967
455.2277
657.3843
689.3004
[M−H]−
1019.5484
0
20
40
60
80
100
400
600
800
1000
1713 : M.findmain = 690.3077 (+/− 20 ppm)
254.1755
299.1967
455.2277
657.3843
689.3004
[M−H]−
1019.5484

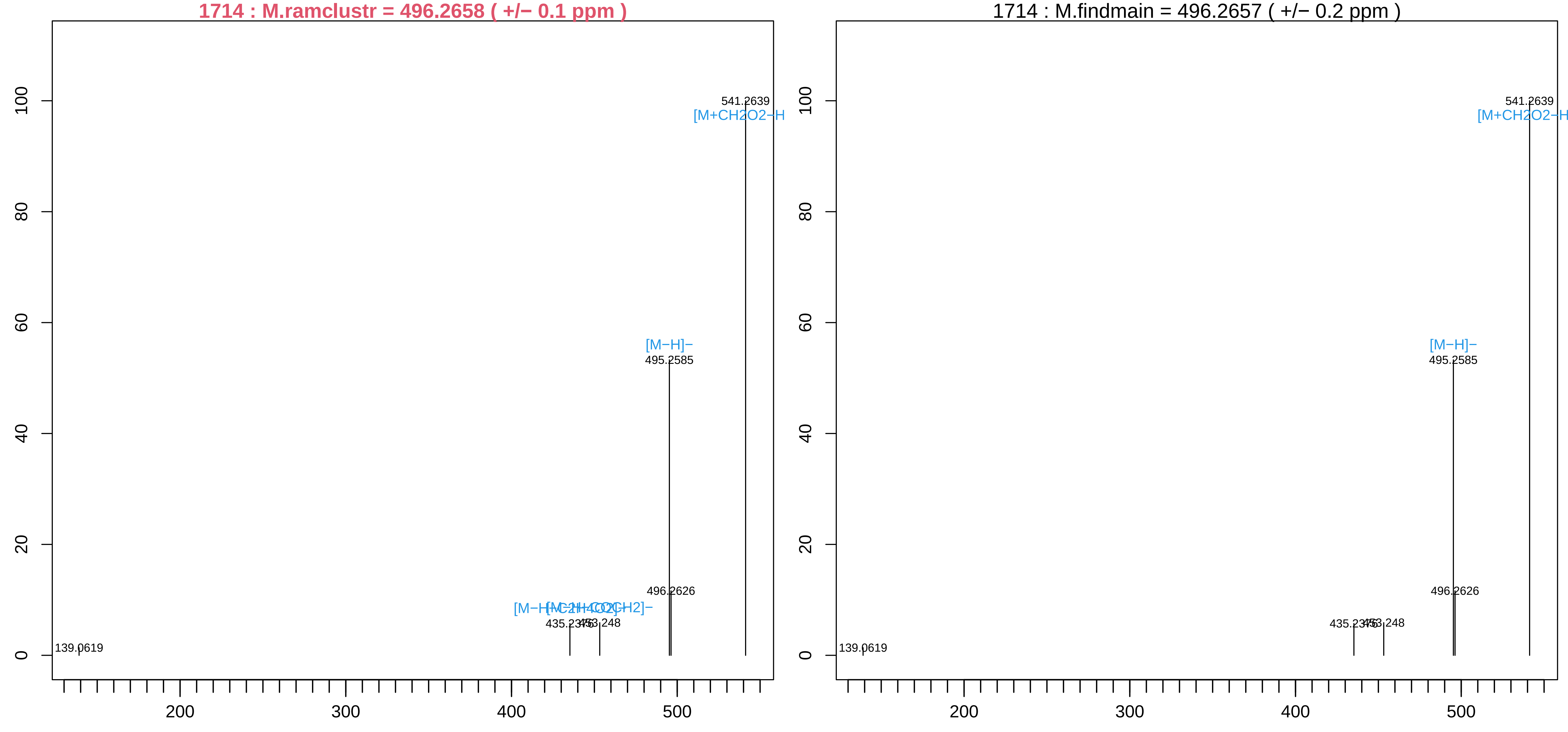
1714 : M.ramclustr = 496.2658 (+/− 0.1 ppm)
541.2639
[M+CH2O2−H
[M−H]−
495.2585
496.2626
435.2376
453.248
139.0619
1714 : M.findmain = 496.2657 (+/− 0.2 ppm)
541.2639
[M+CH2O2−H
[M−H]−
495.2585
496.2626
139.0619
0
20
40
60
80
100
200
300
400
500

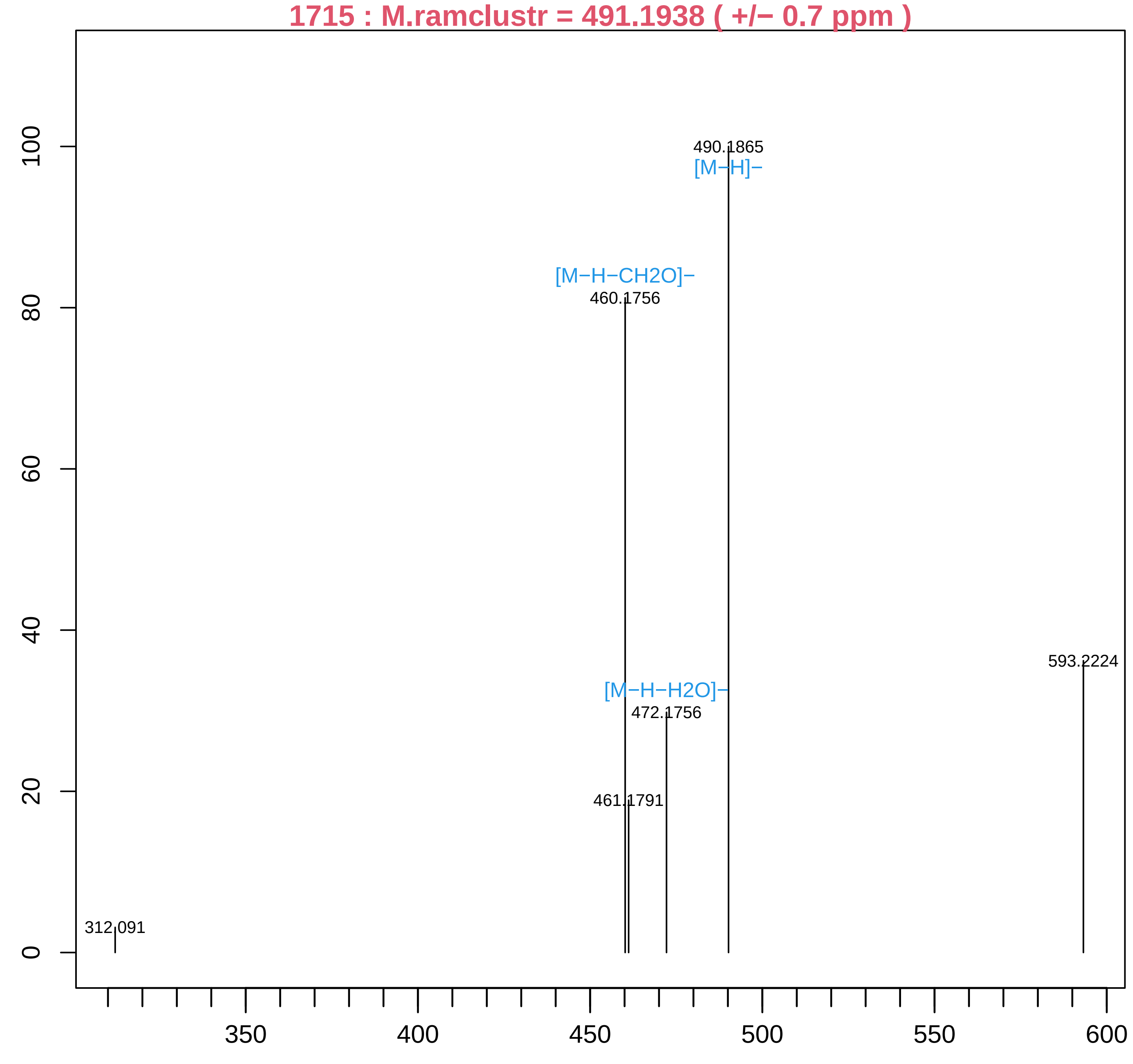
1715 : M.ramclustr = 491.1938 (+/− 0.7 ppm)
490.1865
[M−H]−
[M−H−CH2O]−
460.1756
[M−H−H2O]−
472.1756
461.1791
593.2224
312.091
0
20
40
60
80
100
350
400
450
500
550
600

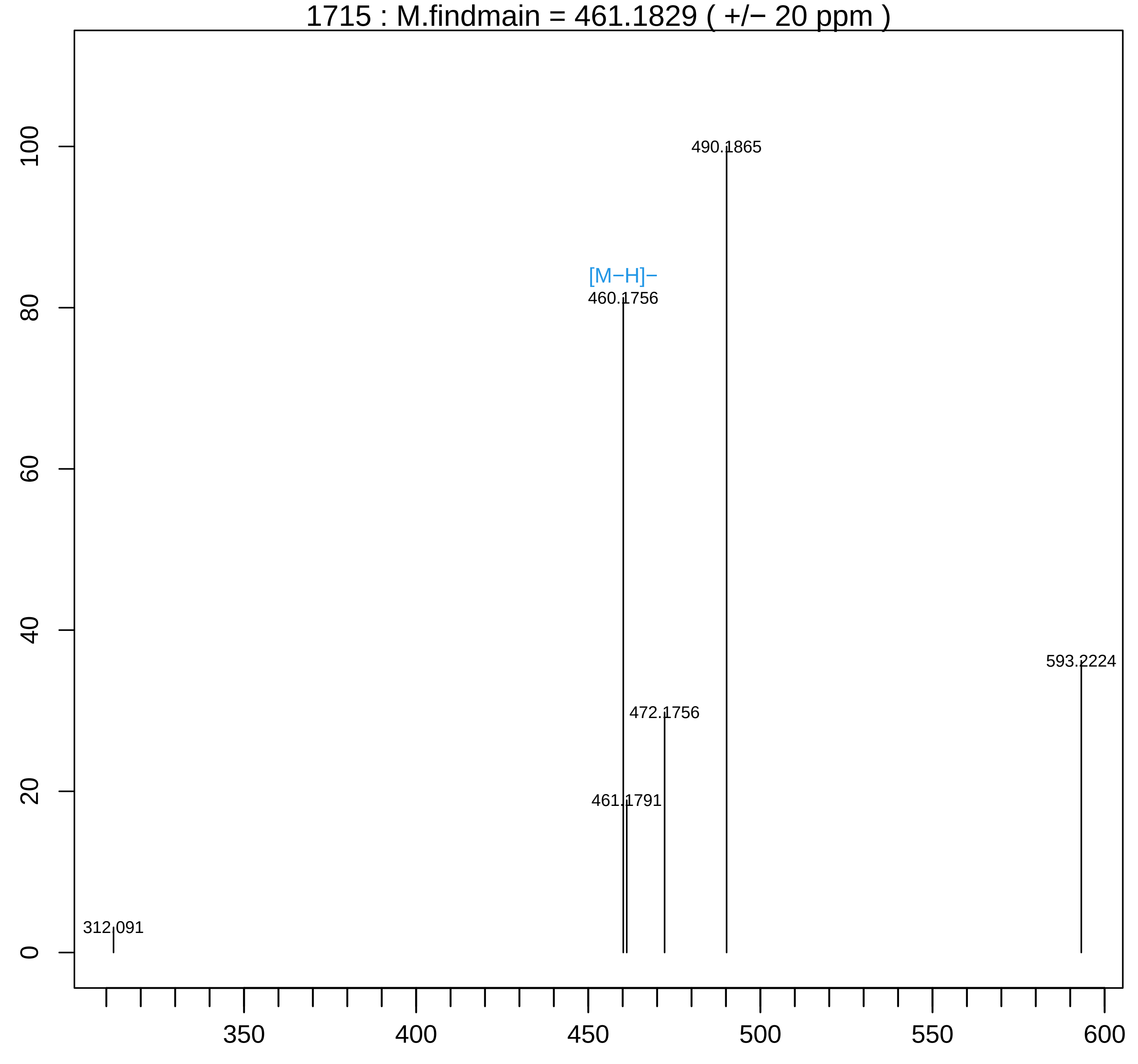
1715 : M.findmain = 461.1829 (+/− 20 ppm)
490.1865
[M−H]−
460.1756
472.1756
461.1791
593.2224
312.091
0
20
40
60
80
100
350
400
450
500
550
600

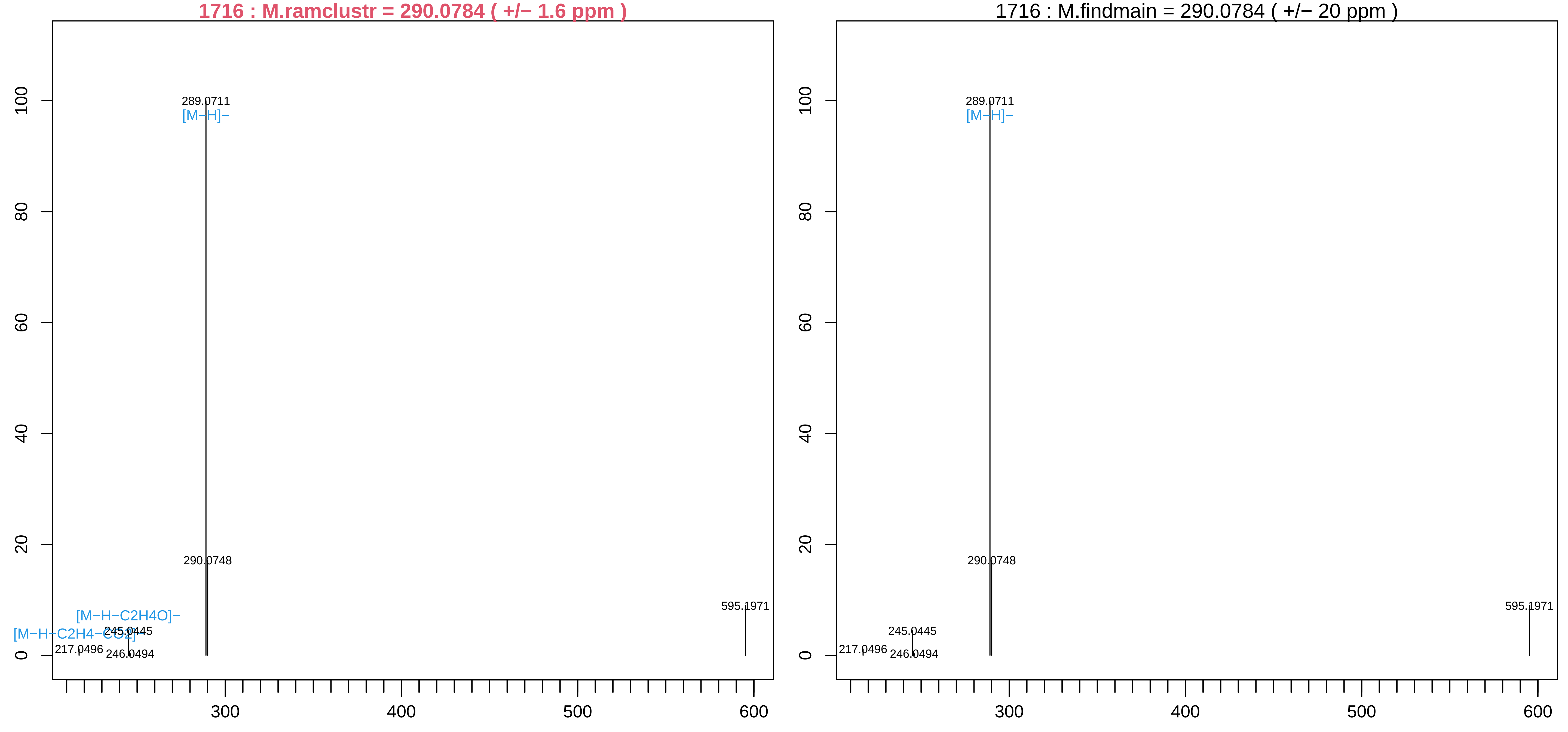
1716 : M.ramclustr = 290.0784 (+/− 1.6 ppm)
289.0711
[M−H]−
290.0748
[M−H−C2H4O]−
[M−H−C2H4−CO2]−
245.0445
217.0496
246.0494
595.1971
1716 : M.findmain = 290.0784 (+/− 20 ppm)
289.0711
[M−H]−
290.0748
245.0445
217.0496
246.0494
595.1971
0
20
40
60
80
100
300
400
500
600

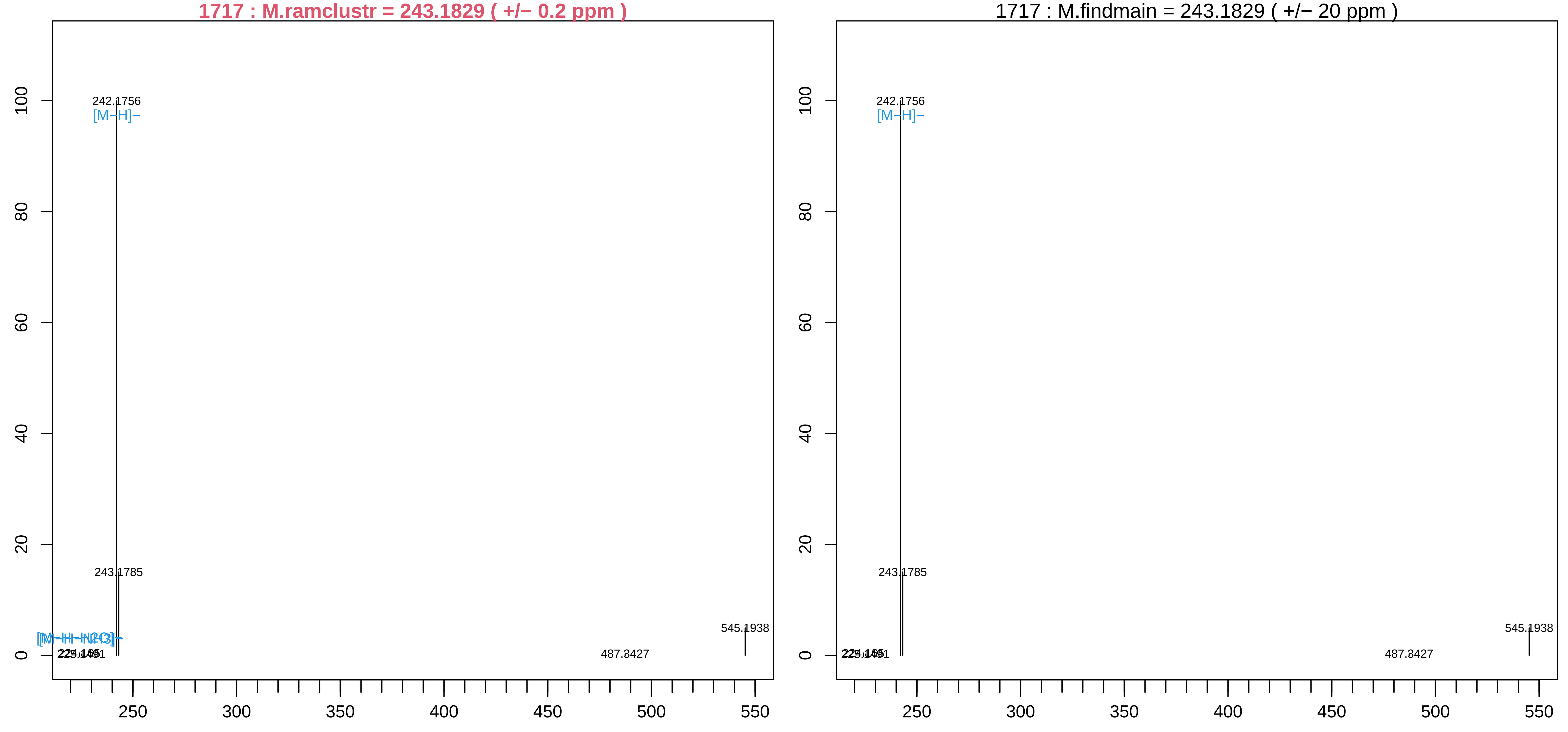
1717 : M.ramclustr = 243.1829 (+/− 0.2 ppm)
242.1756
[M−H]−
243.1785
545.1938
487.8427
1717 : M.findmain = 243.1829 (+/− 20 ppm)
242.1756
[M−H]−
243.1785
545.1938
487.3427
0
20
40
60
80
100
250
300
350
400
450
500
550

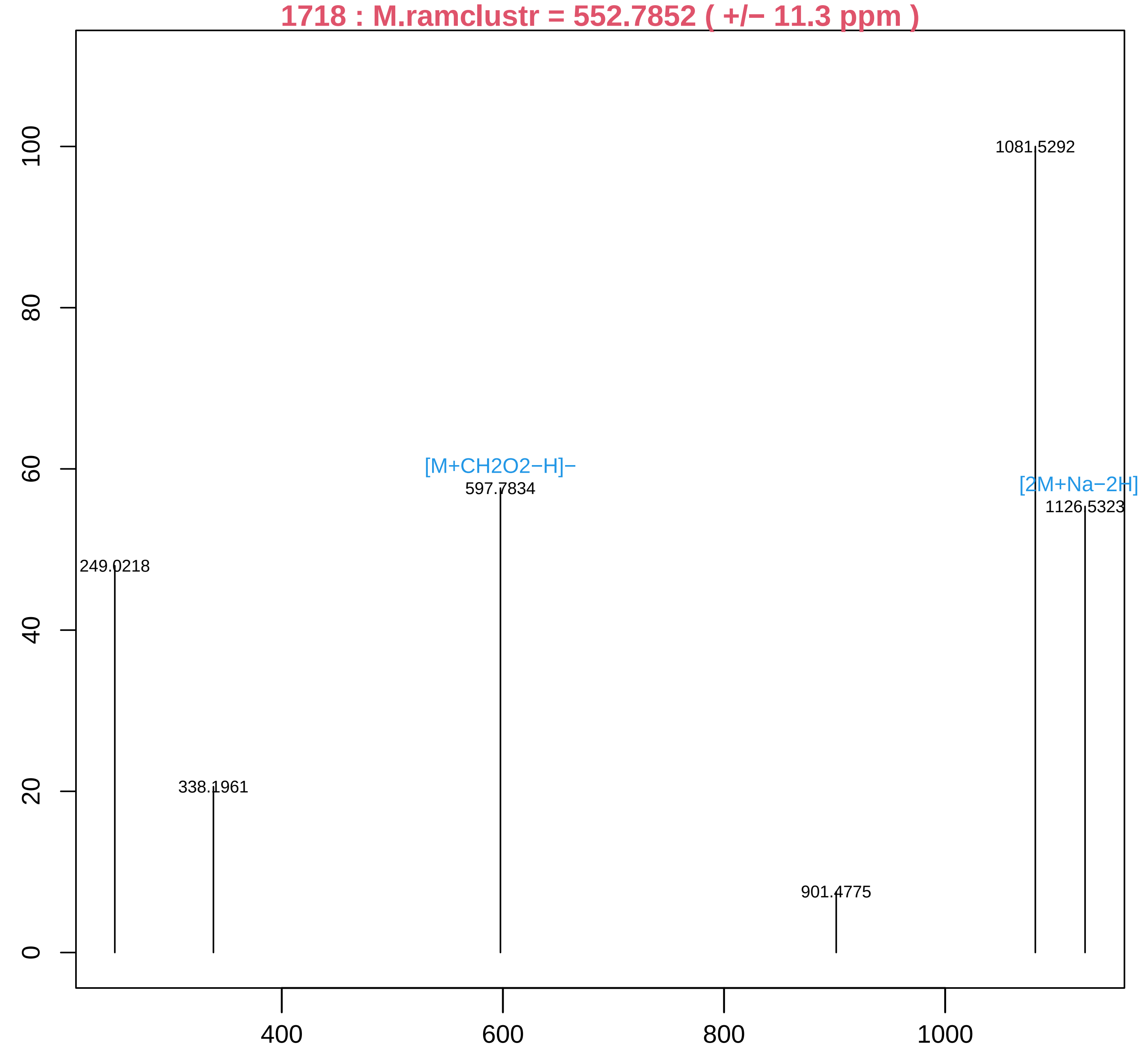
1718 : M.ramclustr = 552.7852 (+/− 11.3 ppm)
100
80
60
40
20
0
400
600
800
1000
1081.5292
[M+CH2O2−H]−
597.7834
[2M+Na−2H]
1126.5323
249.0218
338.1961
901.4775

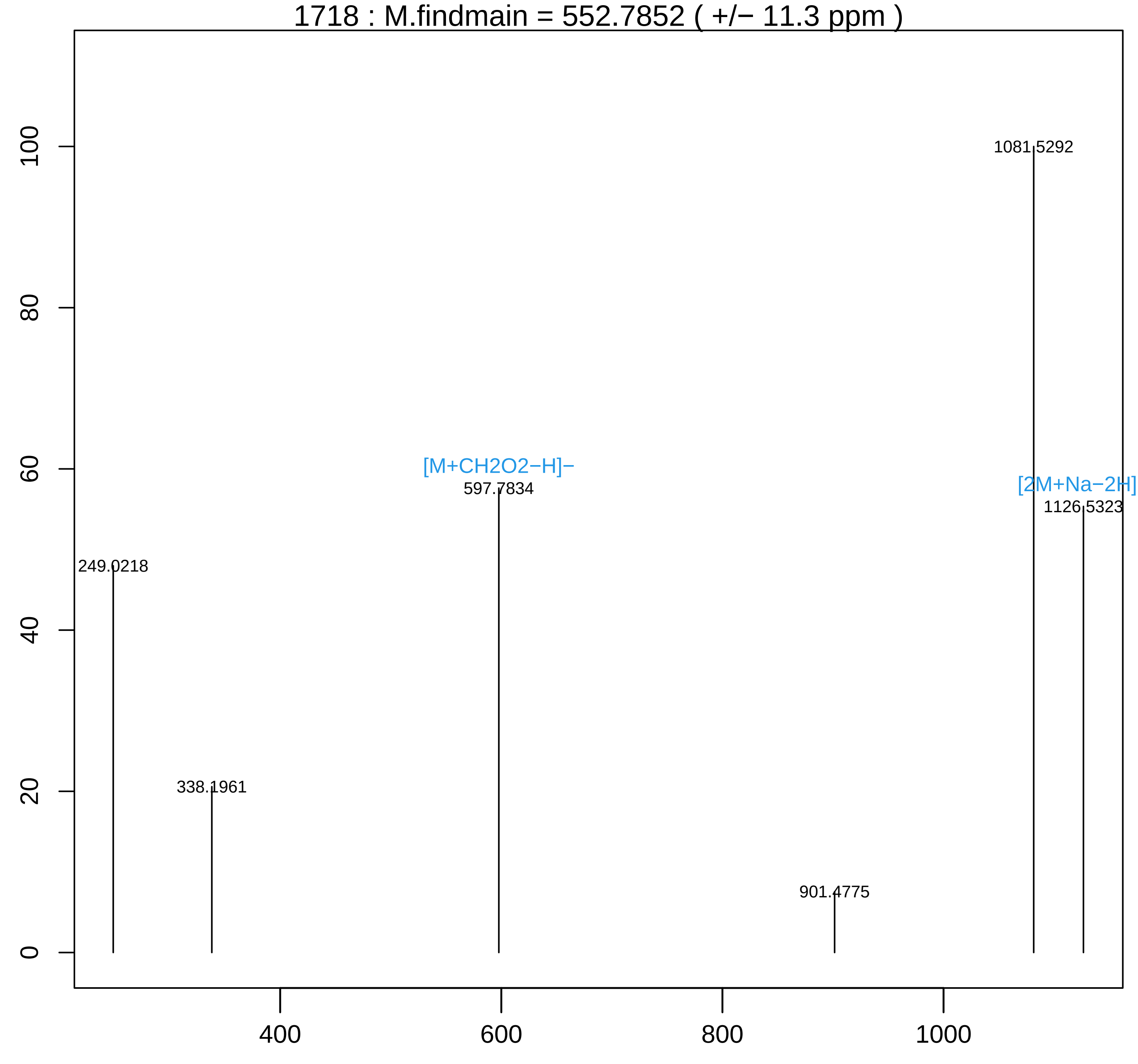
1718 : M.findmain = 552.7852 (+/− 11.3 ppm)
100
80
60
40
20
0
400
600
800
1000
1081.5292
[M+CH2O2−H]−
597.7834
[2M+Na−2H]
1126.5323
249.0218
338.1961
901.4775

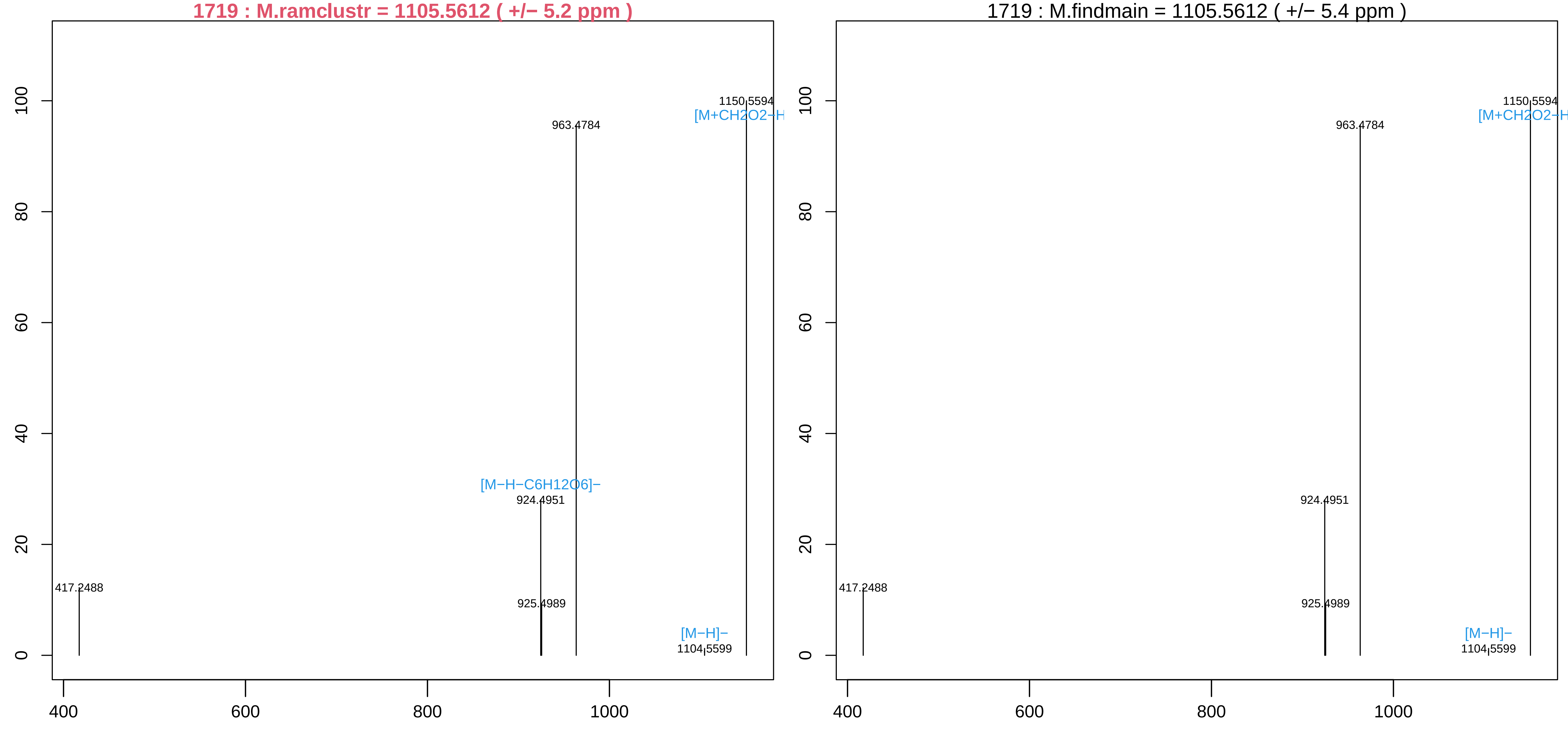
1719 : M.ramclustr = 1105.5612 (+/− 5.2 ppm)
1719 : M.findmain = 1105.5612 (+/− 5.4 ppm)
1150.5594
[M+CH2O2−H
963.4784
[M−H−C6H12O6]−
924.4951
925.4989
417.2488
[M−H]−
1104.5599
0
20
40
60
80
100
400
600
800
1000

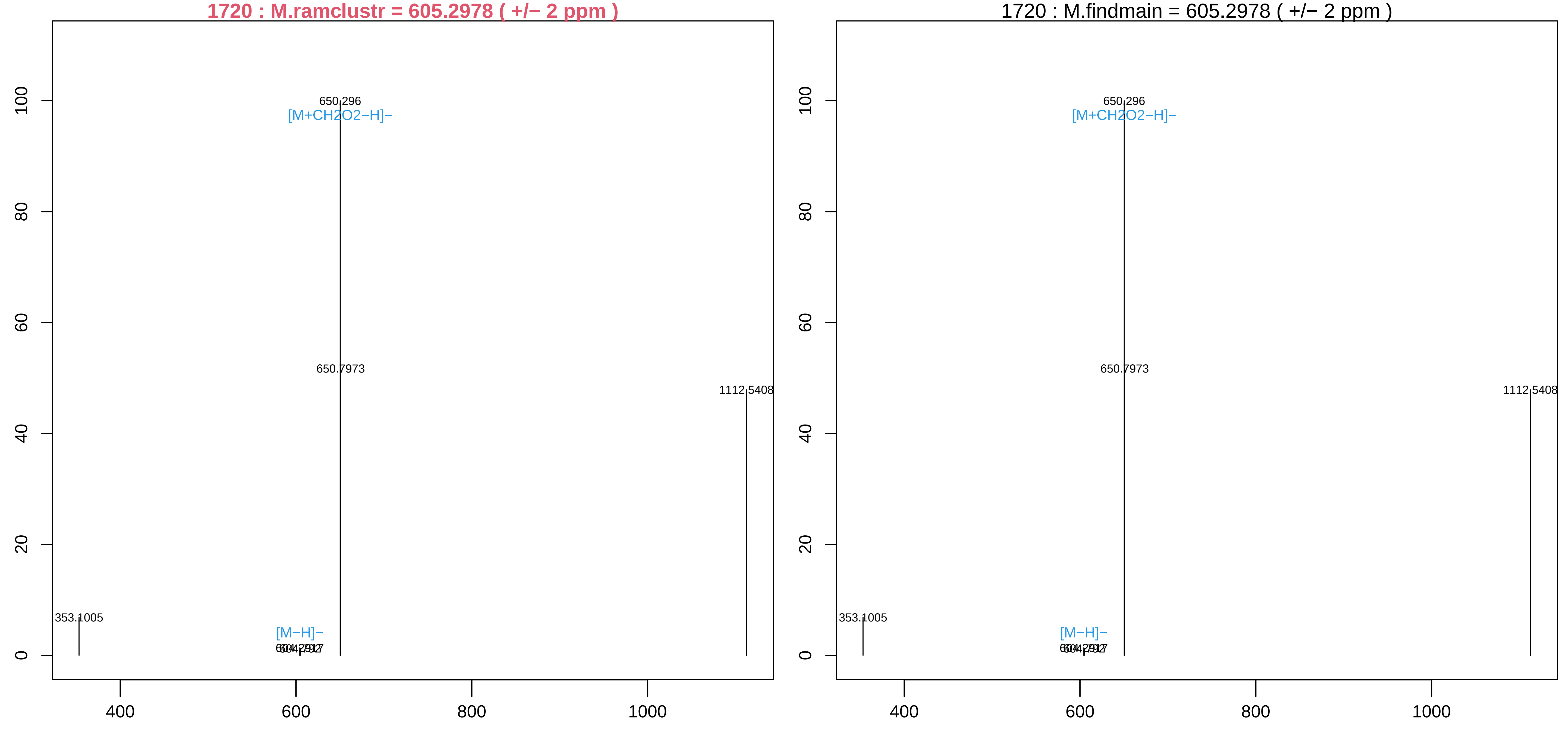
1720 : M.ramclustr = 605.2978 (+/− 2 ppm)
650.296
[M+CH2O2−H]−
650.7973
1112.5408
353.1005
[M−H]−
1720 : M.findmain = 605.2978 (+/− 2 ppm)
650.296
[M+CH2O2−H]−
650.7973
1112.5408
353.1005
[M−H]−

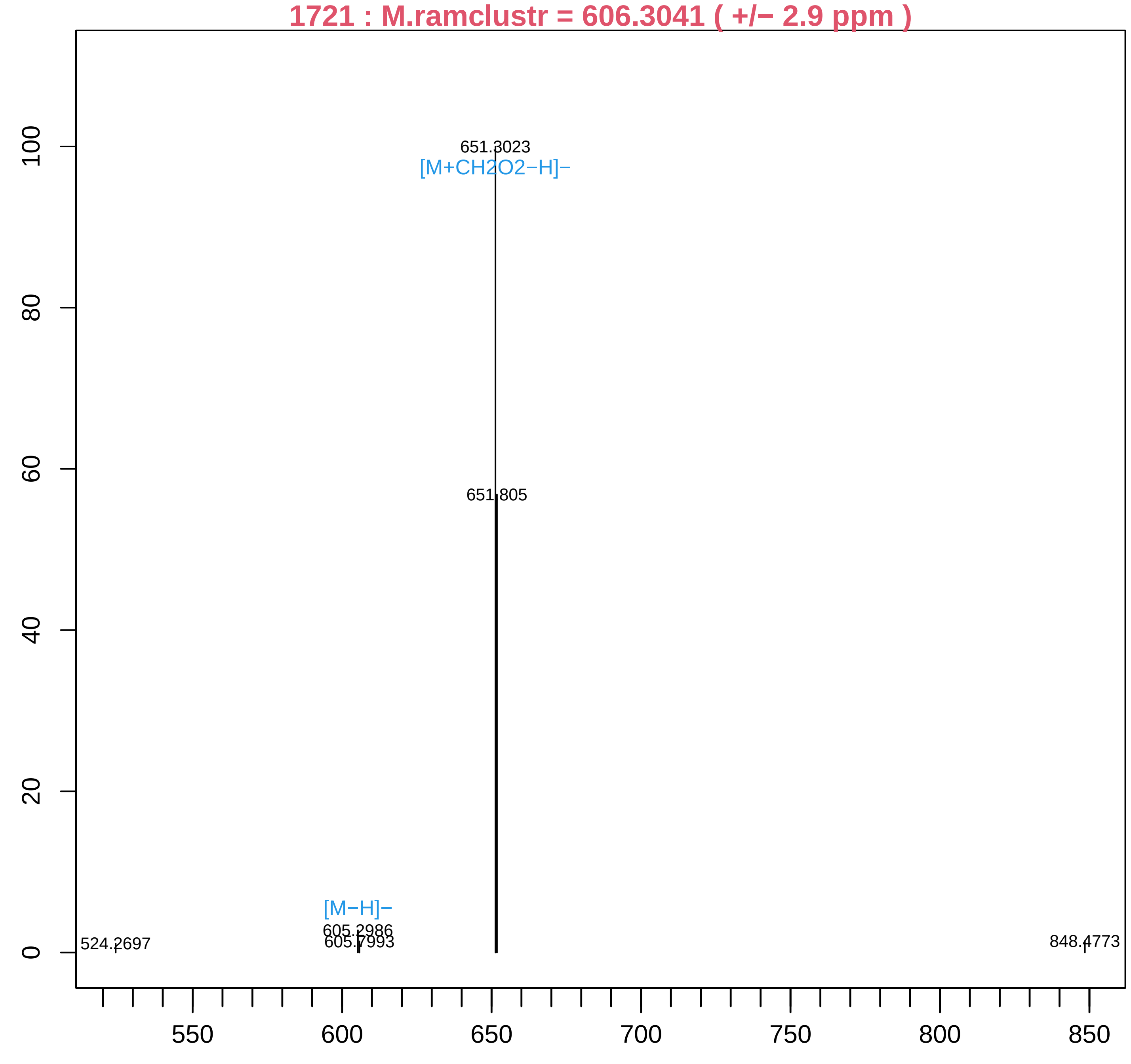
1721 : M.ramclustr = 606.3041 (+/− 2.9 ppm)
651.3023
[M+CH2O2−H]−
651.805
[M−H]−
605.2986
605.7993
524.2697
848.4773
0
20
40
60
80
100
550
600
650
700
750
800
850

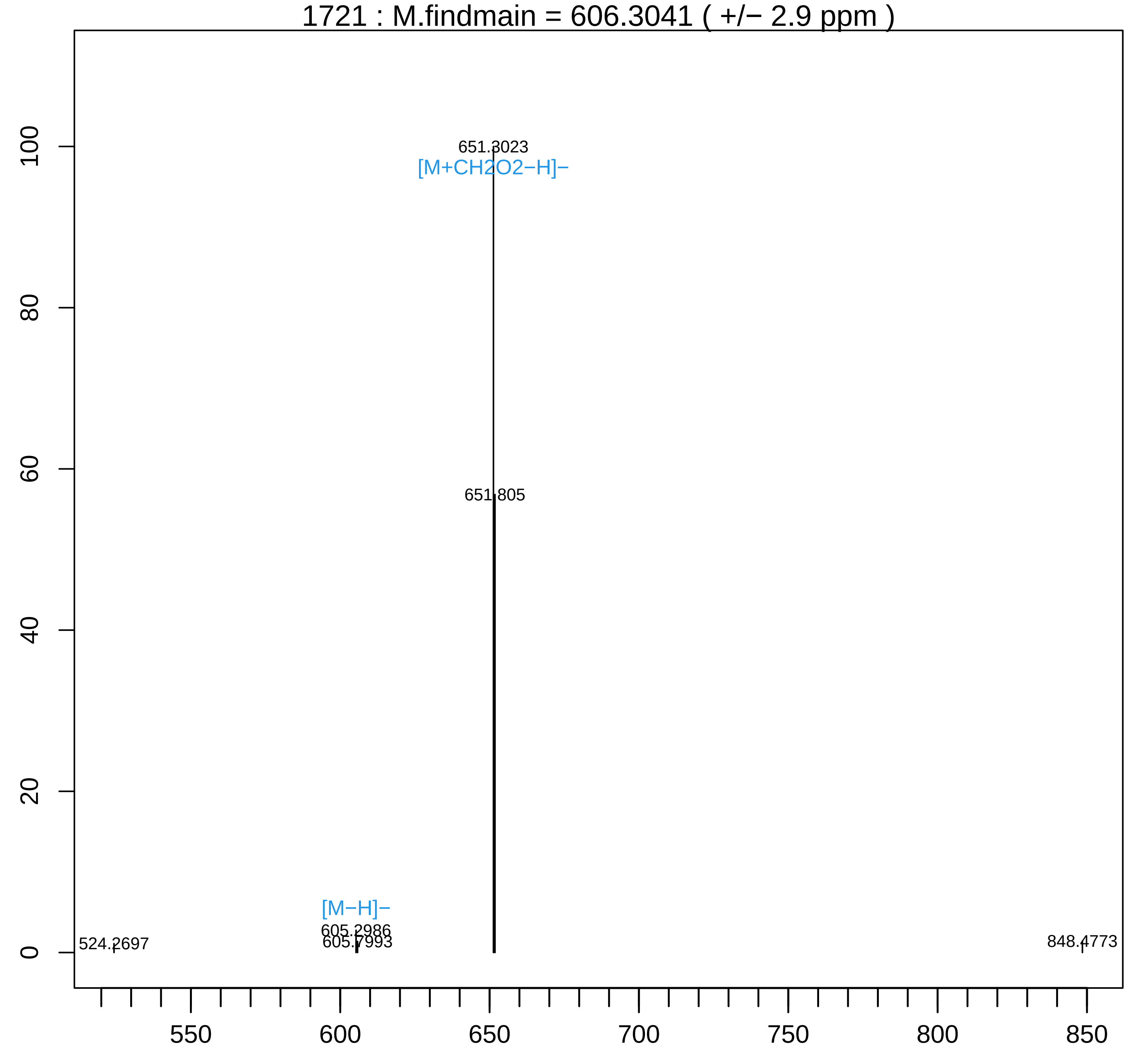
1721 : M.findmain = 606.3041 (+/− 2.9 ppm)
651.3023
[M+CH2O2−H]−
651.805
[M−H]−
605.2986
605.7993
524.2697
848.4773
0
20
40
60
80
100
550
600
650
700
750
800
850

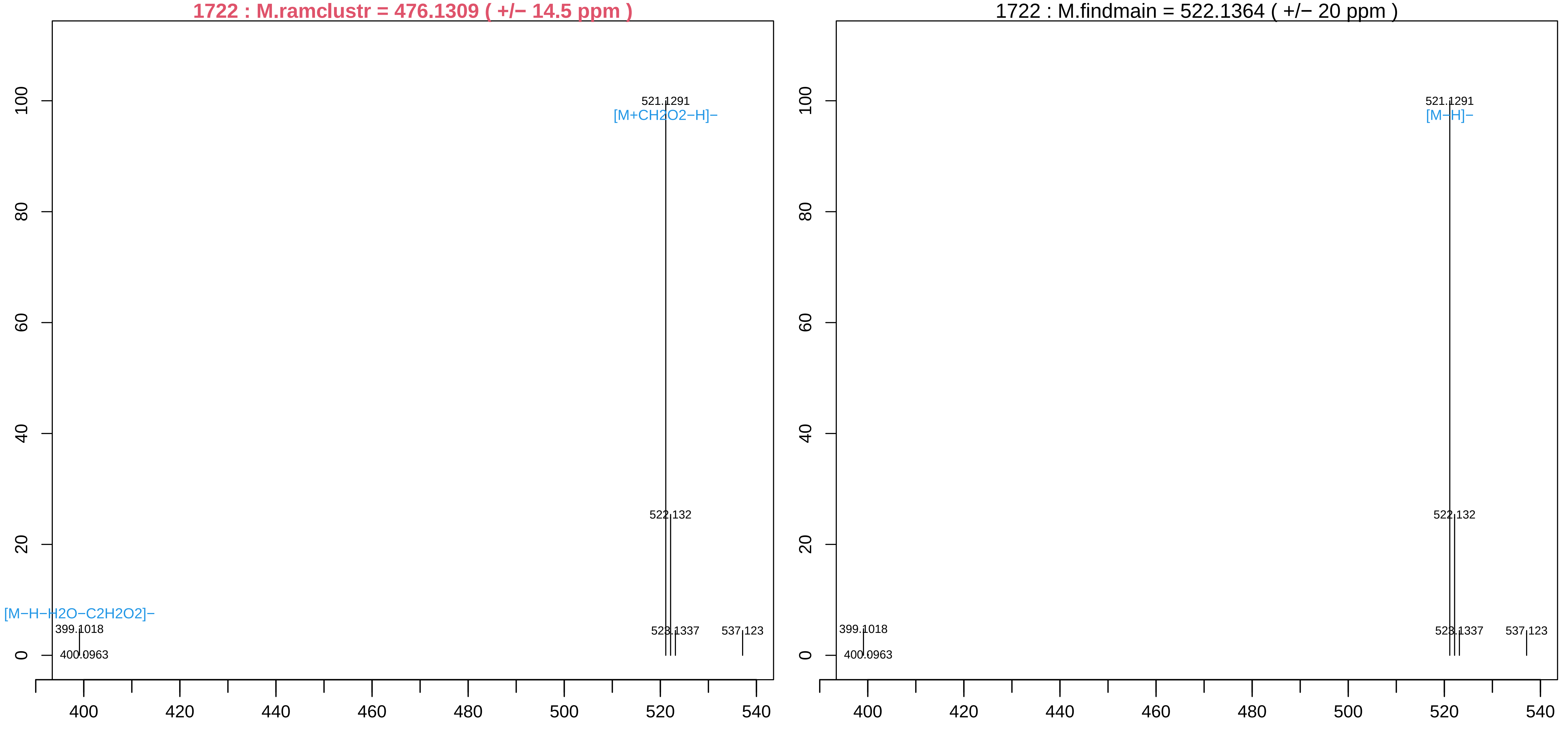
1722 : M.ramclustr = 476.1309 (+/− 14.5 ppm)
521.1291
[M+CH2O2−H]−
522.132
[M−H−H2O−C2H2O2]−
399.1018
523.1337
537.123
400.0963
1722 : M.findmain = 522.1364 (+/− 20 ppm)
521.1291
[M−H]−
522.132
399.1018
523.1337
537.123
400.0963

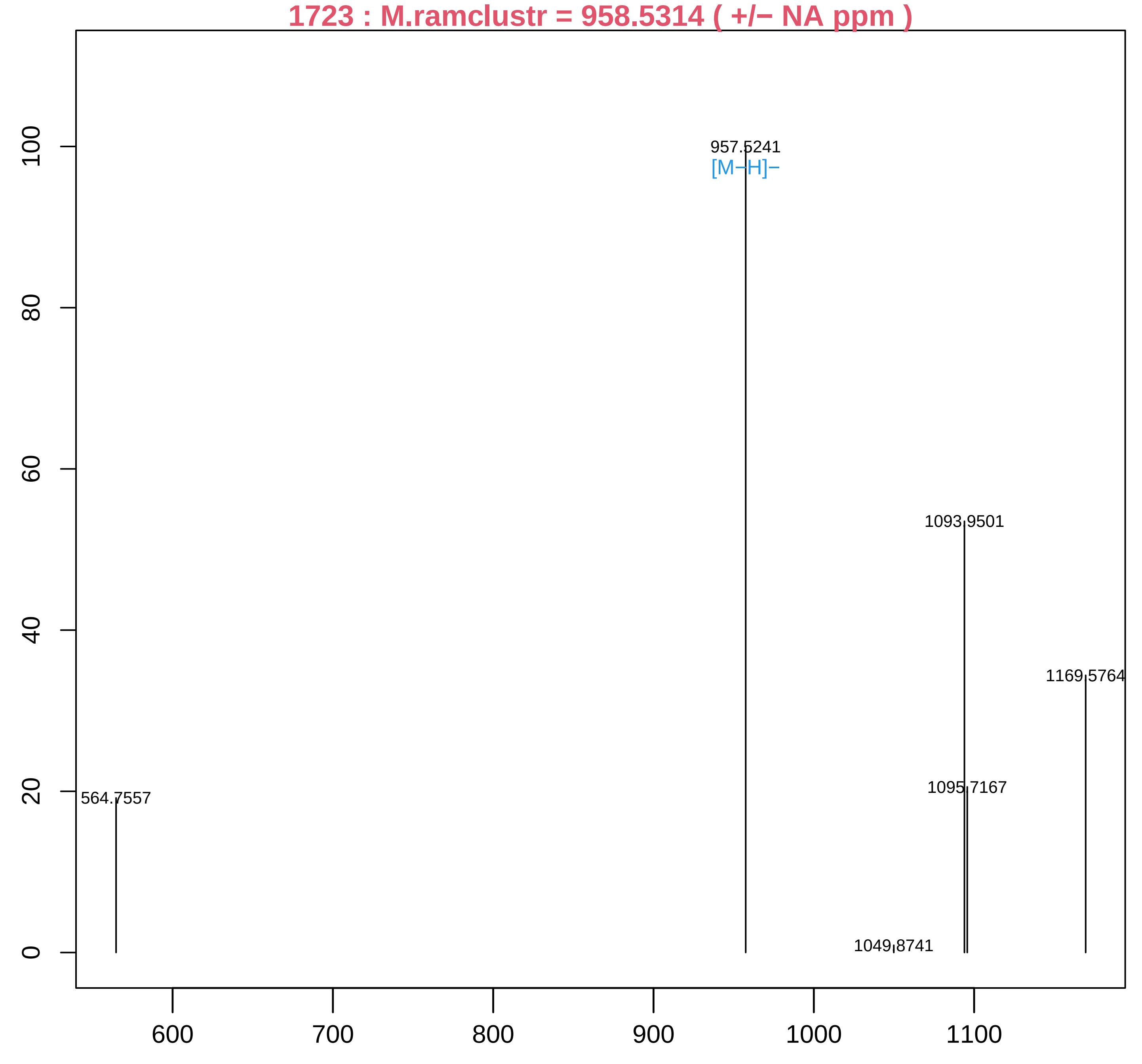
1723 : M.ramclustr = 958.5314 (+/− NA ppm)
957.5241
[M−H]−
1093.9501
1169.5764
1095.7167
564.7557
1049.8741
0
20
40
60
80
100
600
700
800
900
1000
1100

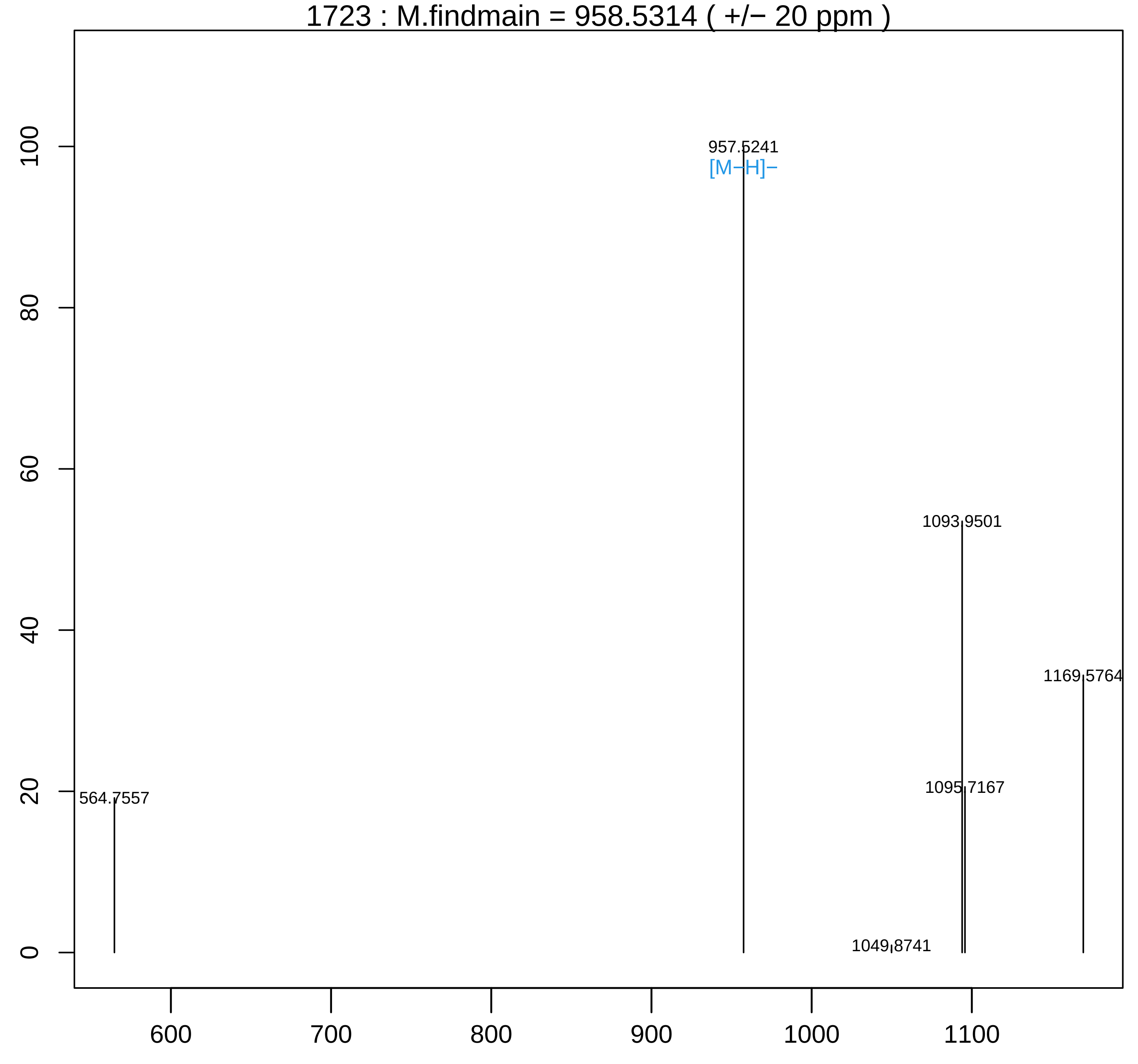
1723 : M.findmain = 958.5314 (+/− 20 ppm)
957.5241
[M−H]−
1093.9501
1169.5764
1095.7167
564.7557
1049.8741
0
20
40
60
80
100
600
700
800
900
1000
1100

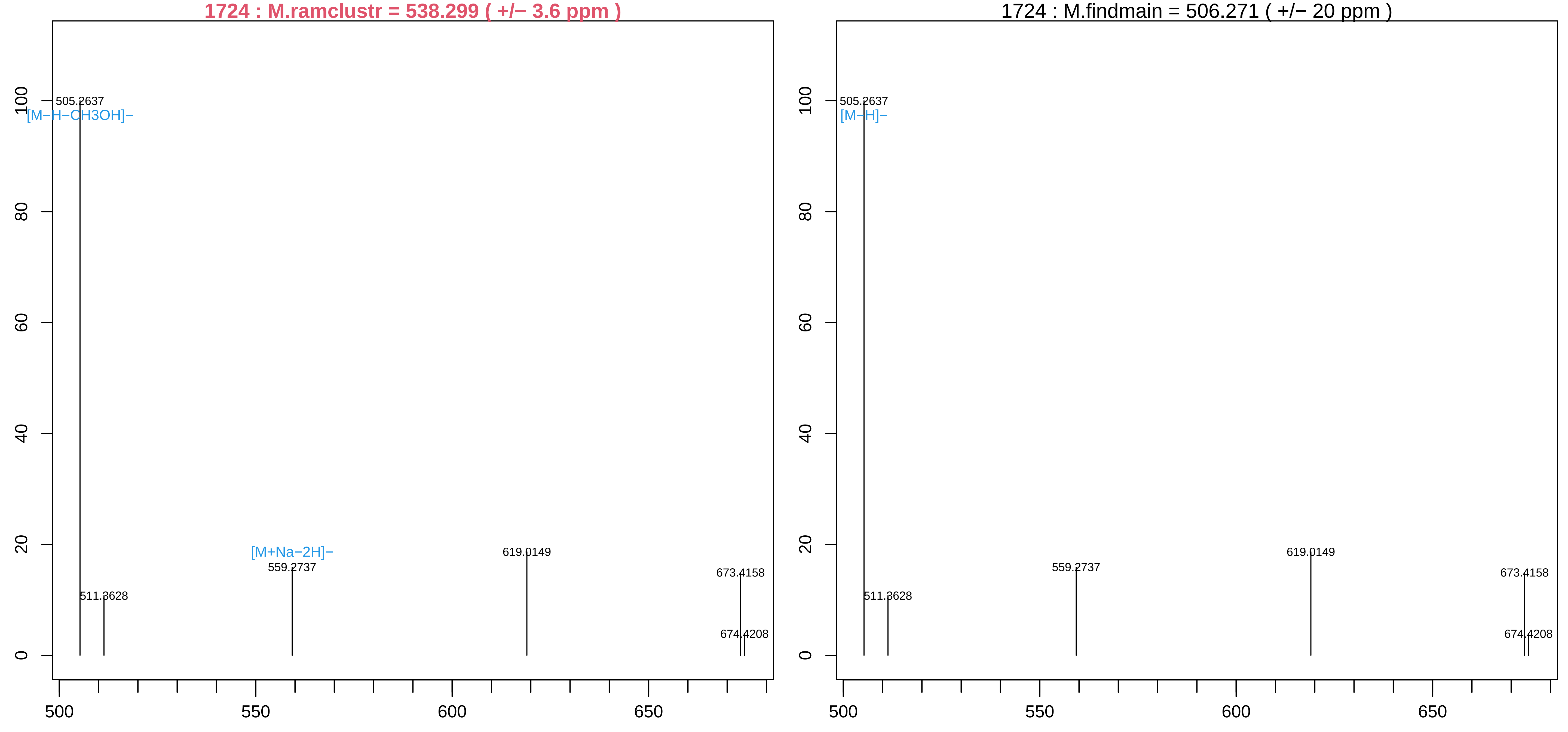
1724 : M.ramclustr = 538.299 (+/− 3.6 ppm)
505.2637
[M−H−CH3OH]−
511.3628
[M+Na−2H]−
559.2737
619.0149
673.4158
674.4208
1724 : M.findmain = 506.271 (+/− 20 ppm)
505.2637
[M−H]−
511.3628
559.2737
619.0149
673.4158
674.4208

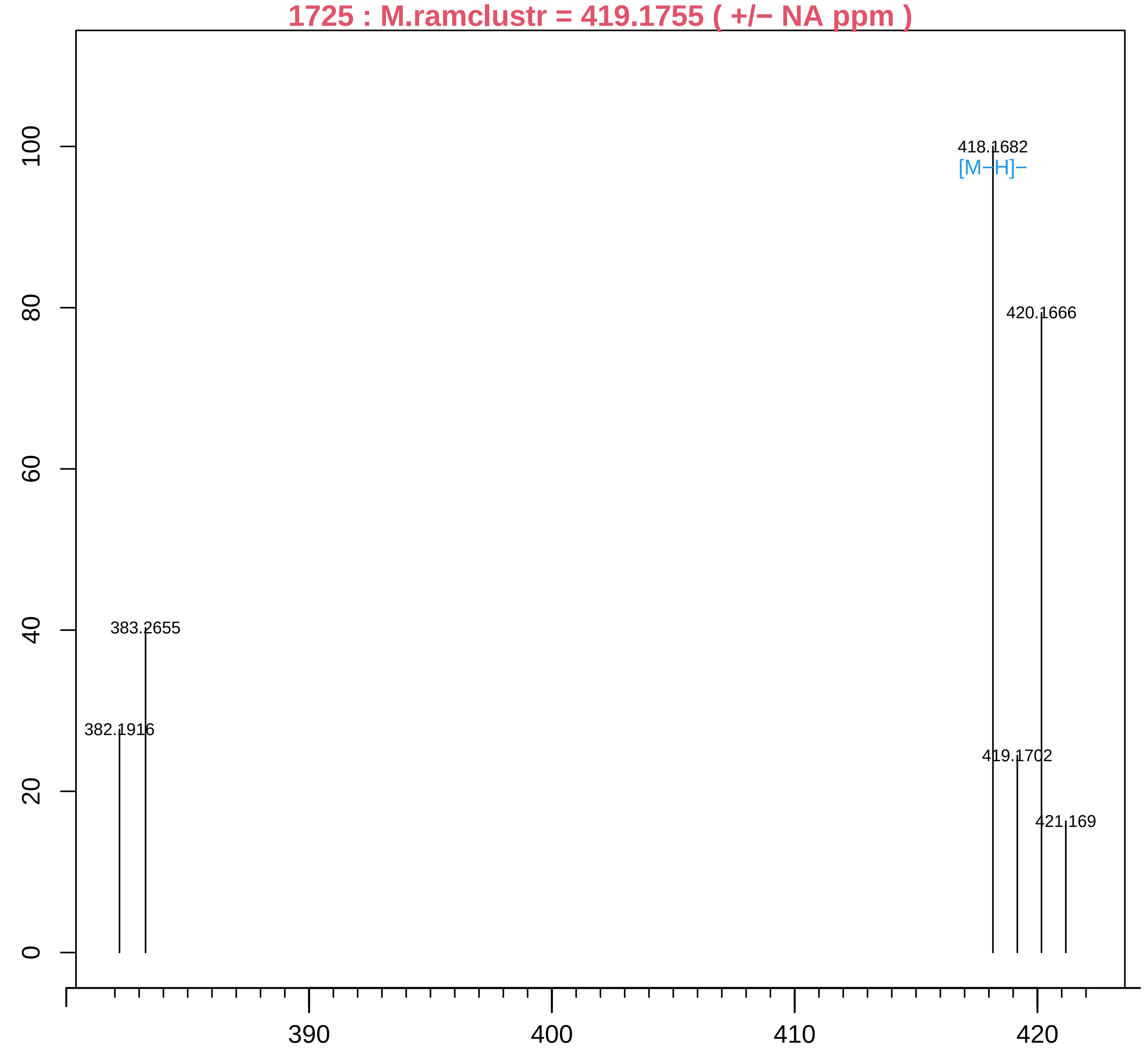
1725 : M.ramclustr = 419.1755 (+/− NA ppm)
418.1682
[M−H]−
420.1666
383.2655
382.1916
419.1702
421.169
0
20
40
60
80
100
390
400
410
420
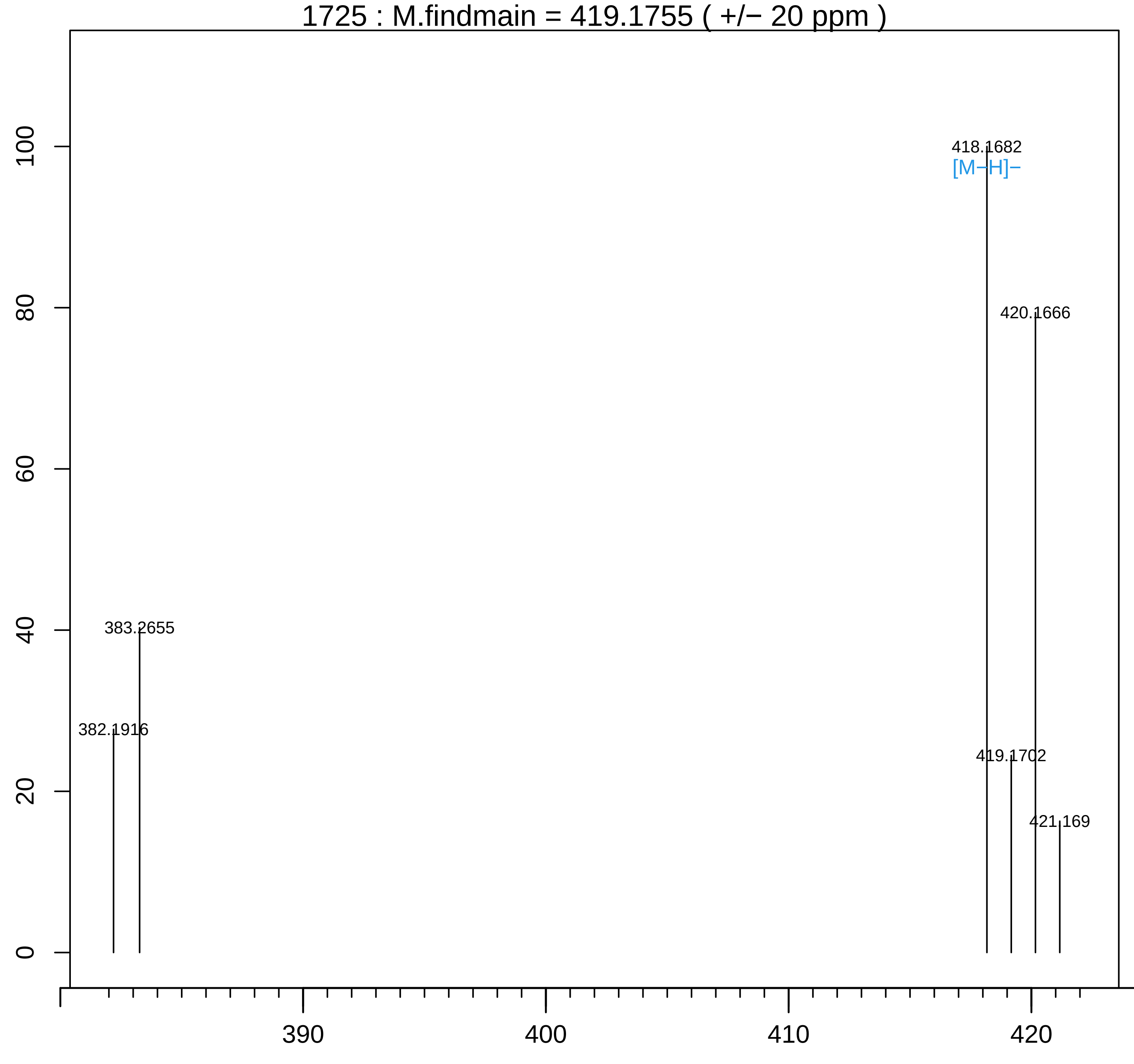
1725 : M.findmain = 419.1755 (+/− 20 ppm)
418.1682
[M−H]−
420.1666
383.2655
382.1916
419.1702
421.169
0
20
40
60
80
100
390
400
410
420

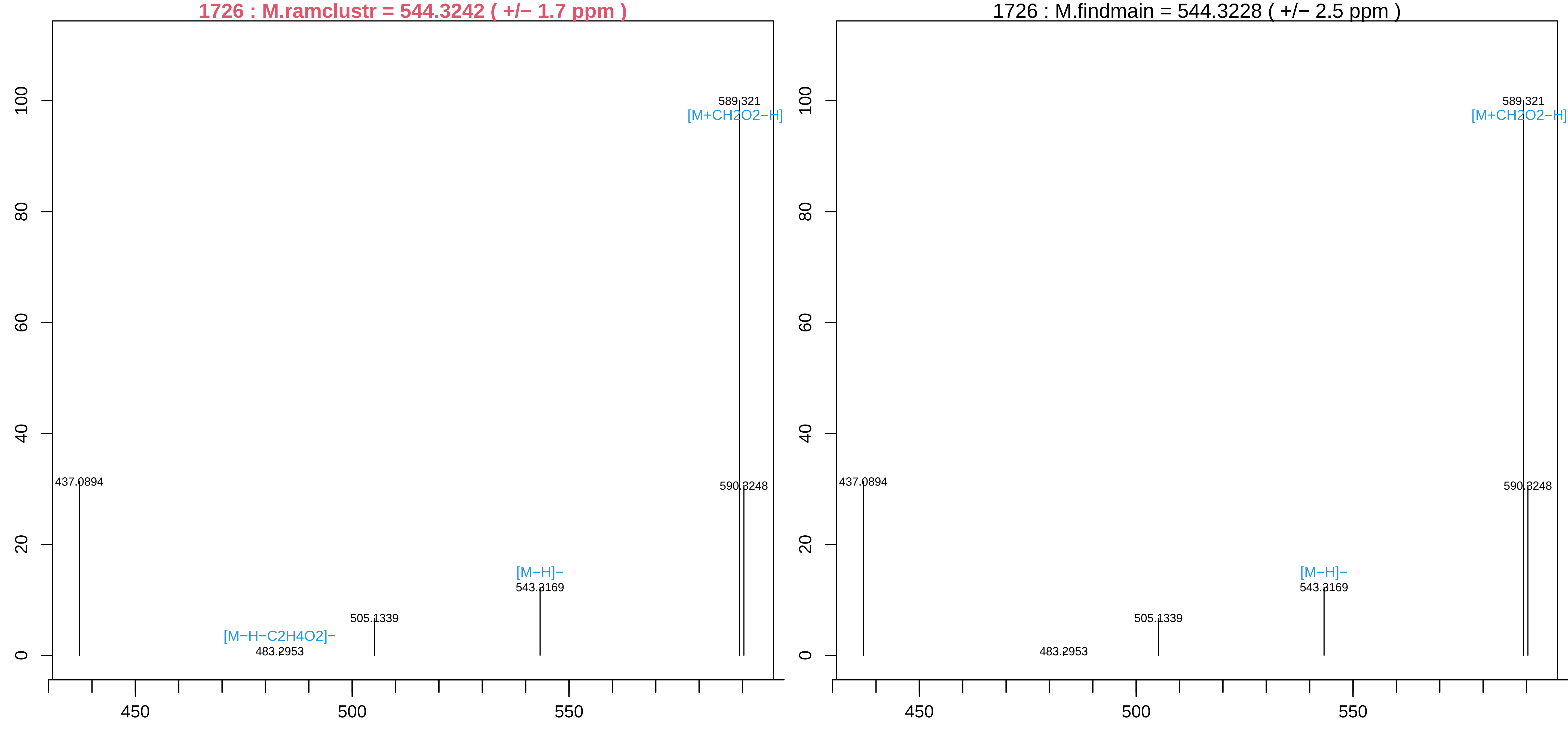
1726 : M.ramclustr = 544.3242 (+/− 1.7 ppm)
589.321
[M+CH2O2−H]
437.0894
590.3248
[M−H]−
543.3169
505.1339
[M−H−C2H4O2]−
483.2953
0
20
40
60
80
100
450
500
550
1726 : M.findmain = 544.3228 (+/− 2.5 ppm)
589.321
[M+CH2O2−H]
437.0894
590.3248
[M−H]−
543.3169
505.1339
483.2953
0
20
40
60
80
100
450
500
550

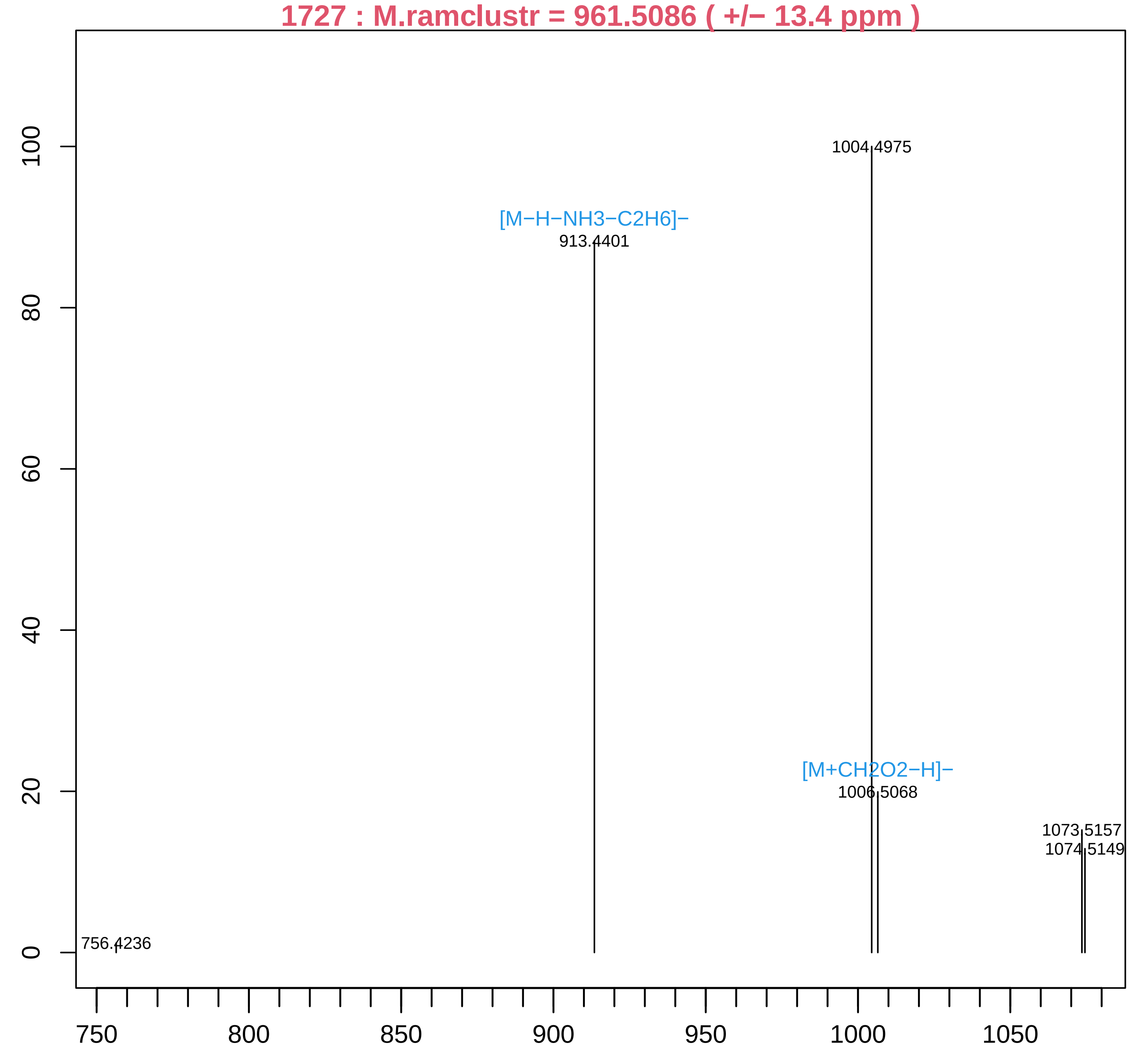

1727 : M.ramclustr = 961.5086 (+/− 13.4 ppm)
[M−H−NH3−C2H6]−
913.4401
1004.4975
[M+CH2O2−H]−
1006.5068
1073.5157
1074.5149
756.4236

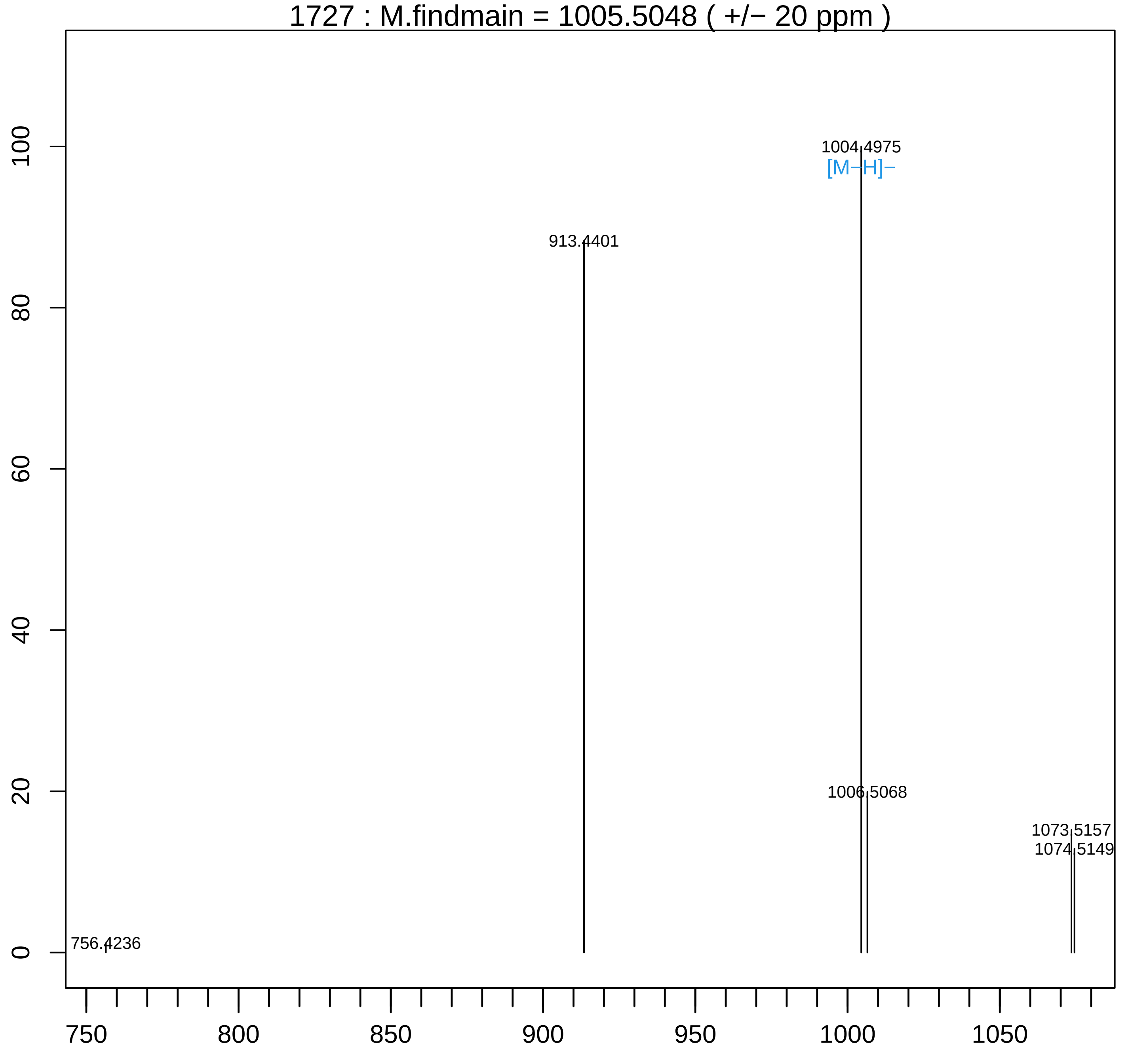

1727 : M.findmain = 1005.5048 (+/− 20 ppm)
1004.4975
[M−H]−
913.4401
1006.5068
1073.5157
1074.5149
756.4236

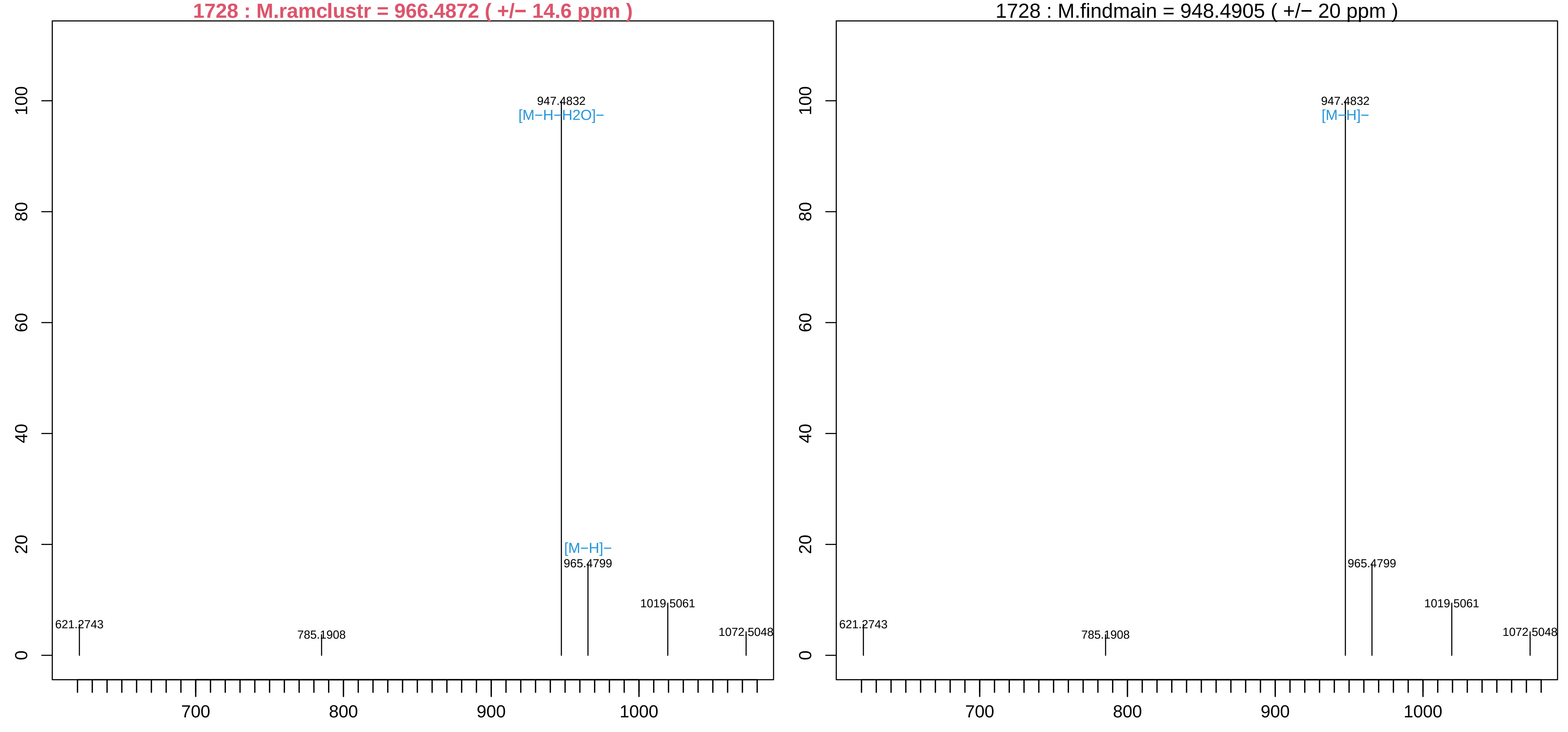
1728 : M.ramclustr = 966.4872 (+/− 14.6 ppm)
947.4832
[M−H−H2O]−
[M−H]−
965.4799
1019.5061
621.2743
785.1908
1072.5048
1728 : M.findmain = 948.4905 (+/− 20 ppm)
947.4832
[M−H]−
965.4799
1019.5061
621.2743
785.1908
1072.5048
0
20
40
60
80
100
700
800
900
1000

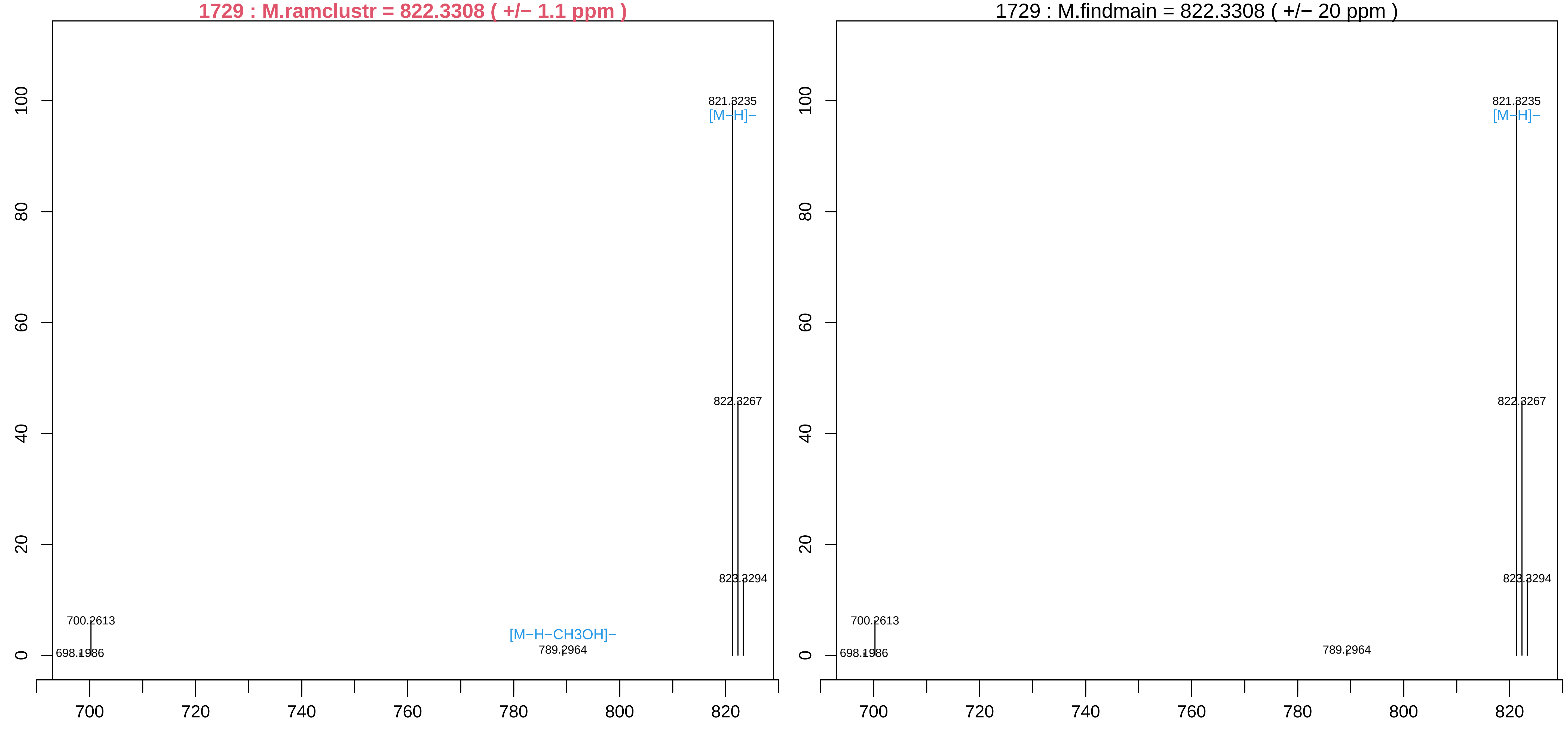
1729 : M.ramclustr = 822.3308 (+/− 1.1 ppm)
821.3235
[M−H]−
822.3267
823.3294
700.2613
698.1986
[M−H−CH3OH]−
789.2964
1729 : M.findmain = 822.3308 (+/− 20 ppm)
821.3235
[M−H]−
822.3267
823.3294
700.2613
698.1986
789.2964
0
20
40
60
80
100
700
720
740
760
780
800
820

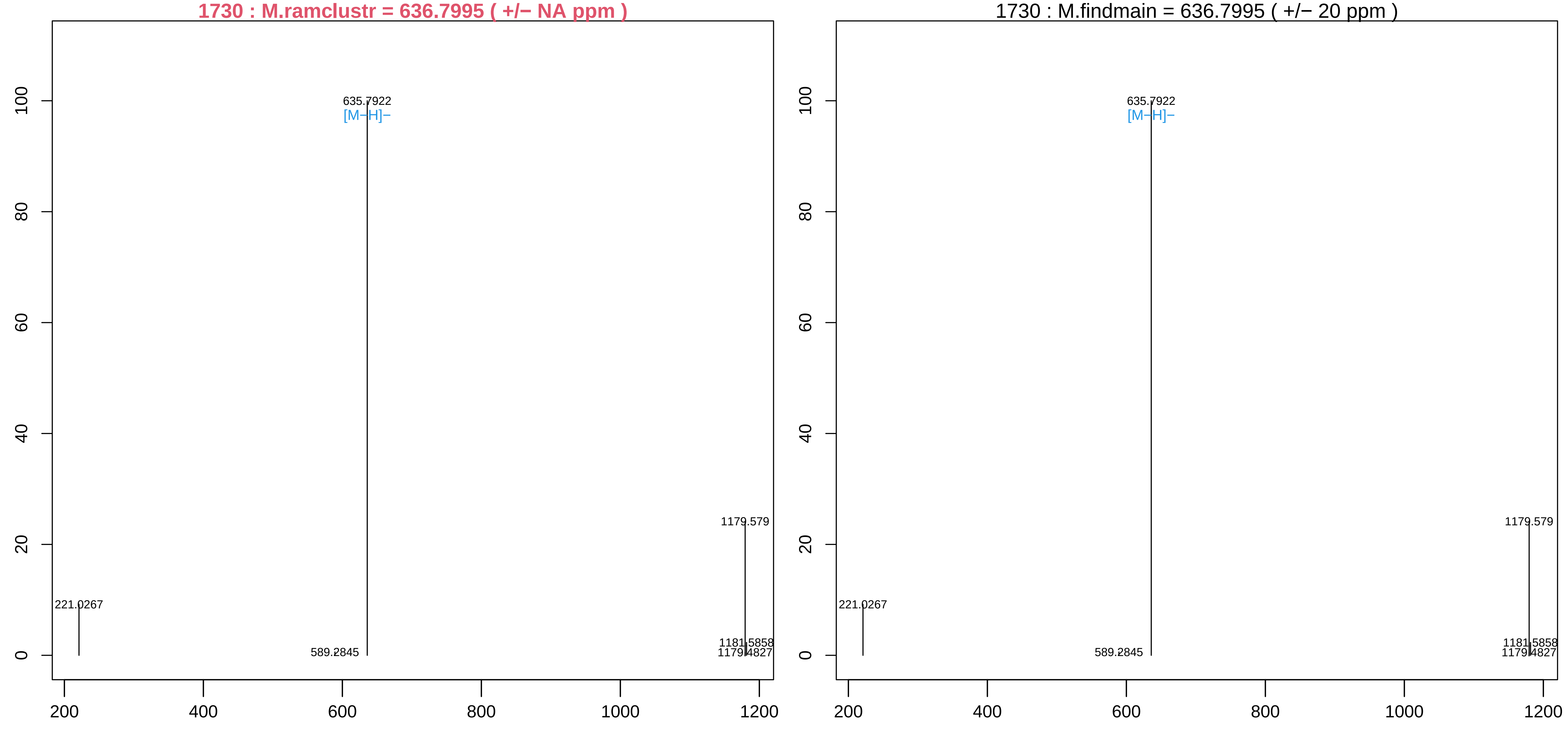

1730 : M.ramclustr = 636.7995 (+/− NA ppm)
635.7922
[M−H]−
1179.579
221.0267
589.2845
1181.5858
1179.4827
1730 : M.findmain = 636.7995 (+/− 20 ppm)
635.7922
[M−H]−
1179.579
221.0267
589.2845
1181.5858
1179.4827

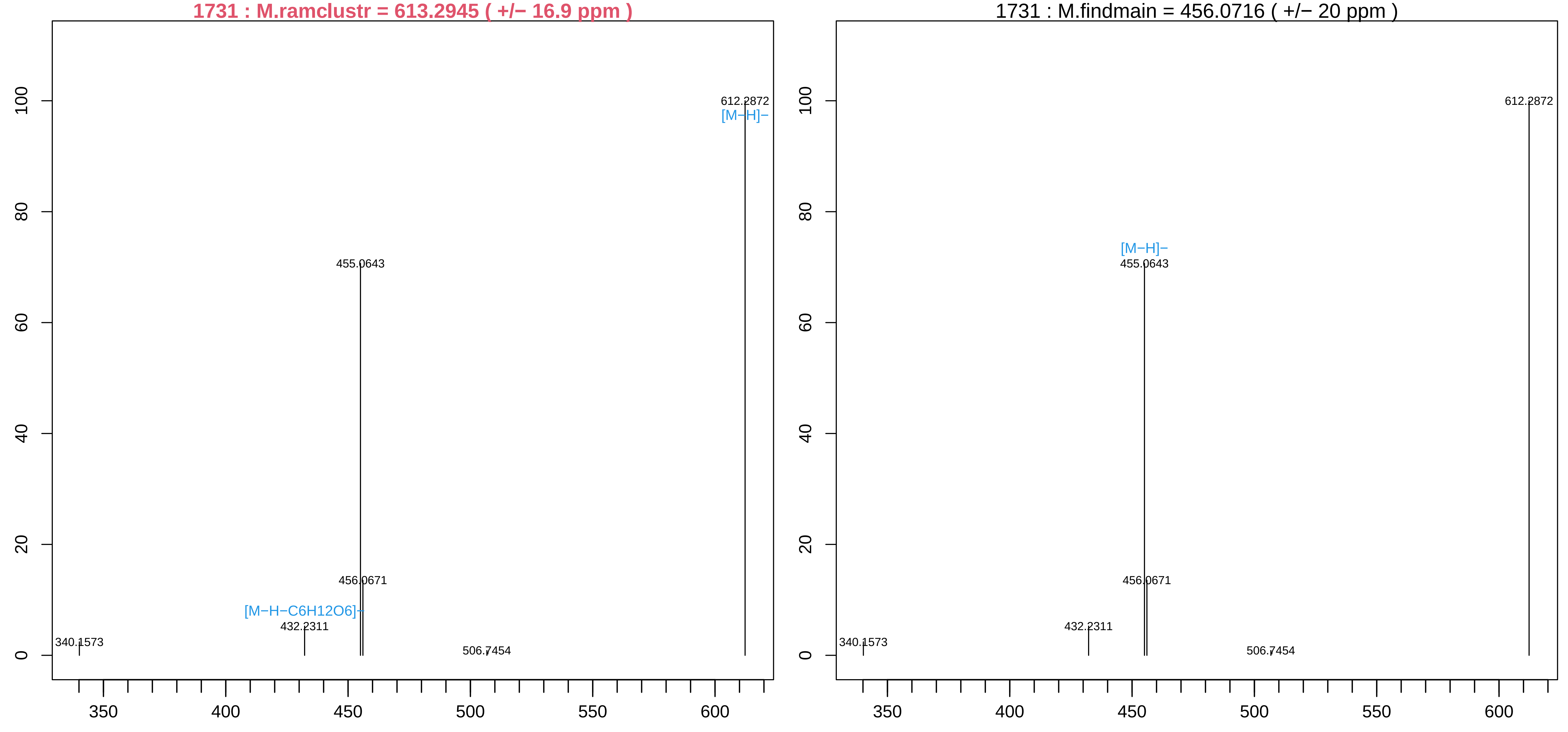
1731 : M.ramclustr = 613.2945 (+/− 16.9 ppm)
612.2872
[M−H]−
455.0643
456.0671
[M−H−C6H12O6]−
432.2311
340.1573
506.7454
1731 : M.findmain = 456.0716 (+/− 20 ppm)
612.2872
[M−H]−
455.0643
456.0671
432.2311
340.1573
506.7454
0
20
40
60
80
100
350
400
450
500
550
600

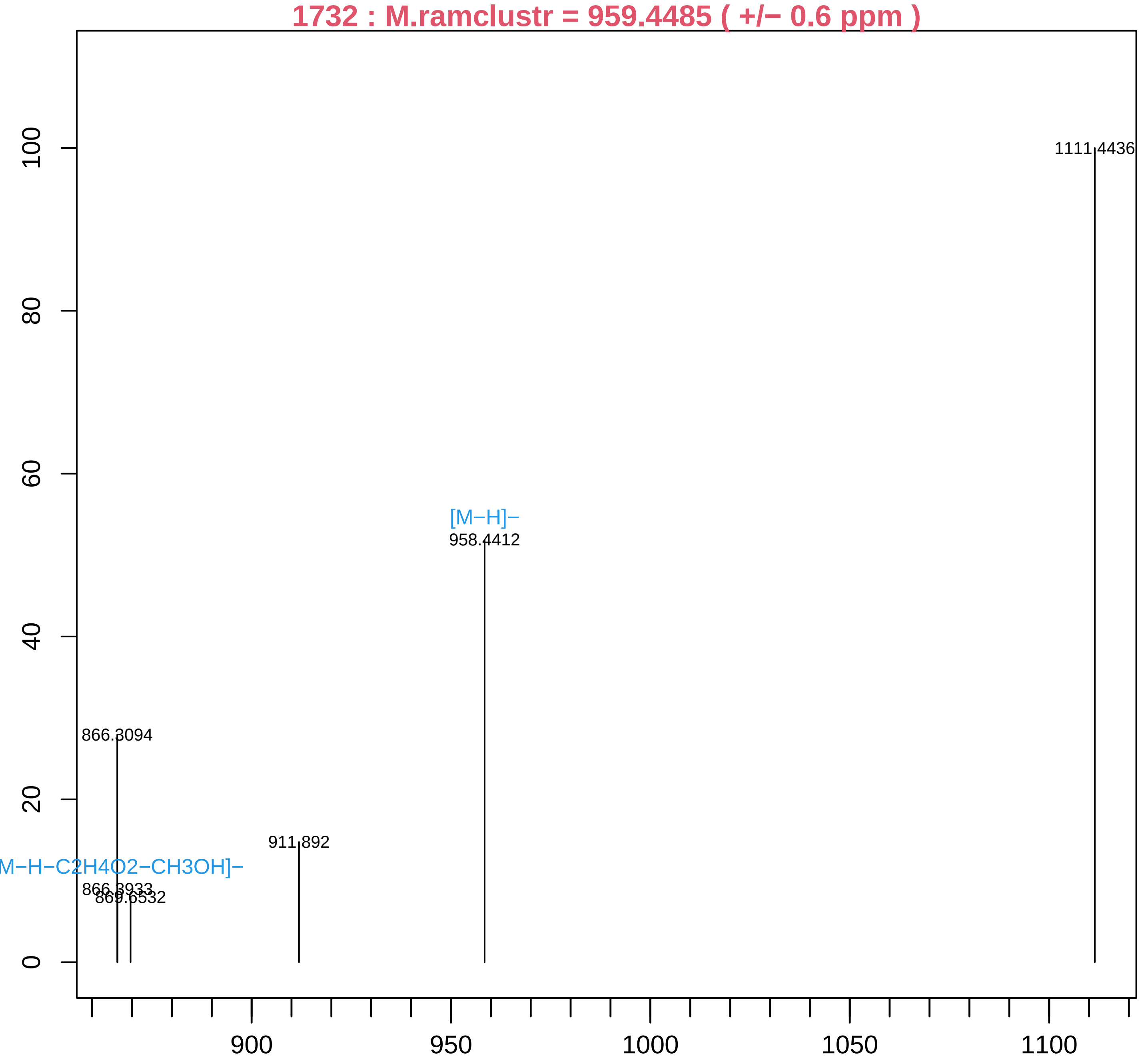

1732 : M.ramclustr = 959.4485 (+/− 0.6 ppm)
1111.4436
[M−H]−
958.4412
866.3094
911.892
[M−H−C2H4O2−CH3OH]−
866.3933
869.6532
0
20
40
60
80
100
900
950
1000
1050
1100

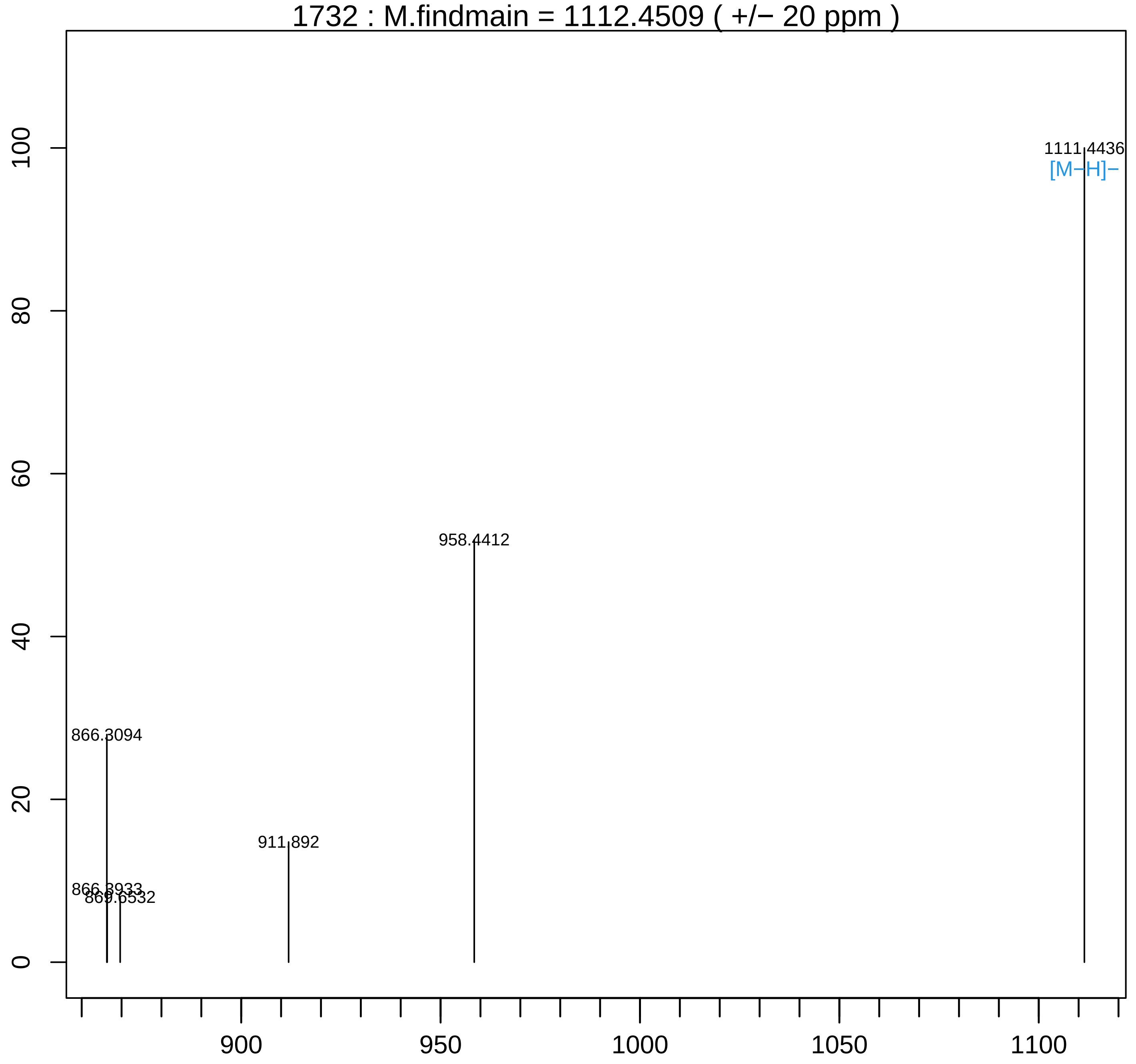

1732 : M.findmain = 1112.4509 (+/− 20 ppm)
1111.4436
[M−H]−
958.4412
866.3094
911.892
866.3933
869.6532
0
20
40
60
80
100
900
950
1000
1050
1100

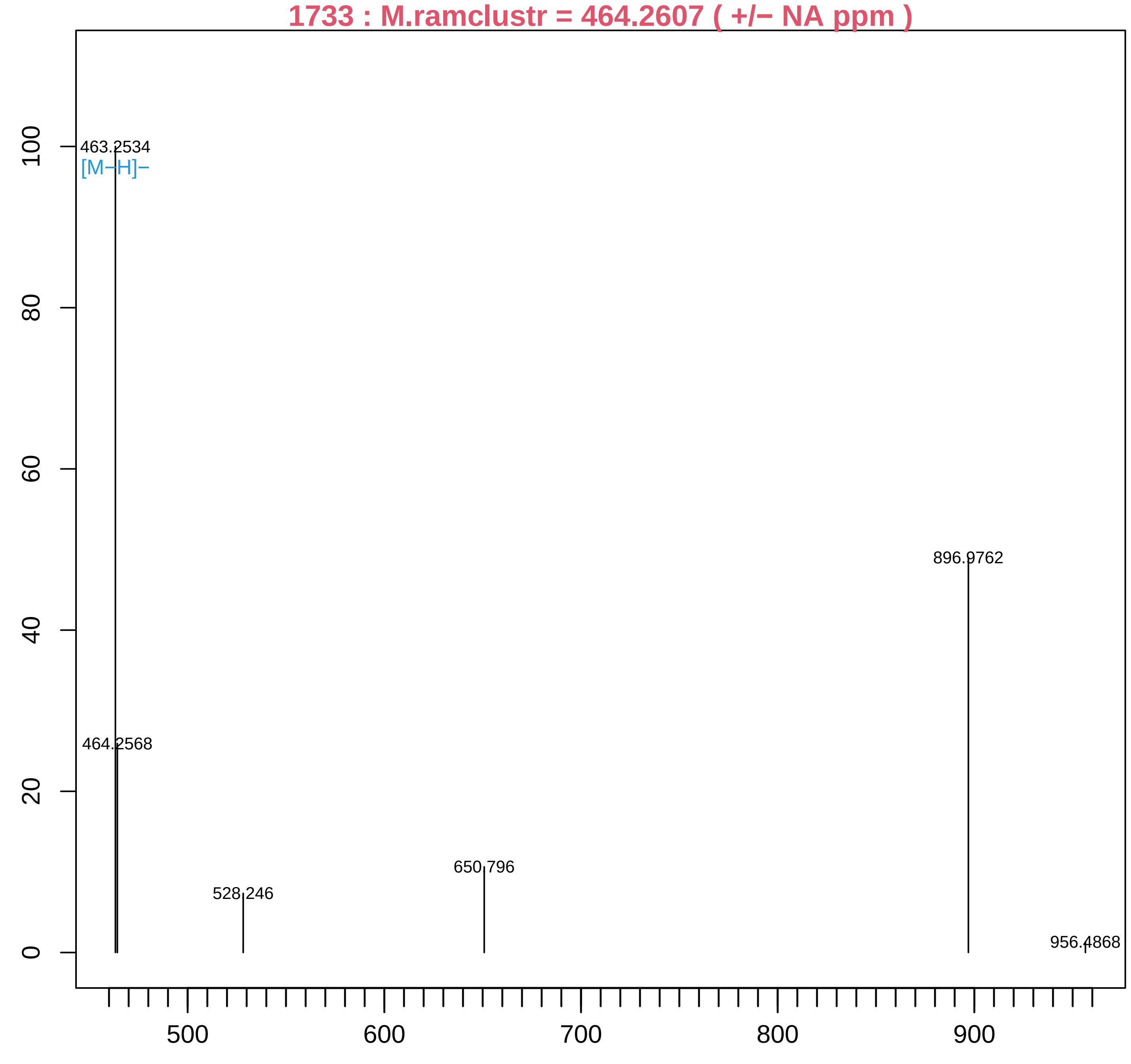

1733 : M.ramclustr = 464.2607 (+/− NA ppm)
463.2534
[M−H]−
464.2568
528.246
650.796
896.9762
956.4868
0
20
40
60
80
100
500
600
700
800
900

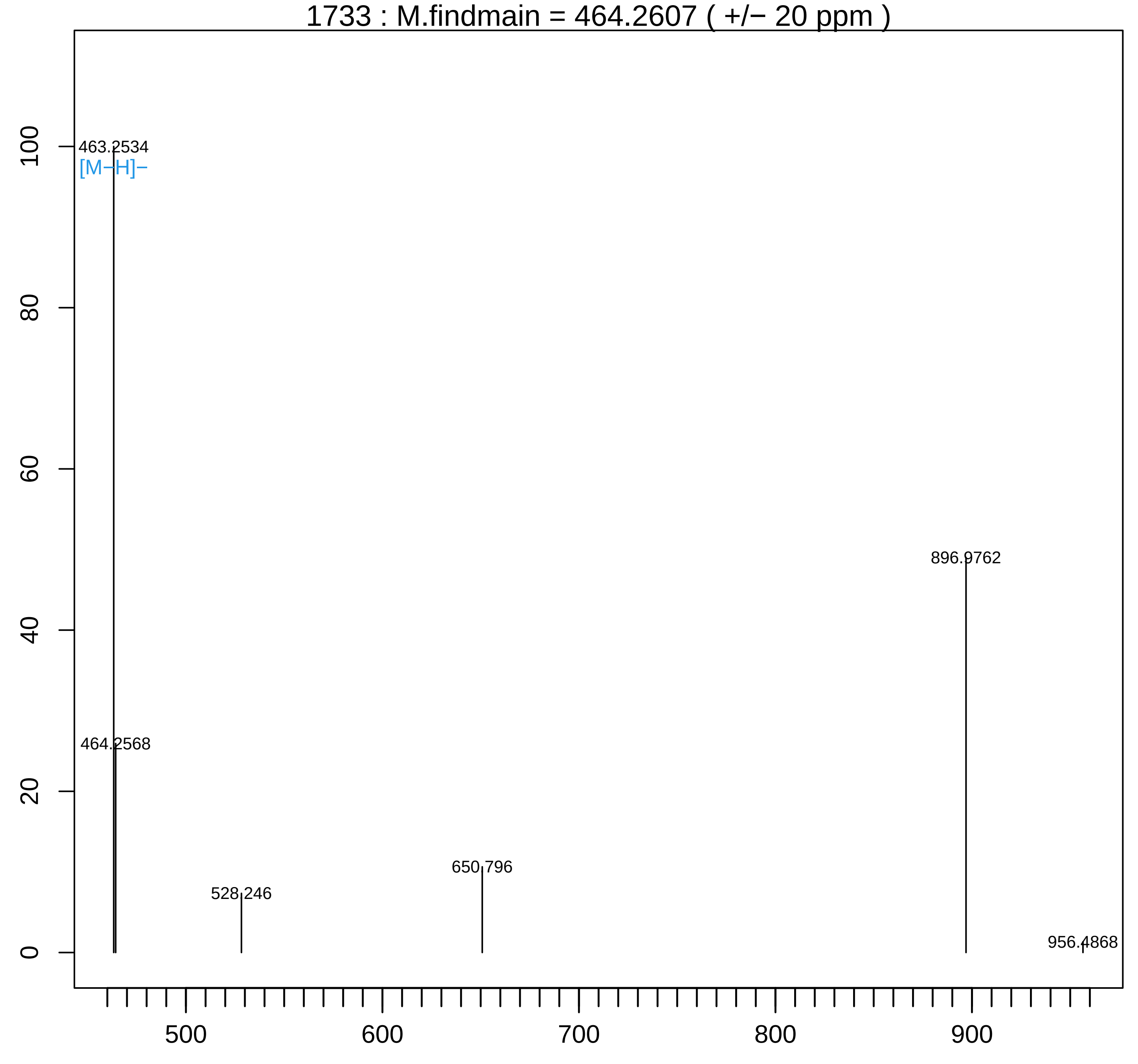

1733 : M.findmain = 464.2607 (+/− 20 ppm)
463.2534
[M−H]−
464.2568
528.246
650.796
896.9762
956.4868
0
20
40
60
80
100
500
600
700
800
900

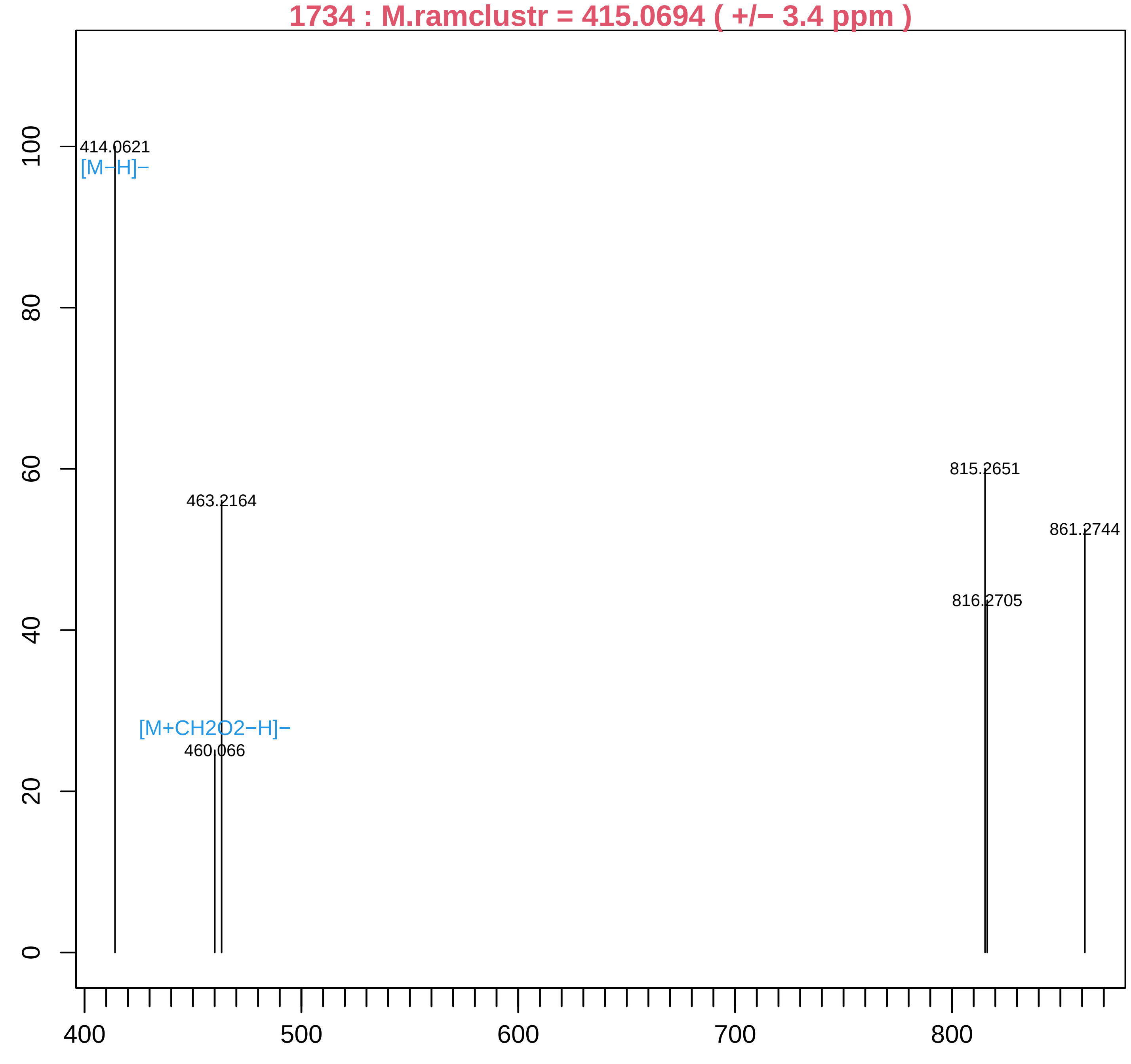
1734 : M.ramclustr = 415.0694 (+/− 3.4 ppm)
414.0621
[M−H]−
463.2164
[M+CH2O2−H]−
460.066
815.2651
816.2705
861.2744
0
20
40
60
80
100
400
500
600
700
800

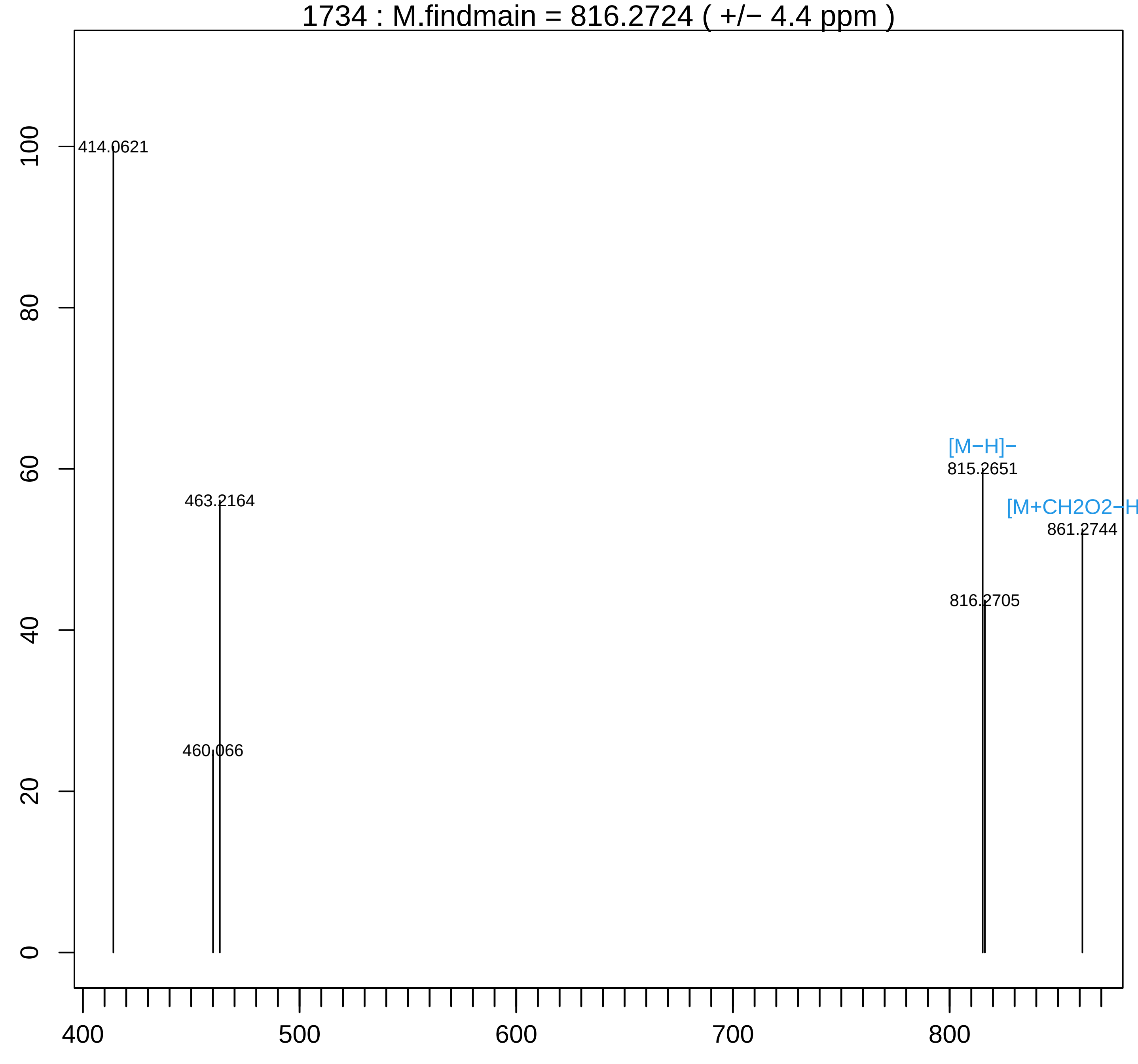
1734 : M.findmain = 816.2724 (+/− 4.4 ppm)
414.0621
463.2164
460.066
[M−H]−
815.2651
816.2705
861.2744
0
20
40
60
80
100
400
500
600
700
800

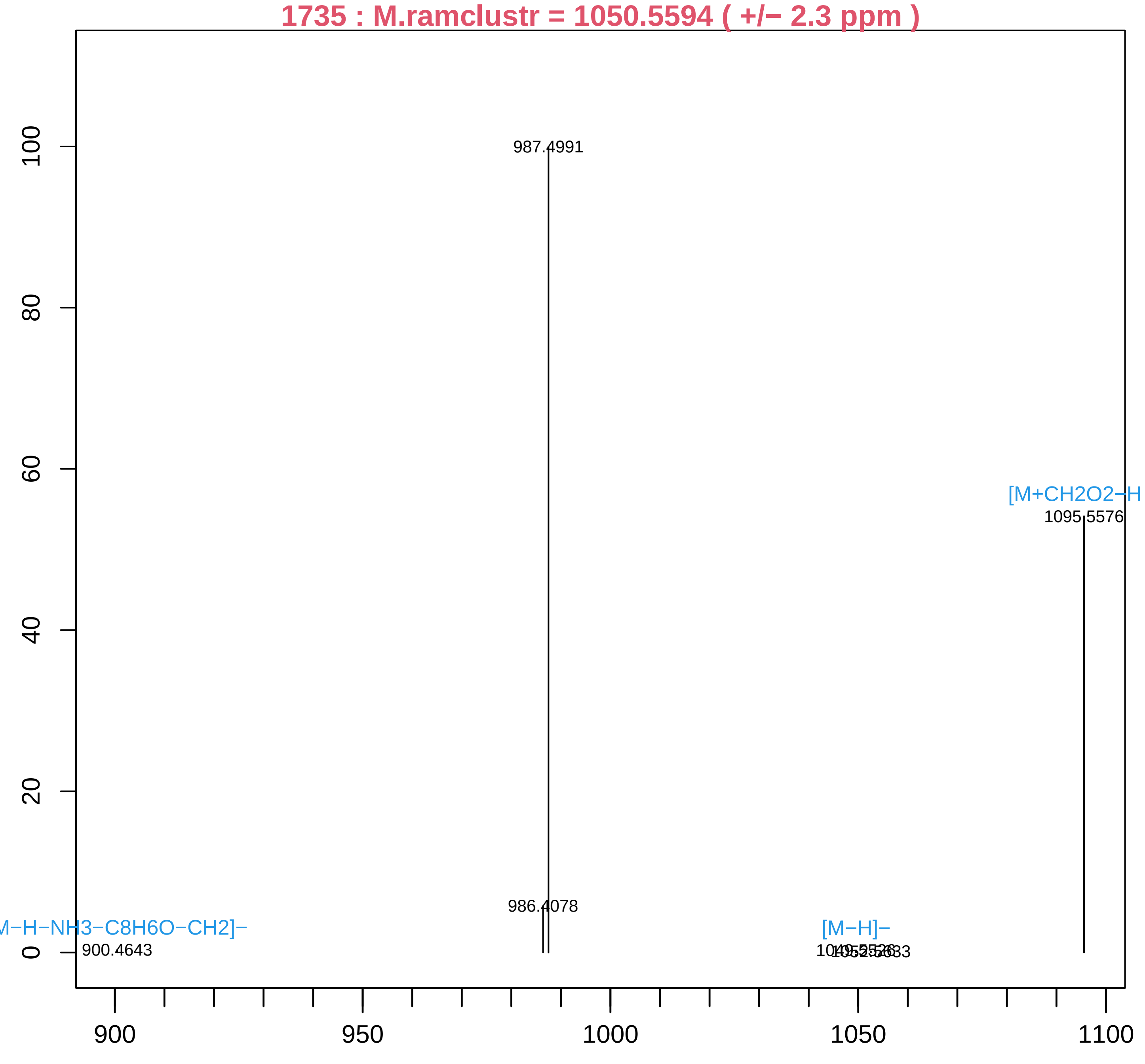
1735 : M.ramclustr = 1050.5594 (+/− 2.3 ppm)
987.4991
[M+CH2O2−H
1095.5576
986.4078
M−H−NH3−C8H6O−CH2]−
900.4643
[M−H]−
0
20
40
60
80
100
900
950
1000
1050
1100

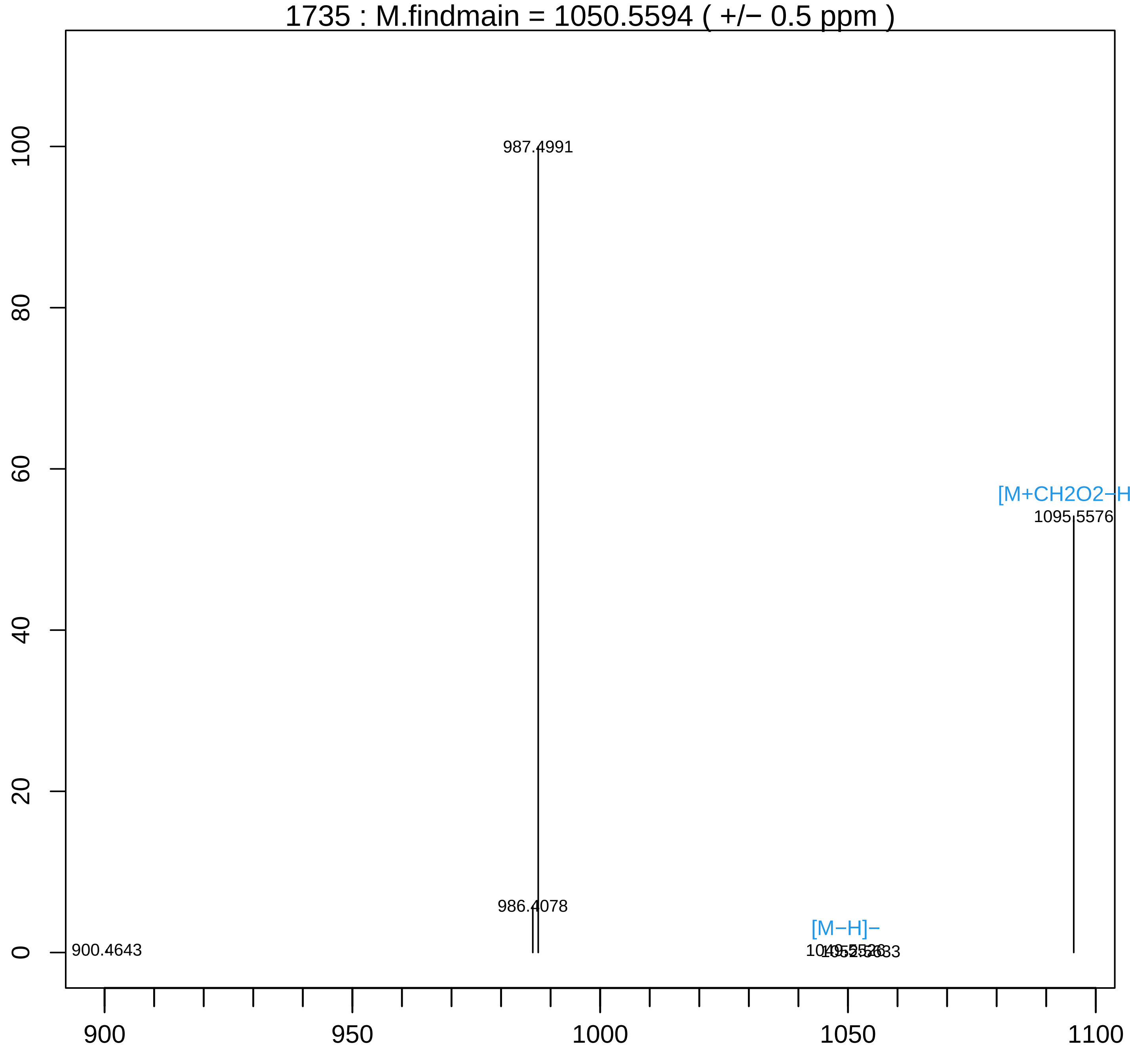
1735 : M.findmain = 1050.5594 (+/− 0.5 ppm)
987.4991
[M+CH2O2−H
1095.5576
986.4078
900.4643
[M−H]−
0
20
40
60
80
100
900
950
1000
1050
1100

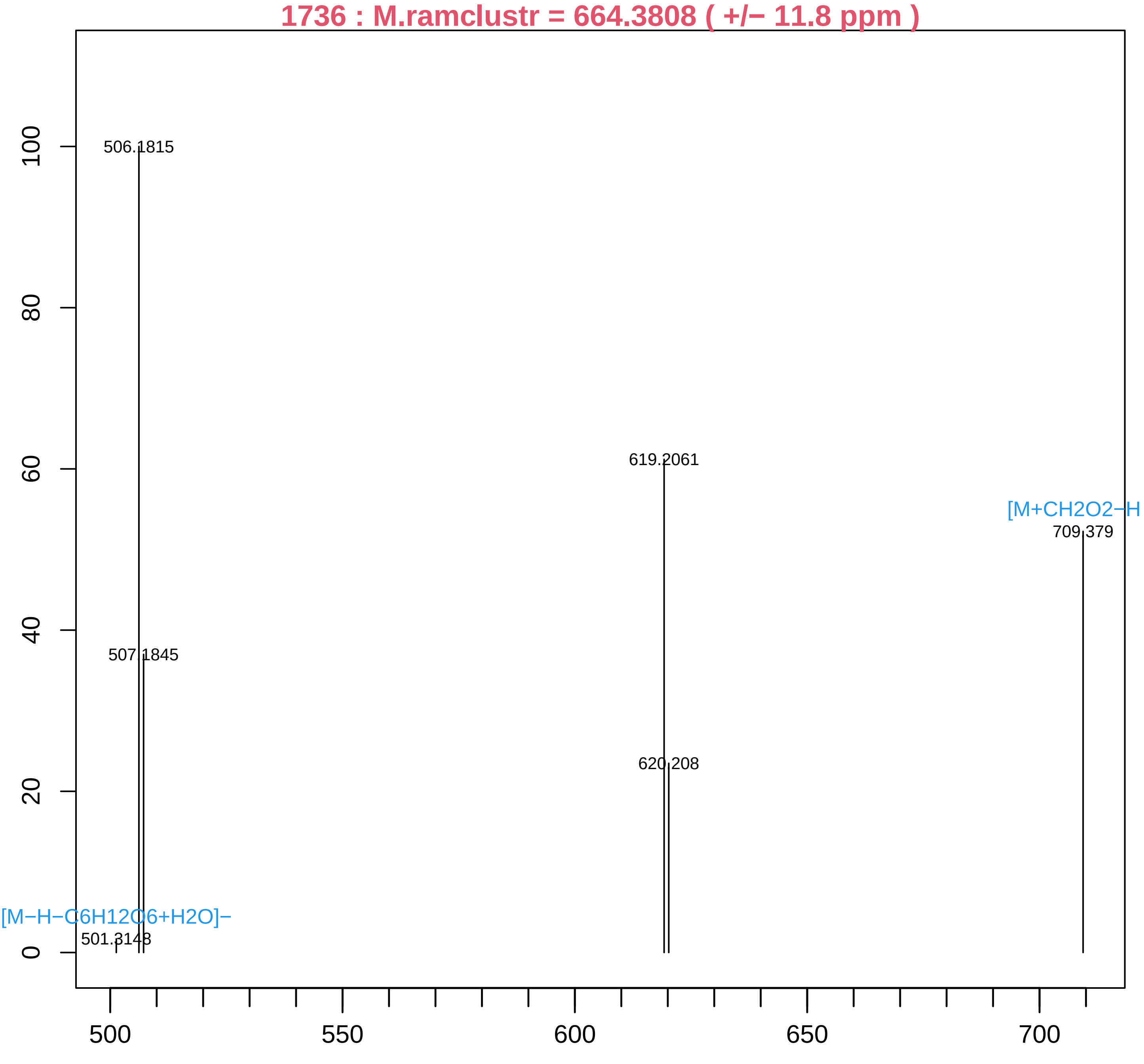
1736 : M.ramclustr = 664.3808 (+/− 11.8 ppm)
506.1815
619.2061
[M+CH2O2−H
709.379
507.1845
620.208
[M−H−C6H12O6+H2O]−
501.3148
0
20
40
60
80
100
500
550
600
650
700
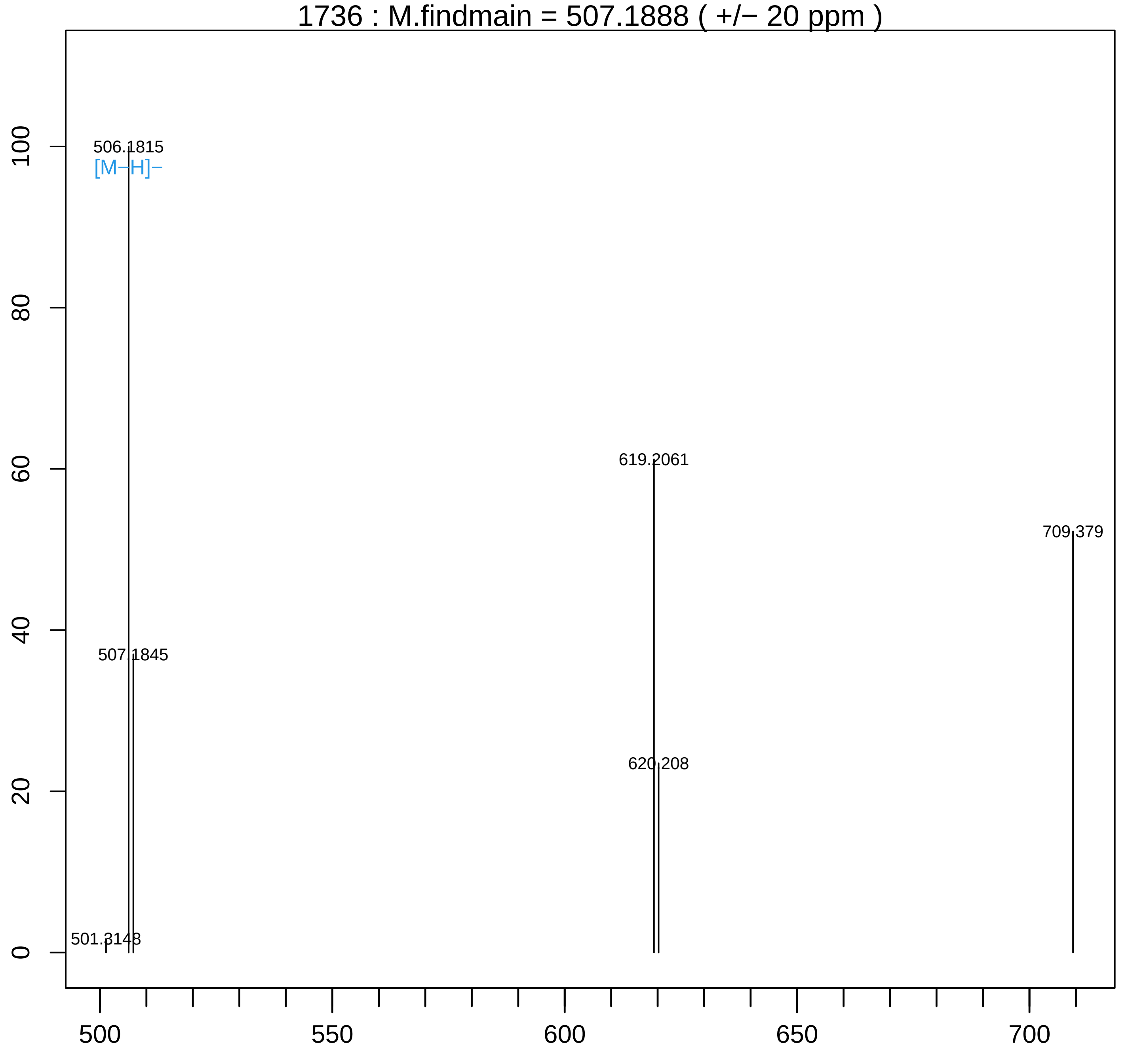
1736 : M.findmain = 507.1888 (+/− 20 ppm)
506.1815
[M−H]−
619.2061
709.379
507.1845
620.208
501.3148
0
20
40
60
80
100
500
550
600
650
700

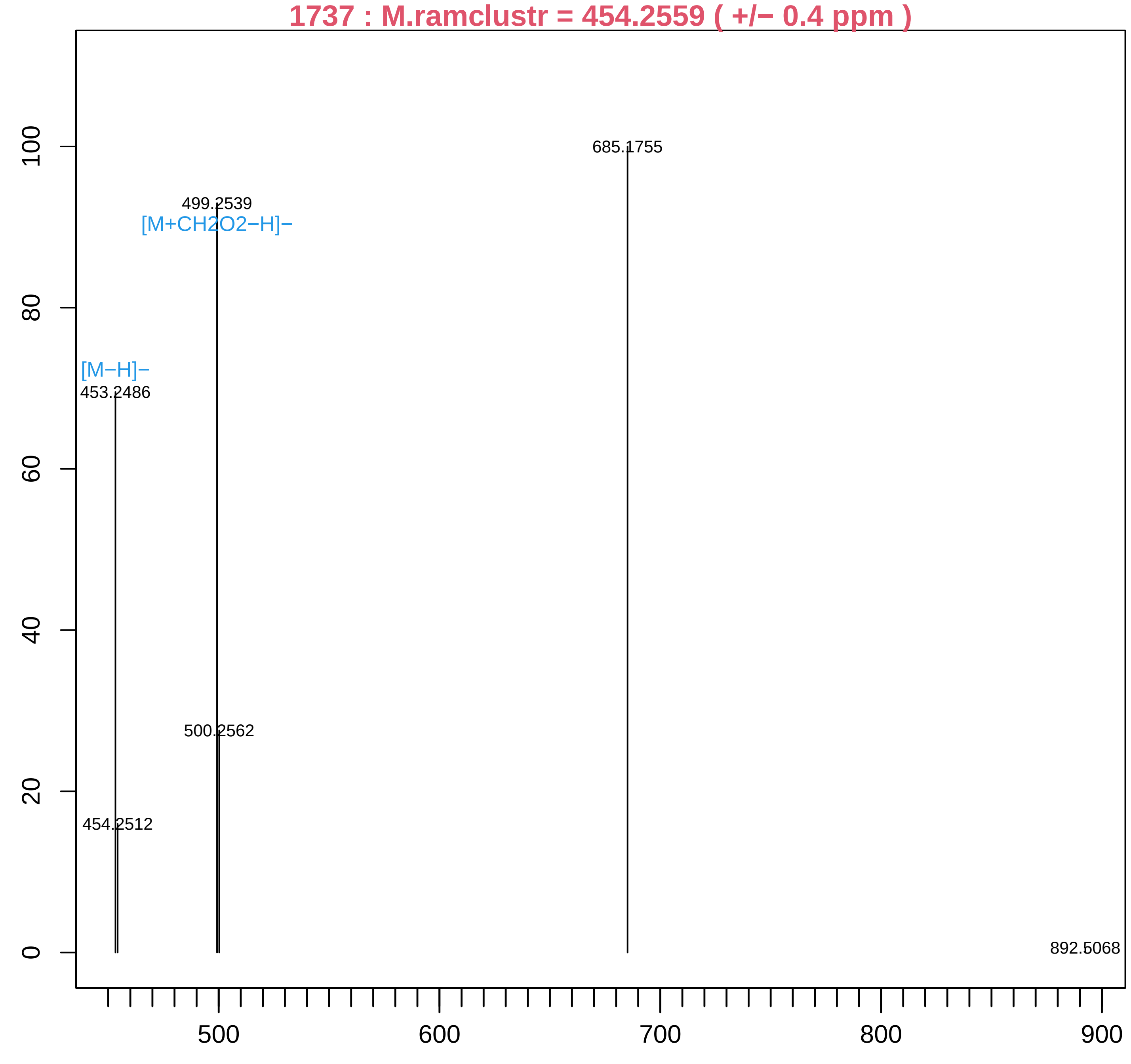
1737 : M.ramclustr = 454.2559 (+/− 0.4 ppm)
685.1755
499.2539
[M+CH2O2−H]−
[M−H]−
453.2486
500.2562
454.2512
892.5068
0
20
40
60
80
100
500
600
700
800
900

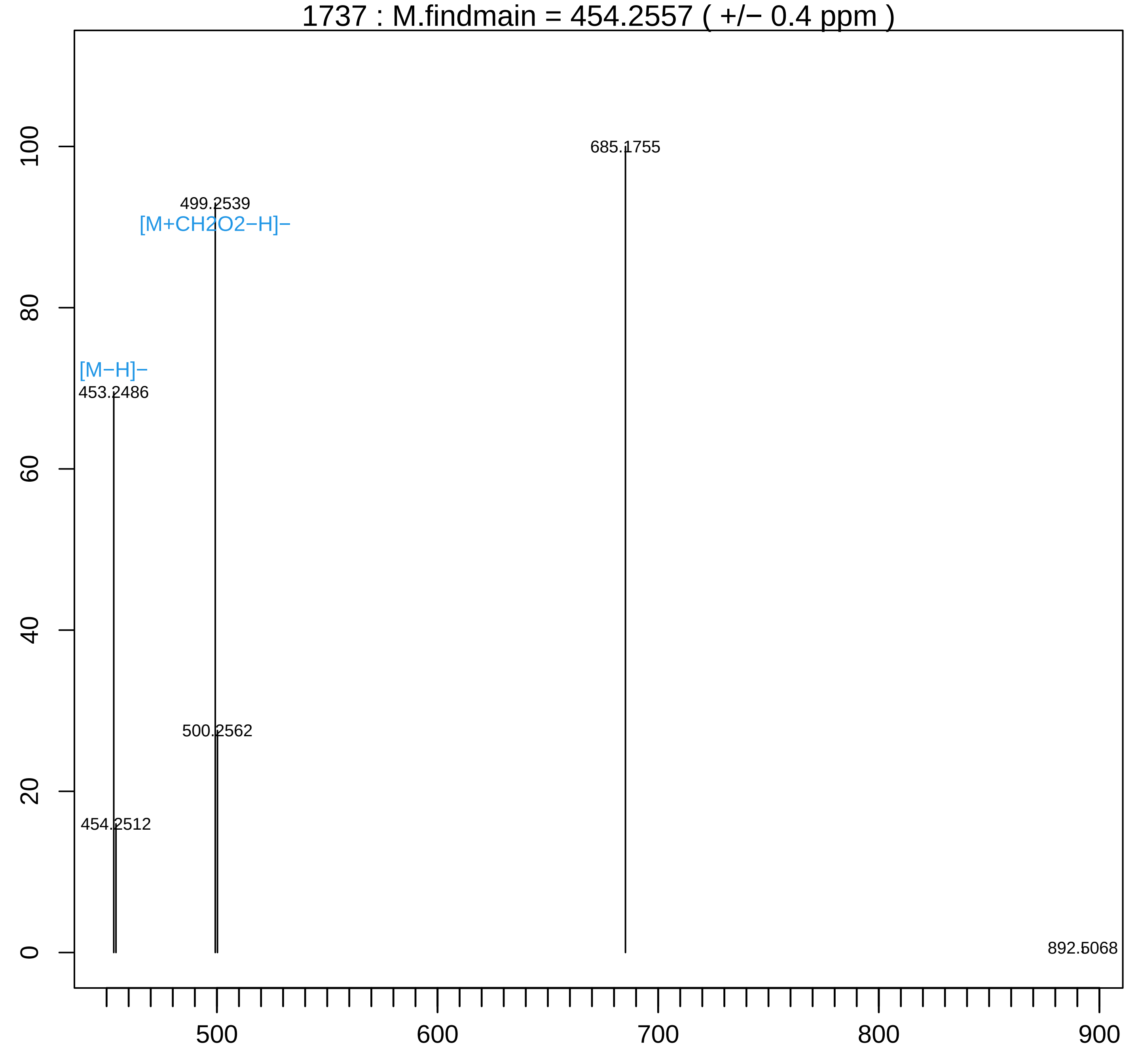
1737 : M.findmain = 454.2557 (+/− 0.4 ppm)
685.1755
499.2539
[M+CH2O2−H]−
[M−H]−
453.2486
500.2562
454.2512
892.5068
0
20
40
60
80
100
500
600
700
800
900

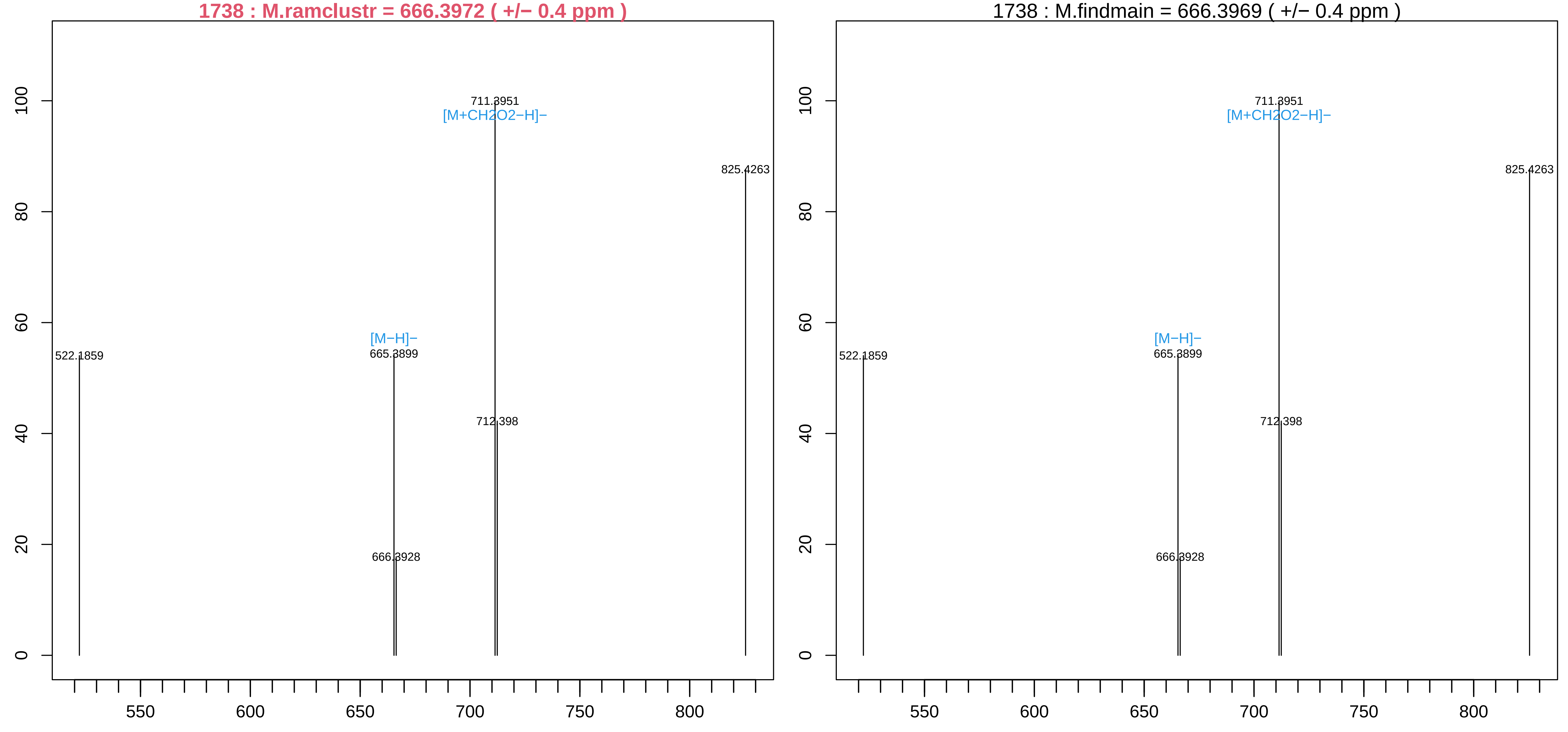
1738 : M.ramclustr = 666.3972 (+/− 0.4 ppm)
1738 : M.findmain = 666.3969 (+/− 0.4 ppm)
711.3951
[M+CH2O2−H]−
825.4263
[M−H]−
665.3899
522.1859
712.398
666.3928
0
20
40
60
80
100
550
600
650
700
750
800

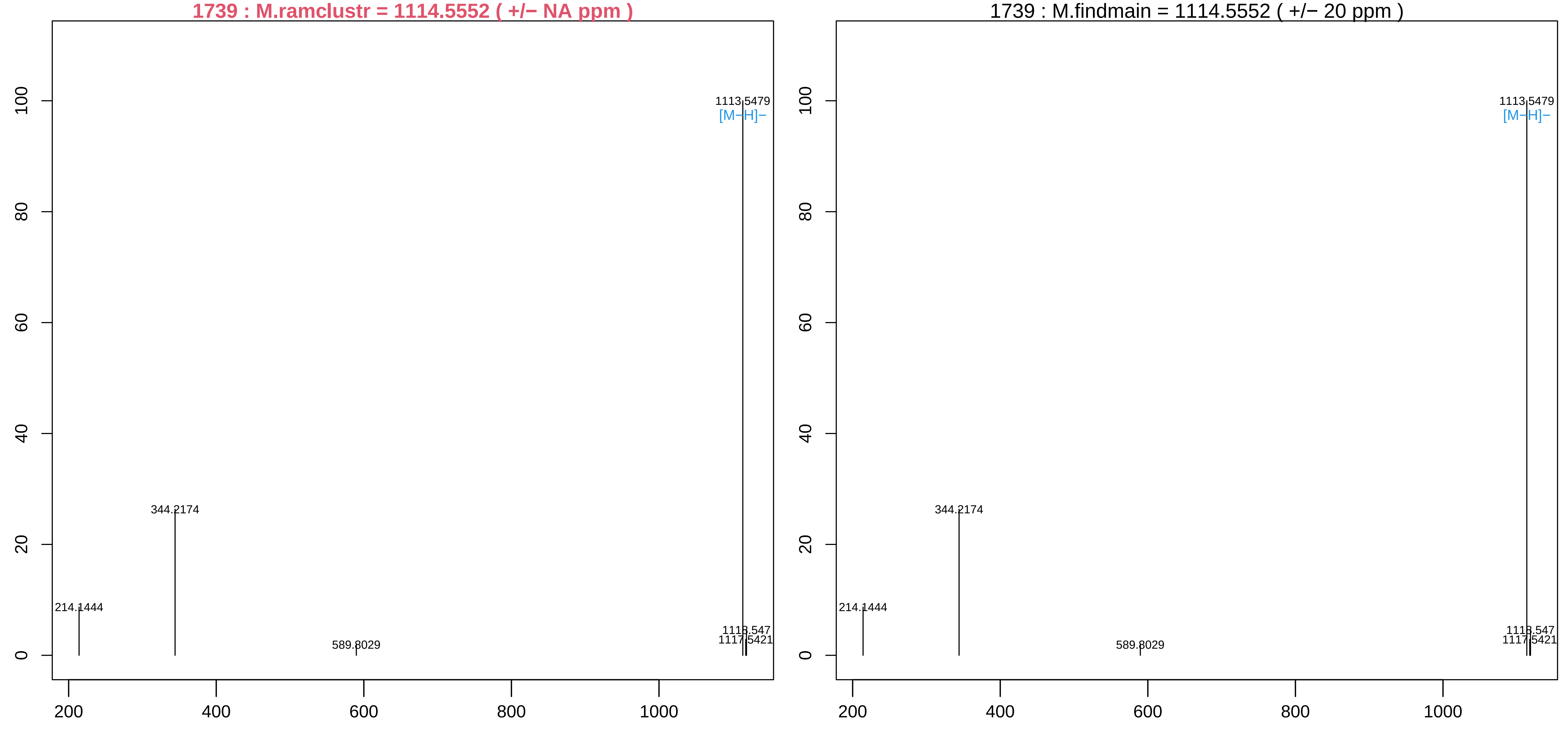
1739 : M.ramclustr = 1114.5552 (+/− NA ppm)
1113.5479
[M−H]−
344.2174
214.1444
589.8029
1118.547
1117.5421
1739 : M.findmain = 1114.5552 (+/− 20 ppm)
1113.5479
[M−H]−
344.2174
214.1444
589.8029
1118.547
1117.5421

1740 : M.ramclustr = 756.262 (+/− 1.9 ppm)

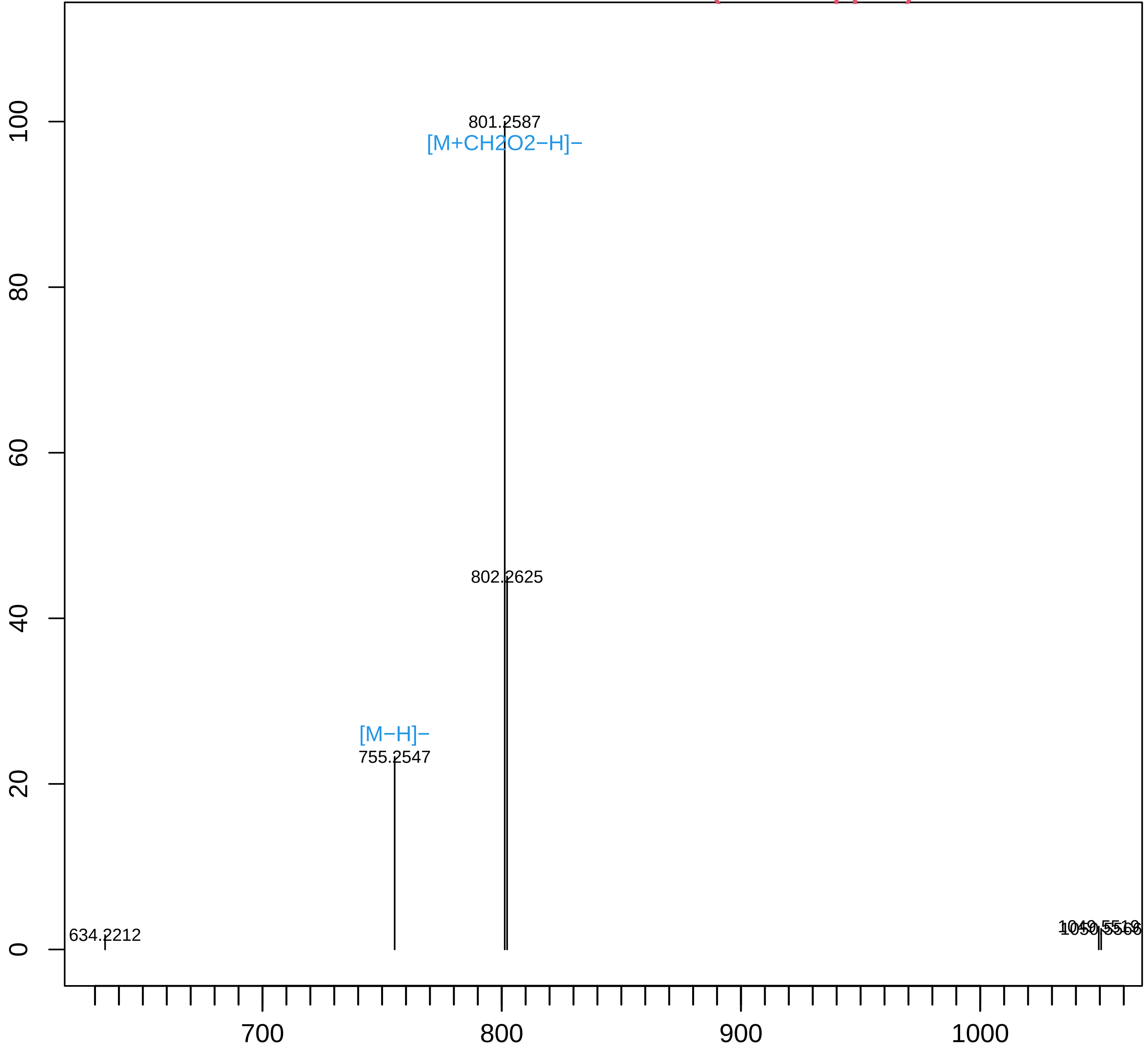

1740 : M.findmain = 756.2605 (+/− 2 ppm)

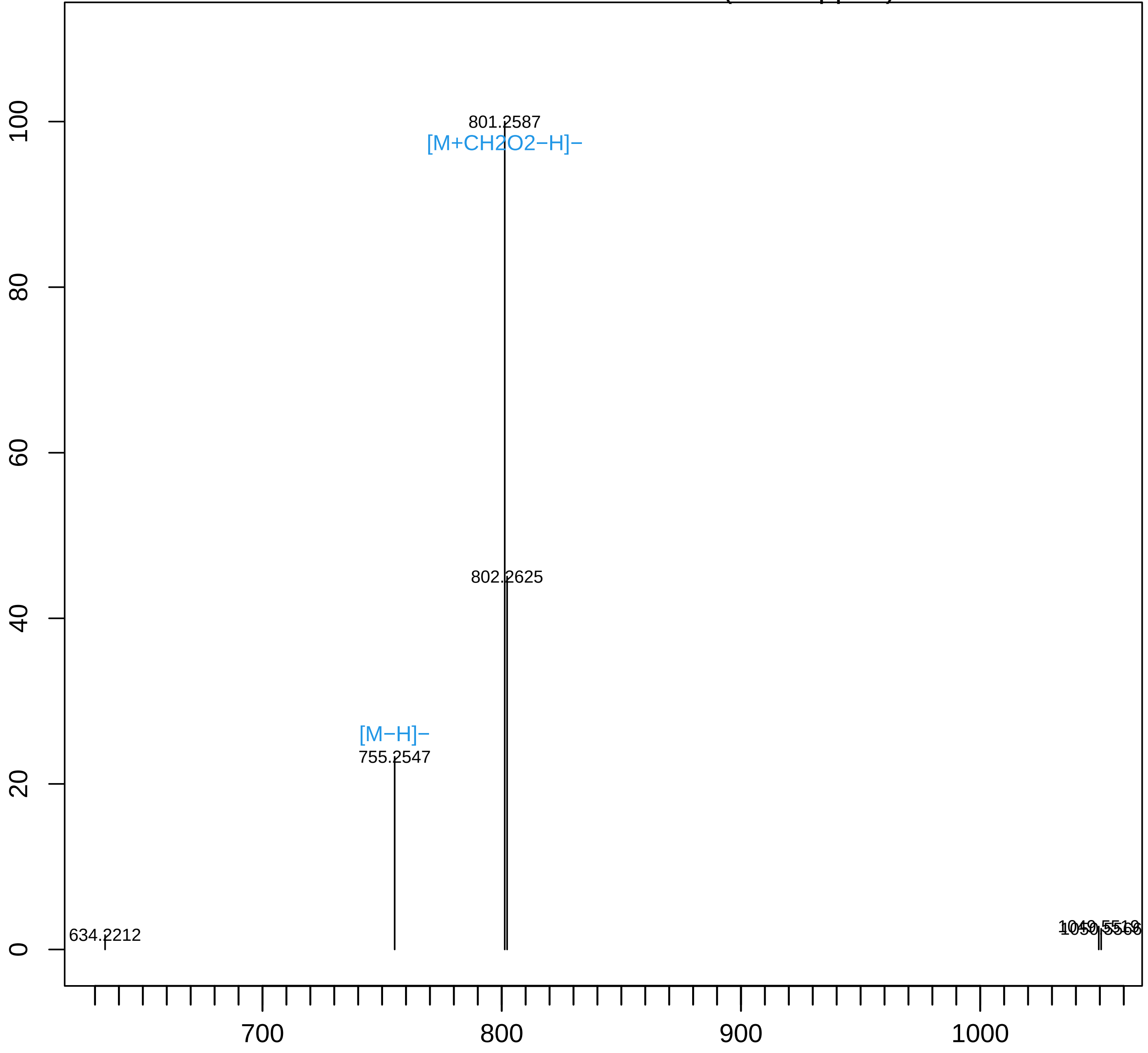

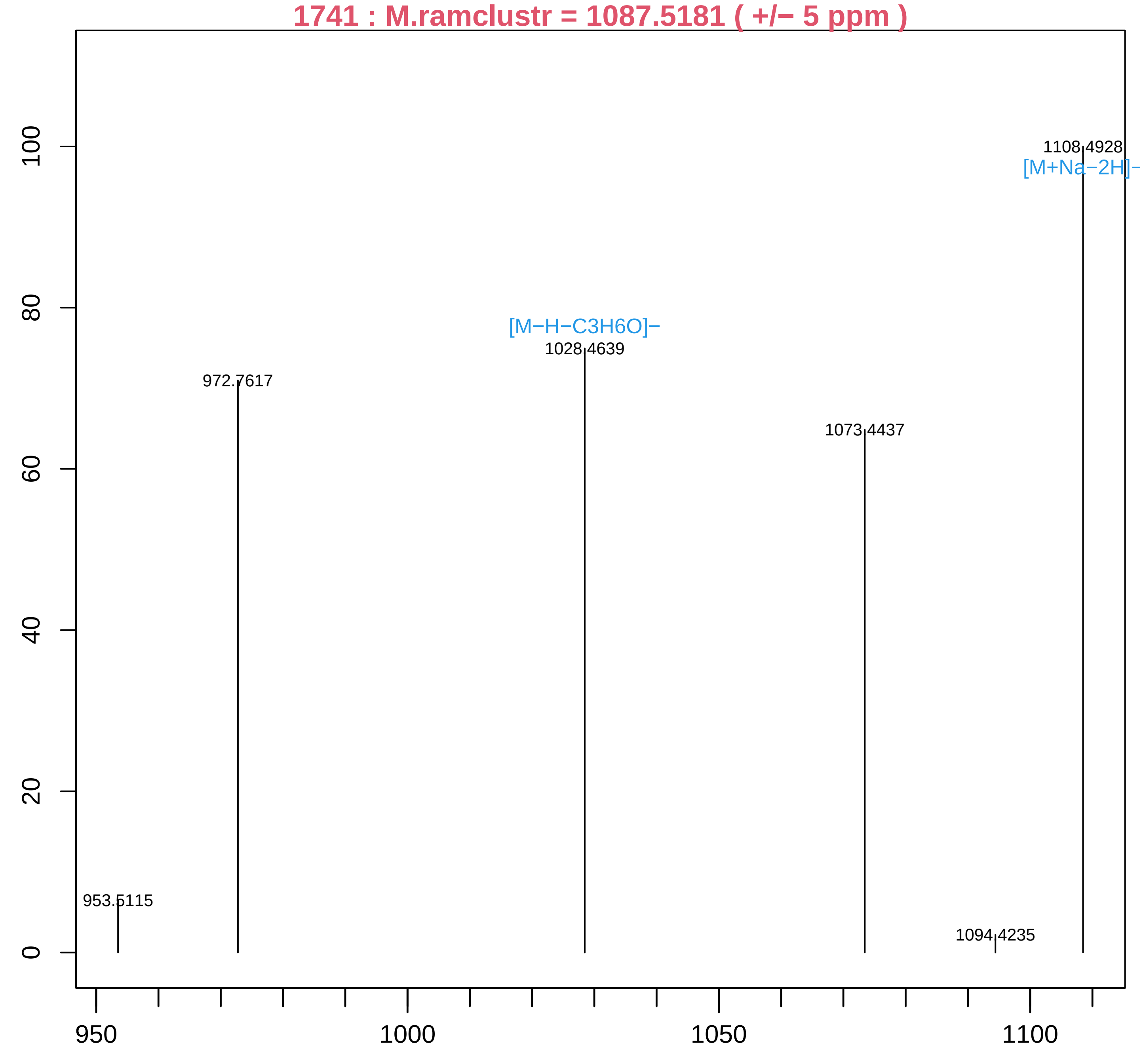
1741 : M.ramclustr = 1087.5181 (+/− 5 ppm)
1108.4928
[M+Na−2H]−
[M−H−C3H6O]−
1028.4639
972.7617
1073.4437
953.5115
1094.4235
0
20
40
60
80
100
950
1000
1050
1100

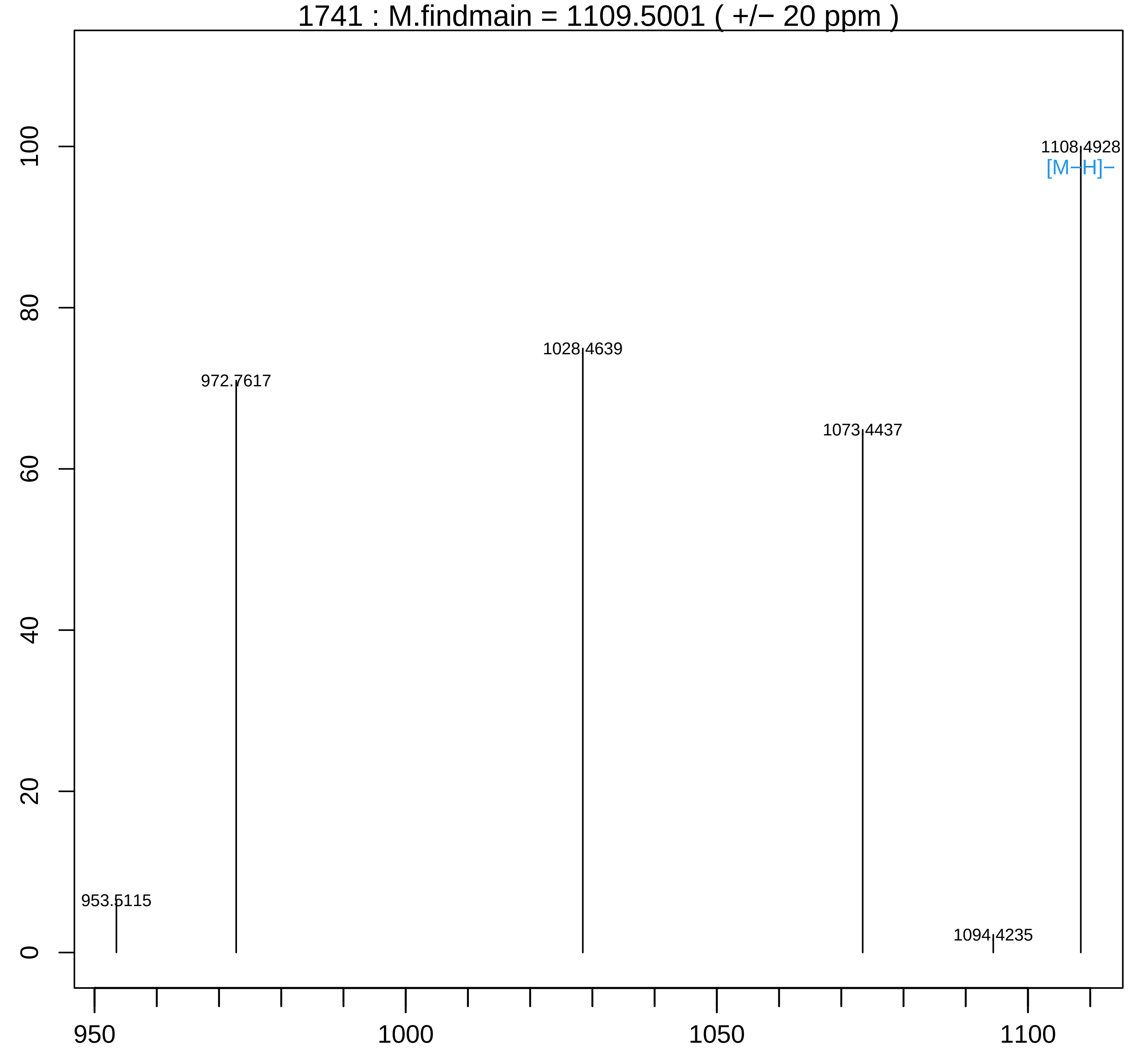
1741 : M.findmain = 1109.5001 (+/− 20 ppm)
1108.4928
[M−H]−
1028.4639
972.7617
1073.4437
953.5115
1094.4235
0
20
40
60
80
100
950
1000
1050
1100

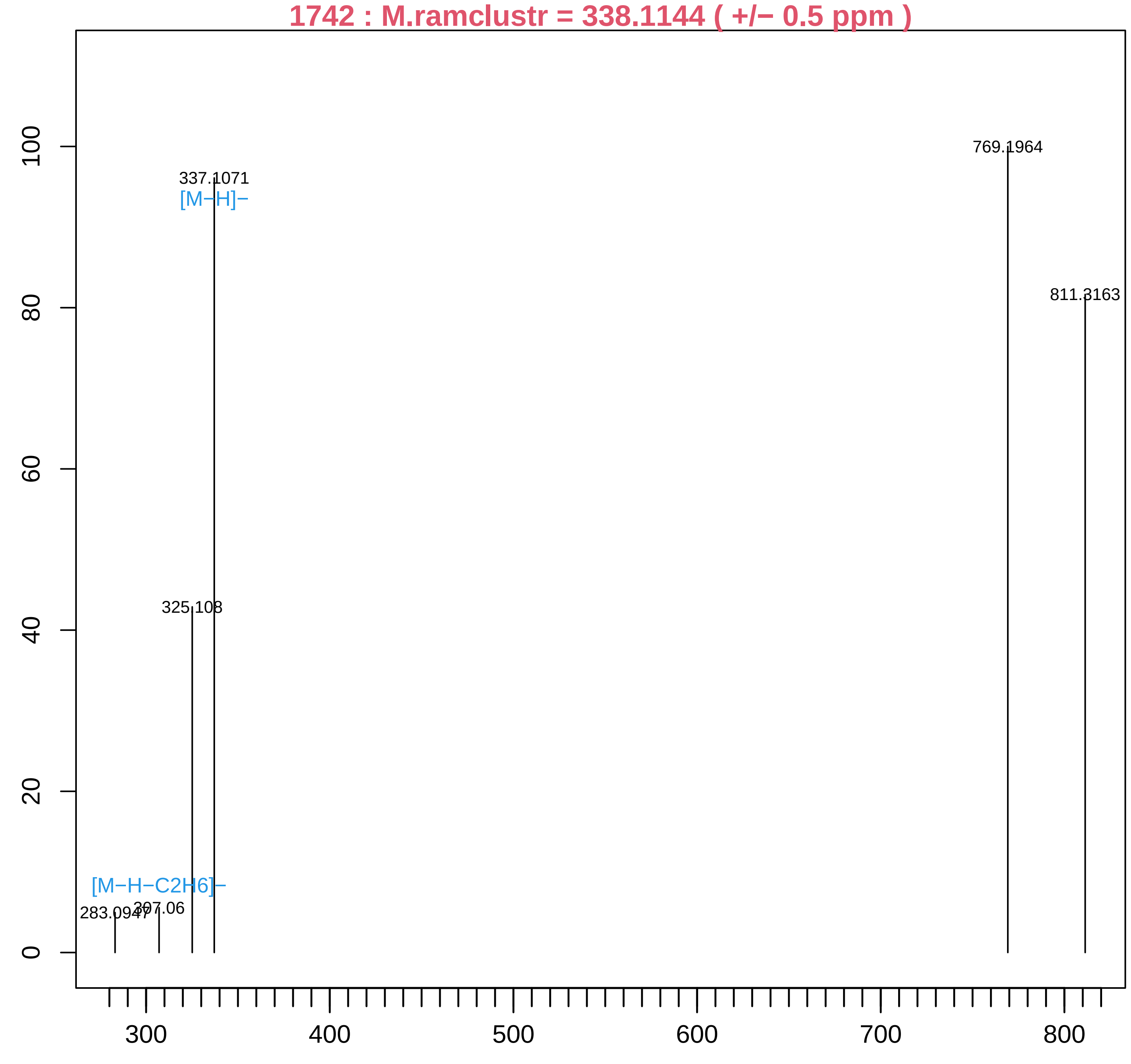
1742 : M.ramclustr = 338.1144 (+/− 0.5 ppm)
337.1071
[M−H]−
769.1964
811.3163
325.108
[M−H−C2H6]−
283.0947
307.06
0
20
40
60
80
100
300
400
500
600
700
800

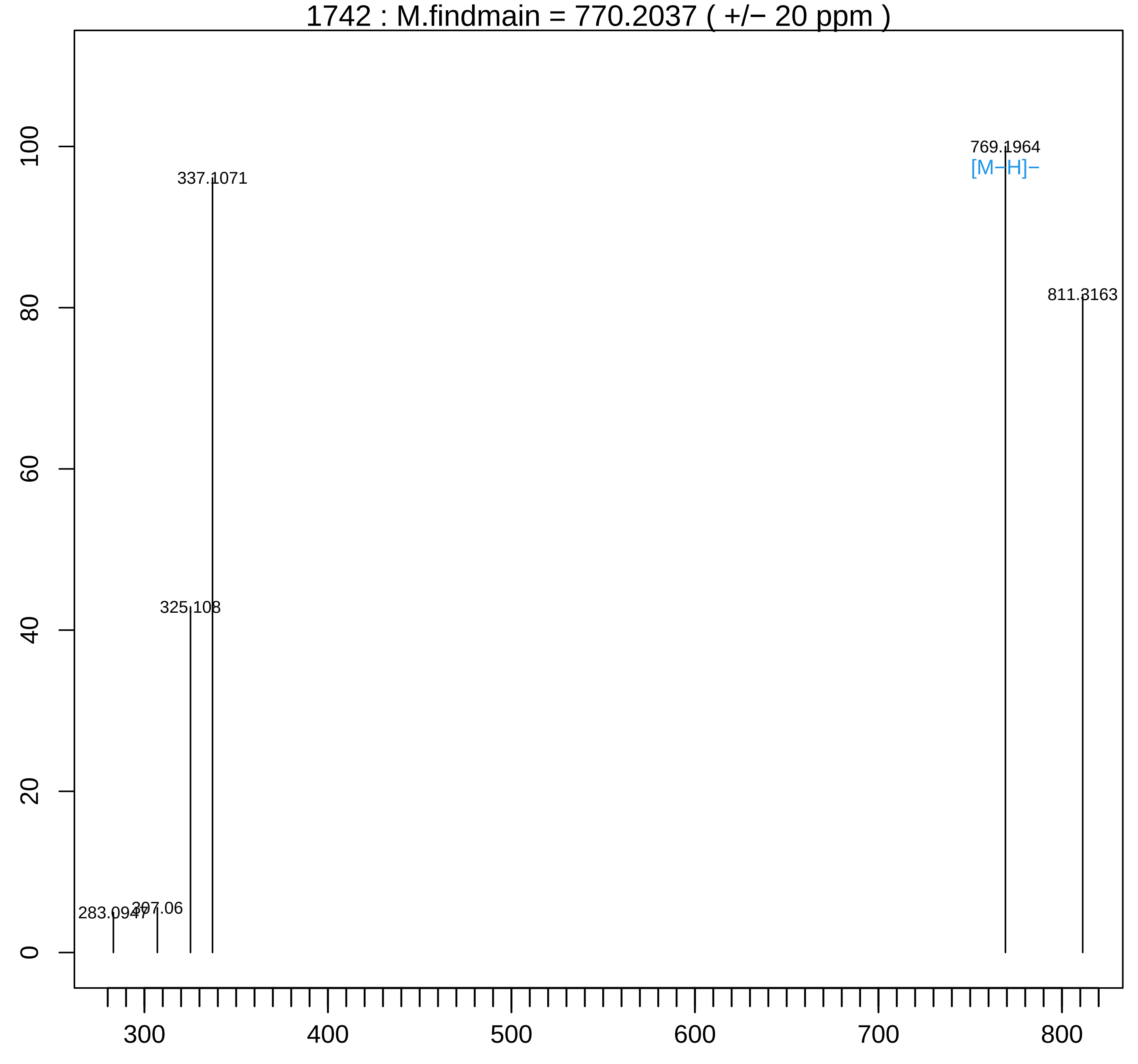
1742 : M.findmain = 770.2037 (+/− 20 ppm)
337.1071
769.1964
[M−H]−
811.3163
325.108
283.0947
307.06
0
20
40
60
80
100
300
400
500
600
700
800

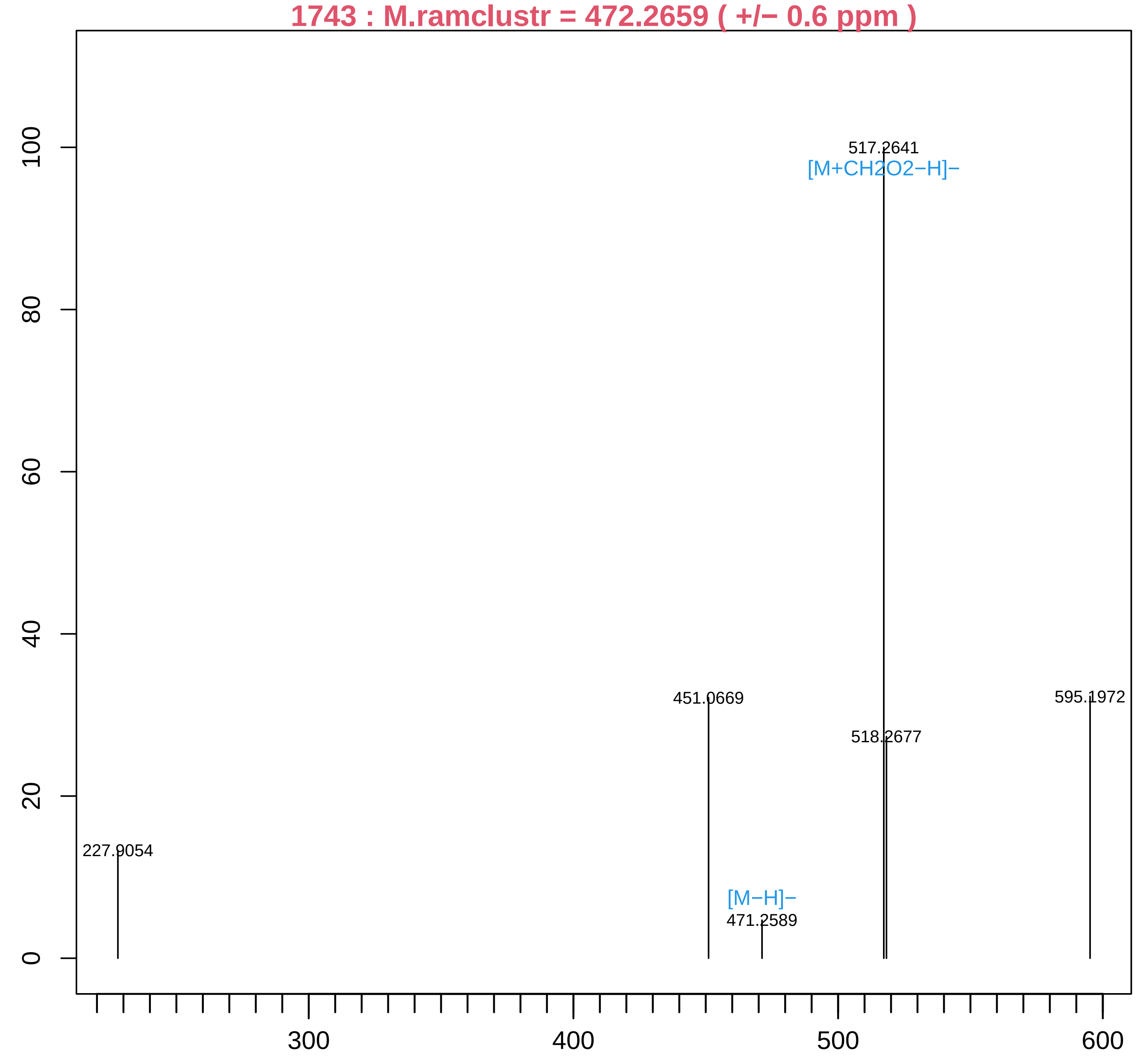
1743 : M.ramclustr = 472.2659 (+/− 0.6 ppm)
517.2641
[M+CH2O2−H]−
451.0669
595.1972
518.2677
227.9054
[M−H]−
471.2589
0
20
40
60
80
100
300
400
500
600

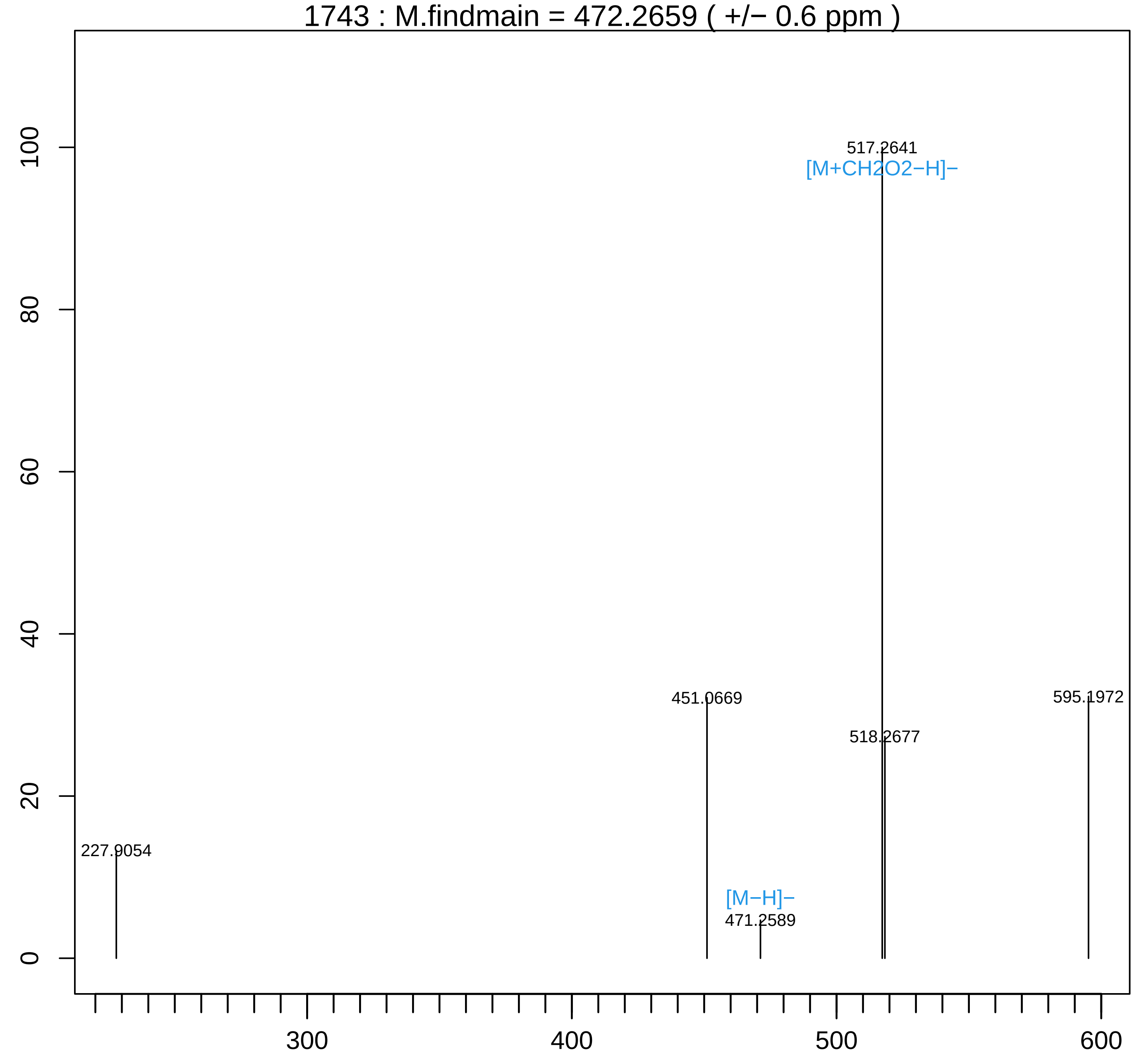
1743 : M.findmain = 472.2659 (+/− 0.6 ppm)
517.2641
[M+CH2O2−H]−
451.0669
595.1972
518.2677
227.9054
[M−H]−
471.2589
0
20
40
60
80
100
300
400
500
600

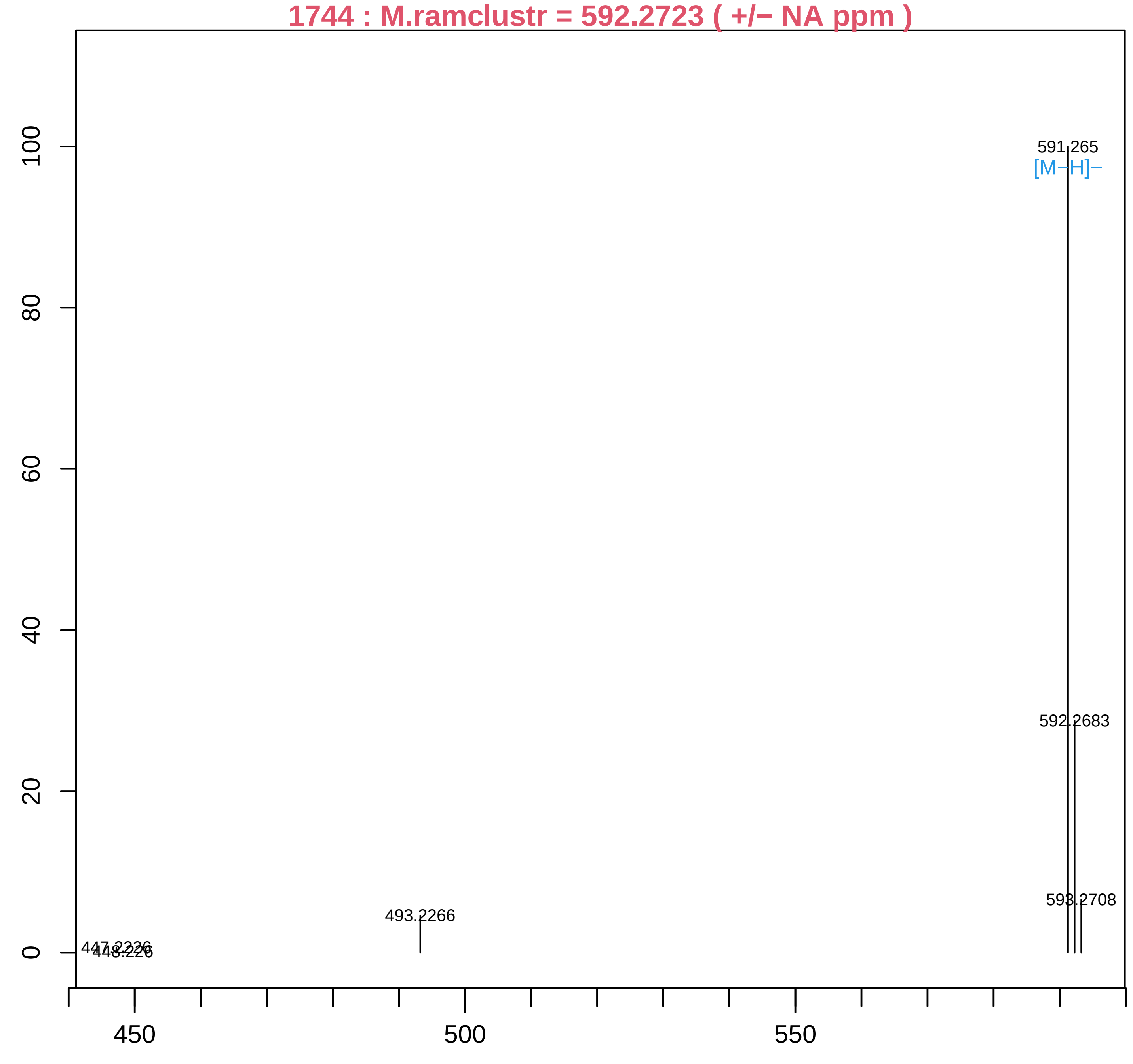
1744 : M.ramclustr = 592.2723 (+/− NA ppm)
591.265
[M−H]−
592.2683
593.2708
493.2266
447.2226
448.226
0
20
40
60
80
100
450
500
550

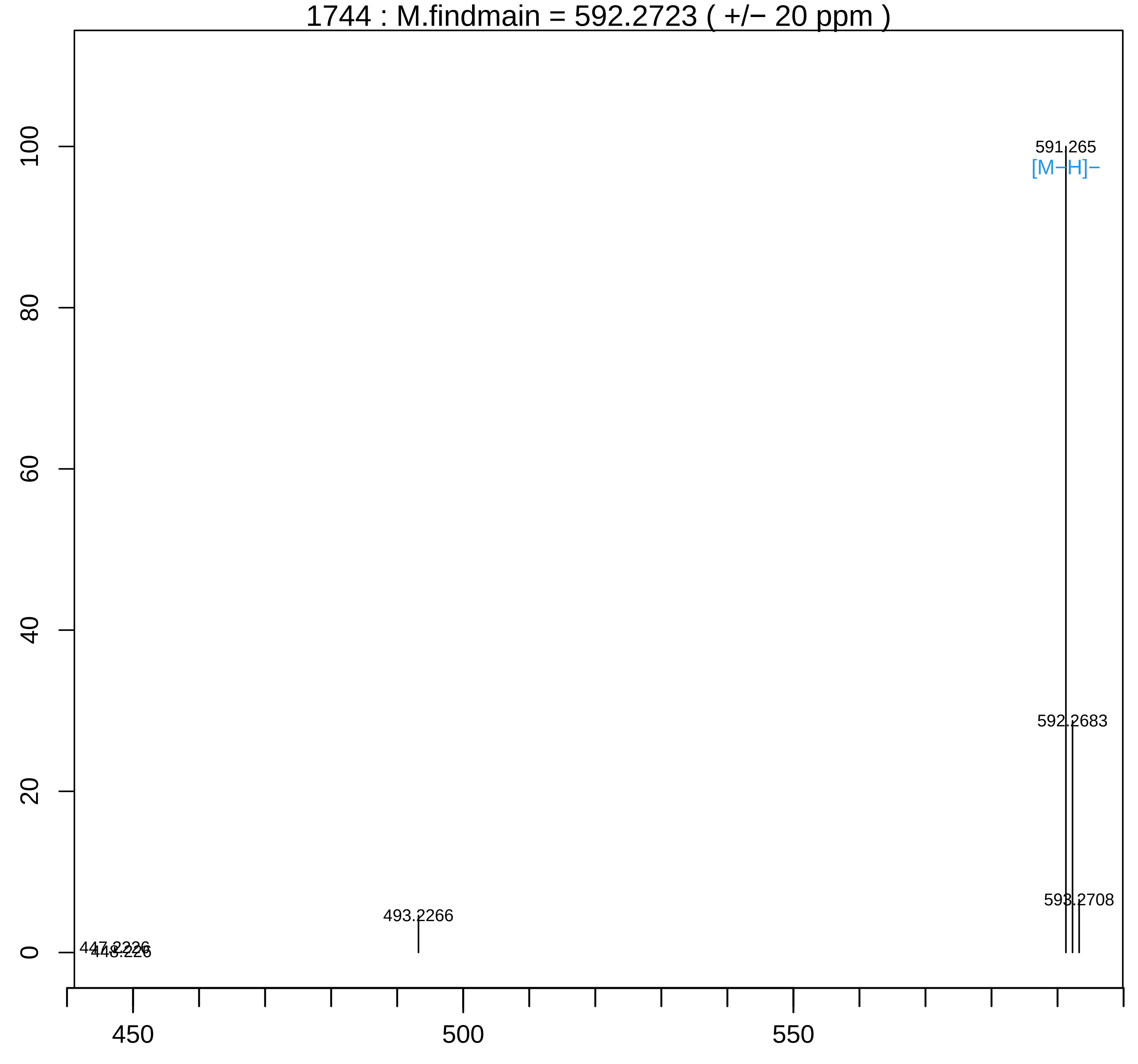
1744 : M.findmain = 592.2723 (+/− 20 ppm)
591.265
[M−H]−
592.2683
593.2708
493.2266
447.2226
448.226
0
20
40
60
80
100
450
500
550

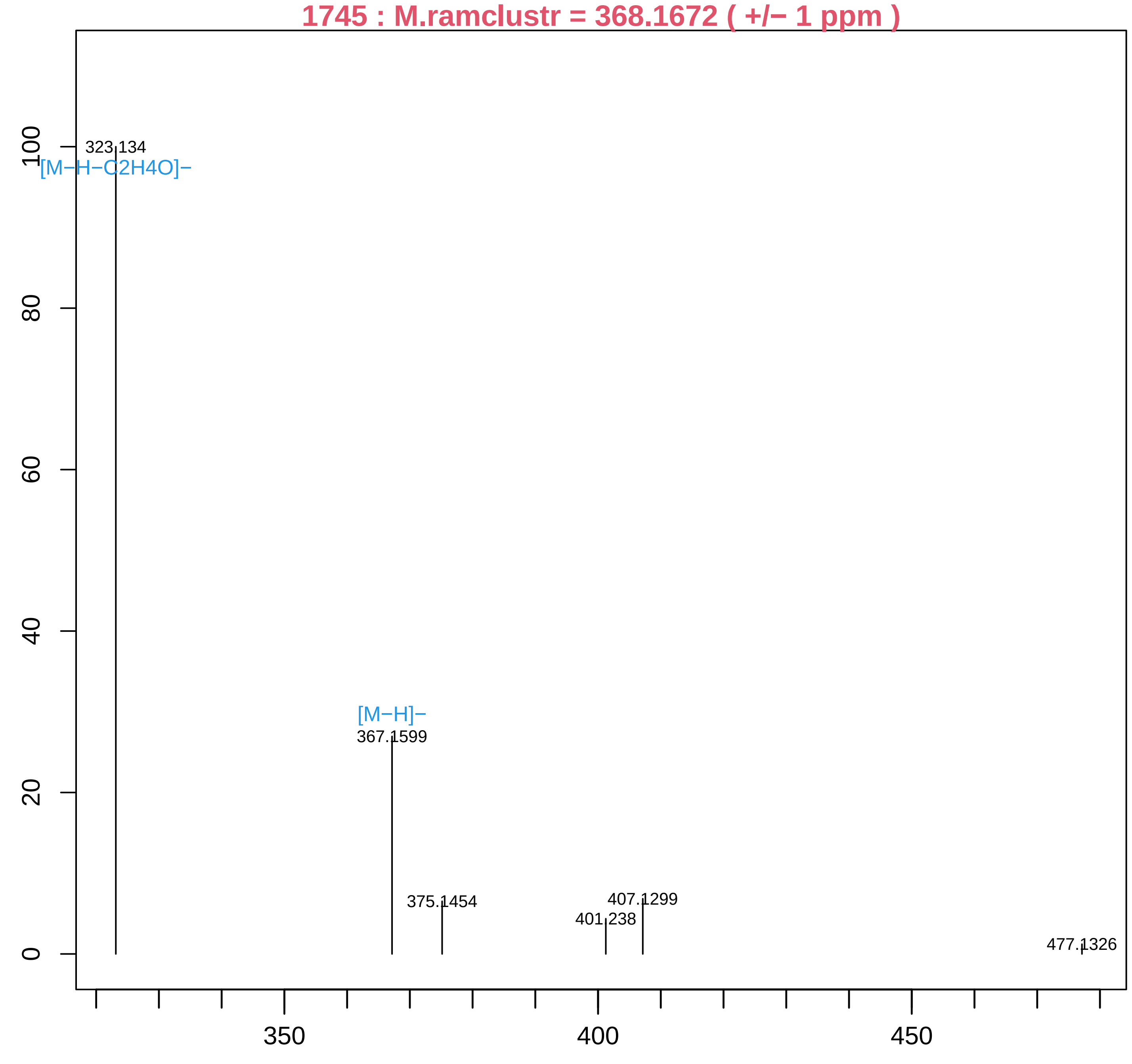
1745 : M.ramclustr = 368.1672 (+/− 1 ppm)
323.134
[M−H−C2H4O]−
[M−H]−
367.1599
375.1454
401.238
407.1299
477.1326
0
20
40
60
80
100
350
400
450

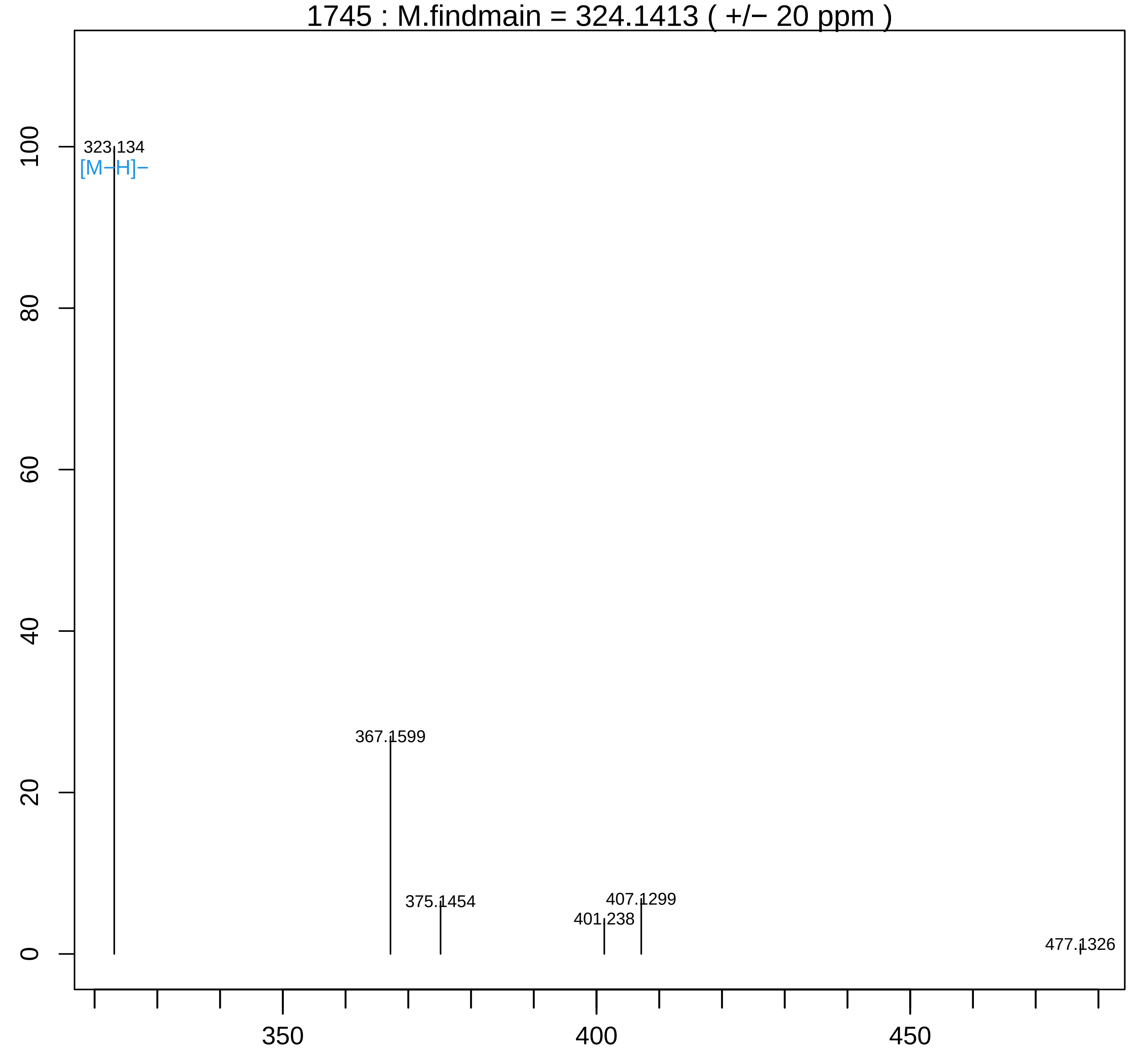
1745 : M.findmain = 324.1413 (+/− 20 ppm)
323.134
[M−H]−
367.1599
375.1454
401.238
407.1299
477.1326
0
20
40
60
80
100
350
400
450

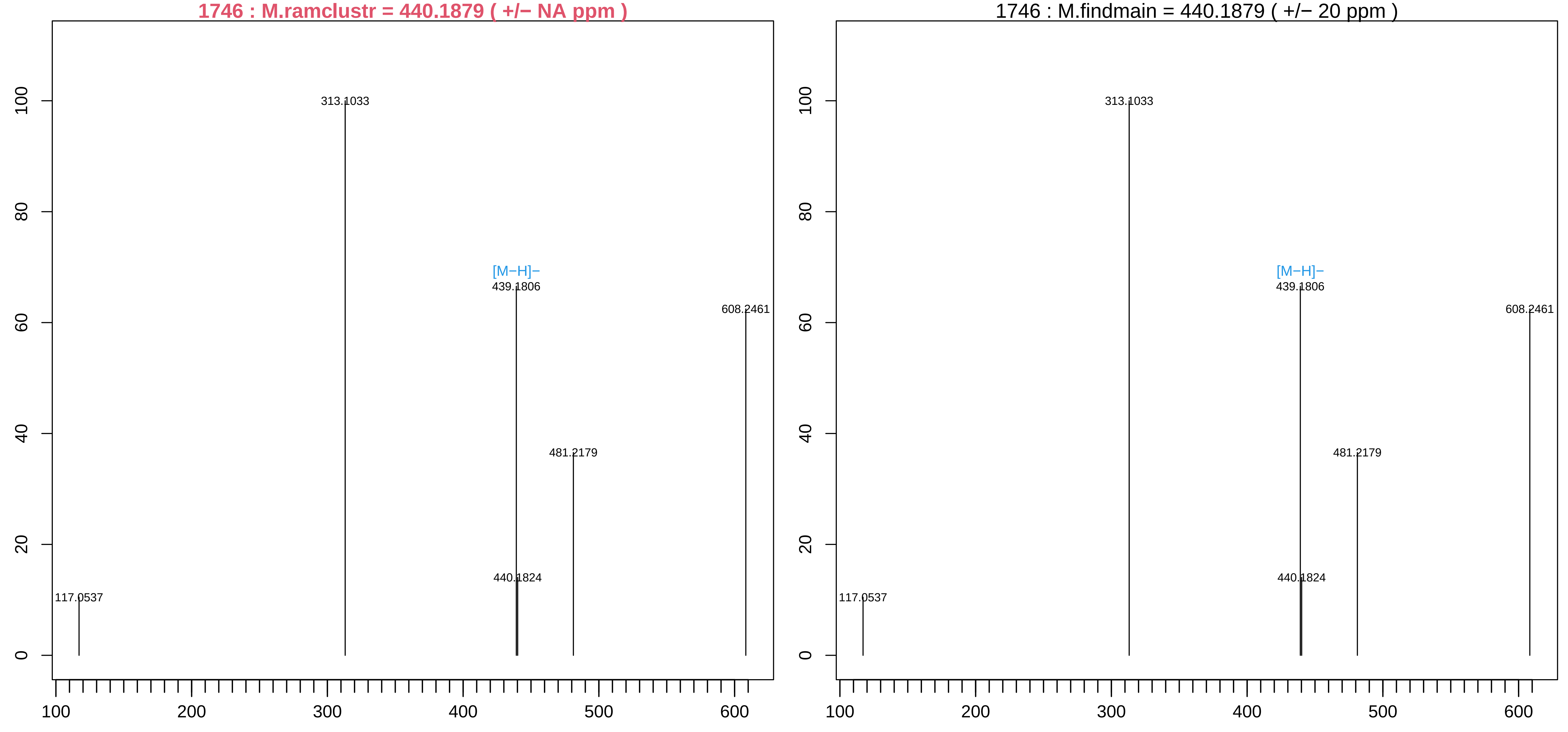

1746 : M.ramclustr = 440.1879 (+/− NA ppm)
313.1033
[M−H]−
439.1806
608.2461
481.2179
440.1824
117.0537
1746 : M.findmain = 440.1879 (+/− 20 ppm)
313.1033
[M−H]−
439.1806
608.2461
481.2179
440.1824
117.0537

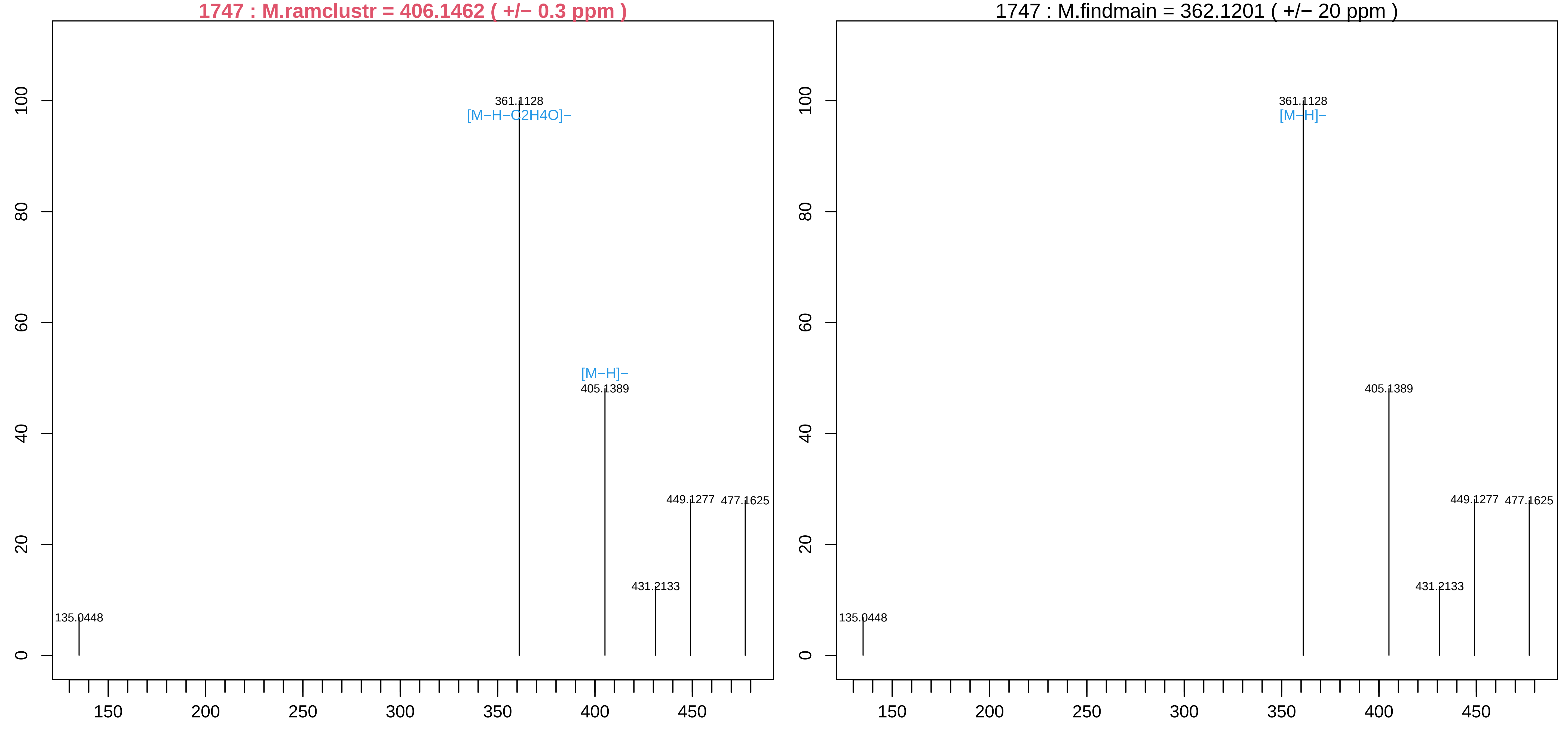
1747 : M.ramclustr = 406.1462 (+/− 0.3 ppm)
361.1128
[M−H−C2H4O]−
[M−H]−
405.1389
449.1277
477.1625
431.2133
135.0448
1747 : M.findmain = 362.1201 (+/− 20 ppm)
361.1128
[M−H]−
405.1389
449.1277
477.1625
431.2133
135.0448

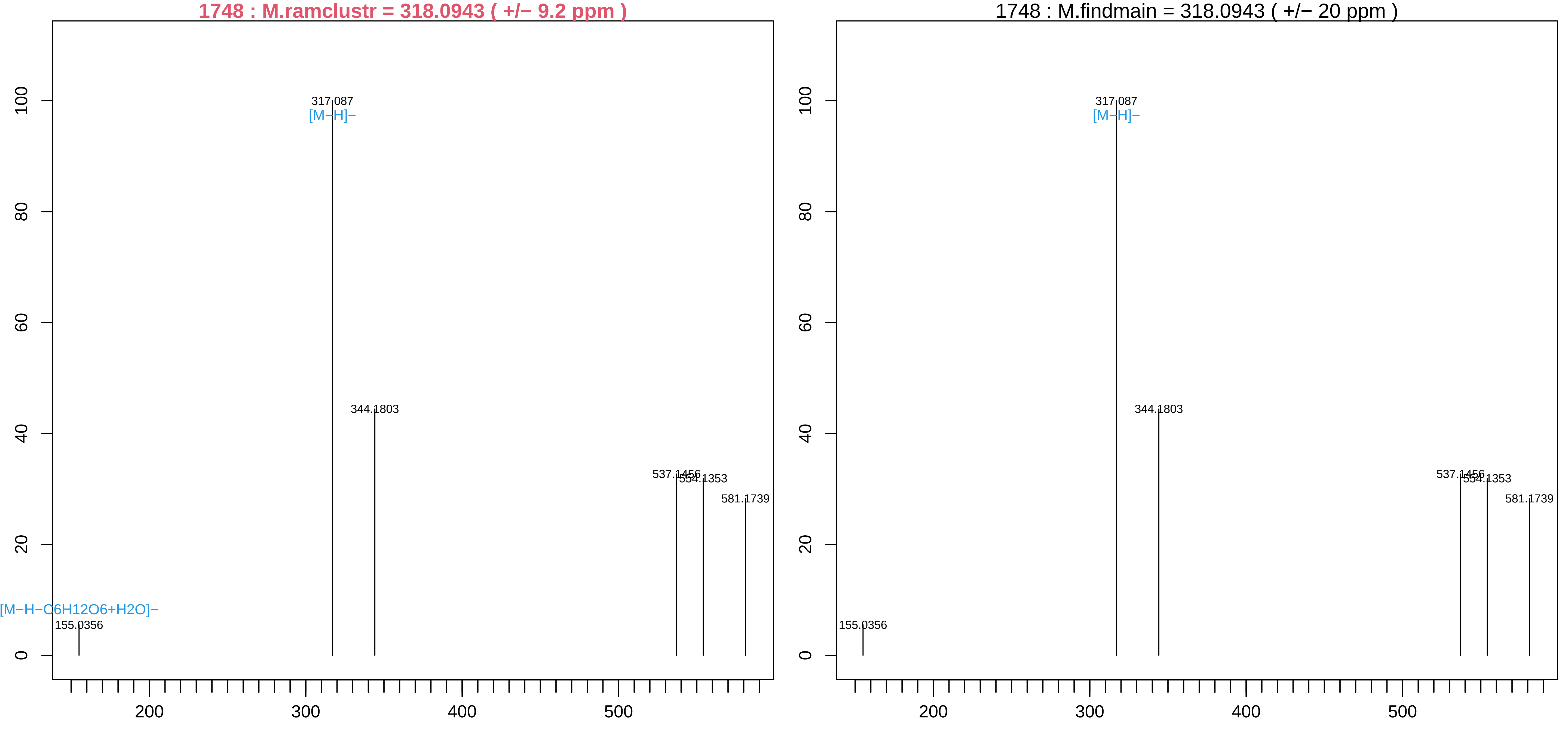
1748 : M.ramclustr = 318.0943 (+/− 9.2 ppm)
317.087
[M−H]−
344.1803
537.1456
554.1353
581.1739
[M−H−C6H12O6+H2O]−
155.0356
0
20
40
60
80
100
200
300
400
500
1748 : M.findmain = 318.0943 (+/− 20 ppm)
317.087
[M−H]−
344.1803
537.1456
554.1353
581.1739
155.0356
0
20
40
60
80
100
200
300
400
500

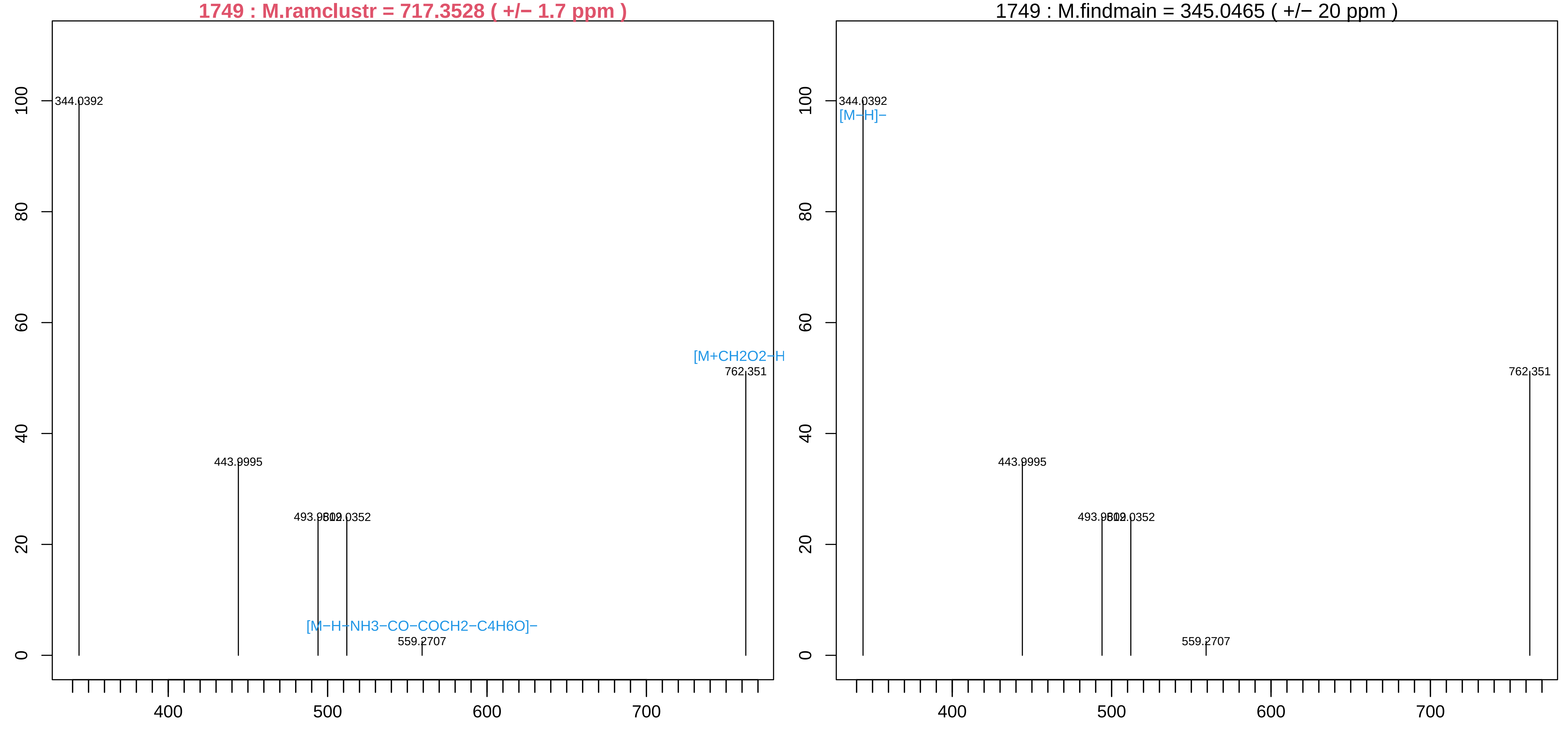

1749 : M.ramclustr = 717.3528 (+/− 1.7 ppm)
344.0392
443.9995
493.9802
512.0352
[M−H−NH3−CO−COCH2−C4H6O]−
559.2707
[M+CH2O2−H
762.351
1749 : M.findmain = 345.0465 (+/− 20 ppm)
344.0392
[M−H]−
443.9995
493.9802
512.0352
559.2707
762.351
0
20
40
60
80
100
400
500
600
700

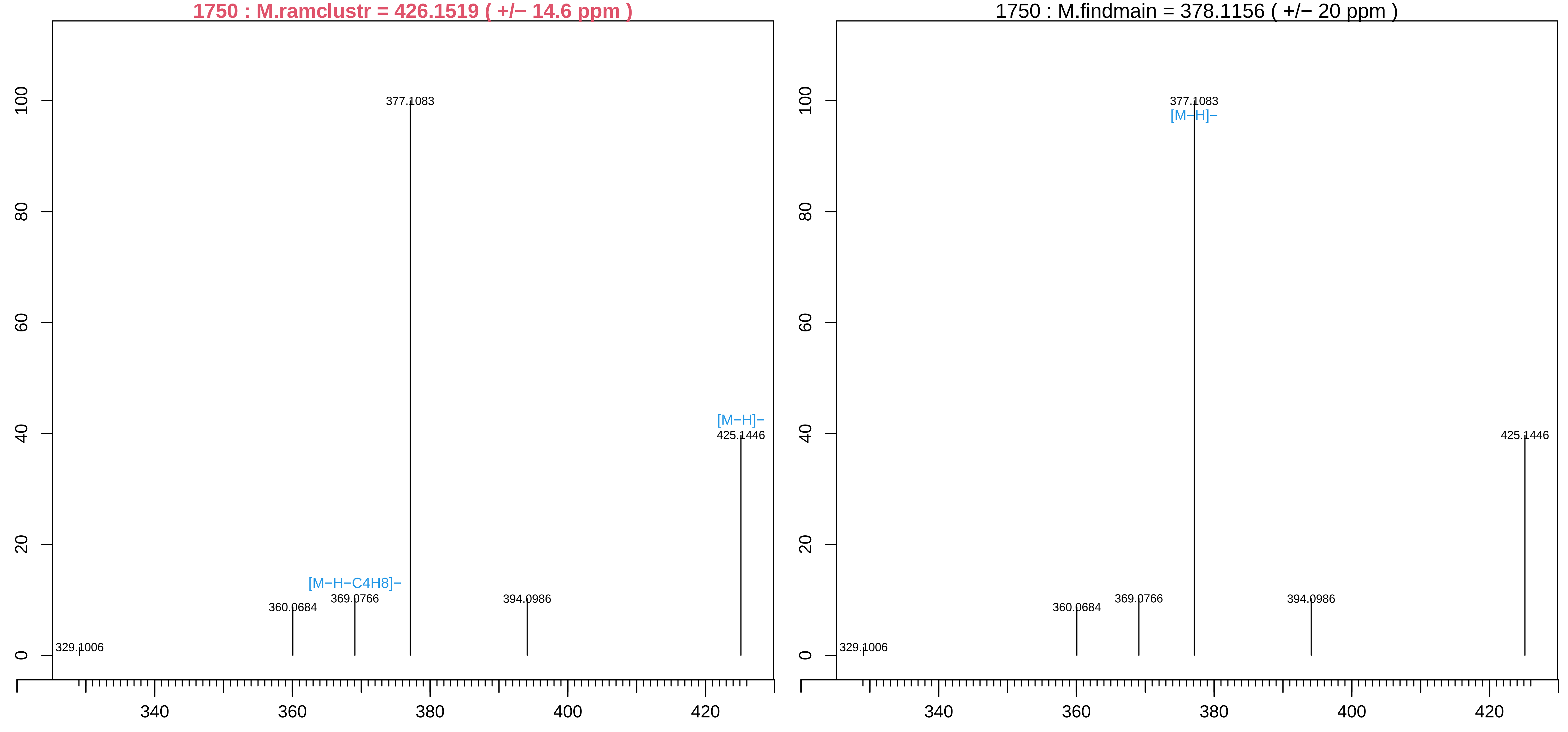

1750 : M.ramclustr = 426.1519 (+/− 14.6 ppm)
377.1083
[M−H]−
425.1446
[M−H−C4H8]−
369.0766
360.0684
394.0986
329.1006
1750 : M.findmain = 378.1156 (+/− 20 ppm)
377.1083
[M−H]−
425.1446
360.0684
369.0766
394.0986
329.1006

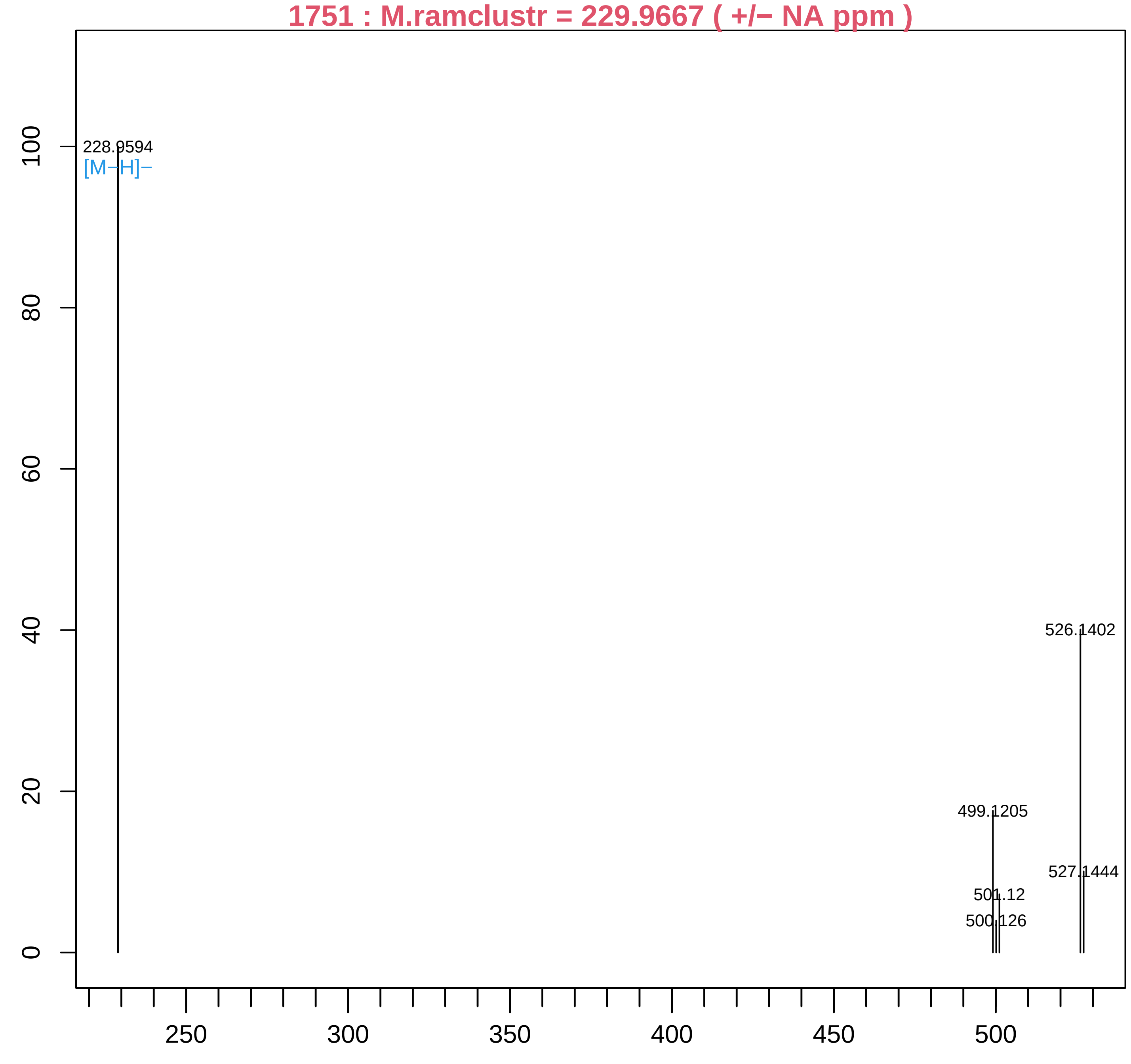
1751 : M.ramclustr = 229.9667 (+/− NA ppm)
228.9594
[M−H]−
526.1402
499.1205
527.1444
501.12
500.126
0
20
40
60
80
100
250
300
350
400
450
500

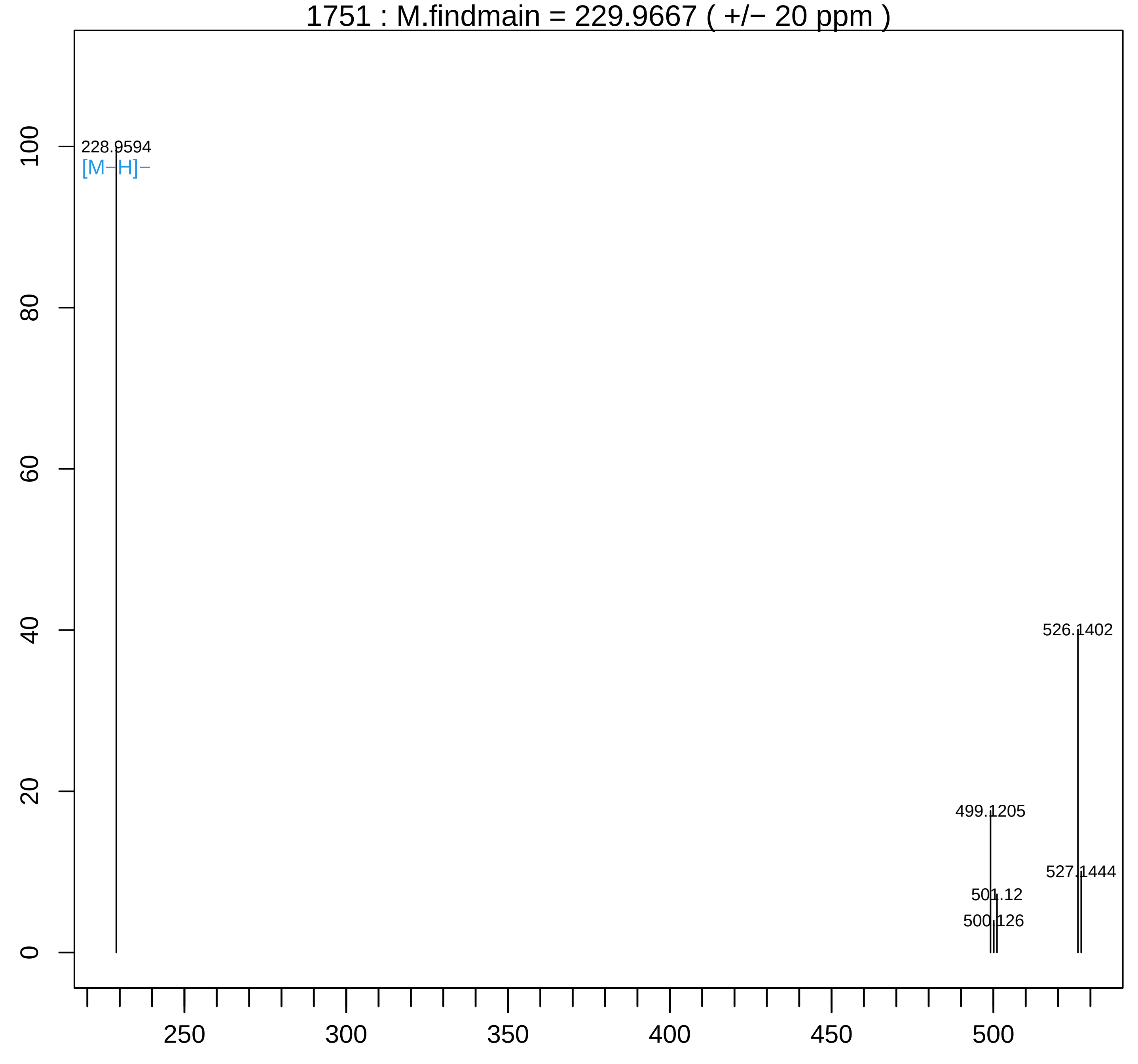
1751 : M.findmain = 229.9667 (+/− 20 ppm)
228.9594
[M−H]−
526.1402
499.1205
527.1444
501.12
500.126
0
20
40
60
80
100
250
300
350
400
450
500

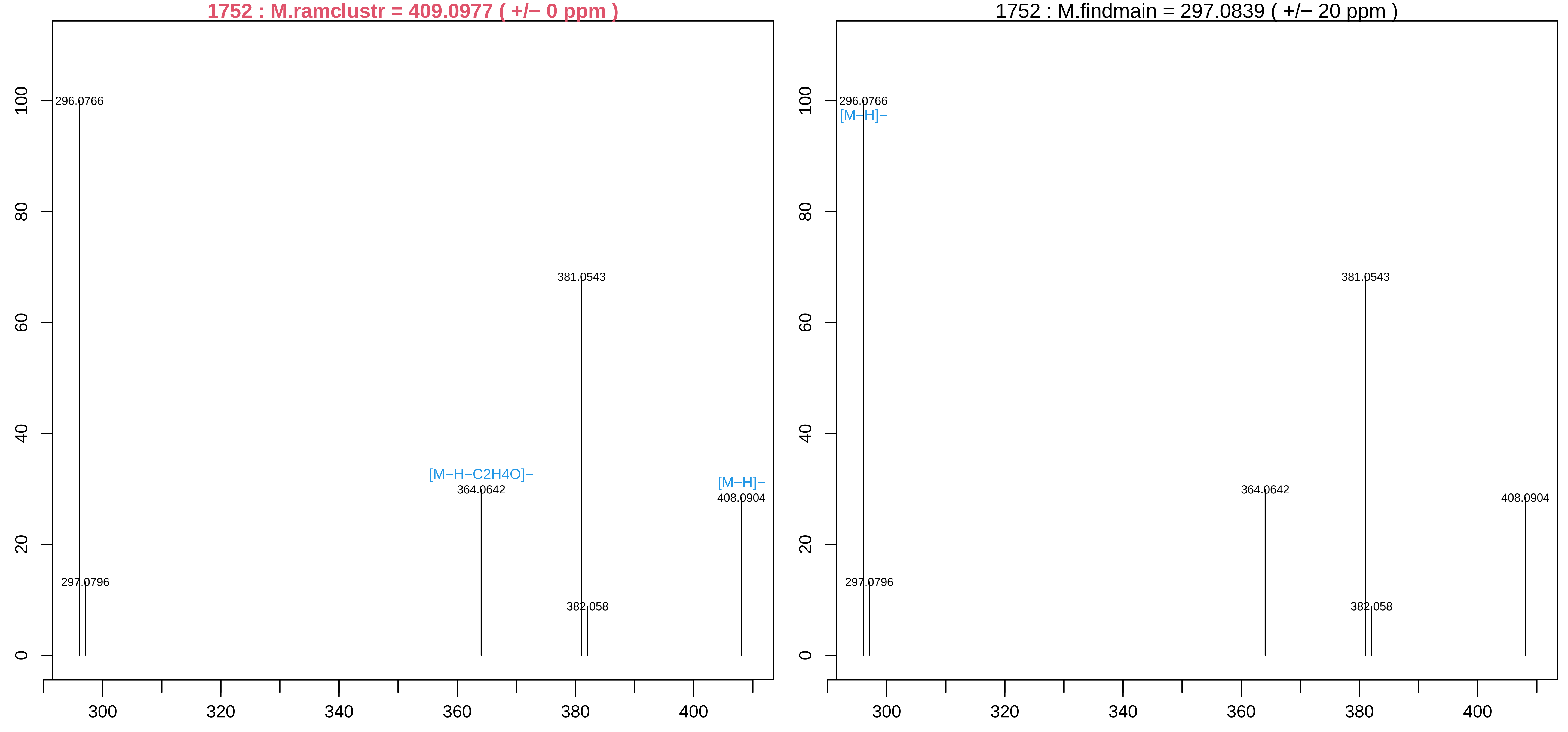
1752 : M.ramclustr = 409.0977 (+/− 0 ppm)
296.0766
297.0796
[M−H−C2H4O]−
364.0642
381.0543
382.058
[M−H]−
408.0904
1752 : M.findmain = 297.0839 (+/− 20 ppm)
296.0766
[M−H]−
297.0796
364.0642
381.0543
382.058
408.0904
0
20
40
60
80
100
300
320
340
360
380
400

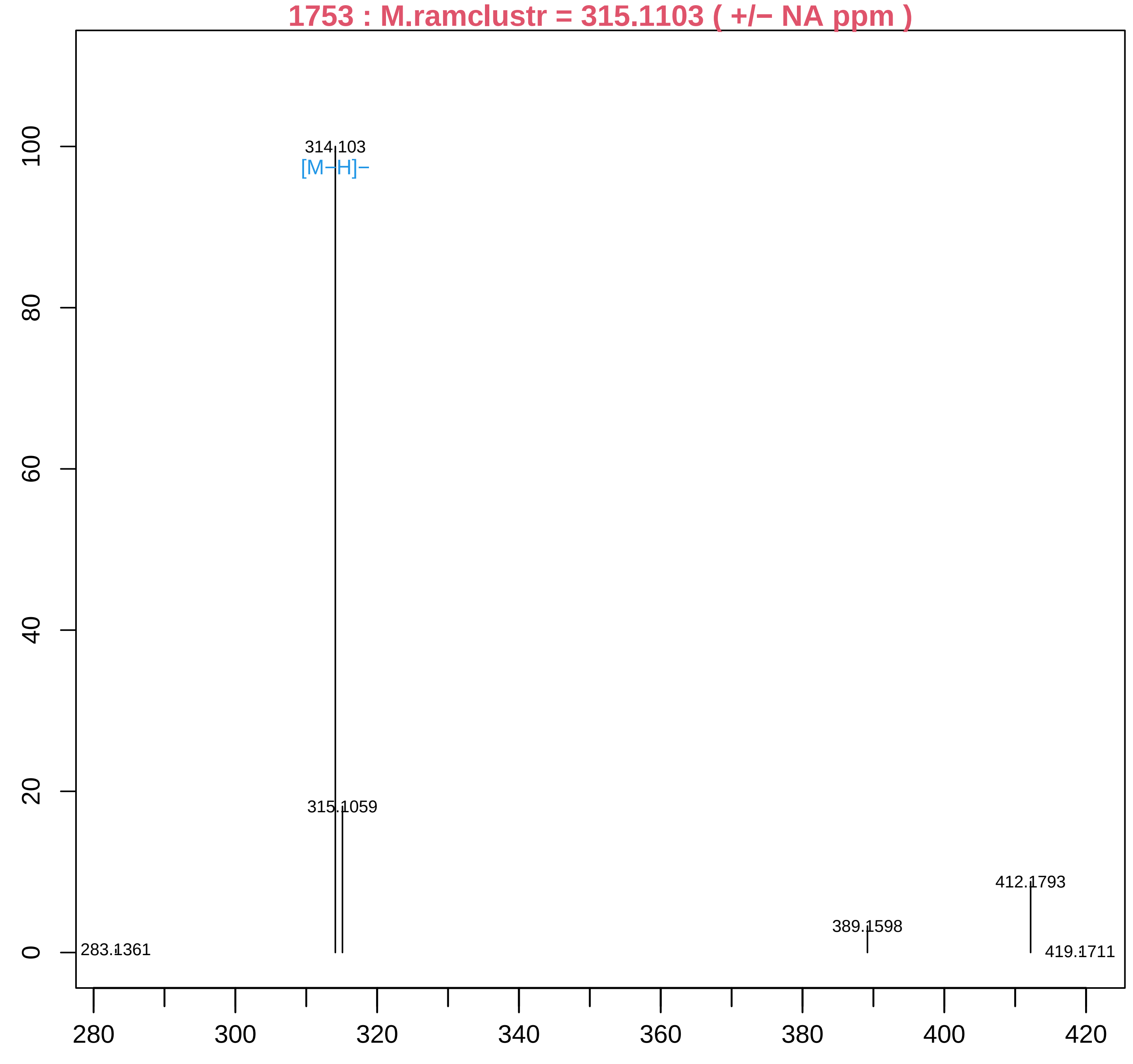
1753 : M.ramclustr = 315.1103 (+/− NA ppm)
314.103
[M−H]−
315.1059
283.1361
389.1598
412.1793
419.1711
0
20
40
60
80
100
280
300
320
340
360
380
400
420

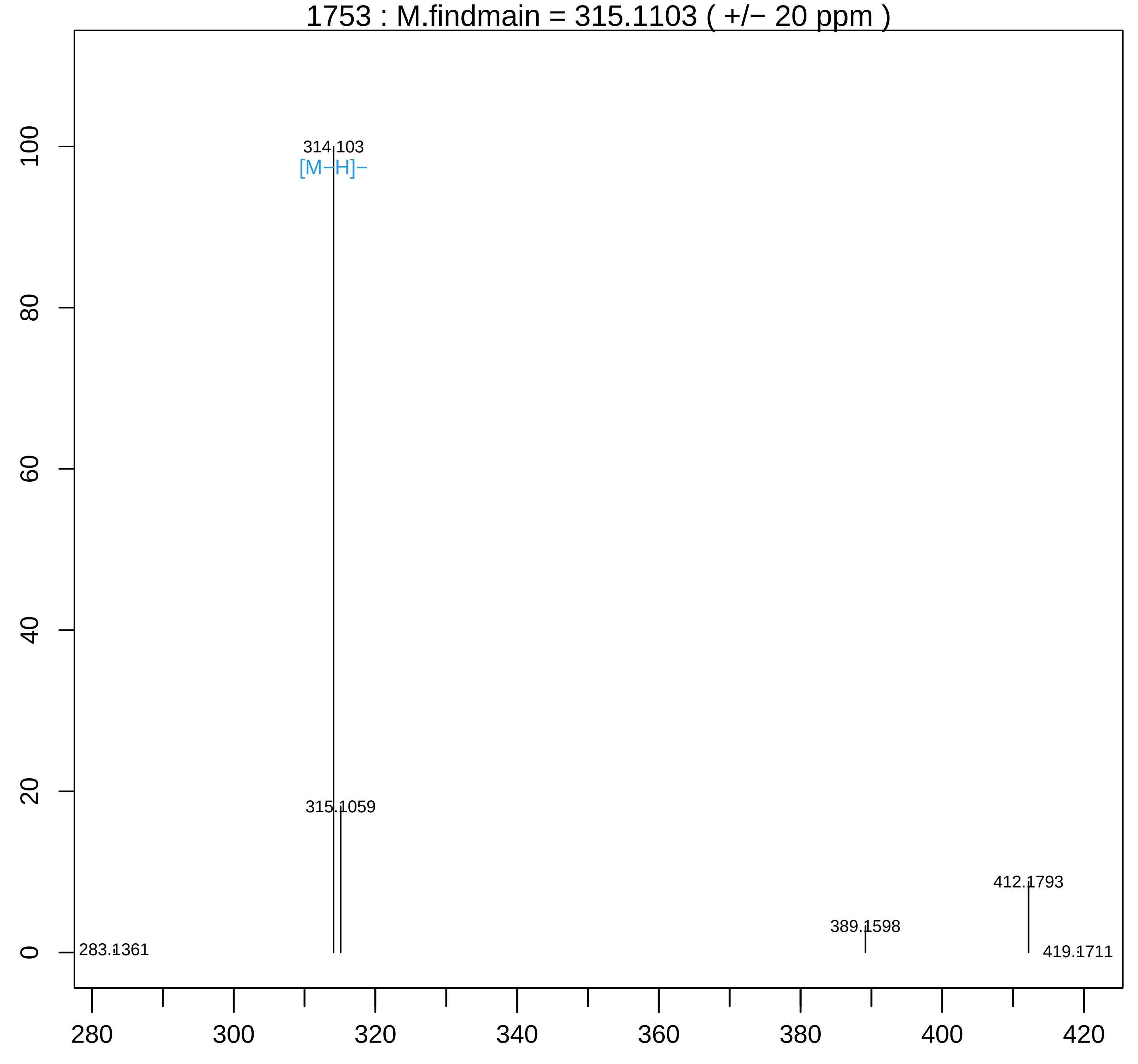
1753 : M.findmain = 315.1103 (+/− 20 ppm)
314.103
[M−H]−
315.1059
283.1361
389.1598
412.1793
419.1711
0
20
40
60
80
100
280
300
320
340
360
380
400
420

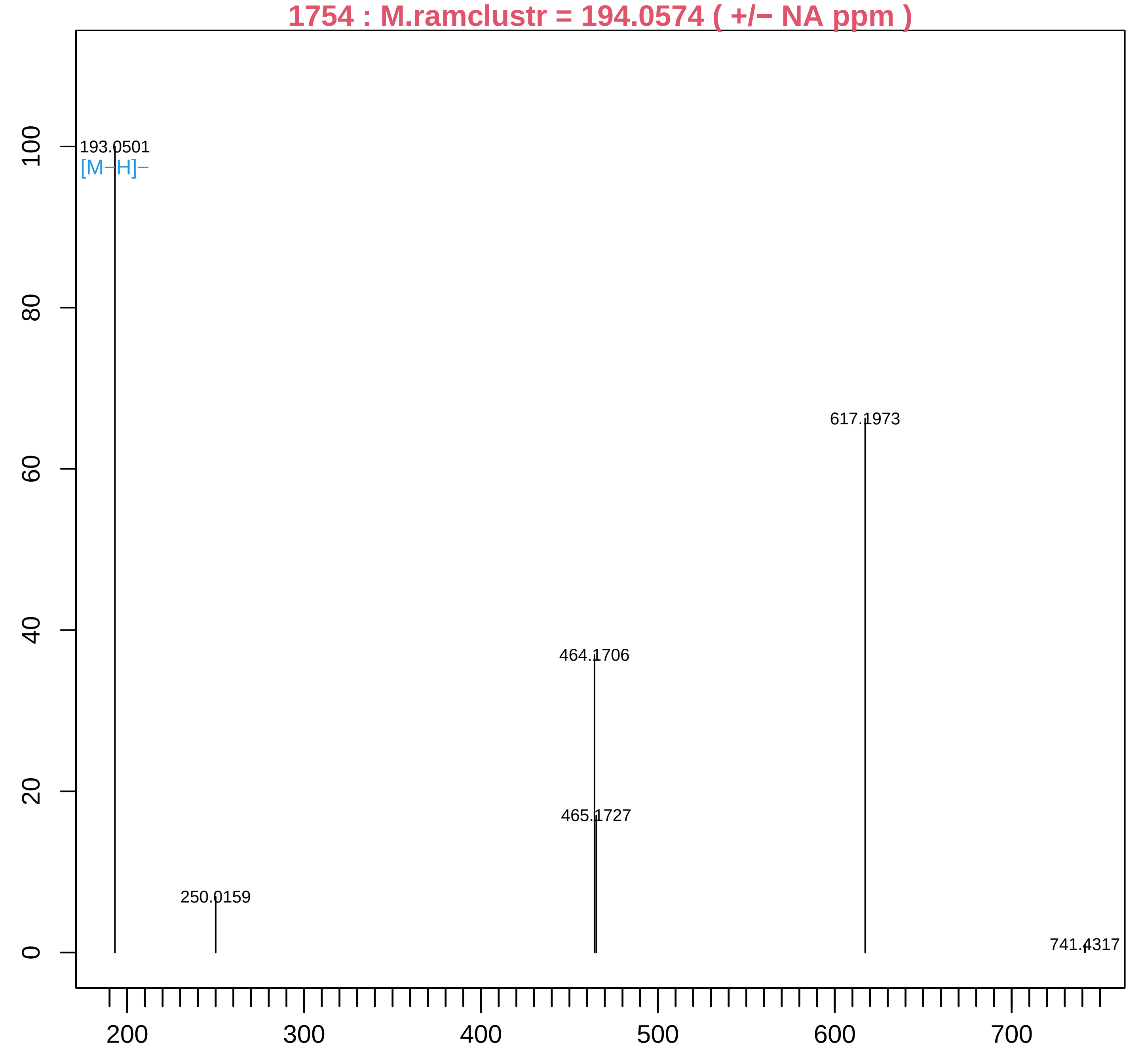

1754 : M.ramclustr = 194.0574 (+/− NA ppm)
193.0501
[M−H]−
250.0159
464.1706
465.1727
617.1973
741.4317
0
20
40
60
80
100
200
300
400
500
600
700

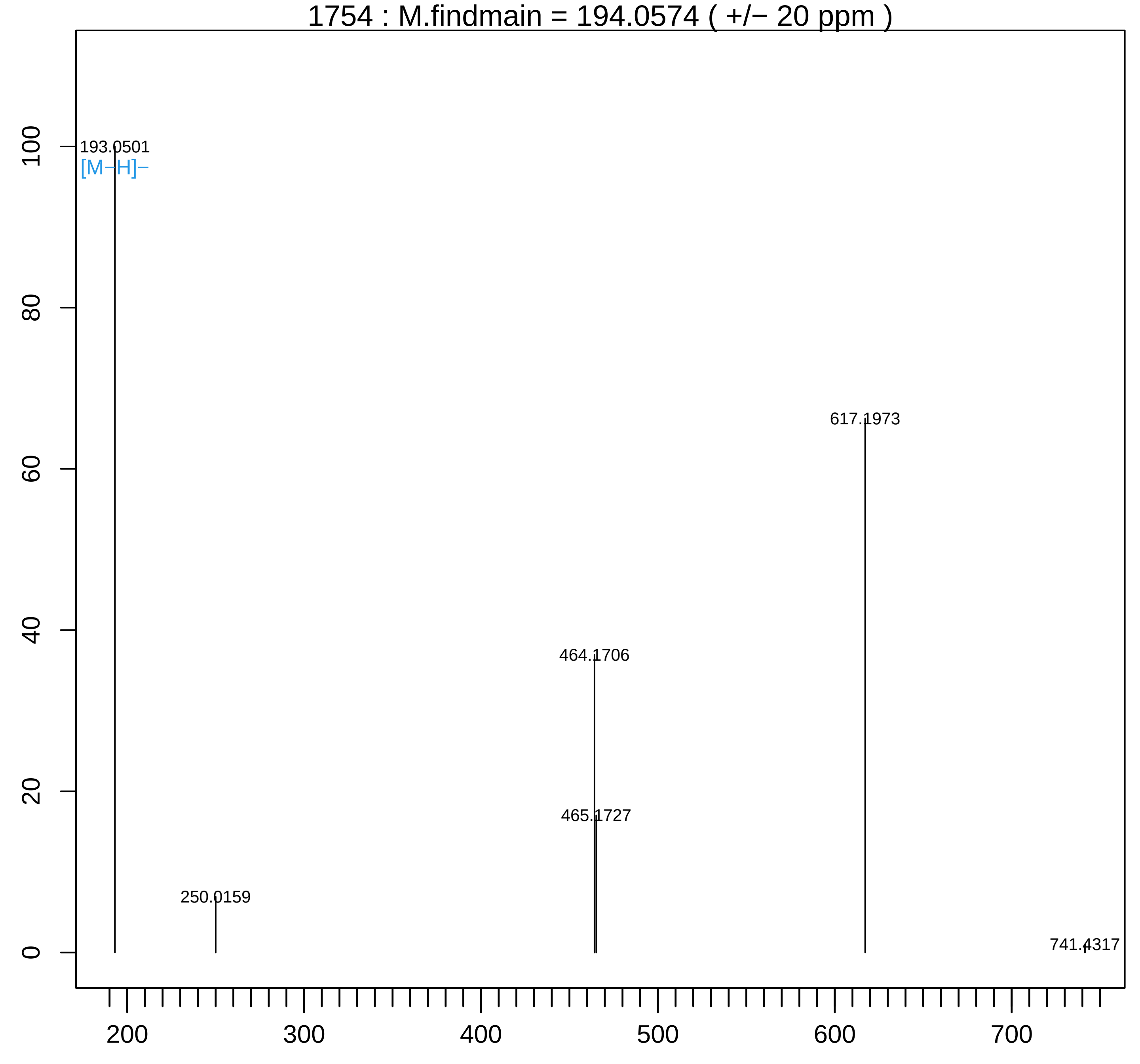

1754 : M.findmain = 194.0574 (+/− 20 ppm)
193.0501
[M−H]−
250.0159
464.1706
465.1727
617.1973
741.4317
0
20
40
60
80
100
200
300
400
500
600
700

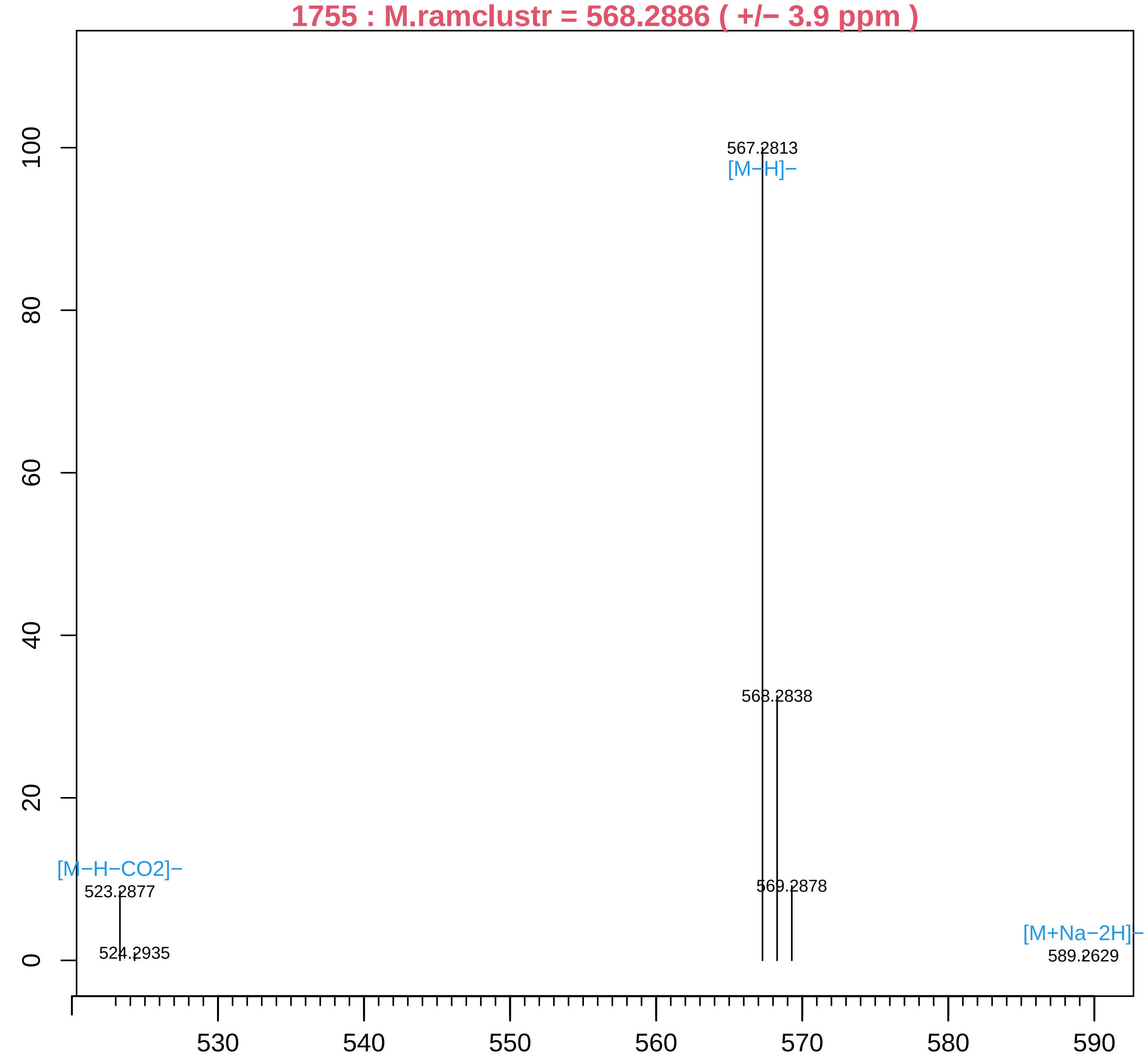
1755 : M.ramclustr = 568.2886 (+/− 3.9 ppm)
567.2813
[M−H]−
568.2838
569.2878
[M−H−CO2]−
523.2877
524.2935
[M+Na−2H]−
589.2629
0
20
40
60
80
100
530
540
550
560
570
580
590

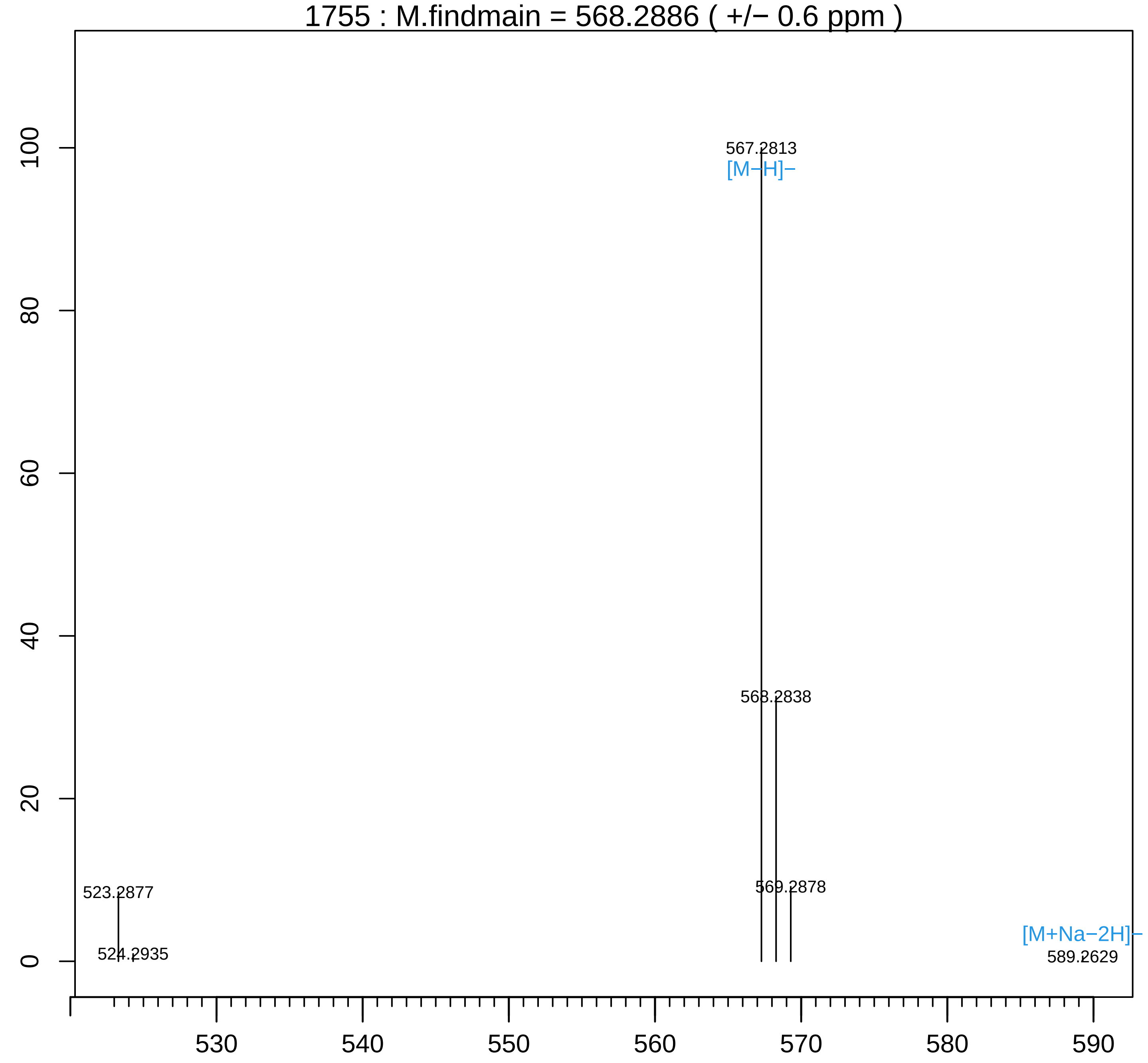
1755 : M.findmain = 568.2886 (+/− 0.6 ppm)
567.2813
[M−H]−
568.2838
569.2878
523.2877
524.2935
[M+Na−2H]−
589.2629
0
20
40
60
80
100
530
540
550
560
570
580
590

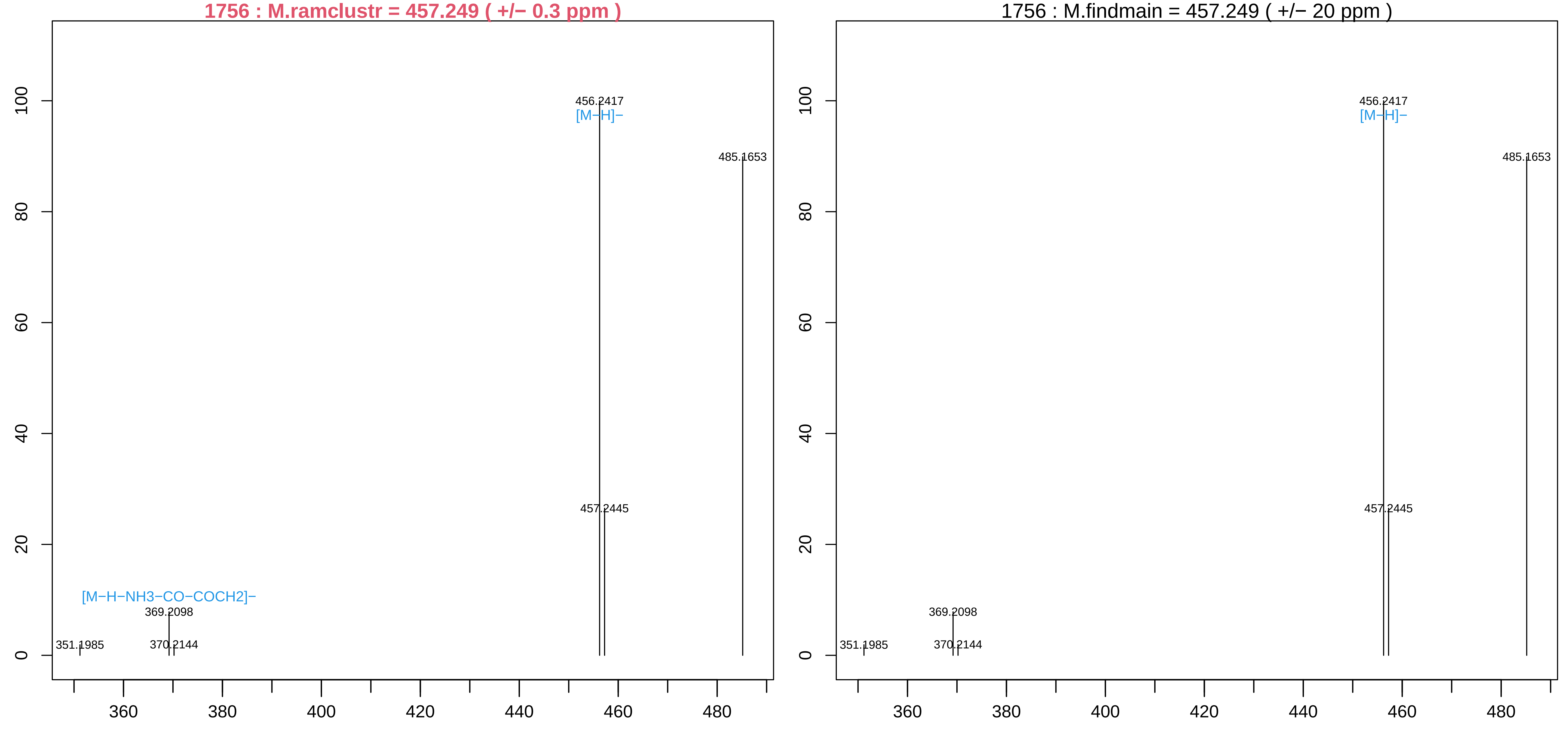
1756 : M.ramclustr = 457.249 (+/− 0.3 ppm)
456.2417
[M−H]−
485.1653
457.2445
[M−H−NH3−CO−COCH2]−
369.2098
351.1985
370.2144
1756 : M.findmain = 457.249 (+/− 20 ppm)
456.2417
[M−H]−
485.1653
457.2445
369.2098
351.1985
370.2144
0
20
40
60
80
100
360
380
400
420
440
460
480

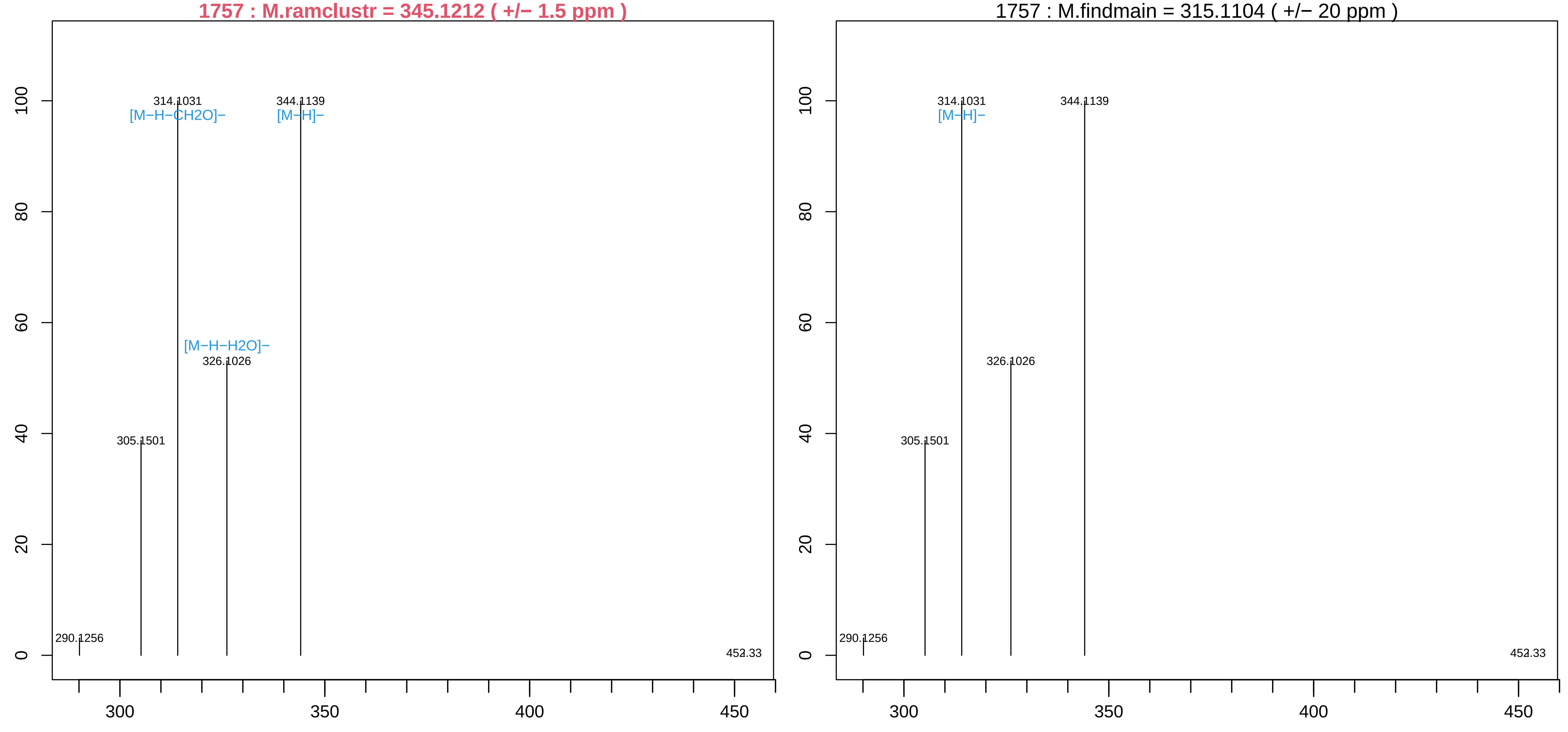
1757 : M.ramclustr = 345.1212 (+/− 1.5 ppm)
314.1031
[M−H−CH2O]−
344.1139
[M−H]−
[M−H−H2O]−
326.1026
305.1501
290.1256
452.33
1757 : M.findmain = 315.1104 (+/− 20 ppm)
314.1031
[M−H]−
344.1139
326.1026
305.1501
290.1256
452.33
0
20
40
60
80
100
300
350
400
450

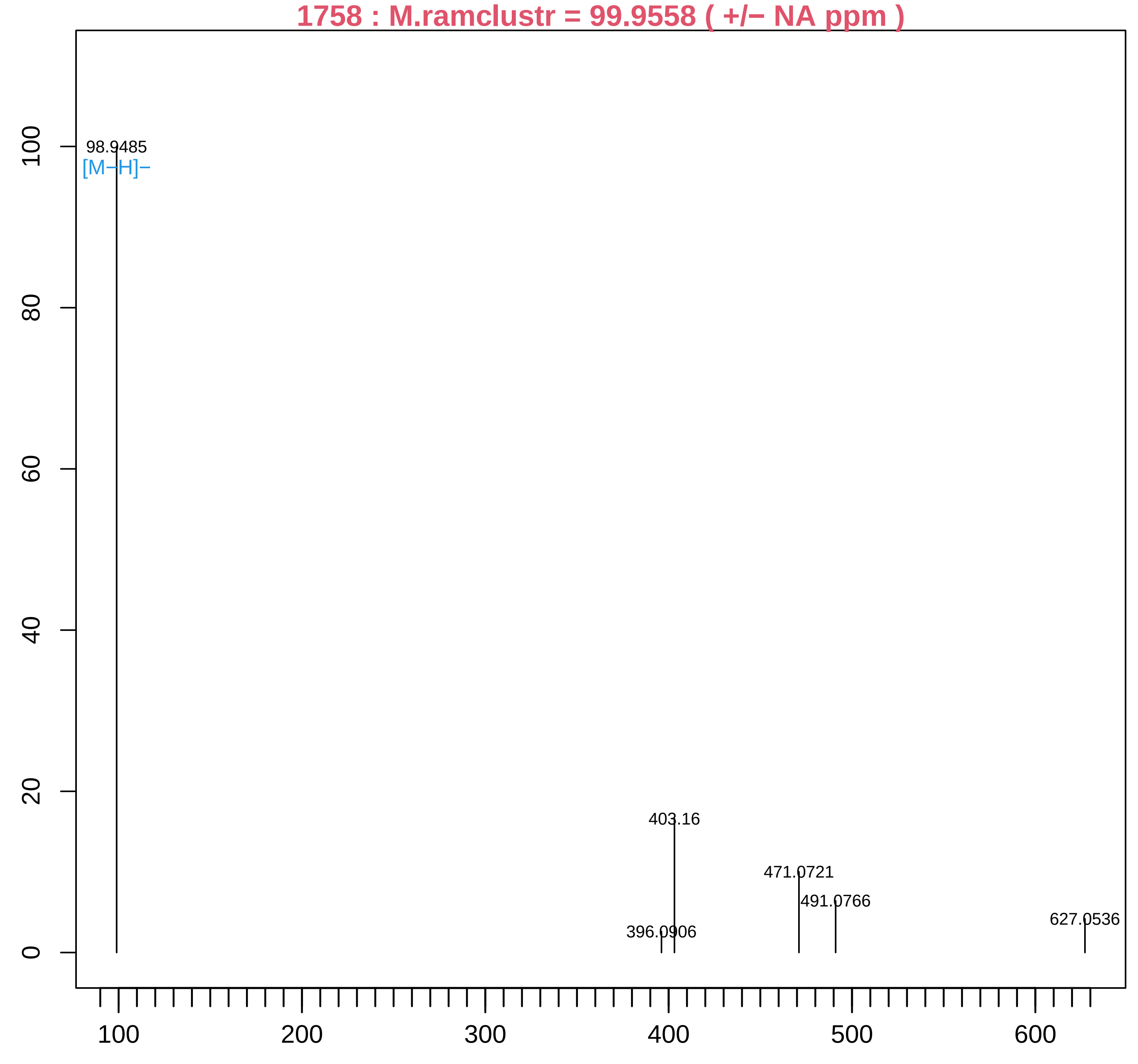
1758 : M.ramclustr = 99.9558 (+/− NA ppm)
98.9485
[M−H]−
403.16
471.0721
491.0766
396.0906
627.0536
0
20
40
60
80
100
100
200
300
400
500
600

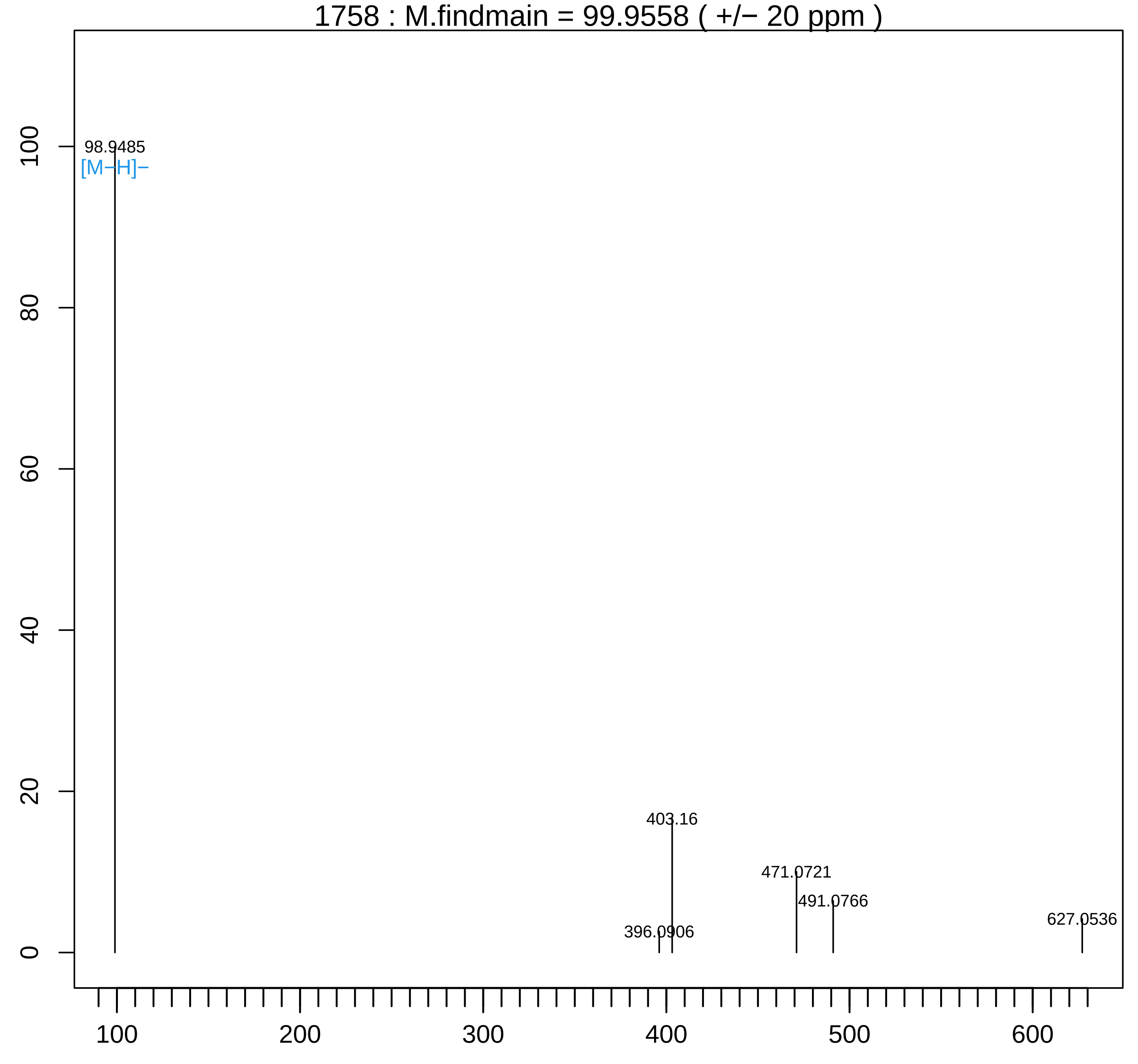
1758 : M.findmain = 99.9558 (+/− 20 ppm)
98.9485
[M−H]−
403.16
471.0721
491.0766
396.0906
627.0536
0
20
40
60
80
100
100
200
300
400
500
600

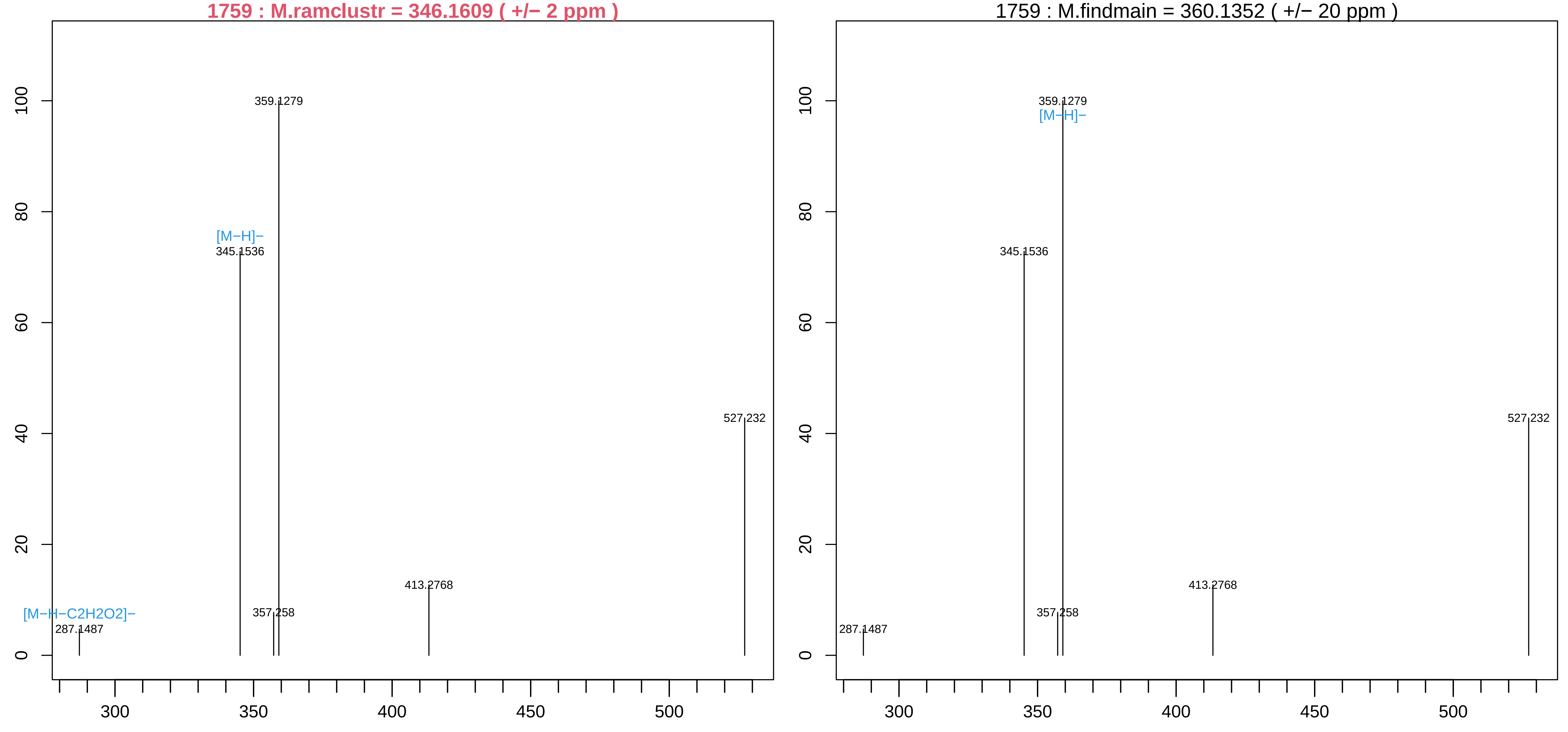
1759 : M.ramclustr = 346.1609 (+/− 2 ppm)
359.1279
[M−H]−
345.1536
527.232
413.2768
[M−H−C2H2O2]−
357.258
287.1487
1759 : M.findmain = 360.1352 (+/− 20 ppm)
359.1279
[M−H]−
345.1536
527.232
413.2768
357.258
287.1487

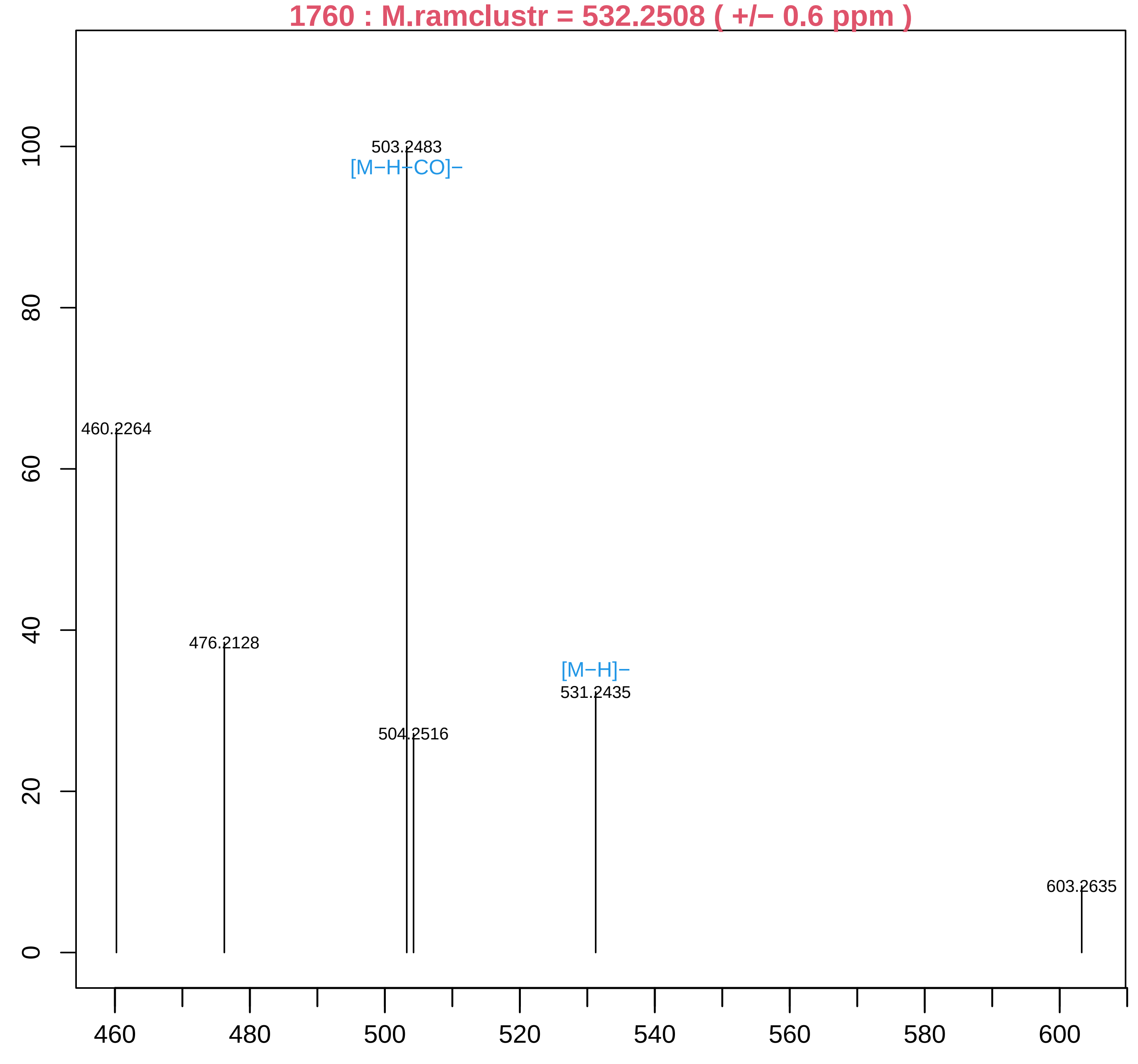
1760 : M.ramclustr = 532.2508 (+/− 0.6 ppm)
503.2483
[M−H−CO]−
460.2264
476.2128
[M−H]−
531.2435
504.2516
603.2635
0
20
40
60
80
100
460
480
500
520
540
560
580
600

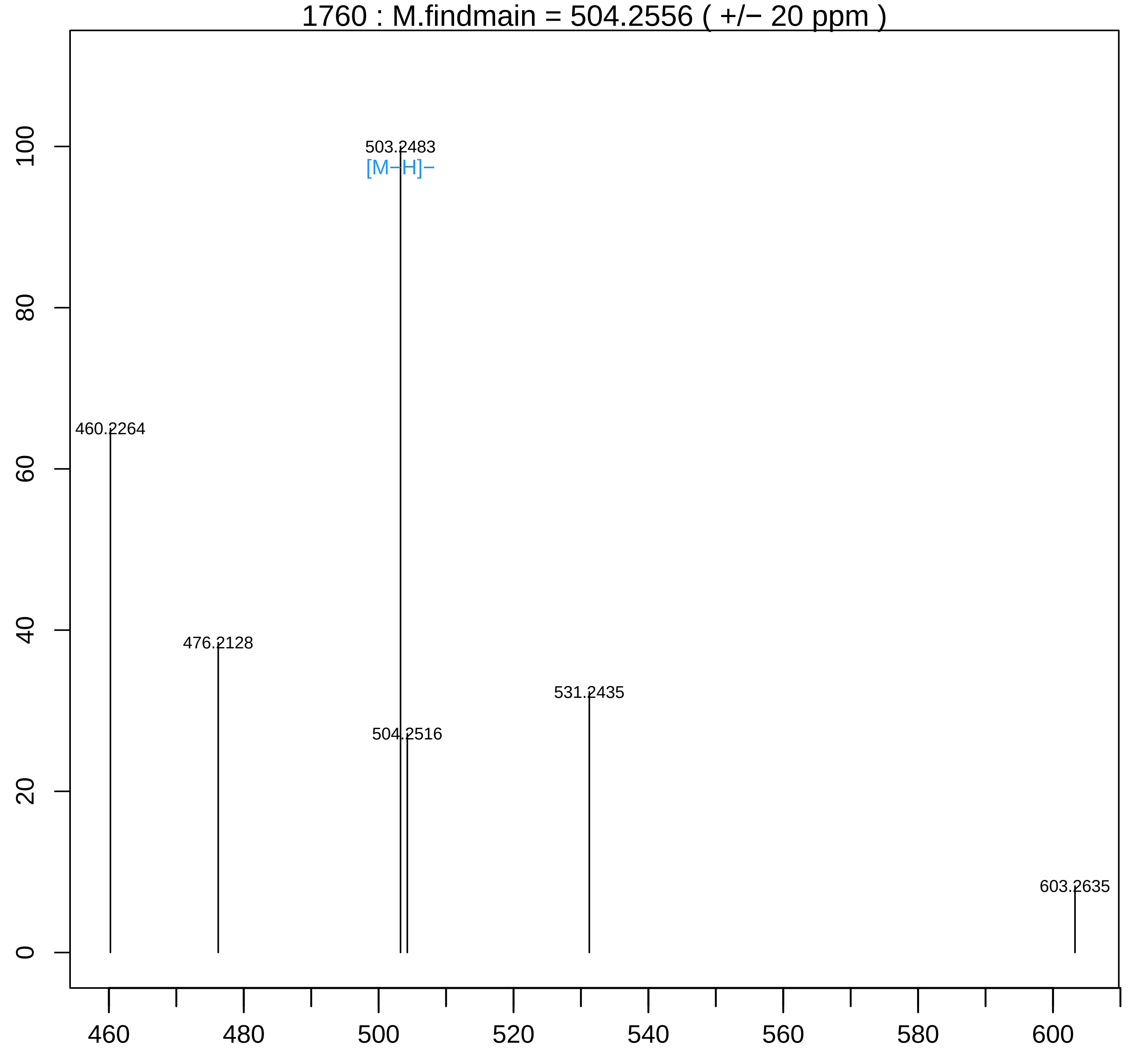
1760 : M.findmain = 504.2556 (+/− 20 ppm)
503.2483
[M−H]−
460.2264
476.2128
531.2435
504.2516
603.2635
0
20
40
60
80
100
460
480
500
520
540
560
580
600

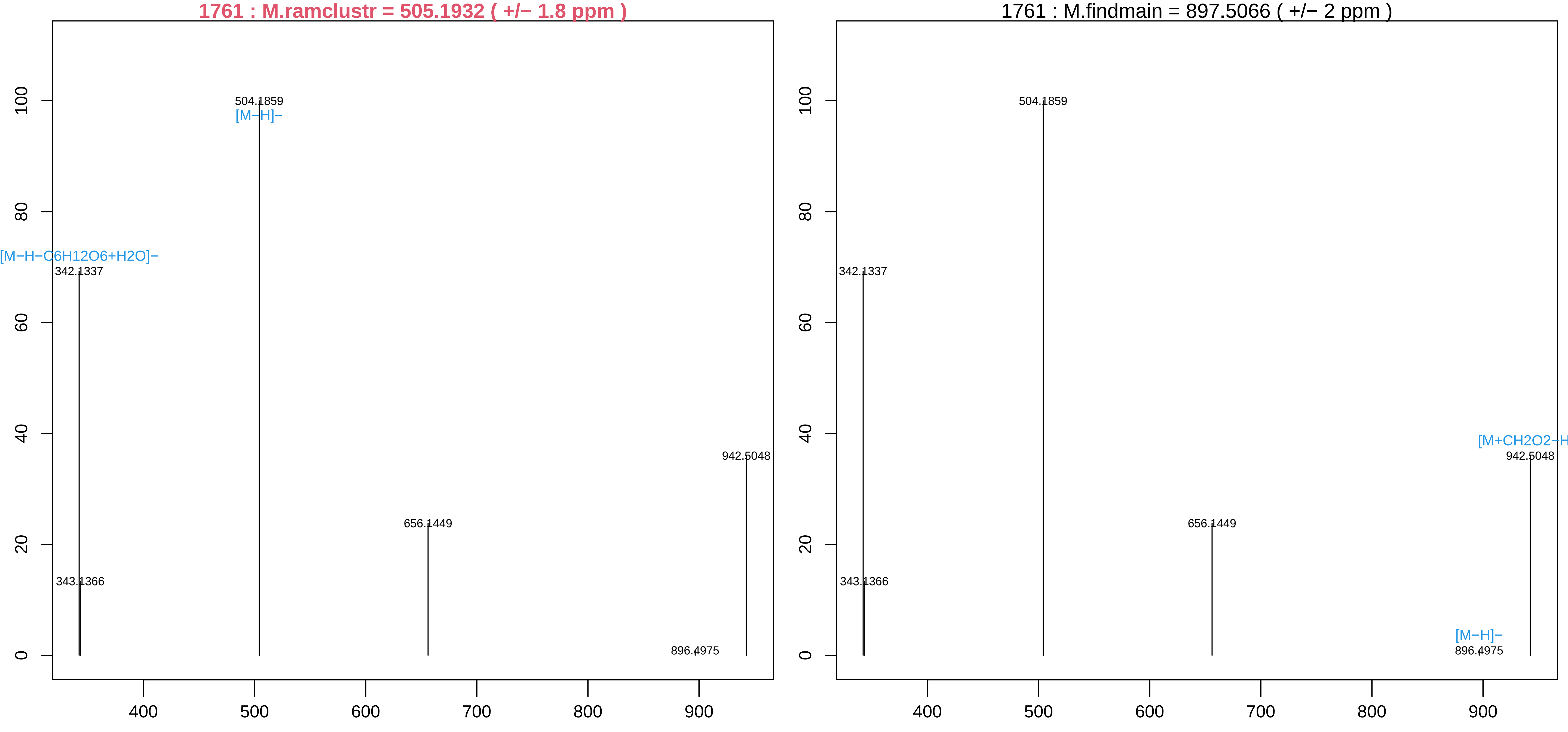

1761 : M.ramclustr = 505.1932 (+/− 1.8 ppm)
504.1859
[M−H]−
[M−H−C6H12O6+H2O]−
342.1337
343.1366
656.1449
942.5048
896.4975
0
20
40
60
80
100
400
500
600
700
800
900
1761 : M.findmain = 897.5066 (+/− 2 ppm)
504.1859
342.1337
343.1366
656.1449
[M+CH2O2−H
942.5048
[M−H]−
896.4975

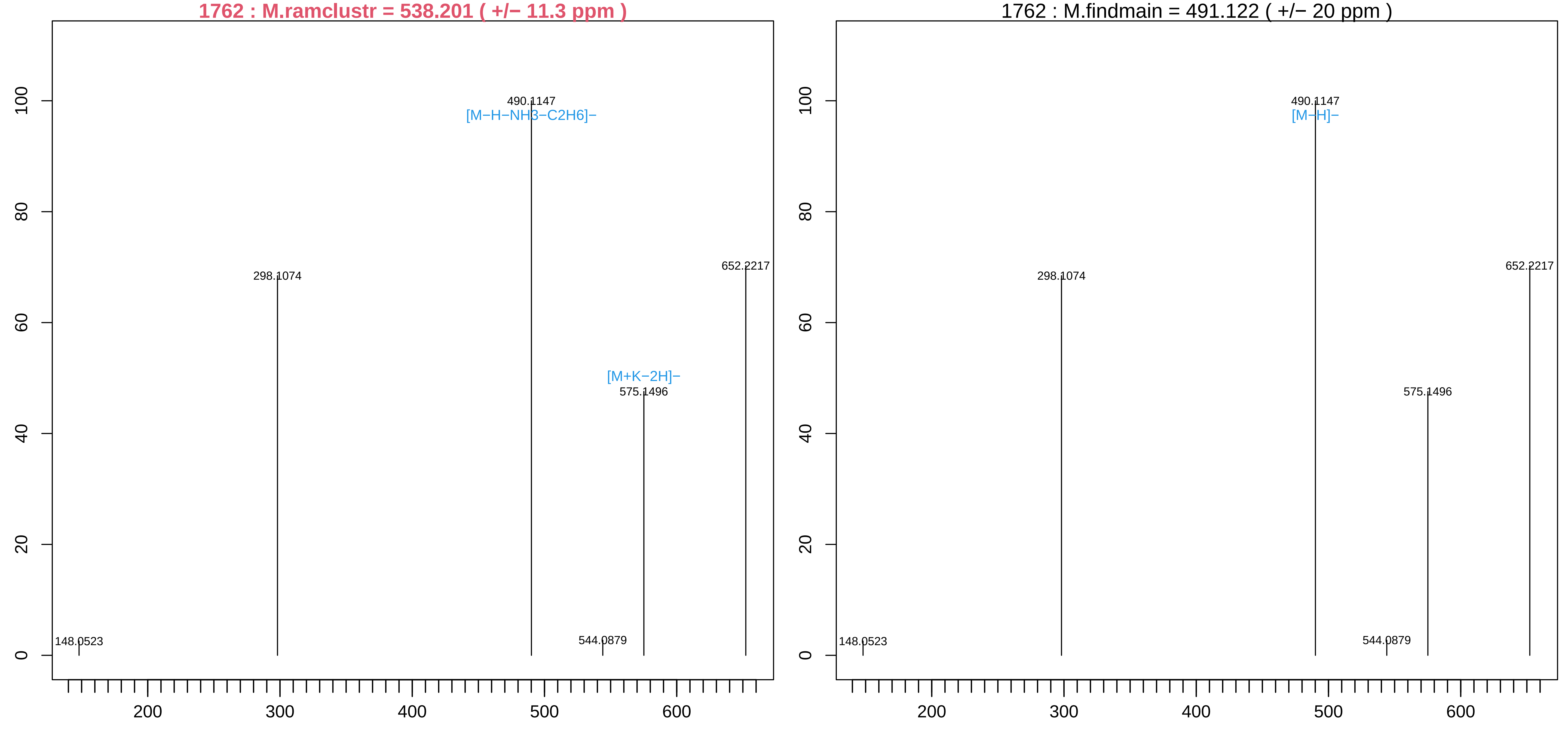

1762 : M.ramclustr = 538.201 (+/− 11.3 ppm)
490.1147
[M−H−NH3−C2H6]−
298.1074
652.2217
[M+K−2H]−
575.1496
148.0523
544.0879
1762 : M.findmain = 491.122 (+/− 20 ppm)
490.1147
[M−H]−
298.1074
652.2217
575.1496
148.0523
544.0879
0
20
40
60
80
100
200
300
400
500
600

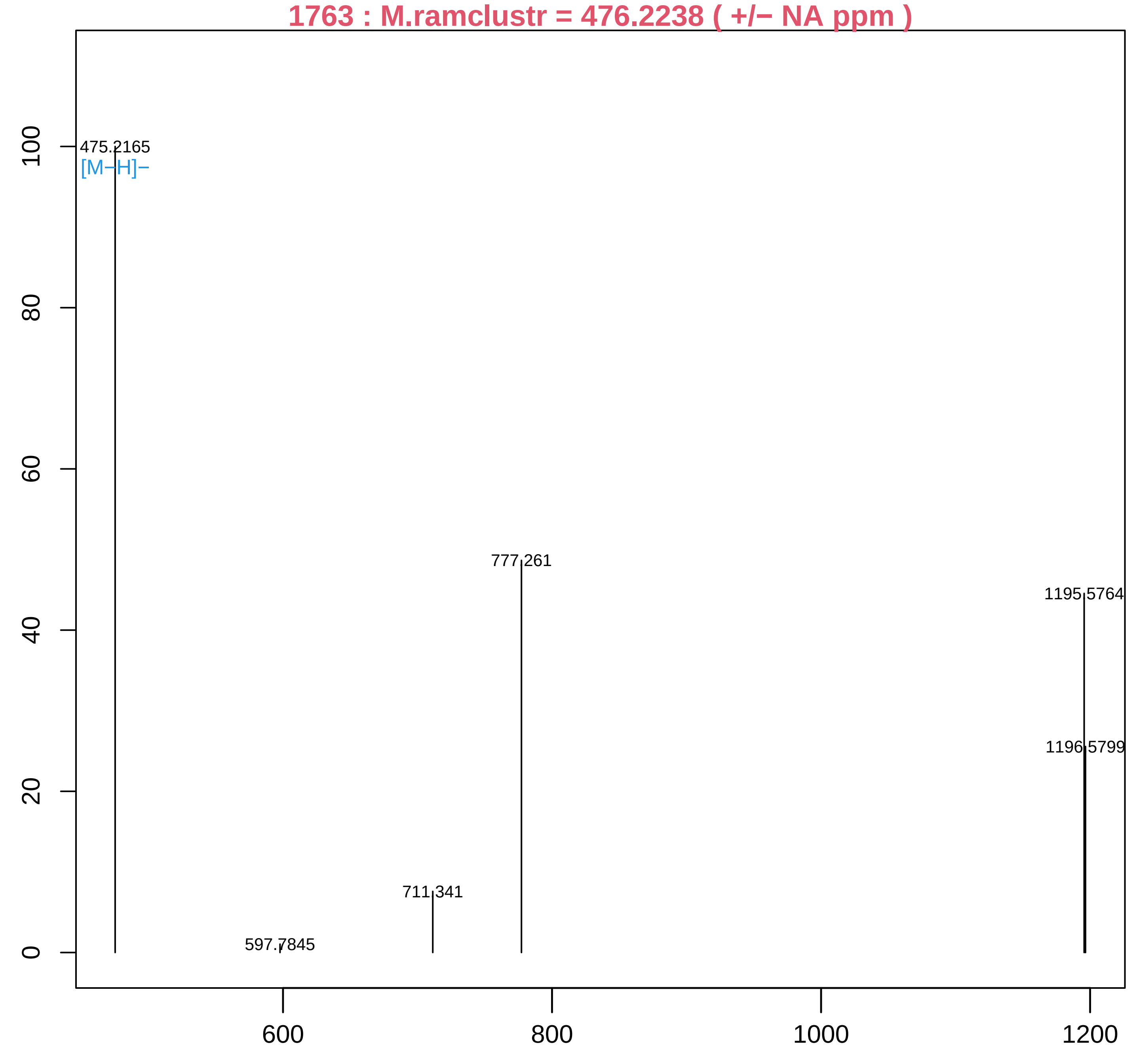

1763 : M.ramclustr = 476.2238 (+/− NA ppm)
475.2165
[M−H]−
597.7845
711.341
777.261
1195.5764
1196.5799
0
20
40
60
80
100
600
800
1000
1200

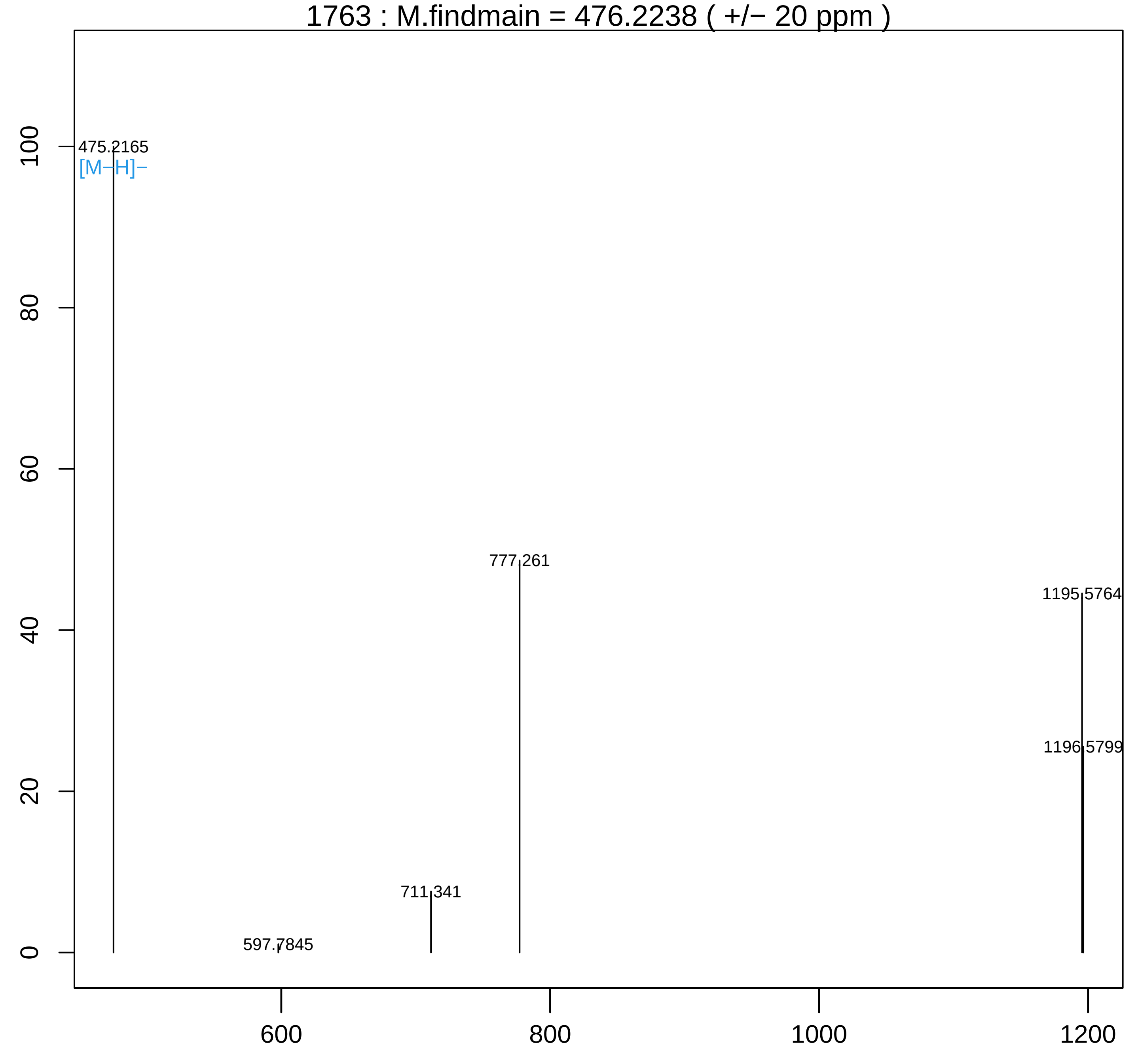

1763 : M.findmain = 476.2238 (+/− 20 ppm)
475.2165
[M−H]−
597.7845
711.341
777.261
1195.5764
1196.5799
0
20
40
60
80
100
600
800
1000
1200

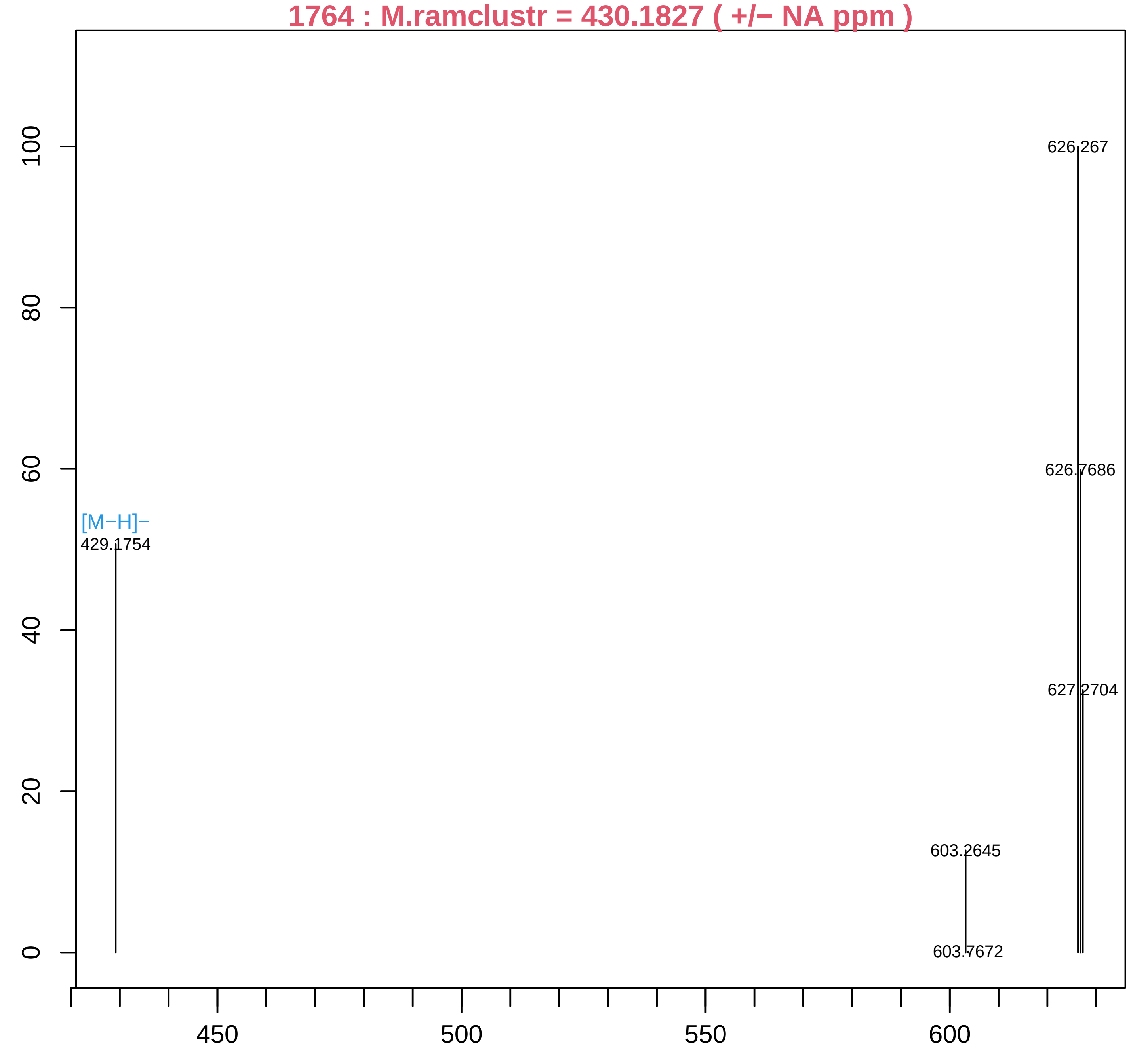
1764 : M.ramclustr = 430.1827 (+/− NA ppm)
[M−H]−
429.1754
626.267
626.7686
627.2704
603.2645
603.7672
0
20
40
60
80
100
450
500
550
600

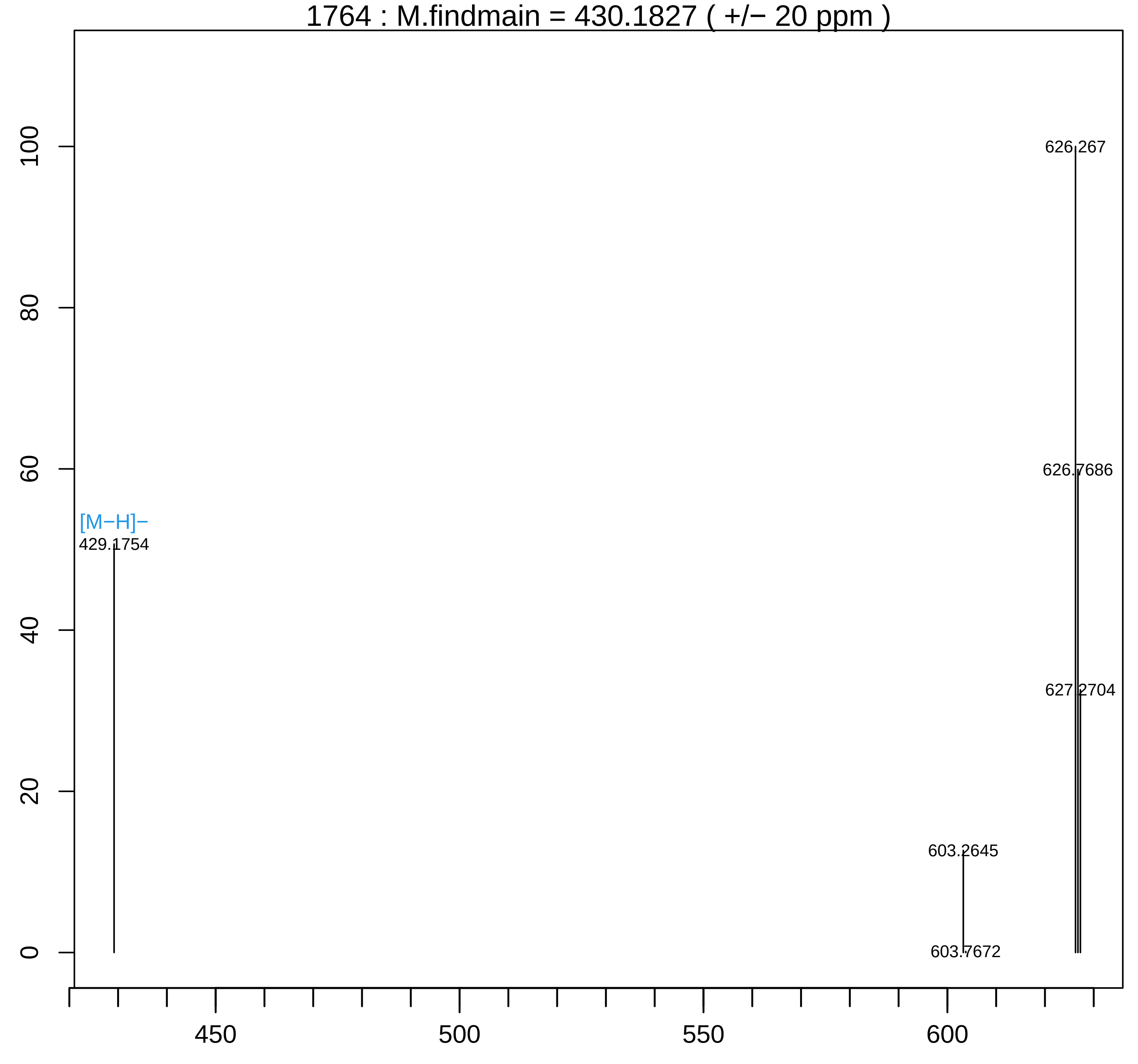
1764 : M.findmain = 430.1827 (+/− 20 ppm)
[M−H]−
429.1754
626.267
626.7686
627.2704
603.2645
603.7672
0
20
40
60
80
100
450
500
550
600

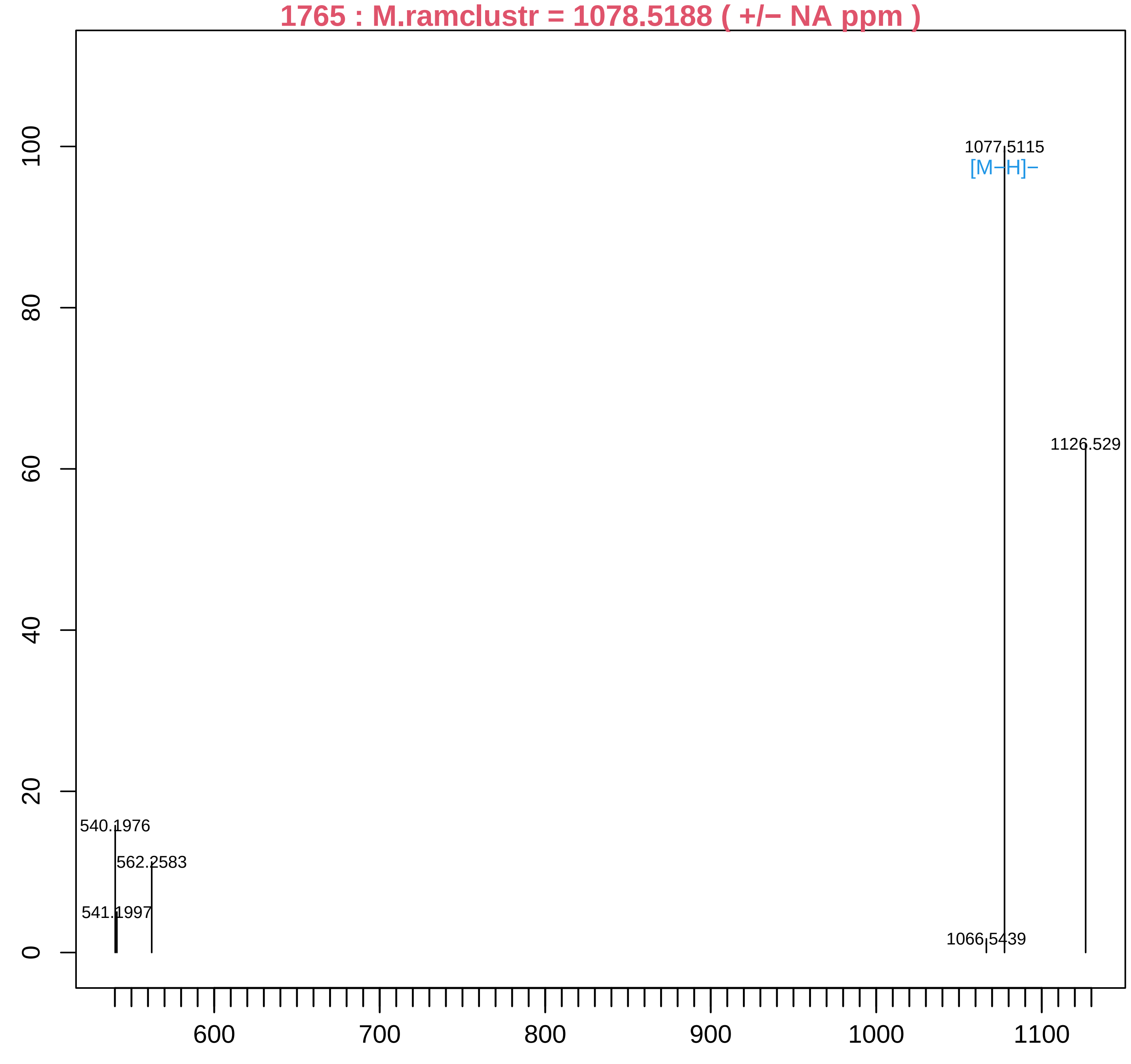
1765 : M.ramclustr = 1078.5188 (+/− NA ppm)
1077.5115
[M−H]−
1126.529
540.1976
562.2583
541.1997
1066.5439
0
20
40
60
80
100
600
700
800
900
1000
1100

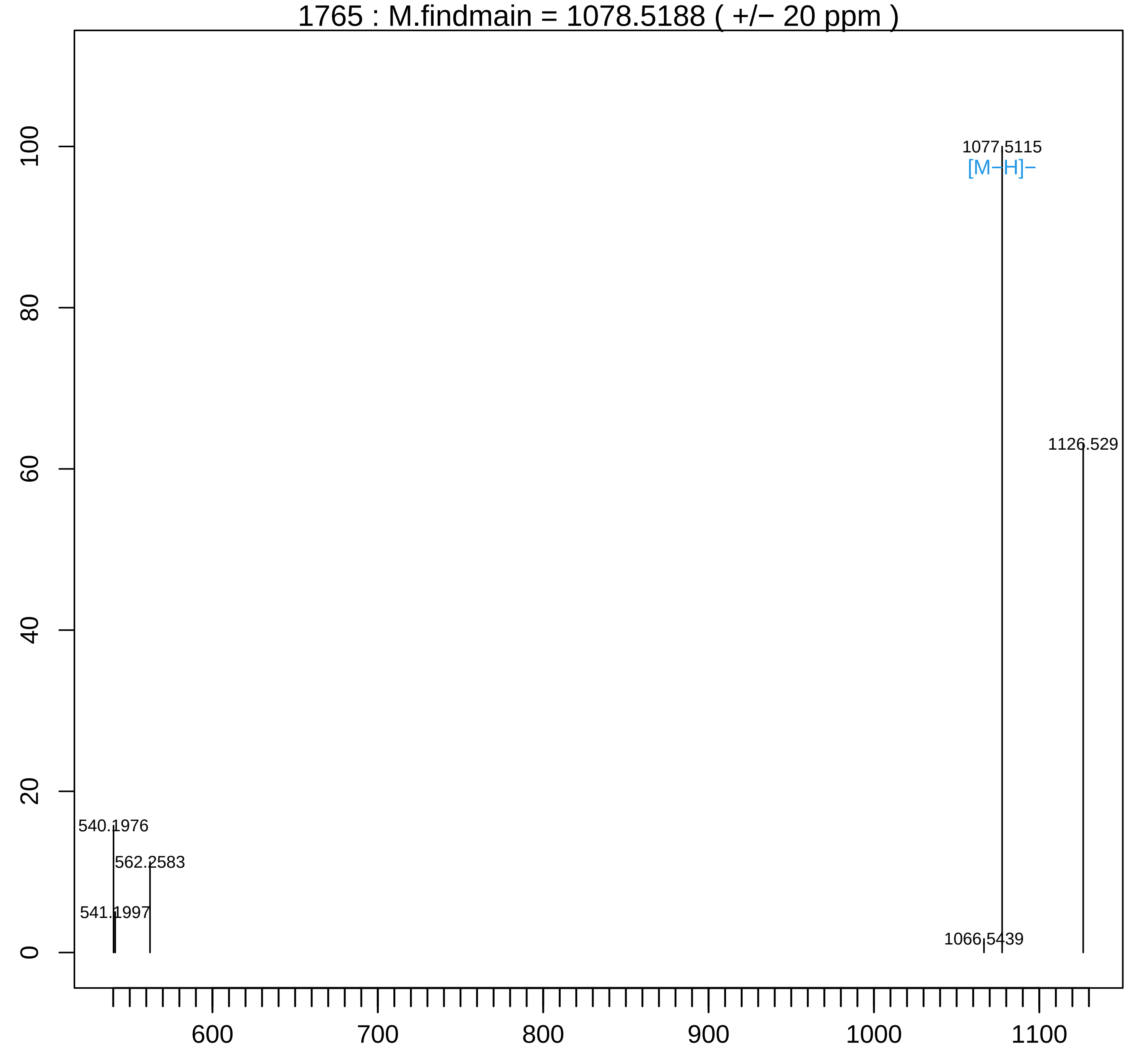
1765 : M.findmain = 1078.5188 (+/− 20 ppm)
1077.5115
[M−H]−
1126.529
540.1976
562.2583
541.1997
1066.5439
0
20
40
60
80
100
600
700
800
900
1000
1100

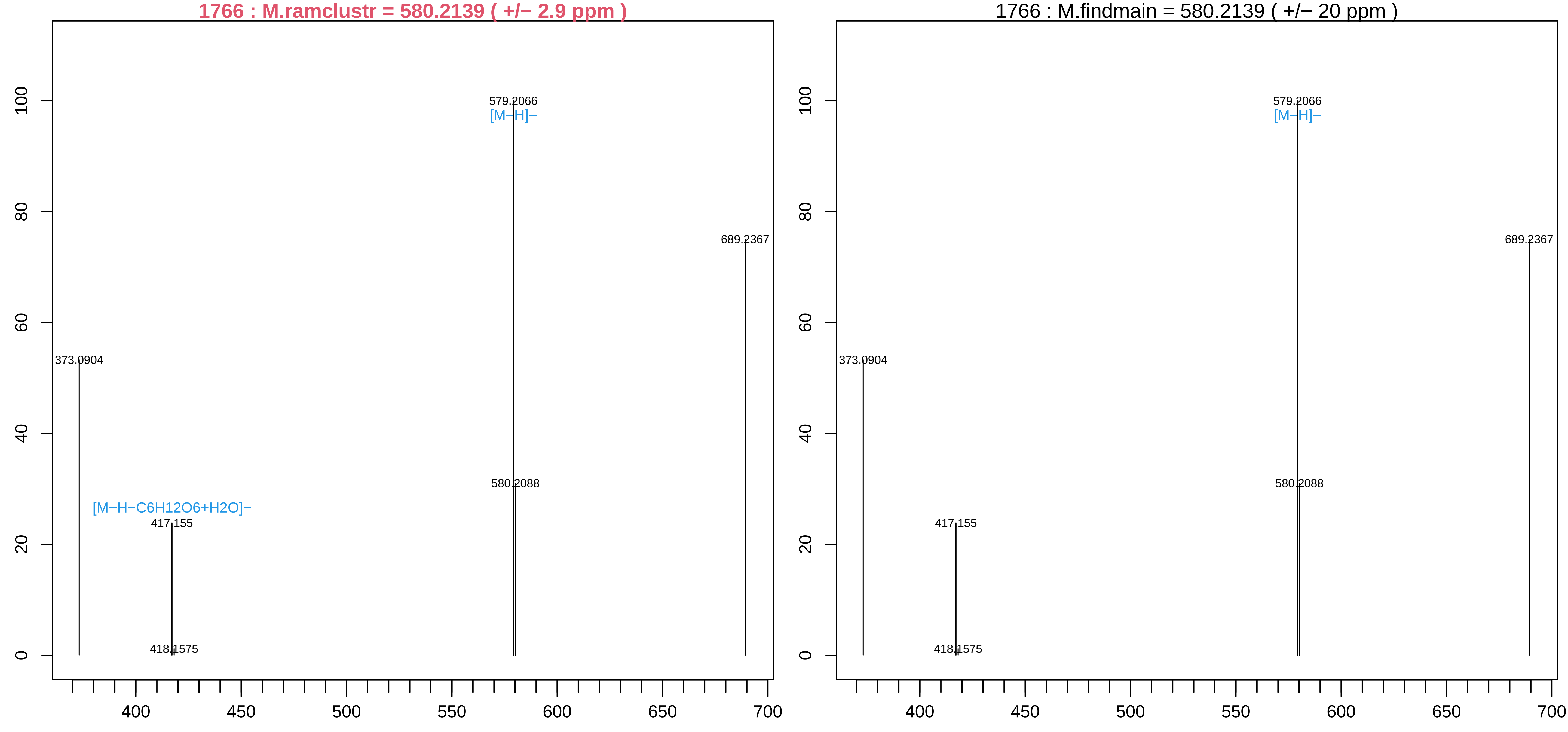
1766 : M.ramclustr = 580.2139 (+/− 2.9 ppm)
579.2066
[M−H]−
689.2367
373.0904
580.2088
[M−H−C6H12O6+H2O]−
417.155
418.1575
0
20
40
60
80
100
400
450
500
550
600
650
700
1766 : M.findmain = 580.2139 (+/− 20 ppm)
579.2066
[M−H]−
689.2367
373.0904
580.2088
417.155
418.1575
0
20
40
60
80
100
400
450
500
550
600
650
700

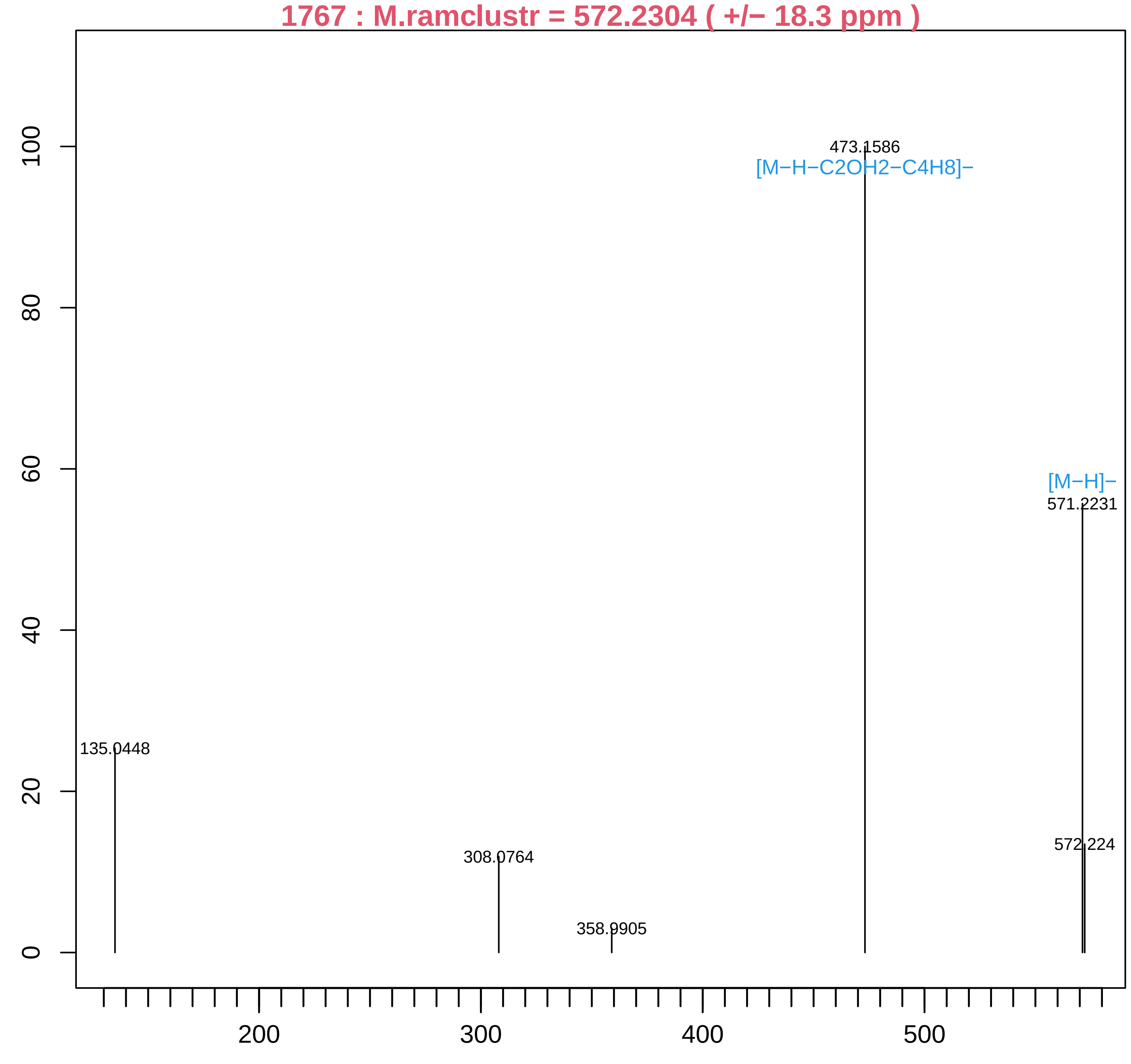
1767 : M.ramclustr = 572.2304 (+/− 18.3 ppm)
473.1586
[M−H−C2OH2−C4H8]−
[M−H]−
571.2231
135.0448
308.0764
572.224
358.9905
0
20
40
60
80
100
200
300
400
500

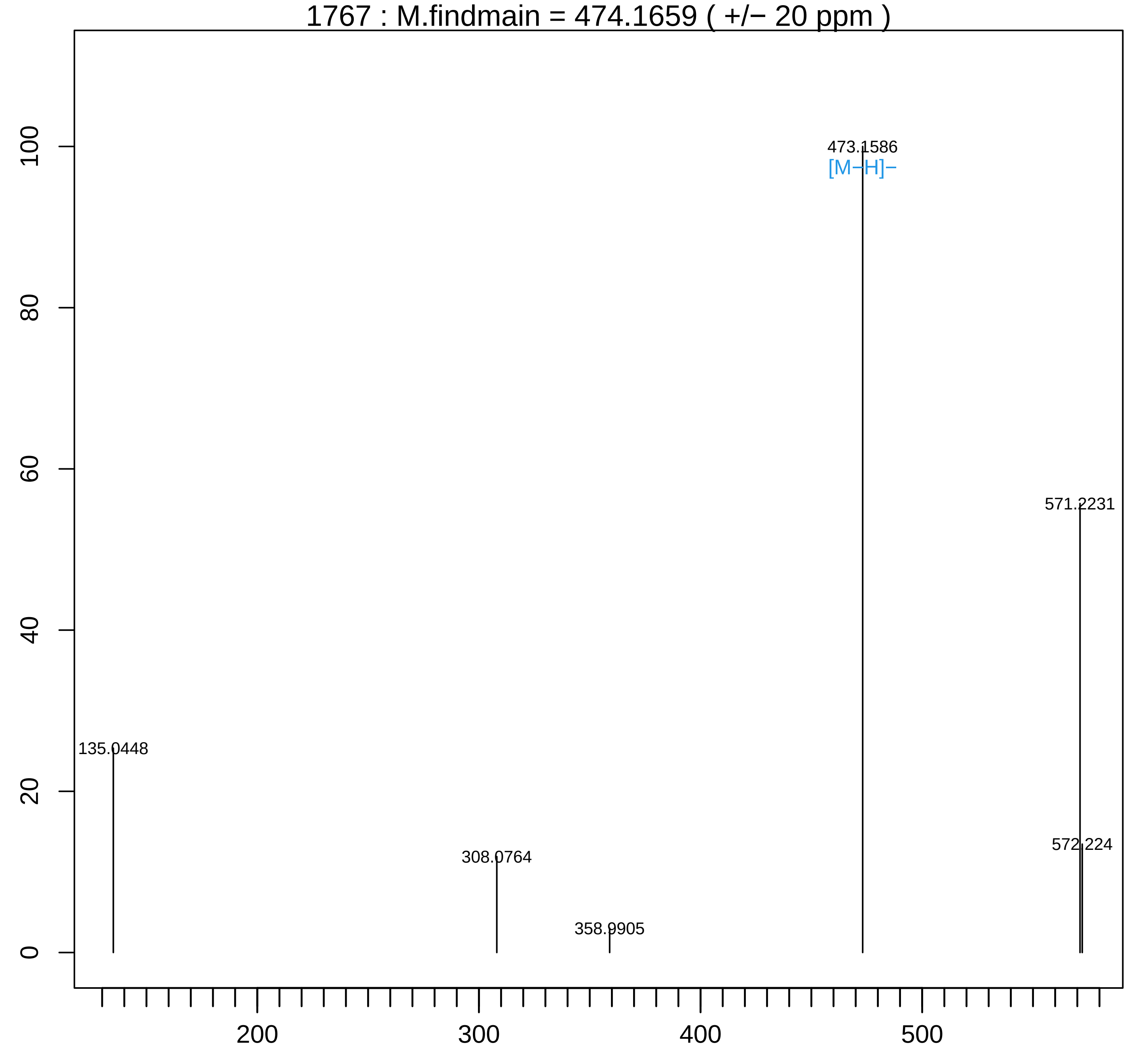
1767 : M.findmain = 474.1659 (+/− 20 ppm)
473.1586
[M−H]−
571.2231
135.0448
308.0764
572.224
358.9905
0
20
40
60
80
100
200
300
400
500

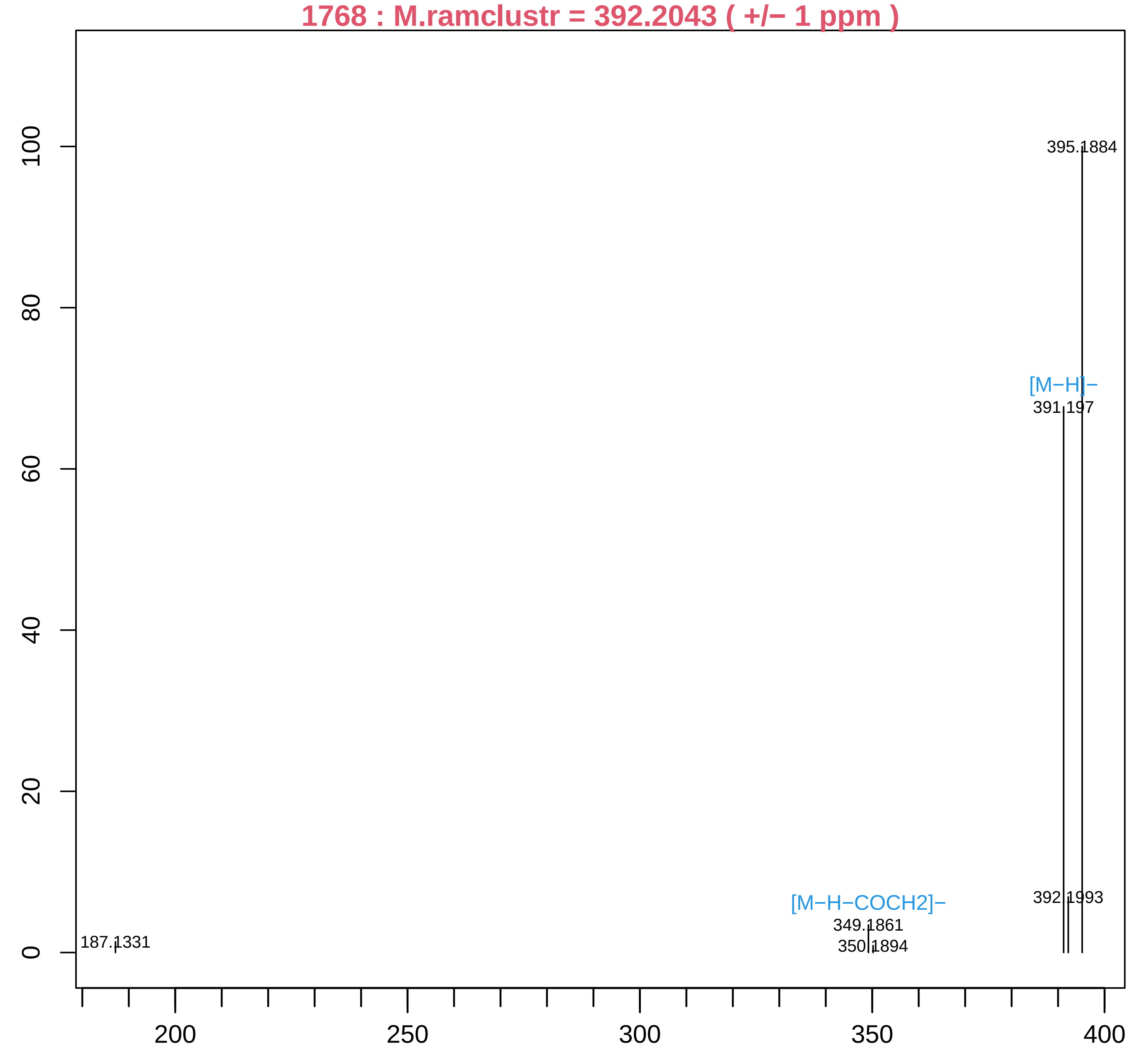
1768 : M.ramclustr = 392.2043 (+/− 1 ppm)
395.1884
[M−H]−
391.197
[M−H−COCH2]−
349.1861
350.1894
392.1993
187.1331
0
20
40
60
80
100
200
250
300
350
400

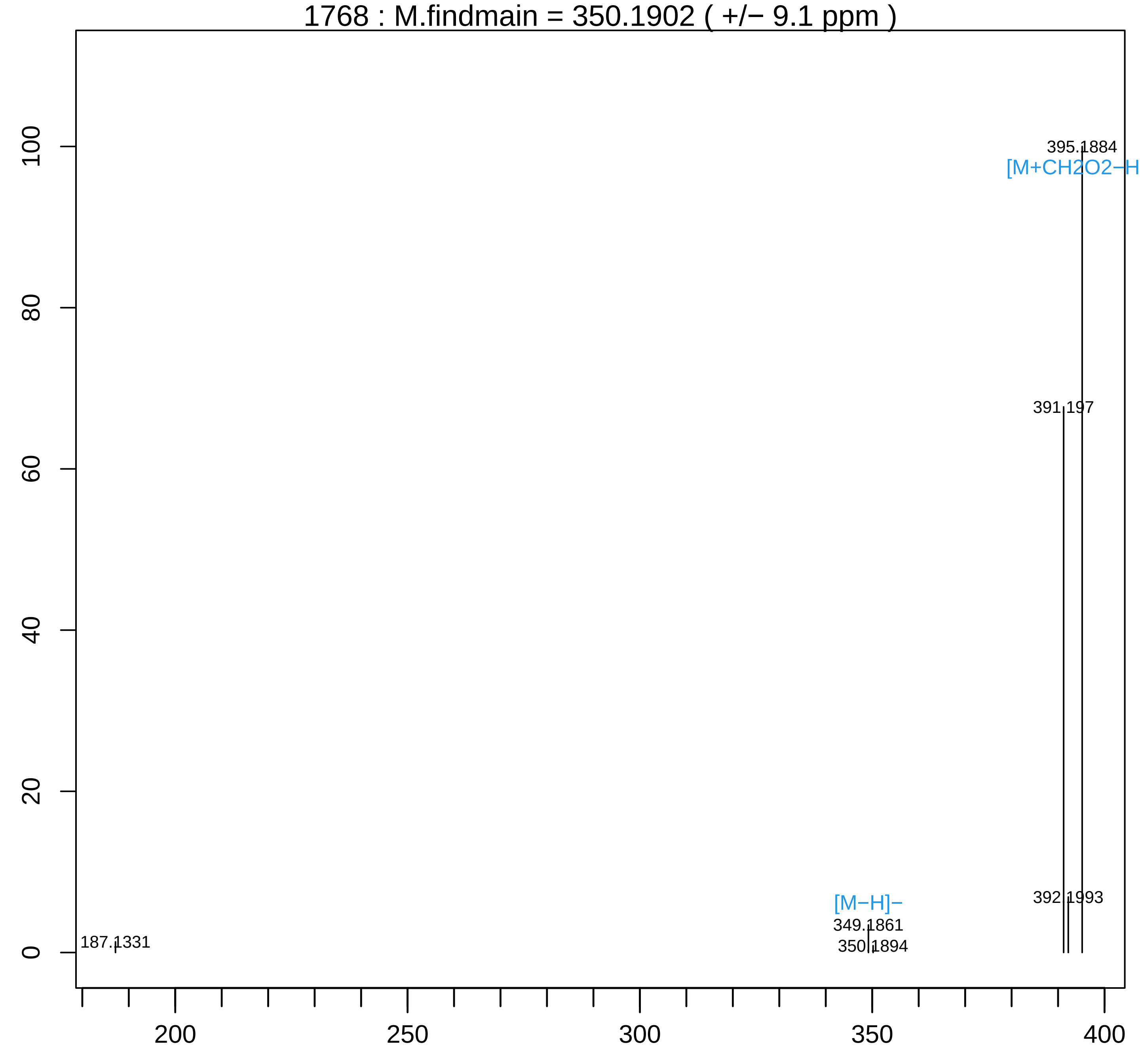
1768 : M.findmain = 350.1902 (+/− 9.1 ppm)
395.1884
[M+CH2O2−H
391.197
[M−H]−
349.1861
350.1894
392.1993
187.1331
0
20
40
60
80
100
200
250
300
350
400

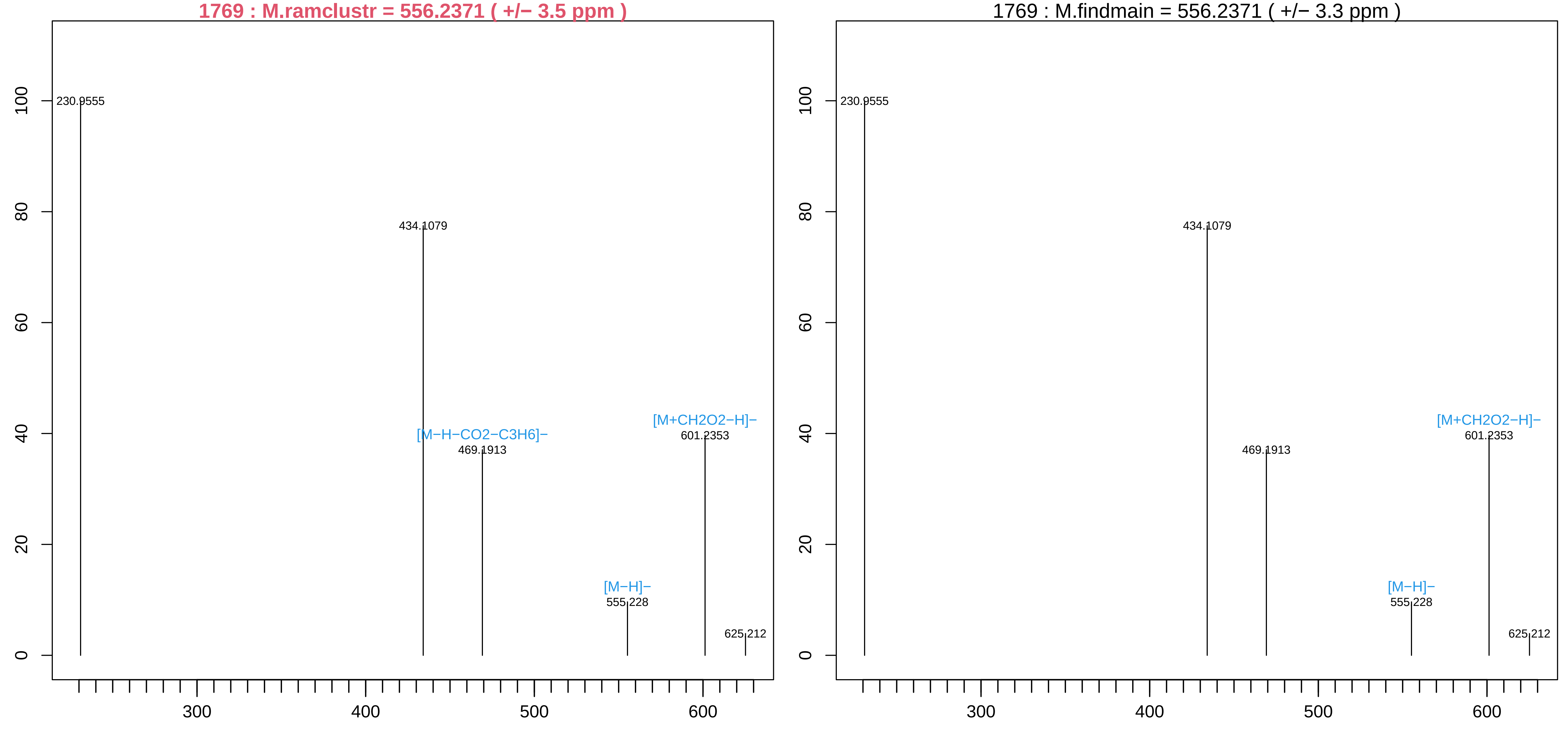
1769 : M.ramclustr = 556.2371 (+/− 3.5 ppm)
230.9555
434.1079
[M−H−CO2−C3H6]−
469.1913
[M−H]−
555.228
[M+CH2O2−H]−
601.2353
625.212
0
20
40
60
80
100
300
400
500
600
1769 : M.findmain = 556.2371 (+/− 3.3 ppm)
230.9555
434.1079
469.1913
[M−H]−
555.228
[M+CH2O2−H]−
601.2353
625.212
0
20
40
60
80
100
300
400
500
600

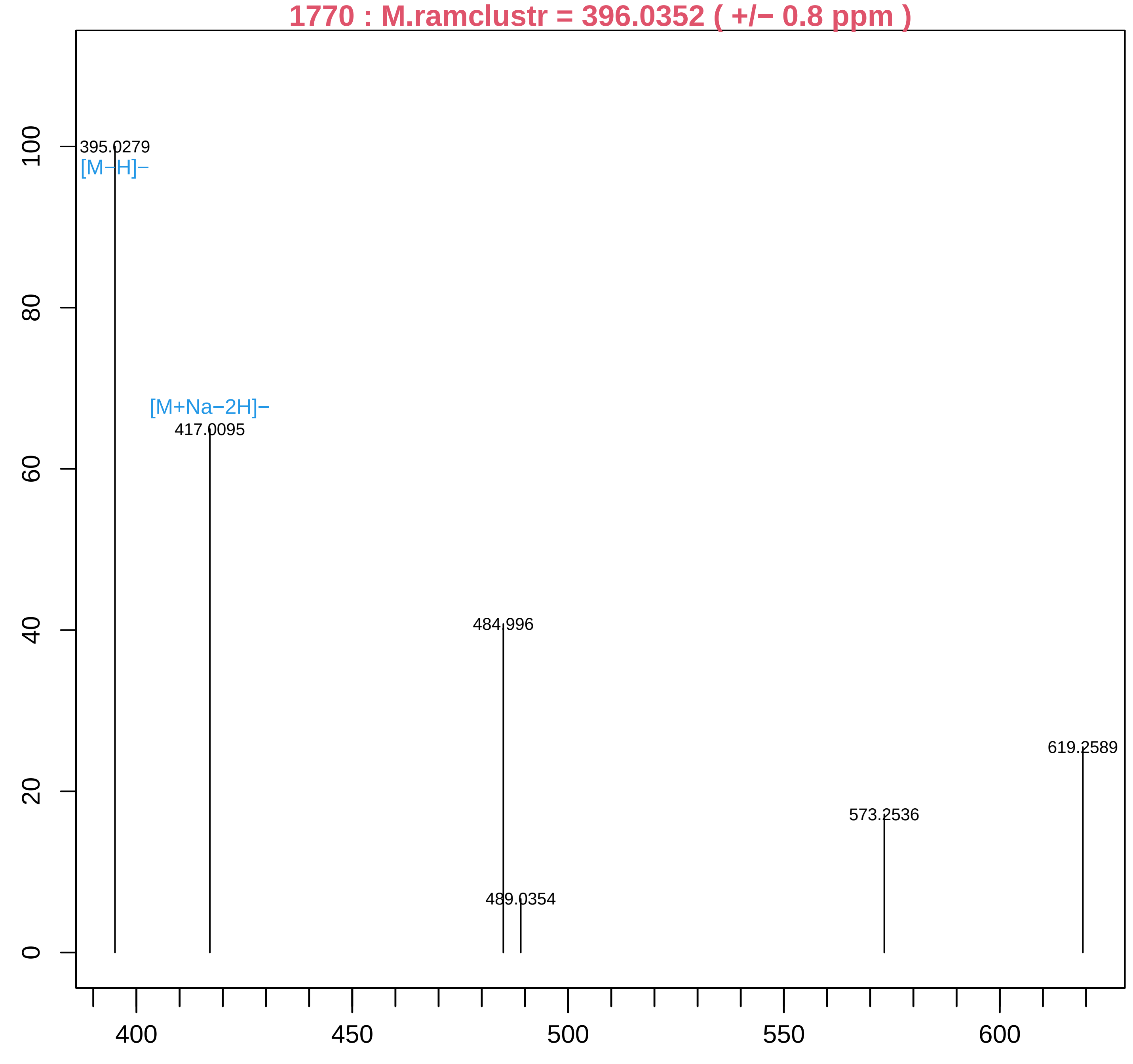
1770 : M.ramclustr = 396.0352 (+/− 0.8 ppm)
395.0279
[M−H]−
[M+Na−2H]−
417.0095
484.996
489.0354
573.2536
619.2589
0
20
40
60
80
100
400
450
500
550
600

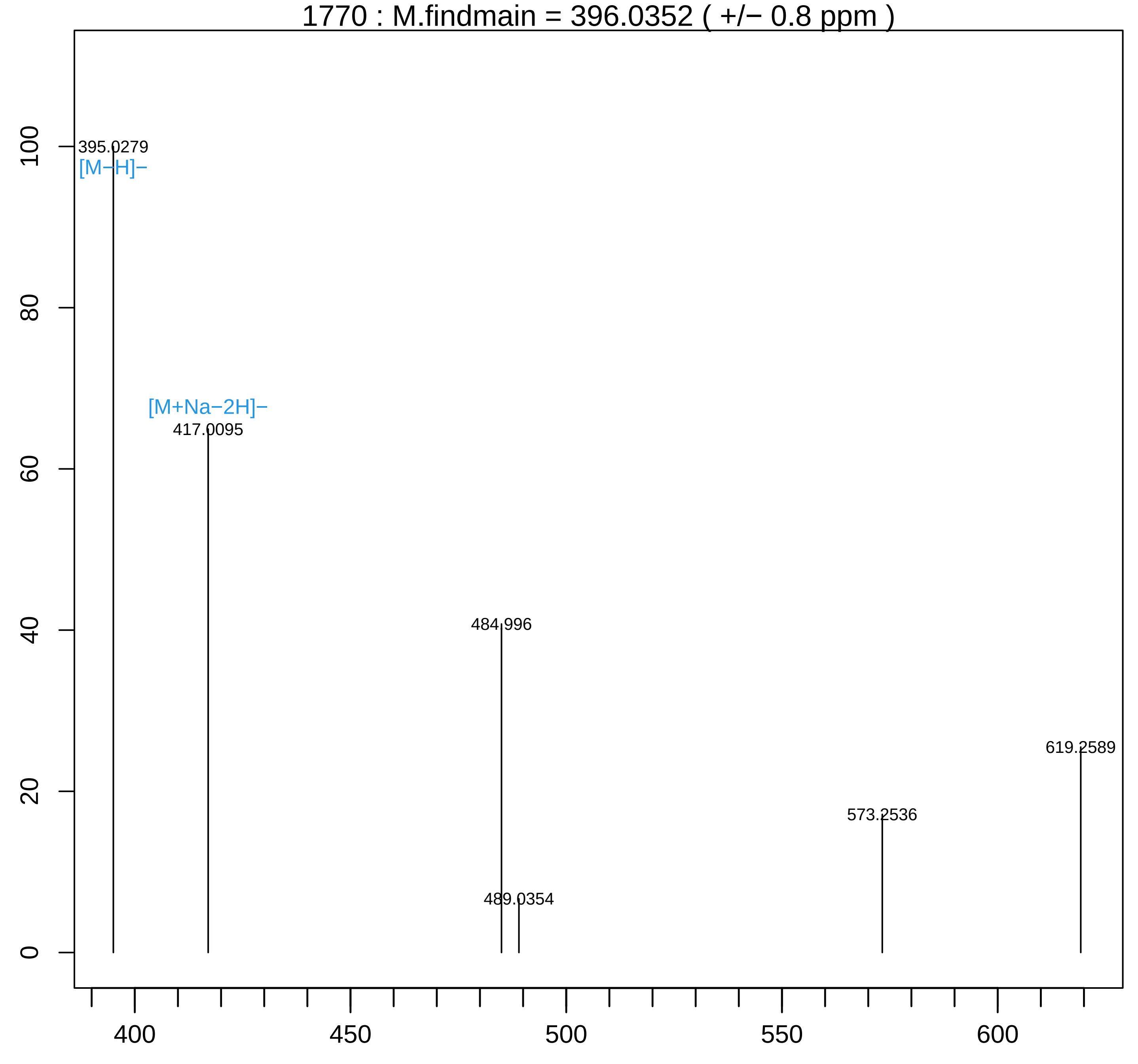
1770 : M.findmain = 396.0352 (+/− 0.8 ppm)
395.0279
[M−H]−
[M+Na−2H]−
417.0095
484.996
489.0354
573.2536
619.2589
0
20
40
60
80
100
400
450
500
550
600

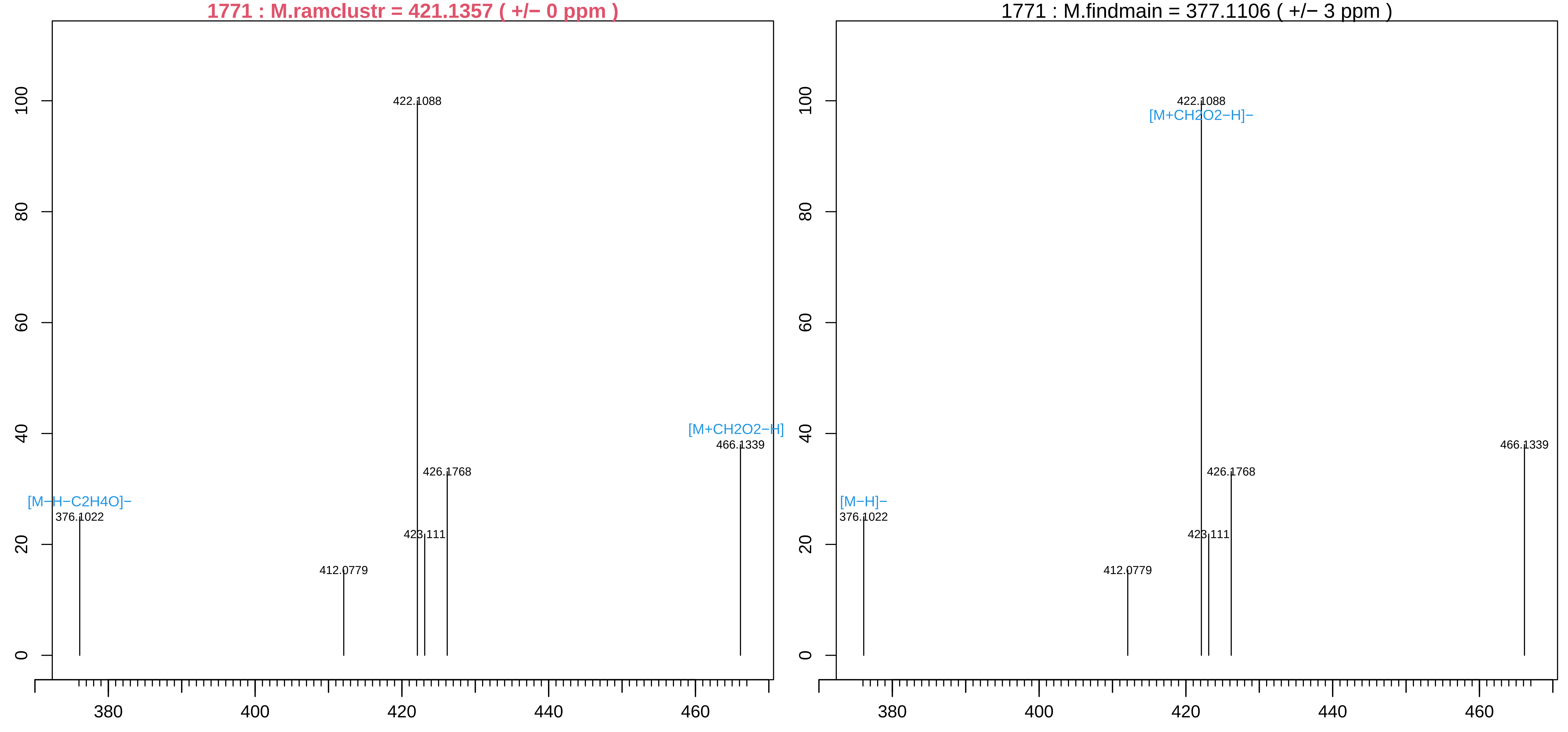

1771 : M.ramclustr = 421.1357 (+/− 0 ppm)
422.1088
[M+CH2O2−H]
466.1339
426.1768
[M−H−C2H4O]−
376.1022
423.111
412.0779
1771 : M.findmain = 377.1106 (+/− 3 ppm)
422.1088
[M+CH2O2−H]−
466.1339
426.1768
[M−H]−
376.1022
423.111
412.0779
0
20
40
60
80
100
380
400
420
440
460

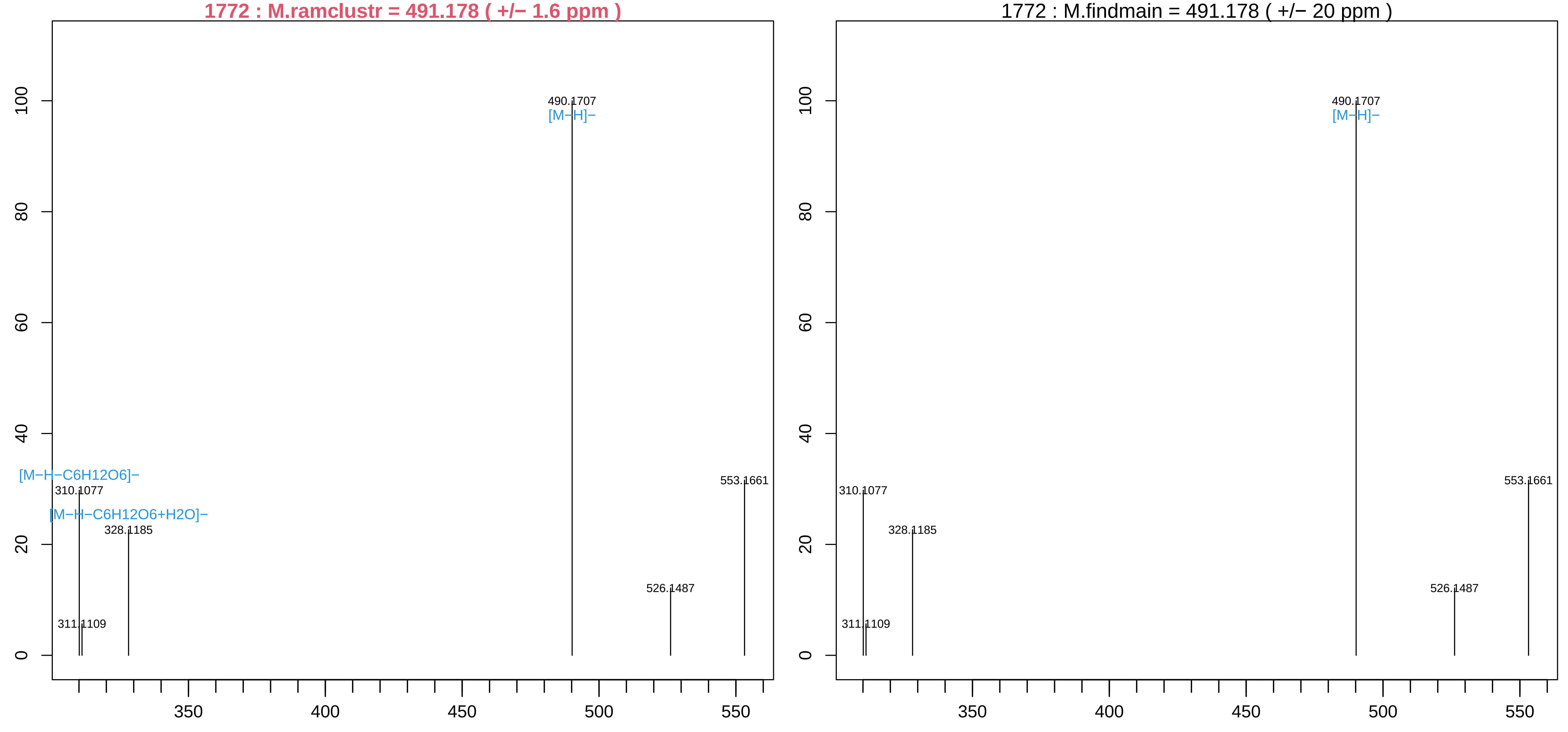

1772 : M.ramclustr = 491.178 (+/− 1.6 ppm)
490.1707
[M−H]−
[M−H−C6H12O6]−
310.1077
[M−H−C6H12O6+H2O]−
328.1185
311.1109
526.1487
553.1661
1772 : M.findmain = 491.178 (+/− 20 ppm)
490.1707
[M−H]−
310.1077
328.1185
311.1109
526.1487
553.1661
0
20
40
60
80
100
350
400
450
500
550

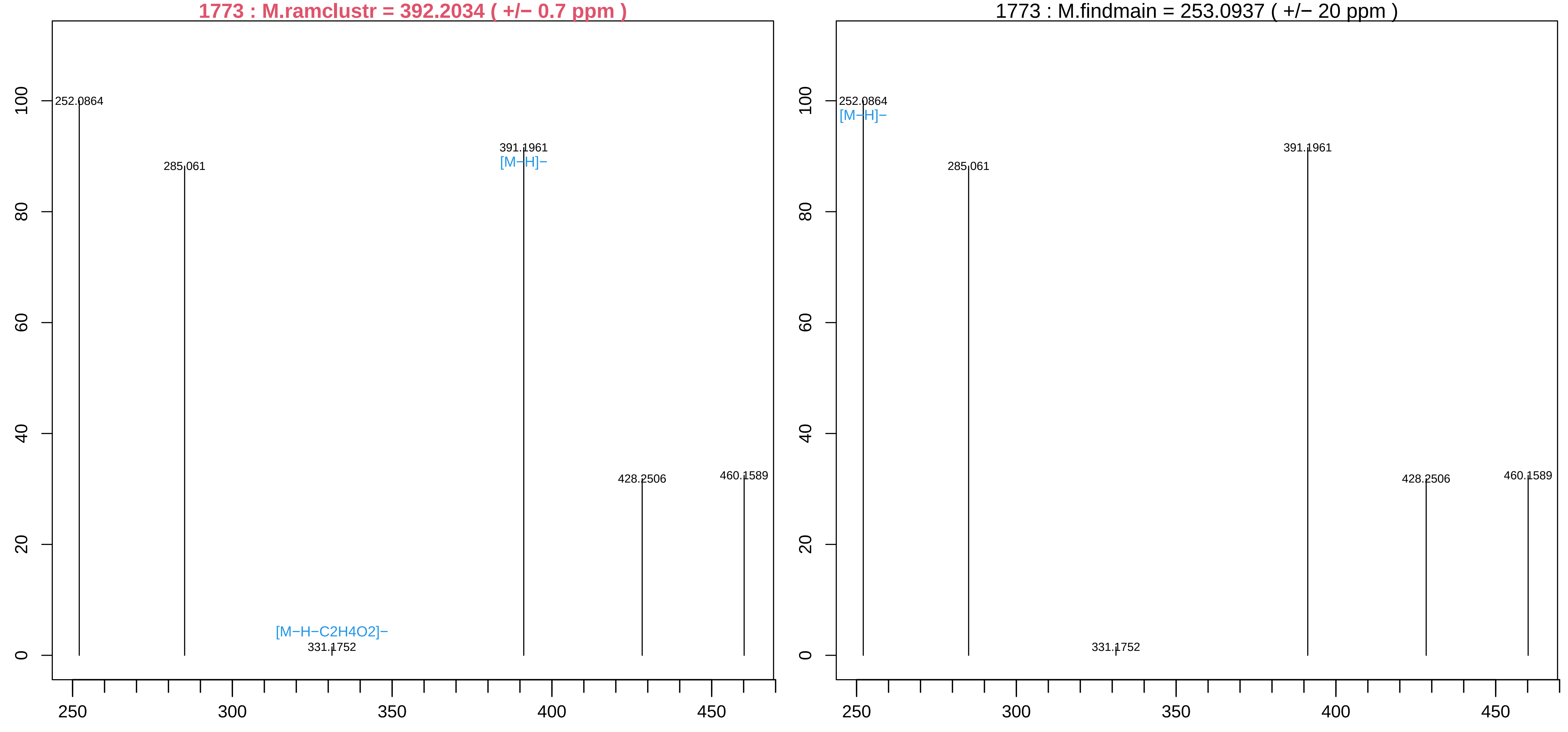

1773 : M.ramclustr = 392.2034 (+/− 0.7 ppm)
252.0864
285.061
391.1961
[M−H]−
428.2506
460.1589
[M−H−C2H4O2]−
331.1752
0
20
40
60
80
100
250
300
350
400
450
1773 : M.findmain = 253.0937 (+/− 20 ppm)
252.0864
[M−H]−
285.061
391.1961
428.2506
460.1589
331.1752
0
20
40
60
80
100
250
300
350
400
450

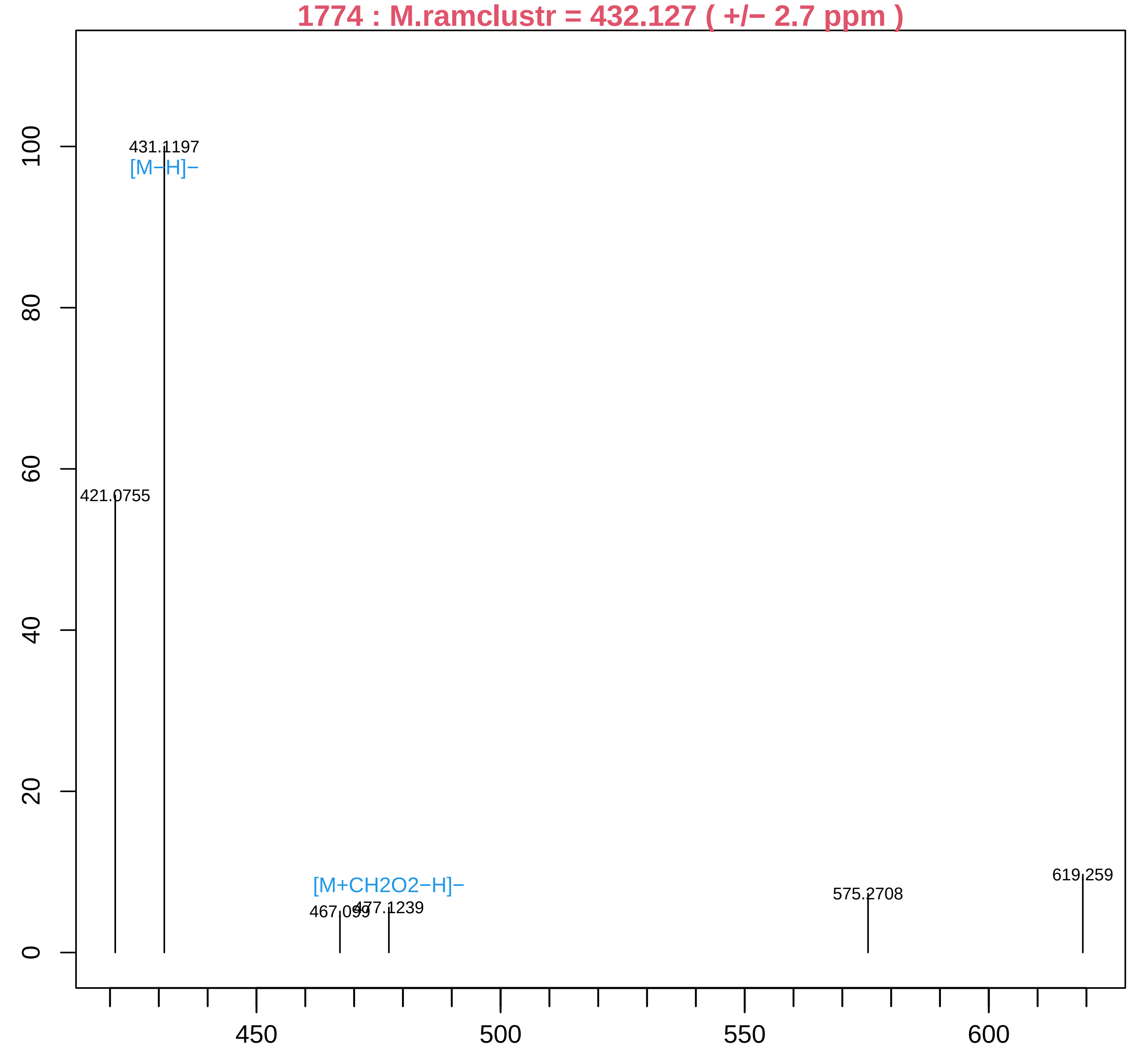
1774 : M.ramclustr = 432.127 (+/− 2.7 ppm)
431.1197
[M−H]−
421.0755
[M+CH2O2−H]−
467.099
477.1239
575.2708
619.259
0
20
40
60
80
100
450
500
550
600

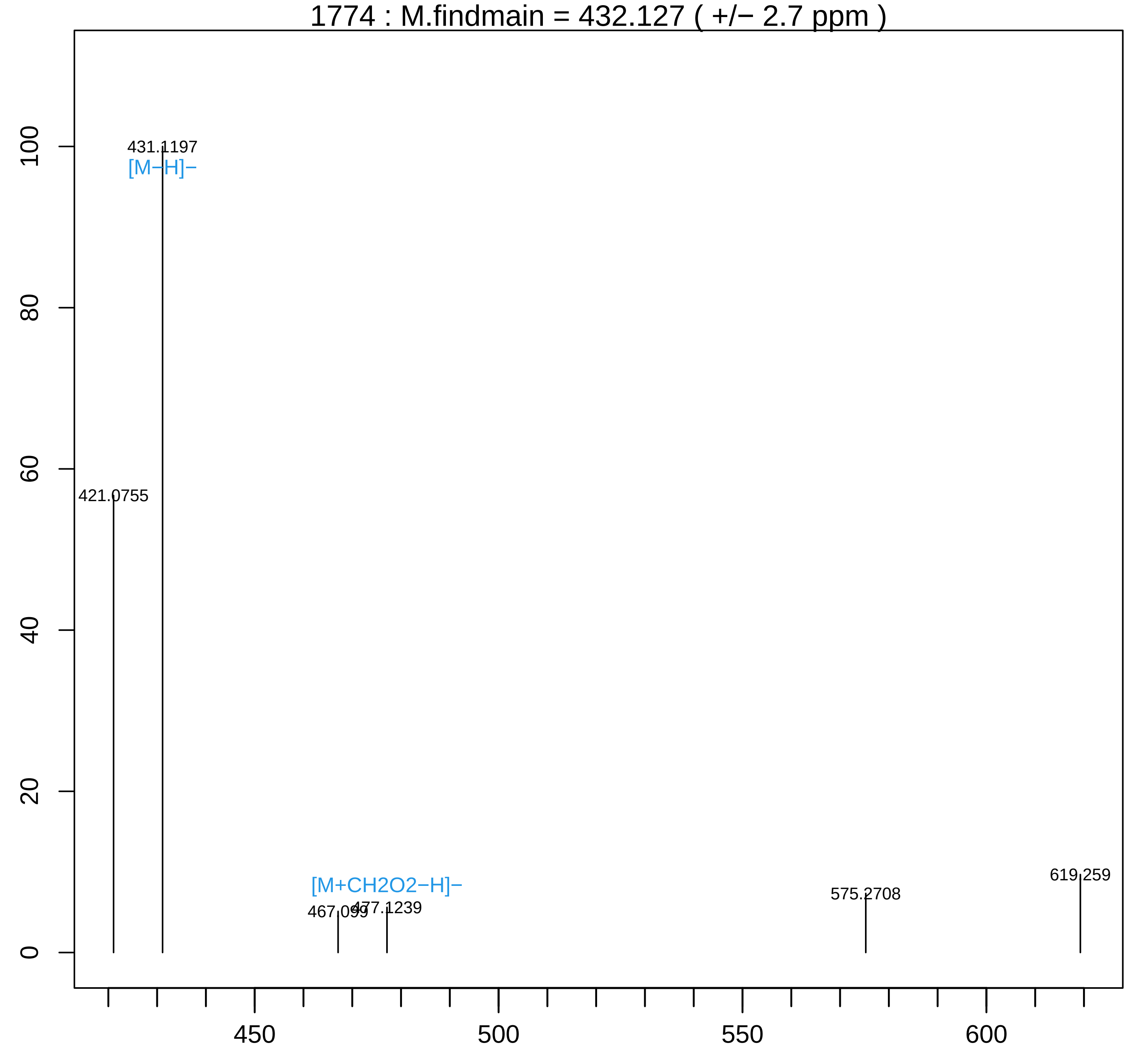
1774 : M.findmain = 432.127 (+/− 2.7 ppm)
431.1197
[M−H]−
421.0755
[M+CH2O2−H]−
467.099
477.1239
575.2708
619.259
0
20
40
60
80
100
450
500
550
600

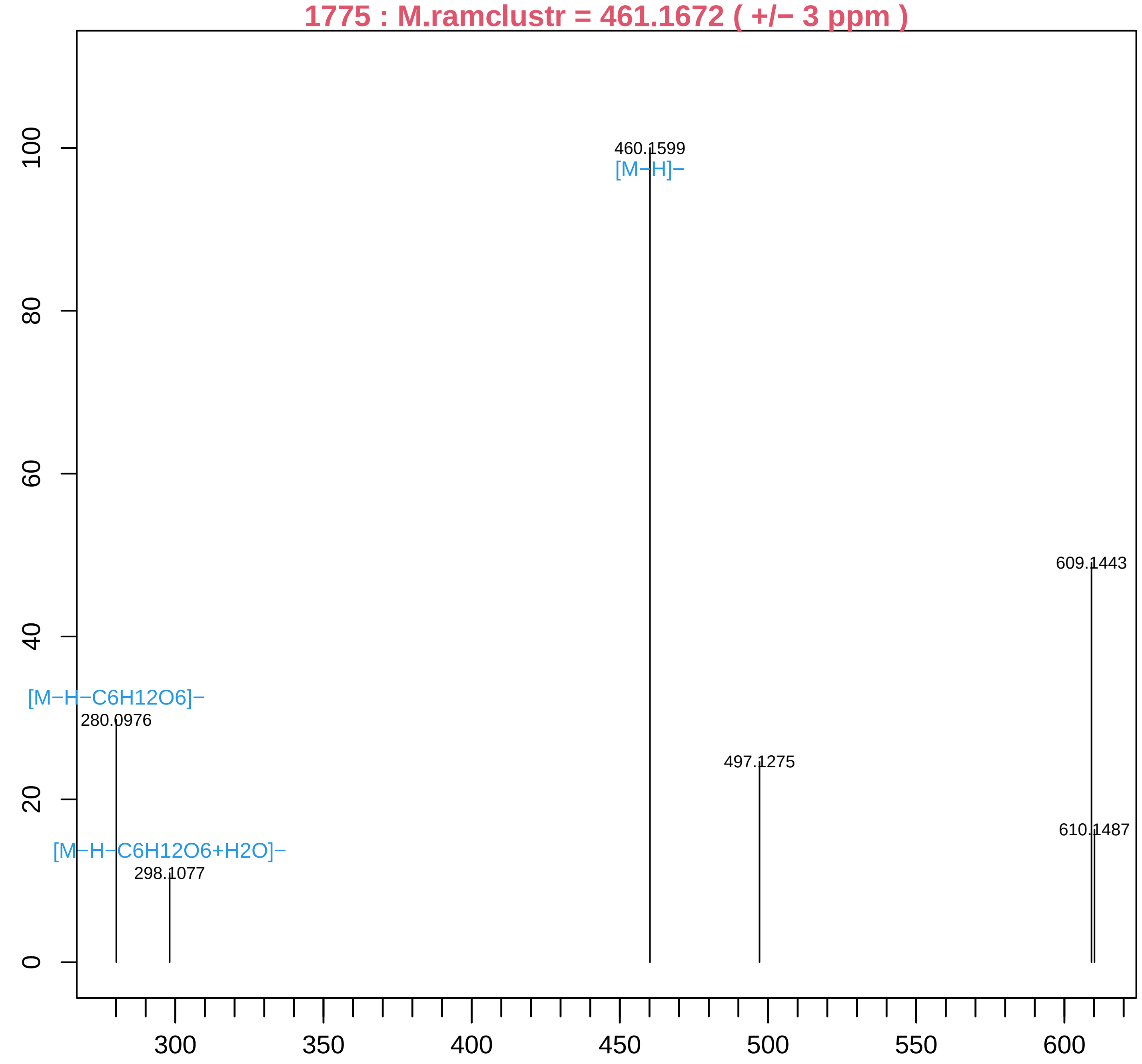
1775 : M.ramclustr = 461.1672 (+/− 3 ppm)
460.1599
[M−H]−
609.1443
[M−H−C6H12O6]−
280.0976
497.1275
610.1487
[M−H−C6H12O6+H2O]−
298.1077
0
20
40
60
80
100
300
350
400
450
500
550
600

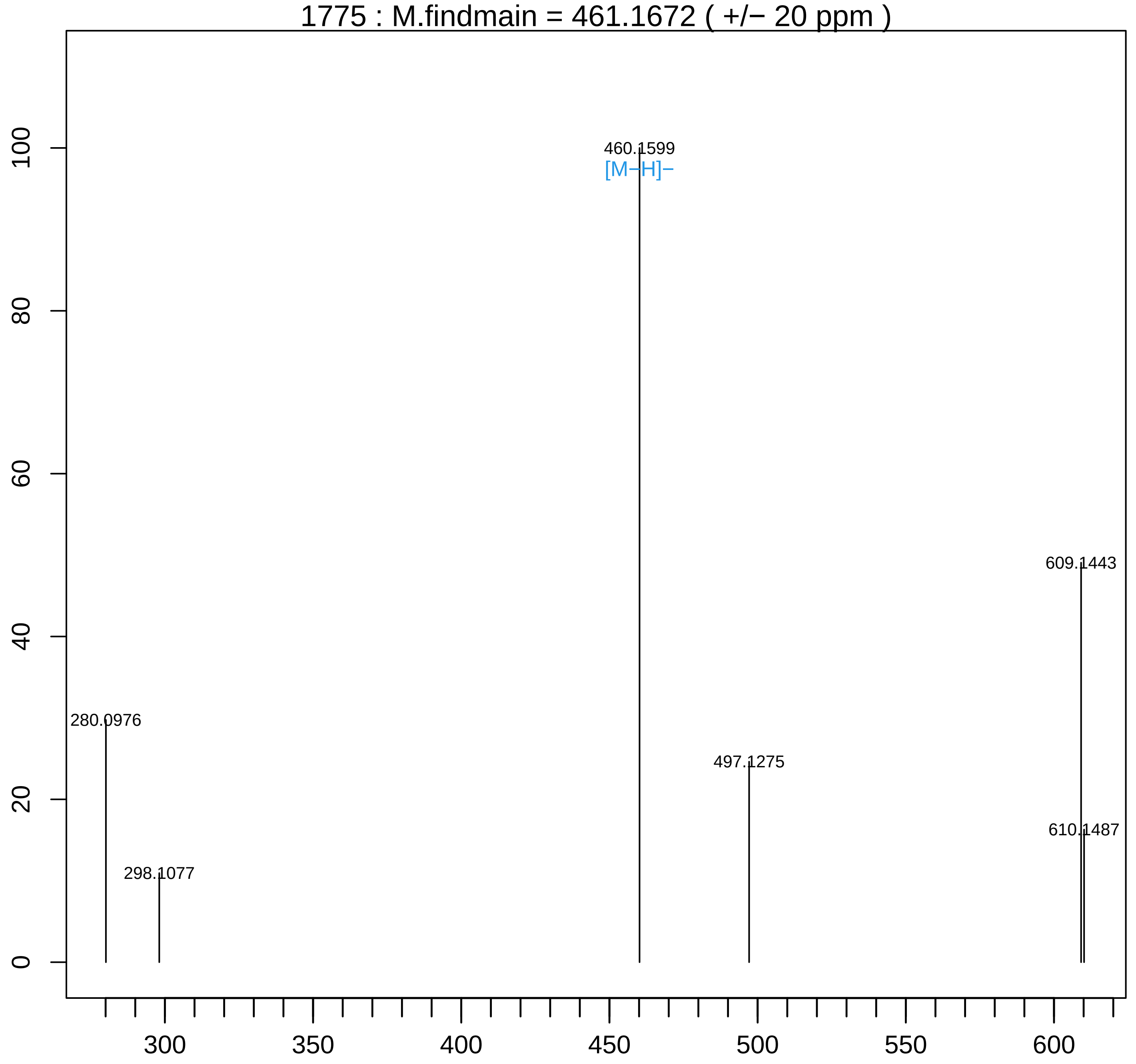
1775 : M.findmain = 461.1672 (+/− 20 ppm)
460.1599
[M−H]−
609.1443
280.0976
497.1275
610.1487
298.1077
0
20
40
60
80
100
300
350
400
450
500
550
600

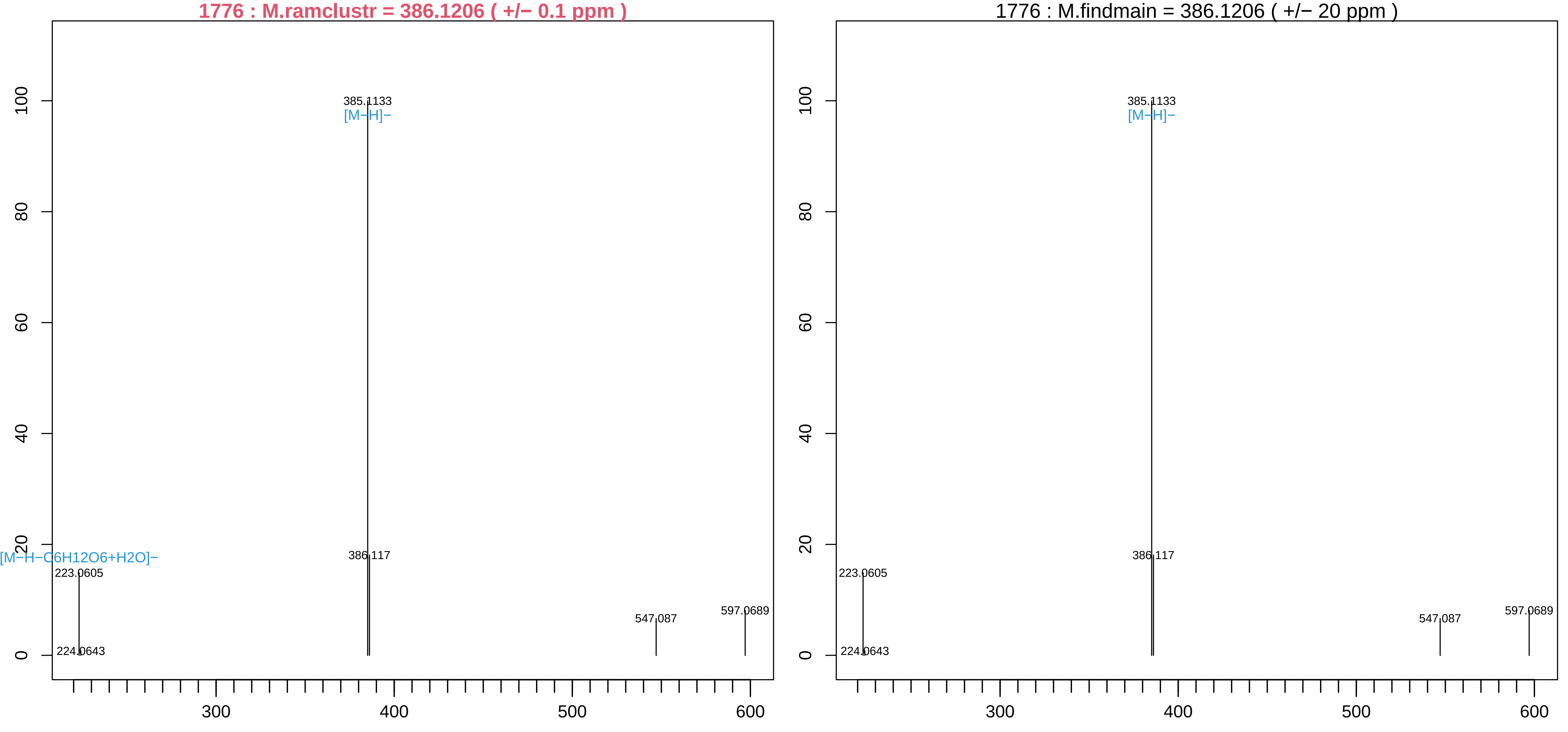

1776 : M.ramclustr = 386.1206 (+/− 0.1 ppm)
1776 : M.findmain = 386.1206 (+/− 20 ppm)
385.1133
[M−H]−
386.117
223.0605
224.0643
547.087
597.0689
[M−H−C6H12O6+H2O]−
0
20
40
60
80
100
300
400
500
600

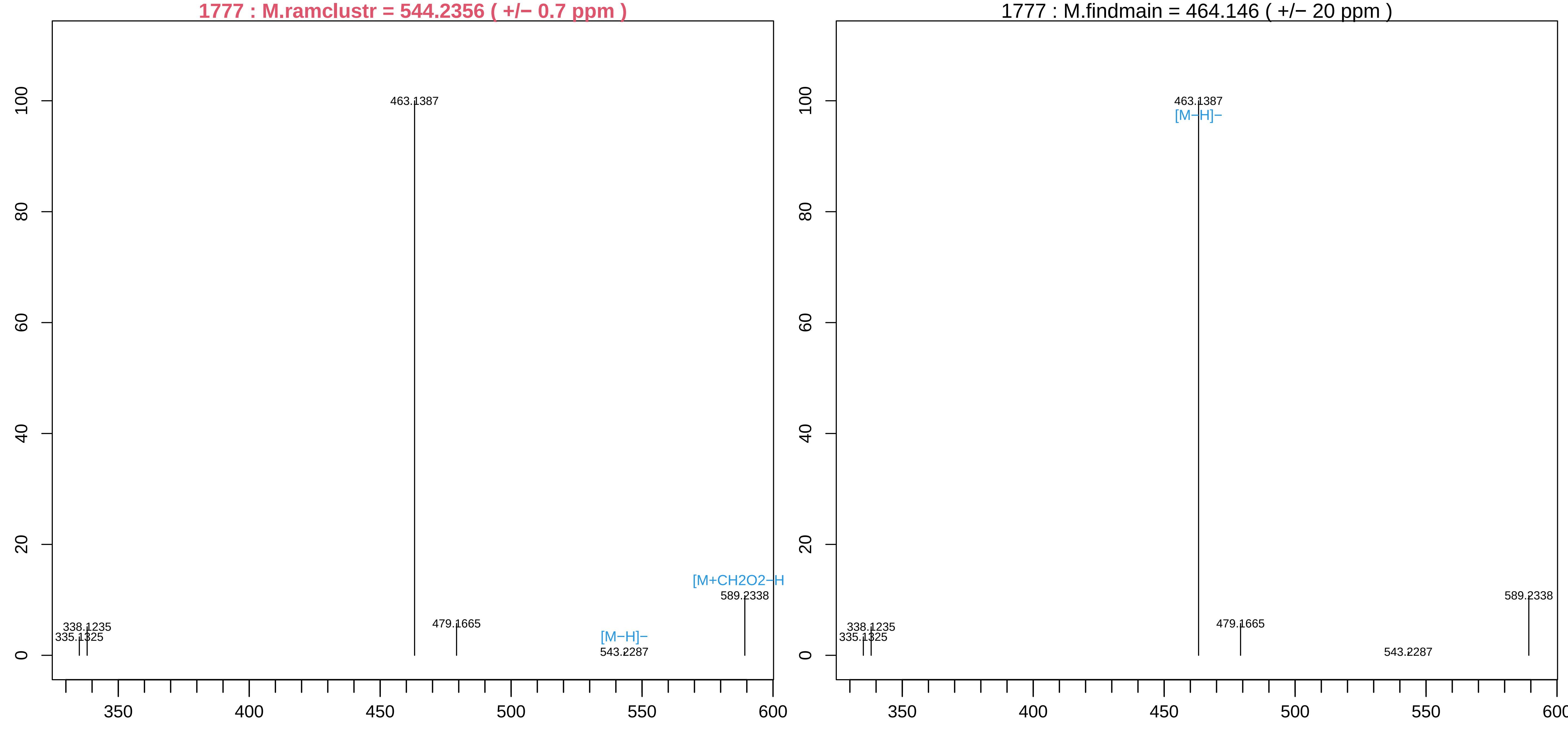
1777 : M.ramclustr = 544.2356 (+/− 0.7 ppm)
463.1387
479.1665
338.1235
335.1325
[M−H]−
543.2287
[M+CH2O2−H
589.2338
0
20
40
60
80
100
350
400
450
500
550
600
1777 : M.findmain = 464.146 (+/− 20 ppm)
463.1387
[M−H]−
479.1665
338.1235
335.1325
543.2287
589.2338

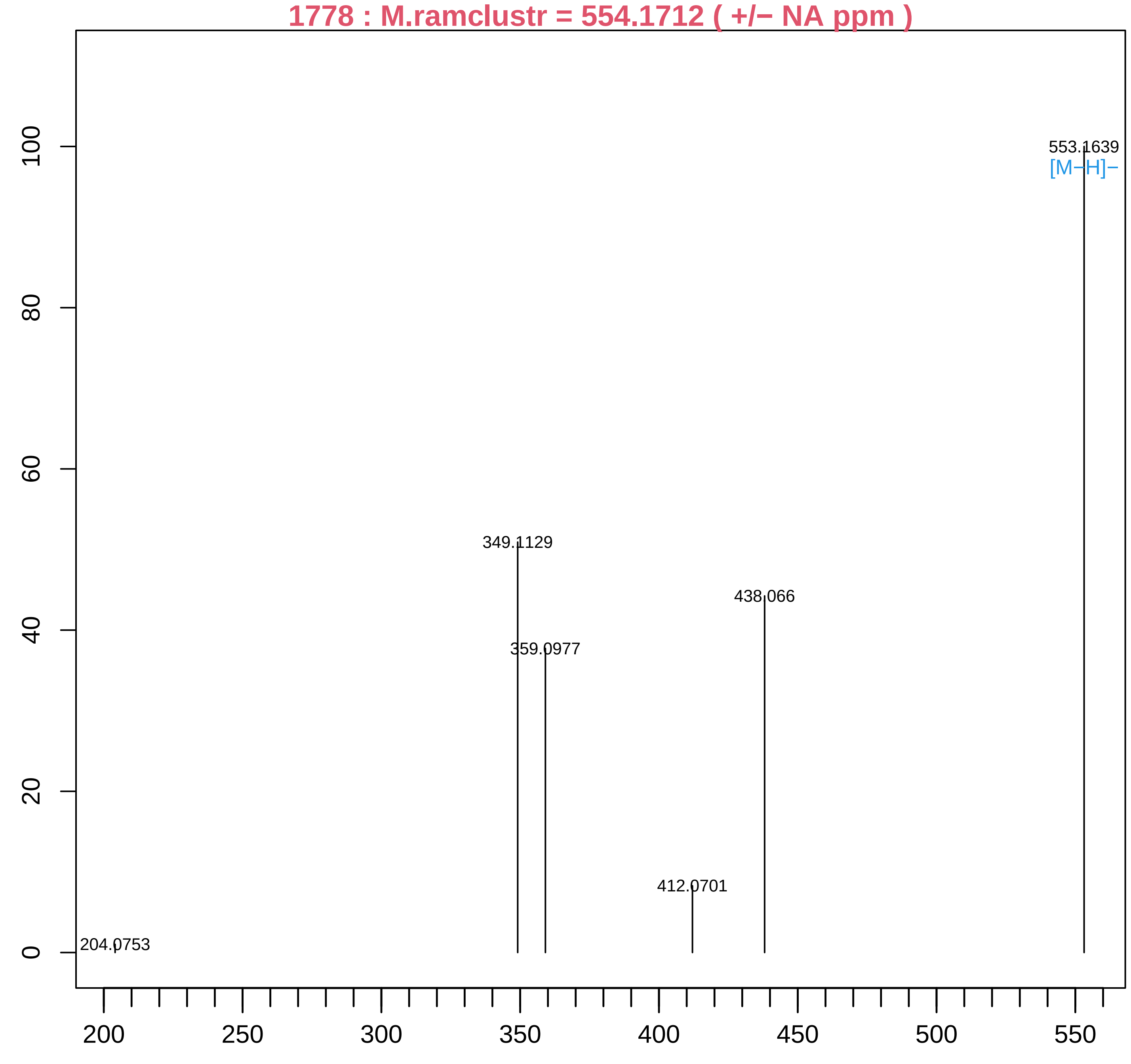
1778 : M.ramclustr = 554.1712 (+/− NA ppm)
553.1639
[M−H]−
349.1129
438.066
359.0977
412.0701
204.0753
0
20
40
60
80
100
200
250
300
350
400
450
500
550

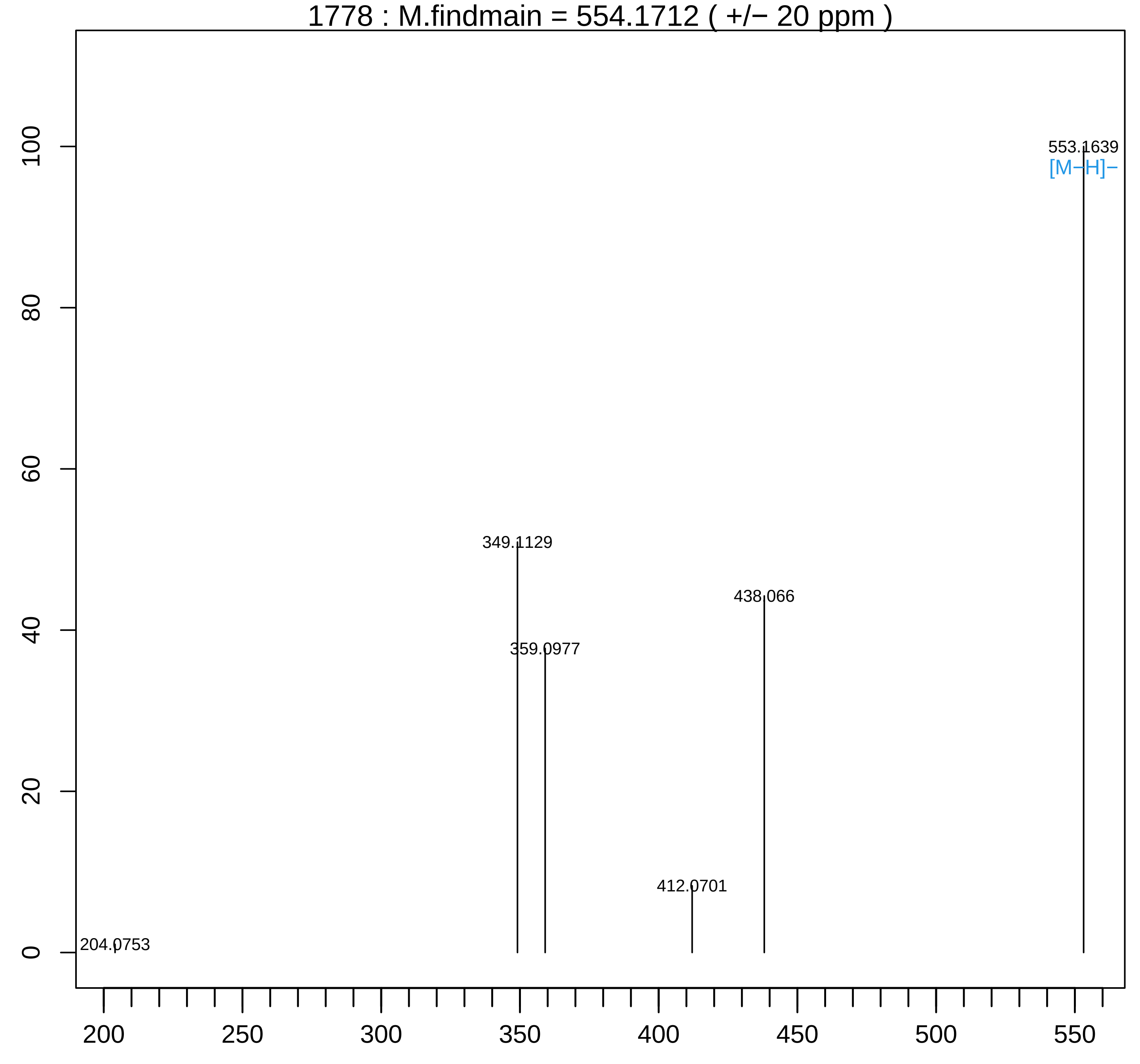
1778 : M.findmain = 554.1712 (+/− 20 ppm)
553.1639
[M−H]−
349.1129
438.066
359.0977
412.0701
204.0753
0
20
40
60
80
100
200
250
300
350
400
450
500
550

1779 : M.ramclustr = 354.0945 (+/− 0.7 ppm)

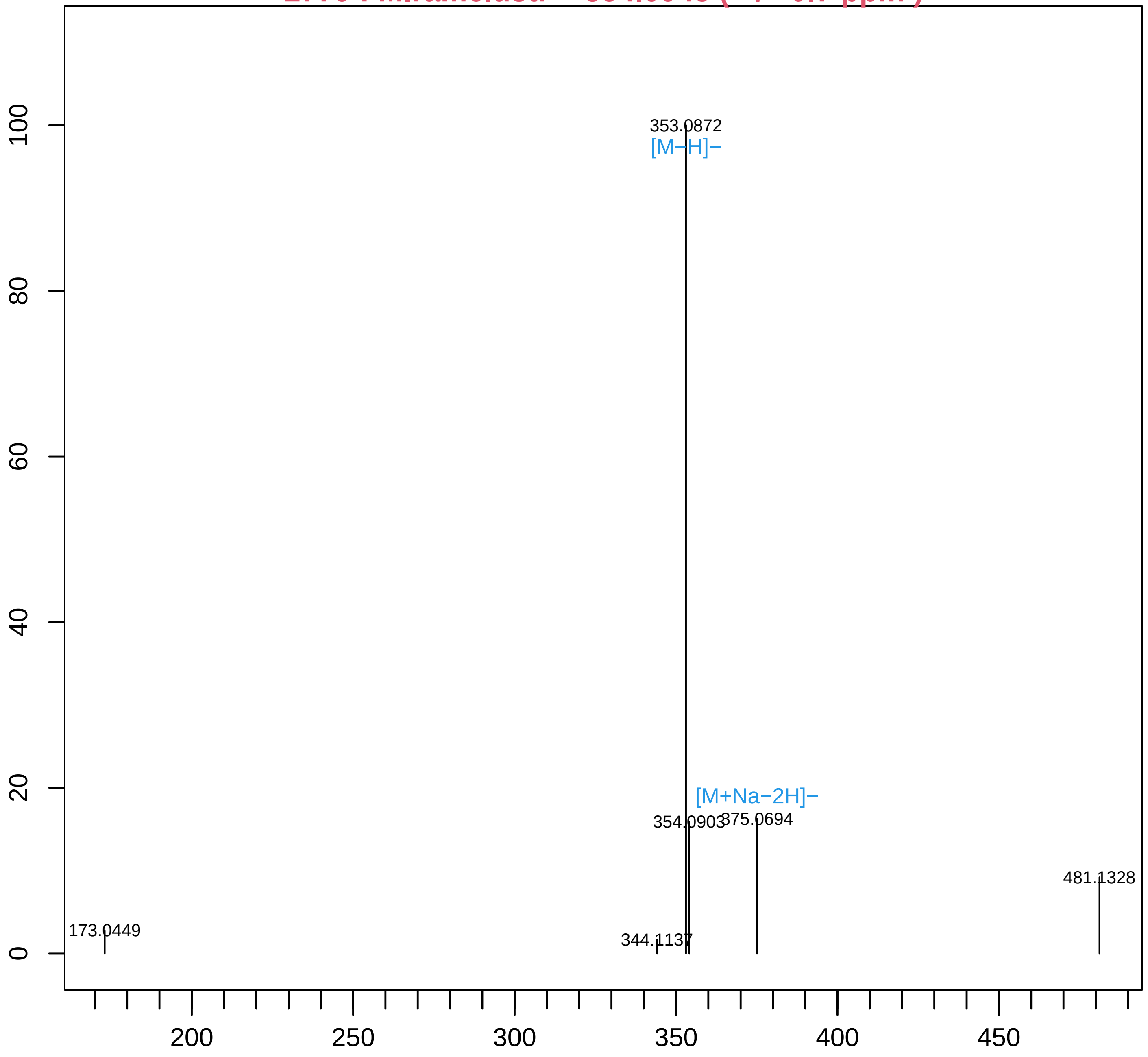

1779 : M.findmain = 354.0945 (+/− 0.7 ppm)

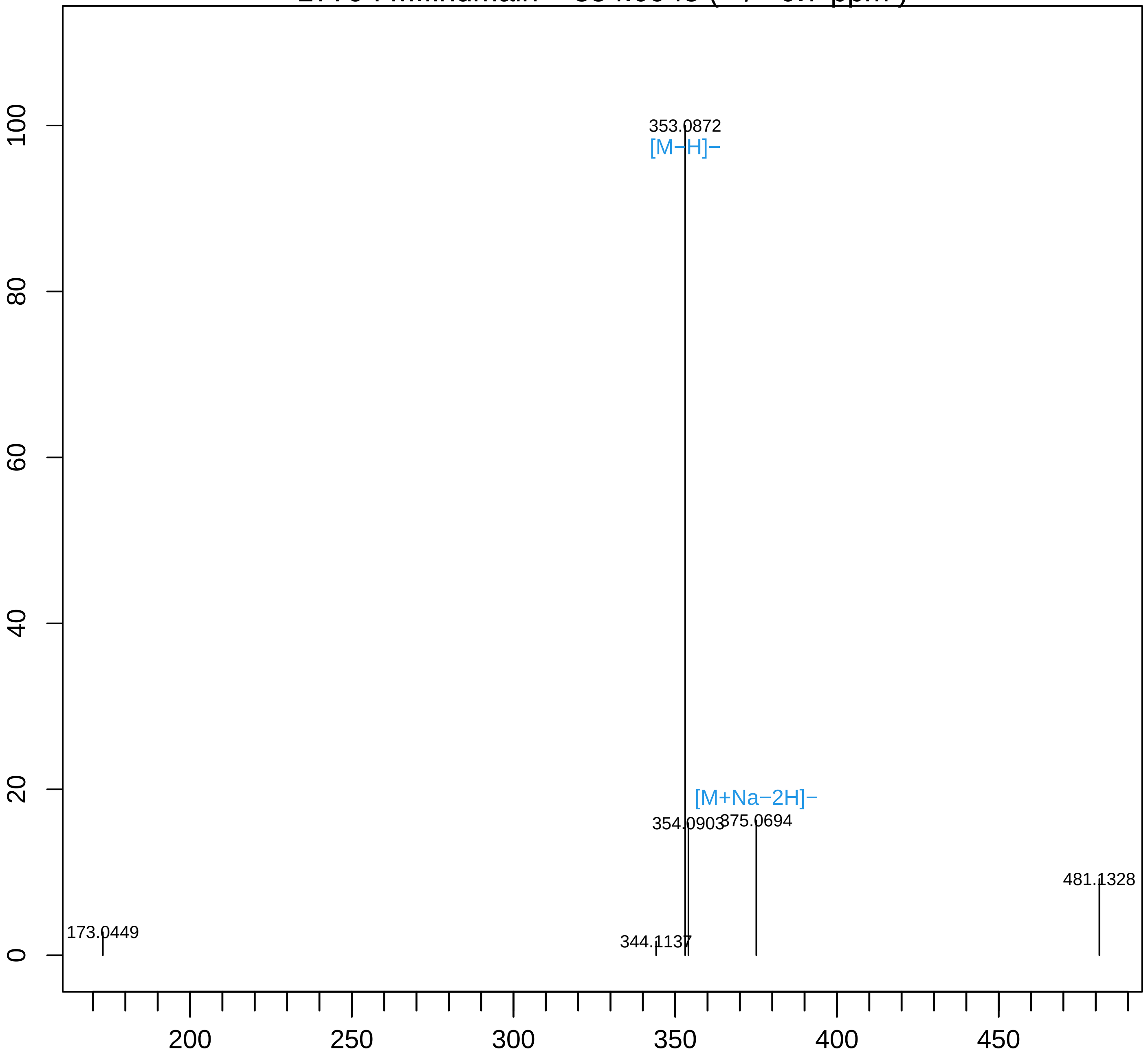

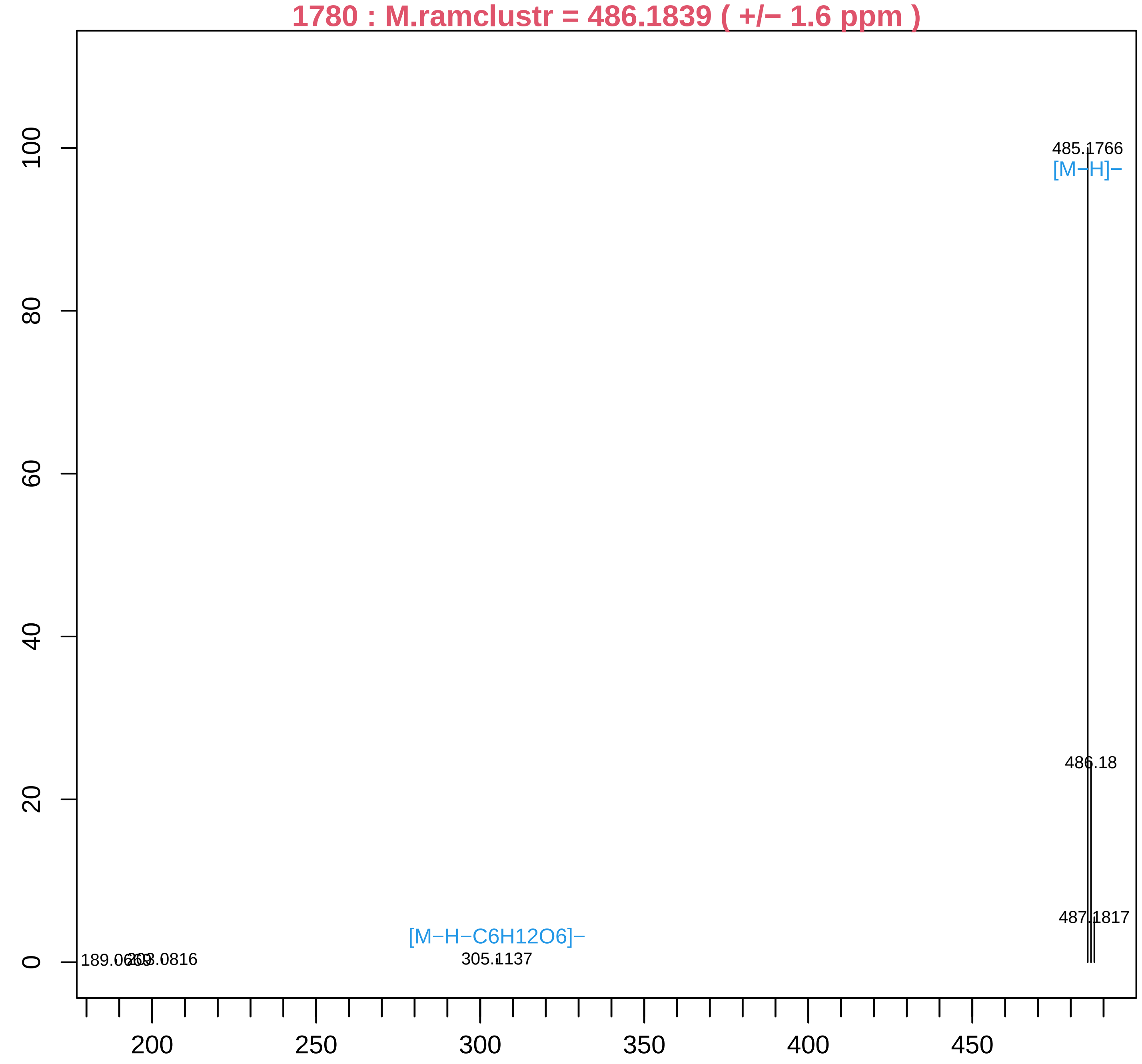

1780 : M.ramclustr = 486.1839 (+/− 1.6 ppm)
485.1766
[M−H]−
486.18
487.1817
[M−H−C6H12O6]−
305.1137
189.0603
203.0816
0
20
40
60
80
100
200
250
300
350
400
450

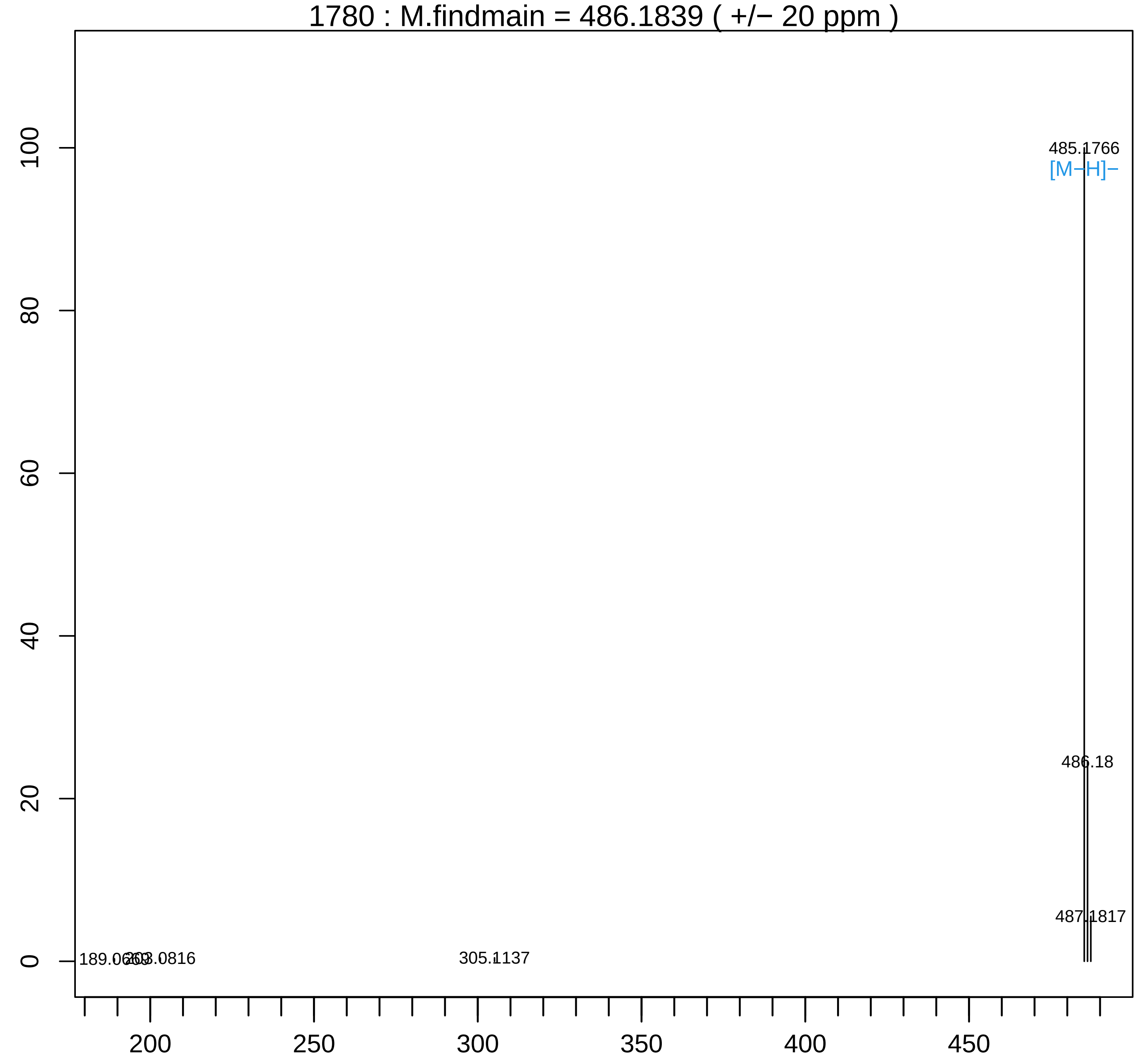

1780 : M.findmain = 486.1839 (+/− 20 ppm)
485.1766
[M−H]−
486.18
487.1817
305.1137
189.0603
203.0816
0
20
40
60
80
100
200
250
300
350
400
450

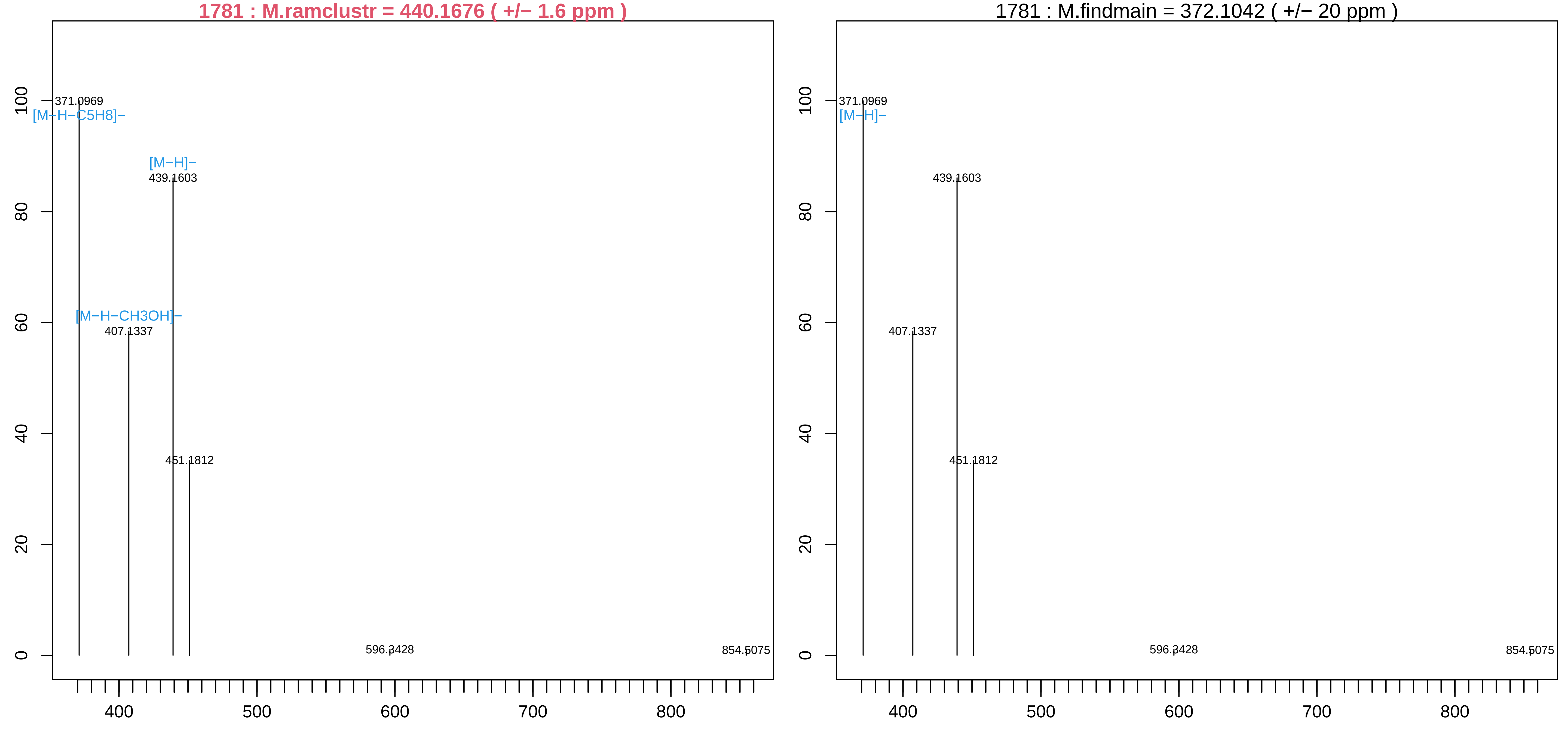

1781 : M.ramclustr = 440.1676 (+/− 1.6 ppm)
371.0969
[M−H−C5H8]−
[M−H]−
439.1603
[M−H−CH3OH]−
407.1337
451.1812
596.3428
854.5075
1781 : M.findmain = 372.1042 (+/− 20 ppm)
371.0969
[M−H]−
439.1603
407.1337
451.1812
596.3428
854.5075
0
20
40
60
80
100
400
500
600
700
800

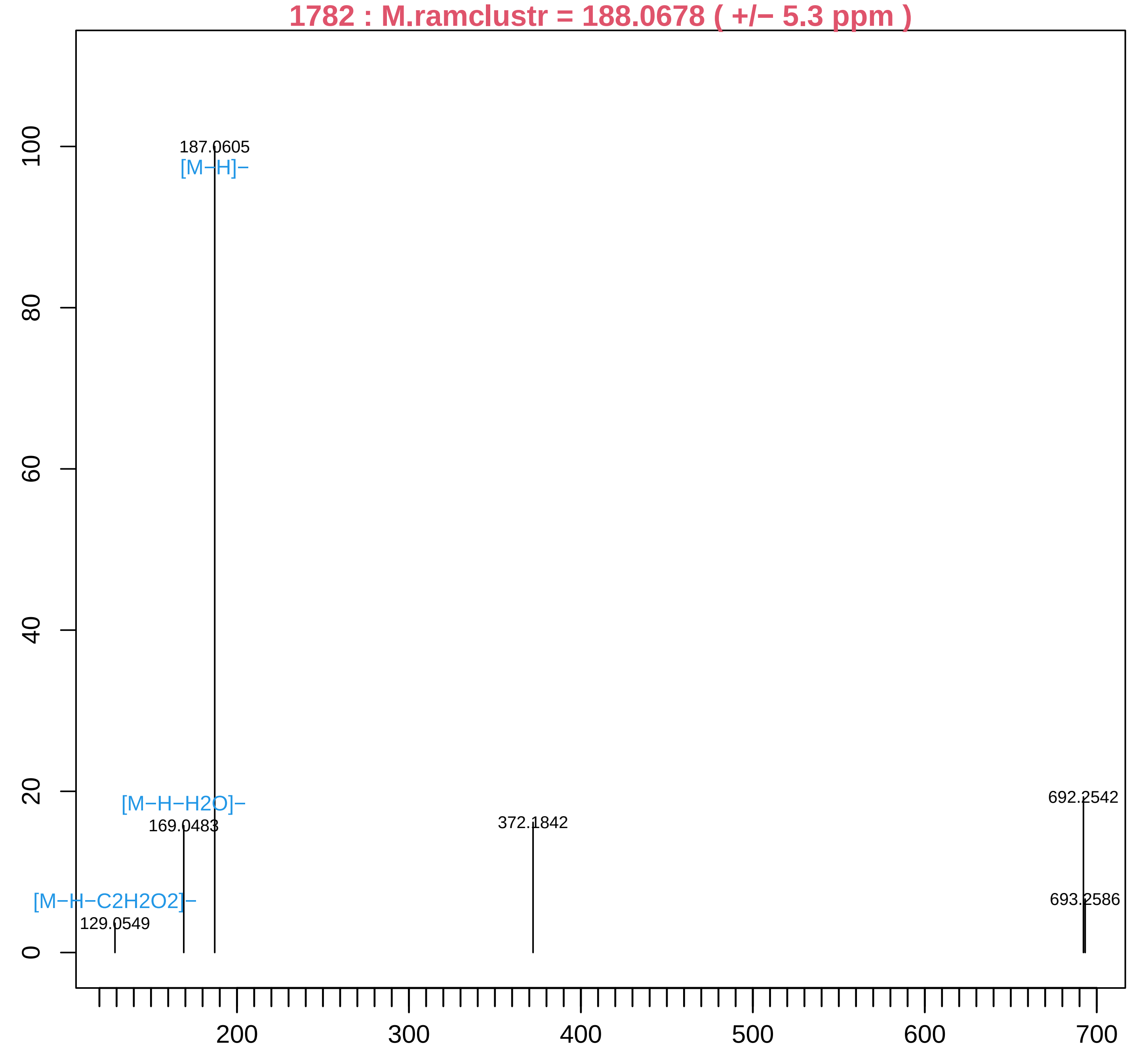
1782 : M.ramclustr = 188.0678 (+/− 5.3 ppm)
187.0605
[M−H]−
[M−H−H2O]−
169.0483
372.1842
692.2542
[M−H−C2H2O2]−
693.2586
129.0549
0
20
40
60
80
100
200
300
400
500
600
700

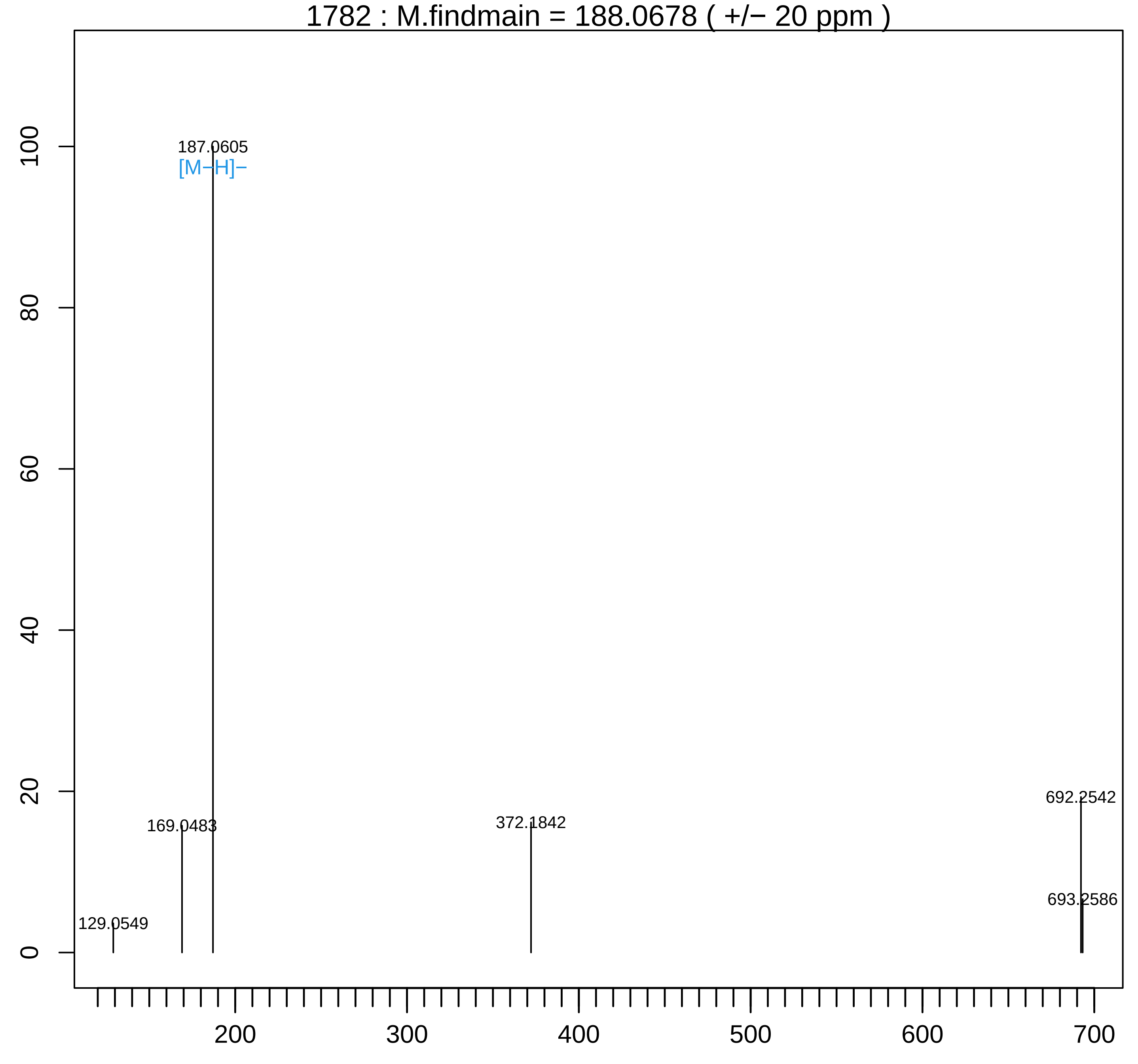
1782 : M.findmain = 188.0678 (+/− 20 ppm)
187.0605
[M−H]−
169.0483
372.1842
692.2542
693.2586
129.0549
0
20
40
60
80
100
200
300
400
500
600
700

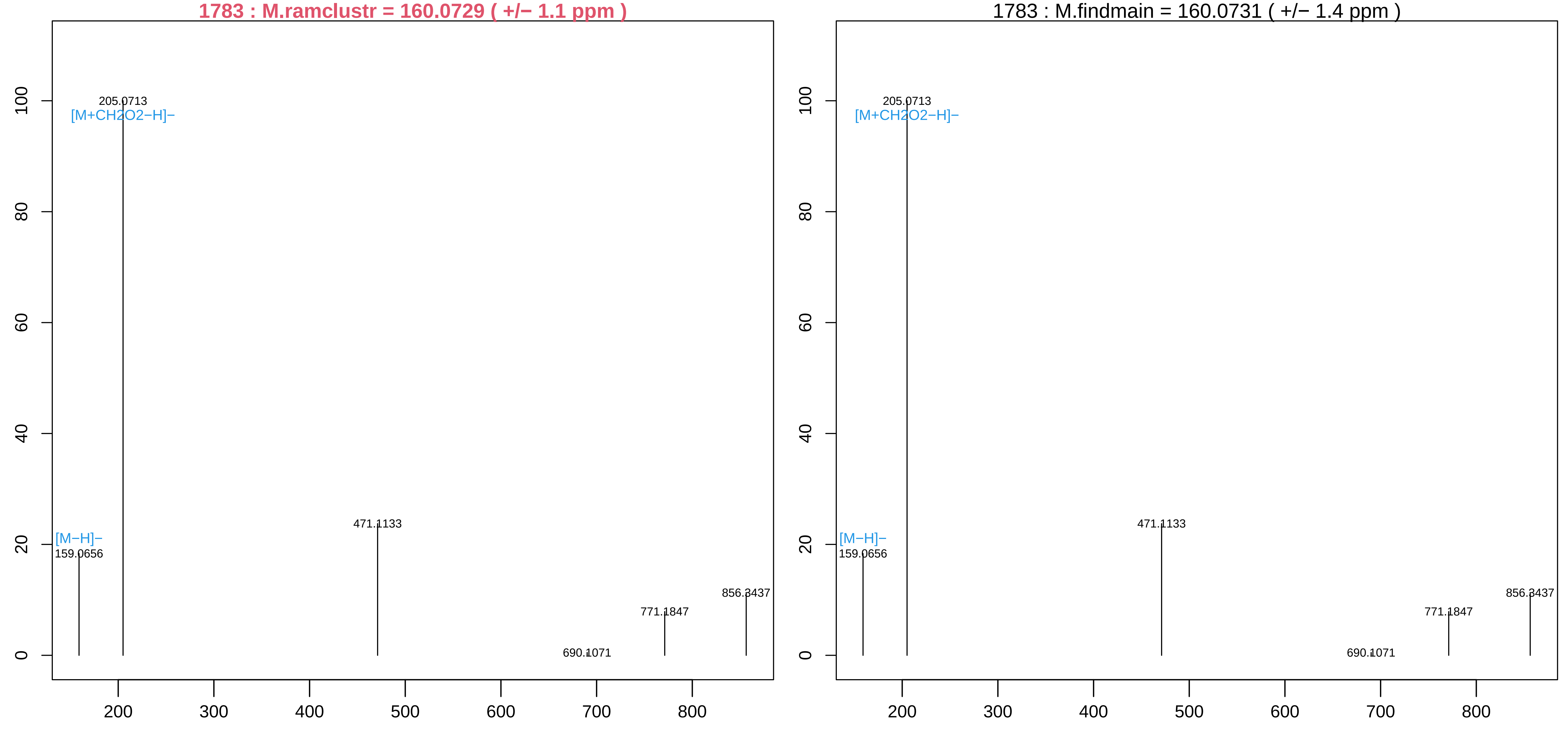
1783 : M.ramclustr = 160.0729 (+/− 1.1 ppm)
205.0713
[M+CH2O2−H]−
[M−H]−
159.0656
471.1133
856.3437
771.1847
690.1071
0
20
40
60
80
100
200
300
400
500
600
700
800
1783 : M.findmain = 160.0731 (+/− 1.4 ppm)
205.0713
[M+CH2O2−H]−
[M−H]−
159.0656
471.1133
856.3437
771.1847
690.1071
0
20
40
60
80
100
200
300
400
500
600
700
800

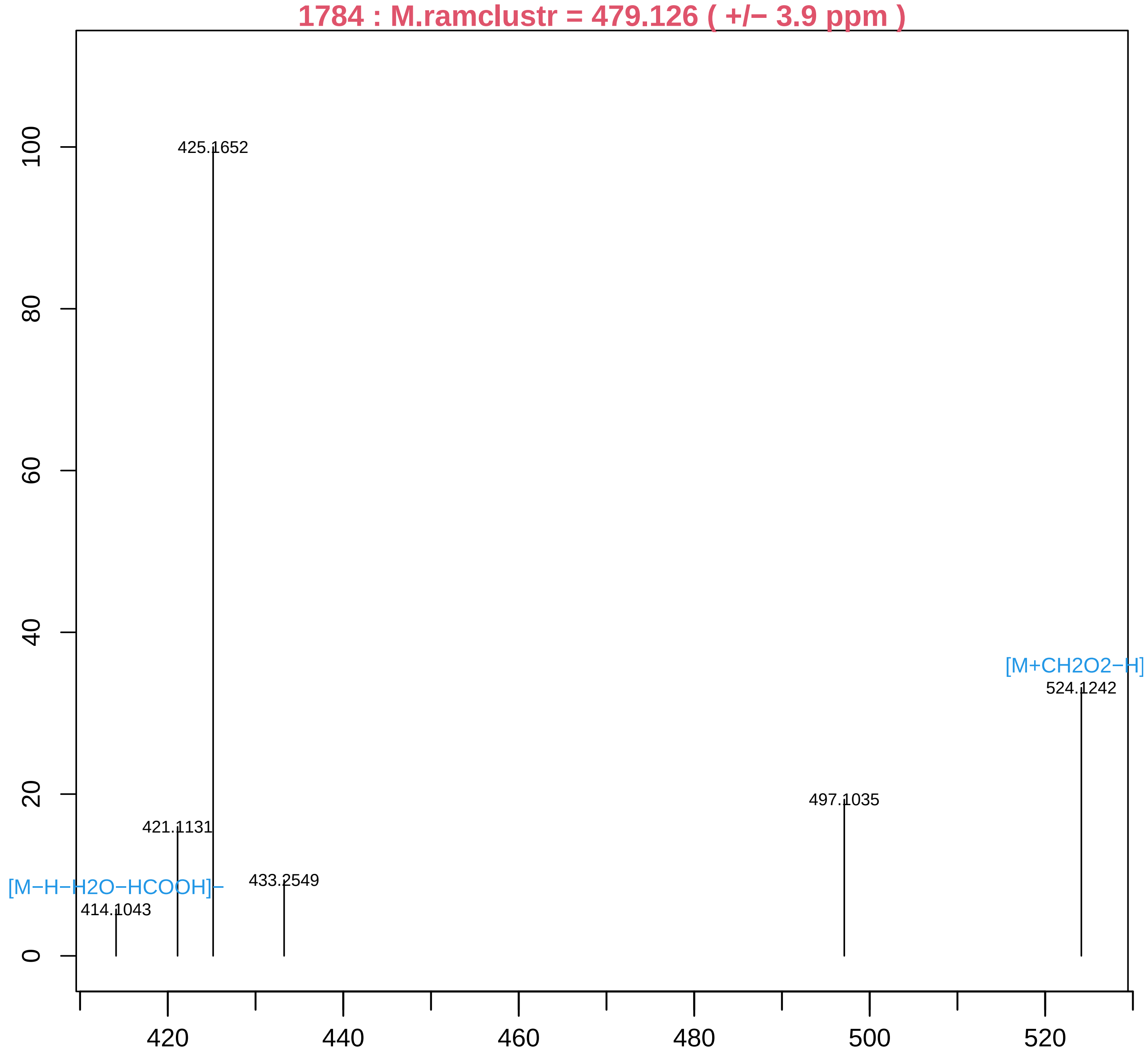
1784 : M.ramclustr = 479.126 (+/− 3.9 ppm)
425.1652
[M+CH2O2−H]
524.1242
497.1035
421.1131
[M−H−H2O−HCOOH]−
433.2549
414.1043
0
20
40
60
80
100
420
440
460
480
500
520

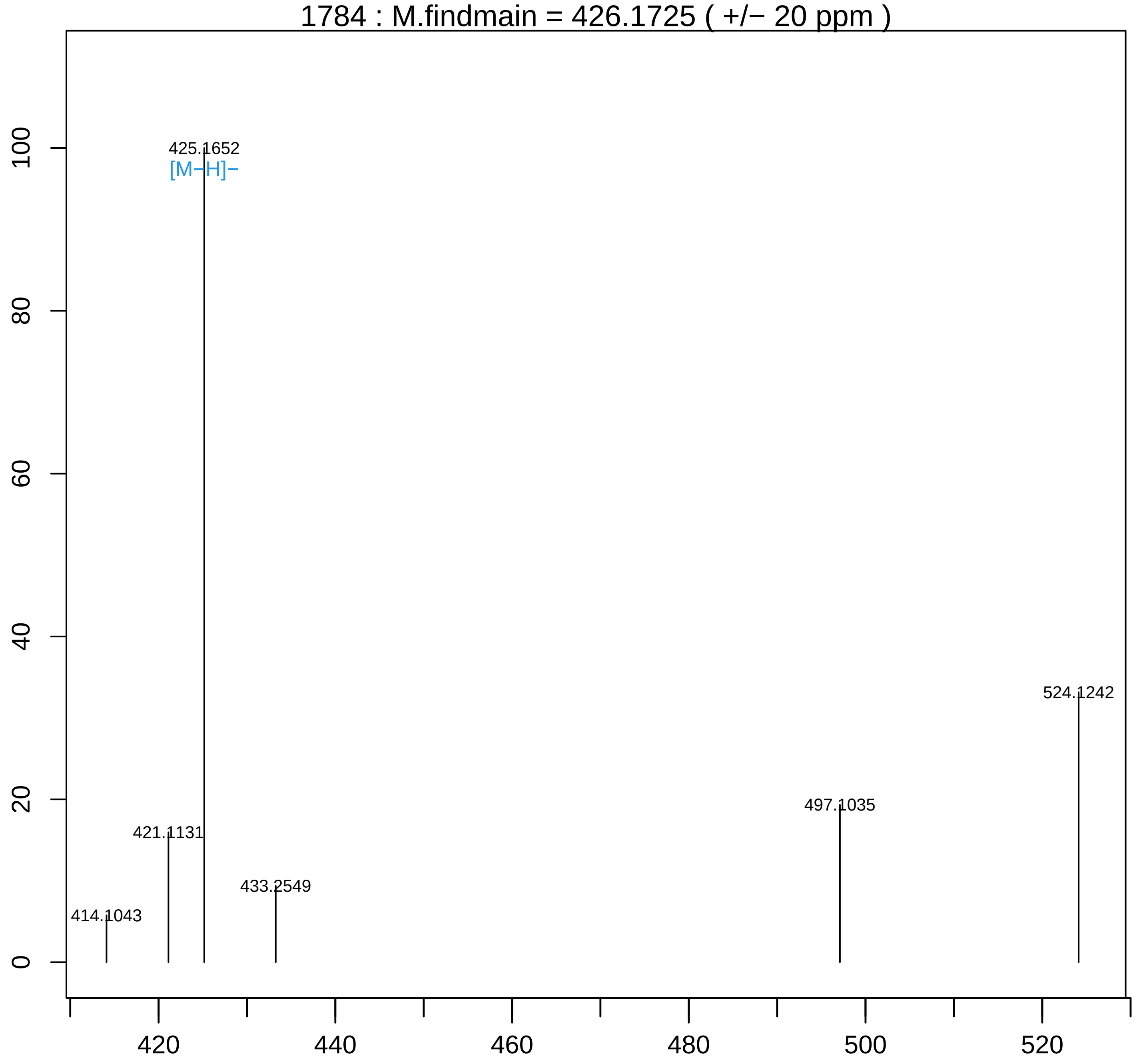
1784 : M.findmain = 426.1725 (+/− 20 ppm)
425.1652
[M−H]−
524.1242
497.1035
421.1131
433.2549
414.1043
0
20
40
60
80
100
420
440
460
480
500
520

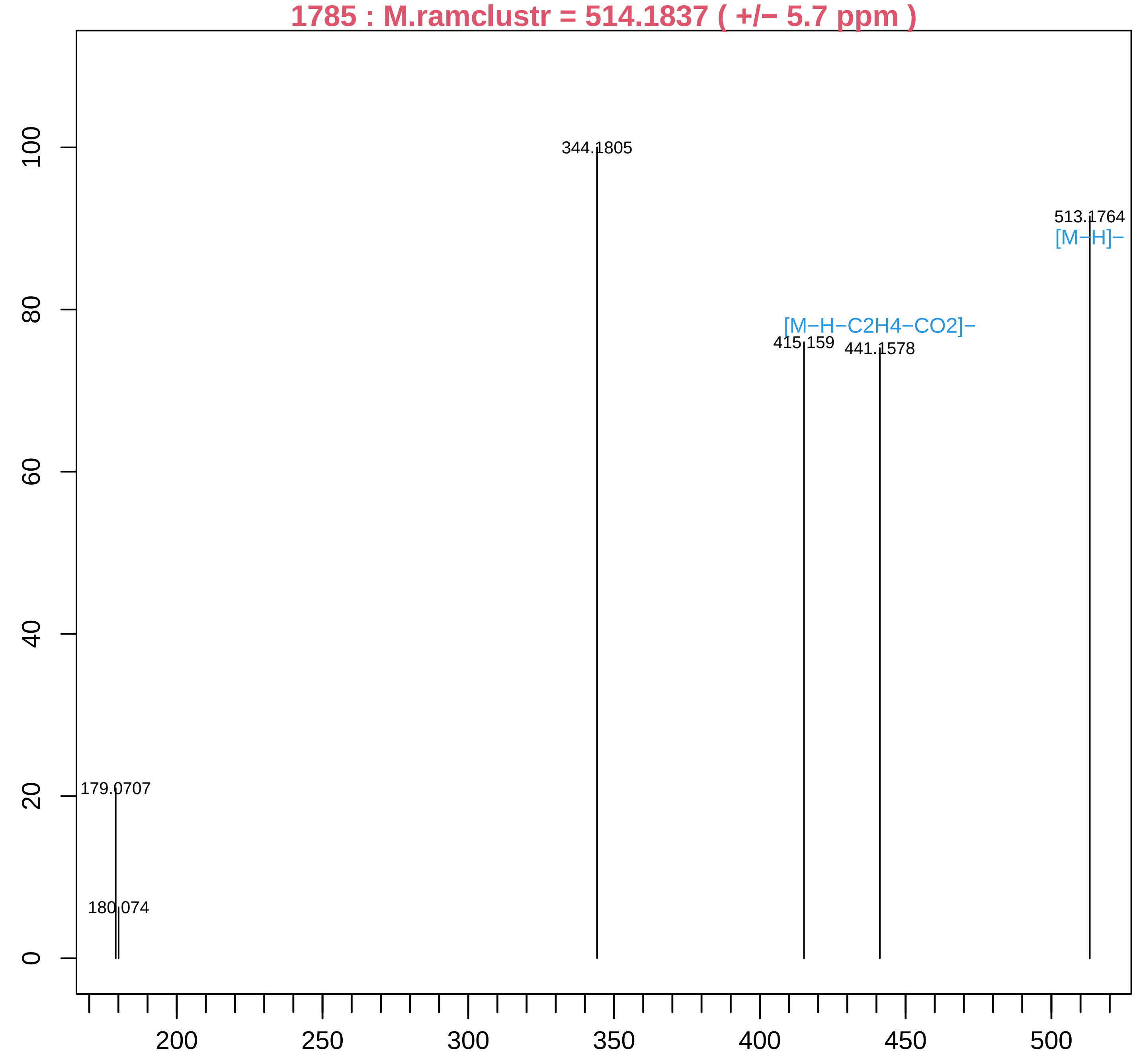
1785 : M.ramclustr = 514.1837 (+/− 5.7 ppm)
344.1805
513.1764
[M−H]−
[M−H−C2H4−CO2]−
415.159
441.1578
179.0707
180.074
0
20
40
60
80
100
200
250
300
350
400
450
500

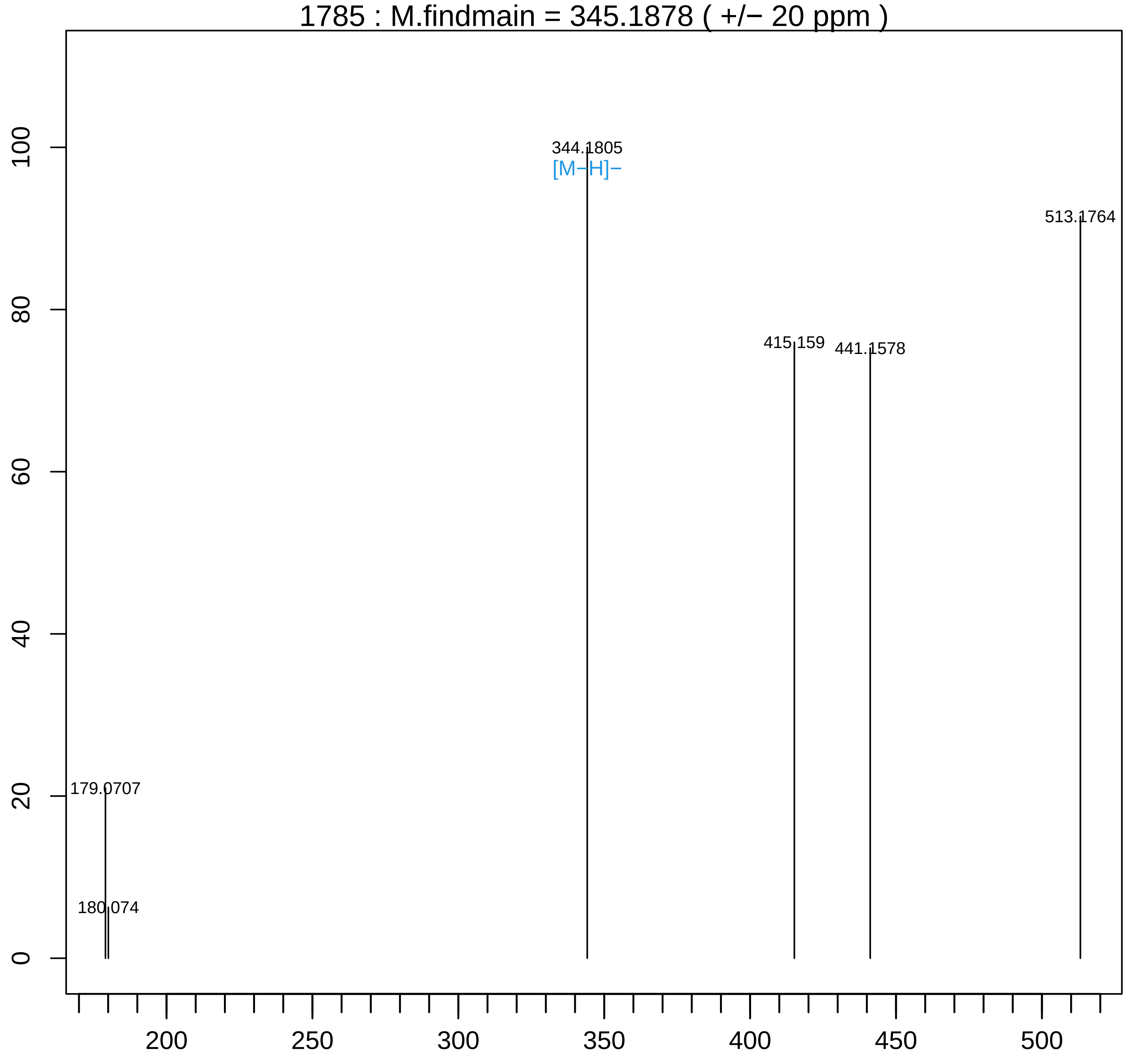
1785 : M.findmain = 345.1878 (+/− 20 ppm)
344.1805
[M−H]−
513.1764
415.159
441.1578
179.0707
180.074
0
20
40
60
80
100
200
250
300
350
400
450
500

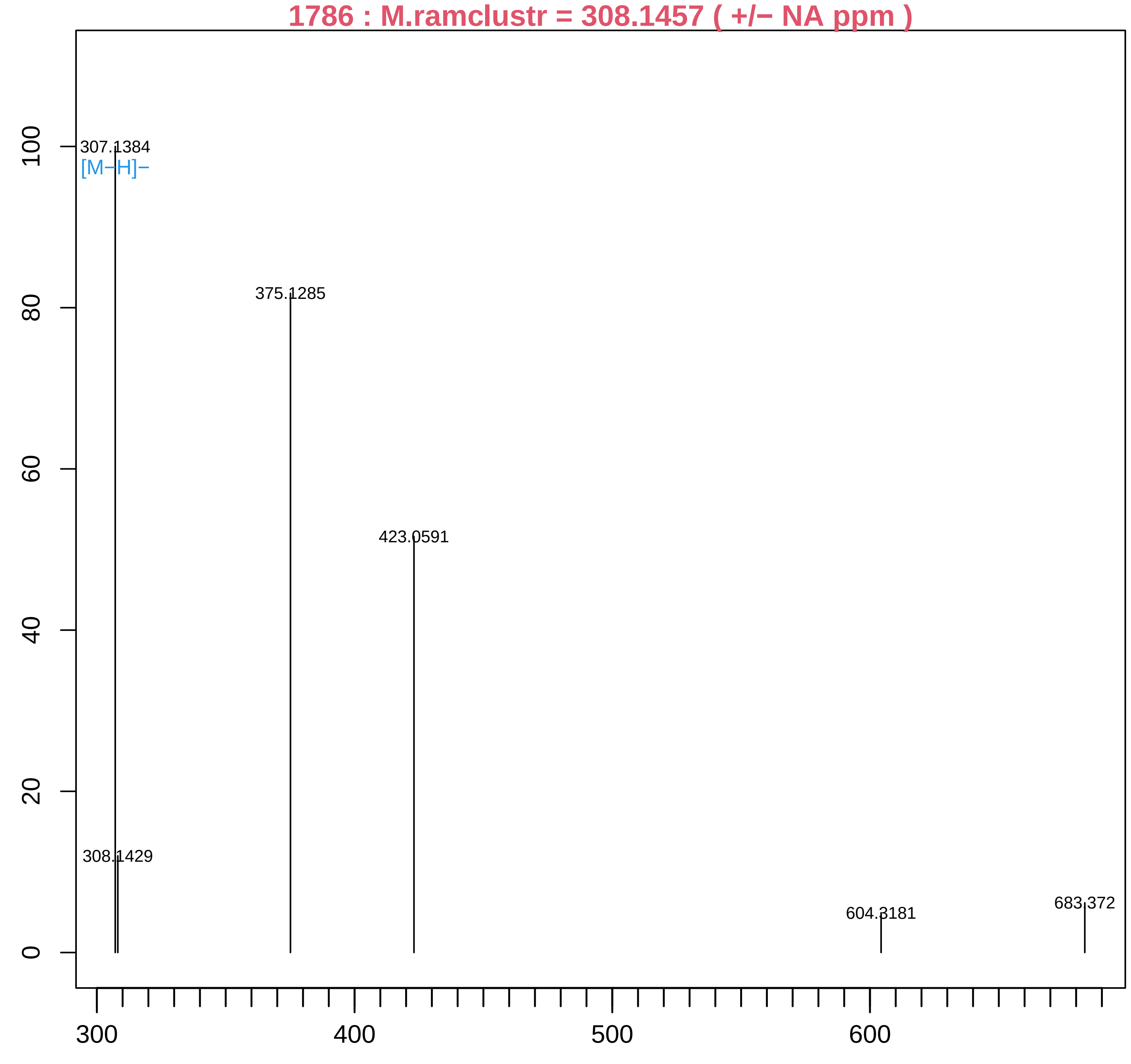
1786 : M.ramclustr = 308.1457 (+/− NA ppm)
307.1384
[M−H]−
375.1285
423.0591
308.1429
604.3181
683.372
0
20
40
60
80
100
300
400
500
600

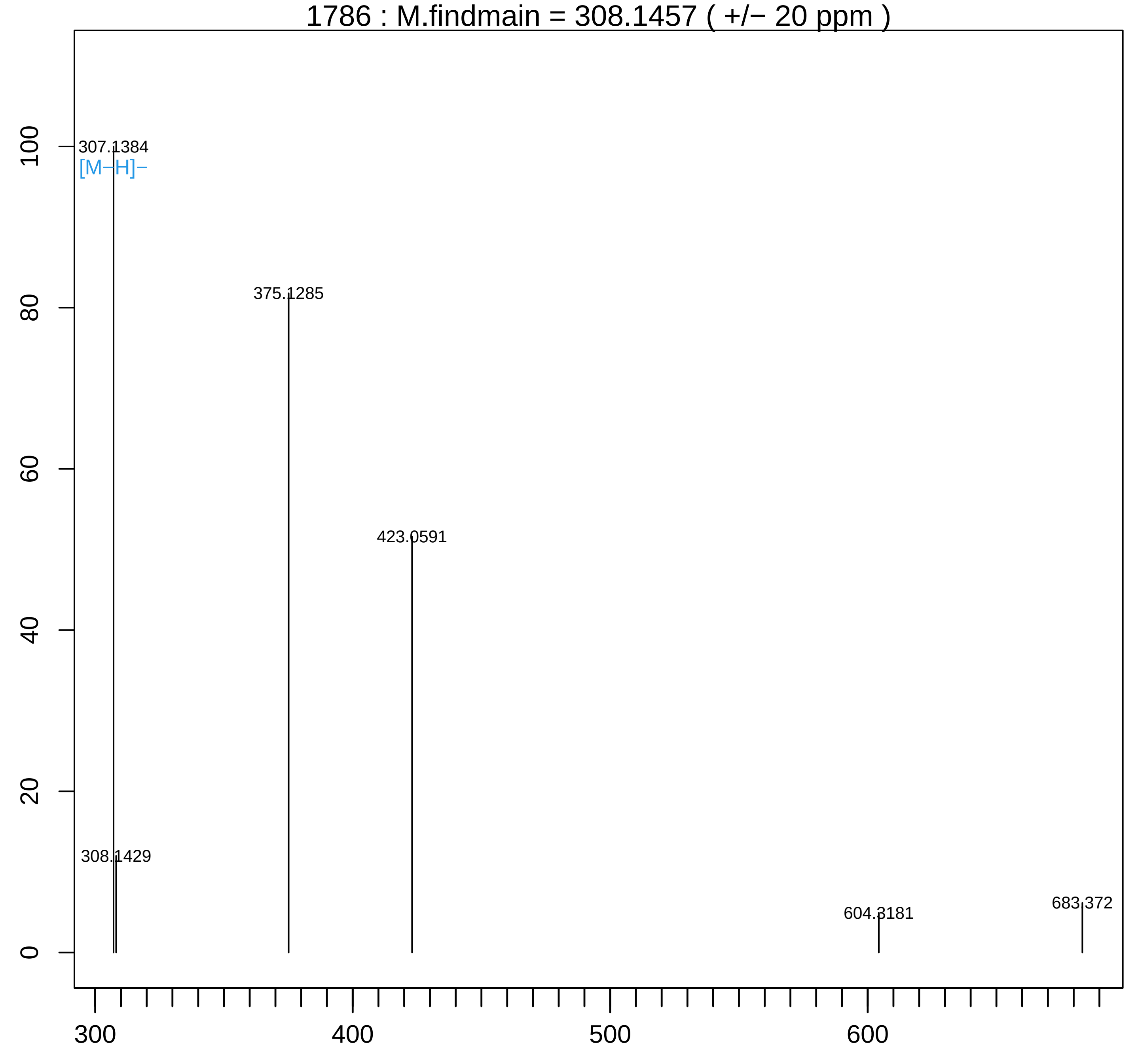
1786 : M.findmain = 308.1457 (+/− 20 ppm)
307.1384
[M−H]−
375.1285
423.0591
308.1429
604.3181
683.372
0
20
40
60
80
100
300
400
500
600

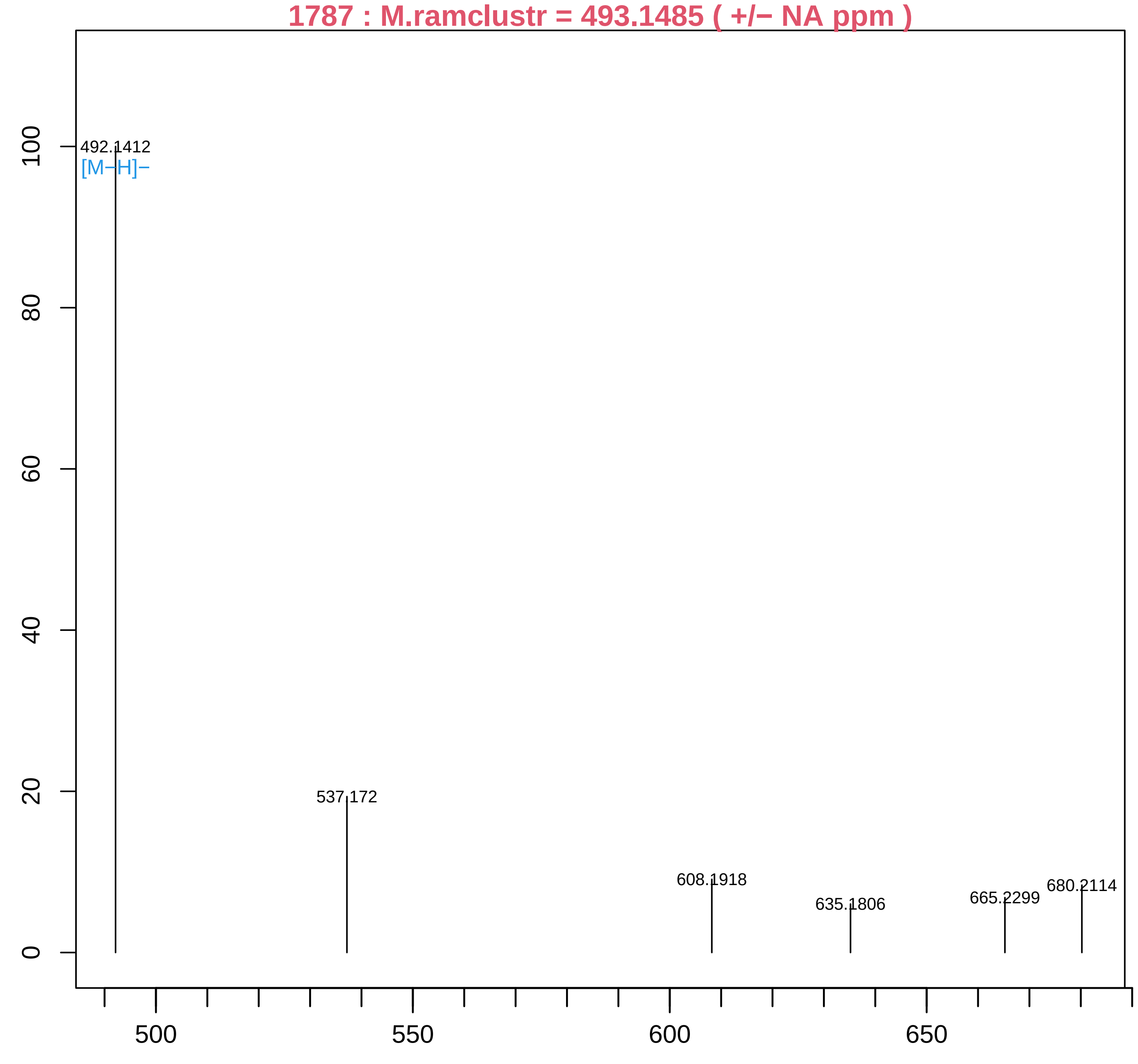
1787 : M.ramclustr = 493.1485 (+/− NA ppm)
492.1412
[M−H]−
537.172
608.1918
635.1806
665.2299
680.2114
0
20
40
60
80
100
500
550
600
650

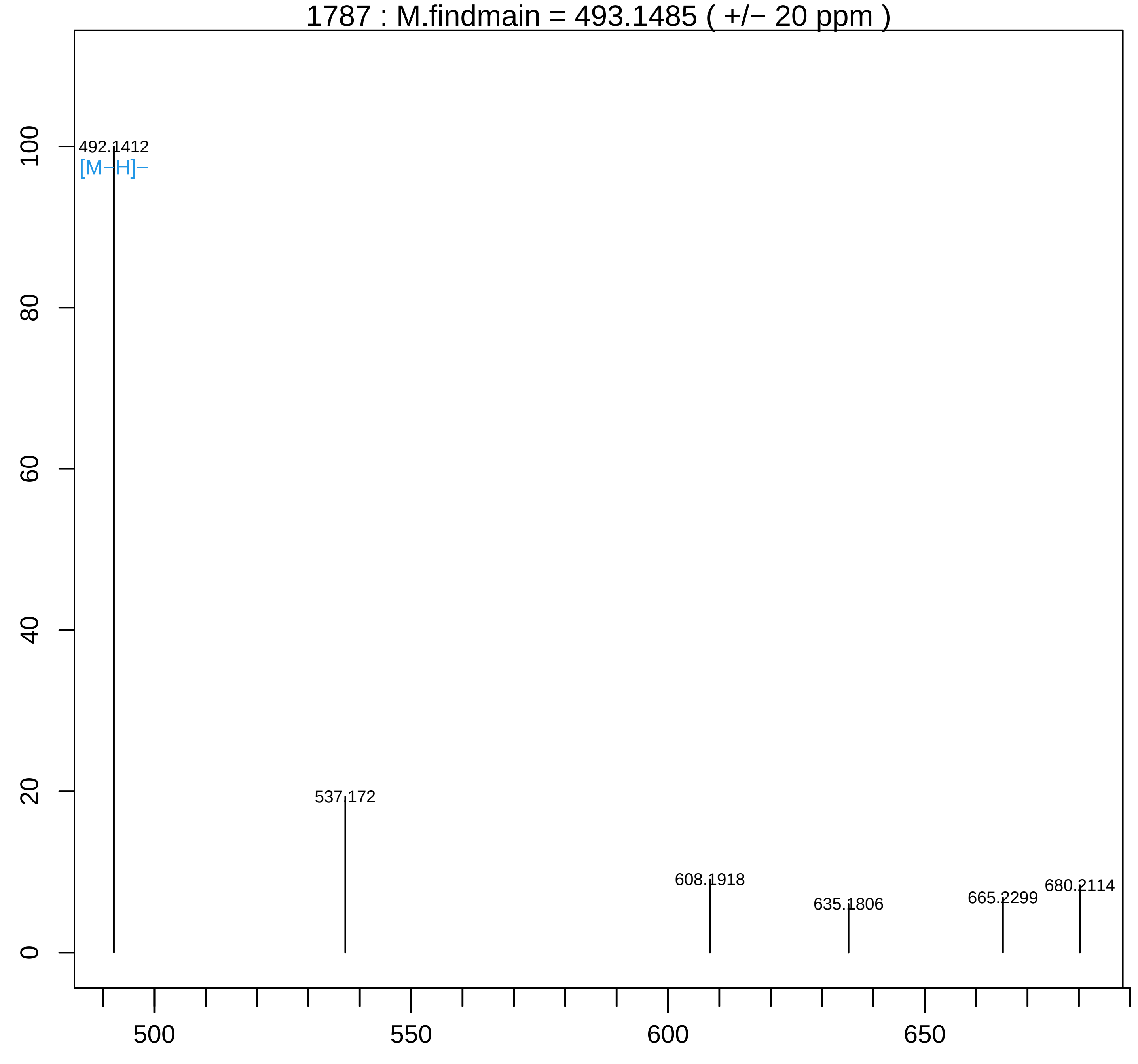
1787 : M.findmain = 493.1485 (+/− 20 ppm)
492.1412
[M−H]−
537.172
608.1918
635.1806
665.2299
680.2114
0
20
40
60
80
100
500
550
600
650

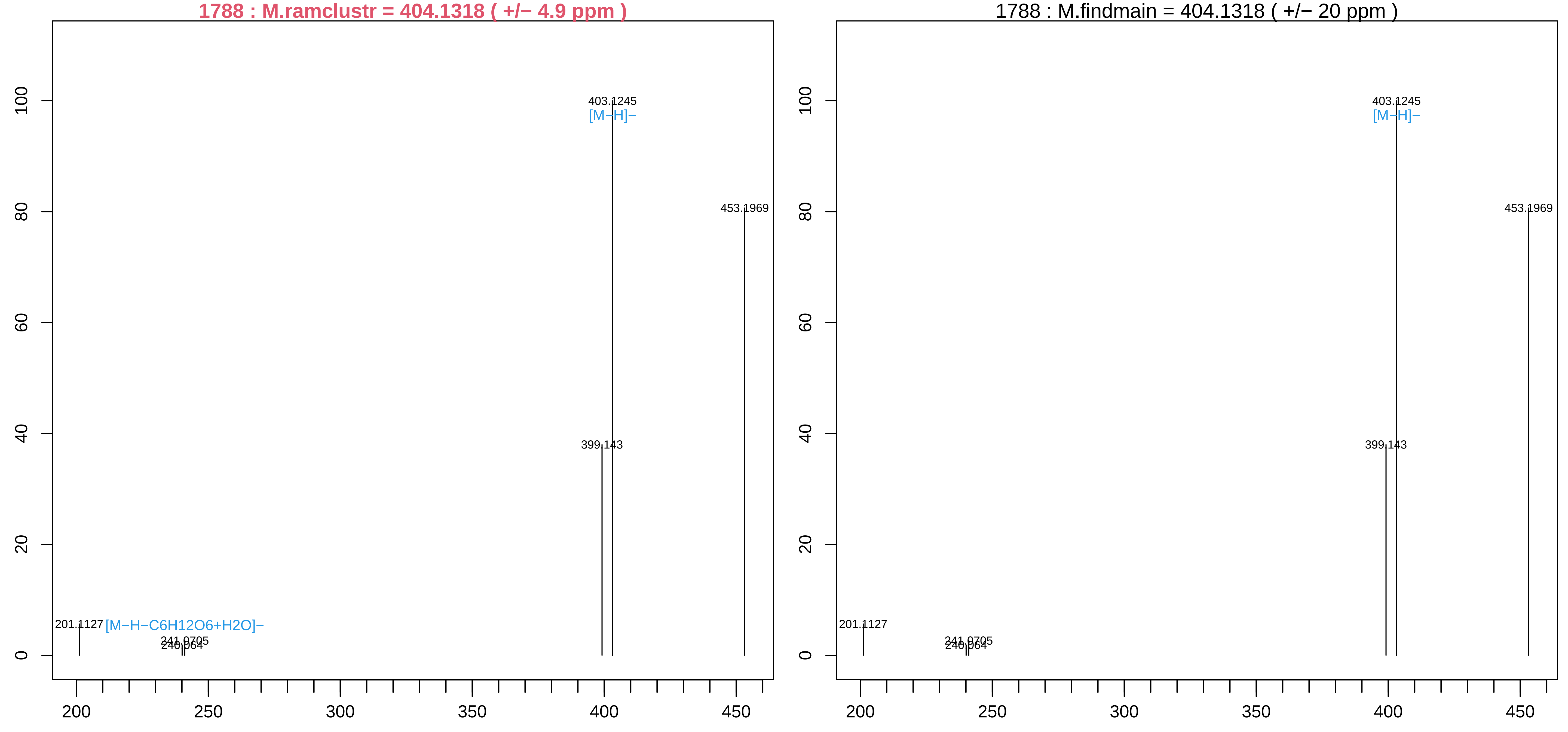

1788 : M.ramclustr = 404.1318 (+/− 4.9 ppm)
403.1245
[M−H]−
453.1969
399.143
201.1127
[M−H−C6H12O6+H2O]−
241.0705
240.064
1788 : M.findmain = 404.1318 (+/− 20 ppm)
403.1245
[M−H]−
453.1969
399.143
201.1127
241.0705
240.064

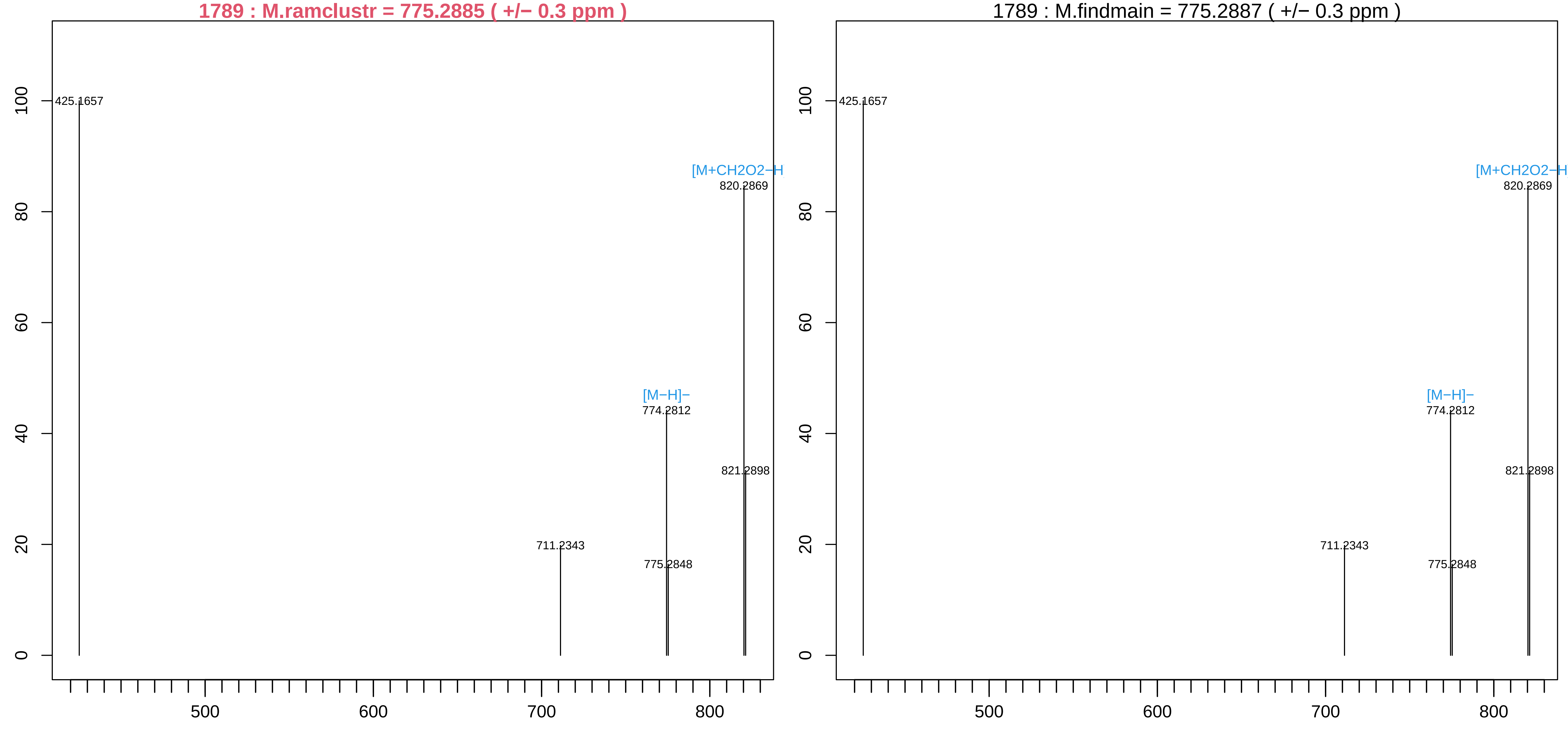
1789 : M.ramclustr = 775.2885 (+/− 0.3 ppm)
1789 : M.findmain = 775.2887 (+/− 0.3 ppm)
425.1657
[M+CH2O2−H
820.2869
[M−H]−
774.2812
821.2898
711.2343
775.2848
0
20
40
60
80
100
500
600
700
800

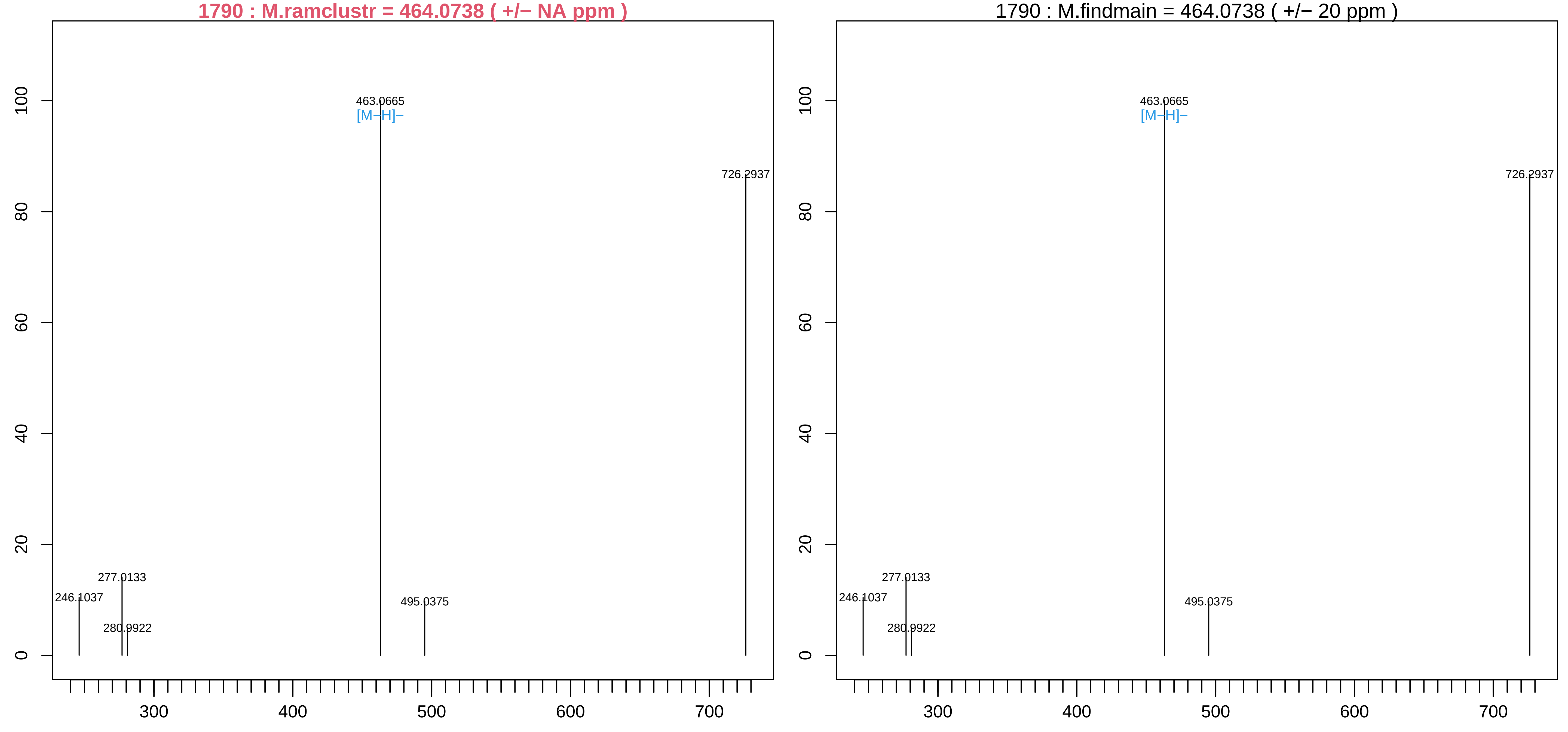

1790 : M.ramclustr = 464.0738 (+/− NA ppm)
463.0665
[M−H]−
726.2937
277.0133
246.1037
280.9922
495.0375
1790 : M.findmain = 464.0738 (+/− 20 ppm)
463.0665
[M−H]−
726.2937
277.0133
246.1037
280.9922
495.0375
0
20
40
60
80
100
300
400
500
600
700

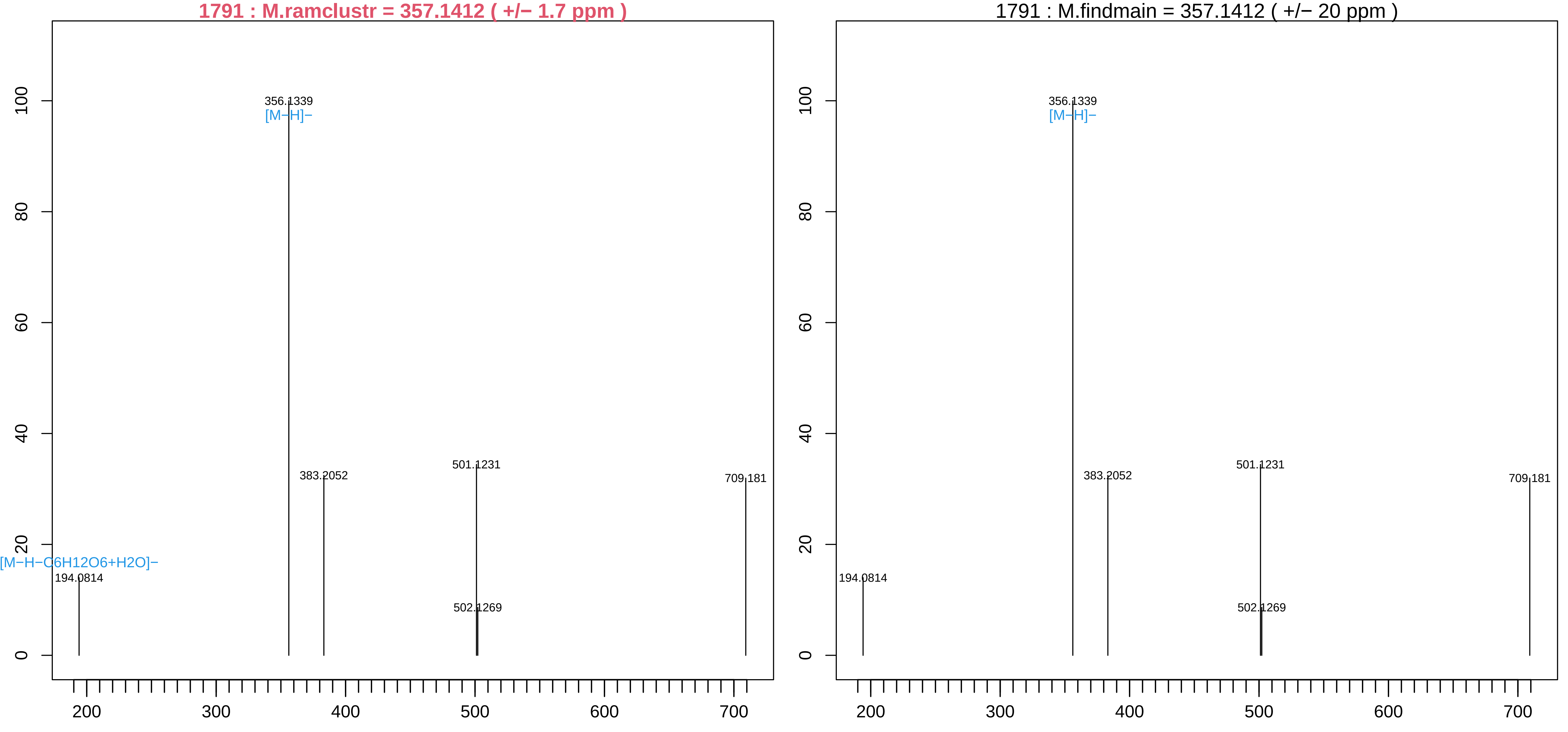
1791 : M.ramclustr = 357.1412 (+/− 1.7 ppm)
356.1339
[M−H]−
383.2052
501.1231
709.181
[M−H−C6H12O6+H2O]−
194.0814
502.1269
1791 : M.findmain = 357.1412 (+/− 20 ppm)
356.1339
[M−H]−
383.2052
501.1231
709.181
194.0814
502.1269

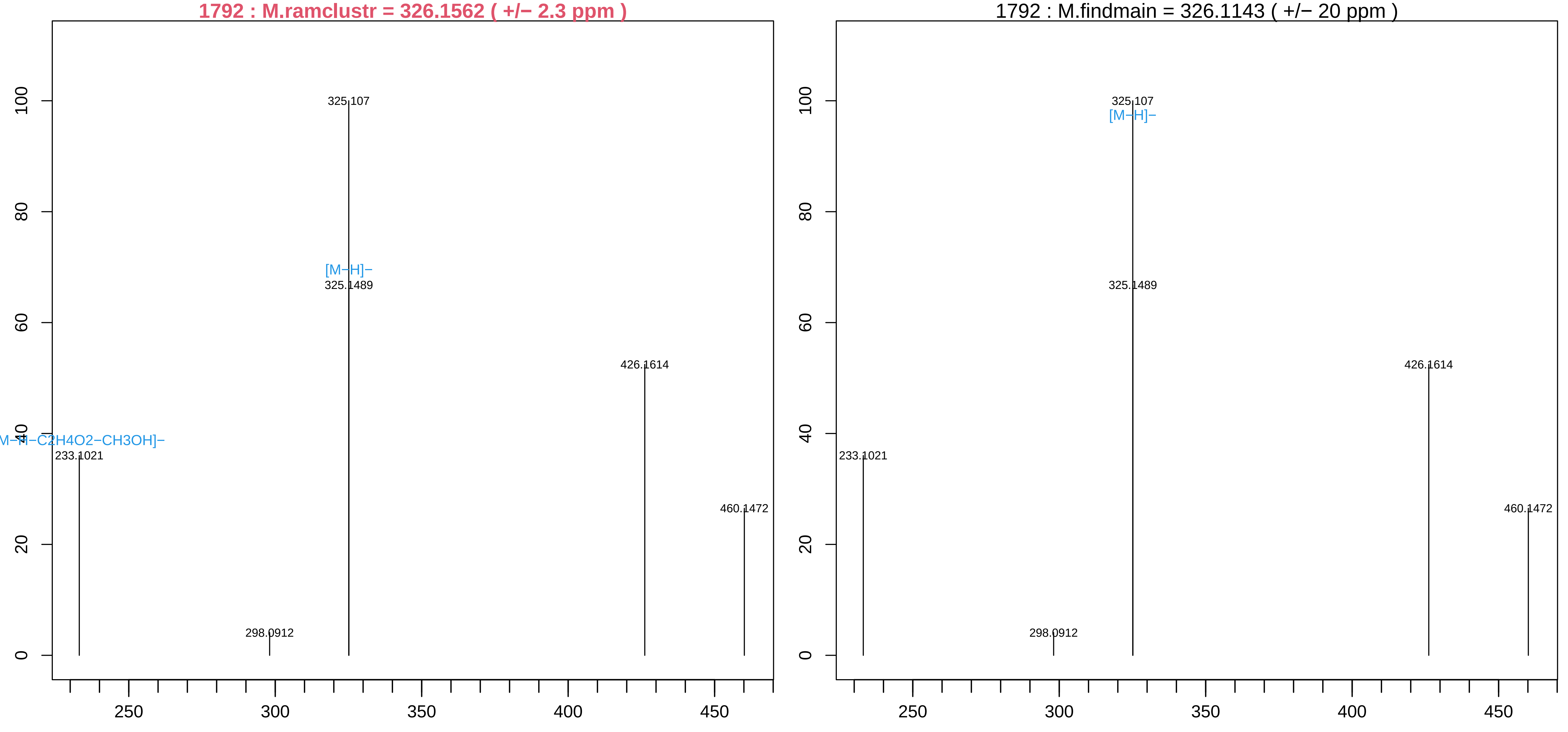
1792 : M.ramclustr = 326.1562 (+/− 2.3 ppm)
325.107
[M−H]−
325.1489
426.1614
[M−H−C2H4O2−CH3OH]−
233.1021
460.1472
298.0912
1792 : M.findmain = 326.1143 (+/− 20 ppm)
325.107
[M−H]−
325.1489
426.1614
233.1021
460.1472
298.0912
0
20
40
60
80
100
250
300
350
400
450

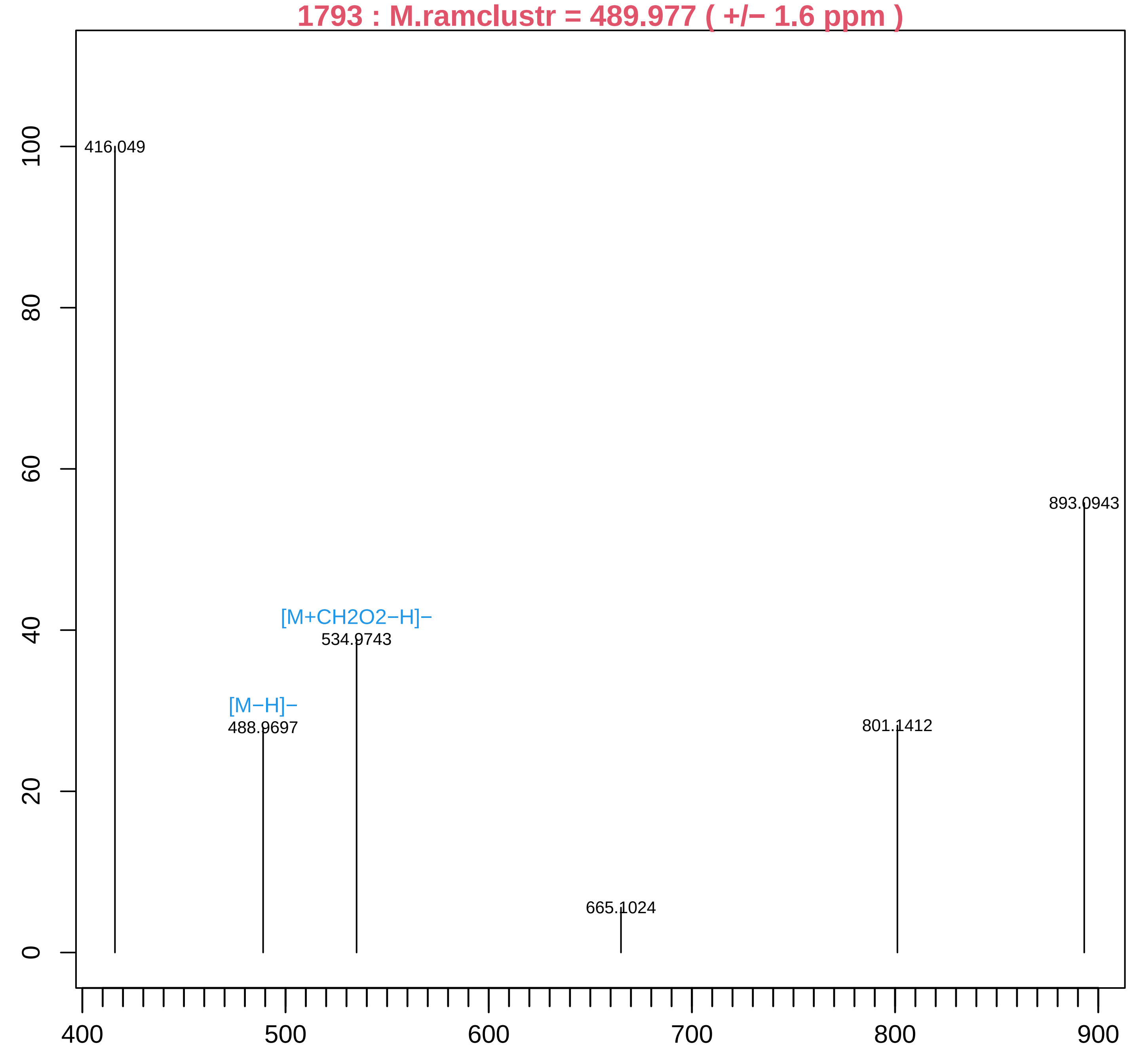
1793 : M.ramclustr = 489.977 (+/− 1.6 ppm)
416.049
893.0943
[M+CH2O2−H]−
534.9743
[M−H]−
488.9697
801.1412
665.1024
0
20
40
60
80
100
400
500
600
700
800
900

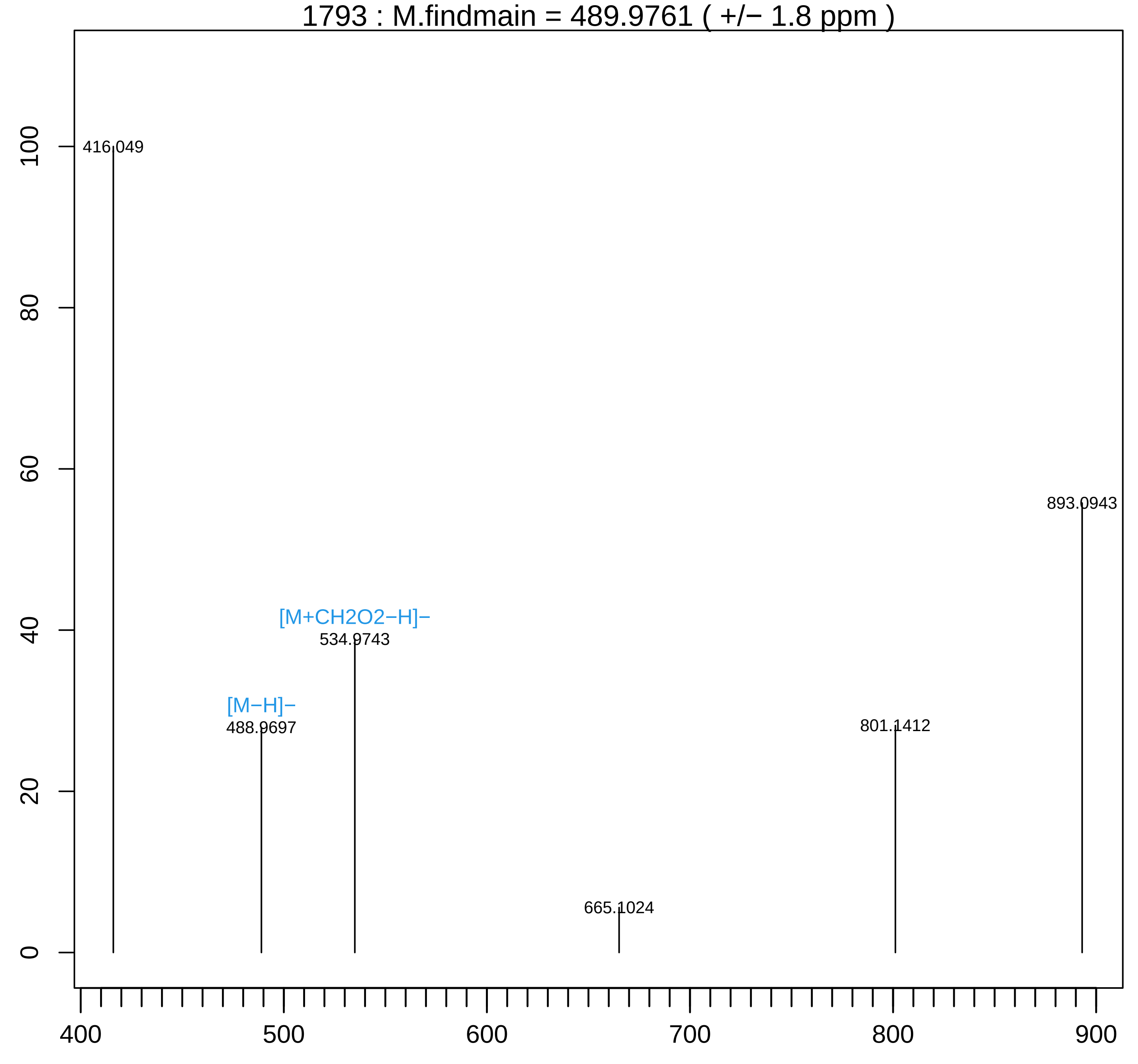
1793 : M.findmain = 489.9761 (+/− 1.8 ppm)
416.049
893.0943
[M+CH2O2−H]−
534.9743
[M−H]−
488.9697
801.1412
665.1024
0
20
40
60
80
100
400
500
600
700
800
900

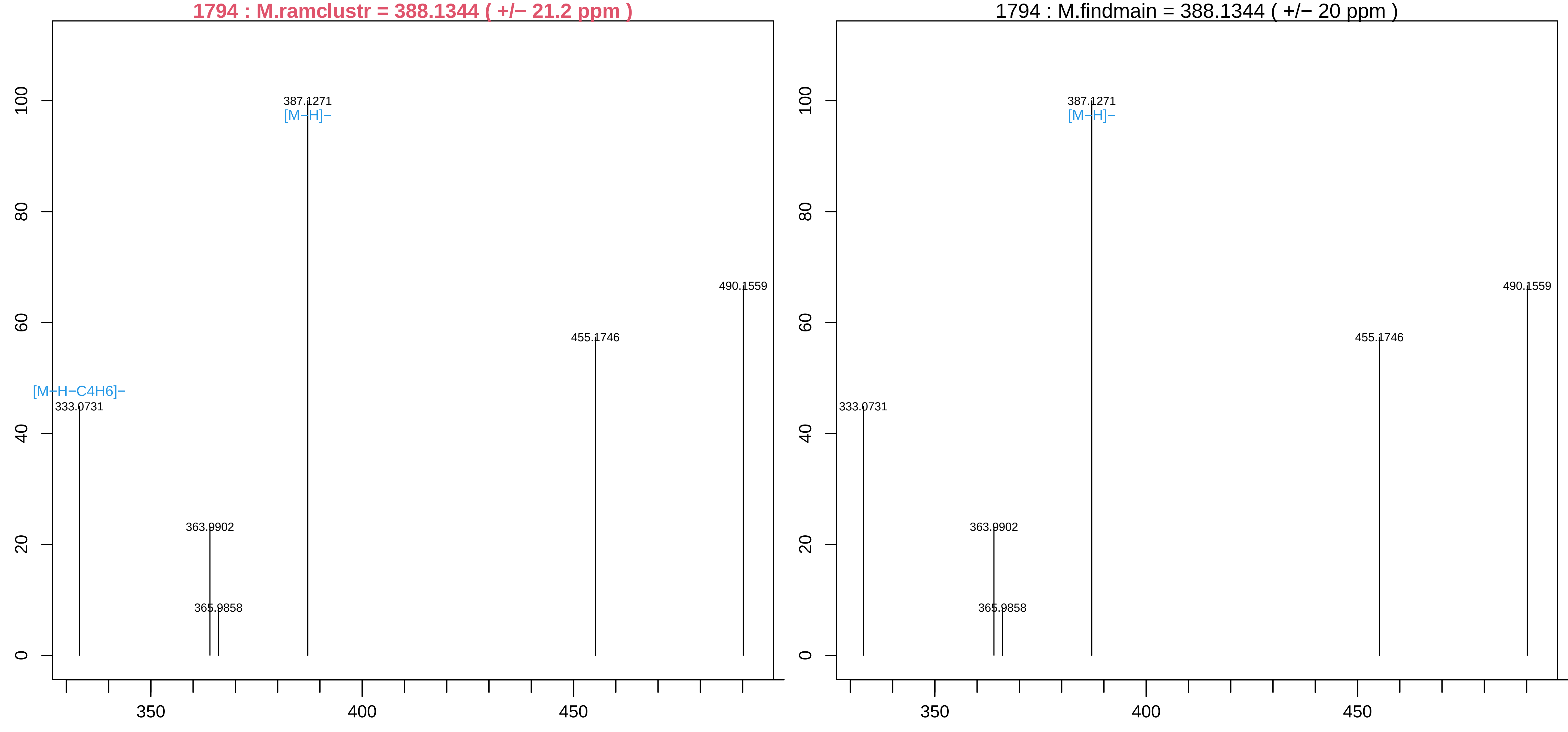
1794 : M.ramclustr = 388.1344 (+/− 21.2 ppm)
387.1271
[M−H]−
490.1559
455.1746
[M−H−C4H6]−
333.0731
363.9902
365.9858
1794 : M.findmain = 388.1344 (+/− 20 ppm)
387.1271
[M−H]−
490.1559
455.1746
333.0731
363.9902
365.9858
0
20
40
60
80
100
350
400
450

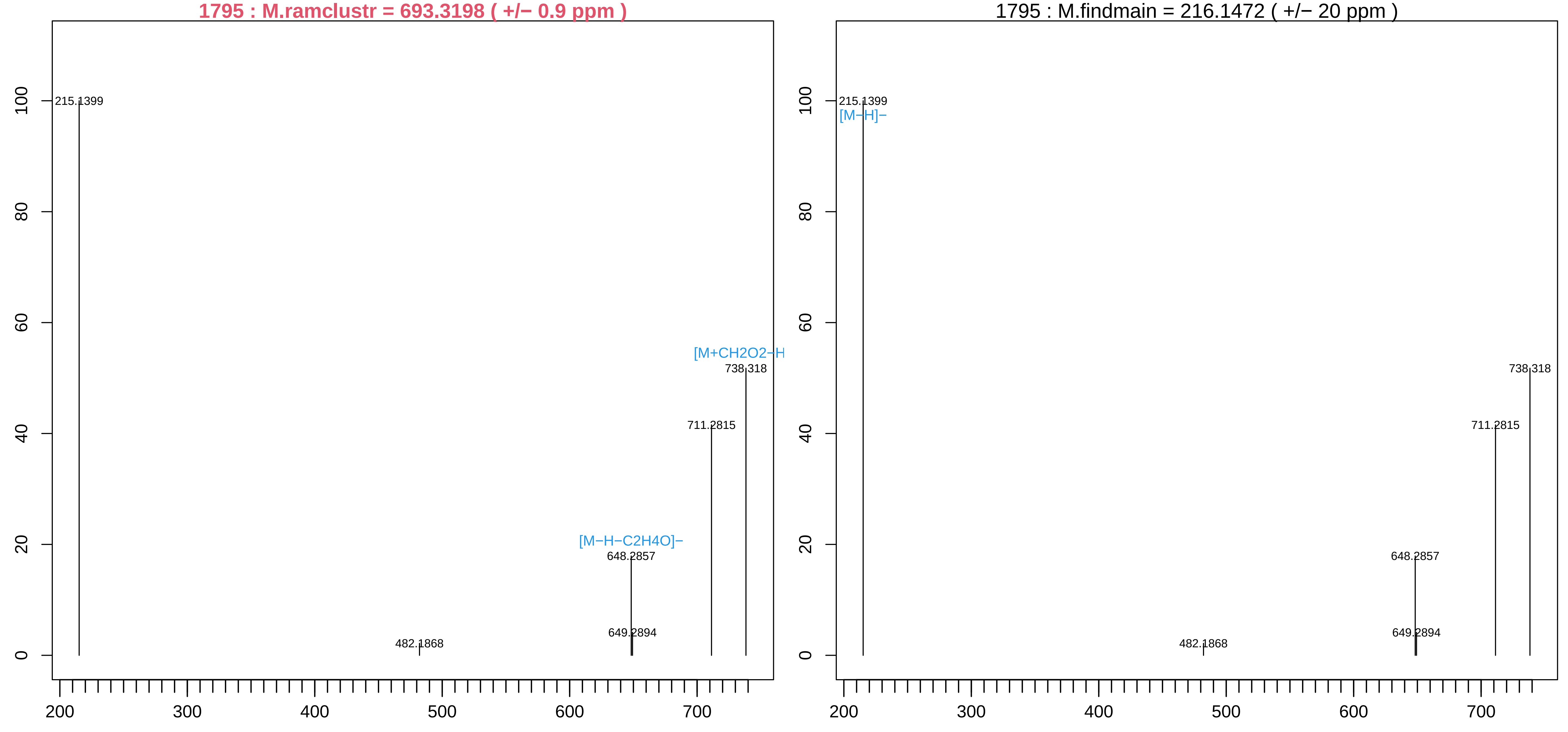
1795 : M.ramclustr = 693.3198 (+/− 0.9 ppm)
215.1399
[M+CH2O2−H
738.318
711.2815
[M−H−C2H4O]−
648.2857
649.2894
482.1868
1795 : M.findmain = 216.1472 (+/− 20 ppm)
215.1399
[M−H]−
738.318
711.2815
648.2857
649.2894
482.1868
0
20
40
60
80
100
200
300
400
500
600
700

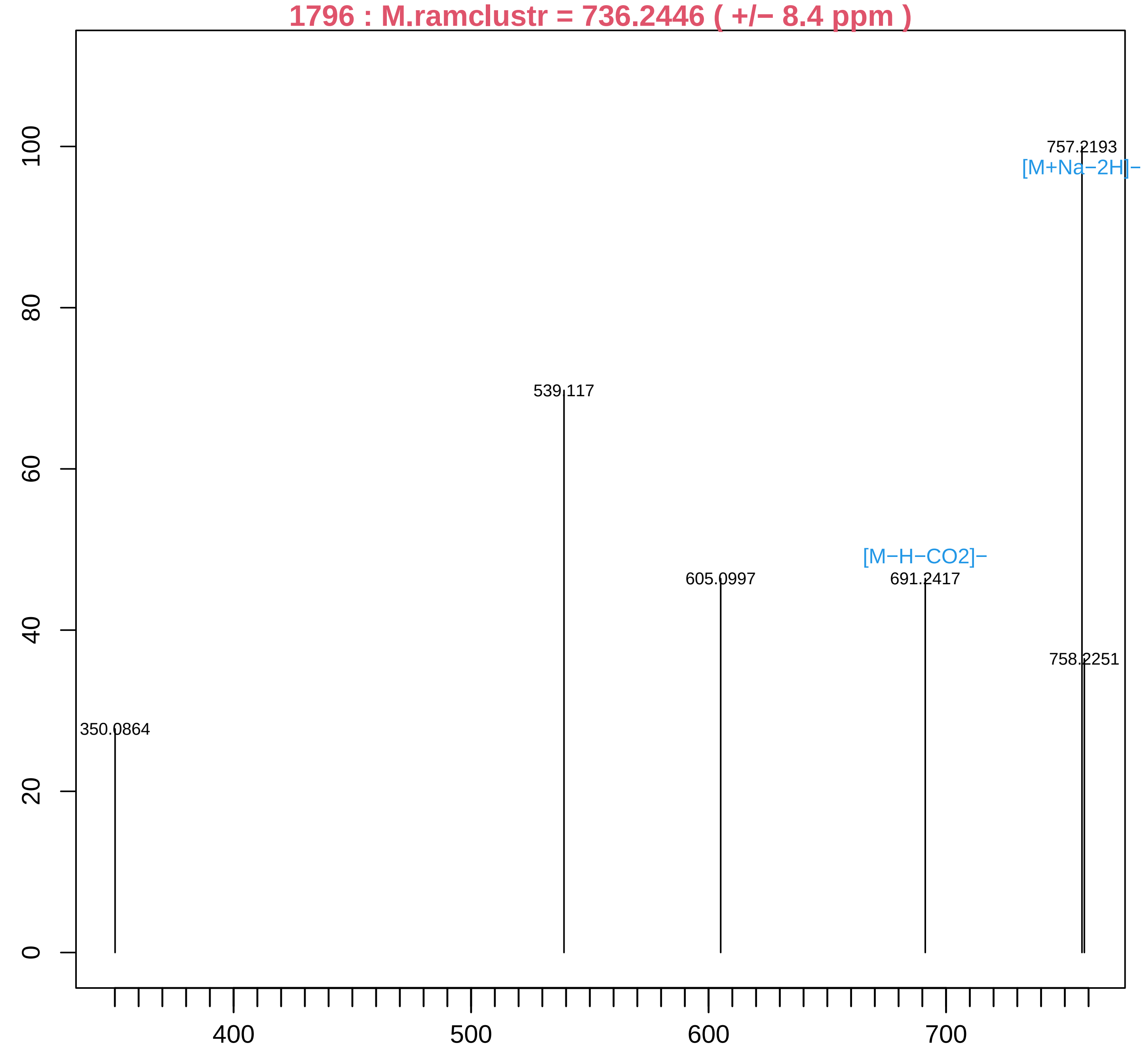
1796 : M.ramclustr = 736.2446 (+/− 8.4 ppm)
757.2193
[M+Na−2H]−
539.117
605.0997
[M−H−CO2]−
691.2417
758.2251
350.0864
0
20
40
60
80
100
400
500
600
700

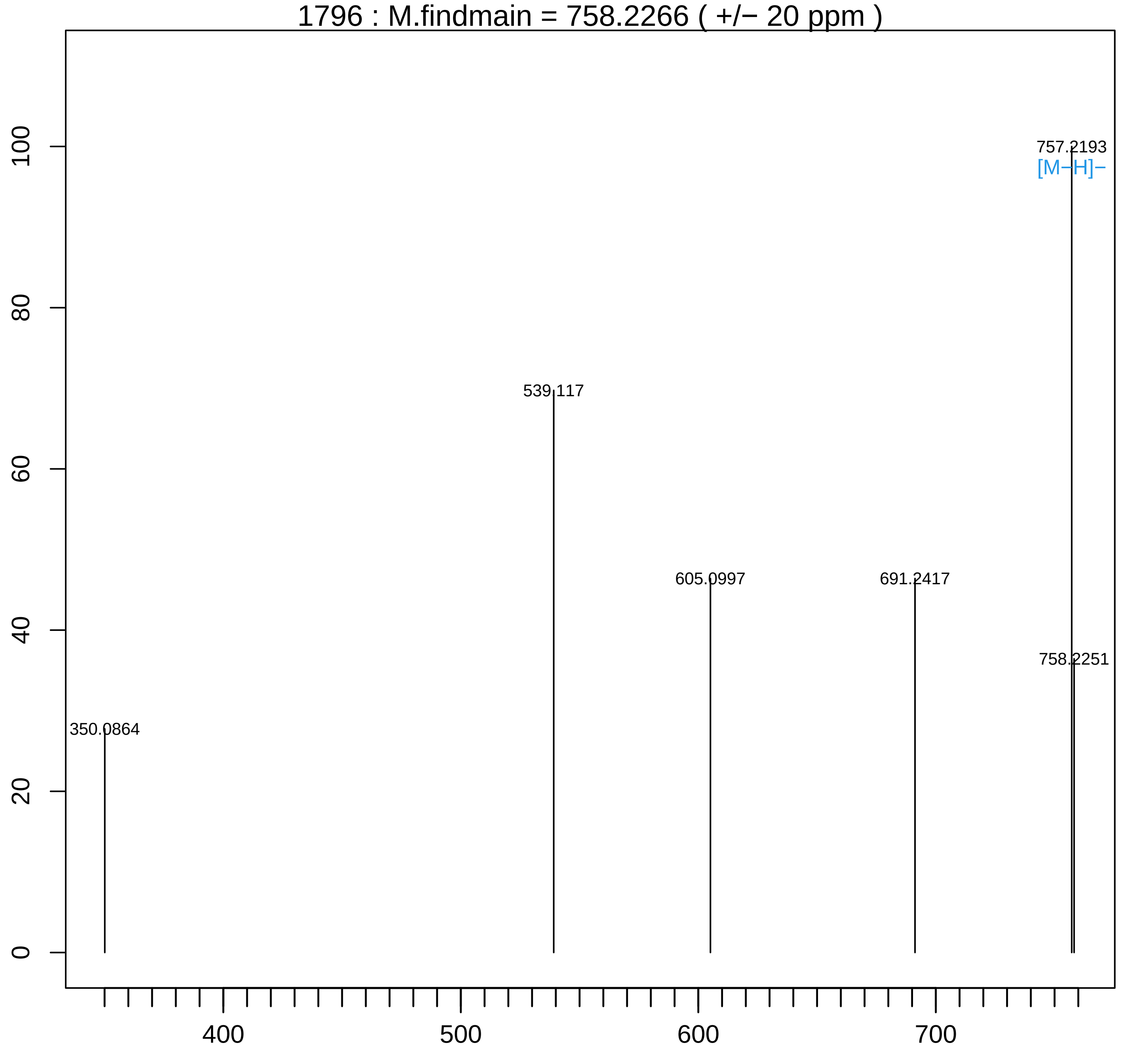
1796 : M.findmain = 758.2266 (+/− 20 ppm)
757.2193
[M−H]−
539.117
605.0997
691.2417
758.2251
350.0864
0
20
40
60
80
100
400
500
600
700

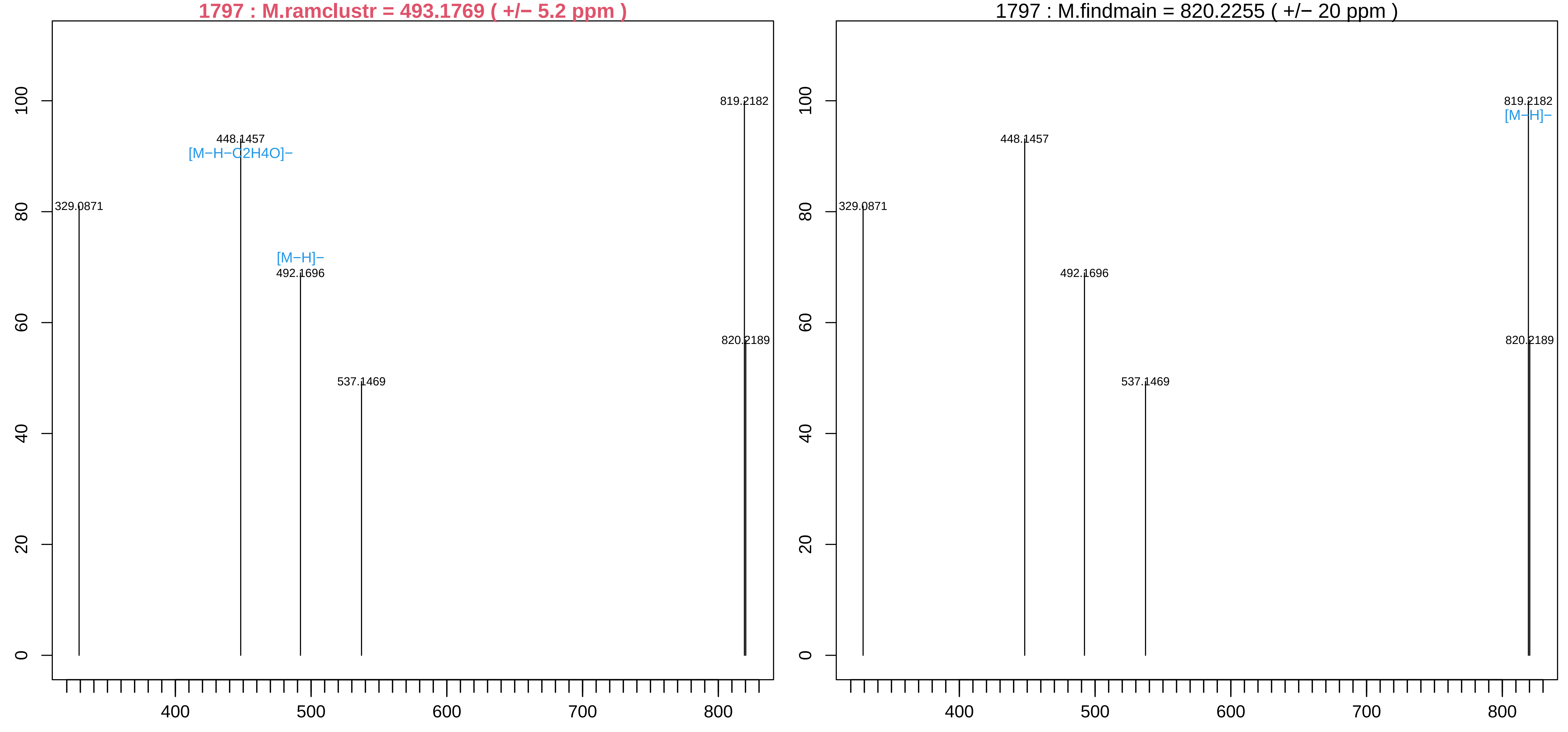

1797 : M.ramclustr = 493.1769 (+/− 5.2 ppm)
329.0871
448.1457
[M−H−C2H4O]−
[M−H]−
492.1696
537.1469
819.2182
820.2189
0
20
40
60
80
100
400
500
600
700
800
1797 : M.findmain = 820.2255 (+/− 20 ppm)
329.0871
448.1457
492.1696
537.1469
819.2182
[M−H]−
820.2189
0
20
40
60
80
100
400
500
600
700
800

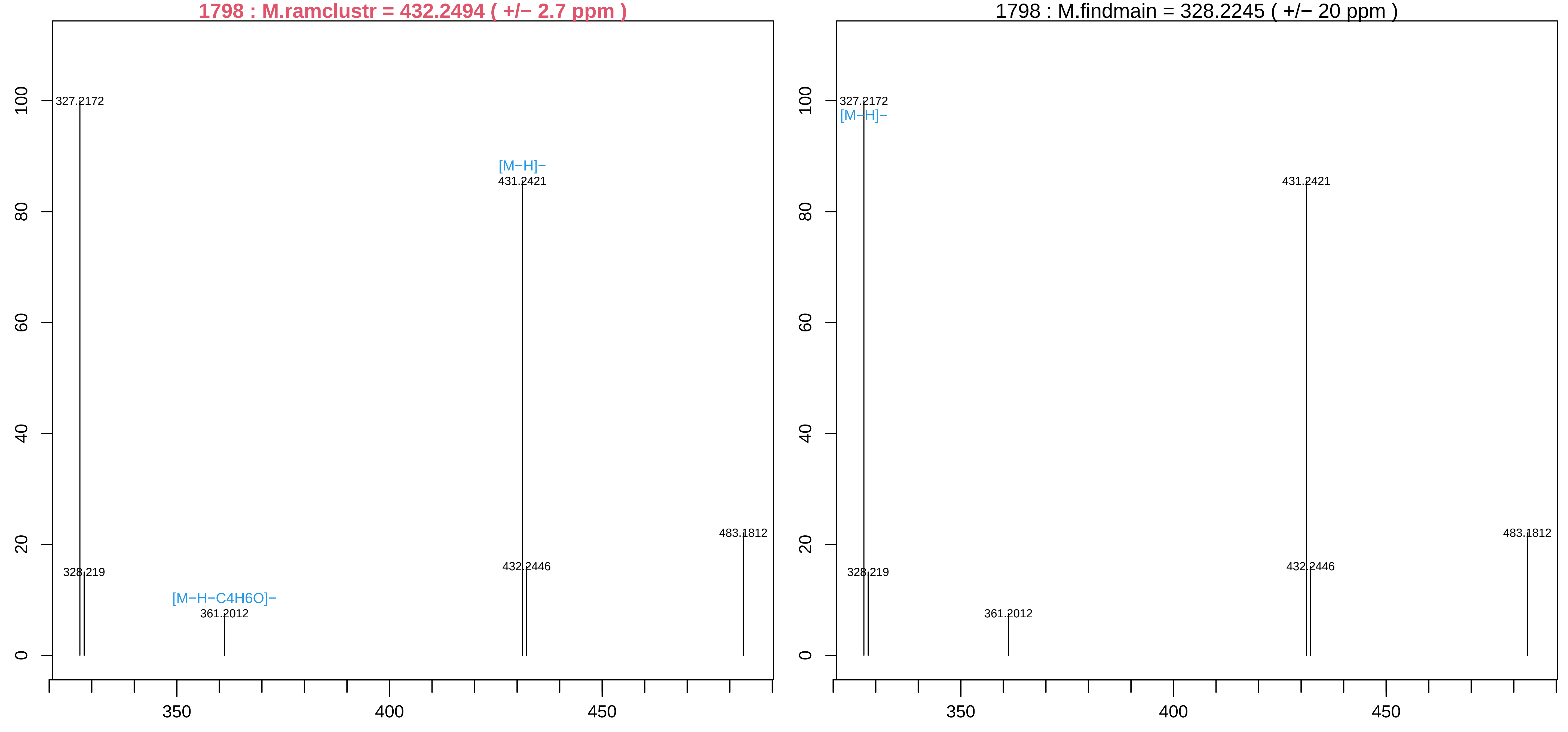
1798 : M.ramclustr = 432.2494 (+/− 2.7 ppm)
327.2172
328.219
[M−H−C4H6O]−
361.2012
[M−H]−
431.2421
432.2446
483.1812
0
20
40
60
80
100
350
400
450
1798 : M.findmain = 328.2245 (+/− 20 ppm)
327.2172
[M−H]−
328.219
361.2012
431.2421
432.2446
483.1812
0
20
40
60
80
100
350
400
450

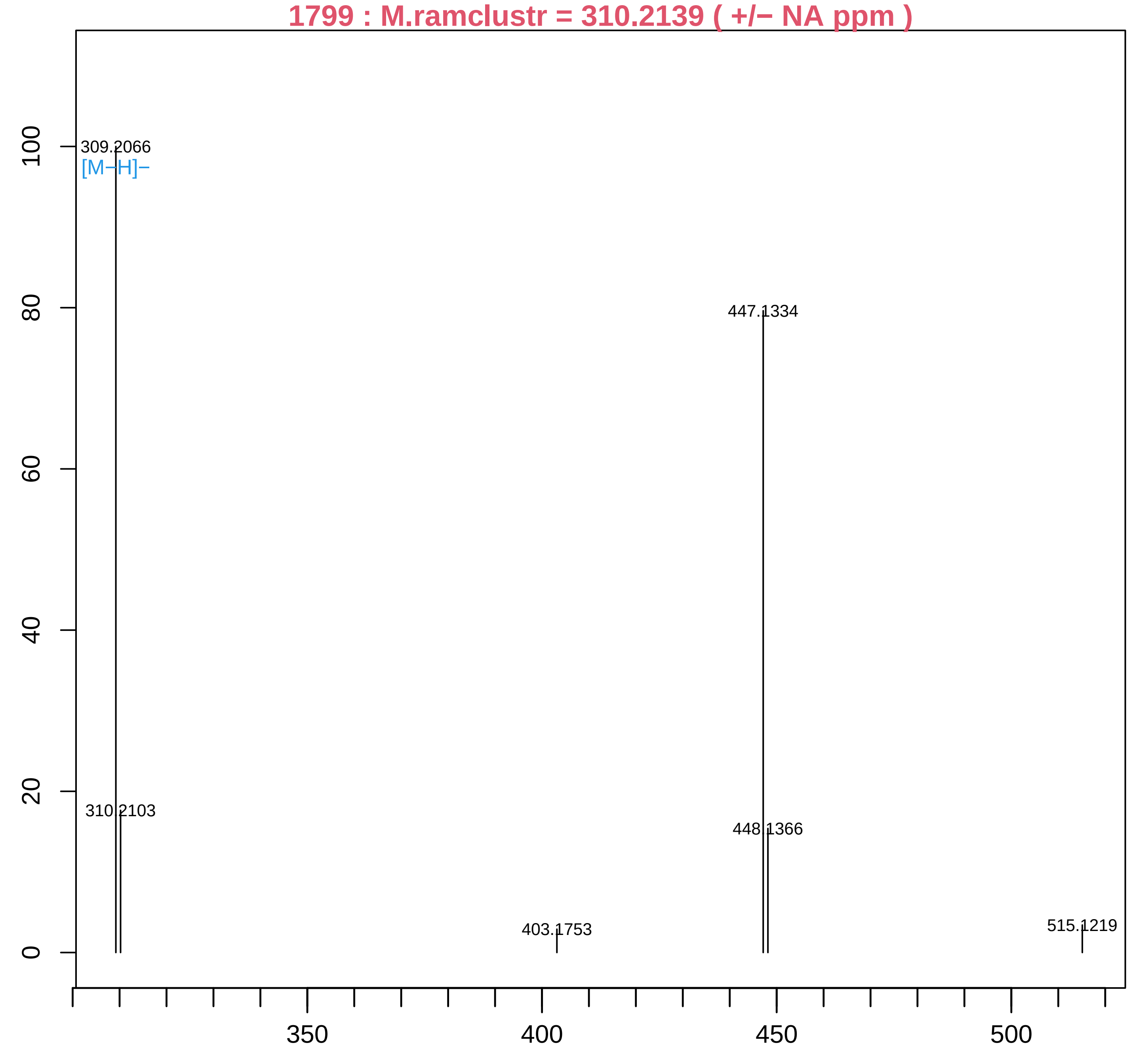
1799 : M.ramclustr = 310.2139 (+/− NA ppm)
309.2066
[M−H]−
447.1334
310.2103
448.1366
403.1753
515.1219
0
20
40
60
80
100
350
400
450
500

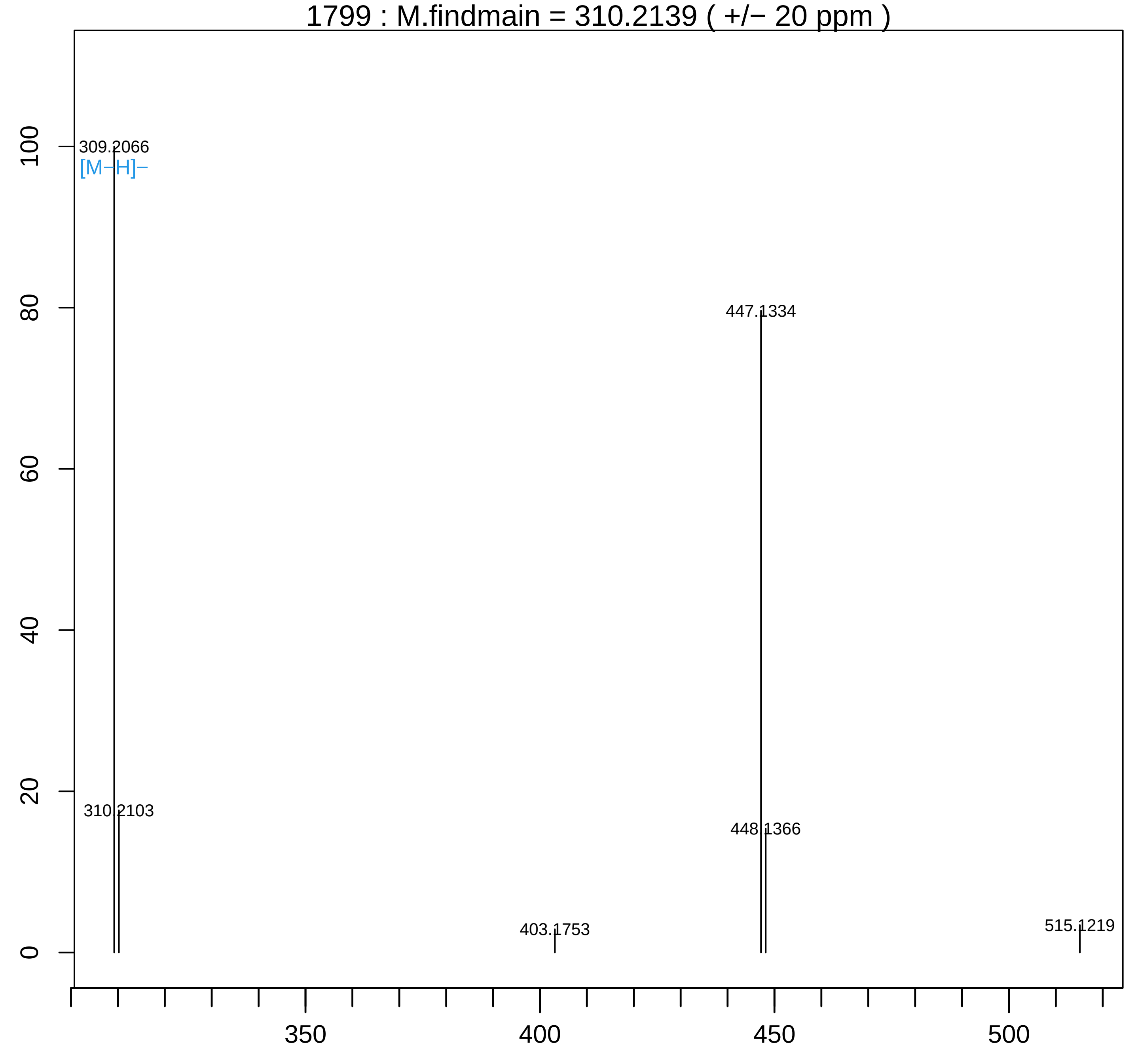
1799 : M.findmain = 310.2139 (+/− 20 ppm)
309.2066
[M−H]−
447.1334
310.2103
448.1366
403.1753
515.1219
0
20
40
60
80
100
350
400
450
500

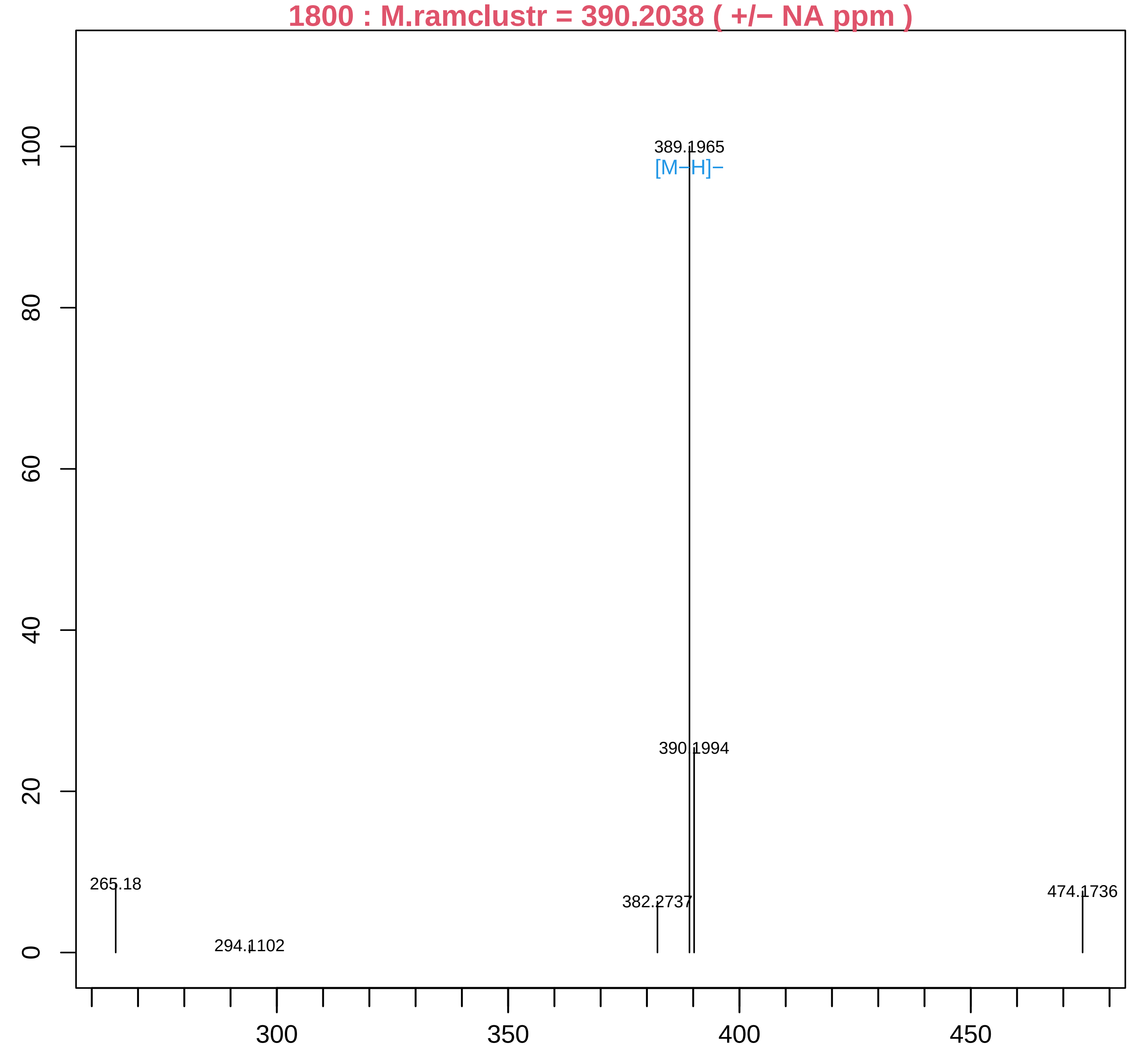
1800 : M.ramclustr = 390.2038 (+/− NA ppm)
389.1965
[M−H]−
390.1994
265.18
294.1102
382.2737
474.1736
0
20
40
60
80
100
300
350
400
450

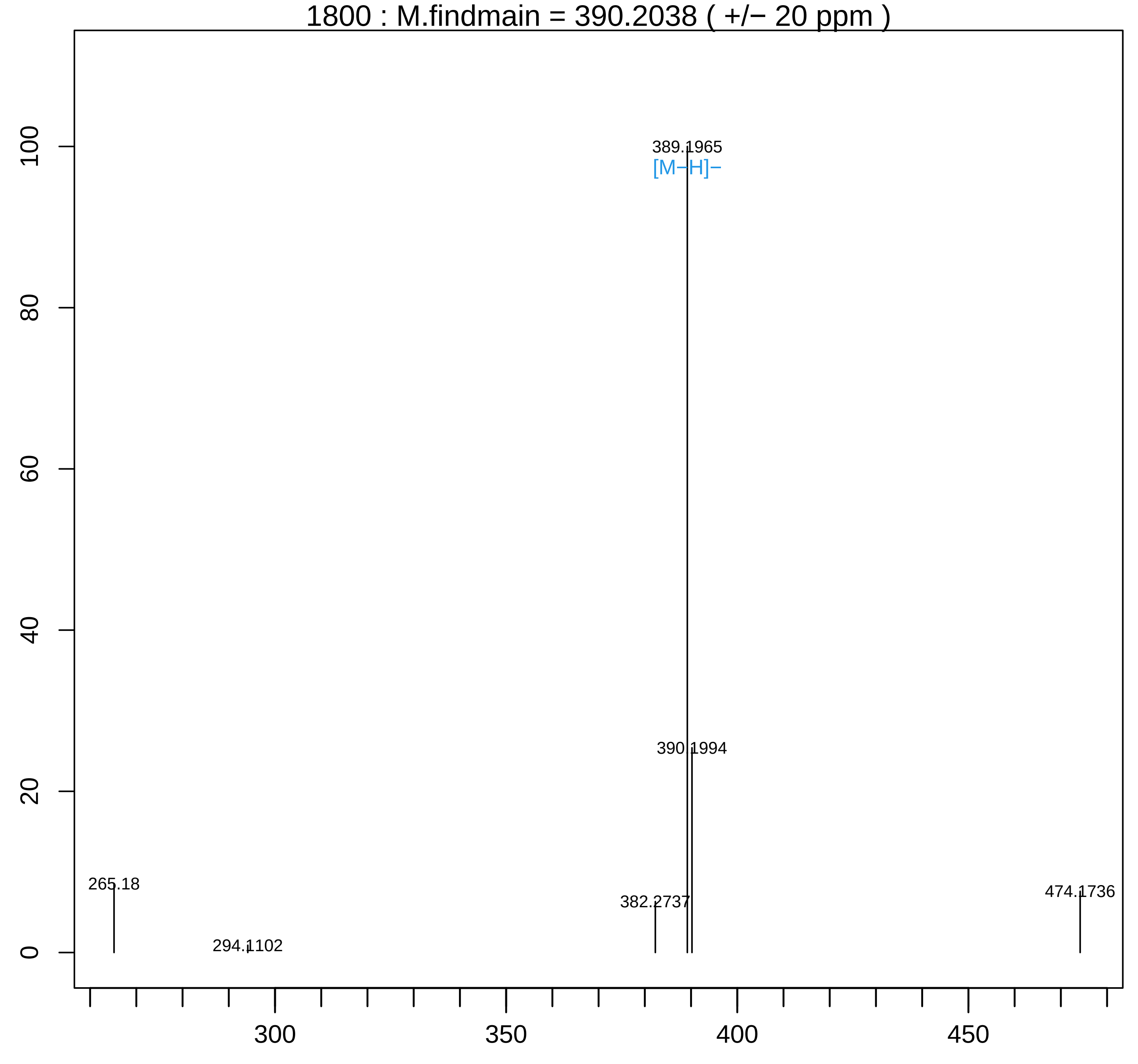
1800 : M.findmain = 390.2038 (+/− 20 ppm)
389.1965
[M−H]−
390.1994
265.18
294.1102
382.2737
474.1736
0
20
40
60
80
100
300
350
400
450

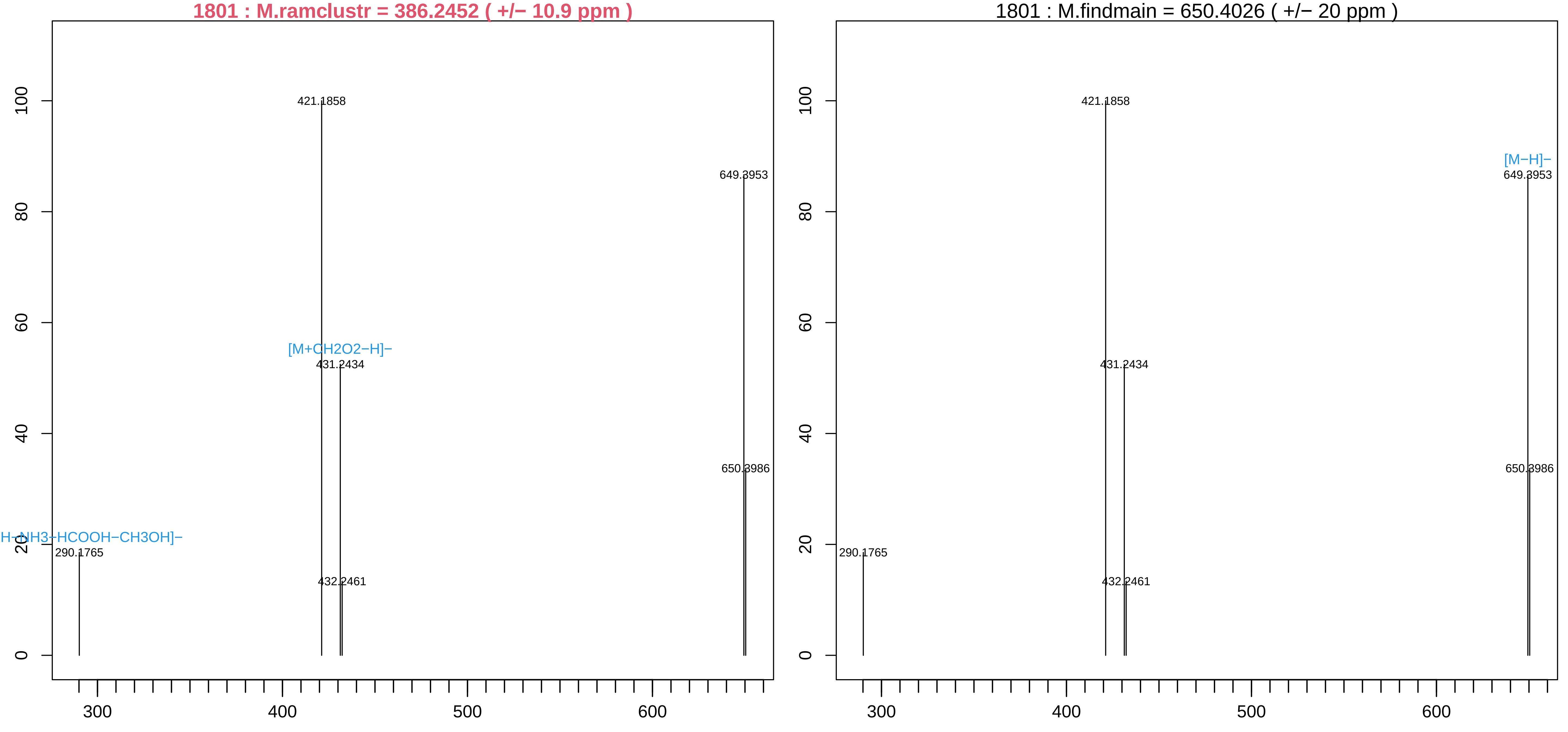

1801 : M.ramclustr = 386.2452 (+/− 10.9 ppm)
421.1858
649.3953
[M+CH2O2−H]−
431.2434
650.3986
H−NH3−HCOOH−CH3OH]−
290.1765
432.2461
1801 : M.findmain = 650.4026 (+/− 20 ppm)
421.1858
[M−H]−
649.3953
431.2434
650.3986
290.1765
432.2461
0
20
40
60
80
100
300
400
500
600

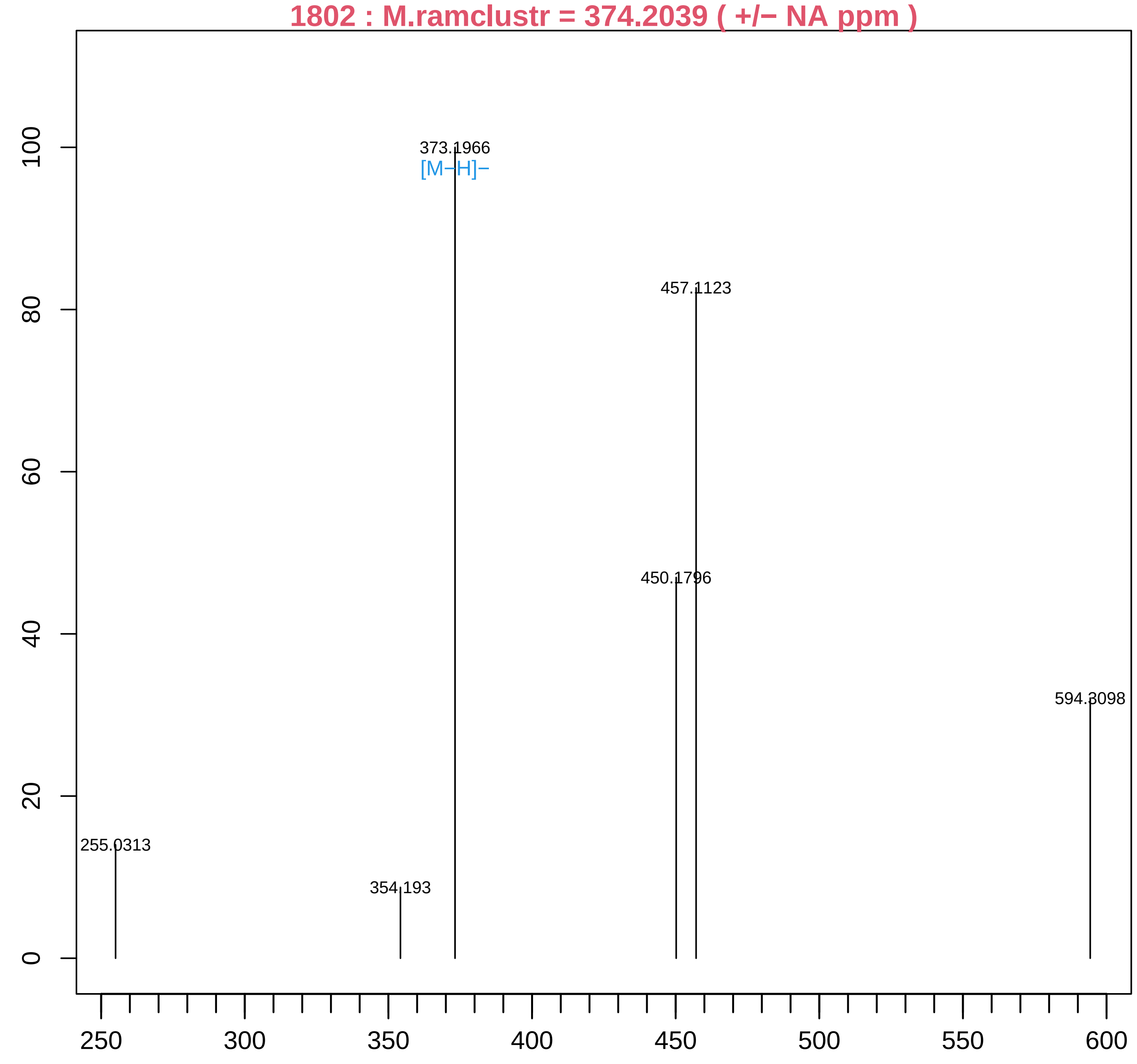
1802 : M.ramclustr = 374.2039 (+/− NA ppm)
373.1966
[M−H]−
457.1123
450.1796
594.3098
255.0313
354.193
0
20
40
60
80
100
250
300
350
400
450
500
550
600

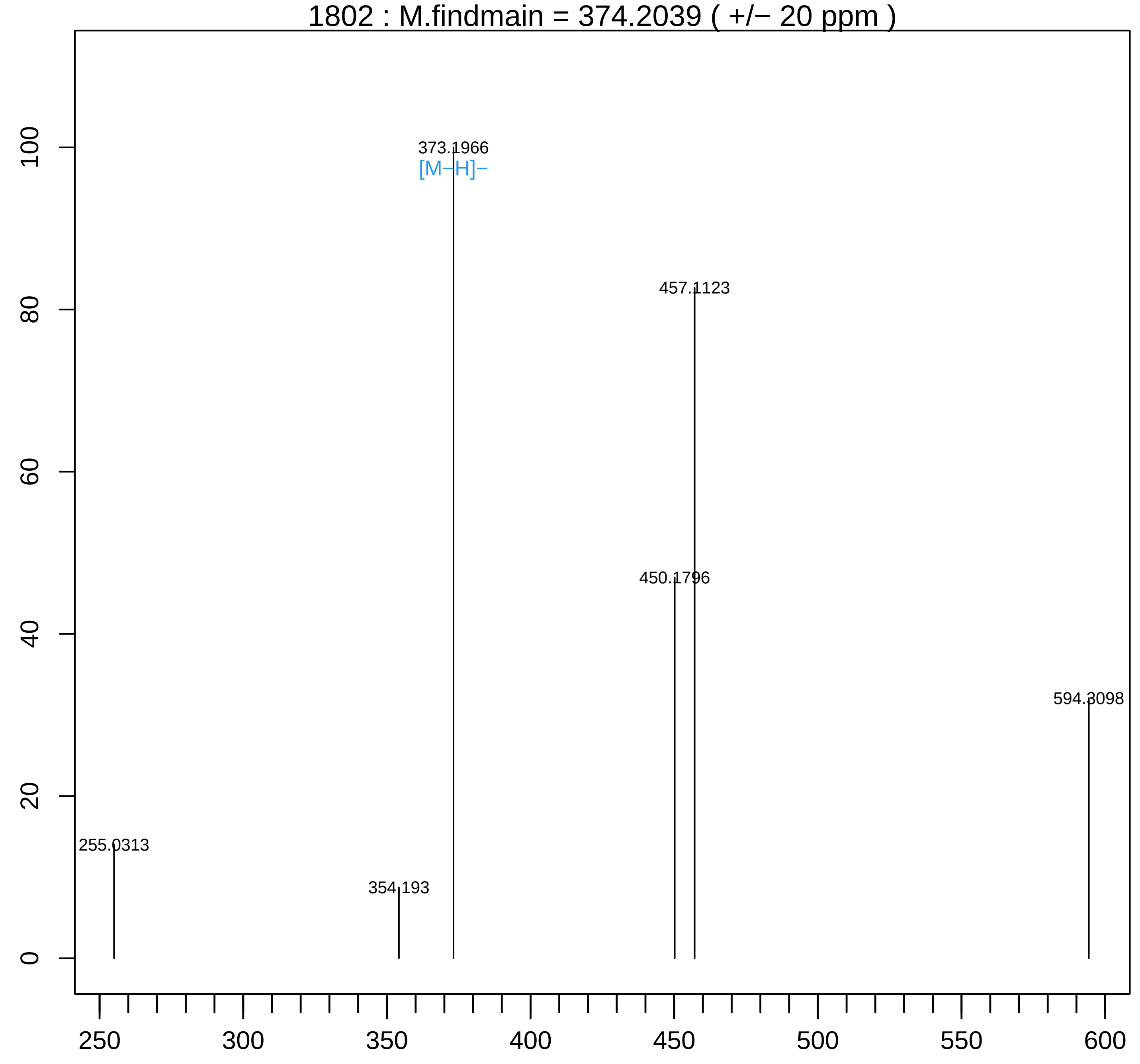
1802 : M.findmain = 374.2039 (+/− 20 ppm)
373.1966
[M−H]−
457.1123
450.1796
594.3098
255.0313
354.193
0
20
40
60
80
100
250
300
350
400
450
500
550
600

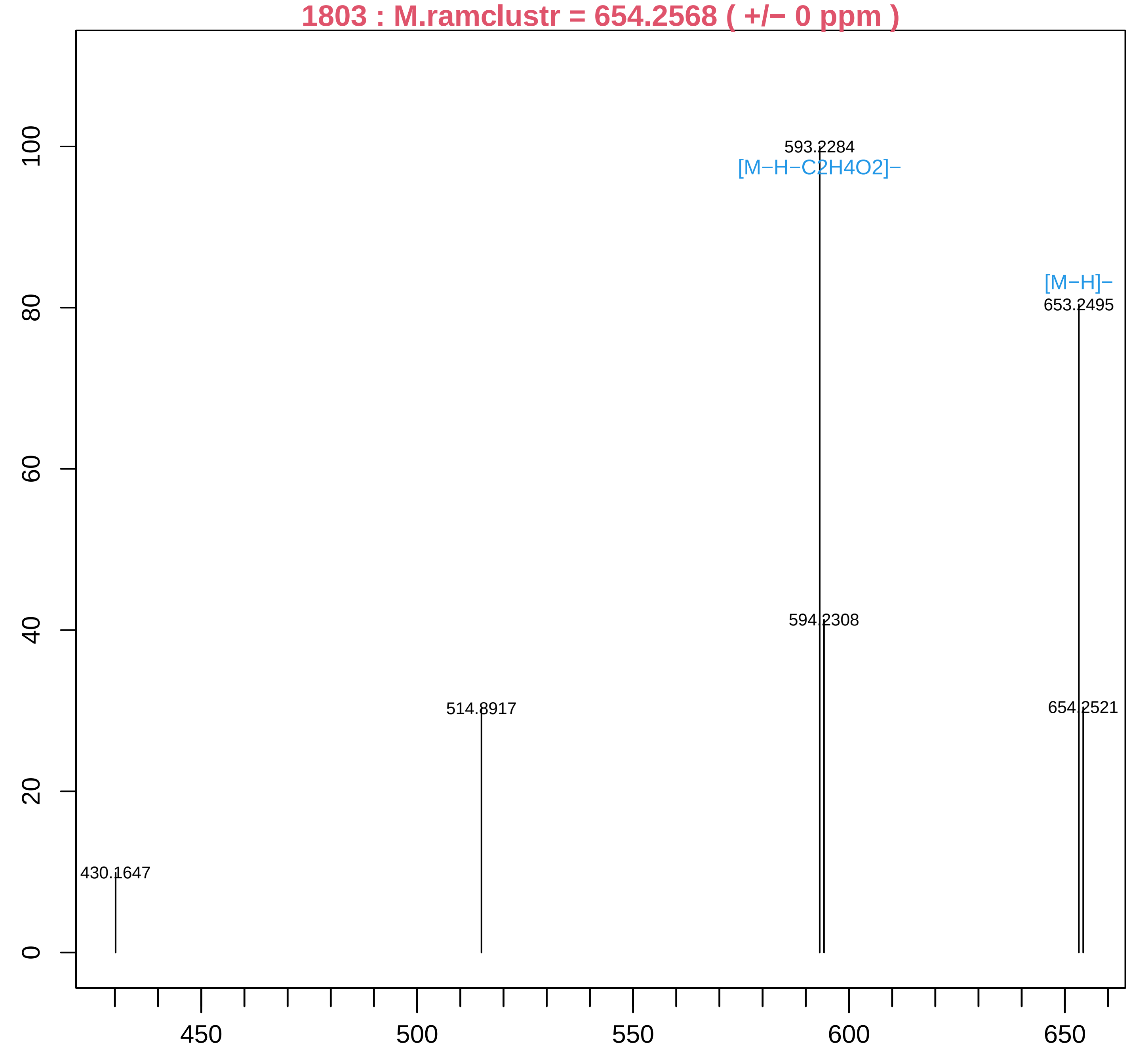
1803 : M.ramclustr = 654.2568 (+/− 0 ppm)
593.2284
[M−H−C2H4O2]−
[M−H]−
653.2495
594.2308
514.8917
654.2521
430.1647
0
20
40
60
80
100
450
500
550
600
650

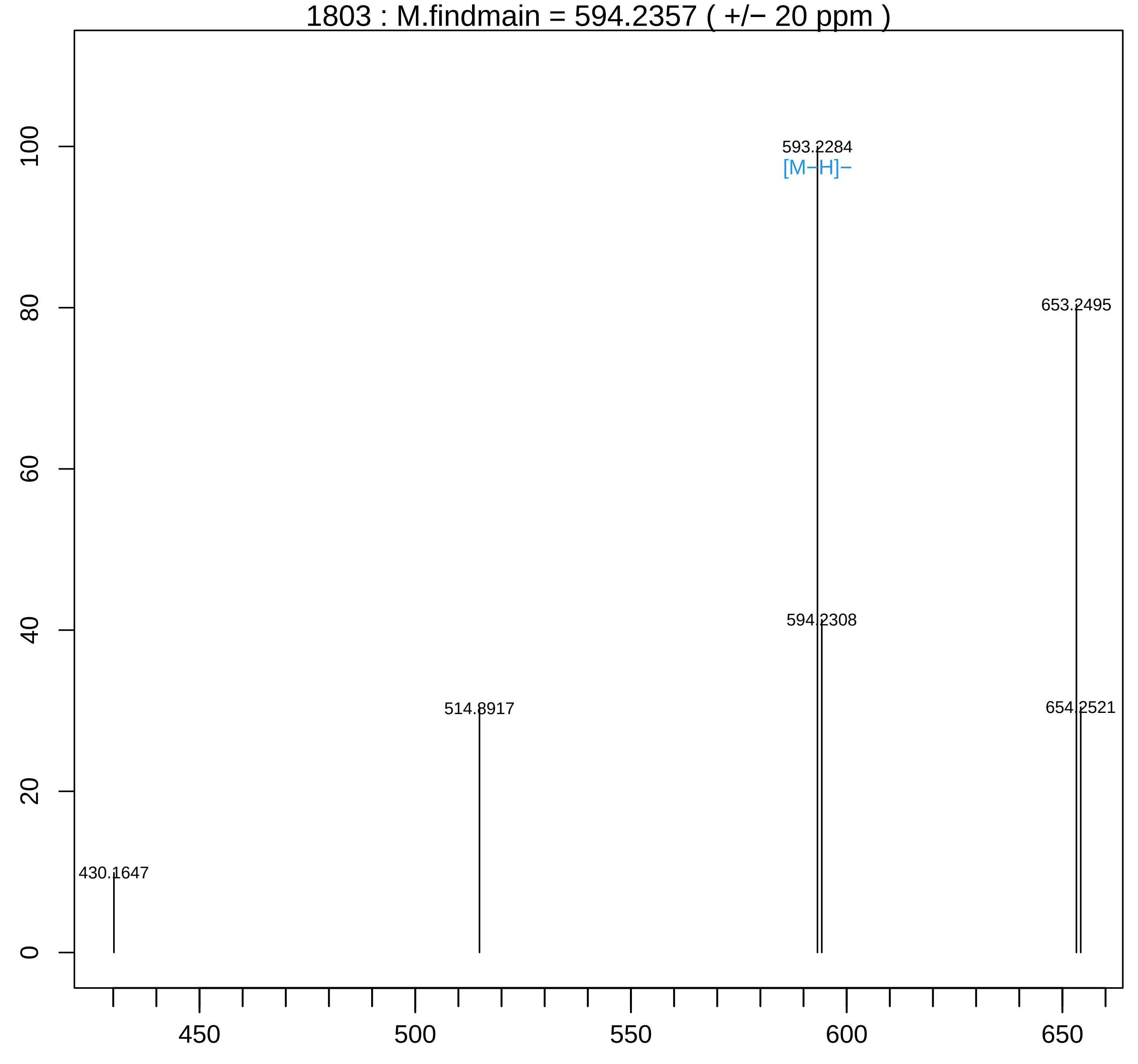
1803 : M.findmain = 594.2357 (+/− 20 ppm)
593.2284
[M−H]−
653.2495
594.2308
514.8917
654.2521
430.1647
0
20
40
60
80
100
450
500
550
600
650

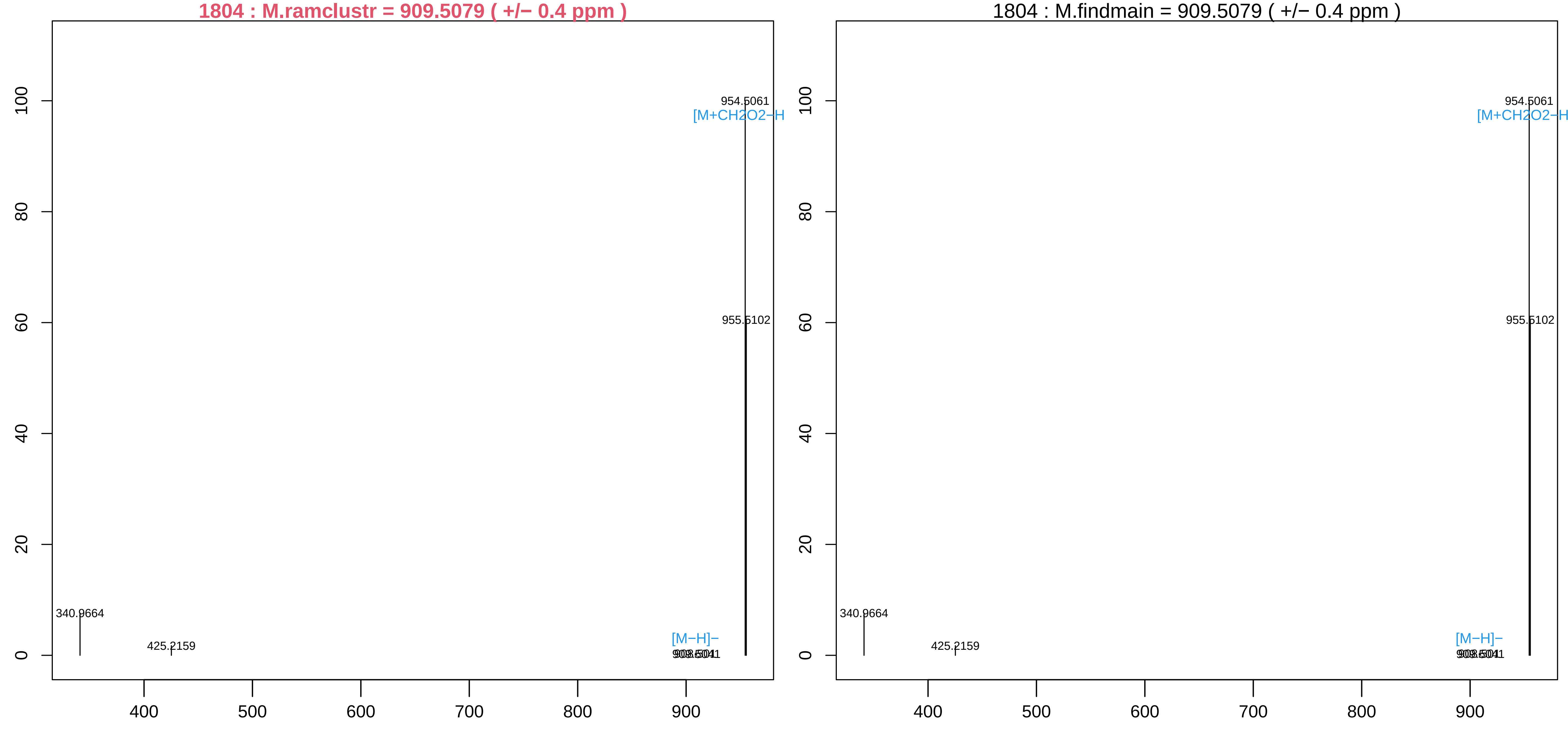
1804 : M.ramclustr = 909.5079 (+/− 0.4 ppm)
954.5061
[M+CH2O2−H
955.5102
340.9664
425.2159
[M−H]−
908.5041
0
20
40
60
80
100
400
500
600
700
800
900
1804 : M.findmain = 909.5079 (+/− 0.4 ppm)
954.5061
[M+CH2O2−H
955.5102
340.9664
425.2159
[M−H]−
908.5041
0
20
40
60
80
100
400
500
600
700
800
900

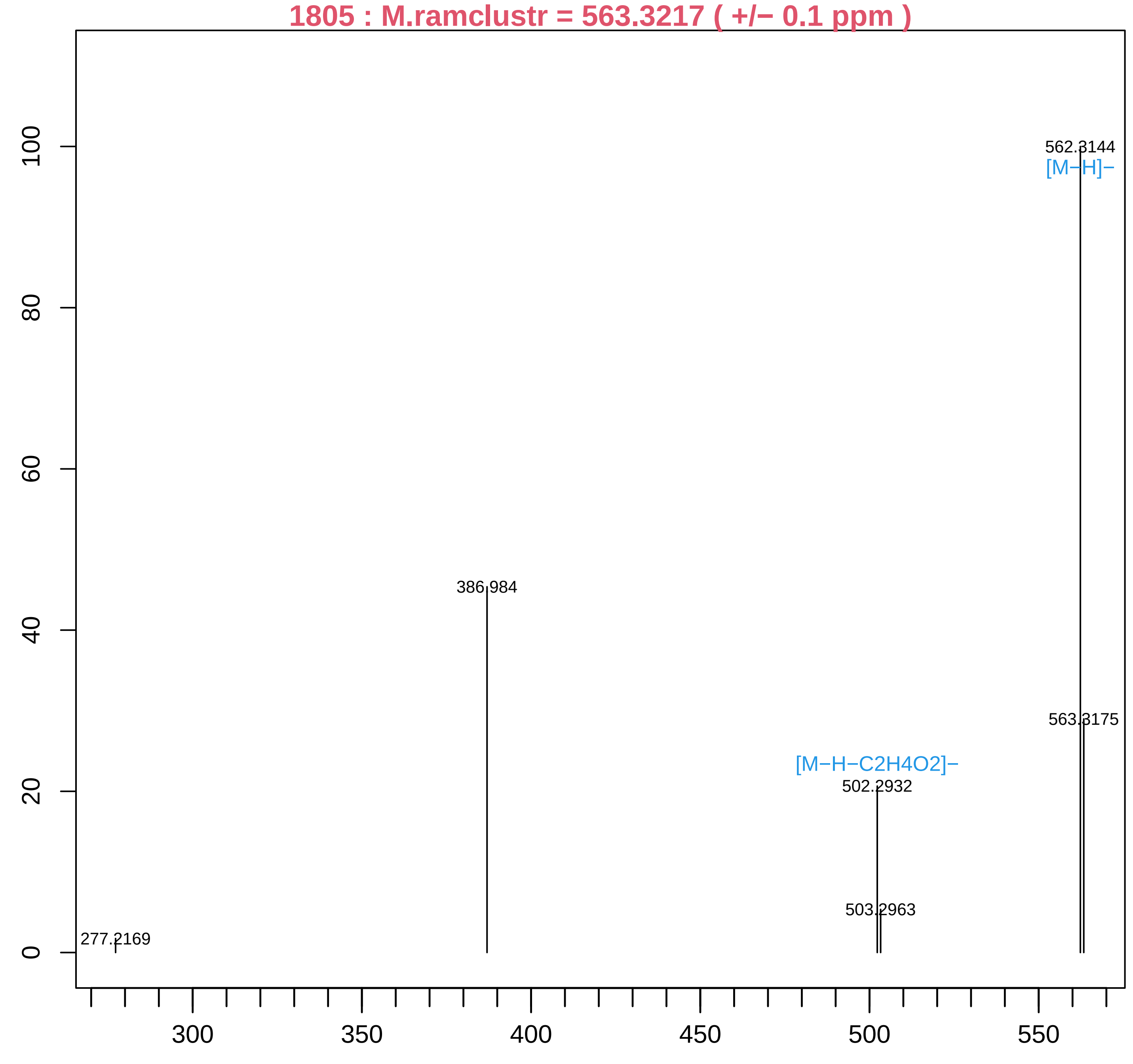

1805 : M.ramclustr = 563.3217 (+/− 0.1 ppm)
562.3144
[M−H]−
386.984
563.3175
[M−H−C2H4O2]−
502.2932
503.2963
277.2169
0
20
40
60
80
100
300
350
400
450
500
550

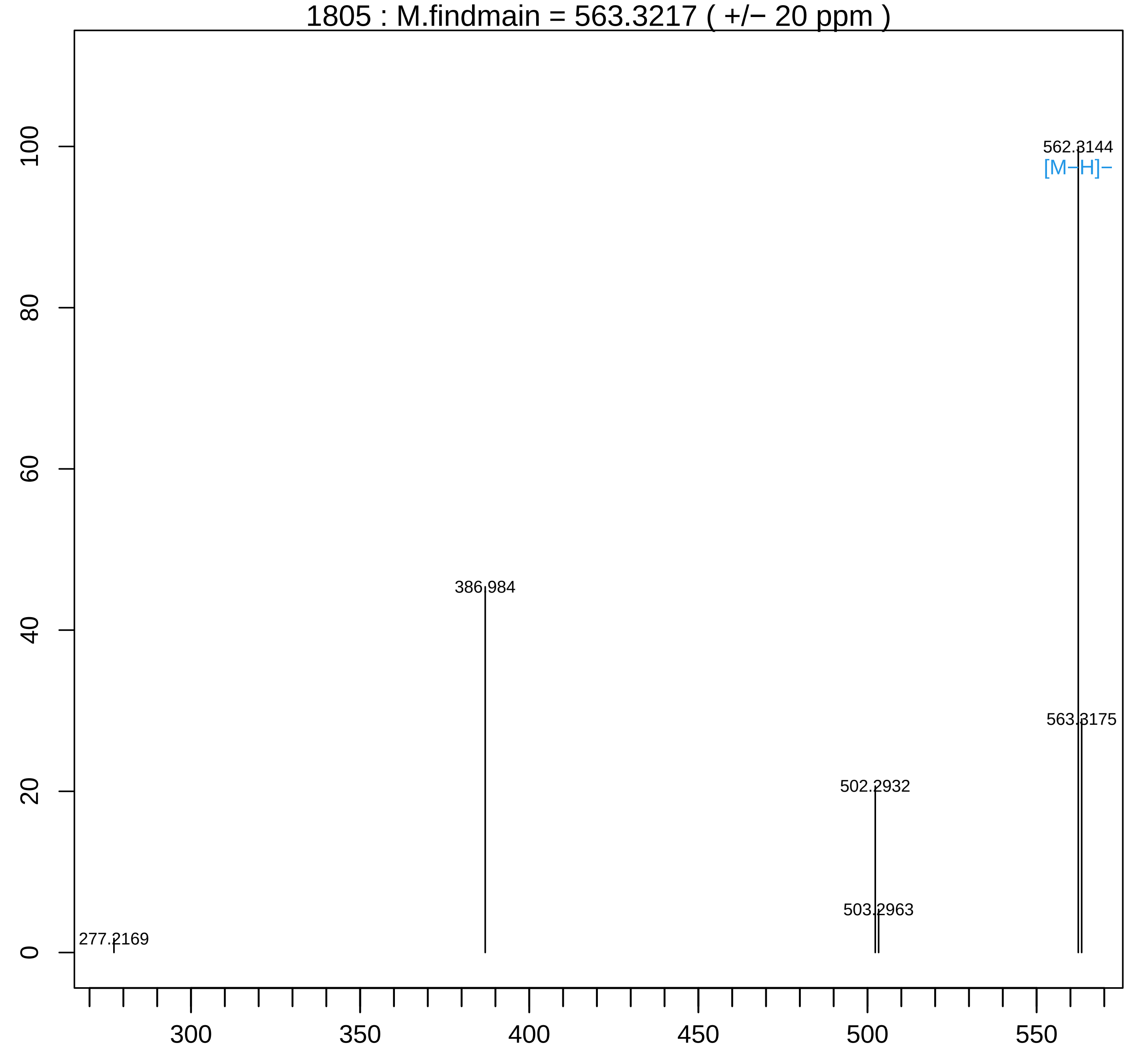

1805 : M.findmain = 563.3217 (+/− 20 ppm)
562.3144
[M−H]−
386.984
563.3175
502.2932
503.2963
277.2169
0
20
40
60
80
100
300
350
400
450
500
550

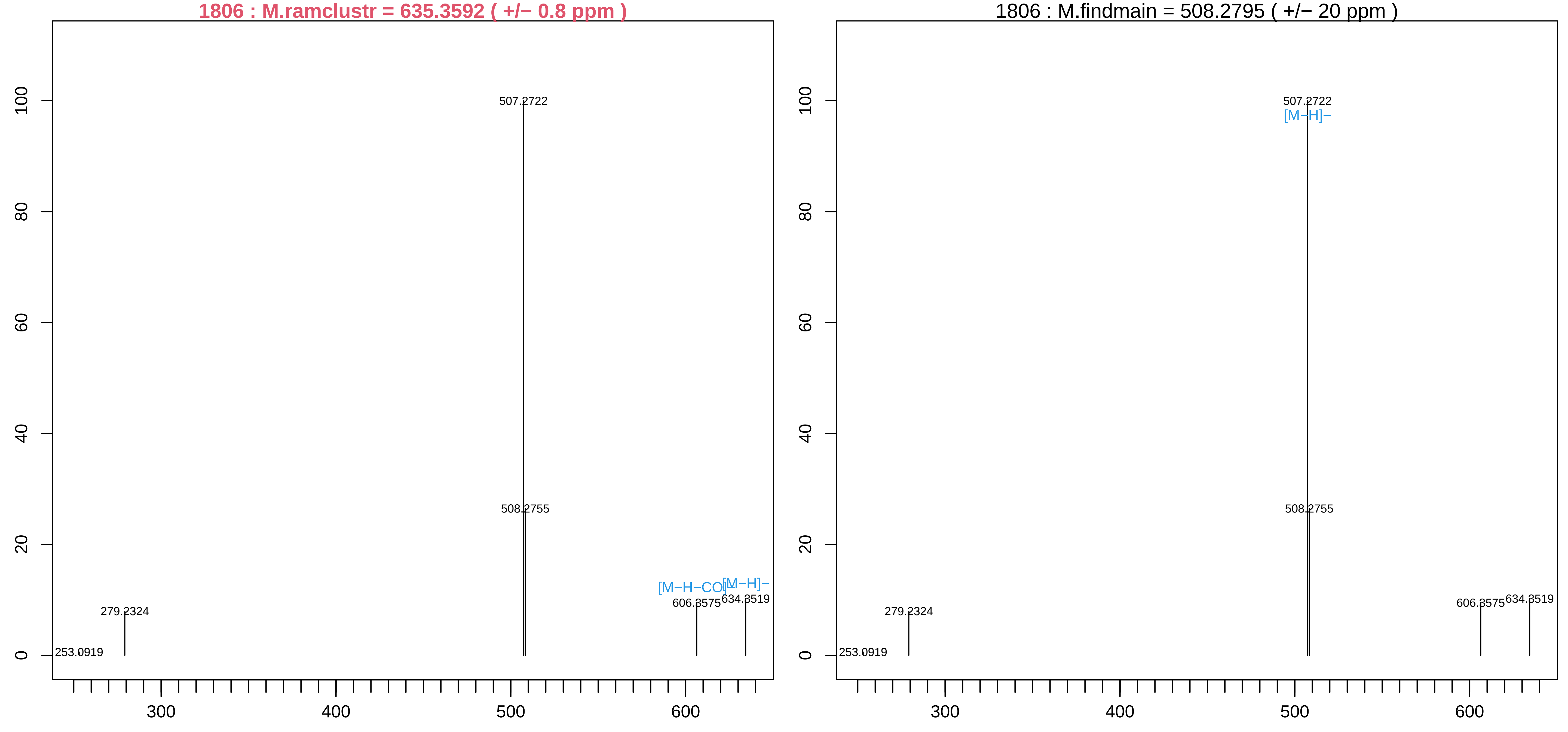
1806 : M.ramclustr = 635.3592 (+/− 0.8 ppm)
507.2722
508.2755
279.2324
253.0919
606.3575
634.3519
[M−H−CO]−
[M−H]−
1806 : M.findmain = 508.2795 (+/− 20 ppm)
507.2722
[M−H]−
508.2755
279.2324
253.0919
606.3575
634.3519
0
20
40
60
80
100
300
400
500
600

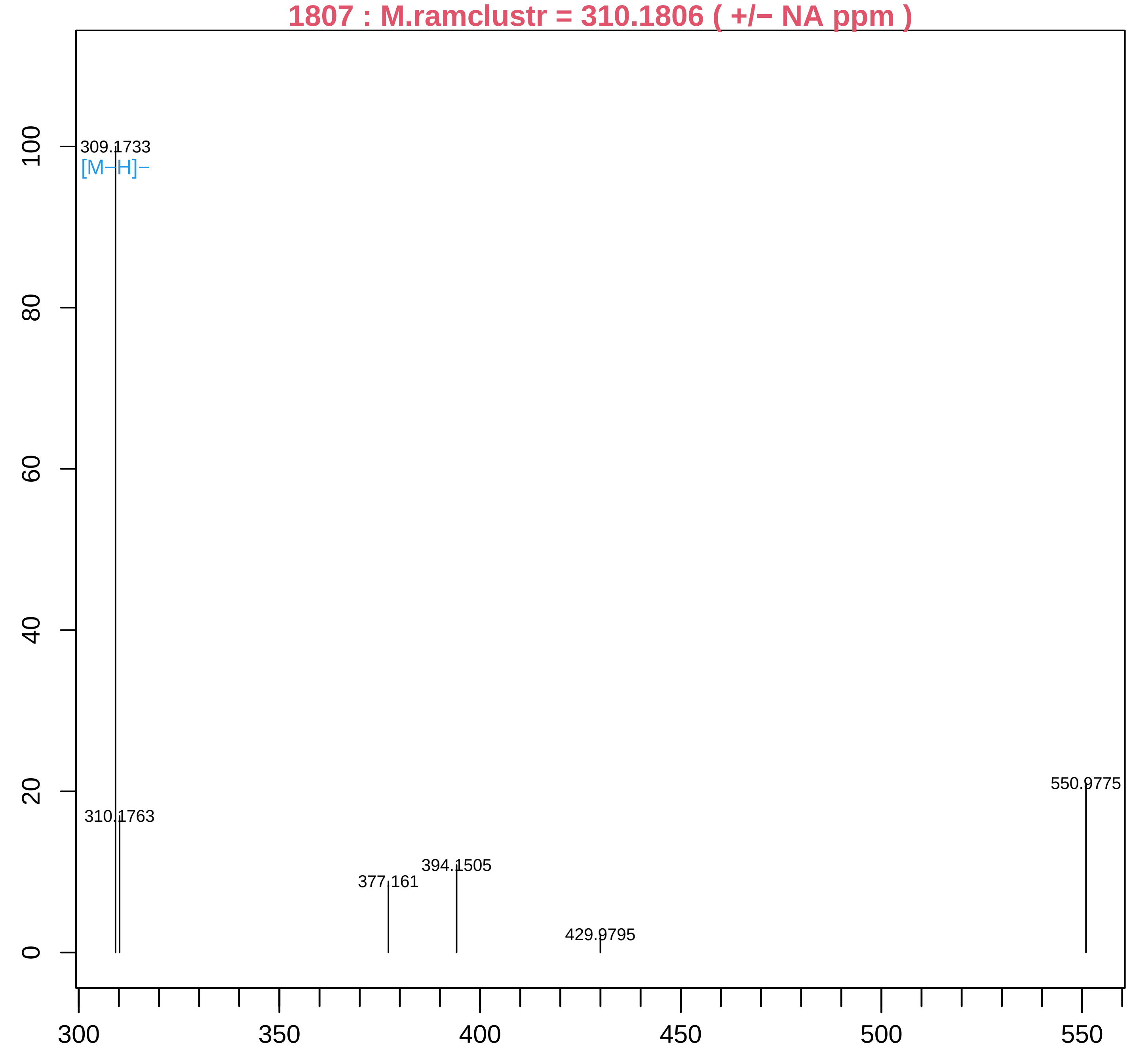

1807 : M.ramclustr = 310.1806 (+/− NA ppm)
309.1733
[M−H]−
310.1763
377.161
394.1505
429.9795
550.9775
0
20
40
60
80
100
300
350
400
450
500
550

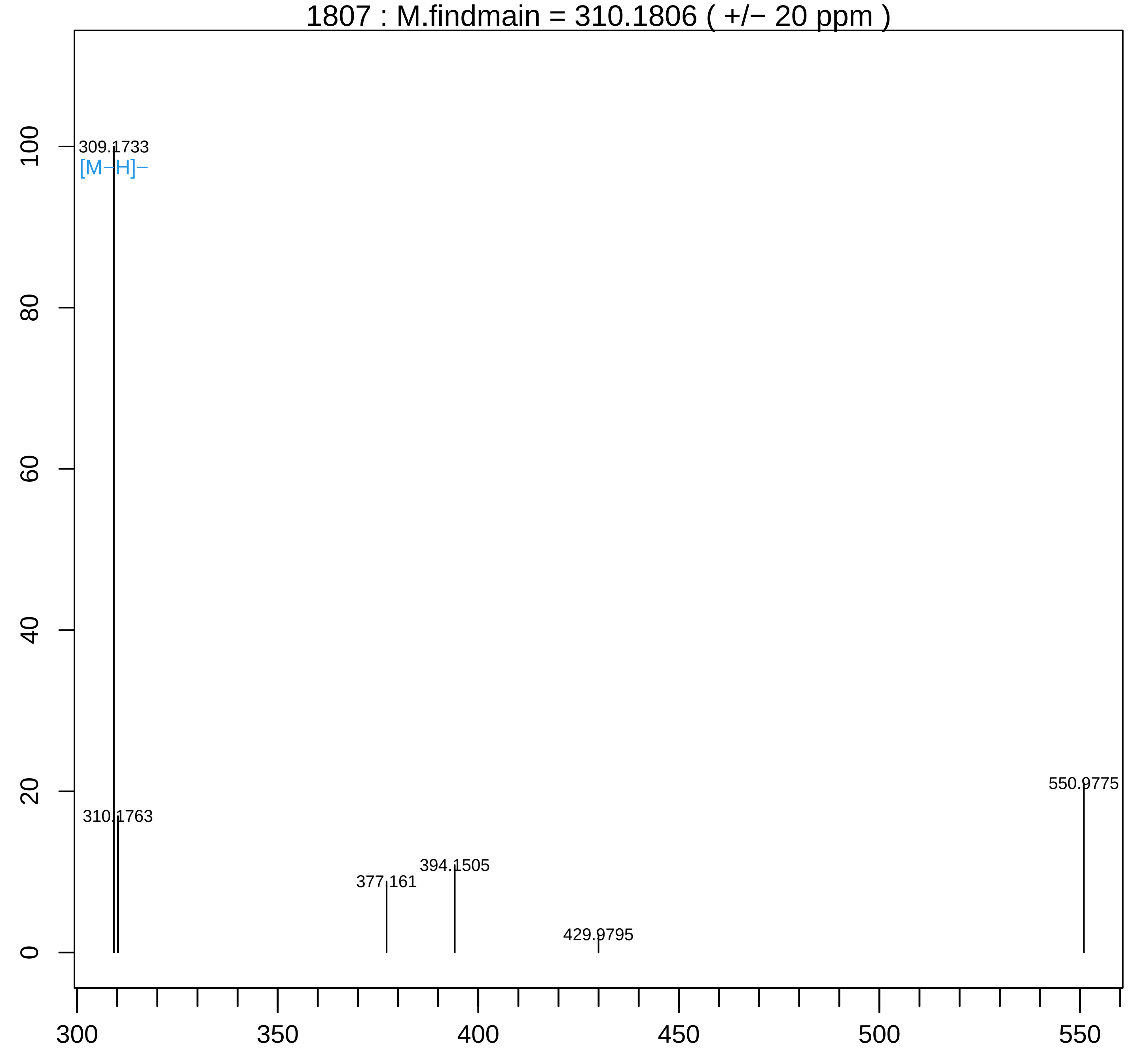

1807 : M.findmain = 310.1806 (+/− 20 ppm)
309.1733
[M−H]−
310.1763
377.161
394.1505
429.9795
550.9775
0
20
40
60
80
100
300
350
400
450
500
550

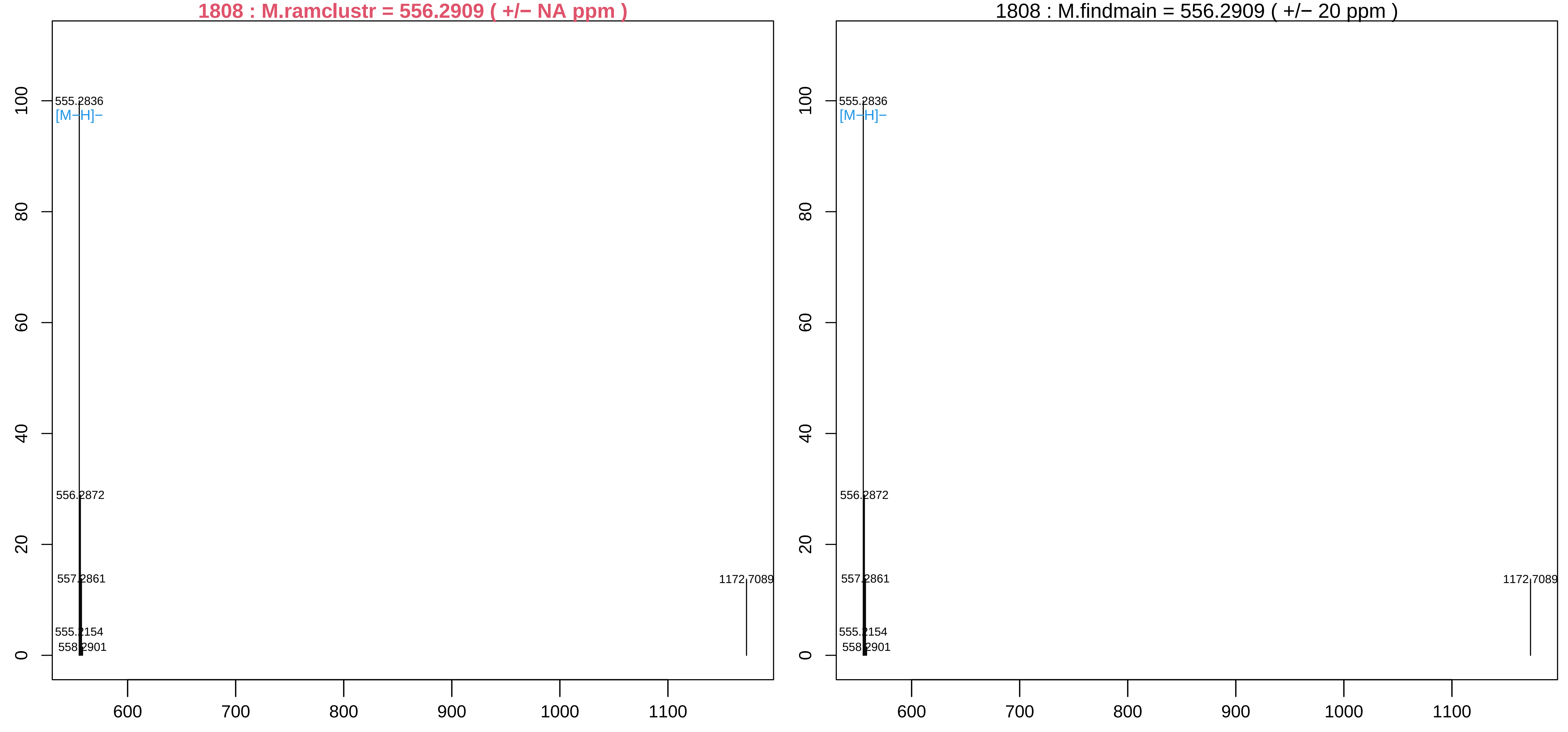
1808 : M.ramclustr = 556.2909 (+/− NA ppm)
555.2836
[M−H]−
556.2872
557.2861
555.2154
558.2901
1172.7089
0
20
40
60
80
100
600
700
800
900
1000
1100
1808 : M.findmain = 556.2909 (+/− 20 ppm)
555.2836
[M−H]−
556.2872
557.2861
555.2154
558.2901
1172.7089

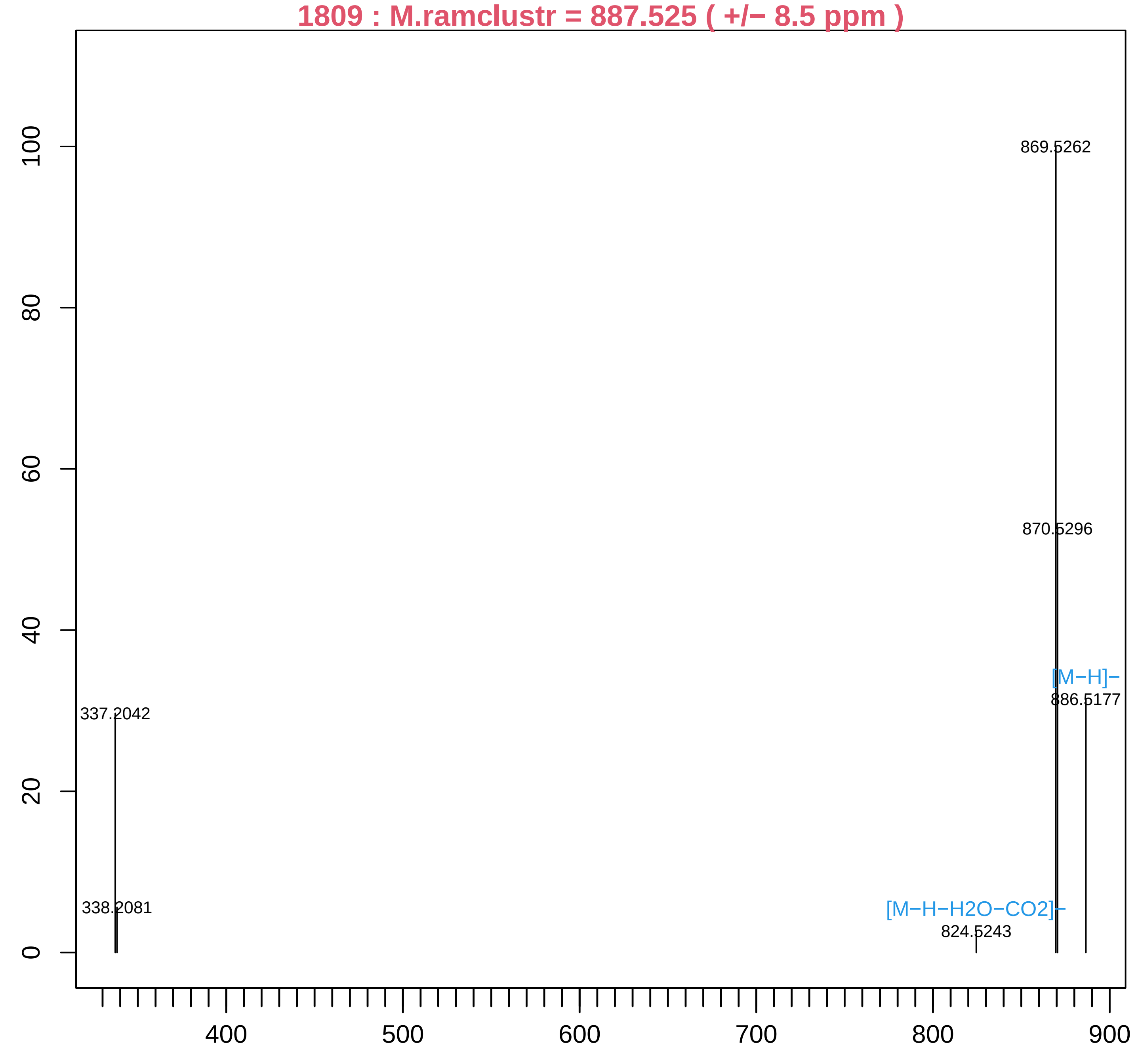

1809 : M.ramclustr = 887.525 (+/− 8.5 ppm)
869.5262
870.5296
[M−H]−
886.5177
337.2042
338.2081
[M−H−H2O−CO2]−
824.5243
0
20
40
60
80
100
400
500
600
700
800
900

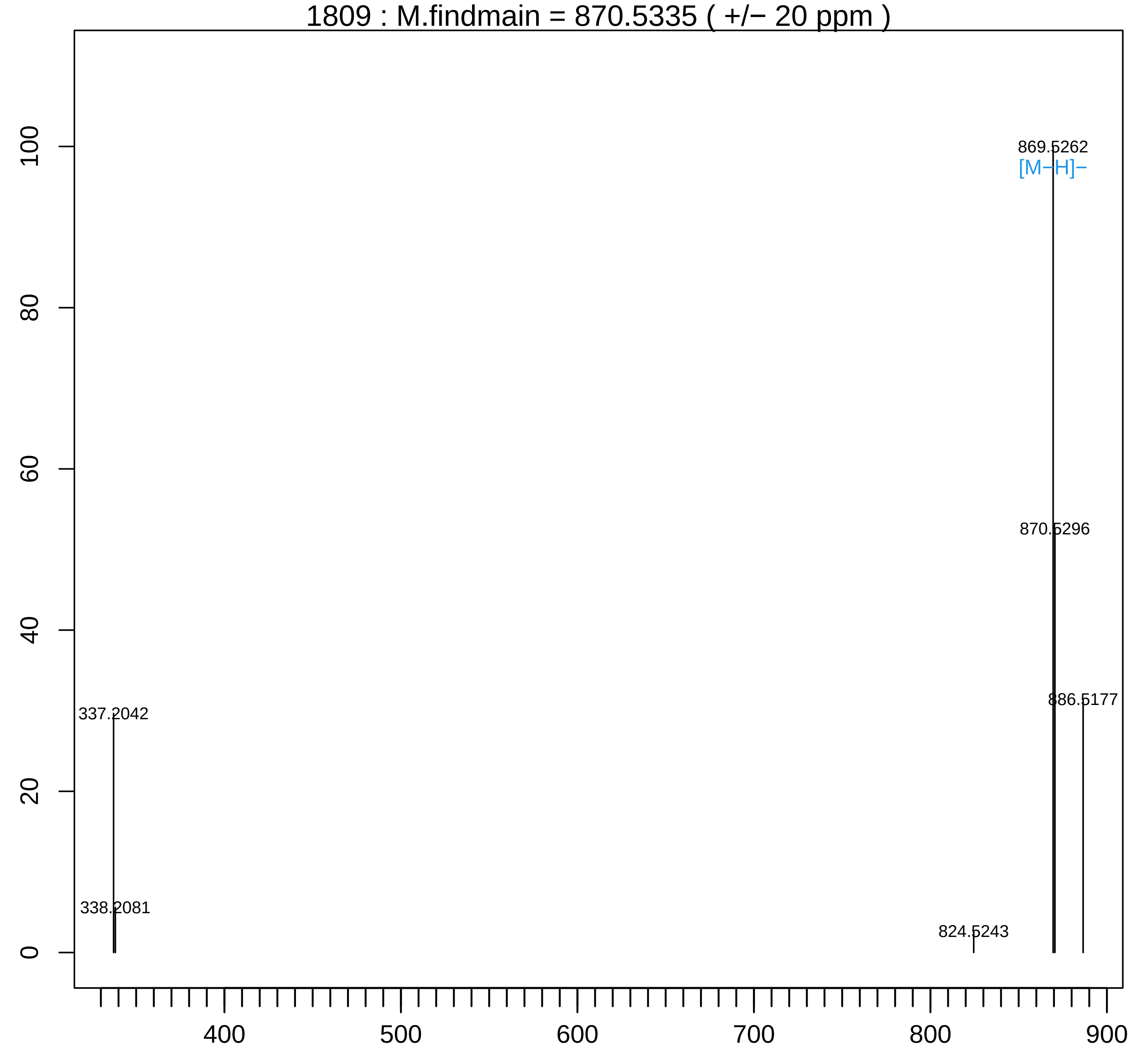

1809 : M.findmain = 870.5335 (+/− 20 ppm)
869.5262
[M−H]−
870.5296
886.5177
337.2042
338.2081
824.5243
0
20
40
60
80
100
400
500
600
700
800
900

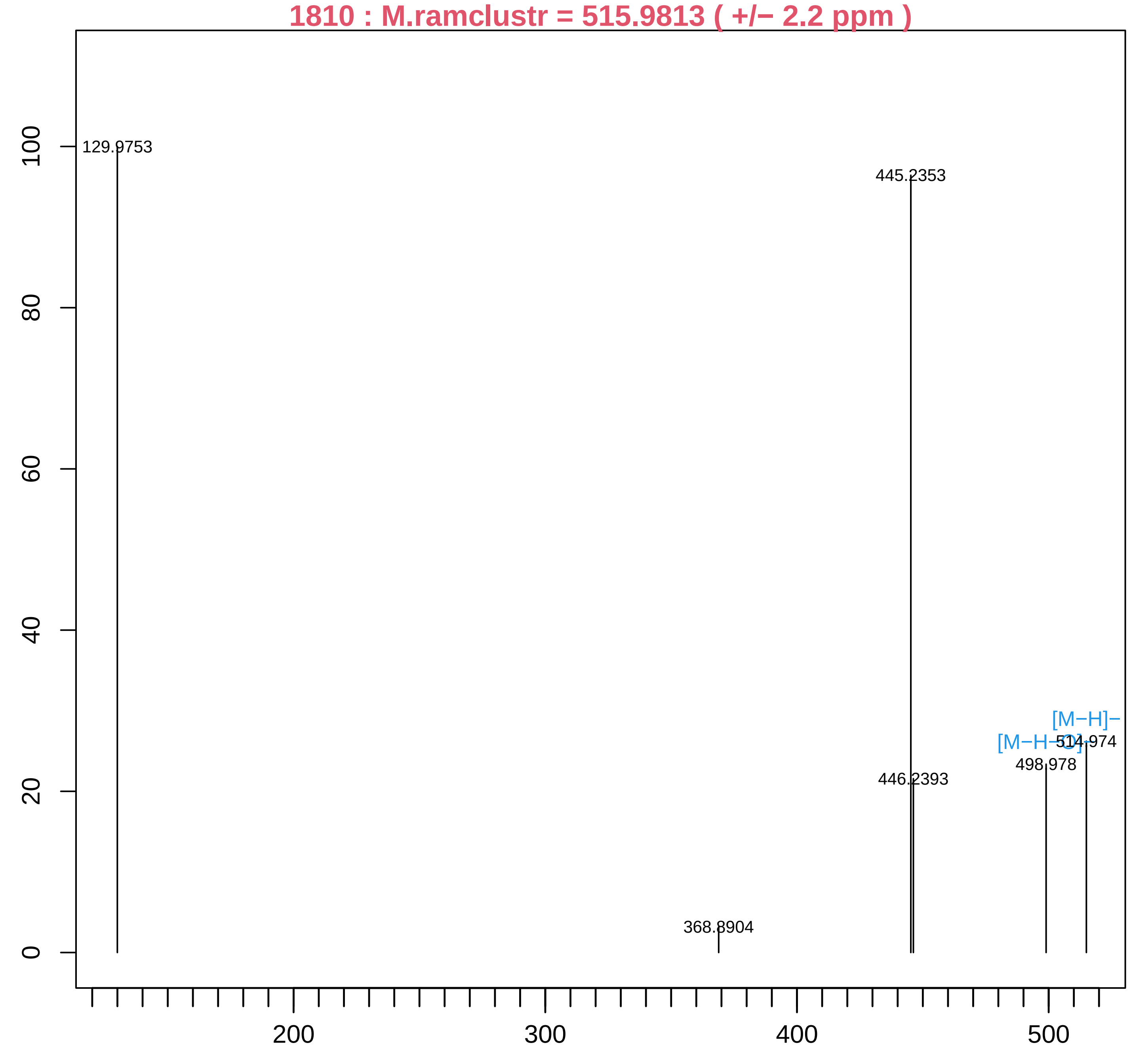
1810 : M.ramclustr = 515.9813 (+/− 2.2 ppm)
129.9753
445.2353
446.2393
368.8904
498.978
514.974
[M−H]−
0
20
40
60
80
100
200
300
400
500

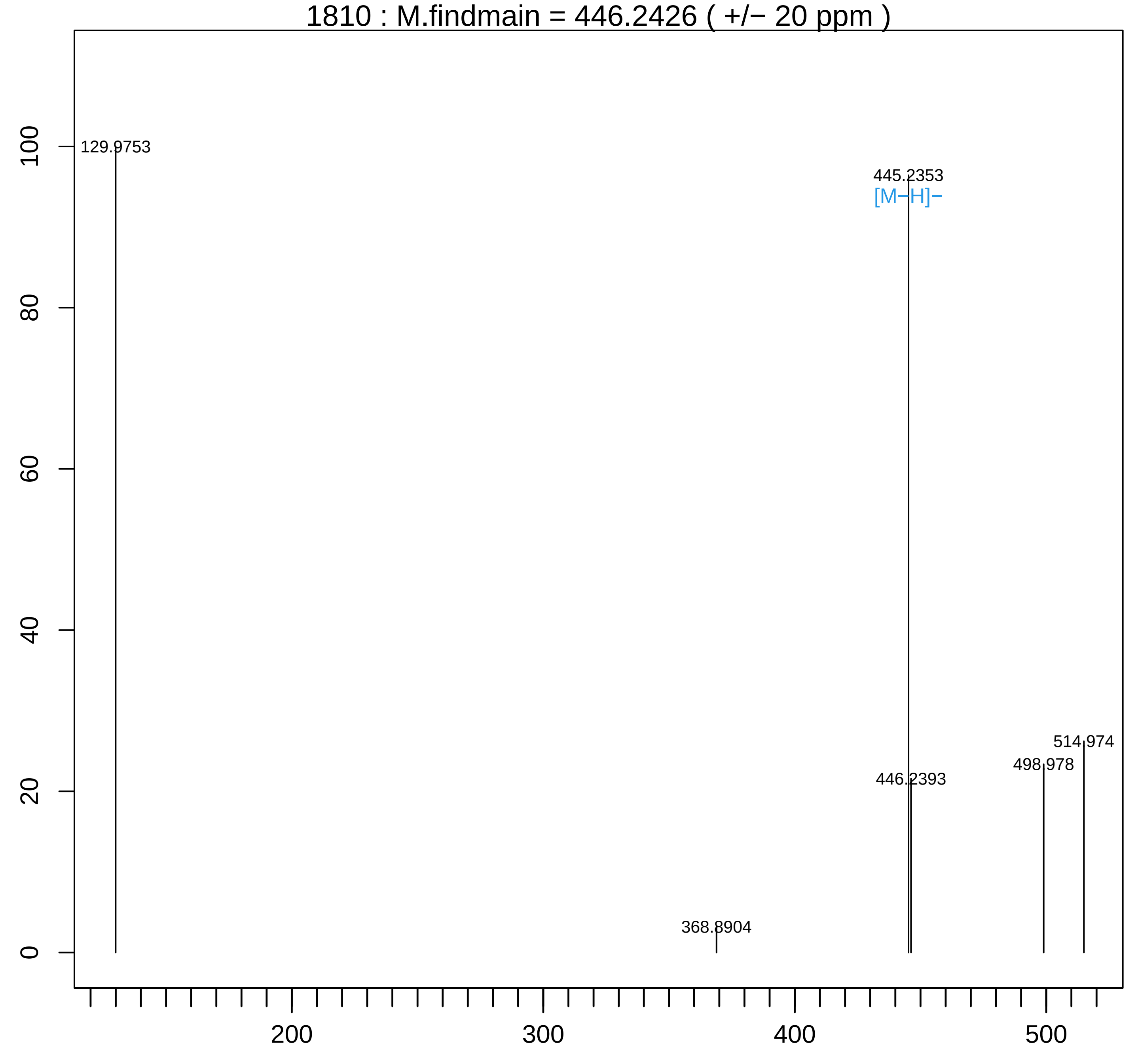
1810 : M.findmain = 446.2426 (+/− 20 ppm)
129.9753
445.2353
[M−H]−
446.2393
368.8904
498.978
514.974
0
20
40
60
80
100
200
300
400
500

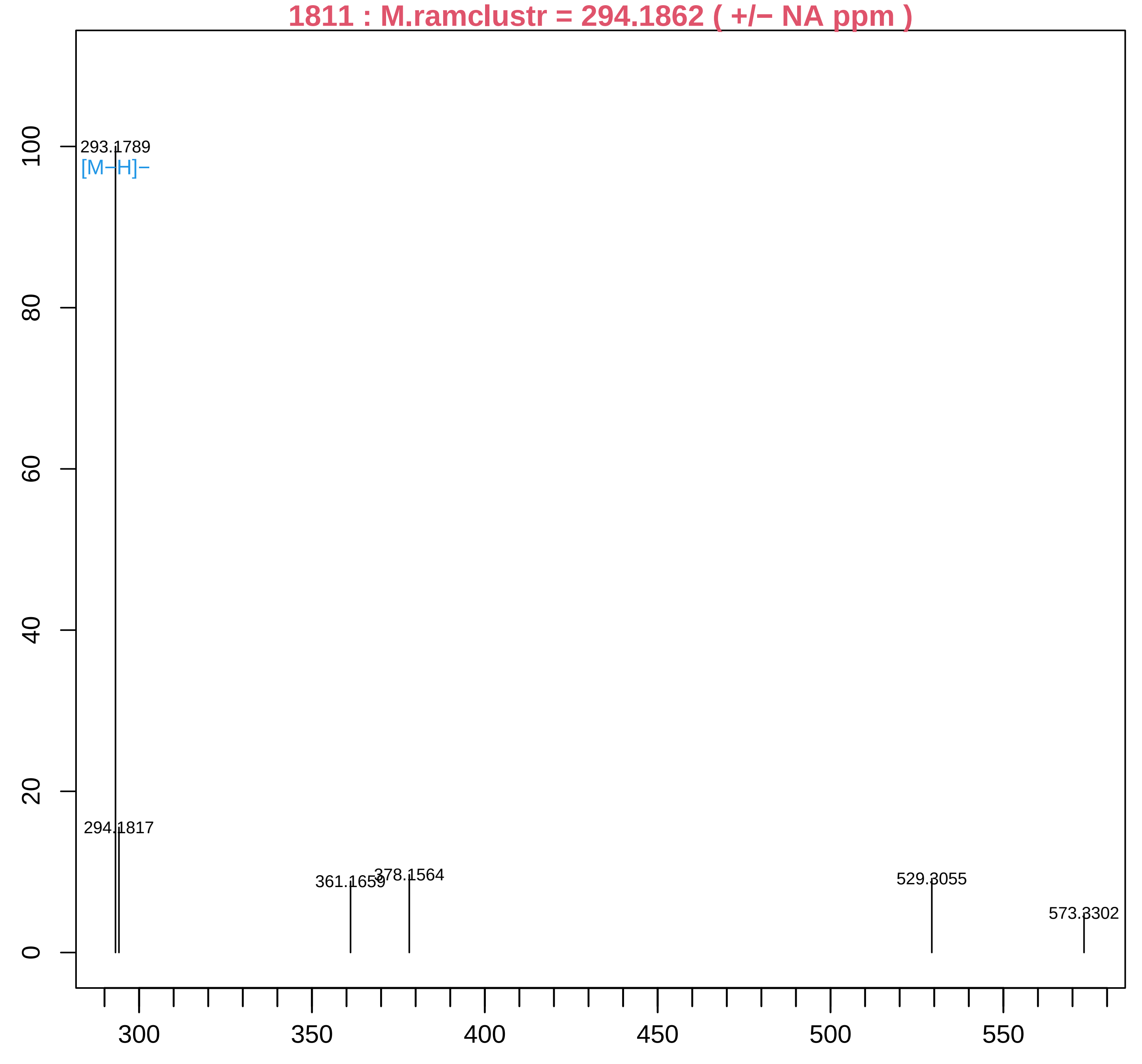

1811 : M.ramclustr = 294.1862 (+/− NA ppm)
293.1789
[M−H]−
294.1817
361.1659
378.1564
529.3055
573.3302

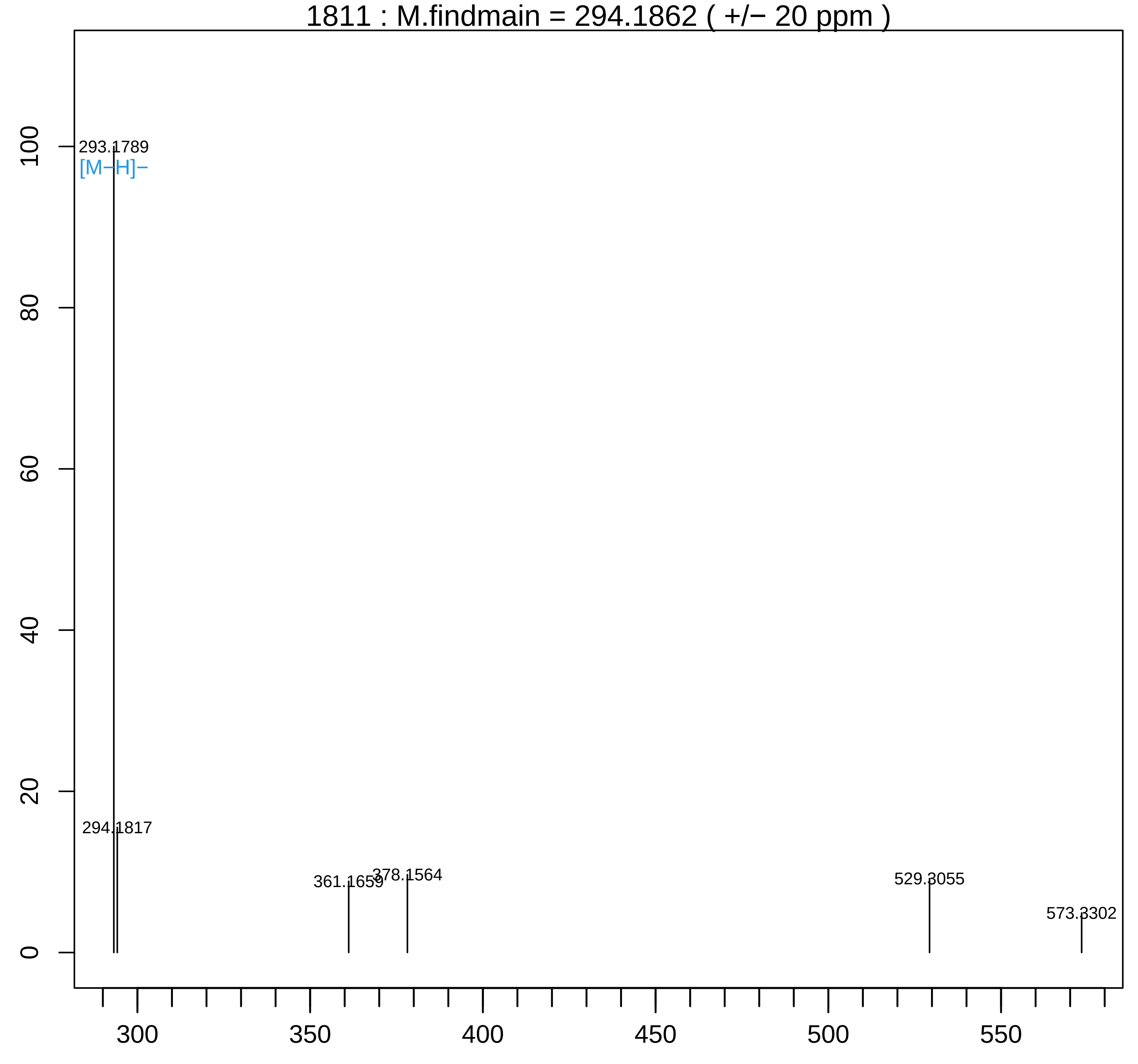

1811 : M.findmain = 294.1862 (+/− 20 ppm)
293.1789
[M−H]−
294.1817
361.1659
378.1564
529.3055
573.3302

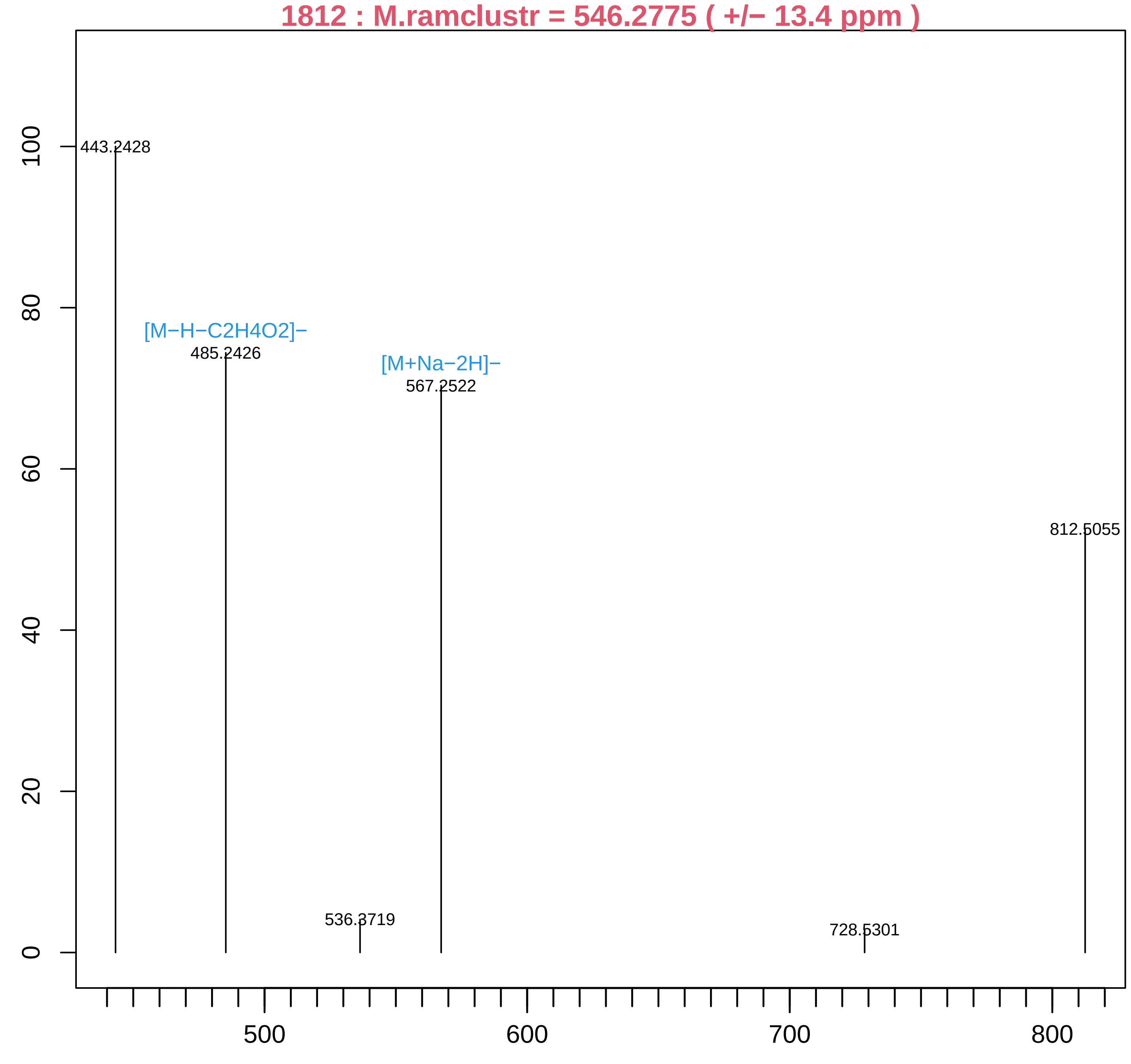

1812 : M.ramclustr = 546.2775 (+/− 13.4 ppm)
443.2428
[M−H−C2H4O2]−
485.2426
[M+Na−2H]−
567.2522
536.3719
728.5301
812.5055
0
20
40
60
80
100
500
600
700
800

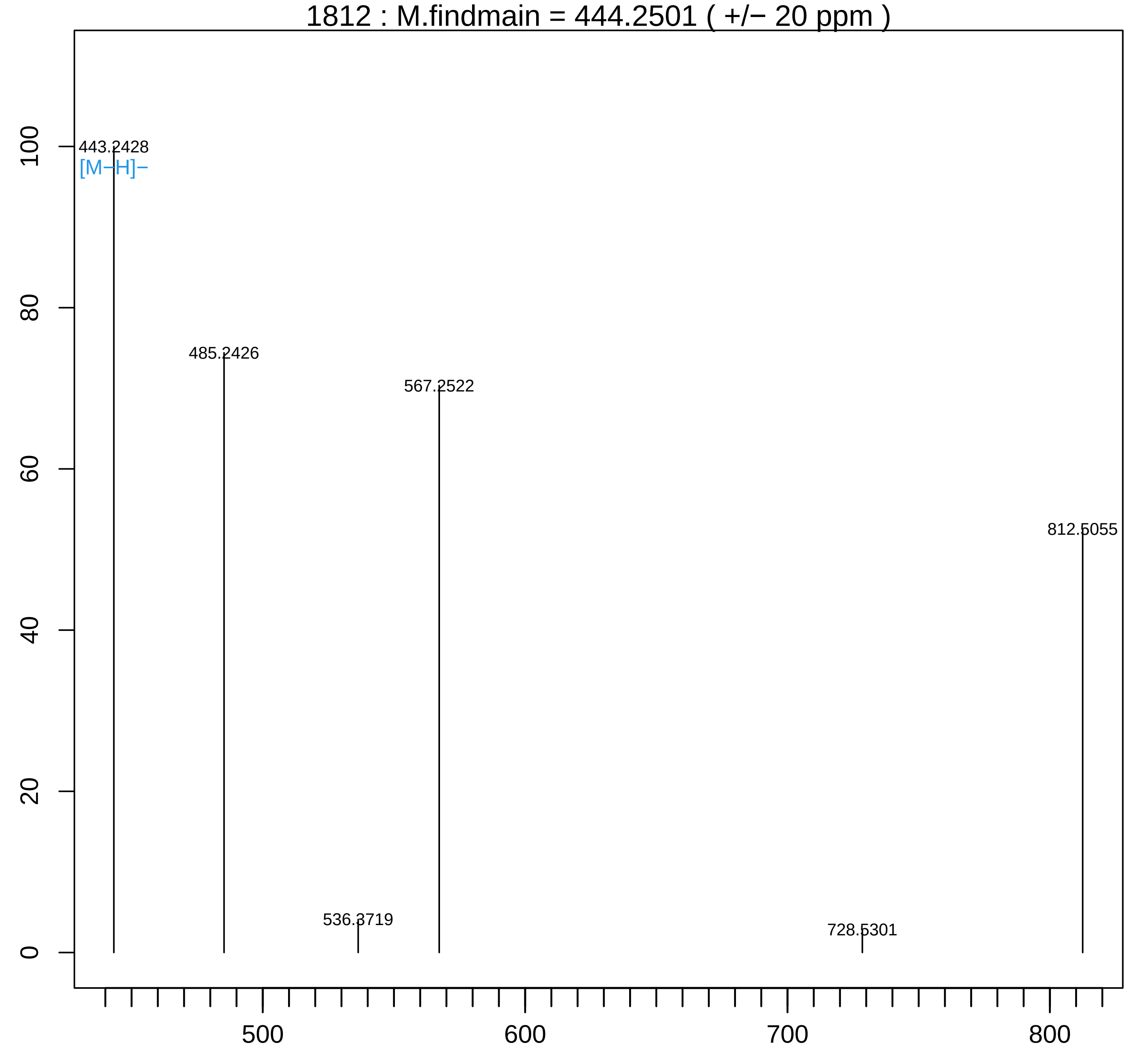

1812 : M.findmain = 444.2501 (+/− 20 ppm)
443.2428
[M−H]−
485.2426
567.2522
536.3719
728.5301
812.5055
0
20
40
60
80
100
500
600
700
800

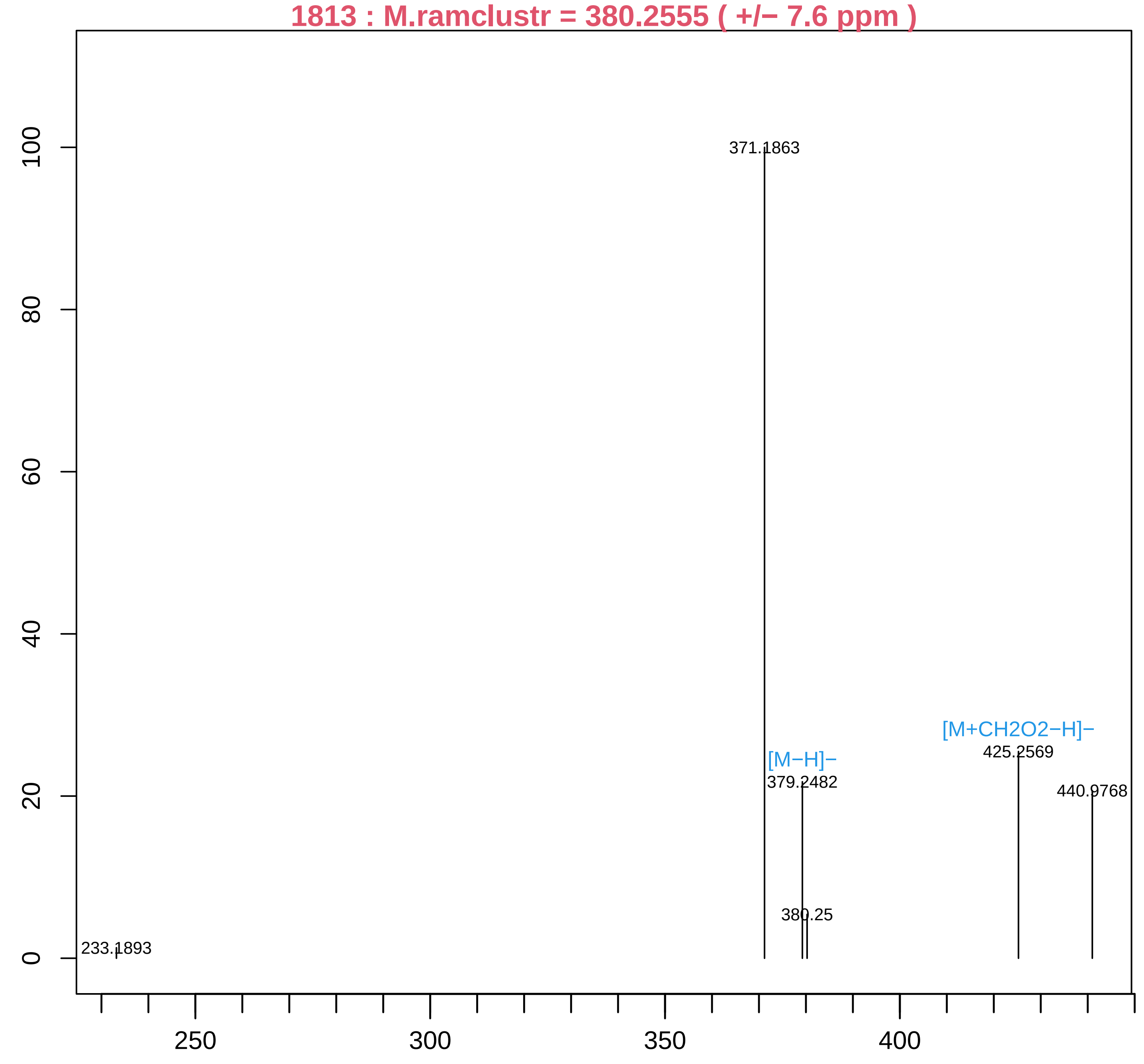
1813 : M.ramclustr = 380.2555 (+/− 7.6 ppm)
371.1863
[M−H]−
379.2482
380.25
[M+CH2O2−H]−
425.2569
440.9768
233.1893
0
20
40
60
80
100
250
300
350
400

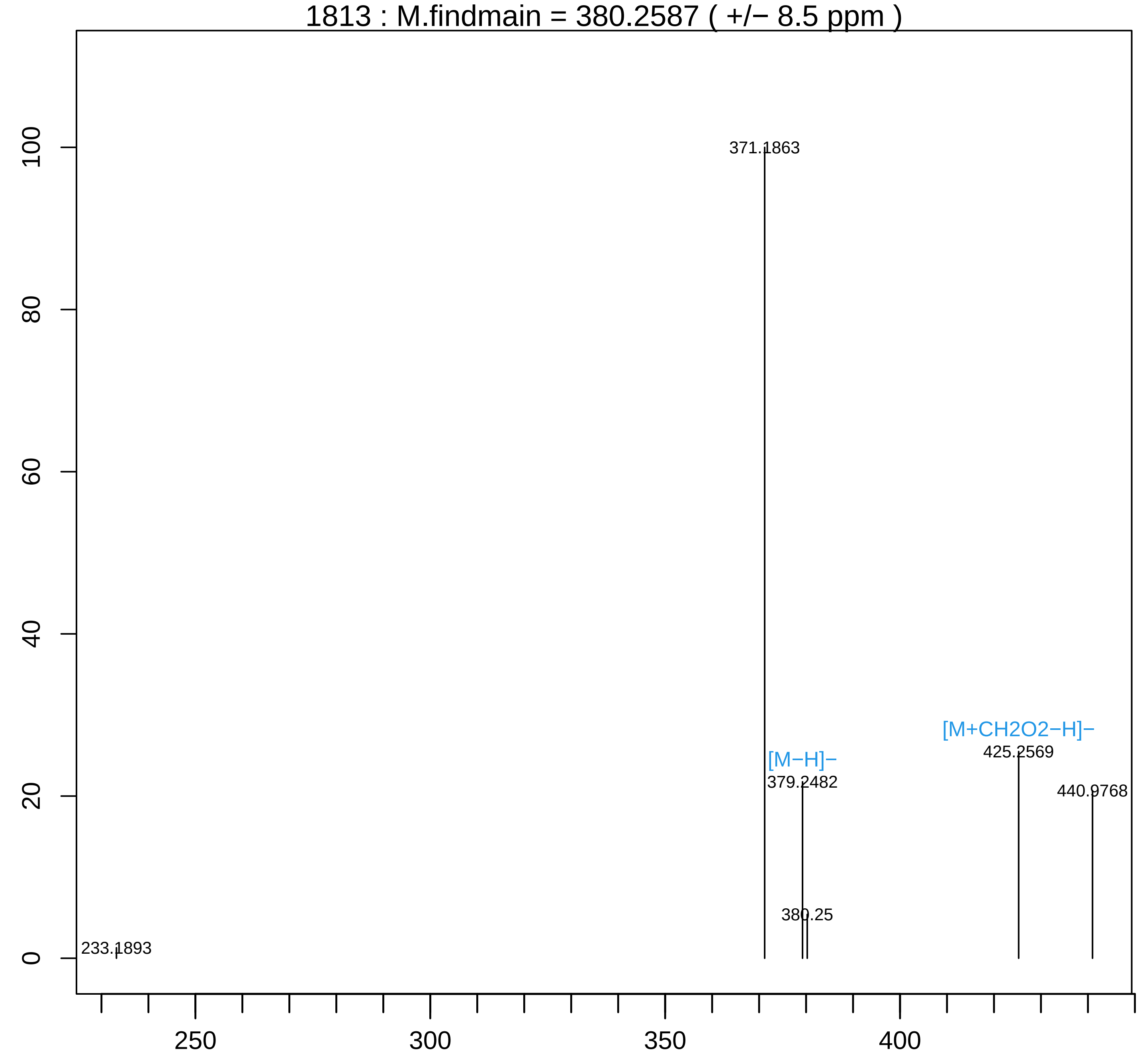
1813 : M.findmain = 380.2587 (+/− 8.5 ppm)
371.1863
[M−H]−
379.2482
380.25
[M+CH2O2−H]−
425.2569
440.9768
233.1893
0
20
40
60
80
100
250
300
350
400

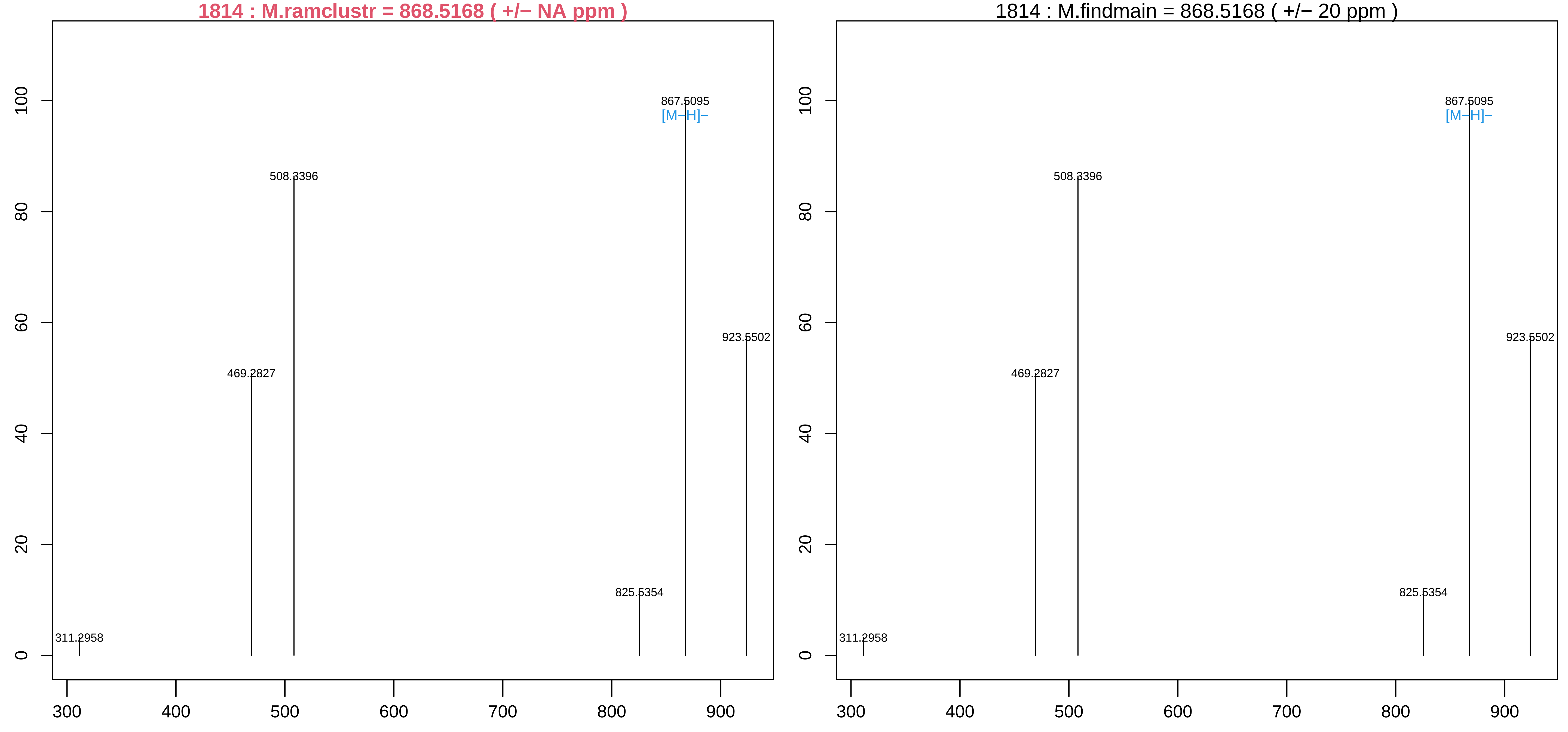
1814 : M.ramclustr = 868.5168 (+/− NA ppm)
867.5095
[M−H]−
508.3396
923.5502
469.2827
825.5354
311.2958
0
20
40
60
80
100
300
400
500
600
700
800
900
1814 : M.findmain = 868.5168 (+/− 20 ppm)
867.5095
[M−H]−
508.3396
923.5502
469.2827
825.5354
311.2958

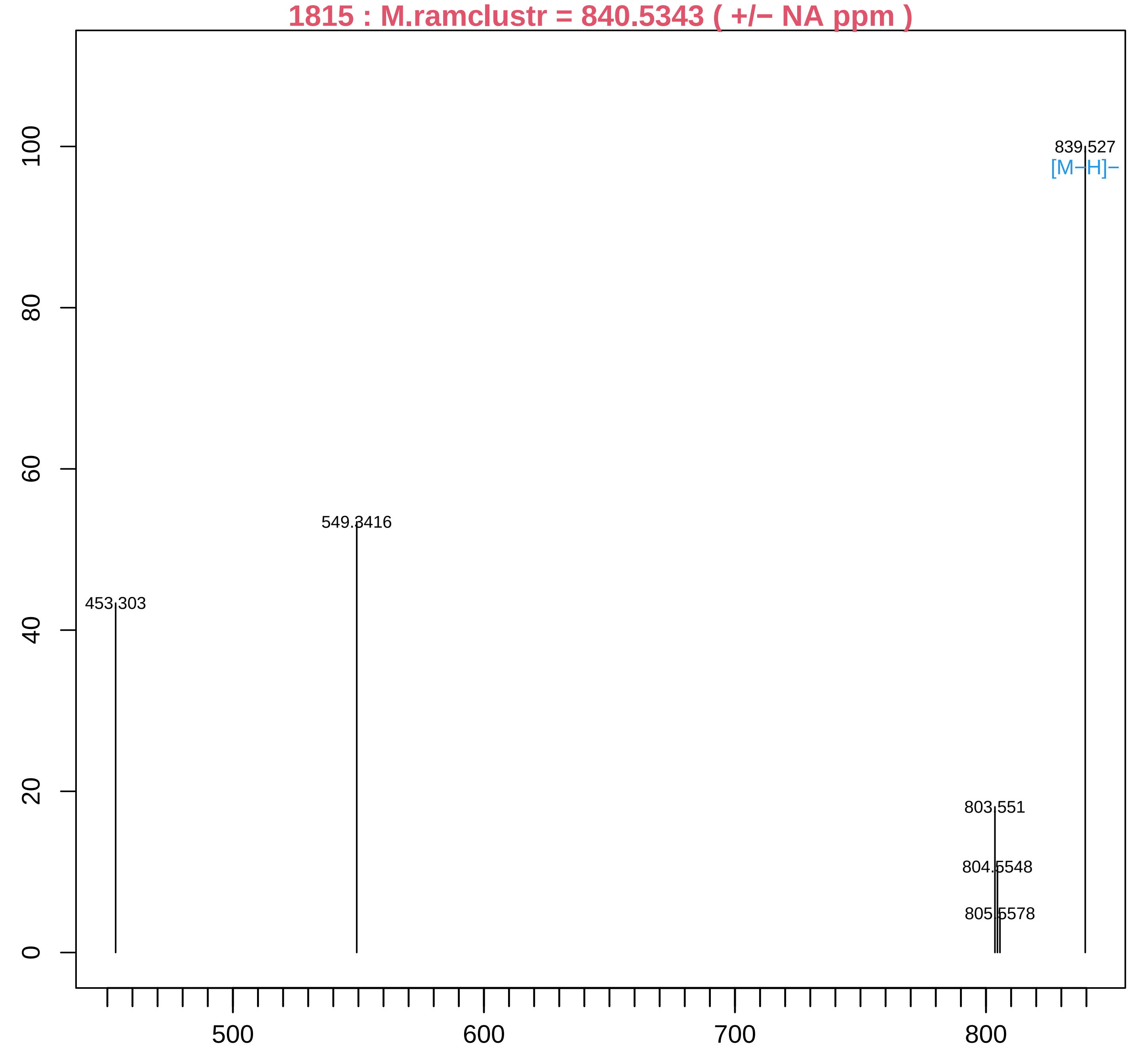
1815 : M.ramclustr = 840.5343 (+/− NA ppm)
839.527
[M−H]−
549.3416
453.303
803.551
804.5548
805.5578
0
20
40
60
80
100
500
600
700
800

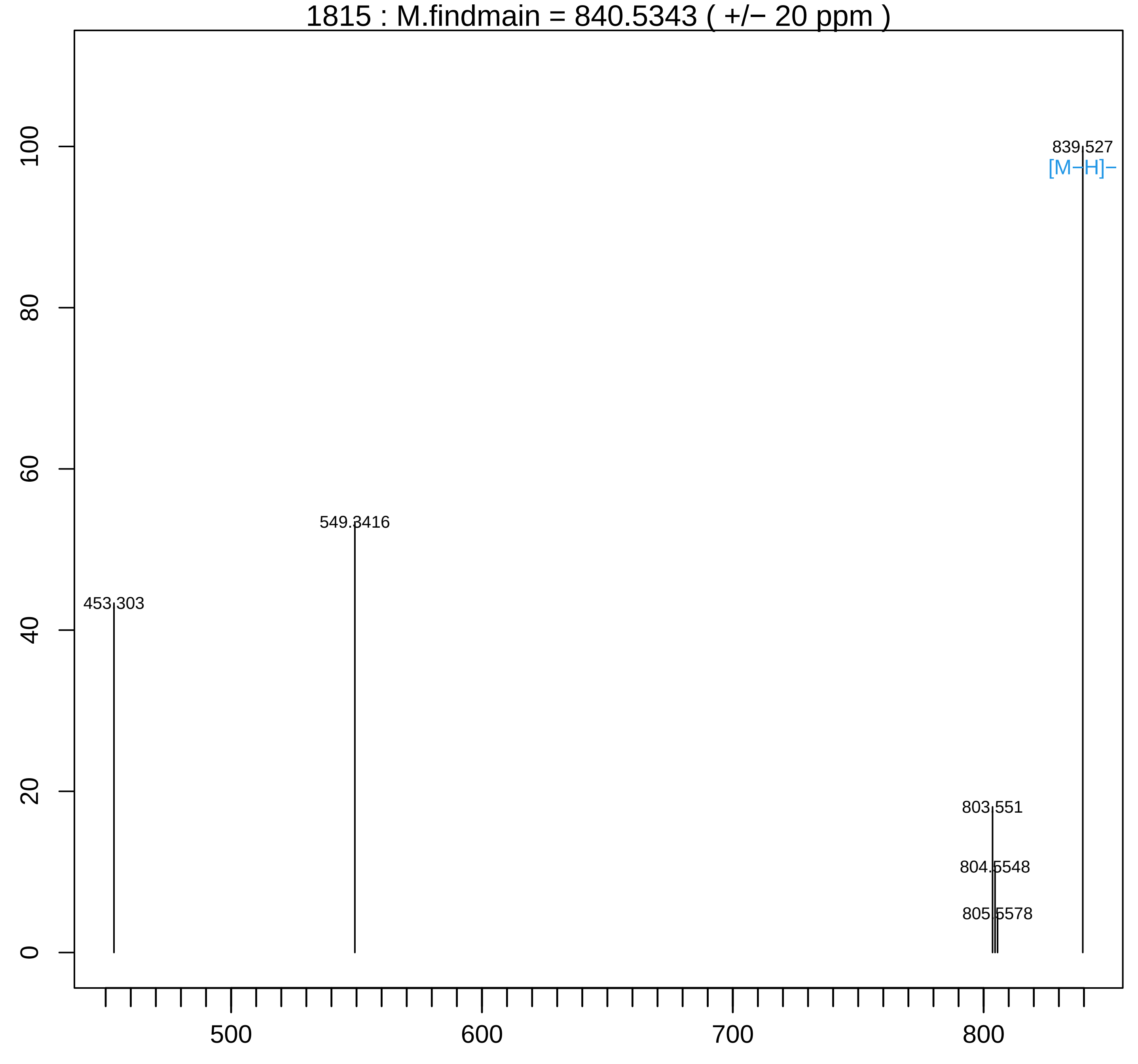
1815 : M.findmain = 840.5343 (+/− 20 ppm)
839.527
[M−H]−
549.3416
453.303
803.551
804.5548
805.5578
0
20
40
60
80
100
500
600
700
800

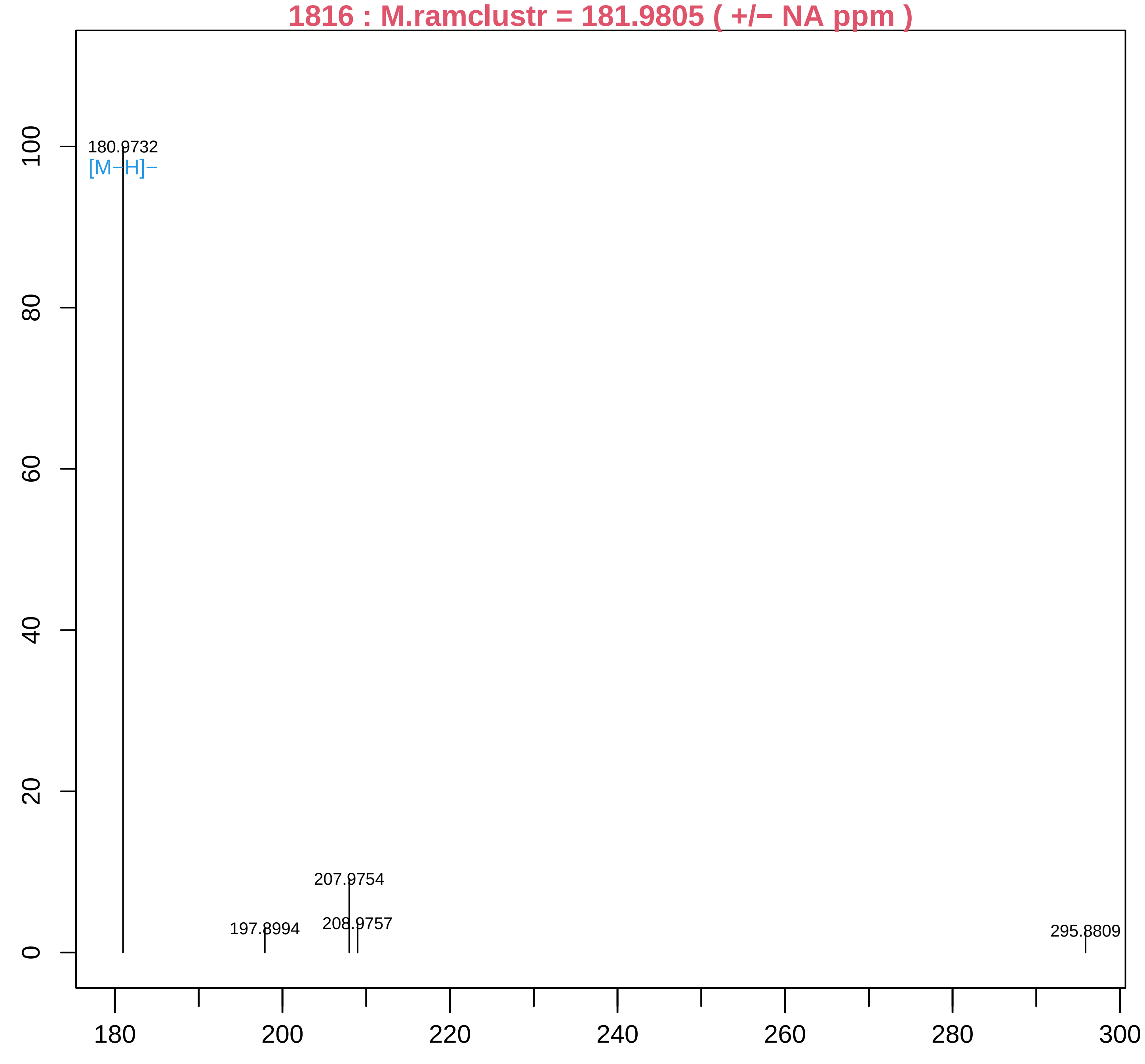
1816 : M.ramclustr = 181.9805 (+/− NA ppm)
180.9732
[M−H]−
207.9754
197.8994
208.9757
295.8809
0
20
40
60
80
100
180
200
220
240
260
280
300

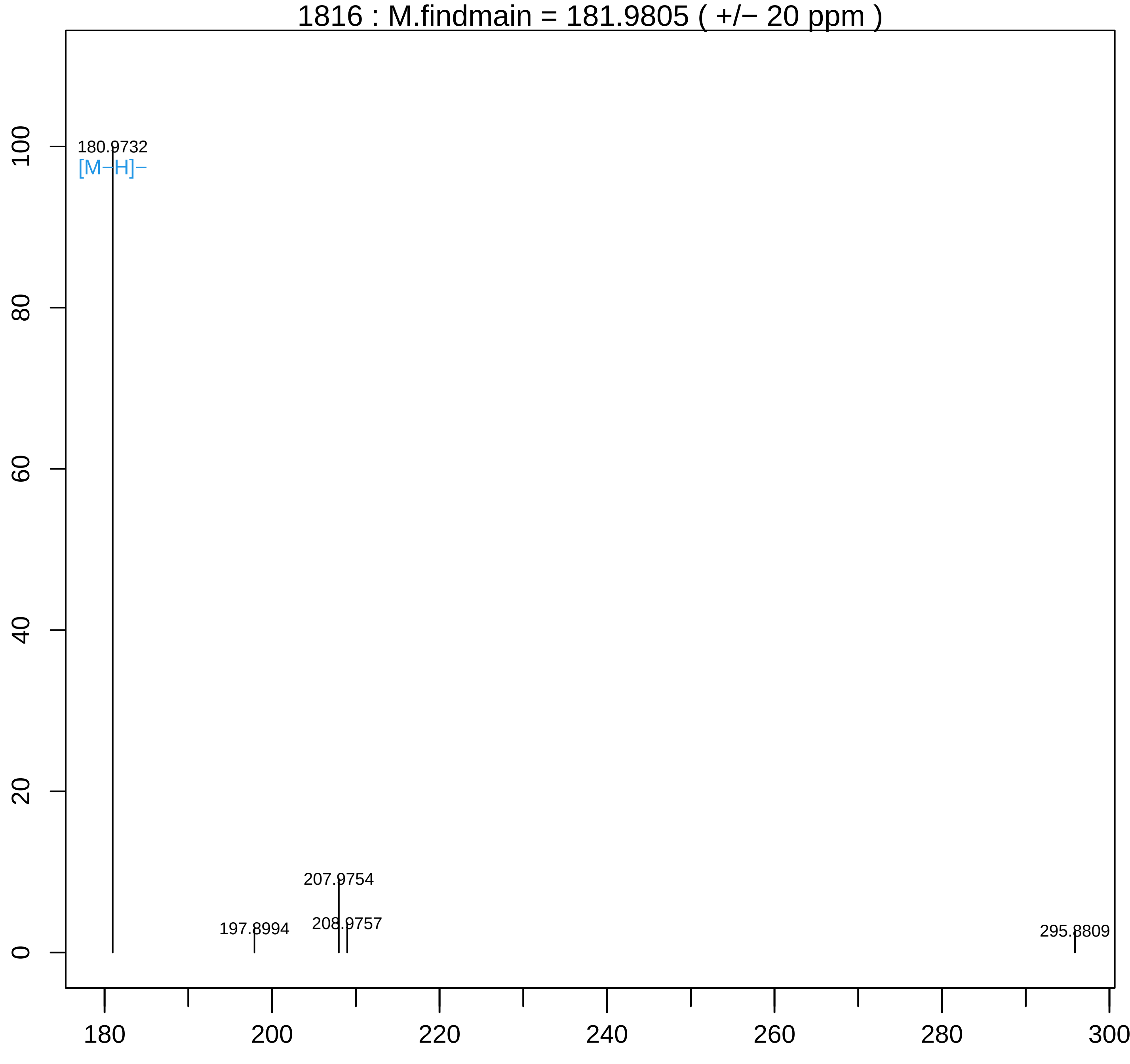
1816 : M.findmain = 181.9805 (+/− 20 ppm)
180.9732
[M−H]−
207.9754
197.8994
208.9757
295.8809
0
20
40
60
80
100
180
200
220
240
260
280
300

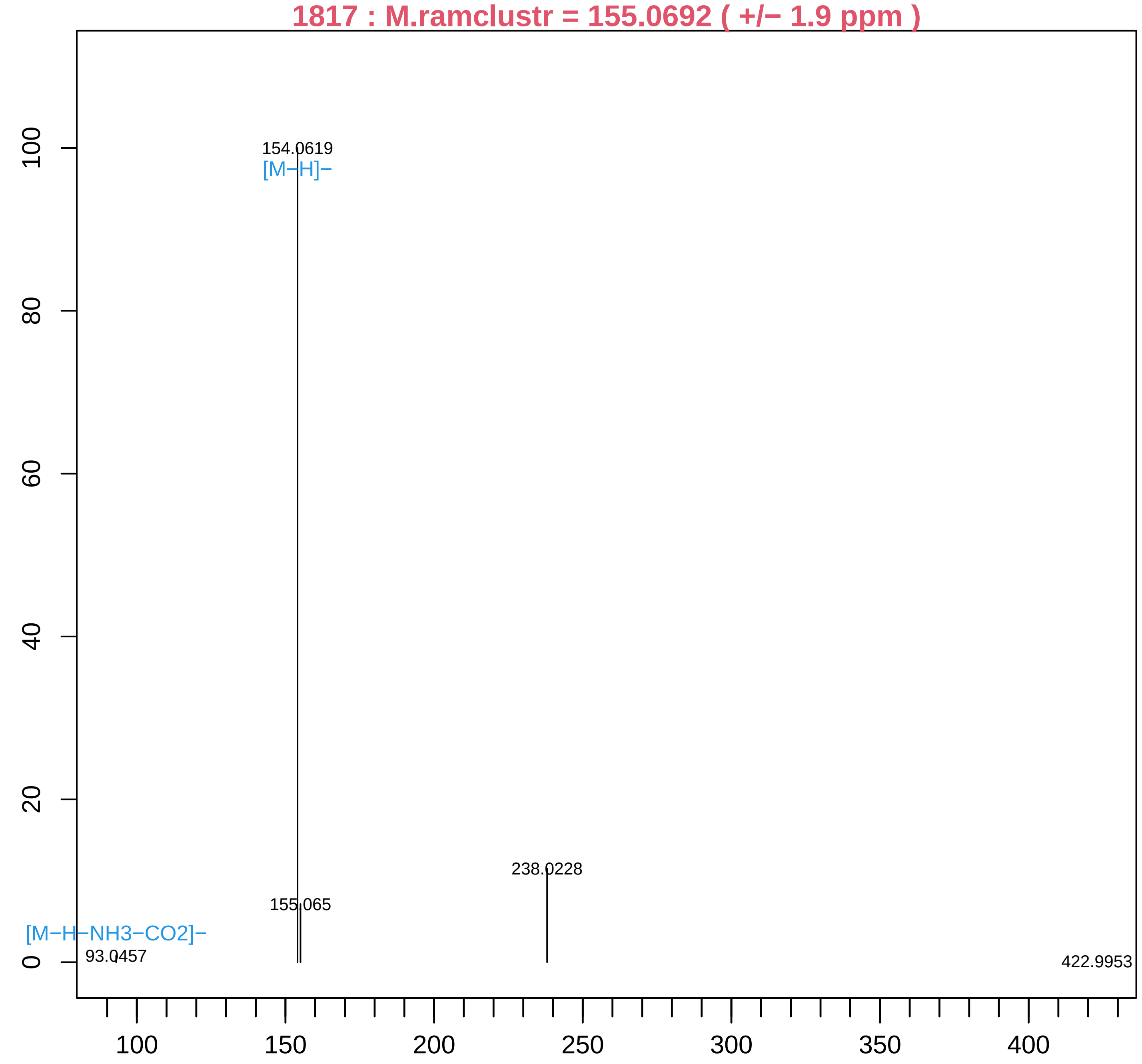
1817 : M.ramclustr = 155.0692 (+/− 1.9 ppm)
154.0619
[M−H]−
155.065
238.0228
[M−H−NH3−CO2]−
93.0457
422.9953
0
20
40
60
80
100
100
150
200
250
300
350
400

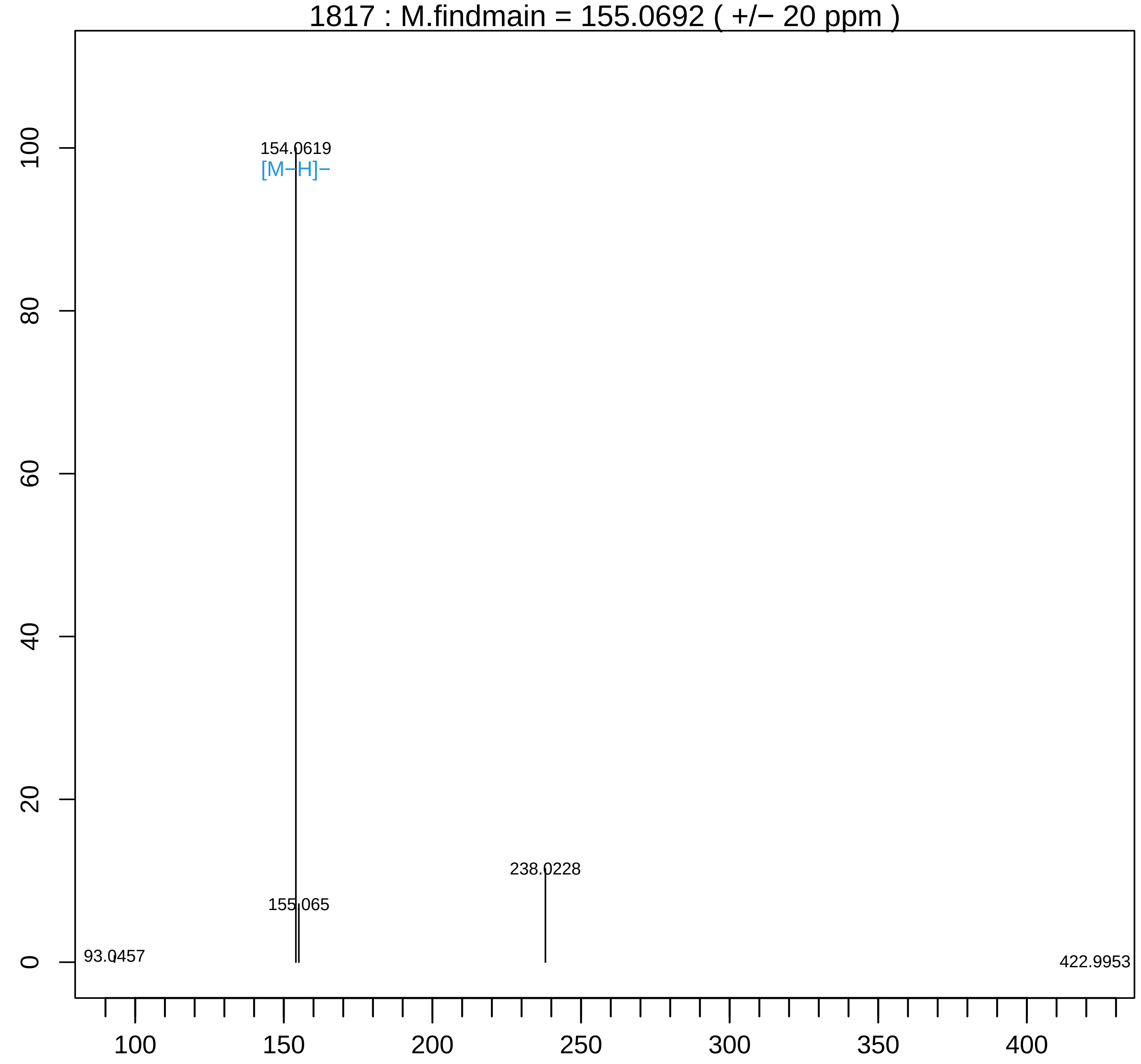
1817 : M.findmain = 155.0692 (+/− 20 ppm)
154.0619
[M−H]−
155.065
238.0228
93.0457
422.9953
0
20
40
60
80
100
100
150
200
250
300
350
400

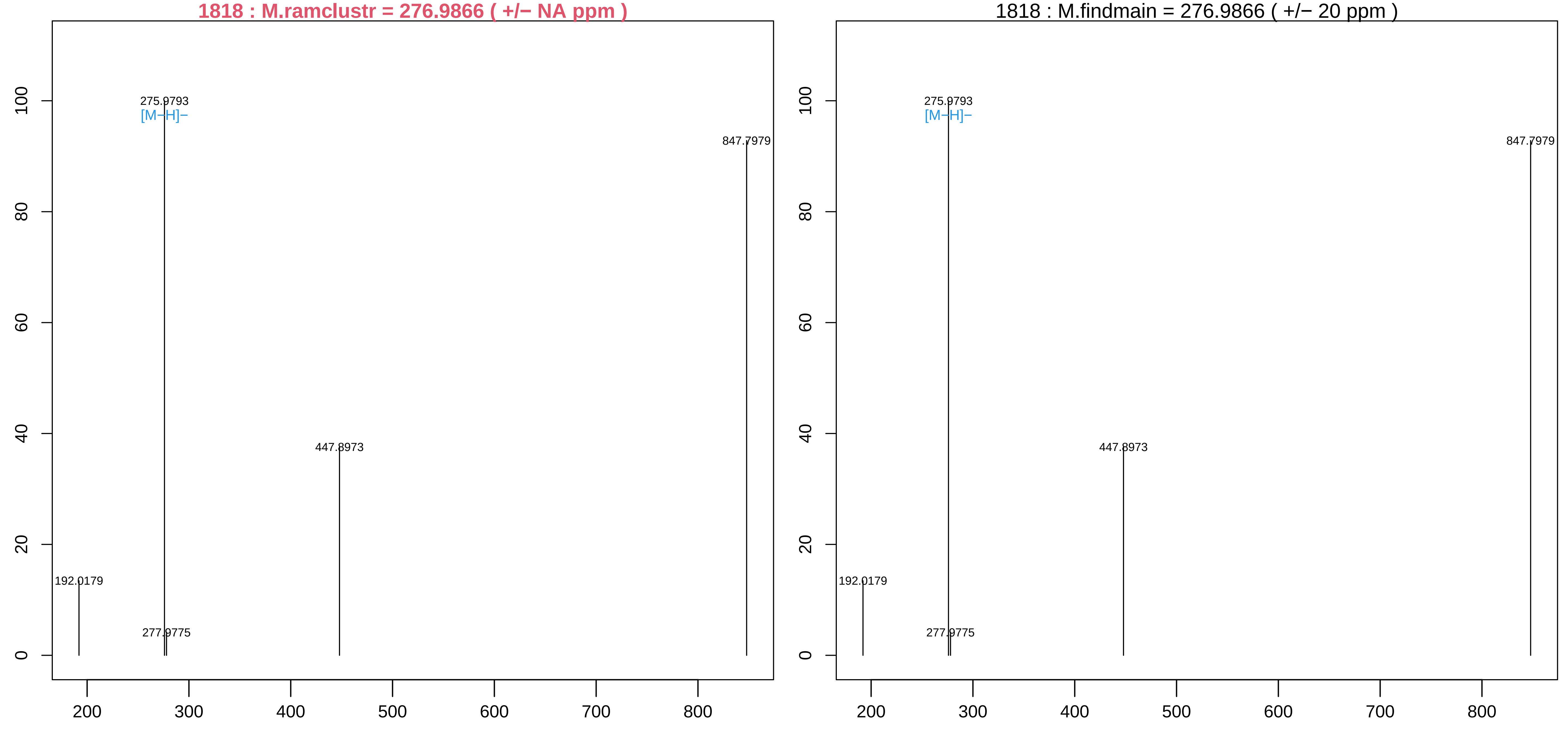
1818 : M.ramclustr = 276.9866 (+/− NA ppm)
275.9793
[M−H]−
847.7979
447.8973
192.0179
277.9775
0
20
40
60
80
100
200
300
400
500
600
700
800
1818 : M.findmain = 276.9866 (+/− 20 ppm)
275.9793
[M−H]−
847.7979
447.8973
192.0179
277.9775

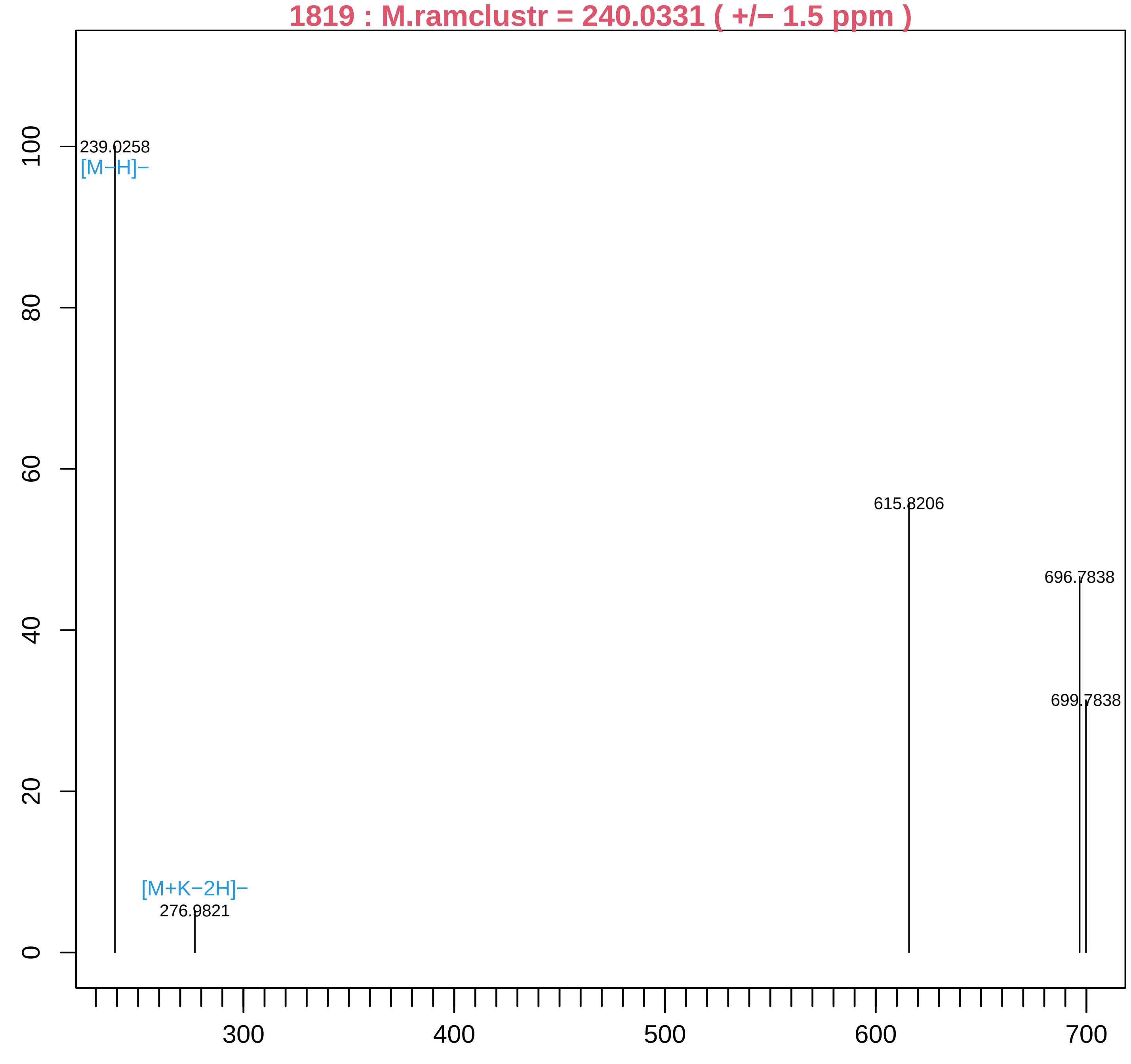
1819 : M.ramclustr = 240.0331 (+/− 1.5 ppm)
239.0258
[M−H]−
[M+K−2H]−
276.9821
615.8206
696.7838
699.7838
0
20
40
60
80
100
300
400
500
600
700
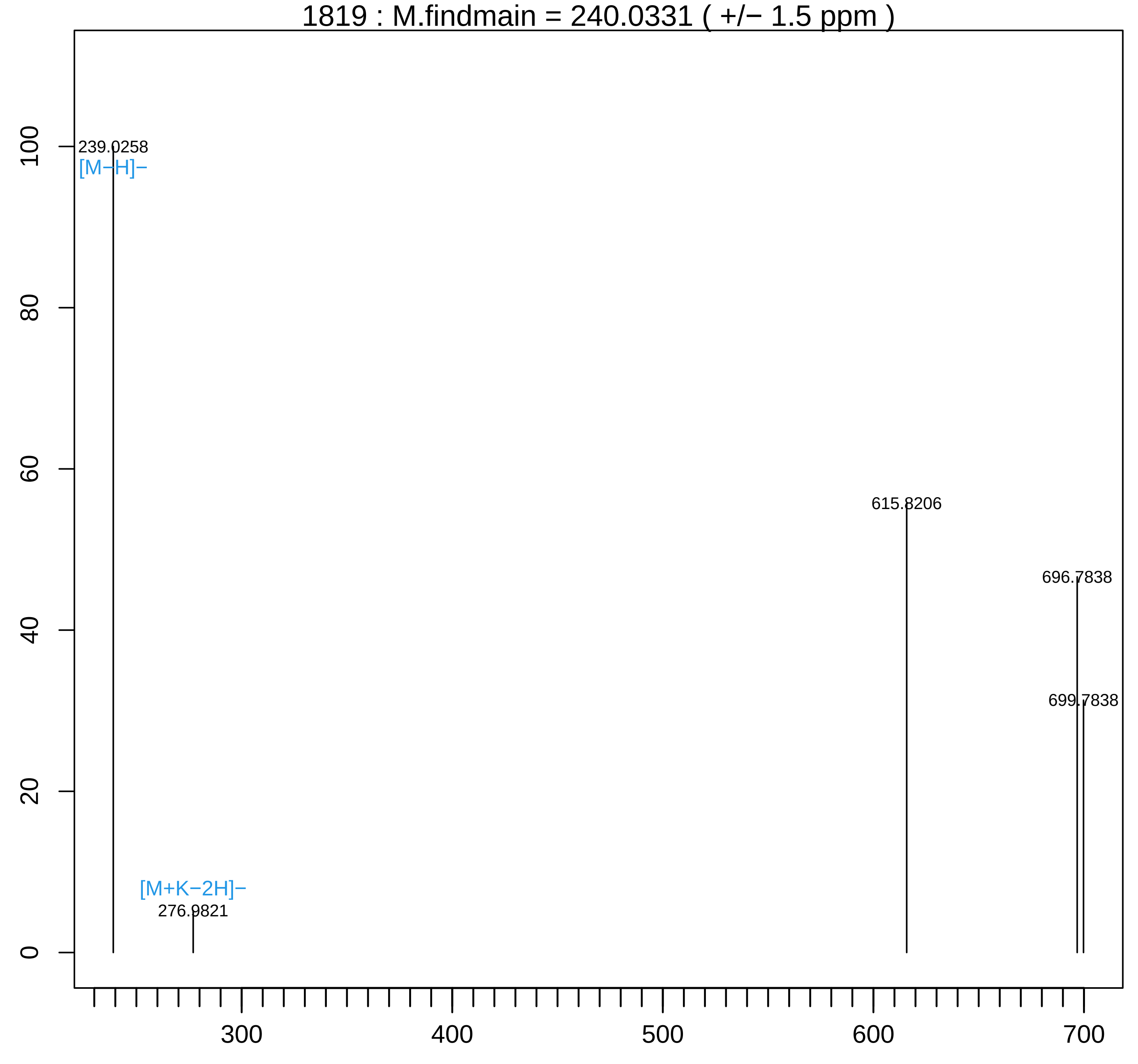
1819 : M.findmain = 240.0331 (+/− 1.5 ppm)
239.0258
[M−H]−
[M+K−2H]−
276.9821
615.8206
696.7838
699.7838
0
20
40
60
80
100
300
400
500
600
700

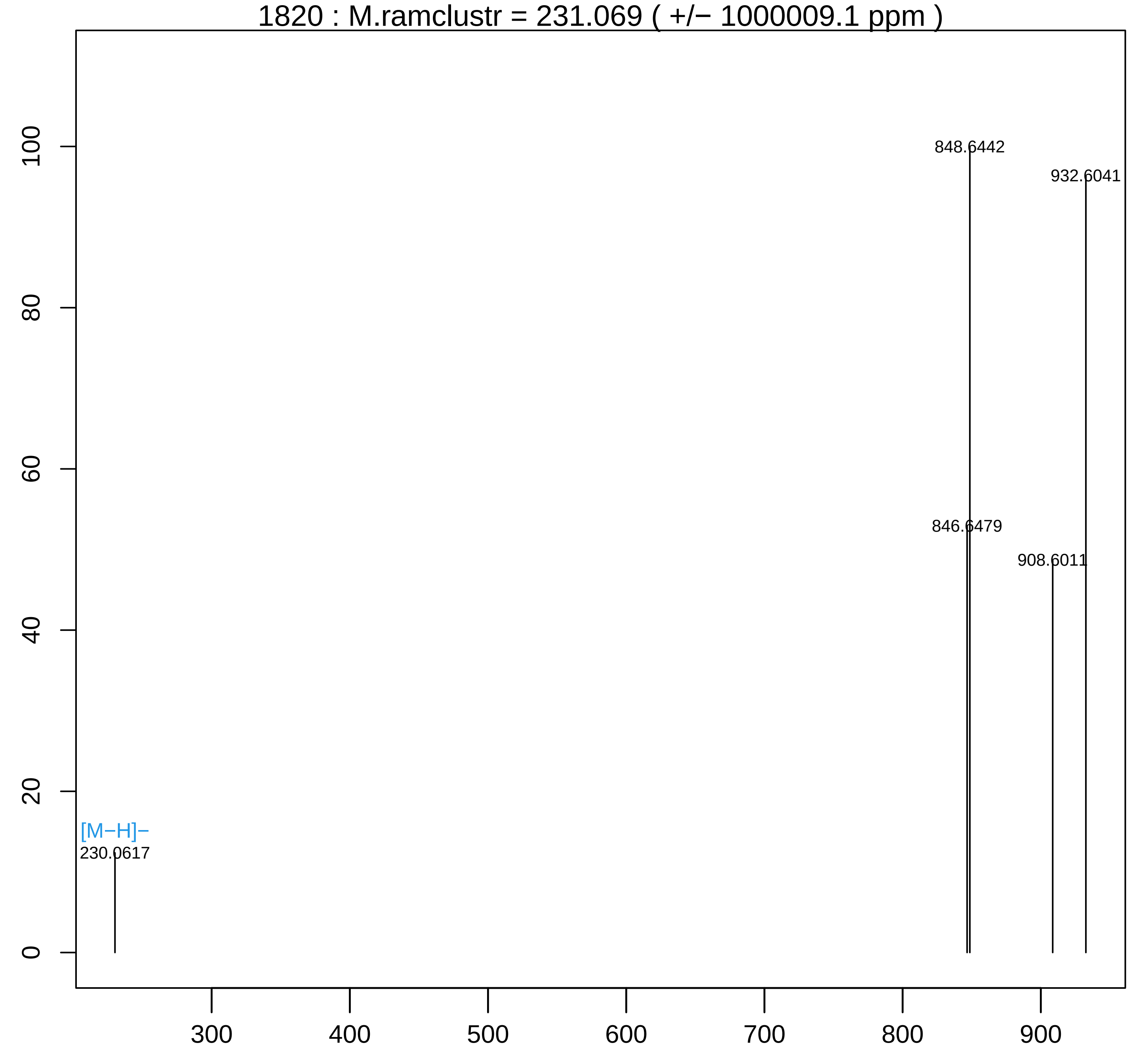

1820 : M.ramclustr = 231.069 (+/− 1000009.1 ppm)
848.6442
932.6041
846.6479
908.6011
[M−H]−
230.0617
0
20
40
60
80
100
300
400
500
600
700
800
900

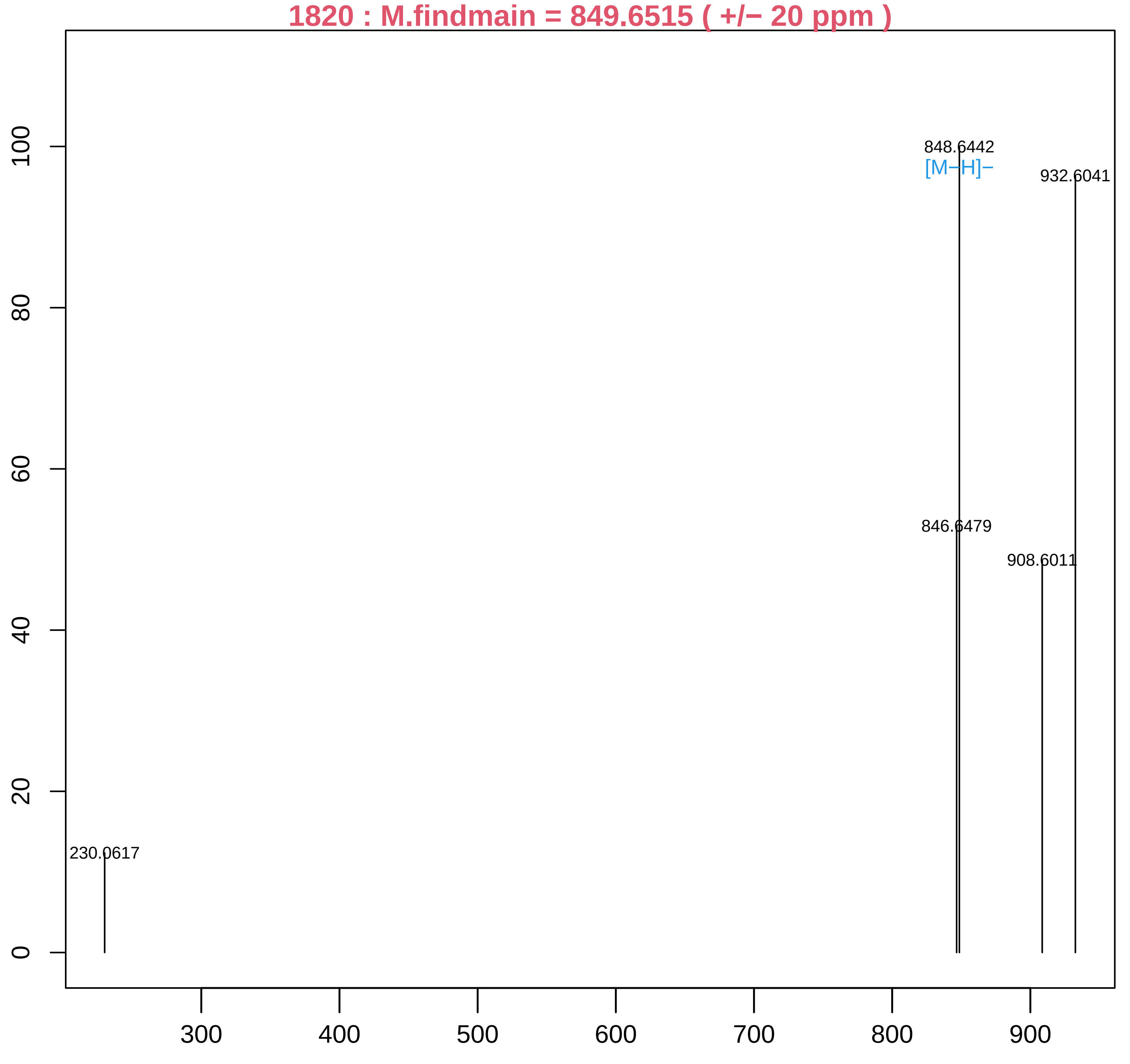

1820 : M.findmain = 849.6515 (+/− 20 ppm)
848.6442
[M−H]−
932.6041
846.6479
908.6011
230.0617
0
20
40
60
80
100
300
400
500
600
700
800
900

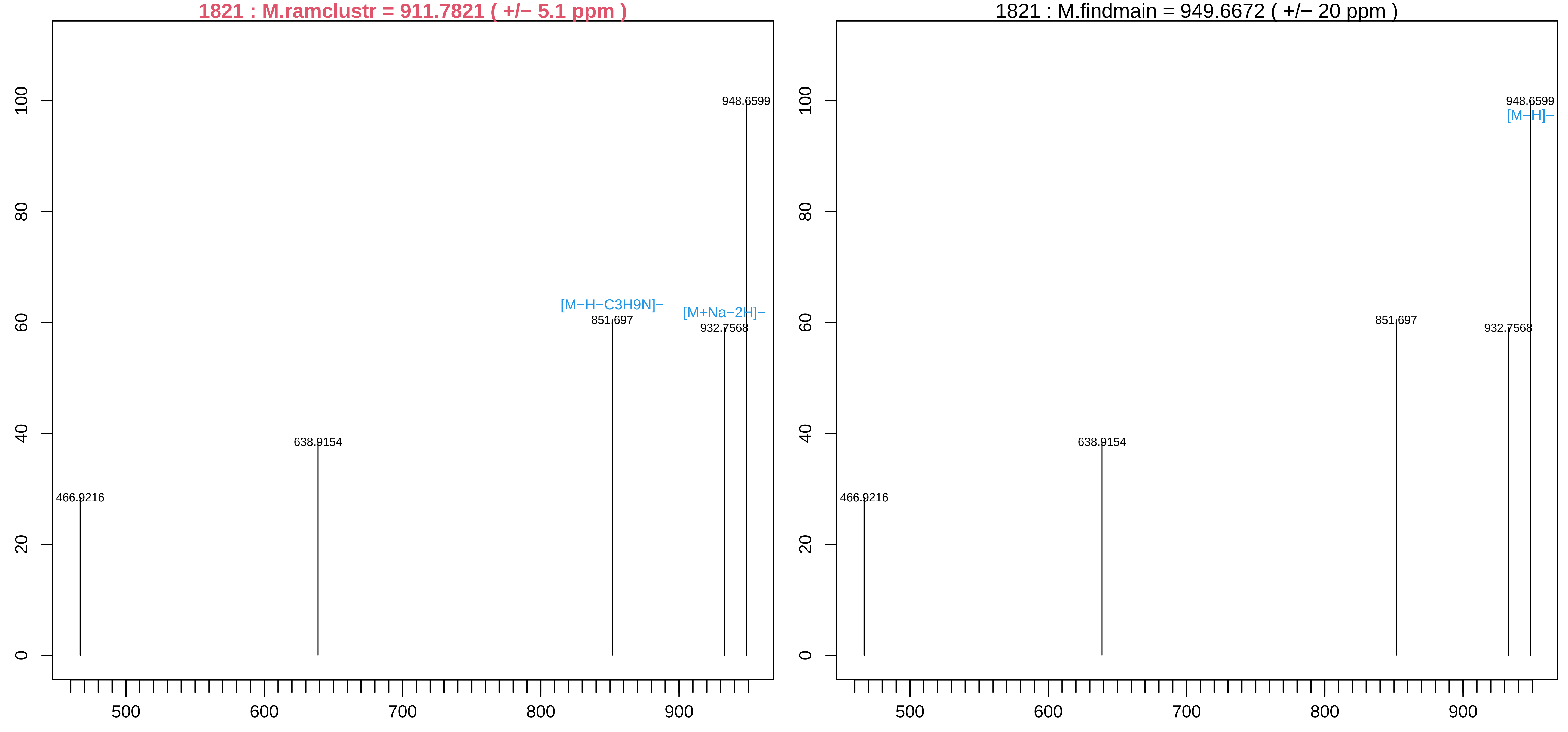
1821 : M.ramclustr = 911.7821 (+/− 5.1 ppm)
948.6599
[M−H−C3H9N]−
851.697
[M+Na−2H]−
932.7568
638.9154
466.9216
1821 : M.findmain = 949.6672 (+/− 20 ppm)
948.6599
[M−H]−
851.697
932.7568
638.9154
466.9216

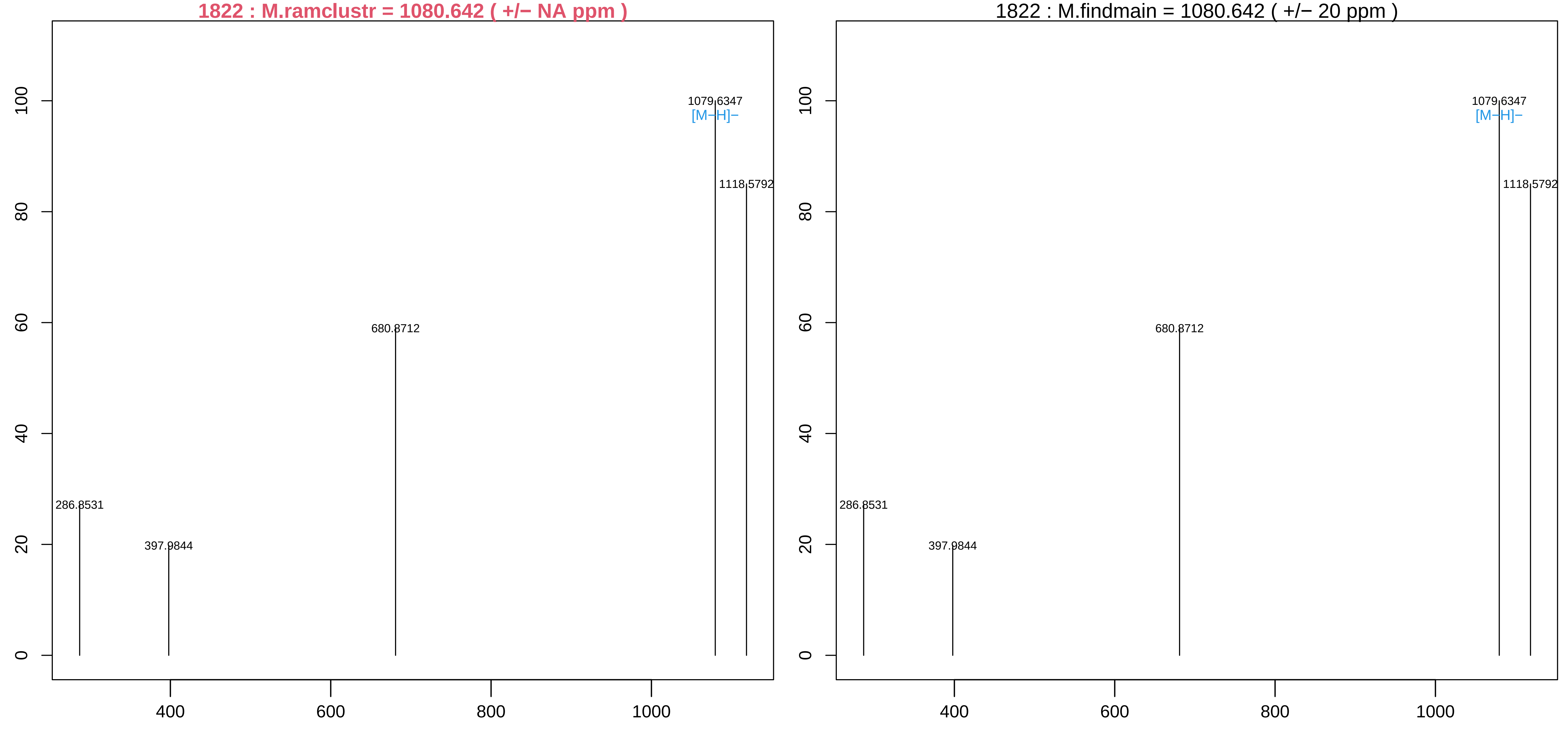
1822 : M.ramclustr = 1080.642 (+/− NA ppm)
1079.6347
[M−H]−
1118.5792
680.8712
286.8531
397.9844
0
20
40
60
80
100
400
600
800
1000
1822 : M.findmain = 1080.642 (+/− 20 ppm)
1079.6347
[M−H]−
1118.5792
680.8712
286.8531
397.9844
0
20
40
60
80
100
400
600
800
1000

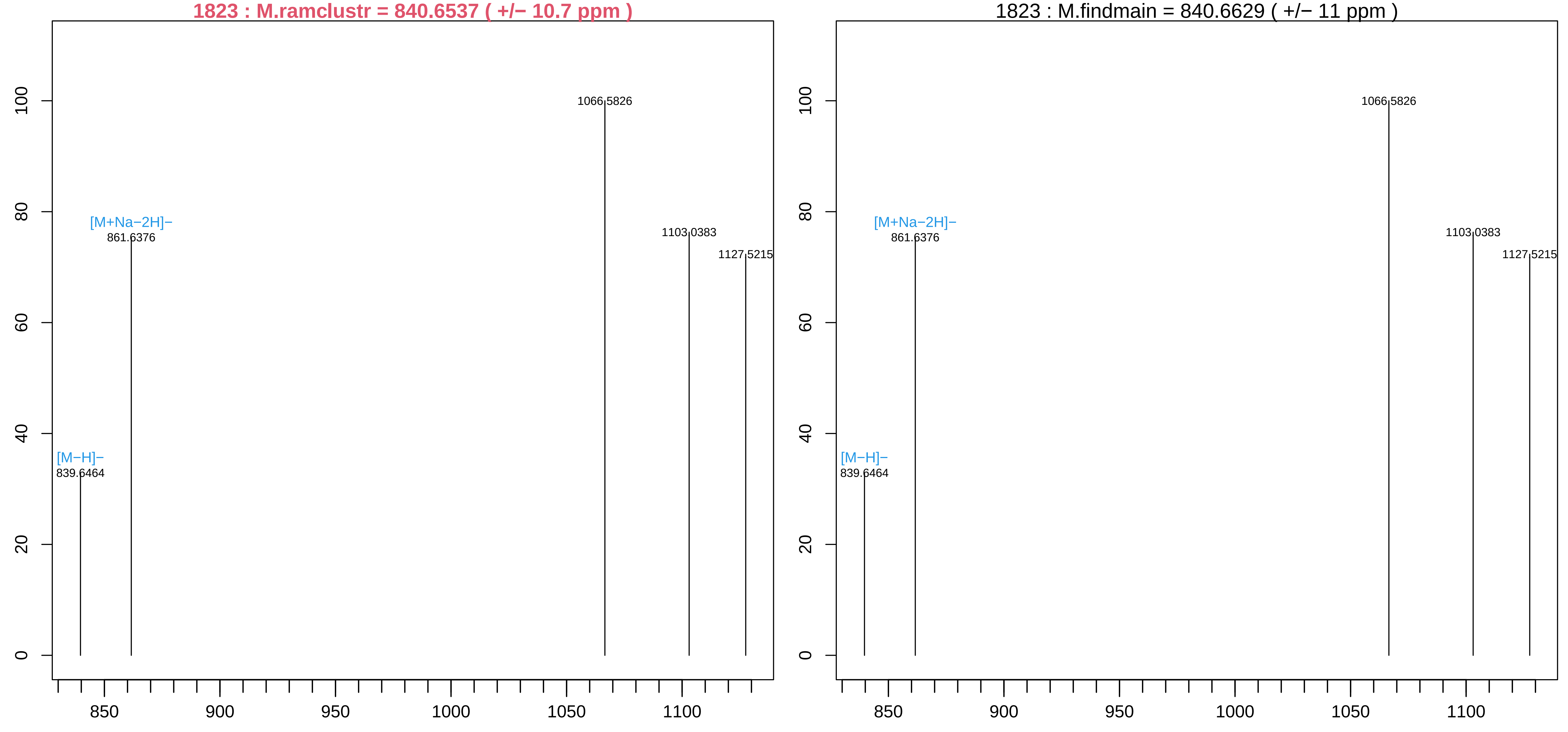

1823 : M.ramclustr = 840.6537 (+/− 10.7 ppm)
1823 : M.findmain = 840.6629 (+/− 11 ppm)
[M−H]−
839.6464
[M+Na−2H]−
861.6376
1066.5826
1103.0383
1127.5215
[M−H]−
839.6464
[M+Na−2H]−
861.6376
1066.5826
1103.0383
1127.5215

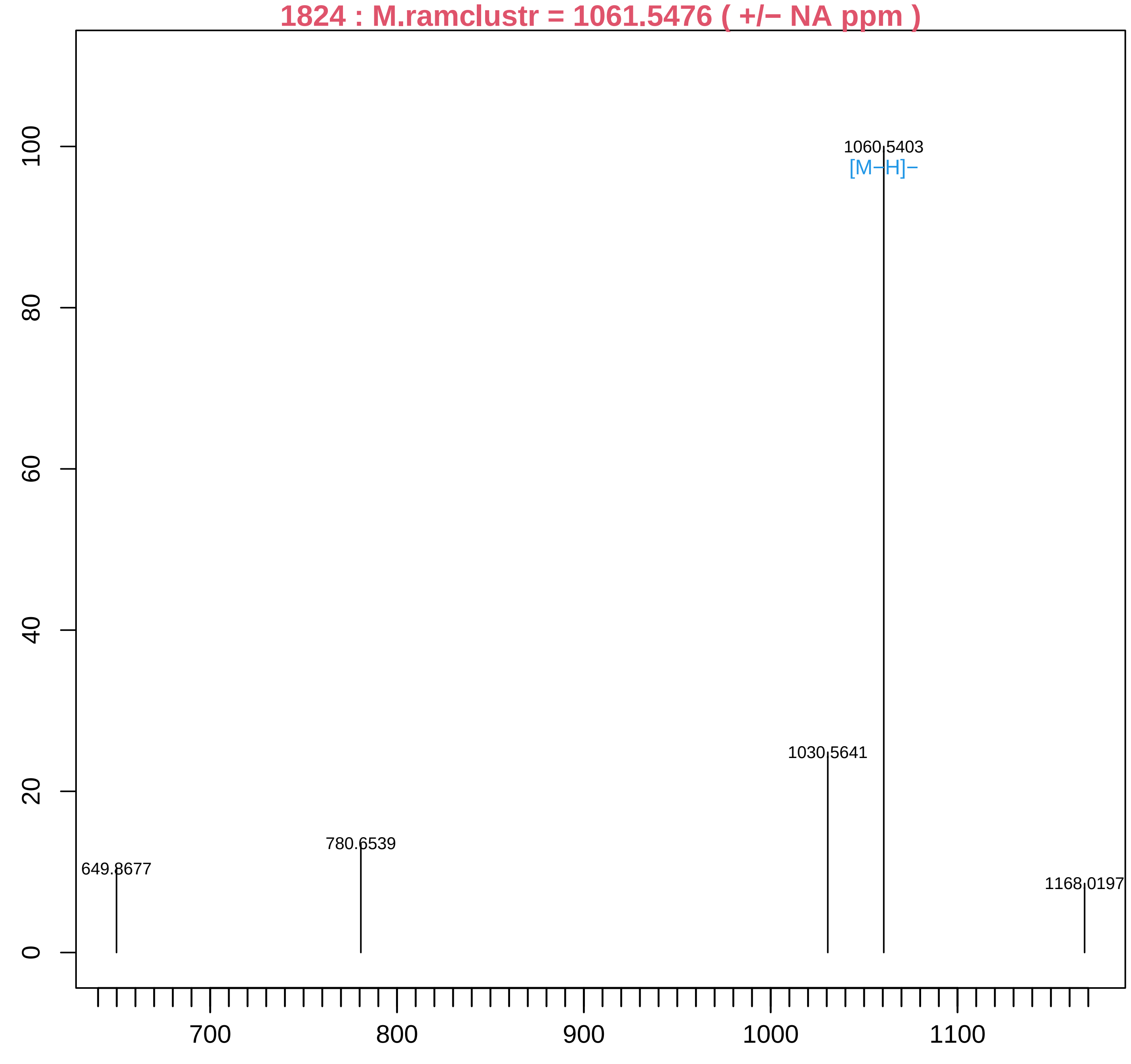
1824 : M.ramclustr = 1061.5476 (+/− NA ppm)
1060.5403
[M−H]−
1030.5641
780.6539
649.8677
1168.0197
0
20
40
60
80
100
700
800
900
1000
1100

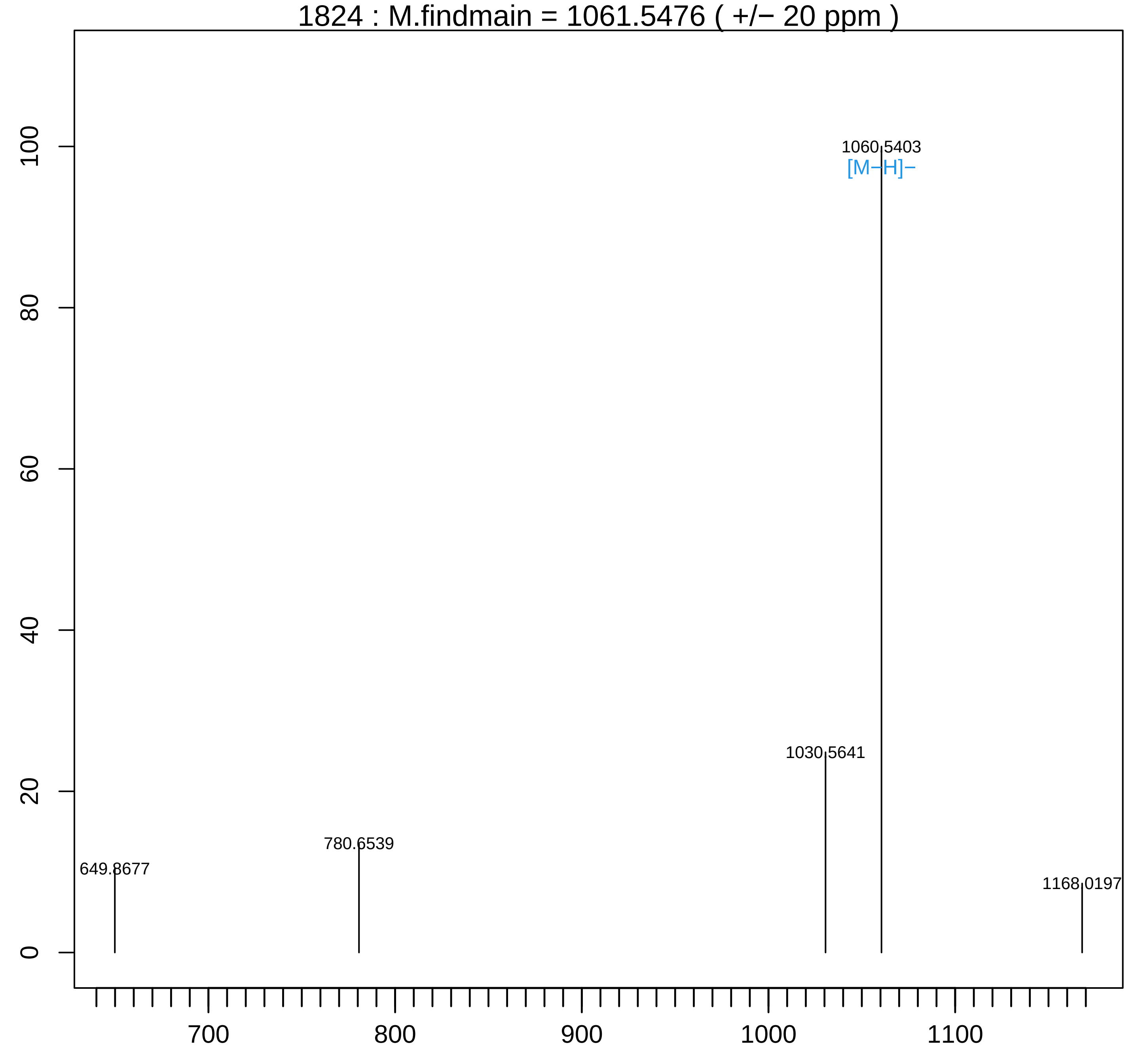
1824 : M.findmain = 1061.5476 (+/− 20 ppm)
1060.5403
[M−H]−
1030.5641
780.6539
649.8677
1168.0197
0
20
40
60
80
100
700
800
900
1000
1100

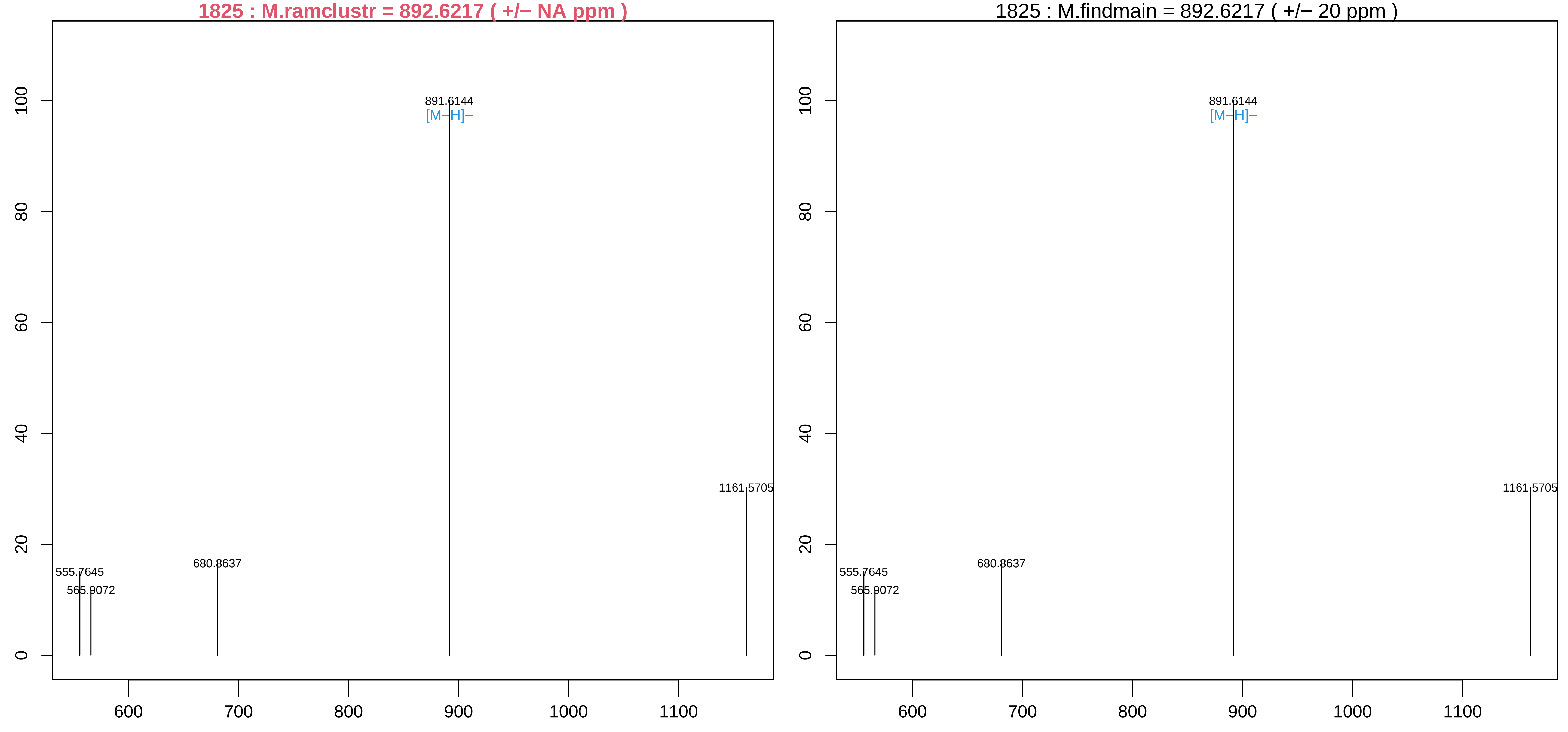

1825 : M.ramclustr = 892.6217 (+/− NA ppm)
1825 : M.findmain = 892.6217 (+/− 20 ppm)
891.6144
[M−H]−
555.7645
565.9072
680.8637
1161.5705
0
20
40
60
80
100
600
700
800
900
1000
1100

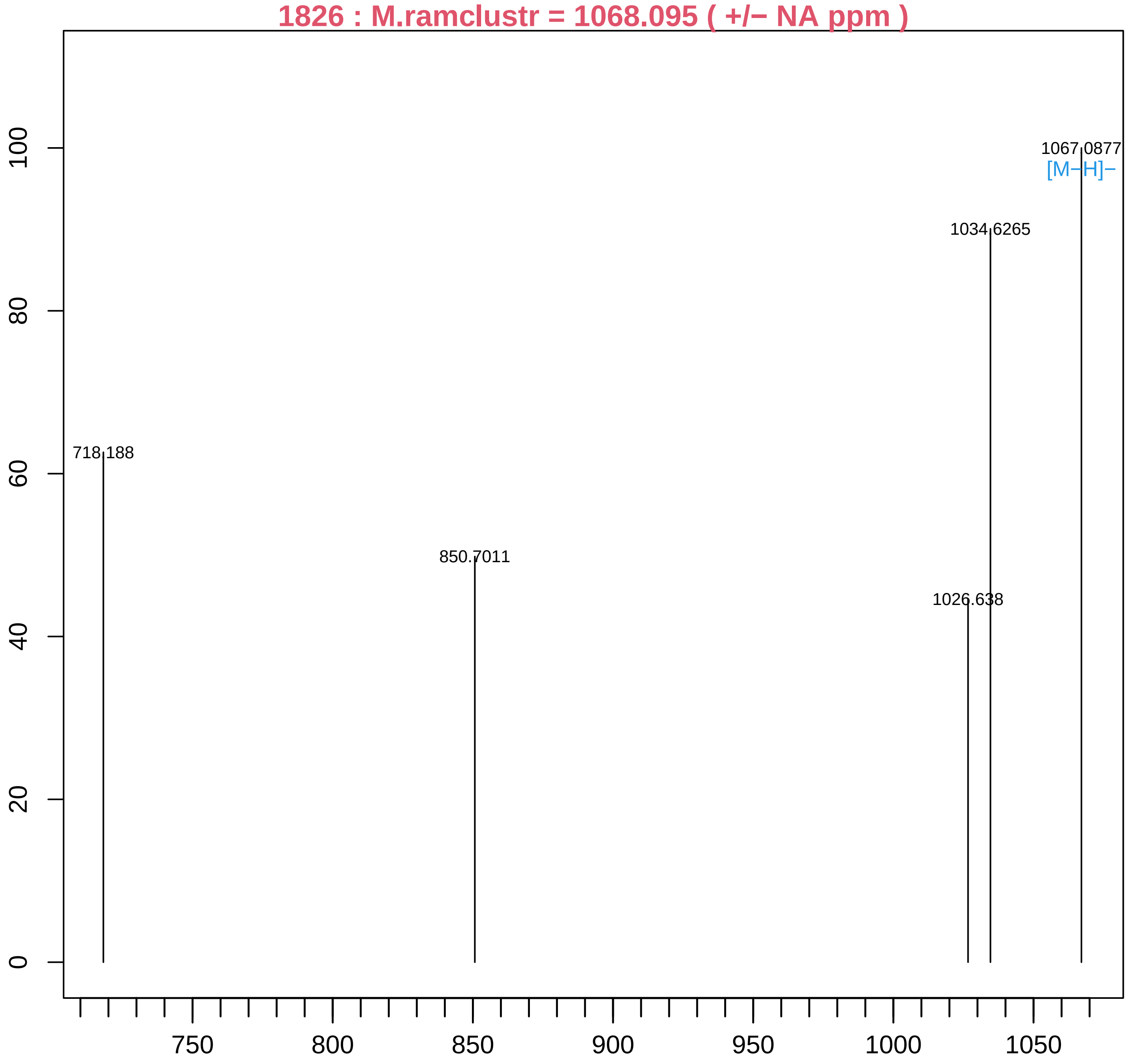
1826 : M.ramclustr = 1068.095 (+/− NA ppm)
718.188
850.7011
1026.638
1034.6265
1067.0877
[M−H]−
0
20
40
60
80
100
750
800
850
900
950
1000
1050

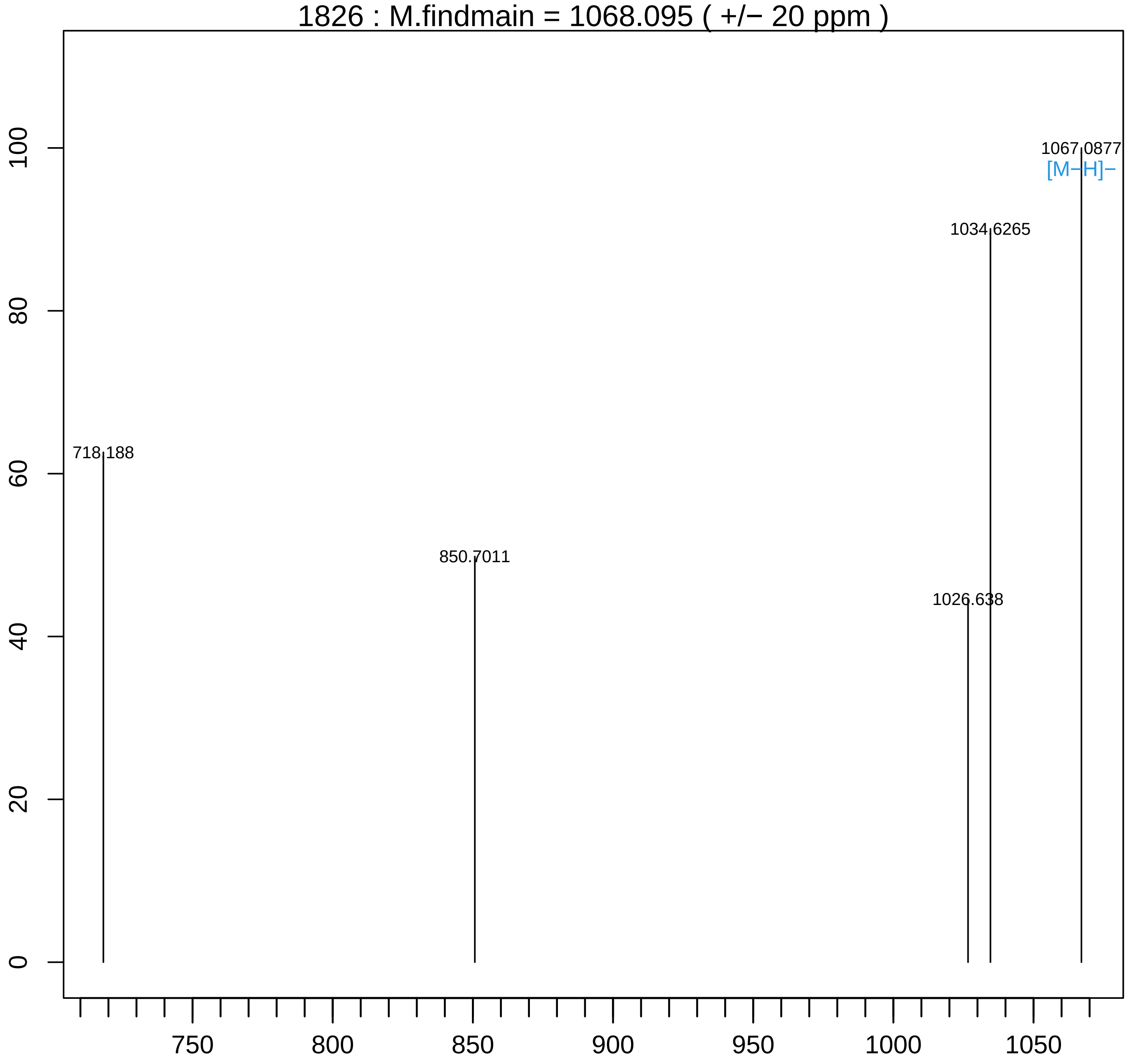
1826 : M.findmain = 1068.095 (+/− 20 ppm)
718.188
850.7011
1026.638
1034.6265
1067.0877
[M−H]−
0
20
40
60
80
100
750
800
850
900
950
1000
1050

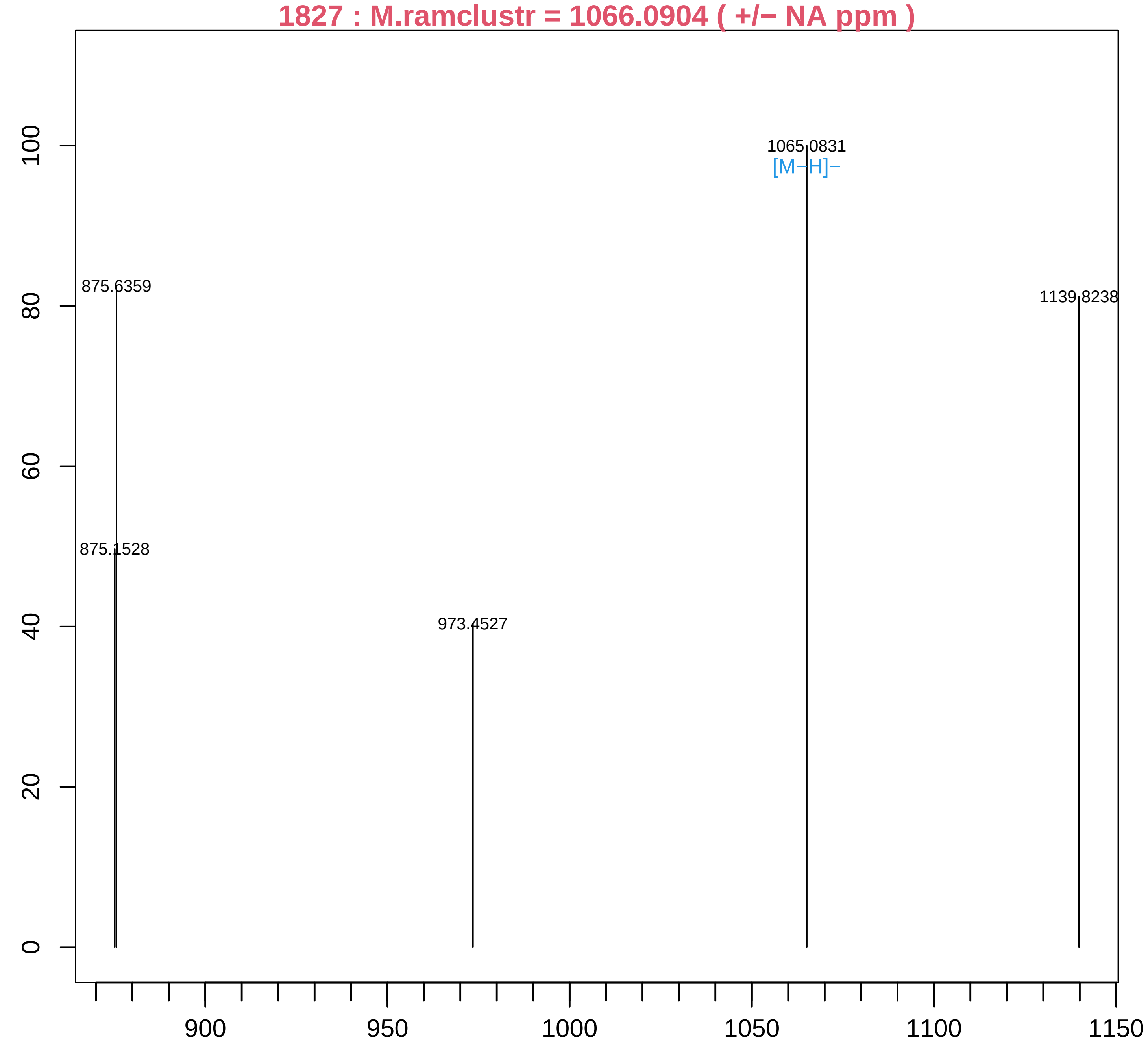
1827 : M.ramclustr = 1066.0904 (+/− NA ppm)
1065.0831
[M−H]−
875.6359
1139.8238
875.1528
973.4527
0
20
40
60
80
100
900
950
1000
1050
1100
1150

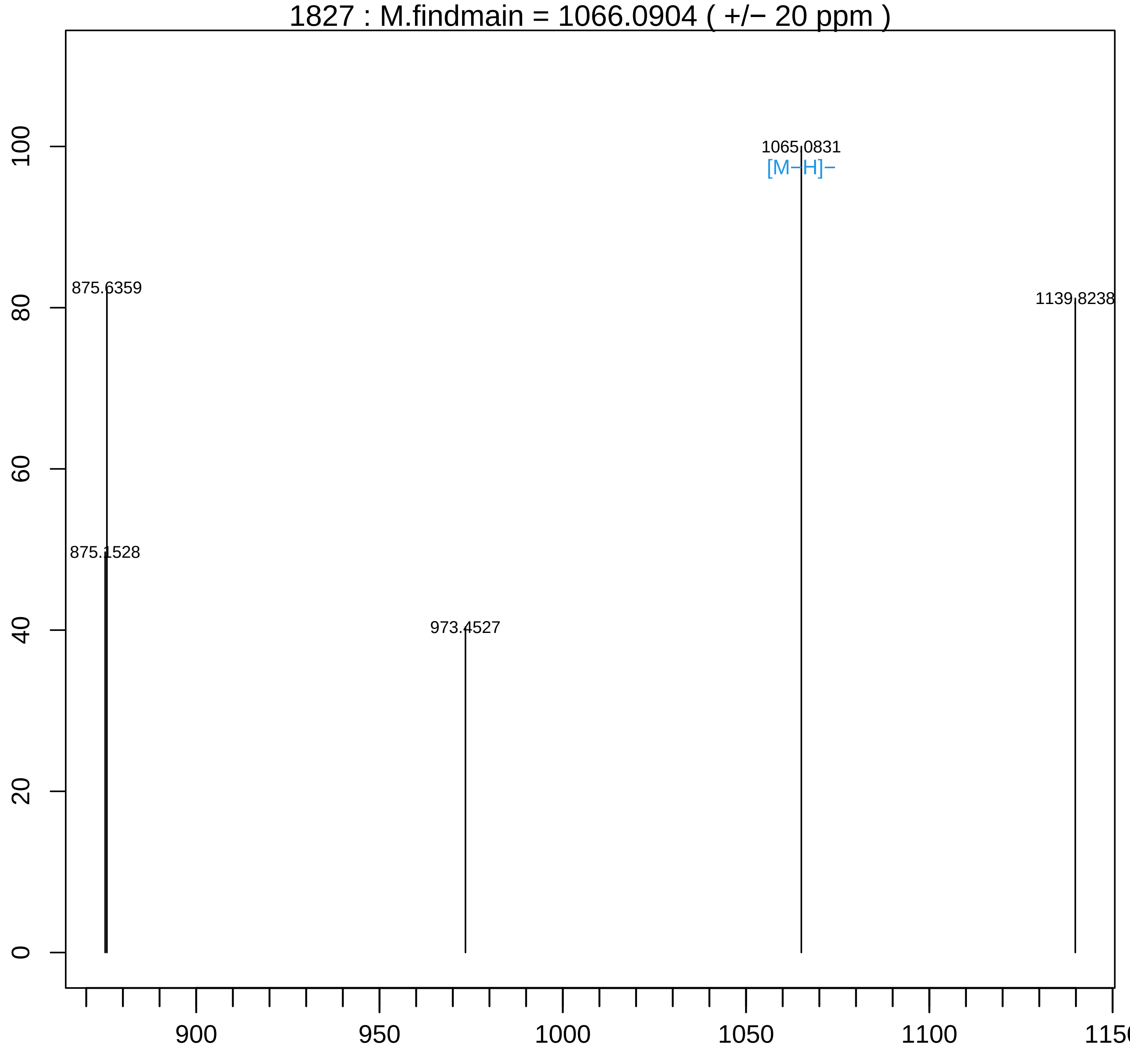
1827 : M.findmain = 1066.0904 (+/− 20 ppm)
1065.0831
[M−H]−
875.6359
1139.8238
875.1528
973.4527
0
20
40
60
80
100
900
950
1000
1050
1100

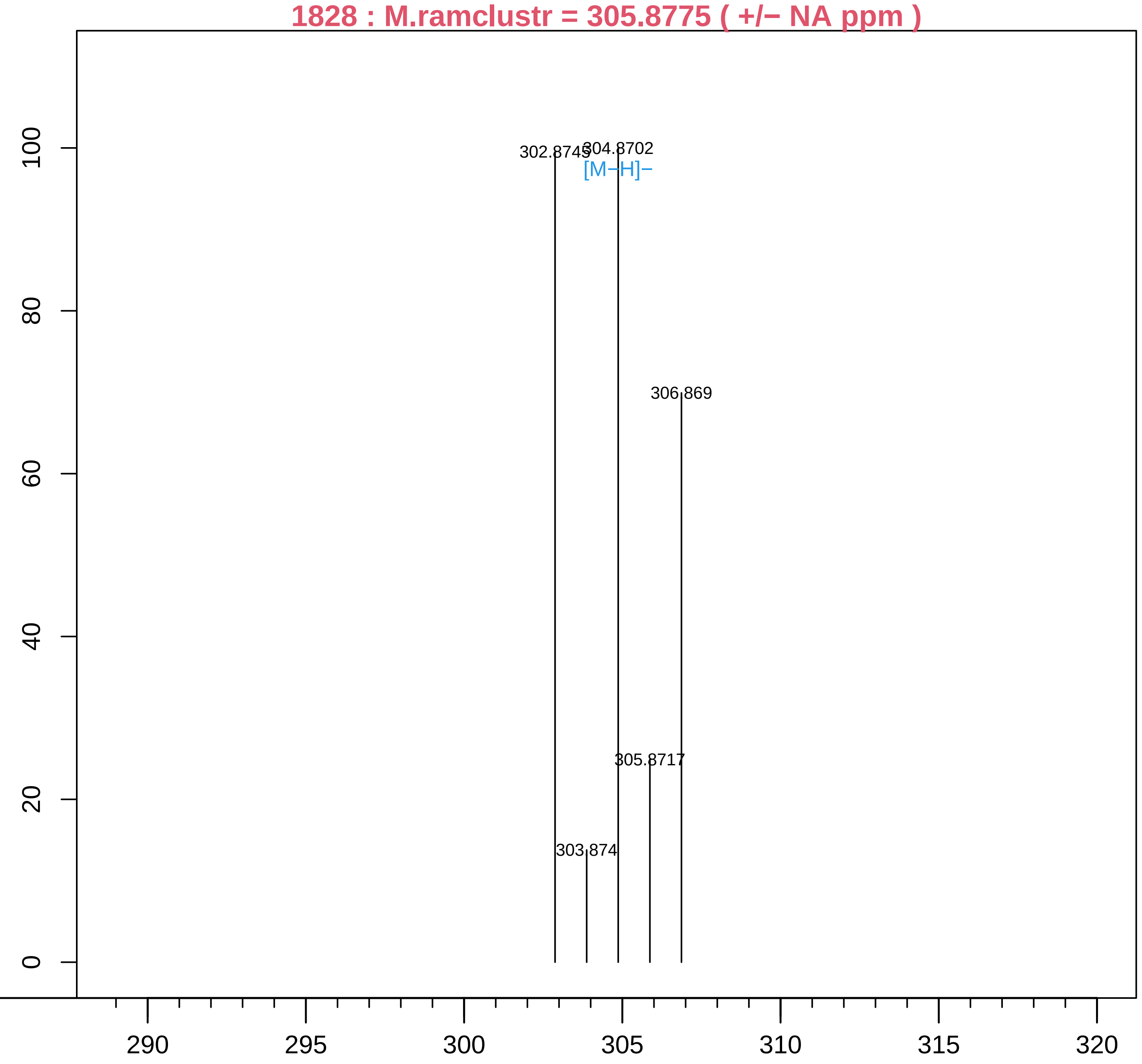
1828 : M.ramclustr = 305.8775 (+/− NA ppm)
302.8743
304.8702
[M−H]−
306.869
305.8717
303.874
0
20
40
60
80
100
290
295
300
305
310
315
320

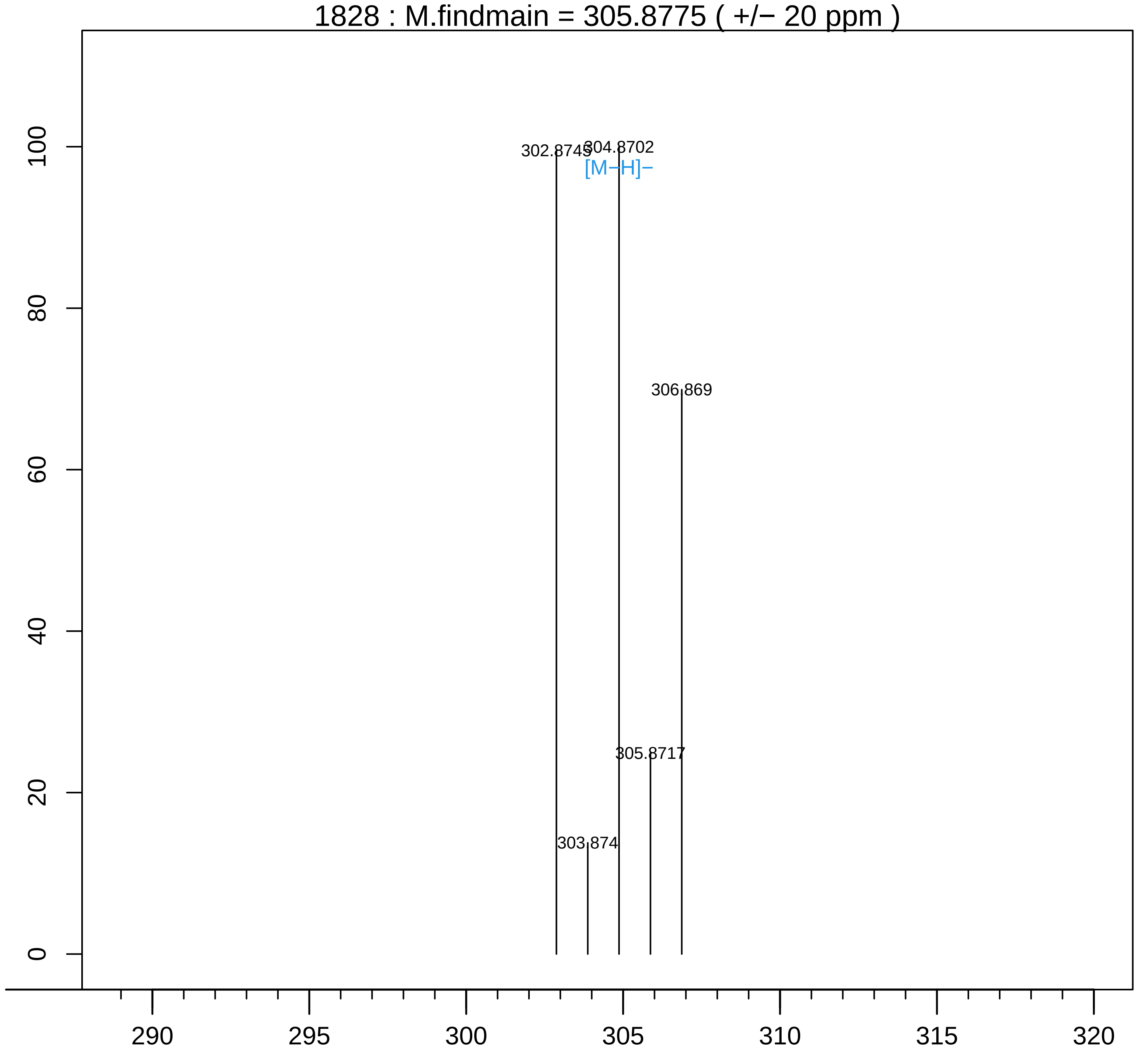
1828 : M.findmain = 305.8775 (+/− 20 ppm)
302.8743
304.8702
[M−H]−
306.869
305.8717
303.874
0
20
40
60
80
100
290
295
300
305
310
315
320

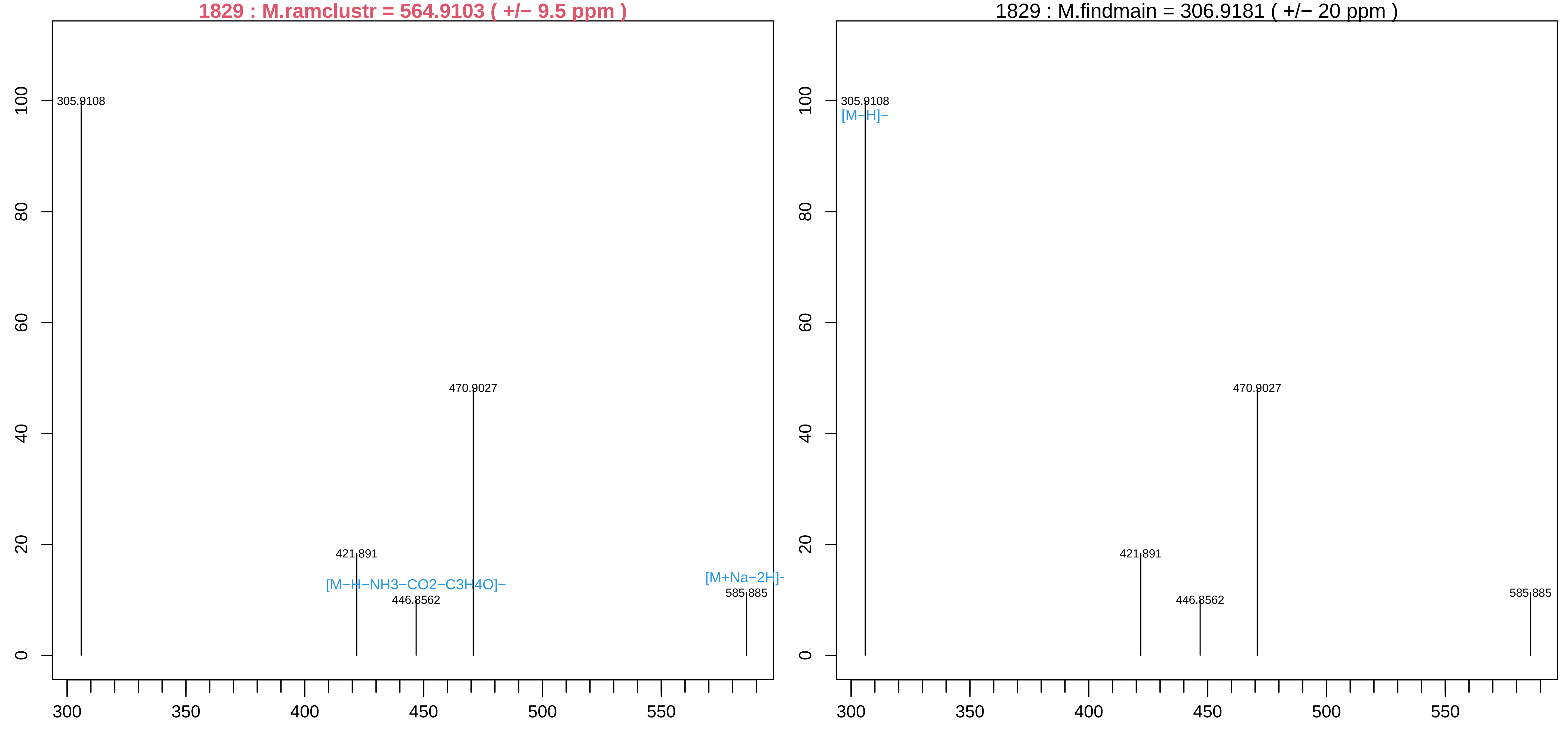
1829 : M.ramclustr = 564.9103 (+/− 9.5 ppm)
305.9108
421.891
[M−H−NH3−CO2−C3H4O]−
446.8562
470.9027
[M+Na−2H]-
585.885
1829 : M.findmain = 306.9181 (+/− 20 ppm)
305.9108
[M−H]−
421.891
446.8562
470.9027
585.885
0
20
40
60
80
100
300
350
400
450
500
550

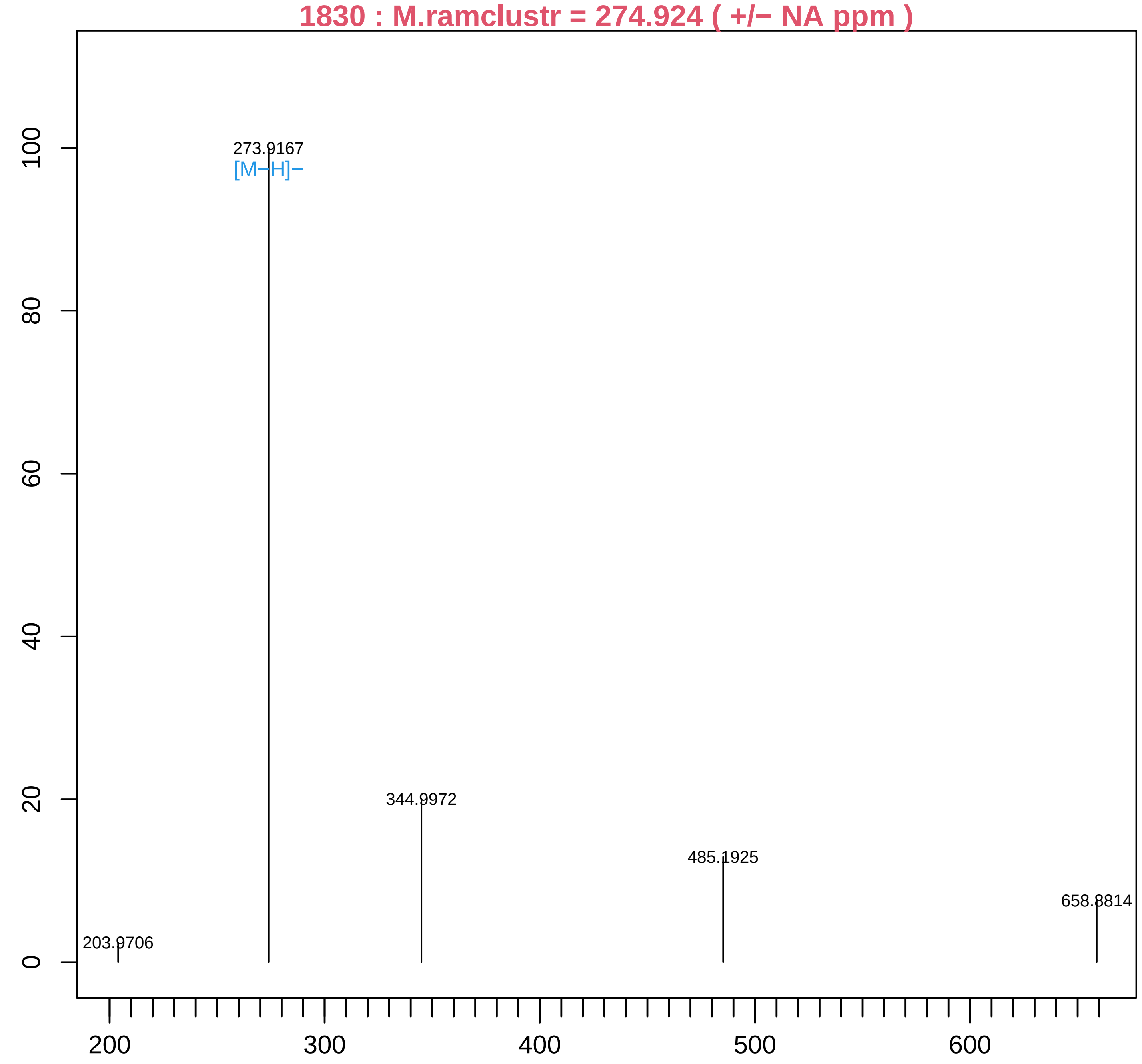

1830 : M.ramclustr = 274.924 (+/− NA ppm)
273.9167
[M−H]−
344.9972
485.1925
658.8814
203.9706

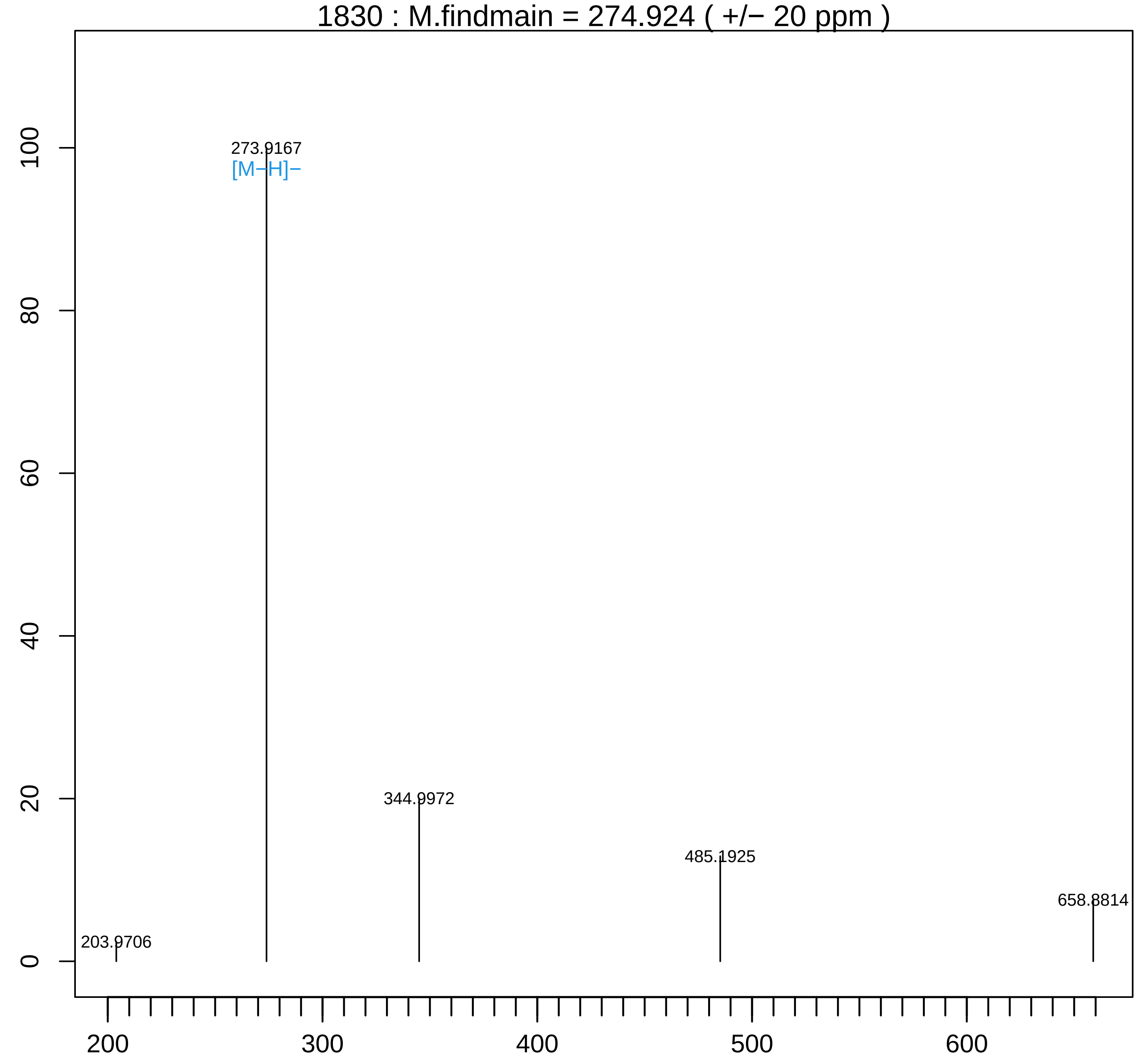

1830 : M.findmain = 274.924 (+/− 20 ppm)
273.9167
[M−H]−
344.9972
485.1925
658.8814
203.9706

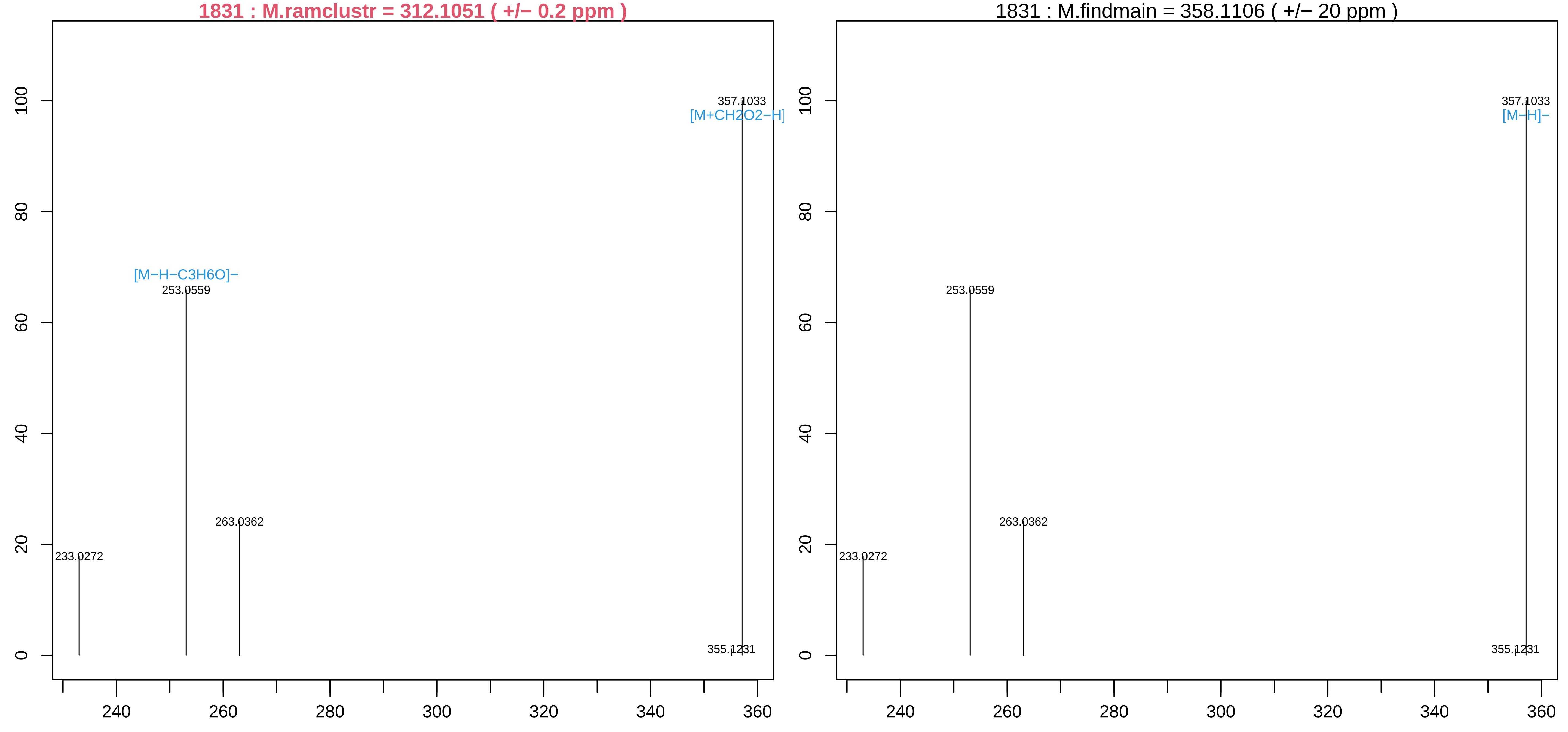

1831 : M.ramclustr = 312.1051 (+/− 0.2 ppm)
1831 : M.findmain = 358.1106 (+/− 20 ppm)
357.1033
[M+CH2O2−H]
[M−H−C3H6O]−
253.0559
263.0362
233.0272
355.1231
[M−H]−
0
20
40
60
80
100
240
260
280
300
320
340
360

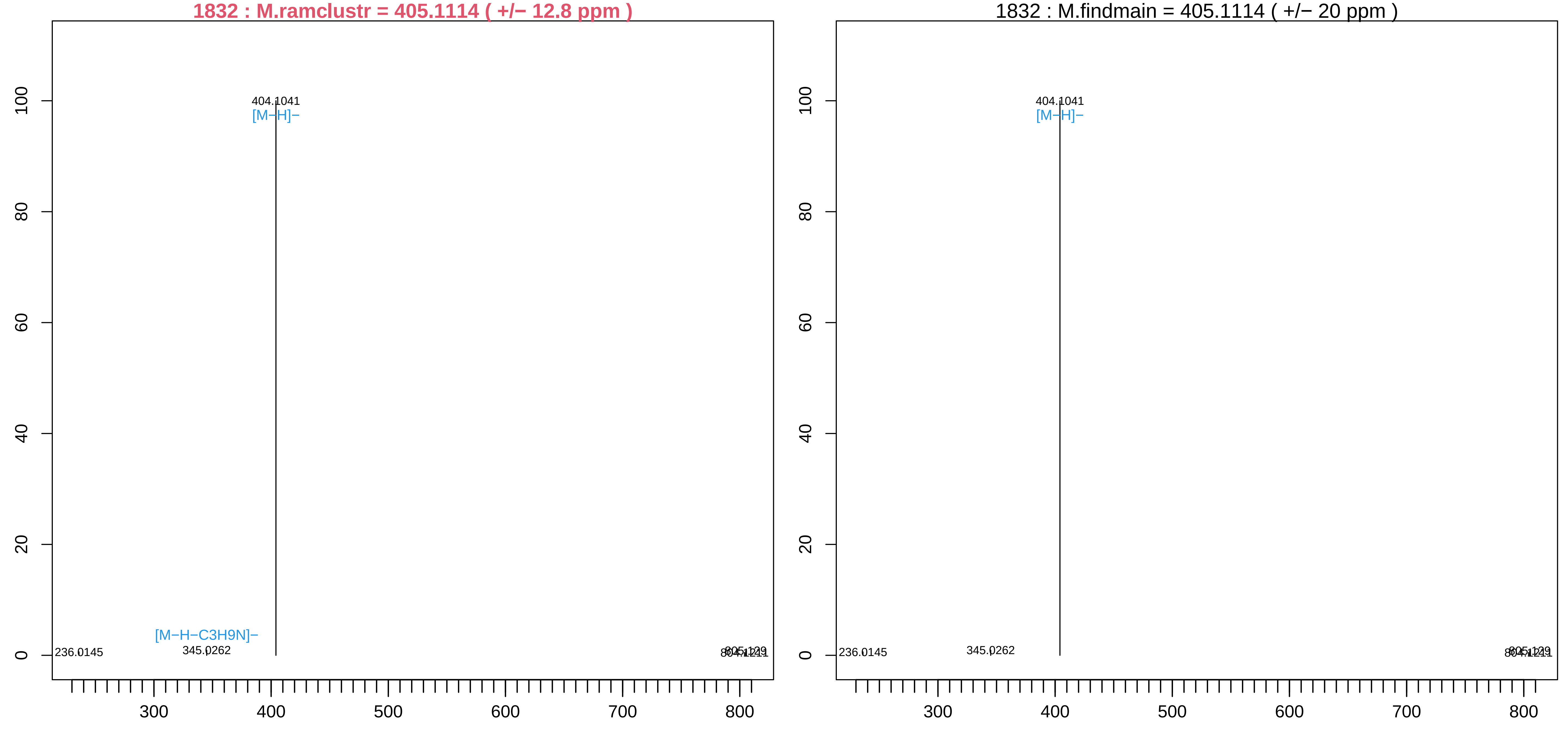

1832 : M.ramclustr = 405.1114 (+/− 12.8 ppm)
404.1041
[M−H]−
[M−H−C3H9N]−
236.0145
345.0262
805.1129
804.1211
0
20
40
60
80
100
300
400
500
600
700
800
1832 : M.findmain = 405.1114 (+/− 20 ppm)
404.1041
[M−H]−
236.0145
345.0262
805.1129
804.1211
0
20
40
60
80
100
300
400
500
600
700
800

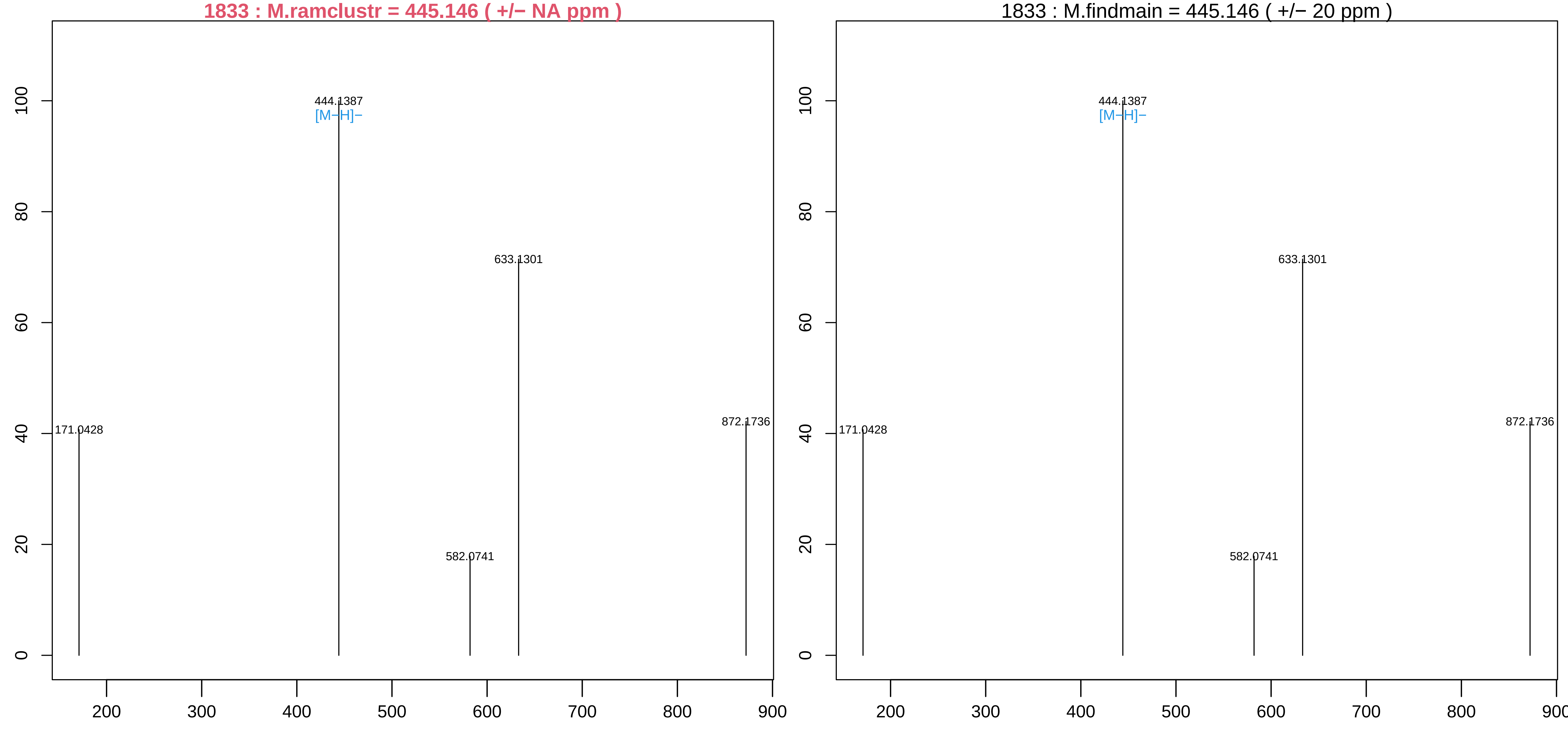

1833 : M.ramclustr = 445.146 (+/− NA ppm)
444.1387
[M−H]−
633.1301
171.0428
872.1736
582.0741
1833 : M.findmain = 445.146 (+/− 20 ppm)
444.1387
[M−H]−
633.1301
171.0428
872.1736
582.0741

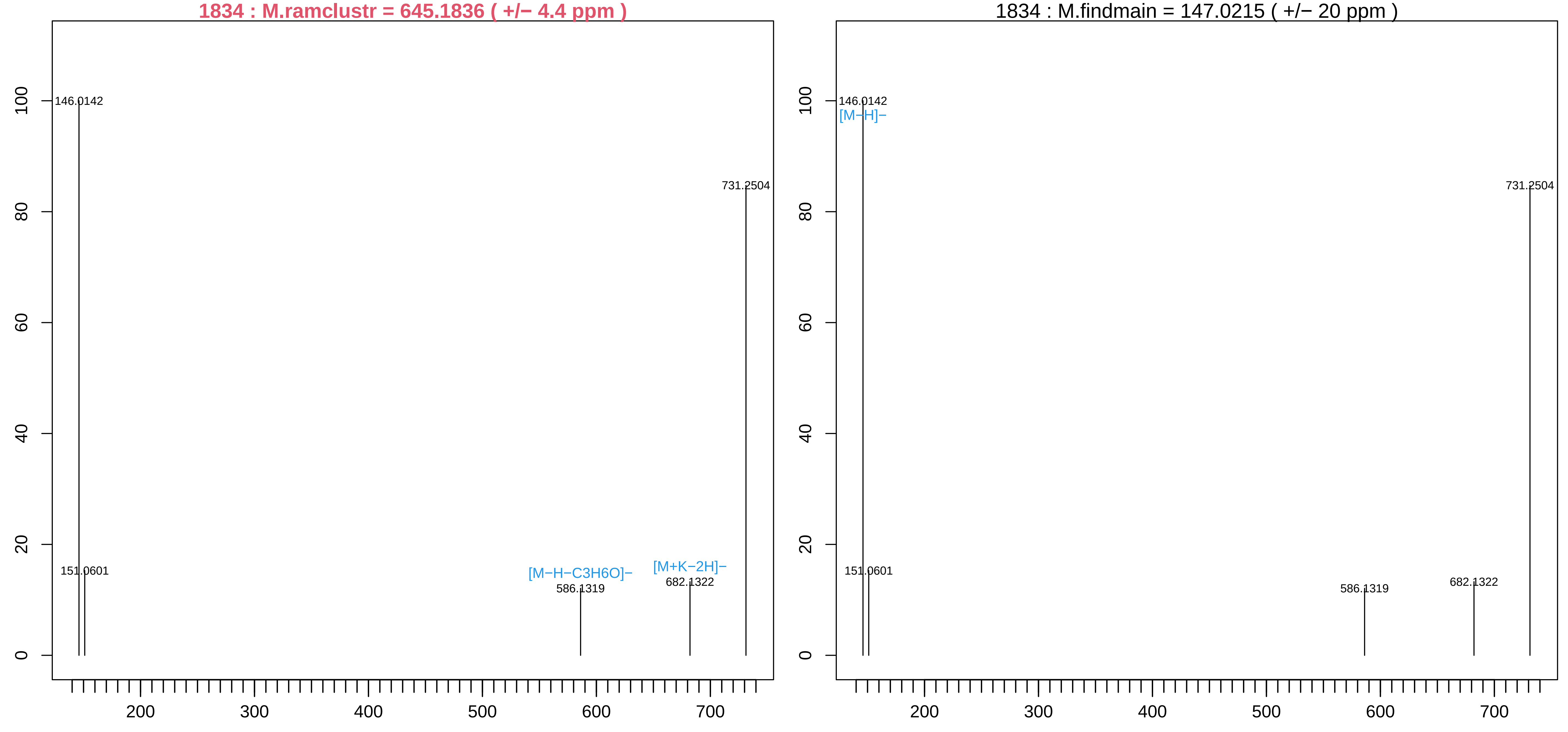
1834 : M.ramclustr = 645.1836 (+/− 4.4 ppm)
146.0142
151.0601
[M−H−C3H6O]−
586.1319
[M+K−2H]−
682.1322
731.2504
1834 : M.findmain = 147.0215 (+/− 20 ppm)
146.0142
[M−H]−
151.0601
586.1319
682.1322
731.2504
0
20
40
60
80
100
200
300
400
500
600
700

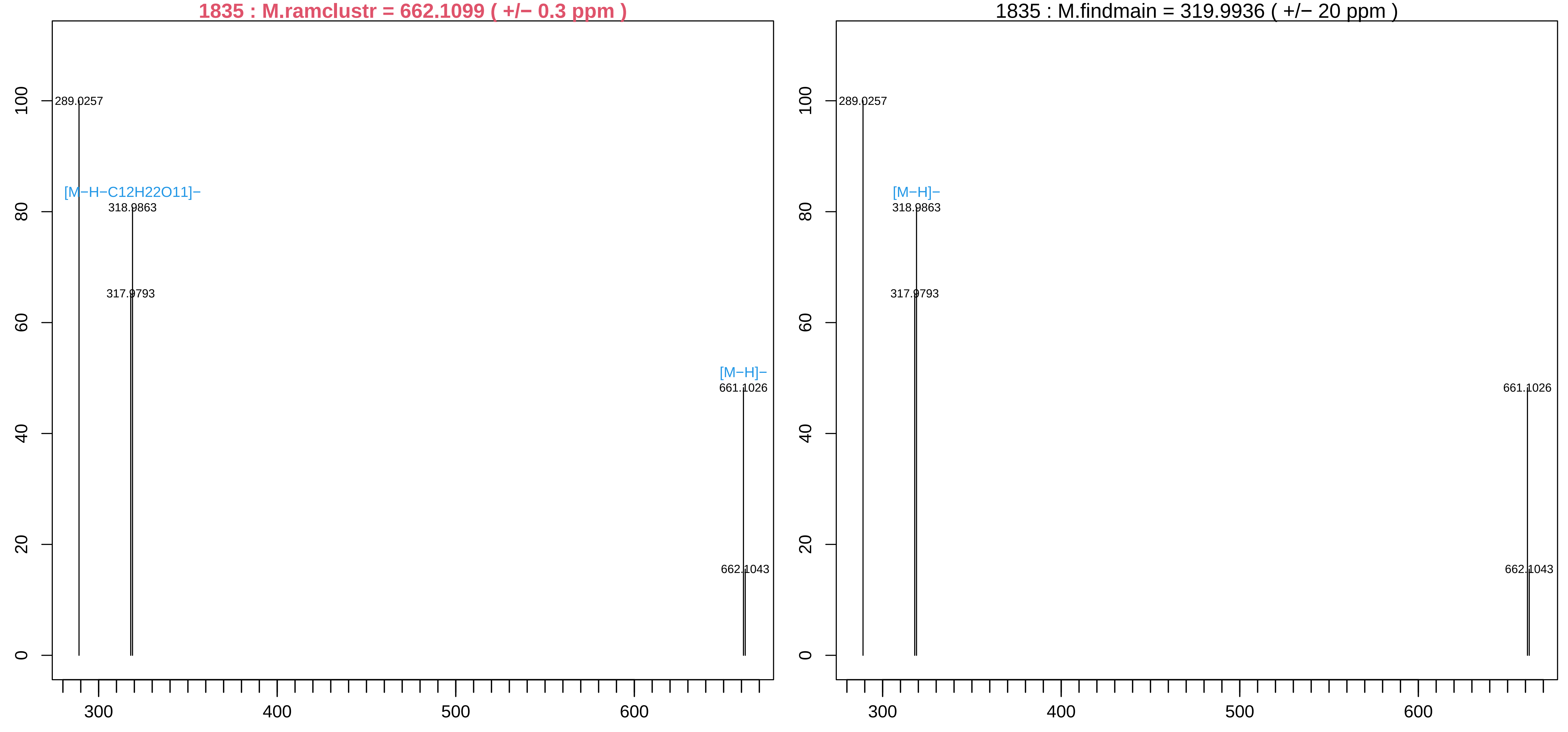

1835 : M.ramclustr = 662.1099 (+/− 0.3 ppm)
289.0257
[M−H−C12H22O11]−
318.9863
317.9793
[M−H]−
661.1026
662.1043
1835 : M.findmain = 319.9936 (+/− 20 ppm)
289.0257
[M−H]−
318.9863
317.9793
661.1026
662.1043
0
20
40
60
80
100
300
400
500
600

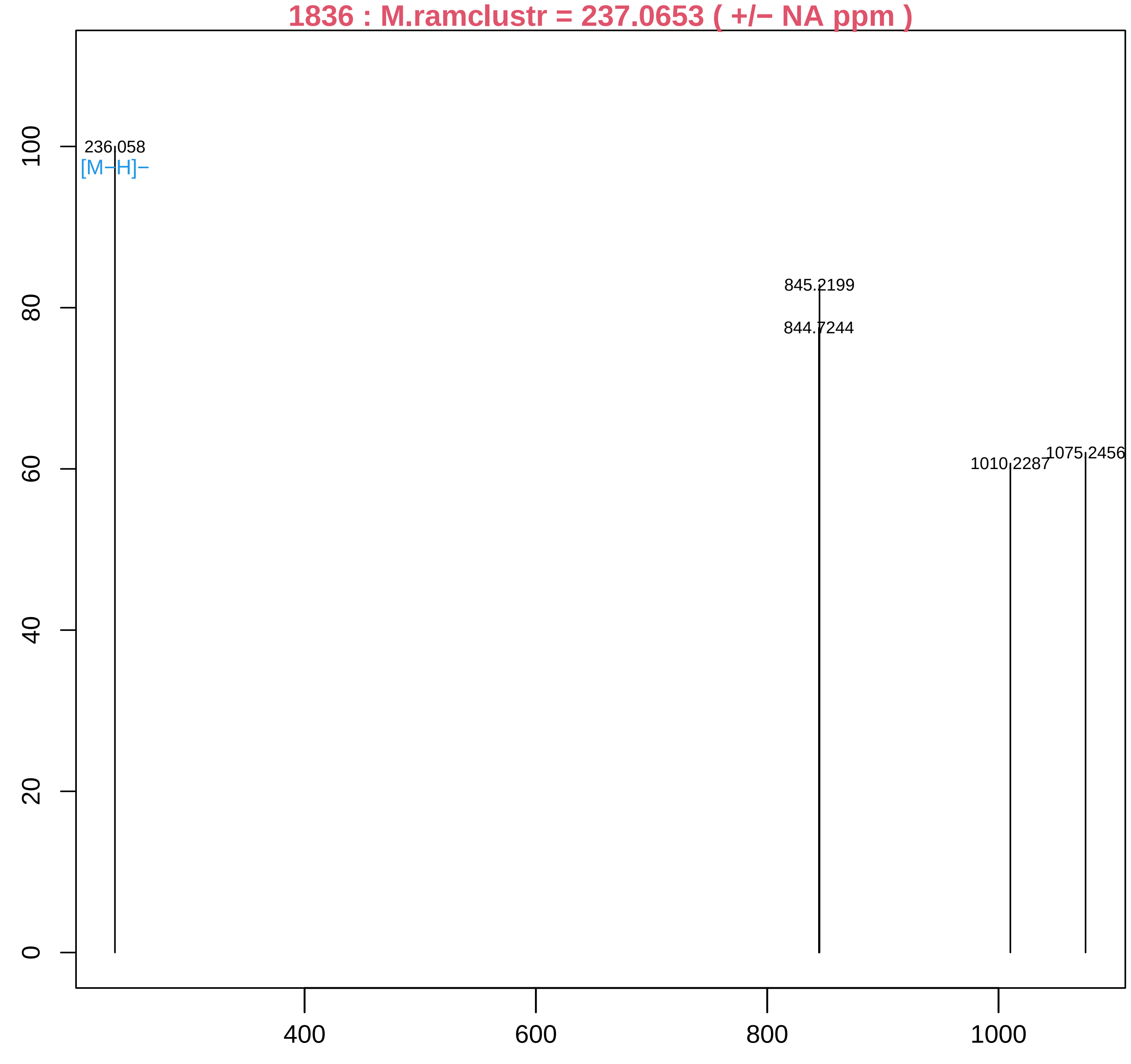
1836 : M.ramclustr = 237.0653 (+/− NA ppm)
236.058
[M−H]−
845.2199
844.7244
1010.2287
1075.2456
0
20
40
60
80
100
400
600
800
1000

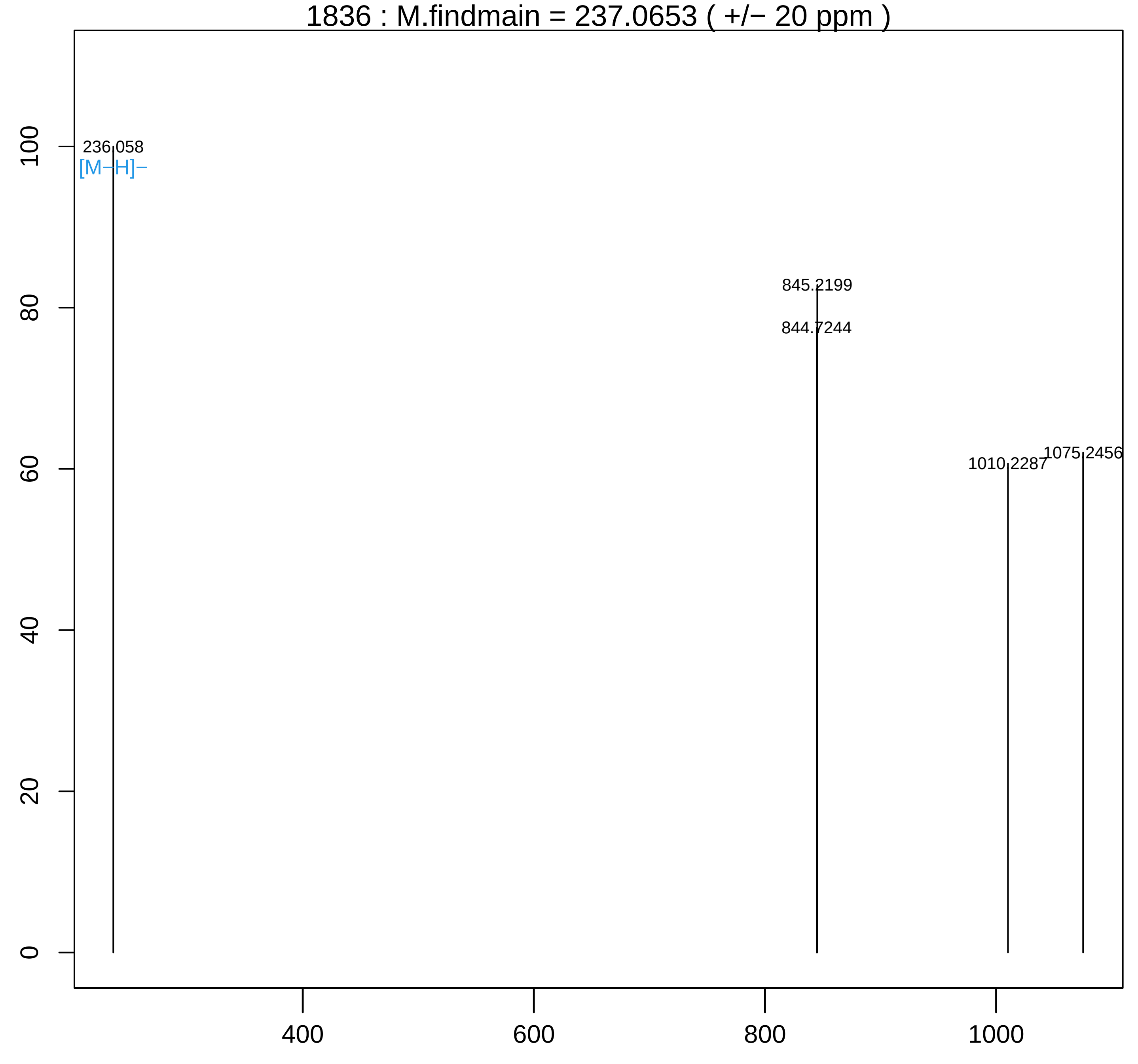
1836 : M.findmain = 237.0653 (+/− 20 ppm)
236.058
[M−H]−
845.2199
844.7244
1010.2287
1075.2456
0
20
40
60
80
100
400
600
800
1000

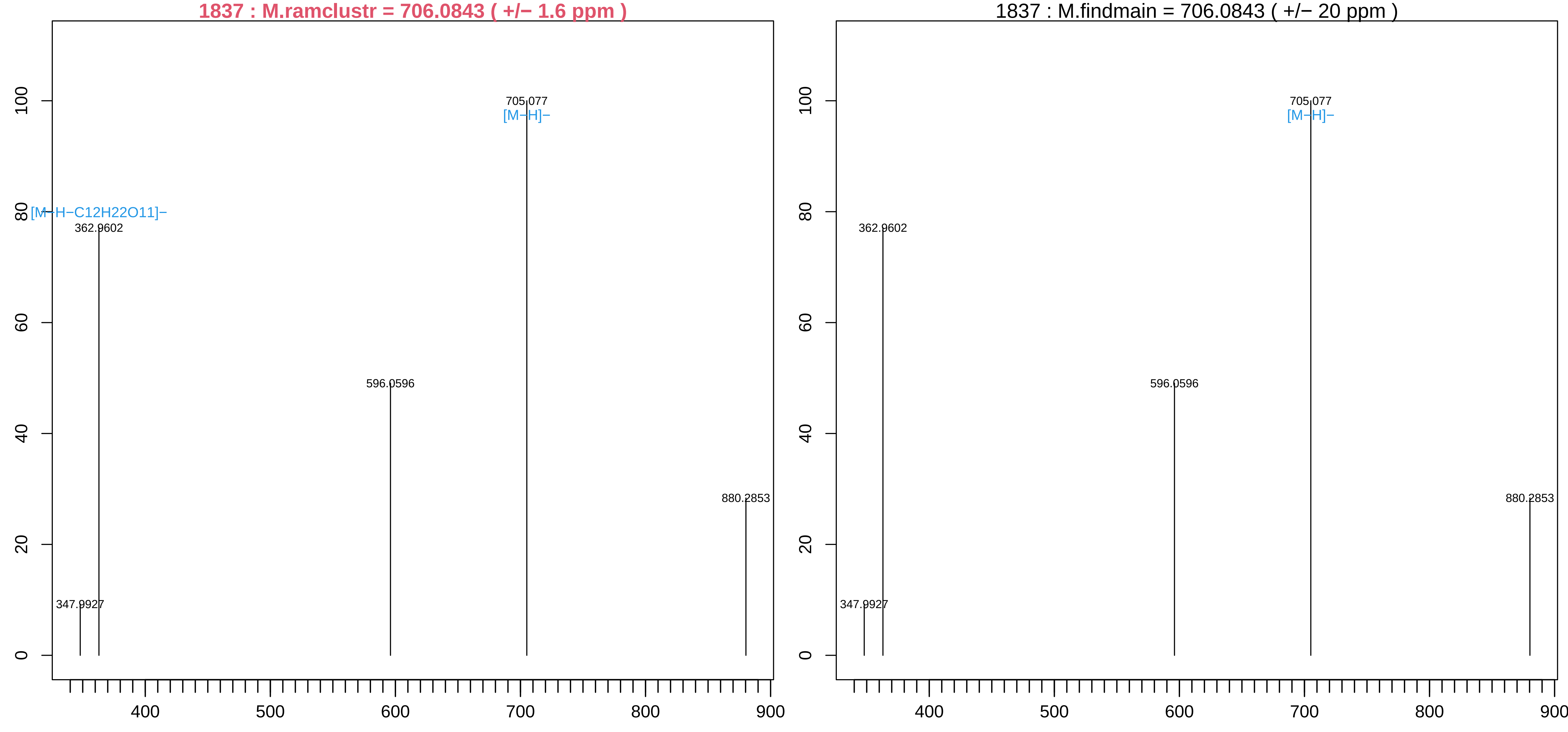
1837 : M.ramclustr = 706.0843 (+/− 1.6 ppm)
705.077
[M−H]−
[M−H−C12H22O11]−
362.9602
596.0596
880.2853
347.9927
1837 : M.findmain = 706.0843 (+/− 20 ppm)
705.077
[M−H]−
362.9602
596.0596
880.2853
347.9927
0
20
40
60
80
100
400
500
600
700
800
900

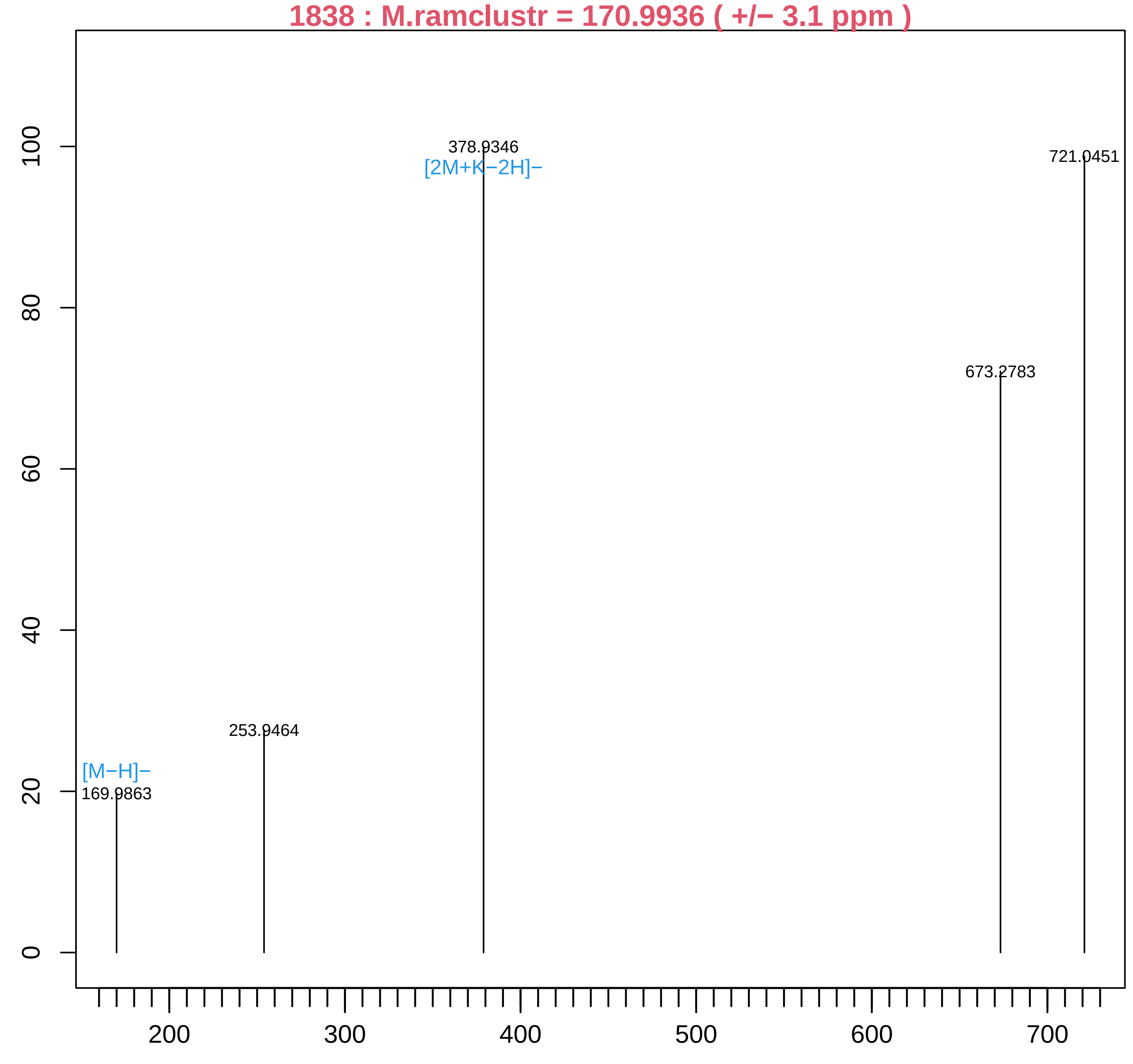
1838 : M.ramclustr = 170.9936 (+/− 3.1 ppm)
378.9346
[2M+K−2H]−
721.0451
673.2783
253.9464
[M−H]−
169.9863
0
20
40
60
80
100
200
300
400
500
600
700
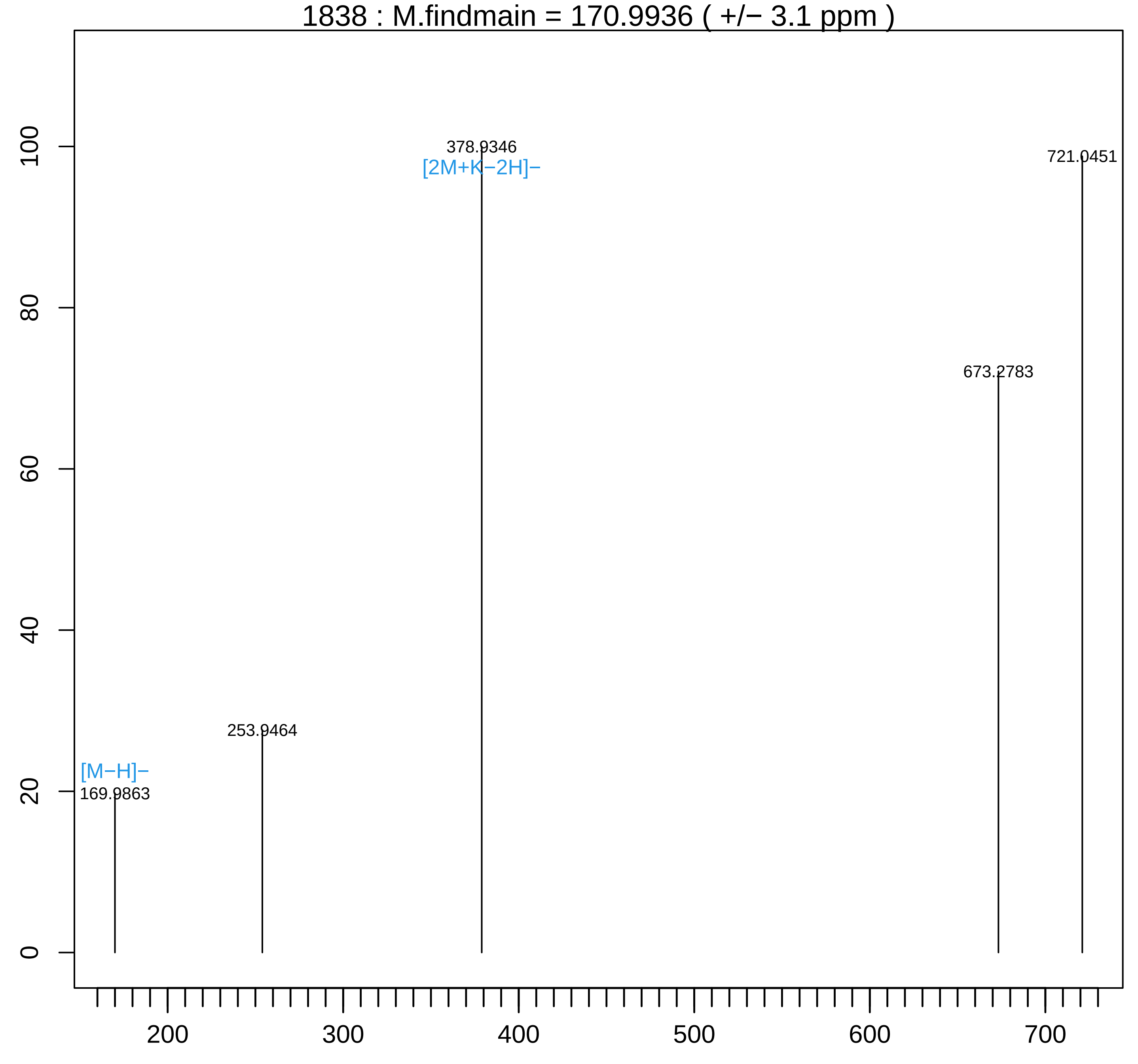
1838 : M.findmain = 170.9936 (+/− 3.1 ppm)
378.9346
[2M+K−2H]−
721.0451
673.2783
253.9464
[M−H]−
169.9863
0
20
40
60
80
100
200
300
400
500
600
700

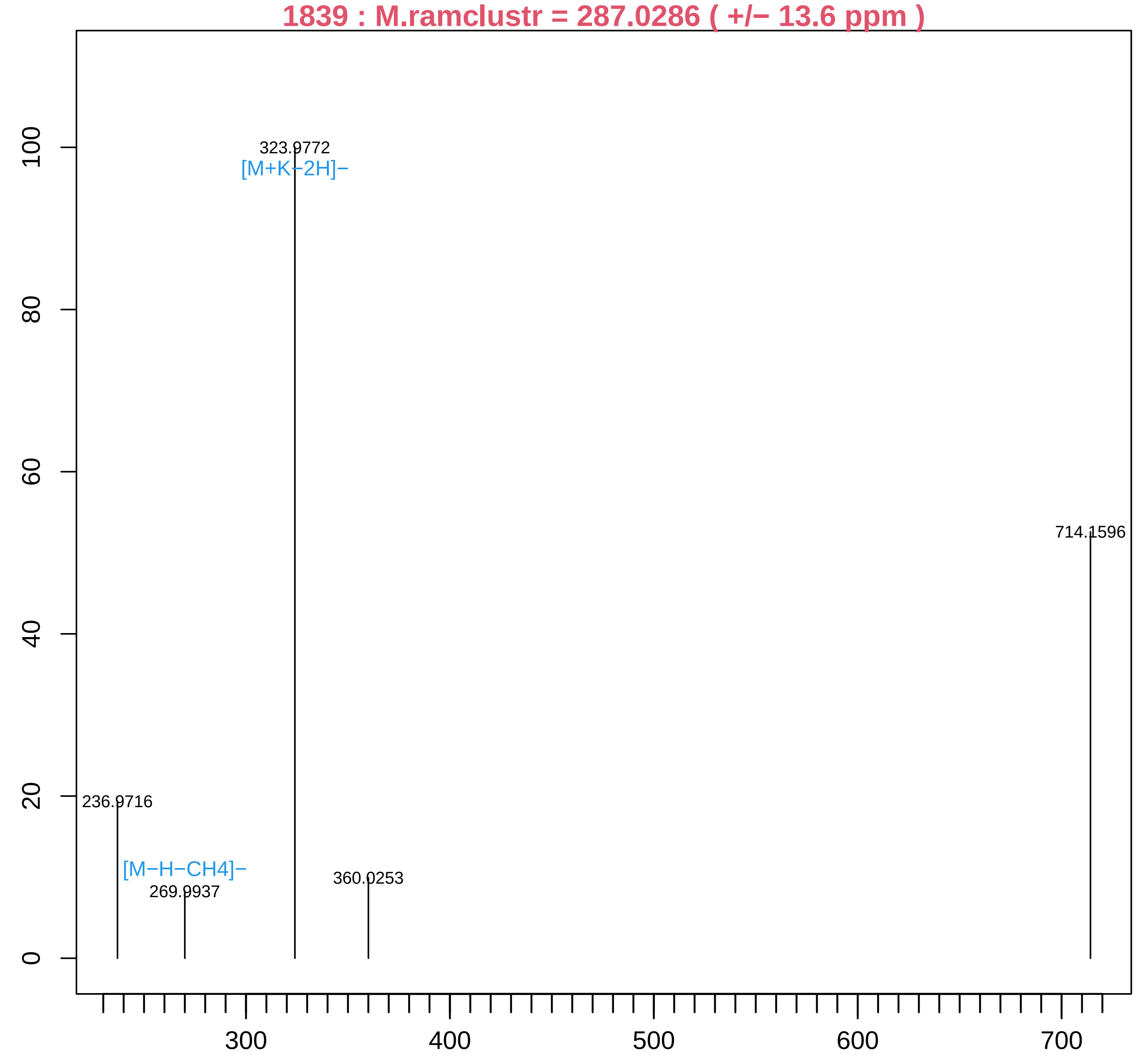
1839 : M.ramclustr = 287.0286 (+/− 13.6 ppm)
323.9772
[M+K−2H]−
714.1596
236.9716
[M−H−CH4]−
269.9937
360.0253
0
20
40
60
80
100
300
400
500
600
700

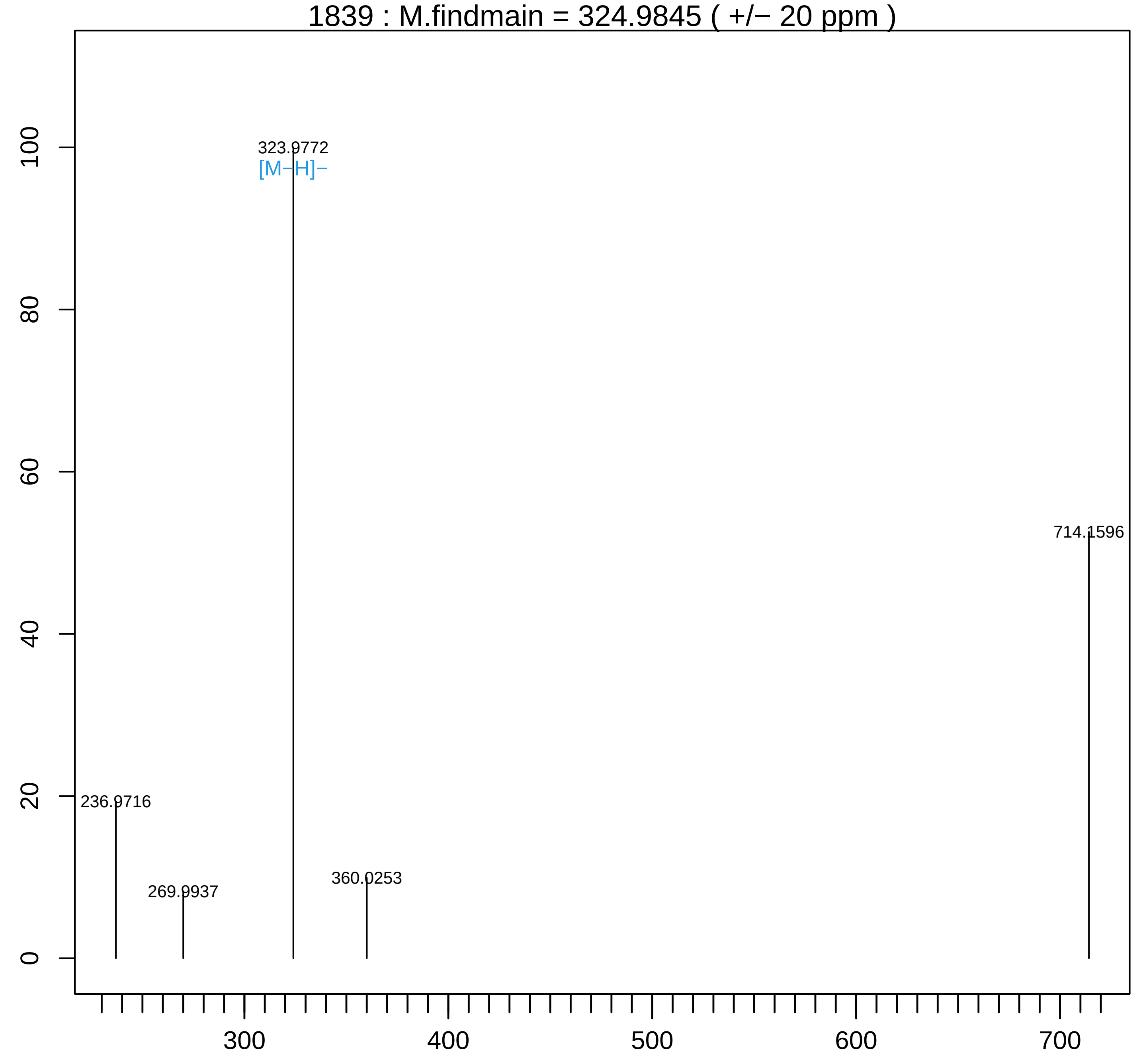
1839 : M.findmain = 324.9845 (+/− 20 ppm)
323.9772
[M−H]−
714.1596
236.9716
269.9937
360.0253
0
20
40
60
80
100
300
400
500
600
700

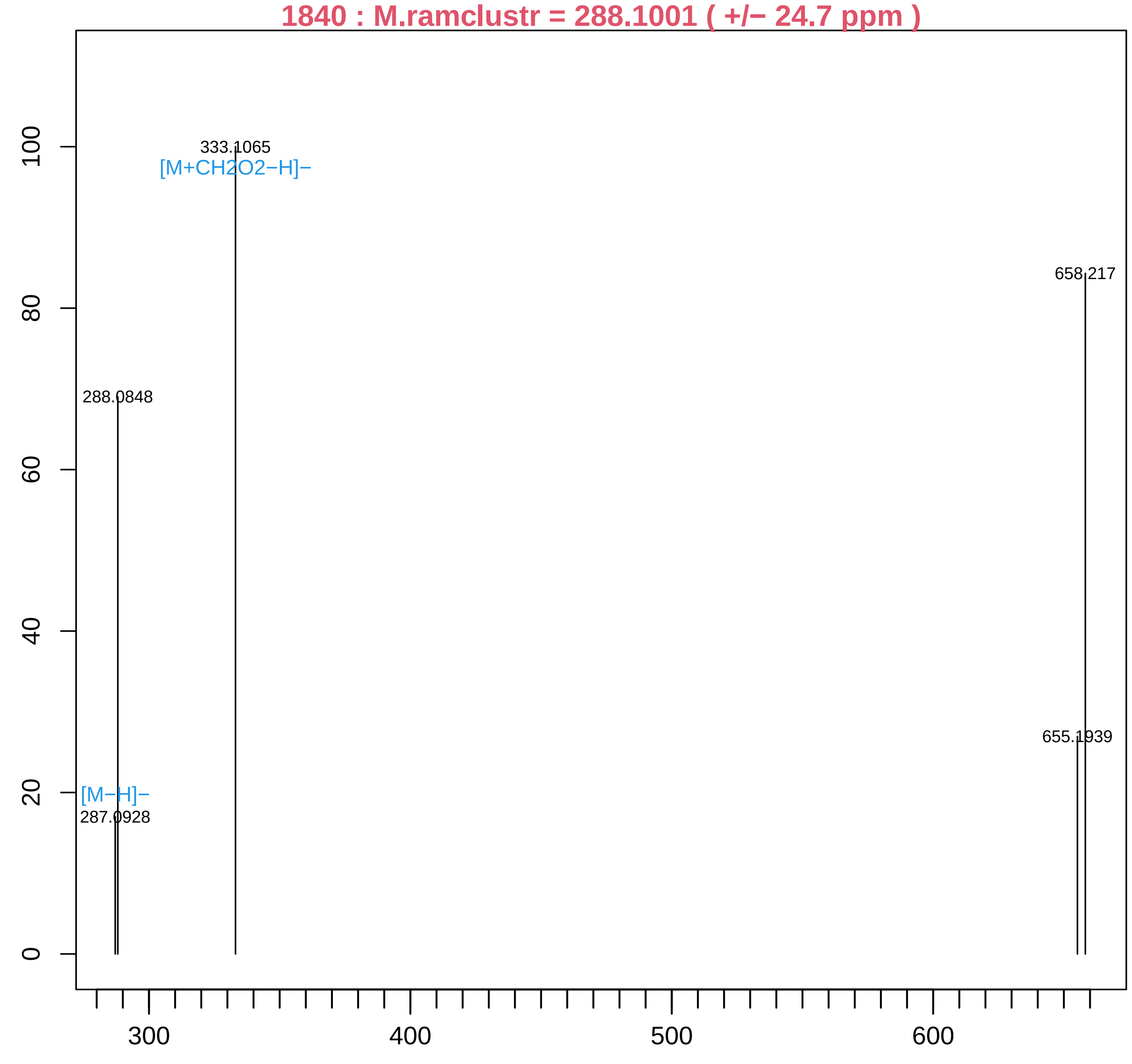
1840 : M.ramclustr = 288.1001 (+/− 24.7 ppm)
333.1065
[M+CH2O2−H]−
658.217
288.0848
655.1939
[M−H]−
287.0928
0
20
40
60
80
100
300
400
500
600

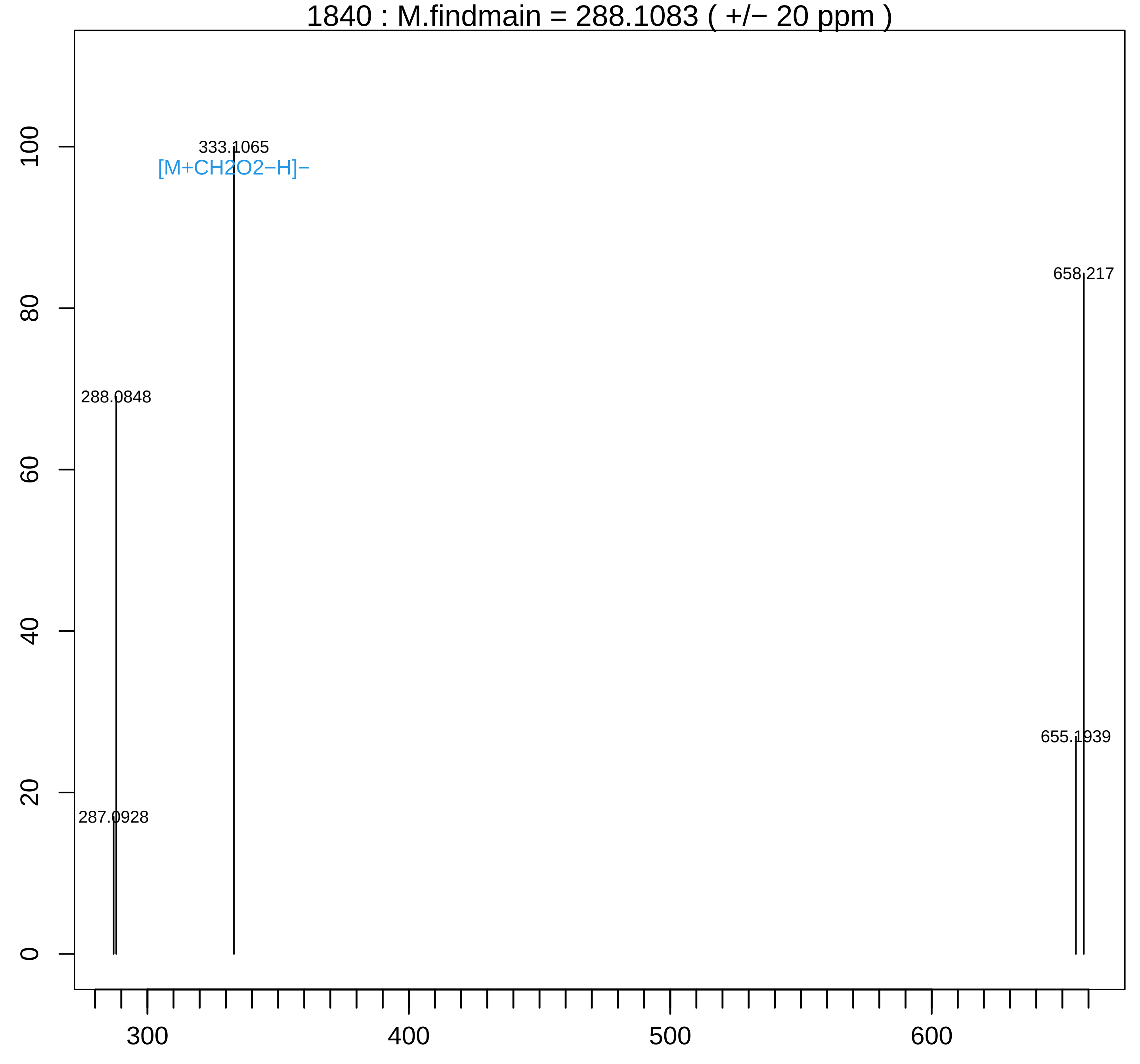
1840 : M.findmain = 288.1083 (+/− 20 ppm)
333.1065
[M+CH2O2−H]−
658.217
288.0848
655.1939
287.0928
0
20
40
60
80
100
300
400
500
600

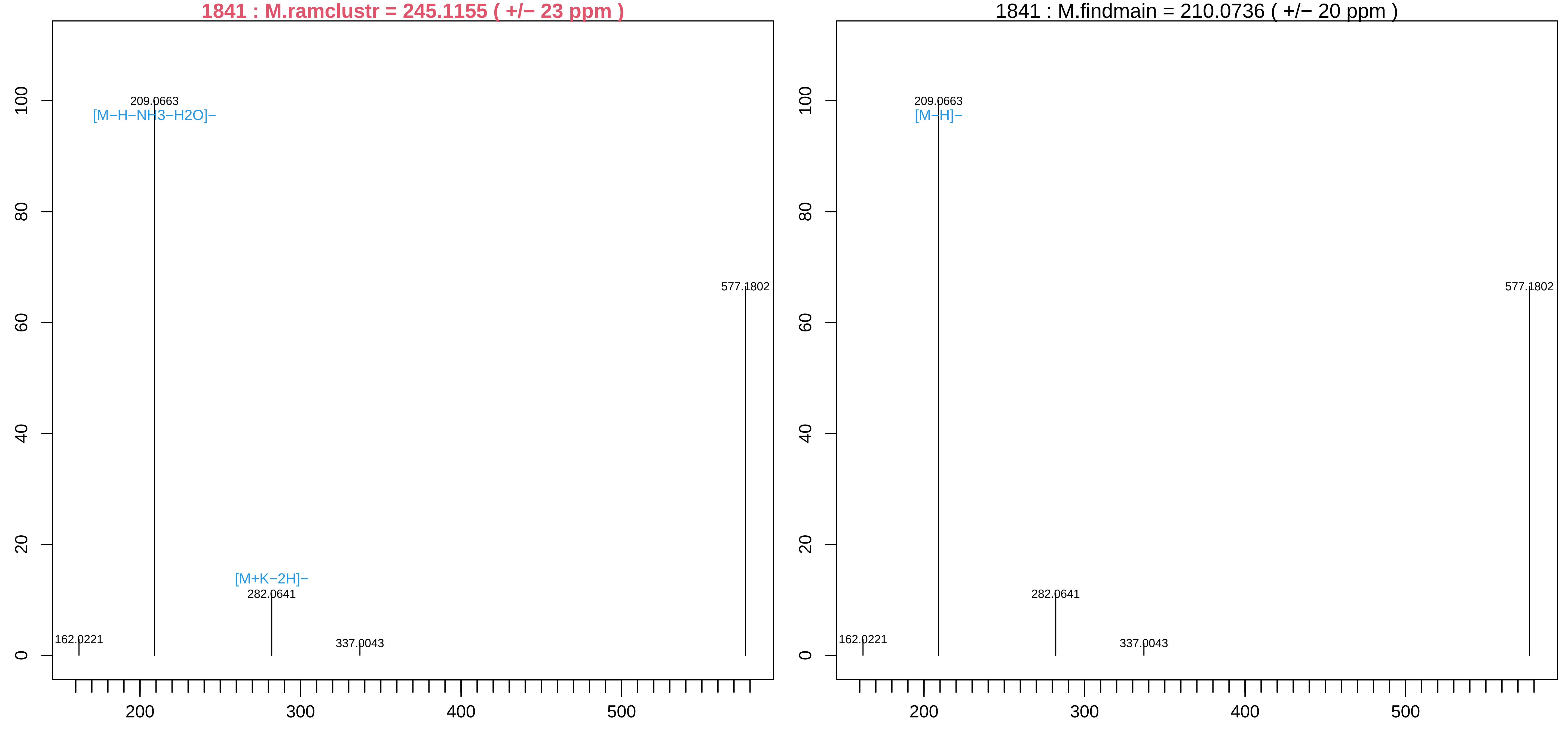
1841 : M.ramclustr = 245.1155 (+/− 23 ppm)
209.0663
[M−H−NH3−H2O]−
577.1802
[M+K−2H]−
282.0641
162.0221
337.0043
0
20
40
60
80
100
200
300
400
500
1841 : M.findmain = 210.0736 (+/− 20 ppm)
209.0663
[M−H]−
577.1802
282.0641
162.0221
337.0043
0
20
40
60
80
100
200
300
400
500

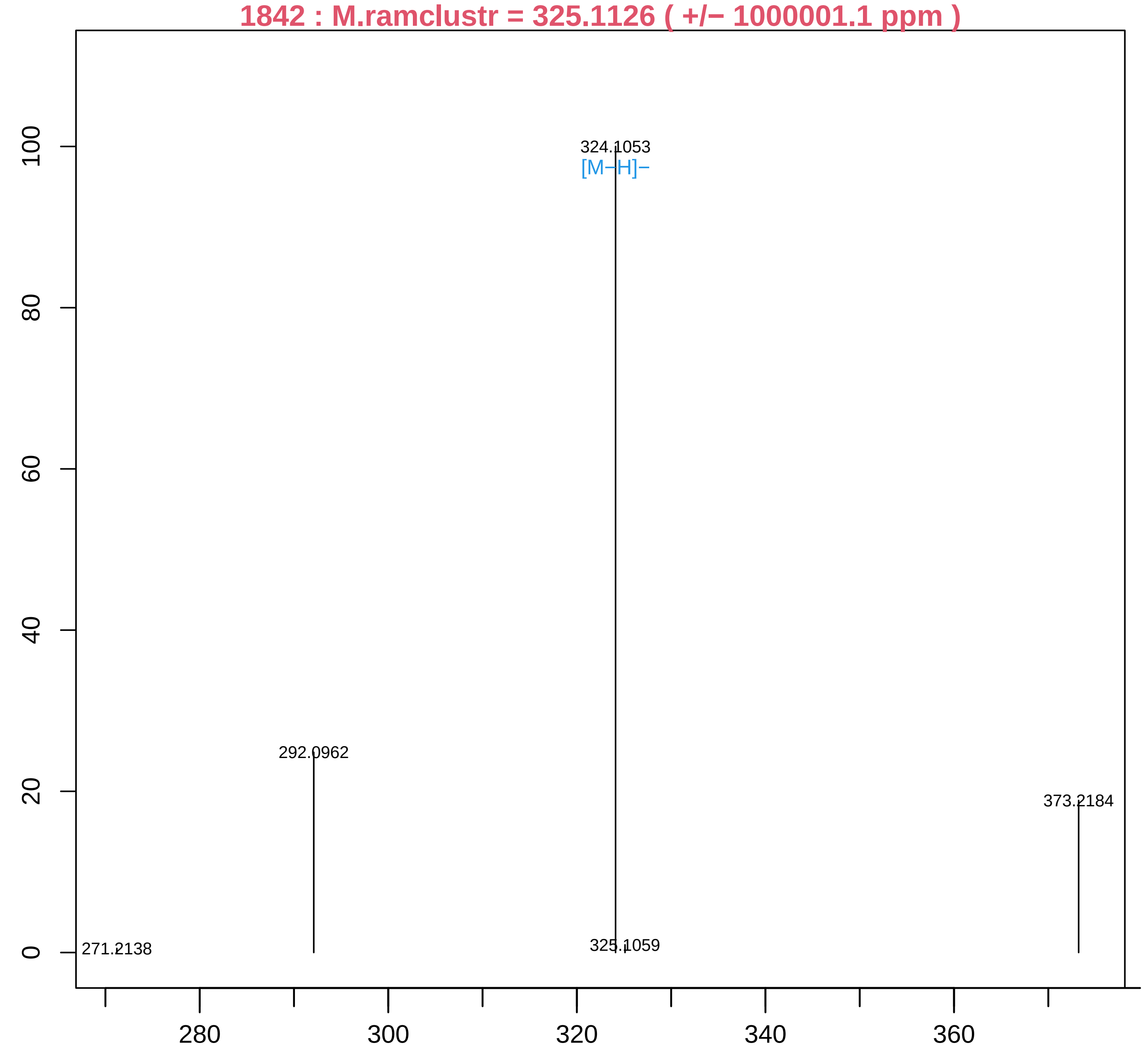
1842 : M.ramclustr = 325.1126 (+/− 1000001.1 ppm)
324.1053
[M−H]−
292.0962
373.2184
271.2138
325.1059
0
20
40
60
80
100
280
300
320
340
360

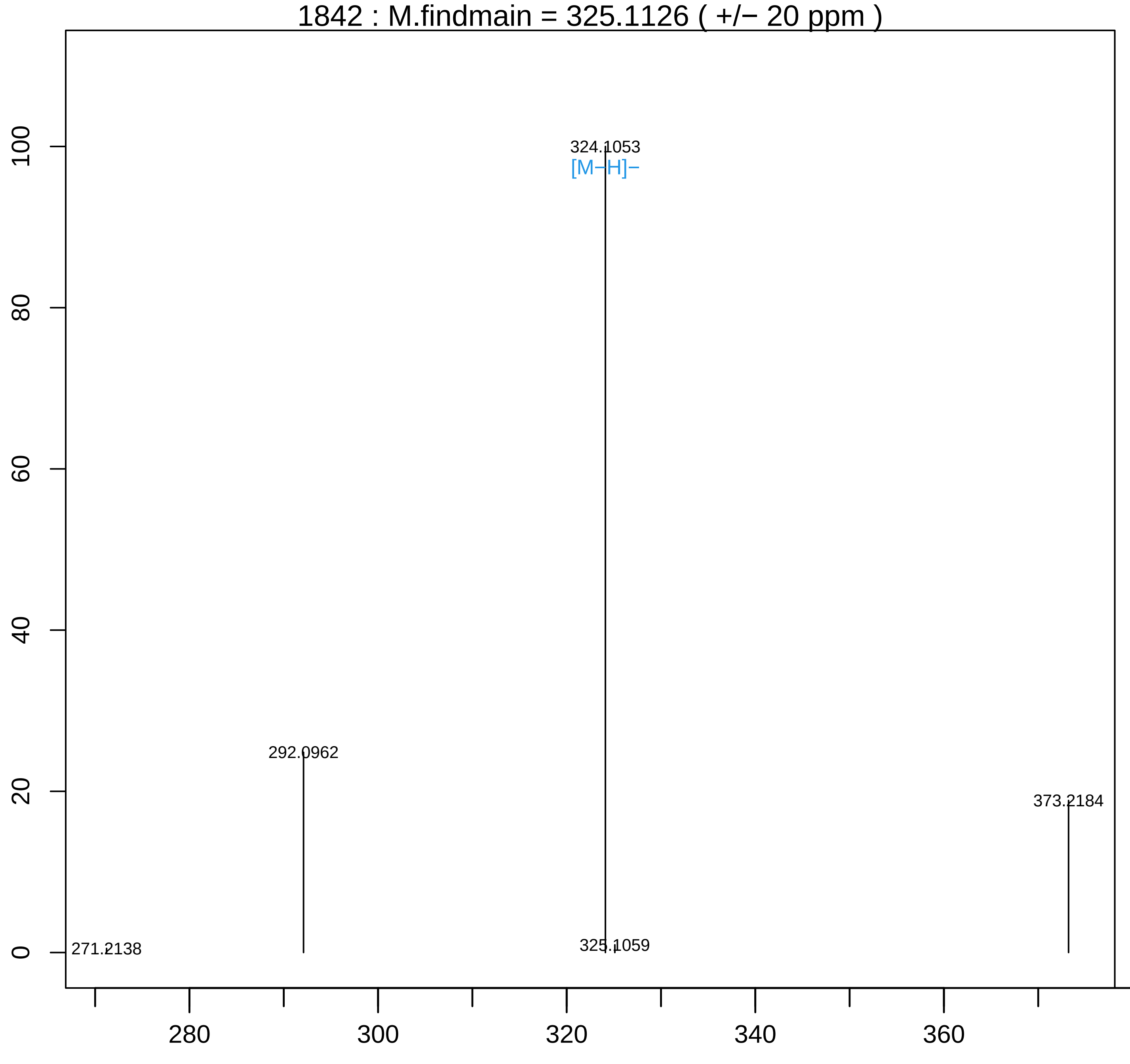
1842 : M.findmain = 325.1126 (+/− 20 ppm)
324.1053
[M−H]−
292.0962
373.2184
271.2138
325.1059
0
20
40
60
80
100
280
300
320
340
360

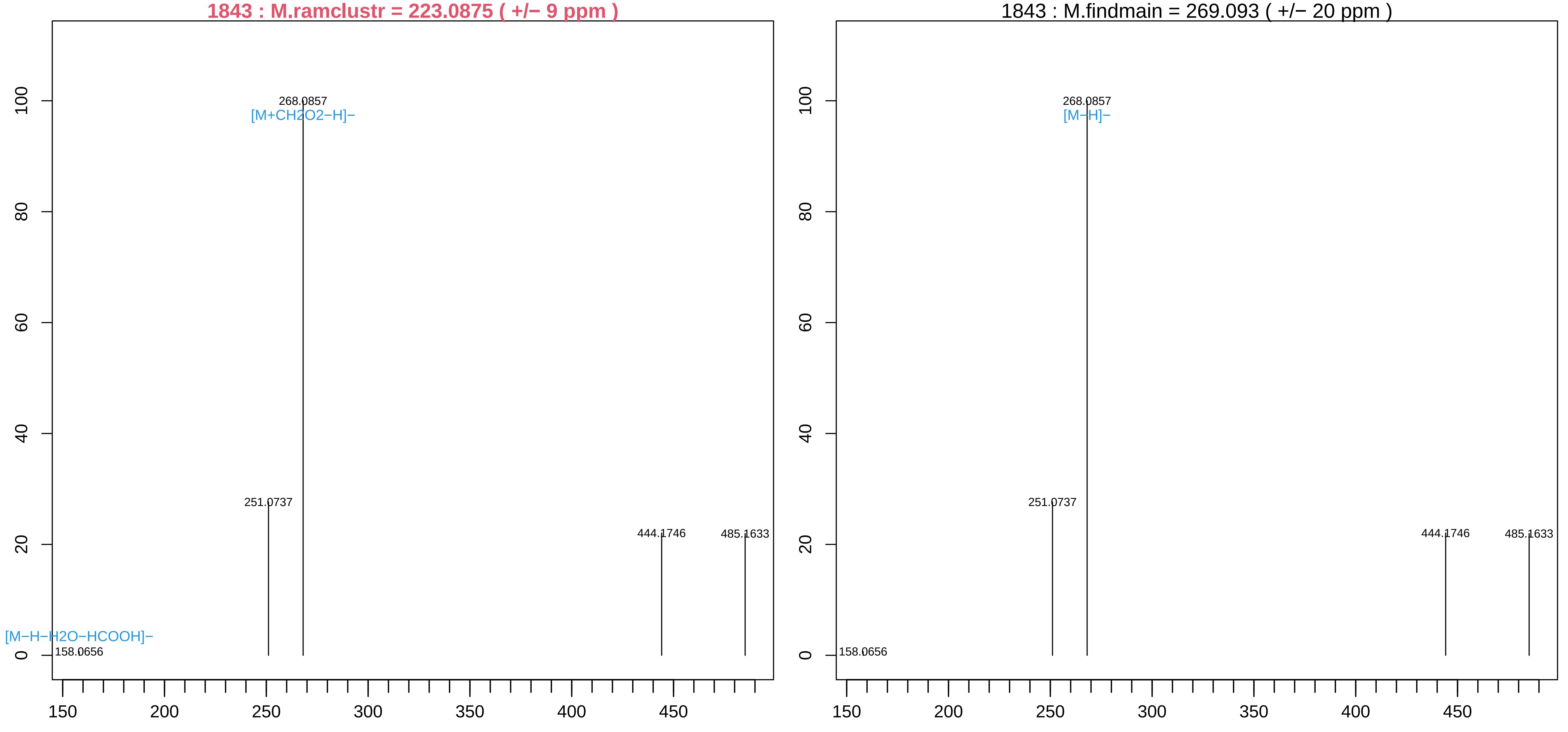

1843 : M.ramclustr = 223.0875 (+/− 9 ppm)
268.0857
[M+CH2O2−H]−
251.0737
444.1746
485.1633
[M−H−H2O−HCOOH]−
158.0656
0
20
40
60
80
100
150
200
250
300
350
400
450
1843 : M.findmain = 269.093 (+/− 20 ppm)
268.0857
[M−H]−
251.0737
444.1746
485.1633
158.0656

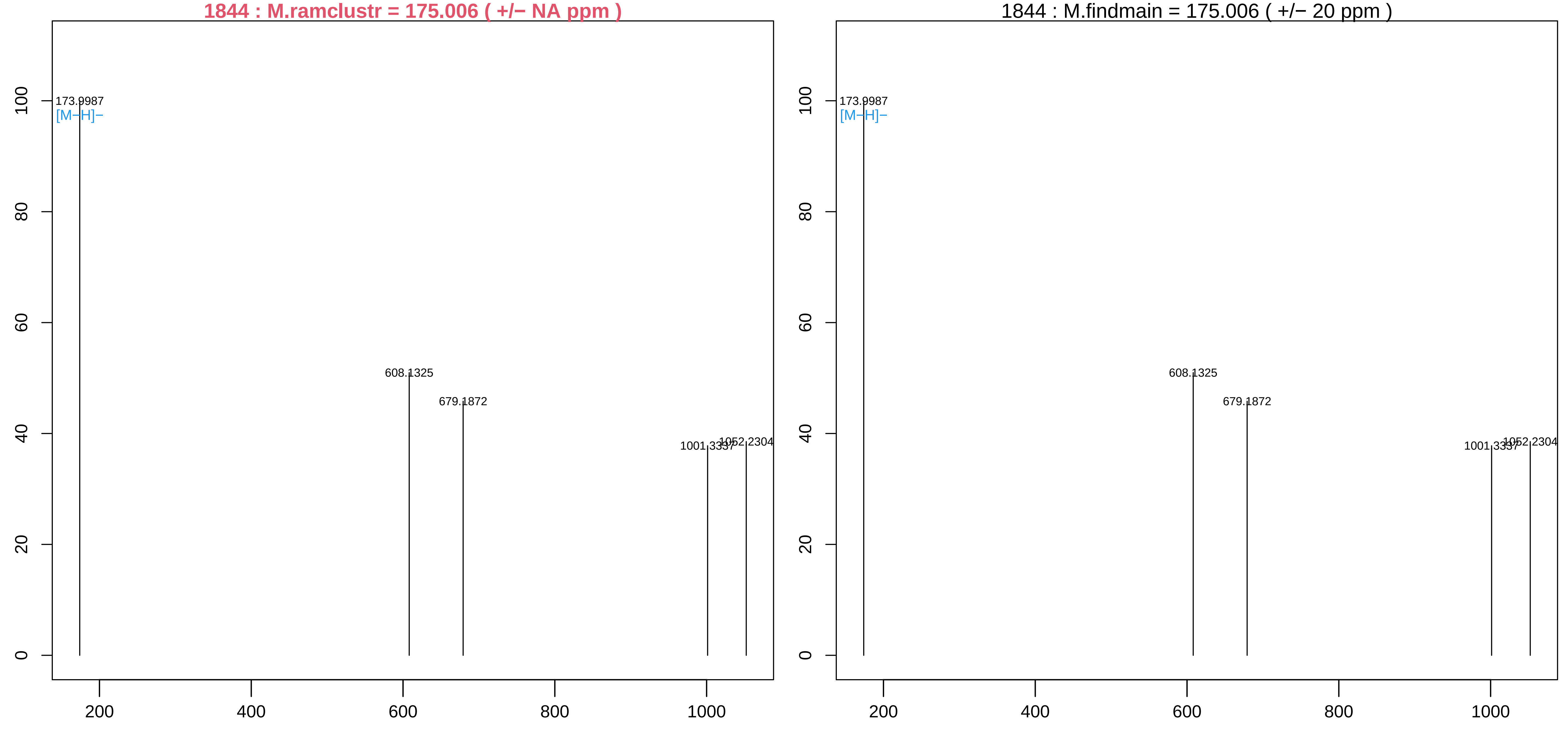
1844 : M.ramclustr = 175.006 (+/− NA ppm)
173.9987
[M−H]−
608.1325
679.1872
1001.3337
1052.2304
0
20
40
60
80
100
200
400
600
800
1000
1844 : M.findmain = 175.006 (+/− 20 ppm)
173.9987
[M−H]−
608.1325
679.1872
1001.3337
1052.2304
0
20
40
60
80
100
200
400
600
800
1000

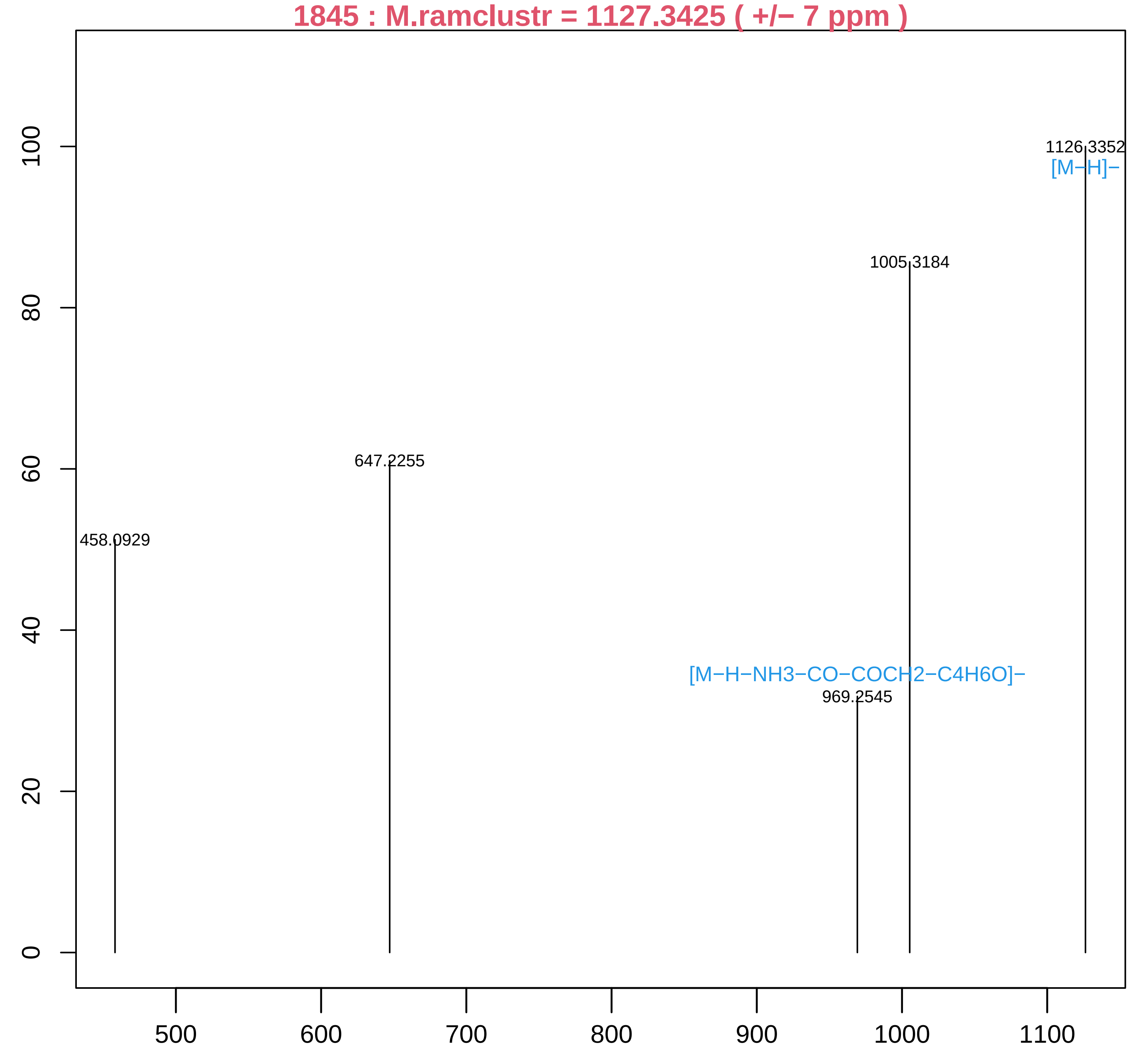
1845 : M.ramclustr = 1127.3425 (+/− 7 ppm)
1126.3352
[M−H]−
1005.3184
647.2255
458.0929
[M−H−NH3−CO−COCH2−C4H6O]−
969.2545
0
20
40
60
80
100
500
600
700
800
900
1000
1100

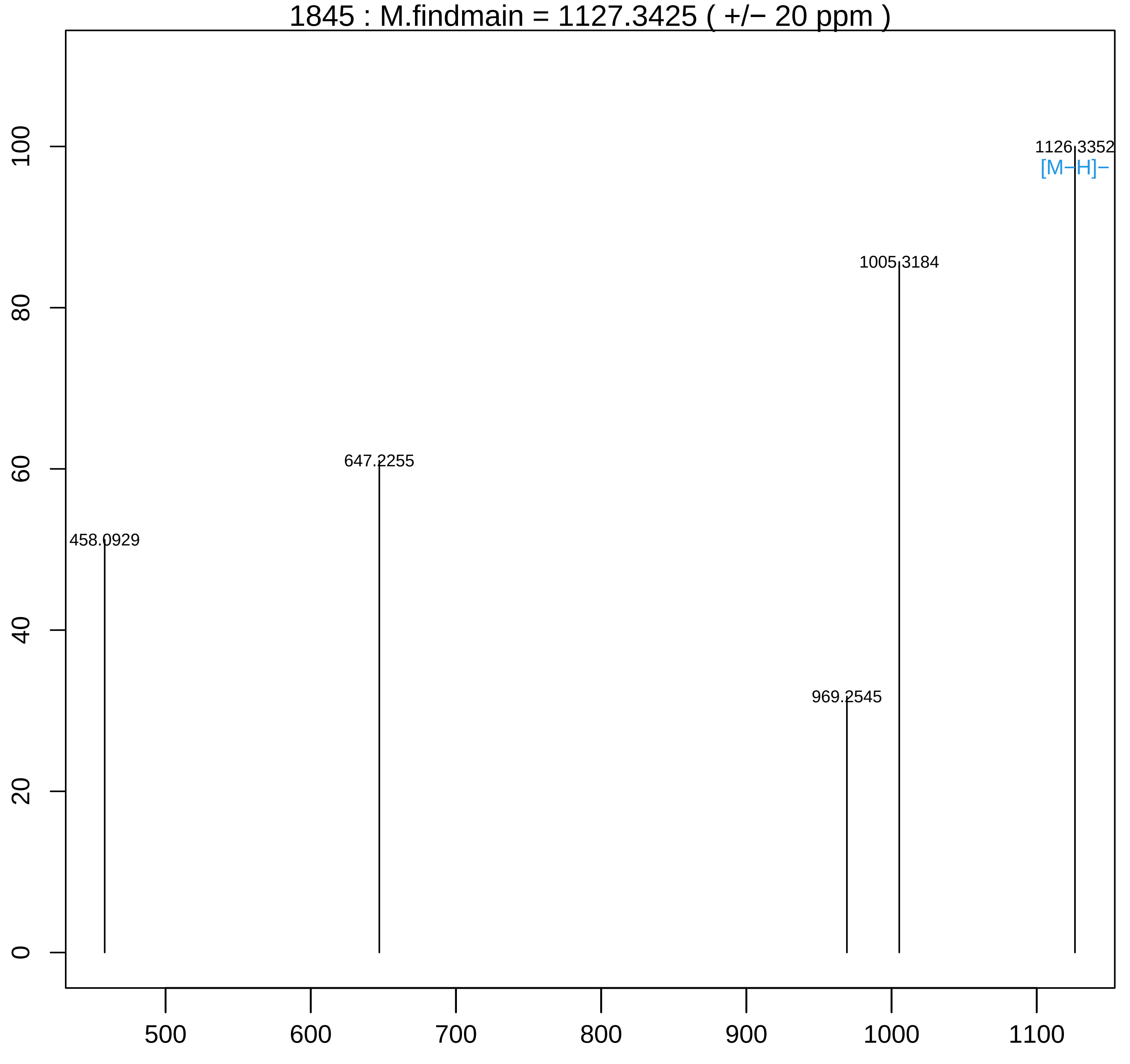
1845 : M.findmain = 1127.3425 (+/− 20 ppm)
1126.3352
[M−H]−
1005.3184
647.2255
458.0929
969.2545
0
20
40
60
80
100
500
600
700
800
900
1000
1100

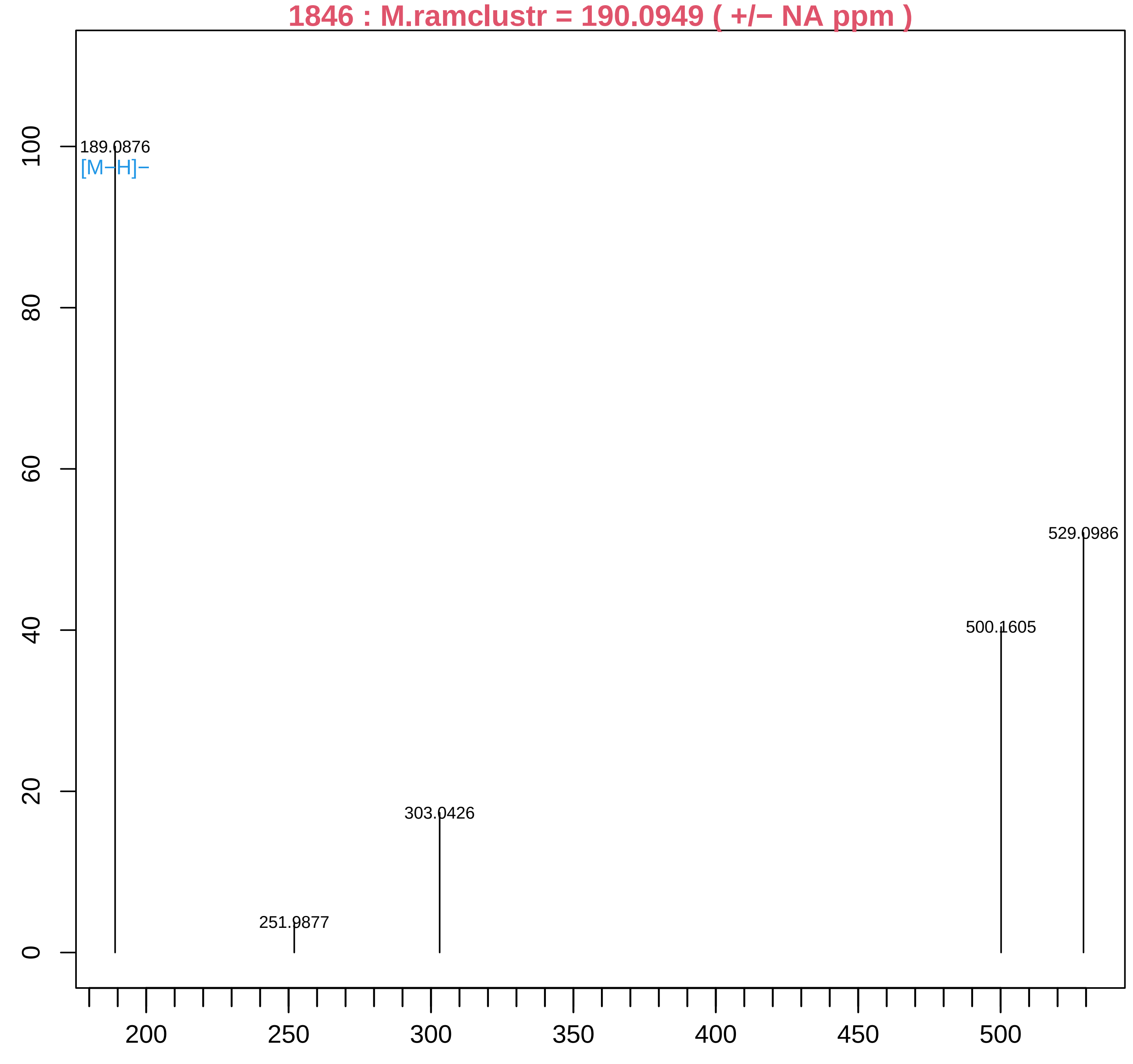

1846 : M.ramclustr = 190.0949 (+/− NA ppm)
189.0876
[M−H]−
251.9877
303.0426
500.1605
529.0986
0
20
40
60
80
100
200
250
300
350
400
450
500

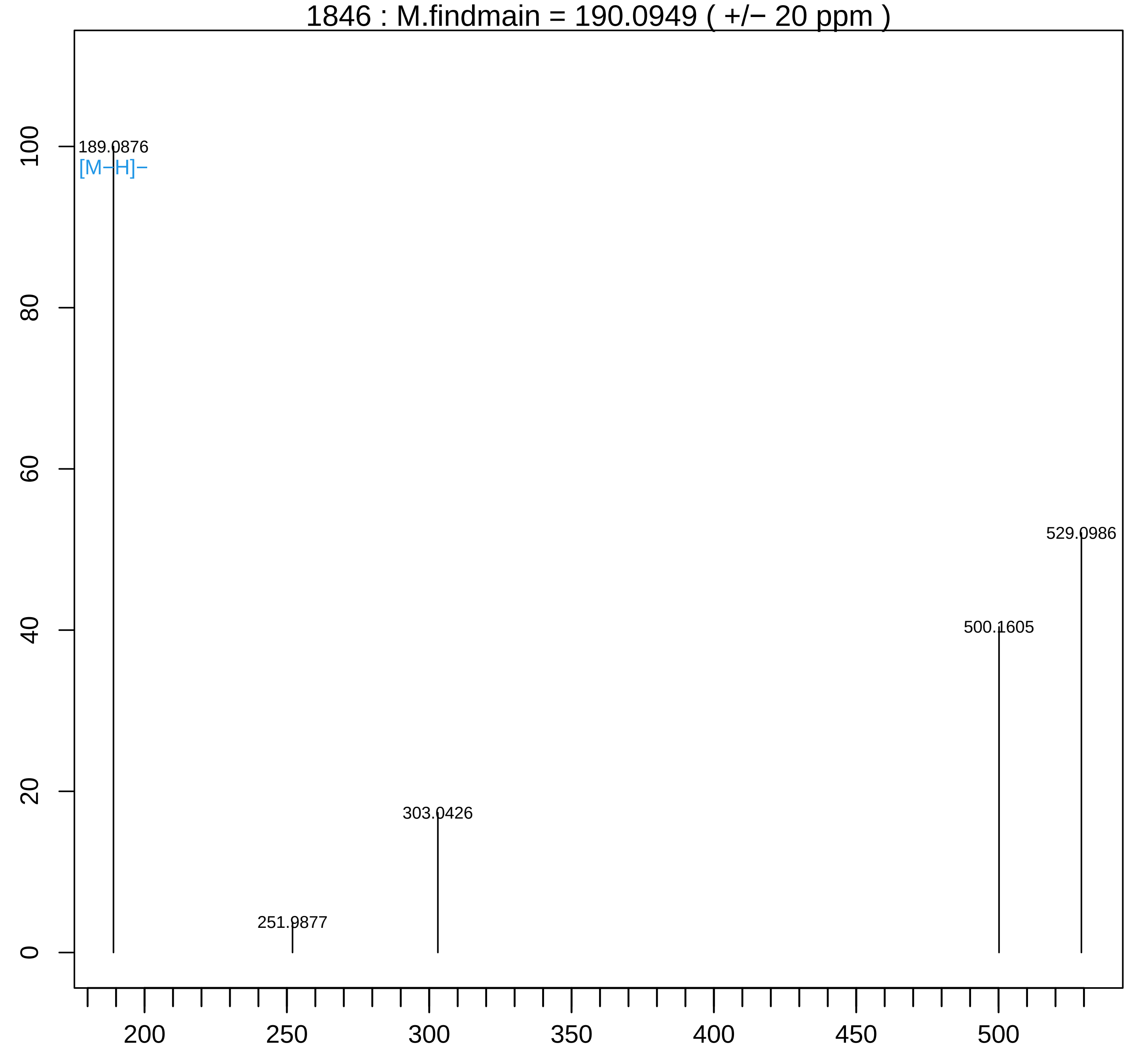

1846 : M.findmain = 190.0949 (+/− 20 ppm)
189.0876
[M−H]−
251.9877
303.0426
500.1605
529.0986
0
20
40
60
80
100
200
250
300
350
400
450
500

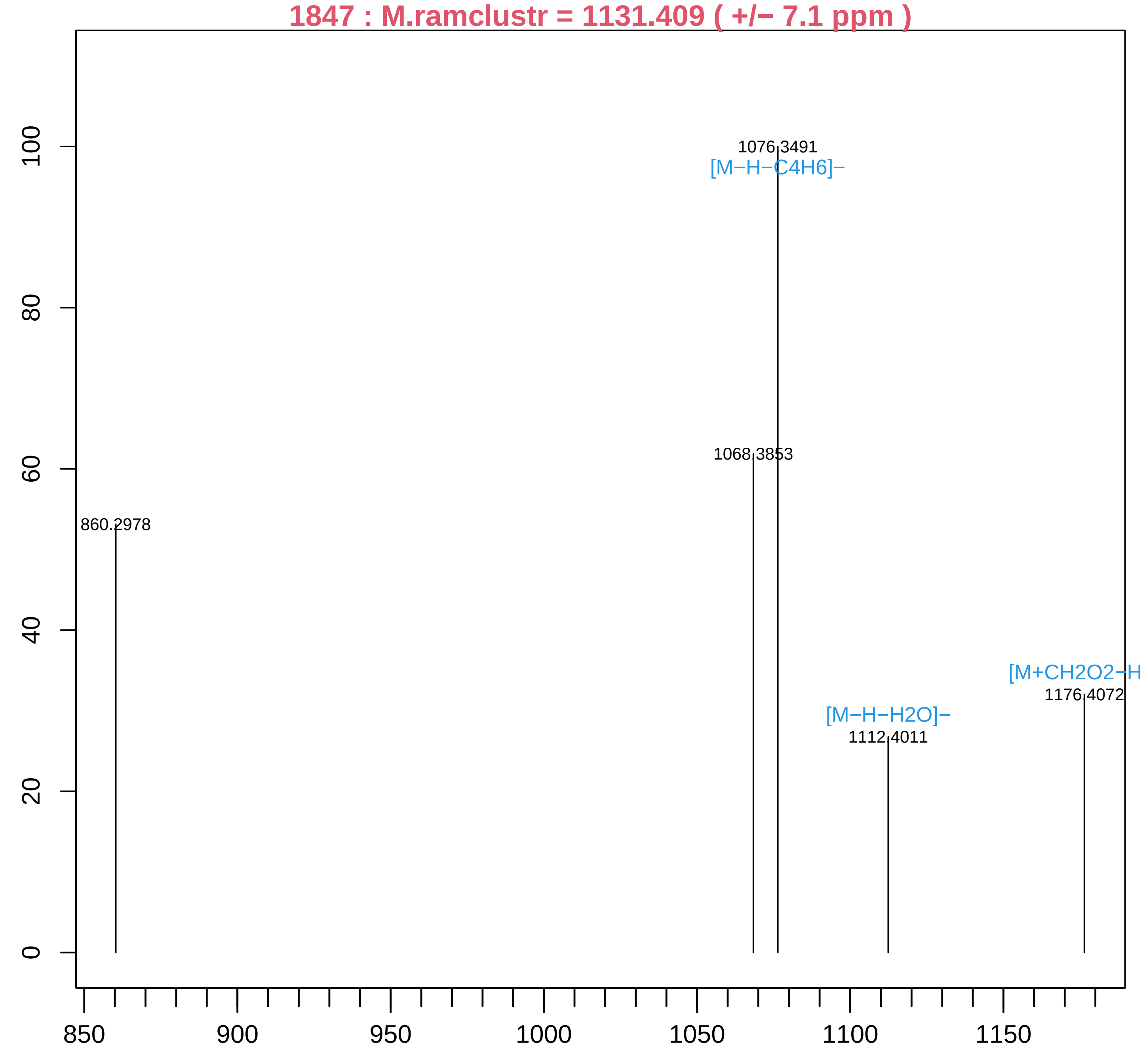

1847 : M.ramclustr = 1131.409 (+/− 7.1 ppm)
1076.3491
[M−H−C4H6]−
1068.3853
860.2978
[M−H−H2O]−
1112.4011
[M+CH2O2−H
1176.4072
0
20
40
60
80
100
850
900
950
1000
1050
1100
1150

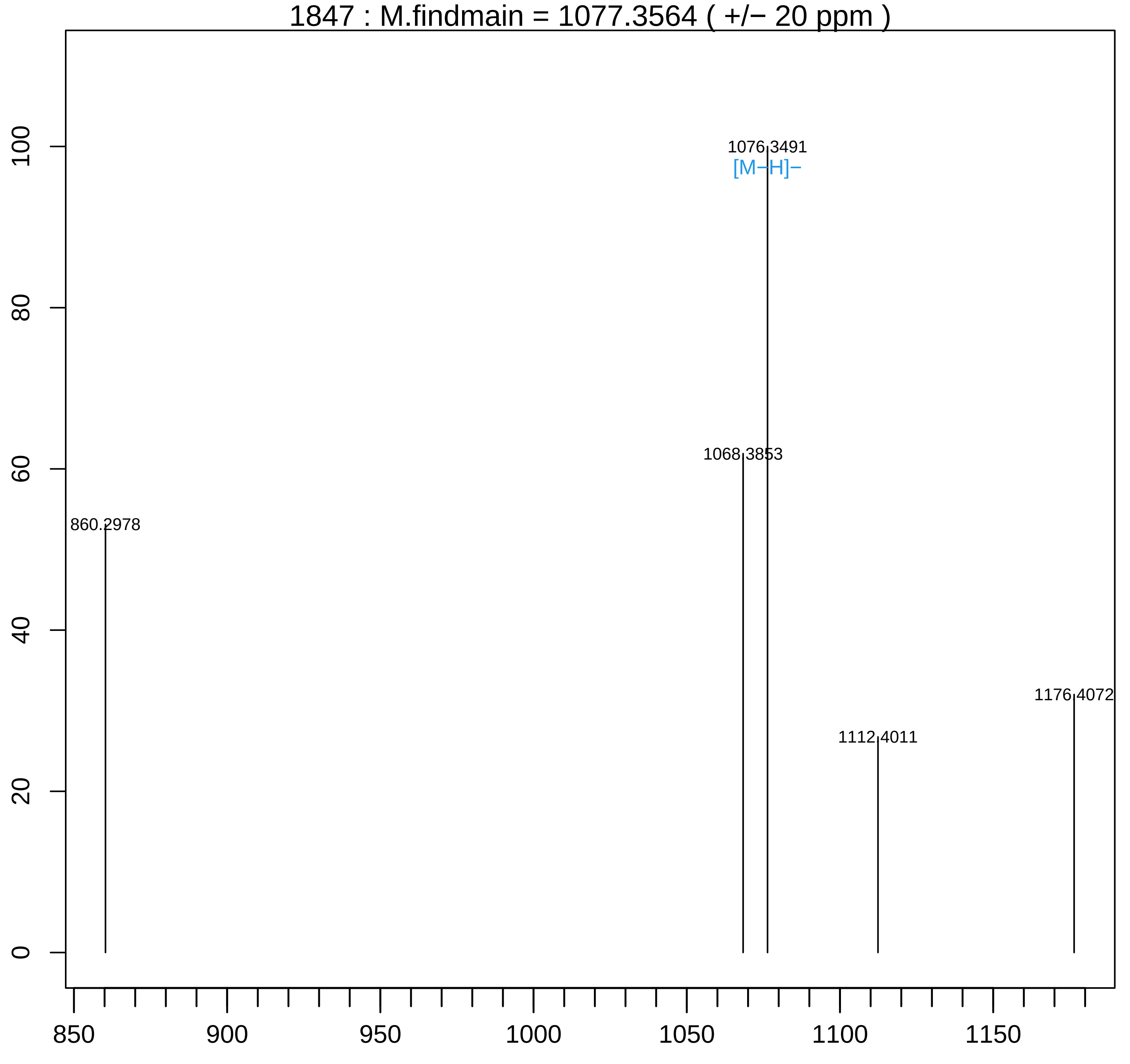

1847 : M.findmain = 1077.3564 (+/− 20 ppm)
1076.3491
[M−H]−
1068.3853
860.2978
1112.4011
1176.4072
0
20
40
60
80
100
850
900
950
1000
1050
1100
1150

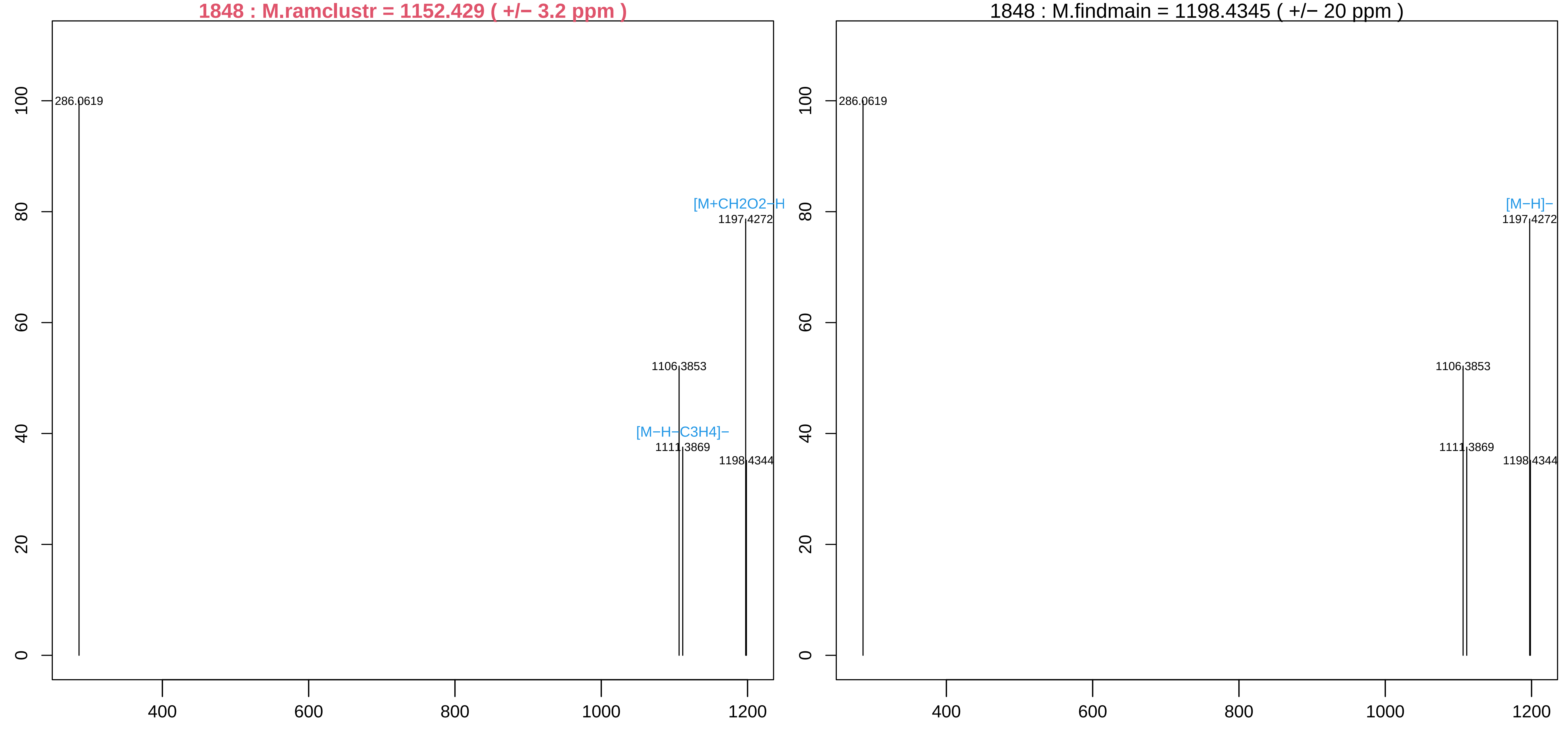

1848 : M.ramclustr = 1152.429 (+/− 3.2 ppm)
286.0619
[M+CH2O2−H
1197.4272
1106.3853
[M−H−C3H4]−
1111.3869
1198.4344
1848 : M.findmain = 1198.4345 (+/− 20 ppm)
286.0619
[M−H]−
1197.4272
1106.3853
1111.3869
1198.4344
0
20
40
60
80
100
400
600
800
1000
1200

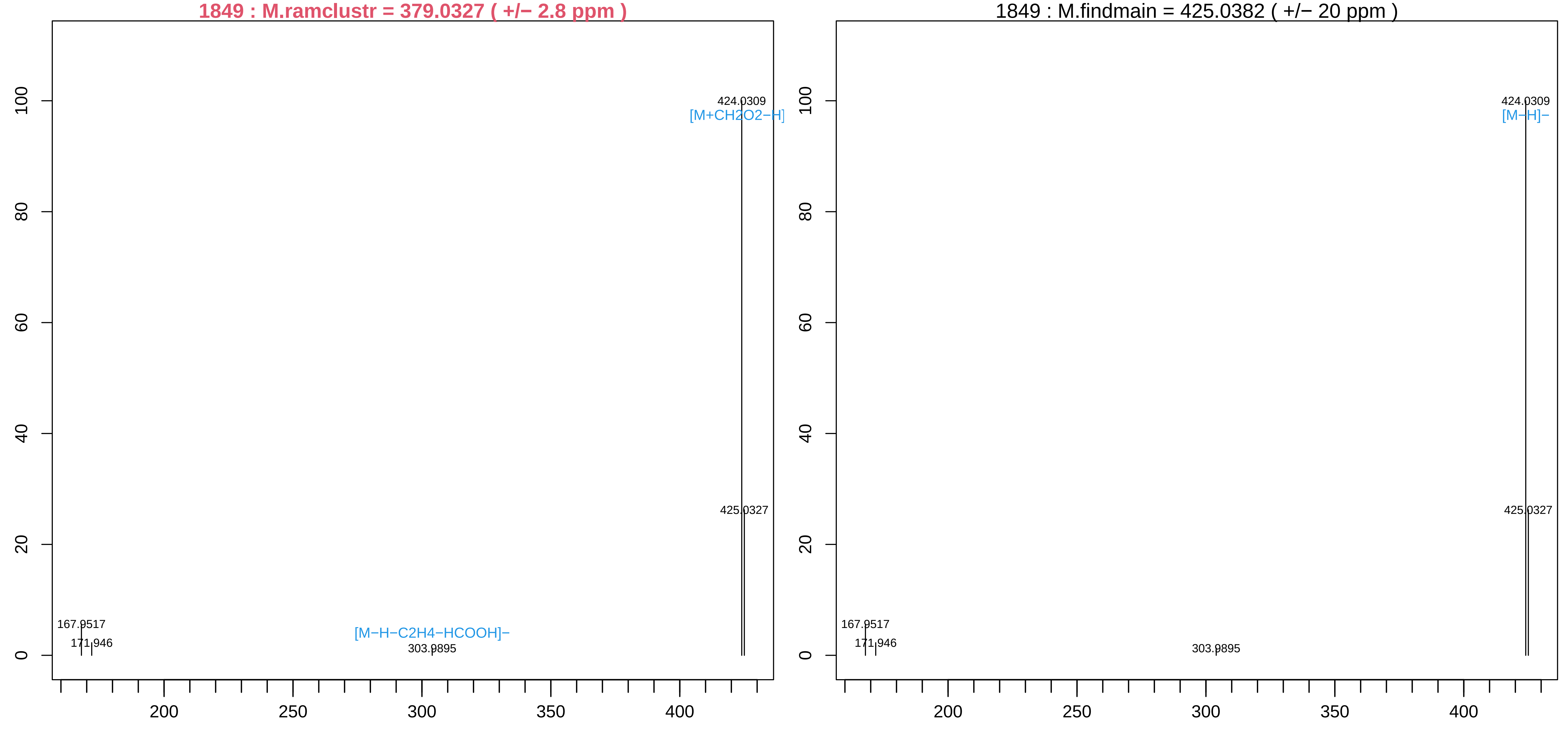

1849 : M.ramclustr = 379.0327 (+/− 2.8 ppm)
424.0309
[M+CH2O2−H]
425.0327
167.9517
171.946
[M−H−C2H4−HCOOH]−
303.9895
0
20
40
60
80
100
200
250
300
350
400
1849 : M.findmain = 425.0382 (+/− 20 ppm)
424.0309
[M−H]−
425.0327
167.9517
171.946
303.9895
0
20
40
60
80
100
200
250
300
350
400

1850 : M.ramclustr = 300.1042 (+/− 0.2 ppm)

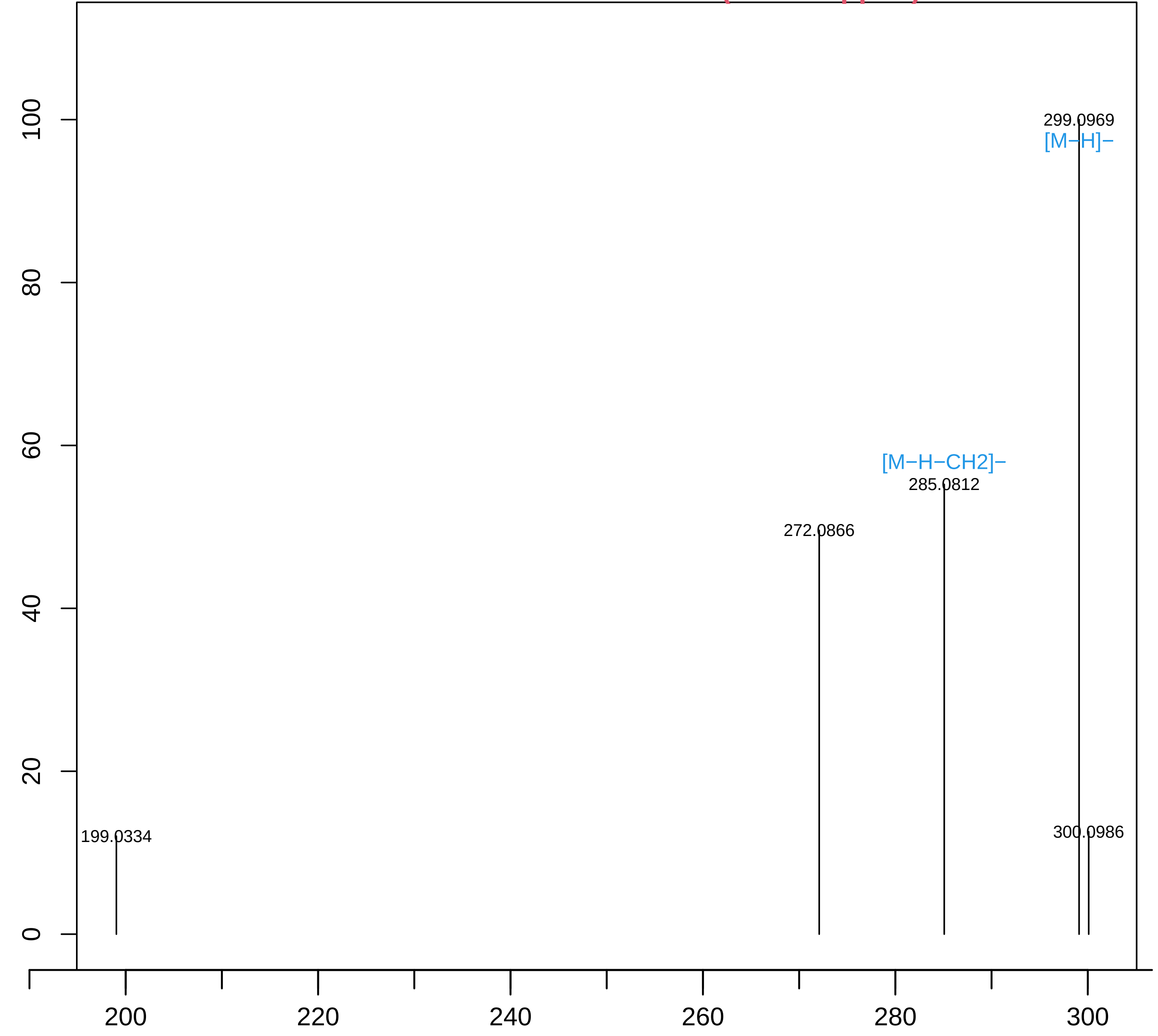

1850 : M.findmain = 300.1042 (+/− 20 ppm)

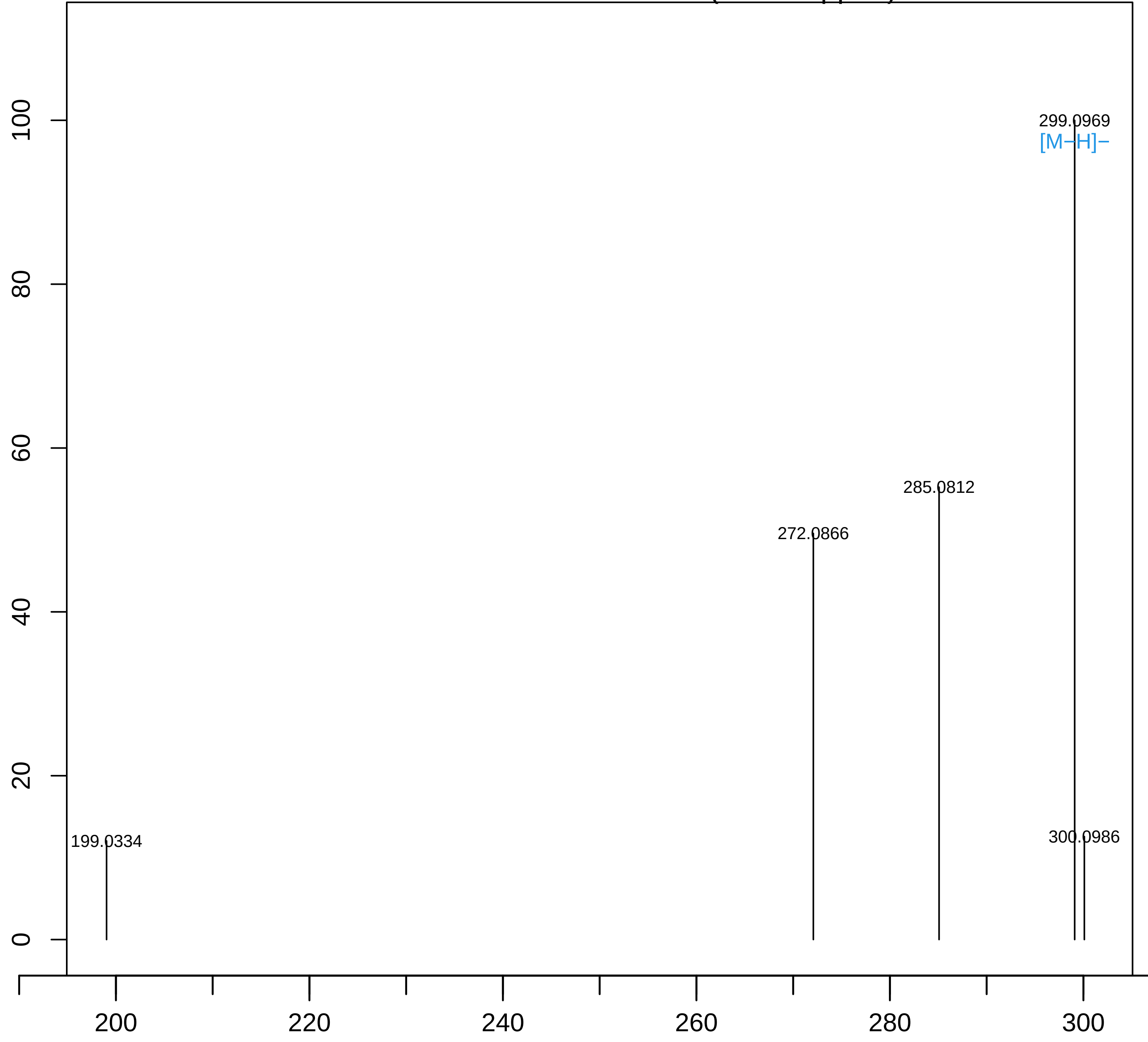

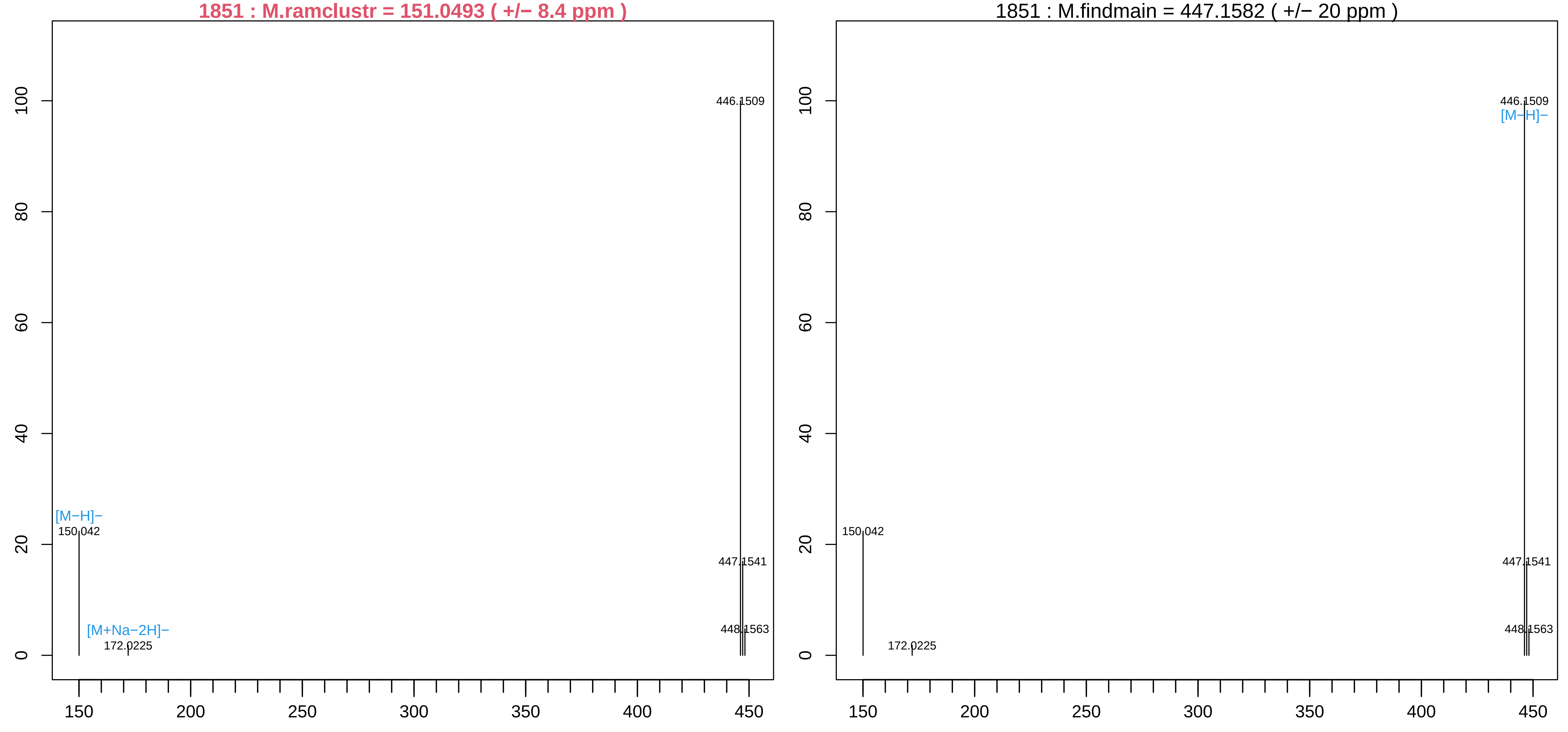
1851 : M.ramclustr = 151.0493 (+/− 8.4 ppm)
[M−H]−
150.042
[M+Na−2H]−
172.0225
446.1509
447.1541
448.1563
1851 : M.findmain = 447.1582 (+/− 20 ppm)
446.1509
[M−H]−
447.1541
448.1563
150.042
172.0225
0
20
40
60
80
100
150
200
250
300
350
400
450

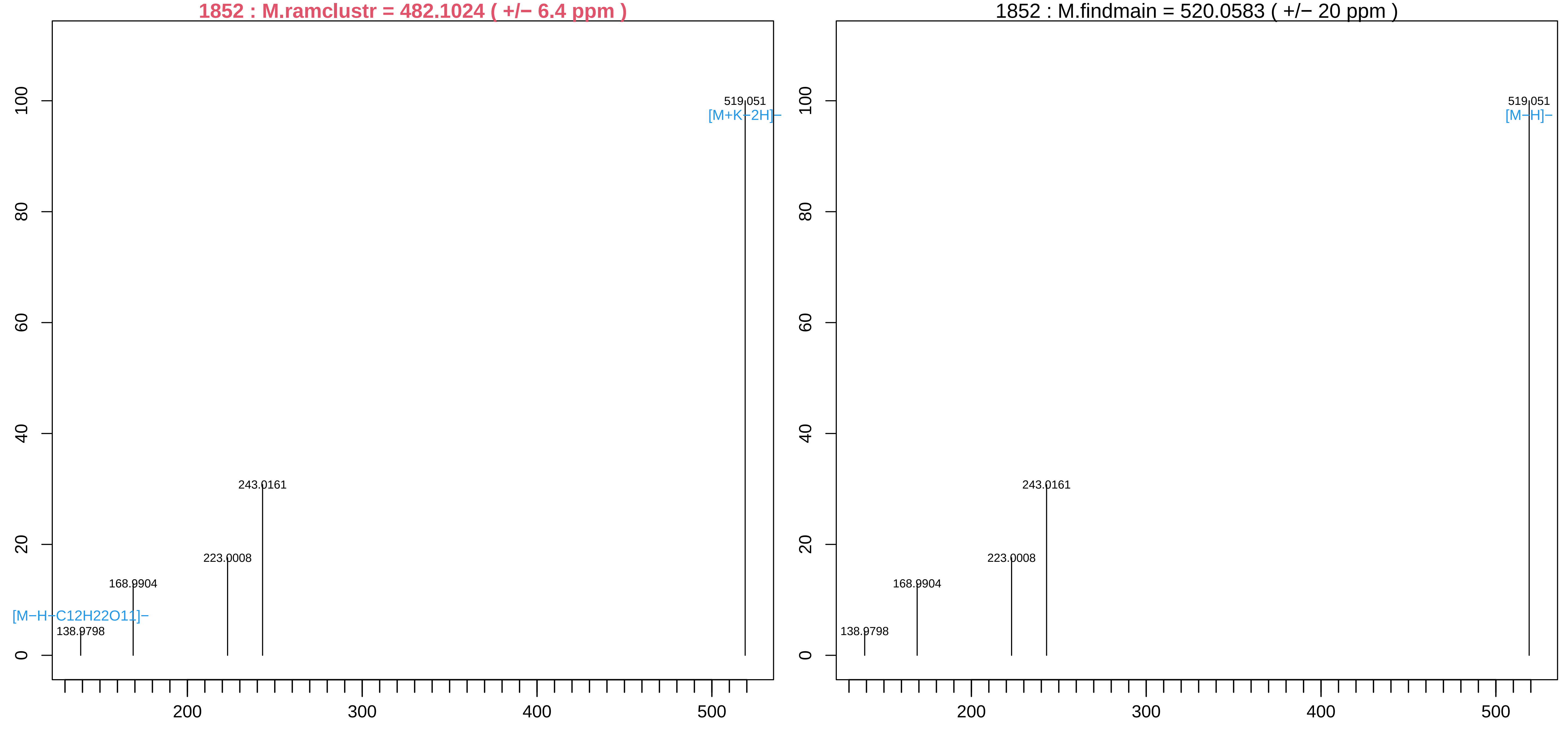
1852 : M.ramclustr = 482.1024 (+/− 6.4 ppm)
519.051
[M+K−2H]−
243.0161
223.0008
168.9904
[M−H−C12H22O11]−
138.9798
1852 : M.findmain = 520.0583 (+/− 20 ppm)
519.051
[M−H]−
243.0161
223.0008
168.9904
138.9798
0
20
40
60
80
100
200
300
400
500

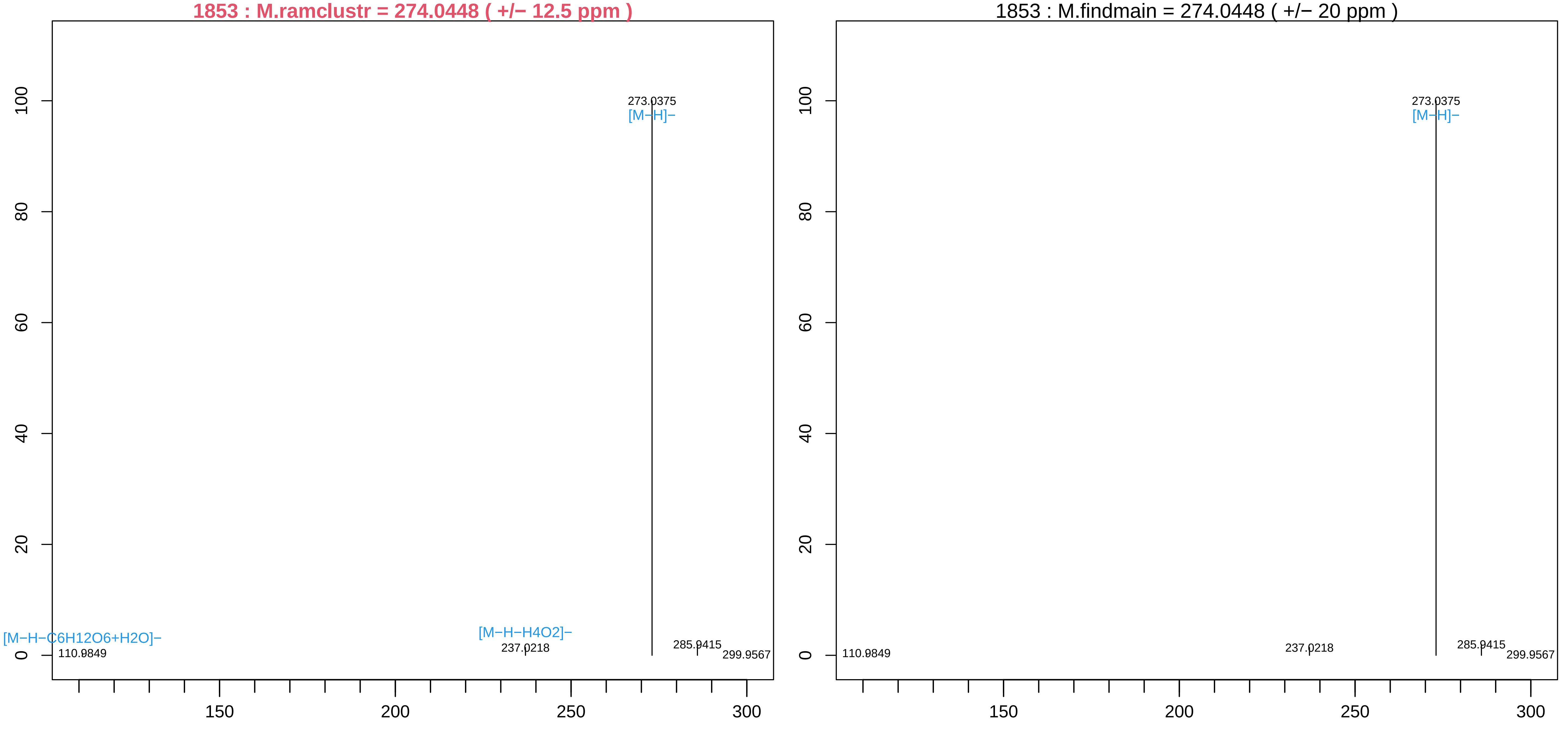

1853 : M.ramclustr = 274.0448 (+/− 12.5 ppm)
273.0375
[M−H]−
[M−H−C6H12O6+H2O]−
110.9849
[M−H−H4O2]−
237.0218
285.9415
299.9567
1853 : M.findmain = 274.0448 (+/− 20 ppm)
273.0375
[M−H]−
110.9849
237.0218
285.9415
299.9567

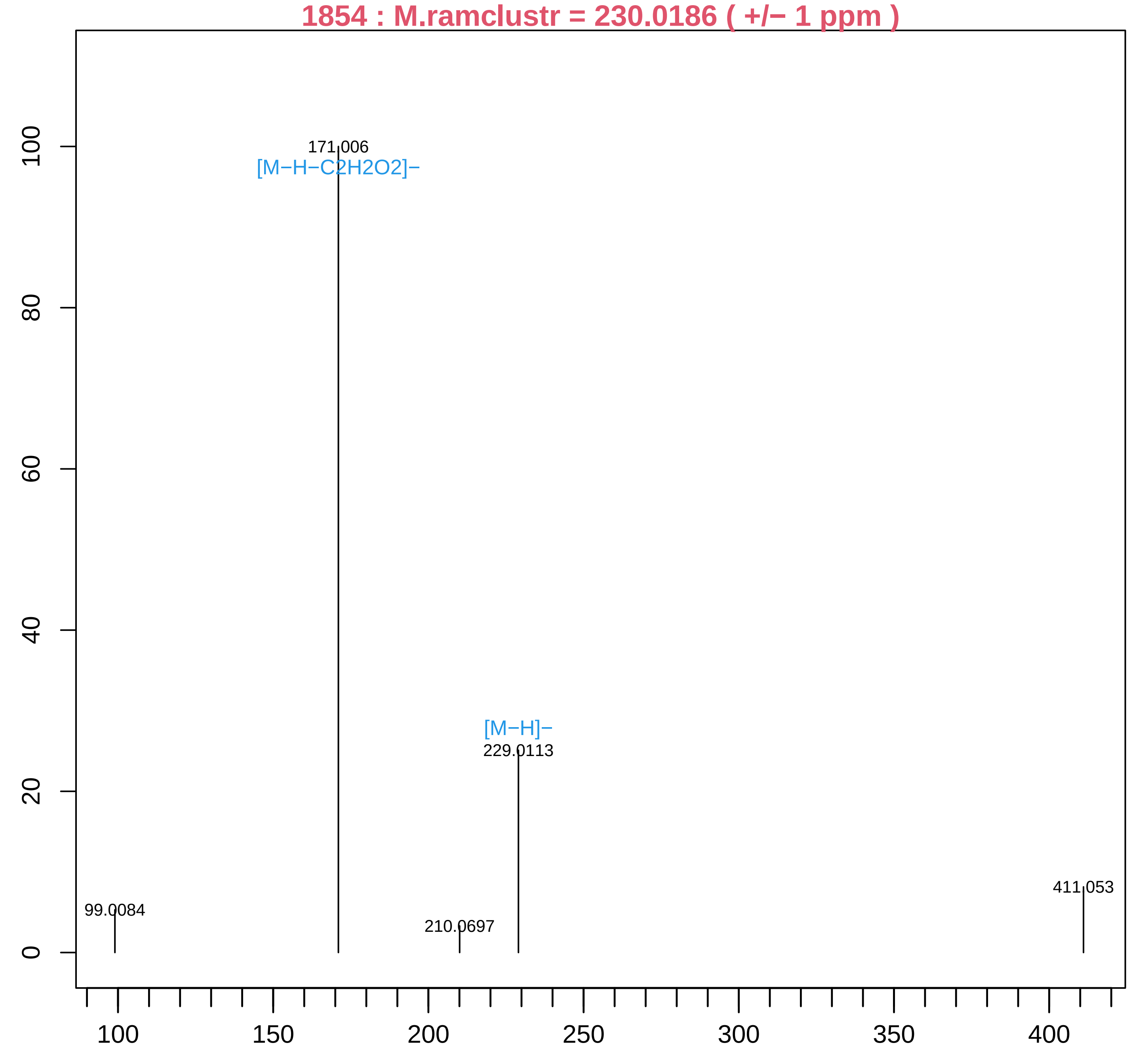
1854 : M.ramclustr = 230.0186 (+/− 1 ppm)
171.006
[M−H−C2H2O2]−
[M−H]−
229.0113
99.0084
210.0697
411.053
0
20
40
60
80
100
100
150
200
250
300
350
400

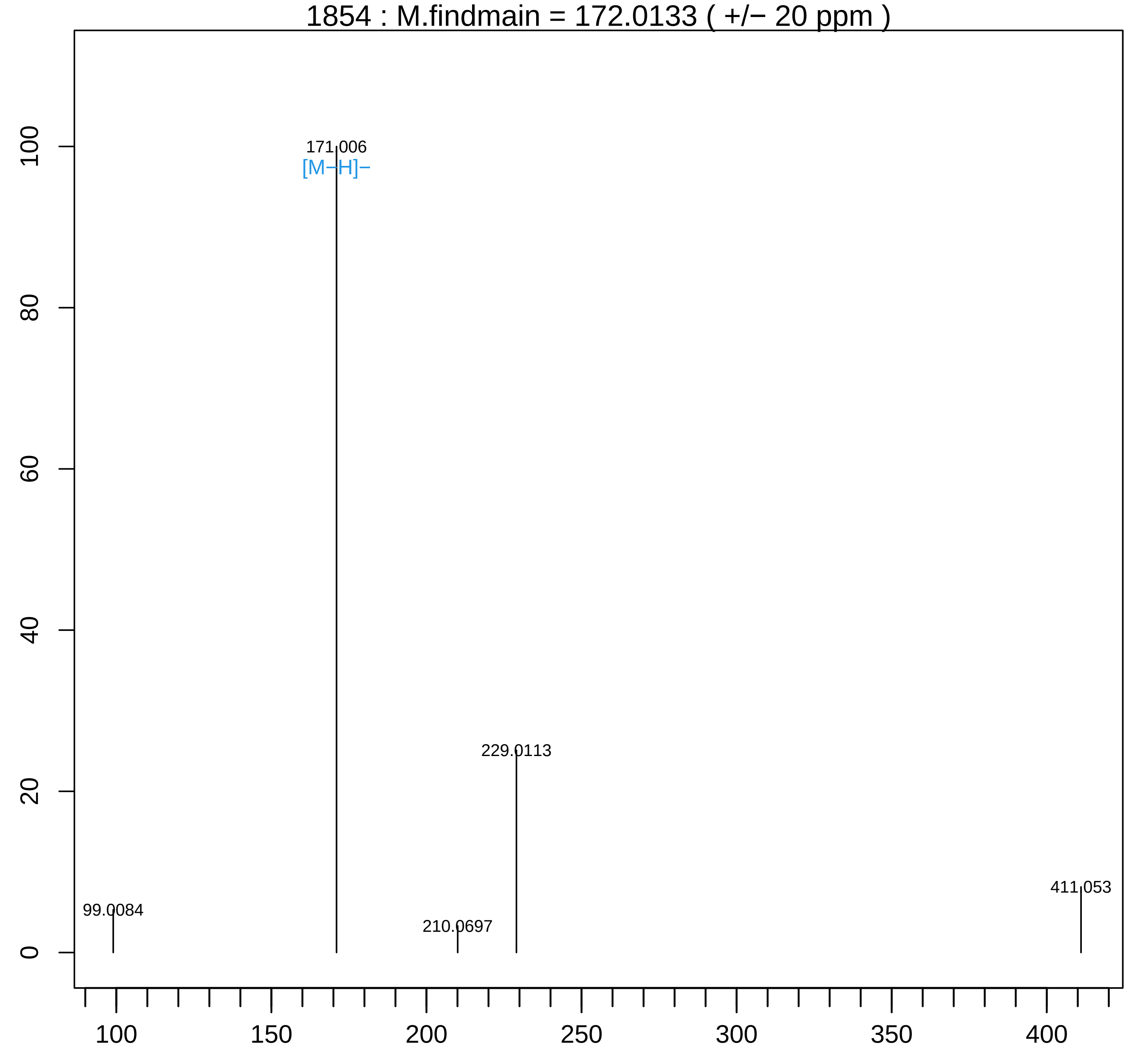
1854 : M.findmain = 172.0133 (+/− 20 ppm)
171.006
[M−H]−
229.0113
99.0084
210.0697
411.053
0
20
40
60
80
100
100
150
200
250
300
350
400

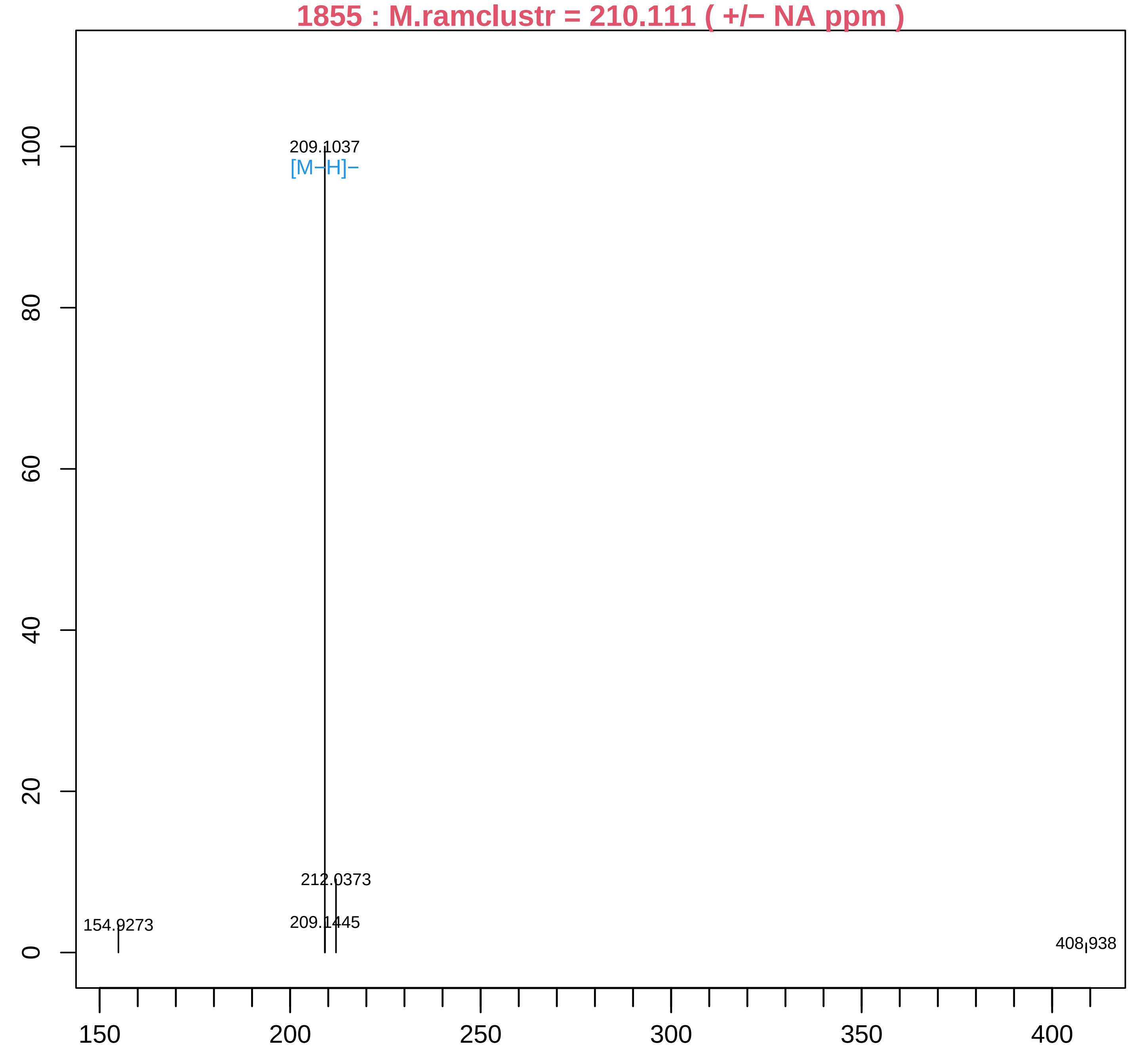
1855 : M.ramclustr = 210.111 (+/− NA ppm)
209.1037
[M−H]−
212.0373
154.9273
209.1445
408.938
0
20
40
60
80
100
150
200
250
300
350
400

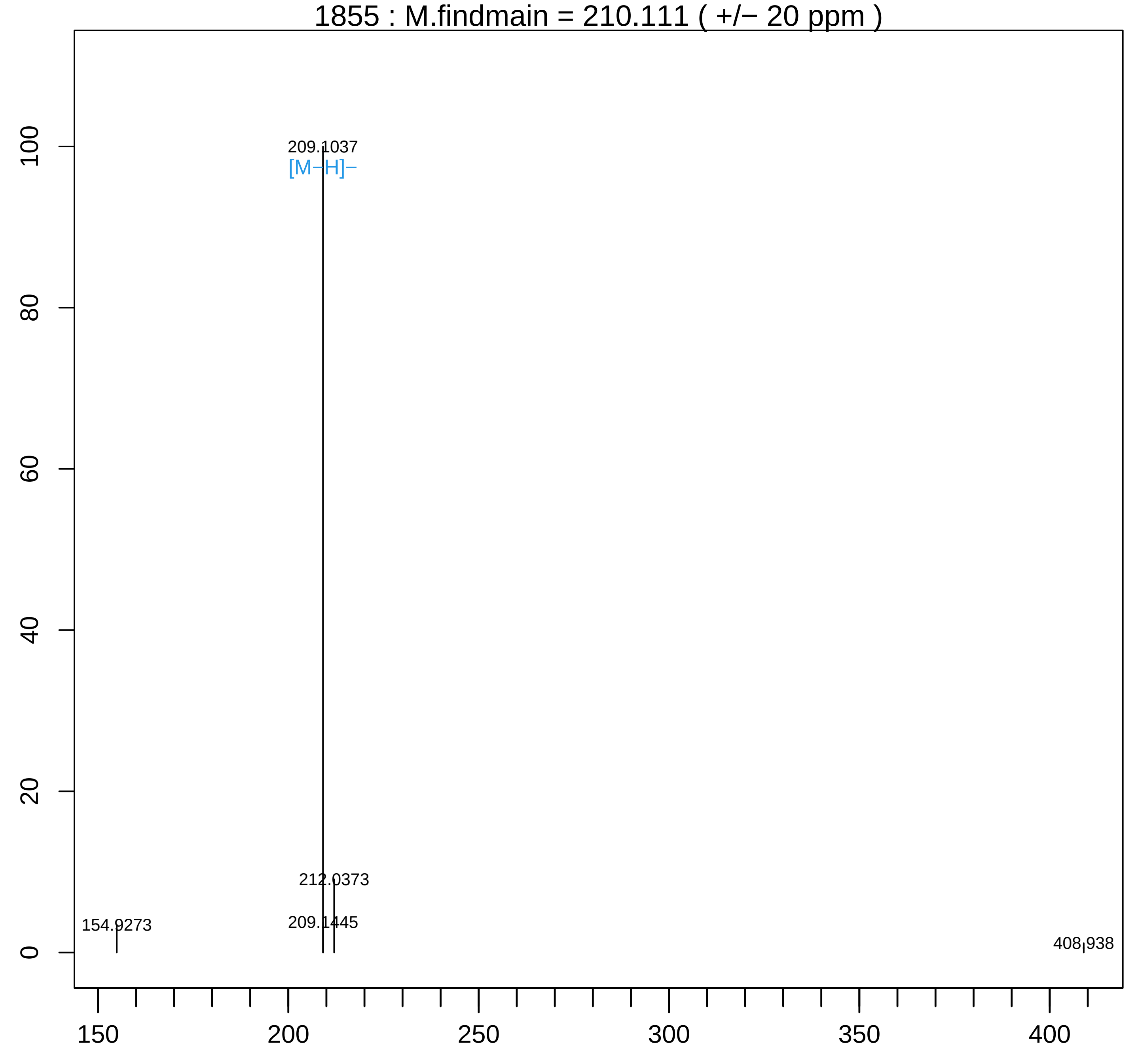
1855 : M.findmain = 210.111 (+/− 20 ppm)
209.1037
[M−H]−
212.0373
154.9273
209.1445
408.938
0
20
40
60
80
100
150
200
250
300
350
400

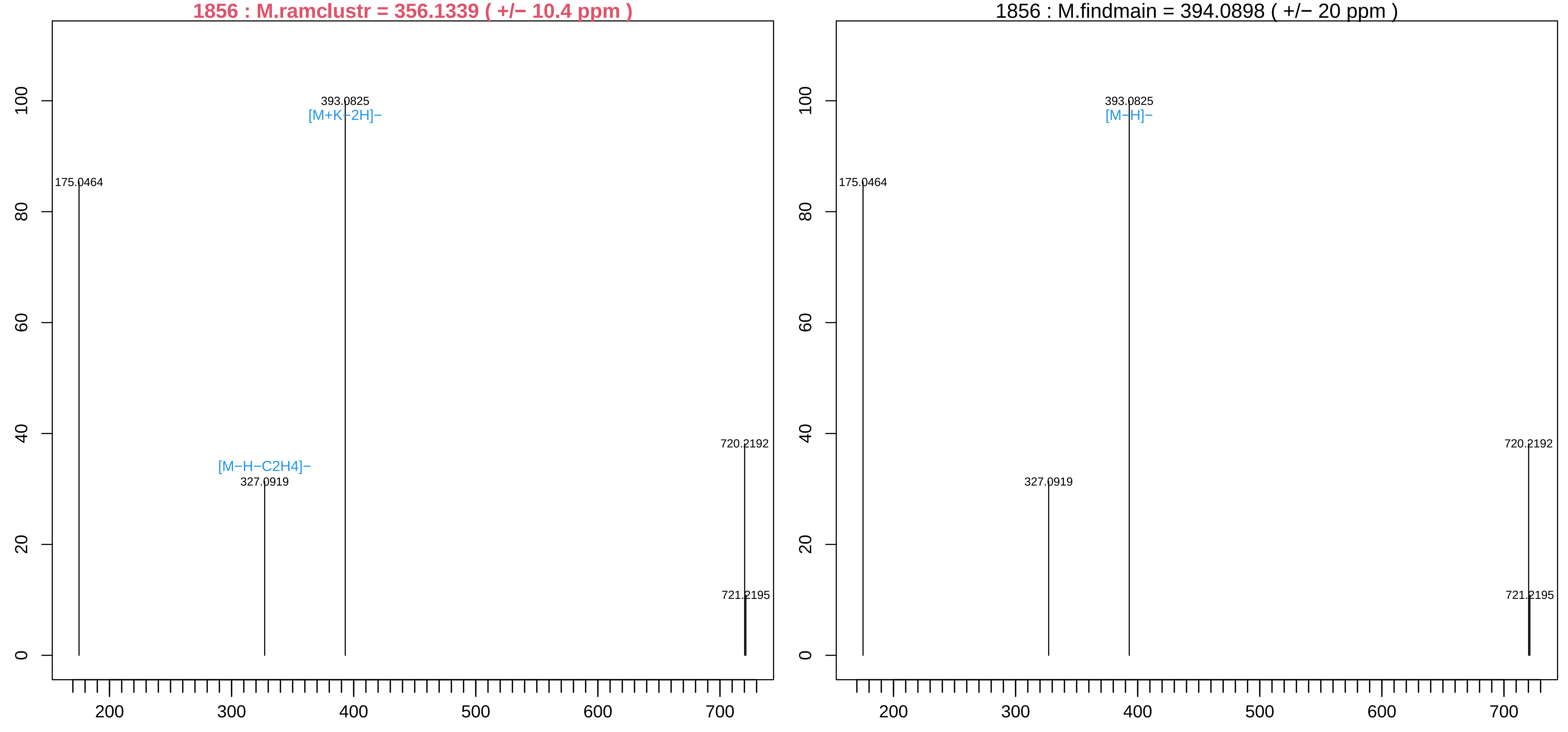
1856 : M.ramclustr = 356.1339 (+/− 10.4 ppm)
393.0825
[M+K−2H]−
175.0464
[M−H−C2H4]−
327.0919
720.2192
721.2195
1856 : M.findmain = 394.0898 (+/− 20 ppm)
393.0825
[M−H]−
175.0464
327.0919
720.2192
721.2195

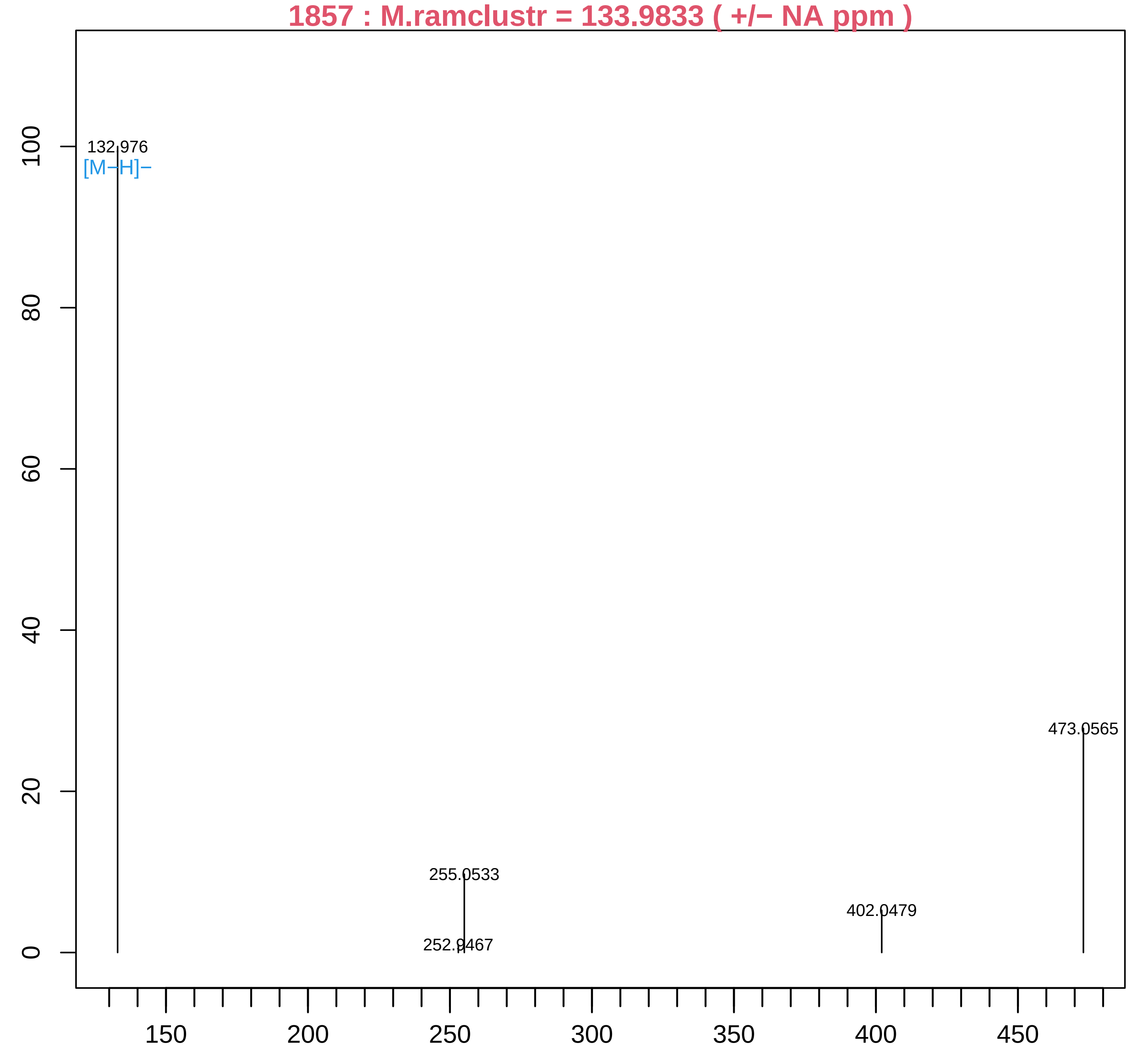
1857 : M.ramclustr = 133.9833 (+/− NA ppm)
132.976
[M−H]−
255.0533
252.9467
402.0479
473.0565
0
20
40
60
80
100
150
200
250
300
350
400
450

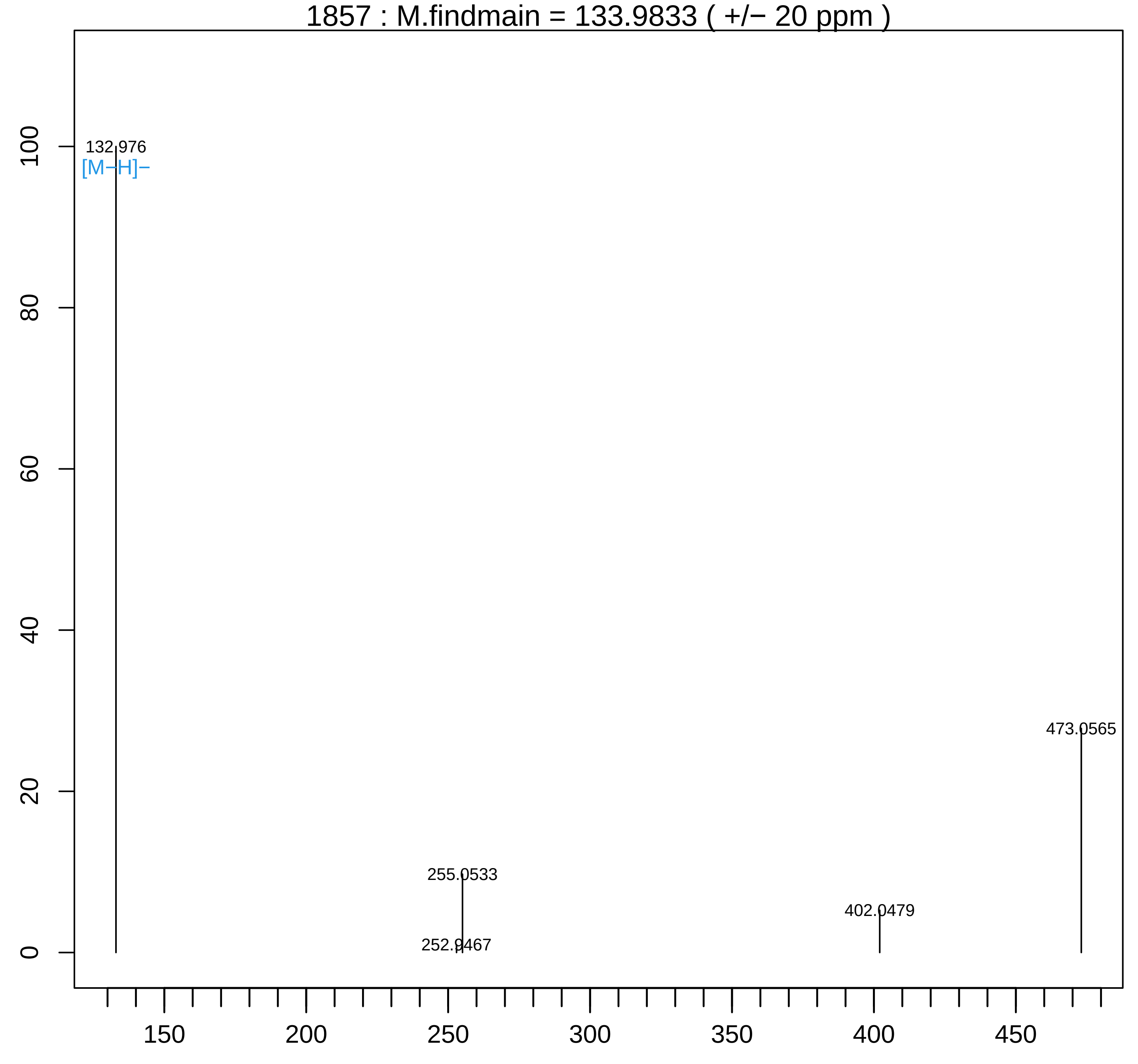
1857 : M.findmain = 133.9833 (+/− 20 ppm)
132.976
[M−H]−
255.0533
252.9467
402.0479
473.0565
0
20
40
60
80
100
150
200
250
300
350
400
450

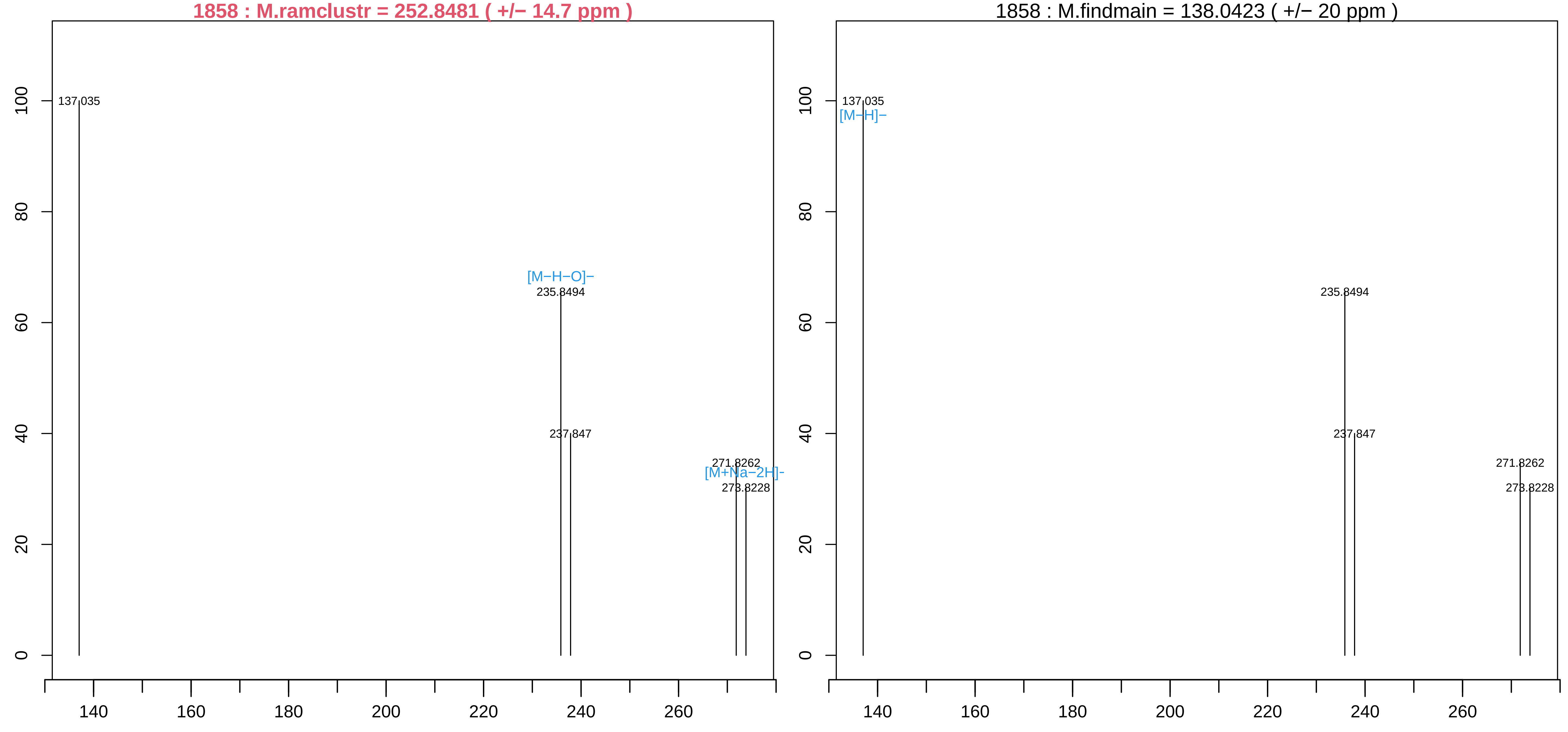
1858 : M.ramclustr = 252.8481 (+/− 14.7 ppm)
137.035
[M−H−O]−
235.8494
237.847
271.8262
[M+Na−2H]-
273.8228
1858 : M.findmain = 138.0423 (+/− 20 ppm)
137.035
[M−H]−
235.8494
237.847
271.8262
273.8228
0
20
40
60
80
100
140
160
180
200
220
240
260

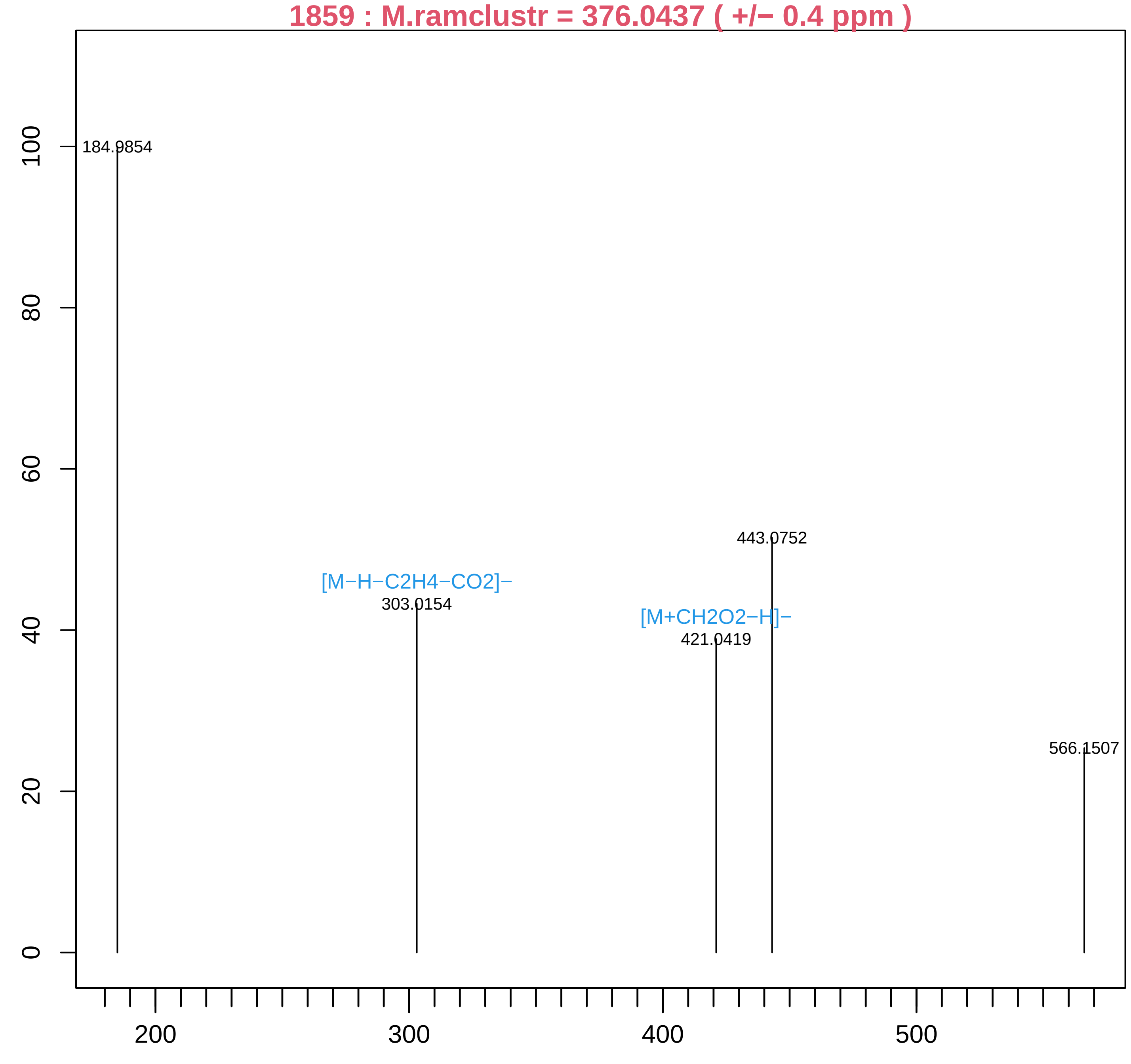
1859 : M.ramclustr = 376.0437 (+/− 0.4 ppm)
184.9854
[M−H−C2H4−CO2]−
303.0154
[M+CH2O2−H]−
421.0419
443.0752
566.1507
0
20
40
60
80
100
200
300
400
500

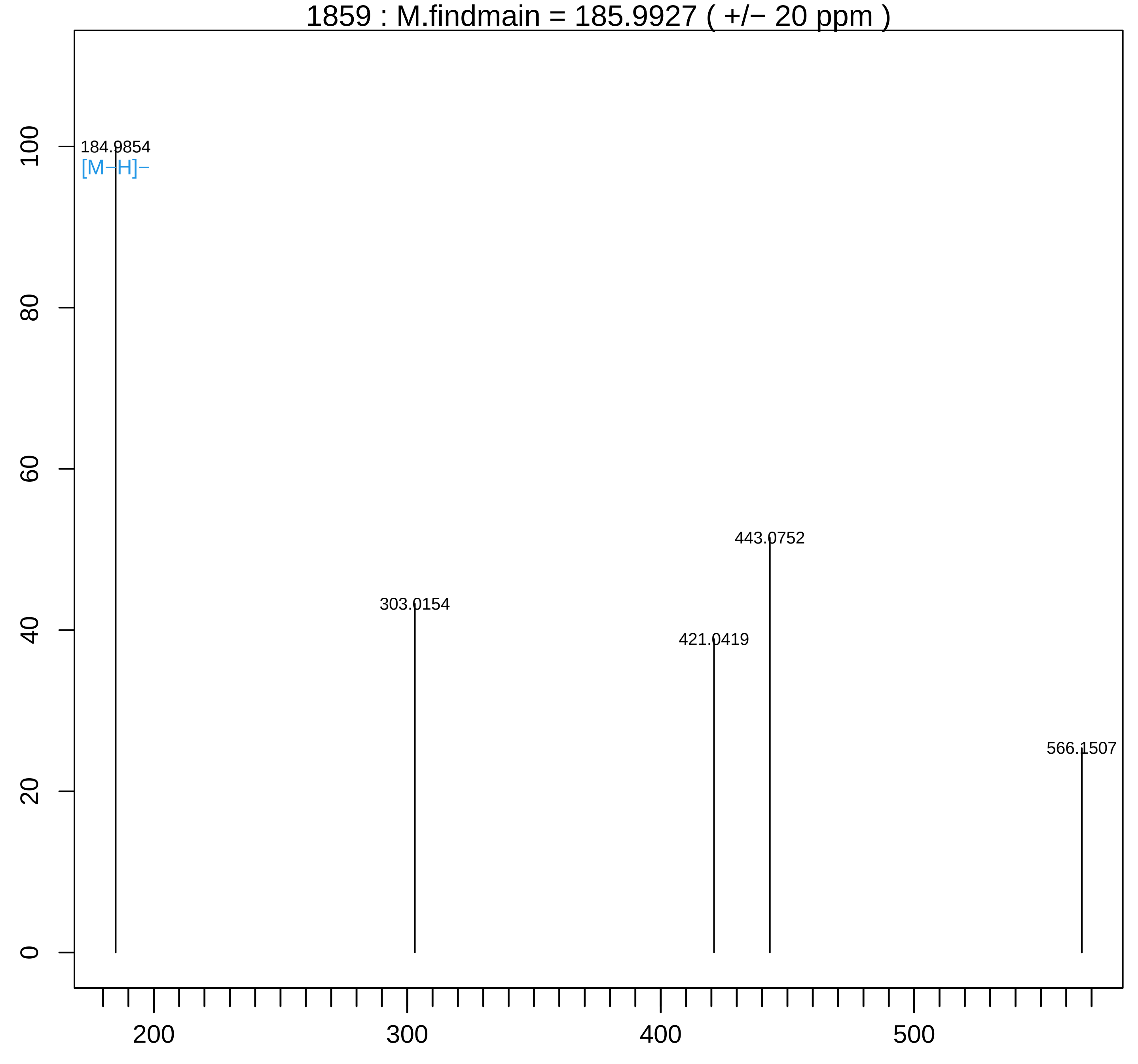
1859 : M.findmain = 185.9927 (+/− 20 ppm)
184.9854
[M−H]−
303.0154
421.0419
443.0752
566.1507
0
20
40
60
80
100
200
300
400
500

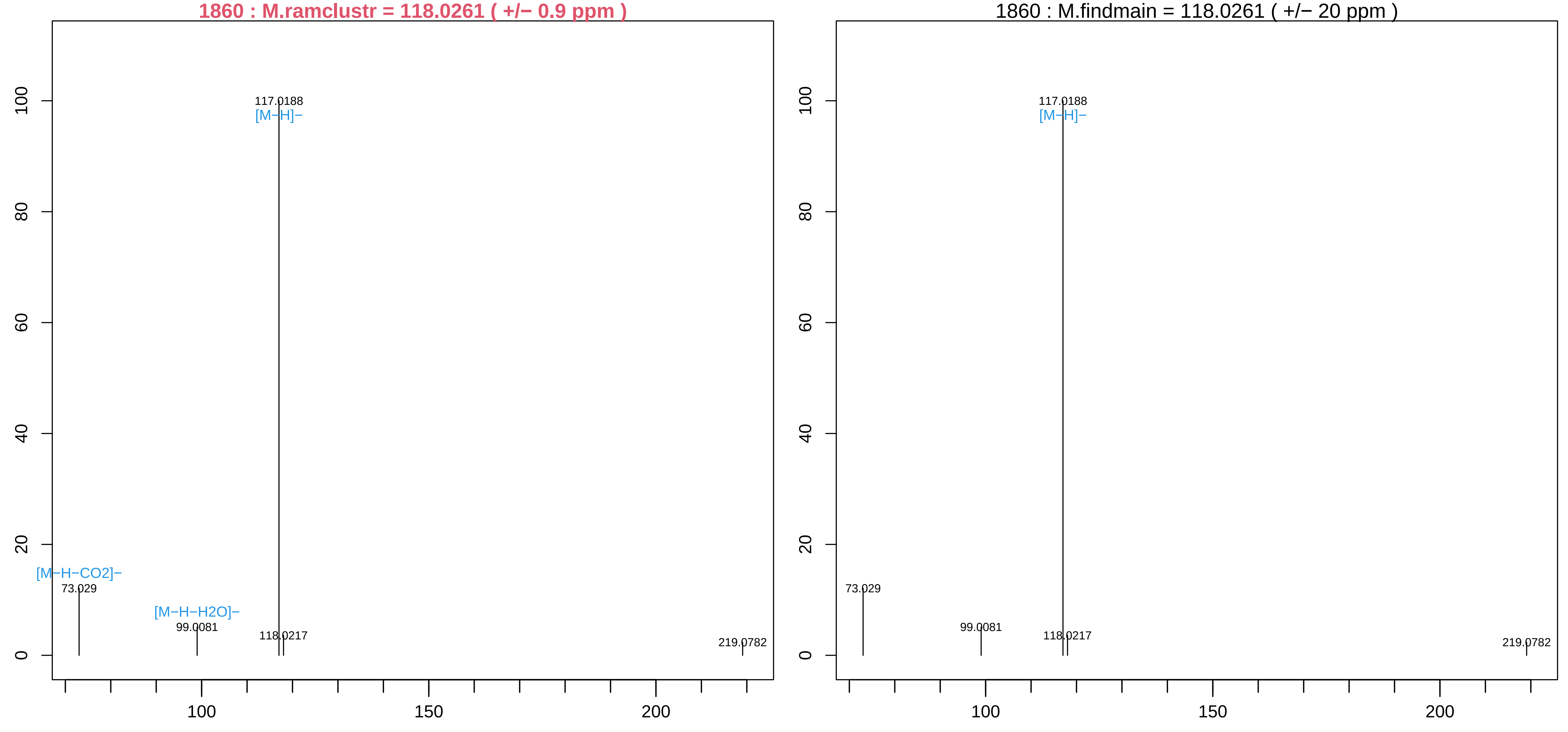
1860 : M.ramclustr = 118.0261 (+/− 0.9 ppm)
117.0188
[M−H]−
[M−H−CO2]−
73.029
[M−H−H2O]−
99.0081
118.0217
219.0782
0
20
40
60
80
100
100
150
200
1860 : M.findmain = 118.0261 (+/− 20 ppm)
117.0188
[M−H]−
73.029
99.0081
118.0217
219.0782
0
20
40
60
80
100
100
150
200

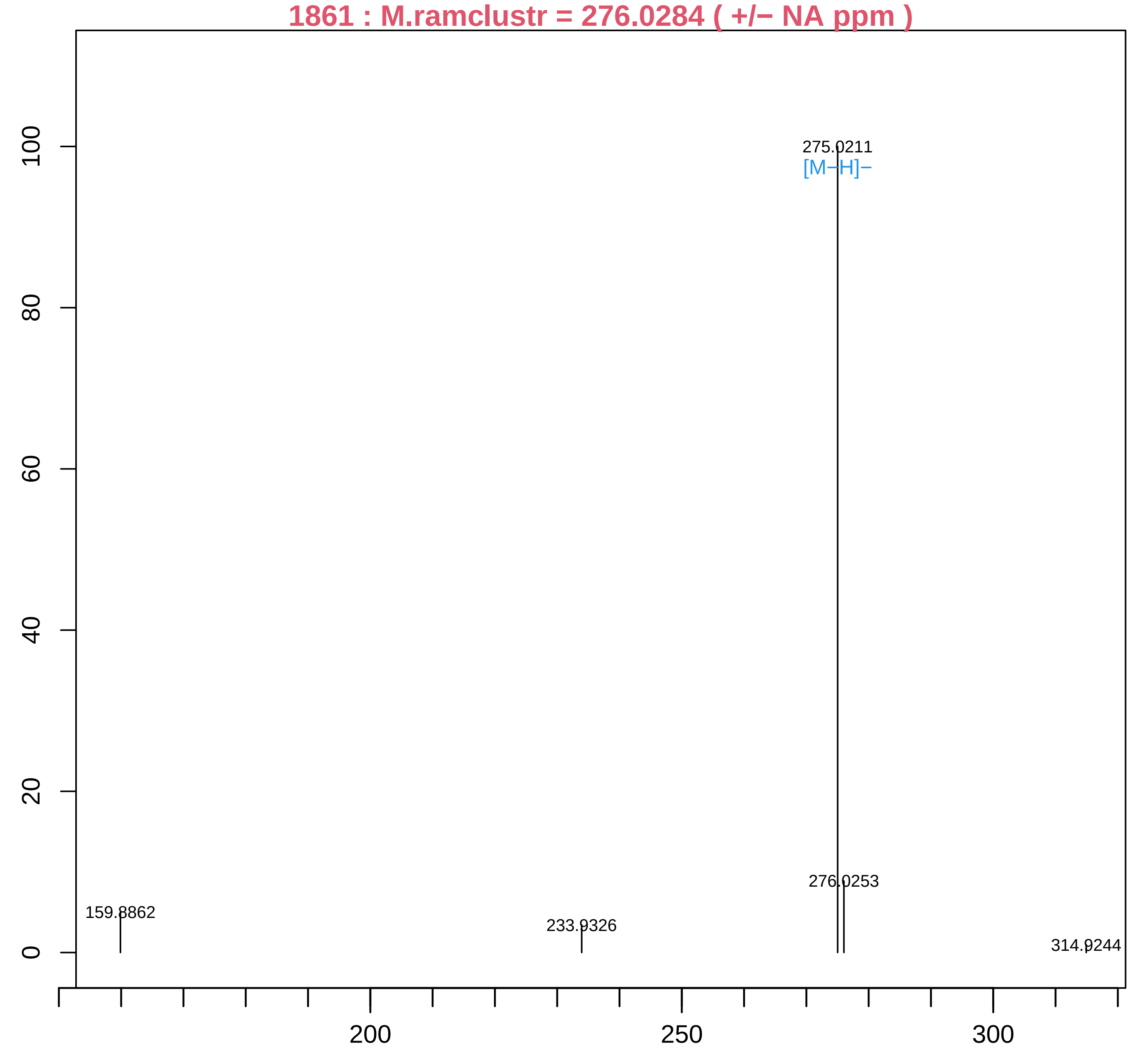
1861 : M.ramclustr = 276.0284 (+/− NA ppm)
275.0211
[M−H]−
276.0253
159.8862
233.9326
314.9244
0
20
40
60
80
100
200
250
300

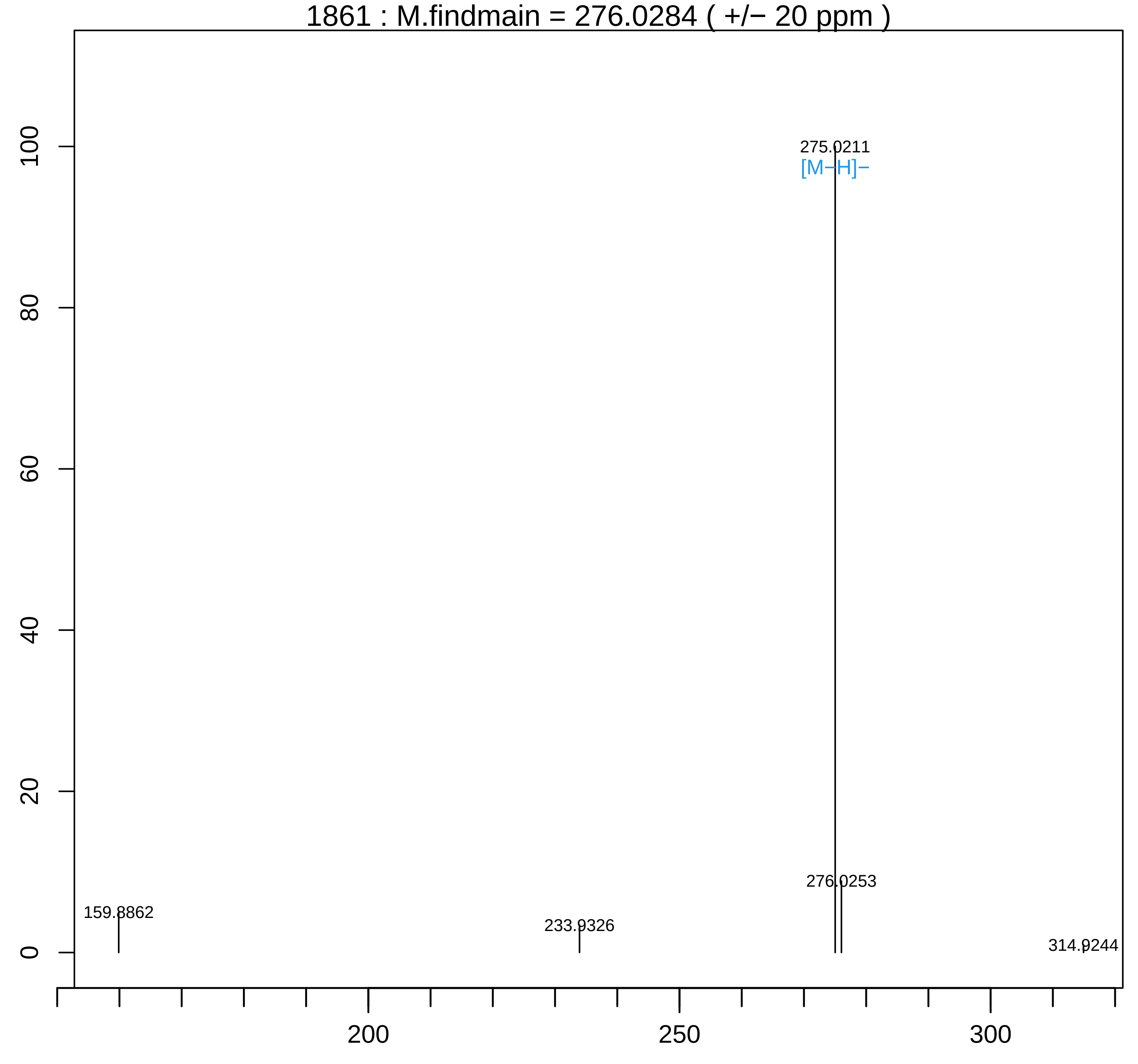
1861 : M.findmain = 276.0284 (+/− 20 ppm)
275.0211
[M−H]−
276.0253
159.8862
233.9326
314.9244
0
20
40
60
80
100
200
250
300

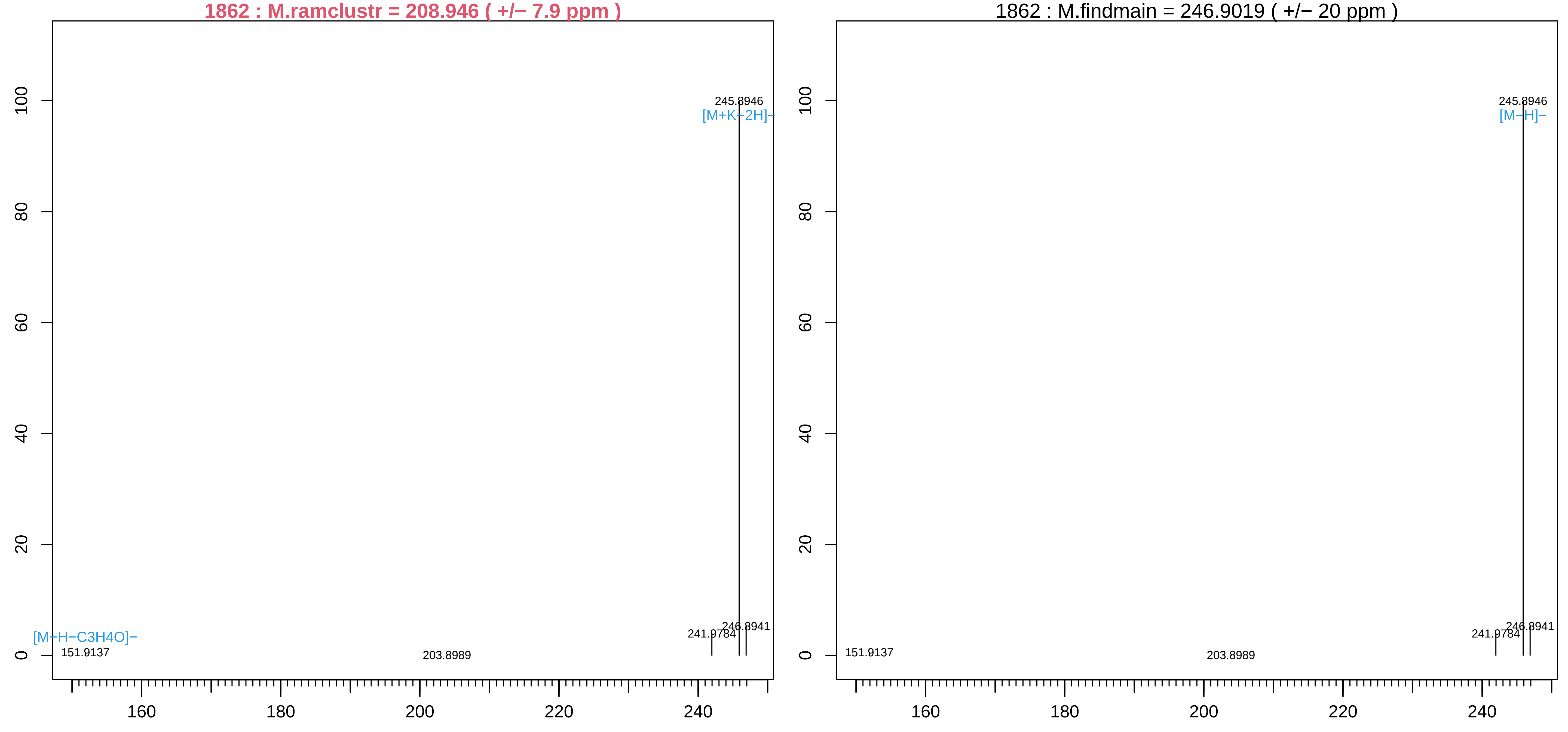
1862 : M.ramclustr = 208.946 (+/− 7.9 ppm)
245.8946
[M+K−2H]−
[M−H−C3H4O]−
151.9137
203.8989
241.9784
246.8941
1862 : M.findmain = 246.9019 (+/− 20 ppm)
245.8946
[M−H]−
151.9137
203.8989
241.9784
246.8941
0
20
40
60
80
100
160
180
200
220
240

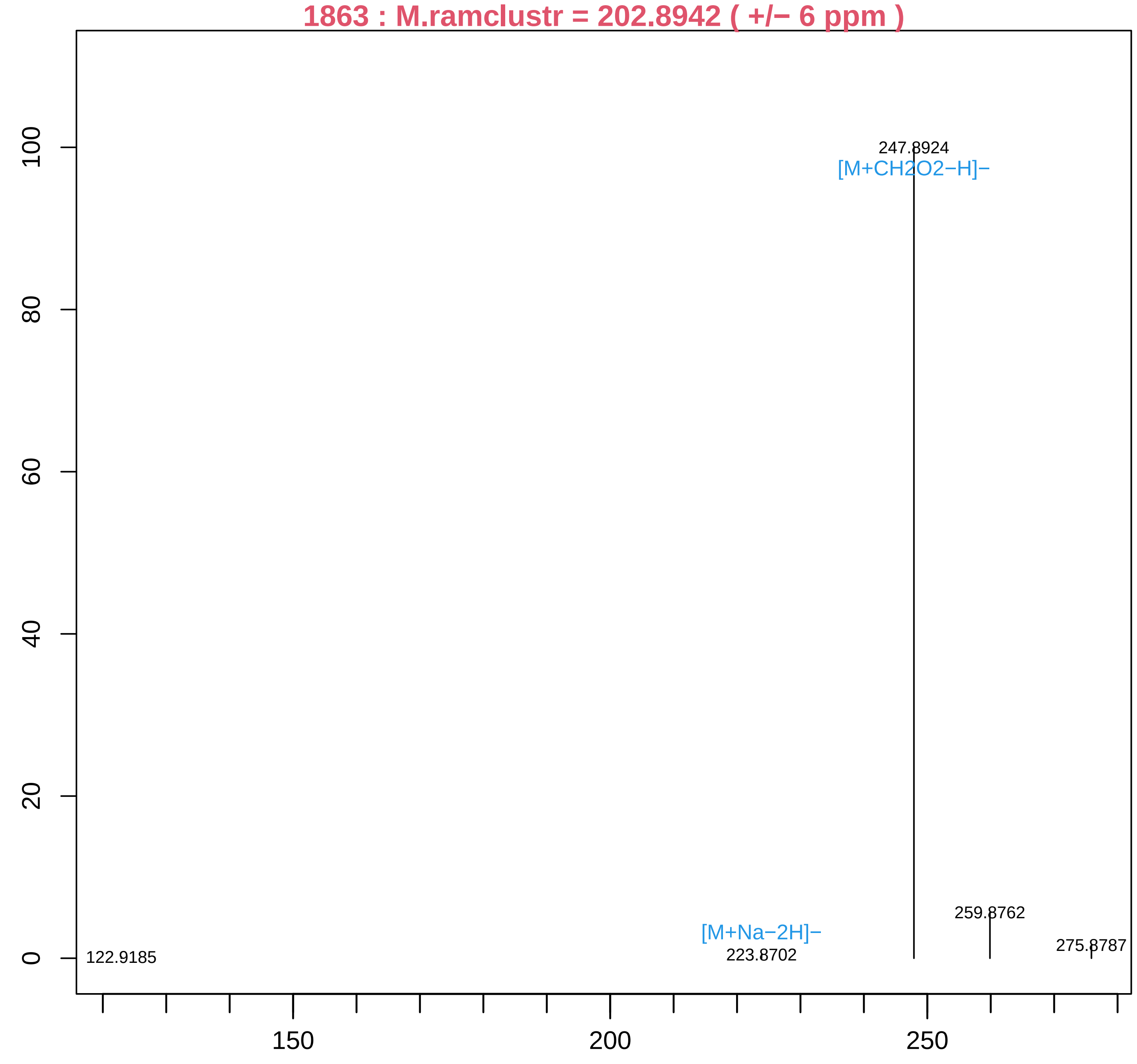
1863 : M.ramclustr = 202.8942 (+/− 6 ppm)
247.8924
[M+CH2O2−H]−
259.8762
275.8787
[M+Na−2H]−
223.8702
122.9185
0
20
40
60
80
100
150
200
250

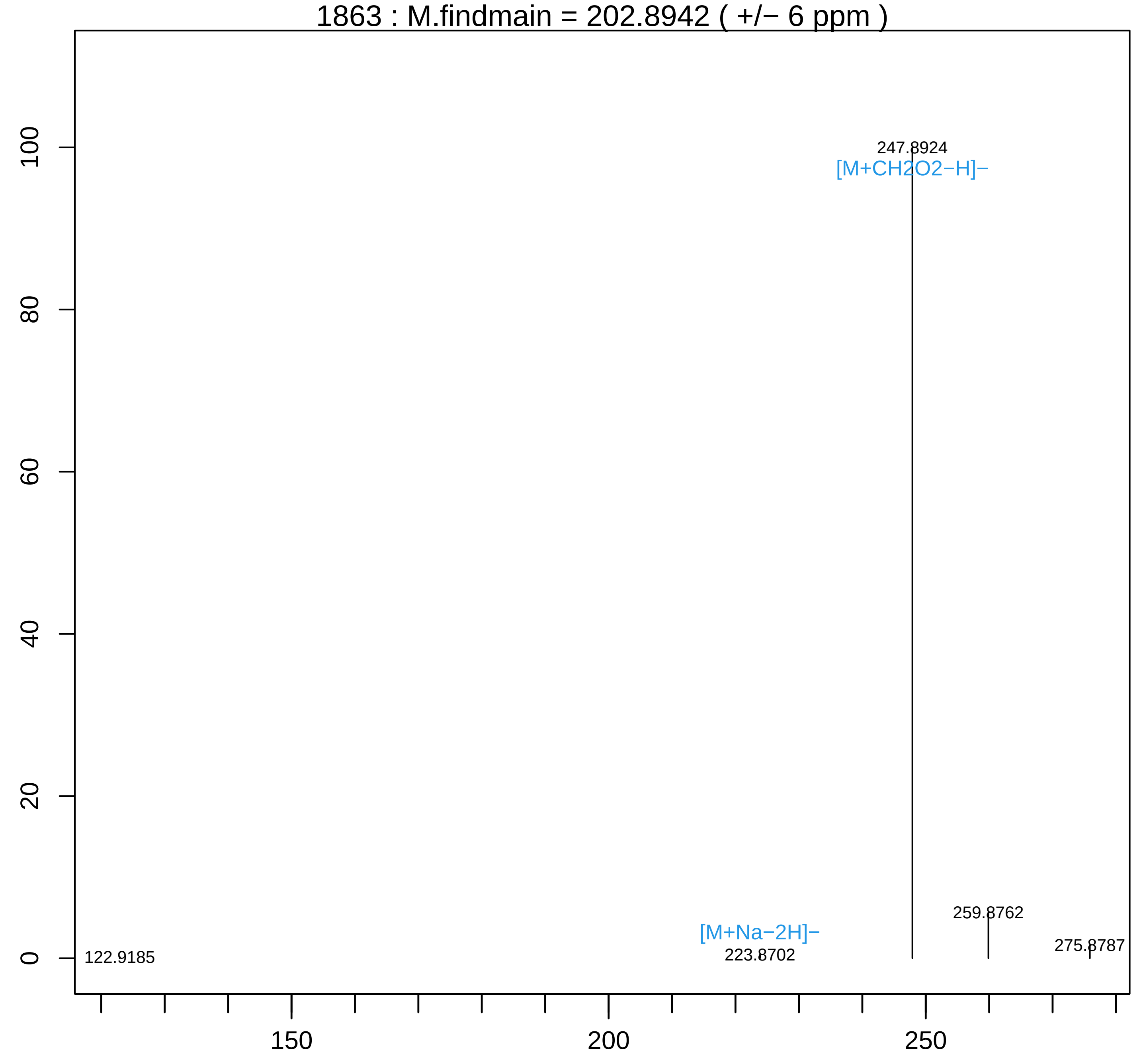
1863 : M.findmain = 202.8942 (+/− 6 ppm)
247.8924
[M+CH2O2−H]−
259.8762
275.8787
[M+Na−2H]−
223.8702
122.9185
0
20
40
60
80
100
150
200
250

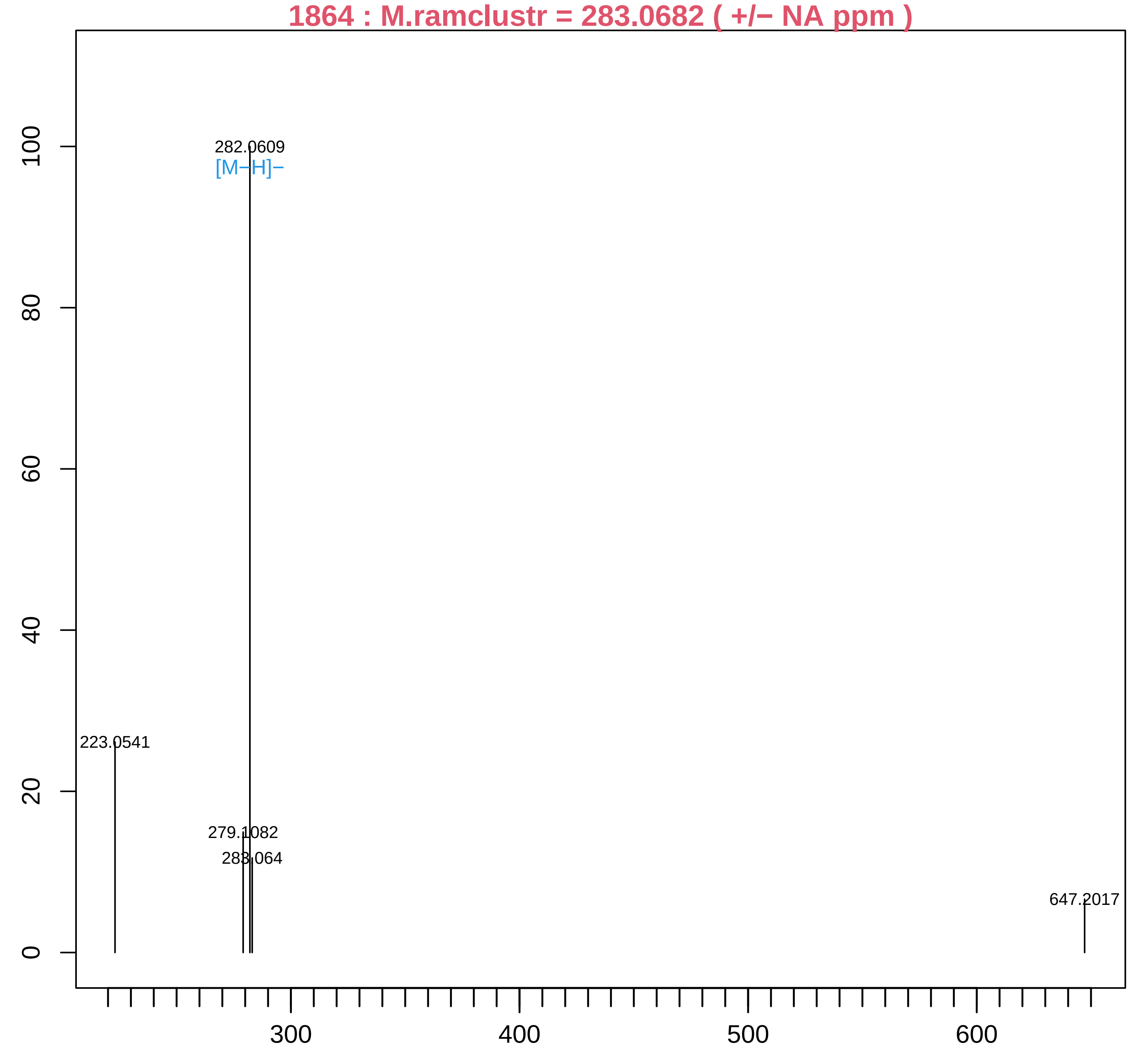
1864 : M.ramclustr = 283.0682 (+/− NA ppm)
282.0609
[M−H]−
223.0541
279.1082
283.064
647.2017
0
20
40
60
80
100
300
400
500
600

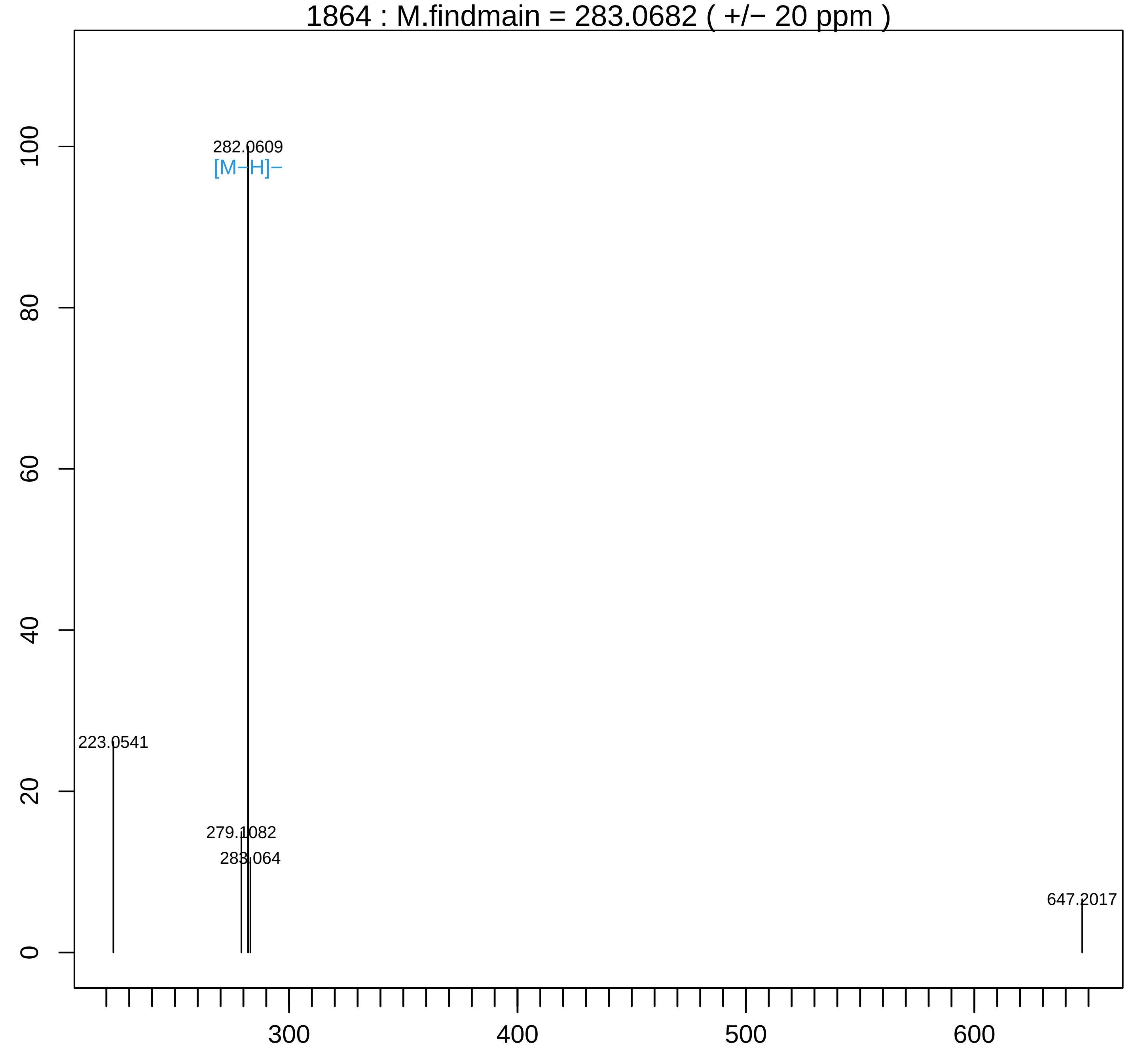
1864 : M.findmain = 283.0682 (+/− 20 ppm)
282.0609
[M−H]−
223.0541
279.1082
283.064
647.2017
0
20
40
60
80
100
300
400
500
600

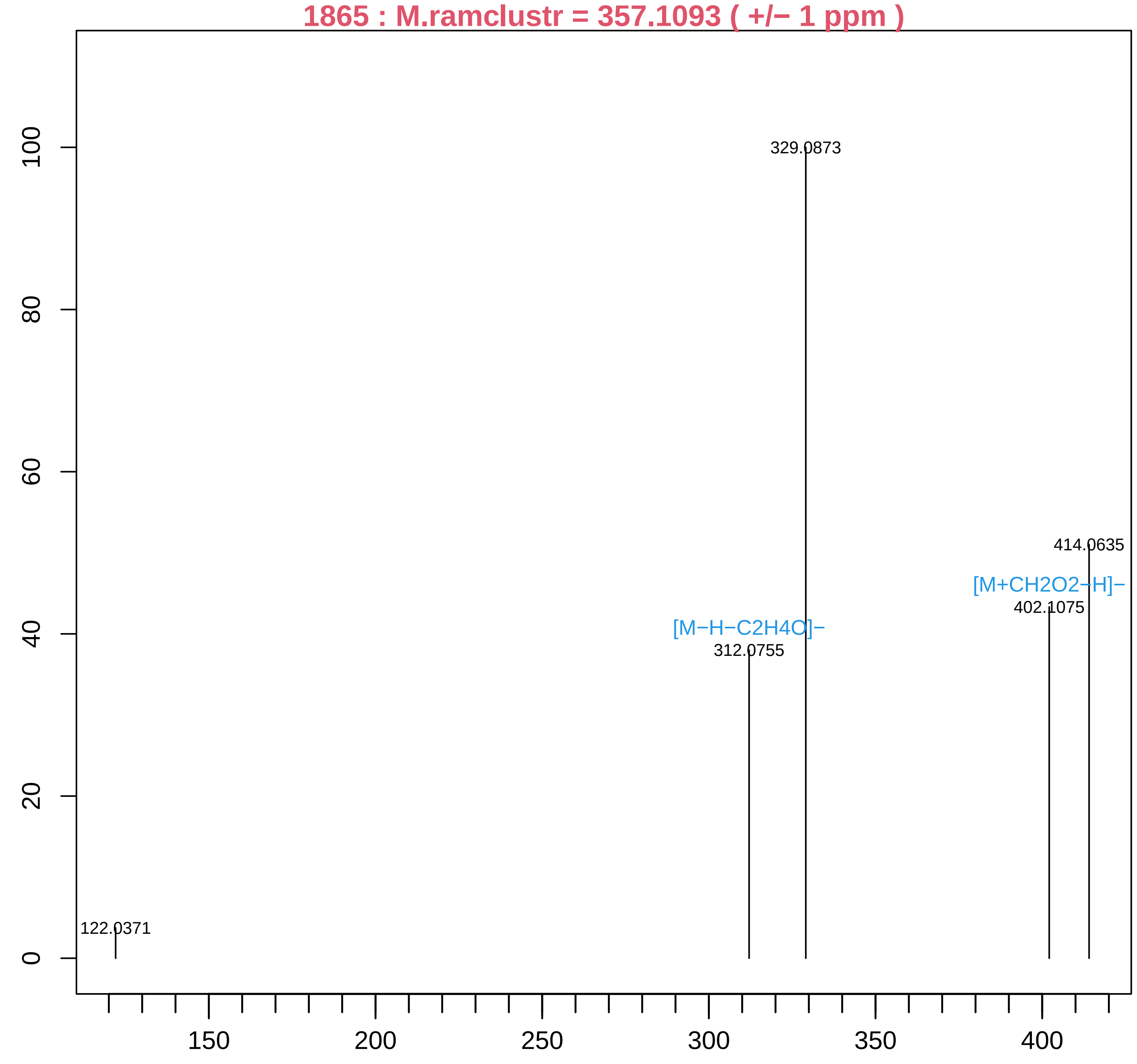
1865 : M.ramclustr = 357.1093 (+/− 1 ppm)
329.0873
414.0635
[M+CH2O2−H]−
402.1075
[M−H−C2H4O]−
312.0755
122.0371
0
20
40
60
80
100
150
200
250
300
350
400

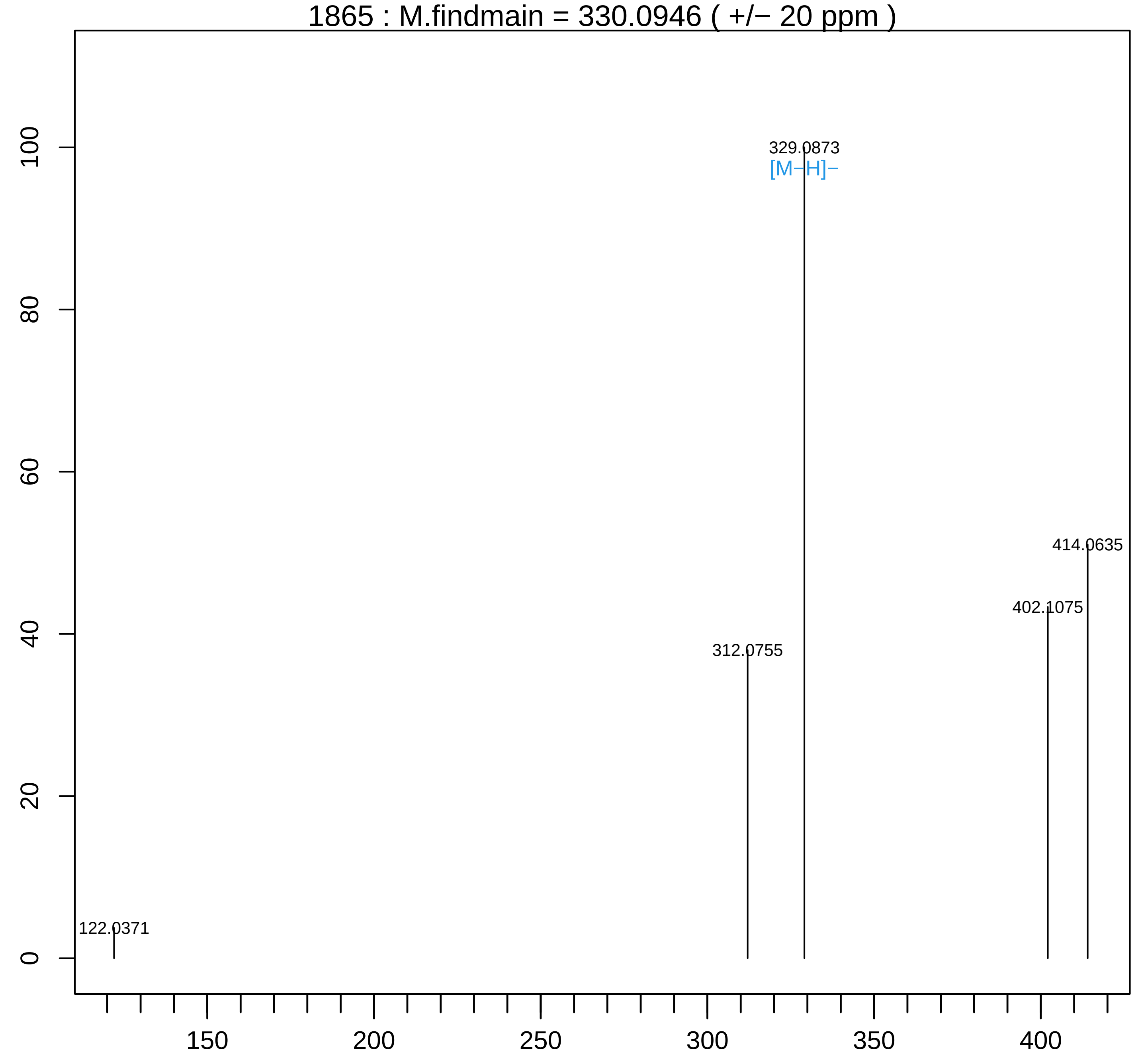
1865 : M.findmain = 330.0946 (+/− 20 ppm)
329.0873
[M−H]−
414.0635
402.1075
312.0755
122.0371
0
20
40
60
80
100
150
200
250
300
350
400

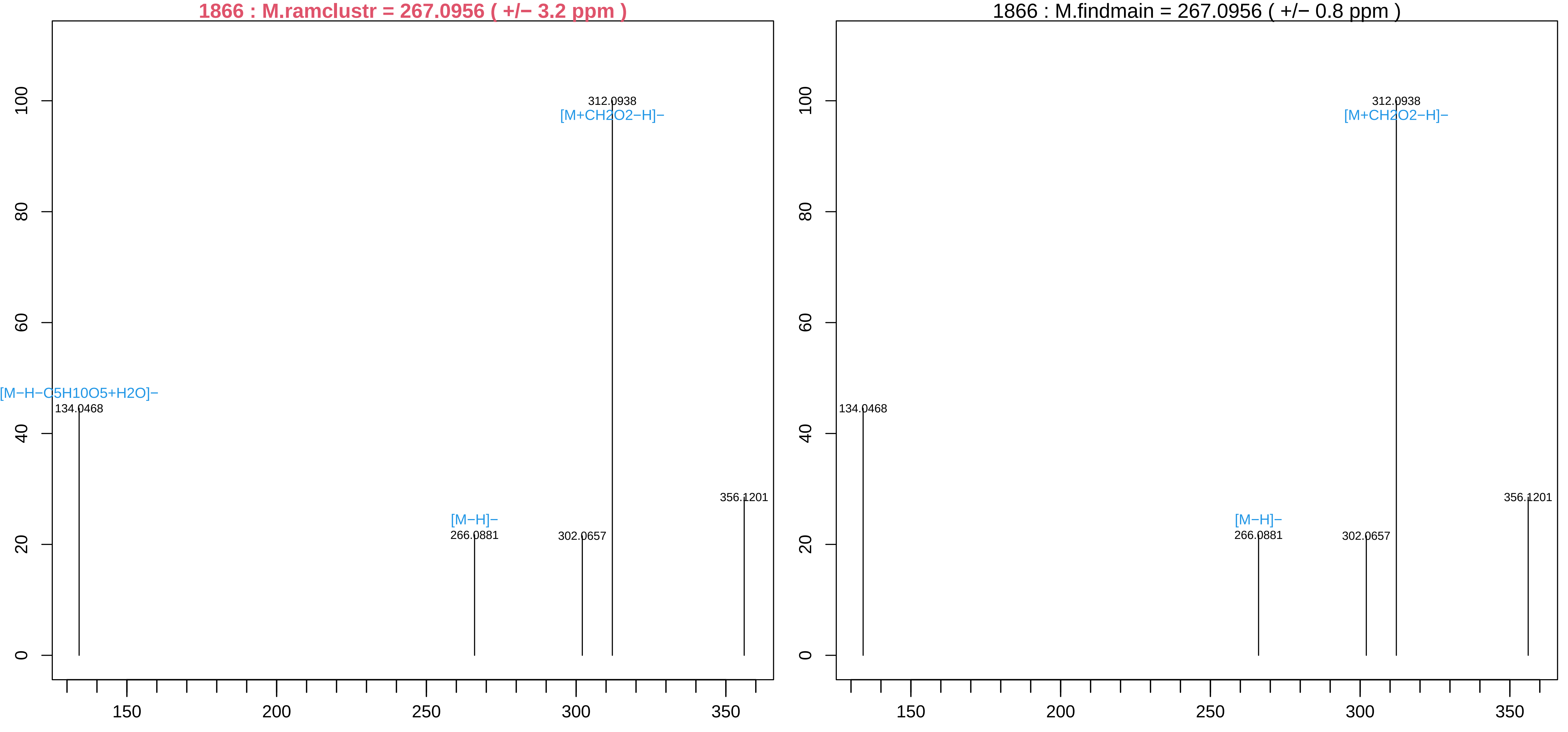

1866 : M.ramclustr = 267.0956 (+/− 3.2 ppm)
312.0938
[M+CH2O2−H]−
[M−H−C5H10O5+H2O]−
134.0468
[M−H]−
266.0881
302.0657
356.1201
1866 : M.findmain = 267.0956 (+/− 0.8 ppm)
312.0938
[M+CH2O2−H]−
134.0468
[M−H]−
266.0881
302.0657
356.1201

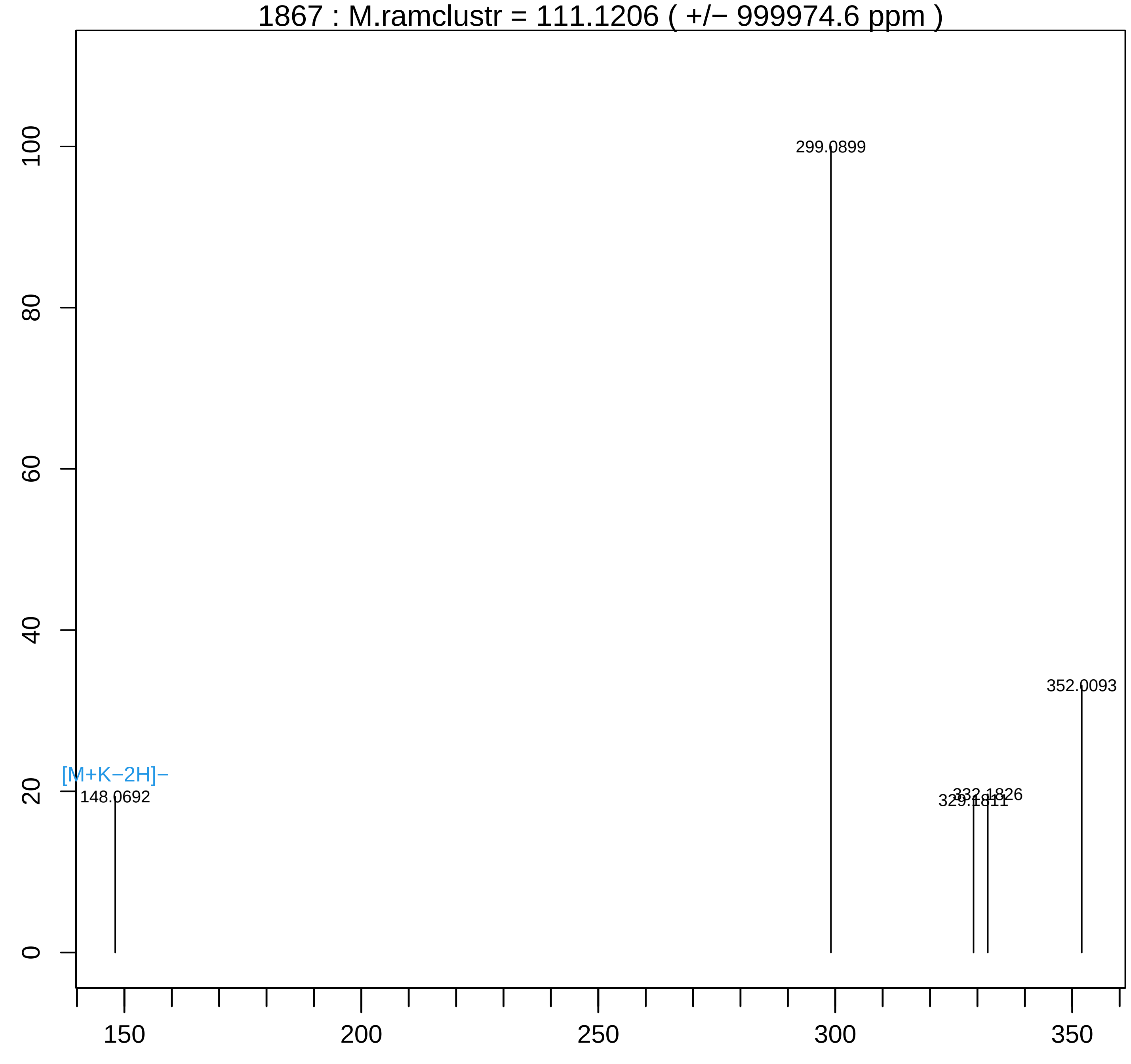
1867 : M.ramclustr = 111.1206 (+/− 999974.6 ppm)
299.0899
352.0093
332.1826
329.1811
[M+K−2H]−
148.0692
0
20
40
60
80
100
150
200
250
300
350

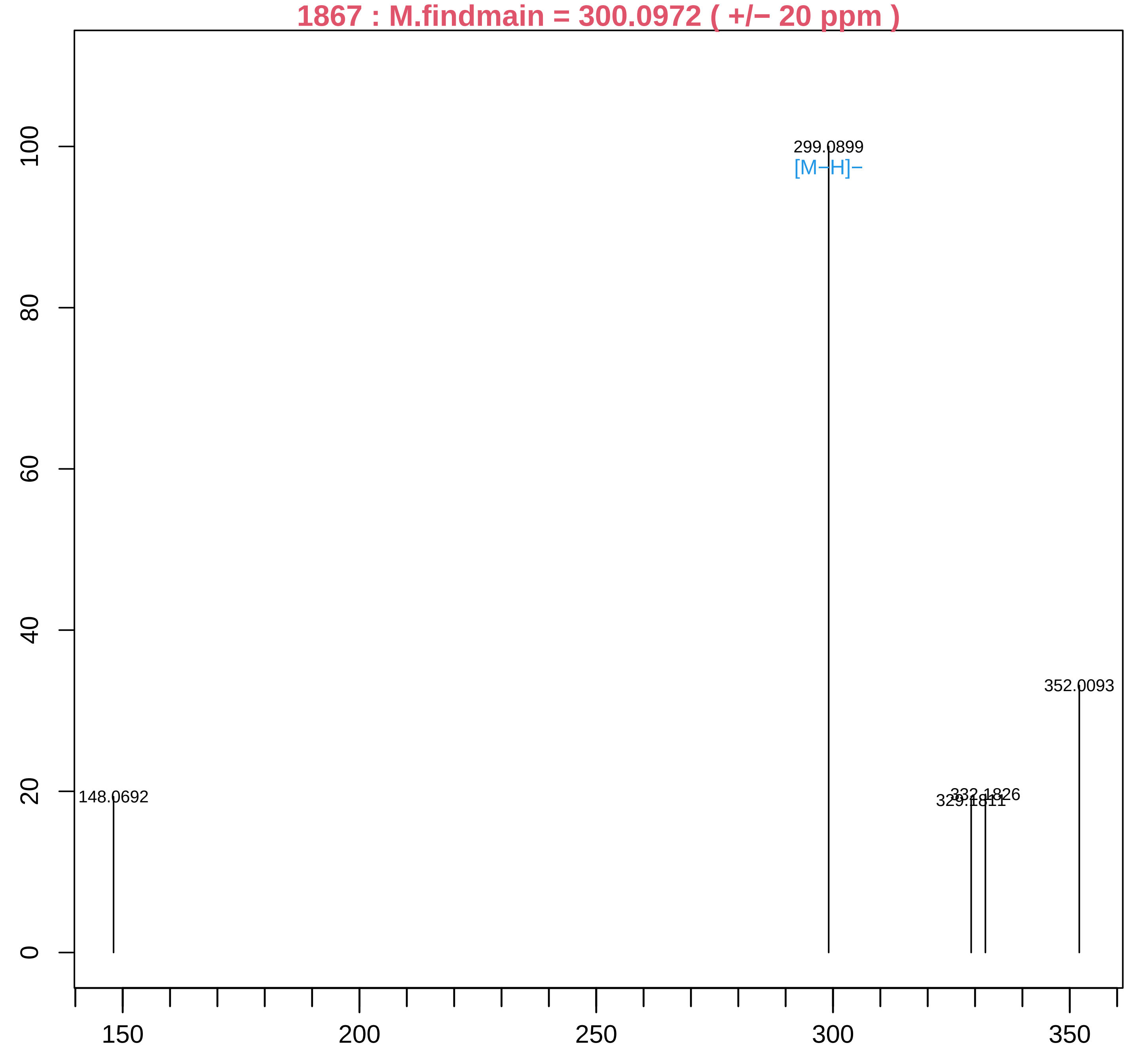
1867 : M.findmain = 300.0972 (+/− 20 ppm)
299.0899
[M−H]−
352.0093
332.1826
329.1811
148.0692
0
20
40
60
80
100
150
200
250
300
350

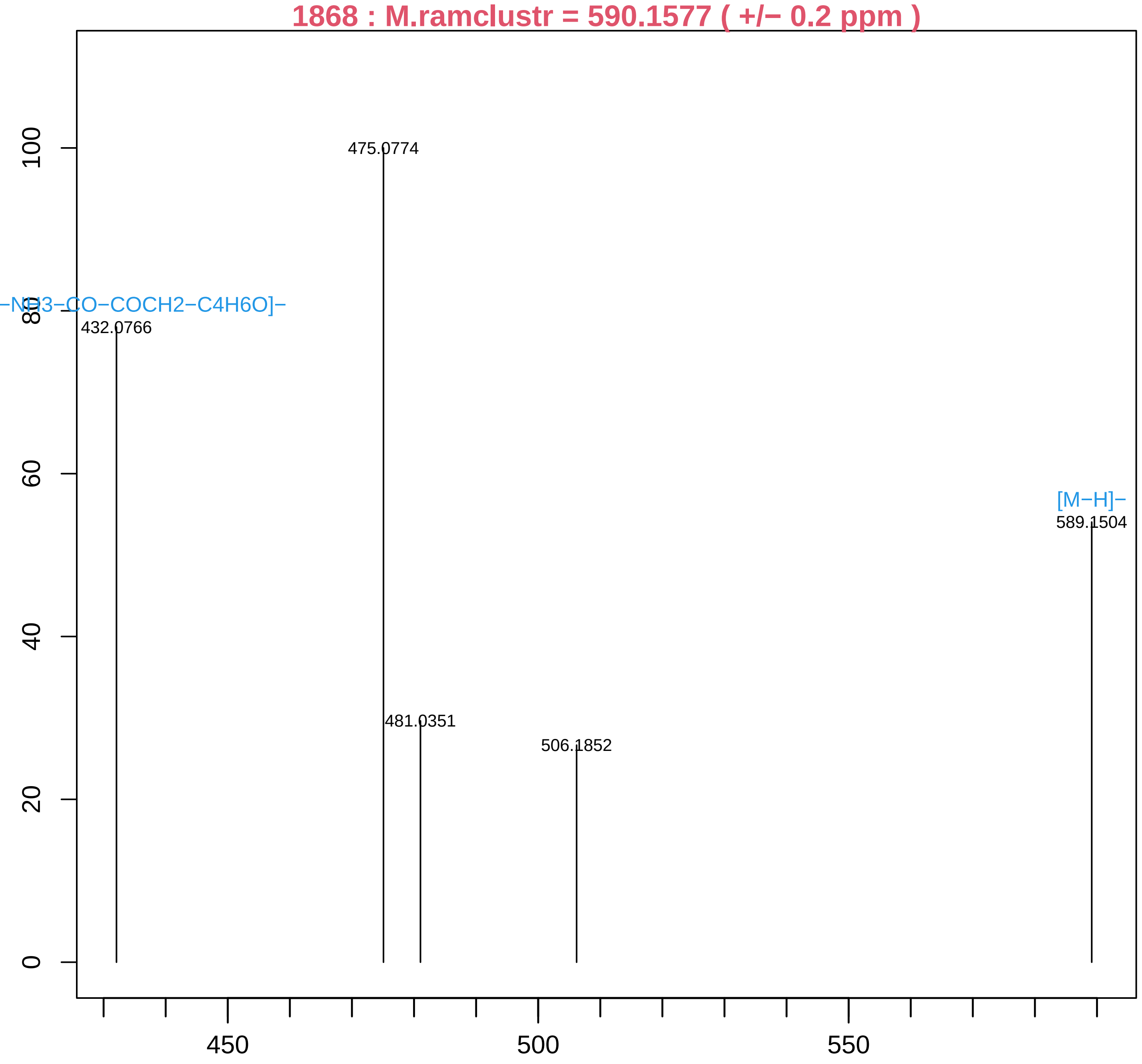
1868 : M.ramclustr = 590.1577 (+/− 0.2 ppm)
−NH3−CO−COCH2−C4H6O]−
432.0766
475.0774
481.0351
506.1852
[M−H]−
589.1504
0
20
40
60
80
100
450
500
550

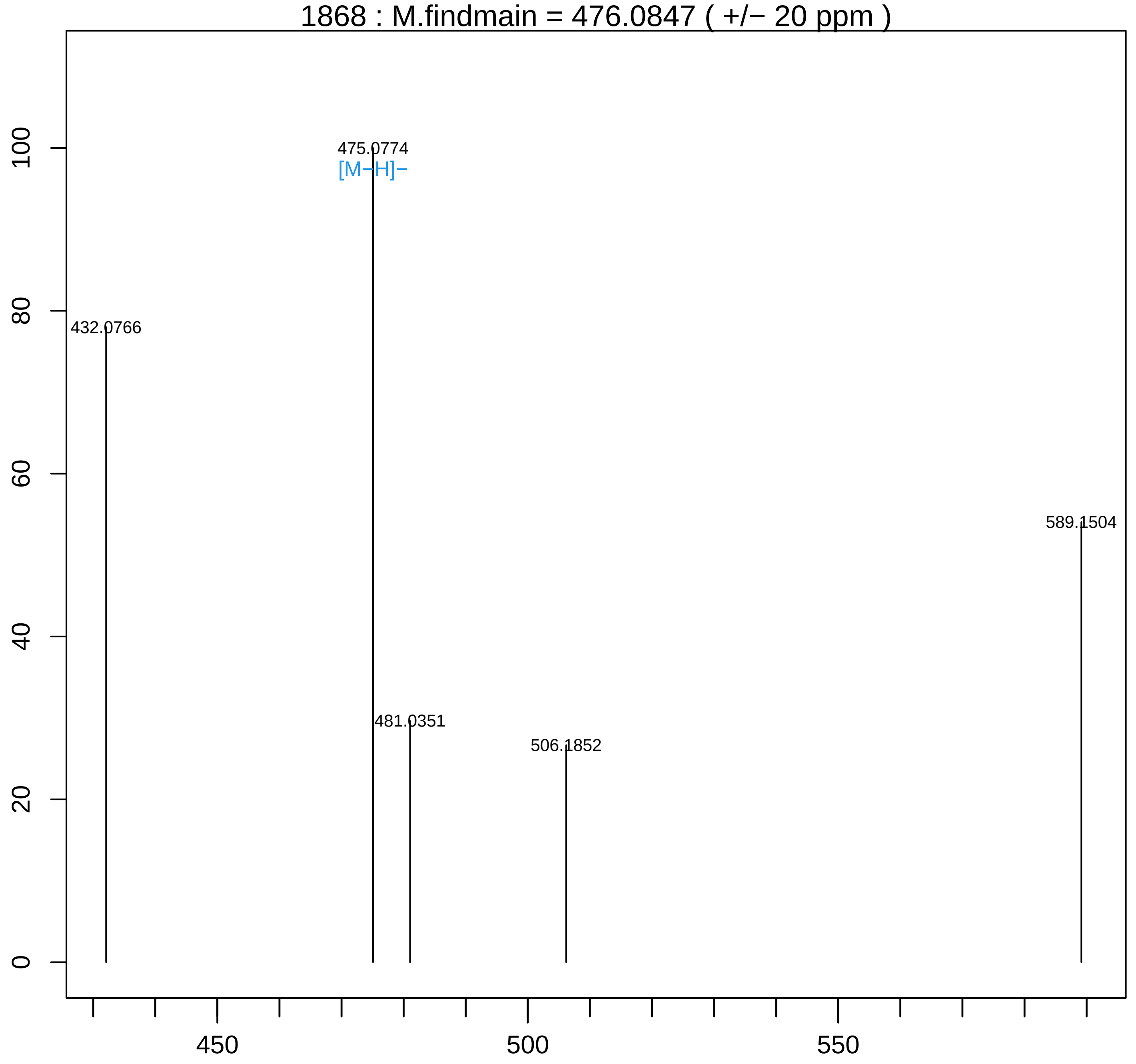
1868 : M.findmain = 476.0847 (+/− 20 ppm)
432.0766
475.0774
[M−H]−
481.0351
506.1852
589.1504
0
20
40
60
80
100
450
500
550

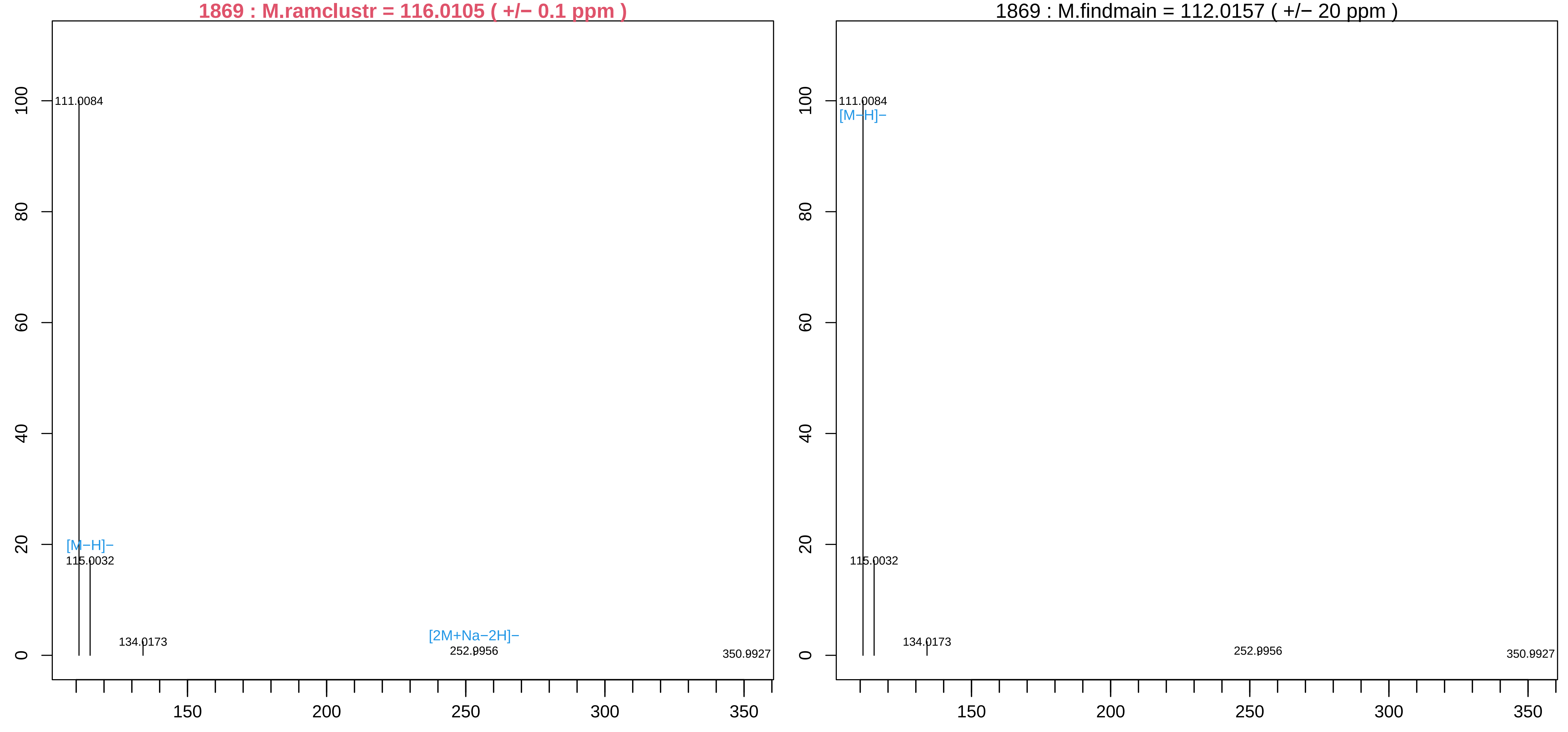

1869 : M.ramclustr = 116.0105 (+/− 0.1 ppm)
111.0084
[M−H]−
115.0032
134.0173
[2M+Na−2H]−
252.9956
350.9927
0
20
40
60
80
100
150
200
250
300
350
1869 : M.findmain = 112.0157 (+/− 20 ppm)
111.0084
[M−H]−
115.0032
134.0173
252.9956
350.9927
0
20
40
60
80
100
150
200
250
300
350

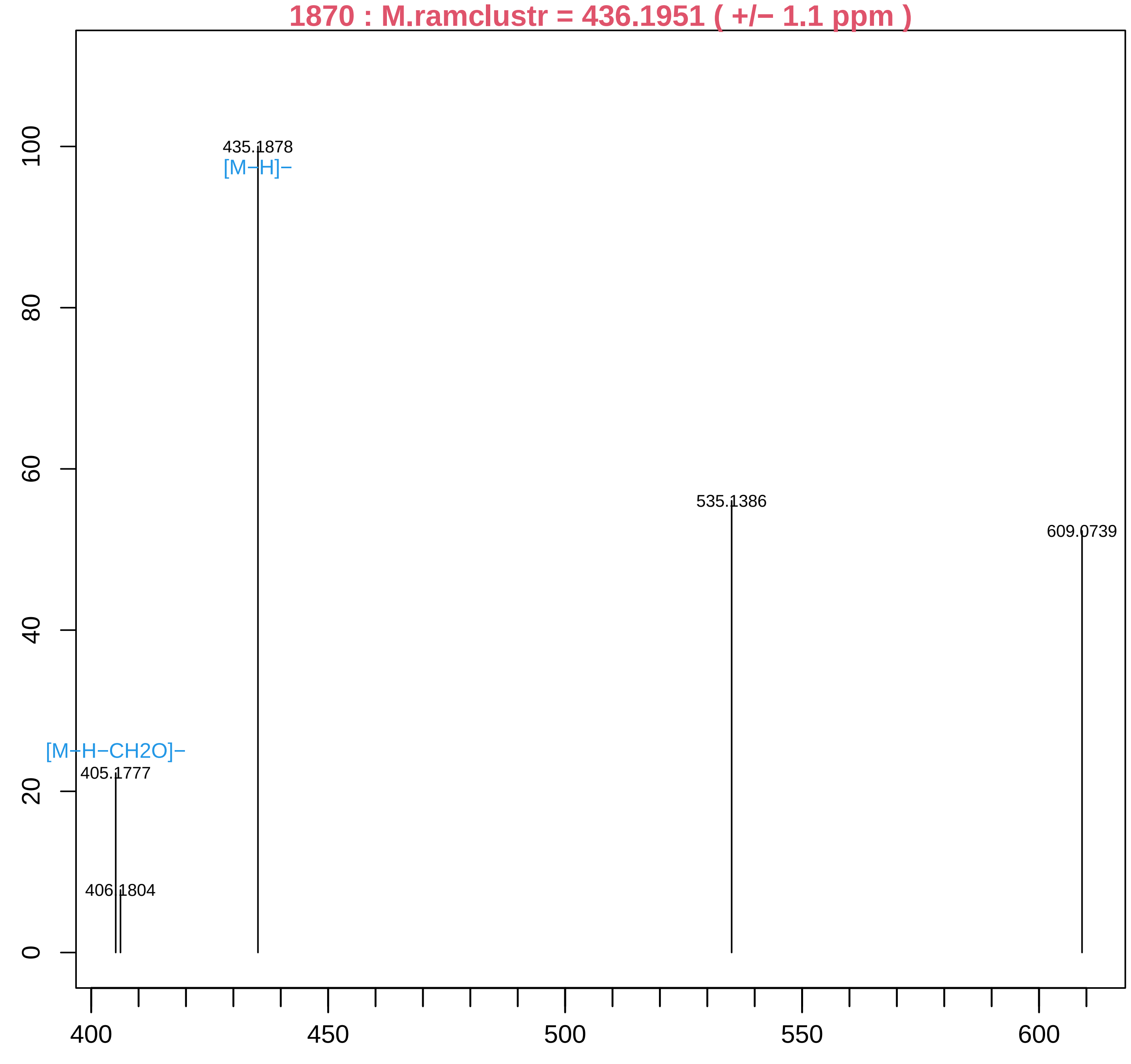
1870 : M.ramclustr = 436.1951 (+/− 1.1 ppm)
435.1878
[M−H]−
535.1386
609.0739
[M−H−CH2O]−
405.1777
406.1804
0
20
40
60
80
100
400
450
500
550
600

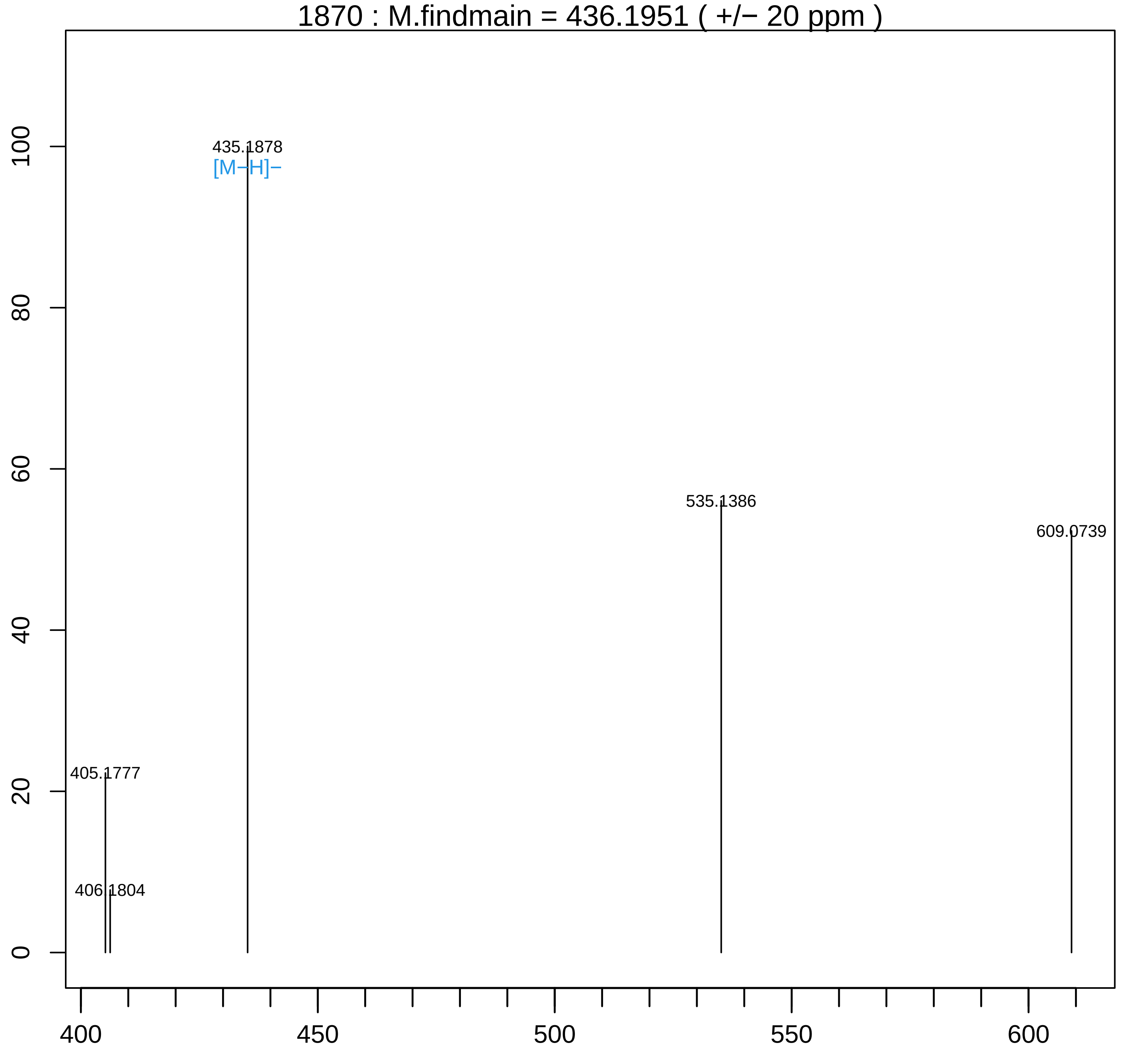
1870 : M.findmain = 436.1951 (+/− 20 ppm)
435.1878
[M−H]−
535.1386
609.0739
405.1777
406.1804
0
20
40
60
80
100
400
450
500
550
600

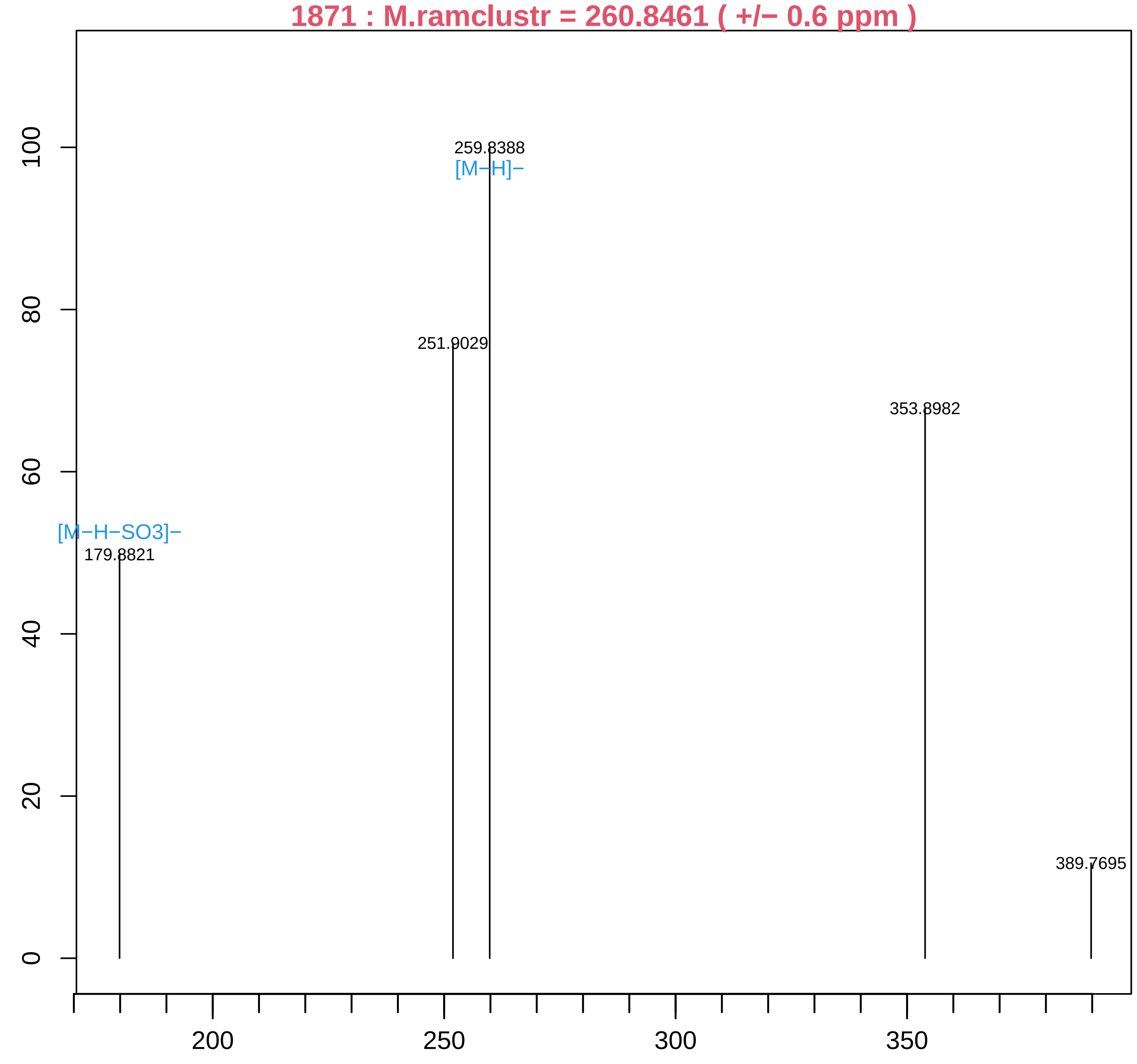
1871 : M.ramclustr = 260.8461 (+/− 0.6 ppm)
259.8388
[M−H]−
251.9029
353.8982
[M−H−SO3]−
179.8821
389.7695
0
20
40
60
80
100
200
250
300
350

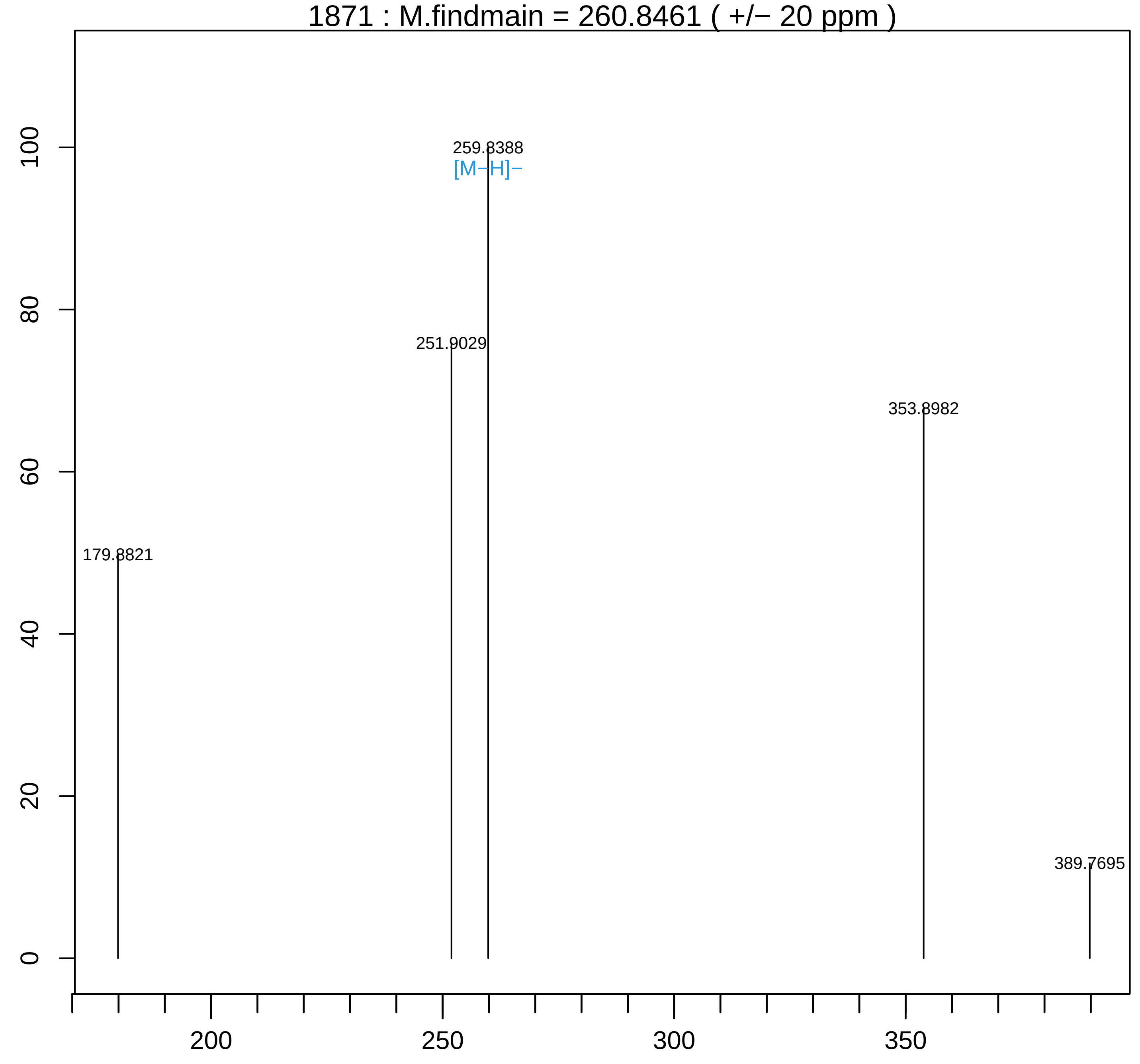
1871 : M.findmain = 260.8461 (+/− 20 ppm)
259.8388
[M−H]−
251.9029
353.8982
179.8821
389.7695
0
20
40
60
80
100
200
250
300
350

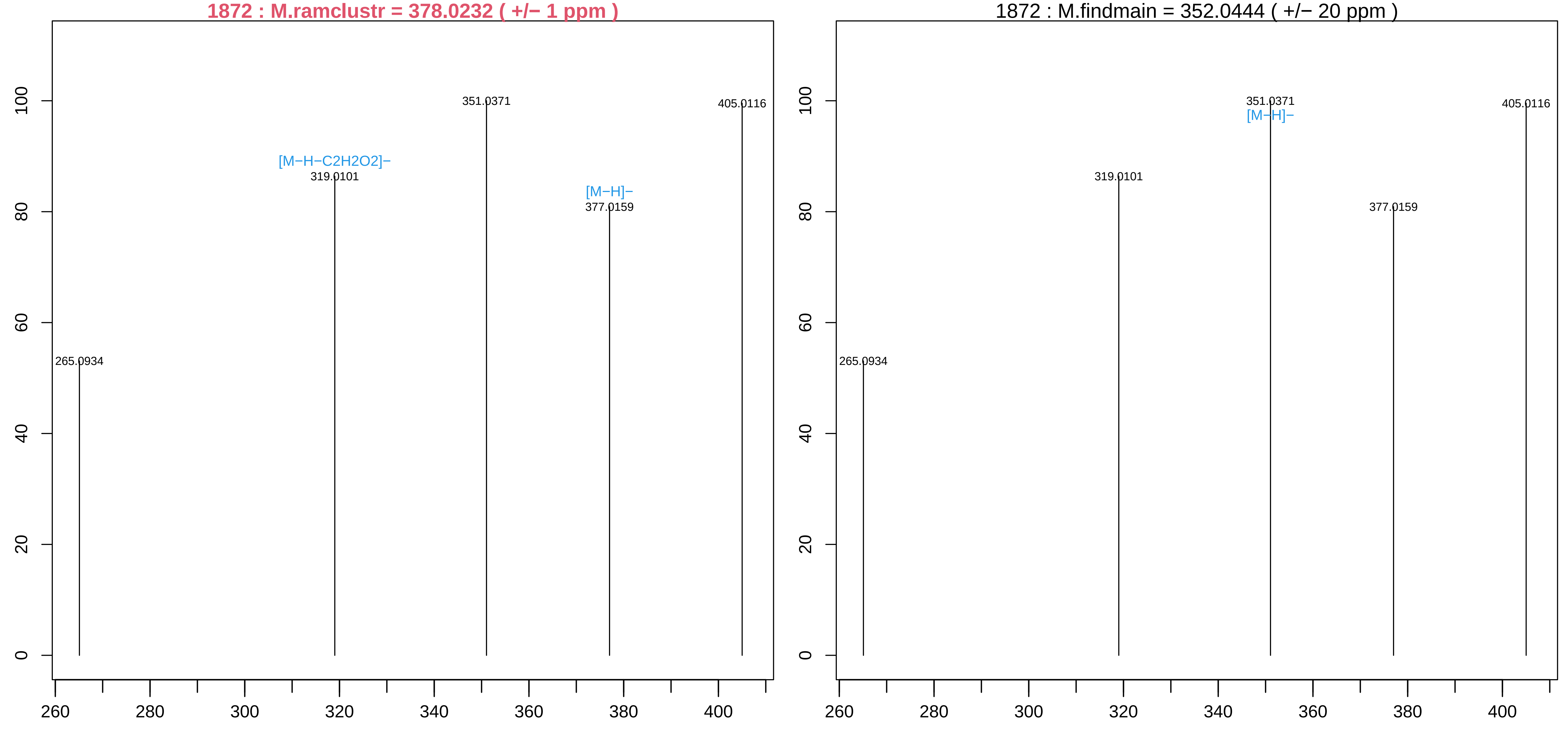

1872 : M.ramclustr = 378.0232 (+/− 1 ppm)
1872 : M.findmain = 352.0444 (+/− 20 ppm)
[M−H−C2H2O2]−
[M−H]−
265.0934
319.0101
351.0371
377.0159
405.0116
0
20
40
60
80
100
260
280
300
320
340
360
380
400

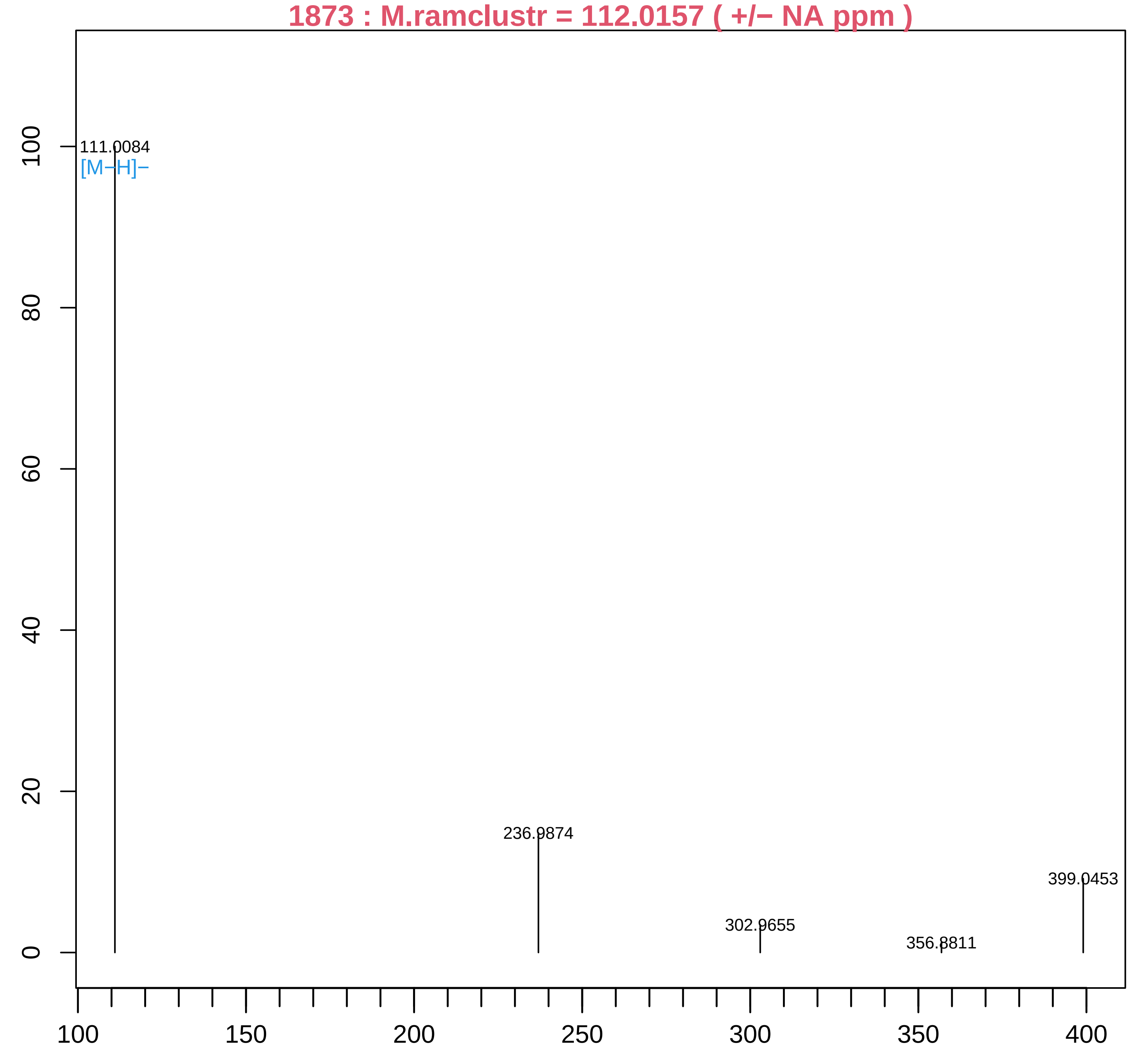

1873 : M.ramclustr = 112.0157 (+/− NA ppm)
111.0084
[M−H]−
236.9874
302.9655
356.8811
399.0453
0
20
40
60
80
100
100
150
200
250
300
350
400

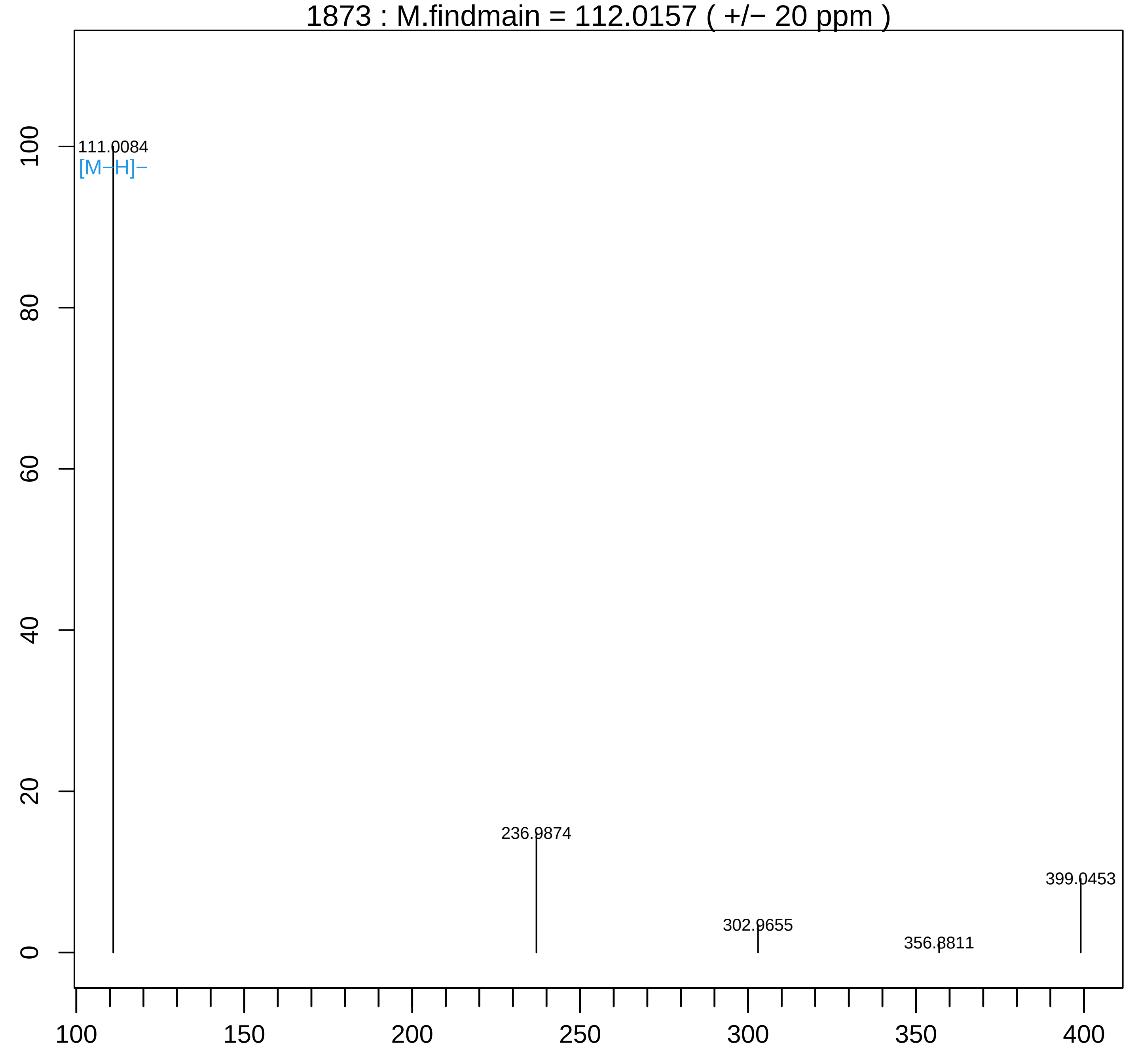

1873 : M.findmain = 112.0157 (+/− 20 ppm)
111.0084
[M−H]−
236.9874
302.9655
356.8811
399.0453
0
20
40
60
80
100
100
150
200
250
300
350
400

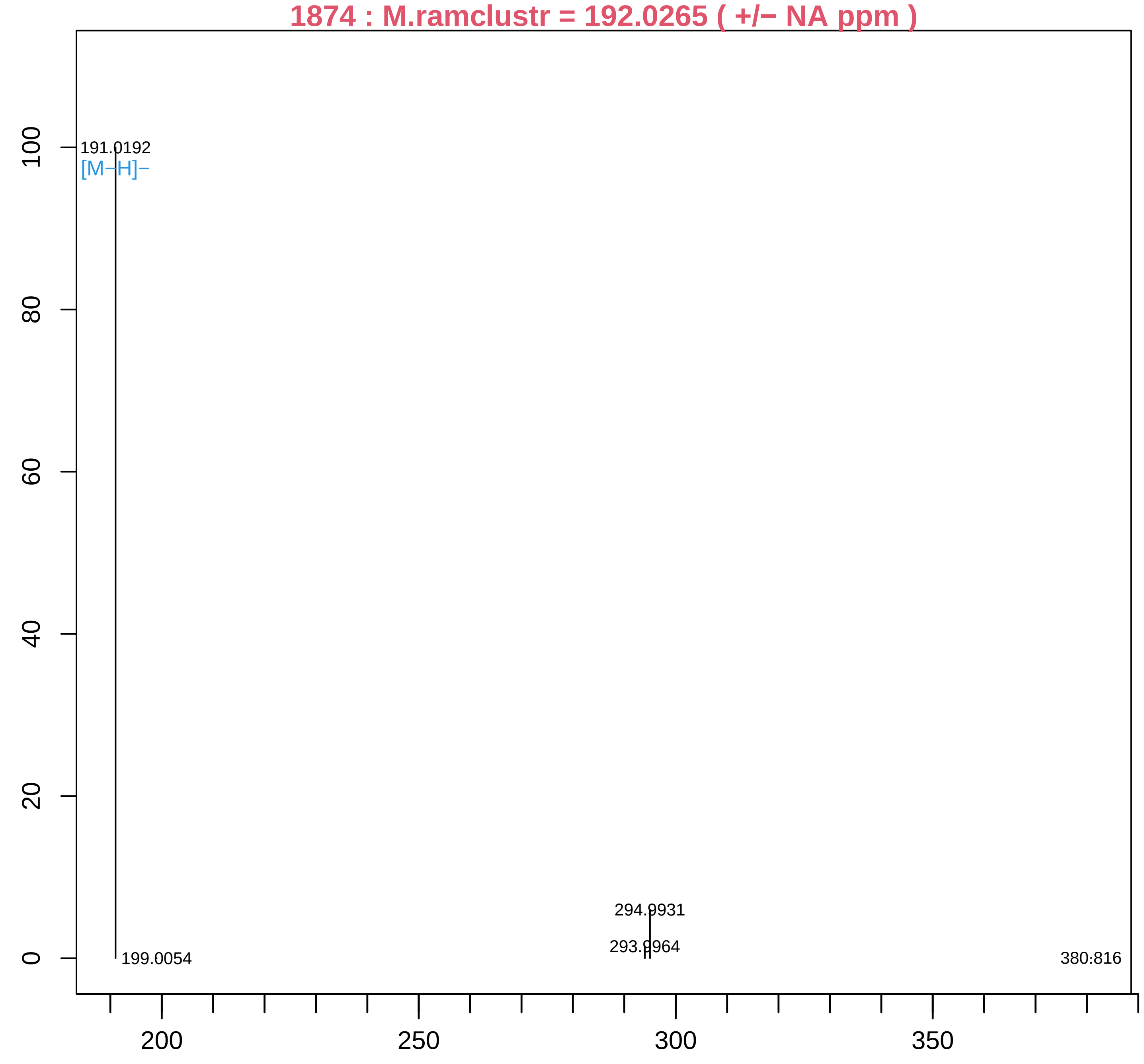
1874 : M.ramclustr = 192.0265 (+/− NA ppm)
191.0192
[M−H]−
294.9931
293.9964
199.0054
380:816
0
20
40
60
80
100
200
250
300
350

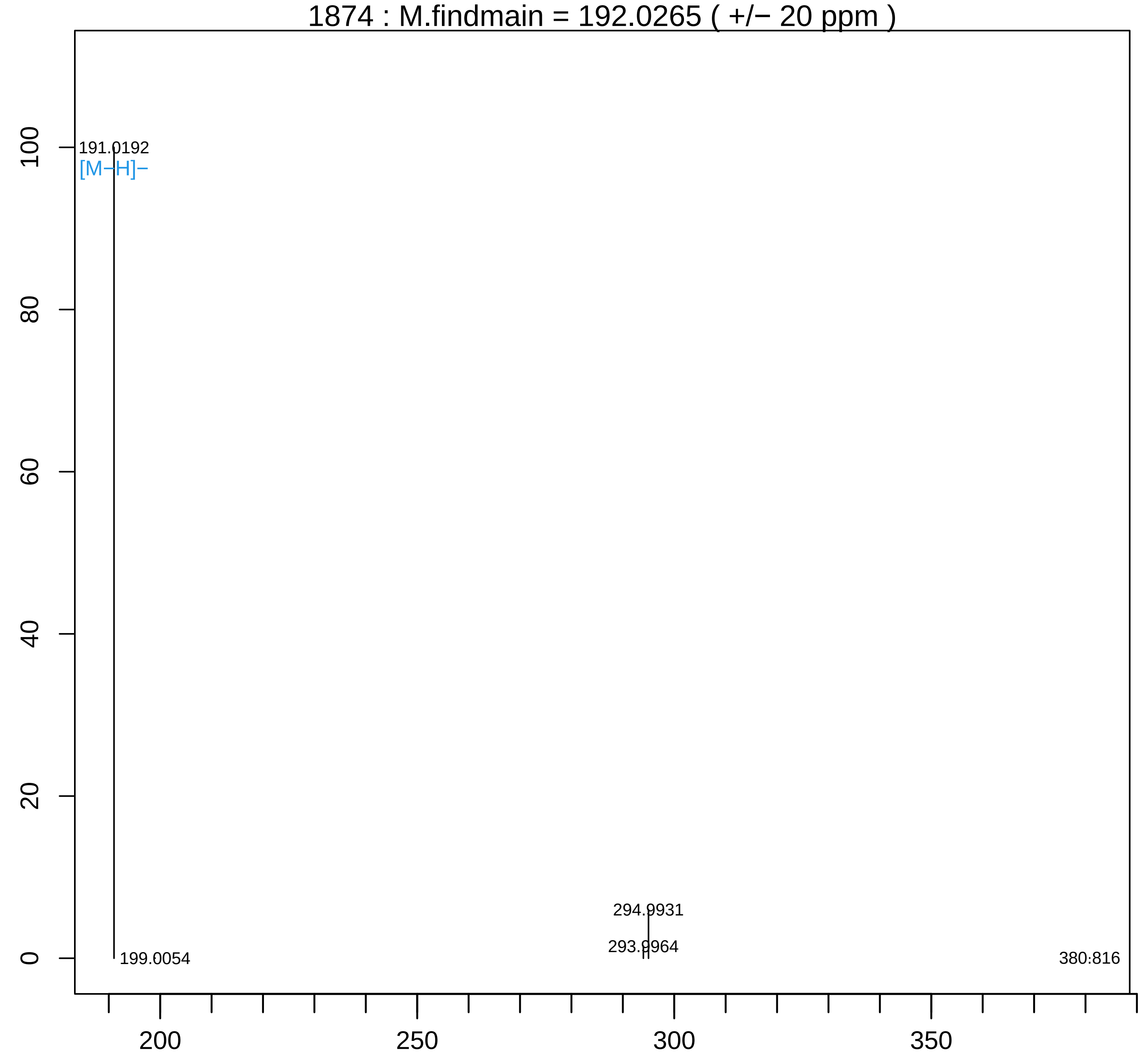
1874 : M.findmain = 192.0265 (+/− 20 ppm)
191.0192
[M−H]−
294.9931
293.9964
199.0054
380:816
0
20
40
60
80
100
200
250
300
350

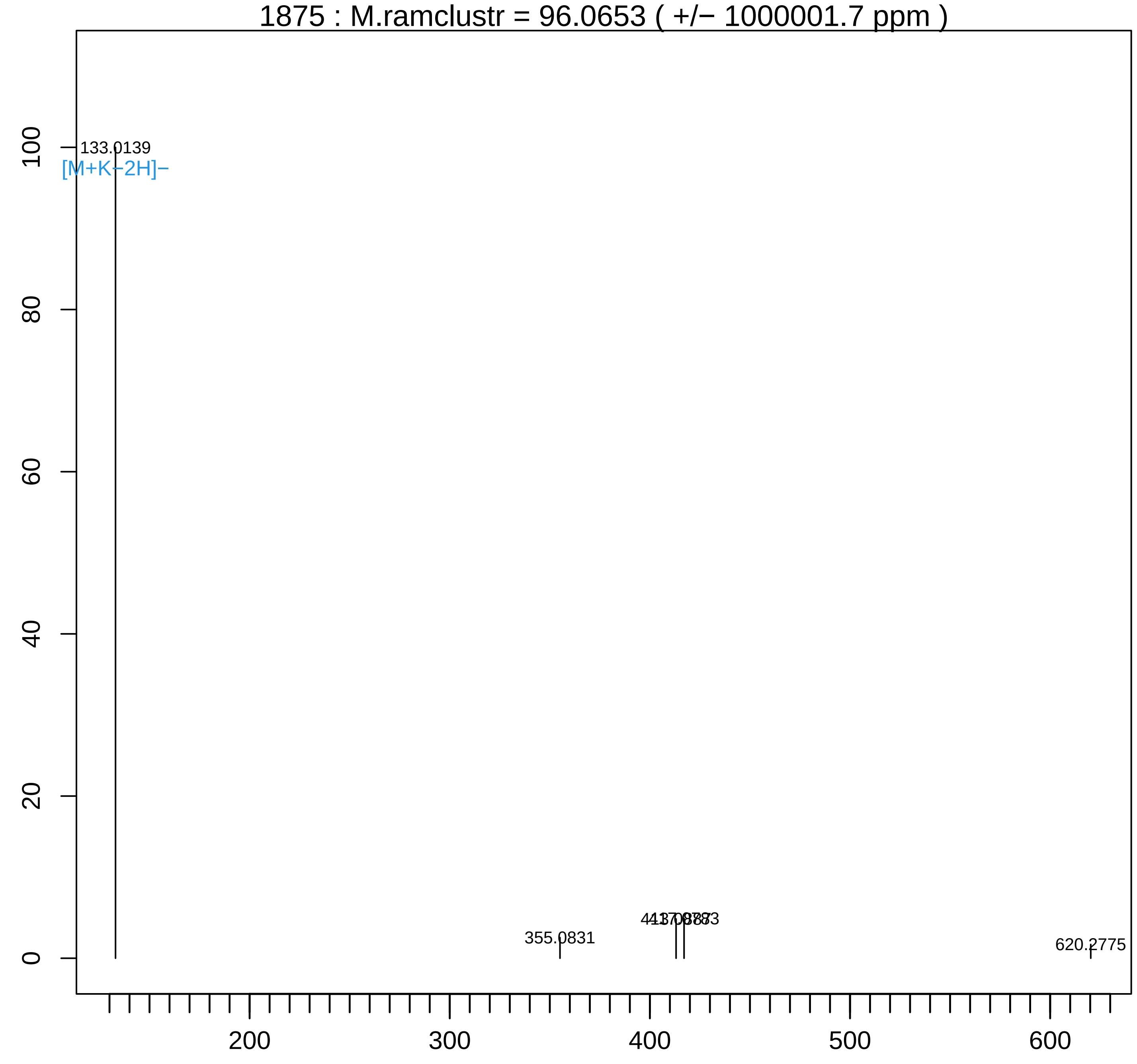
1875 : M.ramclustr = 96.0653 (+/− 1000001.7 ppm)
133.0139
[M+K−2H]−
355.0831
620.2775
0
20
40
60
80
100
200
300
400
500
600

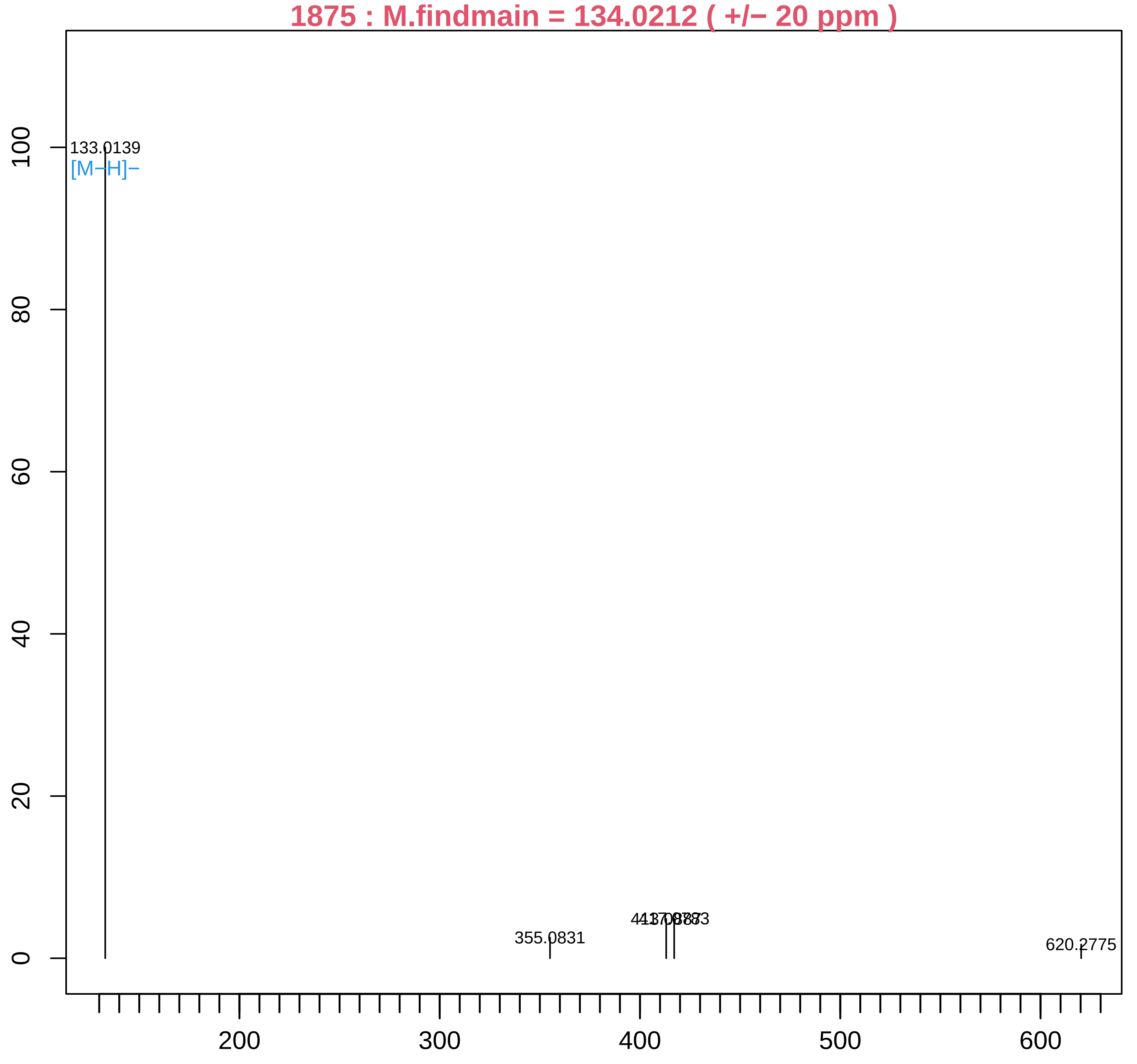
1875 : M.findmain = 134.0212 (+/− 20 ppm)
133.0139
[M−H]−
355.0831
620.2775
0
20
40
60
80
100
200
300
400
500
600

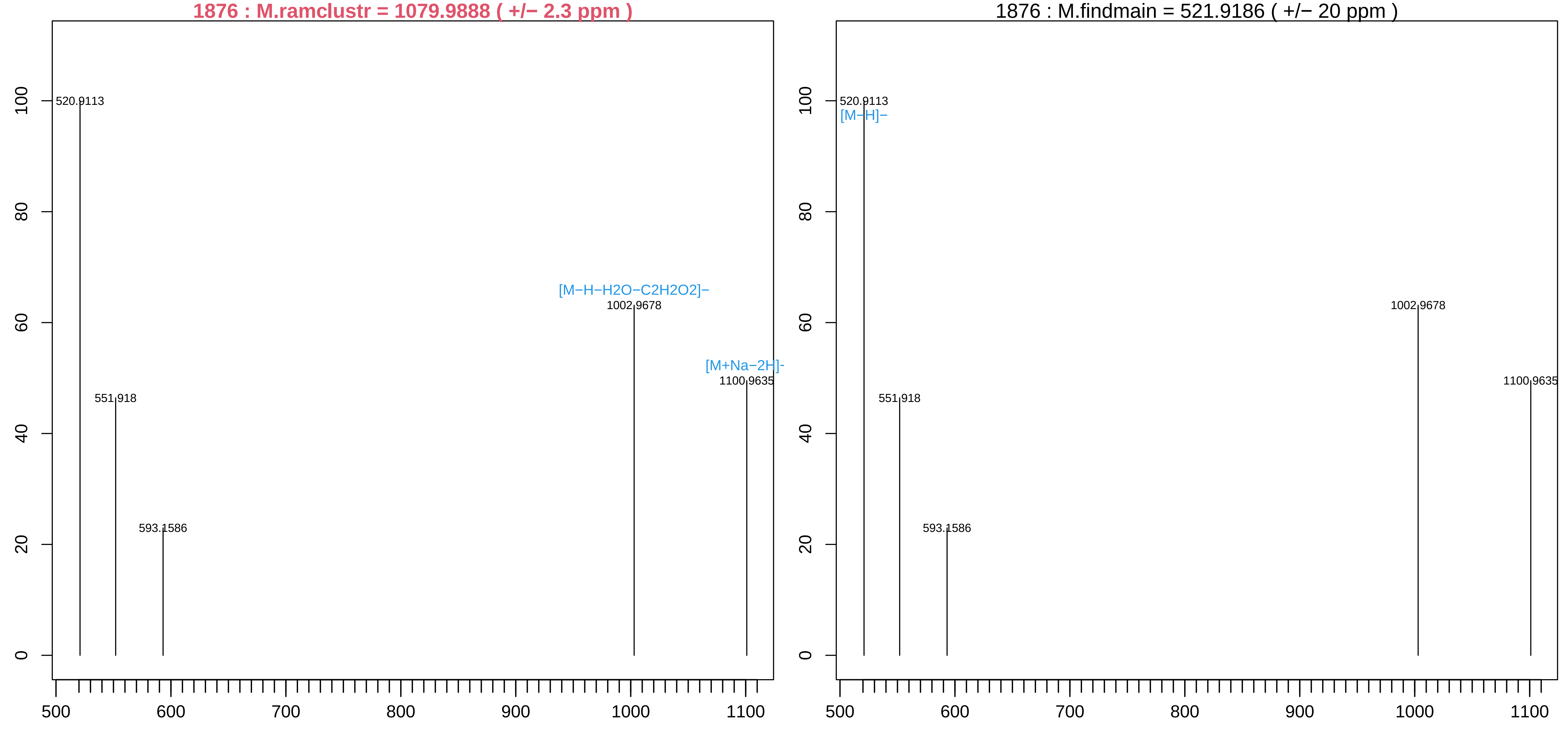
1876 : M.ramclustr = 1079.9888 (+/− 2.3 ppm)
520.9113
551.918
593.1586
[M−H−H2O−C2H2O2]−
1002.9678
[M+Na−2H]-
1100.9635
1876 : M.findmain = 521.9186 (+/− 20 ppm)
520.9113
[M−H]−
551.918
593.1586
1002.9678
1100.9635

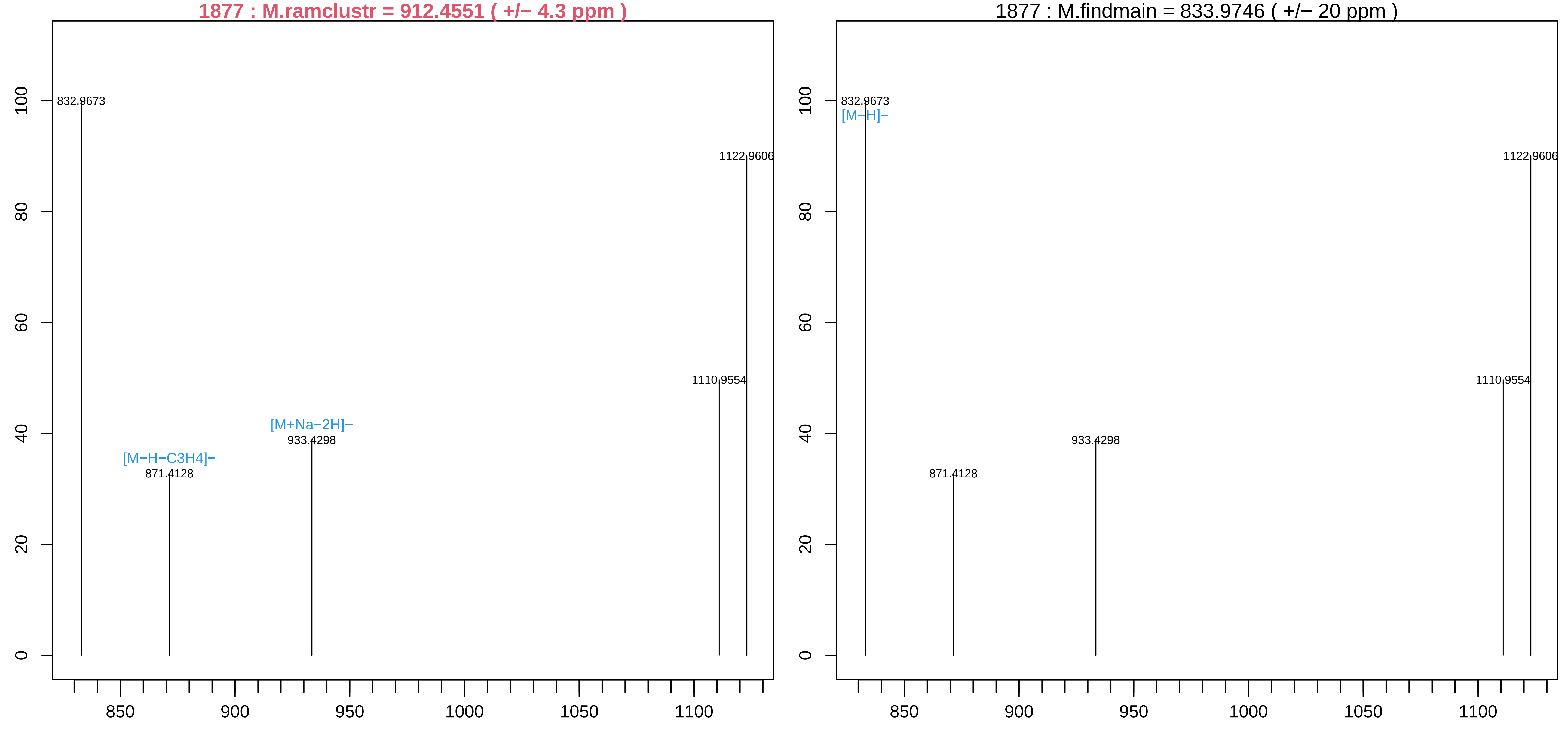
1877 : M.ramclustr = 912.4551 (+/− 4.3 ppm)
832.9673
[M−H−C3H4]−
871.4128
[M+Na−2H]−
933.4298
1110.9554
1122.9606
1877 : M.findmain = 833.9746 (+/− 20 ppm)
832.9673
[M−H]−
871.4128
933.4298
1110.9554
1122.9606
0
20
40
60
80
100
850
900
950
1000
1050
1100

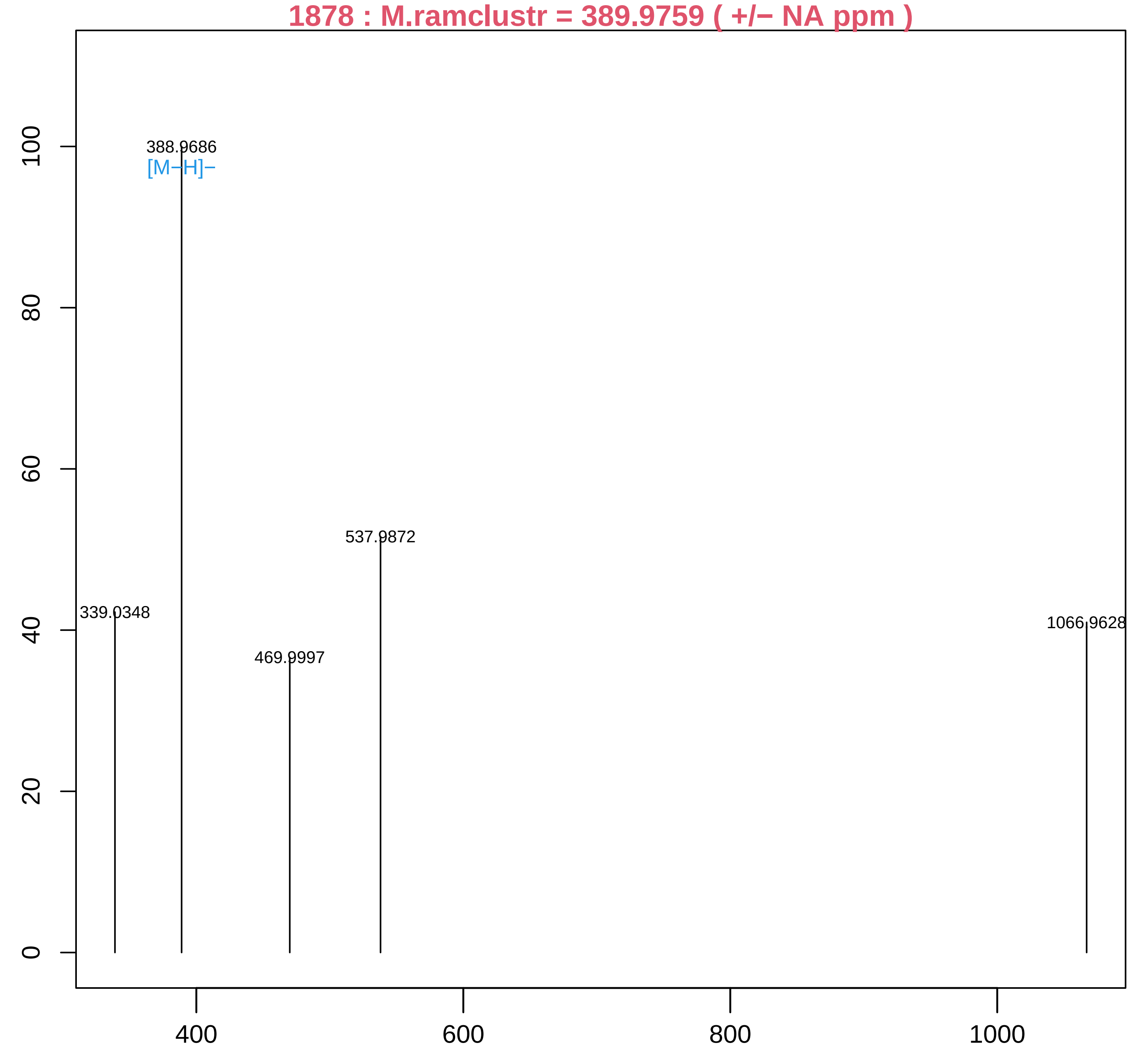
1878 : M.ramclustr = 389.9759 (+/− NA ppm)
388.9686
[M−H]−
537.9872
339.0348
469.9997
1066.9628
0
20
40
60
80
100
400
600
800
1000

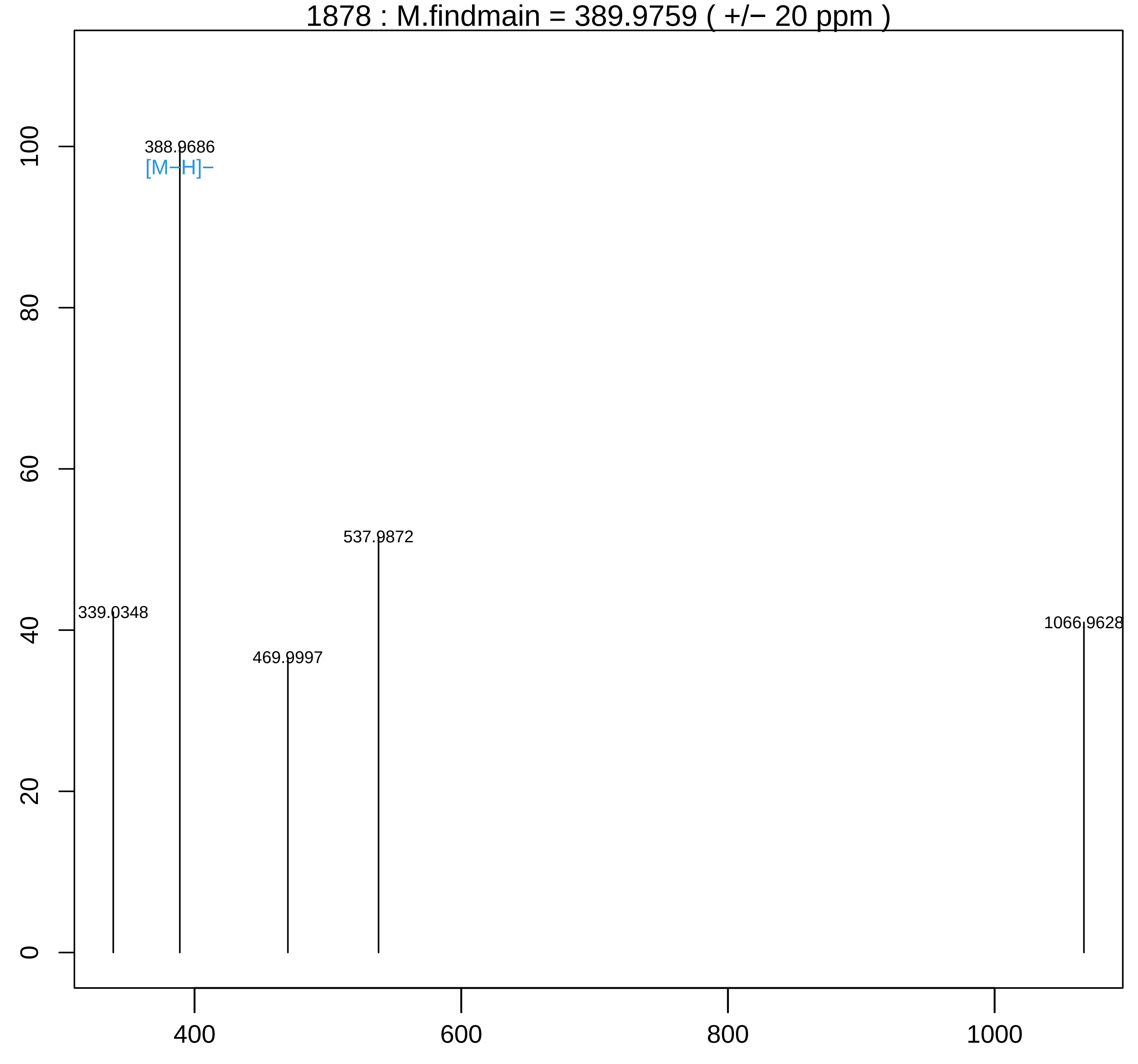
1878 : M.findmain = 389.9759 (+/− 20 ppm)
388.9686
[M−H]−
537.9872
339.0348
469.9997
1066.9628
0
20
40
60
80
100
400
600
800
1000

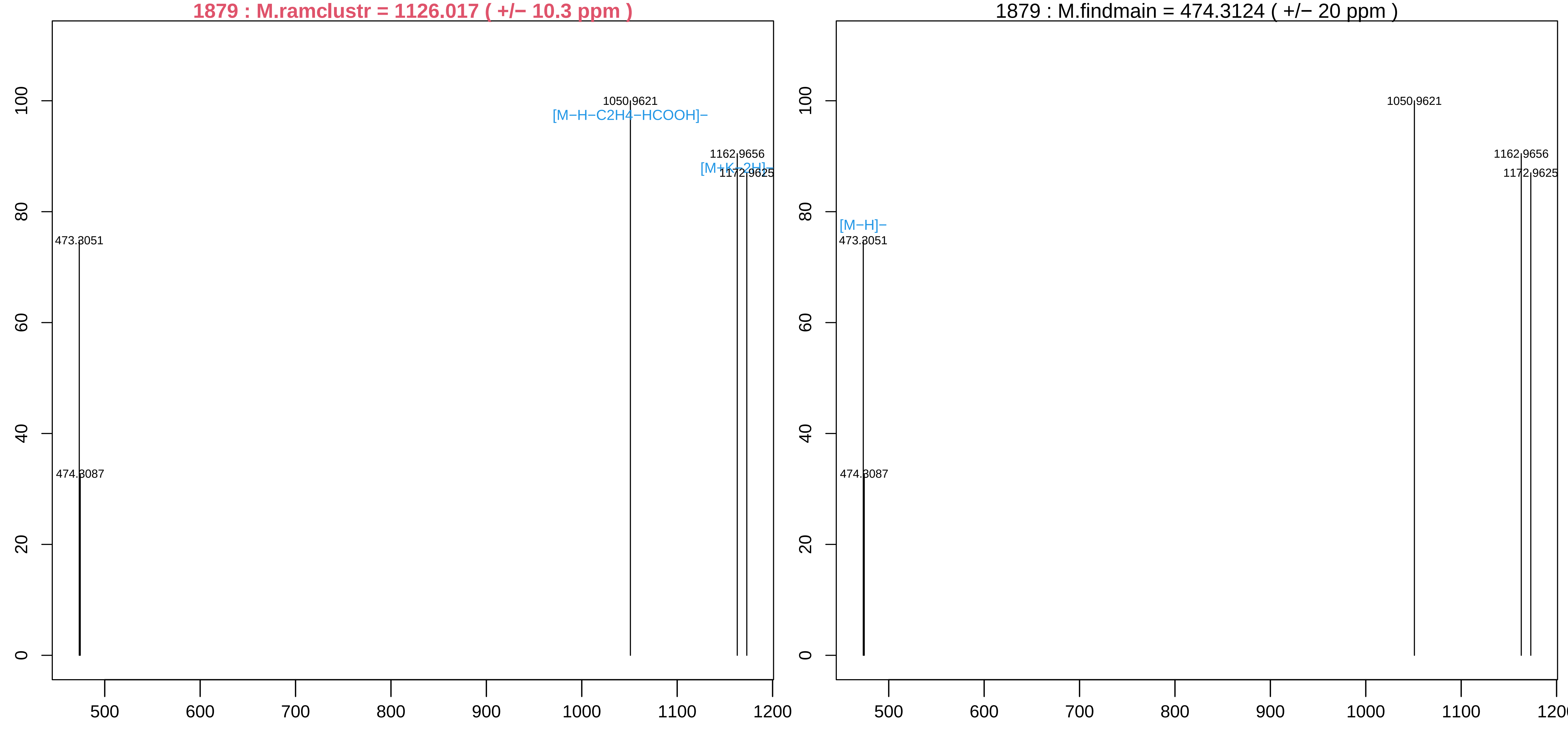

1879 : M.ramclustr = 1126.017 (+/− 10.3 ppm)
1050.9621
[M−H−C2H4−HCOOH]−
1162.9656
[M+K−2H]−
1172.9625
473.3051
474.3087
1879 : M.findmain = 474.3124 (+/− 20 ppm)
1050.9621
1162.9656
1172.9625
[M−H]−
473.3051
474.3087
0
20
40
60
80
100
500
600
700
800
900
1000
1100
1200

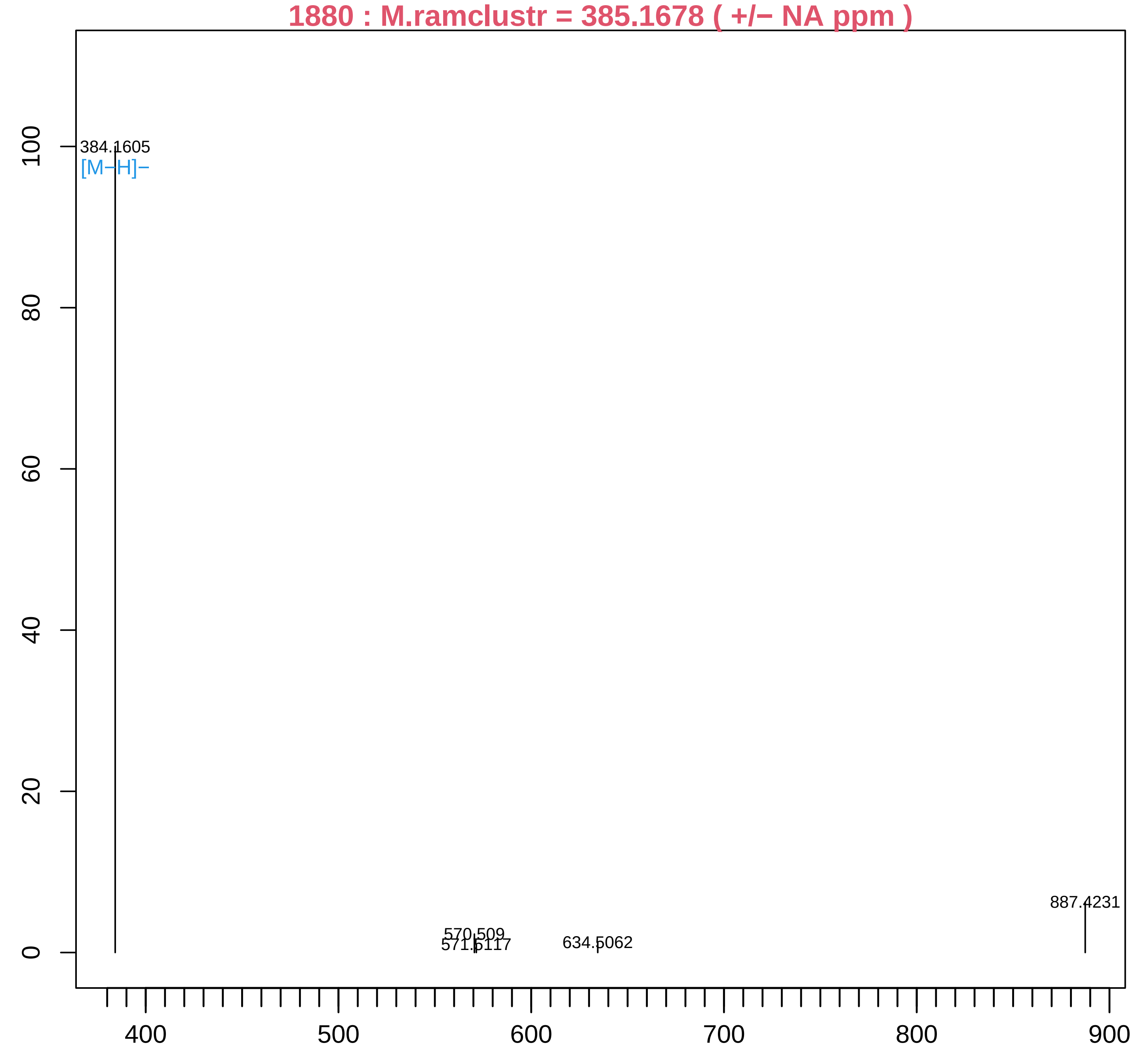
1880 : M.ramclustr = 385.1678 (+/− NA ppm)
384.1605
[M−H]−
570.509
571.5117
634.5062
887.4231
0
20
40
60
80
100
400
500
600
700
800
900

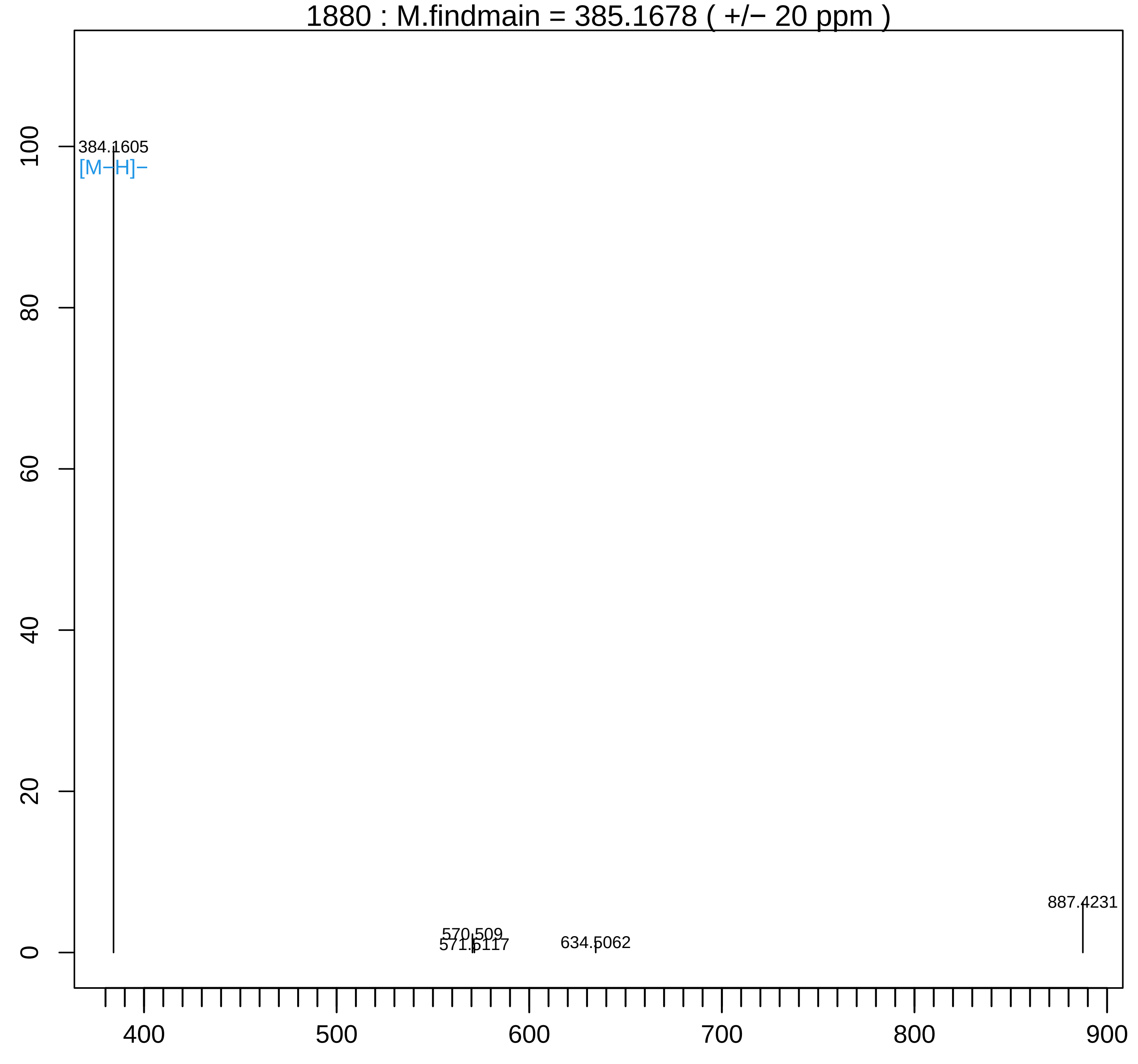
1880 : M.findmain = 385.1678 (+/− 20 ppm)
384.1605
[M−H]−
570.509
571.5117
634.5062
887.4231
0
20
40
60
80
100
400
500
600
700
800
900

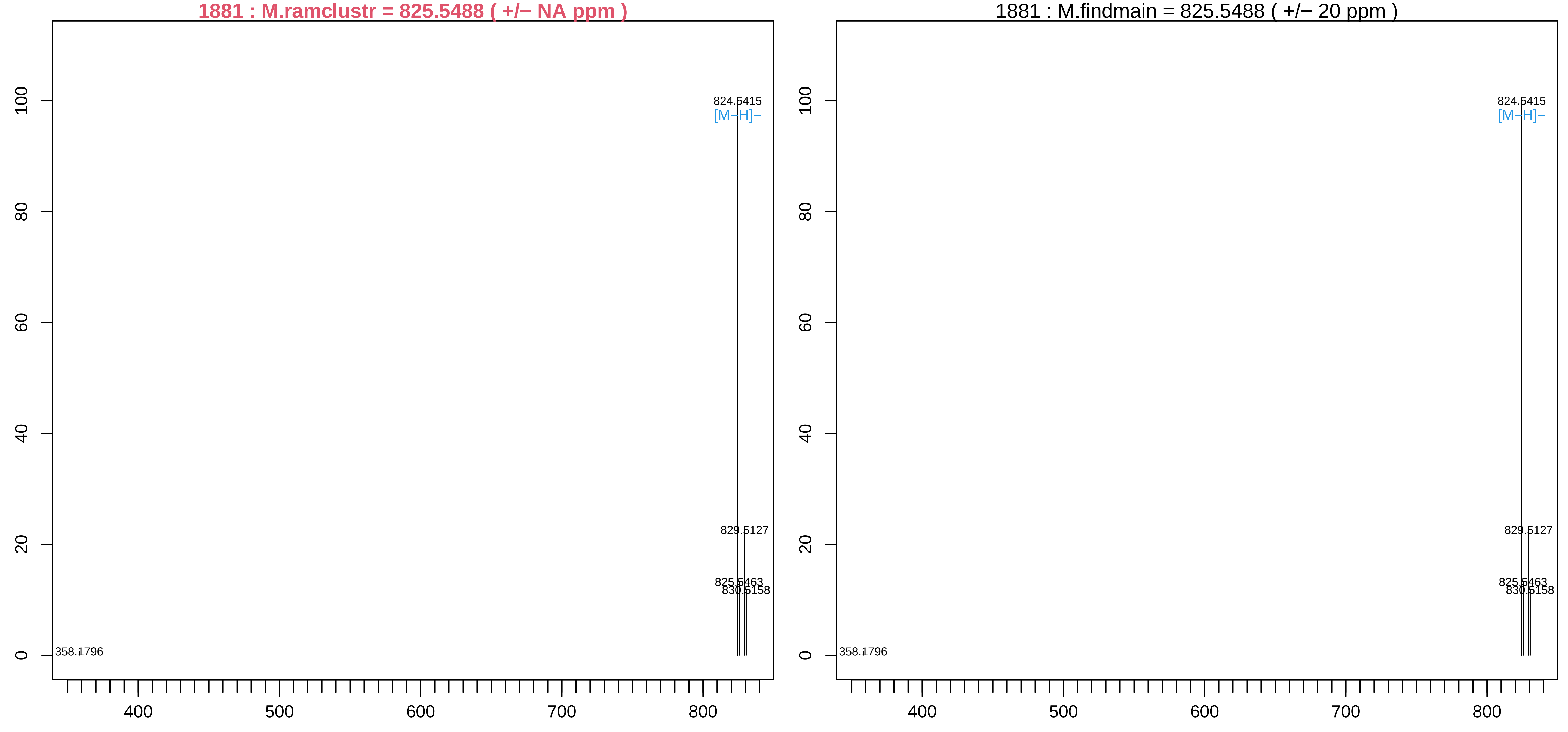
1881 : M.ramclustr = 825.5488 (+/− NA ppm)
824.5415
[M−H]−
829.5127
825.5463
830.5158
358.1796
1881 : M.findmain = 825.5488 (+/− 20 ppm)
824.5415
[M−H]−
829.5127
825.5463
830.5158
358.1796
0
20
40
60
80
100
400
500
600
700
800

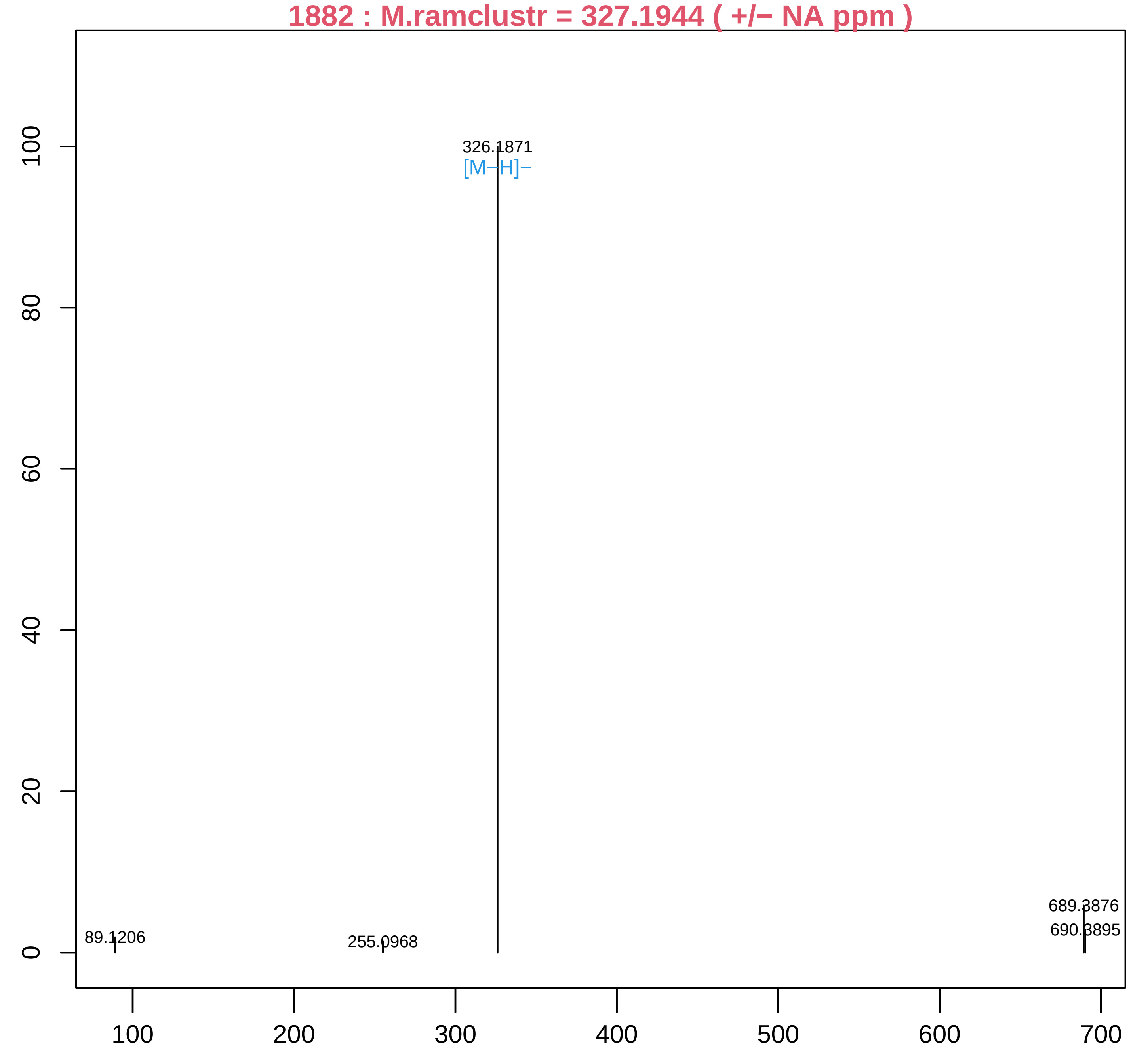
1882 : M.ramclustr = 327.1944 (+/− NA ppm)
326.1871
[M−H]−
89.1206
255.0968
689.3876
690.3895

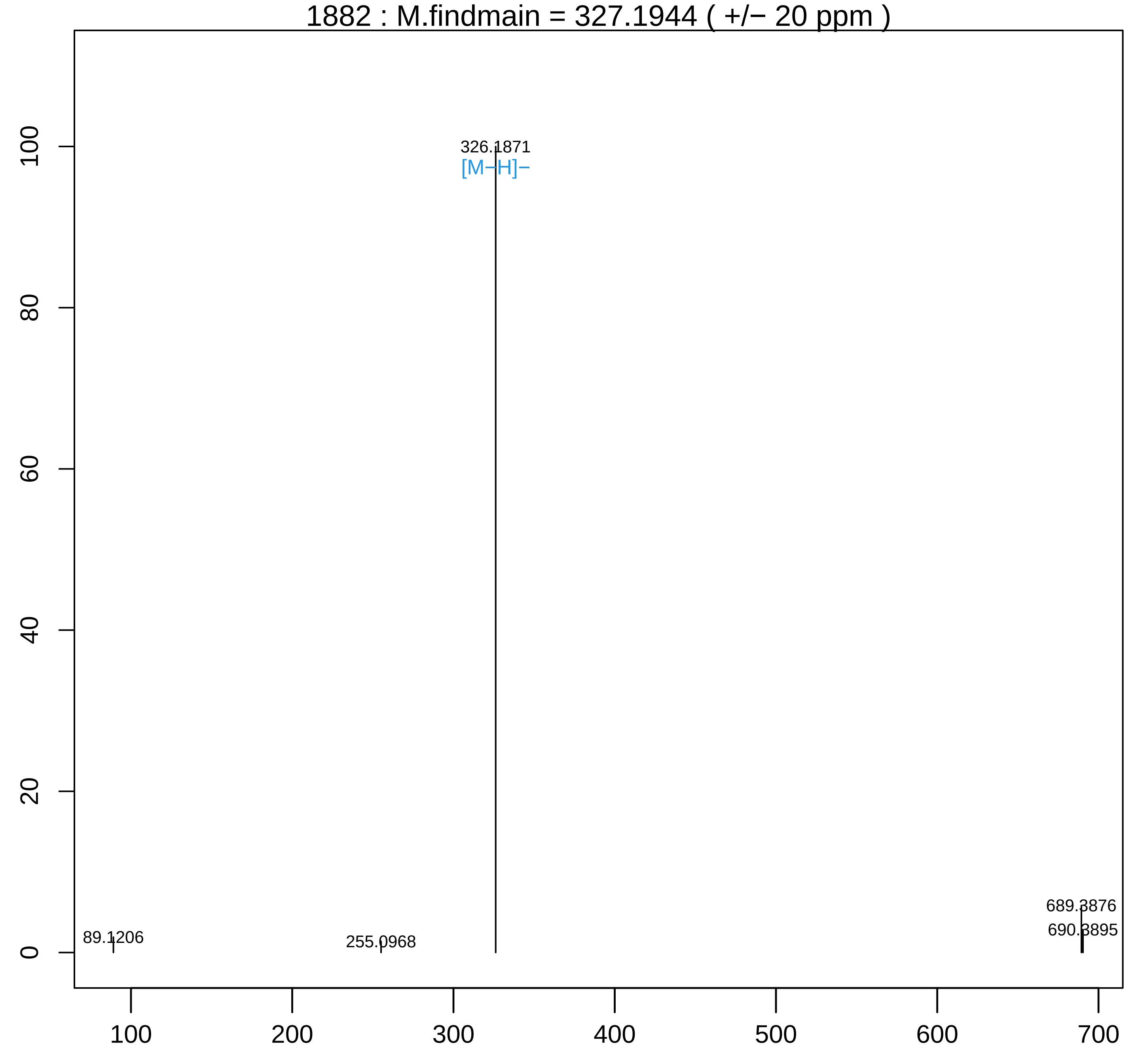
1882 : M.findmain = 327.1944 (+/− 20 ppm)
326.1871
[M−H]−
89.1206
255.0968
689.3876
690.3895

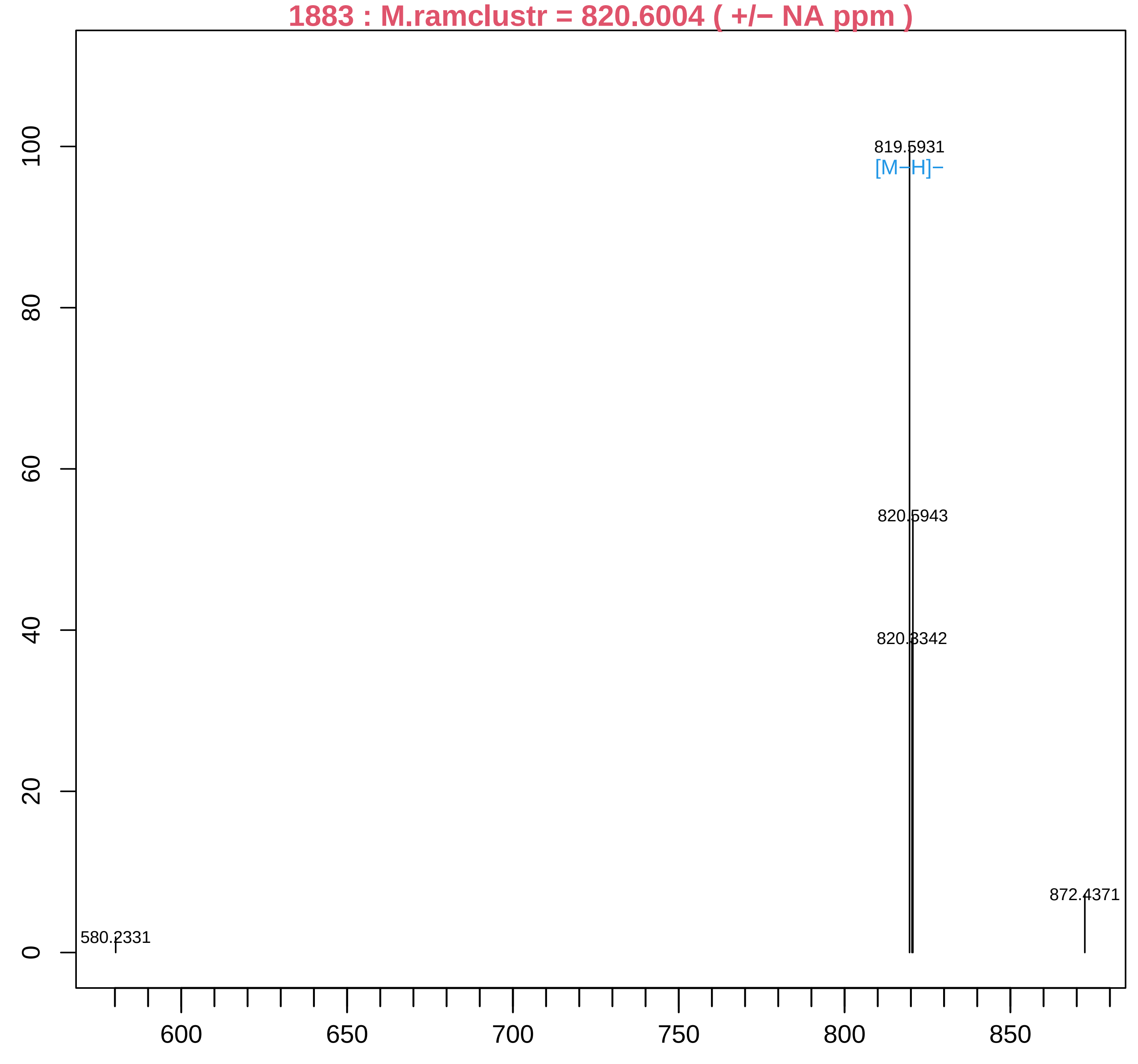

1883 : M.ramclustr = 820.6004 (+/− NA ppm)
819.5931
[M−H]−
820.5943
820.3342
872.4371
580.2331
100
80
60
40
20
0
600
650
700
750
800
850

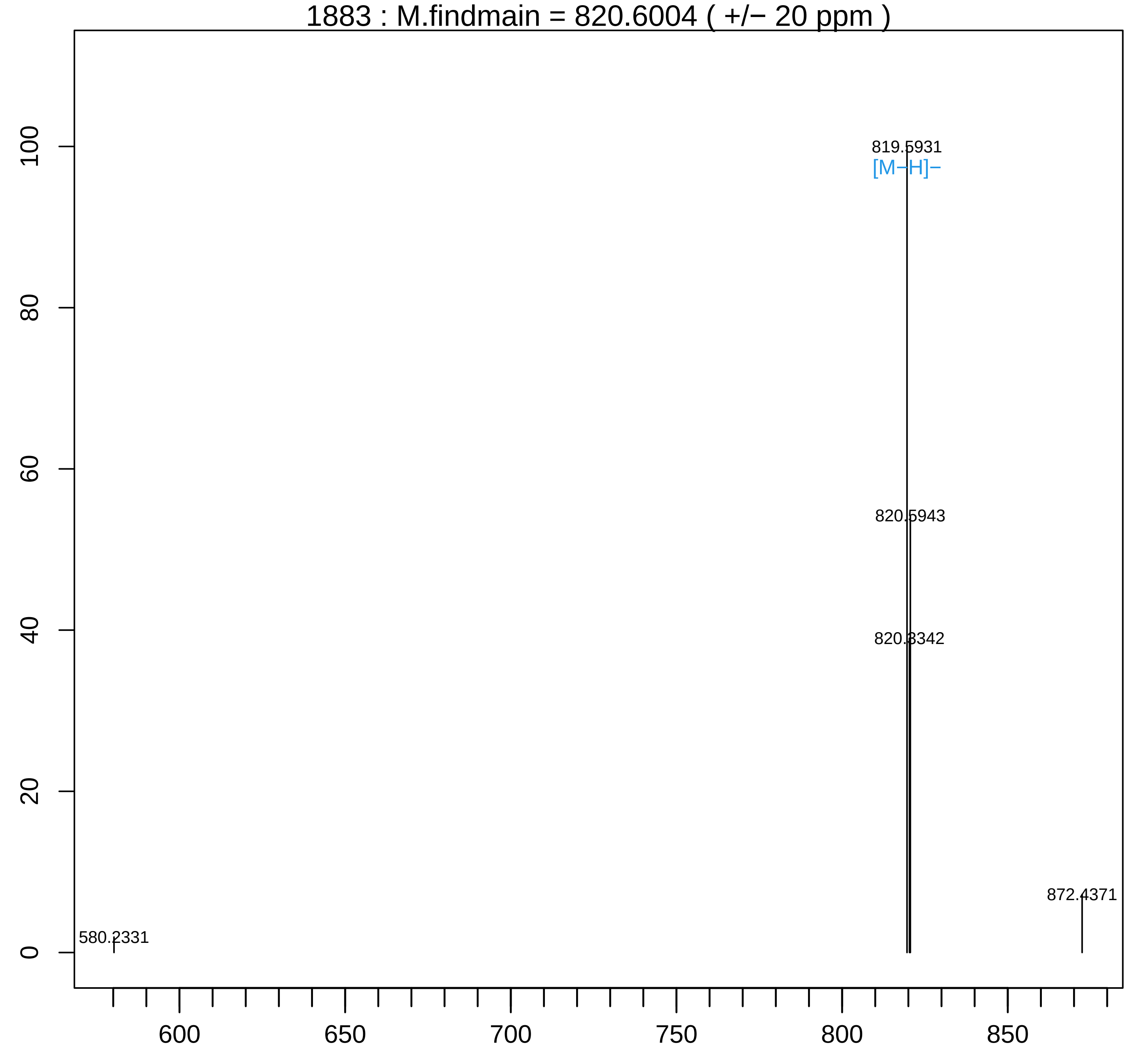

1883 : M.findmain = 820.6004 (+/− 20 ppm)
819.5931
[M−H]−
820.5943
820.3342
872.4371
580.2331
100
80
60
40
20
0
600
650
700
750
800
850

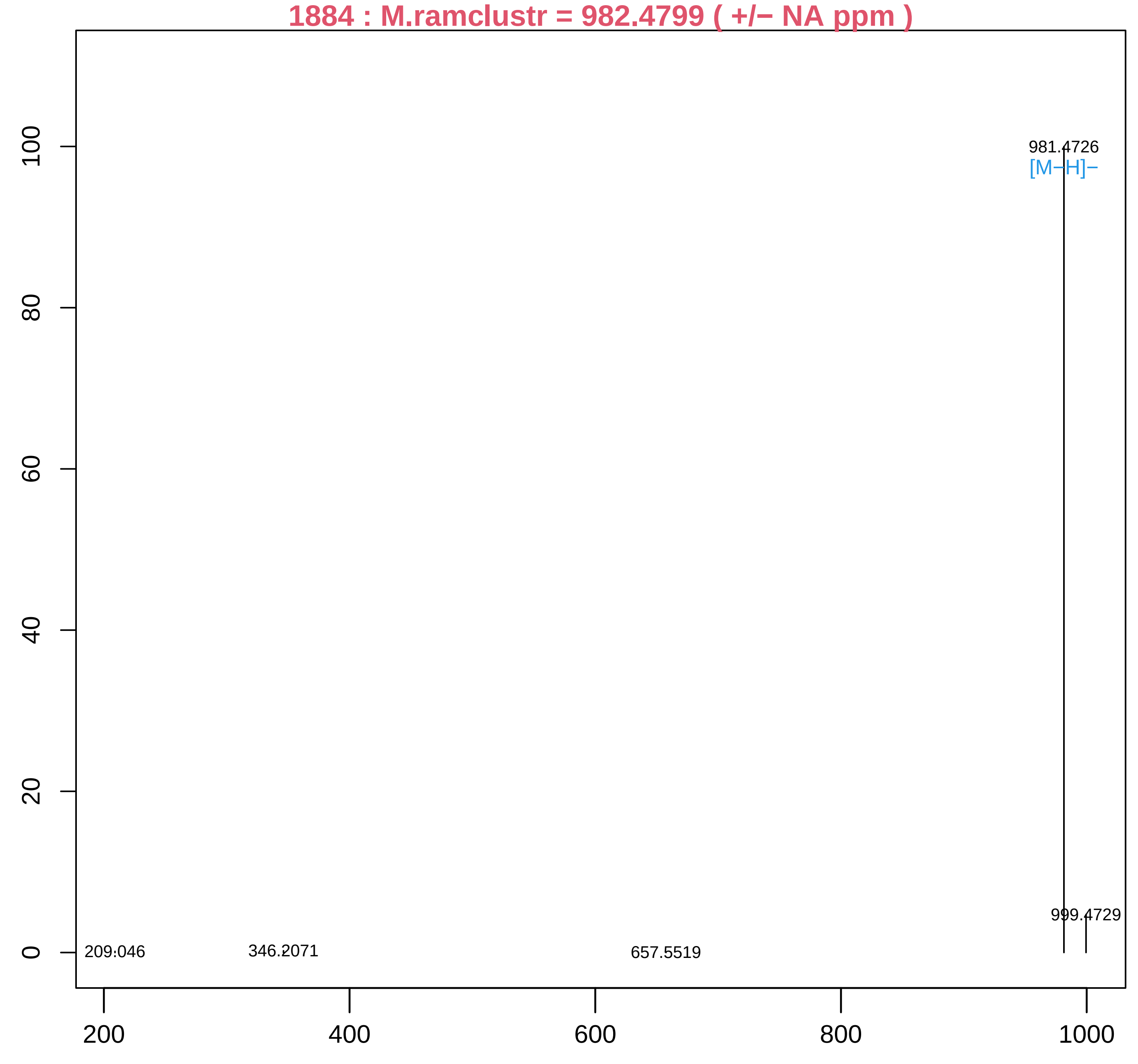
1884 : M.ramclustr = 982.4799 (+/− NA ppm)
981.4726
[M−H]−
999.4729
209:046
346.2071
657.5519
0
20
40
60
80
100
200
400
600
800
1000

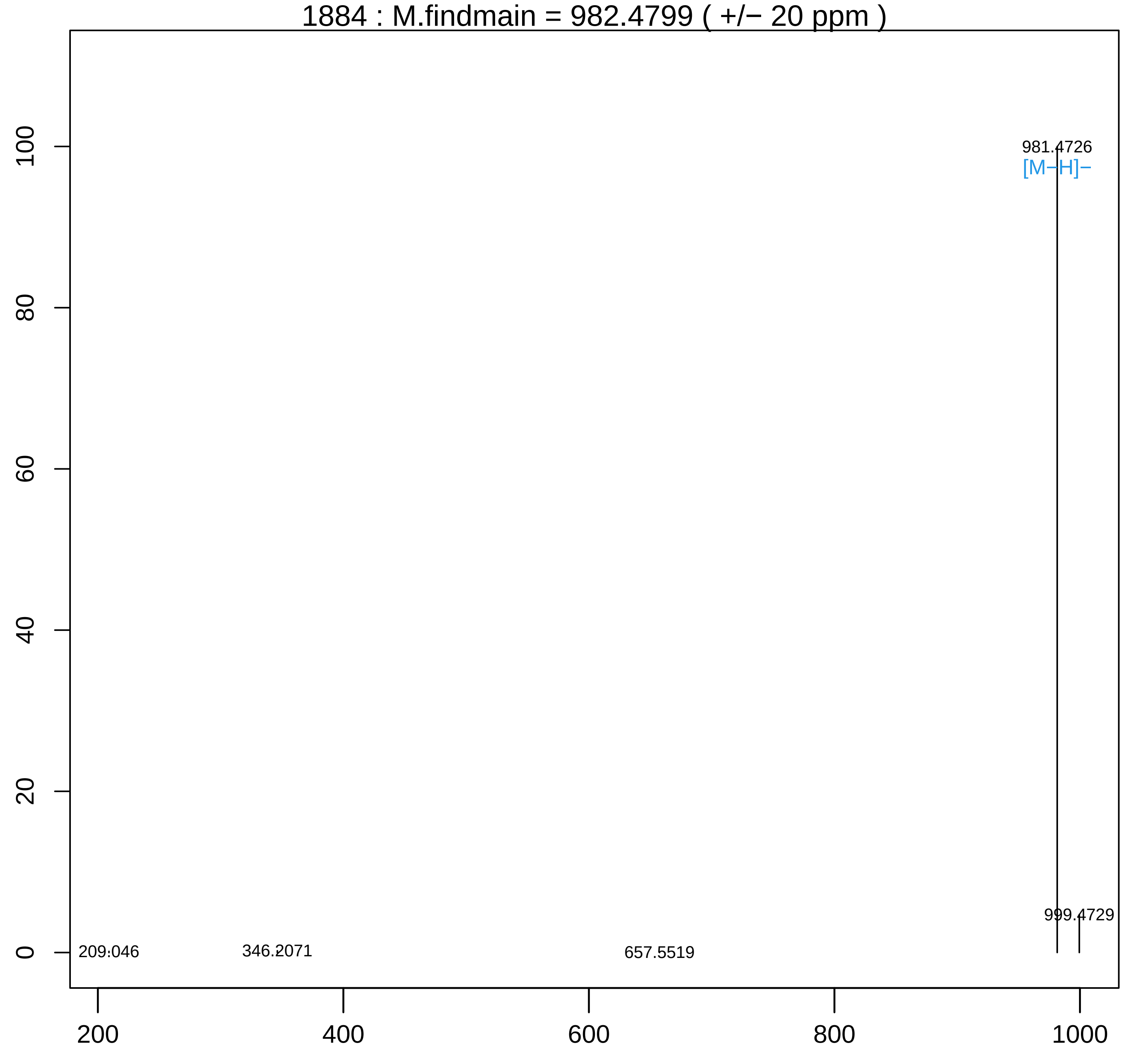
1884 : M.findmain = 982.4799 (+/− 20 ppm)
981.4726
[M−H]−
999.4729
209:046
346.2071
657.5519
0
20
40
60
80
100
200
400
600
800
1000

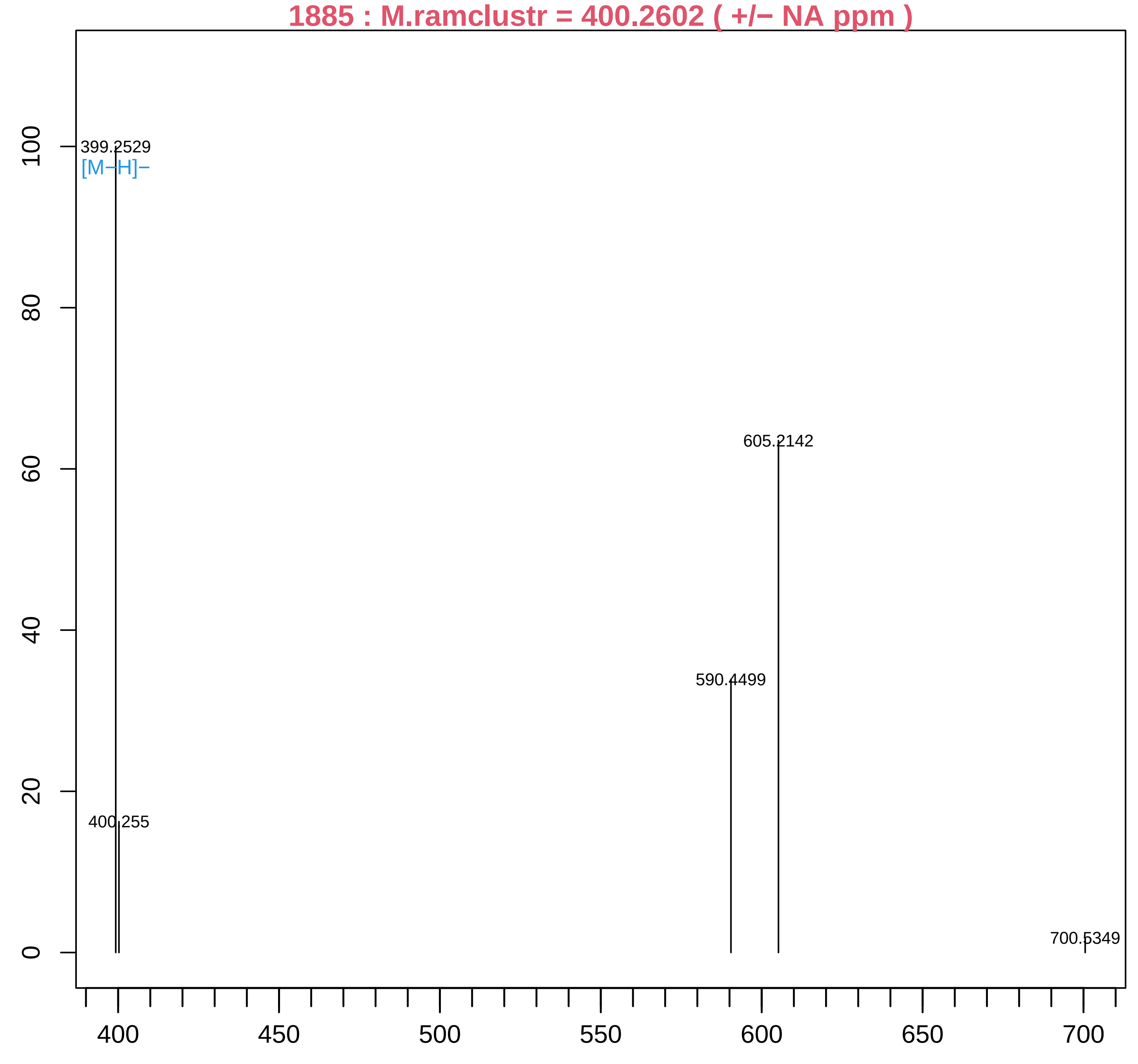
1885 : M.ramclustr = 400.2602 (+/− NA ppm)
399.2529
[M−H]−
400.255
590.4499
605.2142
700.5349
0
20
40
60
80
100
400
450
500
550
600
650
700

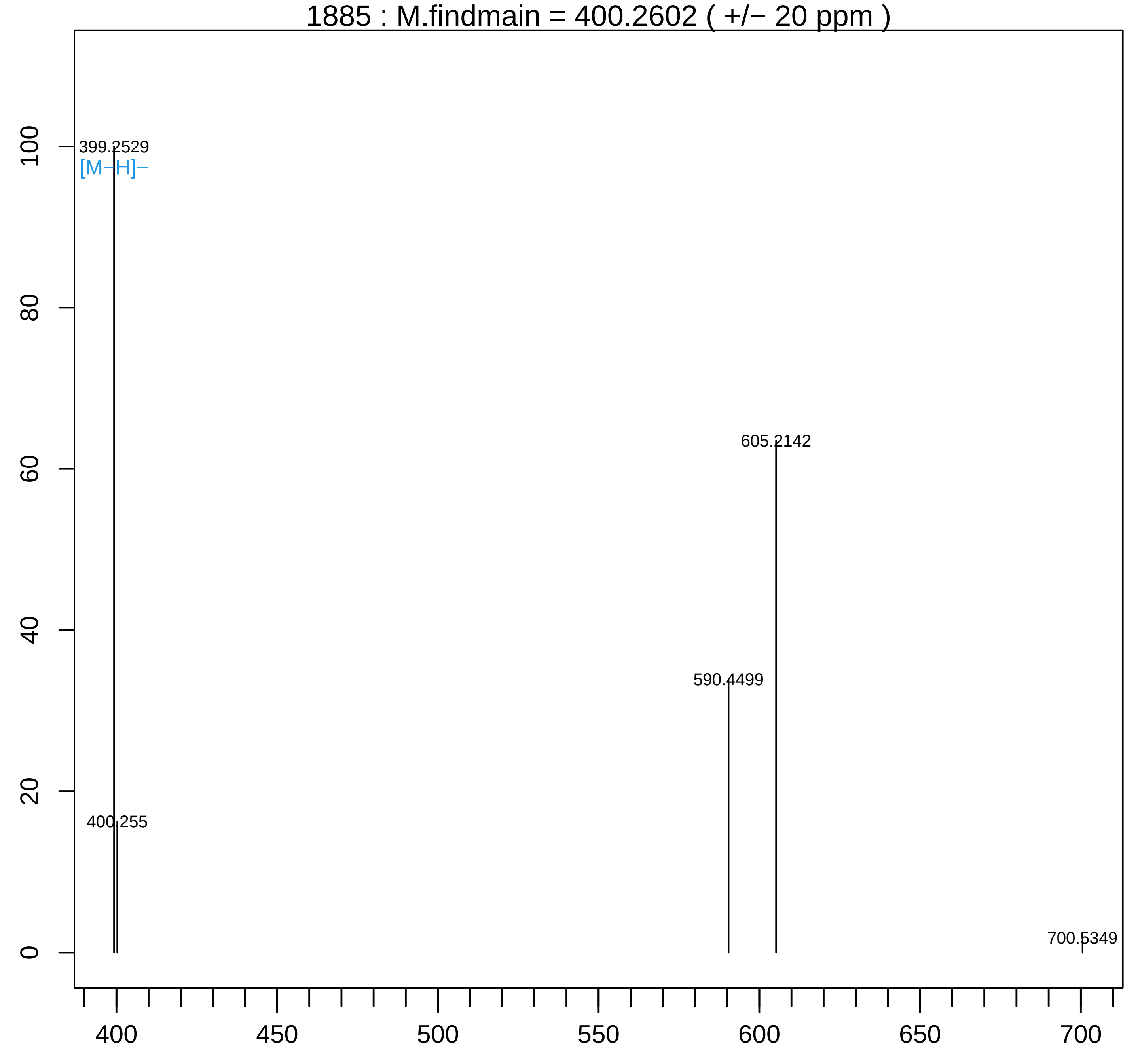
1885 : M.findmain = 400.2602 (+/− 20 ppm)
399.2529
[M−H]−
400.255
590.4499
605.2142
700.5349
0
20
40
60
80
100
400
450
500
550
600
650
700

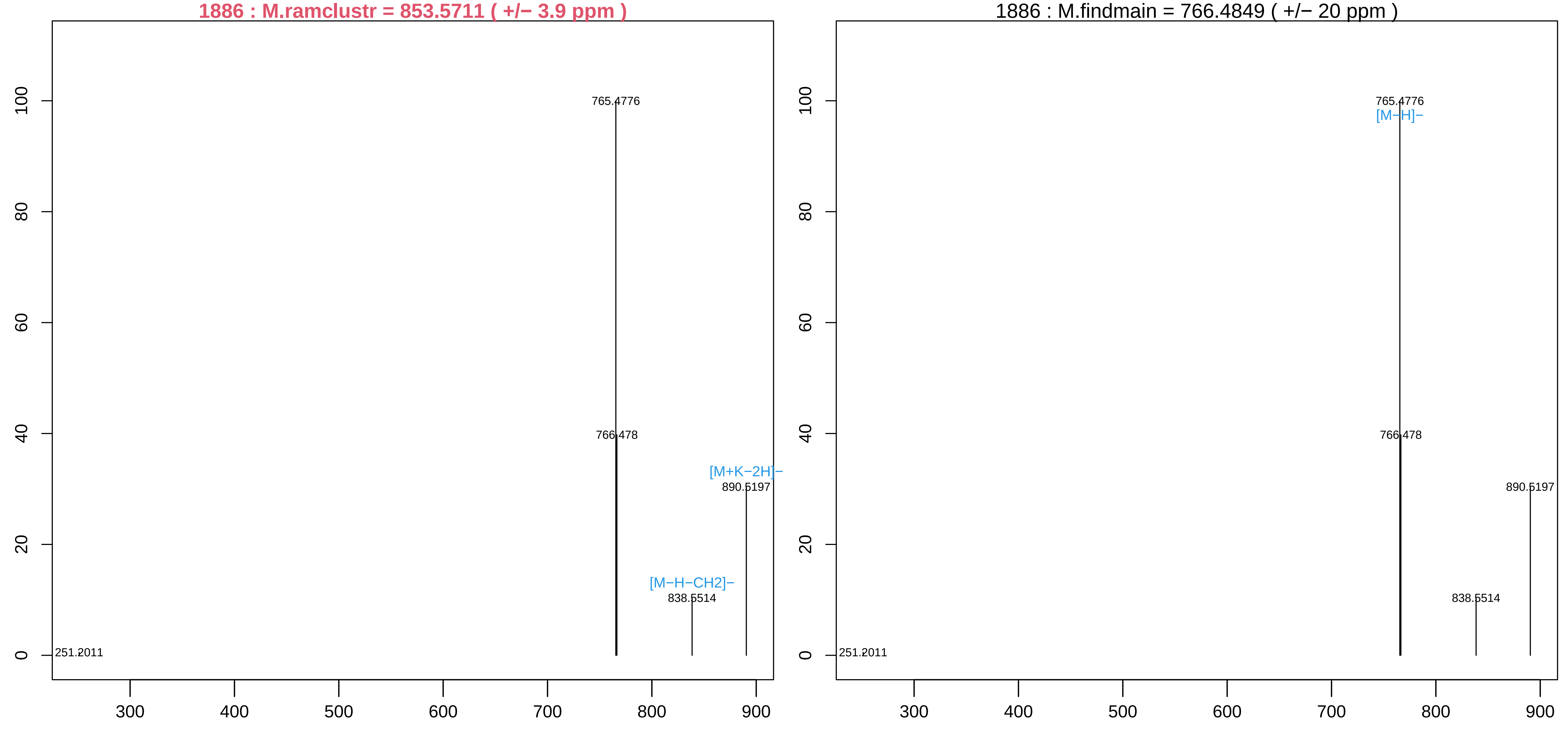
1886 : M.ramclustr = 853.5711 (+/− 3.9 ppm)
765.4776
766.478
[M+K−2H]−
890.5197
[M−H−CH2]−
838.5514
251.2011
1886 : M.findmain = 766.4849 (+/− 20 ppm)
765.4776
[M−H]−
766.478
890.5197
838.5514
251.2011

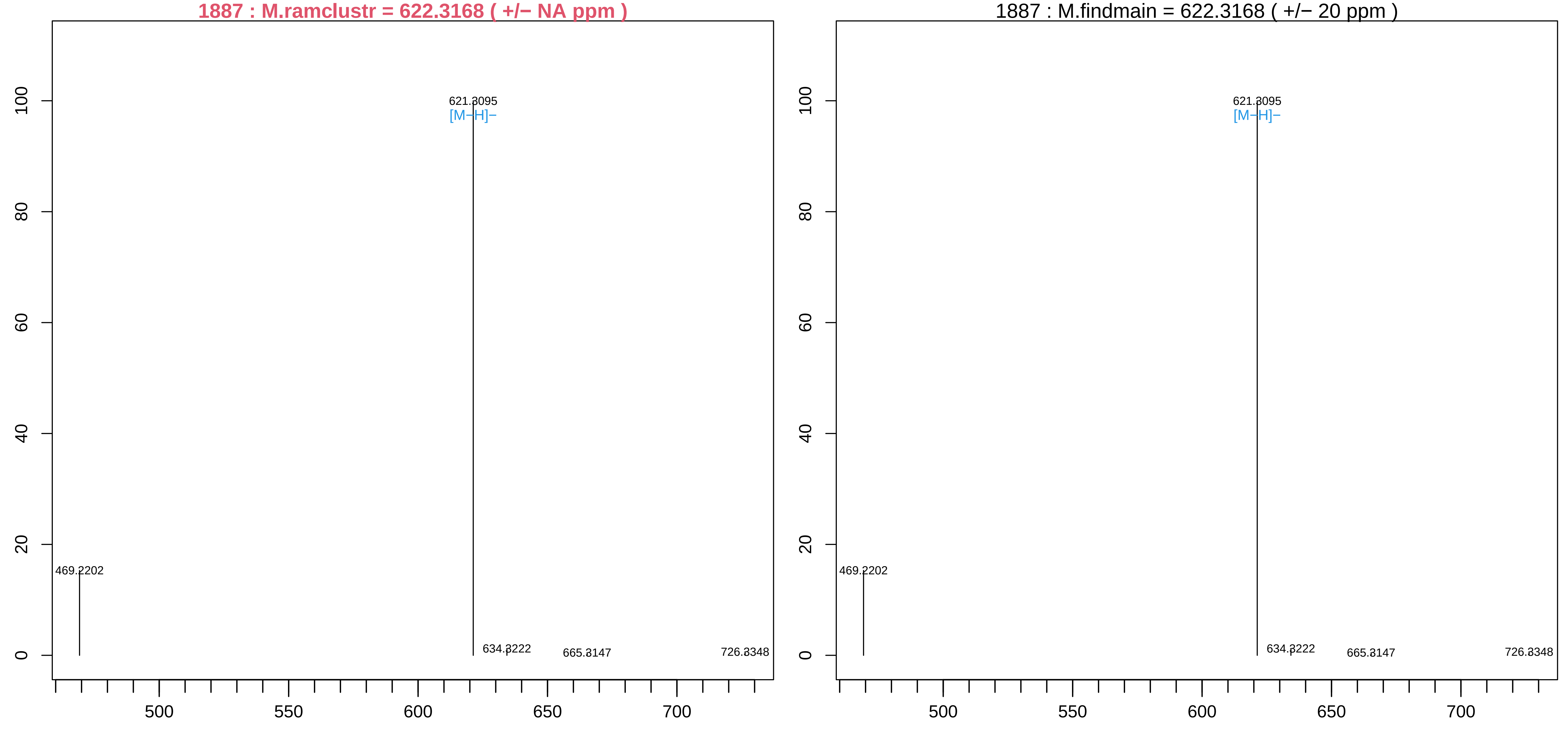
1887 : M.ramclustr = 622.3168 (+/− NA ppm)
1887 : M.findmain = 622.3168 (+/− 20 ppm)
621.3095
[M−H]−
469.2202
634.3222
665.3147
726.3348

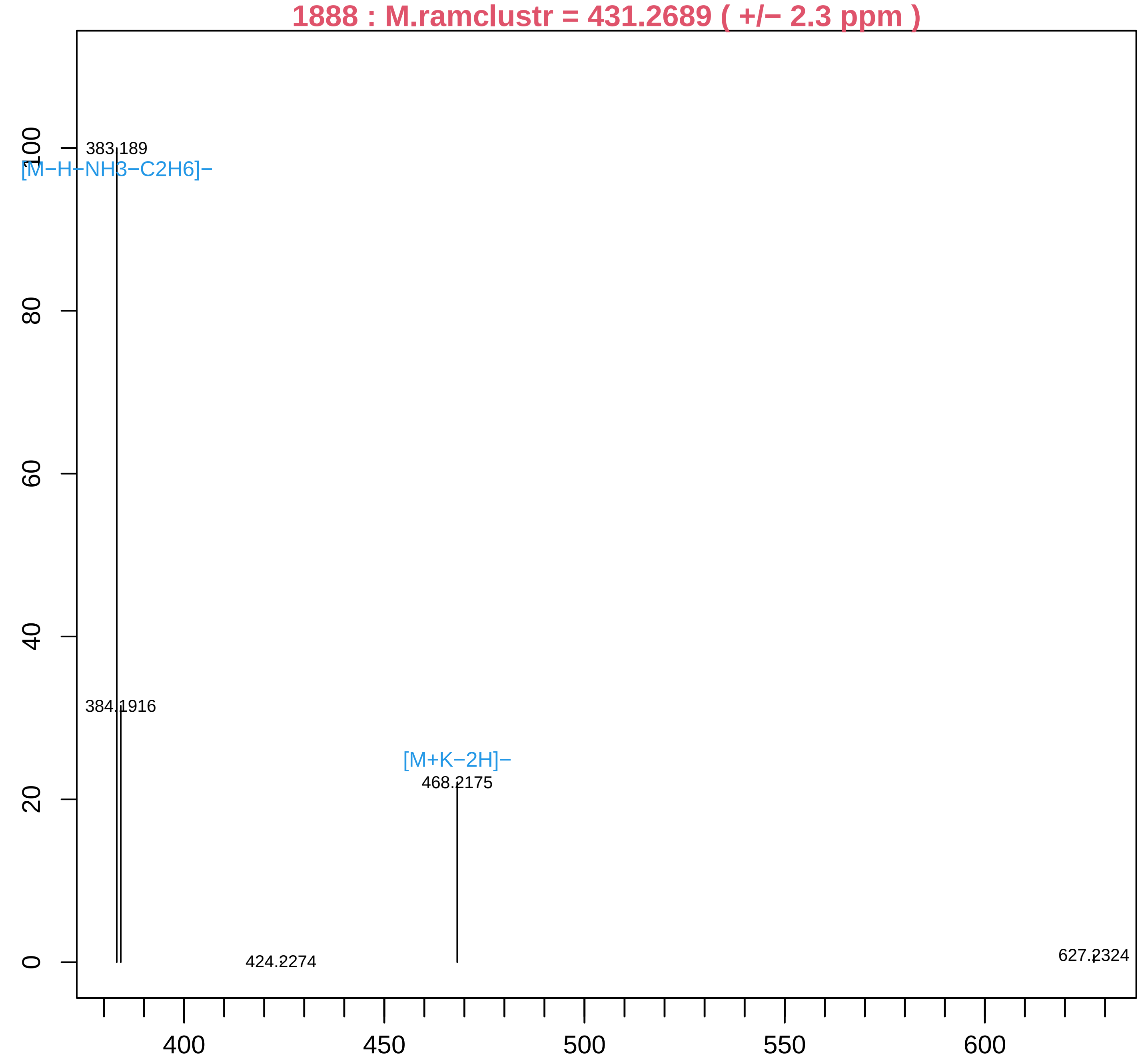
1888 : M.ramclustr = 431.2689 (+/− 2.3 ppm)
383.189
[M−H−NH3−C2H6]−
384.1916
[M+K−2H]−
468.2175
424.2274
627.2324
0
20
40
60
80
100
400
450
500
550
600

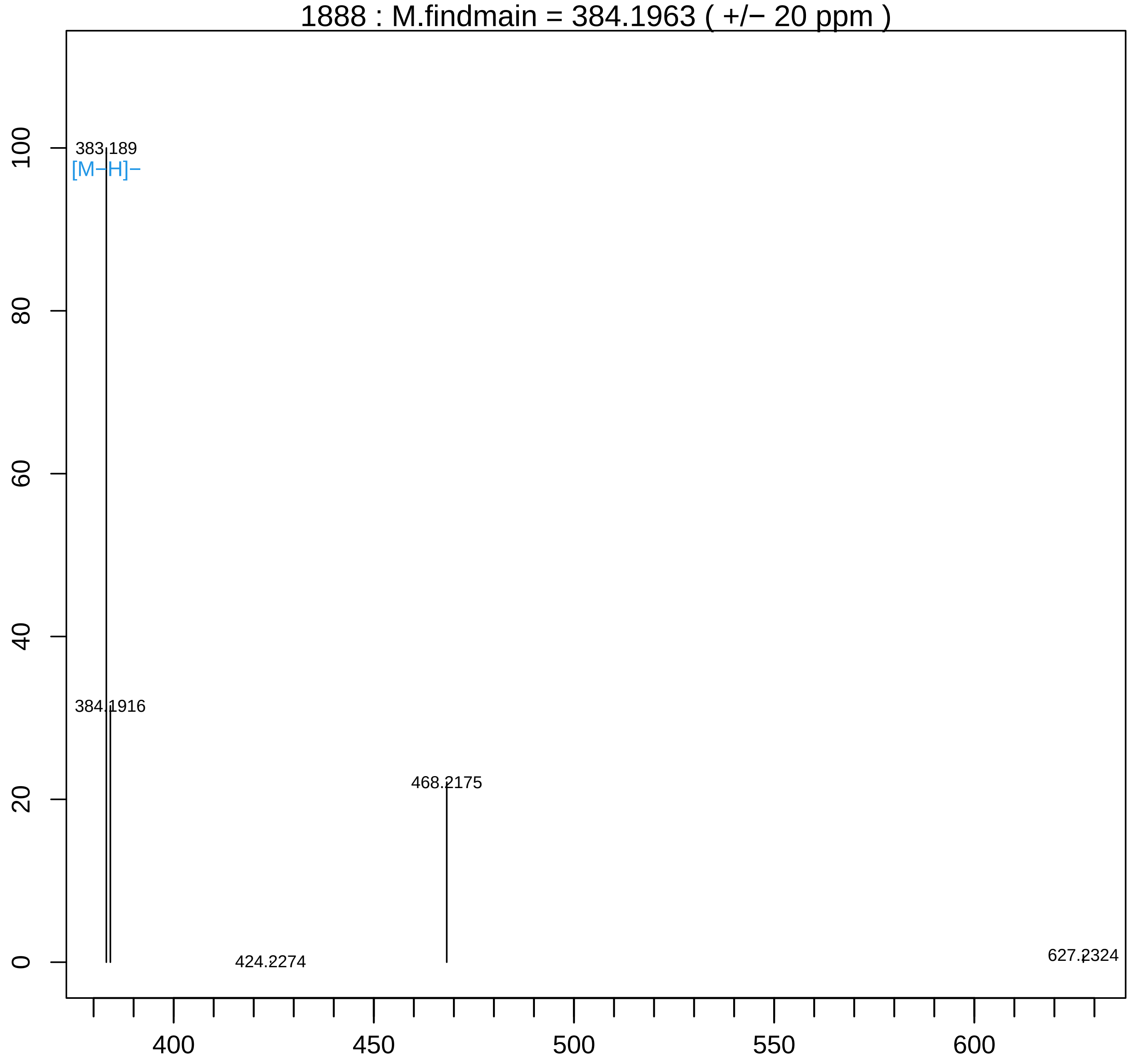
1888 : M.findmain = 384.1963 (+/− 20 ppm)
383.189
[M−H]−
384.1916
468.2175
424.2274
627.2324
0
20
40
60
80
100
400
450
500
550
600

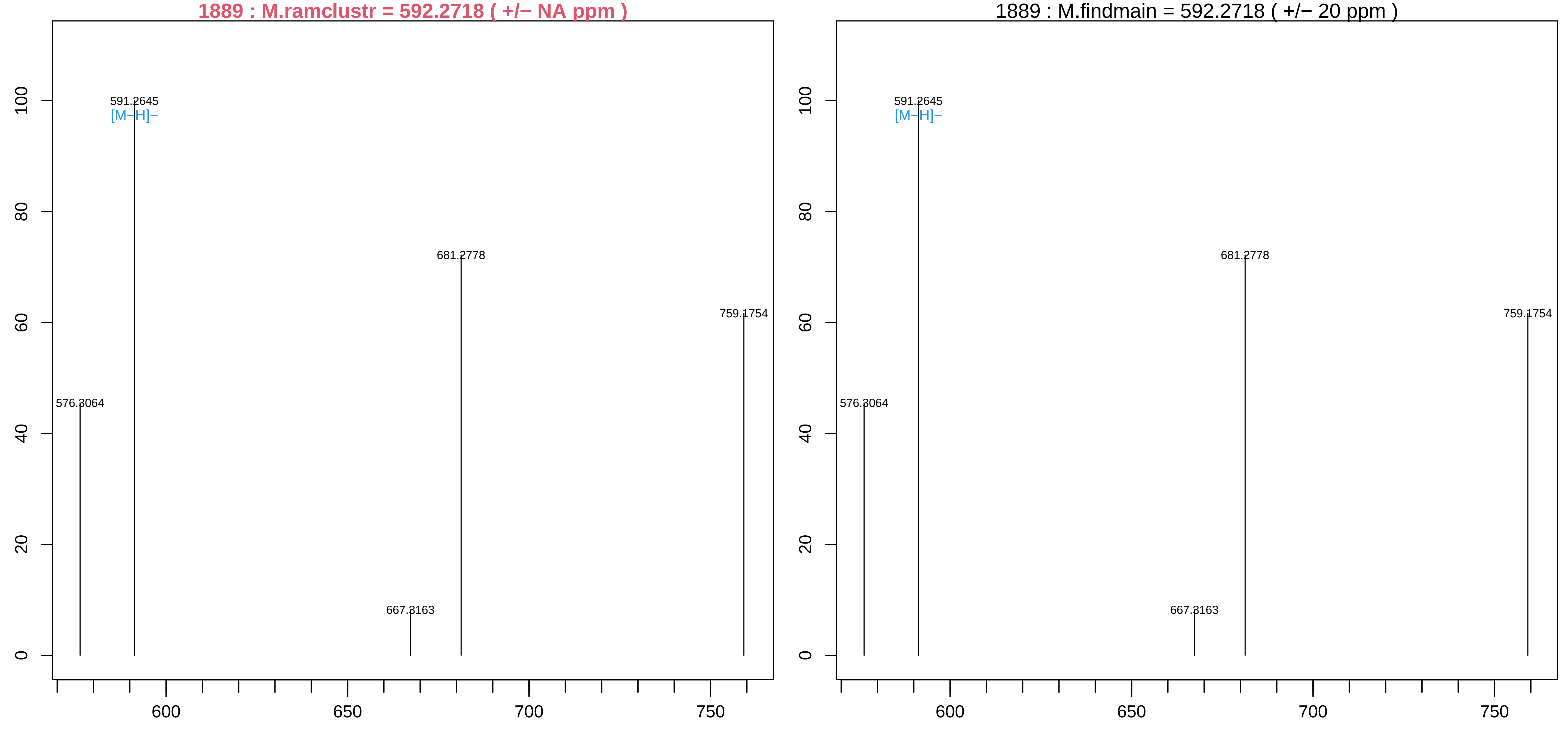
1889 : M.ramclustr = 592.2718 (+/− NA ppm)
591.2645
[M−H]−
681.2778
759.1754
576.3064
667.3163
1889 : M.findmain = 592.2718 (+/− 20 ppm)
591.2645
[M−H]−
681.2778
759.1754
576.3064
667.3163
0
20
40
60
80
100
600
650
700
750

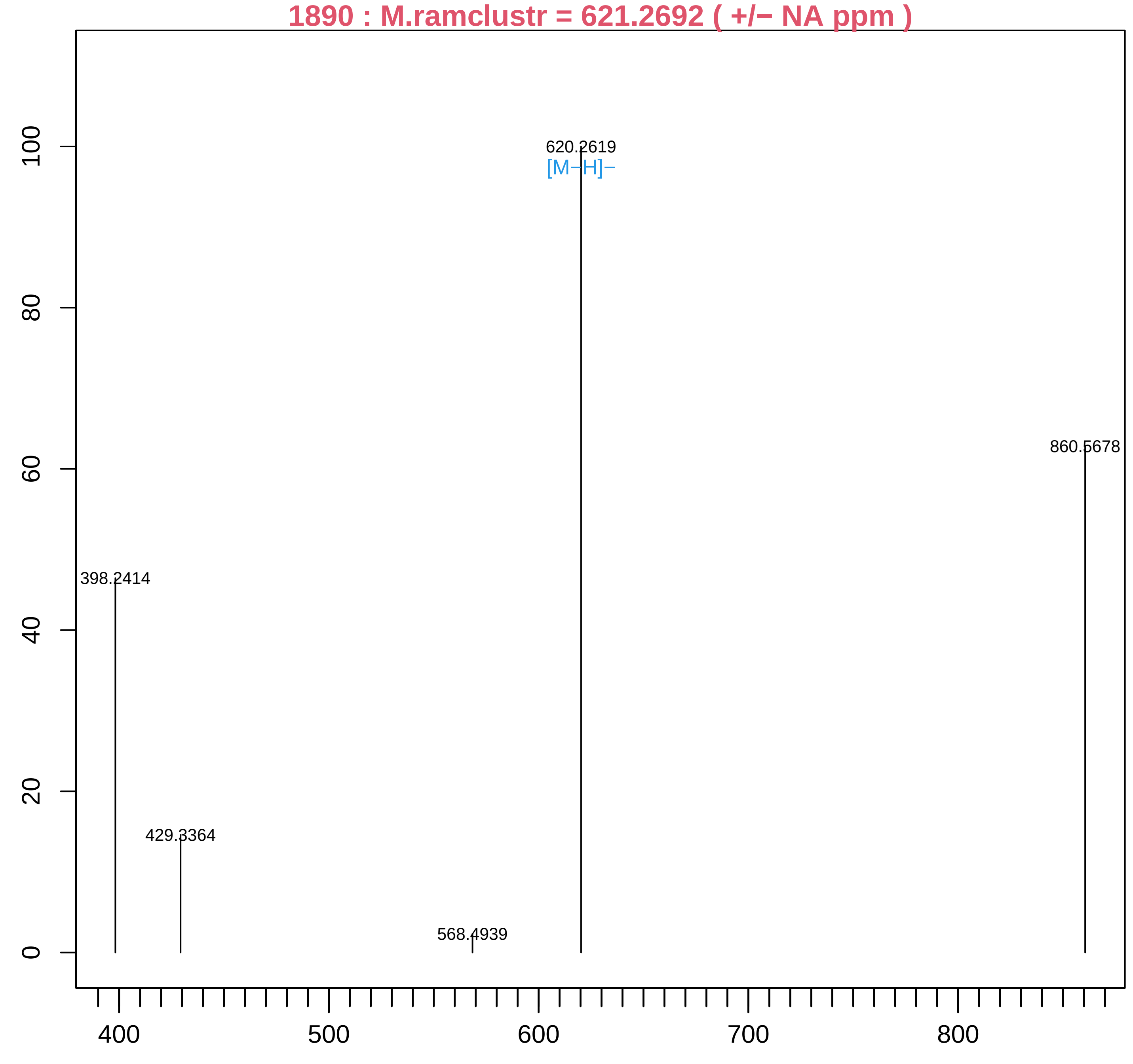
1890 : M.ramclustr = 621.2692 (+/− NA ppm)
620.2619
[M−H]−
860.5678
398.2414
429.3364
568.4939
0
20
40
60
80
100
400
500
600
700
800

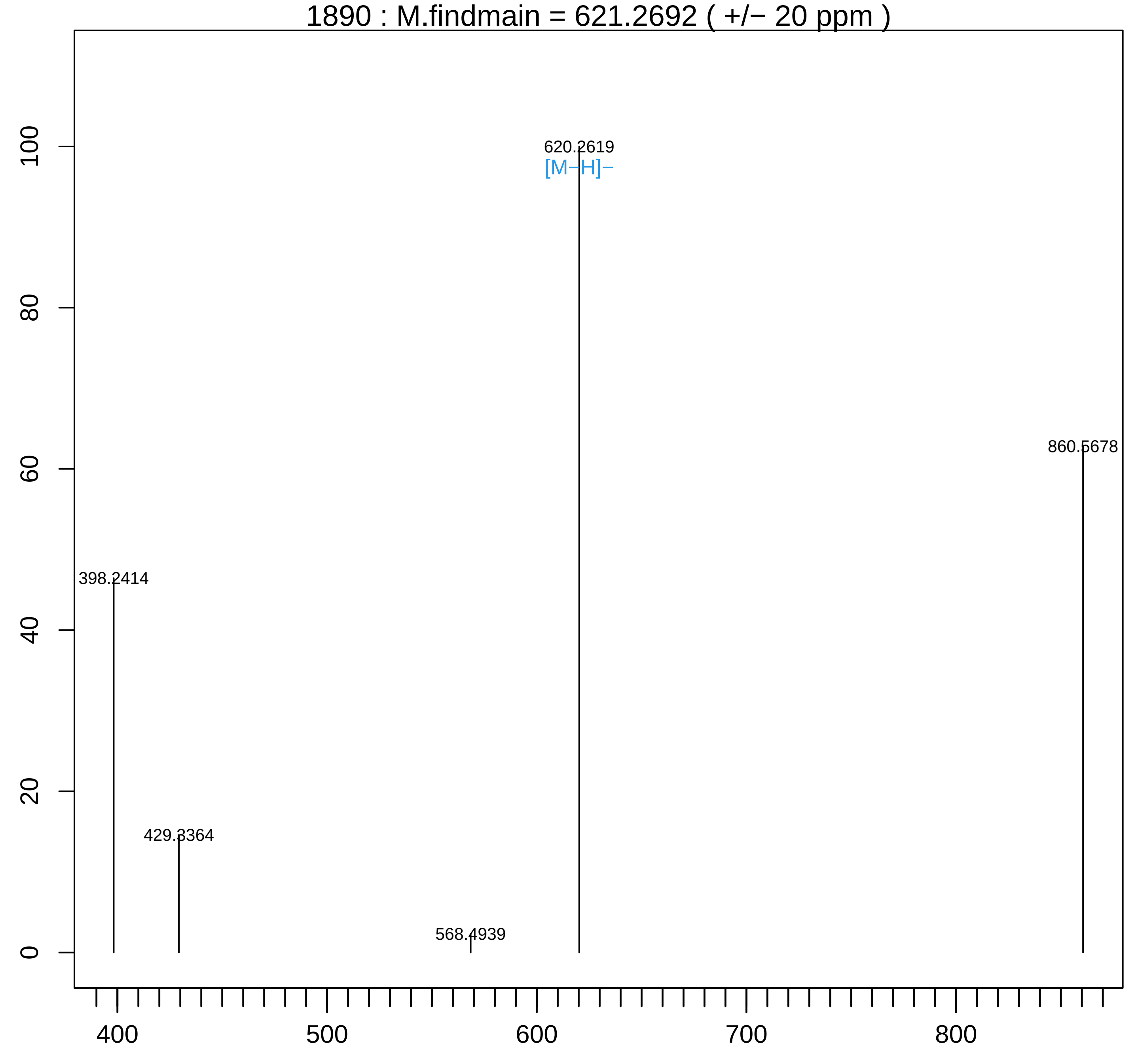
1890 : M.findmain = 621.2692 (+/− 20 ppm)
620.2619
[M−H]−
860.5678
398.2414
429.3364
568.4939
0
20
40
60
80
100
400
500
600
700
800

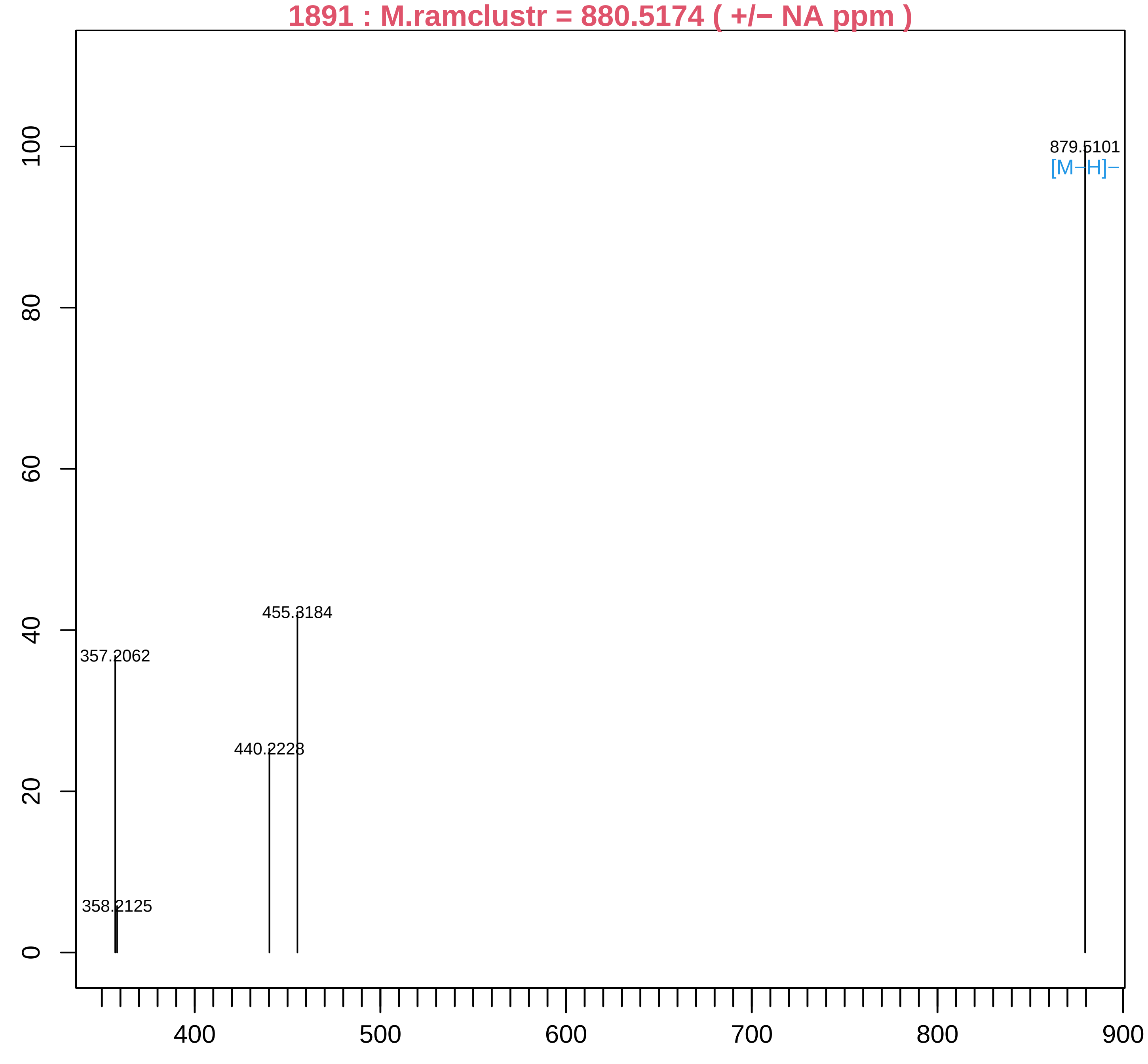
1891 : M.ramclustr = 880.5174 (+/− NA ppm)
879.5101
[M−H]−
455.3184
357.2062
440.2228
358.2125
0
20
40
60
80
100
400
500
600
700
800
900
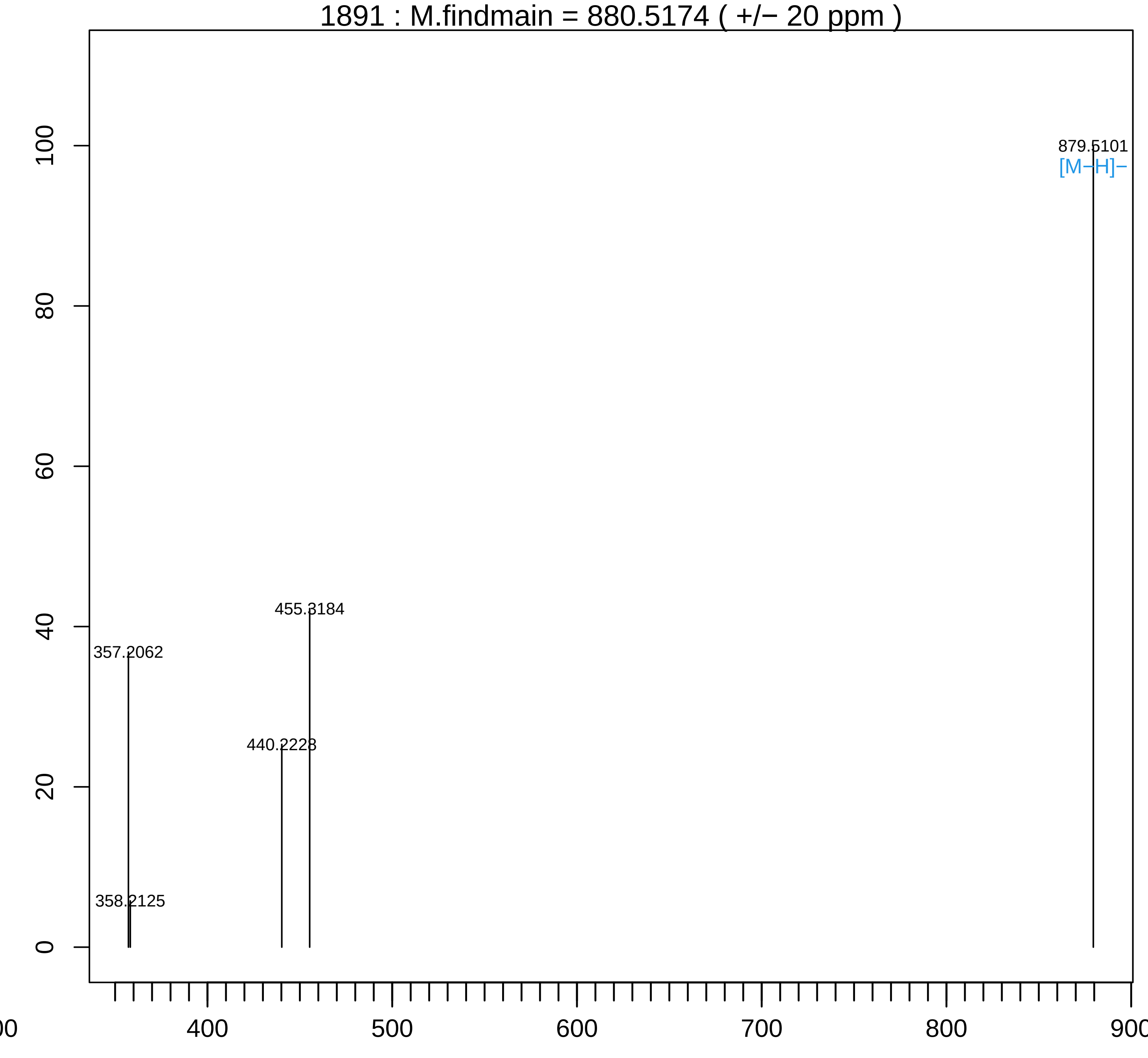
1891 : M.findmain = 880.5174 (+/− 20 ppm)
879.5101
[M−H]−
455.3184
357.2062
440.2228
358.2125
0
20
40
60
80
100
400
500
600
700
800
900

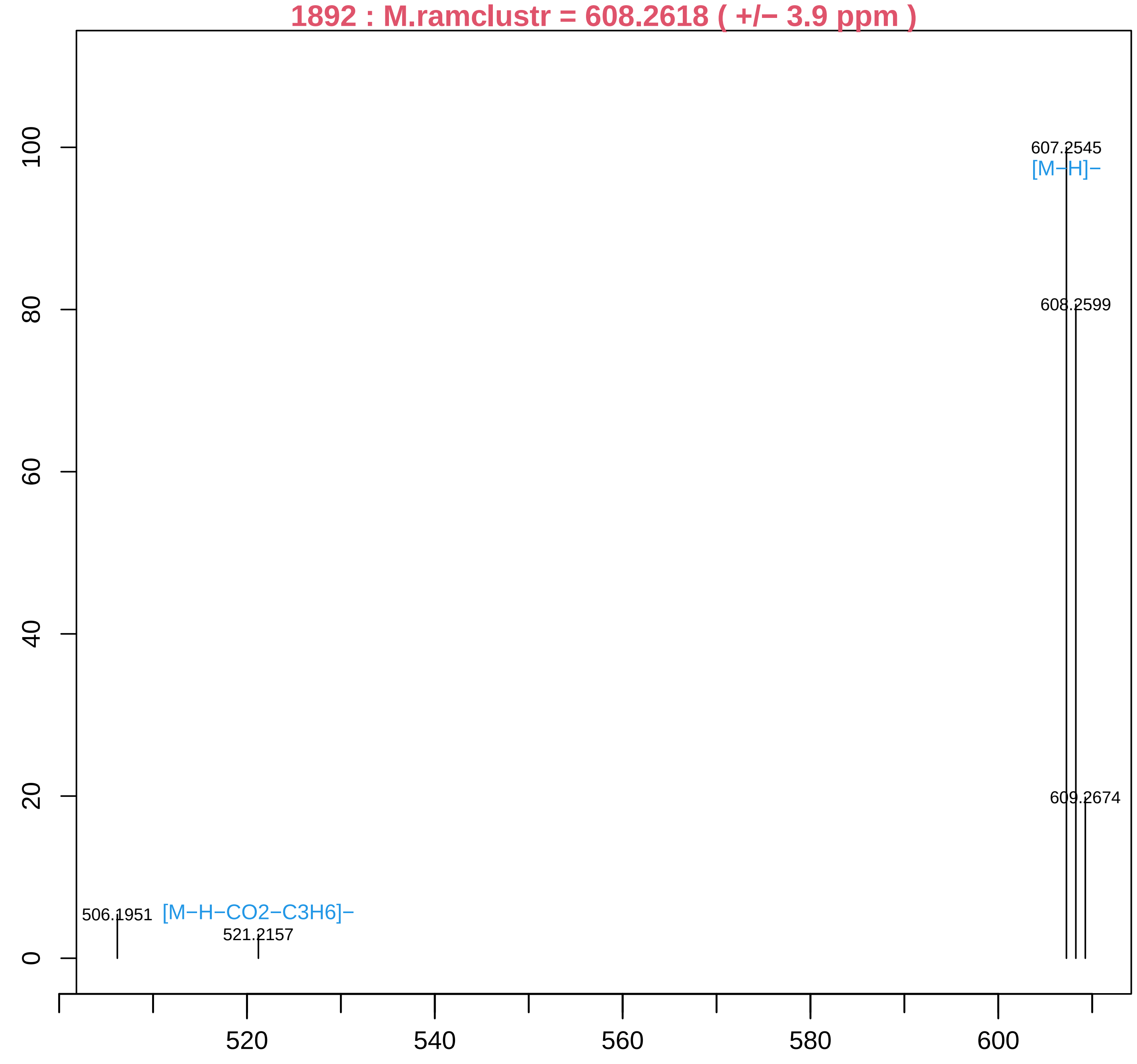
1892 : M.ramclustr = 608.2618 (+/− 3.9 ppm)
607.2545
[M−H]−
608.2599
609.2674
506.1951
[M−H−CO2−C3H6]−
521.2157
0
20
40
60
80
100
520
540
560
580
600

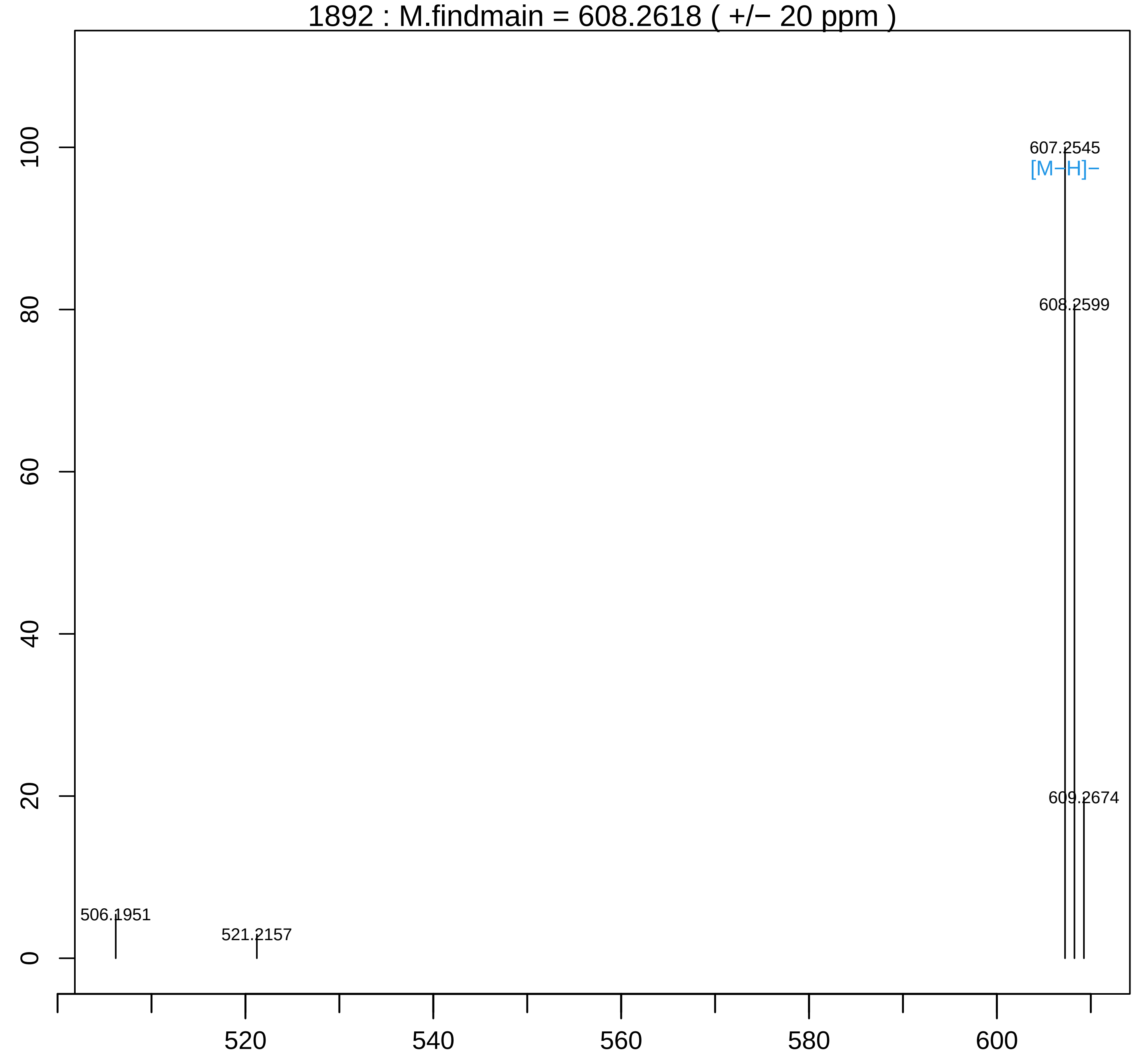
1892 : M.findmain = 608.2618 (+/− 20 ppm)
607.2545
[M−H]−
608.2599
609.2674
506.1951
521.2157
0
20
40
60
80
100
520
540
560
580
600

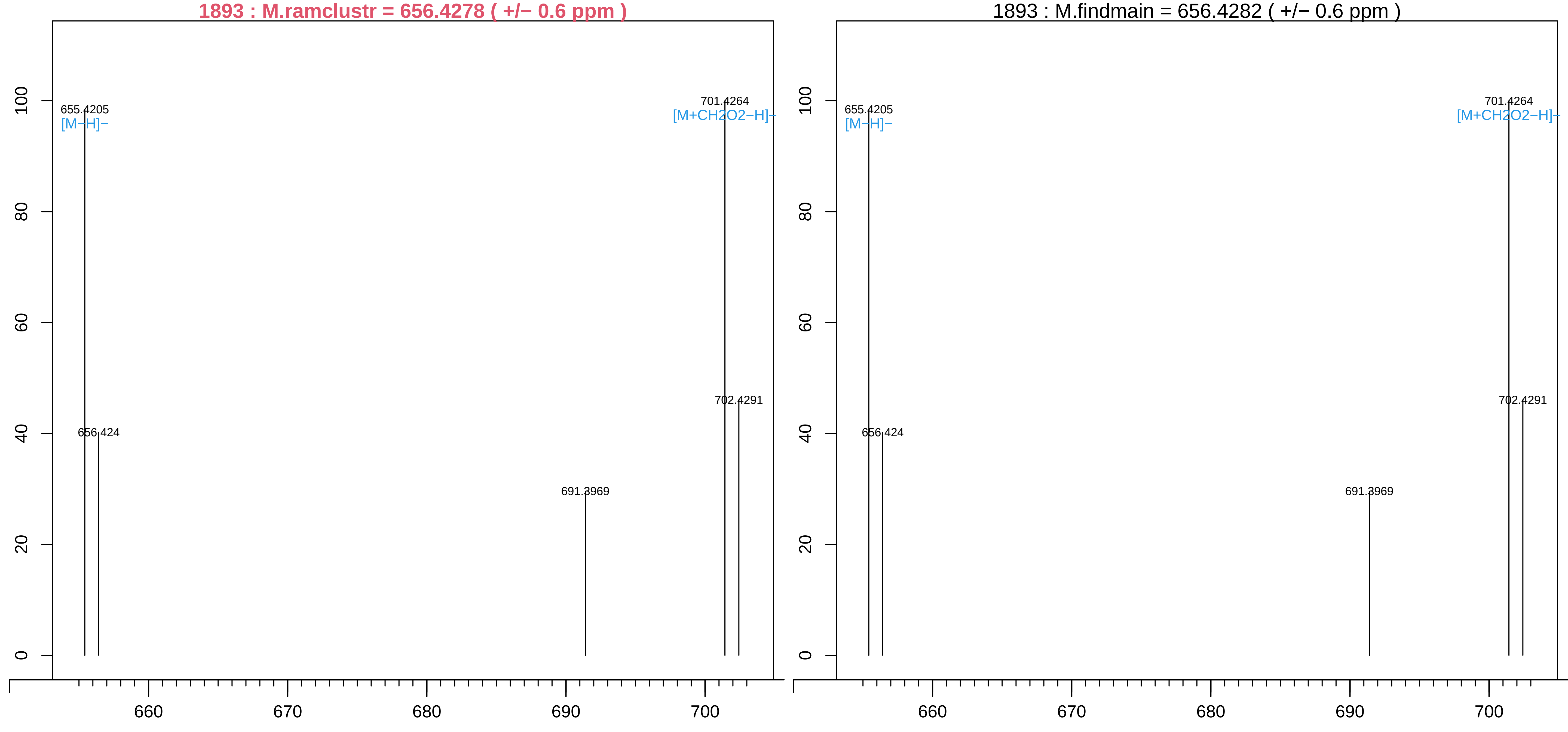
1893 : M.ramclustr = 656.4278 (+/− 0.6 ppm)
655.4205
[M−H]−
656.424
691.3969
701.4264
[M+CH2O2−H]−
702.4291
0
20
40
60
80
100
660
670
680
690
700
1893 : M.findmain = 656.4282 (+/− 0.6 ppm)
655.4205
[M−H]−
656.424
691.3969
701.4264
[M+CH2O2−H]−
702.4291
0
20
40
60
80
100
660
670
680
690
700

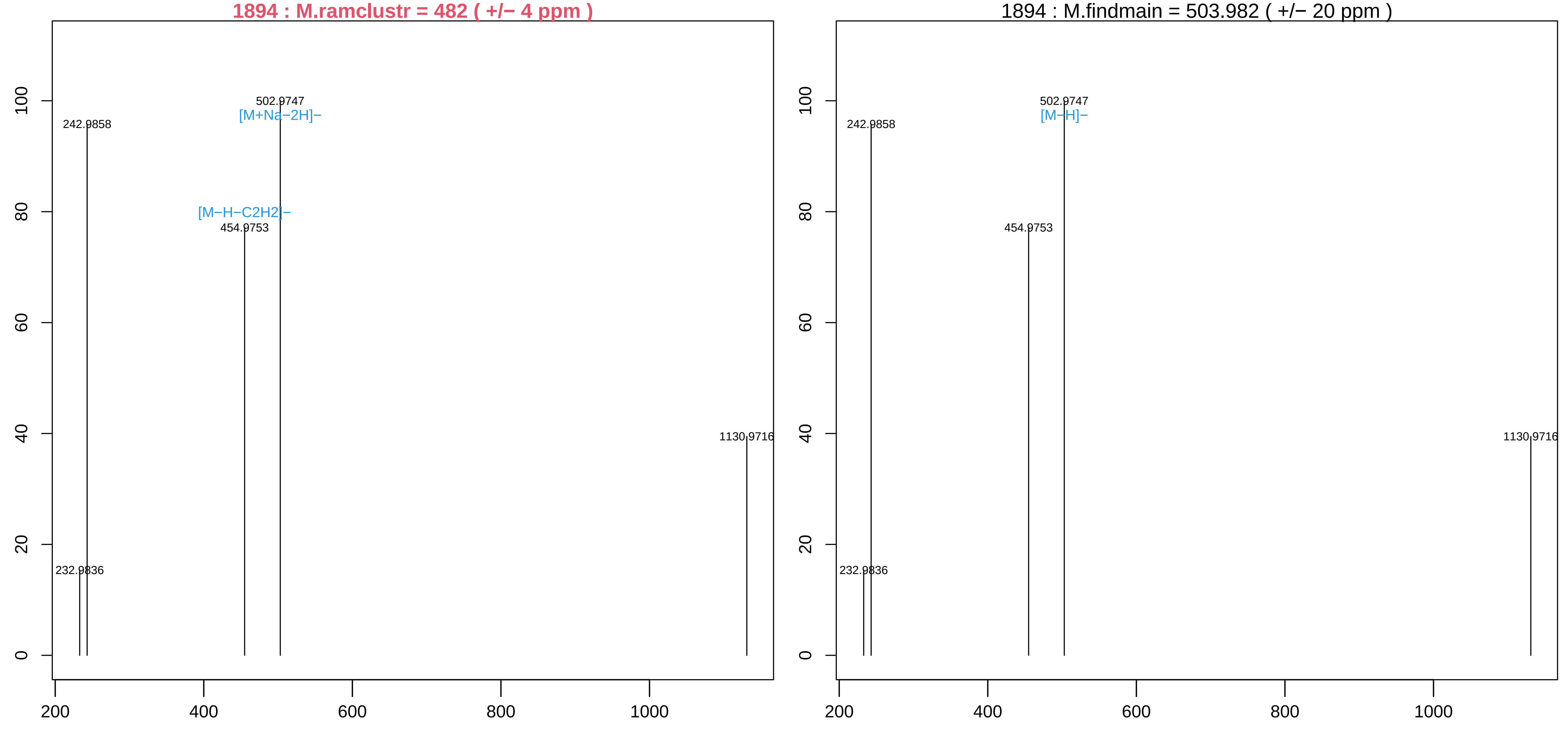
1894 : M.ramclustr = 482 (+/− 4 ppm)
502.9747
[M+Na−2H]−
242.9858
[M−H−C2H2]−
454.9753
232.9836
1130.9716
1894 : M.findmain = 503.982 (+/− 20 ppm)
502.9747
[M−H]−
242.9858
454.9753
232.9836
1130.9716

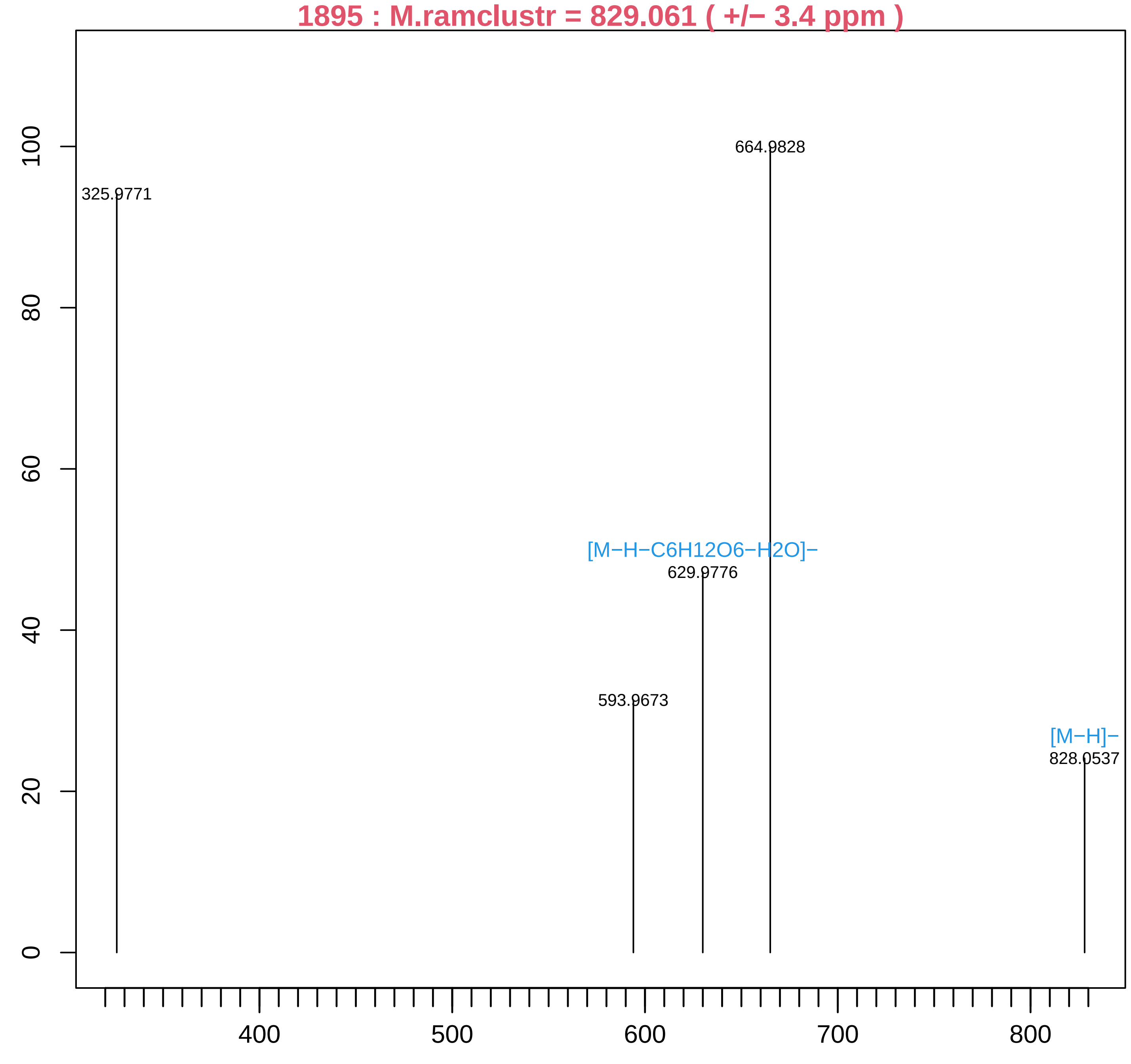
1895 : M.ramclustr = 829.061 (+/− 3.4 ppm)
325.9771
664.9828
[M−H−C6H12O6−H2O]−
629.9776
593.9673
[M−H]−
828.0537
0
20
40
60
80
100
400
500
600
700
800

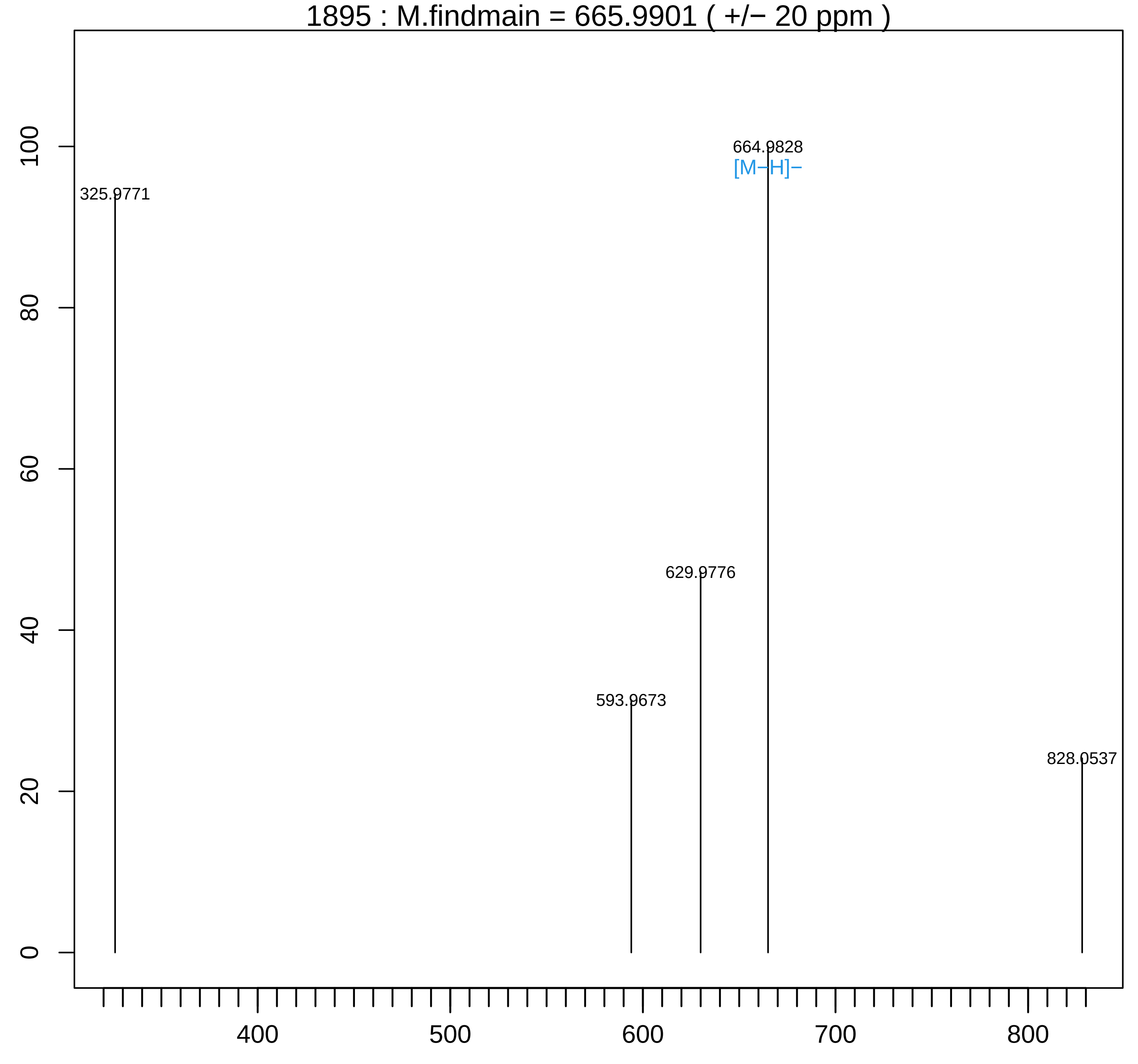
1895 : M.findmain = 665.9901 (+/− 20 ppm)
664.9828
[M−H]−
325.9771
629.9776
593.9673
828.0537
0
20
40
60
80
100
400
500
600
700
800

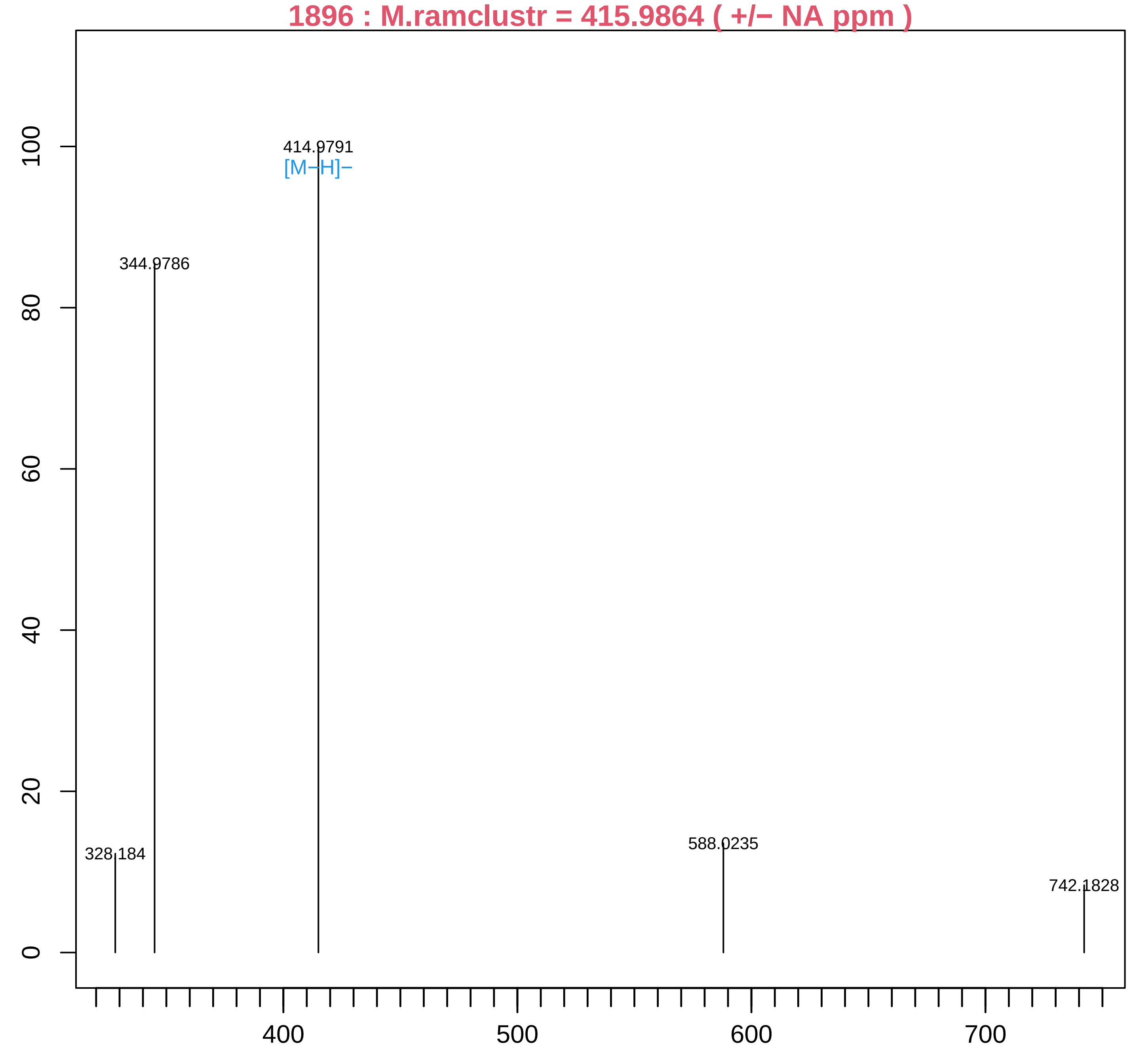
1896 : M.ramclustr = 415.9864 (+/− NA ppm)
414.9791
[M−H]−
344.9786
328.184
588.0235
742.1828
0
20
40
60
80
100
400
500
600
700

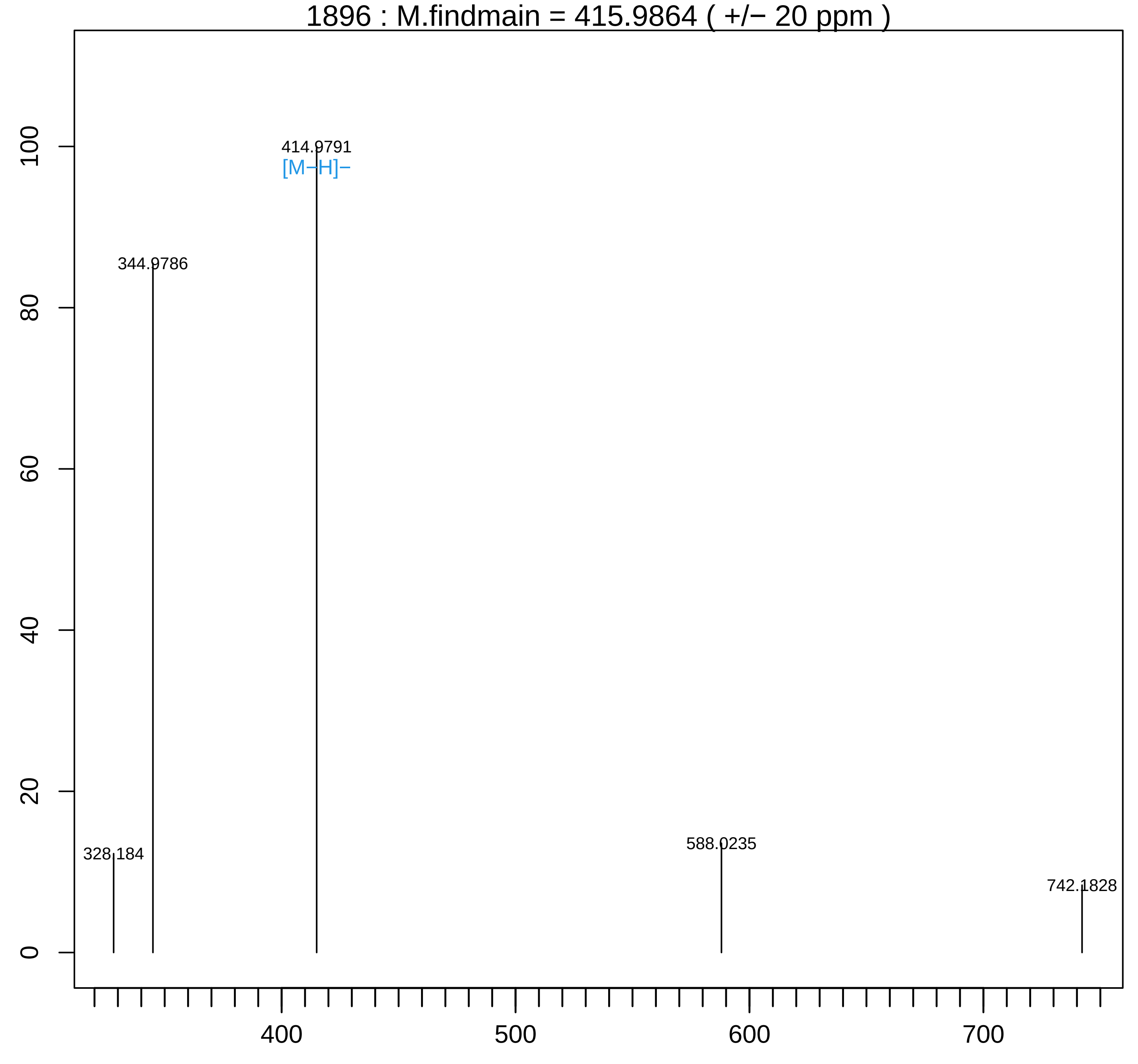
1896 : M.findmain = 415.9864 (+/− 20 ppm)
414.9791
[M−H]−
344.9786
328.184
588.0235
742.1828
0
20
40
60
80
100
400
500
600
700

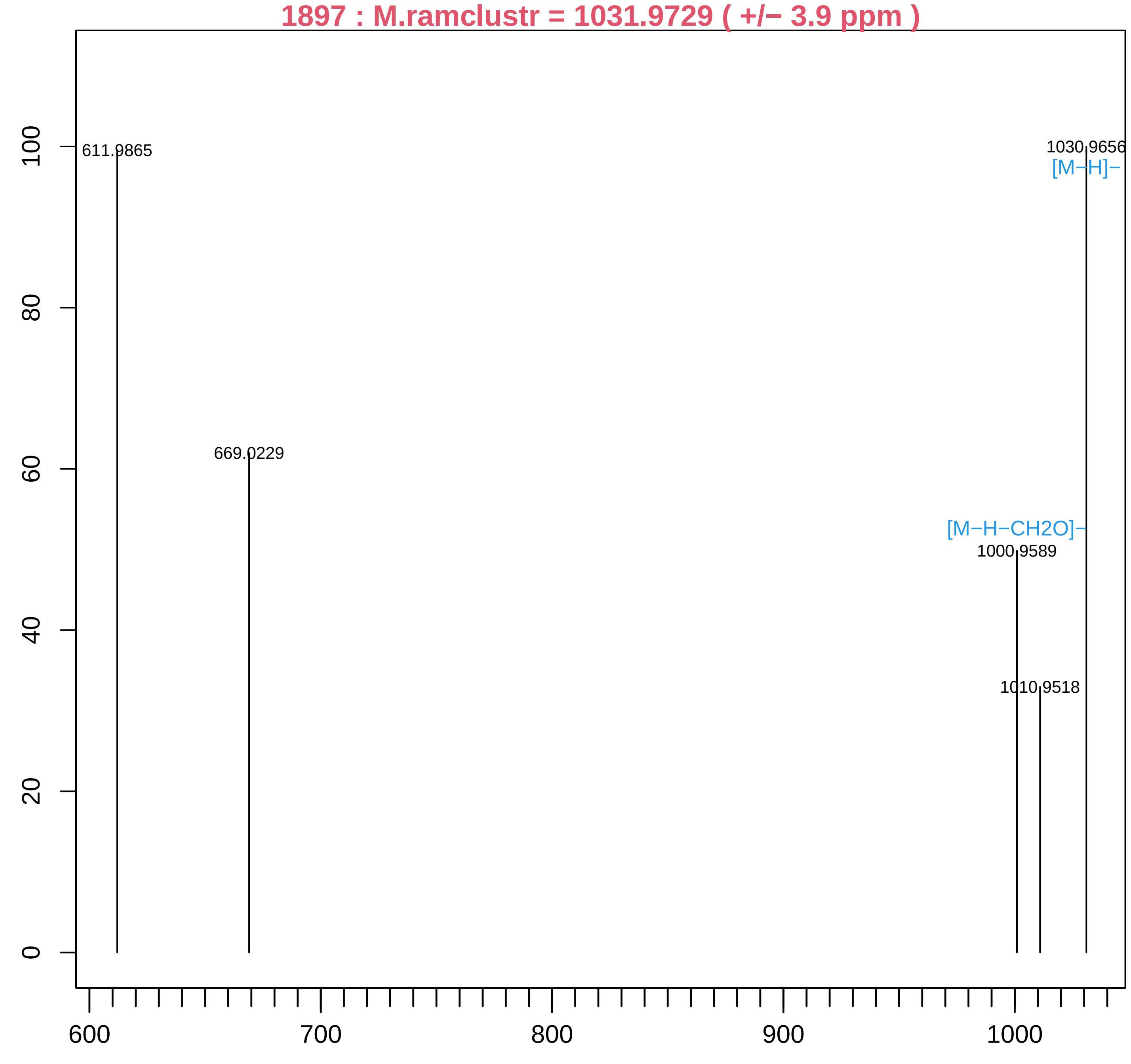
1897 : M.ramclustr = 1031.9729 (+/− 3.9 ppm)
611.9865
669.0229
1030.9656
[M−H]−
[M−H−CH2O]−
1000.9589
1010.9518
0
20
40
60
80
100
600
700
800
900
1000

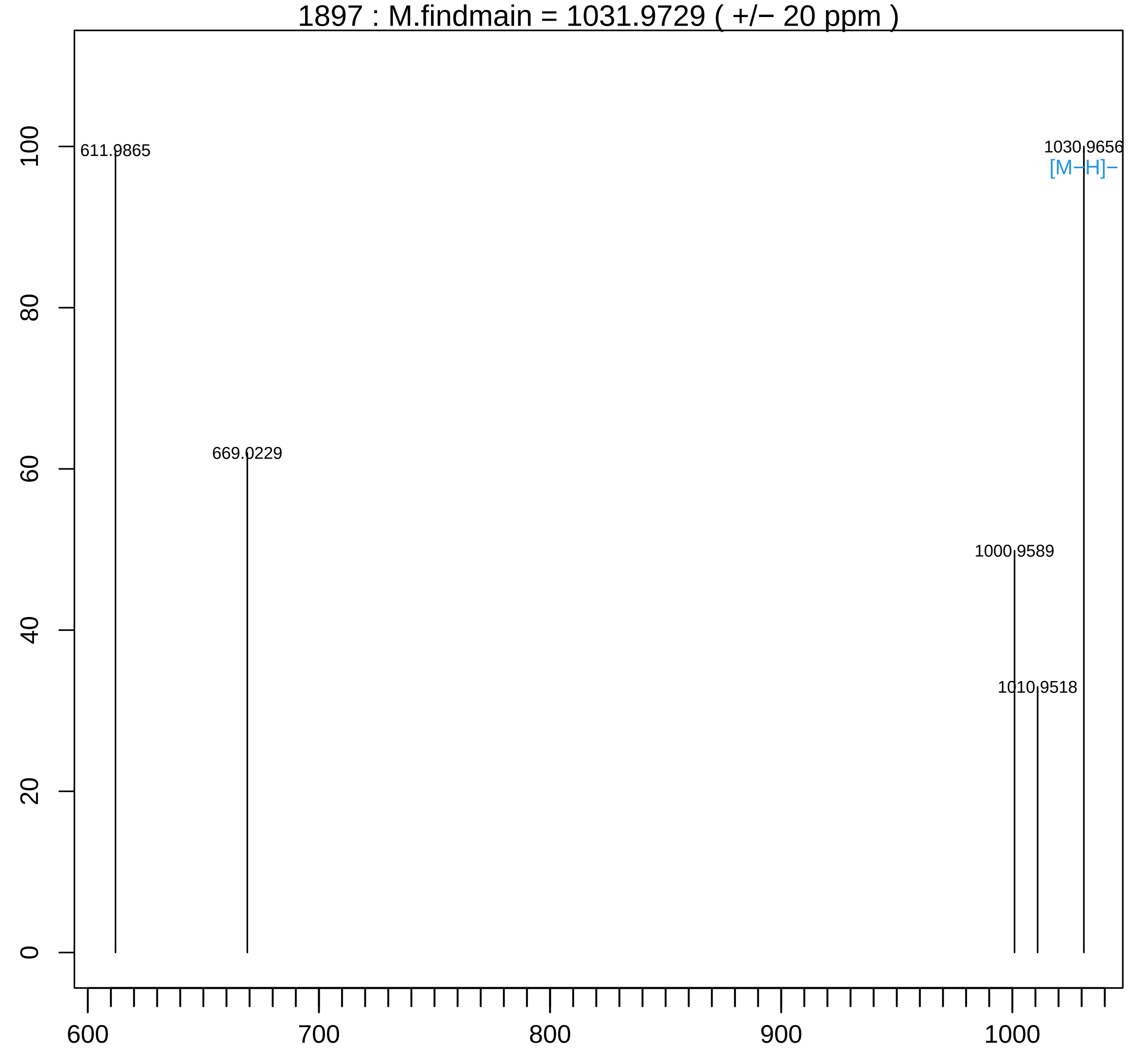
1897 : M.findmain = 1031.9729 (+/− 20 ppm)
611.9865
669.0229
1030.9656
[M−H]−
1000.9589
1010.9518
0
20
40
60
80
100
600
700
800
900
1000

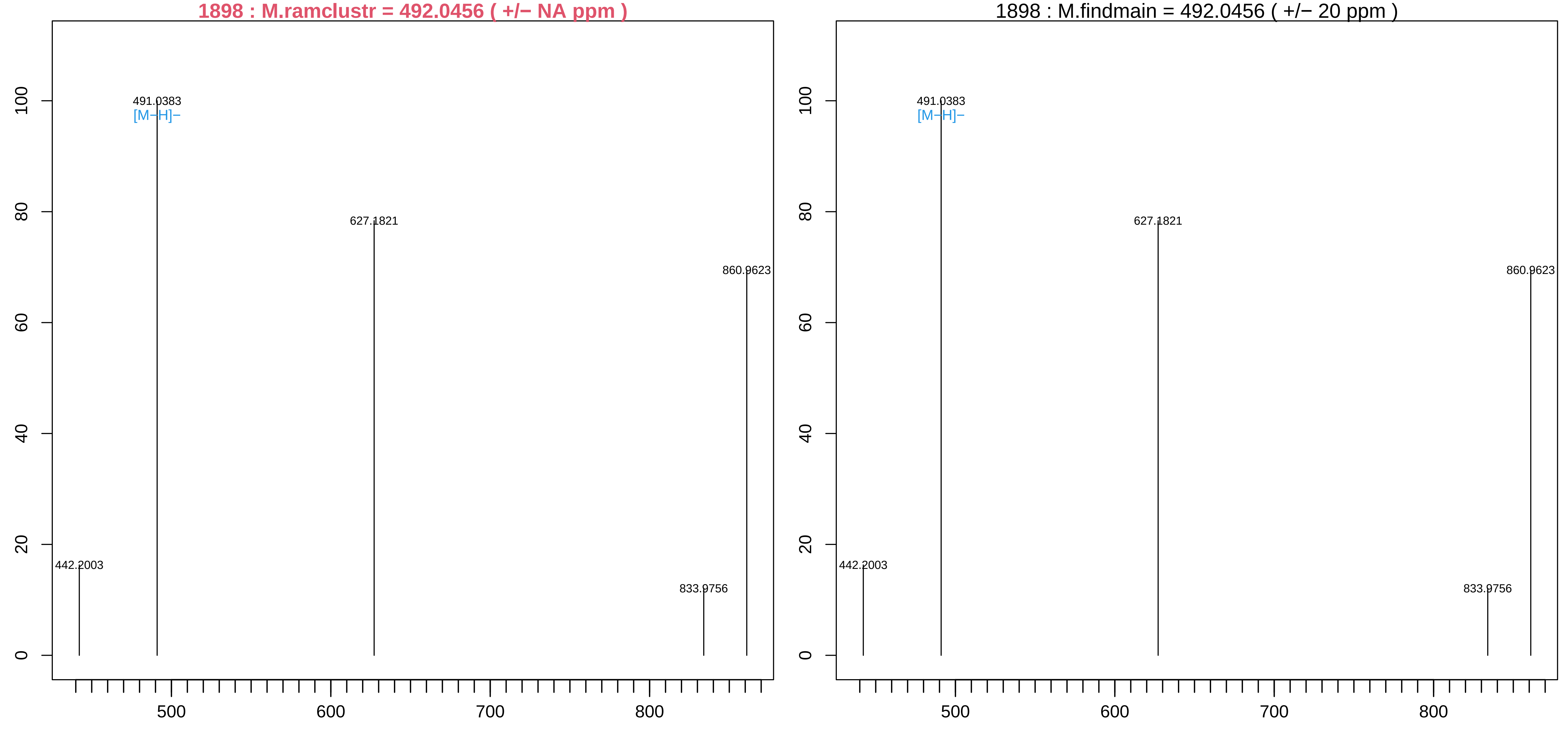

1898 : M.ramclustr = 492.0456 (+/− NA ppm)
1898 : M.findmain = 492.0456 (+/− 20 ppm)
491.0383
[M−H]−
627.1821
860.9623
442.2003
833.9756
0
20
40
60
80
100
500
600
700
800

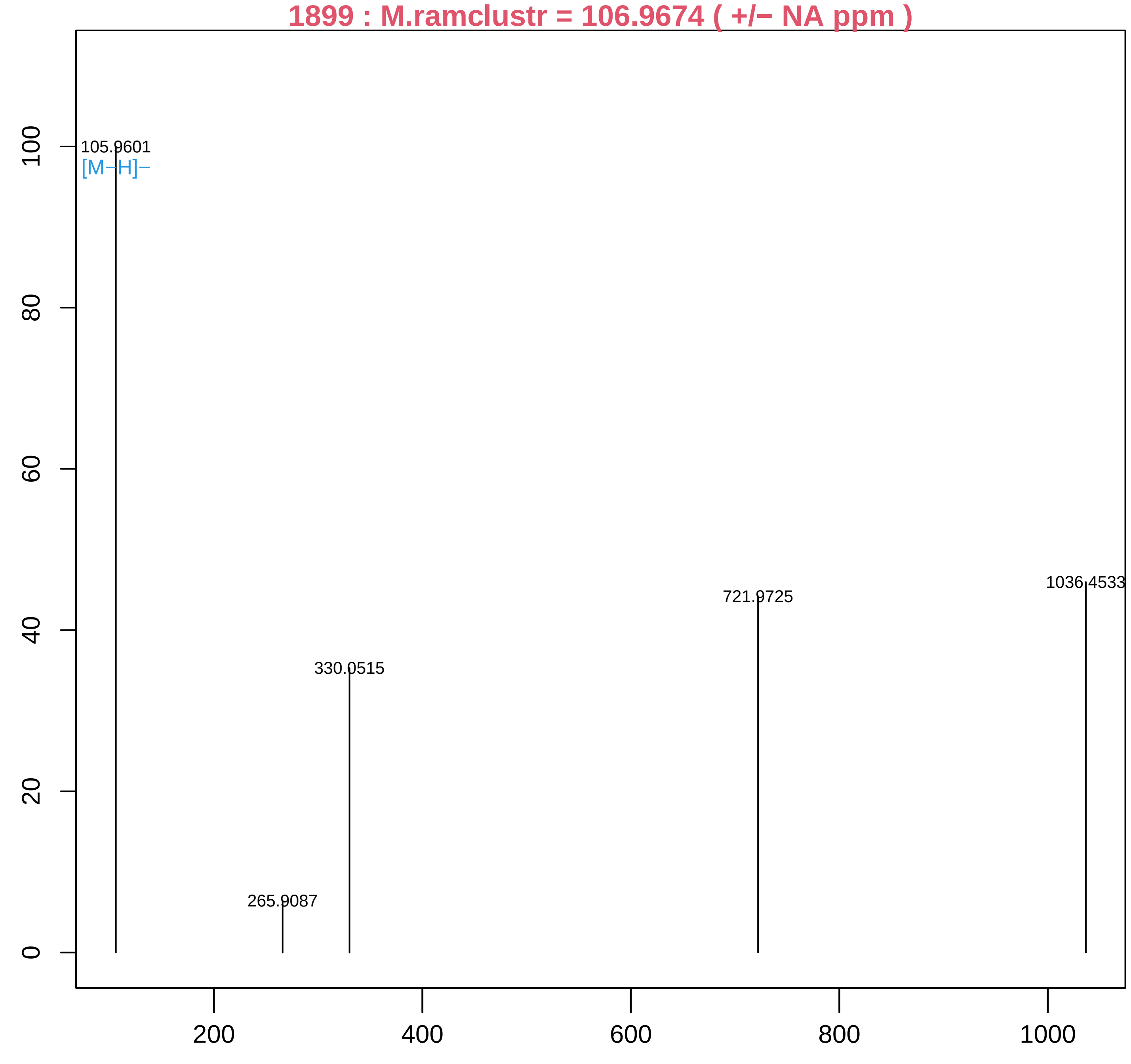
1899 : M.ramclustr = 106.9674 (+/− NA ppm)
105.9601
[M−H]−
265.9087
330.0515
721.9725
1036.4533
0
20
40
60
80
100
200
400
600
800
1000

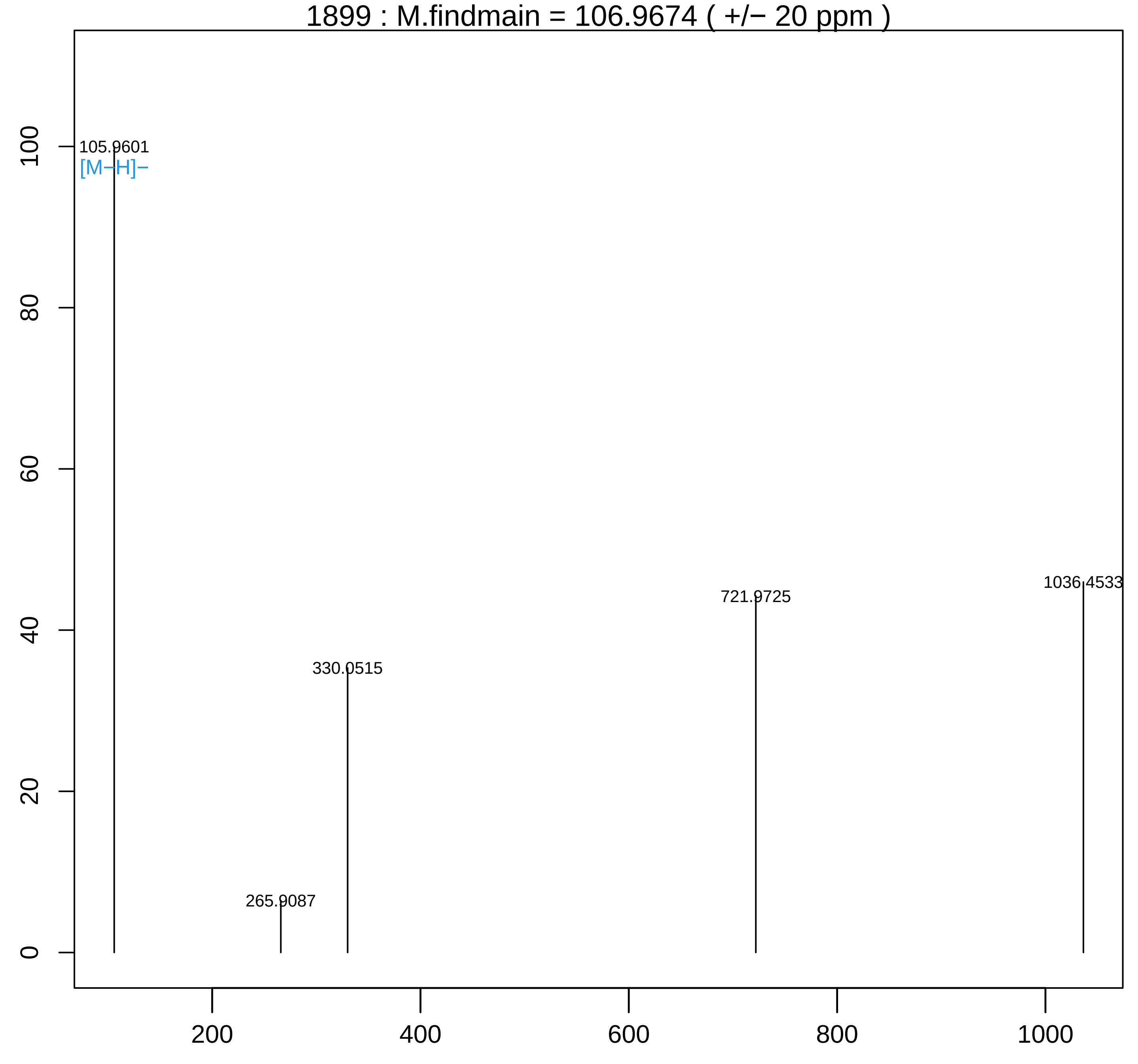
1899 : M.findmain = 106.9674 (+/− 20 ppm)
105.9601
[M−H]−
265.9087
330.0515
721.9725
1036.4533
0
20
40
60
80
100
200
400
600
800
1000

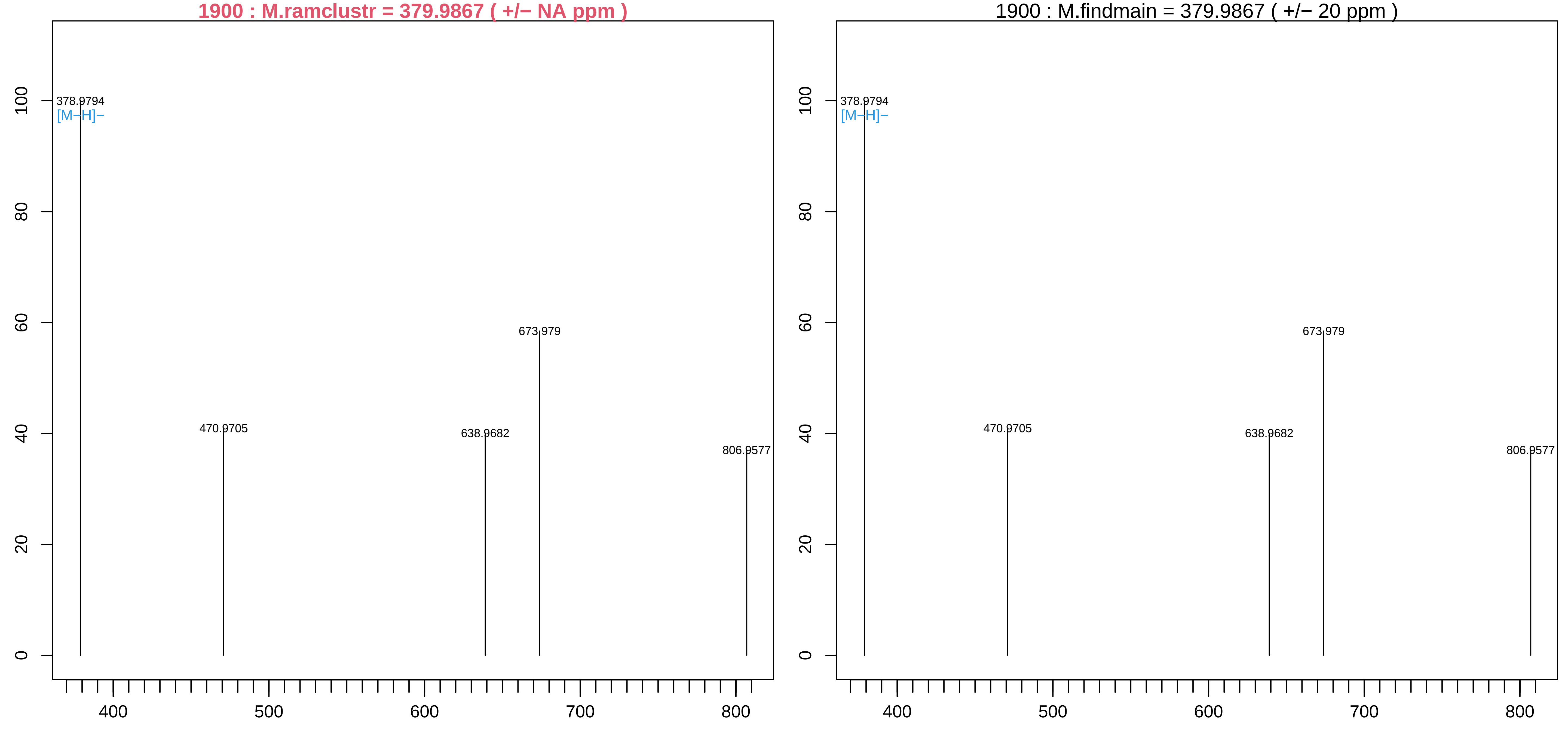

1900 : M.ramclustr = 379.9867 (+/− NA ppm)
378.9794
[M−H]−
470.9705
638.9682
673.979
806.9577
1900 : M.findmain = 379.9867 (+/− 20 ppm)
378.9794
[M−H]−
470.9705
638.9682
673.979
806.9577
0
20
40
60
80
100
400
500
600
700
800

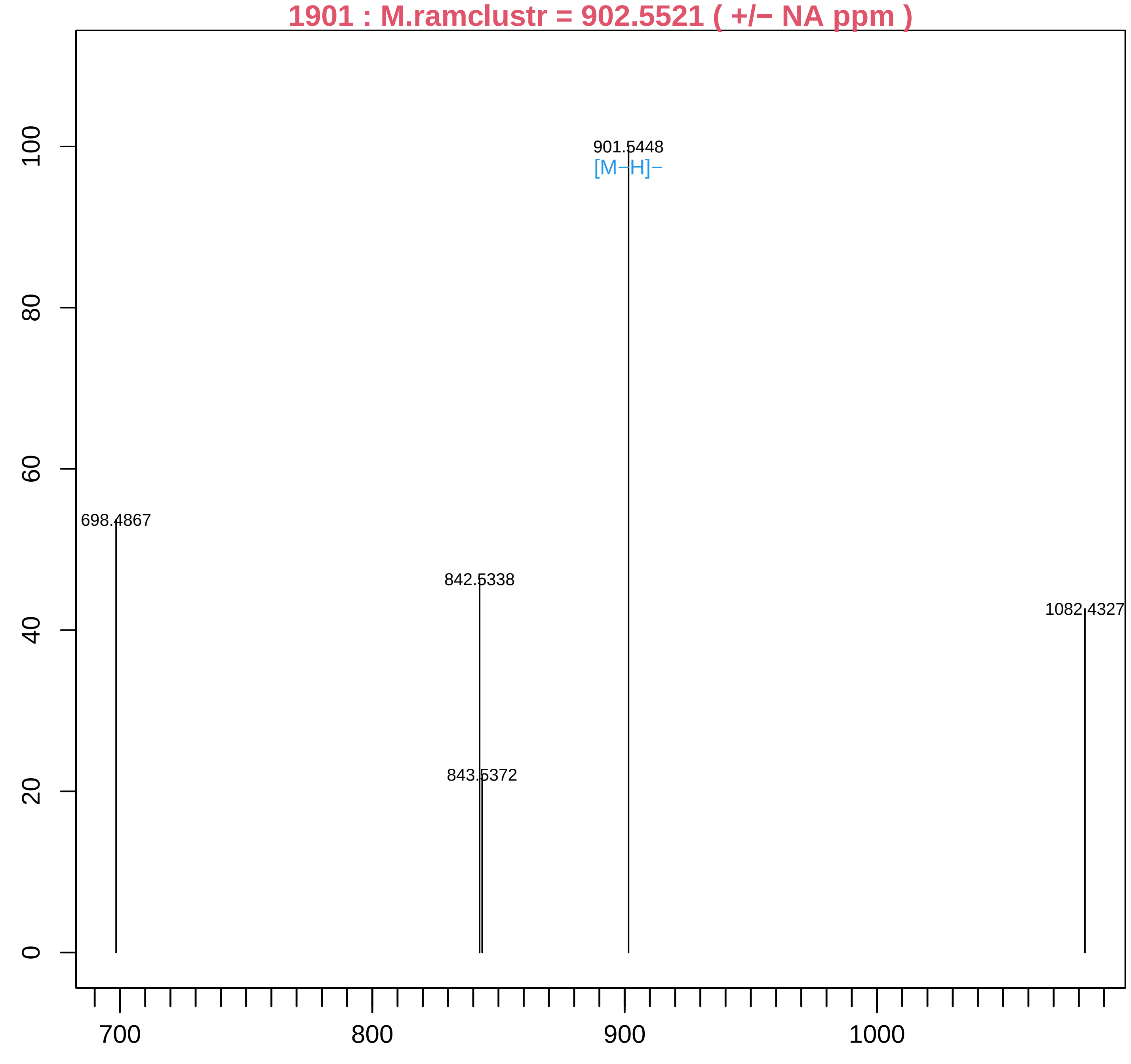
1901 : M.ramclustr = 902.5521 (+/− NA ppm)
901.5448
[M−H]−
698.4867
842.5338
843.5372
1082.4327
0
20
40
60
80
100
700
800
900
1000

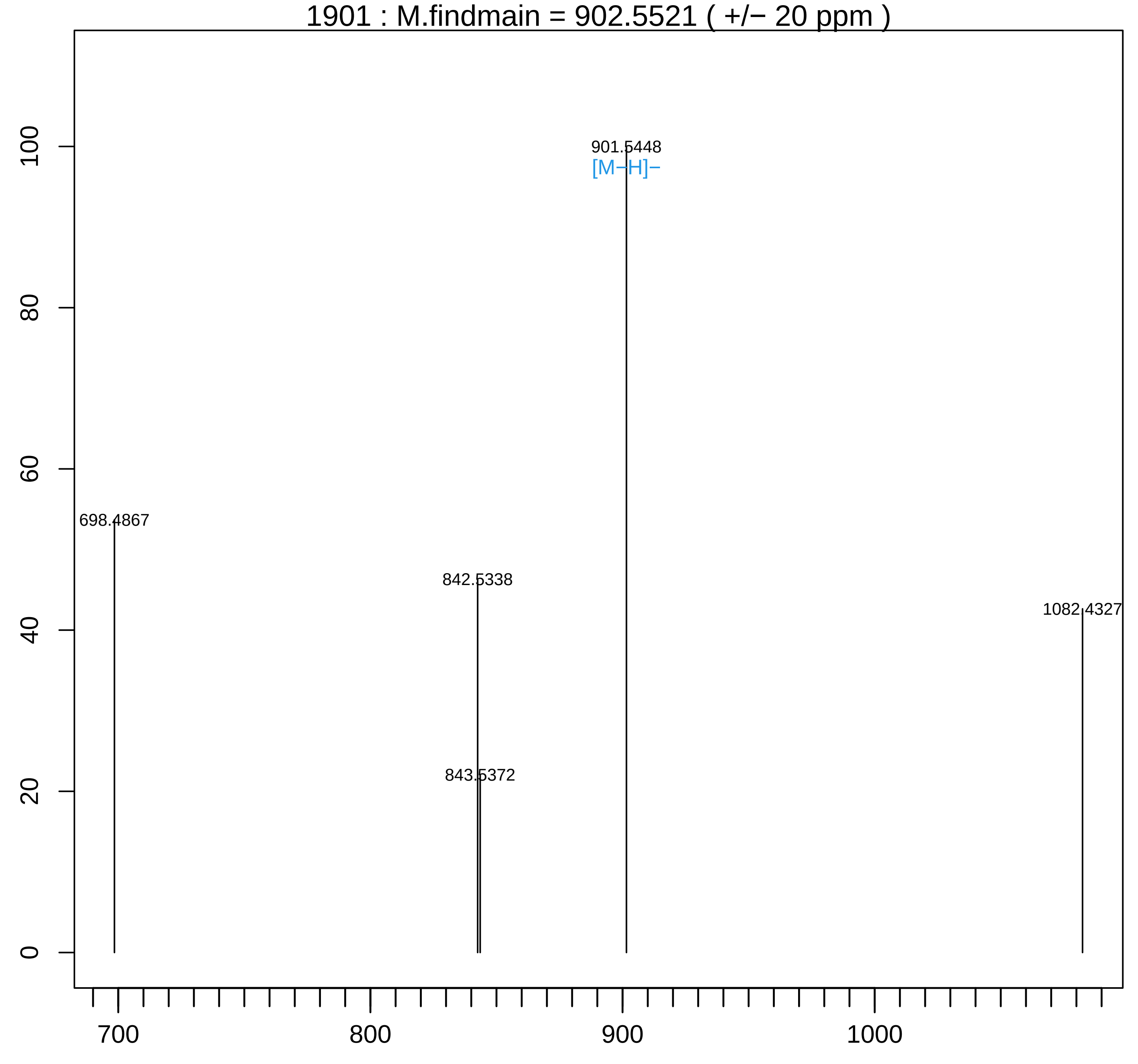
1901 : M.findmain = 902.5521 (+/− 20 ppm)
901.5448
[M−H]−
698.4867
842.5338
843.5372
1082.4327
0
20
40
60
80
100
700
800
900
1000

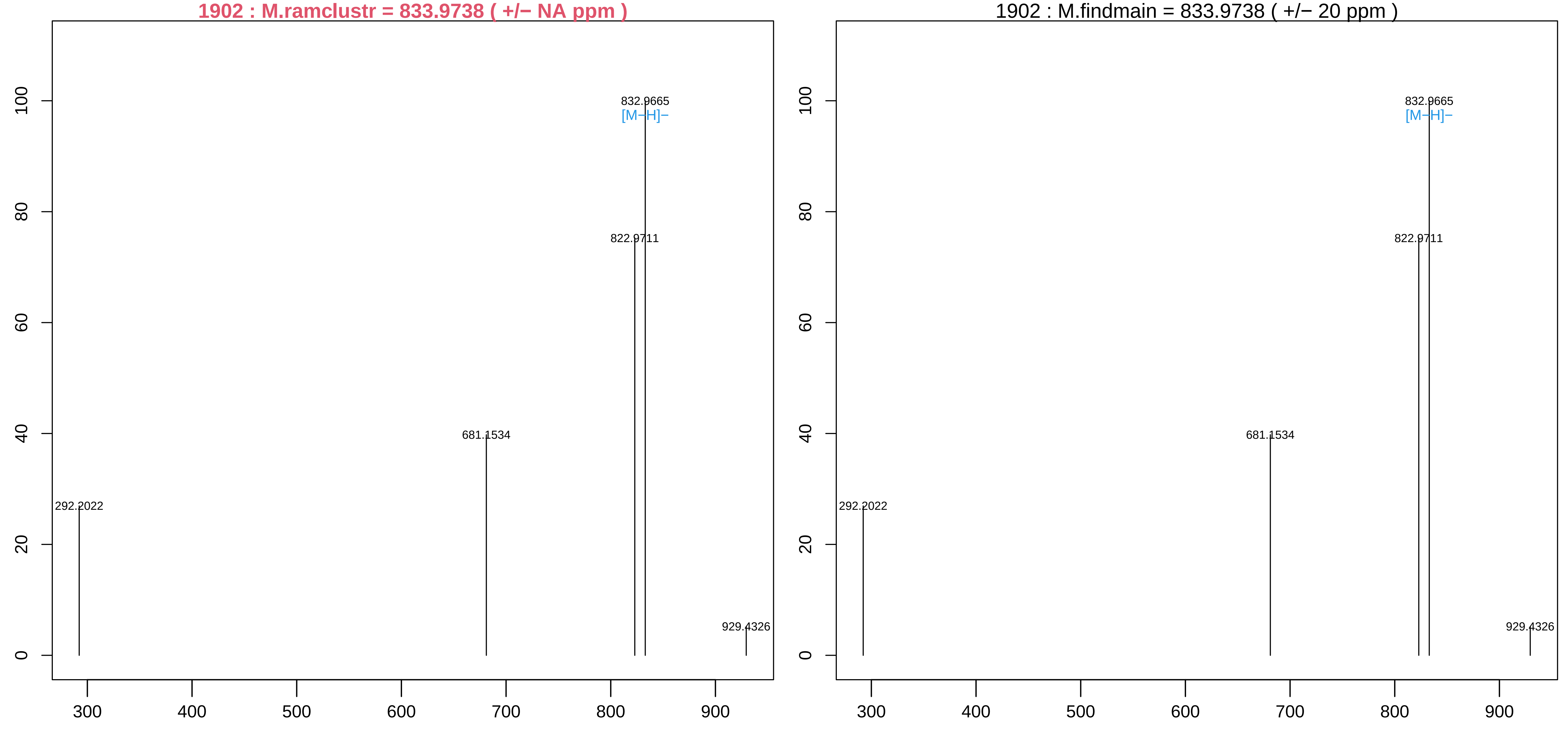
1902 : M.ramclustr = 833.9738 (+/− NA ppm)
832.9665
[M−H]−
822.9711
681.1534
292.2022
929.4326
0
20
40
60
80
100
300
400
500
600
700
800
900
1902 : M.findmain = 833.9738 (+/− 20 ppm)
832.9665
[M−H]−
822.9711
681.1534
292.2022
929.4326
0
20
40
60
80
100
300
400
500
600
700
800
900

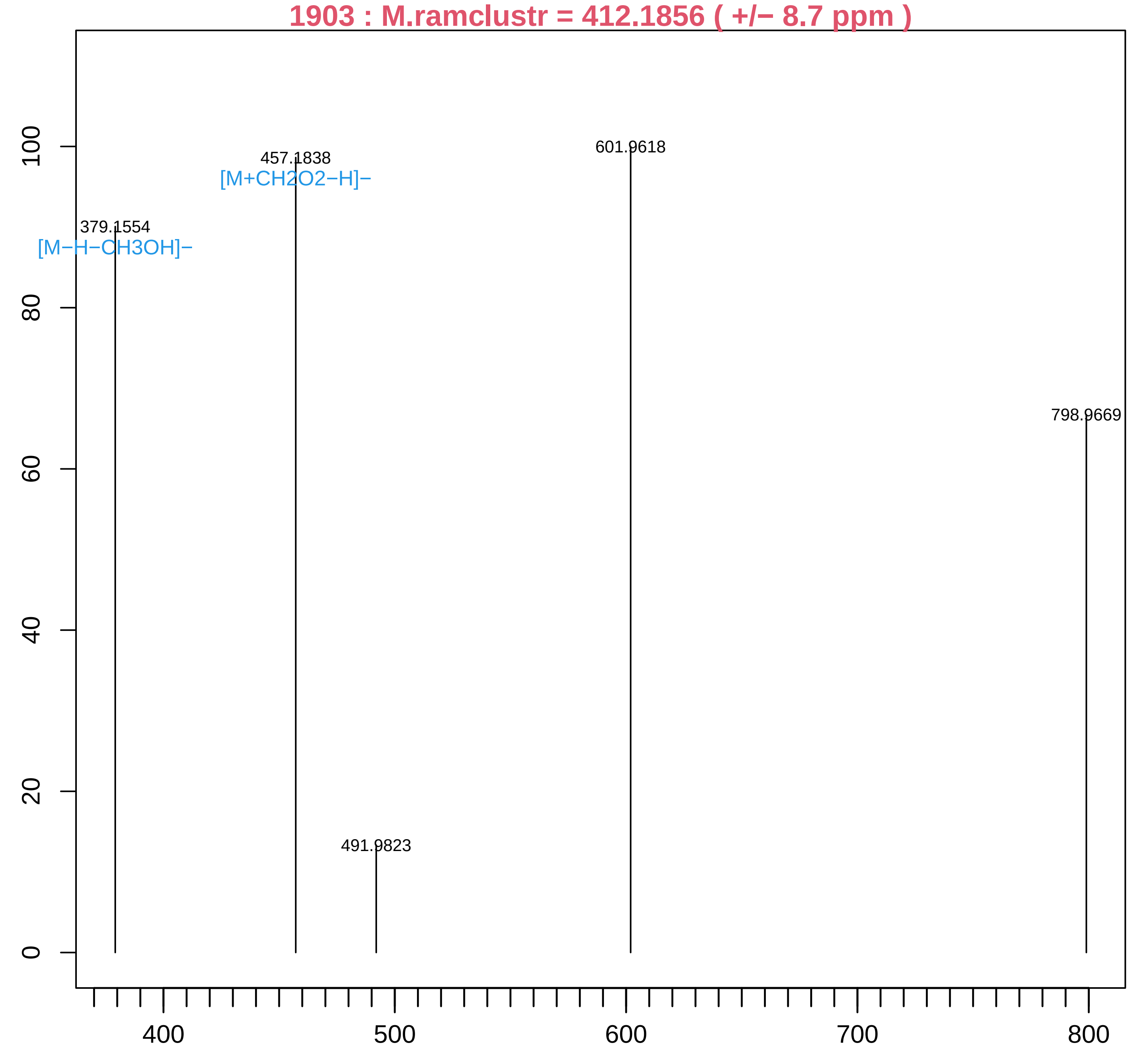
1903 : M.ramclustr = 412.1856 (+/− 8.7 ppm)
379.1554
[M−H−CH3OH]−
457.1838
[M+CH2O2−H]−
491.9823
601.9618
798.9669
0
20
40
60
80
100
400
500
600
700
800

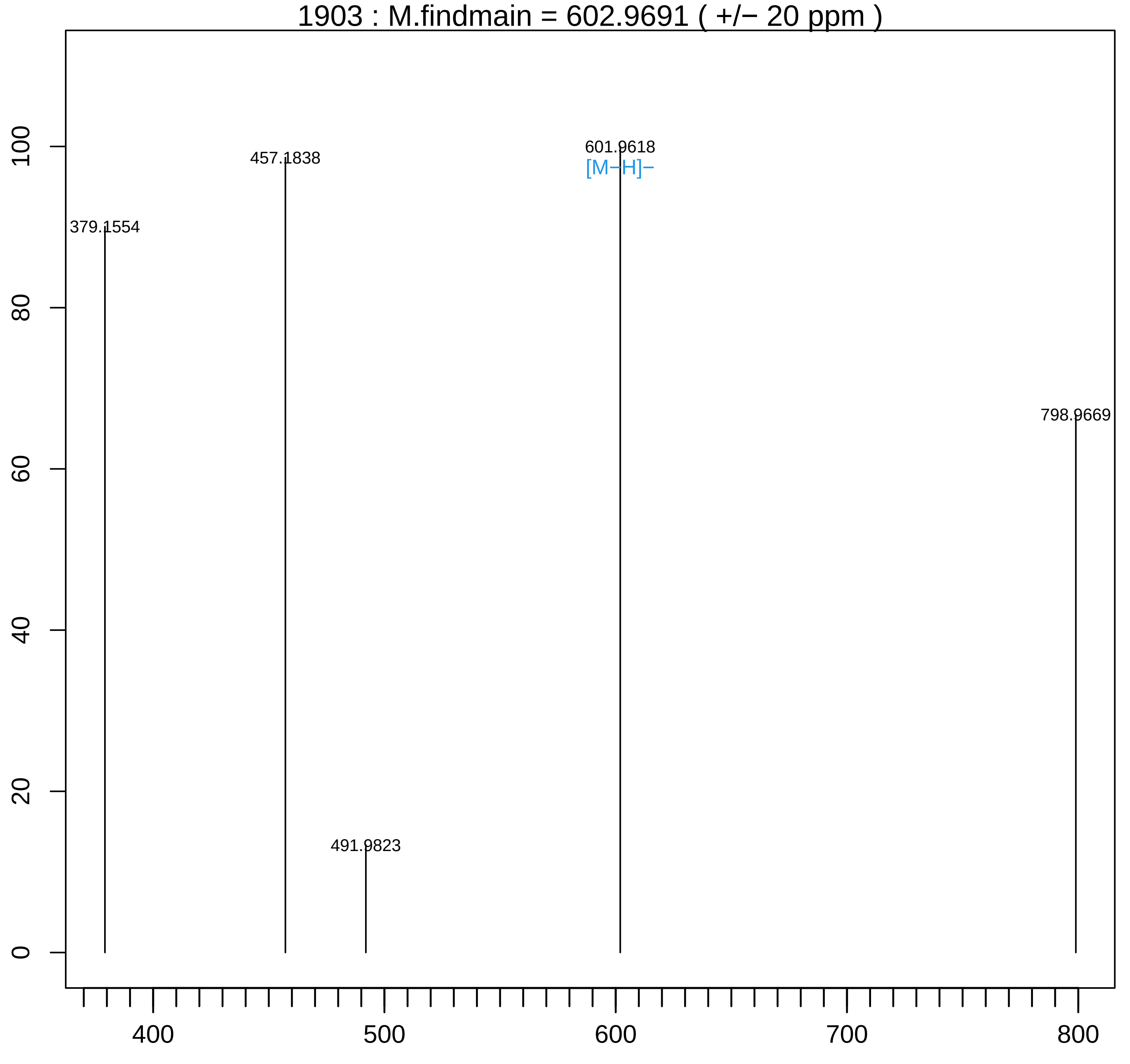
1903 : M.findmain = 602.9691 (+/− 20 ppm)
379.1554
457.1838
491.9823
601.9618
[M−H]−
798.9669
0
20
40
60
80
100
400
500
600
700
800

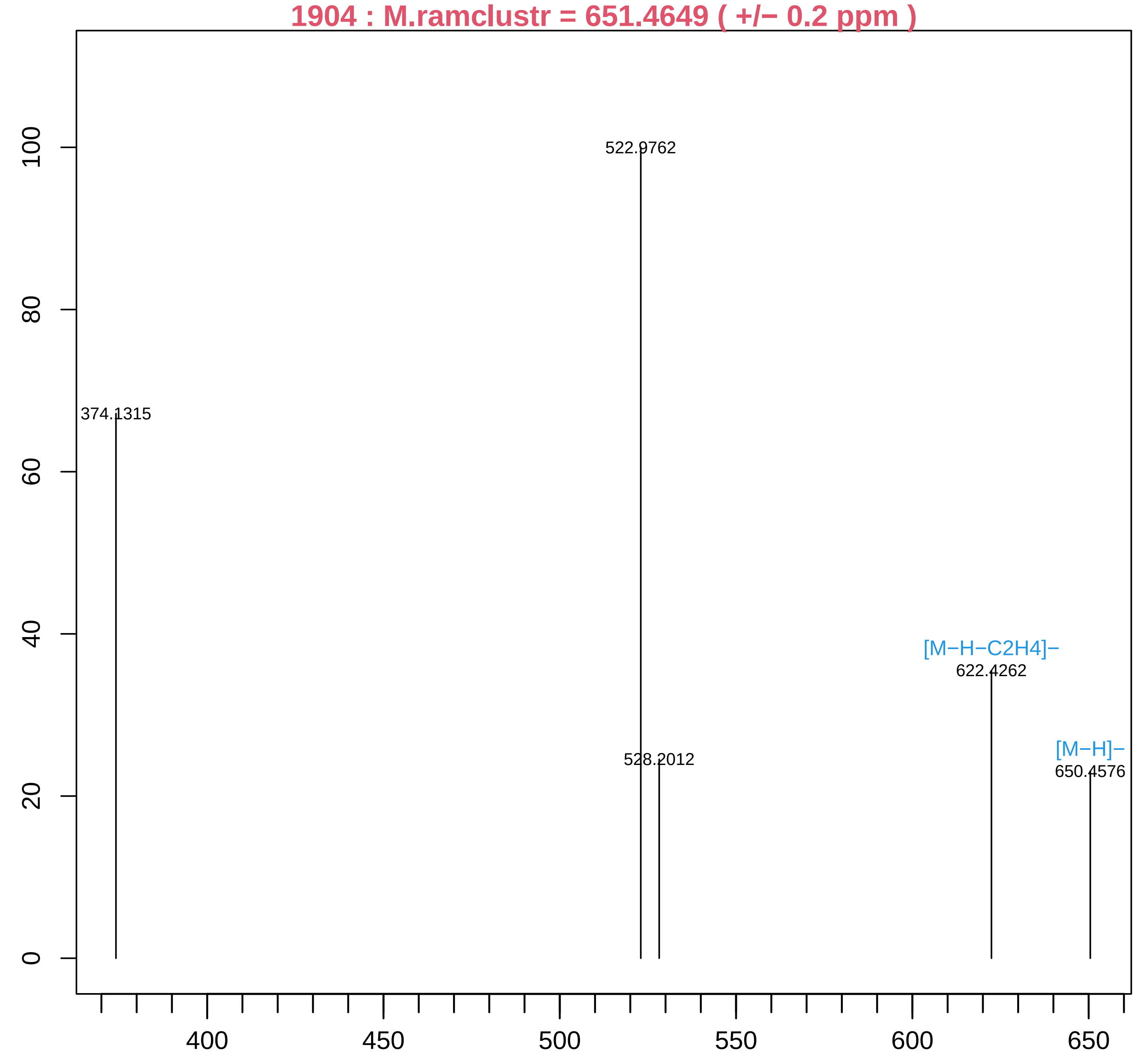
1904 : M.ramclustr = 651.4649 (+/− 0.2 ppm)
522.9762
374.1315
[M−H−C2H4]−
622.4262
[M−H]−
528.2012
650.4576
0
20
40
60
80
100
400
450
500
550
600
650

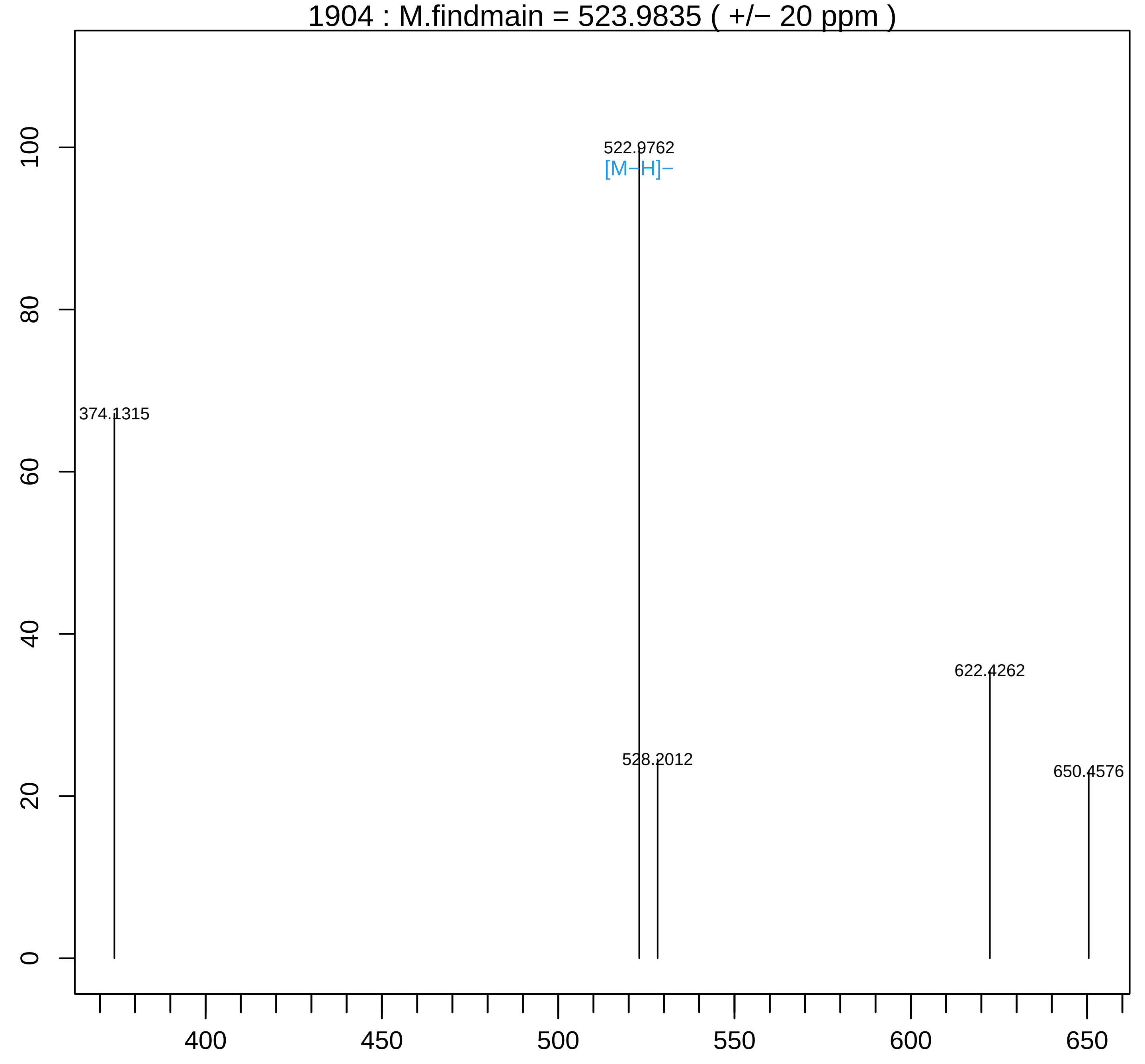
1904 : M.findmain = 523.9835 (+/− 20 ppm)
522.9762
[M−H]−
374.1315
622.4262
528.2012
650.4576
0
20
40
60
80
100
400
450
500
550
600
650

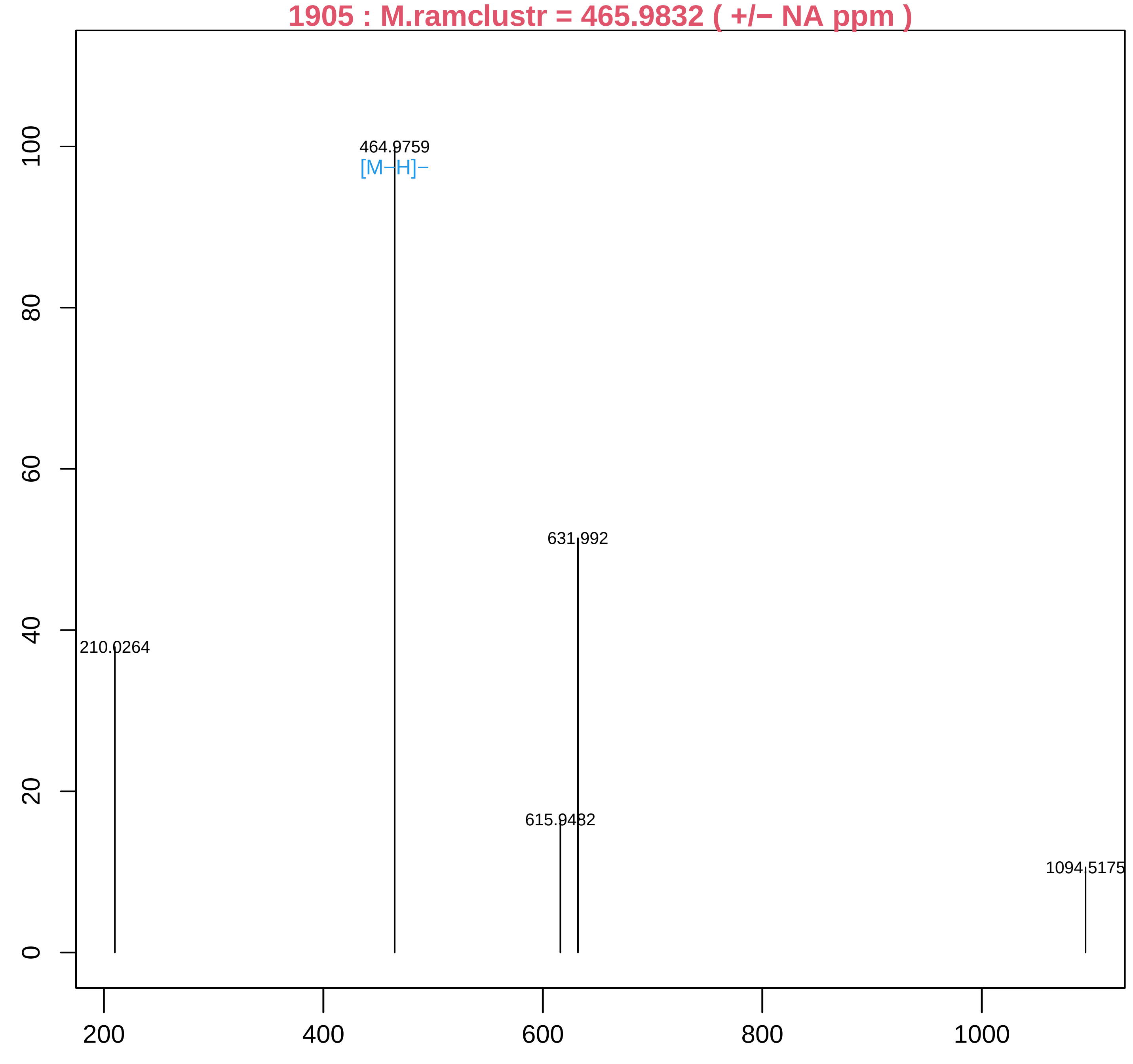
1905 : M.ramclustr = 465.9832 (+/− NA ppm)
464.9759
[M−H]−
631.992
210.0264
615.9482
1094.5175
0
20
40
60
80
100
200
400
600
800
1000

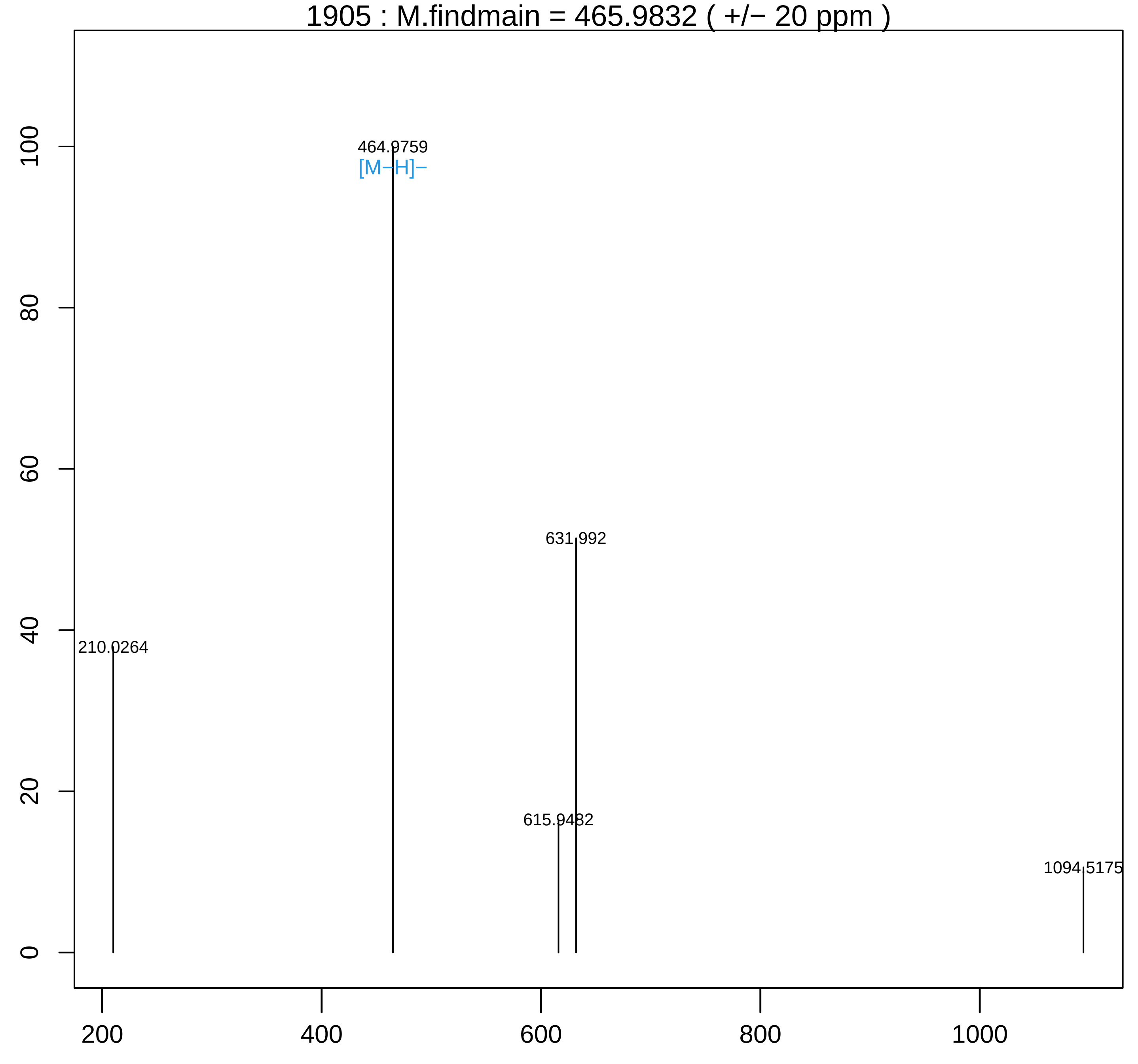
1905 : M.findmain = 465.9832 (+/− 20 ppm)
464.9759
[M−H]−
631.992
210.0264
615.9482
1094.5175
0
20
40
60
80
100
200
400
600
800
1000

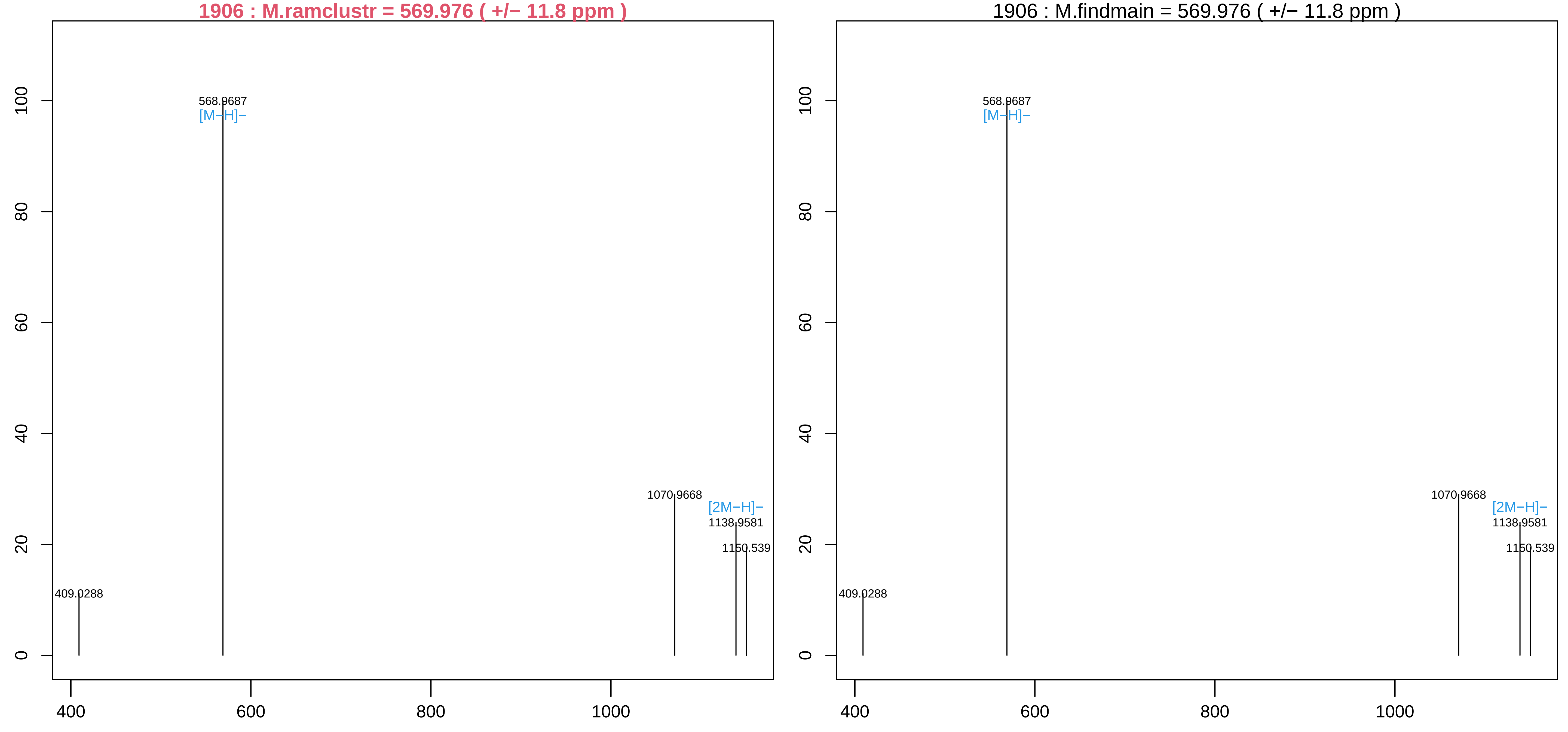
1906 : M.ramclustr = 569.976 (+/− 11.8 ppm)
568.9687
[M−H]−
1070.9668
[2M−H]−
1138.9581
1150.539
409.0288
0
20
40
60
80
100
400
600
800
1000
1906 : M.findmain = 569.976 (+/− 11.8 ppm)
568.9687
[M−H]−
1070.9668
[2M−H]−
1138.9581
1150.539
409.0288
0
20
40
60
80
100
400
600
800
1000

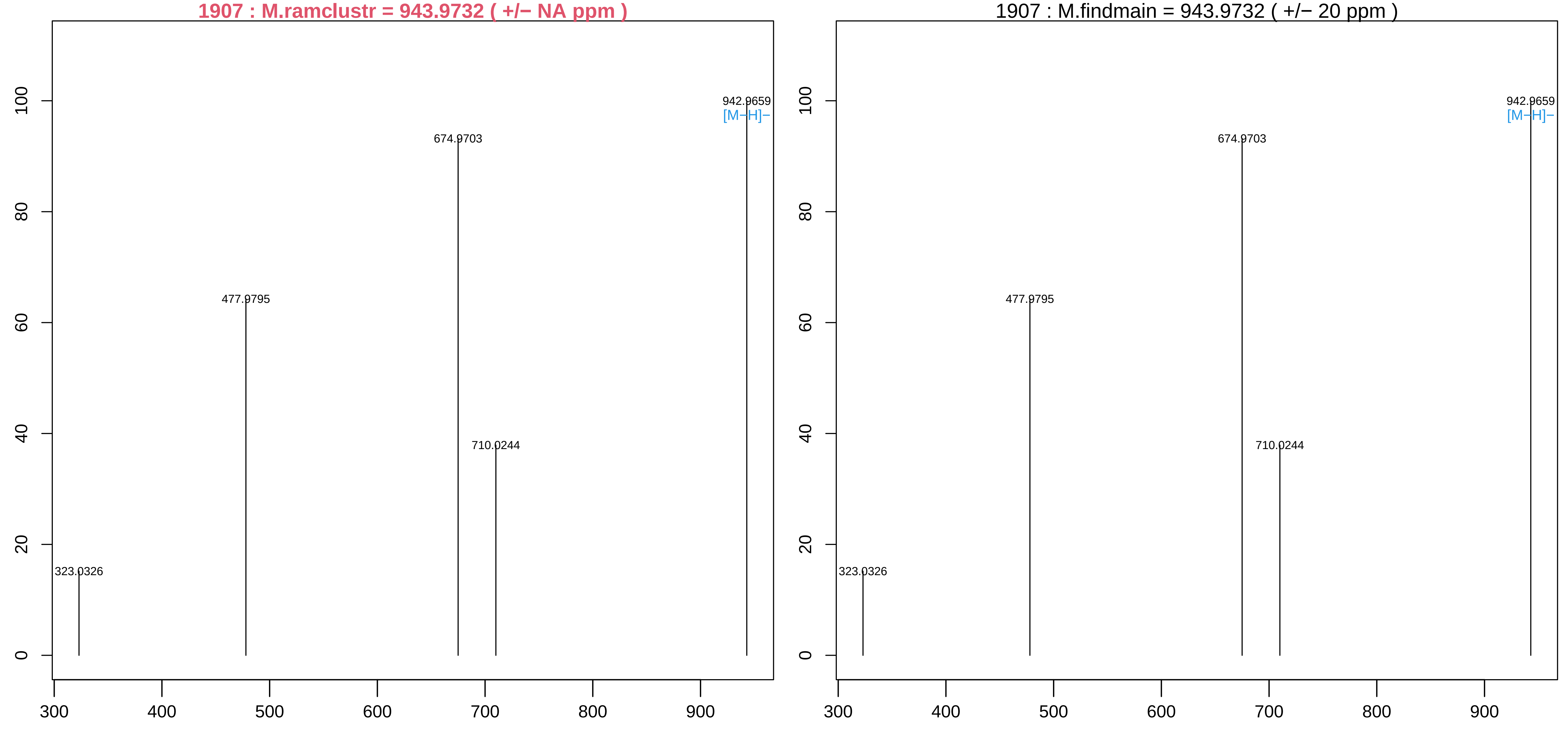
1907 : M.ramclustr = 943.9732 (+/− NA ppm)
942.9659
[M−H]−
674.9703
477.9795
710.0244
323.0326
1907 : M.findmain = 943.9732 (+/− 20 ppm)
942.9659
[M−H]−
674.9703
477.9795
710.0244
323.0326

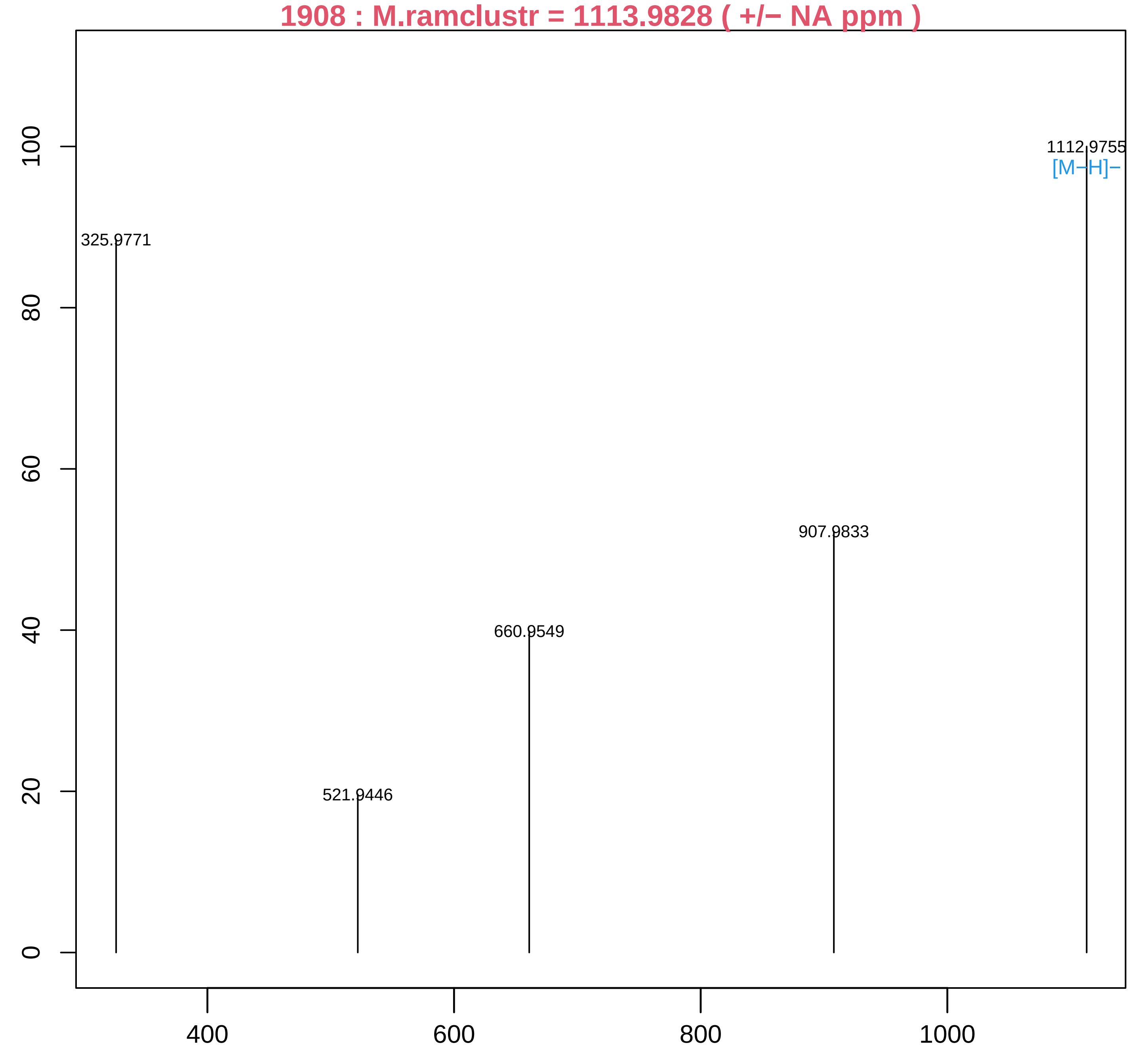
1908 : M.ramclustr = 1113.9828 (+/− NA ppm)
325.9771
521.9446
660.9549
907.9833
1112.9755
[M−H]−
0
20
40
60
80
100
400
600
800
1000

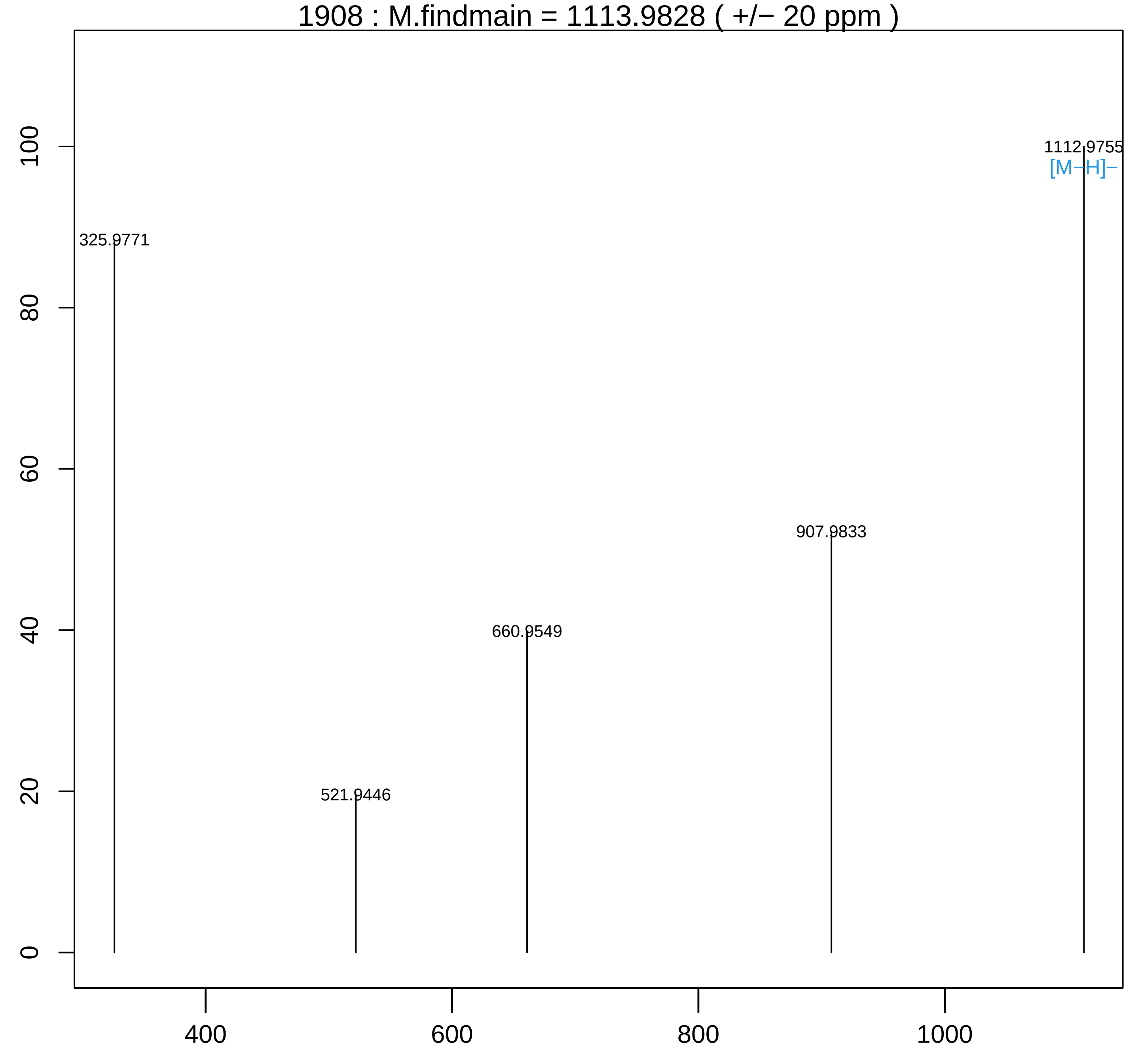
1908 : M.findmain = 1113.9828 (+/− 20 ppm)
325.9771
521.9446
660.9549
907.9833
1112.9755
[M−H]−
0
20
40
60
80
100
400
600
800
1000

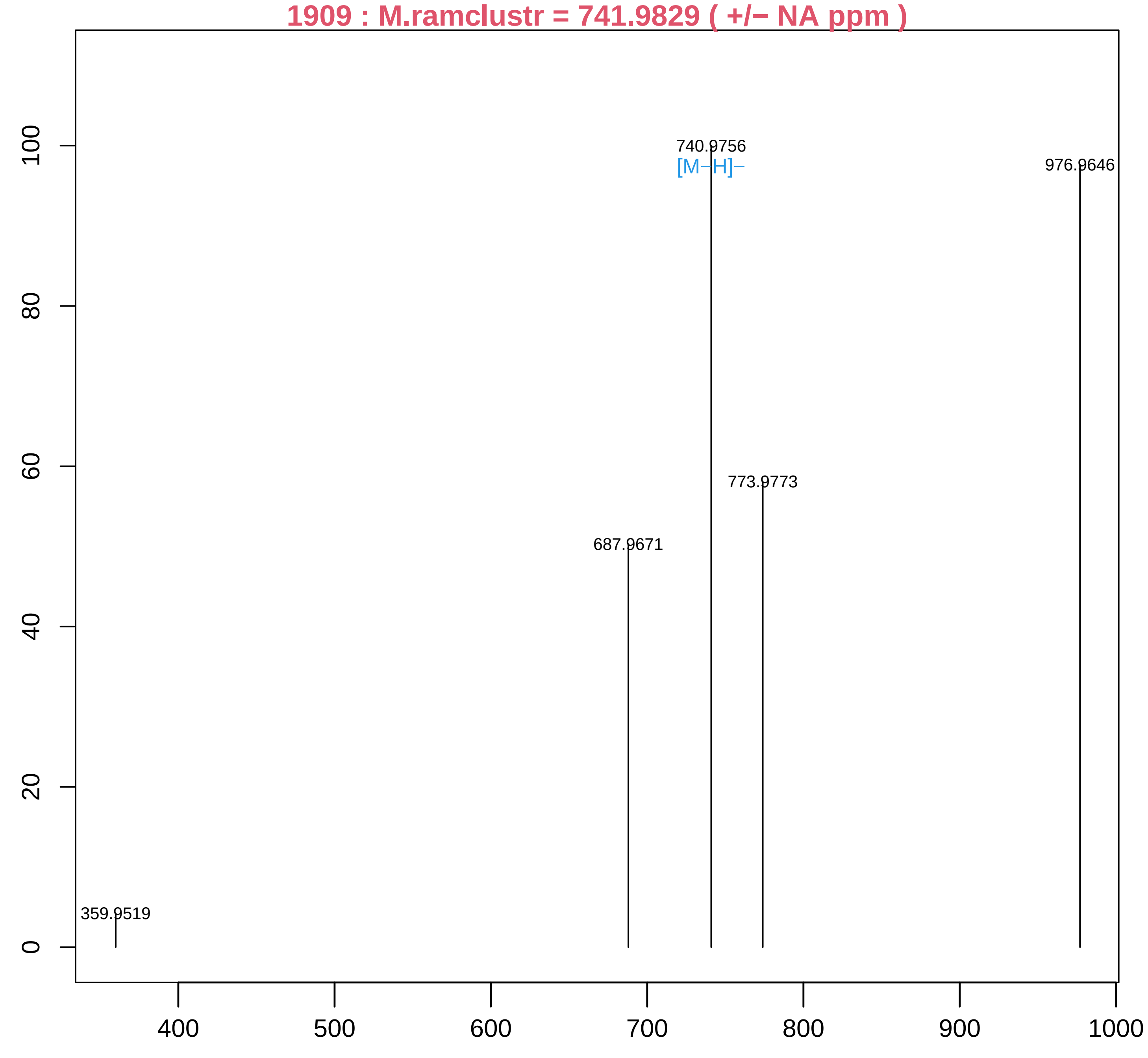
1909 : M.ramclustr = 741.9829 (+/− NA ppm)
740.9756
[M−H]−
976.9646
773.9773
687.9671
359.9519
0
20
40
60
80
100
400
500
600
700
800
900
1000

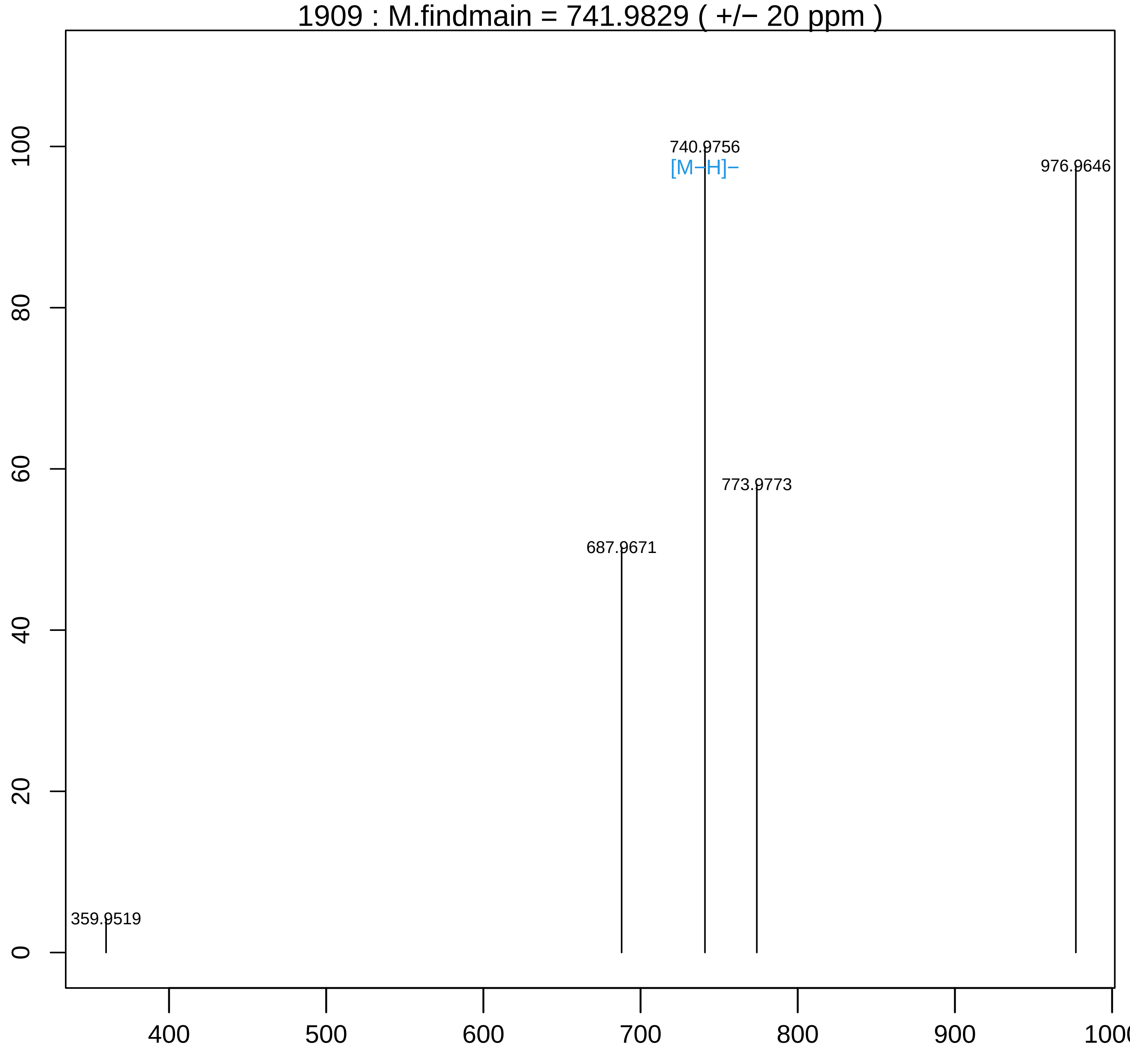
1909 : M.findmain = 741.9829 (+/− 20 ppm)
740.9756
[M−H]−
976.9646
773.9773
687.9671
359.9519
0
20
40
60
80
100
400
500
600
700
800
900
1000

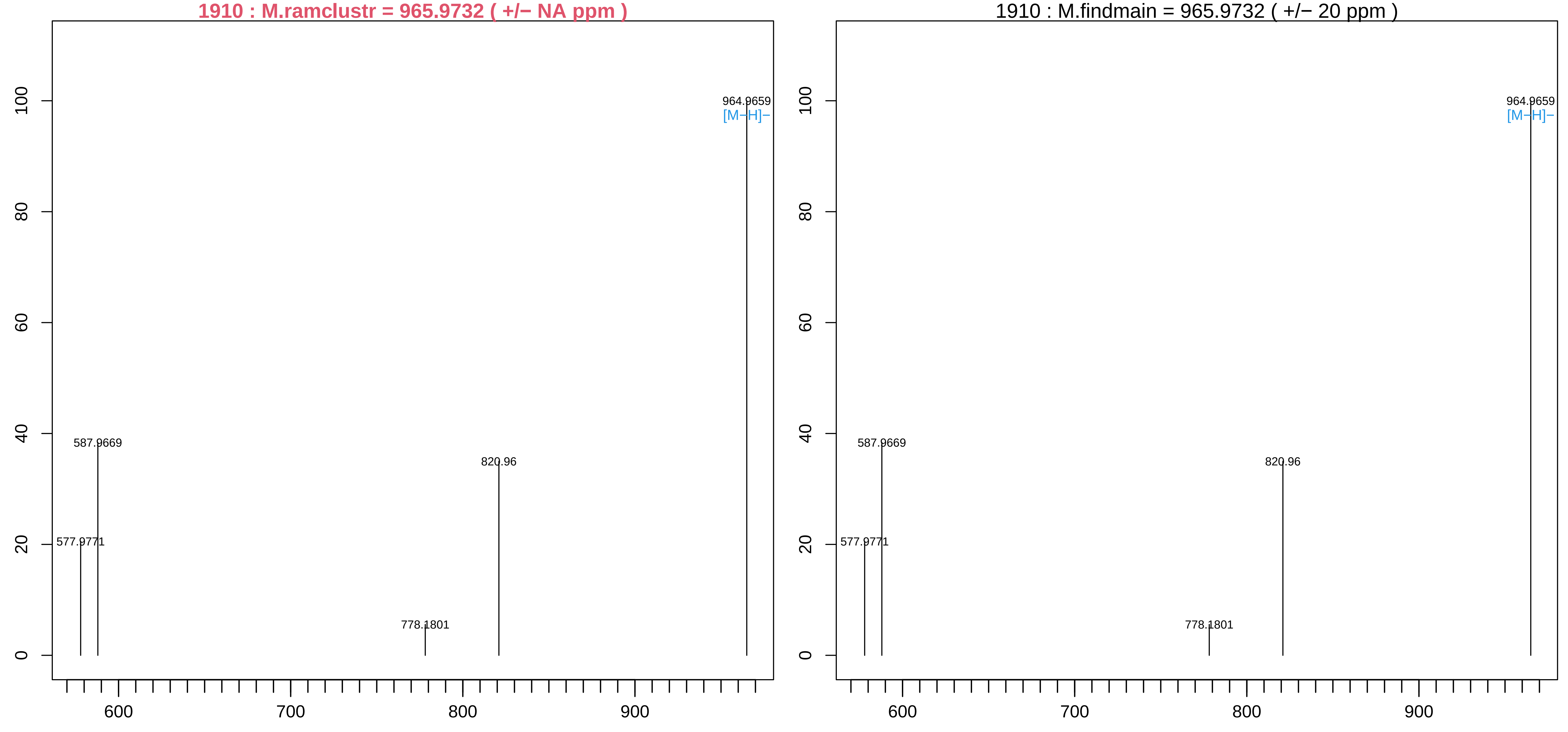
1910 : M.ramclustr = 965.9732 (+/− NA ppm)
964.9659
[M−H]−
587.9669
577.9771
820.96
778.1801
1910 : M.findmain = 965.9732 (+/− 20 ppm)
964.9659
[M−H]−
587.9669
577.9771
820.96
778.1801
0
20
40
60
80
100
600
700
800
900

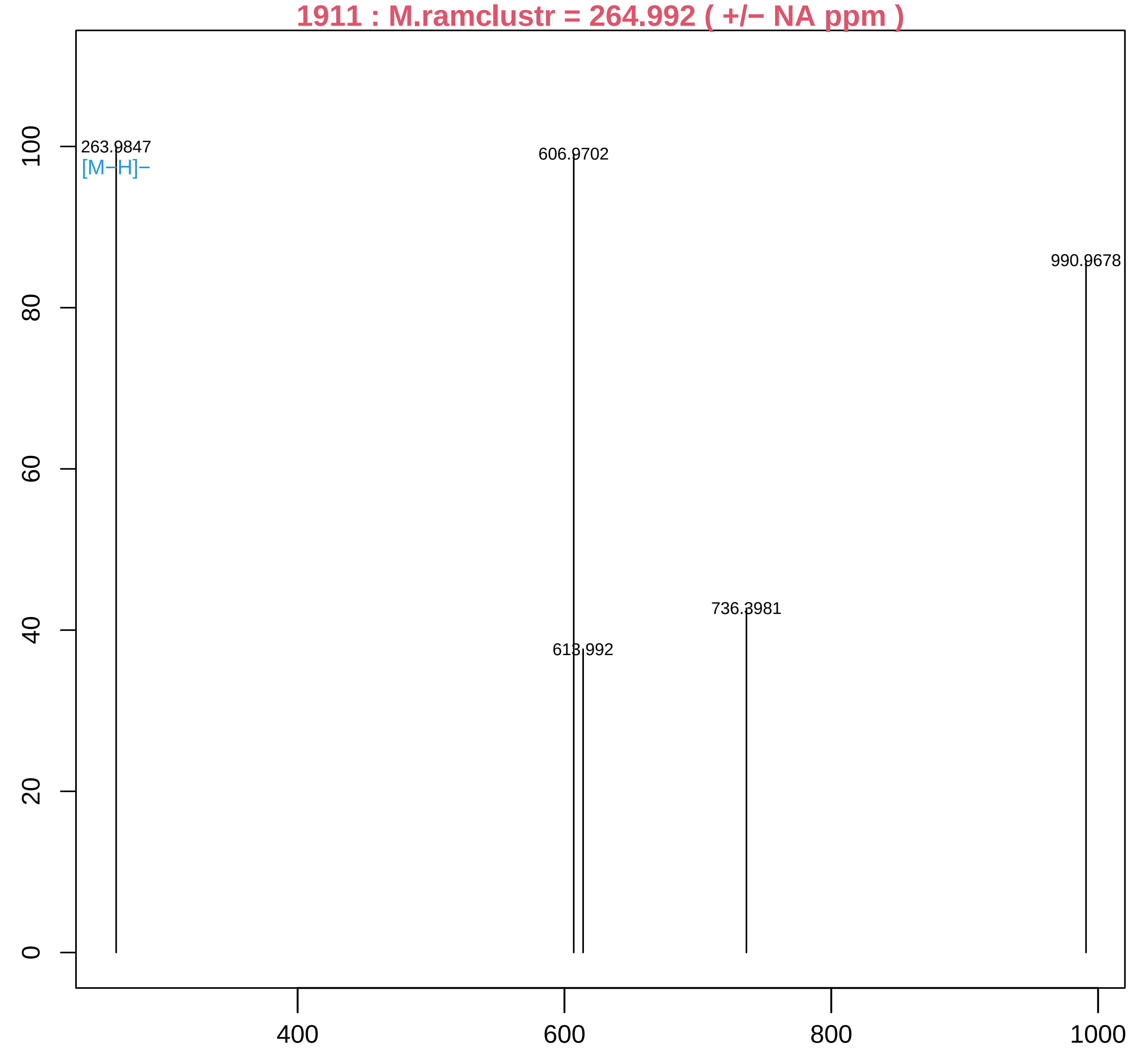
1911 : M.ramclustr = 264.992 (+/− NA ppm)
263.9847
[M−H]−
606.9702
613.992
736.3981
990.9678
0
20
40
60
80
100
400
600
800
1000

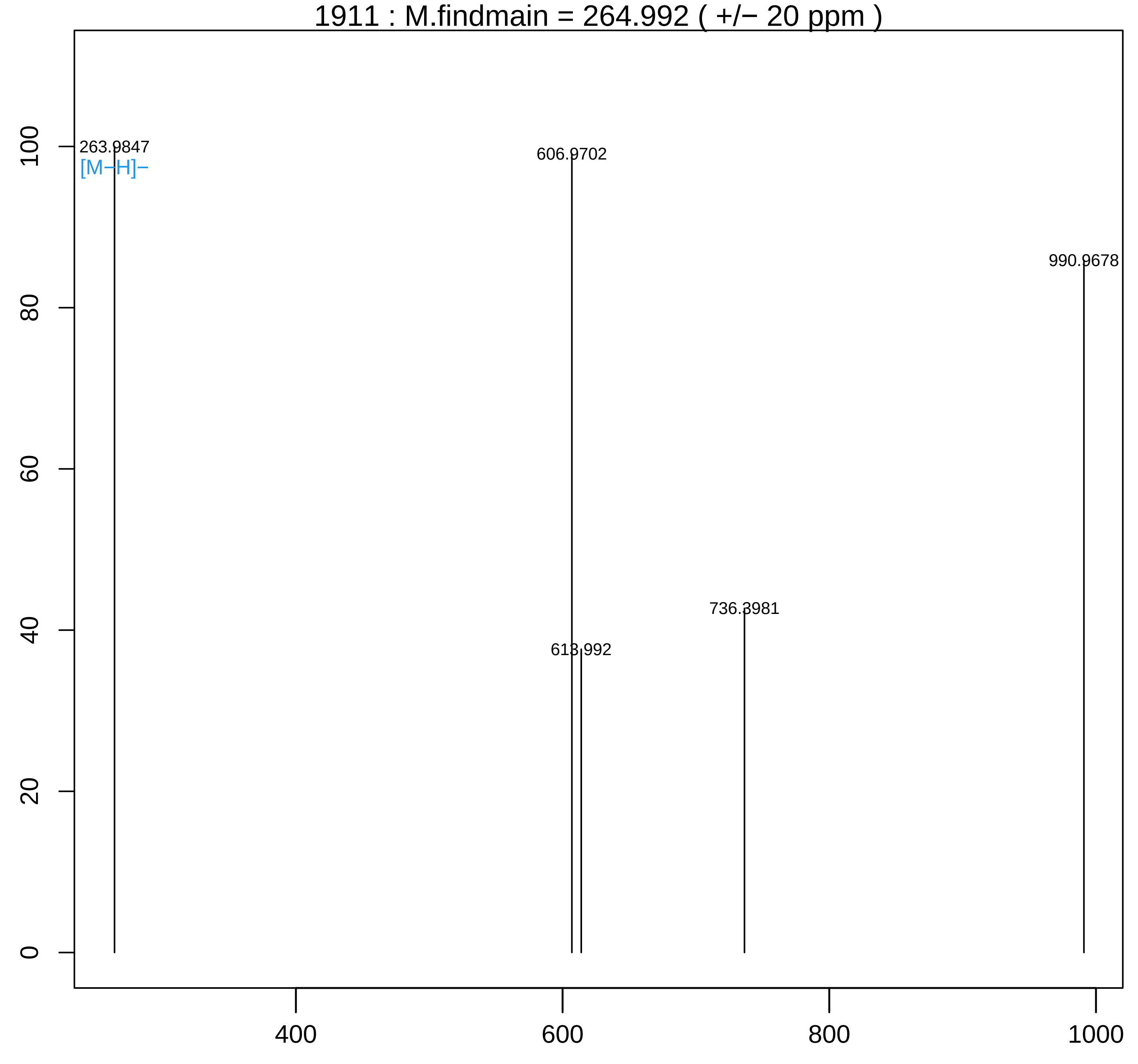
1911 : M.findmain = 264.992 (+/− 20 ppm)
263.9847
[M−H]−
606.9702
613.992
736.3981
990.9678
0
20
40
60
80
100
400
600
800
1000

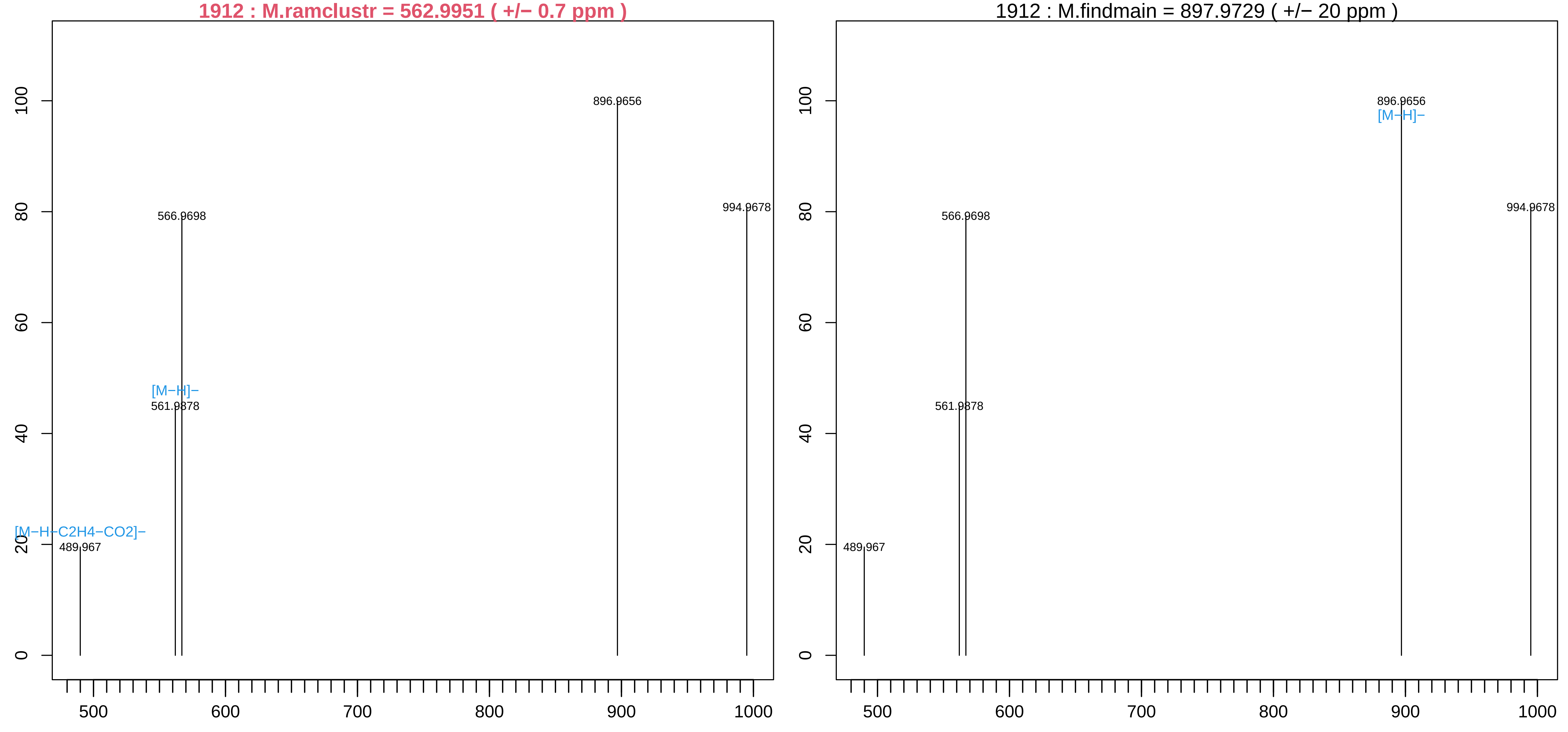

1912 : M.ramclustr = 562.9951 (+/− 0.7 ppm)
896.9656
566.9698
994.9678
[M−H]−
561.9878
[M−H−C2H4−CO2]−
489.967
1912 : M.findmain = 897.9729 (+/− 20 ppm)
896.9656
[M−H]−
566.9698
994.9678
561.9878
489.967

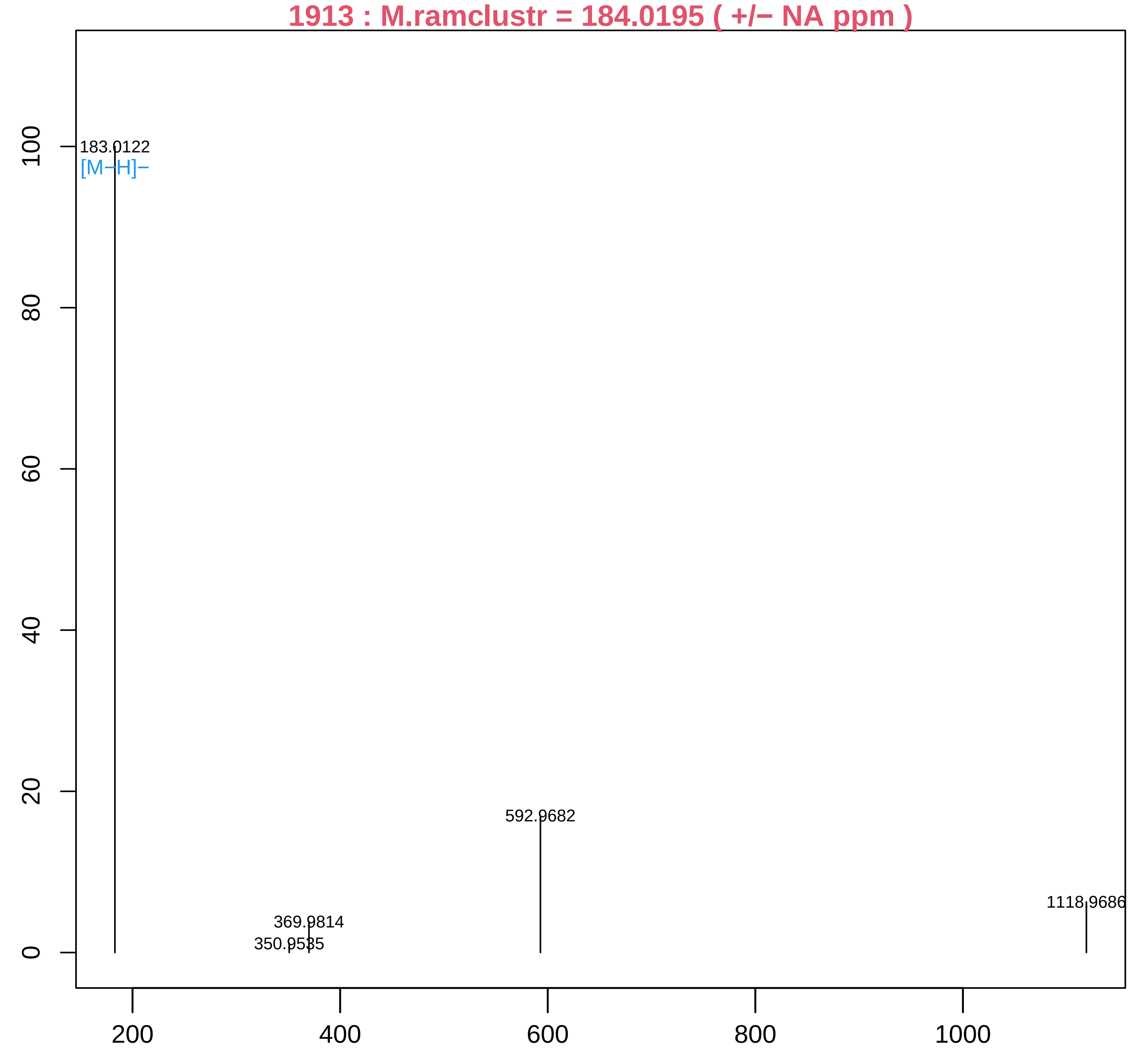
1913 : M.ramclustr = 184.0195 (+/− NA ppm)
183.0122
[M−H]−
369.9814
350.9535
592.9682
1118.9686
0
20
40
60
80
100
200
400
600
800
1000

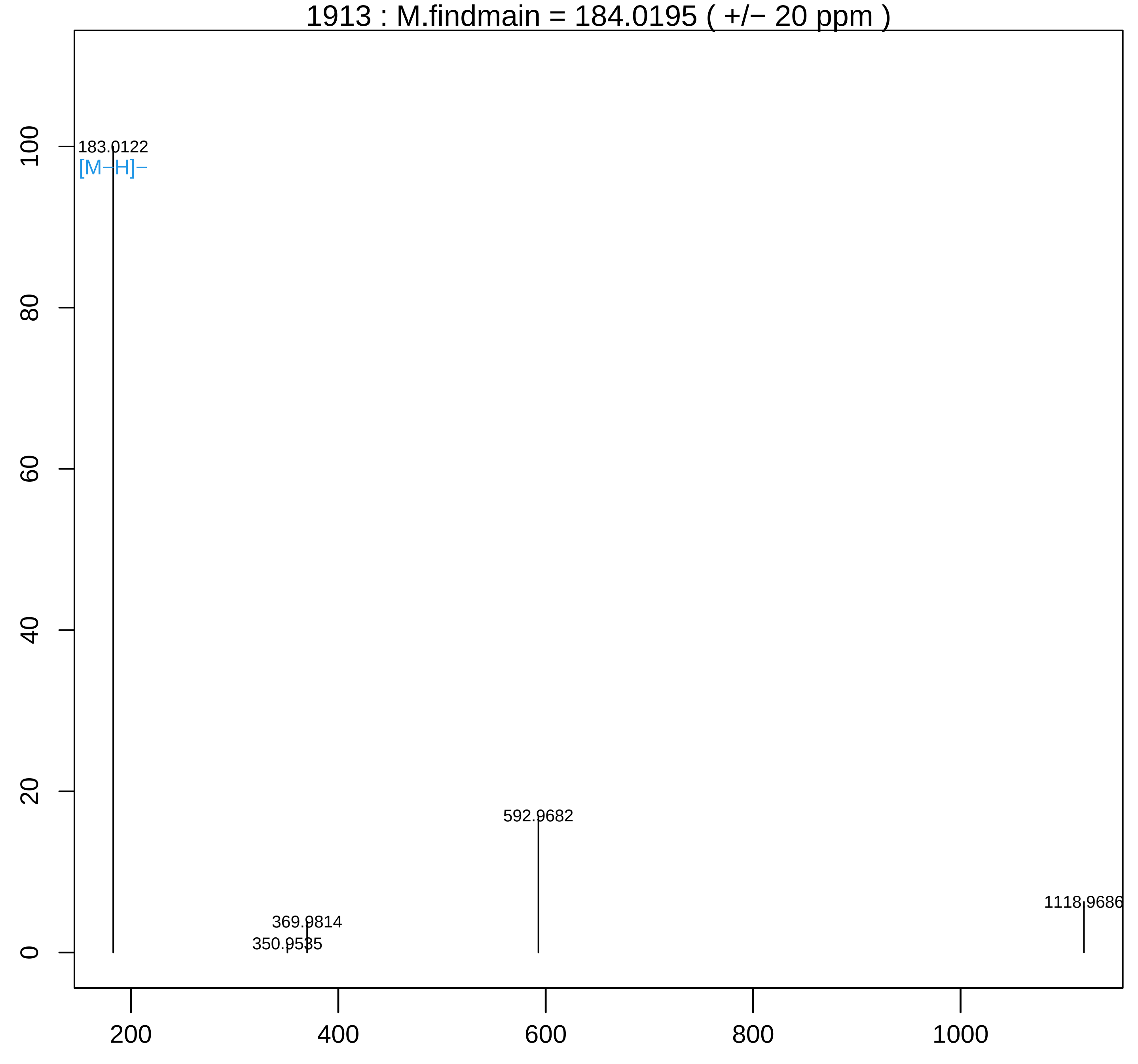
1913 : M.findmain = 184.0195 (+/− 20 ppm)
183.0122
[M−H]−
369.9814
350.9535
592.9682
1118.9686
0
20
40
60
80
100
200
400
600
800
1000

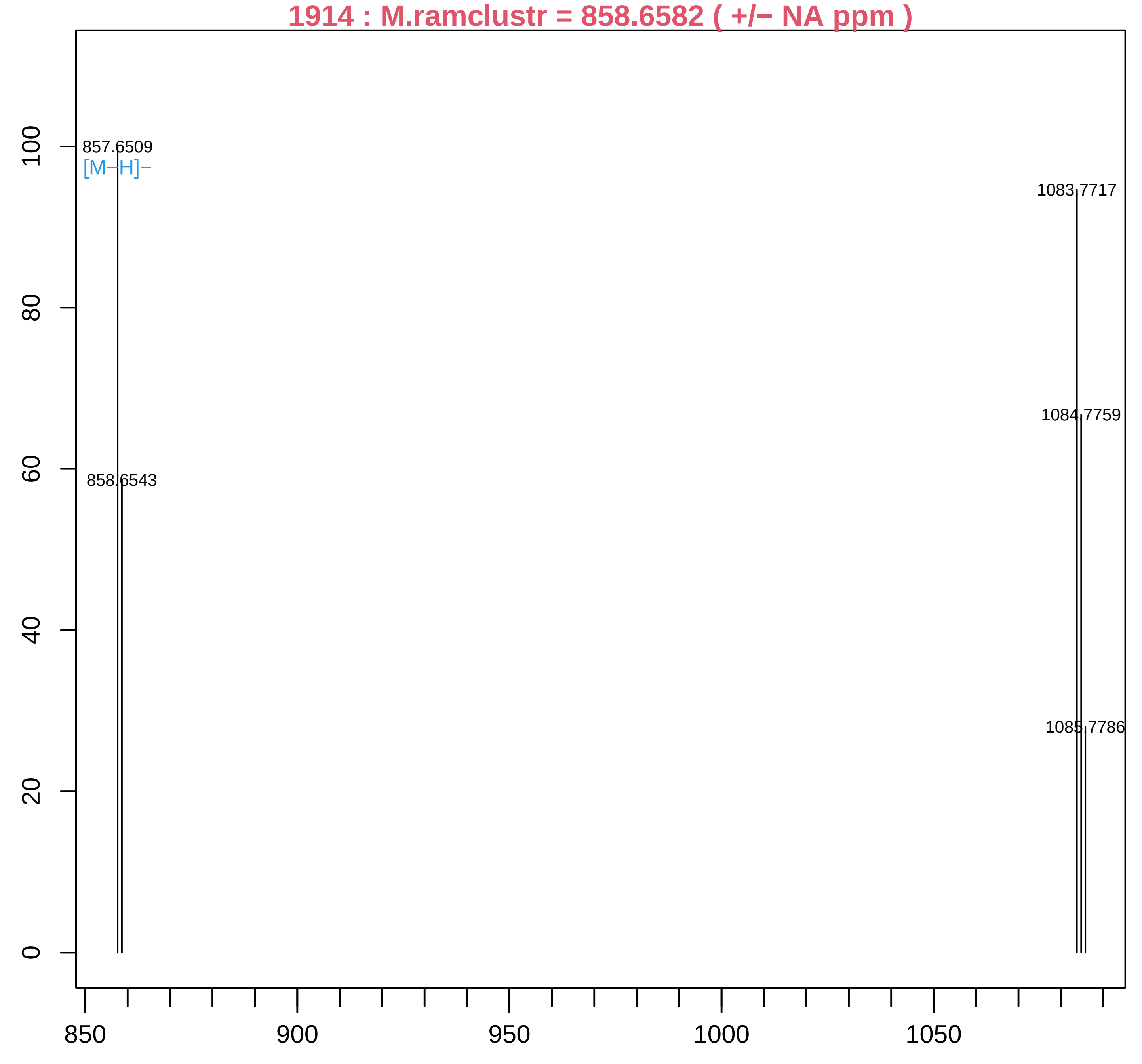

1914 : M.ramclustr = 858.6582 (+/− NA ppm)
857.6509
[M−H]−
858.6543
1083.7717
1084.7759
1085.7786
0
20
40
60
80
100
850
900
950
1000
1050

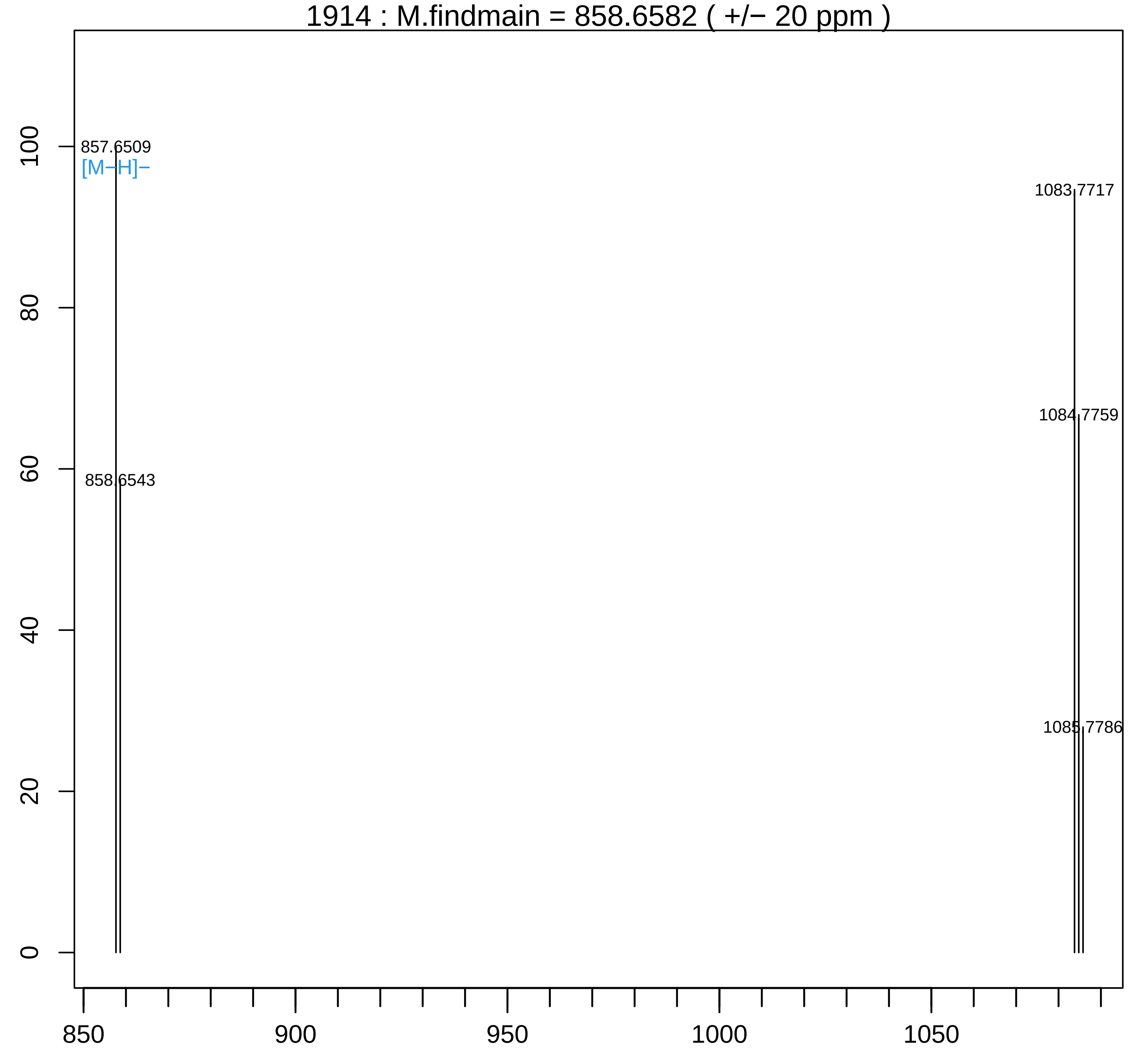

1914 : M.findmain = 858.6582 (+/− 20 ppm)
857.6509
[M−H]−
858.6543
1083.7717
1084.7759
1085.7786
0
20
40
60
80
100
850
900
950
1000
1050

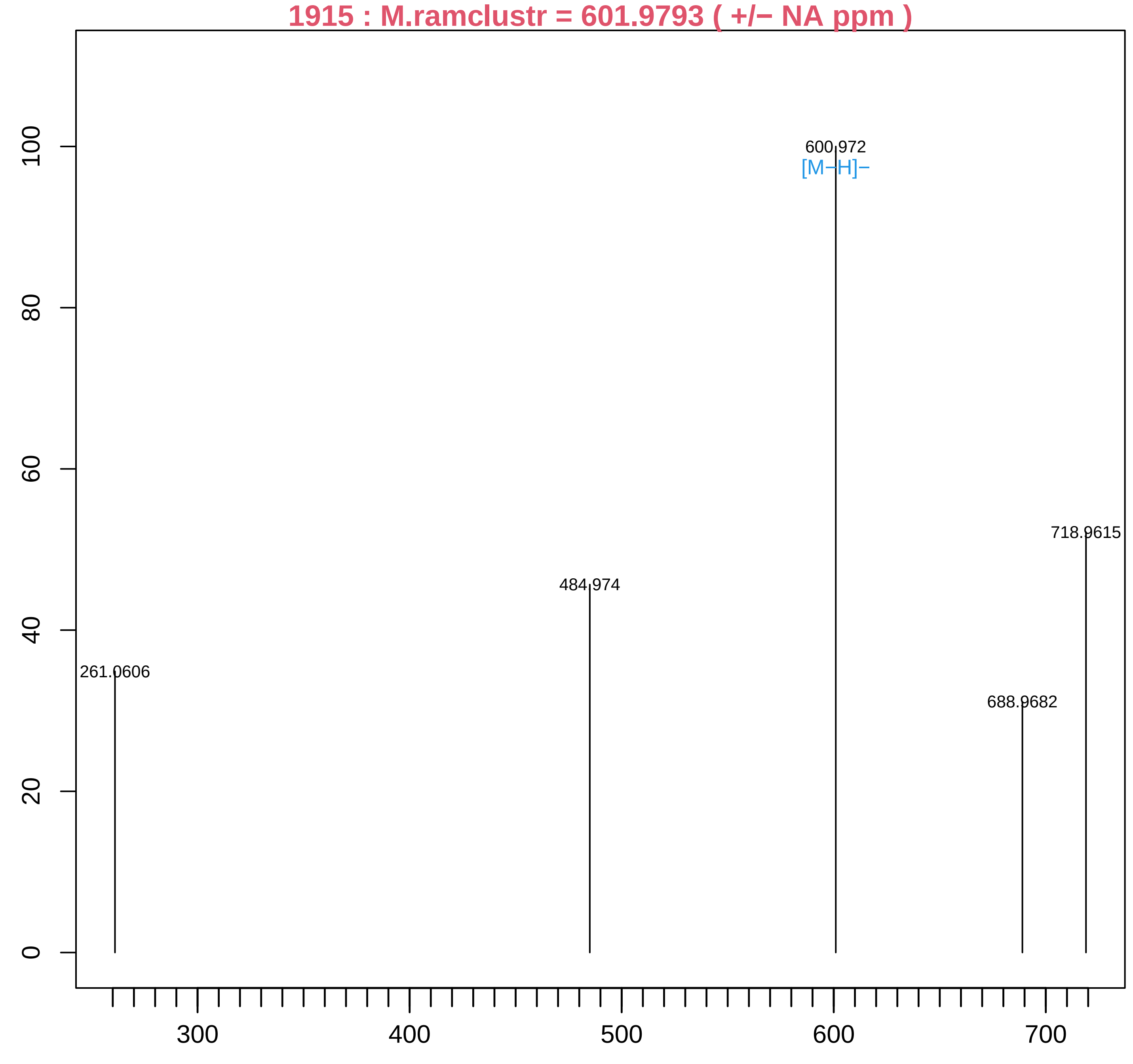
1915 : M.ramclustr = 601.9793 (+/− NA ppm)
600.972
[M−H]−
718.9615
484.974
261.0606
688.9682
0
20
40
60
80
100
300
400
500
600
700

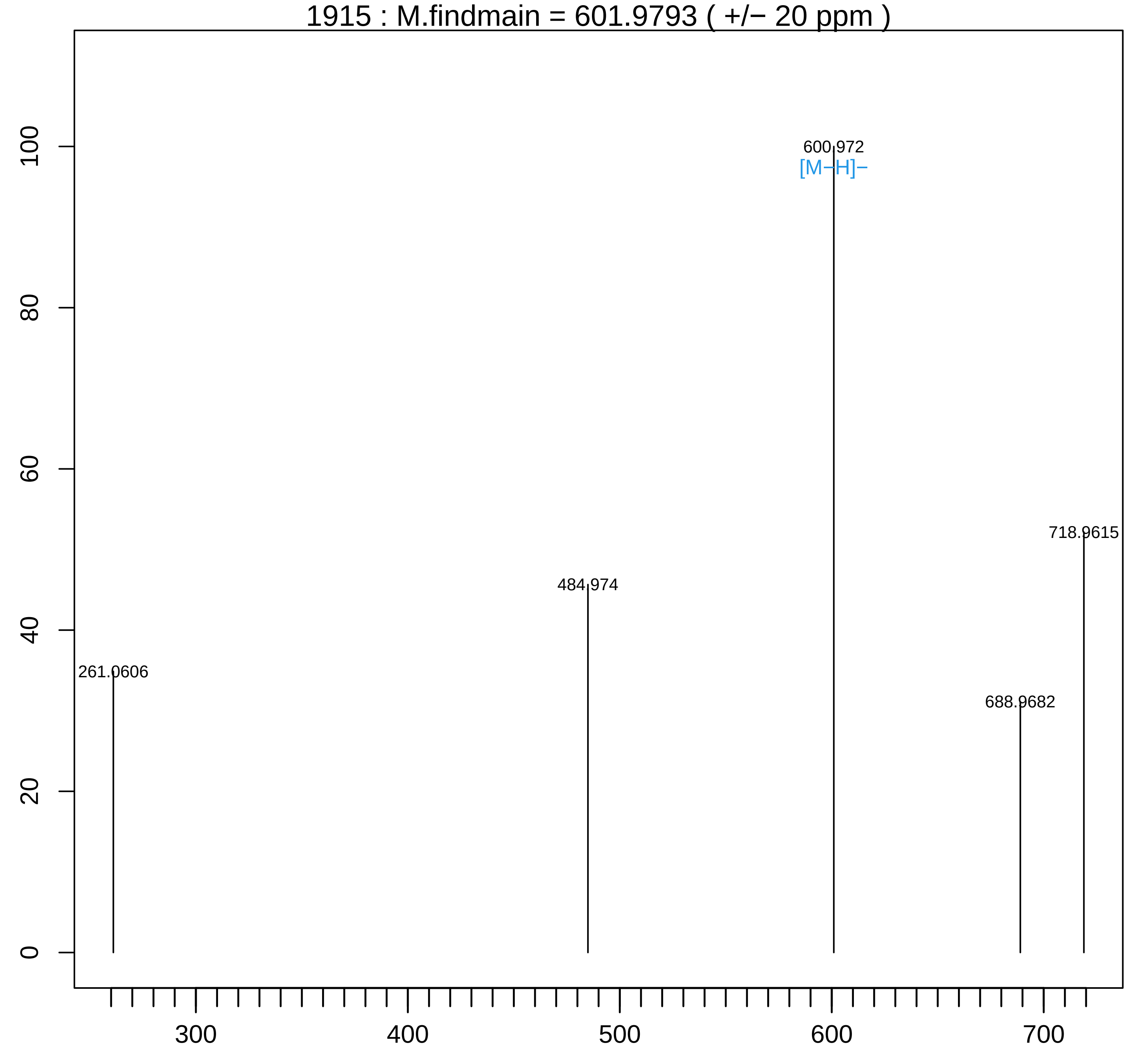
1915 : M.findmain = 601.9793 (+/− 20 ppm)
600.972
[M−H]−
718.9615
484.974
261.0606
688.9682
0
20
40
60
80
100
300
400
500
600
700

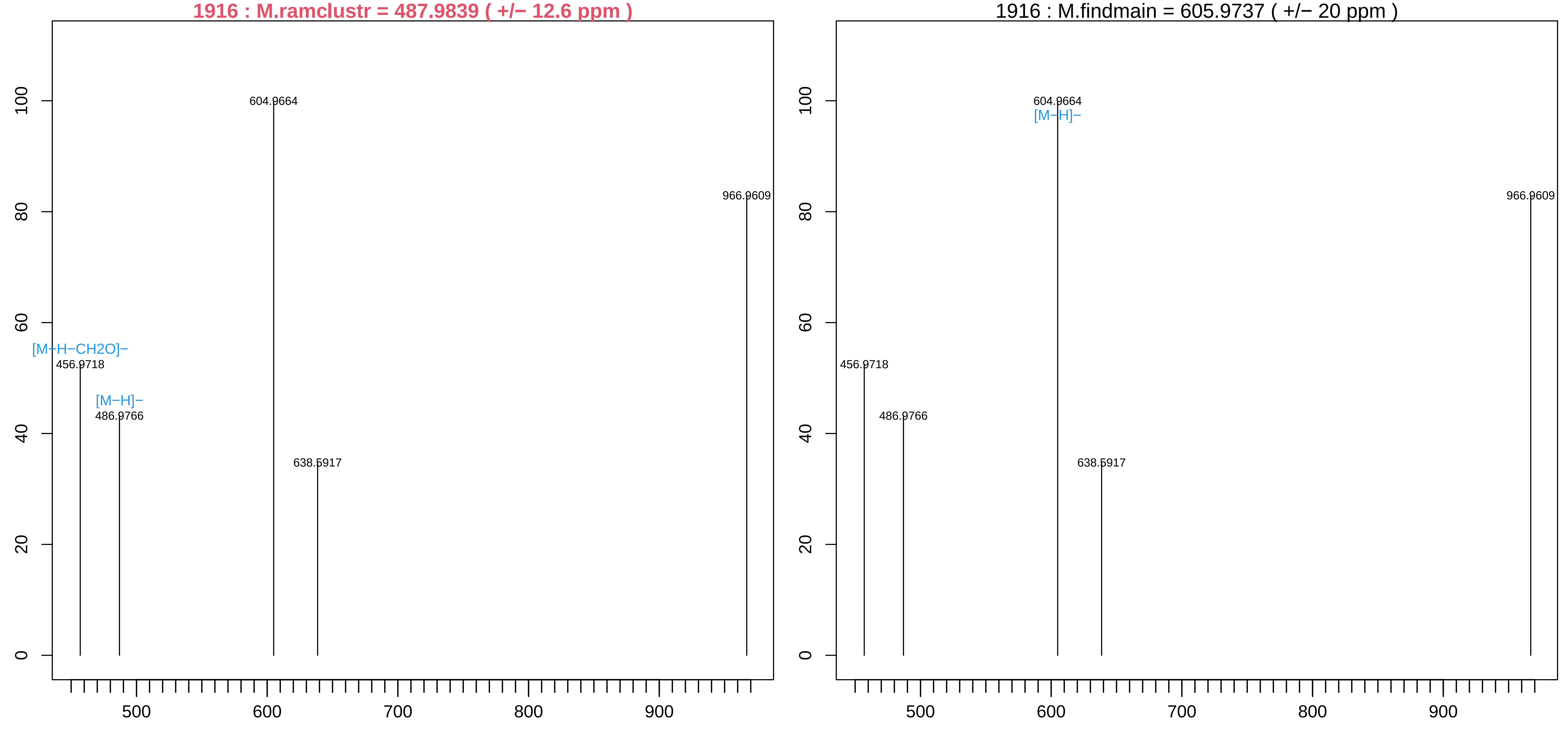

1916 : M.ramclustr = 487.9839 (+/− 12.6 ppm)
604.9664
966.9609
[M−H−CH2O]−
456.9718
[M−H]−
486.9766
638.5917
0
20
40
60
80
100
500
600
700
800
900
1916 : M.findmain = 605.9737 (+/− 20 ppm)
604.9664
[M−H]−
966.9609
456.9718
486.9766
638.5917
0
20
40
60
80
100
500
600
700
800
900

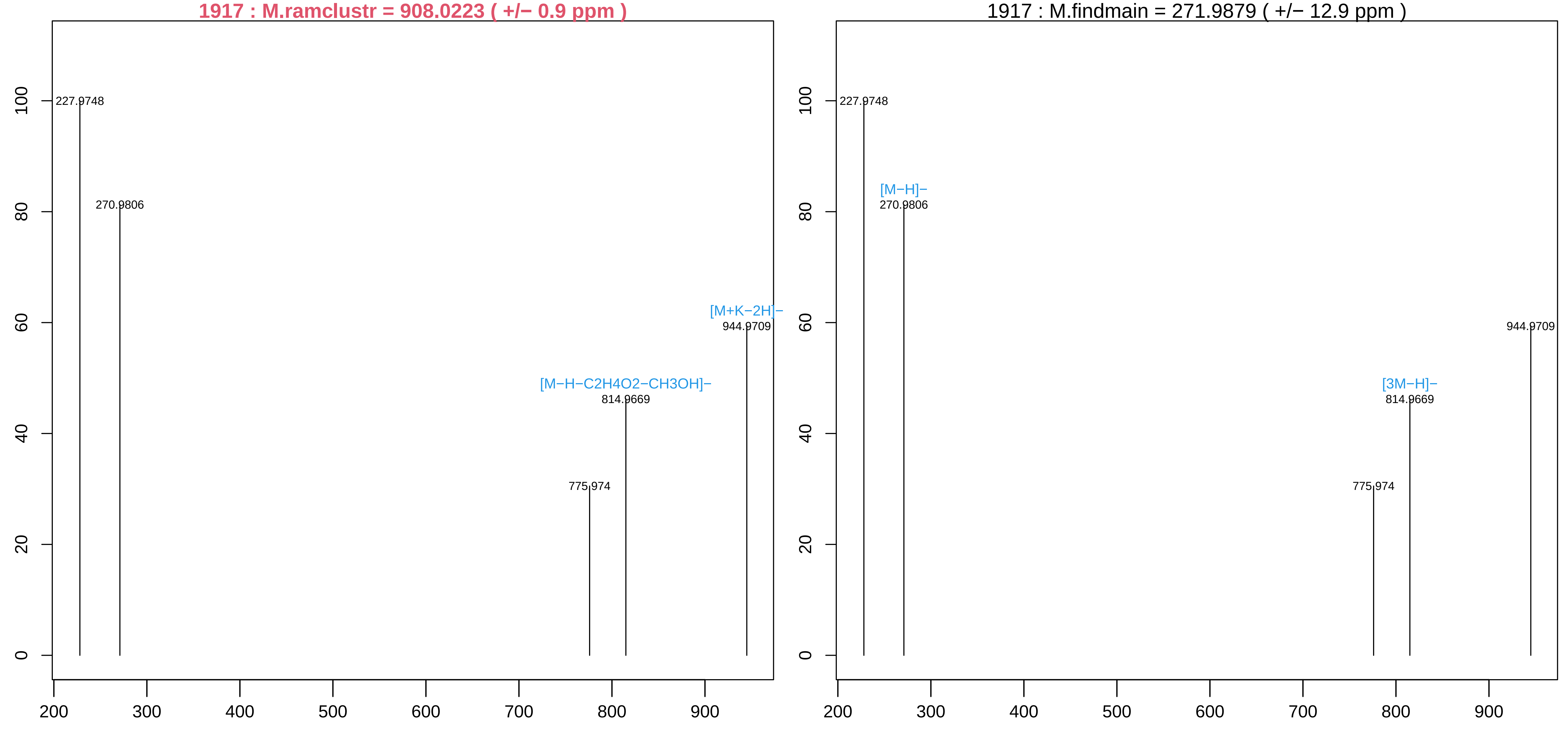

1917 : M.ramclustr = 908.0223 (+/− 0.9 ppm)
227.9748
270.9806
[M+K−2H]−
944.9709
[M−H−C2H4O2−CH3OH]−
814.9669
775.974
1917 : M.findmain = 271.9879 (+/− 12.9 ppm)
227.9748
[M−H]−
270.9806
944.9709
[3M−H]−
814.9669
775.974

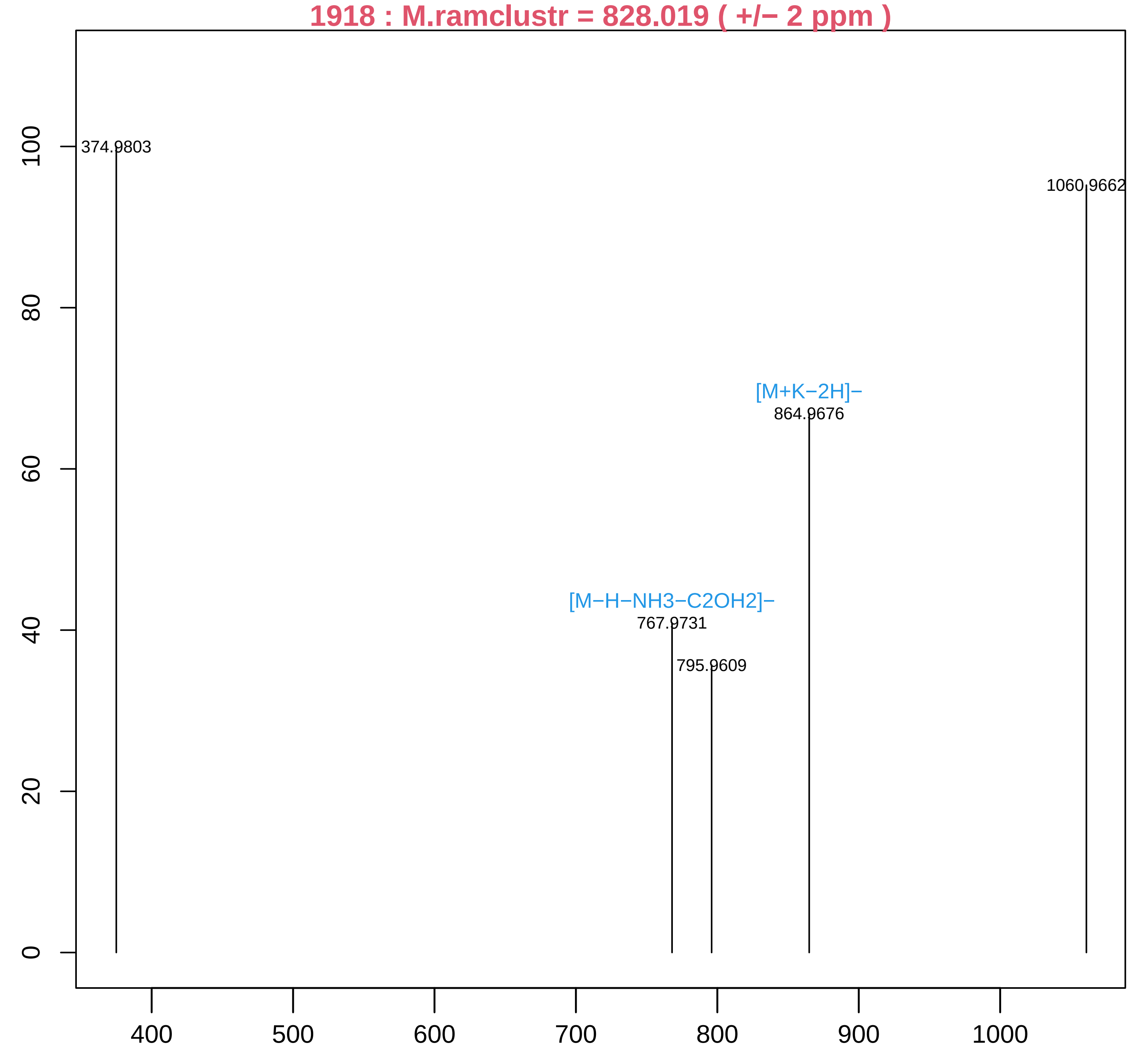

1918 : M.ramclustr = 828.019 (+/− 2 ppm)
374.9803
1060.9662
[M+K−2H]−
864.9676
[M−H−NH3−C2OH2]−
767.9731
795.9609
0
20
40
60
80
100
400
500
600
700
800
900
1000

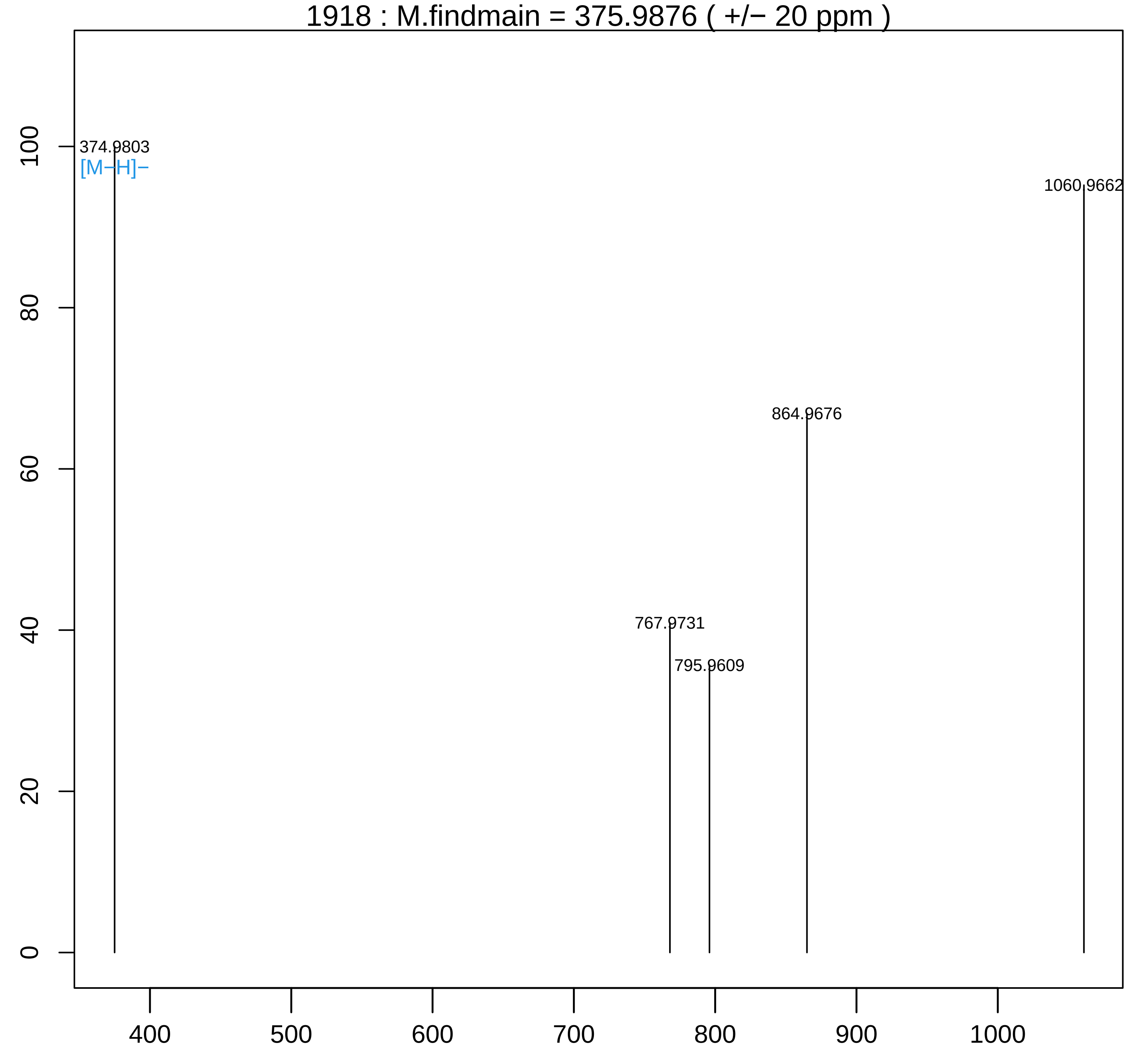

1918 : M.findmain = 375.9876 (+/− 20 ppm)
374.9803
[M−H]−
1060.9662
864.9676
767.9731
795.9609
0
20
40
60
80
100
400
500
600
700
800
900
1000

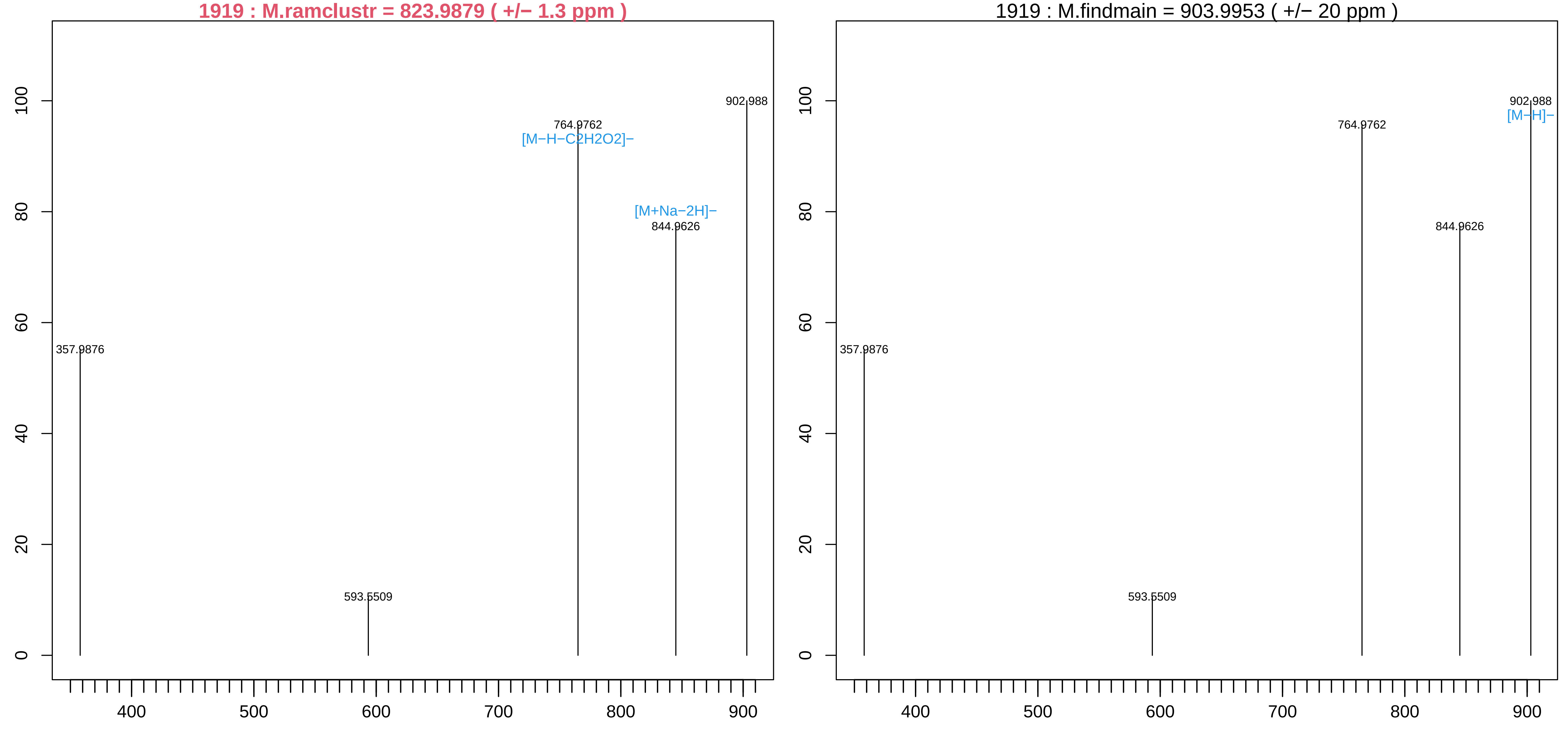
1919 : M.ramclustr = 823.9879 (+/− 1.3 ppm)
357.9876
593.5509
764.9762
[M−H−C2H2O2]−
[M+Na−2H]−
844.9626
902.988
1919 : M.findmain = 903.9953 (+/− 20 ppm)
357.9876
593.5509
764.9762
844.9626
902.988
[M−H]−

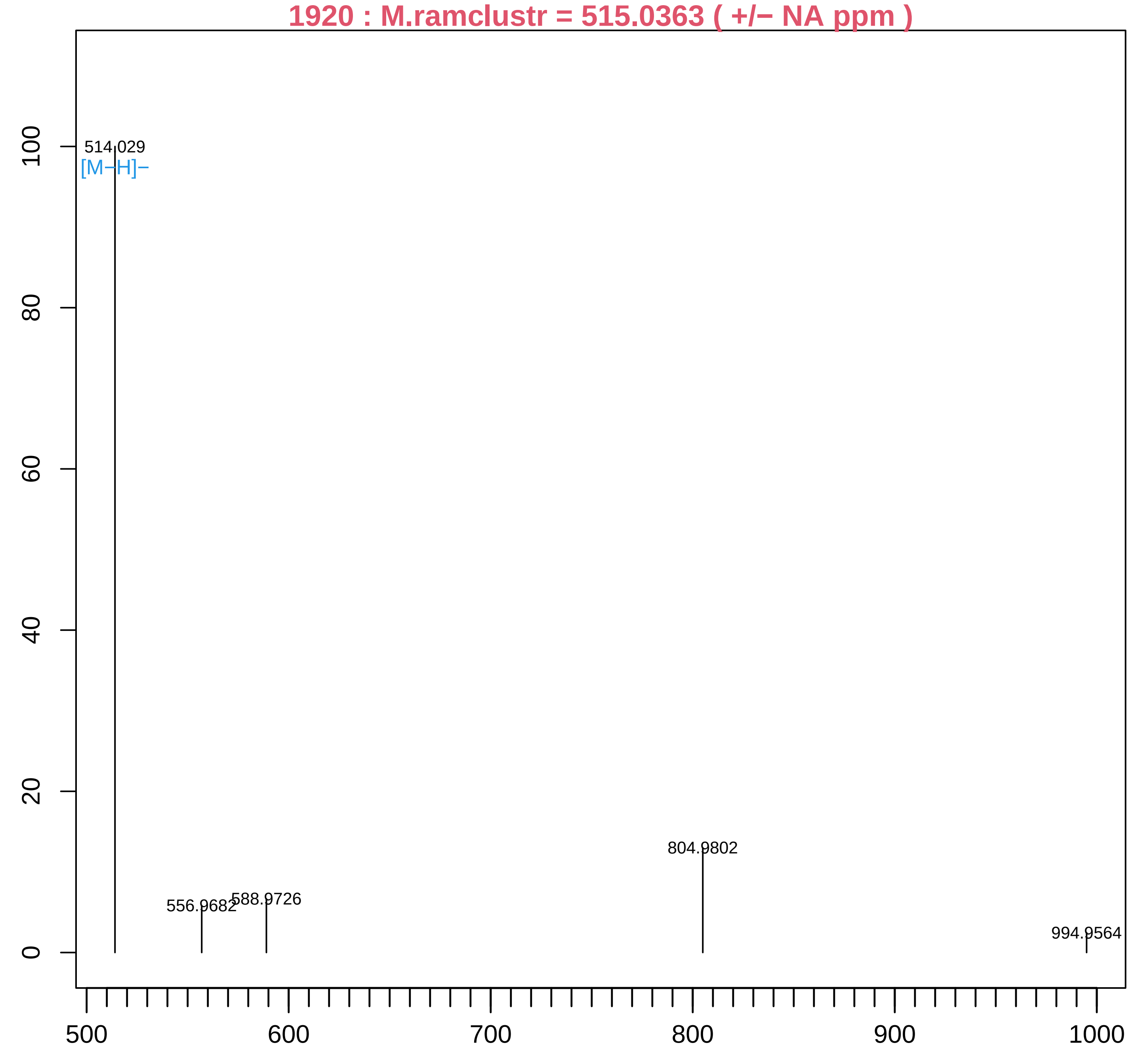

1920 : M.ramclustr = 515.0363 (+/− NA ppm)
514.029
[M−H]−
556.9682
588.9726
804.9802
994.9564
0
20
40
60
80
100
500
600
700
800
900
1000

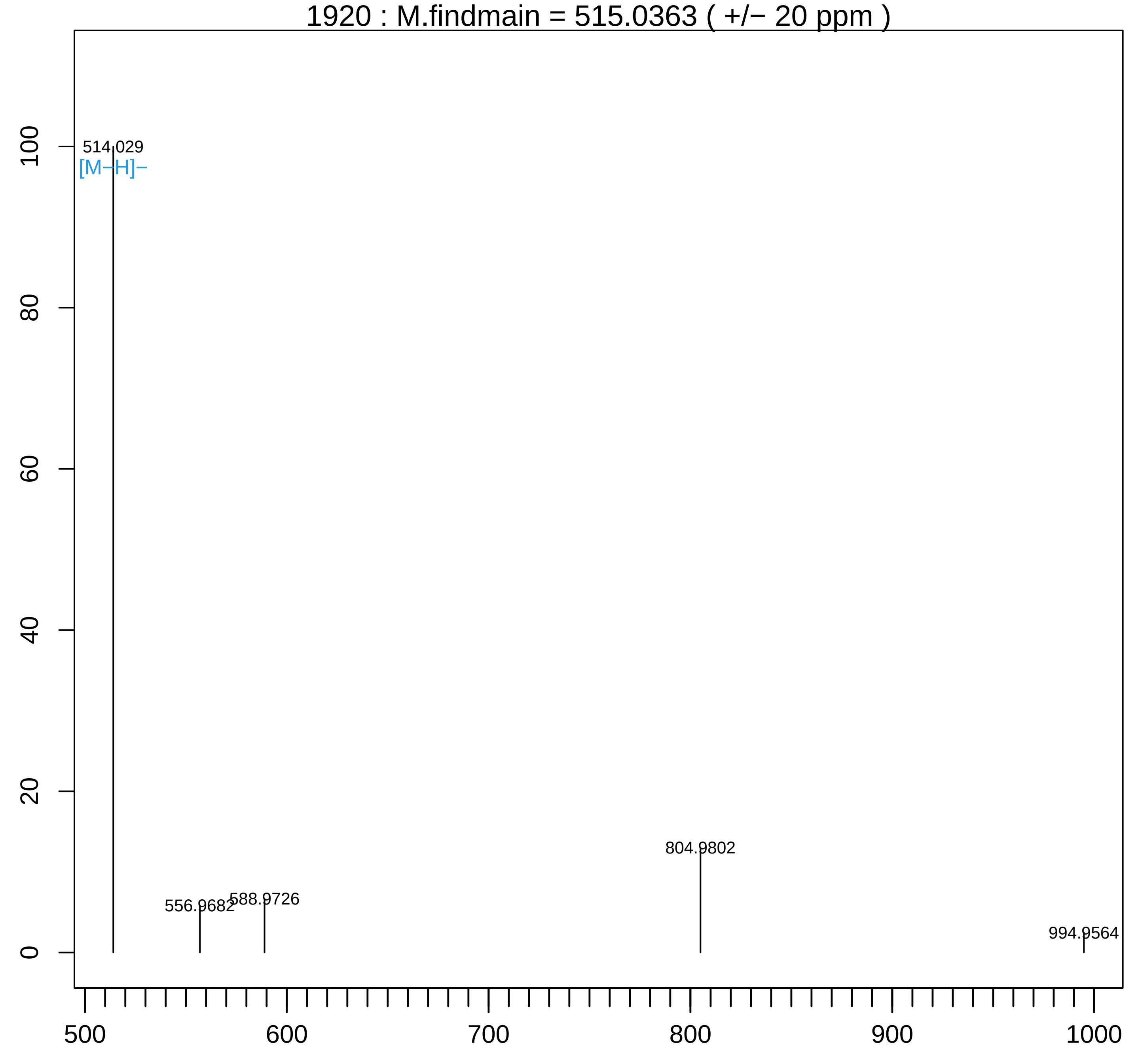

1920 : M.findmain = 515.0363 (+/− 20 ppm)
514.029
[M−H]−
556.9682
588.9726
804.9802
994.9564
0
20
40
60
80
100
500
600
700
800
900
1000

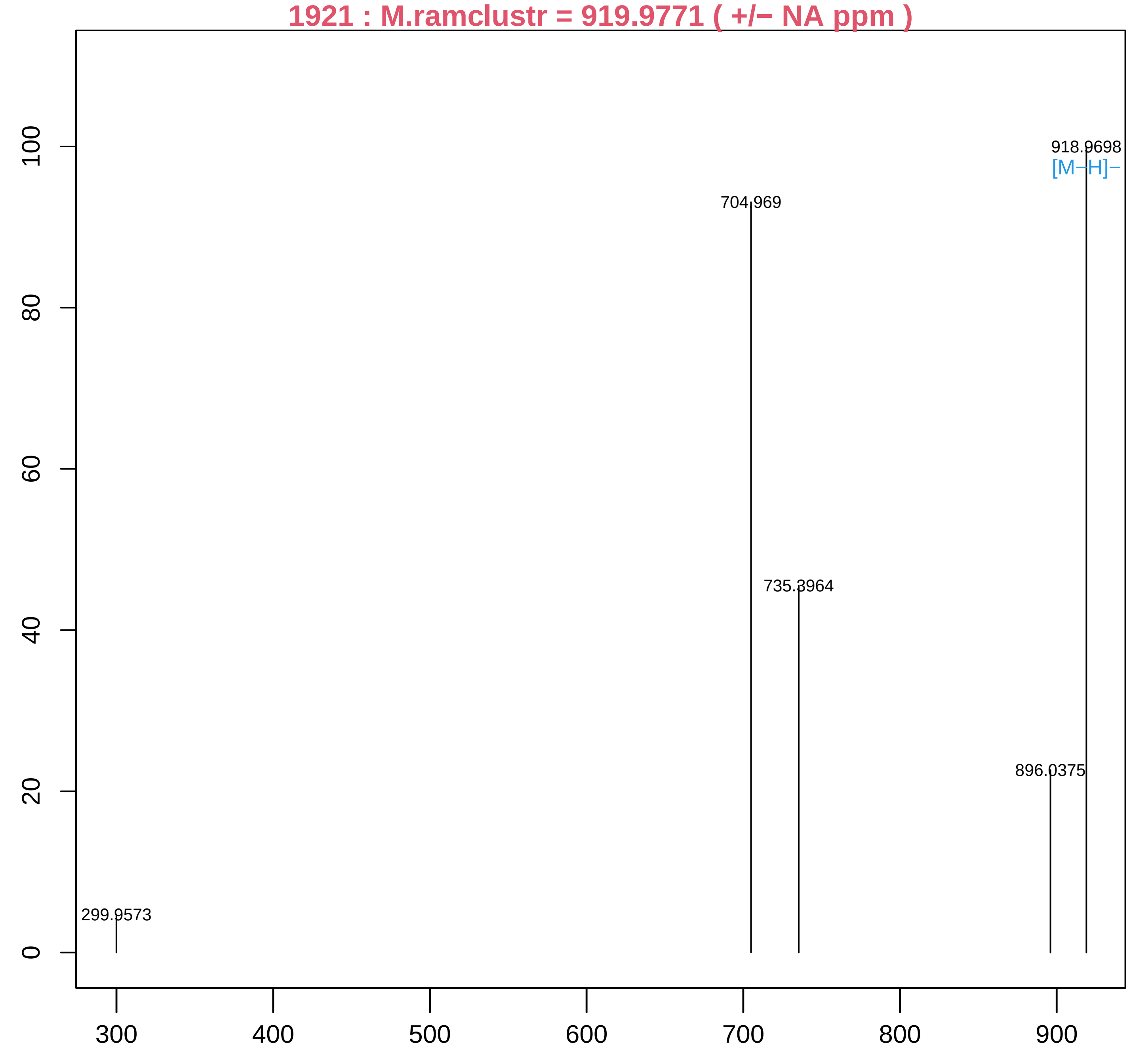
1921 : M.ramclustr = 919.9771 (+/− NA ppm)
918.9698
[M−H]−
704.969
735.3964
896.0375
299.9573
0
20
40
60
80
100
300
400
500
600
700
800
900

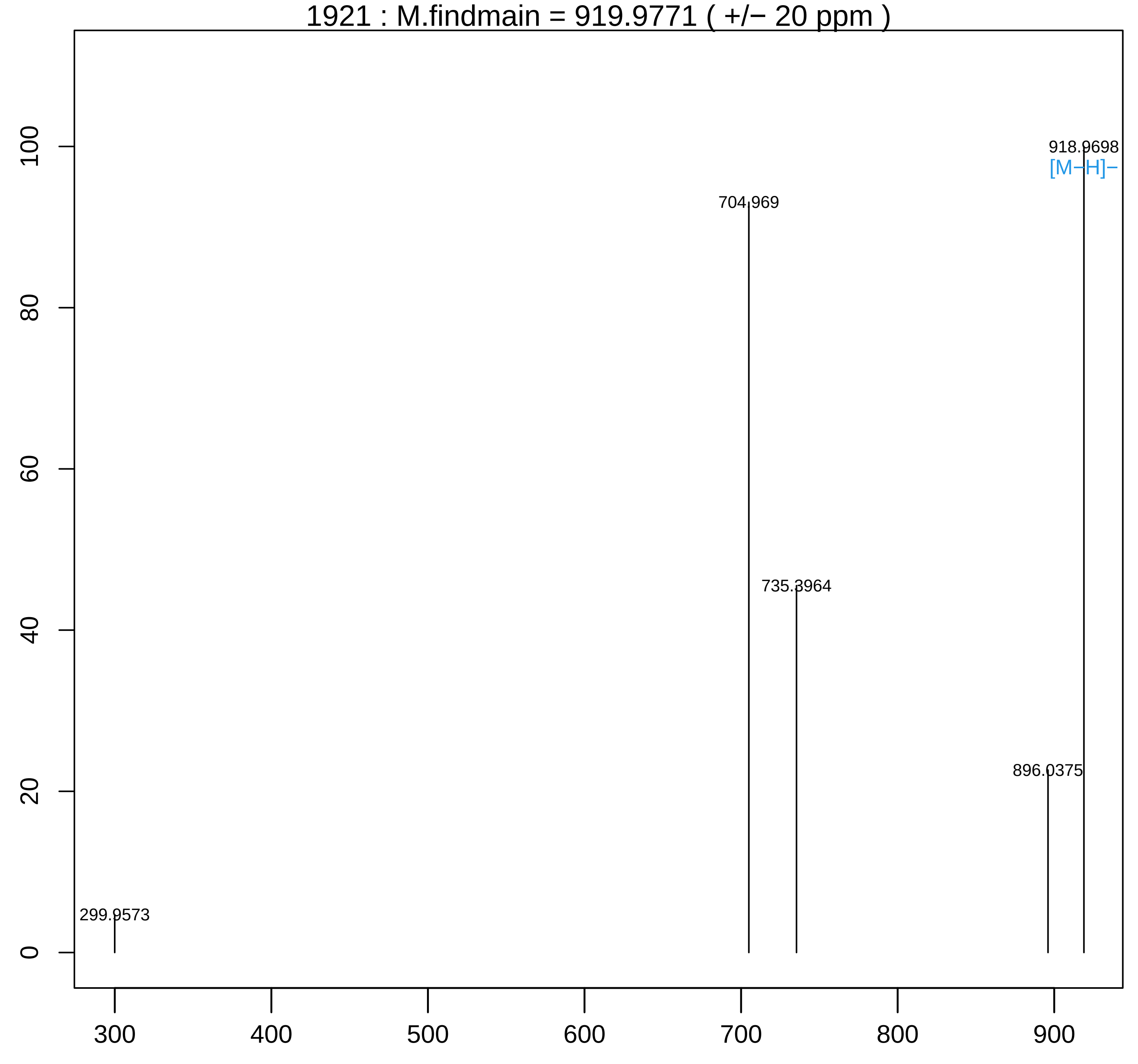
1921 : M.findmain = 919.9771 (+/− 20 ppm)
918.9698
[M−H]−
704.969
735.3964
896.0375
299.9573
0
20
40
60
80
100
300
400
500
600
700
800
900

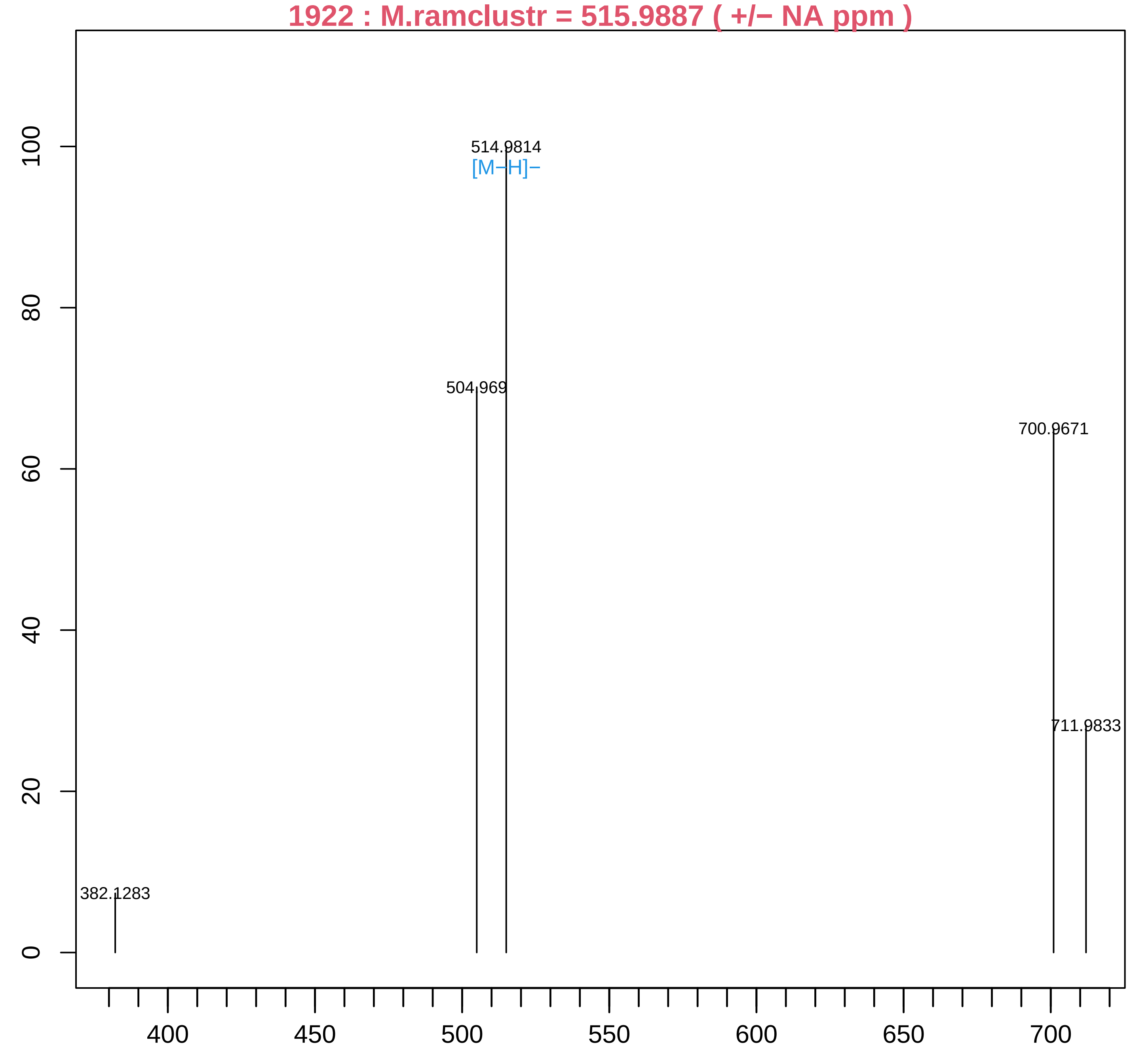

1922 : M.ramclustr = 515.9887 (+/− NA ppm)
514.9814
[M−H]−
504.969
700.9671
711.9833
382.1283
0
20
40
60
80
100
400
450
500
550
600
650
700

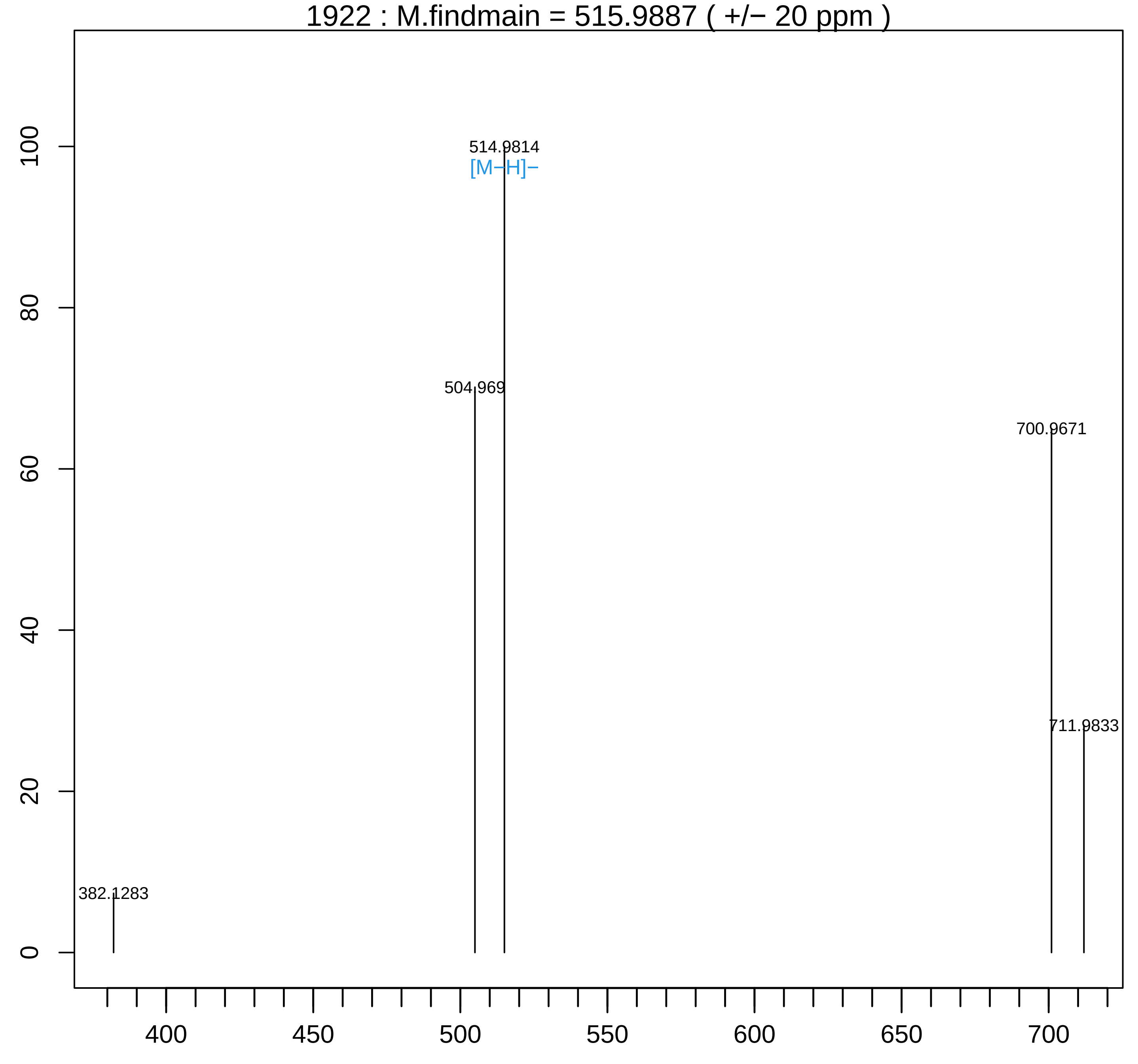

1922 : M.findmain = 515.9887 (+/− 20 ppm)
514.9814
[M−H]−
504.969
700.9671
711.9833
382.1283
0
20
40
60
80
100
400
450
500
550
600
650
700

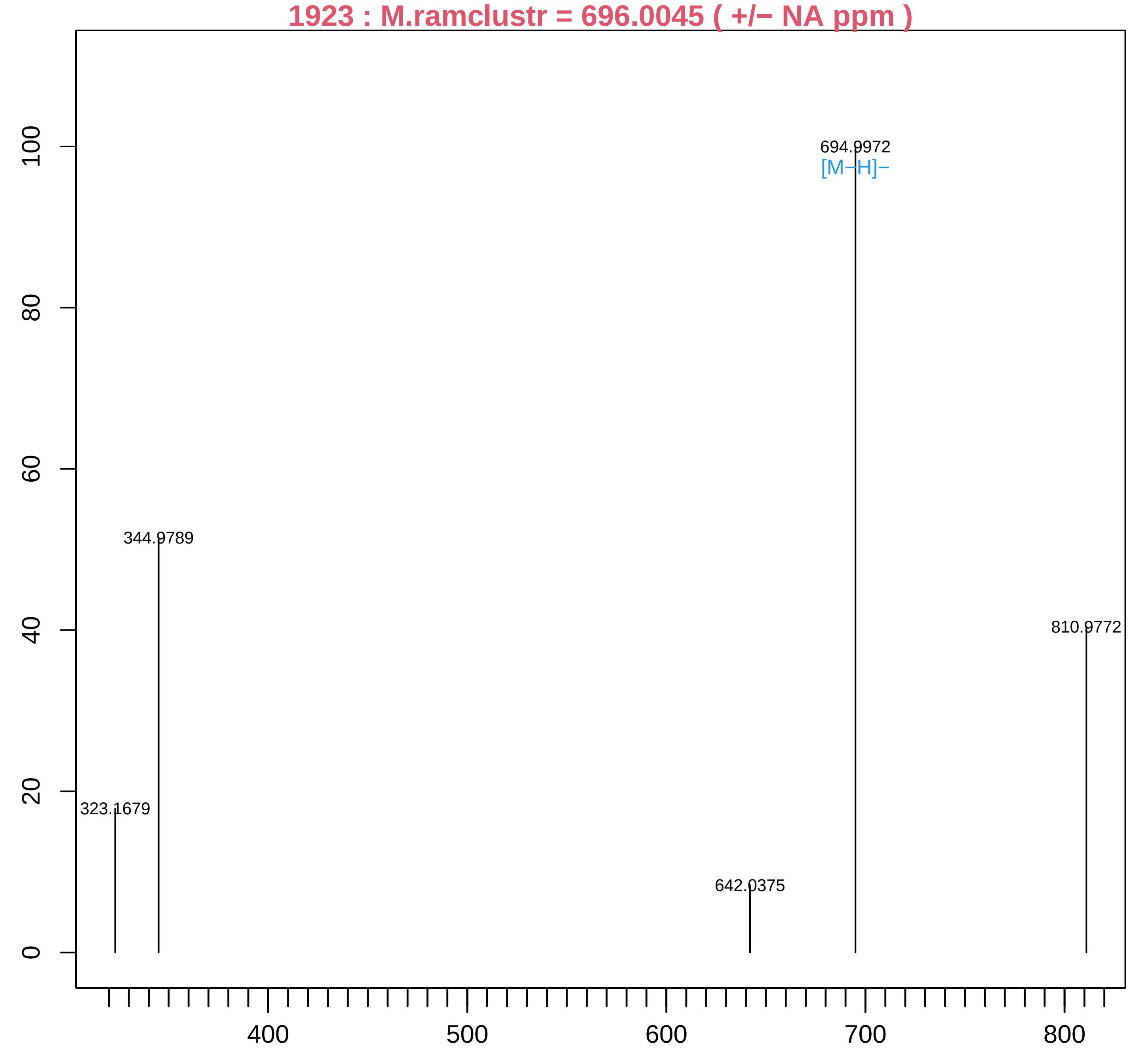
1923 : M.ramclustr = 696.0045 (+/− NA ppm)
694.9972
[M−H]−
344.9789
810.9772
323.1679
642.0375
0
20
40
60
80
100
400
500
600
700
800

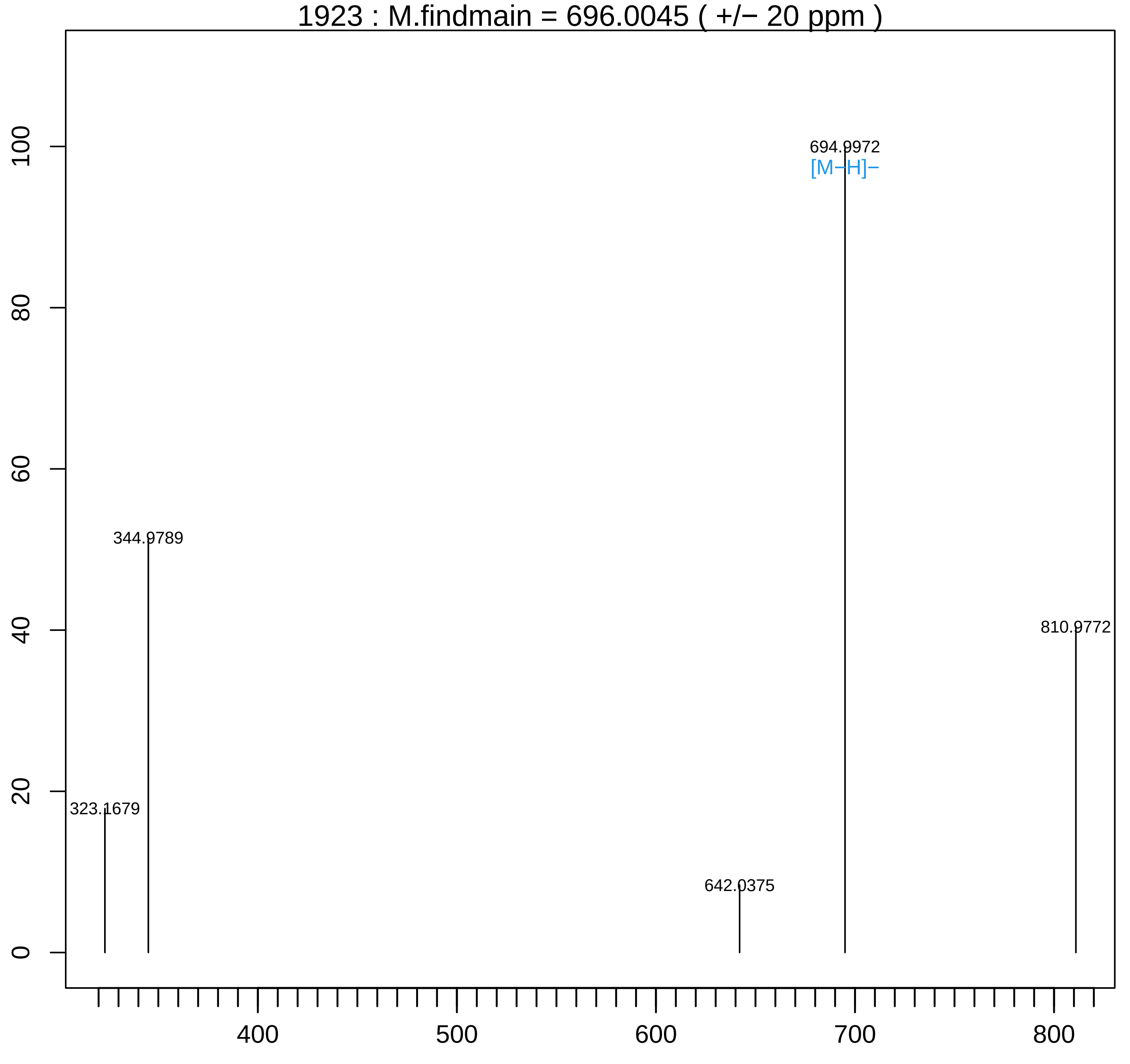
1923 : M.findmain = 696.0045 (+/− 20 ppm)
694.9972
[M−H]−
344.9789
810.9772
323.1679
642.0375
0
20
40
60
80
100
400
500
600
700
800

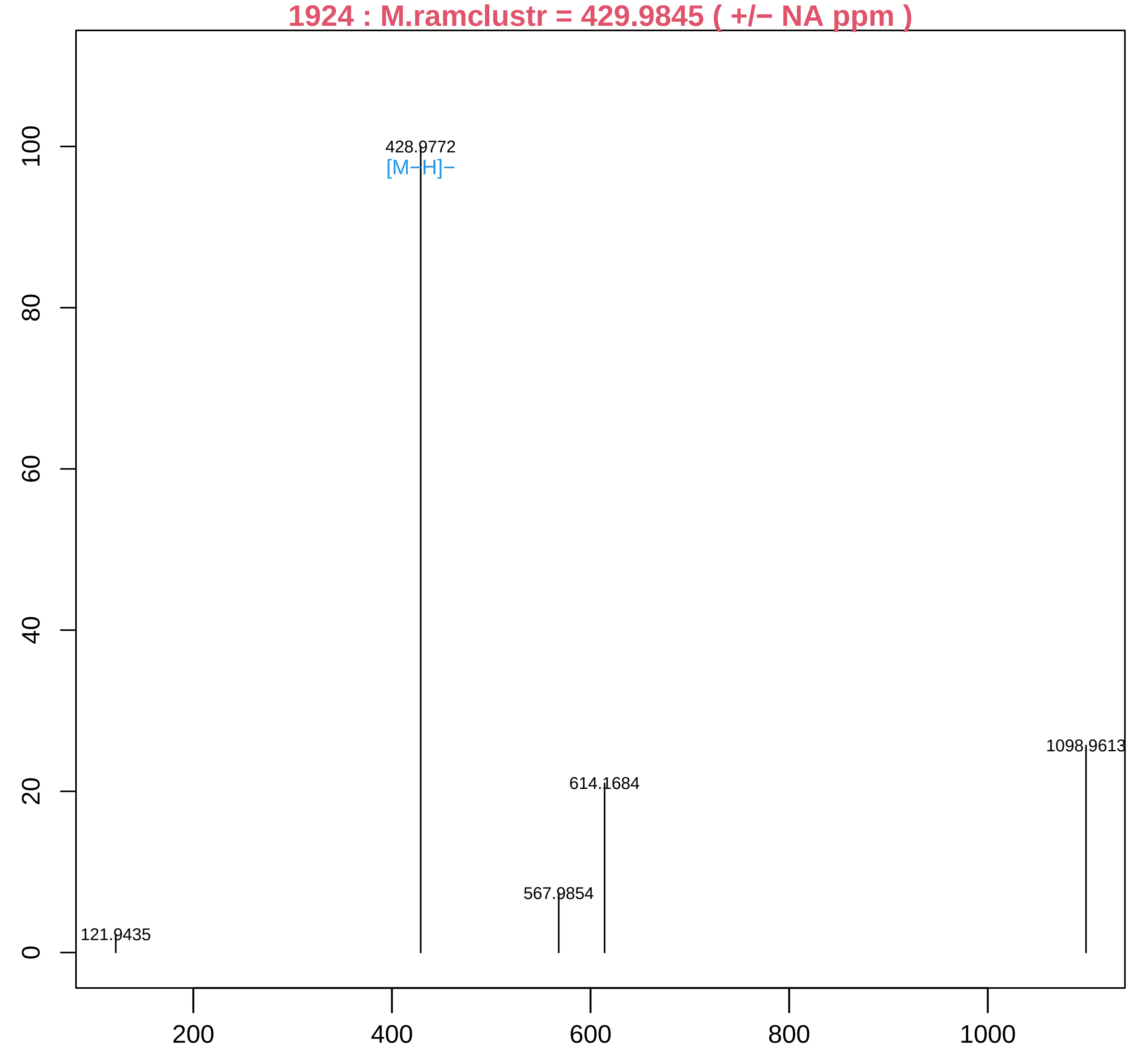
1924 : M.ramclustr = 429.9845 (+/− NA ppm)
428.9772
[M−H]−
614.1684
567.9854
121.9435
1098.9613
0
20
40
60
80
100
200
400
600
800
1000

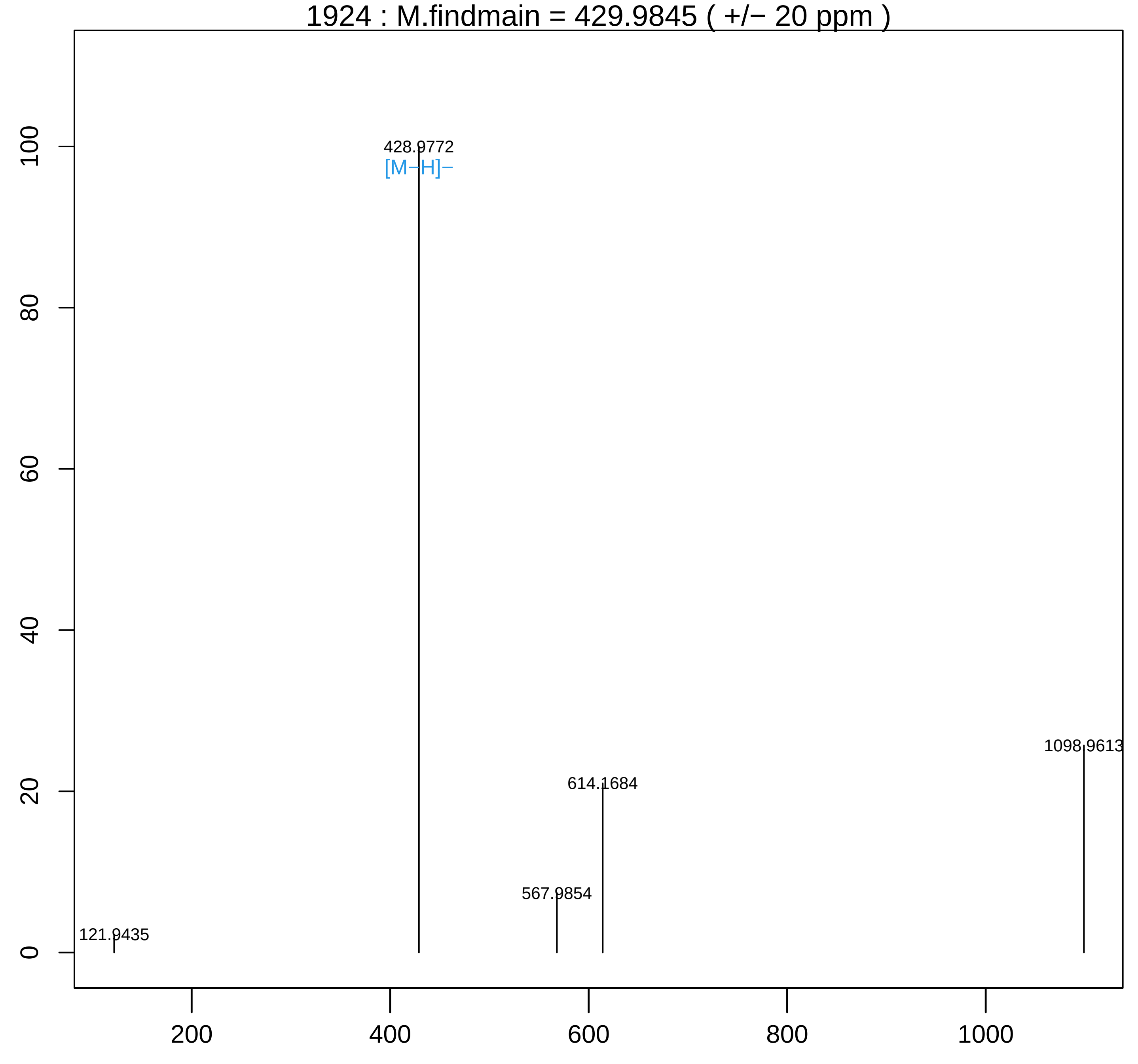
1924 : M.findmain = 429.9845 (+/− 20 ppm)
428.9772
[M−H]−
614.1684
567.9854
121.9435
1098.9613
0
20
40
60
80
100
200
400
600
800
1000

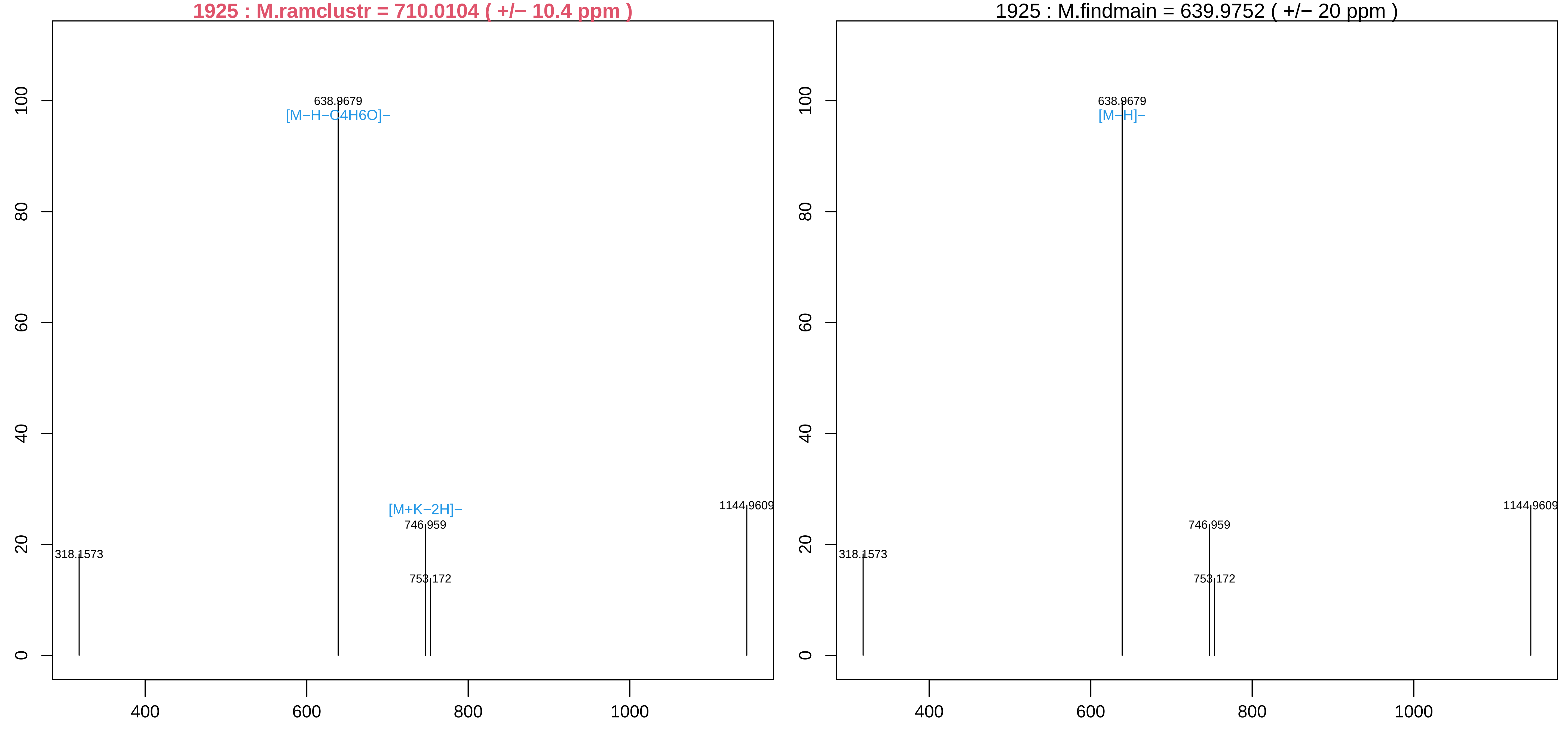

1925 : M.ramclustr = 710.0104 (+/− 10.4 ppm)
638.9679
[M−H−C4H6O]−
[M+K−2H]−
746.959
753.172
318.1573
1144.9609
0
20
40
60
80
100
400
600
800
1000
1925 : M.findmain = 639.9752 (+/− 20 ppm)
638.9679
[M−H]−
746.959
753.172
318.1573
1144.9609

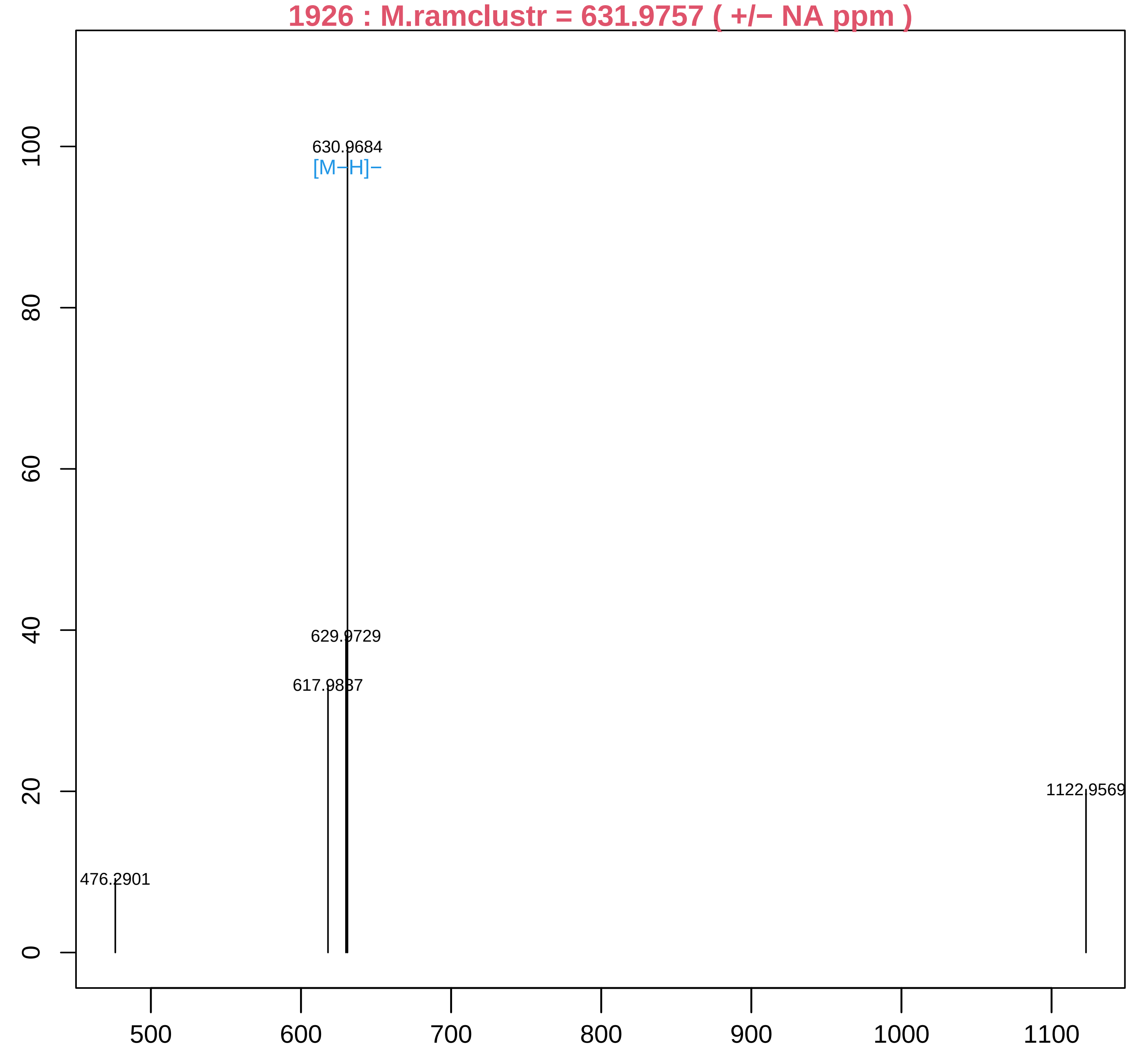

1926 : M.ramclustr = 631.9757 (+/− NA ppm)
630.9684
[M−H]−
629.9729
617.9837
1122.9569
476.2901
0
20
40
60
80
100
500
600
700
800
900
1000
1100

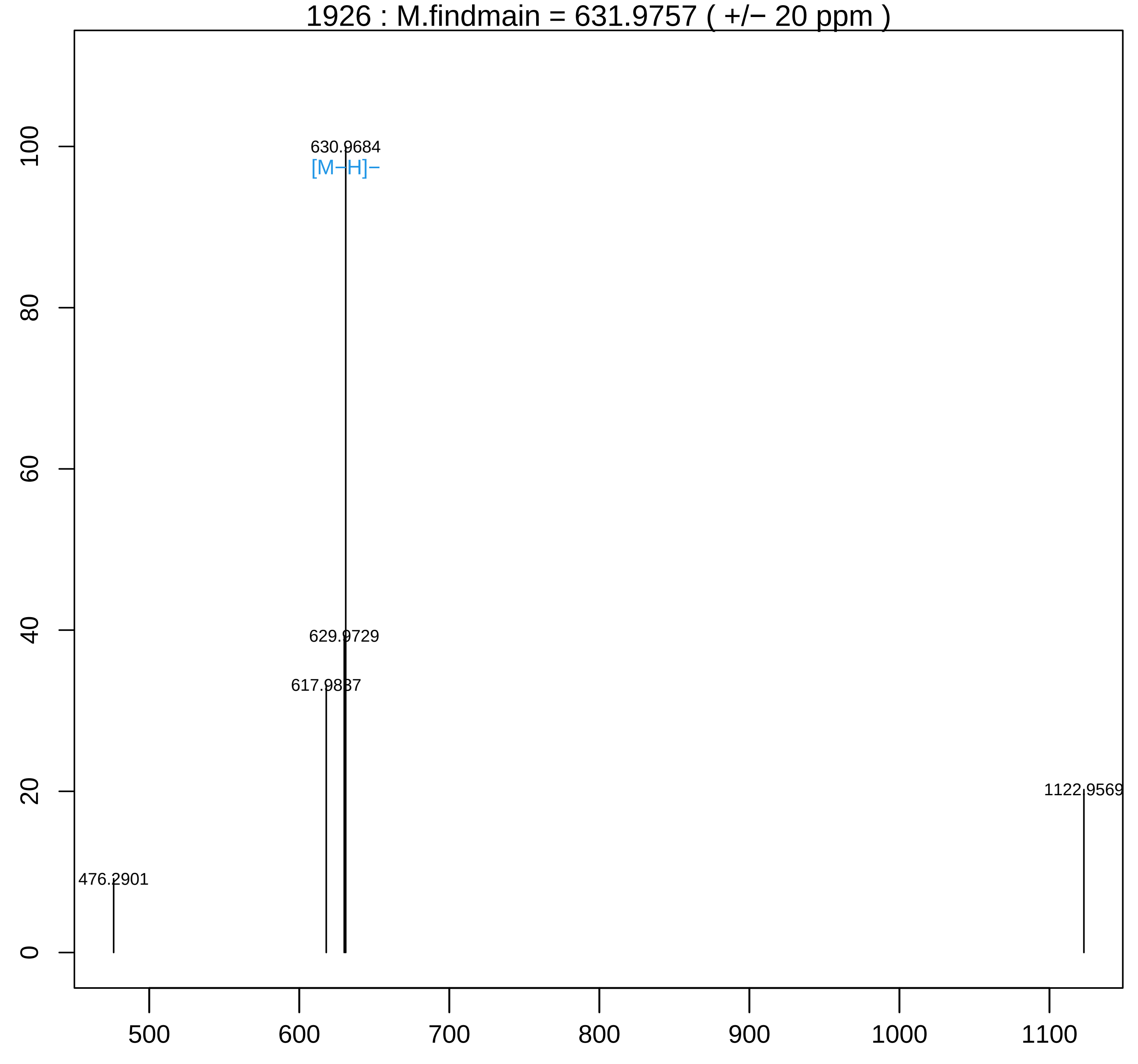

1926 : M.findmain = 631.9757 (+/− 20 ppm)
630.9684
[M−H]−
629.9729
617.9837
1122.9569
476.2901
0
20
40
60
80
100
500
600
700
800
900
1000
1100

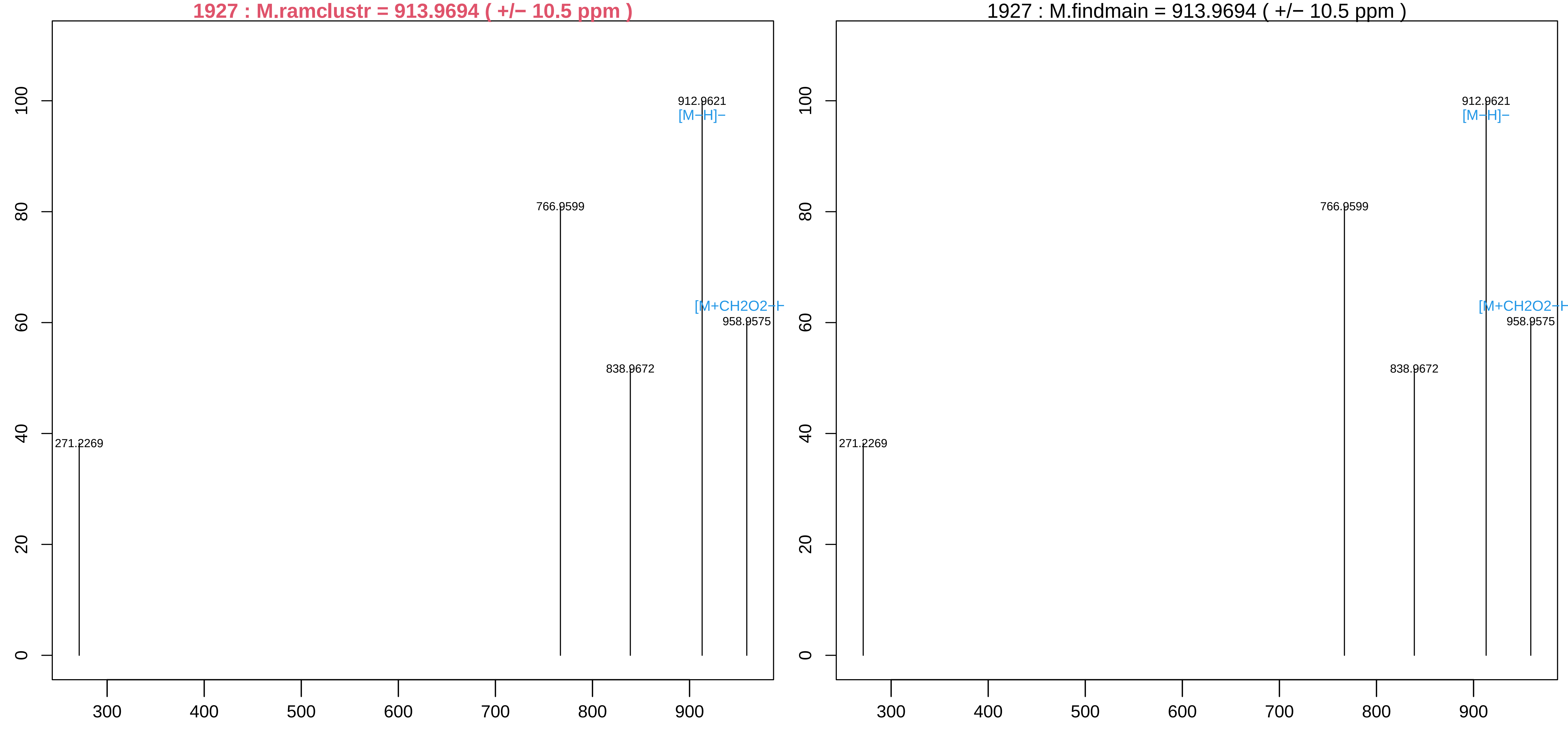
1927 : M.ramclustr = 913.9694 (+/− 10.5 ppm)
912.9621
[M−H]−
766.9599
[M+CH2O2−H
958.9575
838.9672
271.2269
0
20
40
60
80
100
300
400
500
600
700
800
900
1927 : M.findmain = 913.9694 (+/− 10.5 ppm)
912.9621
[M−H]−
766.9599
[M+CH2O2−H
958.9575
838.9672
271.2269
0
20
40
60
80
100
300
400
500
600
700
800
900

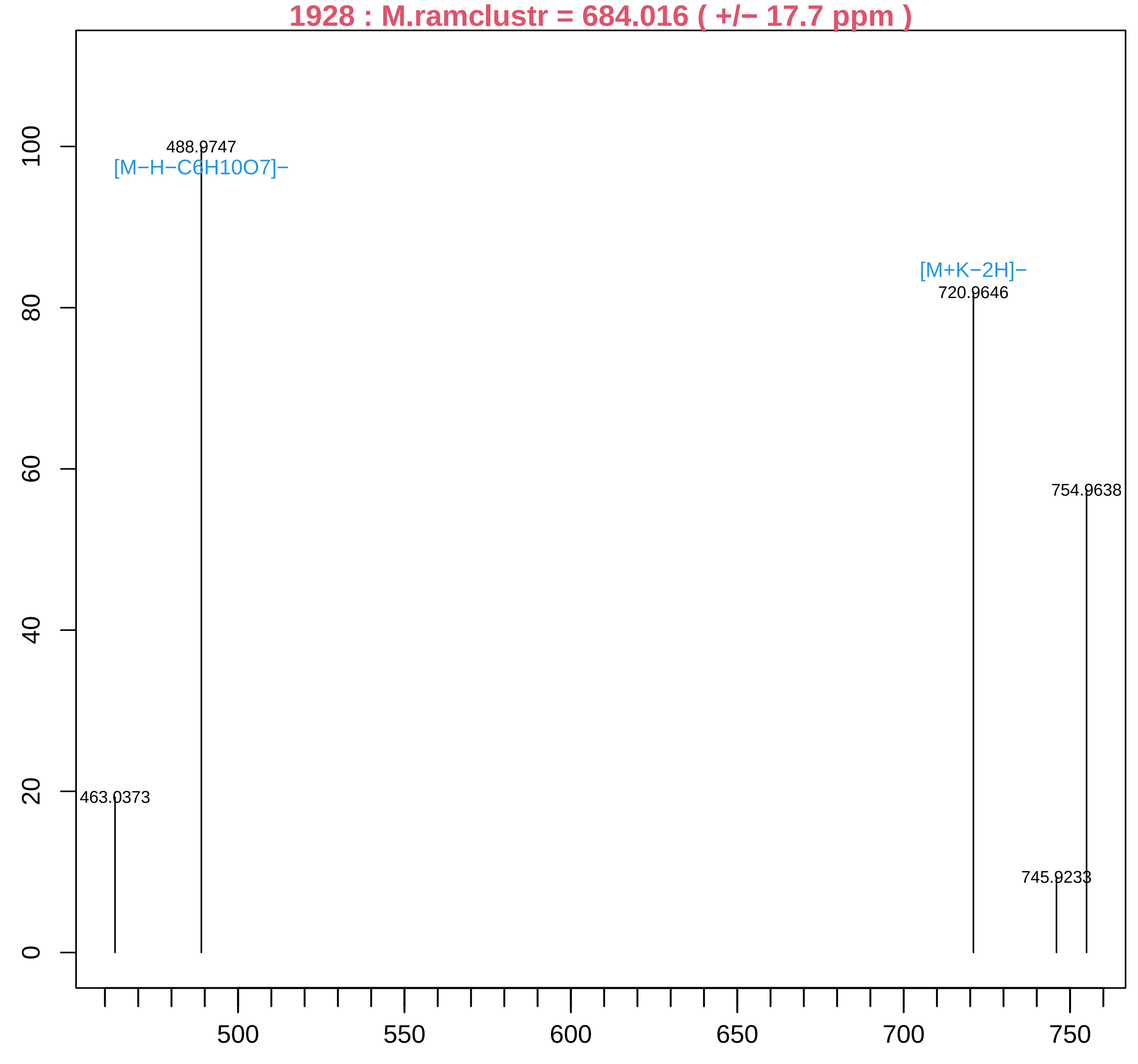

1928 : M.ramclustr = 684.016 (+/− 17.7 ppm)
488.9747
[M−H−C6H10O7]−
[M+K−2H]−
720.9646
754.9638
463.0373
745.9233
0
20
40
60
80
100
500
550
600
650
700
750

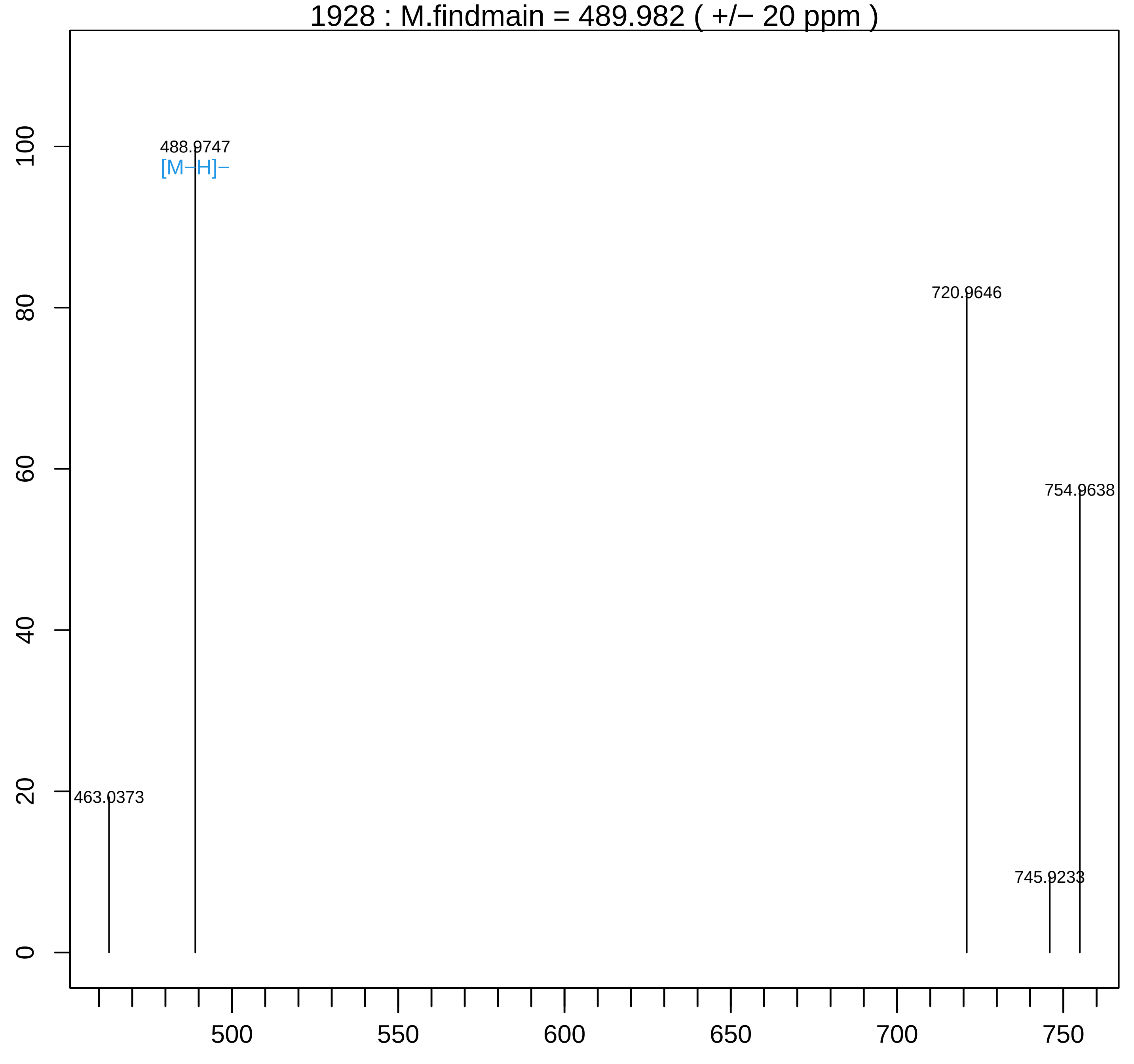

1928 : M.findmain = 489.982 (+/− 20 ppm)
488.9747
[M−H]−
720.9646
754.9638
463.0373
745.9233
0
20
40
60
80
100
500
550
600
650
700
750

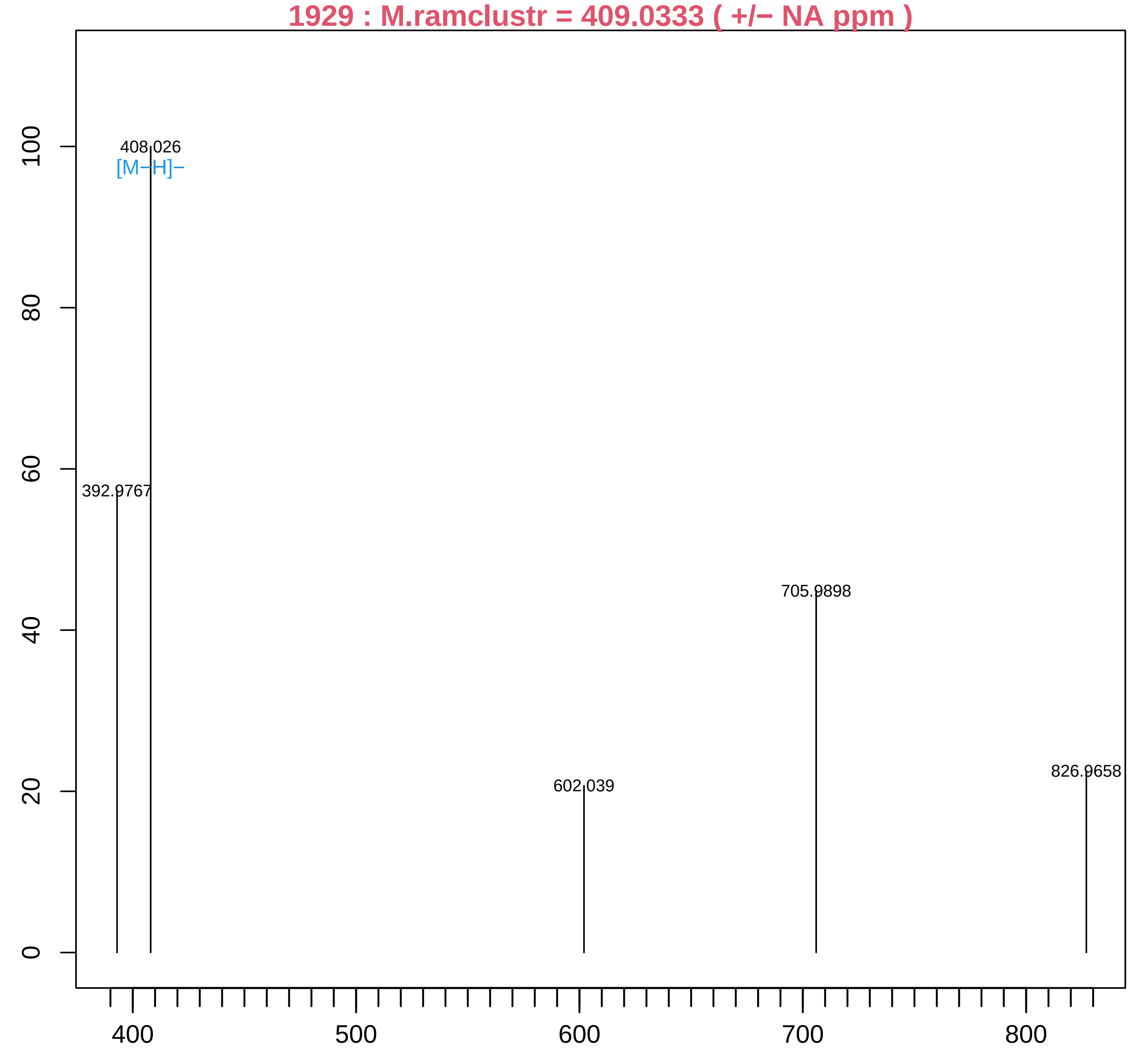
1929 : M.ramclustr = 409.0333 (+/− NA ppm)
408.026
[M−H]−
392.9767
705.9898
602.039
826.9658
0
20
40
60
80
100
400
500
600
700
800

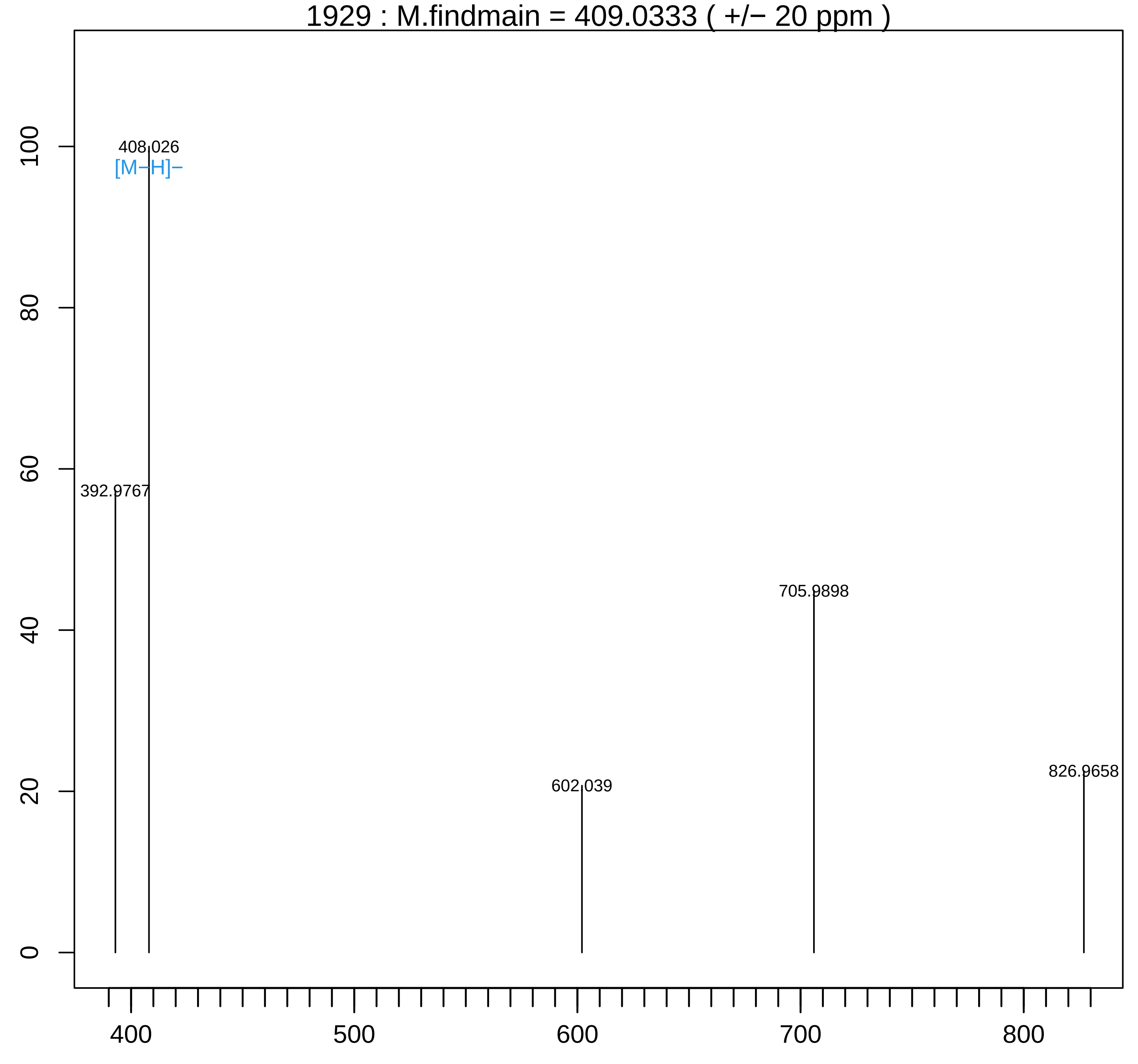
1929 : M.findmain = 409.0333 (+/− 20 ppm)
408.026
[M−H]−
392.9767
705.9898
602.039
826.9658
0
20
40
60
80
100
400
500
600
700
800

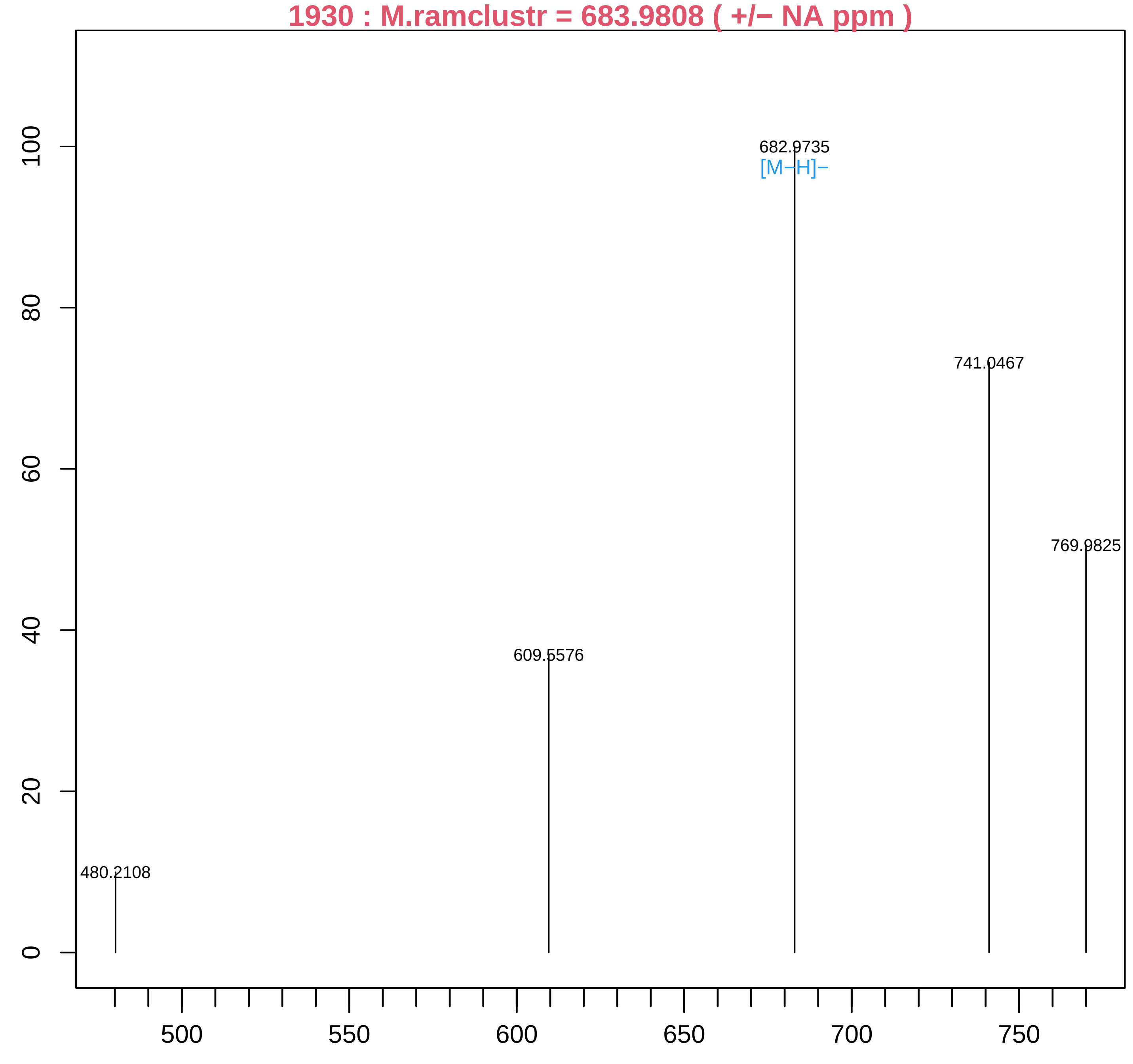

1930 : M.ramclustr = 683.9808 (+/− NA ppm)
682.9735
[M−H]−
741.0467
769.9825
609.5576
480.2108
0
20
40
60
80
100
500
550
600
650
700
750

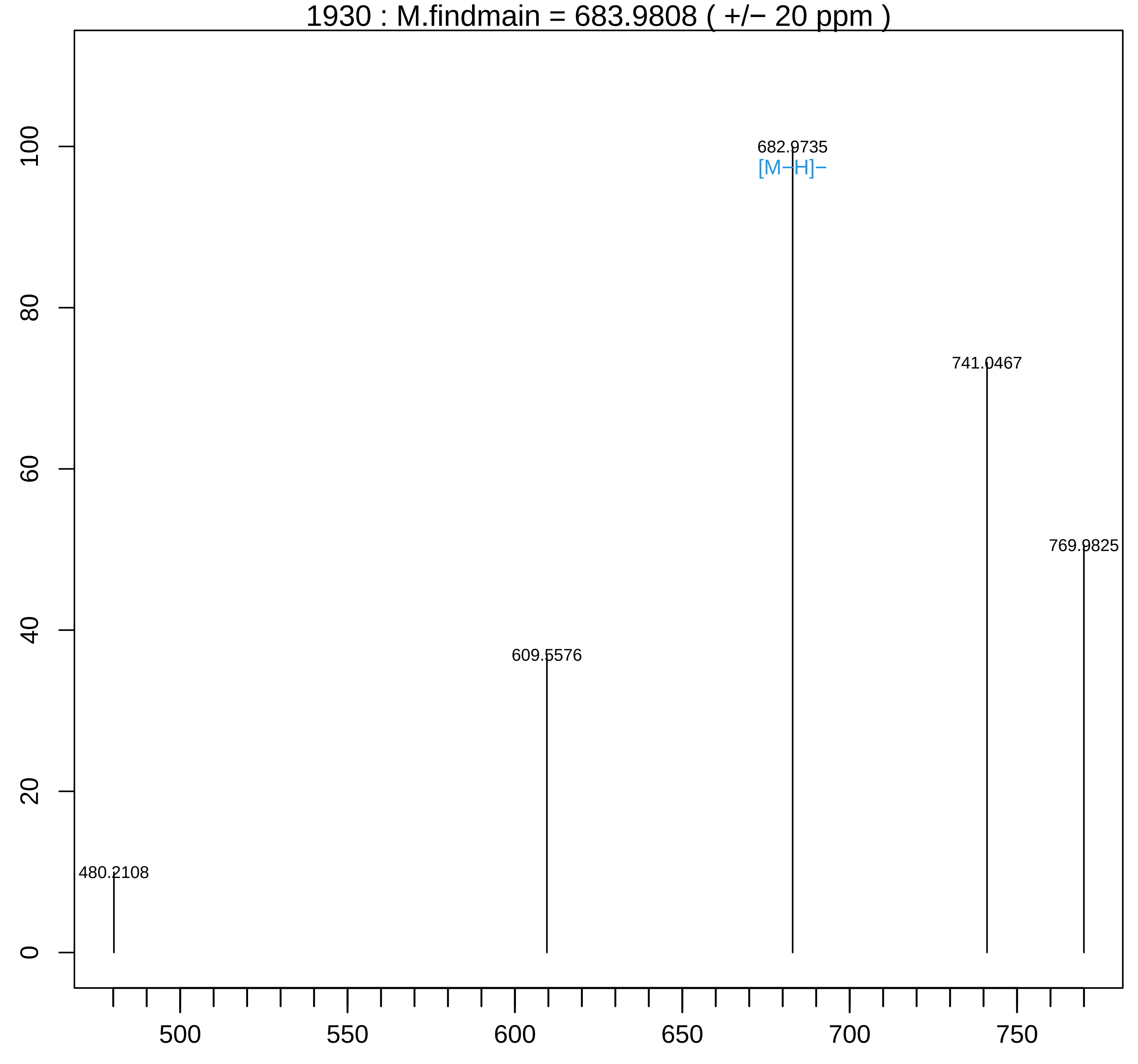

1930 : M.findmain = 683.9808 (+/− 20 ppm)
682.9735
[M−H]−
741.0467
769.9825
609.5576
480.2108
0
20
40
60
80
100
500
550
600
650
700
750

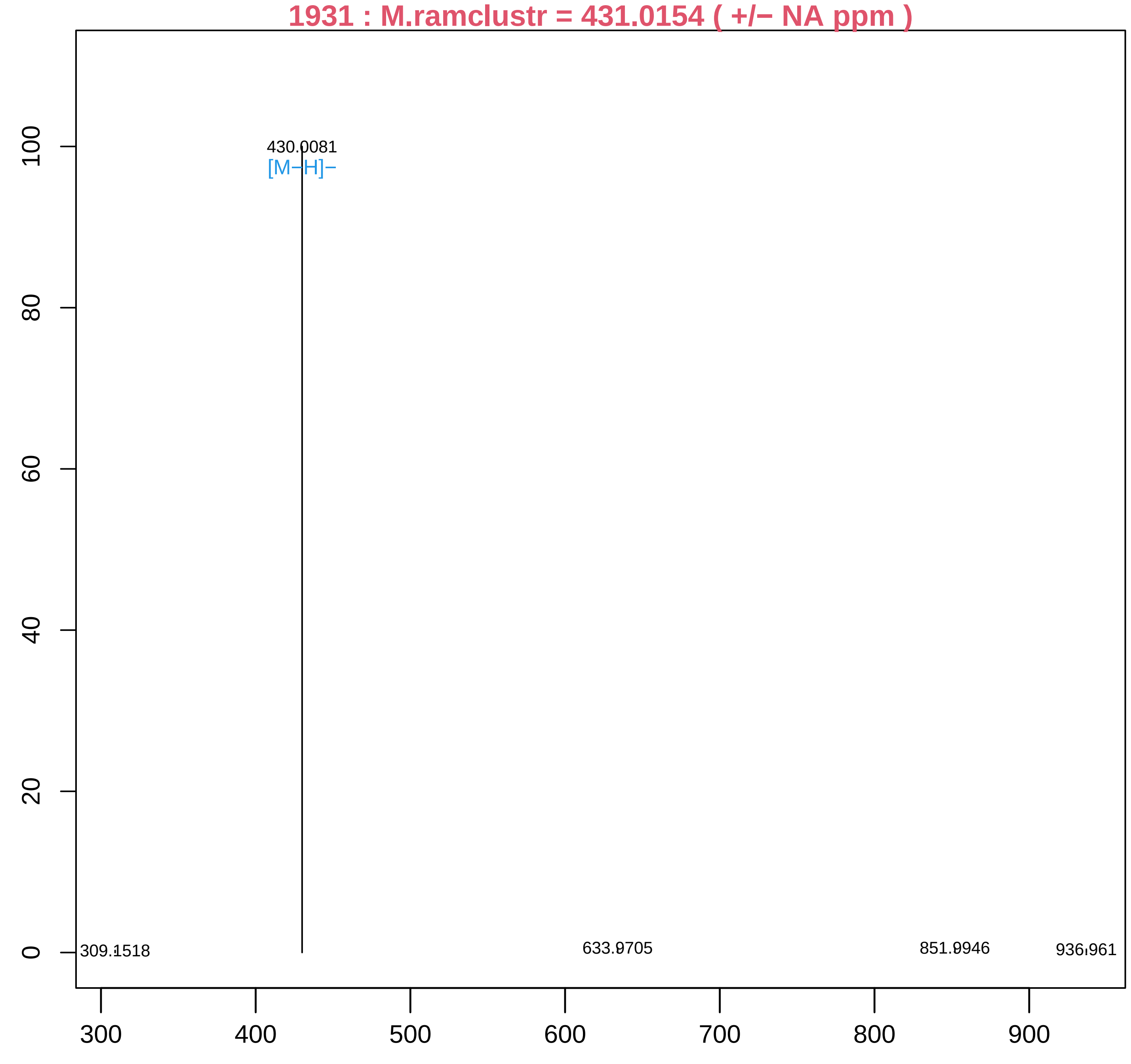

1931 : M.ramclustr = 431.0154 (+/− NA ppm)
430.0081
[M−H]−
309.1518
633.9705
851.9946
936.961
0
20
40
60
80
100
300
400
500
600
700
800
900

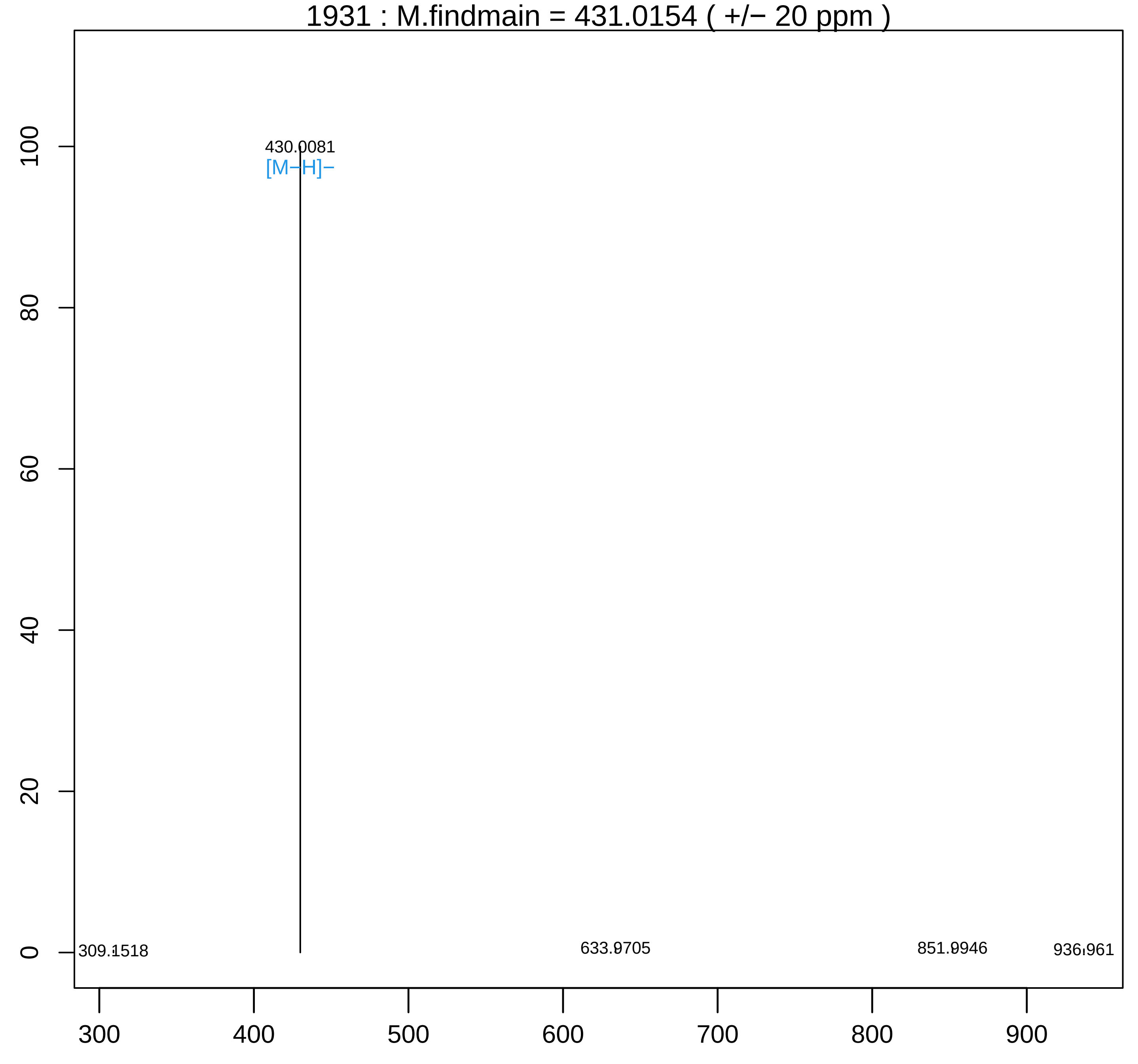

1931 : M.findmain = 431.0154 (+/− 20 ppm)
430.0081
[M−H]−
309.1518
633.9705
851.9946
936.961
0
20
40
60
80
100
300
400
500
600
700
800
900

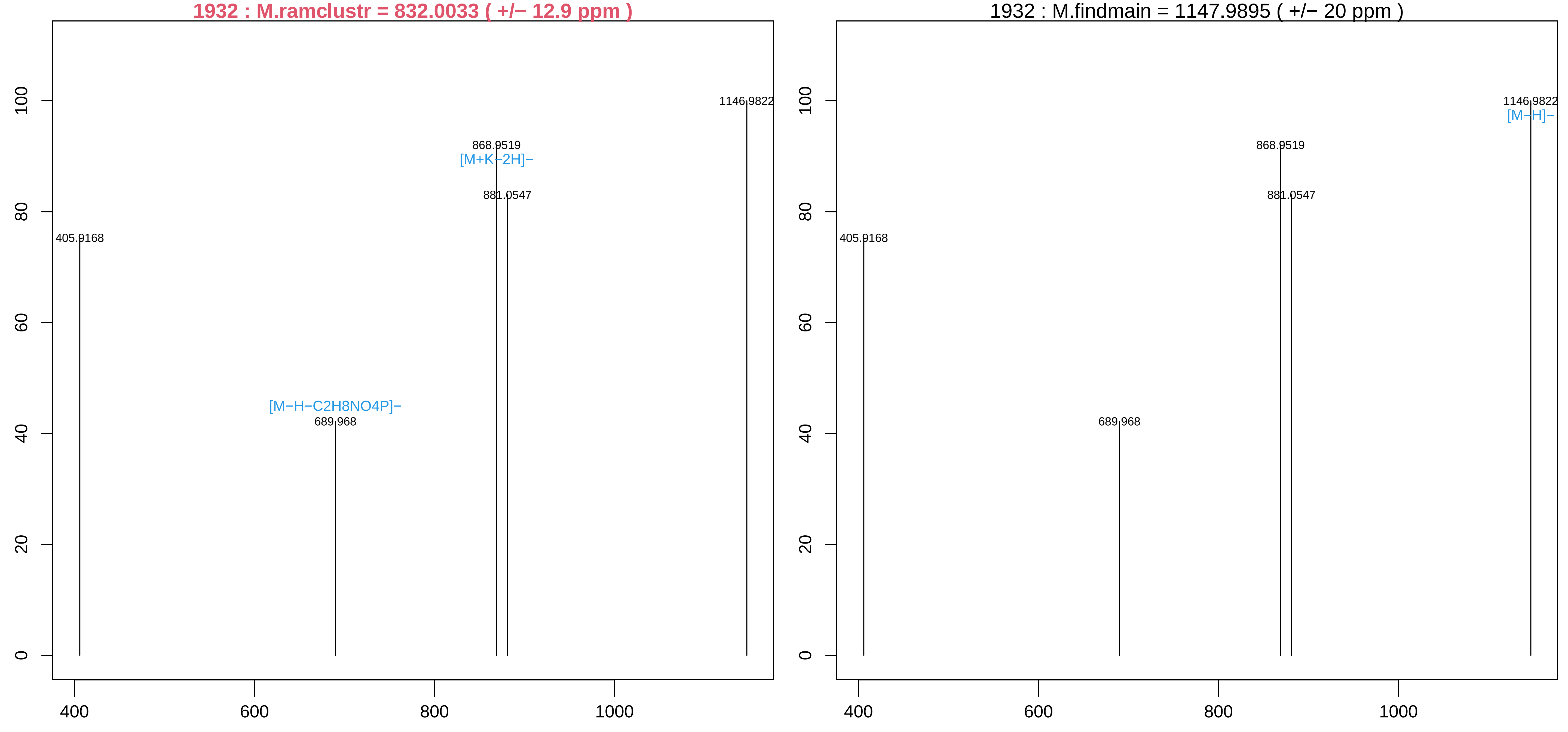

1932 : M.ramclustr = 832.0033 (+/− 12.9 ppm)
1146.9822
868.9519
[M+K−2H]−
881.0547
405.9168
[M−H−C2H8NO4P]−
689.968
1932 : M.findmain = 1147.9895 (+/− 20 ppm)
1146.9822
[M−H]−
868.9519
881.0547
405.9168
689.968
0
20
40
60
80
100
400
600
800
1000

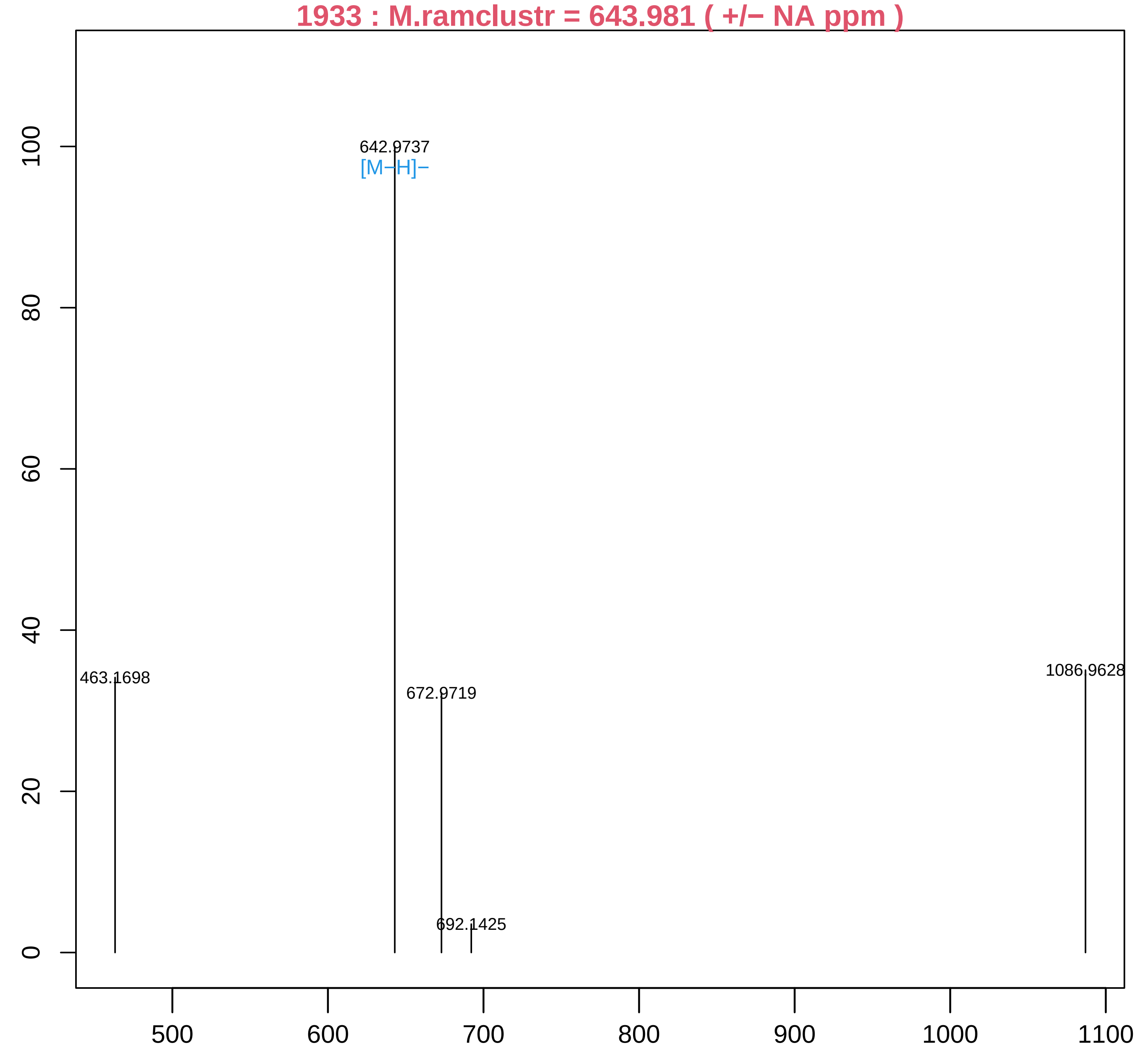
1933 : M.ramclustr = 643.981 (+/− NA ppm)
642.9737
[M−H]−
463.1698
672.9719
692.1425
1086.9628
0
20
40
60
80
100
500
600
700
800
900
1000
1100

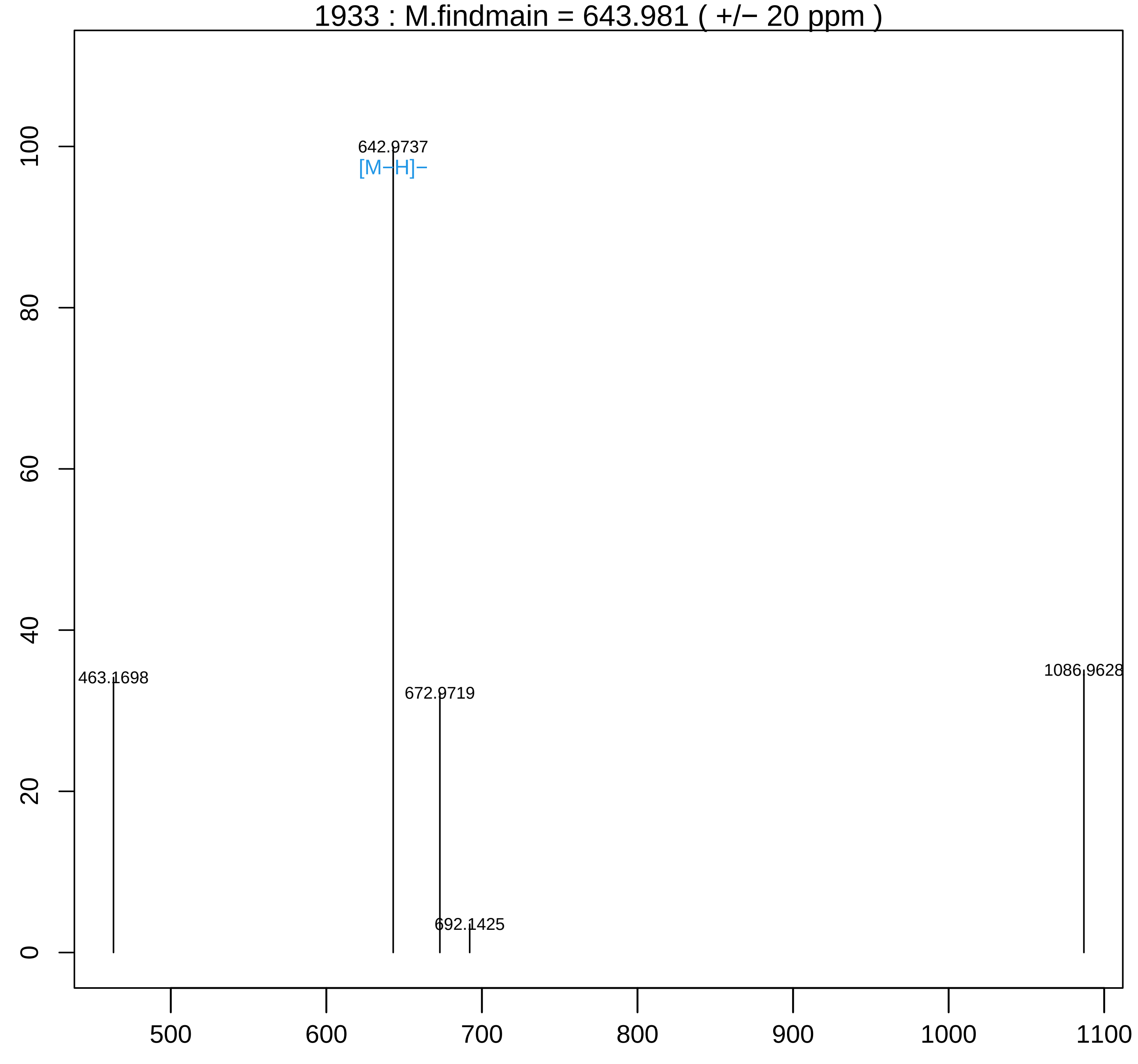
1933 : M.findmain = 643.981 (+/− 20 ppm)
642.9737
[M−H]−
463.1698
672.9719
692.1425
1086.9628
0
20
40
60
80
100
500
600
700
800
900
1000
1100

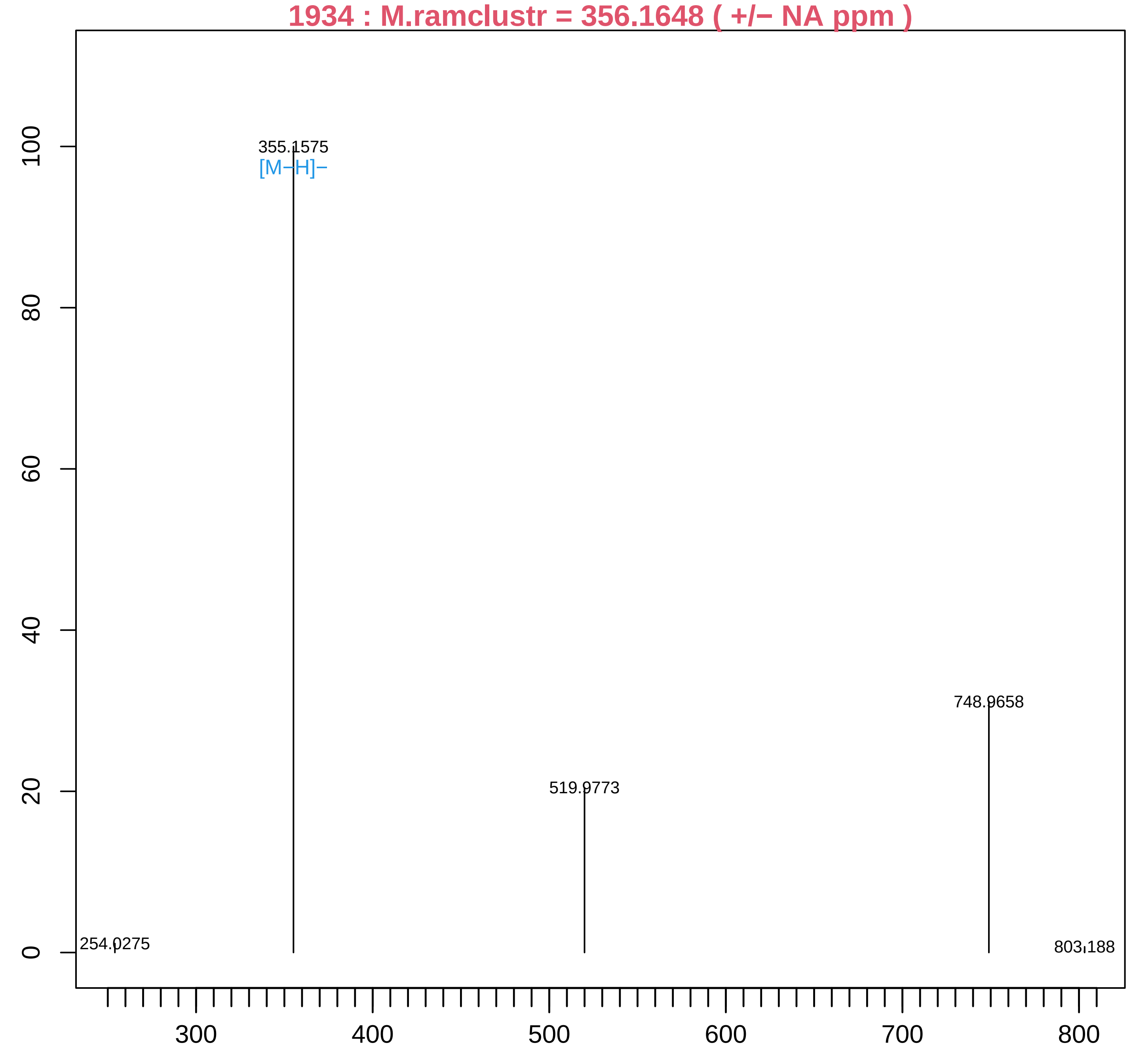
1934 : M.ramclustr = 356.1648 (+/− NA ppm)
355.1575
[M−H]−
748.9658
519.9773
254.0275
803.188
0
20
40
60
80
100
300
400
500
600
700
800

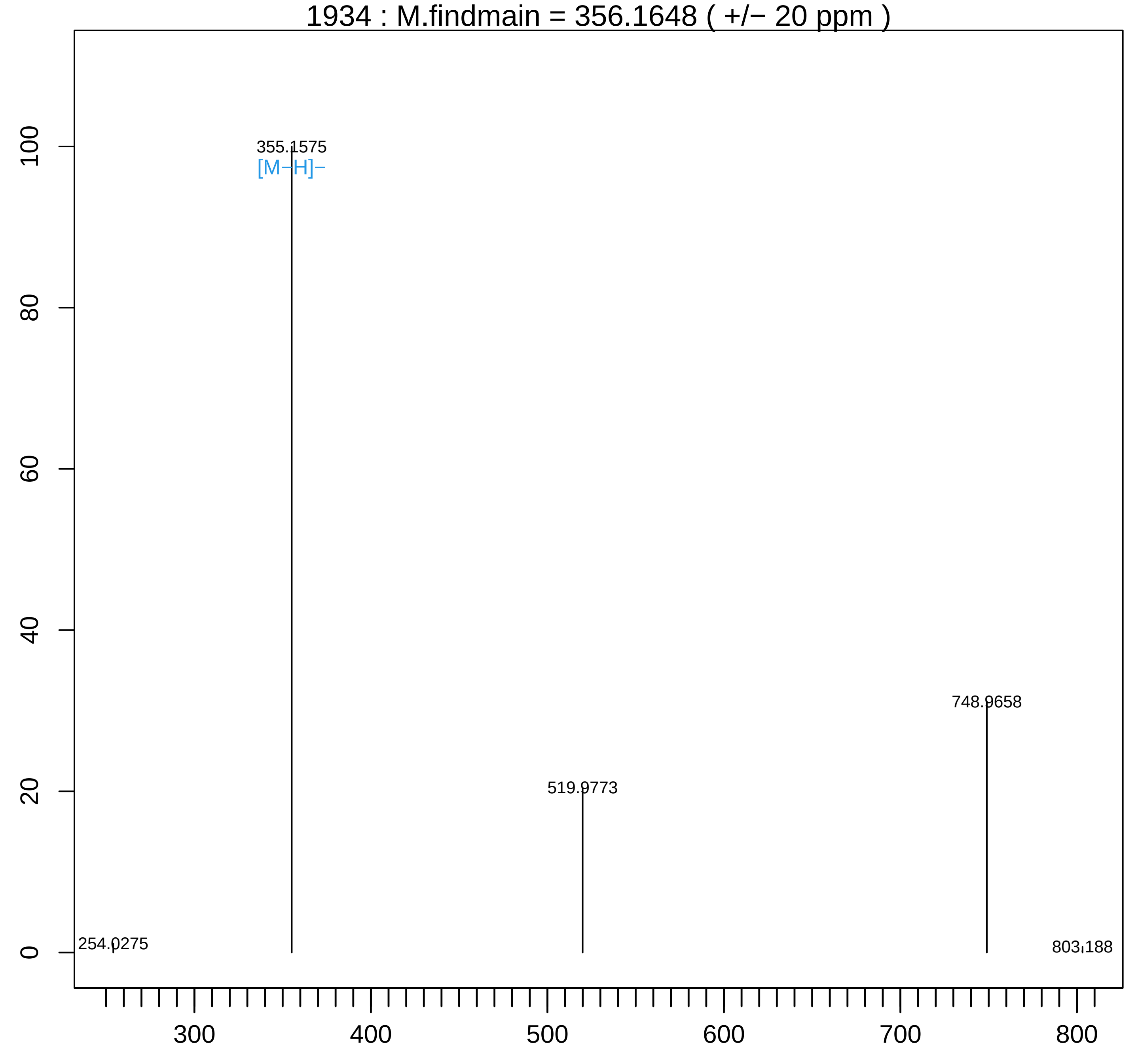
1934 : M.findmain = 356.1648 (+/− 20 ppm)
355.1575
[M−H]−
748.9658
519.9773
254.0275
803.188
0
20
40
60
80
100
300
400
500
600
700
800

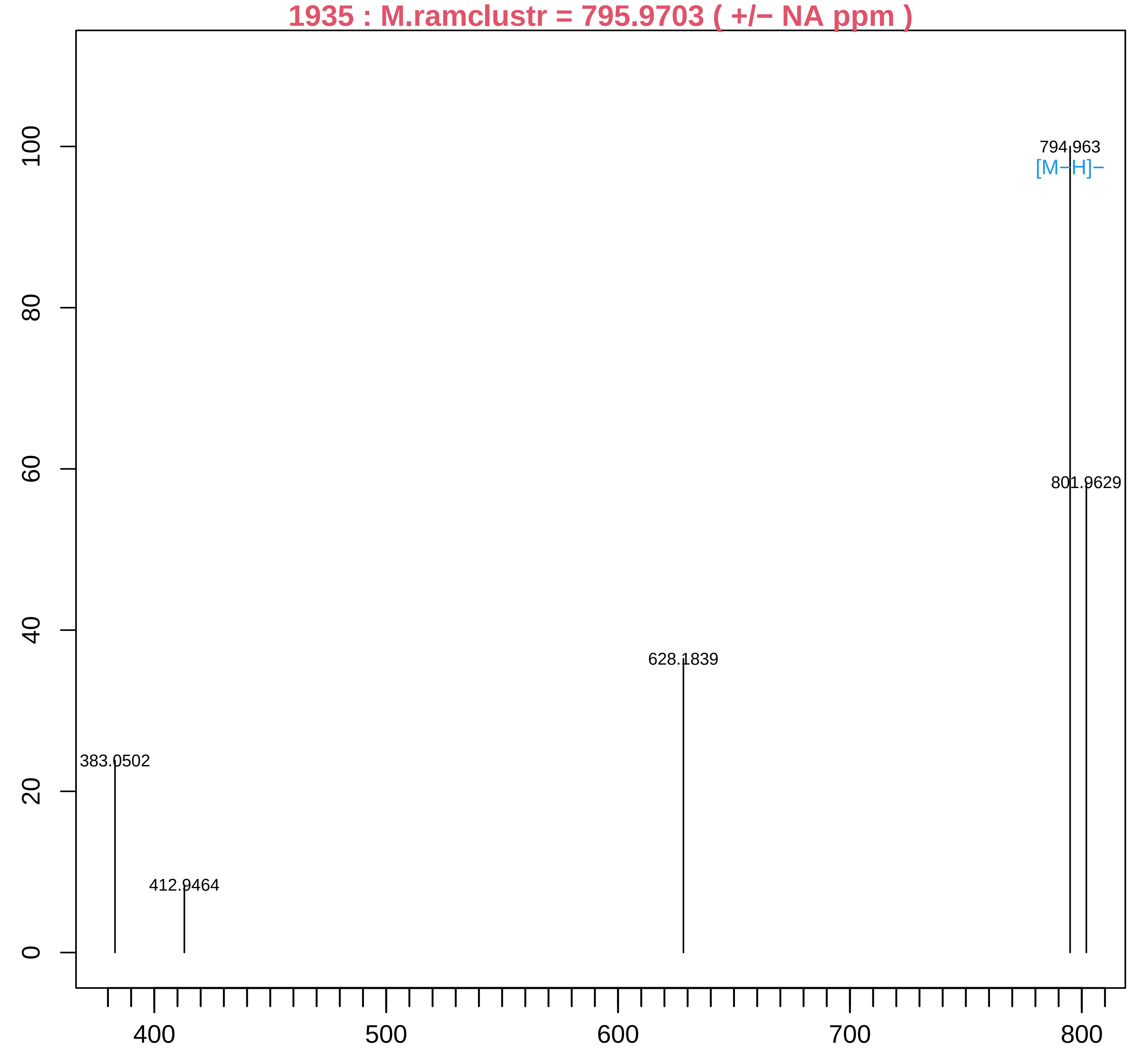
1935 : M.ramclustr = 795.9703 (+/− NA ppm)
794.963
[M−H]−
801.9629
628.1839
383.0502
412.9464
0
20
40
60
80
100
400
500
600
700
800

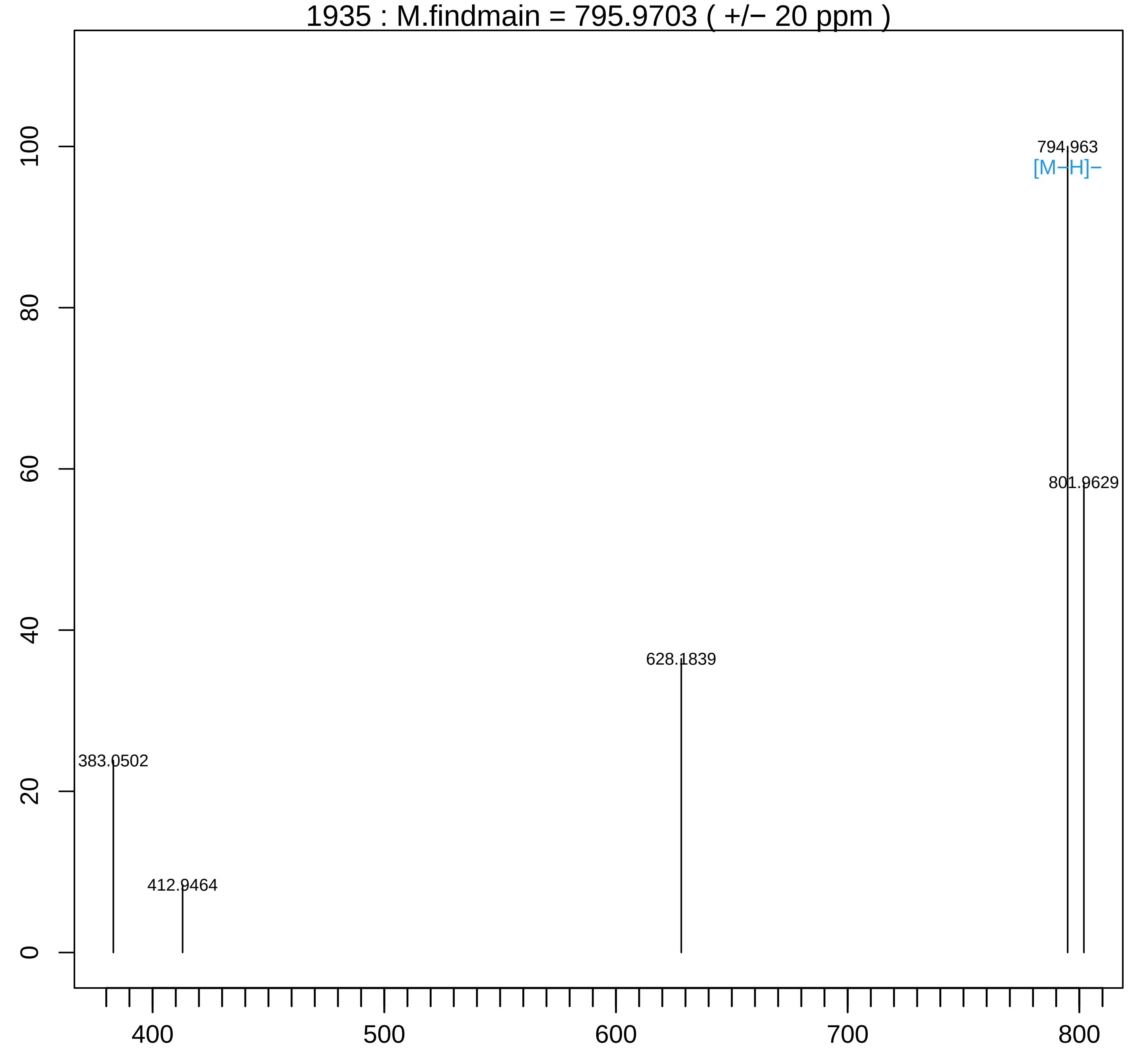
1935 : M.findmain = 795.9703 (+/− 20 ppm)
794.963
[M−H]−
801.9629
628.1839
383.0502
412.9464
0
20
40
60
80
100
400
500
600
700
800

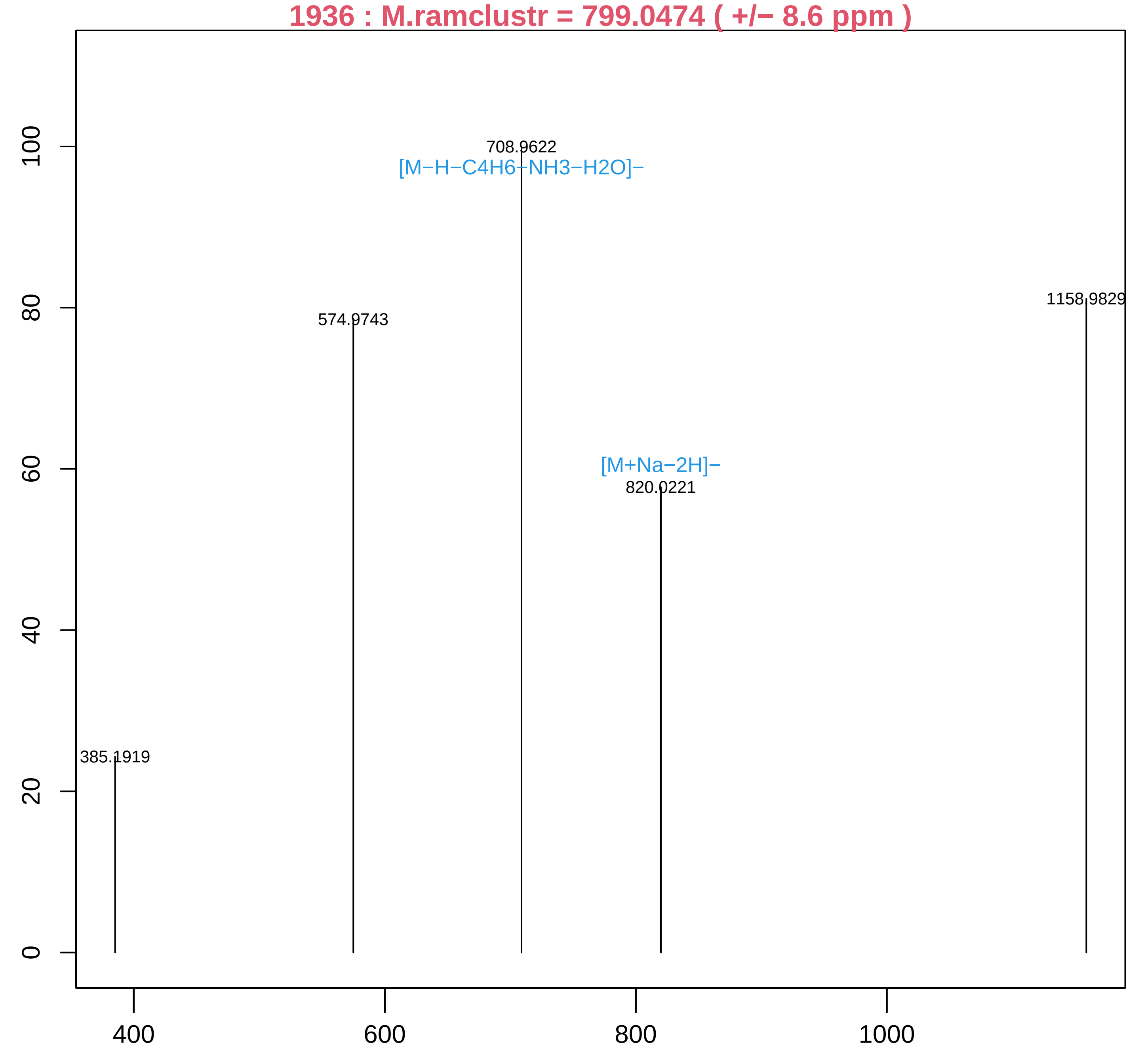

1936 : M.ramclustr = 799.0474 (+/− 8.6 ppm)
708.9622
[M−H−C4H6−NH3−H2O]−
574.9743
1158.9829
[M+Na−2H]−
820.0221
385.1919
0
20
40
60
80
100
400
600
800
1000

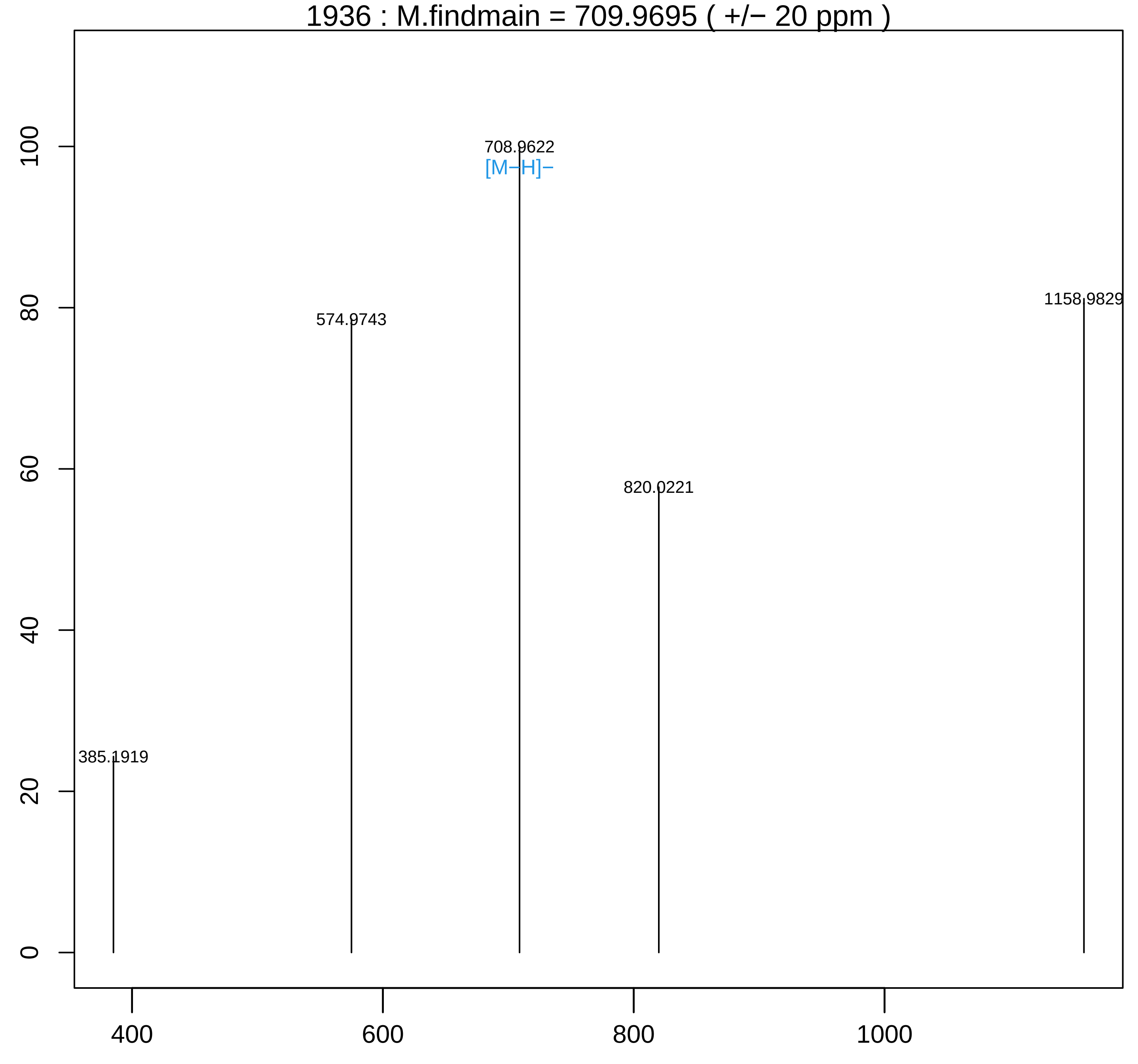

1936 : M.findmain = 709.9695 (+/− 20 ppm)
708.9622
[M−H]−
574.9743
1158.9829
820.0221
385.1919
0
20
40
60
80
100
400
600
800
1000

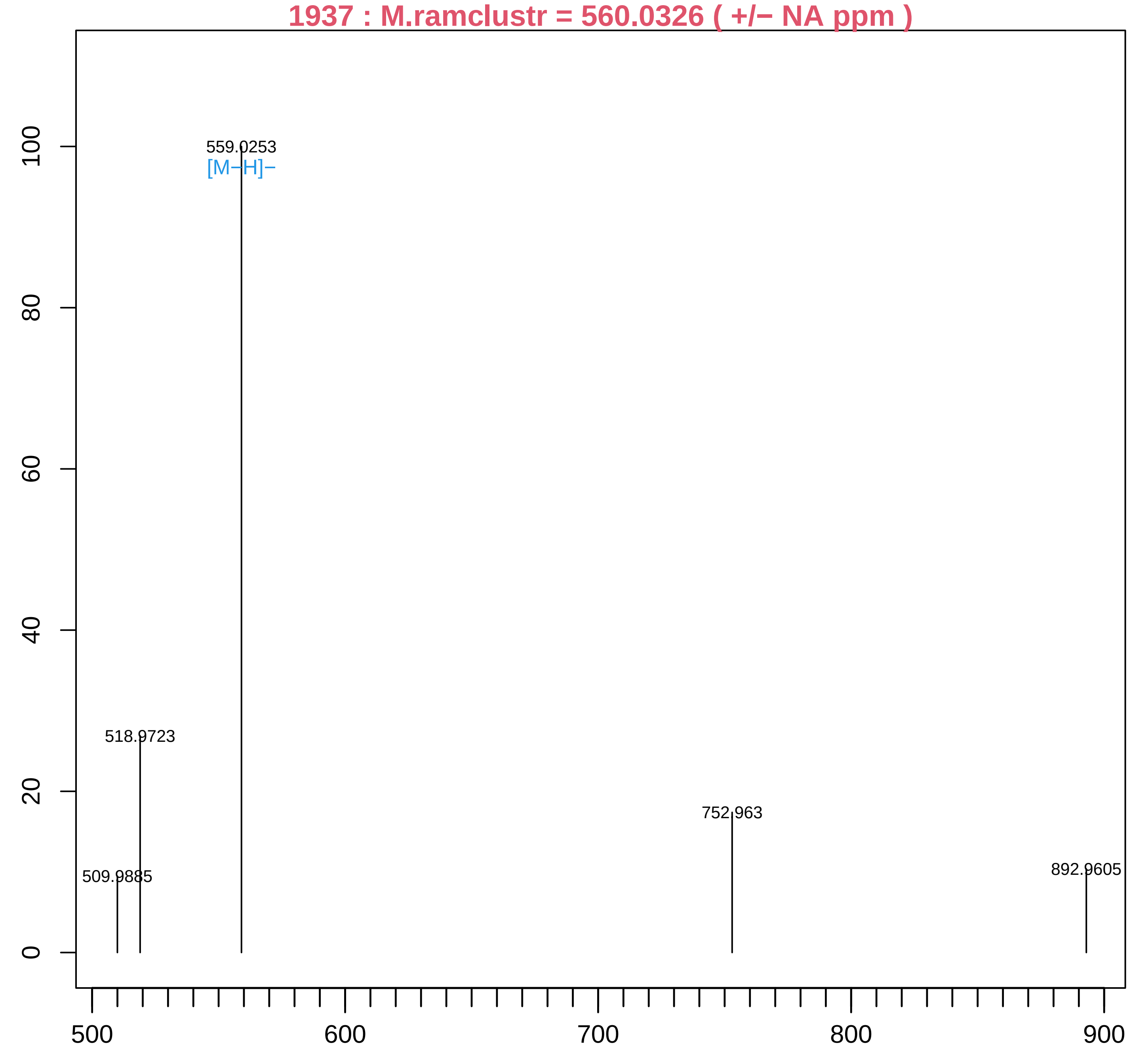
1937 : M.ramclustr = 560.0326 (+/− NA ppm)
559.0253
[M−H]−
518.9723
509.9885
752.963
892.9605
0
20
40
60
80
100
500
600
700
800
900

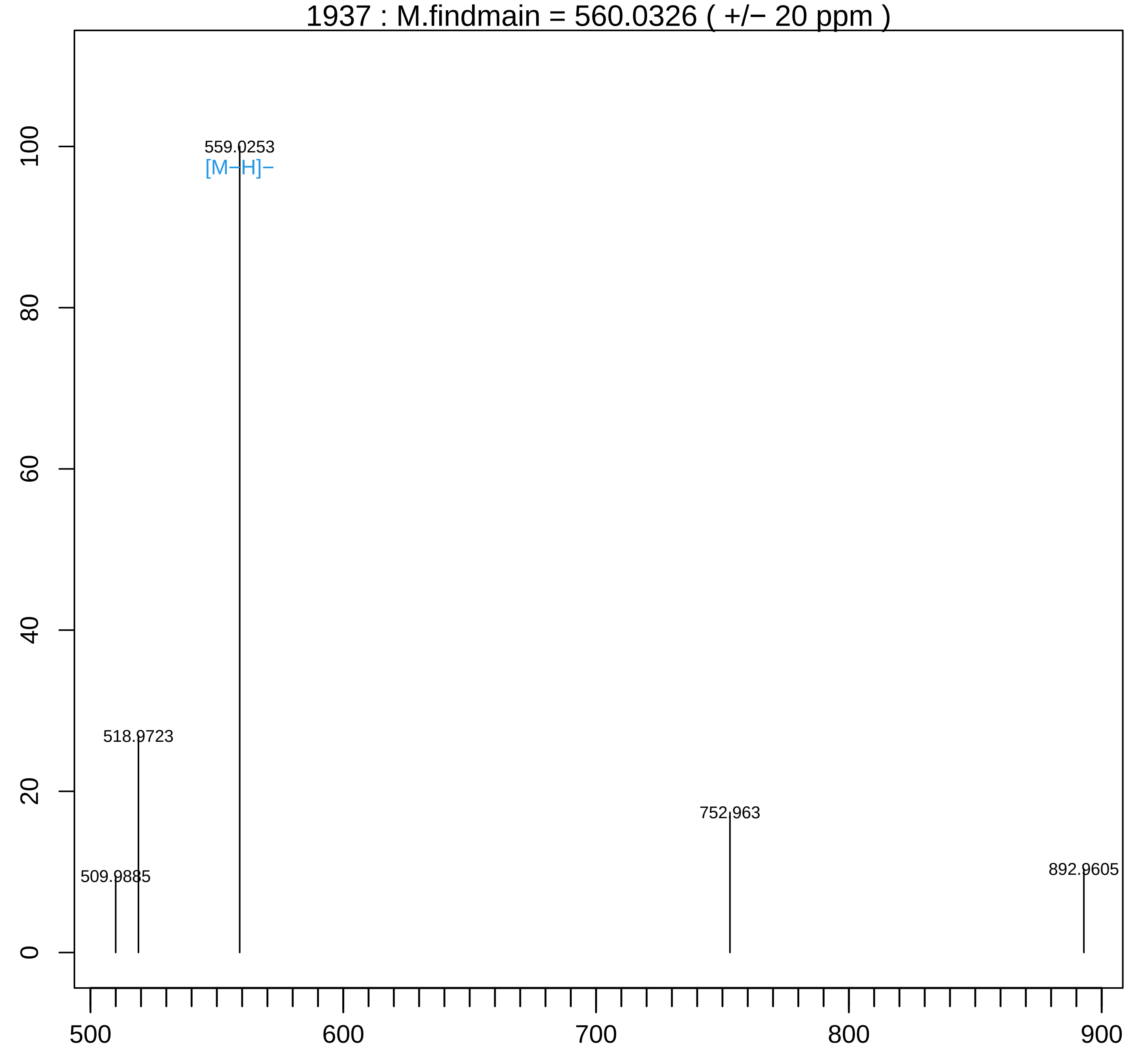
1937 : M.findmain = 560.0326 (+/− 20 ppm)
559.0253
[M−H]−
518.9723
509.9885
752.963
892.9605
0
20
40
60
80
100
500
600
700
800
900

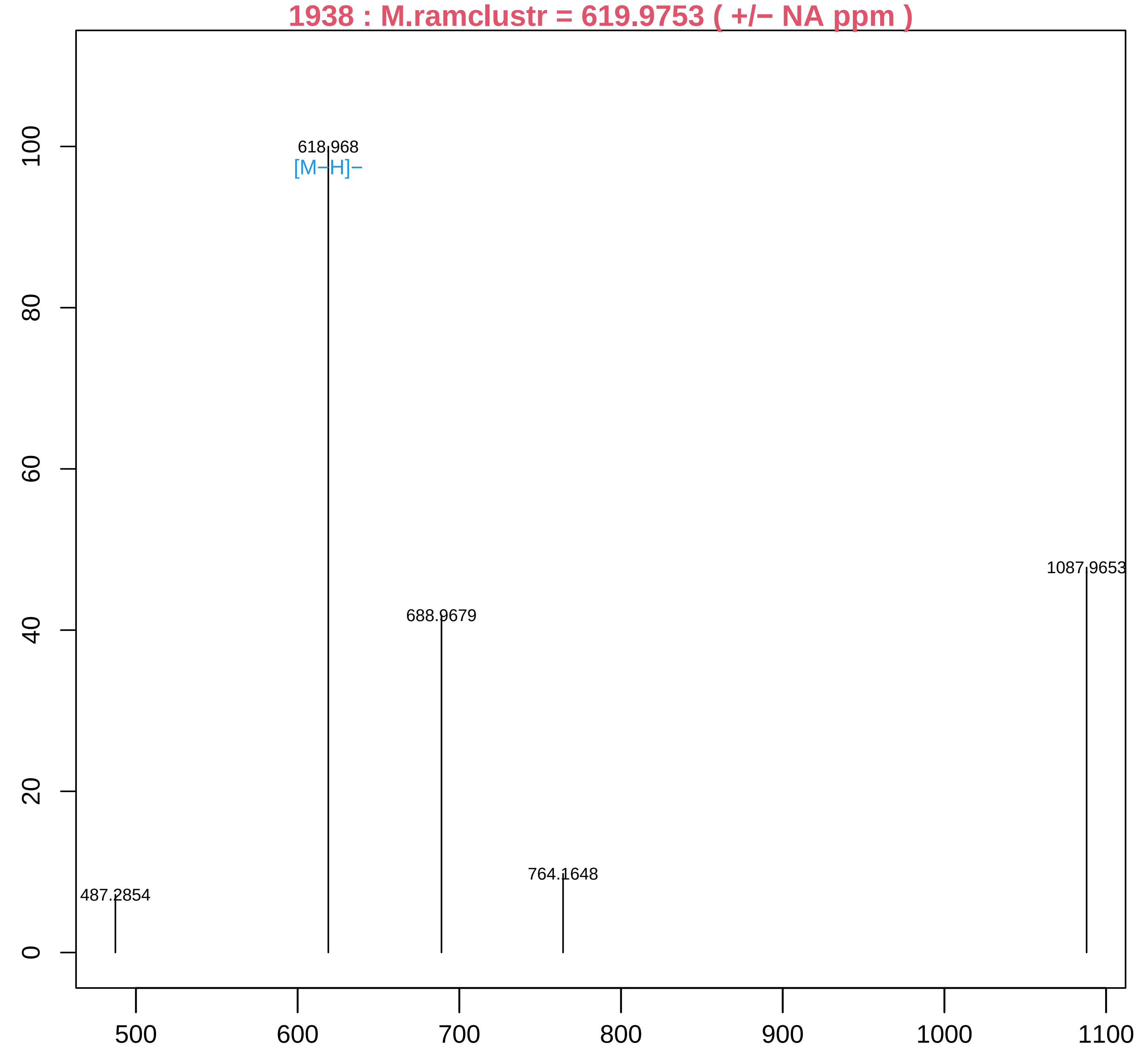
1938 : M.ramclustr = 619.9753 (+/− NA ppm)
618.968
[M−H]−
688.9679
1087.9653
764.1648
487.2854
0
20
40
60
80
100
500
600
700
800
900
1000
1100

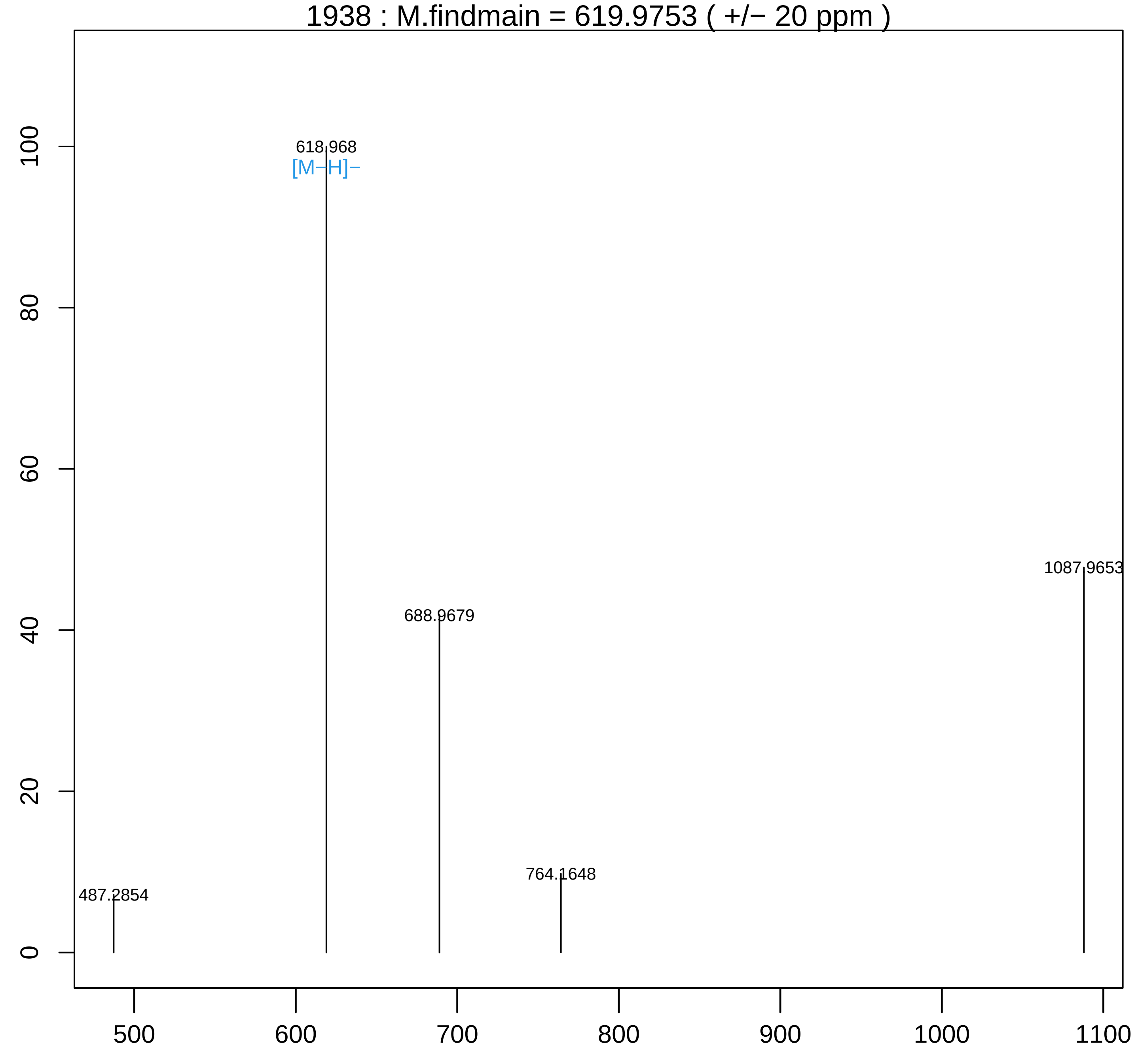
1938 : M.findmain = 619.9753 (+/− 20 ppm)
618.968
[M−H]−
688.9679
1087.9653
764.1648
487.2854
0
20
40
60
80
100
500
600
700
800
900
1000
1100

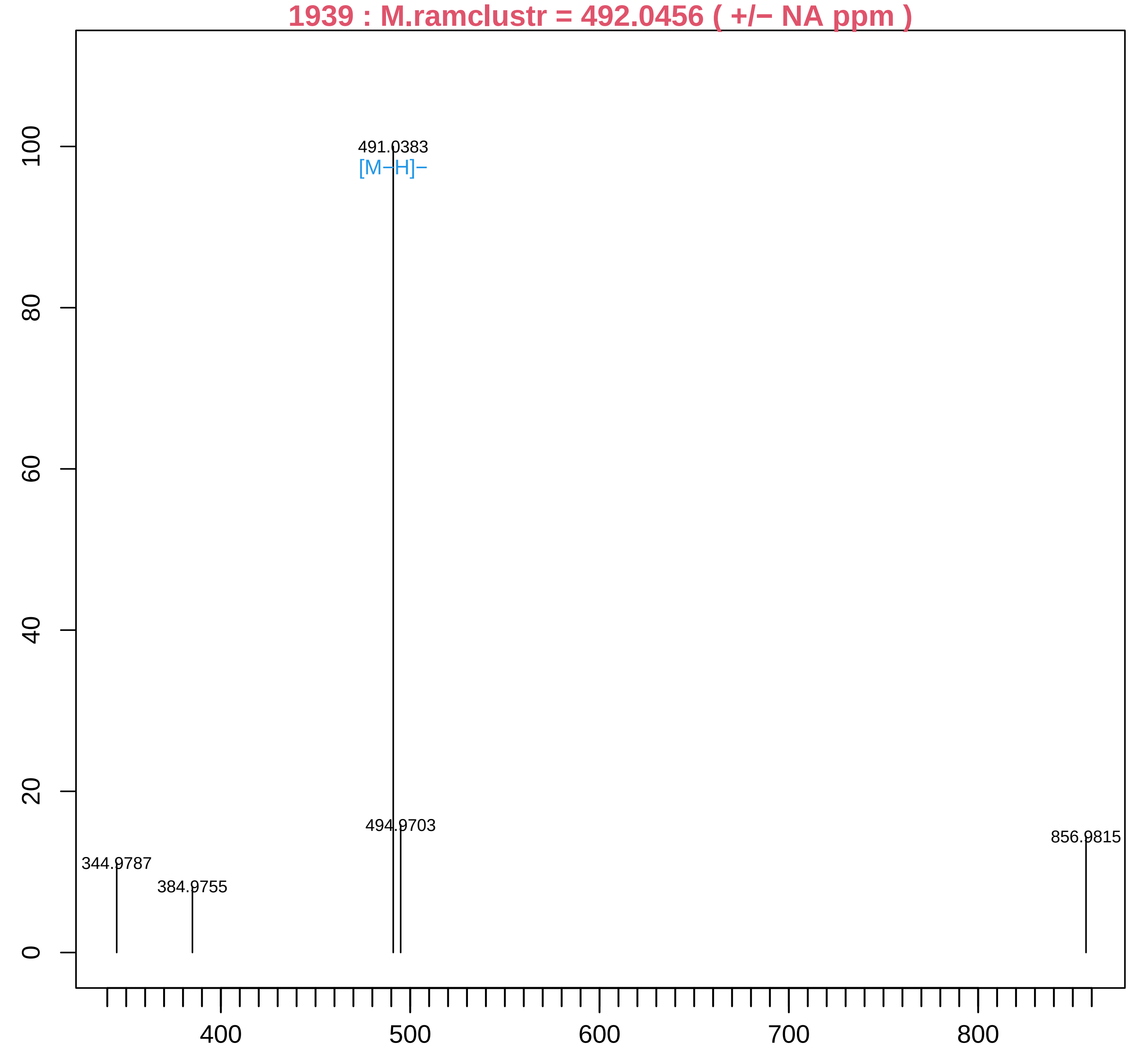

1939 : M.ramclustr = 492.0456 (+/− NA ppm)
491.0383
[M−H]−
494.9703
856.9815
344.9787
384.9755
0
20
40
60
80
100
400
500
600
700
800

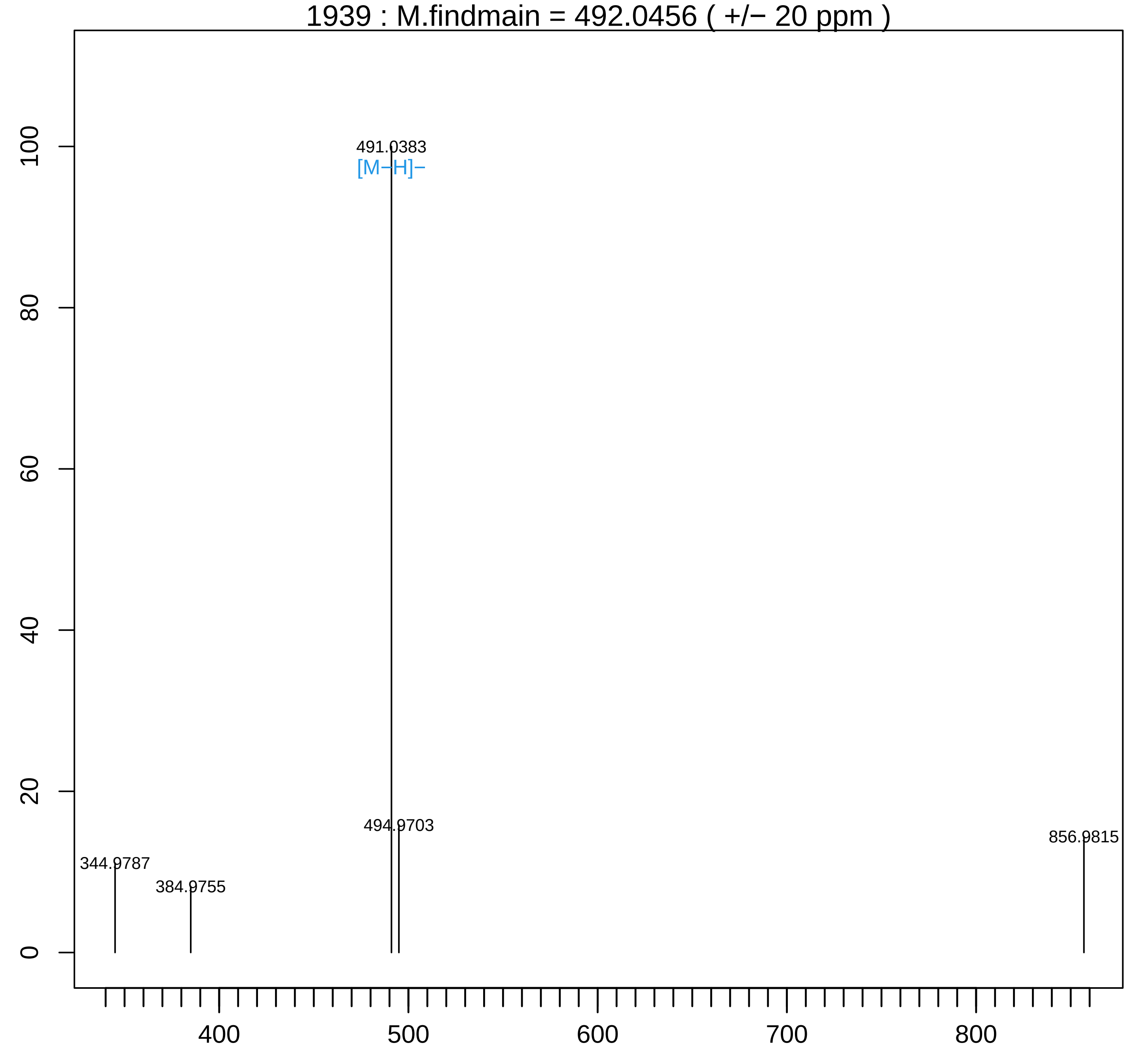

1939 : M.findmain = 492.0456 (+/− 20 ppm)
491.0383
[M−H]−
494.9703
856.9815
344.9787
384.9755
0
20
40
60
80
100
400
500
600
700
800

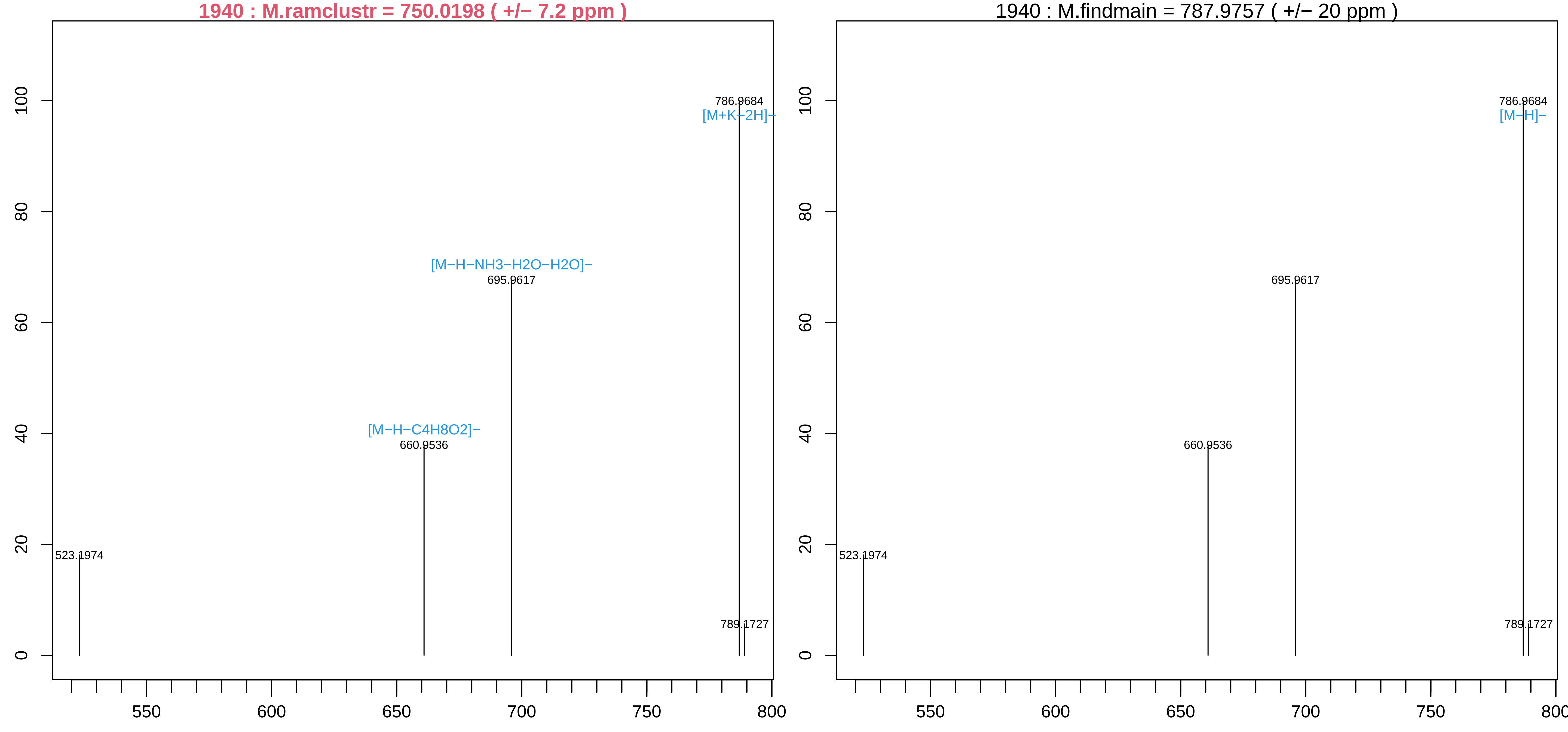
1940 : M.ramclustr = 750.0198 (+/− 7.2 ppm)
786.9684
[M+K−2H]−
[M−H−NH3−H2O−H2O]−
695.9617
[M−H−C4H8O2]−
660.9536
523.1974
789.1727
0
20
40
60
80
100
550
600
650
700
750
800
1940 : M.findmain = 787.9757 (+/− 20 ppm)
786.9684
[M−H]−
695.9617
660.9536
523.1974
789.1727

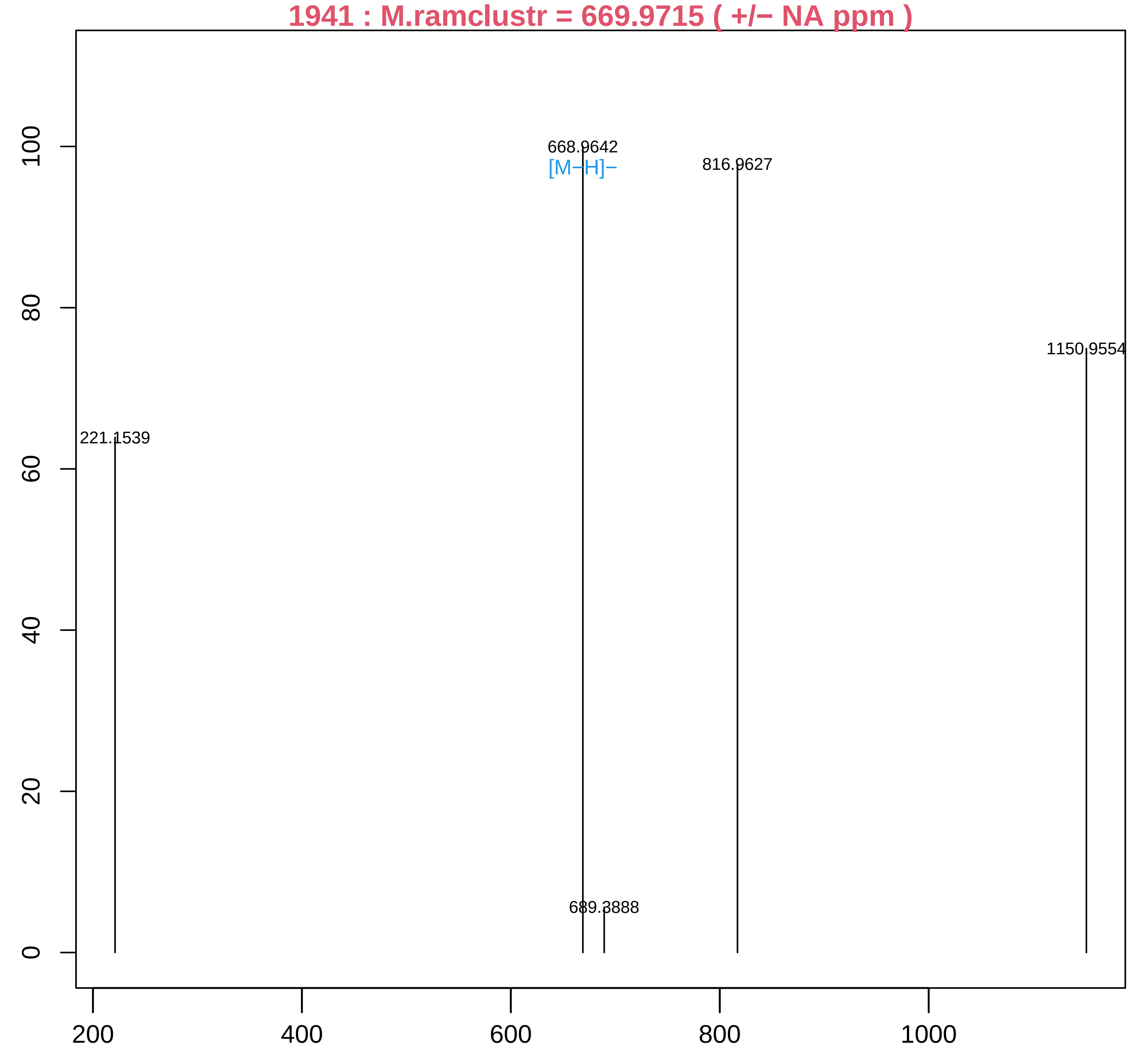
1941 : M.ramclustr = 669.9715 (+/− NA ppm)
668.9642
[M−H]−
816.9627
1150.9554
221.1539
689.3888
0
20
40
60
80
100
200
400
600
800
1000

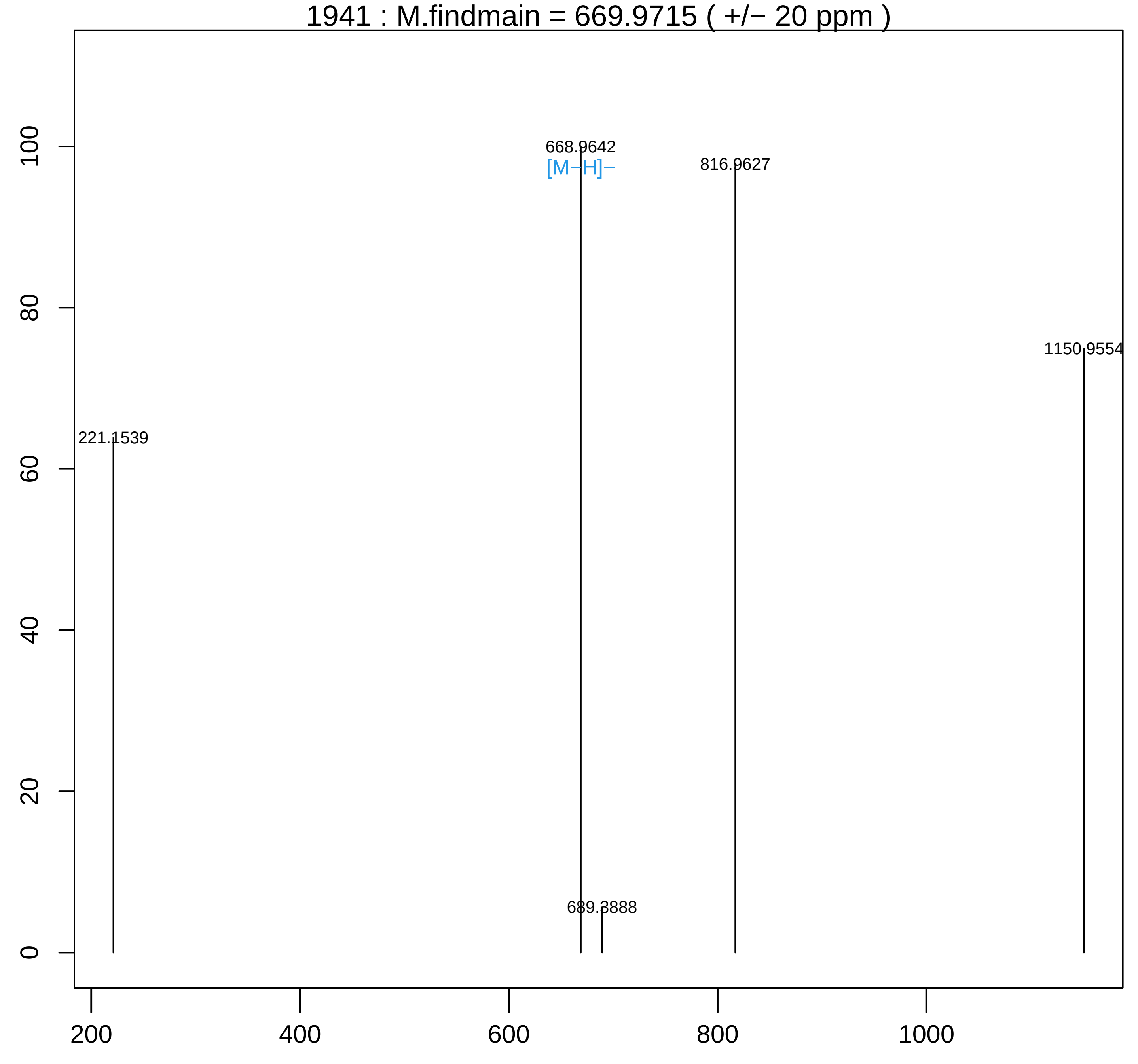
1941 : M.findmain = 669.9715 (+/− 20 ppm)
668.9642
[M−H]−
816.9627
1150.9554
221.1539
689.3888
0
20
40
60
80
100
200
400
600
800
1000

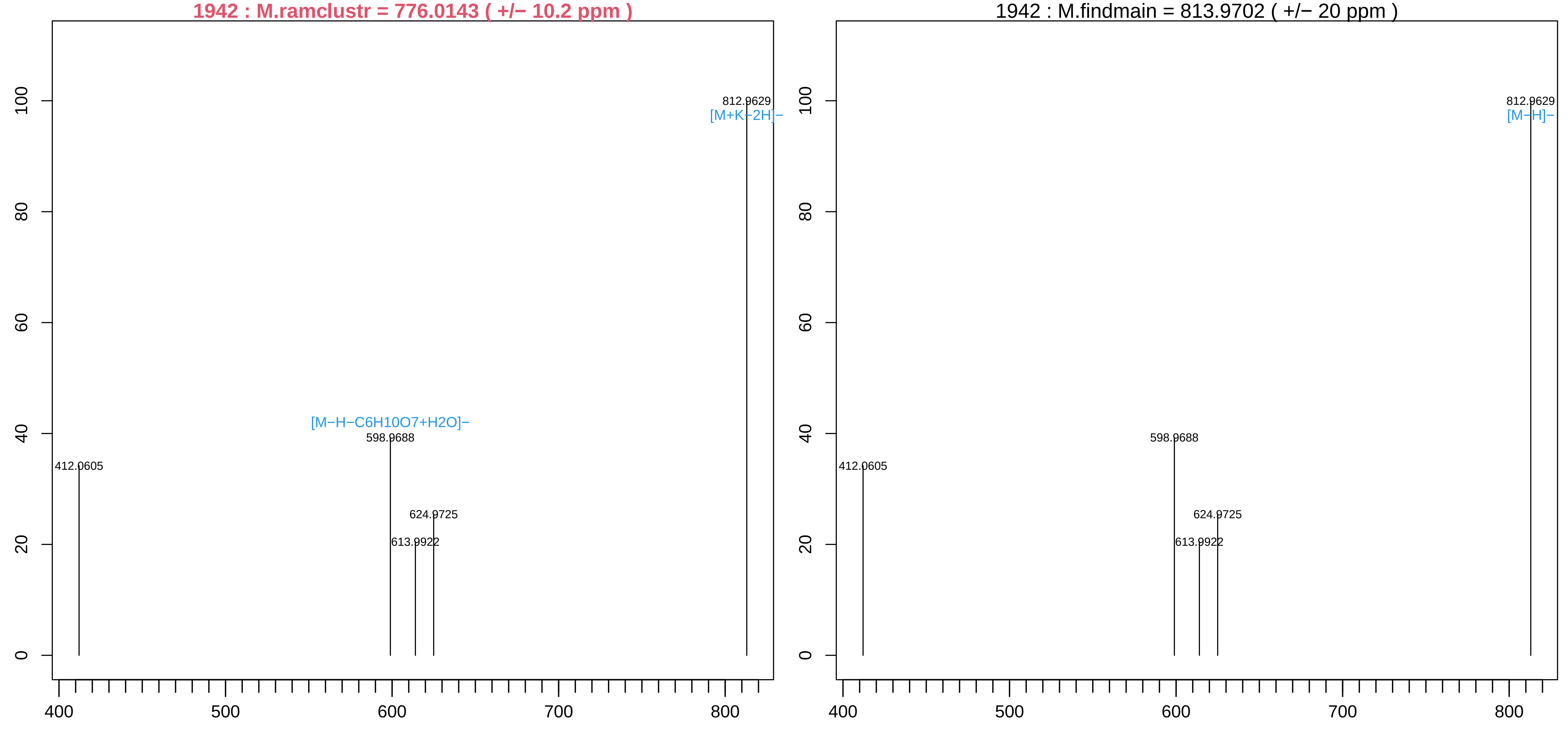
1942 : M.ramclustr = 776.0143 (+/− 10.2 ppm)
812.9629
[M+K−2H]−
[M−H−C6H10O7+H2O]−
598.9688
412.0605
624.9725
613.9922
1942 : M.findmain = 813.9702 (+/− 20 ppm)
812.9629
[M−H]−
598.9688
412.0605
624.9725
613.9922
0
20
40
60
80
100
400
500
600
700
800

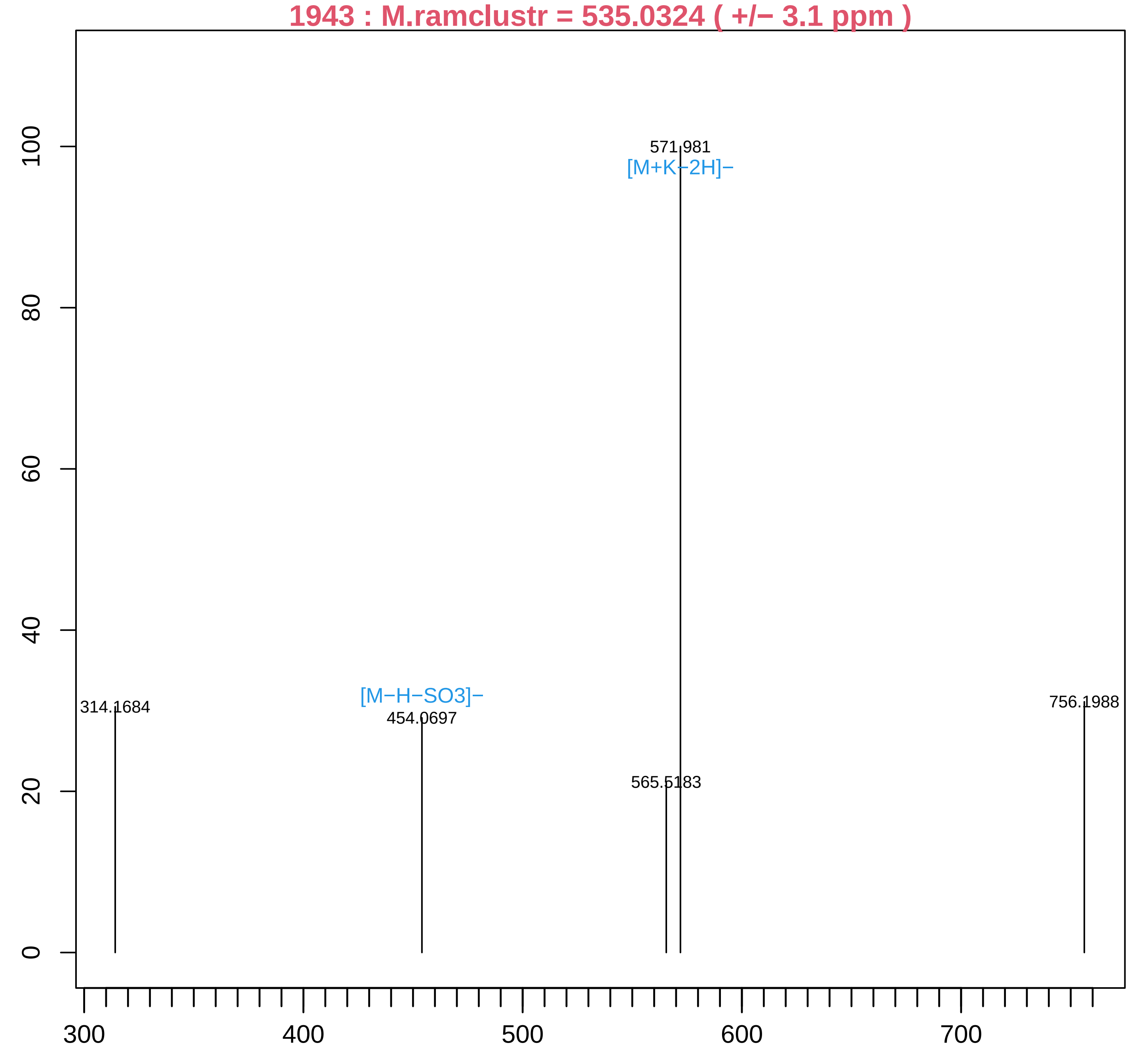

1943 : M.ramclustr = 535.0324 (+/− 3.1 ppm)
571.981
[M+K-2H]−
[M−H−SO3]−
314.1684
454.0697
565.5183
756.1988
0
20
40
60
80
100
300
400
500
600
700

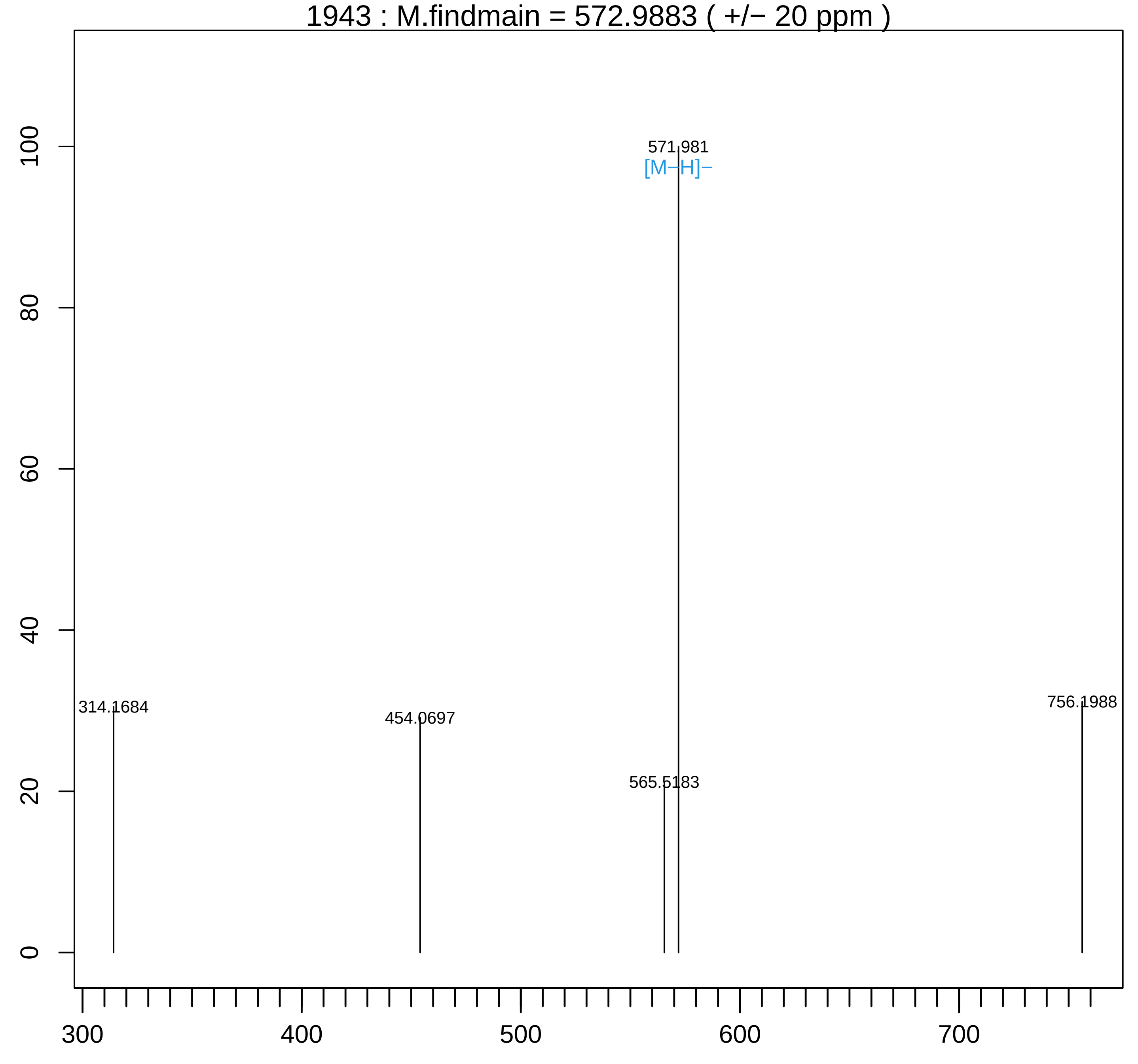

1943 : M.findmain = 572.9883 (+/− 20 ppm)
571.981
[M−H]−
314.1684
454.0697
565.5183
756.1988
0
20
40
60
80
100
300
400
500
600
700

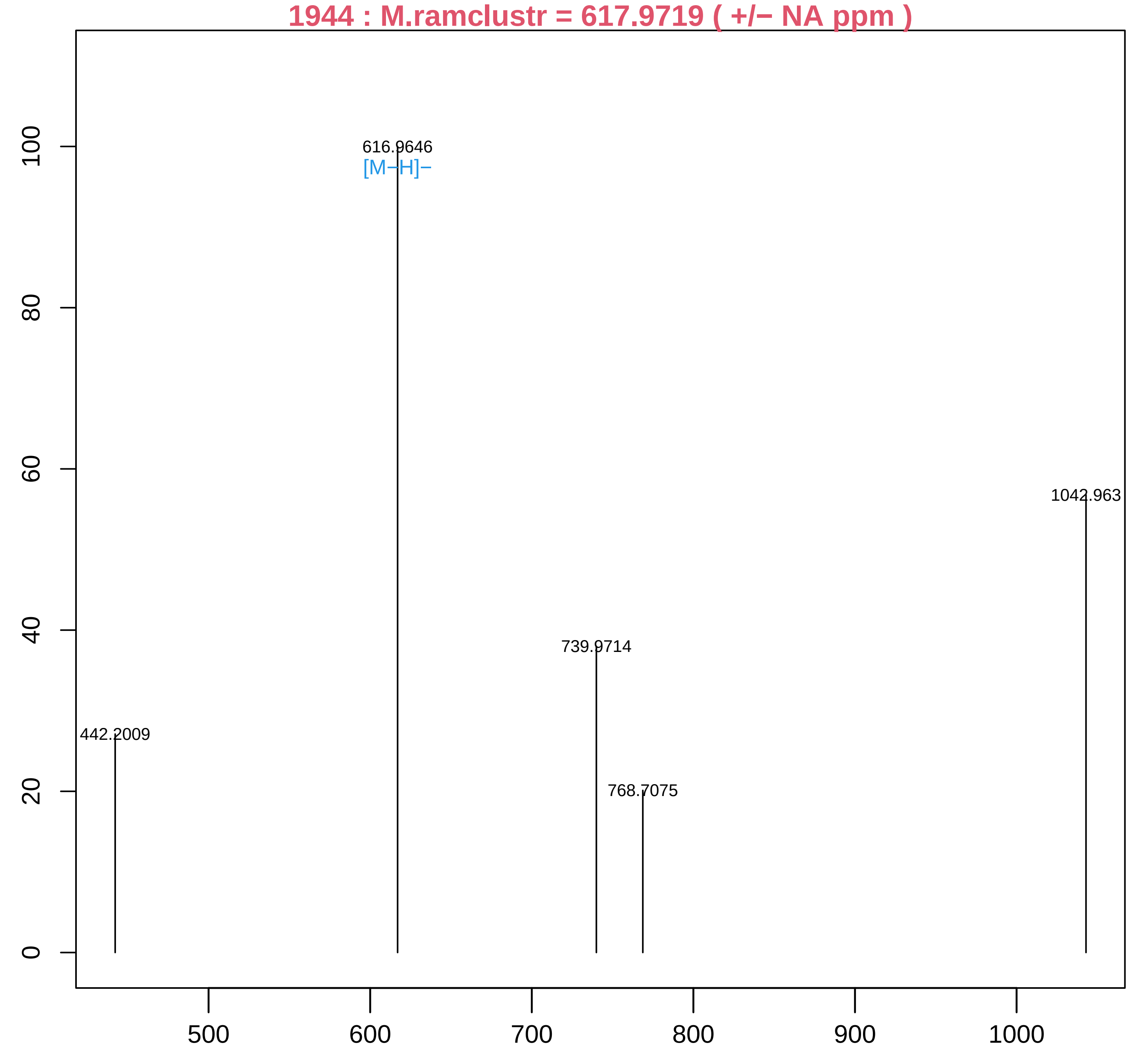
1944 : M.ramclustr = 617.9719 (+/− NA ppm)
616.9646
[M−H]−
1042.963
739.9714
442.2009
768.7075
0
20
40
60
80
100
500
600
700
800
900
1000

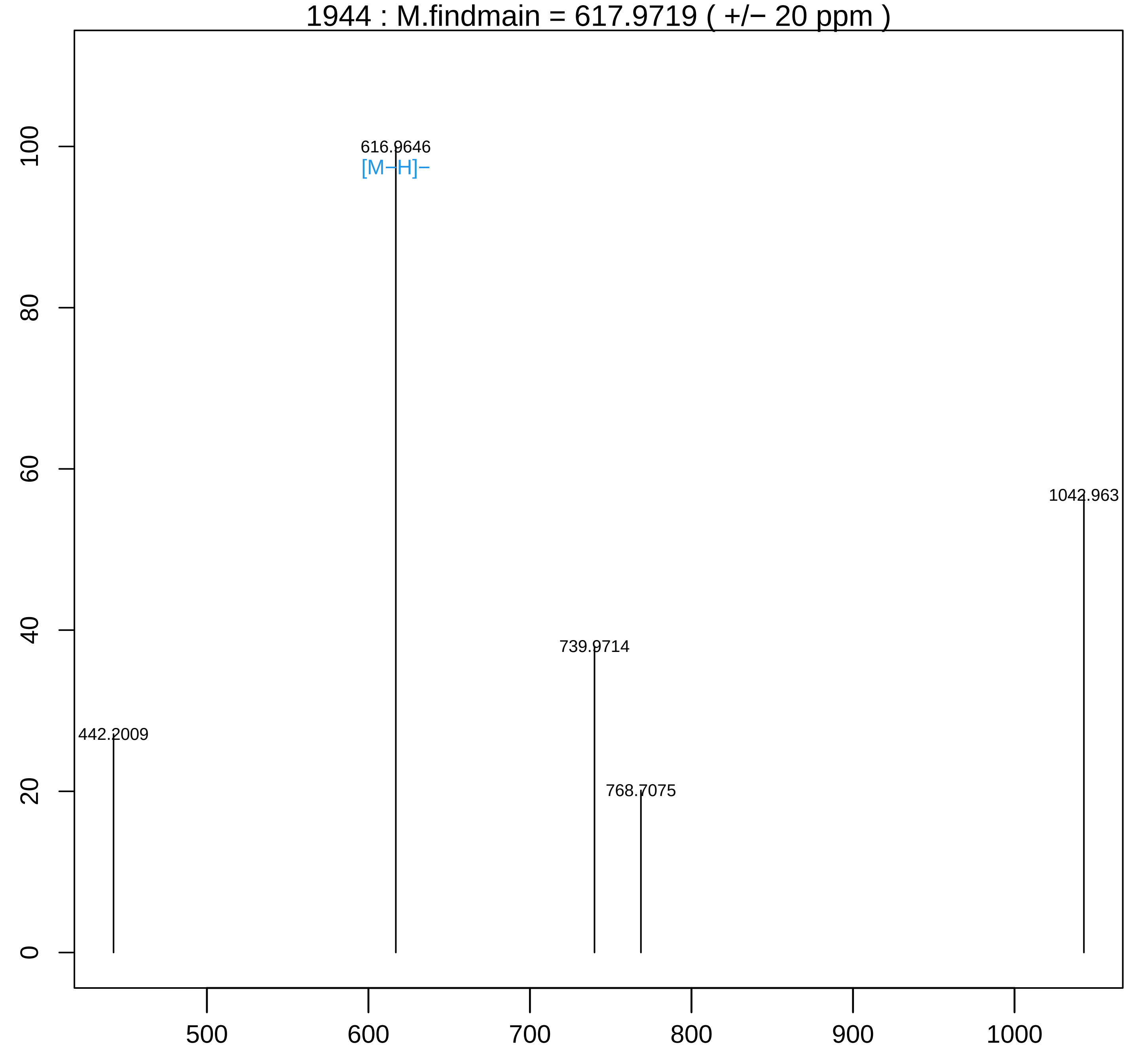
1944 : M.findmain = 617.9719 (+/− 20 ppm)
616.9646
[M−H]−
1042.963
739.9714
442.2009
768.7075
0
20
40
60
80
100
500
600
700
800
900
1000

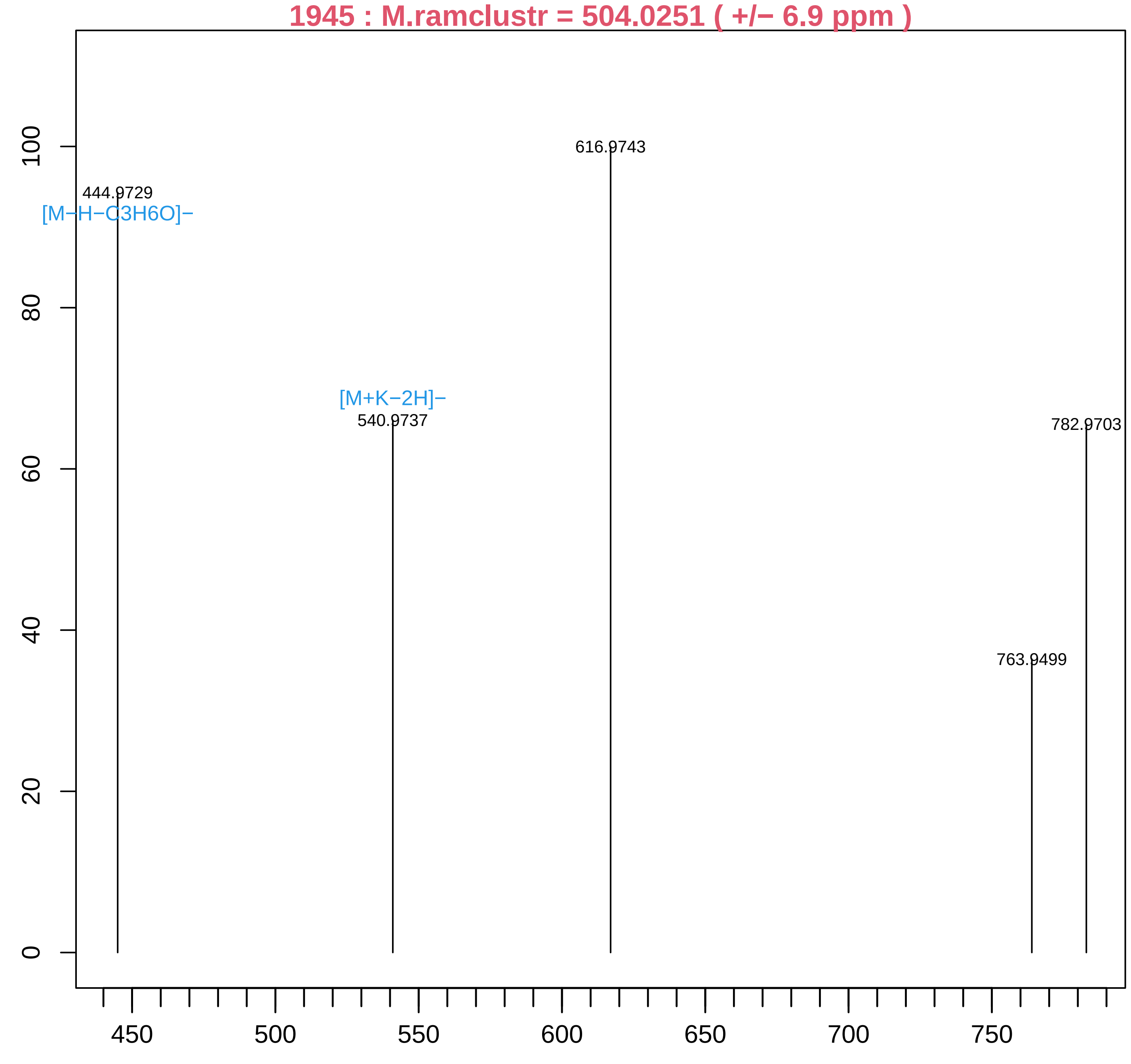
1945 : M.ramclustr = 504.0251 (+/− 6.9 ppm)
444.9729
[M−H−C3H6O]−
[M+K−2H]−
540.9737
616.9743
763.9499
782.9703
0
20
40
60
80
100
450
500
550
600
650
700
750

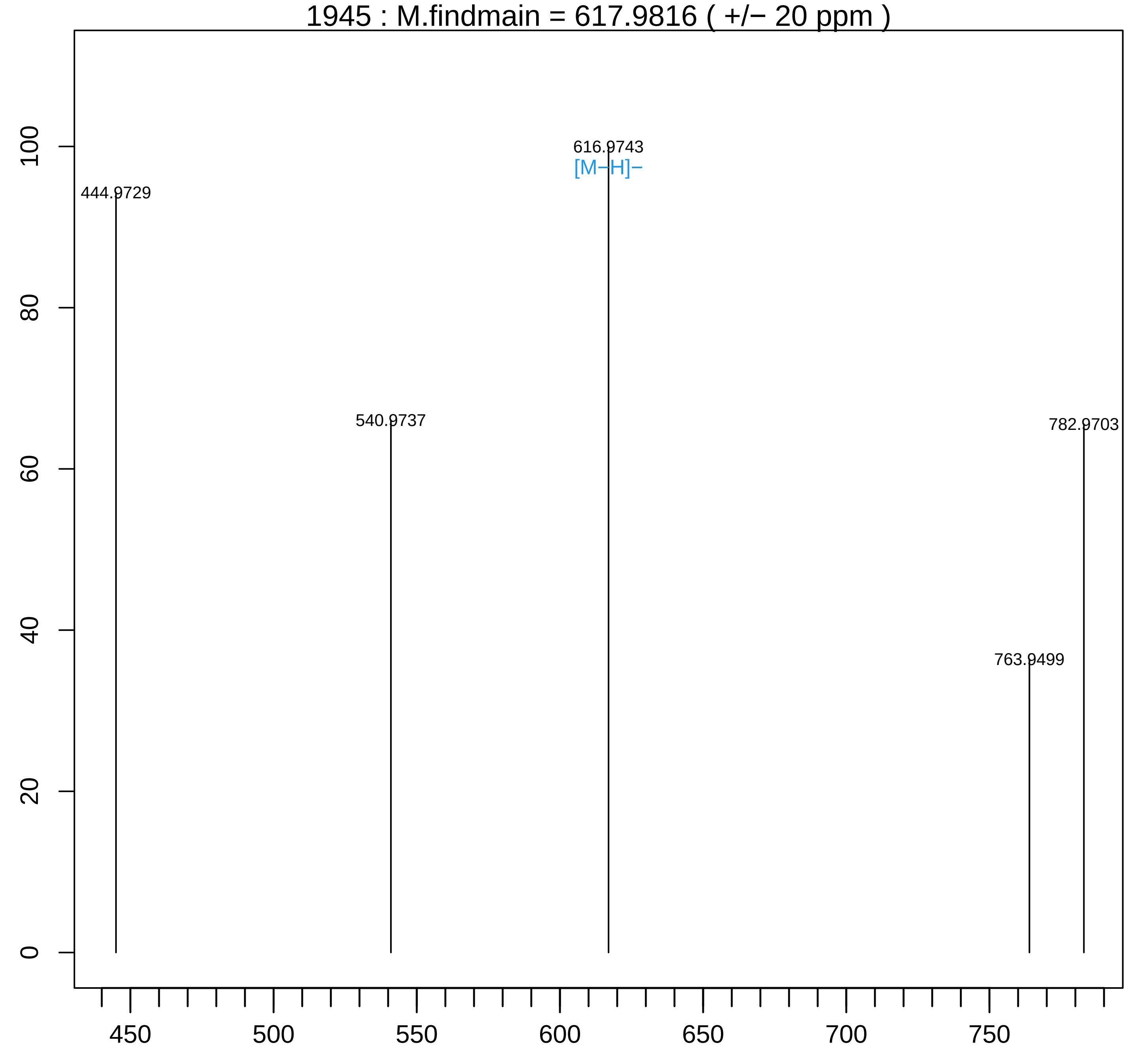
1945 : M.findmain = 617.9816 (+/− 20 ppm)
444.9729
540.9737
616.9743
[M−H]−
763.9499
782.9703
0
20
40
60
80
100
450
500
550
600
650
700
750

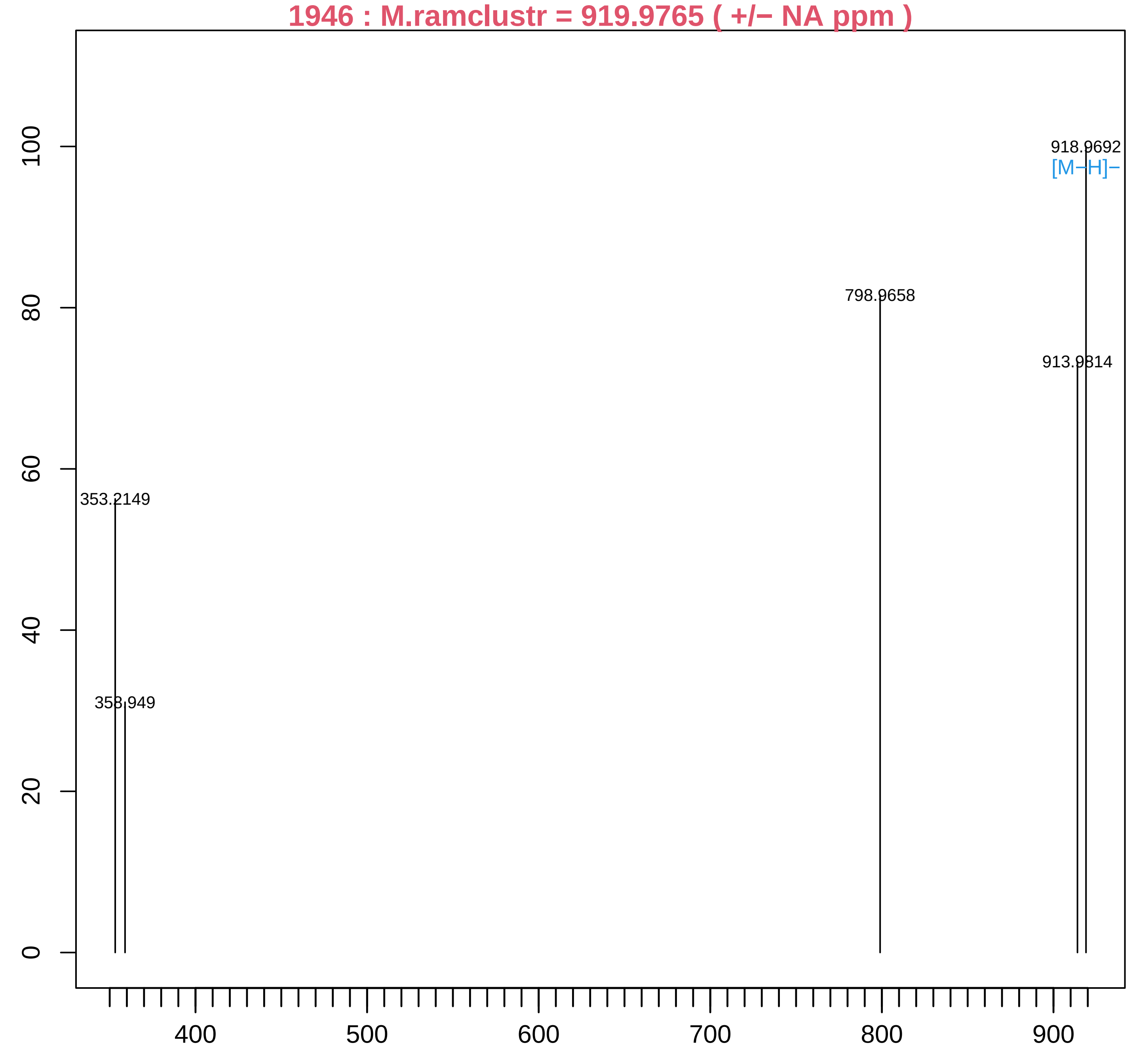
1946 : M.ramclustr = 919.9765 (+/− NA ppm)
918.9692
[M−H]−
798.9658
913.9814
353.2149
358.949
0
20
40
60
80
100
400
500
600
700
800
900

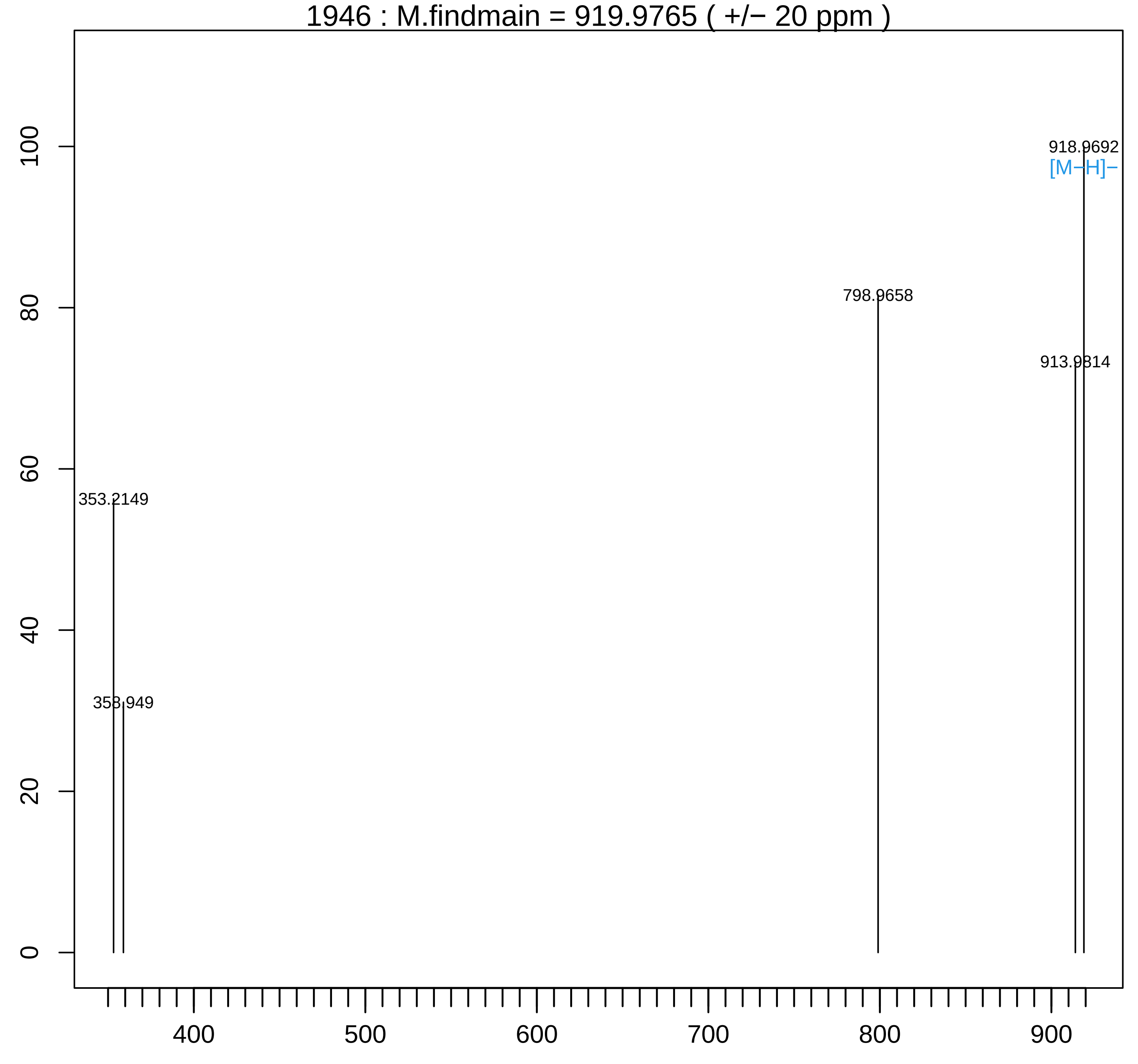
1946 : M.findmain = 919.9765 (+/− 20 ppm)
918.9692
[M−H]−
798.9658
913.9814
353.2149
358.949
0
20
40
60
80
100
400
500
600
700
800
900

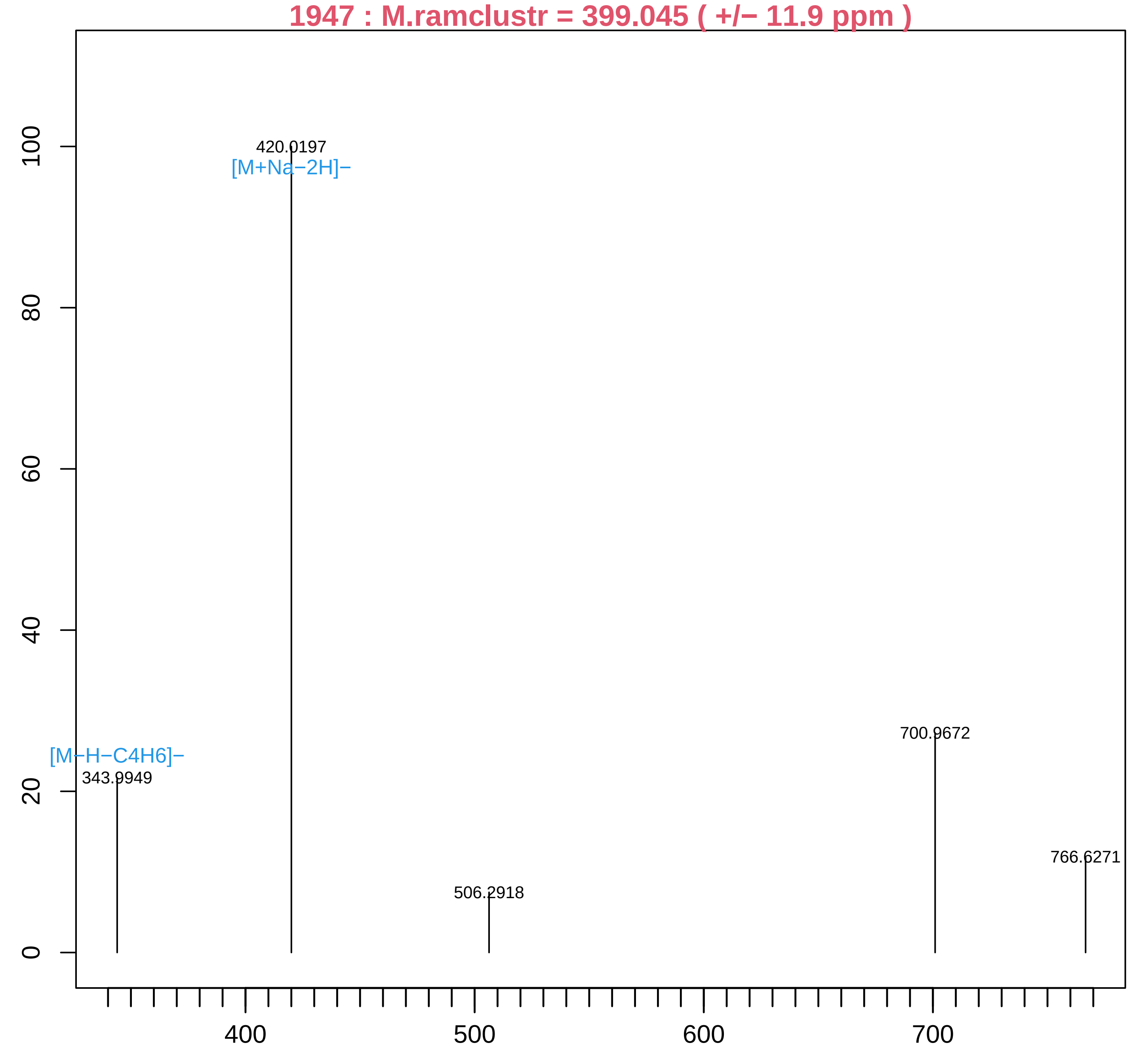

1947 : M.ramclustr = 399.045 (+/− 11.9 ppm)
420.0197
[M+Na−2H]−
[M−H−C4H6]−
343.9949
700.9672
766.6271
506.2918
0
20
40
60
80
100
400
500
600
700

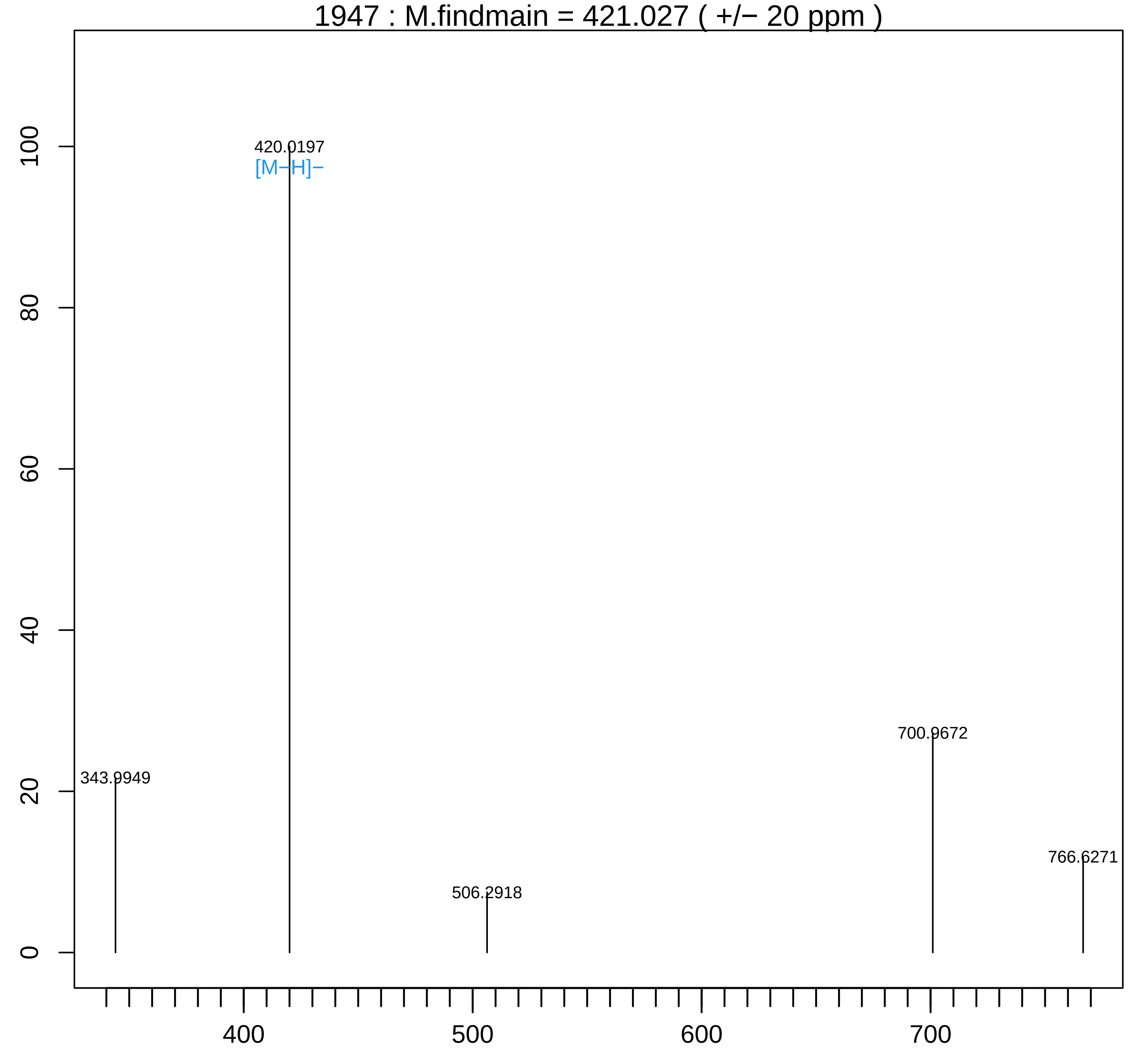

1947 : M.findmain = 421.027 (+/− 20 ppm)
420.0197
[M−H]−
343.9949
700.9672
766.6271
506.2918
0
20
40
60
80
100
400
500
600
700

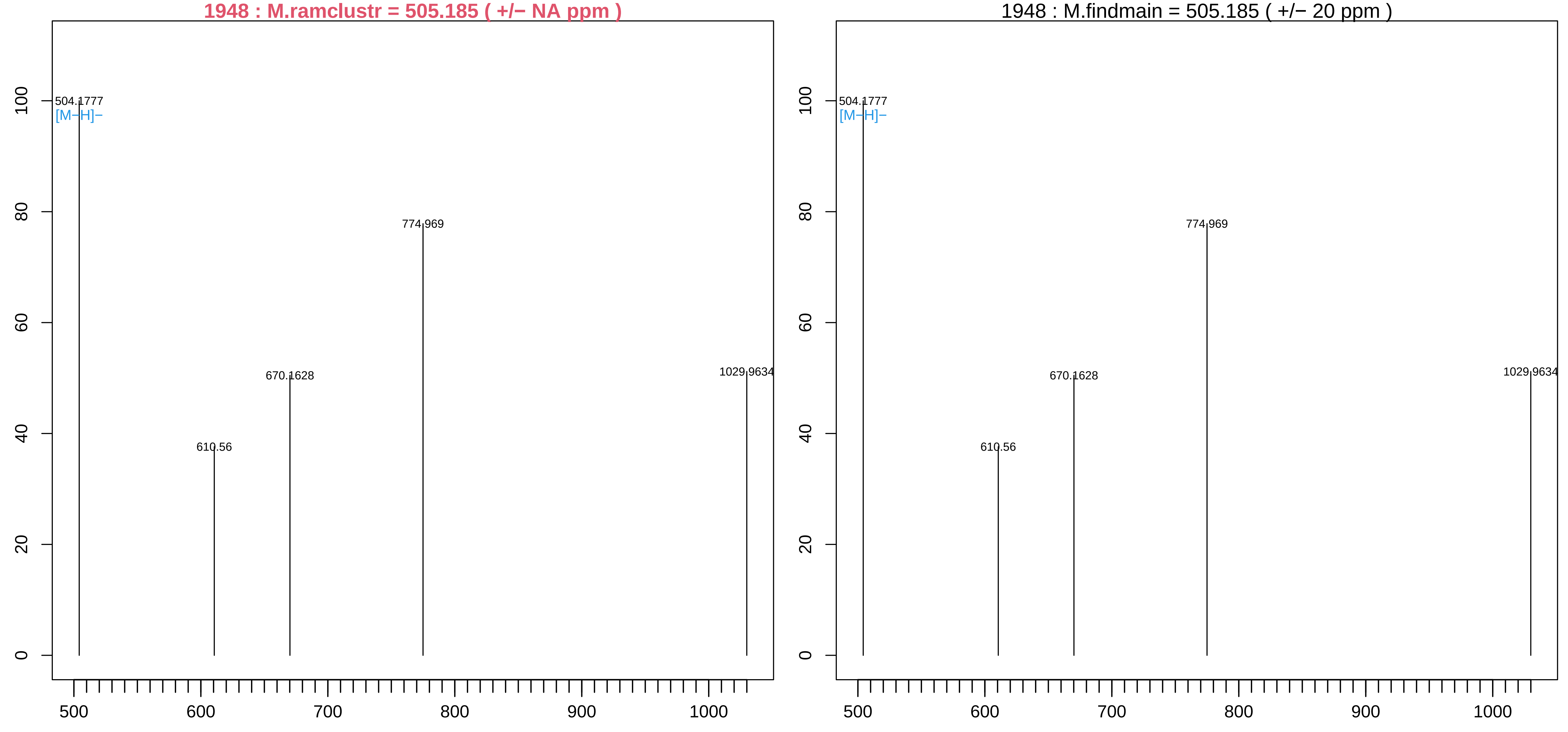

1948 : M.ramclustr = 505.185 (+/− NA ppm)
504.1777
[M−H]−
610.56
670.1628
774.969
1029.9634
1948 : M.findmain = 505.185 (+/− 20 ppm)
504.1777
[M−H]−
610.56
670.1628
774.969
1029.9634
0
20
40
60
80
100
500
600
700
800
900
1000

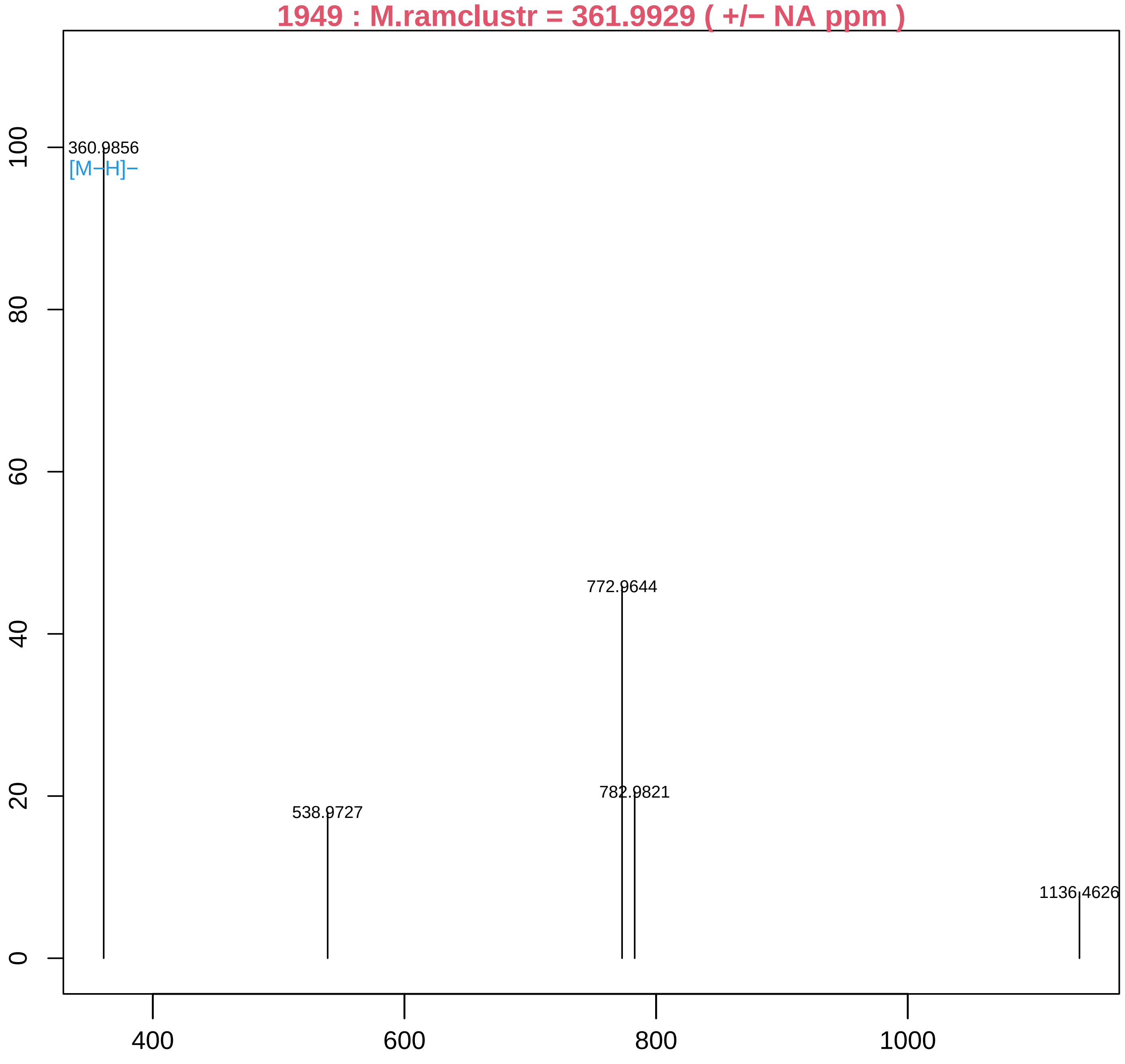
1949 : M.ramclustr = 361.9929 (+/− NA ppm)
360.9856
[M−H]−
772.9644
782.9821
538.9727
1136.4626
0
20
40
60
80
100
400
600
800
1000
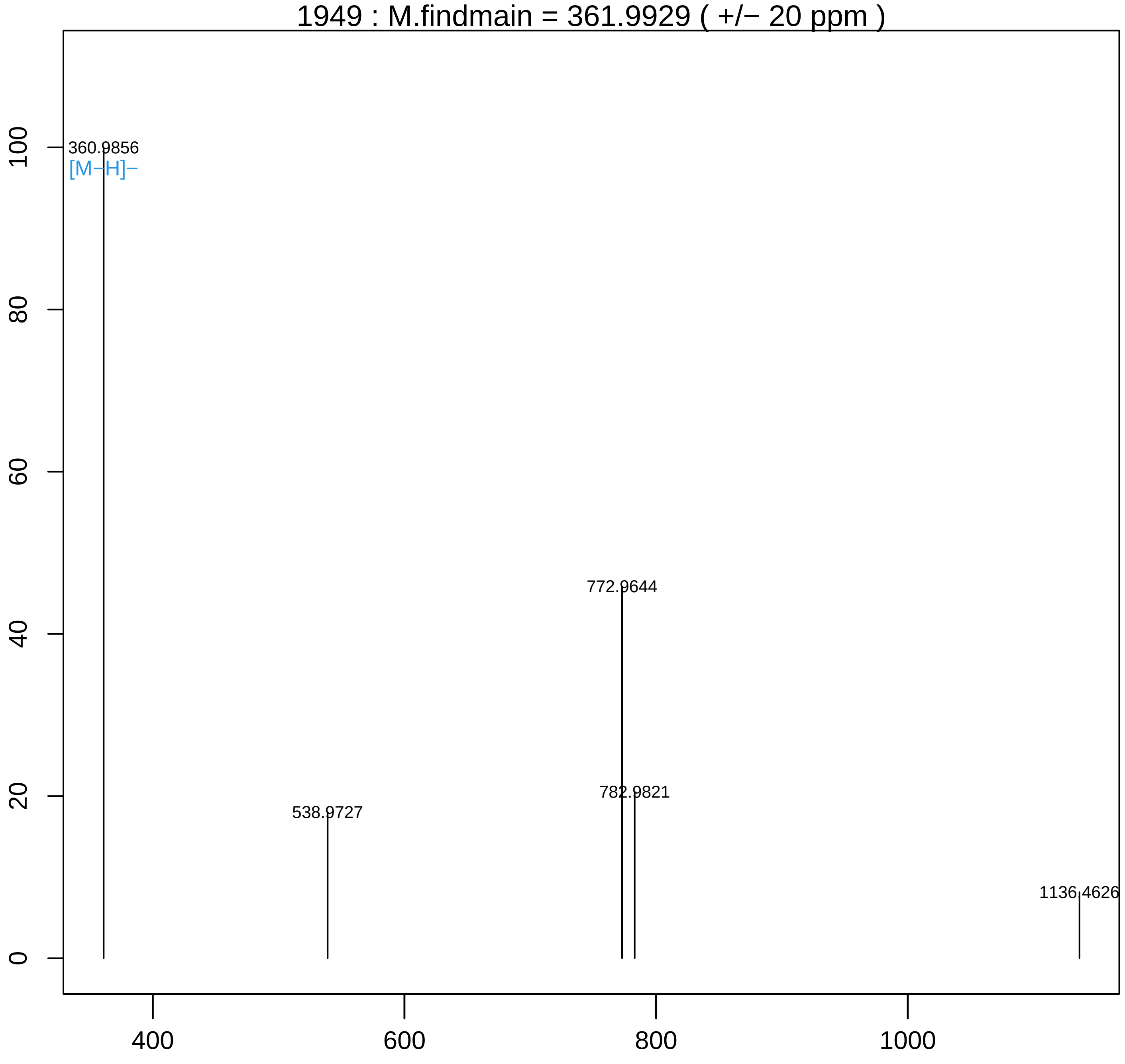
1949 : M.findmain = 361.9929 (+/− 20 ppm)
360.9856
[M−H]−
772.9644
782.9821
538.9727
1136.4626
0
20
40
60
80
100
400
600
800
1000

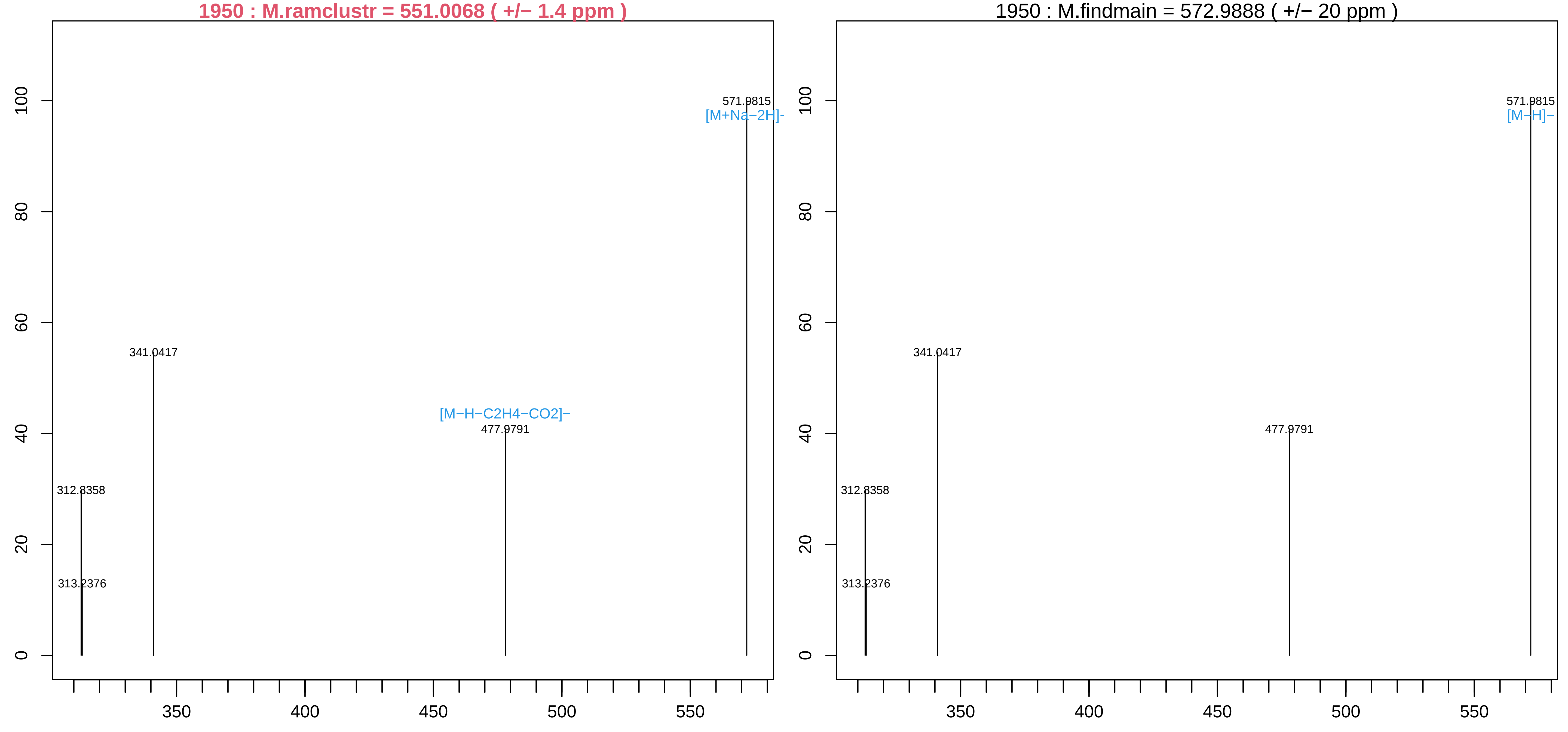
1950 : M.ramclustr = 551.0068 (+/− 1.4 ppm)
571.9815
[M+Na−2H]-
341.0417
[M−H−C2H4−CO2]−
477.9791
312.8358
313.2376
0
20
40
60
80
100
350
400
450
500
550
1950 : M.findmain = 572.9888 (+/− 20 ppm)
571.9815
[M−H]−
341.0417
477.9791
312.8358
313.2376
0
20
40
60
80
100
350
400
450
500
550

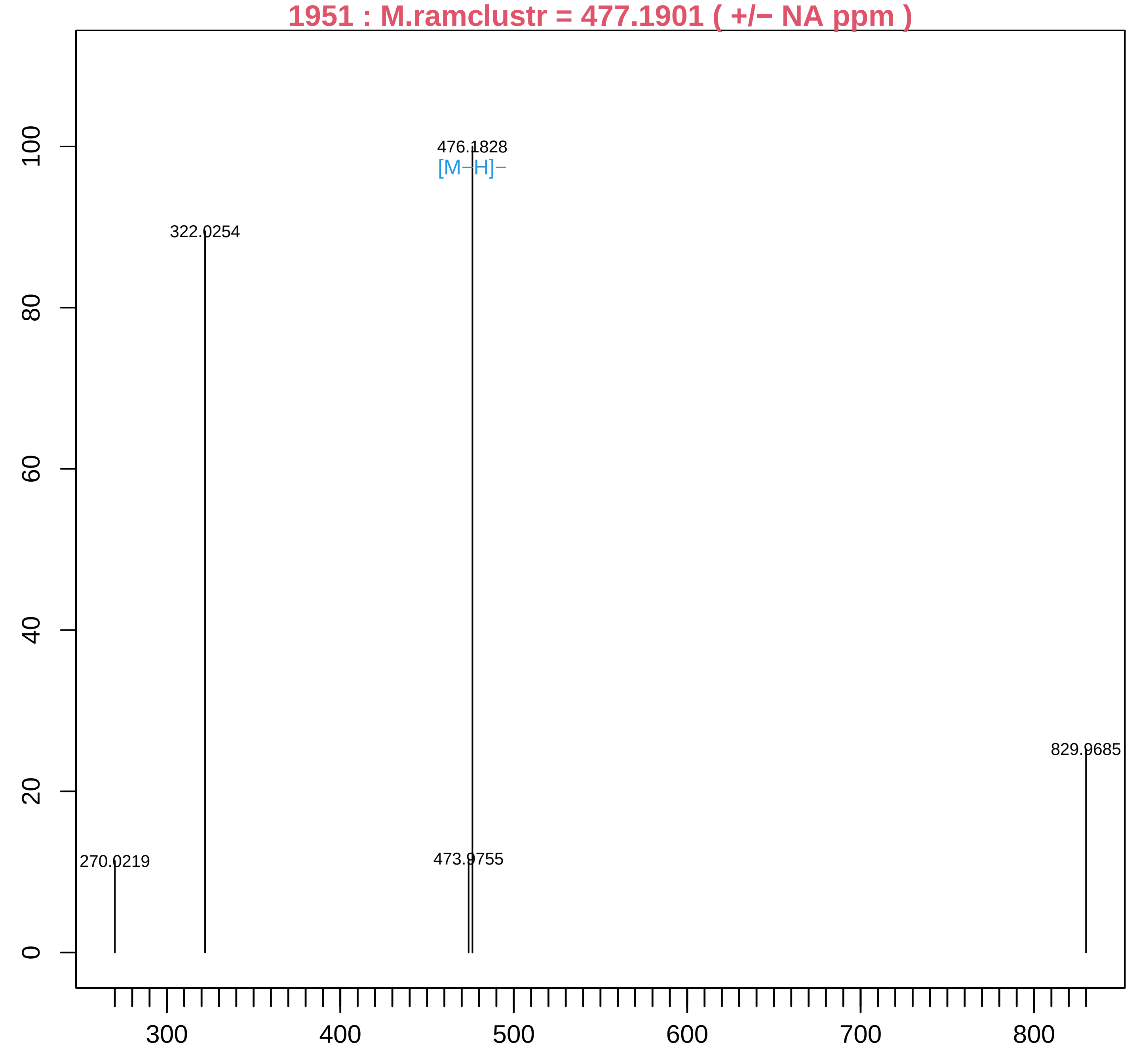
1951 : M.ramclustr = 477.1901 (+/− NA ppm)
476.1828
[M−H]−
322.0254
829.9685
270.0219
473.9755
0
20
40
60
80
100
300
400
500
600
700
800

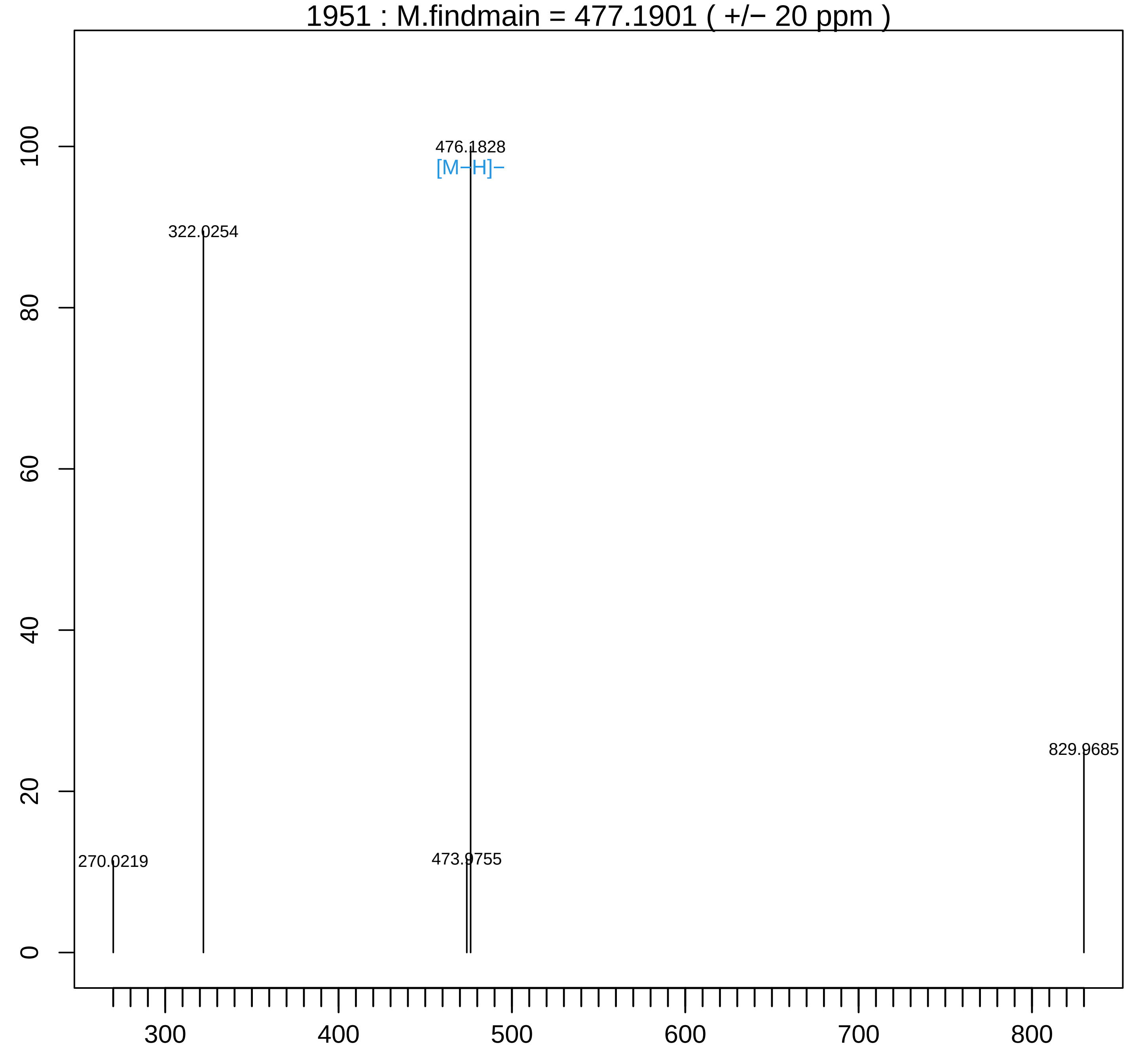
1951 : M.findmain = 477.1901 (+/− 20 ppm)
476.1828
[M−H]−
322.0254
829.9685
270.0219
473.9755
0
20
40
60
80
100
300
400
500
600
700
800

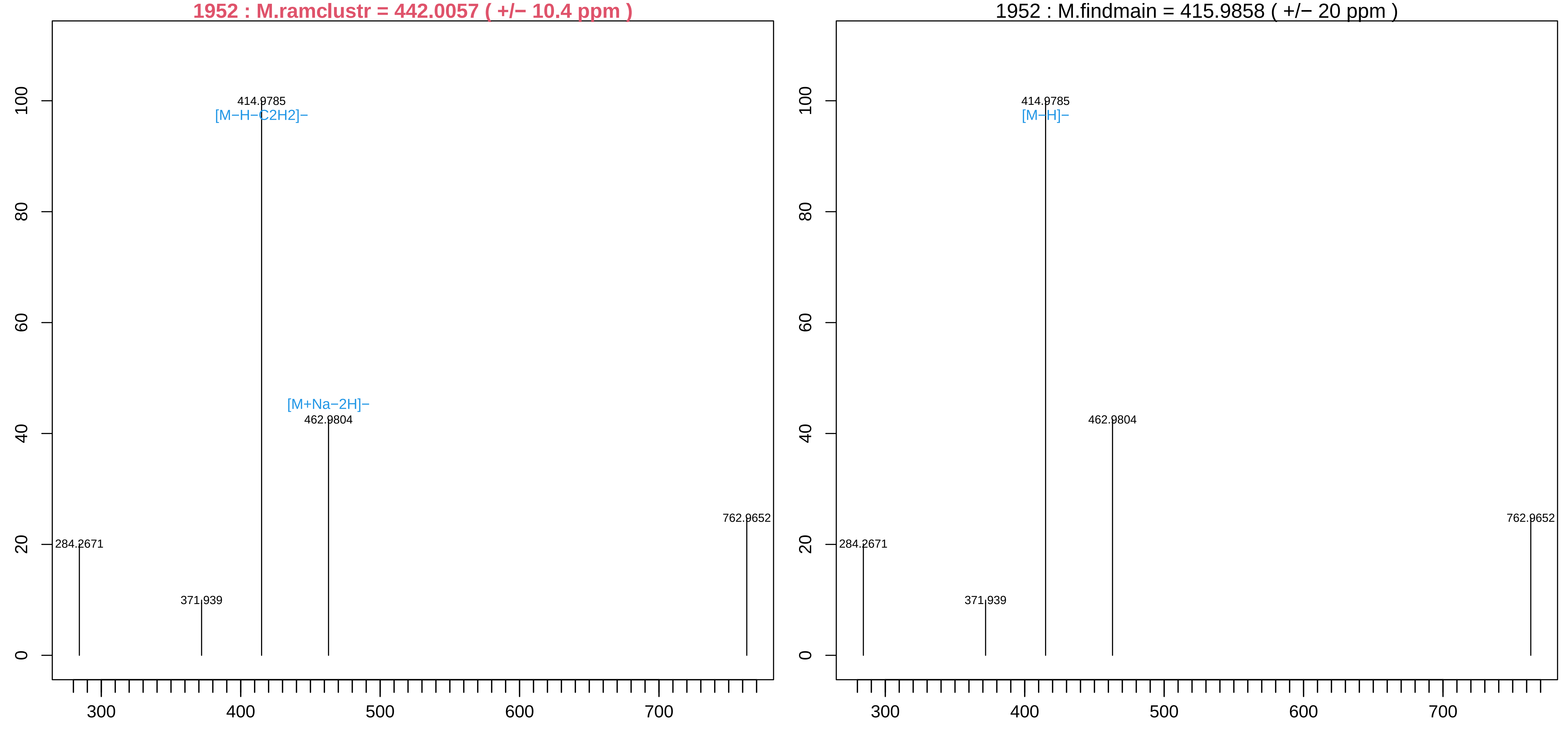
1952 : M.ramclustr = 442.0057 (+/− 10.4 ppm)
414.9785
[M−H−C2H2]−
[M+Na−2H]−
462.9804
762.9652
284.2671
371.939
1952 : M.findmain = 415.9858 (+/− 20 ppm)
414.9785
[M−H]−
462.9804
762.9652
284.2671
371.939
0
20
40
60
80
100
300
400
500
600
700

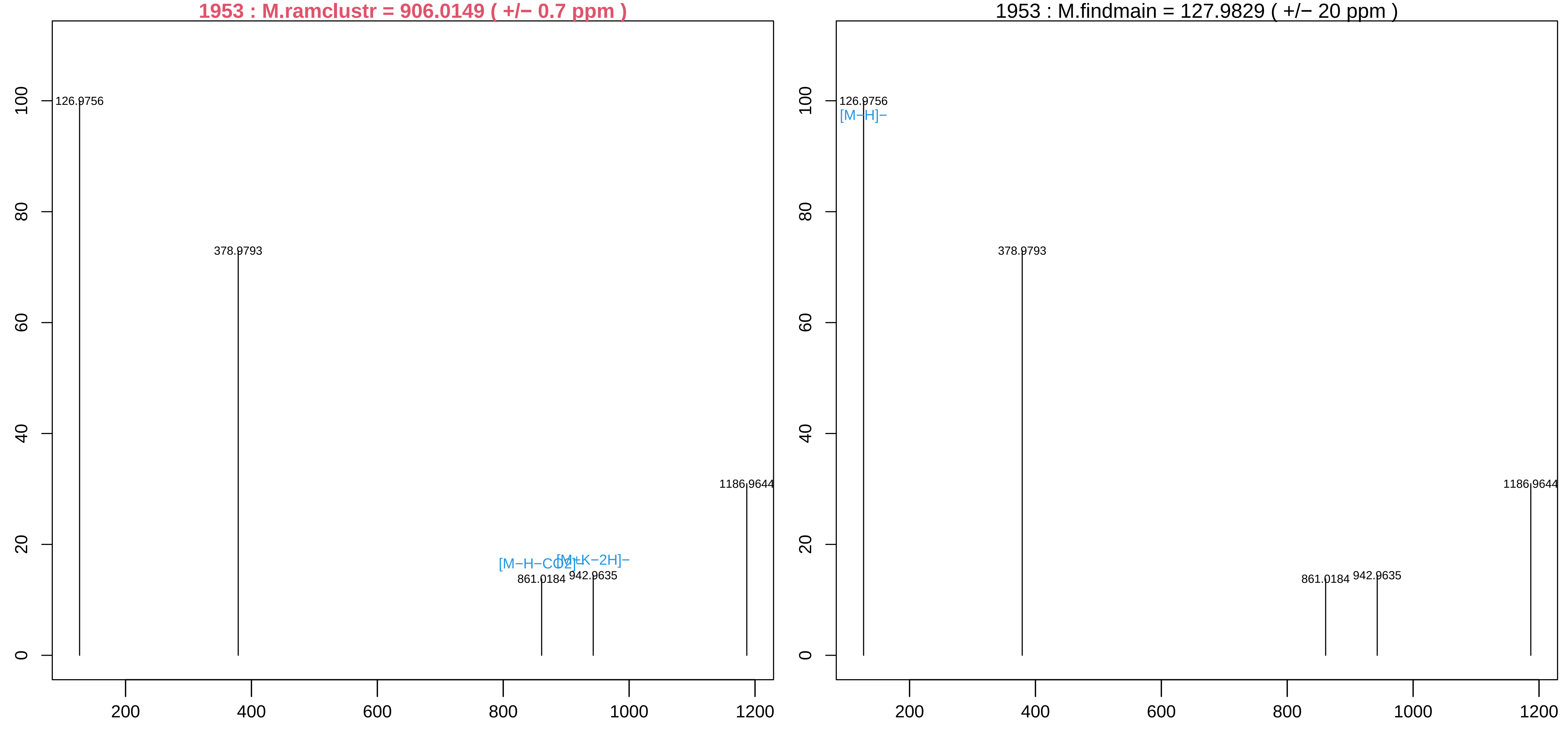

1953 : M.ramclustr = 906.0149 (+/− 0.7 ppm)
126.9756
378.9793
[M−H−CO2]−
[M+K−2H]−
861.0184
942.9635
1186.9644
1953 : M.findmain = 127.9829 (+/− 20 ppm)
126.9756
[M−H]−
378.9793
861.0184
942.9635
1186.9644
0
20
40
60
80
100
200
400
600
800
1000
1200

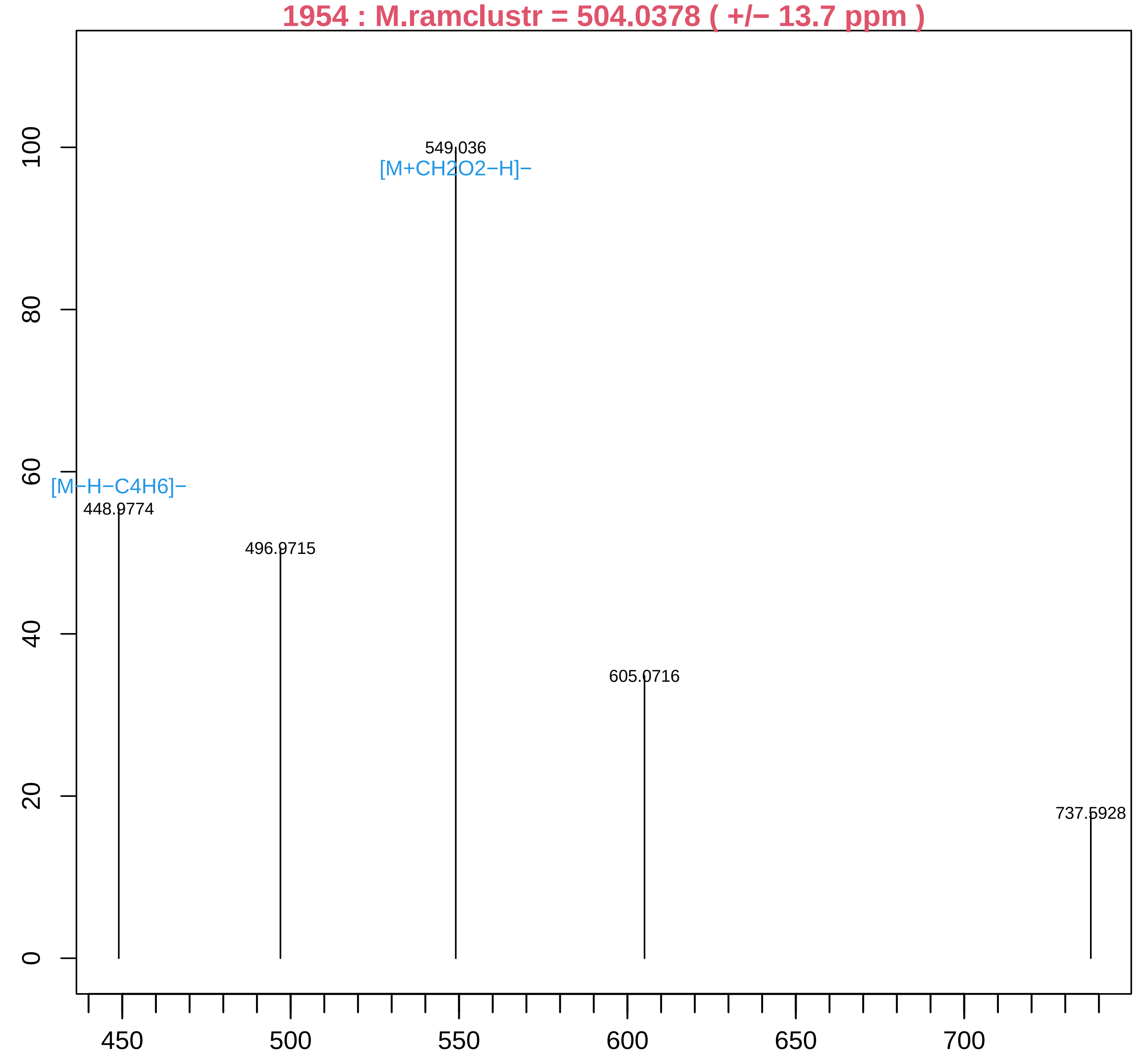

1954 : M.ramclustr = 504.0378 (+/− 13.7 ppm)
549.036
[M+CH2O2−H]−
[M−H−C4H6]−
448.9774
496.9715
605.0716
737.5928
0
20
40
60
80
100
450
500
550
600
650
700

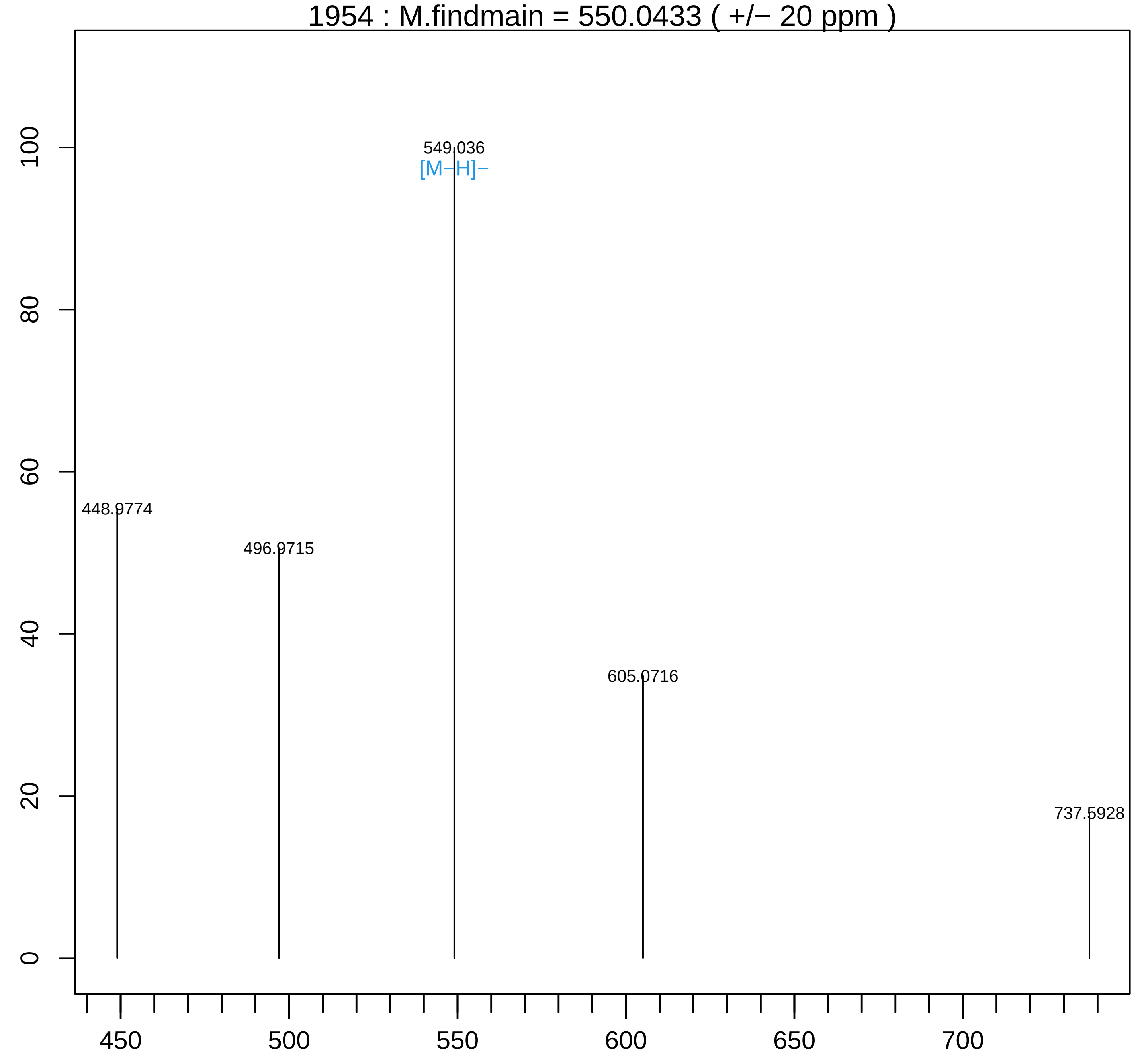

1954 : M.findmain = 550.0433 (+/− 20 ppm)
549.036
[M−H]−
448.9774
496.9715
605.0716
737.5928
0
20
40
60
80
100
450
500
550
600
650
700

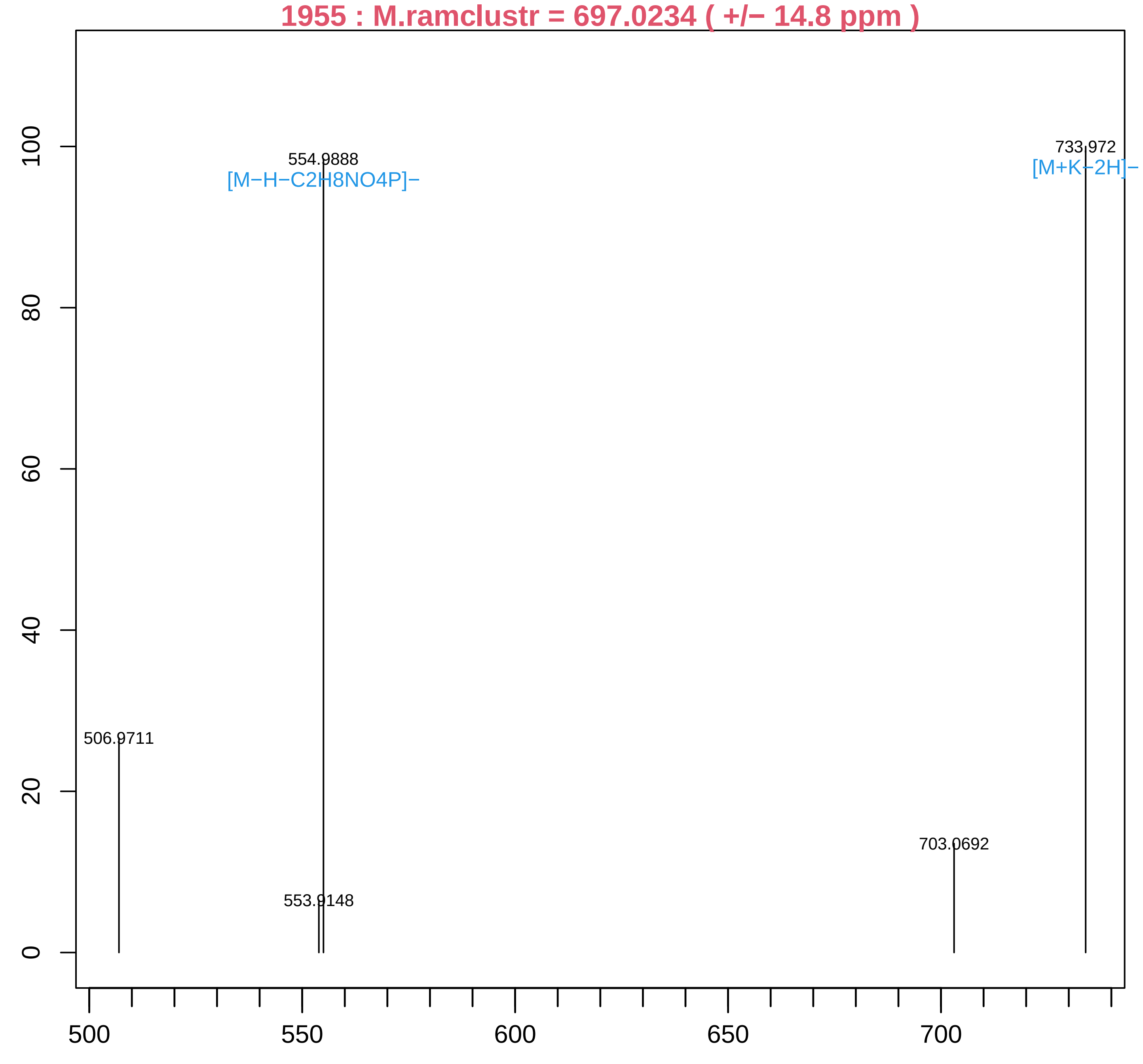
1955 : M.ramclustr = 697.0234 (+/− 14.8 ppm)
733.972
[M+K−2H]−
554.9888
[M−H−C2H8NO4P]−
506.9711
553.9148
703.0692
0
20
40
60
80
100
500
550
600
650
700

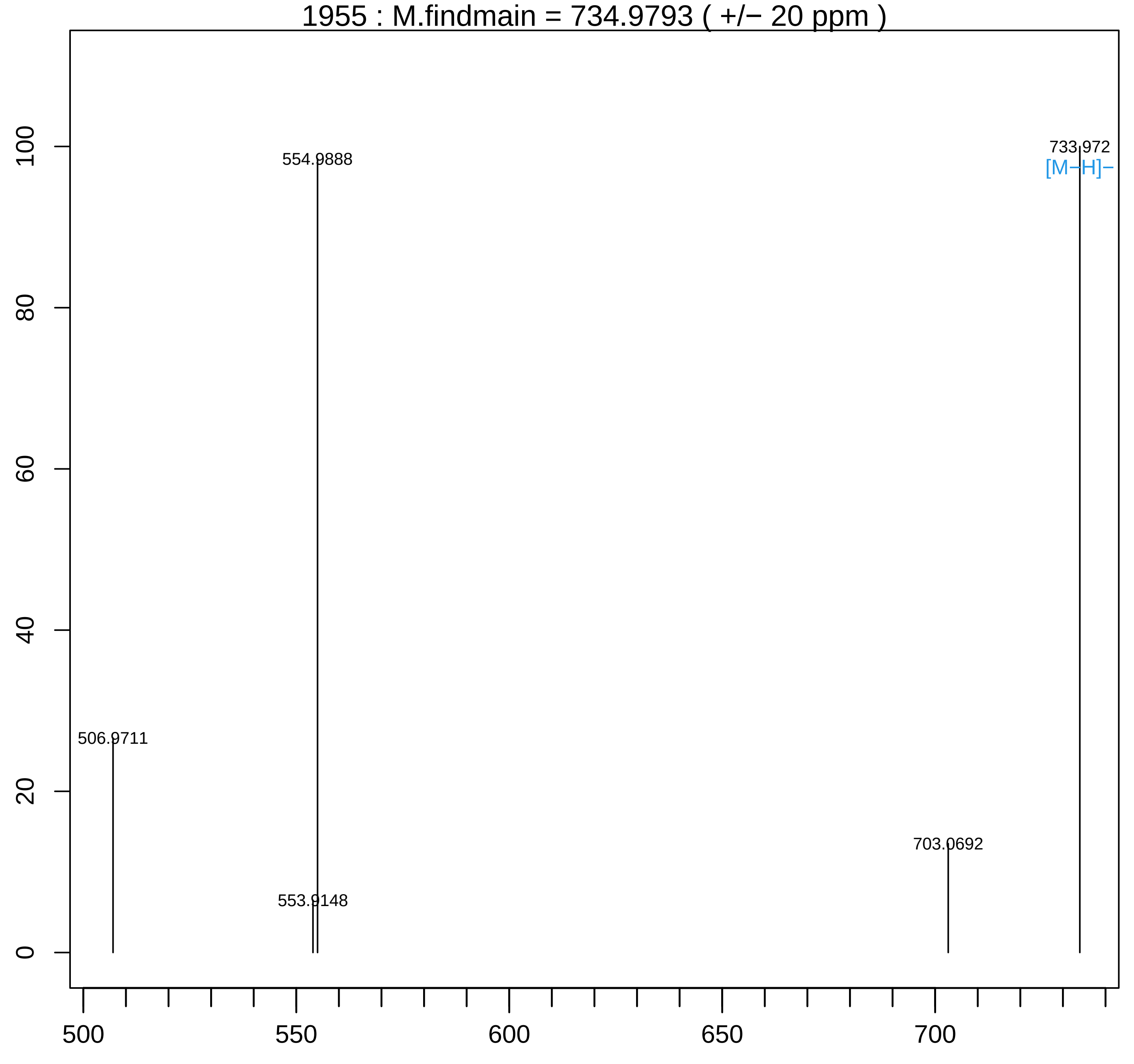
1955 : M.findmain = 734.9793 (+/− 20 ppm)
733.972
[M−H]−
554.9888
506.9711
553.9148
703.0692
0
20
40
60
80
100
500
550
600
650
700

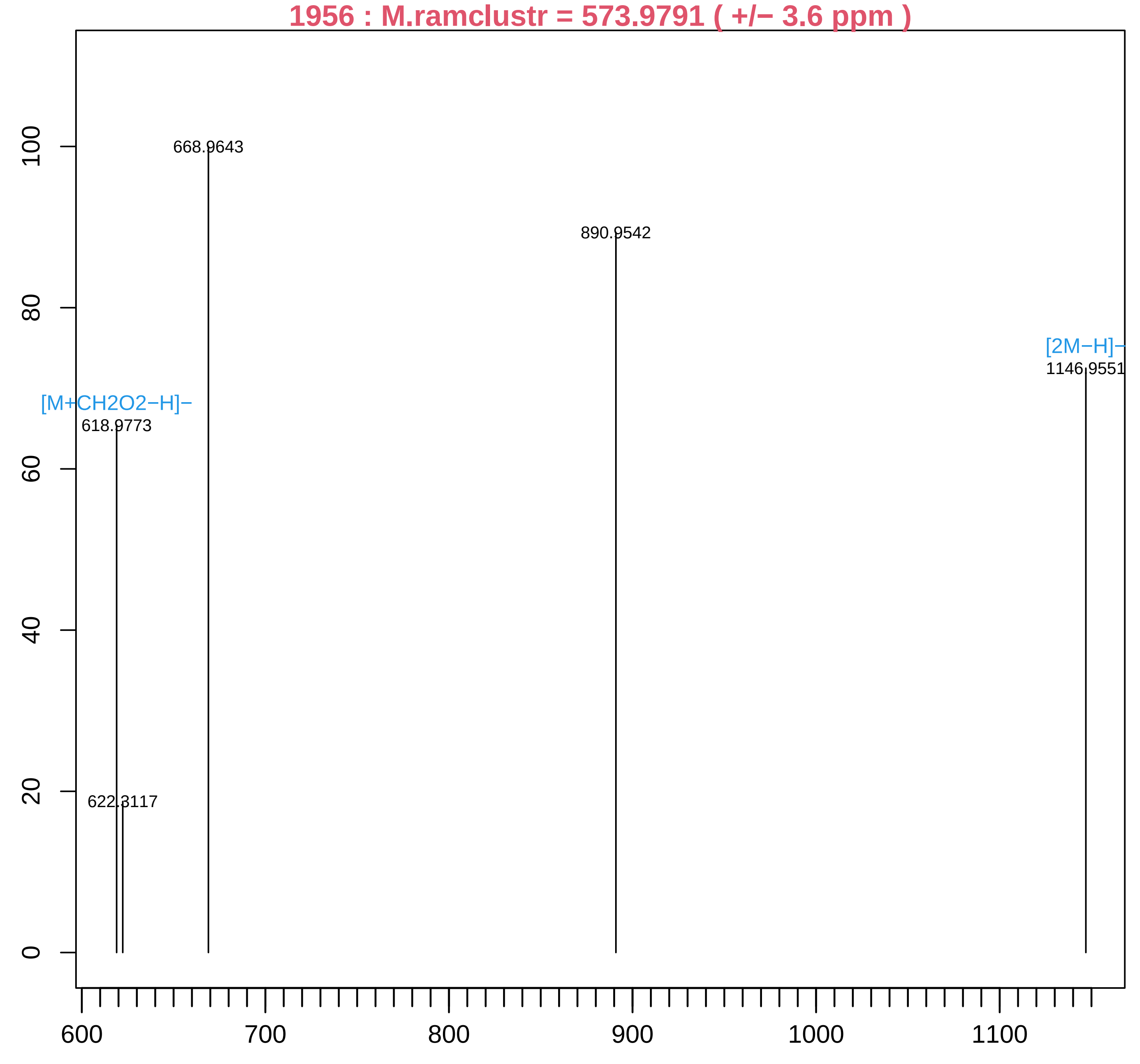
1956 : M.ramclustr = 573.9791 (+/− 3.6 ppm)
668.9643
890.9542
[2M−H]−
1146.9551
[M+CH2O2−H]−
618.9773
622.3117
0
20
40
60
80
100
600
700
800
900
1000
1100

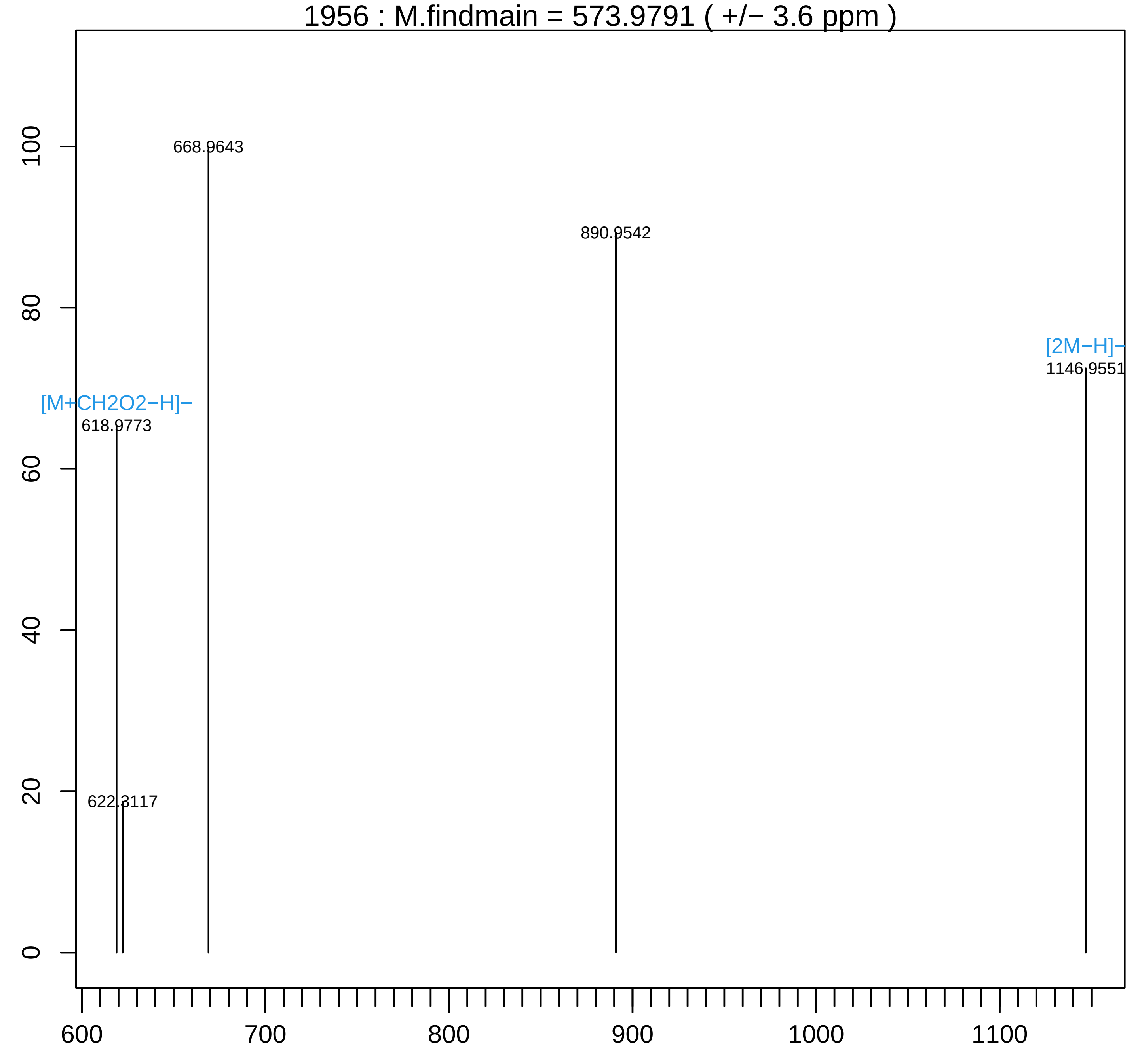
1956 : M.findmain = 573.9791 (+/− 3.6 ppm)
668.9643
890.9542
[2M−H]−
1146.9551
[M+CH2O2−H]−
618.9773
622.3117
0
20
40
60
80
100
600
700
800
900
1000
1100

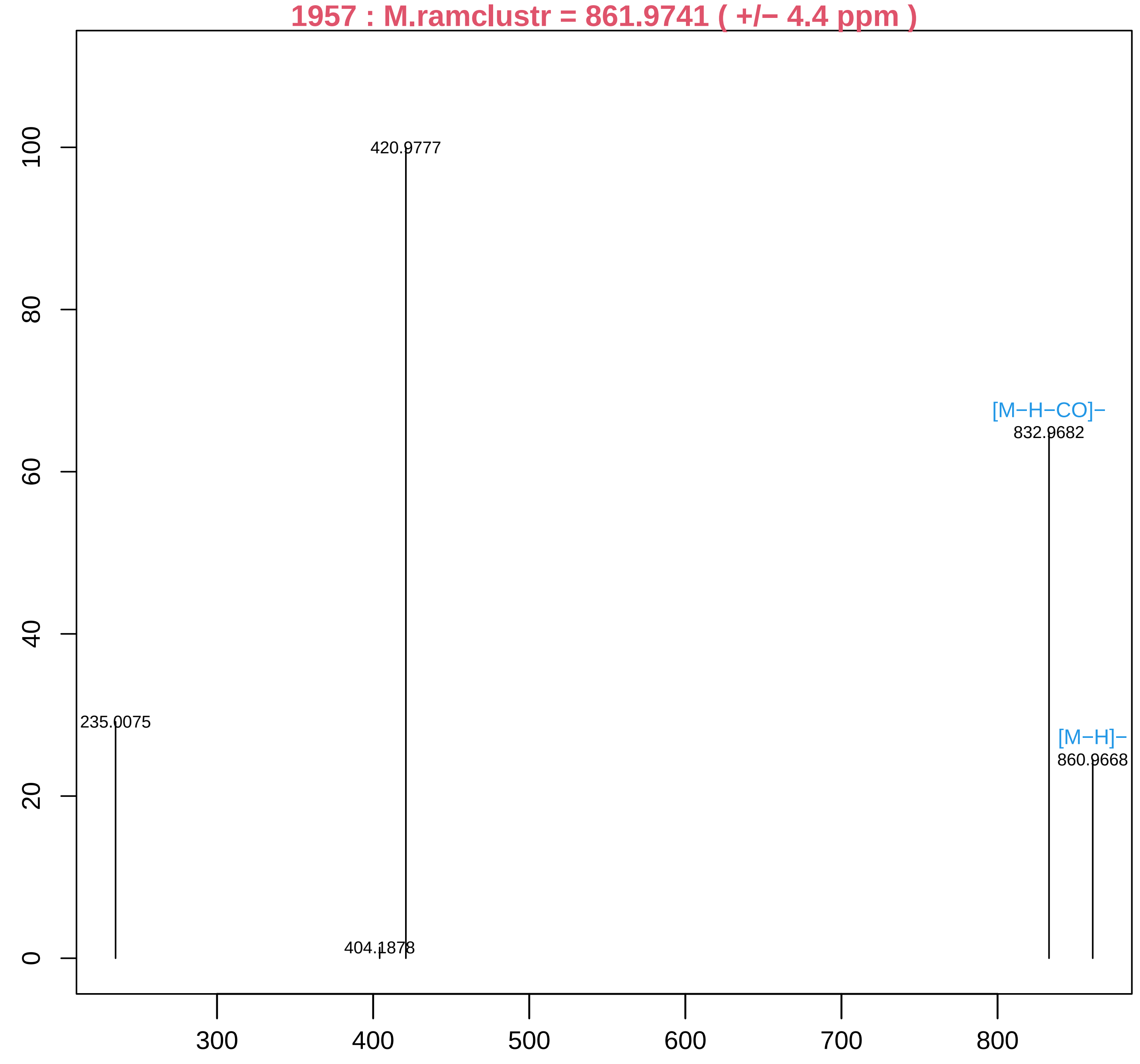
1957 : M.ramclustr = 861.9741 (+/− 4.4 ppm)
420.9777
[M−H−CO]−
832.9682
235.0075
[M−H]−
860.9668
404.1878
0
20
40
60
80
100
300
400
500
600
700
800

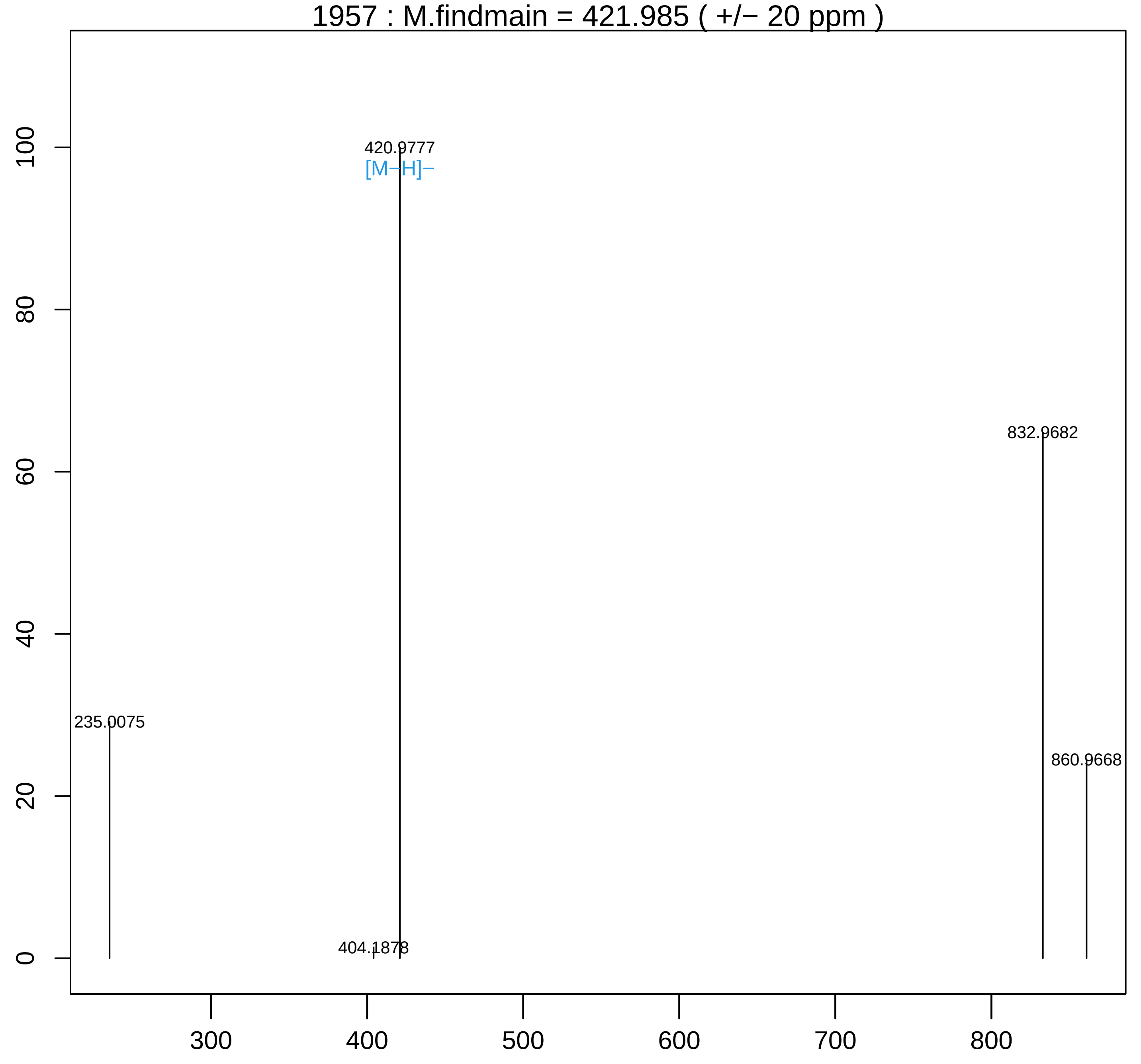
1957 : M.findmain = 421.985 (+/− 20 ppm)
420.9777
[M−H]−
832.9682
235.0075
860.9668
404.1878
0
20
40
60
80
100
300
400
500
600
700
800

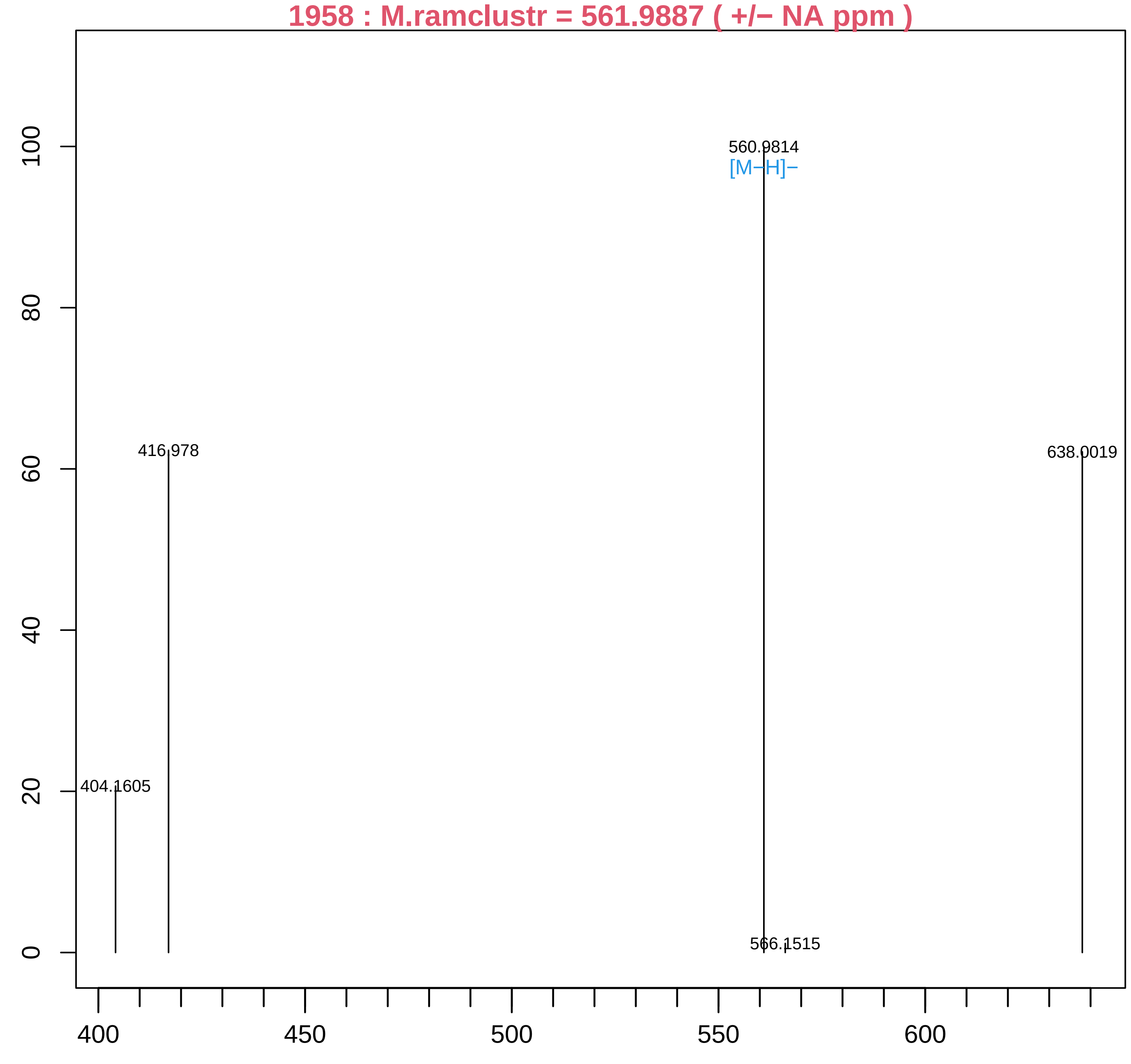
1958 : M.ramclustr = 561.9887 (+/− NA ppm)
560.9814
[M−H]−
416.978
638.0019
404.1605
566.1515
0
20
40
60
80
100
400
450
500
550
600

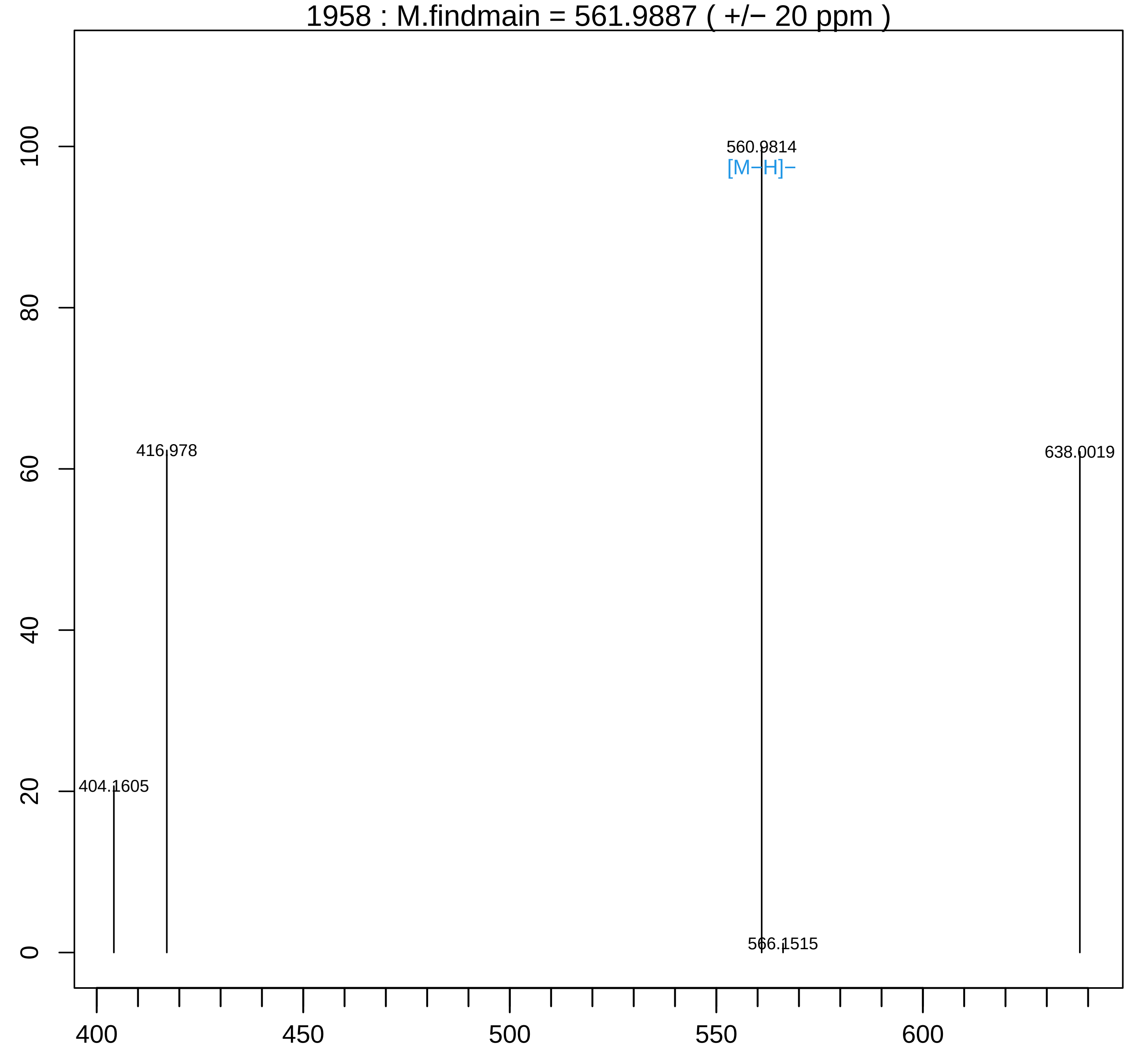
1958 : M.findmain = 561.9887 (+/− 20 ppm)
560.9814
[M−H]−
416.978
638.0019
404.1605
566.1515
0
20
40
60
80
100
400
450
500
550
600

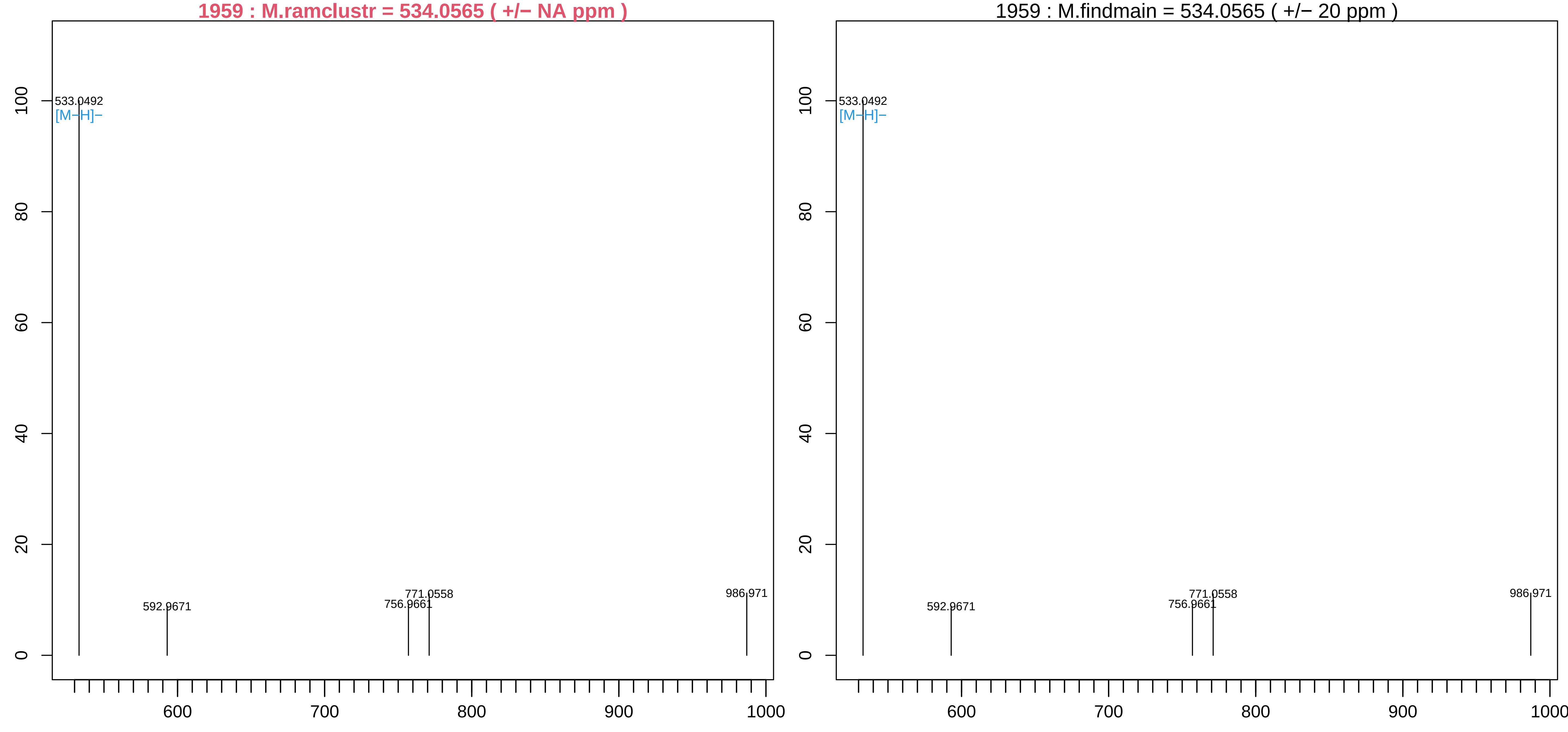
1959 : M.ramclustr = 534.0565 (+/− NA ppm)
533.0492
[M−H]−
592.9671
756.9661
771.0558
986.971
1959 : M.findmain = 534.0565 (+/− 20 ppm)
533.0492
[M−H]−
592.9671
756.9661
771.0558
986.971

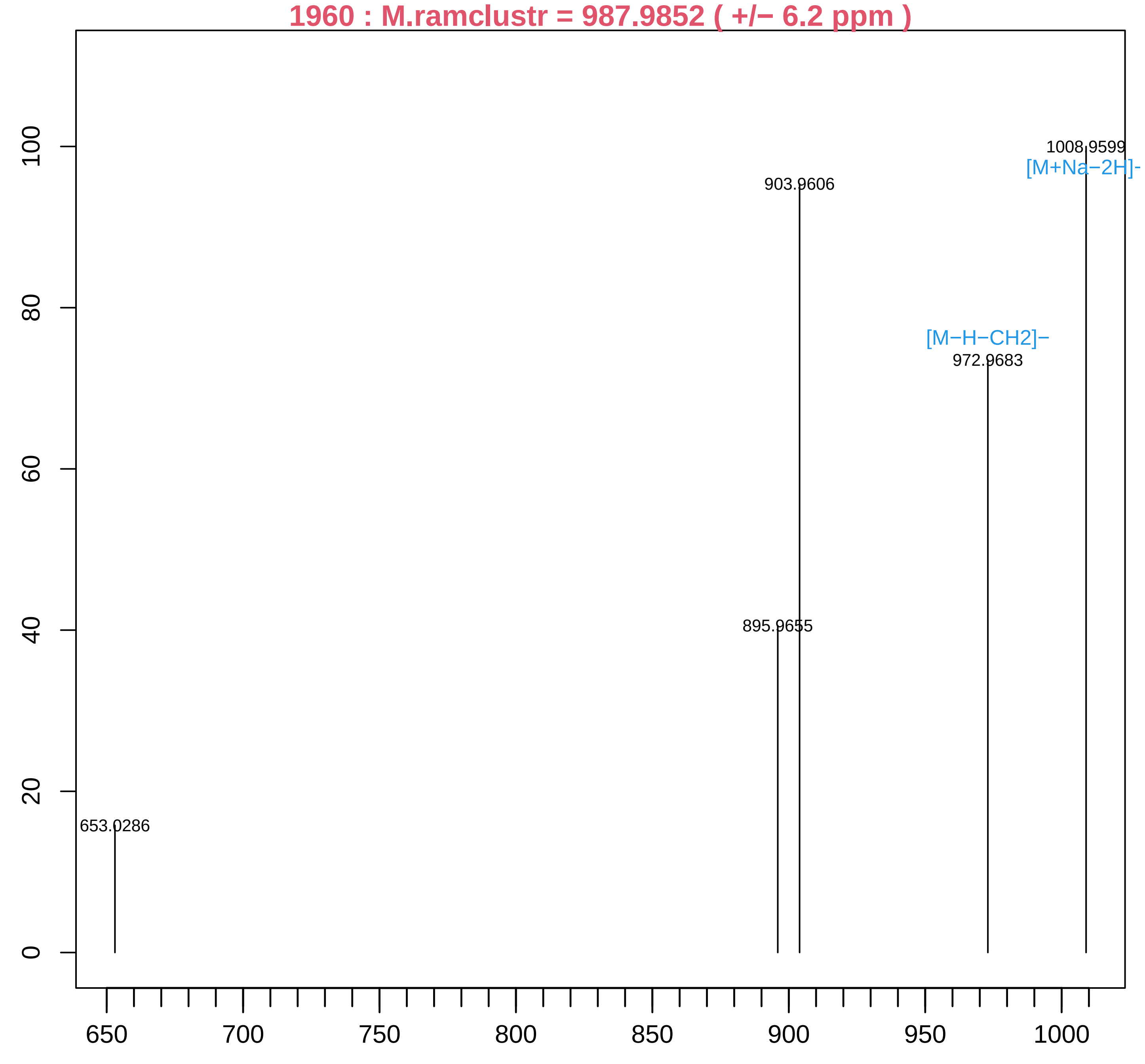
1960 : M.ramclustr = 987.9852 (+/− 6.2 ppm)
1008.9599
[M+Na−2H]-
903.9606
[M−H−CH2]−
972.9683
895.9655
653.0286
0
20
40
60
80
100
650
700
750
800
850
900
950
1000

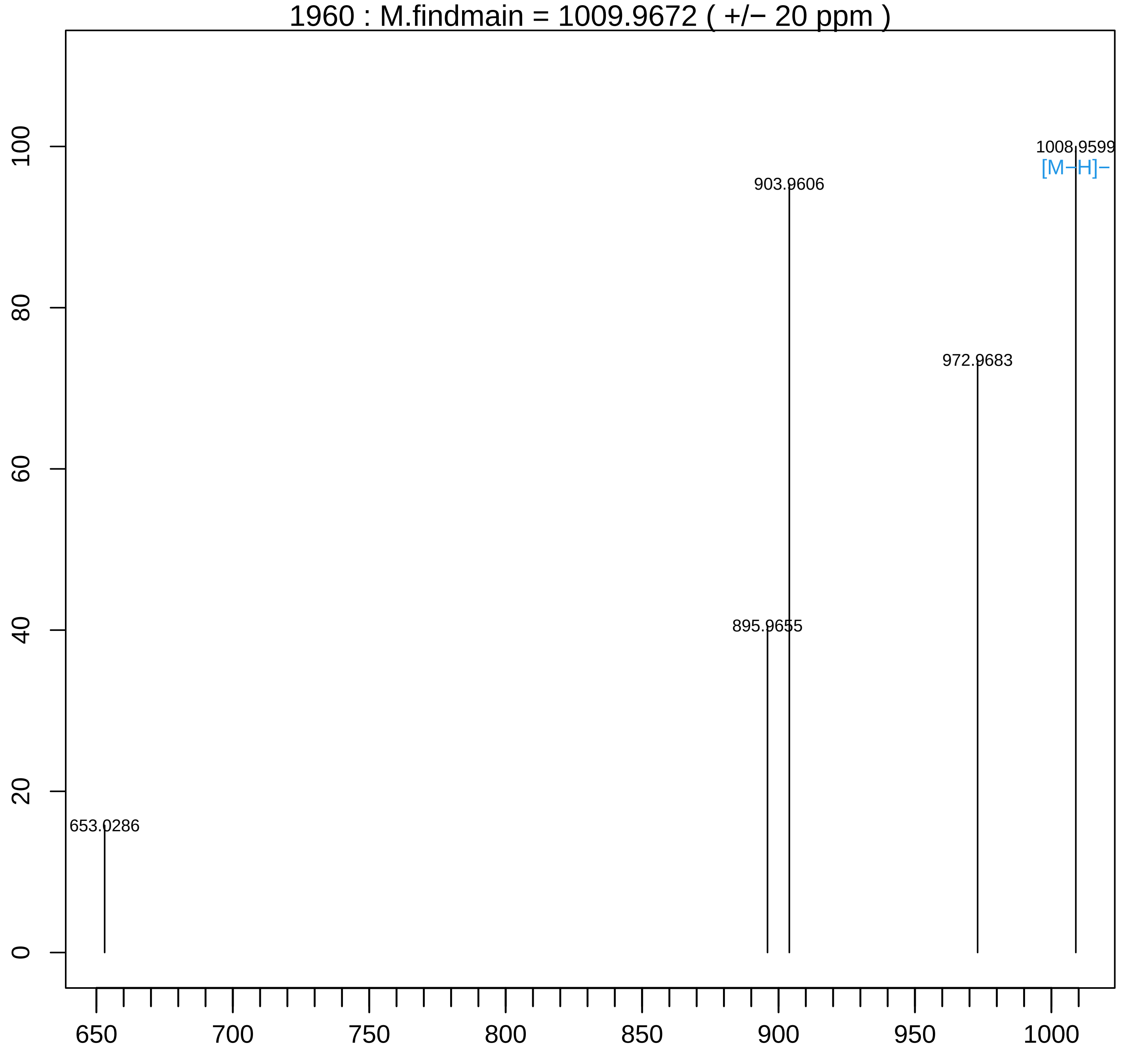
1960 : M.findmain = 1009.9672 (+/− 20 ppm)
1008.9599
[M−H]−
903.9606
972.9683
895.9655
653.0286
0
20
40
60
80
100
650
700
750
800
850
900
950
1000

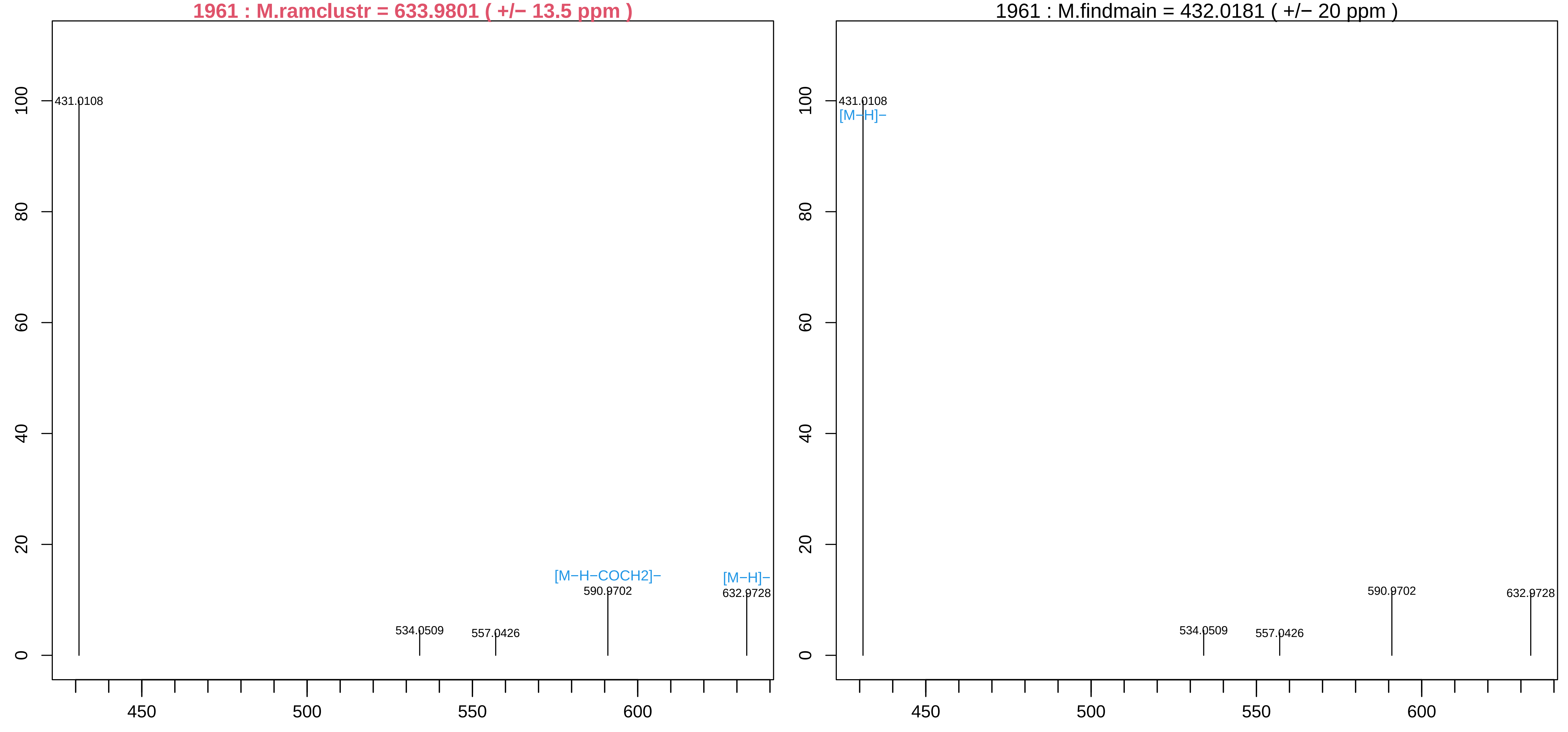
1961 : M.ramclustr = 633.9801 (+/− 13.5 ppm)
431.0108
534.0509
557.0426
[M−H−COCH2]−
590.9702
[M−H]−
632.9728
1961 : M.findmain = 432.0181 (+/− 20 ppm)
431.0108
[M−H]−
534.0509
557.0426
590.9702
632.9728
0
20
40
60
80
100
450
500
550
600

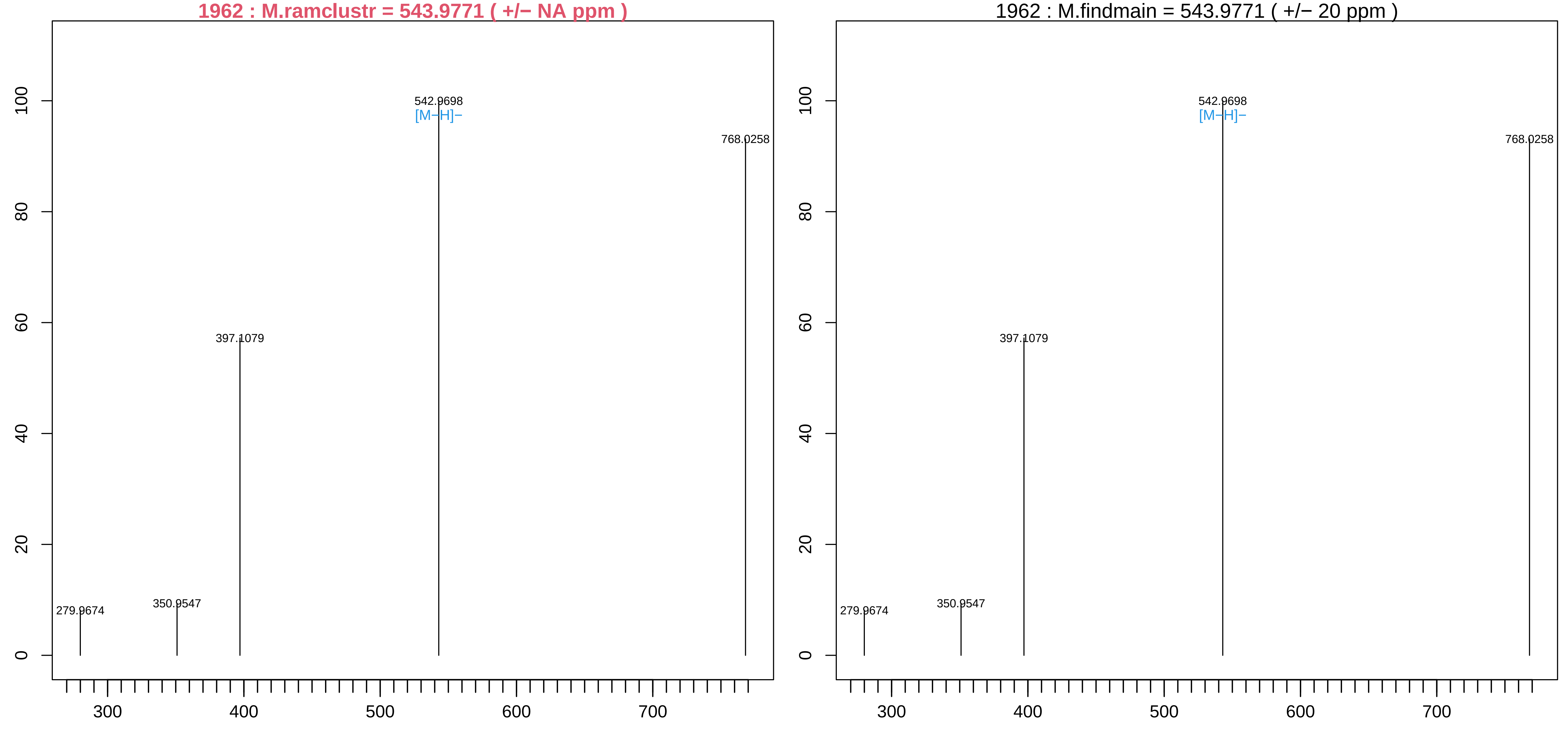
1962 : M.ramclustr = 543.9771 (+/− NA ppm)
1962 : M.findmain = 543.9771 (+/− 20 ppm)
542.9698
[M−H]−
768.0258
397.1079
279.9674
350.9547
0
20
40
60
80
100
300
400
500
600
700

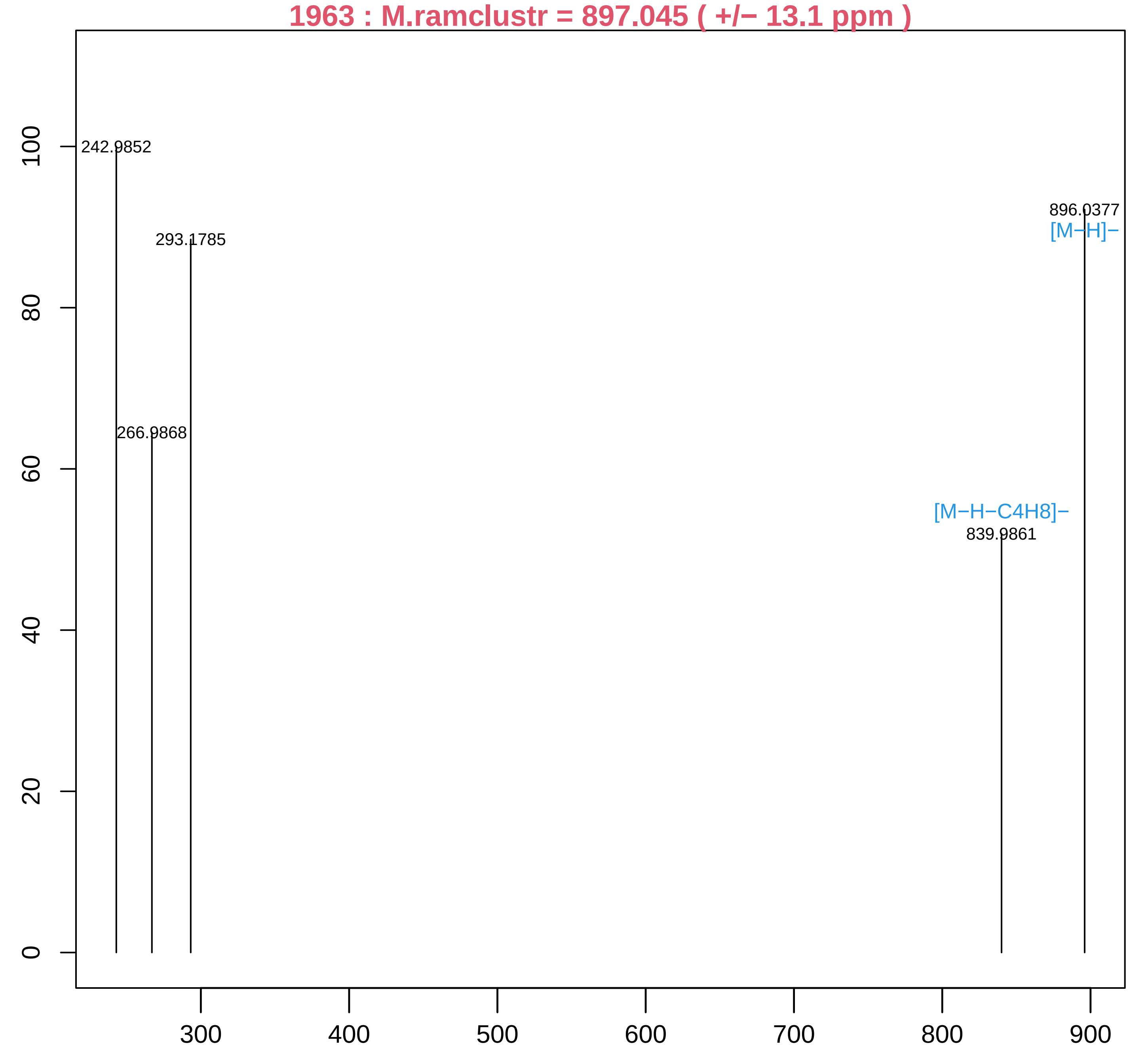
1963 : M.ramclustr = 897.045 (+/− 13.1 ppm)
242.9852
266.9868
293.1785
[M−H−C4H8]−
839.9861
896.0377
[M−H]−
0
20
40
60
80
100
300
400
500
600
700
800
900

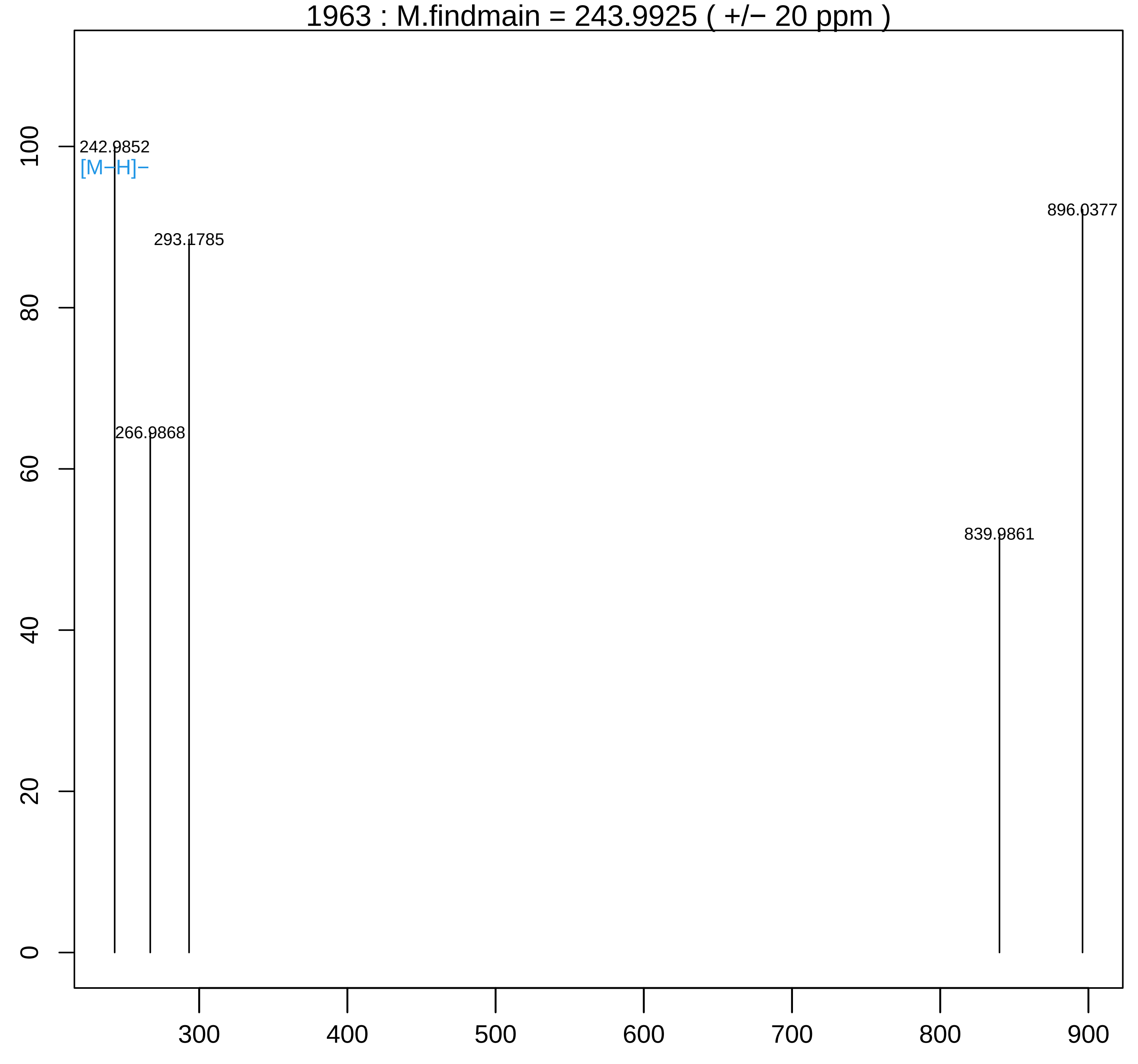
1963 : M.findmain = 243.9925 (+/− 20 ppm)
242.9852
[M−H]−
266.9868
293.1785
839.9861
896.0377
0
20
40
60
80
100
300
400
500
600
700
800
900

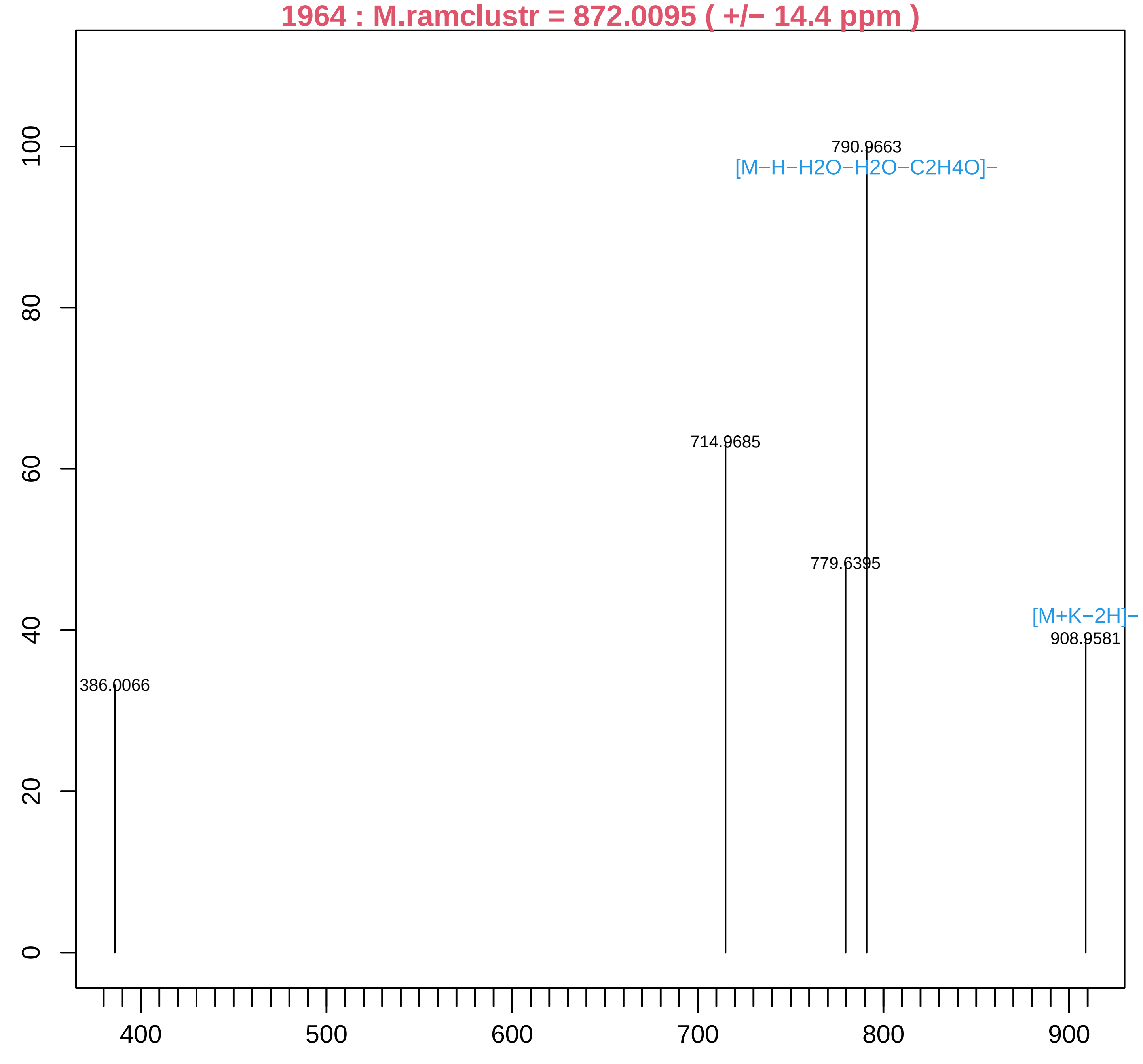
1964 : M.ramclustr = 872.0095 (+/− 14.4 ppm)
790.9663
[M−H−H2O−H2O−C2H4O]−
714.9685
779.6395
[M+K−2H]−
908.9581
386.0066
0
20
40
60
80
100
400
500
600
700
800
900

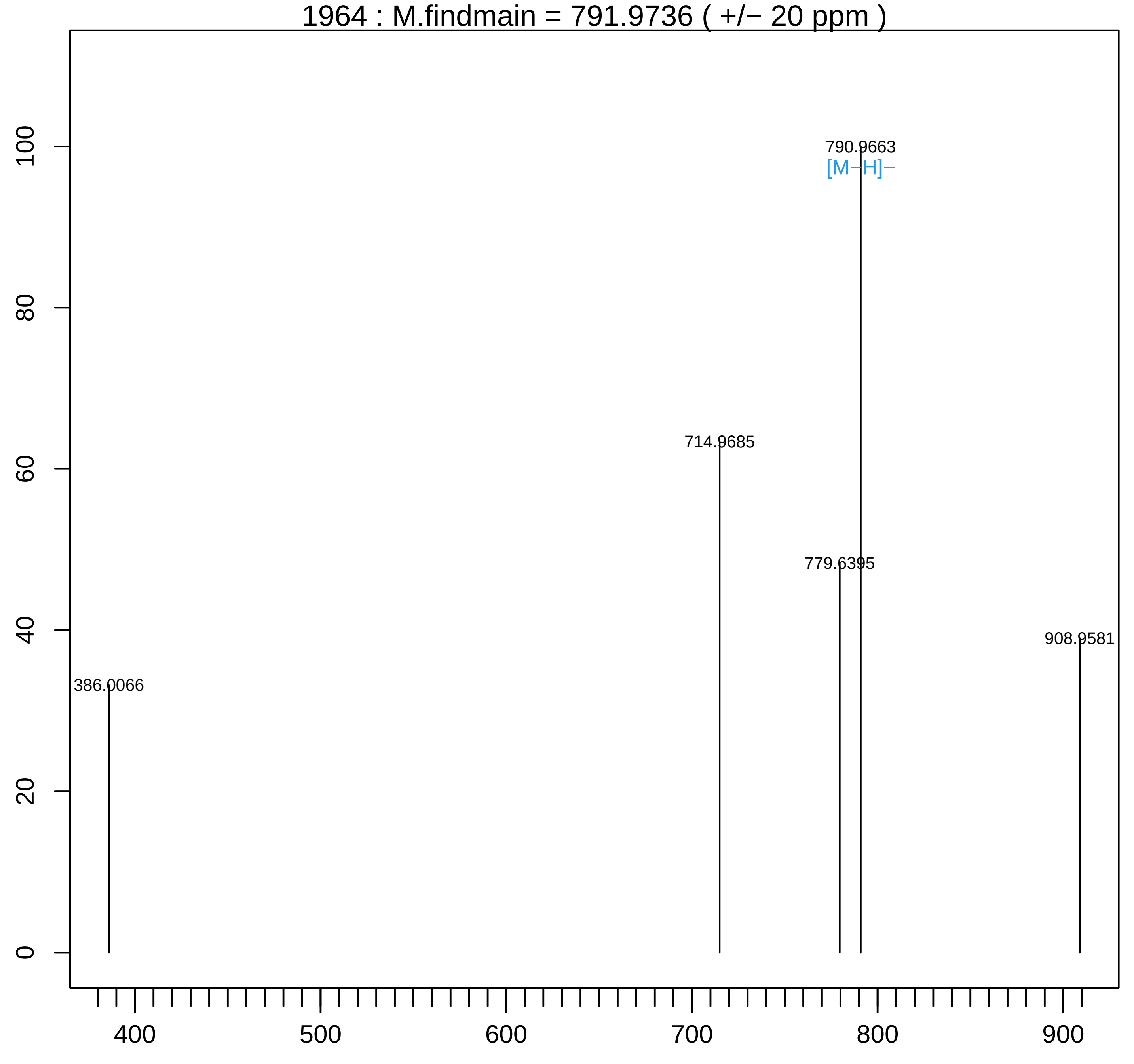
1964 : M.findmain = 791.9736 (+/− 20 ppm)
790.9663
[M−H]−
714.9685
779.6395
908.9581
386.0066
0
20
40
60
80
100
400
500
600
700
800
900

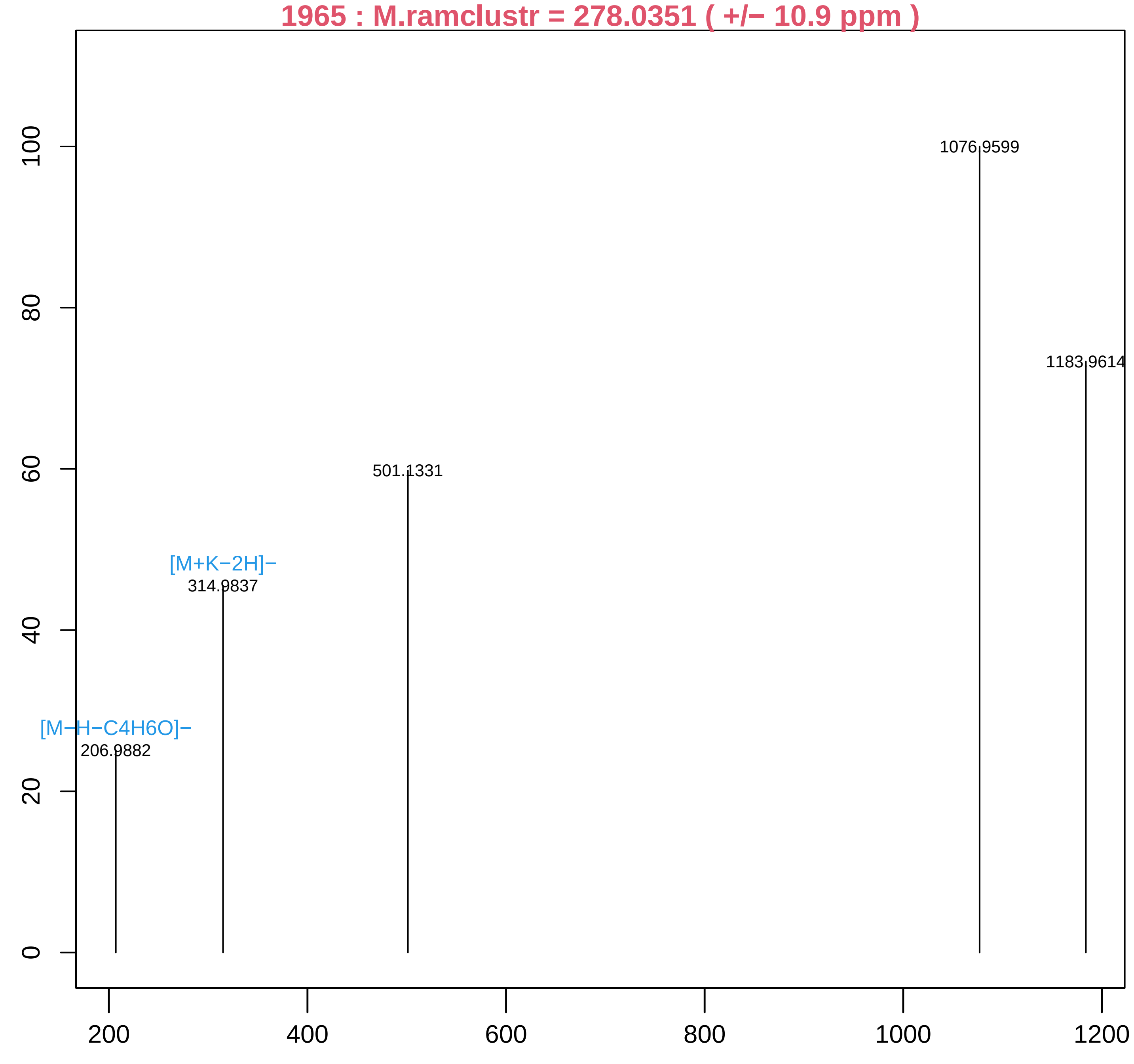

1965 : M.ramclustr = 278.0351 (+/− 10.9 ppm)
1076.9599
1183.9614
501.1331
[M+K−2H]−
314.9837
[M−H−C4H6O]−
206.9882
0
20
40
60
80
100
200
400
600
800
1000
1200

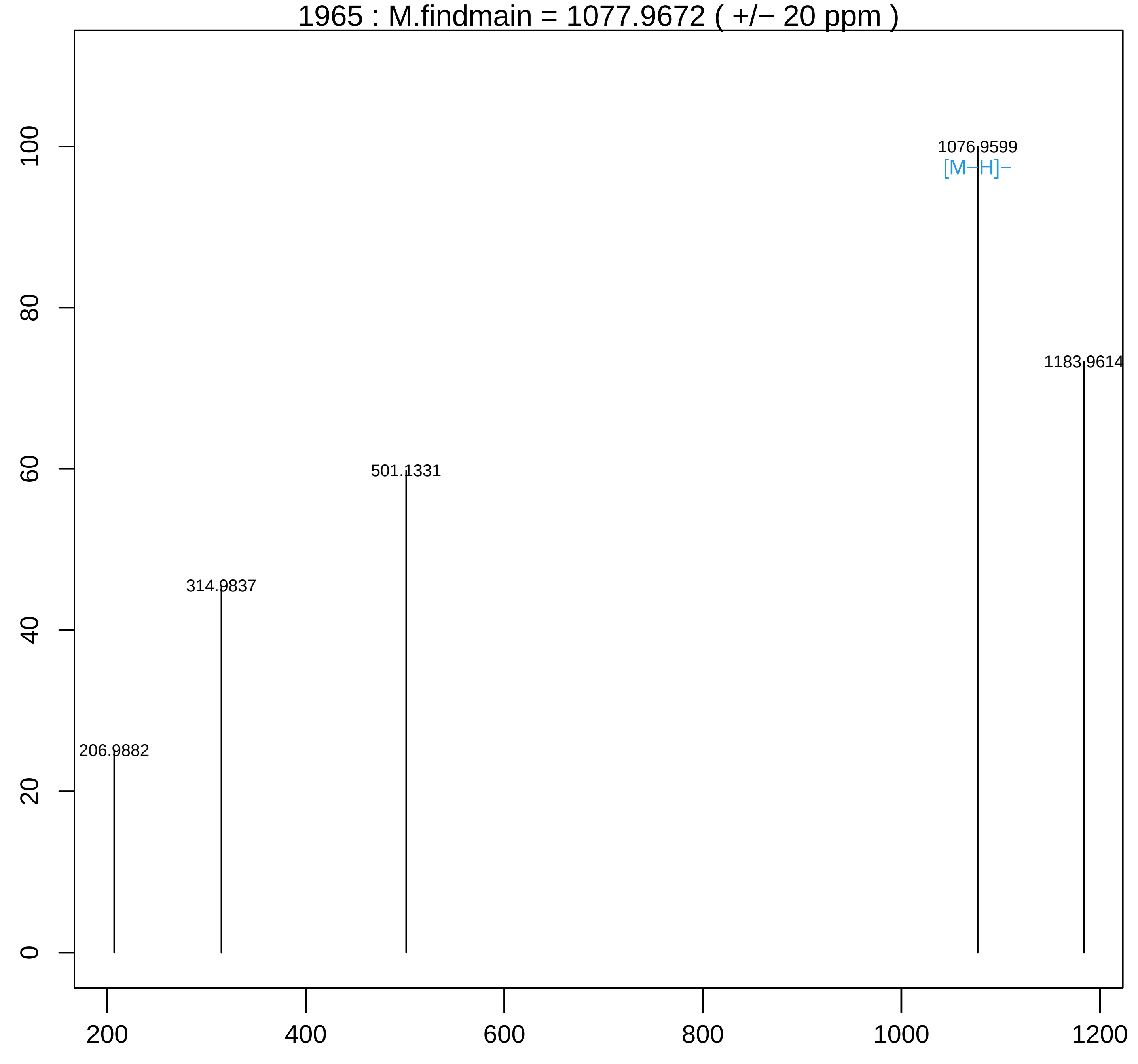

1965 : M.findmain = 1077.9672 (+/− 20 ppm)
1076.9599
[M−H]−
1183.9614
501.1331
314.9837
206.9882
0
20
40
60
80
100
200
400
600
800
1000
1200

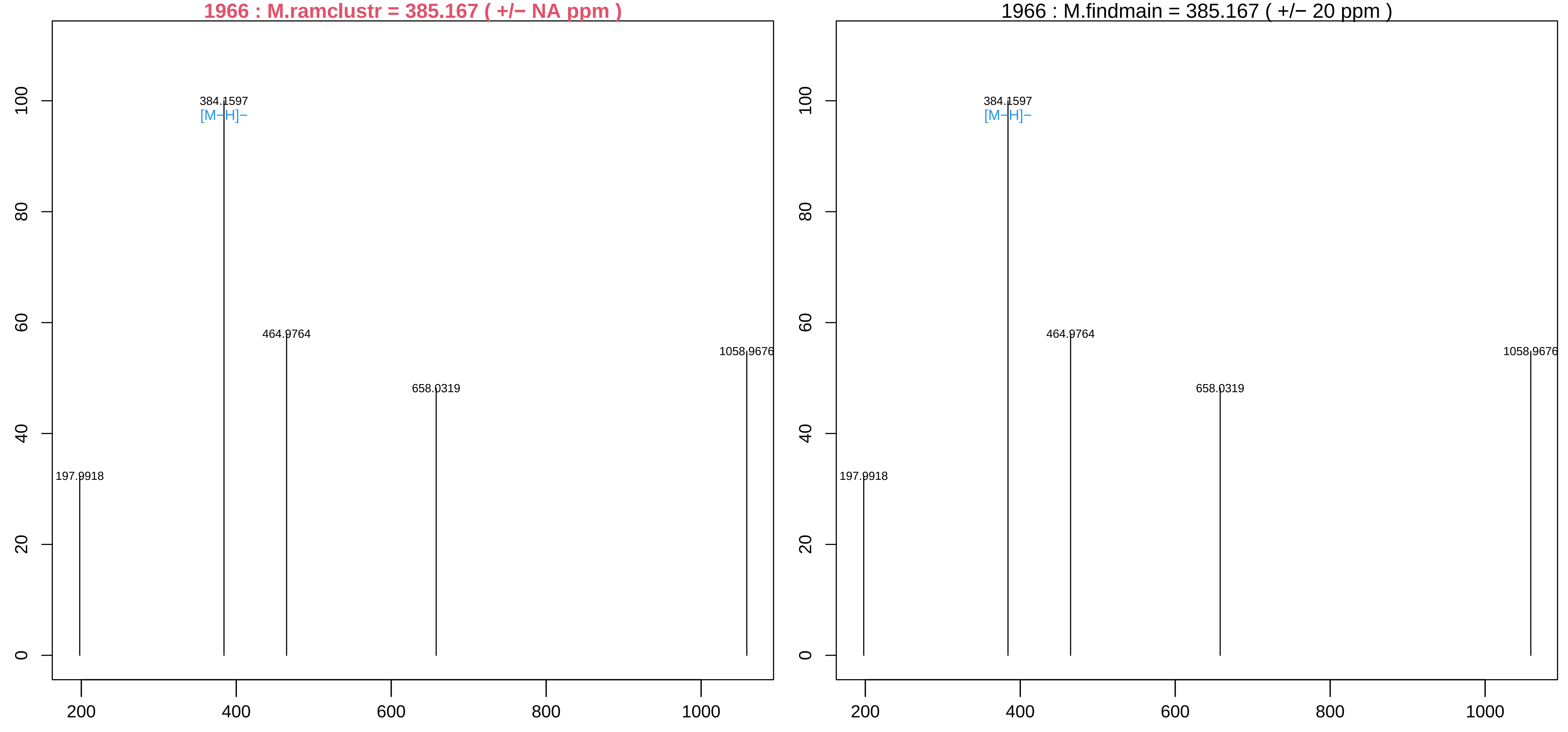
1966 : M.ramclustr = 385.167 (+/− NA ppm)
384.1597
[M−H]−
464.9764
1058.9676
658.0319
197.9918
1966 : M.findmain = 385.167 (+/− 20 ppm)
384.1597
[M−H]−
464.9764
1058.9676
658.0319
197.9918
0
20
40
60
80
100
200
400
600
800
1000

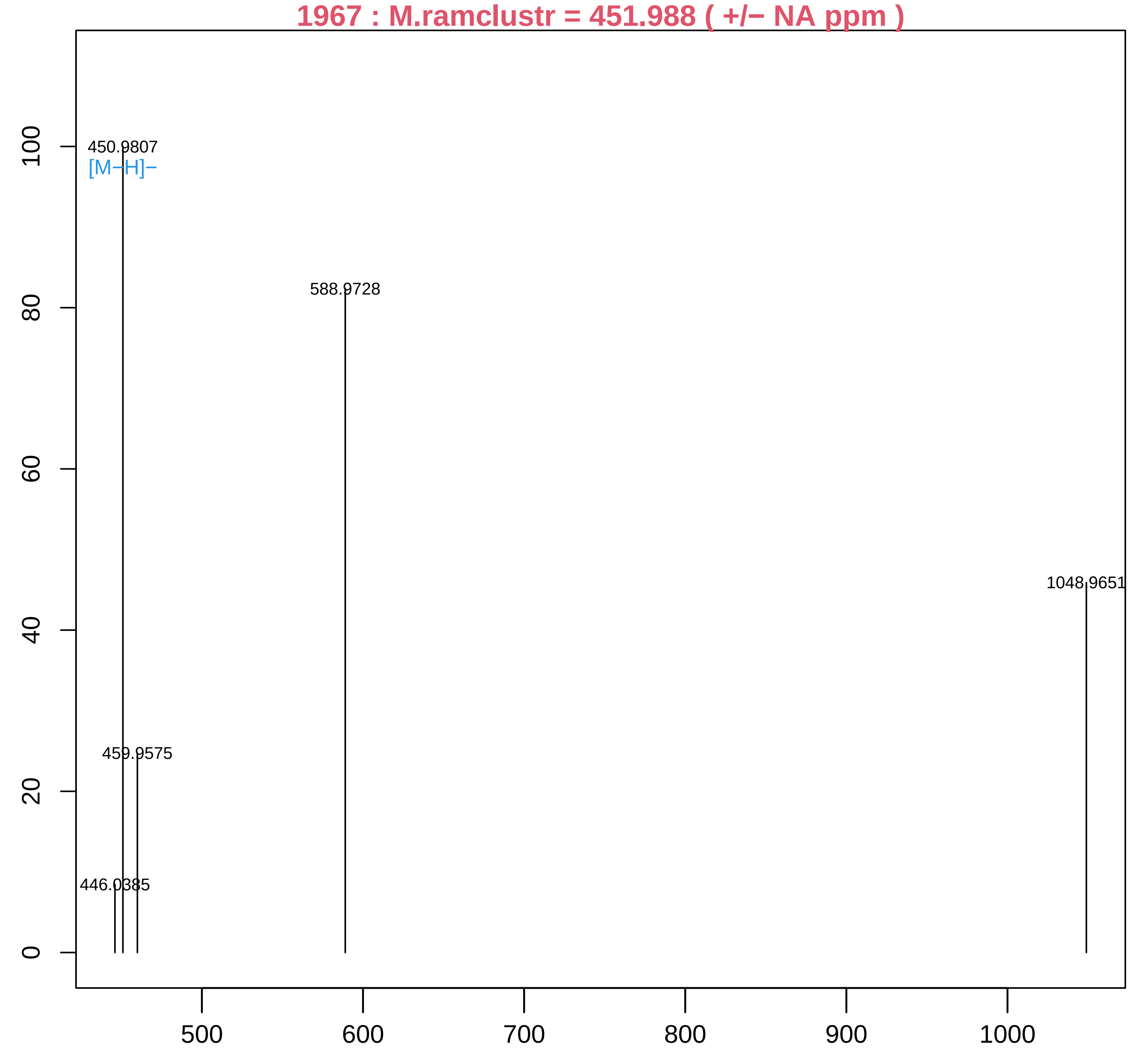
1967 : M.ramclustr = 451.988 (+/− NA ppm)
450.9807
[M−H]−
588.9728
1048.9651
459.9575
446.0385
0
20
40
60
80
100
500
600
700
800
900
1000

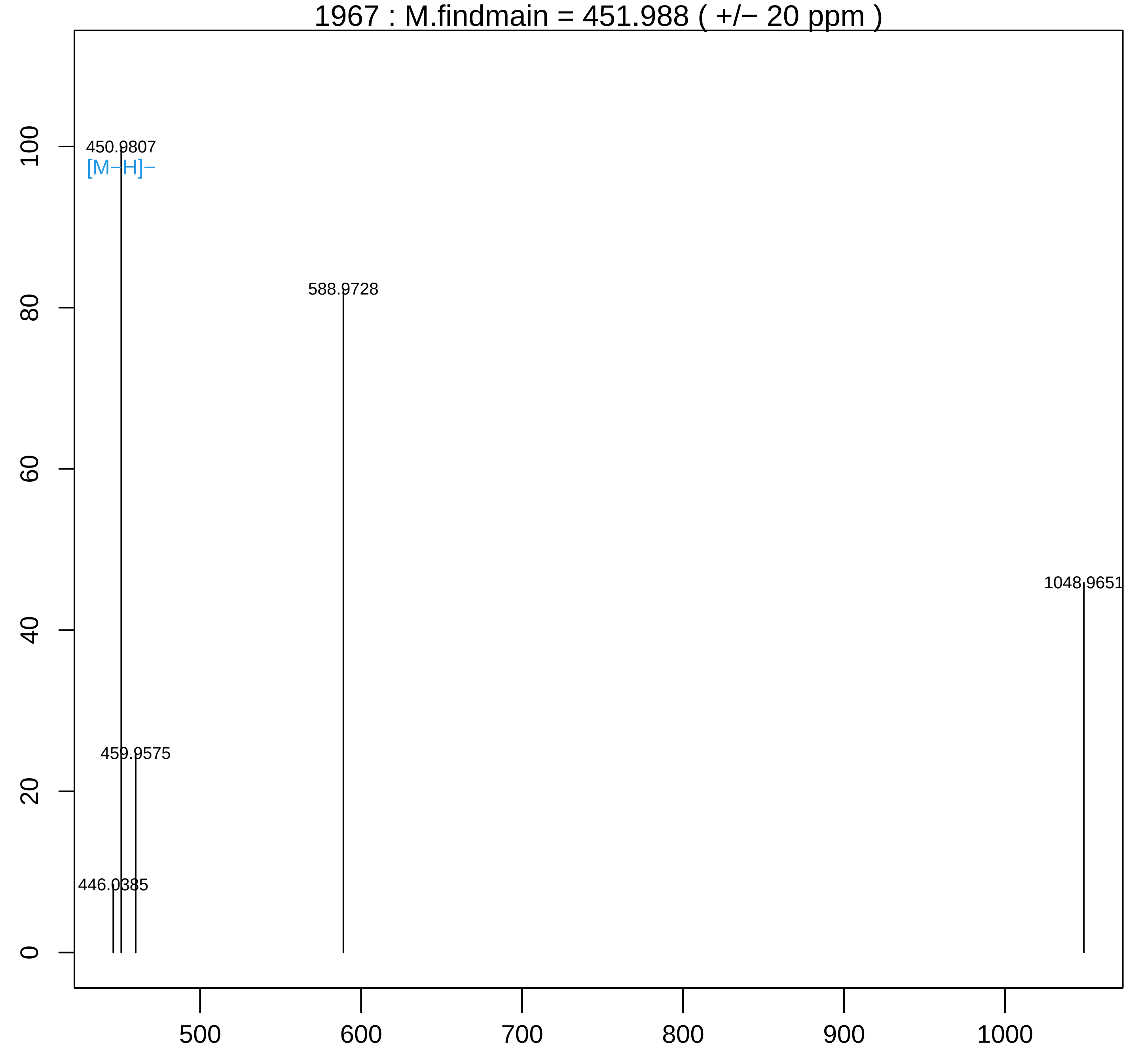
1967 : M.findmain = 451.988 (+/− 20 ppm)
450.9807
[M−H]−
588.9728
1048.9651
459.9575
446.0385
0
20
40
60
80
100
500
600
700
800
900
1000

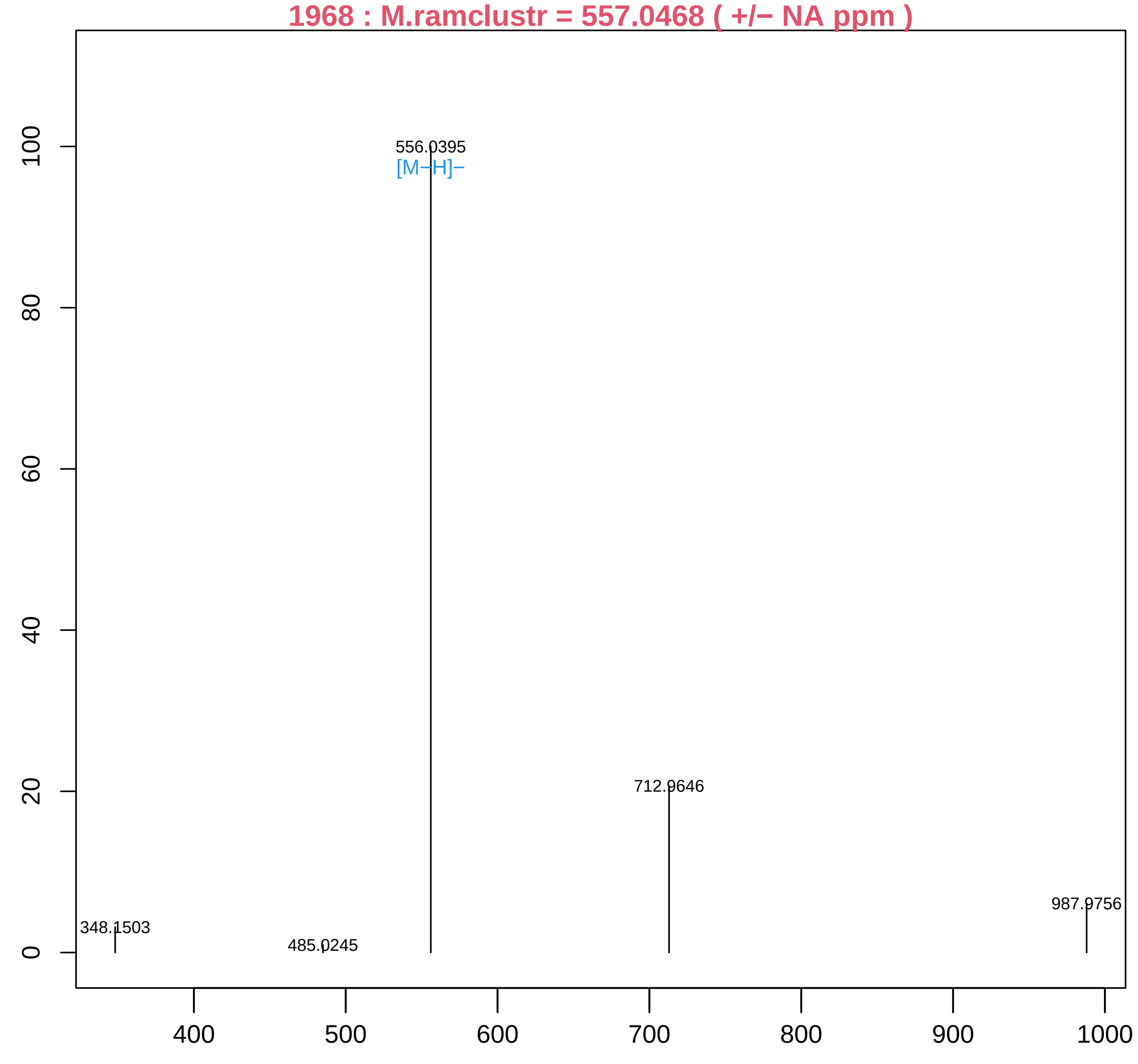
1968 : M.ramclustr = 557.0468 (+/− NA ppm)
556.0395
[M−H]−
712.9646
987.9756
348.1503
485.0245
0
20
40
60
80
100
400
500
600
700
800
900
1000

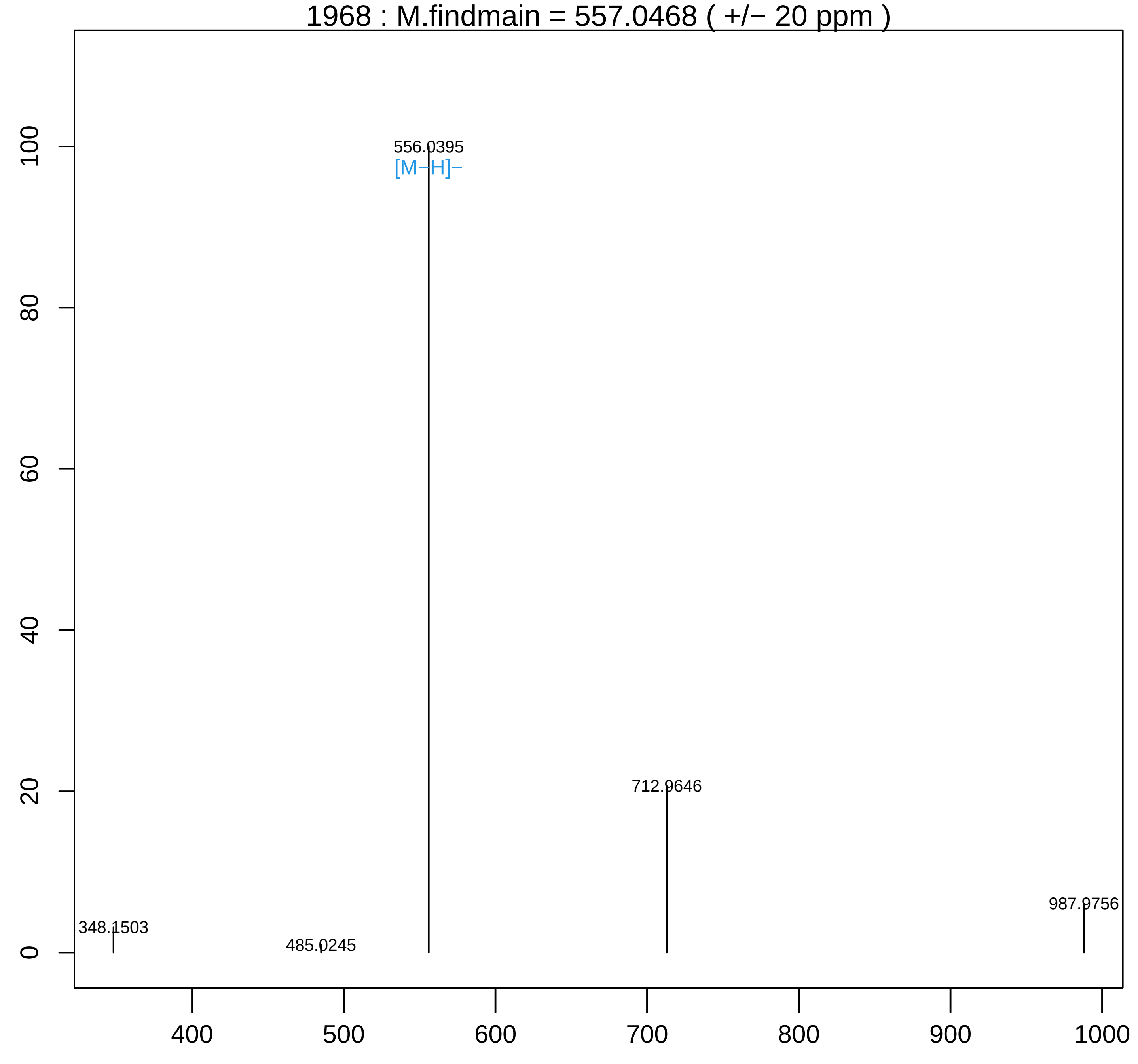
1968 : M.findmain = 557.0468 (+/− 20 ppm)
556.0395
[M−H]−
712.9646
987.9756
348.1503
485.0245
0
20
40
60
80
100
400
500
600
700
800
900
1000

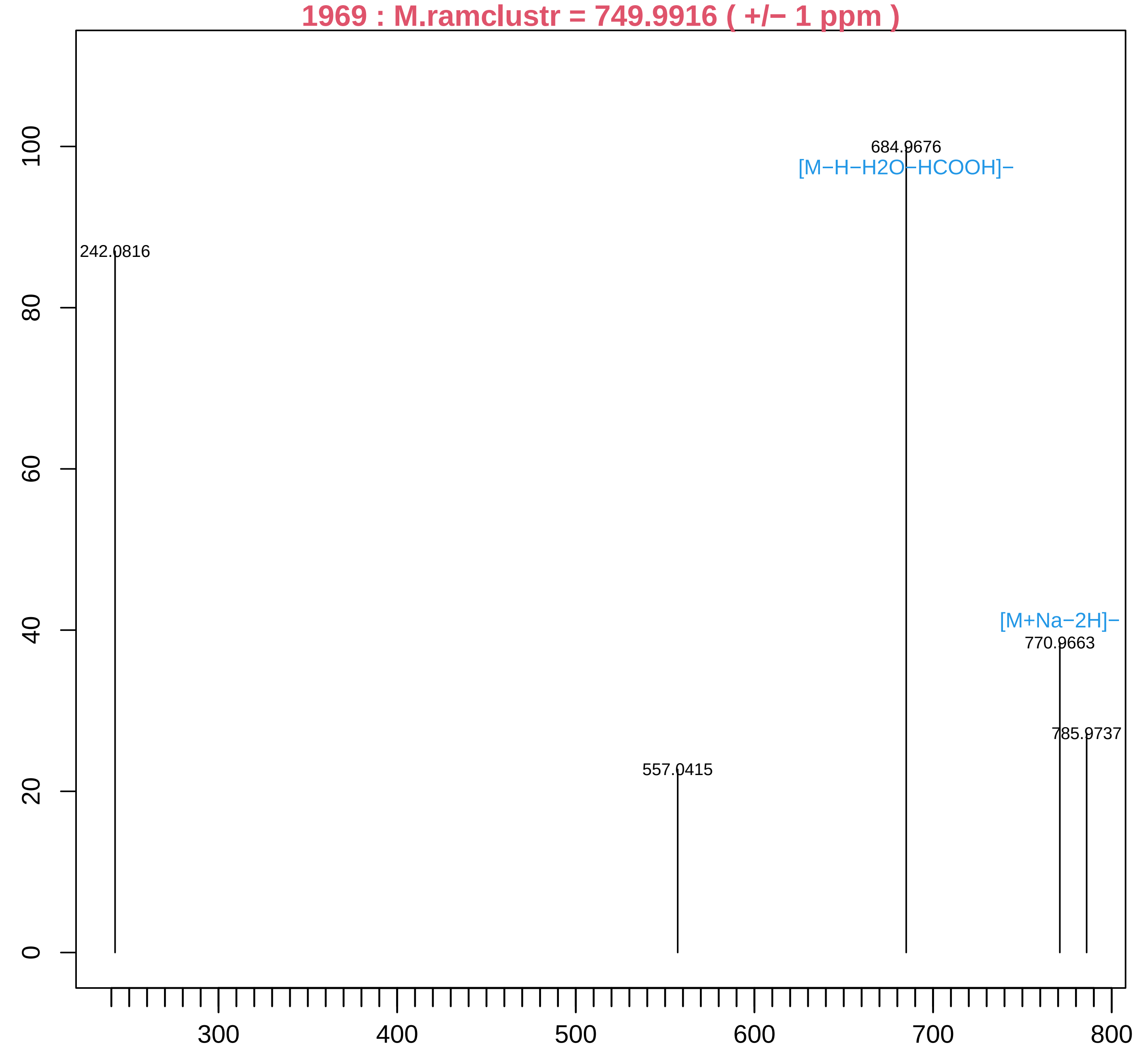
1969 : M.ramclustr = 749.9916 (+/− 1 ppm)
242.0816
557.0415
684.9676
[M−H−H2O−HCOOH]−
[M+Na−2H]−
770.9663
785.9737
0
20
40
60
80
100
300
400
500
600
700
800

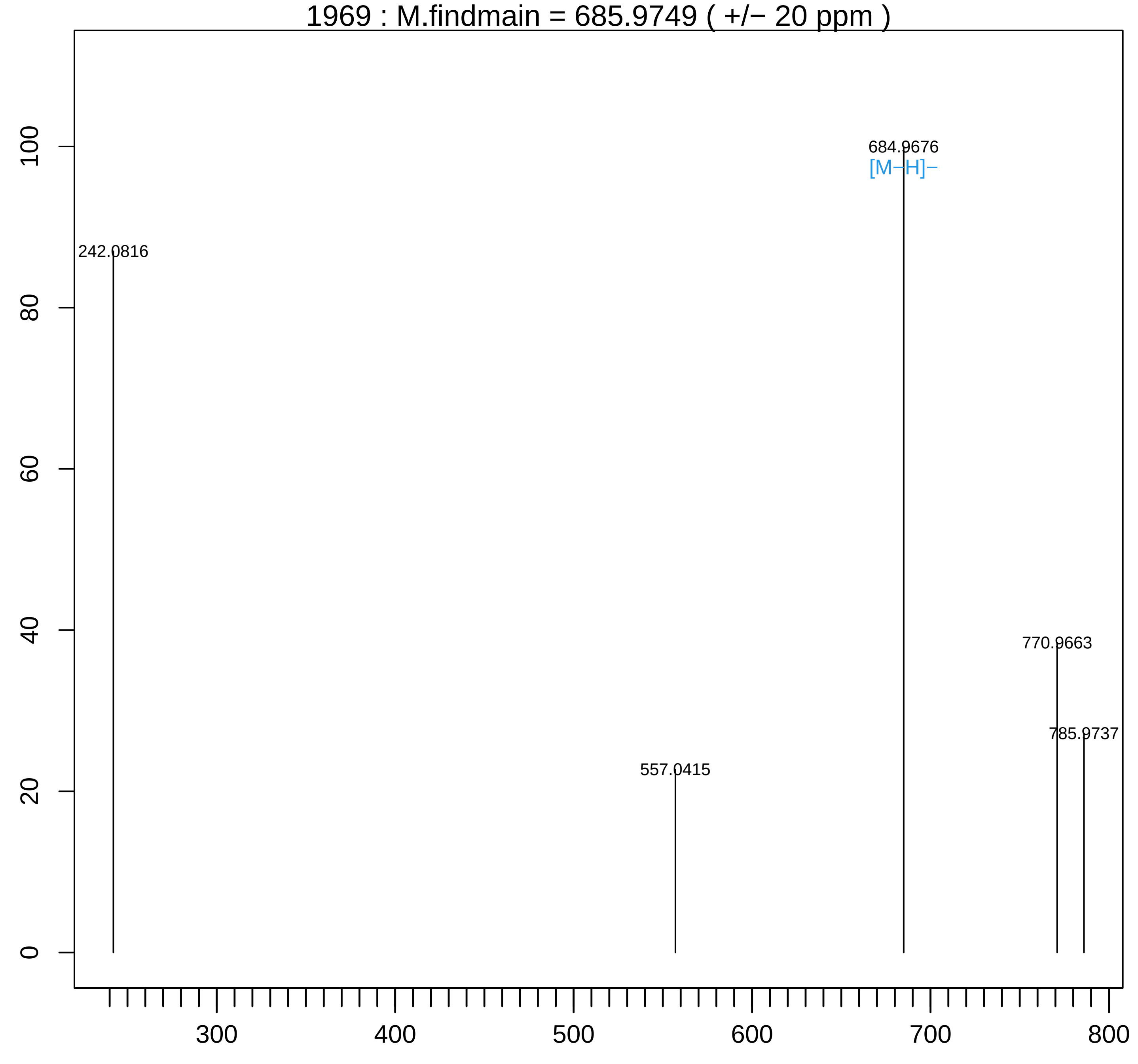
1969 : M.findmain = 685.9749 (+/− 20 ppm)
242.0816
557.0415
684.9676
[M−H]−
770.9663
785.9737
0
20
40
60
80
100
300
400
500
600
700
800

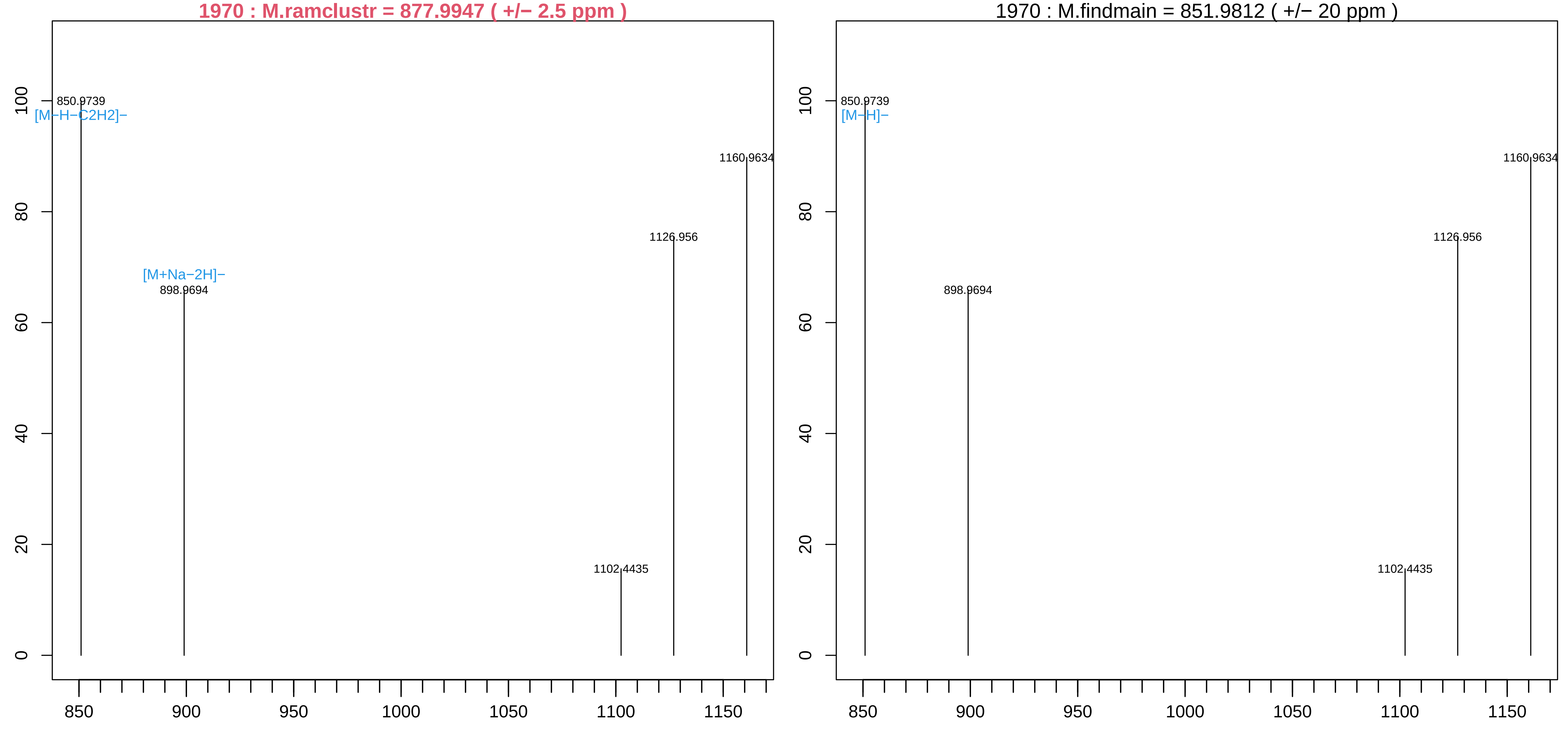

1970 : M.ramclustr = 877.9947 (+/− 2.5 ppm)
850.9739
[M−H−C2H2]−
[M+Na−2H]−
898.9694
1102.4435
1126.956
1160.9634
1970 : M.findmain = 851.9812 (+/− 20 ppm)
850.9739
[M−H]−
898.9694
1102.4435
1126.956
1160.9634

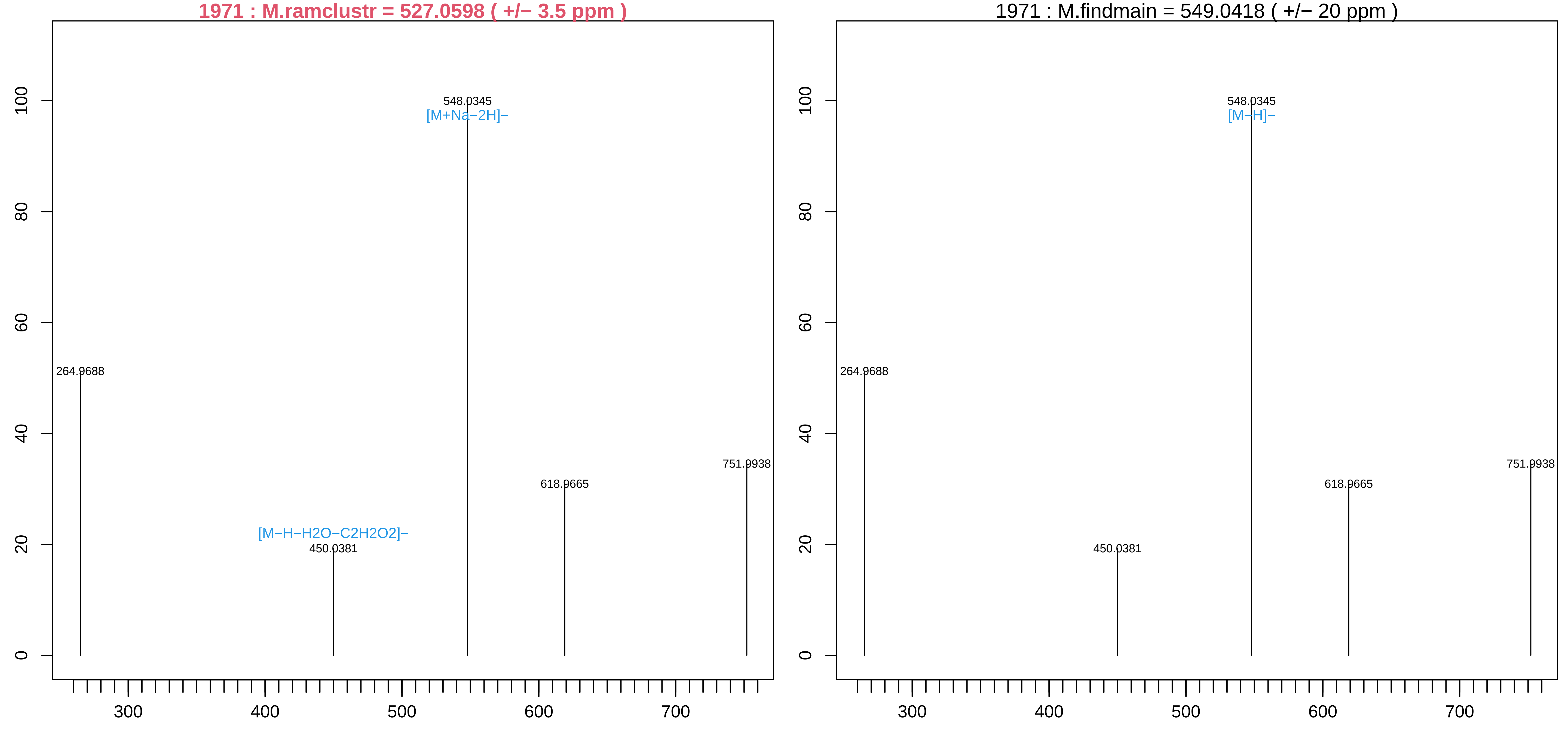
1971 : M.ramclustr = 527.0598 (+/− 3.5 ppm)
548.0345
[M+Na−2H]−
264.9688
[M−H−H2O−C2H2O2]−
450.0381
618.9665
751.9938
1971 : M.findmain = 549.0418 (+/− 20 ppm)
548.0345
[M−H]−
264.9688
450.0381
618.9665
751.9938

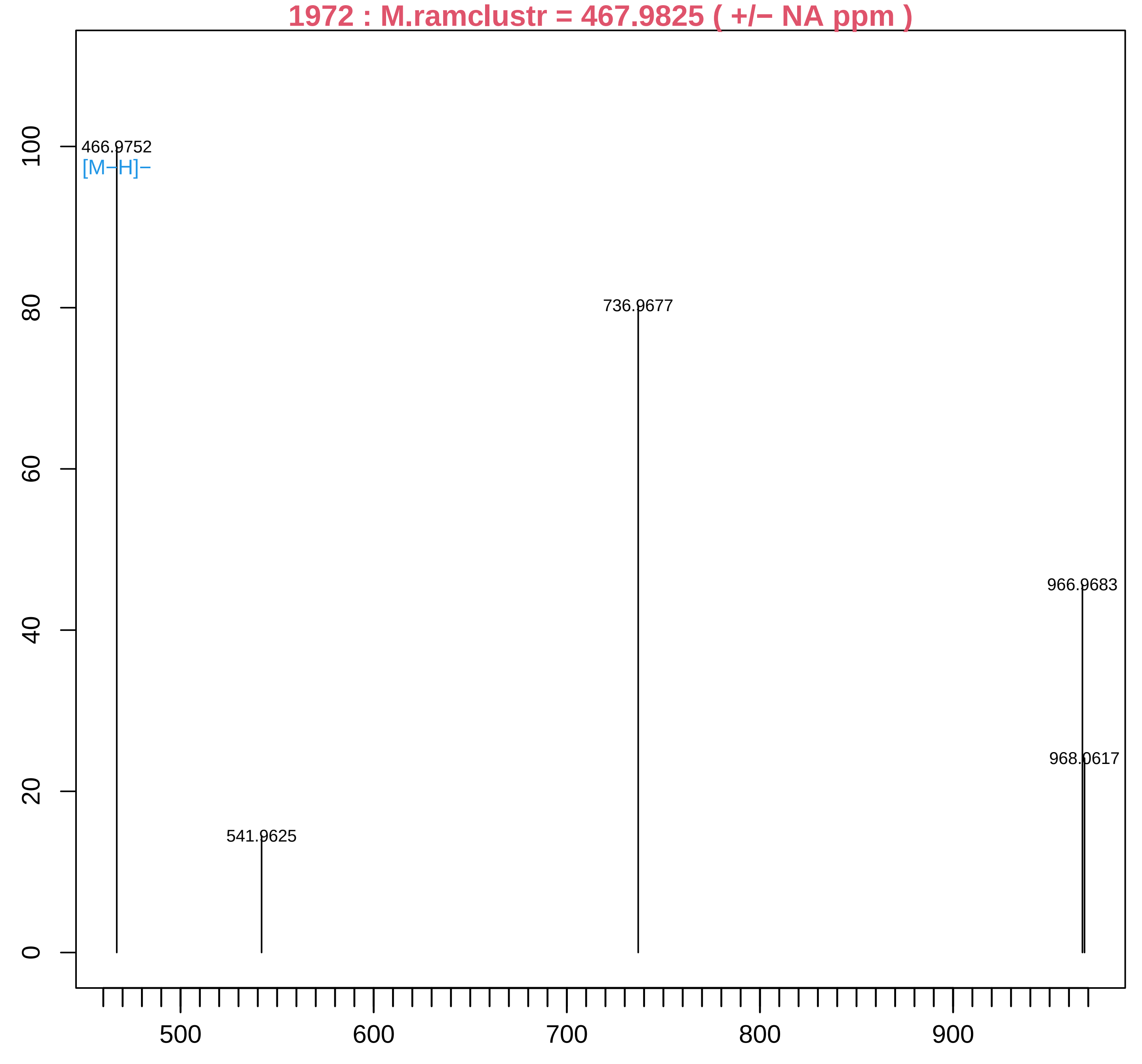
1972 : M.ramclustr = 467.9825 (+/− NA ppm)
466.9752
[M−H]−
736.9677
966.9683
968.0617
541.9625
0
20
40
60
80
100
500
600
700
800
900
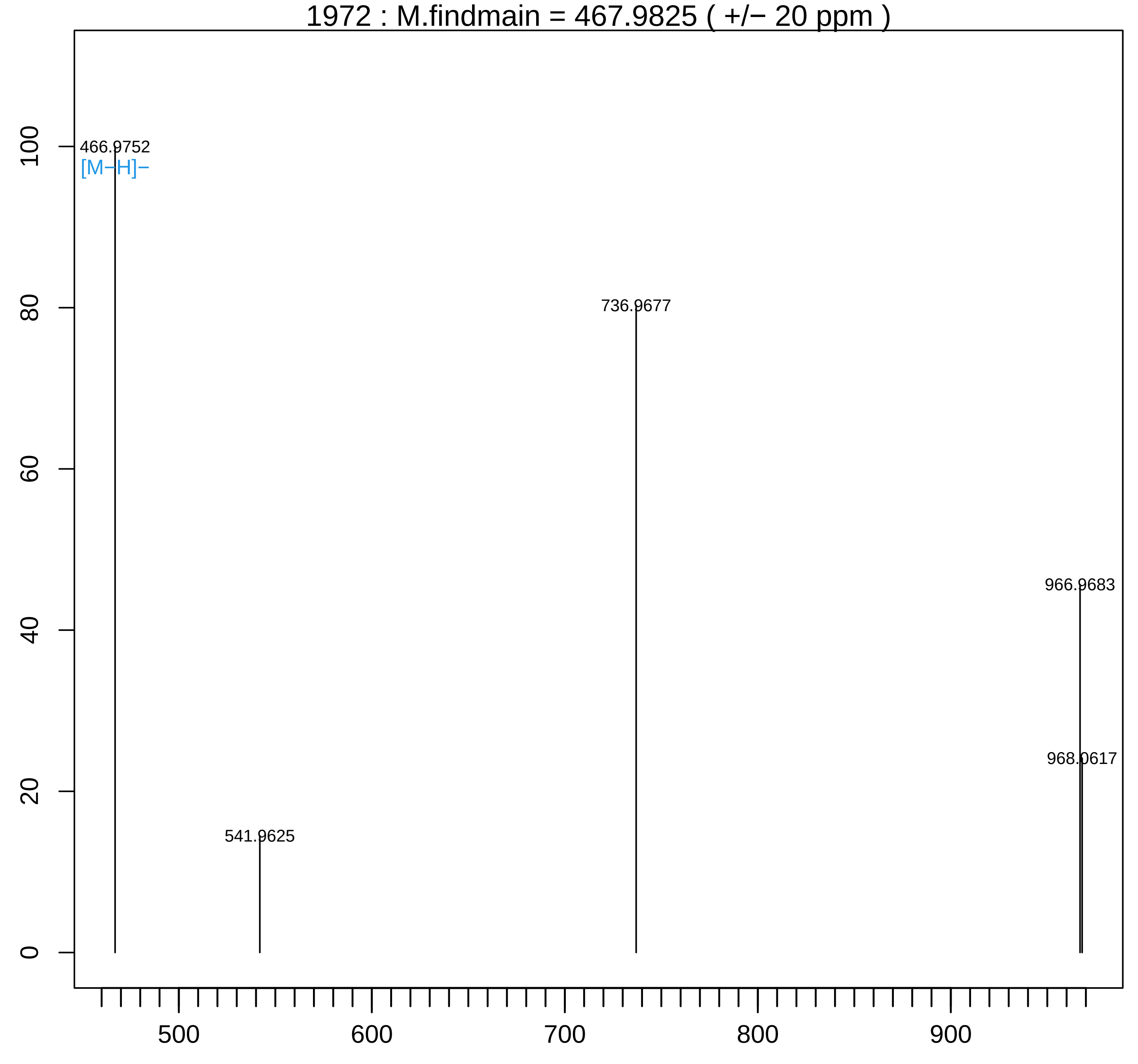
1972 : M.findmain = 467.9825 (+/− 20 ppm)
466.9752
[M−H]−
736.9677
966.9683
968.0617
541.9625
0
20
40
60
80
100
500
600
700
800
900

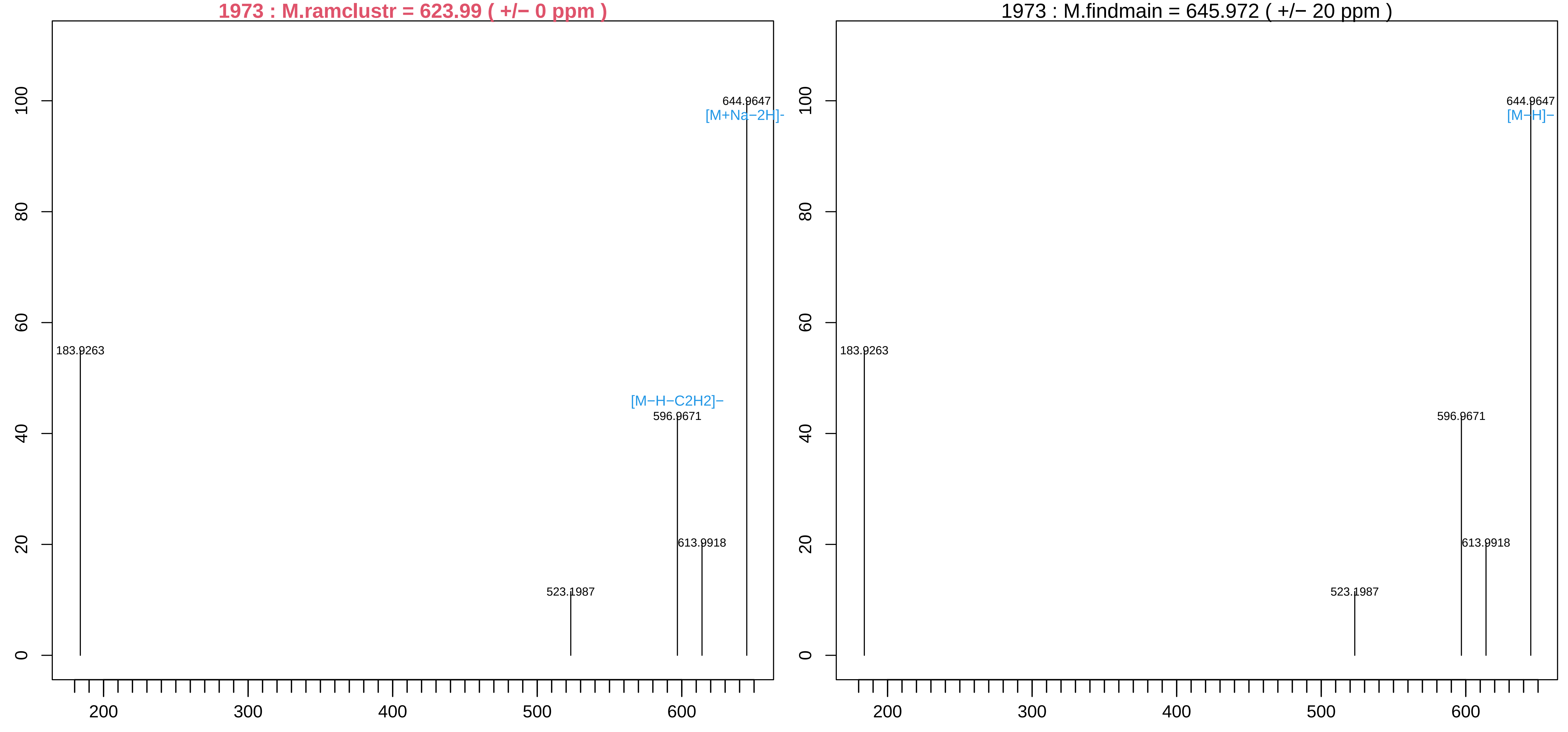
1973 : M.ramclustr = 623.99 (+/− 0 ppm)
644.9647
[M+Na−2H]-
183.9263
[M−H−C2H2]−
596.9671
613.9918
523.1987
0
20
40
60
80
100
200
300
400
500
600
1973 : M.findmain = 645.972 (+/− 20 ppm)
644.9647
[M−H]−
183.9263
596.9671
613.9918
523.1987

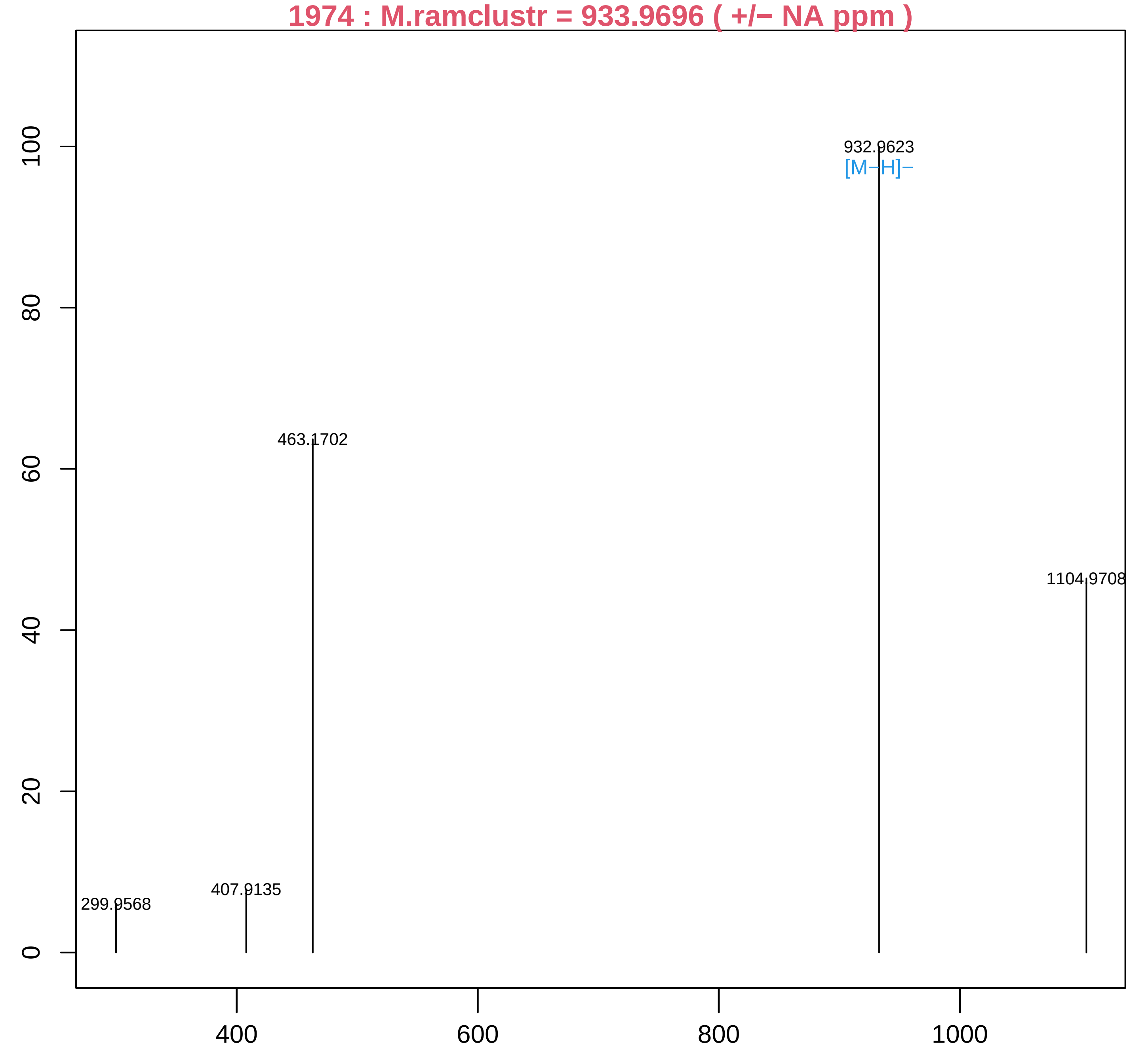
1974 : M.ramclustr = 933.9696 (+/− NA ppm)
932.9623
[M−H]−
463.1702
1104.9708
407.9135
299.9568
0
20
40
60
80
100
400
600
800
1000

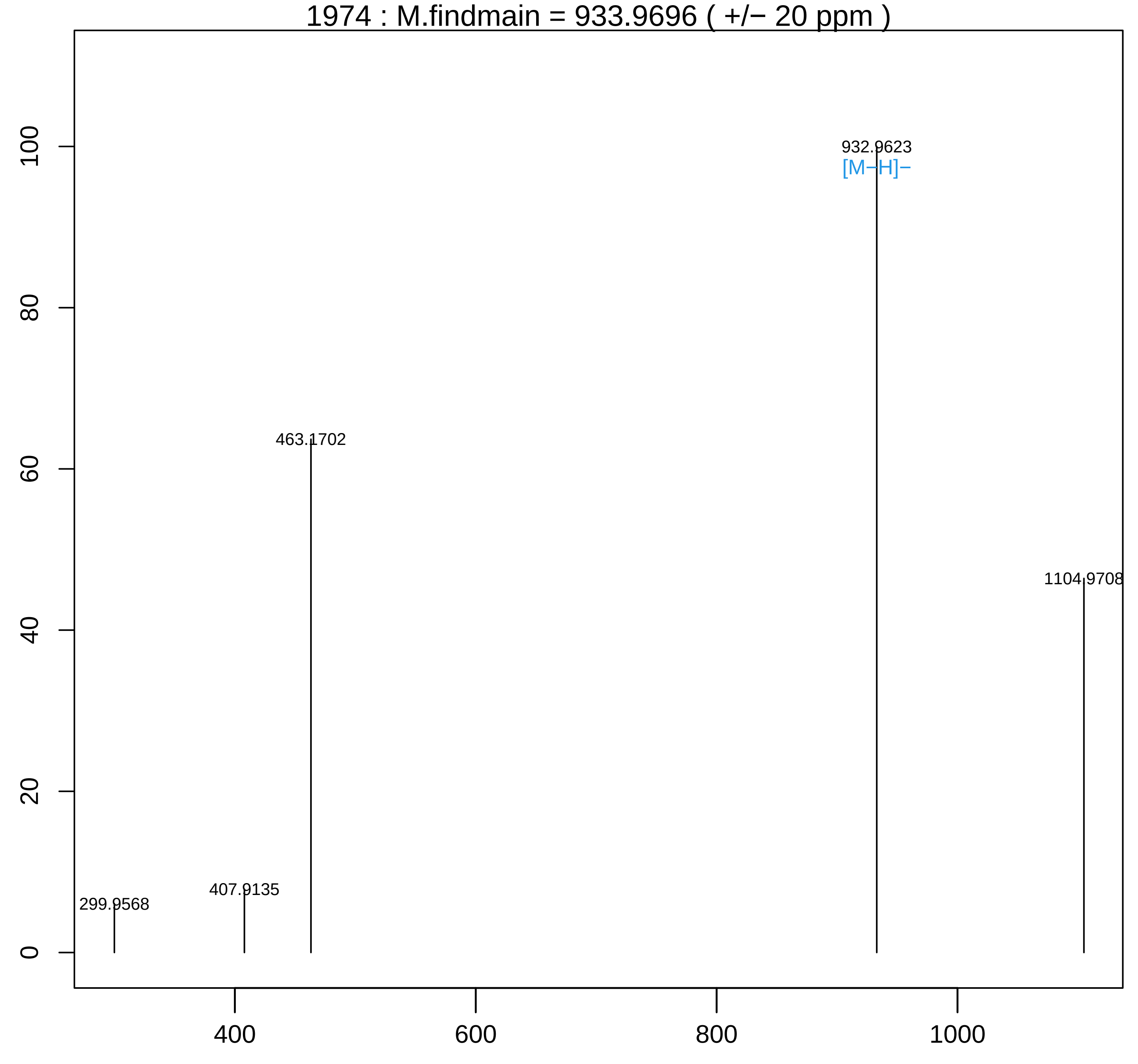
1974 : M.findmain = 933.9696 (+/− 20 ppm)
932.9623
[M−H]−
463.1702
1104.9708
407.9135
299.9568
0
20
40
60
80
100
400
600
800
1000

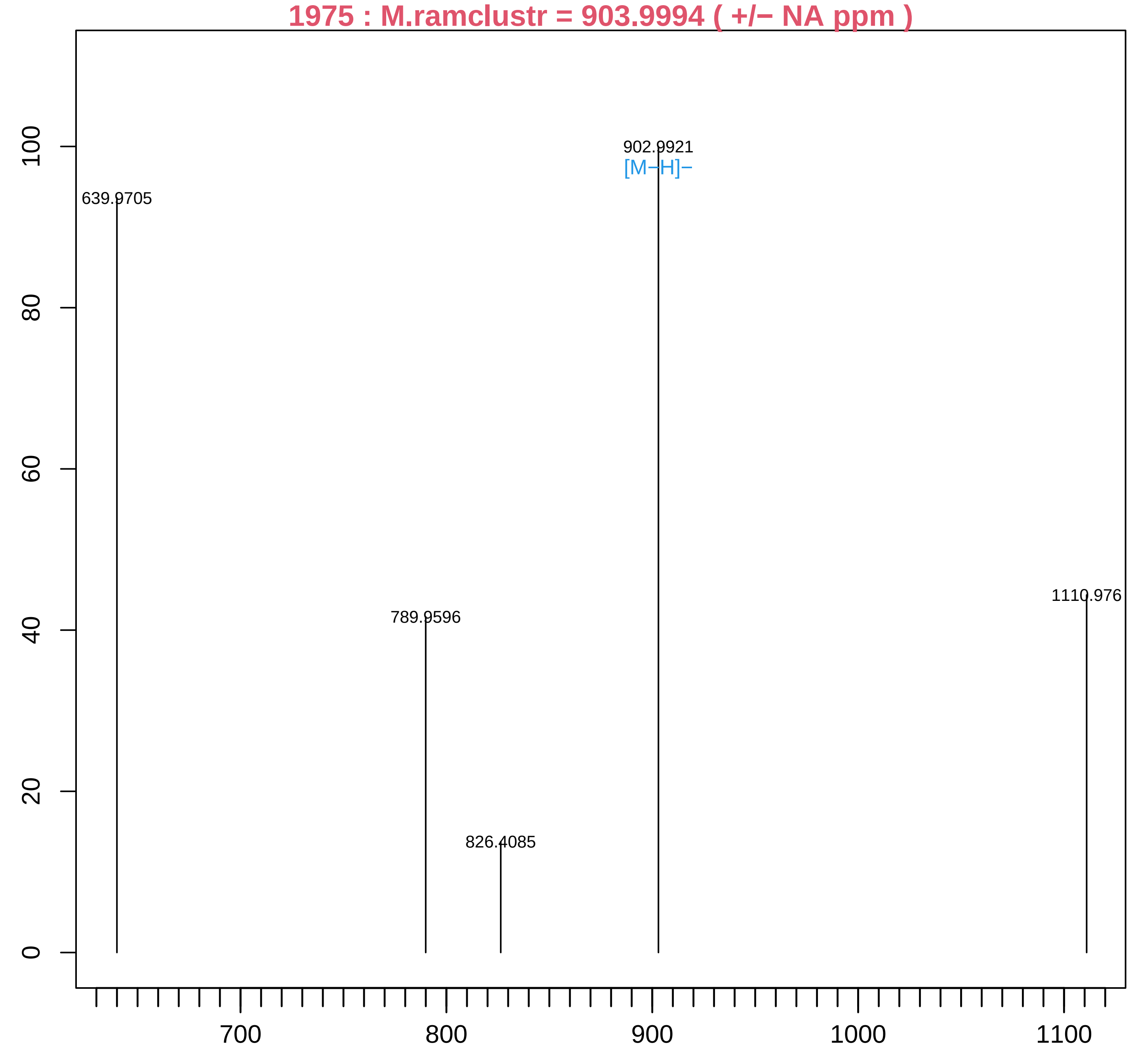

1975 : M.ramclustr = 903.9994 (+/− NA ppm)
639.9705
789.9596
826.4085
902.9921
[M−H]−
1110.976
0
20
40
60
80
100
700
800
900
1000
1100

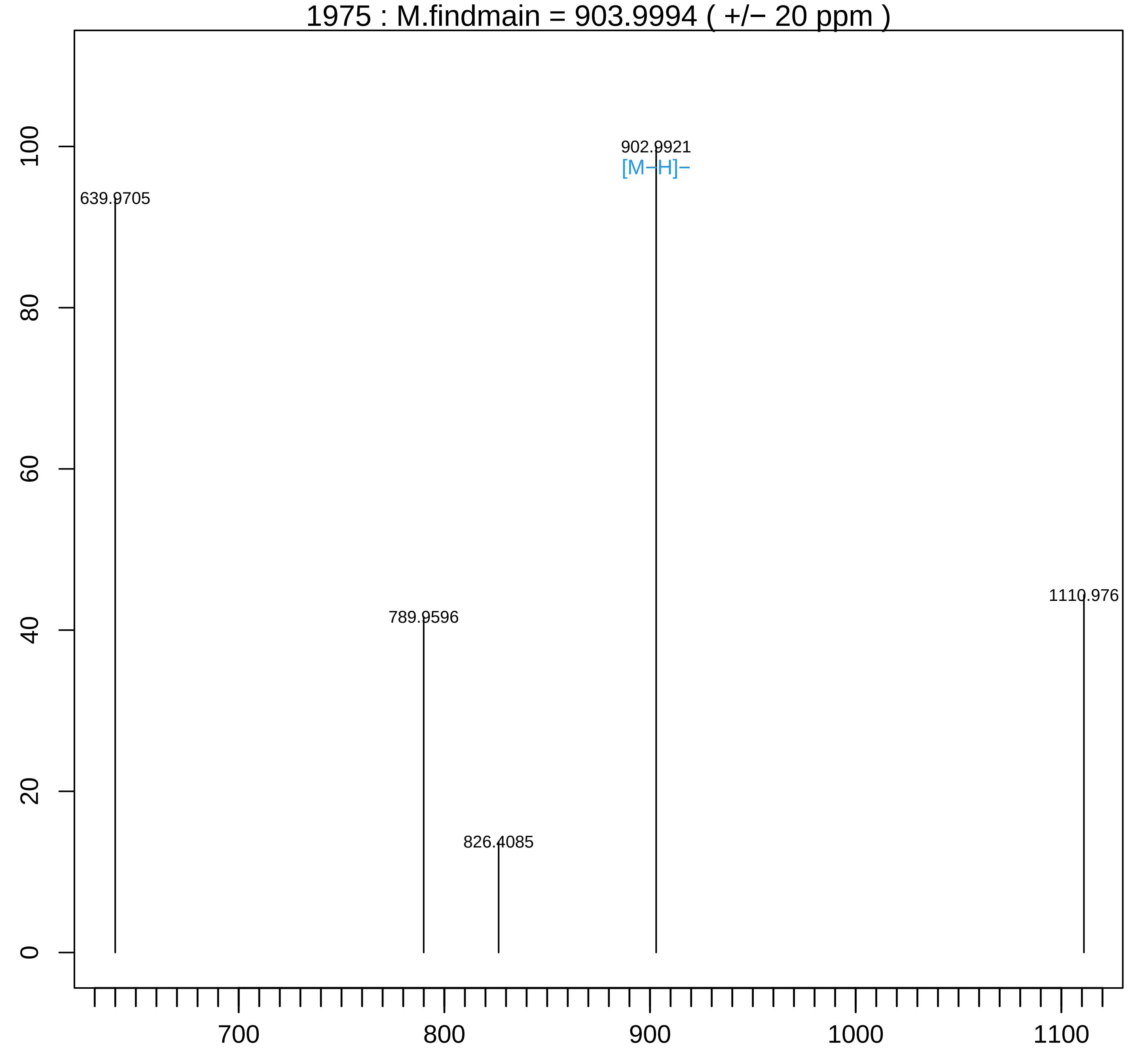

1975 : M.findmain = 903.9994 (+/− 20 ppm)
639.9705
789.9596
826.4085
902.9921
[M−H]−
1110.976
0
20
40
60
80
100
700
800
900
1000
1100

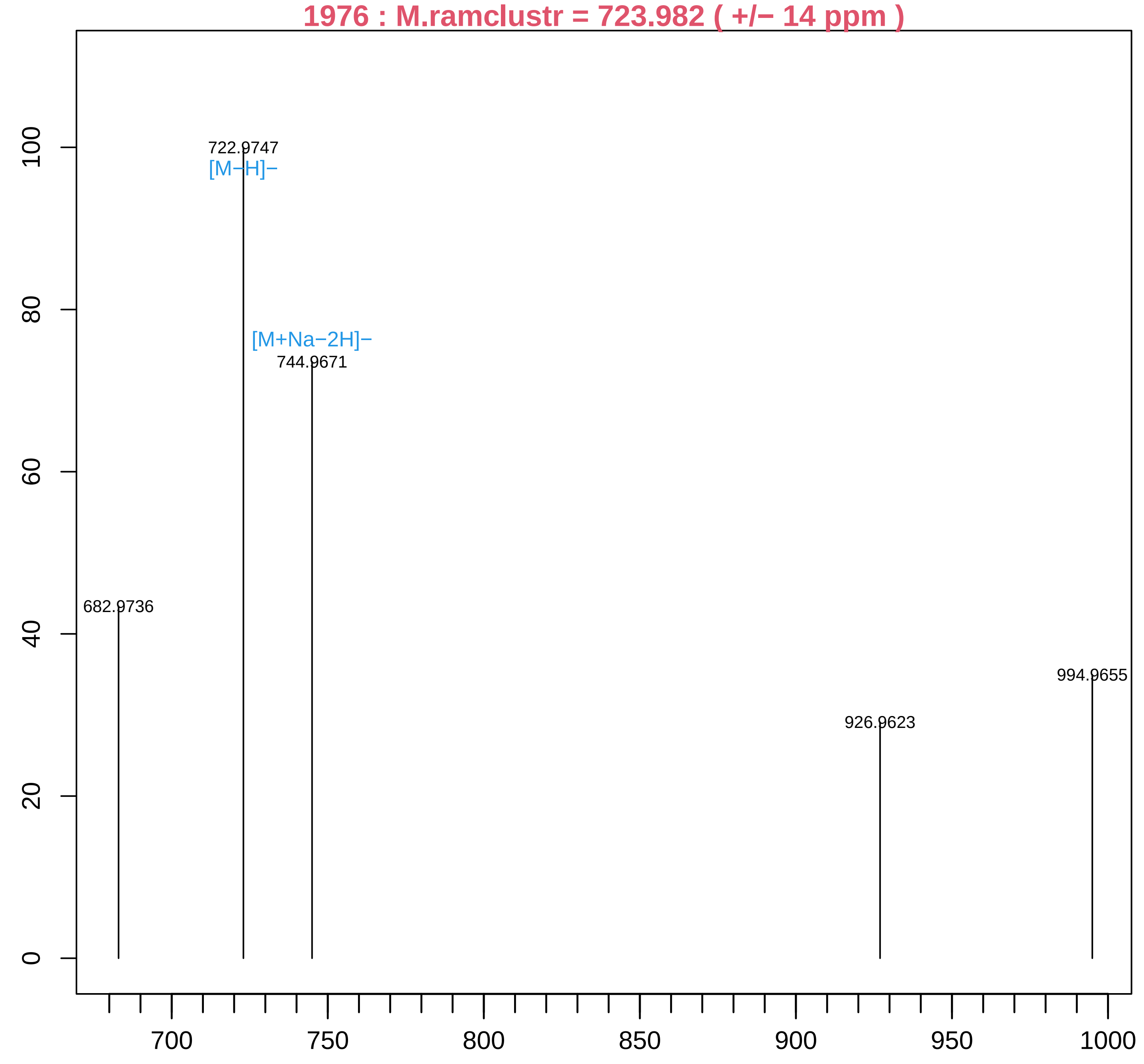
1976 : M.ramclustr = 723.982 (+/− 14 ppm)
722.9747
[M−H]−
[M+Na−2H]−
744.9671
682.9736
994.9655
926.9623
0
20
40
60
80
100
700
750
800
850
900
950
1000

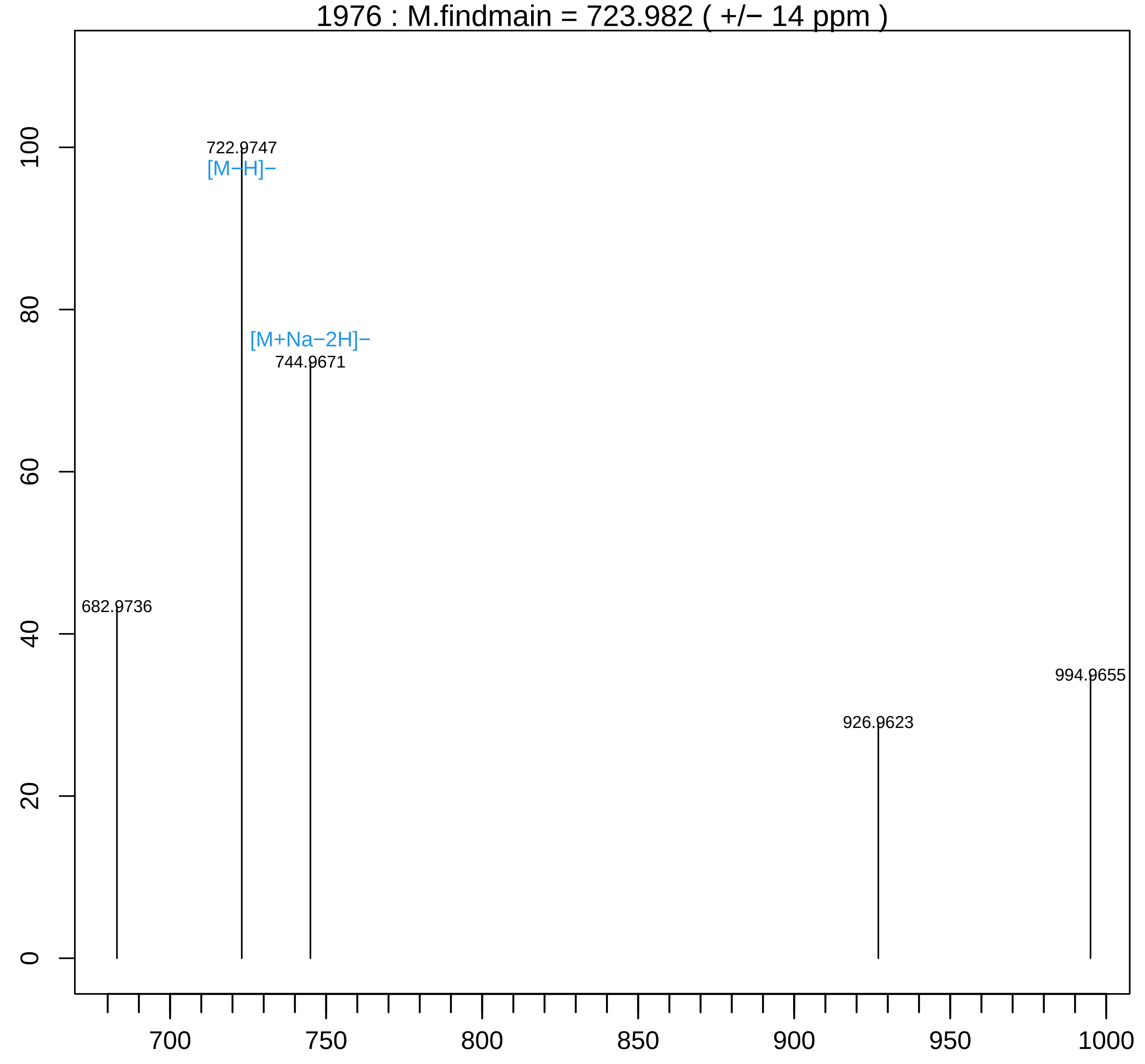
1976 : M.findmain = 723.982 (+/− 14 ppm)
722.9747
[M−H]−
[M+Na−2H]−
744.9671
682.9736
994.9655
926.9623
0
20
40
60
80
100
700
750
800
850
900
950
1000

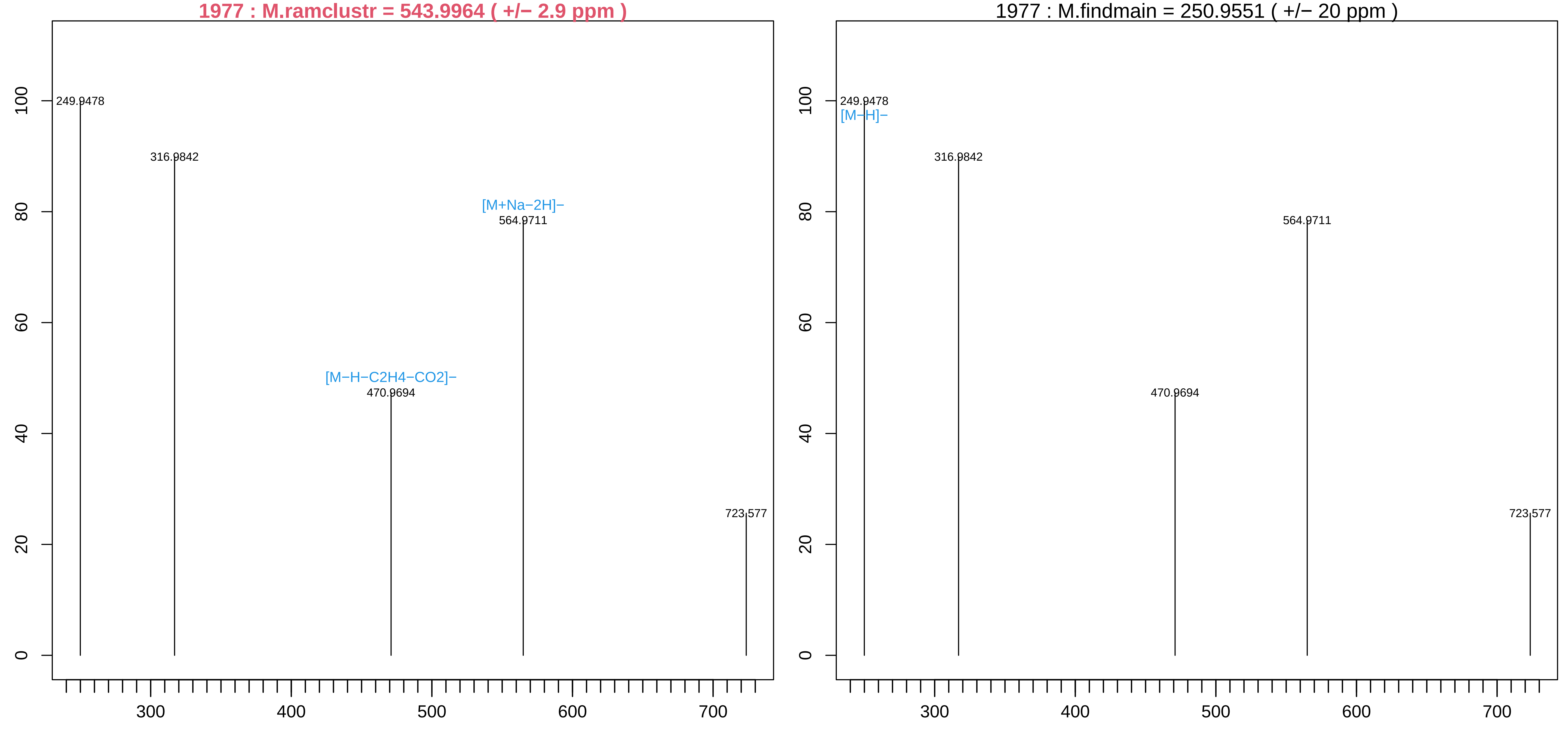
1977 : M.ramclustr = 543.9964 (+/− 2.9 ppm)
249.9478
316.9842
[M+Na−2H]−
564.9711
[M−H−C2H4−CO2]−
470.9694
723.577
1977 : M.findmain = 250.9551 (+/− 20 ppm)
249.9478
[M−H]−
316.9842
564.9711
470.9694
723.577
0
20
40
60
80
100
300
400
500
600
700

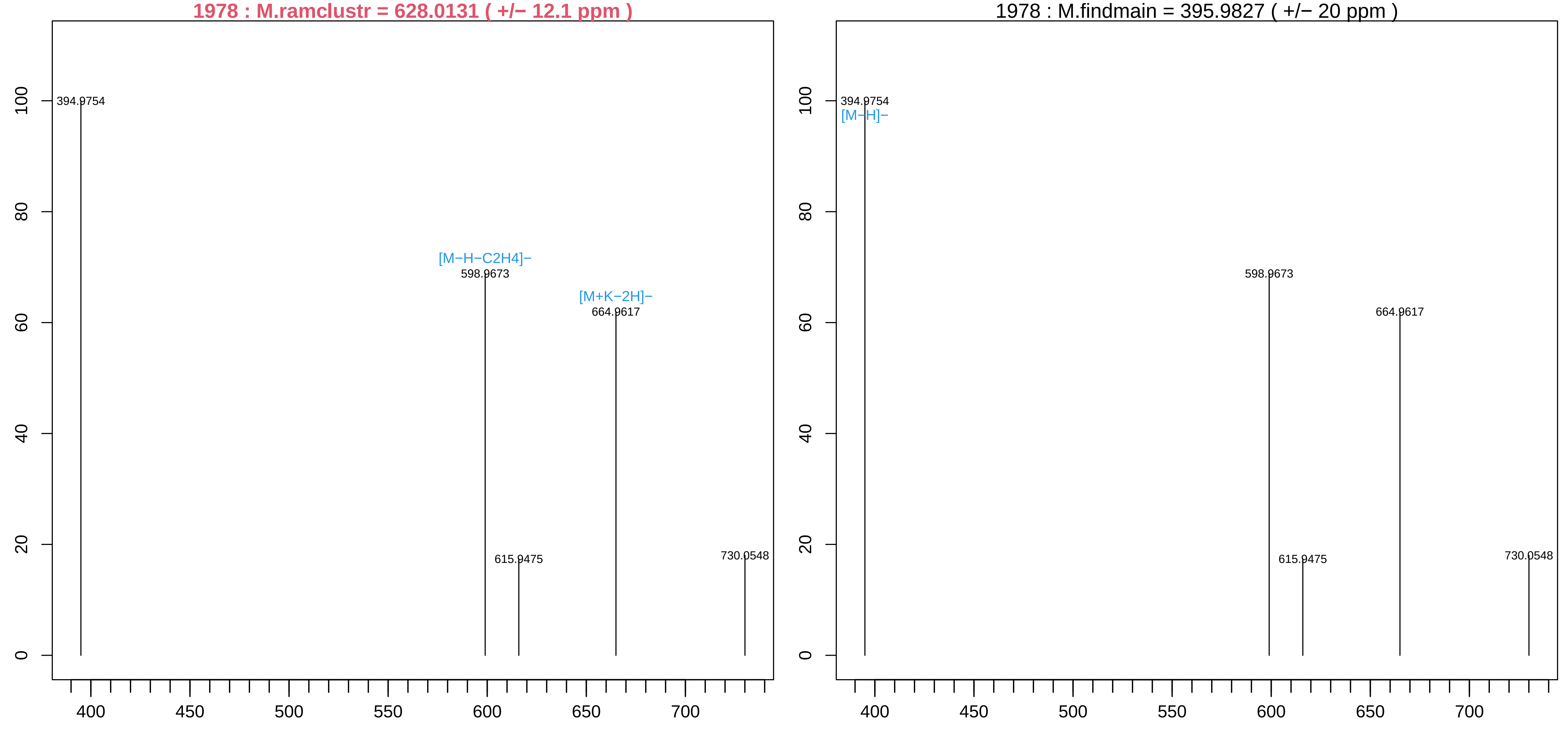
1978 : M.ramclustr = 628.0131 (+/− 12.1 ppm)
394.9754
[M−H−C2H4]−
598.9673
615.9475
[M+K−2H]−
664.9617
730.0548
0
20
40
60
80
100
400
450
500
550
600
650
700
1978 : M.findmain = 395.9827 (+/− 20 ppm)
394.9754
[M−H]−
598.9673
615.9475
664.9617
730.0548
0
20
40
60
80
100
400
450
500
550
600
650
700

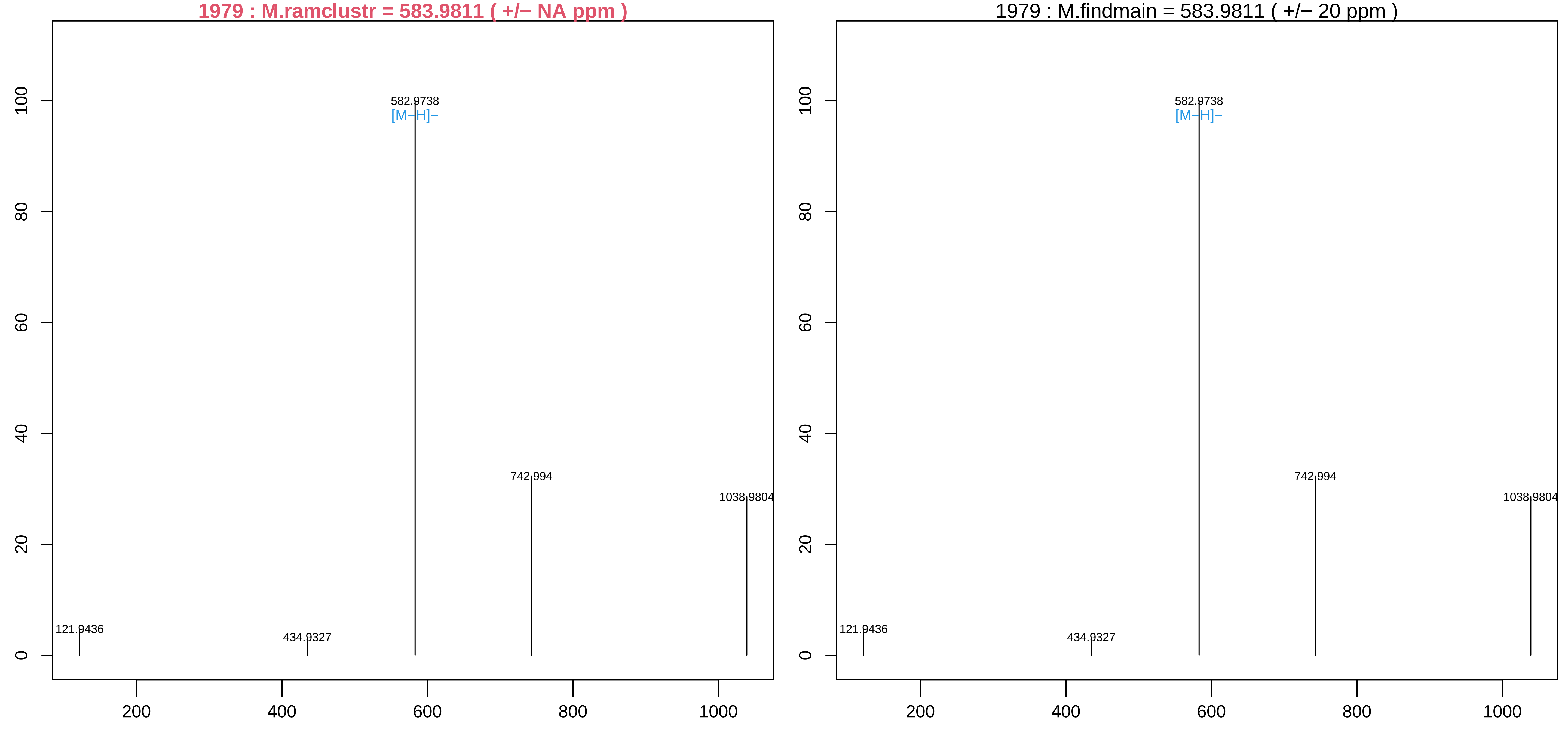
1979 : M.ramclustr = 583.9811 (+/− NA ppm)
582.9738
[M−H]−
742.994
1038.9804
121.9436
434.9327
0
20
40
60
80
100
200
400
600
800
1000
1979 : M.findmain = 583.9811 (+/− 20 ppm)
582.9738
[M−H]−
742.994
1038.9804
121.9436
434.9327
0
20
40
60
80
100
200
400
600
800
1000

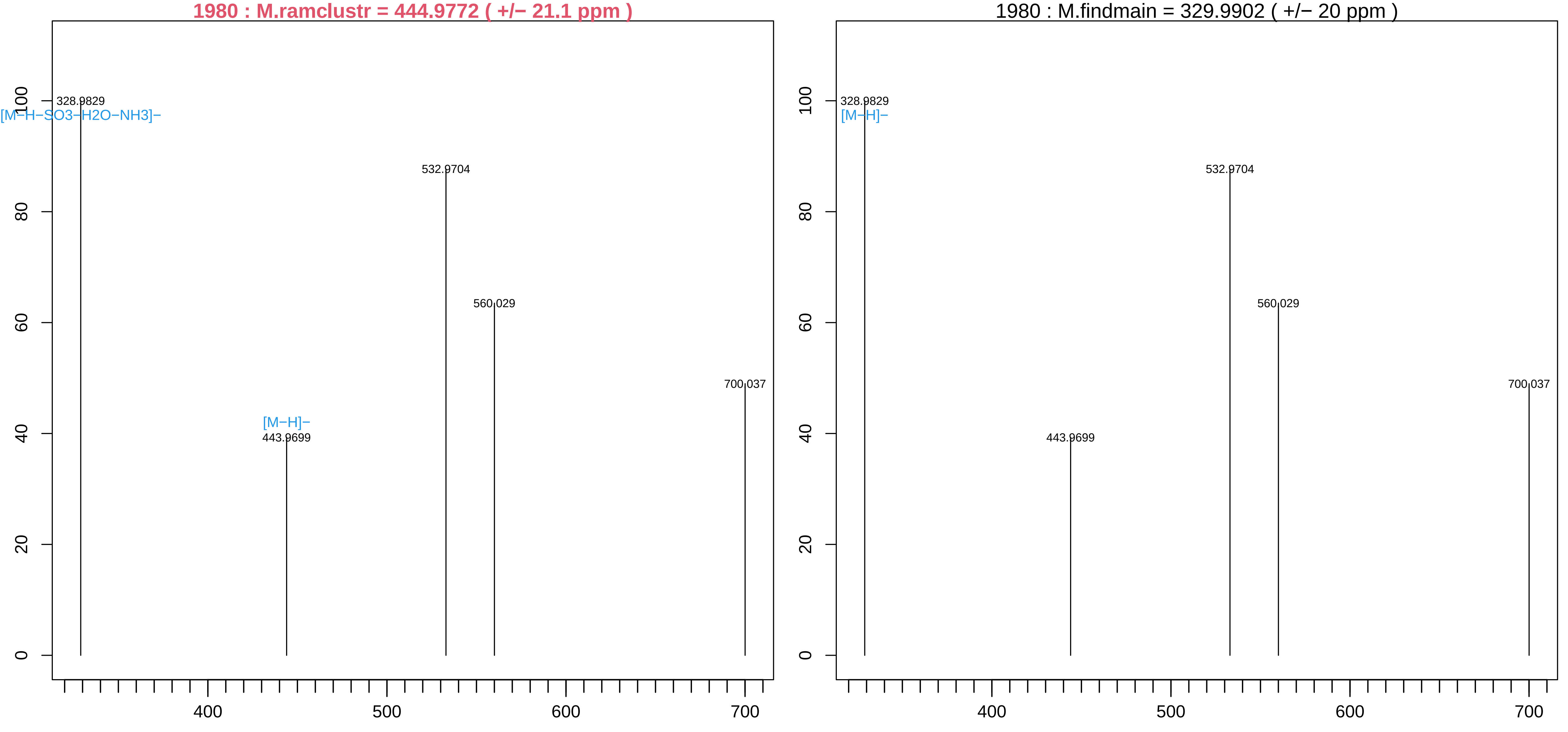
1980 : M.ramclustr = 444.9772 (+/− 21.1 ppm)
328.9829
[M−H−SO3−H2O−NH3]−
532.9704
560.029
700.037
[M−H]−
443.9699
0
20
40
60
80
100
400
500
600
700
1980 : M.findmain = 329.9902 (+/− 20 ppm)
328.9829
[M−H]−
532.9704
560.029
700.037
443.9699
0
20
40
60
80
100
400
500
600
700

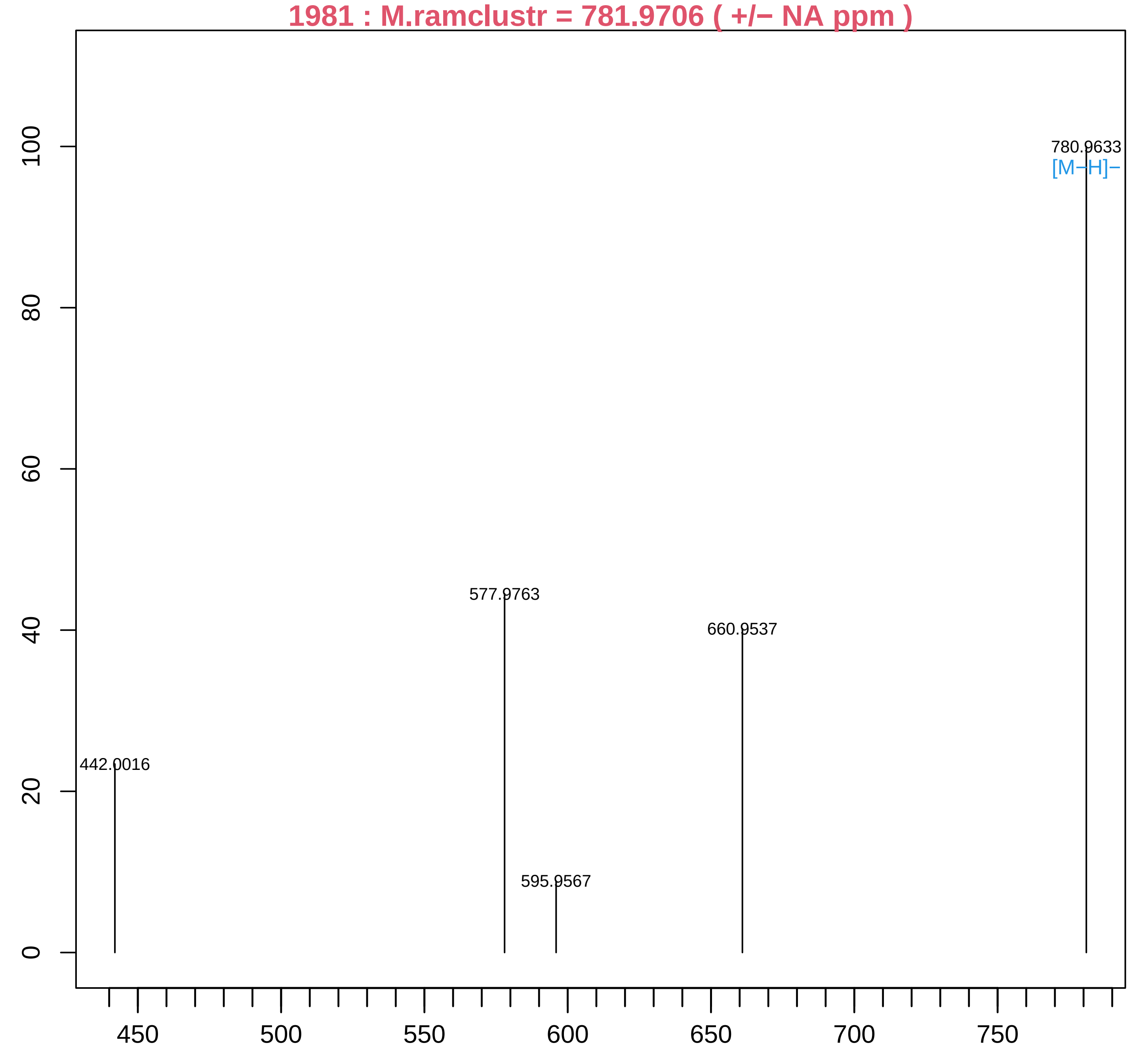
1981 : M.ramclustr = 781.9706 (+/− NA ppm)
780.9633
[M−H]−
577.9763
660.9537
442.0016
595.9567
0
20
40
60
80
100
450
500
550
600
650
700
750

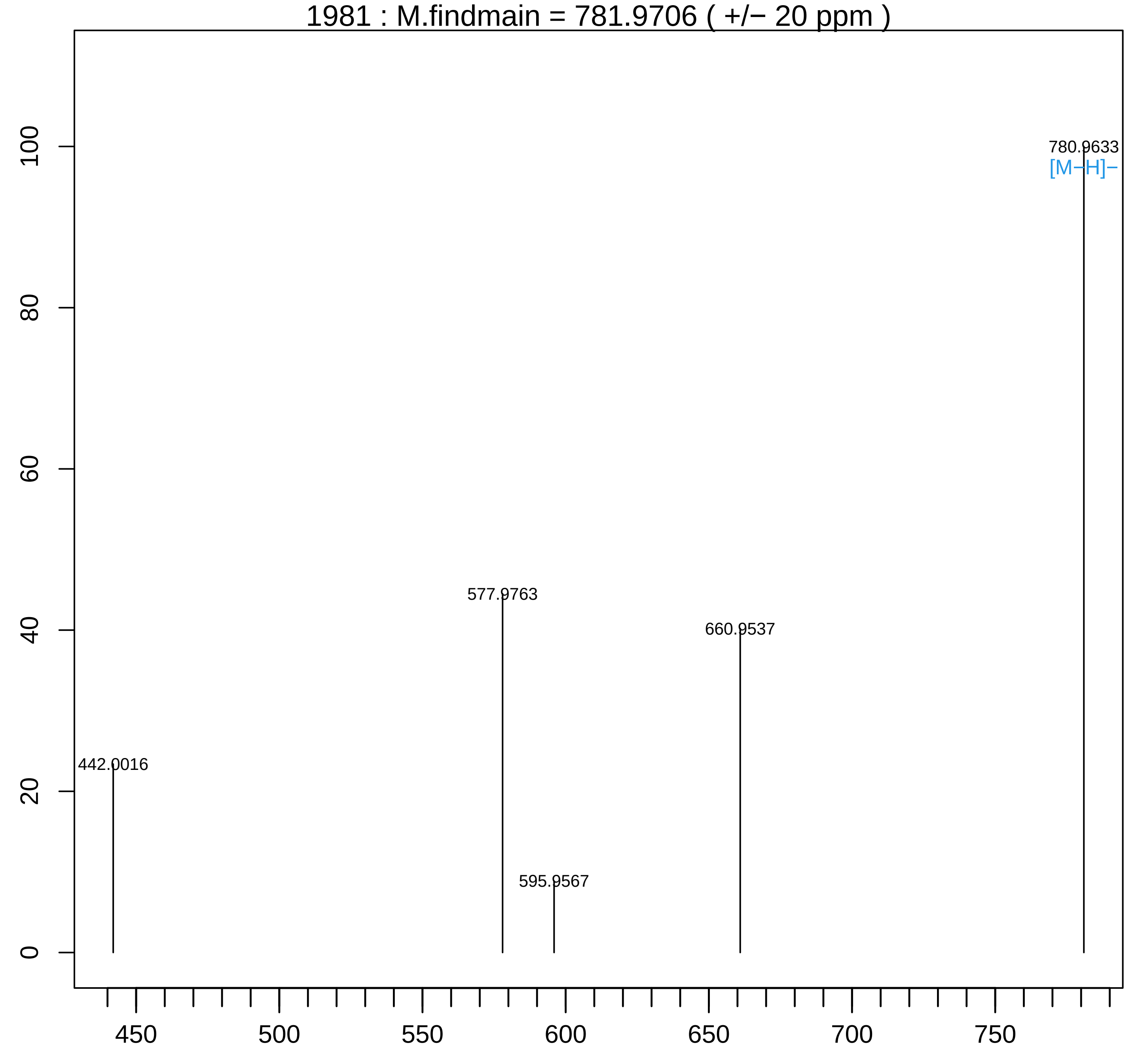
1981 : M.findmain = 781.9706 (+/− 20 ppm)
780.9633
[M−H]−
577.9763
660.9537
442.0016
595.9567
0
20
40
60
80
100
450
500
550
600
650
700
750

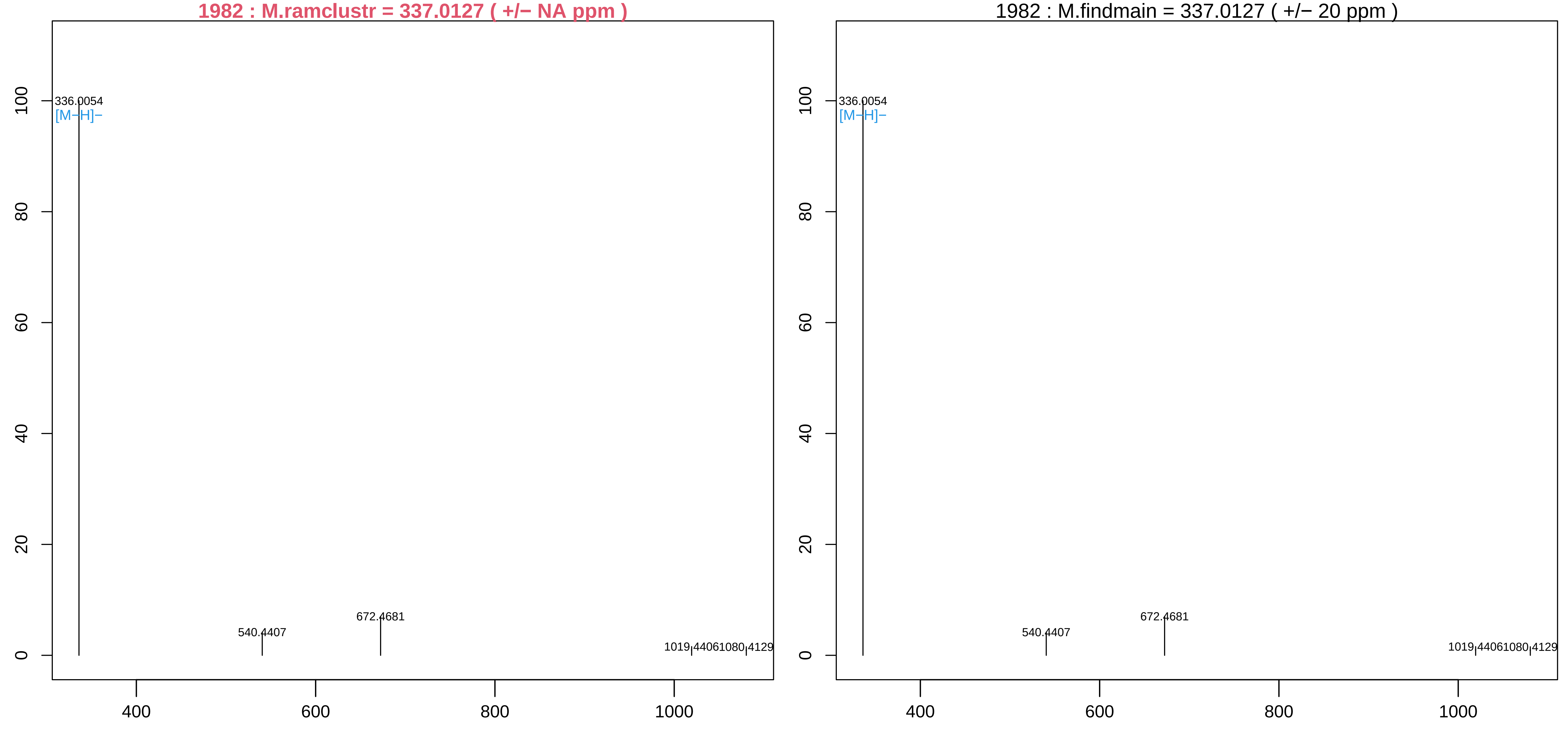
1982 : M.ramclustr = 337.0127 (+/− NA ppm)
1982 : M.findmain = 337.0127 (+/− 20 ppm)
336.0054
[M−H]−
540.4407
672.4681
1019.4406
1080.4129
0
20
40
60
80
100
400
600
800
1000

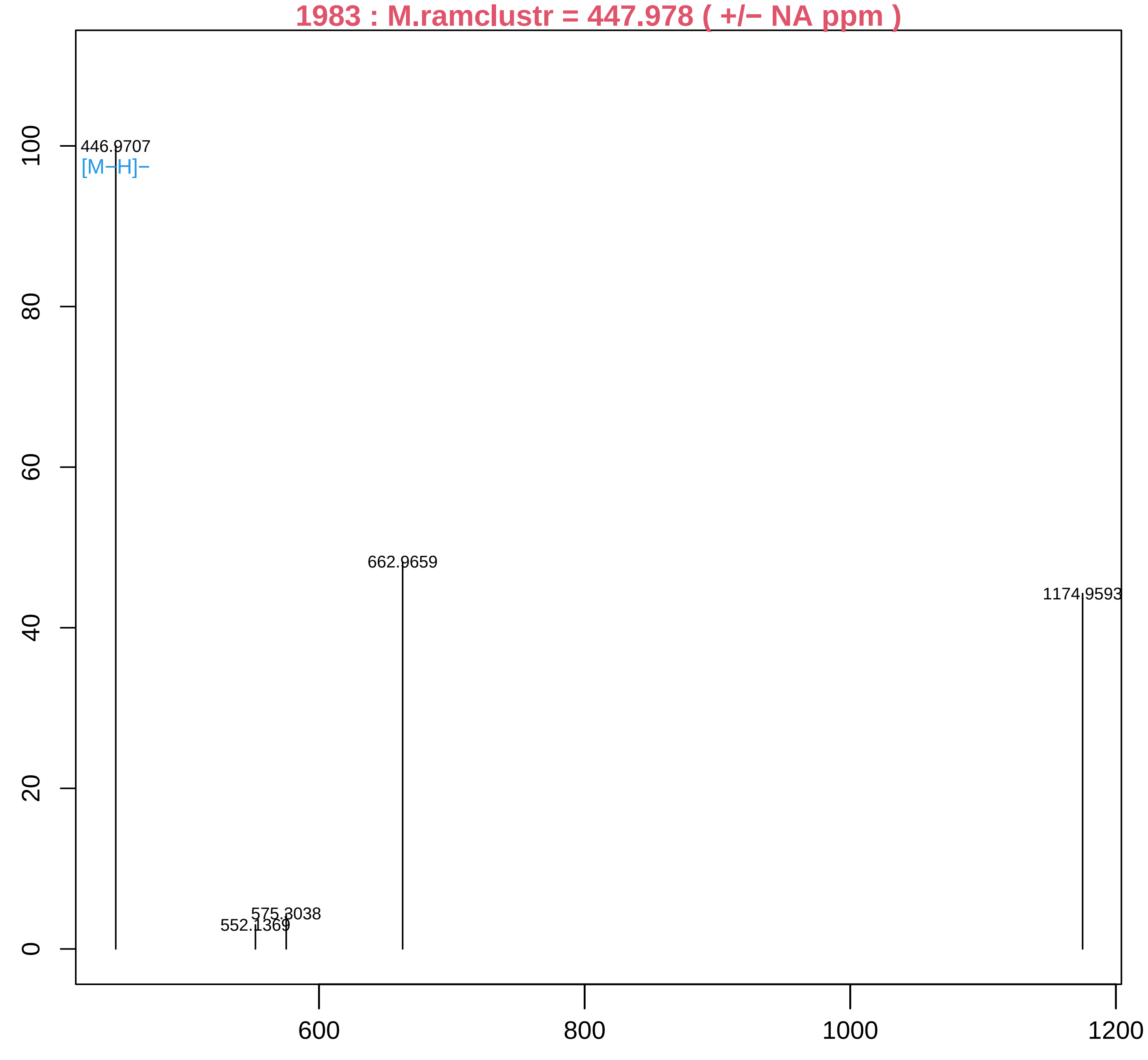
1983 : M.ramclustr = 447.978 (+/− NA ppm)
446.9707
[M−H]−
662.9659
1174.9593
575.3038
552.1369
0
20
40
60
80
100
600
800
1000
1200

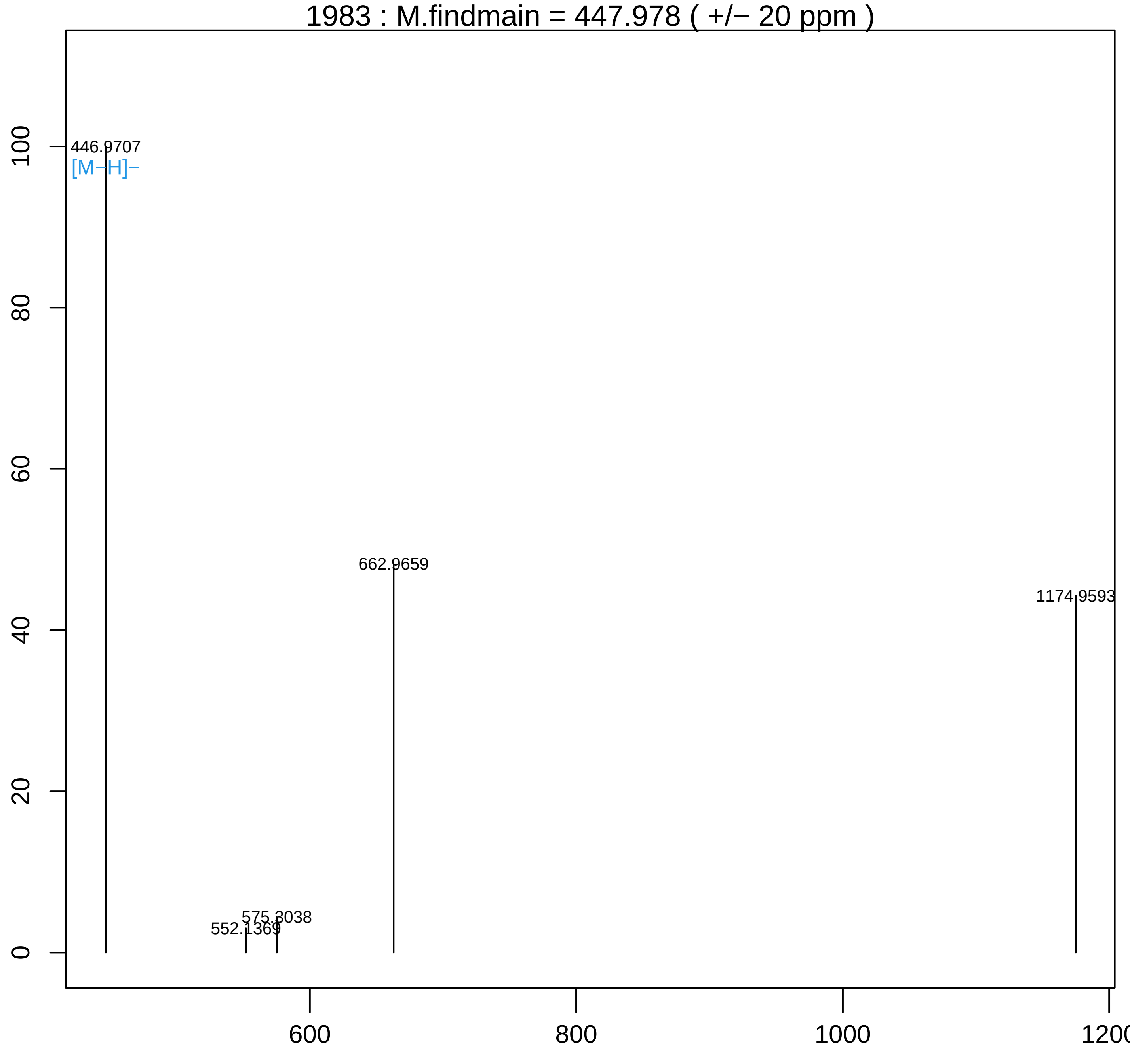
1983 : M.findmain = 447.978 (+/− 20 ppm)
446.9707
[M−H]−
662.9659
1174.9593
575.3038
552.1369
0
20
40
60
80
100
600
800
1000
1200

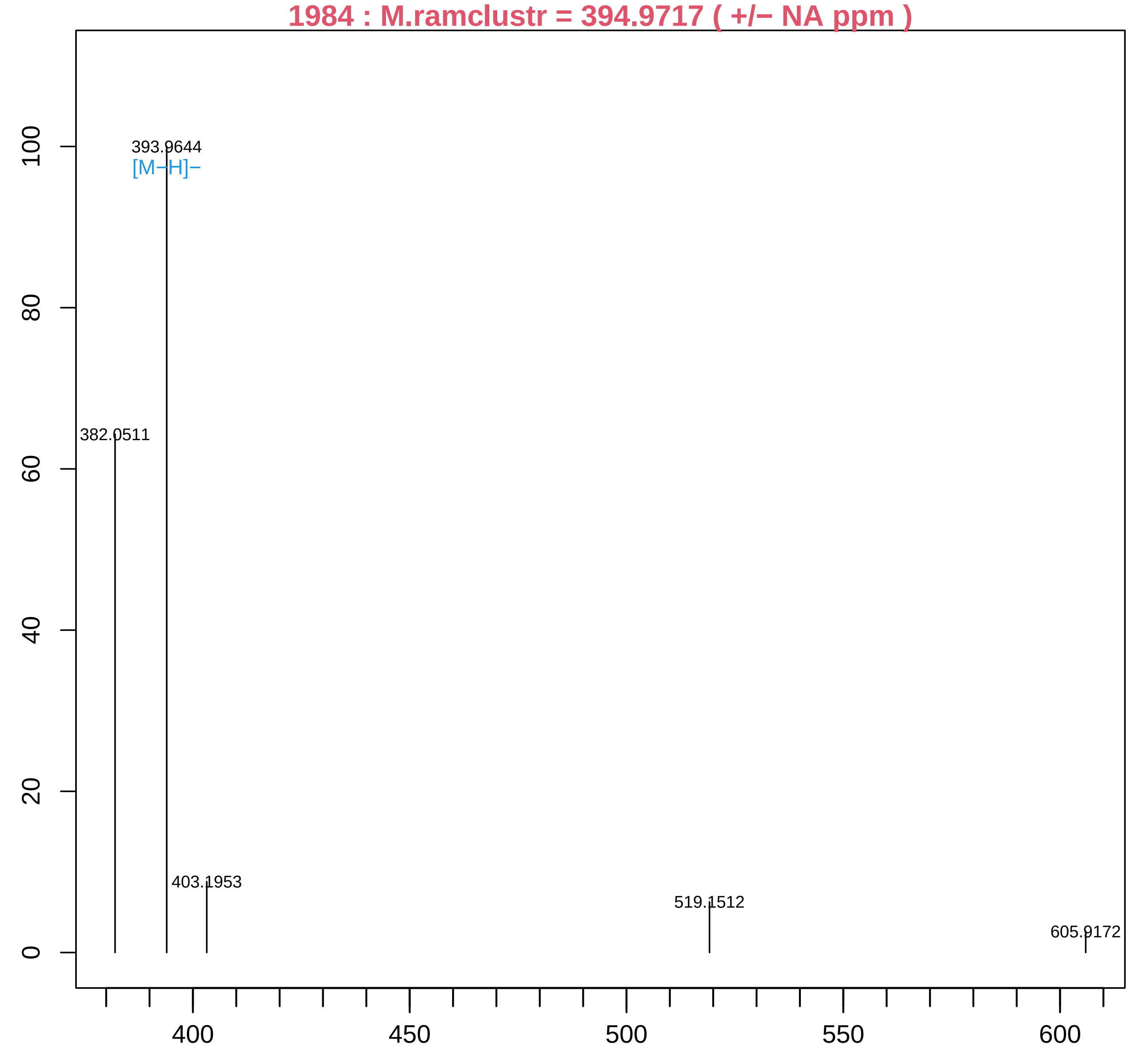

1984 : M.ramclustr = 394.9717 (+/− NA ppm)
393.9644
[M−H]−
382.0511
403.1953
519.1512
605.9172
0
20
40
60
80
100
400
450
500
550
600

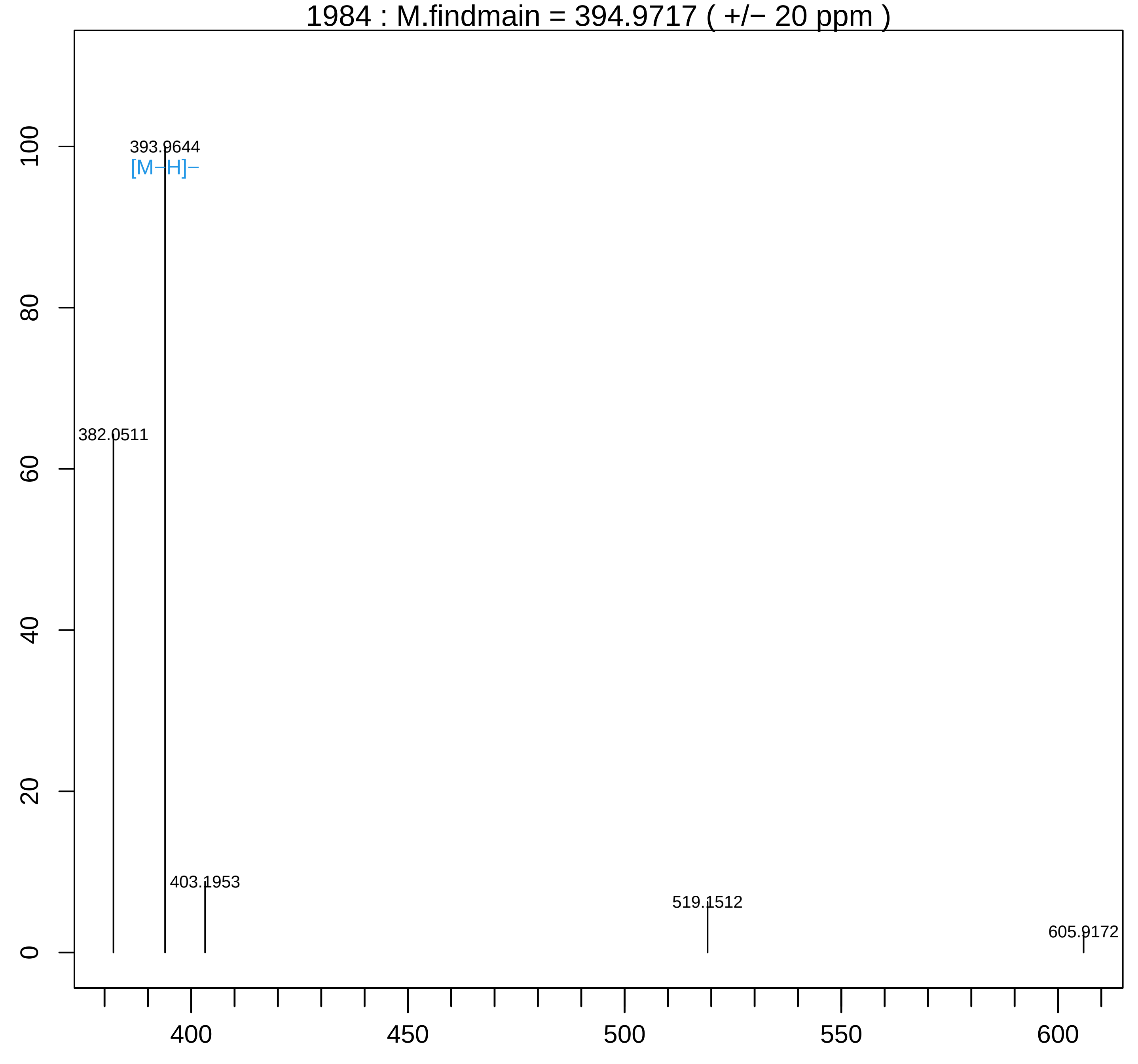

1984 : M.findmain = 394.9717 (+/− 20 ppm)
393.9644
[M−H]−
382.0511
403.1953
519.1512
605.9172
0
20
40
60
80
100
400
450
500
550
600

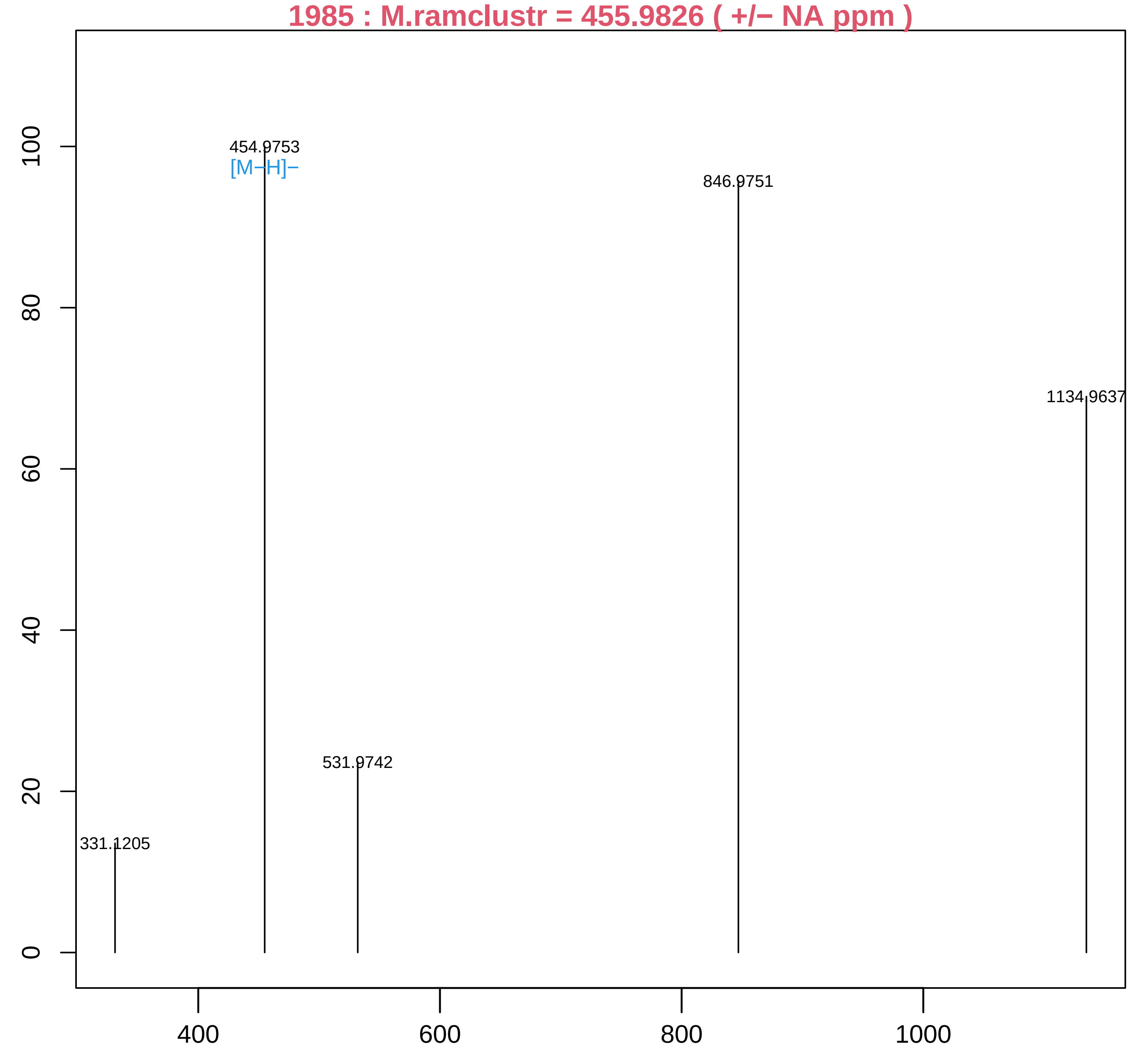
1985 : M.ramclustr = 455.9826 (+/− NA ppm)
454.9753
[M−H]−
846.9751
1134.9637
531.9742
331.1205
0
20
40
60
80
100
400
600
800
1000

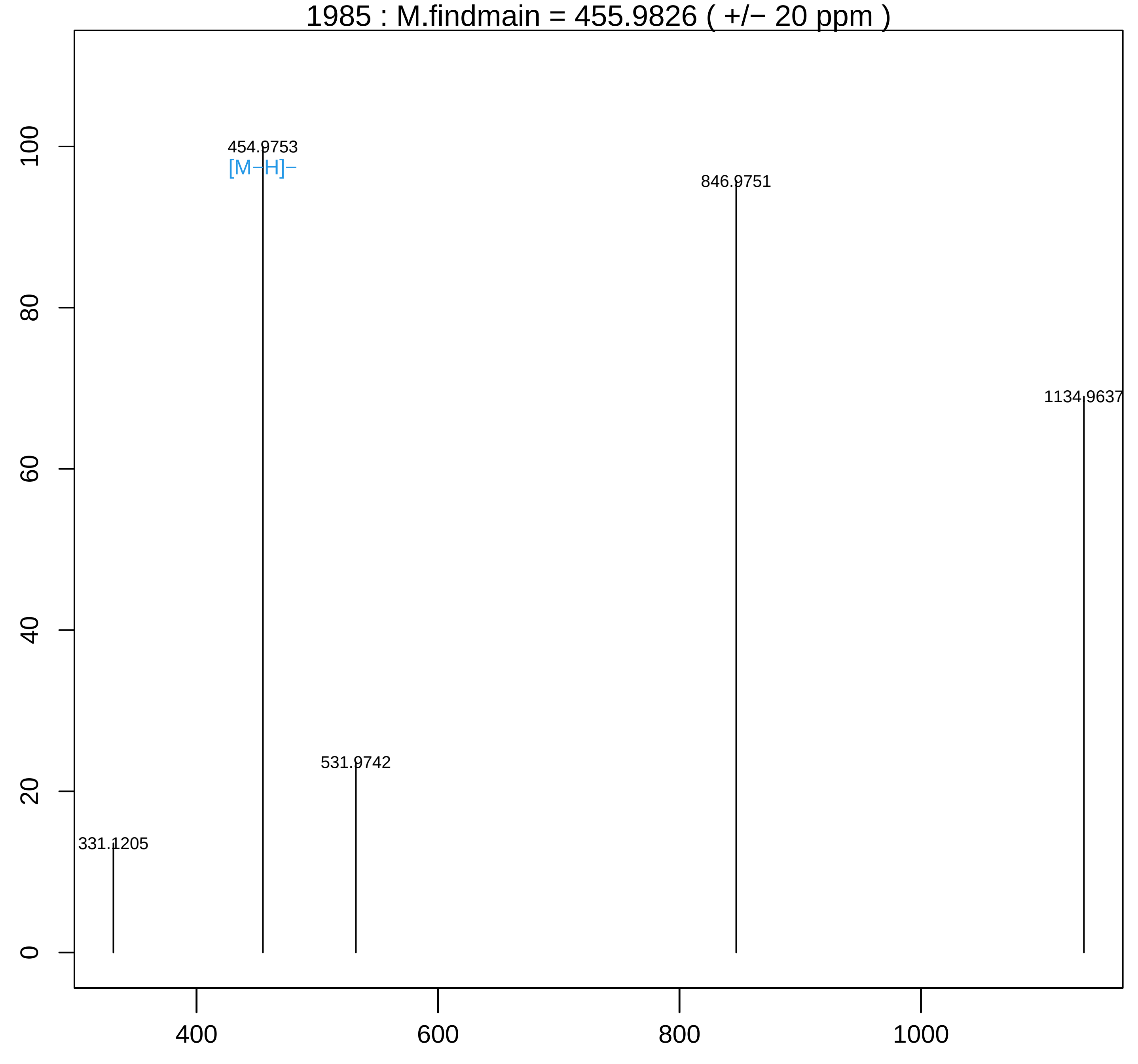
1985 : M.findmain = 455.9826 (+/− 20 ppm)
454.9753
[M−H]−
846.9751
1134.9637
531.9742
331.1205
0
20
40
60
80
100
400
600
800
1000

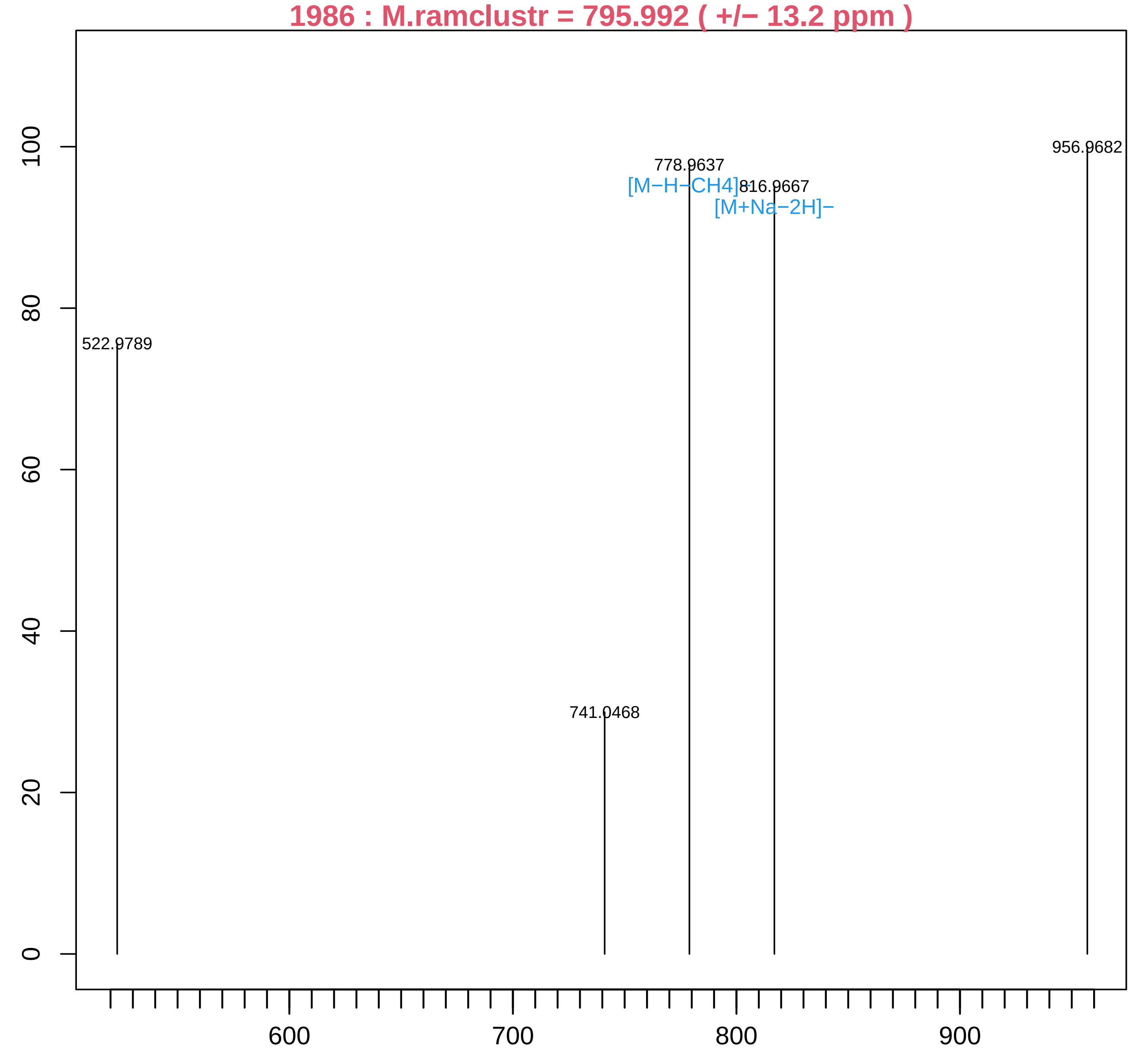
1986 : M.ramclustr = 795.992 (+/− 13.2 ppm)
522.9789
741.0468
778.9637
[M−H−CH4]−
816.9667
[M+Na−2H]−
956.9682
0
20
40
60
80
100
600
700
800
900

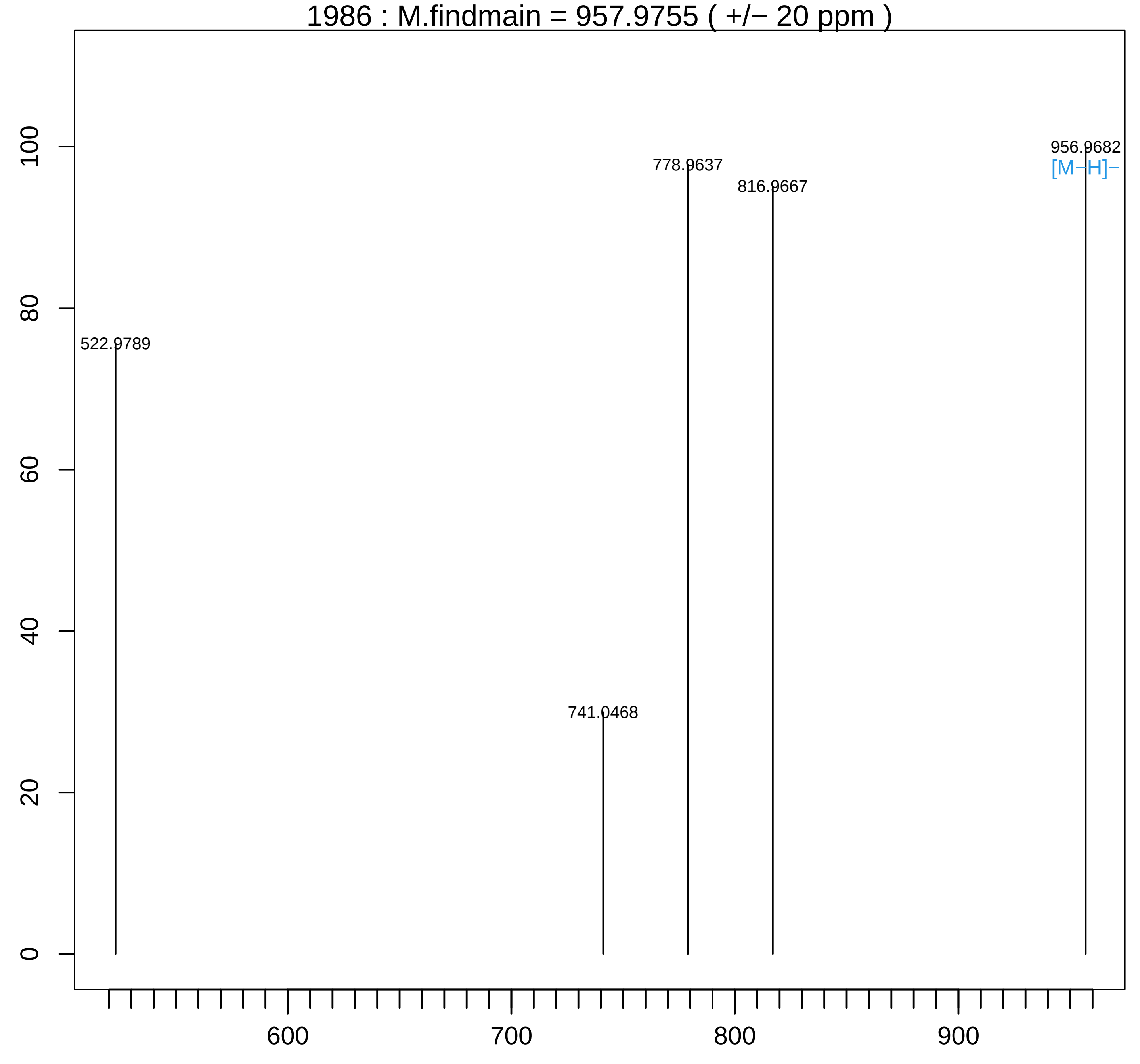
1986 : M.findmain = 957.9755 (+/− 20 ppm)
522.9789
741.0468
778.9637
816.9667
956.9682
[M−H]−
0
20
40
60
80
100
600
700
800
900

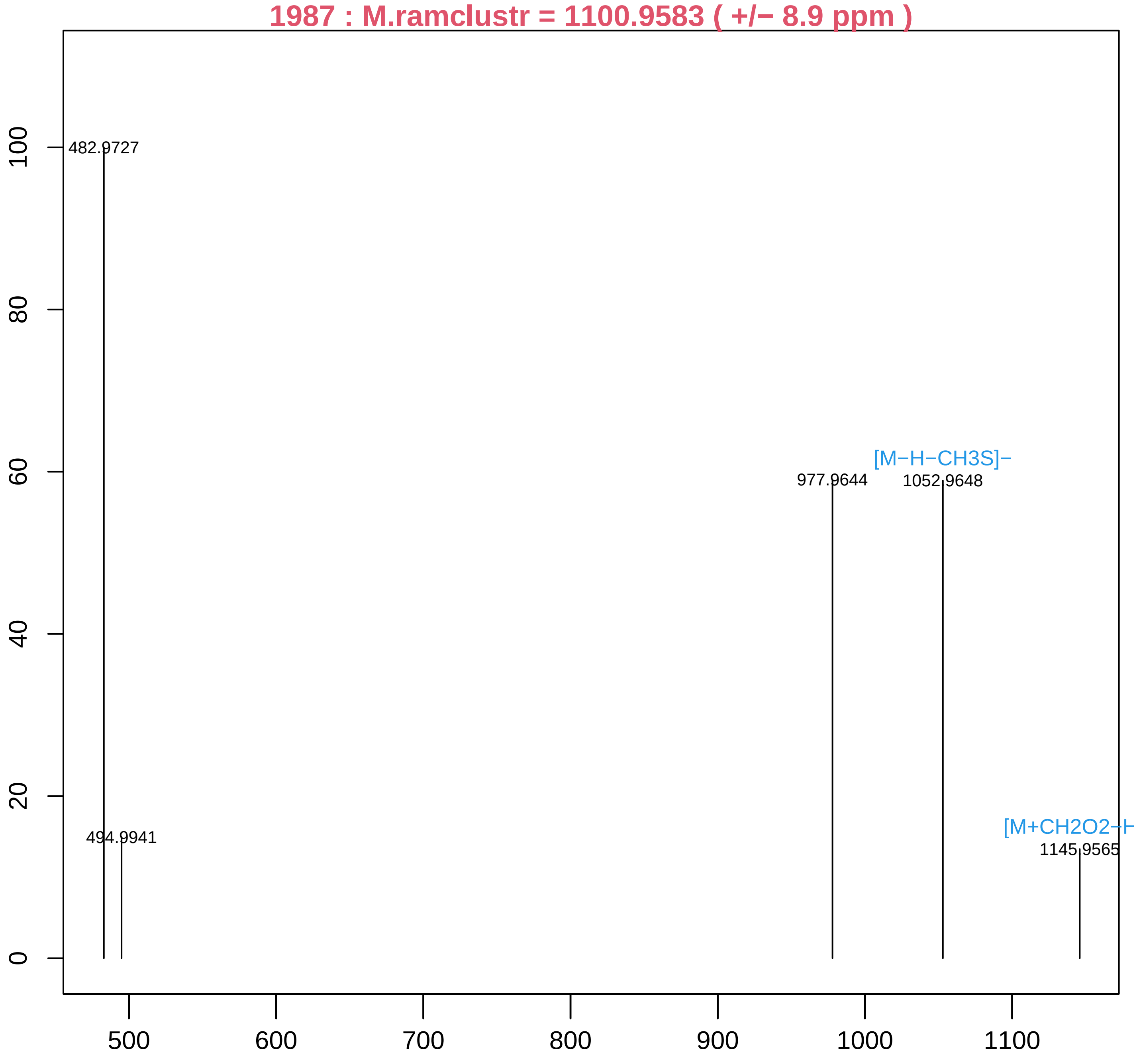
1987 : M.ramclustr = 1100.9583 (+/− 8.9 ppm)
482.9727
494.9941
977.9644
1052.9648
[M−H−CH3S]−
1145.9565
[M+CH2O2−H
0
20
40
60
80
100
500
600
700
800
900
1000
1100

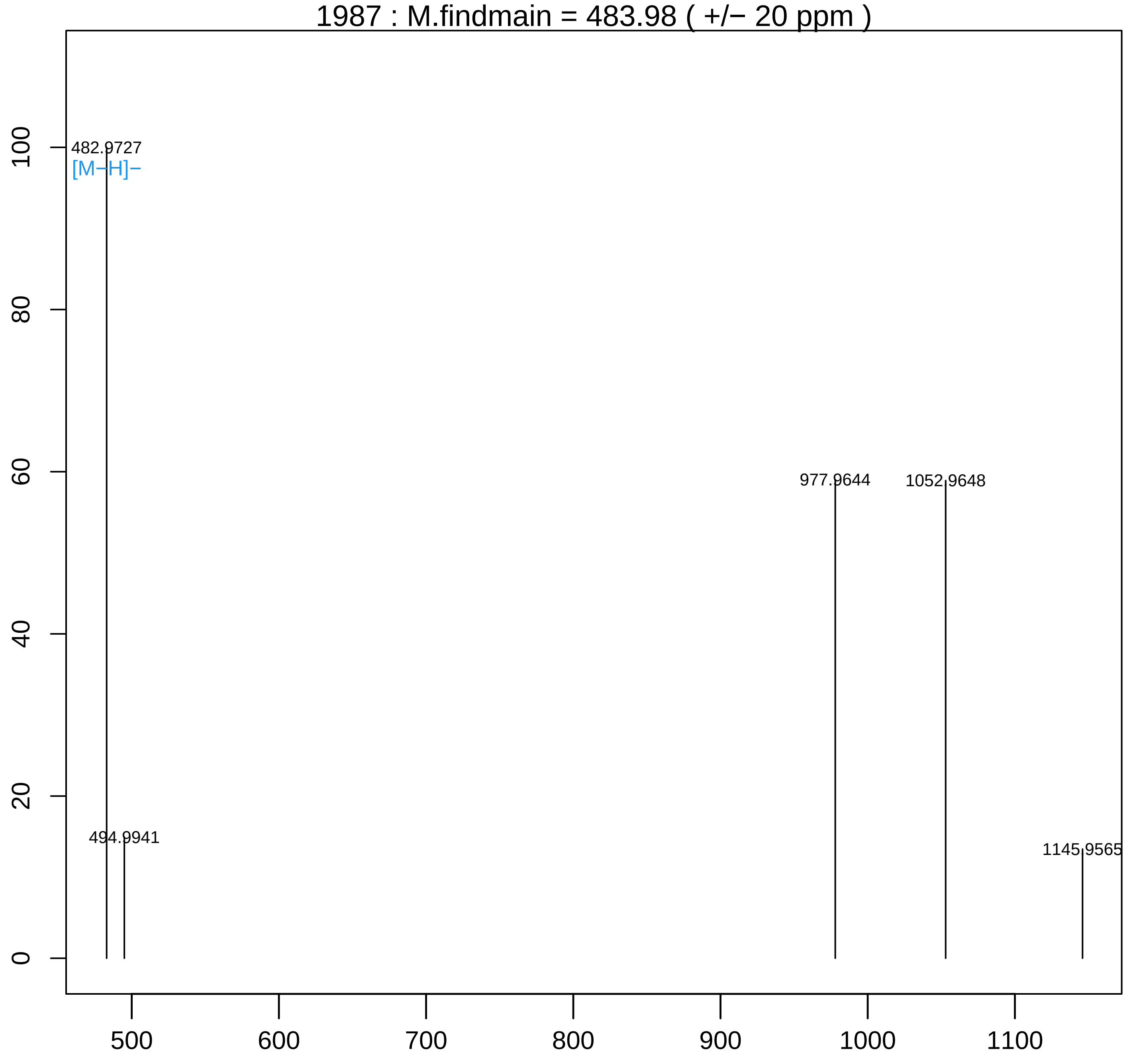
1987 : M.findmain = 483.98 (+/− 20 ppm)
482.9727
[M−H]−
494.9941
977.9644
1052.9648
1145.9565
0
20
40
60
80
100
500
600
700
800
900
1000
1100

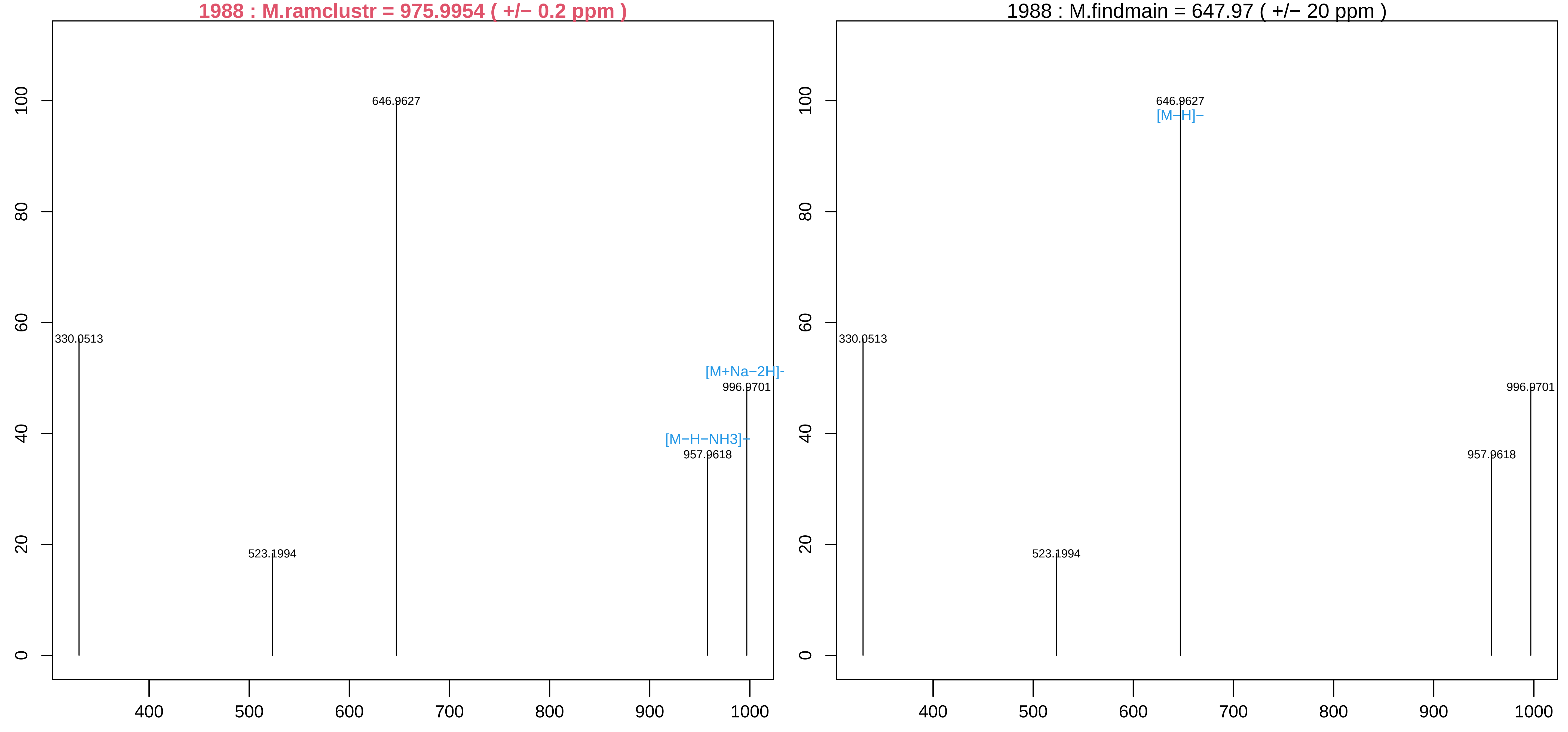
1988 : M.ramclustr = 975.9954 (+/− 0.2 ppm)
646.9627
330.0513
[M+Na−2H]-
996.9701
[M−H−NH3]−
957.9618
523.1994
1988 : M.findmain = 647.97 (+/− 20 ppm)
646.9627
[M−H]−
330.0513
996.9701
957.9618
523.1994

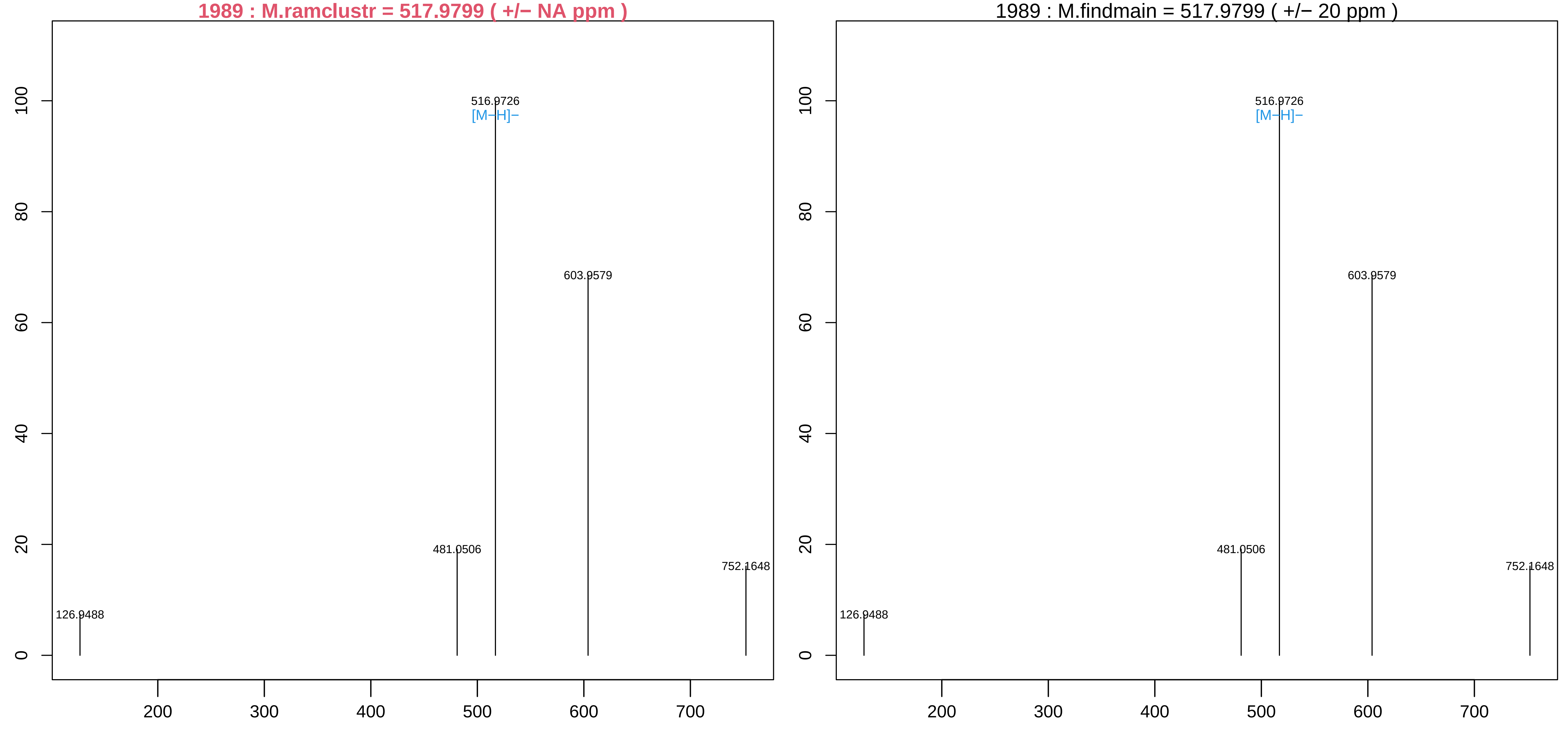
1989 : M.ramclustr = 517.9799 (+/− NA ppm)
516.9726
[M−H]−
603.9579
481.0506
752.1648
126.9488
1989 : M.findmain = 517.9799 (+/− 20 ppm)
516.9726
[M−H]−
603.9579
481.0506
752.1648
126.9488
0
20
40
60
80
100
200
300
400
500
600
700

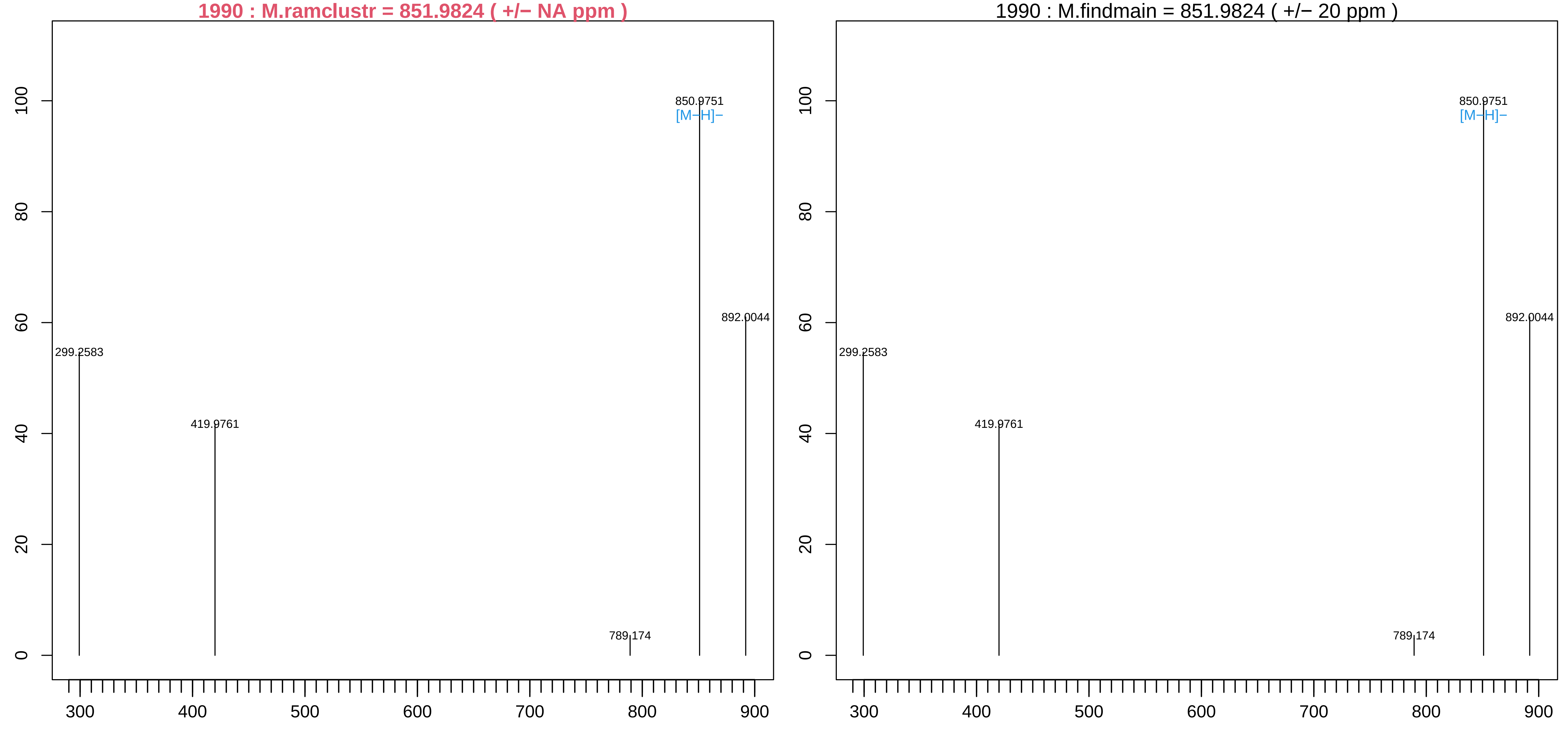

1990 : M.ramclustr = 851.9824 (+/− NA ppm)
850.9751
[M−H]−
892.0044
299.2583
419.9761
789.174
1990 : M.findmain = 851.9824 (+/− 20 ppm)
850.9751
[M−H]−
892.0044
299.2583
419.9761
789.174

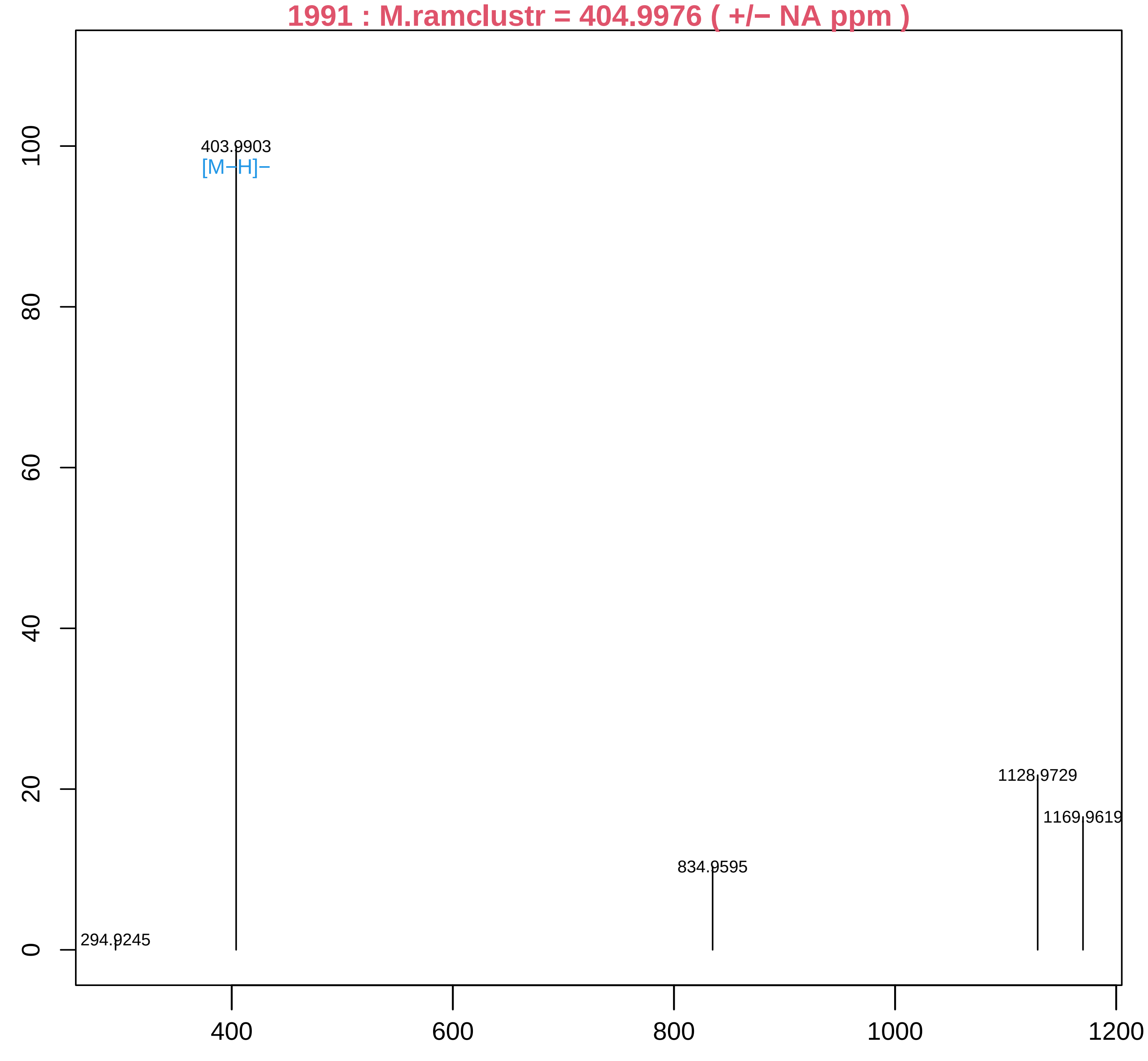

1991 : M.ramclustr = 404.9976 (+/− NA ppm)
403.9903
[M−H]−
294.9245
834.9595
1128.9729
1169.9619
0
20
40
60
80
100
400
600
800
1000
1200

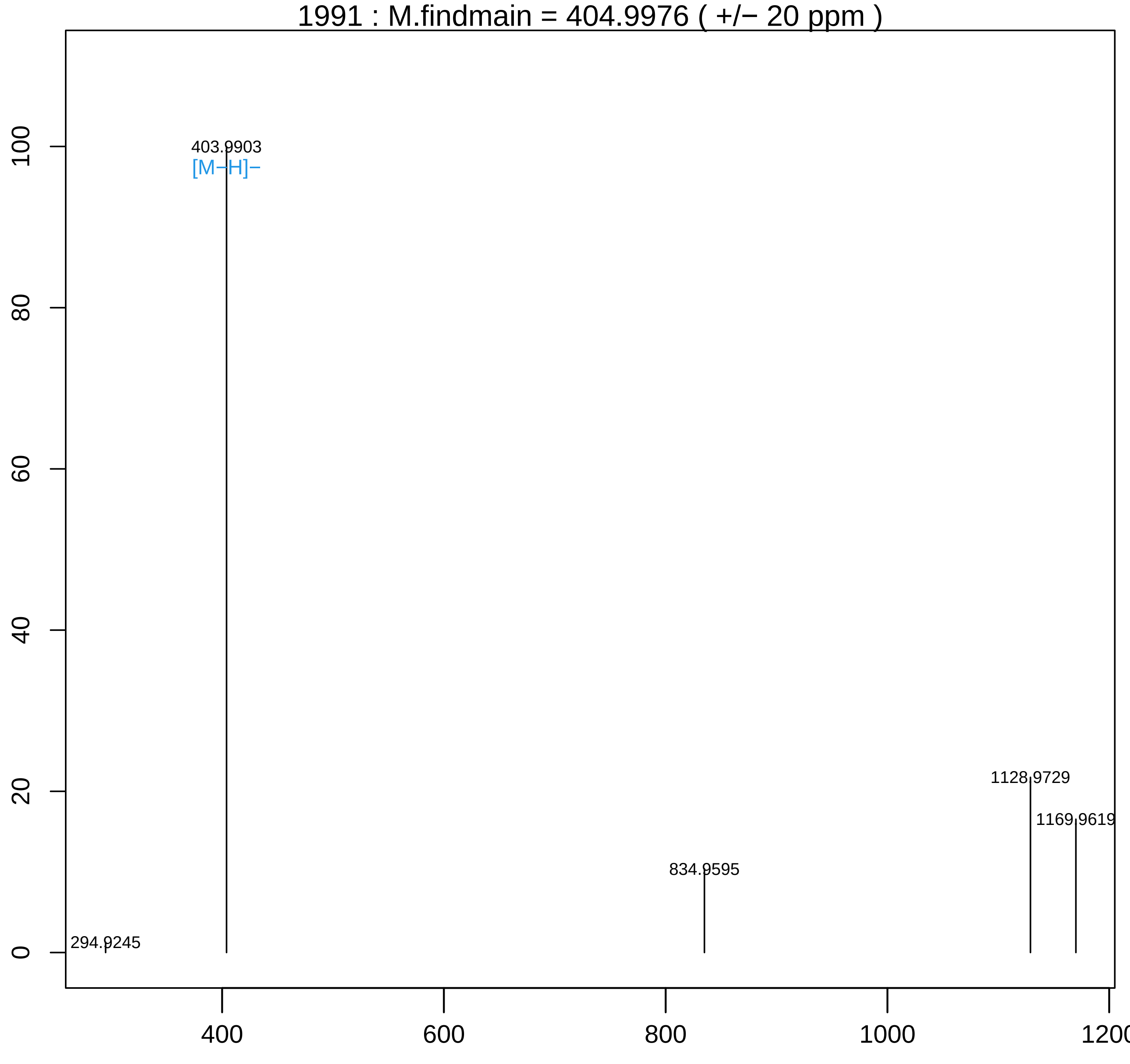

1991 : M.findmain = 404.9976 (+/− 20 ppm)
403.9903
[M−H]−
294.9245
834.9595
1128.9729
1169.9619
0
20
40
60
80
100
400
600
800
1000
1200

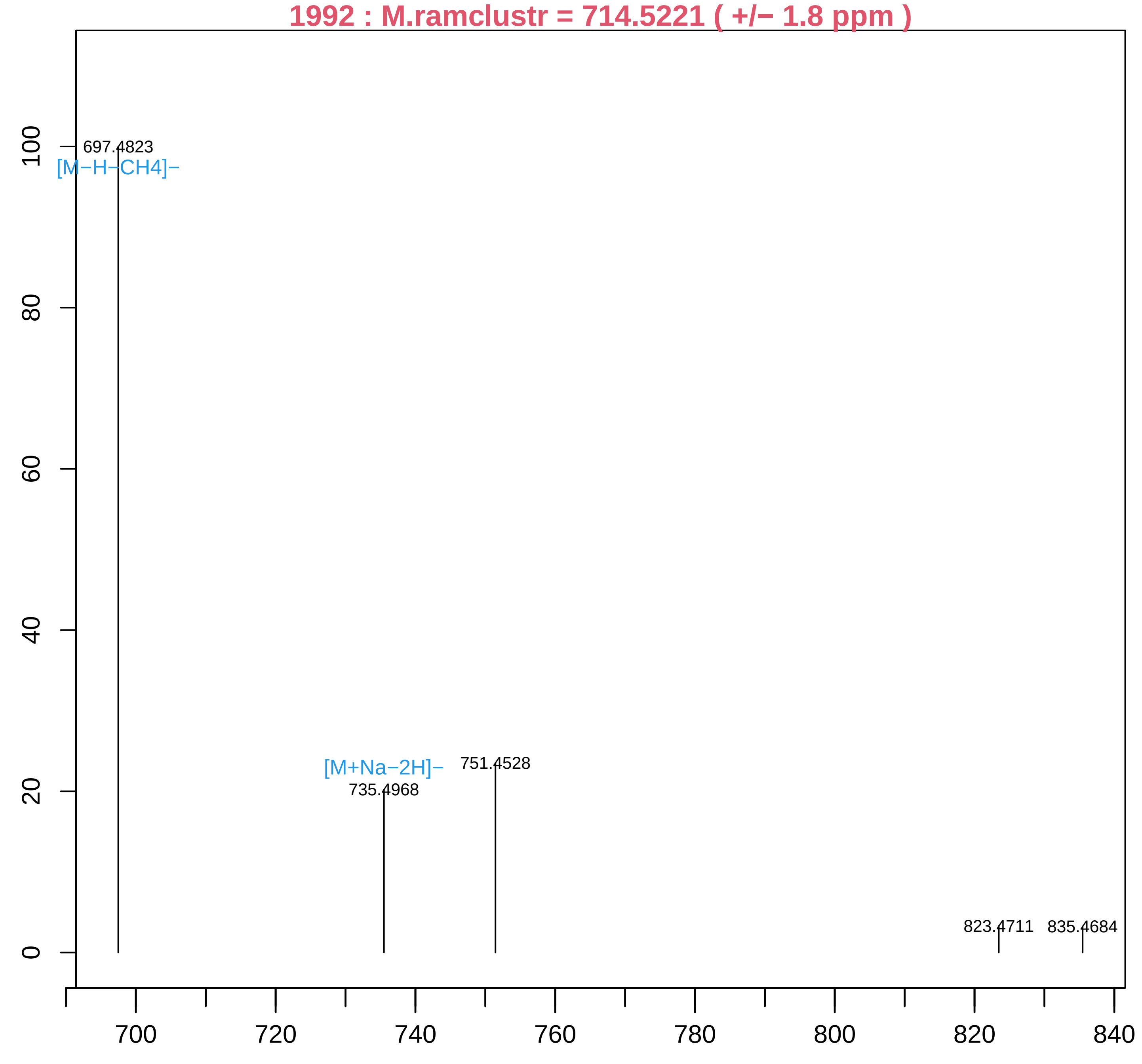
1992 : M.ramclustr = 714.5221 (+/− 1.8 ppm)
697.4823
[M−H−CH4]−
[M+Na−2H]−
735.4968
751.4528
823.4711
835.4684
0
20
40
60
80
100
700
720
740
760
780
800
820
840

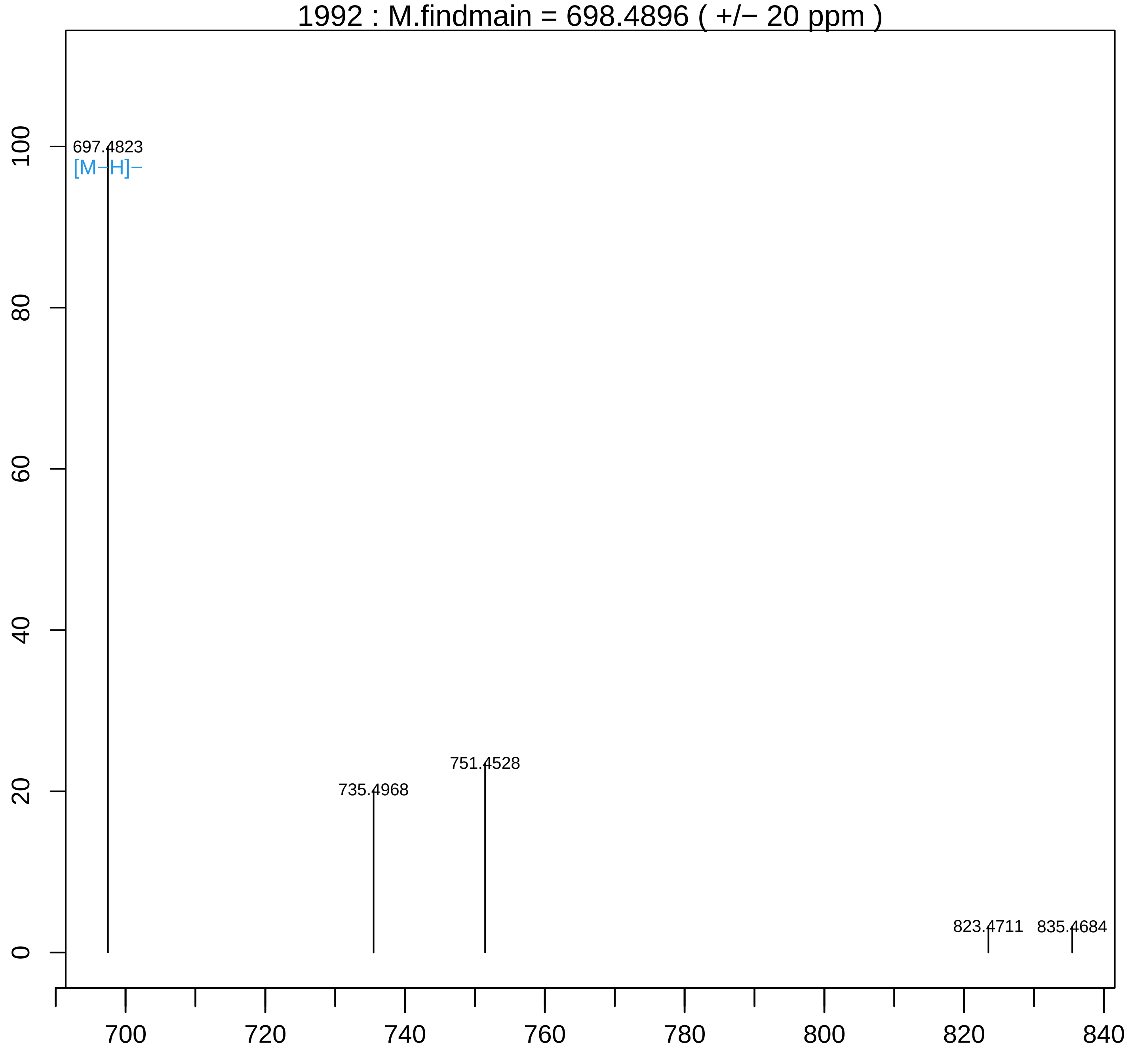
1992 : M.findmain = 698.4896 (+/− 20 ppm)
697.4823
[M−H]−
735.4968
751.4528
823.4711
835.4684
0
20
40
60
80
100
700
720
740
760
780
800
820
840

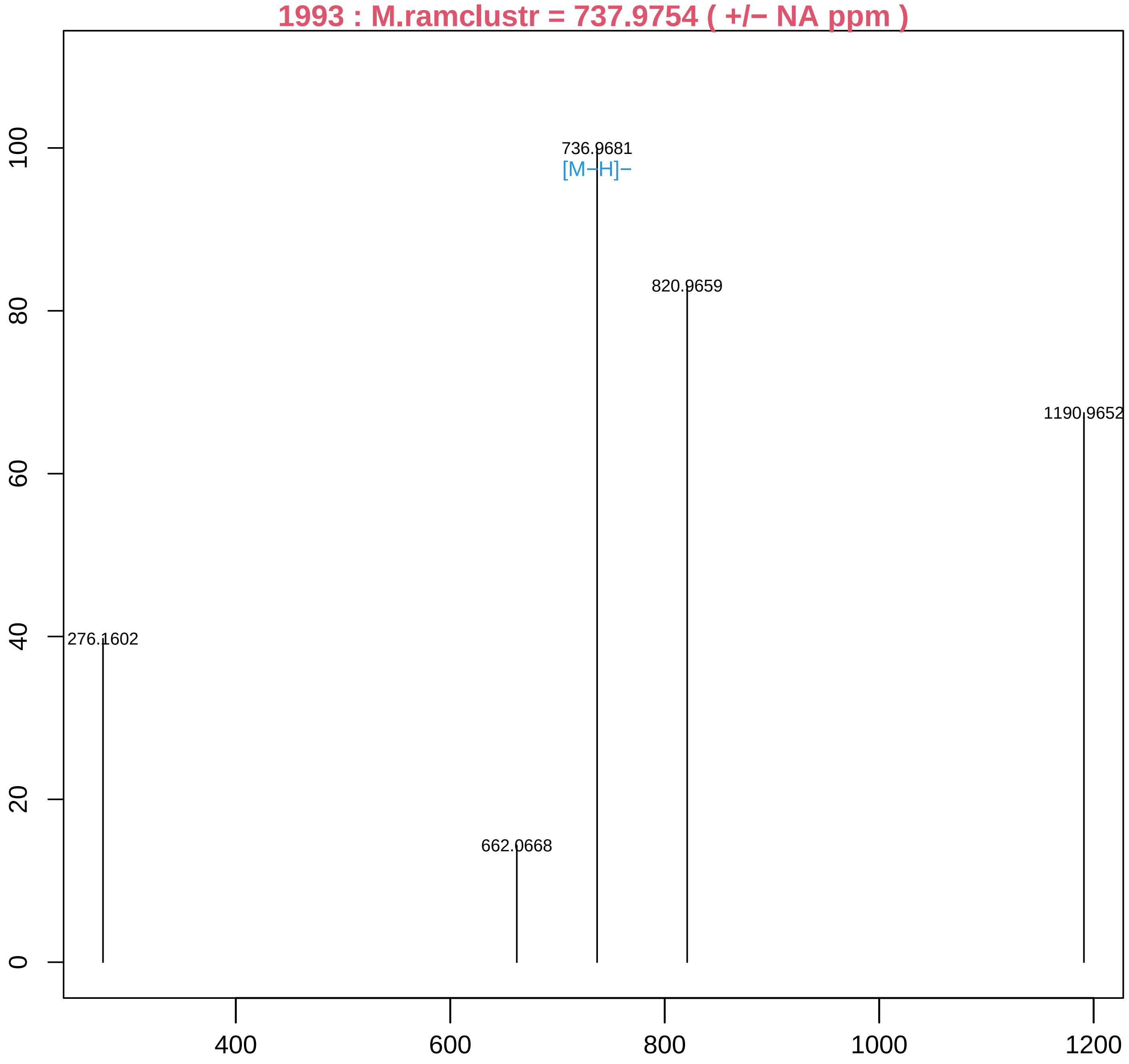
1993 : M.ramclustr = 737.9754 (+/− NA ppm)
736.9681
[M−H]−
820.9659
1190.9652
276.1602
662.0668
0
20
40
60
80
100
400
600
800
1000
1200

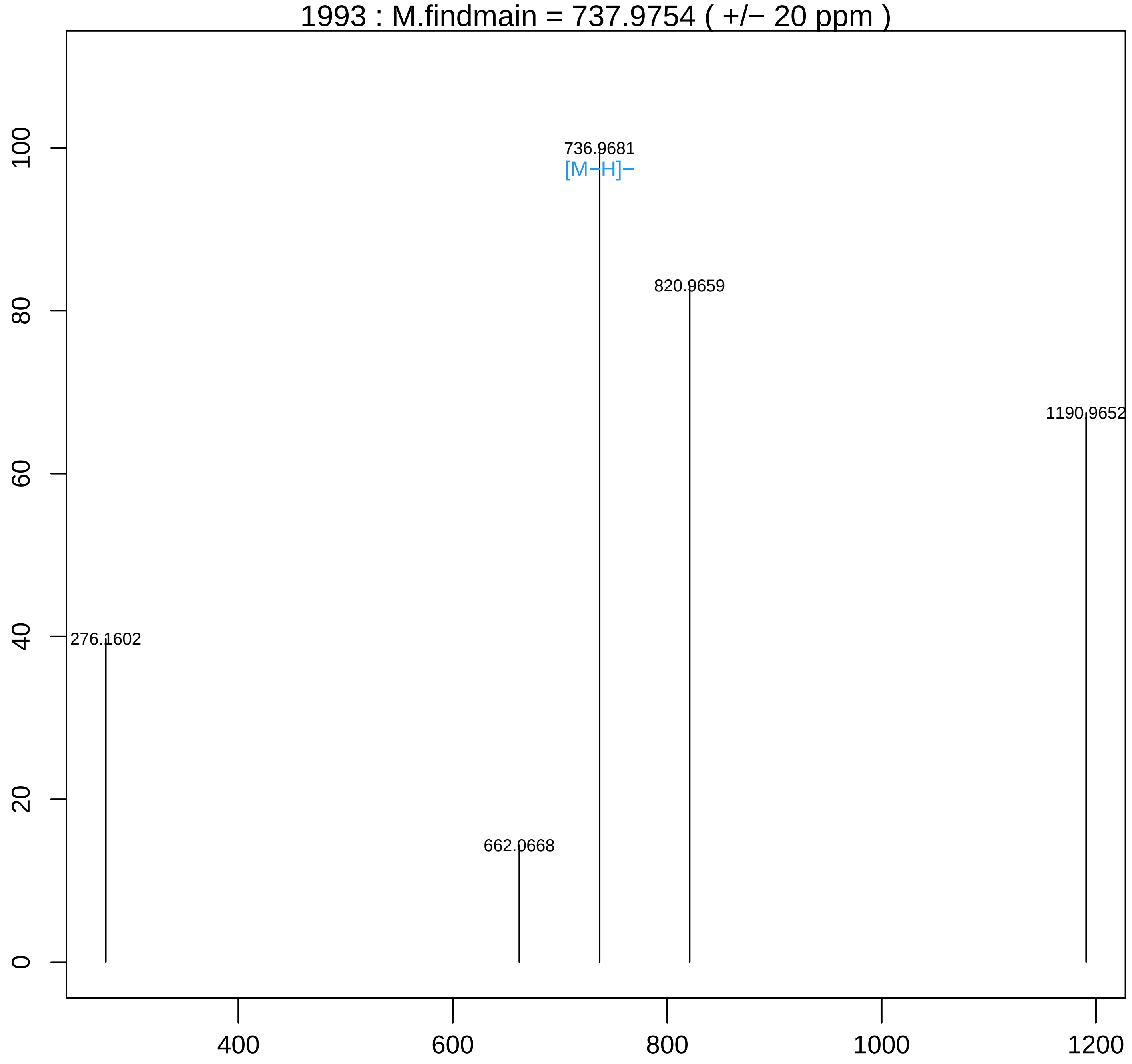
1993 : M.findmain = 737.9754 (+/− 20 ppm)
736.9681
[M−H]−
820.9659
1190.9652
276.1602
662.0668
0
20
40
60
80
100
400
600
800
1000
1200

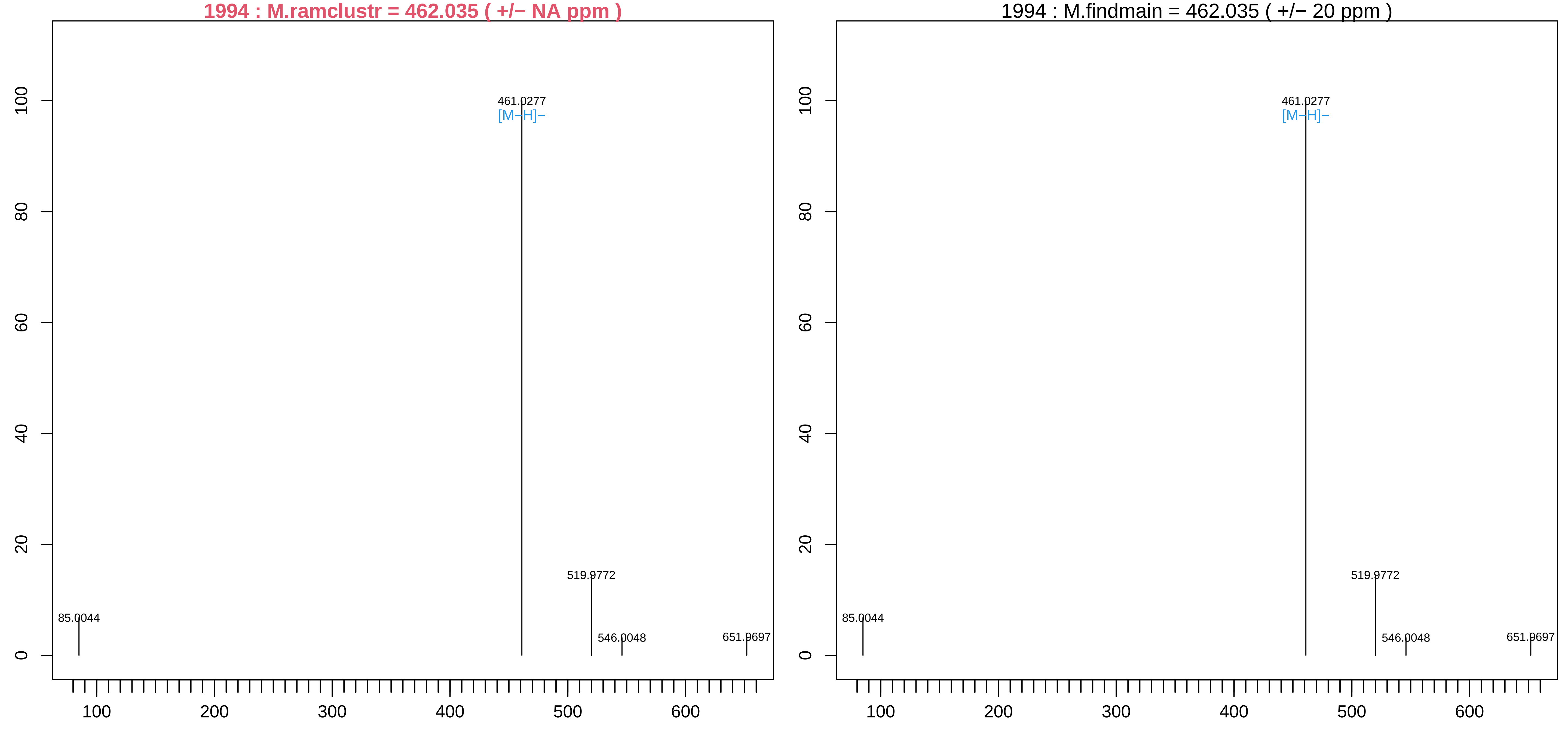

1994 : M.ramclustr = 462.035 (+/− NA ppm)
461.0277
[M−H]−
519.9772
85.0044
546.0048
651.9697
1994 : M.findmain = 462.035 (+/− 20 ppm)
461.0277
[M−H]−
519.9772
85.0044
546.0048
651.9697

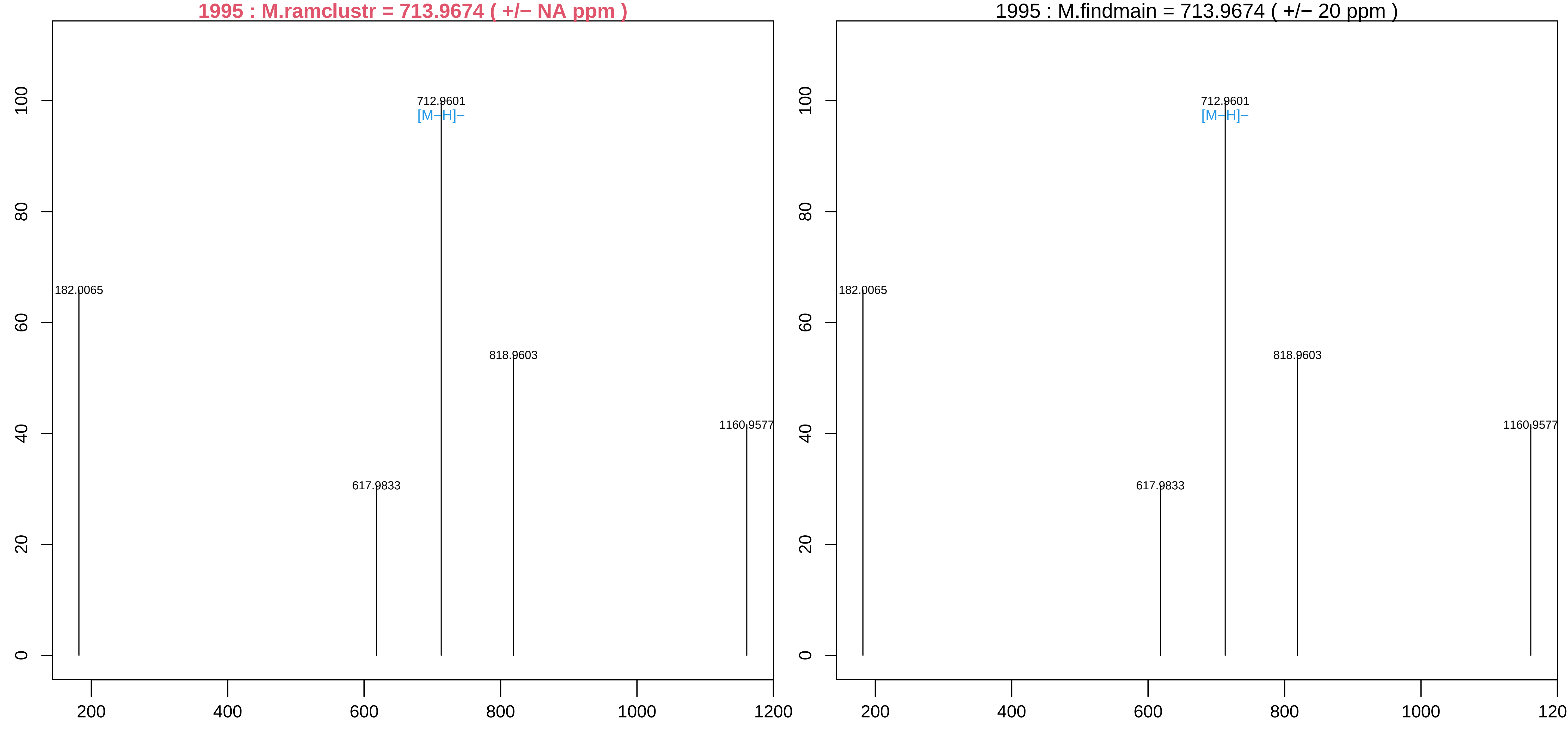

1995 : M.ramclustr = 713.9674 (+/− NA ppm)
1995 : M.findmain = 713.9674 (+/− 20 ppm)
712.9601
[M−H]−
182.0065
818.9603
1160.9577
617.9833
0
20
40
60
80
100
200
400
600
800
1000
1200

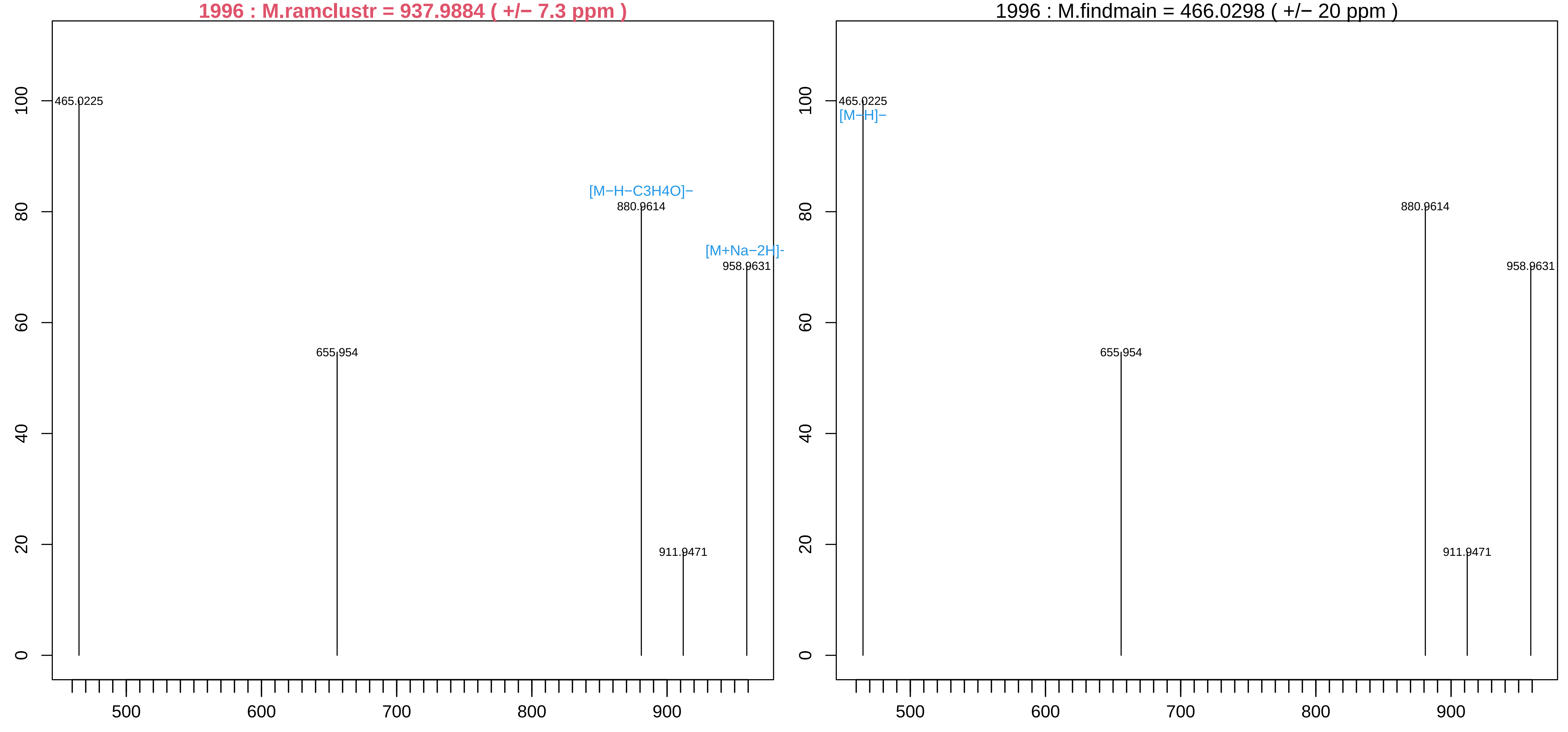
1996 : M.ramclustr = 937.9884 (+/− 7.3 ppm)
465.0225
655.954
[M−H−C3H4O]−
880.9614
911.9471
[M+Na−2H]-
958.9631
1996 : M.findmain = 466.0298 (+/− 20 ppm)
465.0225
[M−H]−
655.954
880.9614
911.9471
958.9631
0
20
40
60
80
100
500
600
700
800
900

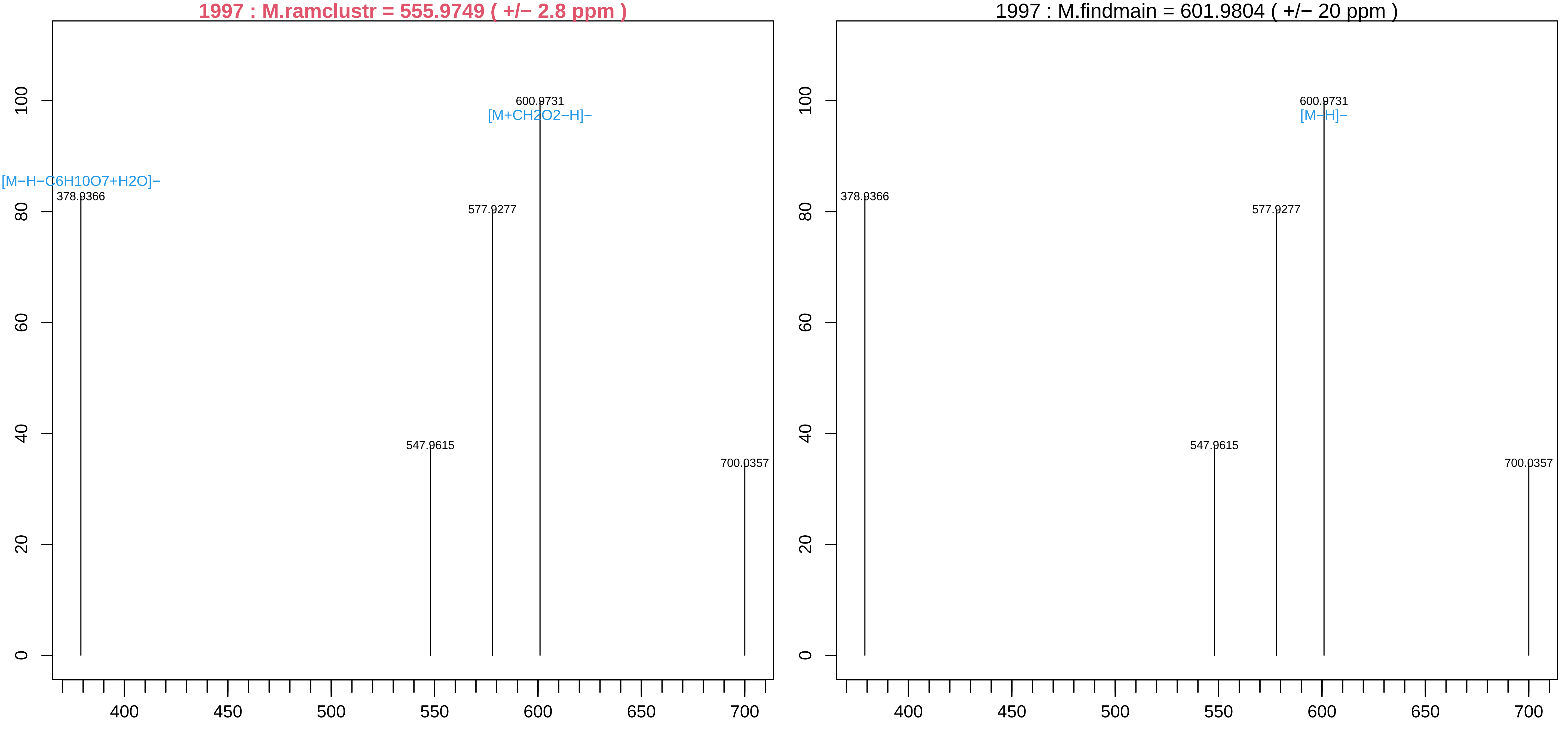
1997 : M.ramclustr = 555.9749 (+/− 2.8 ppm)
[M−H−C6H10O7+H2O]−
378.9366
547.9615
577.9277
600.9731
[M+CH2O2−H]−
700.0357
1997 : M.findmain = 601.9804 (+/− 20 ppm)
378.9366
547.9615
577.9277
600.9731
[M−H]−
700.0357
0
20
40
60
80
100
400
450
500
550
600
650
700

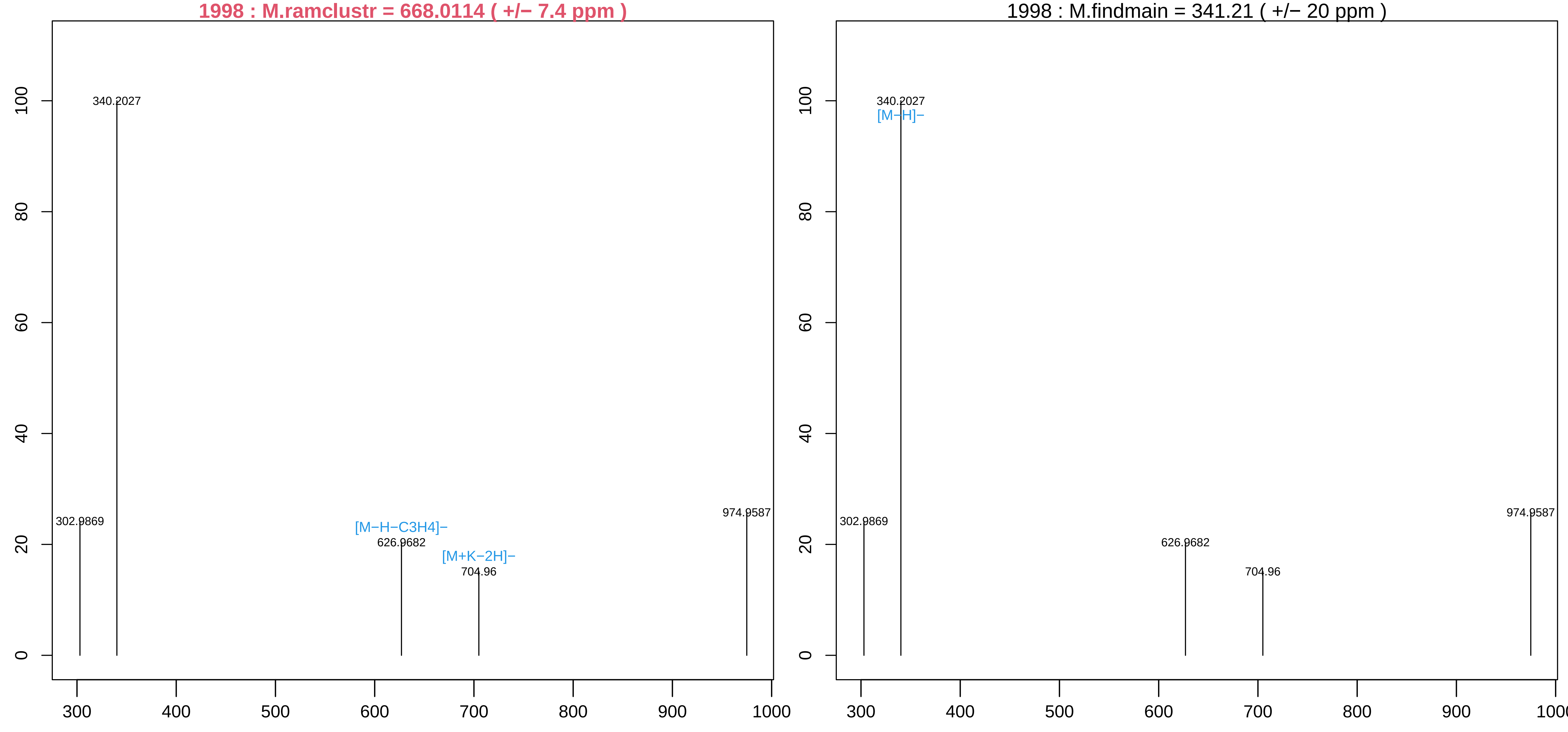

1998 : M.ramclustr = 668.0114 (+/− 7.4 ppm)
340.2027
302.9869
[M−H−C3H4]−
626.9682
[M+K−2H]−
704.96
974.9587
1998 : M.findmain = 341.21 (+/− 20 ppm)
340.2027
[M−H]−
302.9869
626.9682
704.96
974.9587

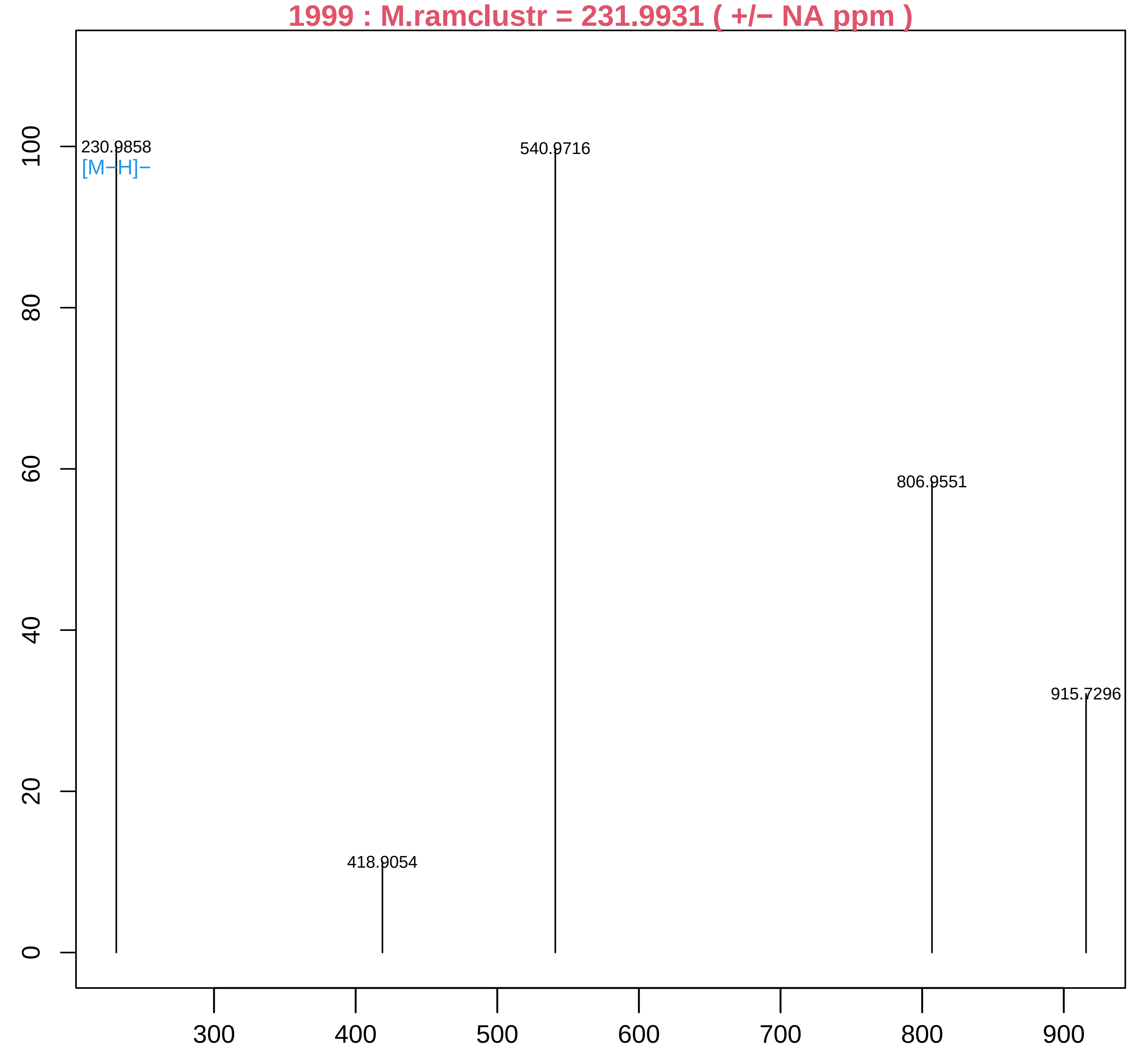
1999 : M.ramclustr = 231.9931 (+/− NA ppm)
230.9858
[M−H]−
540.9716
806.9551
915.7296
418.9054
0
20
40
60
80
100
300
400
500
600
700
800
900

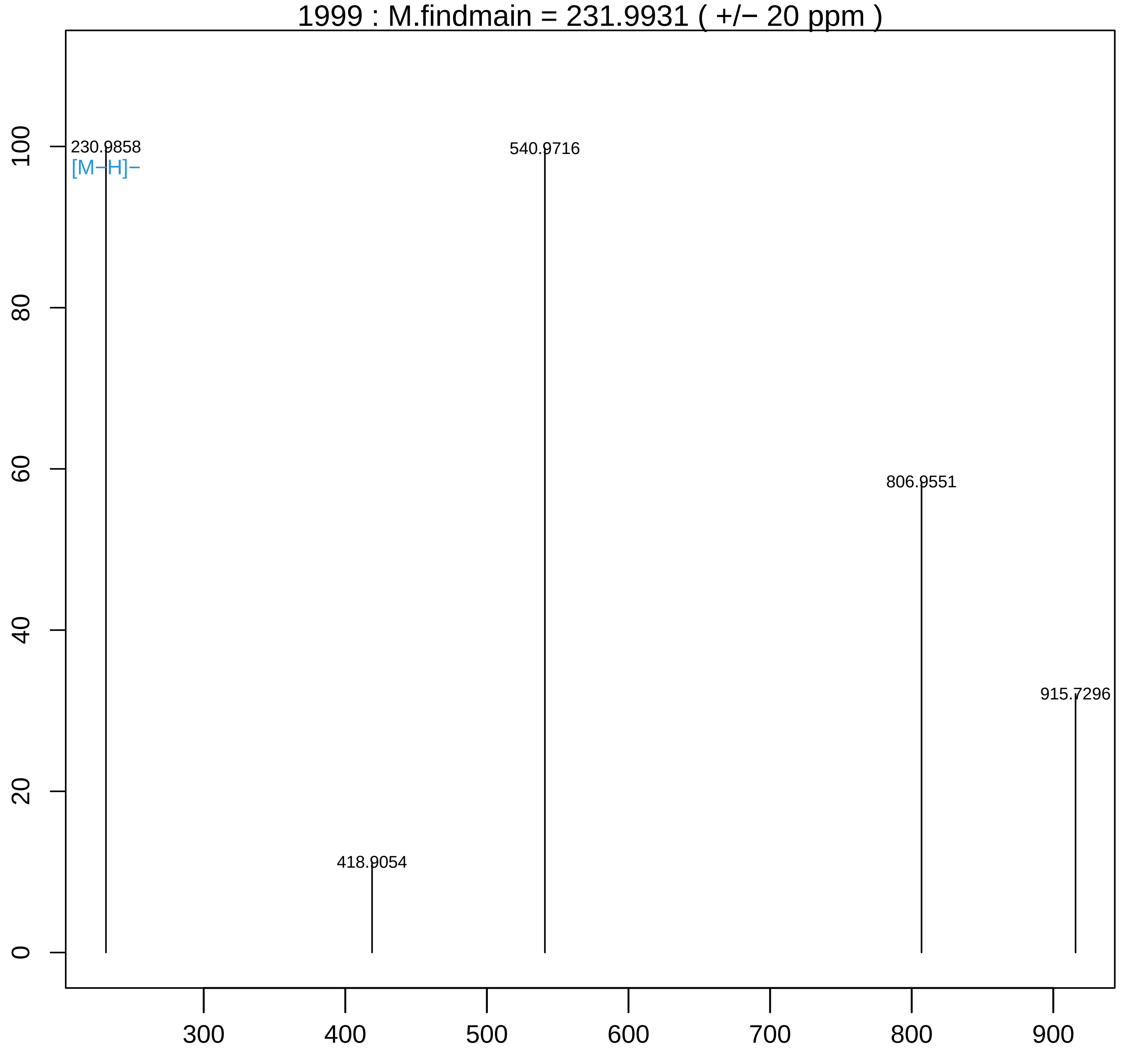
1999 : M.findmain = 231.9931 (+/− 20 ppm)
230.9858
[M−H]−
540.9716
806.9551
915.7296
418.9054
0
20
40
60
80
100
300
400
500
600
700
800
900

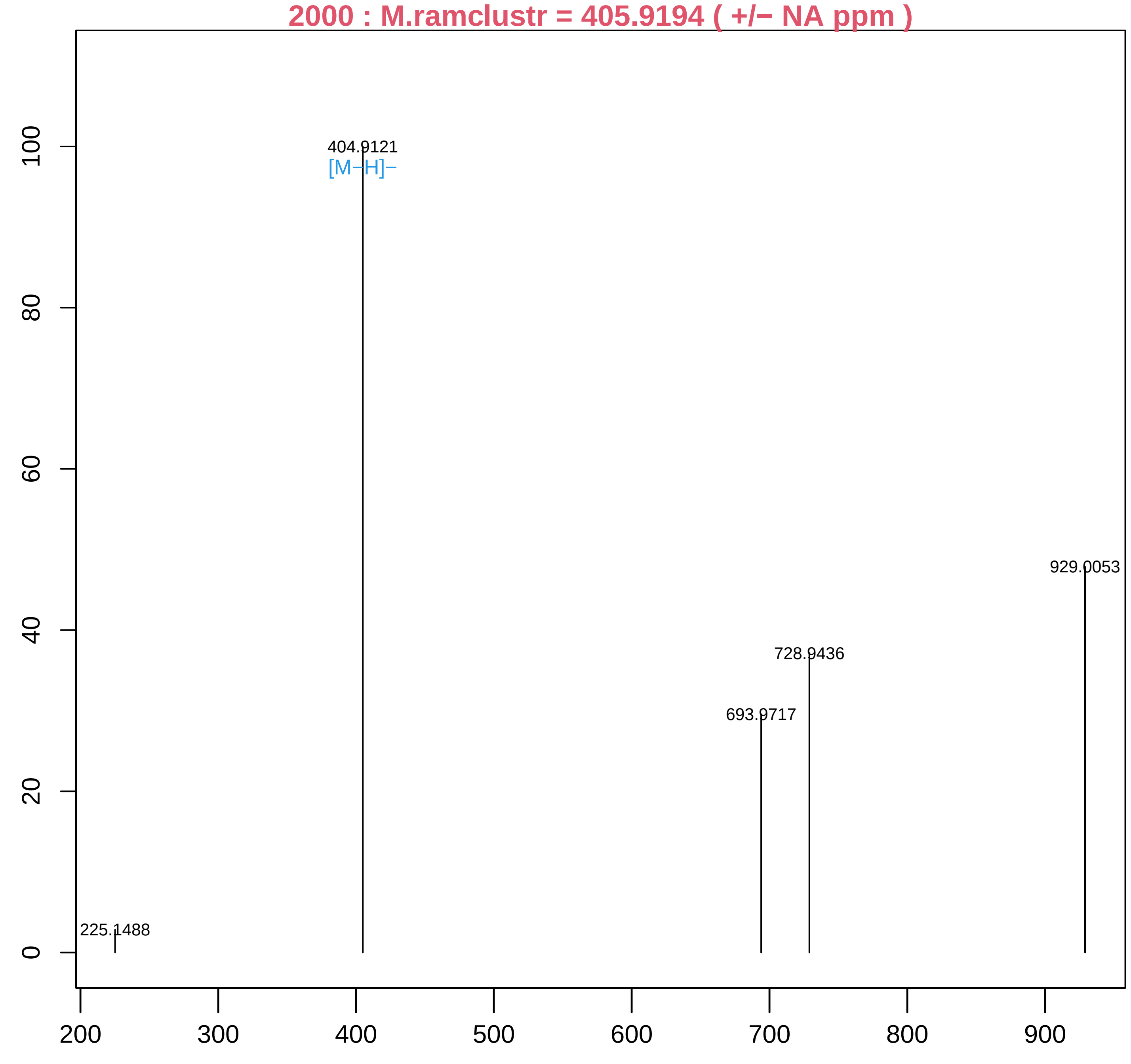
2000 : M.ramclustr = 405.9194 (+/− NA ppm)
404.9121
[M−H]−
929.0053
728.9436
693.9717
225.1488
0
20
40
60
80
100
200
300
400
500
600
700
800
900

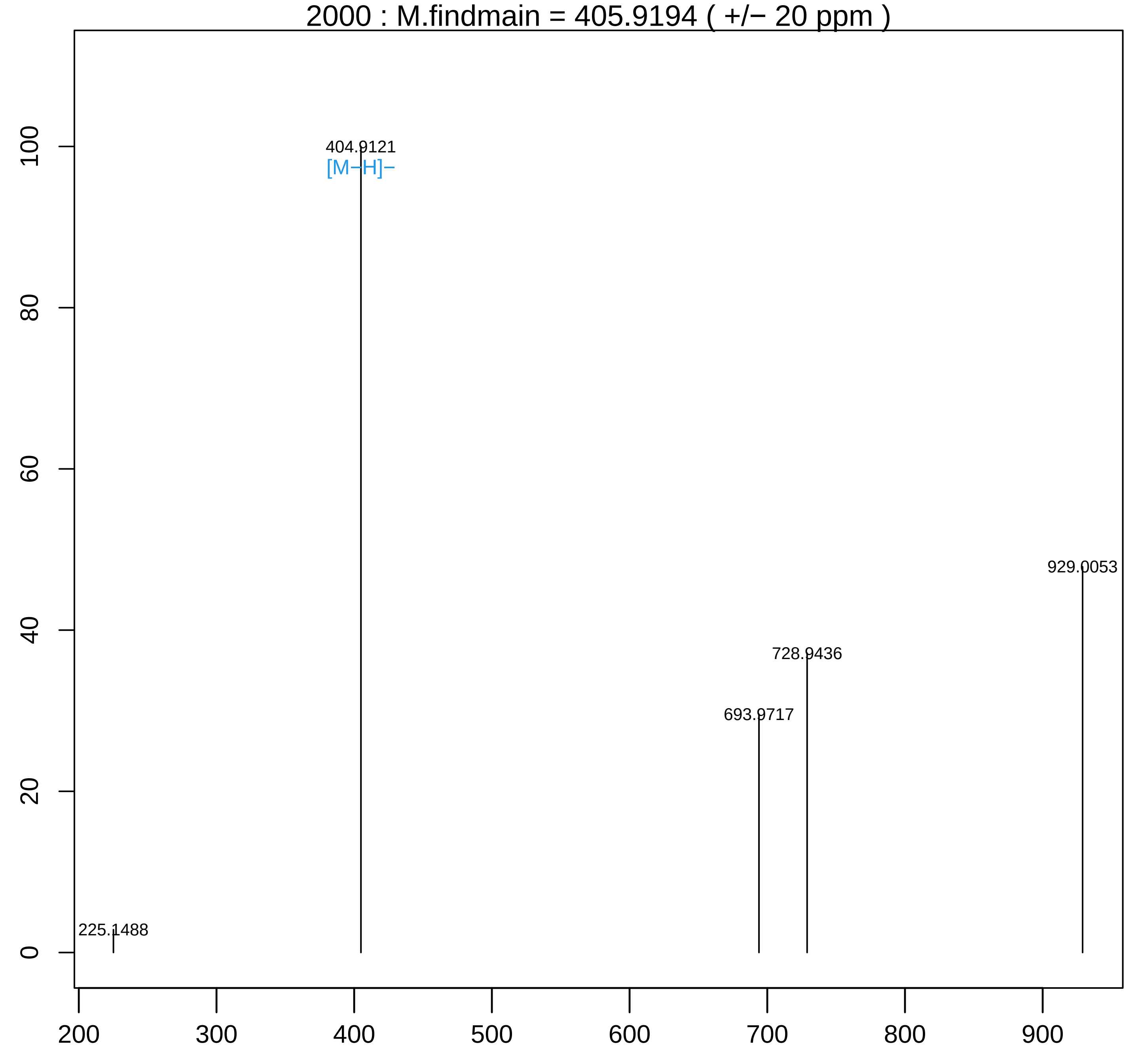
2000 : M.findmain = 405.9194 (+/− 20 ppm)
404.9121
[M−H]−
929.0053
728.9436
693.9717
225.1488
0
20
40
60
80
100
200
300
400
500
600
700
800
900

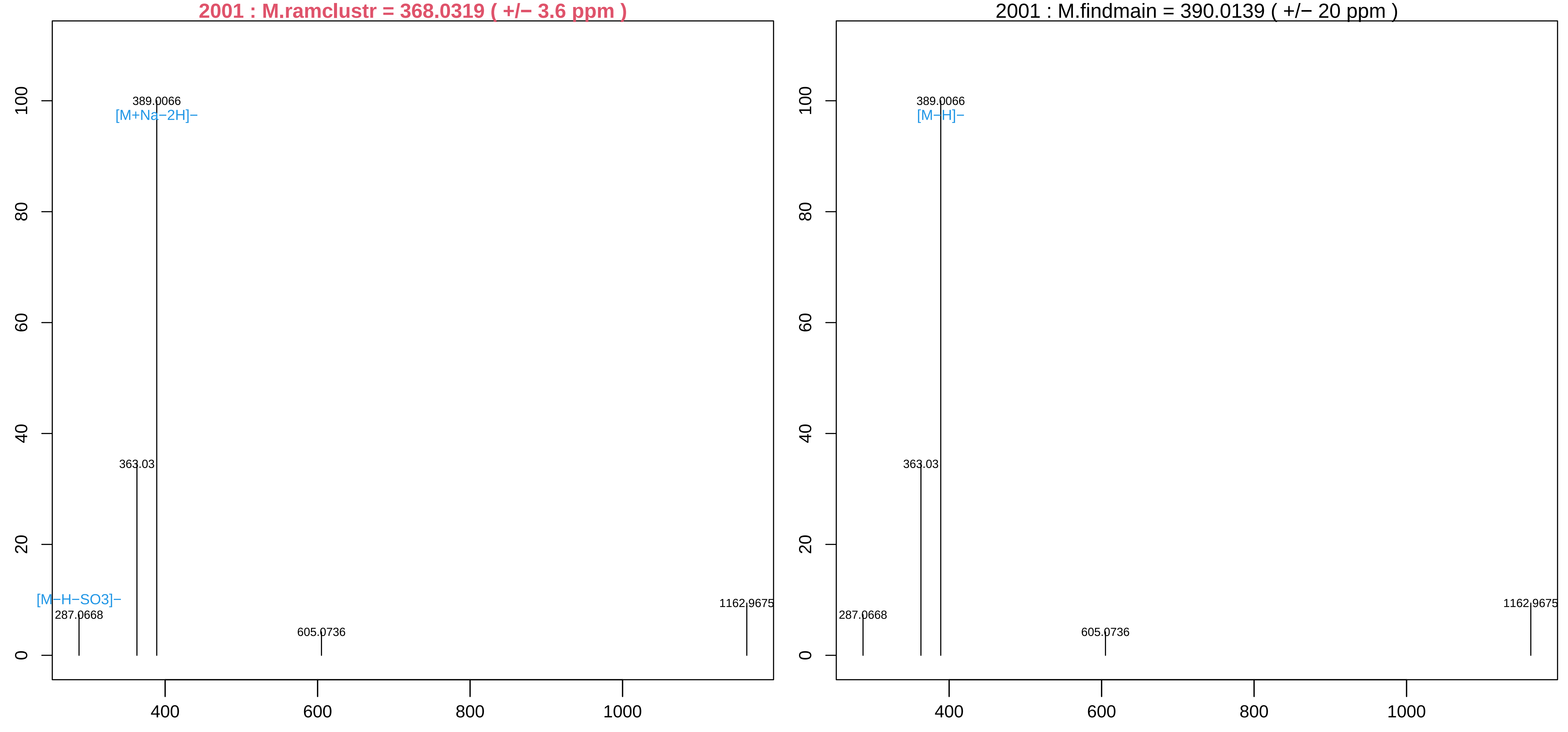
2001 : M.ramclustr = 368.0319 (+/− 3.6 ppm)
389.0066
[M+Na−2H]−
363.03
[M−H−SO3]−
287.0668
605.0736
1162.9675
0
20
40
60
80
100
400
600
800
1000
2001 : M.findmain = 390.0139 (+/− 20 ppm)
389.0066
[M−H]−
363.03
287.0668
605.0736
1162.9675
0
20
40
60
80
100
400
600
800
1000

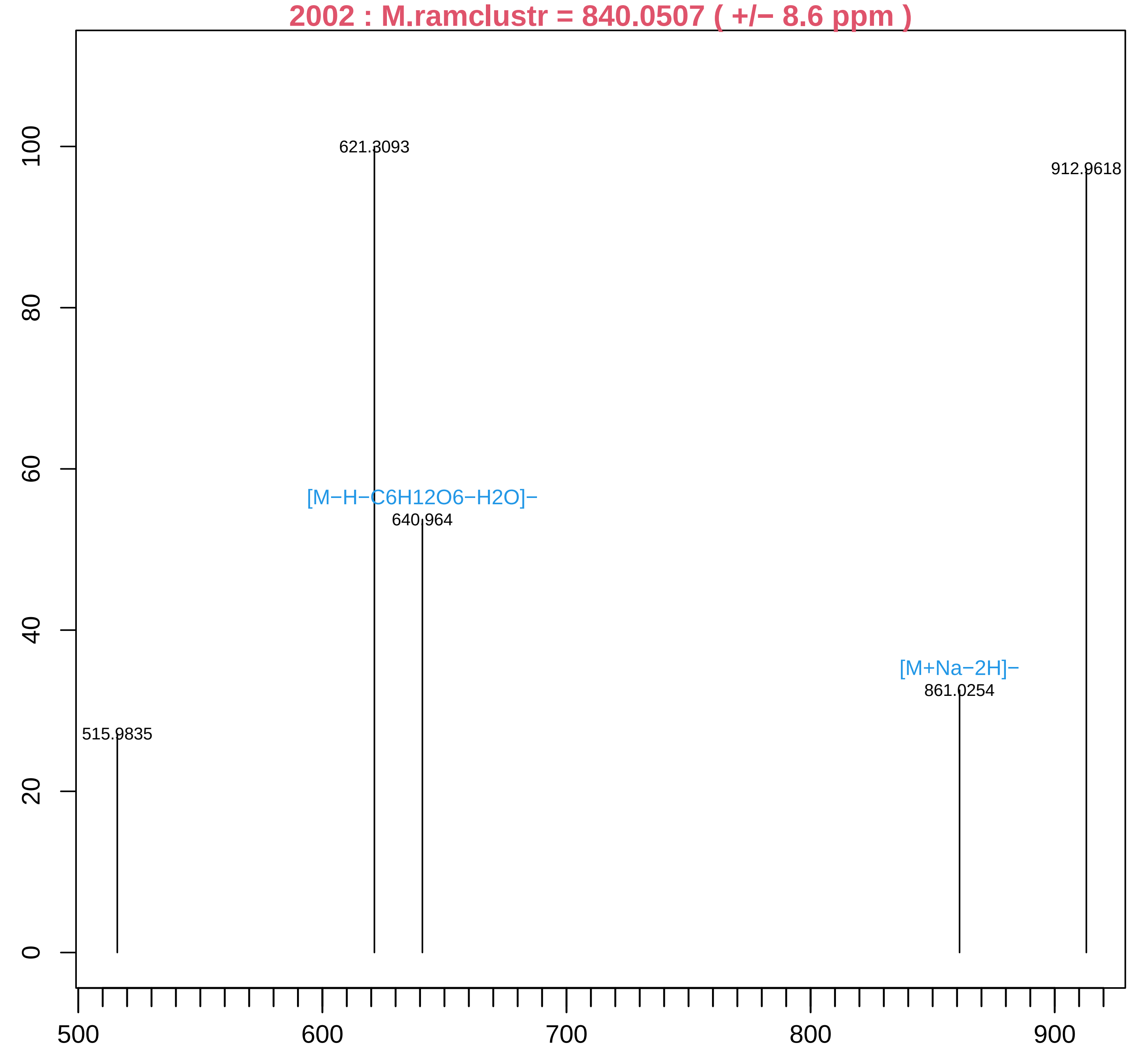
2002 : M.ramclustr = 840.0507 (+/− 8.6 ppm)
621.3093
912.9618
[M−H−C6H12O6−H2O]−
640.964
[M+Na−2H]−
861.0254
515.9835
0
20
40
60
80
100
500
600
700
800
900

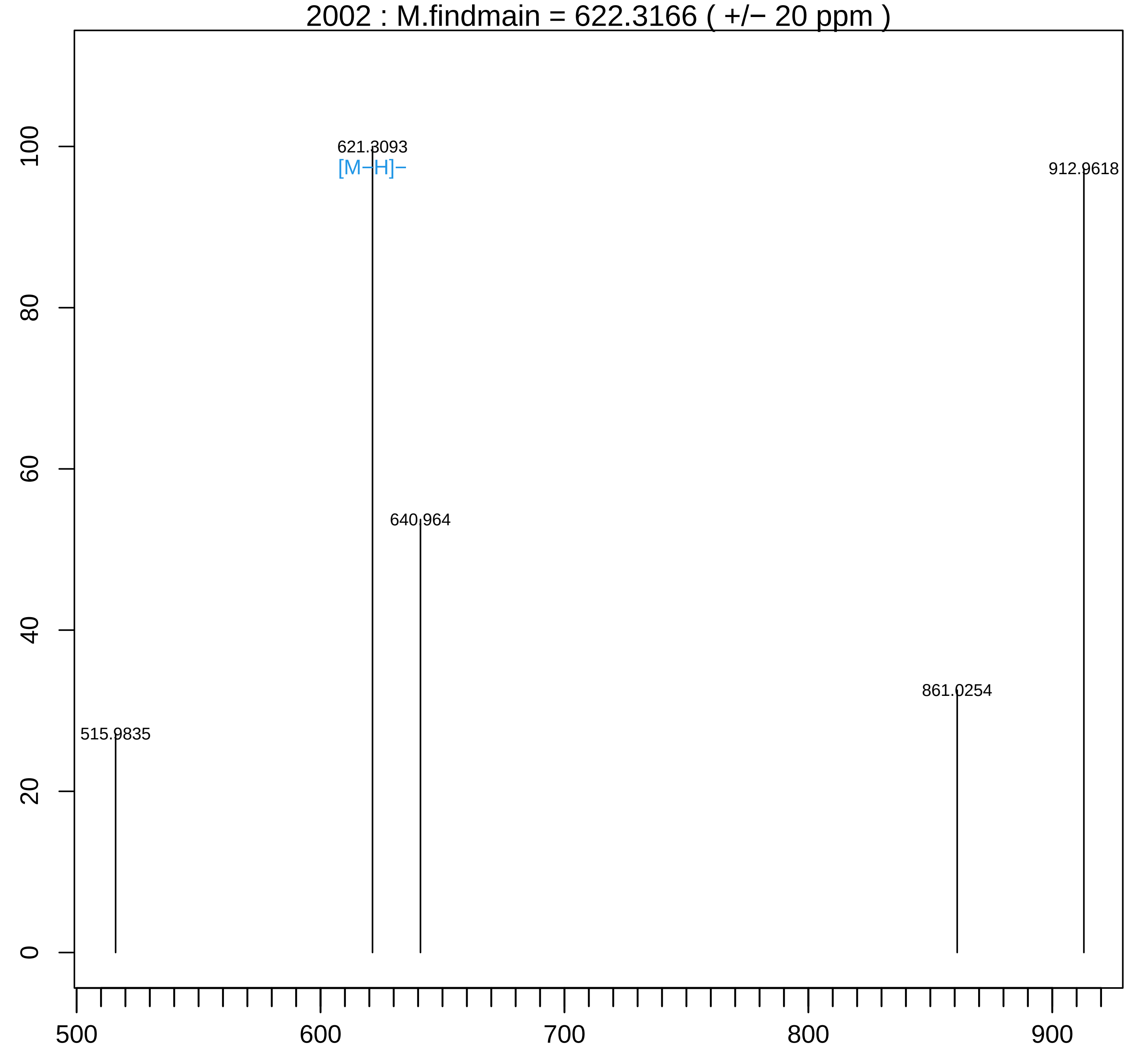
2002 : M.findmain = 622.3166 (+/− 20 ppm)
621.3093
[M−H]−
912.9618
640.964
861.0254
515.9835
0
20
40
60
80
100
500
600
700
800
900

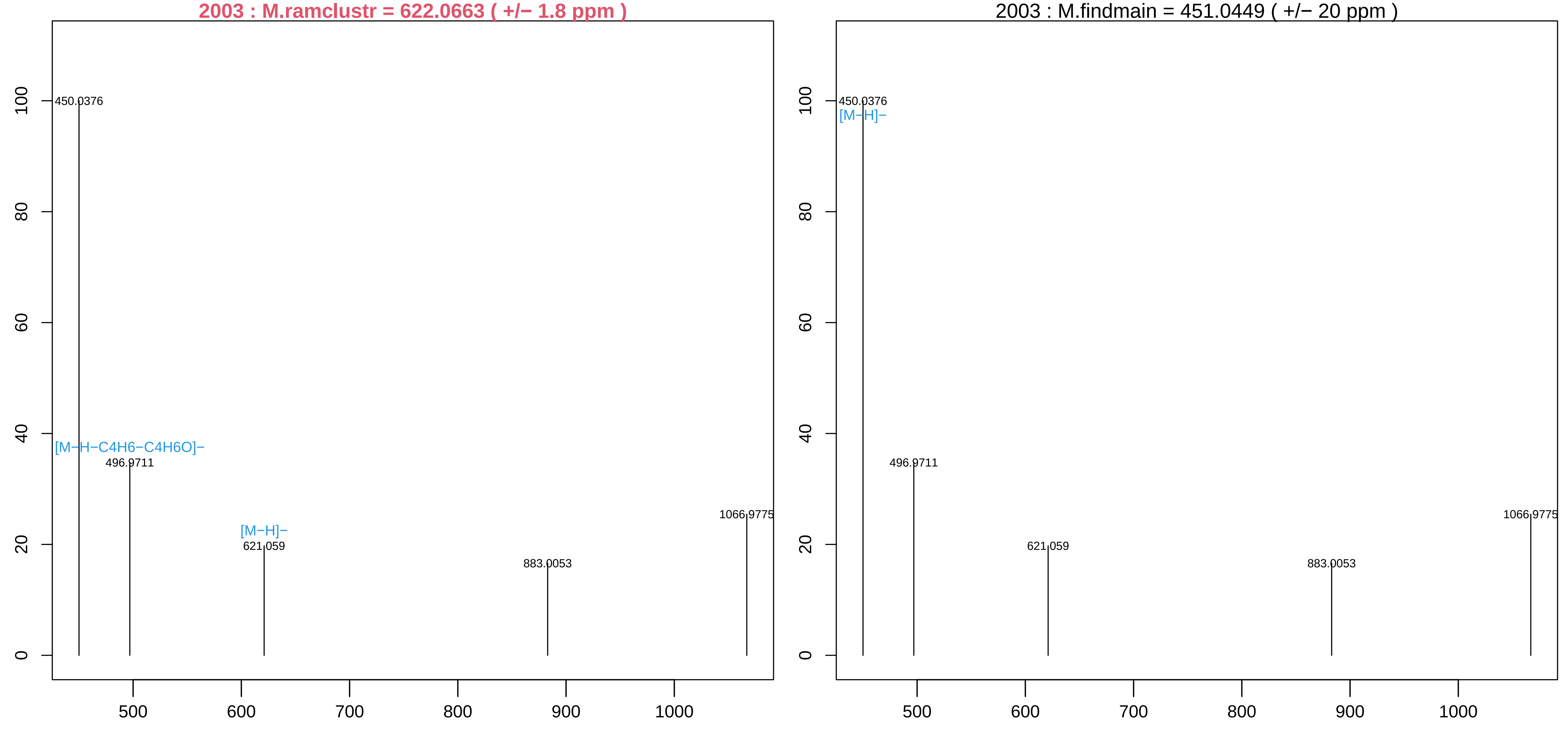

2003 : M.ramclustr = 622.0663 (+/− 1.8 ppm)
450.0376
[M−H−C4H6−C4H6O]−
496.9711
[M−H]−
621.059
883.0053
1066.9775
0
20
40
60
80
100
500
600
700
800
900
1000
2003 : M.findmain = 451.0449 (+/− 20 ppm)
450.0376
[M−H]−
496.9711
621.059
883.0053
1066.9775
0
20
40
60
80
100
500
600
700
800
900
1000

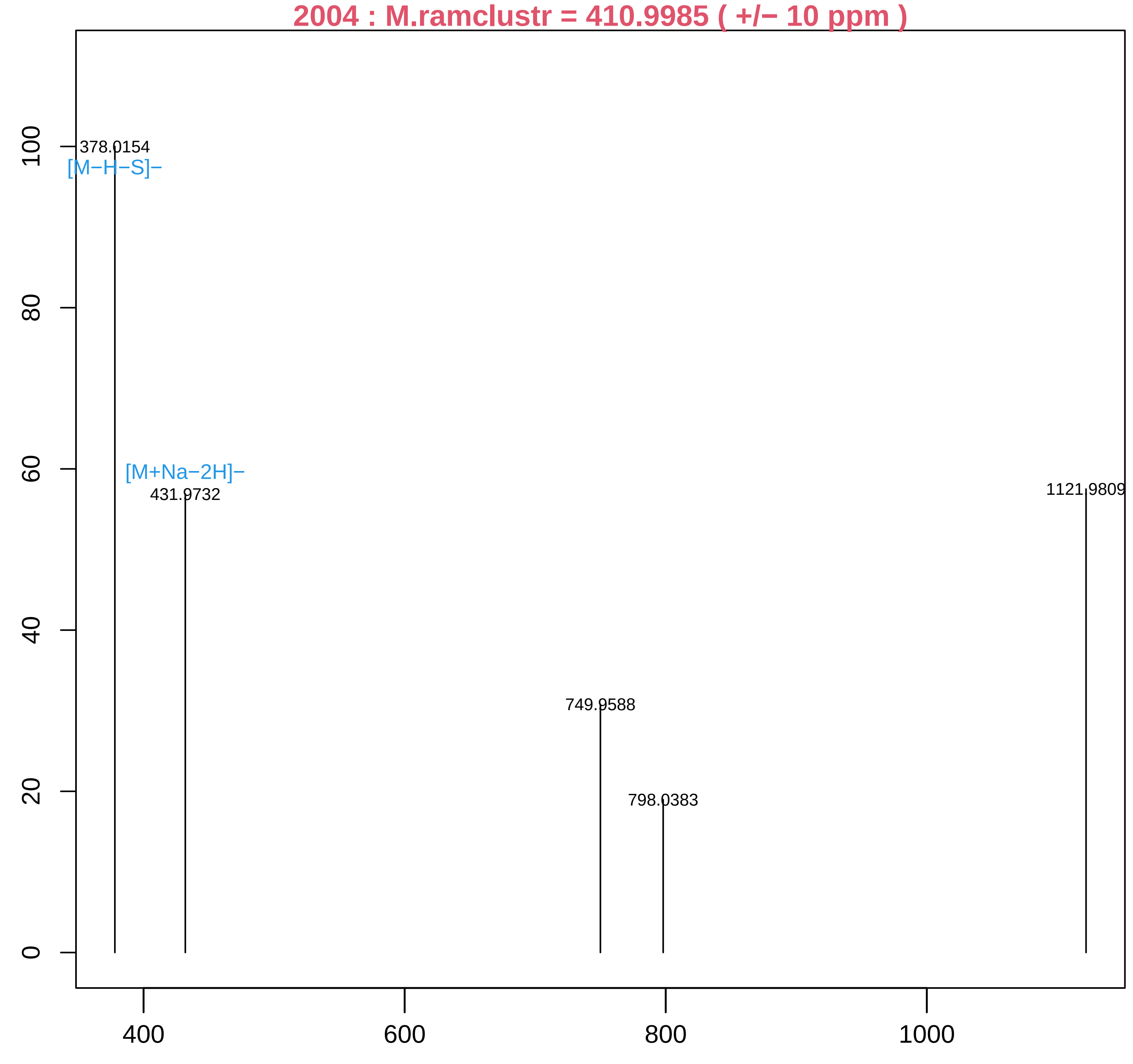
2004 : M.ramclustr = 410.9985 (+/− 10 ppm)
378.0154
[M−H−S]−
[M+Na−2H]−
431.9732
749.9588
798.0383
1121.9809
0
20
40
60
80
100
400
600
800
1000

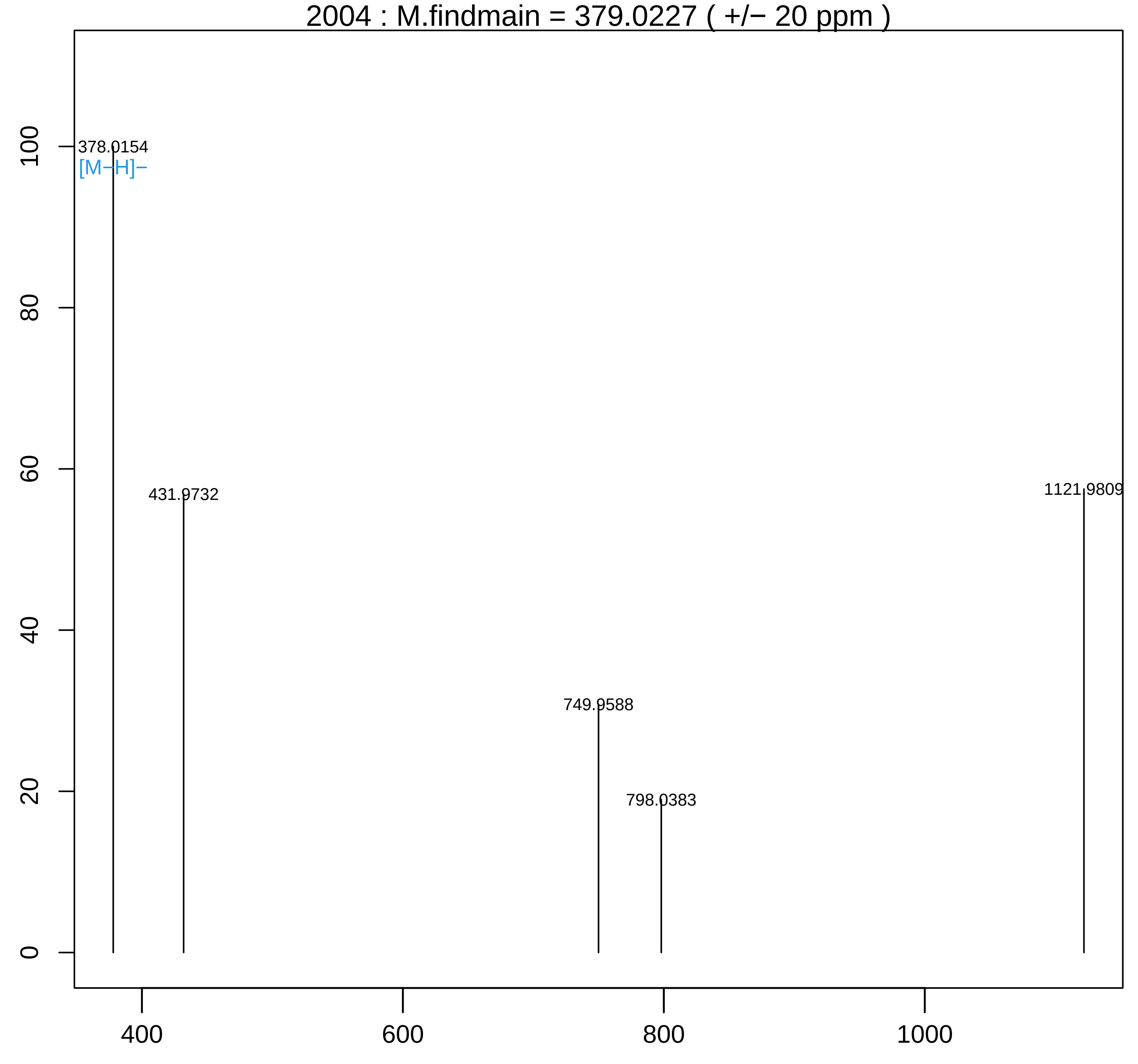
2004 : M.findmain = 379.0227 (+/− 20 ppm)
378.0154
[M−H]−
431.9732
749.9588
798.0383
1121.9809
0
20
40
60
80
100
400
600
800
1000

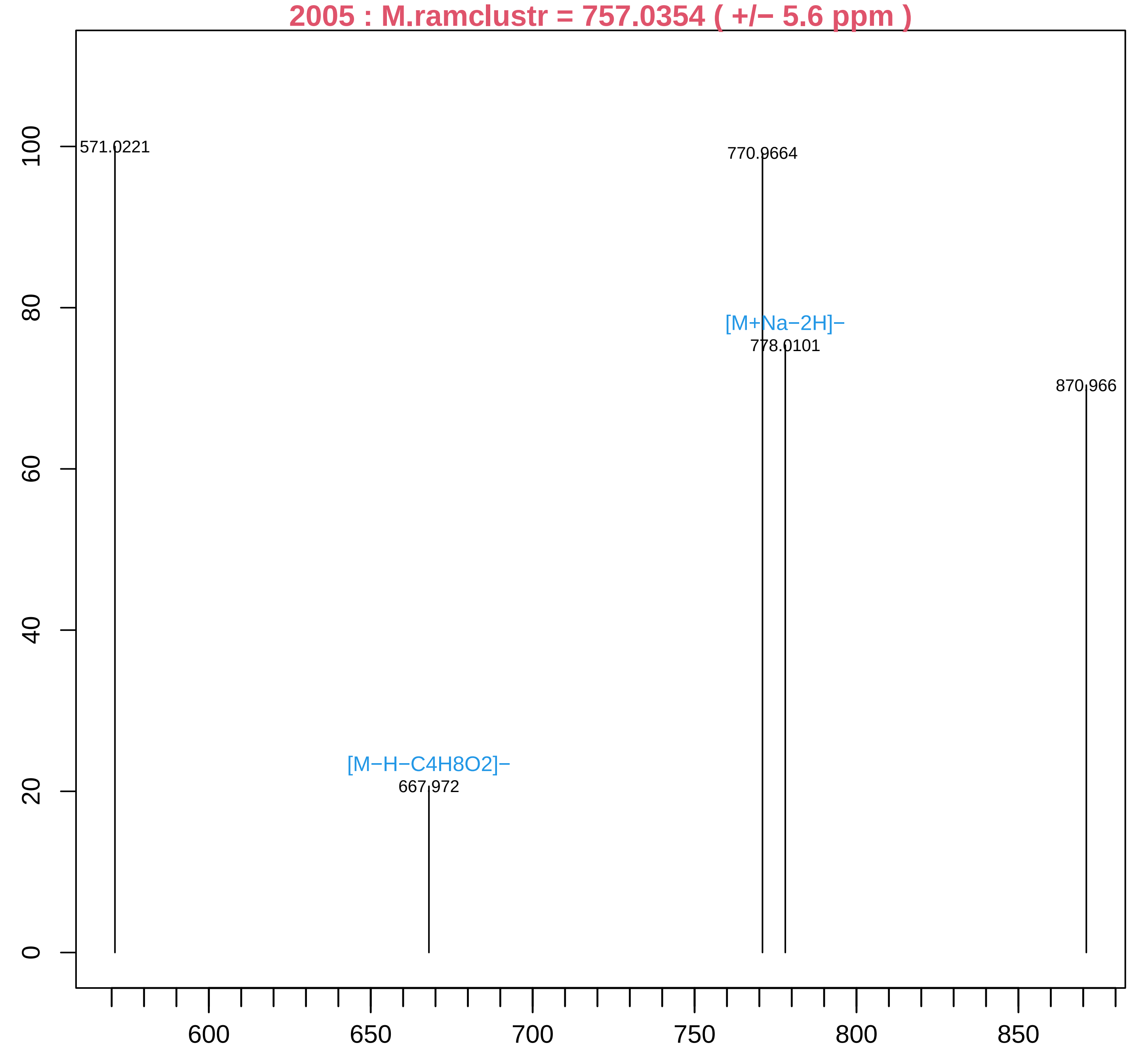
2005 : M.ramclustr = 757.0354 (+/− 5.6 ppm)
571.0221
770.9664
[M+Na−2H]−
778.0101
870.966
[M−H−C4H8O2]−
667.972
0
20
40
60
80
100
600
650
700
750
800
850

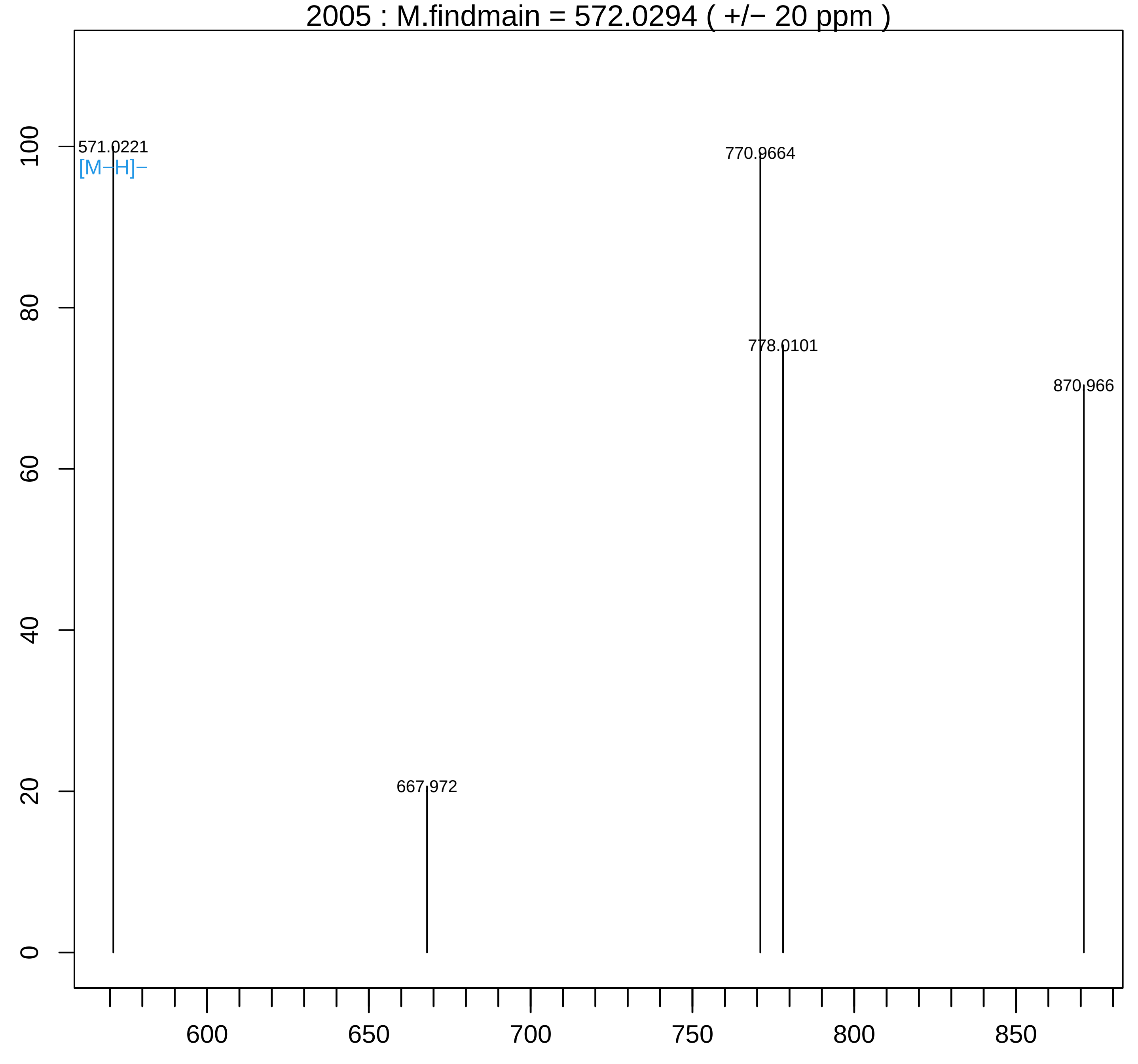
2005 : M.findmain = 572.0294 (+/− 20 ppm)
571.0221
[M−H]−
770.9664
778.0101
870.966
667.972
0
20
40
60
80
100
600
650
700
750
800
850

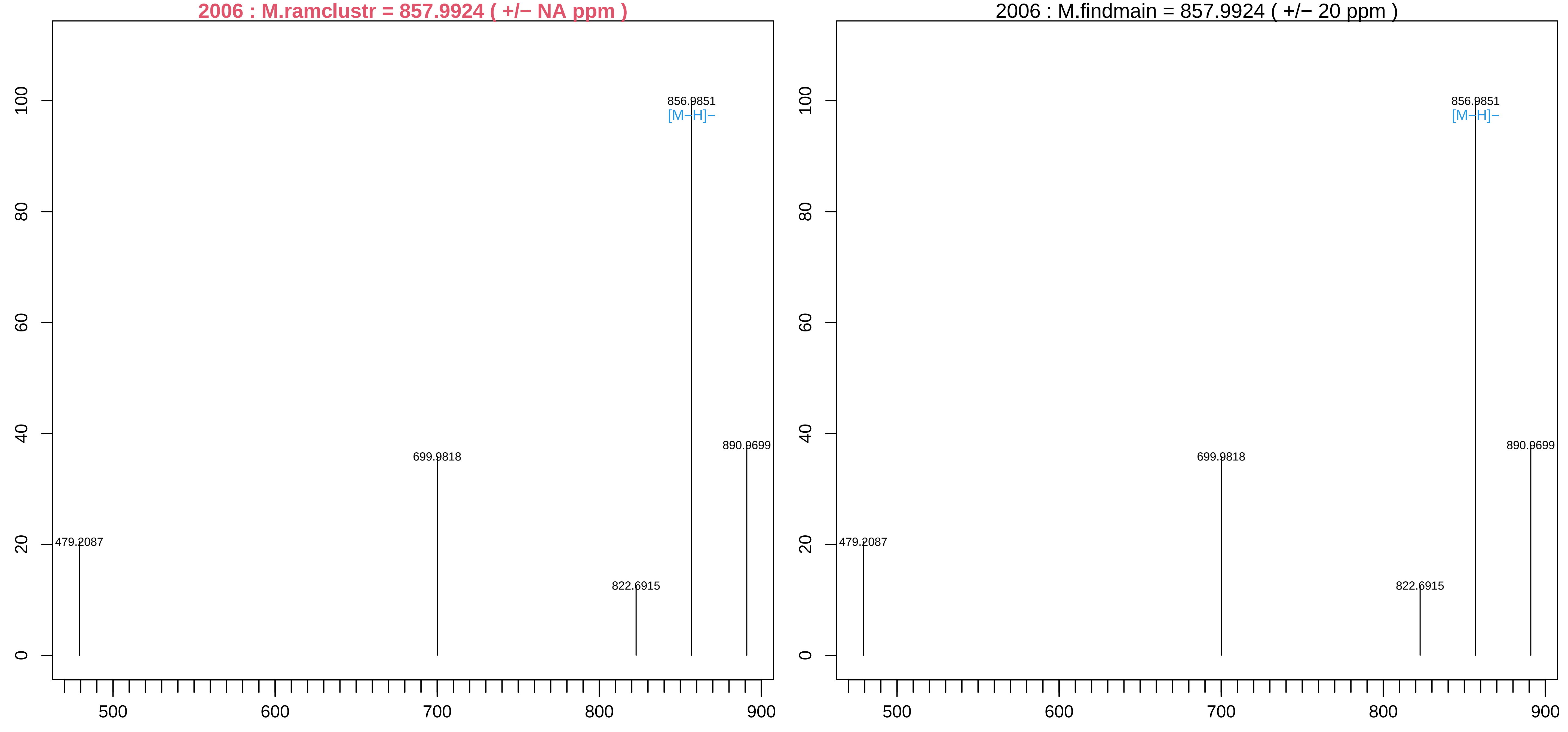
2006 : M.ramclustr = 857.9924 (+/− NA ppm)
856.9851
[M−H]−
699.9818
890.9699
479.2087
822.6915
0
20
40
60
80
100
500
600
700
800
900
2006 : M.findmain = 857.9924 (+/− 20 ppm)
856.9851
[M−H]−
699.9818
890.9699
479.2087
822.6915
0
20
40
60
80
100
500
600
700
800
900

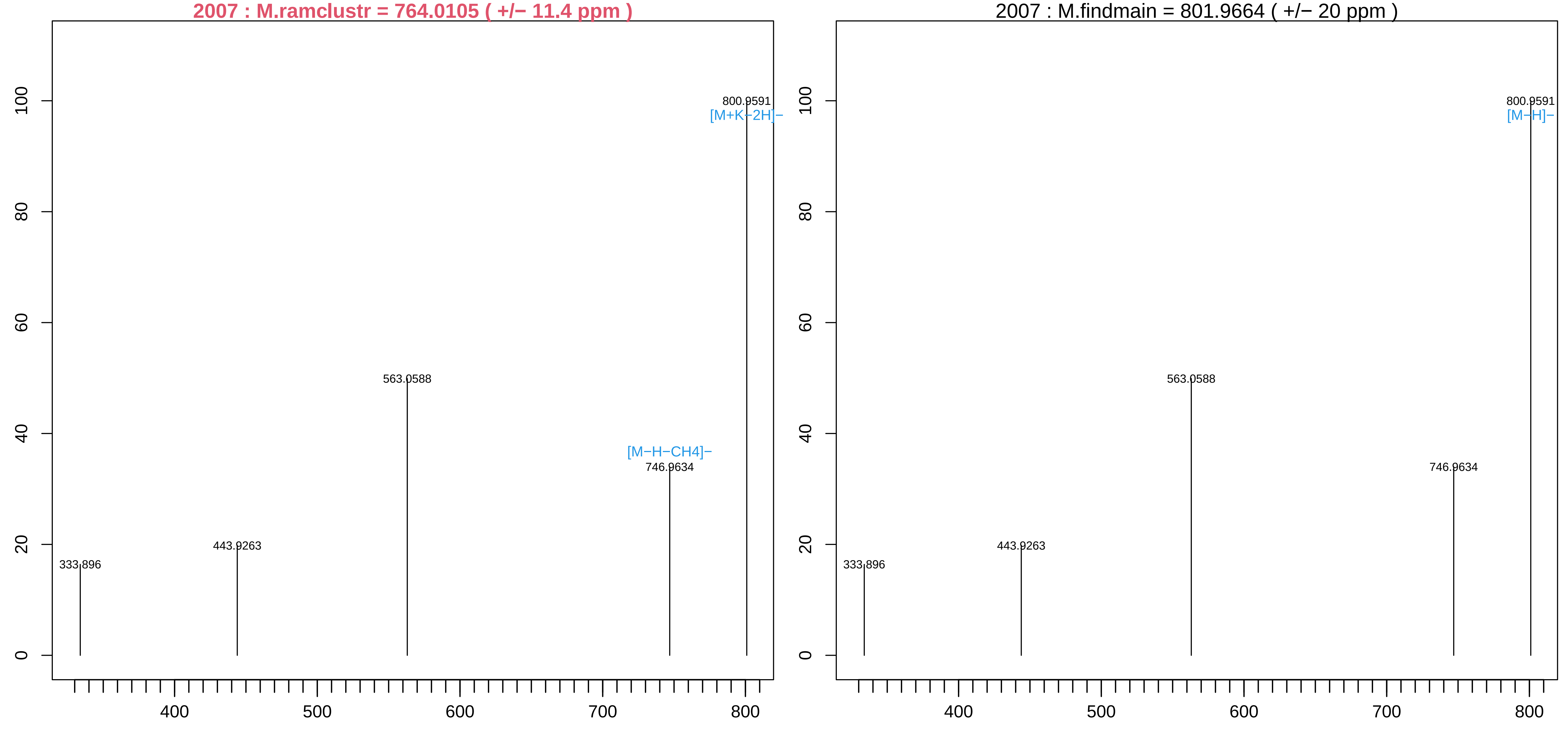
2007 : M.ramclustr = 764.0105 (+/− 11.4 ppm)
800.9591
[M+K−2H]−
563.0588
[M−H−CH4]−
746.9634
443.9263
333.896
0
20
40
60
80
100
400
500
600
700
800
2007 : M.findmain = 801.9664 (+/− 20 ppm)
800.9591
[M−H]−
563.0588
746.9634
443.9263
333.896

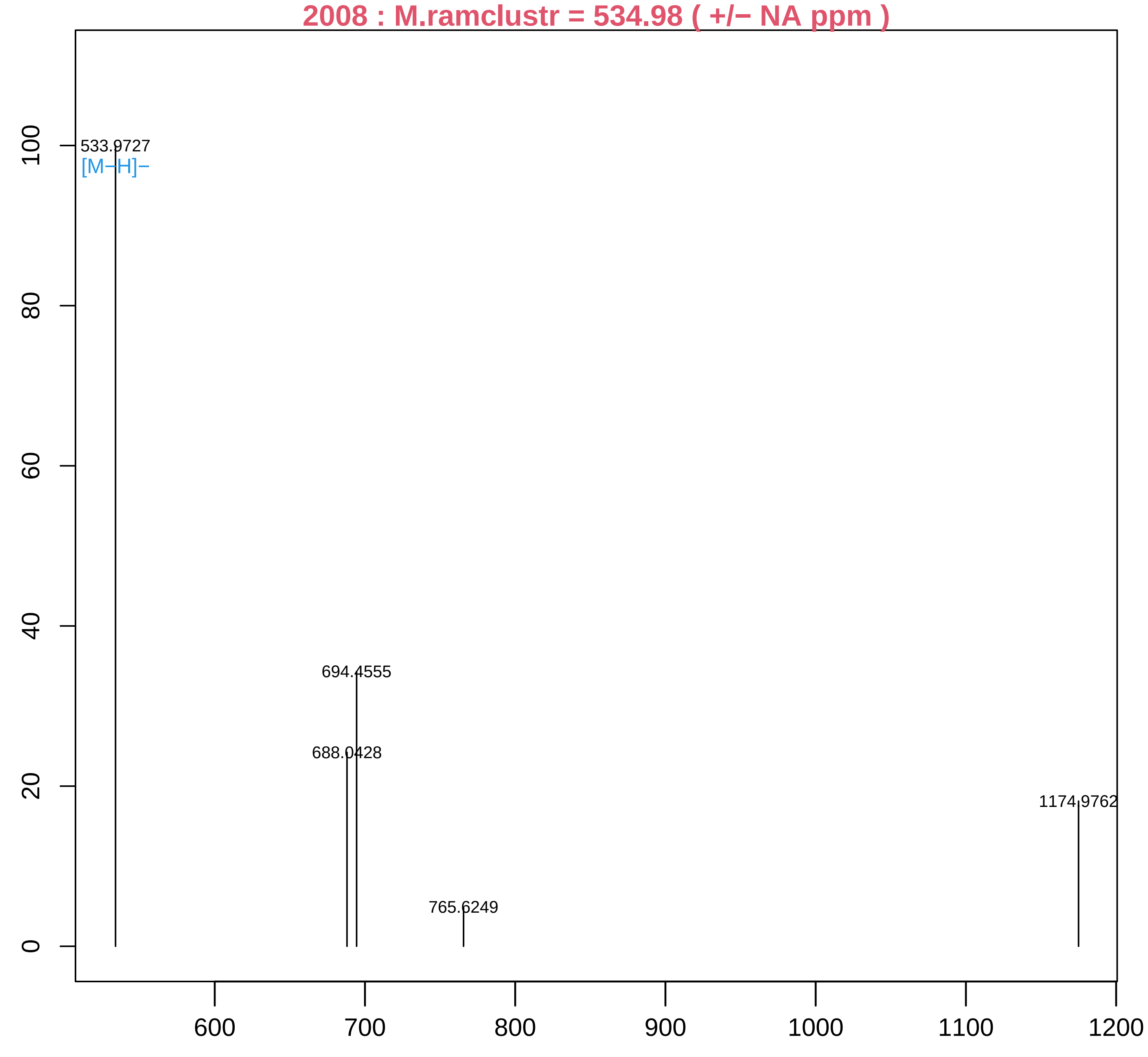
2008 : M.ramclustr = 534.98 (+/− NA ppm)
533.9727
[M−H]−
694.4555
688.0428
765.6249
1174.9762
0
20
40
60
80
100
600
700
800
900
1000
1100
1200

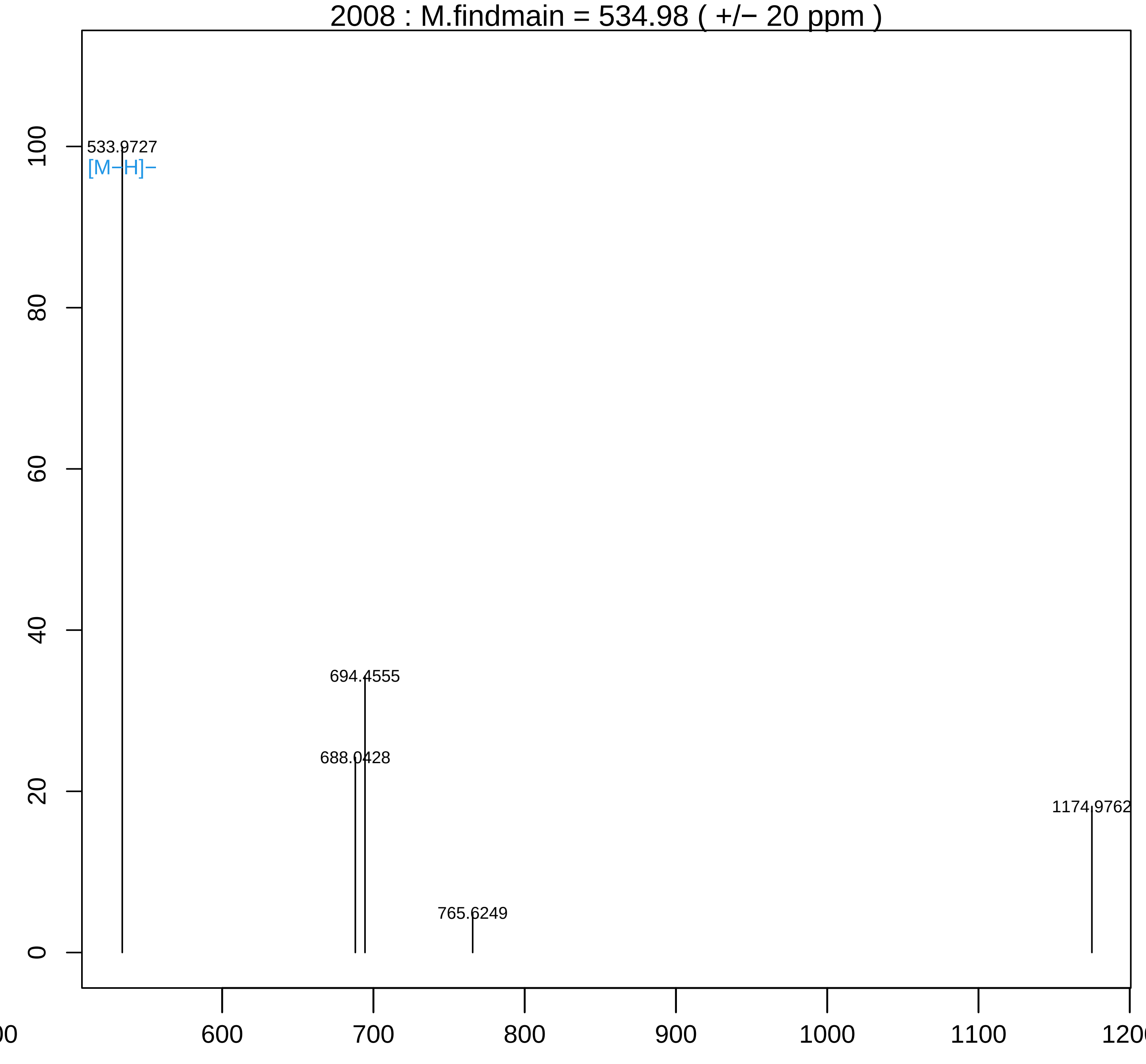
2008 : M.findmain = 534.98 (+/− 20 ppm)
533.9727
[M−H]−
694.4555
688.0428
765.6249
1174.9762
0
20
40
60
80
100
600
700
800
900
1000
1100
1200

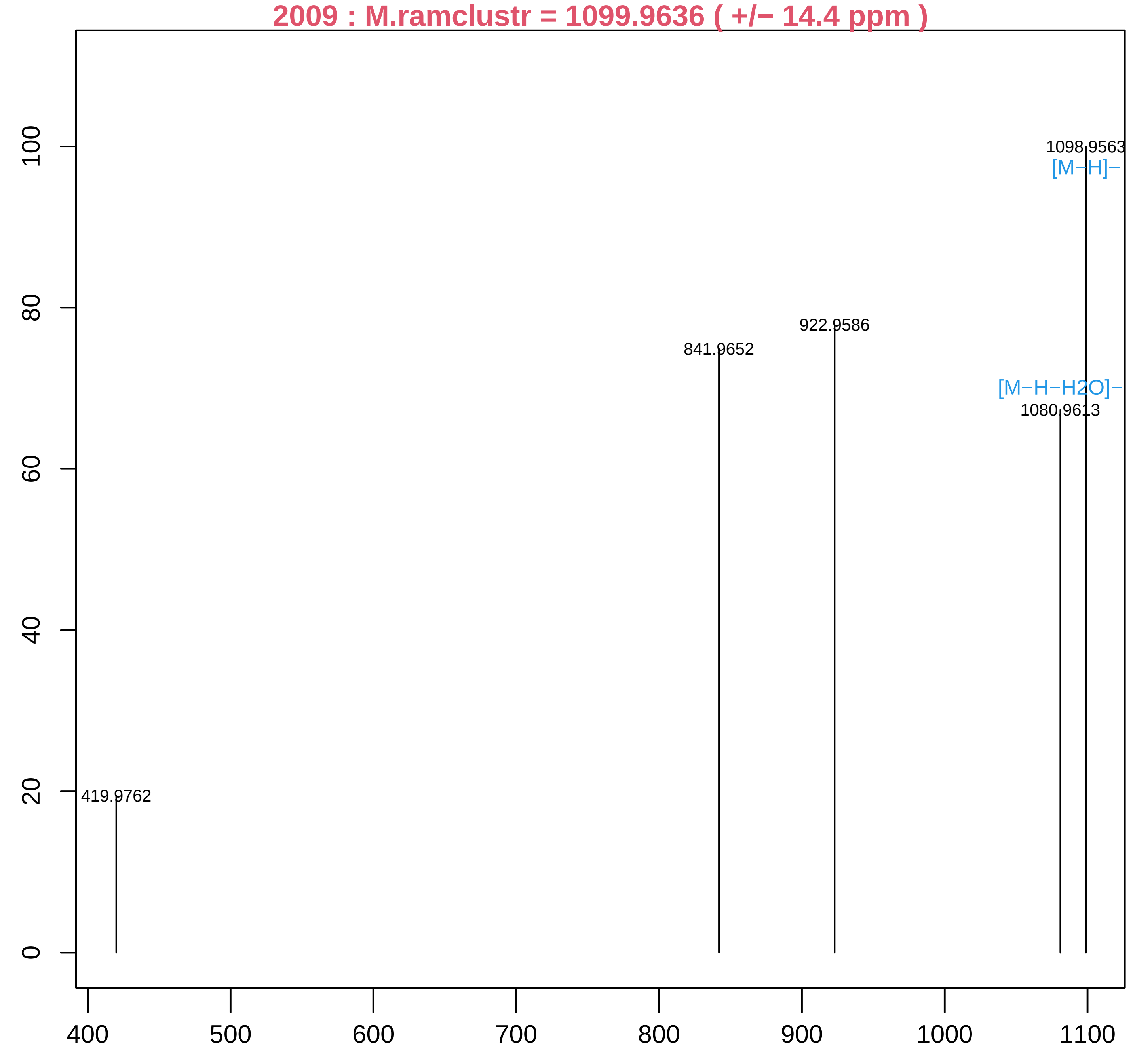

2009 : M.ramclustr = 1099.9636 (+/− 14.4 ppm)
1098.9563
[M−H]−
841.9652
922.9586
[M−H−H2O]−
1080.9613
419.9762
0
20
40
60
80
100
400
500
600
700
800
900
1000
1100

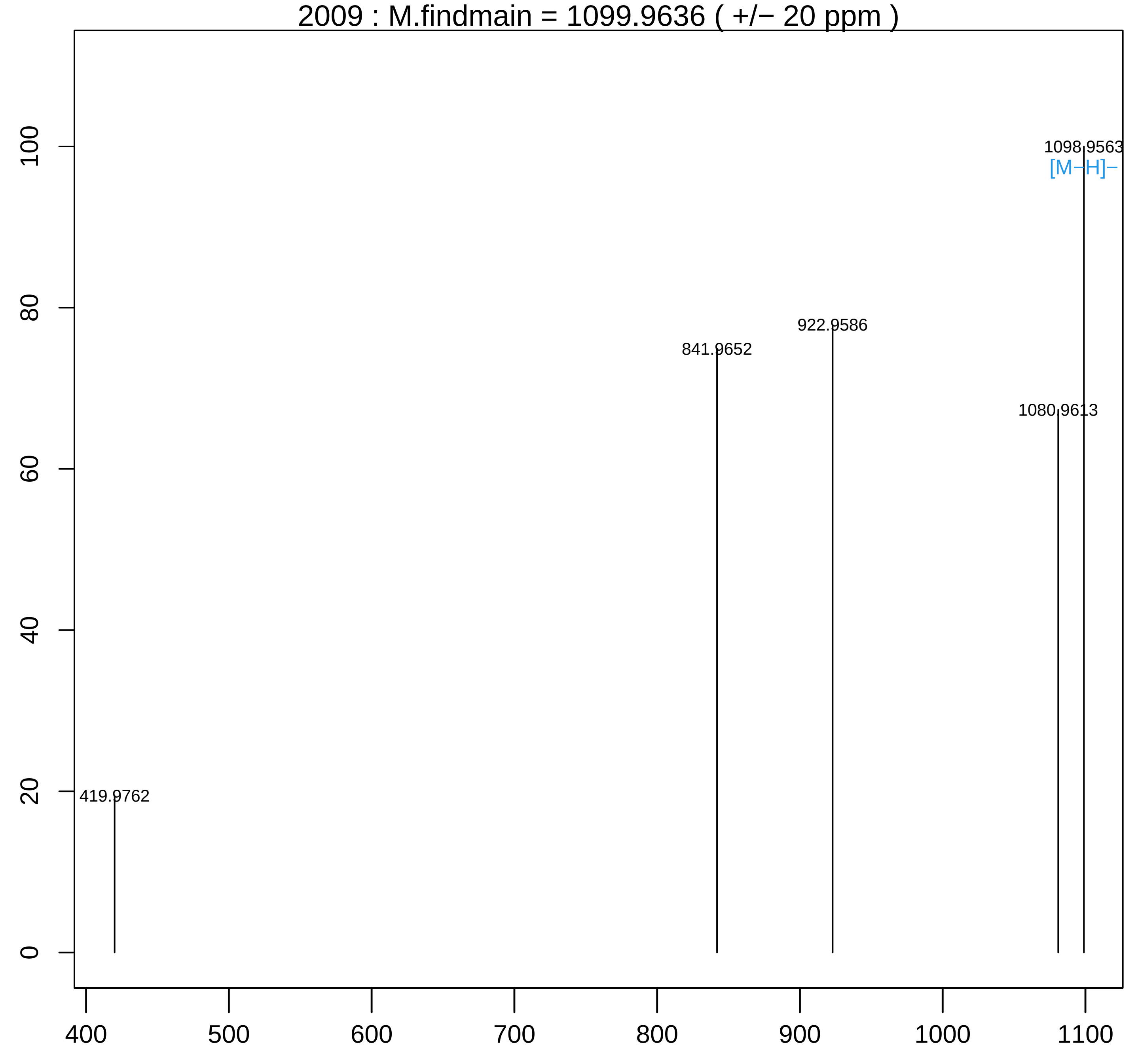

2009 : M.findmain = 1099.9636 (+/− 20 ppm)
1098.9563
[M−H]−
841.9652
922.9586
1080.9613
419.9762
0
20
40
60
80
100
400
500
600
700
800
900
1000
1100

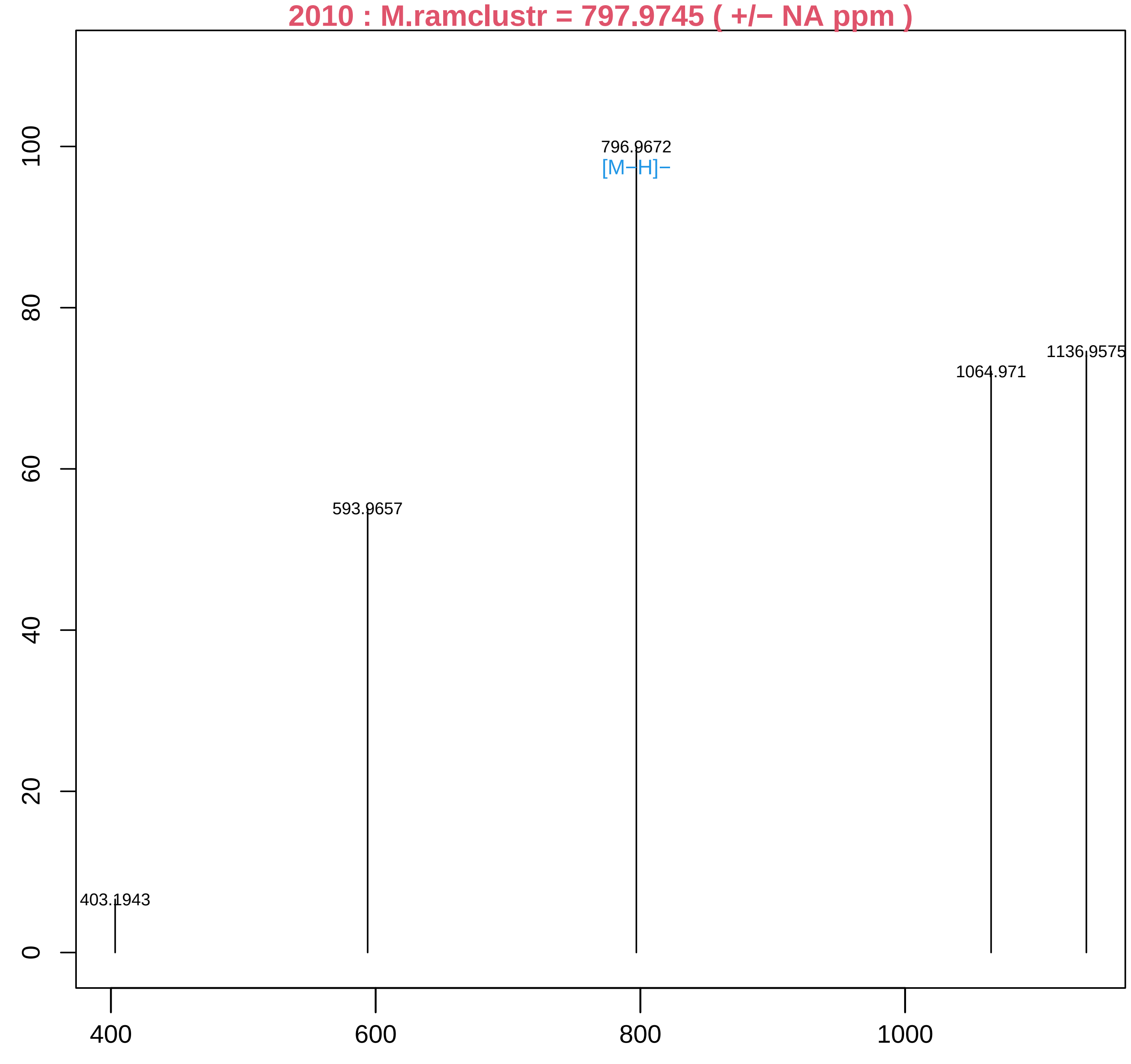
2010 : M.ramclustr = 797.9745 (+/− NA ppm)
796.9672
[M−H]−
1136.9575
1064.971
593.9657
403.1943
0
20
40
60
80
100
400
600
800
1000

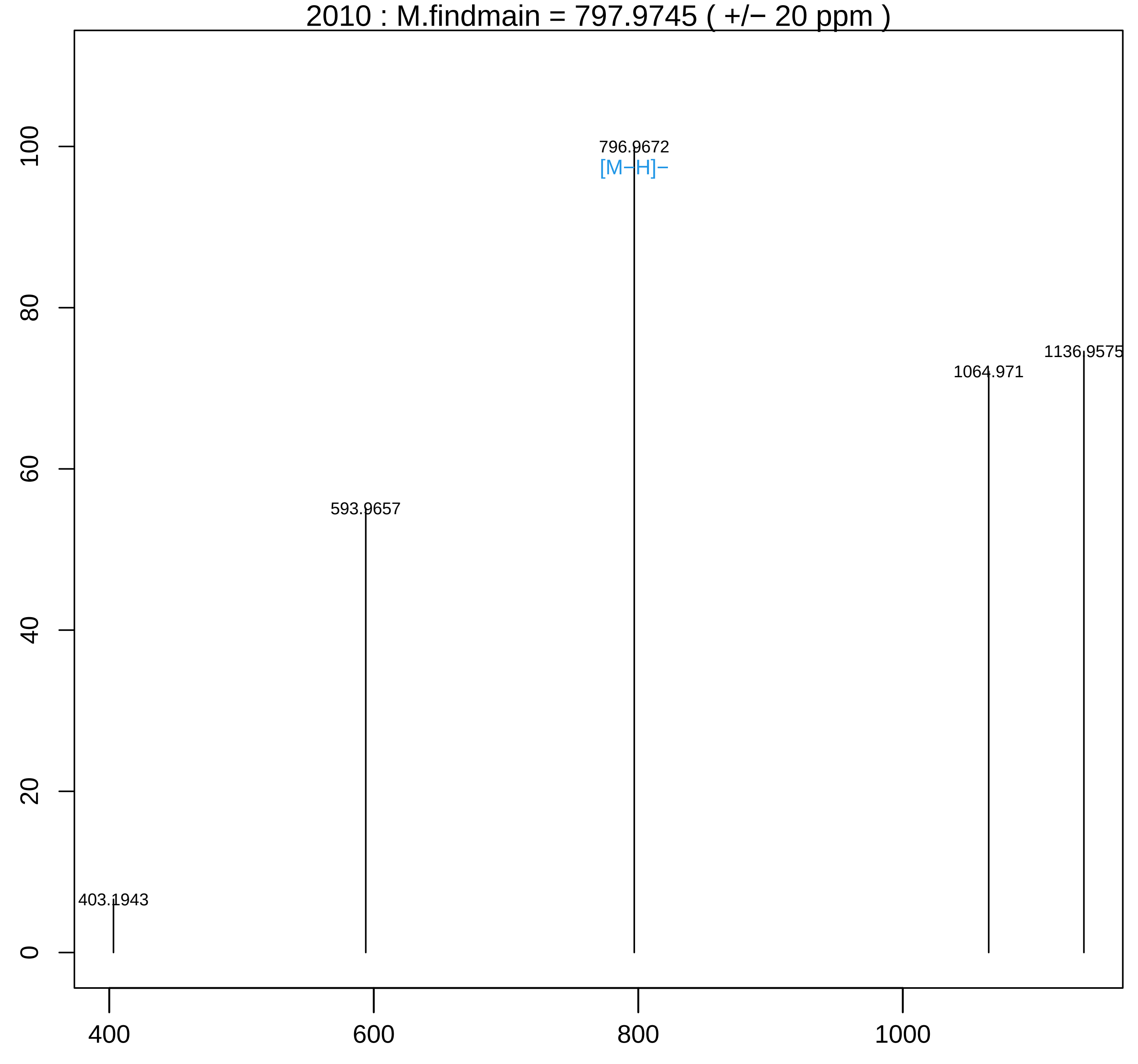
2010 : M.findmain = 797.9745 (+/− 20 ppm)
796.9672
[M−H]−
1136.9575
1064.971
593.9657
403.1943
0
20
40
60
80
100
400
600
800
1000

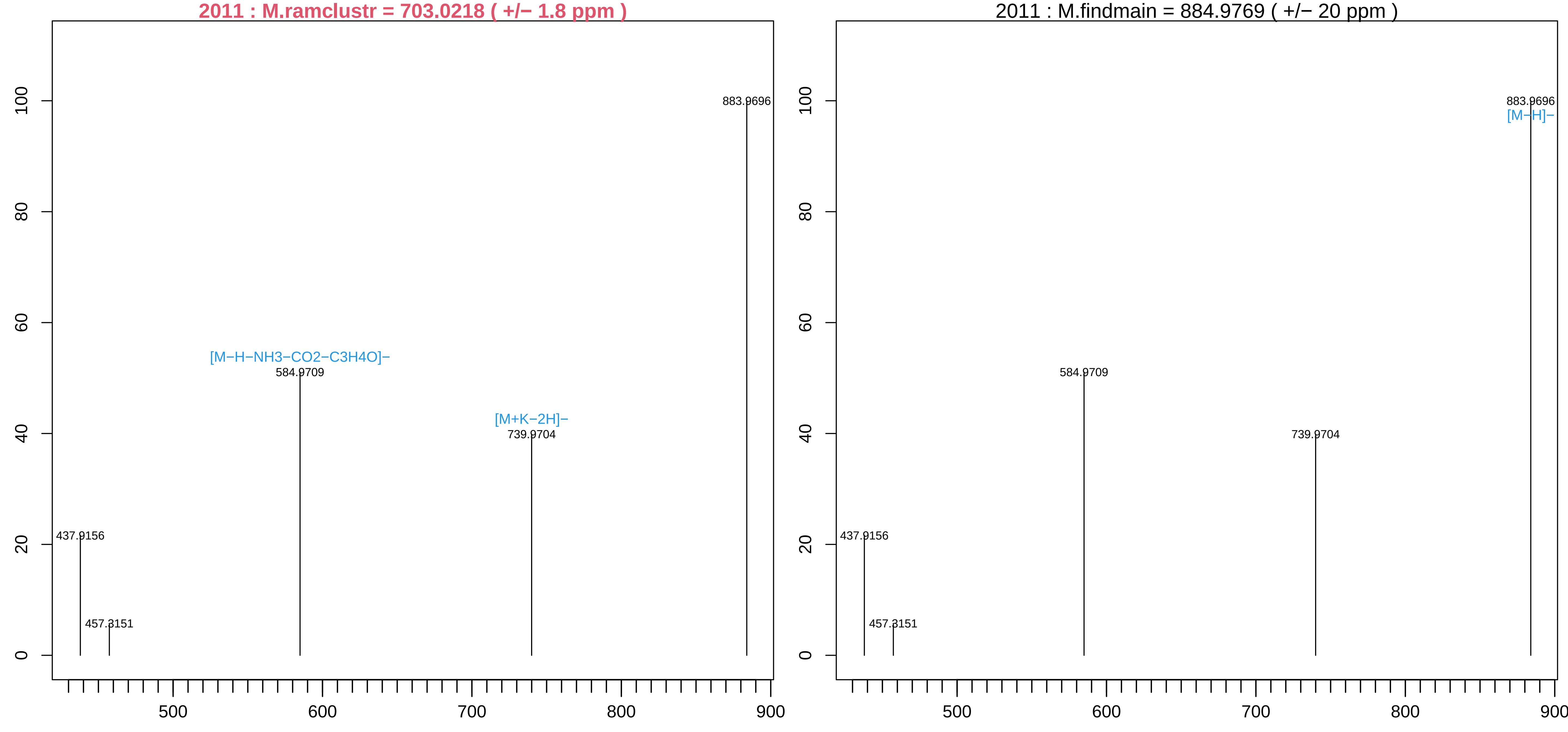
2011 : M.ramclustr = 703.0218 (+/− 1.8 ppm)
883.9696
[M−H−NH3−CO2−C3H4O]−
584.9709
[M+K−2H]−
739.9704
437.9156
457.3151
2011 : M.findmain = 884.9769 (+/− 20 ppm)
883.9696
[M−H]−
584.9709
739.9704
437.9156
457.3151

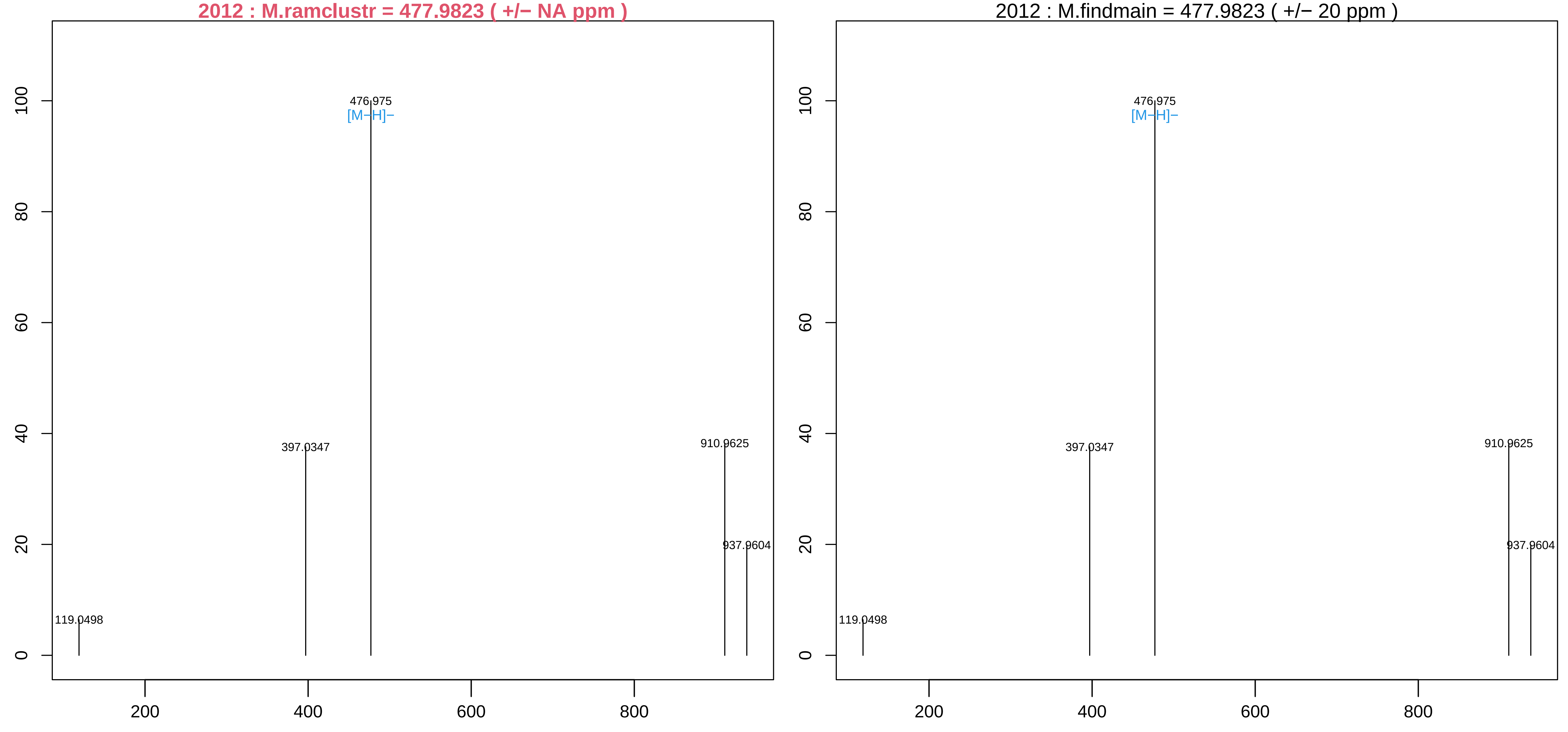

2012 : M.ramclustr = 477.9823 (+/− NA ppm)
2012 : M.findmain = 477.9823 (+/− 20 ppm)
476.975
[M−H]−
397.0347
910.9625
937.9604
119.0498
0
20
40
60
80
100
200
400
600
800

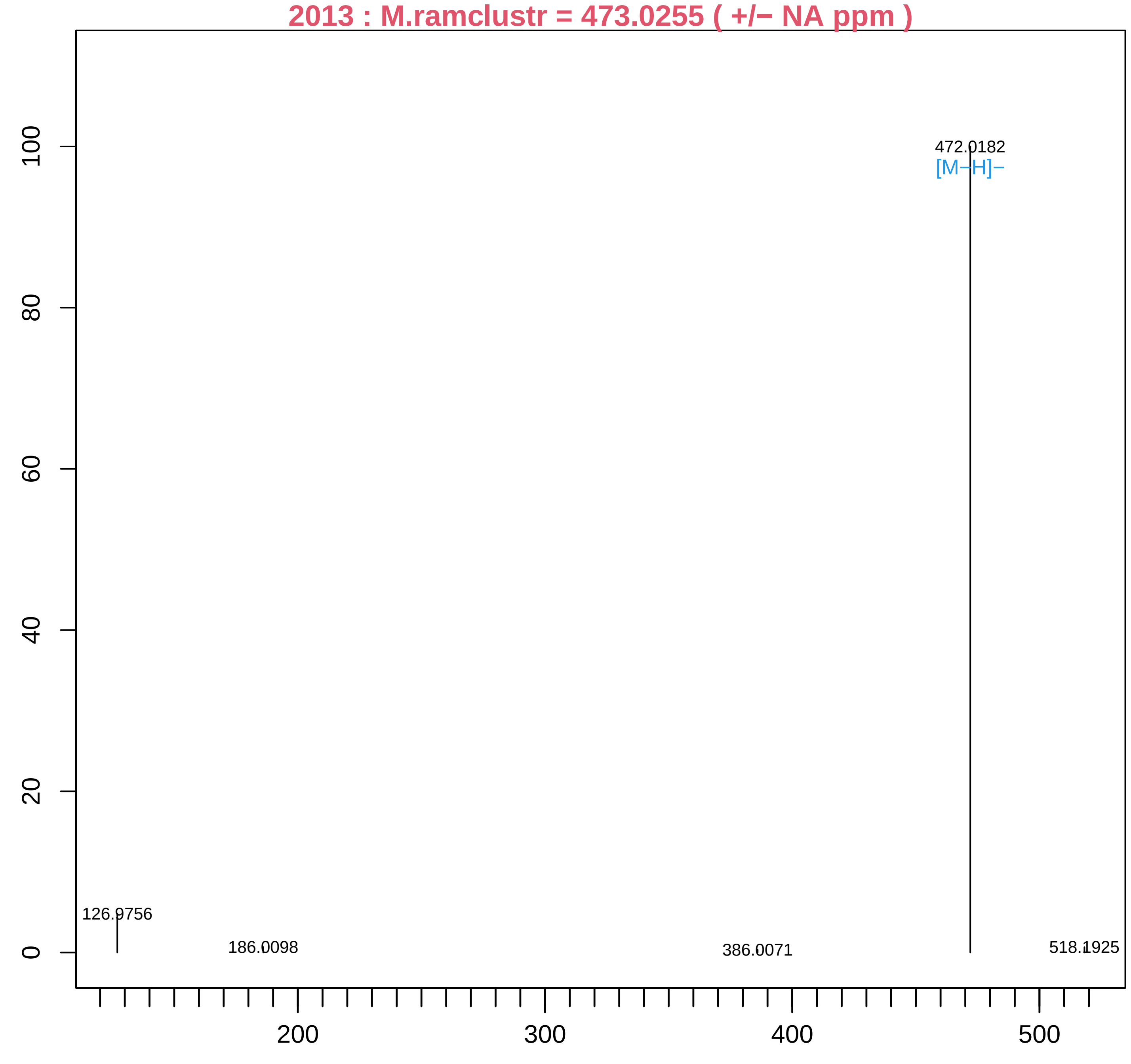

2013 : M.ramclustr = 473.0255 (+/− NA ppm)
472.0182
[M−H]−
126.9756
186.0098
386.0071
518.1925
0
20
40
60
80
100
200
300
400
500

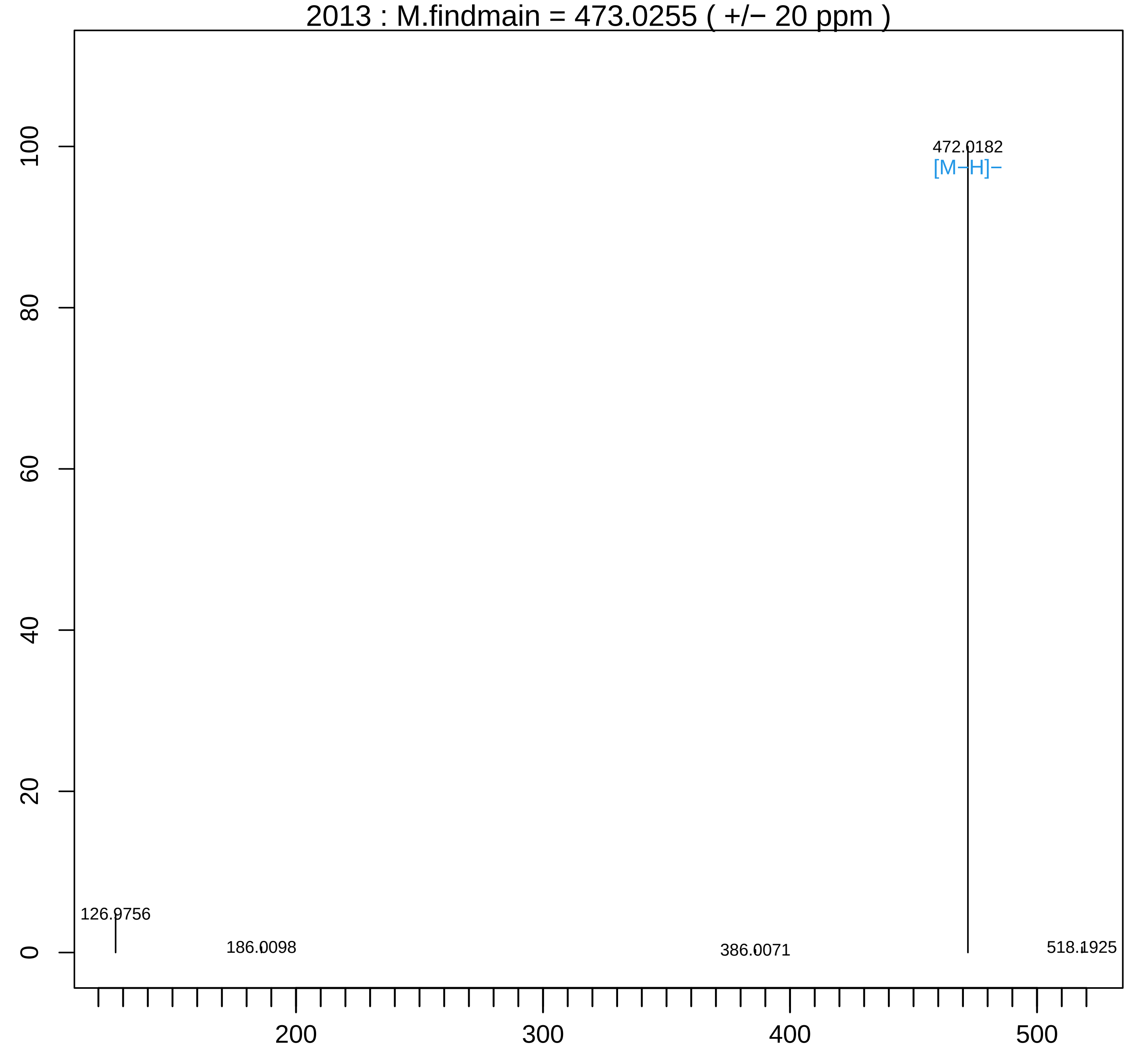

2013 : M.findmain = 473.0255 (+/− 20 ppm)
472.0182
[M−H]−
126.9756
186.0098
386.0071
518.1925
0
20
40
60
80
100
200
300
400
500

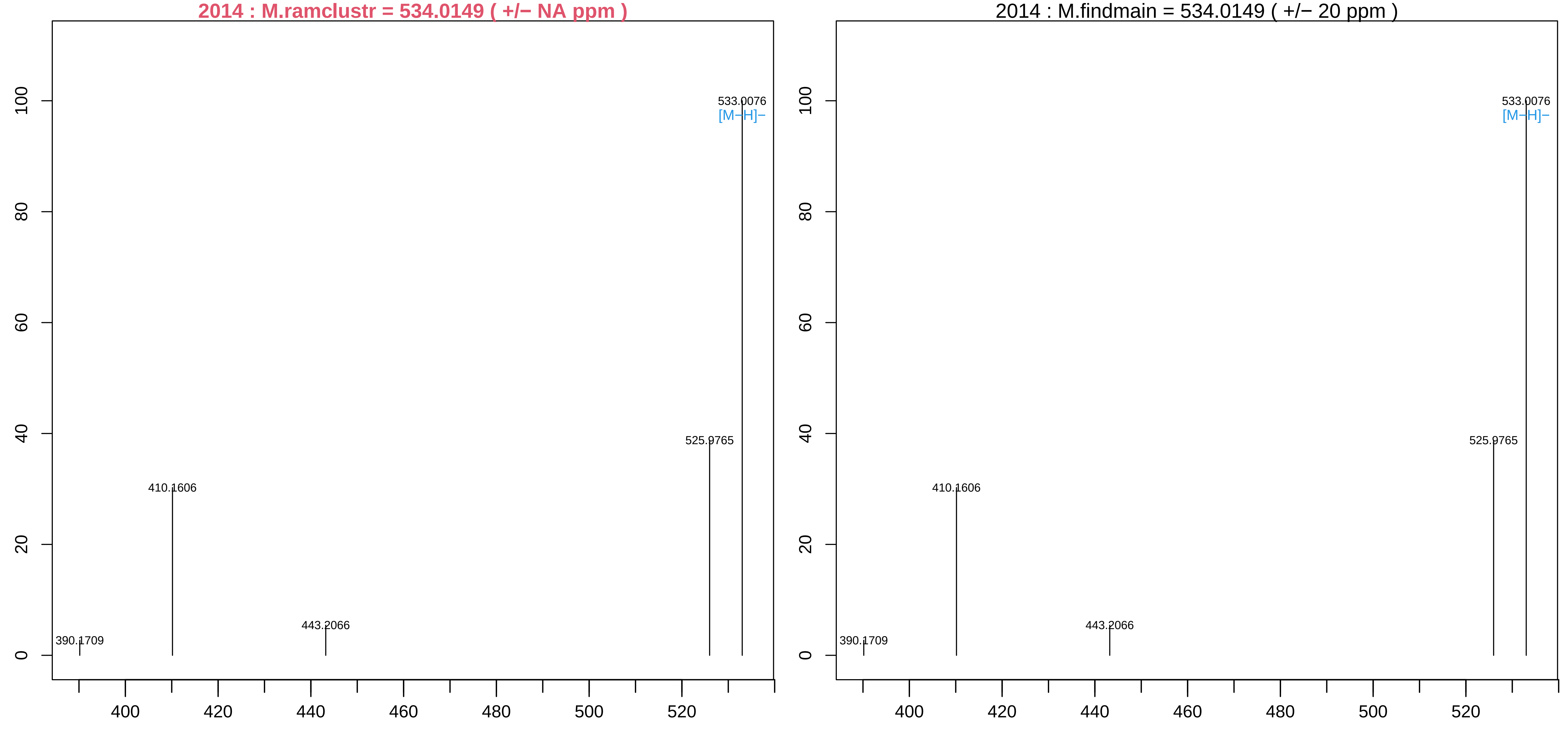
2014 : M.ramclustr = 534.0149 (+/− NA ppm)
533.0076
[M−H]−
525.9765
410.1606
443.2066
390.1709
2014 : M.findmain = 534.0149 (+/− 20 ppm)
533.0076
[M−H]−
525.9765
410.1606
443.2066
390.1709
0
20
40
60
80
100
400
420
440
460
480
500
520

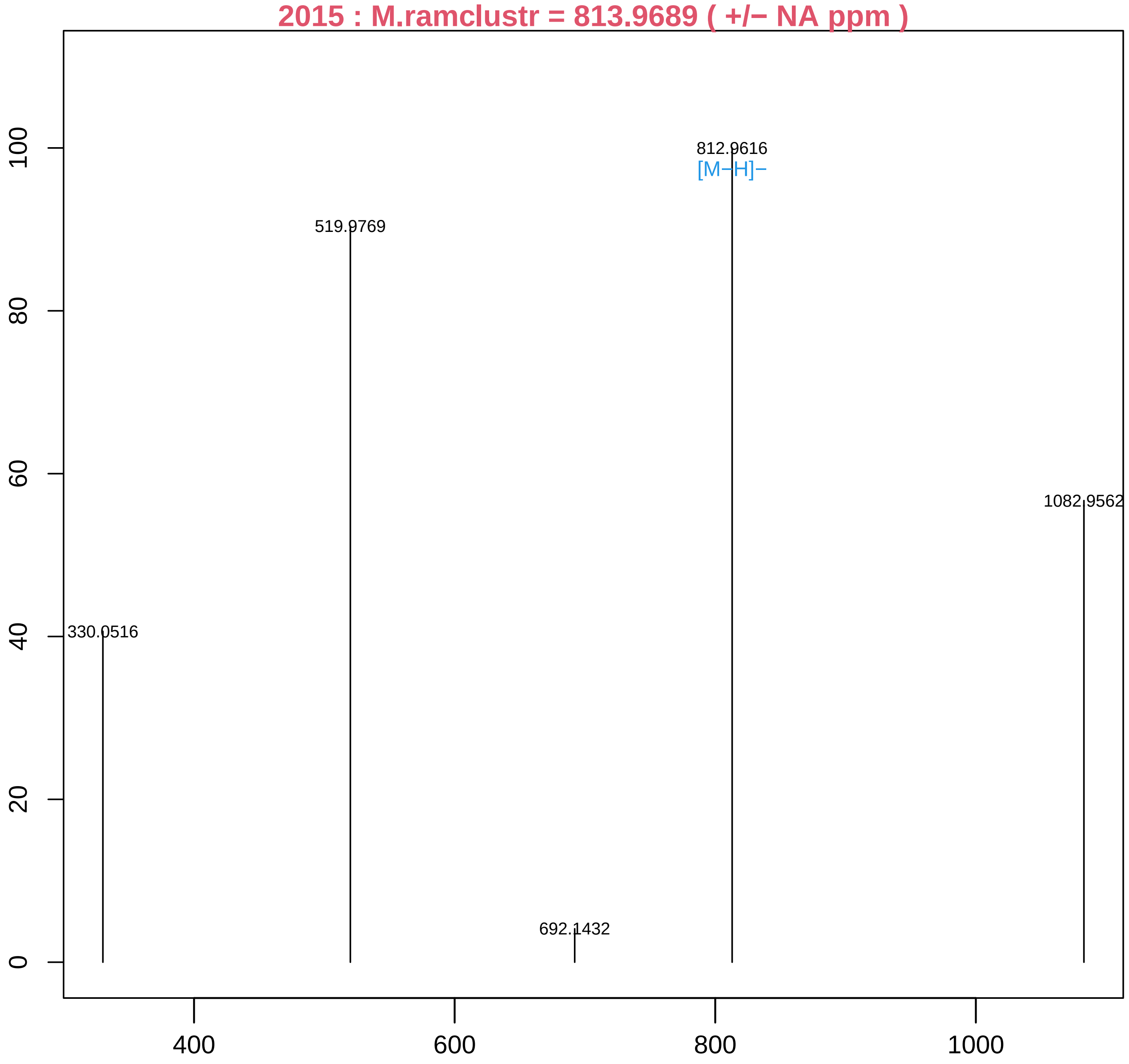
2015 : M.ramclustr = 813.9689 (+/− NA ppm)
812.9616
[M−H]−
519.9769
1082.9562
330.0516
692.1432
0
20
40
60
80
100
400
600
800
1000

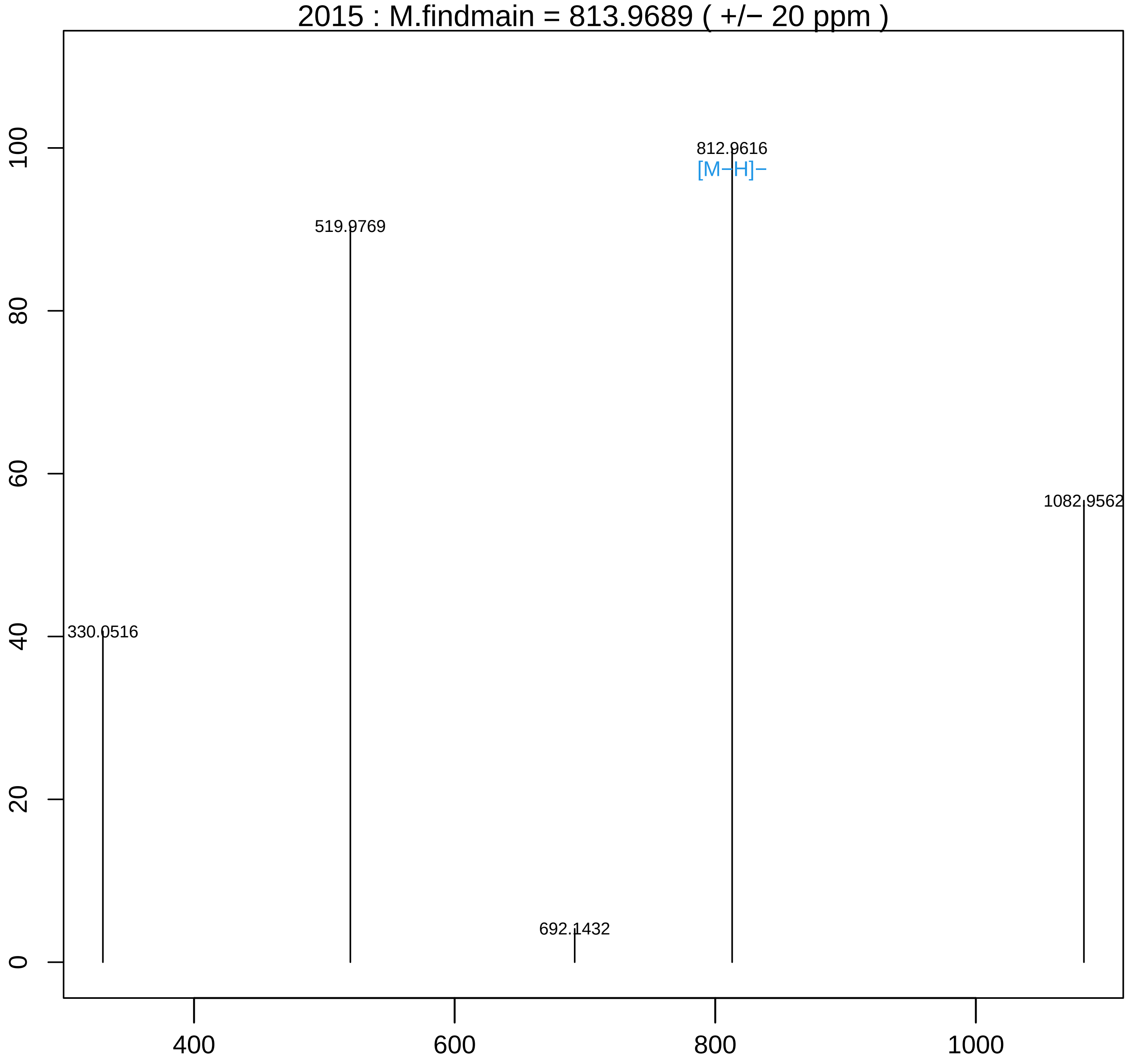
2015 : M.findmain = 813.9689 (+/− 20 ppm)
812.9616
[M−H]−
519.9769
1082.9562
330.0516
692.1432
0
20
40
60
80
100
400
600
800
1000

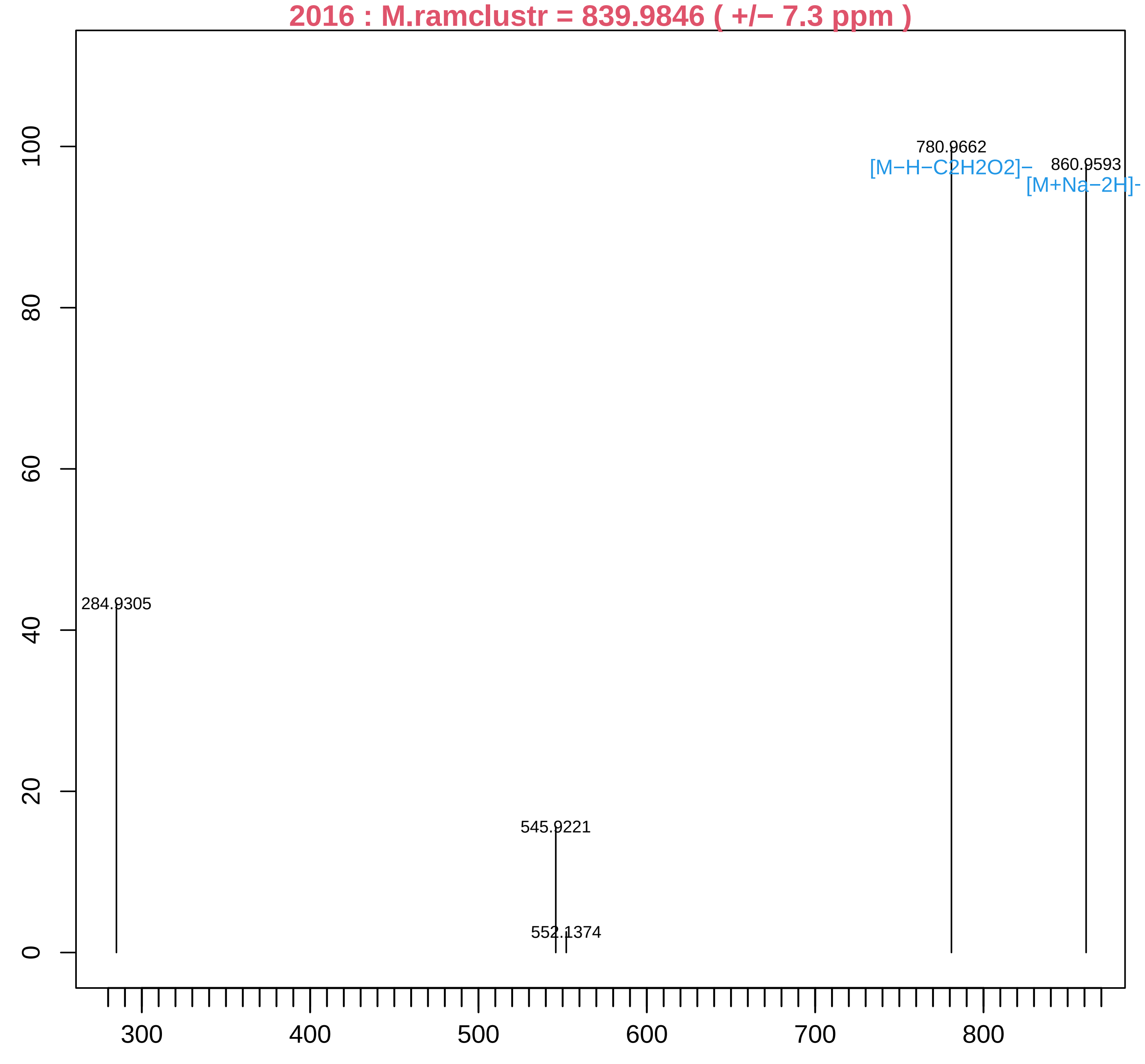

2016 : M.ramclustr = 839.9846 (+/− 7.3 ppm)
780.9662
[M−H−C2H2O2]−
860.9593
[M+Na−2H]-
284.9305
545.9221
552.1374
0
20
40
60
80
100
300
400
500
600
700
800

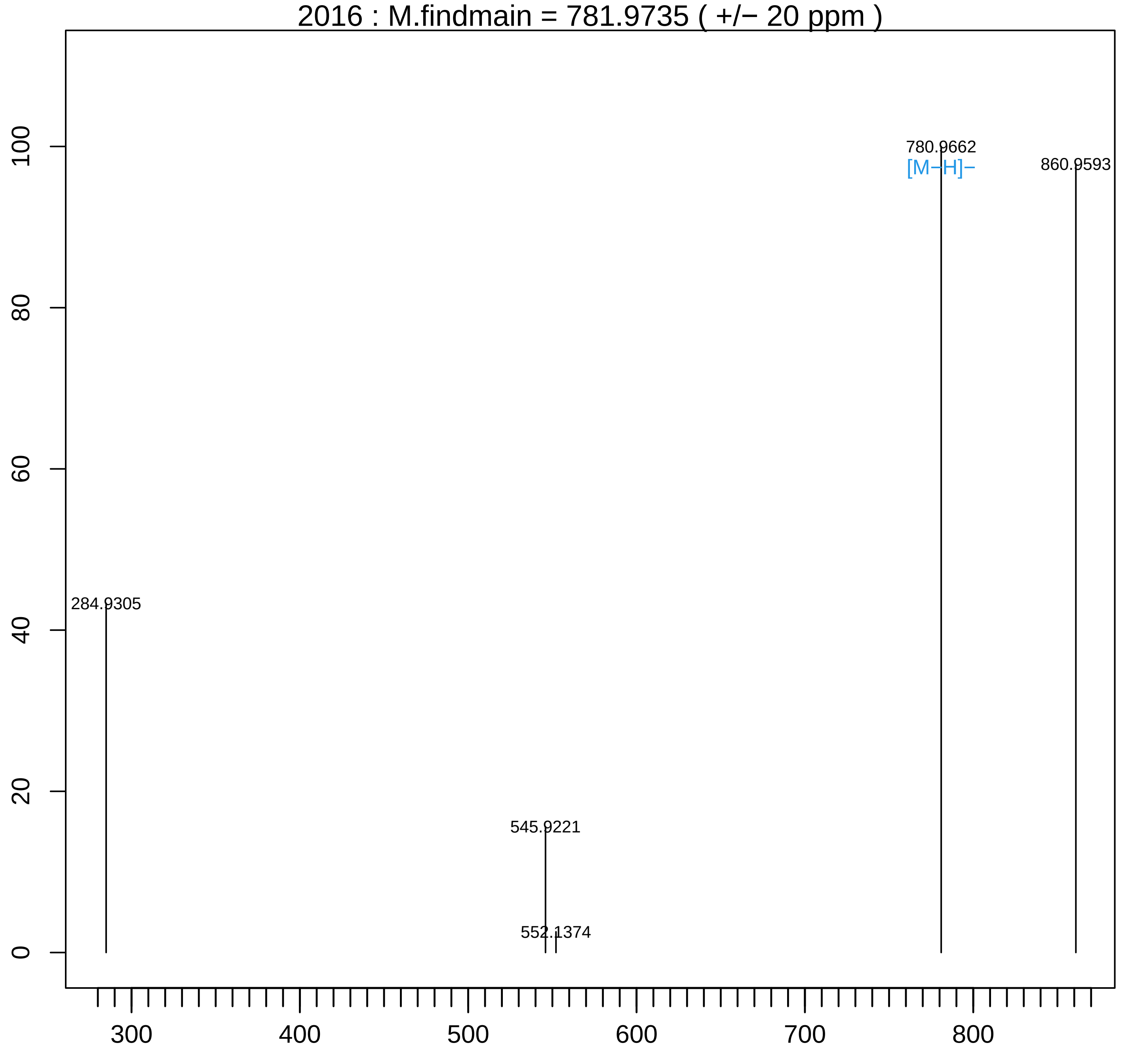

2016 : M.findmain = 781.9735 (+/− 20 ppm)
780.9662
[M−H]−
860.9593
284.9305
545.9221
552.1374
0
20
40
60
80
100
300
400
500
600
700
800

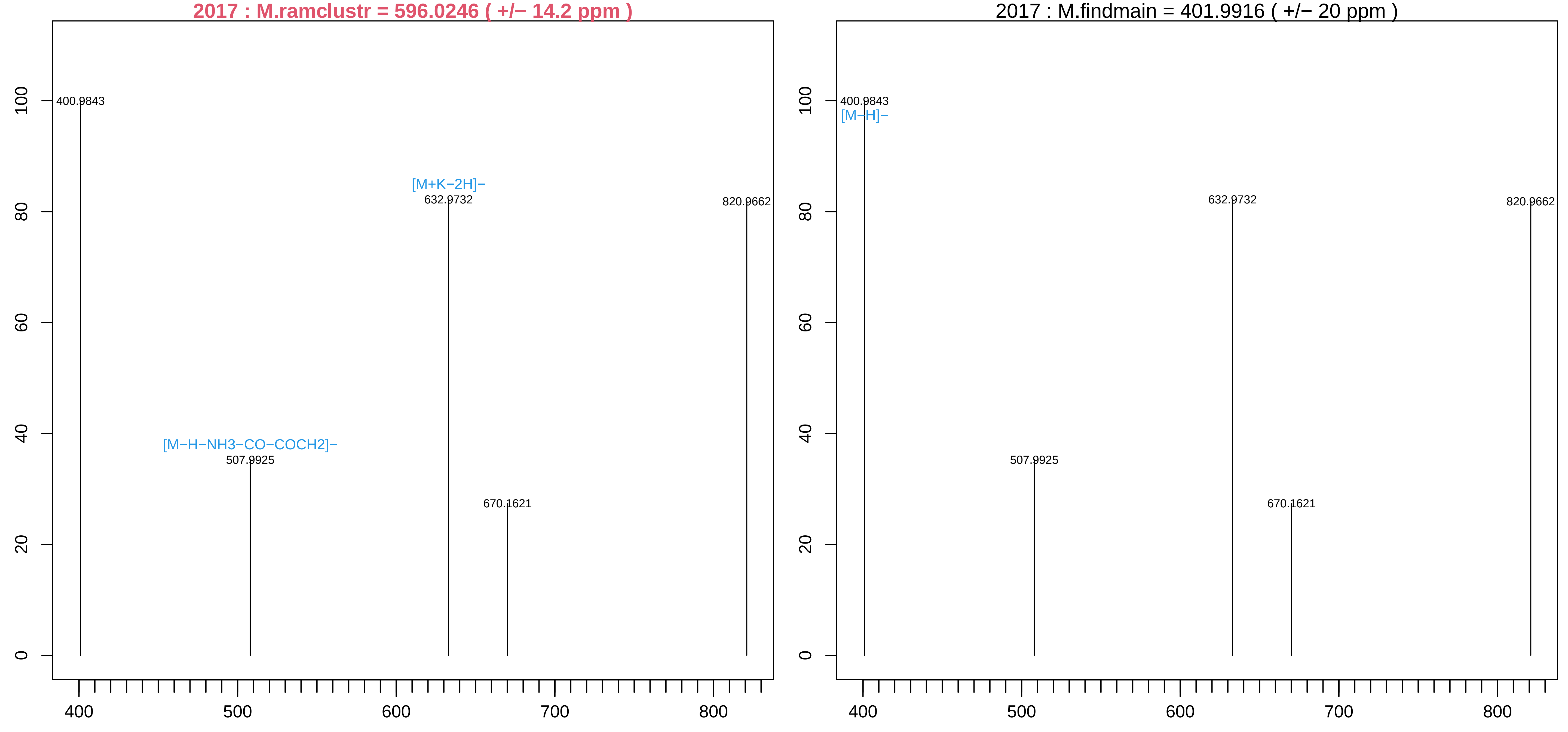
2017 : M.ramclustr = 596.0246 (+/− 14.2 ppm)
400.9843
[M−H−NH3−CO−COCH2]−
507.9925
[M+K−2H]−
632.9732
670.1621
820.9662
2017 : M.findmain = 401.9916 (+/− 20 ppm)
400.9843
[M−H]−
507.9925
632.9732
670.1621
820.9662
0
20
40
60
80
100
400
500
600
700
800

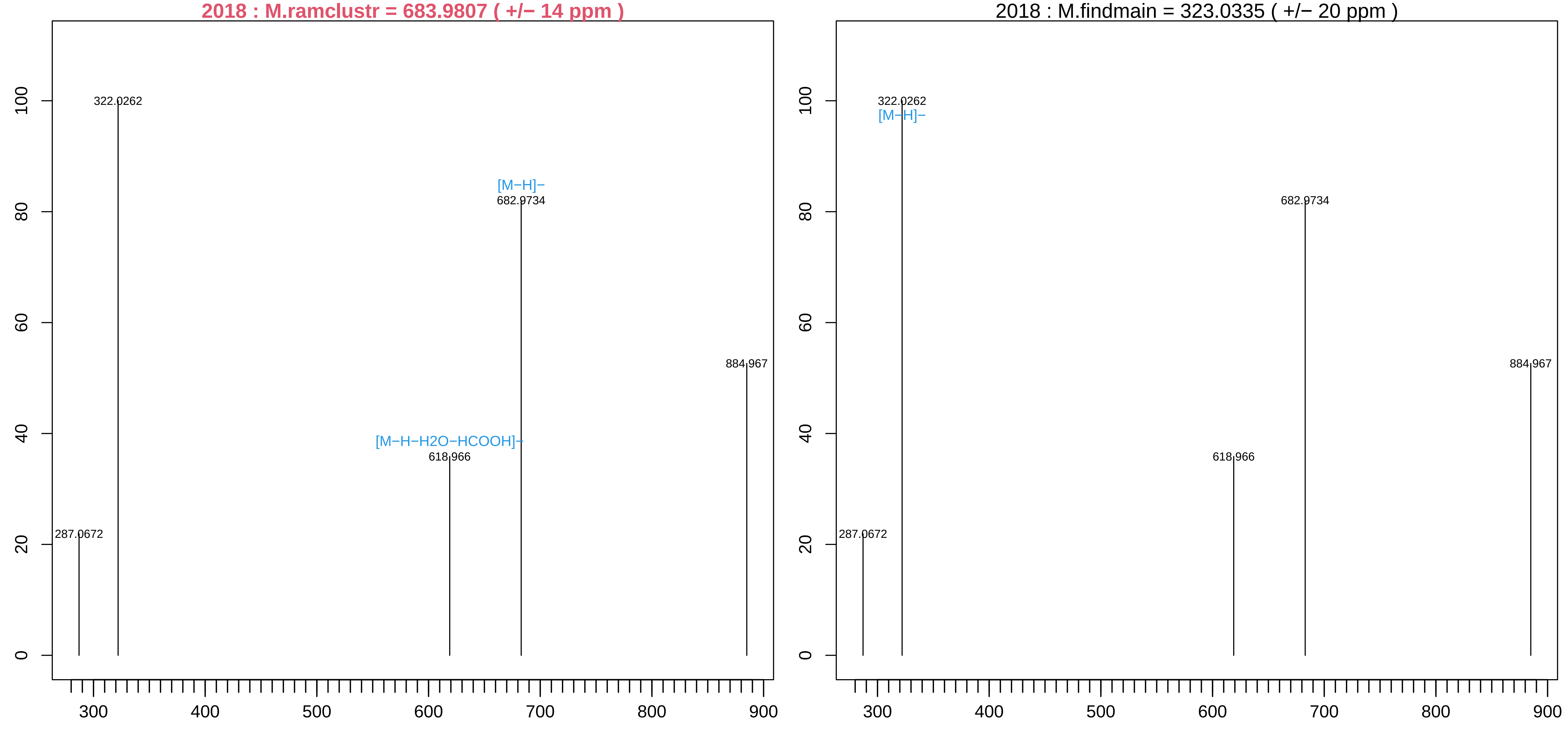
2018 : M.ramclustr = 683.9807 (+/− 14 ppm)
322.0262
[M−H]−
682.9734
884.967
[M−H−H2O−HCOOH]−
618.966
287.0672
0
20
40
60
80
100
300
400
500
600
700
800
900
2018 : M.findmain = 323.0335 (+/− 20 ppm)
322.0262
[M−H]−
682.9734
884.967
618.966
287.0672

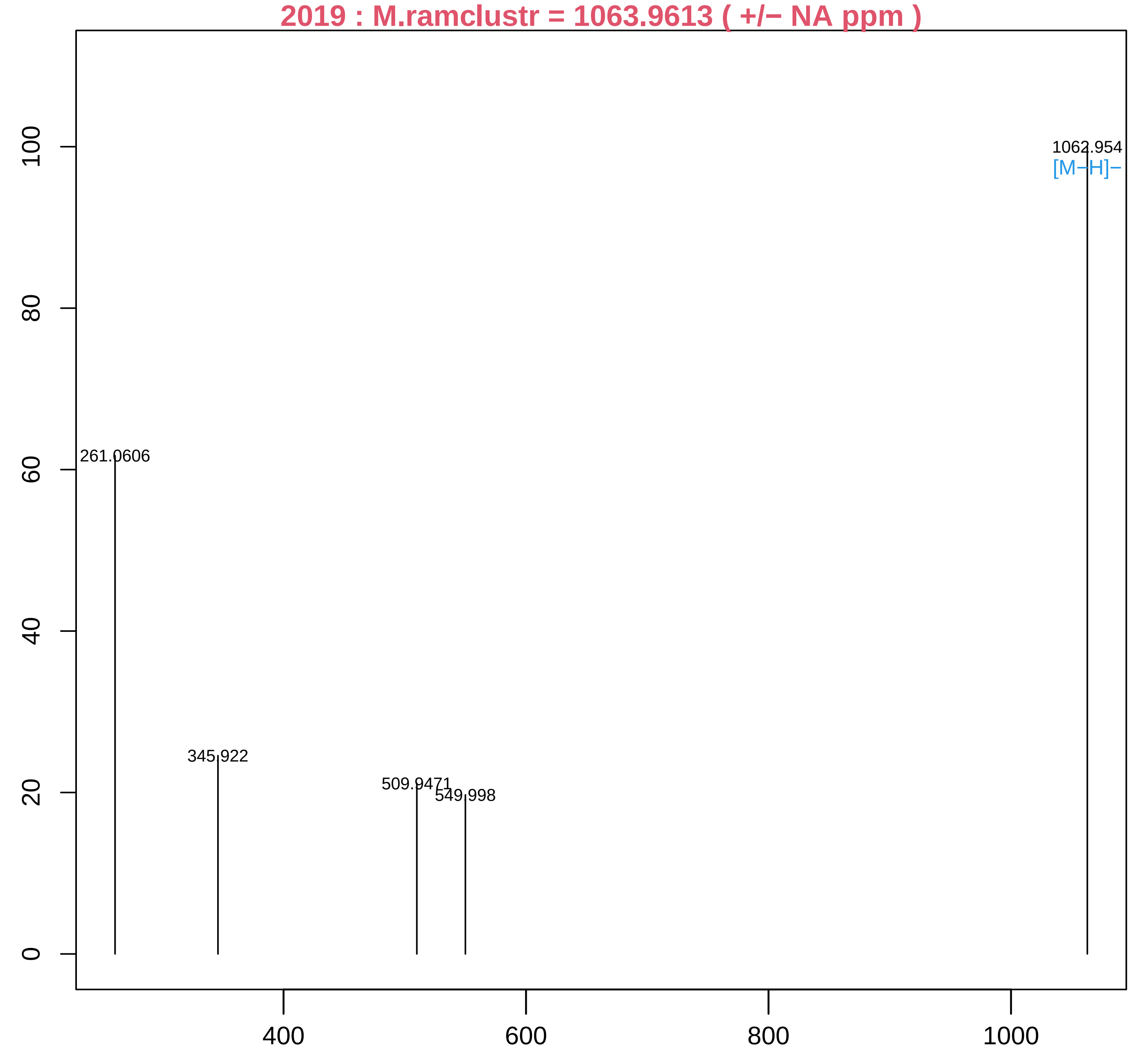

2019 : M.ramclustr = 1063.9613 (+/− NA ppm)
100
80
60
40
20
0
400
600
800
1000
1062.954
[M−H]−
261.0606
345.922
509.9471
549.998

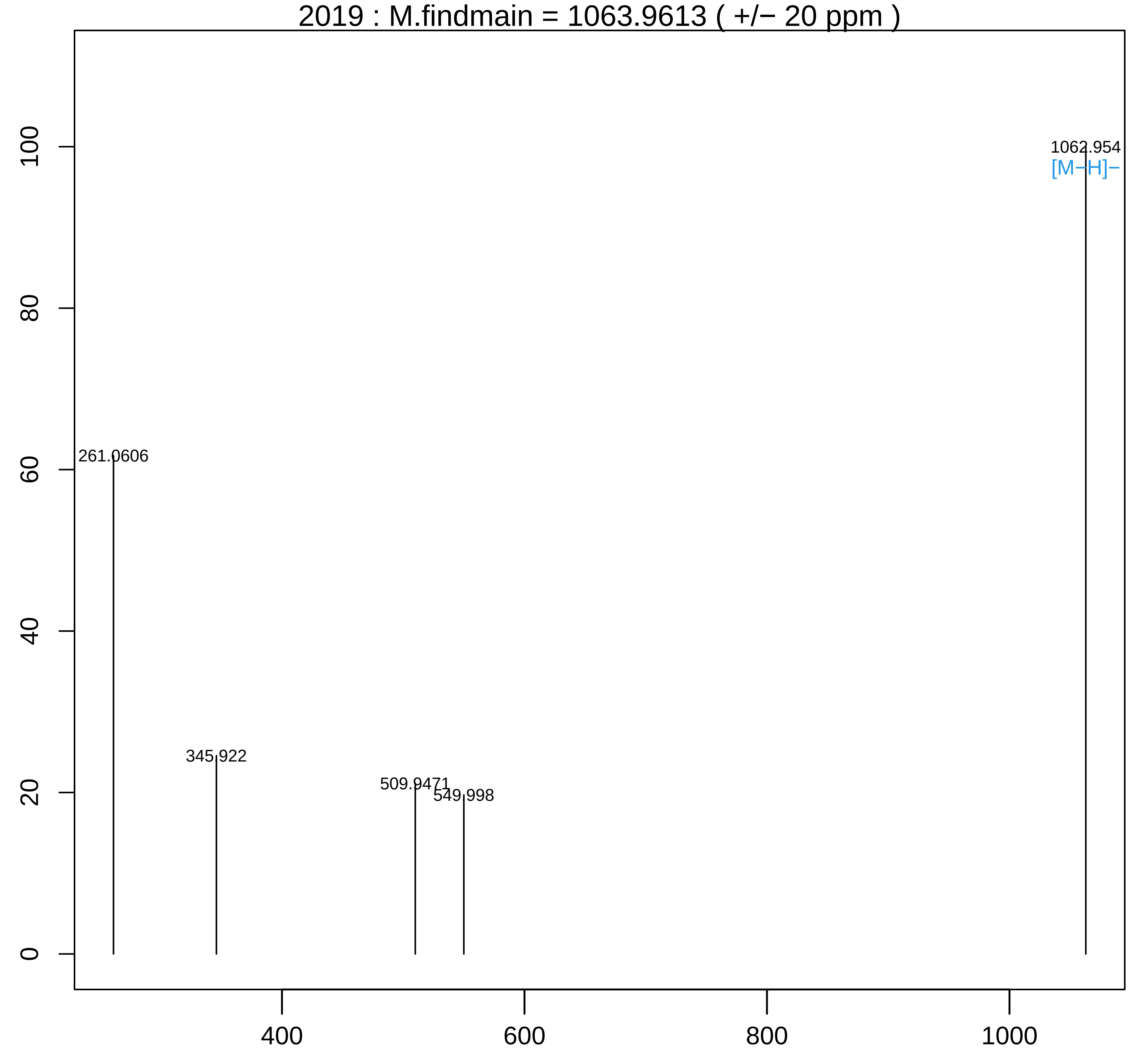

2019 : M.findmain = 1063.9613 (+/− 20 ppm)
100
80
60
40
20
0
400
600
800
1000
1062.954
[M−H]−
261.0606
345.922
509.9471
549.998

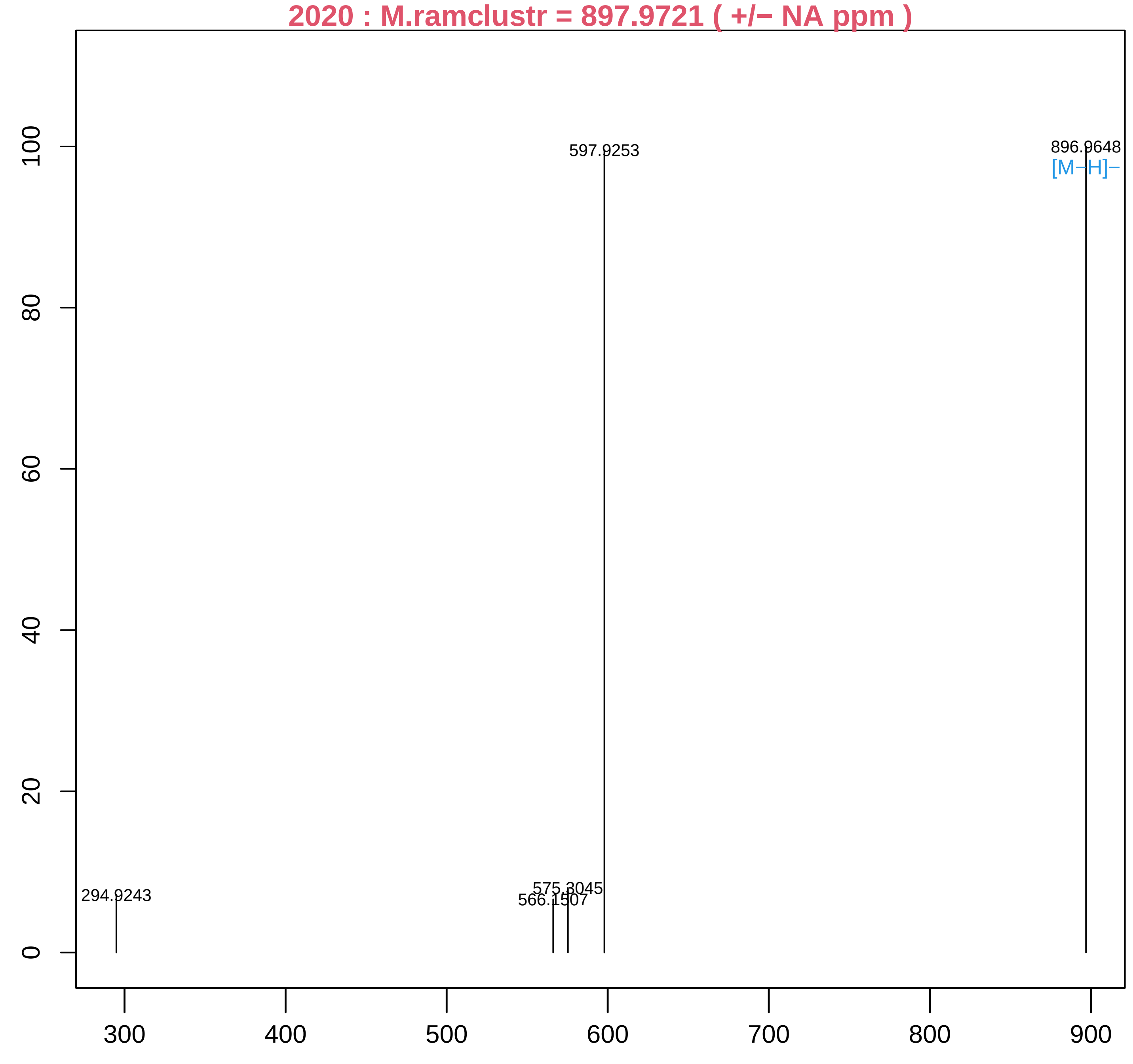
2020 : M.ramclustr = 897.9721 (+/− NA ppm)
597.9253
896.9648
[M−H]−
294.9243
575.3045
566.1507
0
20
40
60
80
100
300
400
500
600
700
800
900

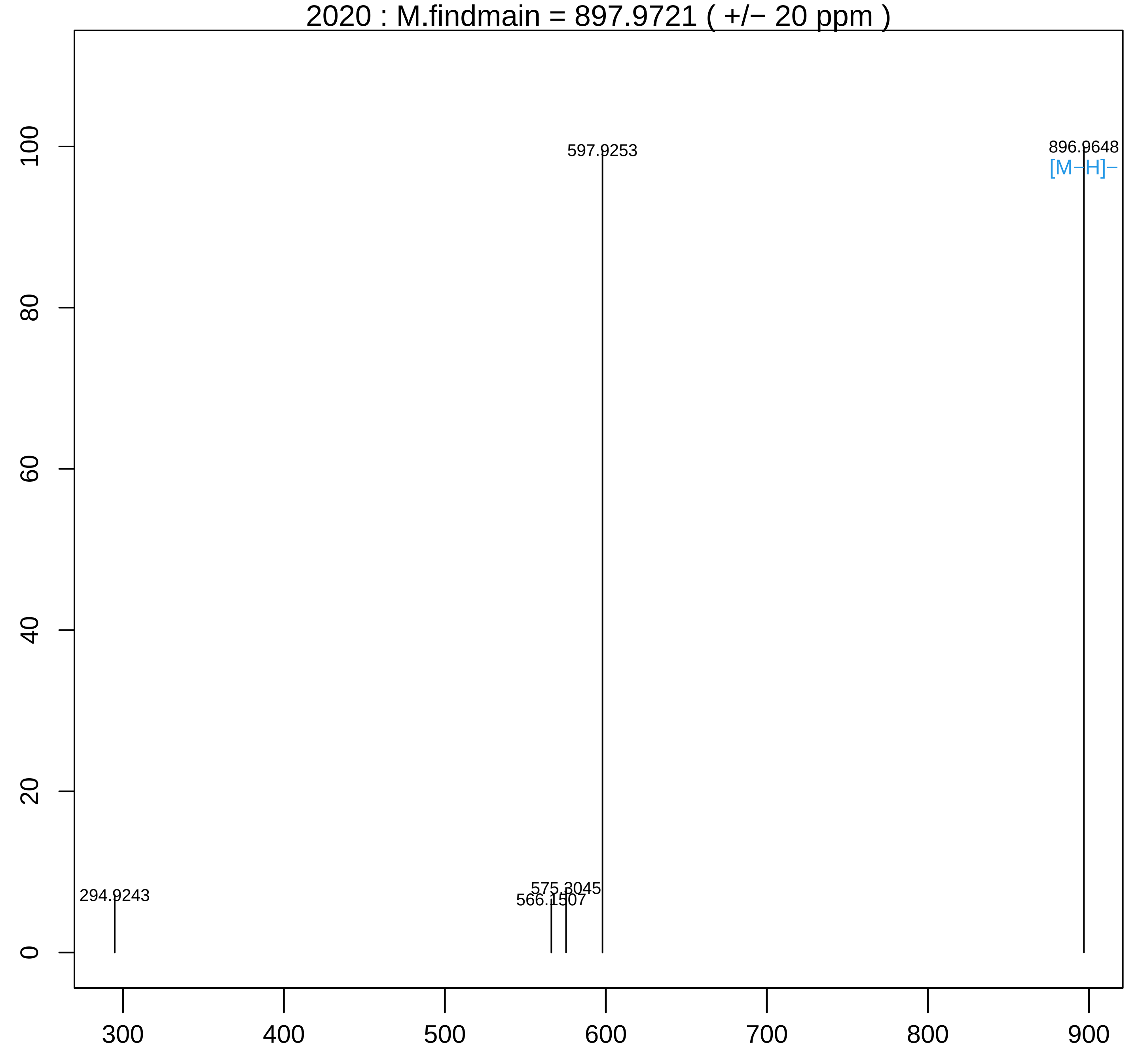
2020 : M.findmain = 897.9721 (+/− 20 ppm)
597.9253
896.9648
[M−H]−
294.9243
575.3045
566.1507
0
20
40
60
80
100
300
400
500
600
700
800
900

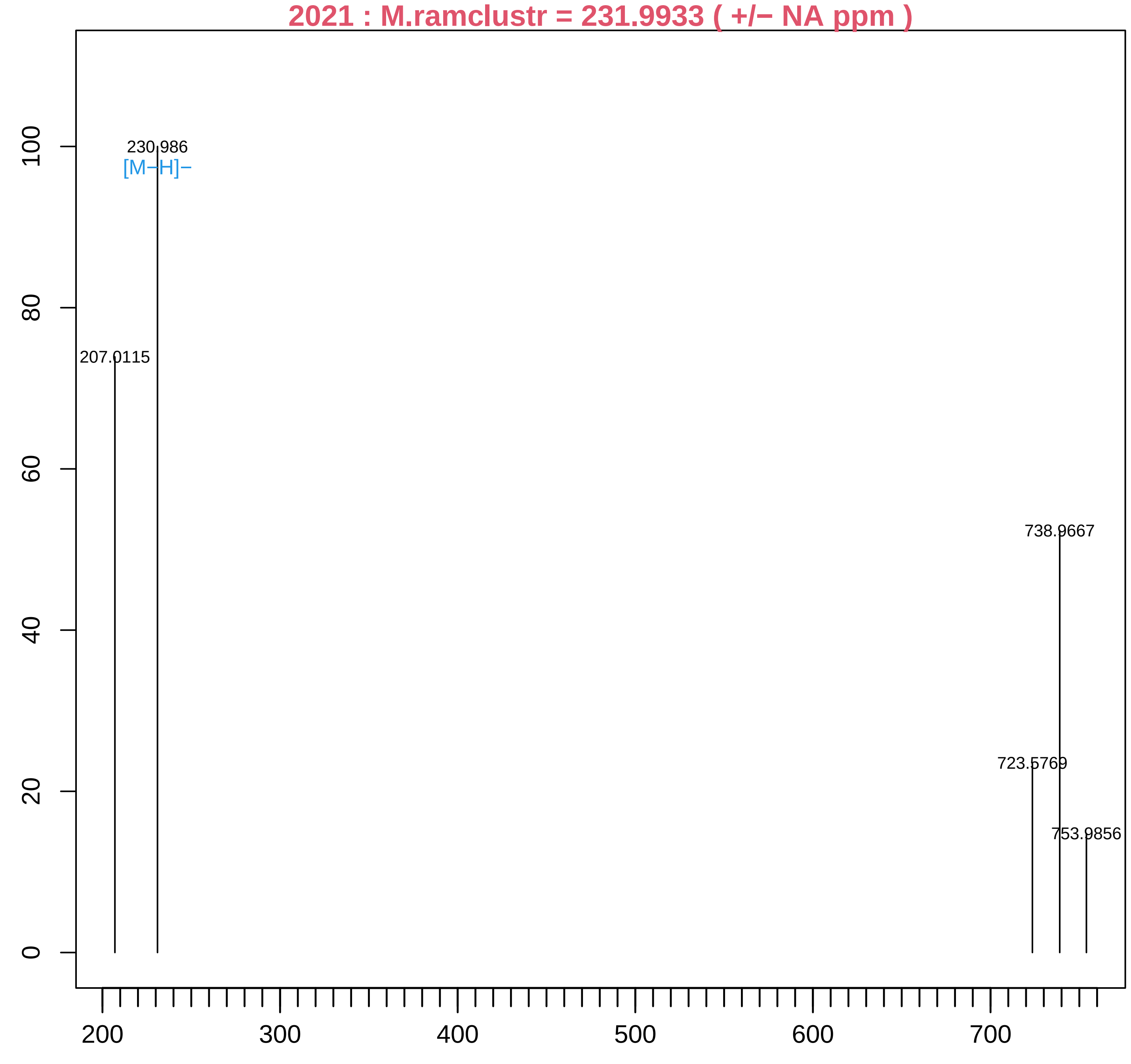
2021 : M.ramclustr = 231.9933 (+/− NA ppm)
230.986
[M−H]−
207.0115
738.9667
723.5769
753.9856
0
20
40
60
80
100
200
300
400
500
600
700

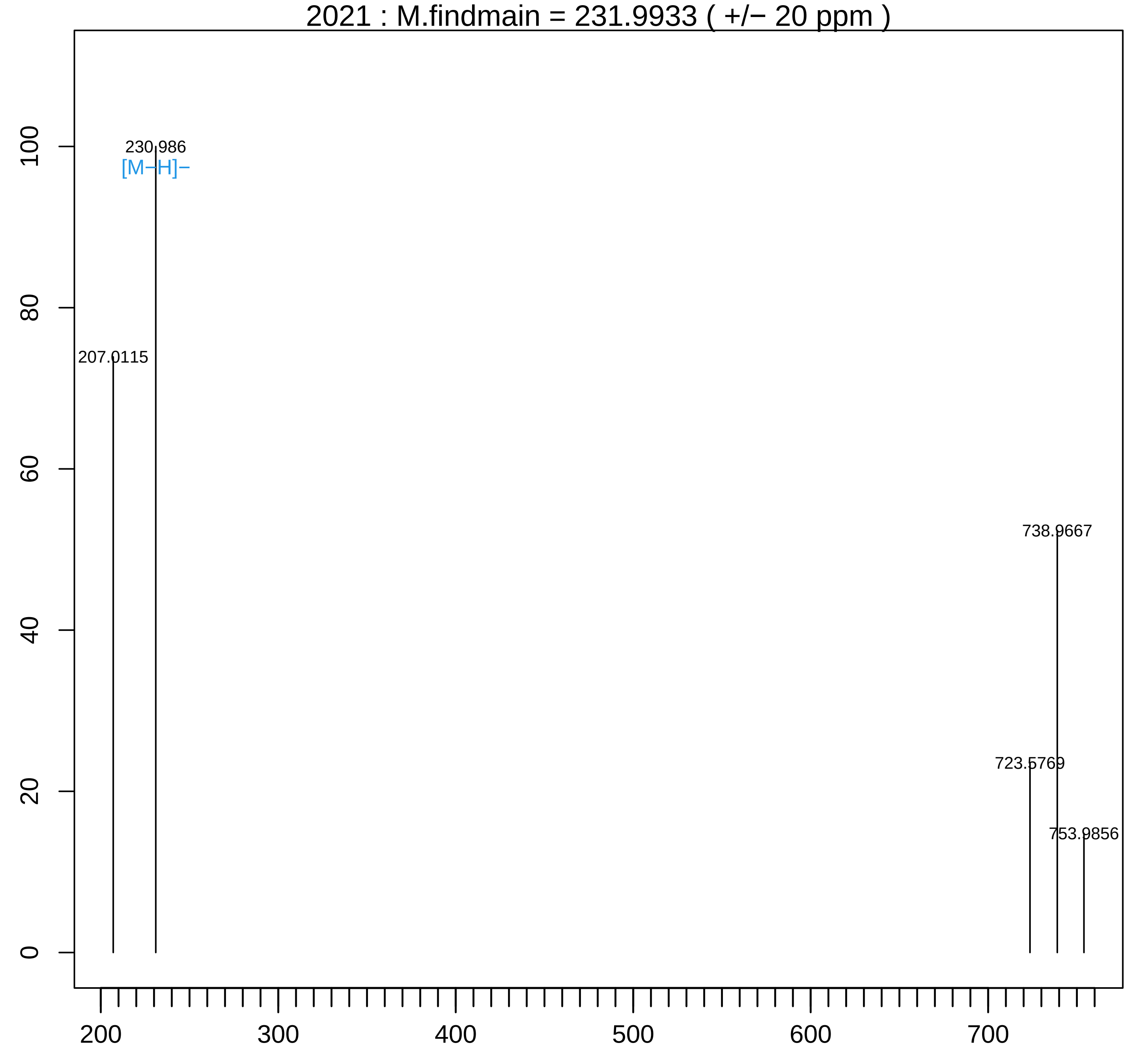
2021 : M.findmain = 231.9933 (+/− 20 ppm)
230.986
[M−H]−
207.0115
738.9667
723.5769
753.9856
0
20
40
60
80
100
200
300
400
500
600
700

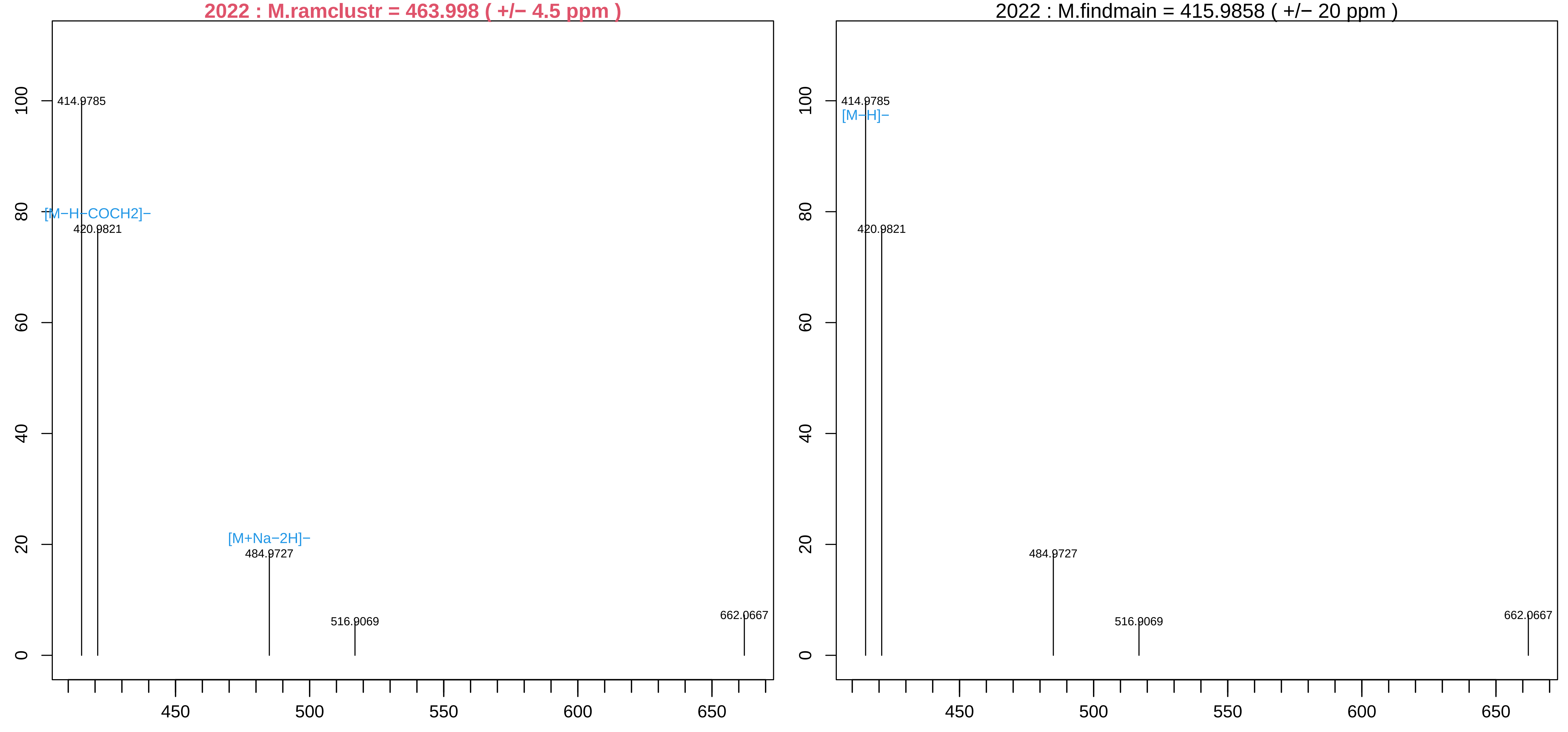

2022 : M.ramclustr = 463.998 (+/− 4.5 ppm)
414.9785
[M−H−COCH2]−
420.9821
[M+Na−2H]−
484.9727
516.9069
662.0667
0
20
40
60
80
100
450
500
550
600
650
2022 : M.findmain = 415.9858 (+/− 20 ppm)
414.9785
[M−H]−
420.9821
484.9727
516.9069
662.0667
0
20
40
60
80
100
450
500
550
600
650

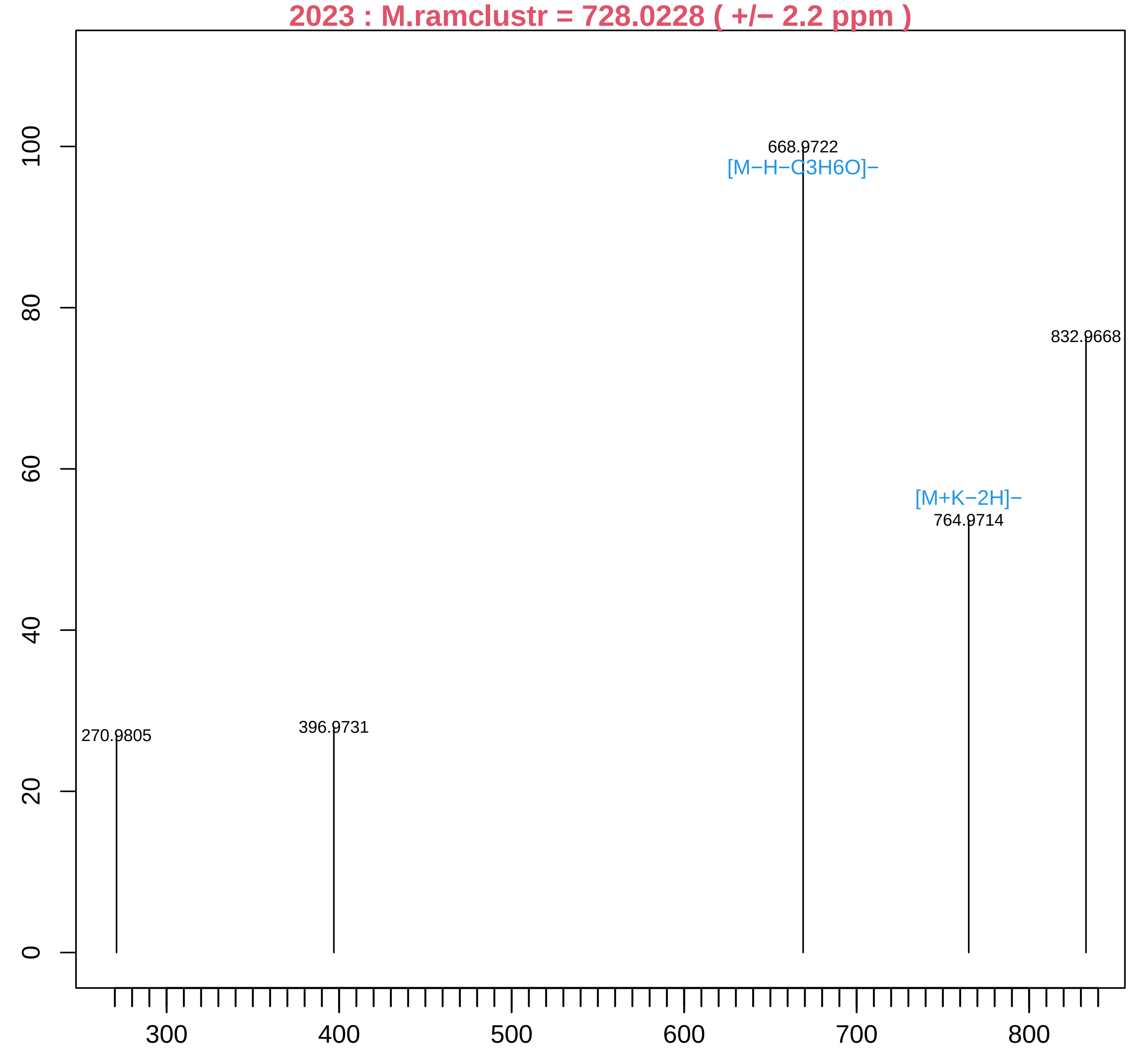

2023 : M.ramclustr = 728.0228 (+/− 2.2 ppm)
668.9722
[M−H−C3H6O]−
832.9668
[M+K−2H]−
764.9714
270.9805
396.9731
0
20
40
60
80
100
300
400
500
600
700
800

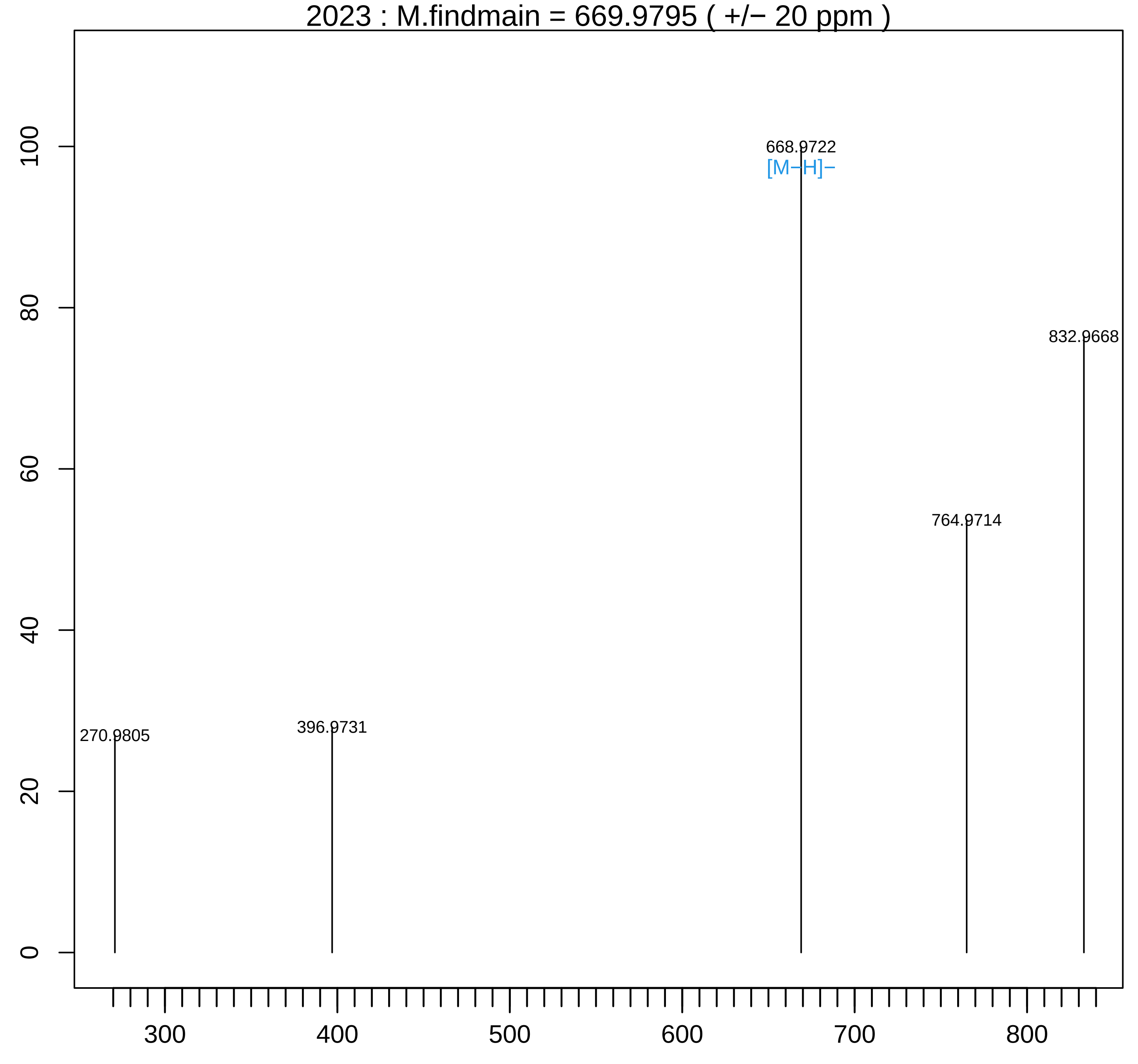

2023 : M.findmain = 669.9795 (+/− 20 ppm)
668.9722
[M−H]−
832.9668
764.9714
270.9805
396.9731
0
20
40
60
80
100
300
400
500
600
700
800

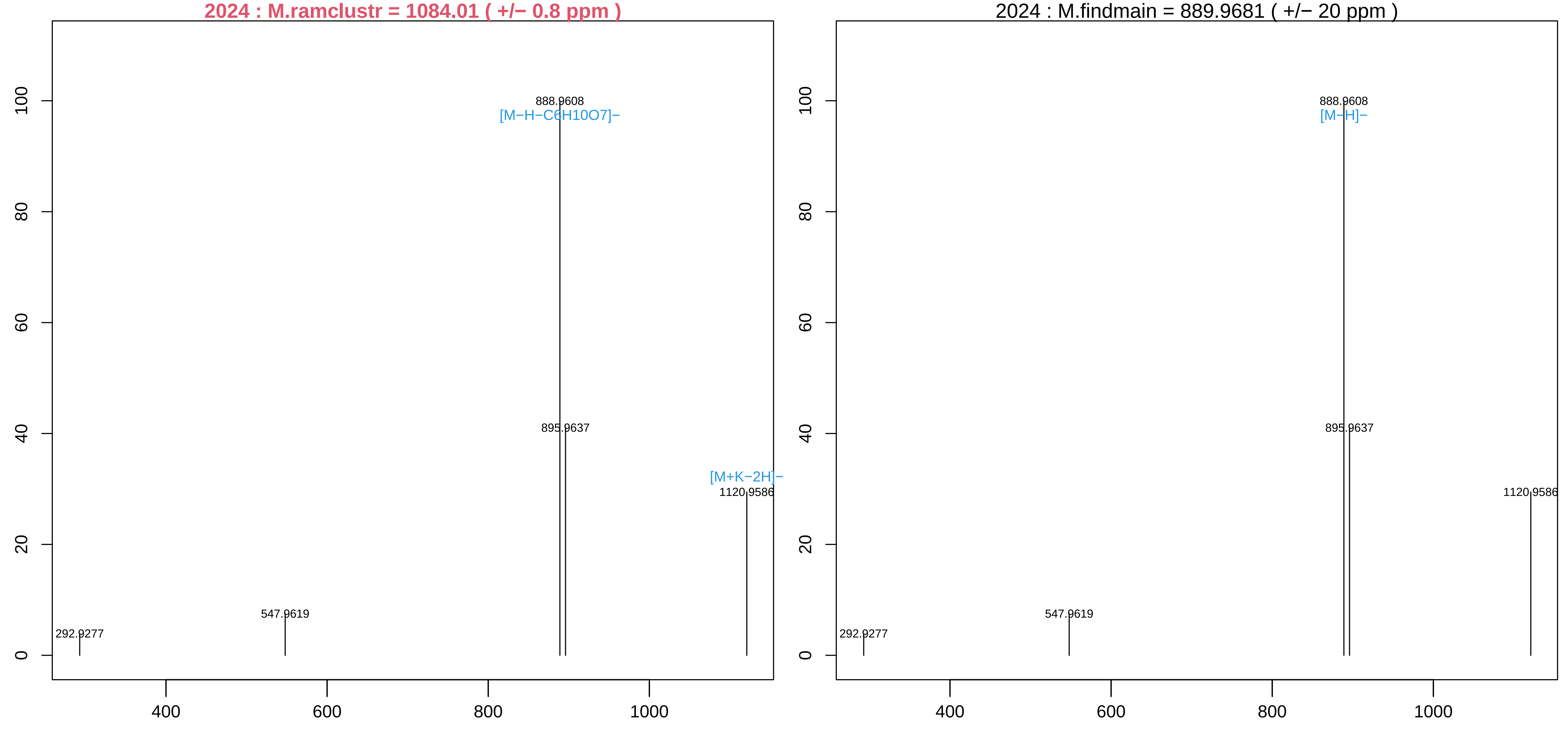

2024 : M.ramclustr = 1084.01 (+/− 0.8 ppm)
888.9608
[M−H−C6H10O7]−
895.9637
[M+K−2H]−
1120.9586
547.9619
292.9277
2024 : M.findmain = 889.9681 (+/− 20 ppm)
888.9608
[M−H]−
895.9637
1120.9586
547.9619
292.9277

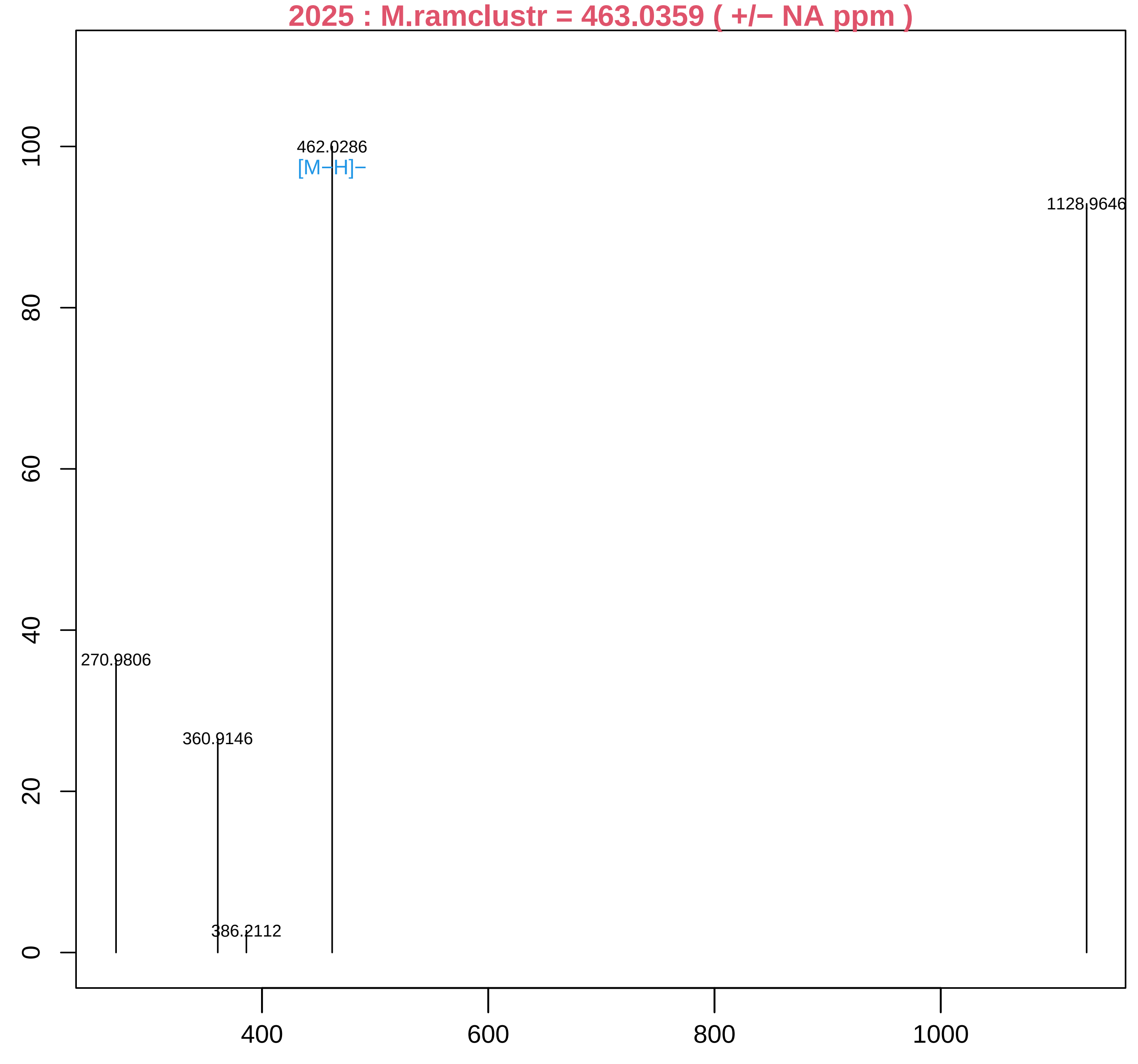
2025 : M.ramclustr = 463.0359 (+/− NA ppm)
462.0286
[M−H]−
1128.9646
270.9806
360.9146
386.2112
0
20
40
60
80
100
400
600
800
1000

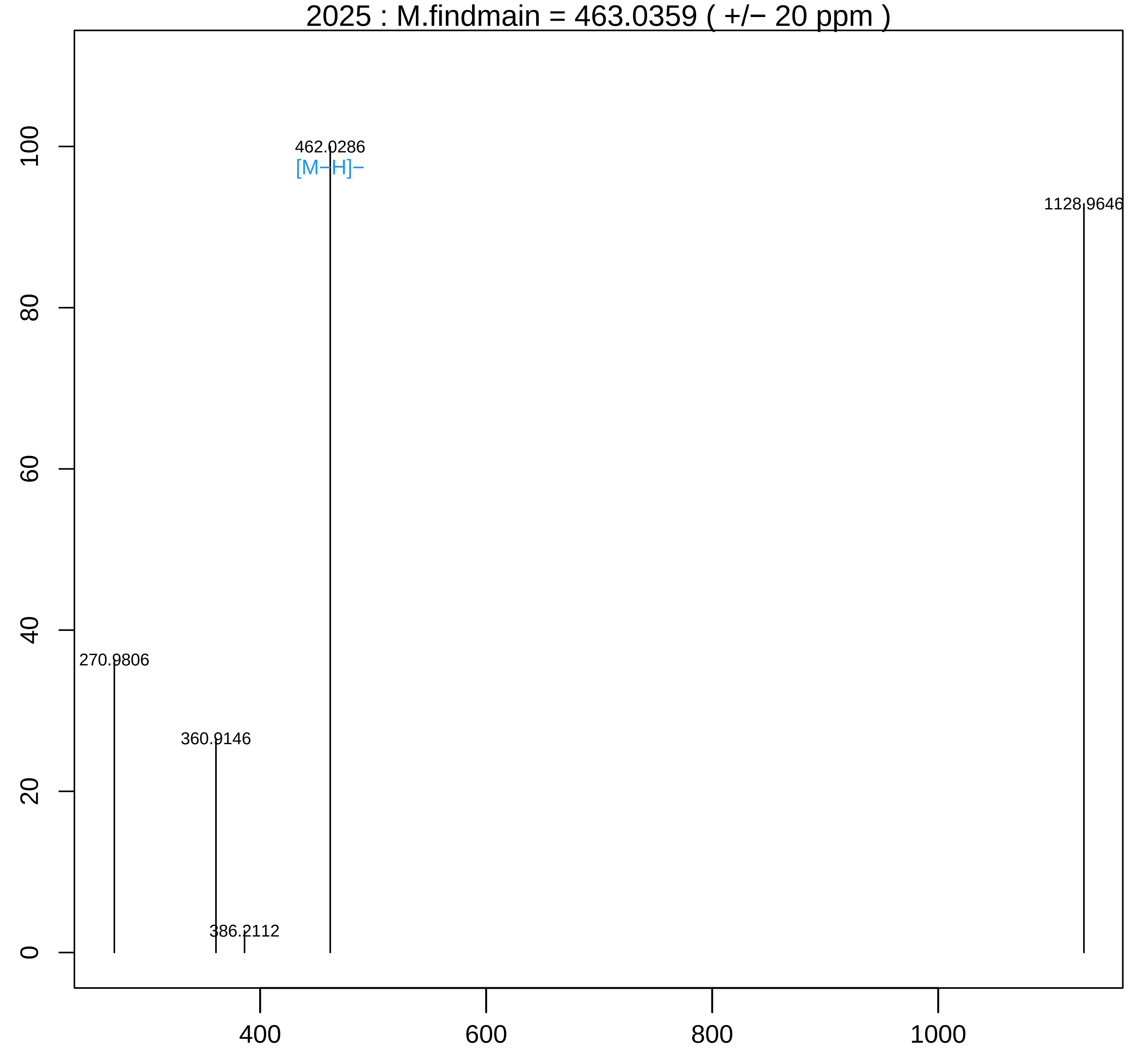
2025 : M.findmain = 463.0359 (+/− 20 ppm)
462.0286
[M−H]−
1128.9646
270.9806
360.9146
386.2112
0
20
40
60
80
100
400
600
800
1000

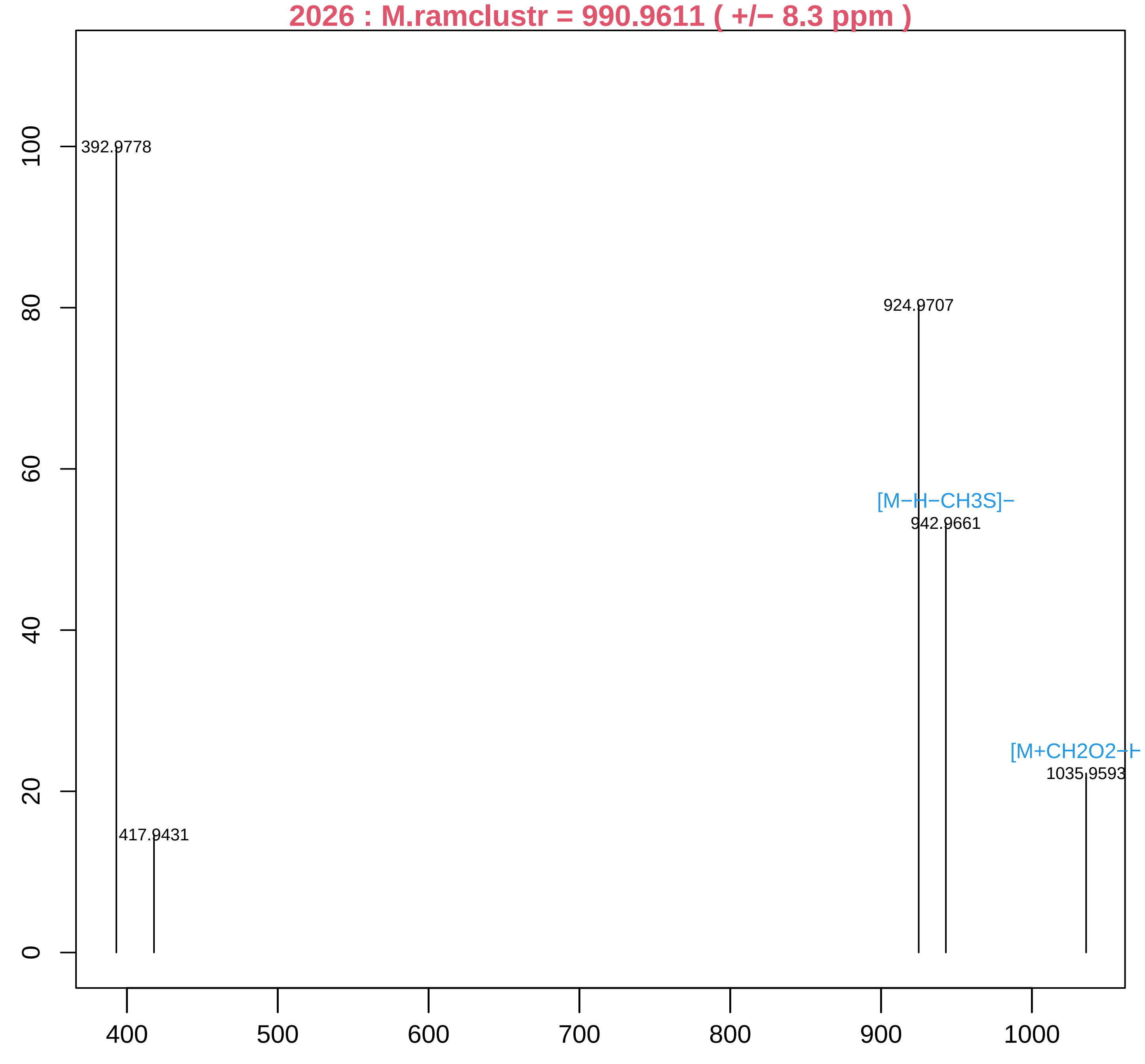
2026 : M.ramclustr = 990.9611 (+/− 8.3 ppm)
392.9778
417.9431
924.9707
[M−H−CH3S]−
942.9661
[M+CH2O2−H
1035.9593
0
20
40
60
80
100
400
500
600
700
800
900
1000

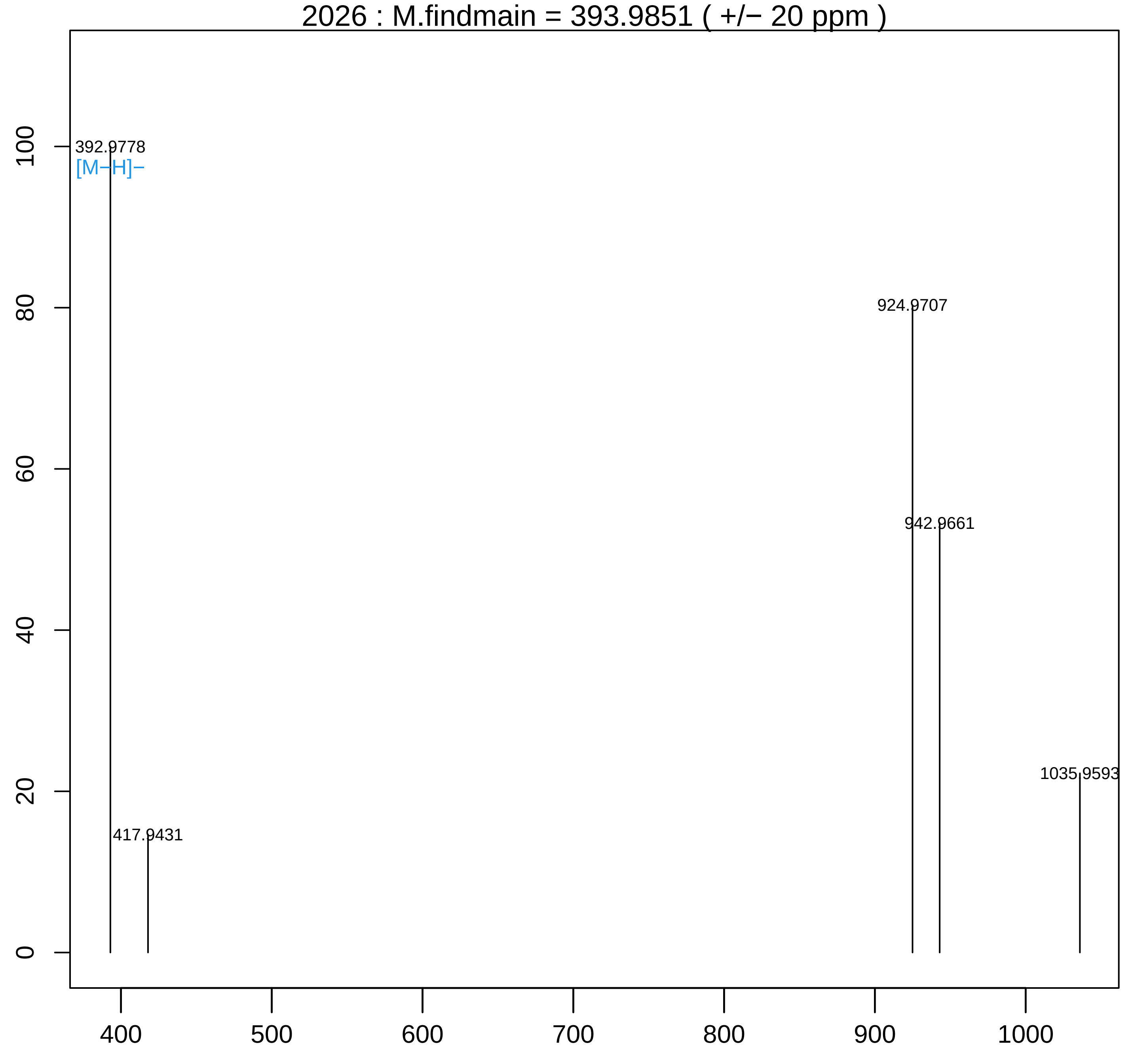
2026 : M.findmain = 393.9851 (+/− 20 ppm)
392.9778
[M−H]−
417.9431
924.9707
942.9661
1035.9593
0
20
40
60
80
100
400
500
600
700
800
900
1000

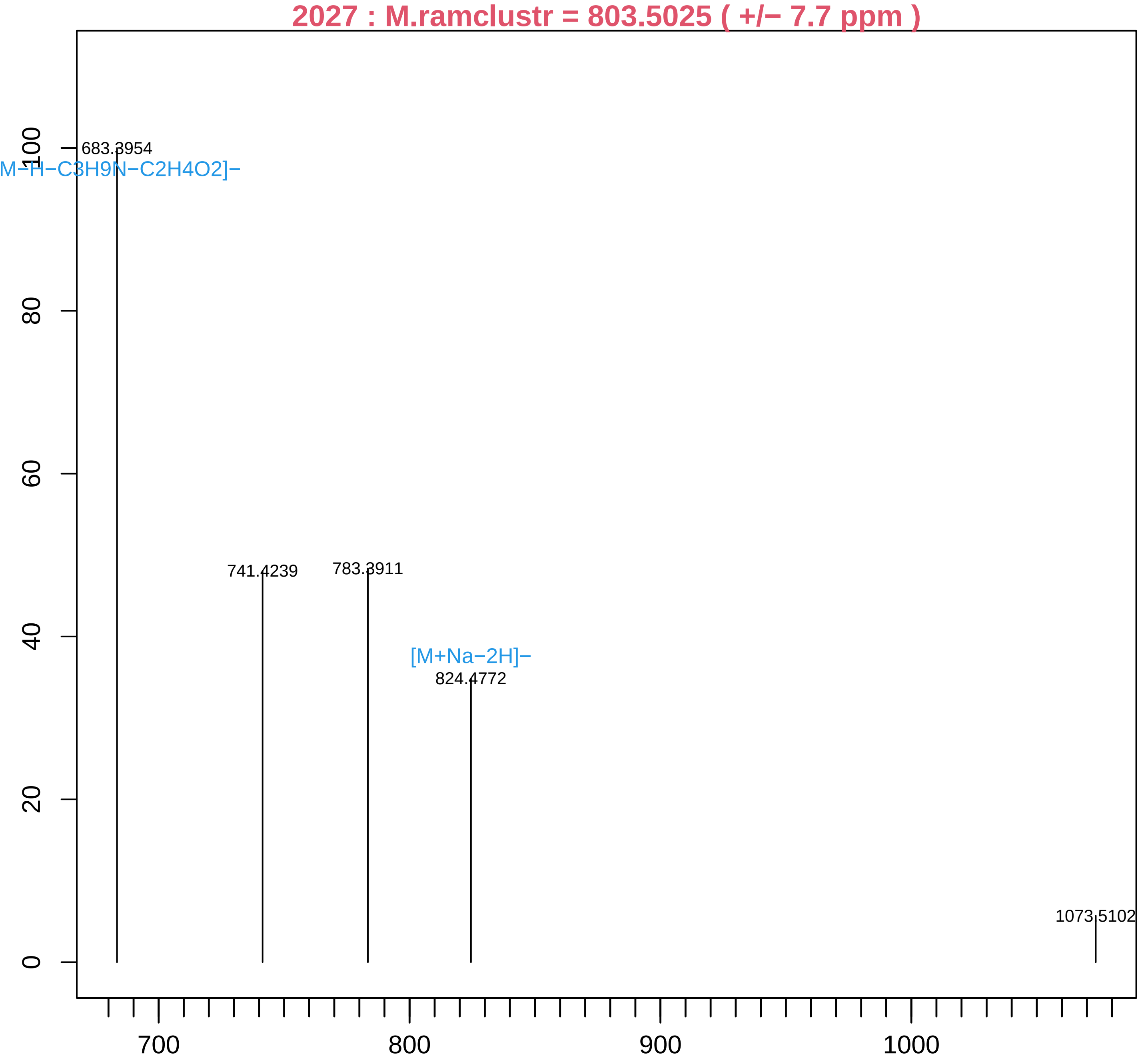
2027 : M.ramclustr = 803.5025 (+/− 7.7 ppm)
683.3954
[M−H−C3H9N−C2H4O2]−
741.4239
783.3911
[M+Na−2H]−
824.4772
1073.5102
0
20
40
60
80
100
700
800
900
1000

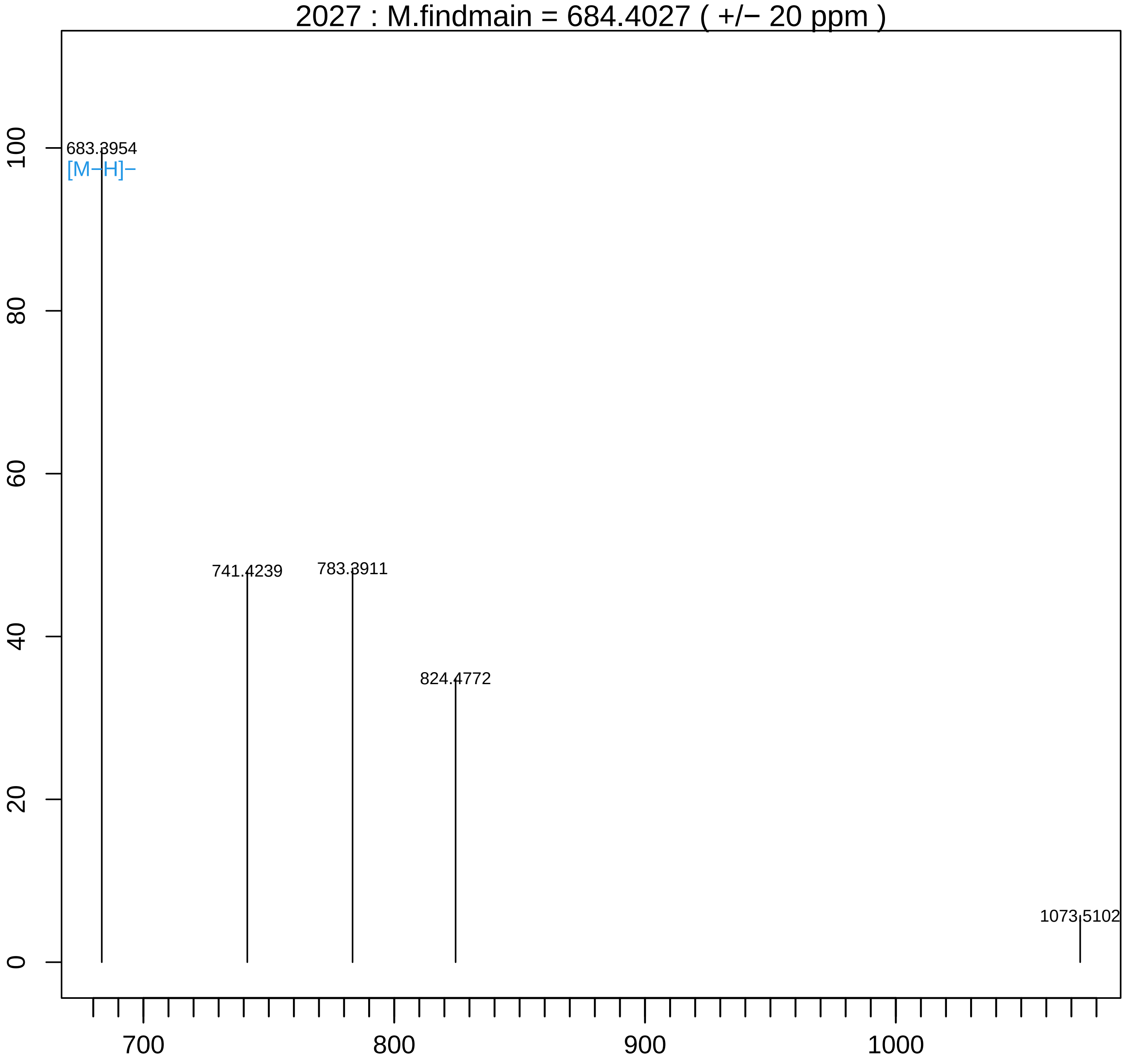
2027 : M.findmain = 684.4027 (+/− 20 ppm)
683.3954
[M−H]−
741.4239
783.3911
824.4772
1073.5102
0
20
40
60
80
100
700
800
900
1000

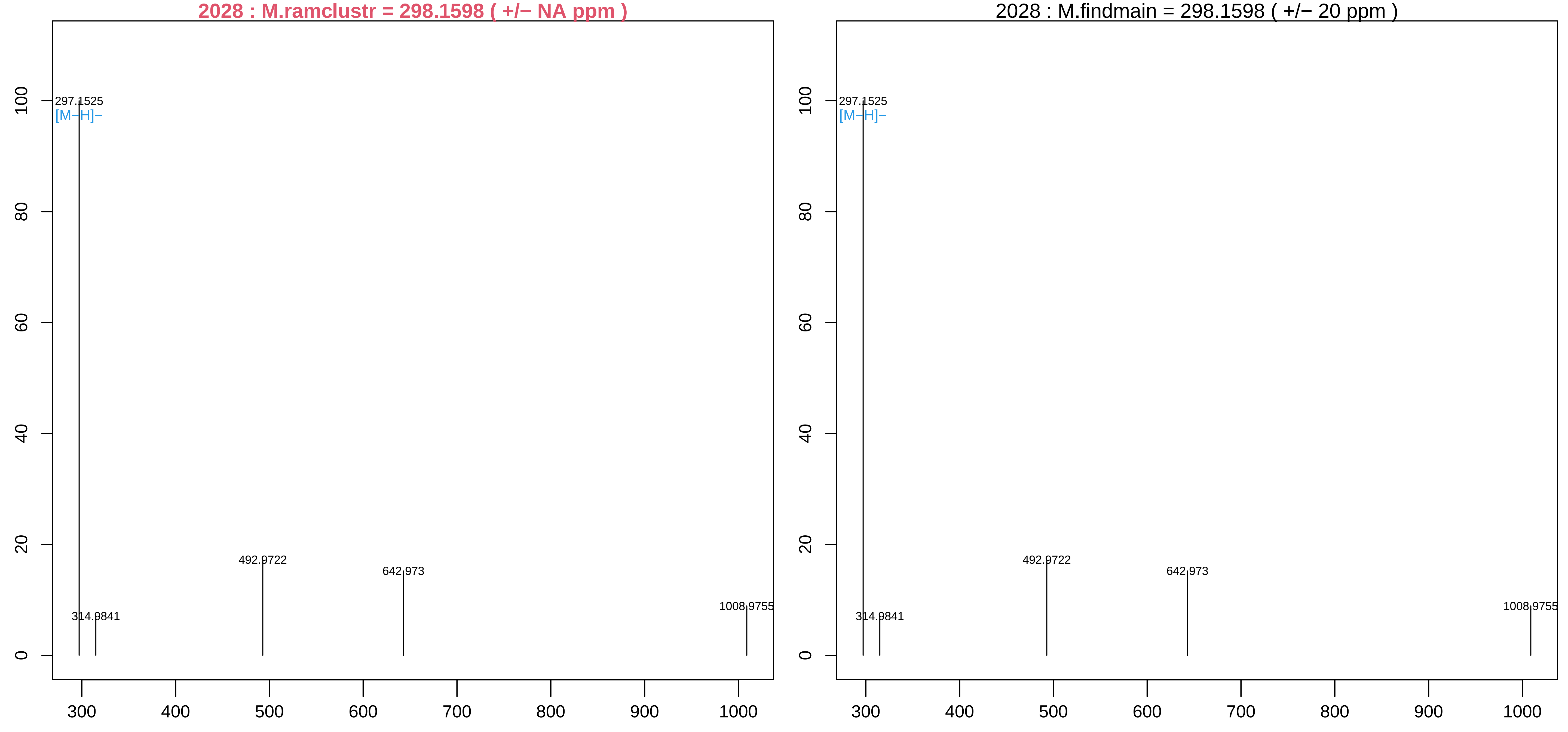
2028 : M.ramclustr = 298.1598 (+/− NA ppm)
297.1525
[M−H]−
314.9841
492.9722
642.973
1008.9755
0
20
40
60
80
100
300
400
500
600
700
800
900
1000
2028 : M.findmain = 298.1598 (+/− 20 ppm)
297.1525
[M−H]−
314.9841
492.9722
642.973
1008.9755
0
20
40
60
80
100
300
400
500
600
700
800
900
1000

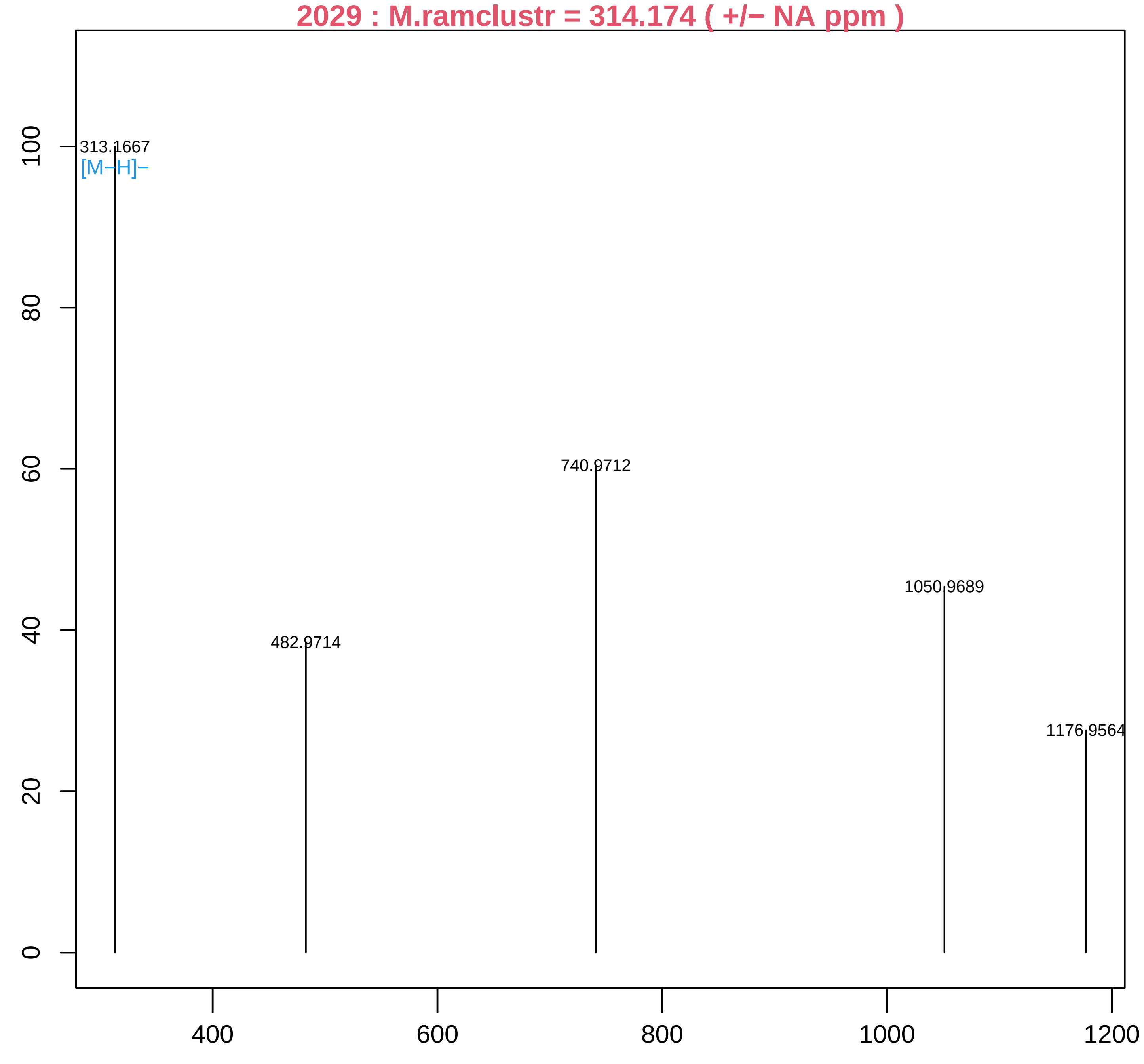
2029 : M.ramclustr = 314.174 (+/− NA ppm)
313.1667
[M−H]−
482.9714
740.9712
1050.9689
1176.9564
0
20
40
60
80
100
400
600
800
1000
1200

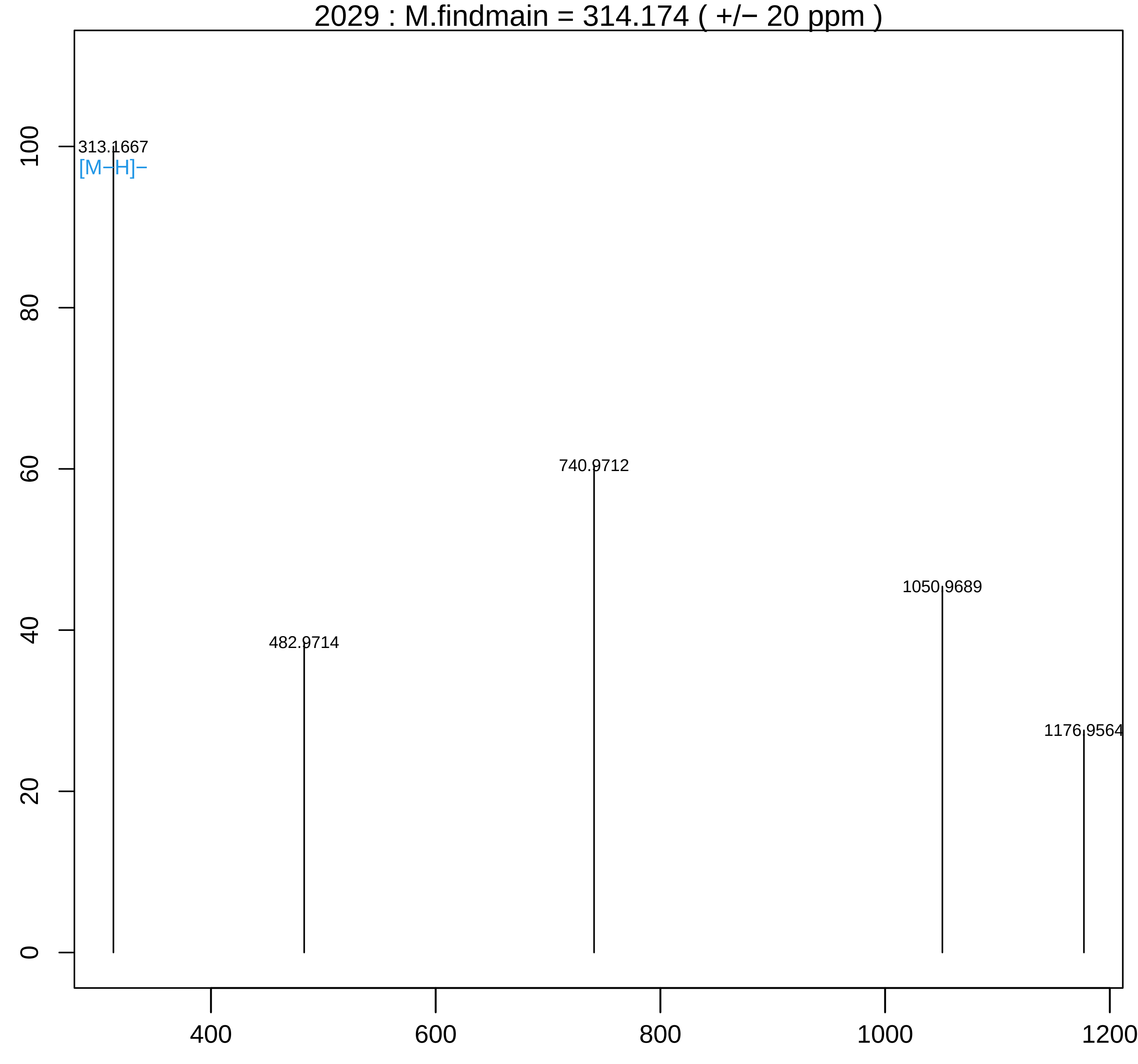
2029 : M.findmain = 314.174 (+/− 20 ppm)
313.1667
[M−H]−
482.9714
740.9712
1050.9689
1176.9564
0
20
40
60
80
100
400
600
800
1000
1200

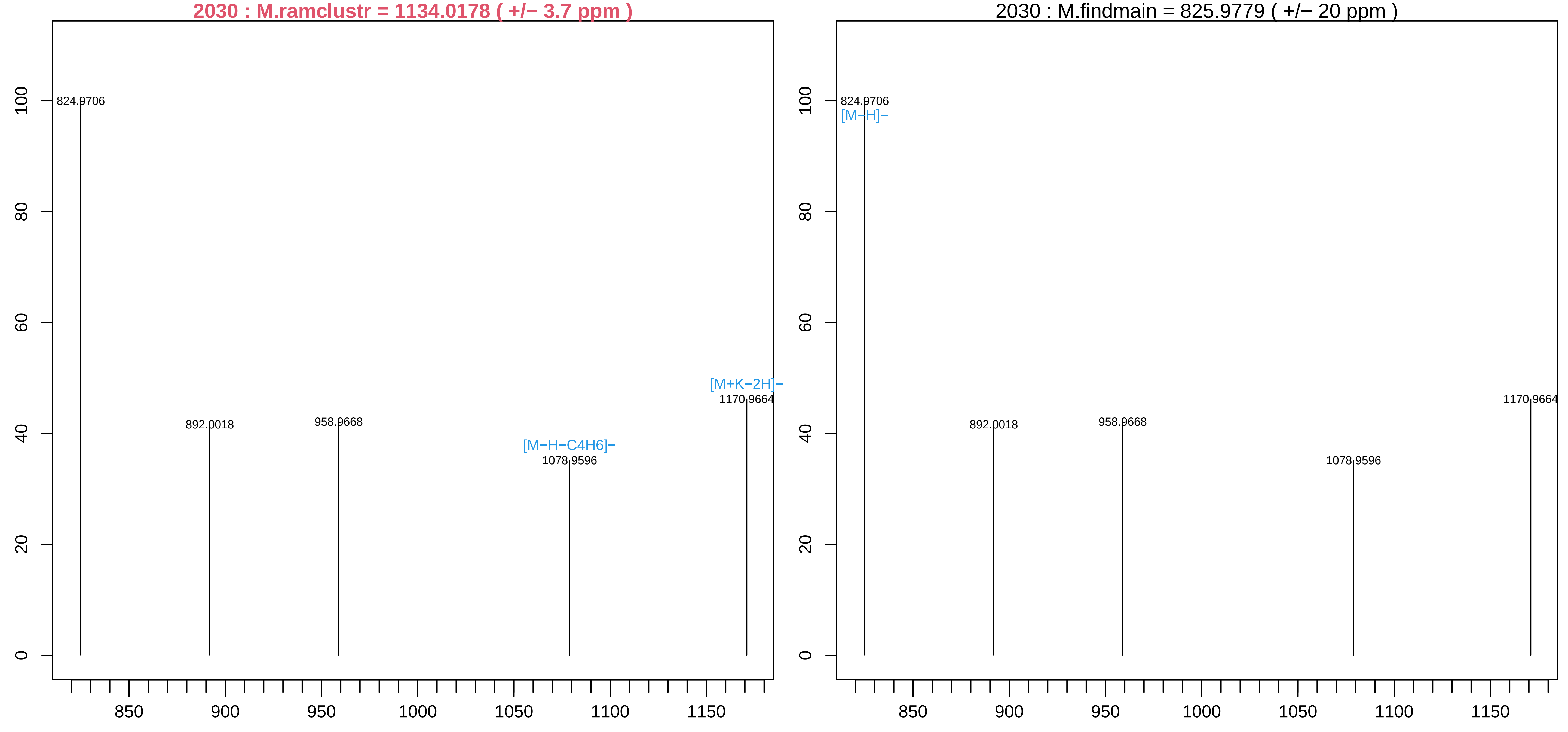

2030 : M.ramclustr = 1134.0178 (+/− 3.7 ppm)
824.9706
892.0018
958.9668
[M−H−C4H6]−
1078.9596
[M+K−2H]−
1170.9664
2030 : M.findmain = 825.9779 (+/− 20 ppm)
824.9706
[M−H]−
892.0018
958.9668
1078.9596
1170.9664
0
20
40
60
80
100
850
900
950
1000
1050
1100
1150

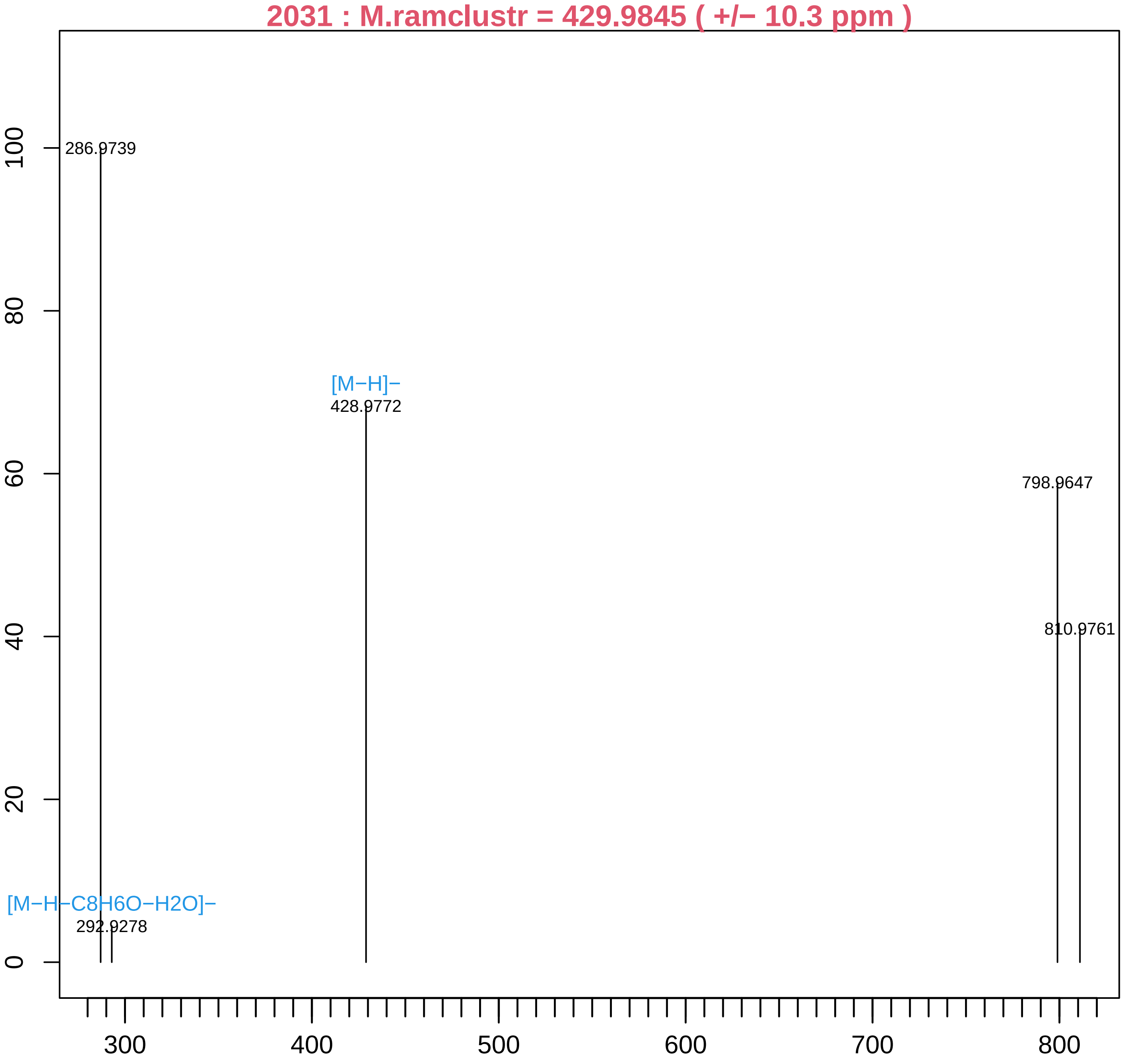
2031 : M.ramclustr = 429.9845 (+/− 10.3 ppm)
286.9739
[M−H]−
428.9772
798.9647
810.9761
[M−H−C8H6O−H2O]−
292.9278
0
20
40
60
80
100
300
400
500
600
700
800

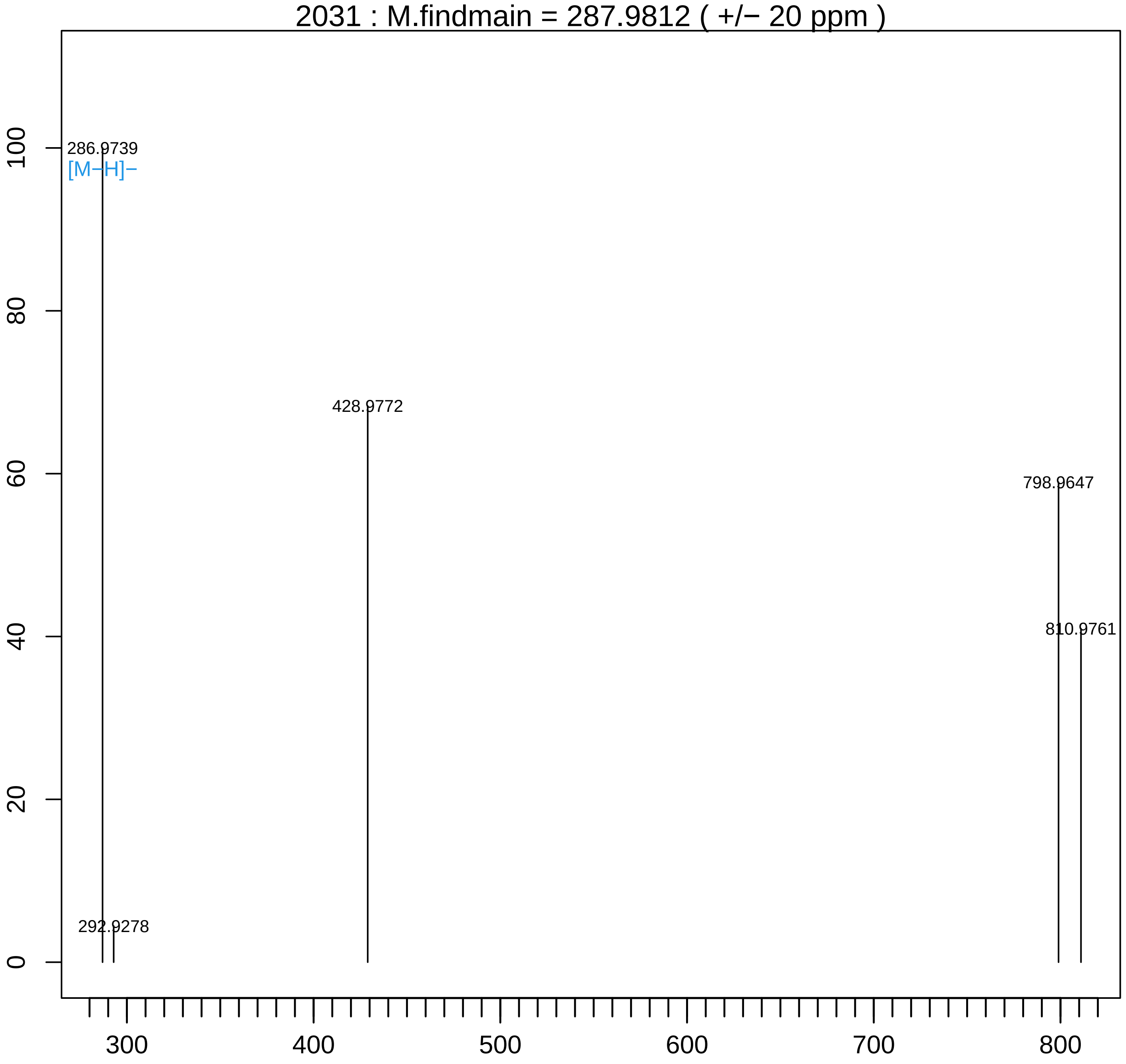
2031 : M.findmain = 287.9812 (+/− 20 ppm)
286.9739
[M−H]−
428.9772
798.9647
810.9761
292.9278
0
20
40
60
80
100
300
400
500
600
700
800

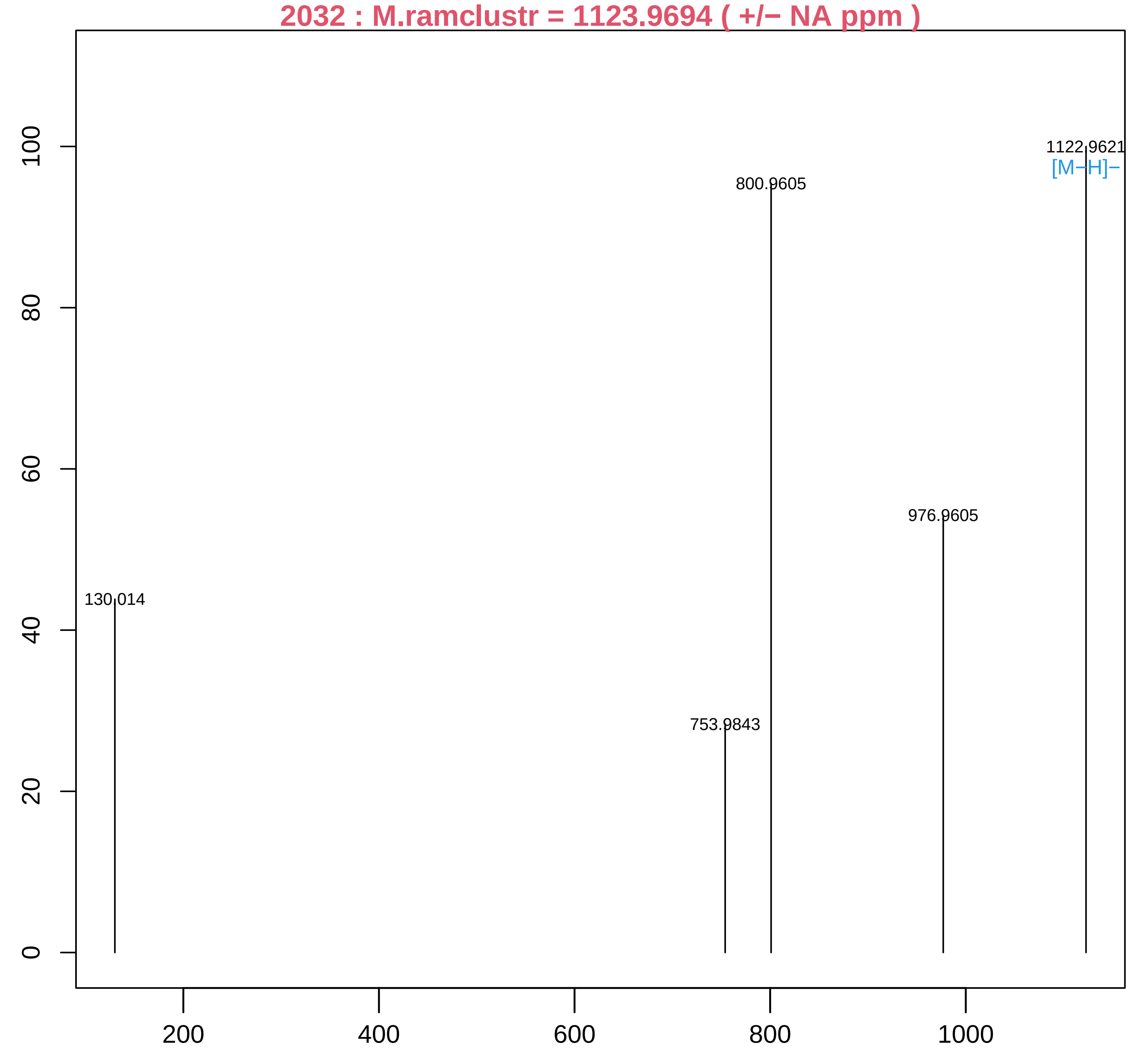
2032 : M.ramclustr = 1123.9694 (+/− NA ppm)
1122.9621
[M−H]−
800.9605
976.9605
753.9843
130.014
0
20
40
60
80
100
200
400
600
800
1000

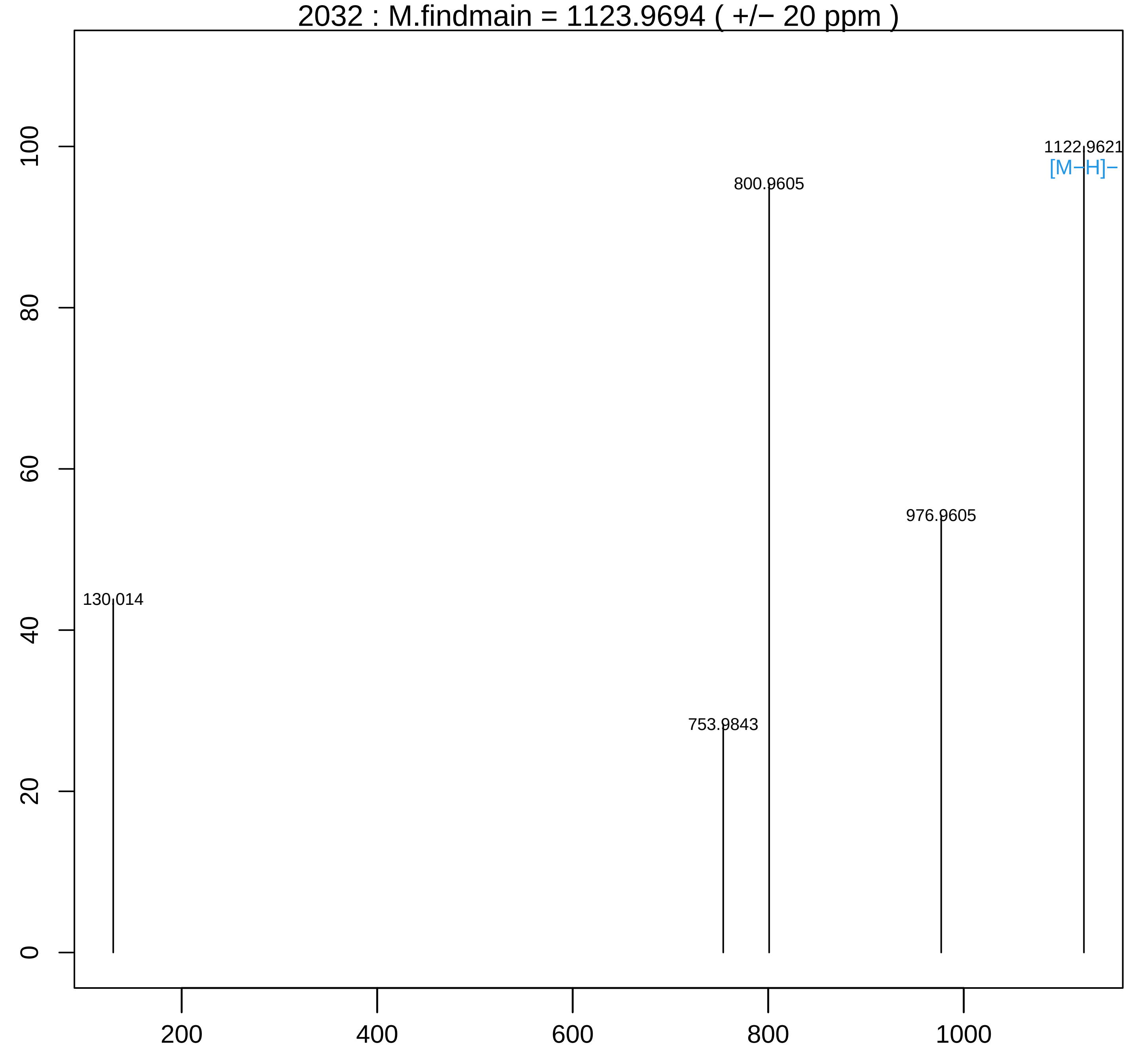
2032 : M.findmain = 1123.9694 (+/− 20 ppm)
1122.9621
[M−H]−
800.9605
976.9605
753.9843
130.014
0
20
40
60
80
100
200
400
600
800
1000

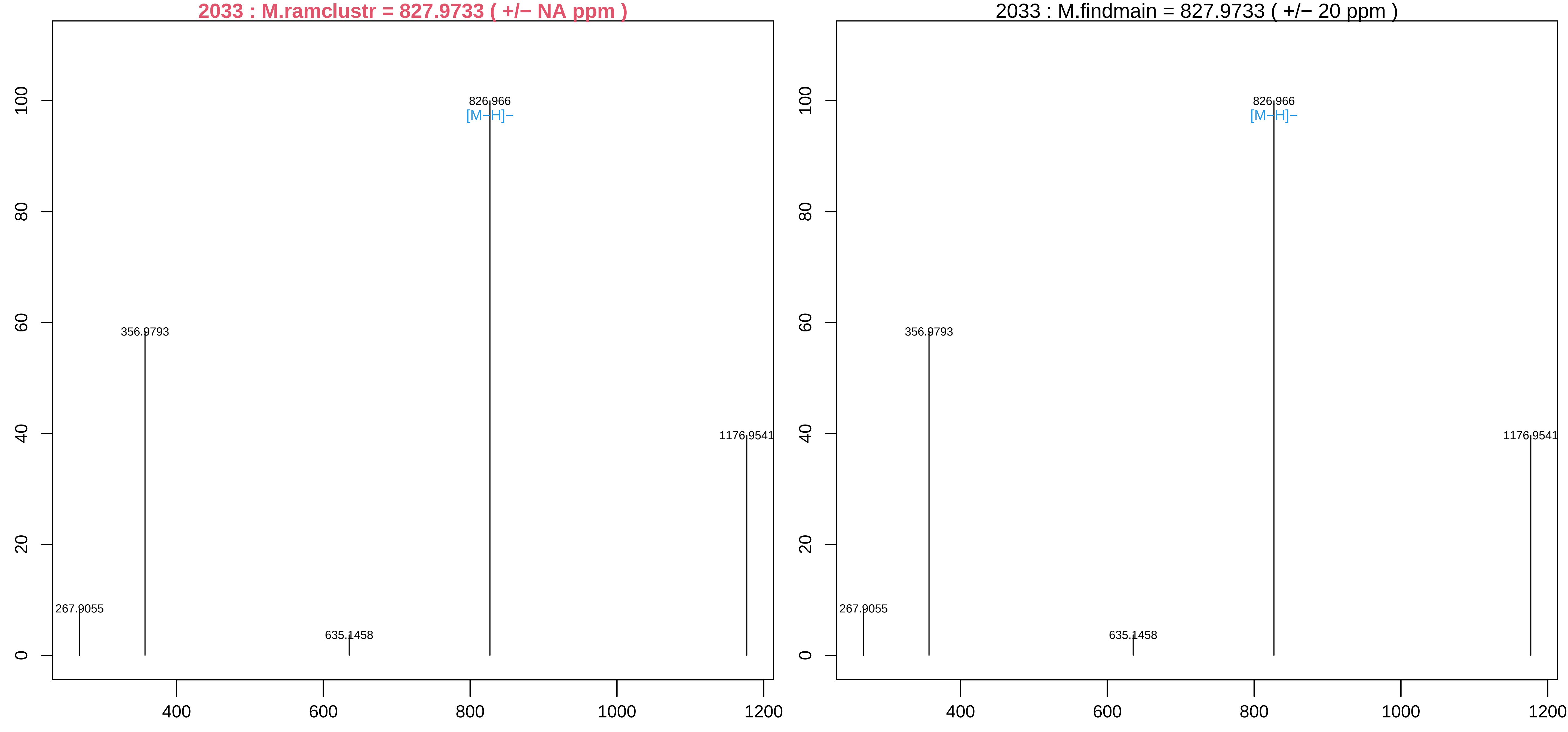
2033 : M.ramclustr = 827.9733 (+/− NA ppm)
826.966
[M−H]−
356.9793
1176.9541
267.9055
635.1458
0
20
40
60
80
100
400
600
800
1000
1200
2033 : M.findmain = 827.9733 (+/− 20 ppm)
826.966
[M−H]−
356.9793
1176.9541
267.9055
635.1458
0
20
40
60
80
100
400
600
800
1000
1200

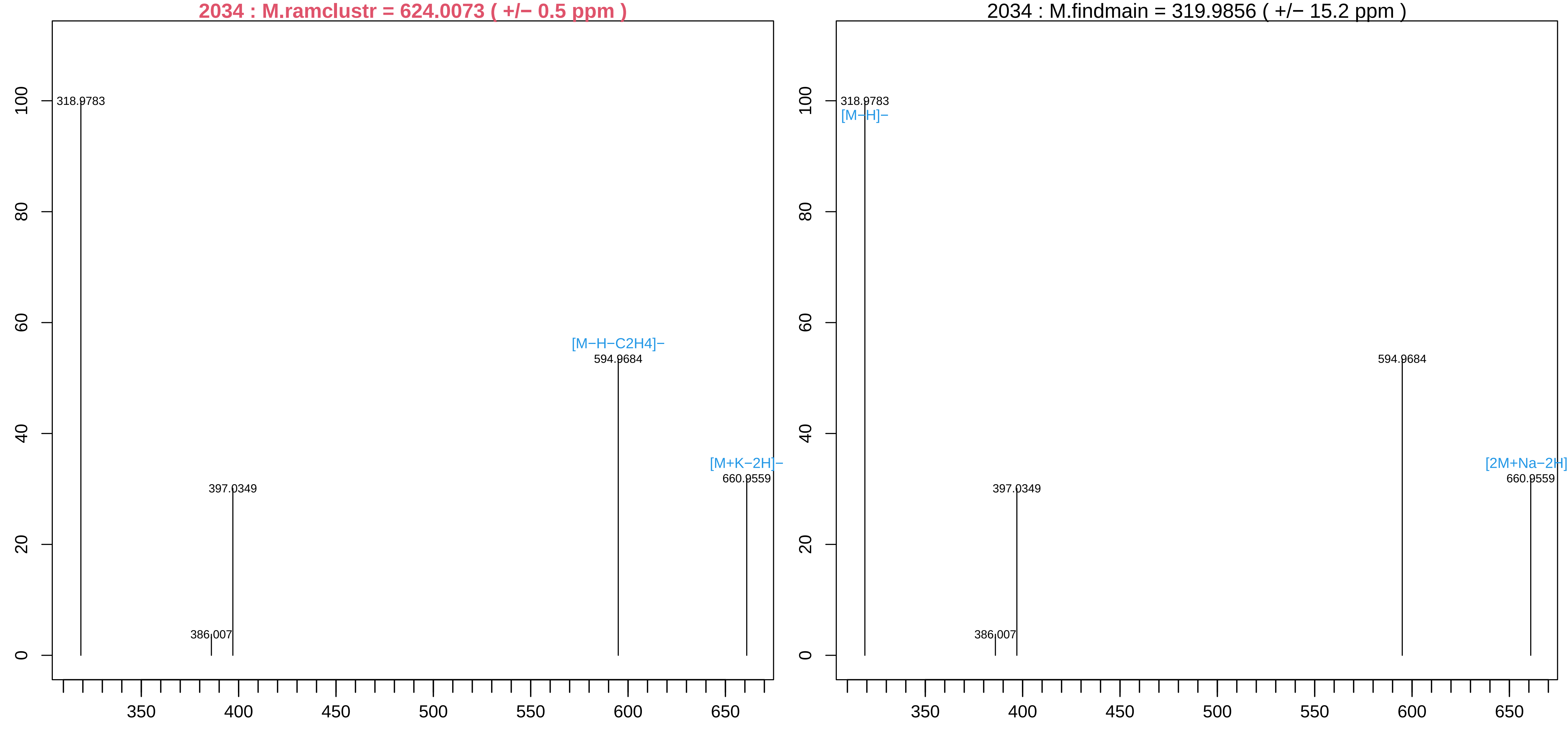

2034 : M.ramclustr = 624.0073 (+/− 0.5 ppm)
318.9783
397.0349
386.007
[M−H−C2H4]−
594.9684
[M+K−2H]−
660.9559
2034 : M.findmain = 319.9856 (+/− 15.2 ppm)
318.9783
[M−H]−
397.0349
386.007
594.9684
[2M+Na−2H]
660.9559
0
20
40
60
80
100
350
400
450
500
550
600
650

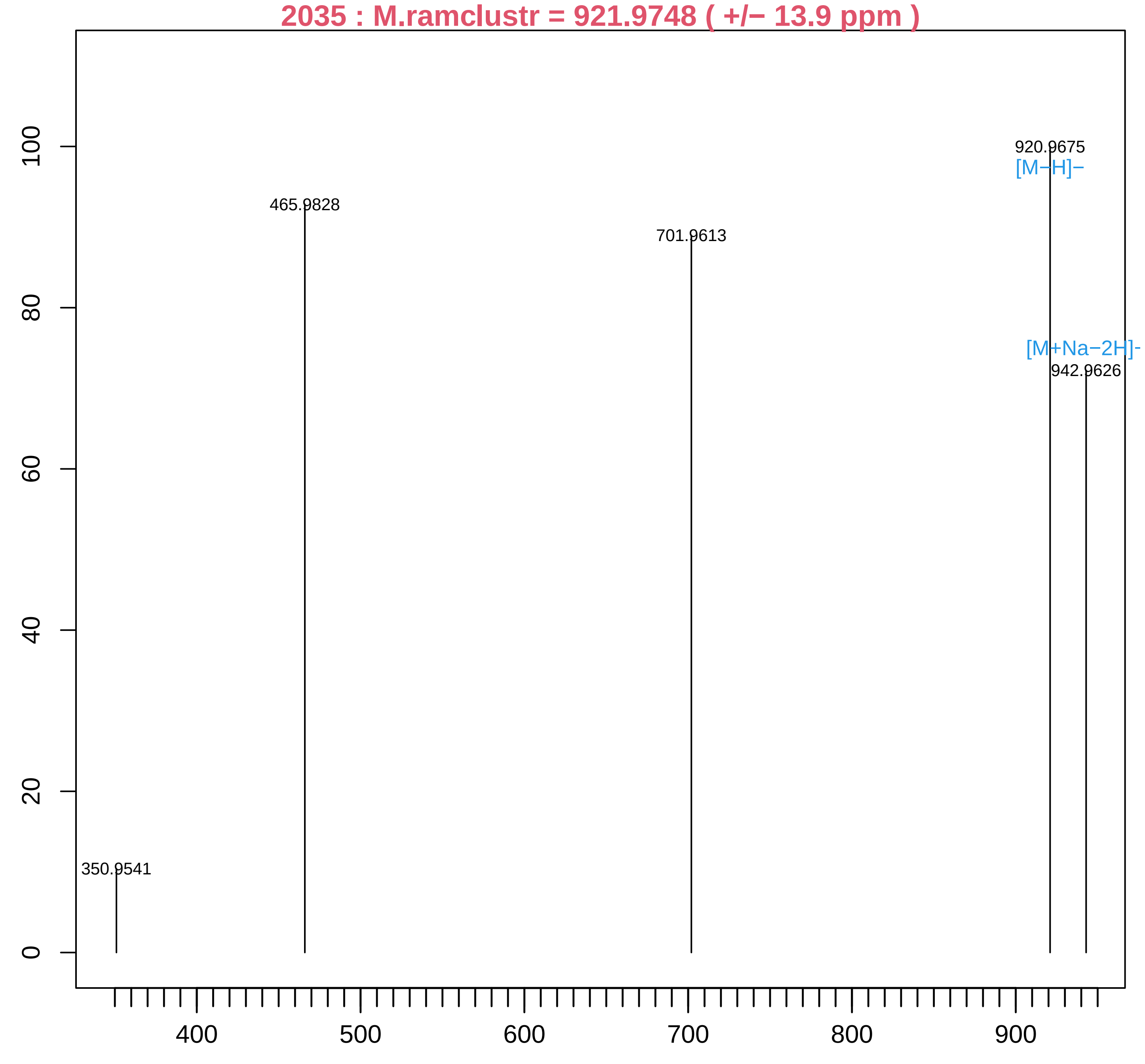

2035 : M.ramclustr = 921.9748 (+/− 13.9 ppm)
920.9675
[M−H]−
465.9828
701.9613
[M+Na−2H]-
942.9626
350.9541
0
20
40
60
80
100
400
500
600
700
800
900

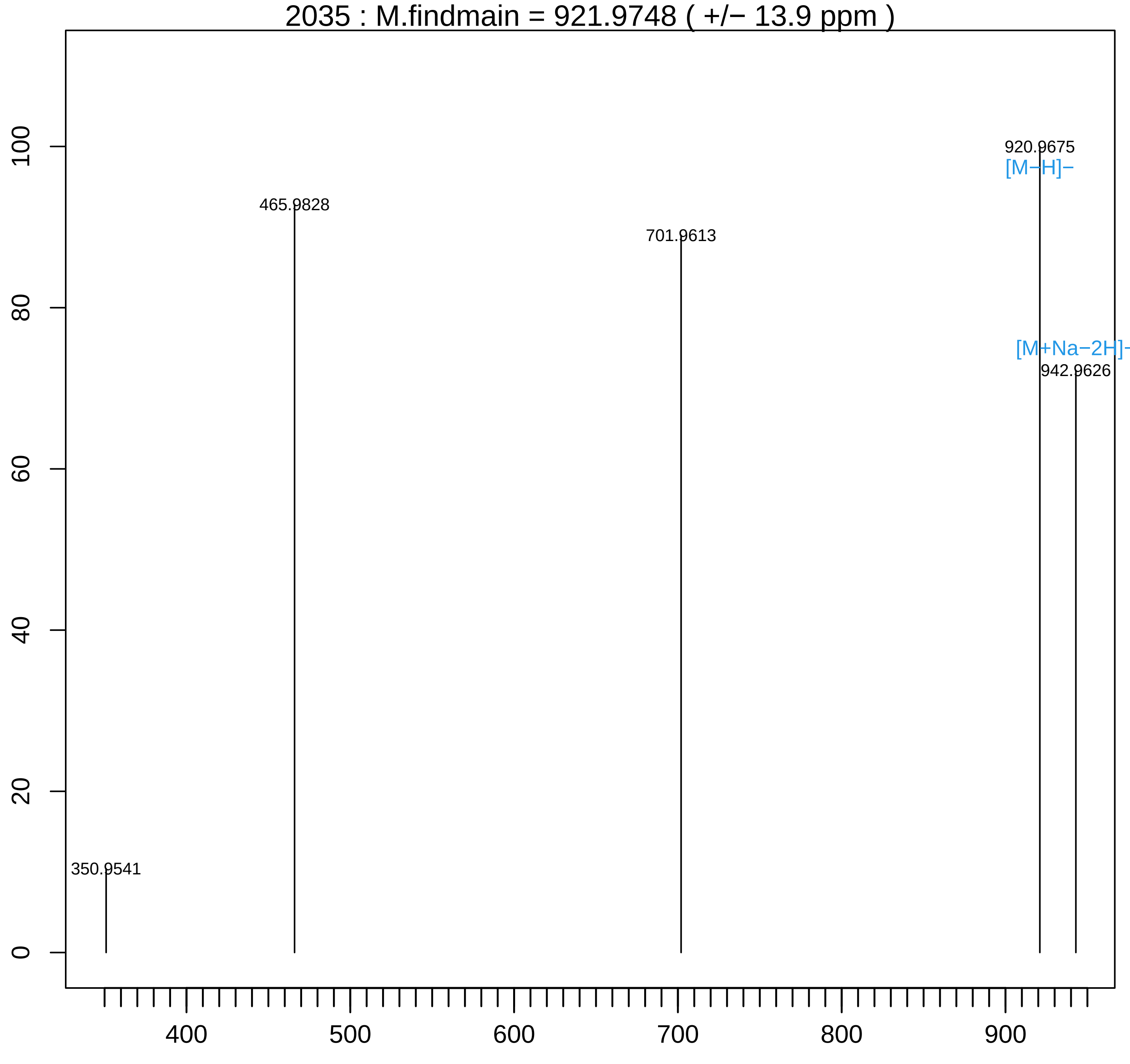

2035 : M.findmain = 921.9748 (+/− 13.9 ppm)
920.9675
[M−H]−
465.9828
701.9613
[M+Na−2H]-
942.9626
350.9541
0
20
40
60
80
100
400
500
600
700
800
900

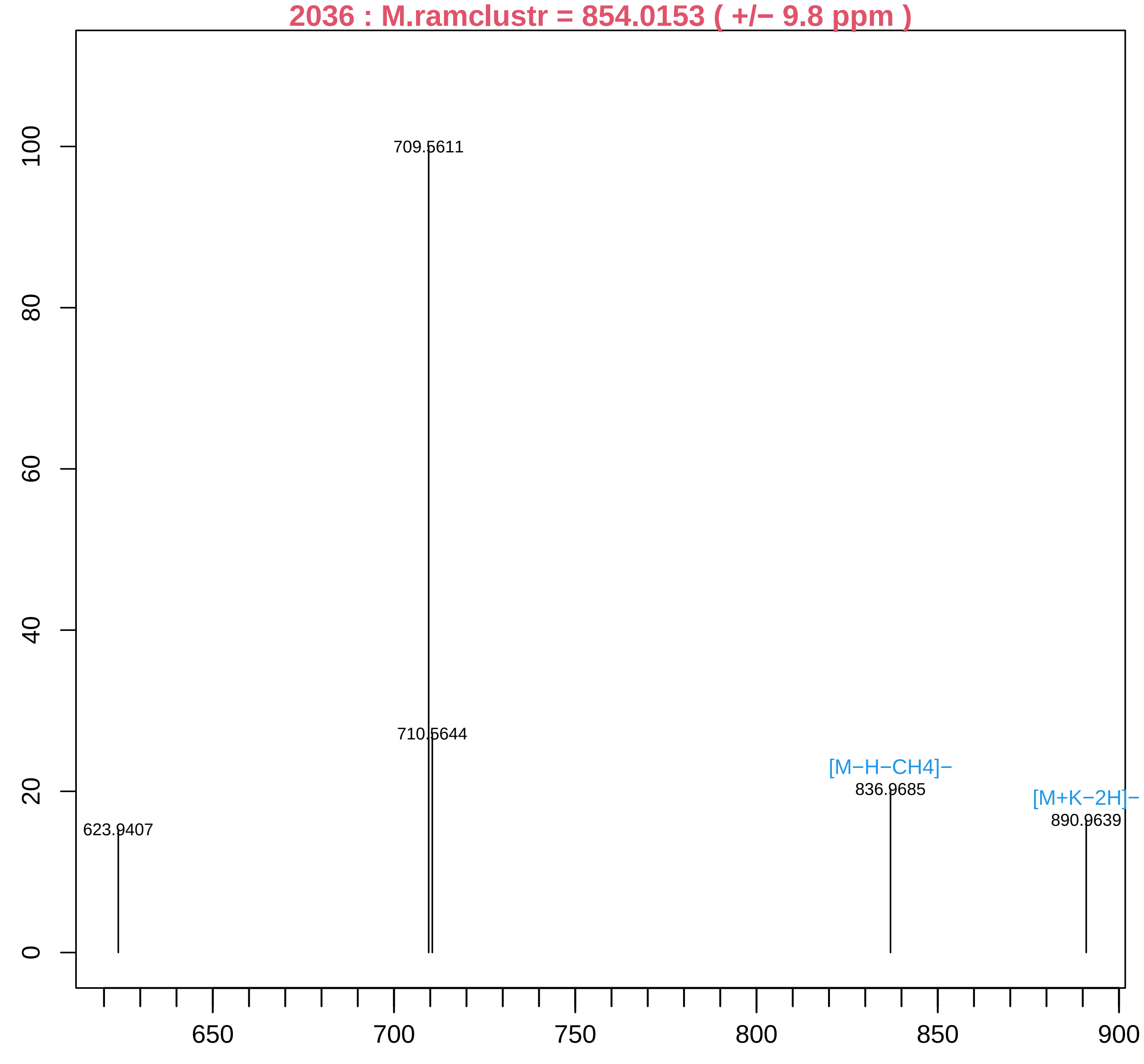

2036 : M.ramclustr = 854.0153 (+/− 9.8 ppm)
709.5611
710.5644
623.9407
[M−H−CH4]−
836.9685
[M+K−2H]−
890.9639
0
20
40
60
80
100
650
700
750
800
850
900

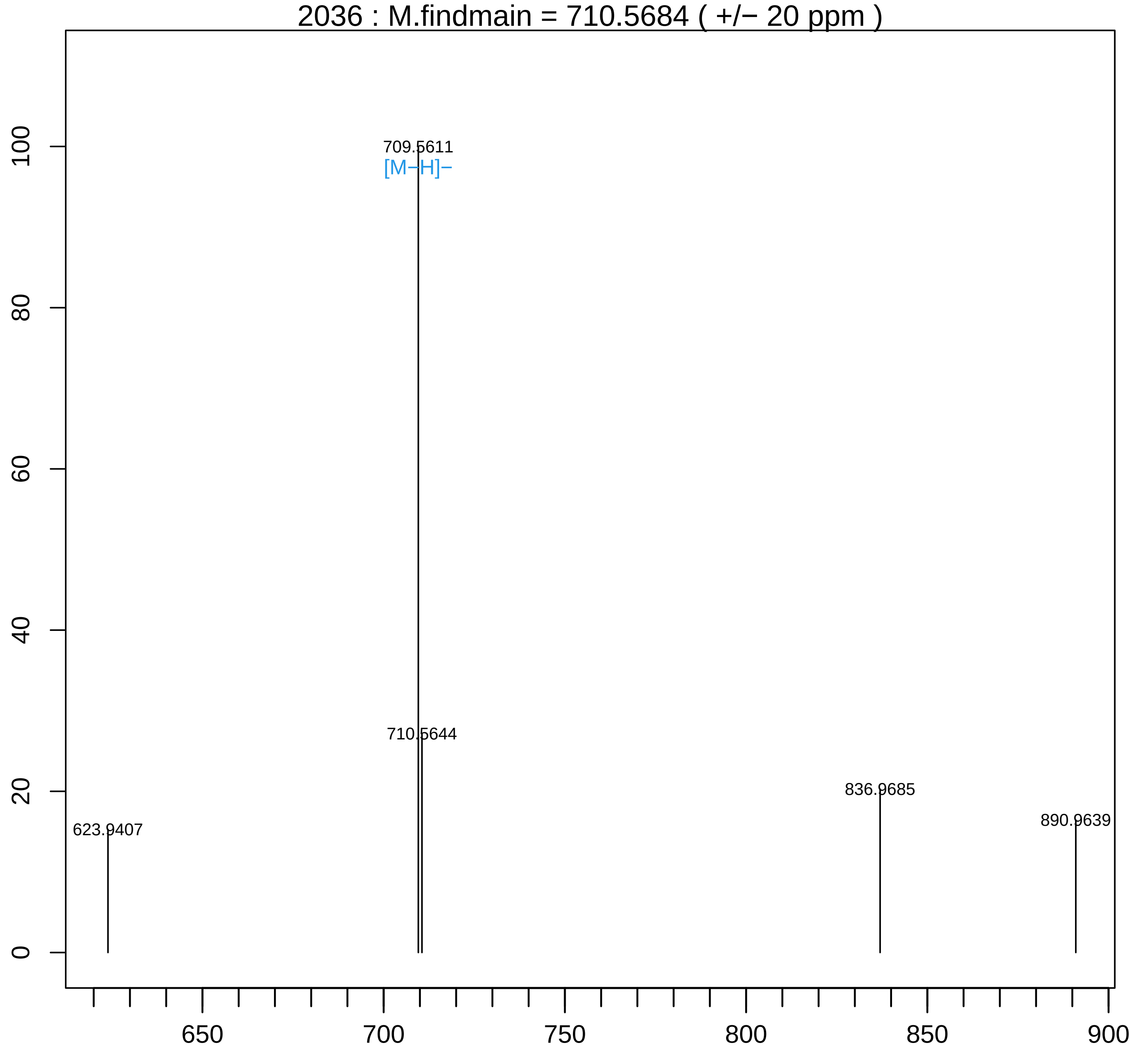

2036 : M.findmain = 710.5684 (+/− 20 ppm)
709.5611
[M−H]−
710.5644
623.9407
836.9685
890.9639
0
20
40
60
80
100
650
700
750
800
850
900

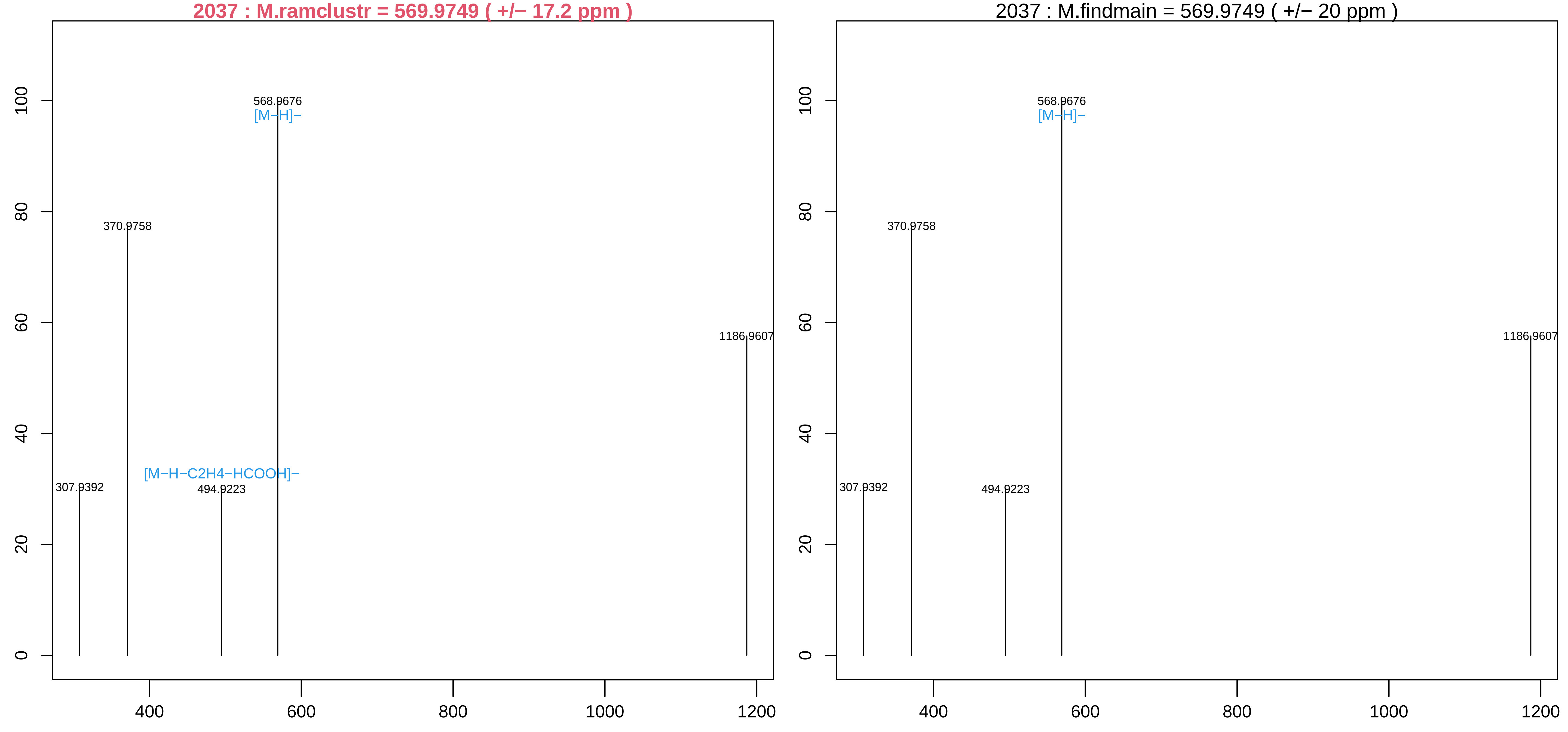
2037 : M.ramclustr = 569.9749 (+/− 17.2 ppm)
568.9676
[M−H]−
370.9758
1186.9607
[M−H−C2H4−HCOOH]−
307.9392
494.9223
2037 : M.findmain = 569.9749 (+/− 20 ppm)
568.9676
[M−H]−
370.9758
1186.9607
307.9392
494.9223
0
20
40
60
80
100
400
600
800
1000
1200

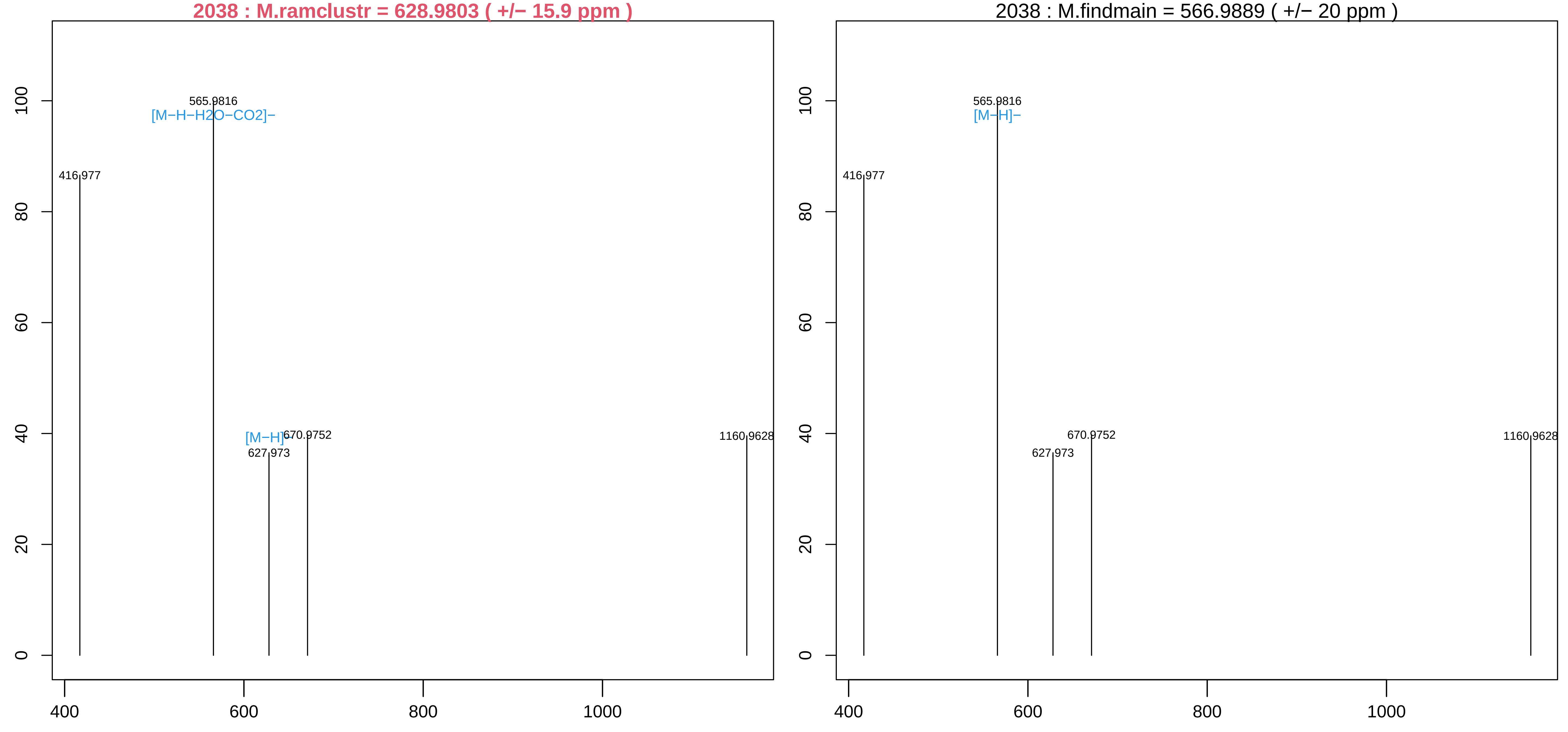

2038 : M.ramclustr = 628.9803 (+/− 15.9 ppm)
565.9816
[M−H−H2O−CO2]−
416.977
[M−H]−
670.9752
627.973
1160.9628
2038 : M.findmain = 566.9889 (+/− 20 ppm)
565.9816
[M−H]−
416.977
670.9752
627.973
1160.9628
0
20
40
60
80
100
400
600
800
1000

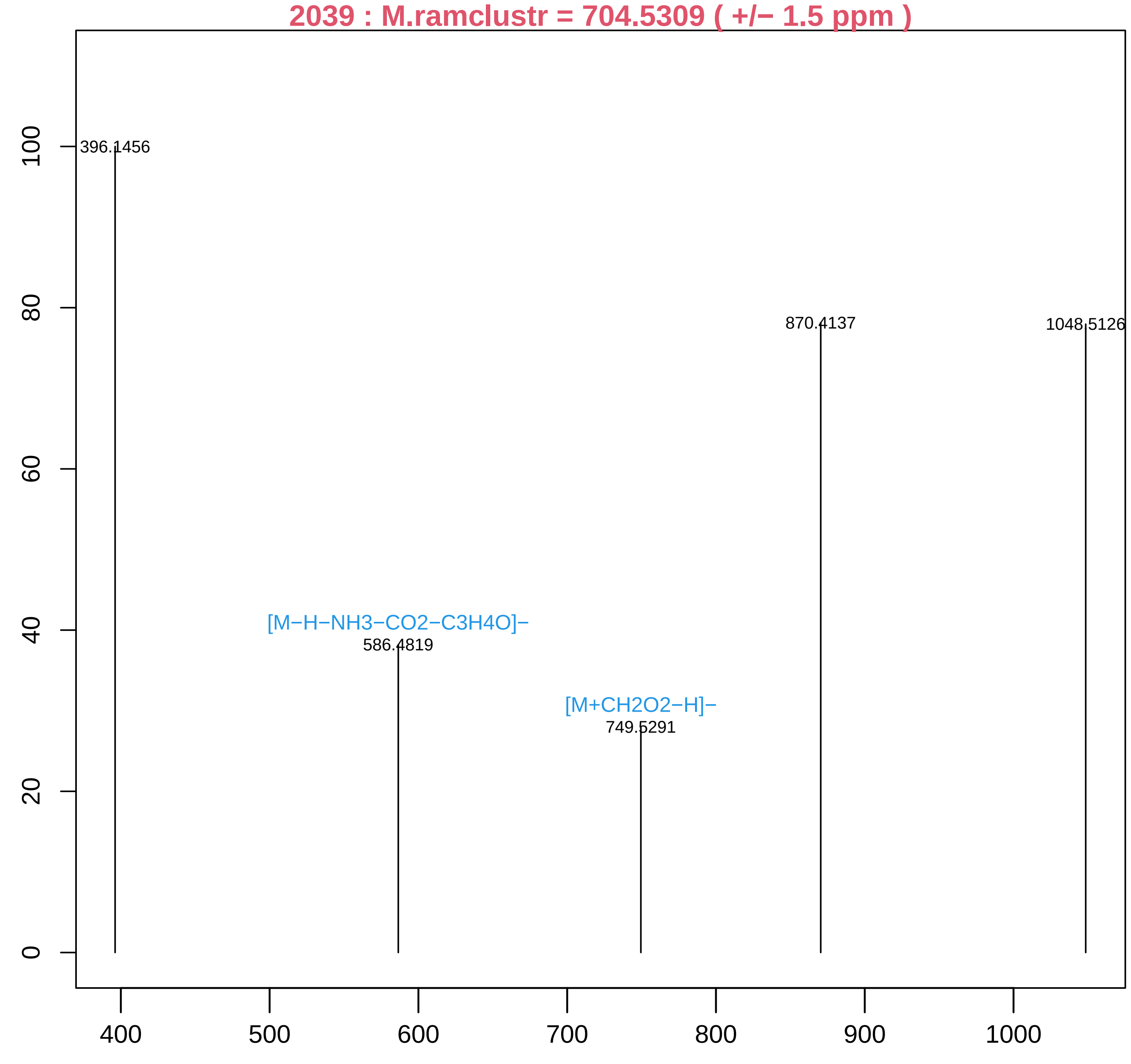
2039 : M.ramclustr = 704.5309 (+/− 1.5 ppm)
396.1456
[M−H−NH3−CO2−C3H4O]−
586.4819
[M+CH2O2−H]−
749.5291
870.4137
1048.5126
0
20
40
60
80
100
400
500
600
700
800
900
1000

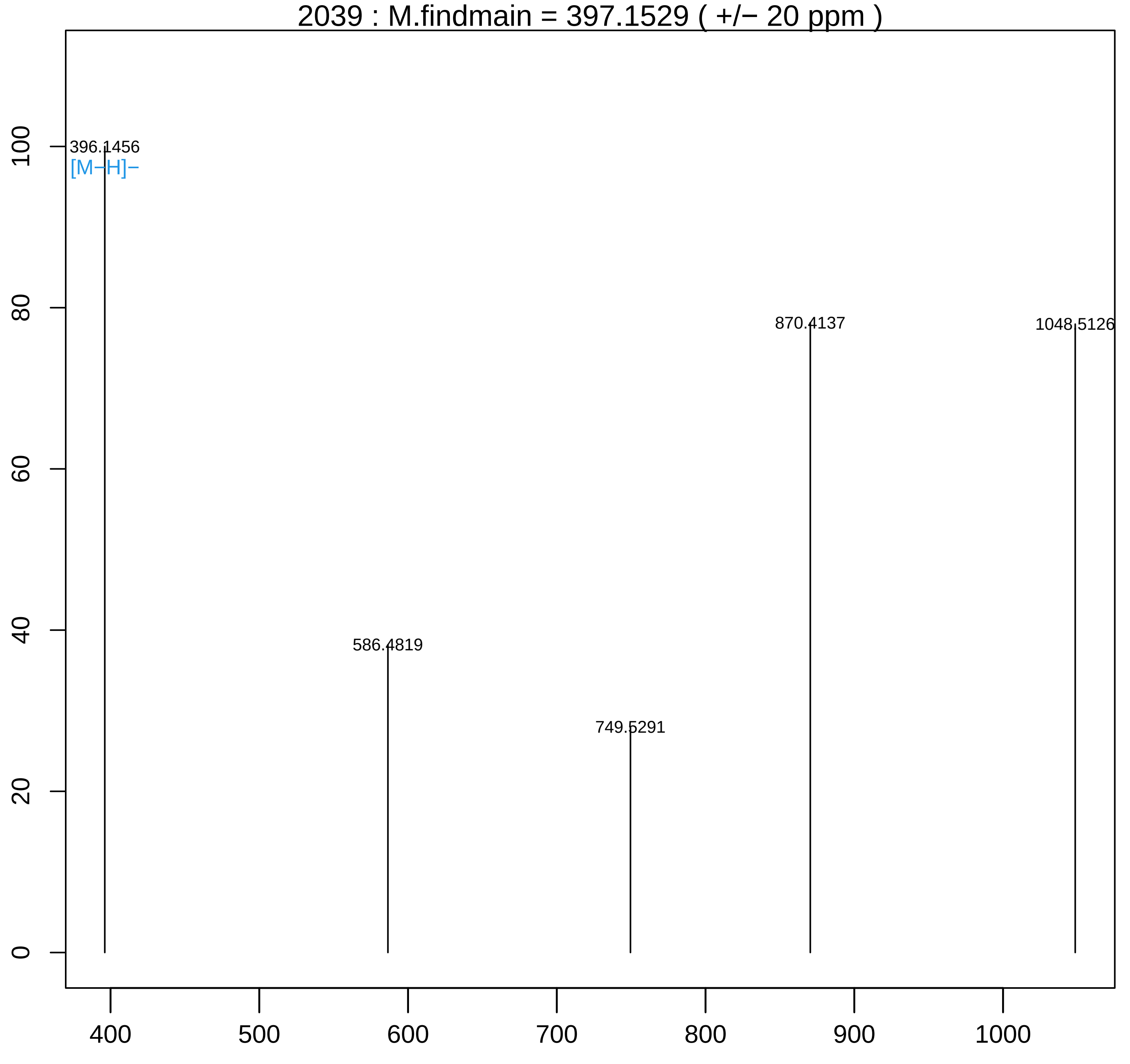
2039 : M.findmain = 397.1529 (+/− 20 ppm)
396.1456
[M−H]−
586.4819
749.5291
870.4137
1048.5126
0
20
40
60
80
100
400
500
600
700
800
900
1000

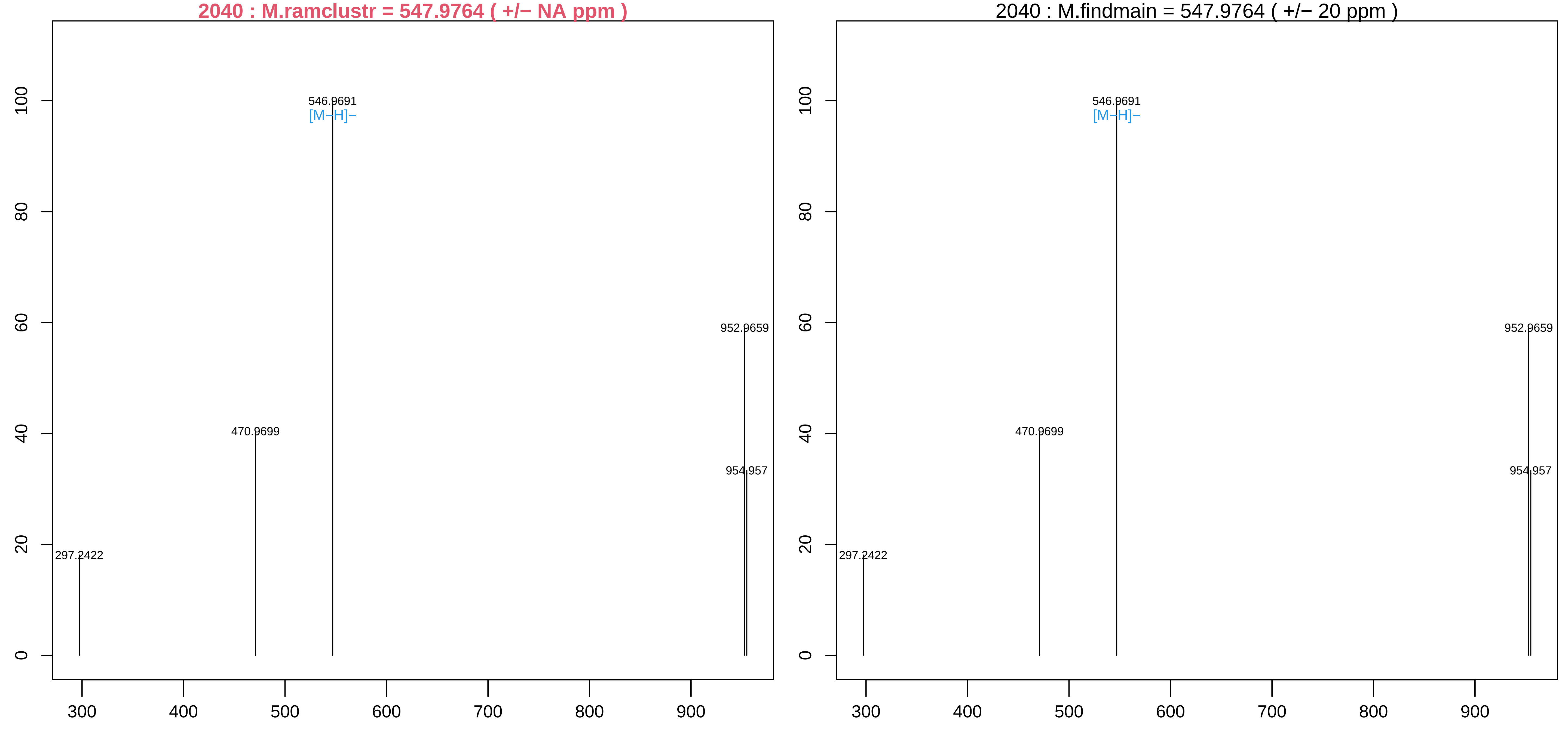
2040 : M.ramclustr = 547.9764 (+/− NA ppm)
2040 : M.findmain = 547.9764 (+/− 20 ppm)
546.9691
[M−H]−
470.9699
297.2422
952.9659
954.957
0
20
40
60
80
100
300
400
500
600
700
800
900

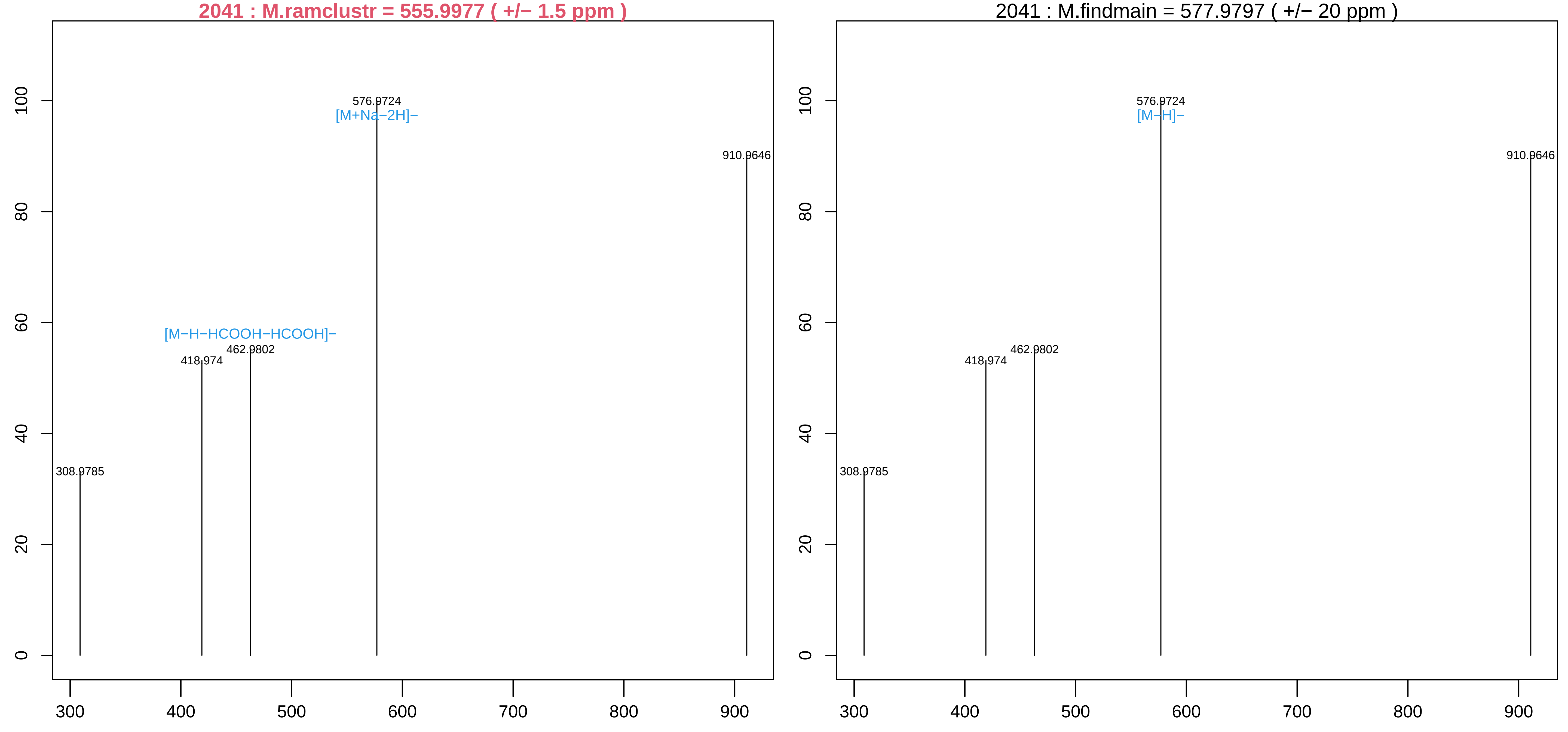
2041 : M.ramclustr = 555.9977 (+/− 1.5 ppm)
576.9724
[M+Na−2H]−
910.9646
[M−H−HCOOH−HCOOH]−
418.974
462.9802
308.9785
2041 : M.findmain = 577.9797 (+/− 20 ppm)
576.9724
[M−H]−
910.9646
418.974
462.9802
308.9785
0
20
40
60
80
100
300
400
500
600
700
800
900

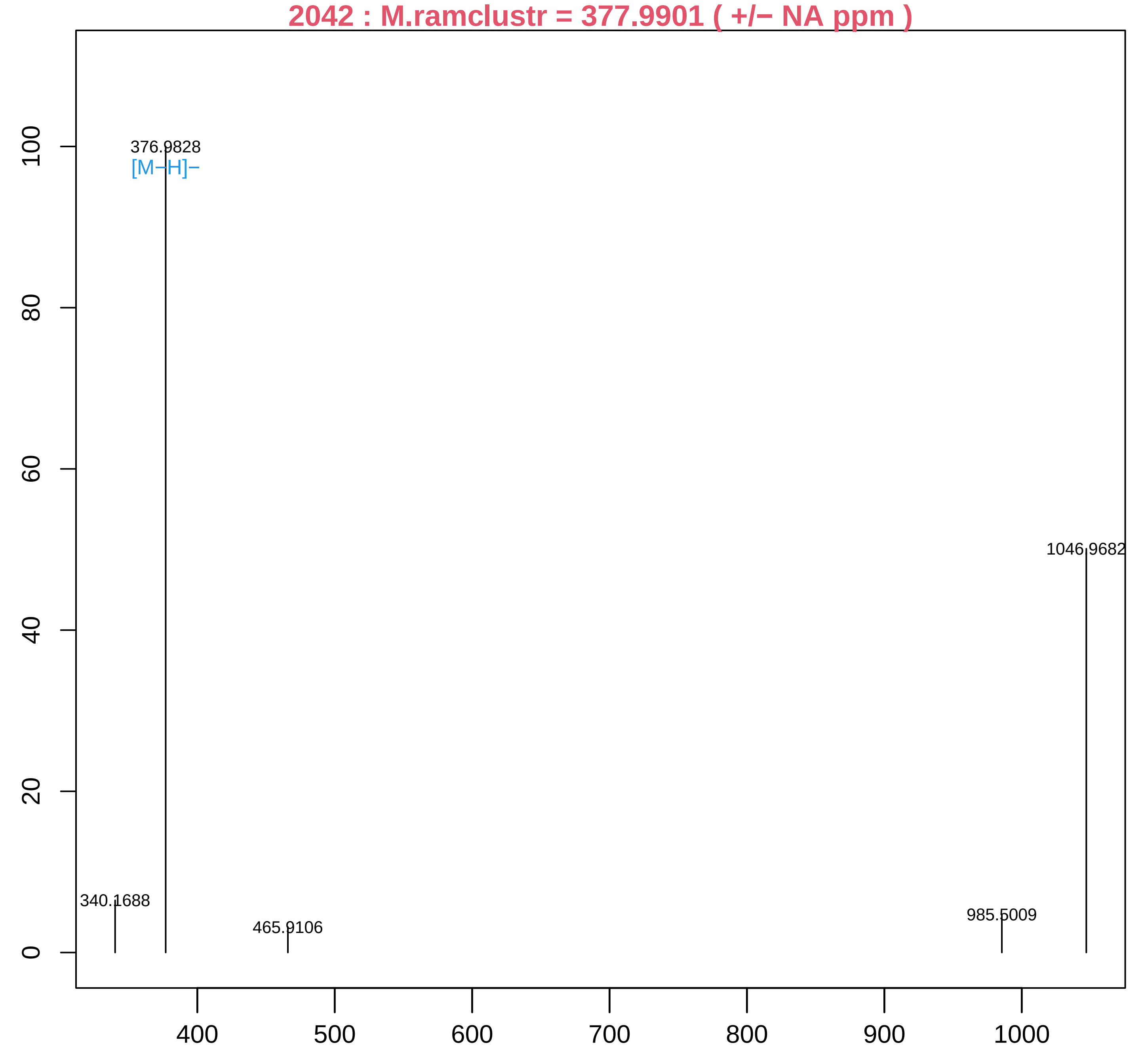

2042 : M.ramclustr = 377.9901 (+/− NA ppm)
376.9828
[M−H]−
1046.9682
340.1688
465.9106
985.5009
0
20
40
60
80
100
400
500
600
700
800
900
1000

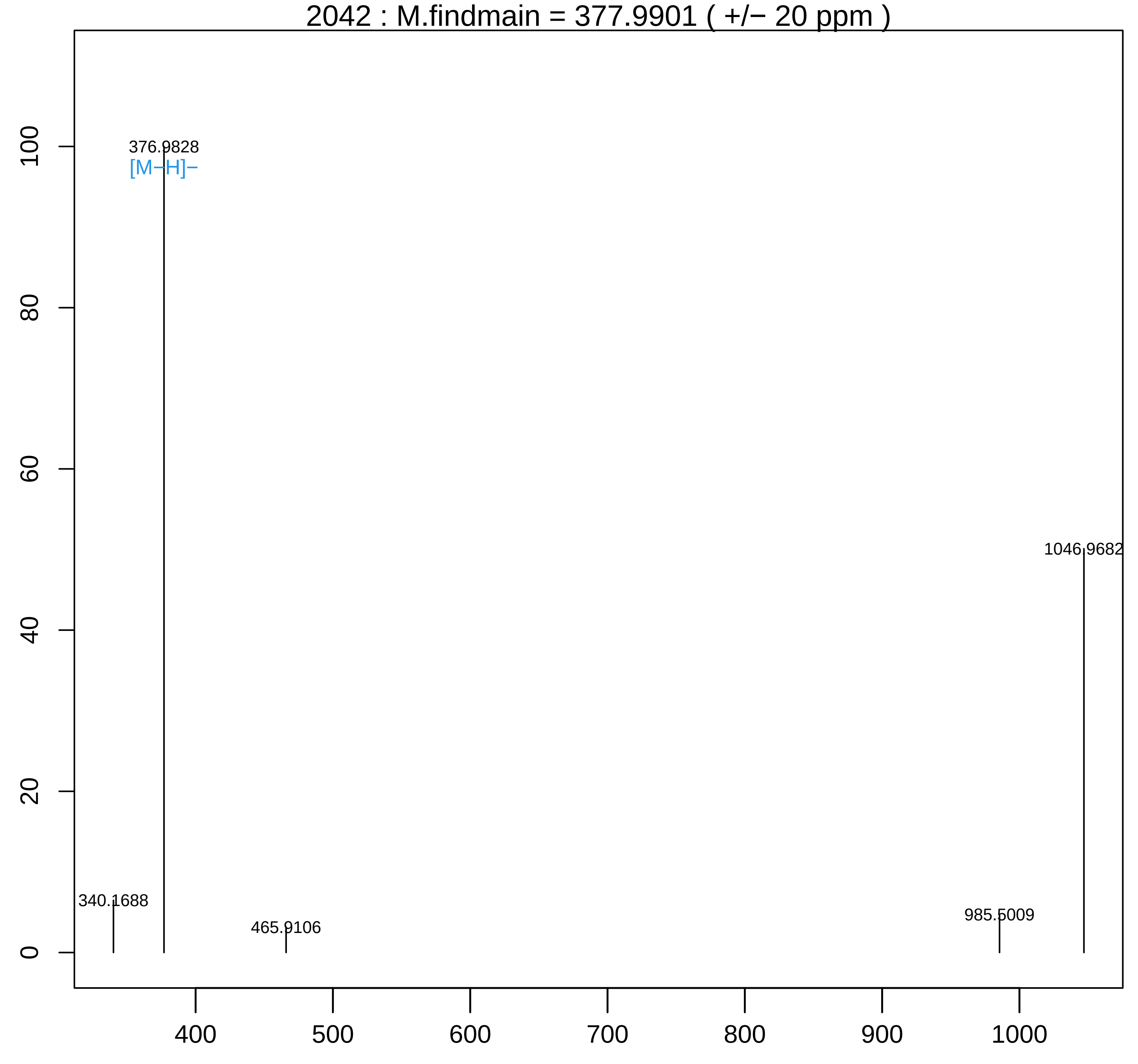

2042 : M.findmain = 377.9901 (+/− 20 ppm)
376.9828
[M−H]−
1046.9682
340.1688
465.9106
985.5009
0
20
40
60
80
100
400
500
600
700
800
900
1000

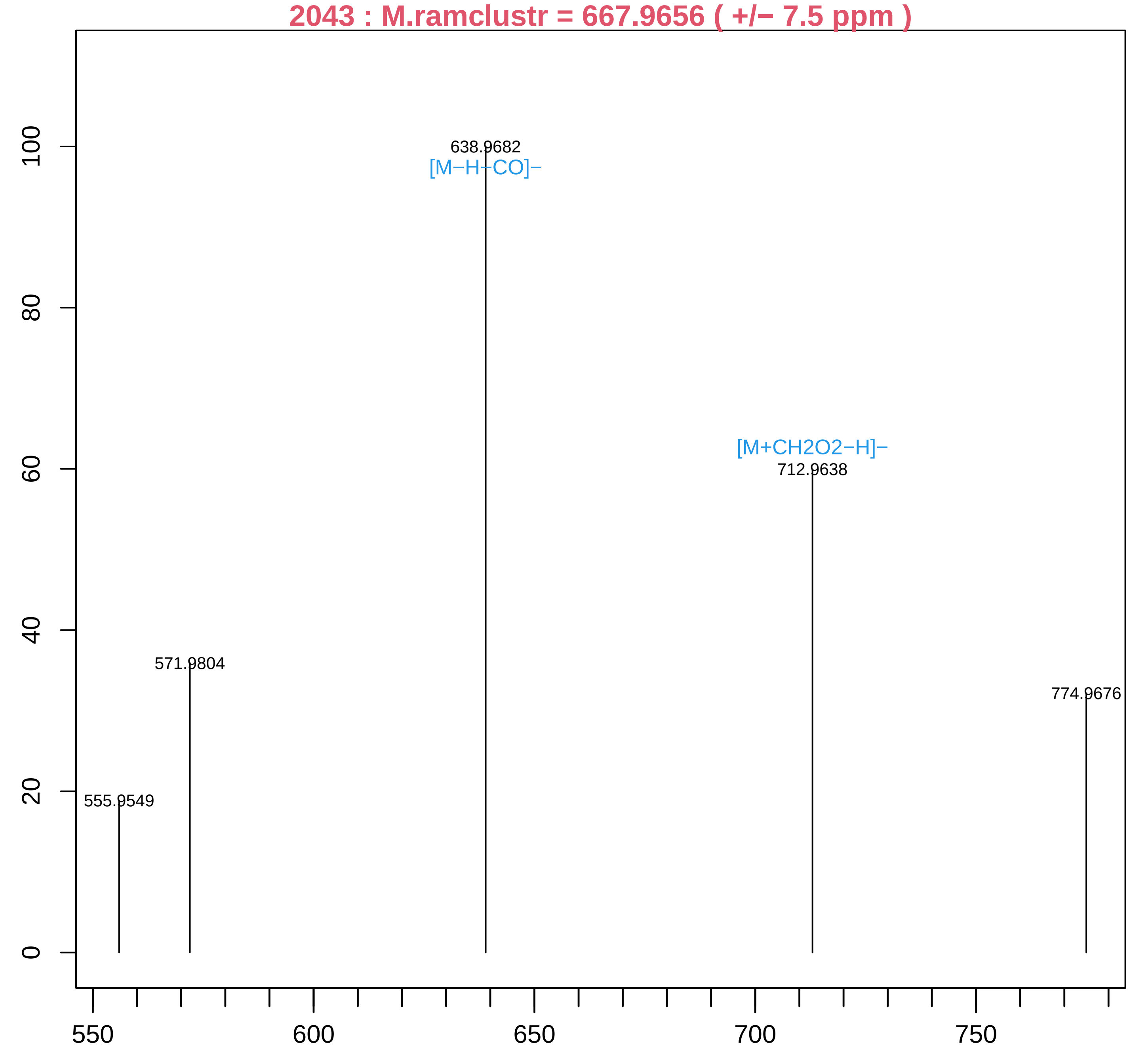
2043 : M.ramclustr = 667.9656 (+/− 7.5 ppm)
638.9682
[M−H−CO]−
[M+CH2O2−H]−
712.9638
571.9804
774.9676
555.9549
0
20
40
60
80
100
550
600
650
700
750

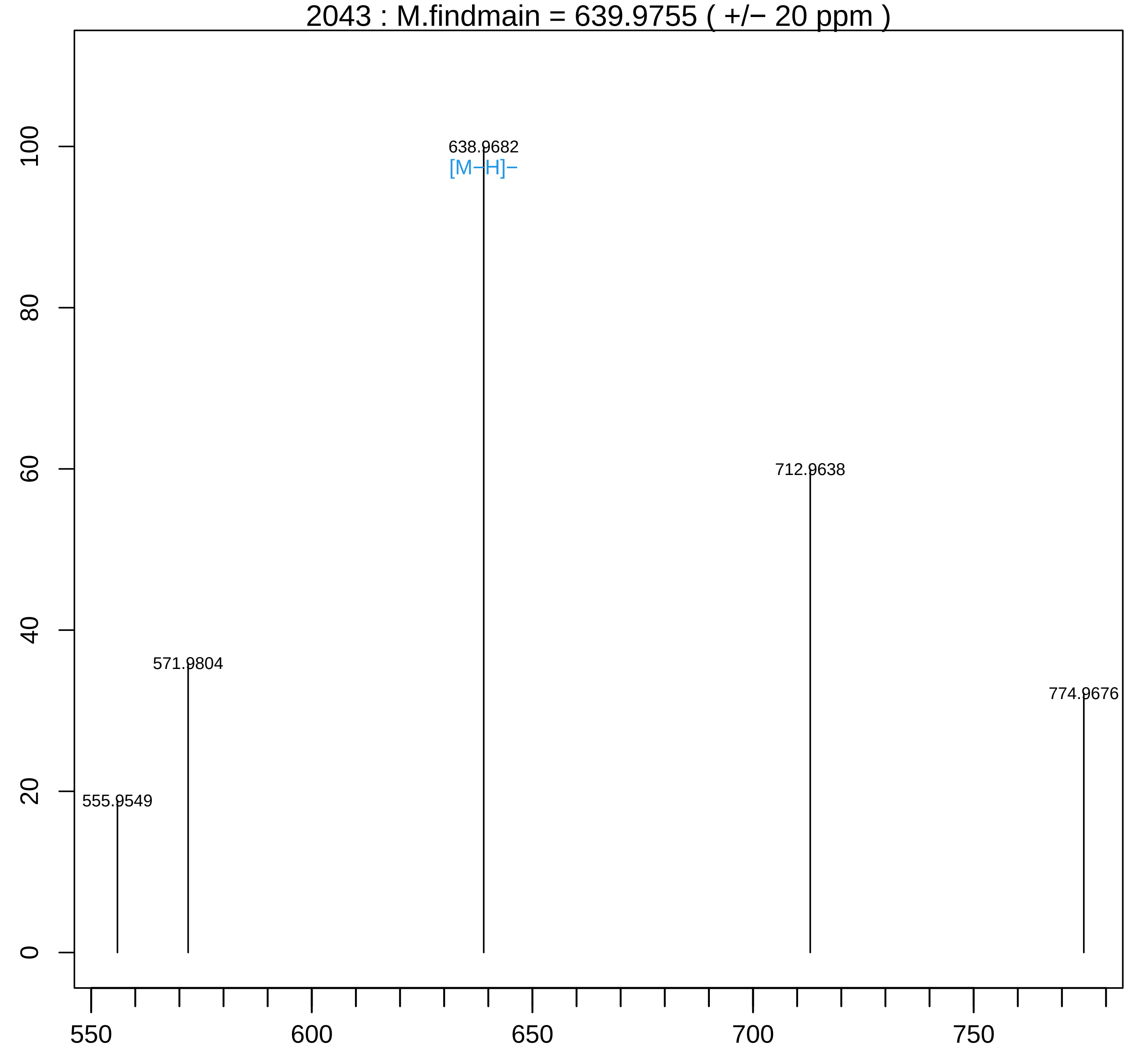
2043 : M.findmain = 639.9755 (+/− 20 ppm)
638.9682
[M−H]−
712.9638
571.9804
774.9676
555.9549
0
20
40
60
80
100
550
600
650
700
750

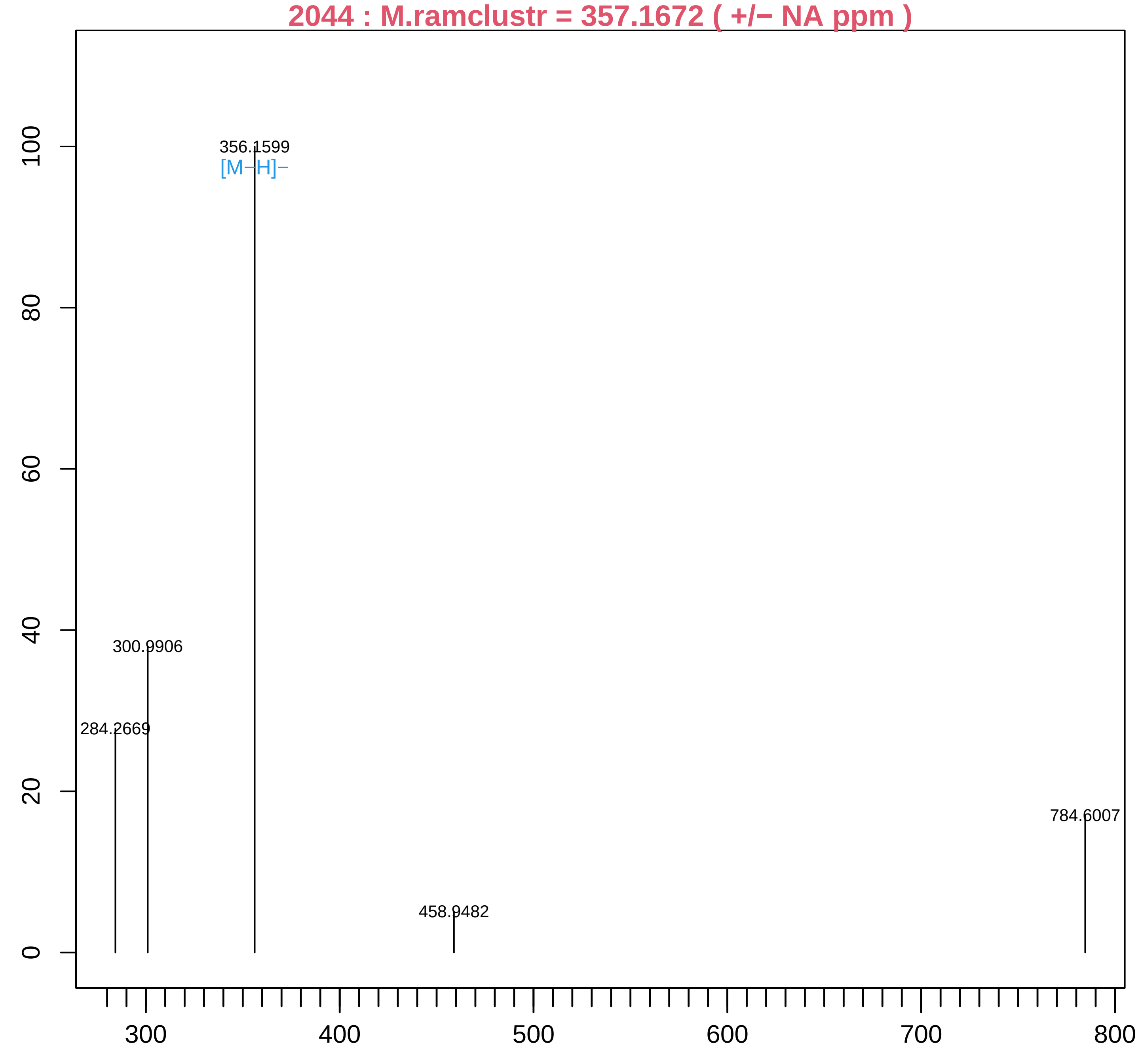
2044 : M.ramclustr = 357.1672 (+/− NA ppm)
356.1599
[M−H]−
300.9906
284.2669
458.9482
784.6007
0
20
40
60
80
100
300
400
500
600
700
800

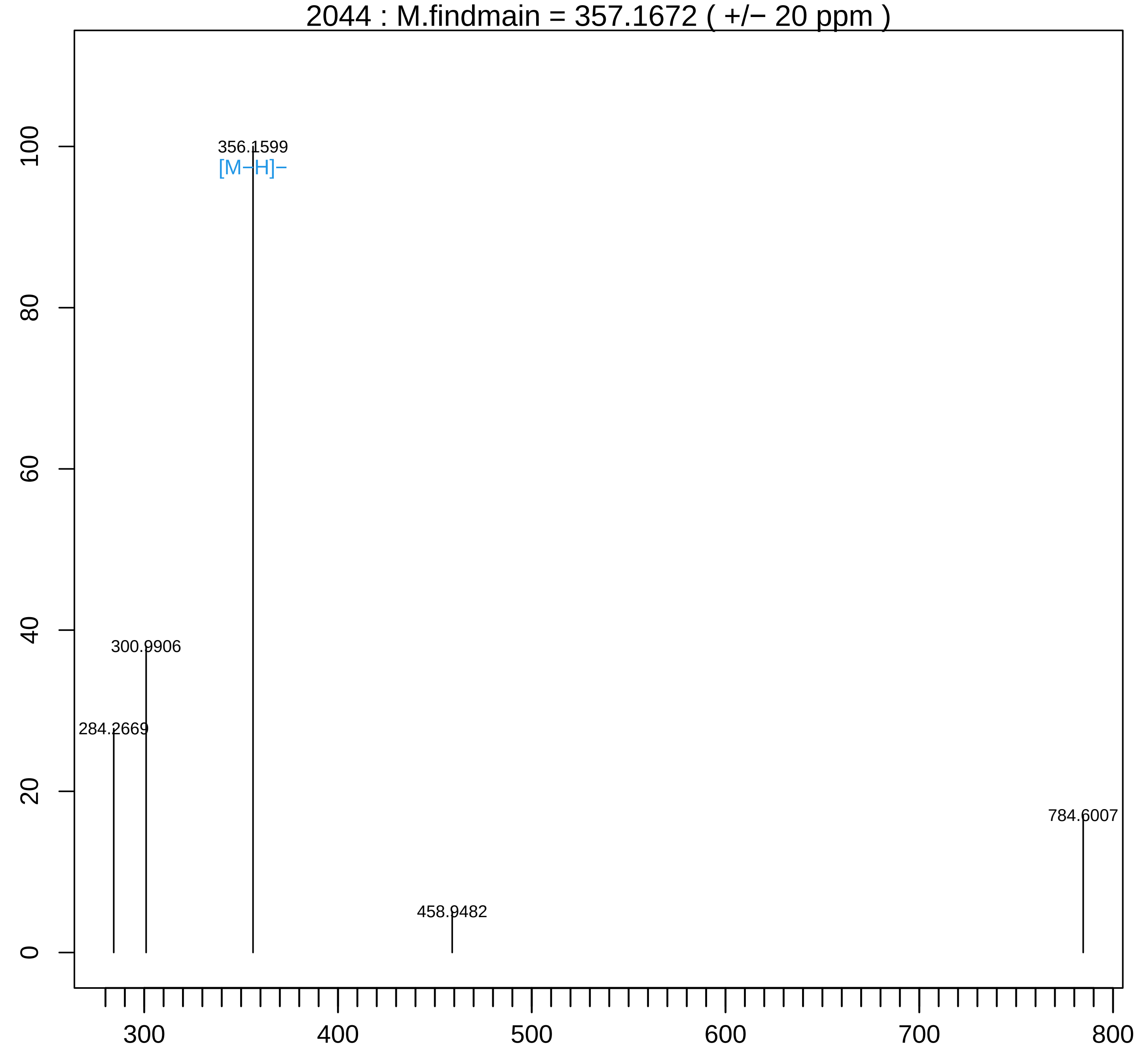
2044 : M.findmain = 357.1672 (+/− 20 ppm)
356.1599
[M−H]−
300.9906
284.2669
458.9482
784.6007
0
20
40
60
80
100
300
400
500
600
700
800

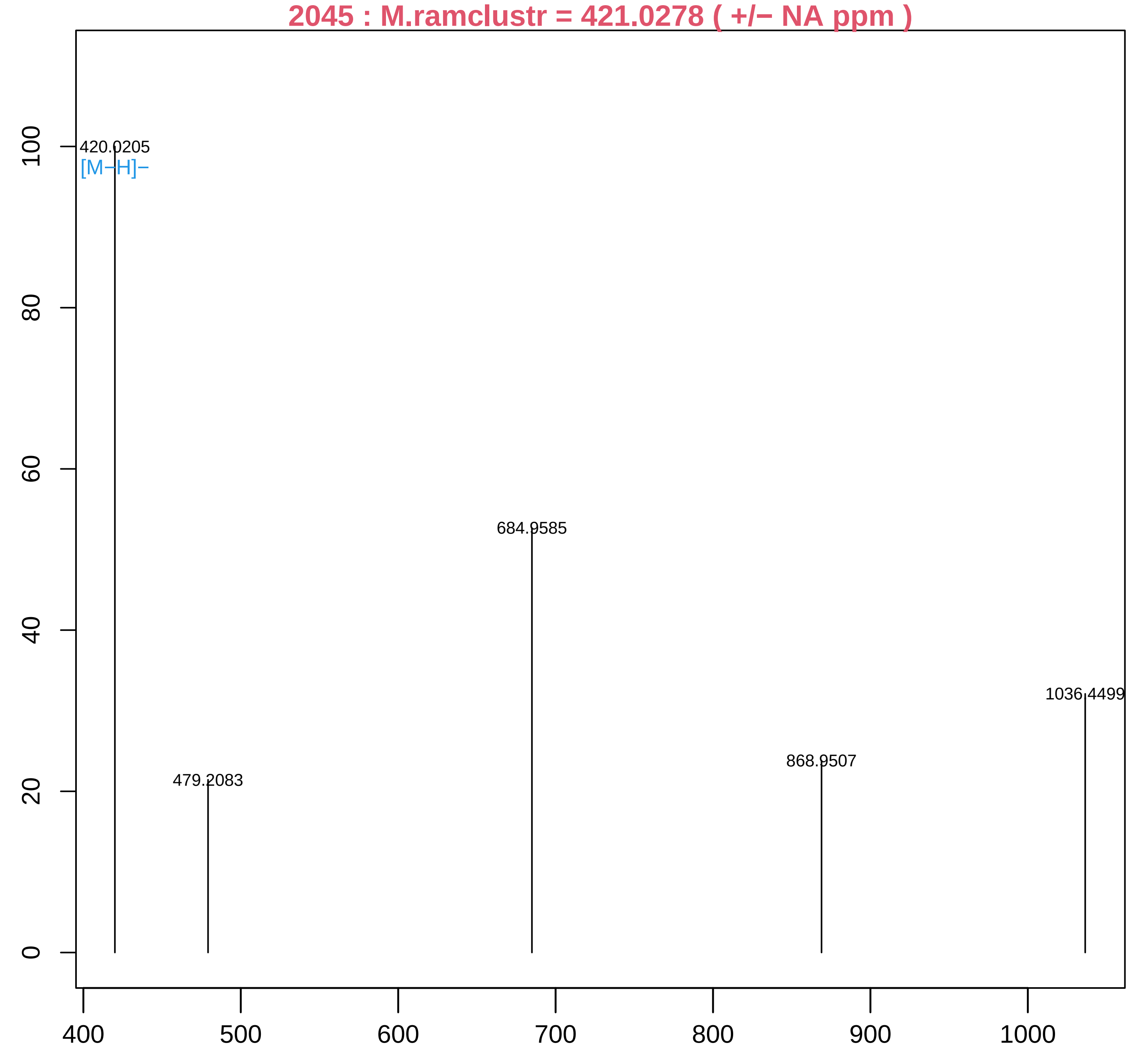

2045 : M.ramclustr = 421.0278 (+/− NA ppm)
420.0205
[M−H]−
479.2083
684.9585
868.9507
1036.4499
0
20
40
60
80
100
400
500
600
700
800
900
1000

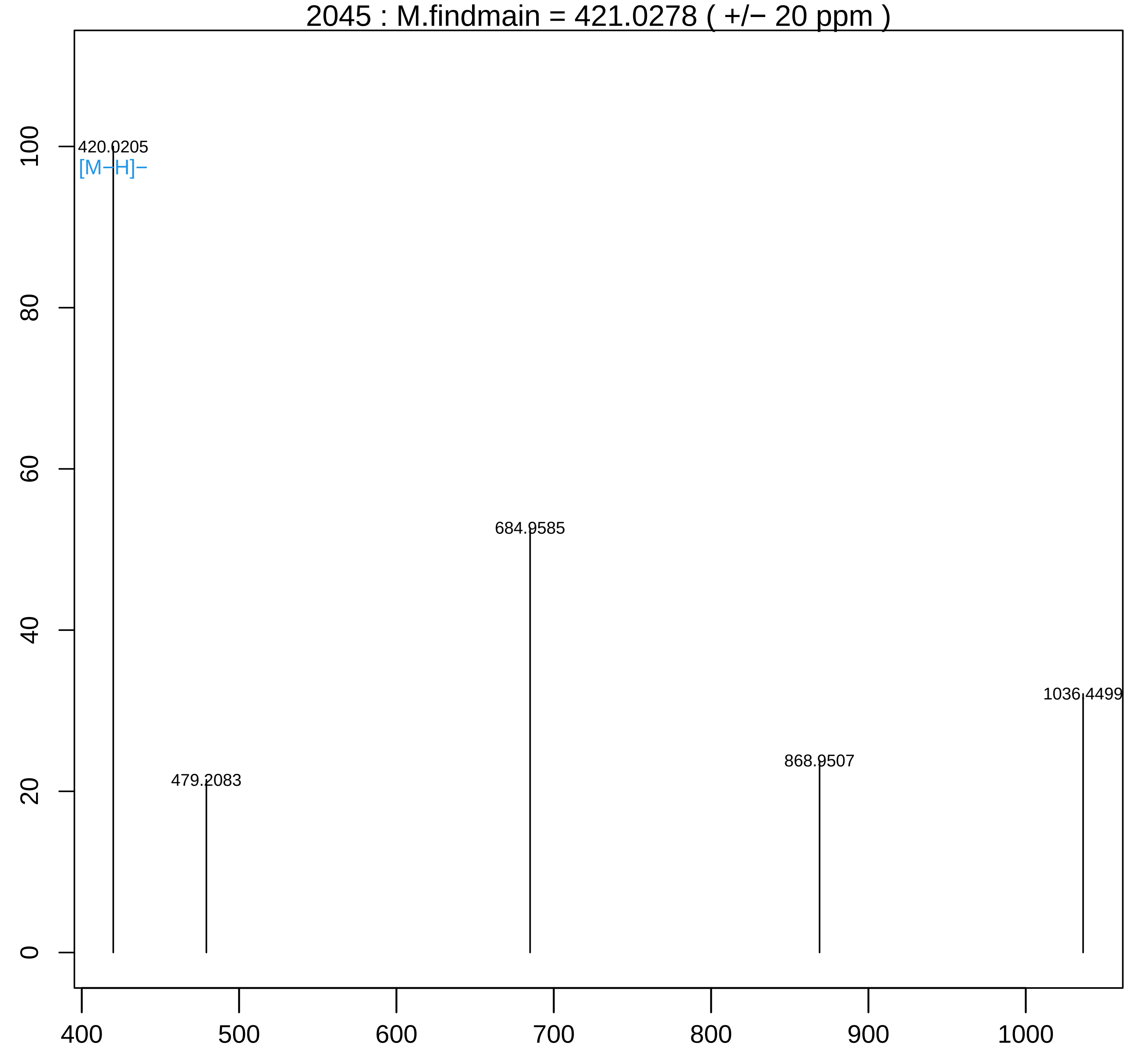

2045 : M.findmain = 421.0278 (+/− 20 ppm)
420.0205
[M−H]−
479.2083
684.9585
868.9507
1036.4499
0
20
40
60
80
100
400
500
600
700
800
900
1000

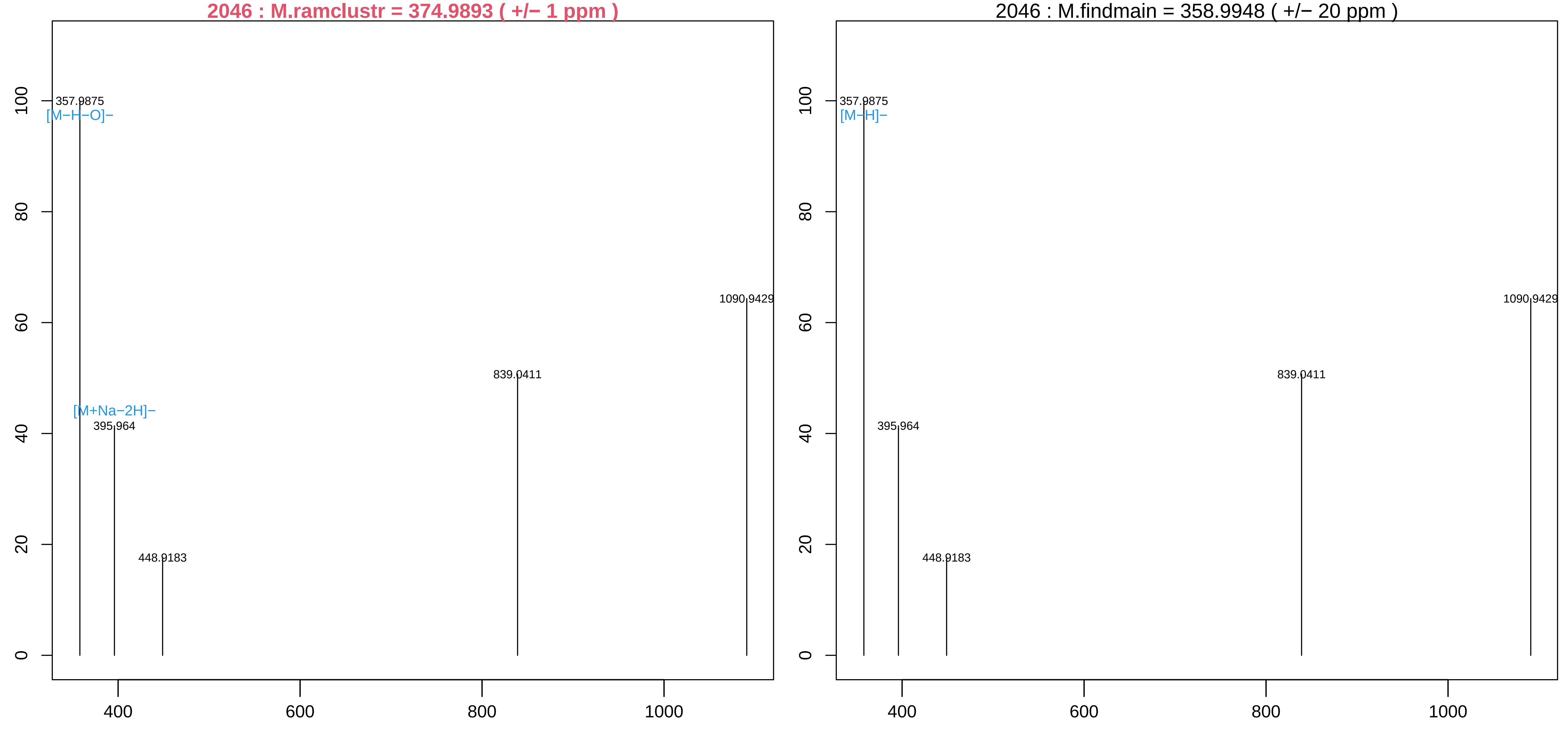
2046 : M.ramclustr = 374.9893 (+/− 1 ppm)
357.9875
[M−H−O]−
[M+Na−2H]−
395.964
448.9183
839.0411
1090.9429
2046 : M.findmain = 358.9948 (+/− 20 ppm)
357.9875
[M−H]−
395.964
448.9183
839.0411
1090.9429
0
20
40
60
80
100
400
600
800
1000

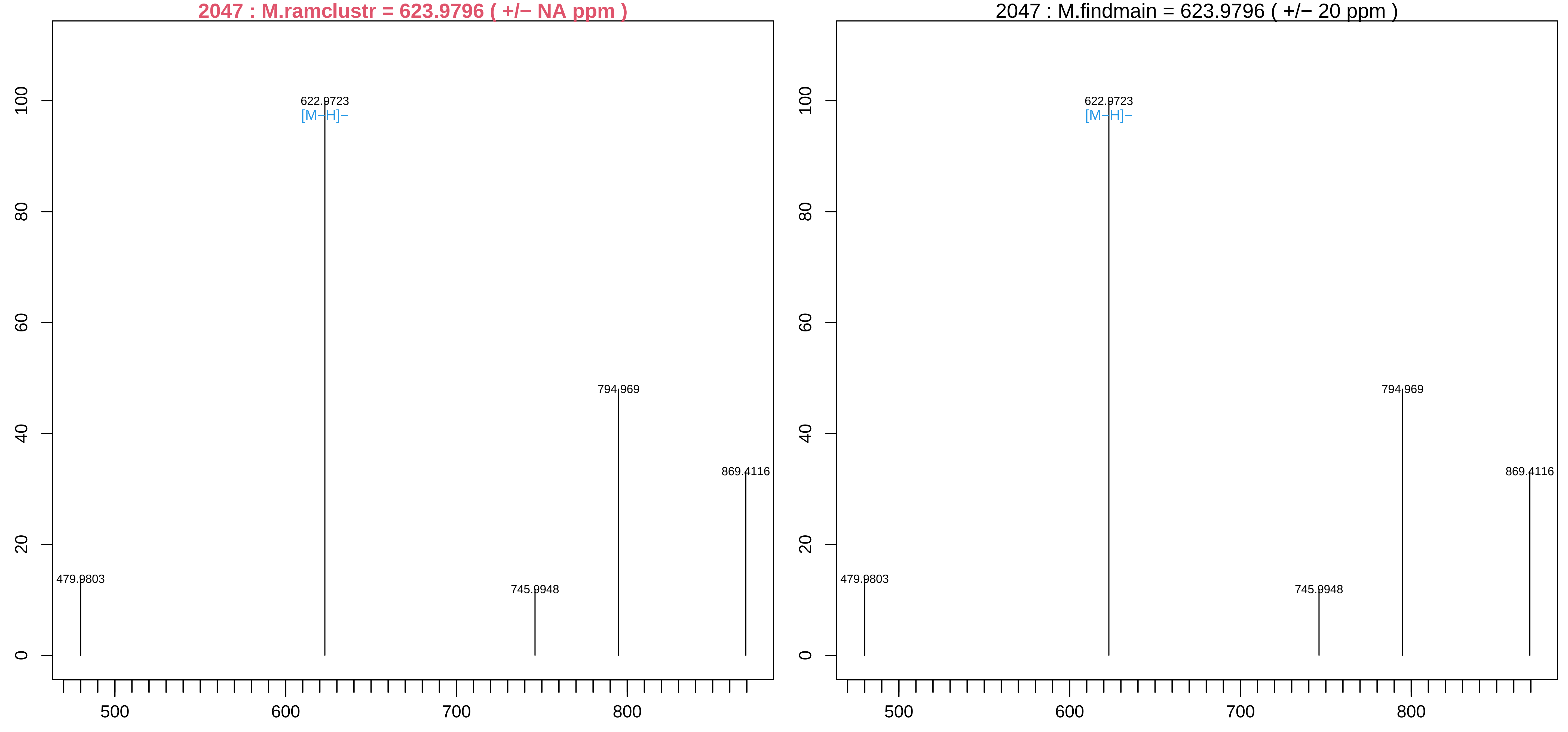

2047 : M.ramclustr = 623.9796 (+/− NA ppm)
622.9723
[M−H]−
794.969
869.4116
479.9803
745.9948
0
20
40
60
80
100
500
600
700
800
2047 : M.findmain = 623.9796 (+/− 20 ppm)
622.9723
[M−H]−
794.969
869.4116
479.9803
745.9948
0
20
40
60
80
100
500
600
700
800

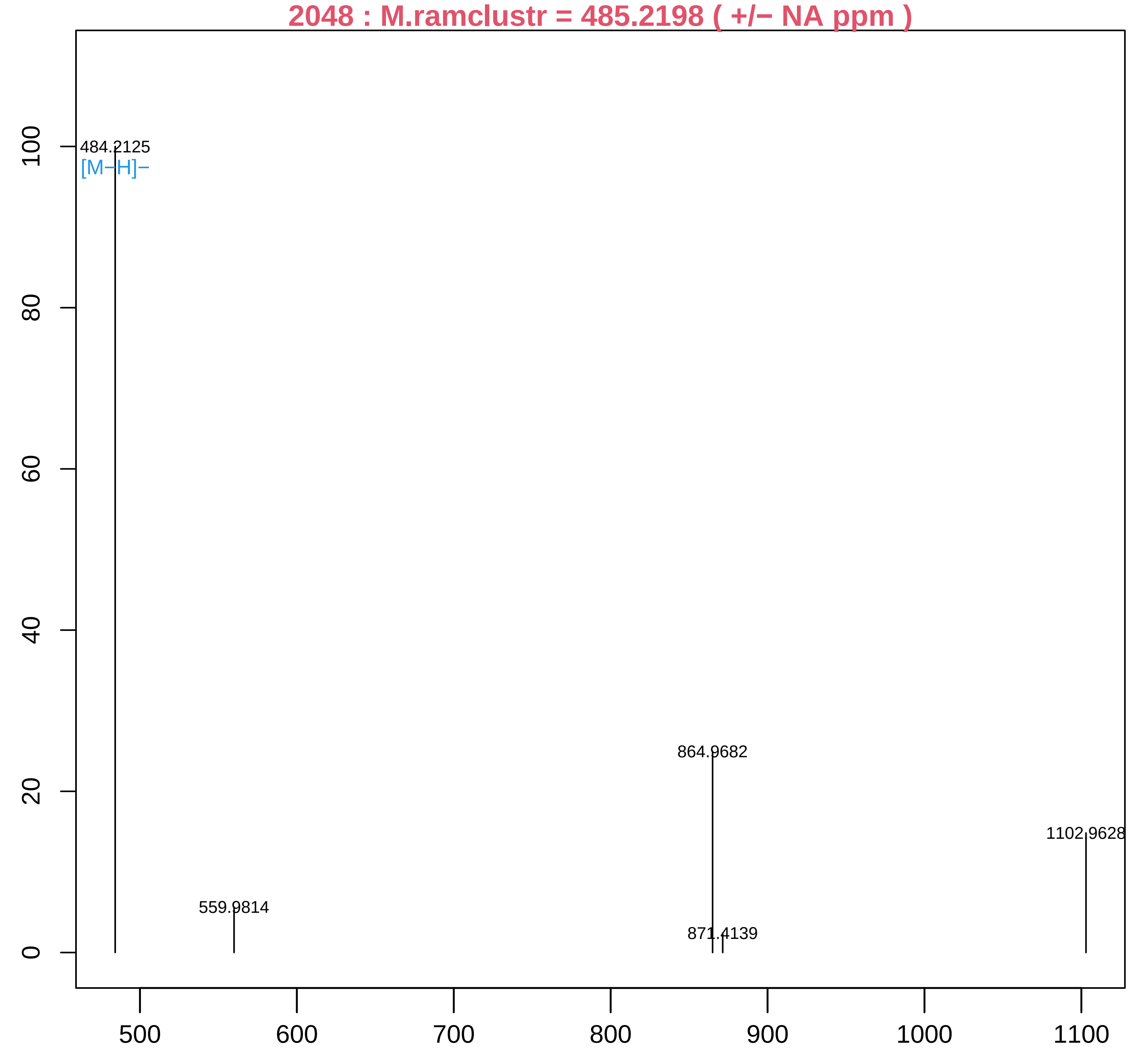
2048 : M.ramclustr = 485.2198 (+/− NA ppm)
484.2125
[M−H]−
559.9814
864.9682
871.4139
1102.9628
0
20
40
60
80
100
500
600
700
800
900
1000
1100

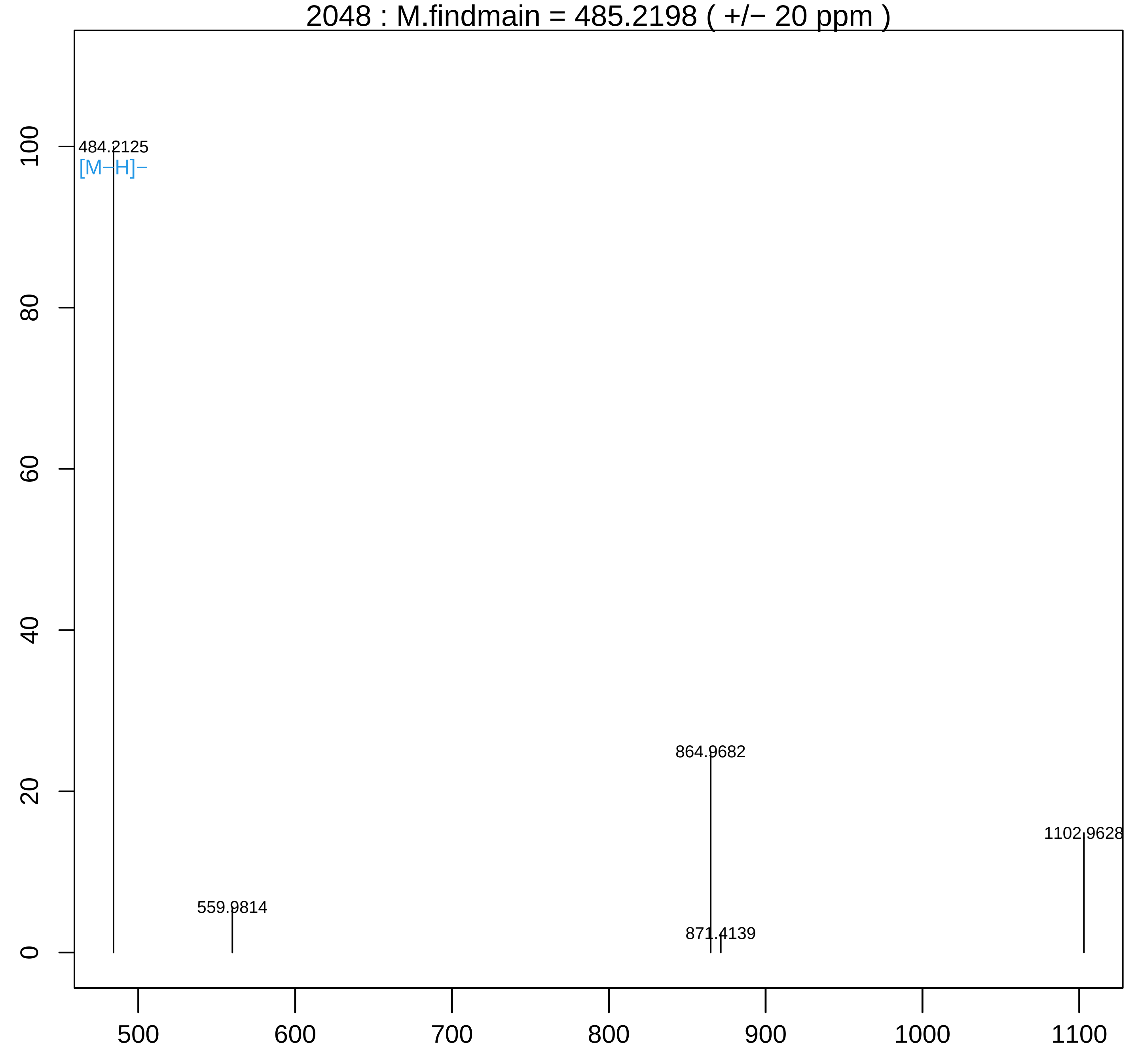
2048 : M.findmain = 485.2198 (+/− 20 ppm)
484.2125
[M−H]−
559.9814
864.9682
871.4139
1102.9628
0
20
40
60
80
100
500
600
700
800
900
1000
1100

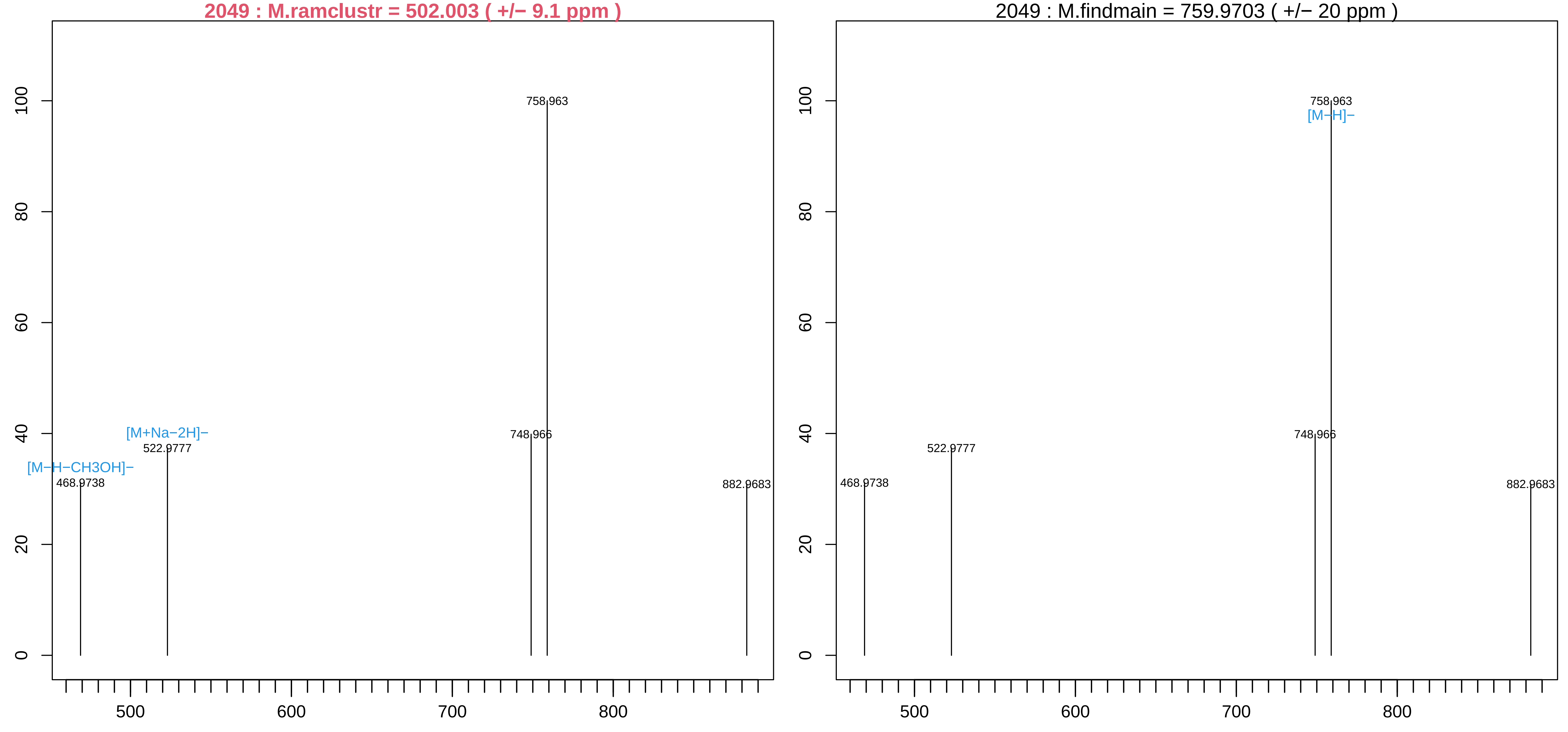

2049 : M.ramclustr = 502.003 (+/− 9.1 ppm)
758.963
748.966
[M+Na−2H]−
522.9777
[M−H−CH3OH]−
468.9738
882.9683
2049 : M.findmain = 759.9703 (+/− 20 ppm)
758.963
[M−H]−
748.966
522.9777
468.9738
882.9683

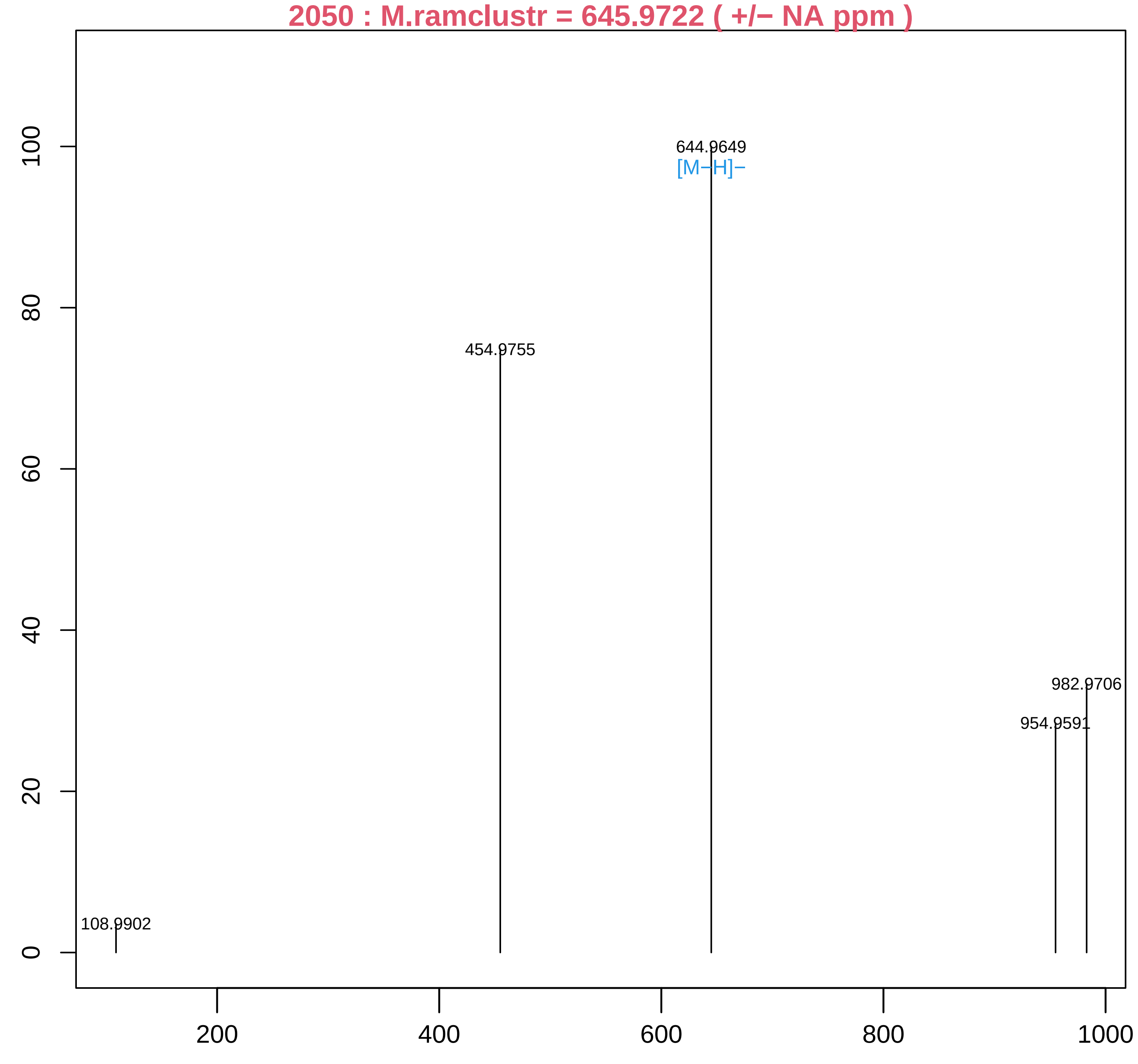
2050 : M.ramclustr = 645.9722 (+/− NA ppm)
644.9649
[M−H]−
454.9755
982.9706
954.9591
108.9902
0
20
40
60
80
100
200
400
600
800
1000

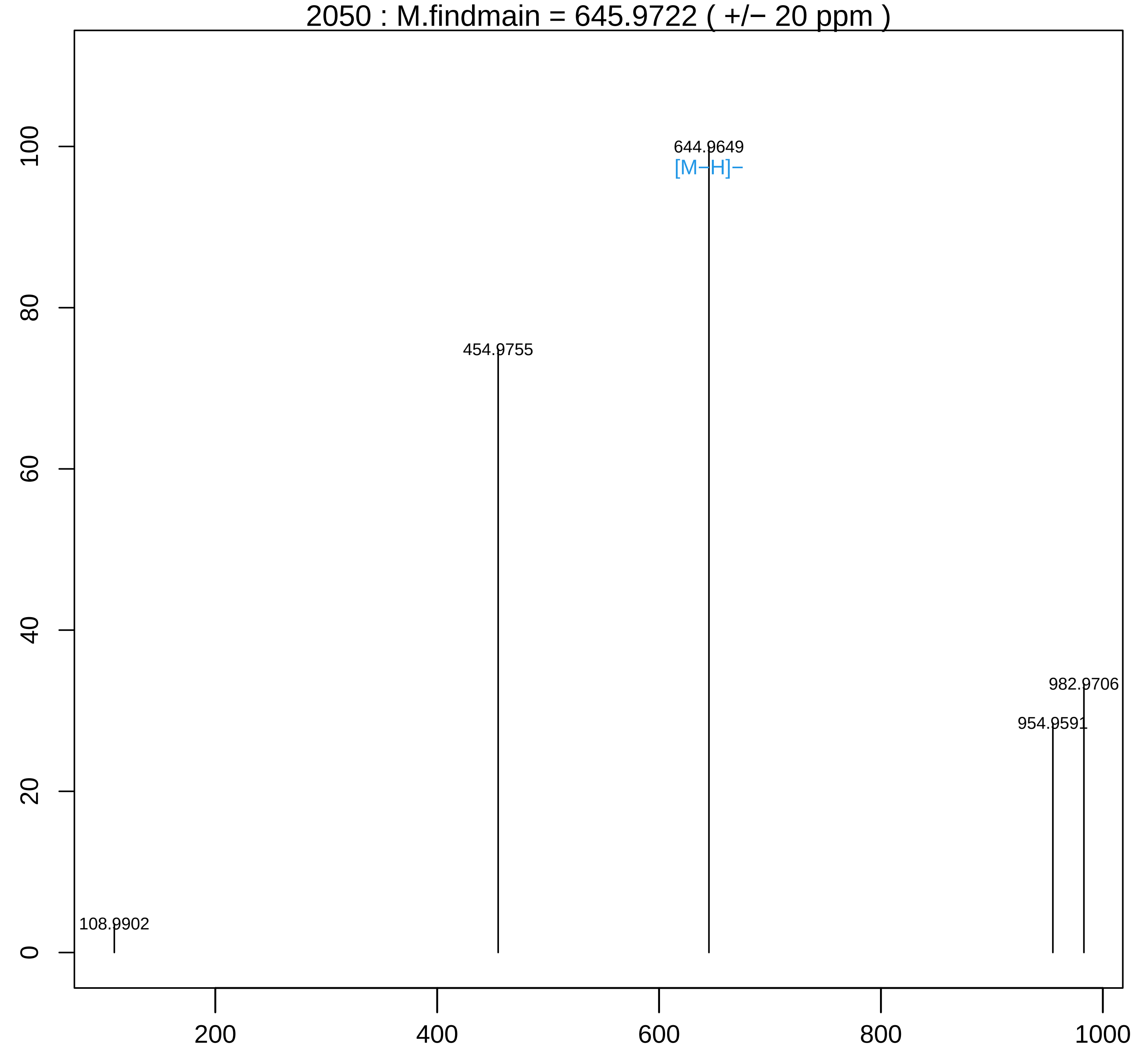
2050 : M.findmain = 645.9722 (+/− 20 ppm)
644.9649
[M−H]−
454.9755
982.9706
954.9591
108.9902
0
20
40
60
80
100
200
400
600
800
1000

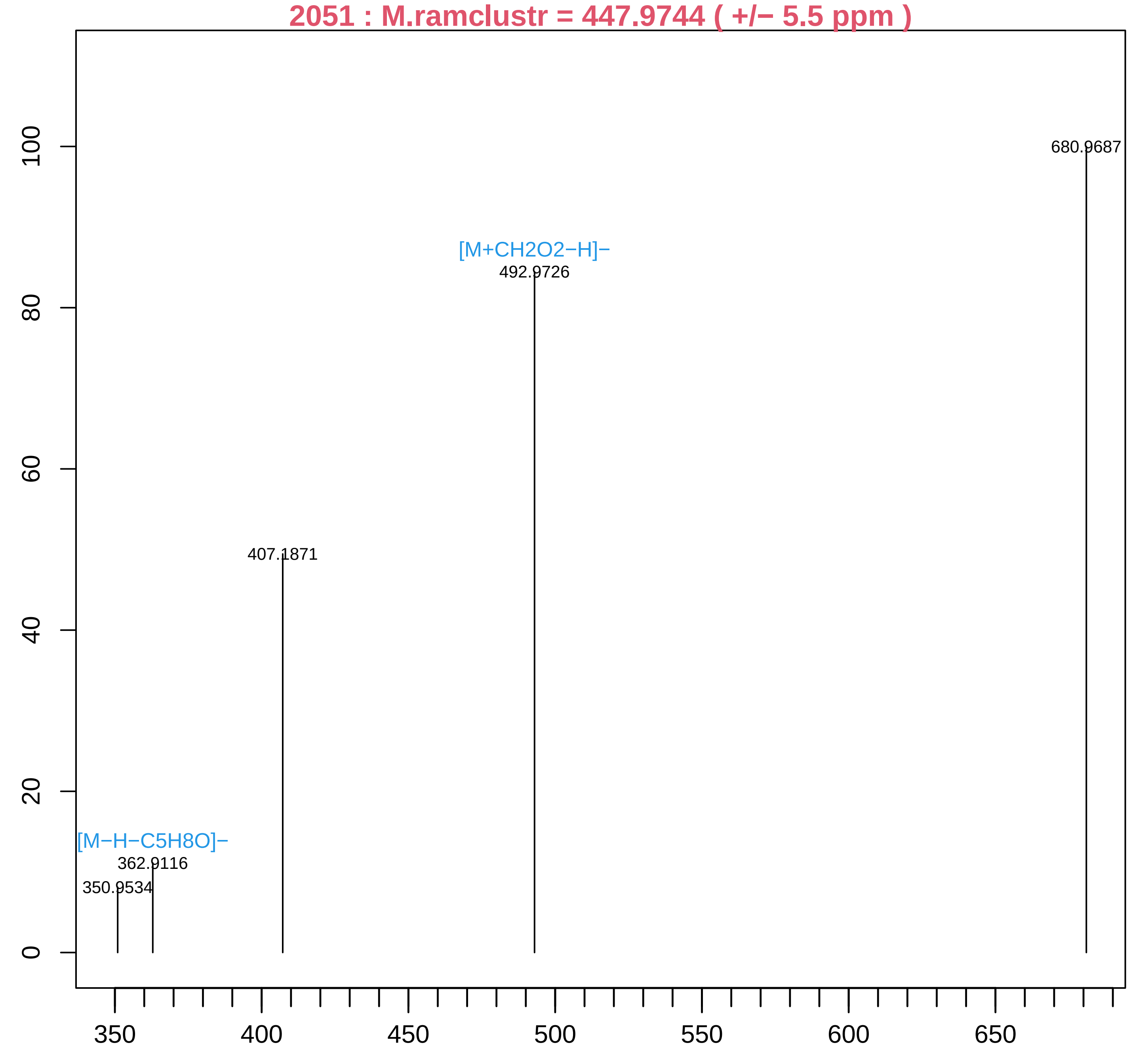
2051 : M.ramclustr = 447.9744 (+/− 5.5 ppm)
680.9687
[M+CH2O2−H]−
492.9726
407.1871
[M−H−C5H8O]−
362.9116
350.9534
0
20
40
60
80
100
350
400
450
500
550
600
650

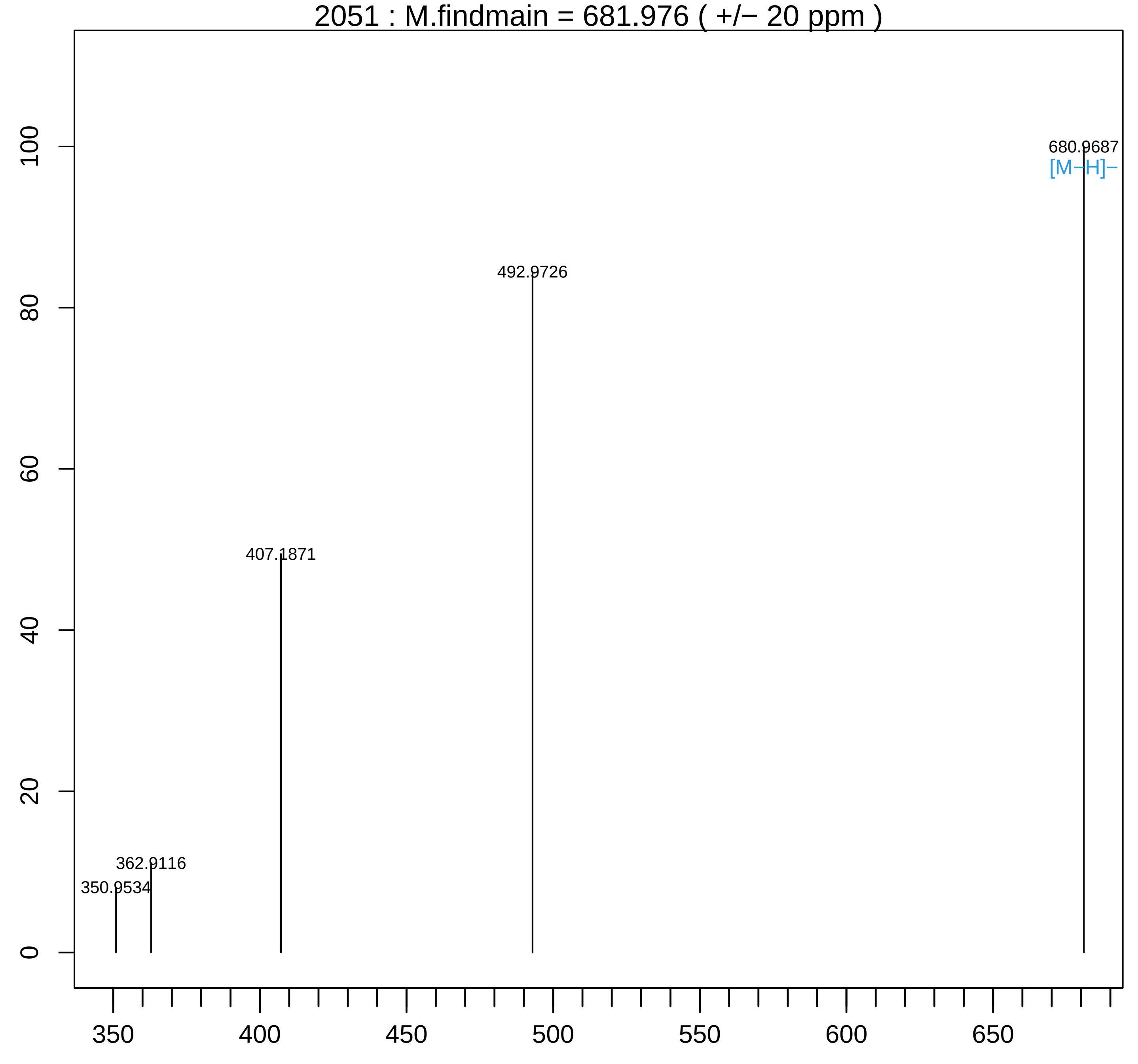
2051 : M.findmain = 681.976 (+/− 20 ppm)
680.9687
[M−H]−
492.9726
407.1871
362.9116
350.9534
0
20
40
60
80
100
350
400
450
500
550
600
650

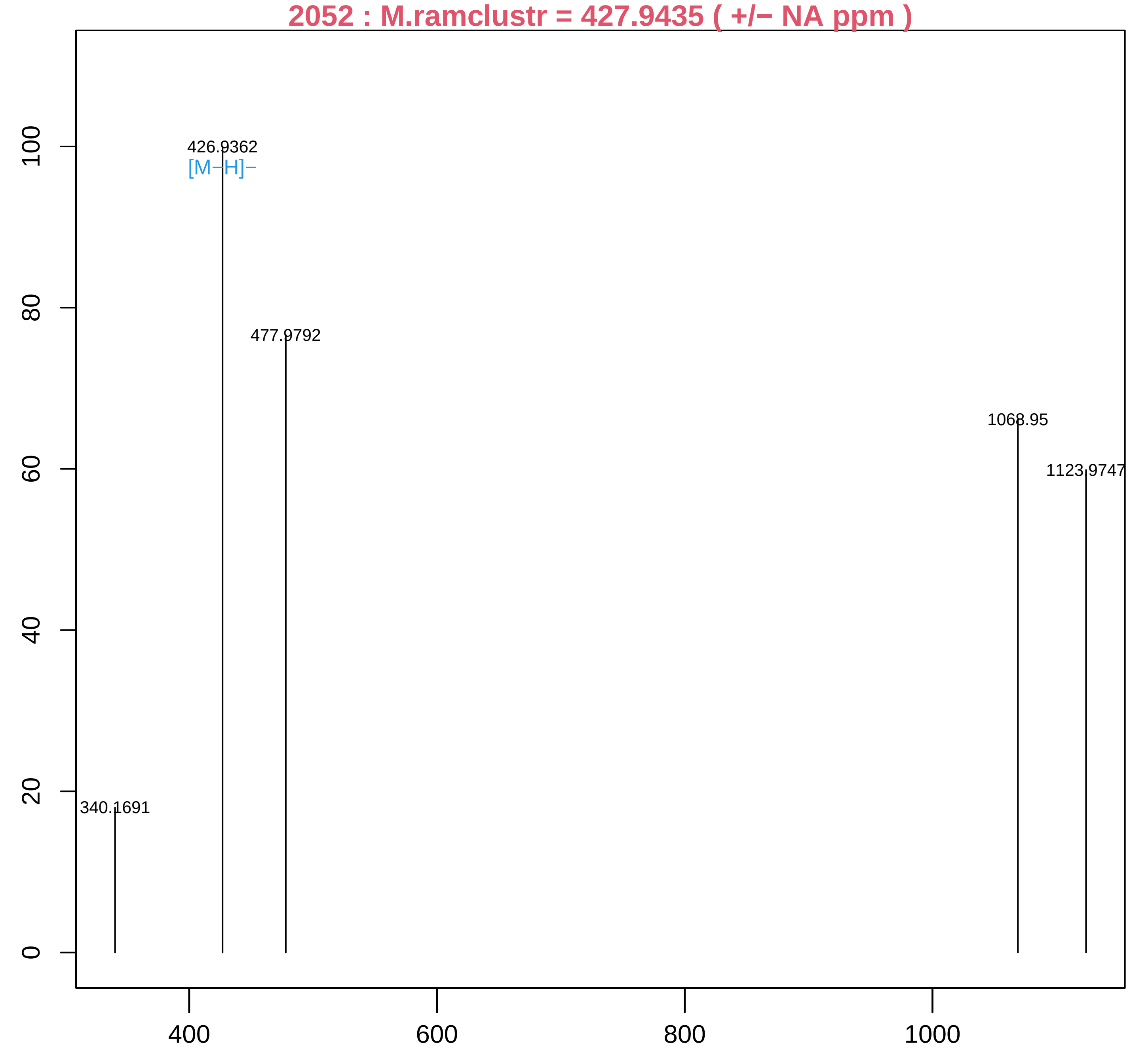

2052 : M.ramclustr = 427.9435 (+/− NA ppm)
426.9362
[M−H]−
477.9792
1068.95
1123.9747
340.1691
0
20
40
60
80
100
400
600
800
1000

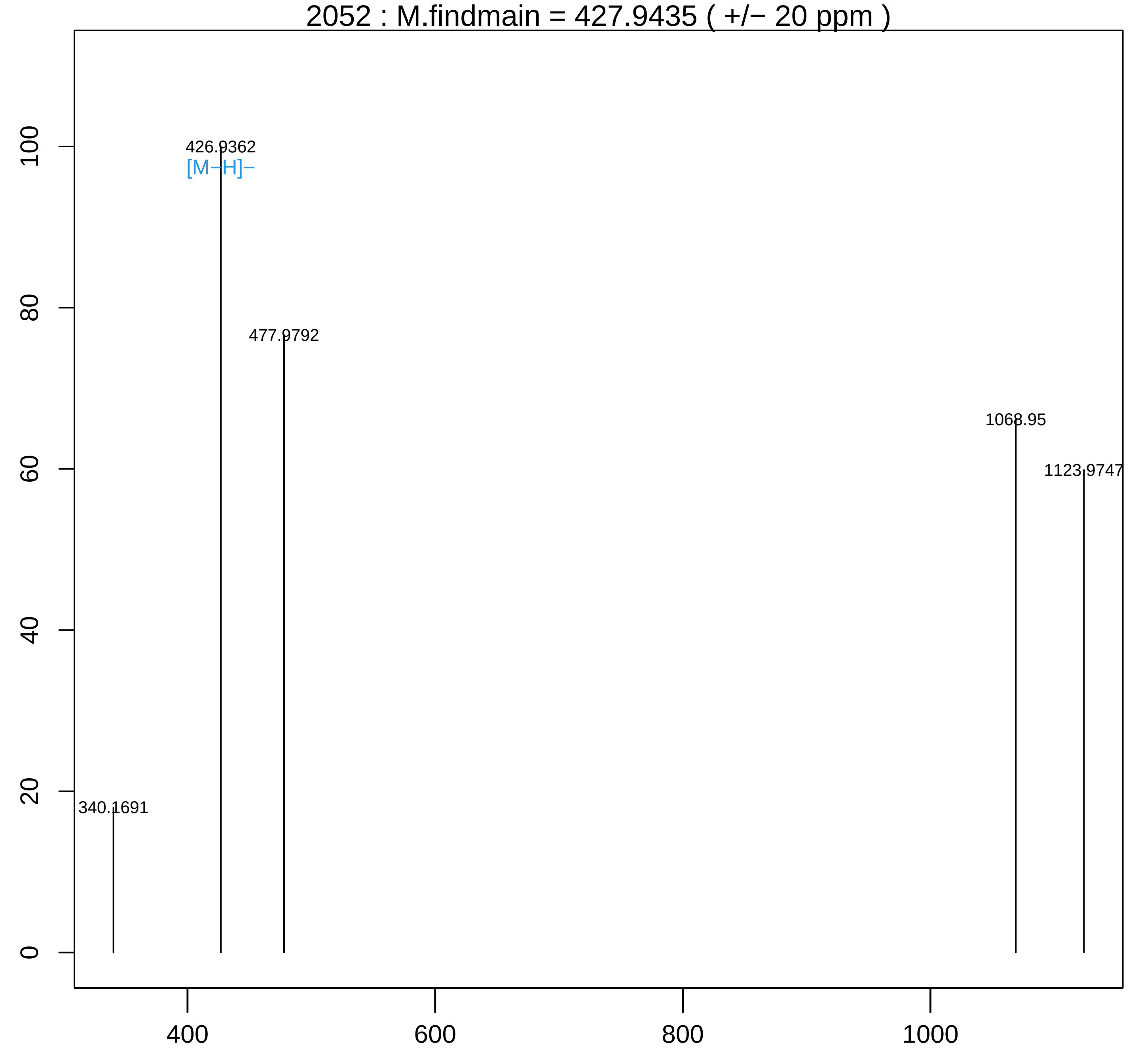

2052 : M.findmain = 427.9435 (+/− 20 ppm)
426.9362
[M−H]−
477.9792
1068.95
1123.9747
340.1691
0
20
40
60
80
100
400
600
800
1000

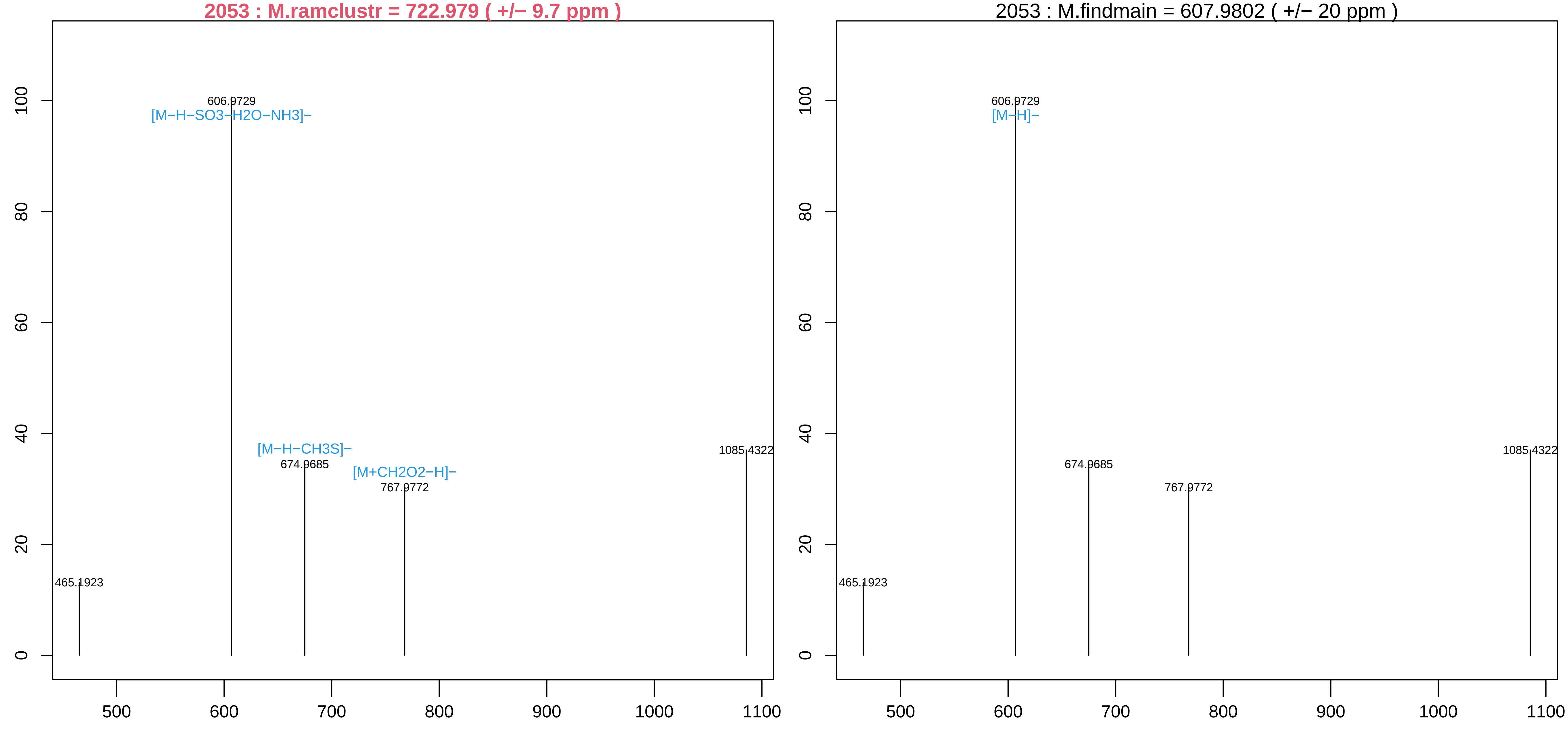

2053 : M.ramclustr = 722.979 (+/− 9.7 ppm)
606.9729
[M−H−SO3−H2O−NH3]−
[M−H−CH3S]−
674.9685
[M+CH2O2−H]−
767.9772
1085.4322
465.1923
0
20
40
60
80
100
500
600
700
800
900
1000
1100
2053 : M.findmain = 607.9802 (+/− 20 ppm)
606.9729
[M−H]−
674.9685
767.9772
1085.4322
465.1923

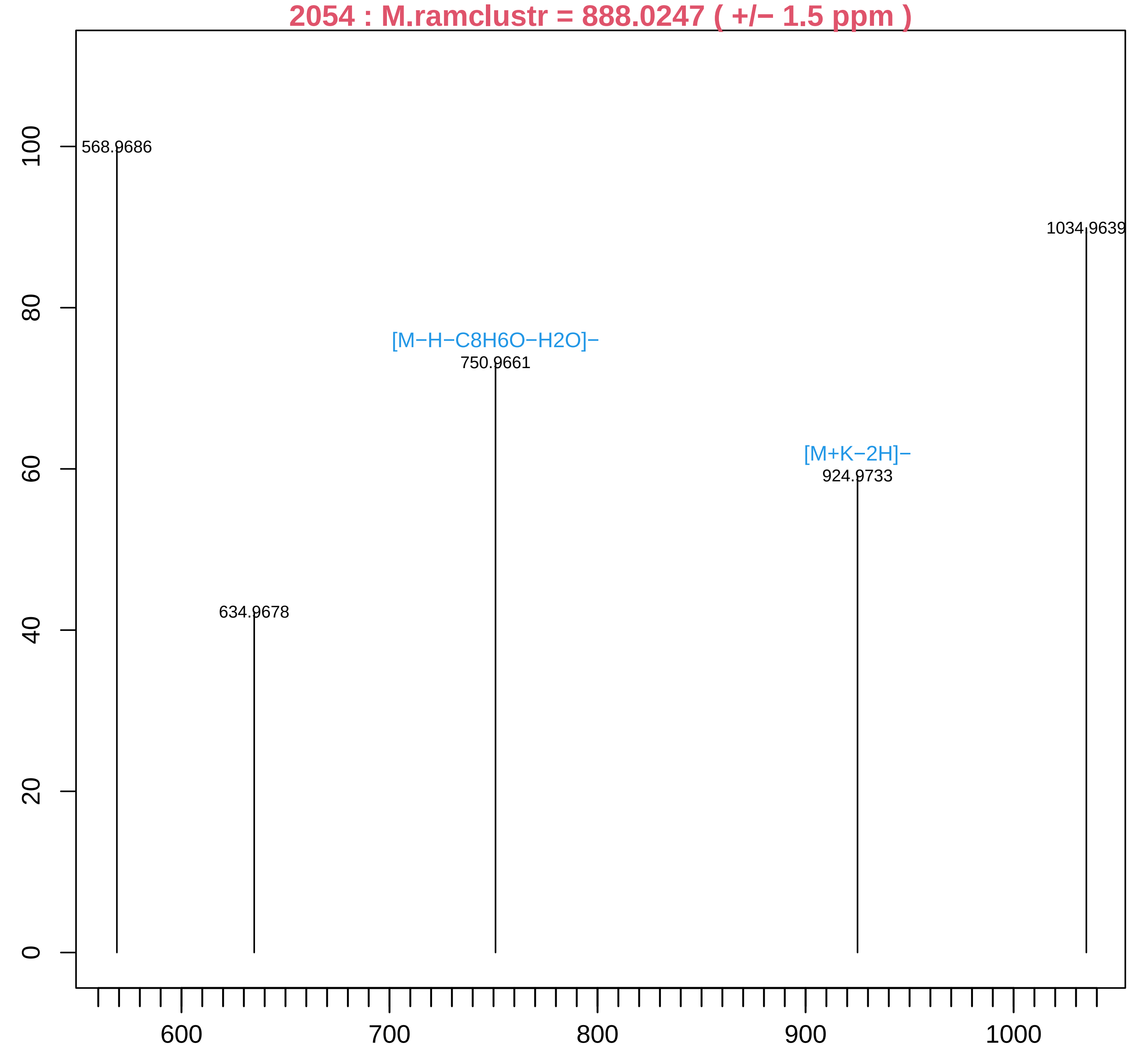

2054 : M.ramclustr = 888.0247 (+/− 1.5 ppm)
568.9686
634.9678
[M−H−C8H6O−H2O]−
750.9661
[M+K−2H]−
924.9733
1034.9639
0
20
40
60
80
100
600
700
800
900
1000

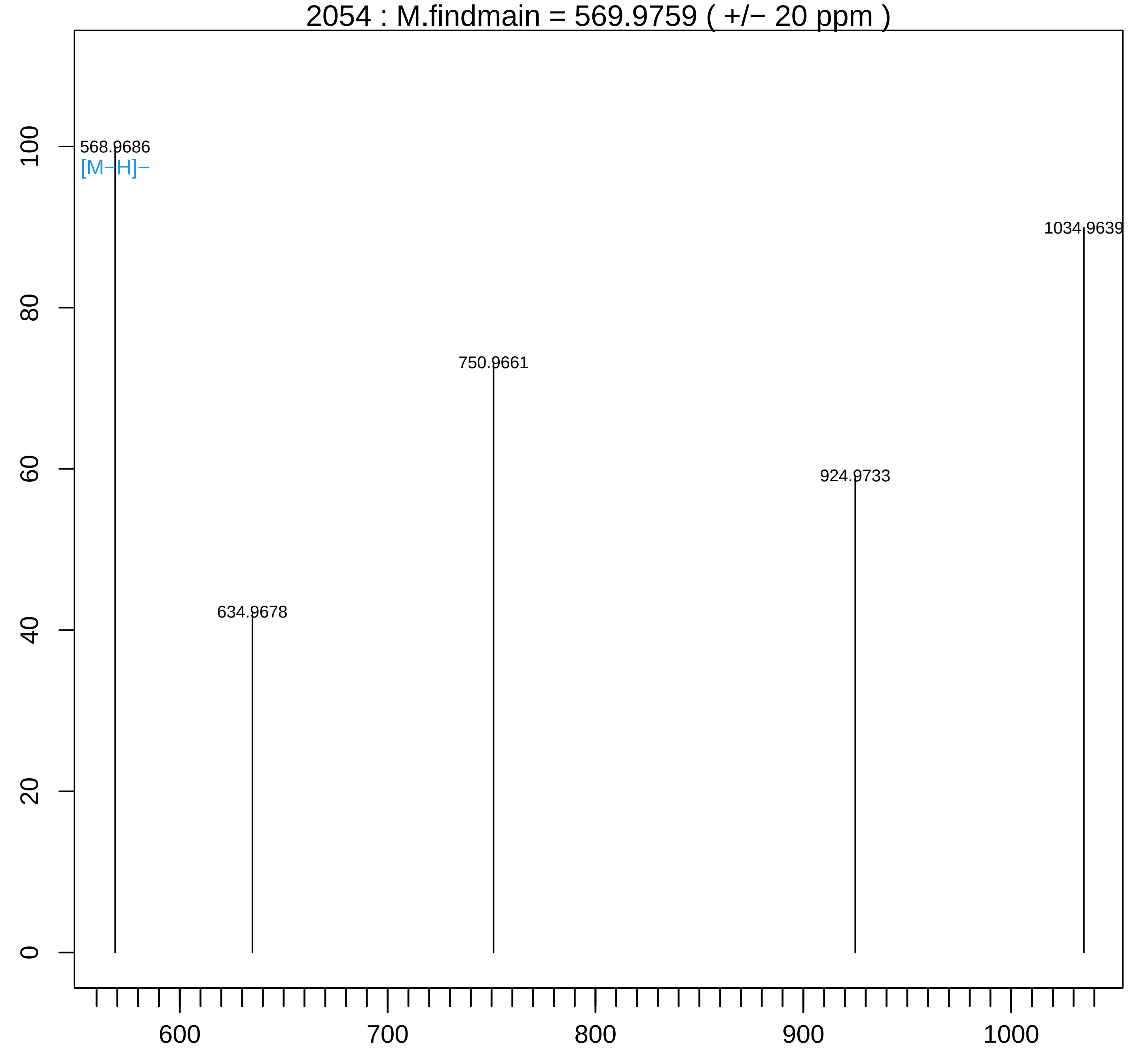

2054 : M.findmain = 569.9759 (+/− 20 ppm)
568.9686
[M−H]−
634.9678
750.9661
924.9733
1034.9639
0
20
40
60
80
100
600
700
800
900
1000

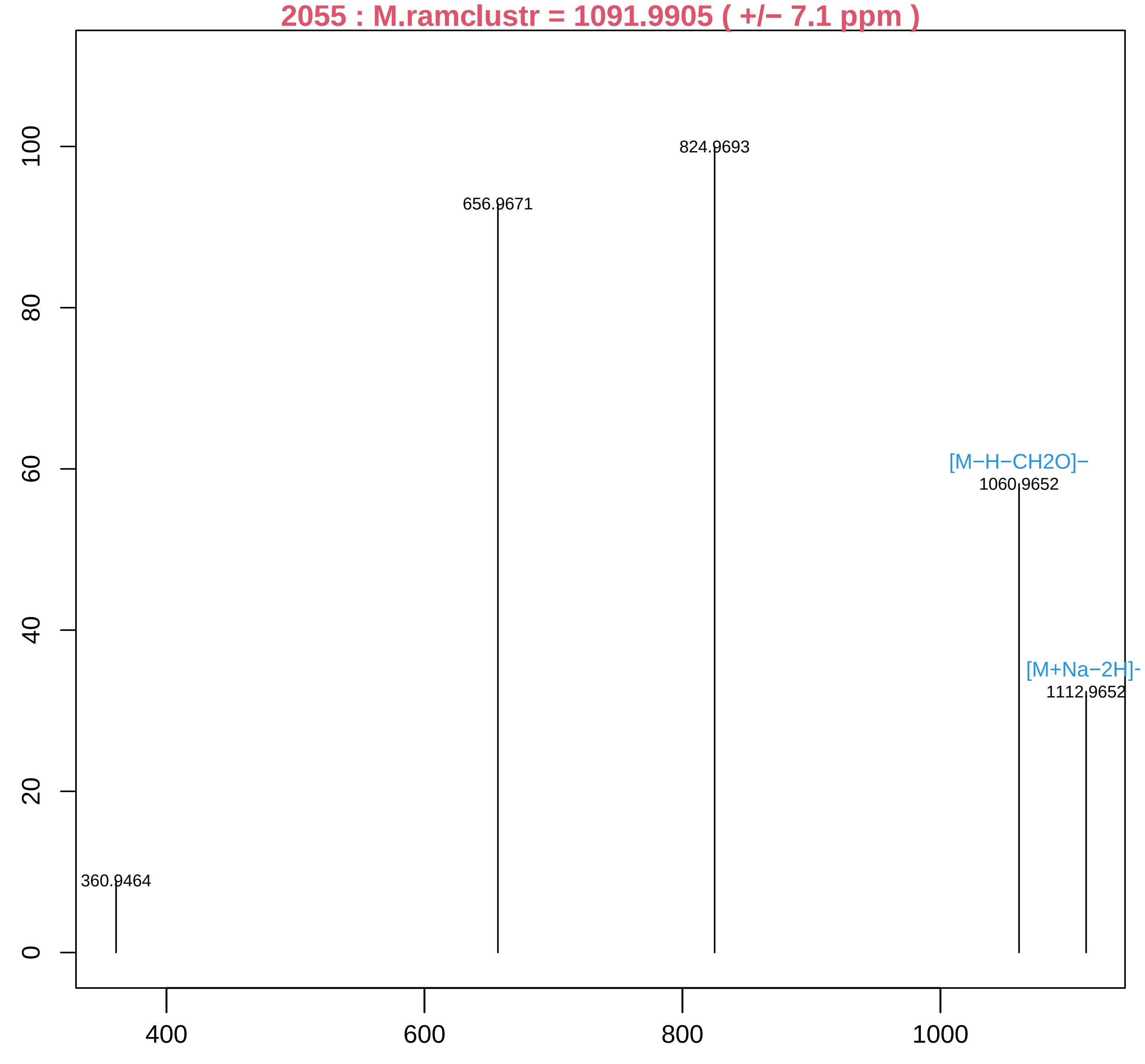

2055 : M.ramclustr = 1091.9905 (+/− 7.1 ppm)
824.9693
656.9671
[M−H−CH2O]−
1060.9652
[M+Na−2H]-
1112.9652
360.9464
0
20
40
60
80
100
400
600
800
1000

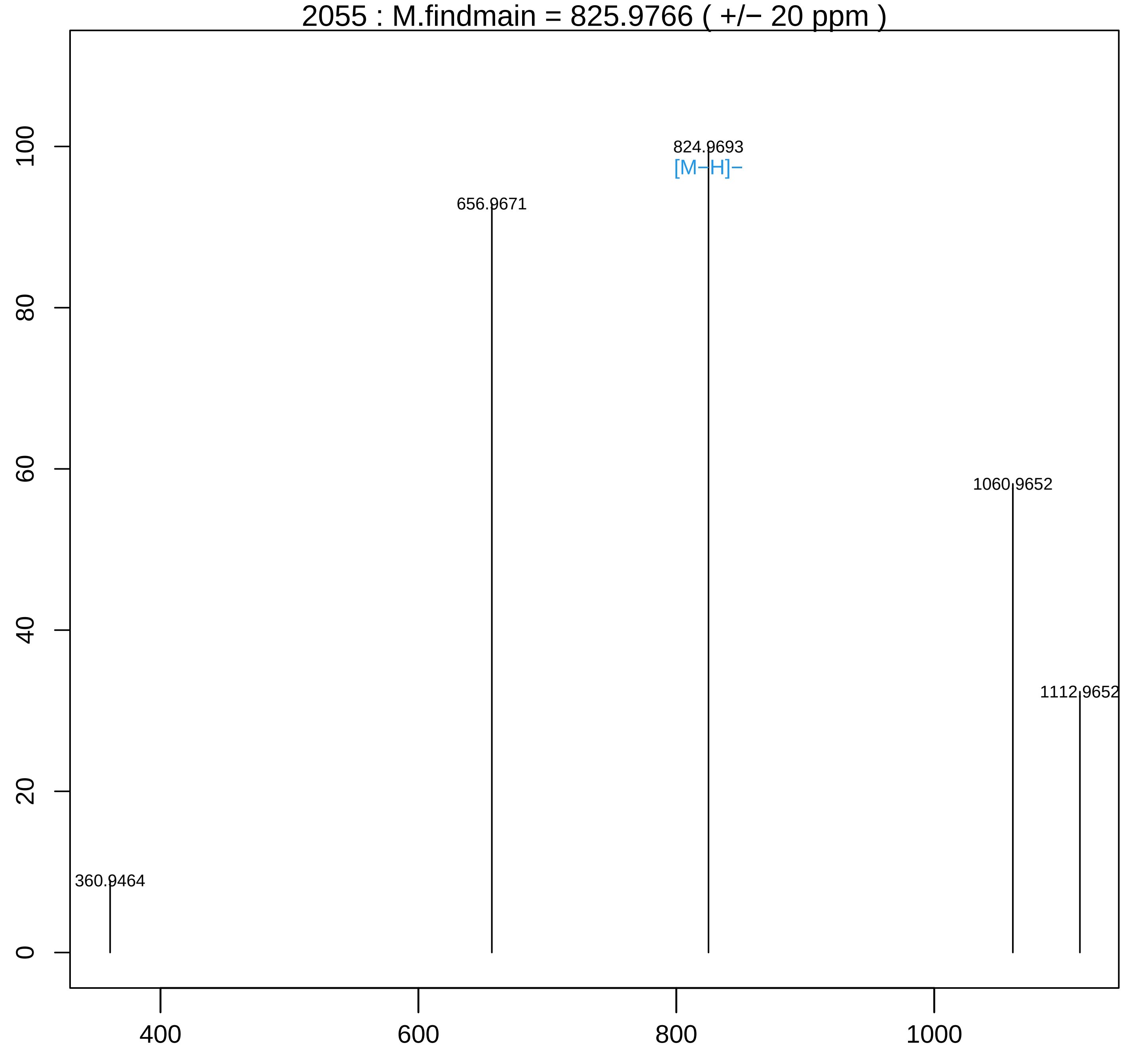

2055 : M.findmain = 825.9766 (+/− 20 ppm)
824.9693
[M−H]−
656.9671
1060.9652
1112.9652
360.9464
0
20
40
60
80
100
400
600
800
1000

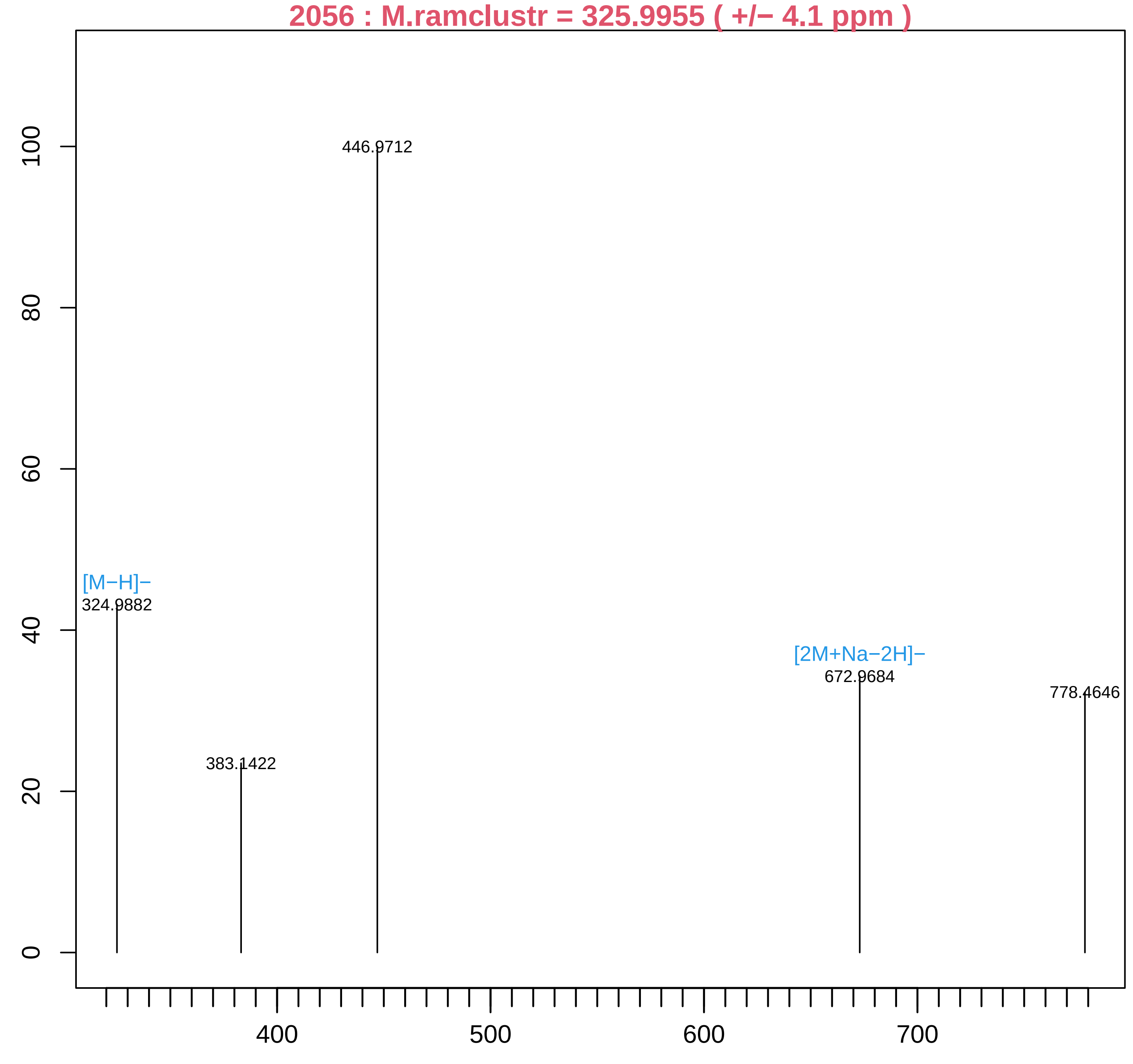
2056 : M.ramclustr = 325.9955 (+/− 4.1 ppm)
[M−H]−
324.9882
383.1422
446.9712
[2M+Na−2H]−
672.9684
778.4646
0
20
40
60
80
100
400
500
600
700

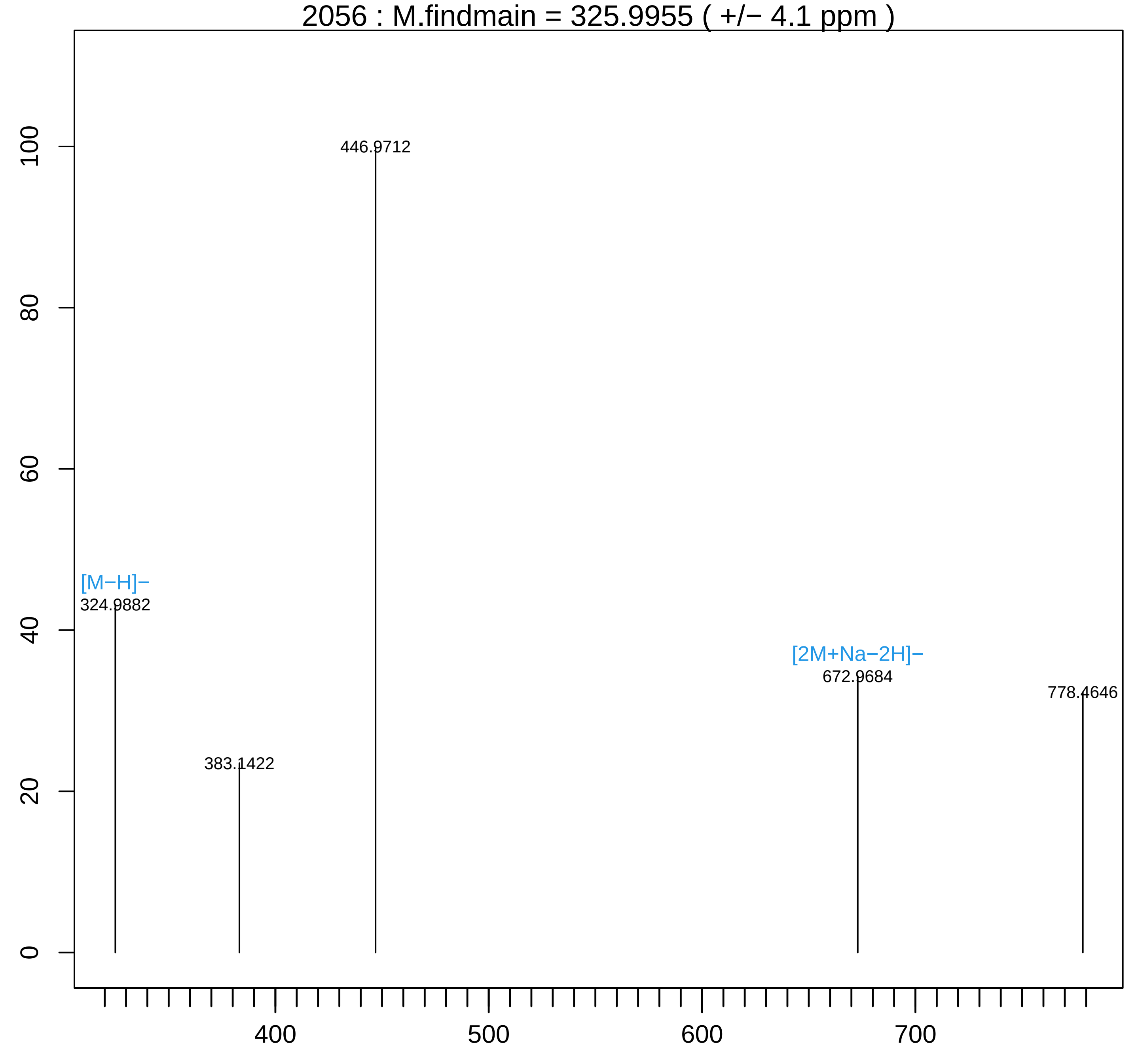
2056 : M.findmain = 325.9955 (+/− 4.1 ppm)
[M−H]−
324.9882
383.1422
446.9712
[2M+Na−2H]−
672.9684
778.4646
0
20
40
60
80
100
400
500
600
700

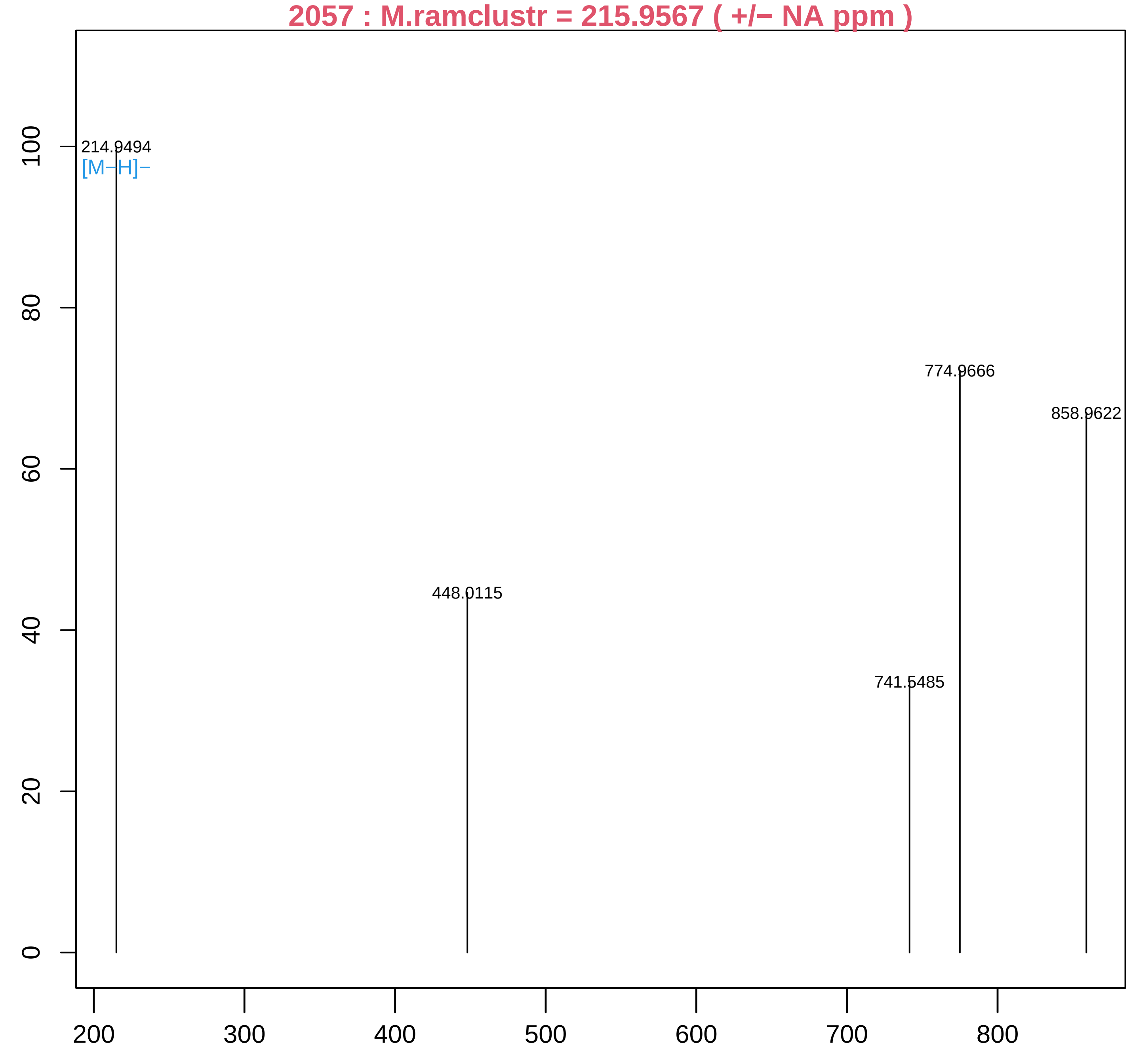
2057 : M.ramclustr = 215.9567 (+/− NA ppm)
214.9494
[M−H]−
448.0115
741.5485
774.9666
858.9622
0
20
40
60
80
100
200
300
400
500
600
700
800

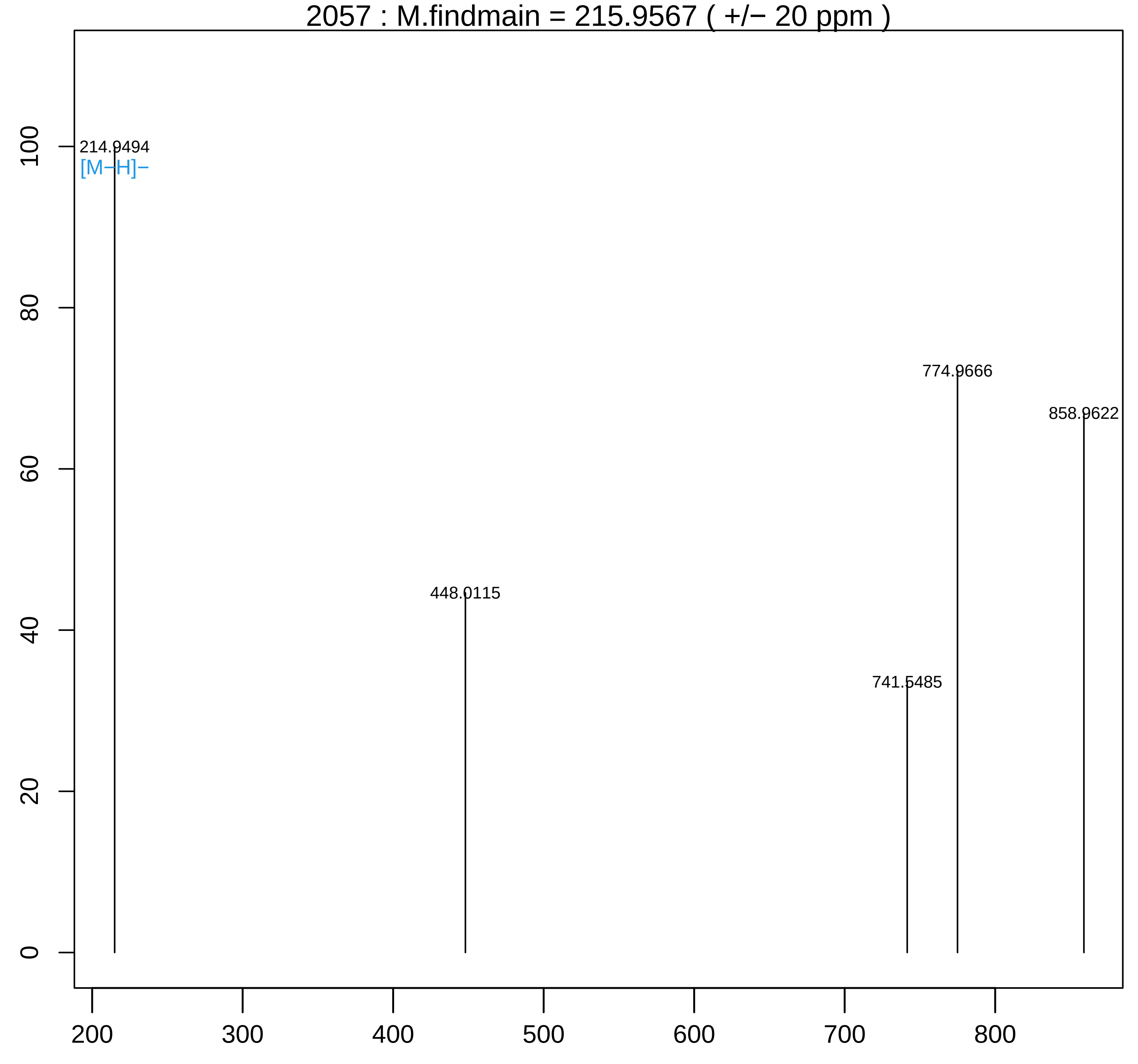
2057 : M.findmain = 215.9567 (+/− 20 ppm)
214.9494
[M−H]−
448.0115
741.5485
774.9666
858.9622
0
20
40
60
80
100
200
300
400
500
600
700
800

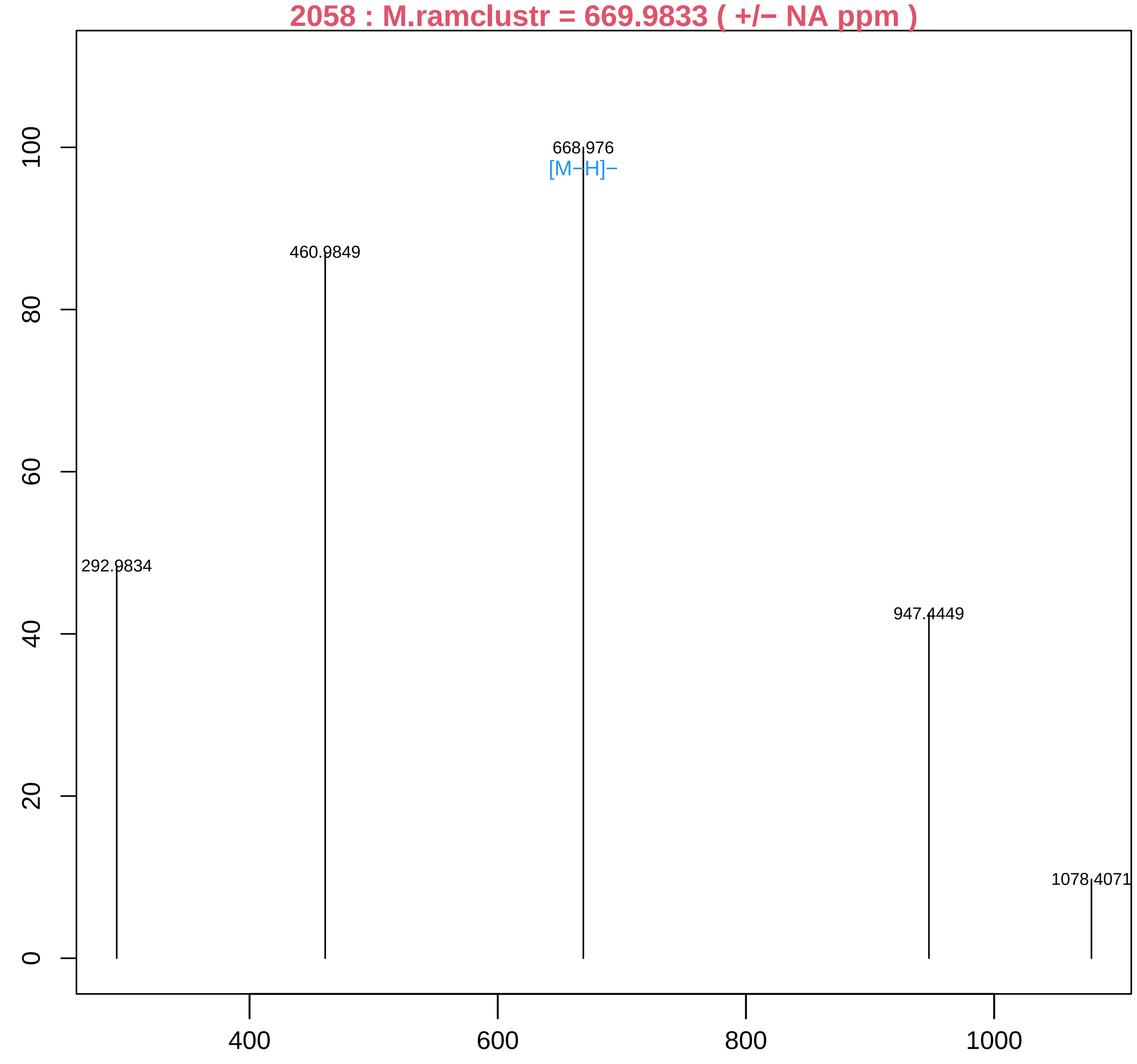
2058 : M.ramclustr = 669.9833 (+/− NA ppm)
0
20
40
60
80
100
400
600
800
1000
292.9834
460.9849
668.976
[M−H]−
947.4449
1078.4071

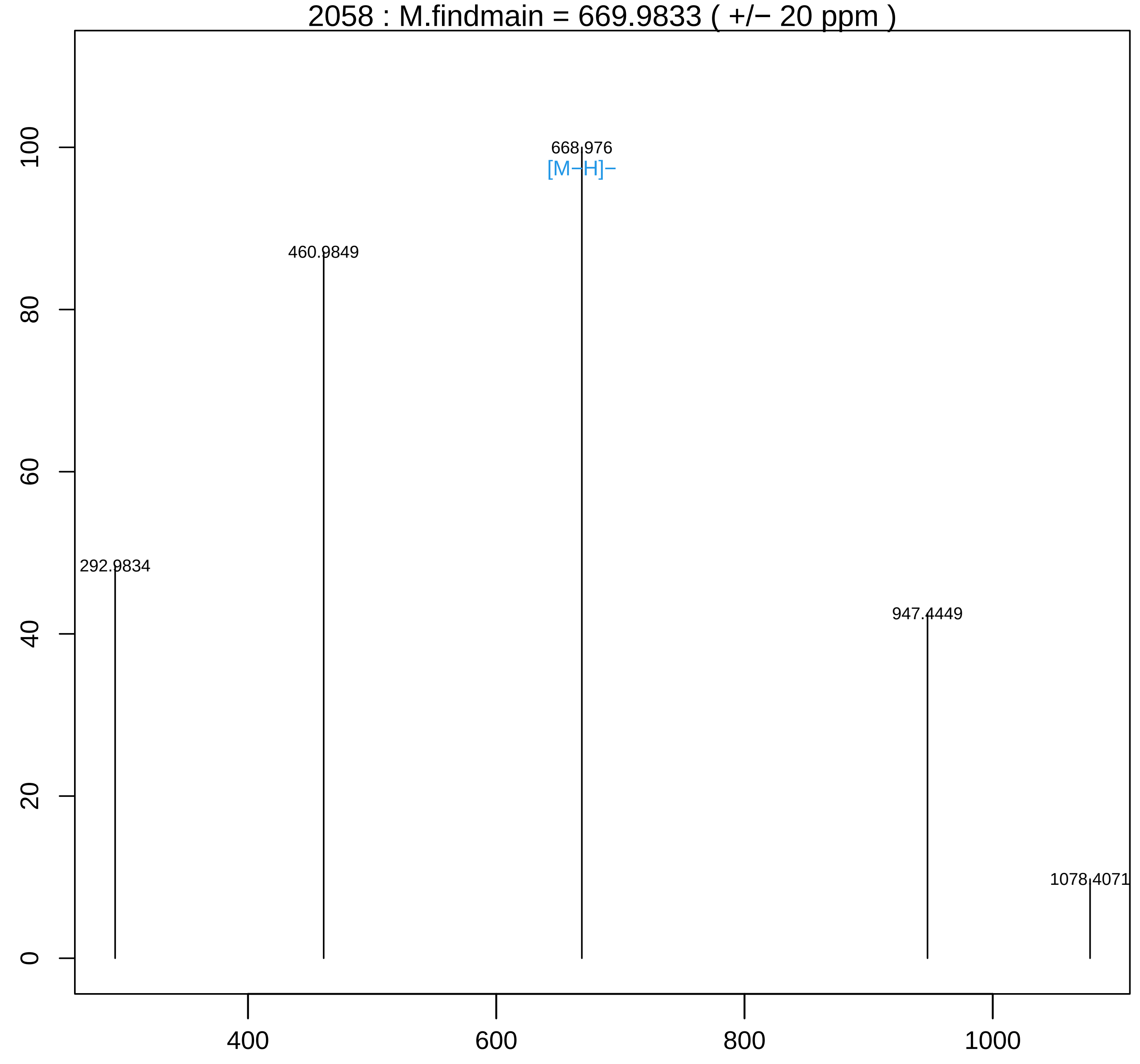
2058 : M.findmain = 669.9833 (+/− 20 ppm)
0
20
40
60
80
100
400
600
800
1000
292.9834
460.9849
668.976
[M−H]−
947.4449
1078.4071

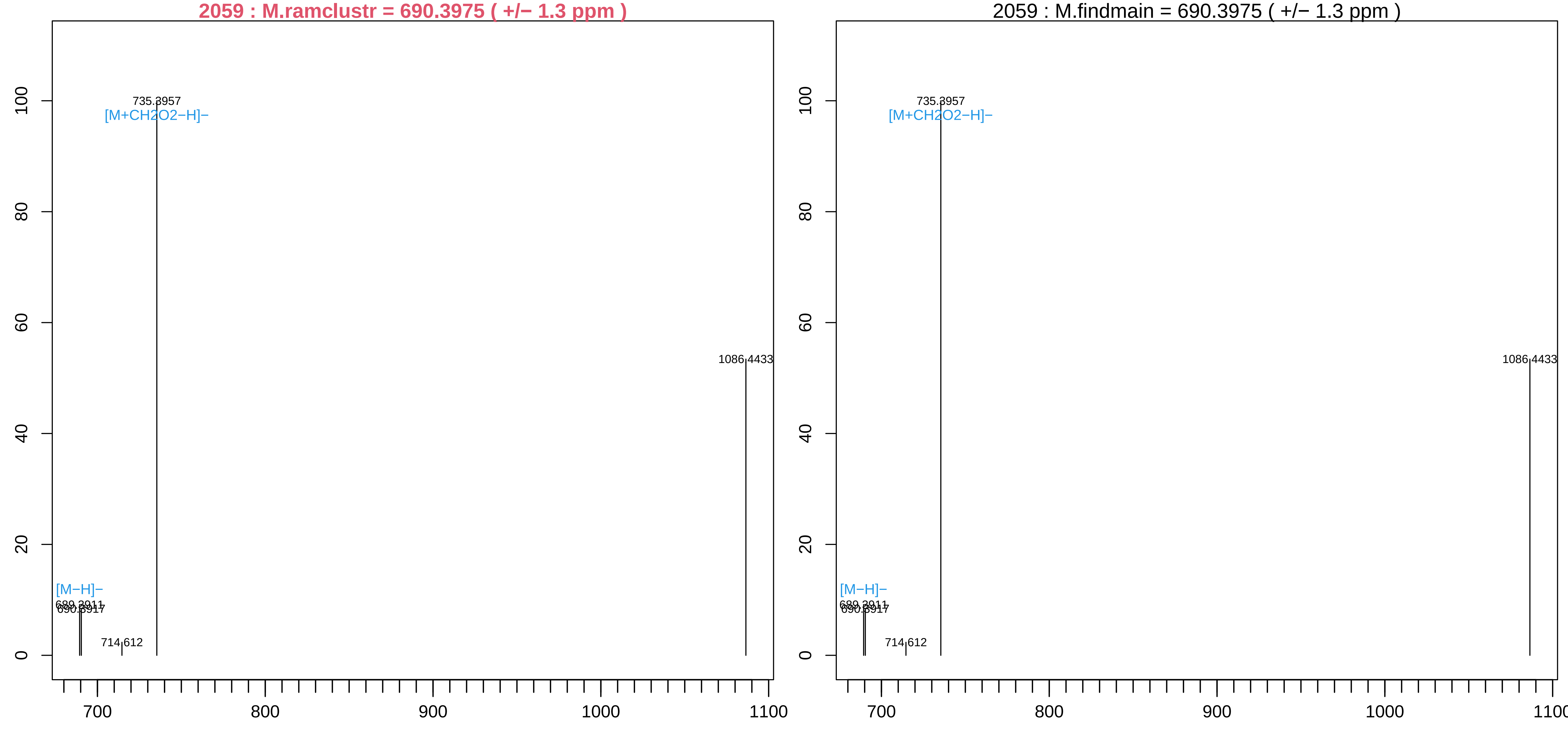
2059 : M.ramclustr = 690.3975 (+/− 1.3 ppm)
735.3957
[M+CH2O2−H]−
1086.4433
[M−H]−
689.3911
690.3917
714.612
2059 : M.findmain = 690.3975 (+/− 1.3 ppm)
735.3957
[M+CH2O2−H]−
1086.4433
[M−H]−
689.3911
690.3917
714.612

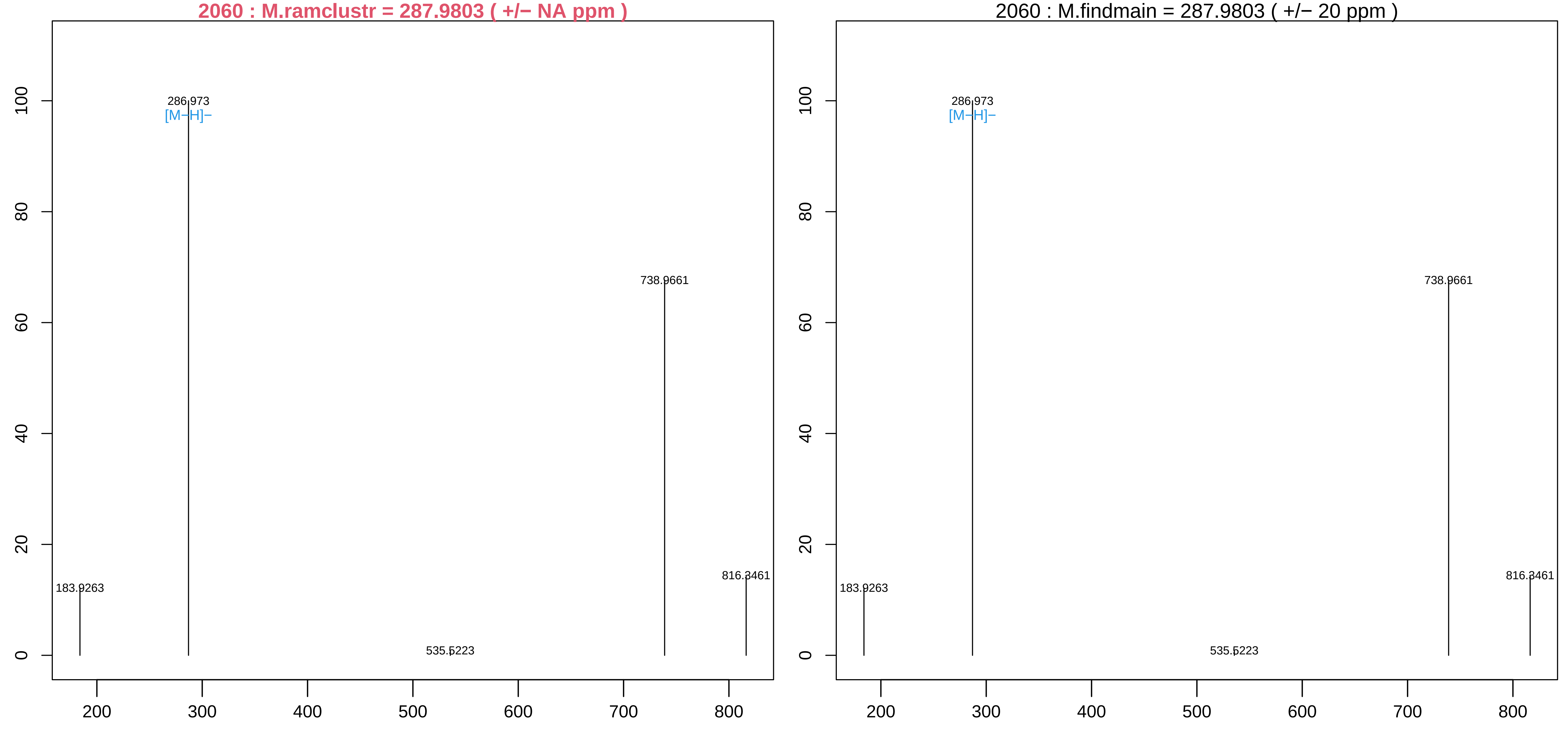
2060 : M.ramclustr = 287.9803 (+/− NA ppm)
286.973
[M−H]−
738.9661
183.9263
816.3461
535.5223
0
20
40
60
80
100
200
300
400
500
600
700
800
2060 : M.findmain = 287.9803 (+/− 20 ppm)
286.973
[M−H]−
738.9661
183.9263
816.3461
535.5223
0
20
40
60
80
100
200
300
400
500
600
700
800

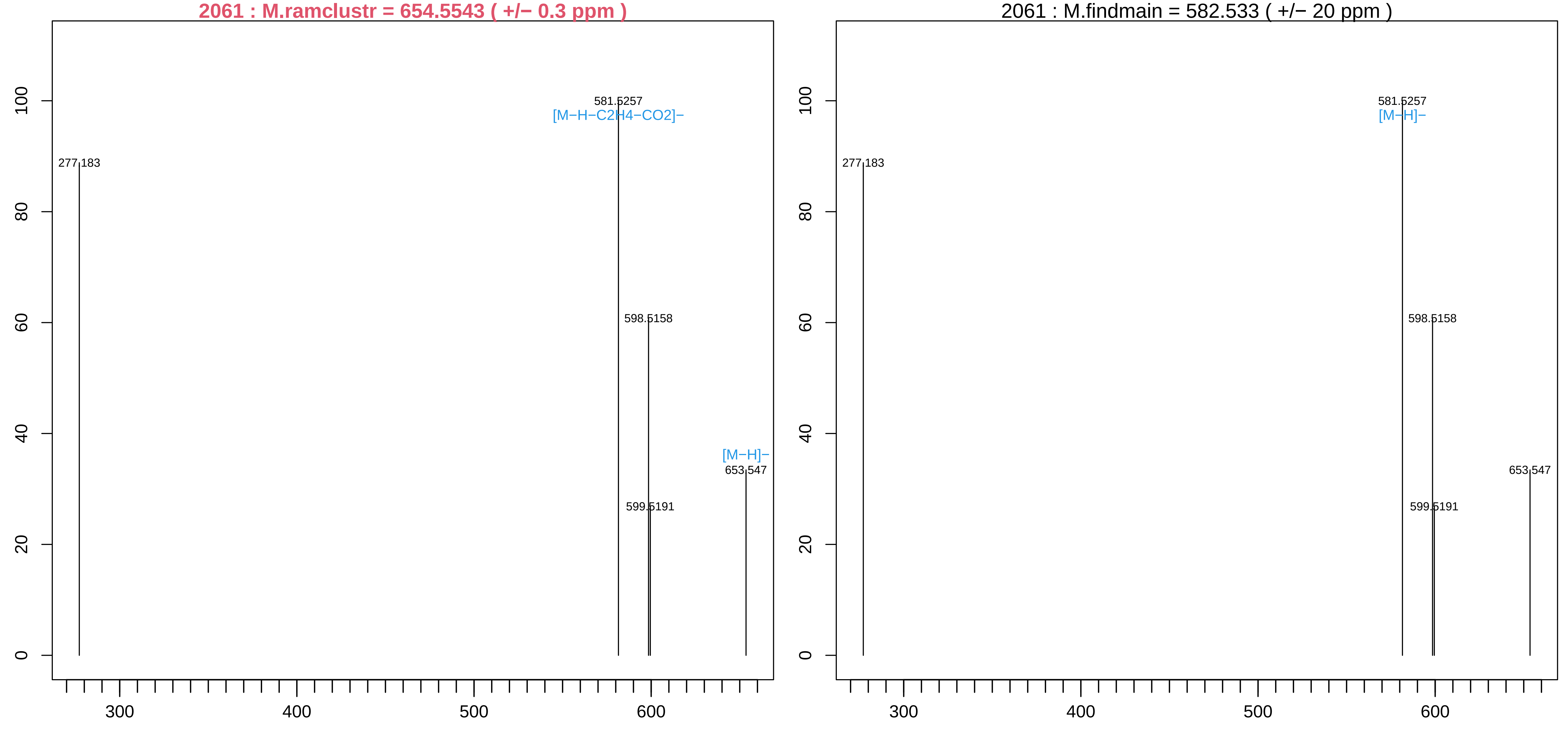

2061 : M.ramclustr = 654.5543 (+/− 0.3 ppm)
581.5257
[M−H−C2H4−CO2]−
277.183
598.5158
[M−H]−
653.547
599.5191
0
20
40
60
80
100
300
400
500
600
2061 : M.findmain = 582.533 (+/− 20 ppm)
581.5257
[M−H]−
277.183
598.5158
653.547
599.5191
0
20
40
60
80
100
300
400
500
600

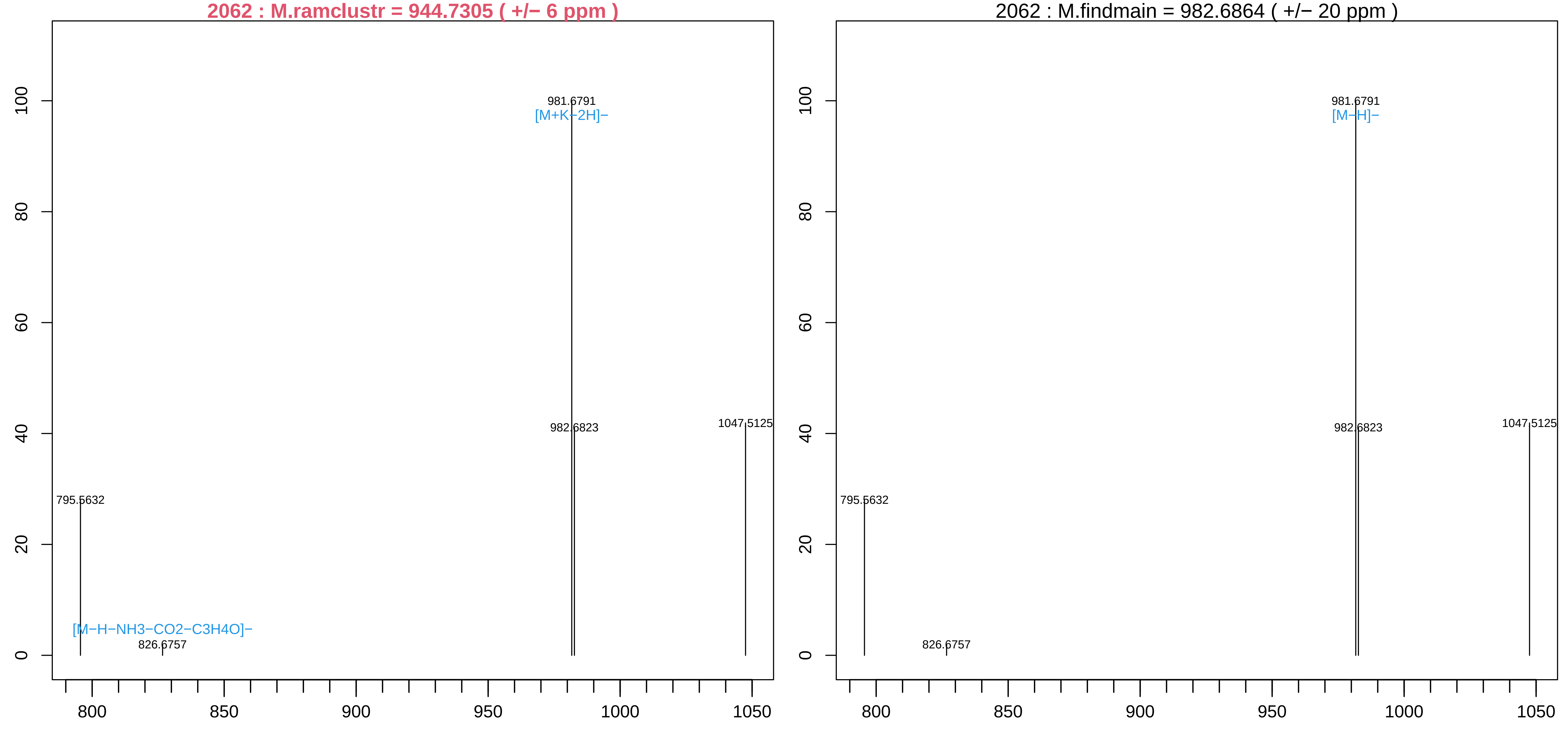
2062 : M.ramclustr = 944.7305 (+/− 6 ppm)
981.6791
[M+K−2H]−
982.6823
1047.5125
795.5632
[M−H−NH3−CO2−C3H4O]−
826.6757
0
20
40
60
80
100
800
850
900
950
1000
1050
2062 : M.findmain = 982.6864 (+/− 20 ppm)
981.6791
[M−H]−
982.6823
1047.5125
795.5632
826.6757

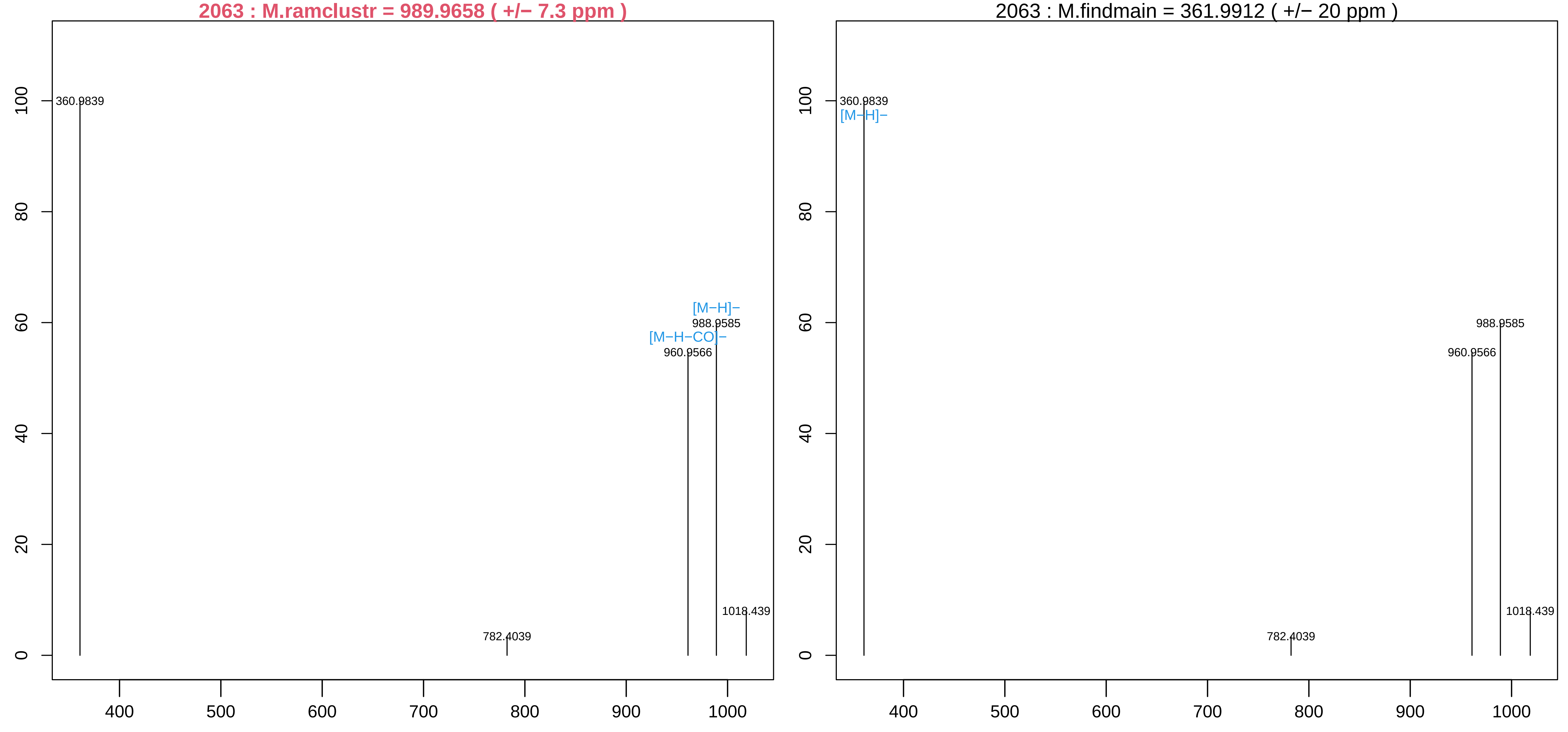

2063 : M.ramclustr = 989.9658 (+/− 7.3 ppm)
360.9839
[M−H]−
988.9585
[M−H−CO]−
960.9566
1018.439
782.4039
0
20
40
60
80
100
400
500
600
700
800
900
1000
2063 : M.findmain = 361.9912 (+/− 20 ppm)
360.9839
[M−H]−
988.9585
960.9566
1018.439
782.4039
0
20
40
60
80
100
400
500
600
700
800
900
1000

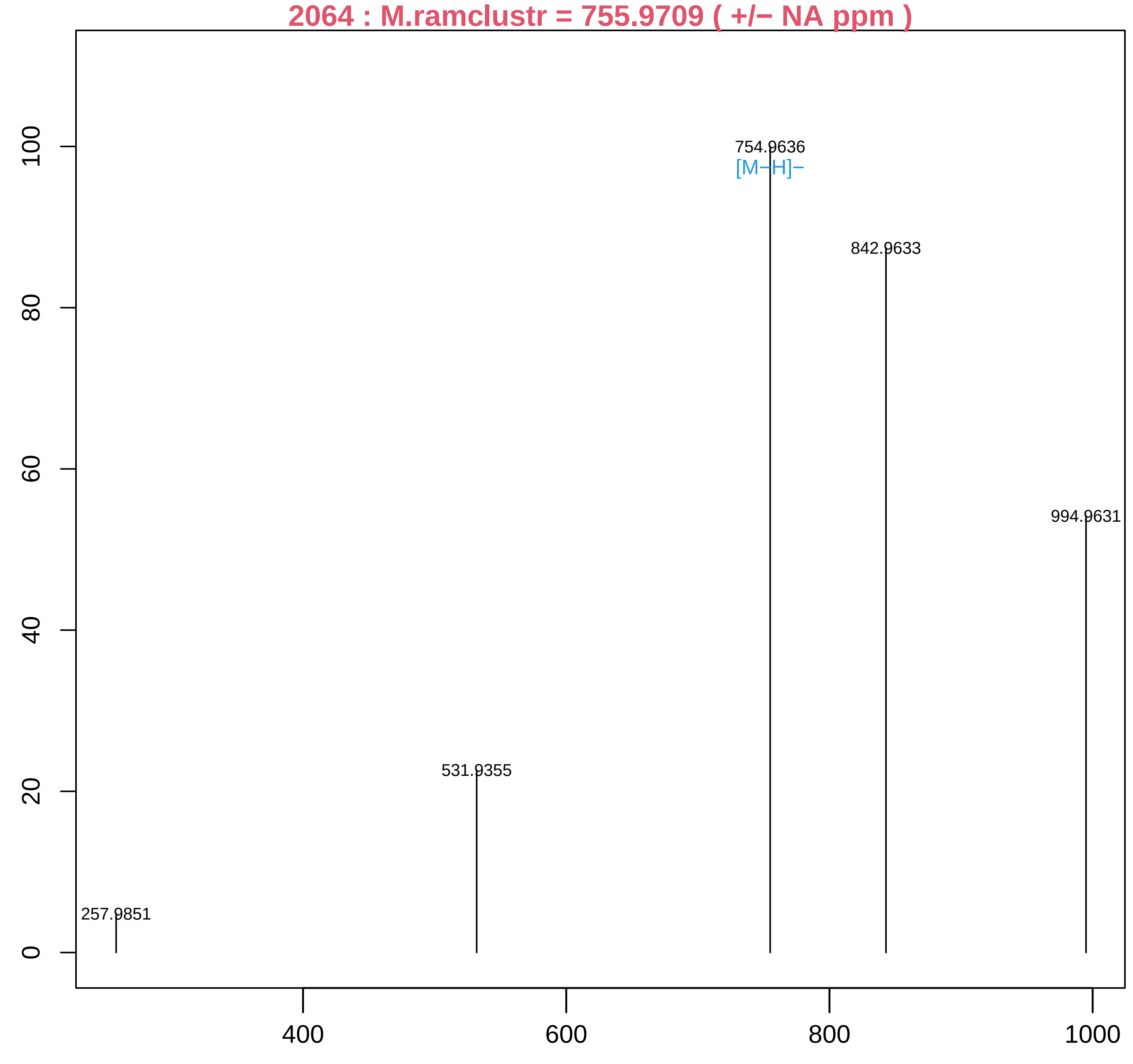
2064 : M.ramclustr = 755.9709 (+/− NA ppm)
754.9636
[M−H]−
842.9633
994.9631
531.9355
257.9851
0
20
40
60
80
100
400
600
800
1000

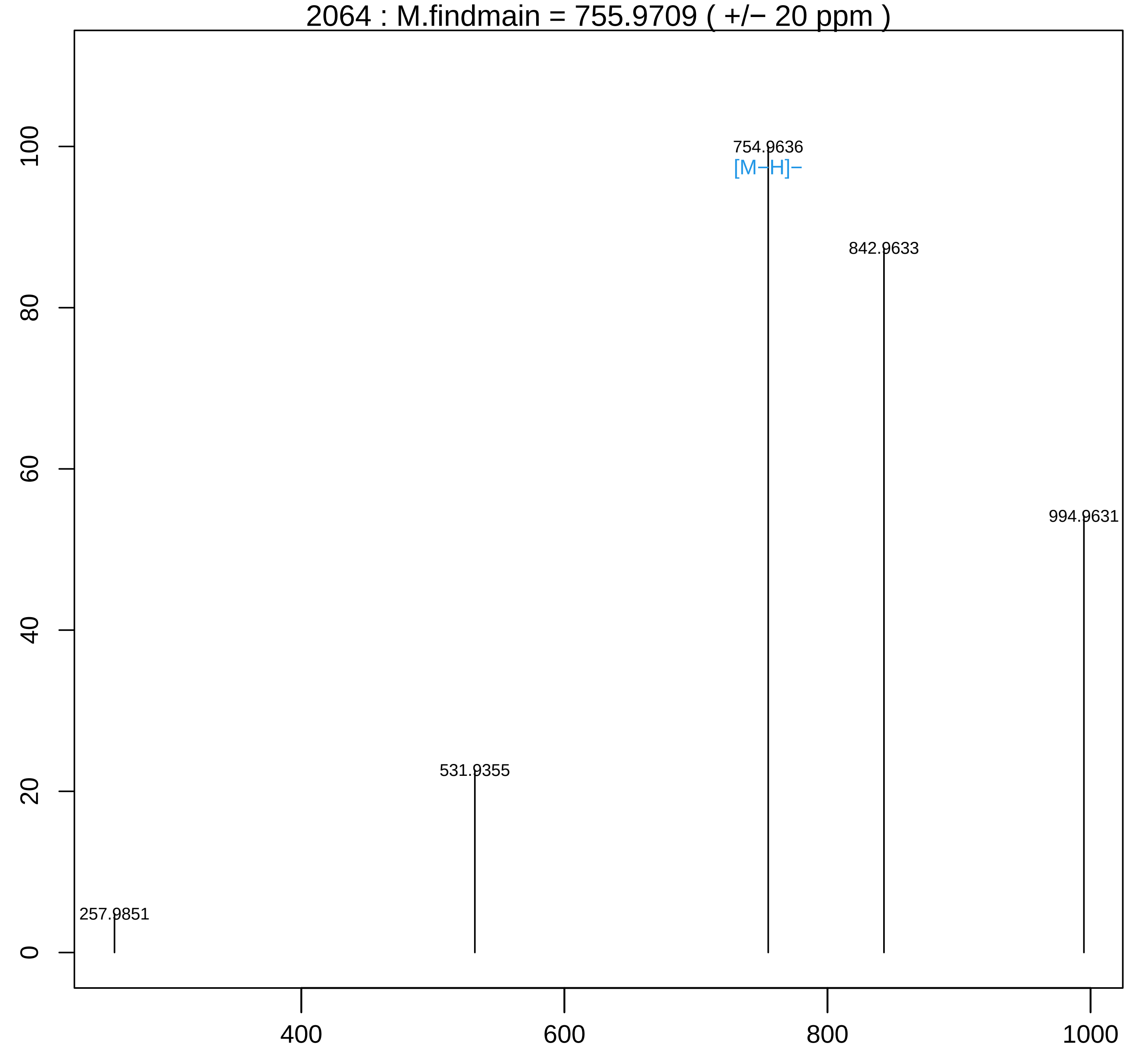
2064 : M.findmain = 755.9709 (+/− 20 ppm)
754.9636
[M−H]−
842.9633
994.9631
531.9355
257.9851
0
20
40
60
80
100
400
600
800
1000

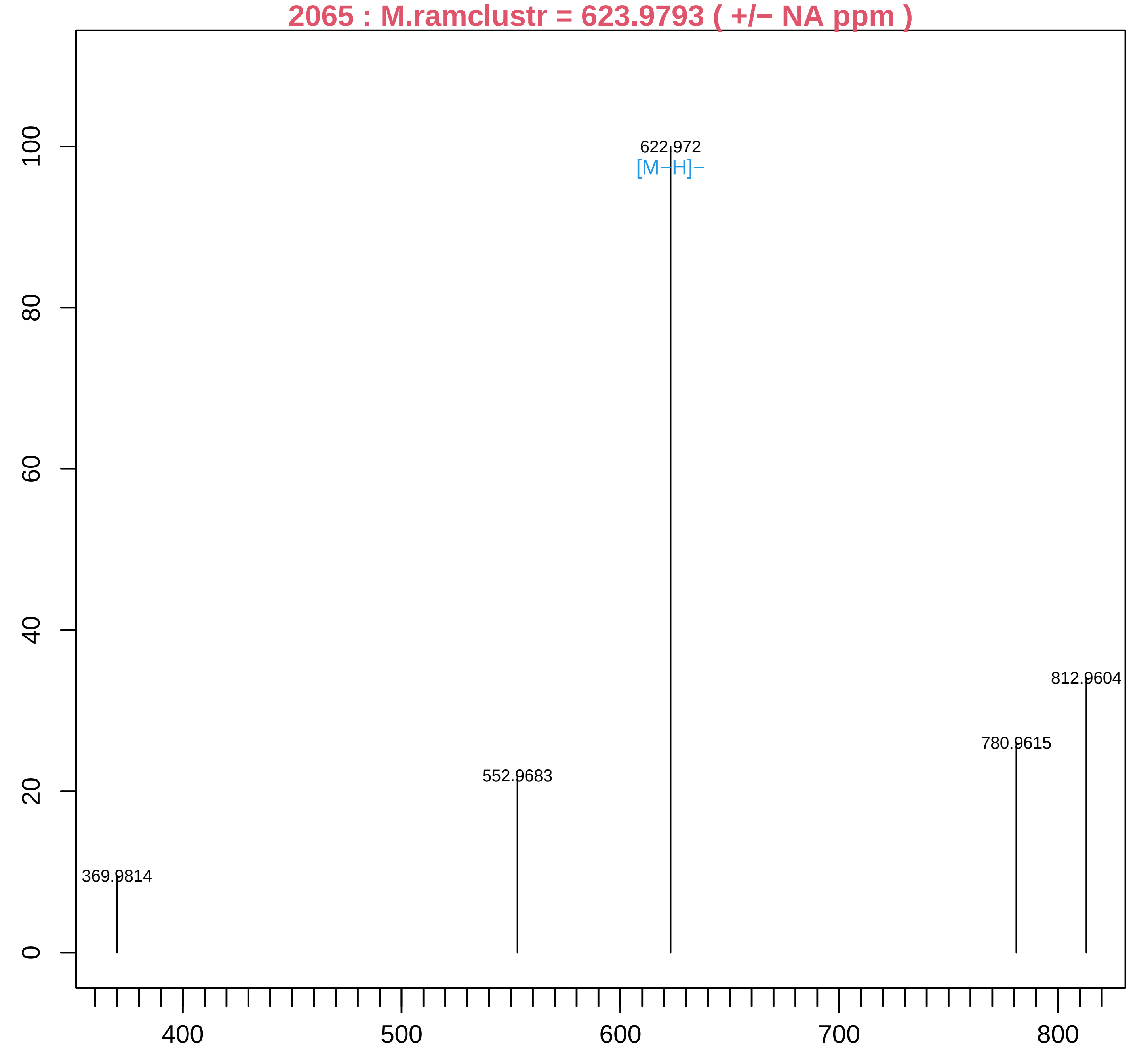
2065 : M.ramclustr = 623.9793 (+/− NA ppm)
622.972
[M−H]−
552.9683
369.9814
780.9615
812.9604
0
20
40
60
80
100
400
500
600
700
800

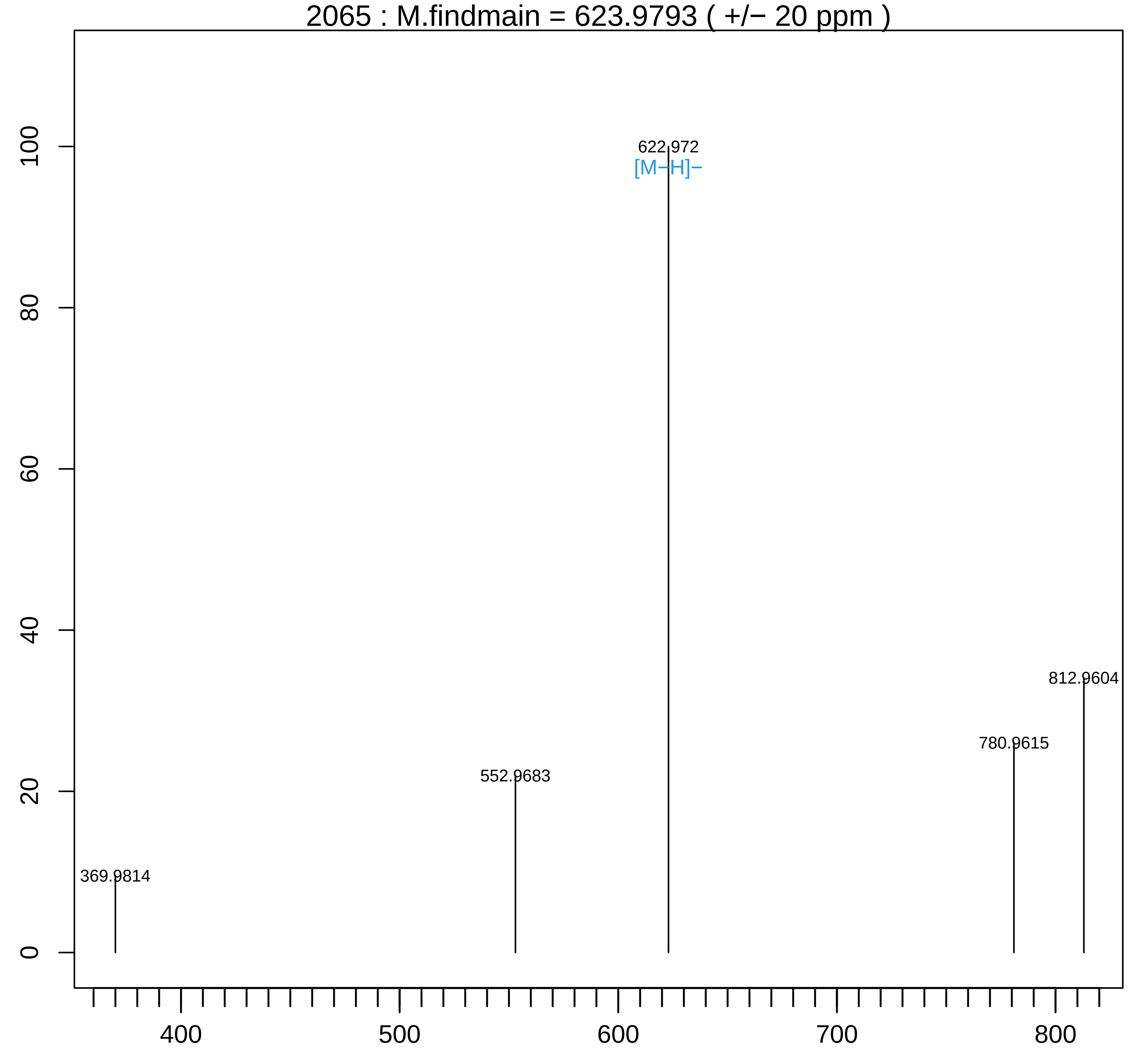
2065 : M.findmain = 623.9793 (+/− 20 ppm)
622.972
[M−H]−
552.9683
369.9814
780.9615
812.9604
0
20
40
60
80
100
400
500
600
700
800

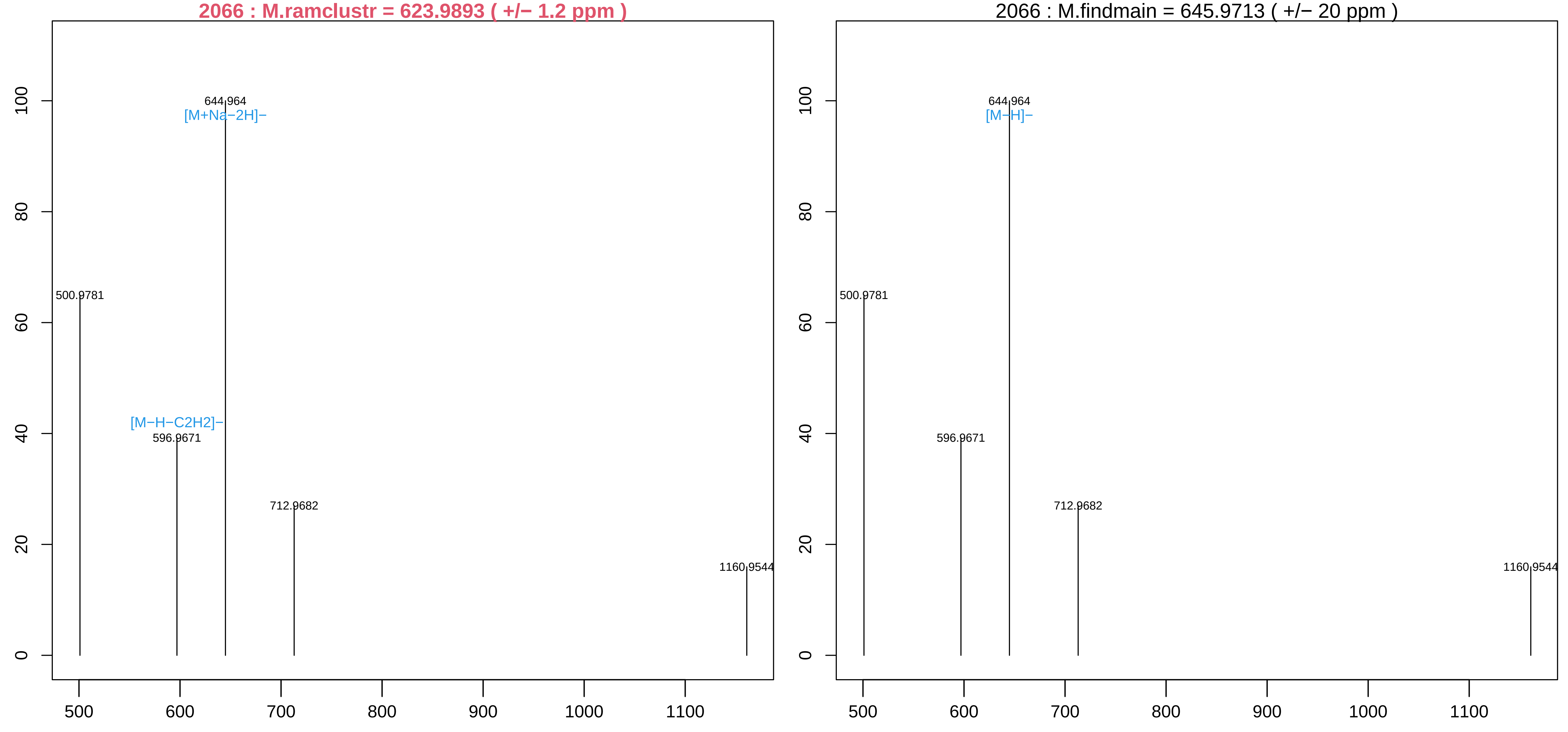

2066 : M.ramclustr = 623.9893 (+/− 1.2 ppm)
644.964
[M+Na−2H]−
500.9781
[M−H−C2H2]−
596.9671
712.9682
1160.9544
2066 : M.findmain = 645.9713 (+/− 20 ppm)
644.964
[M−H]−
500.9781
596.9671
712.9682
1160.9544

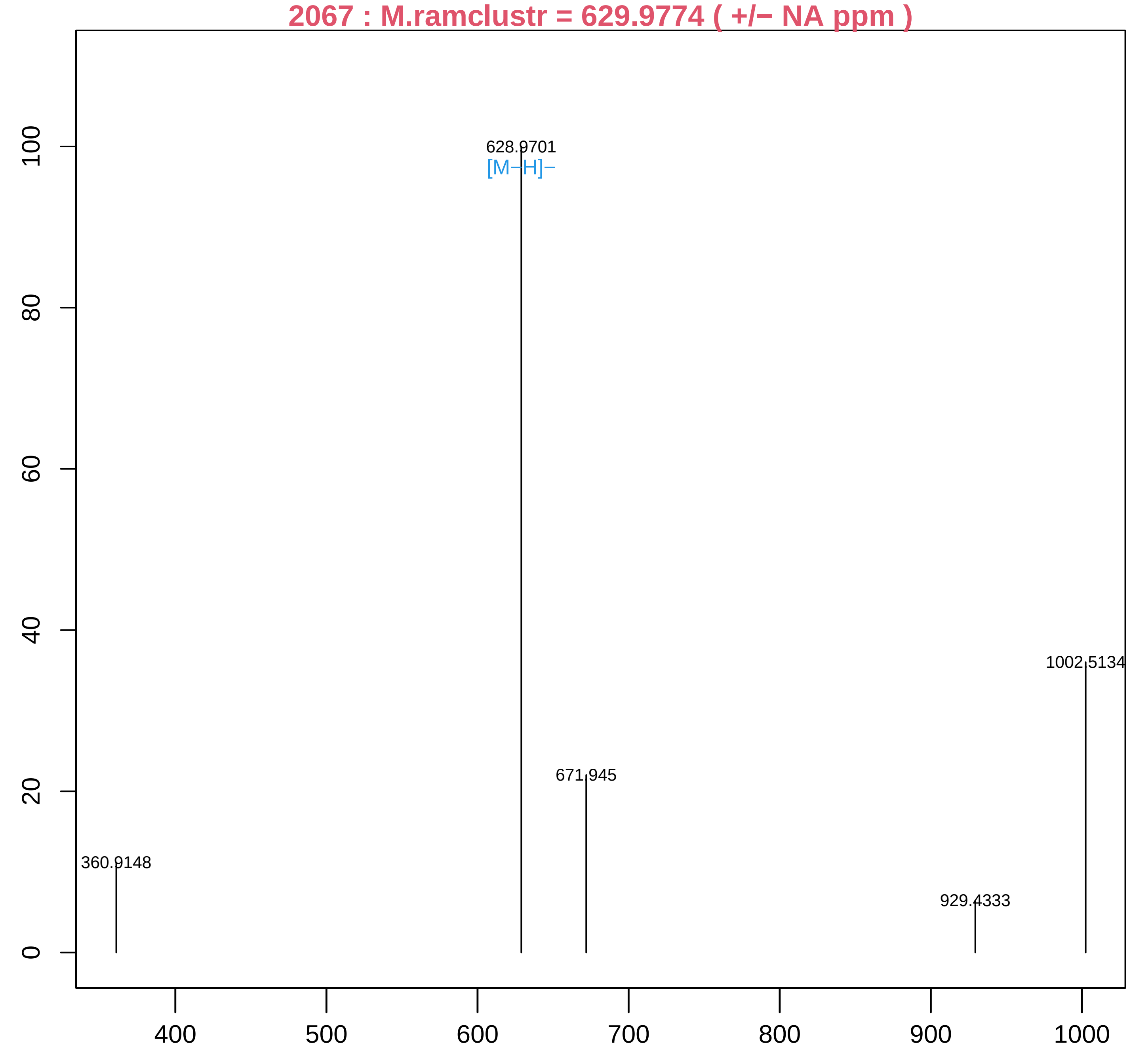
2067 : M.ramclustr = 629.9774 (+/− NA ppm)
628.9701
[M−H]−
671.945
360.9148
929.4333
1002.5134
0
20
40
60
80
100
400
500
600
700
800
900
1000

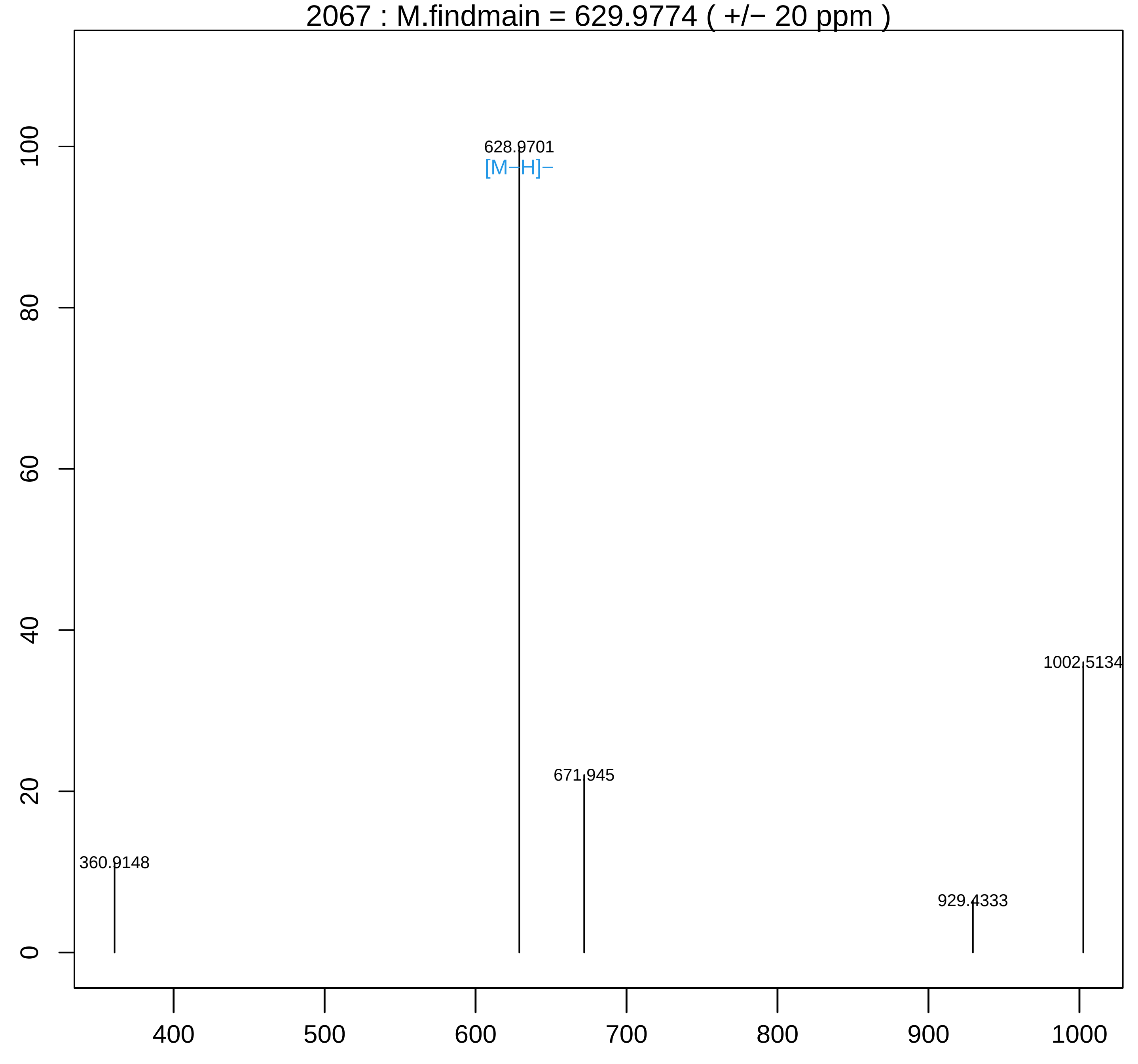
2067 : M.findmain = 629.9774 (+/− 20 ppm)
628.9701
[M−H]−
671.945
360.9148
929.4333
1002.5134
0
20
40
60
80
100
400
500
600
700
800
900
1000

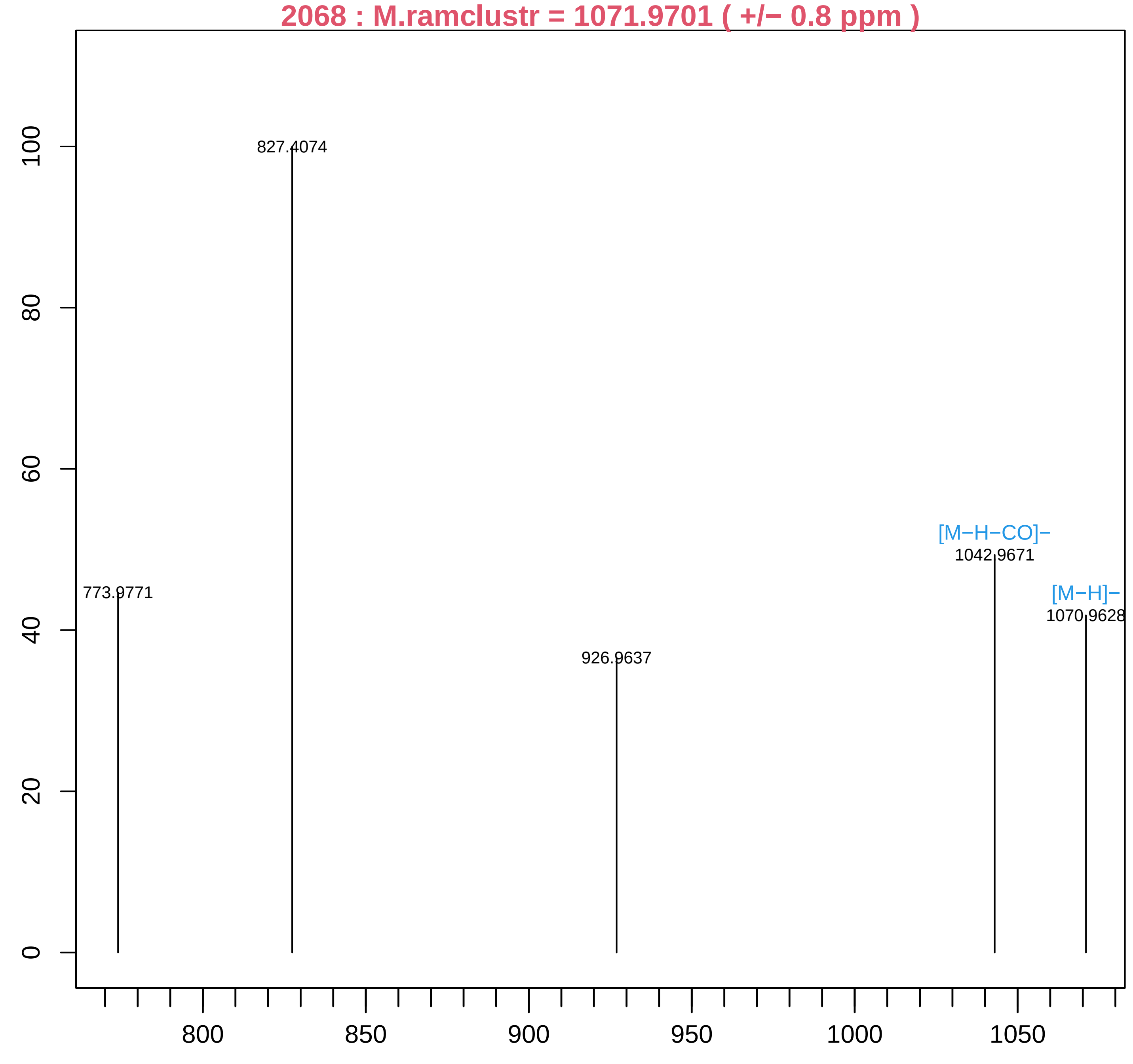

2068 : M.ramclustr = 1071.9701 (+/− 0.8 ppm)
827.4074
773.9771
926.9637
[M−H−CO]−
1042.9671
[M−H]−
1070.9628
0
20
40
60
80
100
800
850
900
950
1000
1050

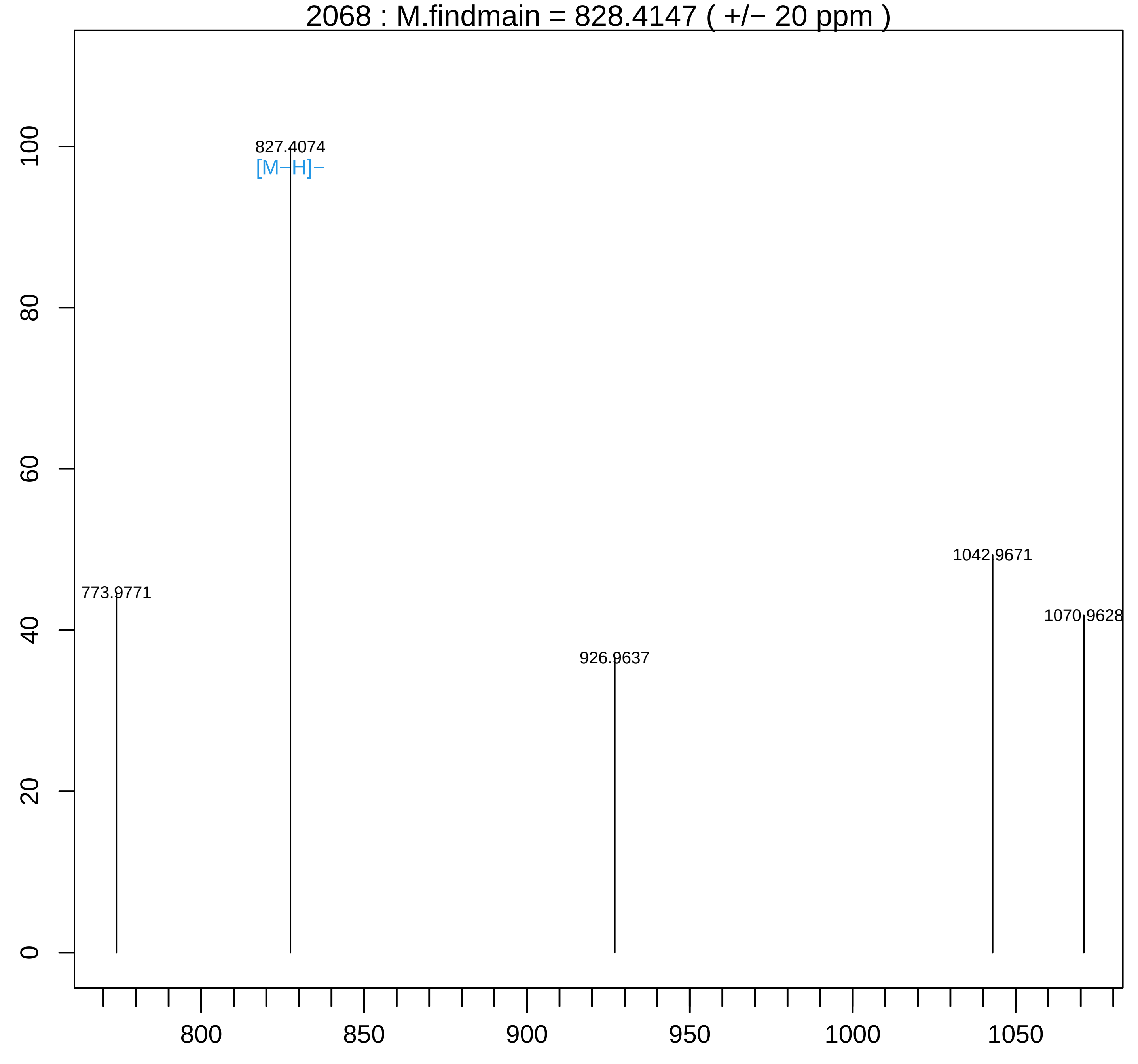

2068 : M.findmain = 828.4147 (+/− 20 ppm)
827.4074
[M−H]−
773.9771
926.9637
1042.9671
1070.9628
0
20
40
60
80
100
800
850
900
950
1000
1050

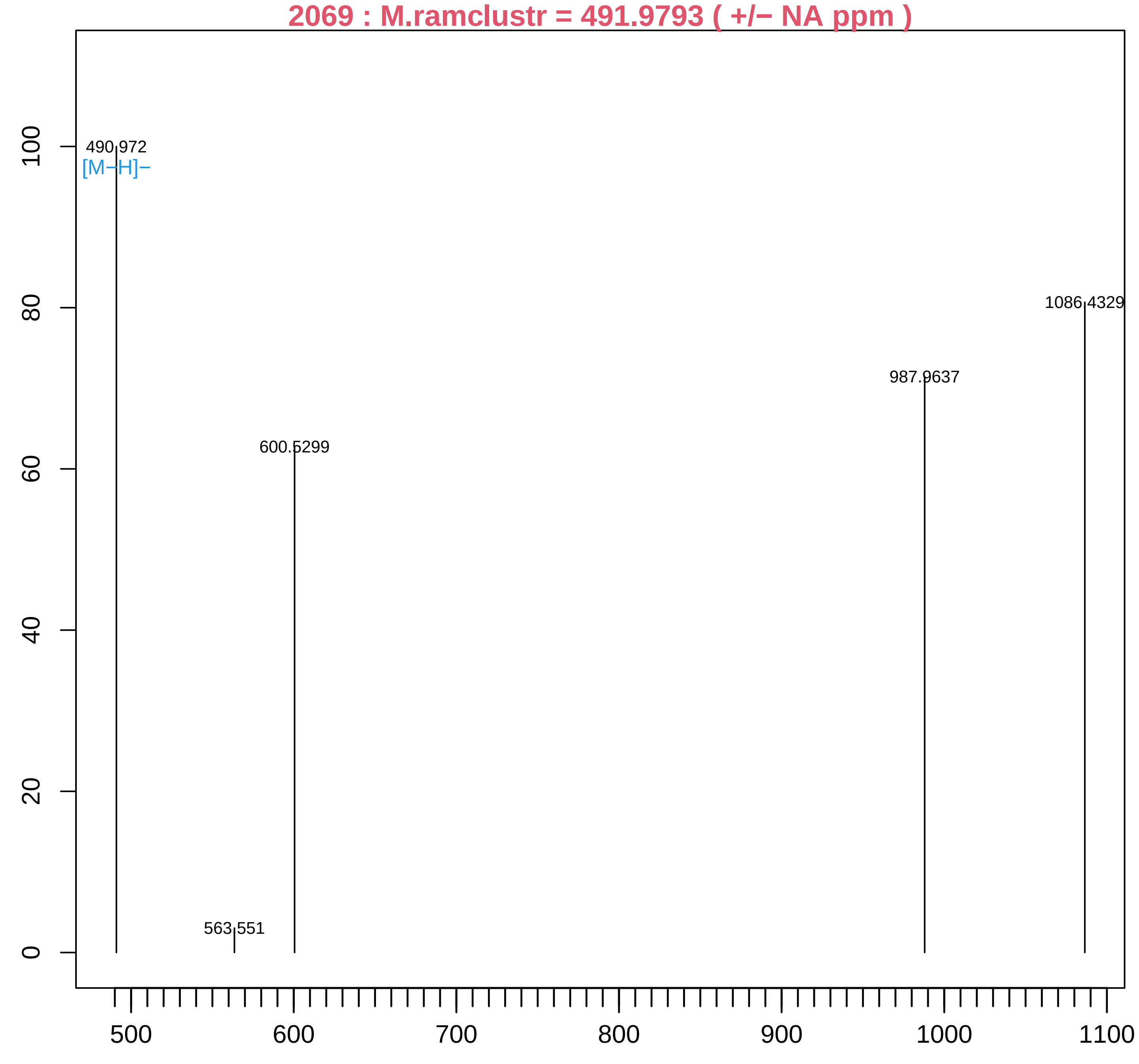

2069 : M.ramclustr = 491.9793 (+/− NA ppm)
490.972
[M−H]−
563.551
600.5299
987.9637
1086.4329

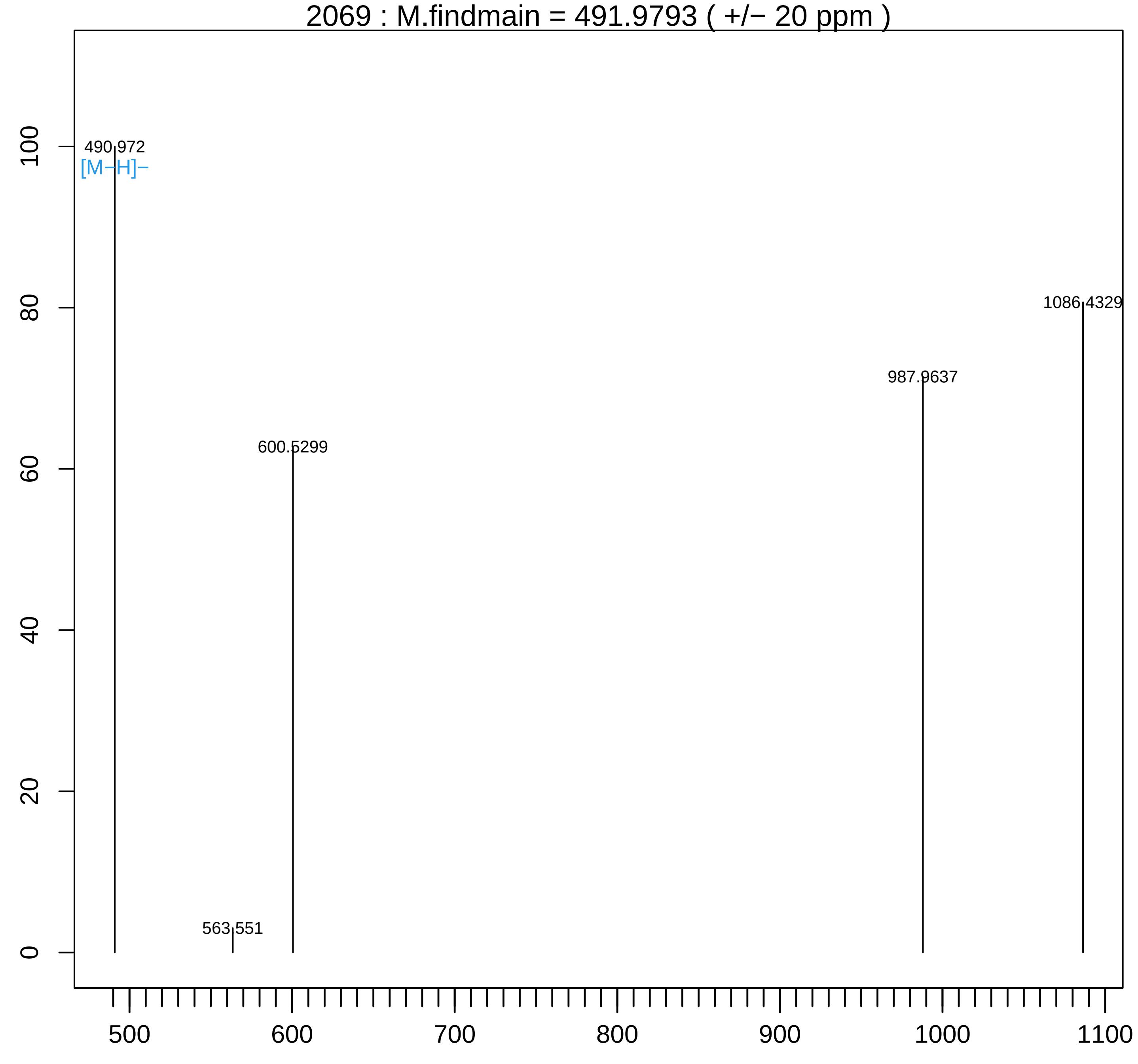

2069 : M.findmain = 491.9793 (+/− 20 ppm)
490.972
[M−H]−
563.551
600.5299
987.9637
1086.4329

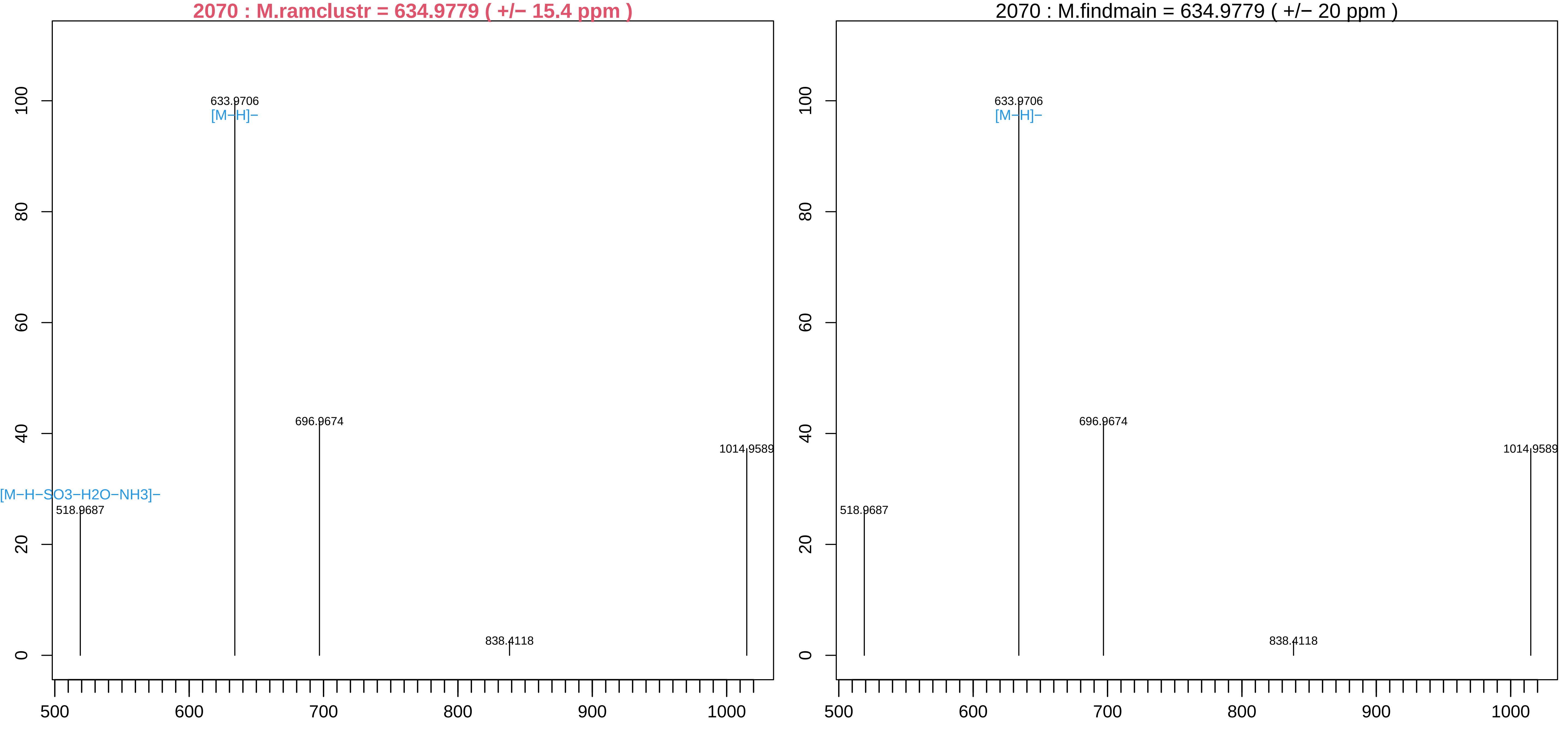
2070 : M.ramclustr = 634.9779 (+/− 15.4 ppm)
633.9706
[M−H]−
696.9674
1014.9589
[M−H−SO3−H2O−NH3]−
518.9687
838.4118
0
20
40
60
80
100
500
600
700
800
900
1000
2070 : M.findmain = 634.9779 (+/− 20 ppm)
633.9706
[M−H]−
696.9674
1014.9589
518.9687
838.4118
0
20
40
60
80
100
500
600
700
800
900
1000

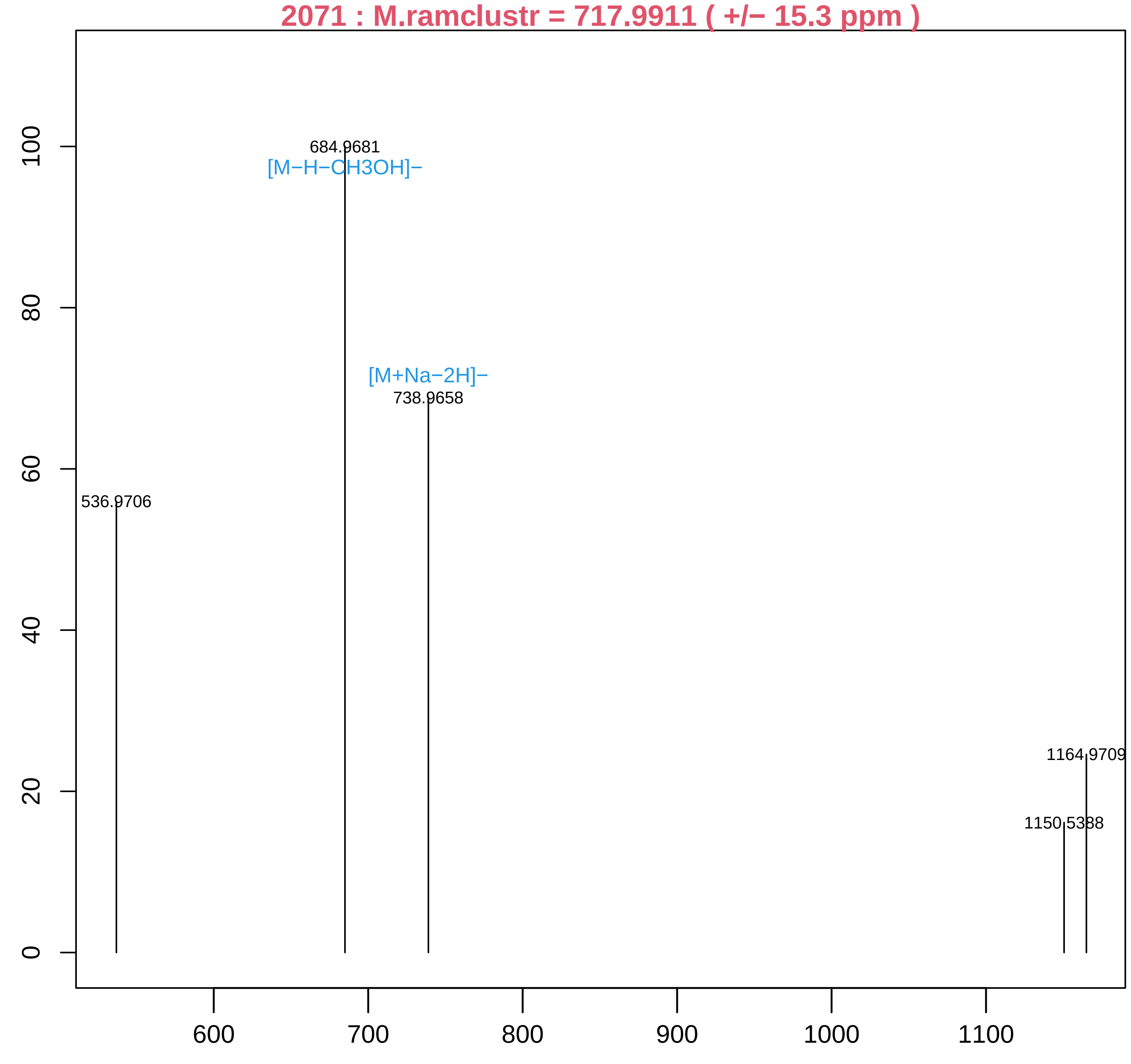
2071 : M.ramclustr = 717.9911 (+/− 15.3 ppm)
684.9681
[M−H−CH3OH]−
[M+Na−2H]−
738.9658
536.9706
1164.9709
1150.5388
0
20
40
60
80
100
600
700
800
900
1000
1100

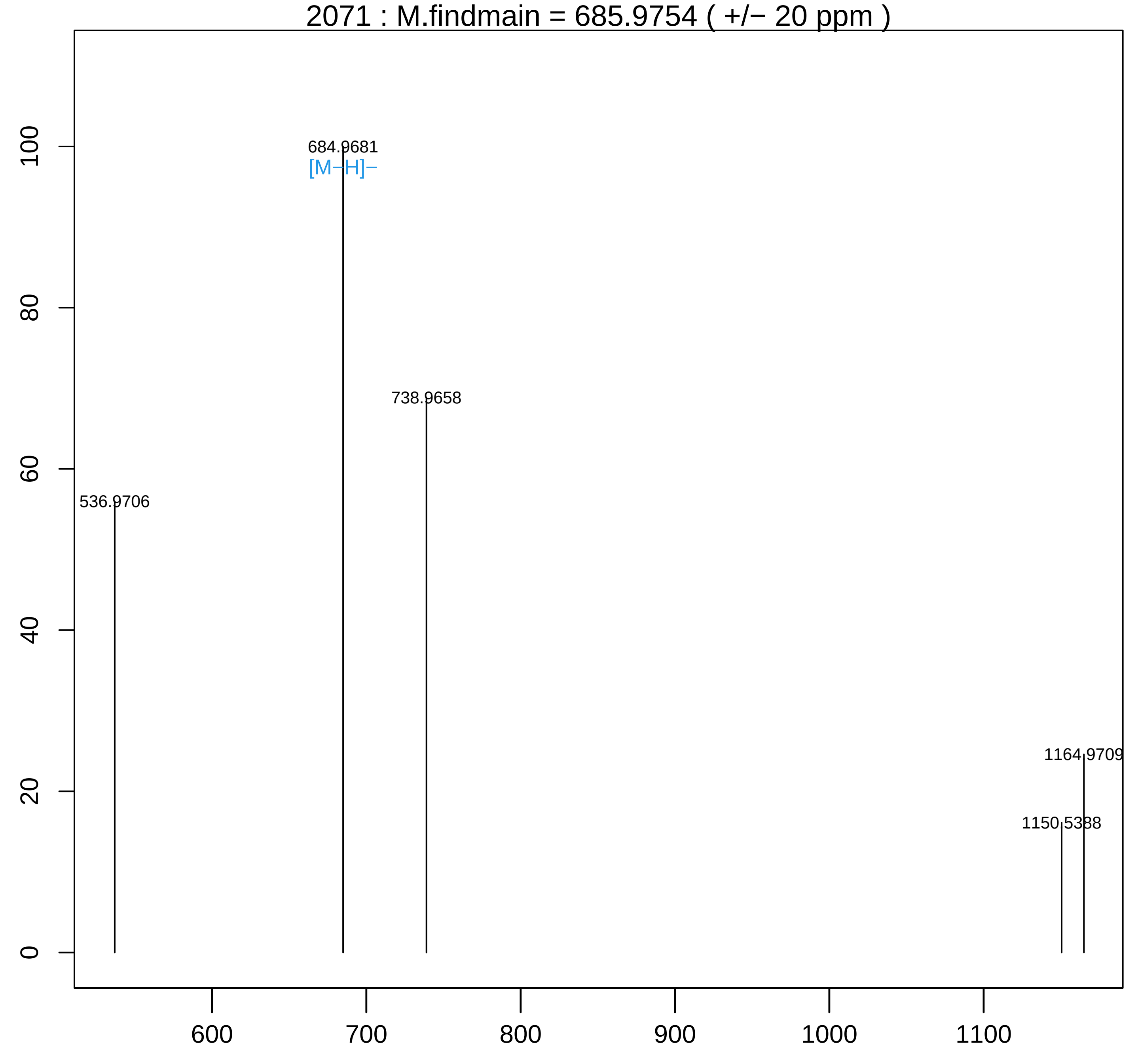
2071 : M.findmain = 685.9754 (+/− 20 ppm)
684.9681
[M−H]−
738.9658
536.9706
1164.9709
1150.5388
0
20
40
60
80
100
600
700
800
900
1000
1100

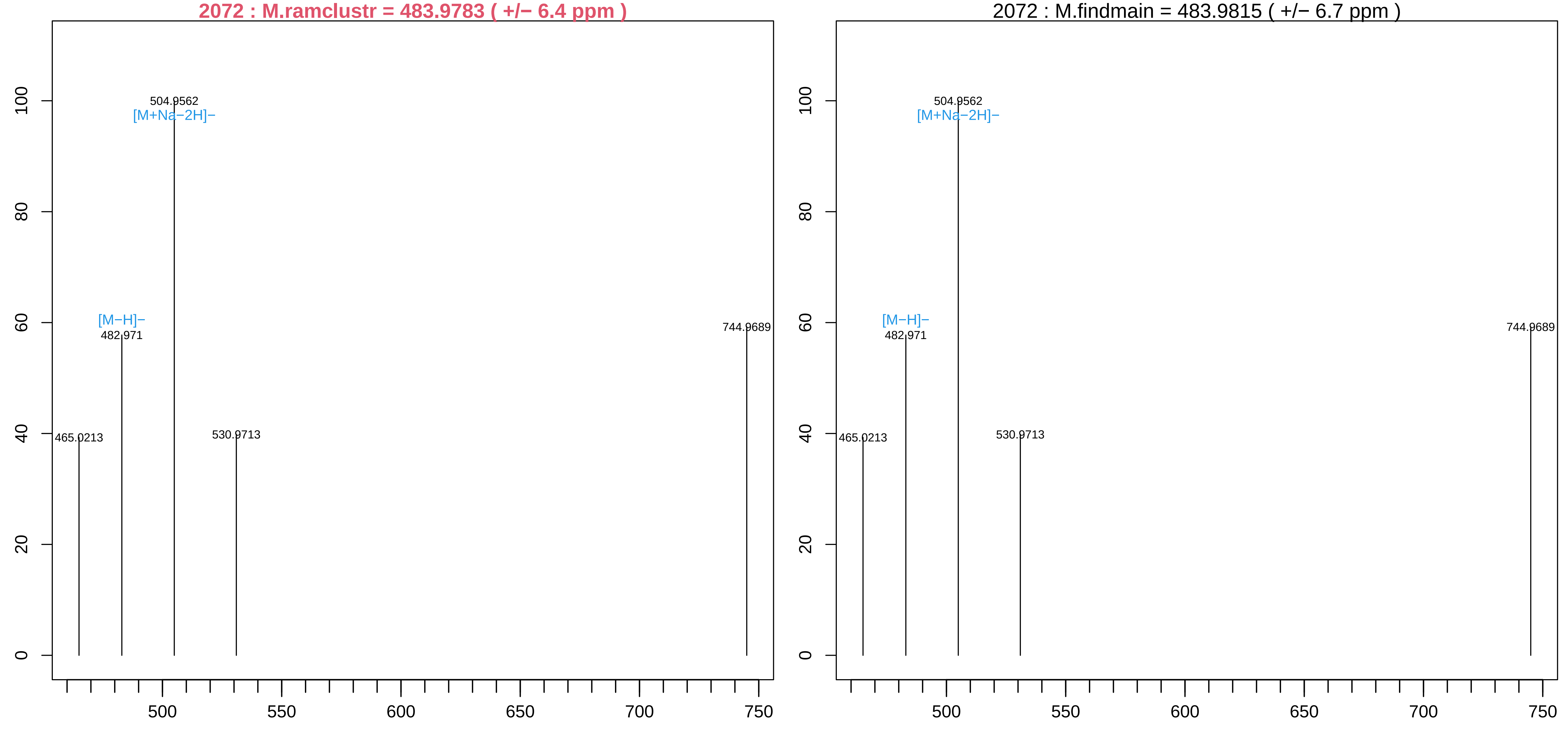
2072 : M.ramclustr = 483.9783 (+/− 6.4 ppm)
504.9562
[M+Na−2H]−
[M−H]−
482.971
744.9689
465.0213
530.9713
2072 : M.findmain = 483.9815 (+/− 6.7 ppm)
504.9562
[M+Na−2H]−
[M−H]−
482.971
744.9689
465.0213
530.9713

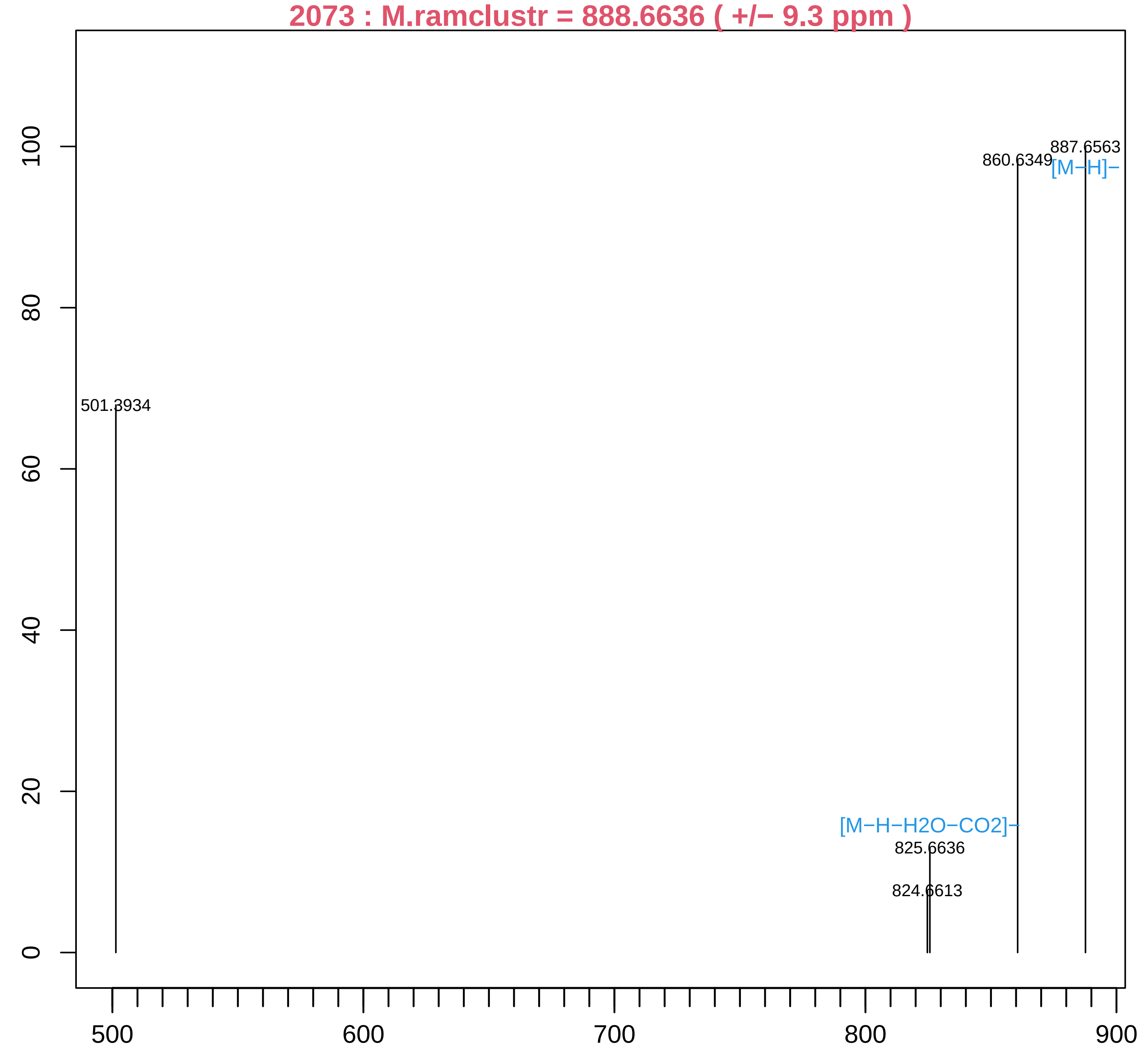
2073 : M.ramclustr = 888.6636 (+/− 9.3 ppm)
501.3934
860.6349
887.6563
[M−H]−
[M−H−H2O−CO2]−
825.6636
824.6613
0
20
40
60
80
100
500
600
700
800
900

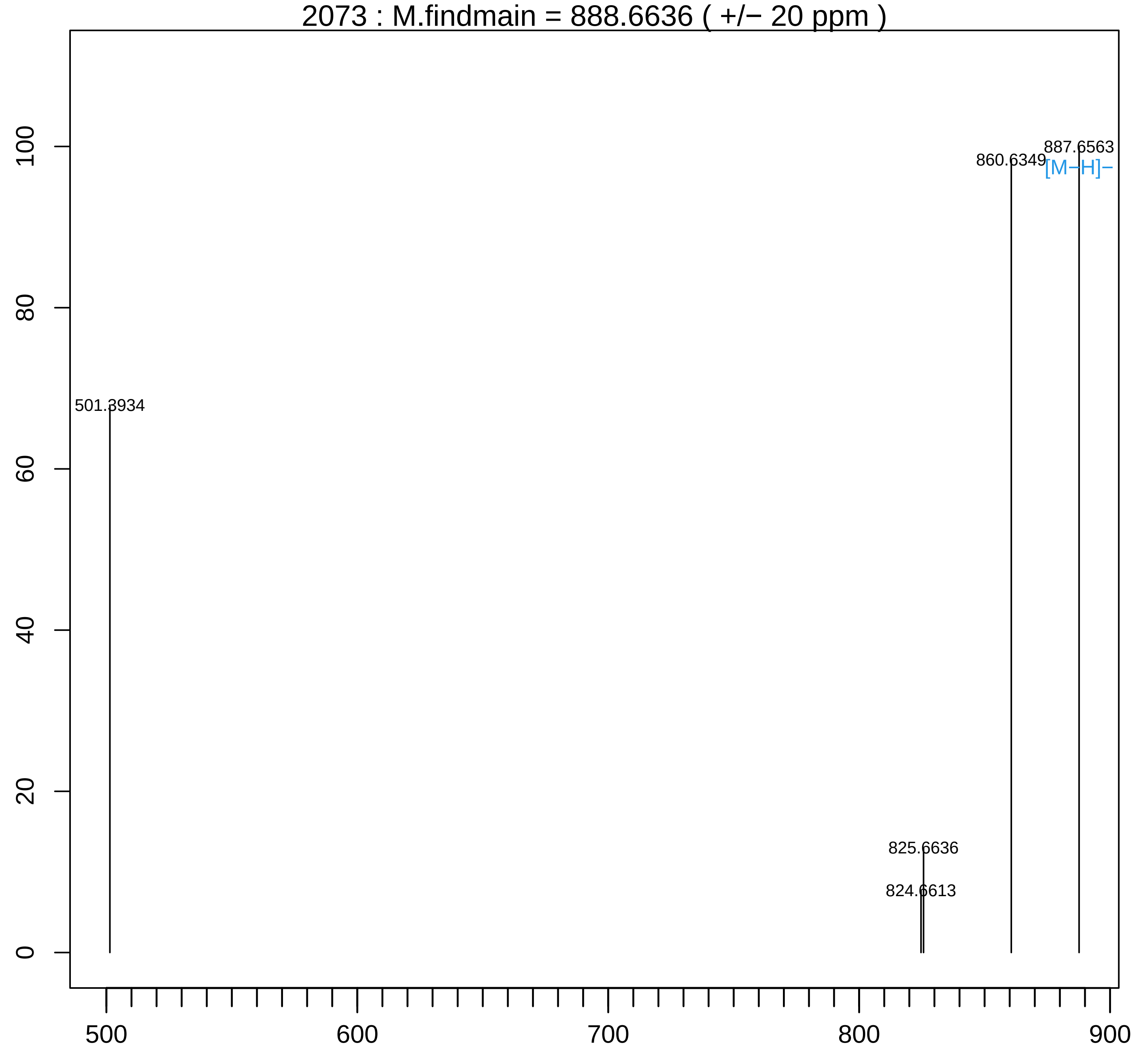
2073 : M.findmain = 888.6636 (+/− 20 ppm)
501.3934
860.6349
887.6563
[M−H]−
825.6636
824.6613
0
20
40
60
80
100
500
600
700
800
900

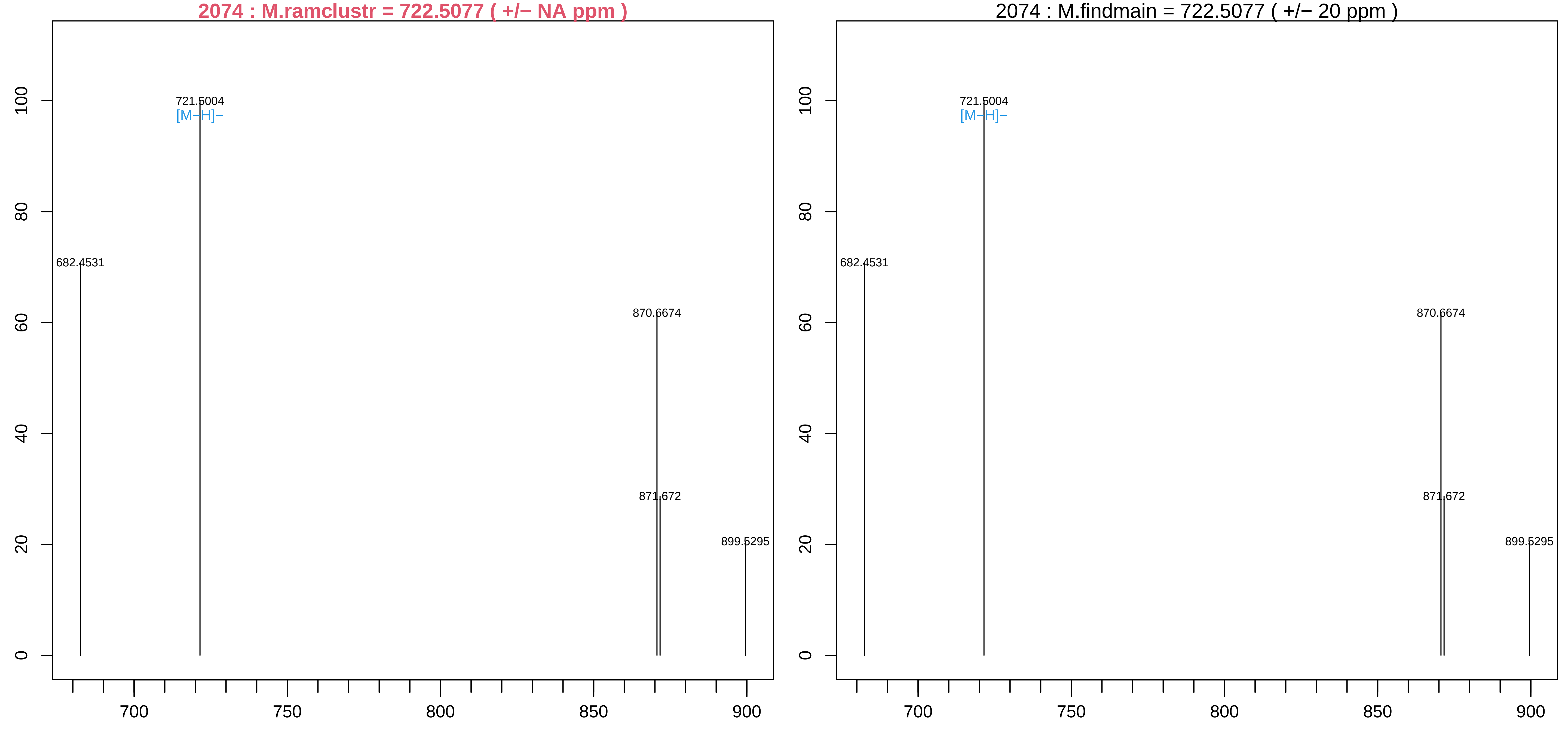
2074 : M.ramclustr = 722.5077 (+/− NA ppm)
682.4531
721.5004
[M−H]−
870.6674
871.672
899.5295
2074 : M.findmain = 722.5077 (+/− 20 ppm)
682.4531
721.5004
[M−H]−
870.6674
871.672
899.5295
0
20
40
60
80
100
700
750
800
850
900

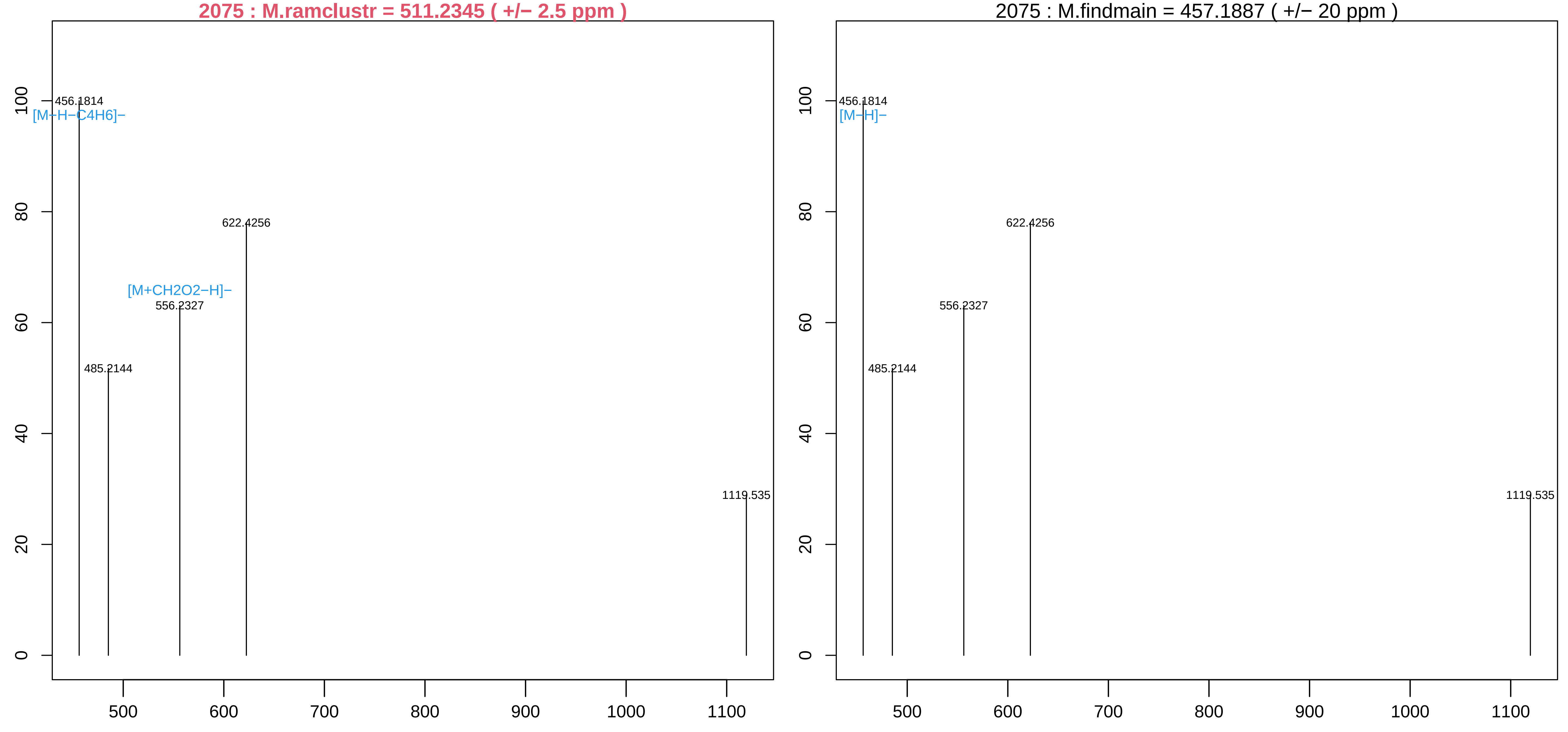

2075 : M.ramclustr = 511.2345 (+/− 2.5 ppm)
456.1814
[M−H−C4H6]−
485.2144
[M+CH2O2−H]−
556.2327
622.4256
1119.535
2075 : M.findmain = 457.1887 (+/− 20 ppm)
456.1814
[M−H]−
485.2144
556.2327
622.4256
1119.535
0
20
40
60
80
100
500
600
700
800
900
1000
1100

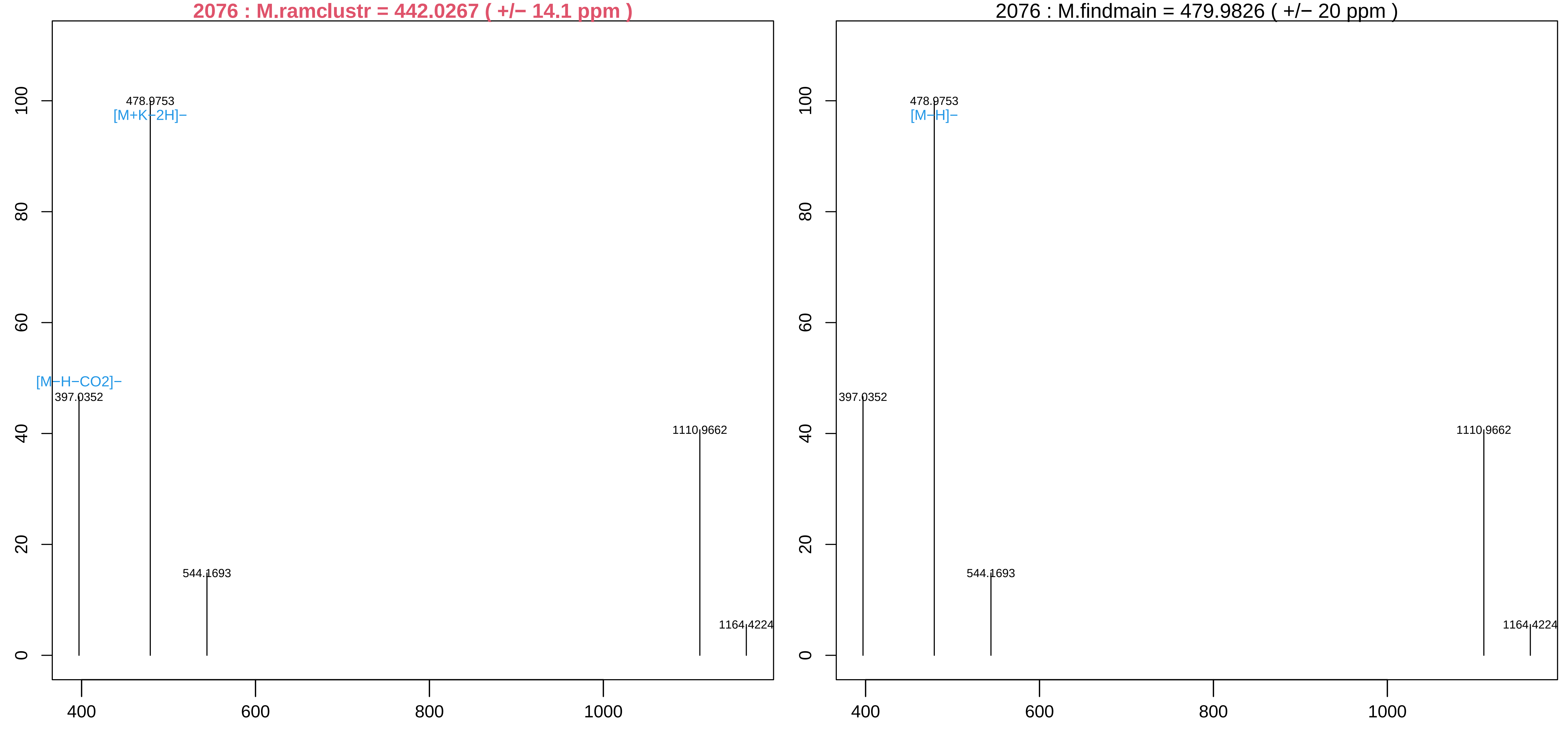

2076 : M.ramclustr = 442.0267 (+/− 14.1 ppm)
478.9753
[M+K−2H]−
[M−H−CO2]−
397.0352
544.1693
1110.9662
1164.4224
2076 : M.findmain = 479.9826 (+/− 20 ppm)
478.9753
[M−H]−
397.0352
544.1693
1110.9662
1164.4224

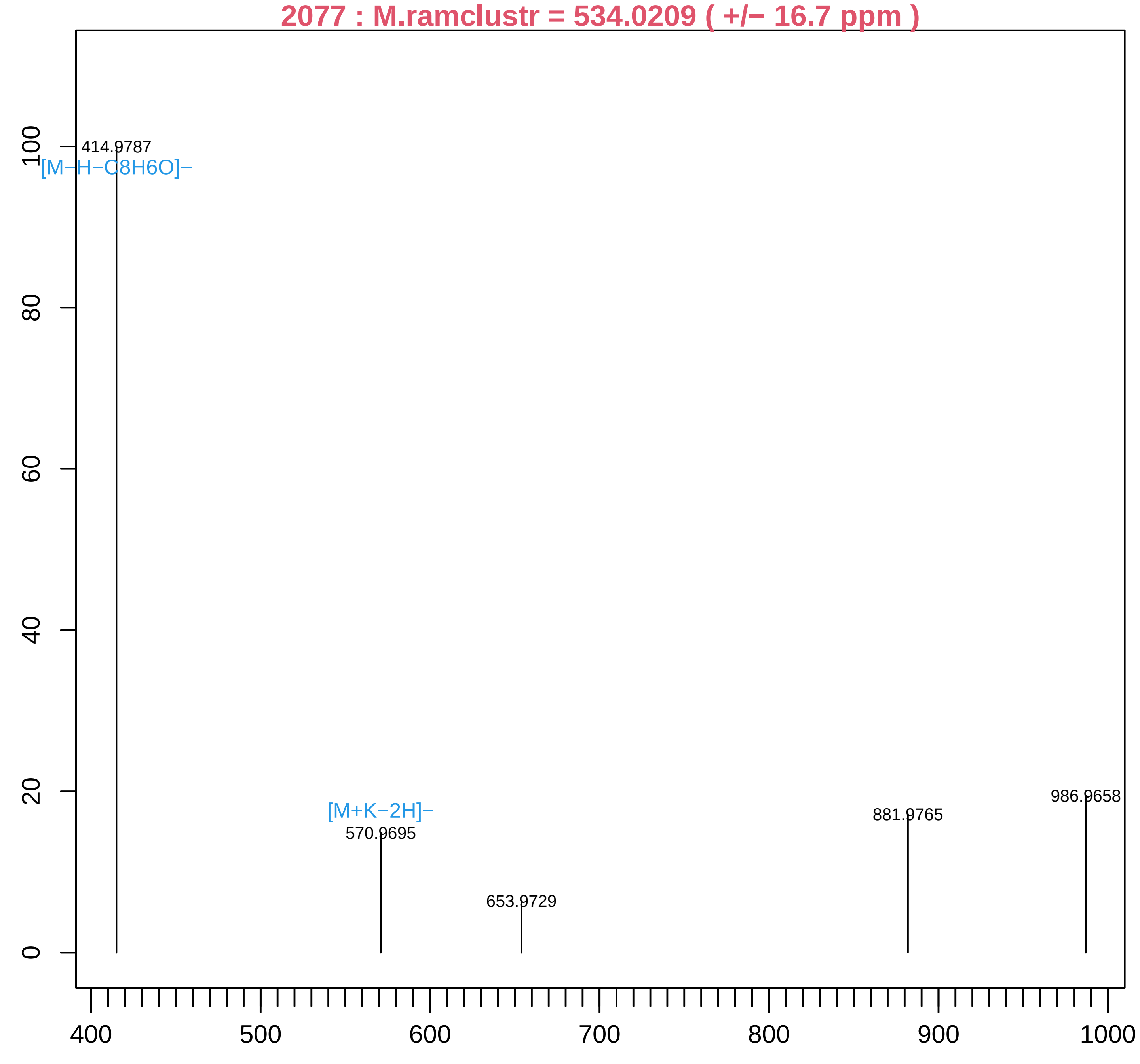

2077 : M.ramclustr = 534.0209 (+/− 16.7 ppm)
414.9787
[M−H−C8H6O]−
[M+K−2H]−
570.9695
653.9729
881.9765
986.9658
0
20
40
60
80
100
400
500
600
700
800
900
1000

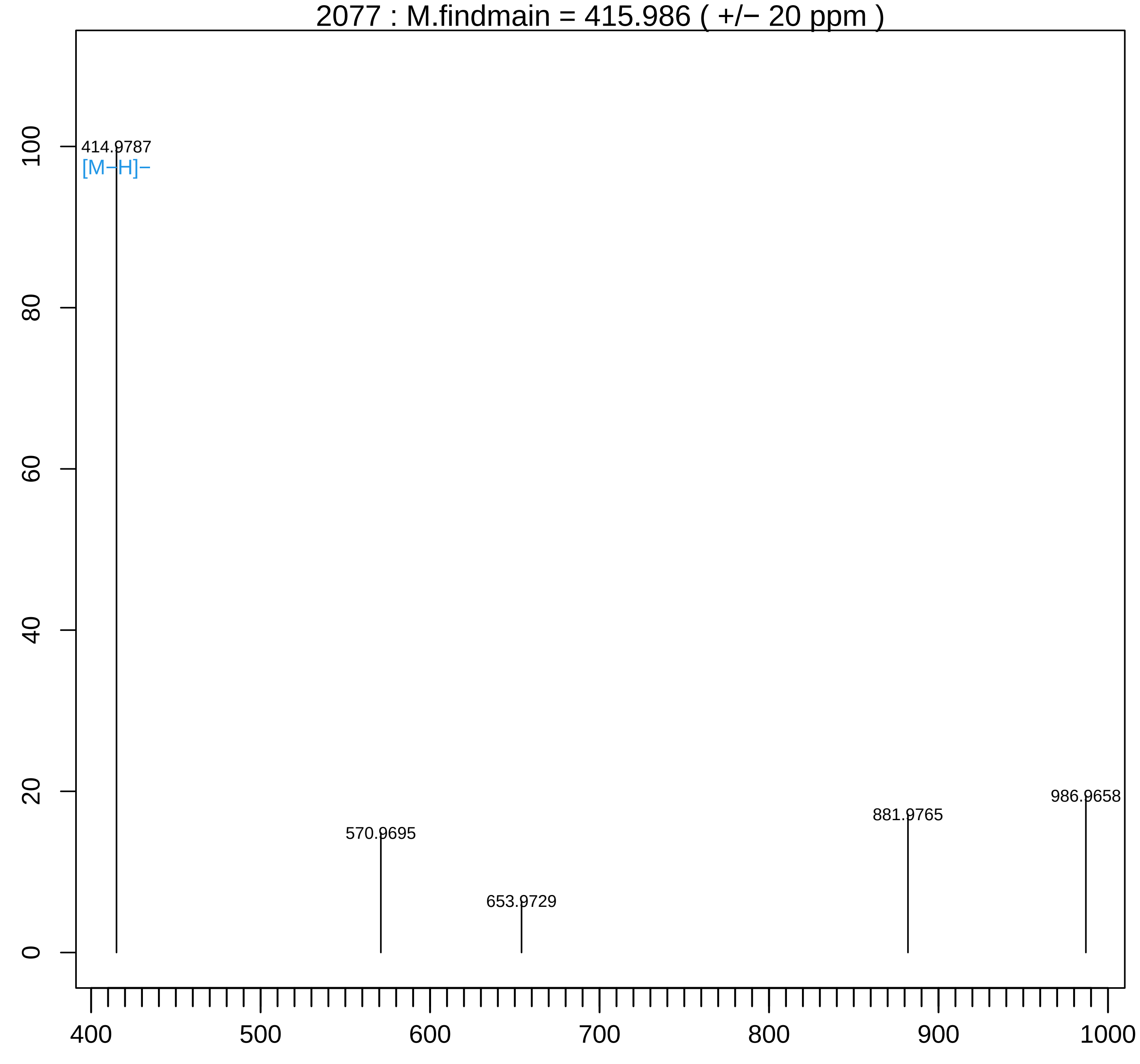

2077 : M.findmain = 415.986 (+/− 20 ppm)
414.9787
[M−H]−
570.9695
653.9729
881.9765
986.9658
0
20
40
60
80
100
400
500
600
700
800
900
1000

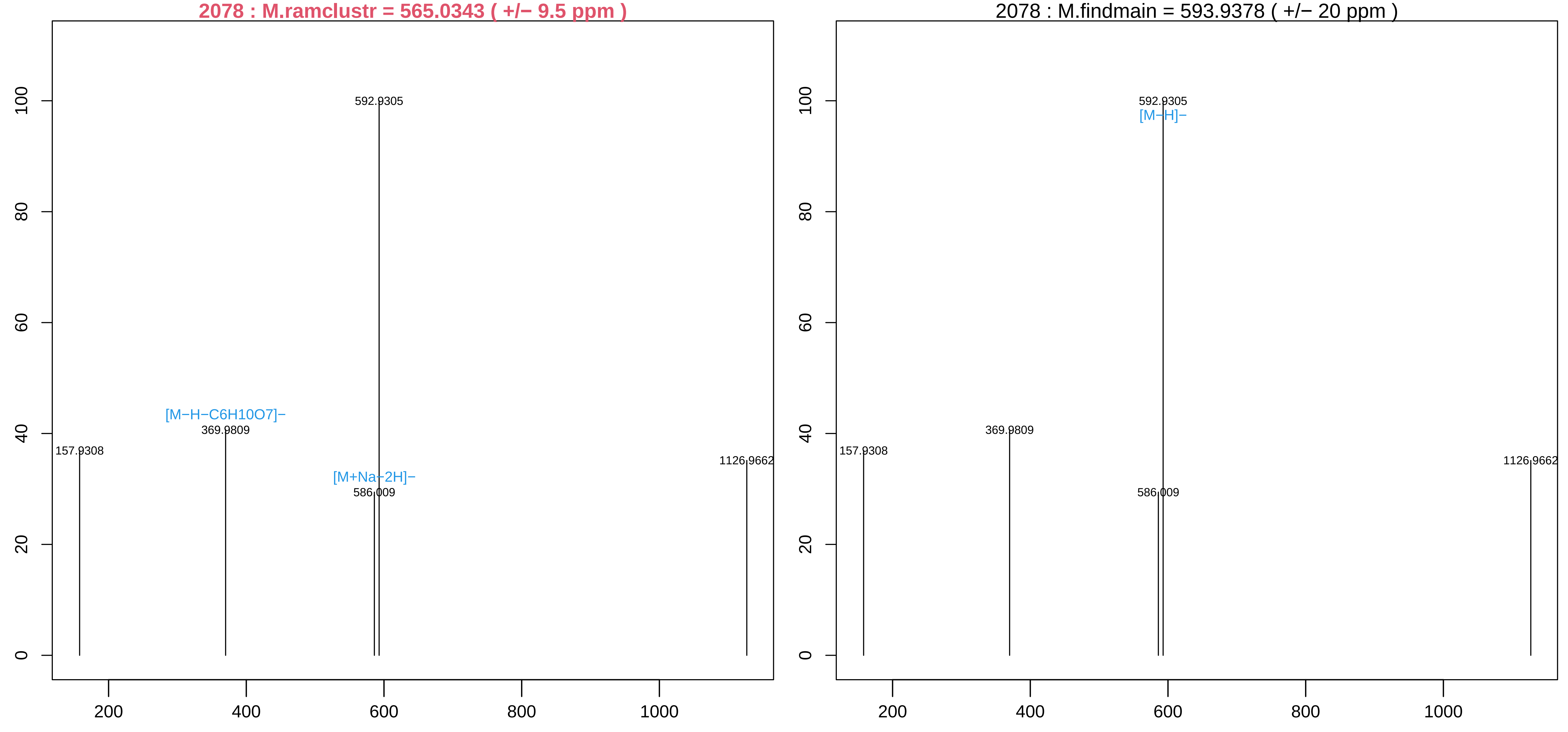

2078 : M.ramclustr = 565.0343 (+/− 9.5 ppm)
592.9305
[M−H−C6H10O7]−
369.9809
157.9308
[M+Na−2H]−
586.009
1126.9662
0
20
40
60
80
100
200
400
600
800
1000
2078 : M.findmain = 593.9378 (+/− 20 ppm)
592.9305
[M−H]−
369.9809
157.9308
586.009
1126.9662

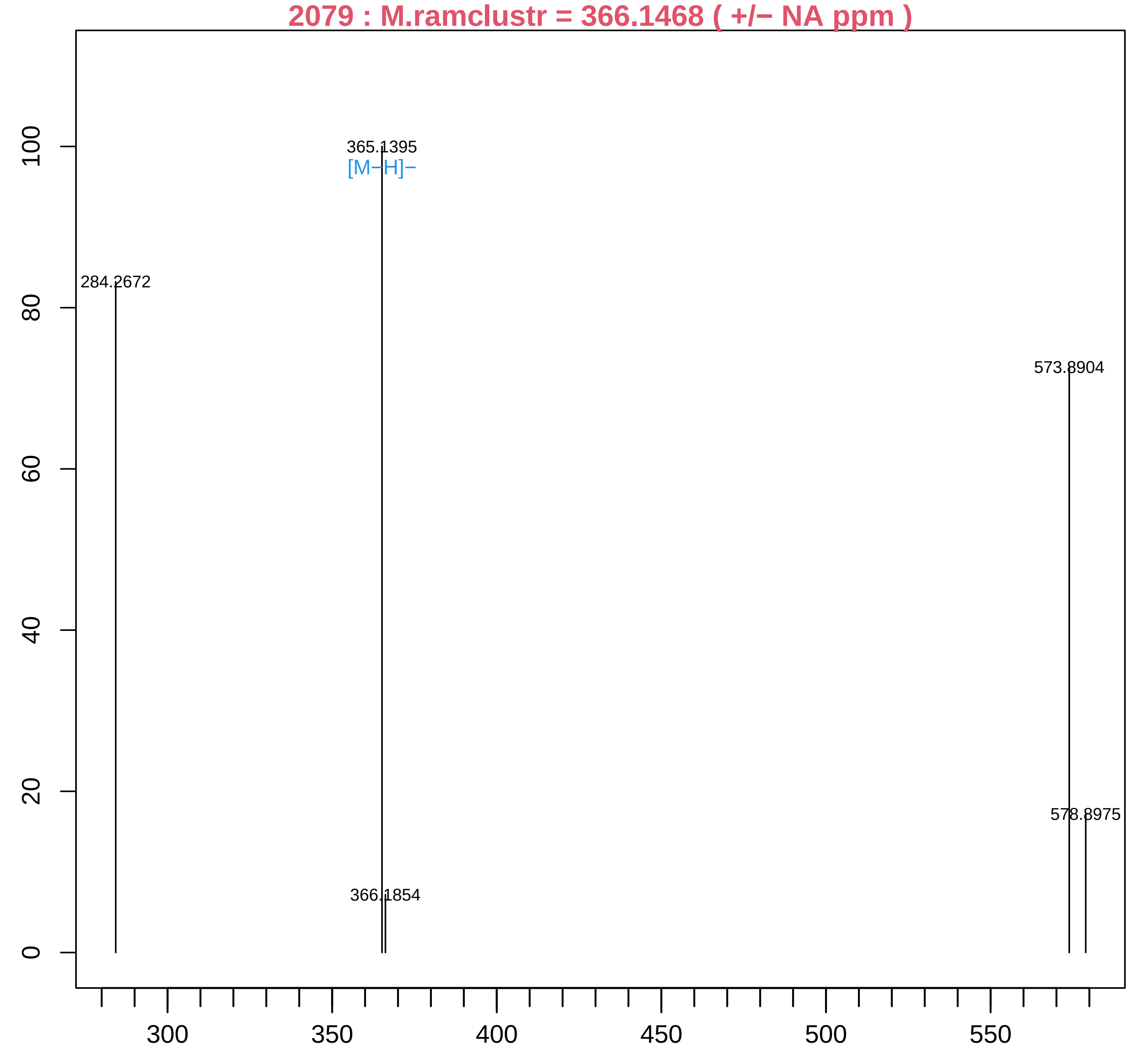
2079 : M.ramclustr = 366.1468 (+/− NA ppm)
284.2672
365.1395
[M−H]−
366.1854
573.8904
578.8975
0
20
40
60
80
100
300
350
400
450
500
550

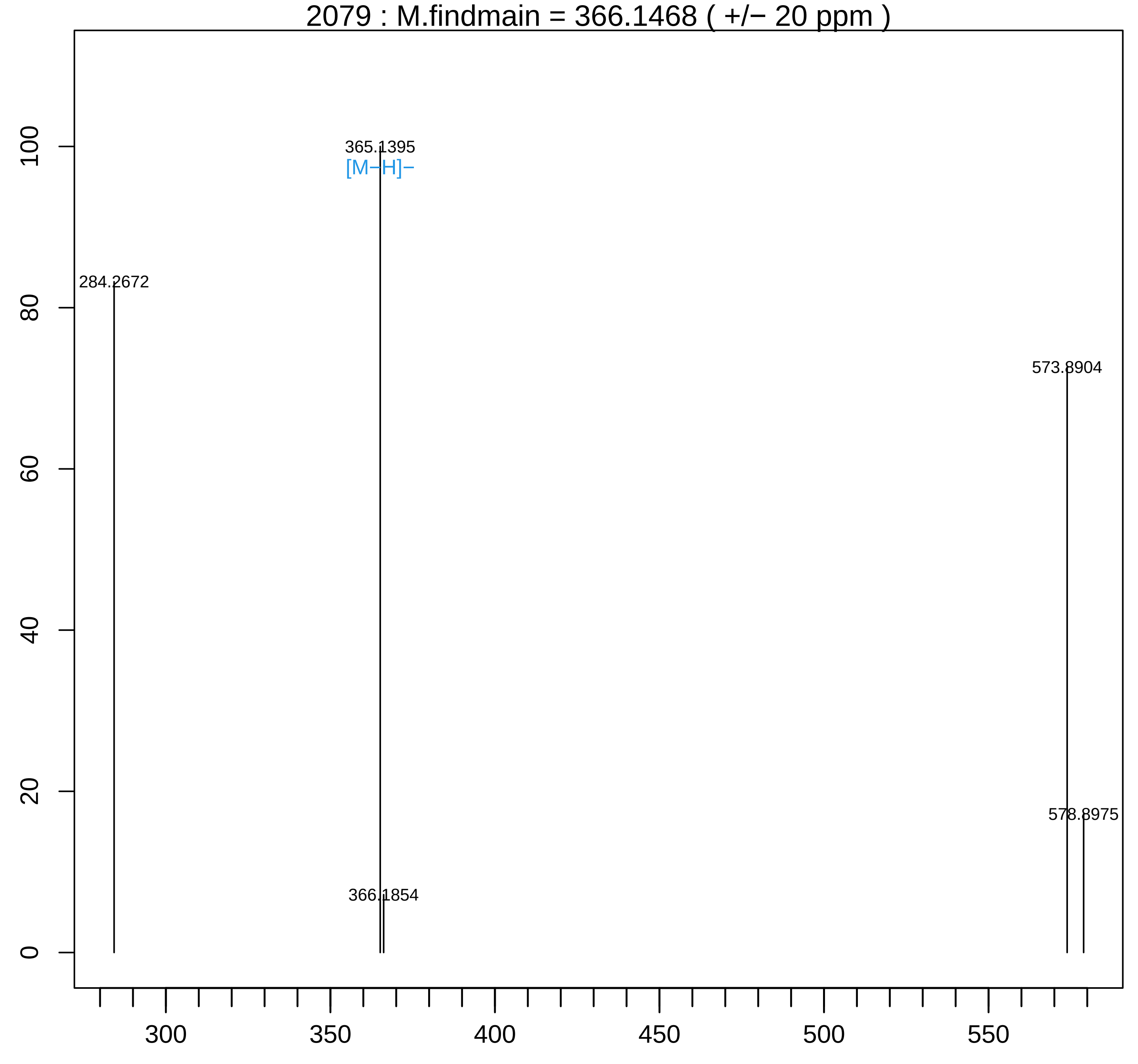
2079 : M.findmain = 366.1468 (+/− 20 ppm)
284.2672
365.1395
[M−H]−
366.1854
573.8904
578.8975
0
20
40
60
80
100
300
350
400
450
500
550

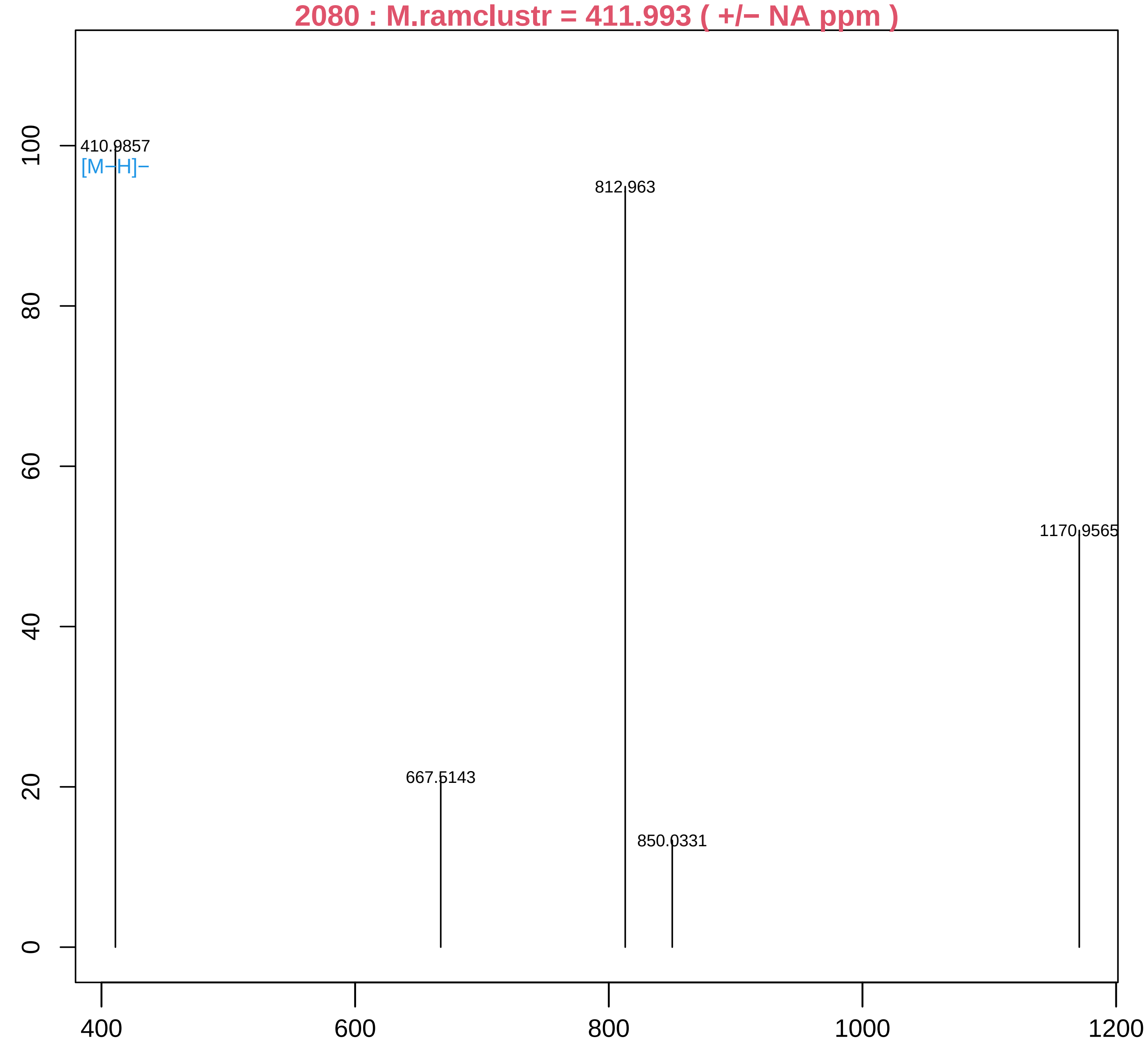
2080 : M.ramclustr = 411.993 (+/− NA ppm)
410.9857
[M−H]−
812.963
667.5143
850.0331
1170.9565
0
20
40
60
80
100
400
600
800
1000
1200

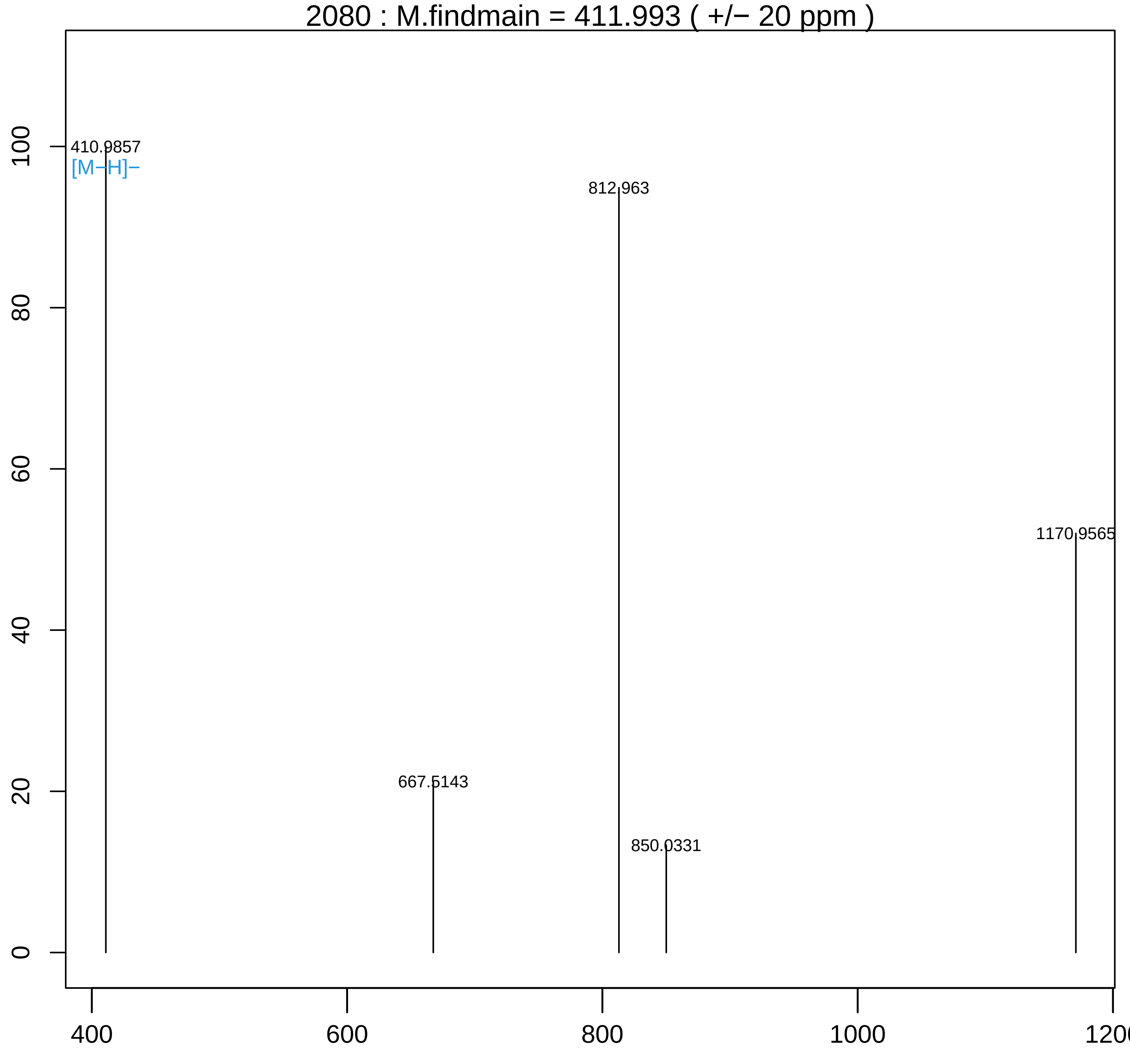
2080 : M.findmain = 411.993 (+/− 20 ppm)
410.9857
[M−H]−
812.963
667.5143
850.0331
1170.9565
0
20
40
60
80
100
400
600
800
1000

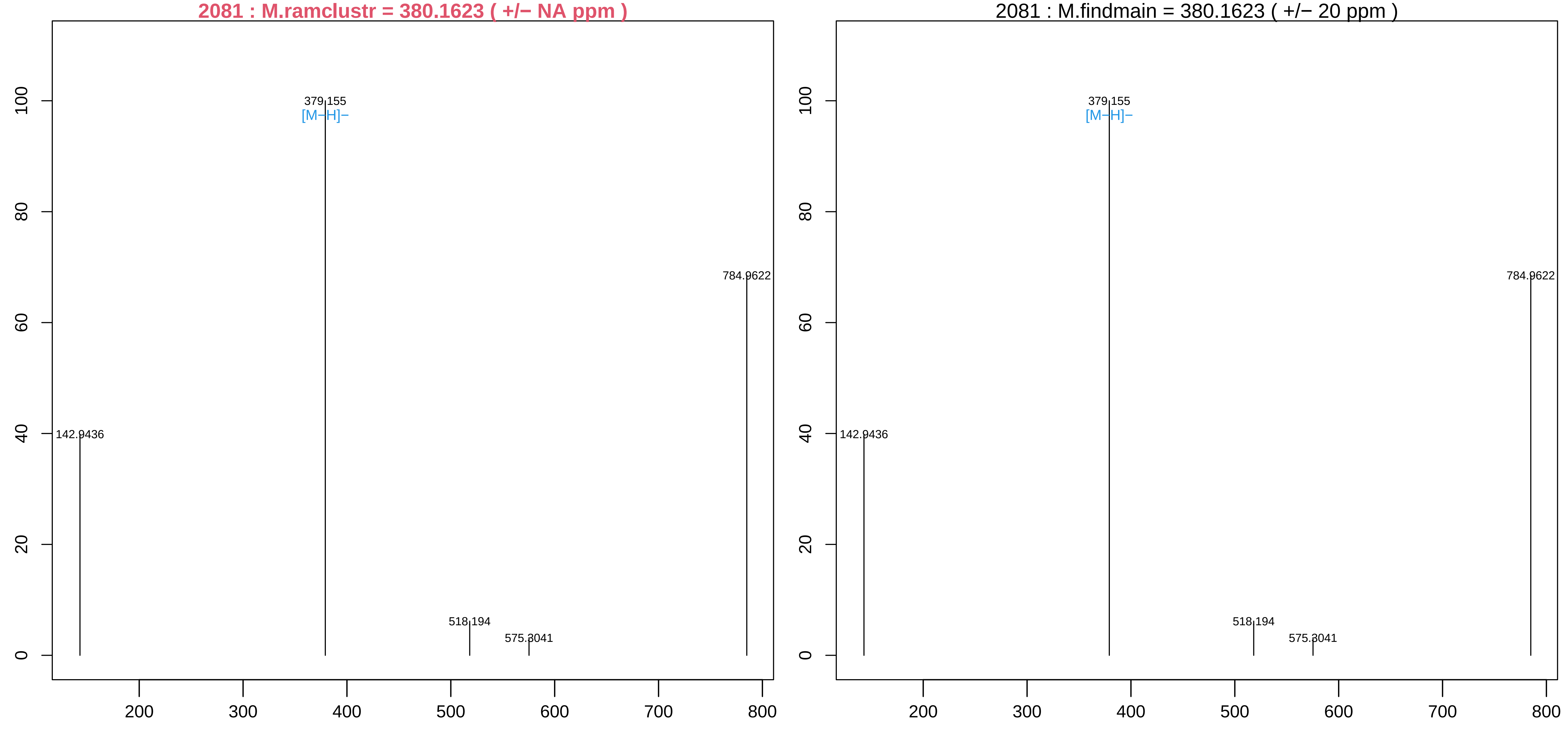

2081 : M.ramclustr = 380.1623 (+/− NA ppm)
379.155
[M−H]−
784.9622
142.9436
518.194
575.3041
0
20
40
60
80
100
200
300
400
500
600
700
800
2081 : M.findmain = 380.1623 (+/− 20 ppm)
379.155
[M−H]−
784.9622
142.9436
518.194
575.3041
0
20
40
60
80
100
200
300
400
500
600
700
800

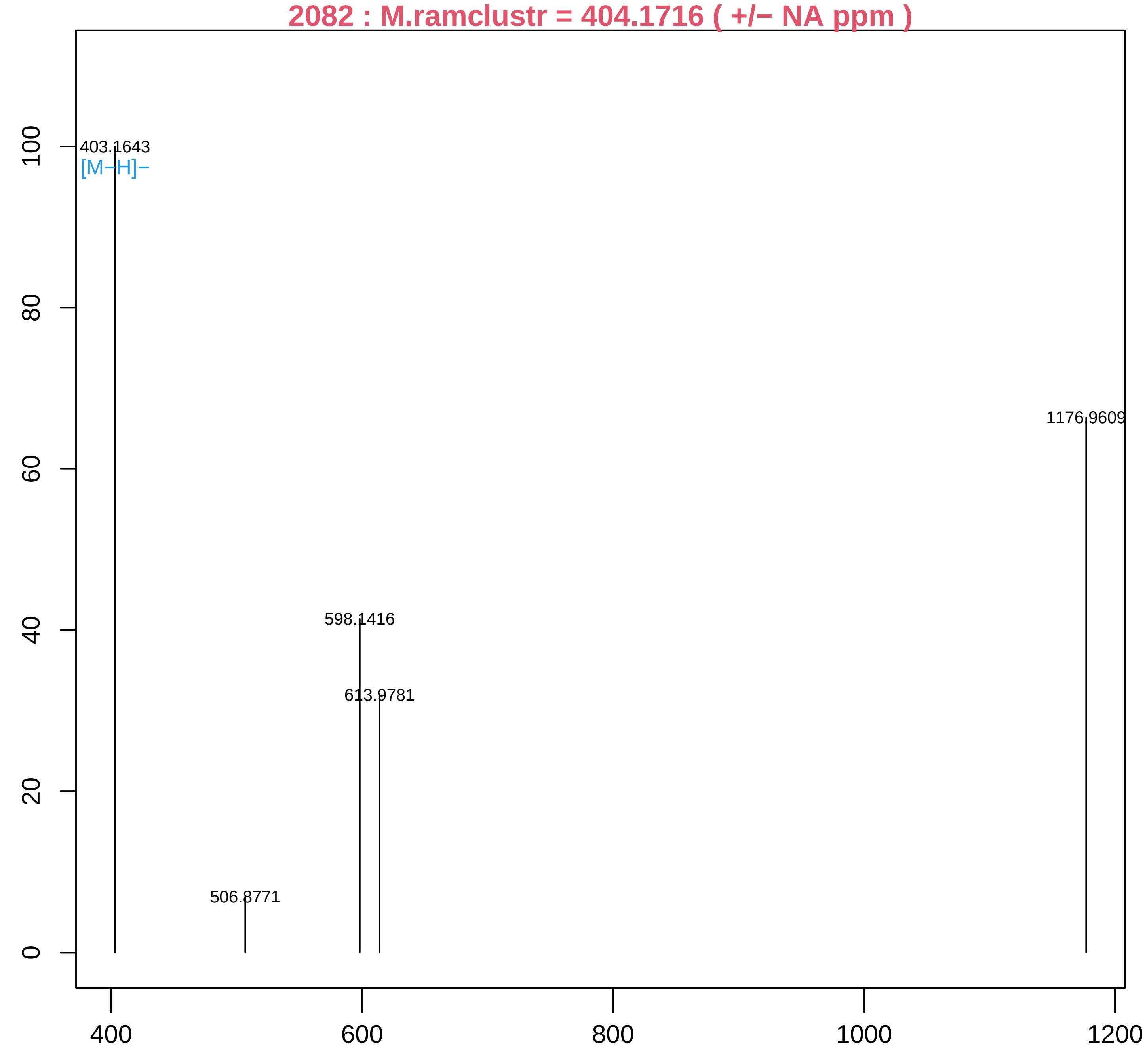
2082 : M.ramclustr = 404.1716 (+/− NA ppm)
403.1643
[M−H]−
1176.9609
598.1416
613.9781
506.8771
0
20
40
60
80
100
400
600
800
1000
1200

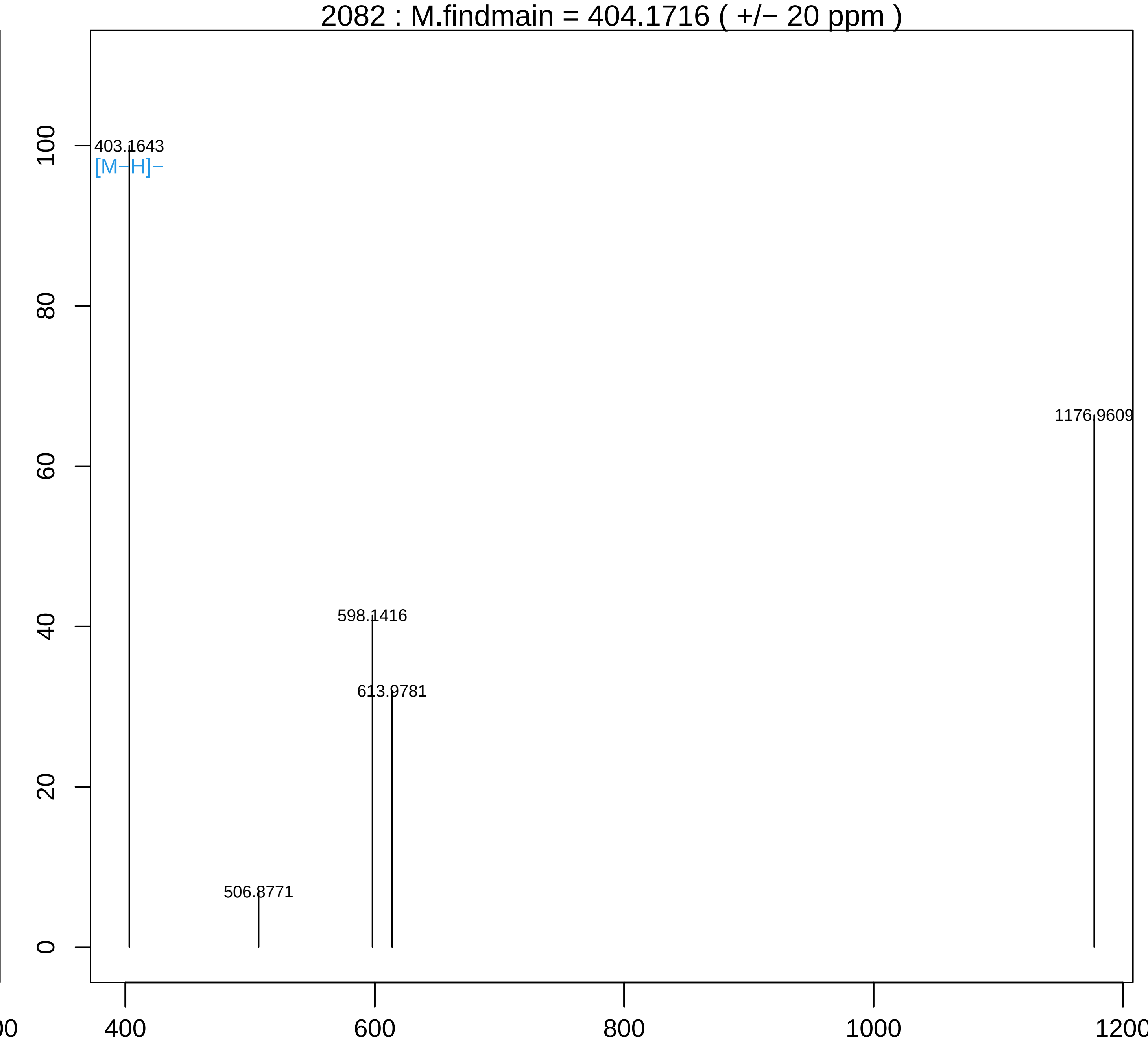
2082 : M.findmain = 404.1716 (+/− 20 ppm)
403.1643
[M−H]−
1176.9609
598.1416
613.9781
506.8771
0
20
40
60
80
100
400
600
800
1000
1200

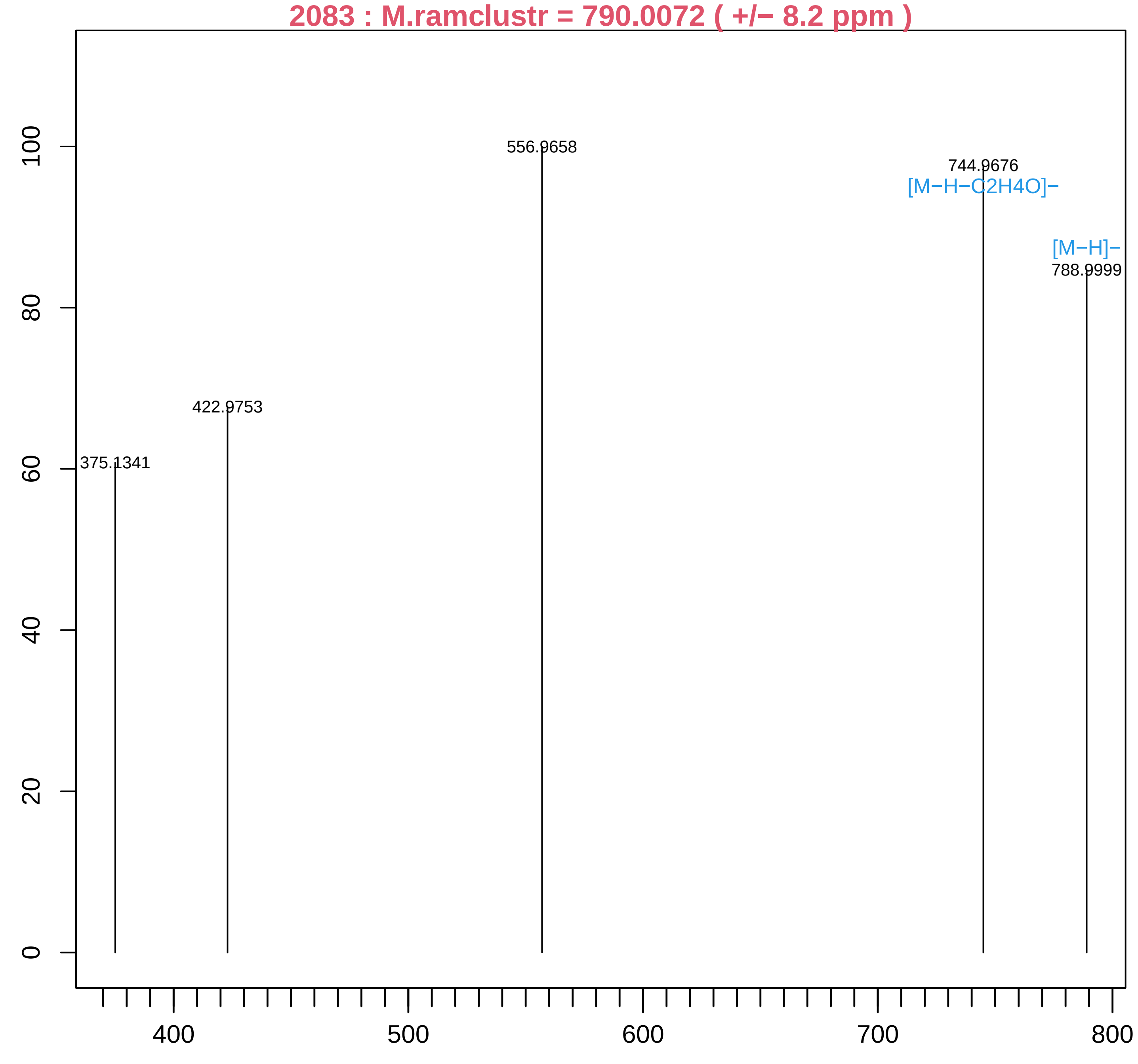
2083 : M.ramclustr = 790.0072 (+/− 8.2 ppm)
556.9658
744.9676
[M−H−C2H4O]−
[M−H]−
788.9999
422.9753
375.1341
0
20
40
60
80
100
400
500
600
700
800

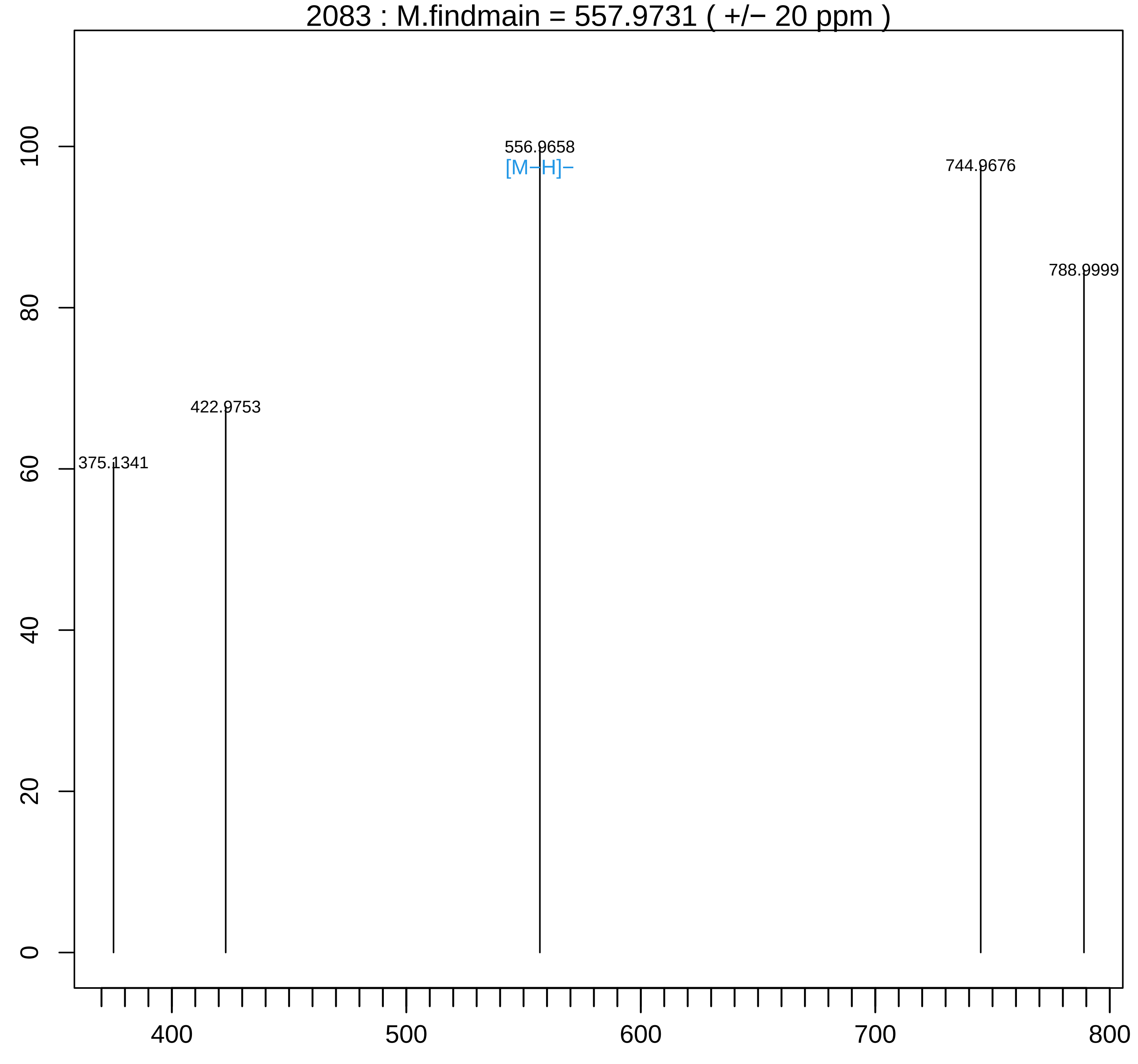
2083 : M.findmain = 557.9731 (+/− 20 ppm)
556.9658
[M−H]−
744.9676
788.9999
422.9753
375.1341
0
20
40
60
80
100
400
500
600
700
800

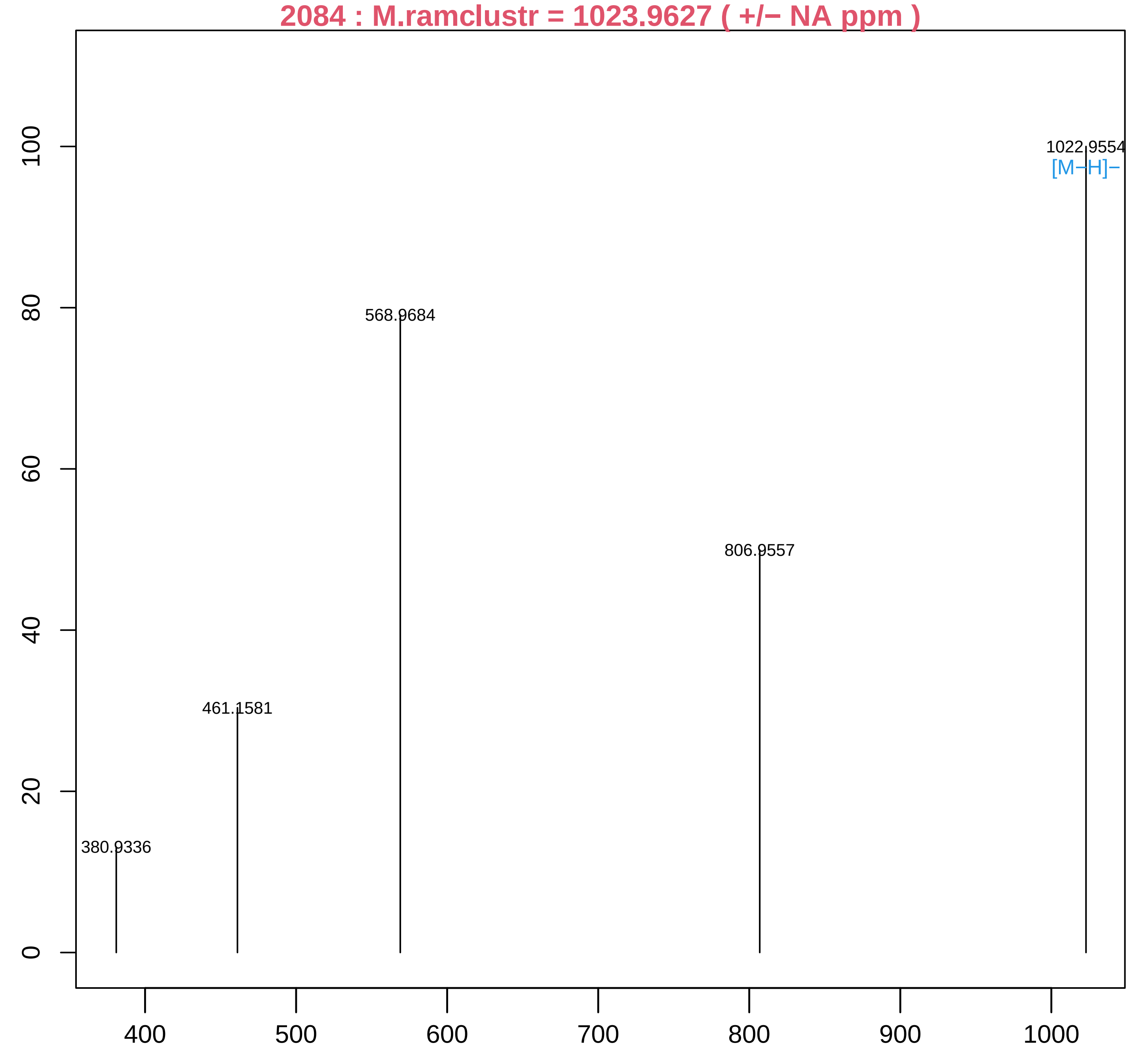
2084 : M.ramclustr = 1023.9627 (+/− NA ppm)
1022.9554
[M−H]−
568.9684
806.9557
461.1581
380.9336
0
20
40
60
80
100
400
500
600
700
800
900
1000

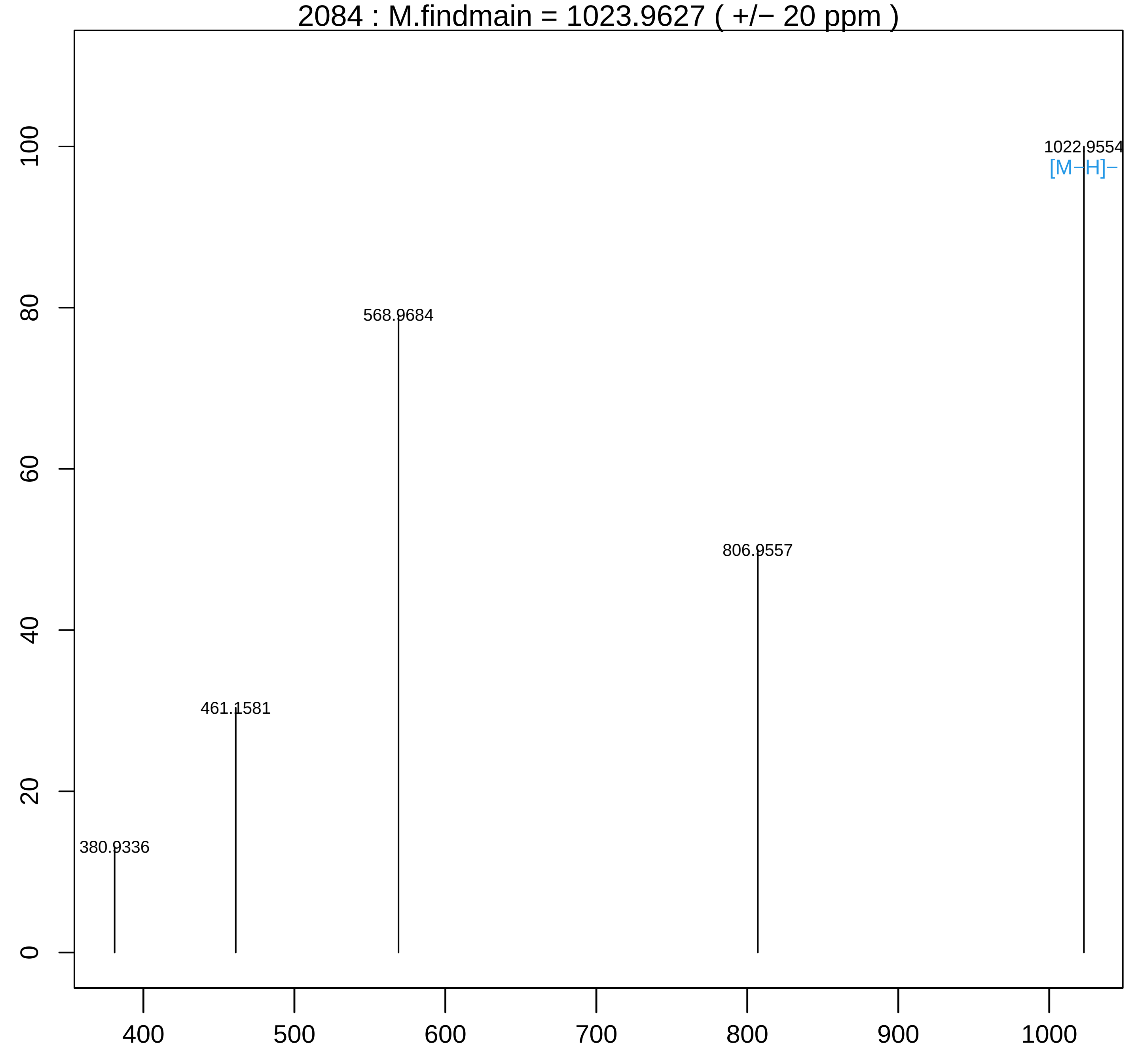
2084 : M.findmain = 1023.9627 (+/− 20 ppm)
1022.9554
[M−H]−
568.9684
806.9557
461.1581
380.9336
0
20
40
60
80
100
400
500
600
700
800
900
1000

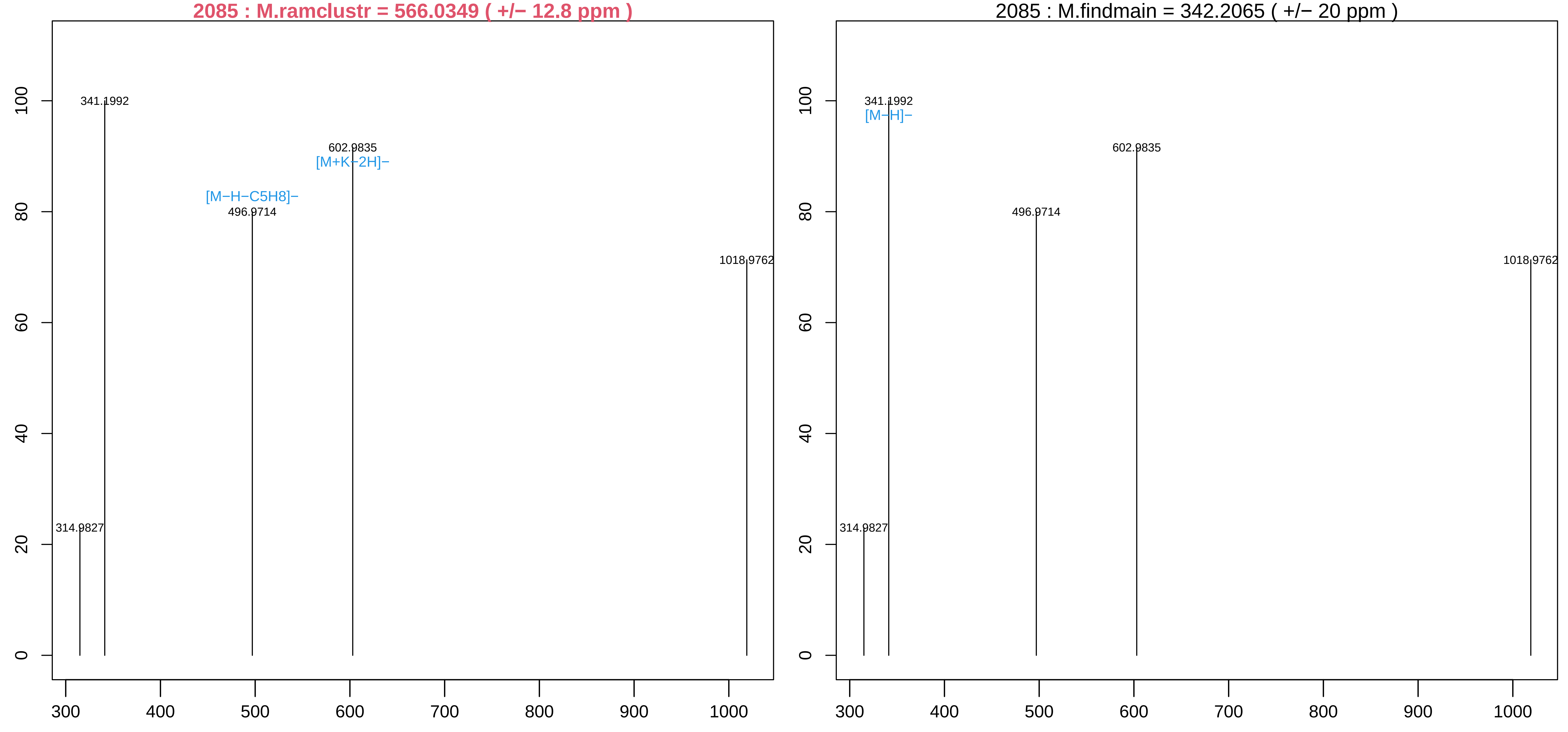
2085 : M.ramclustr = 566.0349 (+/− 12.8 ppm)
341.1992
602.9835
[M+K−2H]−
[M−H−C5H8]−
496.9714
1018.9762
314.9827
2085 : M.findmain = 342.2065 (+/− 20 ppm)
341.1992
[M−H]−
602.9835
496.9714
1018.9762
314.9827
0
20
40
60
80
100
300
400
500
600
700
800
900
1000

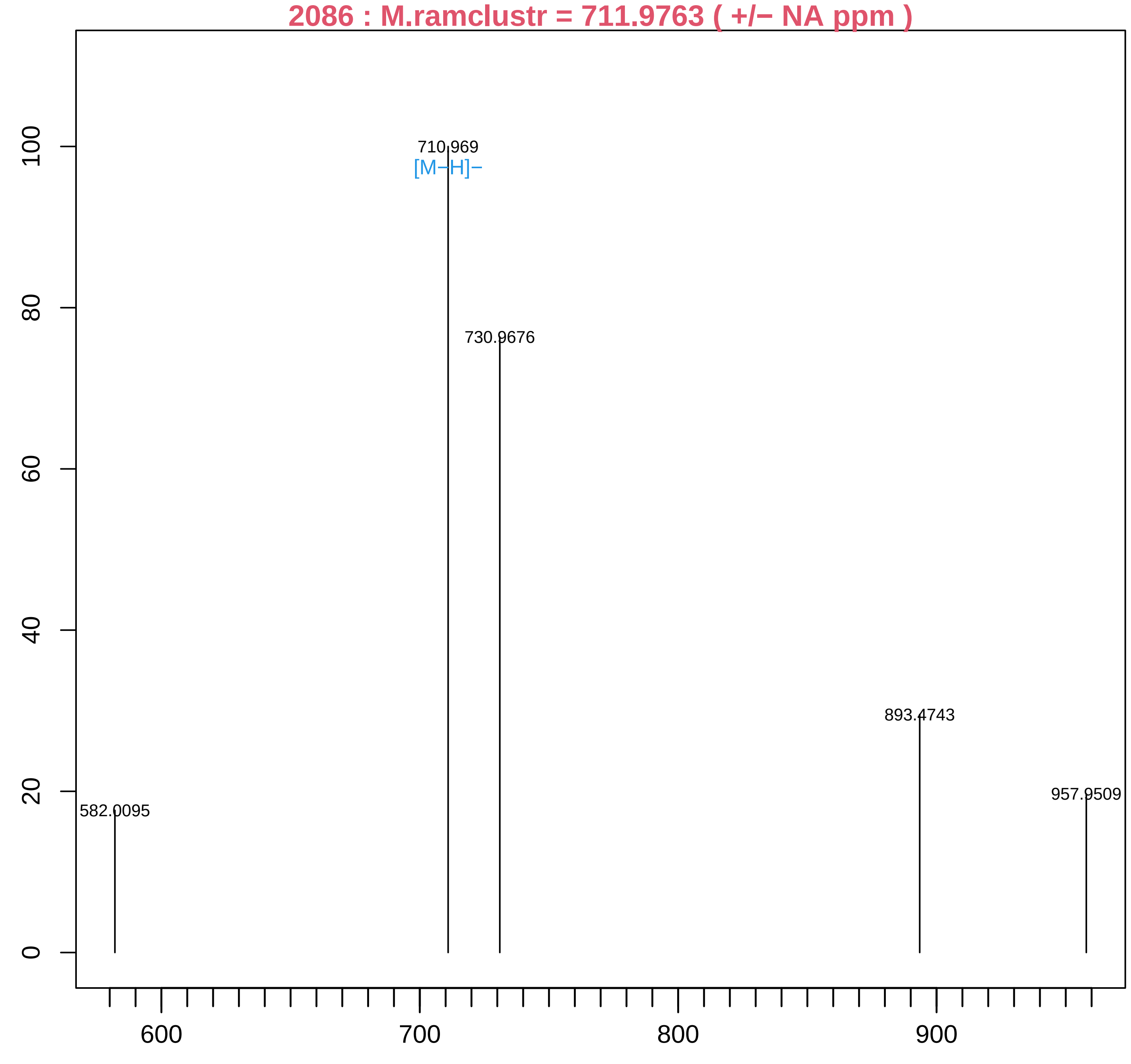
2086 : M.ramclustr = 711.9763 (+/− NA ppm)
710.969
[M−H]−
730.9676
893.4743
957.9509
582.0095
0
20
40
60
80
100
600
700
800
900

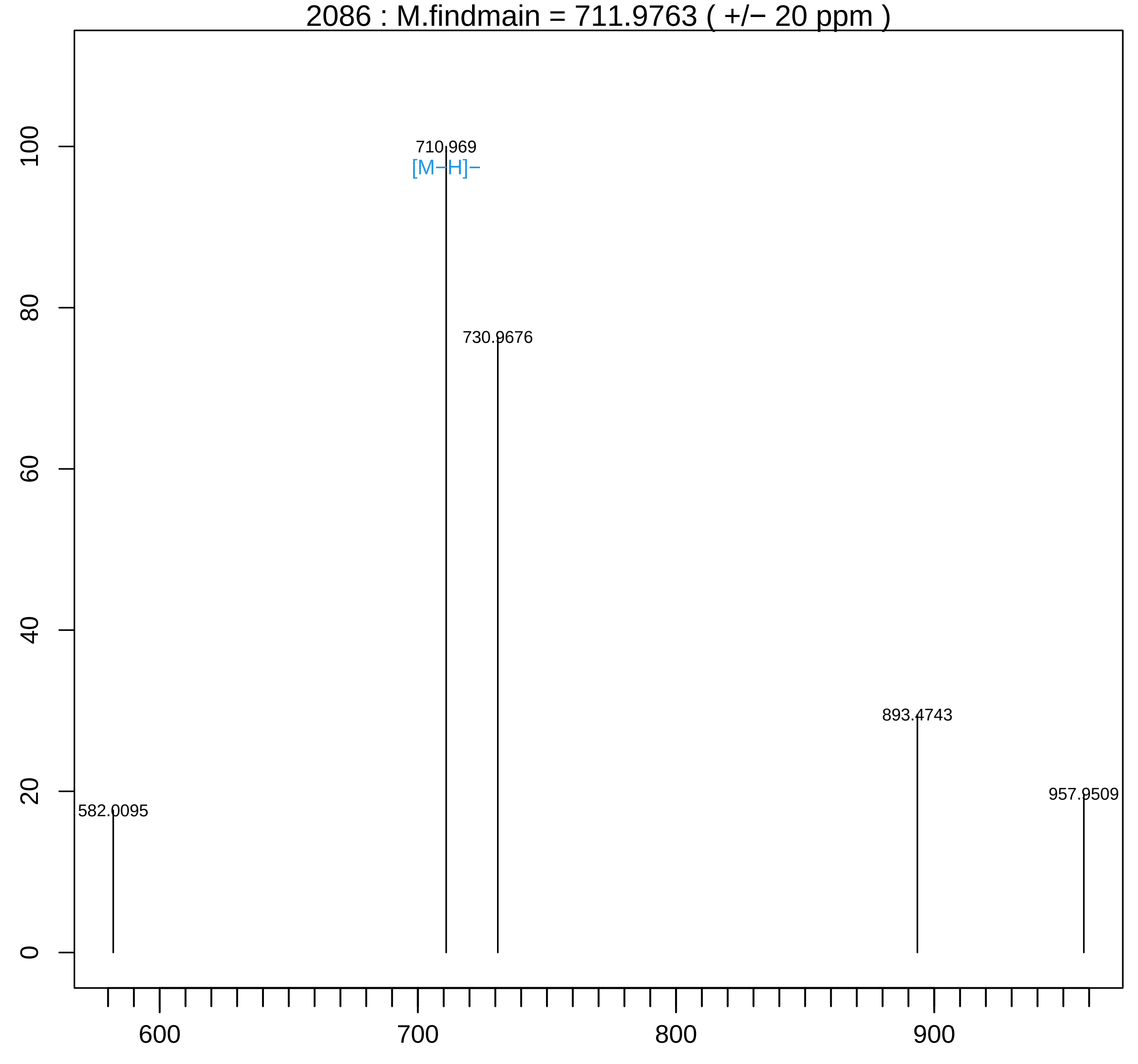
2086 : M.findmain = 711.9763 (+/− 20 ppm)
710.969
[M−H]−
730.9676
893.4743
957.9509
582.0095
0
20
40
60
80
100
600
700
800
900

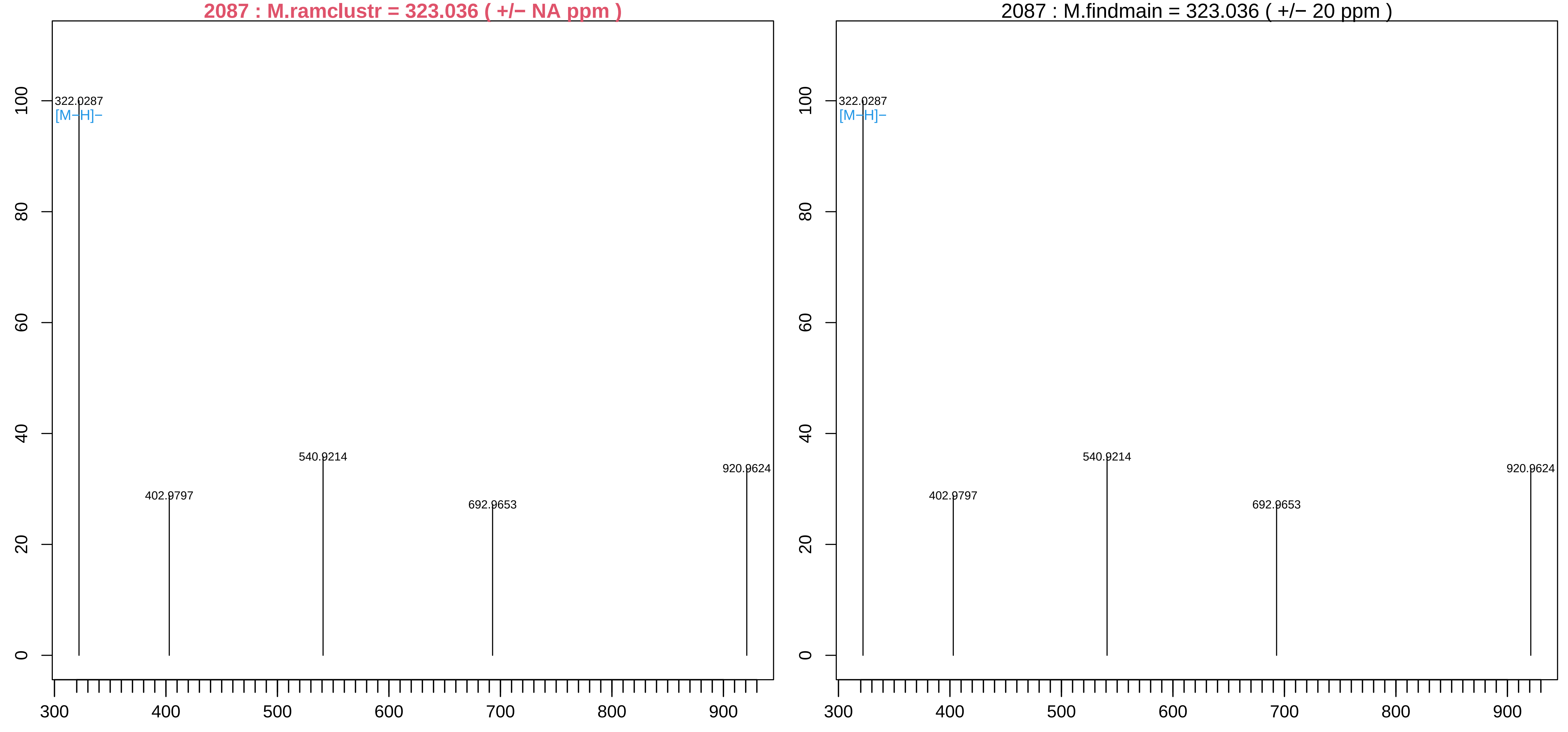
2087 : M.ramclustr = 323.036 (+/− NA ppm)
322.0287
[M−H]−
402.9797
540.9214
692.9653
920.9624
0
20
40
60
80
100
300
400
500
600
700
800
900
2087 : M.findmain = 323.036 (+/− 20 ppm)
322.0287
[M−H]−
402.9797
540.9214
692.9653
920.9624
0
20
40
60
80
100
300
400
500
600
700
800
900

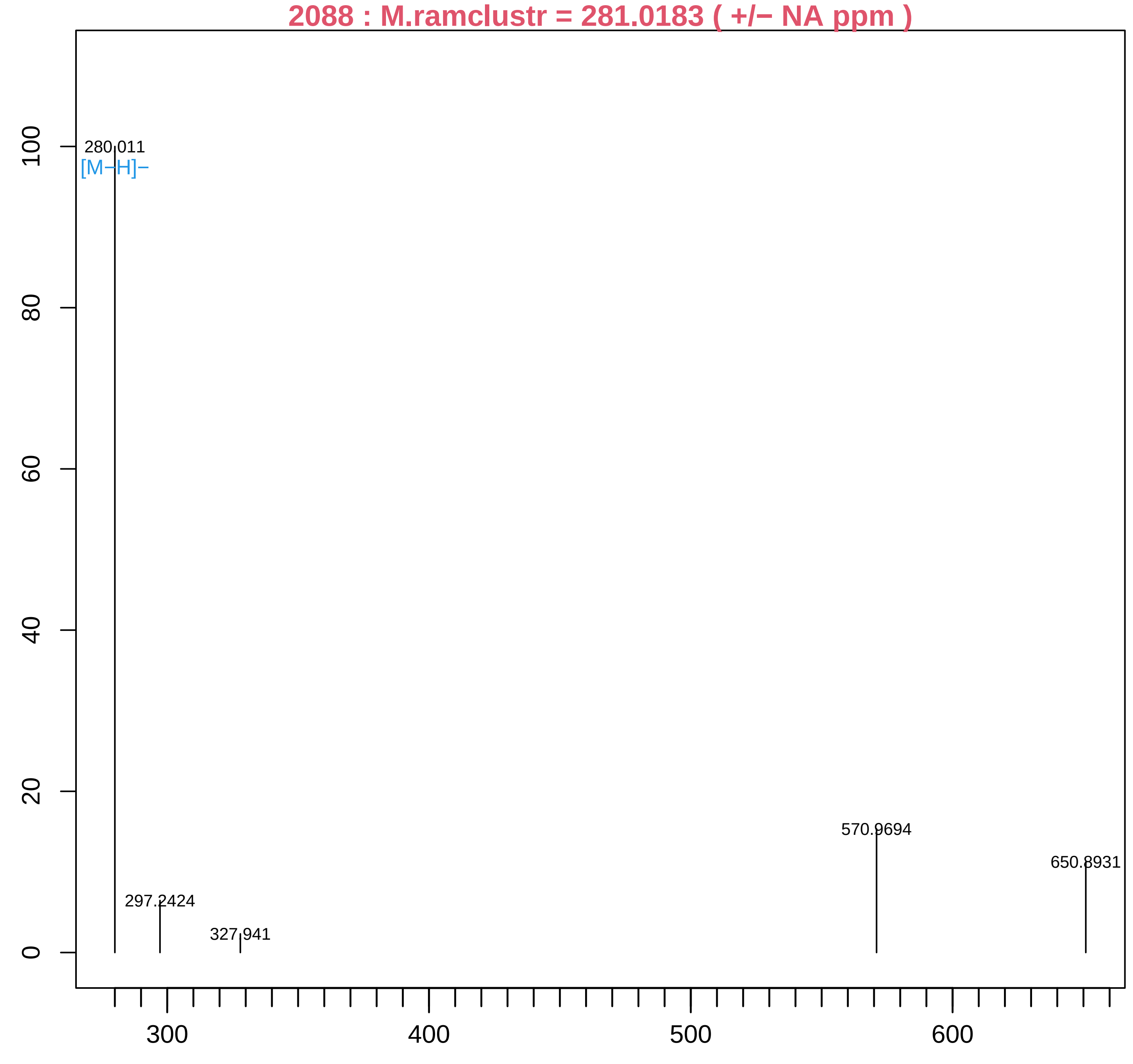
2088 : M.ramclustr = 281.0183 (+/− NA ppm)
280.011
[M−H]−
297.2424
327.941
570.9694
650.8931
0
20
40
60
80
100
300
400
500
600
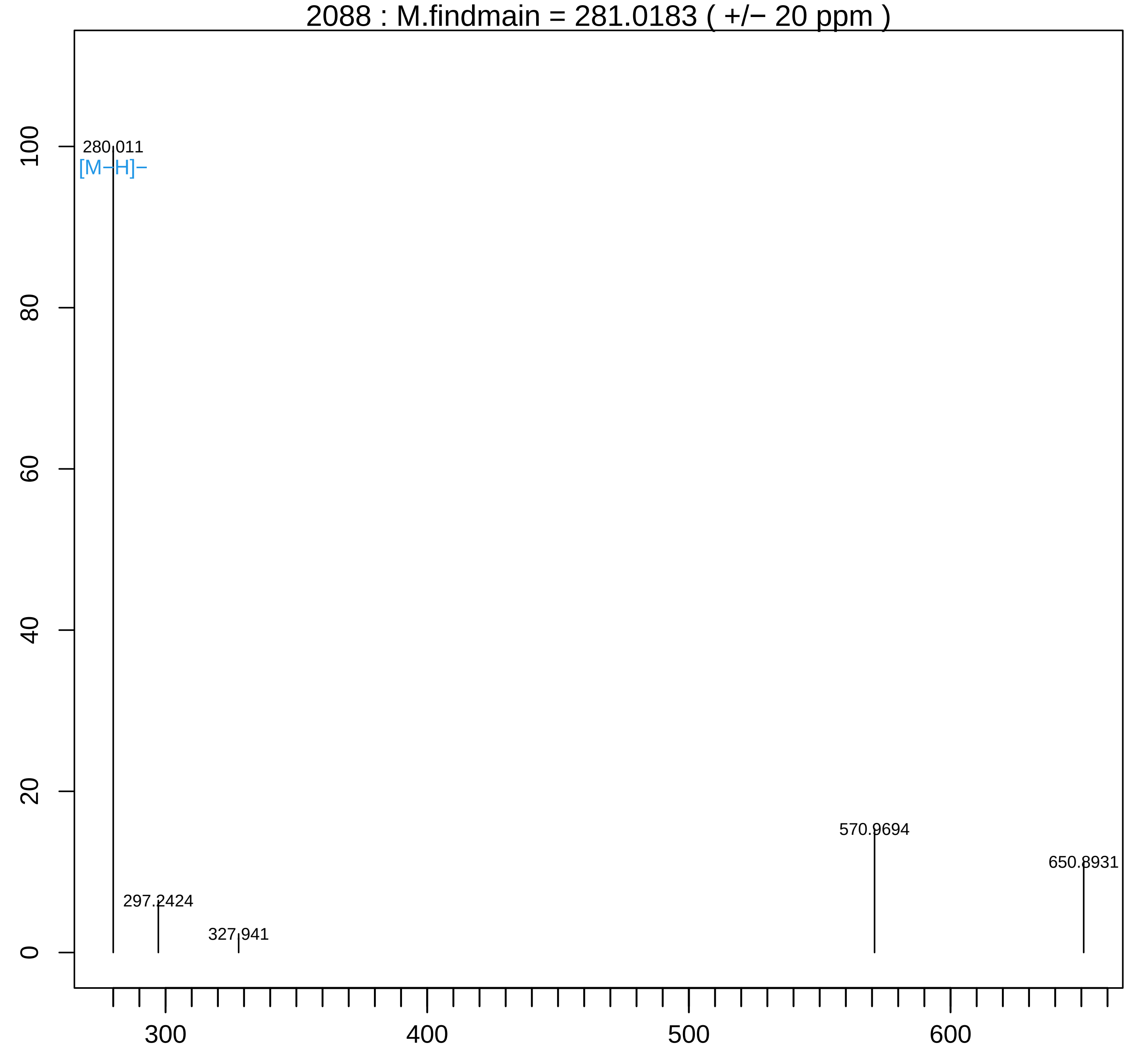
2088 : M.findmain = 281.0183 (+/− 20 ppm)
280.011
[M−H]−
297.2424
327.941
570.9694
650.8931
0
20
40
60
80
100
300
400
500
600

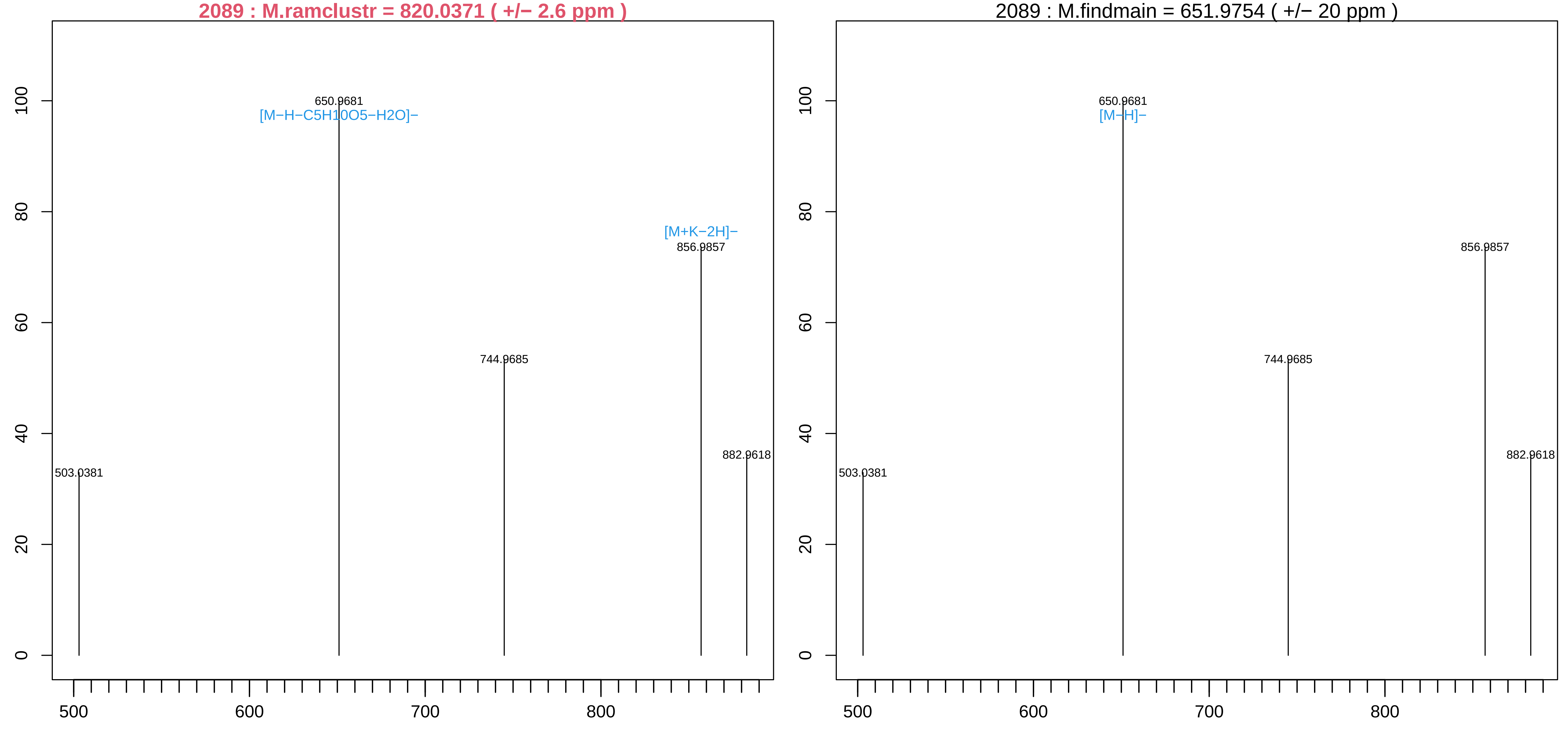

2089 : M.ramclustr = 820.0371 (+/− 2.6 ppm)
650.9681
[M−H−C5H10O5−H2O]−
[M+K−2H]−
856.9857
744.9685
882.9618
503.0381
2089 : M.findmain = 651.9754 (+/− 20 ppm)
650.9681
[M−H]−
856.9857
744.9685
882.9618
503.0381
0
20
40
60
80
100
500
600
700
800

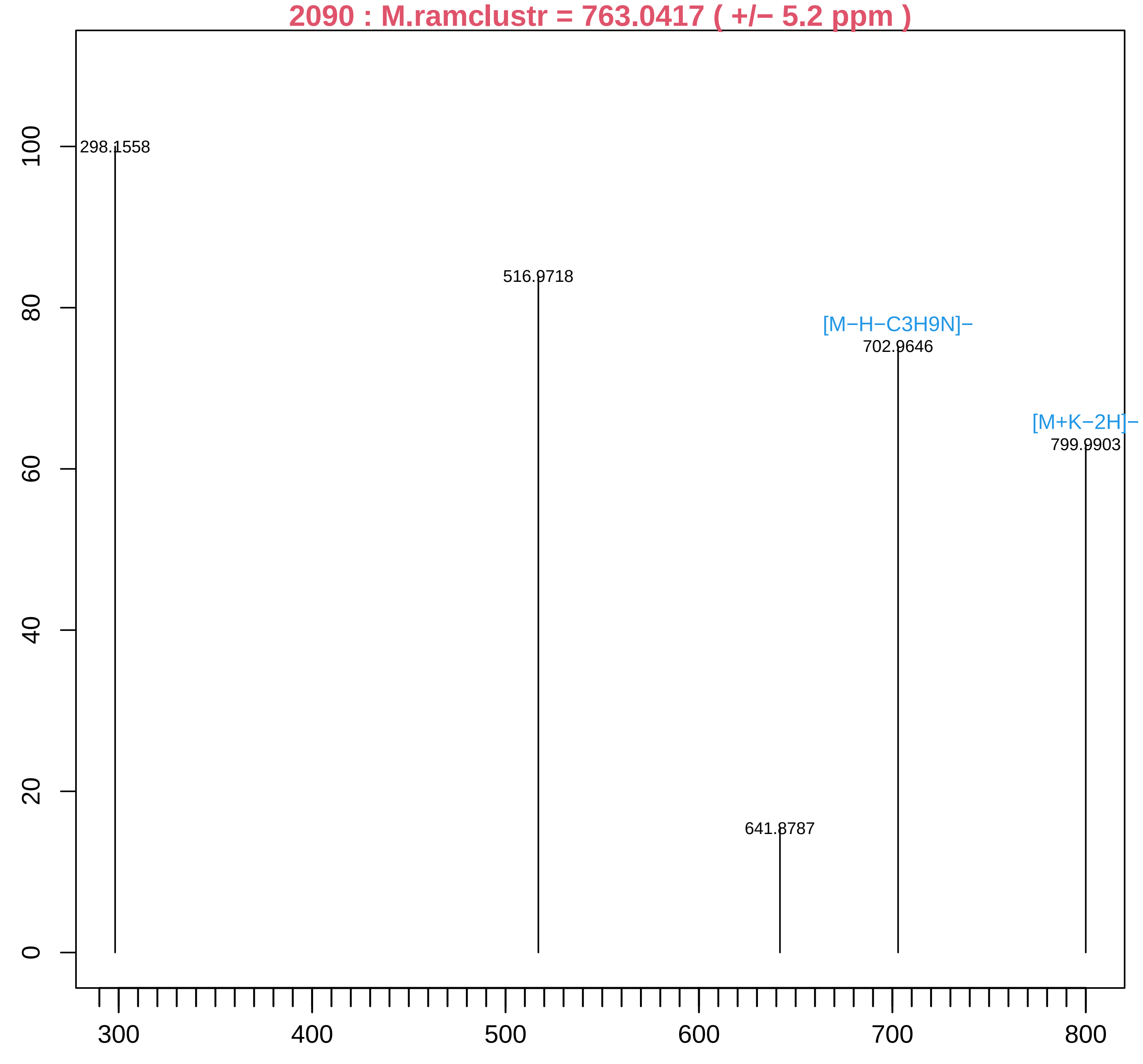
2090 : M.ramclustr = 763.0417 (+/− 5.2 ppm)
298.1558
516.9718
[M−H−C3H9N]−
702.9646
[M+K−2H]−
799.9903
641.8787
0
20
40
60
80
100
300
400
500
600
700
800
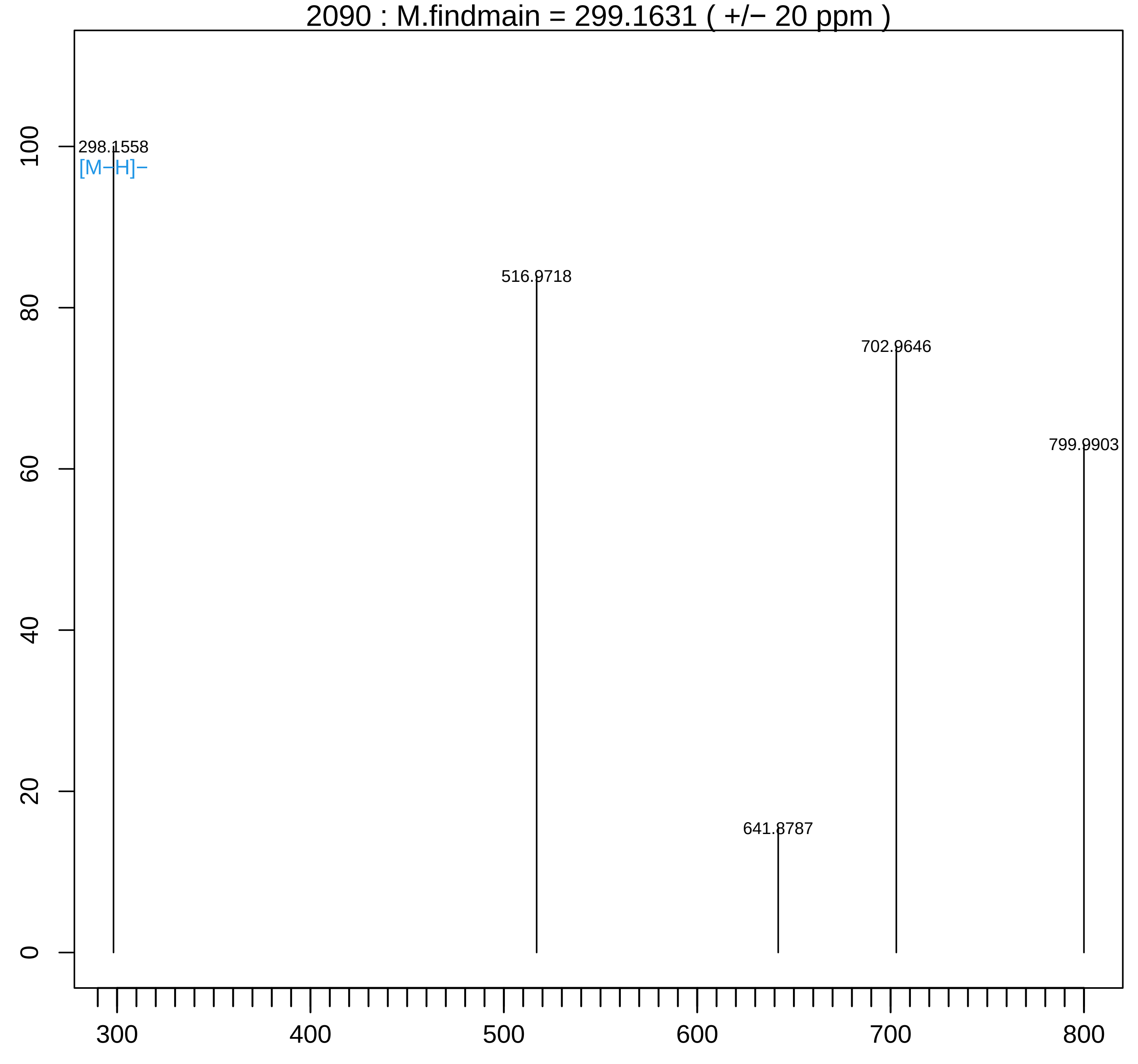
2090 : M.findmain = 299.1631 (+/− 20 ppm)
298.1558
[M−H]−
516.9718
702.9646
799.9903
641.8787
0
20
40
60
80
100
300
400
500
600
700
800

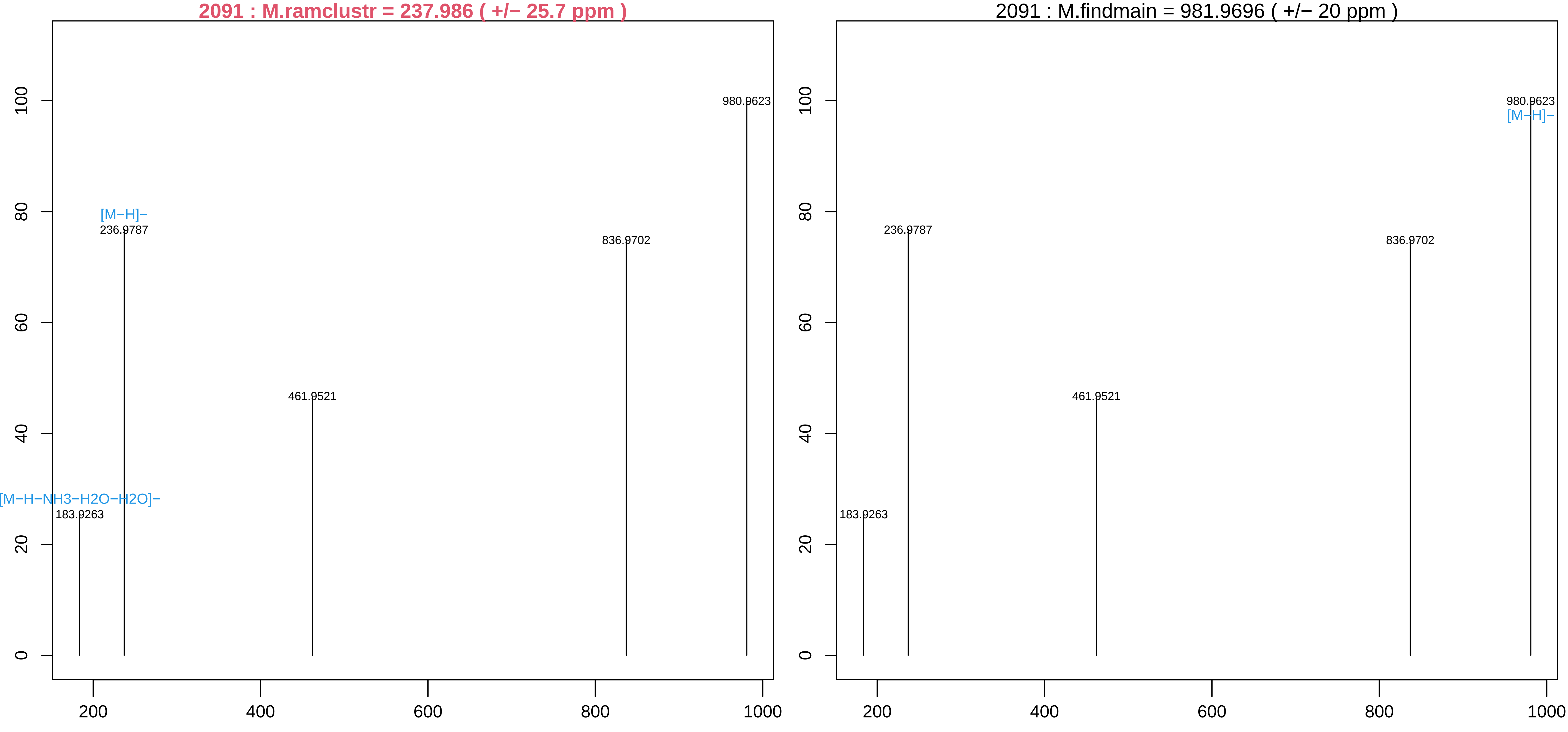
2091 : M.ramclustr = 237.986 (+/− 25.7 ppm)
[M−H]−
236.9787
[M−H−NH3−H2O−H2O]−
183.9263
461.9521
836.9702
980.9623
2091 : M.findmain = 981.9696 (+/− 20 ppm)
980.9623
[M−H]−
236.9787
183.9263
461.9521
836.9702
0
20
40
60
80
100
200
400
600
800
1000

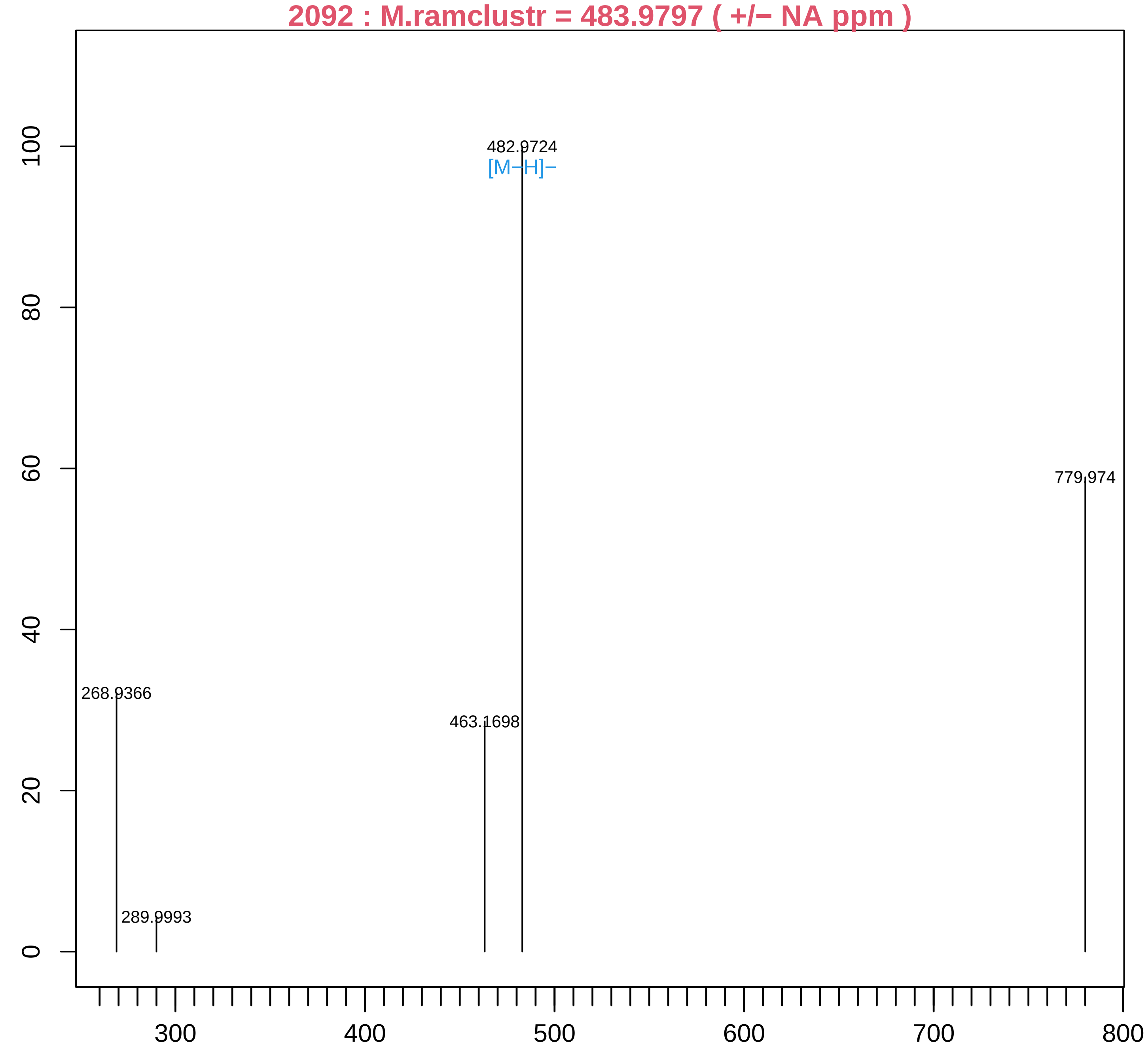
2092 : M.ramclustr = 483.9797 (+/− NA ppm)
482.9724
[M−H]−
779.974
268.9366
463.1698
289.9993
0
20
40
60
80
100
300
400
500
600
700
800

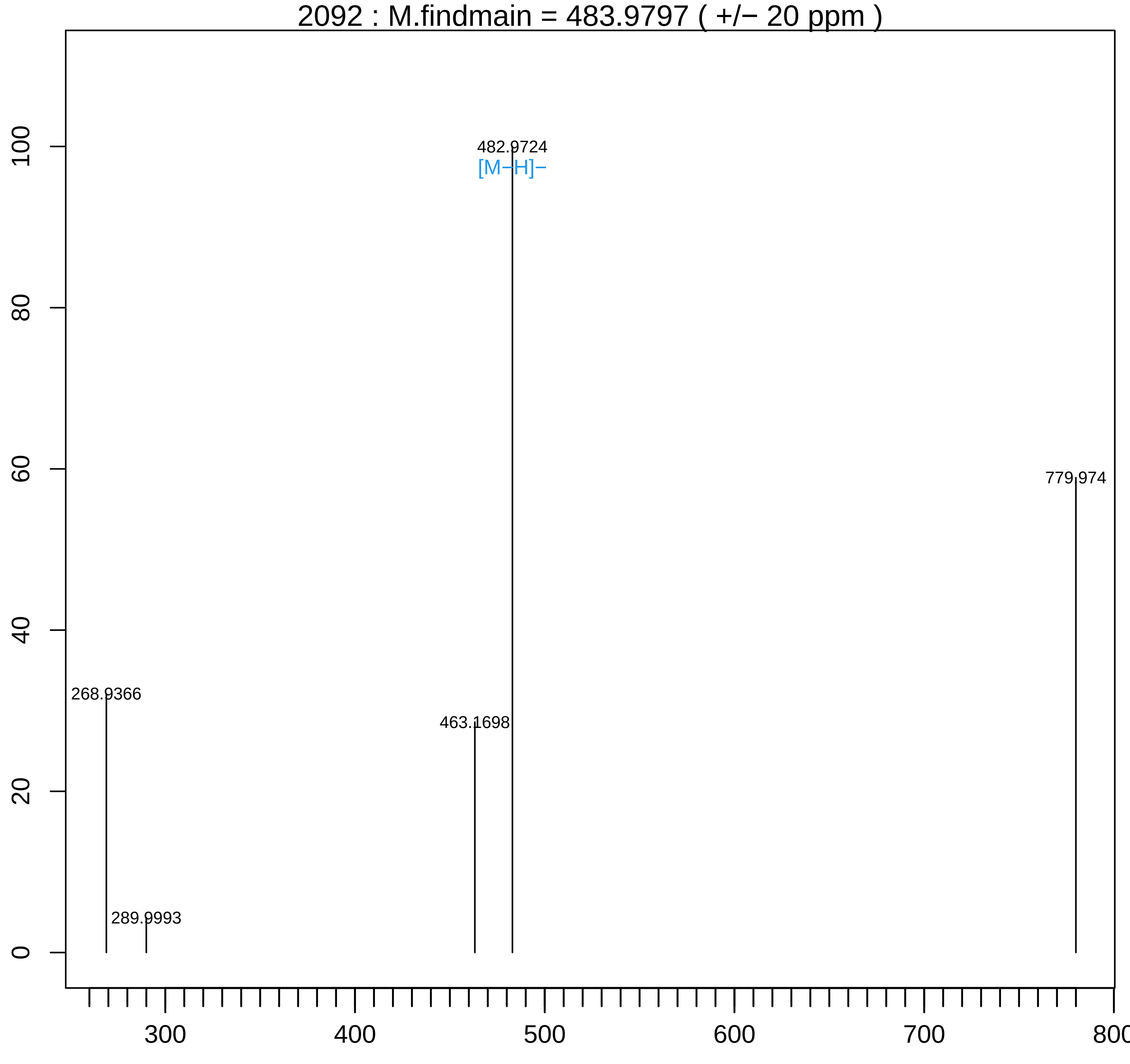
2092 : M.findmain = 483.9797 (+/− 20 ppm)
482.9724
[M−H]−
779.974
268.9366
463.1698
289.9993
0
20
40
60
80
100
300
400
500
600
700
800

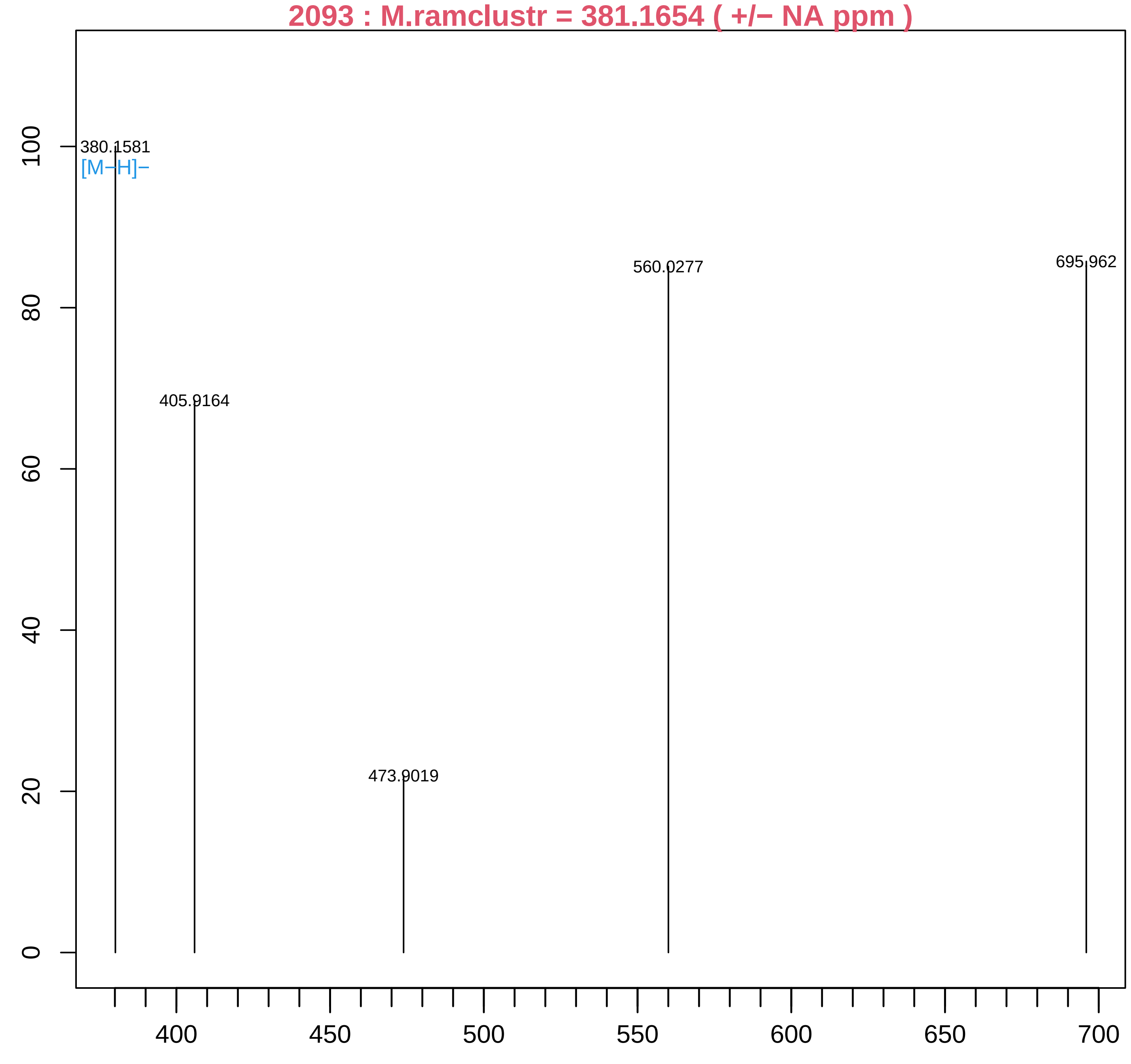
2093 : M.ramclustr = 381.1654 (+/− NA ppm)
380.1581
[M−H]−
405.9164
473.9019
560.0277
695.962
0
20
40
60
80
100
400
450
500
550
600
650
700

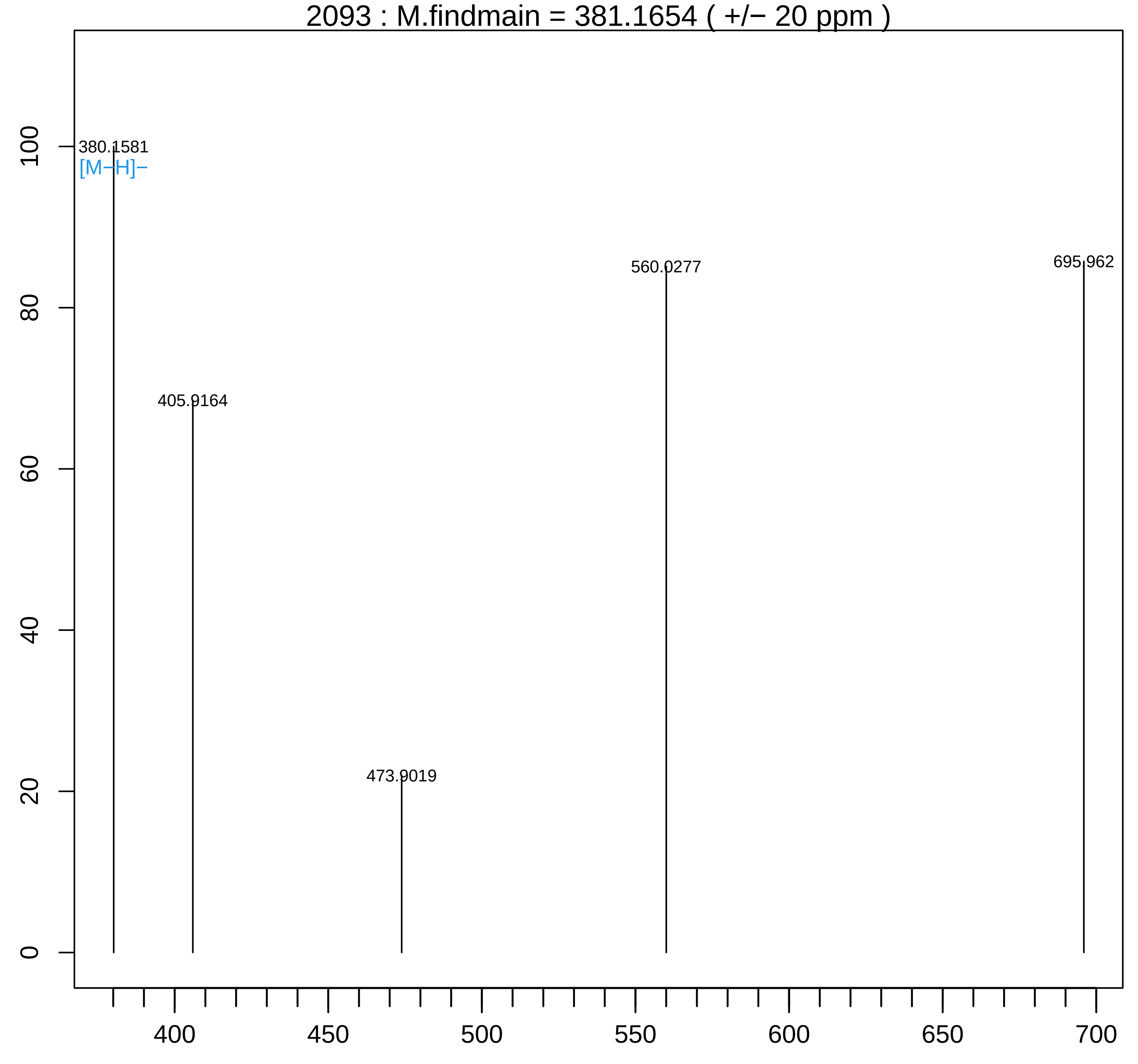
2093 : M.findmain = 381.1654 (+/− 20 ppm)
380.1581
[M−H]−
405.9164
473.9019
560.0277
695.962
0
20
40
60
80
100
400
450
500
550
600
650
700

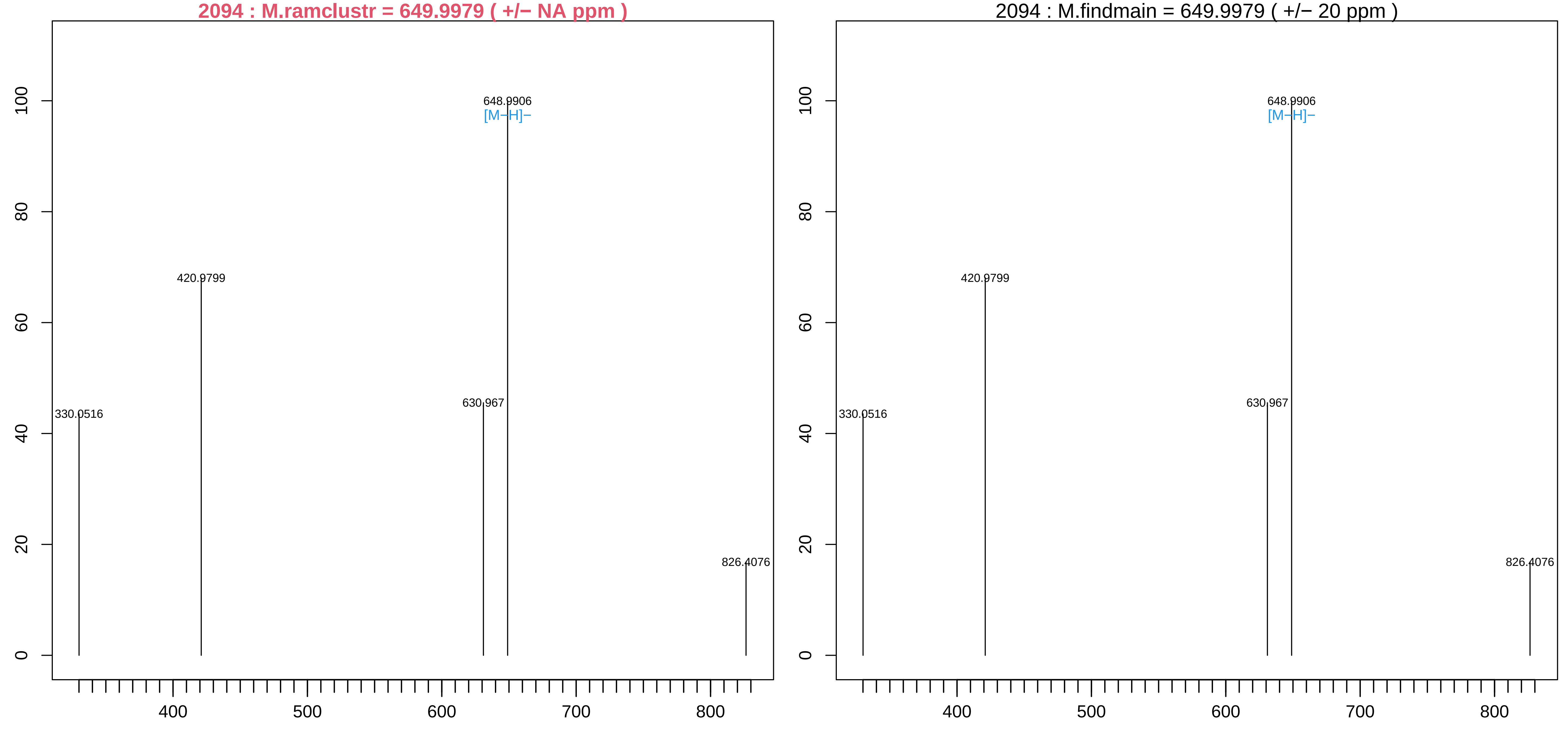

2094 : M.ramclustr = 649.9979 (+/− NA ppm)
648.9906
[M−H]−
420.9799
630.967
330.0516
826.4076
2094 : M.findmain = 649.9979 (+/− 20 ppm)
648.9906
[M−H]−
420.9799
630.967
330.0516
826.4076

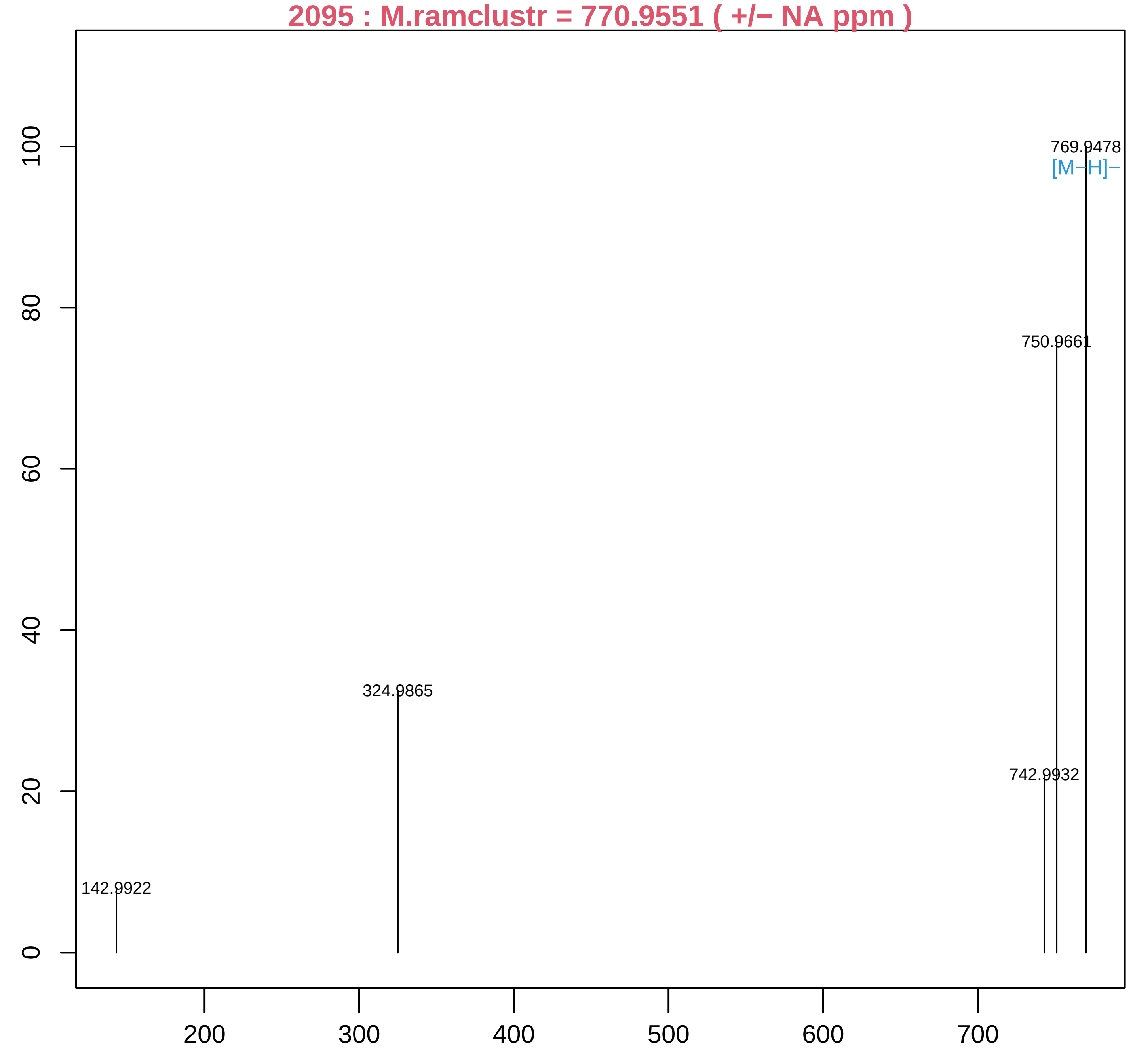
2095 : M.ramclustr = 770.9551 (+/− NA ppm)
769.9478
[M−H]−
750.9661
742.9932
324.9865
142.9922
0
20
40
60
80
100
200
300
400
500
600
700

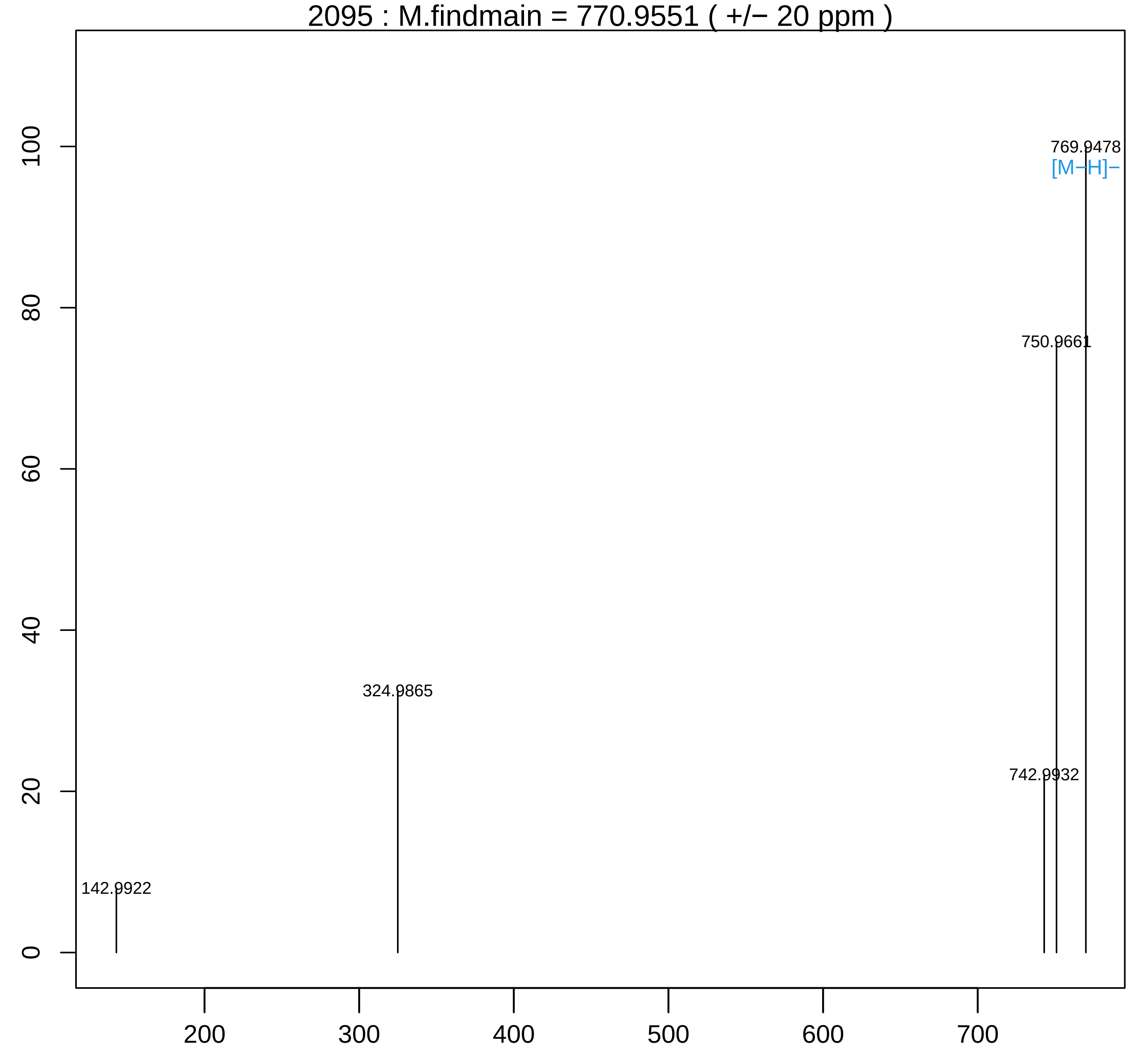
2095 : M.findmain = 770.9551 (+/− 20 ppm)
769.9478
[M−H]−
750.9661
742.9932
324.9865
142.9922
0
20
40
60
80
100
200
300
400
500
600
700

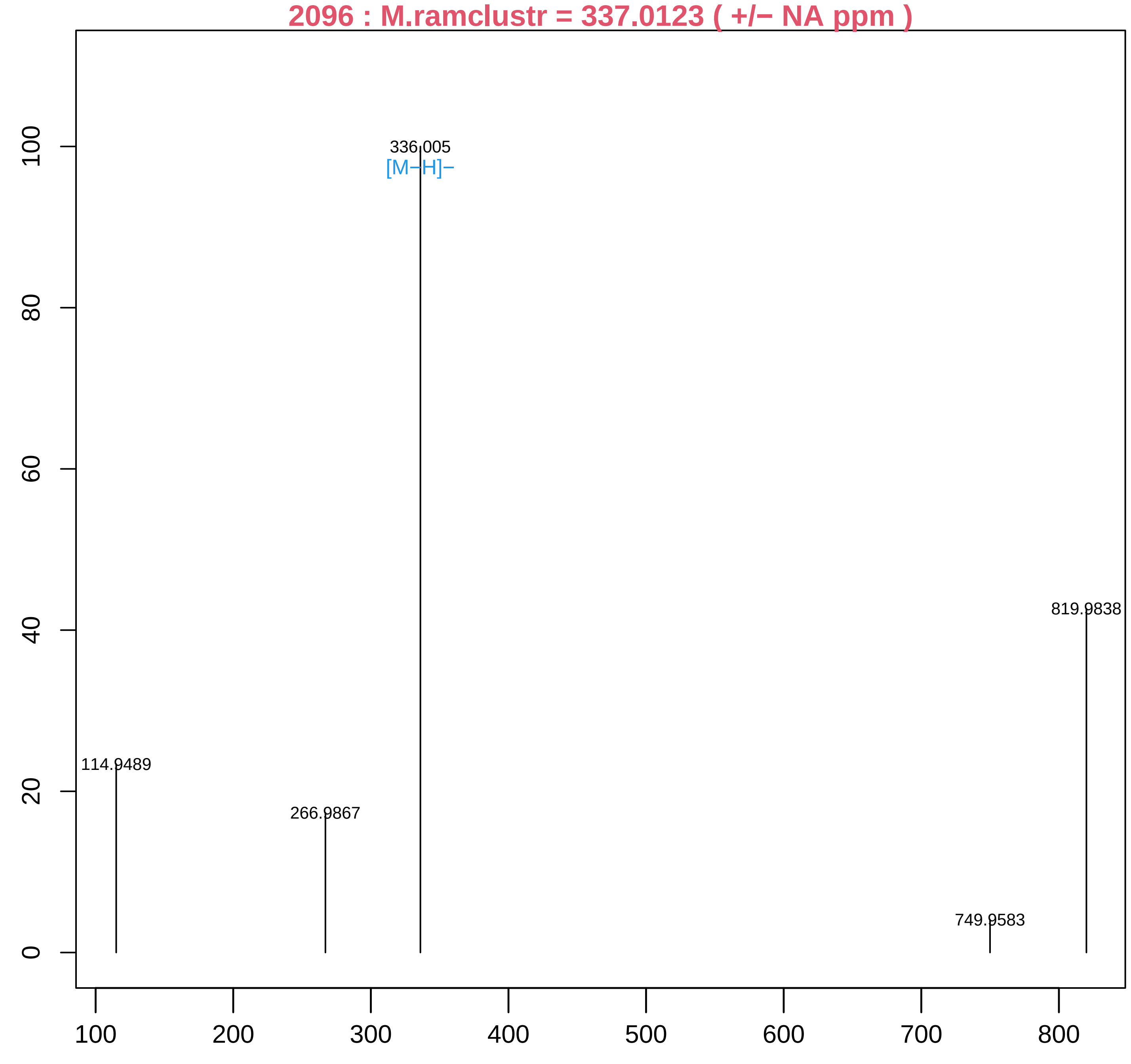
2096 : M.ramclustr = 337.0123 (+/− NA ppm)
336.005
[M−H]−
819.9838
114.9489
266.9867
749.9583
0
20
40
60
80
100
100
200
300
400
500
600
700
800

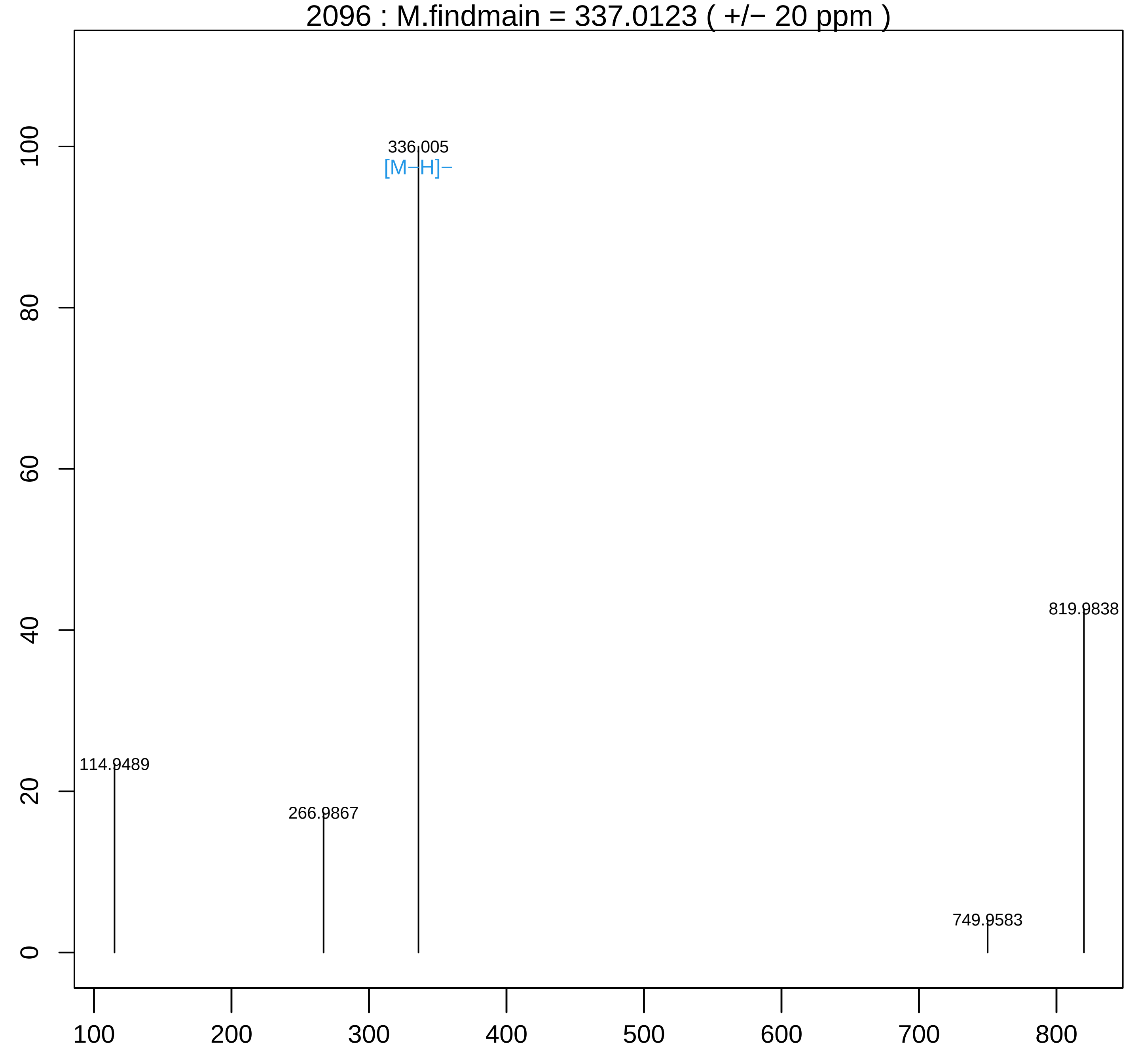
2096 : M.findmain = 337.0123 (+/− 20 ppm)
336.005
[M−H]−
819.9838
114.9489
266.9867
749.9583
0
20
40
60
80
100
100
200
300
400
500
600
700
800

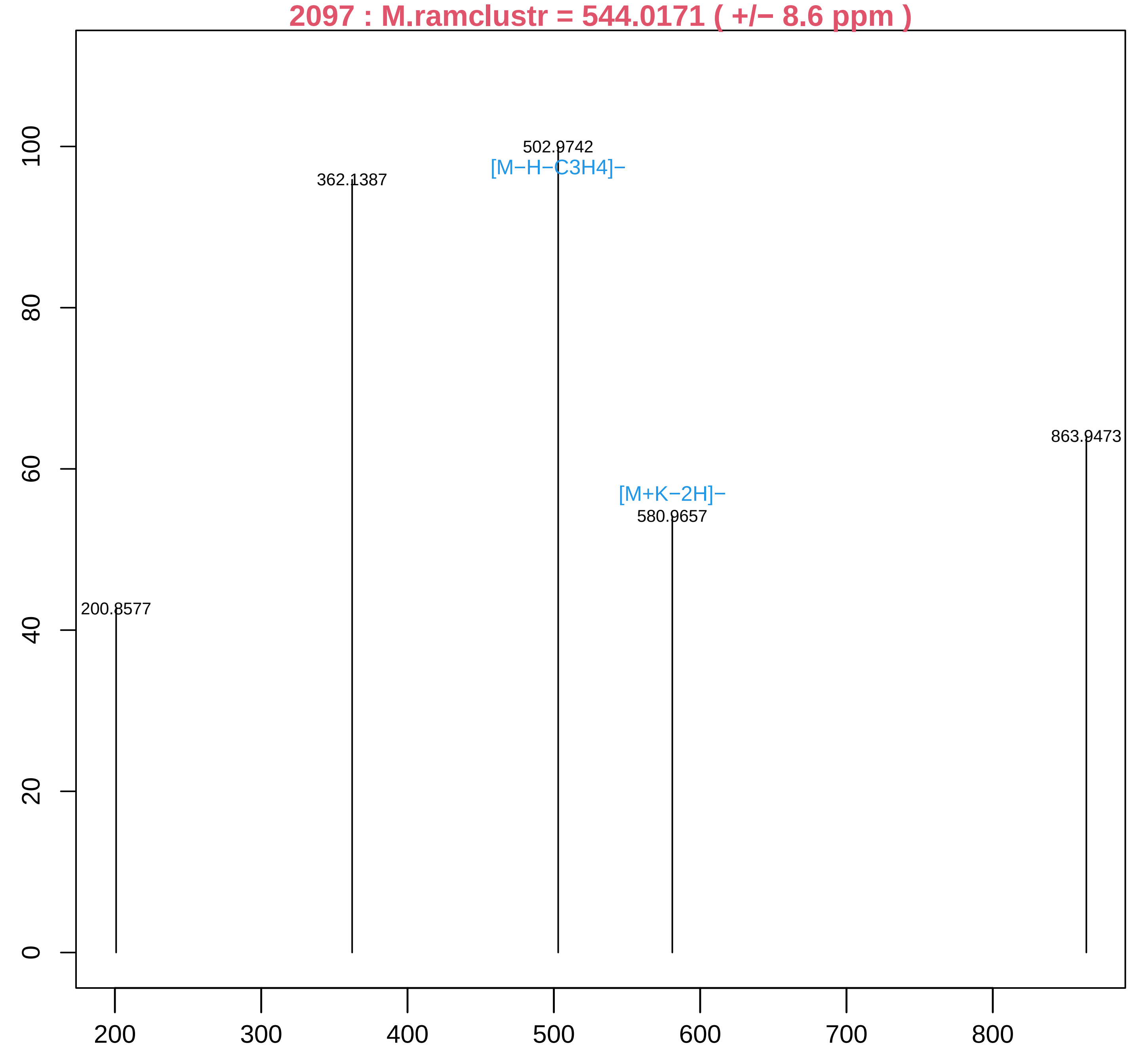

2097 : M.ramclustr = 544.0171 (+/− 8.6 ppm)
502.9742
[M−H−C3H4]−
362.1387
863.9473
[M+K−2H]−
580.9657
200.8577
0
20
40
60
80
100
200
300
400
500
600
700
800

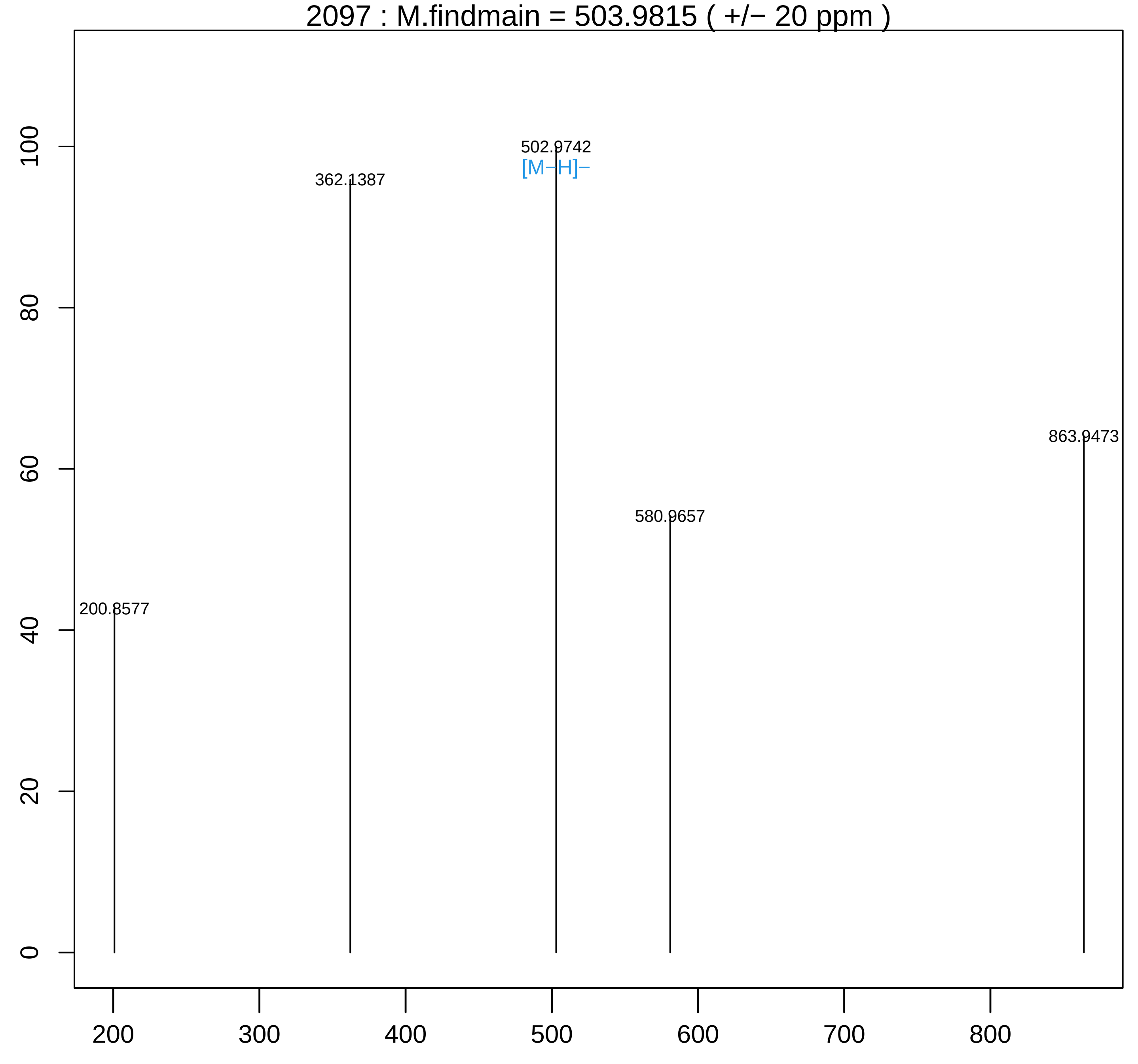

2097 : M.findmain = 503.9815 (+/− 20 ppm)
502.9742
[M−H]−
362.1387
863.9473
580.9657
200.8577
0
20
40
60
80
100
200
300
400
500
600
700
800

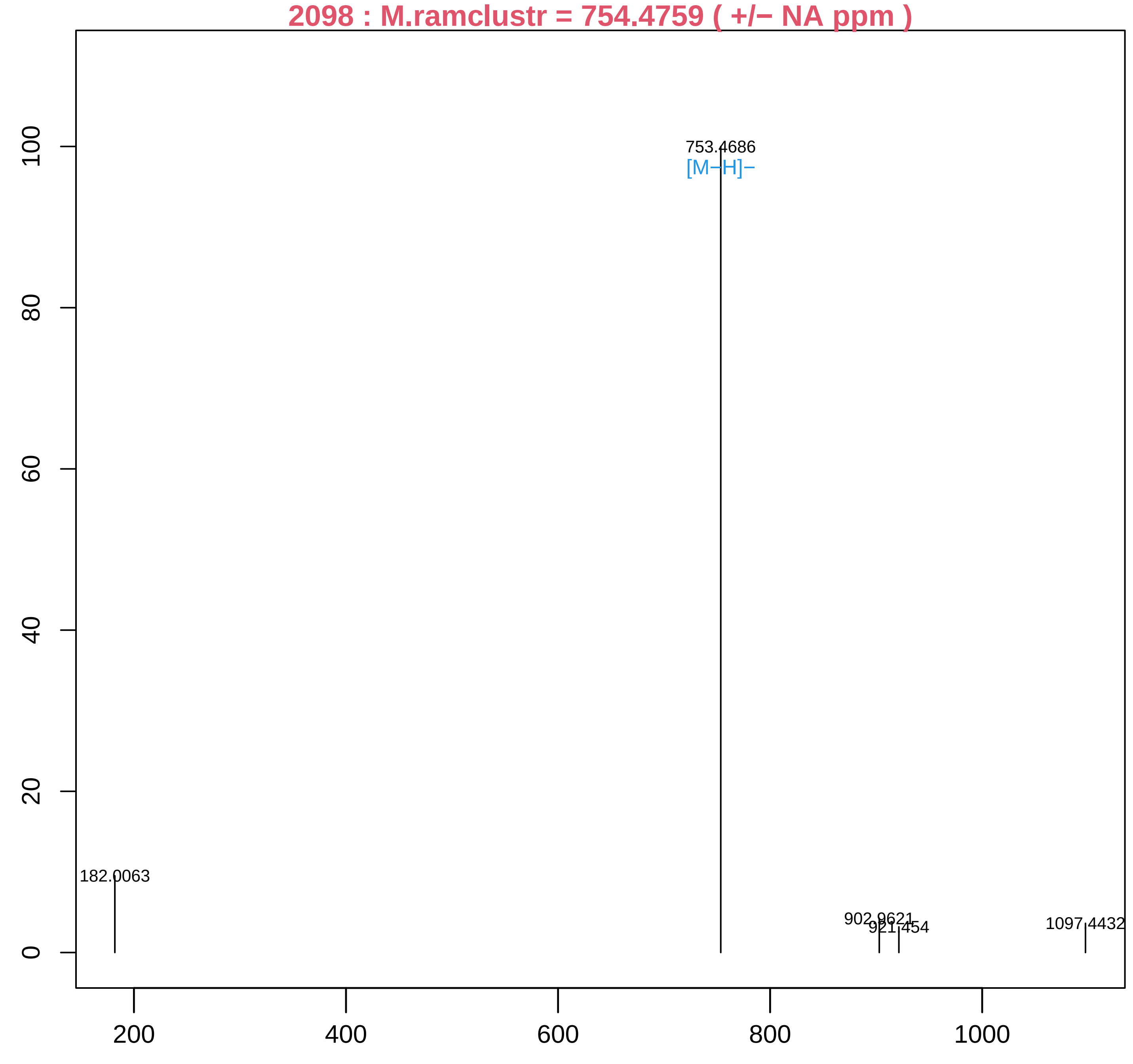
2098 : M.ramclustr = 754.4759 (+/− NA ppm)
753.4686
[M−H]−
182.0063
902.9621
921.454
1097.4432
0
20
40
60
80
100
200
400
600
800
1000

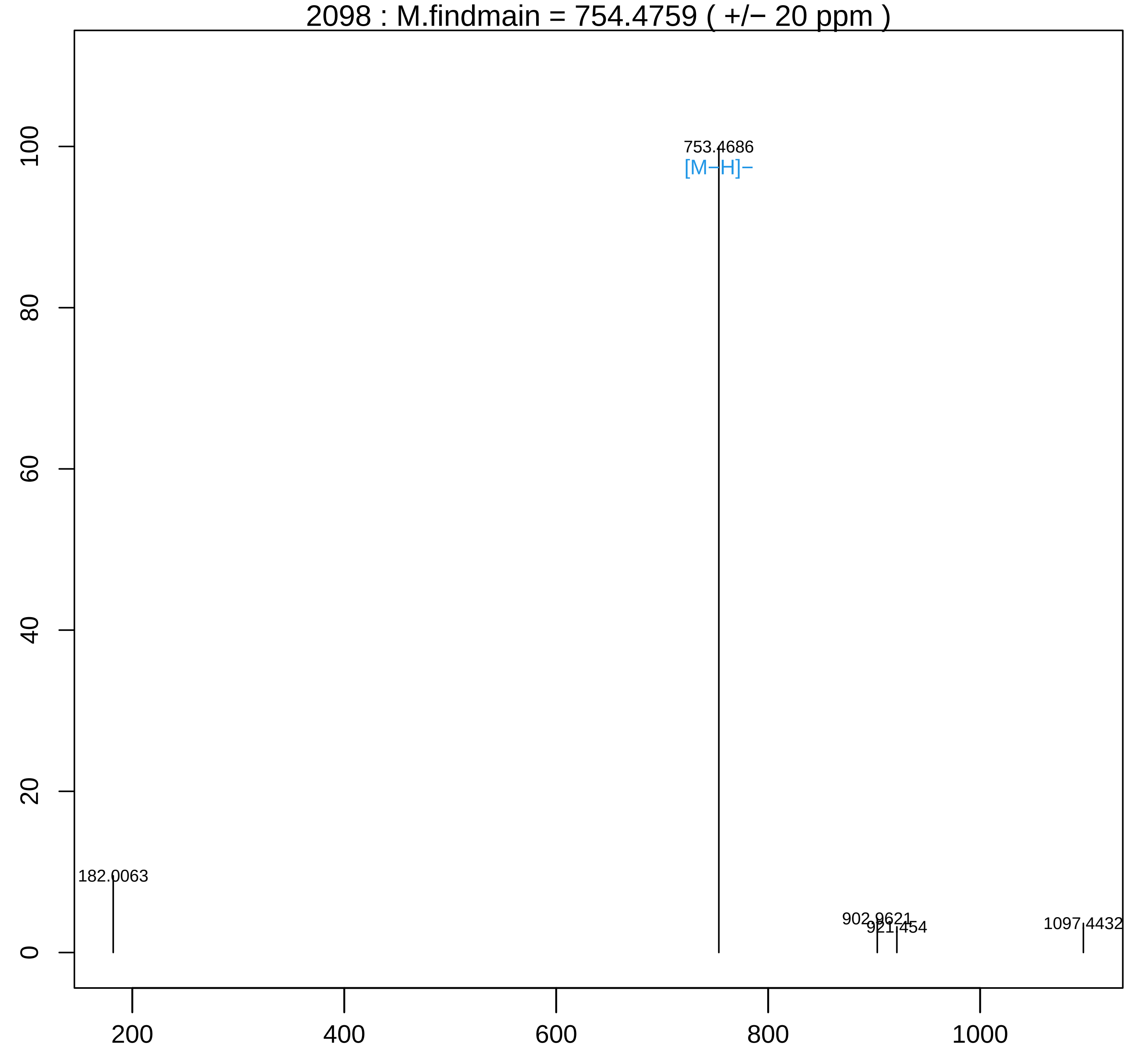
2098 : M.findmain = 754.4759 (+/− 20 ppm)
753.4686
[M−H]−
182.0063
902.9621
921.454
1097.4432
0
20
40
60
80
100
200
400
600
800
1000

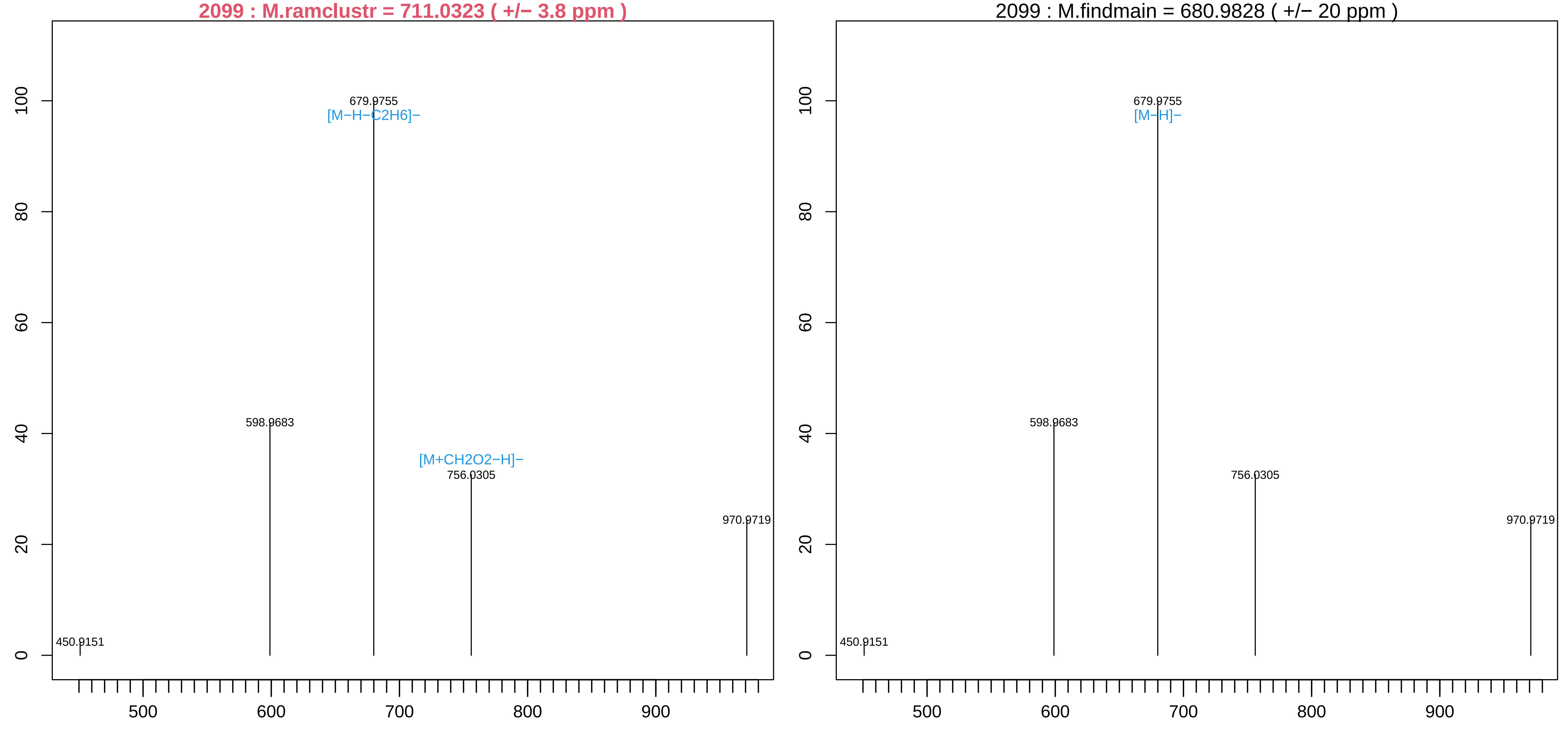
2099 : M.ramclustr = 711.0323 (+/− 3.8 ppm)
679.9755
[M−H−C2H6]−
598.9683
[M+CH2O2−H]−
756.0305
970.9719
450.9151
2099 : M.findmain = 680.9828 (+/− 20 ppm)
679.9755
[M−H]−
598.9683
756.0305
970.9719
450.9151
0
20
40
60
80
100
500
600
700
800
900

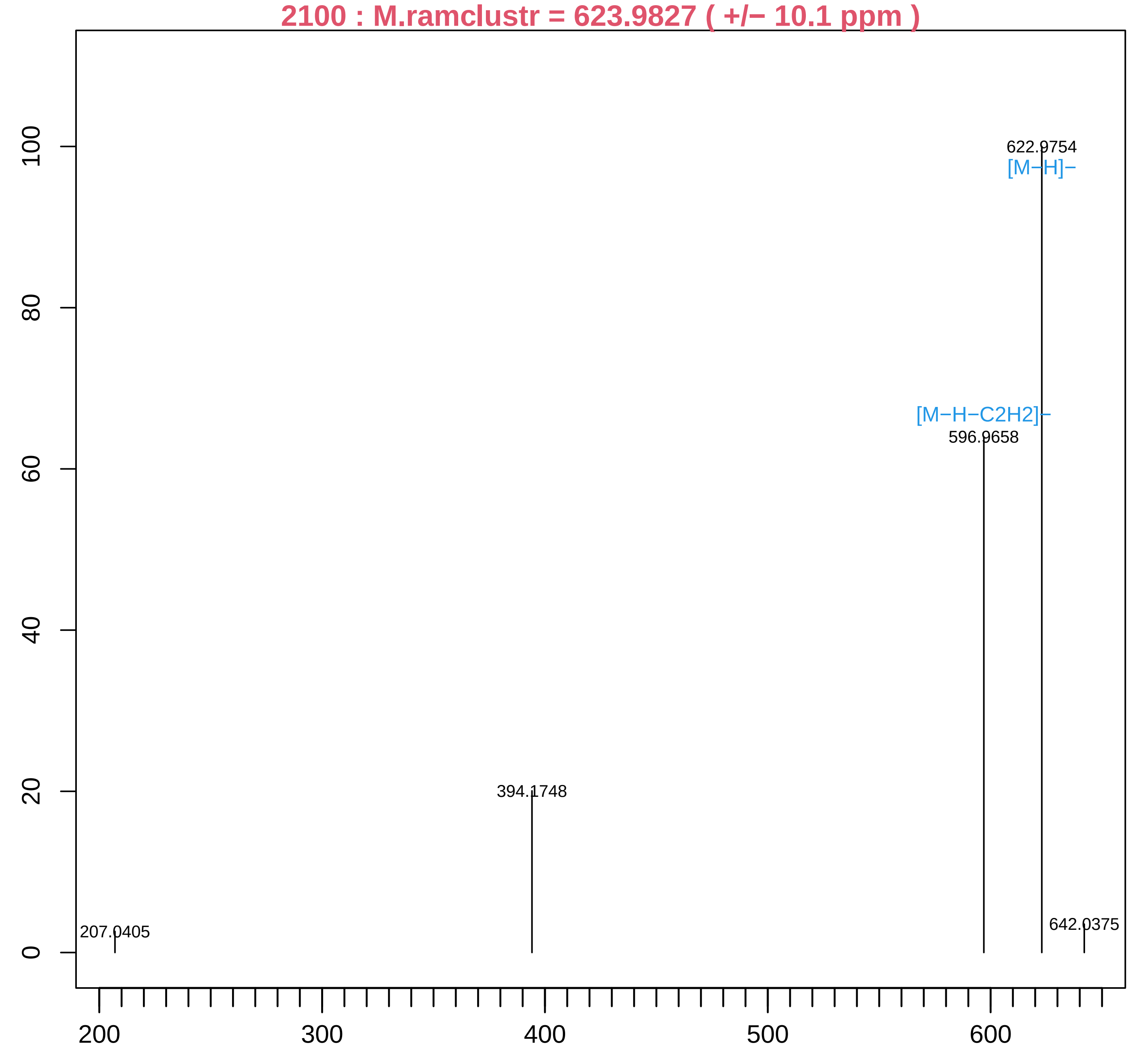
2100 : M.ramclustr = 623.9827 (+/− 10.1 ppm)
622.9754
[M−H]−
[M−H−C2H2]−
596.9658
394.1748
207.0405
642.0375
0
20
40
60
80
100
200
300
400
500
600

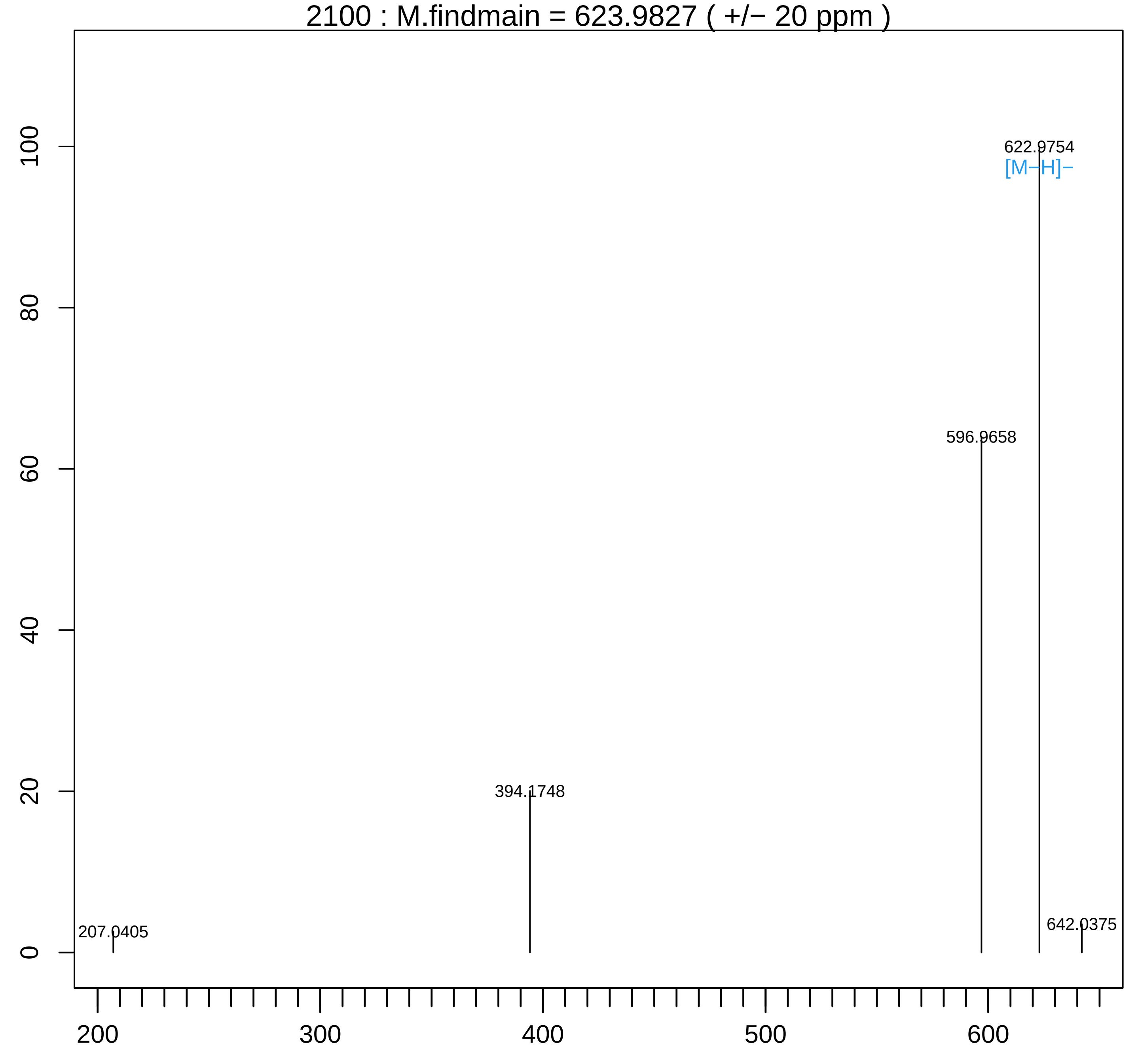
2100 : M.findmain = 623.9827 (+/− 20 ppm)
622.9754
[M−H]−
596.9658
394.1748
207.0405
642.0375
0
20
40
60
80
100
200
300
400
500
600

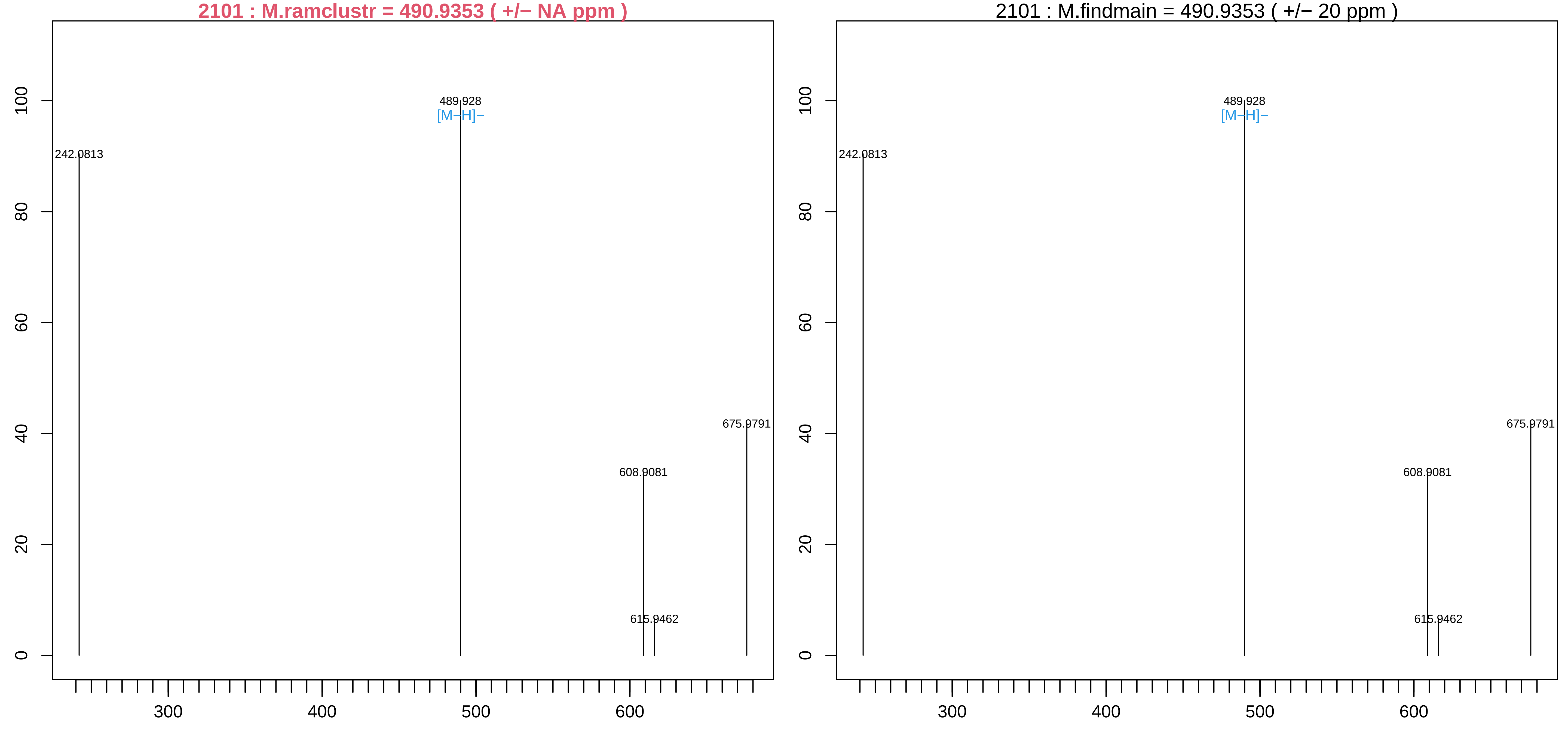
2101 : M.ramclustr = 490.9353 (+/− NA ppm)
489.928
[M−H]−
242.0813
675.9791
608.9081
615.9462
2101 : M.findmain = 490.9353 (+/− 20 ppm)
489.928
[M−H]−
242.0813
675.9791
608.9081
615.9462
0
20
40
60
80
100
300
400
500
600

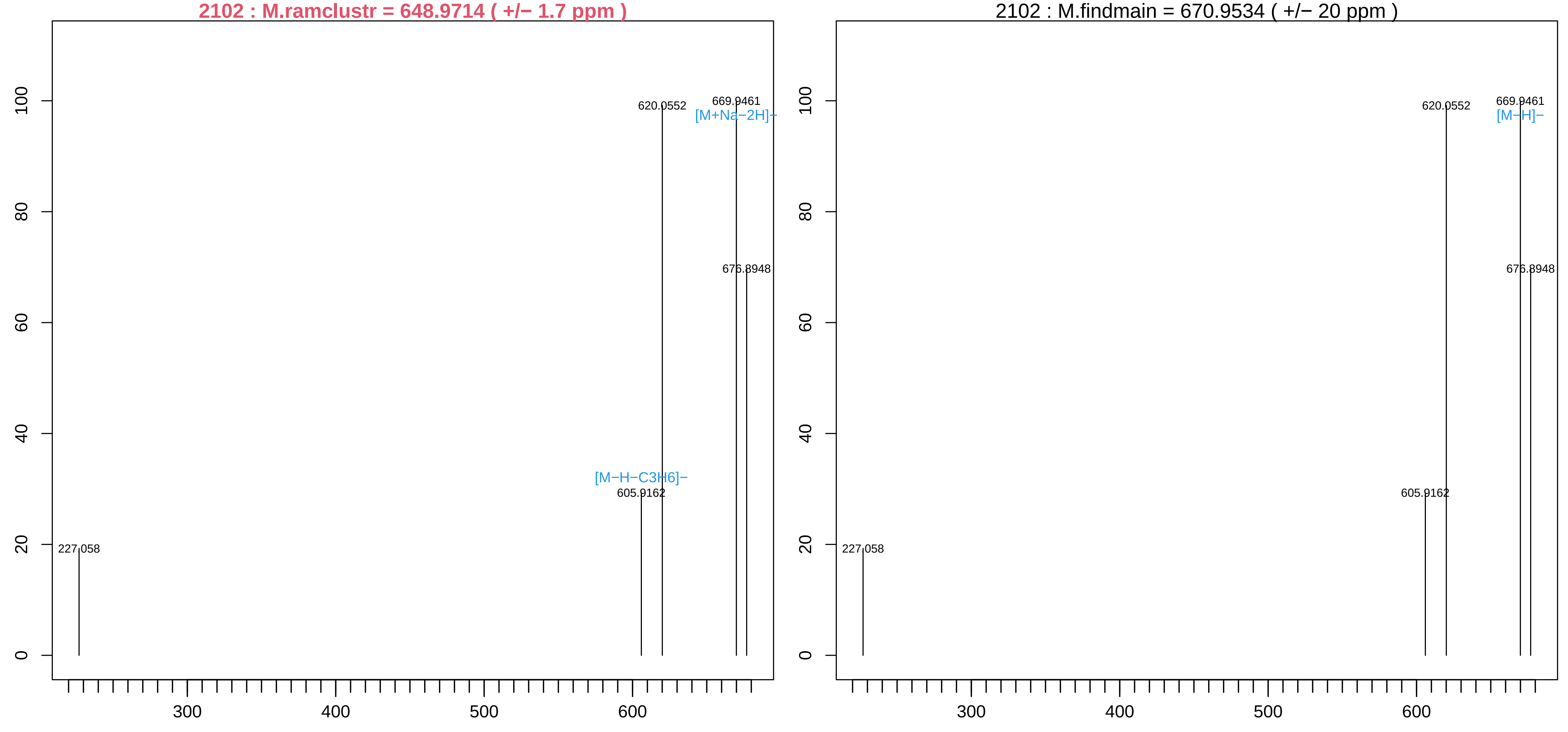

2102 : M.ramclustr = 648.9714 (+/− 1.7 ppm)
227.058
605.9162
[M−H−C3H6]−
620.0552
669.9461
[M+Na−2H]−
676.8948
2102 : M.findmain = 670.9534 (+/− 20 ppm)
227.058
605.9162
620.0552
669.9461
[M−H]−
676.8948
0
20
40
60
80
100
300
400
500
600

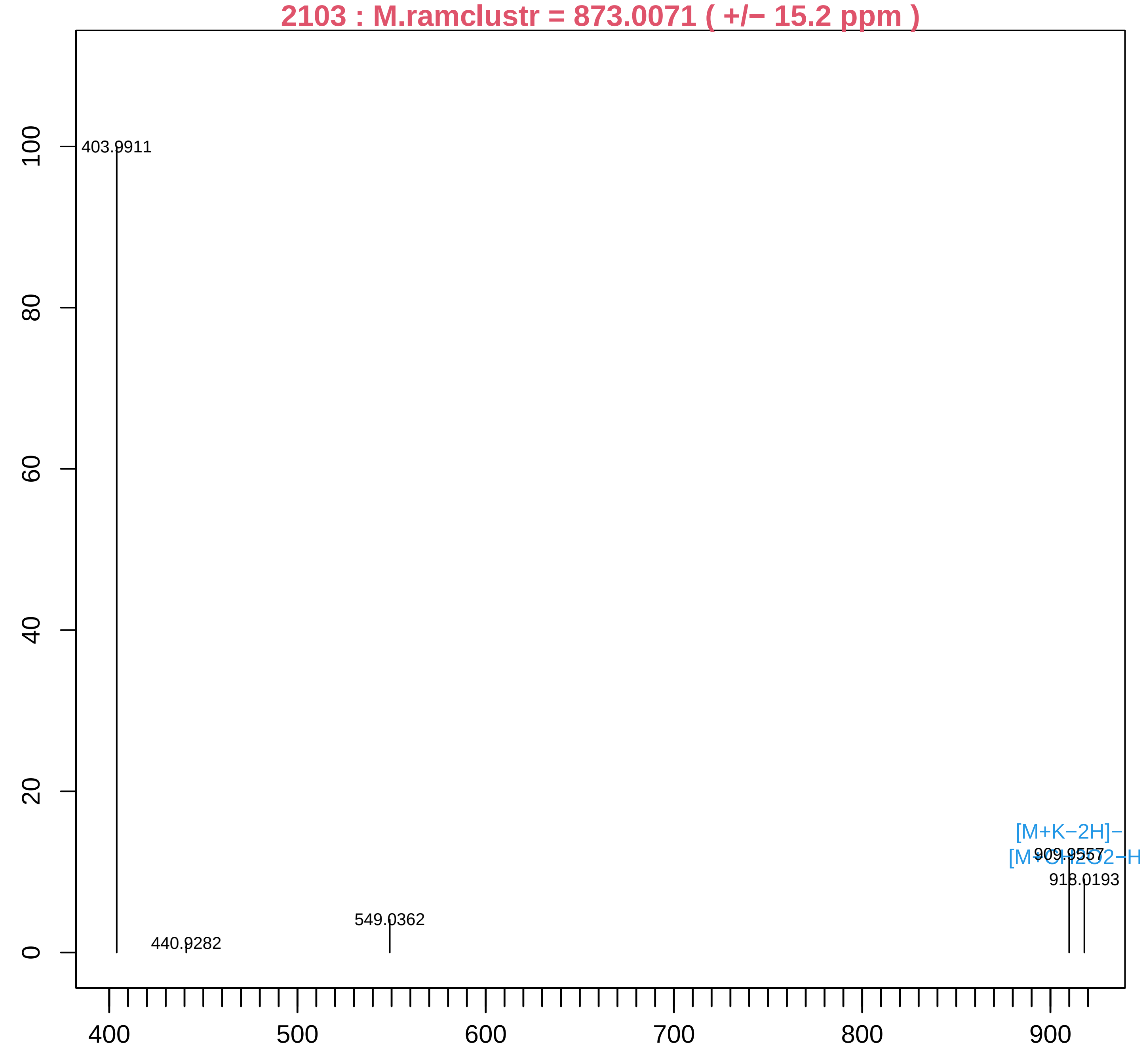
2103 : M.ramclustr = 873.0071 (+/− 15.2 ppm)
403.9911
440.9282
549.0362
[M+K−2H]−
909.9557
918.0193
0
20
40
60
80
100
400
500
600
700
800
900

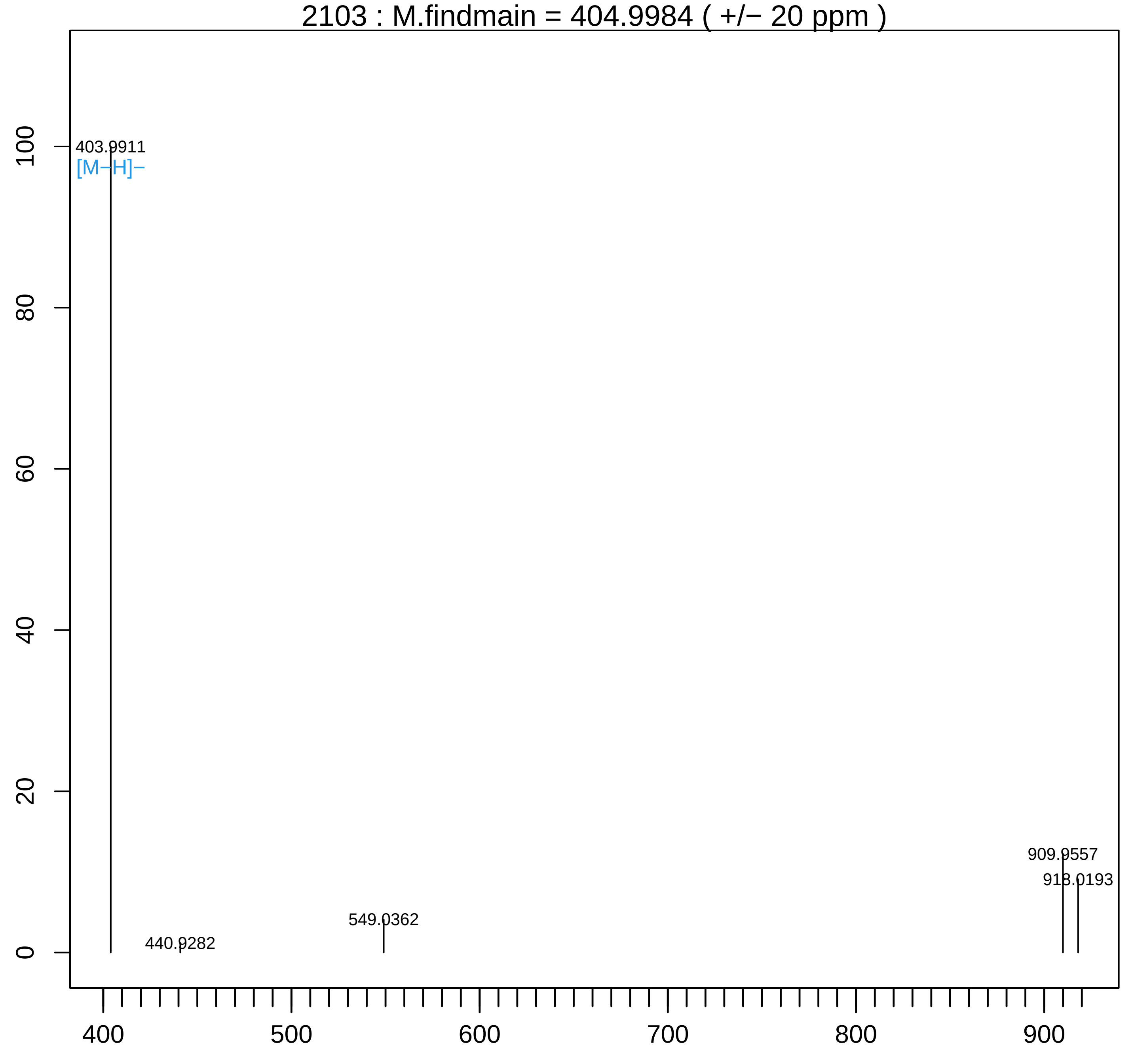
2103 : M.findmain = 404.9984 (+/− 20 ppm)
403.9911
[M−H]−
440.9282
549.0362
909.9557
918.0193
0
20
40
60
80
100
400
500
600
700
800
900

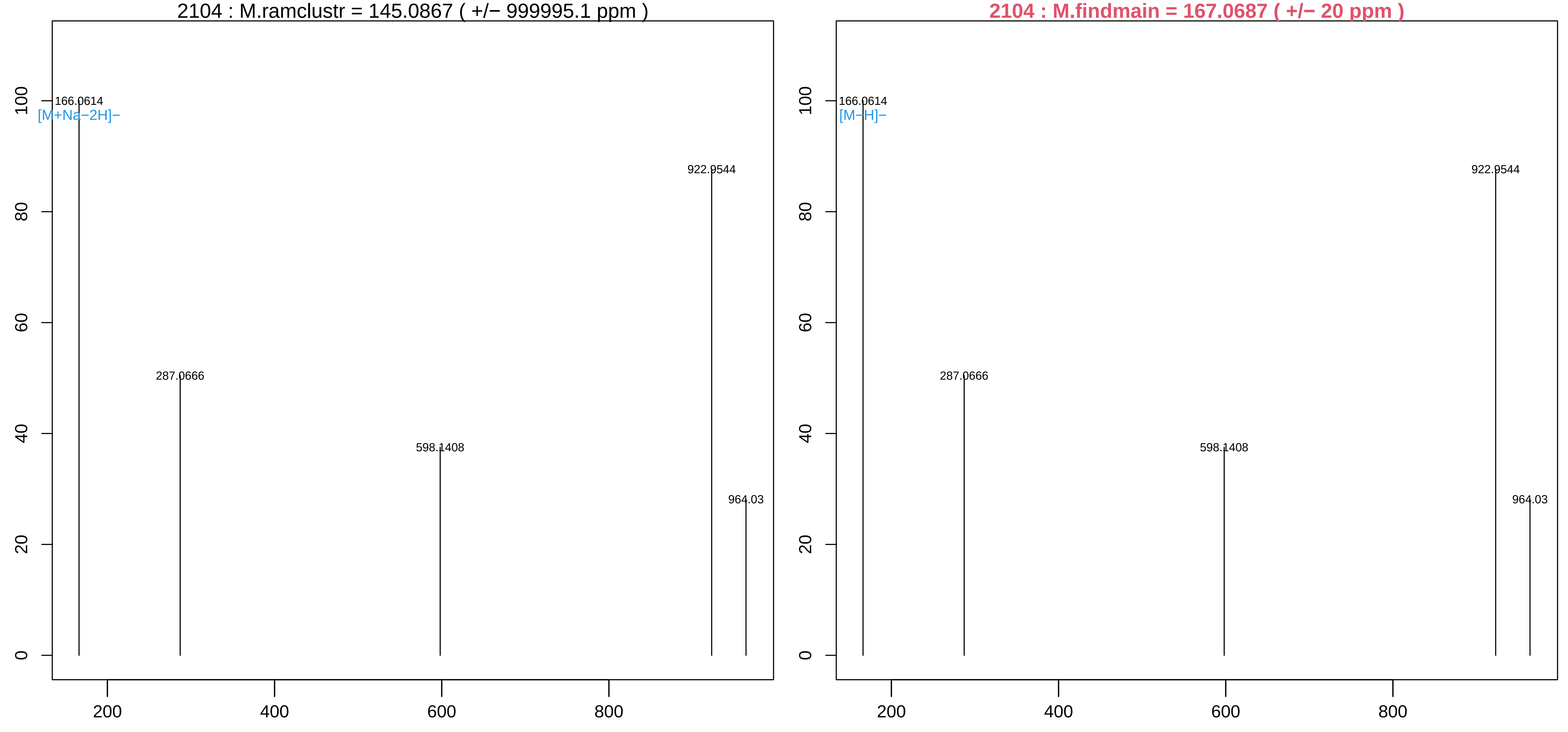
2104 : M.ramclustr = 145.0867 (+/− 999995.1 ppm)
166.0614
[M+Na−2H]−
287.0666
598.1408
922.9544
964.03
0
20
40
60
80
100
200
400
600
800
2104 : M.findmain = 167.0687 (+/− 20 ppm)
166.0614
[M−H]−
287.0666
598.1408
922.9544
964.03
0
20
40
60
80
100
200
400
600
800

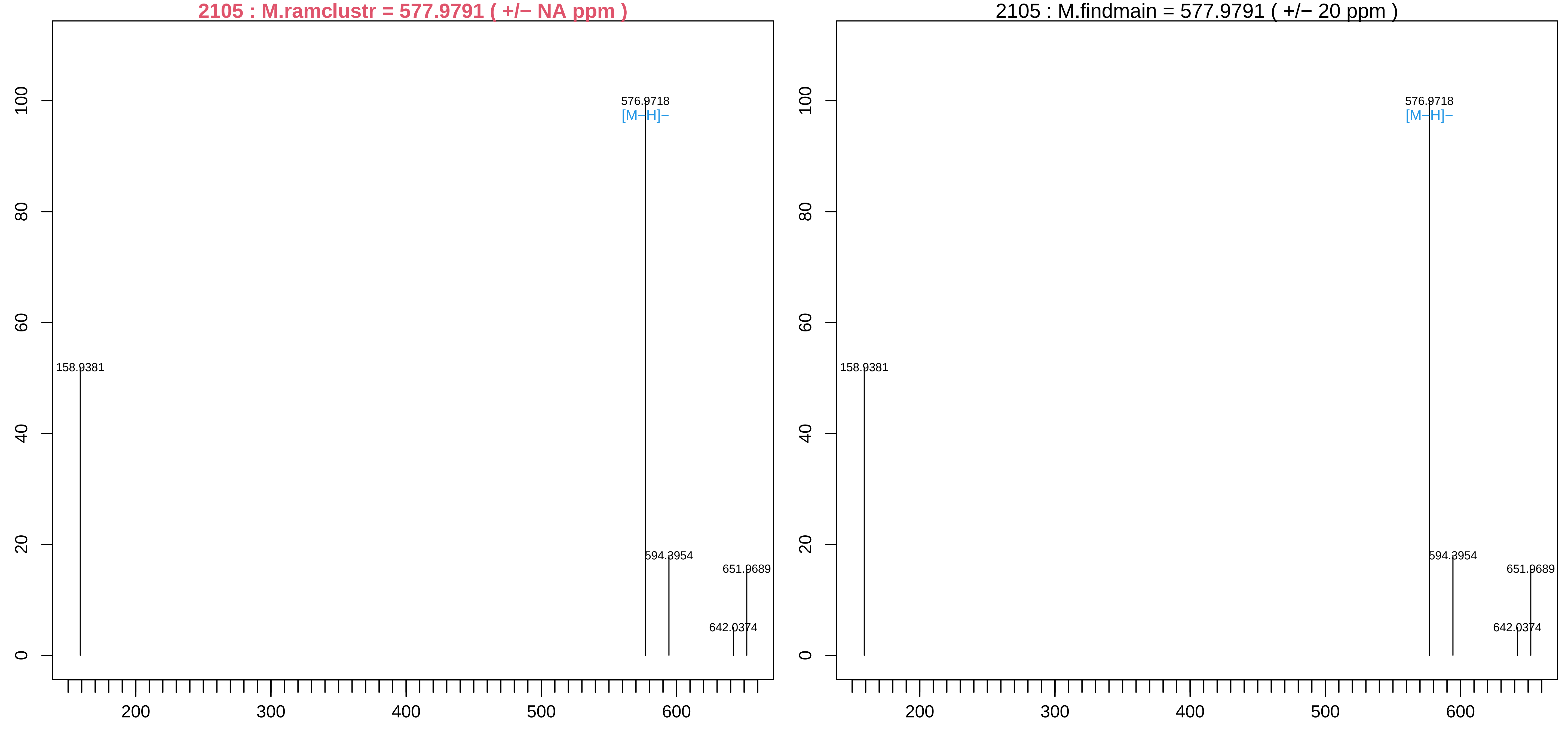
2105 : M.ramclustr = 577.9791 (+/− NA ppm)
576.9718
[M−H]−
158.9381
594.3954
651.9689
642.0374
0
20
40
60
80
100
200
300
400
500
600
2105 : M.findmain = 577.9791 (+/− 20 ppm)
576.9718
[M−H]−
158.9381
594.3954
651.9689
642.0374
0
20
40
60
80
100
200
300
400
500
600

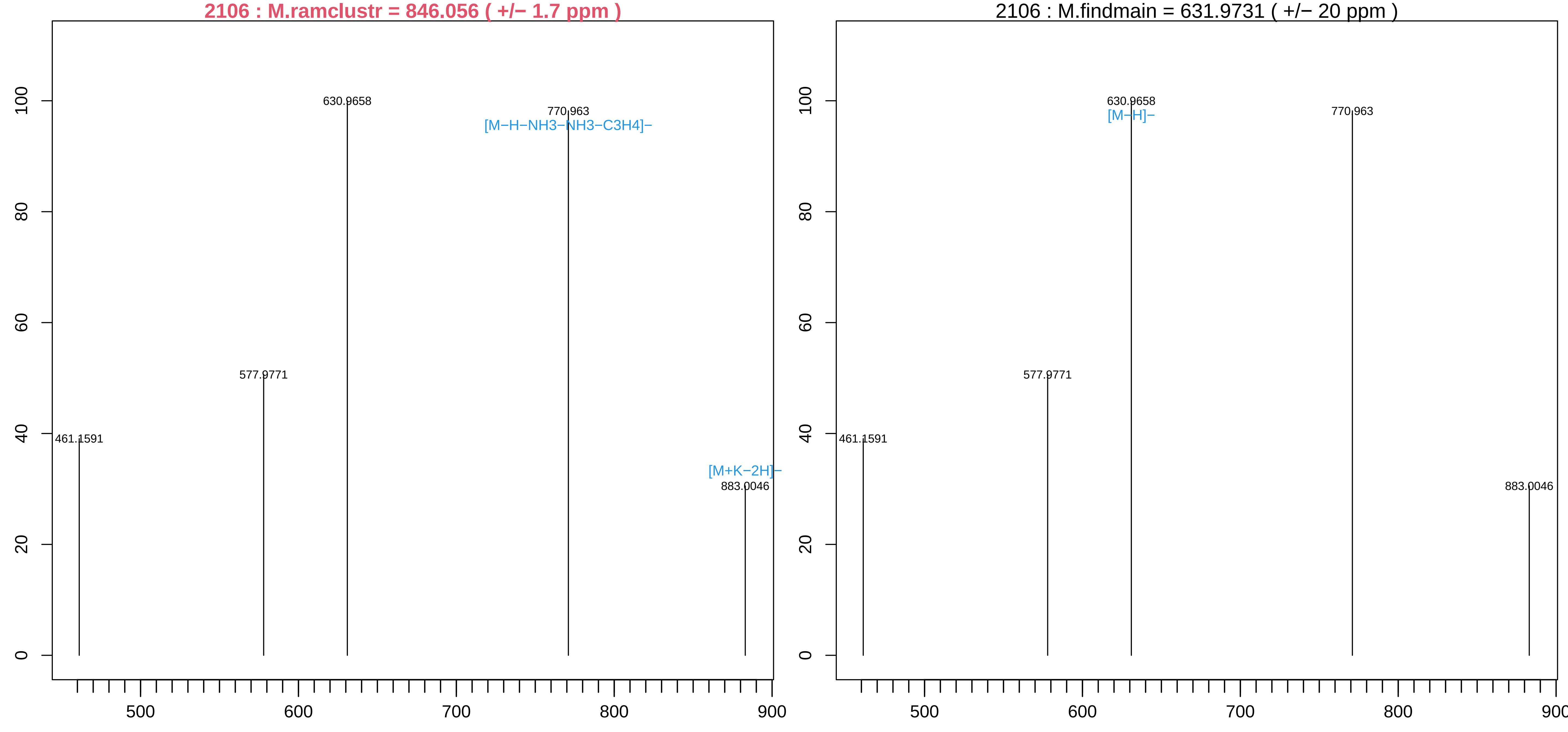
2106 : M.ramclustr = 846.056 (+/− 1.7 ppm)
630.9658
770.963
[M−H−NH3−NH3−C3H4]−
577.9771
461.1591
[M+K−2H]−
883.0046
2106 : M.findmain = 631.9731 (+/− 20 ppm)
630.9658
[M−H]−
770.963
577.9771
461.1591
883.0046
0
20
40
60
80
100
500
600
700
800
900

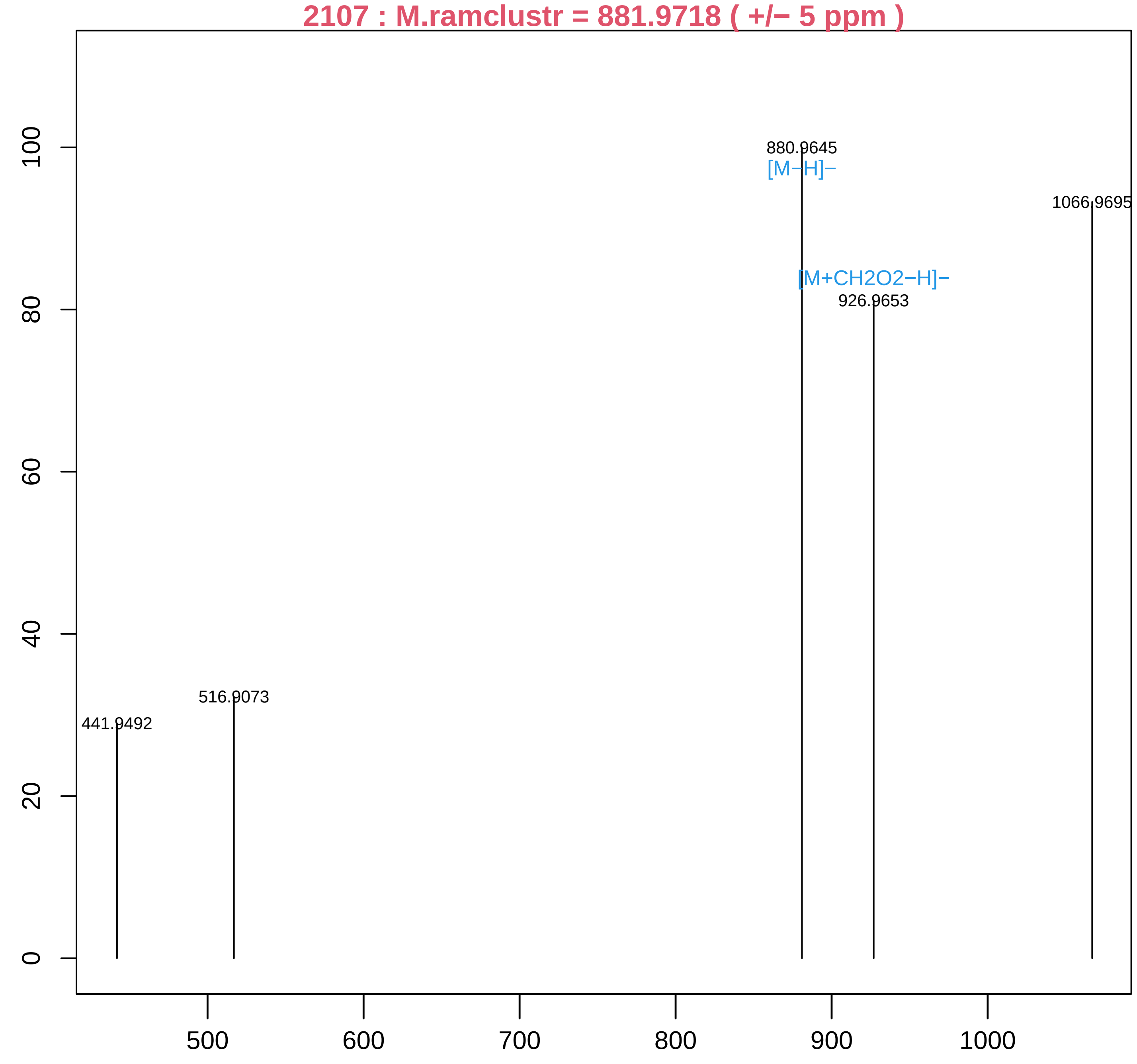
2107 : M.ramclustr = 881.9718 (+/− 5 ppm)
880.9645
[M−H]−
1066.9695
[M+CH2O2−H]−
926.9653
516.9073
441.9492
0
20
40
60
80
100
500
600
700
800
900
1000

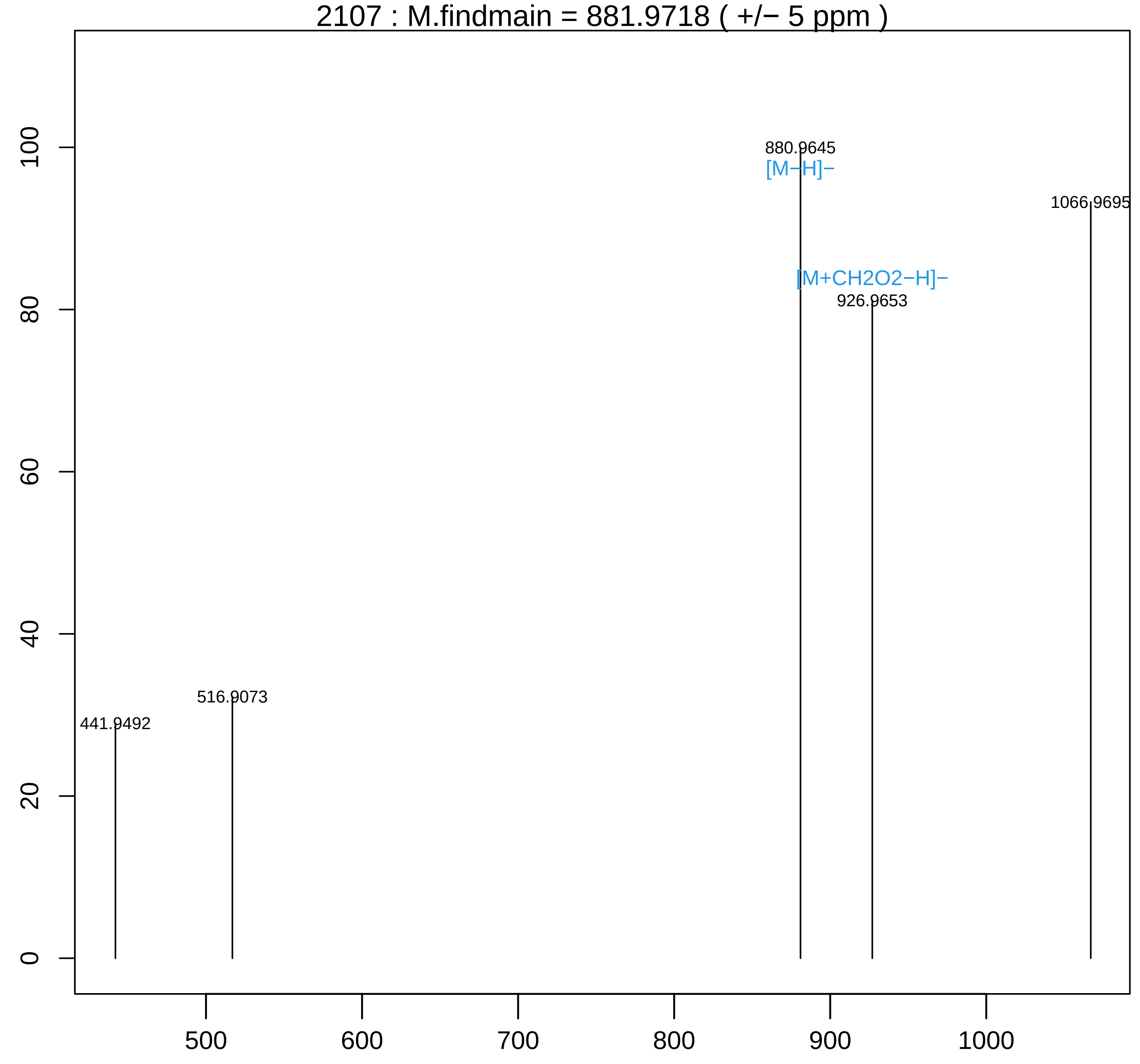
2107 : M.findmain = 881.9718 (+/− 5 ppm)
880.9645
[M−H]−
1066.9695
[M+CH2O2−H]−
926.9653
516.9073
441.9492
0
20
40
60
80
100
500
600
700
800
900
1000

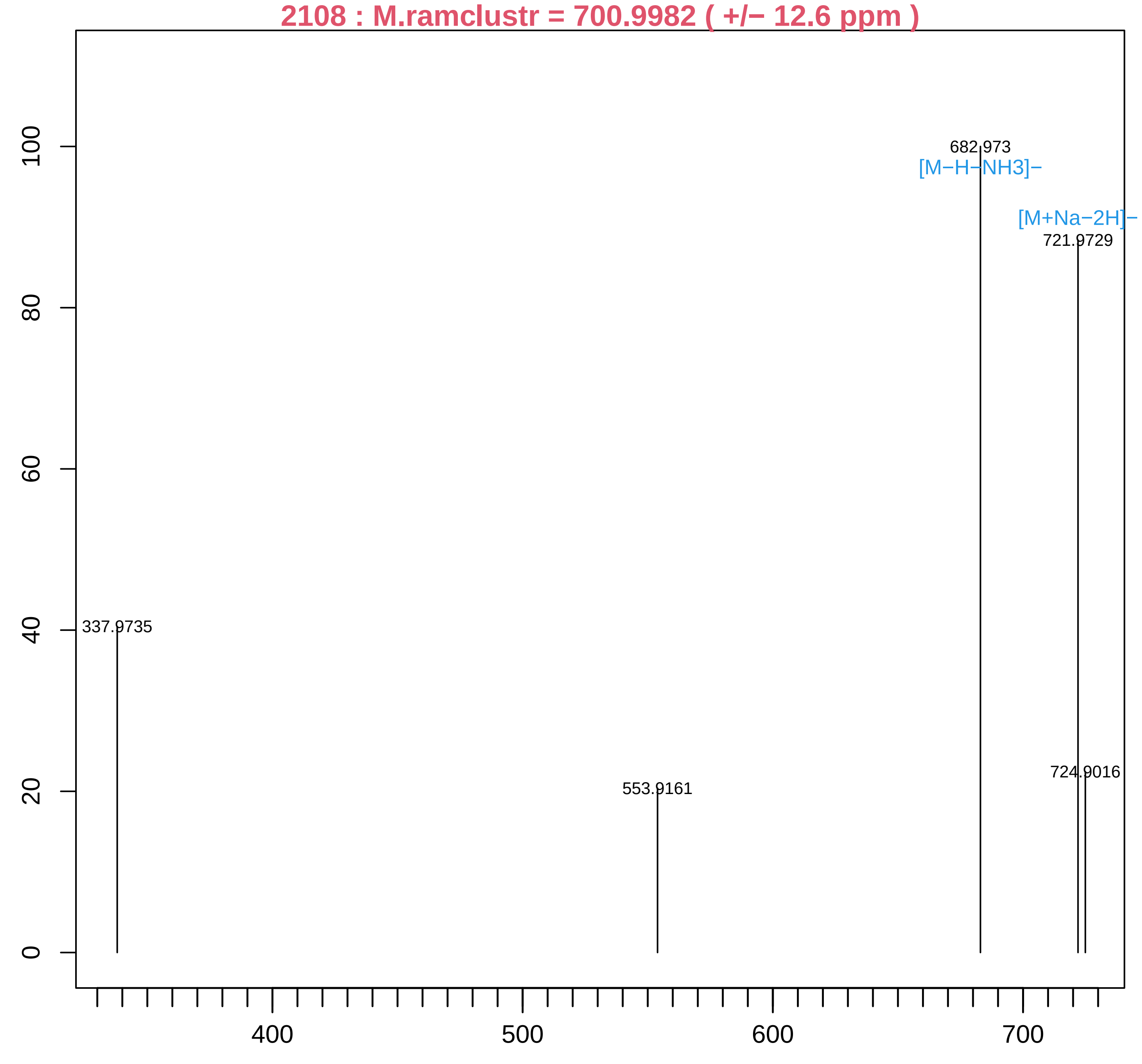
2108 : M.ramclustr = 700.9982 (+/− 12.6 ppm)
682.973
[M−H−NH3]−
[M+Na−2H]−
721.9729
337.9735
553.9161
724.9016
0
20
40
60
80
100
400
500
600
700

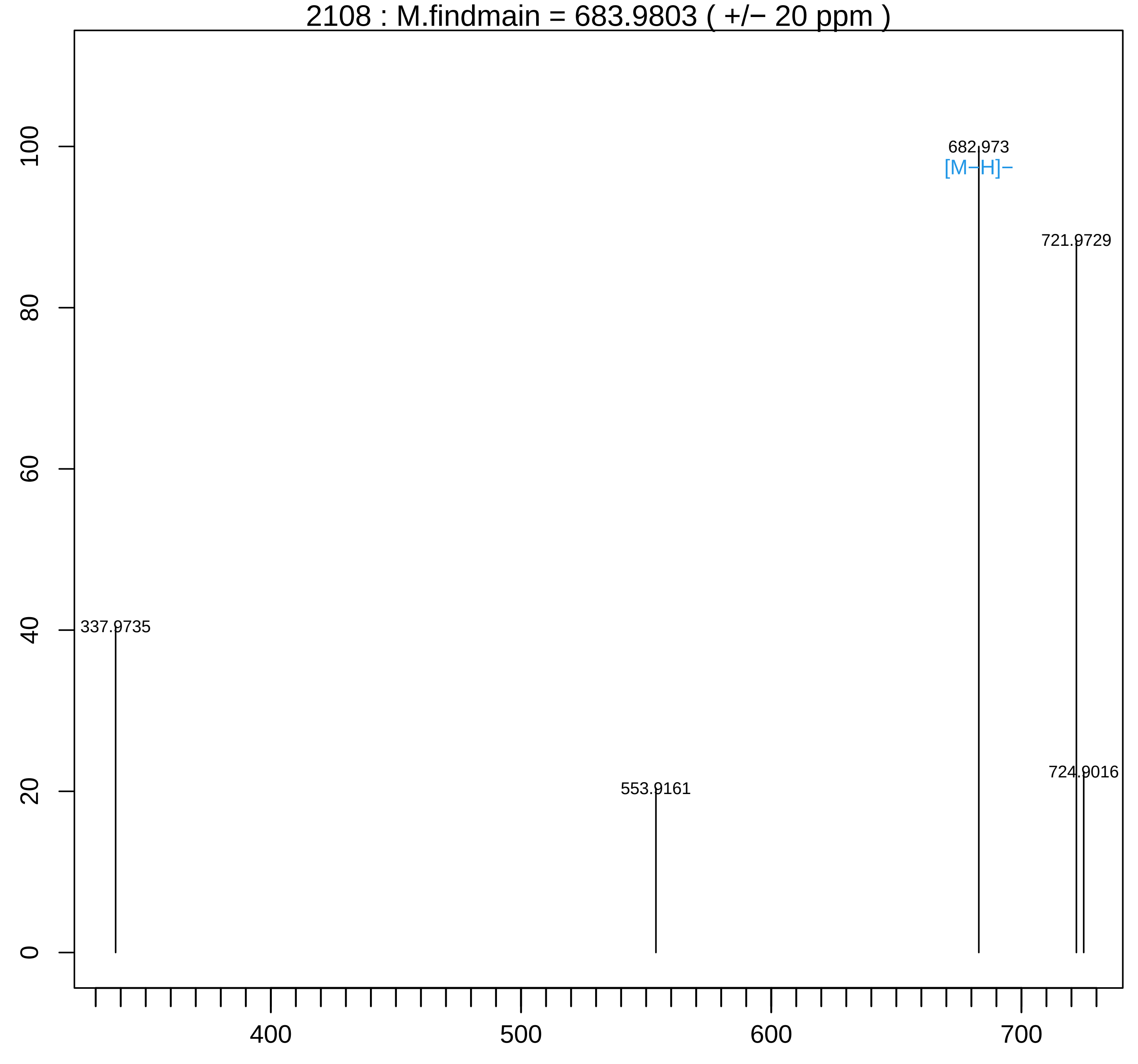
2108 : M.findmain = 683.9803 (+/− 20 ppm)
682.973
[M−H]−
721.9729
337.9735
553.9161
724.9016
0
20
40
60
80
100
400
500
600
700

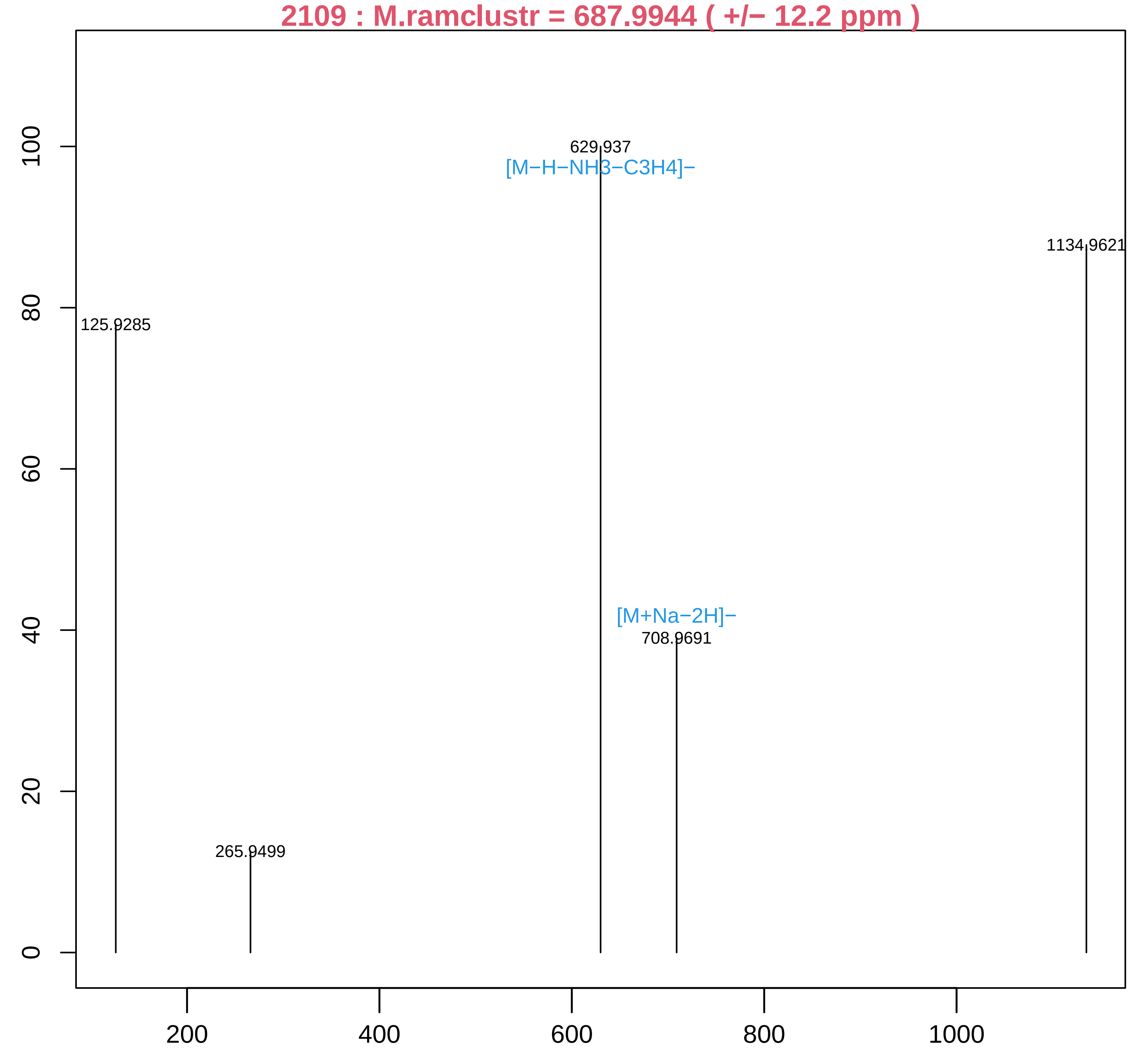
2109 : M.ramclustr = 687.9944 (+/− 12.2 ppm)
629.937
[M−H−NH3−C3H4]−
1134.9621
125.9285
[M+Na−2H]−
708.9691
265.9499
0
20
40
60
80
100
200
400
600
800
1000

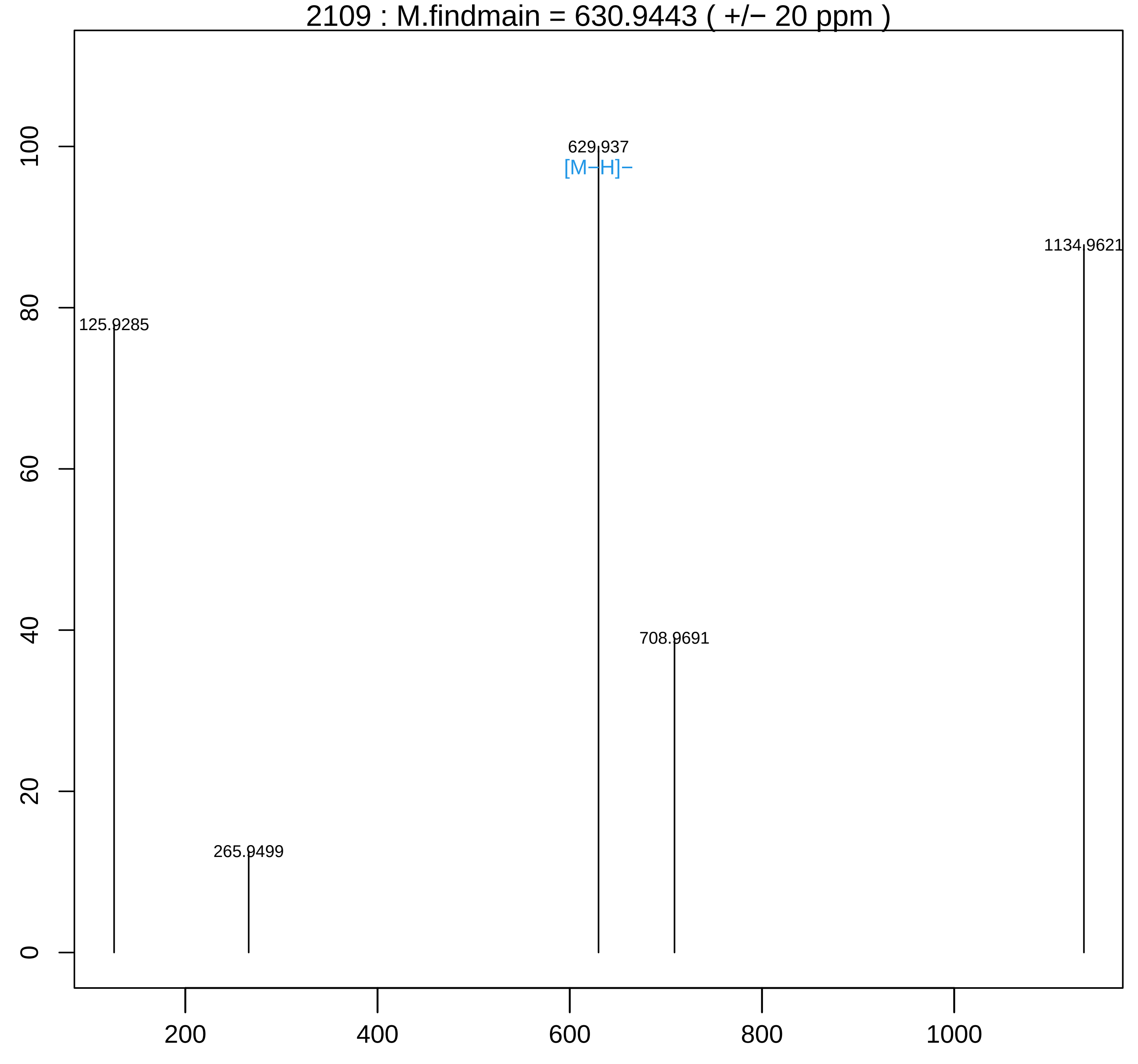
2109 : M.findmain = 630.9443 (+/− 20 ppm)
629.937
[M−H]−
1134.9621
125.9285
708.9691
265.9499
0
20
40
60
80
100
200
400
600
800
1000

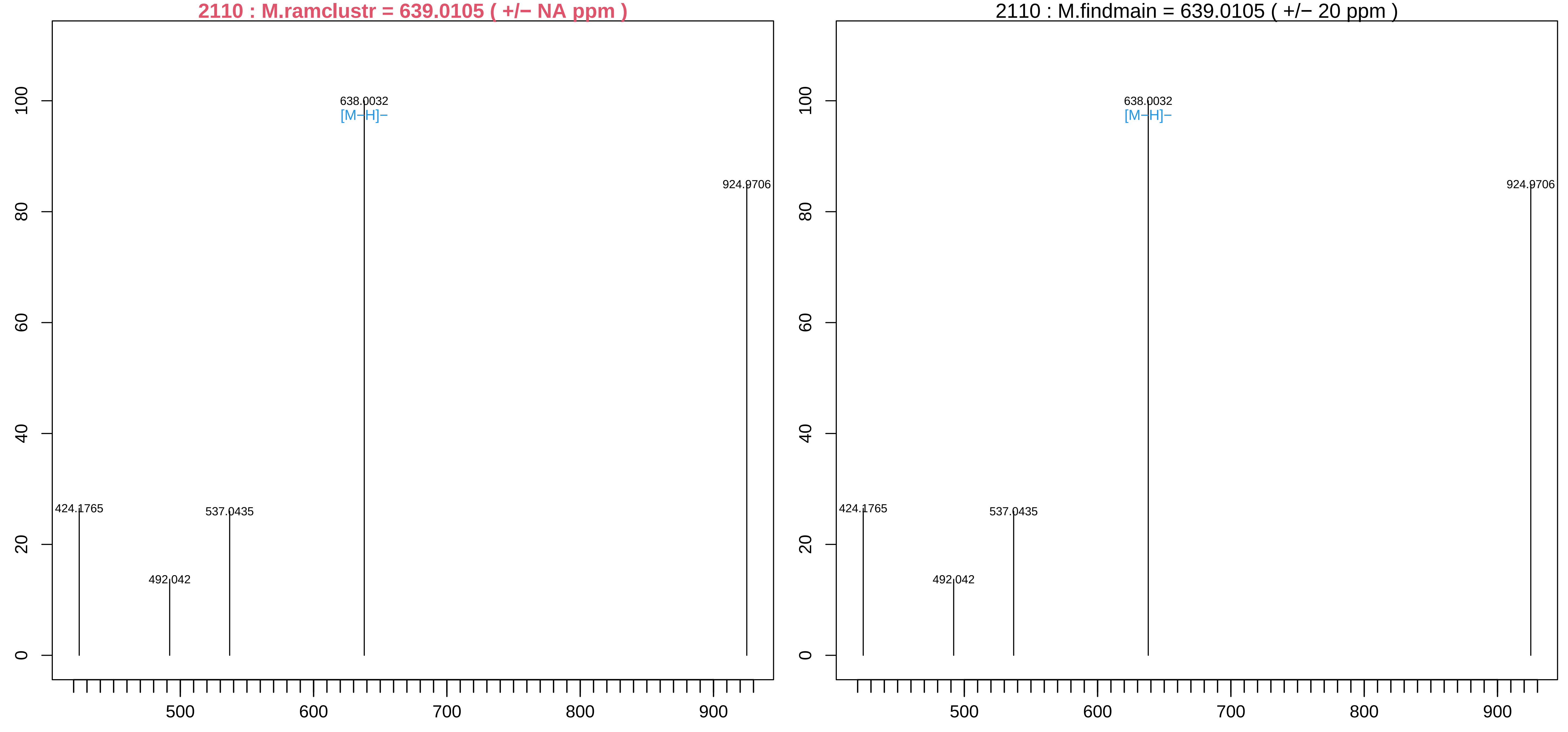

2110 : M.ramclustr = 639.0105 (+/− NA ppm)
638.0032
[M−H]−
924.9706
424.1765
537.0435
492.042
0
20
40
60
80
100
500
600
700
800
900
2110 : M.findmain = 639.0105 (+/− 20 ppm)
638.0032
[M−H]−
924.9706
424.1765
537.0435
492.042
0
20
40
60
80
100
500
600
700
800
900

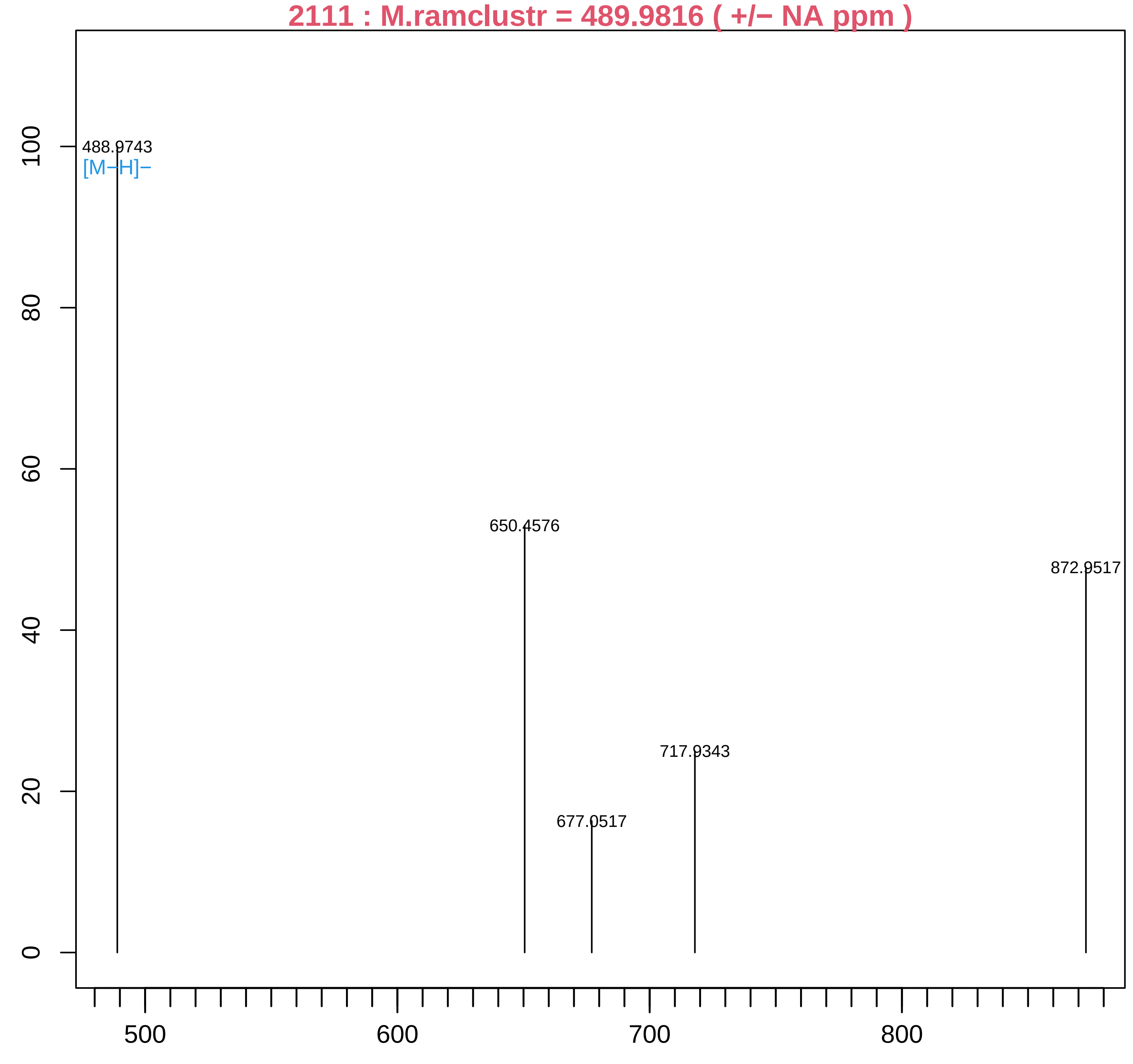
2111 : M.ramclustr = 489.9816 (+/− NA ppm)
488.9743
[M−H]−
650.4576
677.0517
717.9343
872.9517
0
20
40
60
80
100
500
600
700
800

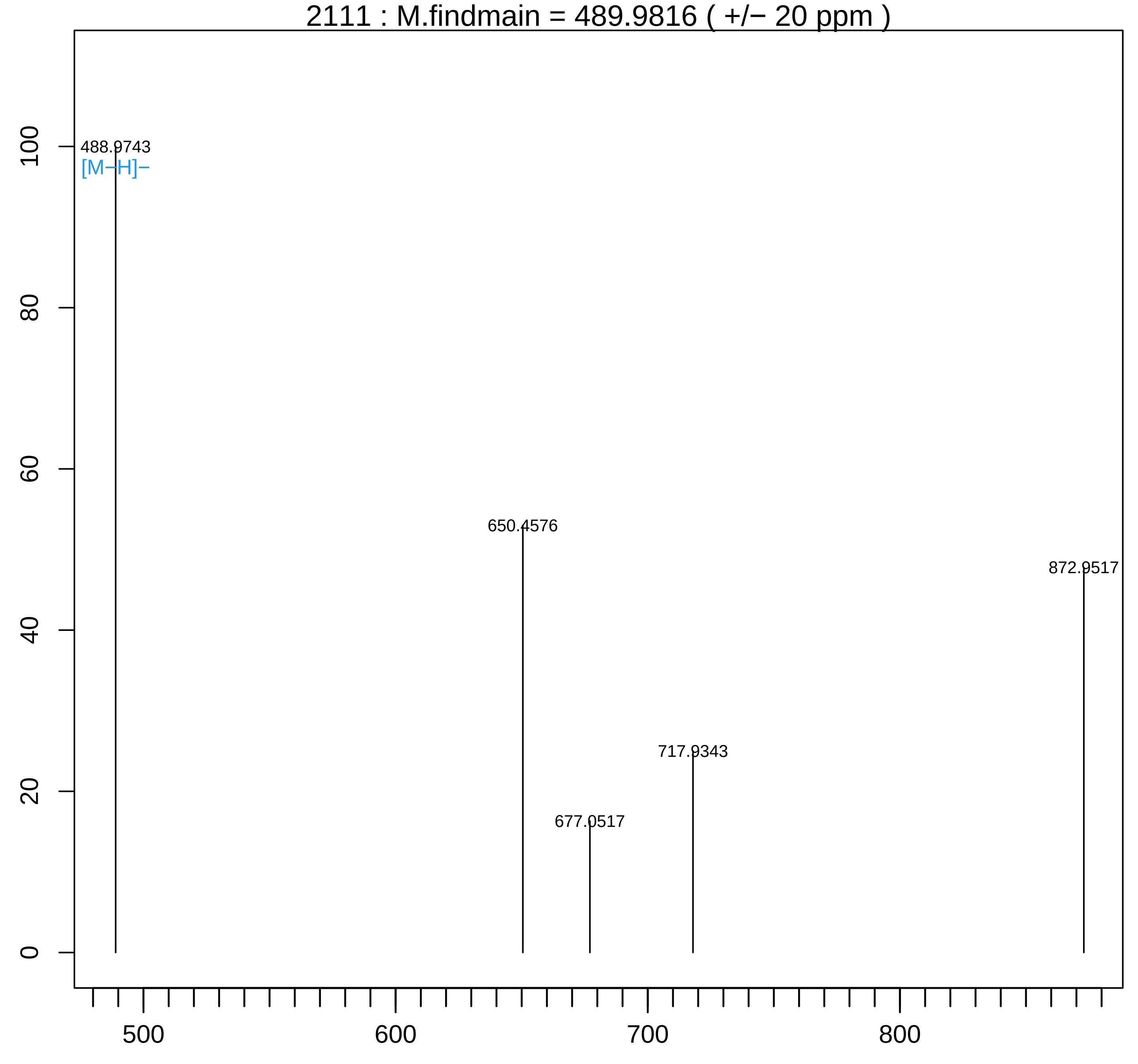
2111 : M.findmain = 489.9816 (+/− 20 ppm)
488.9743
[M−H]−
650.4576
677.0517
717.9343
872.9517
0
20
40
60
80
100
500
600
700
800

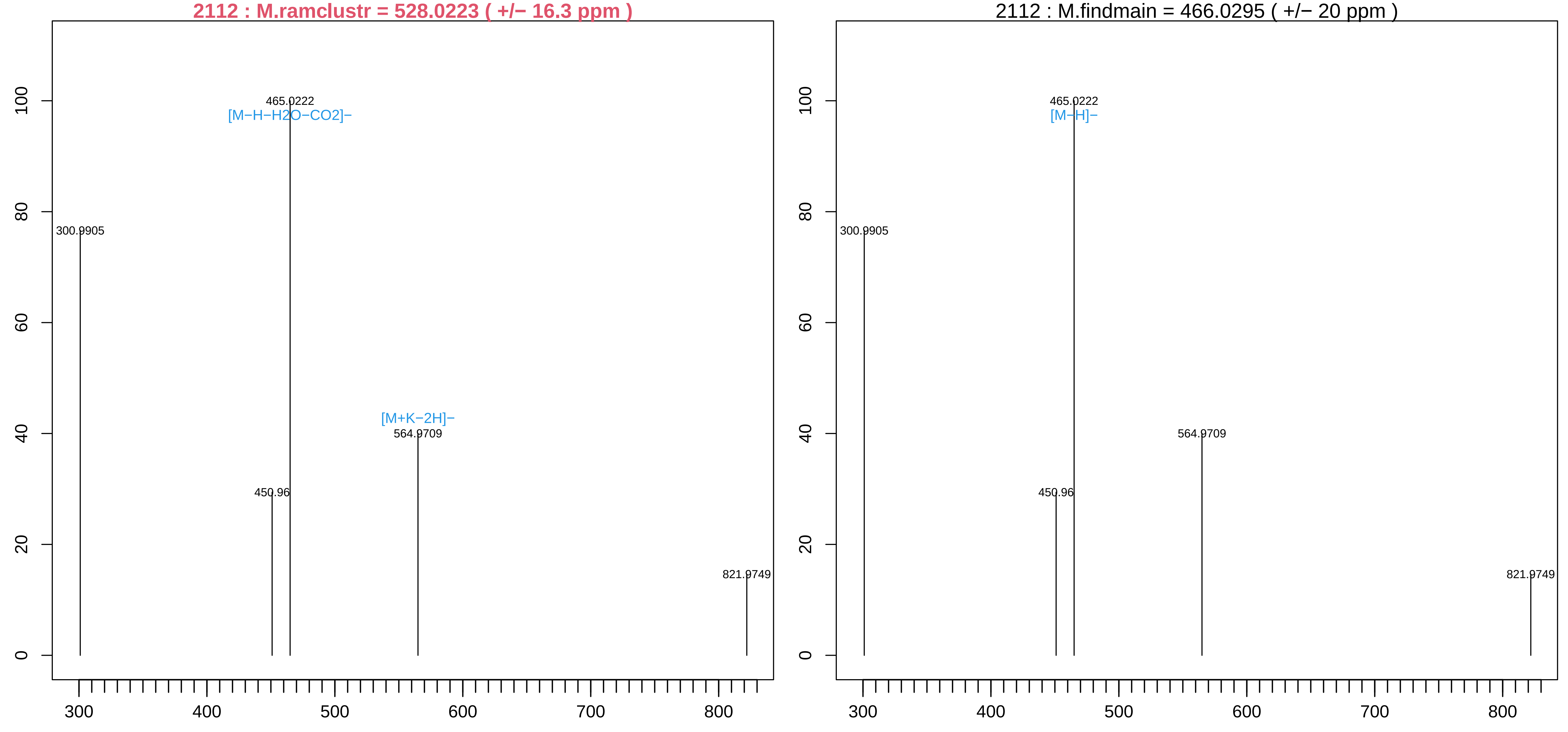

2112 : M.ramclustr = 528.0223 (+/− 16.3 ppm)
465.0222
[M−H−H2O−CO2]−
300.9905
[M+K−2H]−
564.9709
450.96
821.9749
2112 : M.findmain = 466.0295 (+/− 20 ppm)
465.0222
[M−H]−
300.9905
564.9709
450.96
821.9749
0
20
40
60
80
100
300
400
500
600
700
800

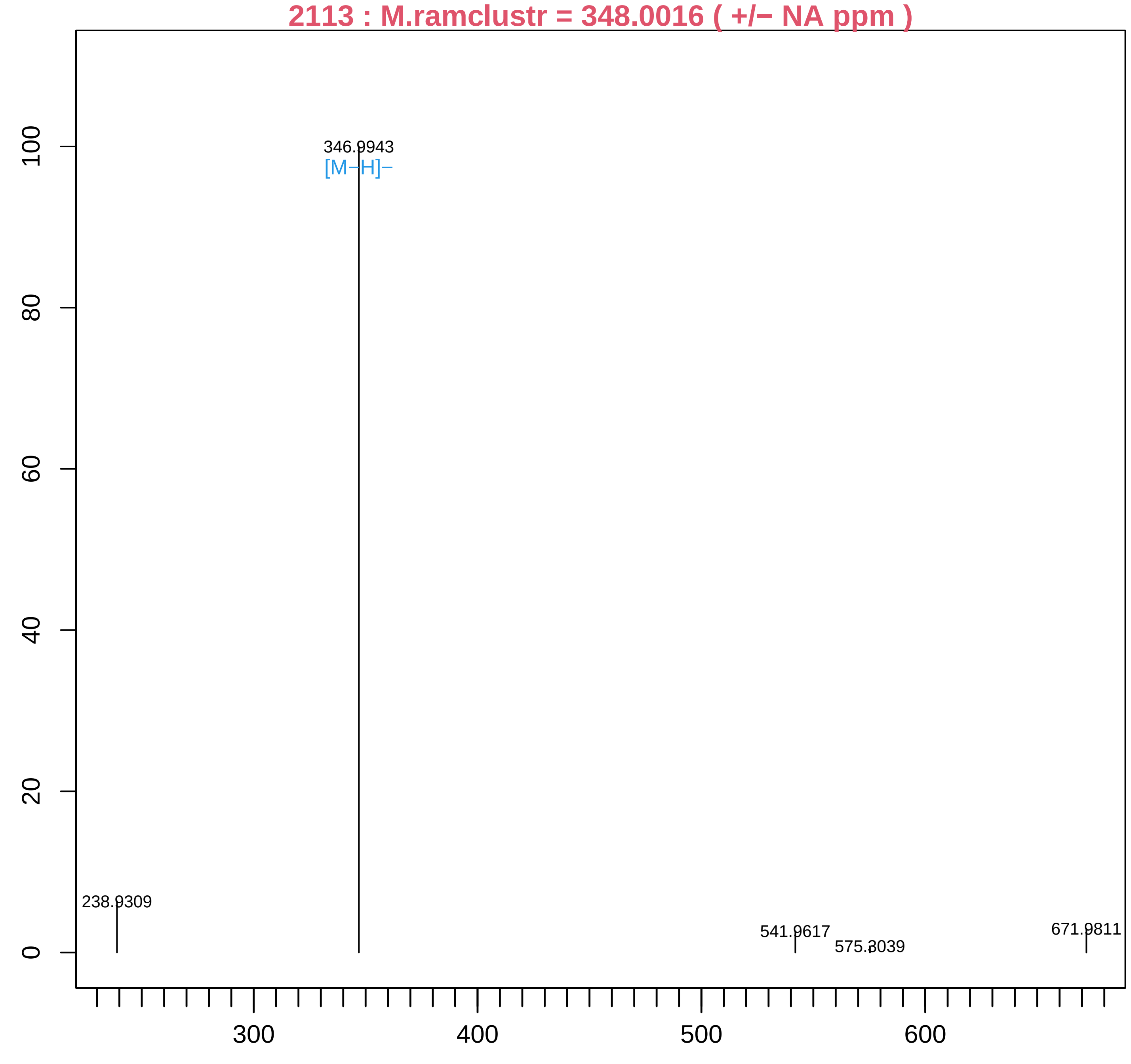
2113 : M.ramclustr = 348.0016 (+/− NA ppm)
346.9943
[M−H]−
238.9309
541.9617
575.3039
671.9811
0
20
40
60
80
100
300
400
500
600

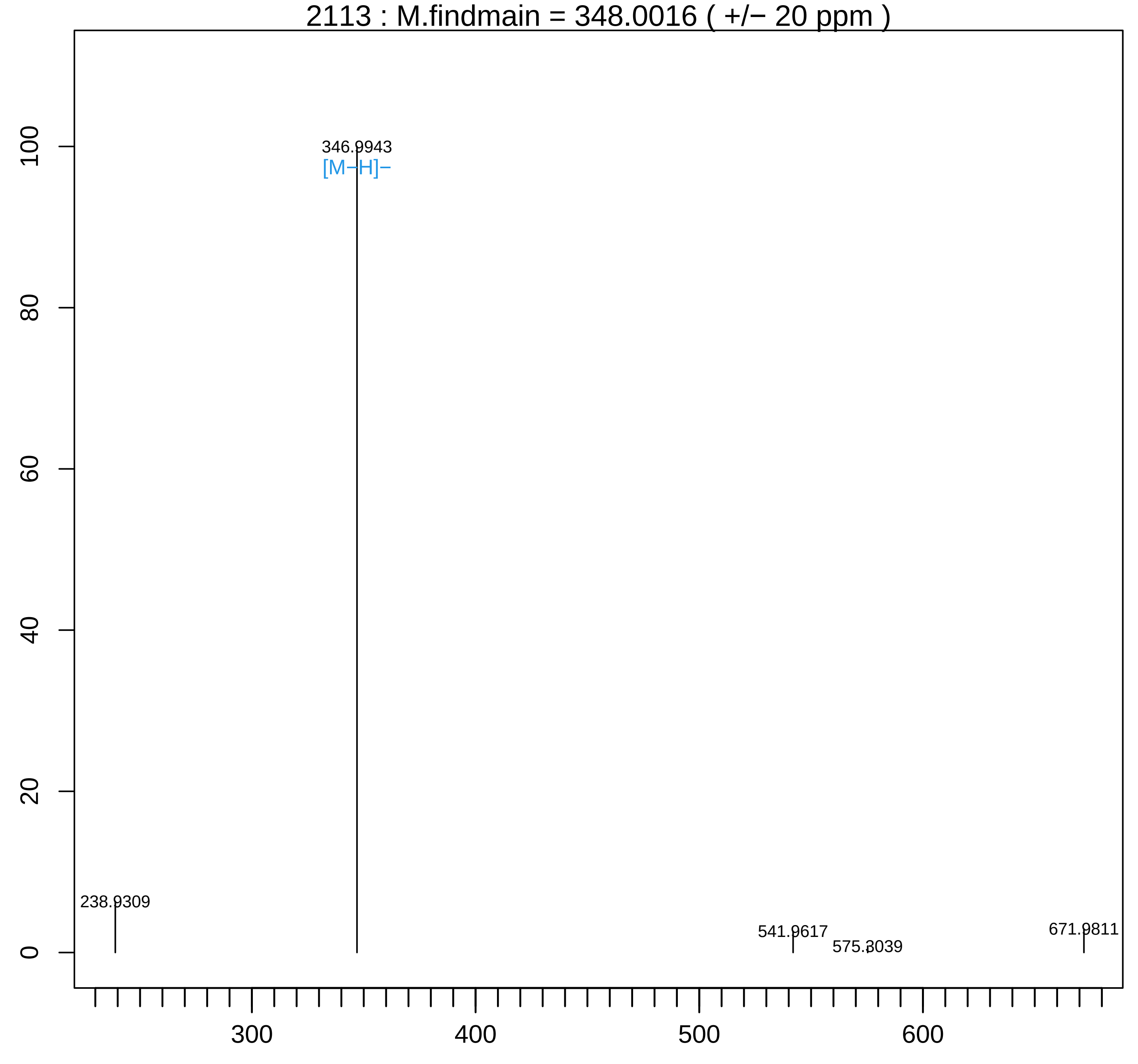
2113 : M.findmain = 348.0016 (+/− 20 ppm)
346.9943
[M−H]−
238.9309
541.9617
575.3039
671.9811
0
20
40
60
80
100
300
400
500
600

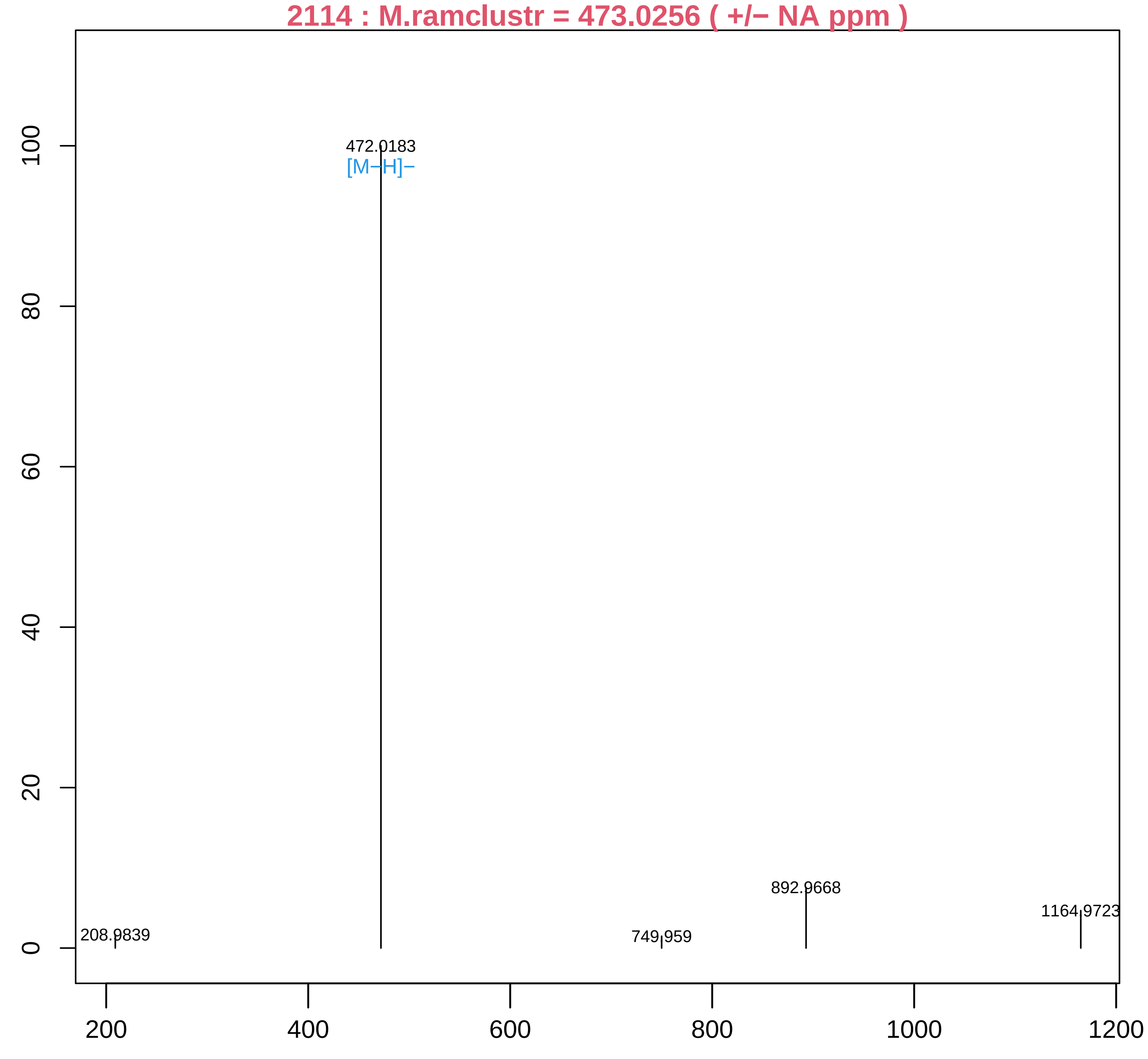
2114 : M.ramclustr = 473.0256 (+/− NA ppm)
472.0183
[M−H]−
208.9839
749.959
892.9668
1164.9723
0
20
40
60
80
100
200
400
600
800
1000
1200

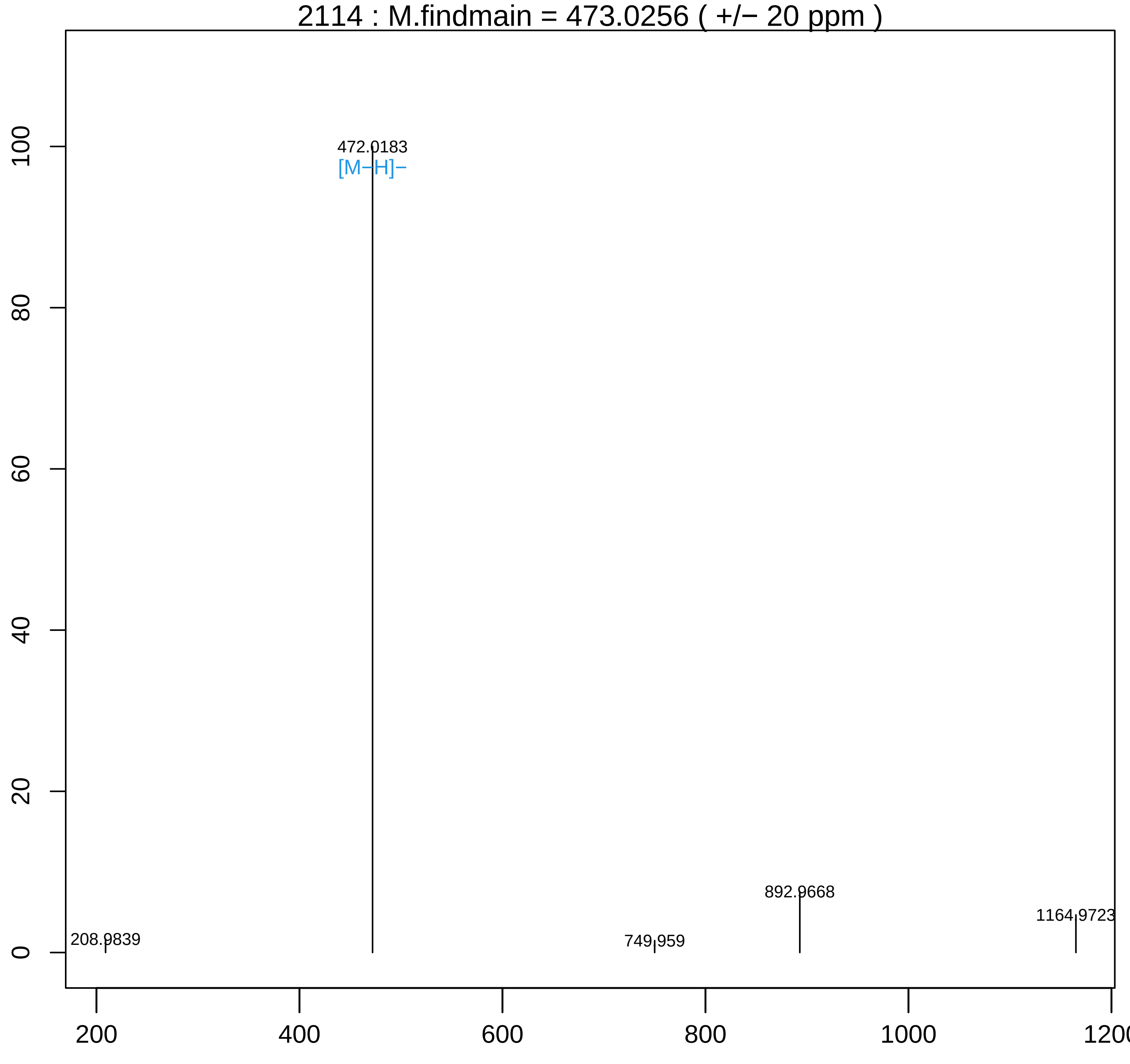
2114 : M.findmain = 473.0256 (+/− 20 ppm)
472.0183
[M−H]−
208.9839
749.959
892.9668
1164.9723
0
20
40
60
80
100
200
400
600
800
1000
1200

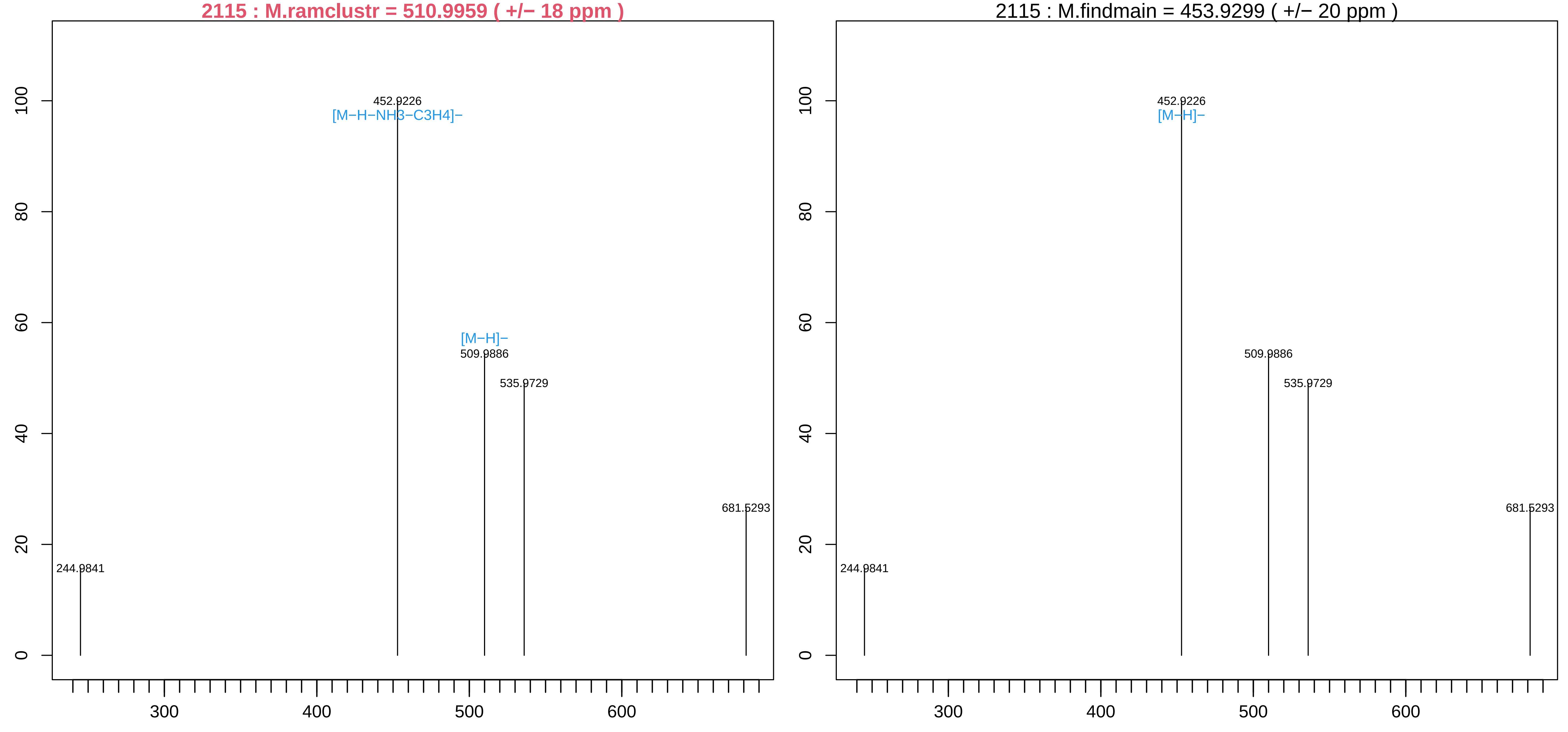

2115 : M.ramclustr = 510.9959 (+/− 18 ppm)
452.9226
[M−H−NH3−C3H4]−
[M−H]−
509.9886
535.9729
681.5293
244.9841
2115 : M.findmain = 453.9299 (+/− 20 ppm)
452.9226
[M−H]−
509.9886
535.9729
681.5293
244.9841

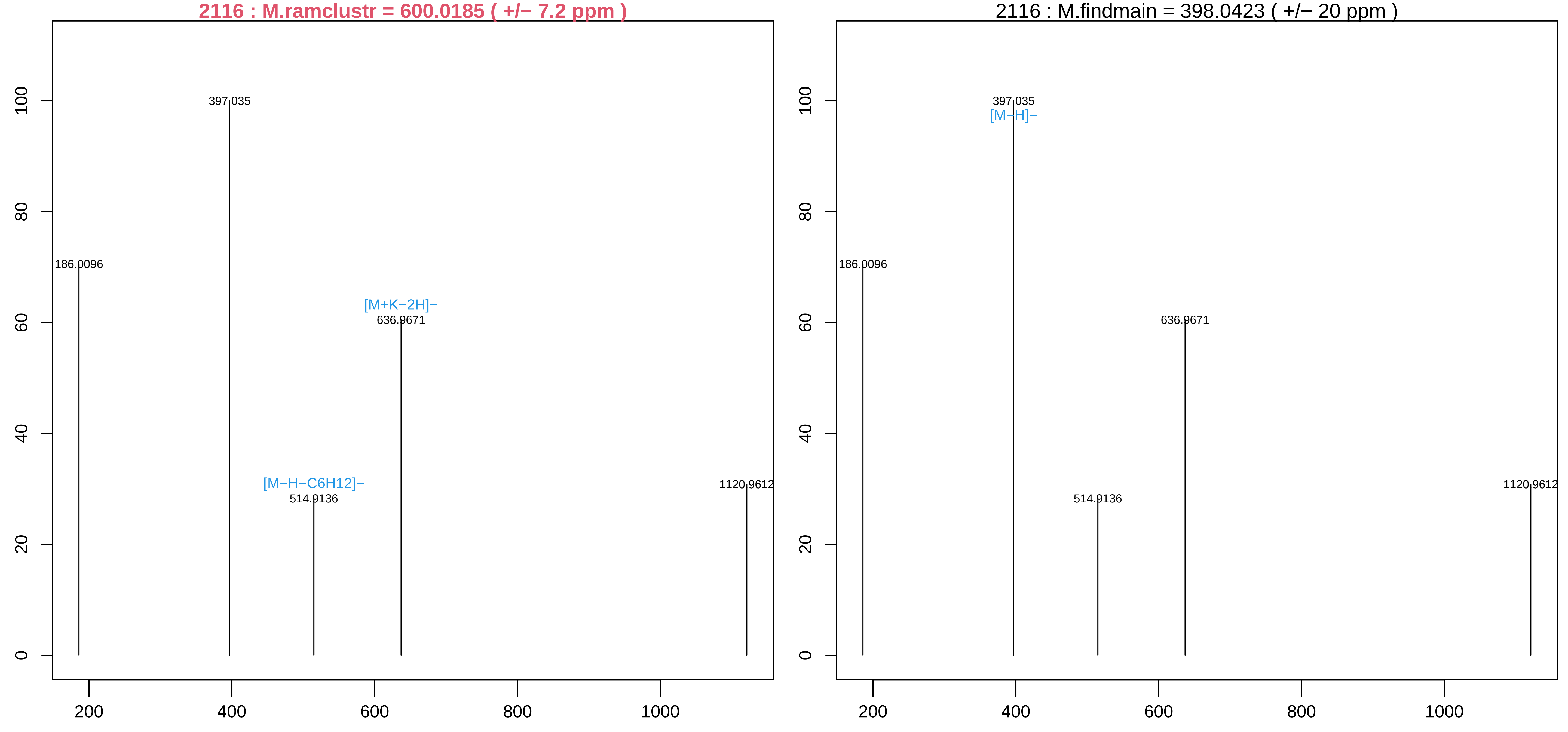
2116 : M.ramclustr = 600.0185 (+/− 7.2 ppm)
397.035
186.0096
[M+K−2H]−
636.9671
[M−H−C6H12]−
514.9136
1120.9612
0
20
40
60
80
100
200
400
600
800
1000
2116 : M.findmain = 398.0423 (+/− 20 ppm)
397.035
[M−H]−
186.0096
636.9671
514.9136
1120.9612

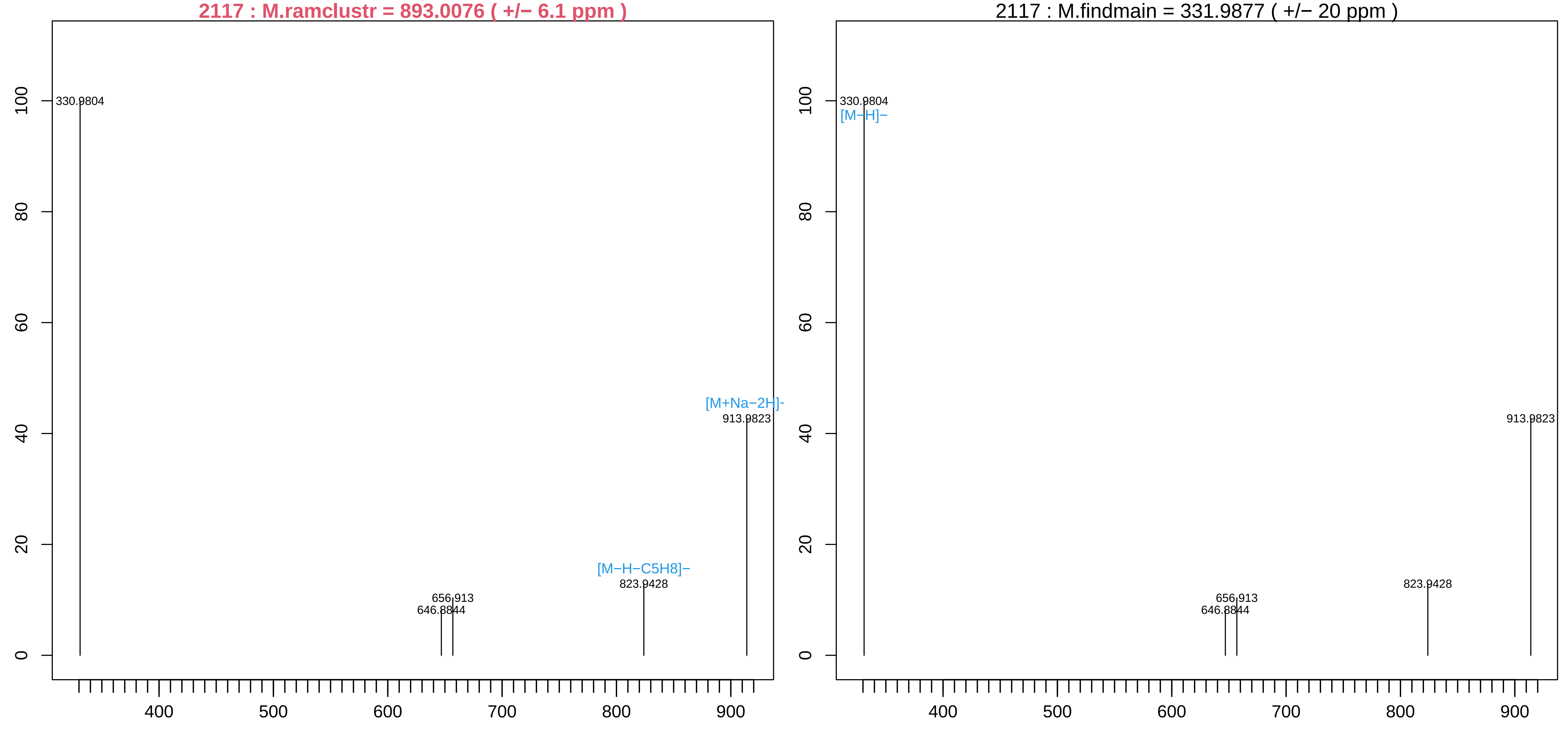

2117 : M.ramclustr = 893.0076 (+/− 6.1 ppm)
330.9804
656.913
646.8844
[M−H−C5H8]−
823.9428
[M+Na−2H]-
913.9823
2117 : M.findmain = 331.9877 (+/− 20 ppm)
330.9804
[M−H]−
656.913
646.8844
823.9428
913.9823
0
20
40
60
80
100
400
500
600
700
800
900

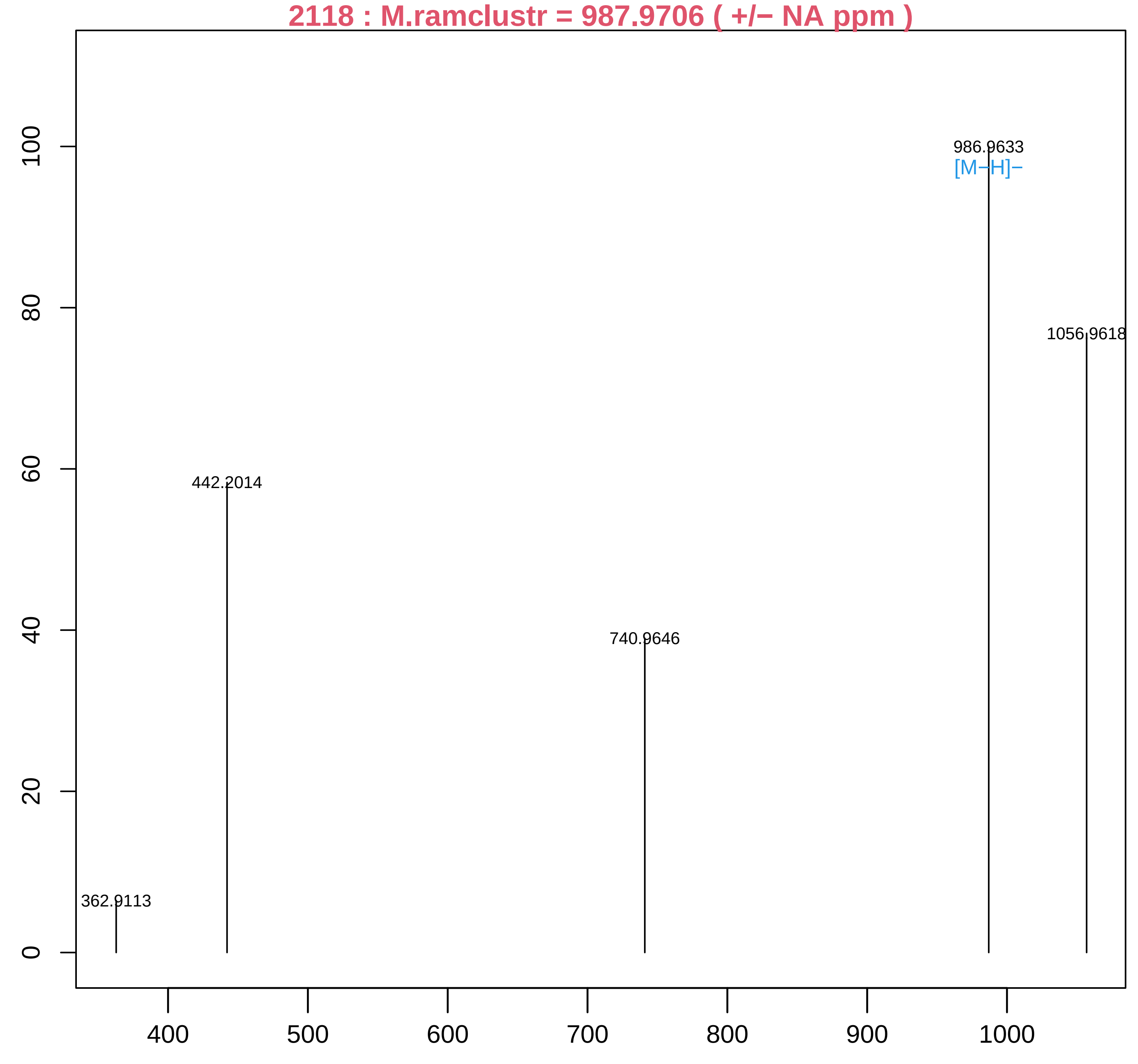
2118 : M.ramclustr = 987.9706 (+/− NA ppm)
986.9633
[M−H]−
1056.9618
442.2014
740.9646
362.9113
0
20
40
60
80
100
400
500
600
700
800
900
1000

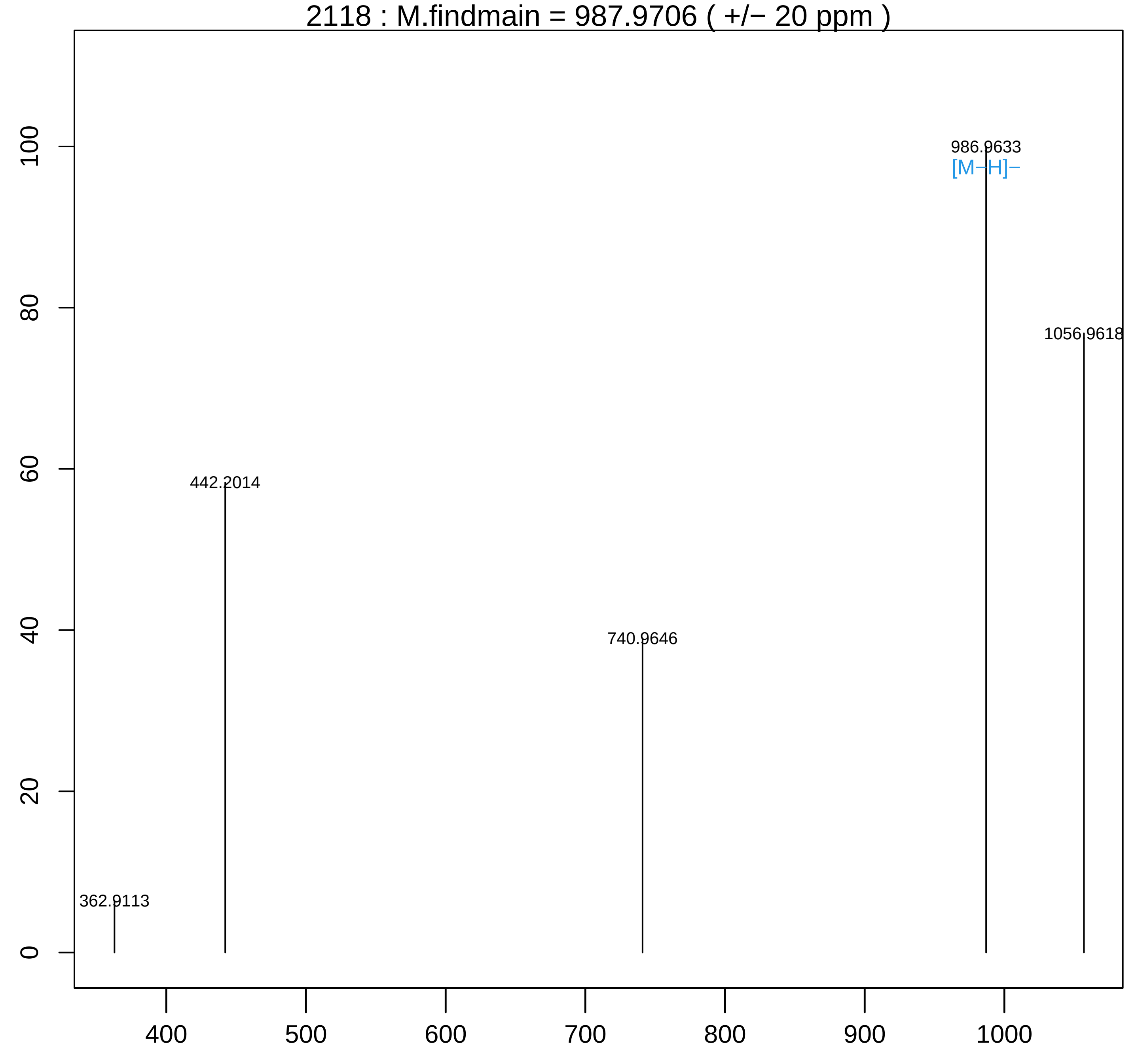
2118 : M.findmain = 987.9706 (+/− 20 ppm)
986.9633
[M−H]−
1056.9618
442.2014
740.9646
362.9113
0
20
40
60
80
100
400
500
600
700
800
900
1000

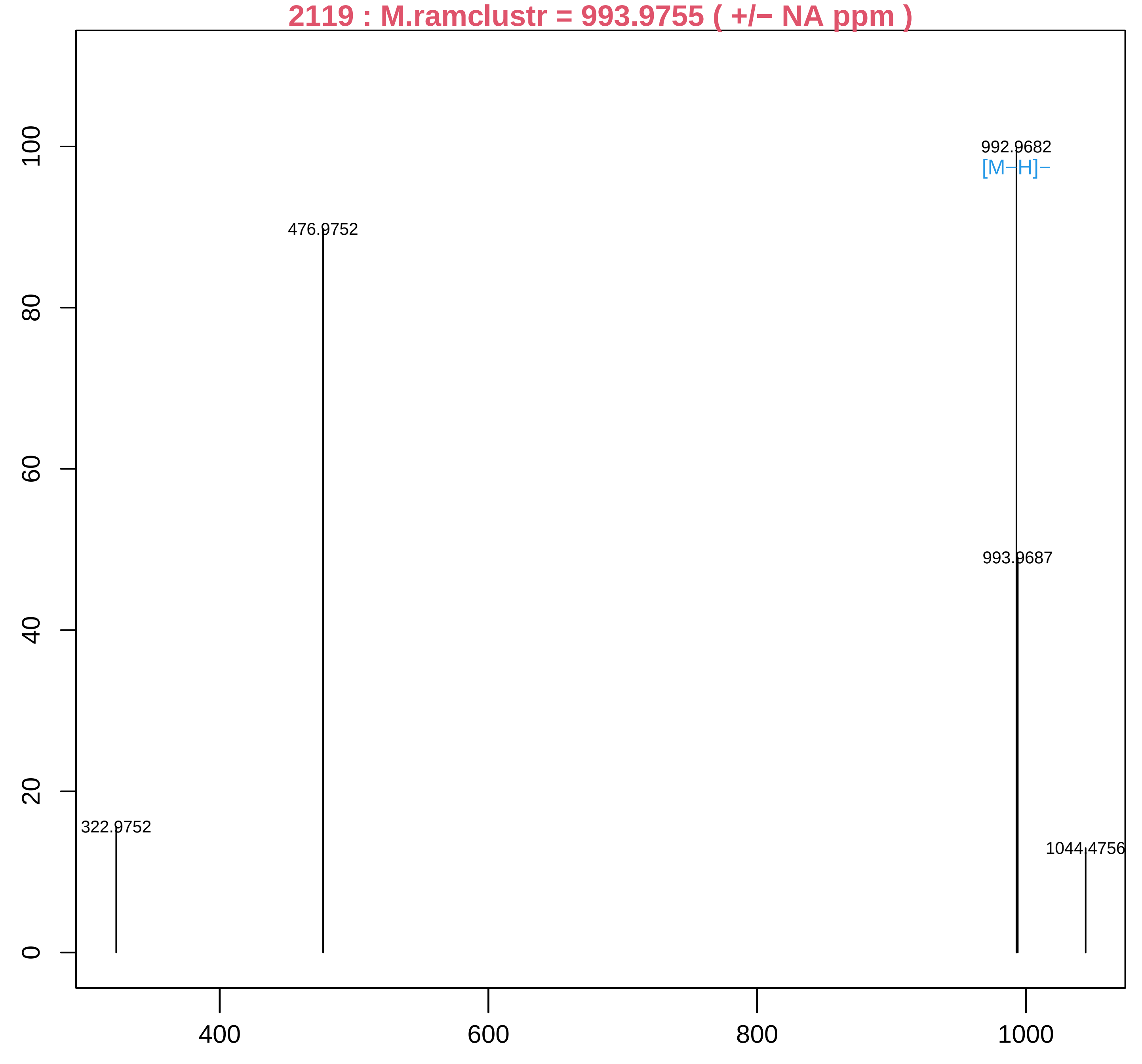

2119 : M.ramclustr = 993.9755 (+/− NA ppm)
992.9682
[M−H]−
476.9752
993.9687
322.9752
1044.4756
0
20
40
60
80
100
400
600
800
1000

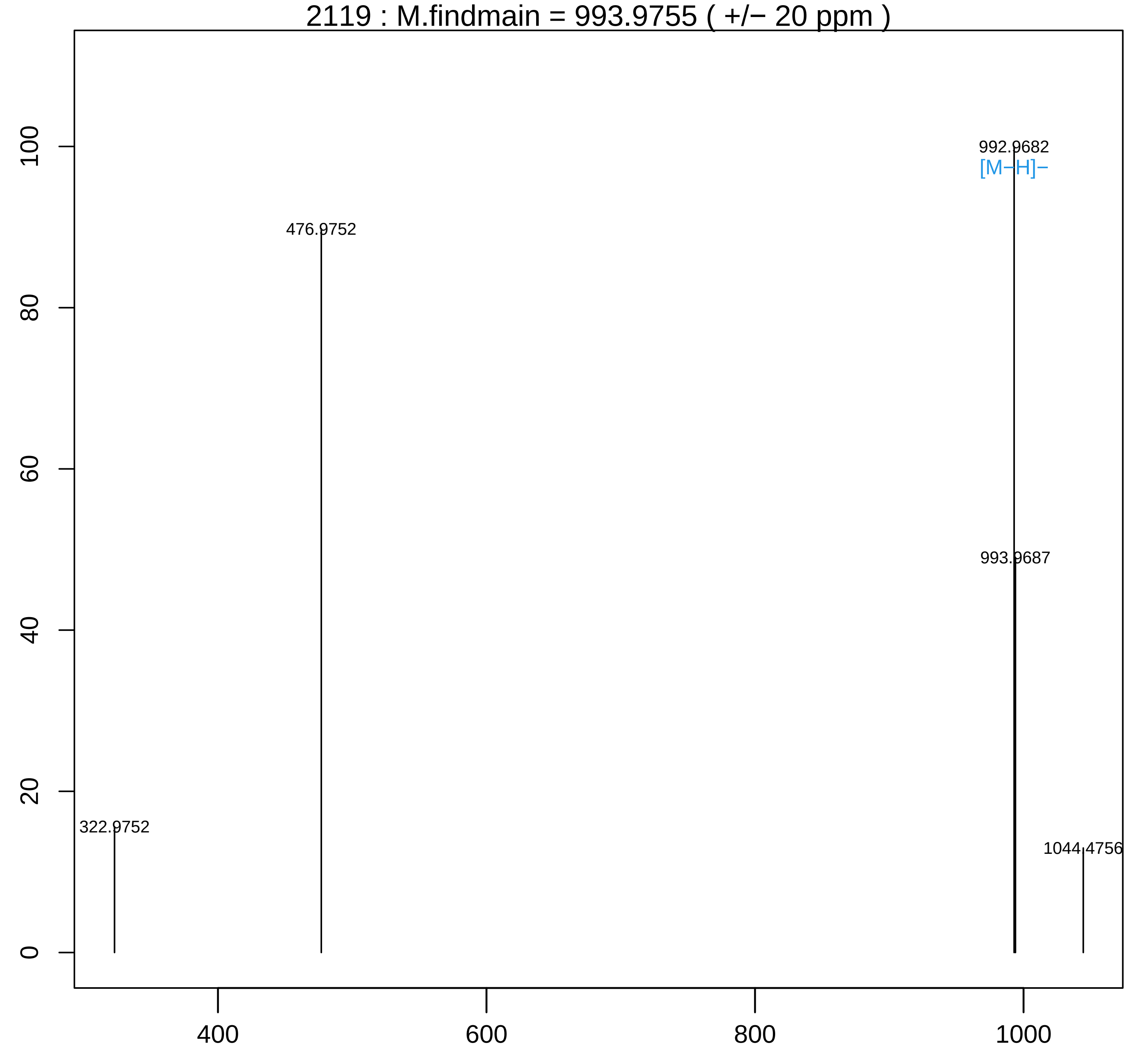

2119 : M.findmain = 993.9755 (+/− 20 ppm)
992.9682
[M−H]−
476.9752
993.9687
322.9752
1044.4756
0
20
40
60
80
100
400
600
800
1000

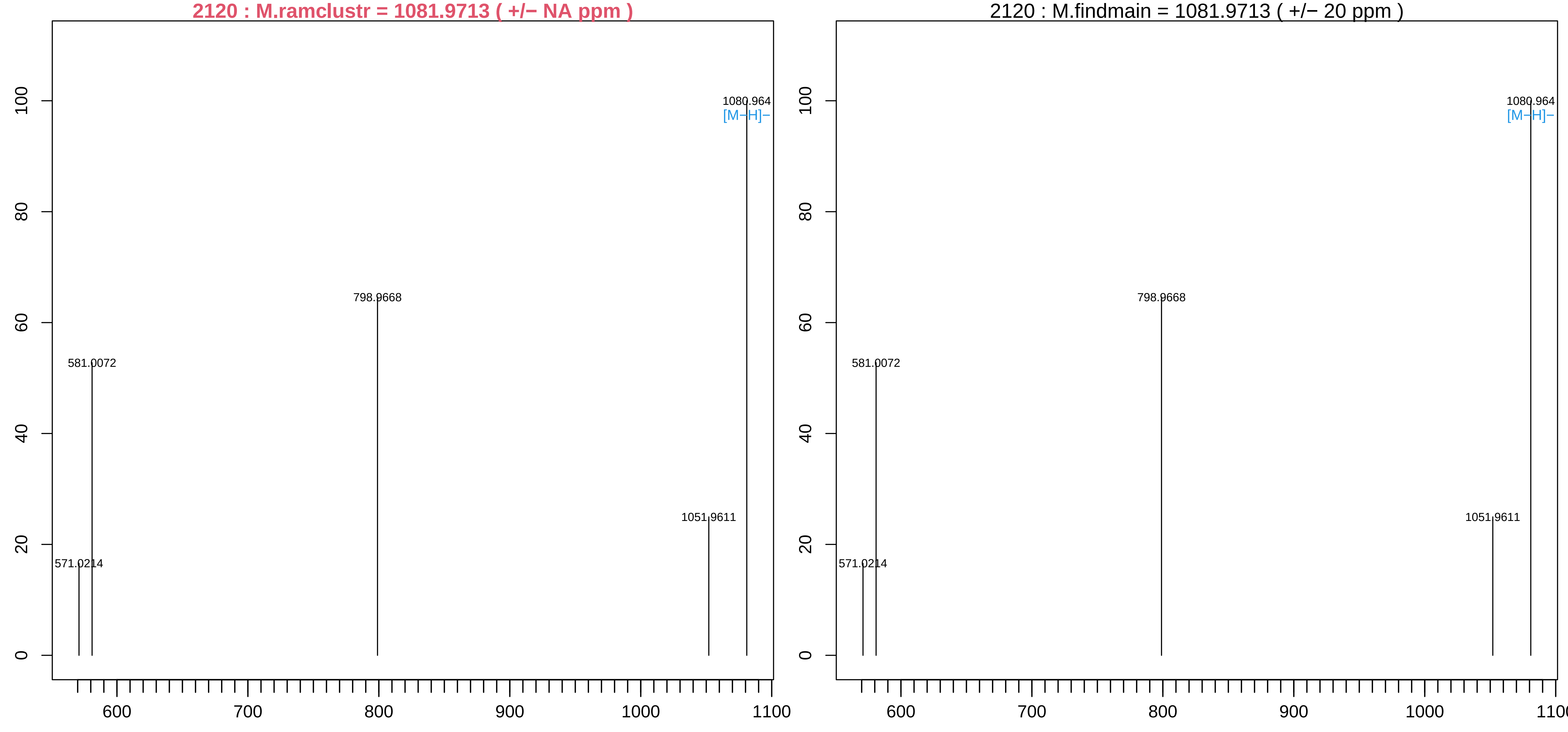
2120 : M.ramclustr = 1081.9713 (+/− NA ppm)
2120 : M.findmain = 1081.9713 (+/− 20 ppm)
1080.964
[M−H]−
798.9668
581.0072
571.0214
1051.9611
0
20
40
60
80
100
600
700
800
900
1000
1100

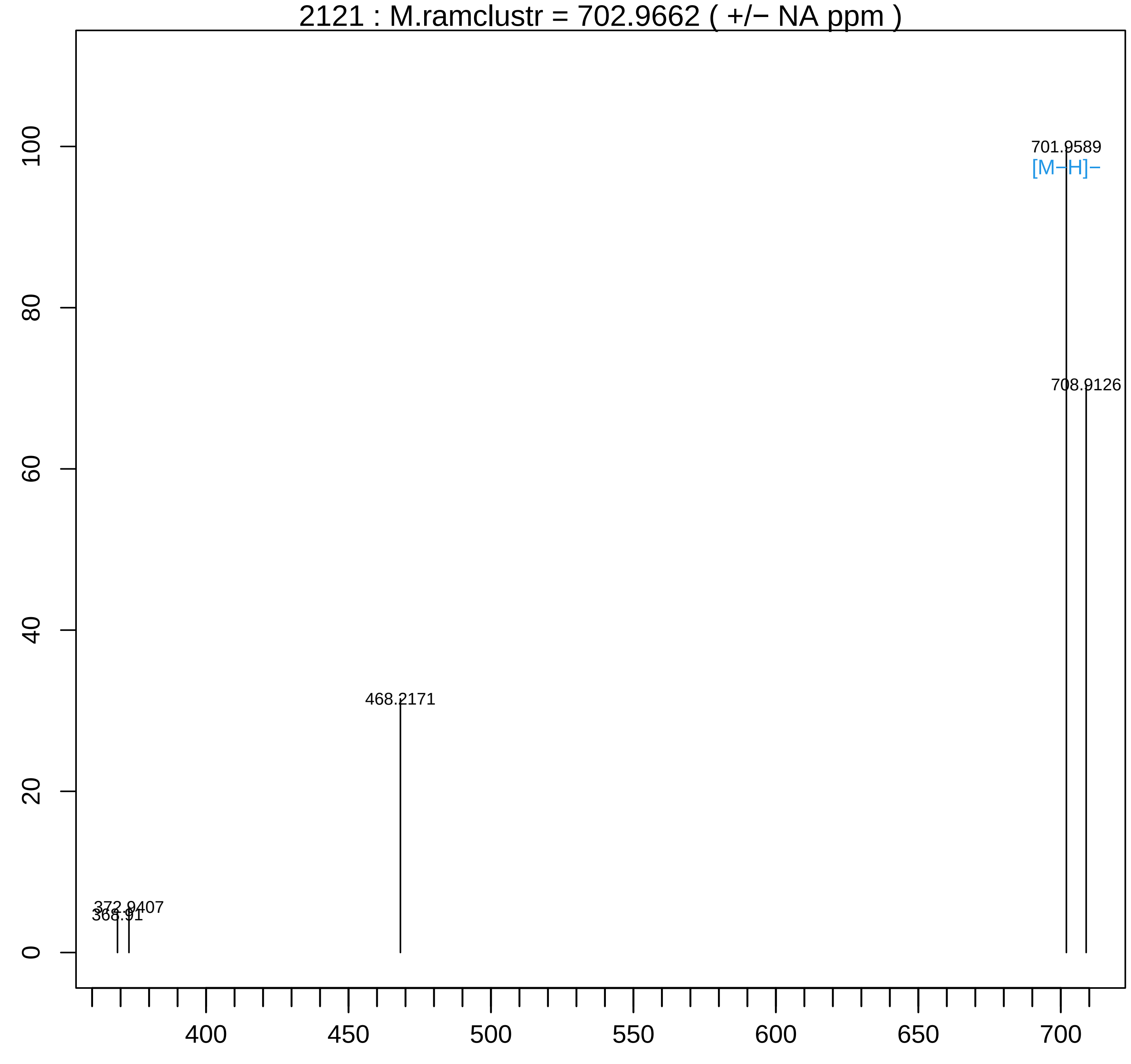

2121 : M.ramclustr = 702.9662 (+/− NA ppm)
701.9589
[M−H]−
708.9126
468.2171
372.9407
368.91
0
20
40
60
80
100
400
450
500
550
600
650
700

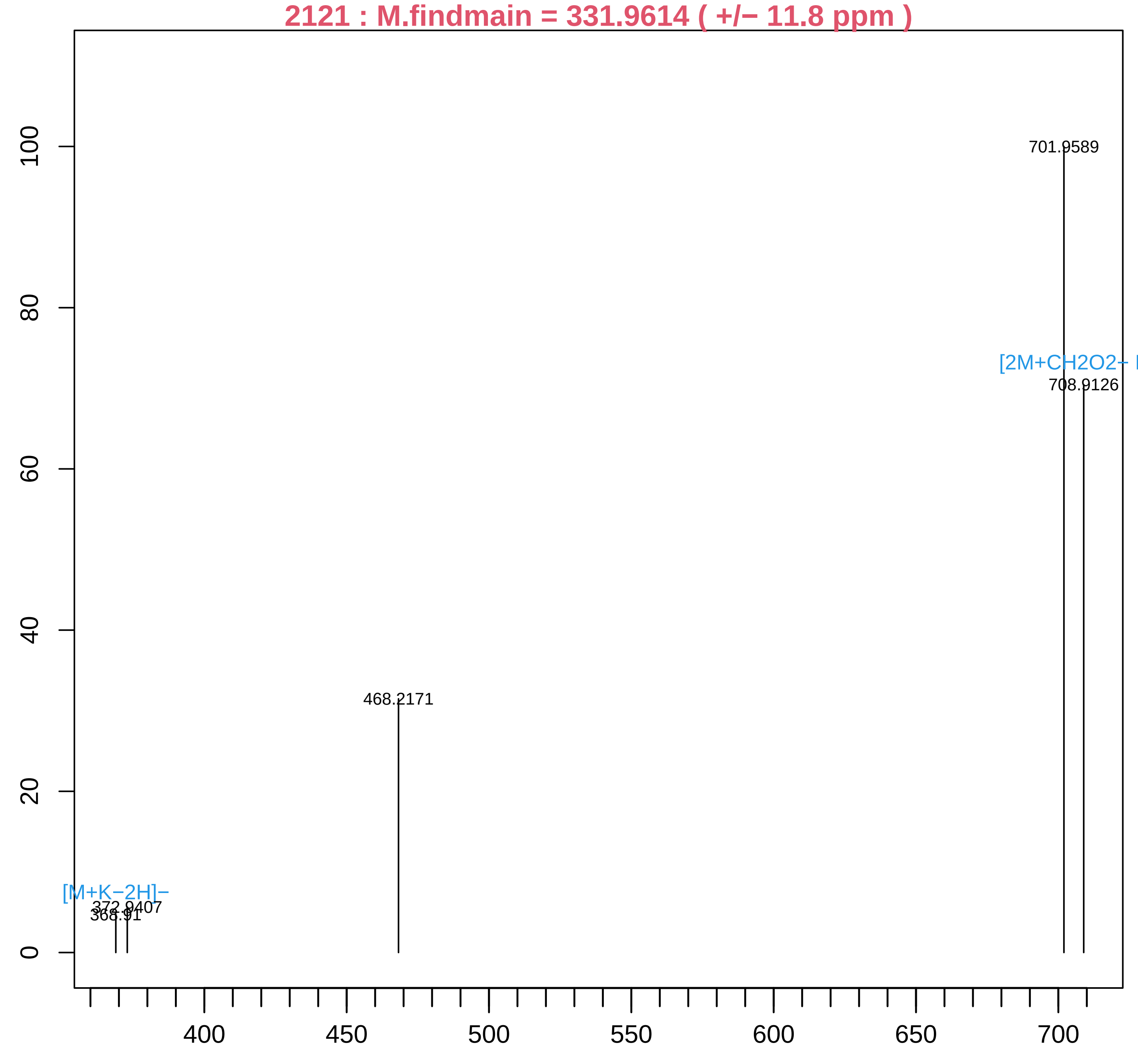

2121 : M.findmain = 331.9614 (+/− 11.8 ppm)
701.9589
[2M+CH2O2−
708.9126
468.2171
[M+K−2H]−
372.9407
368.91
0
20
40
60
80
100
400
450
500
550
600
650
700

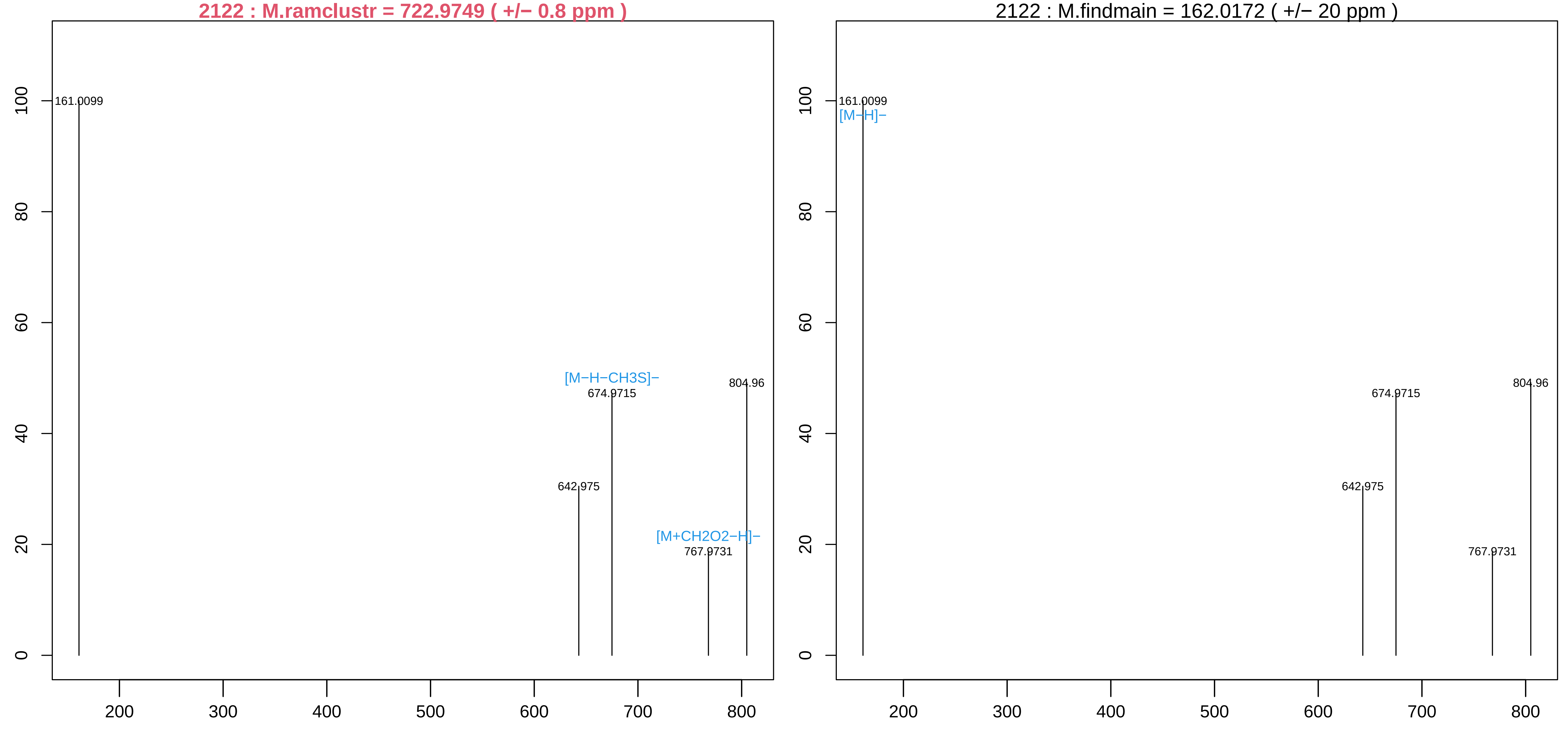
2122 : M.ramclustr = 722.9749 (+/− 0.8 ppm)
161.0099
[M−H−CH3S]−
674.9715
804.96
642.975
[M+CH2O2−H]−
767.9731
0
20
40
60
80
100
200
300
400
500
600
700
800
2122 : M.findmain = 162.0172 (+/− 20 ppm)
161.0099
[M−H]−
674.9715
804.96
642.975
767.9731

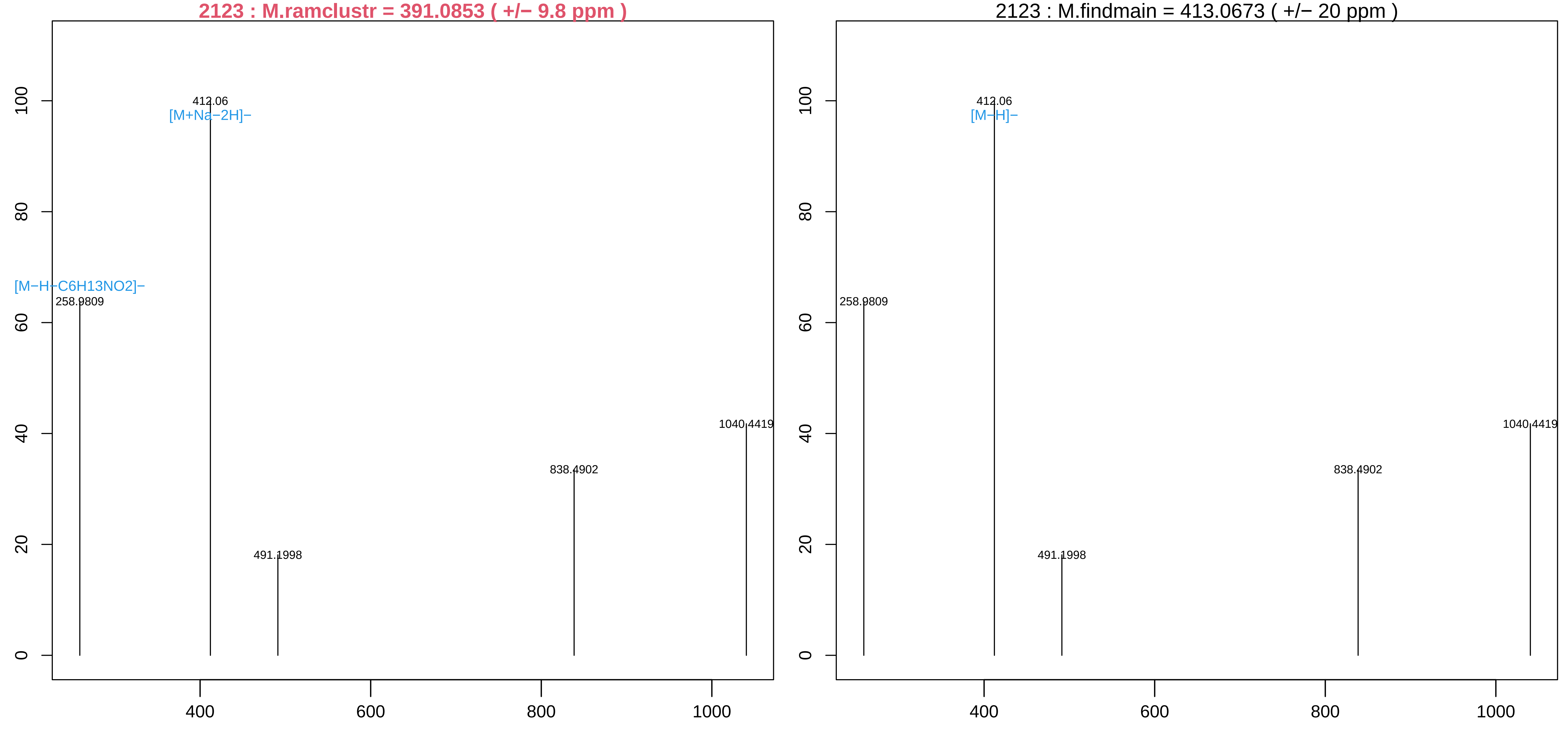
2123 : M.ramclustr = 391.0853 (+/− 9.8 ppm)
412.06
[M+Na−2H]−
[M−H−C6H13NO2]−
258.9809
491.1998
838.4902
1040.4419
2123 : M.findmain = 413.0673 (+/− 20 ppm)
412.06
[M−H]−
258.9809
491.1998
838.4902
1040.4419
0
20
40
60
80
100
400
600
800
1000

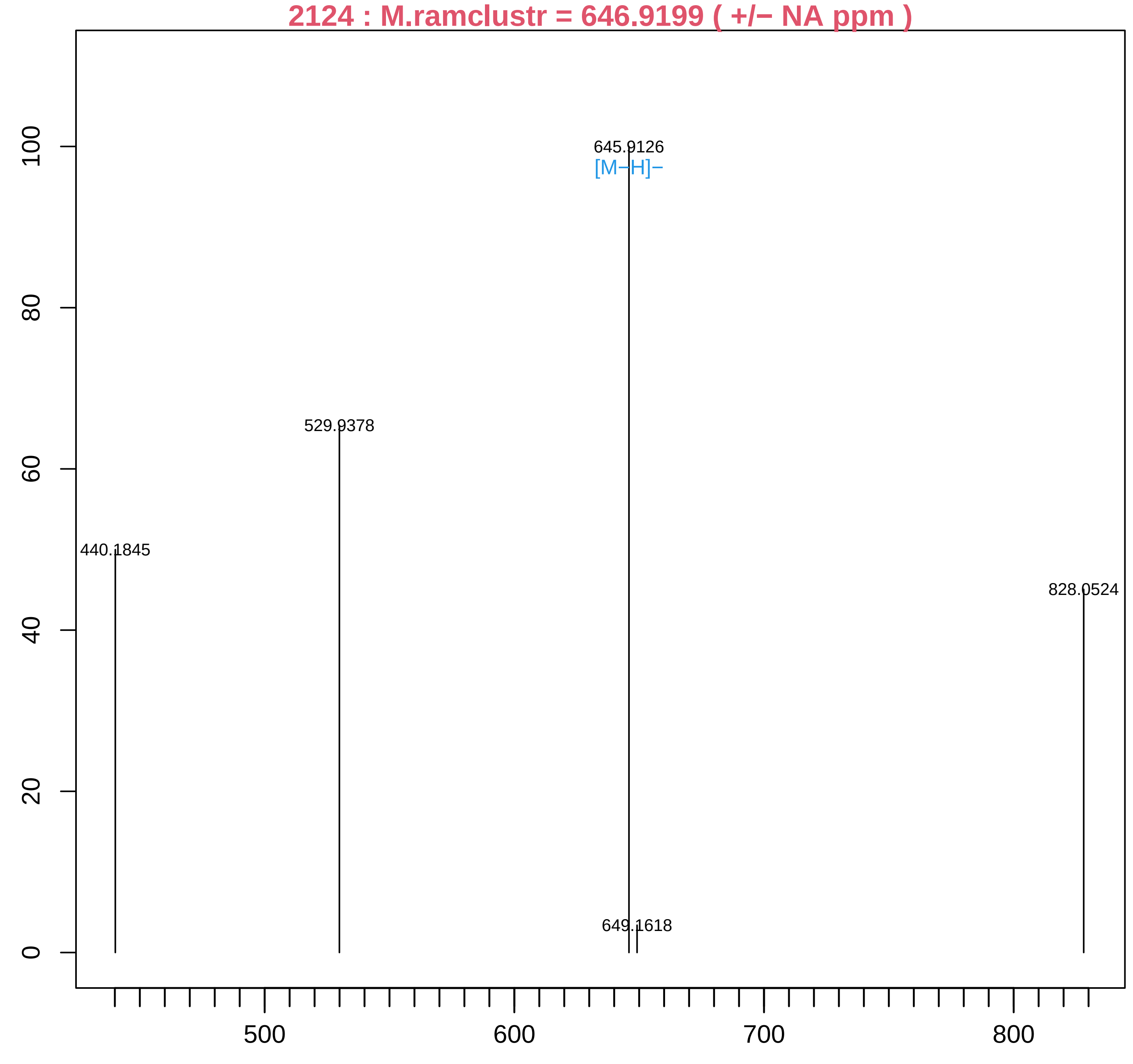

2124 : M.ramclustr = 646.9199 (+/− NA ppm)
645.9126
[M−H]−
529.9378
440.1845
828.0524
649.1618
0
20
40
60
80
100
500
600
700
800

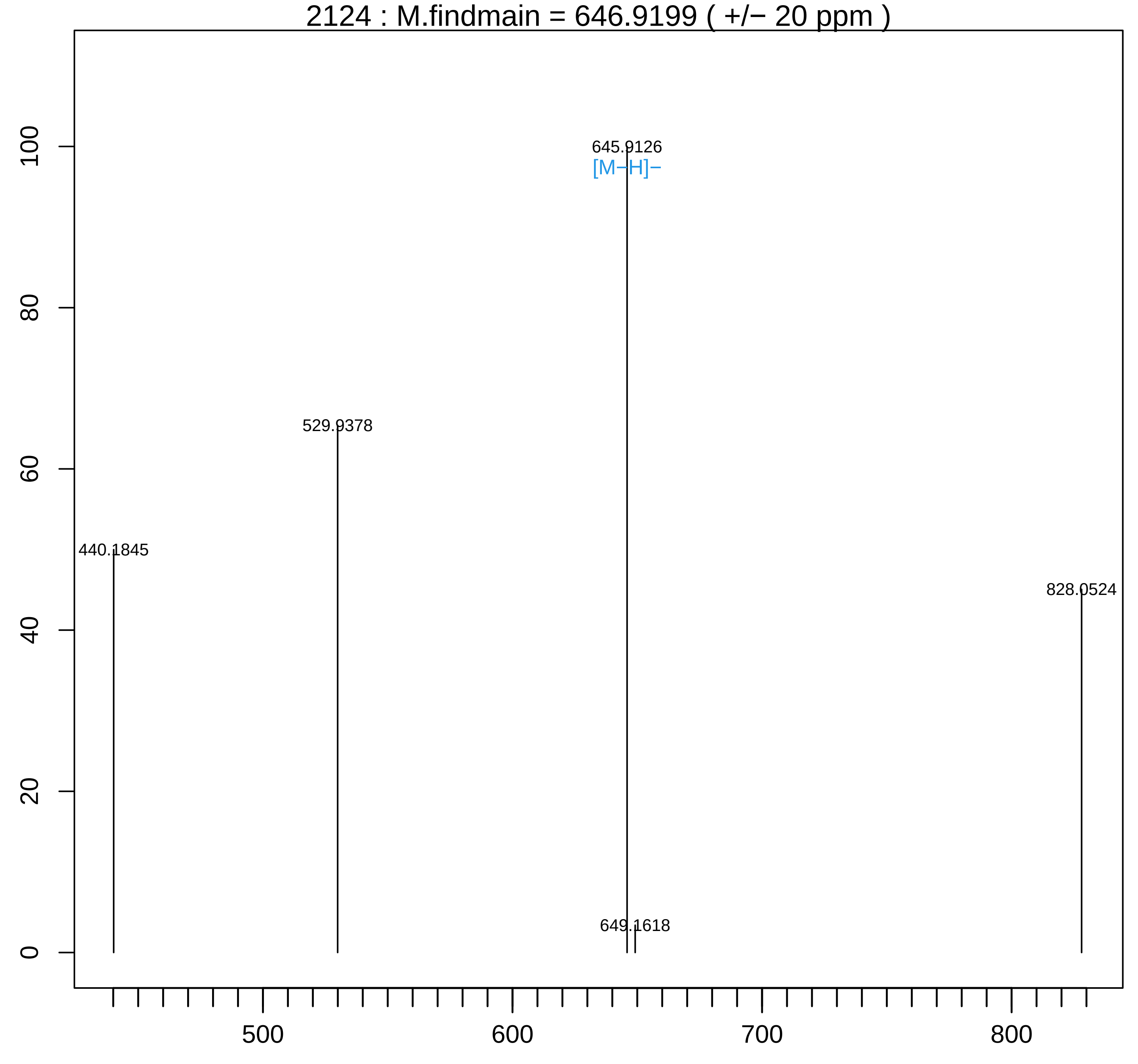

2124 : M.findmain = 646.9199 (+/− 20 ppm)
645.9126
[M−H]−
529.9378
440.1845
828.0524
649.1618
0
20
40
60
80
100
500
600
700
800

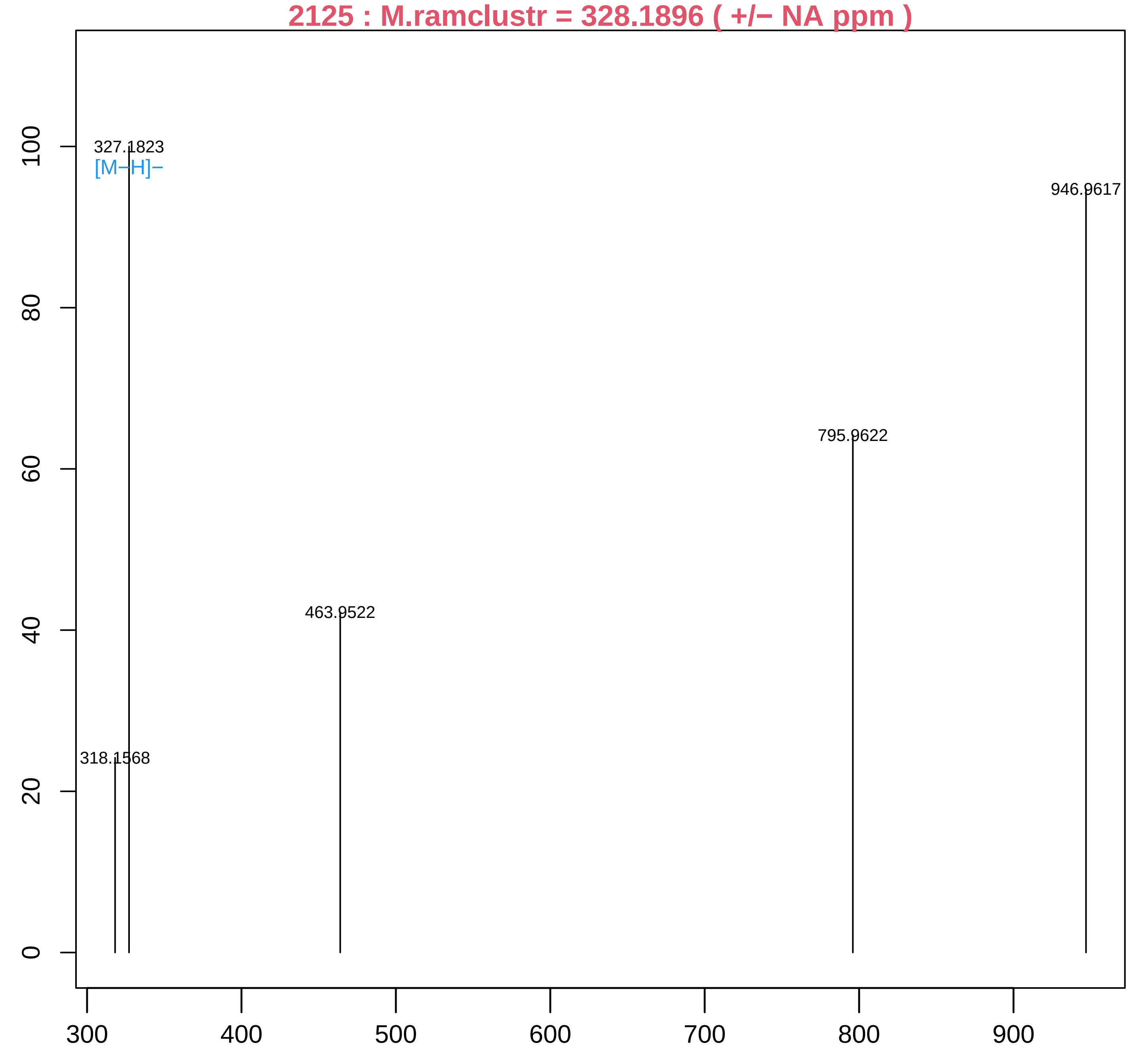
2125 : M.ramclustr = 328.1896 (+/− NA ppm)
327.1823
[M−H]−
946.9617
795.9622
463.9522
318.1568
0
20
40
60
80
100
300
400
500
600
700
800
900

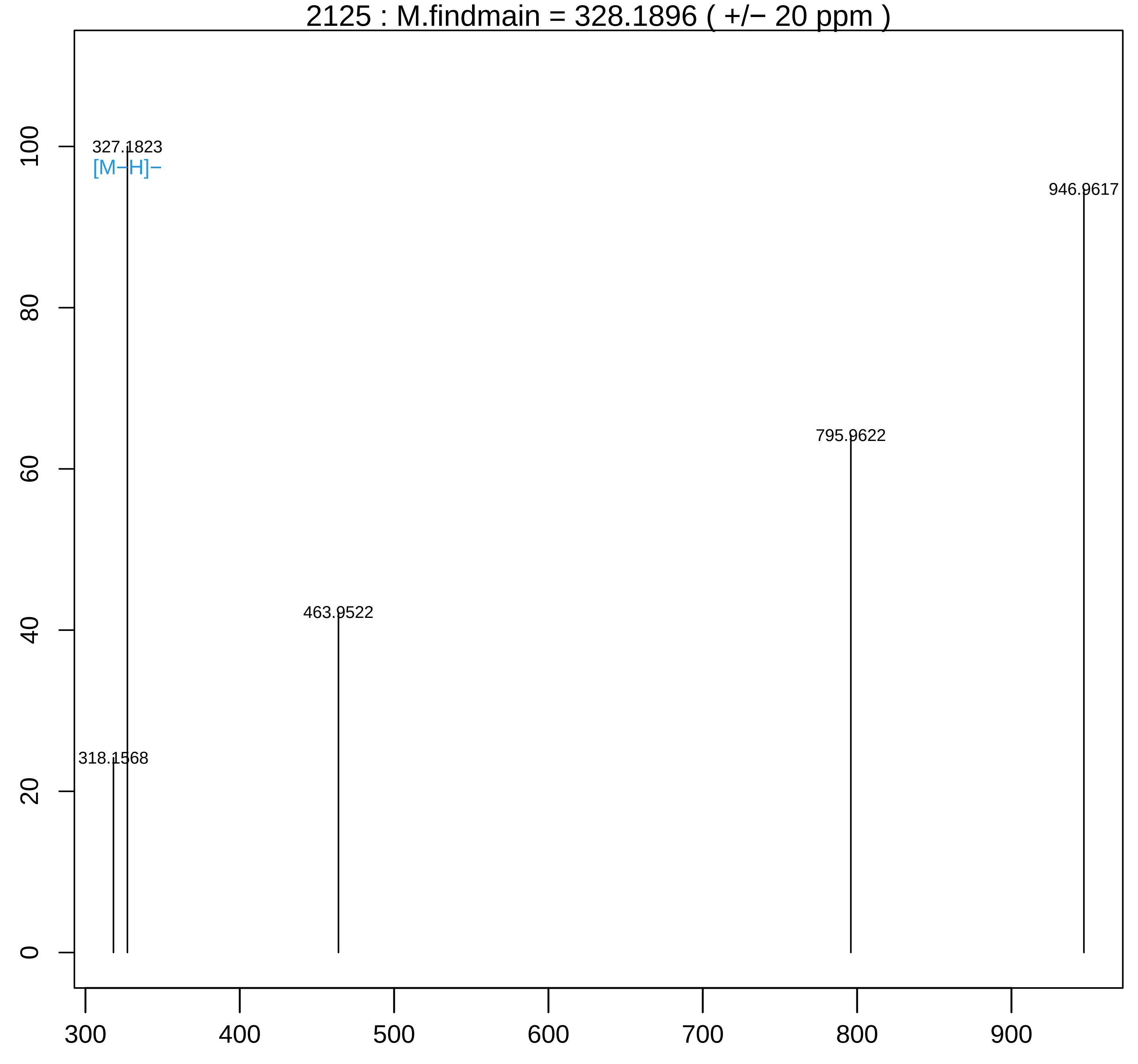
2125 : M.findmain = 328.1896 (+/− 20 ppm)
327.1823
[M−H]−
946.9617
795.9622
463.9522
318.1568
0
20
40
60
80
100
300
400
500
600
700
800
900

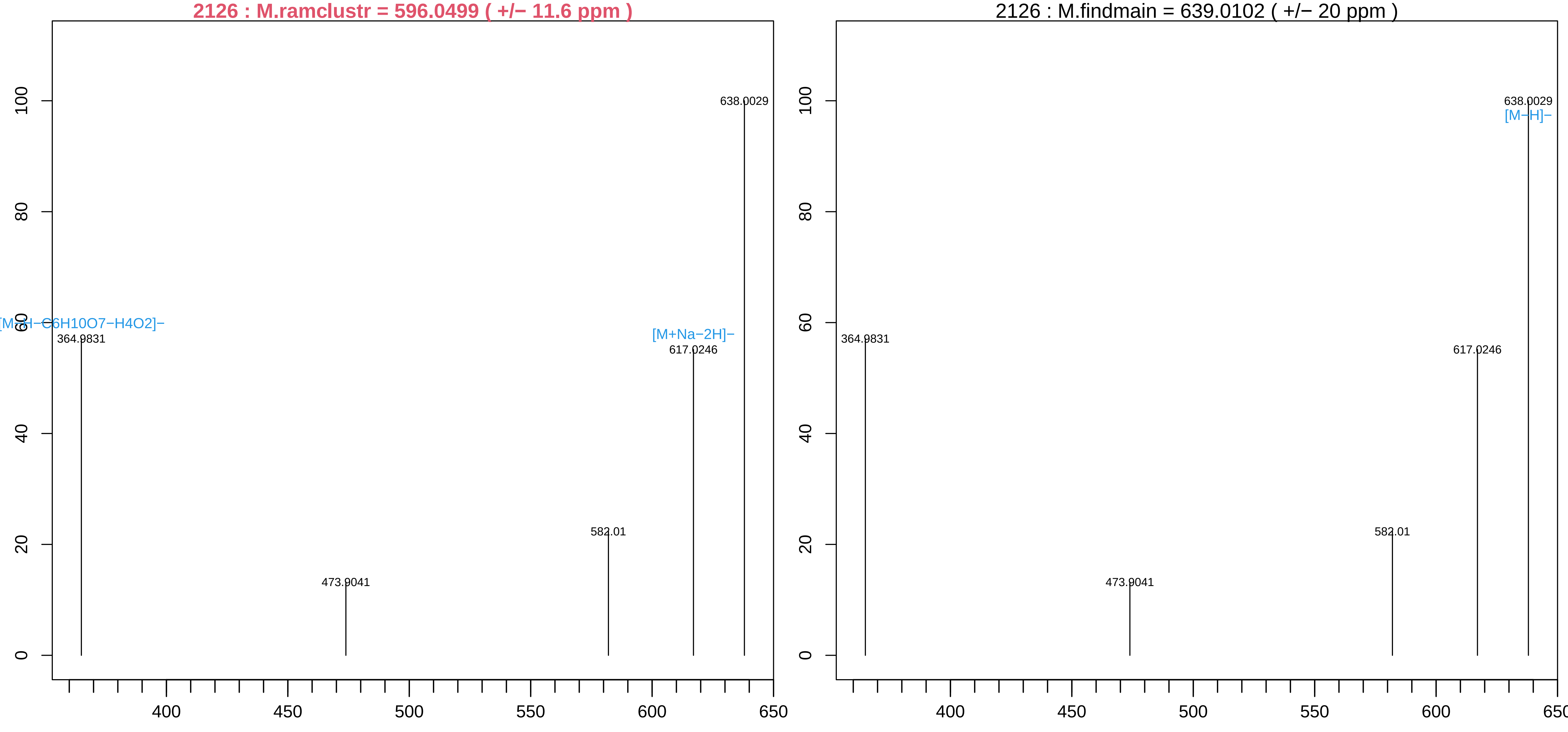
2126 : M.ramclustr = 596.0499 (+/− 11.6 ppm)
[M-H−C6H10O7−H4O2]−
364.9831
473.9041
582.01
[M+Na−2H]−
617.0246
638.0029
2126 : M.findmain = 639.0102 (+/− 20 ppm)
364.9831
473.9041
582.01
617.0246
638.0029
[M−H]−

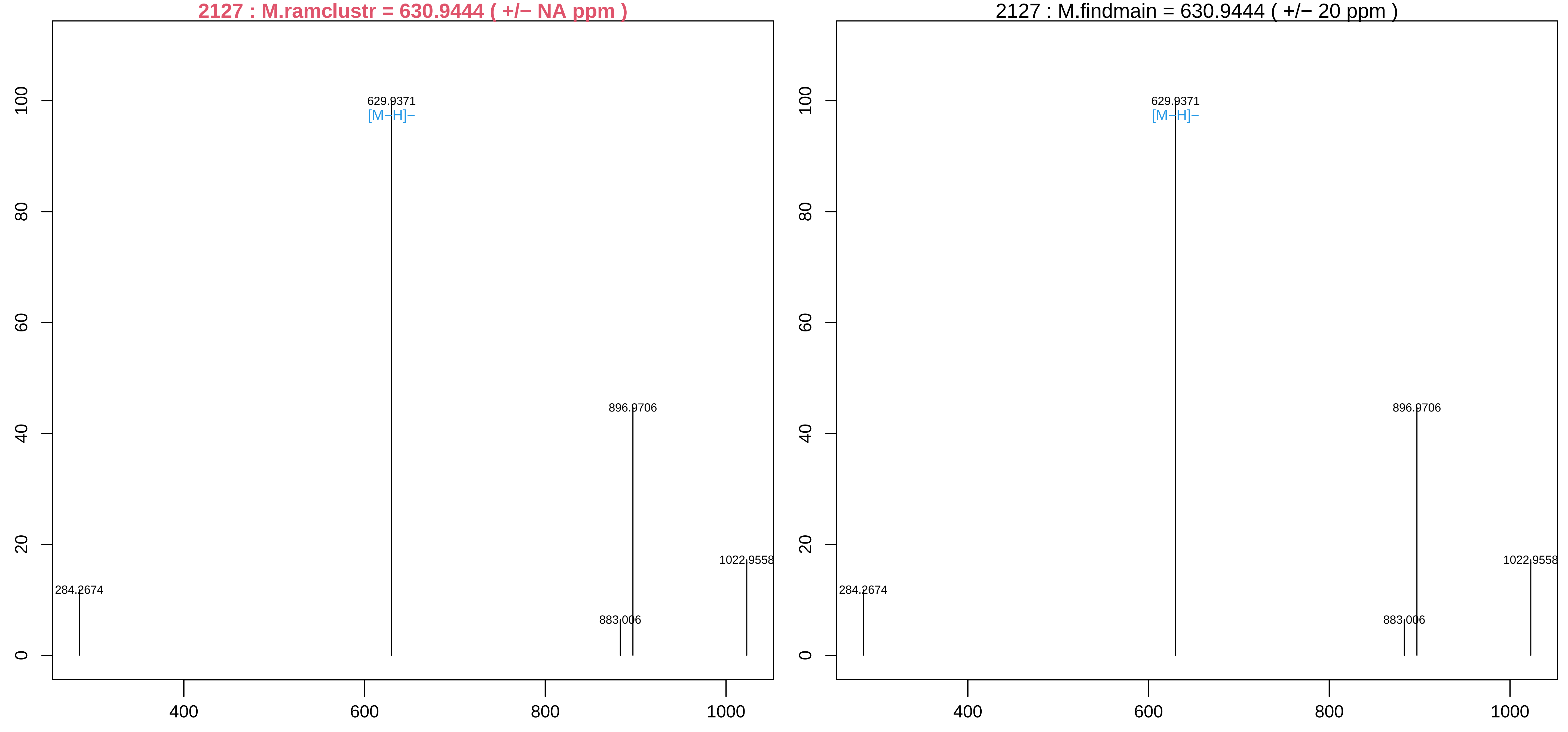

2127 : M.ramclustr = 630.9444 (+/− NA ppm)
629.9371
[M−H]−
896.9706
1022.9558
284.2674
883.006
0
20
40
60
80
100
400
600
800
1000
2127 : M.findmain = 630.9444 (+/− 20 ppm)
629.9371
[M−H]−
896.9706
1022.9558
284.2674
883.006
0
20
40
60
80
100
400
600
800
1000

2128 : M.ramclustr = 485.2199 (+/− NA ppm)

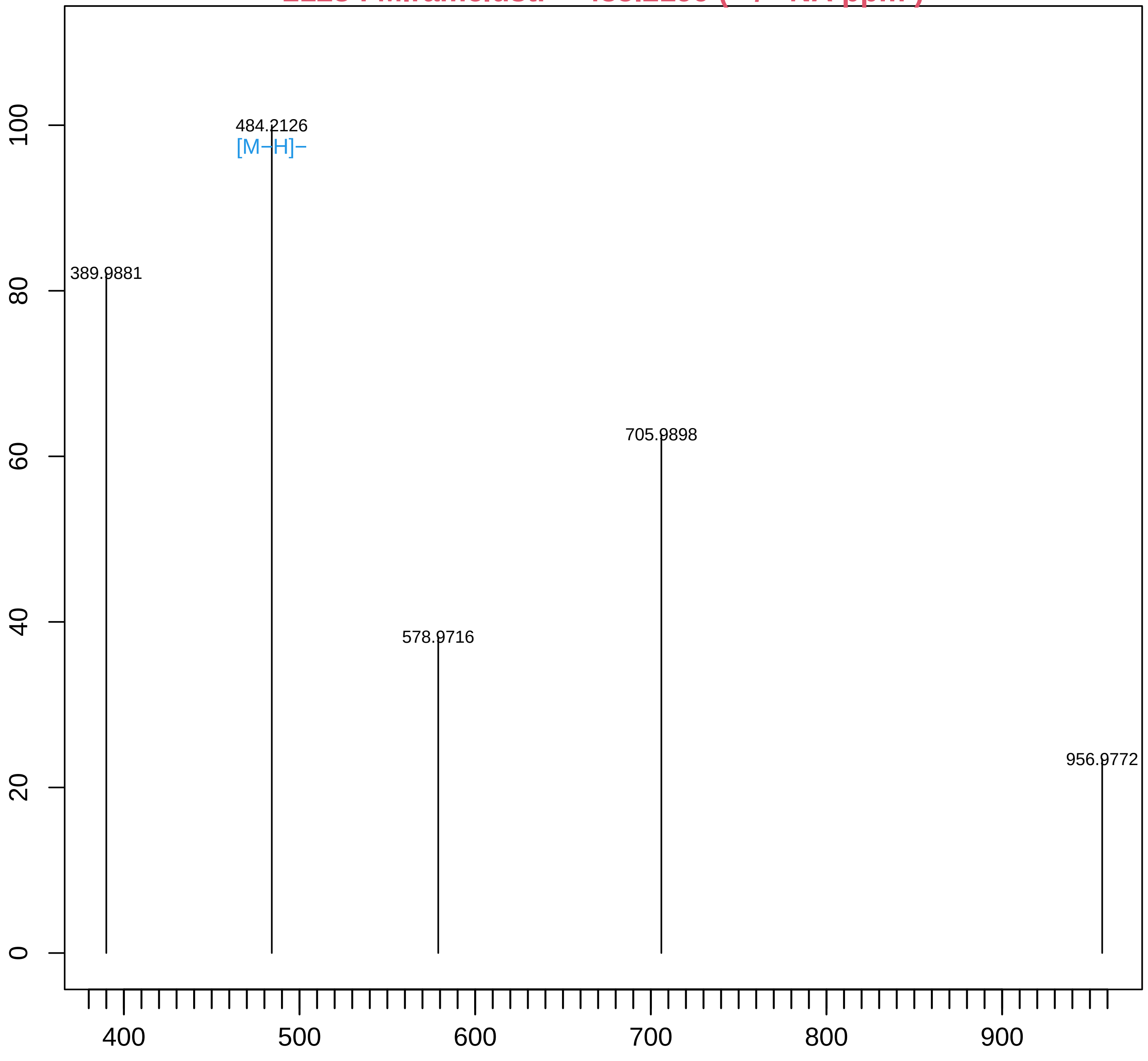

2128 : M.findmain = 485.2199 (+/− 20 ppm)

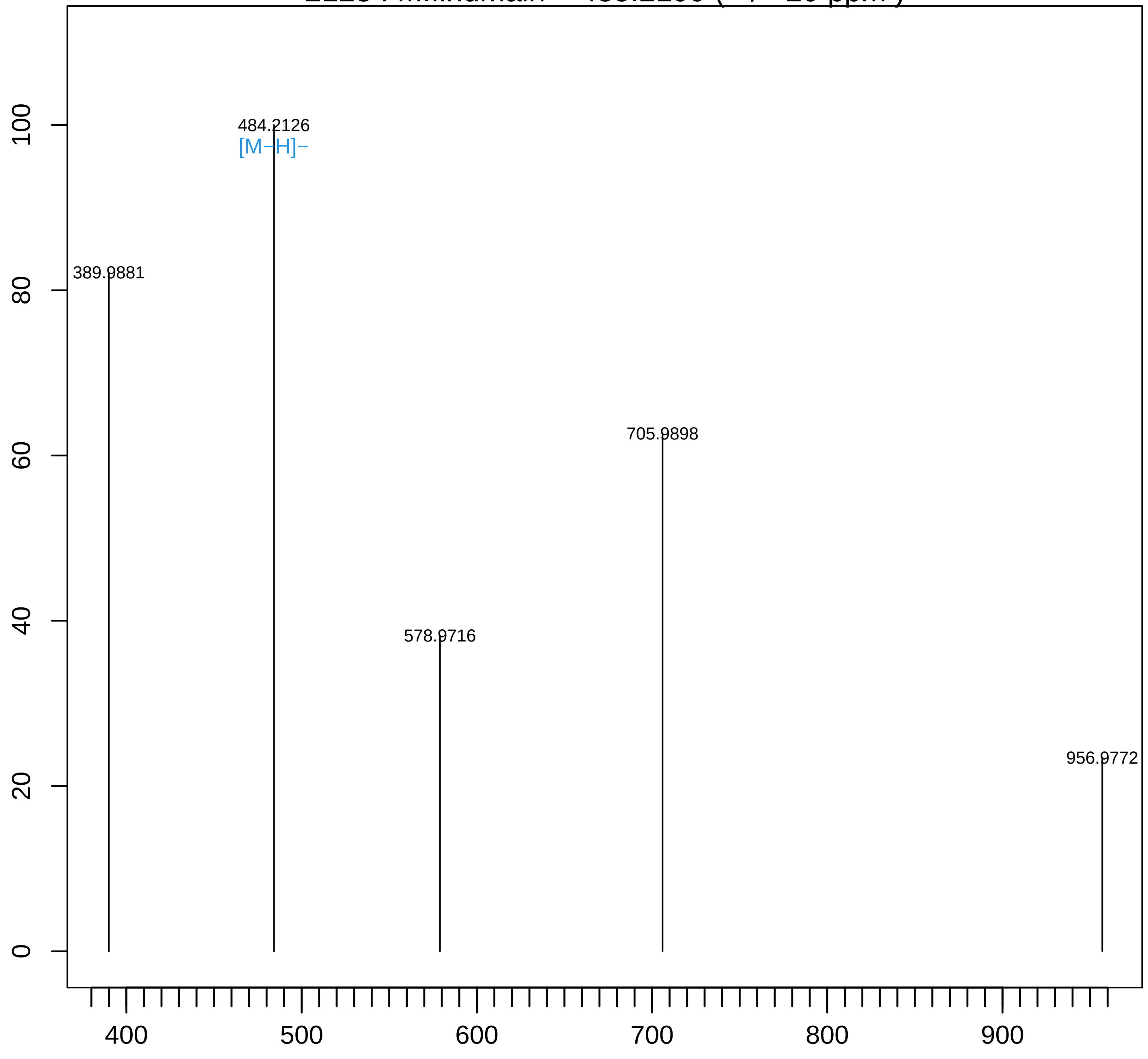

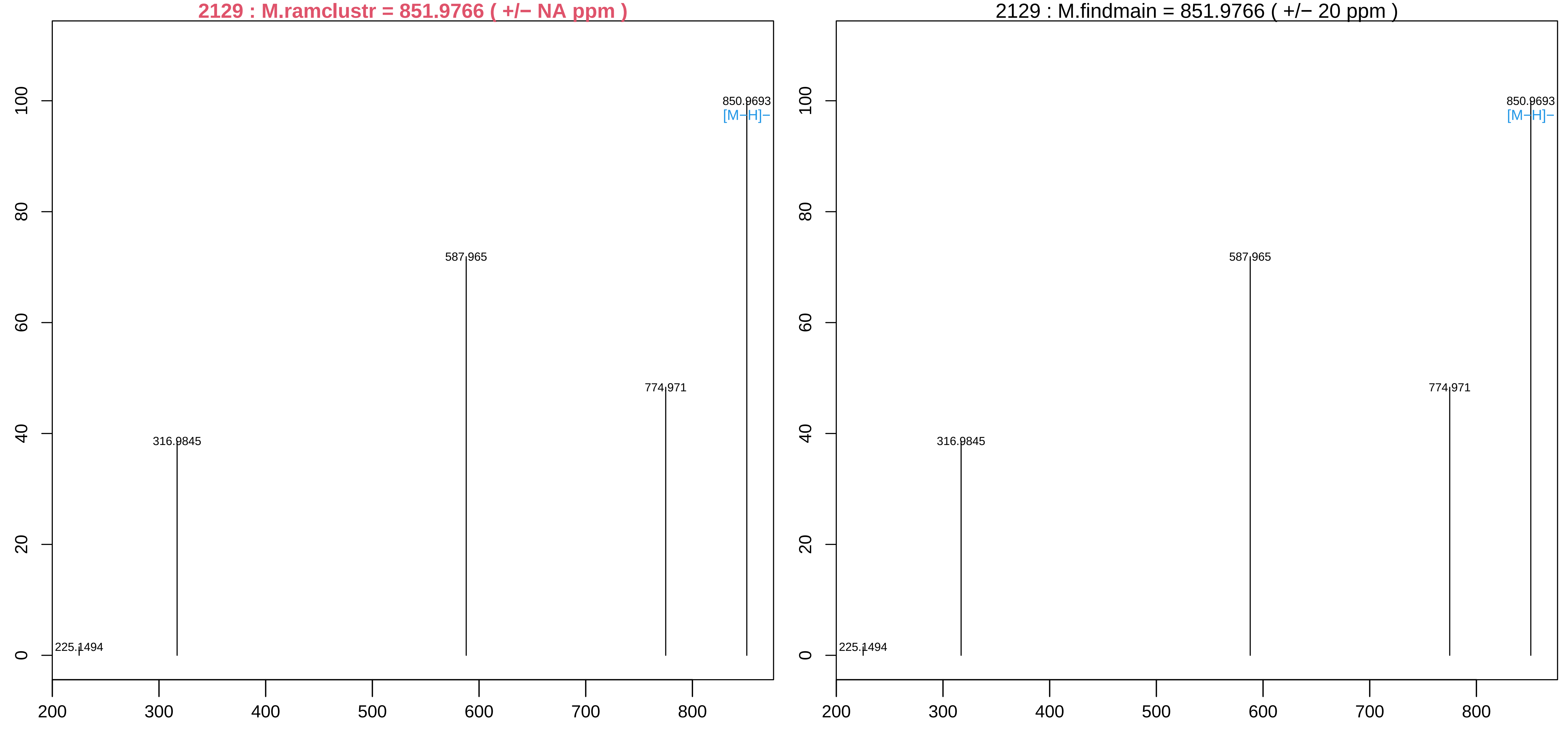

2129 : M.ramclustr = 851.9766 (+/− NA ppm)
850.9693
[M−H]−
587.965
774.971
316.9845
225.1494
2129 : M.findmain = 851.9766 (+/− 20 ppm)
850.9693
[M−H]−
587.965
774.971
316.9845
225.1494

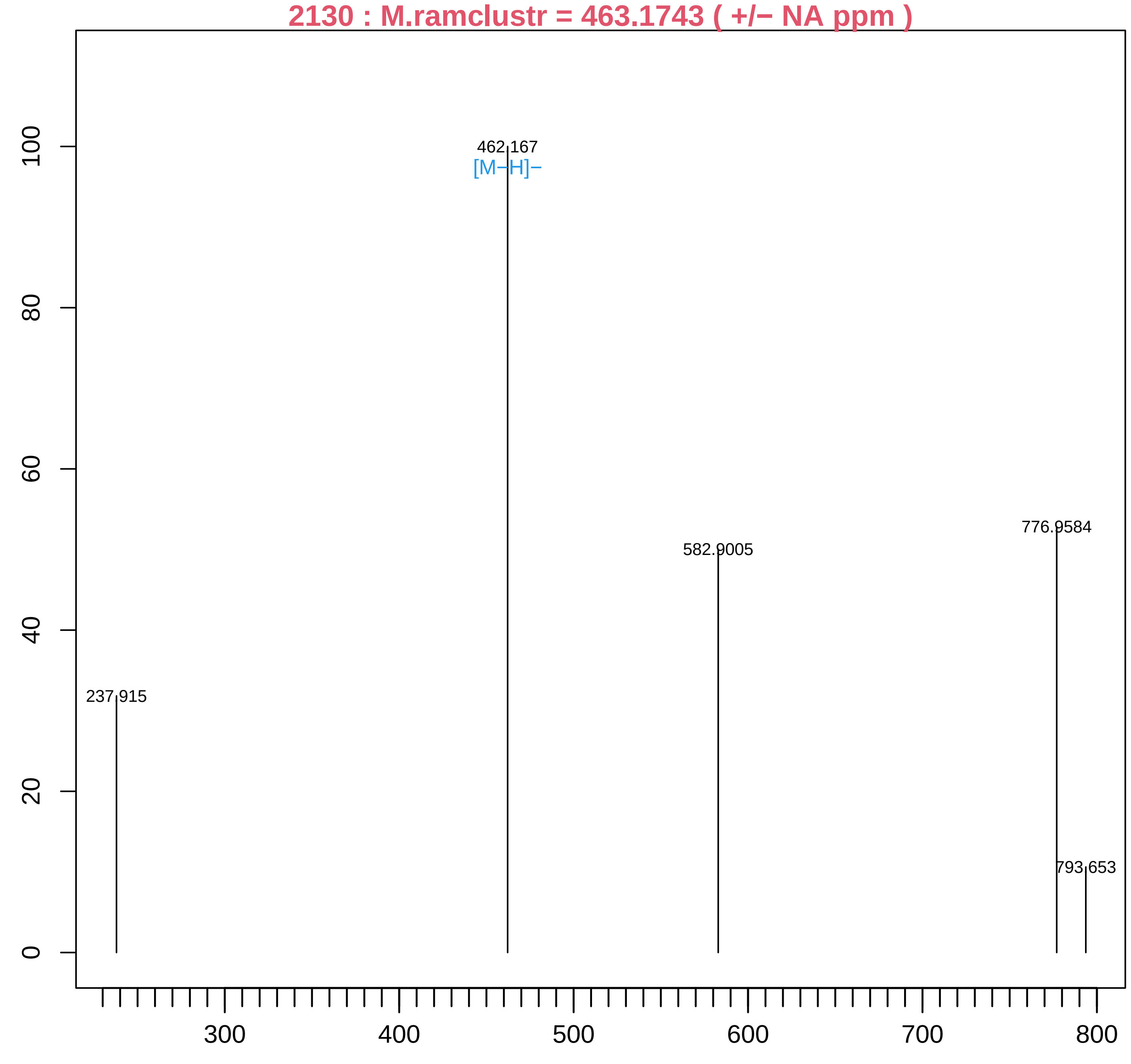
2130 : M.ramclustr = 463.1743 (+/− NA ppm)
462.167
[M−H]−
582.9005
776.9584
237.915
793.653
0
20
40
60
80
100
300
400
500
600
700
800

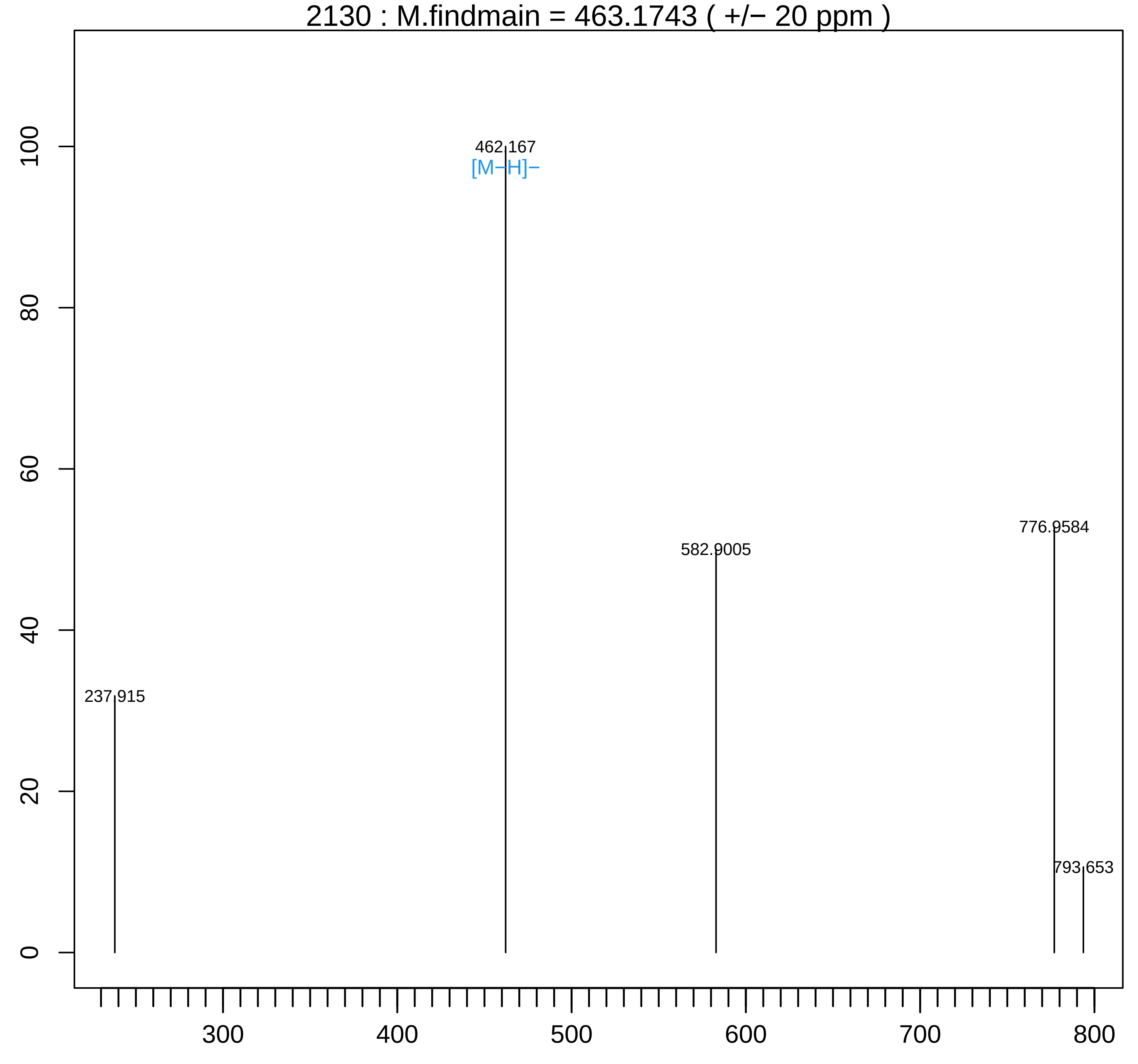
2130 : M.findmain = 463.1743 (+/− 20 ppm)
462.167
[M−H]−
582.9005
776.9584
237.915
793.653
0
20
40
60
80
100
300
400
500
600
700
800

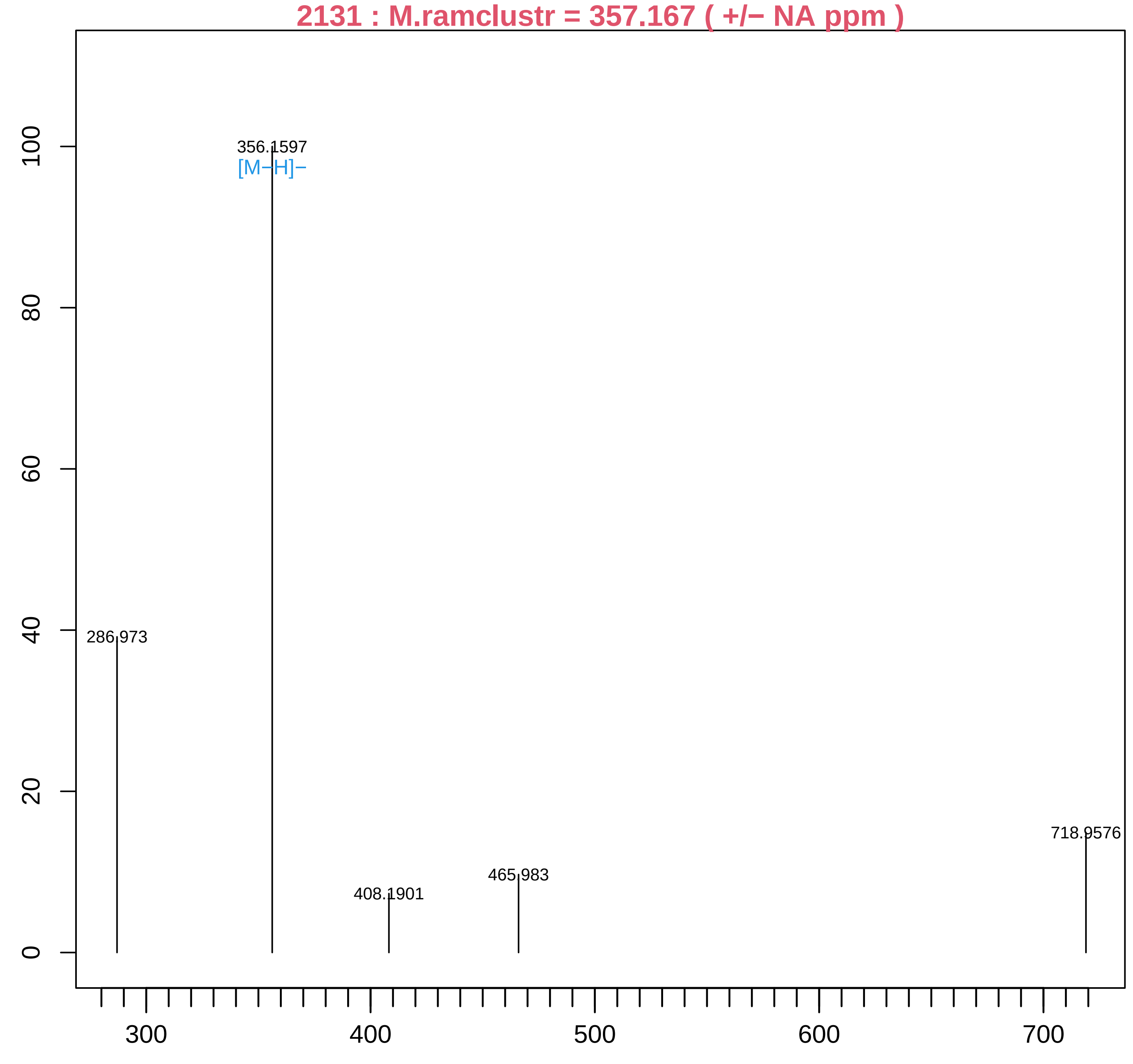
2131 : M.ramclustr = 357.167 (+/− NA ppm)
356.1597
[M−H]−
286.973
408.1901
465.983
718.9576
0
20
40
60
80
100
300
400
500
600
700

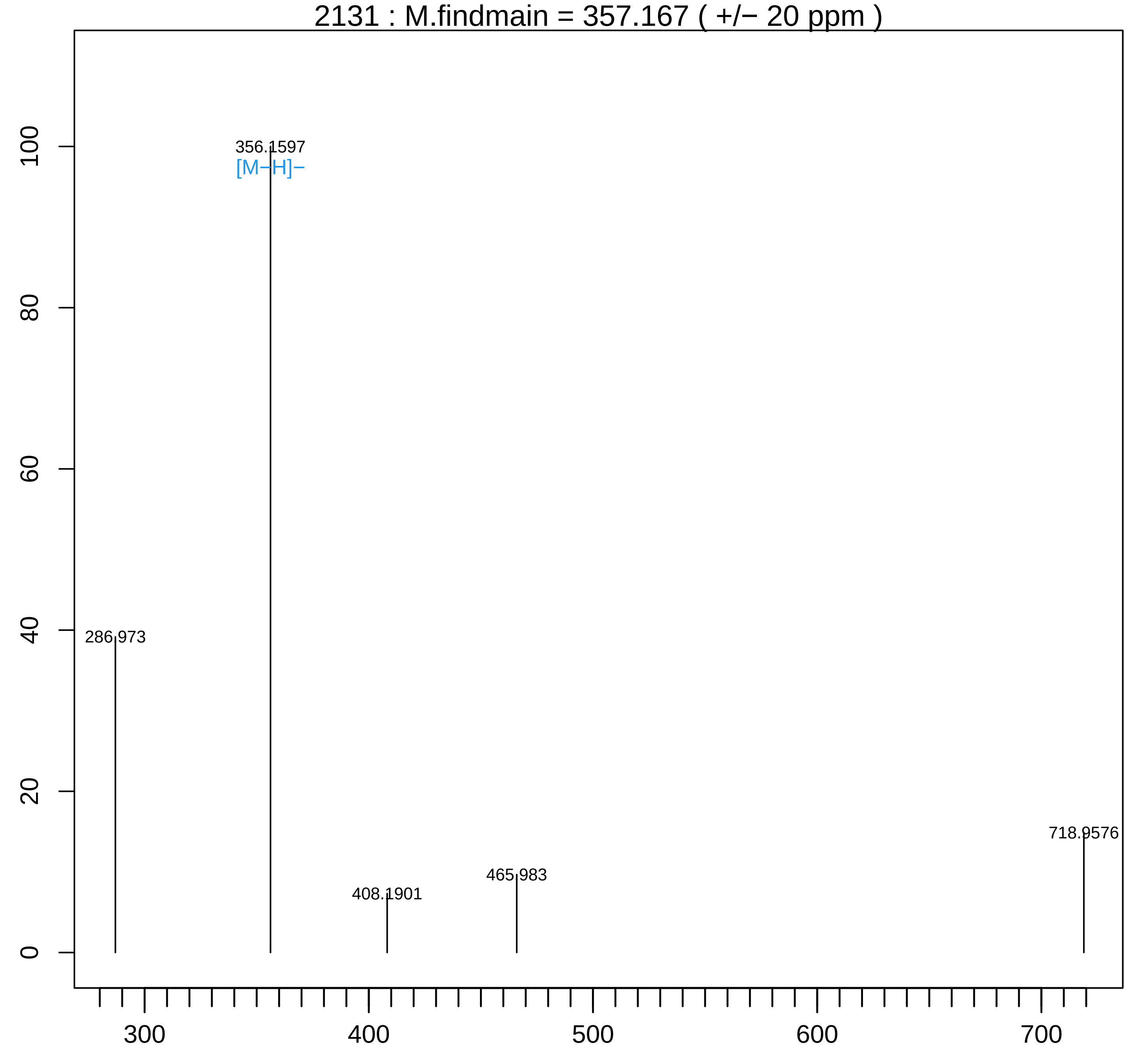
2131 : M.findmain = 357.167 (+/− 20 ppm)
356.1597
[M−H]−
286.973
408.1901
465.983
718.9576
0
20
40
60
80
100
300
400
500
600
700

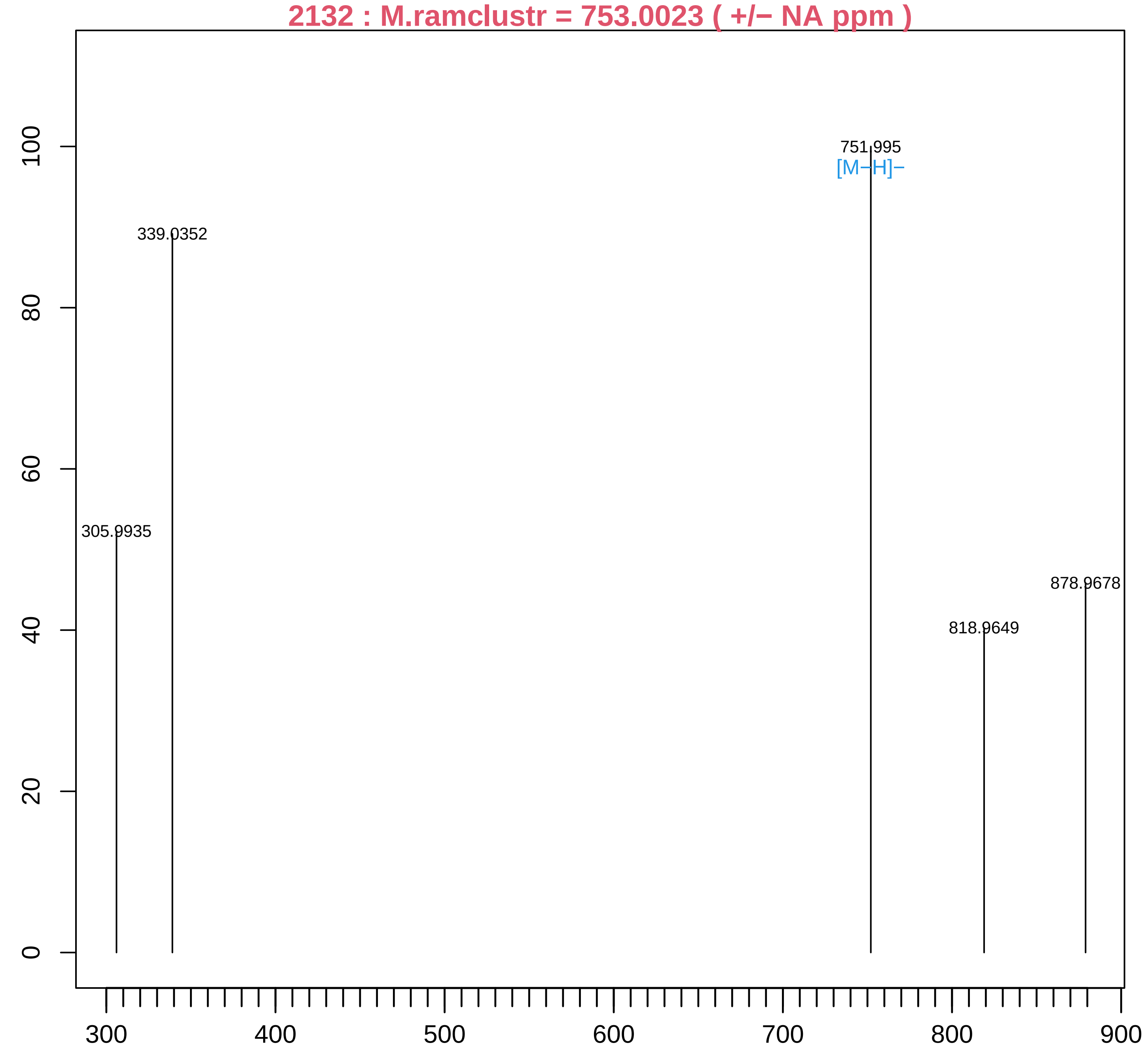
2132 : M.ramclustr = 753.0023 (+/− NA ppm)
751.995
[M−H]−
339.0352
305.9935
878.9678
818.9649
0
20
40
60
80
100
300
400
500
600
700
800
900
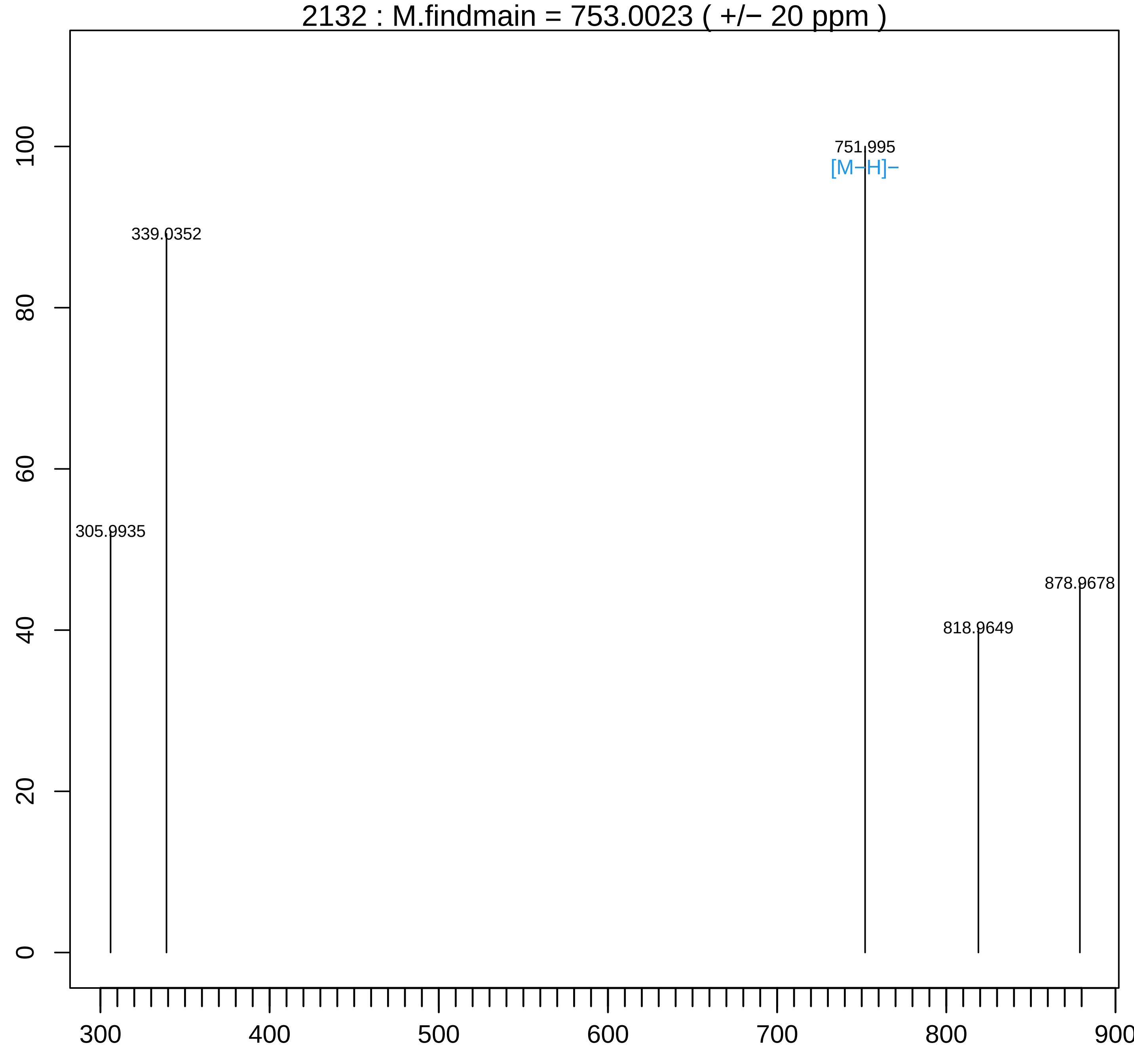
2132 : M.findmain = 753.0023 (+/− 20 ppm)
751.995
[M−H]−
339.0352
305.9935
878.9678
818.9649
0
20
40
60
80
100
300
400
500
600
700
800
900

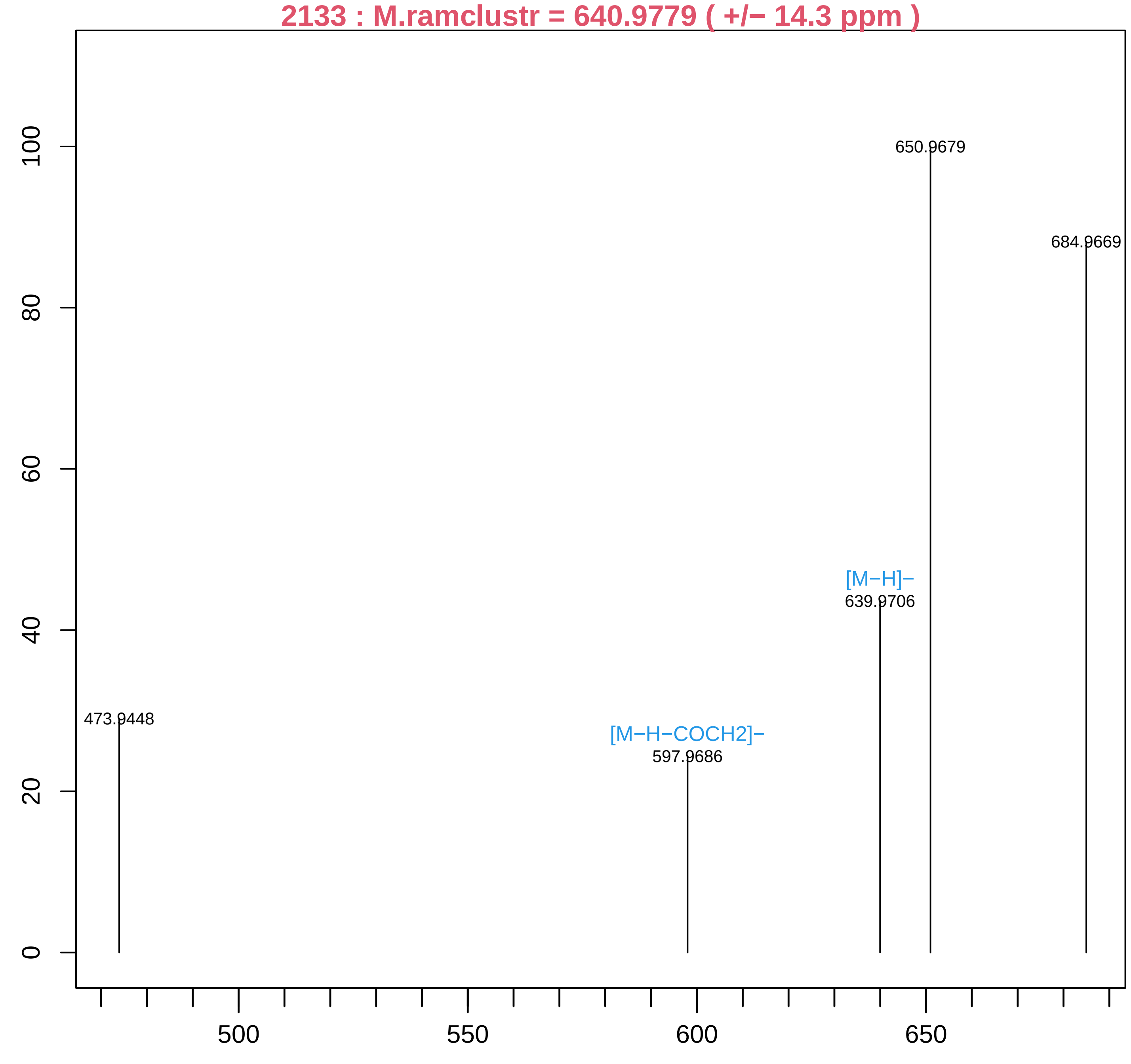

2133 : M.ramclustr = 640.9779 (+/− 14.3 ppm)
650.9679
684.9669
[M−H]−
639.9706
473.9448
[M−H−COCH2]−
597.9686
0
20
40
60
80
100
500
550
600
650

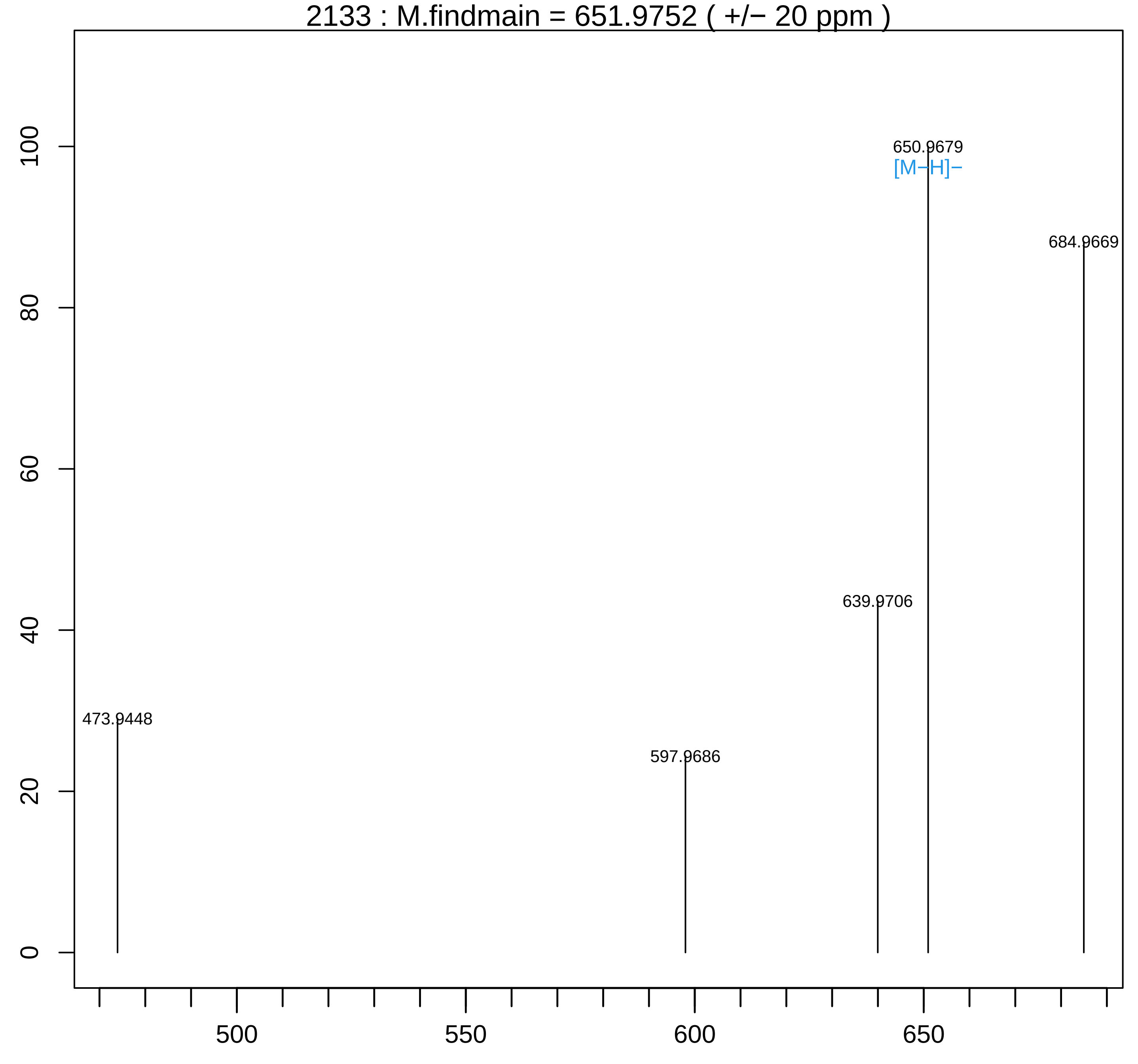

2133 : M.findmain = 651.9752 (+/− 20 ppm)
650.9679
[M−H]−
684.9669
639.9706
473.9448
597.9686
0
20
40
60
80
100
500
550
600
650

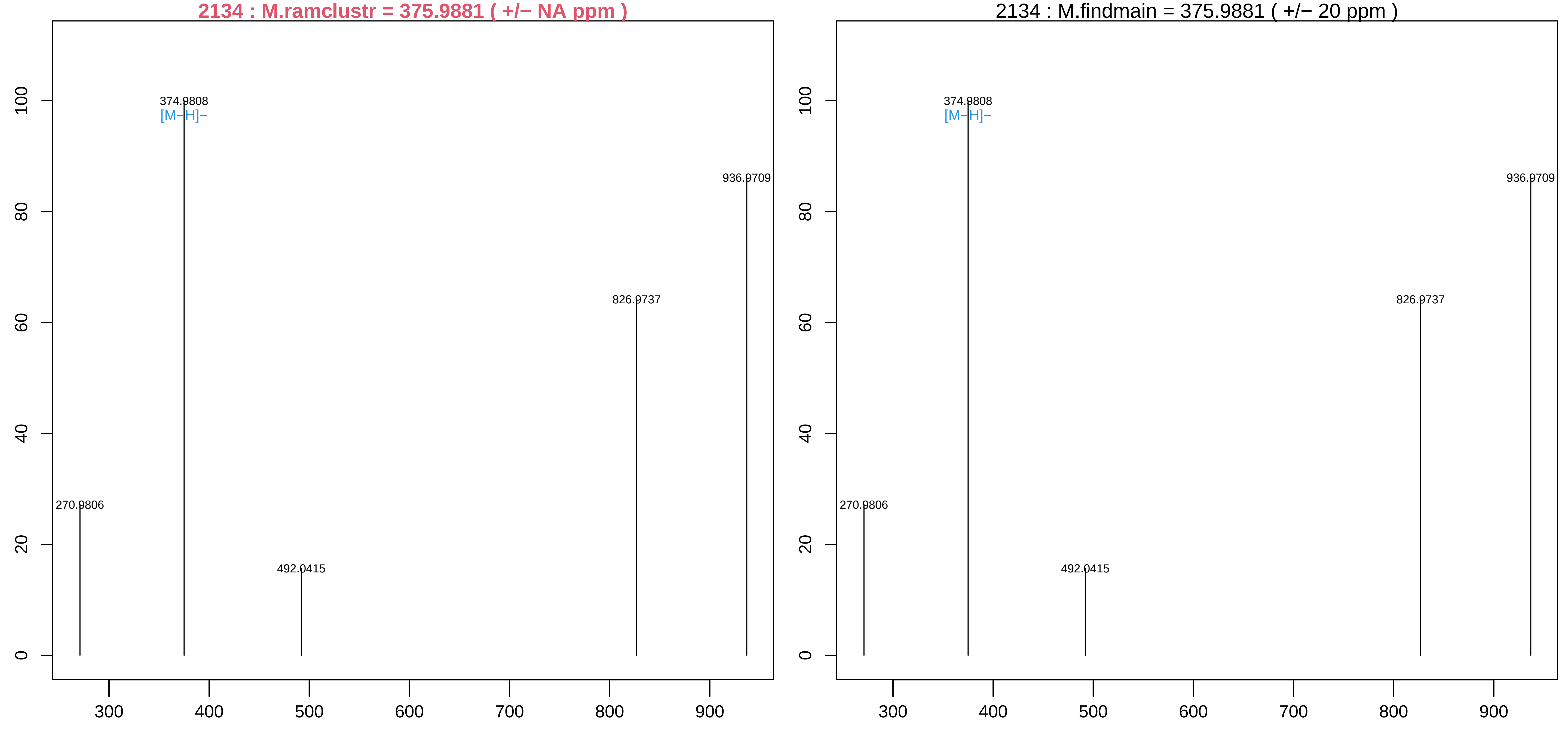
2134 : M.ramclustr = 375.9881 (+/− NA ppm)
2134 : M.findmain = 375.9881 (+/− 20 ppm)
374.9808
[M−H]−
936.9709
826.9737
270.9806
492.0415
0
20
40
60
80
100
300
400
500
600
700
800
900

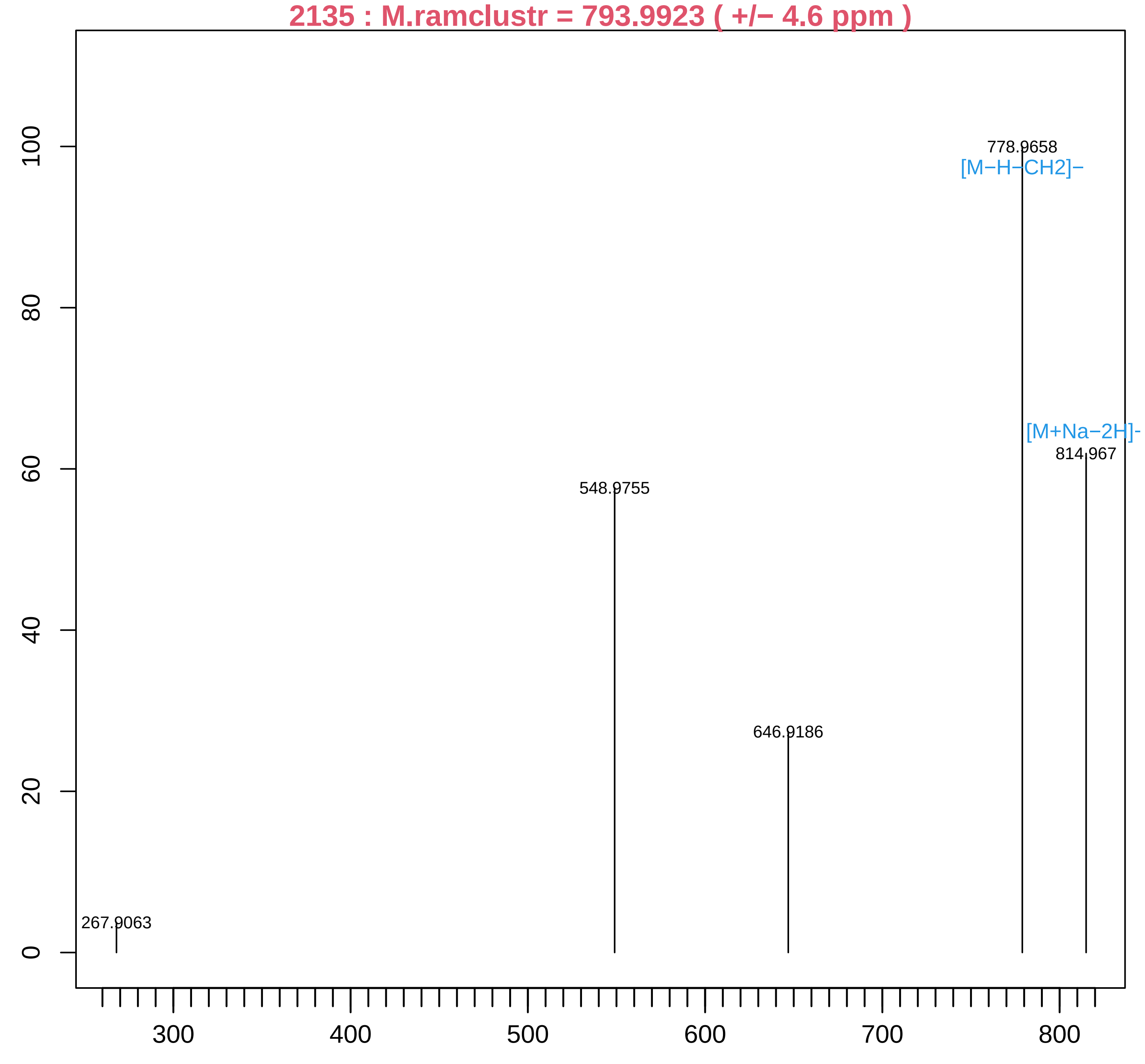
2135 : M.ramclustr = 793.9923 (+/− 4.6 ppm)
778.9658
[M−H−CH2]−
[M+Na−2H]-
814.967
548.9755
646.9186
267.9063
0
20
40
60
80
100
300
400
500
600
700
800

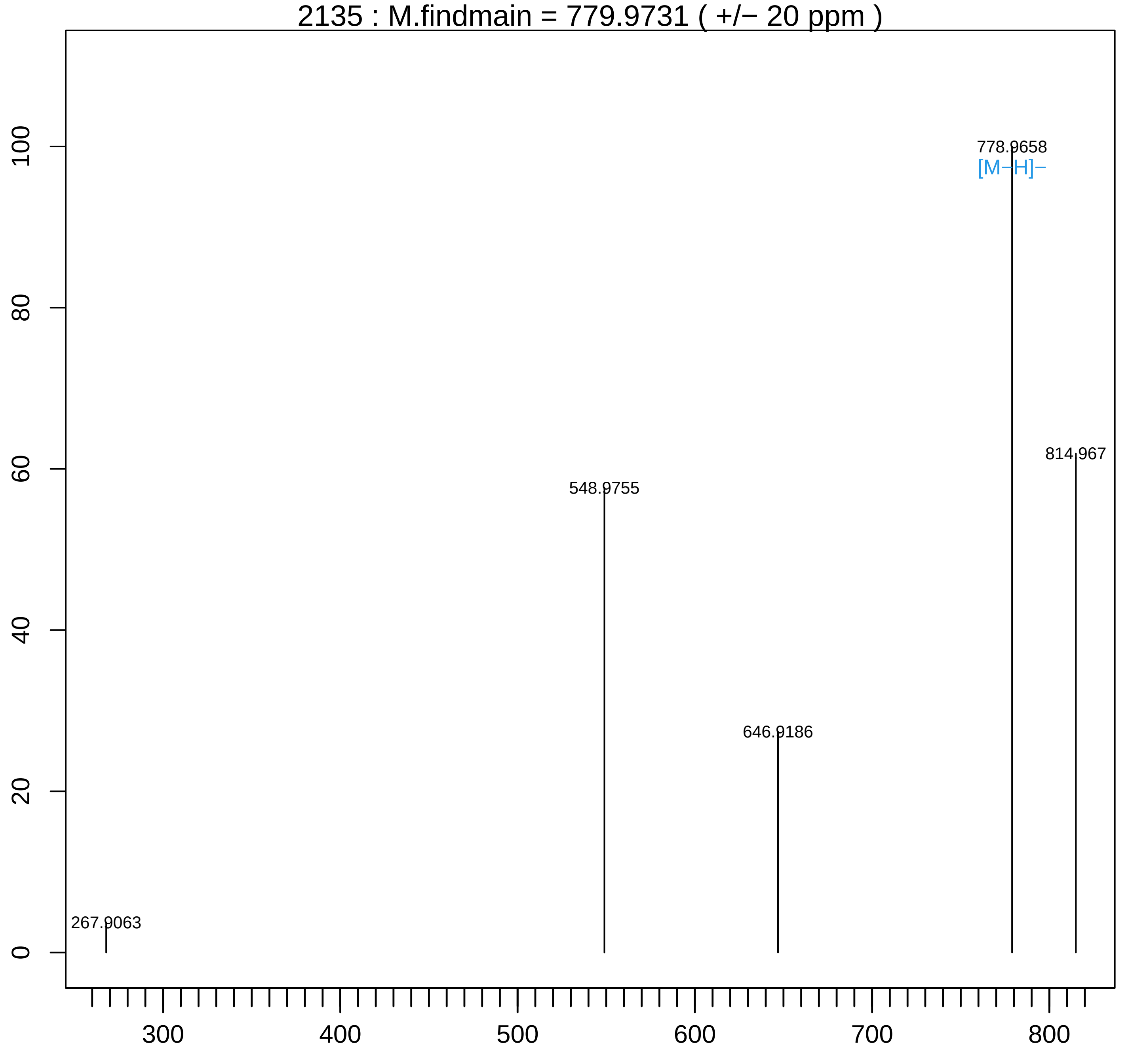
2135 : M.findmain = 779.9731 (+/− 20 ppm)
778.9658
[M−H]−
814.967
548.9755
646.9186
267.9063
0
20
40
60
80
100
300
400
500
600
700
800

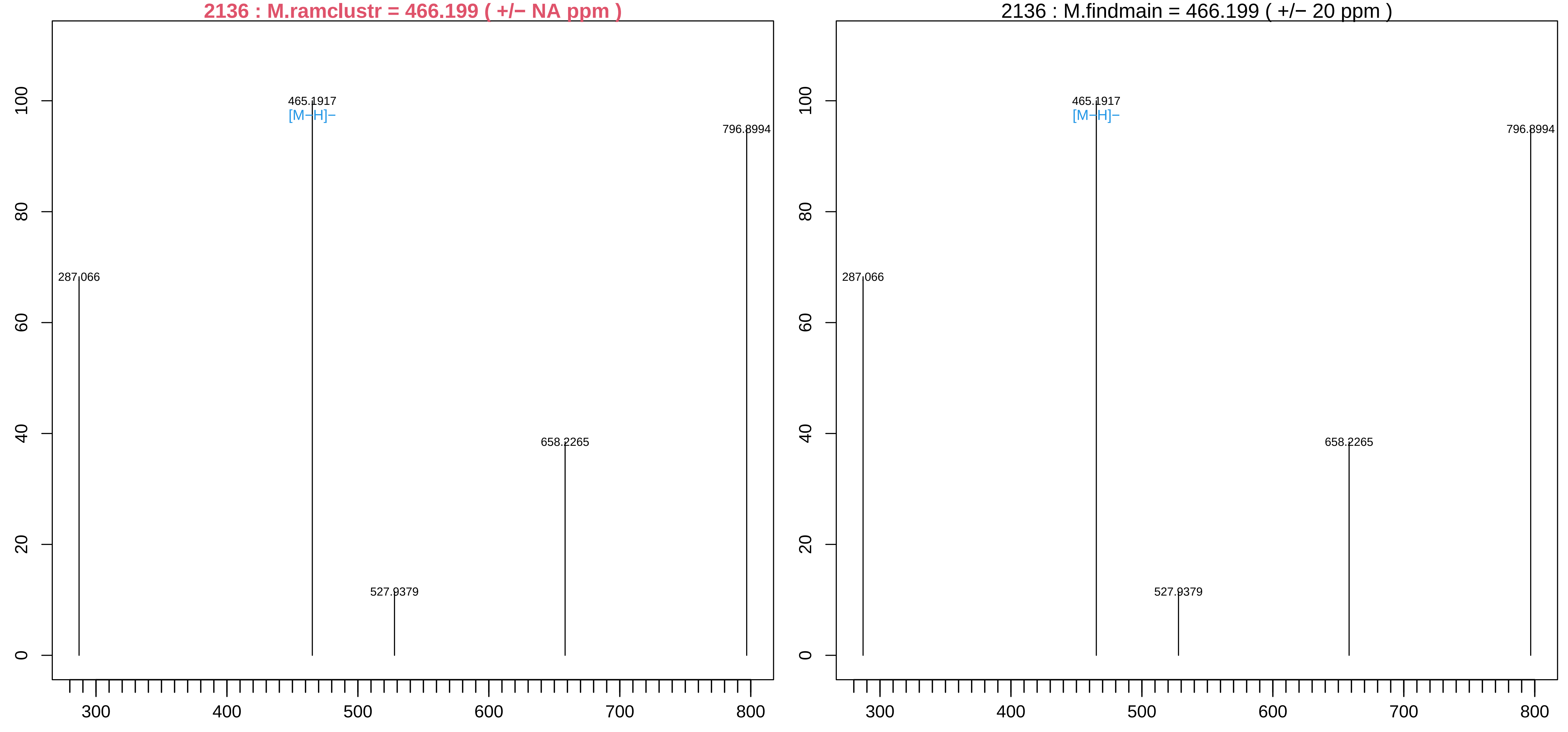
2136 : M.ramclustr = 466.199 (+/− NA ppm)
2136 : M.findmain = 466.199 (+/− 20 ppm)
287.066
465.1917
[M−H]−
527.9379
658.2265
796.8994
0
20
40
60
80
100
300
400
500
600
700
800

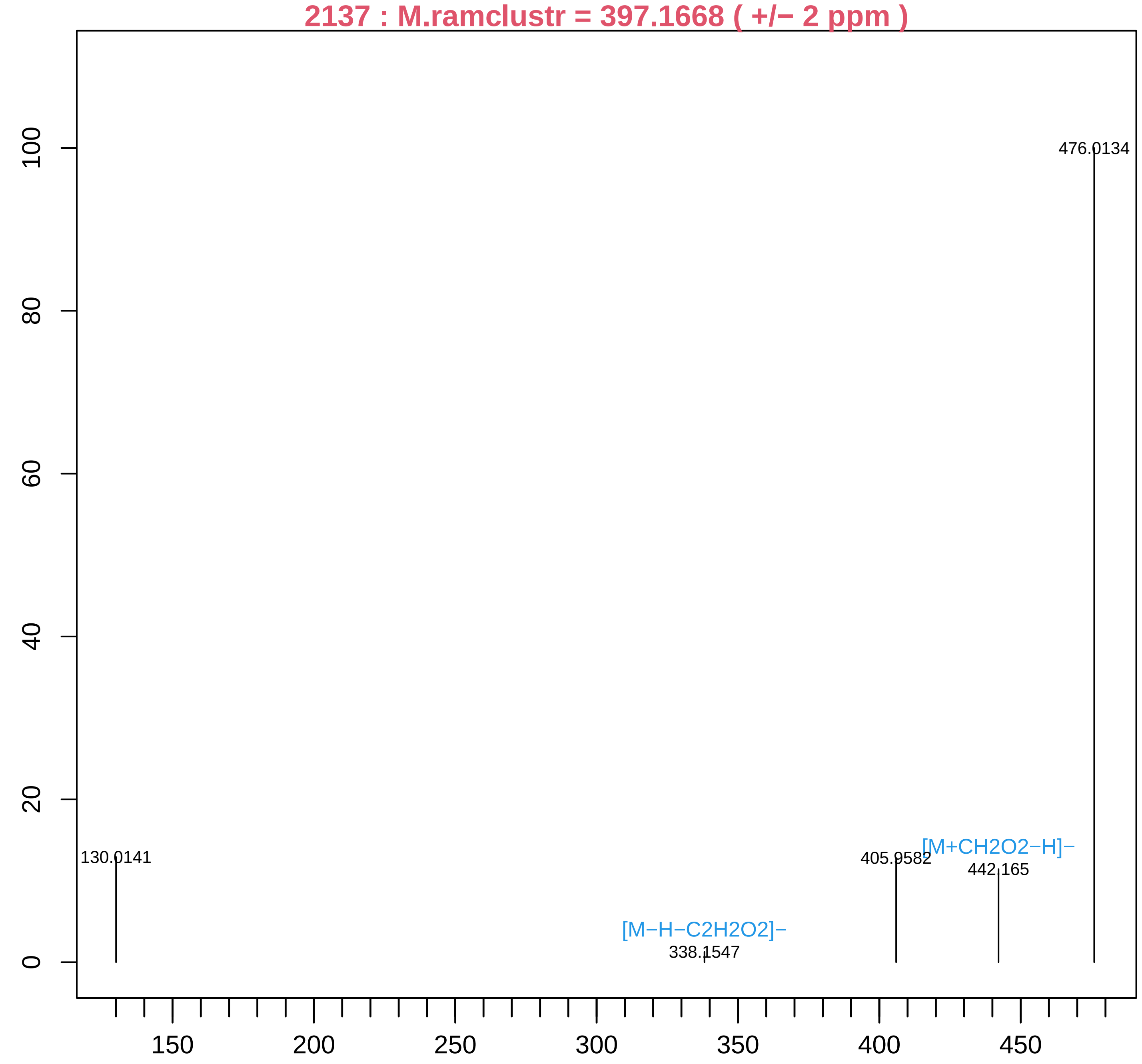

2137 : M.ramclustr = 397.1668 (+/− 2 ppm)
476.0134
130.0141
405.9582
[M+CH2O2−H]−
442.165
[M−H−C2H2O2]−
338.1547
0
20
40
60
80
100
150
200
250
300
350
400
450

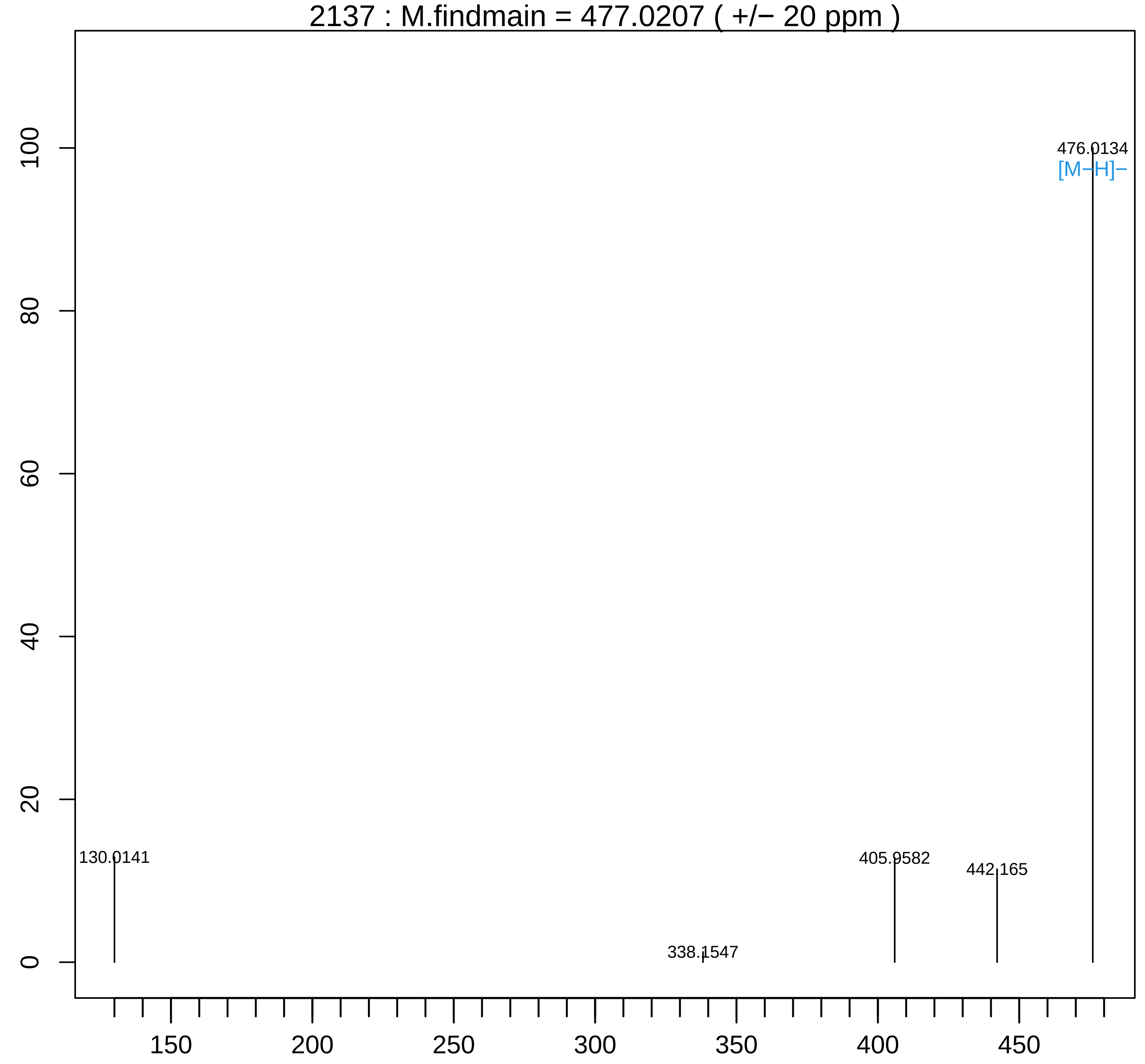

2137 : M.findmain = 477.0207 (+/− 20 ppm)
476.0134
[M−H]−
130.0141
405.9582
442.165
338.1547
0
20
40
60
80
100
150
200
250
300
350
400
450

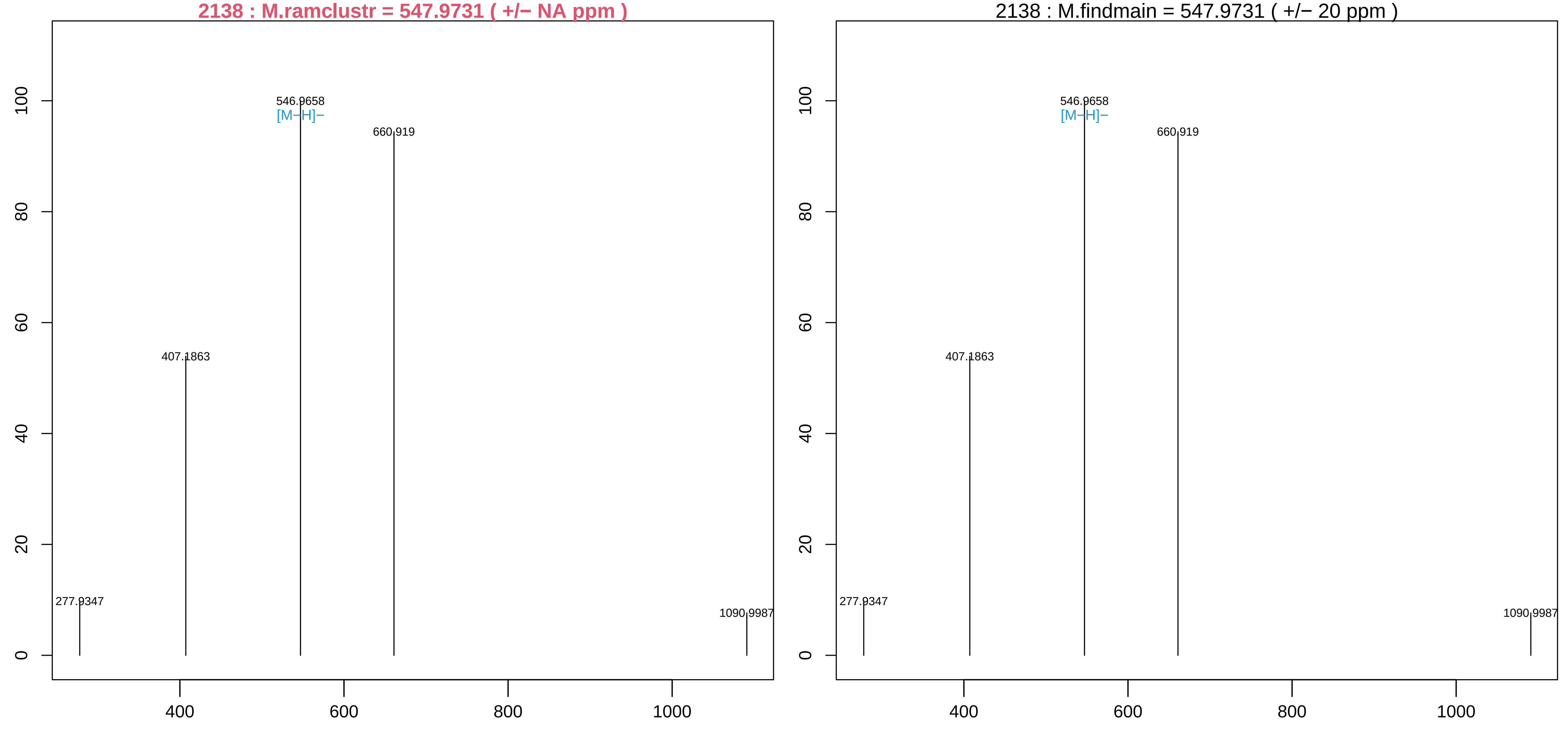

2138 : M.ramclustr = 547.9731 (+/− NA ppm)
2138 : M.findmain = 547.9731 (+/− 20 ppm)
546.9658
[M−H]−
660.919
407.1863
277.9347
1090.9987
0
20
40
60
80
100
400
600
800
1000

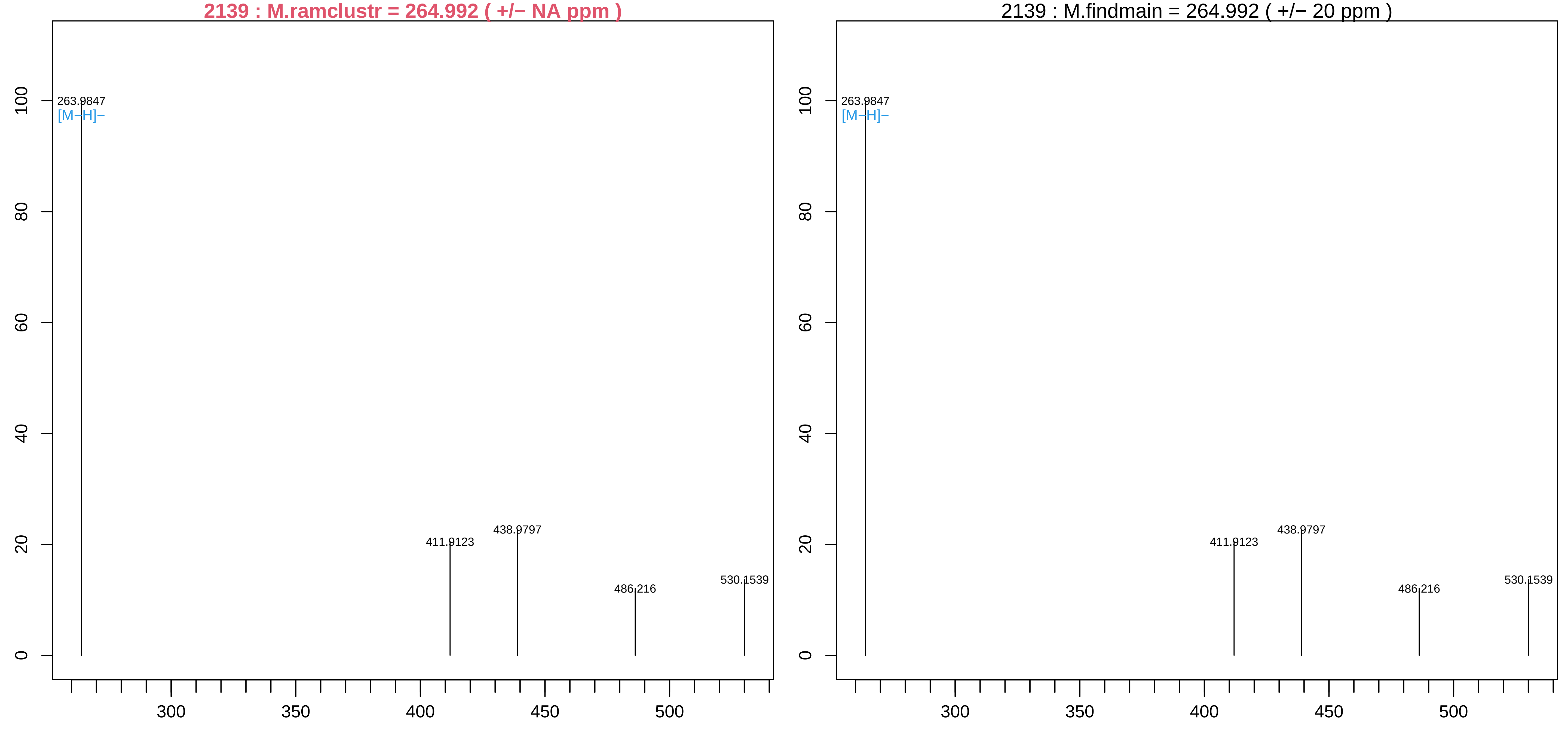

2139 : M.ramclustr = 264.992 (+/− NA ppm)
263.9847
[M−H]−
411.9123
438.9797
486.216
530.1539
0
20
40
60
80
100
300
350
400
450
500
2139 : M.findmain = 264.992 (+/− 20 ppm)
263.9847
[M−H]−
411.9123
438.9797
486.216
530.1539
0
20
40
60
80
100
300
350
400
450
500

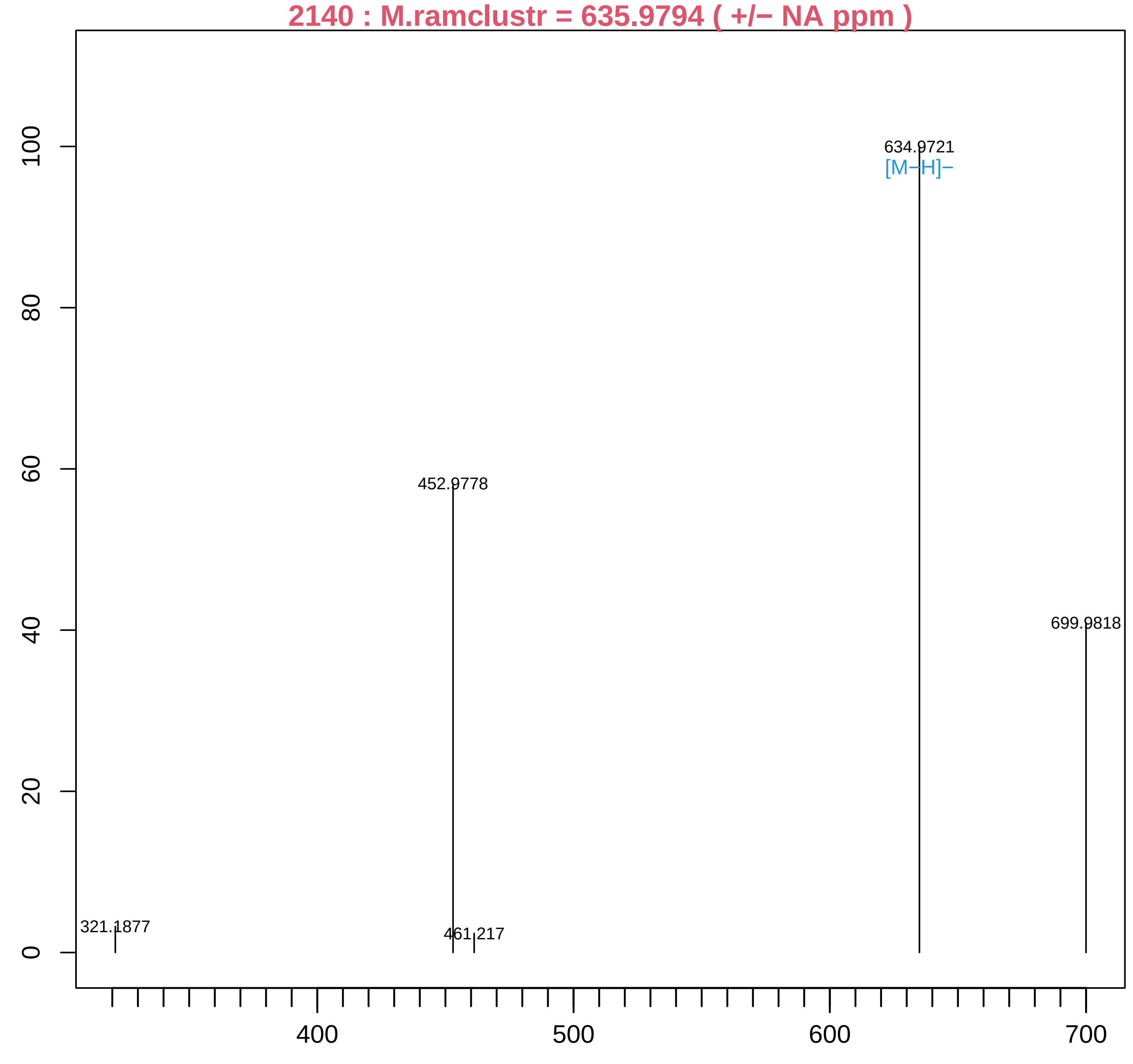
2140 : M.ramclustr = 635.9794 (+/− NA ppm)
634.9721
[M−H]−
452.9778
699.9818
321.1877
461.217
0
20
40
60
80
100
400
500
600
700

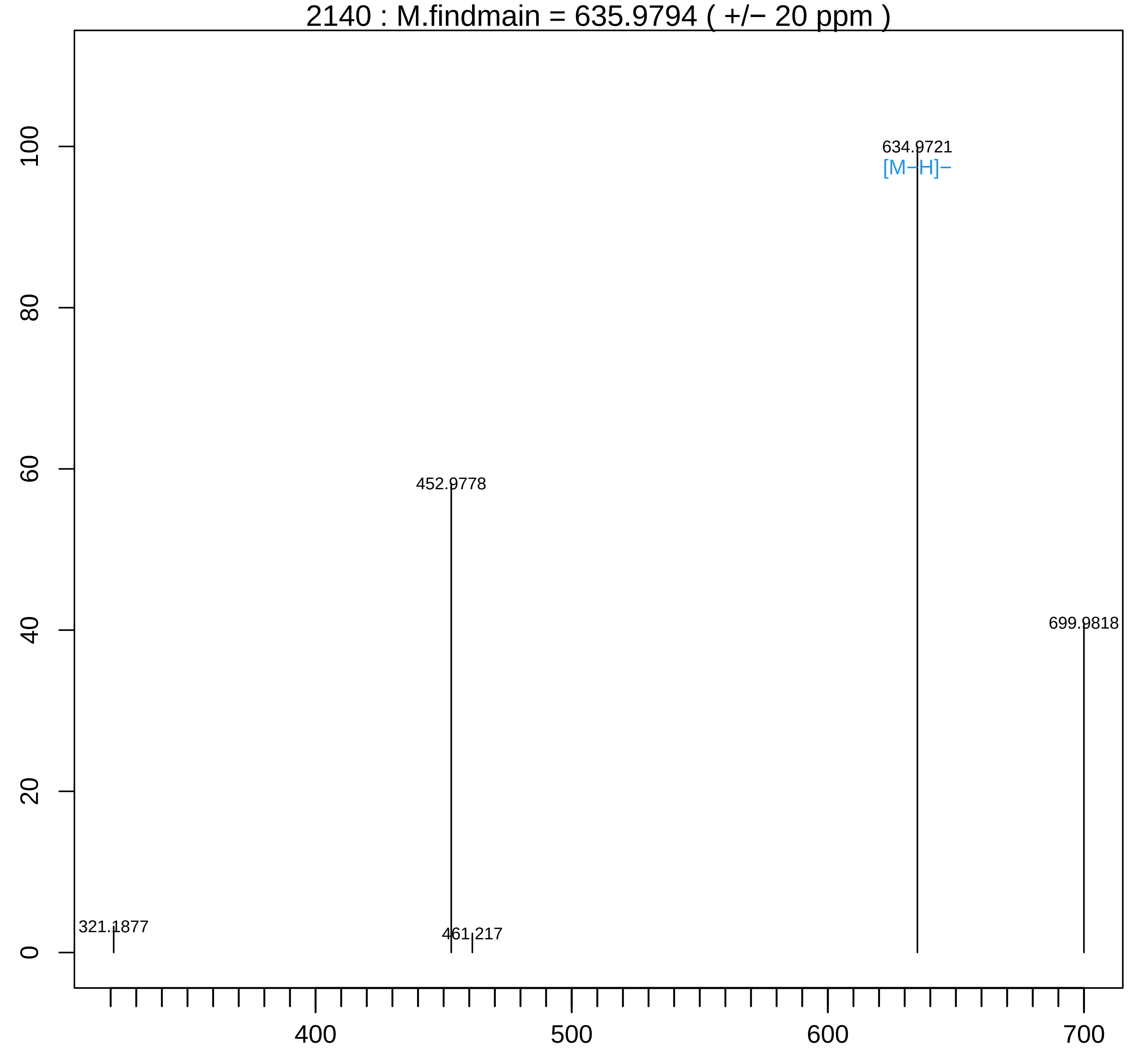
2140 : M.findmain = 635.9794 (+/− 20 ppm)
634.9721
[M−H]−
452.9778
699.9818
321.1877
461.217
0
20
40
60
80
100
400
500
600
700

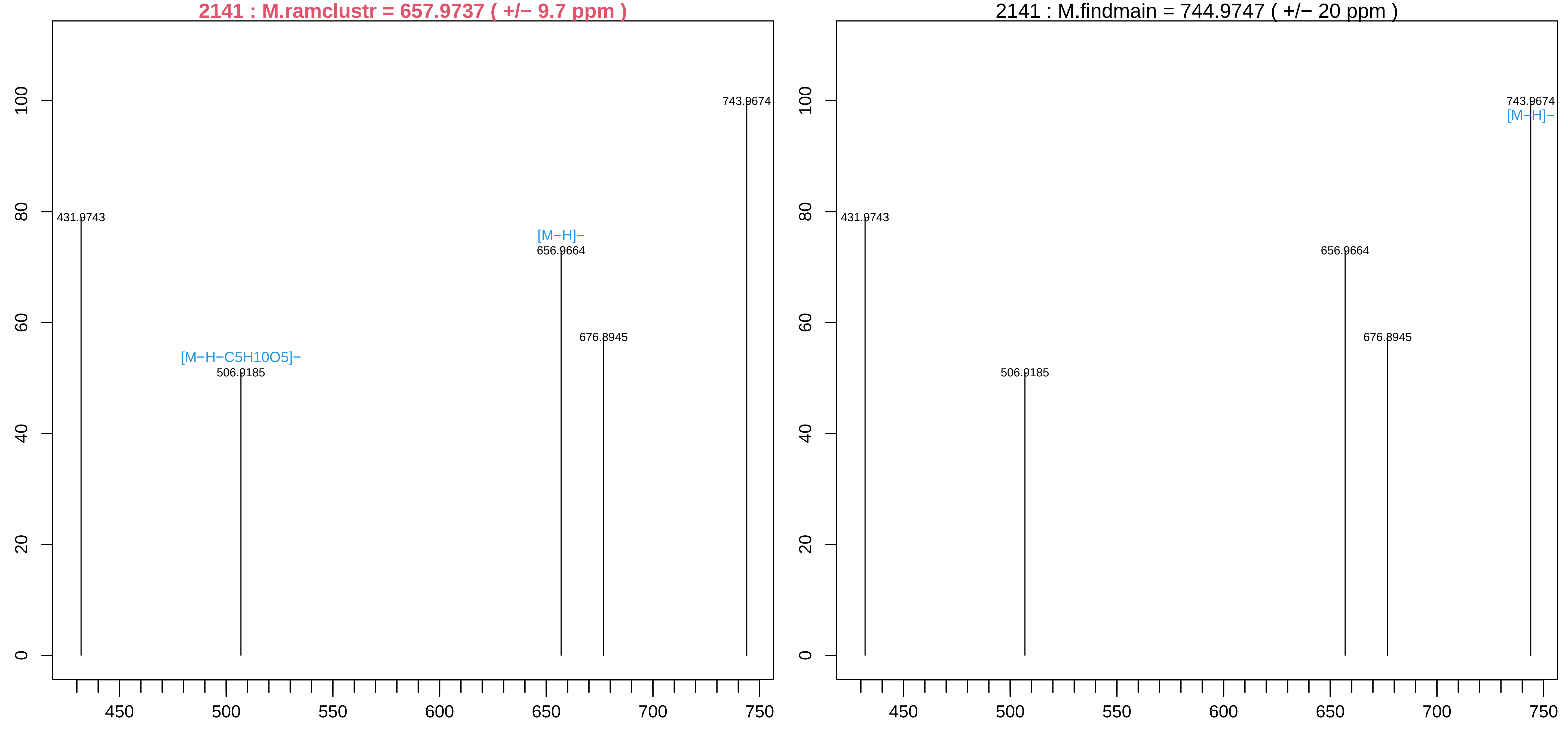
2141 : M.ramclustr = 657.9737 (+/− 9.7 ppm)
431.9743
[M−H−C5H10O5]−
506.9185
[M−H]−
656.9664
676.8945
743.9674
2141 : M.findmain = 744.9747 (+/− 20 ppm)
431.9743
506.9185
656.9664
676.8945
743.9674
[M−H]−

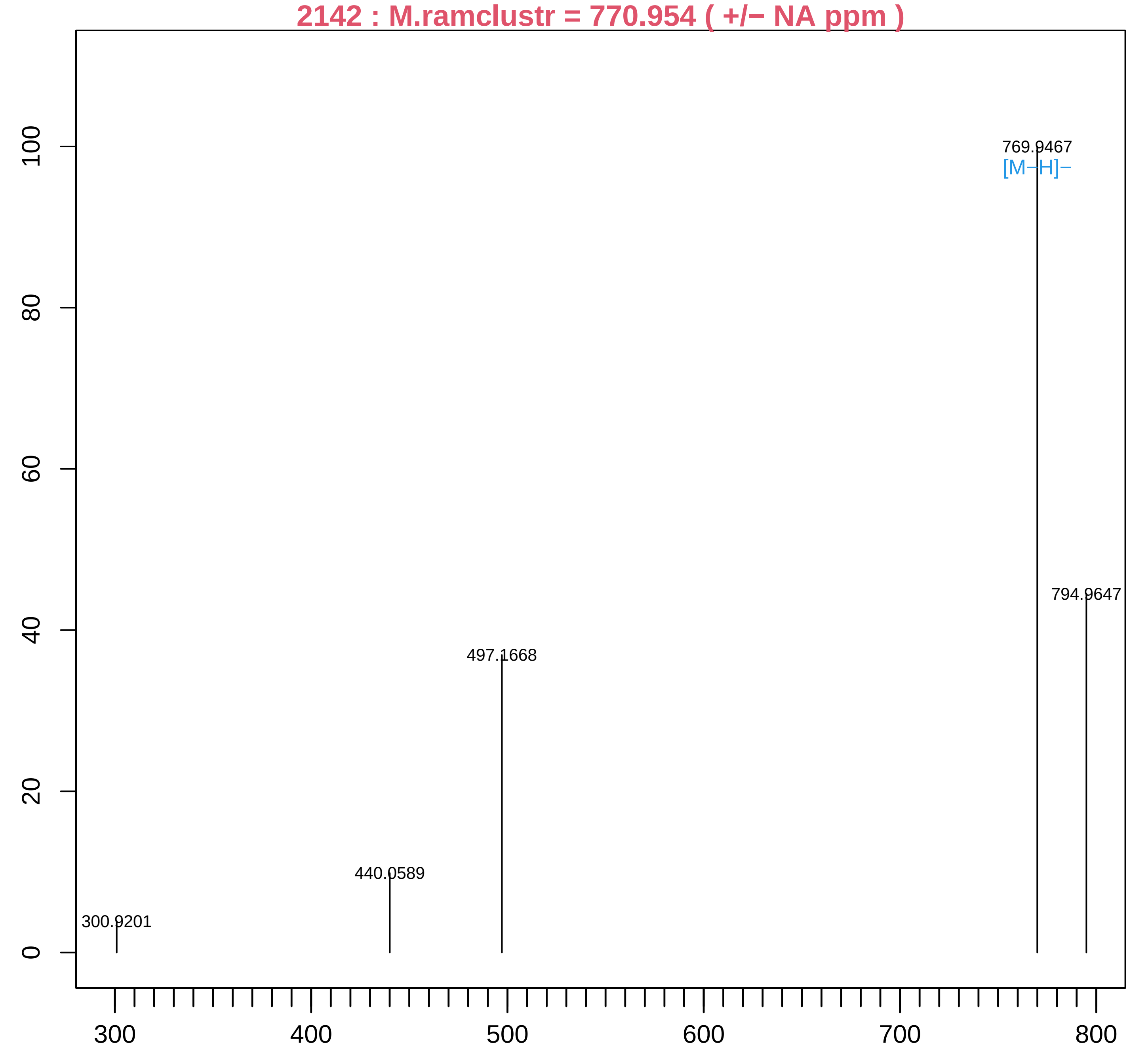
2142 : M.ramclustr = 770.954 (+/− NA ppm)
0
20
40
60
80
100
300
400
500
600
700
800
300.9201
440.0589
497.1668
769.9467
[M−H]−
794.9647

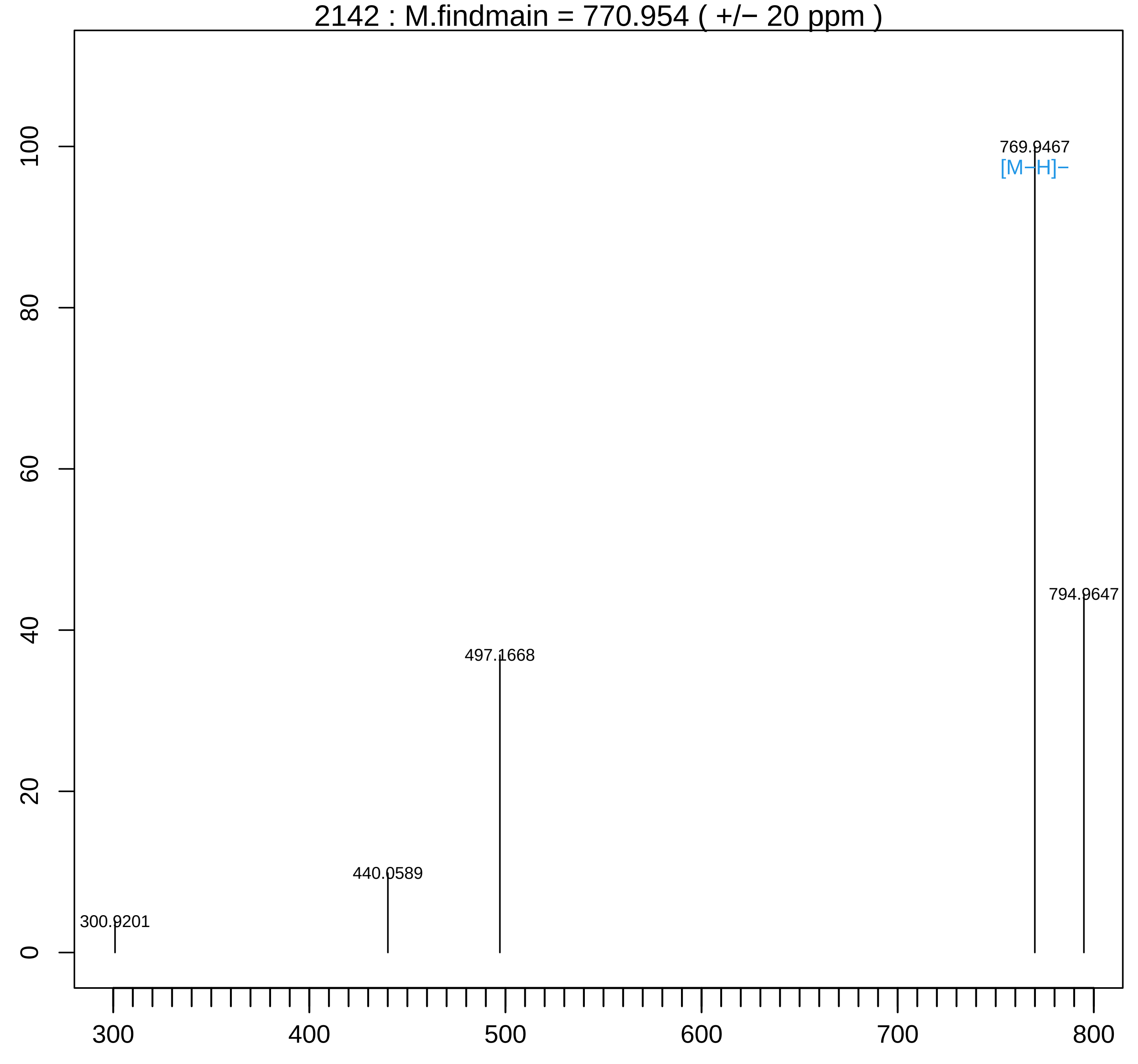
2142 : M.findmain = 770.954 (+/− 20 ppm)
0
20
40
60
80
100
300
400
500
600
700
800
300.9201
440.0589
497.1668
769.9467
[M−H]−
794.9647

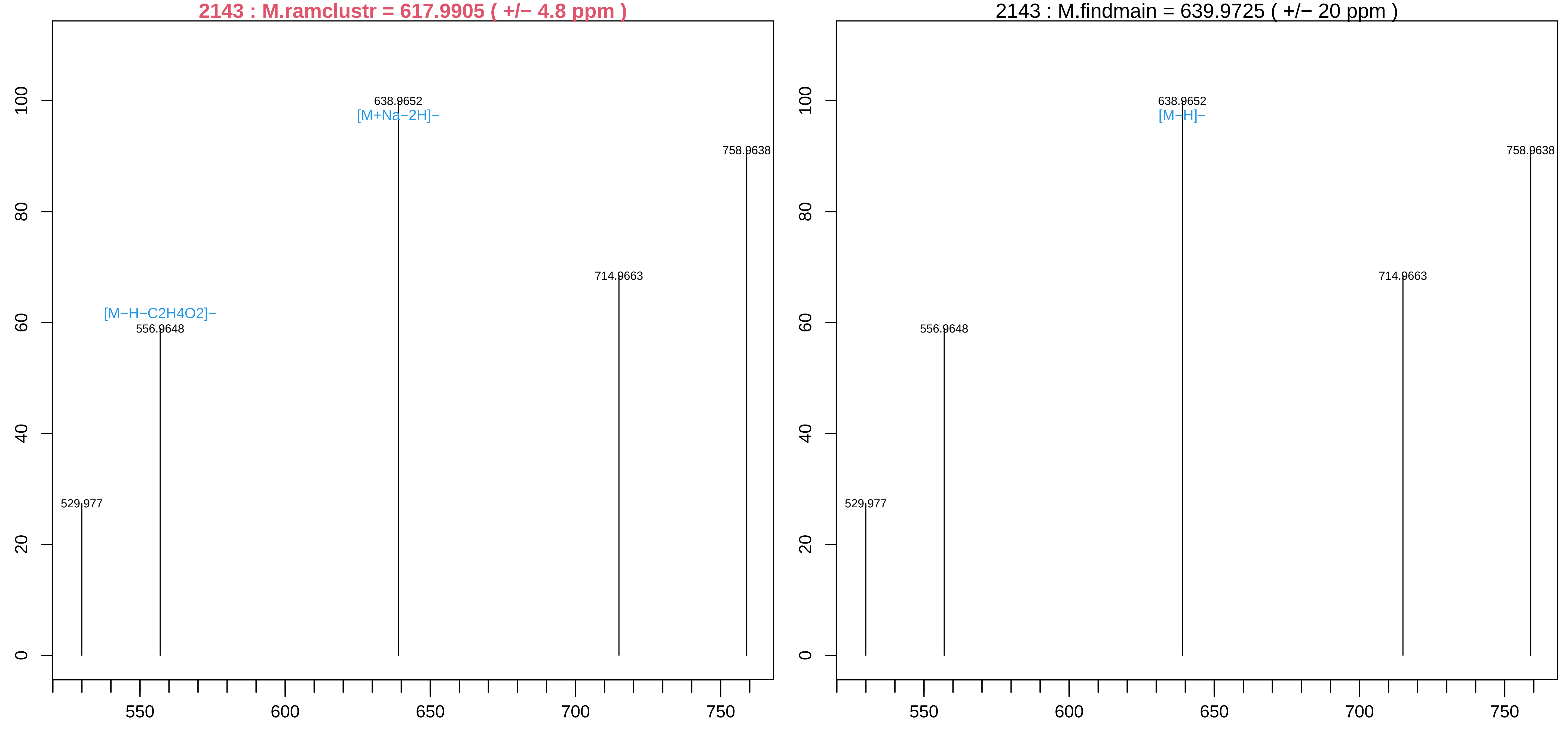

2143 : M.ramclustr = 617.9905 (+/− 4.8 ppm)
[M+Na−2H]−
638.9652
758.9638
714.9663
[M−H−C2H4O2]−
556.9648
529.977
0
20
40
60
80
100
550
600
650
700
750
2143 : M.findmain = 639.9725 (+/− 20 ppm)
638.9652
[M−H]−
758.9638
714.9663
556.9648
529.977

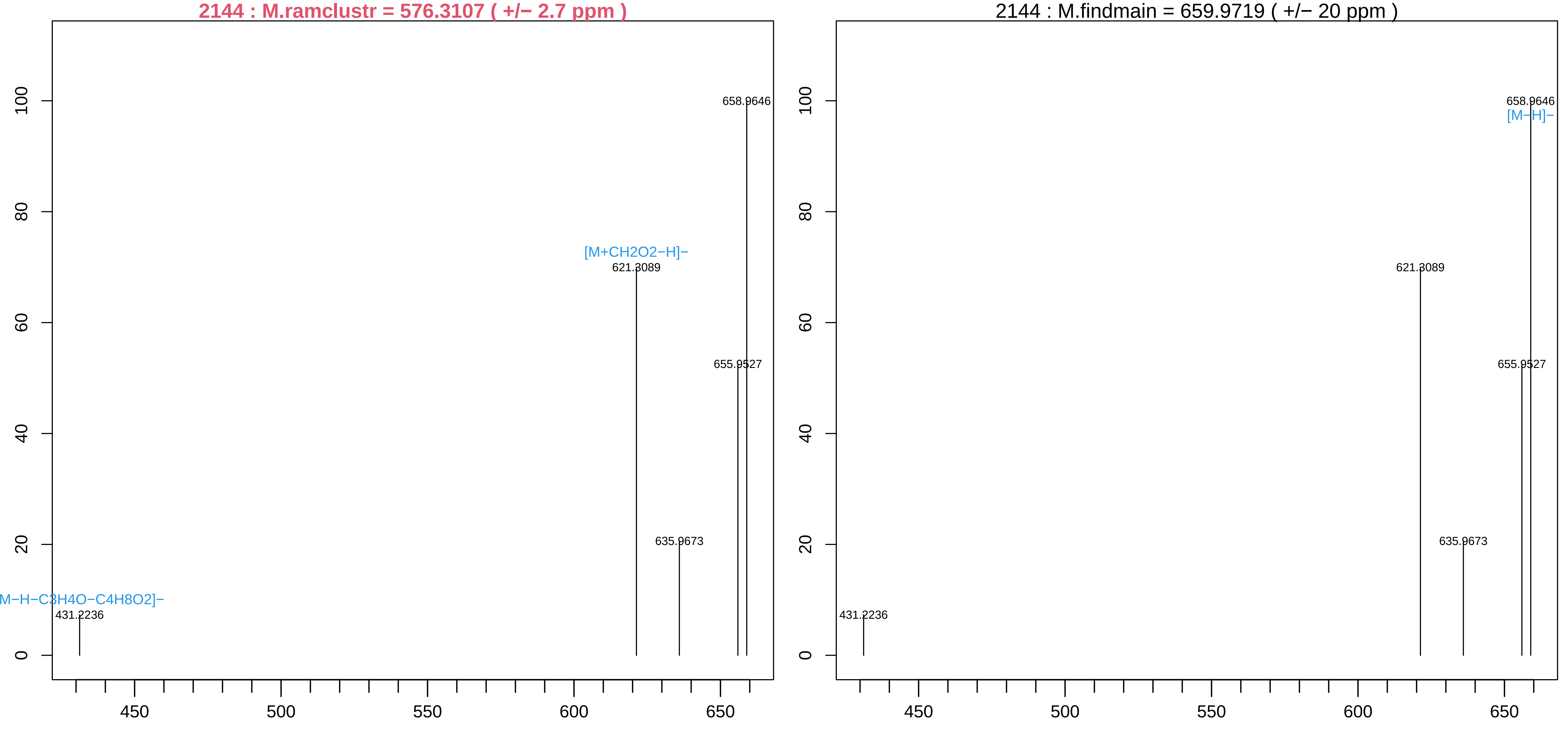
2144 : M.ramclustr = 576.3107 (+/− 2.7 ppm)
2144 : M.findmain = 659.9719 (+/− 20 ppm)
658.9646
[M−H]−
[M+CH2O2−H]−
621.3089
655.9527
635.9673
M−H−C3H4O−C4H8O2]−
431.2236
0
20
40
60
80
100
450
500
550
600
650

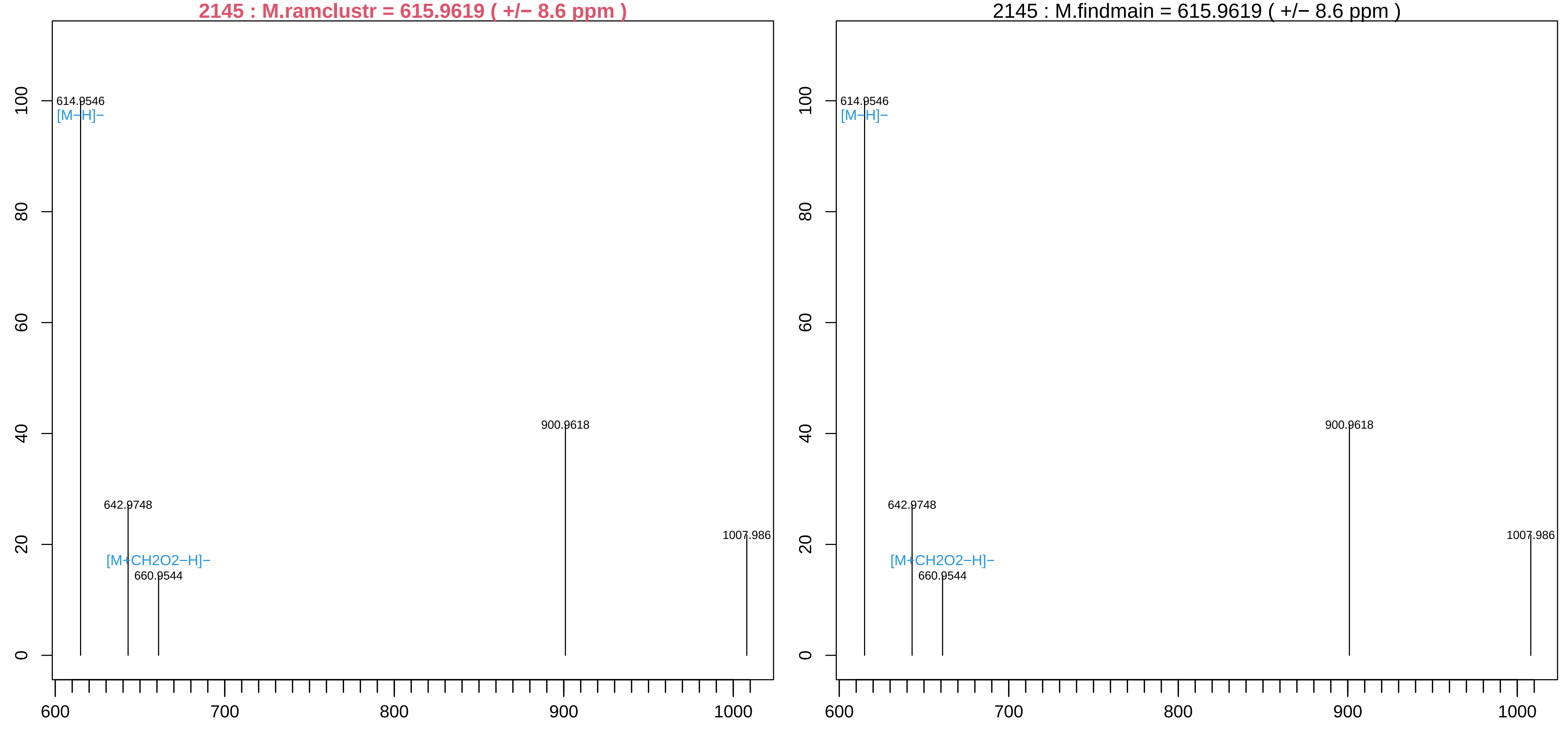
2145 : M.ramclustr = 615.9619 (+/− 8.6 ppm)
614.9546
[M−H]−
642.9748
[M+CH2O2−H]−
660.9544
900.9618
1007.986
0
20
40
60
80
100
600
700
800
900
1000
2145 : M.findmain = 615.9619 (+/− 8.6 ppm)
614.9546
[M−H]−
642.9748
[M+CH2O2−H]−
660.9544
900.9618
1007.986
0
20
40
60
80
100
600
700
800
900
1000

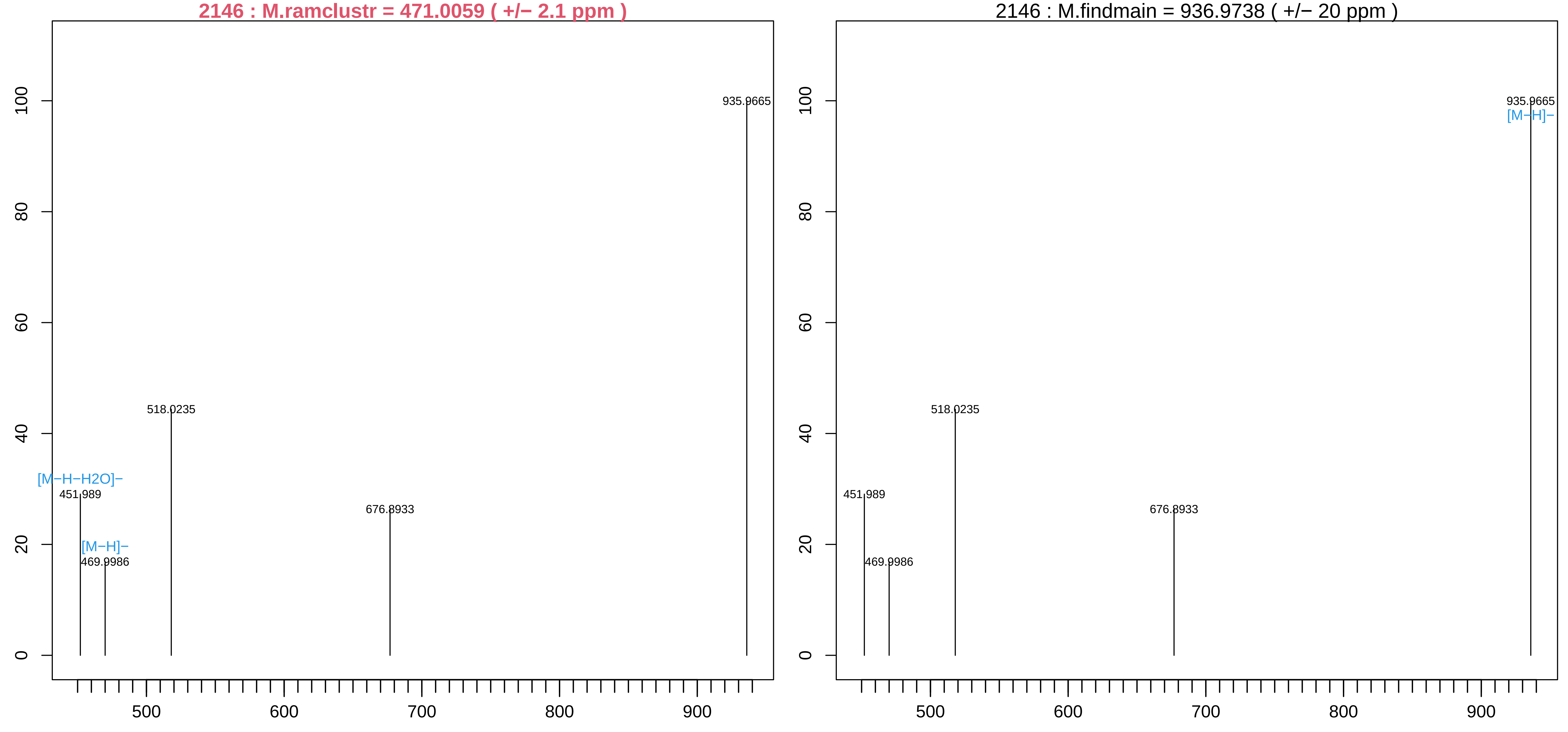

2146 : M.ramclustr = 471.0059 (+/− 2.1 ppm)
935.9665
518.0235
[M−H−H2O]−
451.989
[M−H]−
469.9986
676.8933
2146 : M.findmain = 936.9738 (+/− 20 ppm)
935.9665
[M−H]−
518.0235
451.989
469.9986
676.8933
0
20
40
60
80
100
500
600
700
800
900

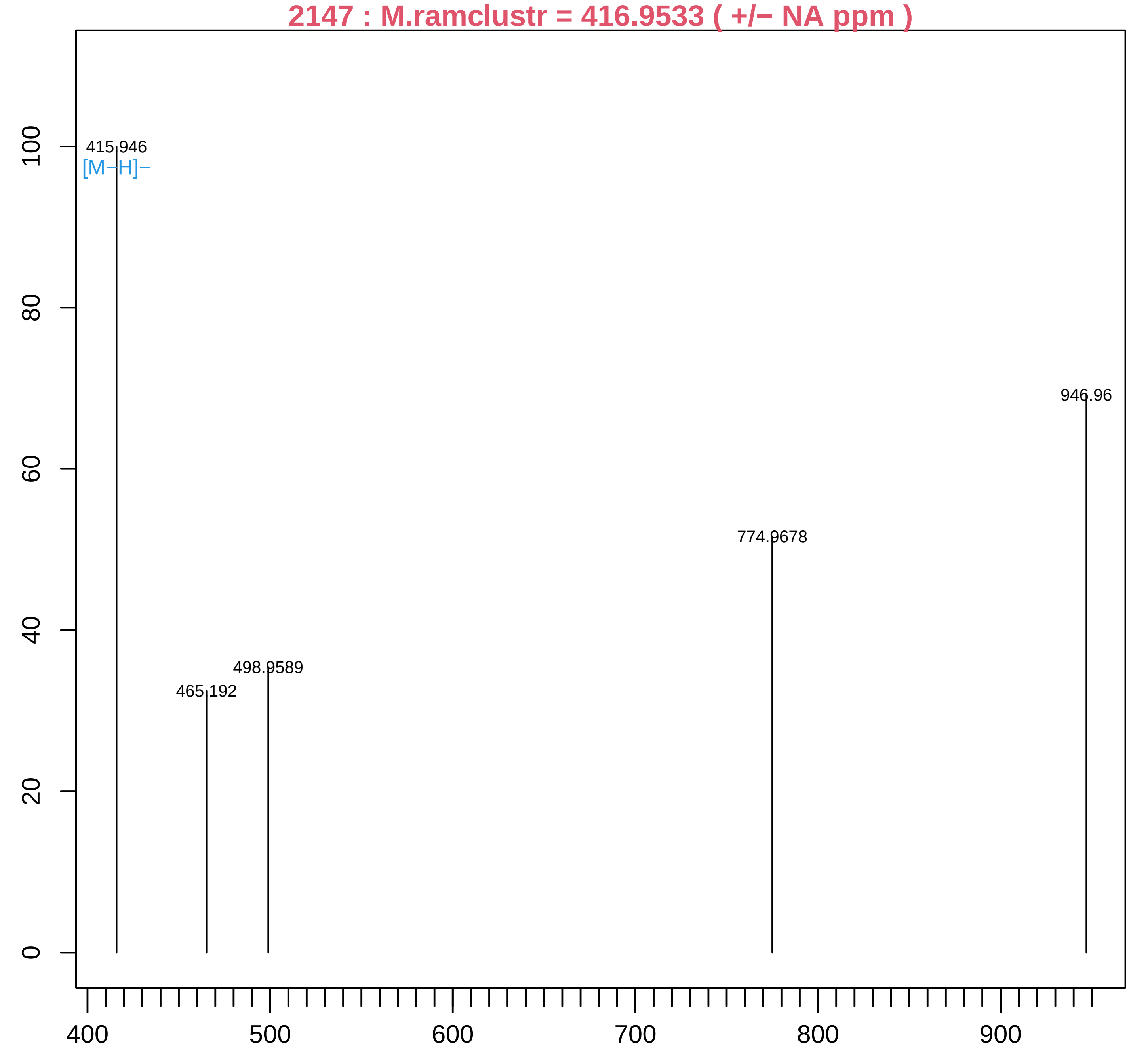
2147 : M.ramclustr = 416.9533 (+/− NA ppm)
415.946
[M−H]−
465.192
498.9589
774.9678
946.96
0
20
40
60
80
100
400
500
600
700
800
900

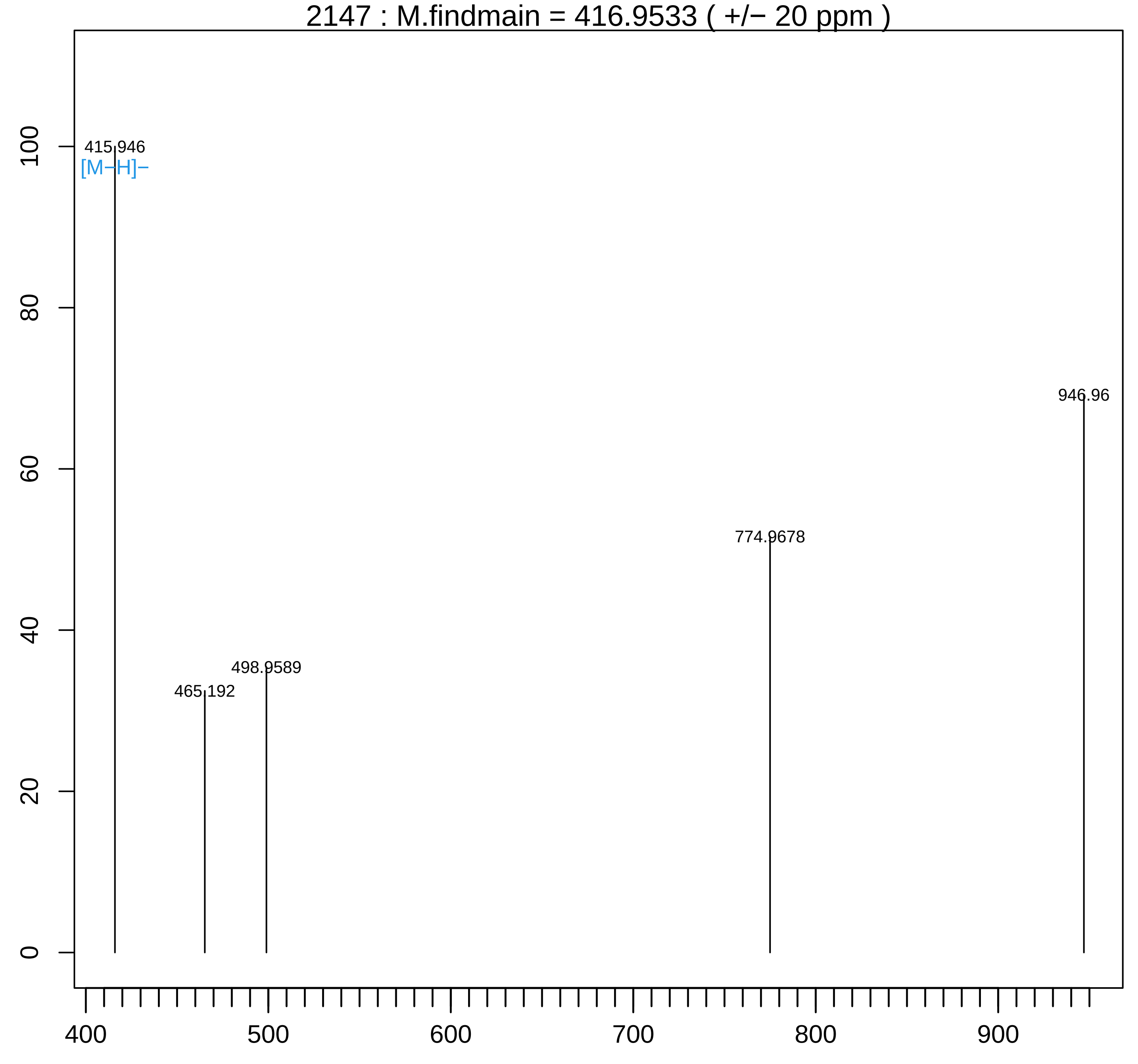
2147 : M.findmain = 416.9533 (+/− 20 ppm)
415.946
[M−H]−
465.192
498.9589
774.9678
946.96
0
20
40
60
80
100
400
500
600
700
800
900

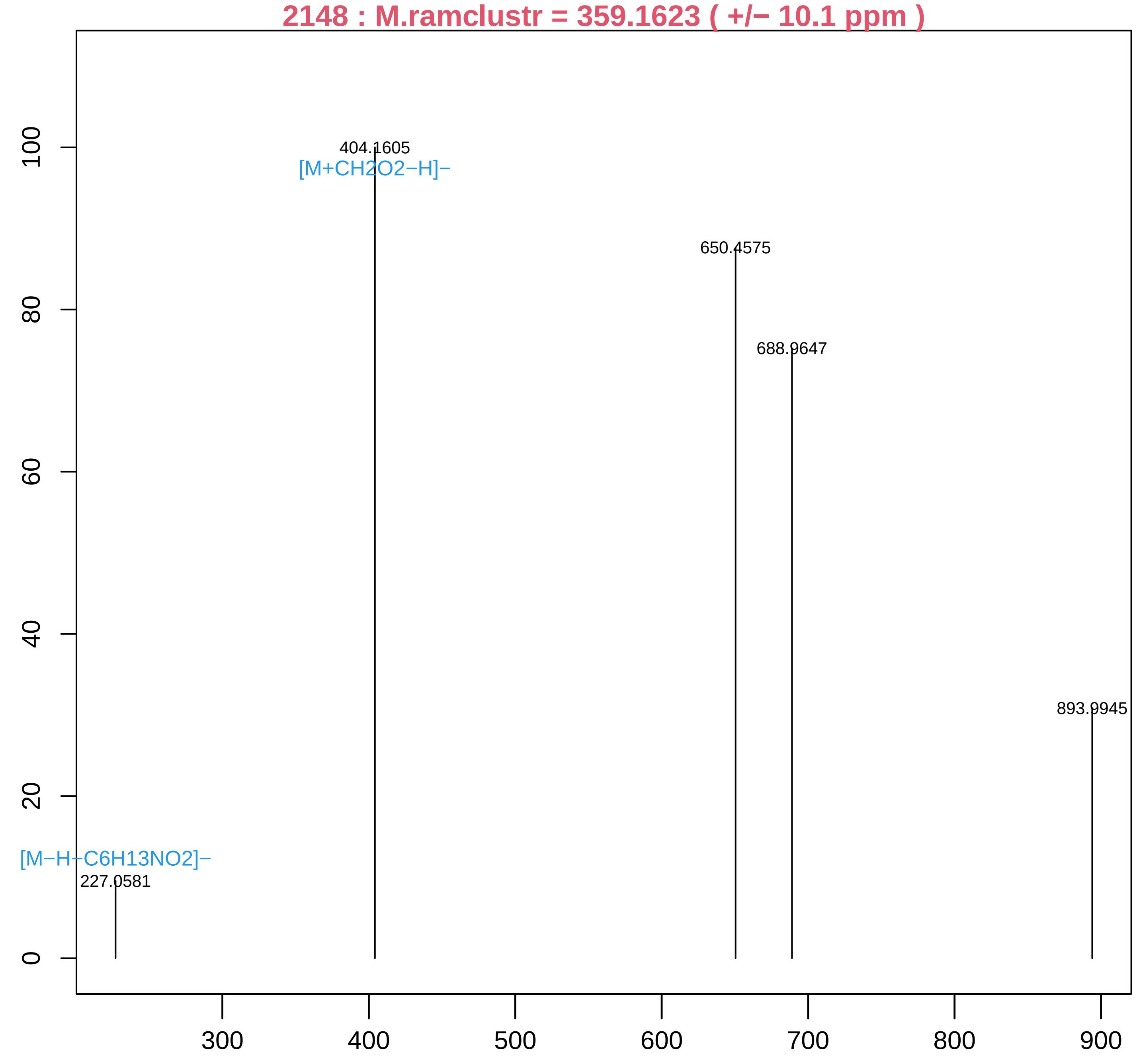
2148 : M.ramclustr = 359.1623 (+/− 10.1 ppm)
404.1605
[M+CH2O2−H]−
650.4575
688.9647
893.9945
[M−H−C6H13NO2]−
227.0581
0
20
40
60
80
100
300
400
500
600
700
800
900
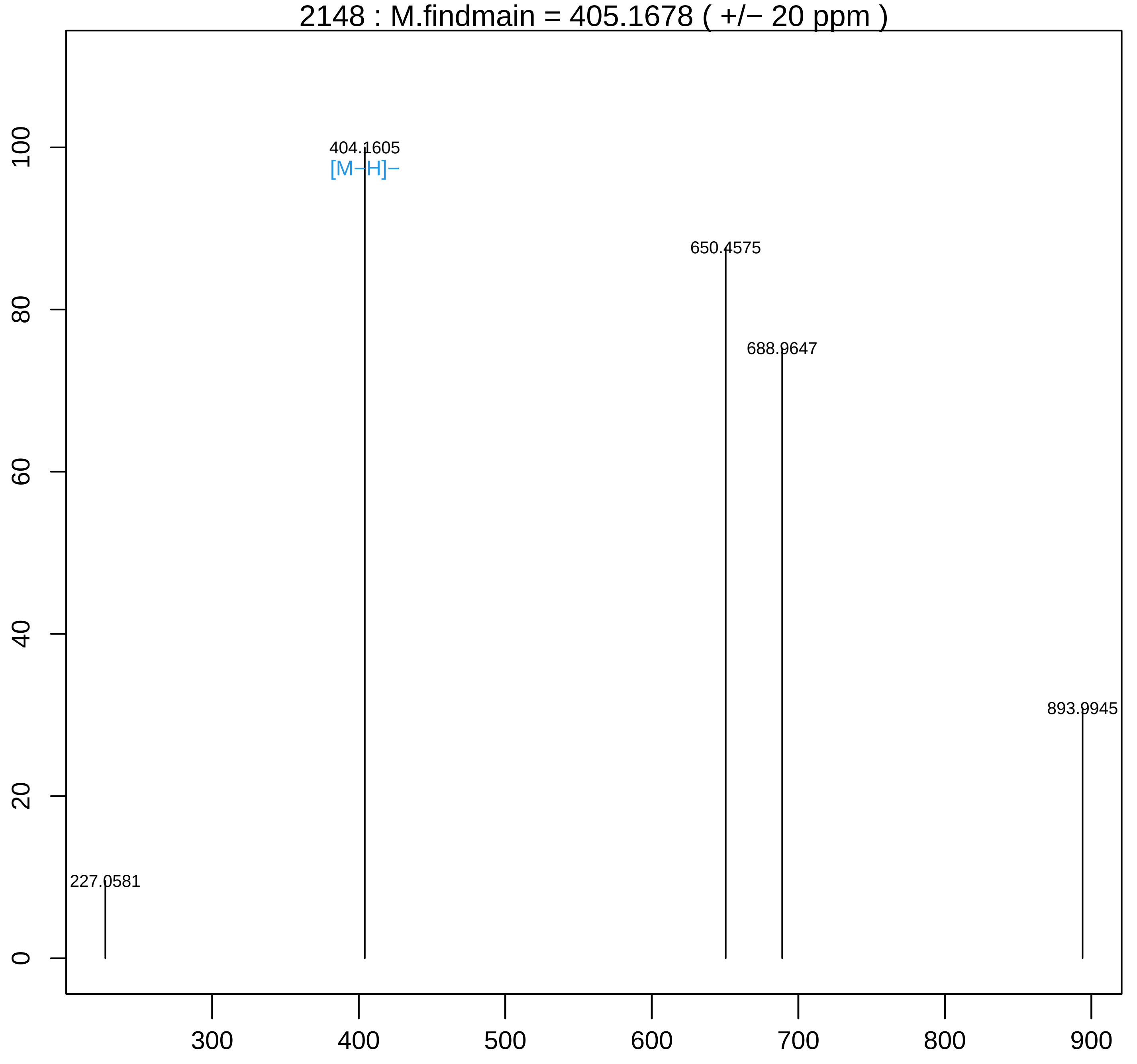
2148 : M.findmain = 405.1678 (+/− 20 ppm)
404.1605
[M−H]−
650.4575
688.9647
893.9945
227.0581
0
20
40
60
80
100
300
400
500
600
700
800
900

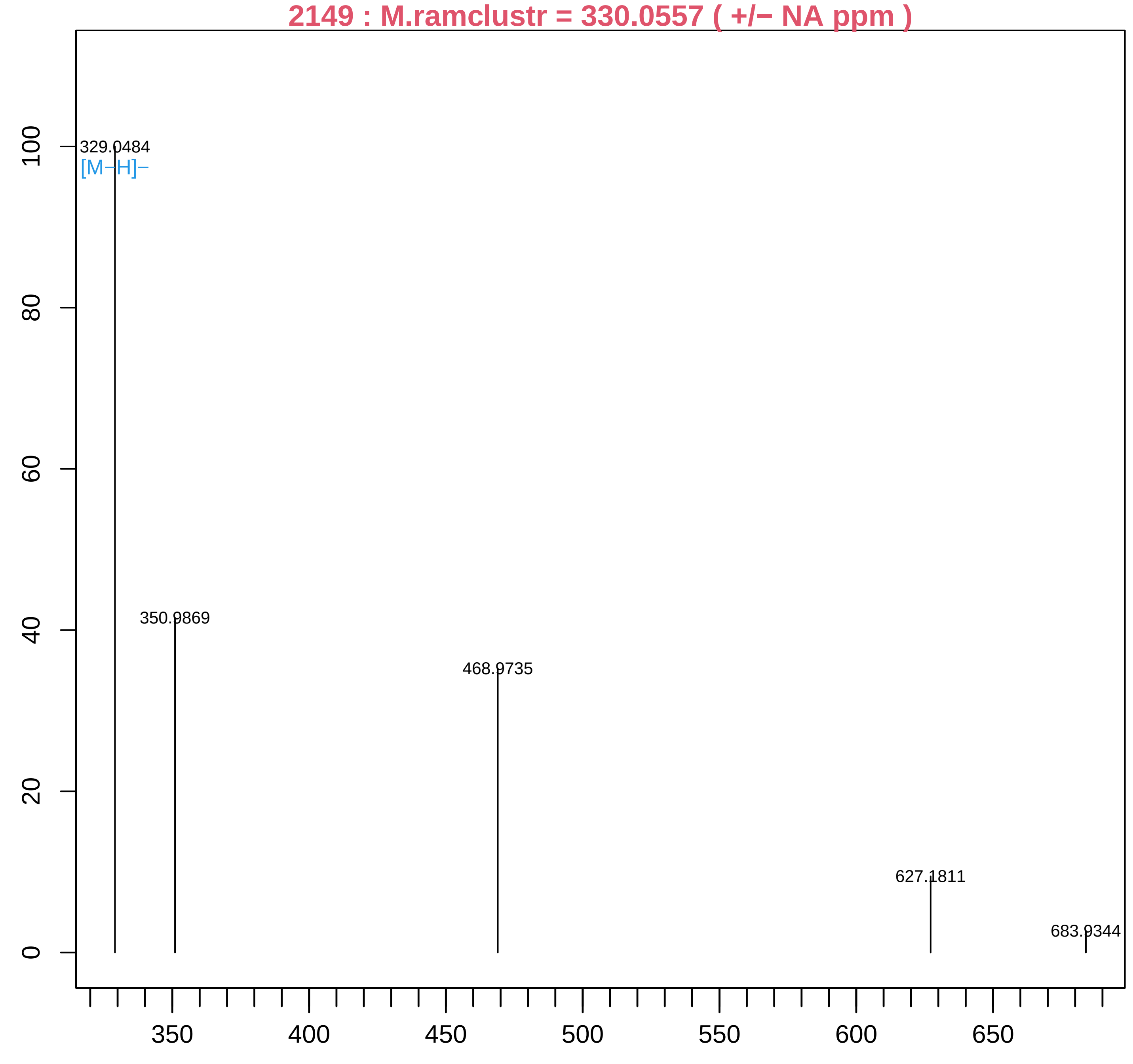

2149 : M.ramclustr = 330.0557 (+/− NA ppm)
329.0484
[M−H]−
350.9869
468.9735
627.1811
683.9344
0
20
40
60
80
100
350
400
450
500
550
600
650

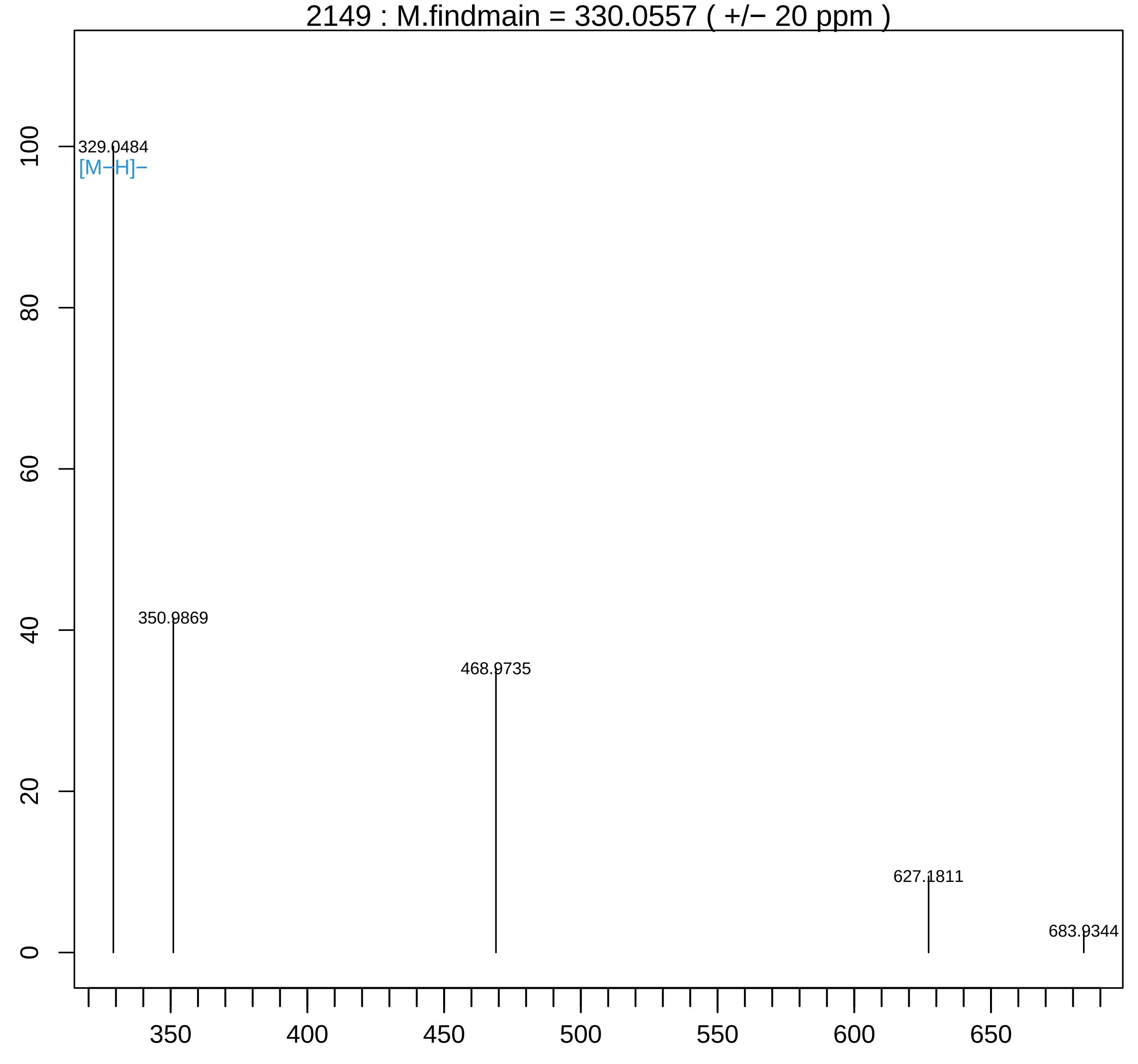

2149 : M.findmain = 330.0557 (+/− 20 ppm)
329.0484
[M−H]−
350.9869
468.9735
627.1811
683.9344
0
20
40
60
80
100
350
400
450
500
550
600
650

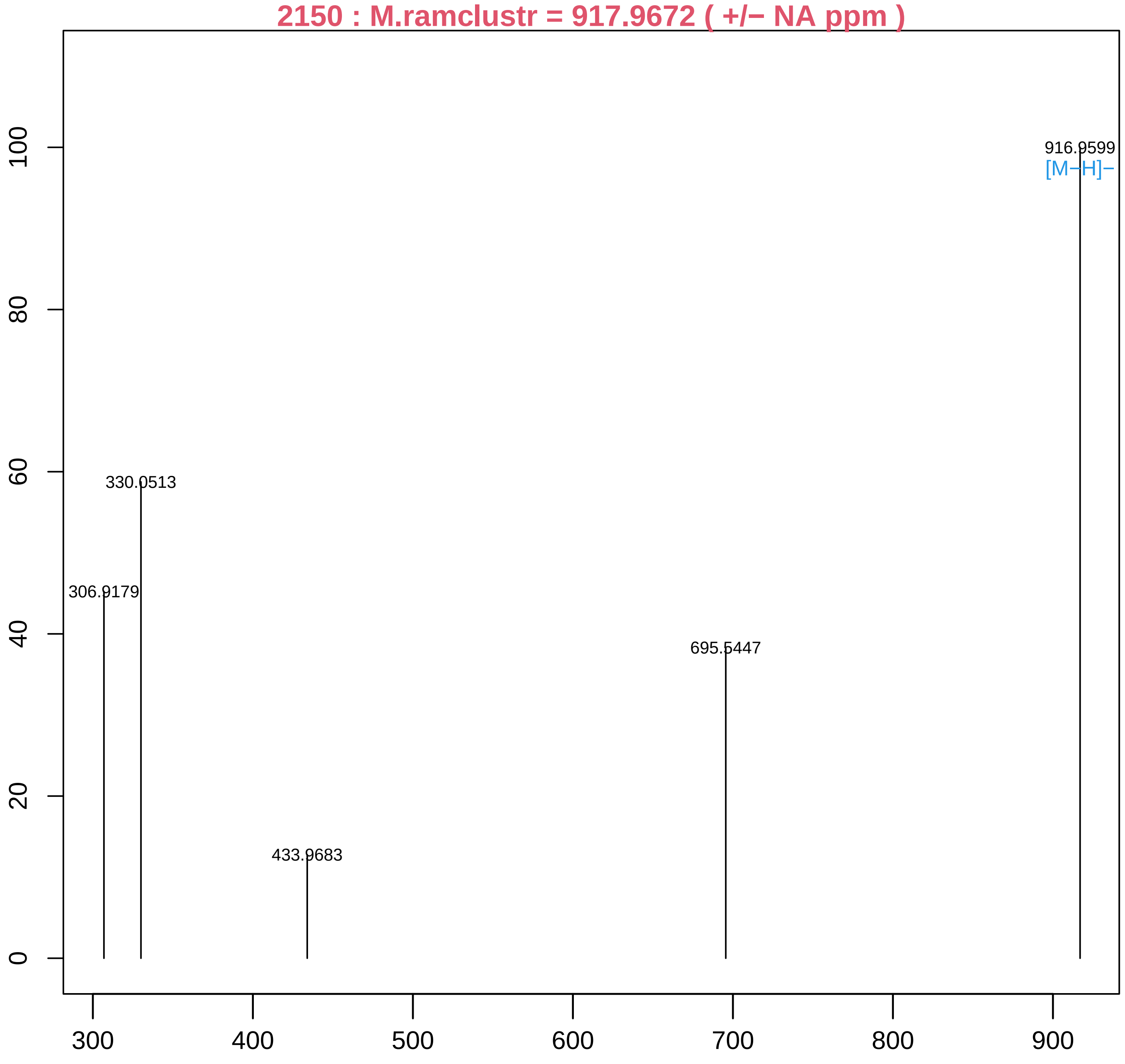
2150 : M.ramclustr = 917.9672 (+/− NA ppm)
916.9599
[M−H]−
330.0513
306.9179
695.5447
433.9683
0
20
40
60
80
100
300
400
500
600
700
800
900

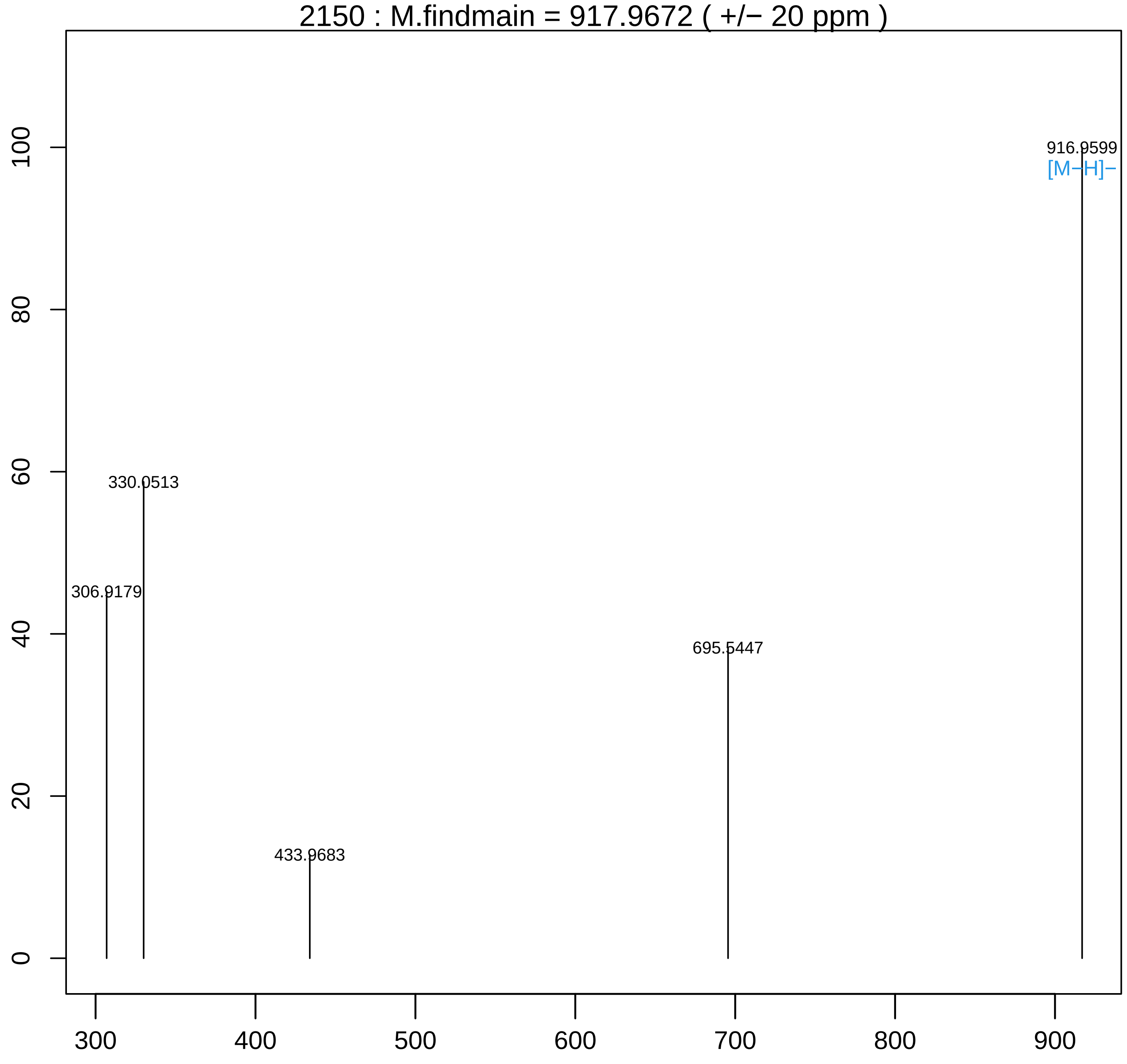
2150 : M.findmain = 917.9672 (+/− 20 ppm)
916.9599
[M−H]−
330.0513
306.9179
695.5447
433.9683
0
20
40
60
80
100
300
400
500
600
700
800
900

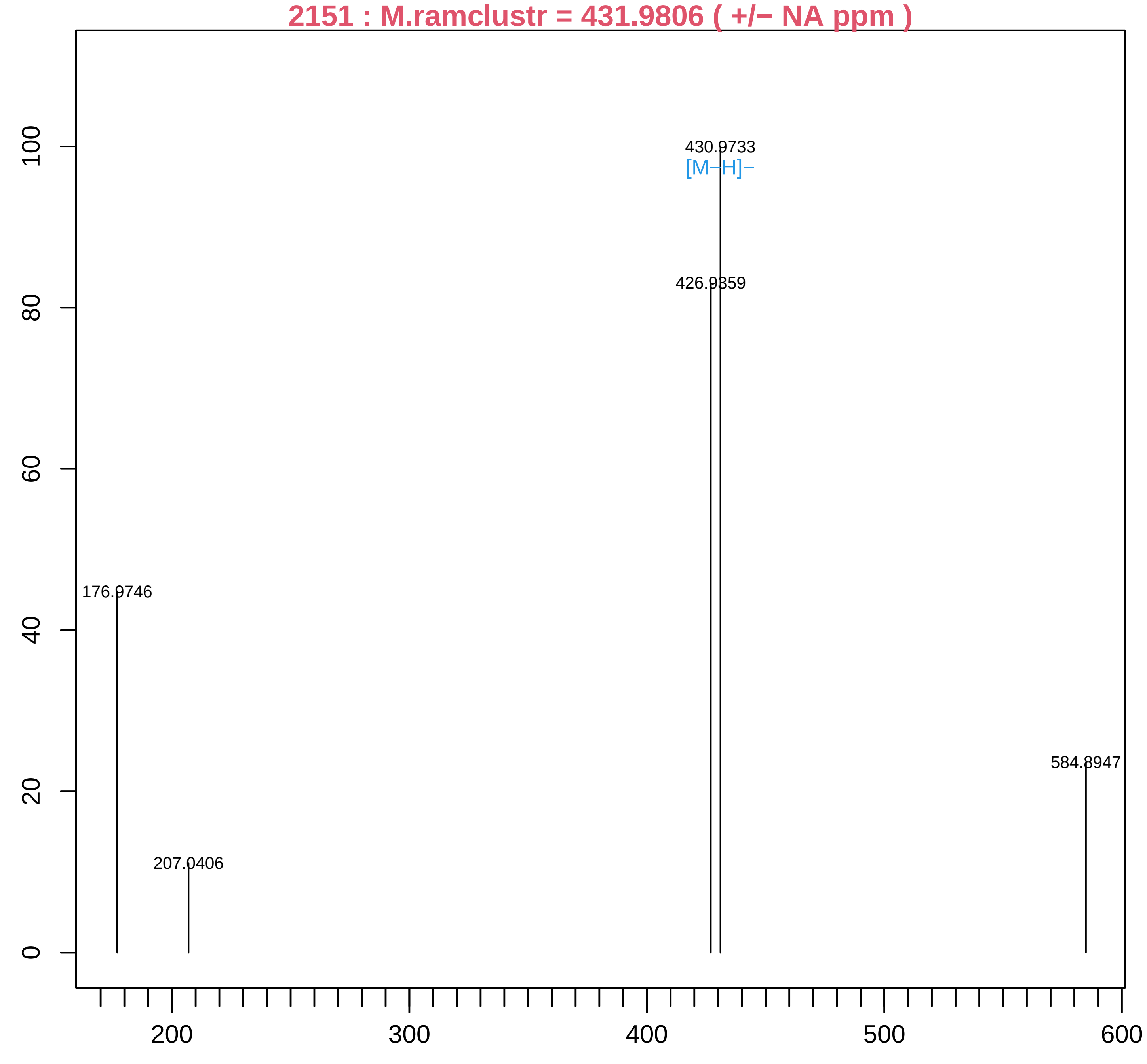
2151 : M.ramclustr = 431.9806 (+/− NA ppm)
430.9733
[M−H]−
426.9359
176.9746
207.0406
584.8947
0
20
40
60
80
100
200
300
400
500
600

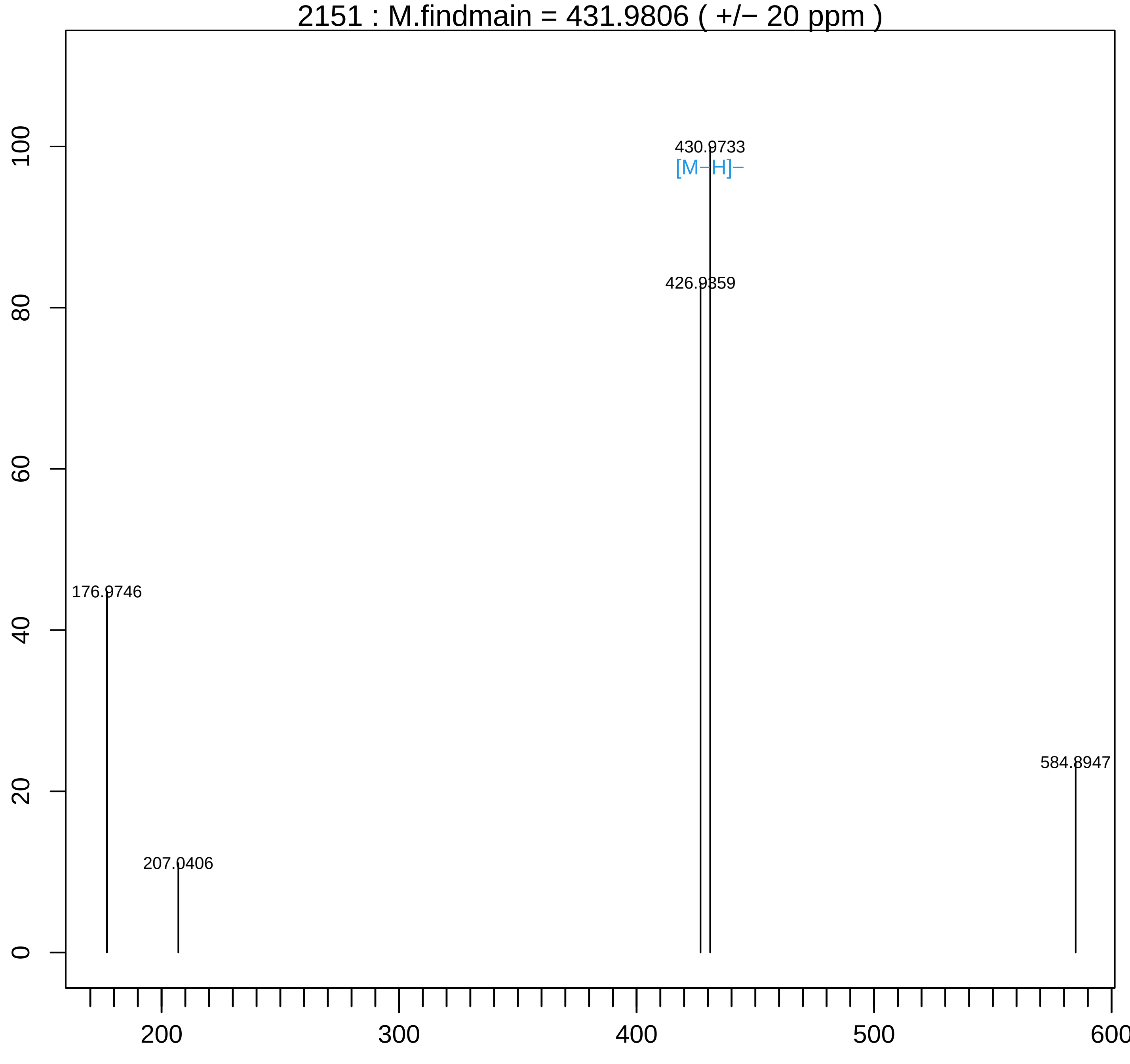
2151 : M.findmain = 431.9806 (+/− 20 ppm)
430.9733
[M−H]−
426.9359
176.9746
207.0406
584.8947
0
20
40
60
80
100
200
300
400
500
600

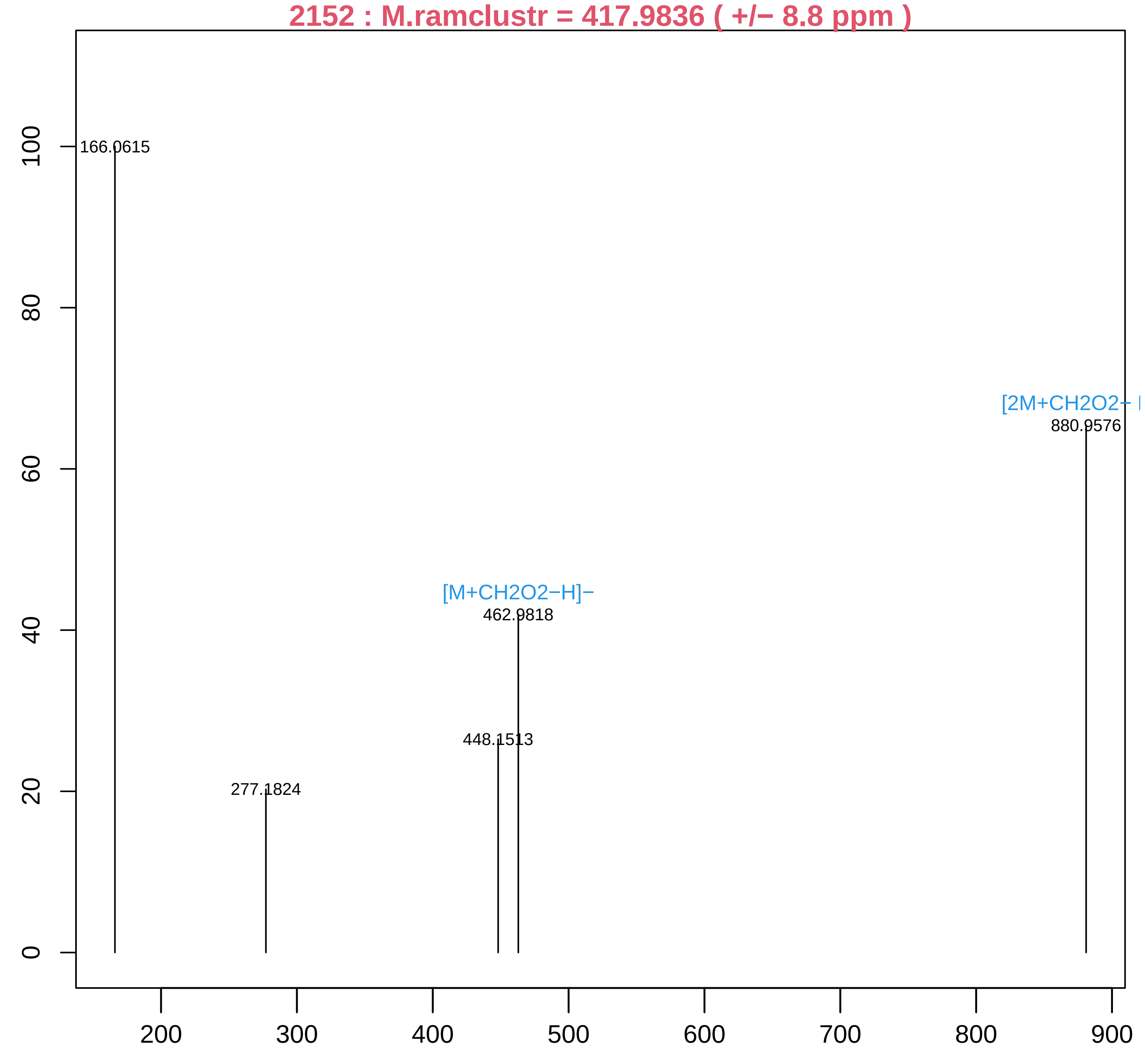
2152 : M.ramclustr = 417.9836 (+/− 8.8 ppm)
166.0615
277.1824
448.1513
[M+CH2O2−H]−
462.9818
[2M+CH2O2−
880.9576
0
20
40
60
80
100
200
300
400
500
600
700
800
900

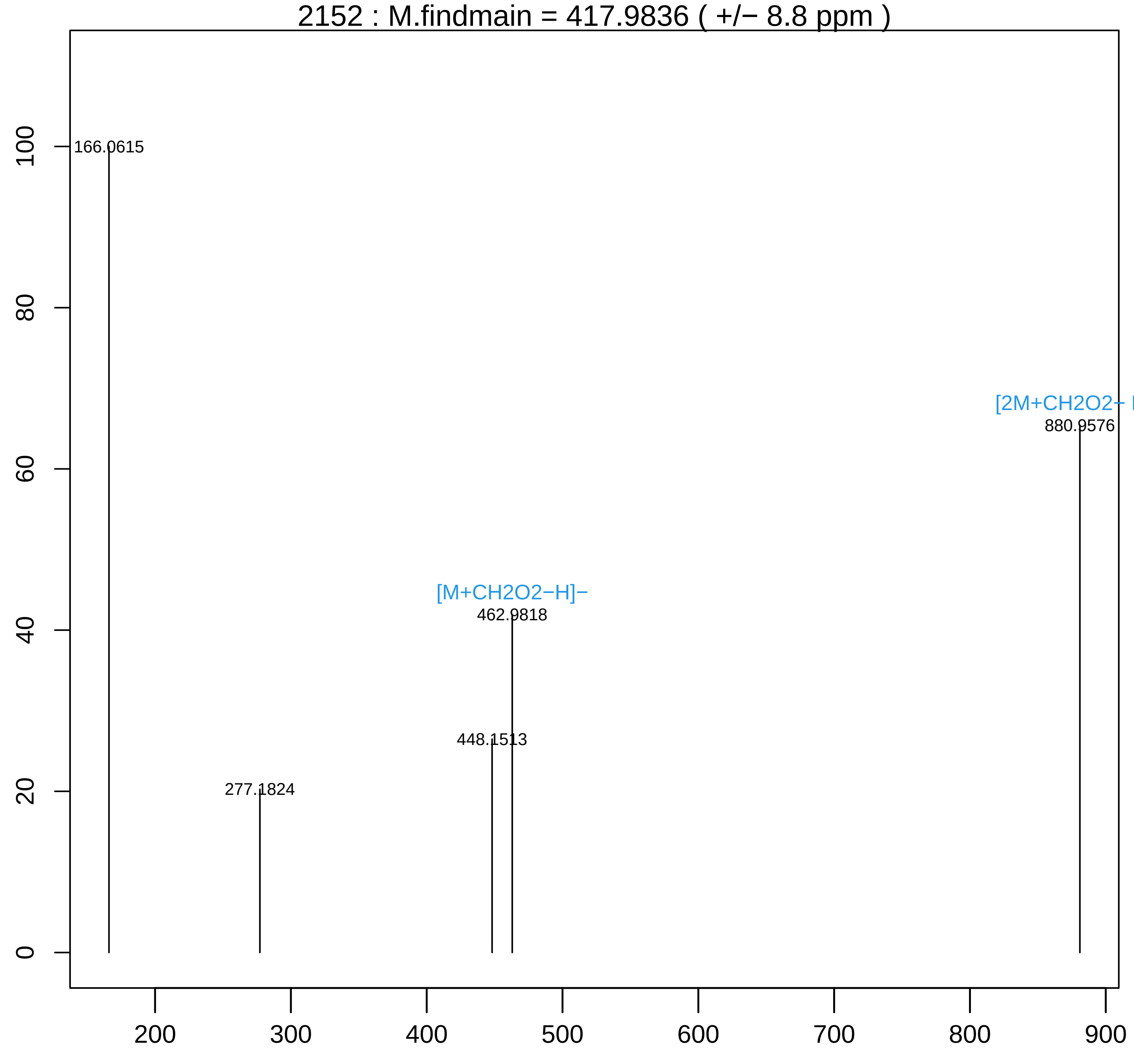
2152 : M.findmain = 417.9836 (+/− 8.8 ppm)
166.0615
277.1824
448.1513
[M+CH2O2−H]−
462.9818
[2M+CH2O2−
880.9576
0
20
40
60
80
100
200
300
400
500
600
700
800
900

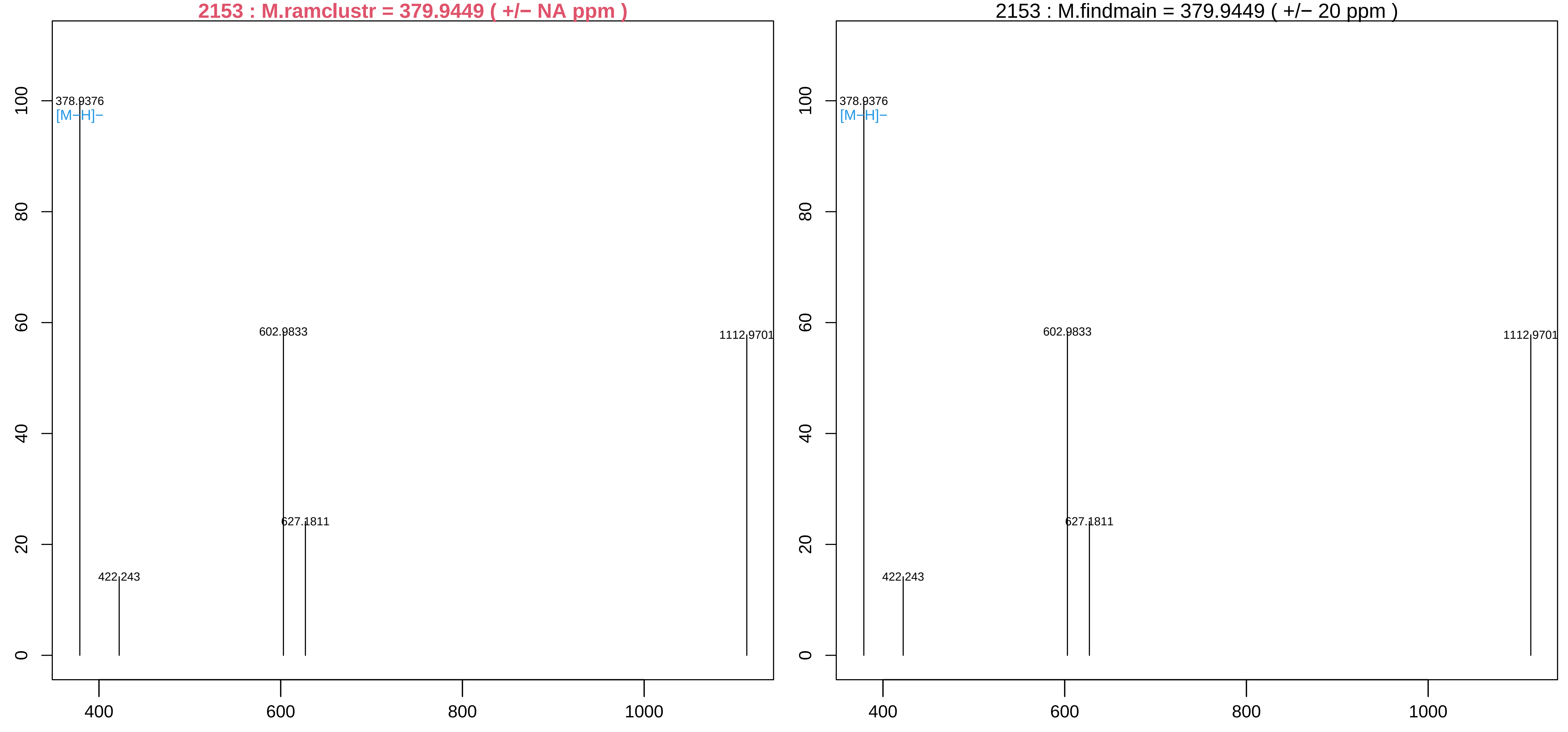

2153 : M.ramclustr = 379.9449 (+/− NA ppm)
378.9376
[M−H]−
422.243
602.9833
627.1811
1112.9701
2153 : M.findmain = 379.9449 (+/− 20 ppm)
378.9376
[M−H]−
422.243
602.9833
627.1811
1112.9701
0
20
40
60
80
100
400
600
800
1000

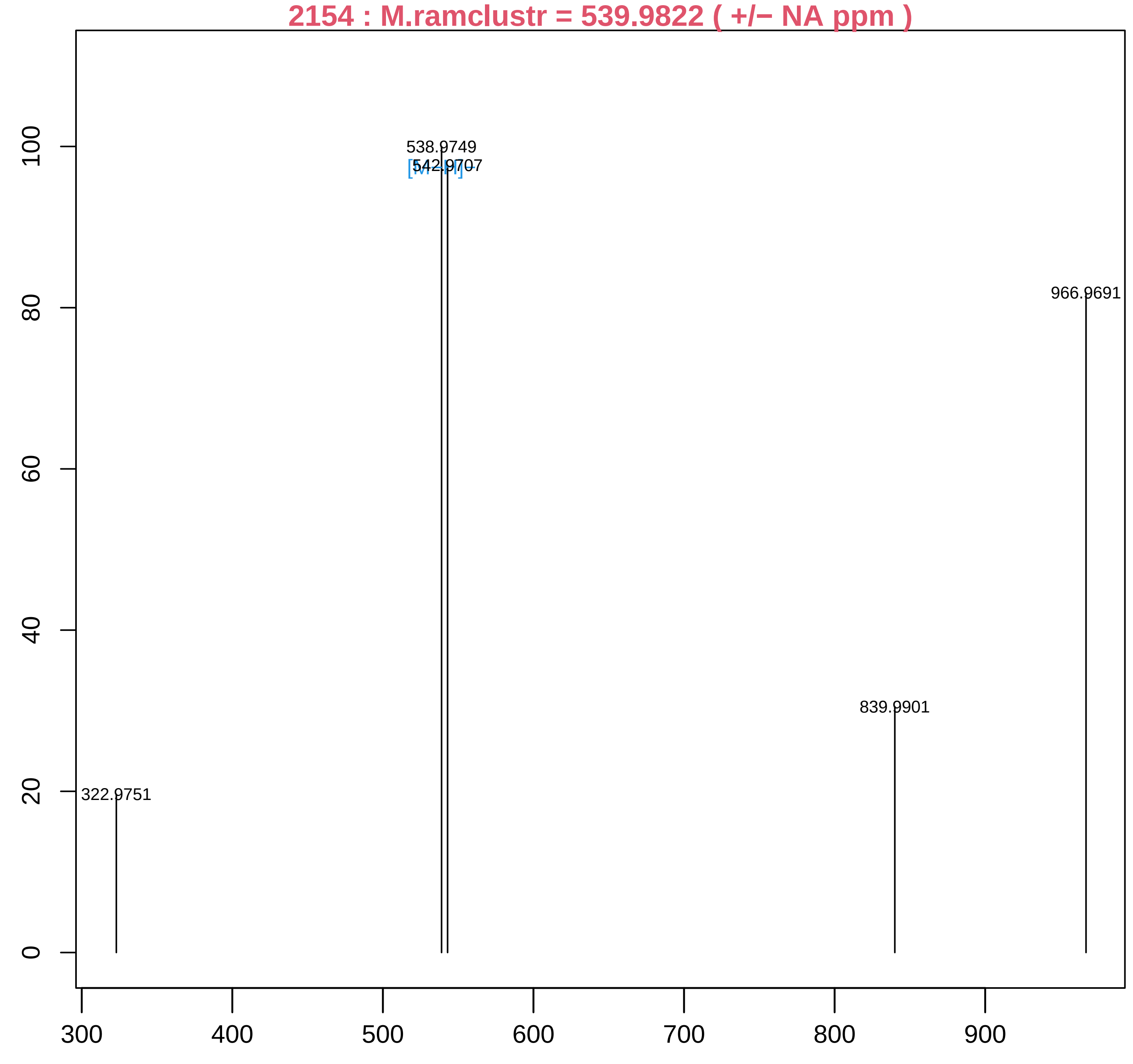

2154 : M.ramclustr = 539.9822 (+/− NA ppm)
538.9749
[M−H]−
542.9707
966.9691
839.9901
322.9751

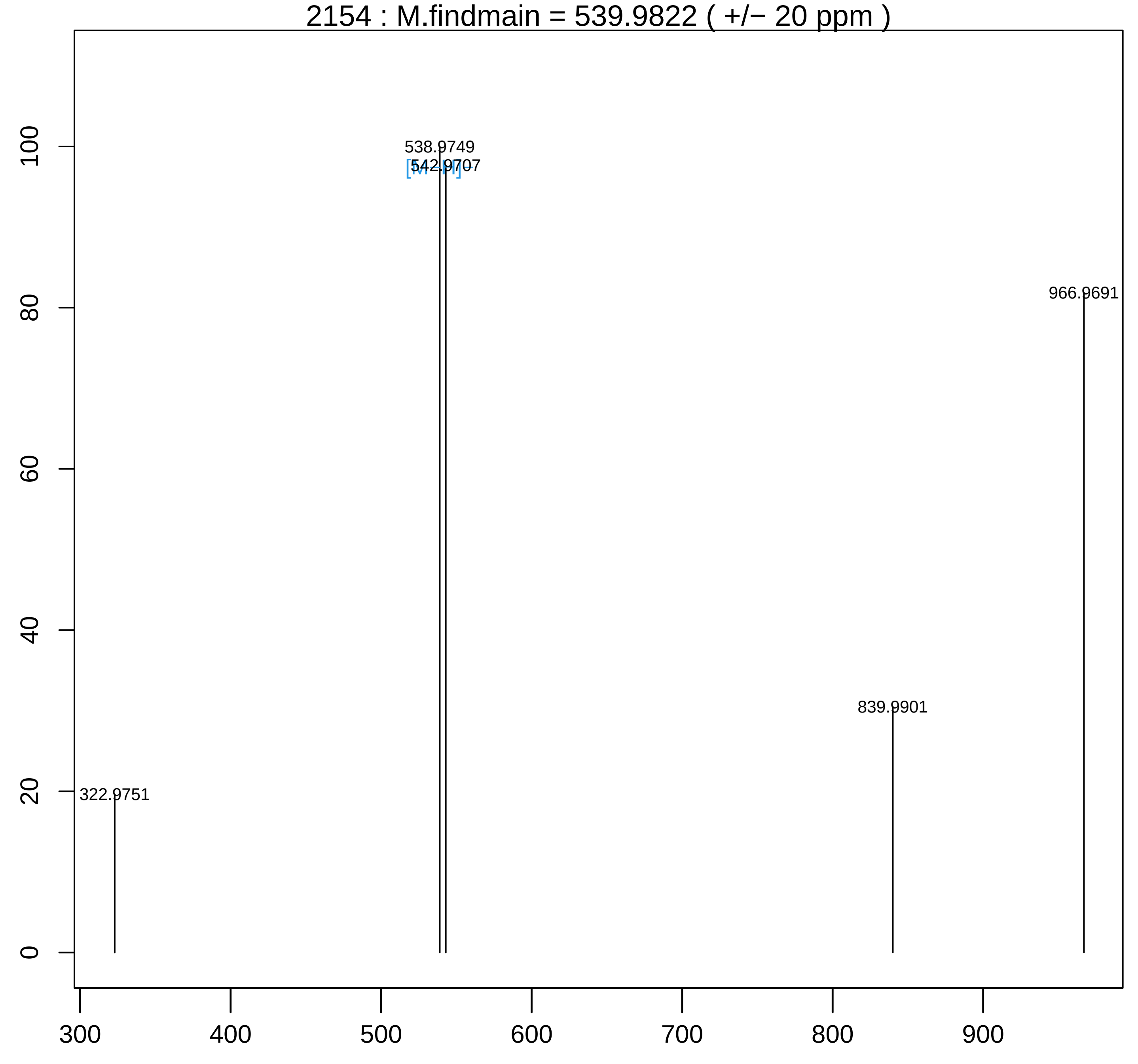

2154 : M.findmain = 539.9822 (+/− 20 ppm)
538.9749
[M−H]−
542.9707
966.9691
839.9901
322.9751

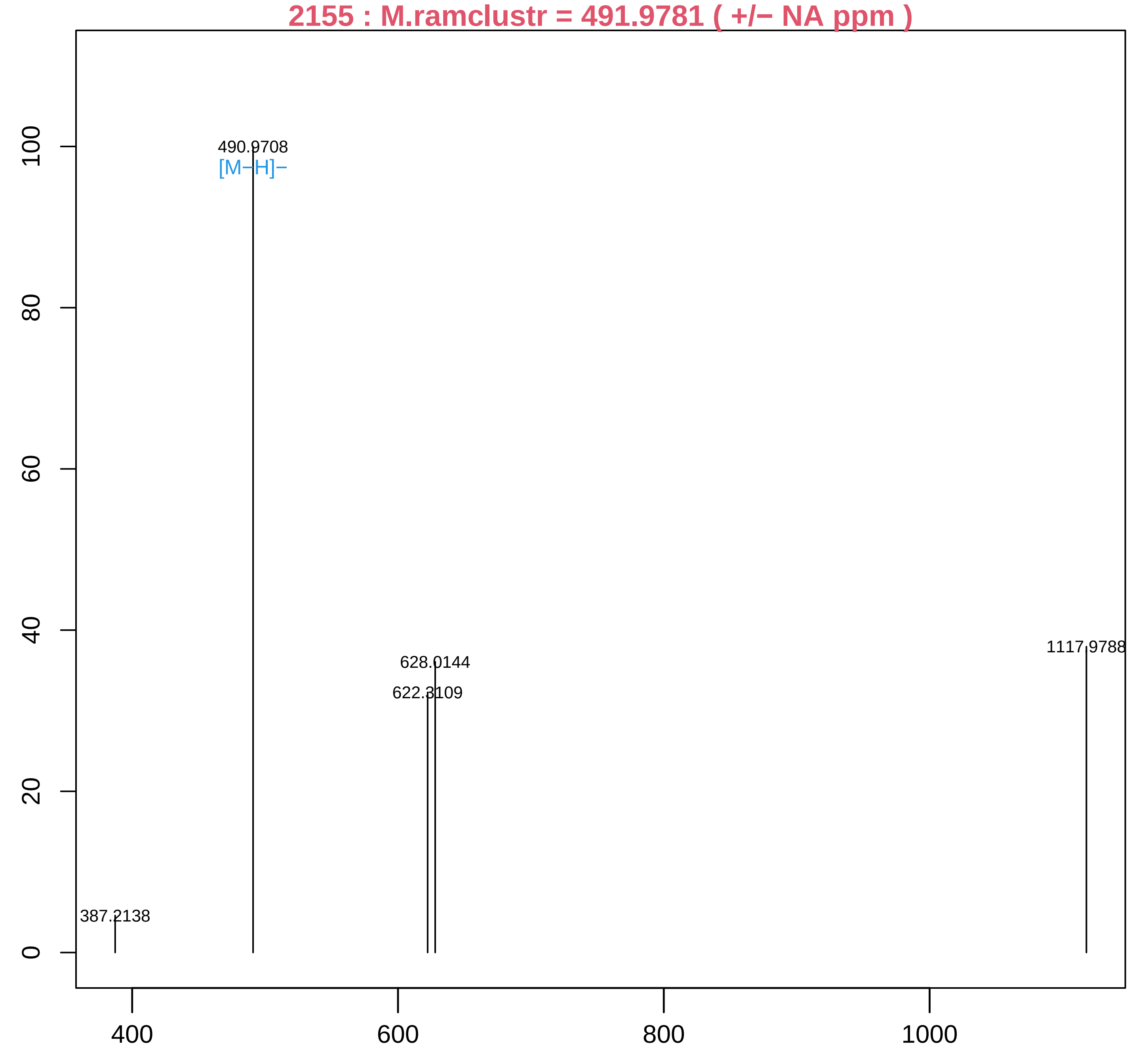
2155 : M.ramclustr = 491.9781 (+/− NA ppm)
490.9708
[M−H]−
628.0144
622.3109
1117.9788
387.2138
0
20
40
60
80
100
400
600
800
1000

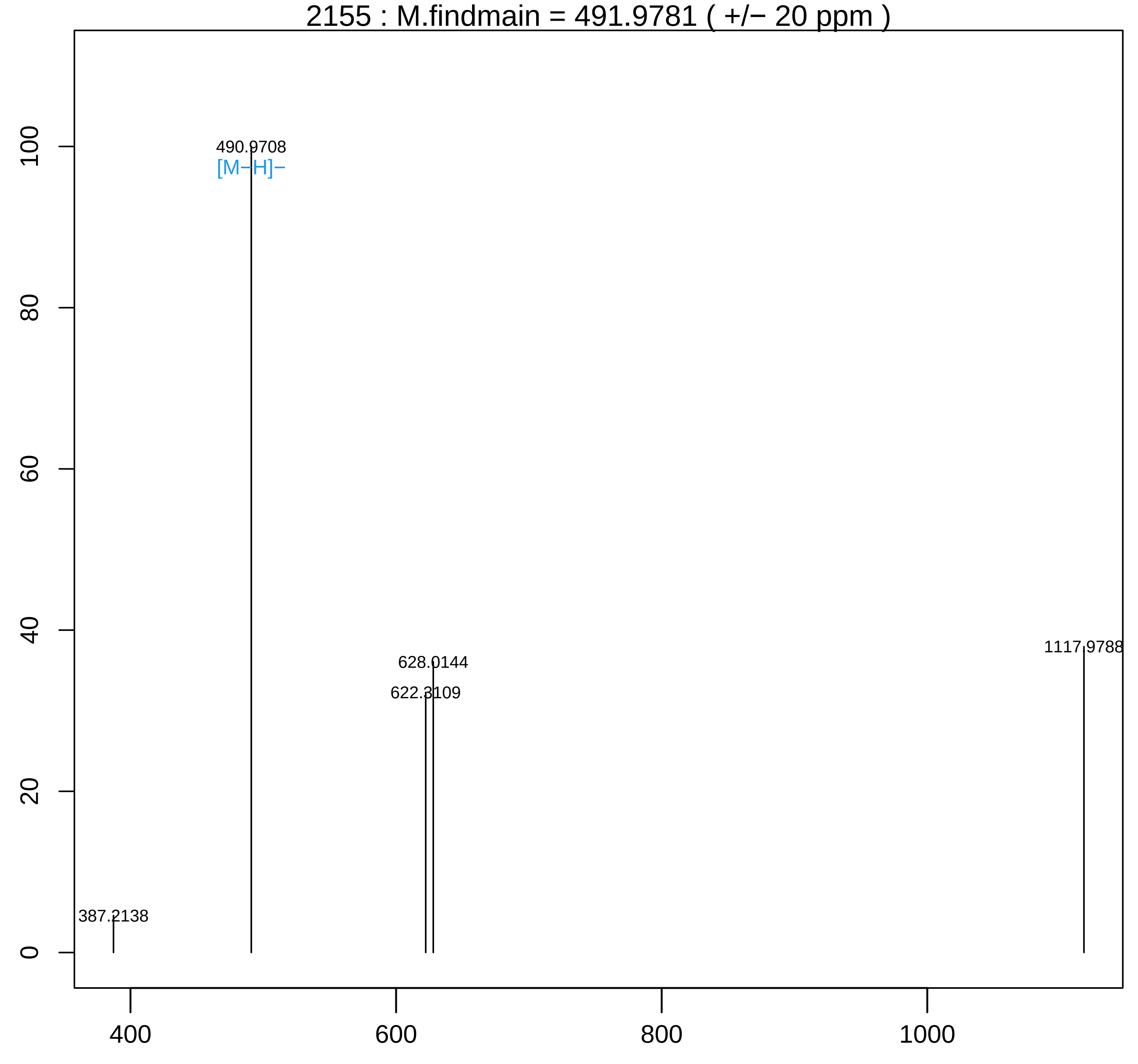
2155 : M.findmain = 491.9781 (+/− 20 ppm)
490.9708
[M−H]−
628.0144
622.3109
1117.9788
387.2138
0
20
40
60
80
100
400
600
800
1000

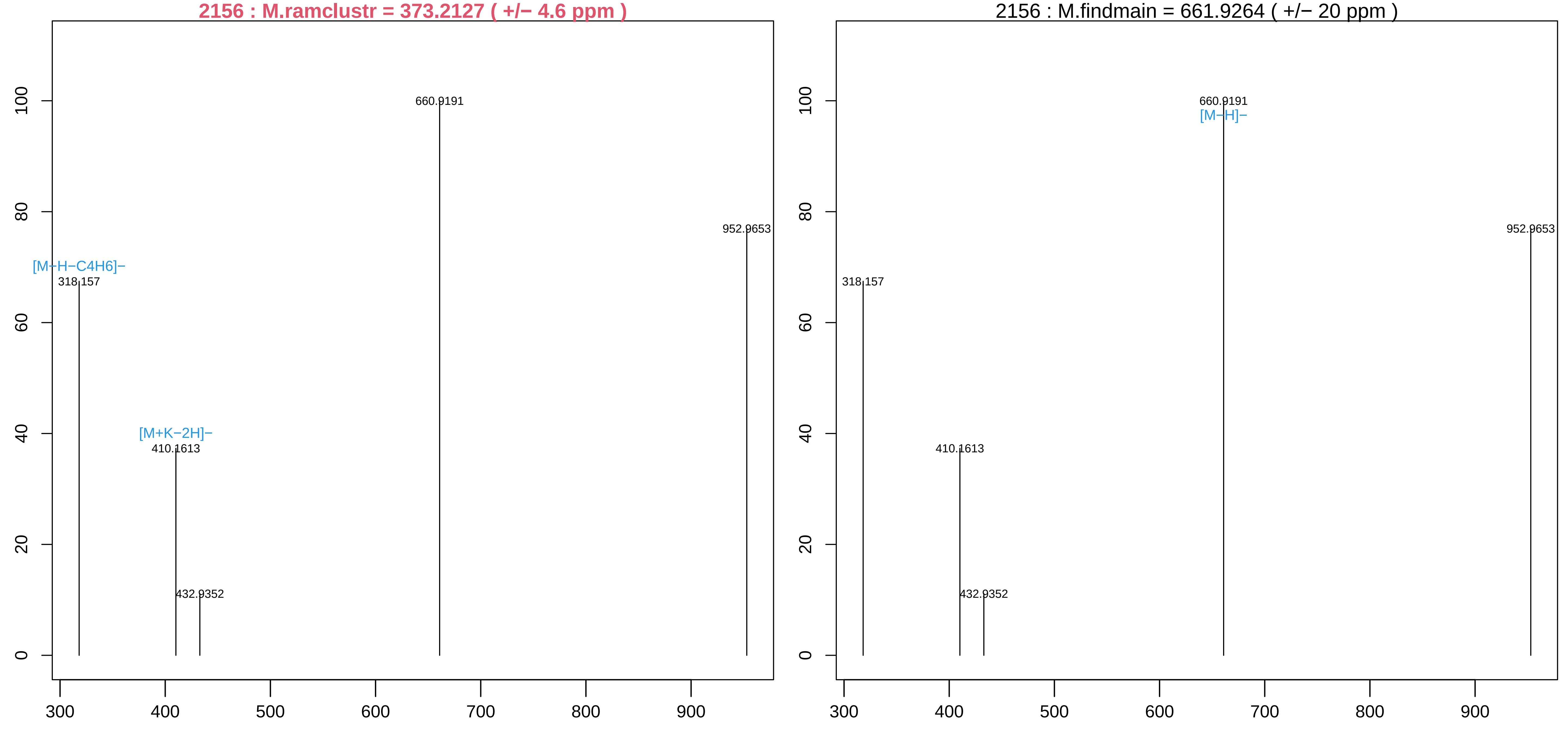

2156 : M.ramclustr = 373.2127 (+/− 4.6 ppm)
[M−H−C4H6]−
318.157
[M+K−2H]−
410.1613
432.9352
660.9191
952.9653
2156 : M.findmain = 661.9264 (+/− 20 ppm)
660.9191
[M−H]−
318.157
410.1613
432.9352
952.9653

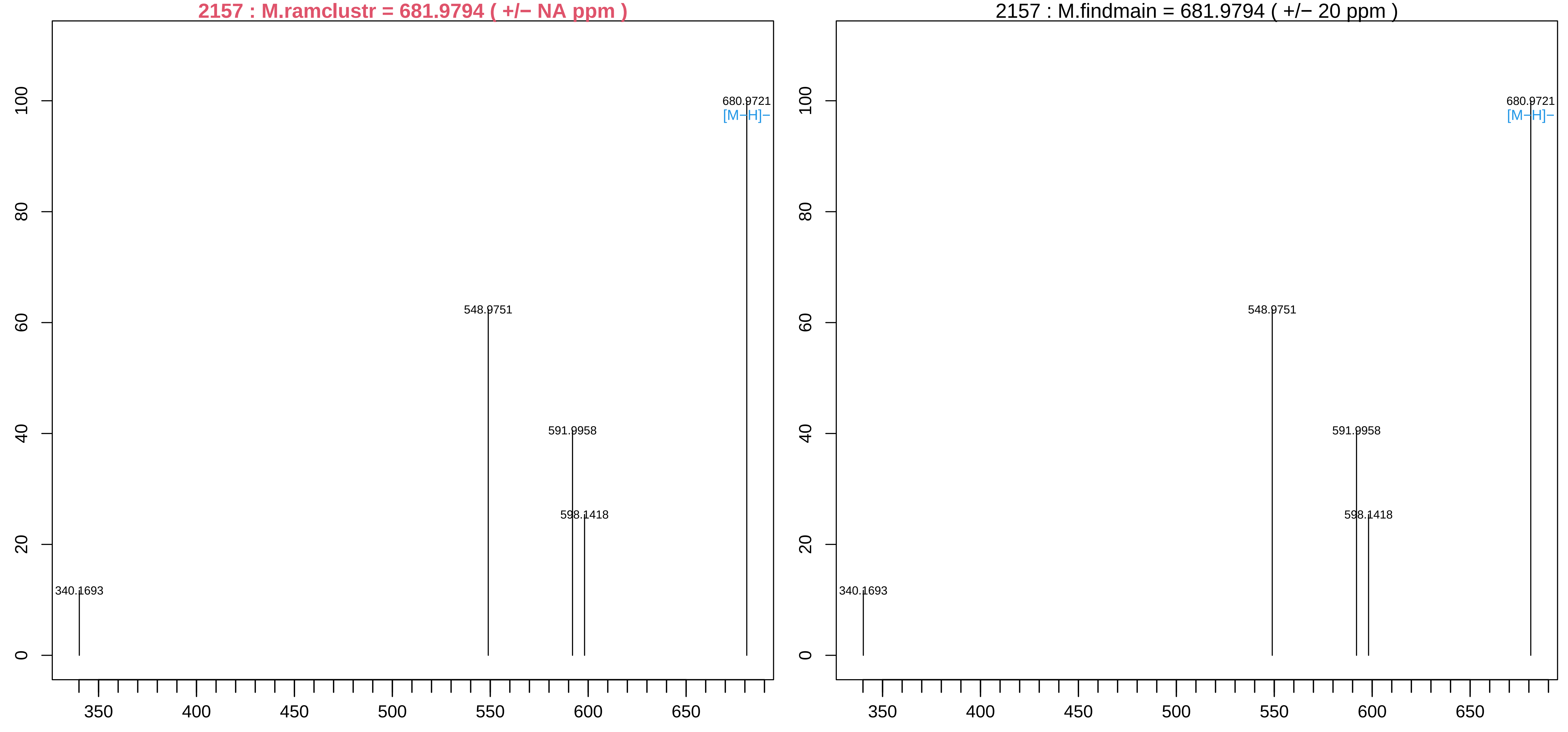

2157 : M.ramclustr = 681.9794 (+/− NA ppm)
680.9721
[M−H]−
548.9751
591.9958
598.1418
340.1693
0
20
40
60
80
100
350
400
450
500
550
600
650
2157 : M.findmain = 681.9794 (+/− 20 ppm)
680.9721
[M−H]−
548.9751
591.9958
598.1418
340.1693
0
20
40
60
80
100
350
400
450
500
550
600
650

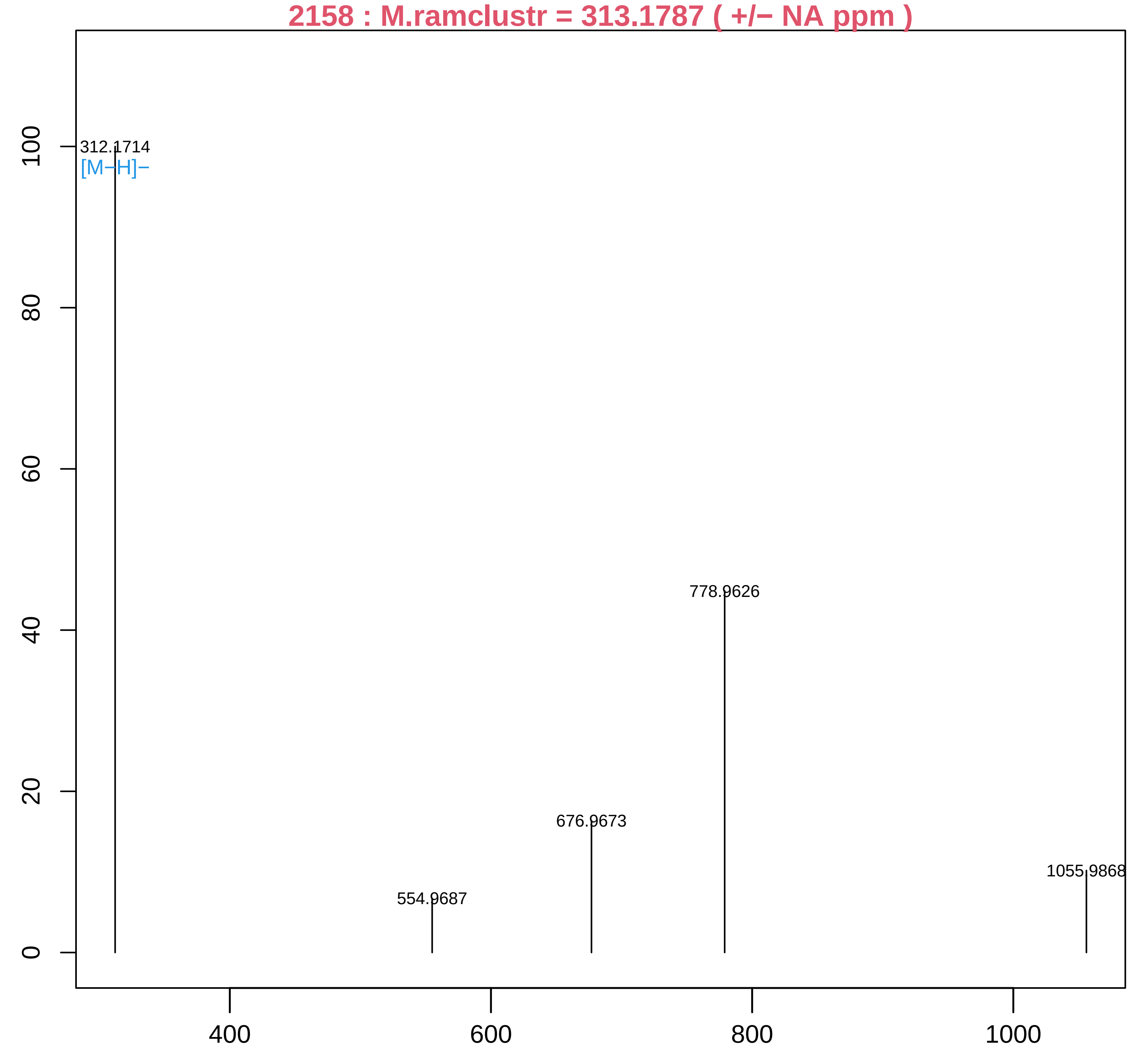

2158 : M.ramclustr = 313.1787 (+/− NA ppm)
312.1714
[M−H]−
778.9626
676.9673
554.9687
1055.9868
0
20
40
60
80
100
400
600
800
1000

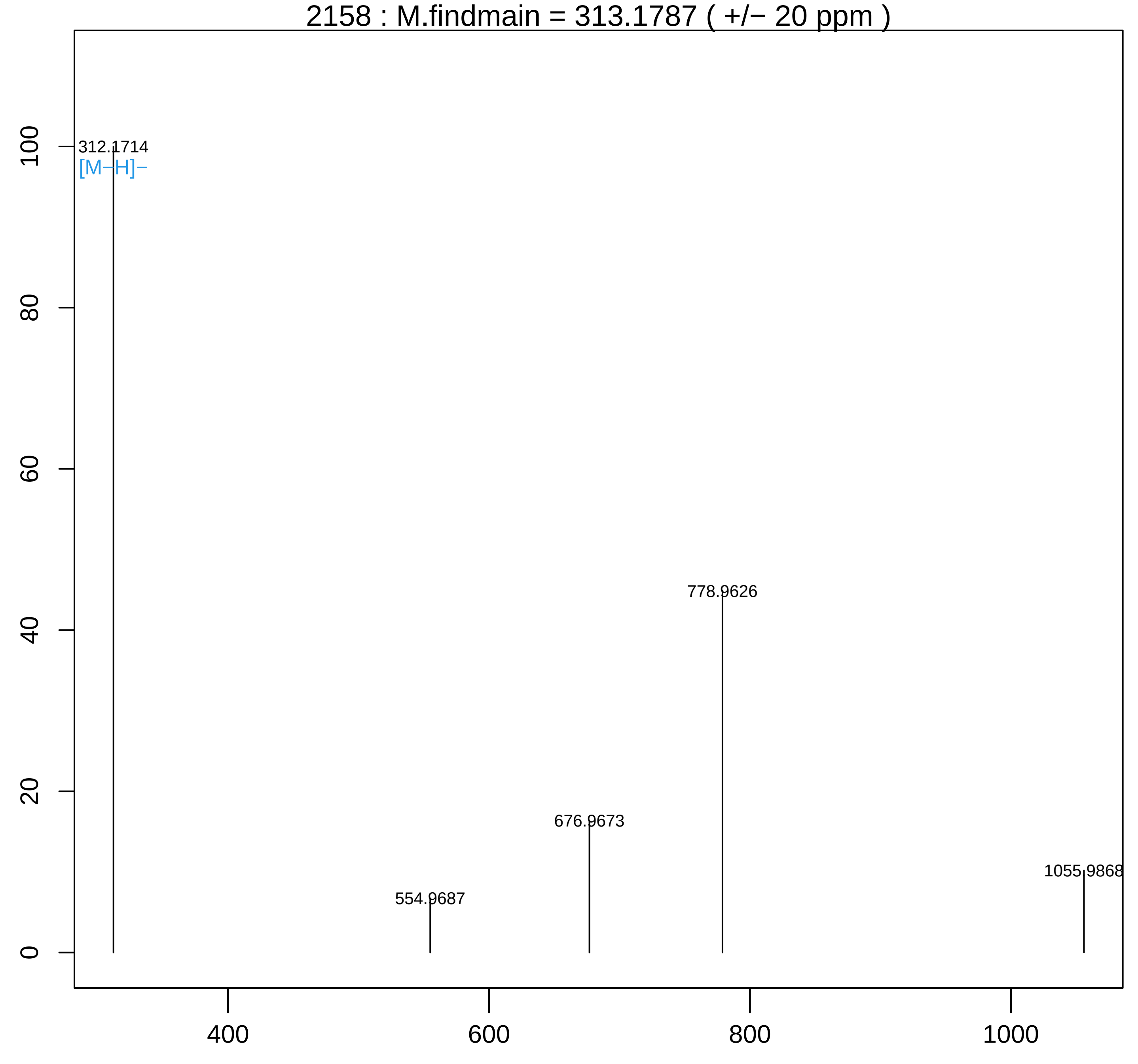

2158 : M.findmain = 313.1787 (+/− 20 ppm)
312.1714
[M−H]−
778.9626
676.9673
554.9687
1055.9868
0
20
40
60
80
100
400
600
800
1000

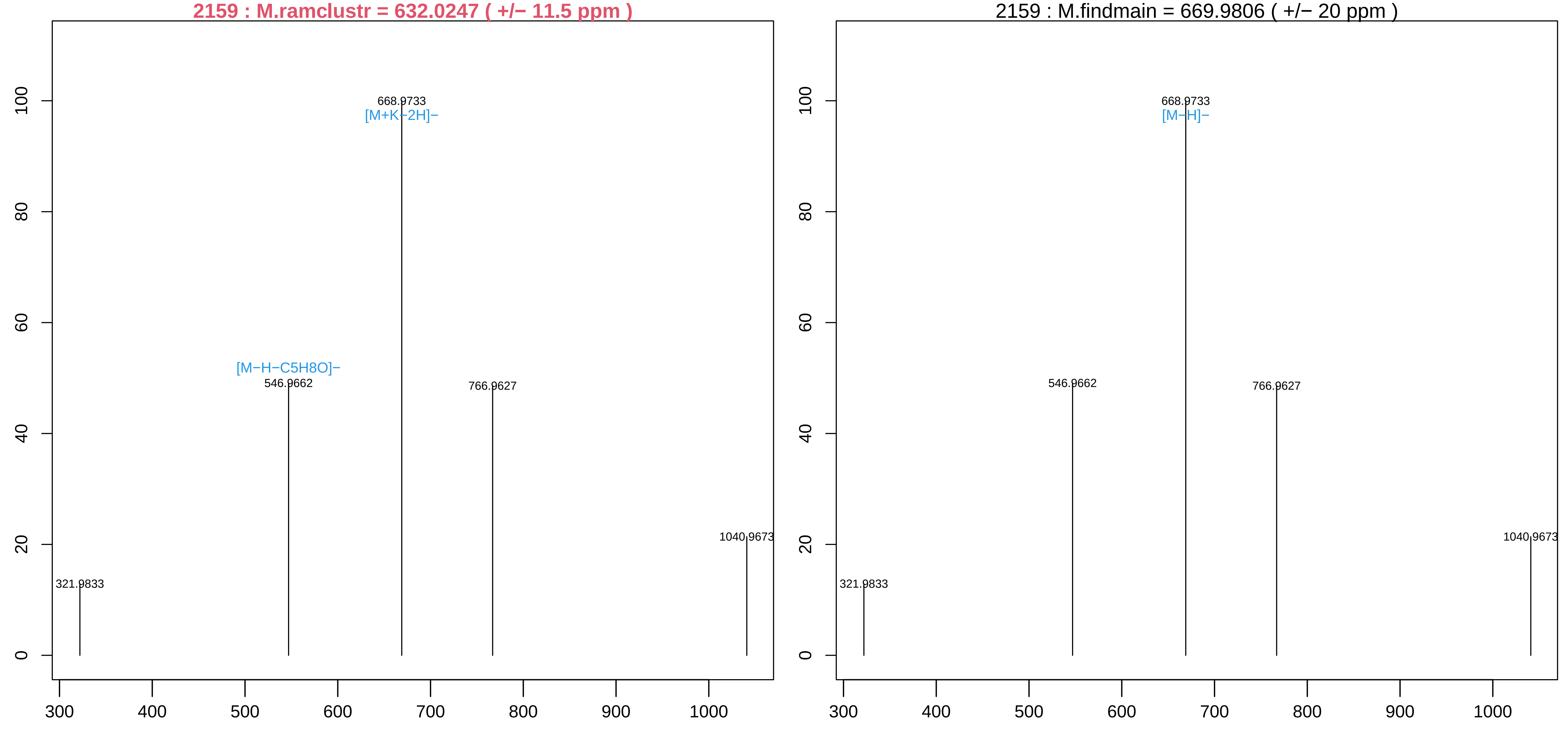
2159 : M.ramclustr = 632.0247 (+/− 11.5 ppm)
668.9733
[M+K−2H]−
[M−H−C5H8O]−
546.9662
766.9627
1040.9673
321.9833
2159 : M.findmain = 669.9806 (+/− 20 ppm)
668.9733
[M−H]−
546.9662
766.9627
1040.9673
321.9833

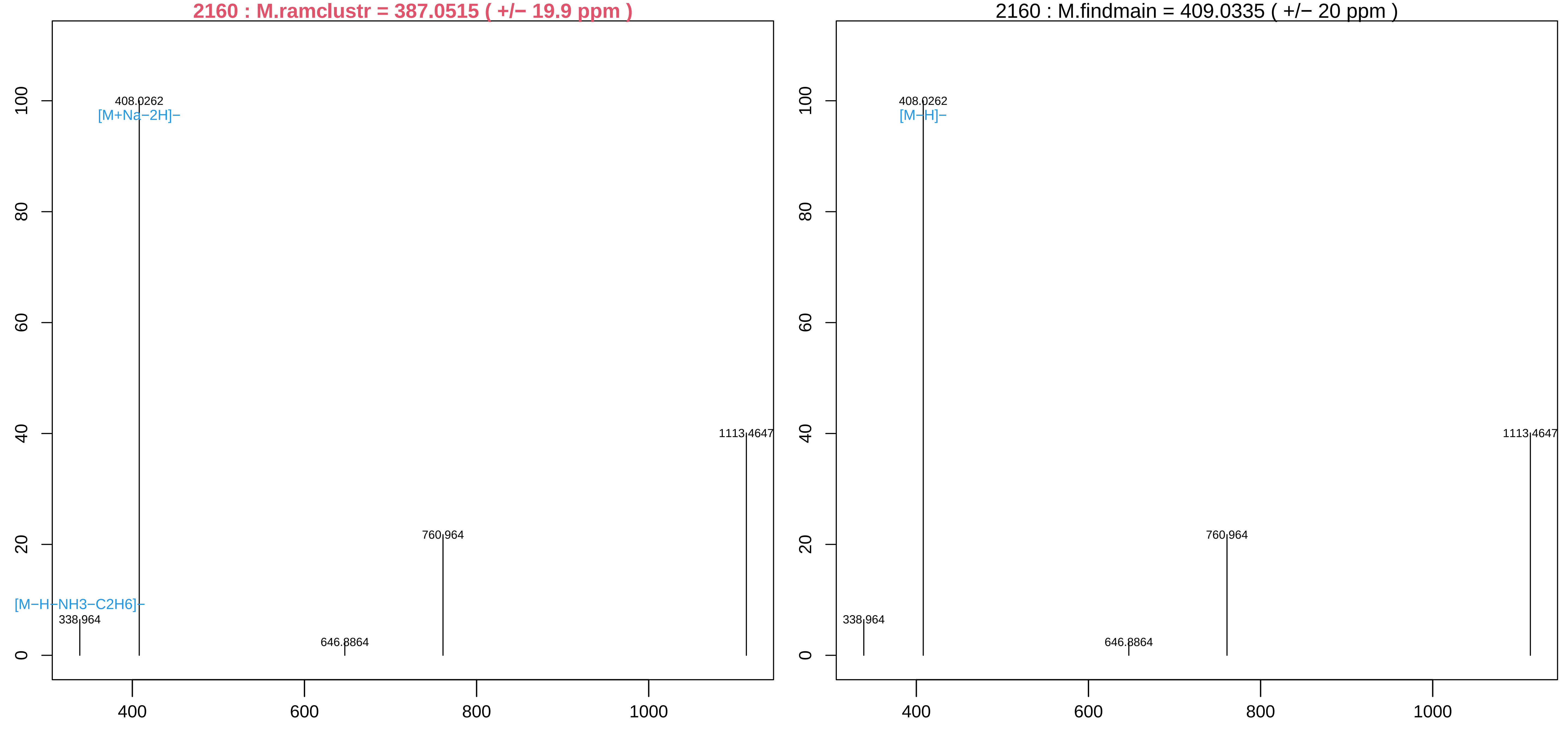
2160 : M.ramclustr = 387.0515 (+/− 19.9 ppm)
408.0262
[M+Na−2H]−
[M−H−NH3−C2H6]−
338.964
646.8864
760.964
1113.4647
2160 : M.findmain = 409.0335 (+/− 20 ppm)
408.0262
[M−H]−
338.964
646.8864
760.964
1113.4647
0
20
40
60
80
100
400
600
800
1000

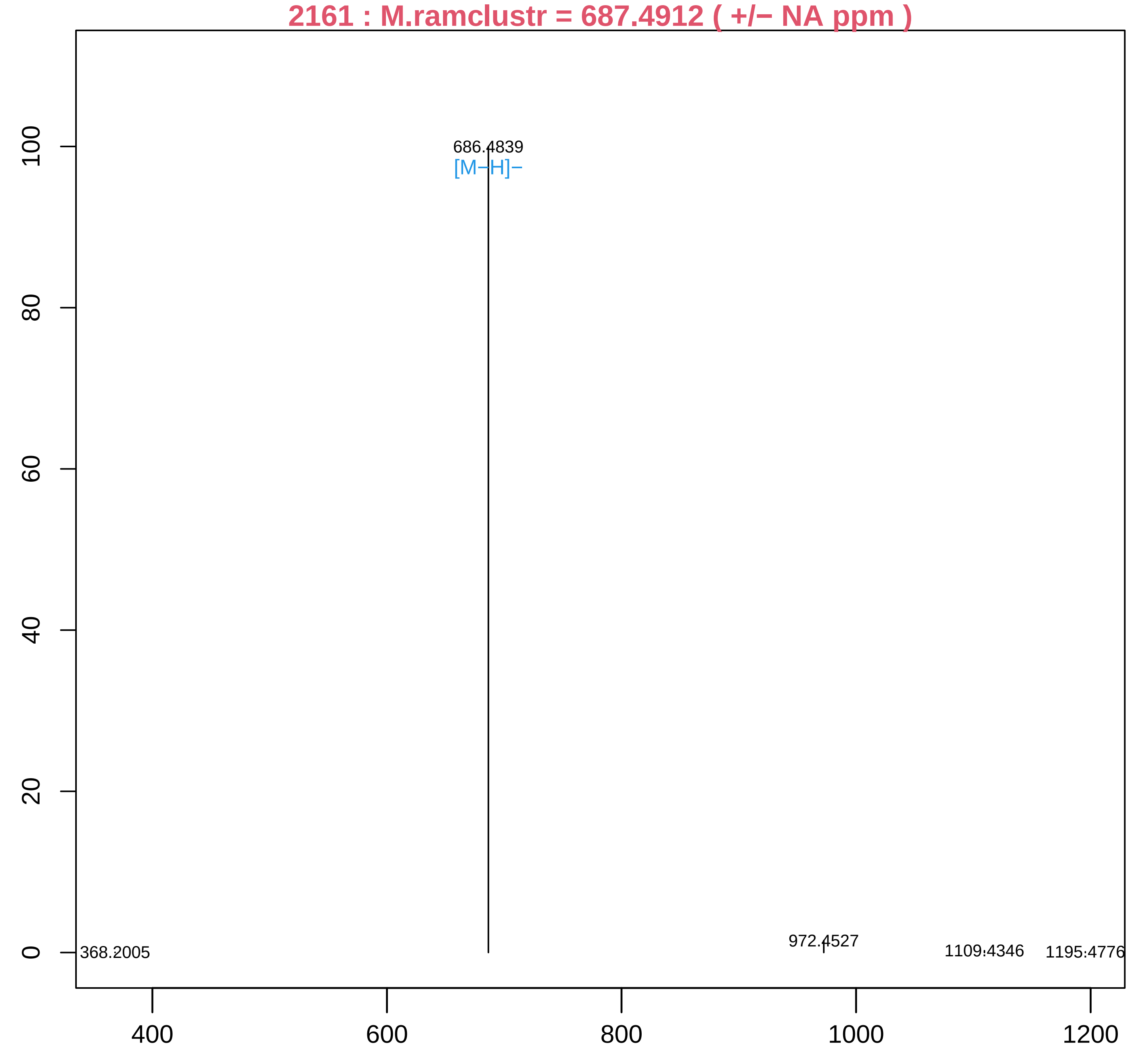

2161 : M.ramclustr = 687.4912 (+/− NA ppm)
686.4839
[M−H]−
972.4527
1109.4346
1195.4776
368.2005
0
20
40
60
80
100
400
600
800
1000
1200

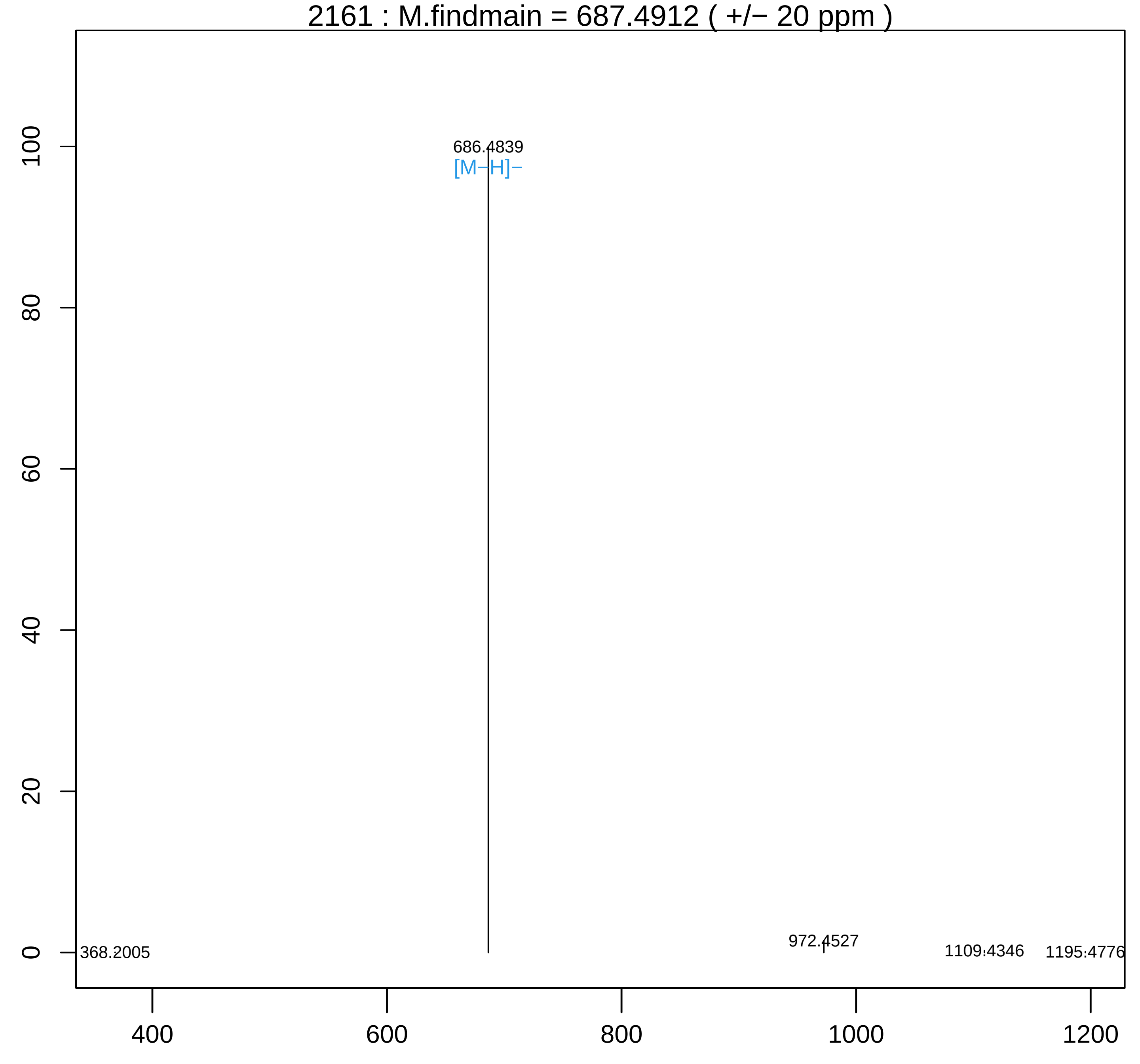

2161 : M.findmain = 687.4912 (+/− 20 ppm)
686.4839
[M−H]−
972.4527
1109.4346
1195.4776
368.2005
0
20
40
60
80
100
400
600
800
1000
1200

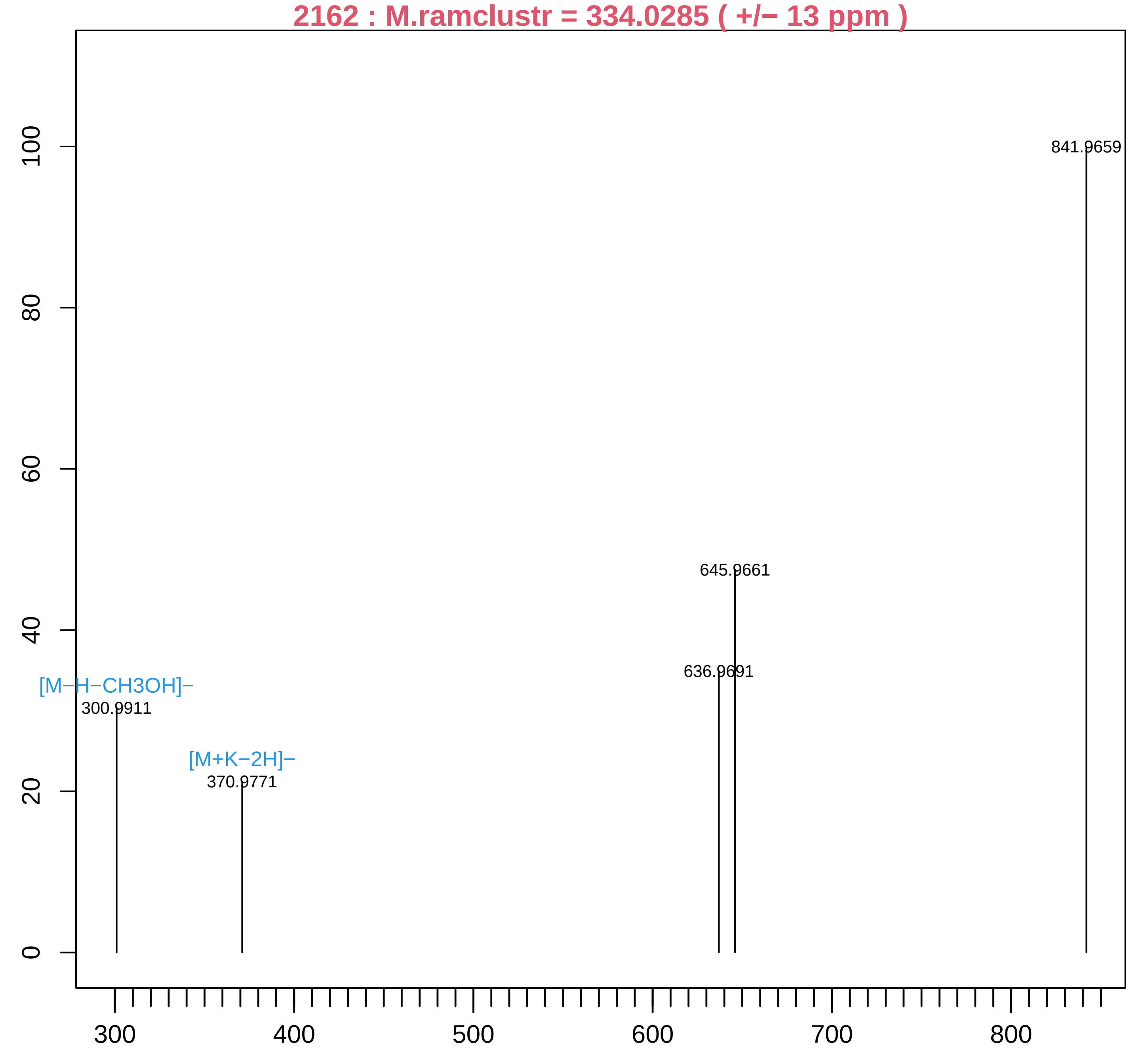
2162 : M.ramclustr = 334.0285 (+/− 13 ppm)
841.9659
645.9661
636.9691
[M−H−CH3OH]−
300.9911
[M+K−2H]−
370.9771
0
20
40
60
80
100
300
400
500
600
700
800

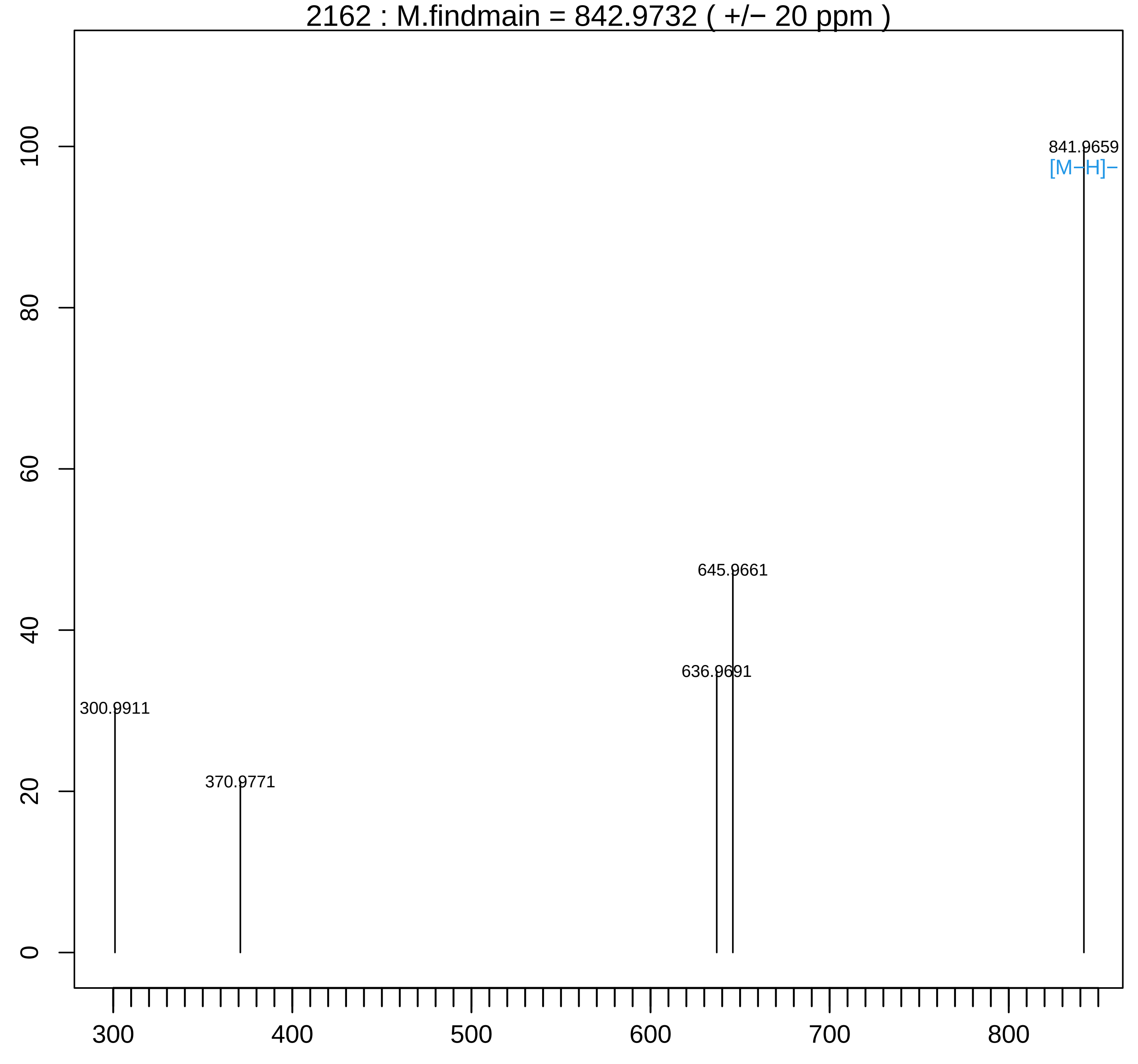
2162 : M.findmain = 842.9732 (+/− 20 ppm)
841.9659
[M−H]−
645.9661
636.9691
300.9911
370.9771
0
20
40
60
80
100
300
400
500
600
700
800

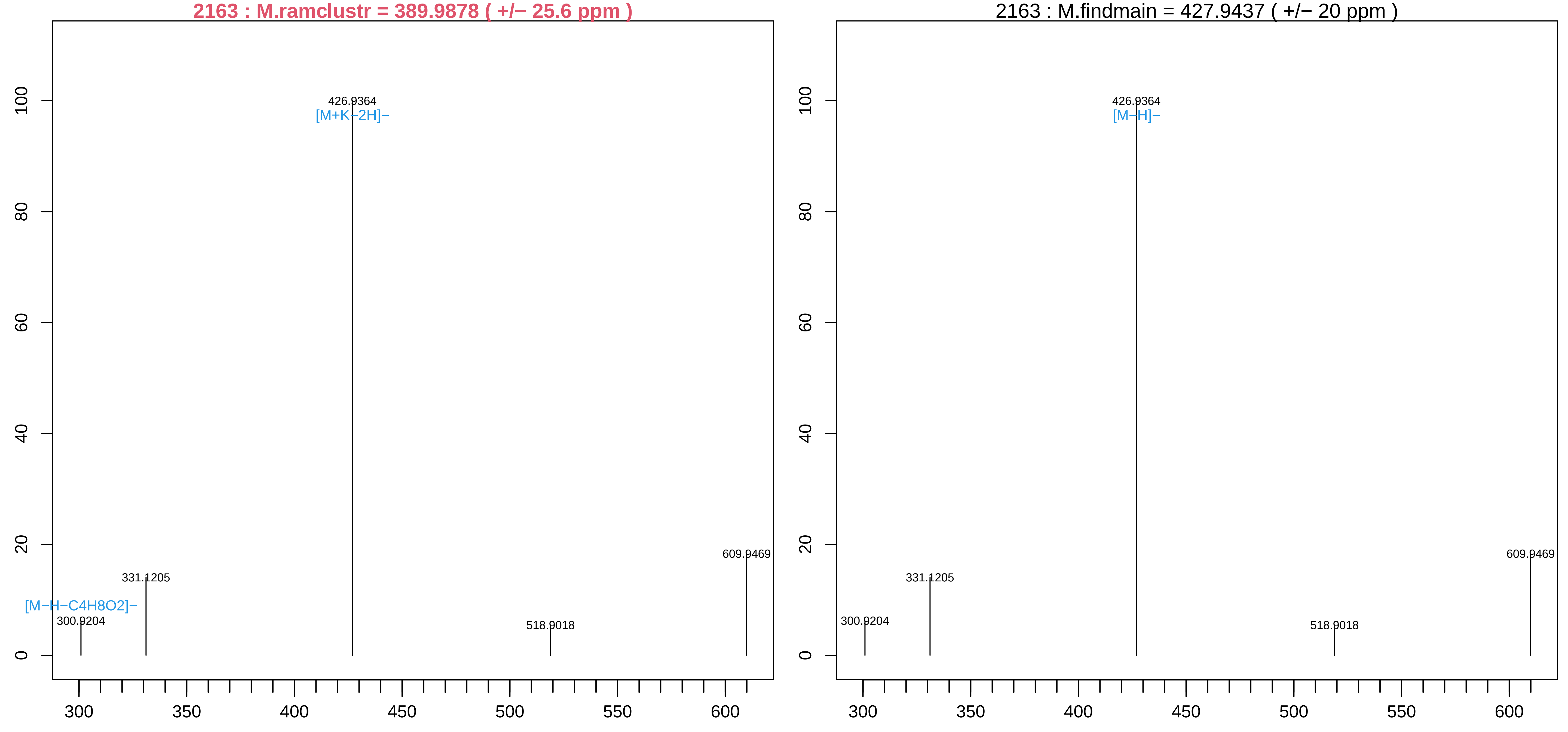
2163 : M.ramclustr = 389.9878 (+/− 25.6 ppm)
426.9364
[M+K−2H]−
331.1205
[M−H−C4H8O2]−
300.9204
518.9018
609.9469
2163 : M.findmain = 427.9437 (+/− 20 ppm)
426.9364
[M−H]−
331.1205
300.9204
518.9018
609.9469

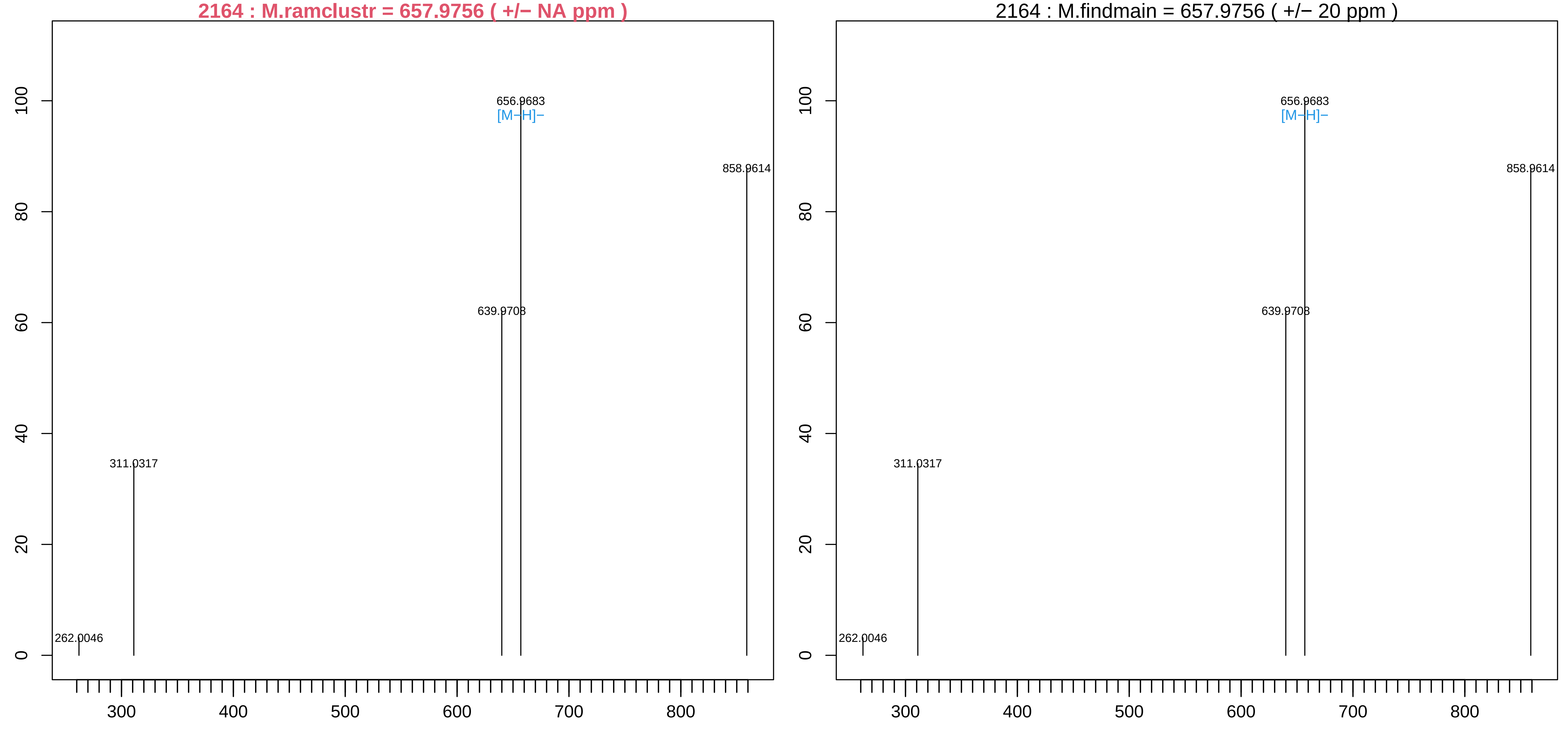
2164 : M.ramclustr = 657.9756 (+/− NA ppm)
656.9683
[M−H]−
858.9614
639.9703
311.0317
262.0046
2164 : M.findmain = 657.9756 (+/− 20 ppm)
656.9683
[M−H]−
858.9614
639.9703
311.0317
262.0046

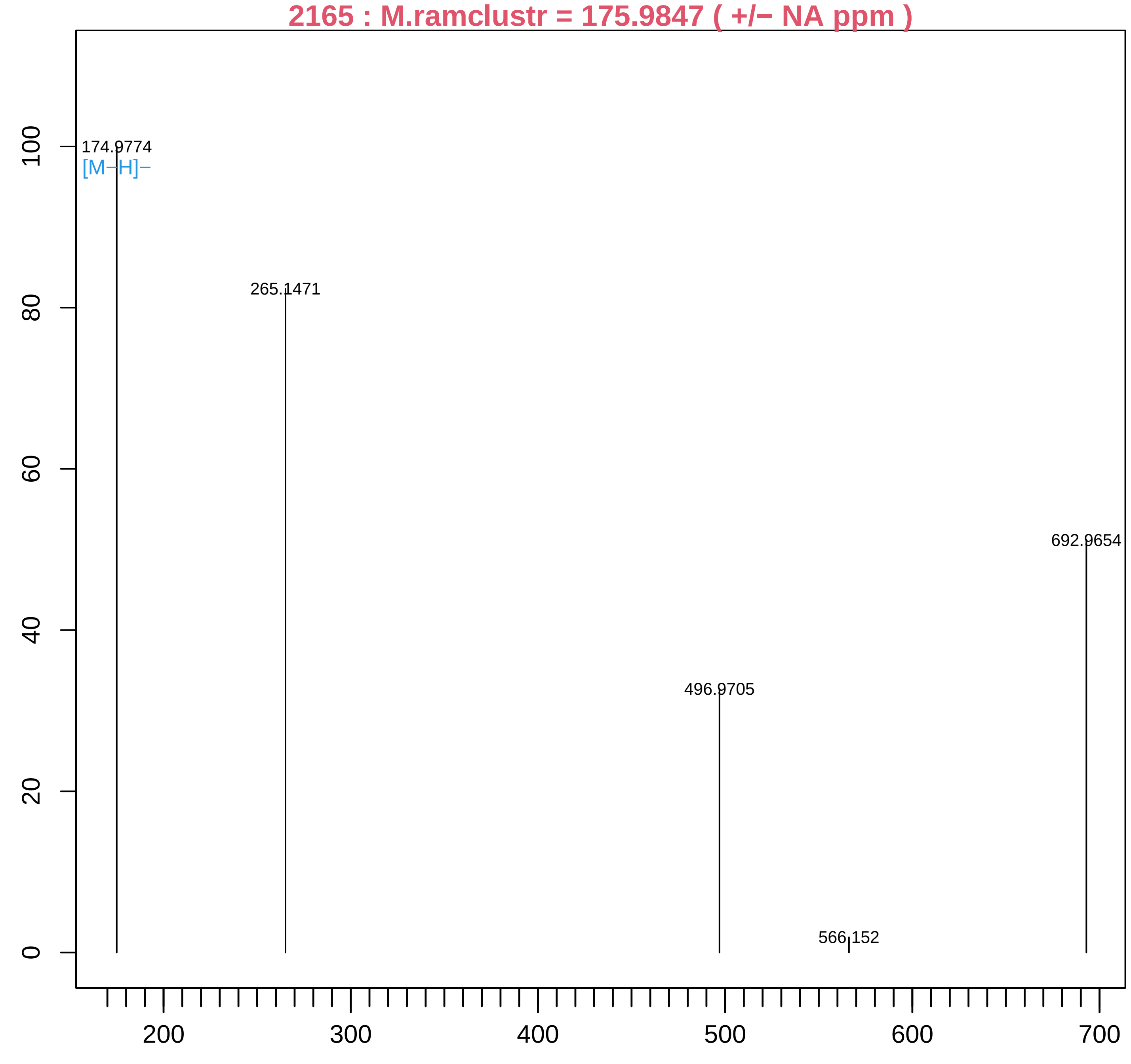
2165 : M.ramclustr = 175.9847 (+/− NA ppm)
174.9774
[M−H]−
265.1471
496.9705
566.152
692.9654
0
20
40
60
80
100
200
300
400
500
600
700

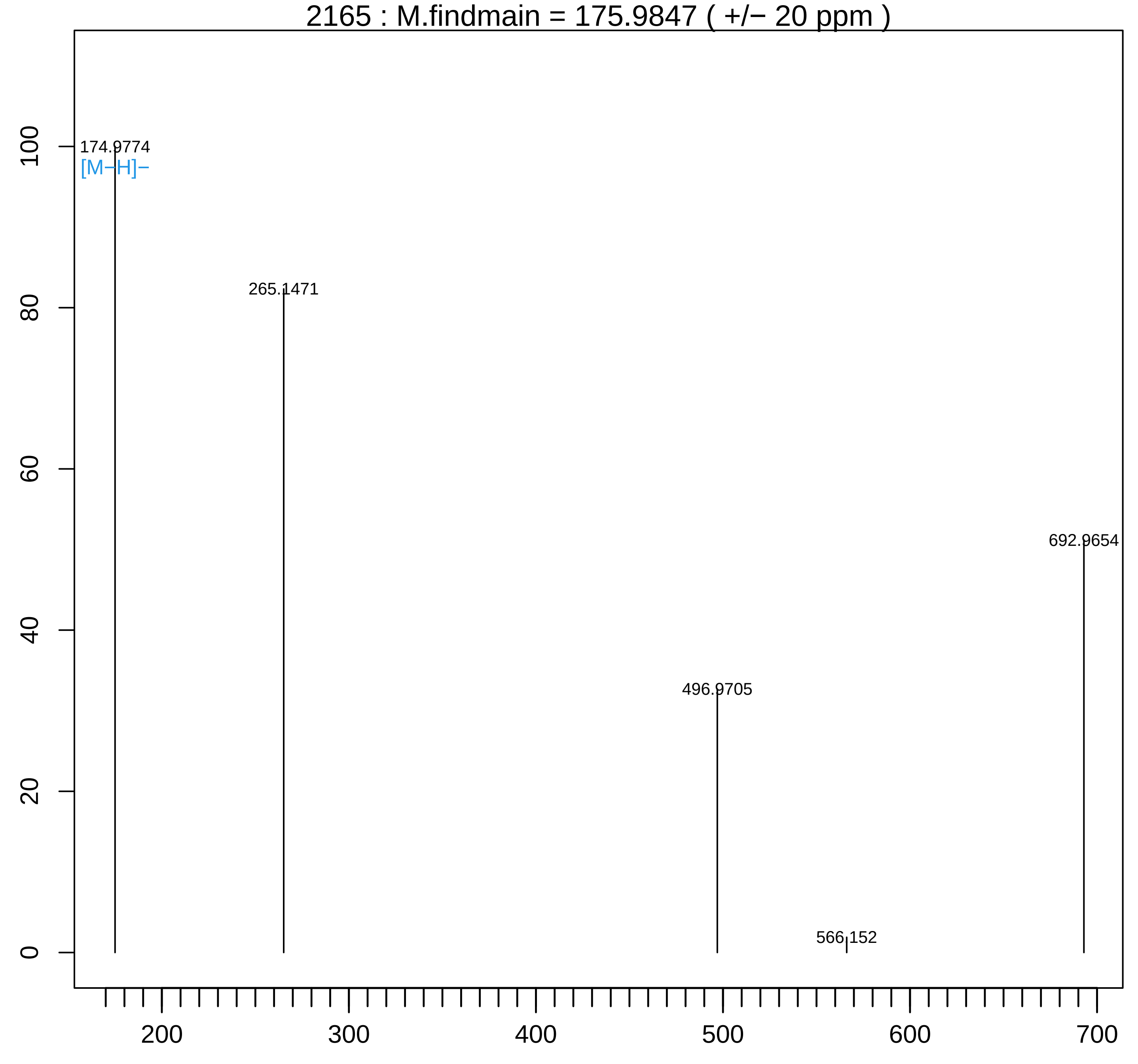
2165 : M.findmain = 175.9847 (+/− 20 ppm)
174.9774
[M−H]−
265.1471
496.9705
566.152
692.9654
0
20
40
60
80
100
200
300
400
500
600
700

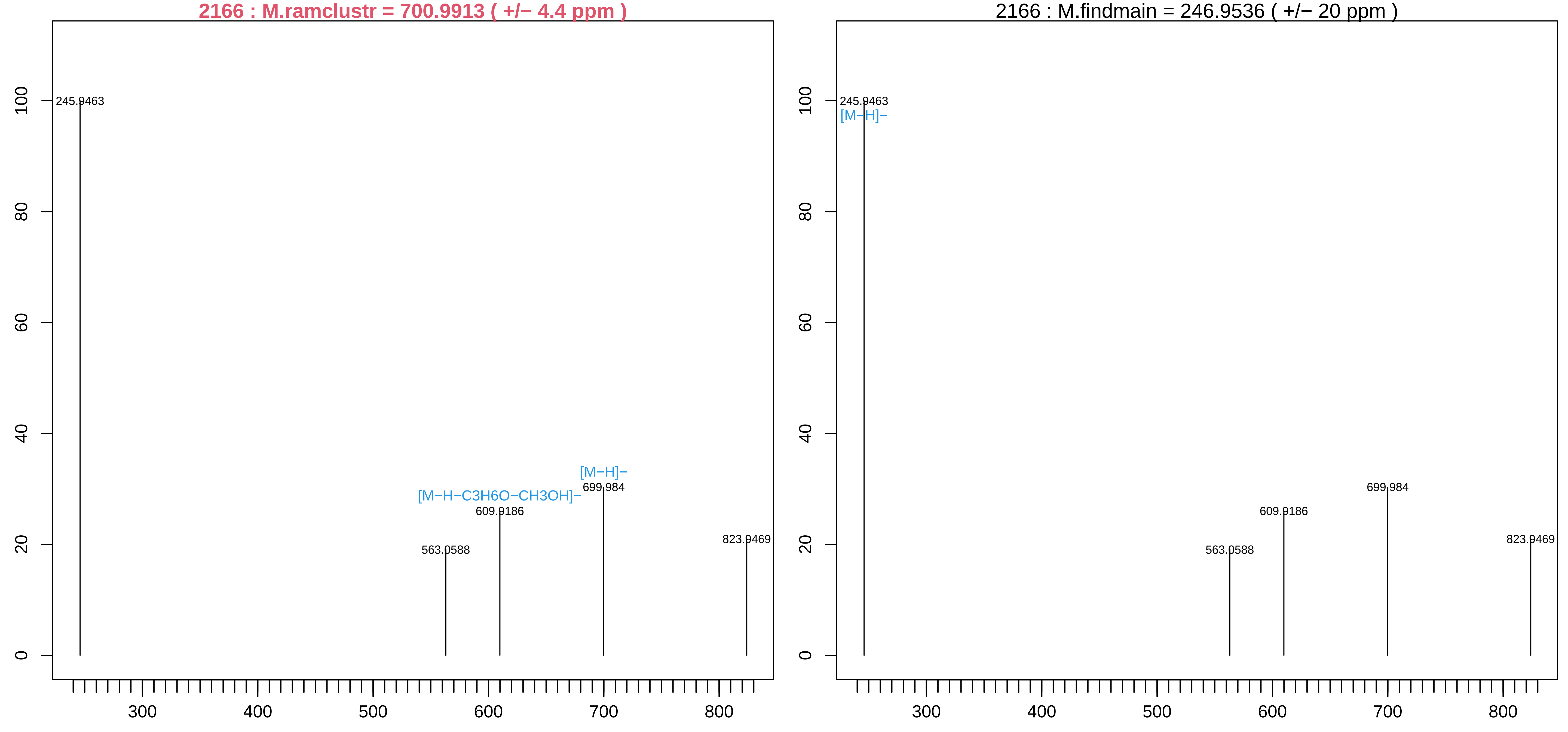

2166 : M.ramclustr = 700.9913 (+/− 4.4 ppm)
245.9463
[M−H−C3H6O−CH3OH]−
609.9186
[M−H]−
699.984
563.0588
823.9469
2166 : M.findmain = 246.9536 (+/− 20 ppm)
245.9463
[M−H]−
563.0588
609.9186
699.984
823.9469
0
20
40
60
80
100
300
400
500
600
700
800

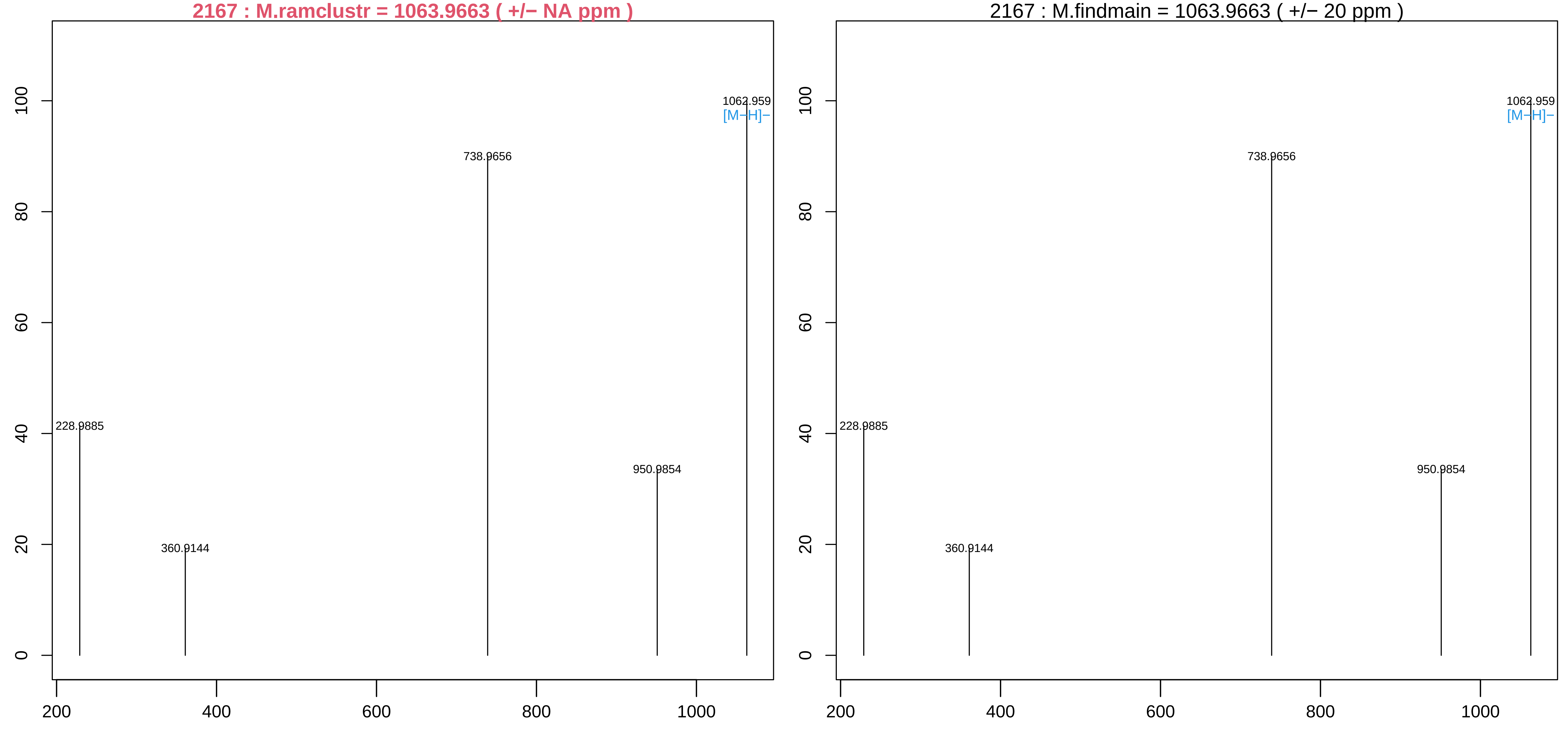
2167 : M.ramclustr = 1063.9663 (+/− NA ppm)
2167 : M.findmain = 1063.9663 (+/− 20 ppm)
1062.959
[M−H]−
738.9656
228.9885
950.9854
360.9144
0
20
40
60
80
100
200
400
600
800
1000

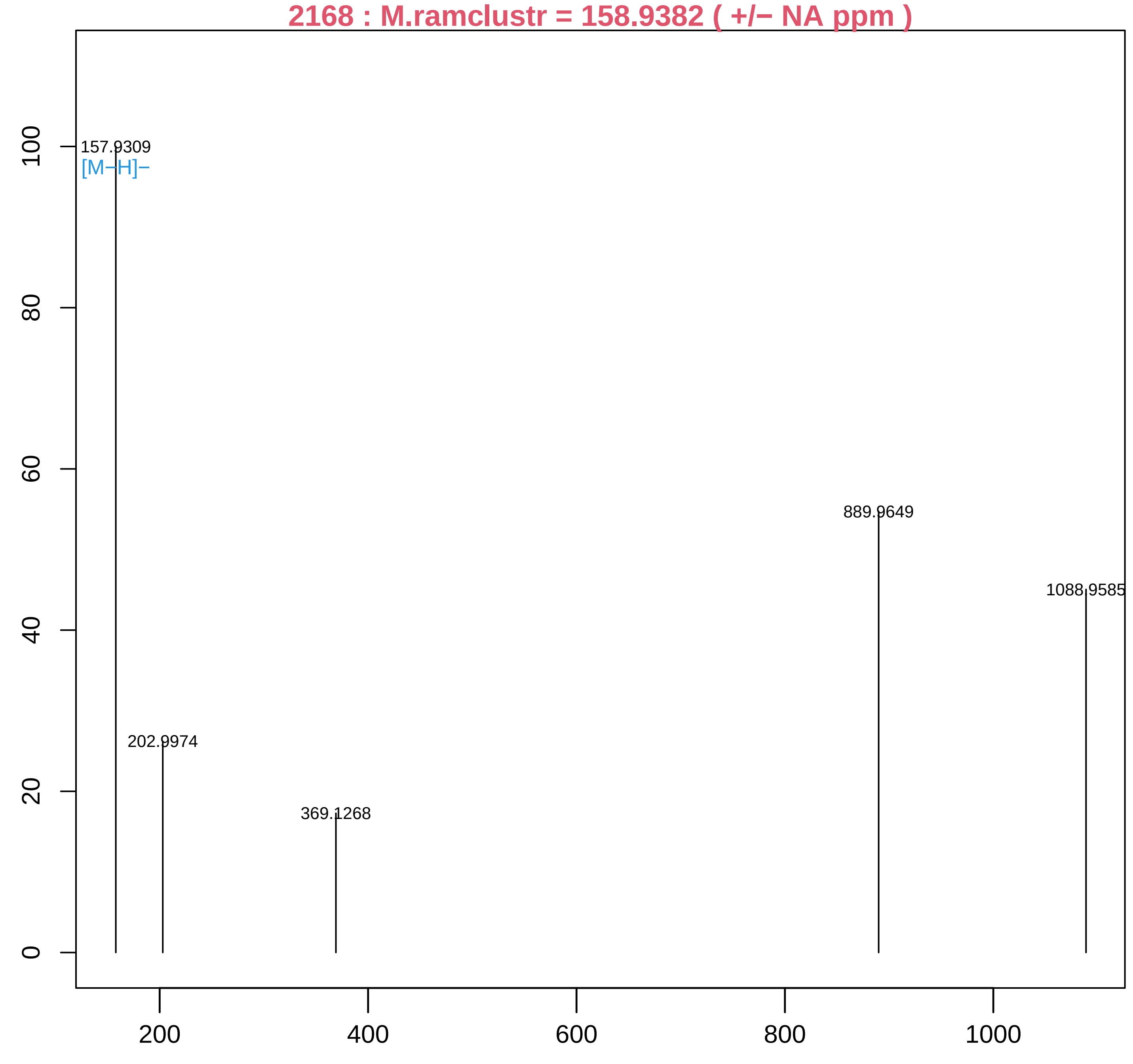

2168 : M.ramclustr = 158.9382 (+/− NA ppm)
157.9309
[M−H]−
202.9974
369.1268
889.9649
1088.9585
0
20
40
60
80
100
200
400
600
800
1000

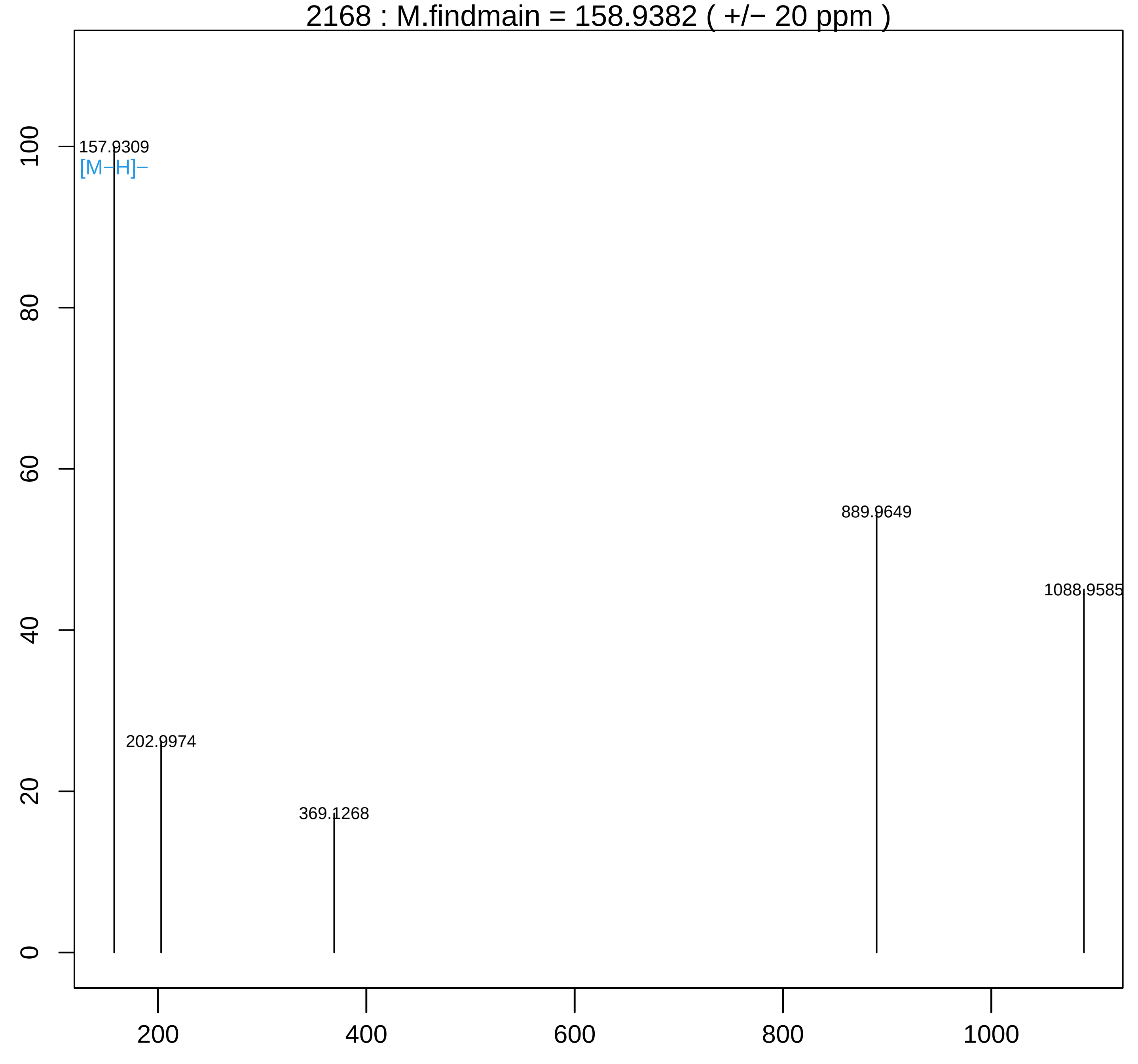

2168 : M.findmain = 158.9382 (+/− 20 ppm)
157.9309
[M−H]−
202.9974
369.1268
889.9649
1088.9585
0
20
40
60
80
100
200
400
600
800
1000

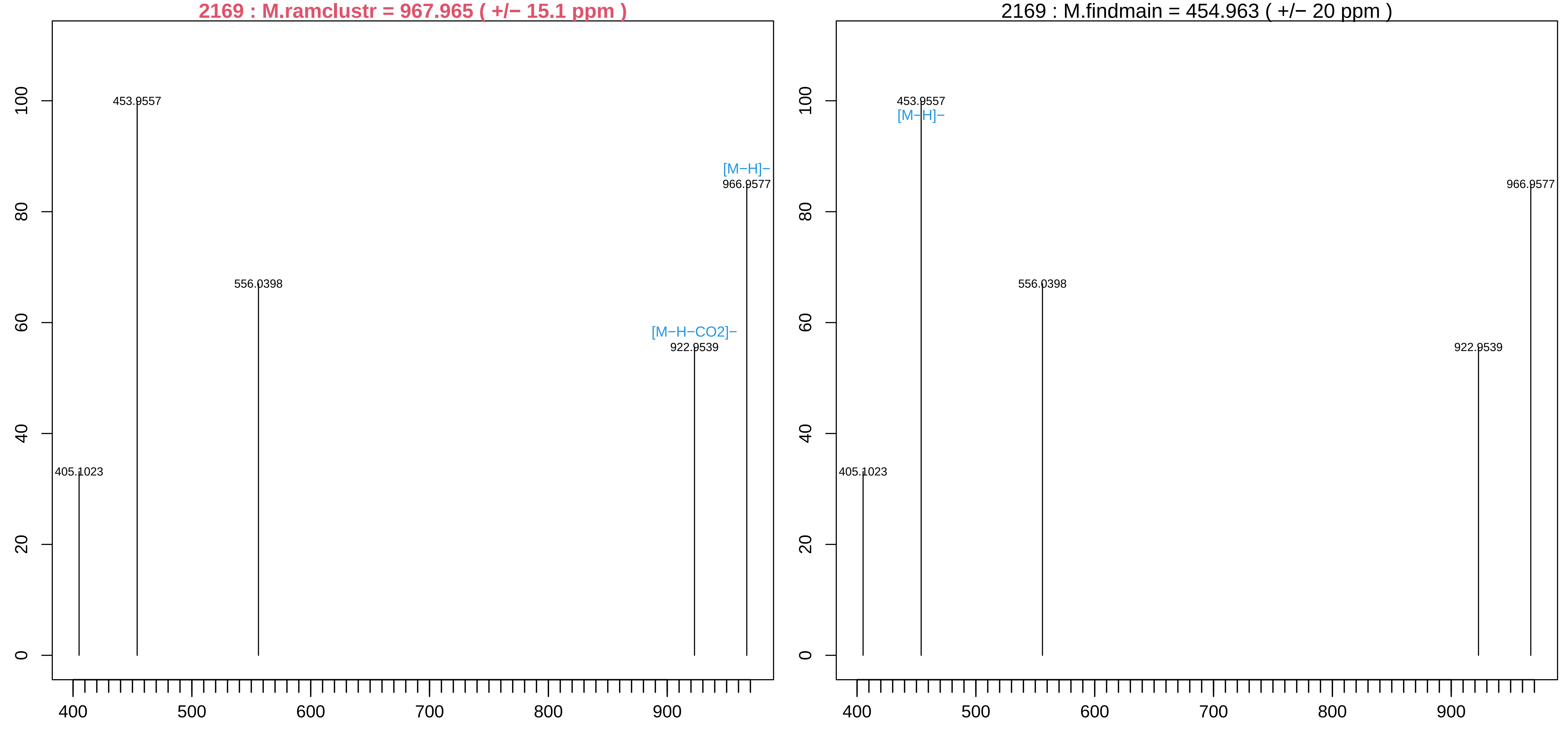
2169 : M.ramclustr = 967.965 (+/− 15.1 ppm)
453.9557
556.0398
405.1023
[M−H]−
966.9577
[M−H−CO2]−
922.9539
2169 : M.findmain = 454.963 (+/− 20 ppm)
453.9557
[M−H]−
556.0398
405.1023
966.9577
922.9539
0
20
40
60
80
100
400
500
600
700
800
900

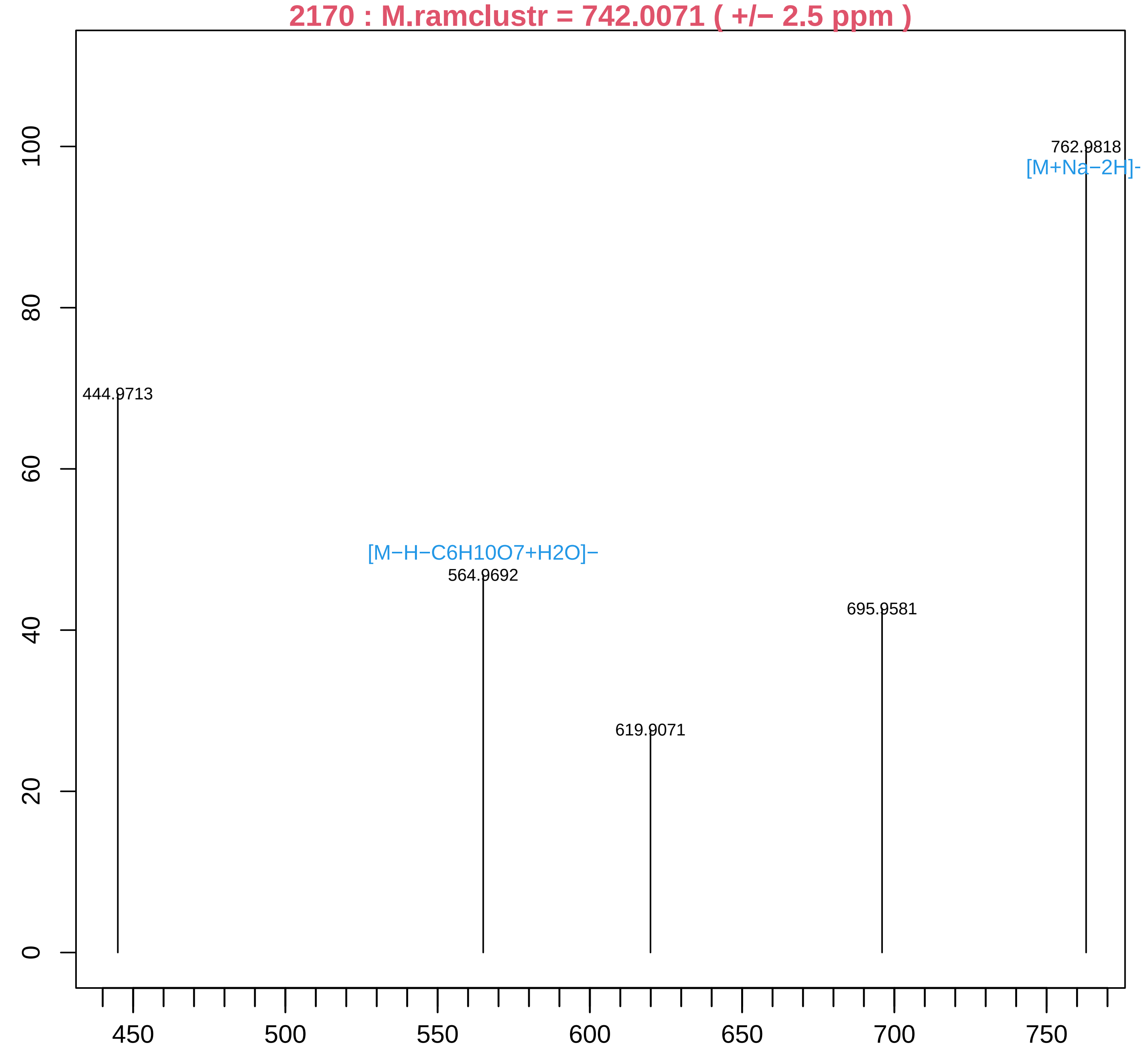
2170 : M.ramclustr = 742.0071 (+/− 2.5 ppm)
762.9818
[M+Na−2H]-
444.9713
[M−H−C6H10O7+H2O]−
564.9692
619.9071
695.9581
0
20
40
60
80
100
450
500
550
600
650
700
750

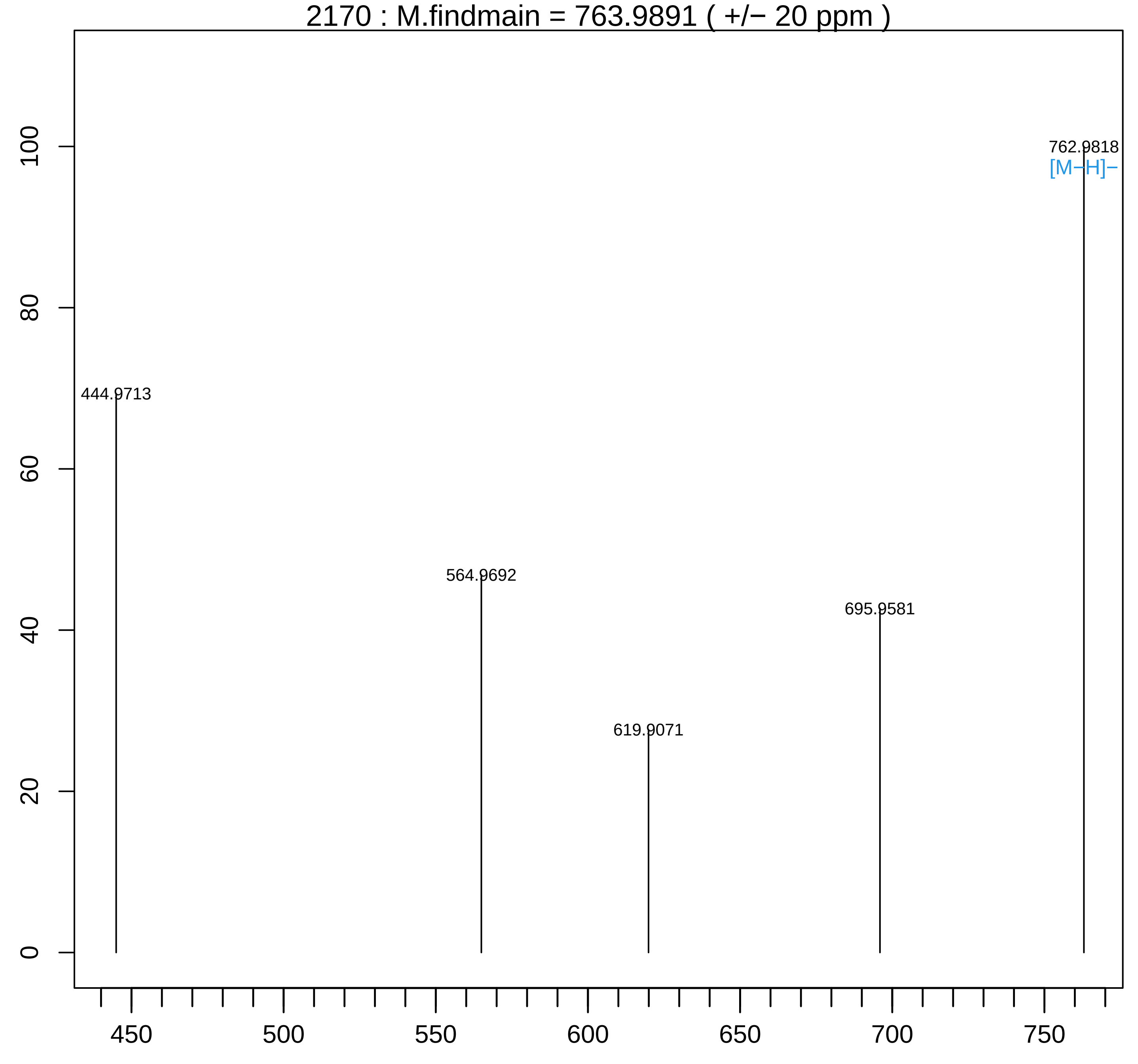
2170 : M.findmain = 763.9891 (+/− 20 ppm)
762.9818
[M−H]−
444.9713
564.9692
619.9071
695.9581
0
20
40
60
80
100
450
500
550
600
650
700
750

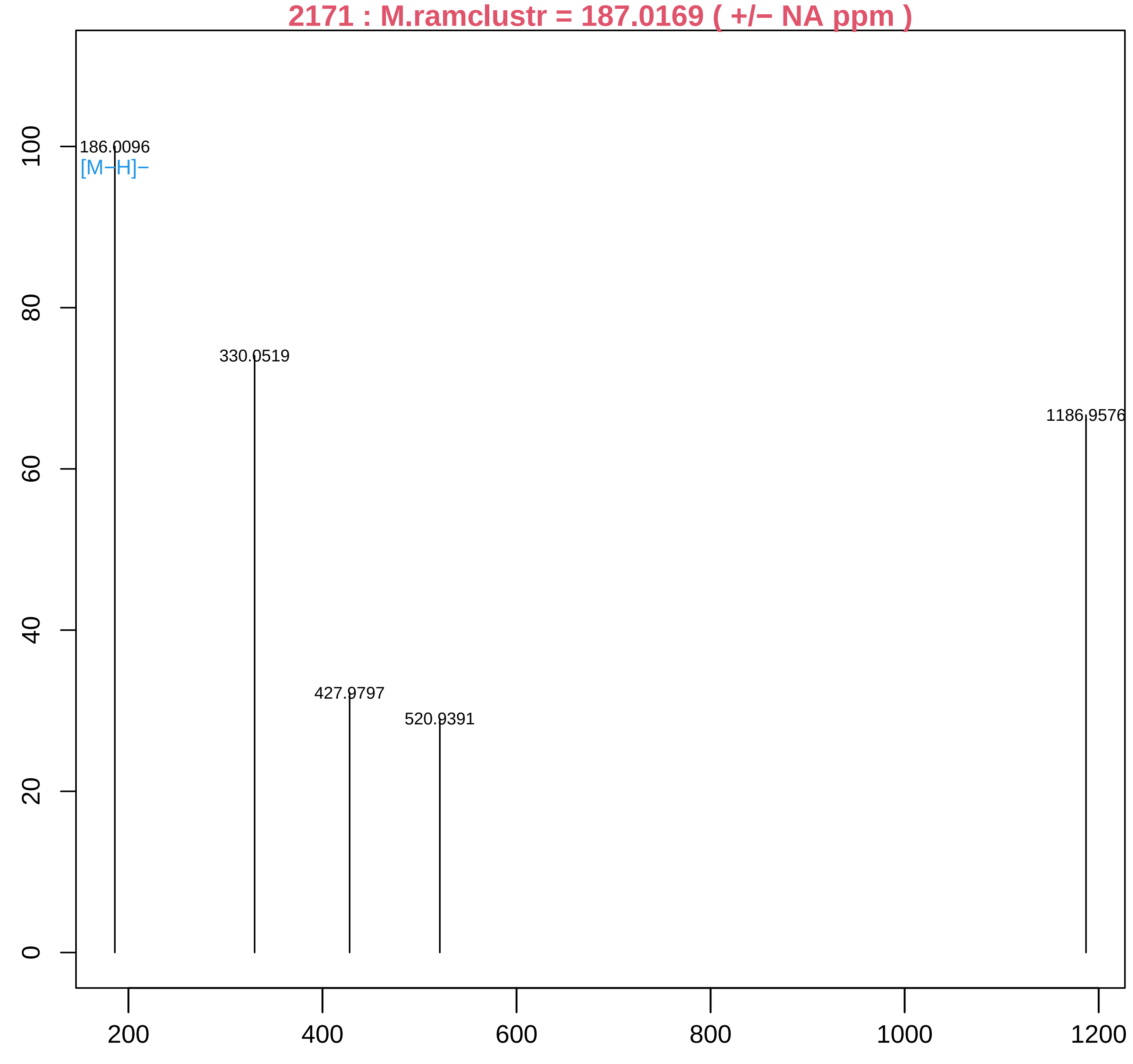
2171 : M.ramclustr = 187.0169 (+/− NA ppm)
186.0096
[M−H]−
330.0519
427.9797
520.9391
1186.9576
0
20
40
60
80
100
200
400
600
800
1000
1200

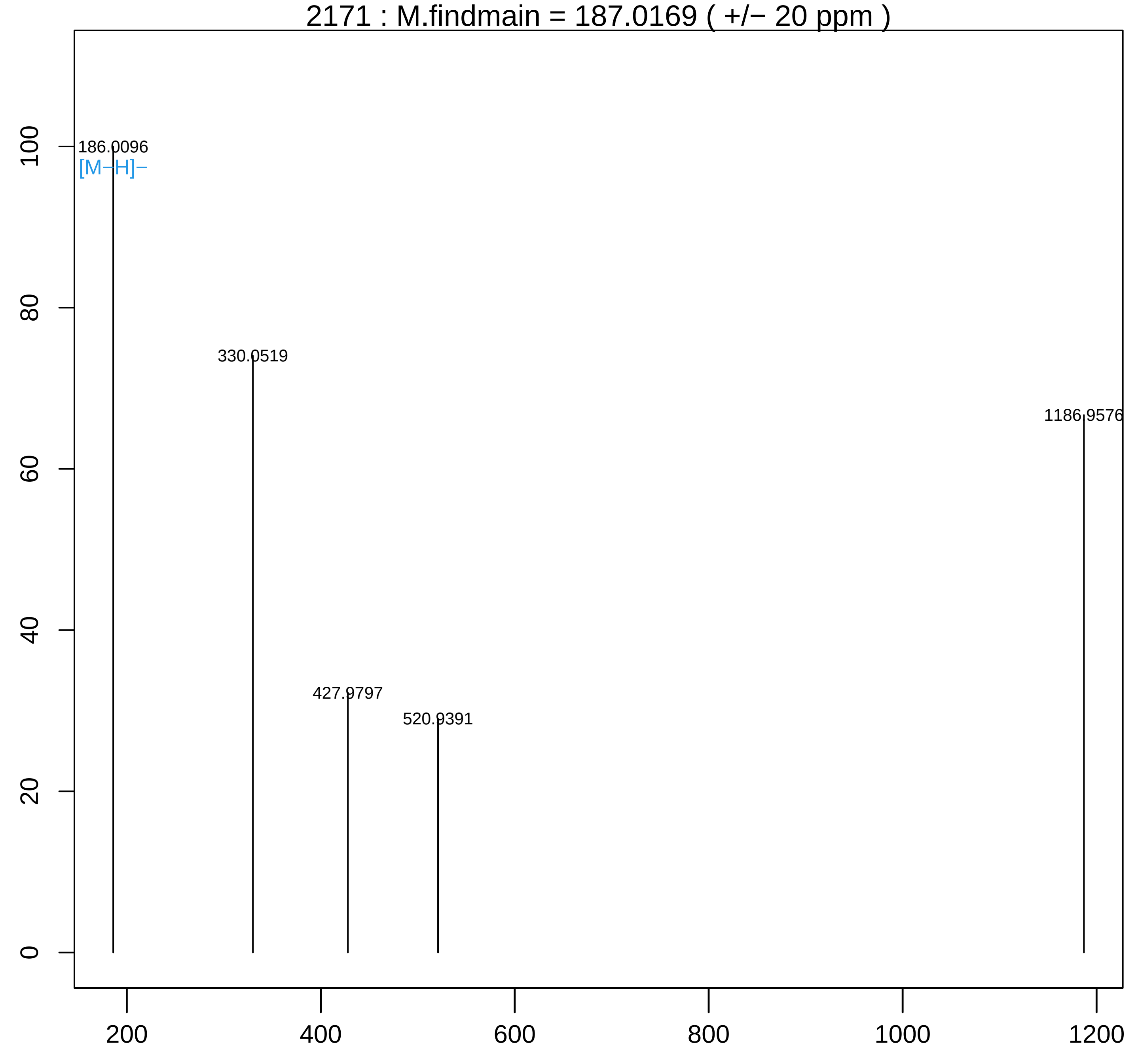
2171 : M.findmain = 187.0169 (+/− 20 ppm)
186.0096
[M−H]−
330.0519
427.9797
520.9391
1186.9576
0
20
40
60
80
100
200
400
600
800
1000
1200

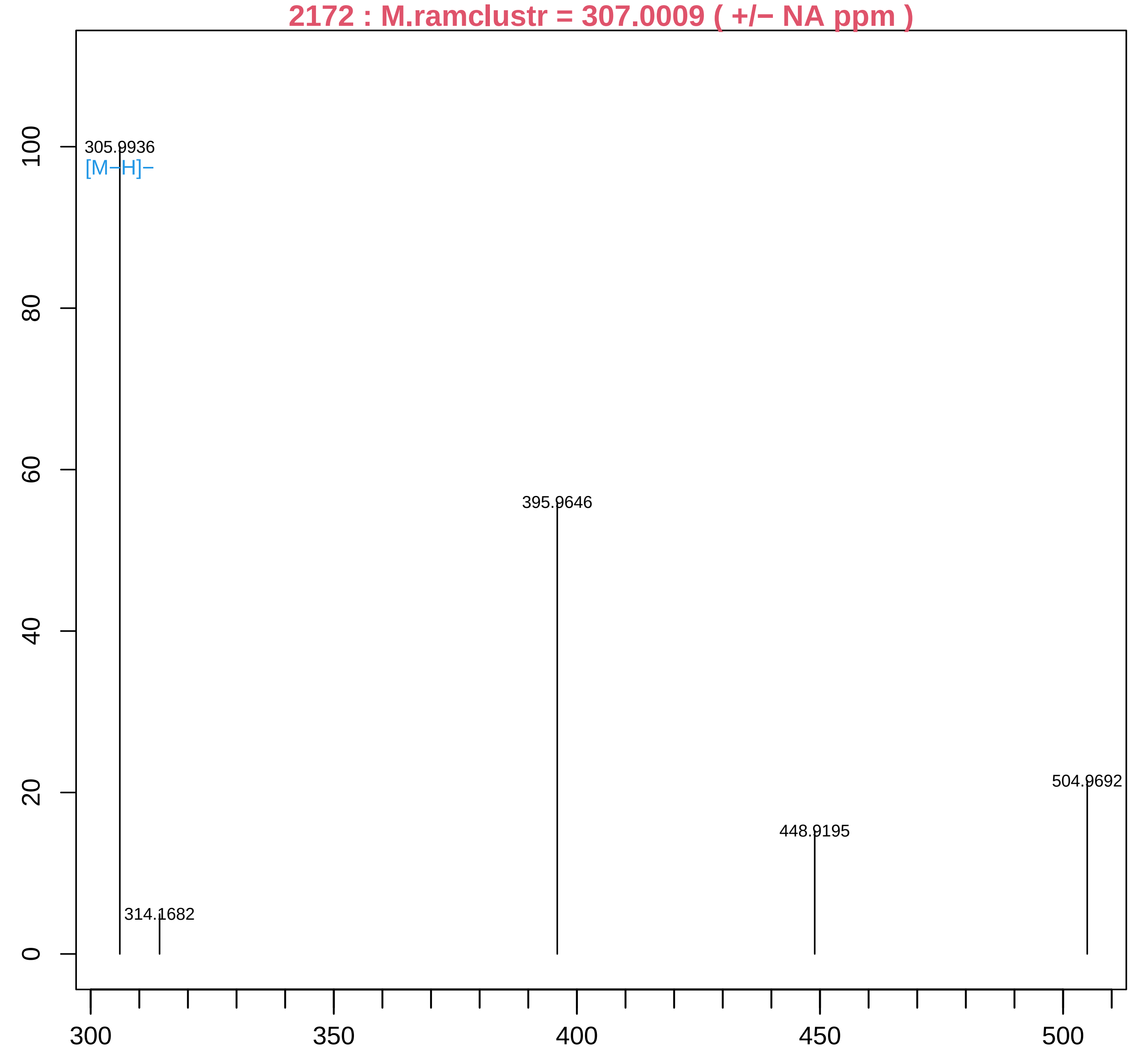
2172 : M.ramclustr = 307.0009 (+/− NA ppm)
305.9936
[M−H]−
395.9646
504.9692
448.9195
314.1682
0
20
40
60
80
100
300
350
400
450
500

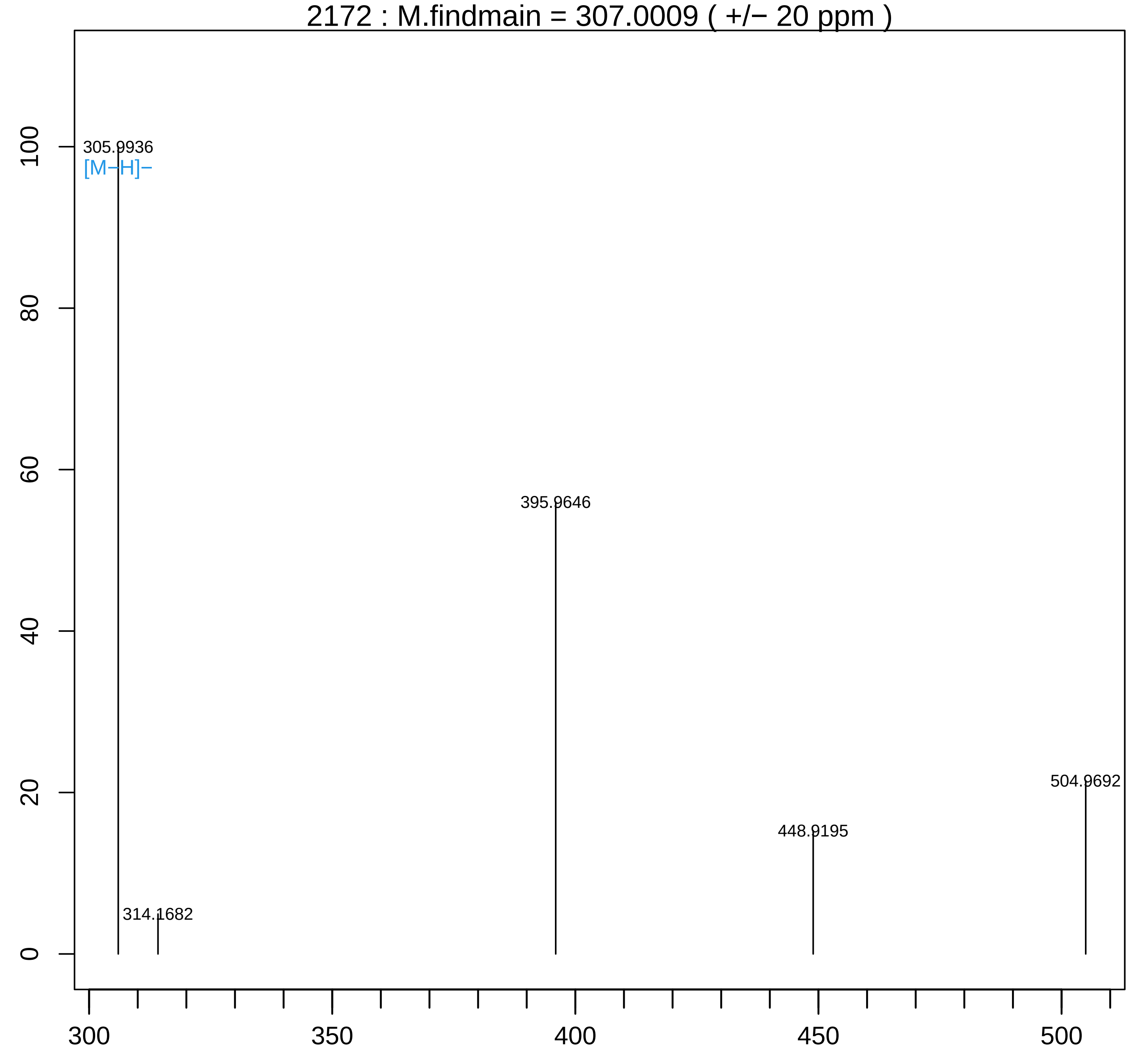
2172 : M.findmain = 307.0009 (+/− 20 ppm)
305.9936
[M−H]−
395.9646
504.9692
448.9195
314.1682
0
20
40
60
80
100
300
350
400
450
500

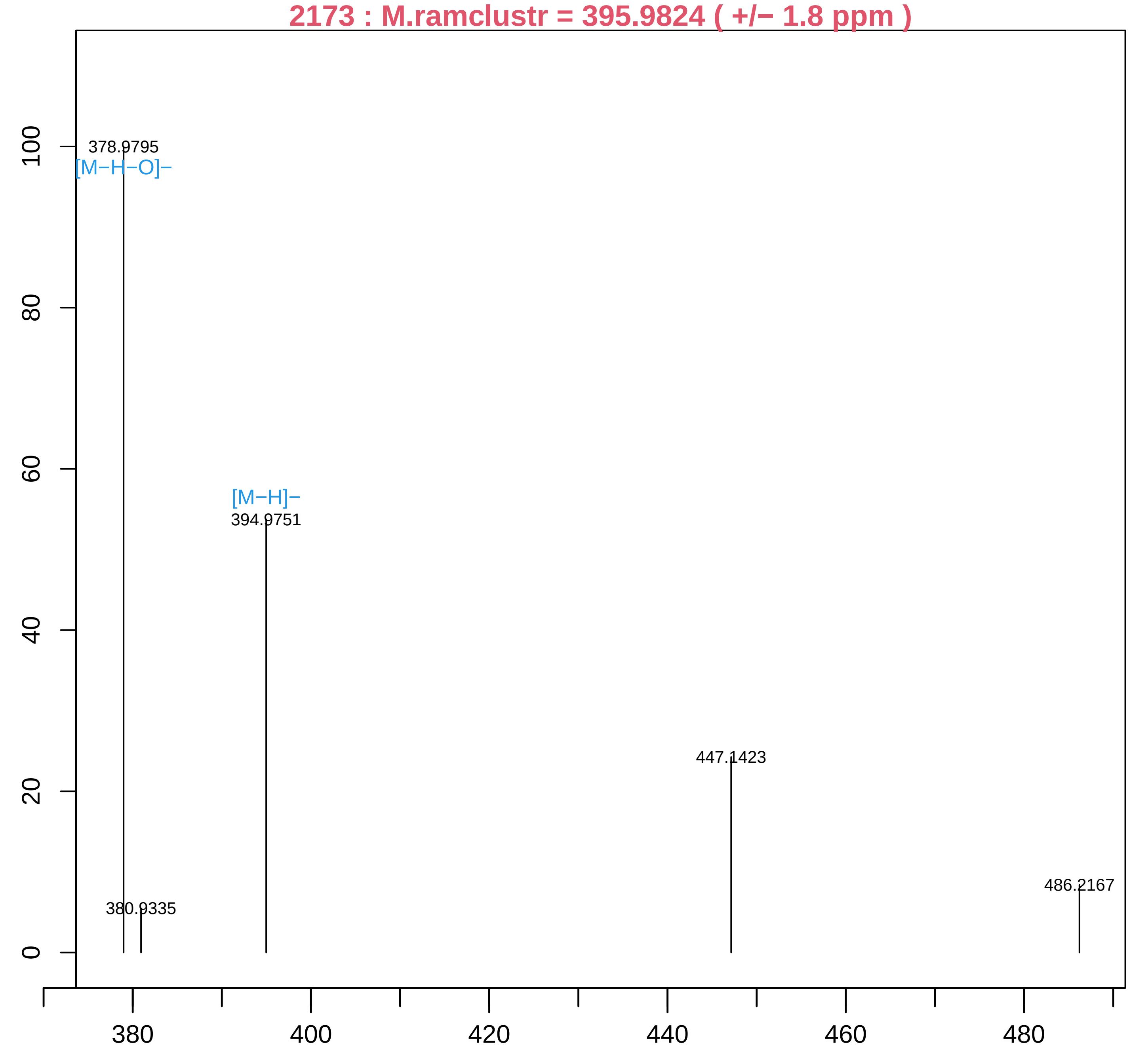
2173 : M.ramclustr = 395.9824 (+/− 1.8 ppm)
378.9795
[M−H−O]−
[M−H]−
394.9751
447.1423
486.2167
380.9335
0
20
40
60
80
100
380
400
420
440
460
480

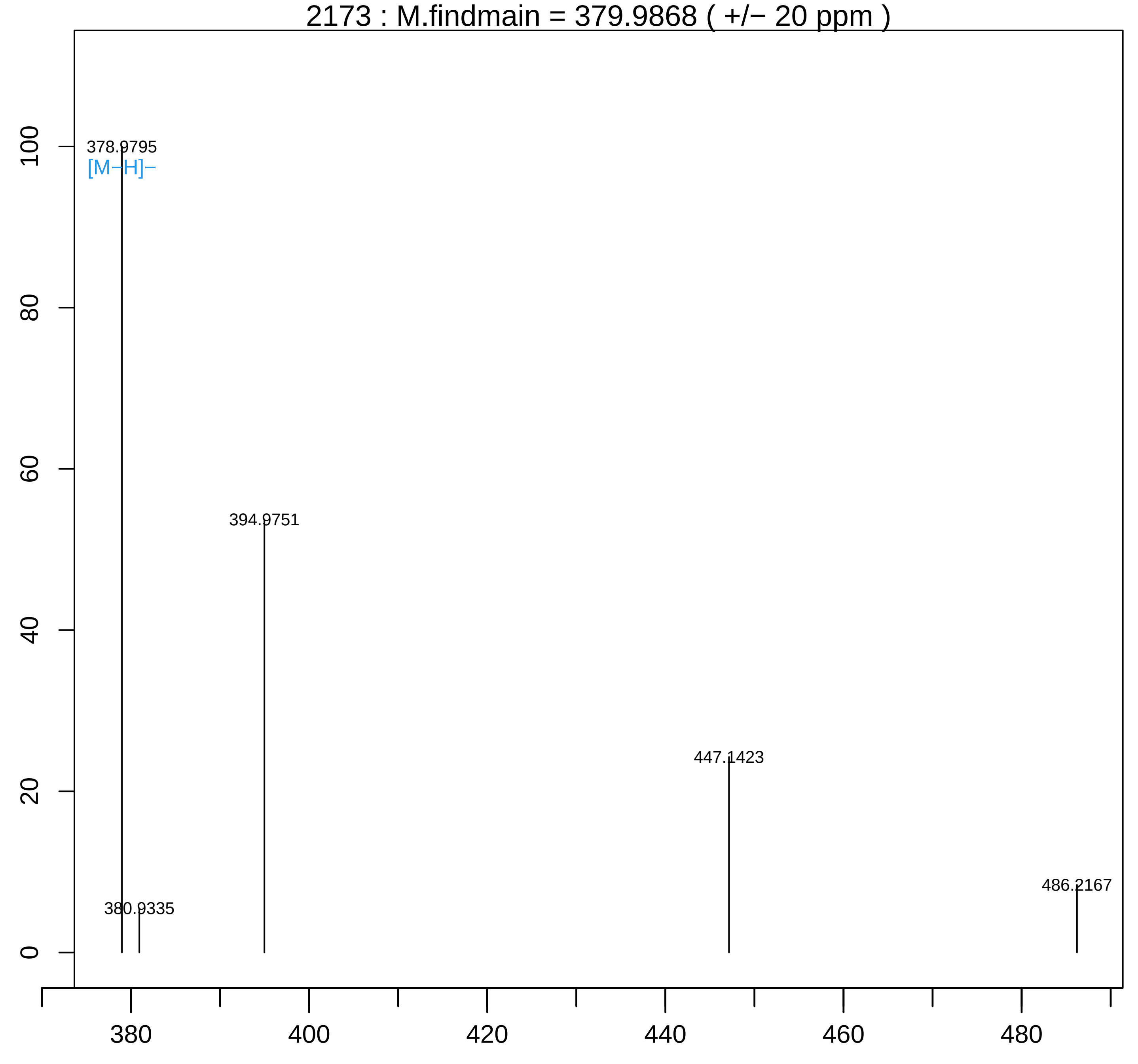
2173 : M.findmain = 379.9868 (+/− 20 ppm)
378.9795
[M−H]−
394.9751
447.1423
486.2167
380.9335
0
20
40
60
80
100
380
400
420
440
460
480

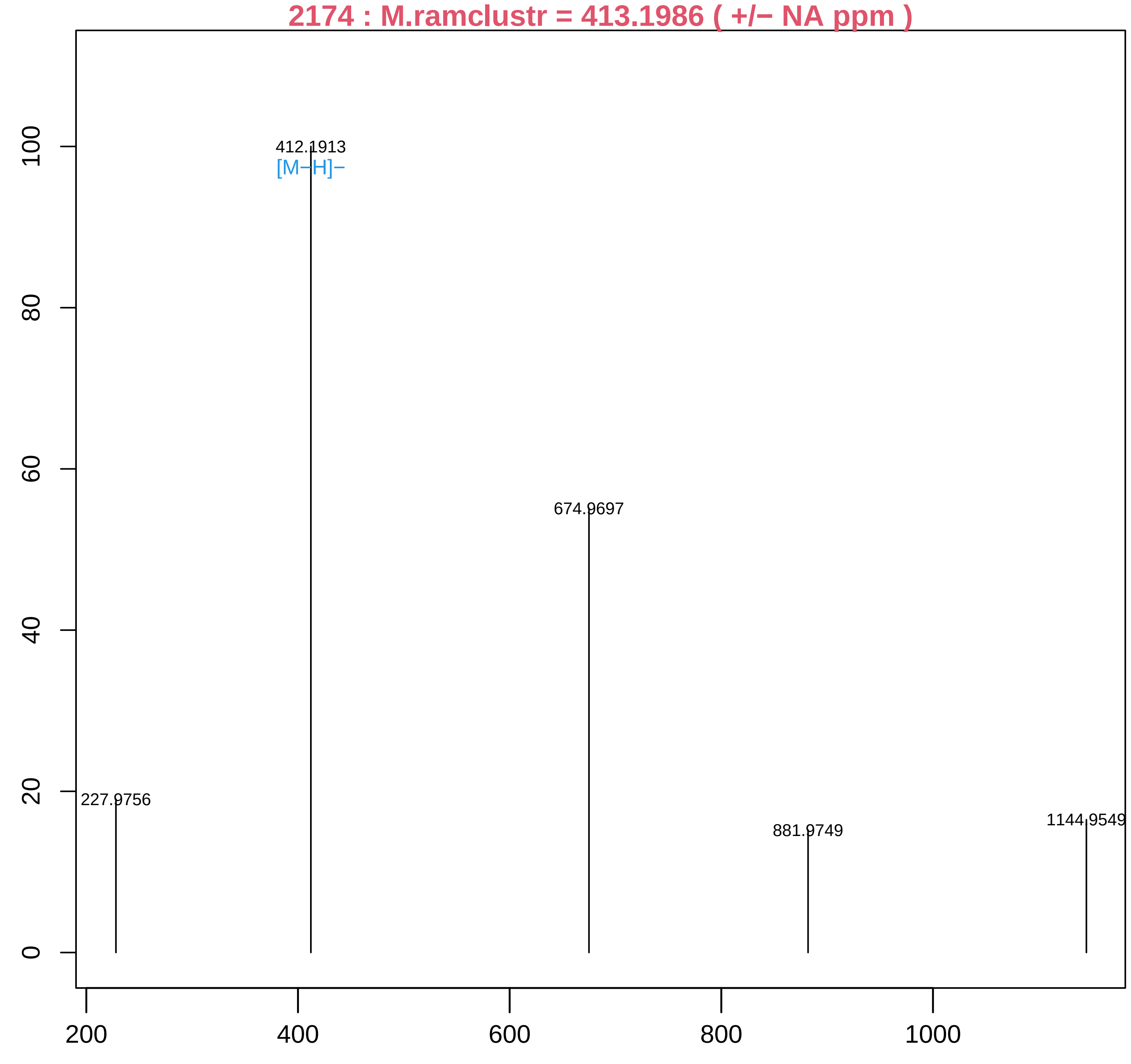
2174 : M.ramclustr = 413.1986 (+/− NA ppm)
412.1913
[M−H]−
674.9697
227.9756
881.9749
1144.9549
0
20
40
60
80
100
200
400
600
800
1000

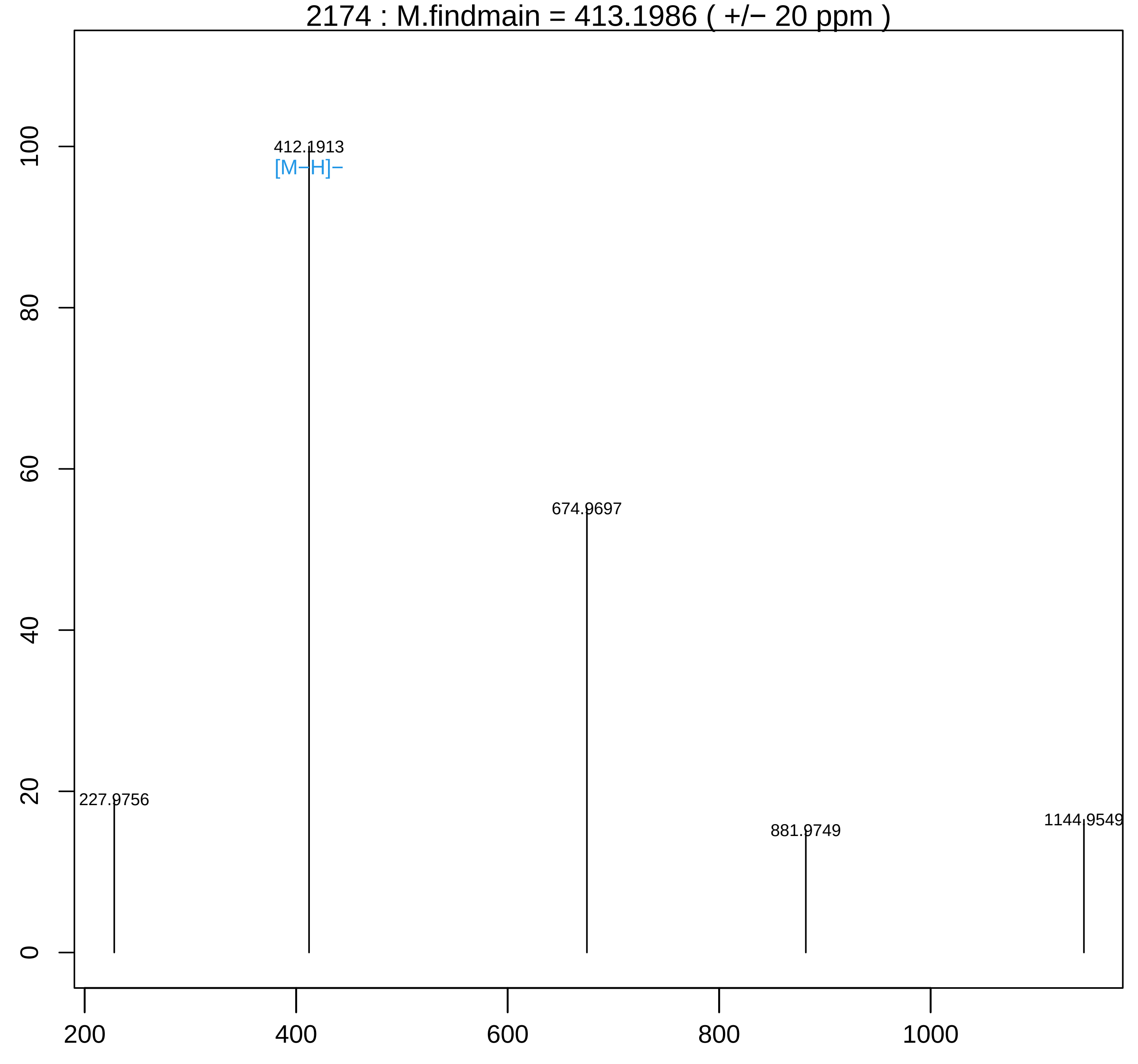
2174 : M.findmain = 413.1986 (+/− 20 ppm)
412.1913
[M−H]−
674.9697
227.9756
881.9749
1144.9549
0
20
40
60
80
100
200
400
600
800
1000

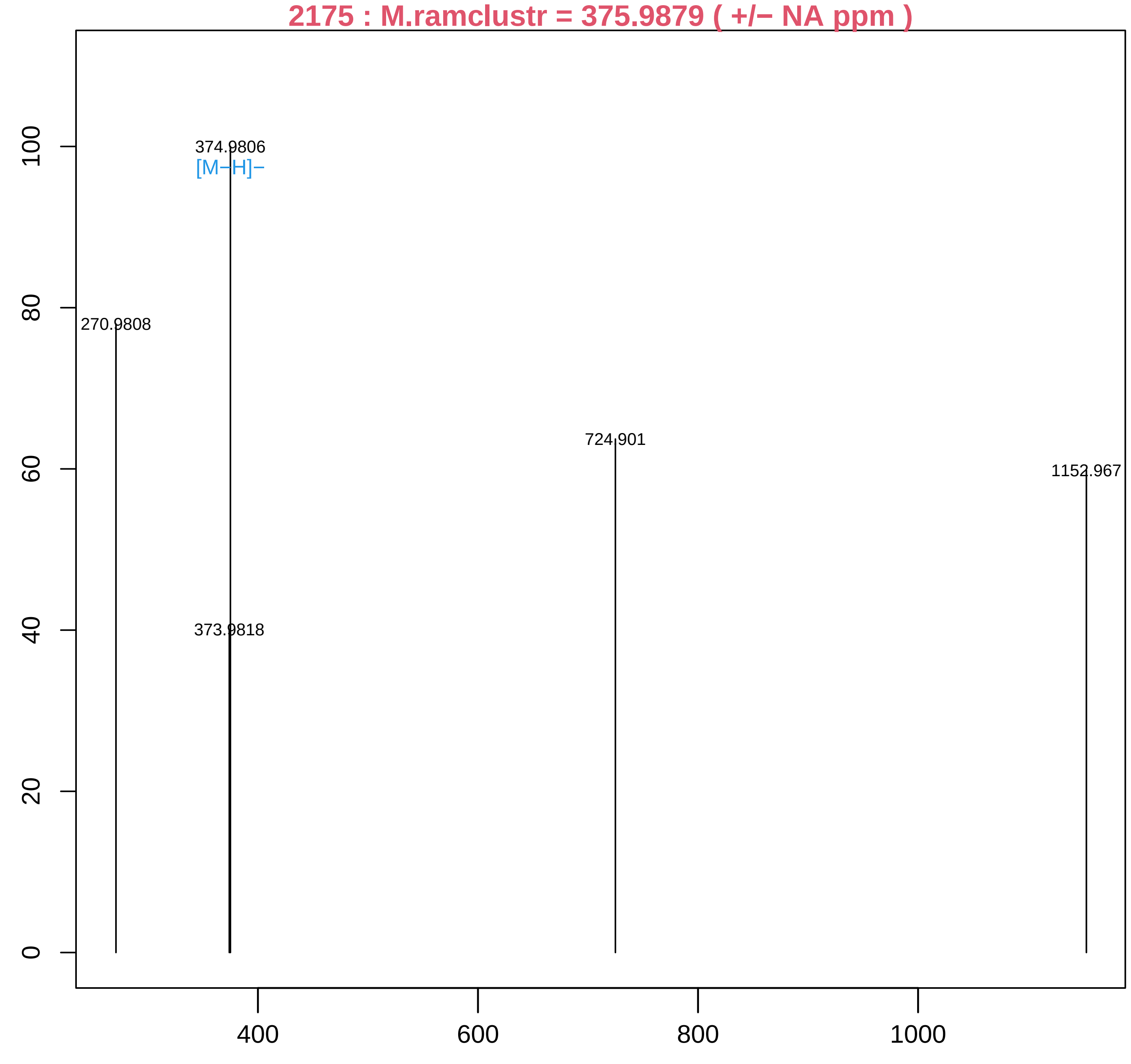
2175 : M.ramclustr = 375.9879 (+/− NA ppm)
374.9806
[M−H]−
270.9808
724.901
1152.967
373.9818
0
20
40
60
80
100
400
600
800
1000

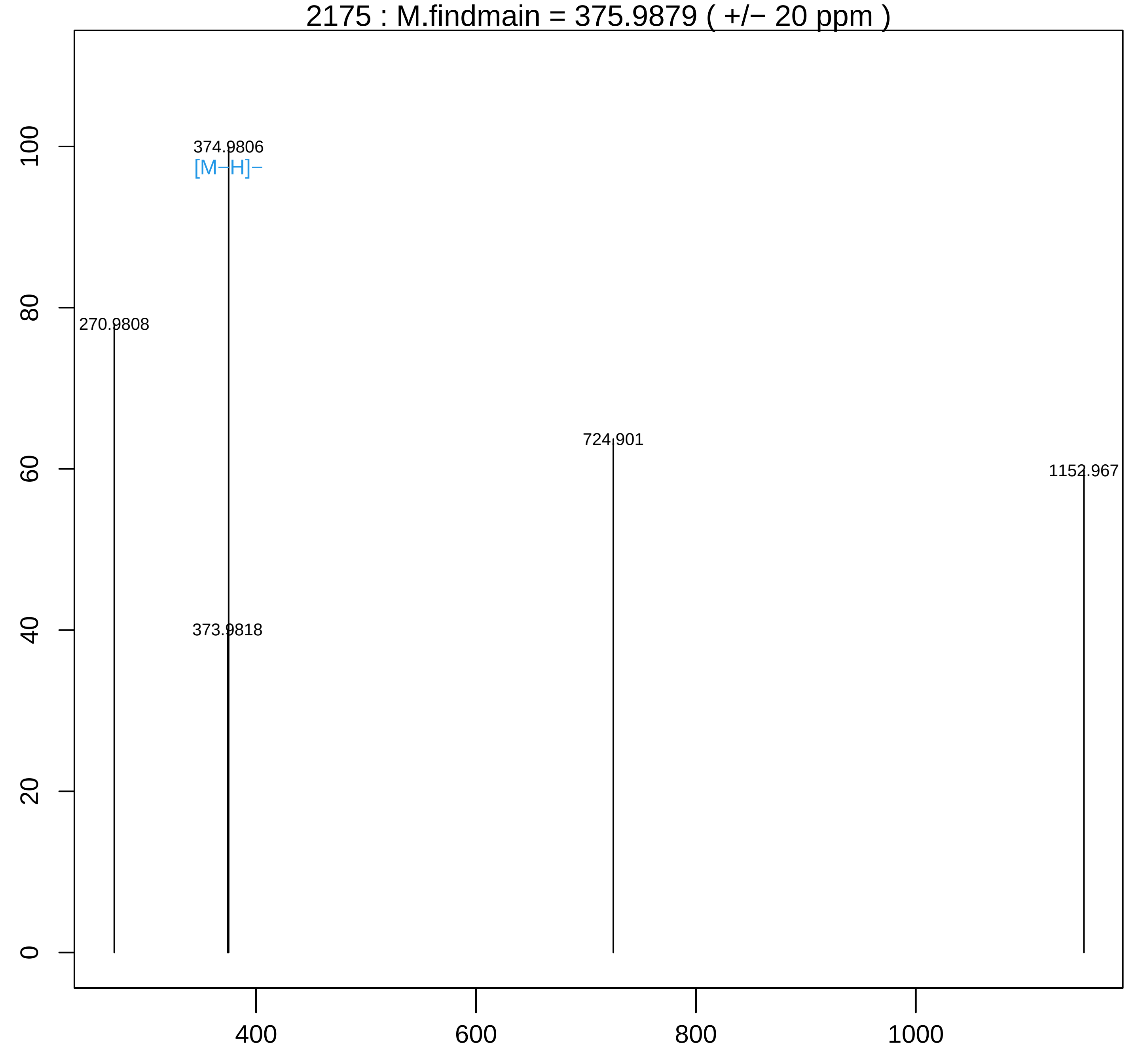
2175 : M.findmain = 375.9879 (+/− 20 ppm)
374.9806
[M−H]−
270.9808
724.901
1152.967
373.9818
0
20
40
60
80
100
400
600
800
1000

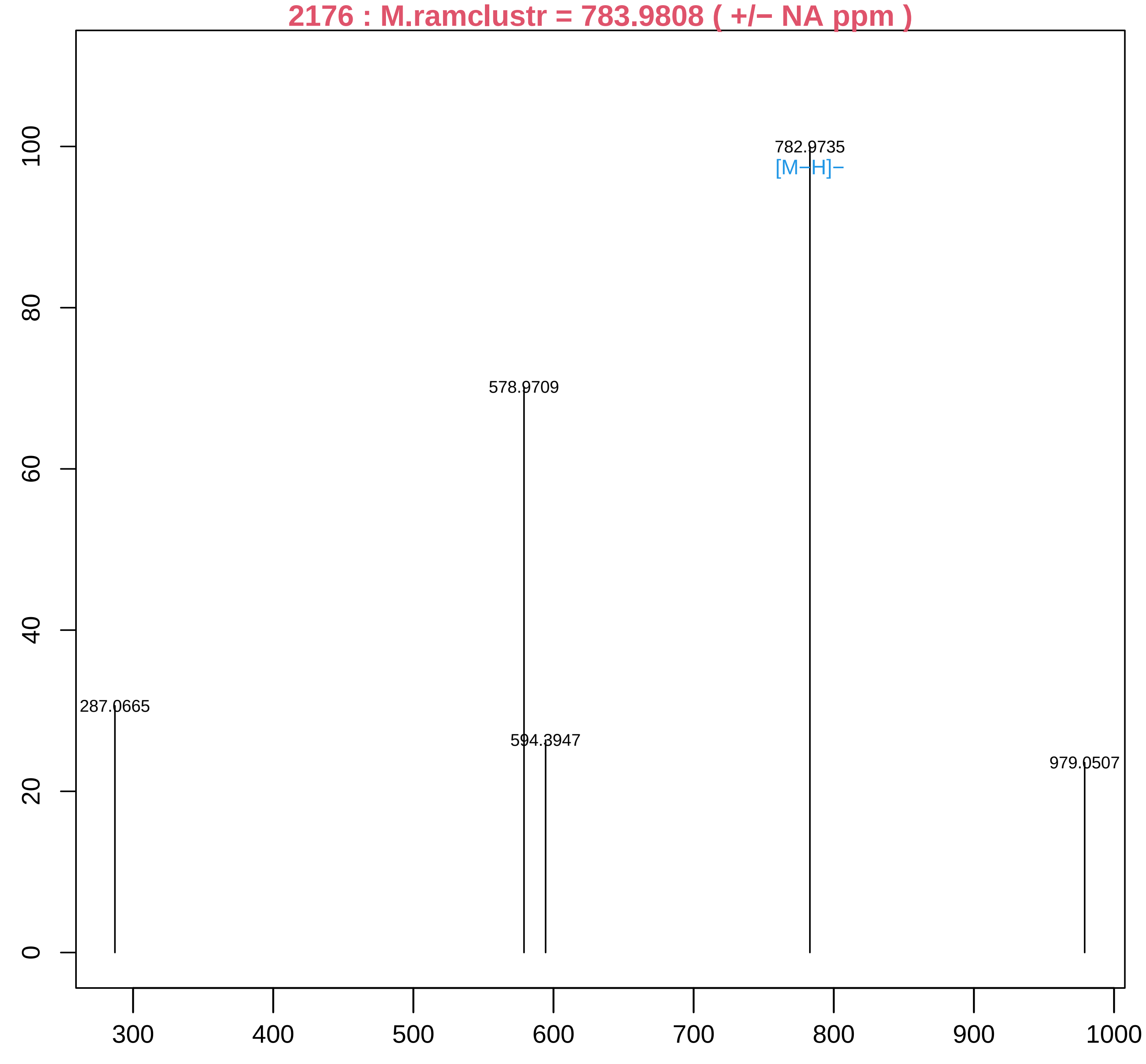
2176 : M.ramclustr = 783.9808 (+/− NA ppm)
782.9735
[M−H]−
578.9709
287.0665
594.3947
979.0507
0
20
40
60
80
100
300
400
500
600
700
800
900
1000

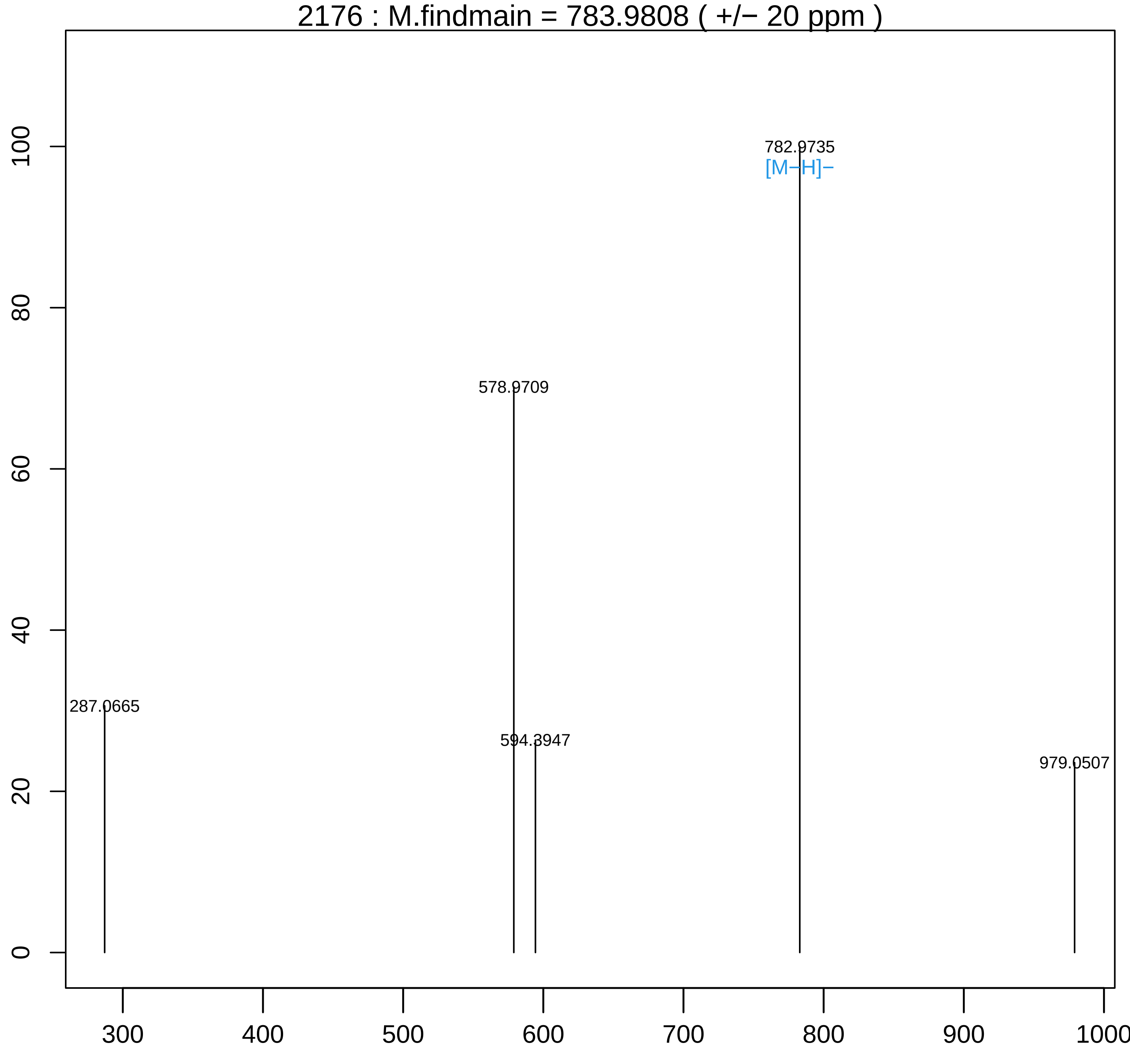
2176 : M.findmain = 783.9808 (+/− 20 ppm)
782.9735
[M−H]−
578.9709
287.0665
594.3947
979.0507
0
20
40
60
80
100
300
400
500
600
700
800
900
1000

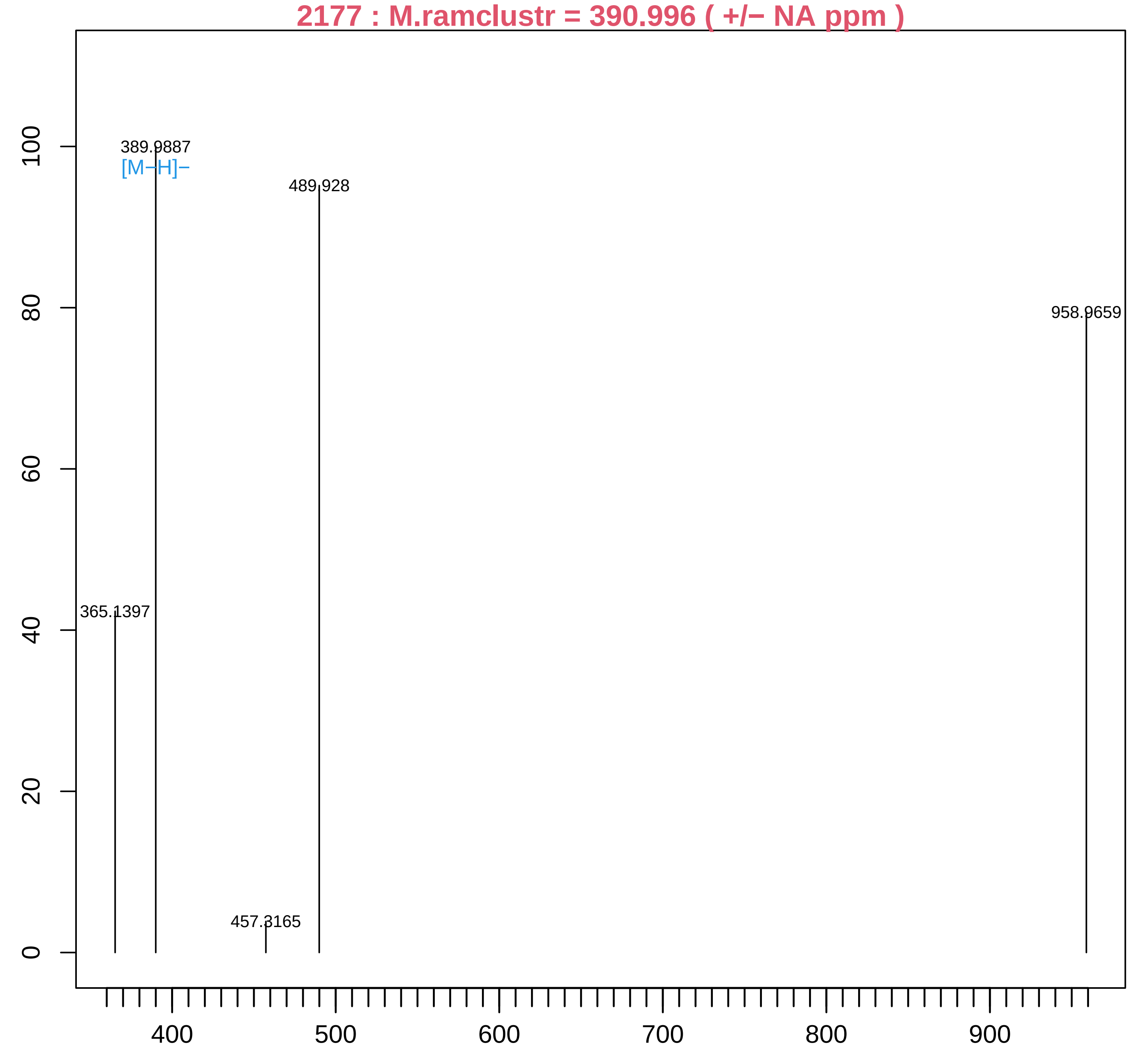
2177 : M.ramclustr = 390.996 (+/− NA ppm)
389.9887
[M−H]−
489.928
958.9659
365.1397
457.3165
0
20
40
60
80
100
400
500
600
700
800
900

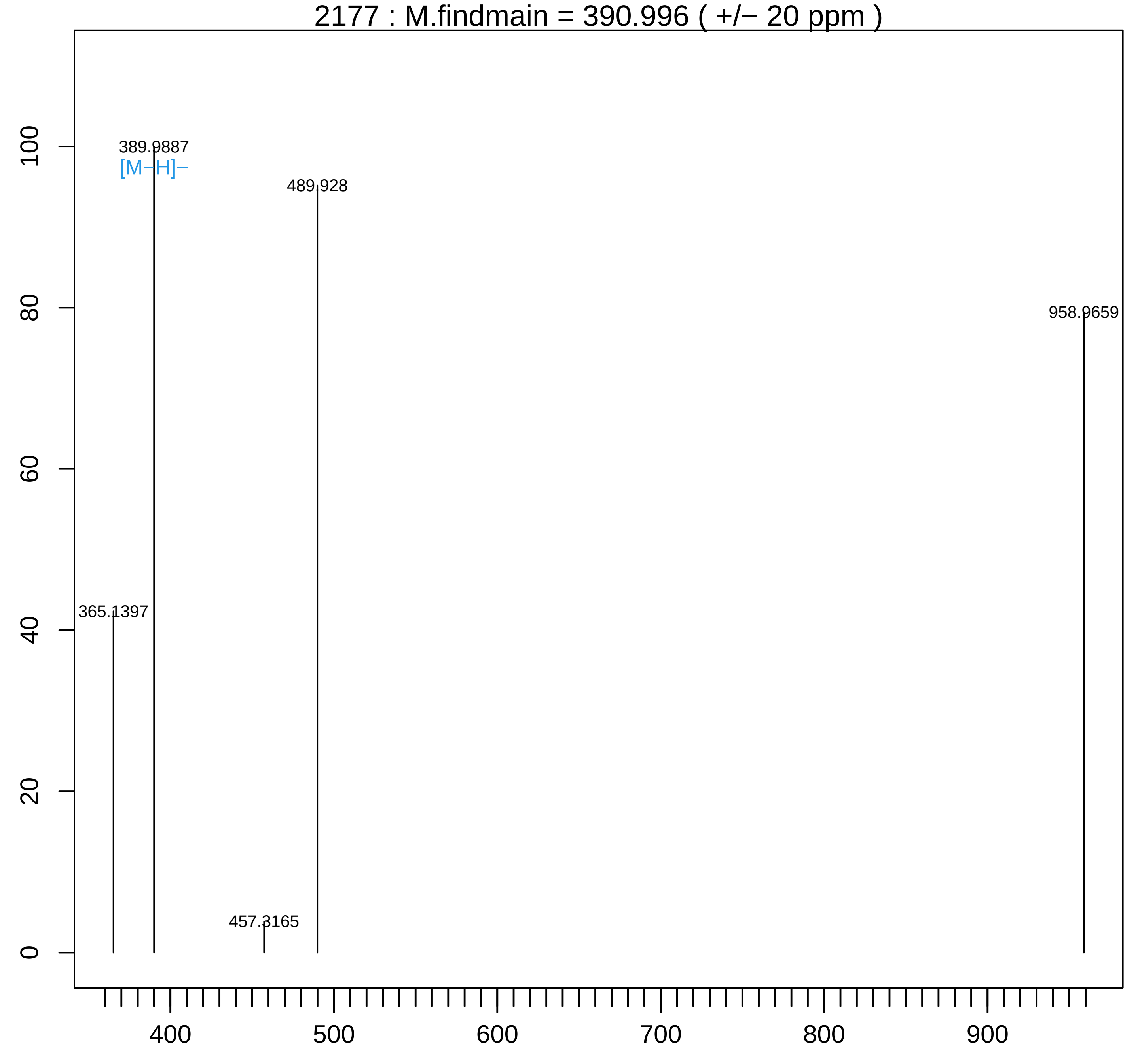
2177 : M.findmain = 390.996 (+/− 20 ppm)
389.9887
[M−H]−
489.928
958.9659
365.1397
457.3165
0
20
40
60
80
100
400
500
600
700
800
900

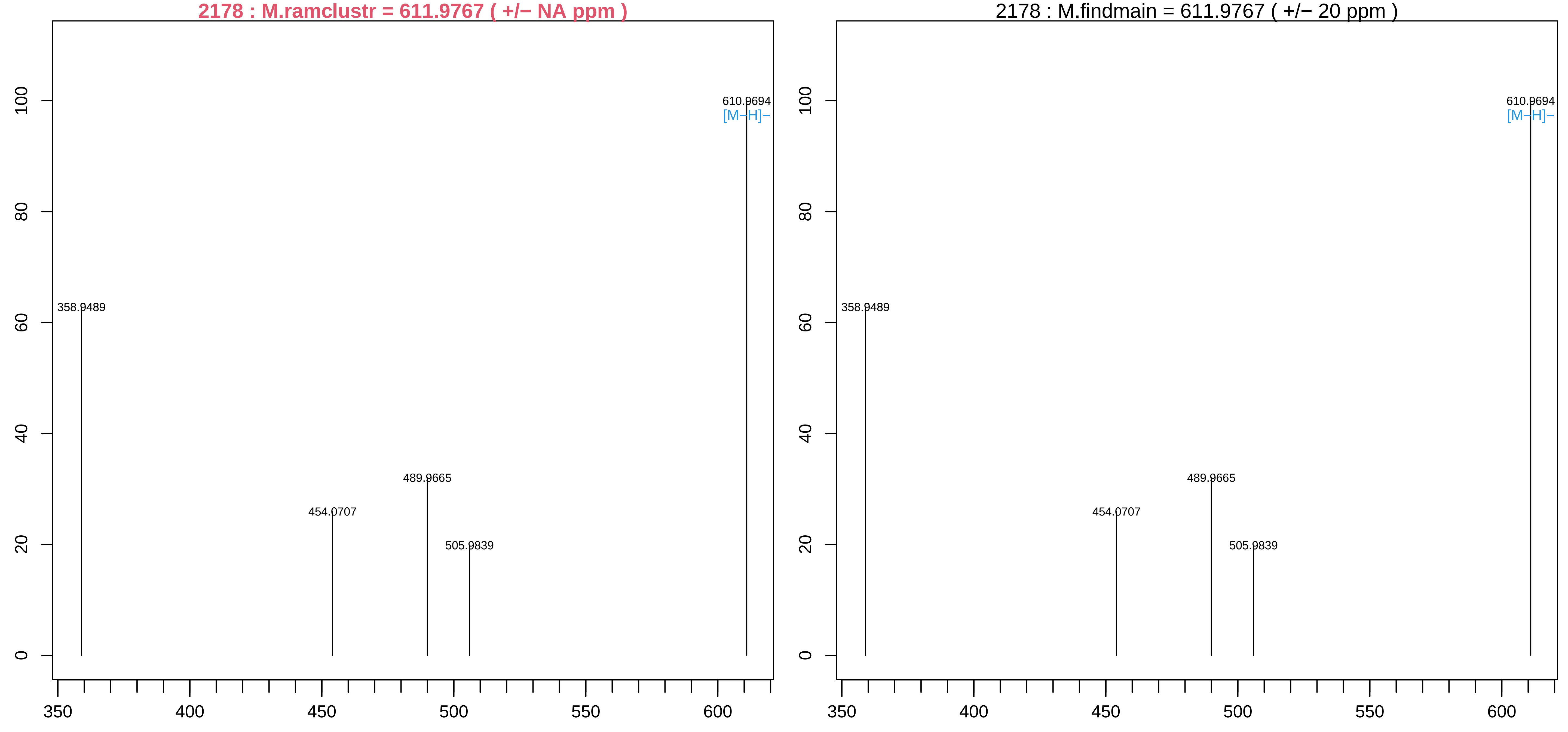
2178 : M.ramclustr = 611.9767 (+/− NA ppm)
2178 : M.findmain = 611.9767 (+/− 20 ppm)
610.9694
[M−H]−
358.9489
454.0707
489.9665
505.9839
0
20
40
60
80
100
350
400
450
500
550
600

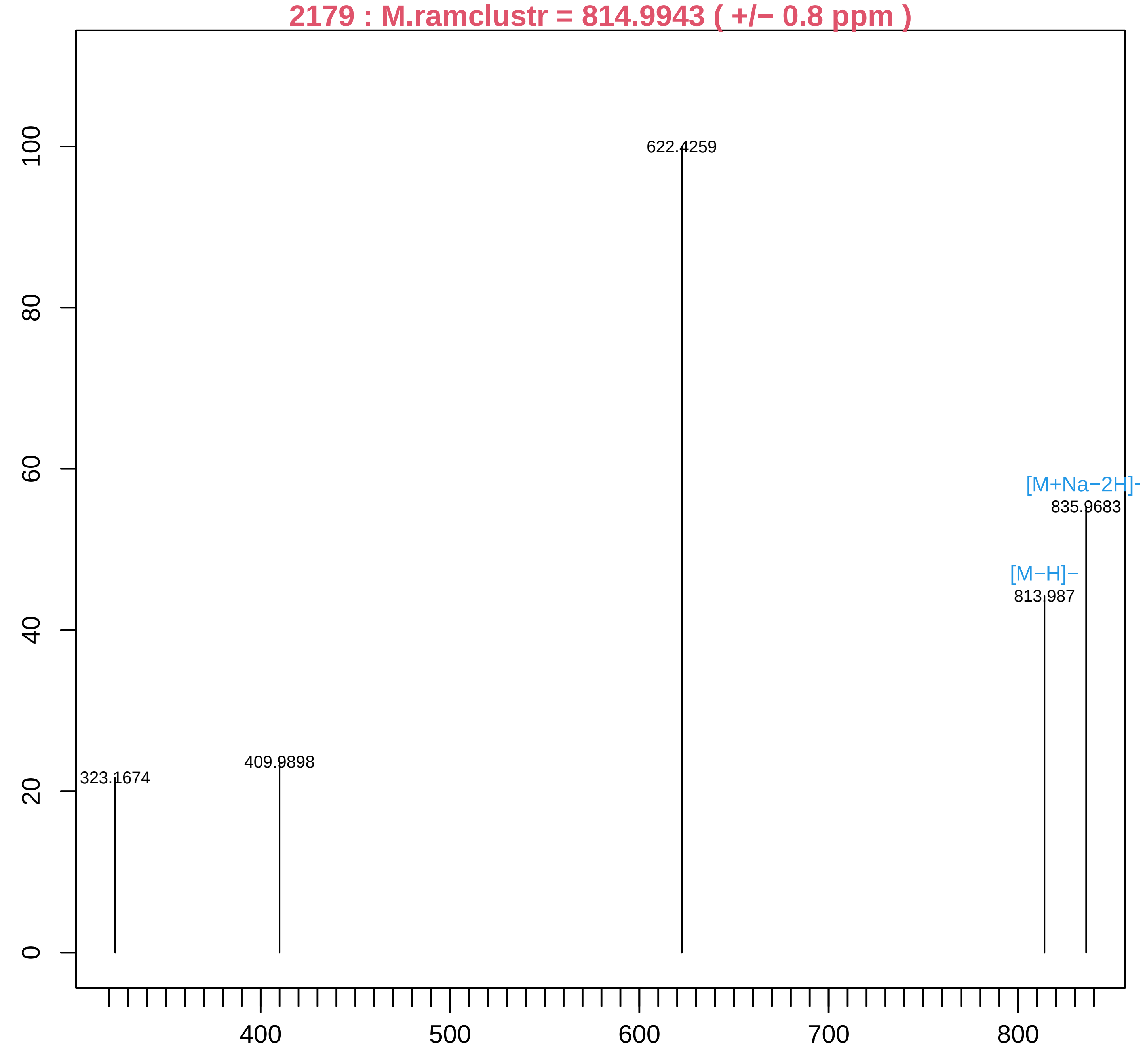
2179 : M.ramclustr = 814.9943 (+/− 0.8 ppm)
622.4259
[M+Na−2H]-
835.9683
[M−H]−
813.987
409.9898
323.1674
0
20
40
60
80
100
400
500
600
700
800

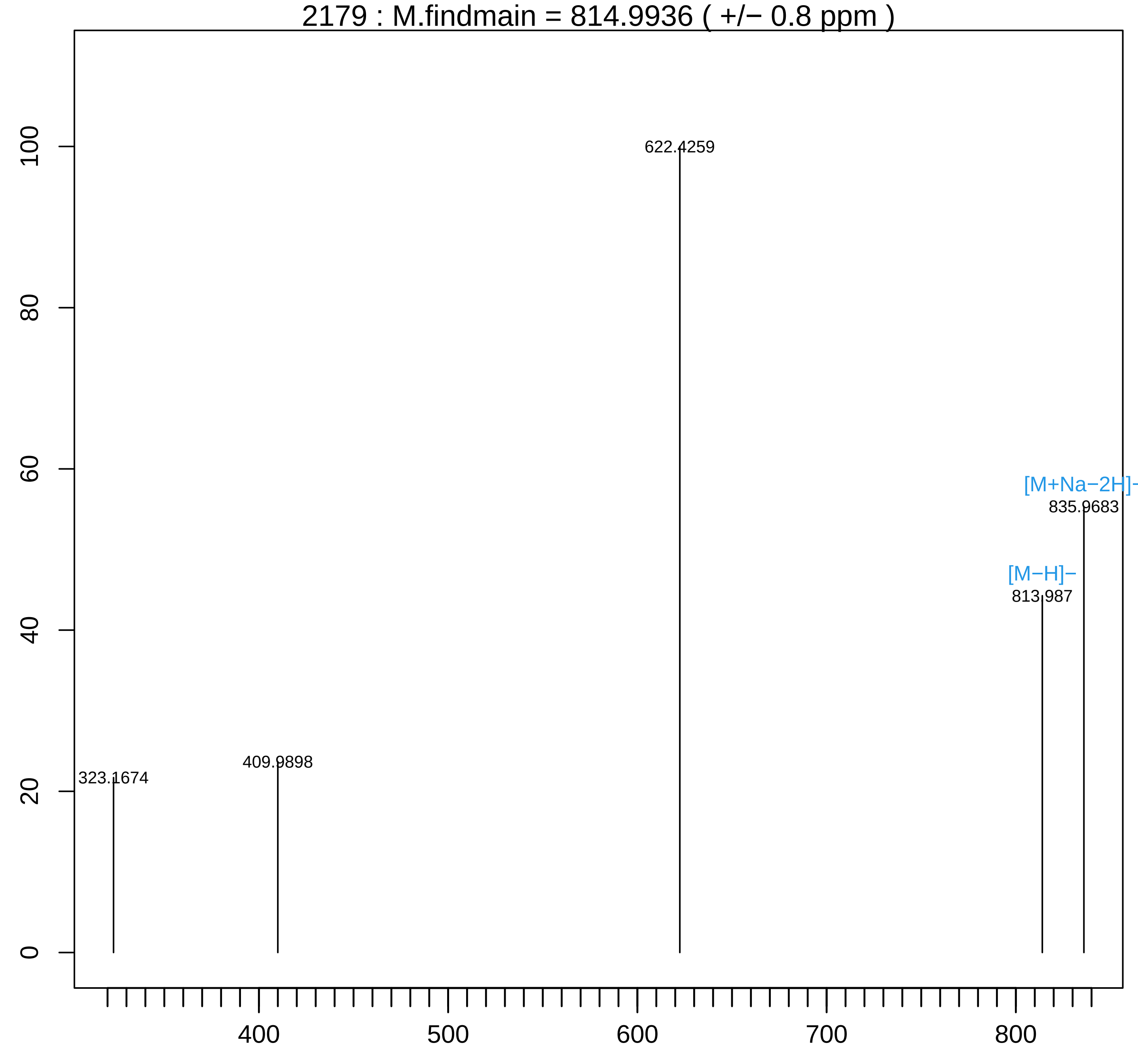
2179 : M.findmain = 814.9936 (+/− 0.8 ppm)
622.4259
[M+Na−2H]-
835.9683
[M−H]−
813.987
409.9898
323.1674
0
20
40
60
80
100
400
500
600
700
800

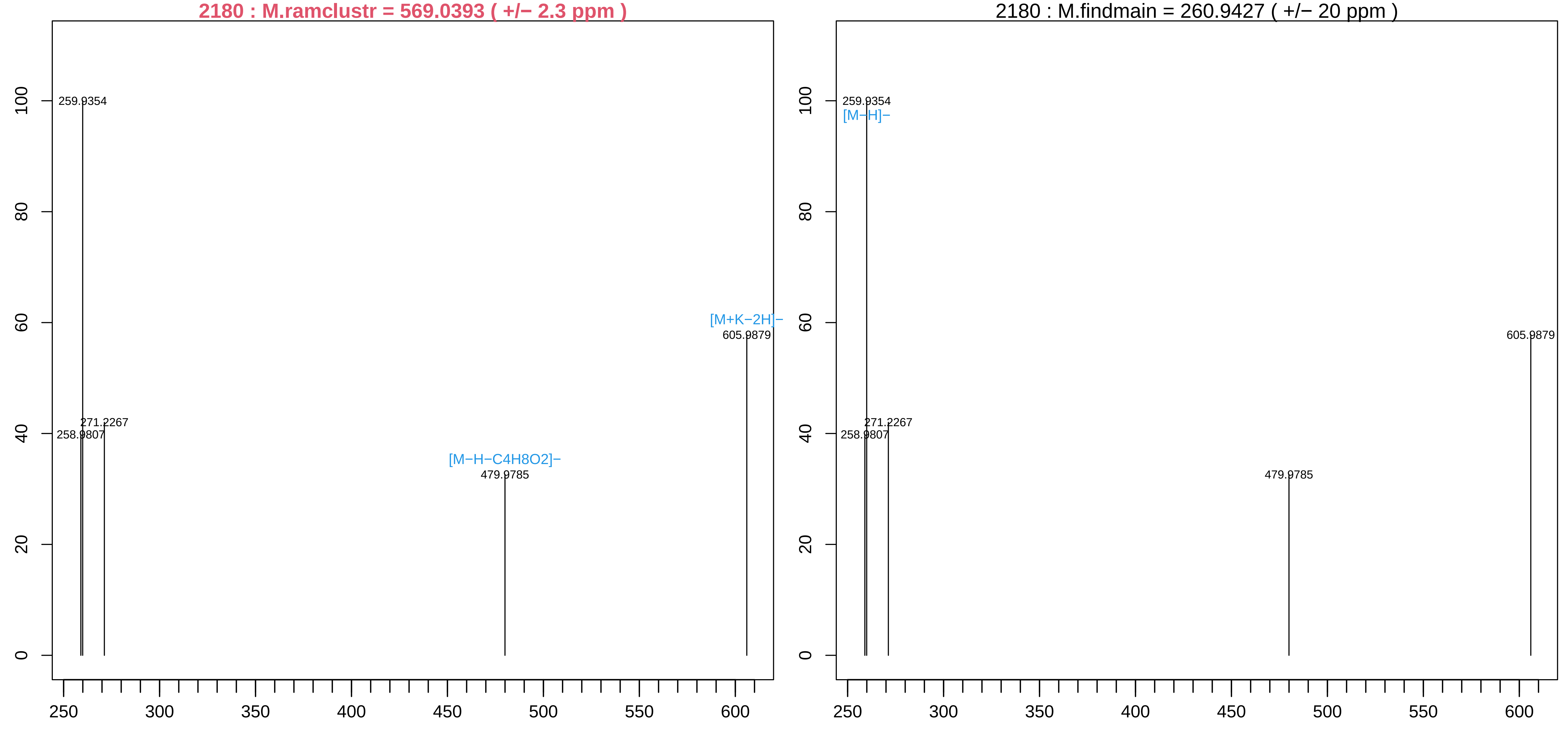
2180 : M.ramclustr = 569.0393 (+/− 2.3 ppm)
259.9354
271.2267
258.9807
[M−H−C4H8O2]−
479.9785
[M+K−2H]−
605.9879
2180 : M.findmain = 260.9427 (+/− 20 ppm)
259.9354
[M−H]−
271.2267
258.9807
479.9785
605.9879

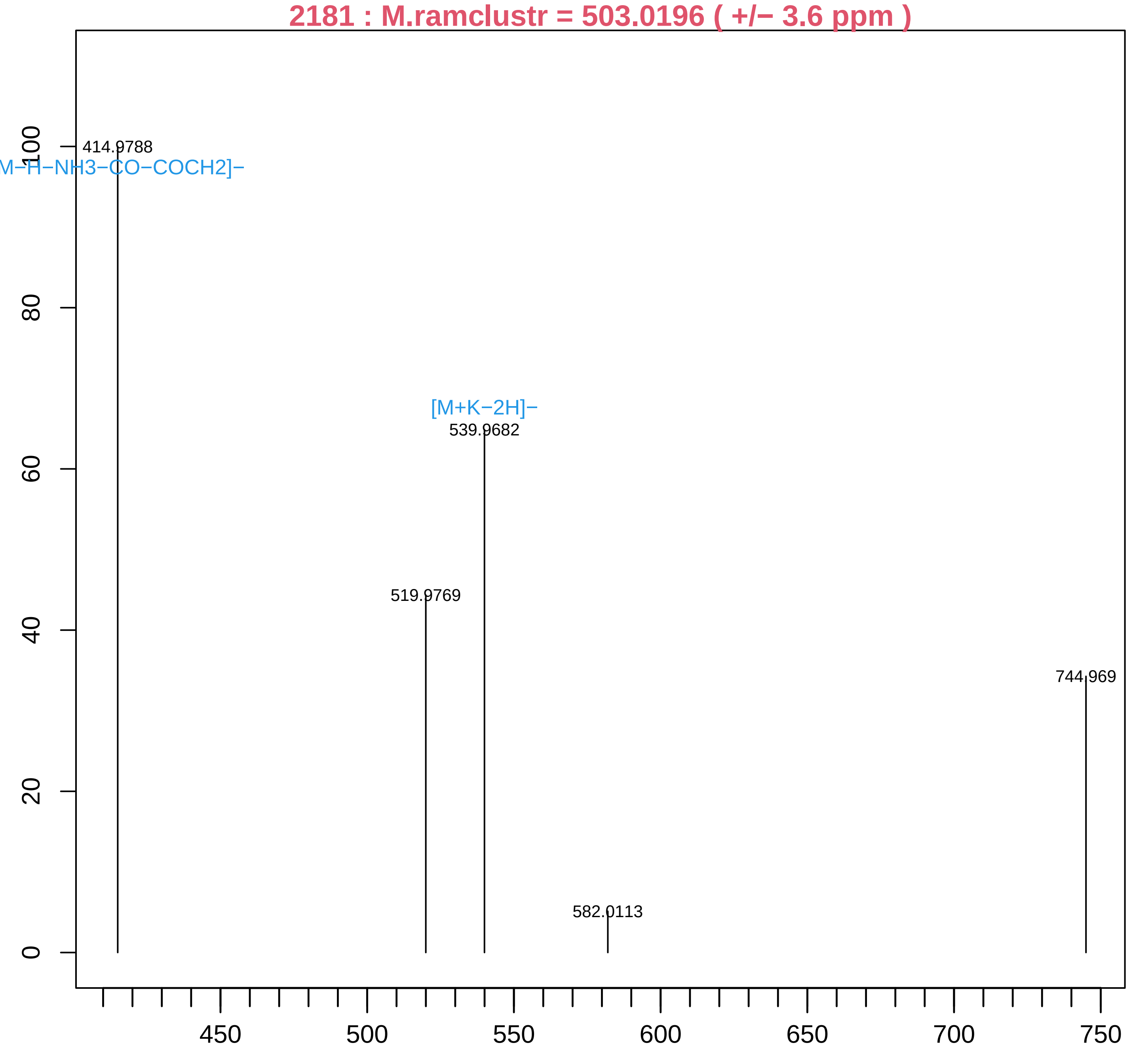

2181 : M.ramclustr = 503.0196 (+/− 3.6 ppm)
414.9788
[M−H−NH3−CO−COCH2]−
[M+K−2H]−
539.9682
519.9769
582.0113
744.969
0
20
40
60
80
100
450
500
550
600
650
700
750

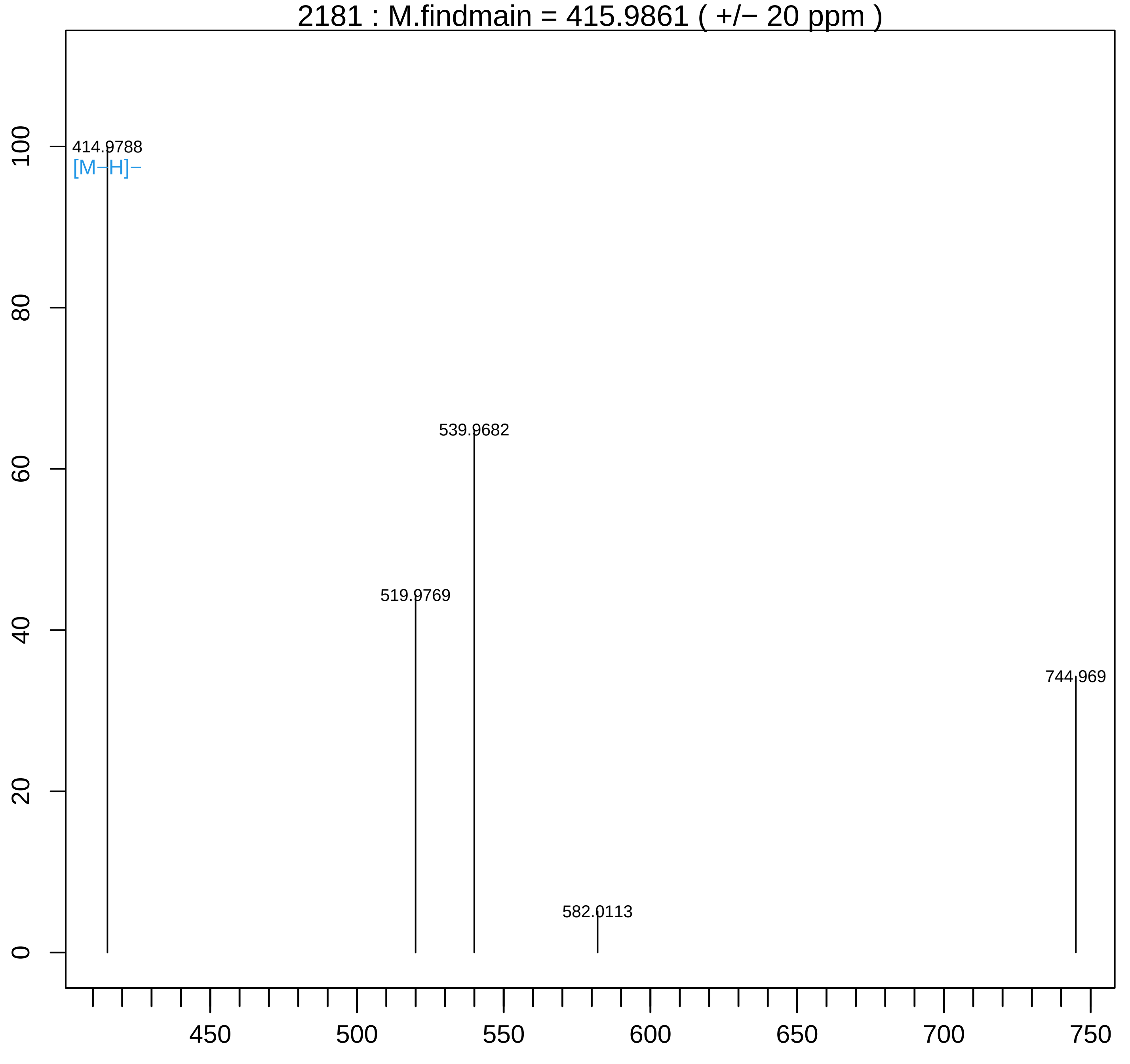

2181 : M.findmain = 415.9861 (+/− 20 ppm)
414.9788
[M−H]−
539.9682
519.9769
582.0113
744.969
0
20
40
60
80
100
450
500
550
600
650
700
750

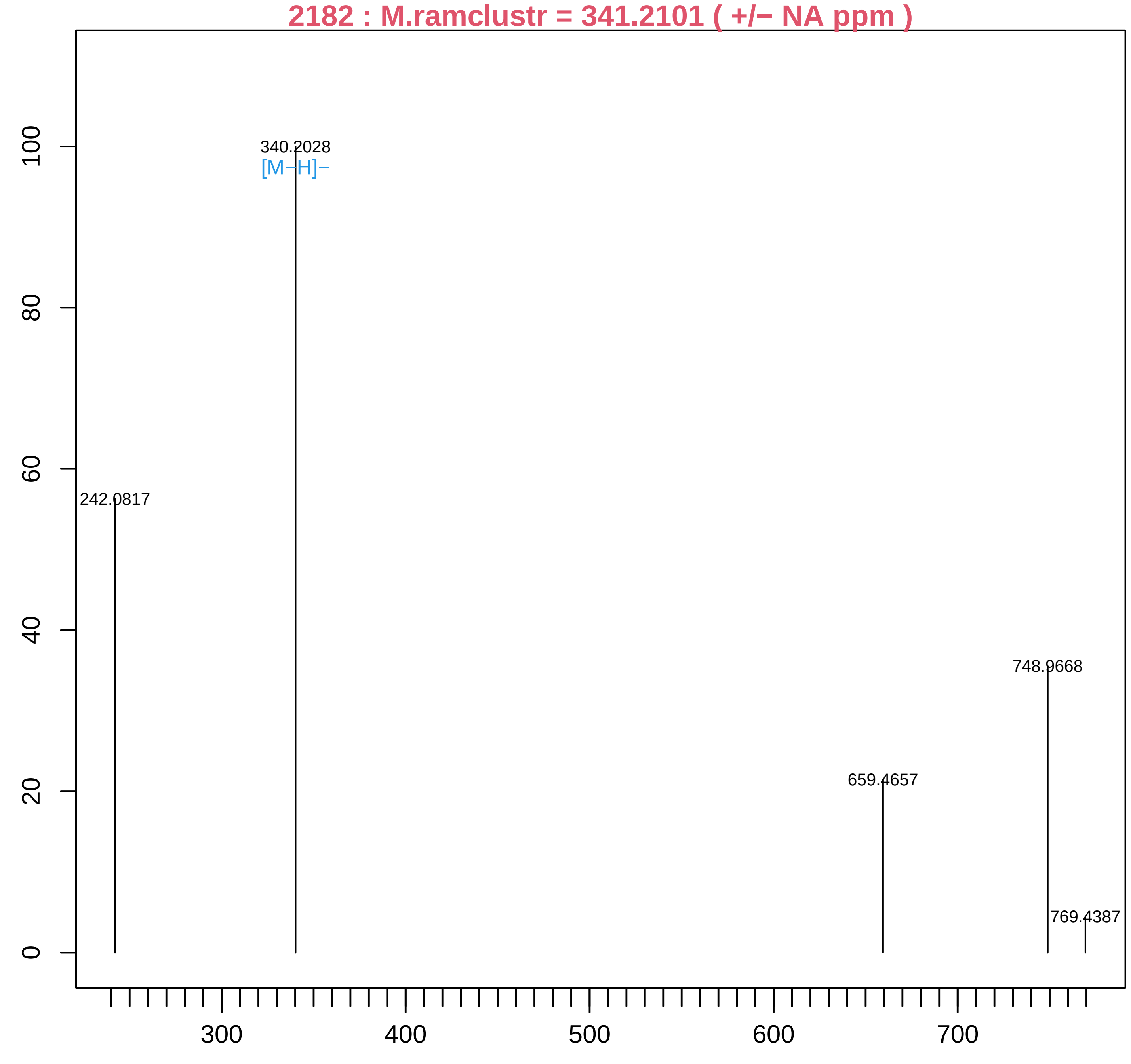
2182 : M.ramclustr = 341.2101 (+/− NA ppm)
340.2028
[M−H]−
242.0817
748.9668
659.4657
769.4387
0
20
40
60
80
100
300
400
500
600
700

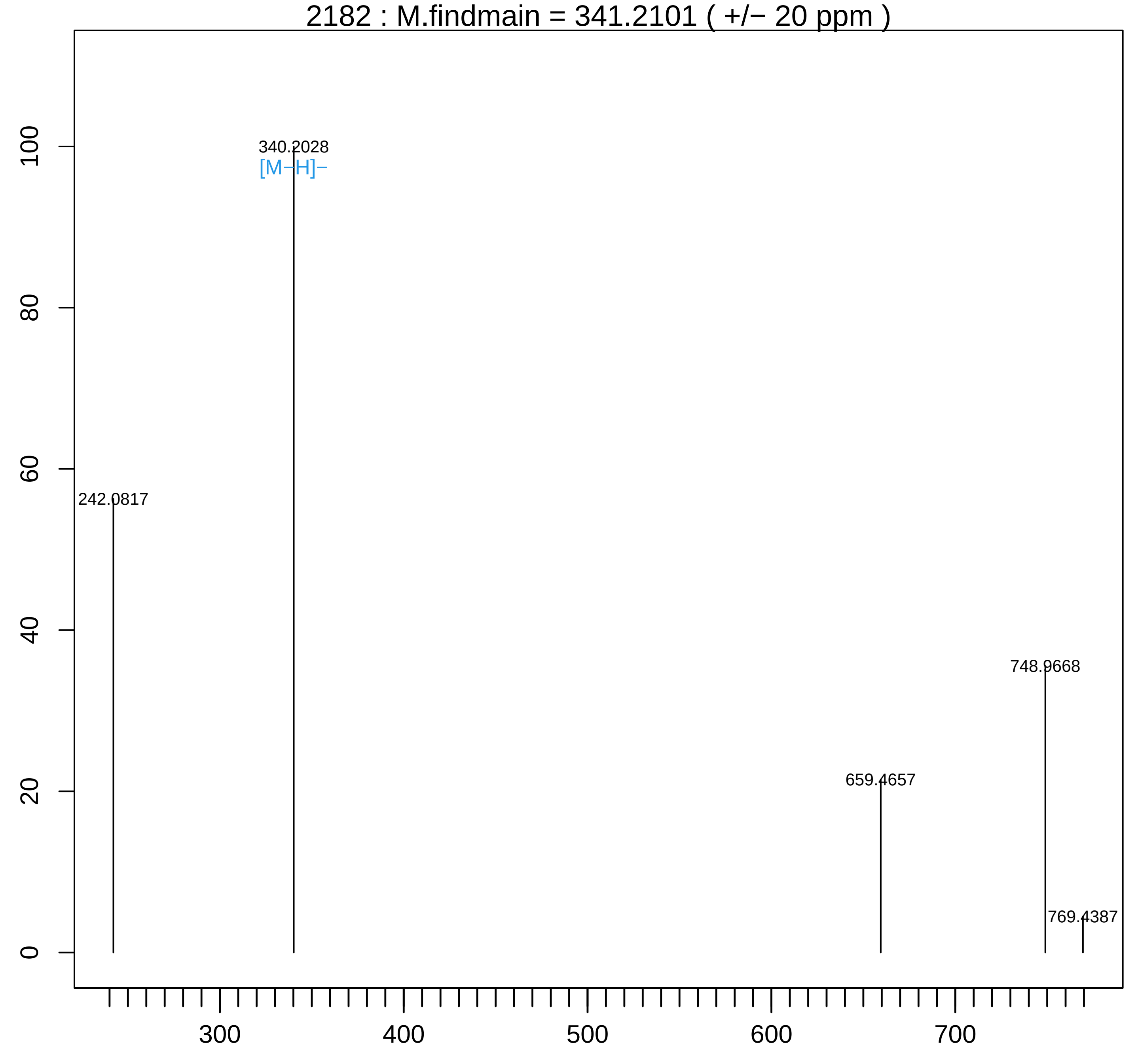
2182 : M.findmain = 341.2101 (+/− 20 ppm)
340.2028
[M−H]−
242.0817
748.9668
659.4657
769.4387
0
20
40
60
80
100
300
400
500
600
700

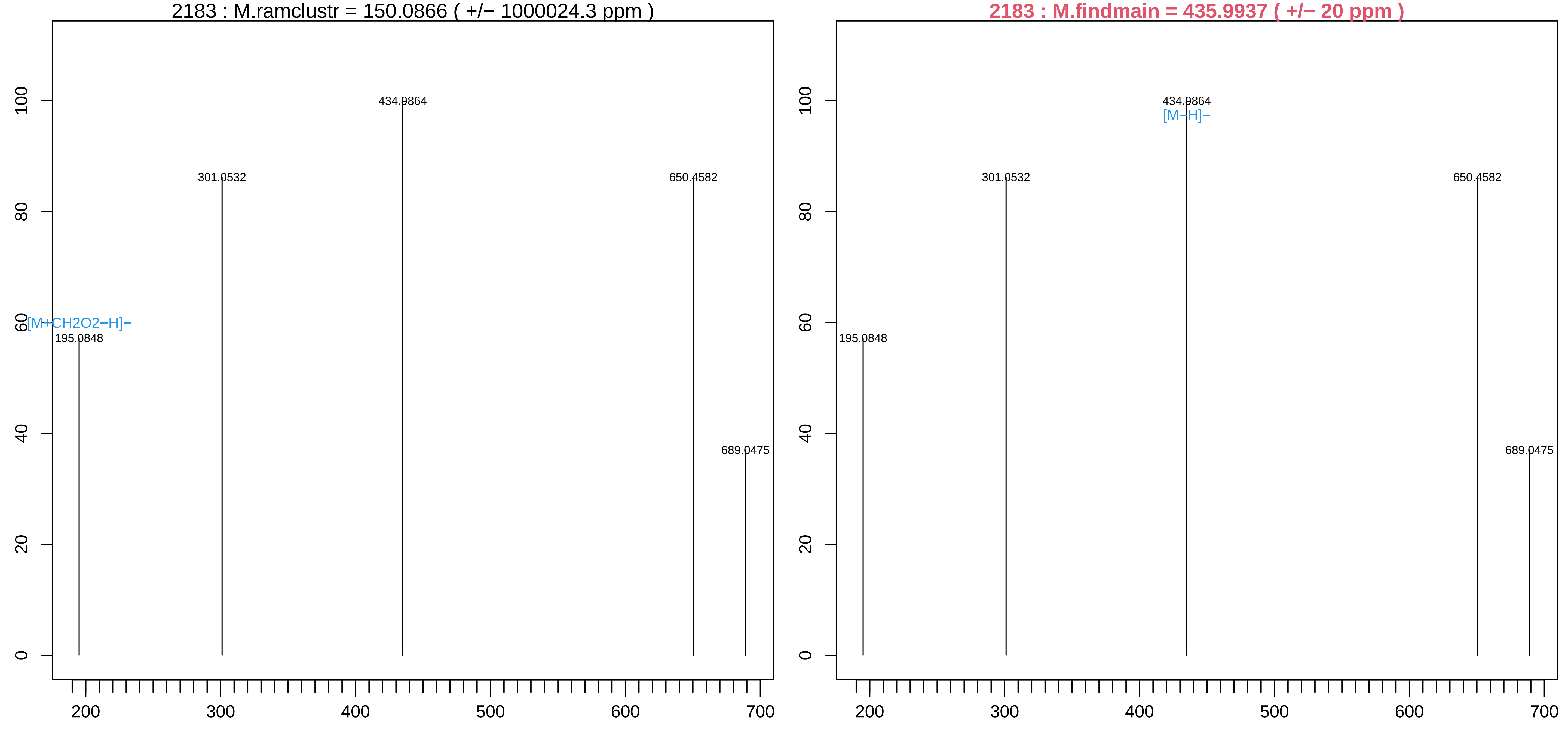

2183 : M.ramclustr = 150.0866 (+/− 1000024.3 ppm)
[M+CH2O2−H]−
195.0848
301.0532
434.9864
650.4582
689.0475
2183 : M.findmain = 435.9937 (+/− 20 ppm)
195.0848
301.0532
434.9864
[M−H]−
650.4582
689.0475

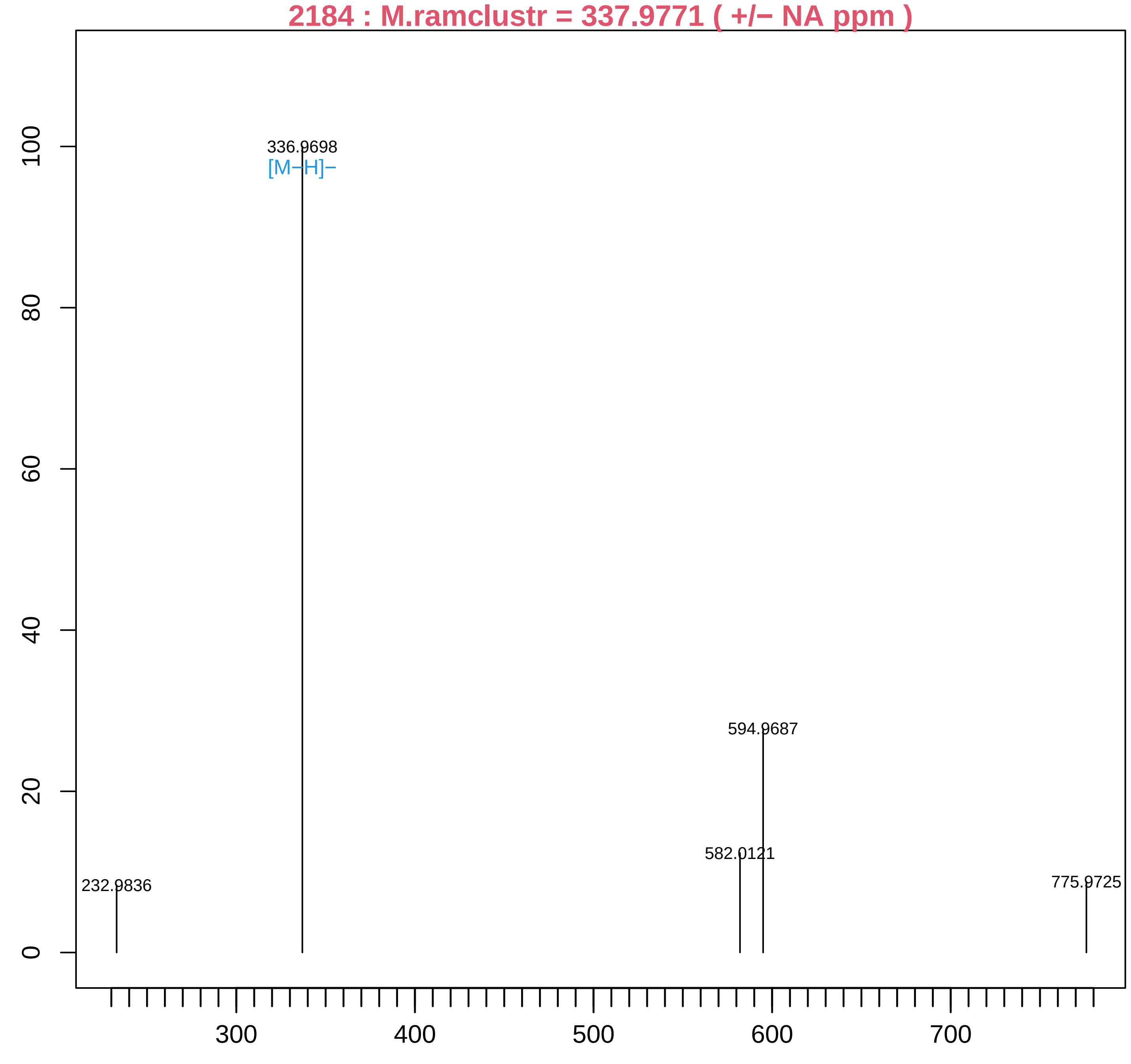
2184 : M.ramclustr = 337.9771 (+/− NA ppm)
336.9698
[M−H]−
594.9687
582.0121
232.9836
775.9725
0
20
40
60
80
100
300
400
500
600
700

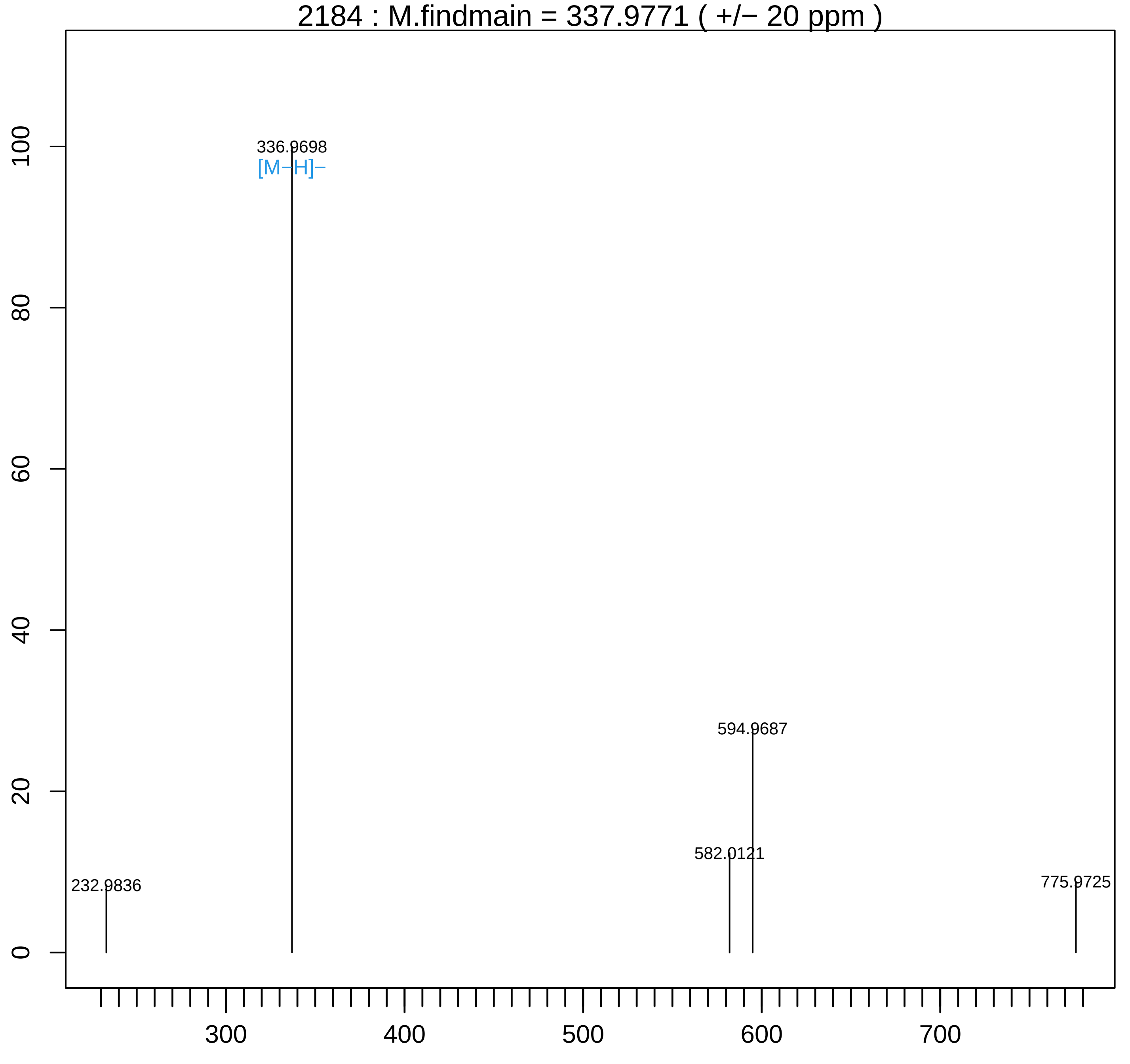
2184 : M.findmain = 337.9771 (+/− 20 ppm)
336.9698
[M−H]−
594.9687
582.0121
232.9836
775.9725
0
20
40
60
80
100
300
400
500
600
700

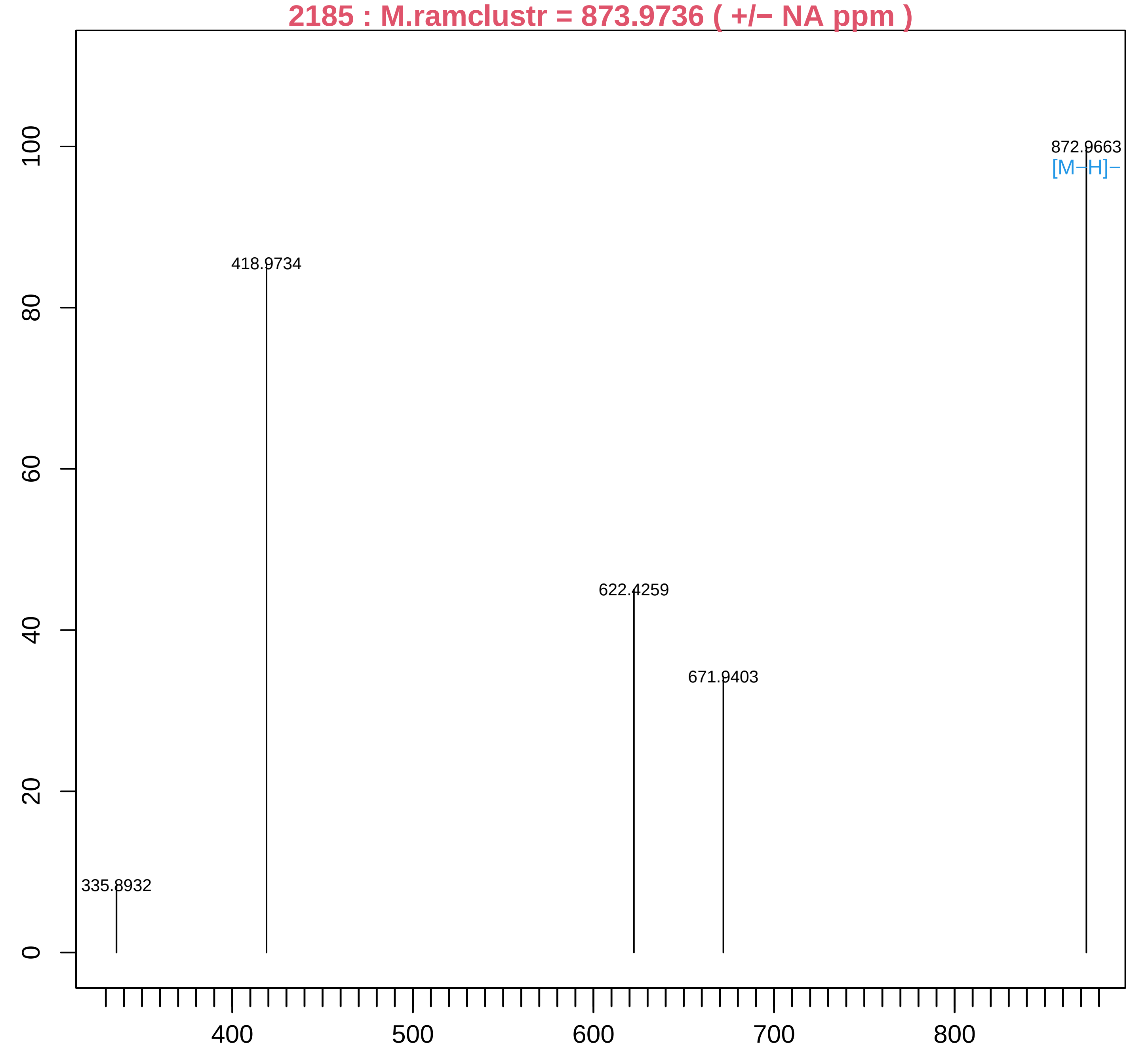
2185 : M.ramclustr = 873.9736 (+/− NA ppm)
872.9663
[M−H]−
418.9734
622.4259
671.9403
335.8932
0
20
40
60
80
100
400
500
600
700
800

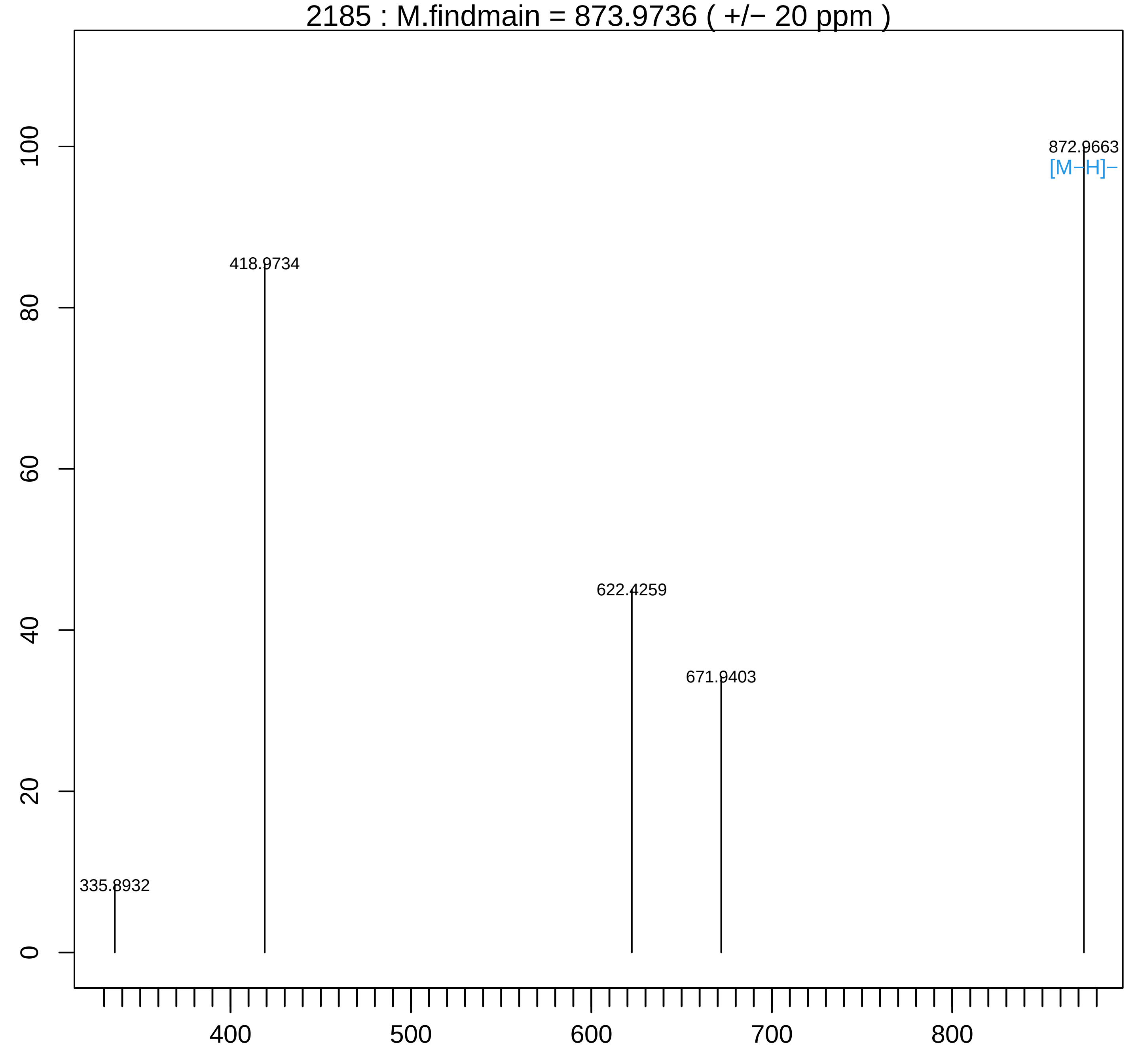
2185 : M.findmain = 873.9736 (+/− 20 ppm)
872.9663
[M−H]−
418.9734
622.4259
671.9403
335.8932
0
20
40
60
80
100
400
500
600
700
800

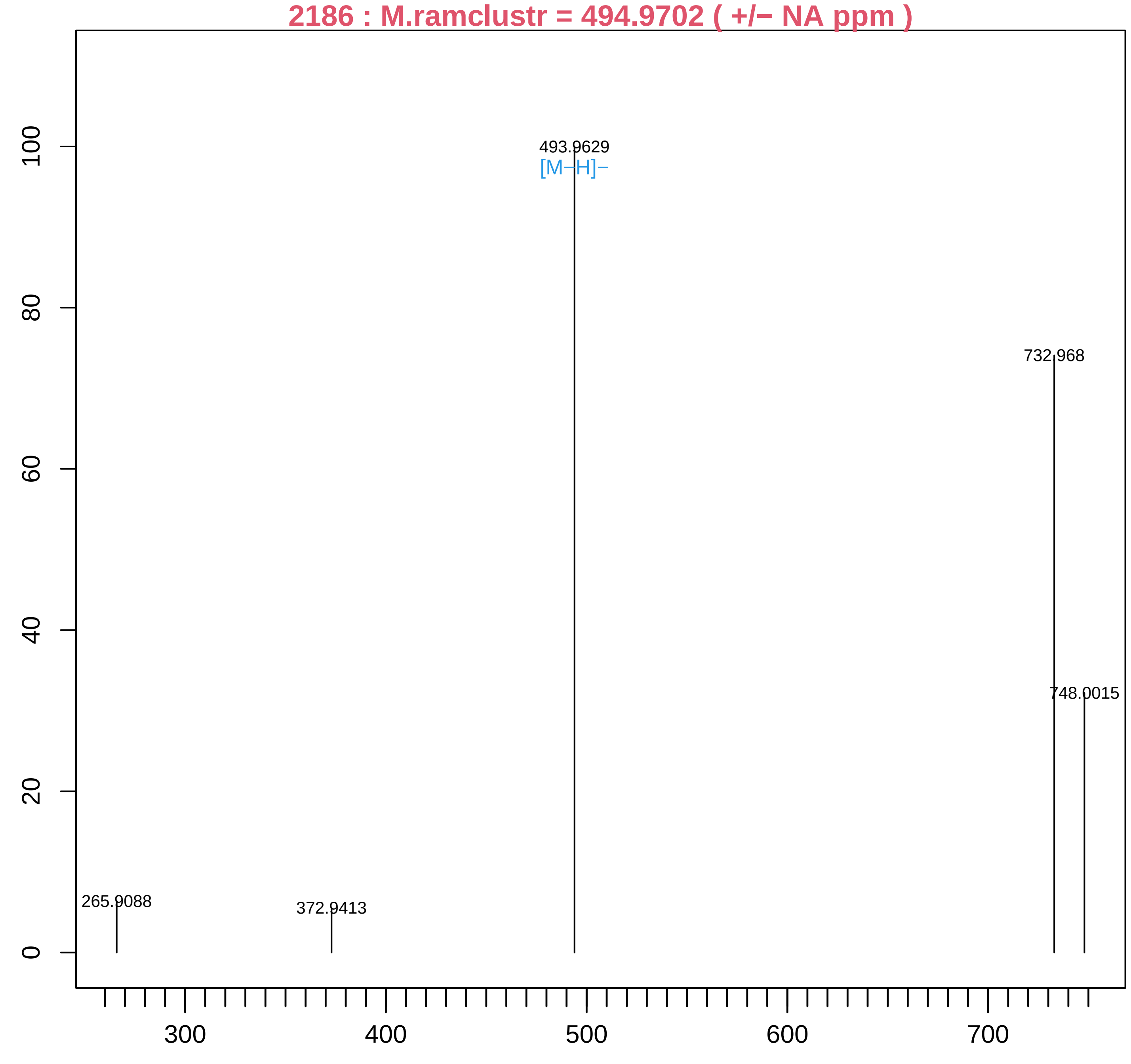
2186 : M.ramclustr = 494.9702 (+/− NA ppm)
493.9629
[M−H]−
732.968
748.0015
265.9088
372.9413
0
20
40
60
80
100
300
400
500
600
700

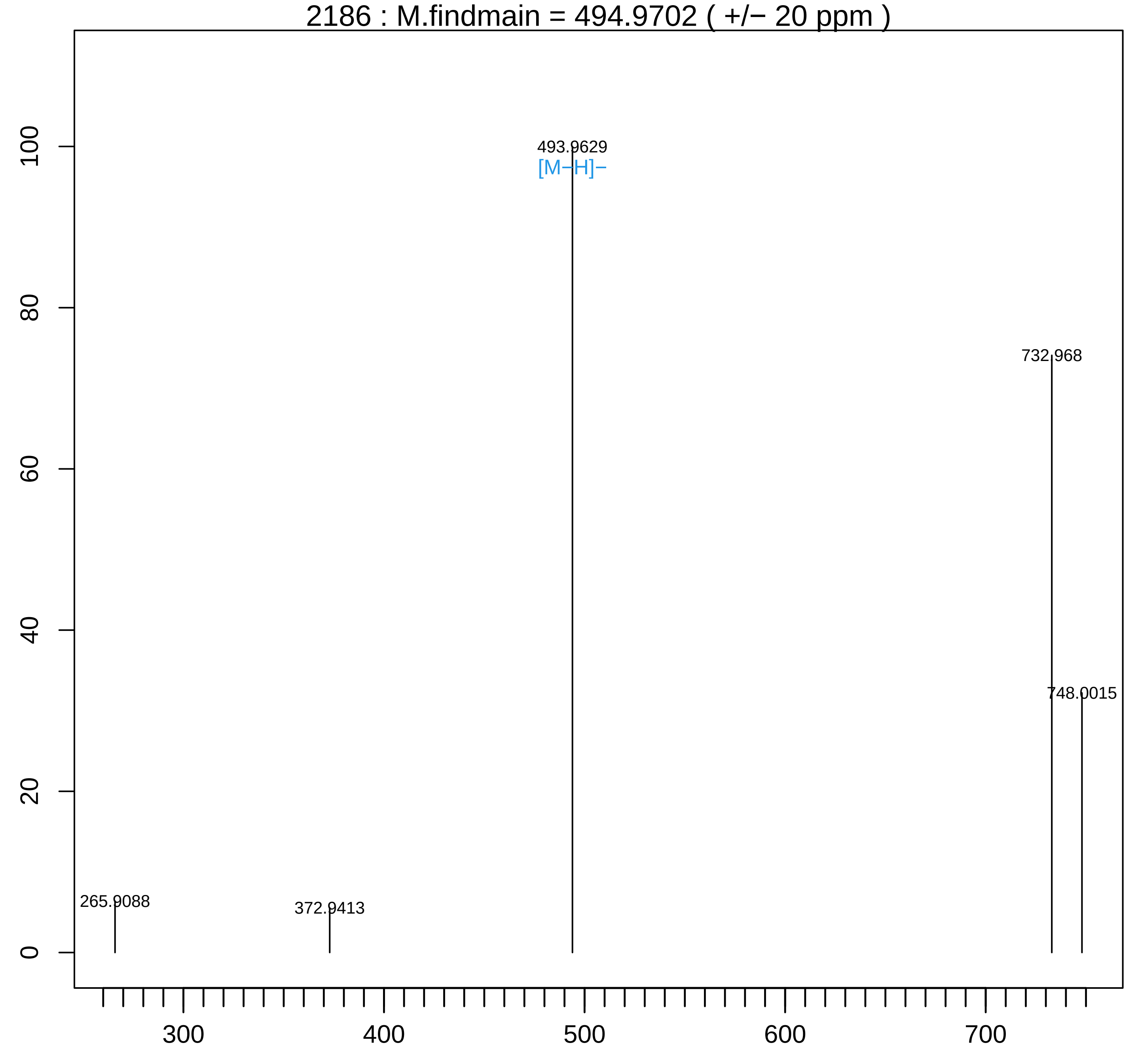
2186 : M.findmain = 494.9702 (+/− 20 ppm)
493.9629
[M−H]−
732.968
748.0015
265.9088
372.9413
0
20
40
60
80
100
300
400
500
600
700

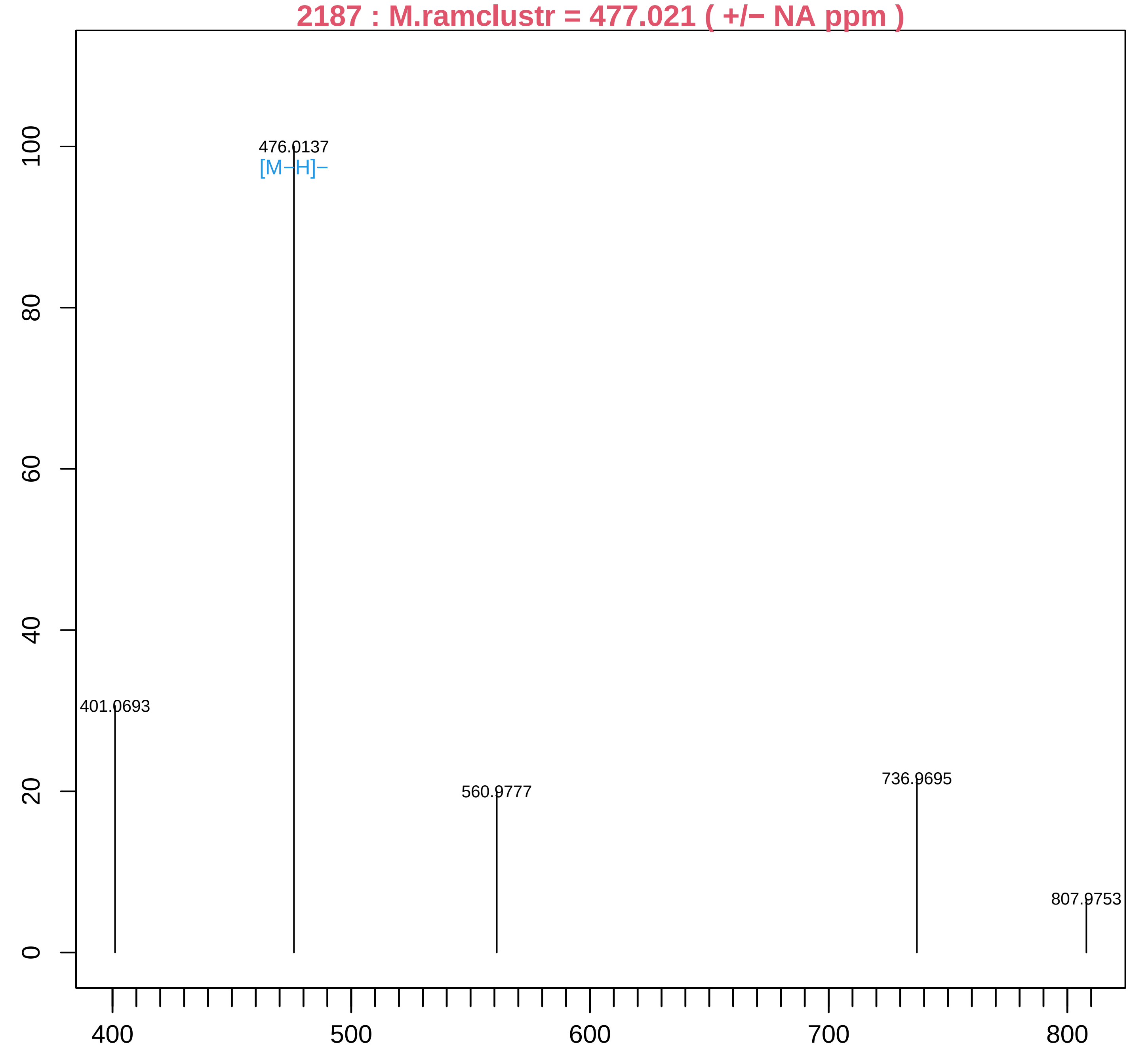
2187 : M.ramclustr = 477.021 (+/− NA ppm)
476.0137
[M−H]−
401.0693
560.9777
736.9695
807.9753
0
20
40
60
80
100
400
500
600
700
800

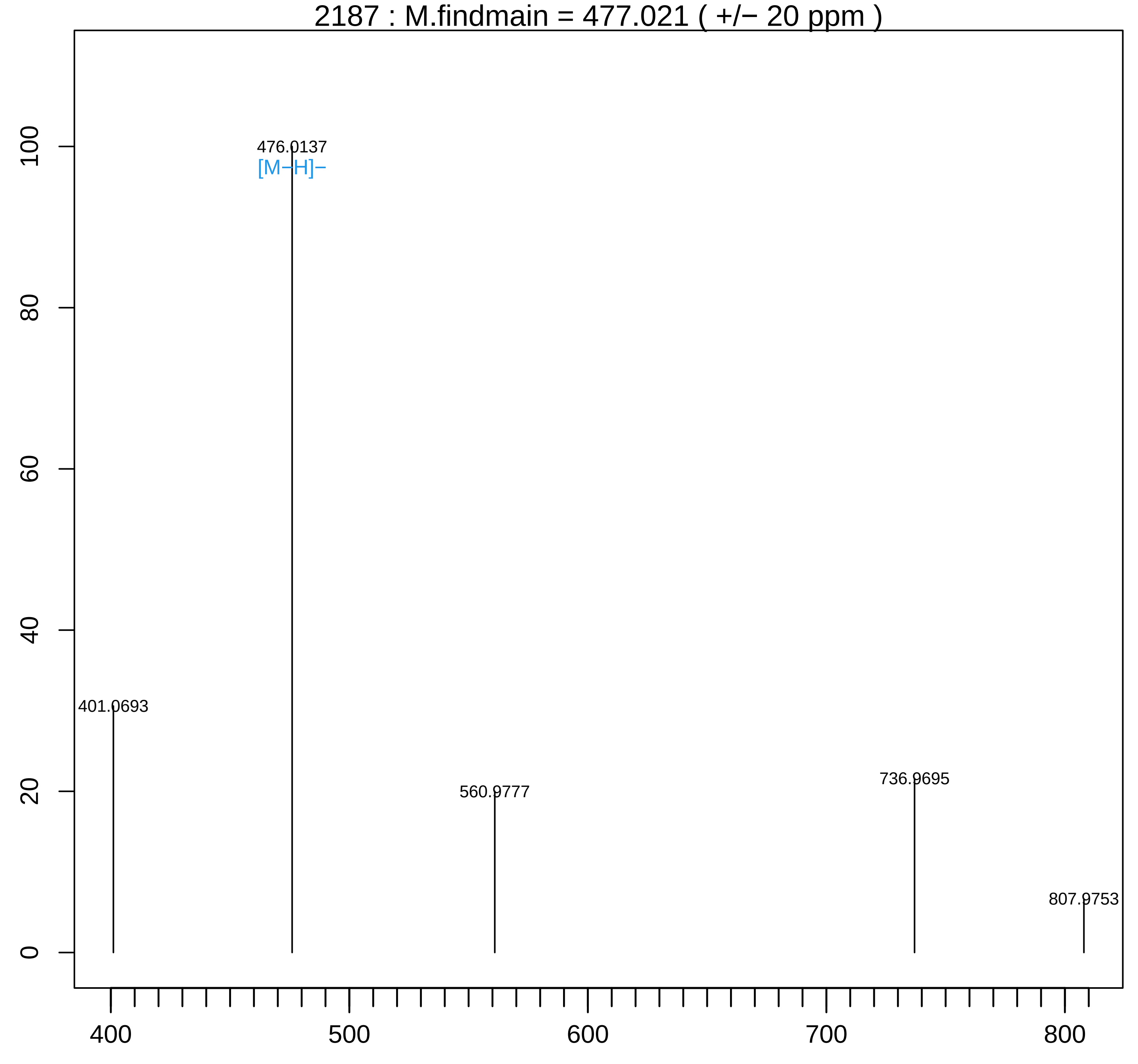
2187 : M.findmain = 477.021 (+/− 20 ppm)
476.0137
[M−H]−
401.0693
560.9777
736.9695
807.9753
0
20
40
60
80
100
400
500
600
700
800

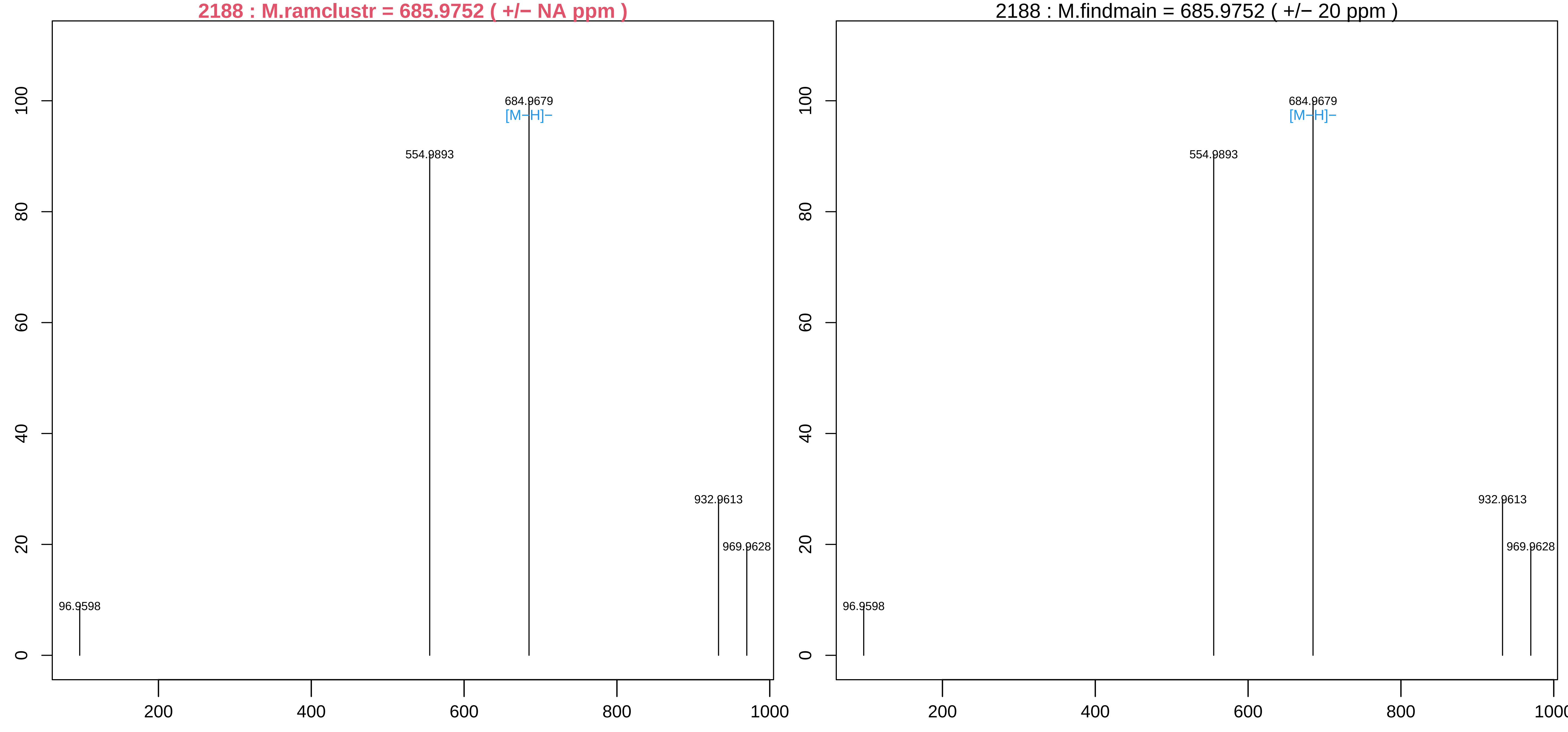
2188 : M.ramclustr = 685.9752 (+/− NA ppm)
2188 : M.findmain = 685.9752 (+/− 20 ppm)
684.9679
[M−H]−
554.9893
932.9613
969.9628
96.9598
0
20
40
60
80
100
200
400
600
800
1000

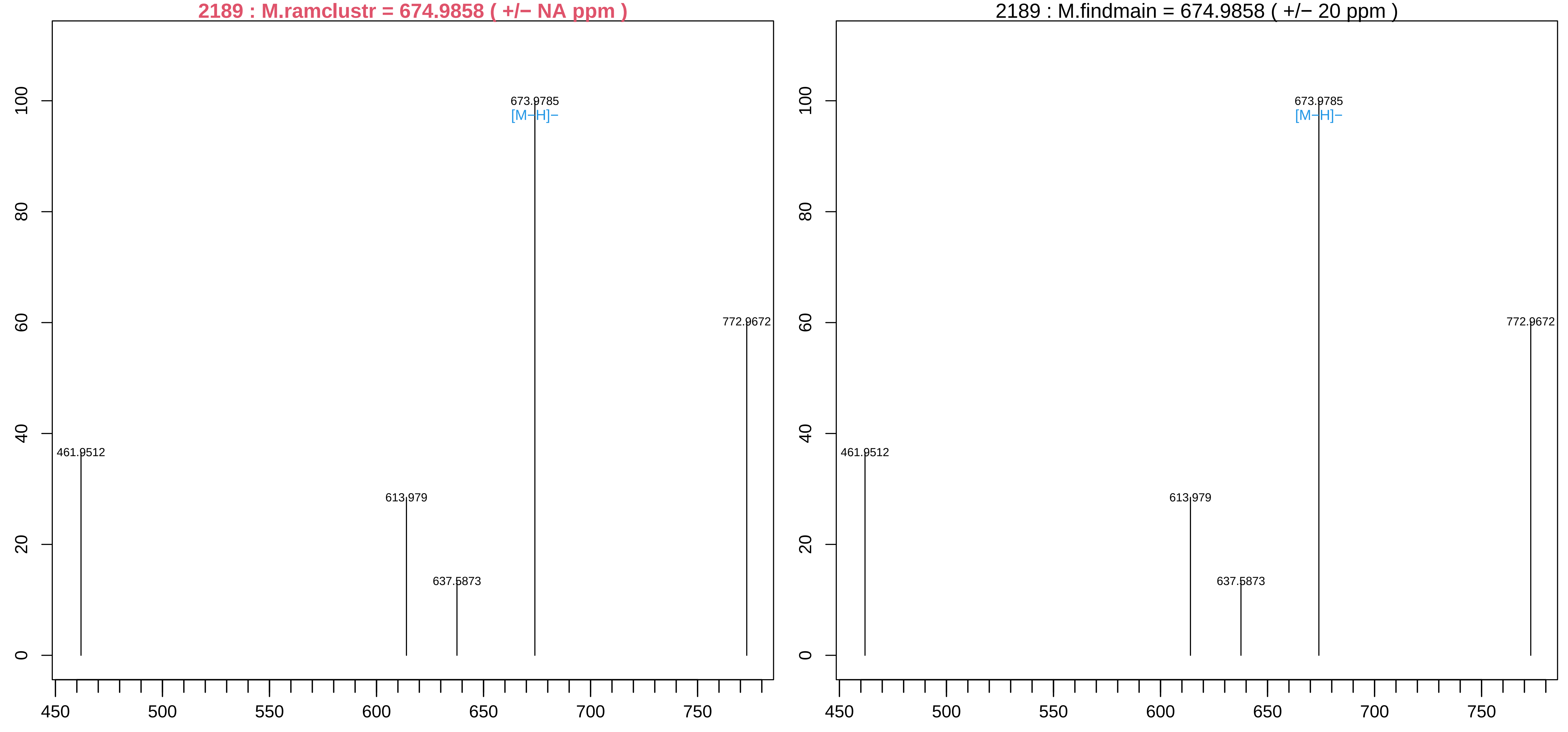
2189 : M.ramclustr = 674.9858 (+/− NA ppm)
673.9785
[M−H]−
772.9672
461.9512
613.979
637.5873
2189 : M.findmain = 674.9858 (+/− 20 ppm)
673.9785
[M−H]−
772.9672
461.9512
613.979
637.5873
0
20
40
60
80
100
450
500
550
600
650
700
750

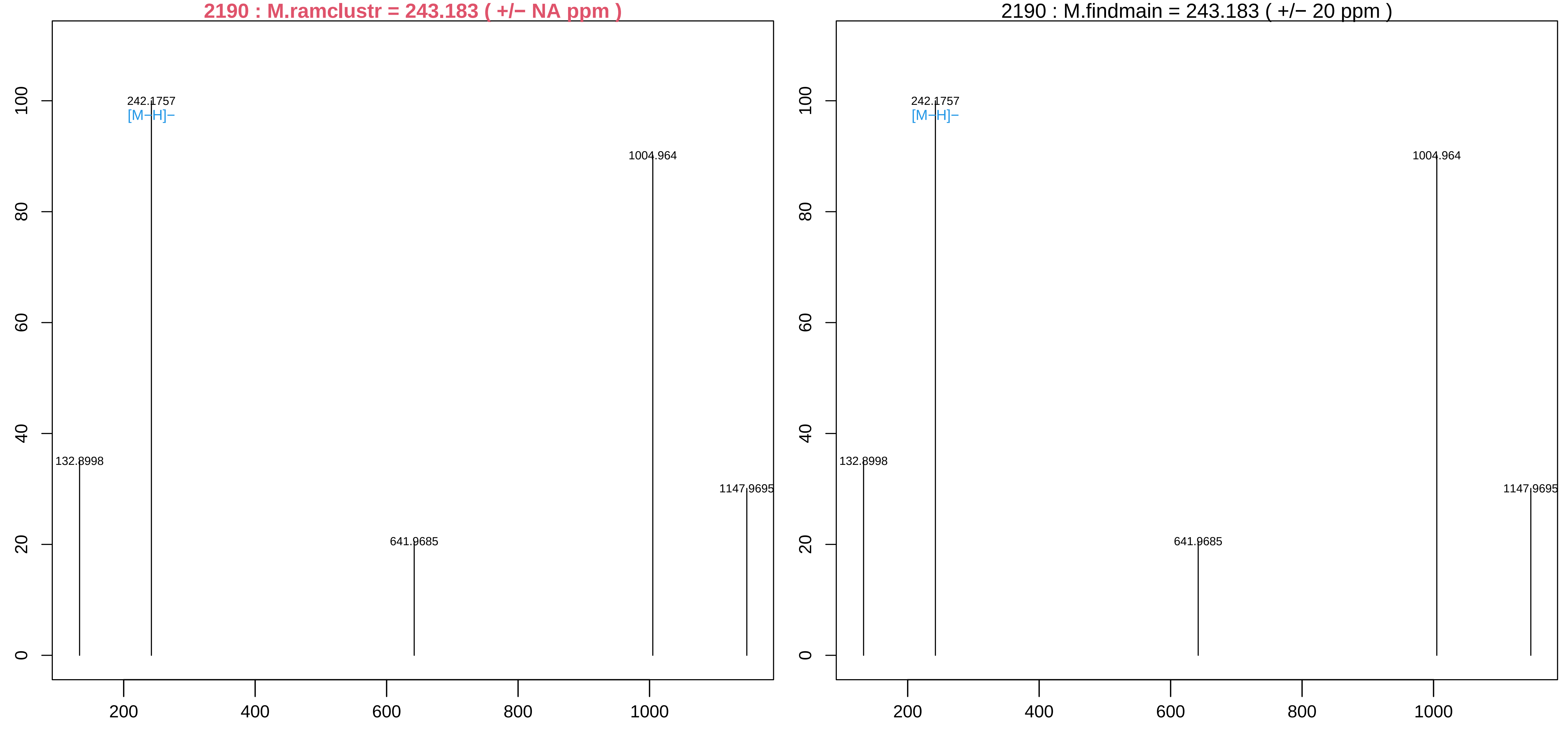
2190 : M.ramclustr = 243.183 (+/− NA ppm)
2190 : M.findmain = 243.183 (+/− 20 ppm)
242.1757
[M−H]−
1004.964
132.8998
1147.9695
641.9685
0
20
40
60
80
100
200
400
600
800
1000

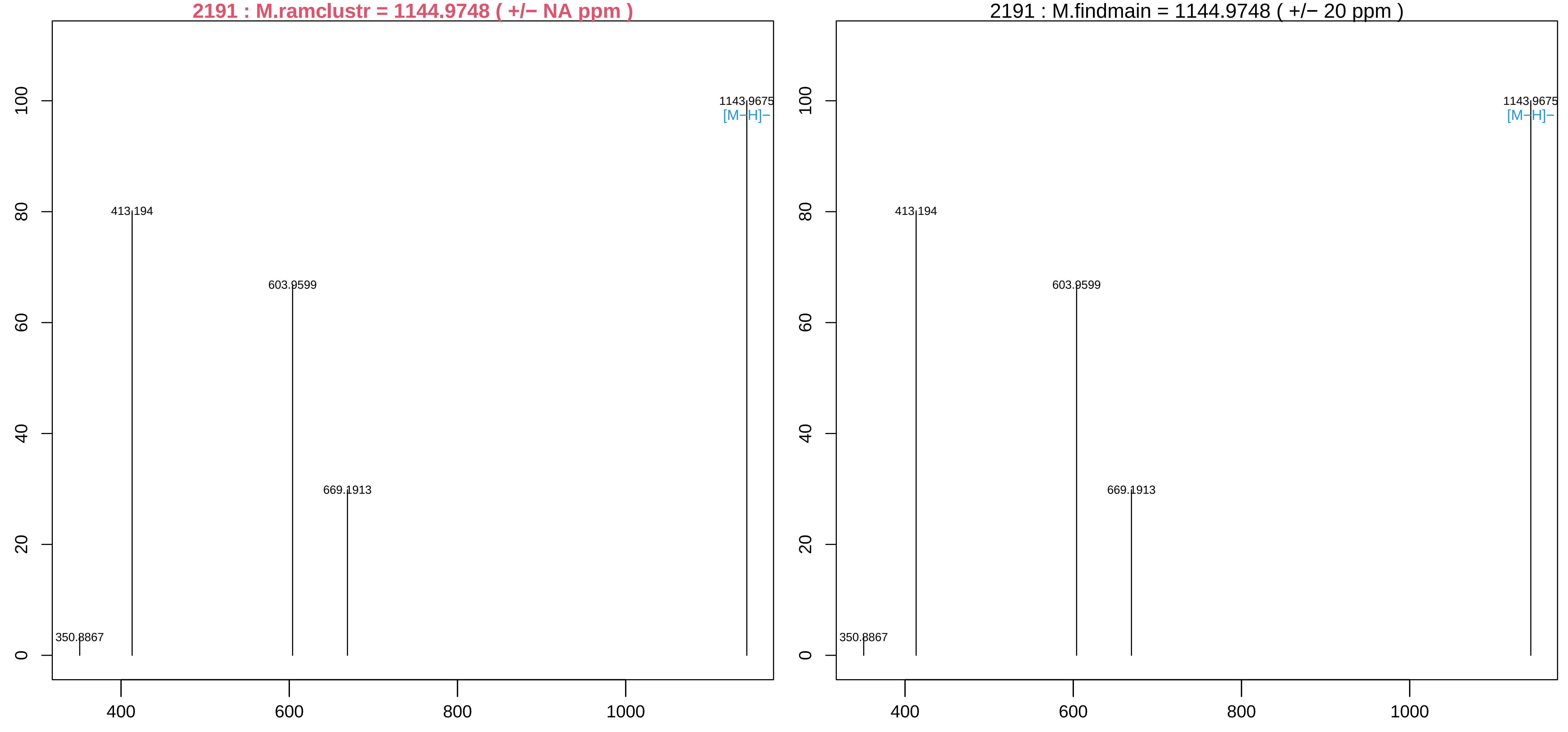
2191 : M.ramclustr = 1144.9748 (+/− NA ppm)
1143.9675
[M−H]−
413.194
603.9599
669.1913
350.8867
2191 : M.findmain = 1144.9748 (+/− 20 ppm)
1143.9675
[M−H]−
413.194
603.9599
669.1913
350.8867

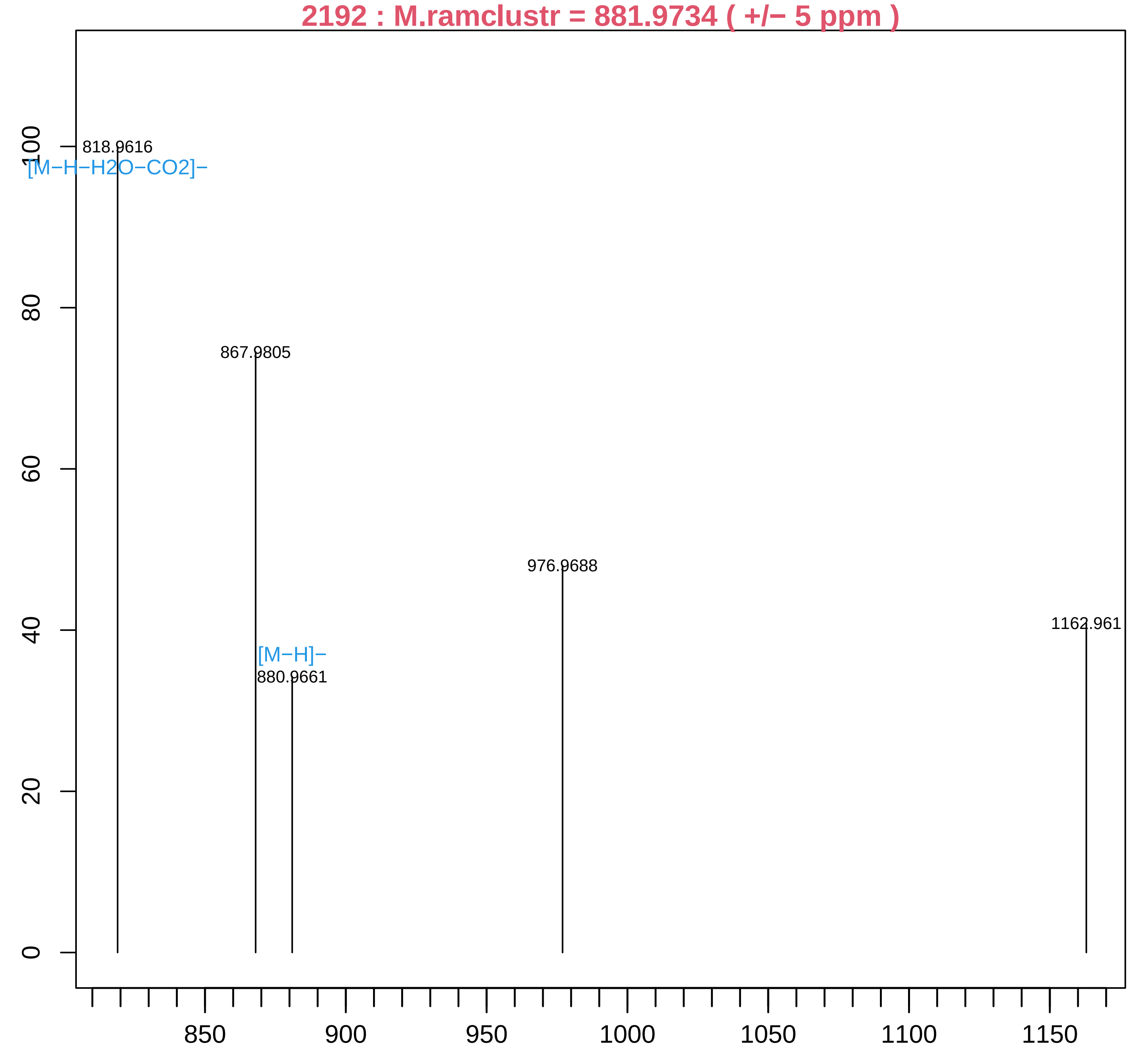
2192 : M.ramclustr = 881.9734 (+/− 5 ppm)
818.9616
[M−H−H2O−CO2]−
867.9805
[M−H]−
880.9661
976.9688
1162.961
0
20
40
60
80
100
850
900
950
1000
1050
1100
1150

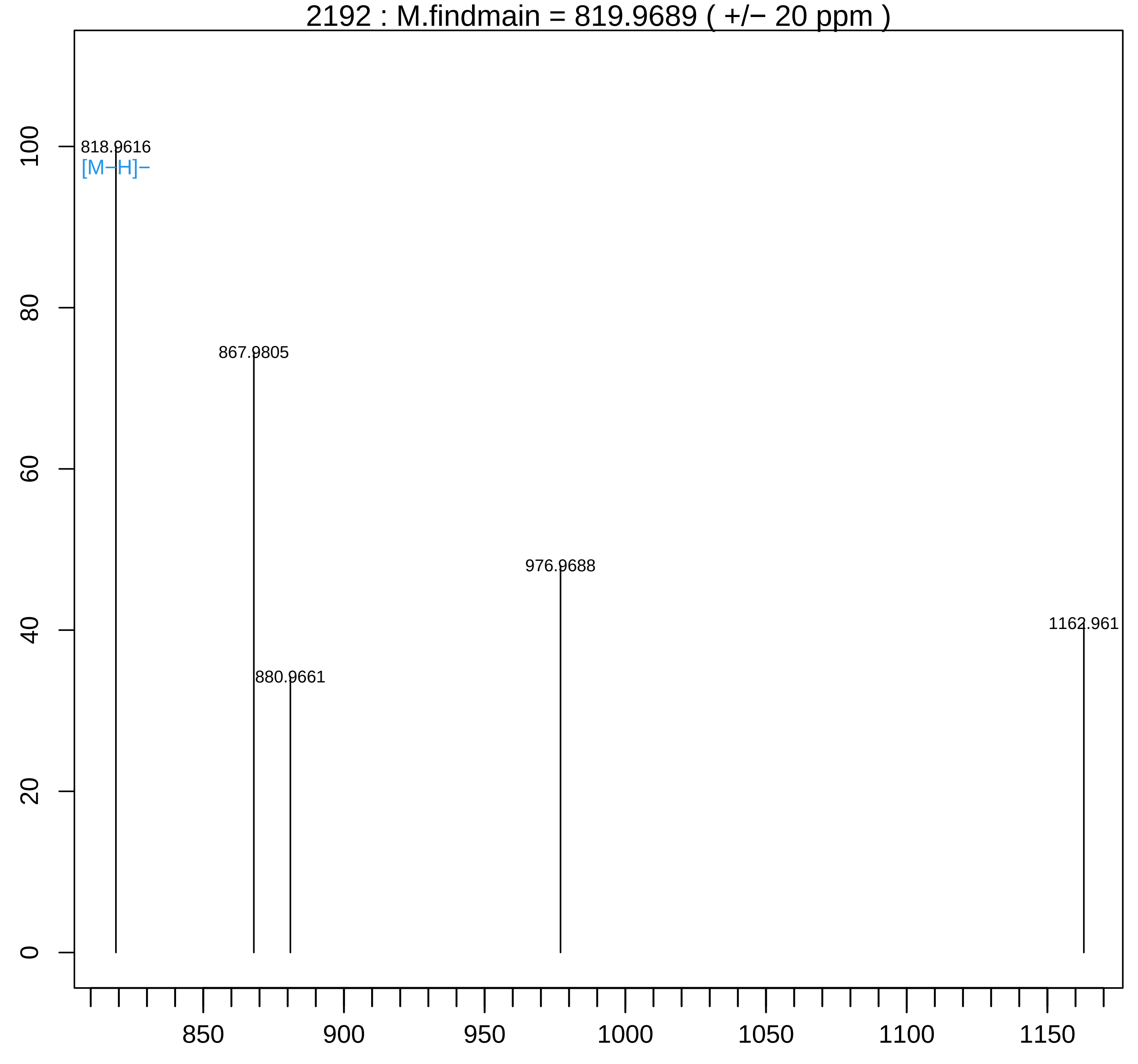
2192 : M.findmain = 819.9689 (+/− 20 ppm)
818.9616
[M−H]−
867.9805
880.9661
976.9688
1162.961
0
20
40
60
80
100
850
900
950
1000
1050
1100
1150

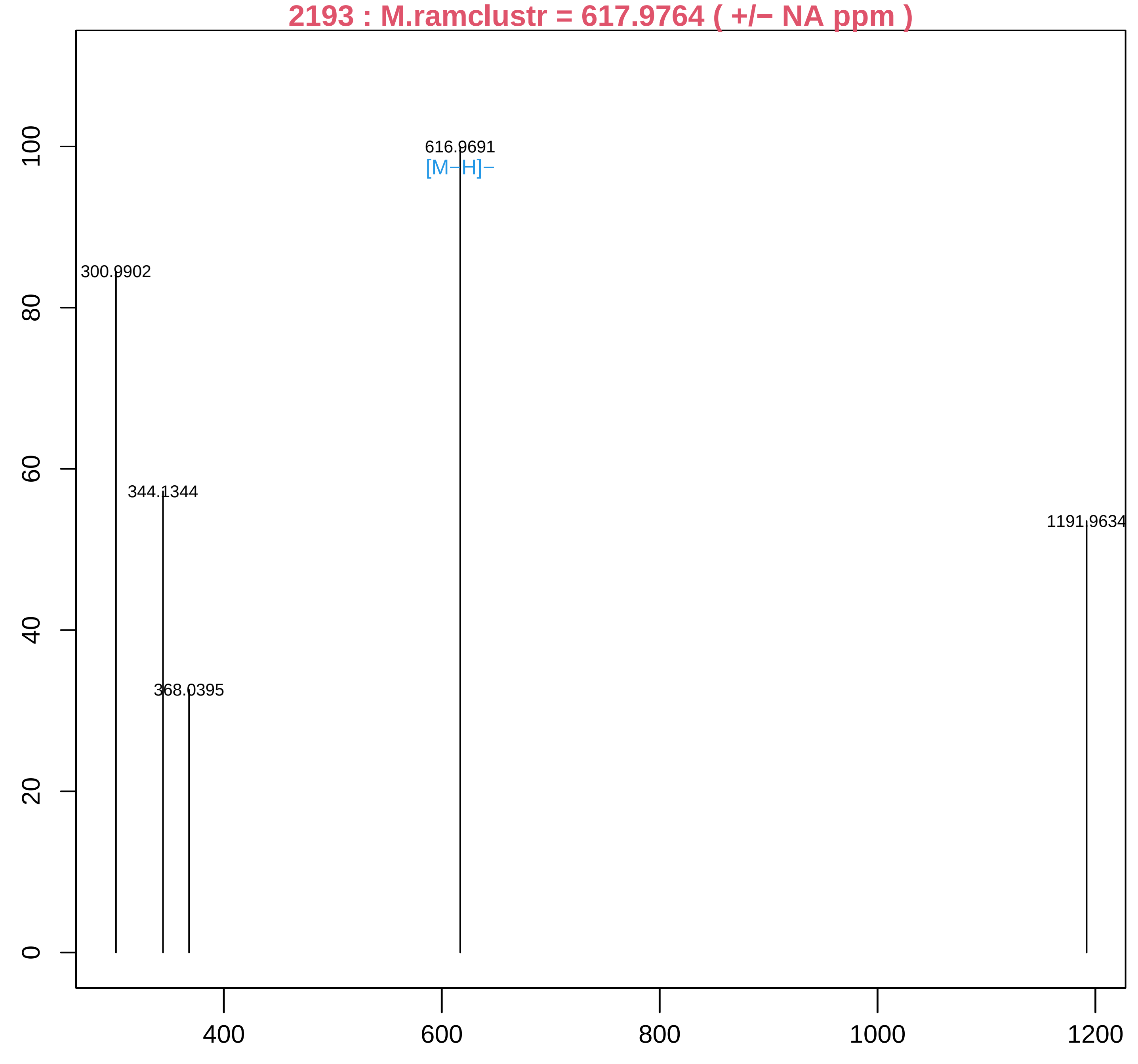
2193 : M.ramclustr = 617.9764 (+/− NA ppm)
616.9691
[M−H]−
300.9902
344.1344
368.0395
1191.9634
0
20
40
60
80
100
400
600
800
1000
1200

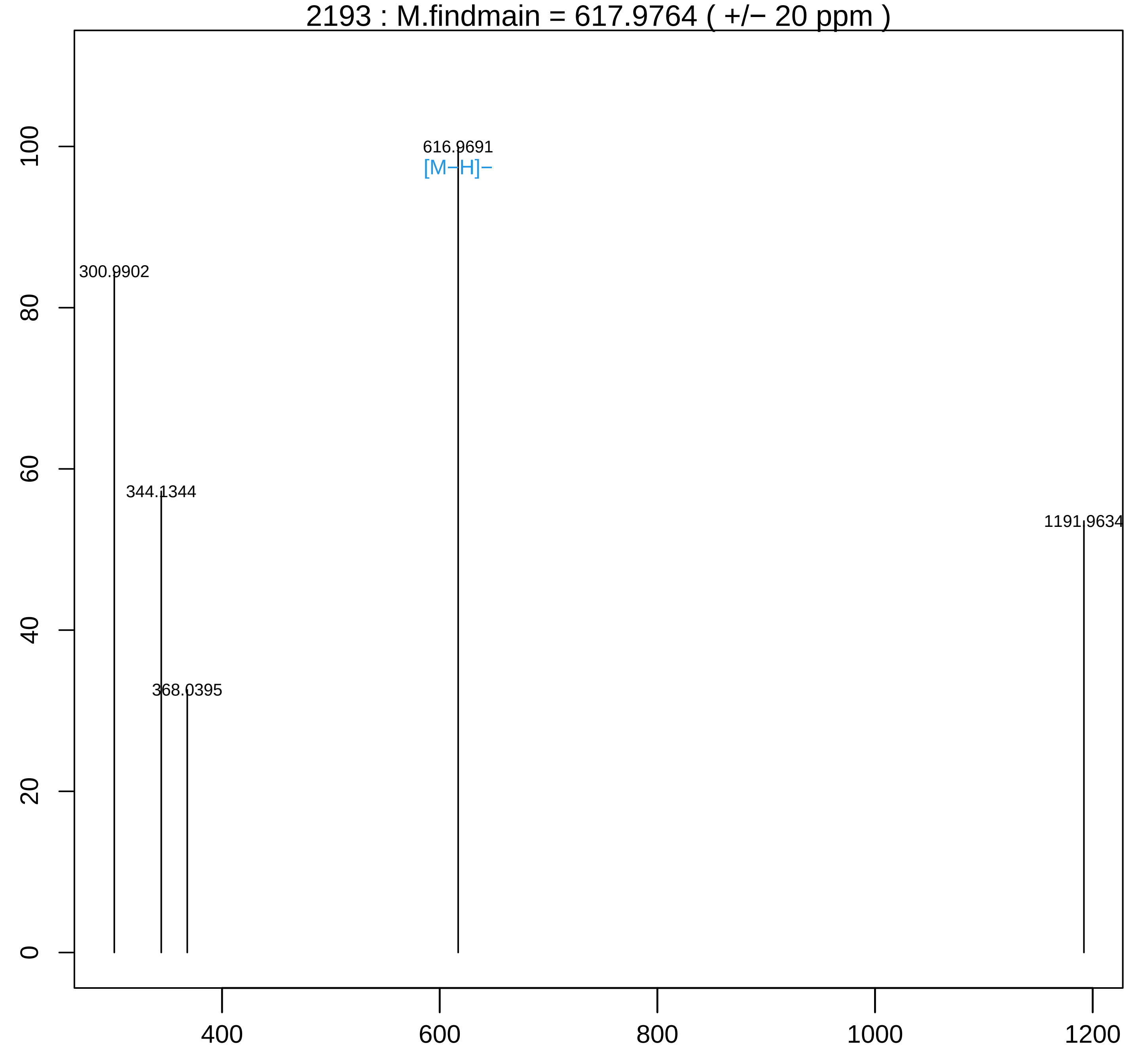
2193 : M.findmain = 617.9764 (+/− 20 ppm)
616.9691
[M−H]−
300.9902
344.1344
368.0395
1191.9634
0
20
40
60
80
100
400
600
800
1000
1200

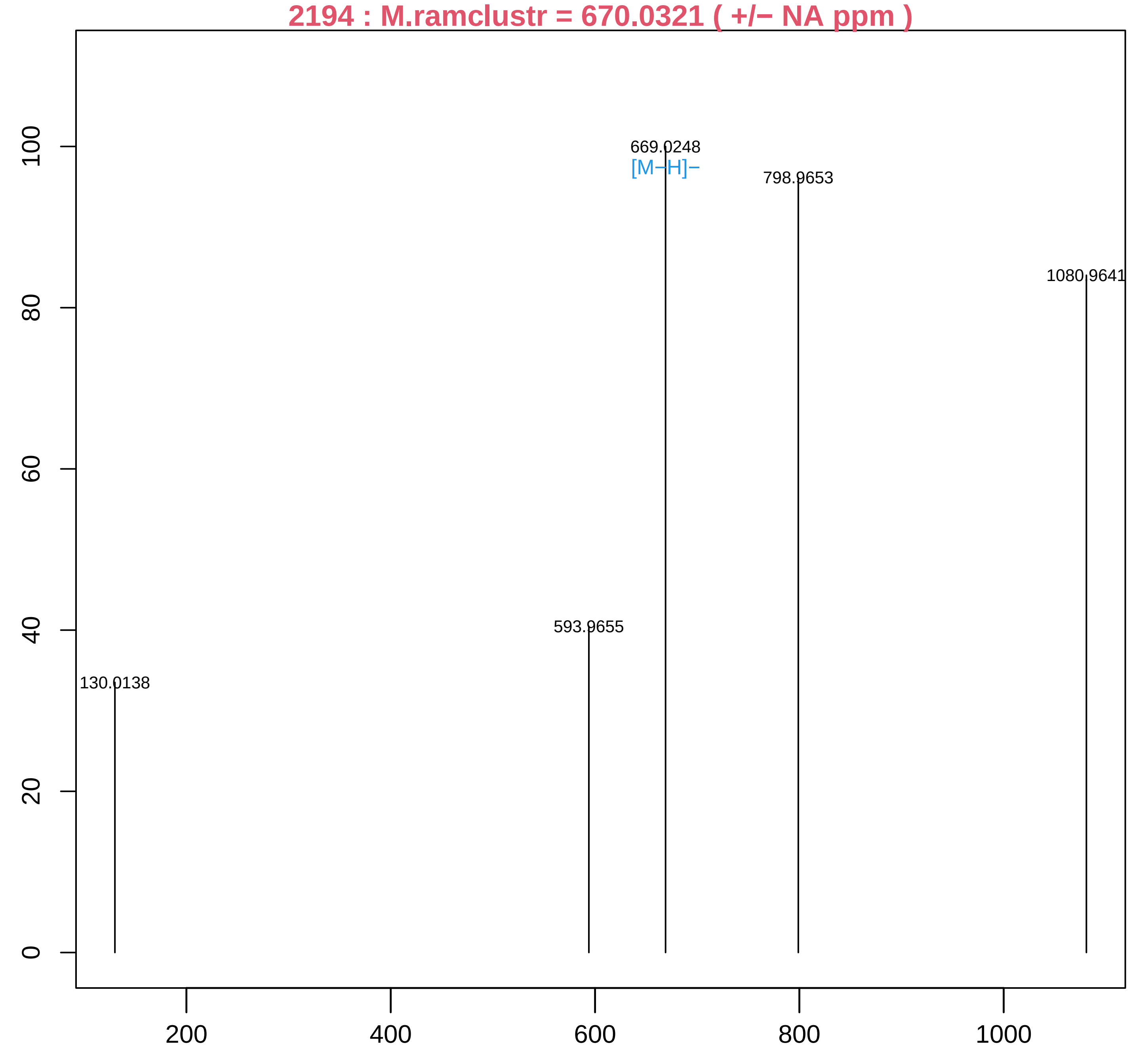
2194 : M.ramclustr = 670.0321 (+/− NA ppm)
669.0248
[M−H]−
798.9653
1080.9641
593.9655
130.0138
0
20
40
60
80
100
200
400
600
800
1000

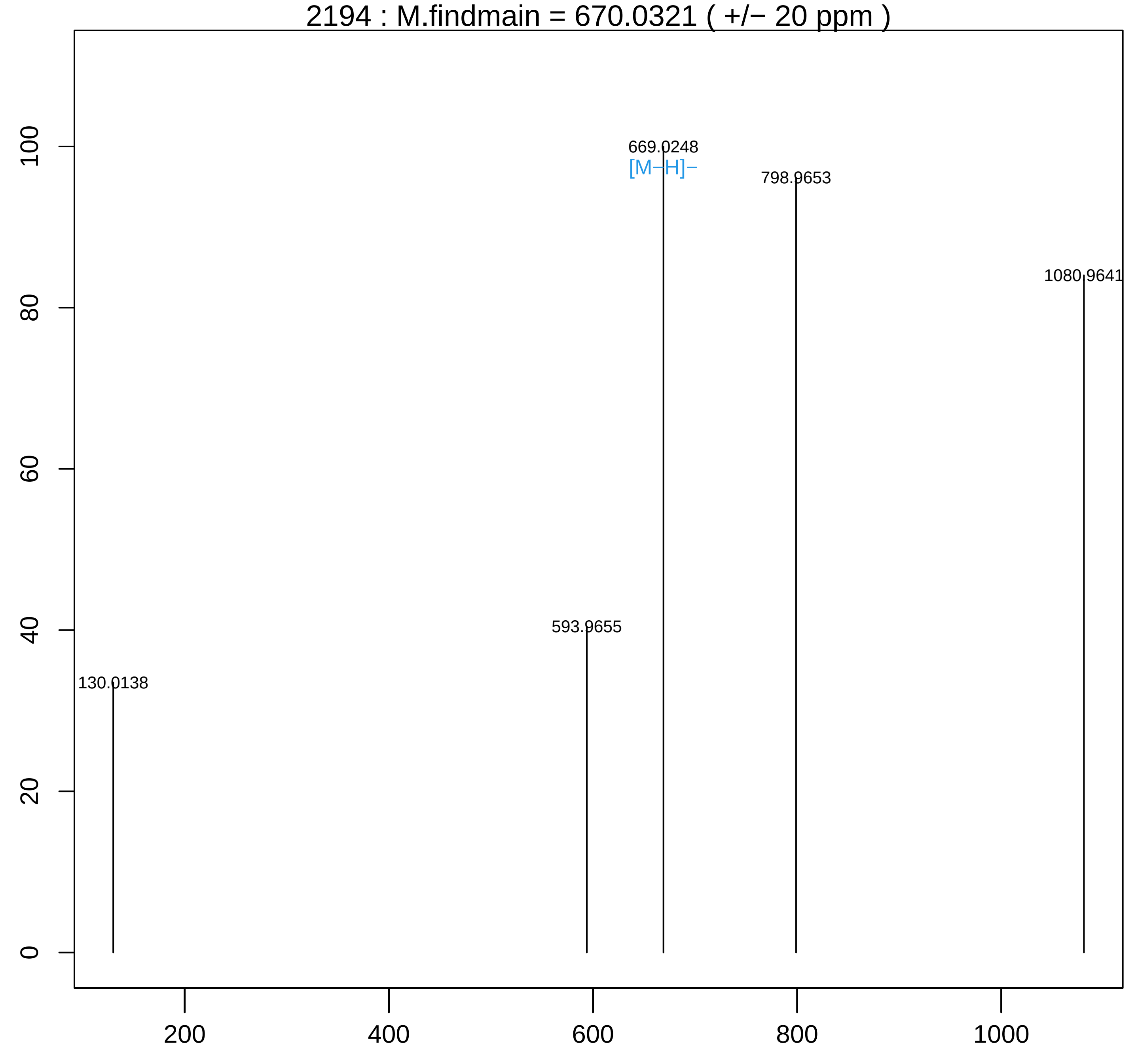
2194 : M.findmain = 670.0321 (+/− 20 ppm)
669.0248
[M−H]−
798.9653
1080.9641
593.9655
130.0138
0
20
40
60
80
100
200
400
600
800
1000

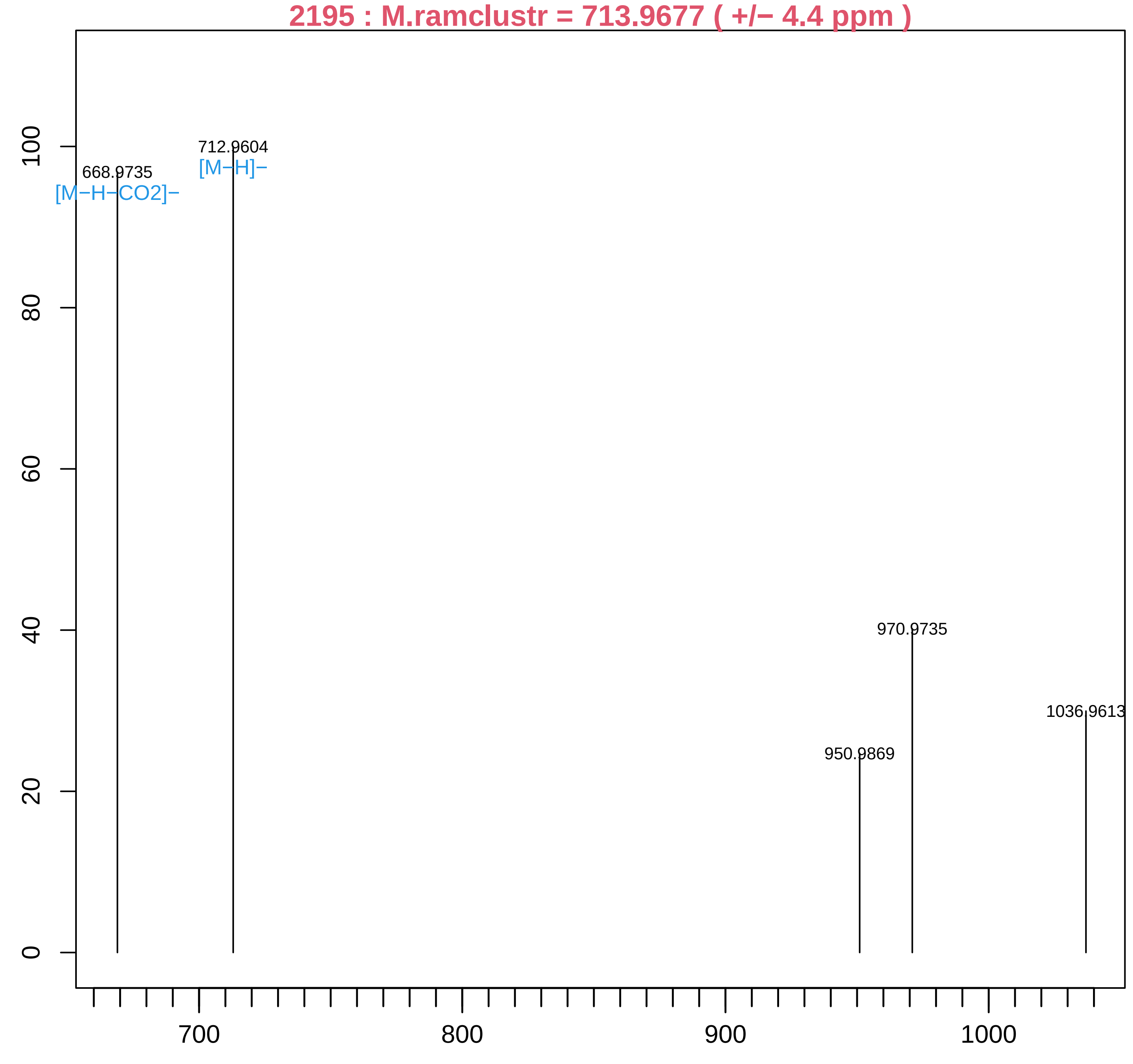
2195 : M.ramclustr = 713.9677 (+/− 4.4 ppm)
668.9735
[M−H−CO2]−
712.9604
[M−H]−
950.9869
970.9735
1036.9613
0
20
40
60
80
100
700
800
900
1000

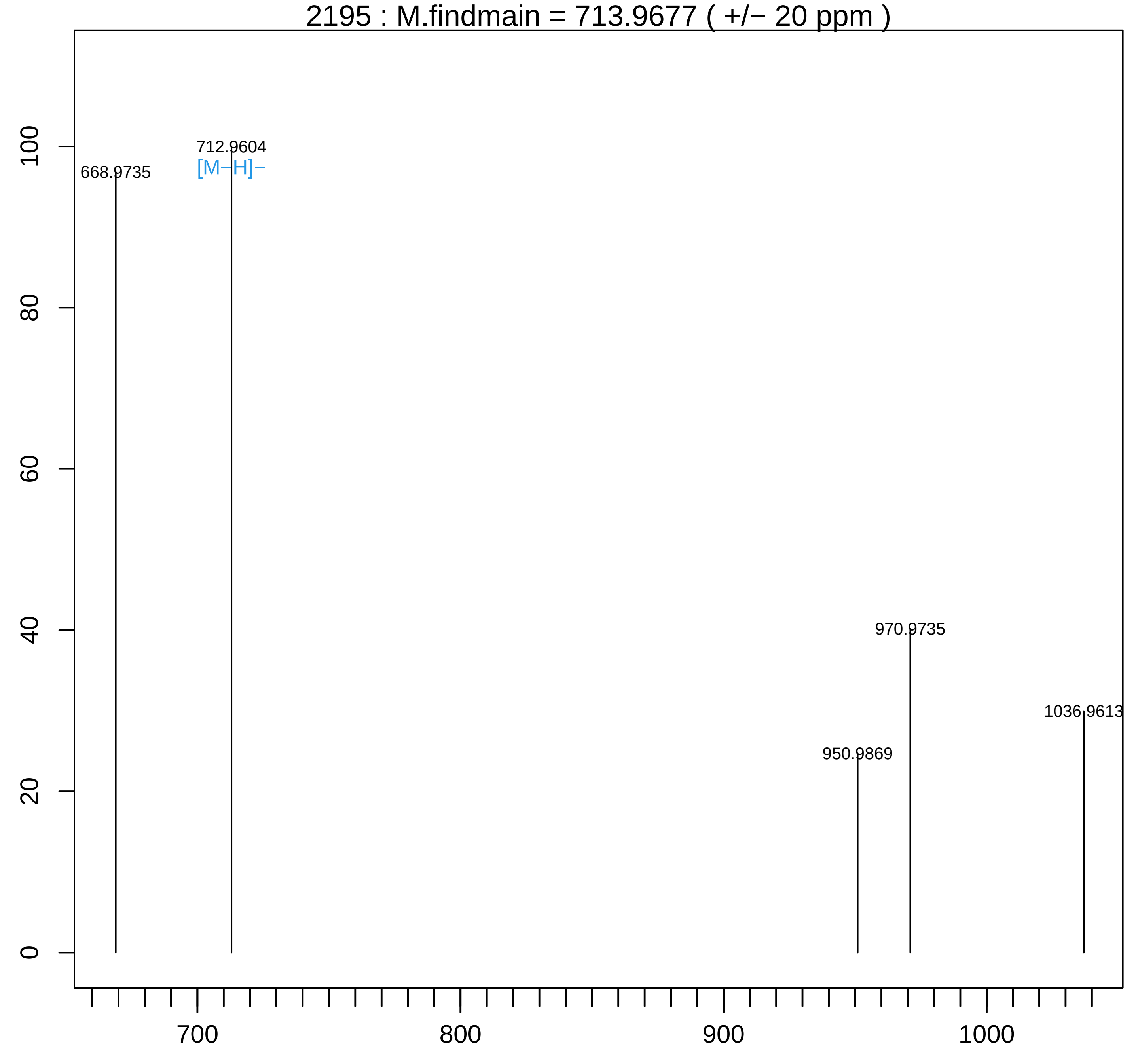
2195 : M.findmain = 713.9677 (+/− 20 ppm)
668.9735
712.9604
[M−H]−
950.9869
970.9735
1036.9613
0
20
40
60
80
100
700
800
900
1000

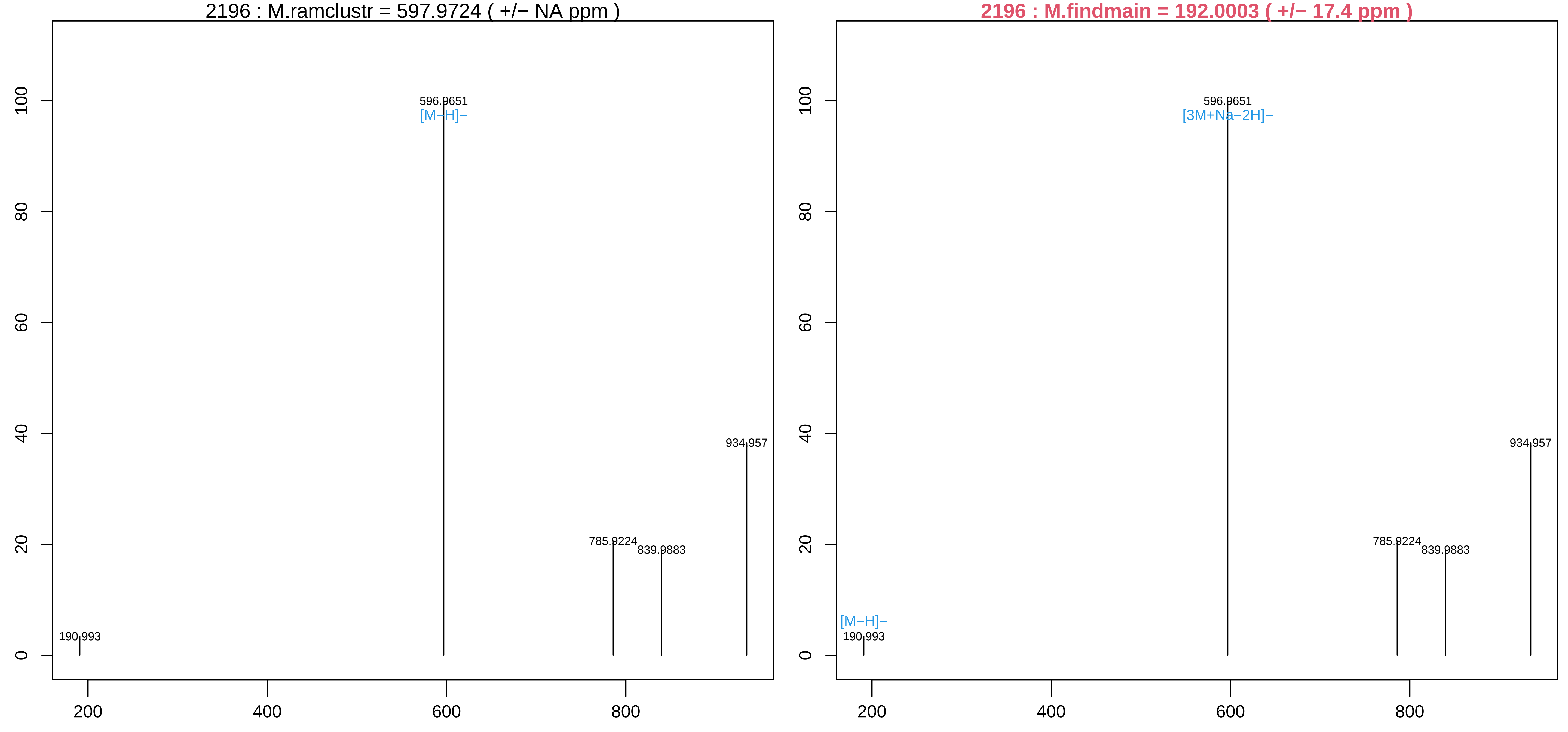
2196 : M.ramclustr = 597.9724 (+/− NA ppm)
596.9651
[M−H]−
934.957
785.9224
839.9883
190.993
2196 : M.findmain = 192.0003 (+/− 17.4 ppm)
596.9651
[3M+Na−2H]−
934.957
785.9224
839.9883
[M−H]−
190.993
0
20
40
60
80
100
200
400
600
800

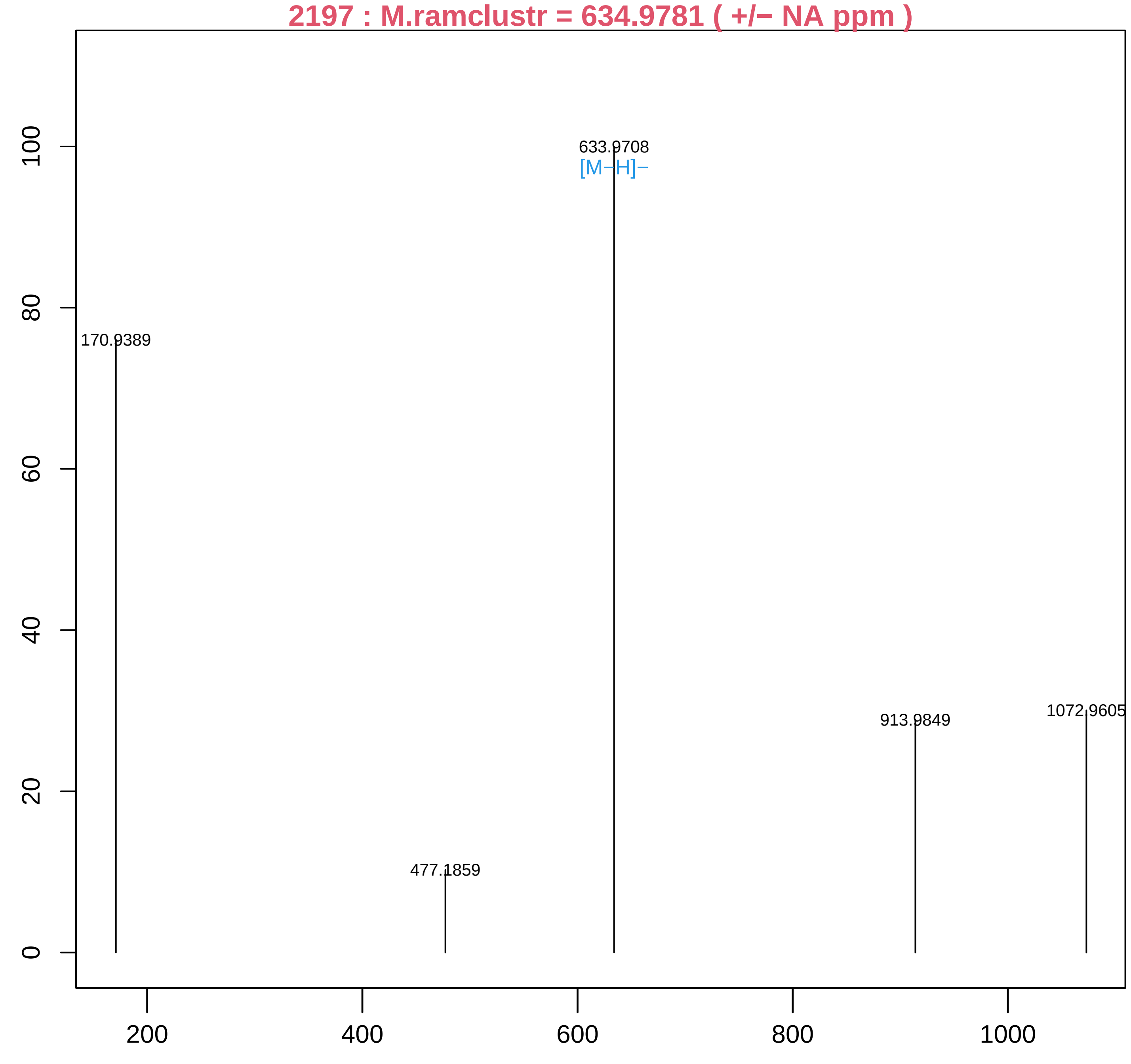
2197 : M.ramclustr = 634.9781 (+/− NA ppm)
170.9389
477.1859
633.9708
[M−H]−
913.9849
1072.9605
0
20
40
60
80
100
200
400
600
800
1000

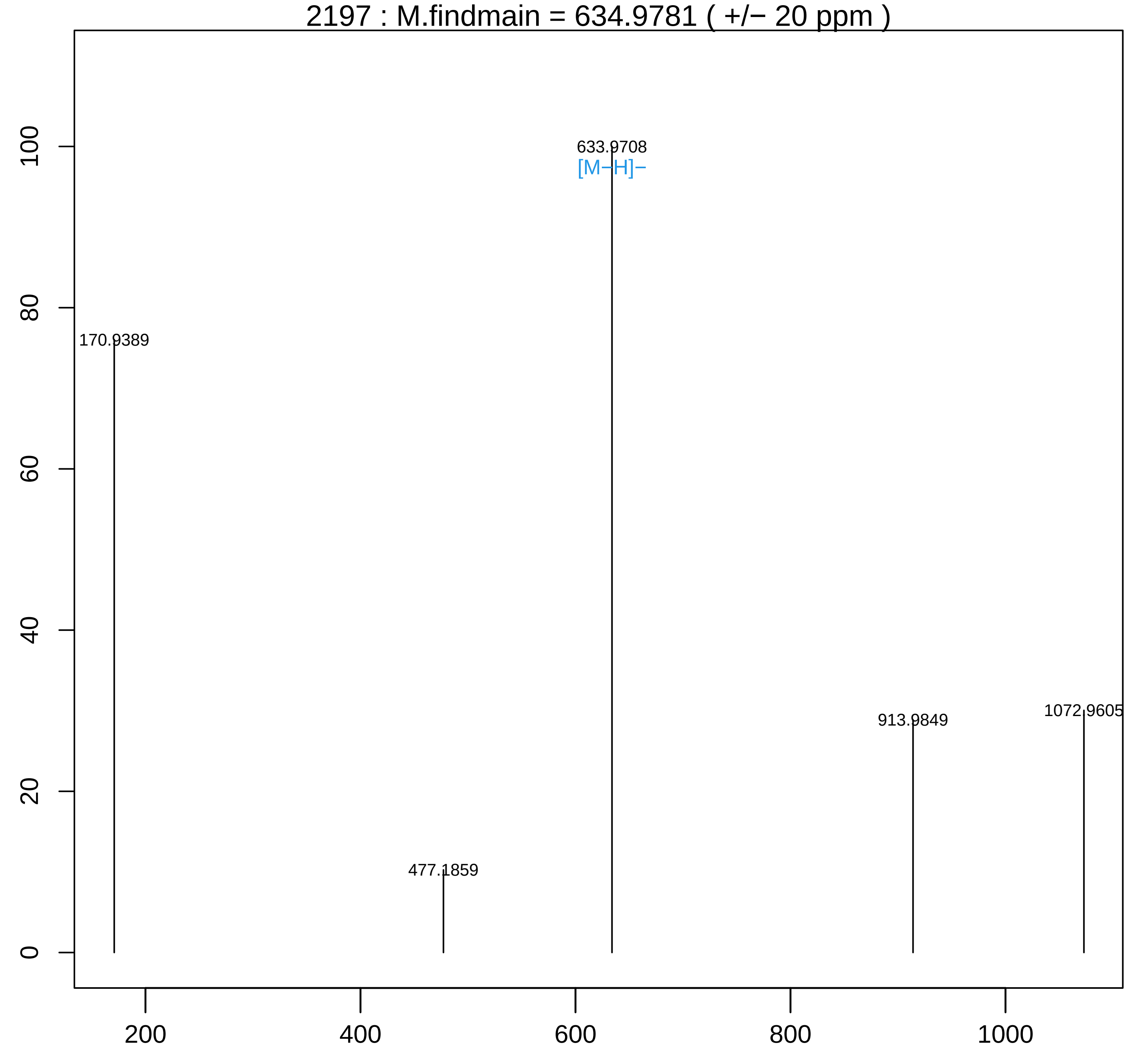
2197 : M.findmain = 634.9781 (+/− 20 ppm)
170.9389
477.1859
633.9708
[M−H]−
913.9849
1072.9605
0
20
40
60
80
100
200
400
600
800
1000

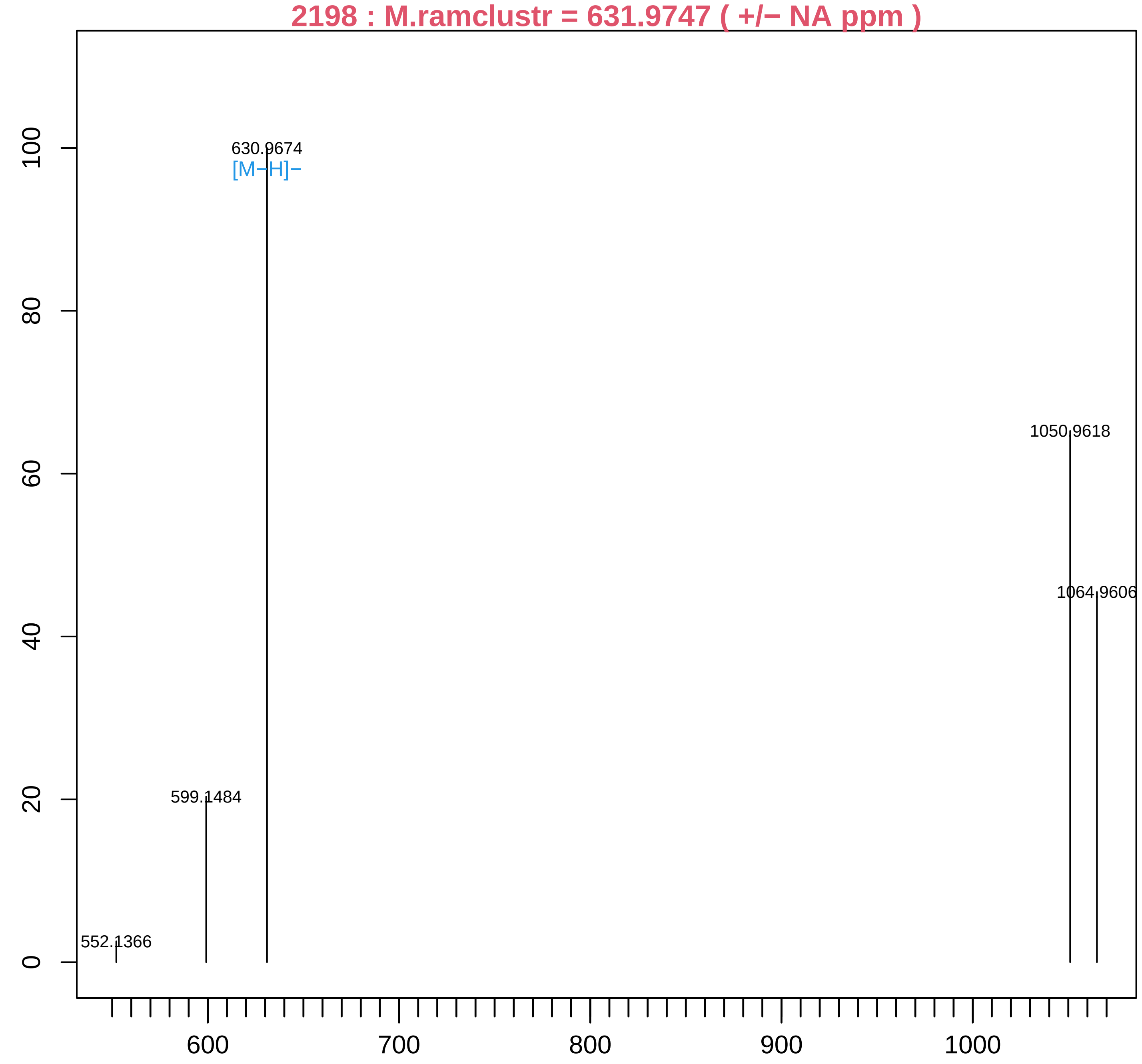

2198 : M.ramclustr = 631.9747 (+/− NA ppm)
630.9674
[M−H]−
1050.9618
1064.9606
599.1484
552.1366
0
20
40
60
80
100
600
700
800
900
1000

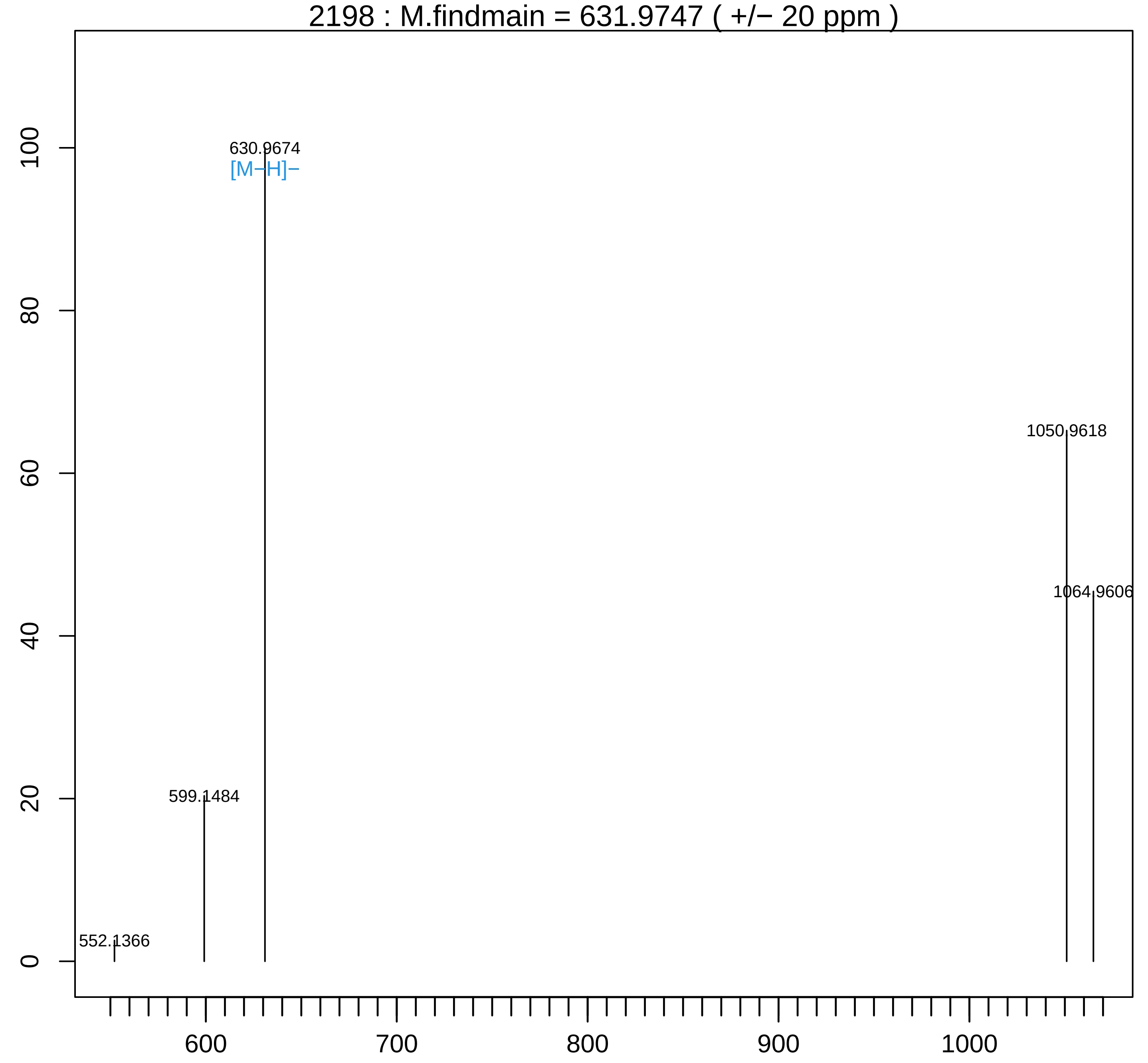

2198 : M.findmain = 631.9747 (+/− 20 ppm)
630.9674
[M−H]−
1050.9618
1064.9606
599.1484
552.1366
0
20
40
60
80
100
600
700
800
900
1000

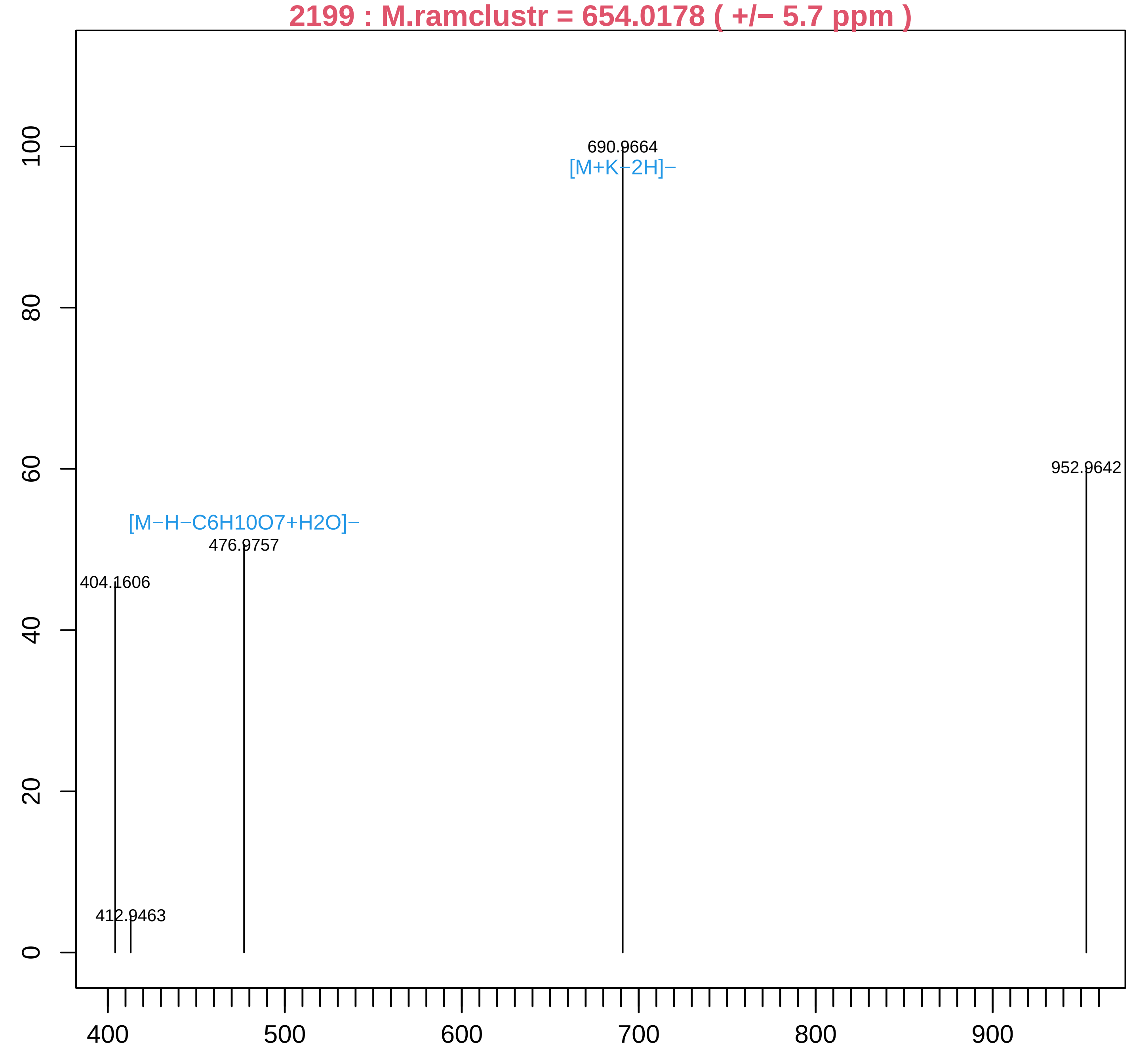
2199 : M.ramclustr = 654.0178 (+/− 5.7 ppm)
690.9664
[M+K−2H]−
952.9642
[M−H−C6H10O7+H2O]−
476.9757
404.1606
412.9463
0
20
40
60
80
100
400
500
600
700
800
900

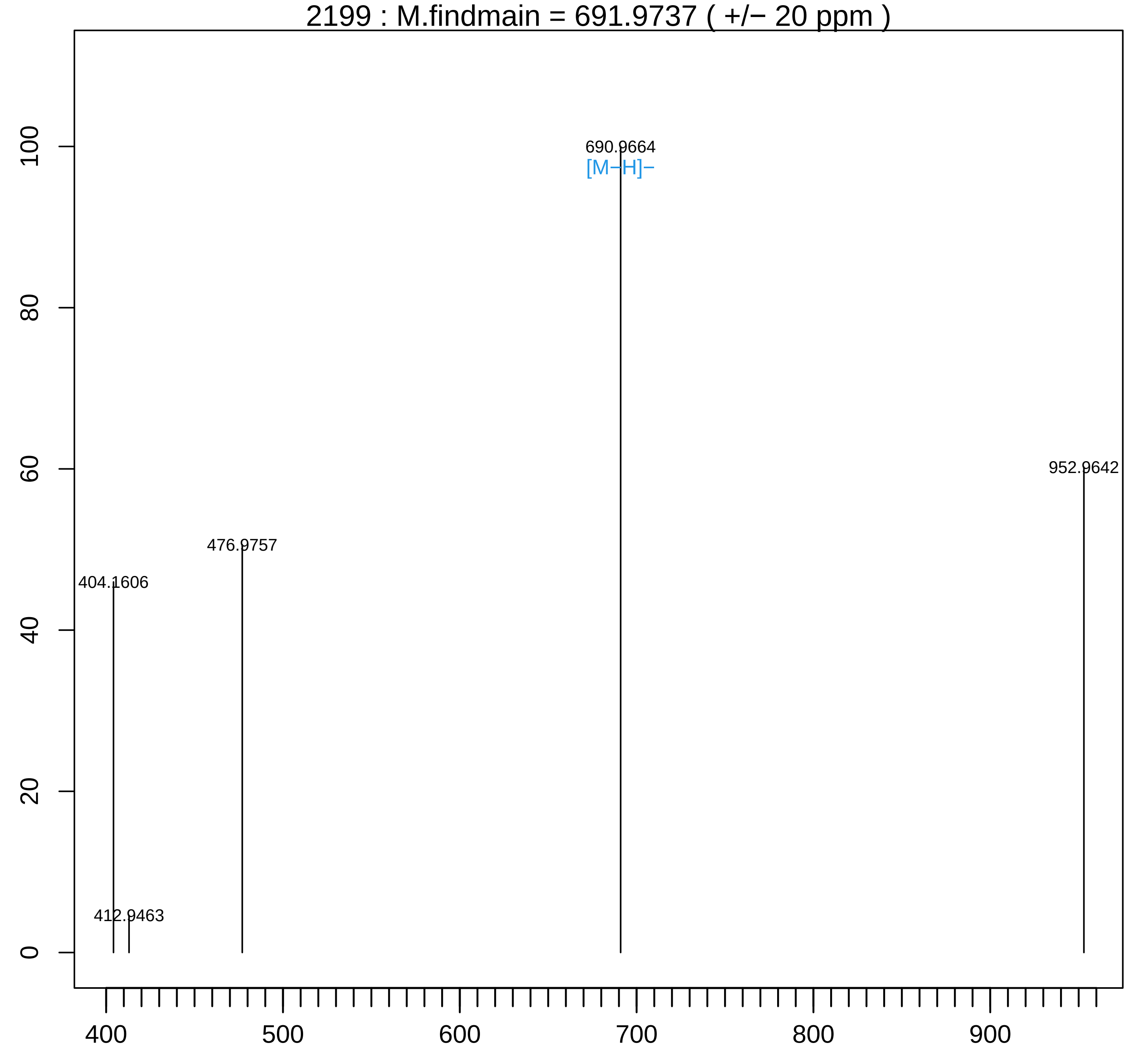
2199 : M.findmain = 691.9737 (+/− 20 ppm)
690.9664
[M−H]−
952.9642
476.9757
404.1606
412.9463
0
20
40
60
80
100
400
500
600
700
800
900

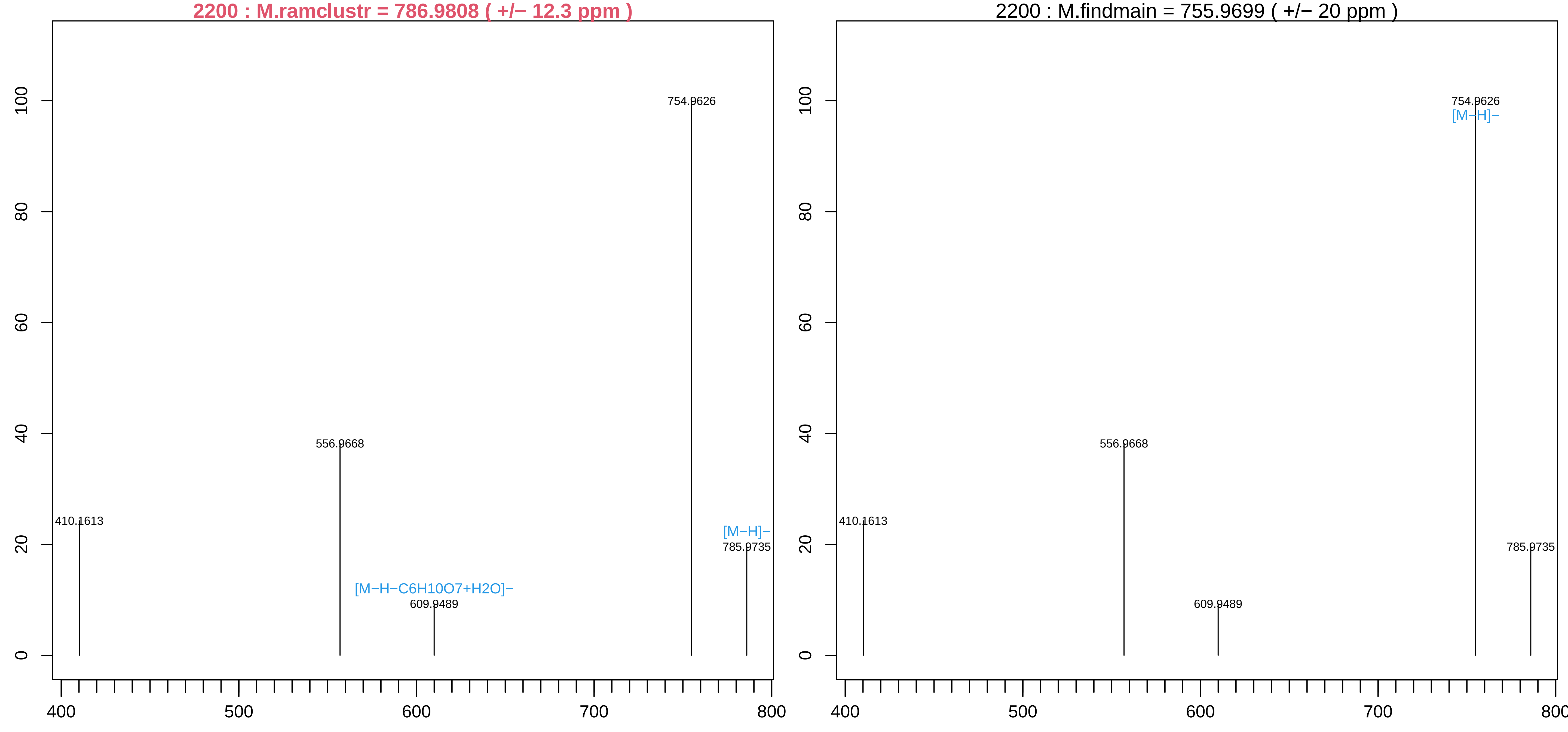
2200 : M.ramclustr = 786.9808 (+/− 12.3 ppm)
754.9626
556.9668
410.1613
[M−H−C6H10O7+H2O]−
609.9489
[M−H]−
785.9735
2200 : M.findmain = 755.9699 (+/− 20 ppm)
754.9626
[M−H]−
556.9668
410.1613
609.9489
785.9735
0
20
40
60
80
100
400
500
600
700
800

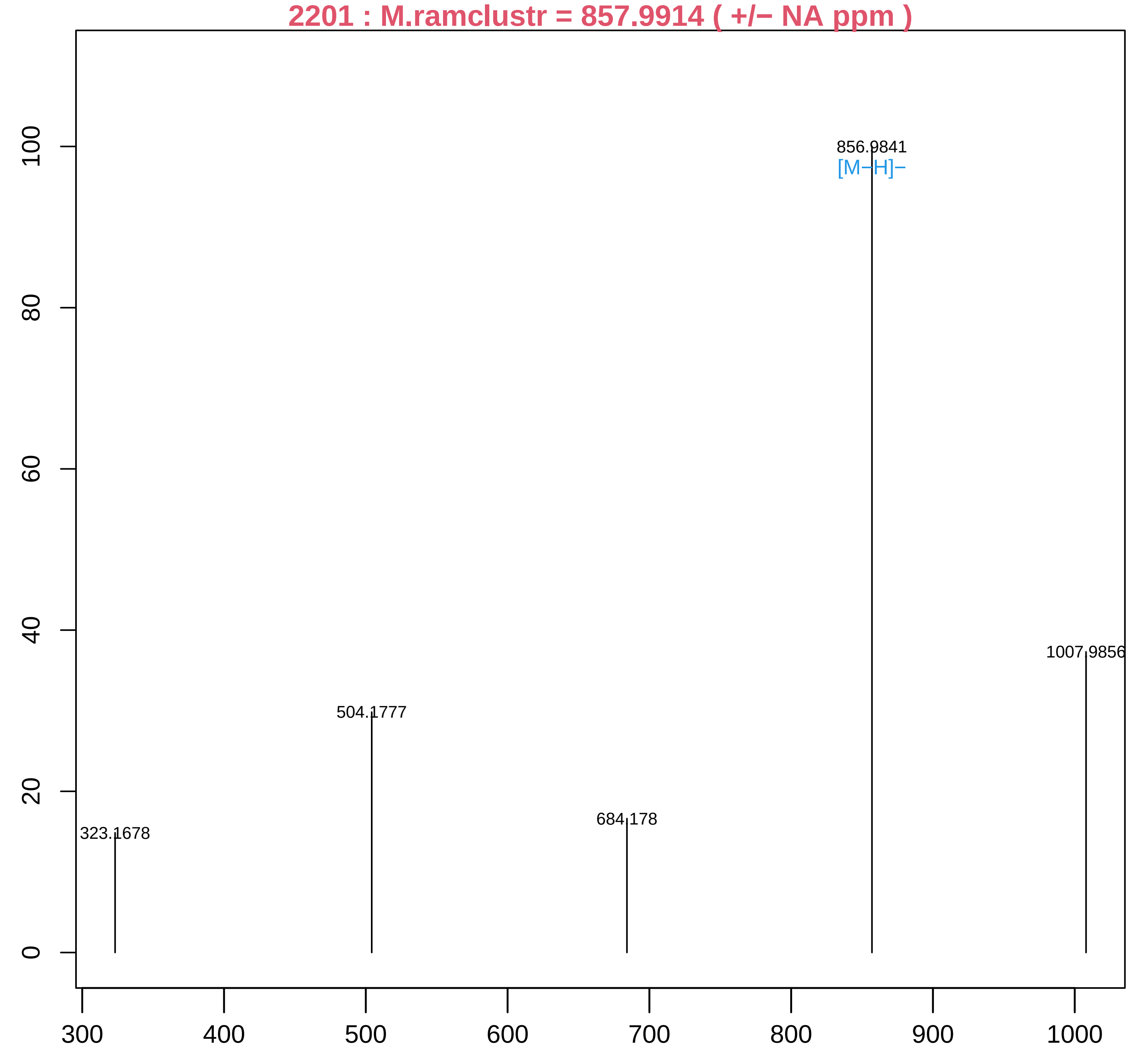
2201 : M.ramclustr = 857.9914 (+/− NA ppm)
856.9841
[M−H]−
1007.9856
504.1777
684.178
323.1678
0
20
40
60
80
100
300
400
500
600
700
800
900
1000

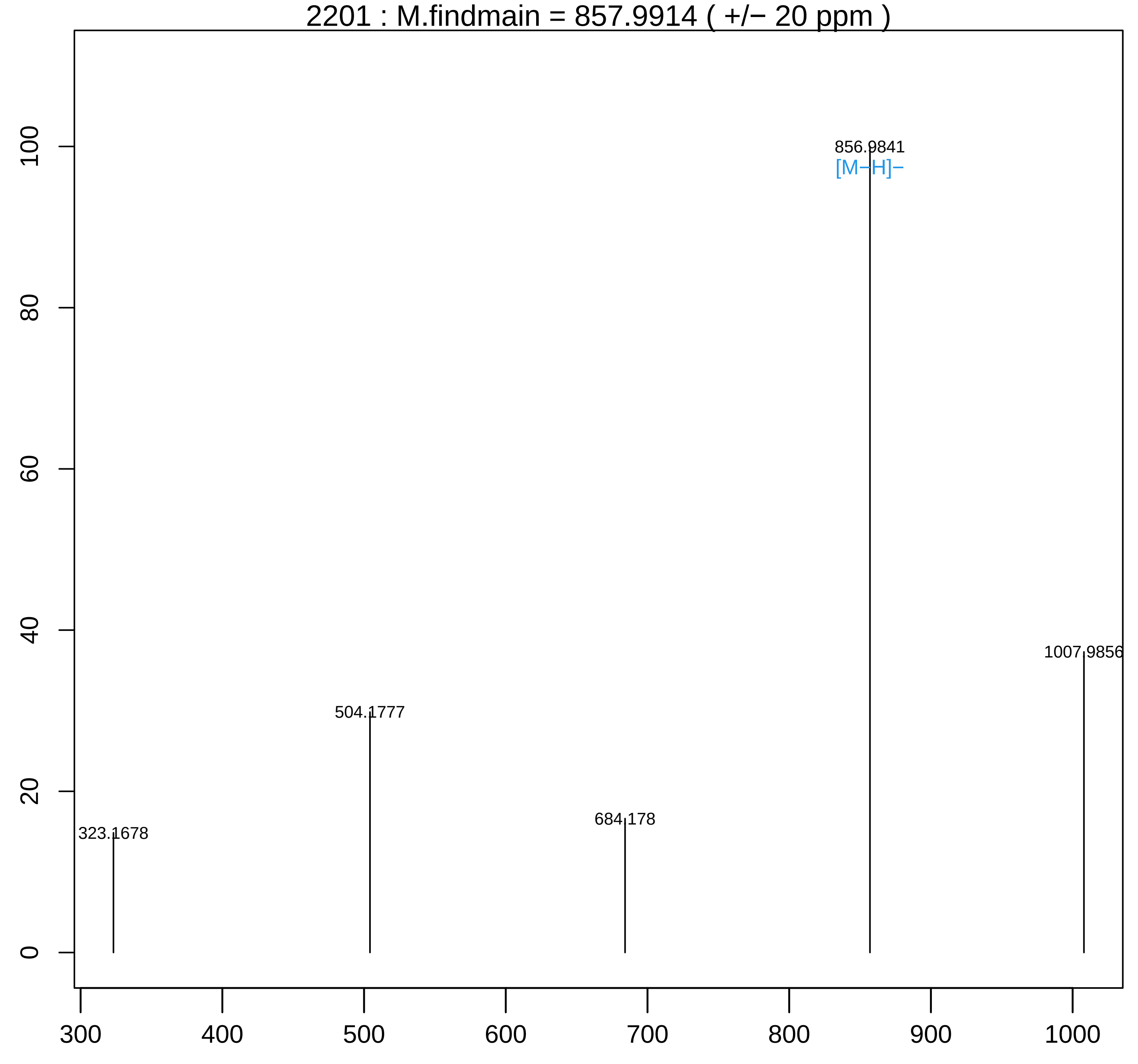
2201 : M.findmain = 857.9914 (+/− 20 ppm)
856.9841
[M−H]−
1007.9856
504.1777
684.178
323.1678
0
20
40
60
80
100
300
400
500
600
700
800
900
1000

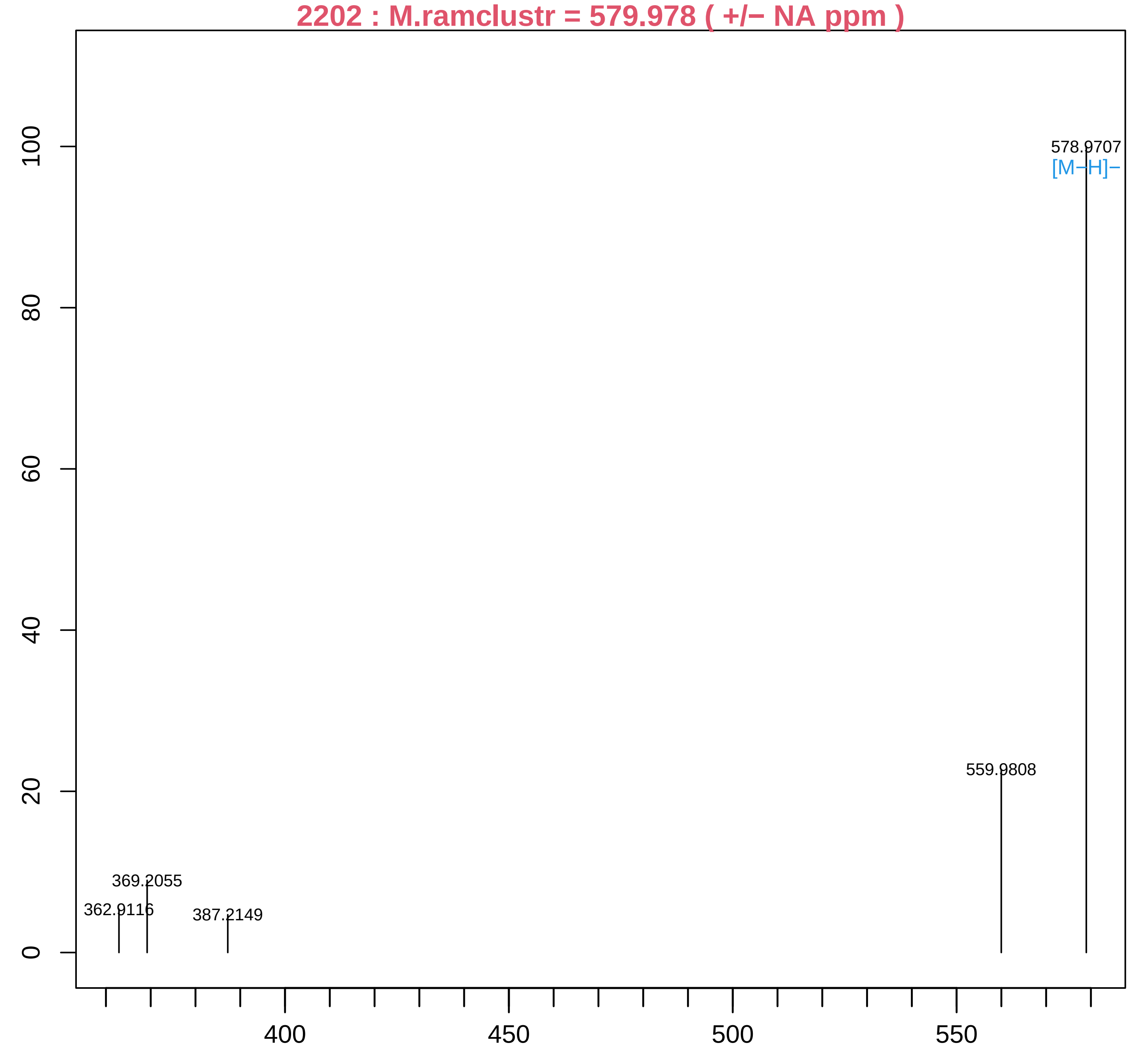
2202 : M.ramclustr = 579.978 (+/− NA ppm)
578.9707
[M−H]−
559.9808
369.2055
362.9116
387.2149
0
20
40
60
80
100
400
450
500
550

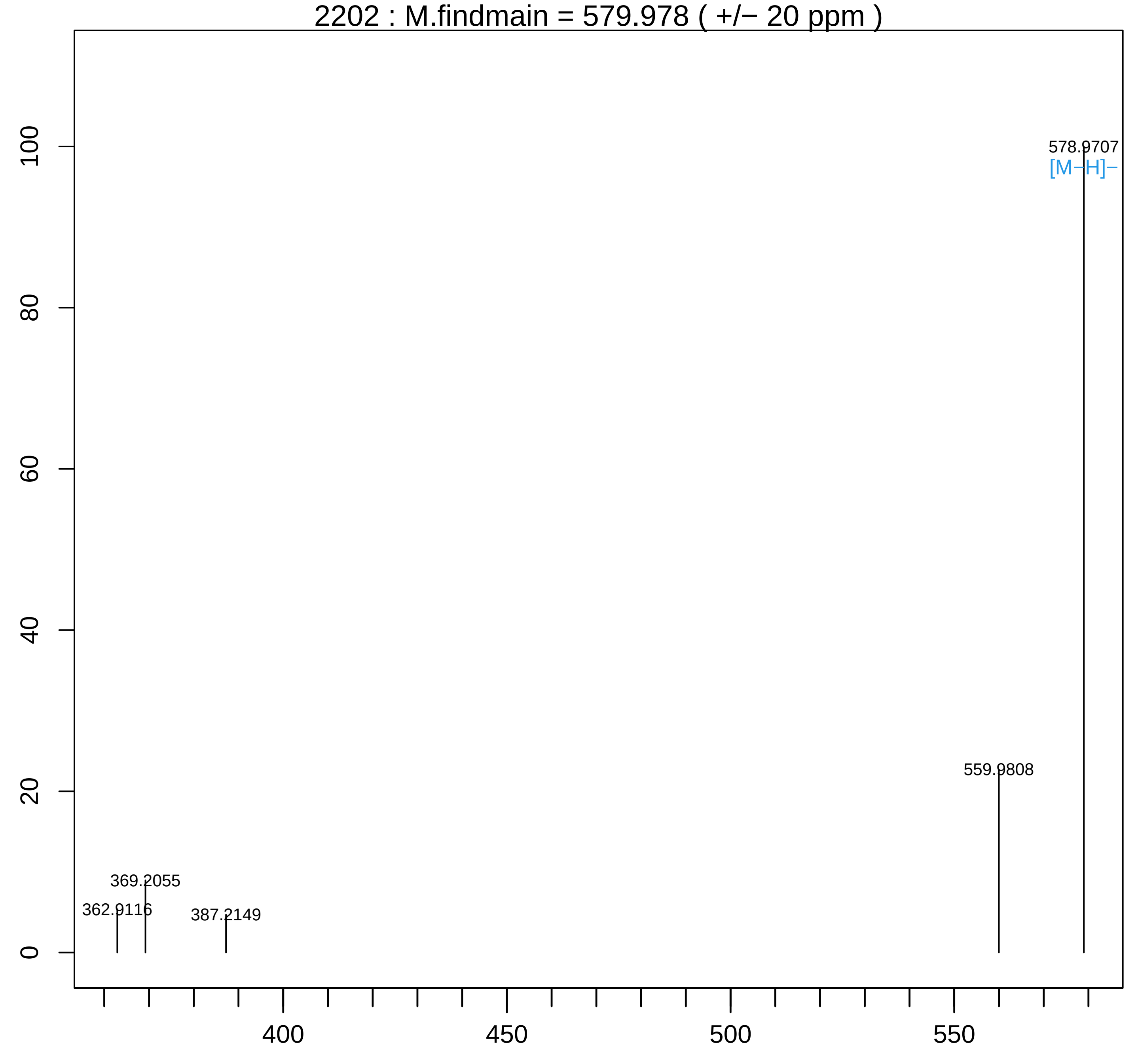
2202 : M.findmain = 579.978 (+/− 20 ppm)
578.9707
[M−H]−
559.9808
369.2055
362.9116
387.2149
0
20
40
60
80
100
400
450
500
550

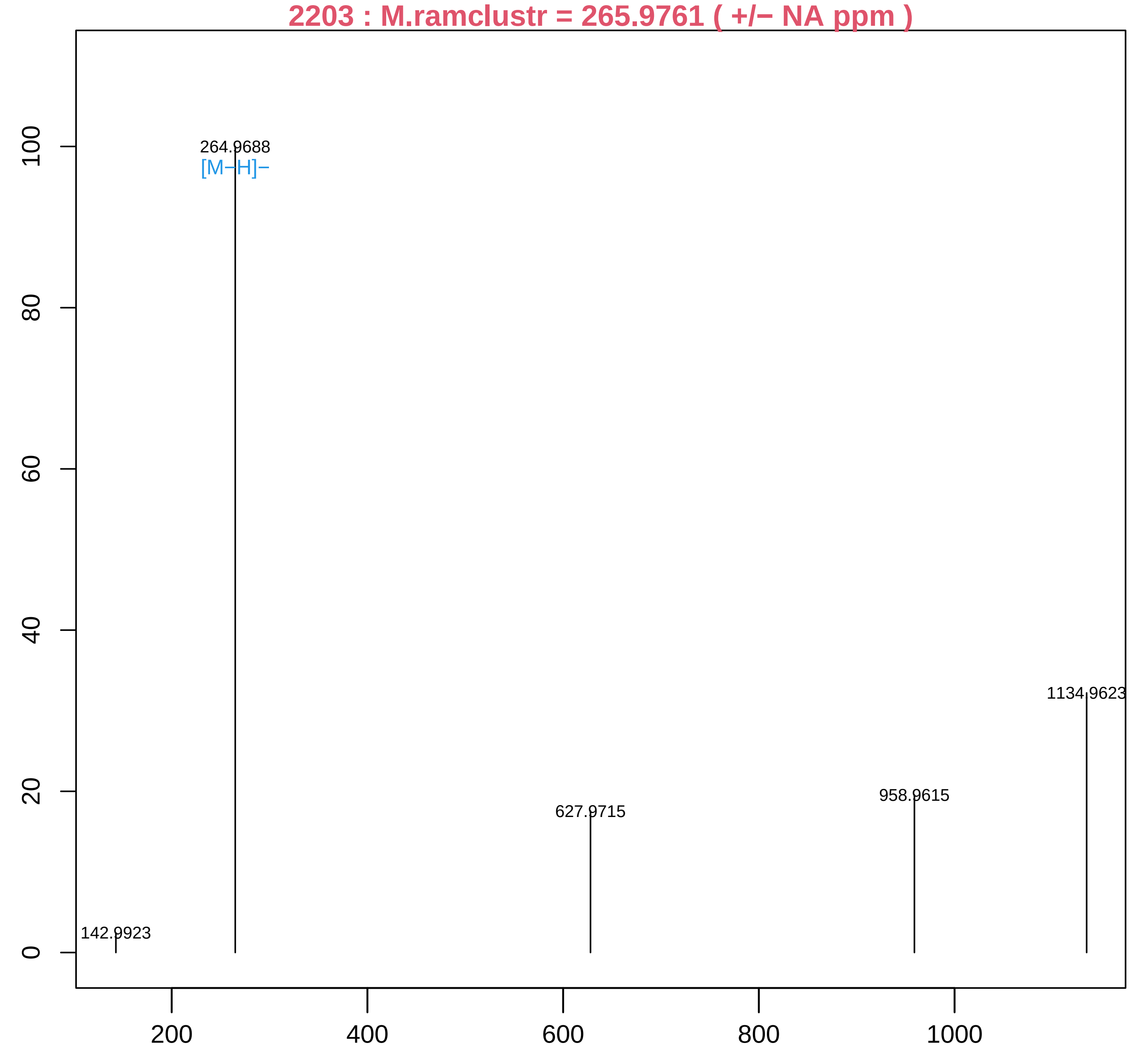

2203 : M.ramclustr = 265.9761 (+/− NA ppm)
264.9688
[M−H]−
142.9923
627.9715
958.9615
1134.9623
0
20
40
60
80
100
200
400
600
800
1000

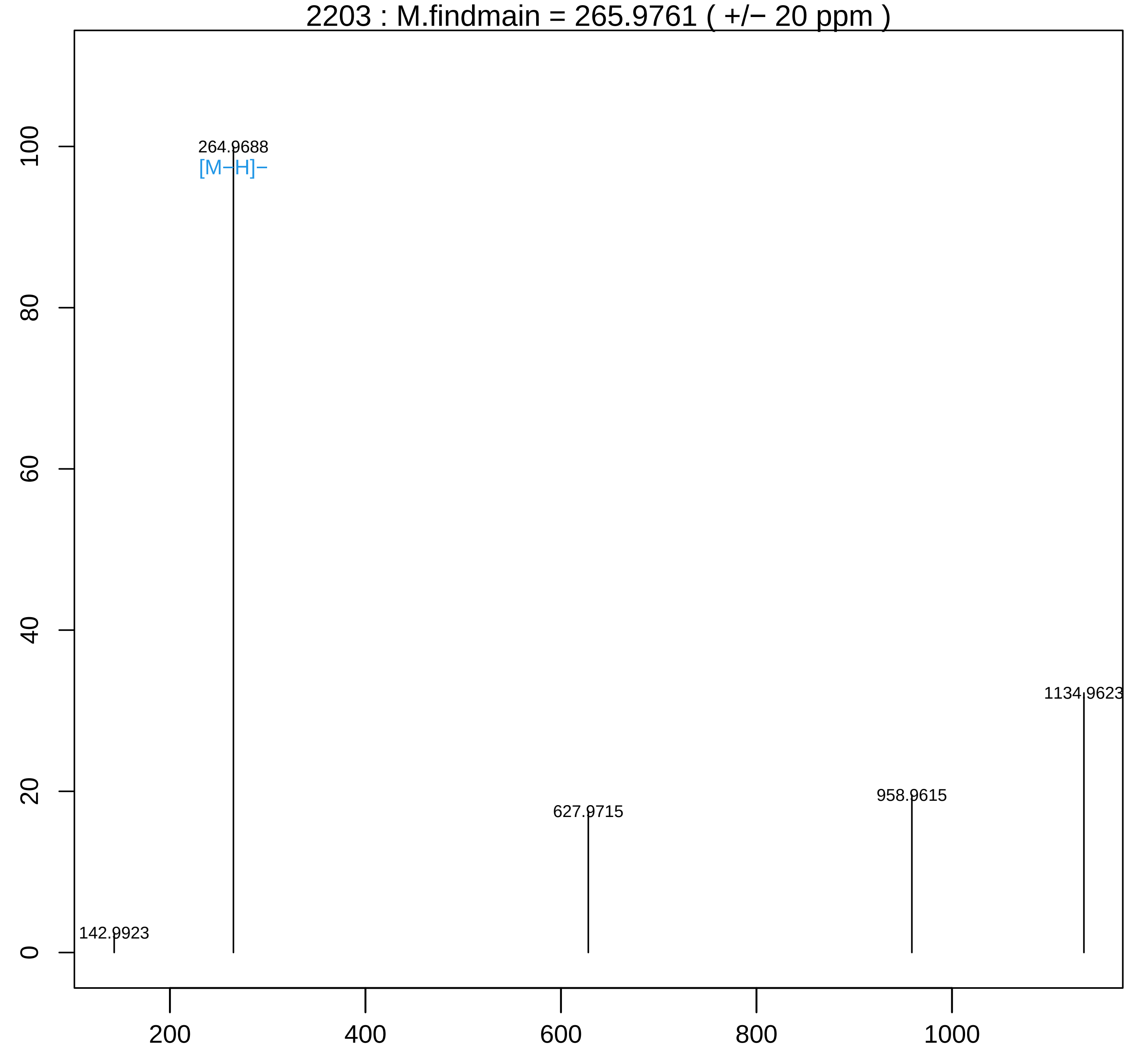

2203 : M.findmain = 265.9761 (+/− 20 ppm)
264.9688
[M−H]−
142.9923
627.9715
958.9615
1134.9623
0
20
40
60
80
100
200
400
600
800
1000

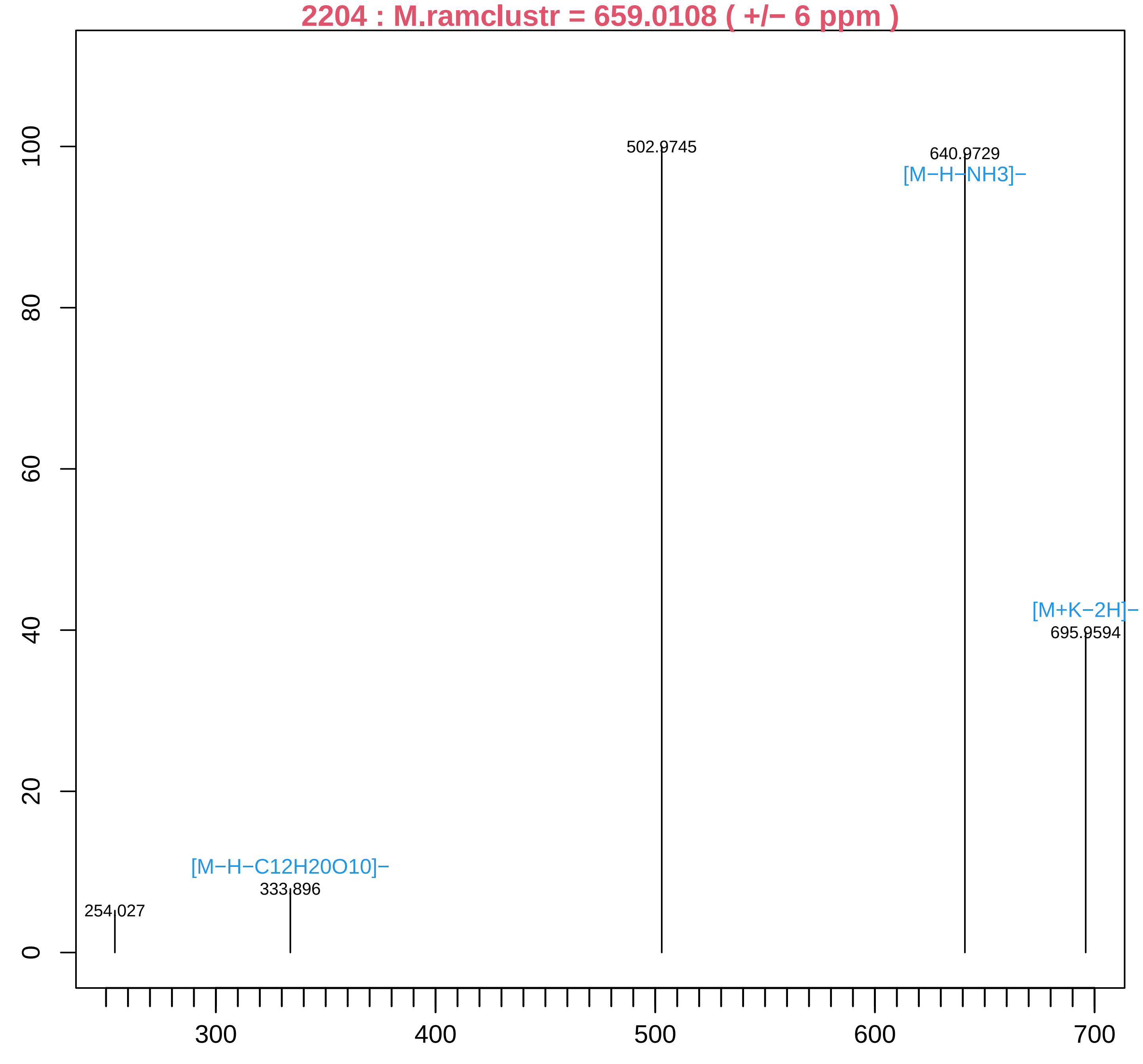
2204 : M.ramclustr = 659.0108 (+/− 6 ppm)
502.9745
640.9729
[M−H−NH3]−
[M+K−2H]−
695.9594
[M−H−C12H20O10]−
333.896
254.027
0
20
40
60
80
100
300
400
500
600
700

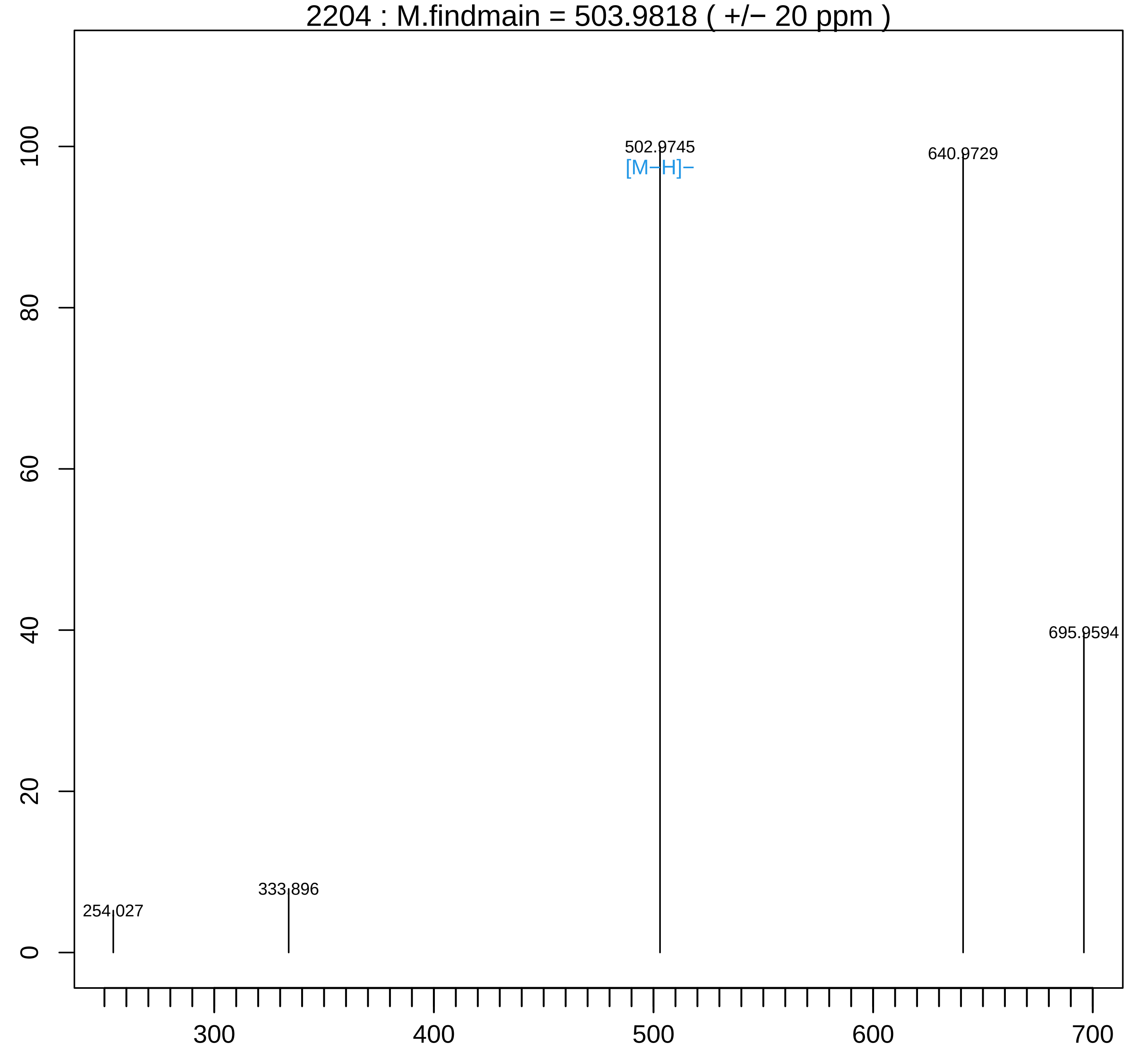
2204 : M.findmain = 503.9818 (+/− 20 ppm)
502.9745
[M−H]−
640.9729
695.9594
333.896
254.027
0
20
40
60
80
100
300
400
500
600
700

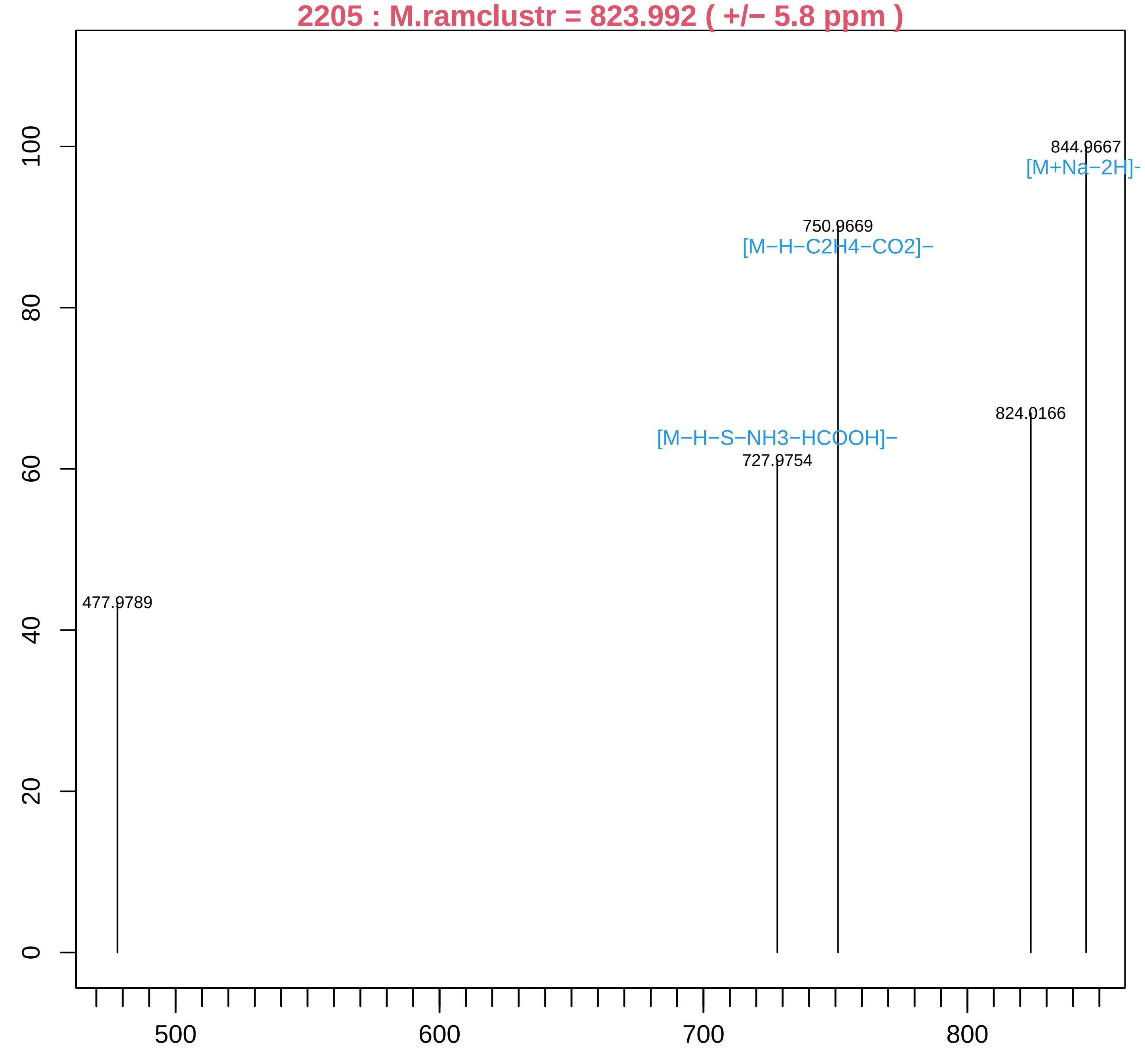

2205 : M.ramclustr = 823.992 (+/− 5.8 ppm)
844.9667
[M+Na−2H]-
750.9669
[M−H−C2H4−CO2]−
824.0166
[M−H−S−NH3−HCOOH]−
727.9754
477.9789
0
20
40
60
80
100
500
600
700
800

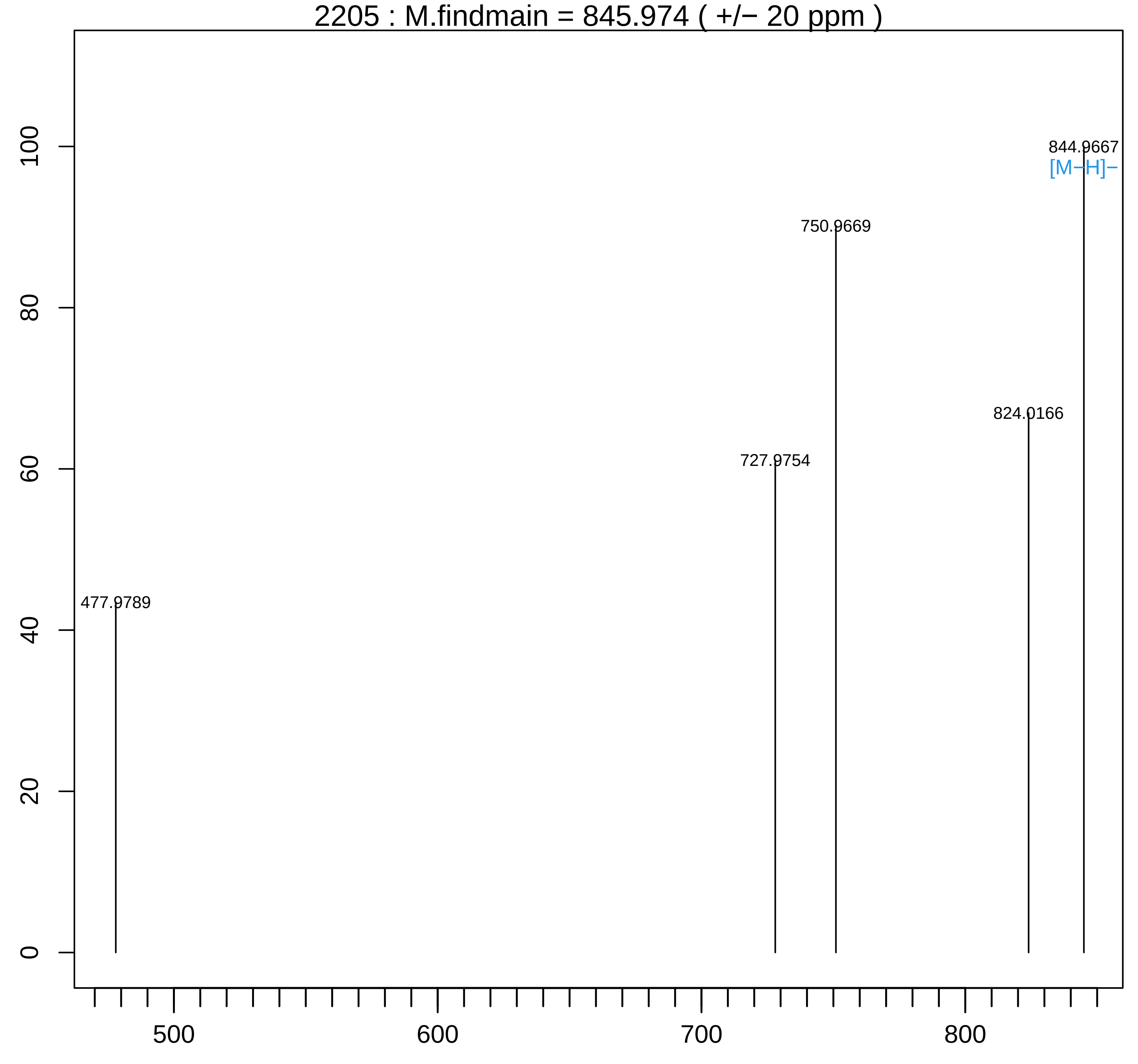

2205 : M.findmain = 845.974 (+/− 20 ppm)
844.9667
[M−H]−
750.9669
824.0166
727.9754
477.9789
0
20
40
60
80
100
500
600
700
800

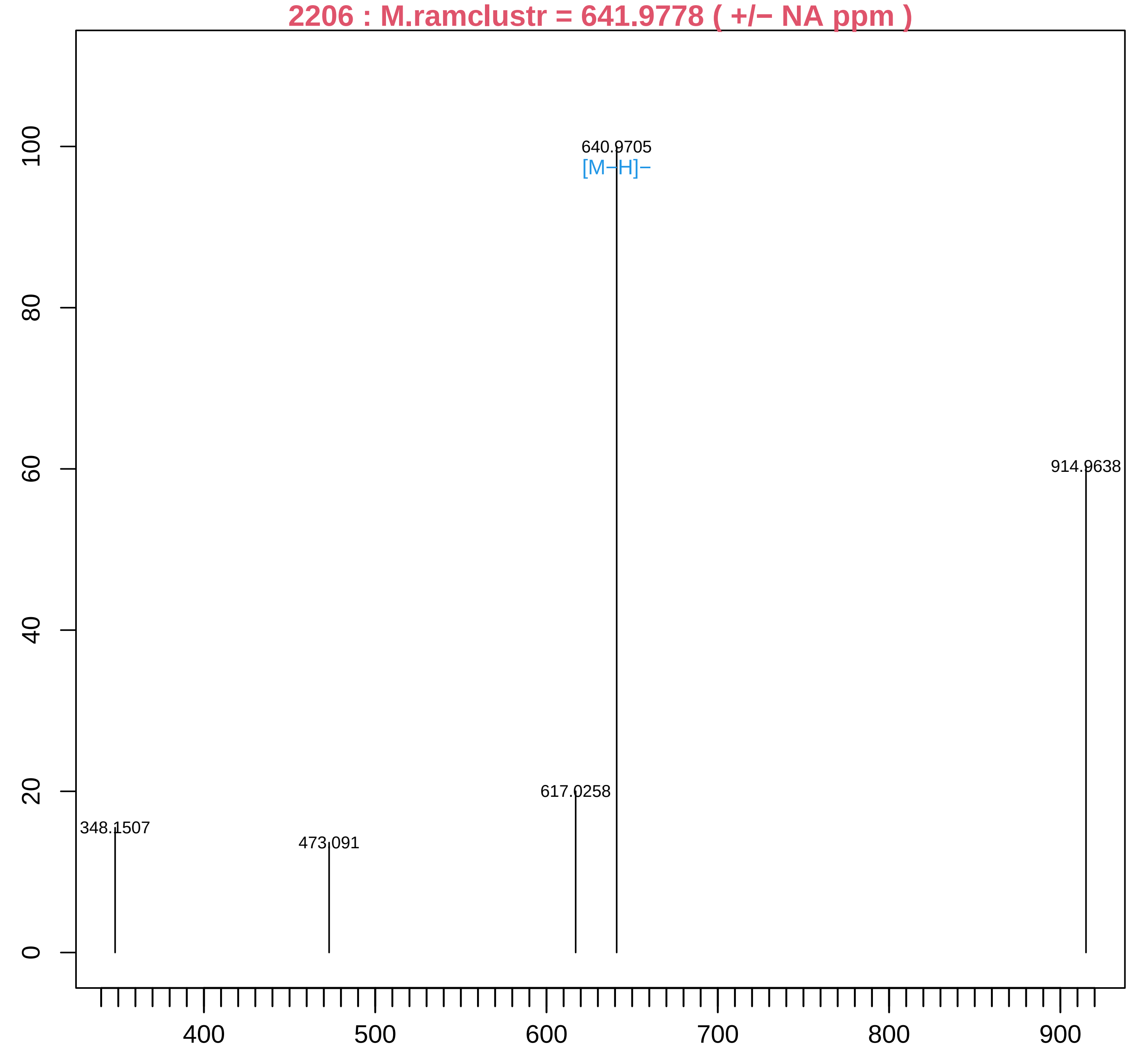

2206 : M.ramclustr = 641.9778 (+/− NA ppm)
640.9705
[M−H]−
914.9638
617.0258
348.1507
473.091
0
20
40
60
80
100
400
500
600
700
800
900

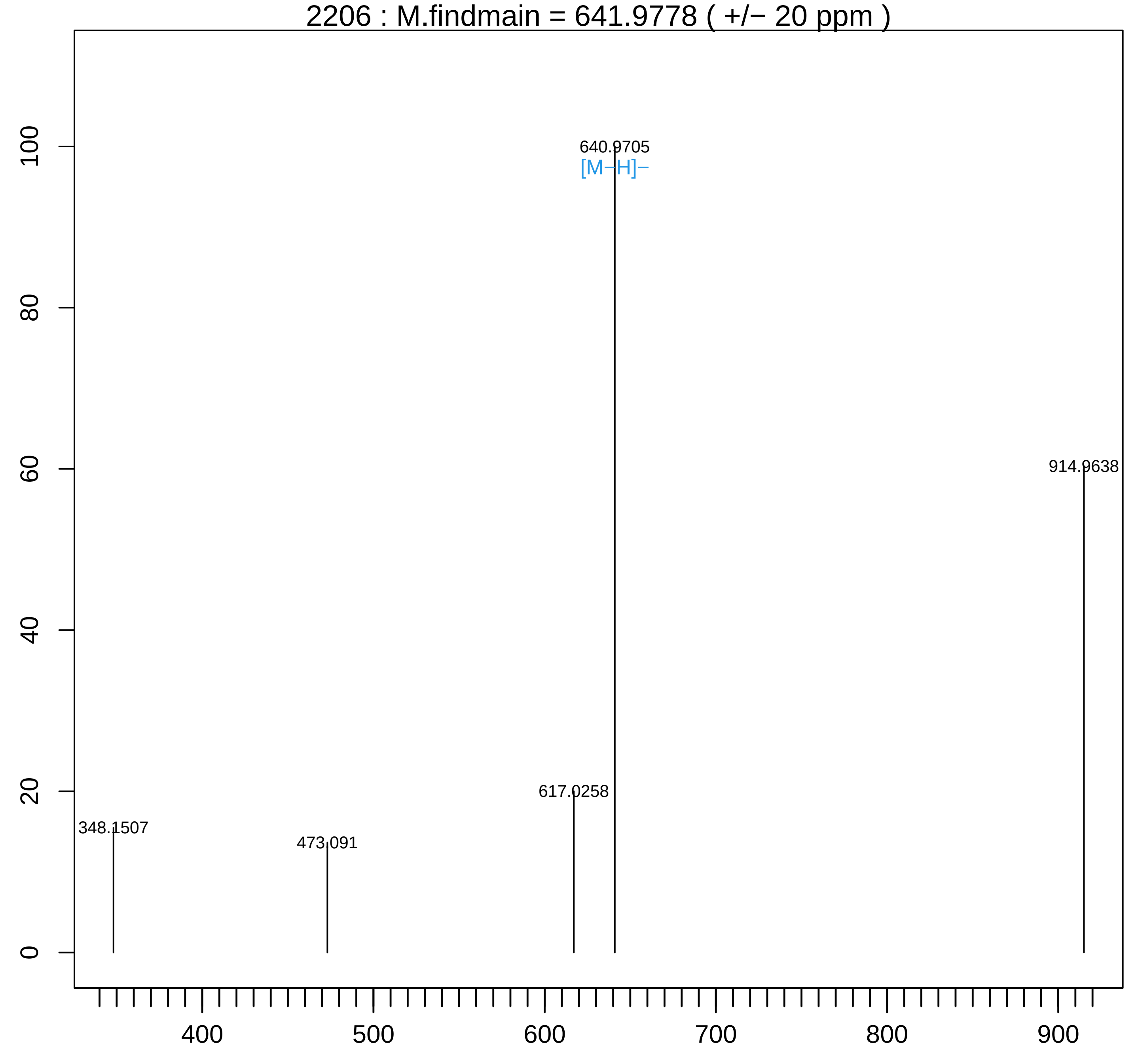

2206 : M.findmain = 641.9778 (+/− 20 ppm)
640.9705
[M−H]−
914.9638
617.0258
348.1507
473.091
0
20
40
60
80
100
400
500
600
700
800
900

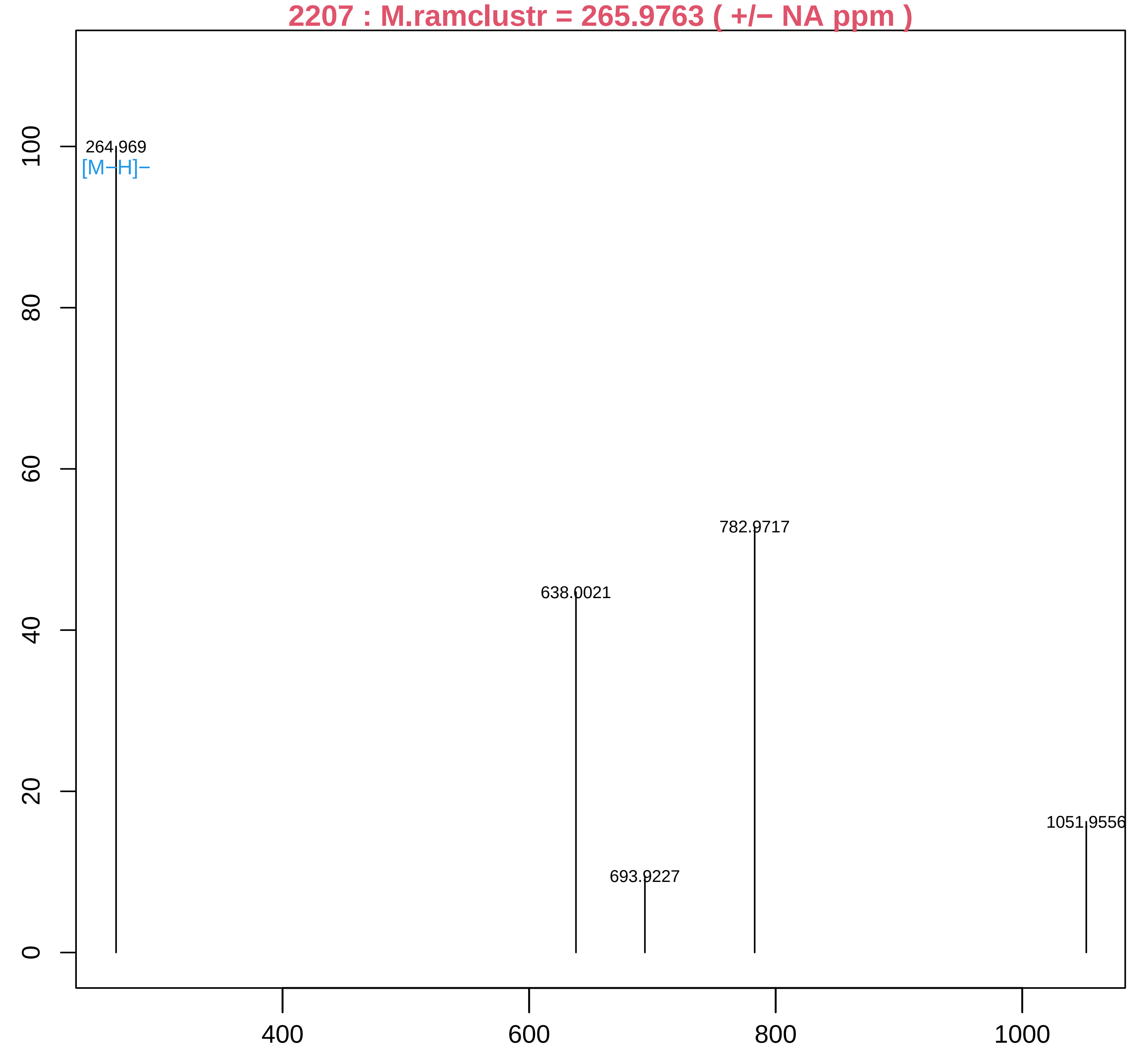
2207 : M.ramclustr = 265.9763 (+/− NA ppm)
264.969
[M−H]−
638.0021
693.9227
782.9717
1051.9556
0
20
40
60
80
100
400
600
800
1000

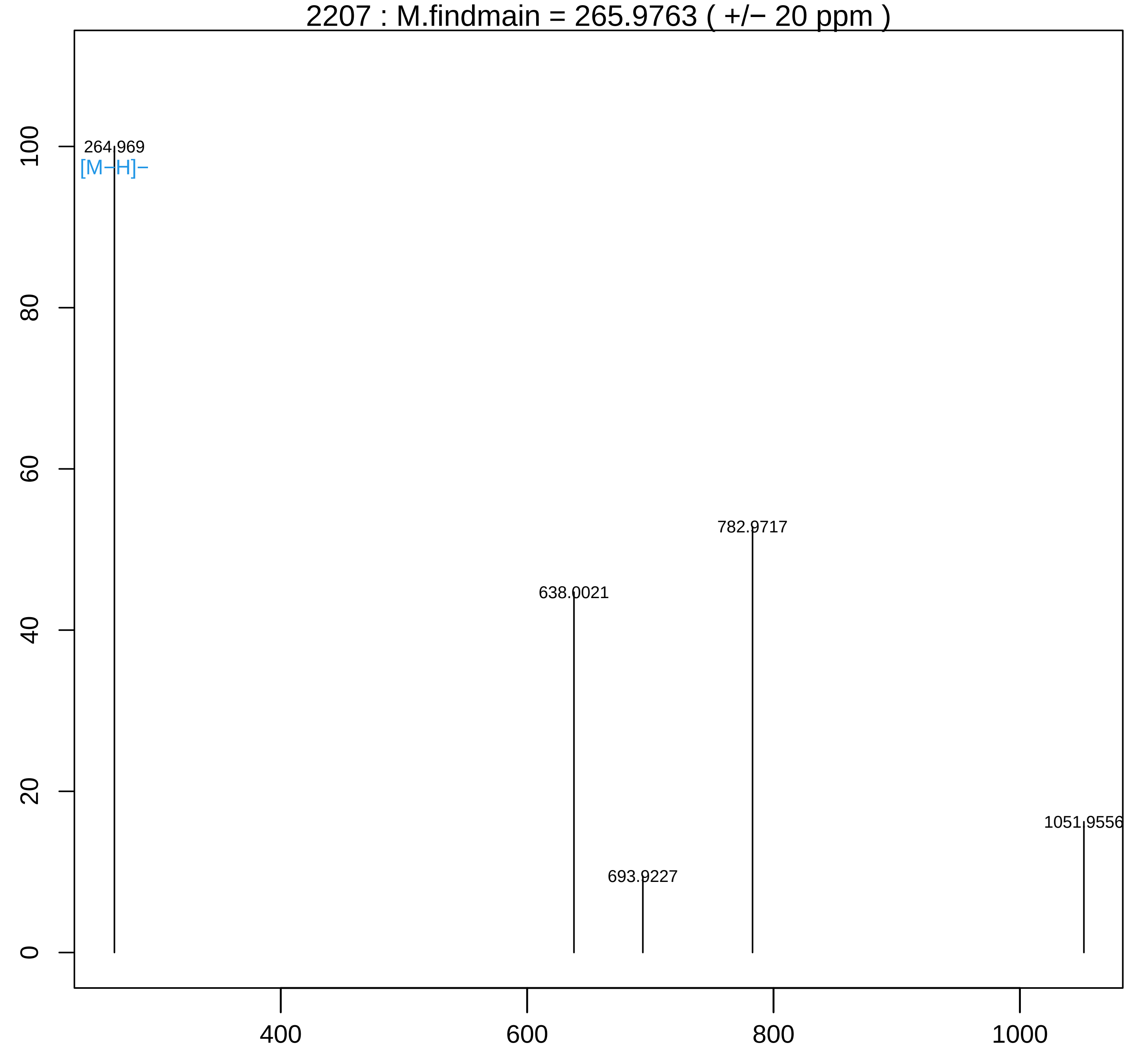
2207 : M.findmain = 265.9763 (+/− 20 ppm)
264.969
[M−H]−
638.0021
693.9227
782.9717
1051.9556
0
20
40
60
80
100
400
600
800
1000

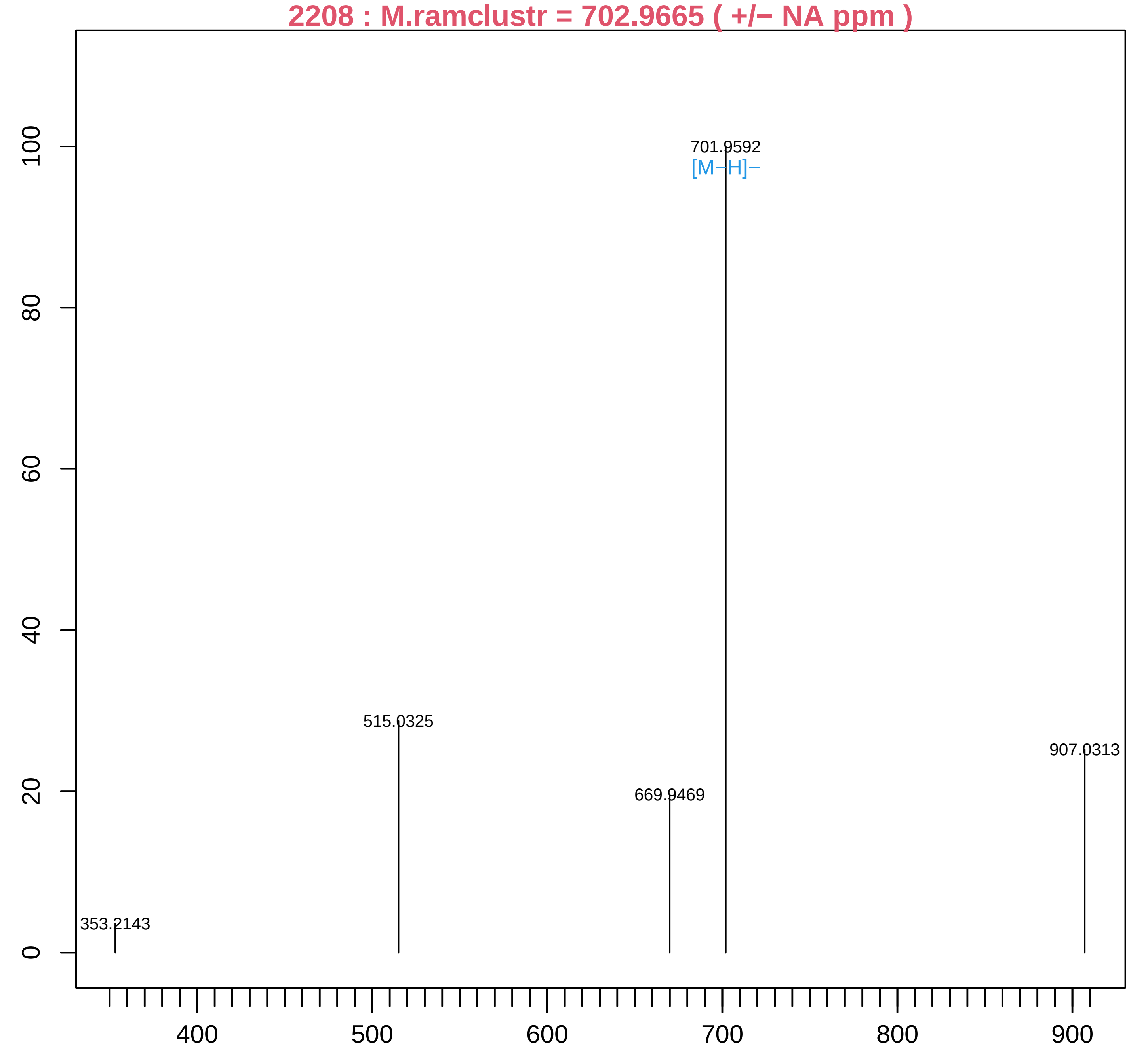
2208 : M.ramclustr = 702.9665 (+/− NA ppm)
701.9592
[M−H]−
515.0325
669.9469
907.0313
353.2143
0
20
40
60
80
100
400
500
600
700
800
900

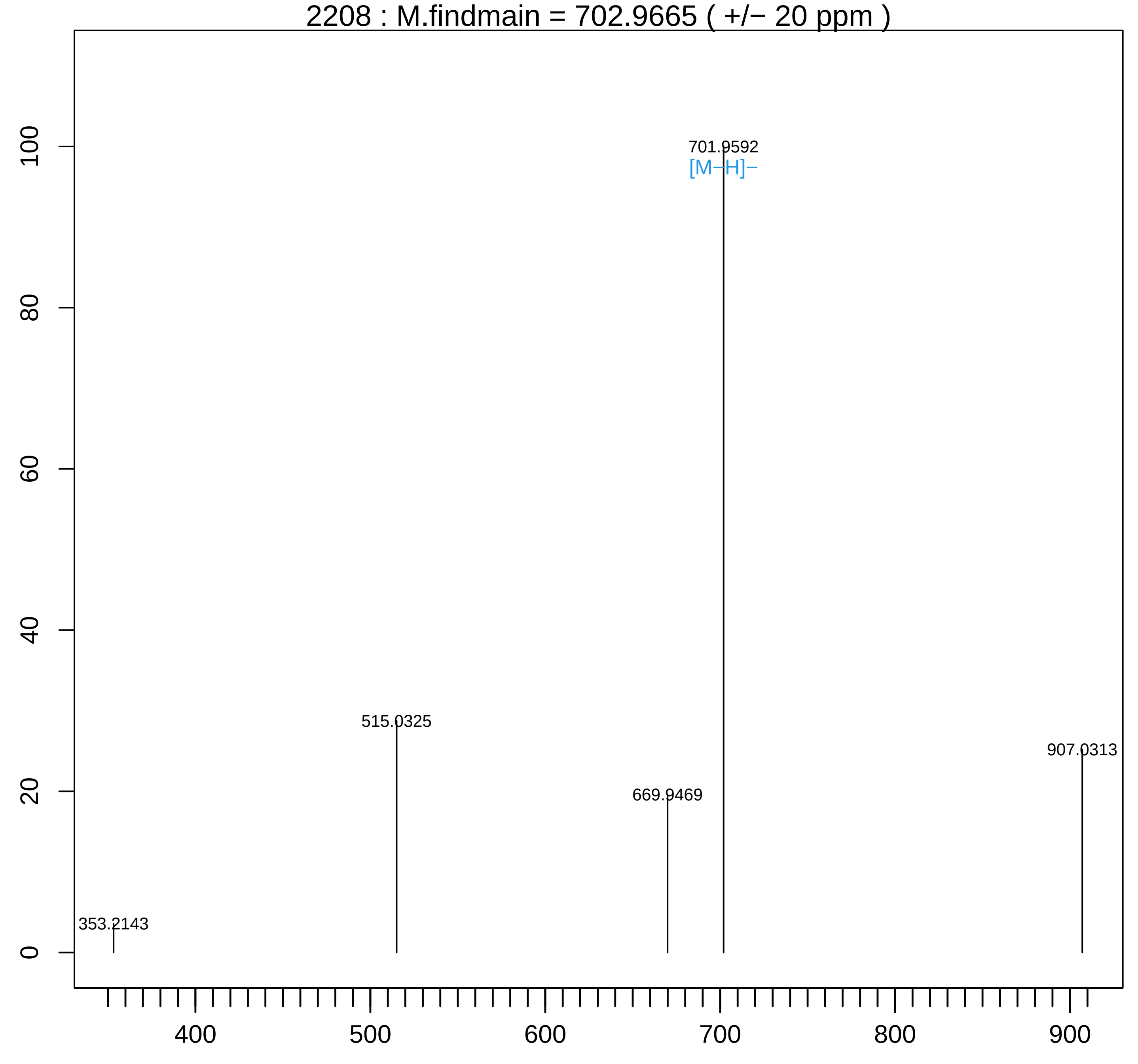
2208 : M.findmain = 702.9665 (+/− 20 ppm)
701.9592
[M−H]−
515.0325
669.9469
907.0313
353.2143
0
20
40
60
80
100
400
500
600
700
800
900

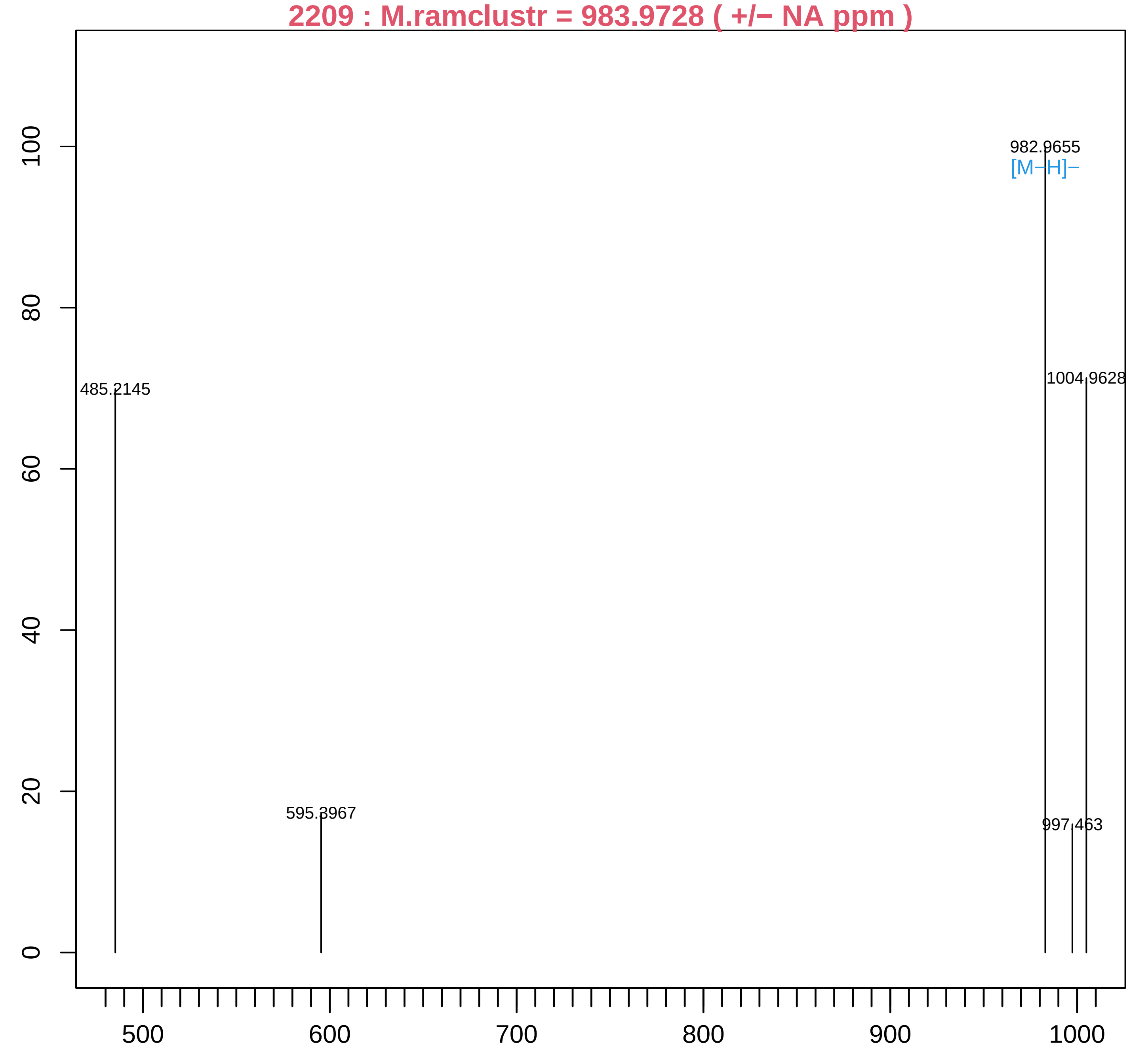

2209 : M.ramclustr = 983.9728 (+/− NA ppm)
982.9655
[M−H]−
485.2145
1004.9628
595.3967
997.463
0
20
40
60
80
100
500
600
700
800
900
1000

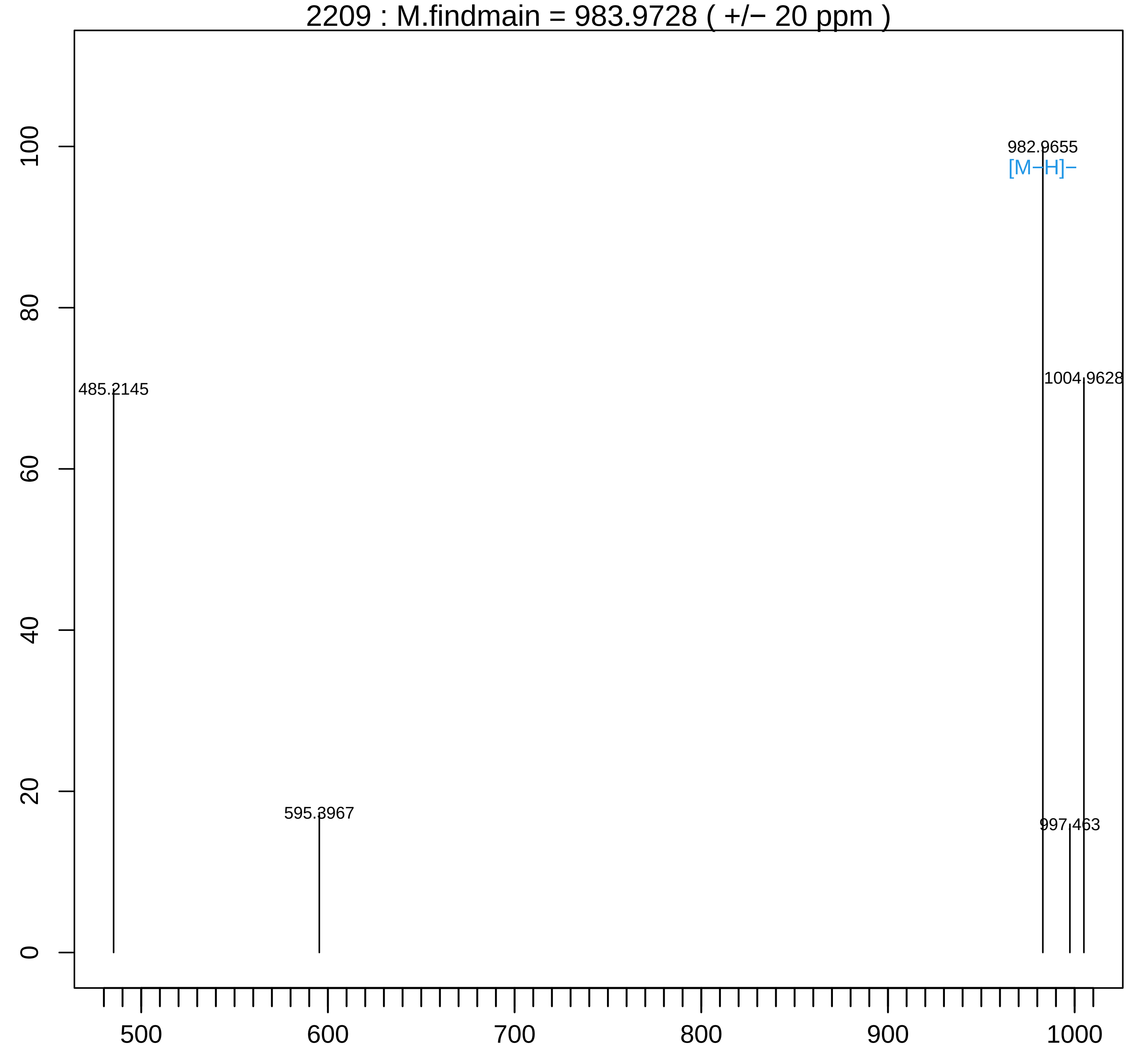

2209 : M.findmain = 983.9728 (+/− 20 ppm)
982.9655
[M−H]−
485.2145
1004.9628
595.3967
997.463
0
20
40
60
80
100
500
600
700
800
900
1000

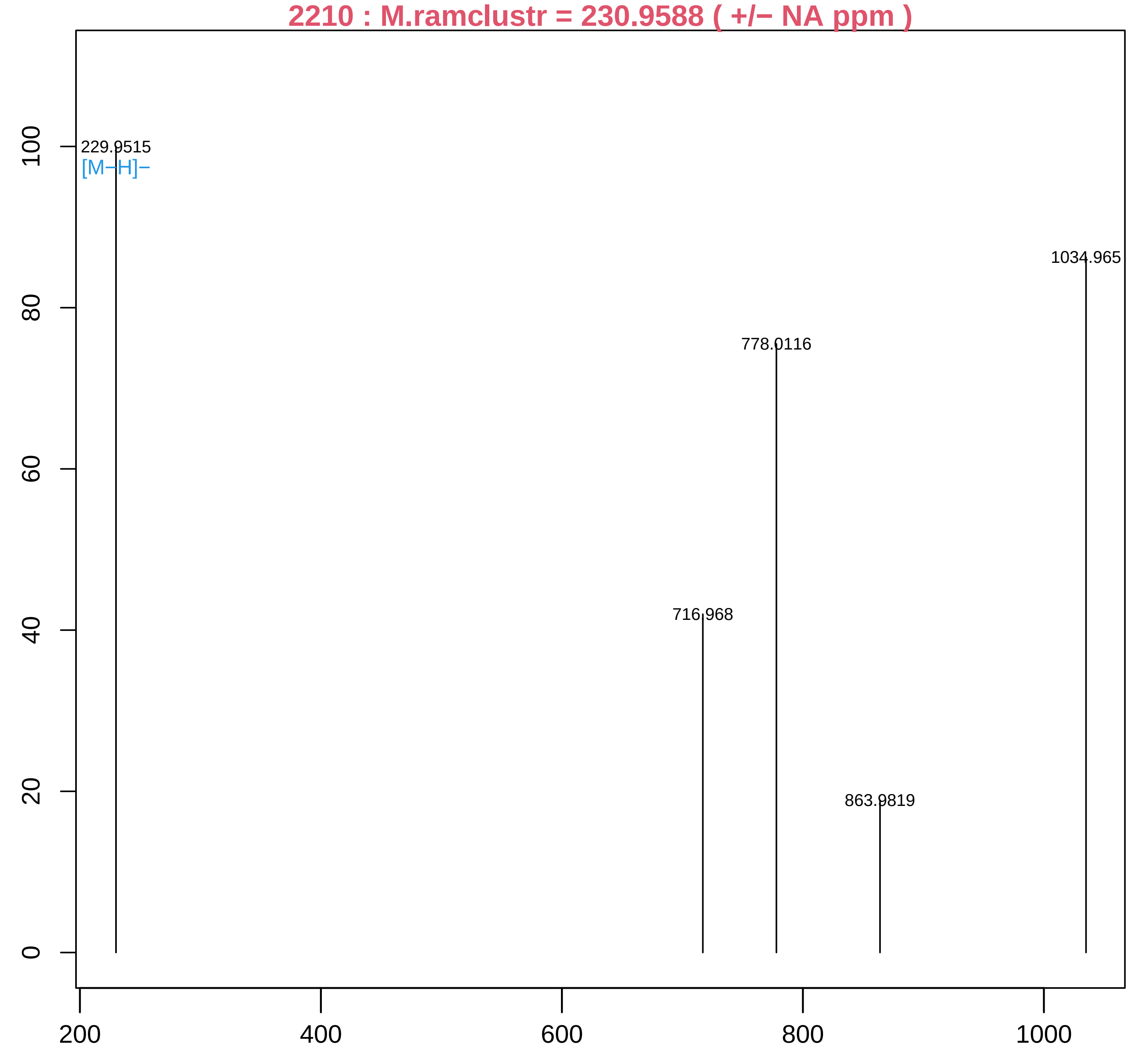
2210 : M.ramclustr = 230.9588 (+/− NA ppm)
229.9515
[M−H]−
1034.965
778.0116
716.968
863.9819
0
20
40
60
80
100
200
400
600
800
1000

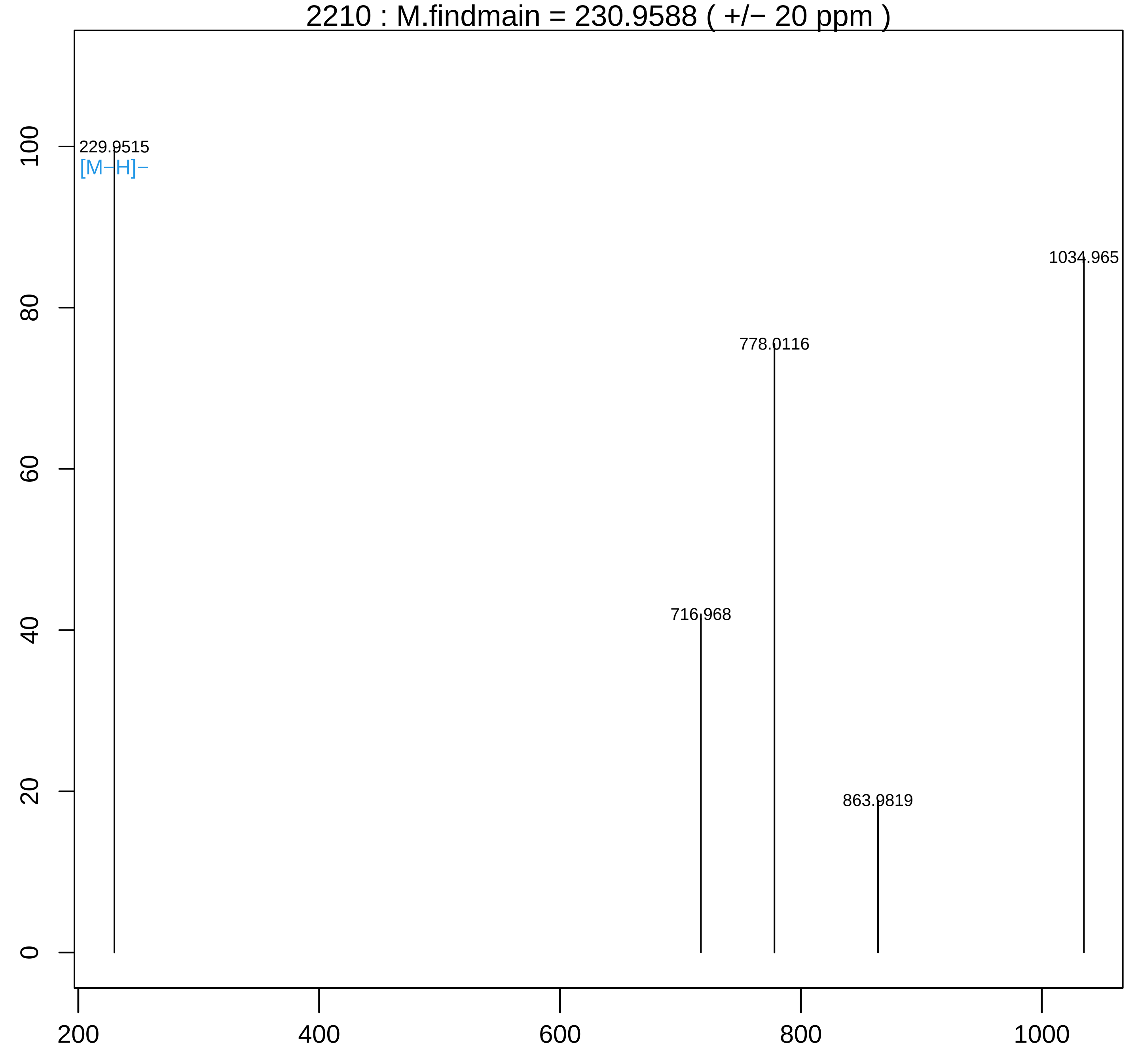
2210 : M.findmain = 230.9588 (+/− 20 ppm)
229.9515
[M−H]−
1034.965
778.0116
716.968
863.9819
0
20
40
60
80
100
200
400
600
800
1000

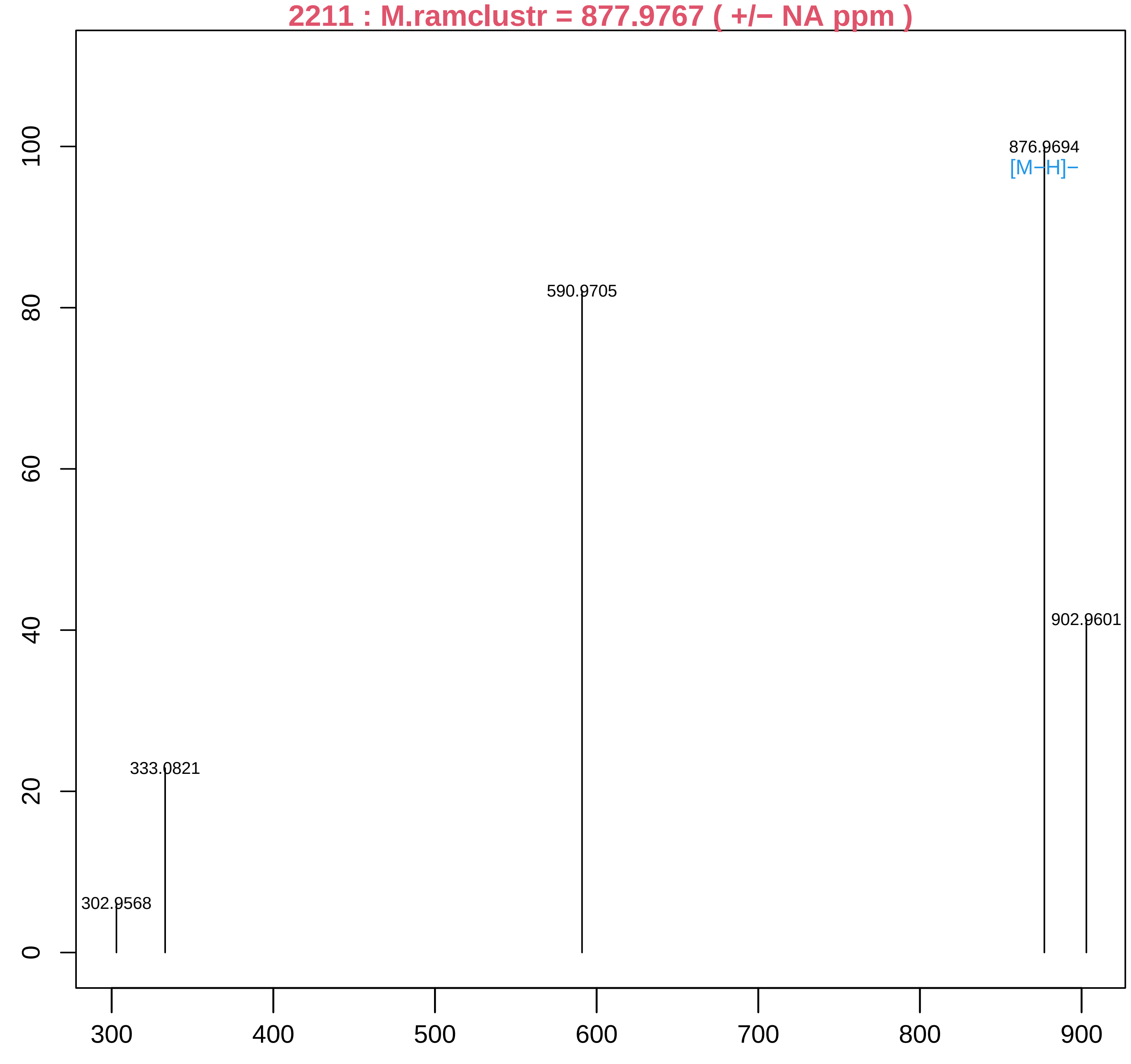
2211 : M.ramclustr = 877.9767 (+/− NA ppm)
876.9694
[M−H]−
590.9705
902.9601
333.0821
302.9568
0
20
40
60
80
100
300
400
500
600
700
800
900

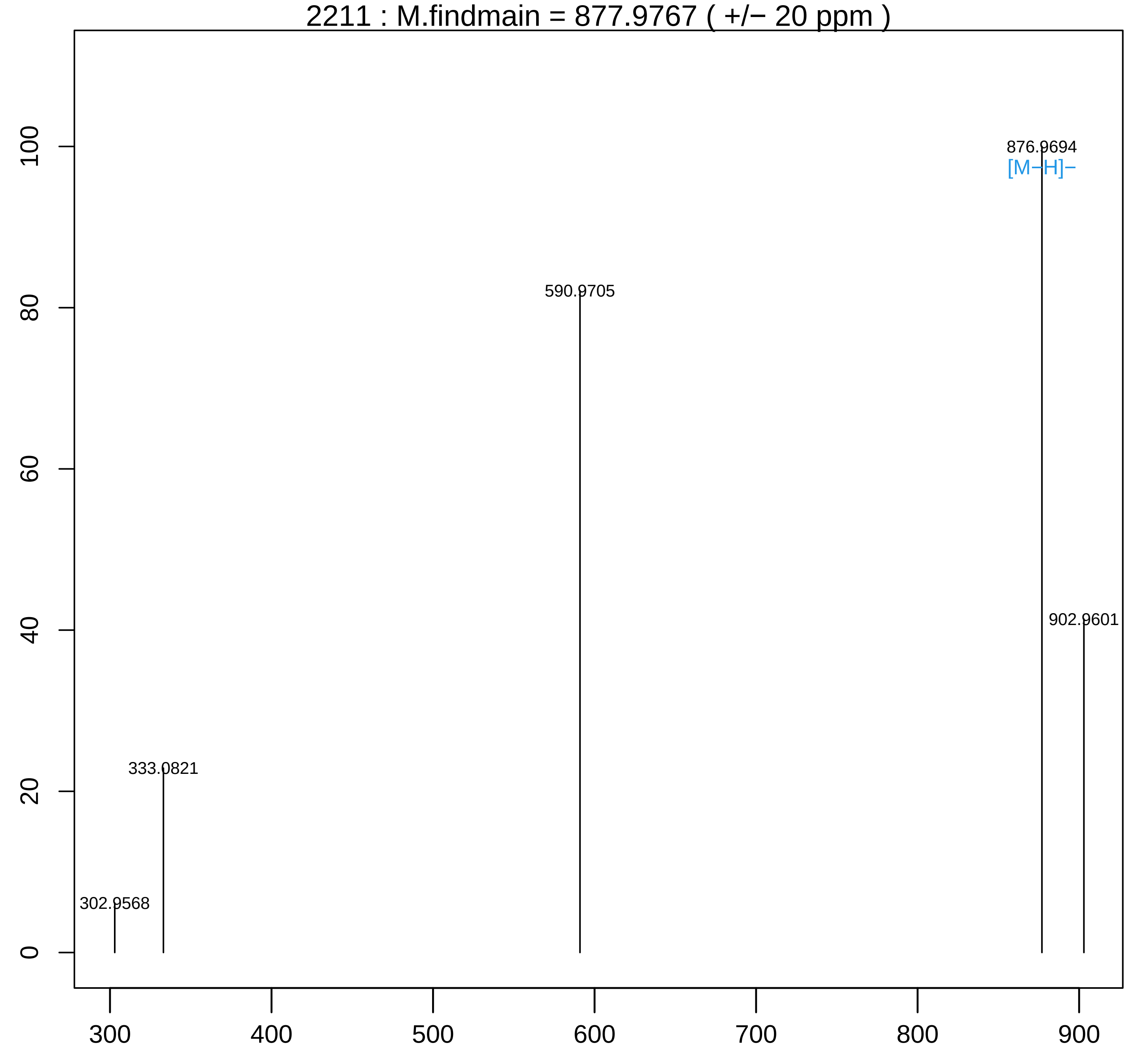
2211 : M.findmain = 877.9767 (+/− 20 ppm)
876.9694
[M−H]−
590.9705
902.9601
333.0821
302.9568
0
20
40
60
80
100
300
400
500
600
700
800
900

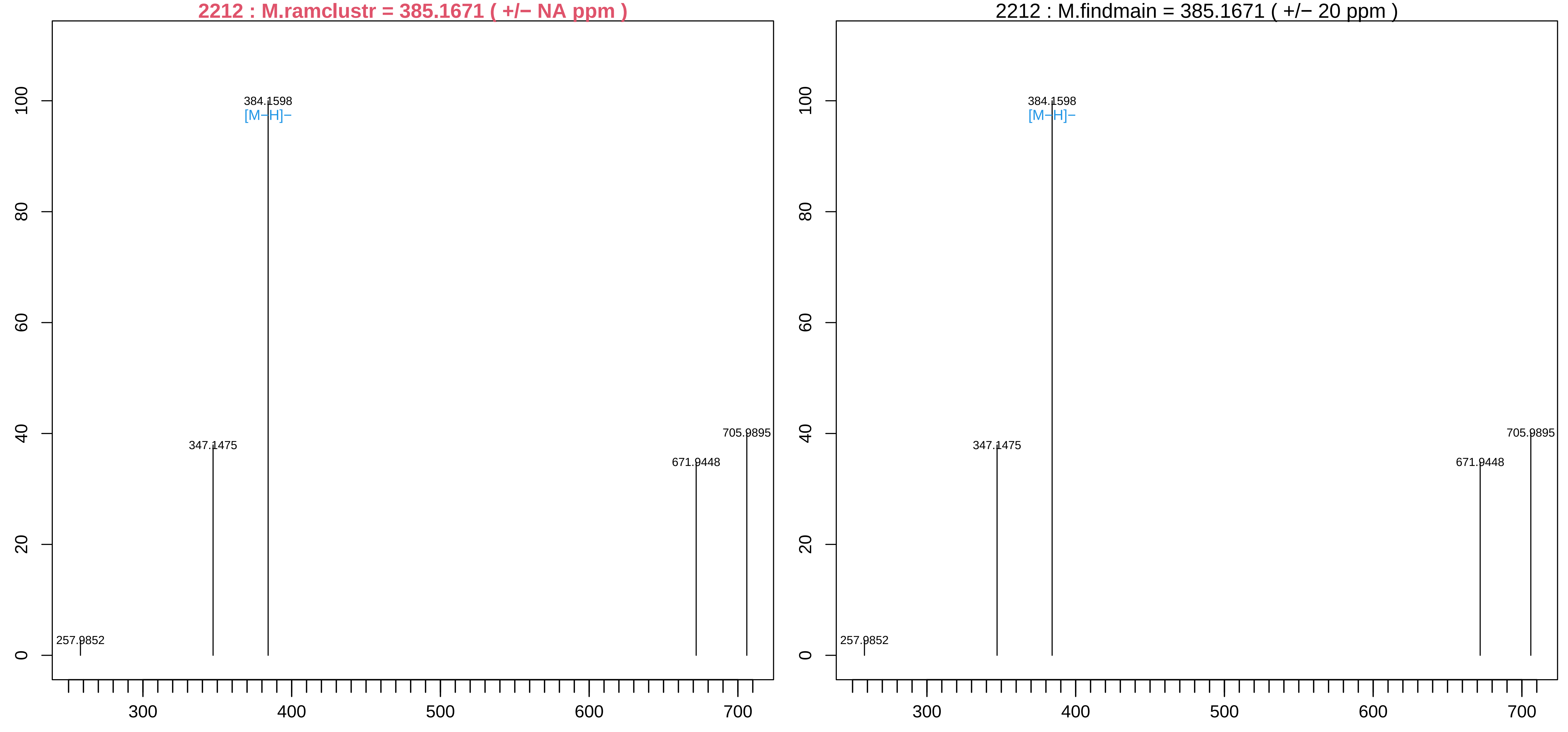
2212 : M.ramclustr = 385.1671 (+/− NA ppm)
384.1598
[M−H]−
347.1475
671.9448
705.9895
257.9852
2212 : M.findmain = 385.1671 (+/− 20 ppm)
384.1598
[M−H]−
347.1475
671.9448
705.9895
257.9852
0
20
40
60
80
100
300
400
500
600
700

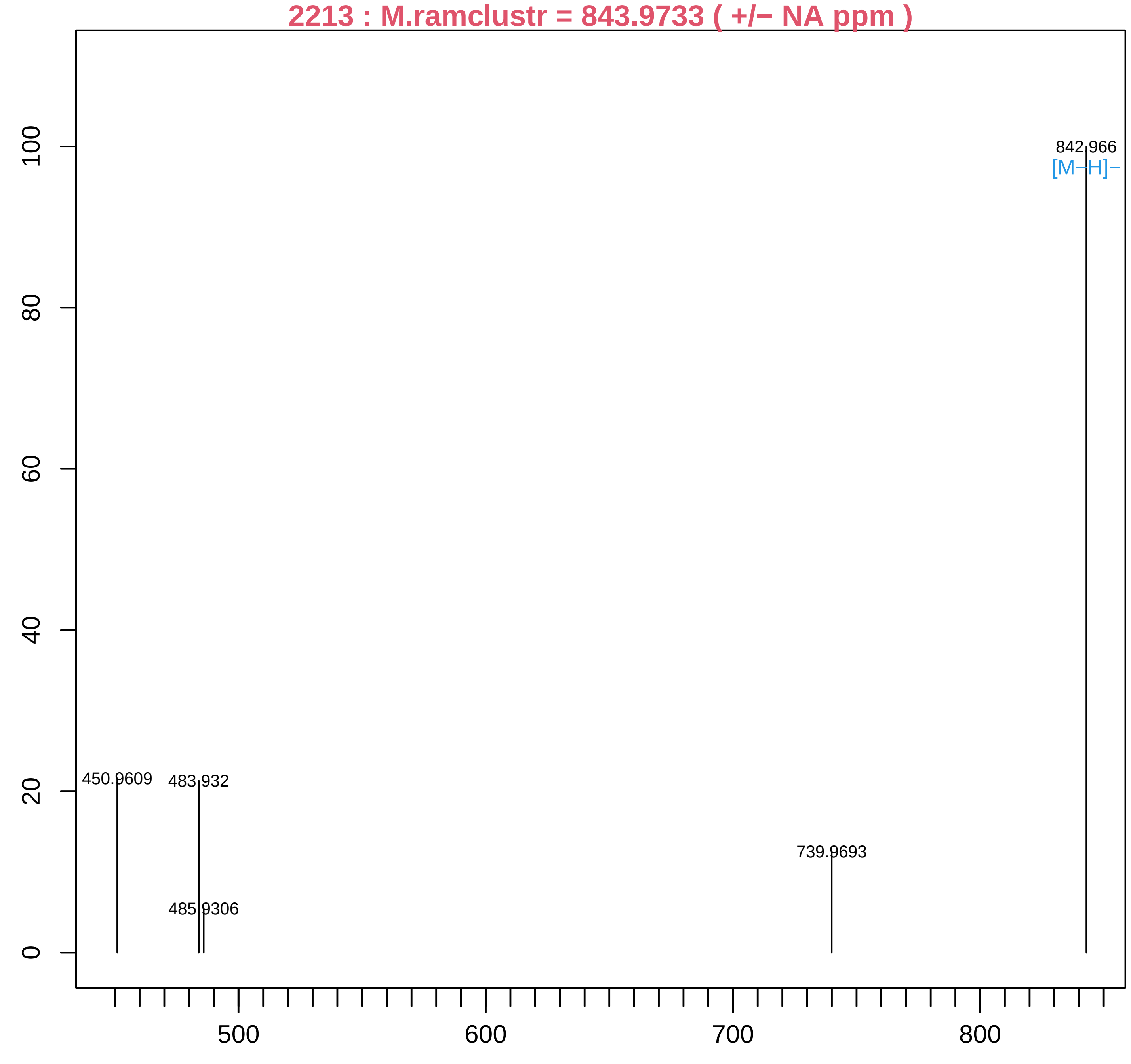
2213 : M.ramclustr = 843.9733 (+/− NA ppm)
842.966
[M−H]−
450.9609
483.932
485.9306
739.9693
0
20
40
60
80
100
500
600
700
800

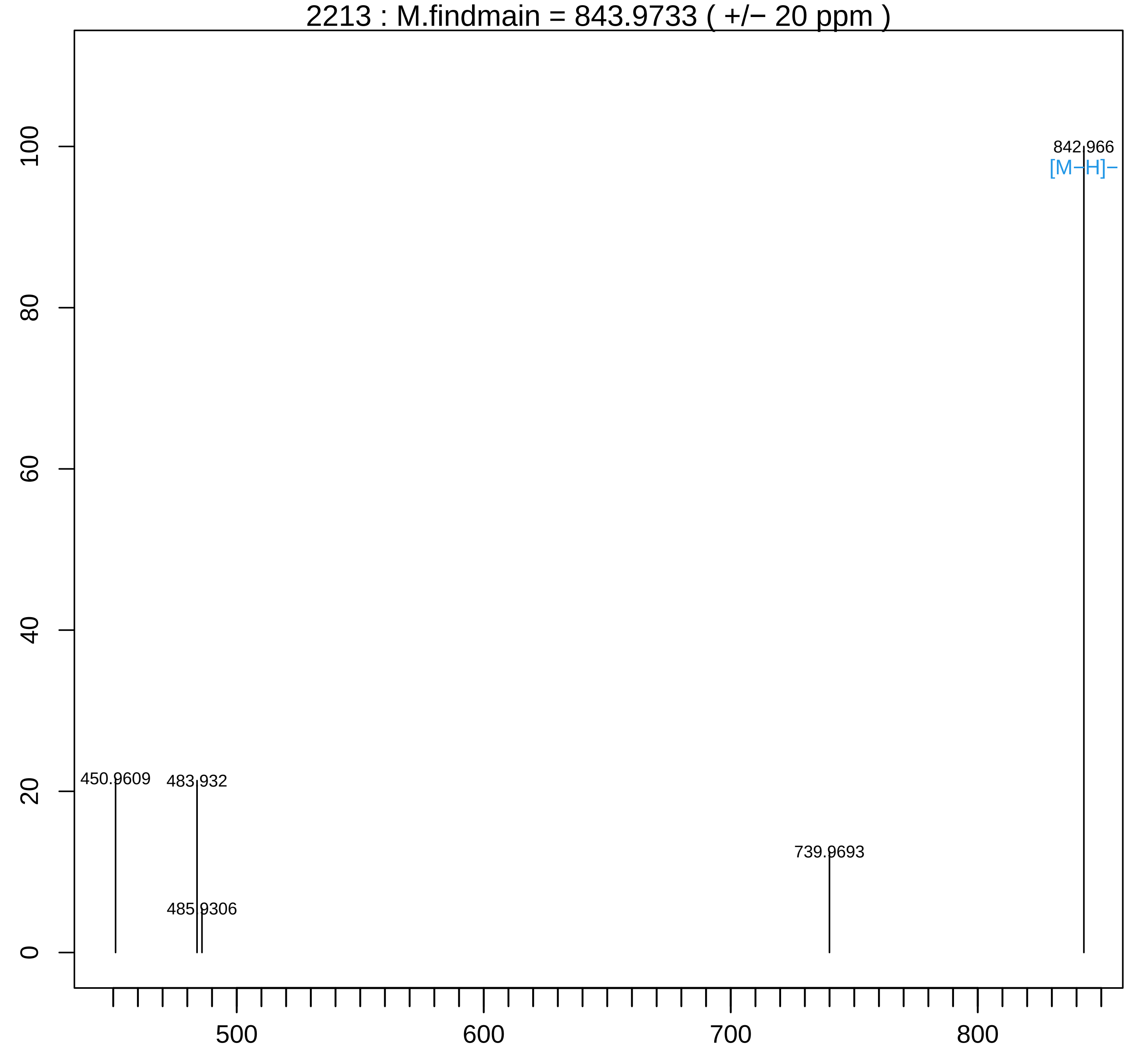
2213 : M.findmain = 843.9733 (+/− 20 ppm)
842.966
[M−H]−
450.9609
483.932
485.9306
739.9693
0
20
40
60
80
100
500
600
700
800

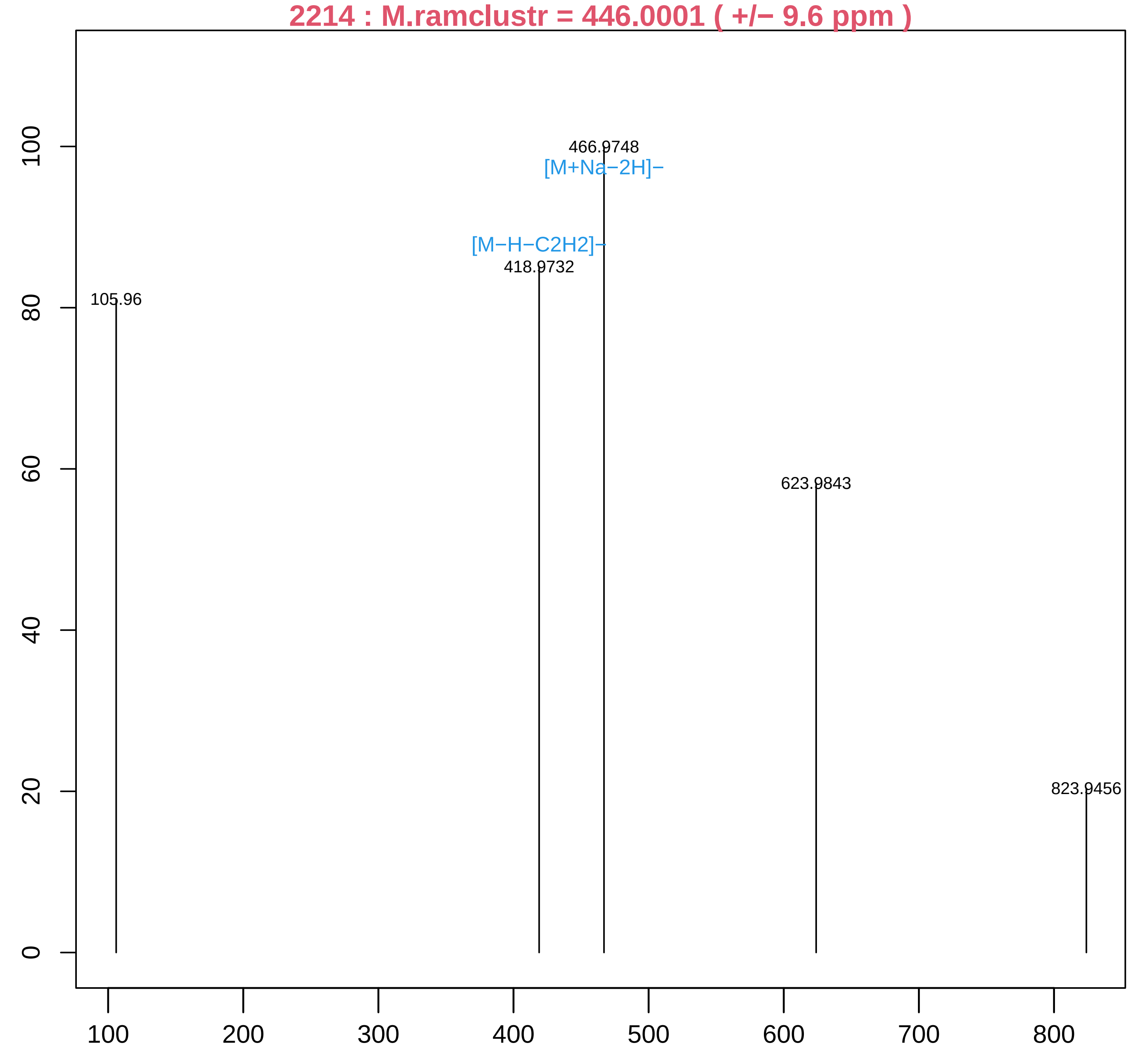
2214 : M.ramclustr = 446.0001 (+/− 9.6 ppm)
466.9748
[M+Na−2H]−
[M−H−C2H2]−
418.9732
105.96
623.9843
823.9456
0
20
40
60
80
100
100
200
300
400
500
600
700
800

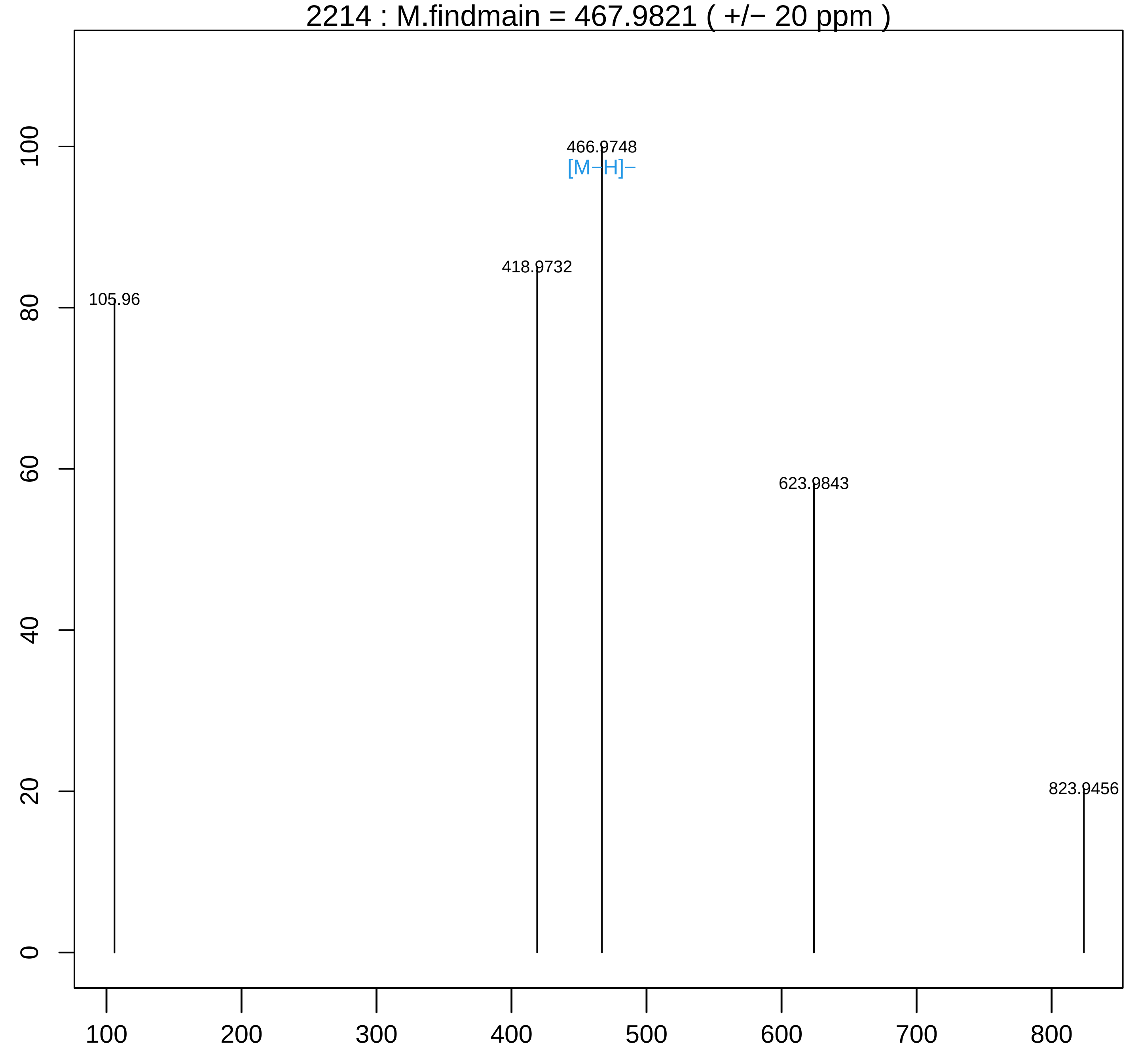
2214 : M.findmain = 467.9821 (+/− 20 ppm)
466.9748
[M−H]−
418.9732
105.96
623.9843
823.9456
0
20
40
60
80
100
100
200
300
400
500
600
700
800

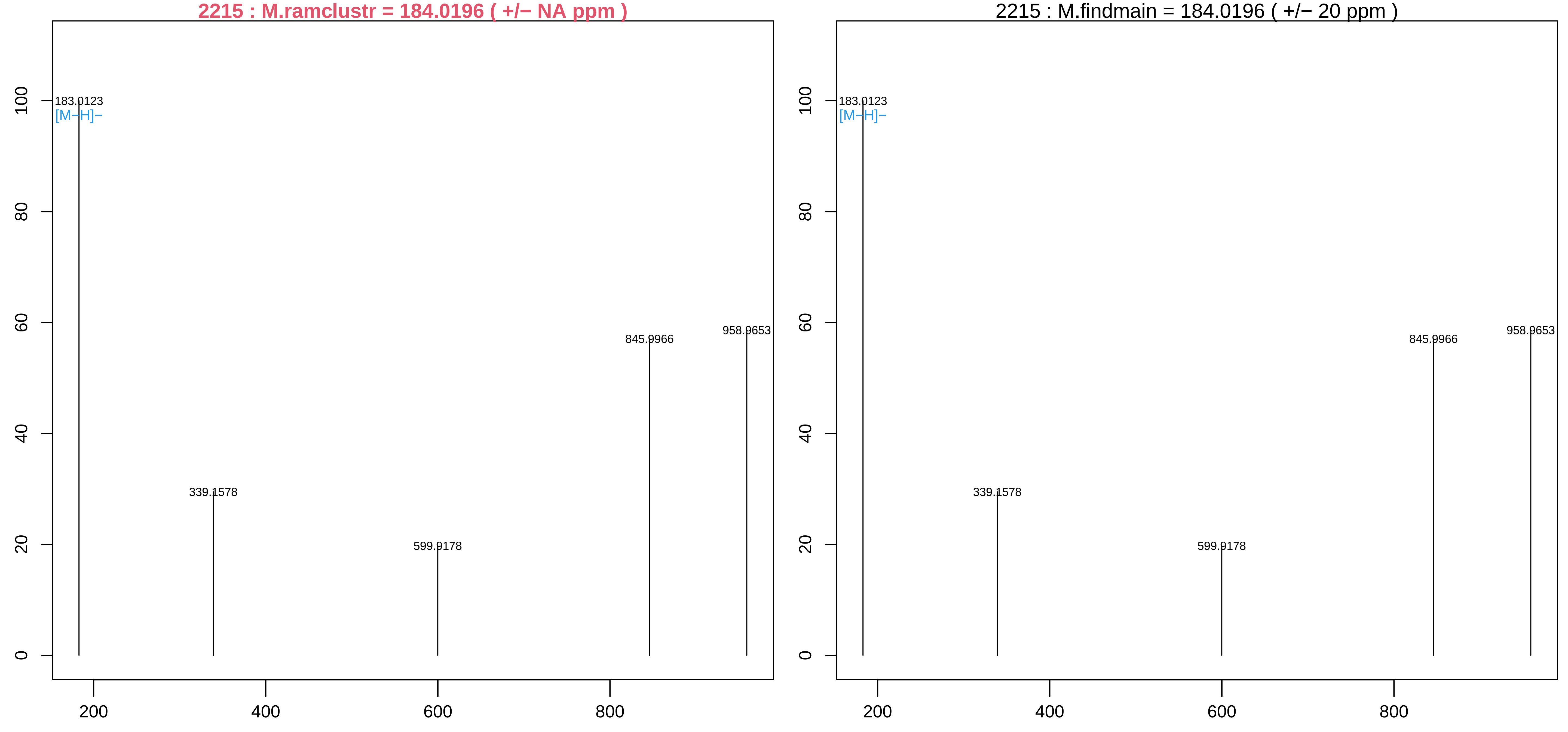
2215 : M.ramclustr = 184.0196 (+/− NA ppm)
183.0123
[M−H]−
339.1578
599.9178
845.9966
958.9653
2215 : M.findmain = 184.0196 (+/− 20 ppm)
183.0123
[M−H]−
339.1578
599.9178
845.9966
958.9653
0
20
40
60
80
100
200
400
600
800

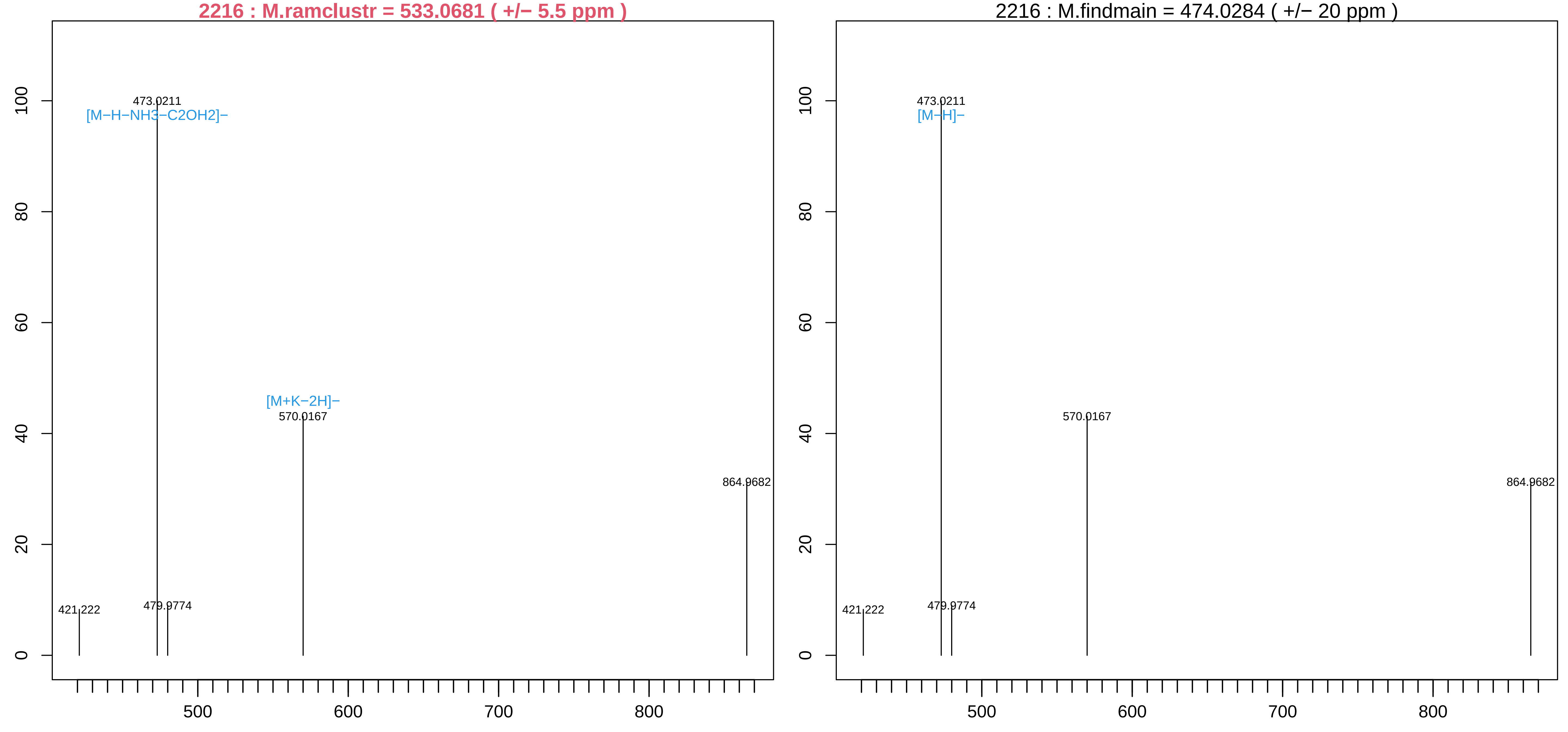
2216 : M.ramclustr = 533.0681 (+/− 5.5 ppm)
473.0211
[M−H−NH3−C2OH2]−
[M+K−2H]−
570.0167
864.9682
421.222
479.9774
2216 : M.findmain = 474.0284 (+/− 20 ppm)
473.0211
[M−H]−
570.0167
864.9682
421.222
479.9774
0
20
40
60
80
100
500
600
700
800

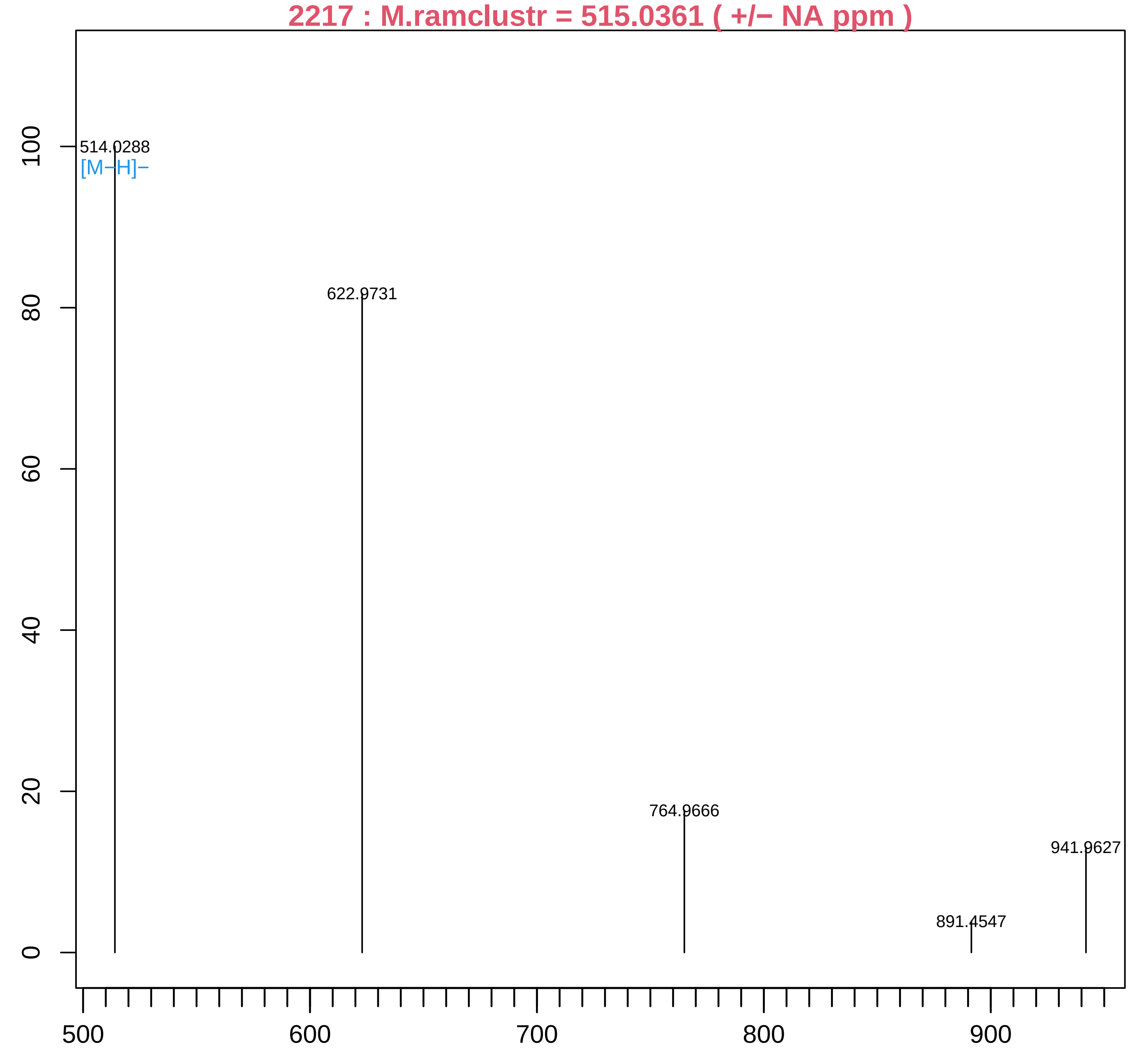
2217 : M.ramclustr = 515.0361 (+/− NA ppm)
514.0288
[M−H]−
622.9731
764.9666
891.4547
941.9627
0
20
40
60
80
100
500
600
700
800
900

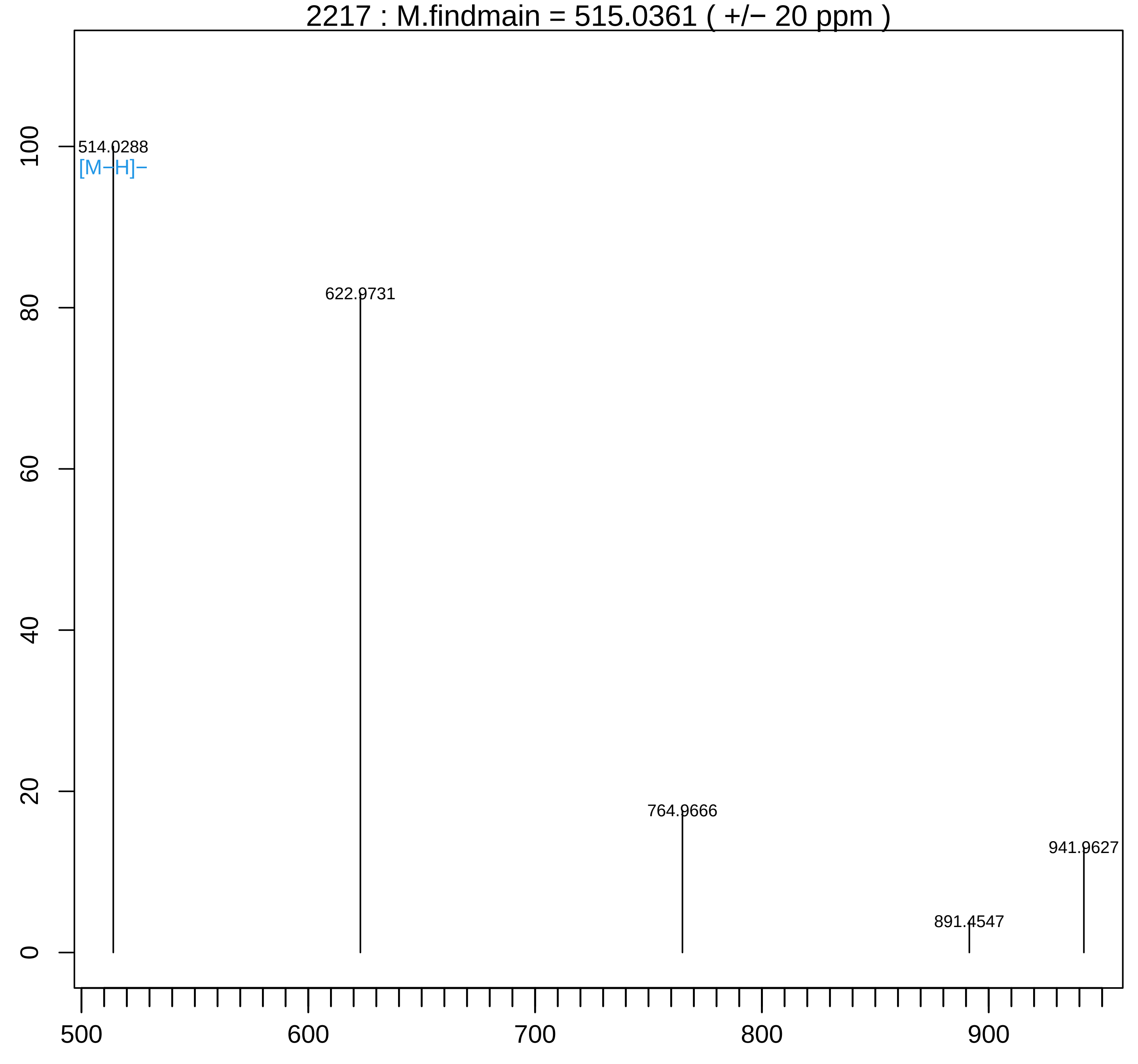
2217 : M.findmain = 515.0361 (+/− 20 ppm)
514.0288
[M−H]−
622.9731
764.9666
891.4547
941.9627
0
20
40
60
80
100
500
600
700
800
900

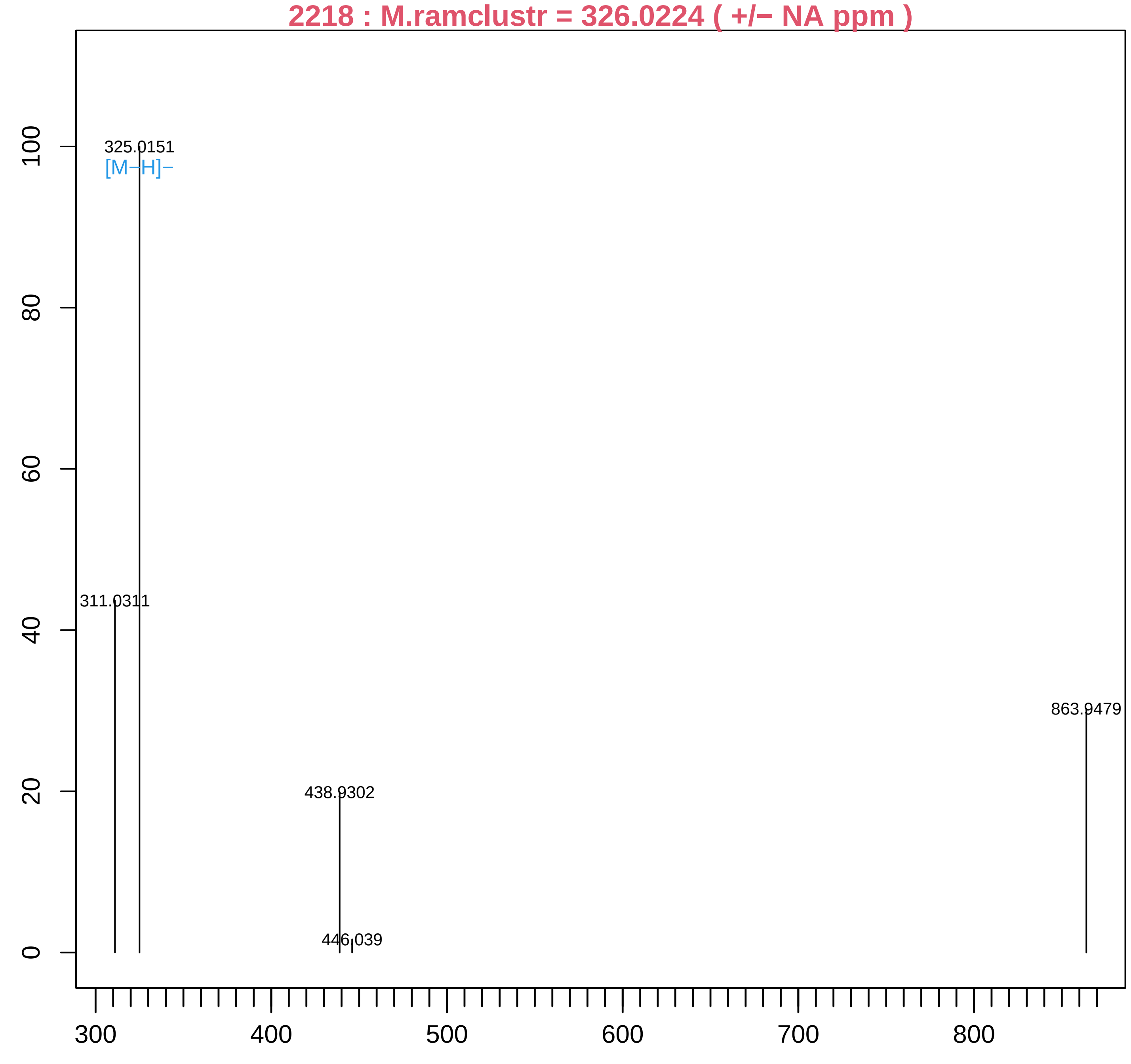
2218 : M.ramclustr = 326.0224 (+/− NA ppm)
325.0151
[M−H]−
311.0311
438.9302
446.039
863.9479
0
20
40
60
80
100
300
400
500
600
700
800

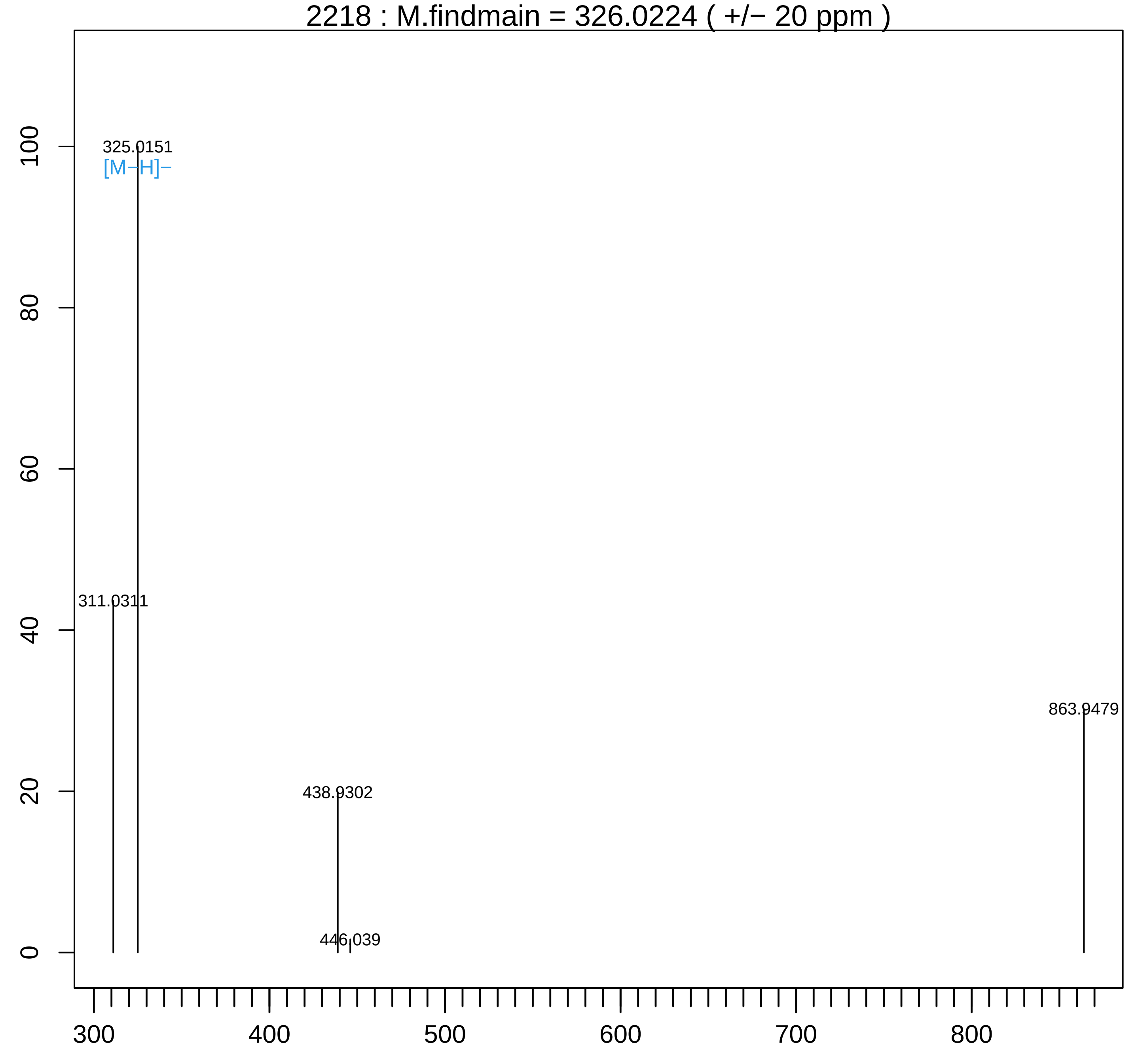
2218 : M.findmain = 326.0224 (+/− 20 ppm)
325.0151
[M−H]−
311.0311
438.9302
446.039
863.9479
0
20
40
60
80
100
300
400
500
600
700
800

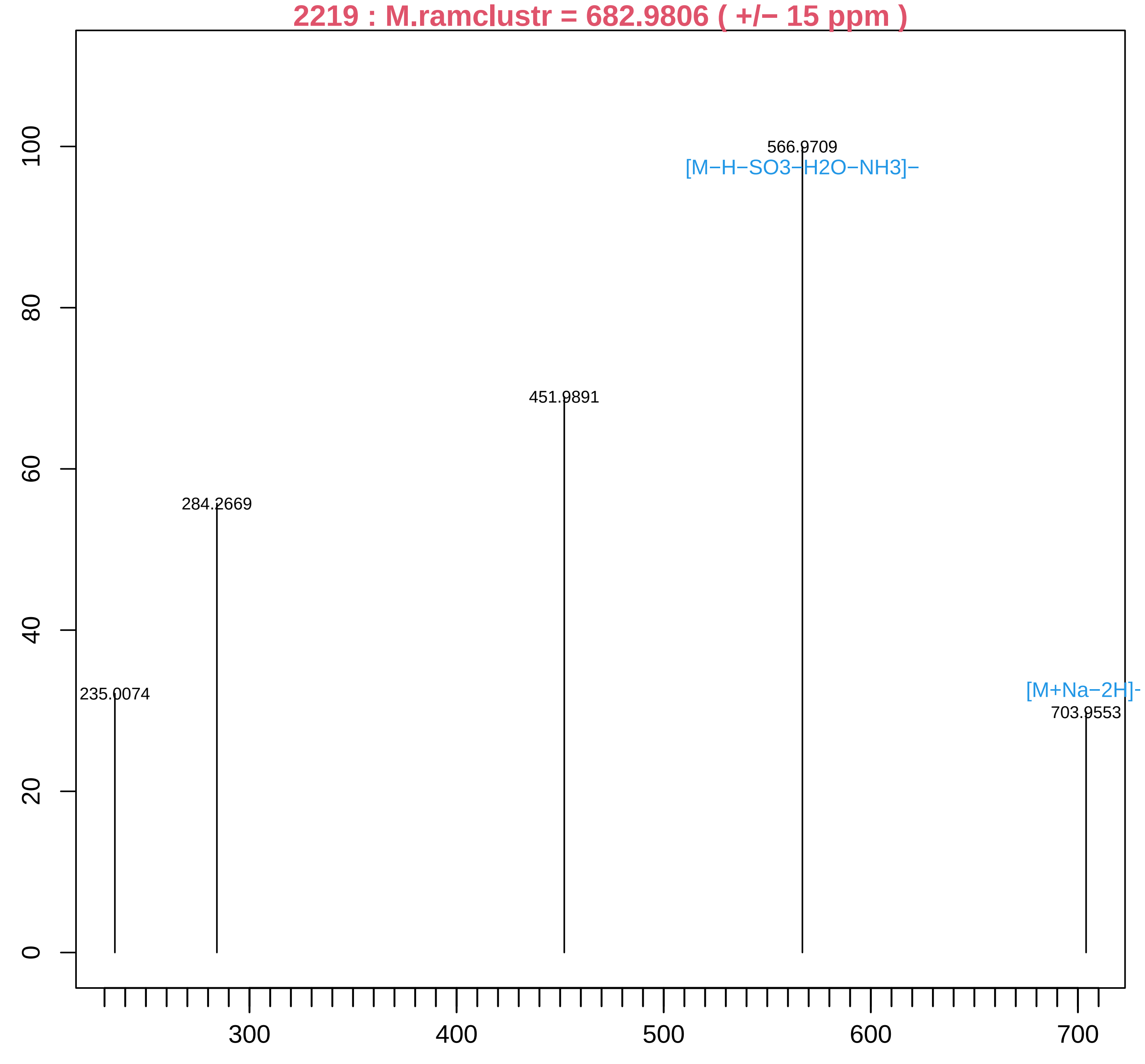
2219 : M.ramclustr = 682.9806 (+/− 15 ppm)
566.9709
[M−H−SO3−H2O−NH3]−
451.9891
284.2669
235.0074
[M+Na−2H]-
703.9553
0
20
40
60
80
100
300
400
500
600
700

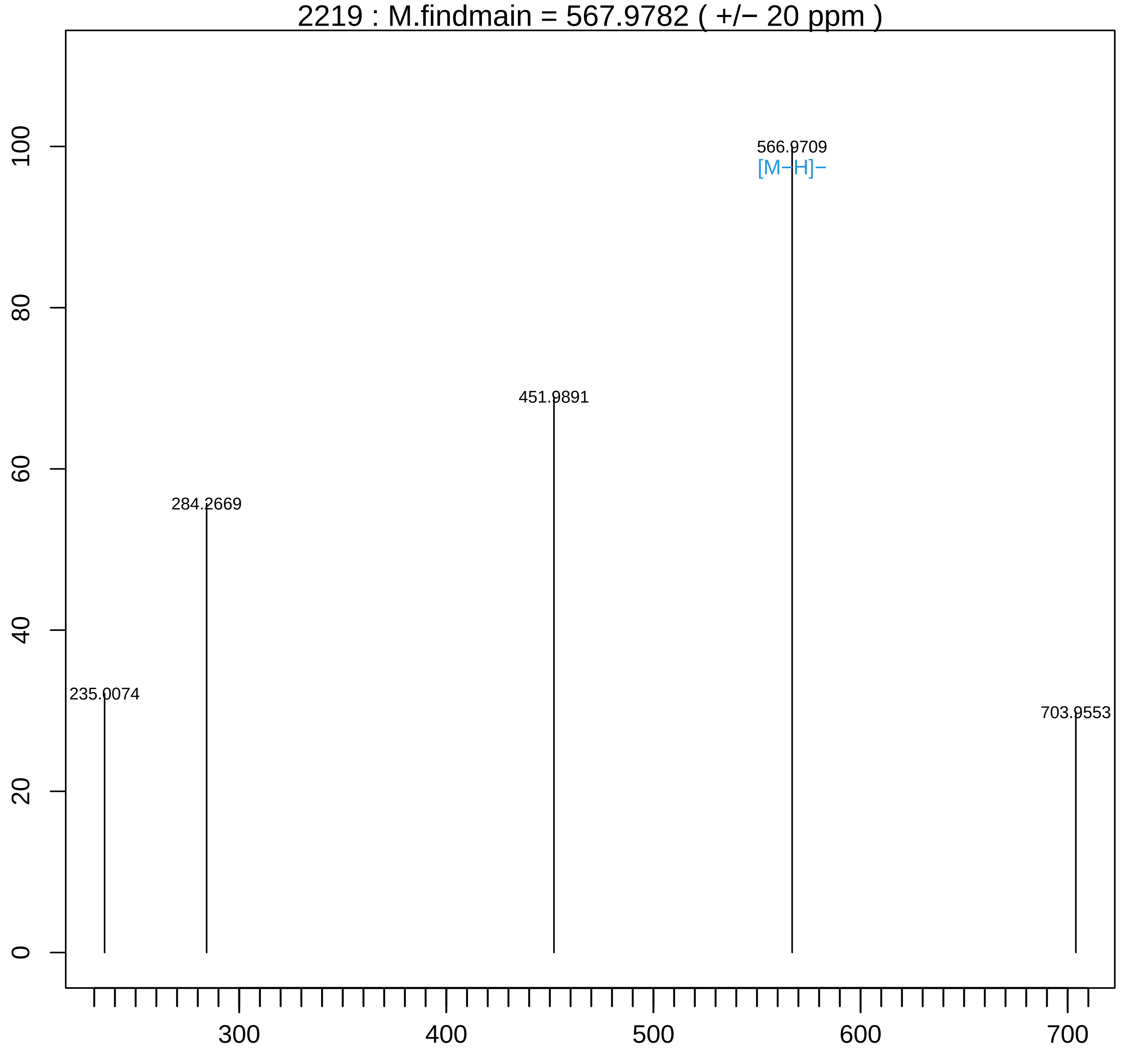
2219 : M.findmain = 567.9782 (+/− 20 ppm)
566.9709
[M−H]−
451.9891
284.2669
235.0074
703.9553
0
20
40
60
80
100
300
400
500
600
700

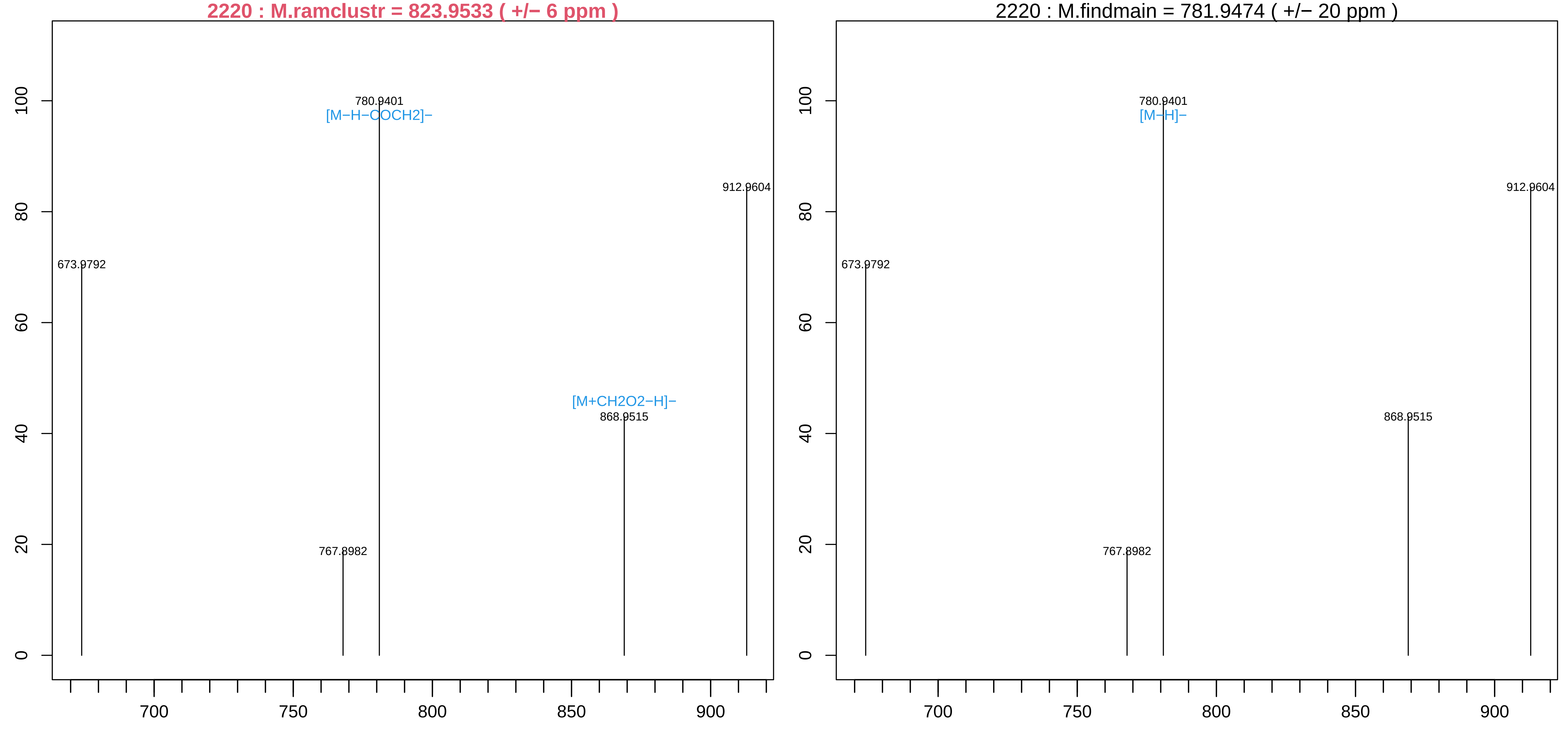
2220 : M.ramclustr = 823.9533 (+/− 6 ppm)
780.9401
[M−H−COCH2]−
912.9604
673.9792
[M+CH2O2−H]−
868.9515
767.8982
2220 : M.findmain = 781.9474 (+/− 20 ppm)
780.9401
[M−H]−
912.9604
673.9792
868.9515
767.8982
0
20
40
60
80
100
700
750
800
850
900

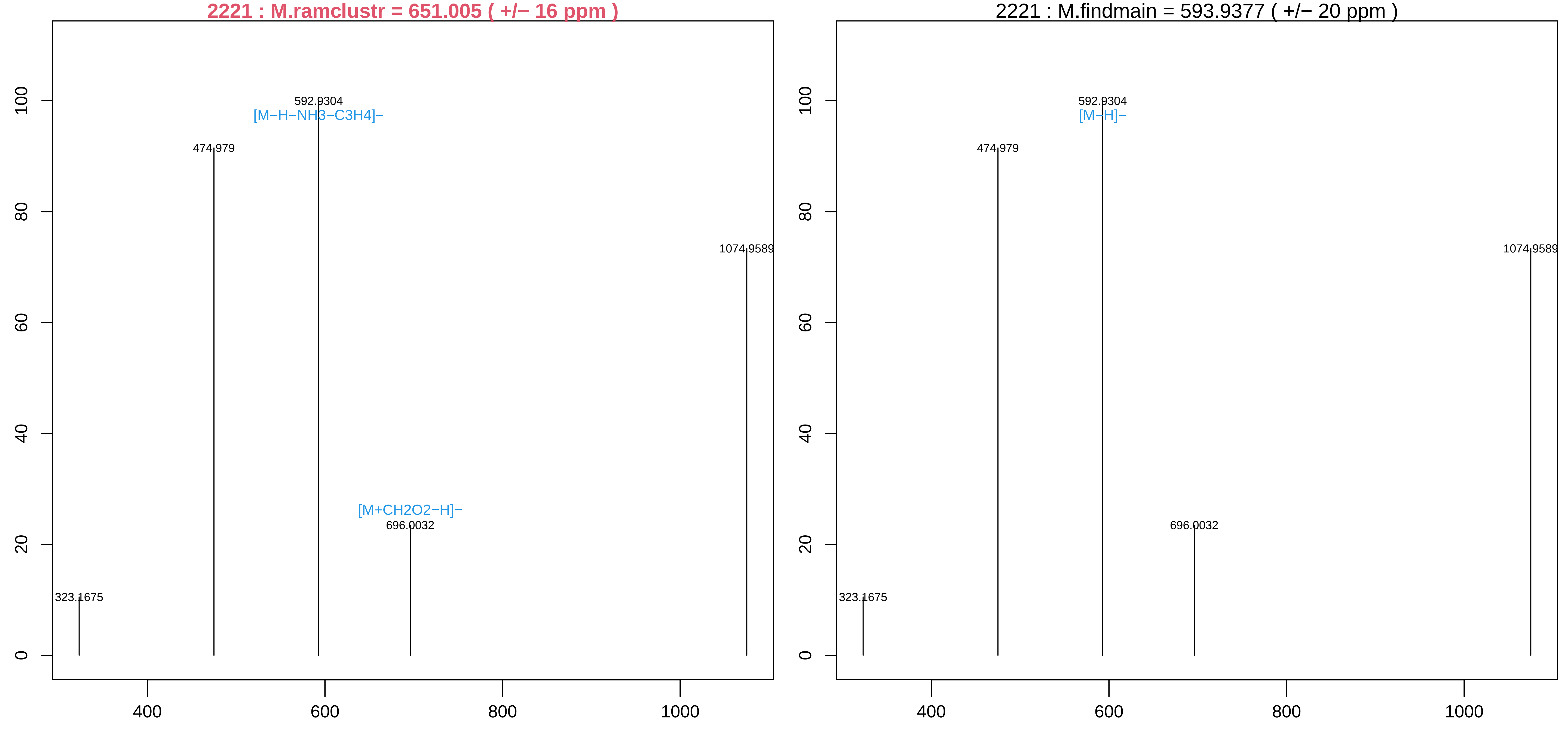
2221 : M.ramclustr = 651.005 (+/− 16 ppm)
592.9304
[M−H−NH3−C3H4]−
474.979
1074.9589
[M+CH2O2−H]−
696.0032
323.1675
2221 : M.findmain = 593.9377 (+/− 20 ppm)
592.9304
[M−H]−
474.979
1074.9589
696.0032
323.1675
0
20
40
60
80
100
400
600
800
1000

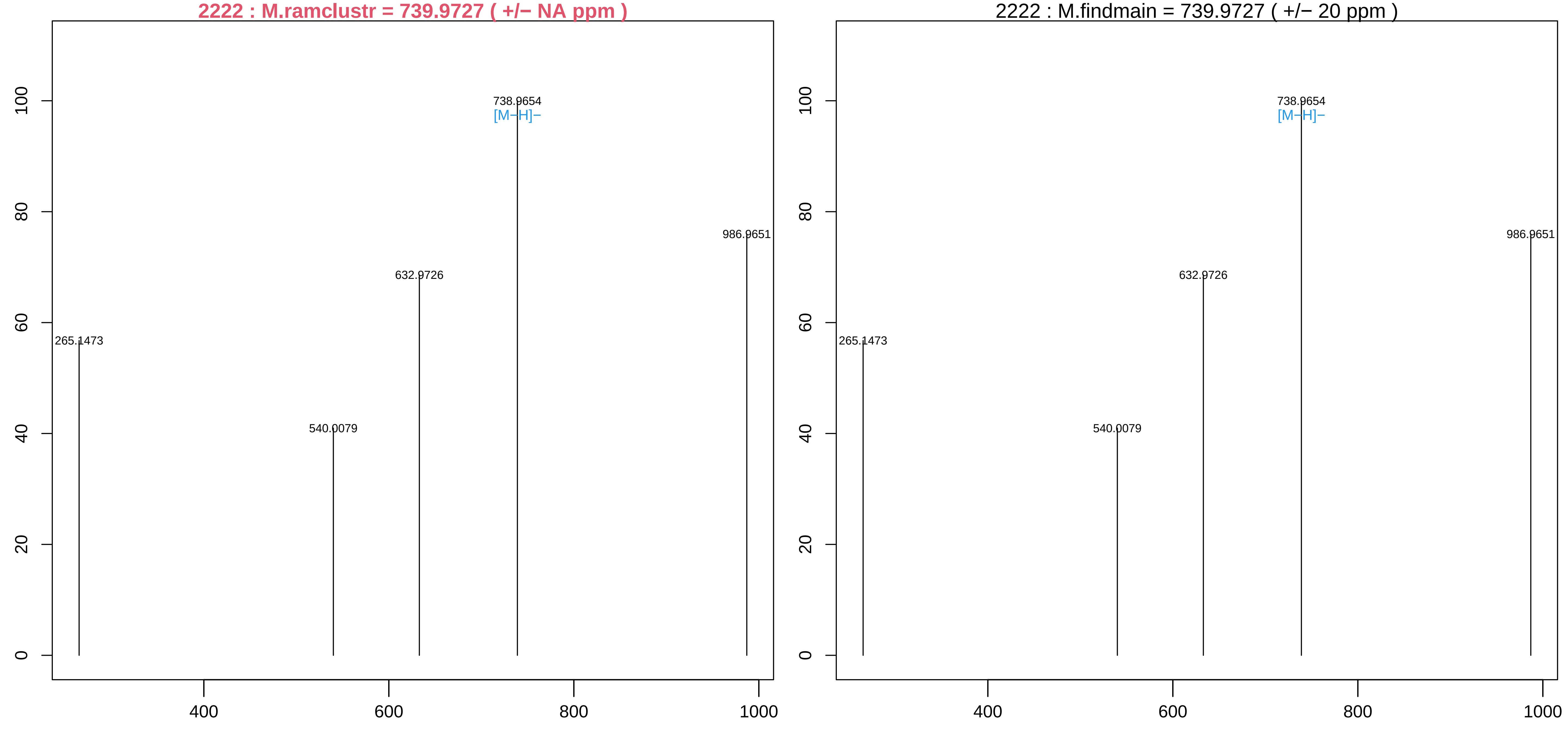

2222 : M.ramclustr = 739.9727 (+/− NA ppm)
738.9654
[M−H]−
986.9651
632.9726
265.1473
540.0079
2222 : M.findmain = 739.9727 (+/− 20 ppm)
738.9654
[M−H]−
986.9651
632.9726
265.1473
540.0079
0
20
40
60
80
100
400
600
800
1000

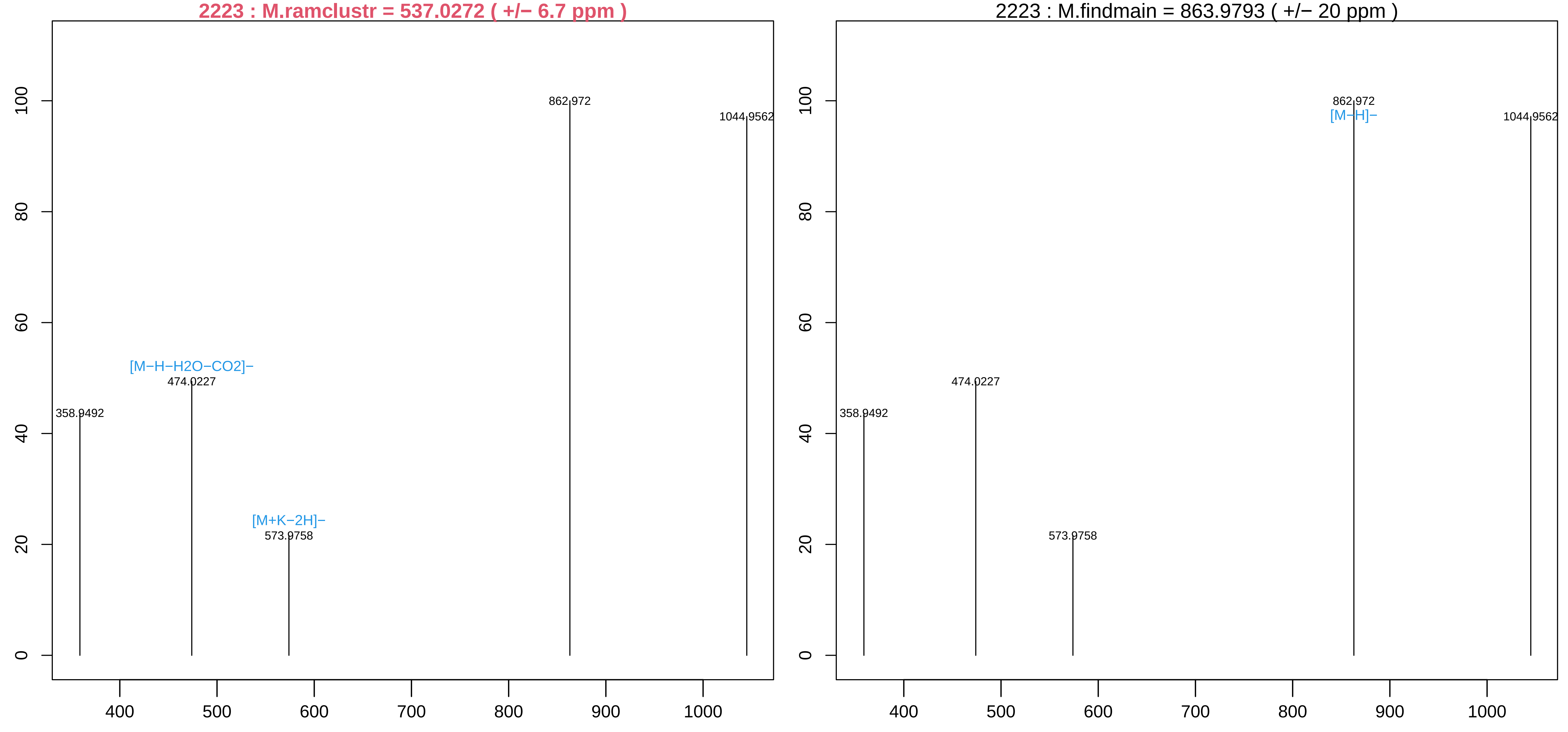
2223 : M.ramclustr = 537.0272 (+/− 6.7 ppm)
862.972
1044.9562
[M−H−H2O−CO2]−
474.0227
358.9492
[M+K−2H]−
573.9758
0
20
40
60
80
100
400
500
600
700
800
900
1000
2223 : M.findmain = 863.9793 (+/− 20 ppm)
862.972
[M−H]−
1044.9562
474.0227
358.9492
573.9758

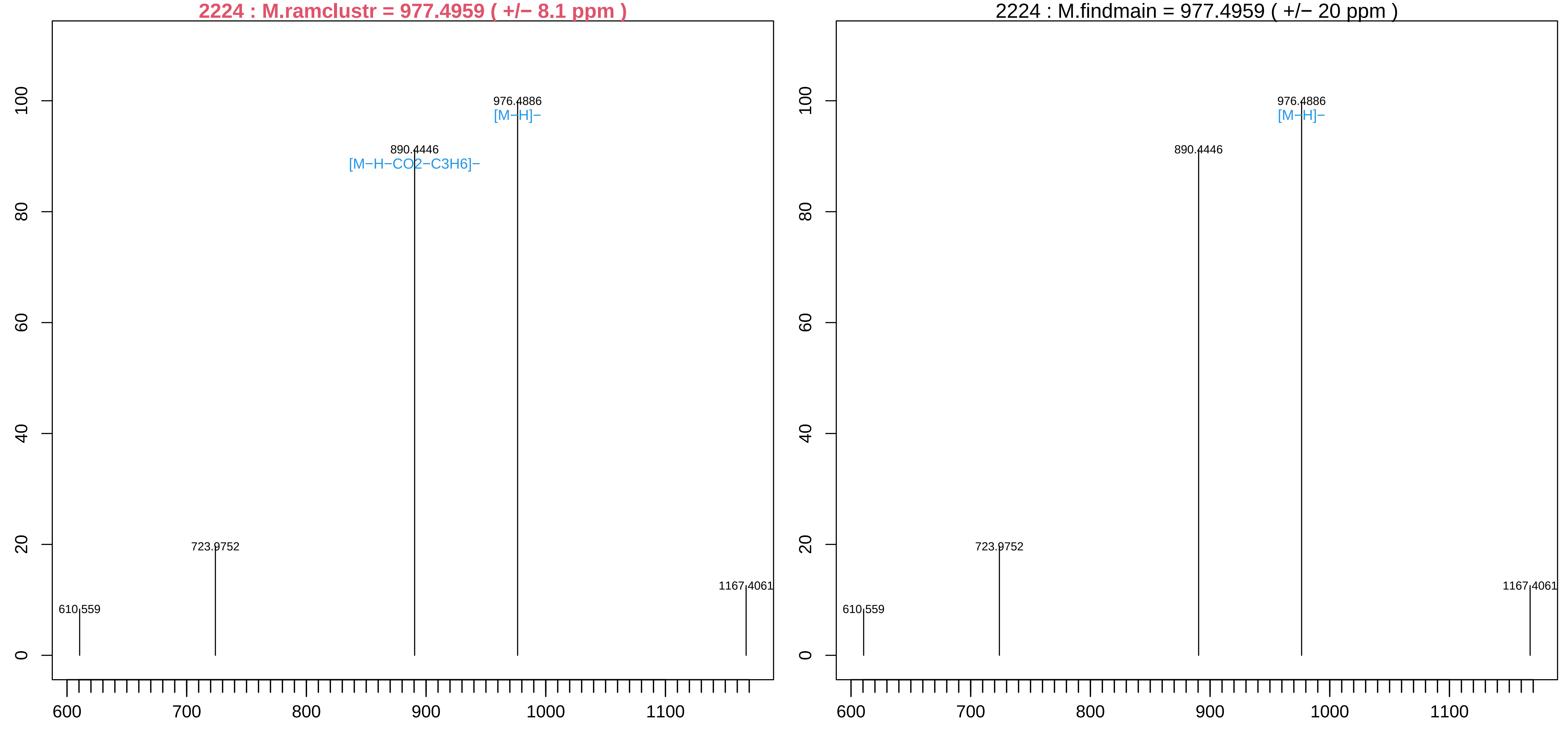

2224 : M.ramclustr = 977.4959 (+/− 8.1 ppm)
976.4886
[M−H]−
890.4446
[M−H−CO2−C3H6]−
723.9752
1167.4061
610.559
2224 : M.findmain = 977.4959 (+/− 20 ppm)
976.4886
[M−H]−
890.4446
723.9752
1167.4061
610.559

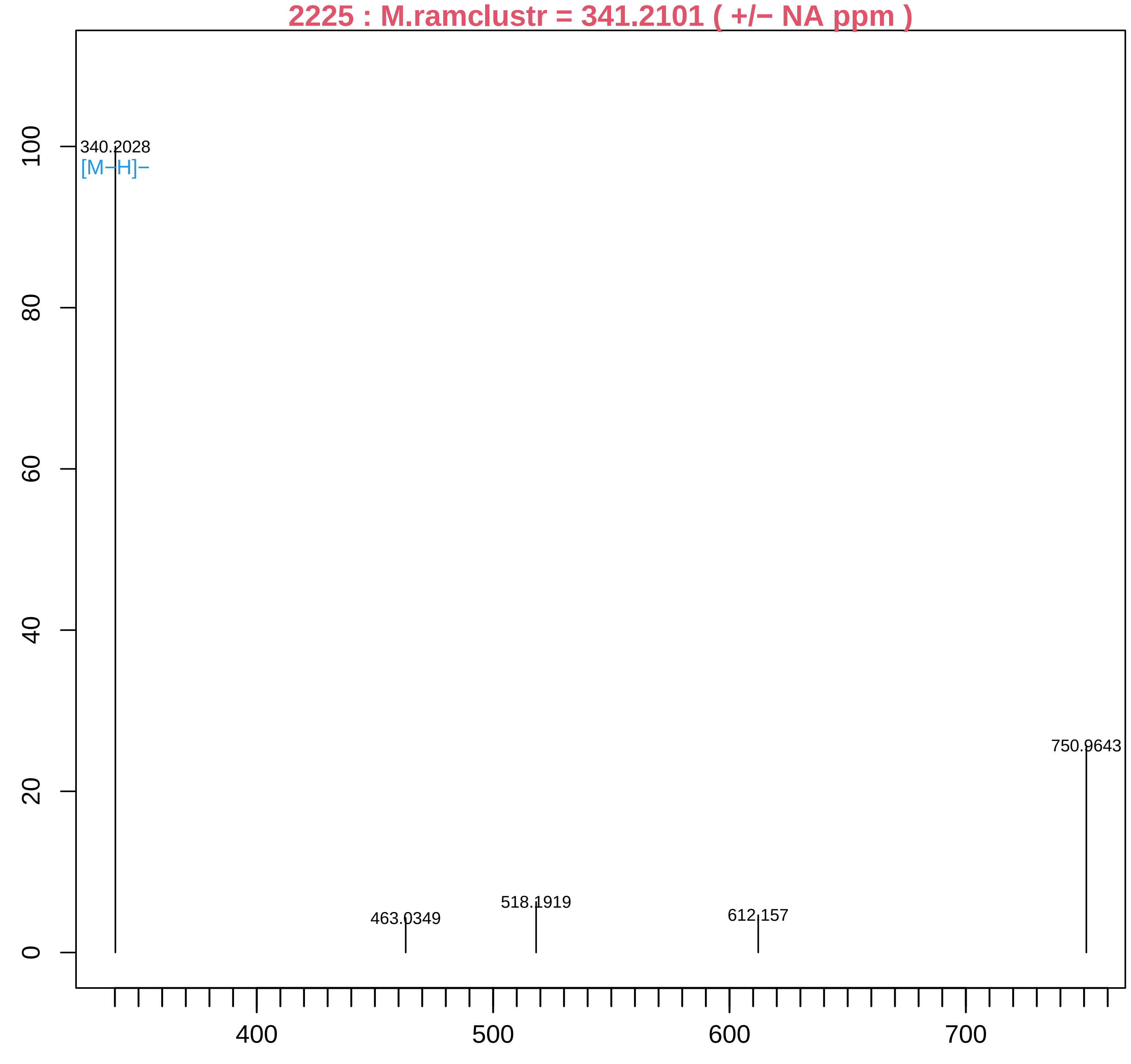

2225 : M.ramclustr = 341.2101 (+/− NA ppm)
340.2028
[M−H]−
463.0349
518.1919
612.157
750.9643
0
20
40
60
80
100
400
500
600
700

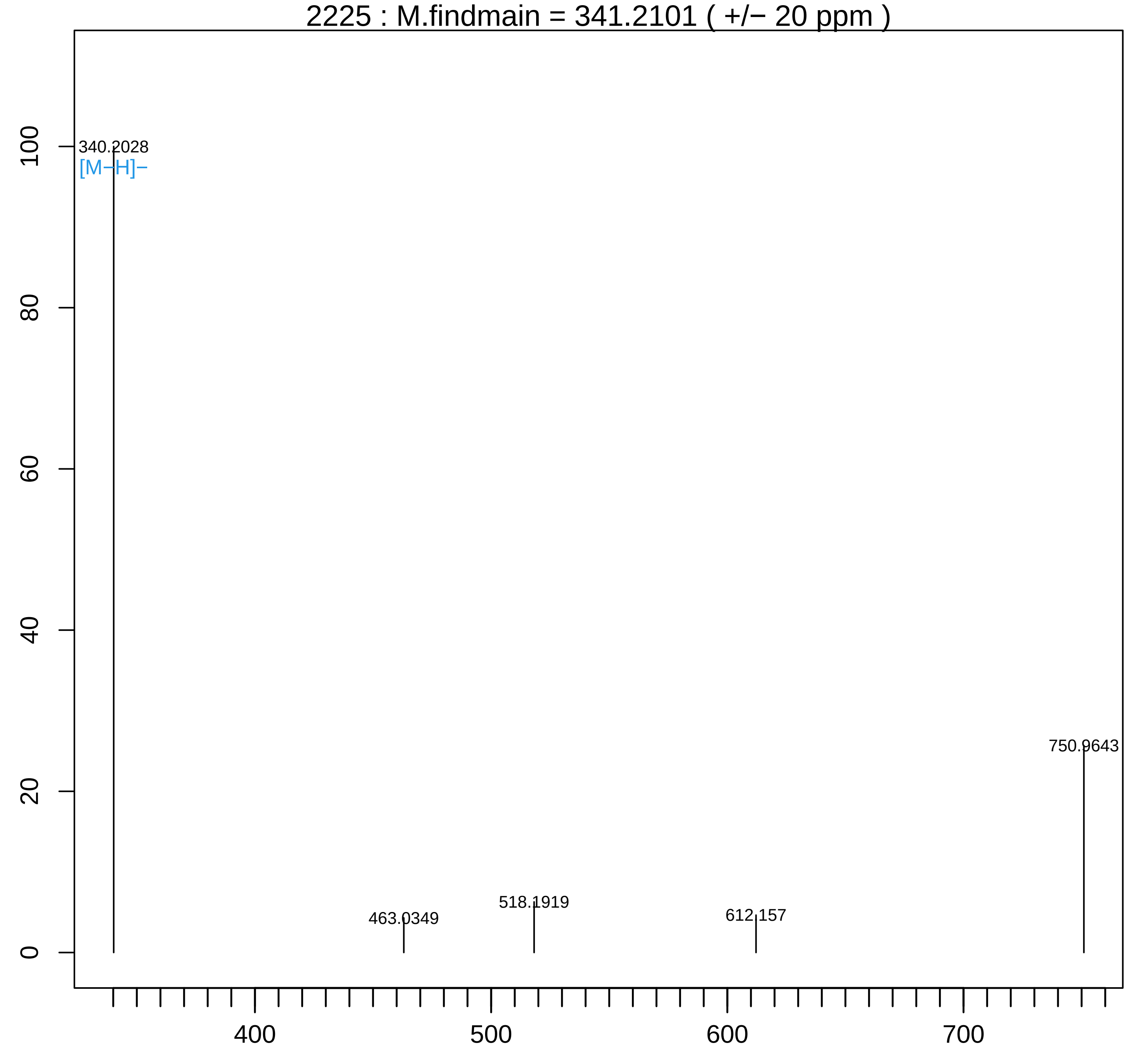

2225 : M.findmain = 341.2101 (+/− 20 ppm)
340.2028
[M−H]−
463.0349
518.1919
612.157
750.9643
0
20
40
60
80
100
400
500
600
700

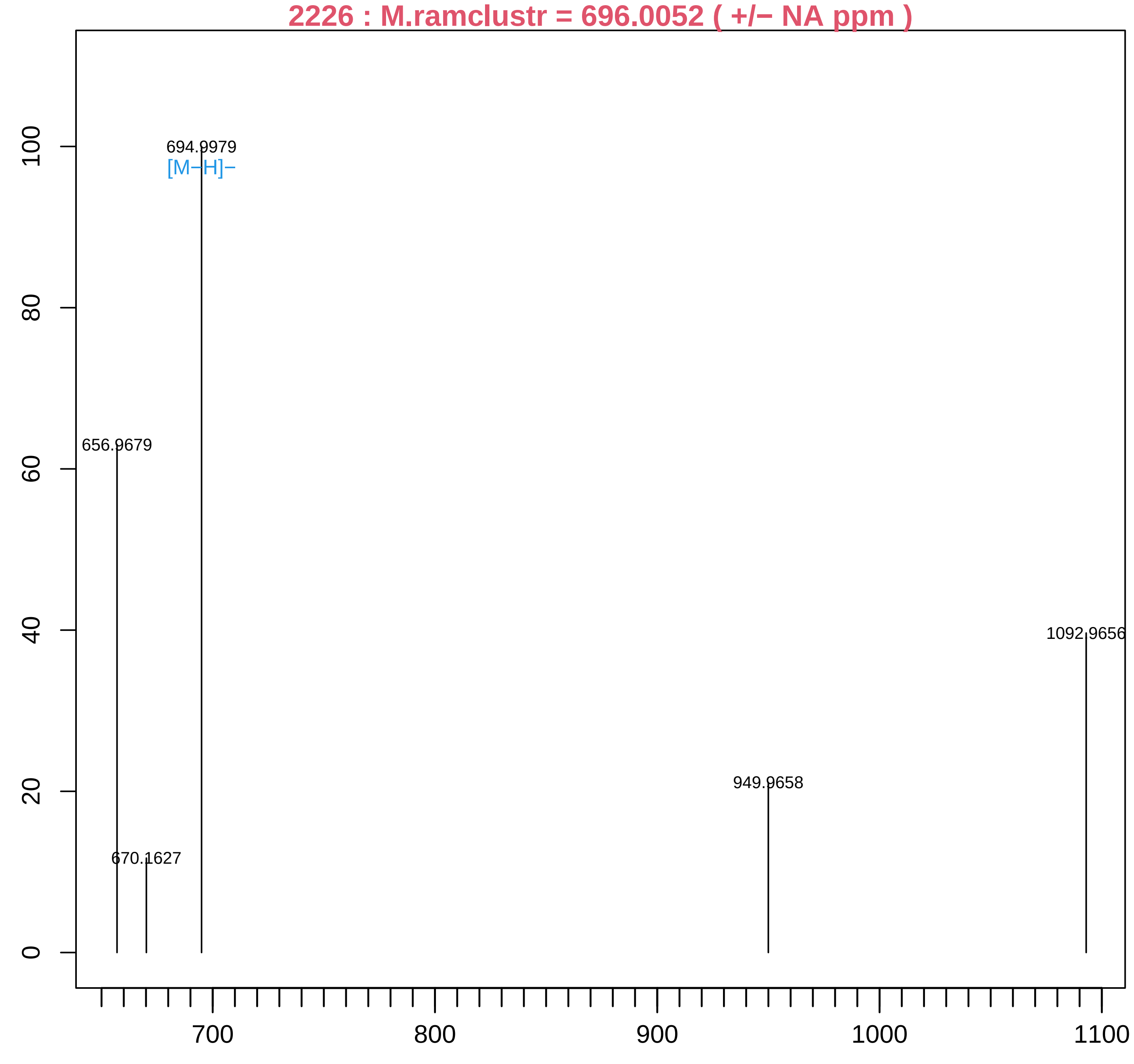
2226 : M.ramclustr = 696.0052 (+/− NA ppm)
694.9979
[M−H]−
656.9679
670.1627
949.9658
1092.9656
0
20
40
60
80
100
700
800
900
1000
1100

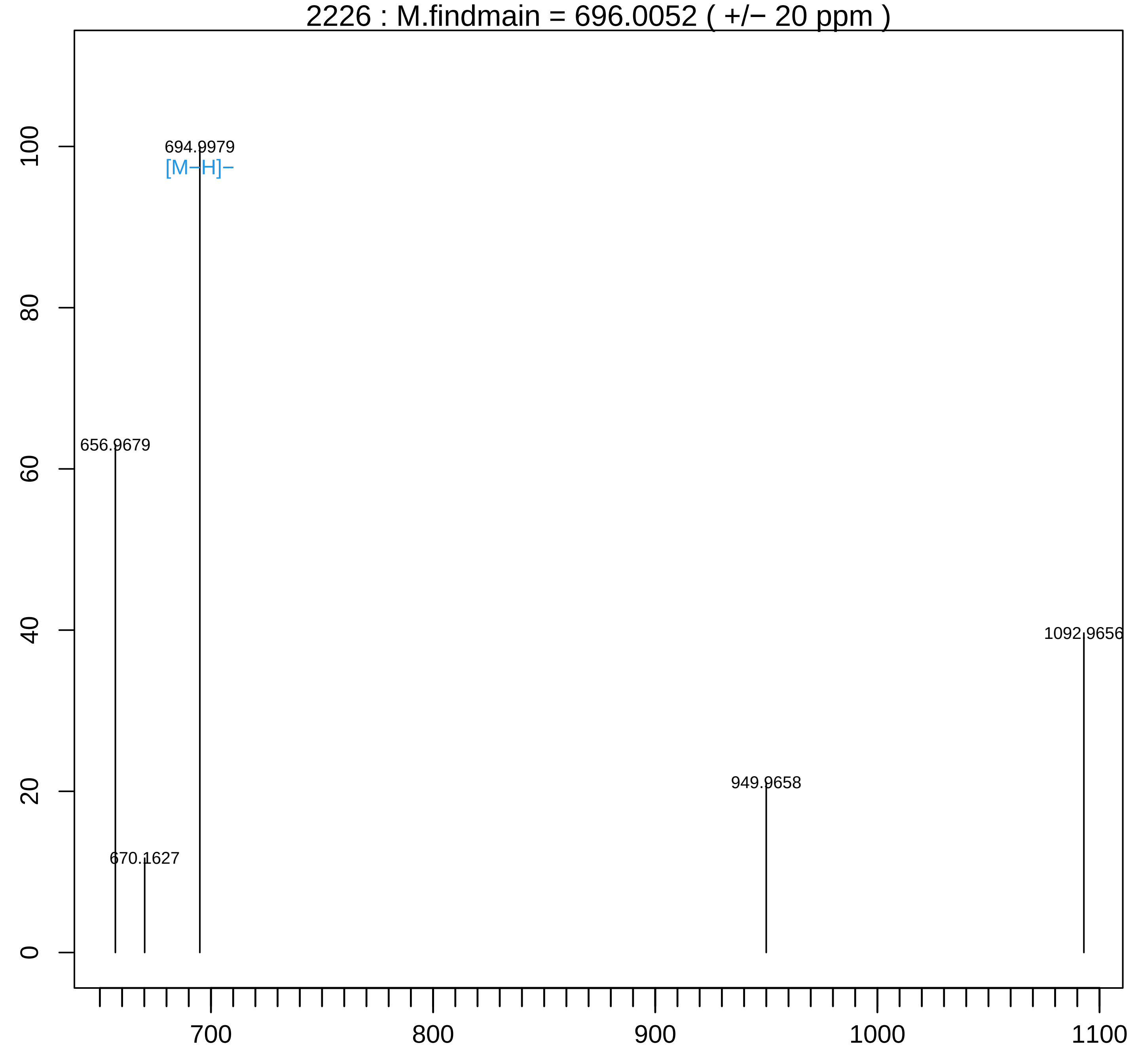
2226 : M.findmain = 696.0052 (+/− 20 ppm)
694.9979
[M−H]−
656.9679
670.1627
949.9658
1092.9656
0
20
40
60
80
100
700
800
900
1000
1100

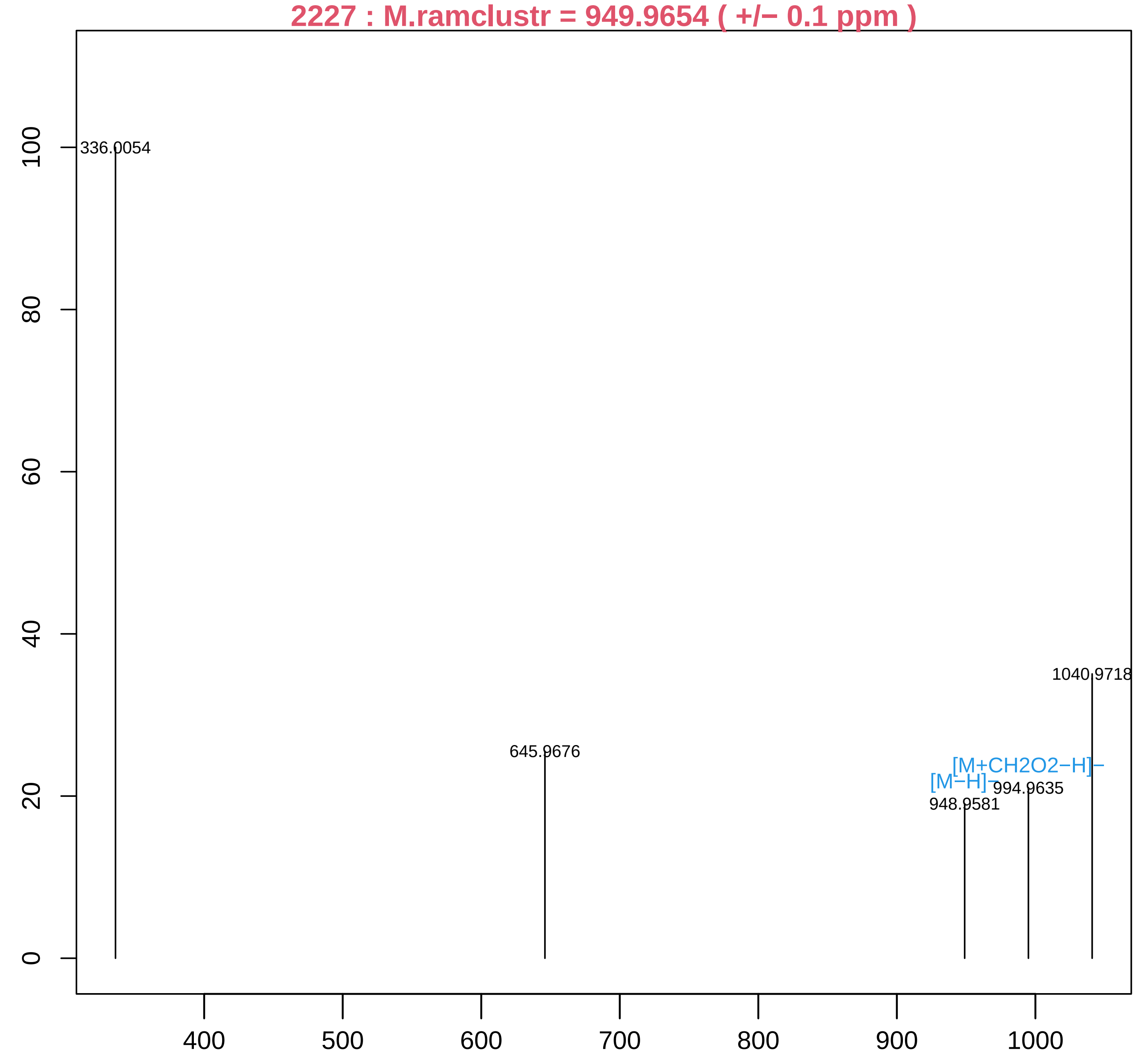
2227 : M.ramclustr = 949.9654 (+/− 0.1 ppm)
336.0054
645.9676
[M−H]−
948.9581
[M+CH2O2−H]−
994.9635
1040.9718
0
20
40
60
80
100
400
500
600
700
800
900
1000
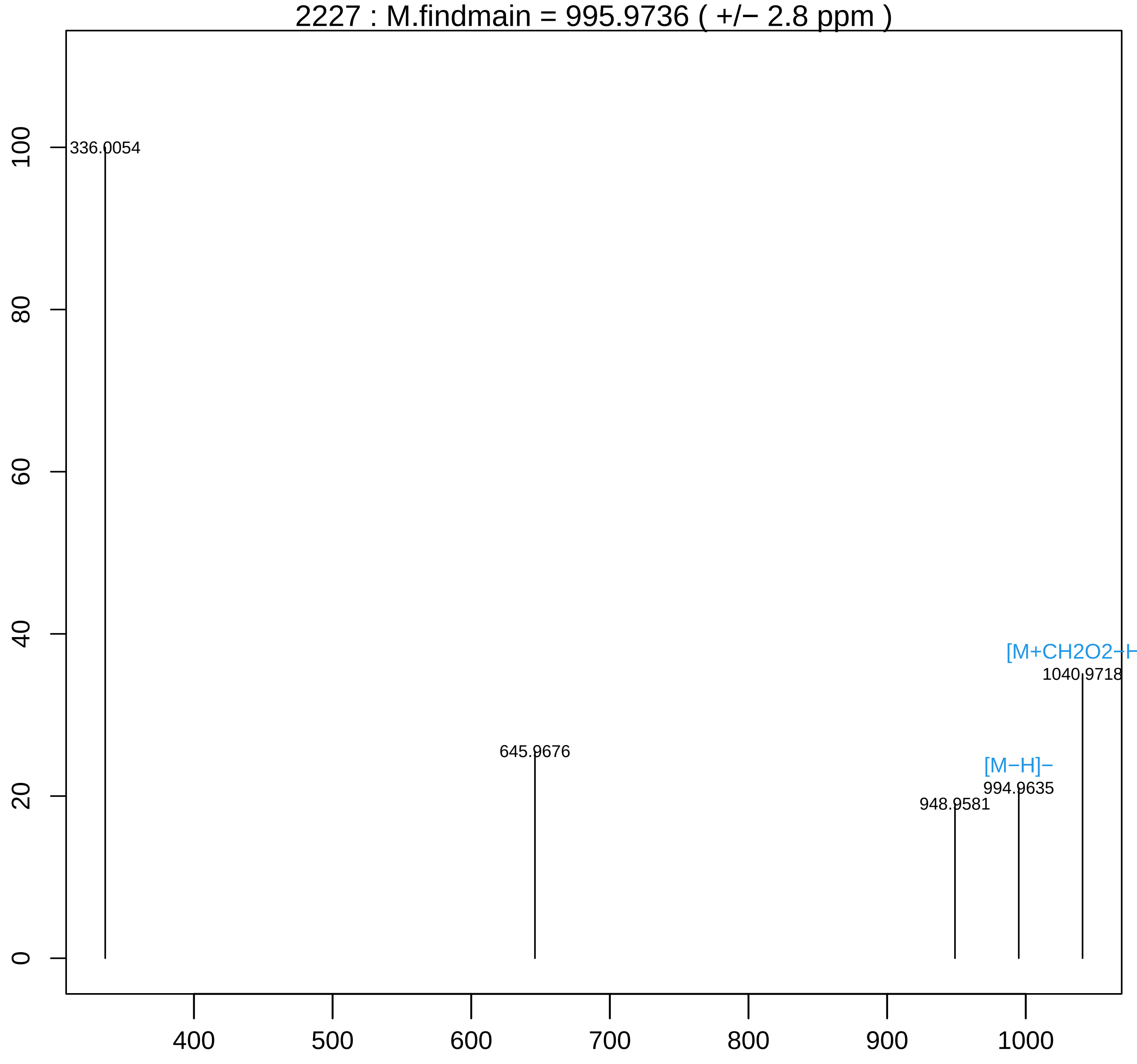
2227 : M.findmain = 995.9736 (+/− 2.8 ppm)
336.0054
645.9676
948.9581
[M−H]−
994.9635
[M+CH2O2−H
1040.9718
0
20
40
60
80
100
400
500
600
700
800
900
1000

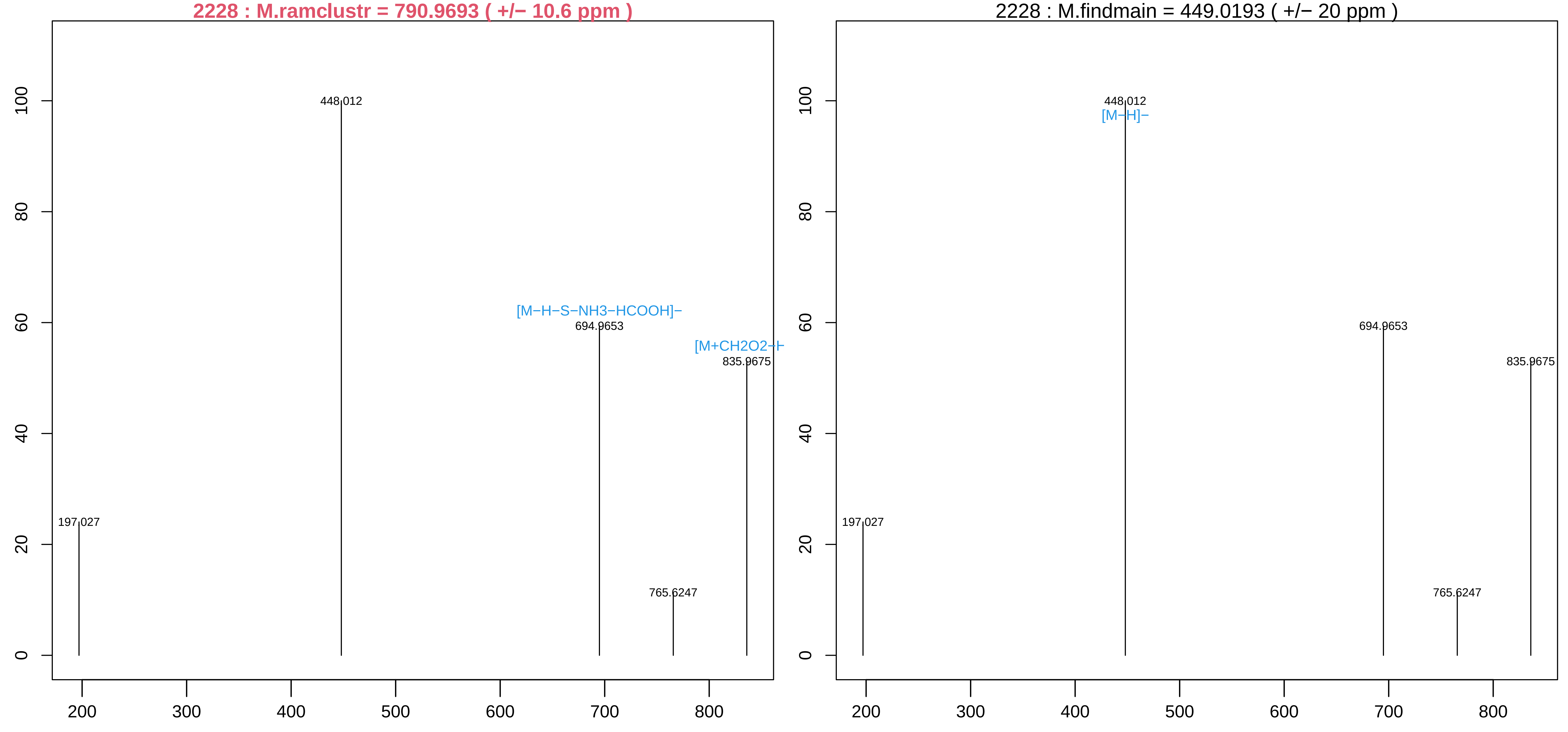
2228 : M.ramclustr = 790.9693 (+/− 10.6 ppm)
448.012
[M−H−S−NH3−HCOOH]−
694.9653
[M+CH2O2−H
835.9675
765.6247
197.027
2228 : M.findmain = 449.0193 (+/− 20 ppm)
448.012
[M−H]−
694.9653
835.9675
765.6247
197.027

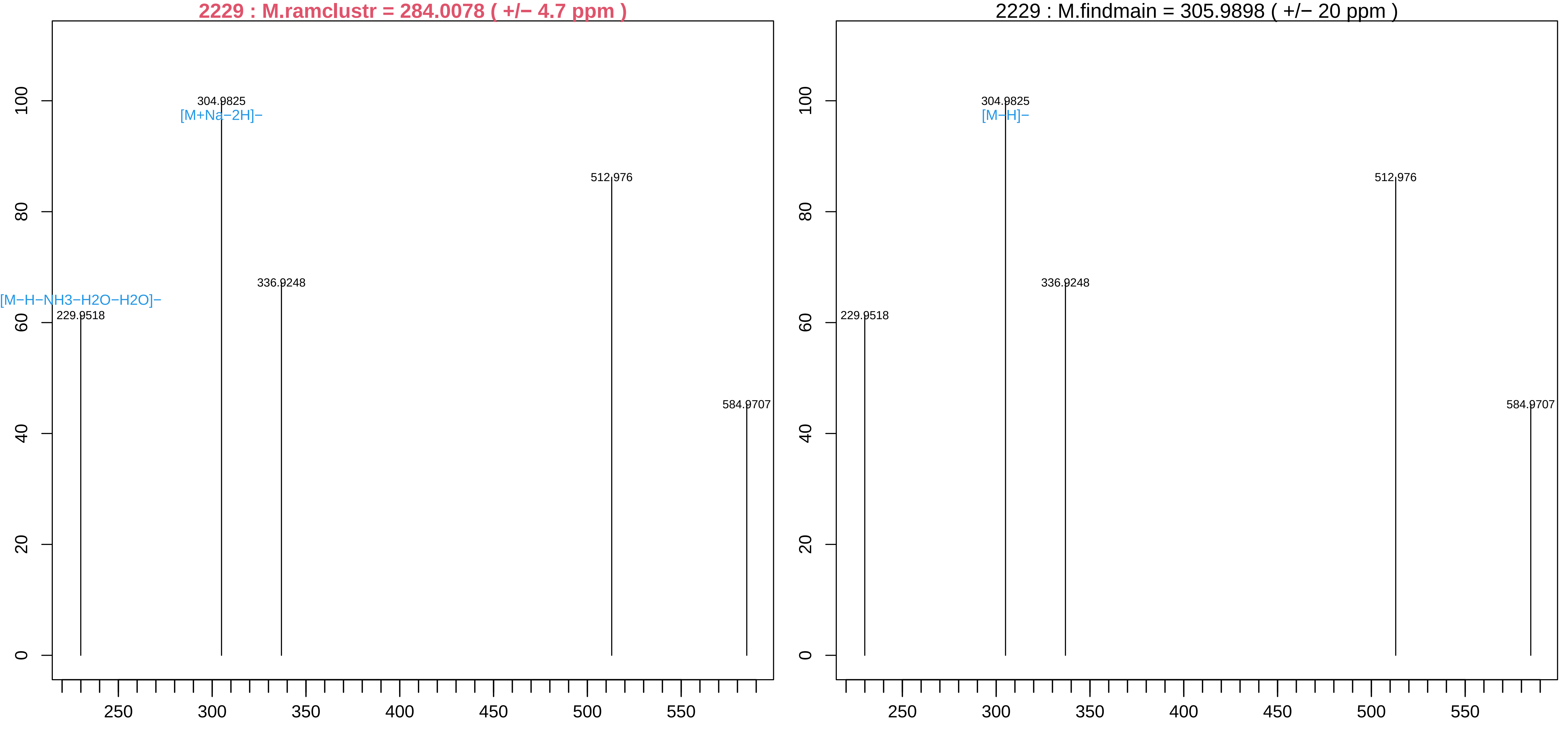

2229 : M.ramclustr = 284.0078 (+/− 4.7 ppm)
304.9825
[M+Na−2H]−
336.9248
512.976
584.9707
229.9518
[M−H−NH3−H2O−H2O]−
2229 : M.findmain = 305.9898 (+/− 20 ppm)
304.9825
[M−H]−
336.9248
512.976
584.9707
229.9518
0
20
40
60
80
100
250
300
350
400
450
500
550

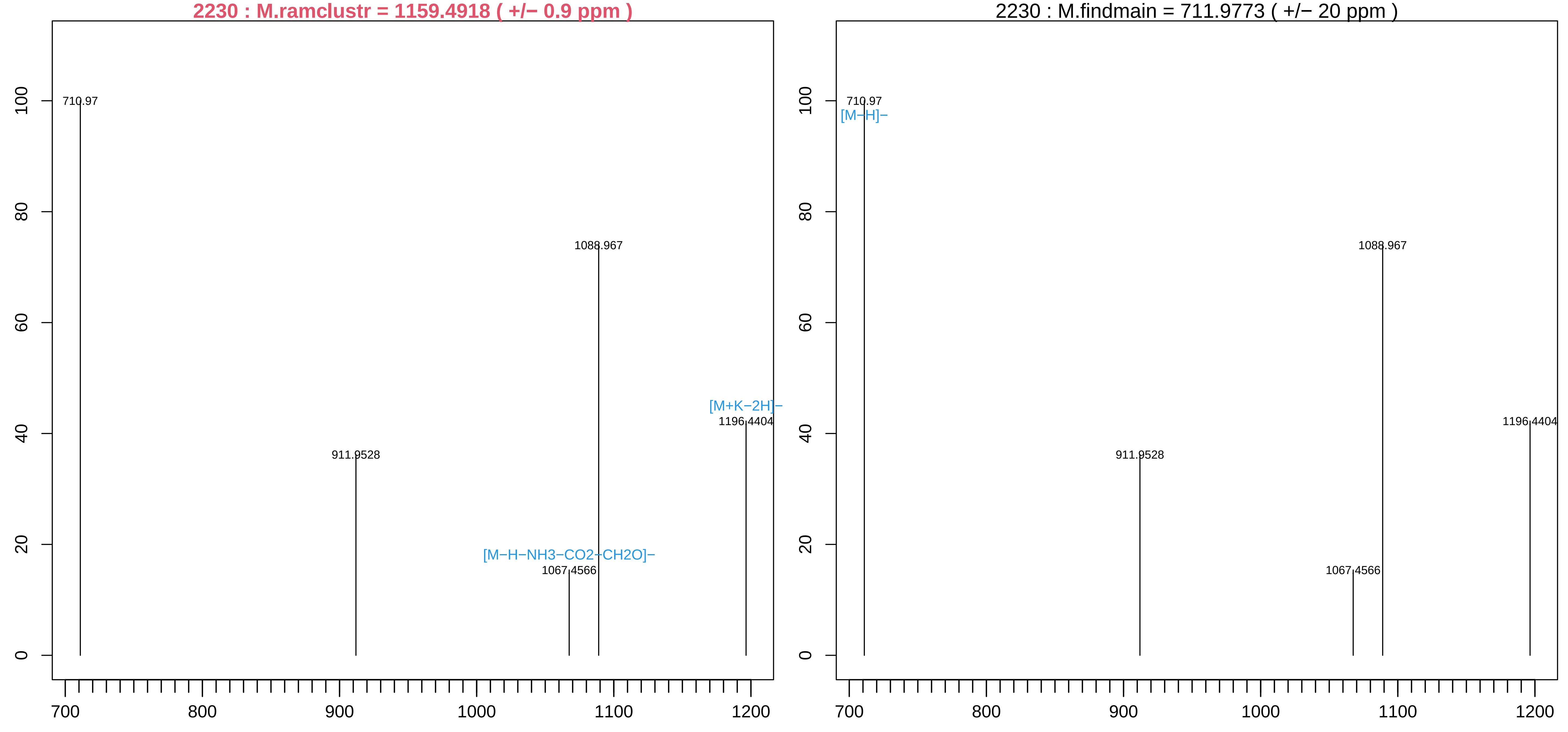
2230 : M.ramclustr = 1159.4918 (+/− 0.9 ppm)
710.97
911.9528
1088.967
[M−H−NH3−CO2−CH2O]−
1067.4566
[M+K−2H]−
1196.4404
2230 : M.findmain = 711.9773 (+/− 20 ppm)
710.97
[M−H]−
911.9528
1088.967
1067.4566
1196.4404
0
20
40
60
80
100
700
800
900
1000
1100
1200

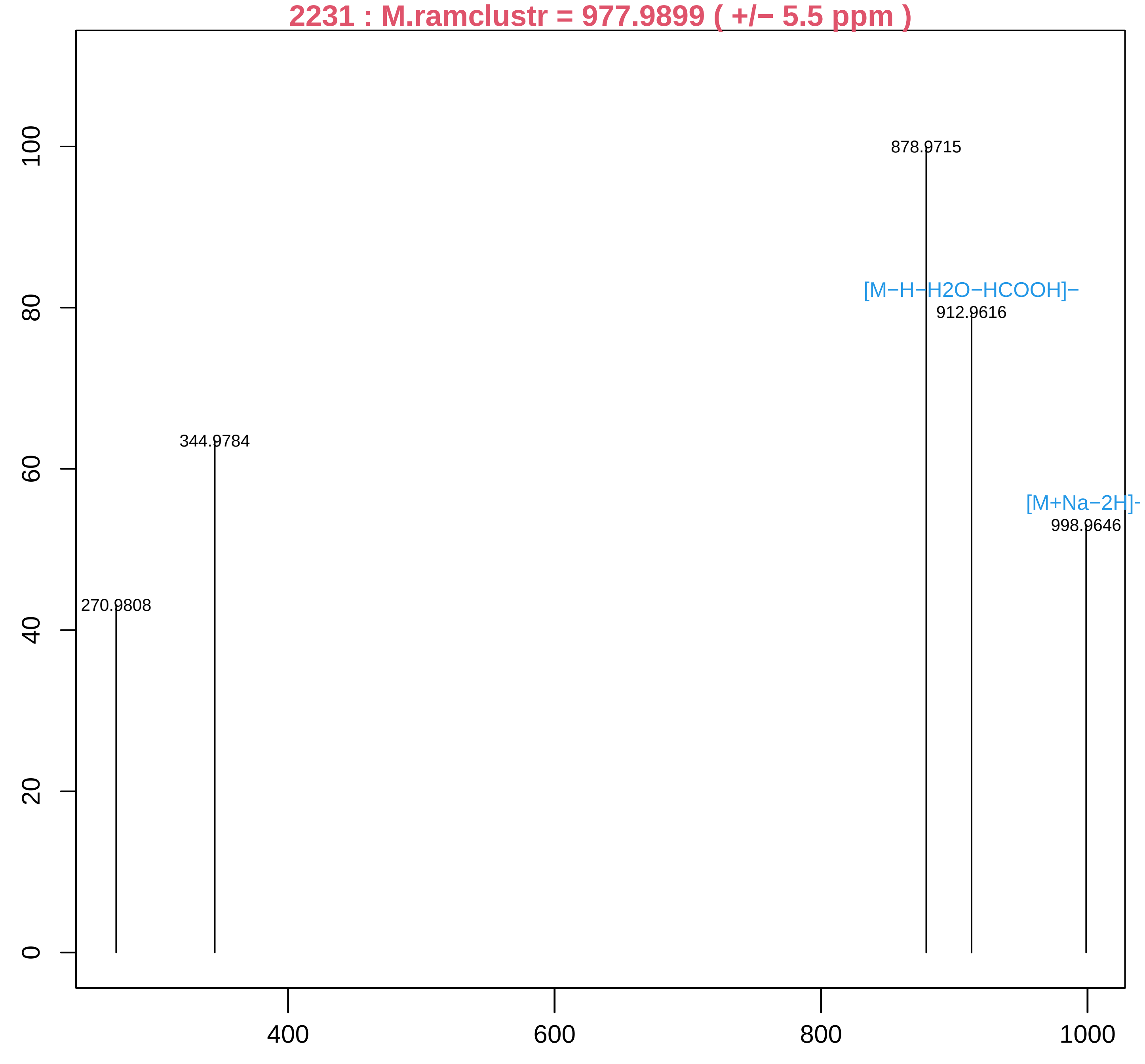
2231 : M.ramclustr = 977.9899 (+/− 5.5 ppm)
878.9715
[M−H−H2O−HCOOH]−
912.9616
344.9784
[M+Na−2H]-
998.9646
270.9808
0
20
40
60
80
100
400
600
800
1000

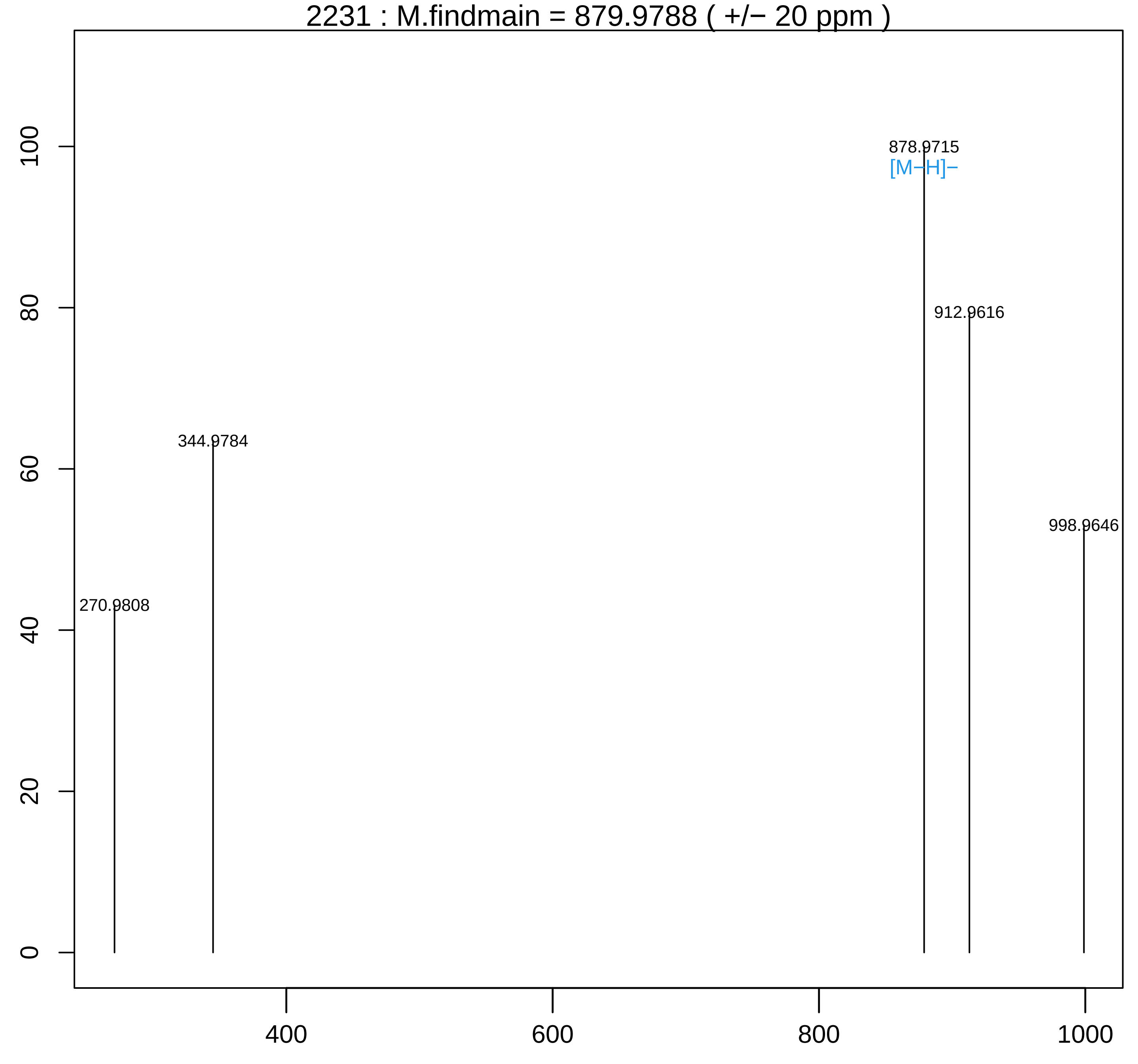
2231 : M.findmain = 879.9788 (+/− 20 ppm)
878.9715
[M−H]−
912.9616
344.9784
998.9646
270.9808
0
20
40
60
80
100
400
600
800
1000

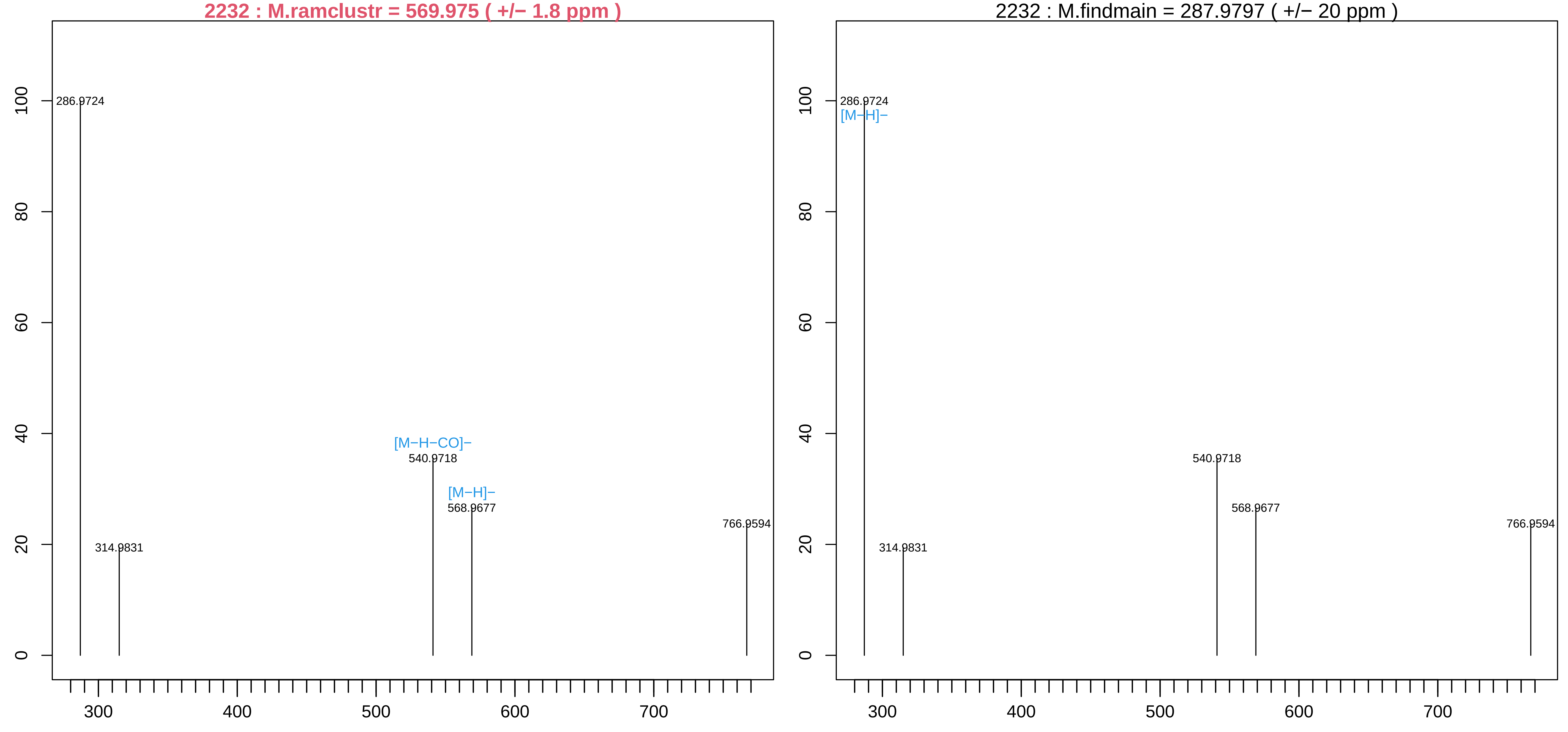
2232 : M.ramclustr = 569.975 (+/− 1.8 ppm)
286.9724
314.9831
[M−H−CO]−
540.9718
[M−H]−
568.9677
766.9594
2232 : M.findmain = 287.9797 (+/− 20 ppm)
286.9724
[M−H]−
314.9831
540.9718
568.9677
766.9594
0
20
40
60
80
100
300
400
500
600
700

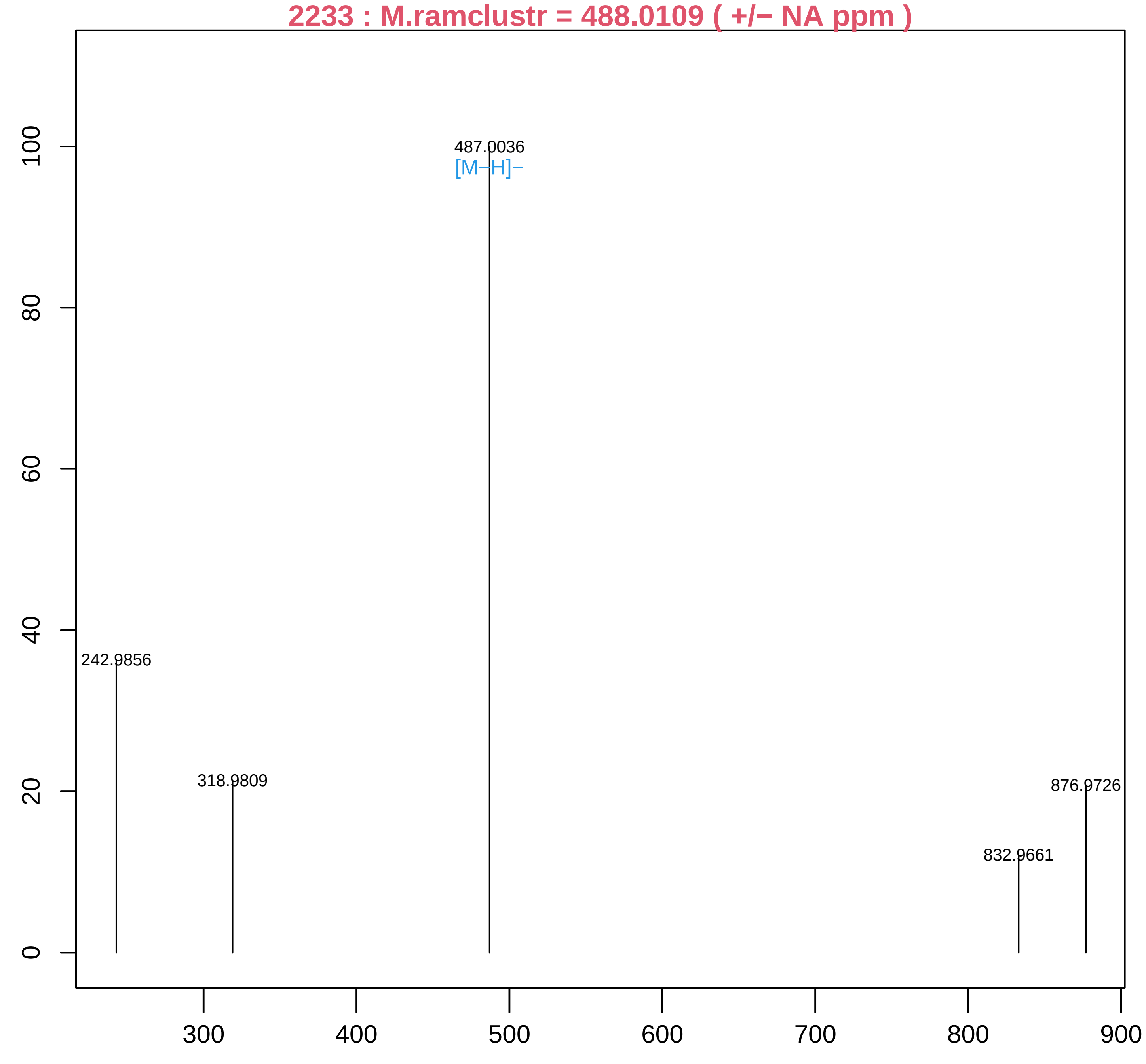
2233 : M.ramclustr = 488.0109 (+/− NA ppm)
487.0036
[M−H]−
242.9856
318.9809
832.9661
876.9726
0
20
40
60
80
100
300
400
500
600
700
800
900

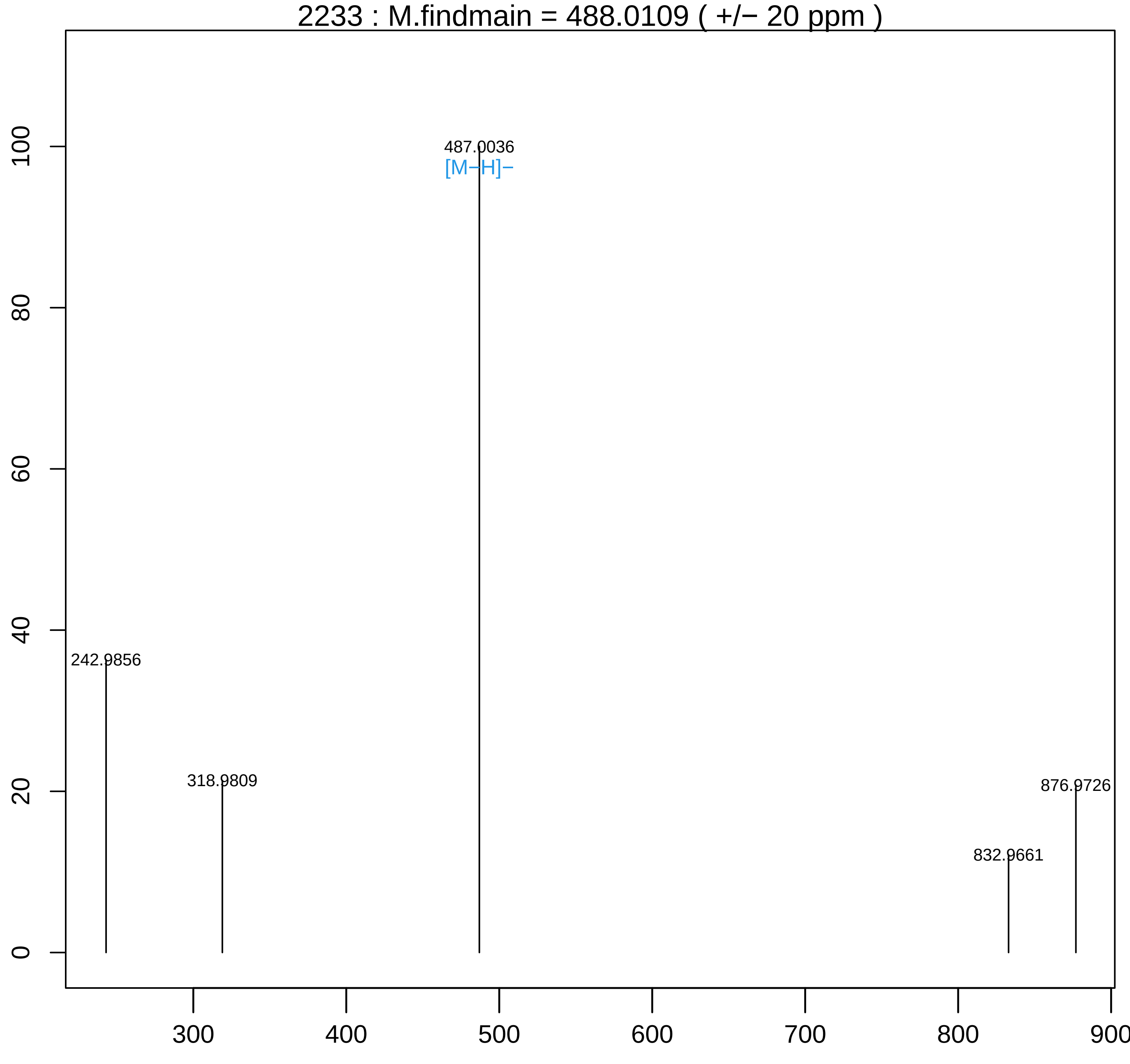
2233 : M.findmain = 488.0109 (+/− 20 ppm)
487.0036
[M−H]−
242.9856
318.9809
832.9661
876.9726
0
20
40
60
80
100
300
400
500
600
700
800
900

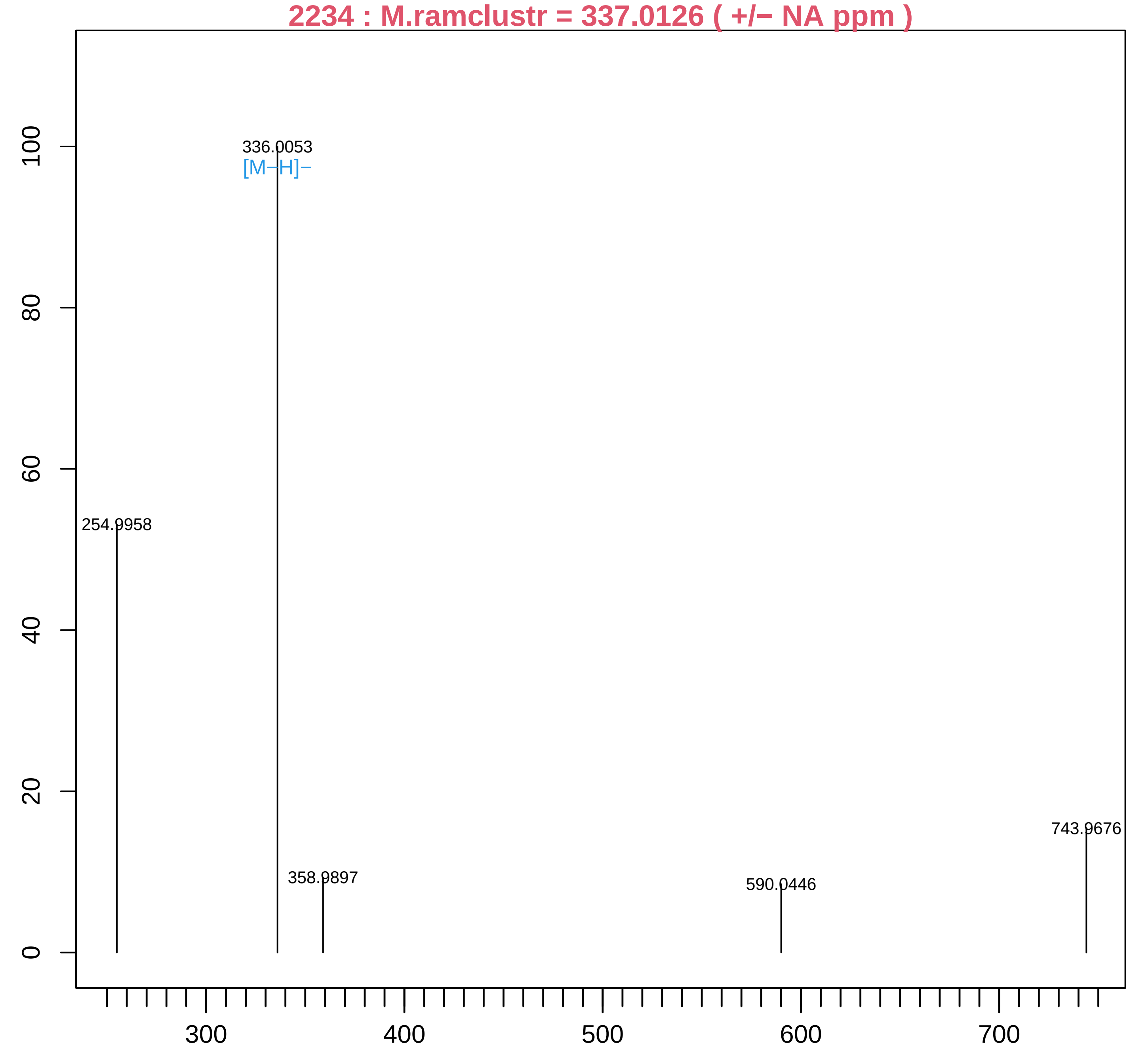
2234 : M.ramclustr = 337.0126 (+/− NA ppm)
336.0053
[M−H]−
254.9958
358.9897
590.0446
743.9676
0
20
40
60
80
100
300
400
500
600
700

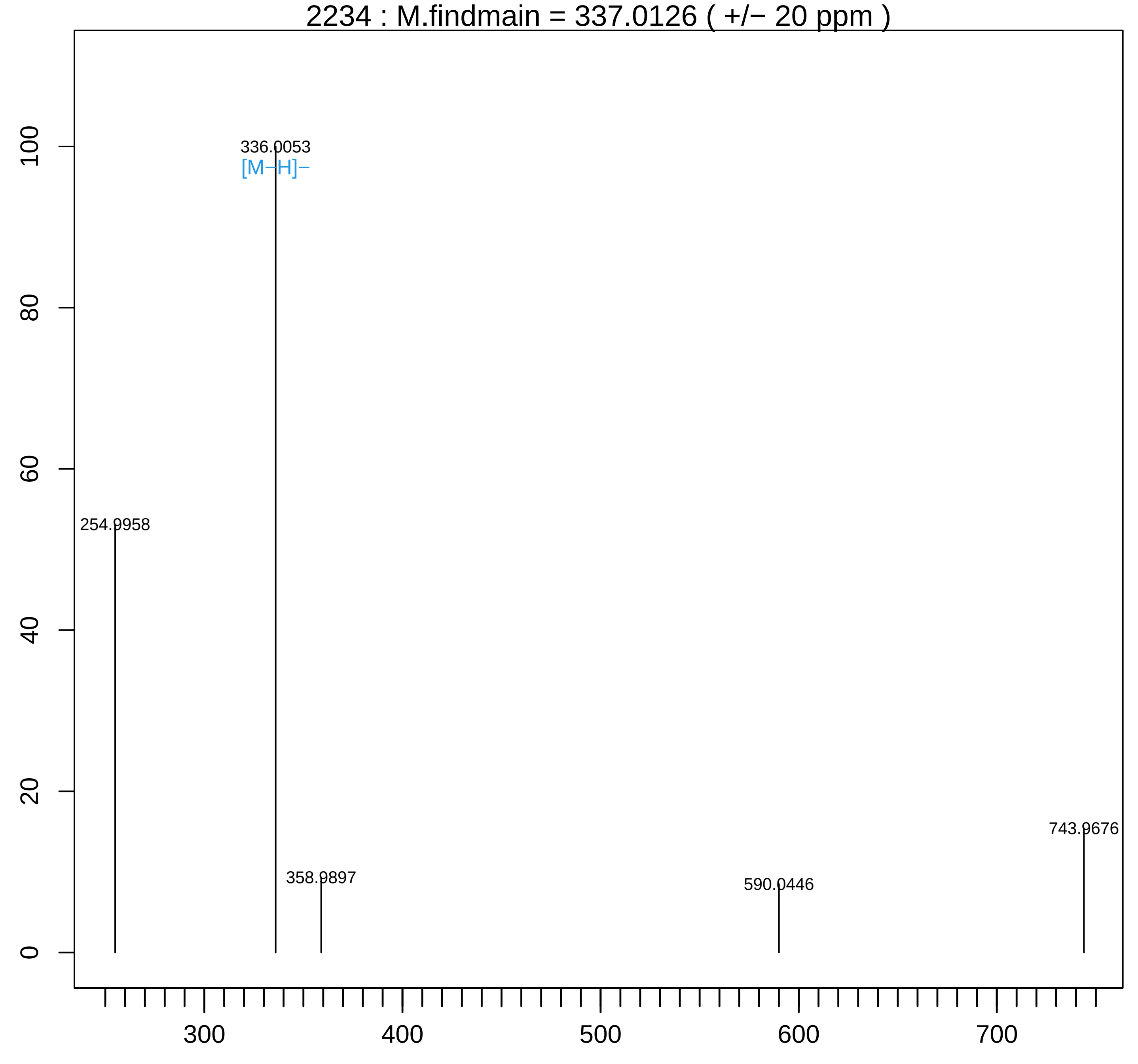
2234 : M.findmain = 337.0126 (+/− 20 ppm)
336.0053
[M−H]−
254.9958
358.9897
590.0446
743.9676
0
20
40
60
80
100
300
400
500
600
700

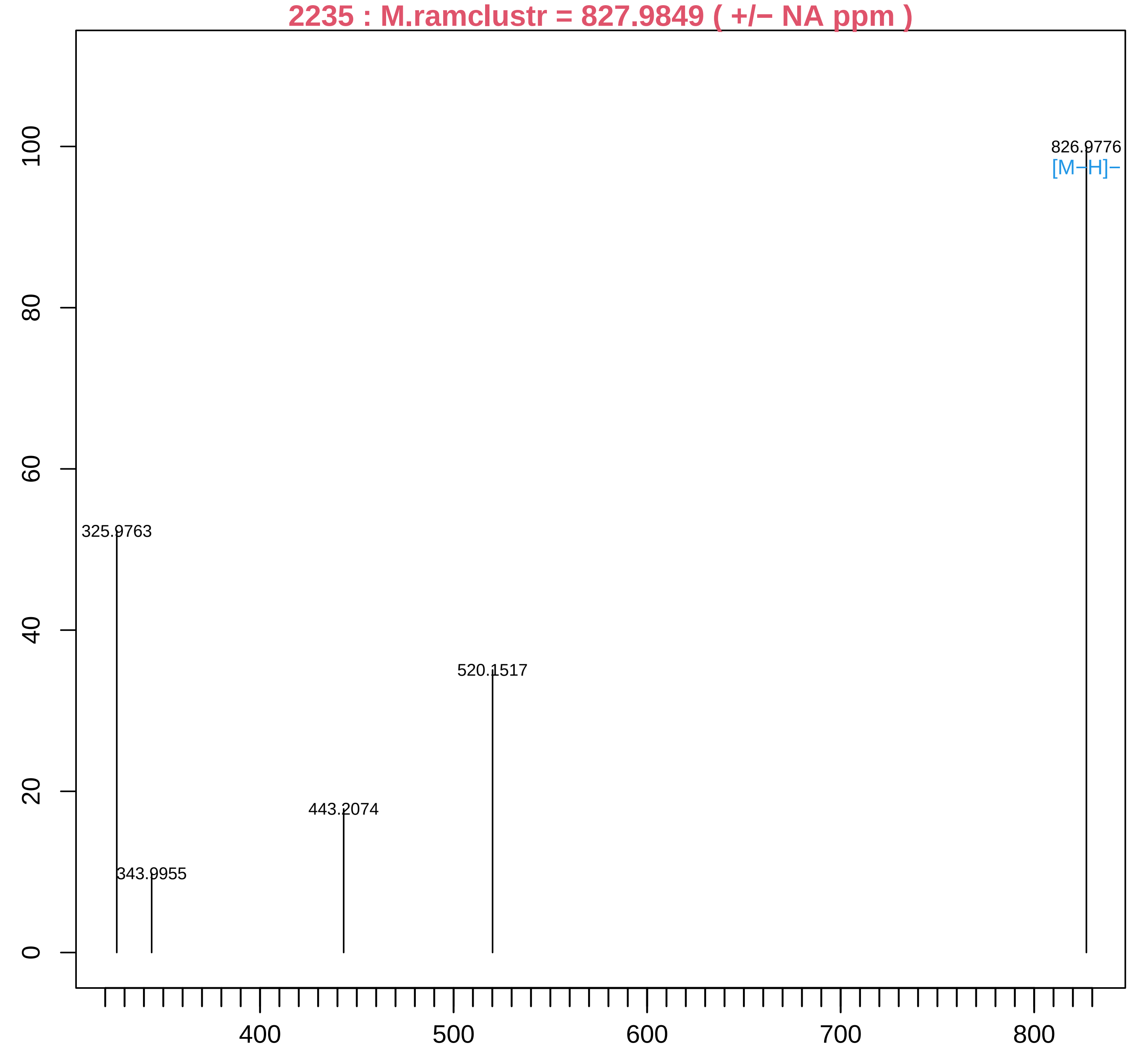
2235 : M.ramclustr = 827.9849 (+/− NA ppm)
826.9776
[M−H]−
325.9763
343.9955
443.2074
520.1517
0
20
40
60
80
100
400
500
600
700
800

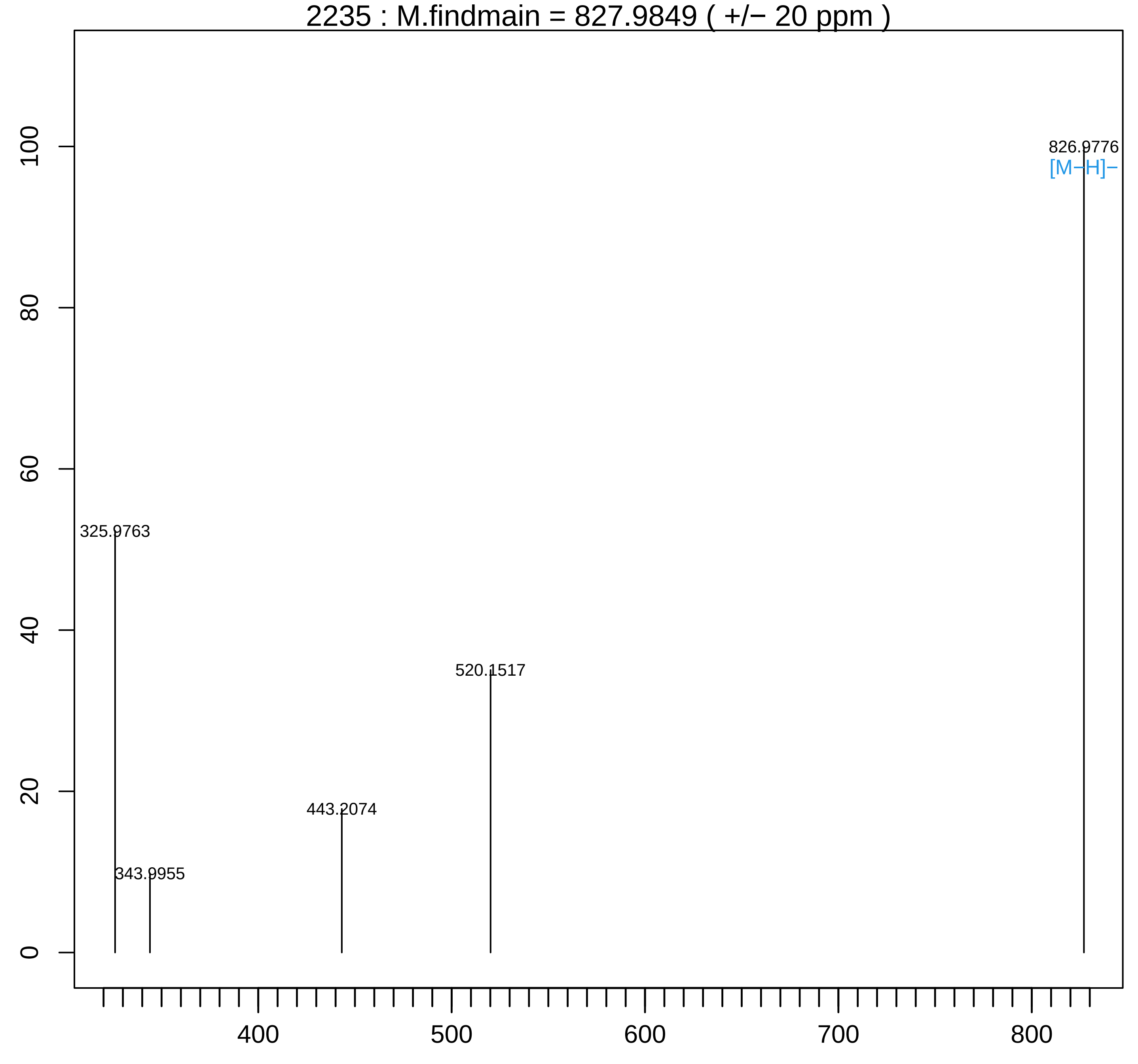
2235 : M.findmain = 827.9849 (+/− 20 ppm)
826.9776
[M−H]−
325.9763
343.9955
443.2074
520.1517
0
20
40
60
80
100
400
500
600
700
800

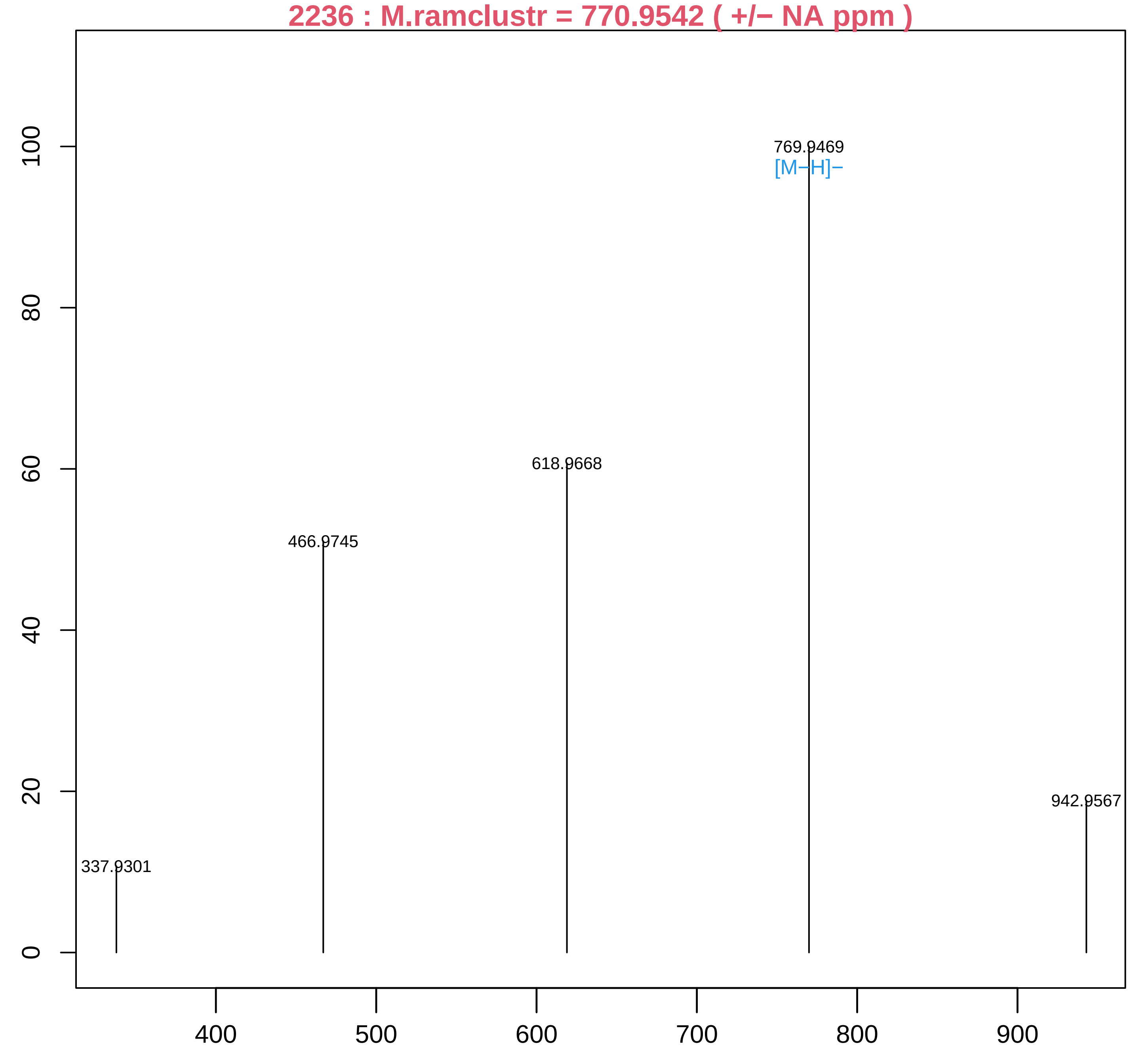
2236 : M.ramclustr = 770.9542 (+/− NA ppm)
769.9469
[M−H]−
618.9668
466.9745
942.9567
337.9301
0
20
40
60
80
100
400
500
600
700
800
900

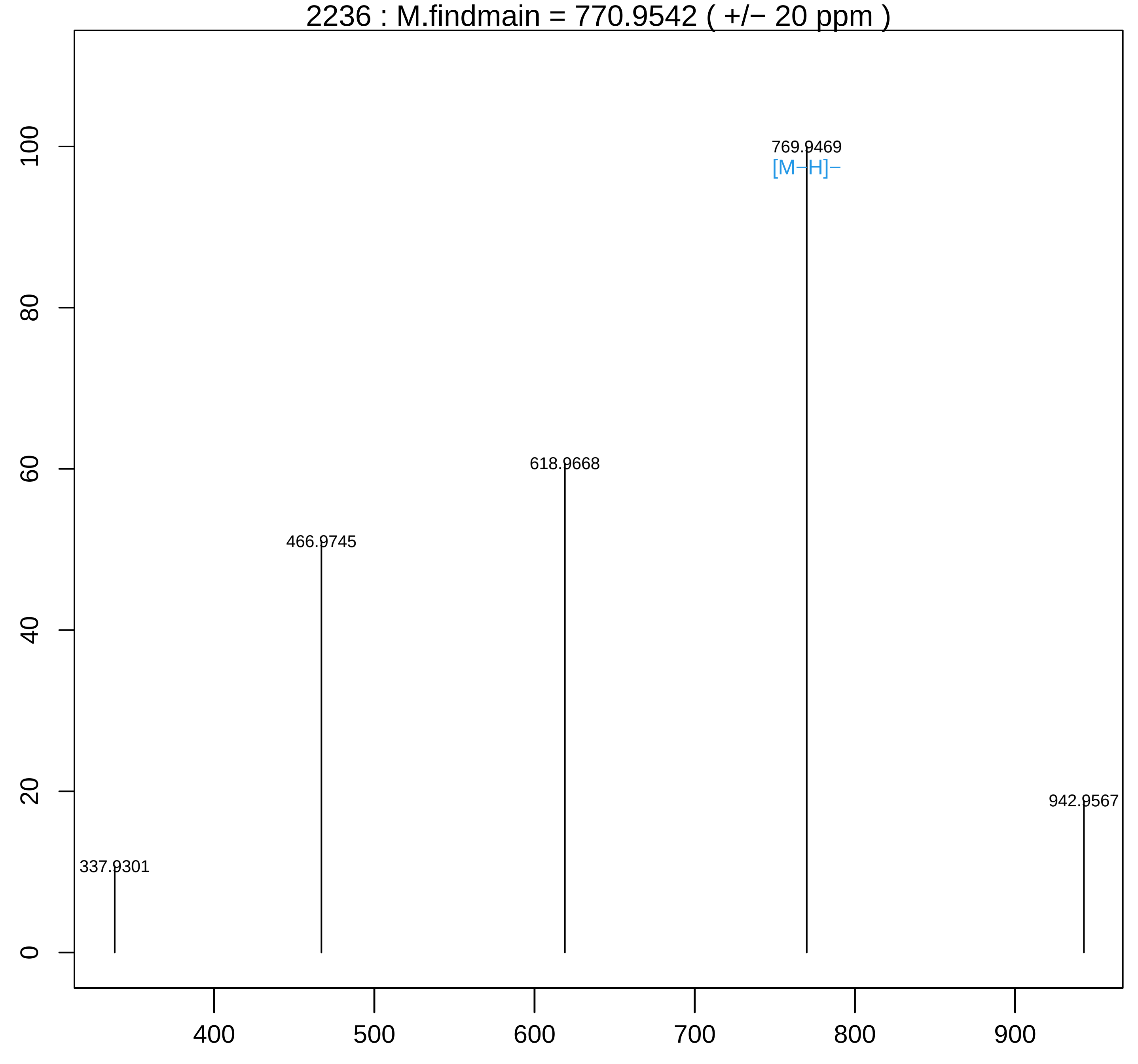
2236 : M.findmain = 770.9542 (+/− 20 ppm)
769.9469
[M−H]−
618.9668
466.9745
942.9567
337.9301
0
20
40
60
80
100
400
500
600
700
800
900

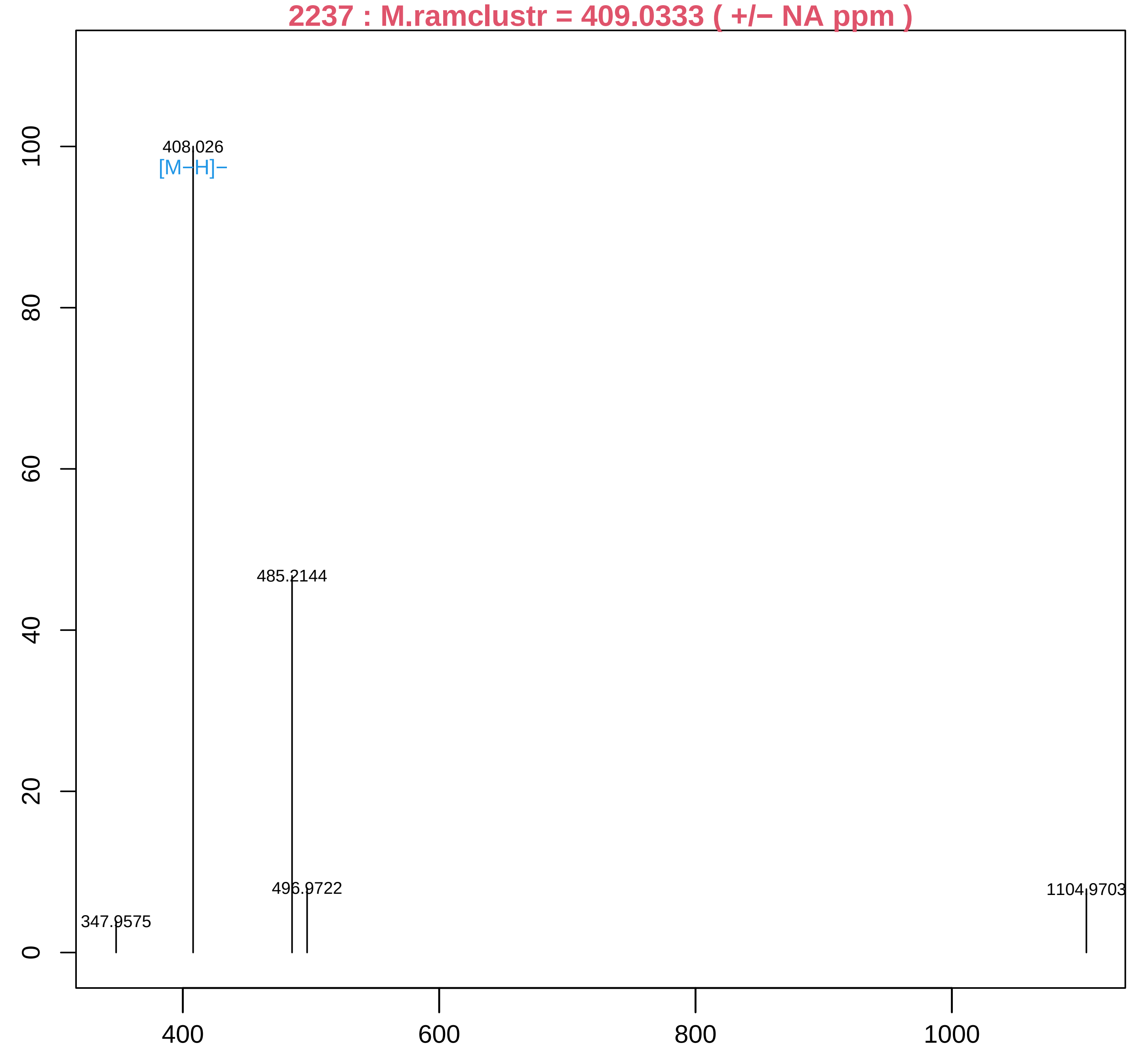
2237 : M.ramclustr = 409.0333 (+/− NA ppm)
408.026
[M−H]−
485.2144
496.9722
347.9575
1104.9703
0
20
40
60
80
100
400
600
800
1000

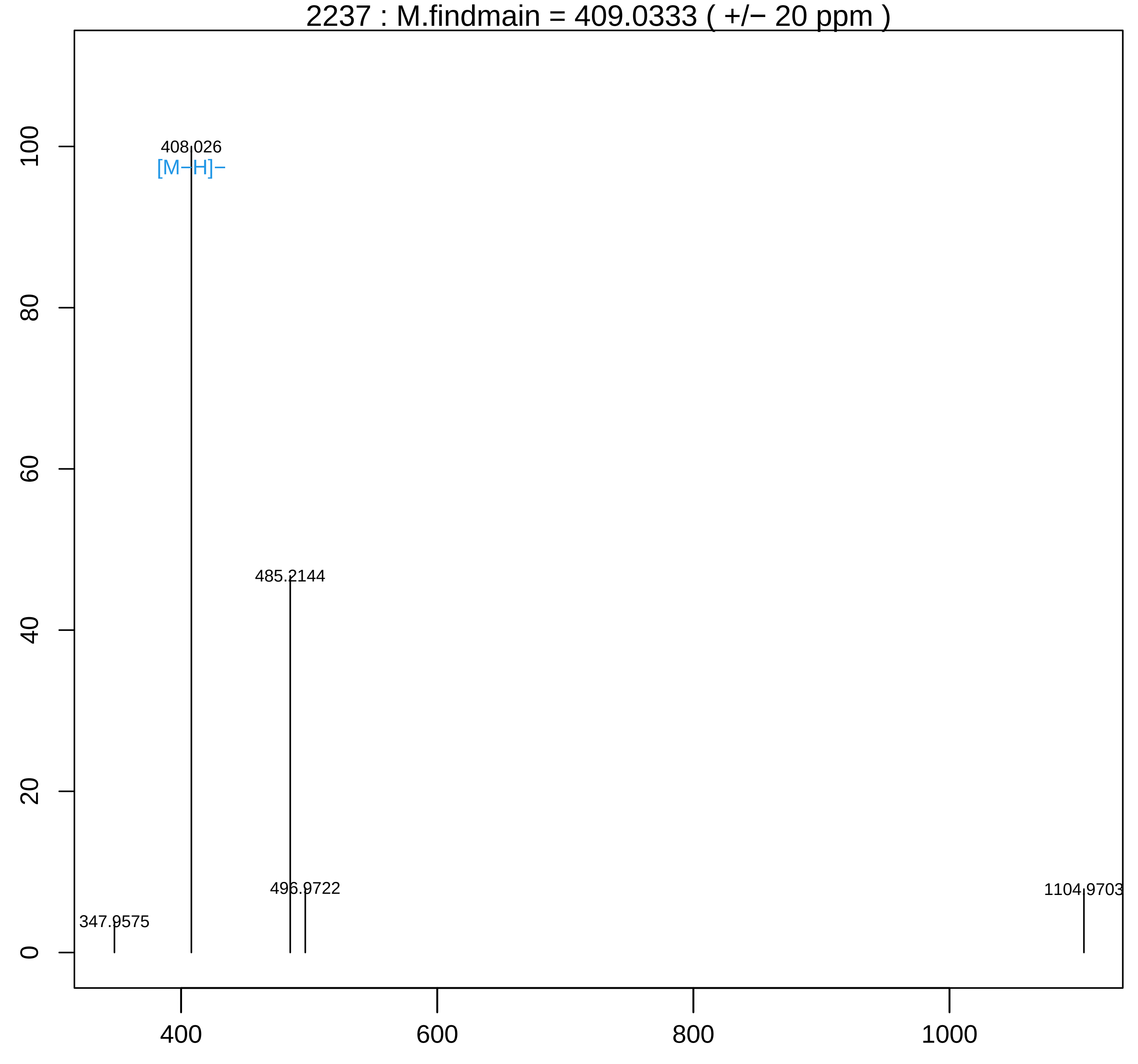
2237 : M.findmain = 409.0333 (+/− 20 ppm)
408.026
[M−H]−
485.2144
496.9722
347.9575
1104.9703
0
20
40
60
80
100
400
600
800
1000

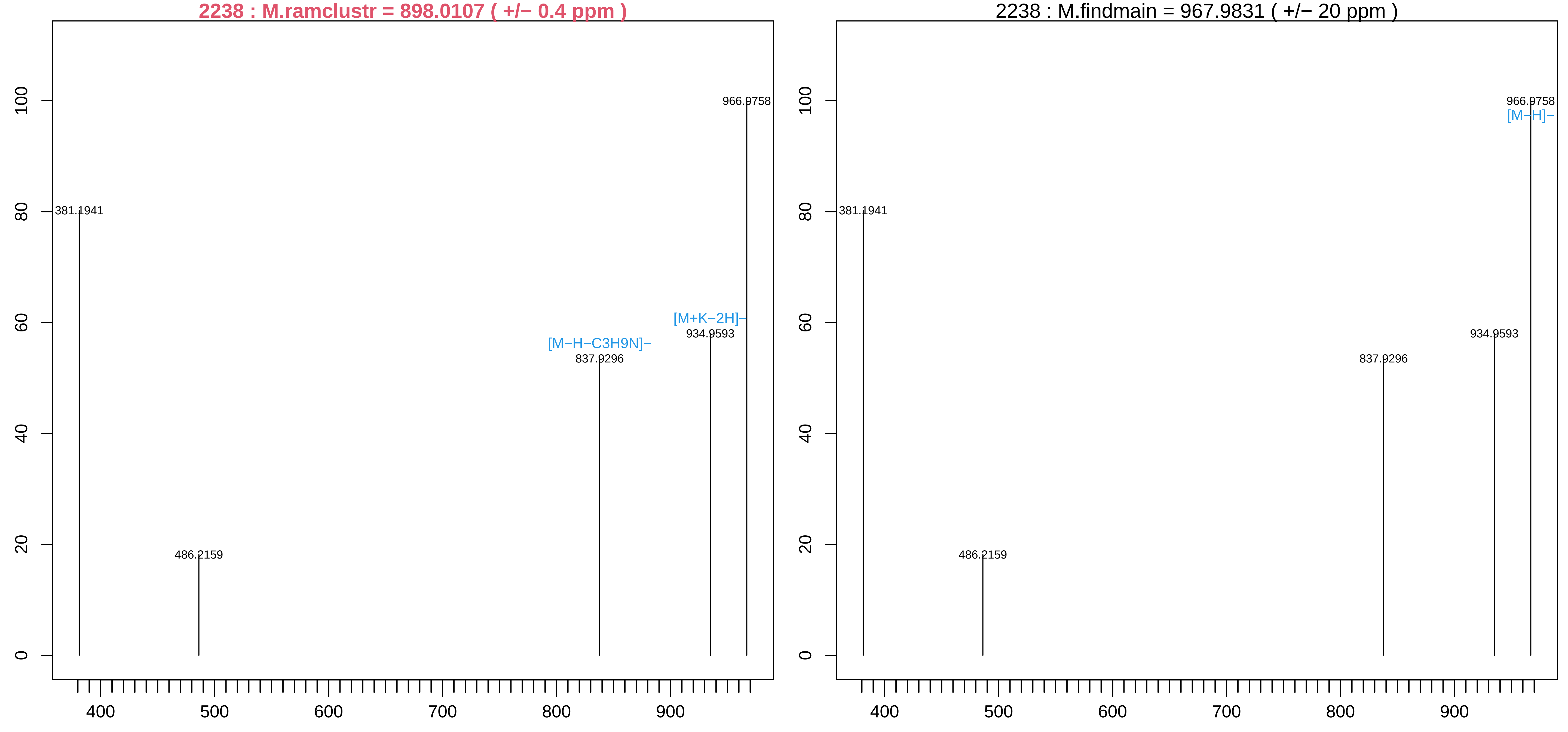
2238 : M.ramclustr = 898.0107 (+/− 0.4 ppm)
966.9758
381.1941
[M+K−2H]−
934.9593
[M−H−C3H9N]−
837.9296
486.2159
2238 : M.findmain = 967.9831 (+/− 20 ppm)
966.9758
[M−H]−
381.1941
934.9593
837.9296
486.2159
0
20
40
60
80
100
400
500
600
700
800
900

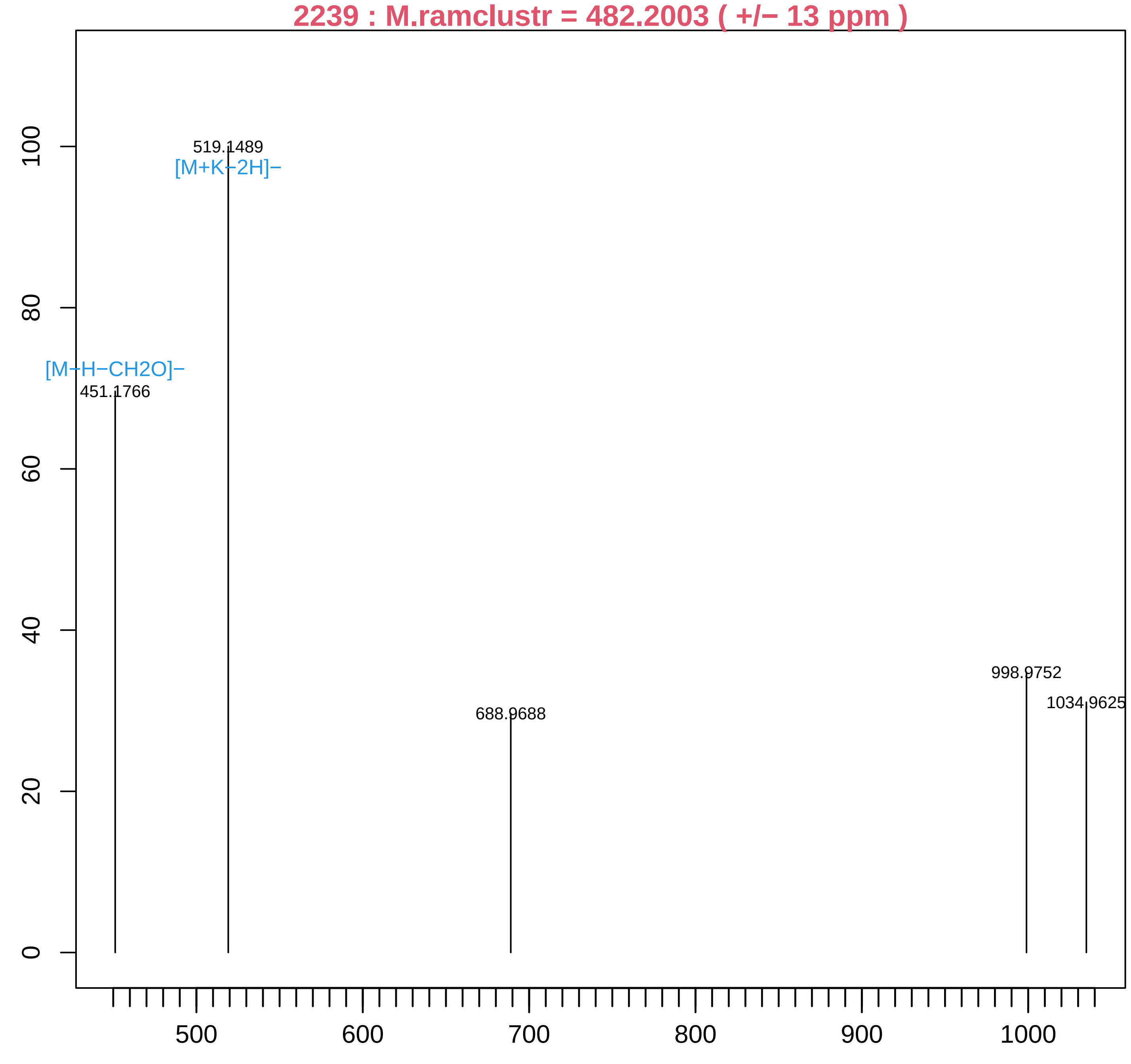
2239 : M.ramclustr = 482.2003 (+/− 13 ppm)
519.1489
[M+K−2H]−
[M+H−CH2O]−
451.1766
688.9688
998.9752
1034.9625
0
20
40
60
80
100
500
600
700
800
900
1000

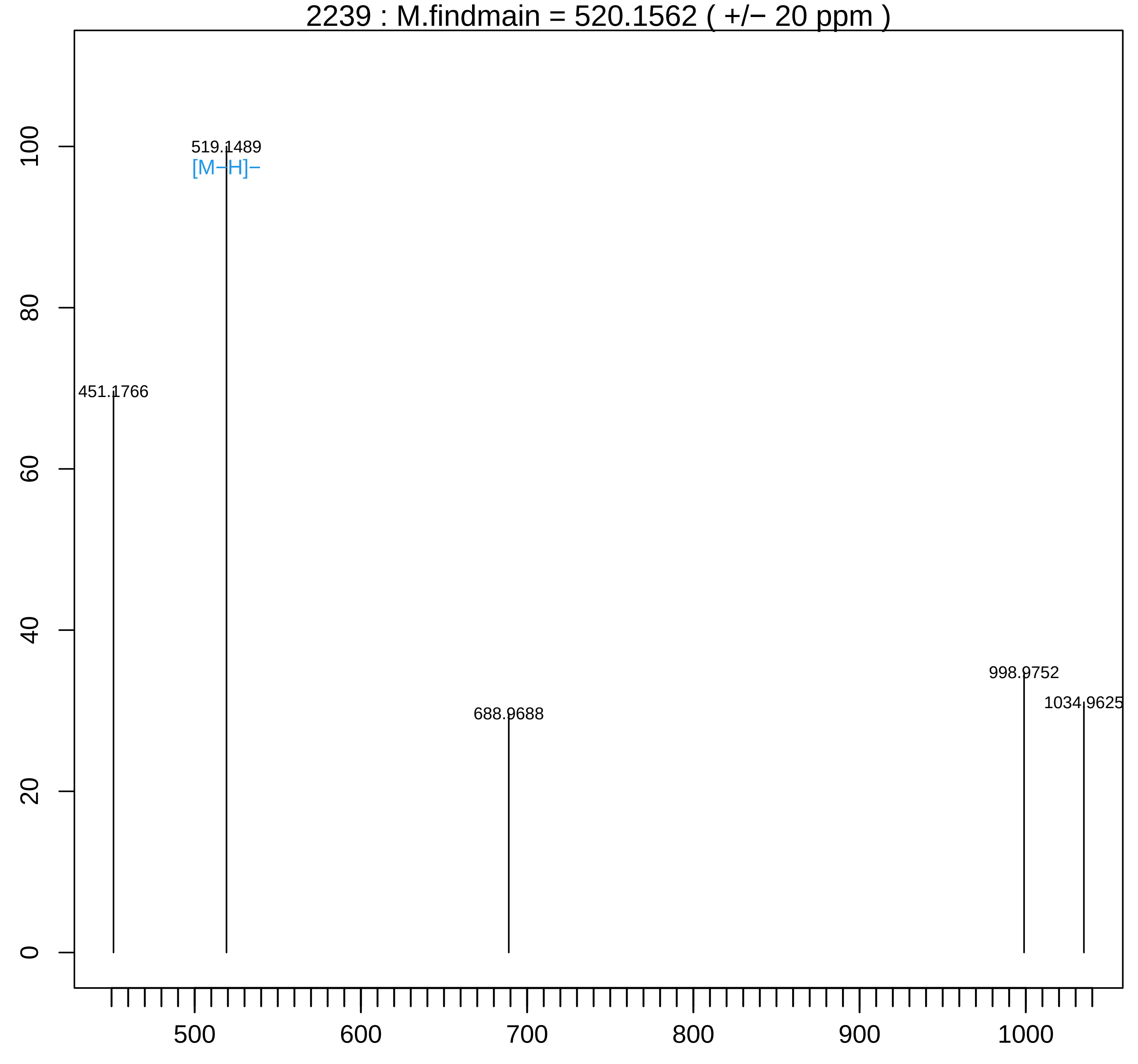
2239 : M.findmain = 520.1562 (+/− 20 ppm)
519.1489
[M−H]−
451.1766
688.9688
998.9752
1034.9625
0
20
40
60
80
100
500
600
700
800
900
1000

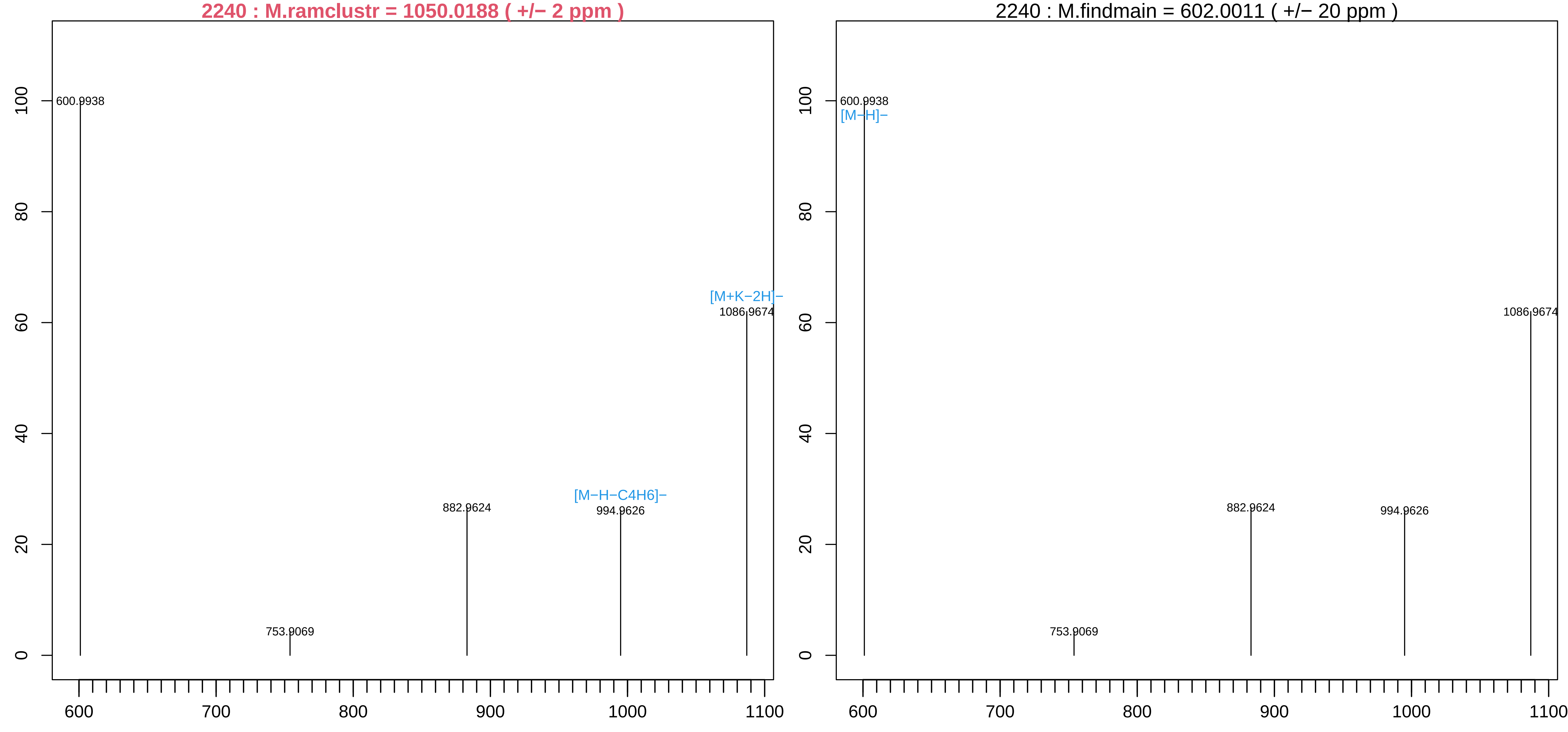

2240 : M.ramclustr = 1050.0188 (+/− 2 ppm)
600.9938
753.9069
882.9624
994.9626
[M−H−C4H6]−
1086.9674
[M+K−2H]−
2240 : M.findmain = 602.0011 (+/− 20 ppm)
600.9938
[M−H]−
753.9069
882.9624
994.9626
1086.9674
0
20
40
60
80
100
600
700
800
900
1000
1100

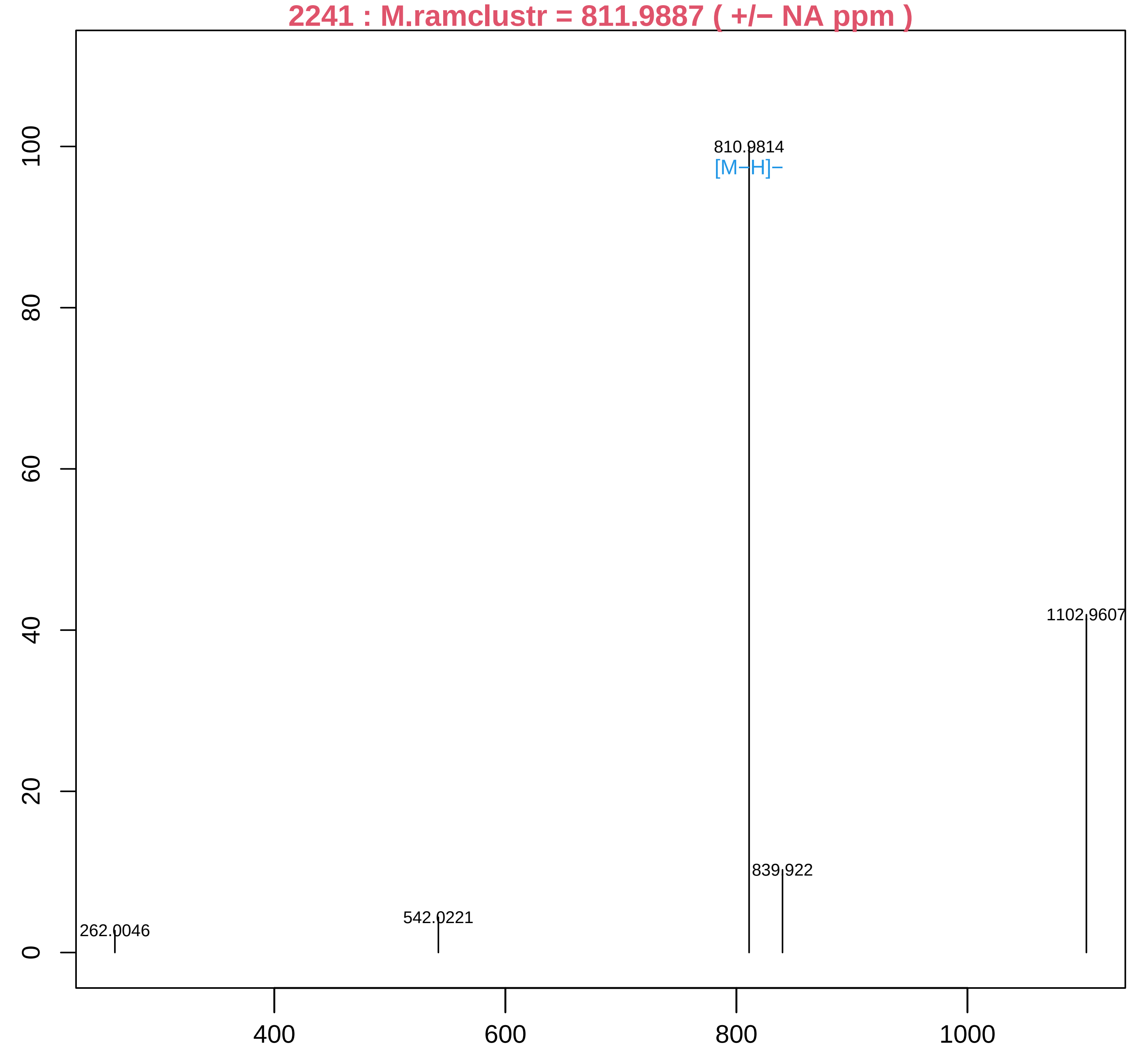
2241 : M.ramclustr = 811.9887 (+/− NA ppm)
810.9814
[M−H]−
1102.9607
839.922
542.0221
262.0046
0
20
40
60
80
100
400
600
800
1000

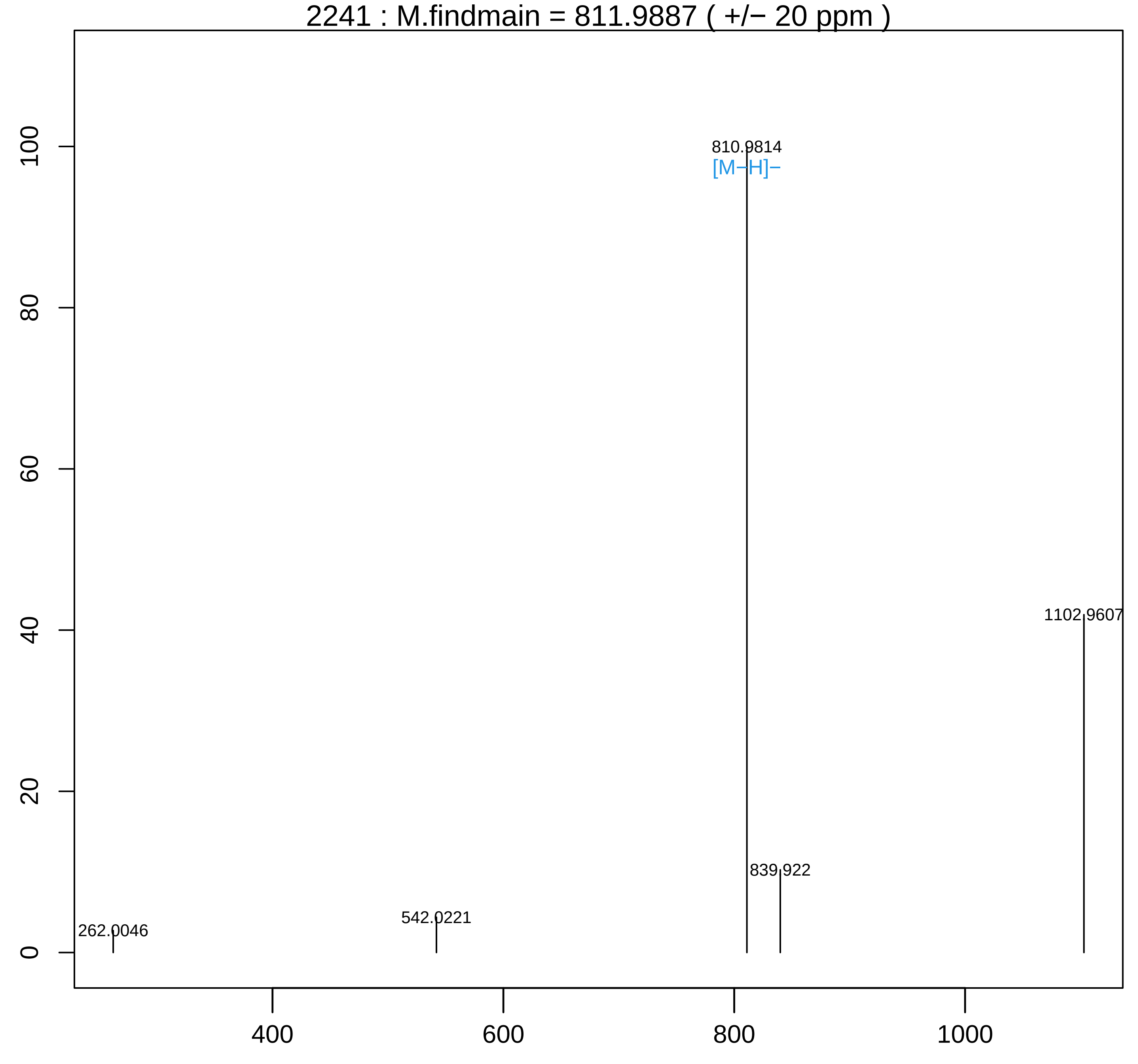
2241 : M.findmain = 811.9887 (+/− 20 ppm)
810.9814
[M−H]−
1102.9607
839.922
542.0221
262.0046
0
20
40
60
80
100
400
600
800
1000

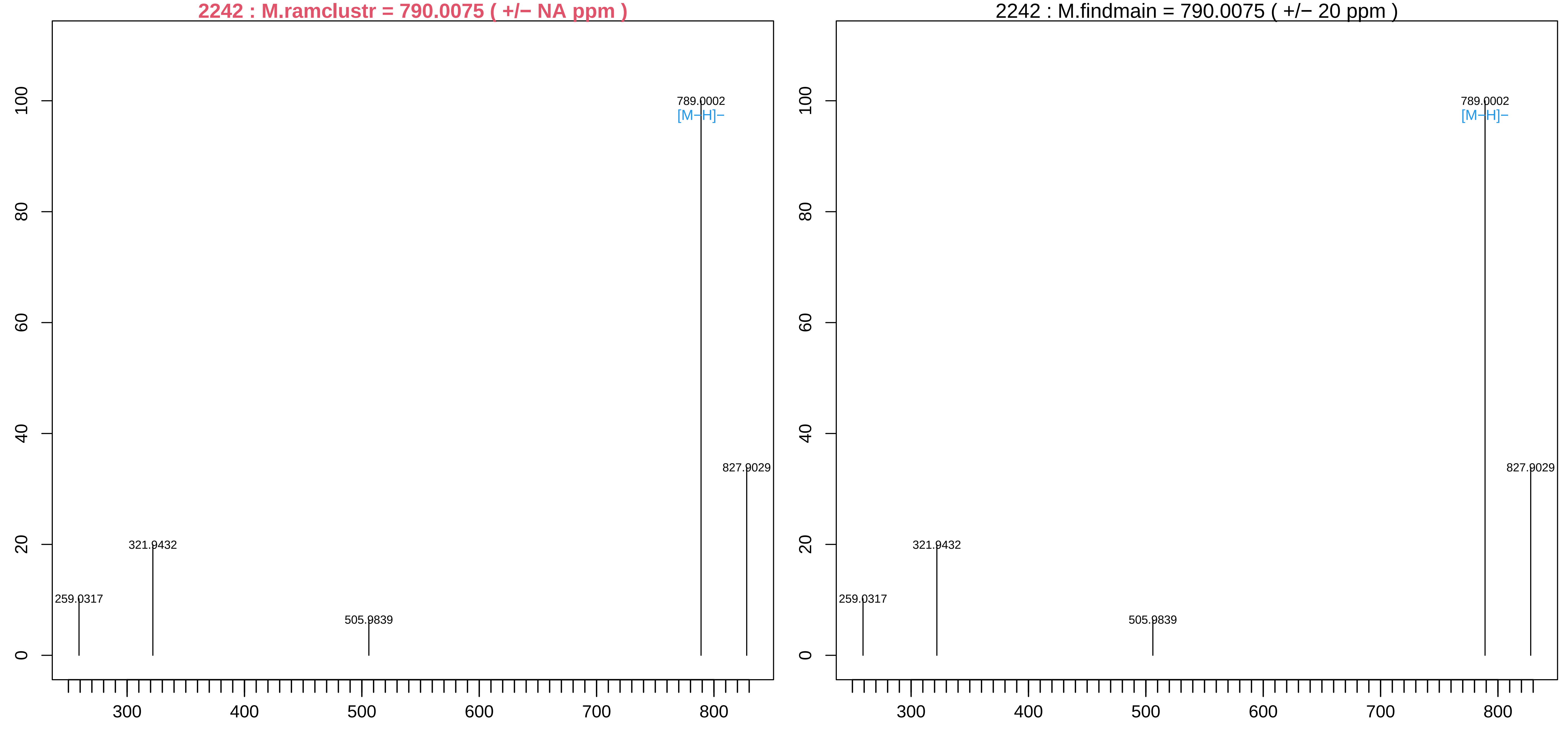

2242 : M.ramclustr = 790.0075 (+/− NA ppm)
789.0002
[M−H]−
827.9029
321.9432
259.0317
505.9839
2242 : M.findmain = 790.0075 (+/− 20 ppm)
789.0002
[M−H]−
827.9029
321.9432
259.0317
505.9839

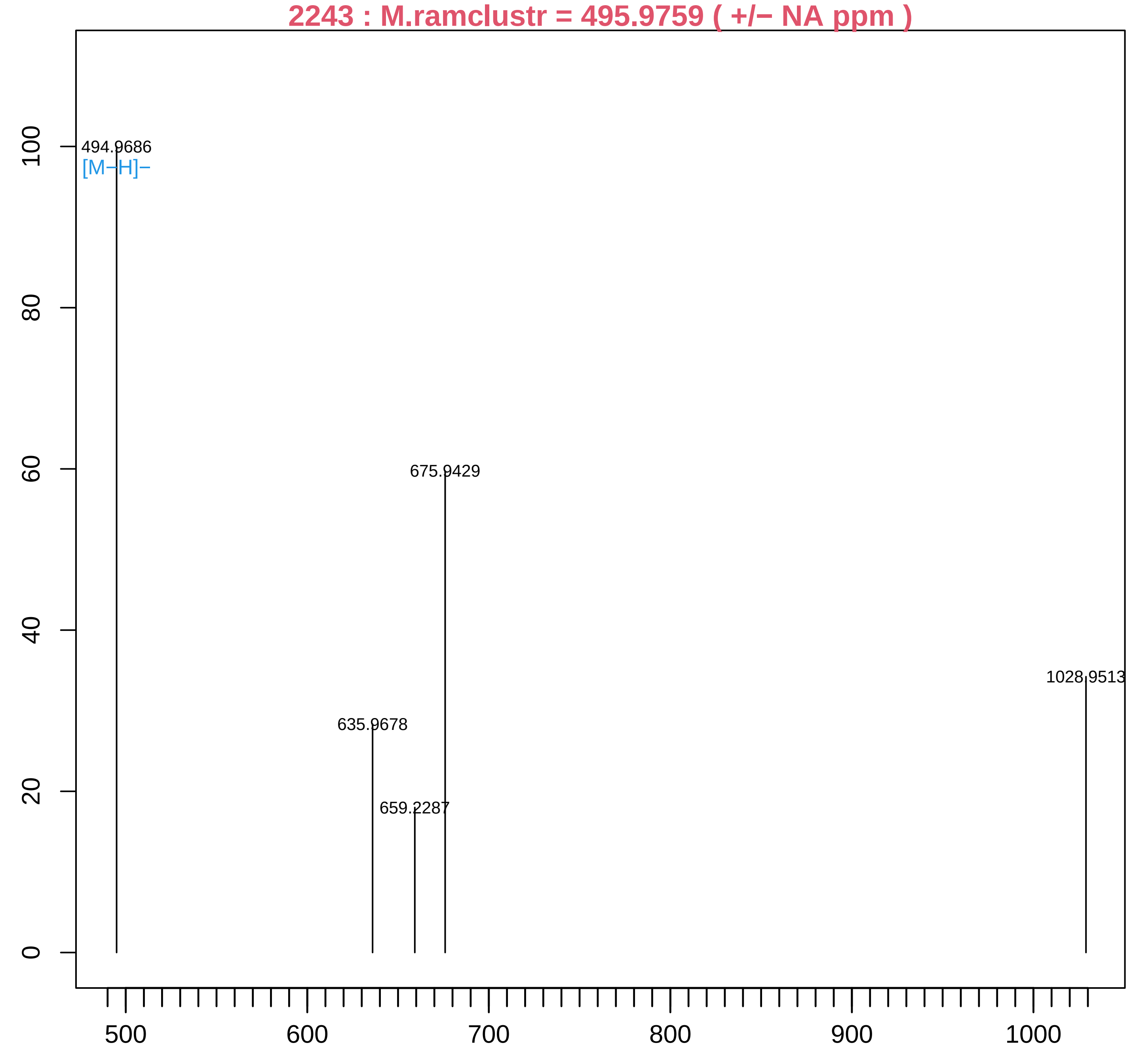
2243 : M.ramclustr = 495.9759 (+/− NA ppm)
494.9686
[M−H]−
675.9429
1028.9513
635.9678
659.2287
0
20
40
60
80
100
500
600
700
800
900
1000

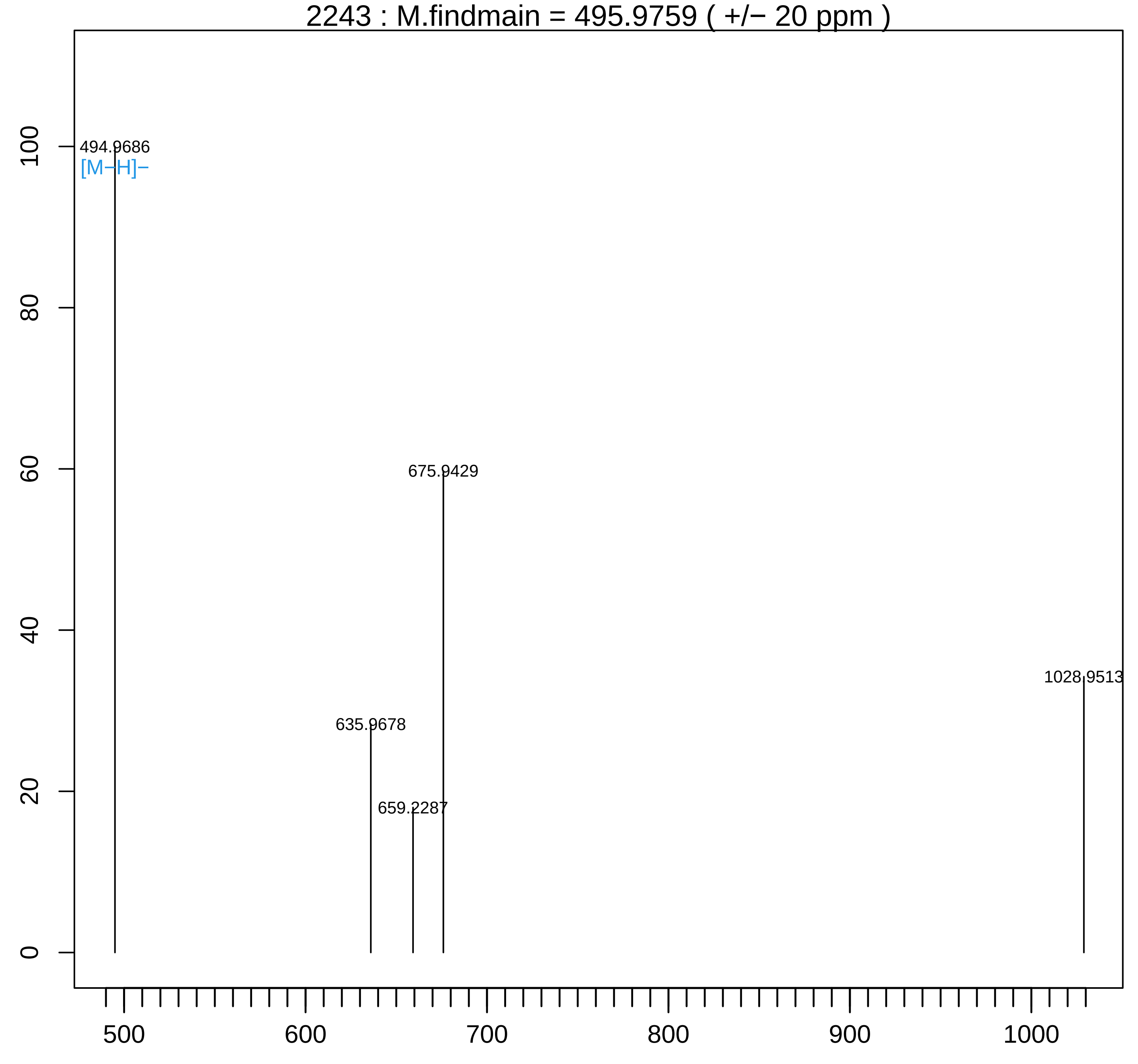
2243 : M.findmain = 495.9759 (+/− 20 ppm)
494.9686
[M−H]−
675.9429
1028.9513
635.9678
659.2287
0
20
40
60
80
100
500
600
700
800
900
1000

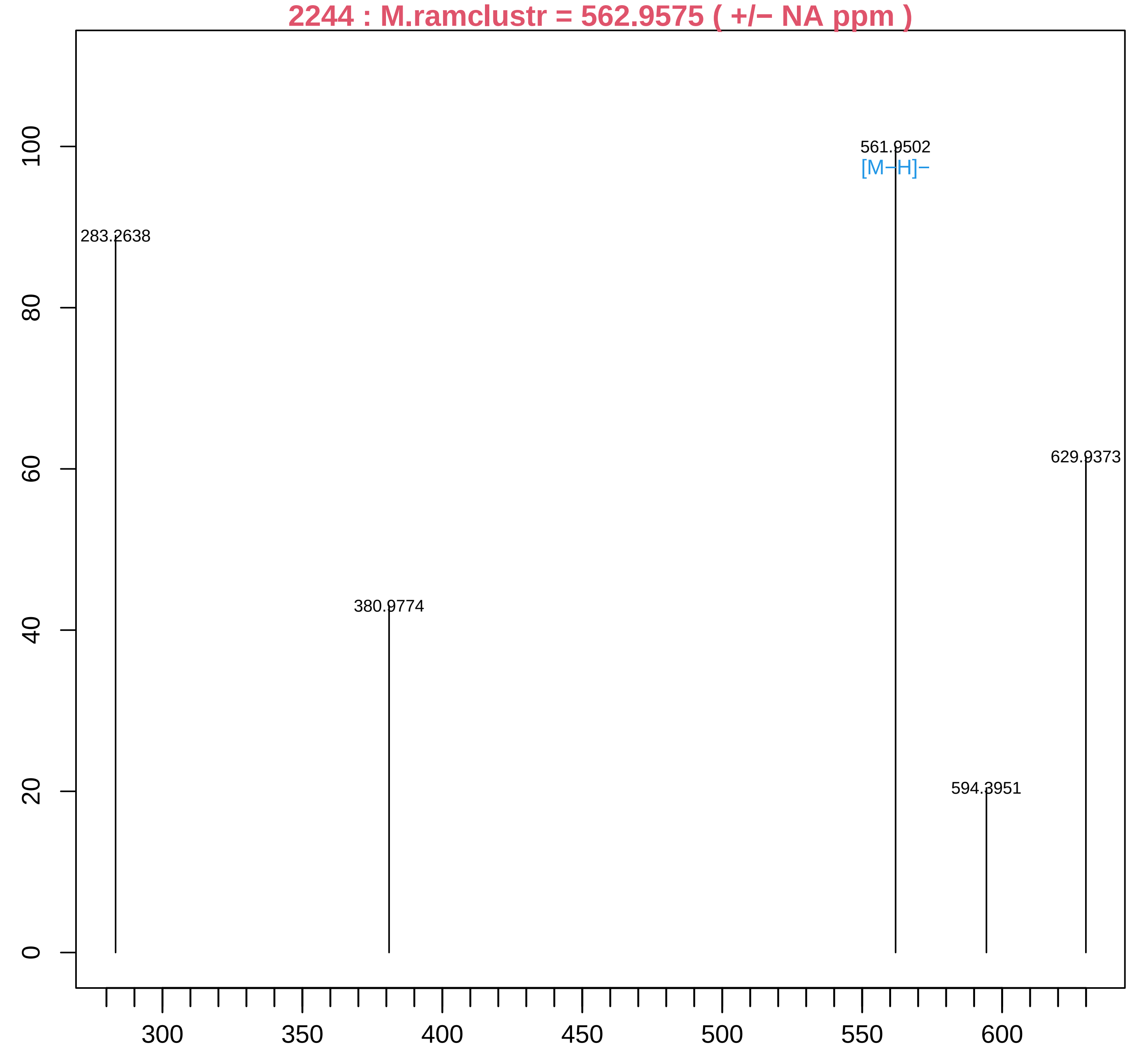

2244 : M.ramclustr = 562.9575 (+/− NA ppm)
561.9502
[M−H]−
283.2638
629.9373
380.9774
594.3951
0
20
40
60
80
100
300
350
400
450
500
550
600

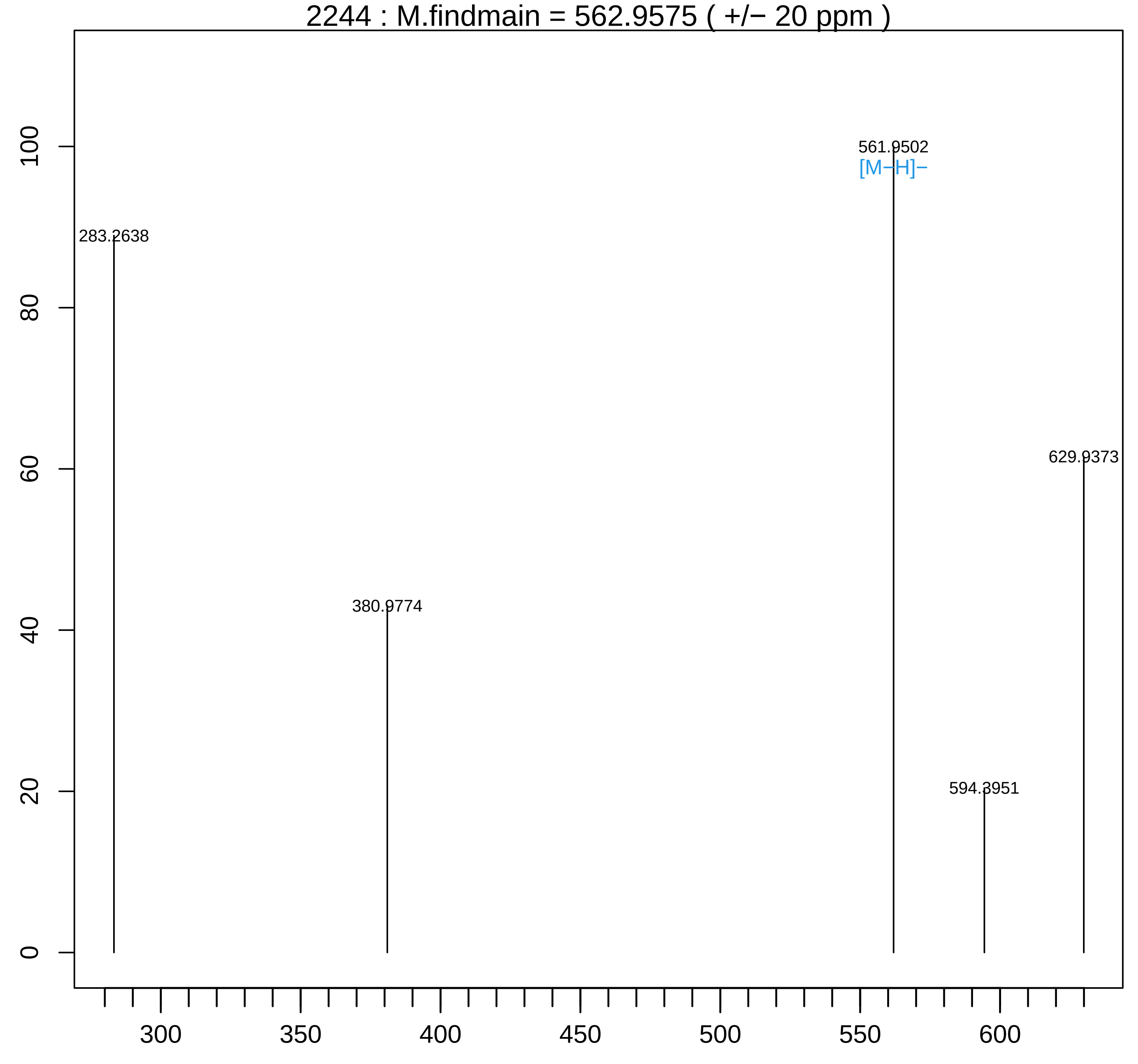

2244 : M.findmain = 562.9575 (+/− 20 ppm)
561.9502
[M−H]−
283.2638
629.9373
380.9774
594.3951
0
20
40
60
80
100
300
350
400
450
500
550
600

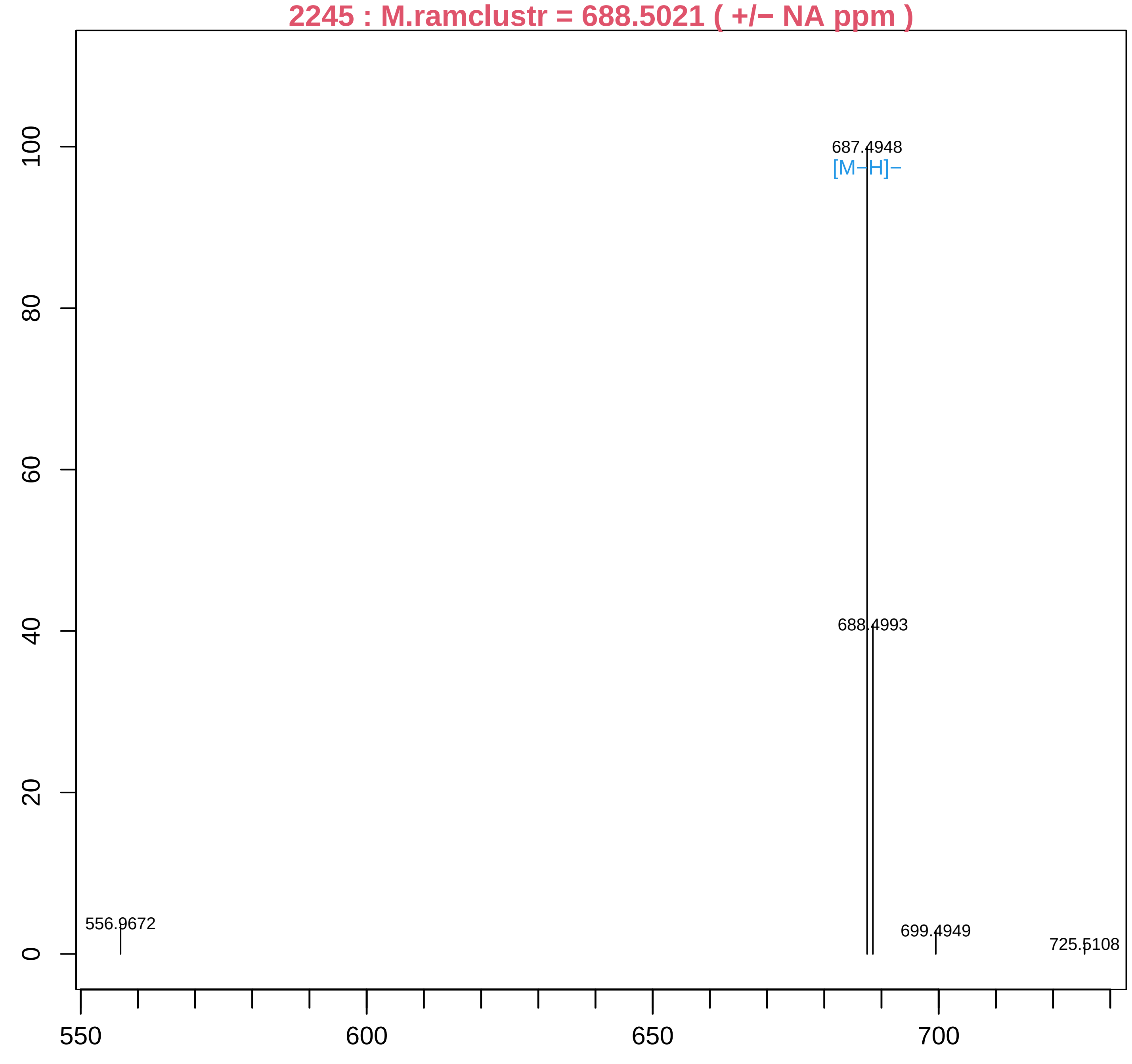

2245 : M.ramclustr = 688.5021 (+/− NA ppm)
687.4948
[M−H]−
688.4993
556.9672
699.4949
725.5108
0
20
40
60
80
100
550
600
650
700

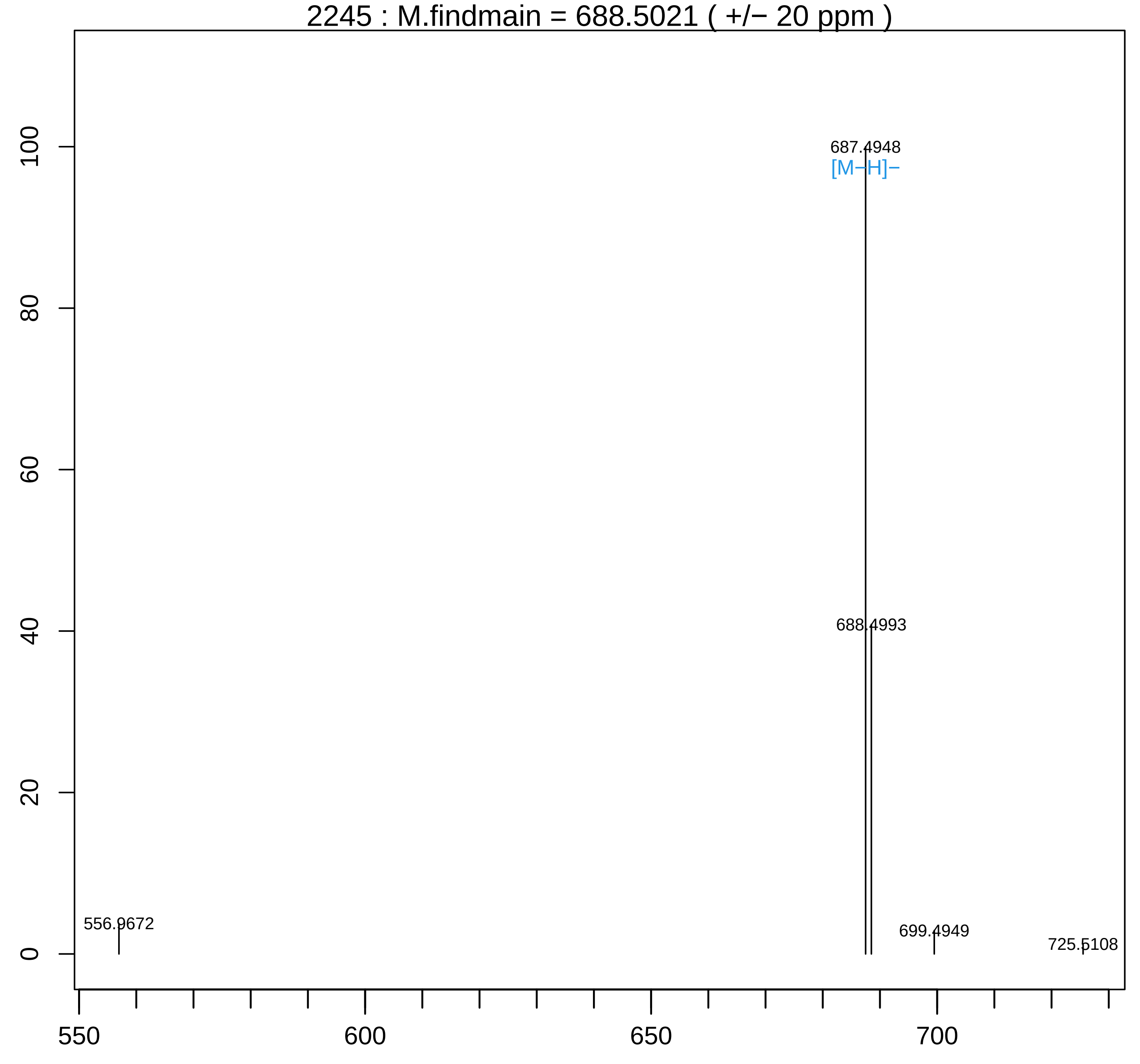

2245 : M.findmain = 688.5021 (+/− 20 ppm)
687.4948
[M−H]−
688.4993
556.9672
699.4949
725.5108
0
20
40
60
80
100
550
600
650
700

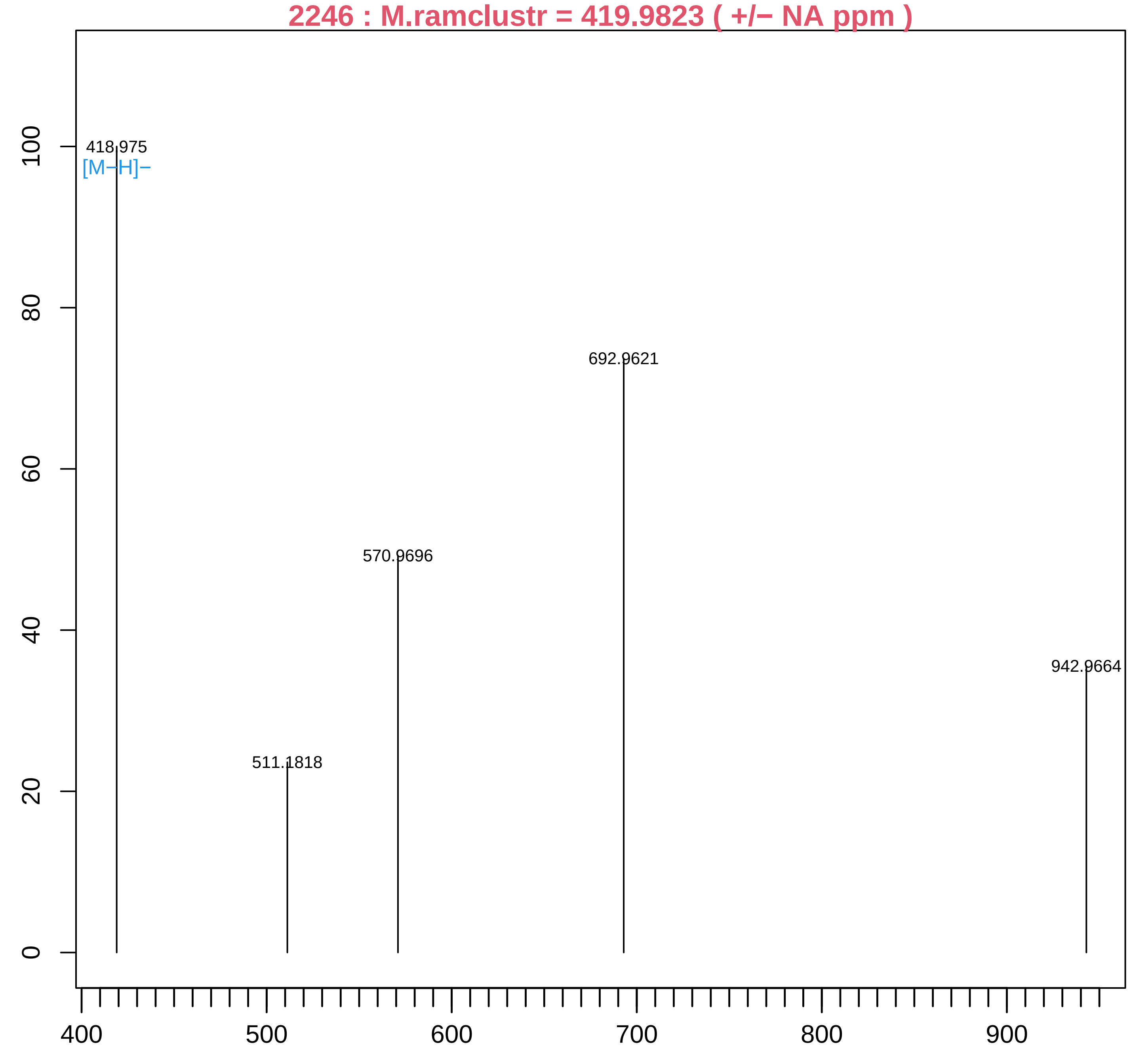

2246 : M.ramclustr = 419.9823 (+/− NA ppm)
418.975
[M−H]−
511.1818
570.9696
692.9621
942.9664
0
20
40
60
80
100
400
500
600
700
800
900

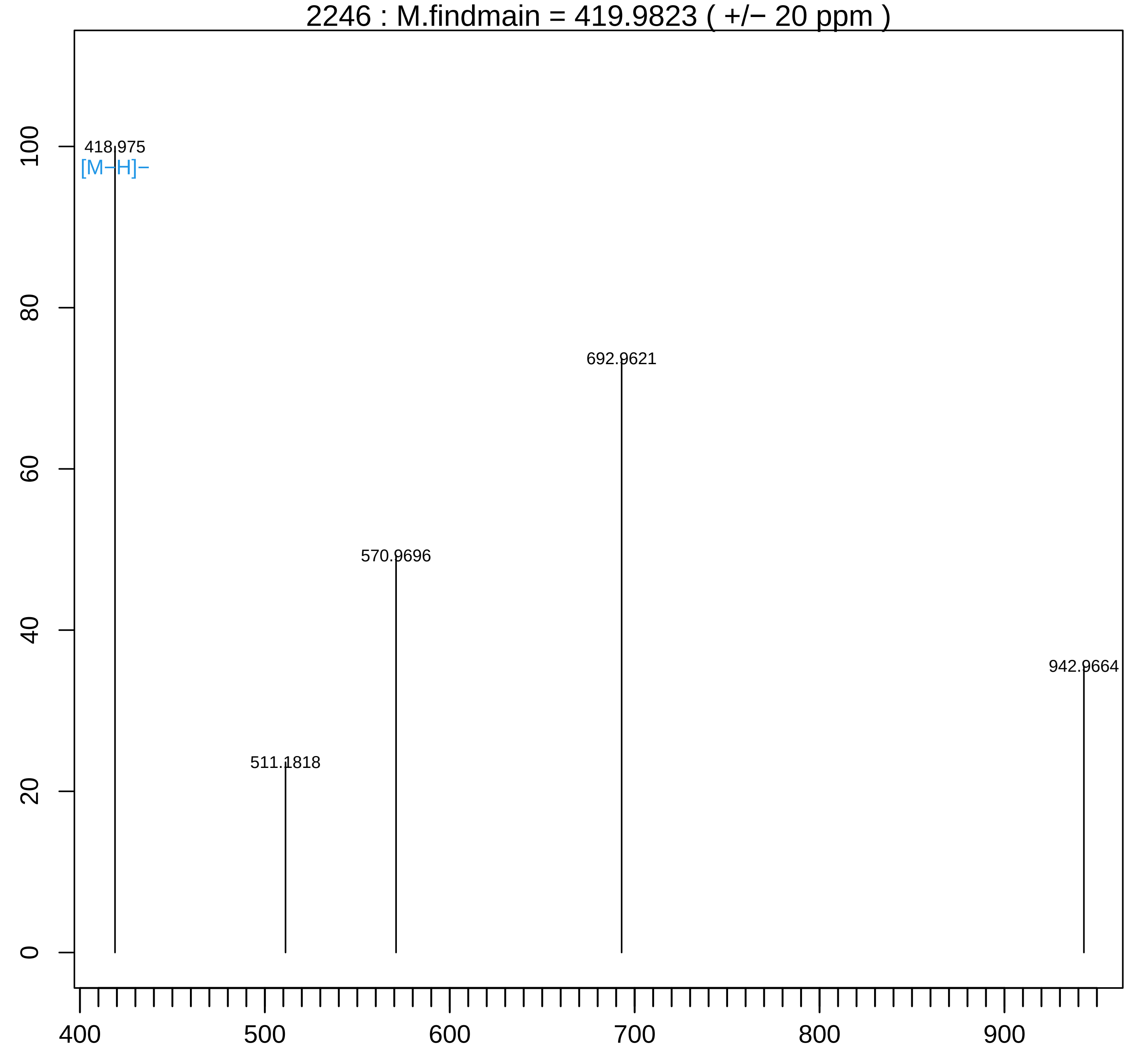

2246 : M.findmain = 419.9823 (+/− 20 ppm)
418.975
[M−H]−
511.1818
570.9696
692.9621
942.9664
0
20
40
60
80
100
400
500
600
700
800
900

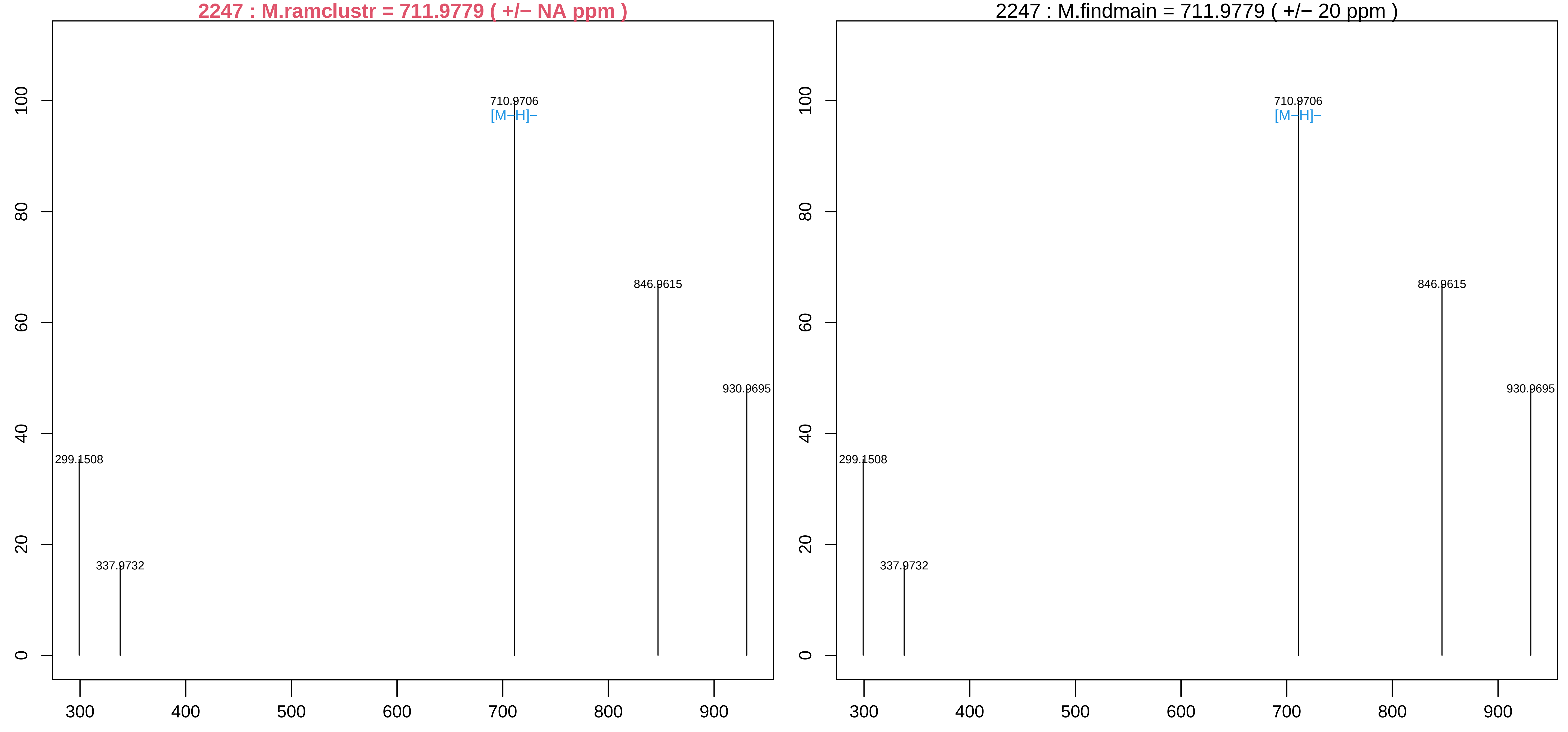

2247 : M.ramclustr = 711.9779 (+/− NA ppm)
710.9706
[M−H]−
846.9615
930.9695
299.1508
337.9732
2247 : M.findmain = 711.9779 (+/− 20 ppm)
710.9706
[M−H]−
846.9615
930.9695
299.1508
337.9732

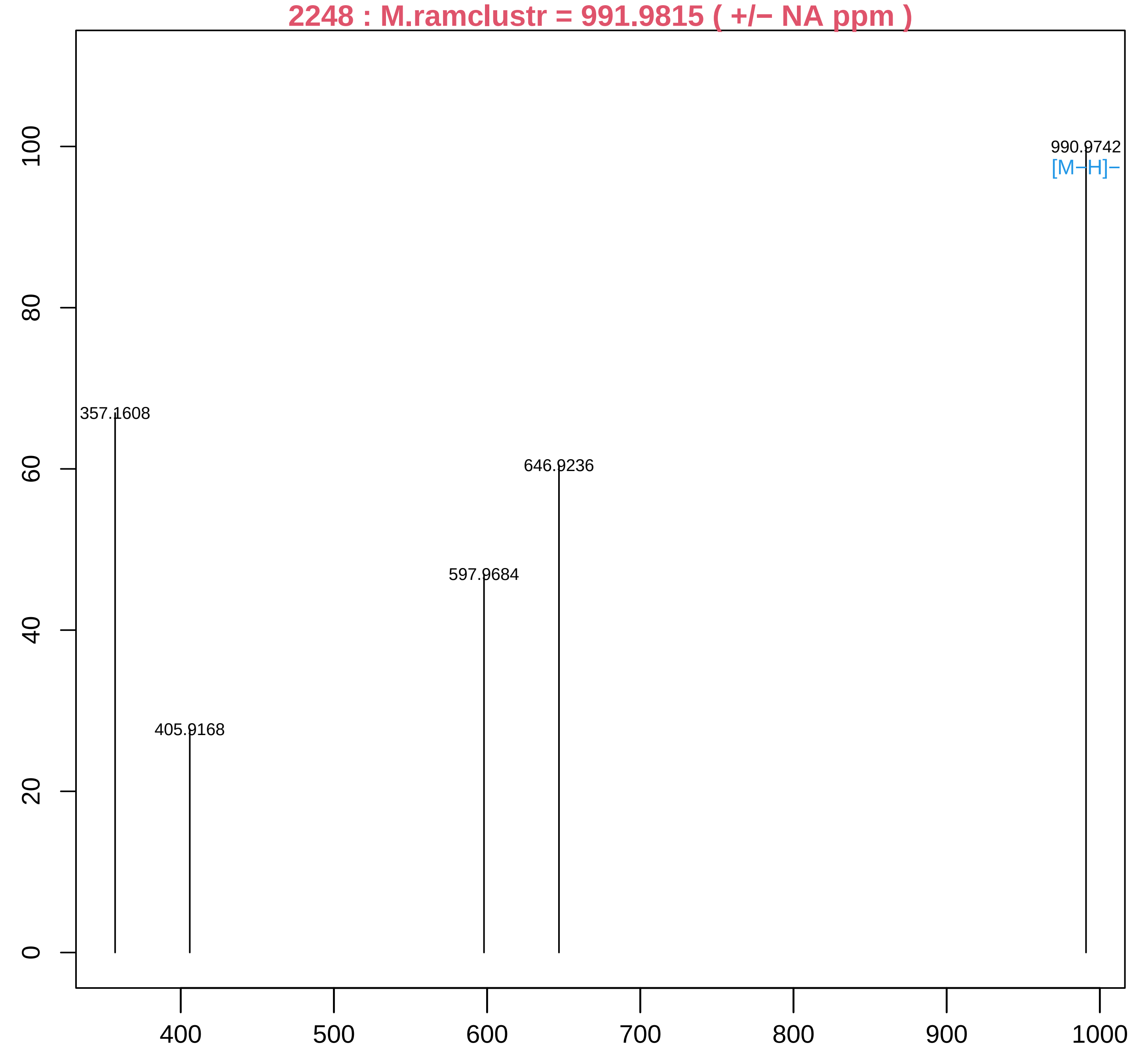

2248 : M.ramclustr = 991.9815 (+/− NA ppm)
990.9742
[M−H]−
357.1608
646.9236
597.9684
405.9168
0
20
40
60
80
100
400
500
600
700
800
900
1000

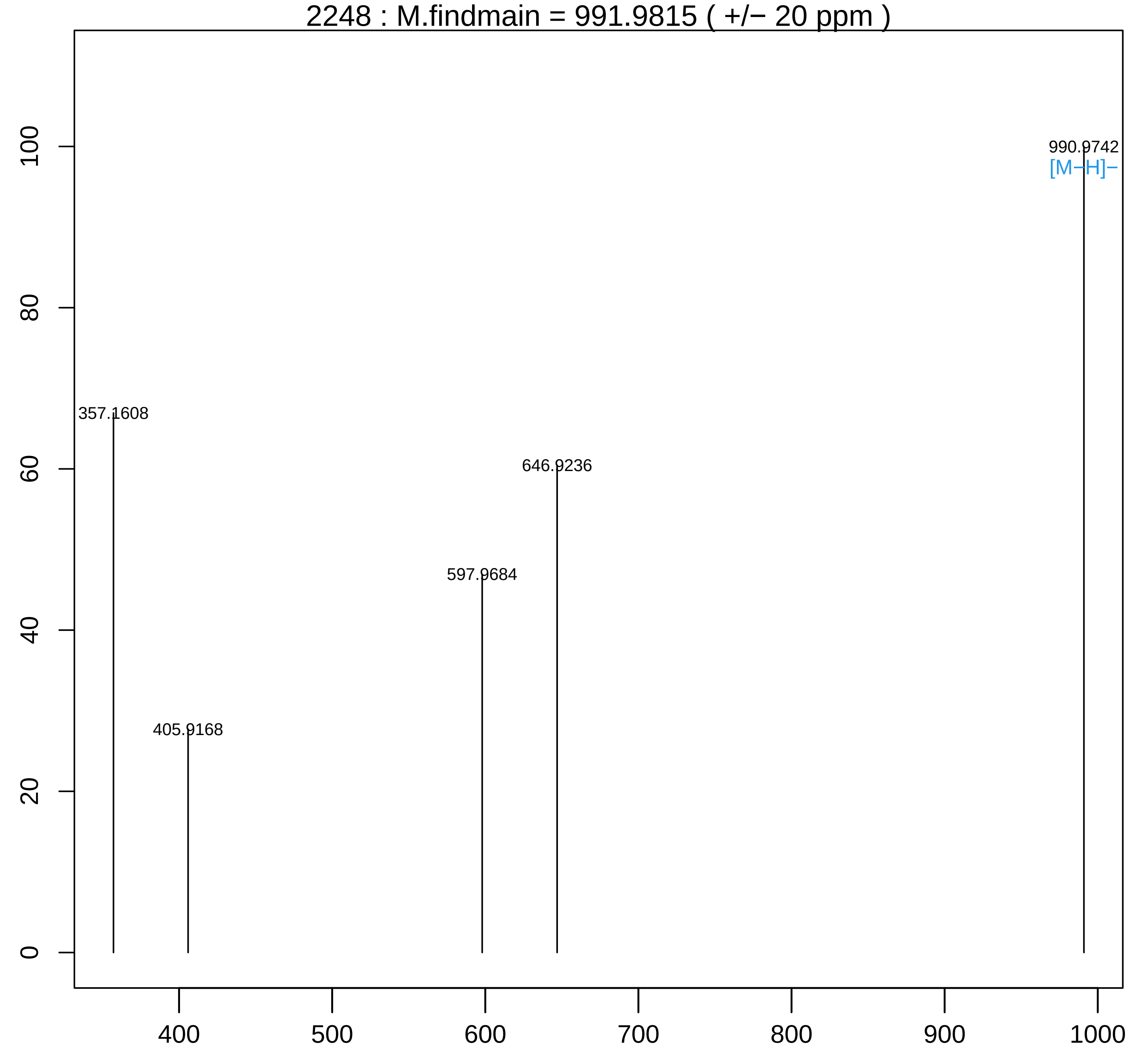

2248 : M.findmain = 991.9815 (+/− 20 ppm)
990.9742
[M−H]−
357.1608
646.9236
597.9684
405.9168
0
20
40
60
80
100
400
500
600
700
800
900
1000

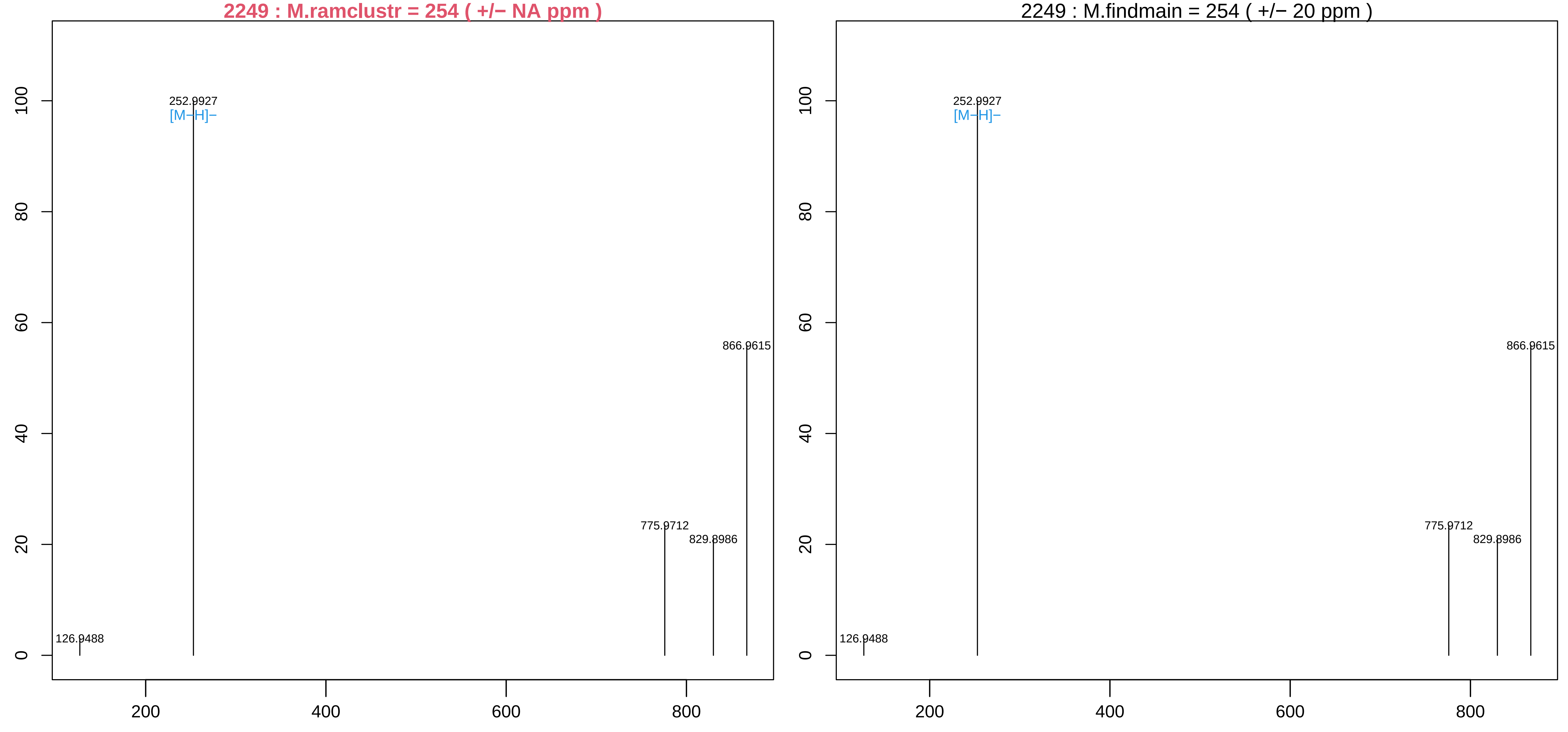
2249 : M.ramclustr = 254 (+/− NA ppm)
252.9927
[M−H]−
866.9615
775.9712
829.8986
126.9488
2249 : M.findmain = 254 (+/− 20 ppm)
252.9927
[M−H]−
866.9615
775.9712
829.8986
126.9488

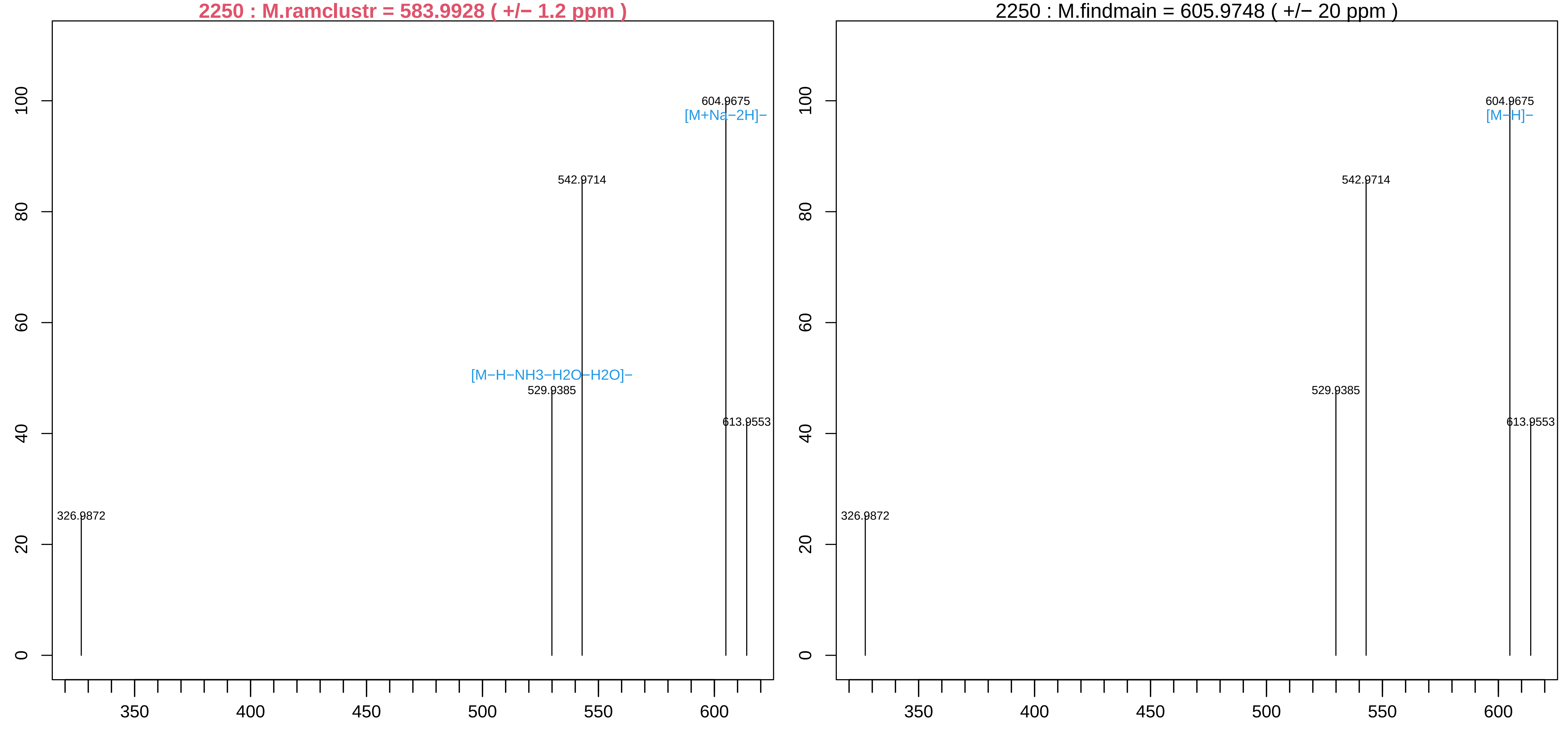
2250 : M.ramclustr = 583.9928 (+/− 1.2 ppm)
604.9675
[M+Na−2H]−
542.9714
[M−H−NH3−H2O−H2O]−
529.9385
613.9553
326.9872
2250 : M.findmain = 605.9748 (+/− 20 ppm)
604.9675
[M−H]−
542.9714
529.9385
613.9553
326.9872
0
20
40
60
80
100
350
400
450
500
550
600

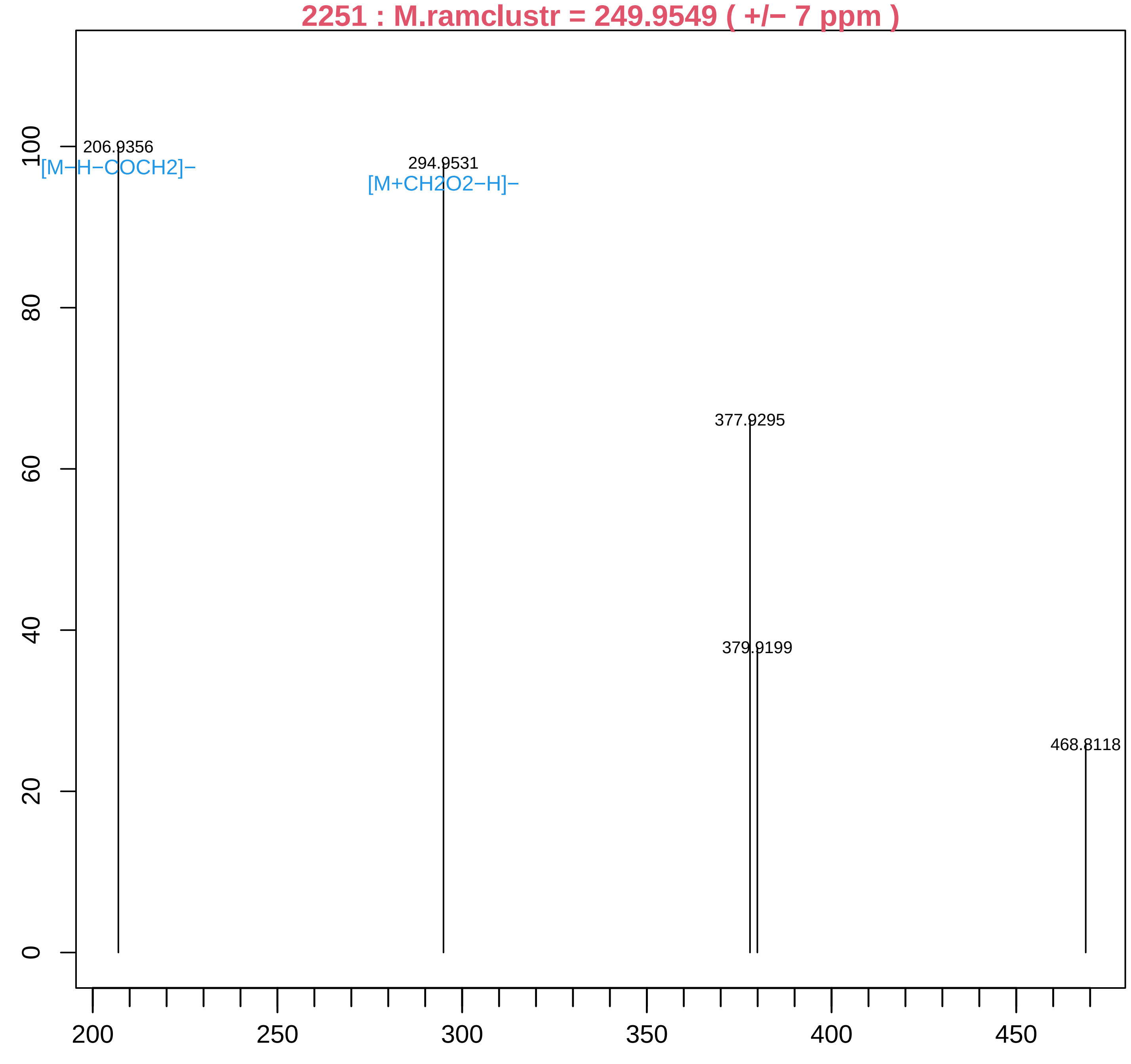

2251 : M.ramclustr = 249.9549 (+/− 7 ppm)
206.9356
[M−H−COCH2]−
294.9531
[M+CH2O2−H]−
377.9295
379.9199
468.8118
0
20
40
60
80
100
200
250
300
350
400
450

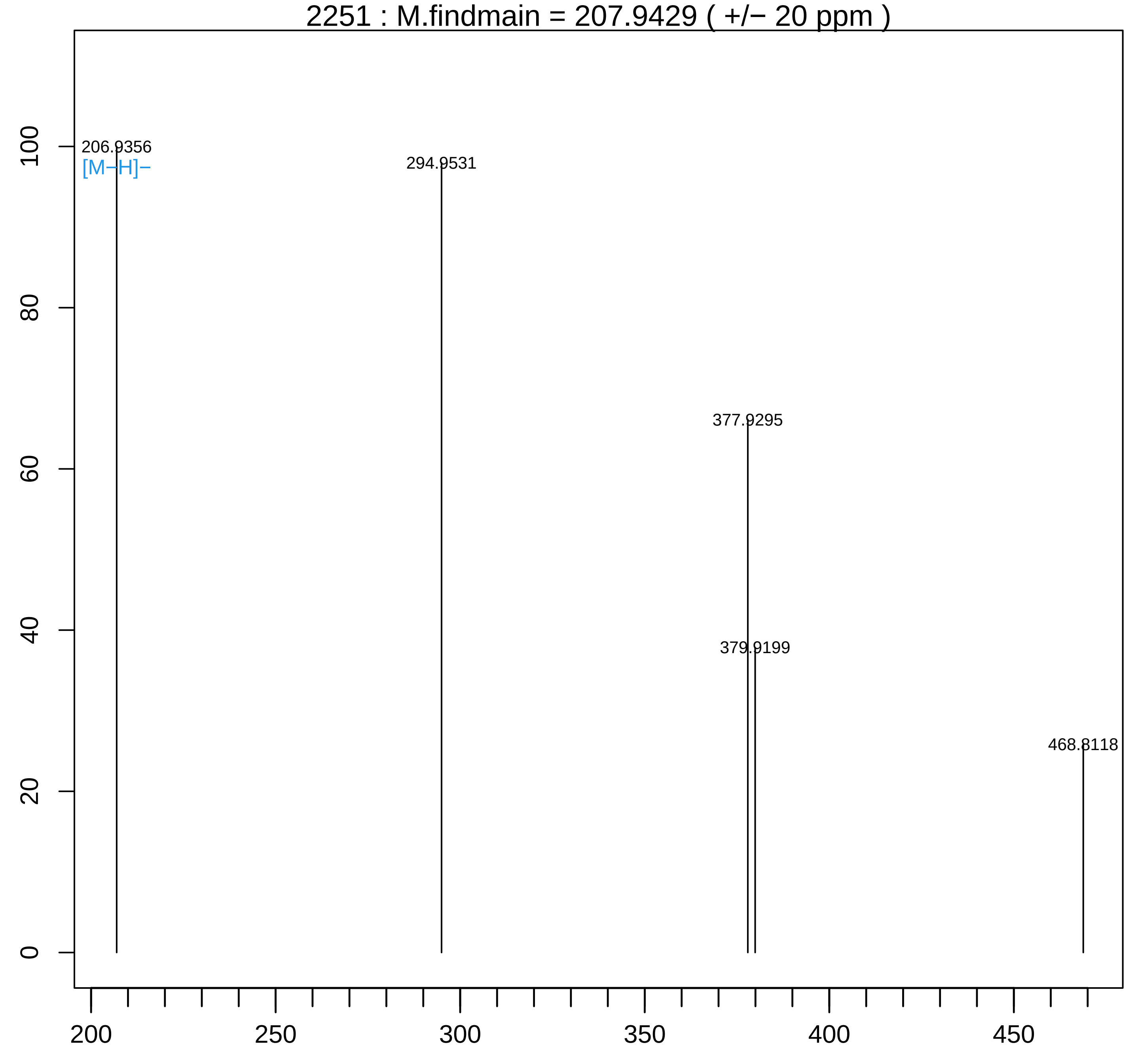

2251 : M.findmain = 207.9429 (+/− 20 ppm)
206.9356
[M−H]−
294.9531
377.9295
379.9199
468.8118
0
20
40
60
80
100
200
250
300
350
400
450

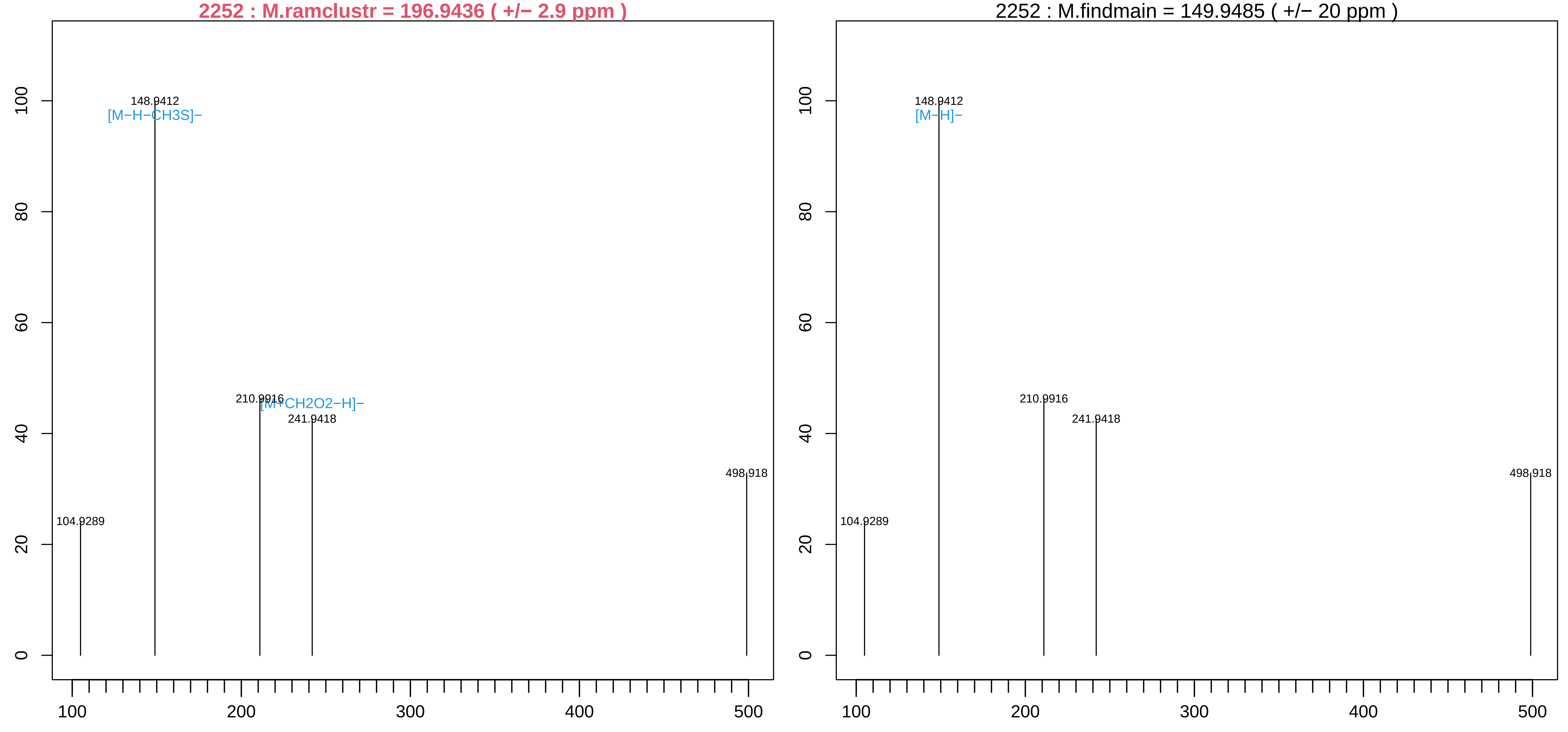

2252 : M.ramclustr = 196.9436 (+/− 2.9 ppm)
148.9412
[M−H−CH3S]−
210.9916
[M+CH2O2−H]−
241.9418
498.918
104.9289
0
20
40
60
80
100
100
200
300
400
500
2252 : M.findmain = 149.9485 (+/− 20 ppm)
148.9412
[M−H]−
210.9916
241.9418
498.918
104.9289
0
20
40
60
80
100
100
200
300
400
500

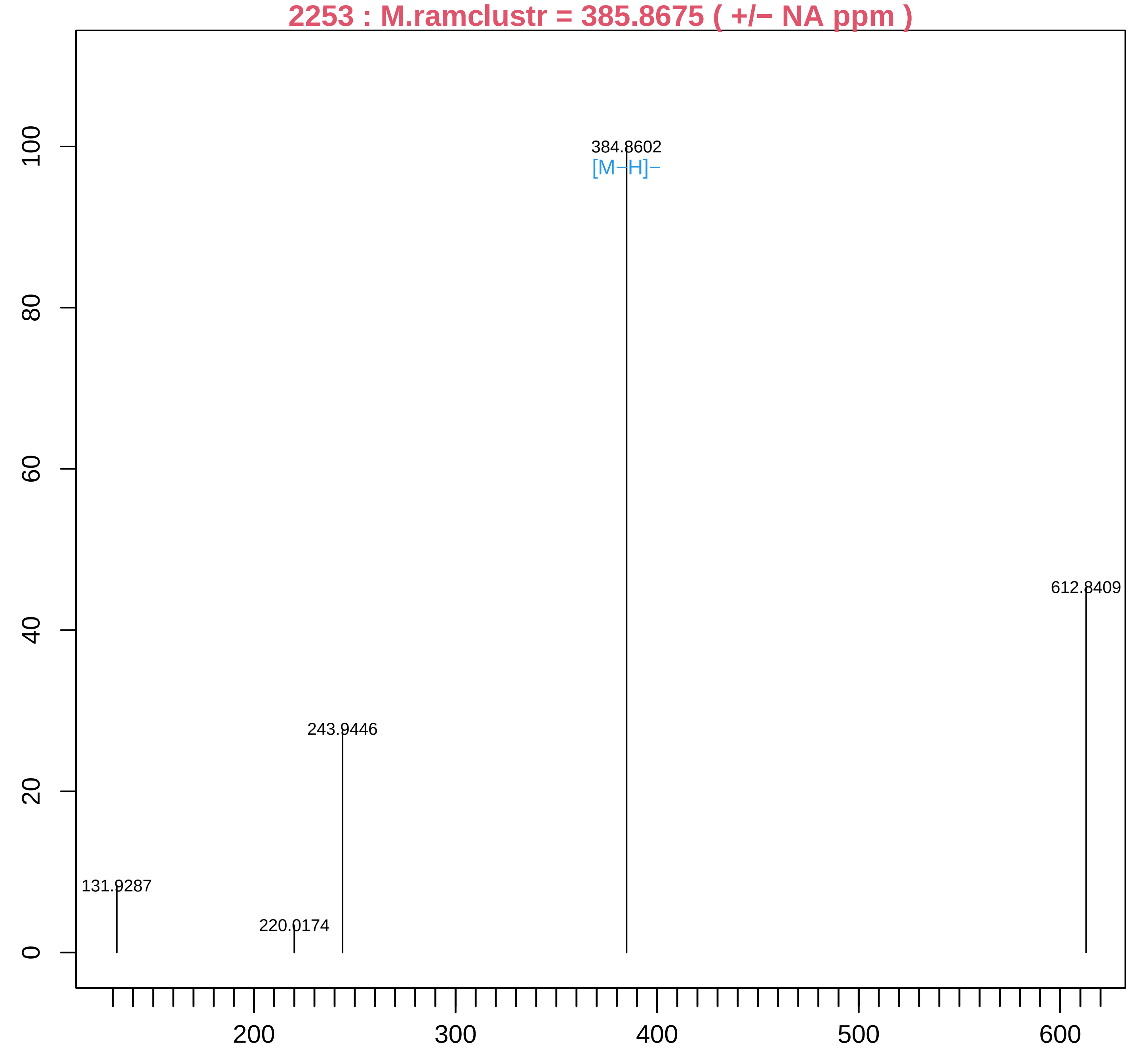

2253 : M.ramclustr = 385.8675 (+/− NA ppm)
384.8602
[M−H]−
612.8409
243.9446
131.9287
220.0174
0
20
40
60
80
100
200
300
400
500
600

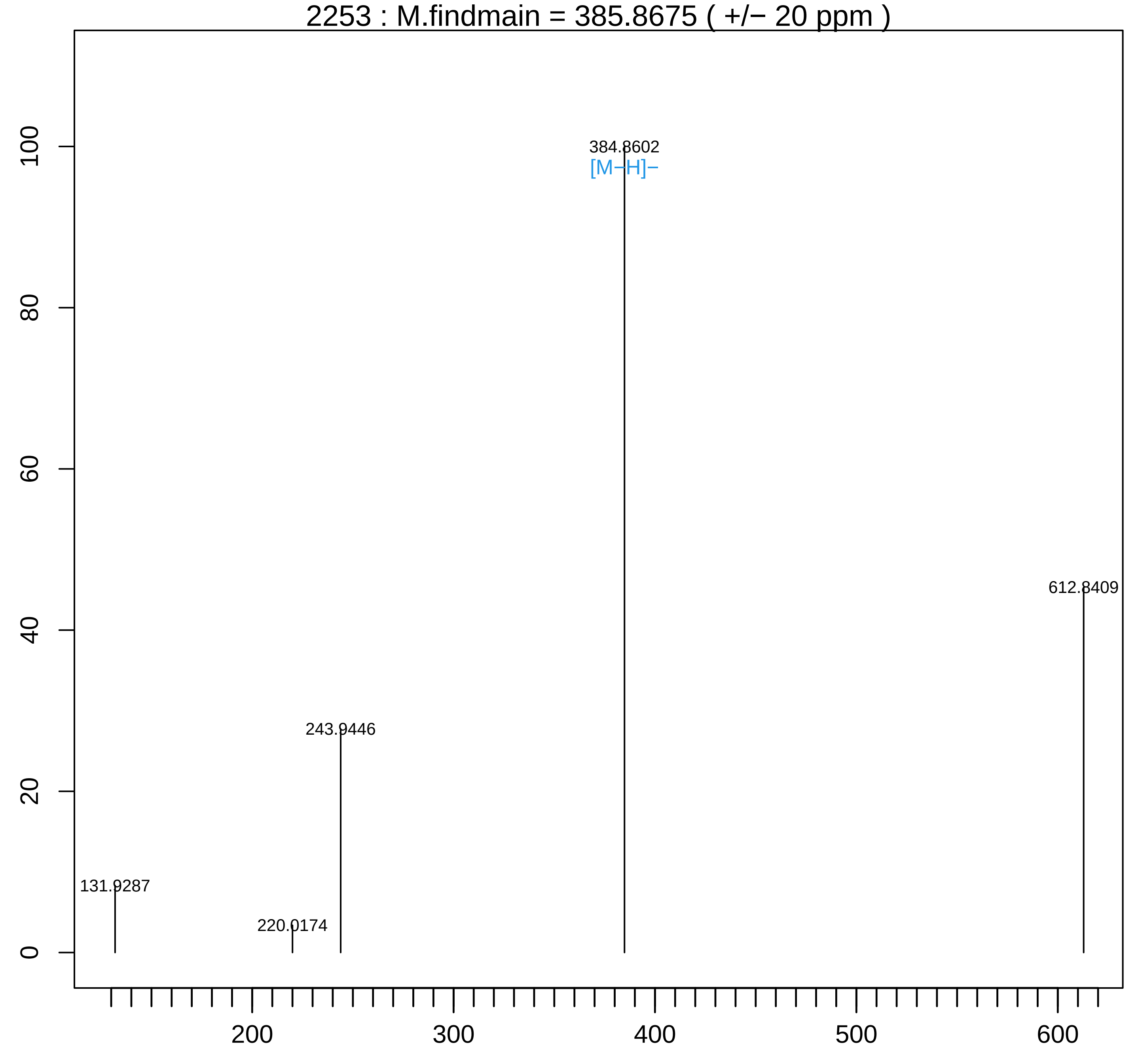

2253 : M.findmain = 385.8675 (+/− 20 ppm)
384.8602
[M−H]−
612.8409
243.9446
131.9287
220.0174
0
20
40
60
80
100
200
300
400
500
600

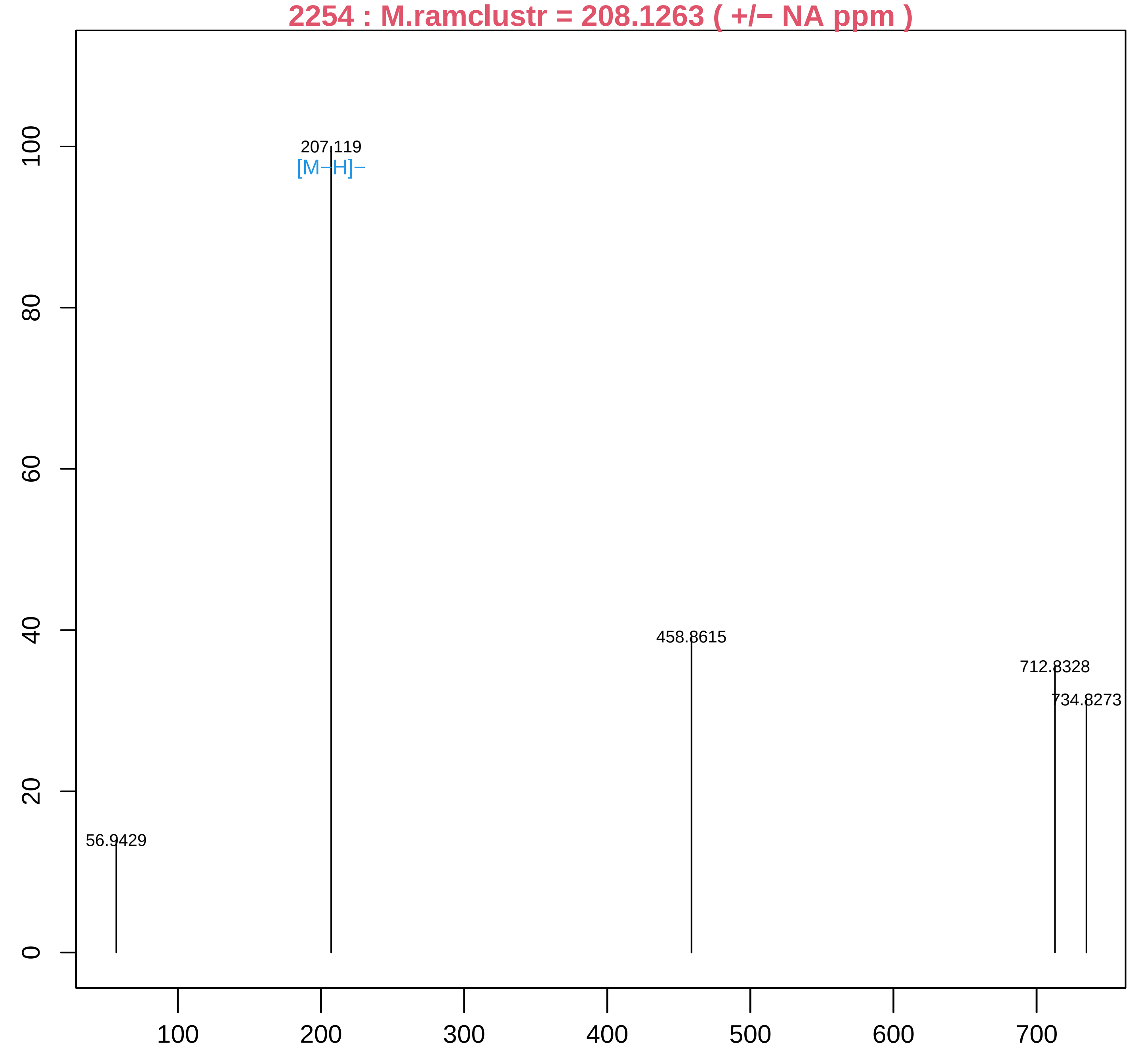
2254 : M.ramclustr = 208.1263 (+/− NA ppm)
207.119
[M−H]−
458.8615
712.8328
734.8273
56.9429
0
20
40
60
80
100
100
200
300
400
500
600
700

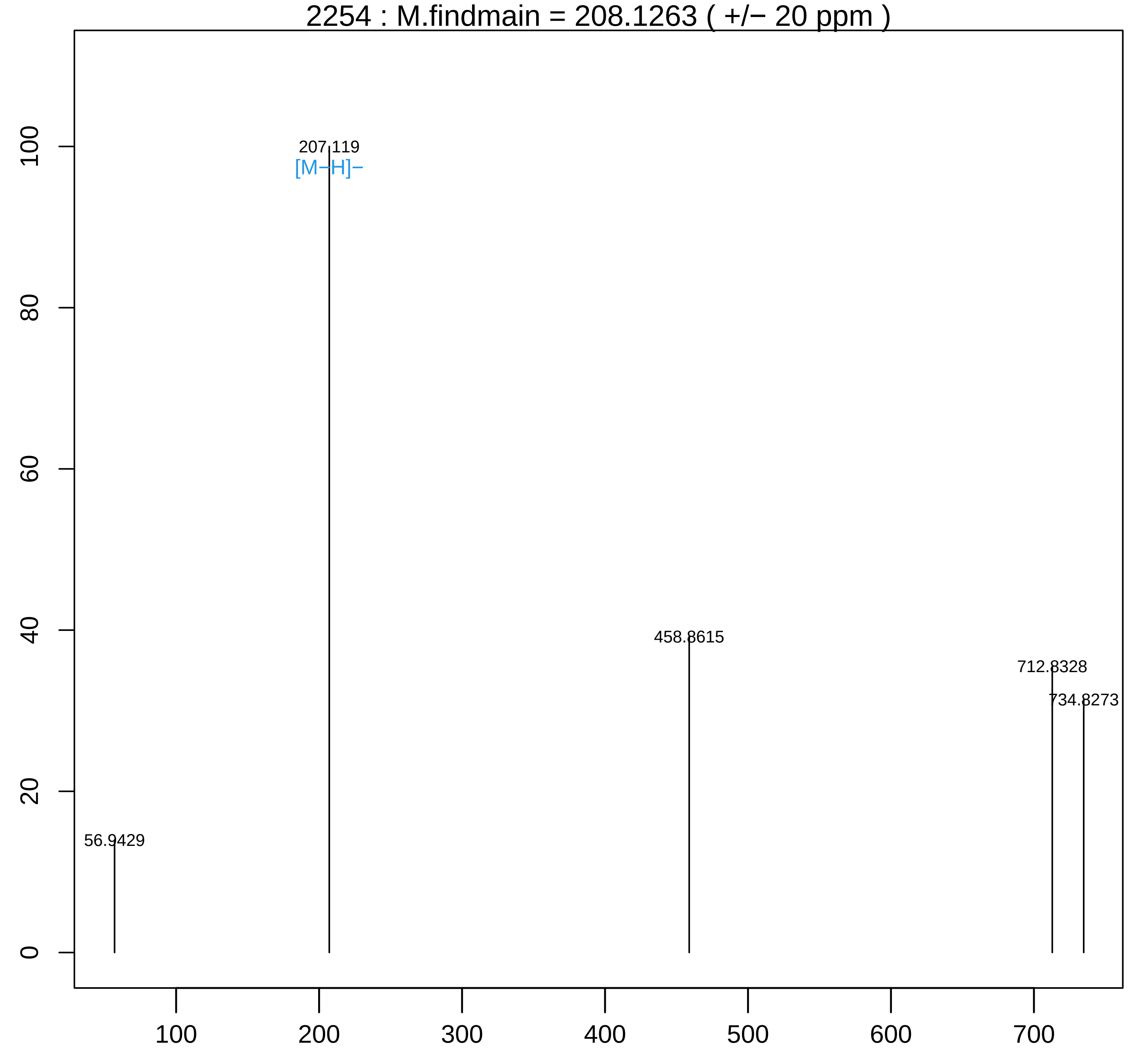
2254 : M.findmain = 208.1263 (+/− 20 ppm)
207.119
[M−H]−
458.8615
712.8328
734.8273
56.9429
0
20
40
60
80
100
100
200
300
400
500
600
700

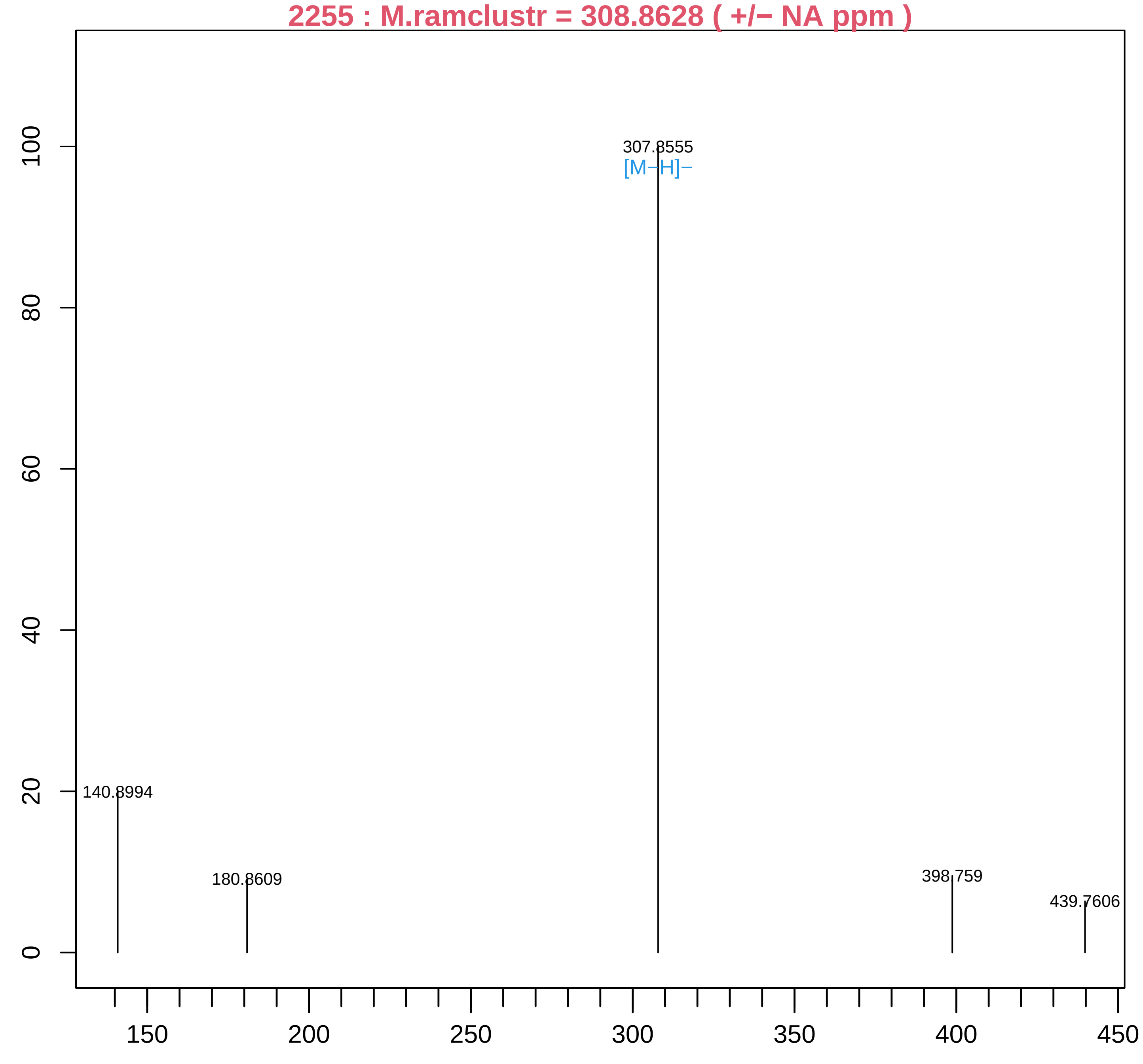
2255 : M.ramclustr = 308.8628 (+/− NA ppm)
307.8555
[M−H]−
140.8994
180.8609
398.759
439.7606
0
20
40
60
80
100
150
200
250
300
350
400
450

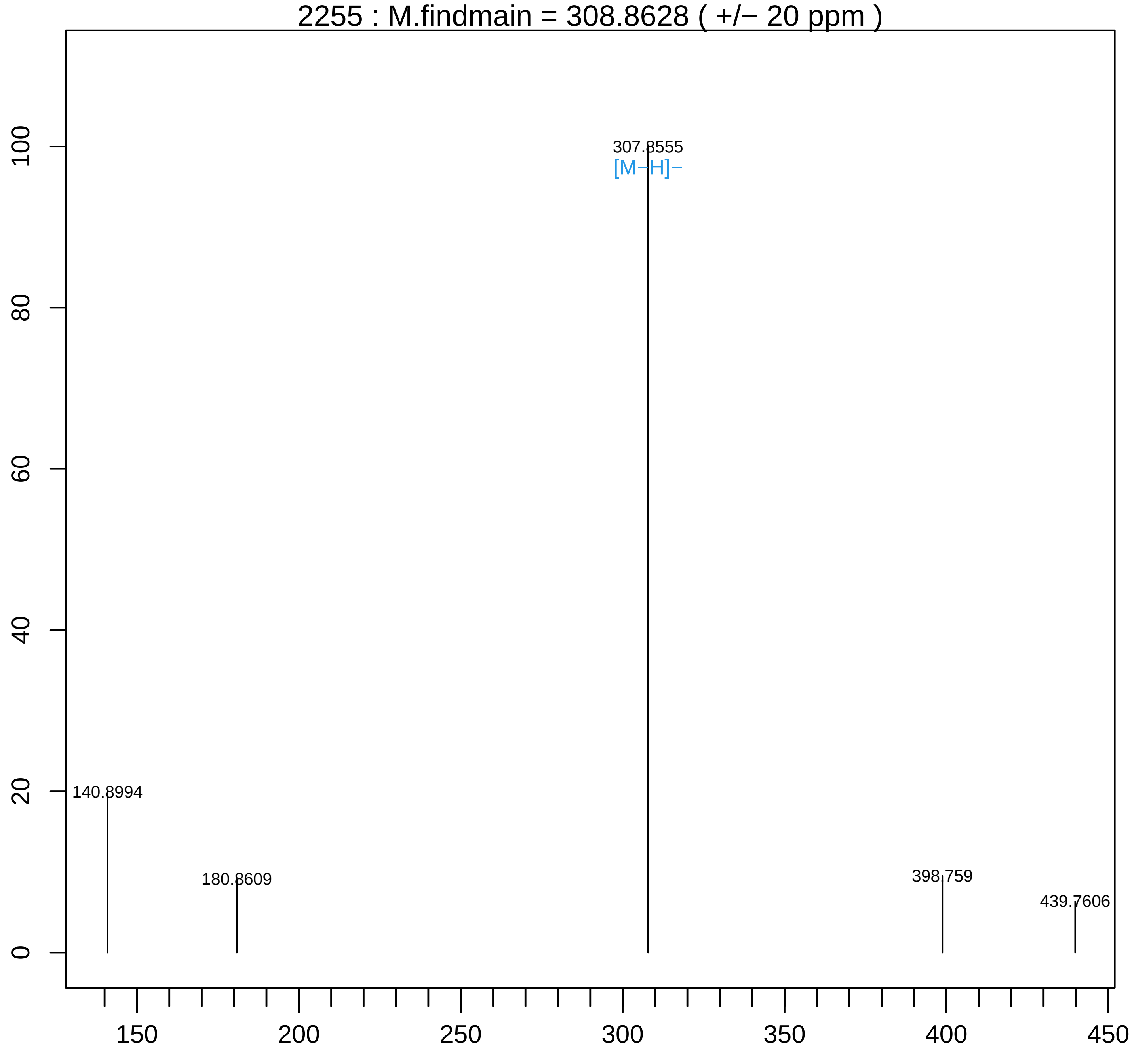
2255 : M.findmain = 308.8628 (+/− 20 ppm)
307.8555
[M−H]−
140.8994
180.8609
398.759
439.7606
0
20
40
60
80
100
150
200
250
300
350
400
450

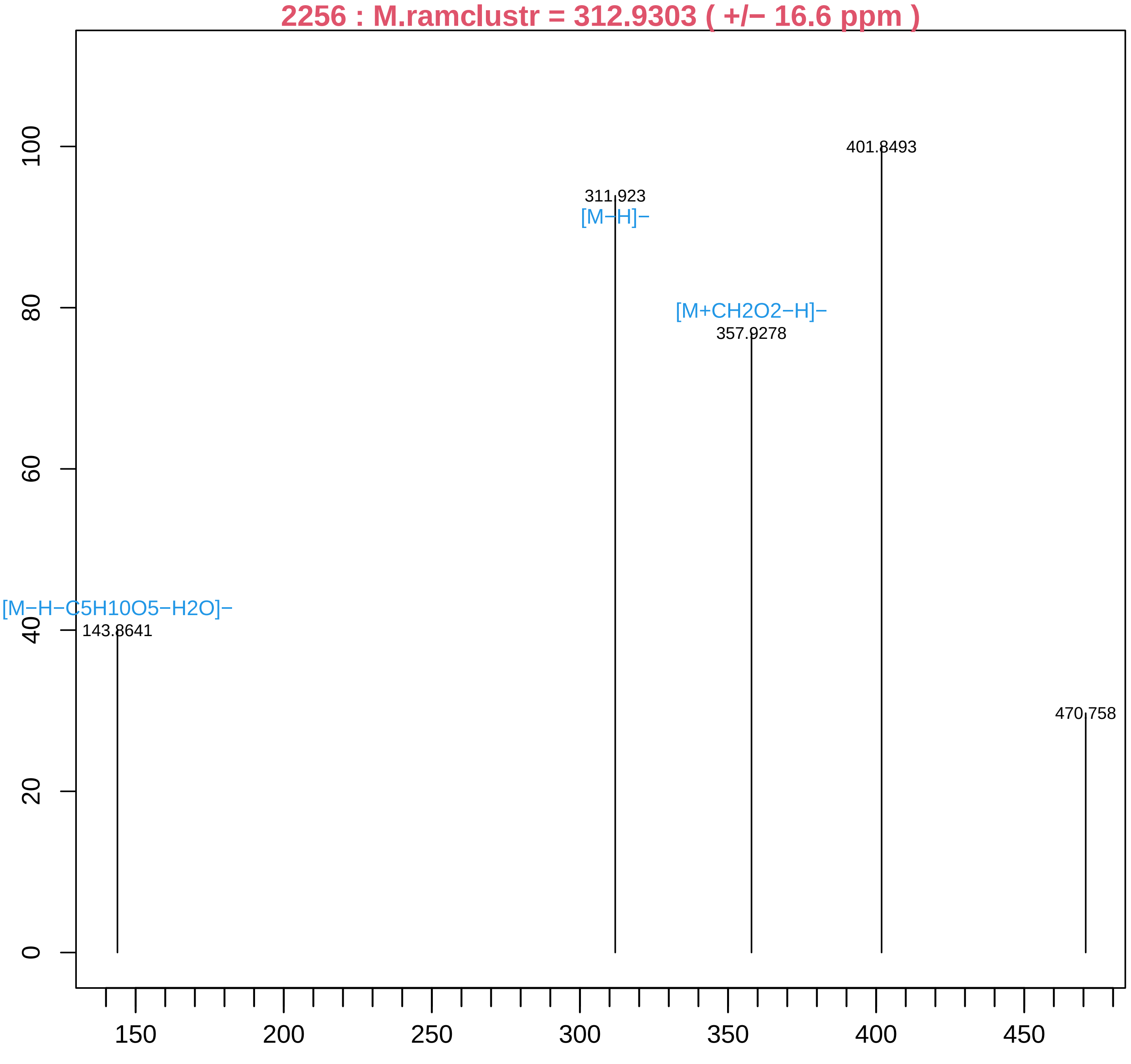
2256 : M.ramclustr = 312.9303 (+/− 16.6 ppm)
311.923
[M−H]−
401.8493
[M+CH2O2−H]−
357.9278
[M−H−C5H10O5−H2O]−
143.8641
470.758
0
20
40
60
80
100
150
200
250
300
350
400
450

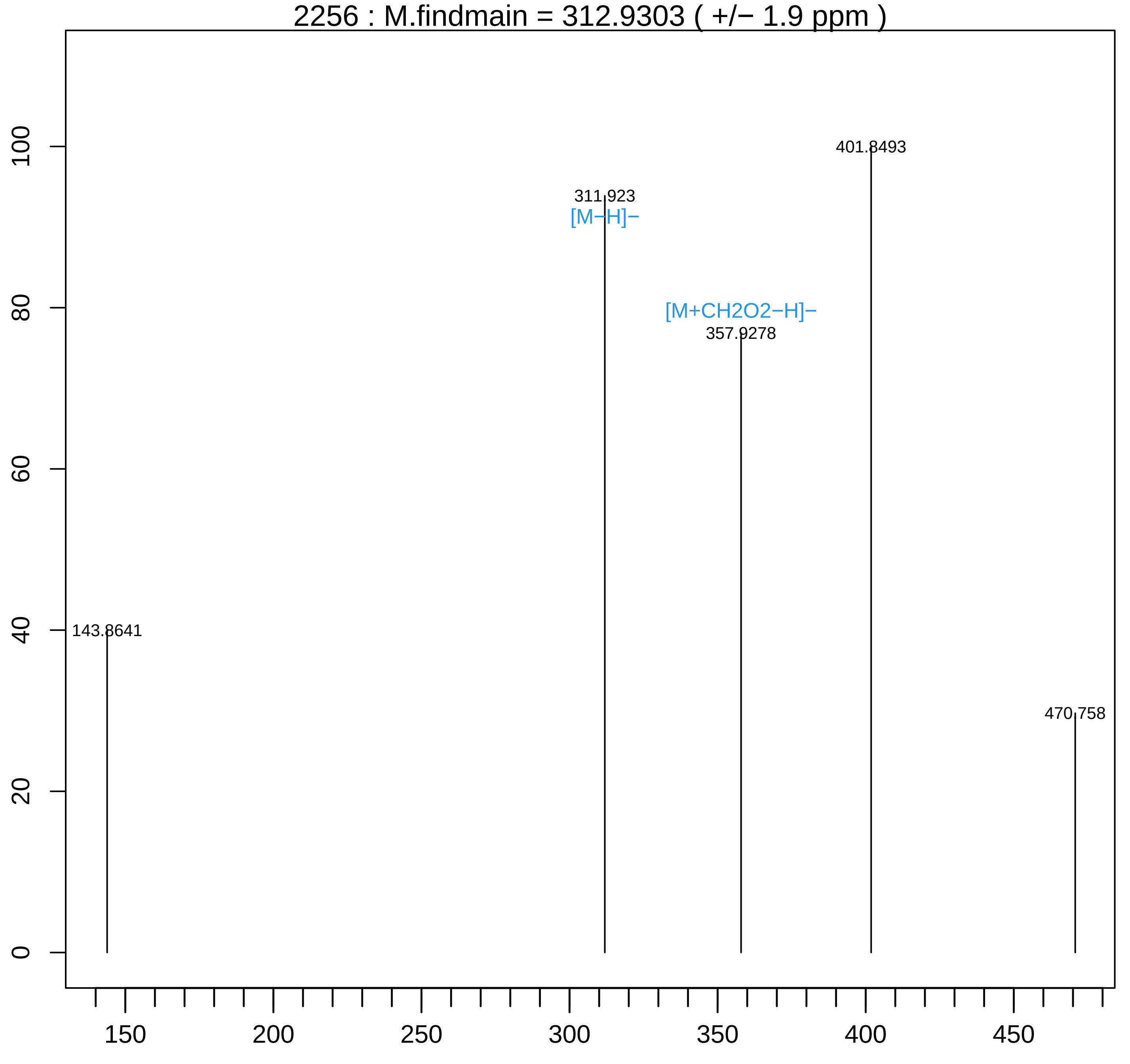
2256 : M.findmain = 312.9303 (+/− 1.9 ppm)
311.923
[M−H]−
401.8493
[M+CH2O2−H]−
357.9278
143.8641
470.758
0
20
40
60
80
100
150
200
250
300
350
400
450

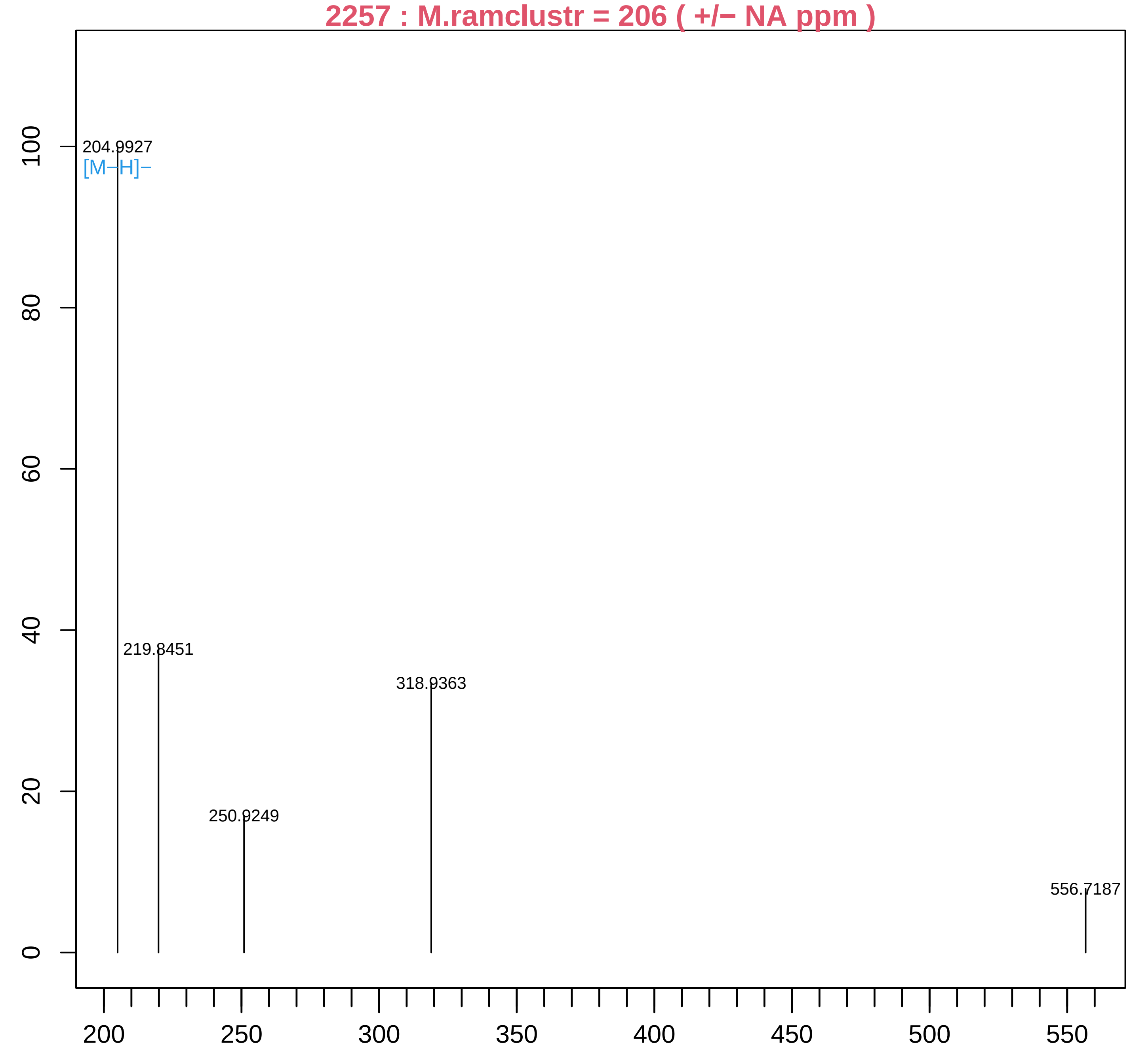

2257 : M.ramclustr = 206 (+/− NA ppm)
204.9927
[M−H]−
219.8451
250.9249
318.9363
556.7187
0
20
40
60
80
100
200
250
300
350
400
450
500
550

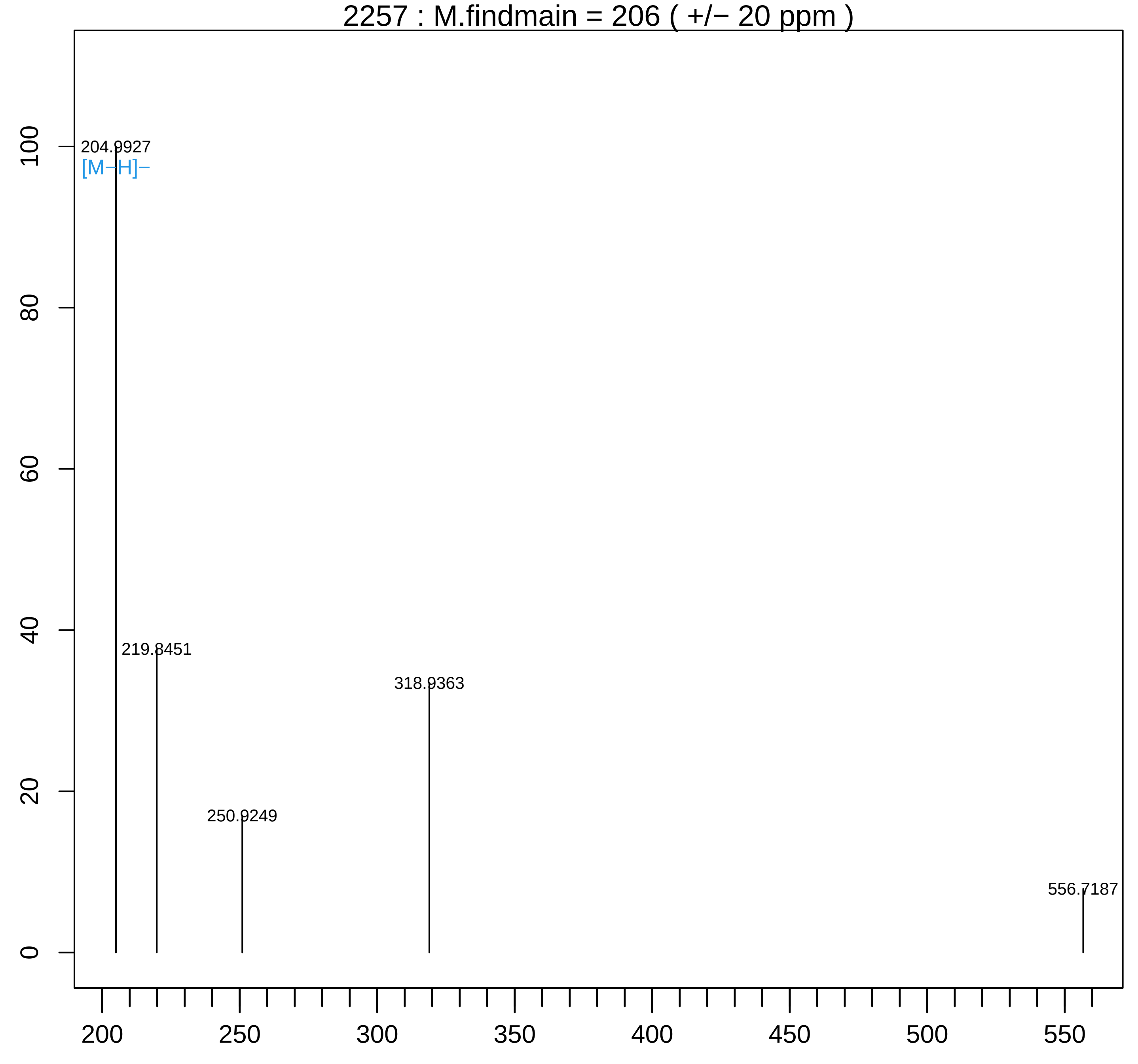

2257 : M.findmain = 206 (+/− 20 ppm)
204.9927
[M−H]−
219.8451
250.9249
318.9363
556.7187
0
20
40
60
80
100
200
250
300
350
400
450
500
550

2258 : M.ramclustr = 80.9661 (+/− 999980 ppm)

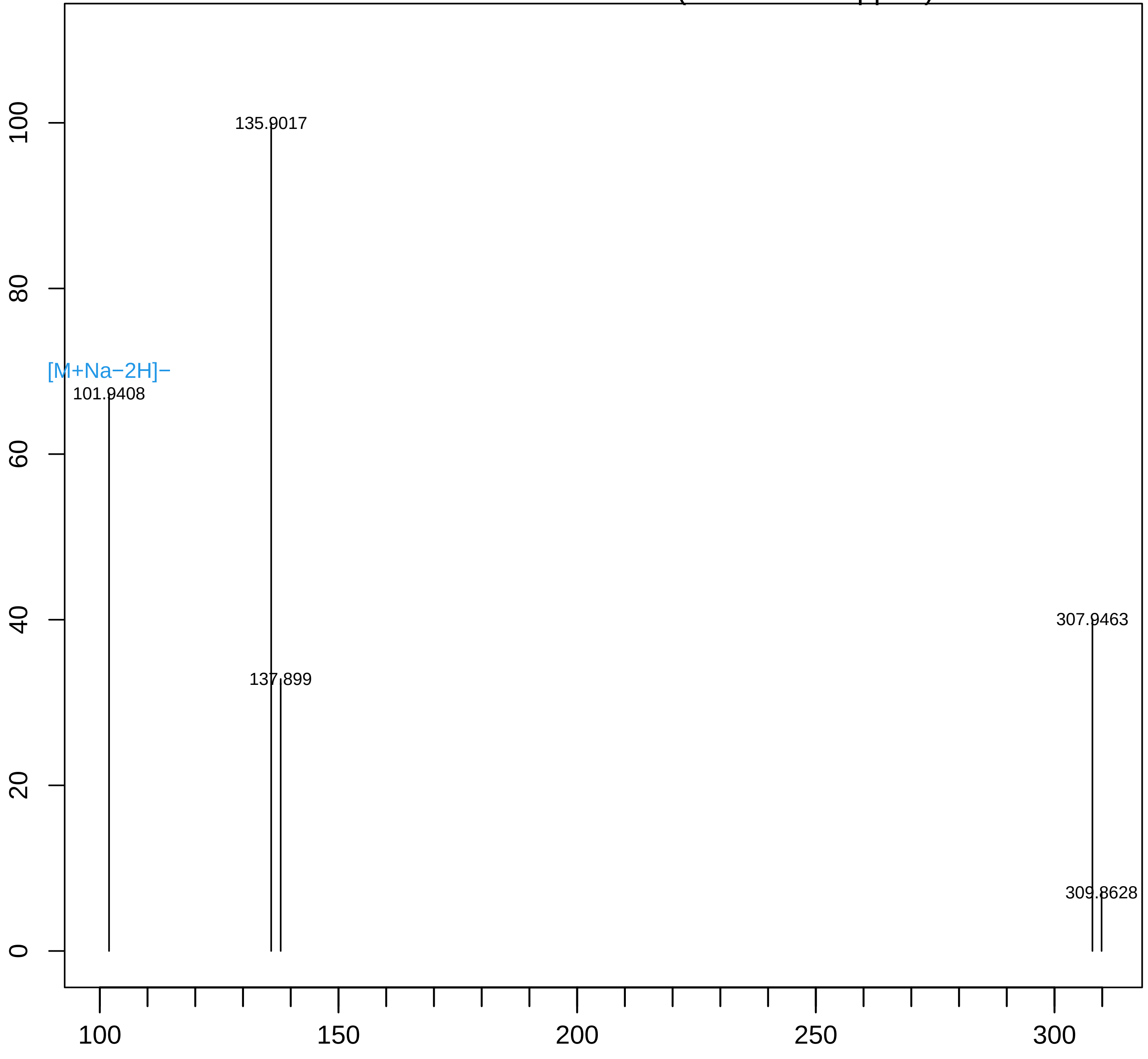

2258 : M.findmain = 136.909 (+/− 20 ppm)

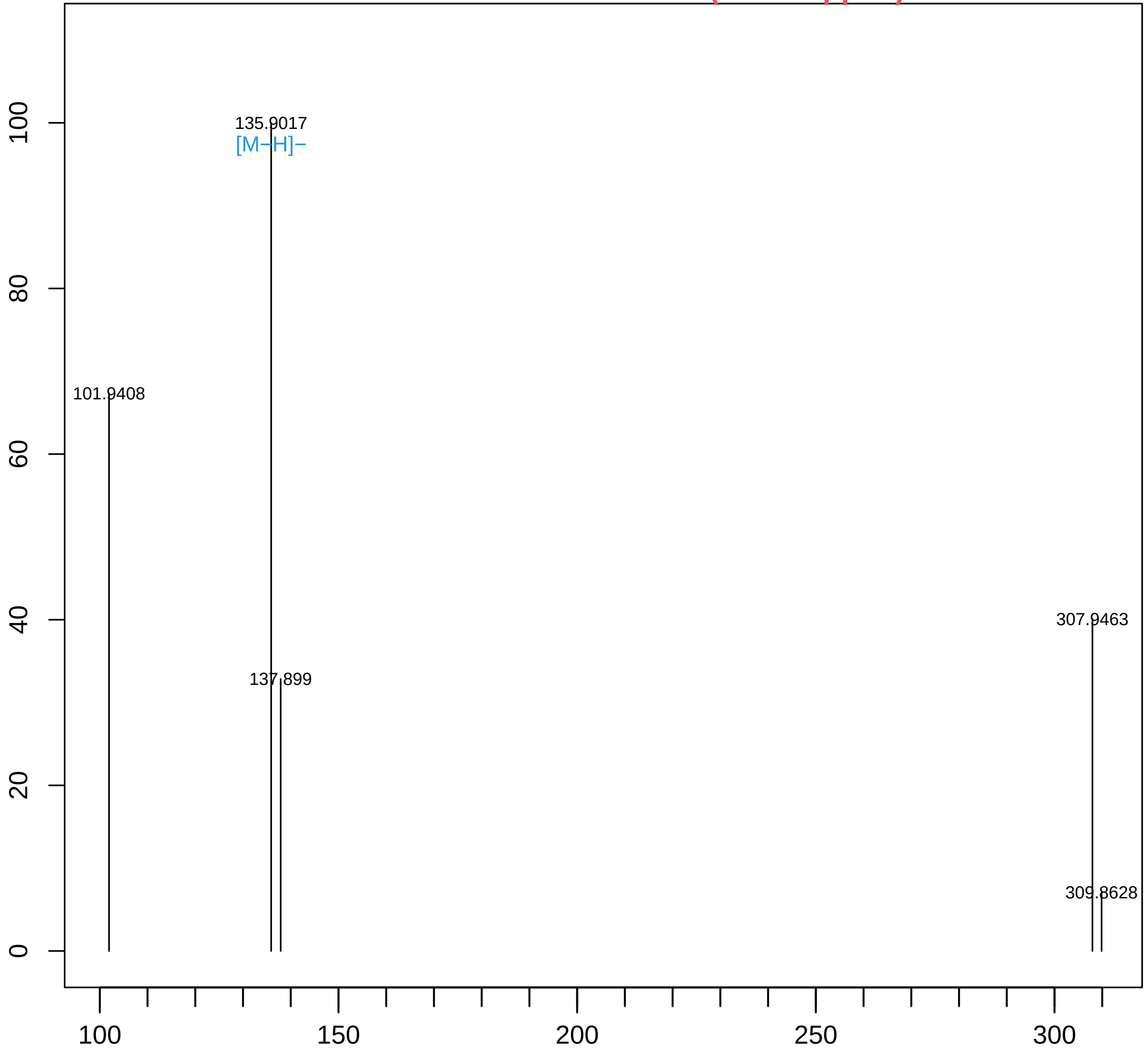

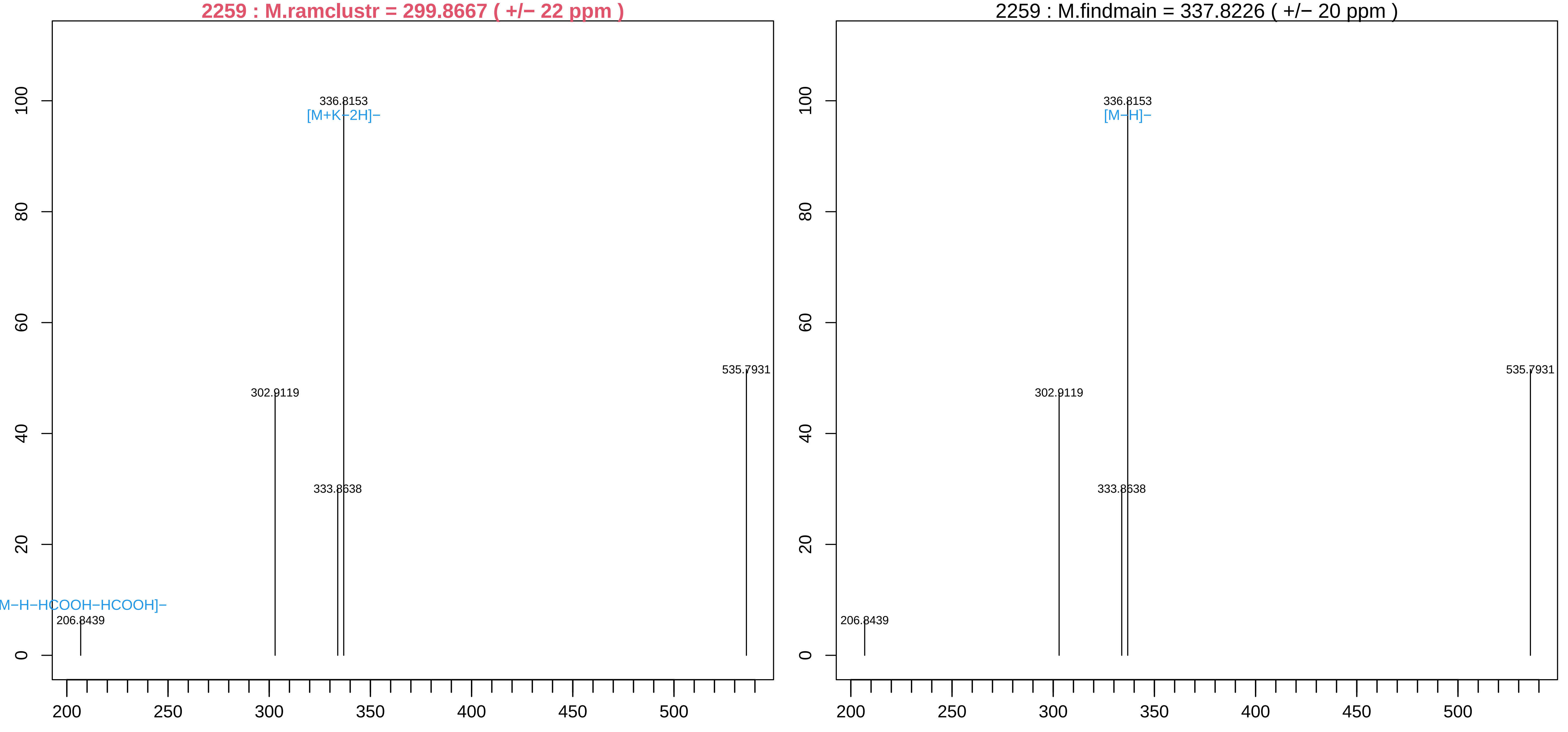

2259 : M.ramclustr = 299.8667 (+/− 22 ppm)
336.8153
[M+K−2H]−
302.9119
333.8638
535.7931
[M−H−HCOOH−HCOOH]−
206.8439
2259 : M.findmain = 337.8226 (+/− 20 ppm)
336.8153
[M−H]−
302.9119
333.8638
535.7931
206.8439

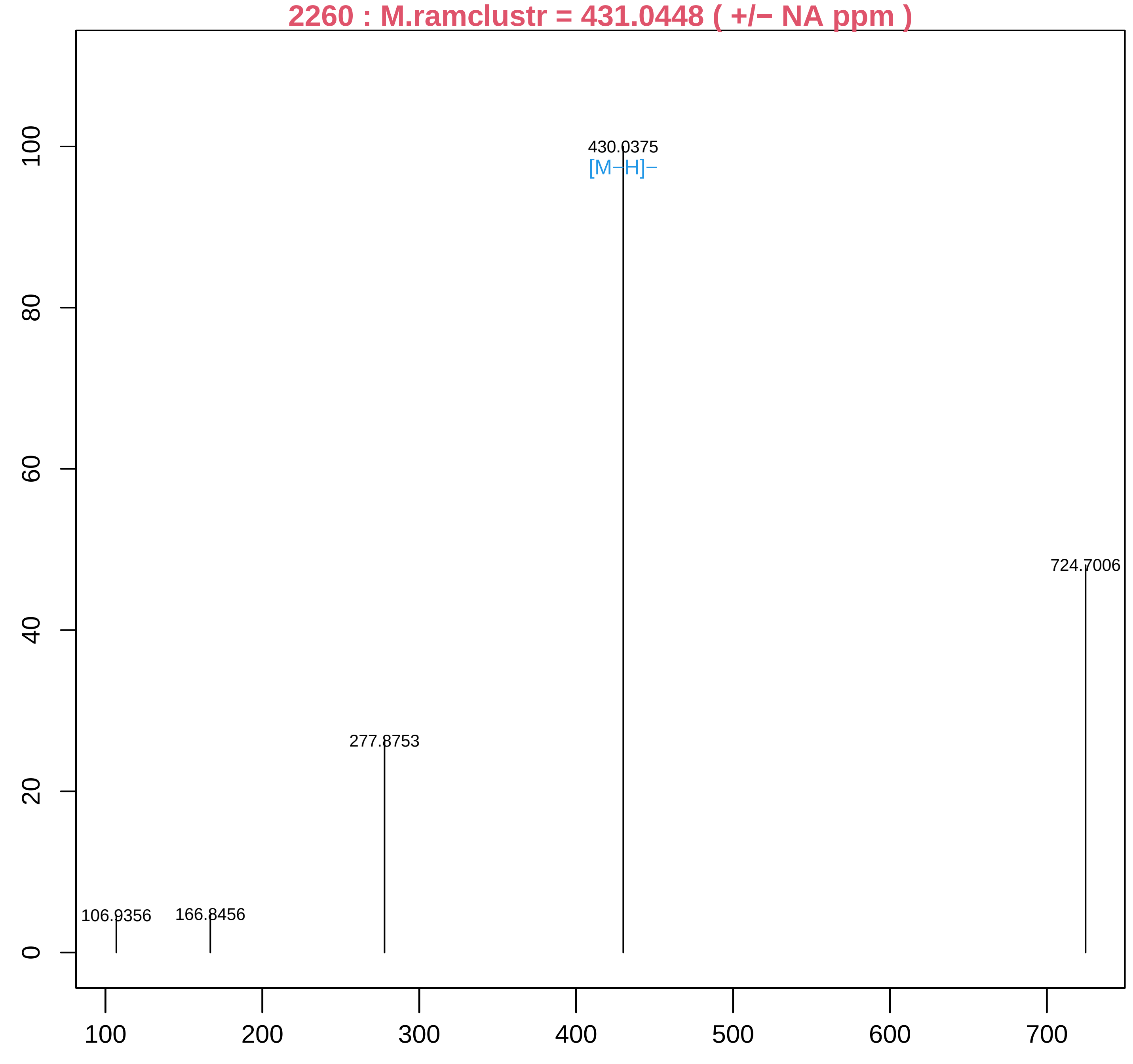

2260 : M.ramclustr = 431.0448 (+/− NA ppm)
430.0375
[M−H]−
724.7006
277.8753
106.9356
166.8456
0
20
40
60
80
100
100
200
300
400
500
600
700

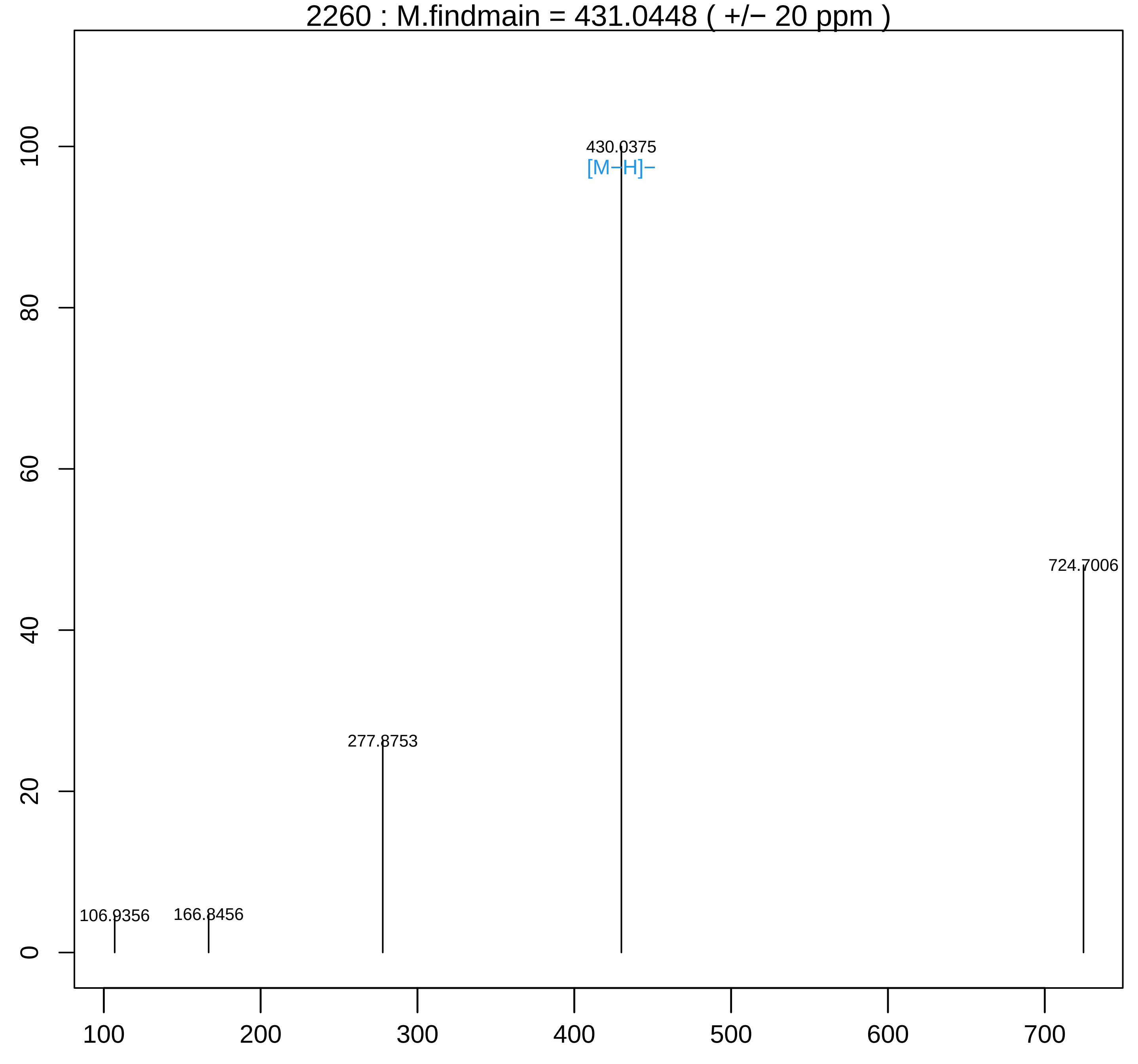

2260 : M.findmain = 431.0448 (+/− 20 ppm)
430.0375
[M−H]−
724.7006
277.8753
106.9356
166.8456
0
20
40
60
80
100
100
200
300
400
500
600
700

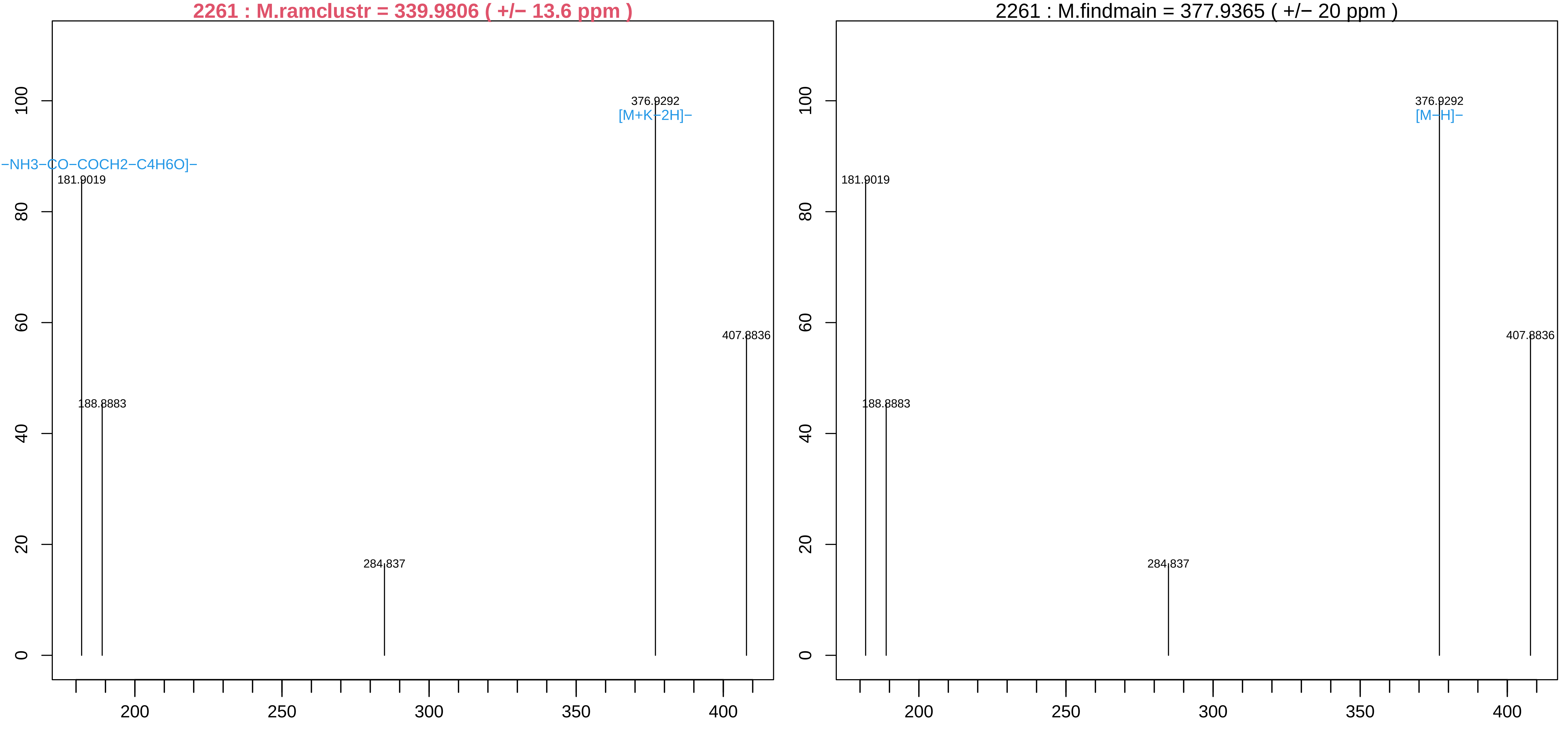
2261 : M.ramclustr = 339.9806 (+/− 13.6 ppm)
−NH3−CO−COCH2−C4H6O]−
181.9019
188.8883
284.837
376.9292
[M+K−2H]−
407.8836
2261 : M.findmain = 377.9365 (+/− 20 ppm)
181.9019
188.8883
284.837
376.9292
[M−H]−
407.8836
0
20
40
60
80
100
200
250
300
350
400

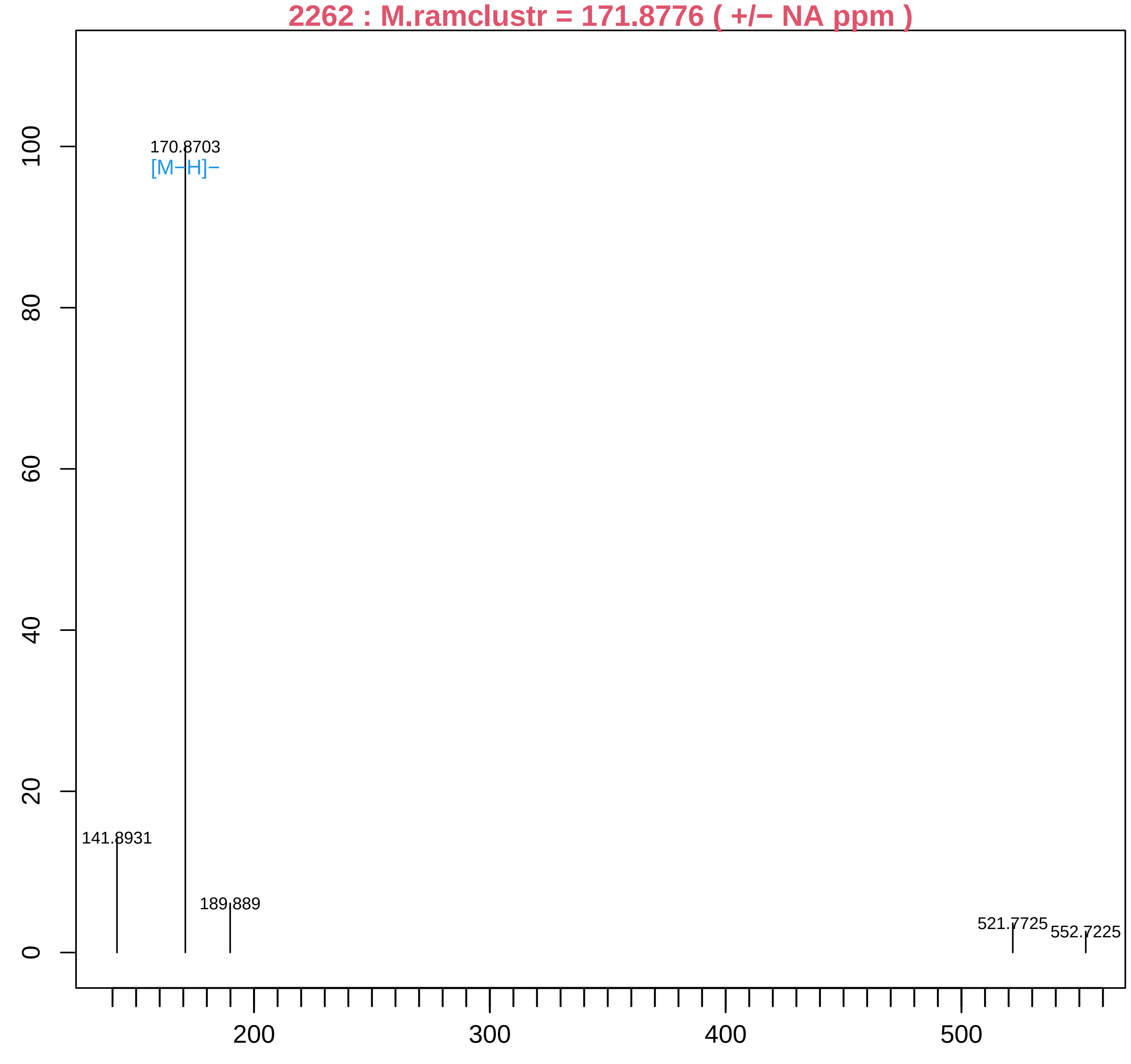
2262 : M.ramclustr = 171.8776 (+/− NA ppm)
170.8703
[M−H]−
141.8931
189.889
521.7725
552.7225
0
20
40
60
80
100
200
300
400
500

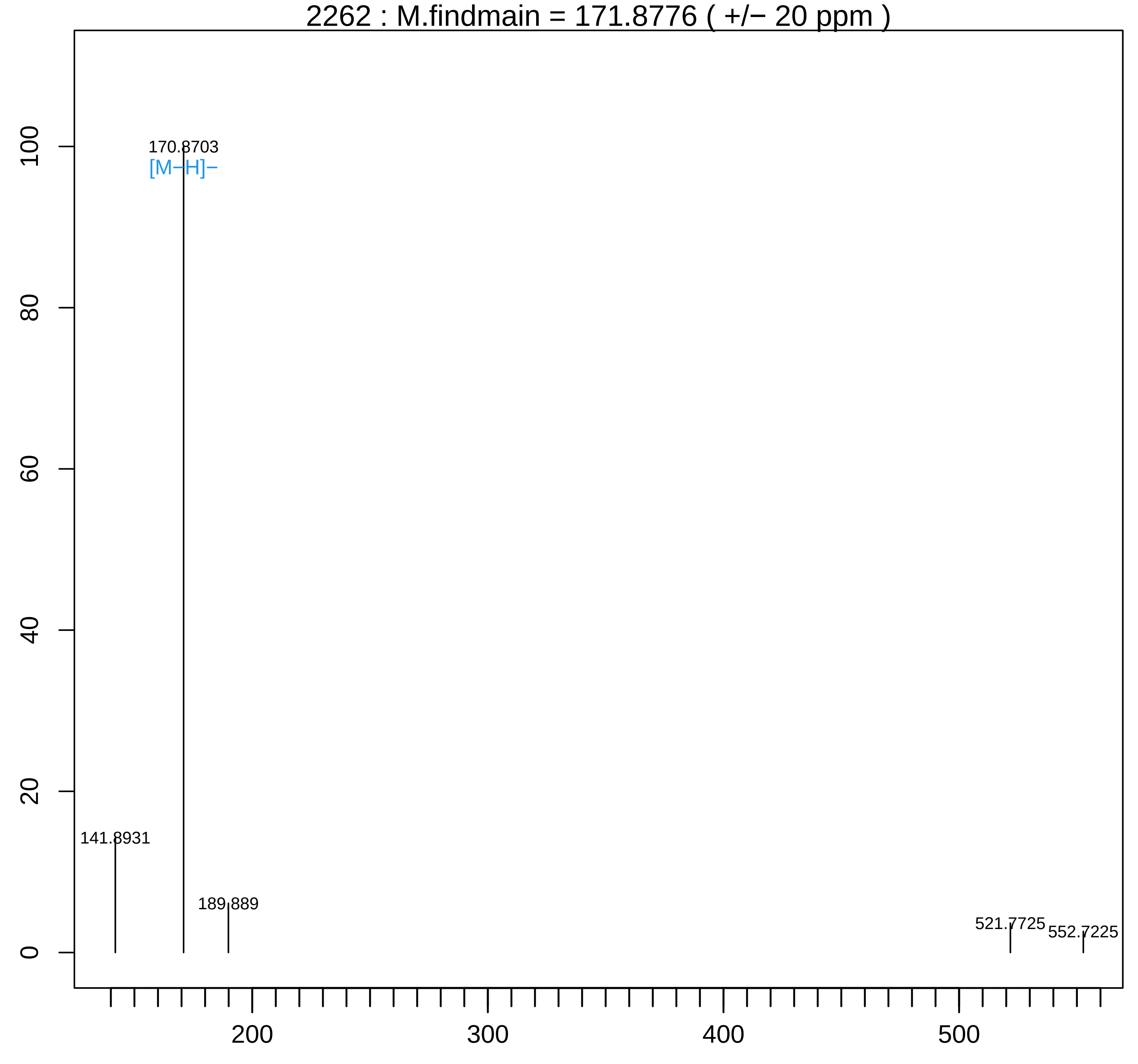
2262 : M.findmain = 171.8776 (+/− 20 ppm)
170.8703
[M−H]−
141.8931
189.889
521.7725
552.7225
0
20
40
60
80
100
200
300
400
500

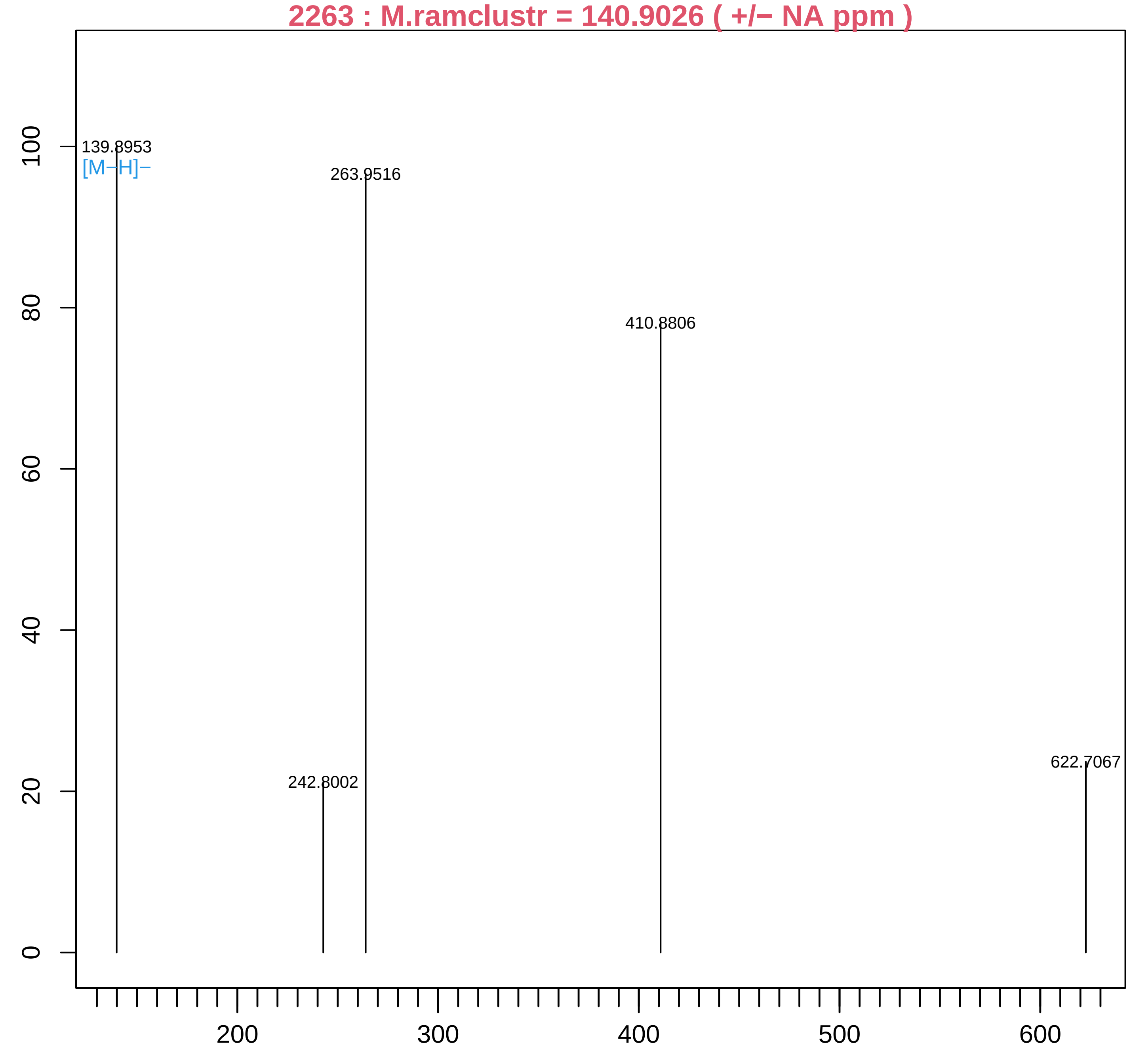

2263 : M.ramclustr = 140.9026 (+/− NA ppm)
139.8953
[M−H]−
263.9516
410.8806
242.8002
622.7067
0
20
40
60
80
100
200
300
400
500
600

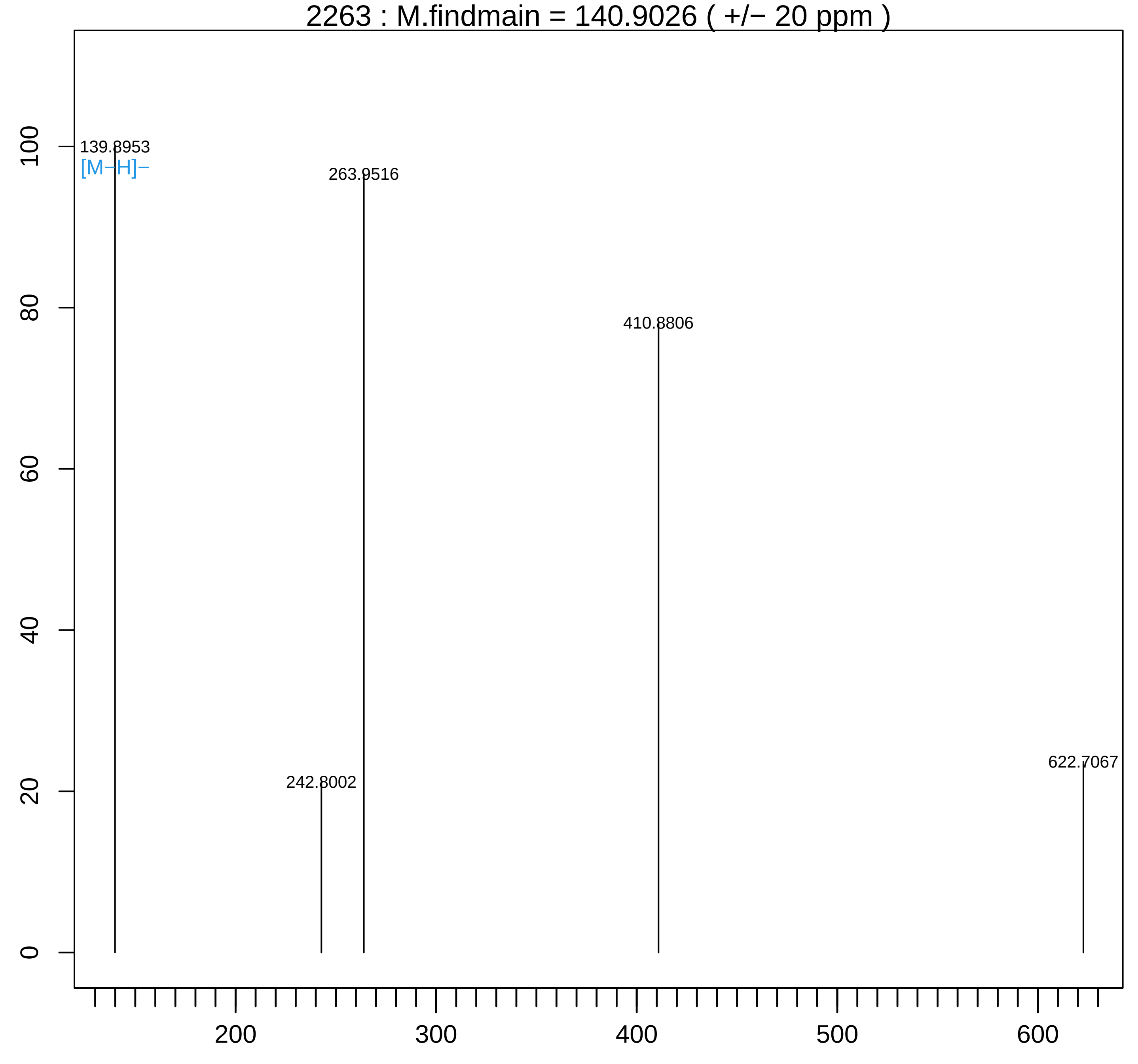

2263 : M.findmain = 140.9026 (+/− 20 ppm)
139.8953
[M−H]−
263.9516
410.8806
242.8002
622.7067
0
20
40
60
80
100
200
300
400
500
600

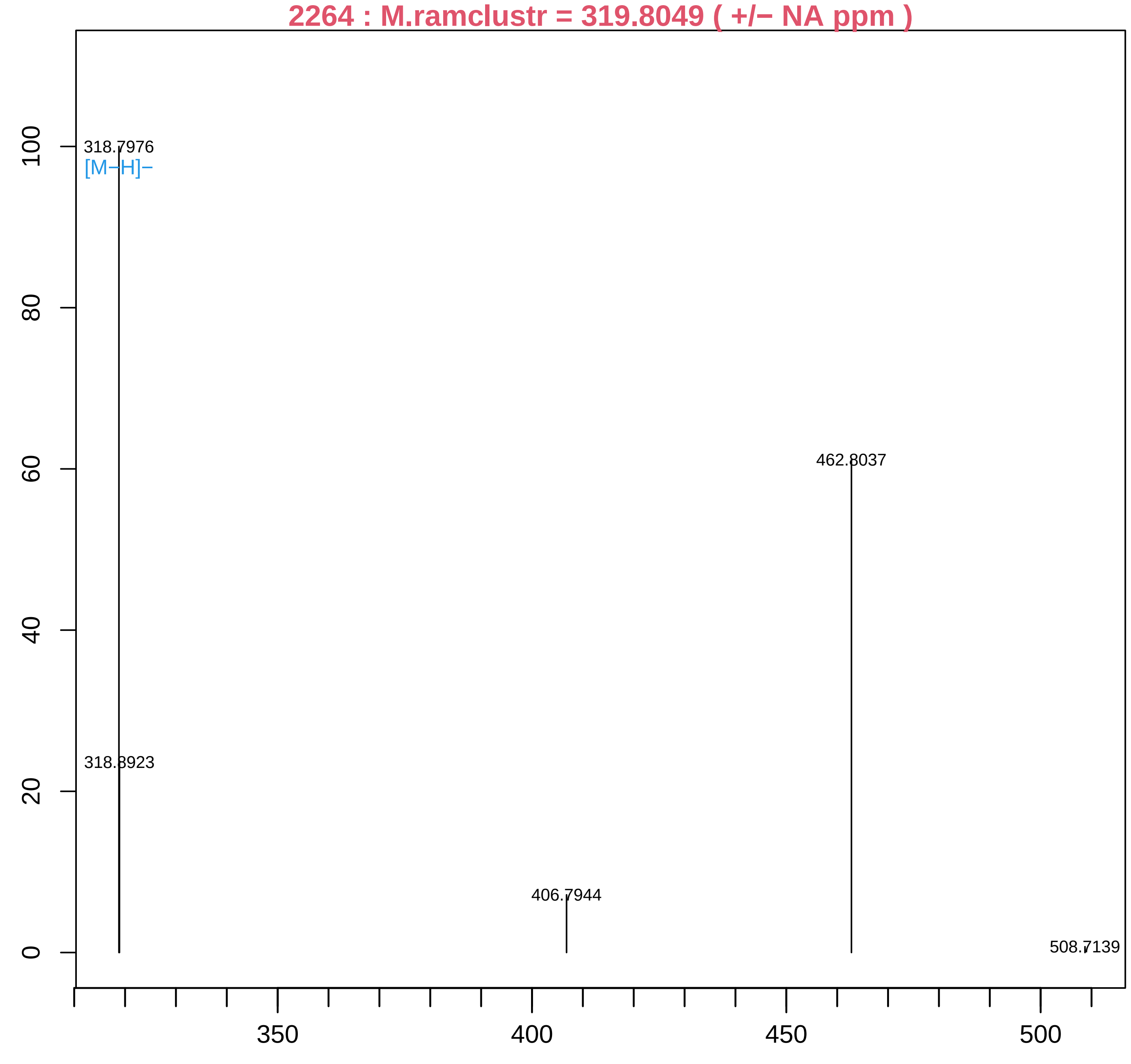
2264 : M.ramclustr = 319.8049 (+/− NA ppm)
318.7976
[M−H]−
318.8923
406.7944
462.8037
508.7139
0
20
40
60
80
100
350
400
450
500

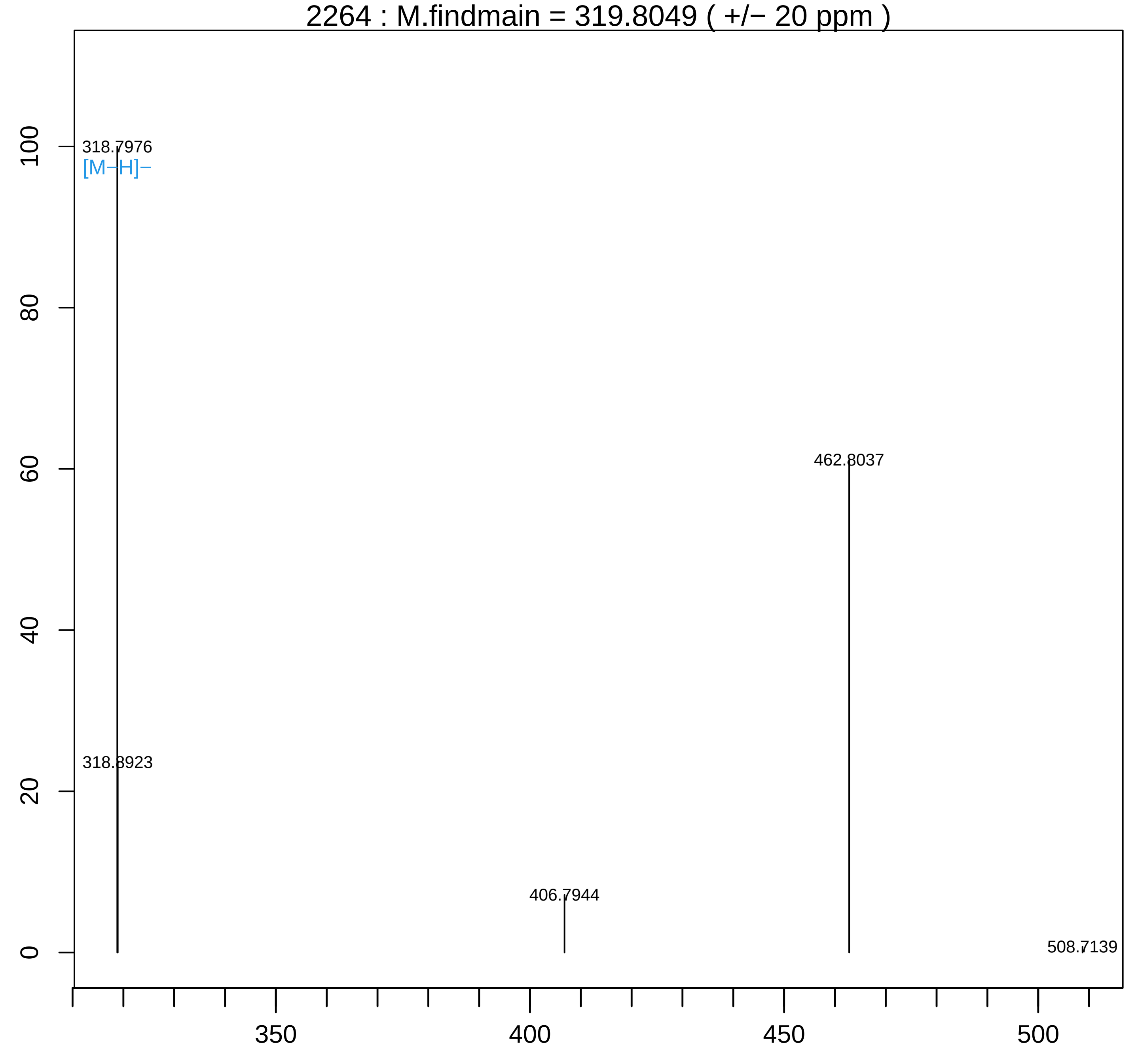
2264 : M.findmain = 319.8049 (+/− 20 ppm)
318.7976
[M−H]−
318.8923
406.7944
462.8037
508.7139
0
20
40
60
80
100
350
400
450
500

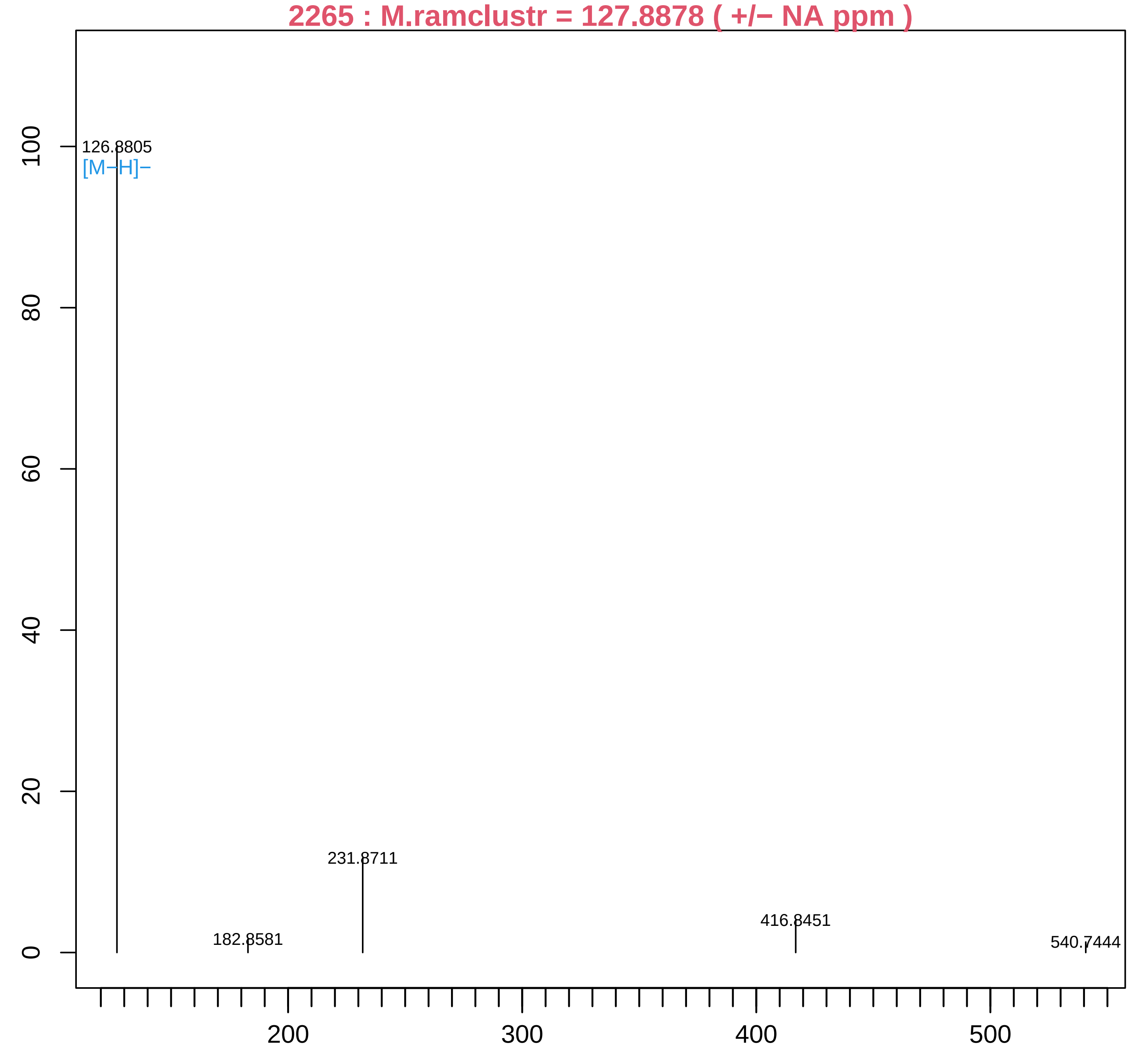
2265 : M.ramclustr = 127.8878 (+/− NA ppm)
126.8805
[M−H]−
182.8581
231.8711
416.8451
540.7444
0
20
40
60
80
100
200
300
400
500

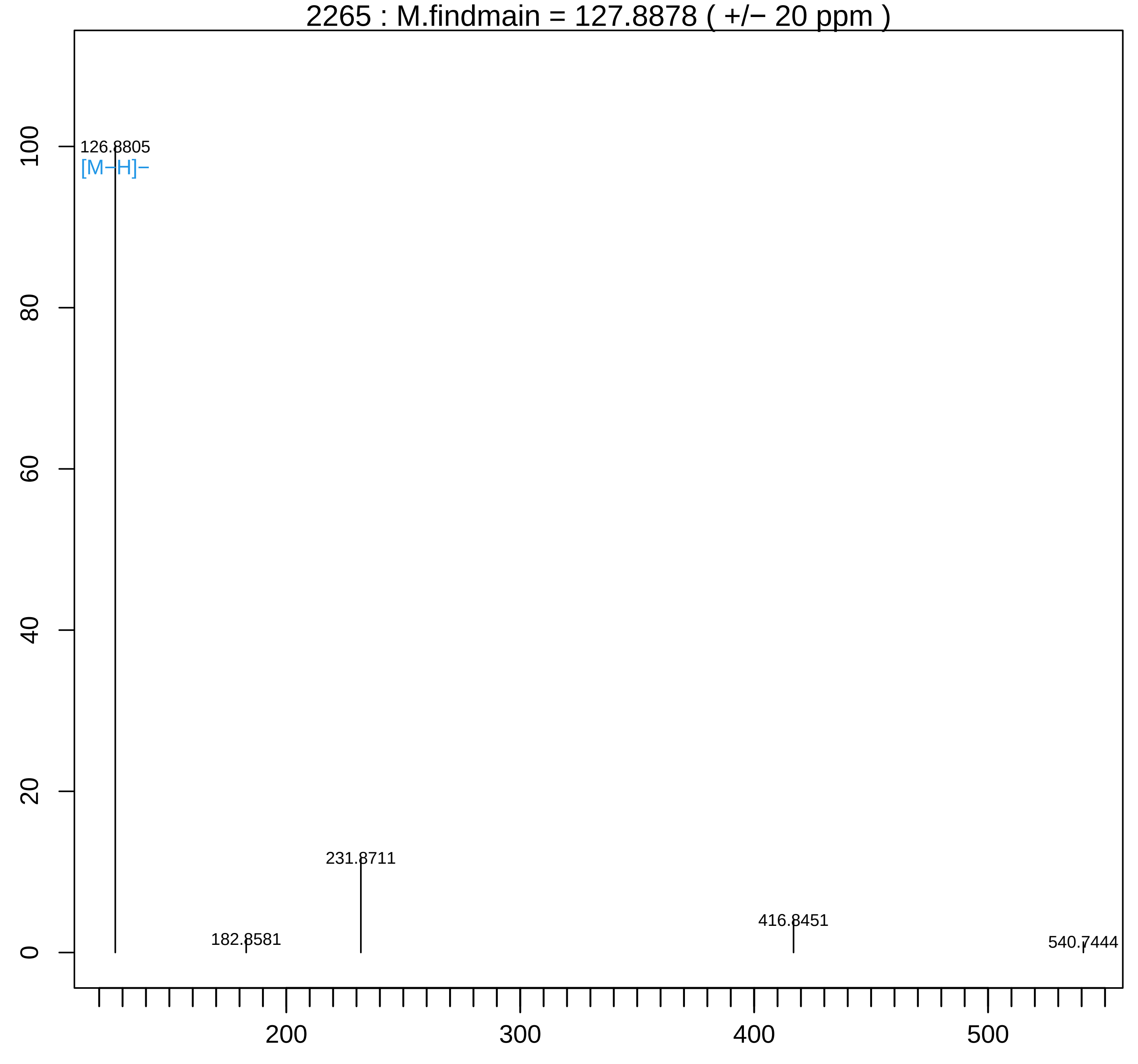
2265 : M.findmain = 127.8878 (+/− 20 ppm)
126.8805
[M−H]−
182.8581
231.8711
416.8451
540.7444
0
20
40
60
80
100
200
300
400
500

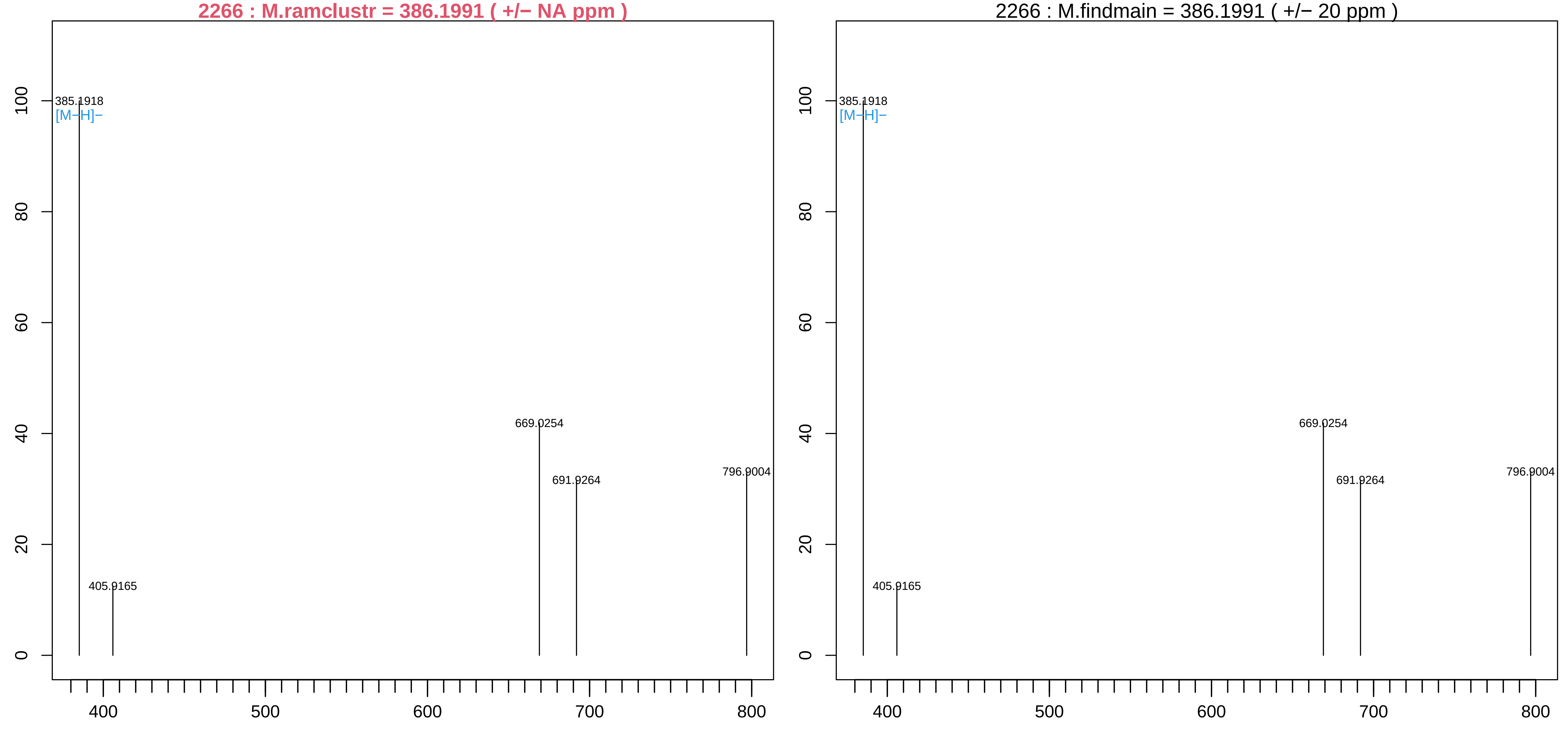
2266 : M.ramclustr = 386.1991 (+/− NA ppm)
385.1918
[M−H]−
405.9165
669.0254
691.9264
796.9004
0
20
40
60
80
100
400
500
600
700
800
2266 : M.findmain = 386.1991 (+/− 20 ppm)
385.1918
[M−H]−
405.9165
669.0254
691.9264
796.9004
0
20
40
60
80
100
400
500
600
700
800

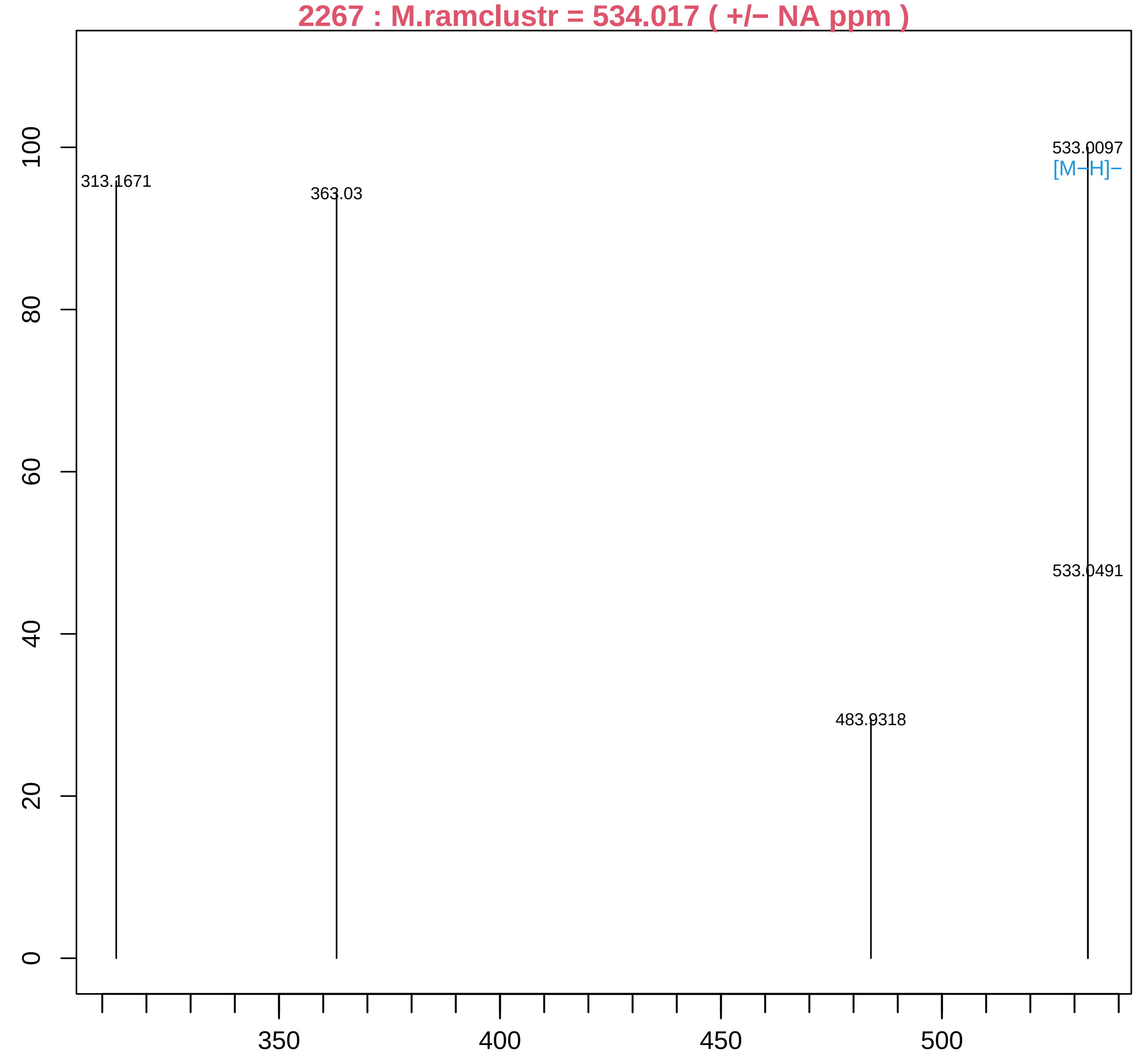
2267 : M.ramclustr = 534.017 (+/− NA ppm)
313.1671
363.03
483.9318
533.0097
[M−H]−
533.0491
0
20
40
60
80
100
350
400
450
500
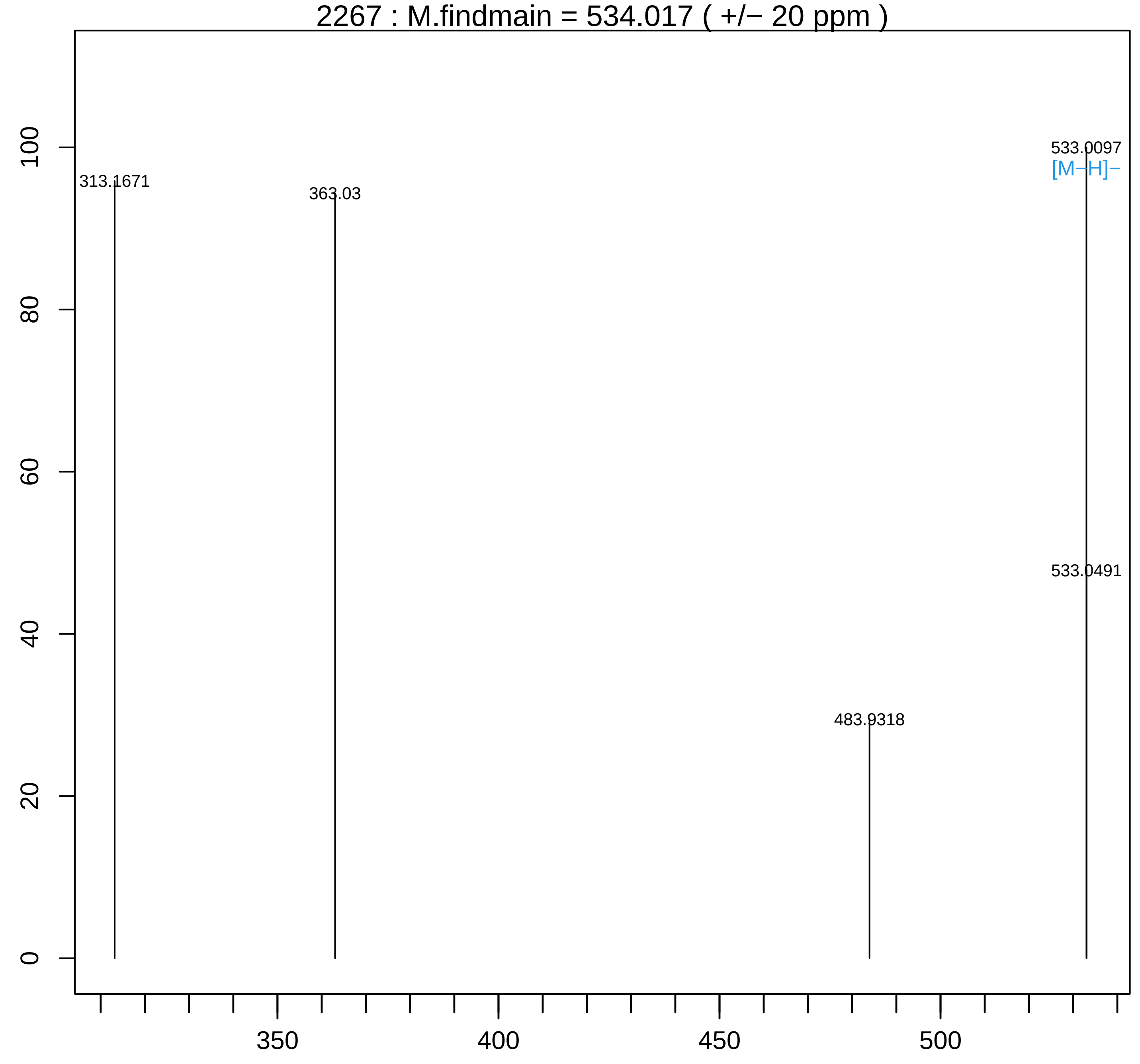
2267 : M.findmain = 534.017 (+/− 20 ppm)
313.1671
363.03
483.9318
533.0097
[M−H]−
533.0491
0
20
40
60
80
100
350
400
450
500

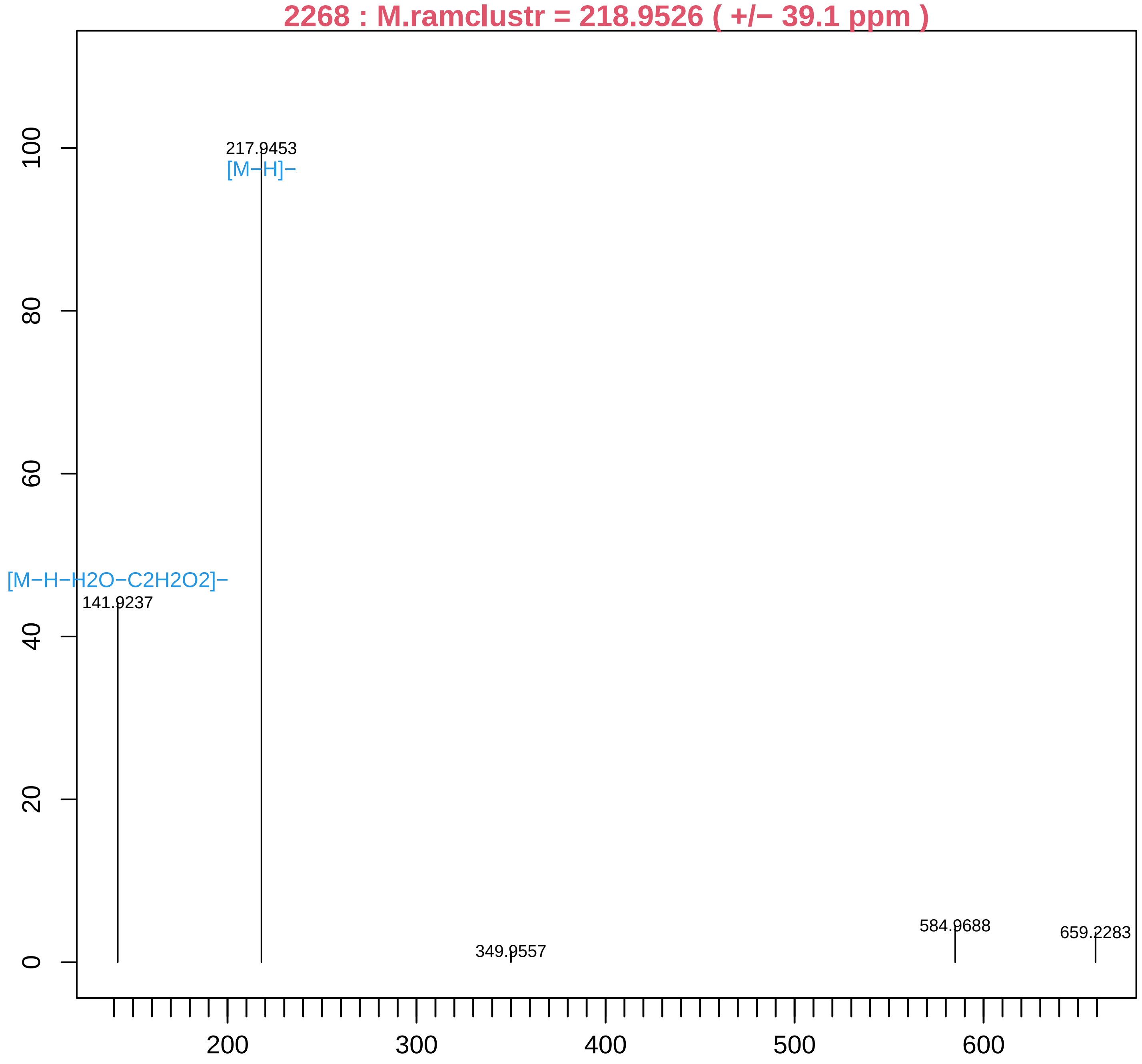
2268 : M.ramclustr = 218.9526 (+/− 39.1 ppm)
217.9453
[M−H]−
[M−H−H2O−C2H2O2]−
141.9237
349.9557
584.9688
659.2283
0
20
40
60
80
100
200
300
400
500
600

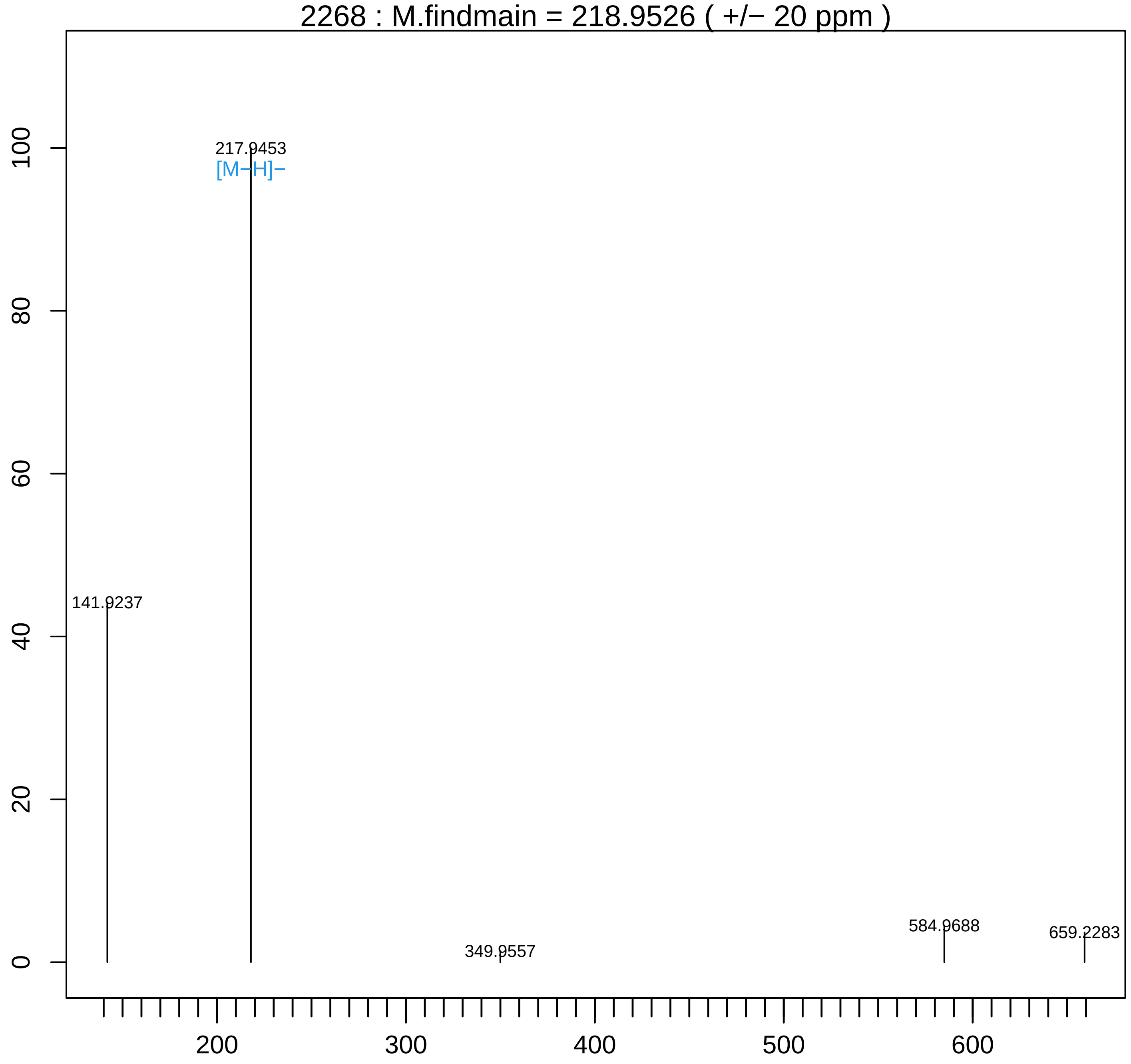
2268 : M.findmain = 218.9526 (+/− 20 ppm)
217.9453
[M−H]−
141.9237
349.9557
584.9688
659.2283
0
20
40
60
80
100
200
300
400
500
600

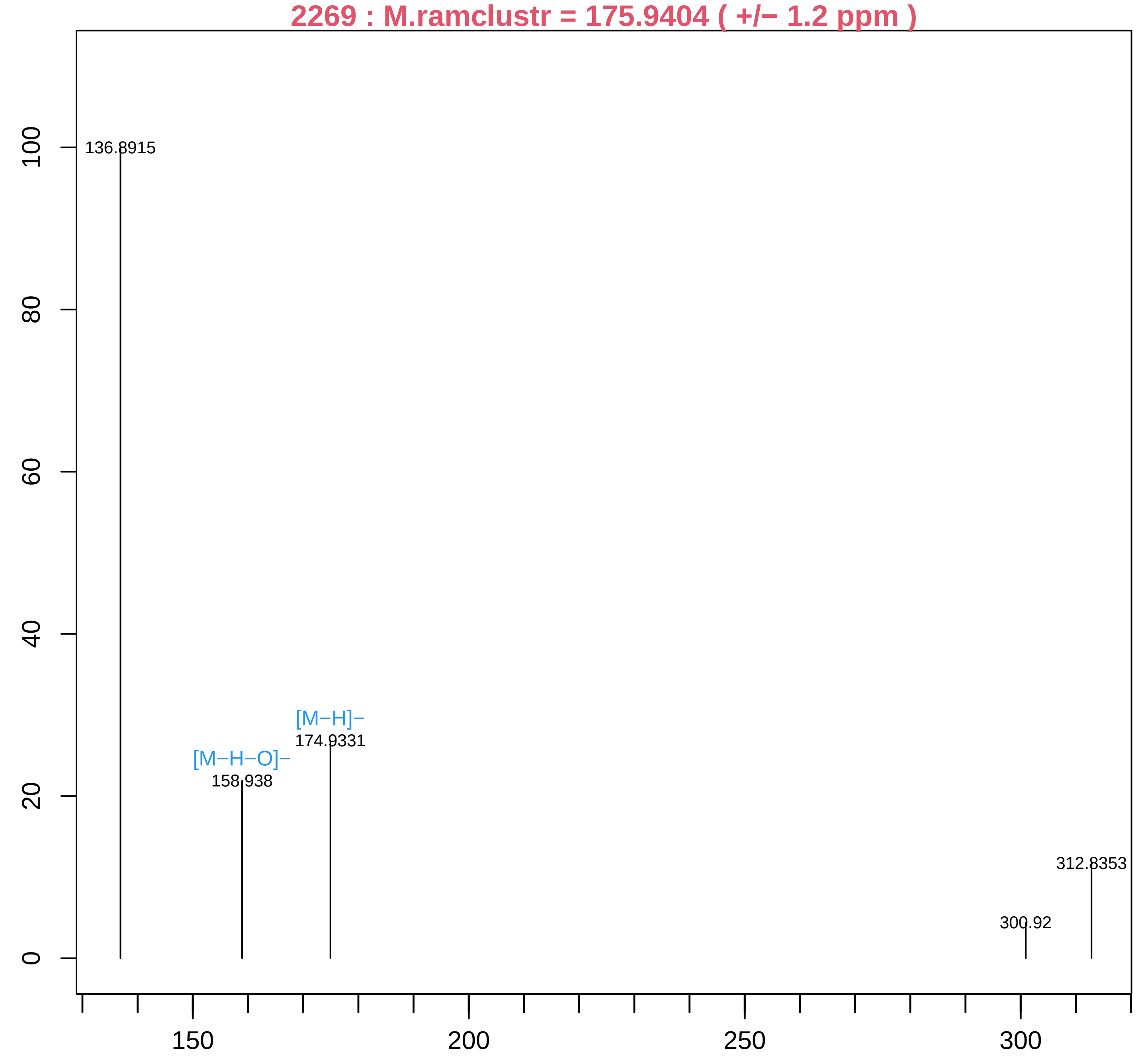
2269 : M.ramclustr = 175.9404 (+/− 1.2 ppm)
136.8915
[M−H−O]−
158.938
[M−H]−
174.9331
300.92
312.8353
0
20
40
60
80
100
150
200
250
300

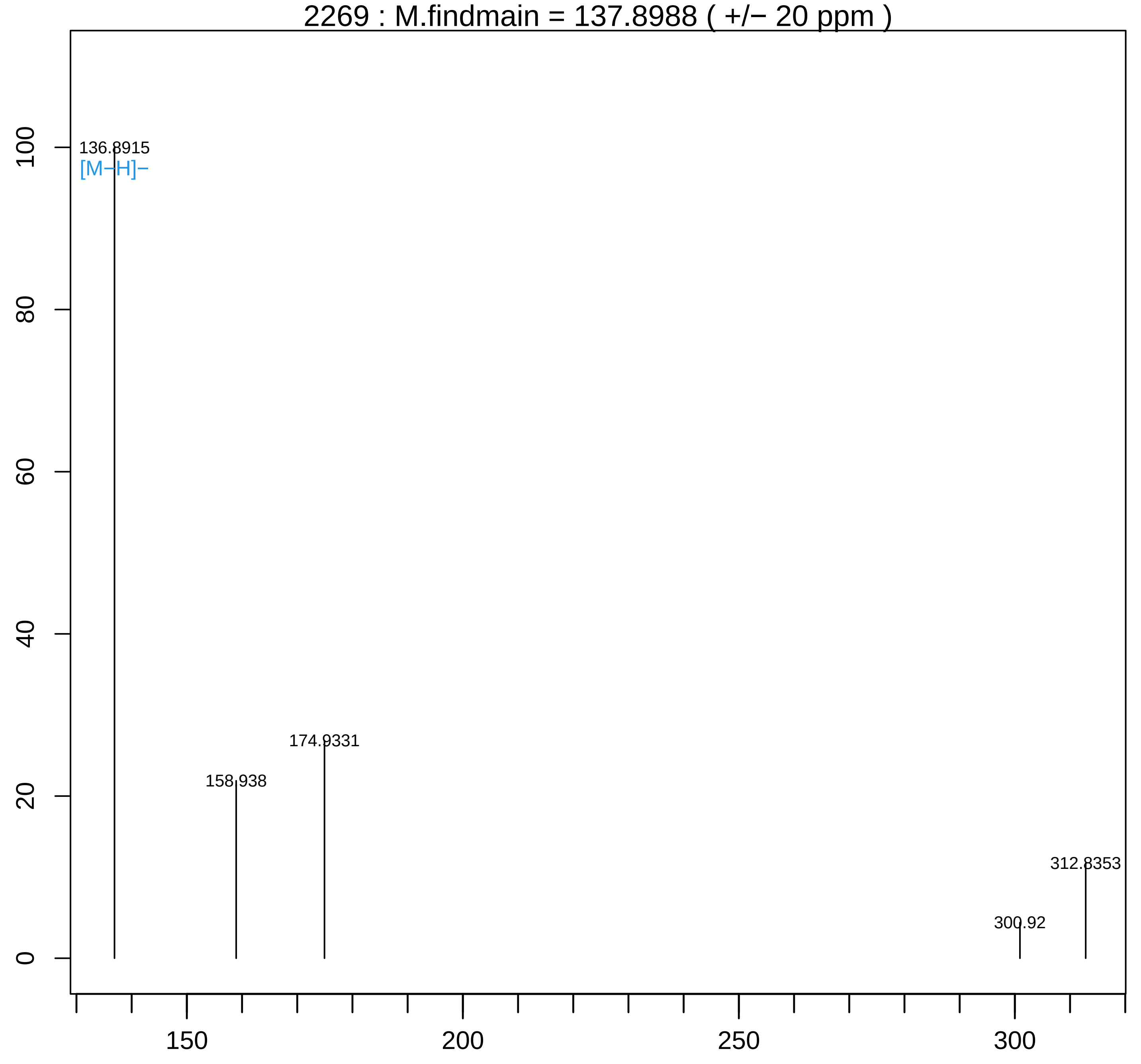
2269 : M.findmain = 137.8988 (+/− 20 ppm)
136.8915
[M−H]−
158.938
174.9331
300.92
312.8353
0
20
40
60
80
100
150
200
250
300

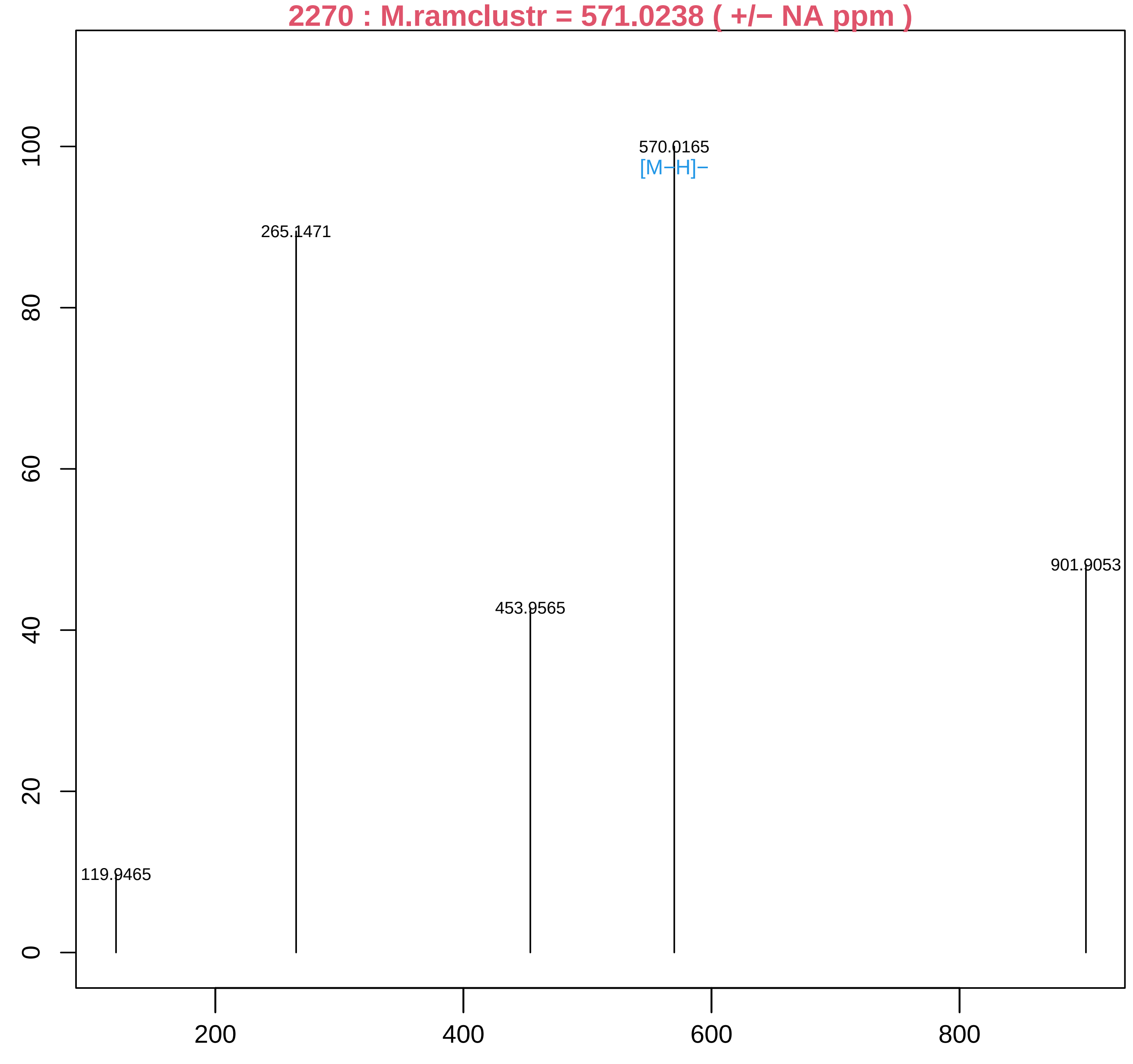

2270 : M.ramclustr = 571.0238 (+/− NA ppm)
570.0165
[M−H]−
265.1471
453.9565
901.9053
119.9465
0
20
40
60
80
100
200
400
600
800

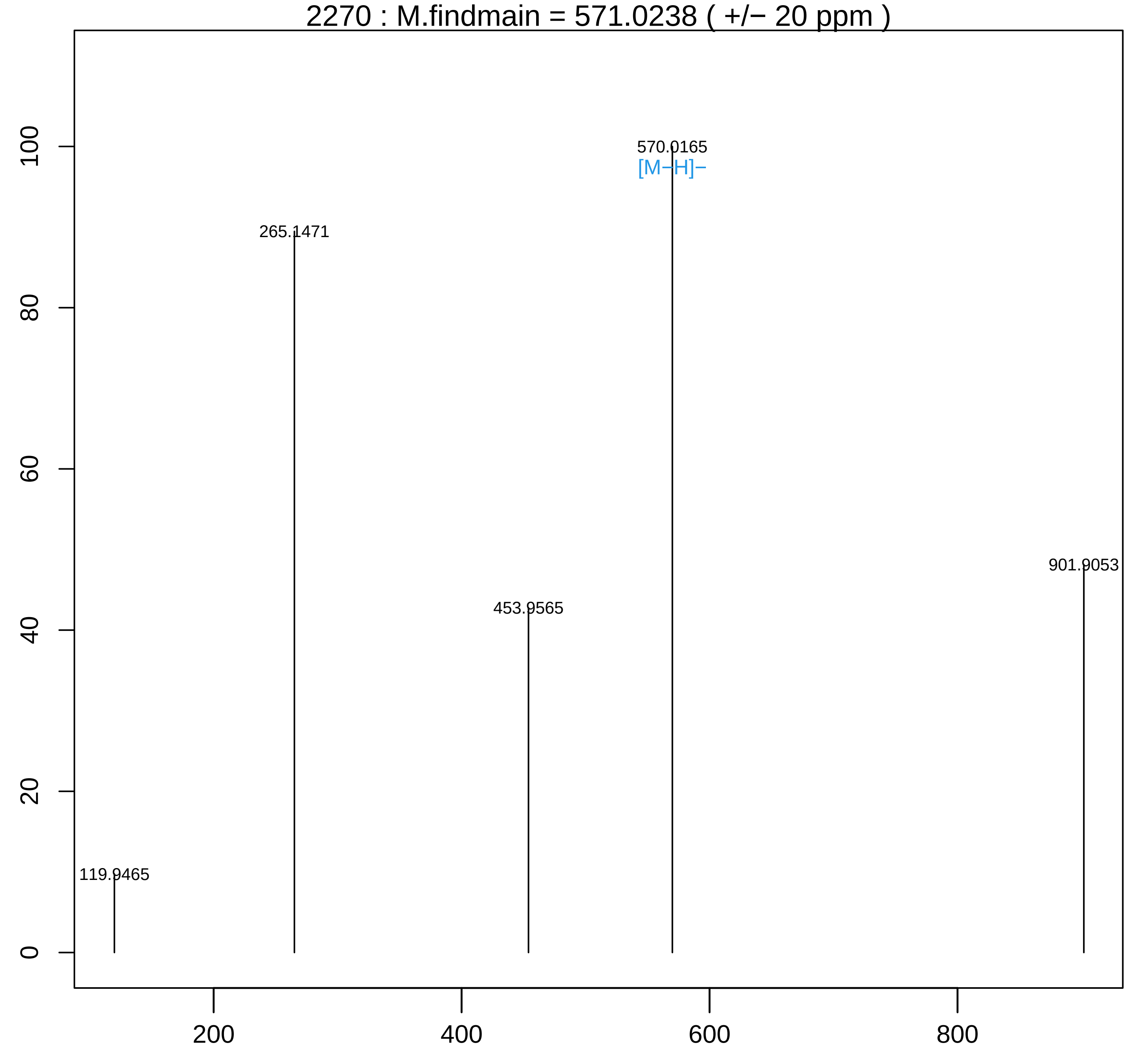

2270 : M.findmain = 571.0238 (+/− 20 ppm)
570.0165
[M−H]−
265.1471
453.9565
901.9053
119.9465
0
20
40
60
80
100
200
400
600
800

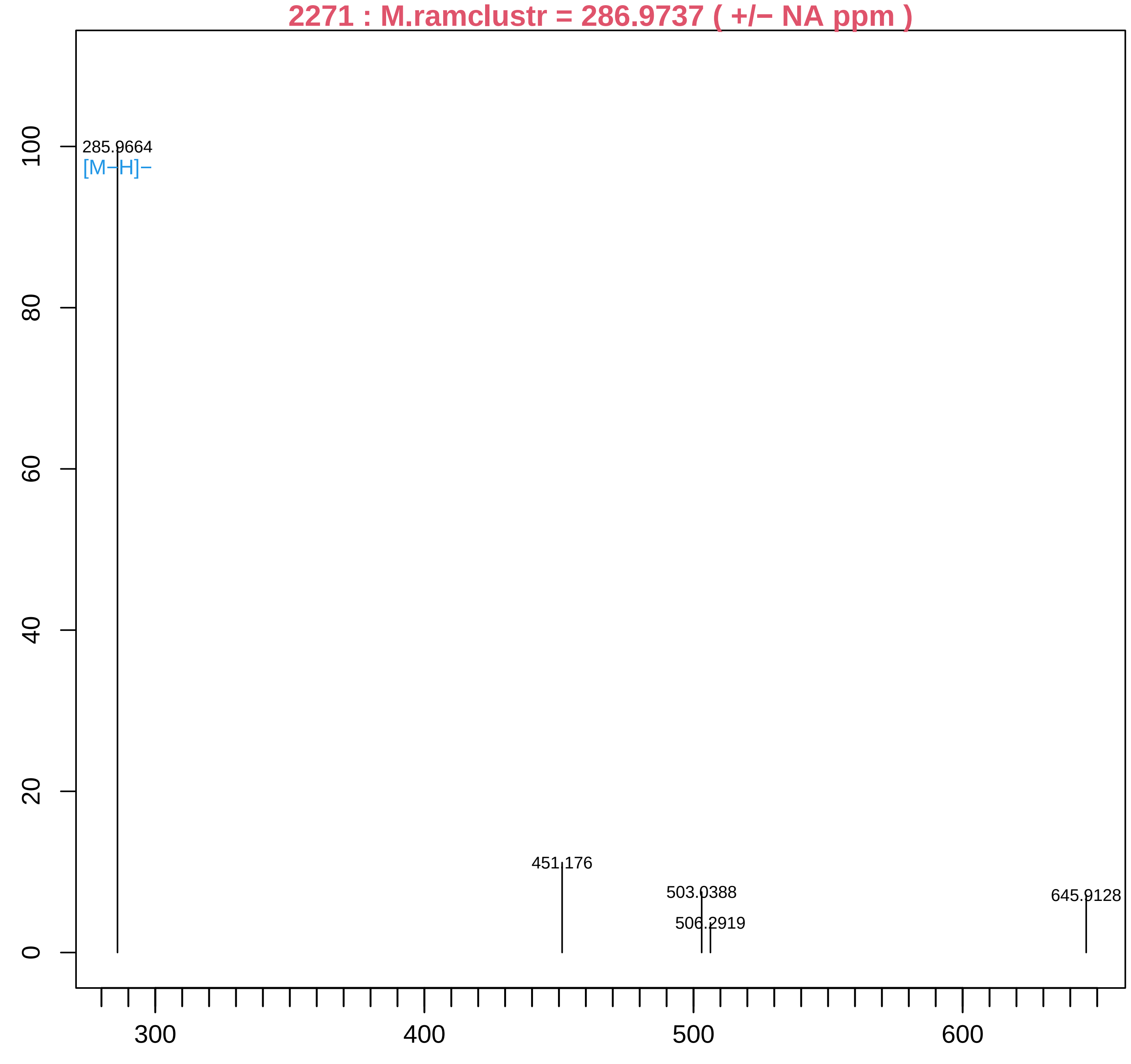
2271 : M.ramclustr = 286.9737 (+/− NA ppm)
285.9664
[M−H]−
451.176
503.0388
506.2919
645.9128
0
20
40
60
80
100
300
400
500
600

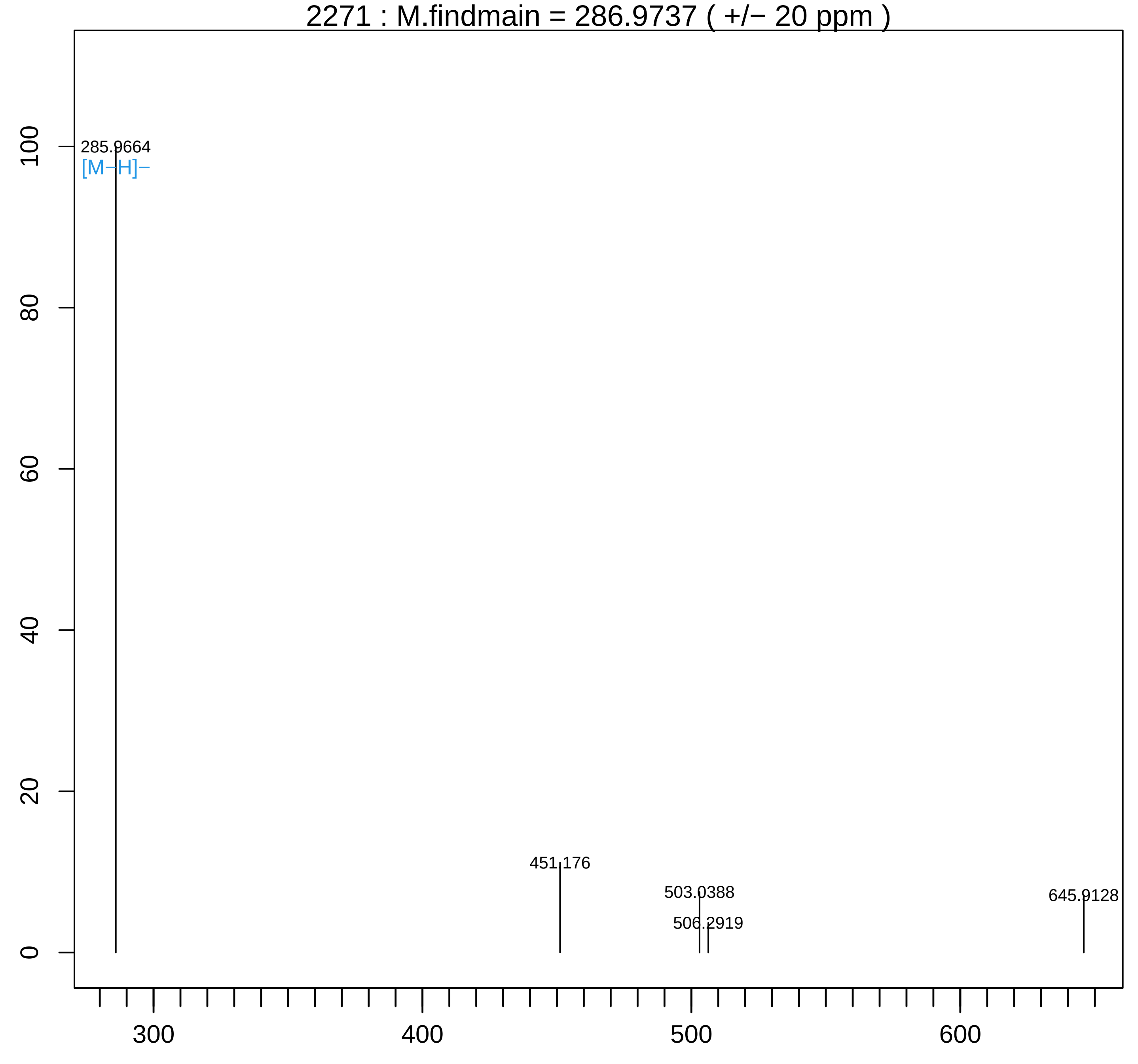
2271 : M.findmain = 286.9737 (+/− 20 ppm)
285.9664
[M−H]−
451.176
503.0388
506.2919
645.9128
0
20
40
60
80
100
300
400
500
600

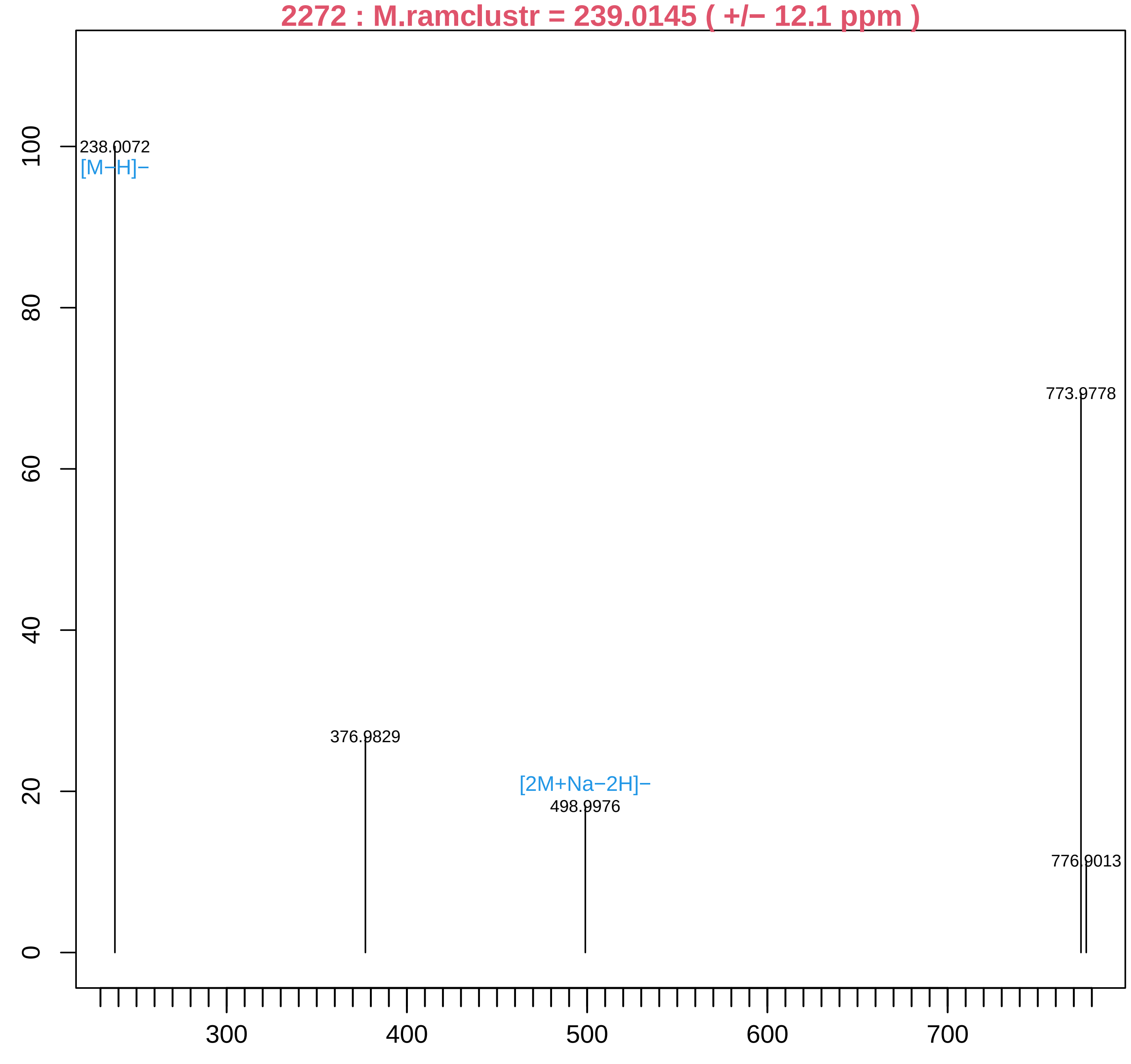

2272 : M.ramclustr = 239.0145 (+/− 12.1 ppm)
238.0072
[M−H]−
376.9829
[2M+Na−2H]−
498.9976
773.9778
776.9013
0
20
40
60
80
100
300
400
500
600
700

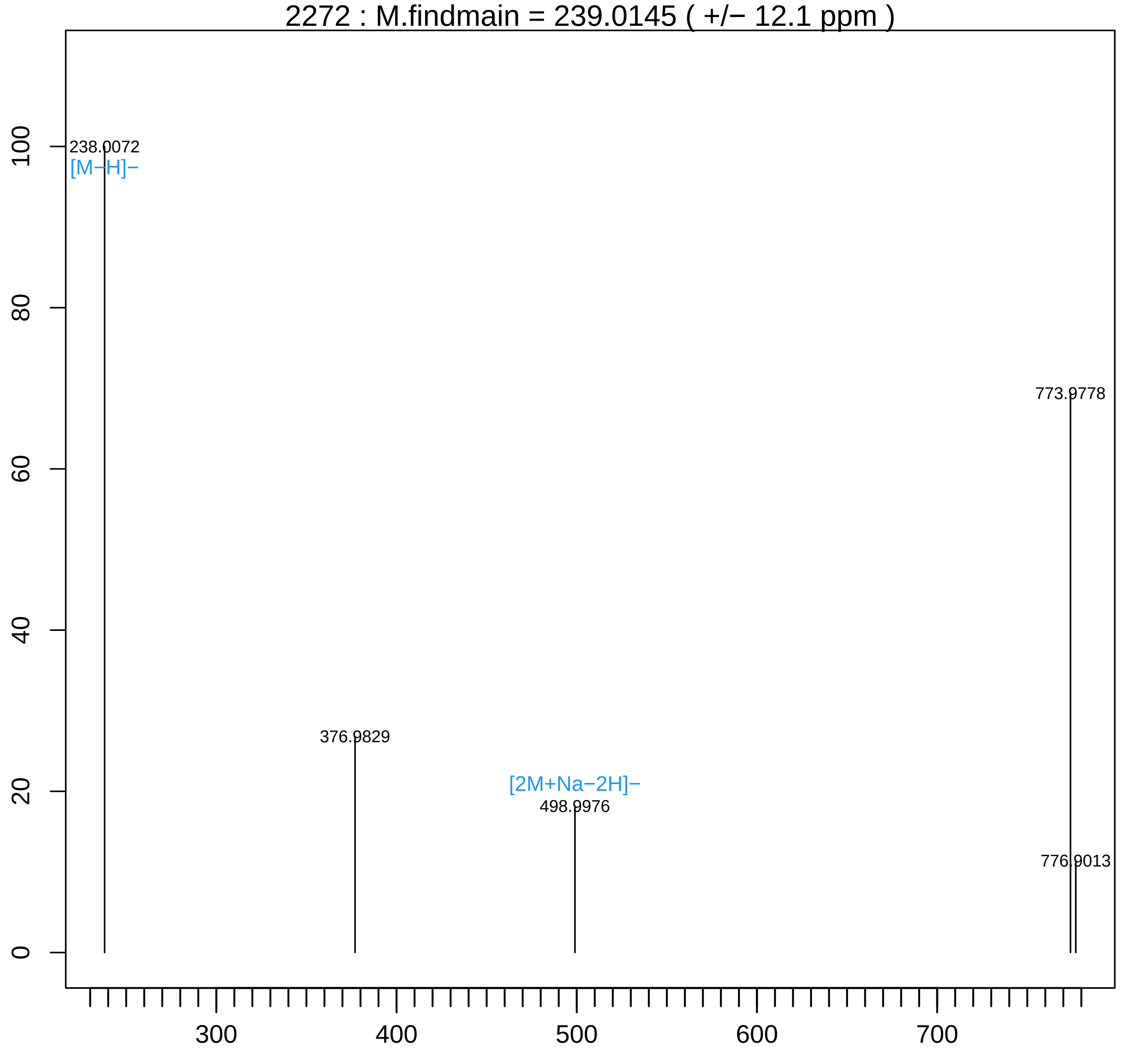

2272 : M.findmain = 239.0145 (+/− 12.1 ppm)
238.0072
[M−H]−
376.9829
[2M+Na−2H]−
498.9976
773.9778
776.9013
0
20
40
60
80
100
300
400
500
600
700

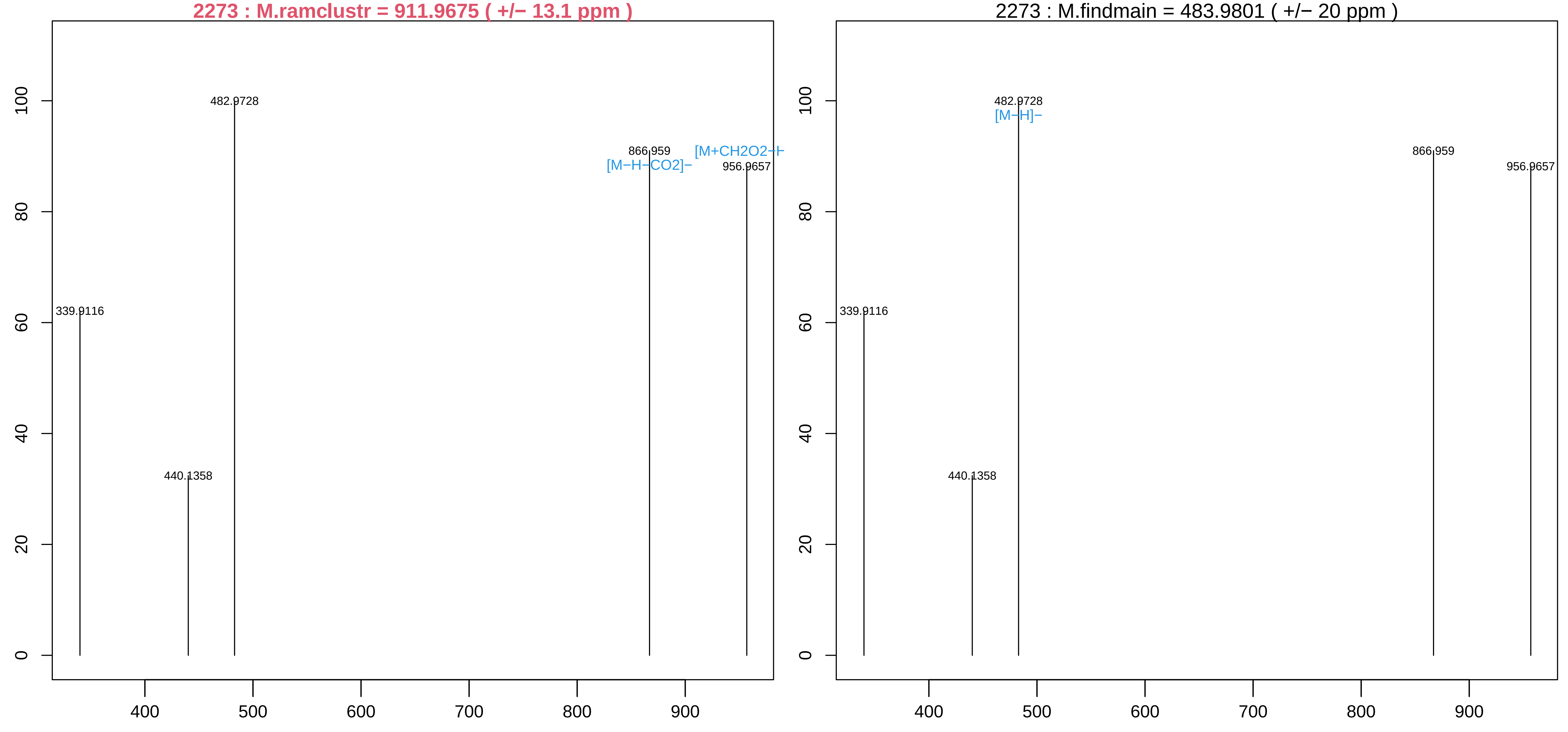
2273 : M.ramclustr = 911.9675 (+/− 13.1 ppm)
482.9728
866.959
[M−H−CO2]−
[M+CH2O2−H
956.9657
339.9116
440.1358
2273 : M.findmain = 483.9801 (+/− 20 ppm)
482.9728
[M−H]−
866.959
956.9657
339.9116
440.1358
0
20
40
60
80
100
400
500
600
700
800
900

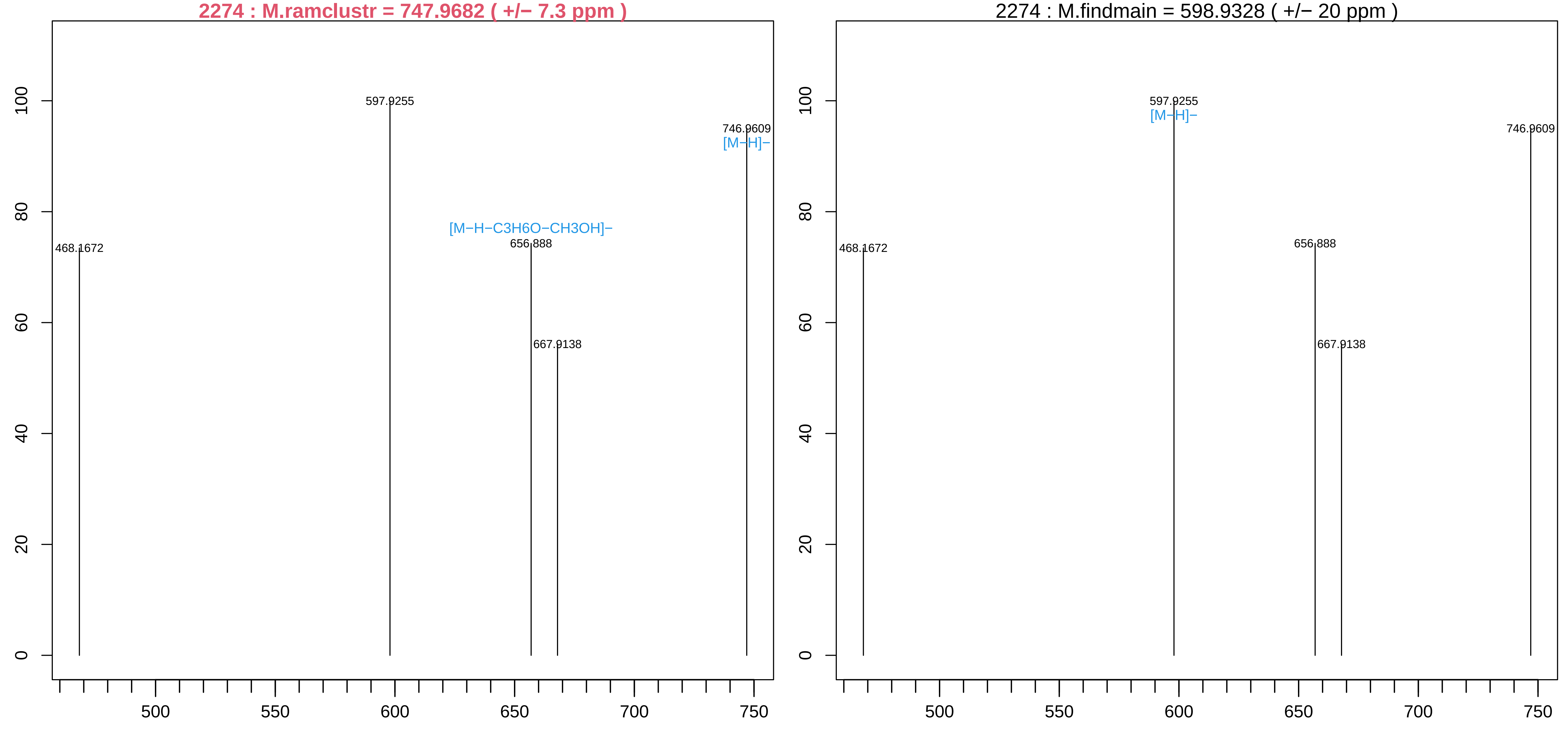
2274 : M.ramclustr = 747.9682 (+/− 7.3 ppm)
597.9255
746.9609
[M−H]−
[M−H−C3H6O−CH3OH]−
656.888
468.1672
667.9138
2274 : M.findmain = 598.9328 (+/− 20 ppm)
597.9255
[M−H]−
746.9609
468.1672
656.888
667.9138
0
20
40
60
80
100
500
550
600
650
700
750

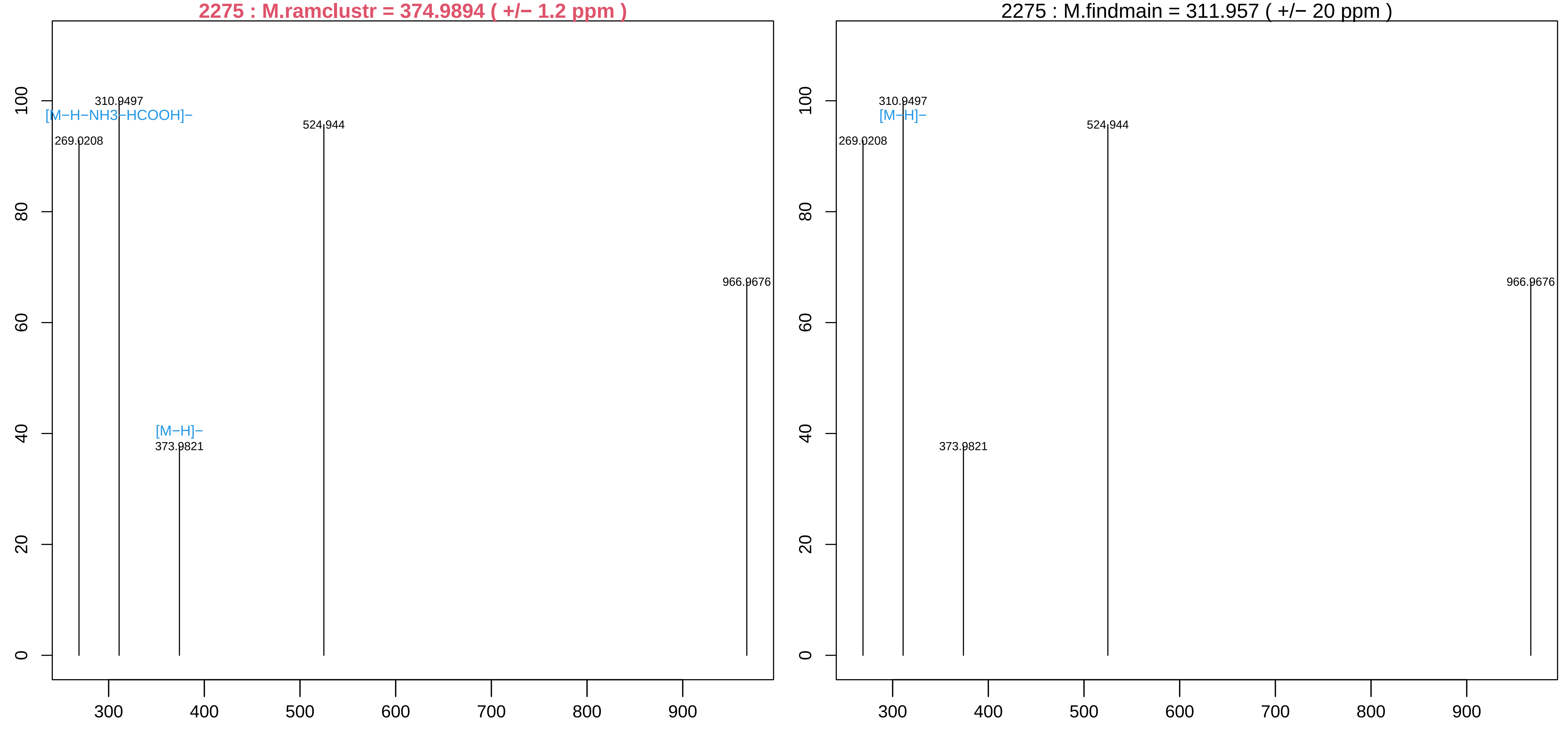
2275 : M.ramclustr = 374.9894 (+/− 1.2 ppm)
310.9497
[M−H−NH3−HCOOH]−
269.0208
524.944
966.9676
[M−H]−
373.9821
2275 : M.findmain = 311.957 (+/− 20 ppm)
310.9497
[M−H]−
269.0208
524.944
966.9676
373.9821
0
20
40
60
80
100
300
400
500
600
700
800
900

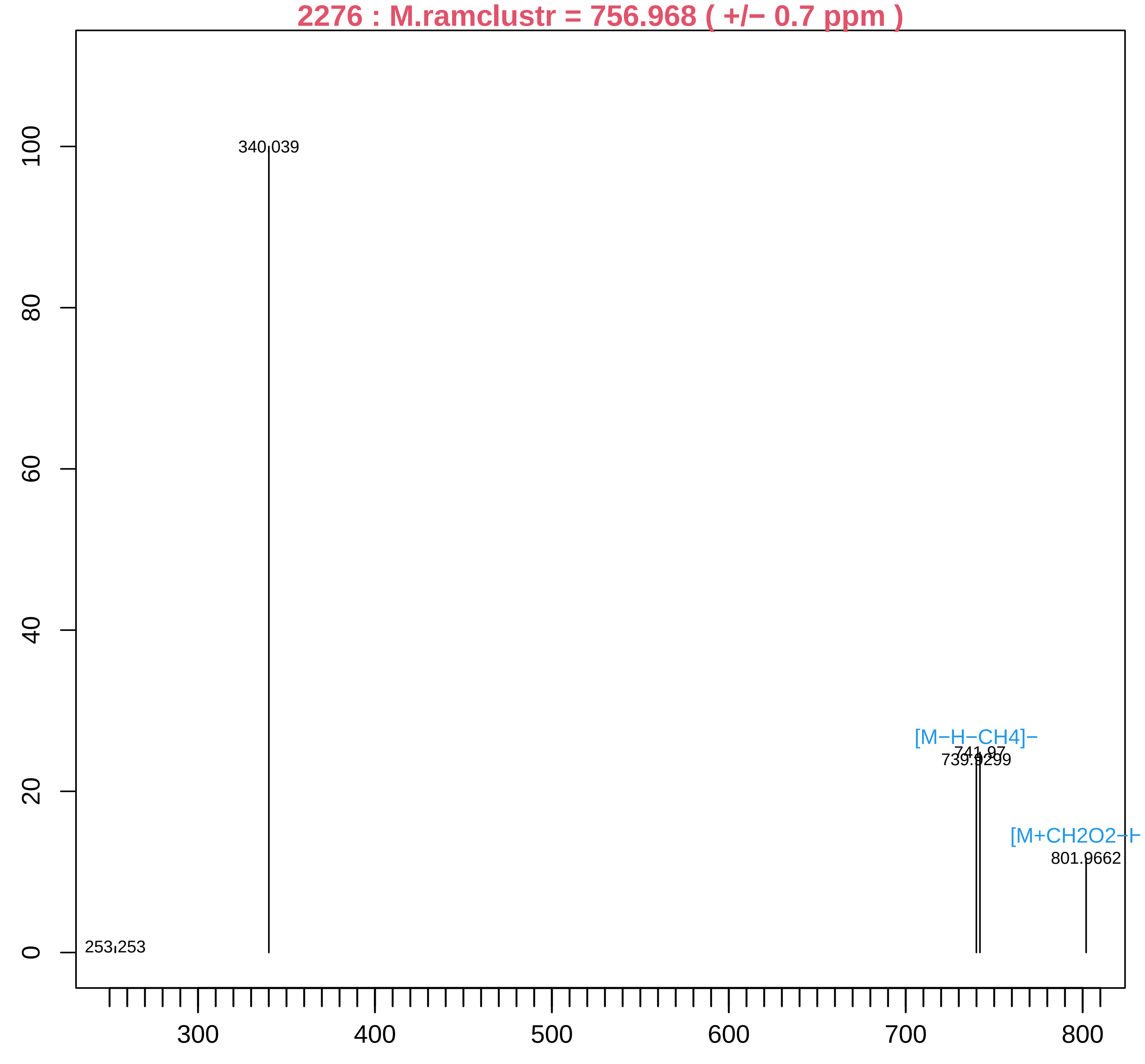
2276 : M.ramclustr = 756.968 (+/− 0.7 ppm)
340.039
[M−H−CH4]−
741.97
739.9299
[M+CH2O2−H
801.9662
253.253
0
20
40
60
80
100
300
400
500
600
700
800

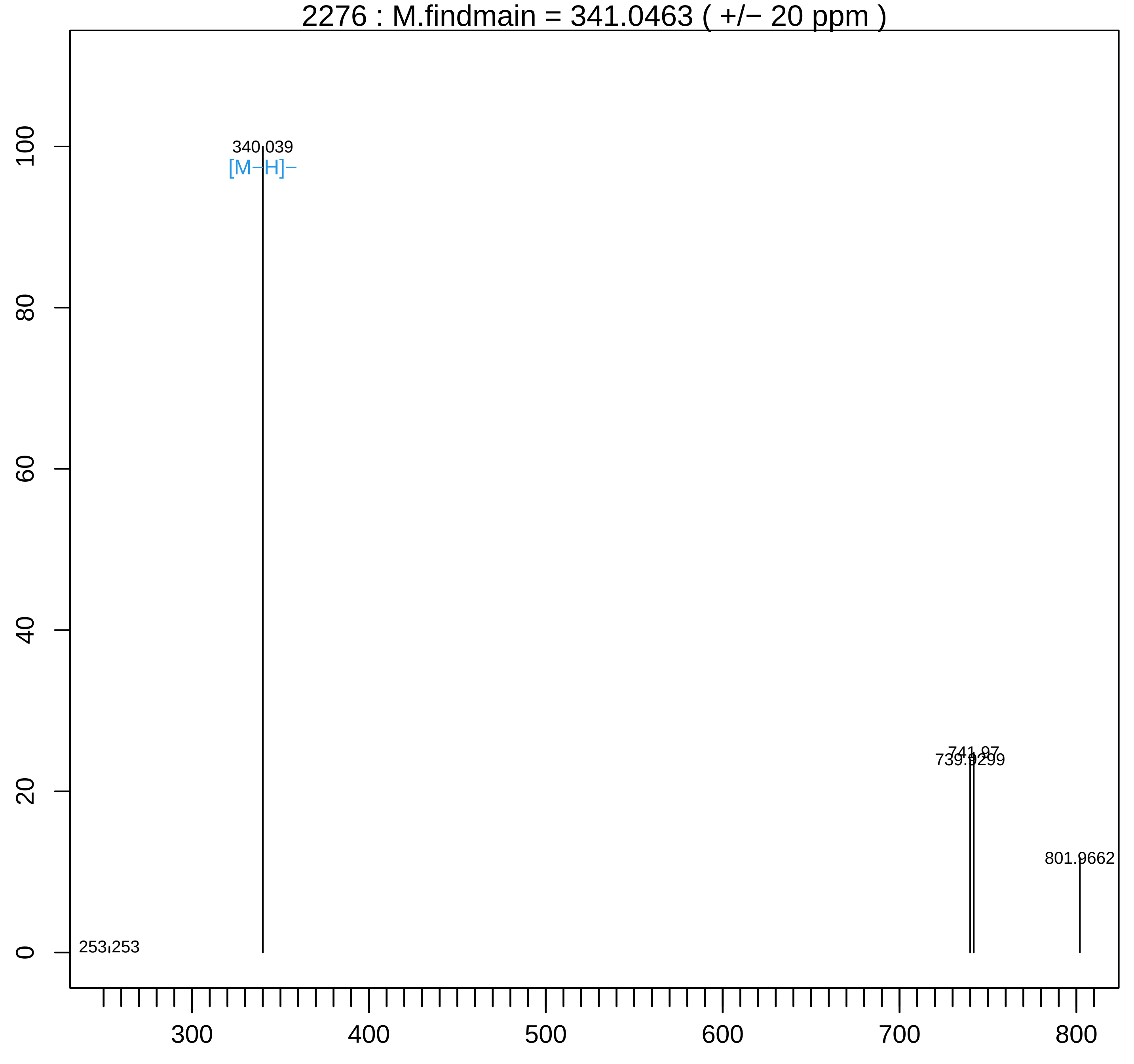
2276 : M.findmain = 341.0463 (+/− 20 ppm)
340.039
[M−H]−
741.97
739.9299
801.9662
253.253
0
20
40
60
80
100
300
400
500
600
700
800

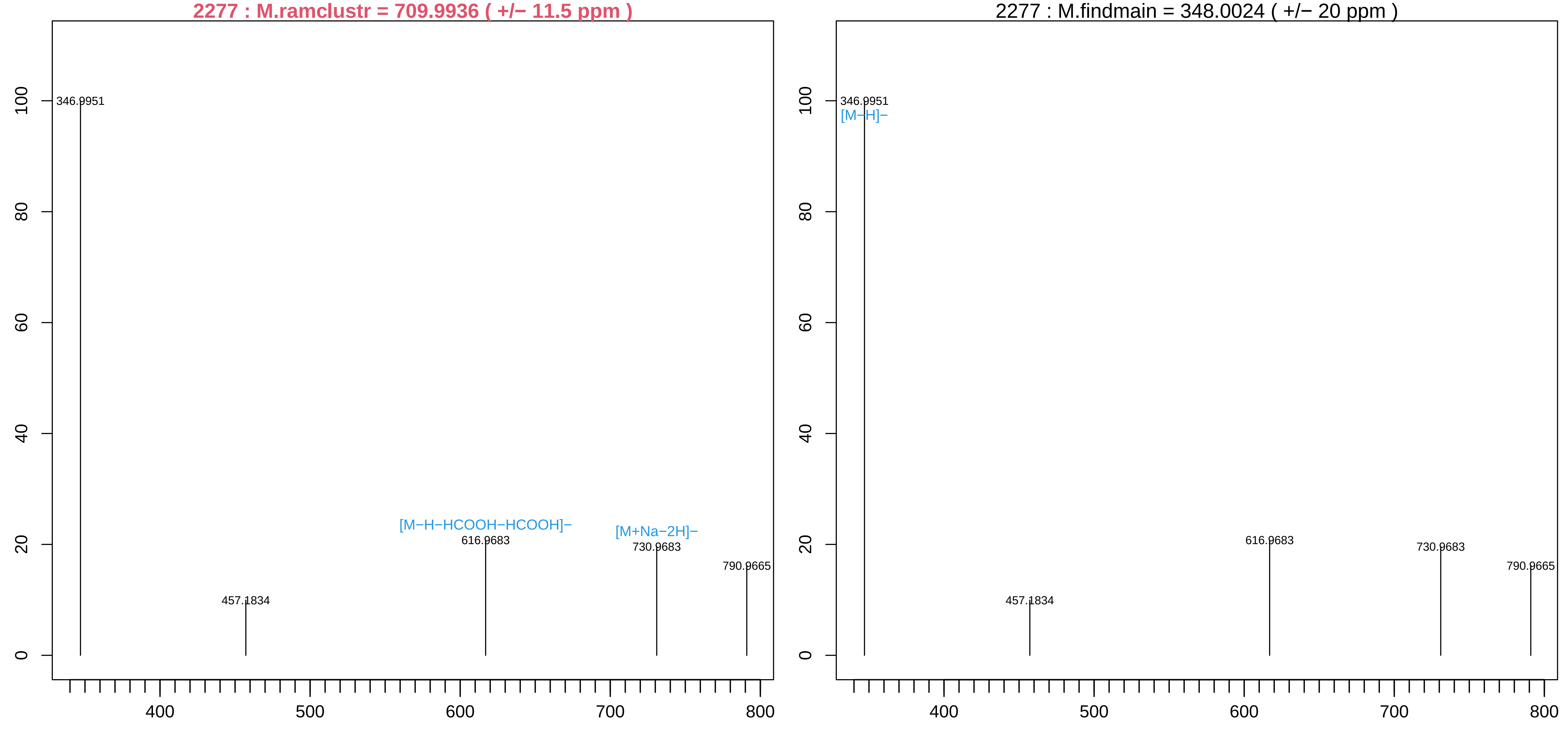

2277 : M.ramclustr = 709.9936 (+/− 11.5 ppm)
346.9951
457.1834
[M−H−HCOOH−HCOOH]−
616.9683
[M+Na−2H]−
730.9683
790.9665
2277 : M.findmain = 348.0024 (+/− 20 ppm)
346.9951
[M−H]−
457.1834
616.9683
730.9683
790.9665
0
20
40
60
80
100
400
500
600
700
800

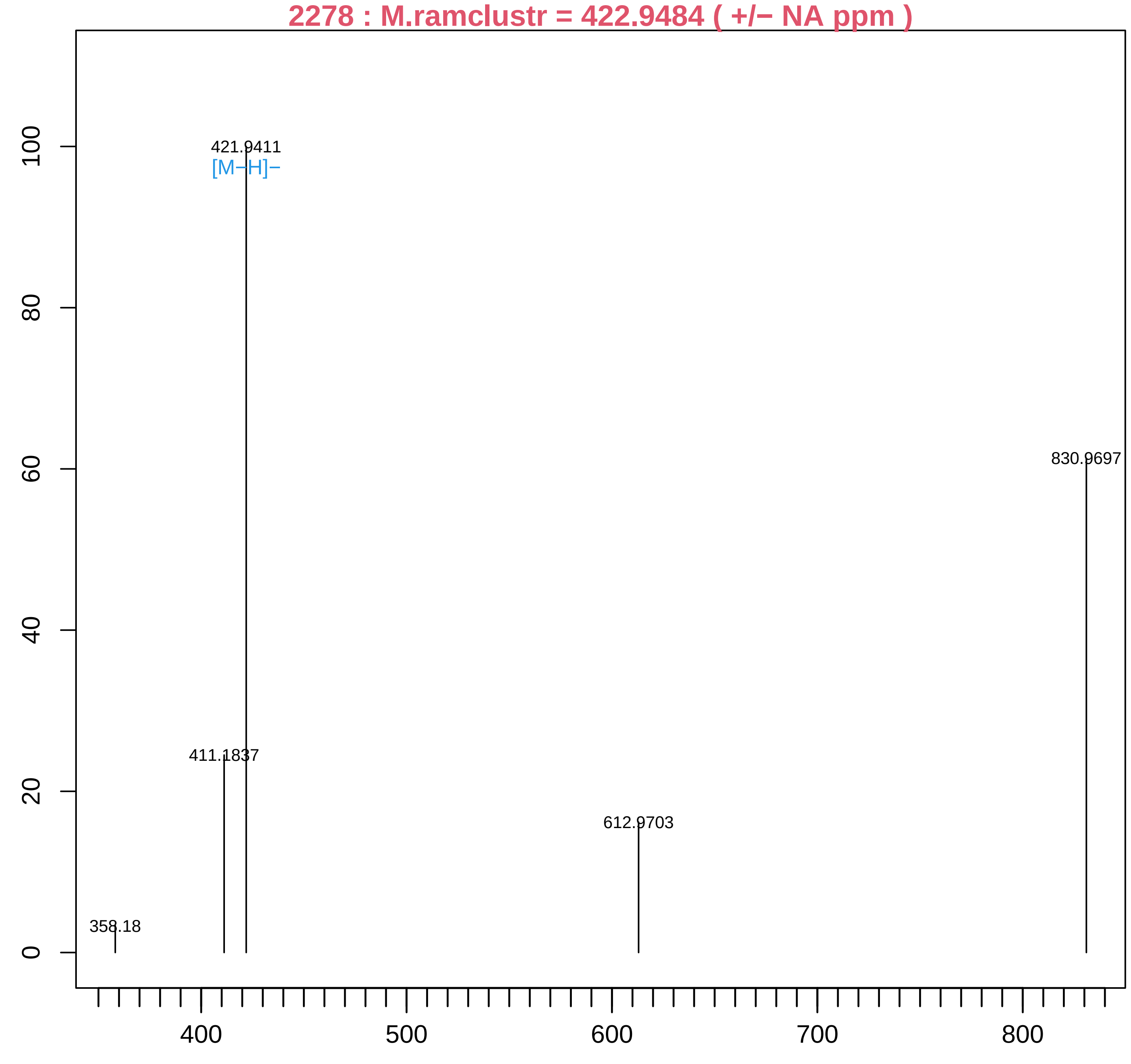
2278 : M.ramclustr = 422.9484 (+/− NA ppm)
421.9411
[M−H]−
830.9697
411.1837
612.9703
358.18
0
20
40
60
80
100
400
500
600
700
800

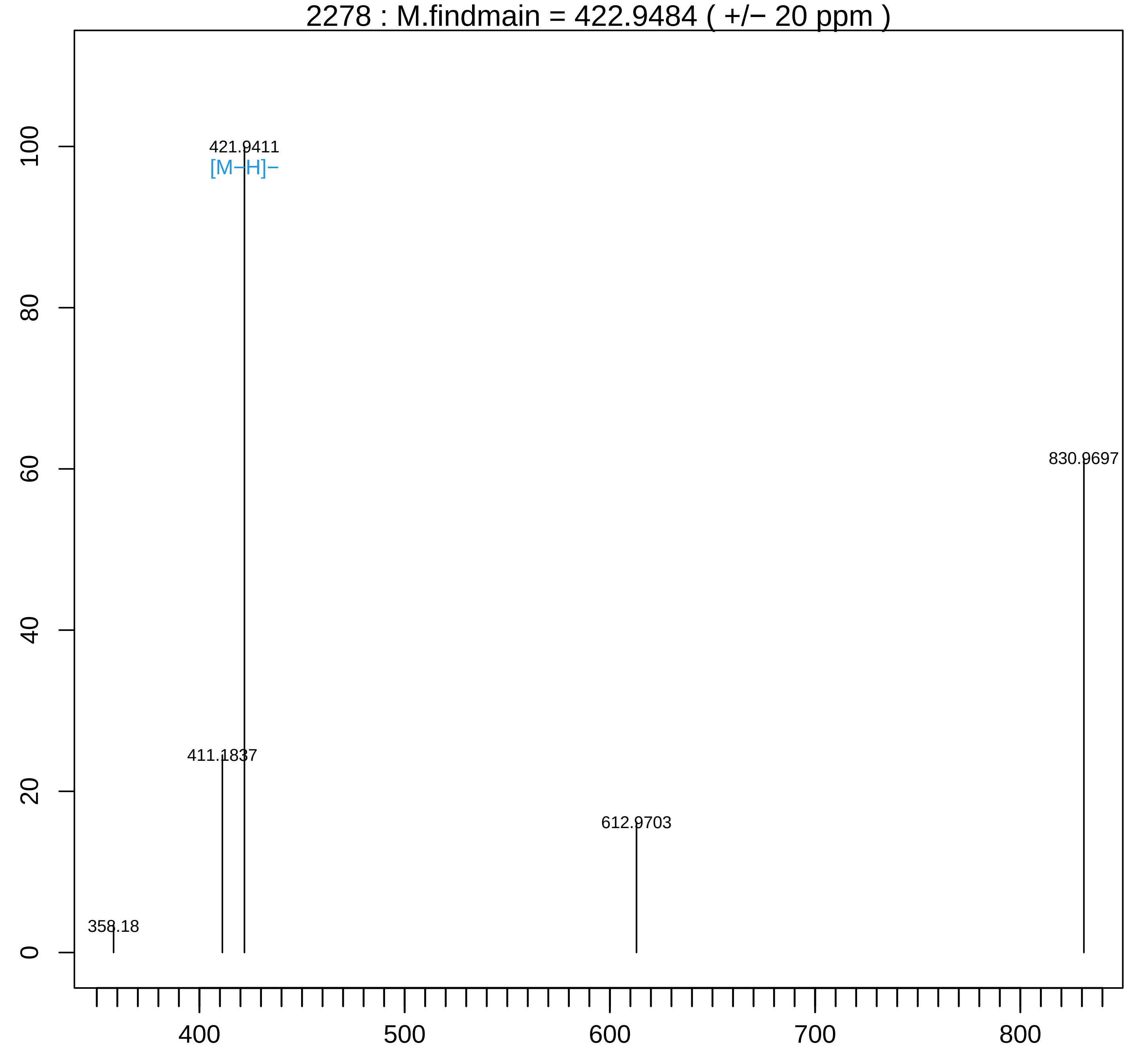
2278 : M.findmain = 422.9484 (+/− 20 ppm)
421.9411
[M−H]−
830.9697
411.1837
612.9703
358.18
0
20
40
60
80
100
400
500
600
700
800

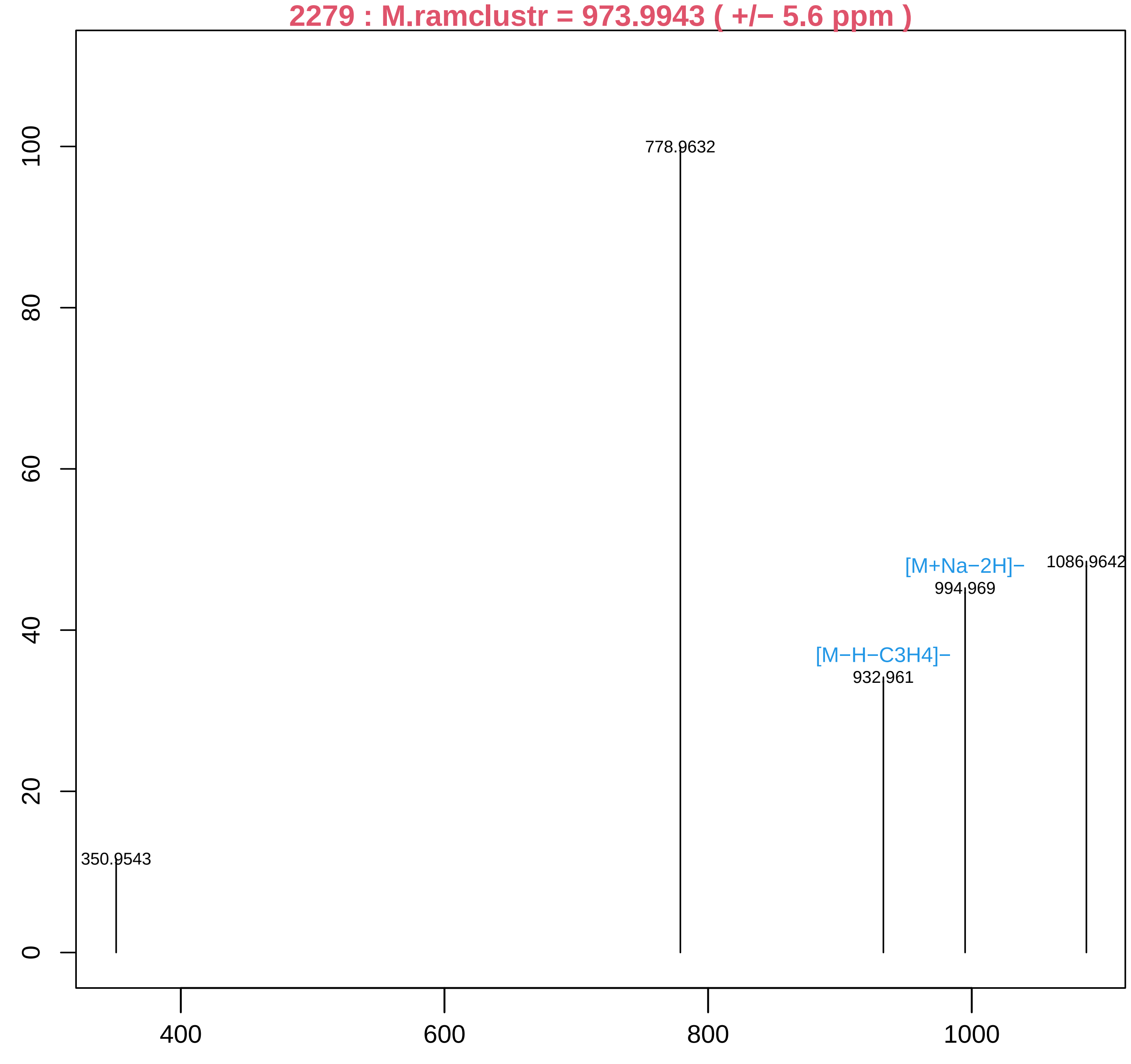
2279 : M.ramclustr = 973.9943 (+/− 5.6 ppm)
778.9632
[M+Na−2H]−
994.969
1086.9642
[M−H−C3H4]−
932.961
350.9543
0
20
40
60
80
100
400
600
800
1000

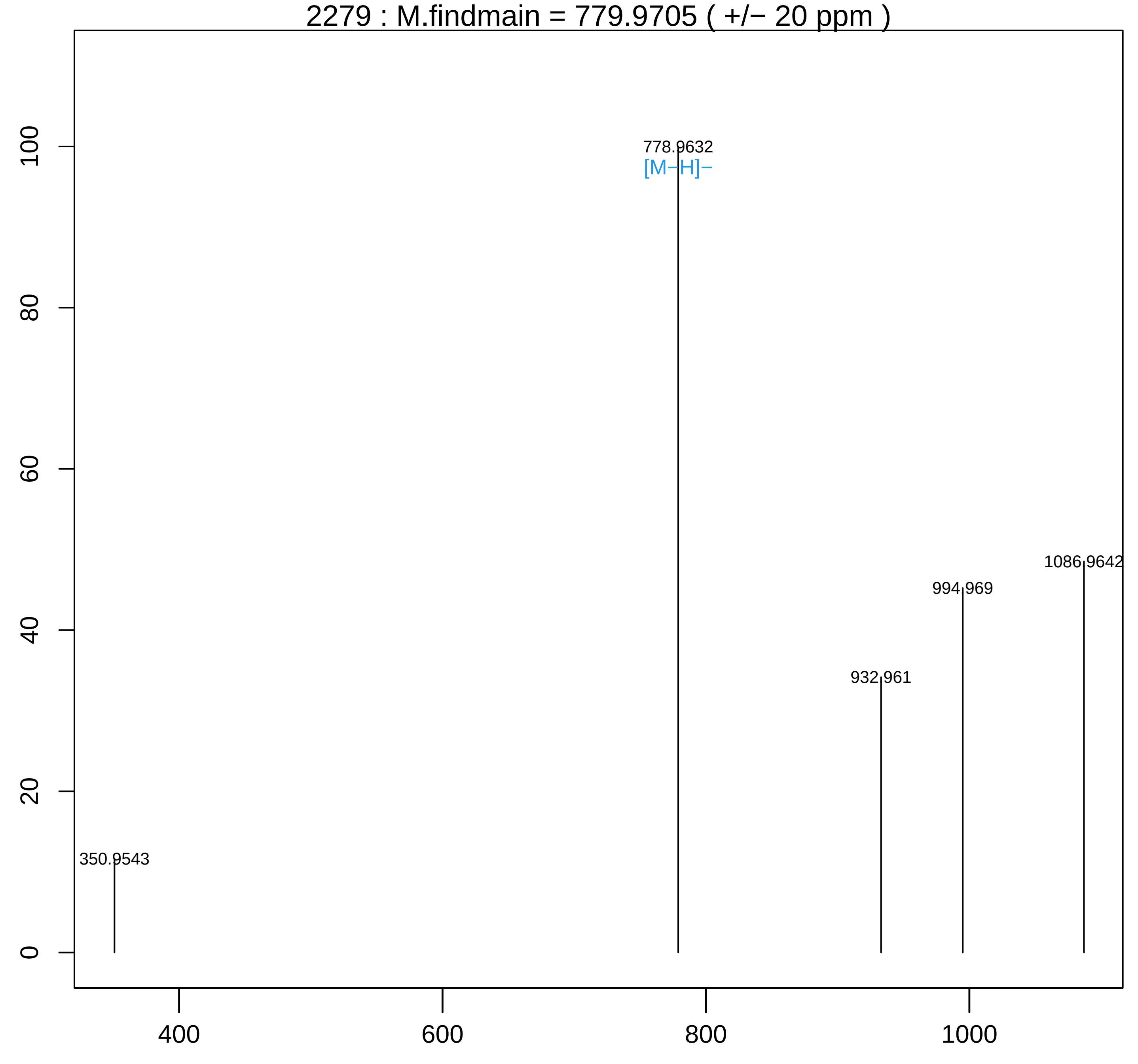
2279 : M.findmain = 779.9705 (+/− 20 ppm)
778.9632
[M−H]−
994.969
1086.9642
932.961
350.9543
0
20
40
60
80
100
400
600
800
1000

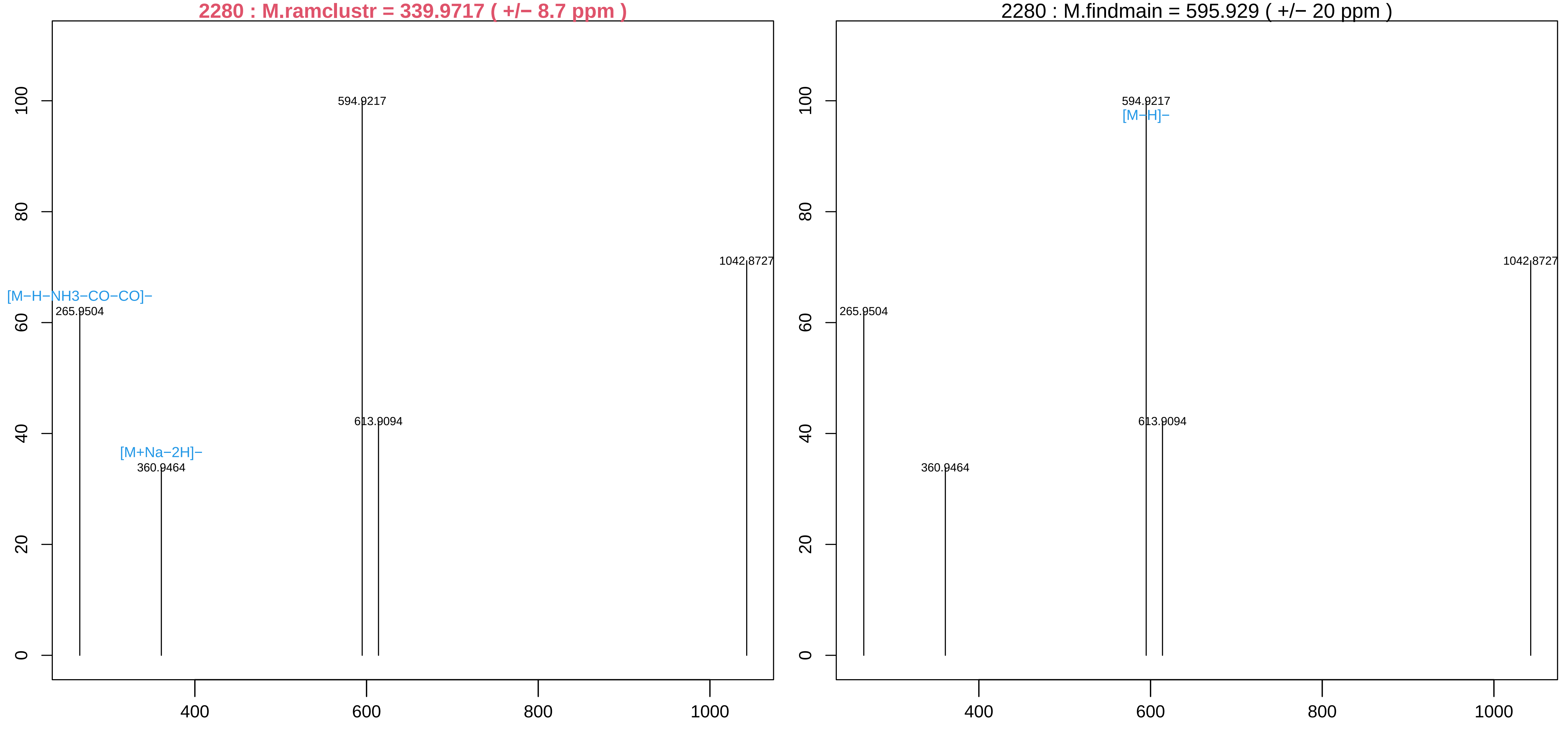
2280 : M.ramclustr = 339.9717 (+/− 8.7 ppm)
594.9217
1042.8727
[M−H−NH3−CO−CO]−
265.9504
613.9094
[M+Na−2H]−
360.9464
0
20
40
60
80
100
400
600
800
1000
2280 : M.findmain = 595.929 (+/− 20 ppm)
594.9217
[M−H]−
1042.8727
265.9504
613.9094
360.9464
0
20
40
60
80
100
400
600
800
1000

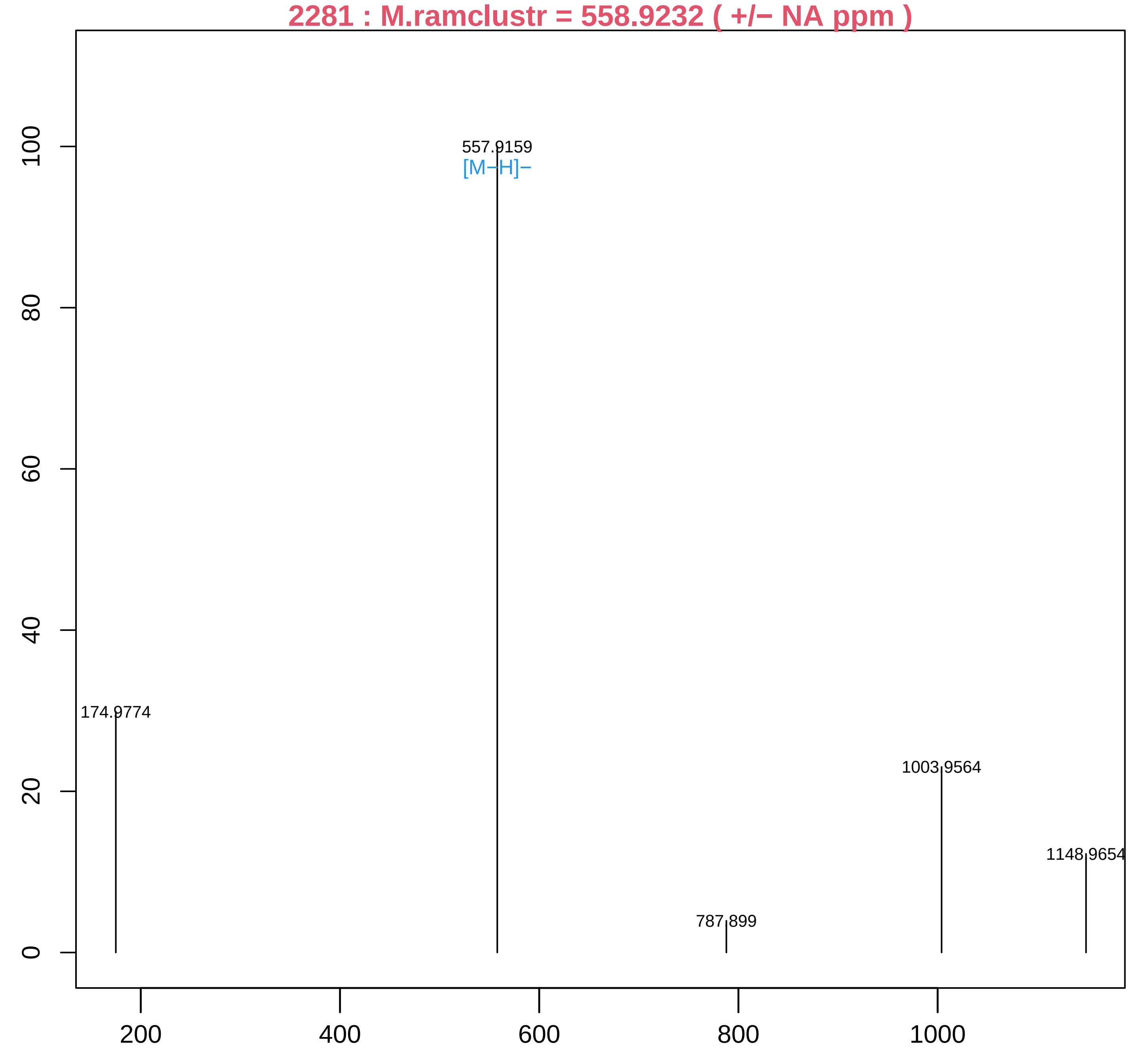

2281 : M.ramclustr = 558.9232 (+/− NA ppm)
557.9159
[M−H]−
174.9774
1003.9564
1148.9654
787.899
0
20
40
60
80
100
200
400
600
800
1000

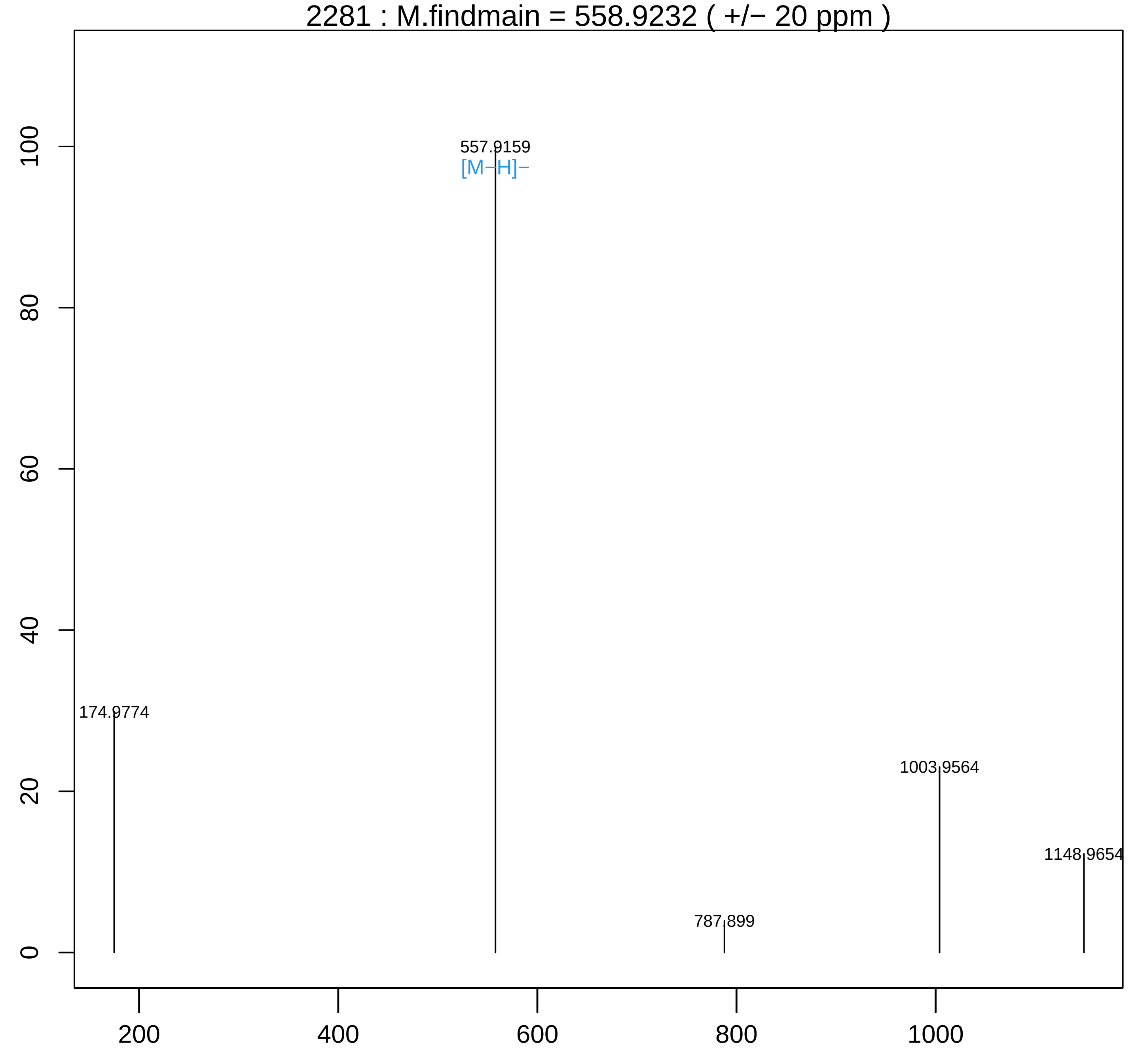

2281 : M.findmain = 558.9232 (+/− 20 ppm)
557.9159
[M−H]−
174.9774
1003.9564
1148.9654
787.899
0
20
40
60
80
100
200
400
600
800
1000

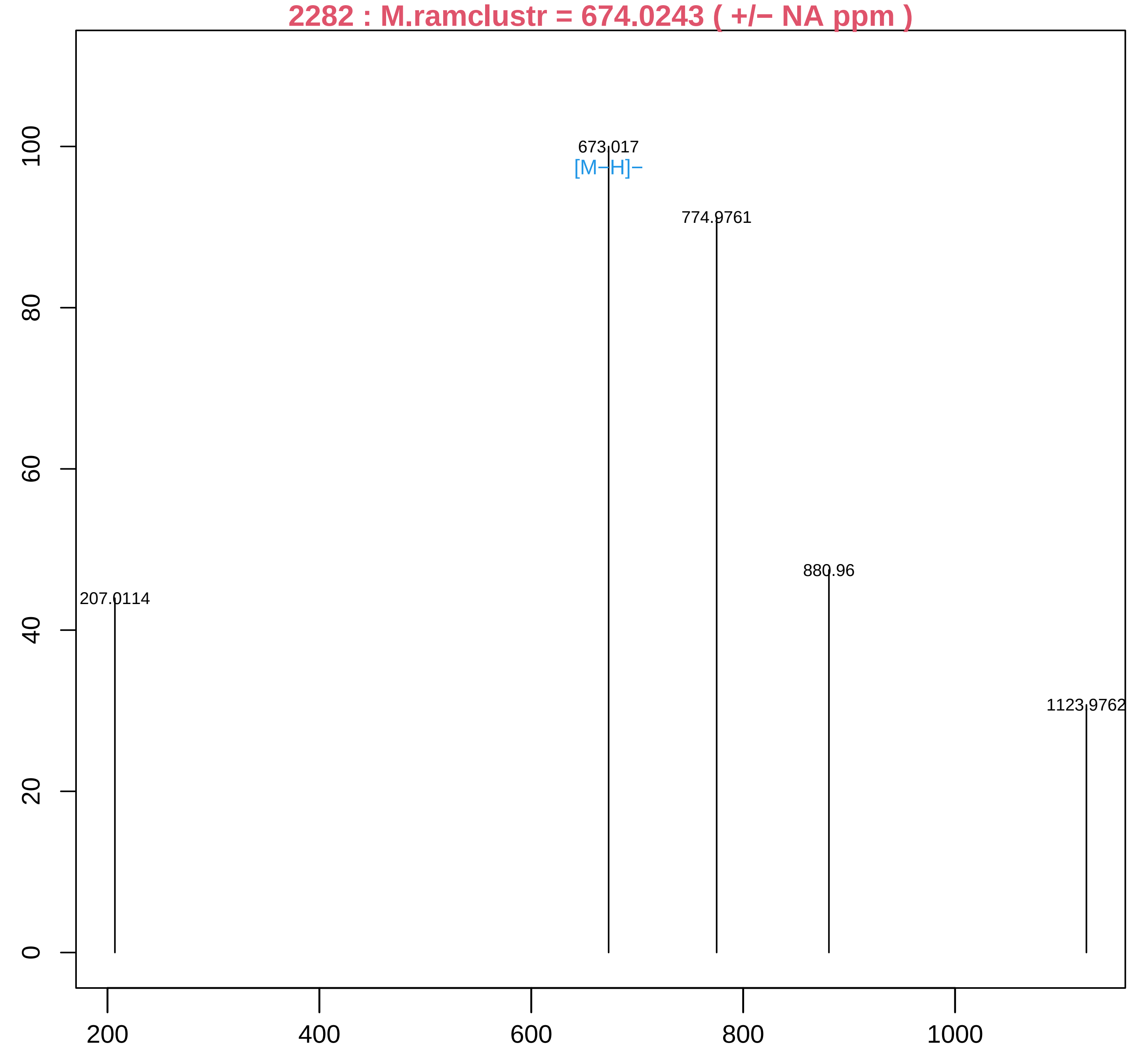
2282 : M.ramclustr = 674.0243 (+/− NA ppm)
673.017
[M−H]−
774.9761
880.96
207.0114
1123.9762
0
20
40
60
80
100
200
400
600
800
1000

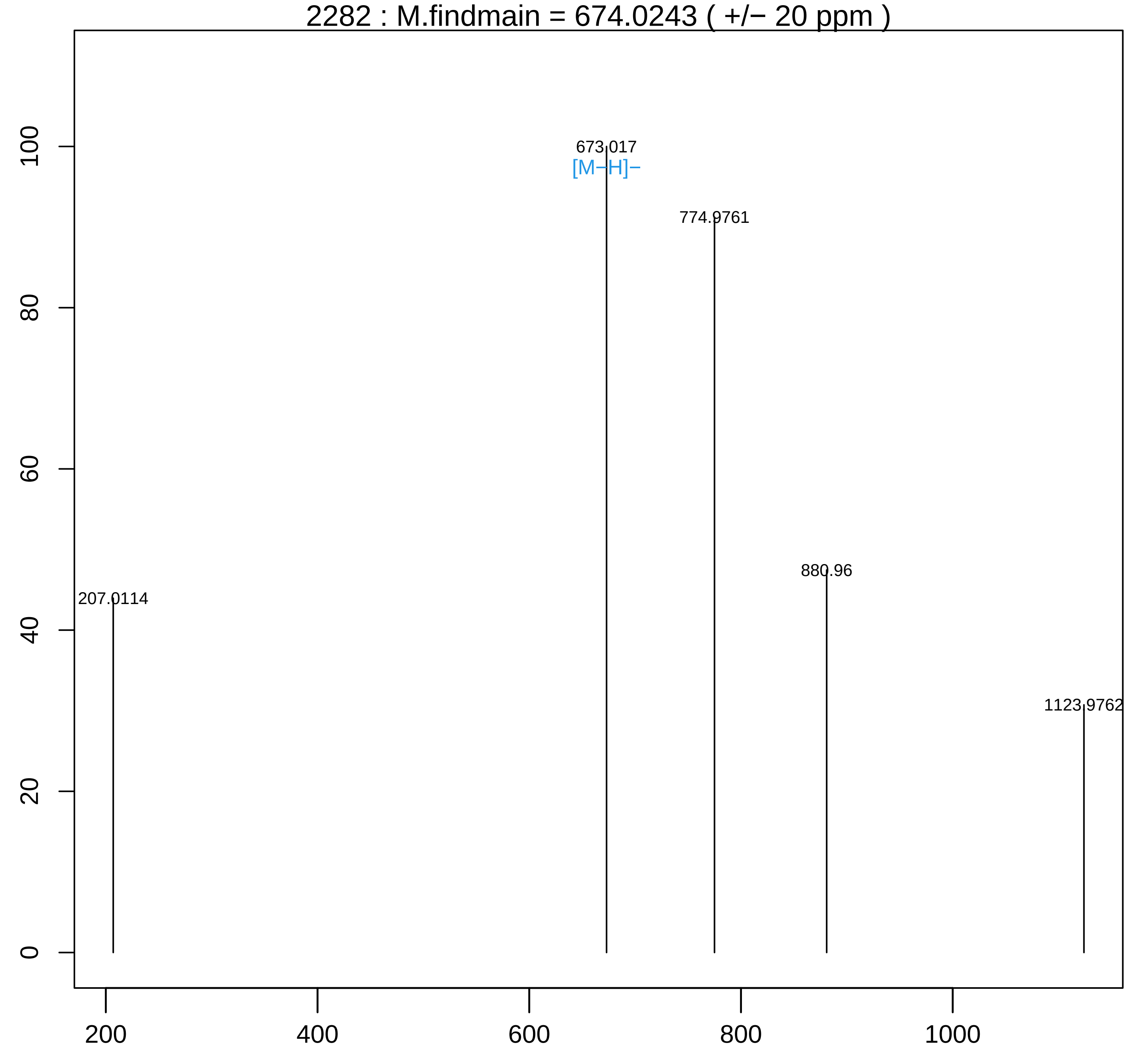
2282 : M.findmain = 674.0243 (+/− 20 ppm)
673.017
[M−H]−
774.9761
880.96
207.0114
1123.9762
0
20
40
60
80
100
200
400
600
800
1000

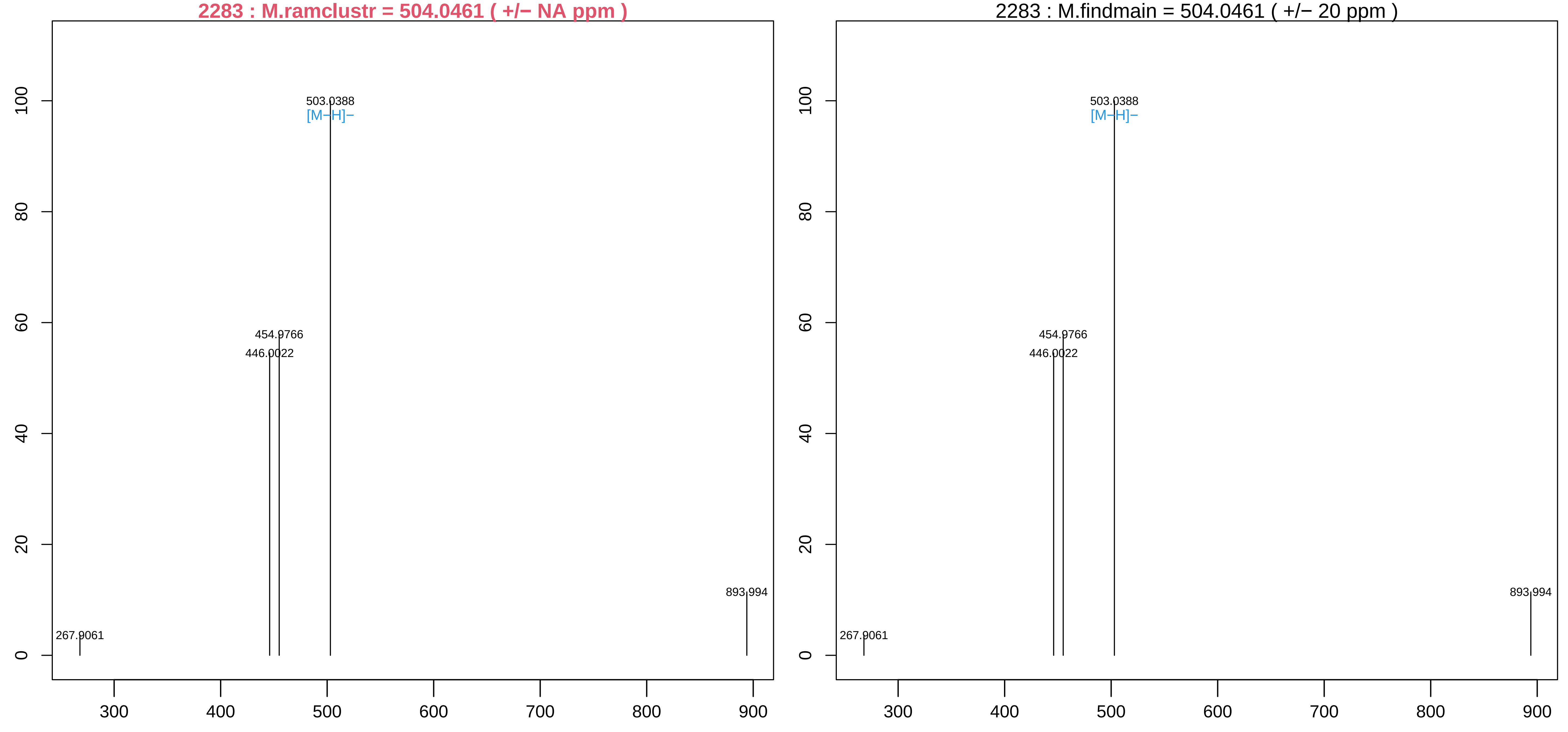

2283 : M.ramclustr = 504.0461 (+/− NA ppm)
503.0388
[M−H]−
454.9766
446.0022
893.994
267.9061
0
20
40
60
80
100
300
400
500
600
700
800
900
2283 : M.findmain = 504.0461 (+/− 20 ppm)
503.0388
[M−H]−
454.9766
446.0022
893.994
267.9061

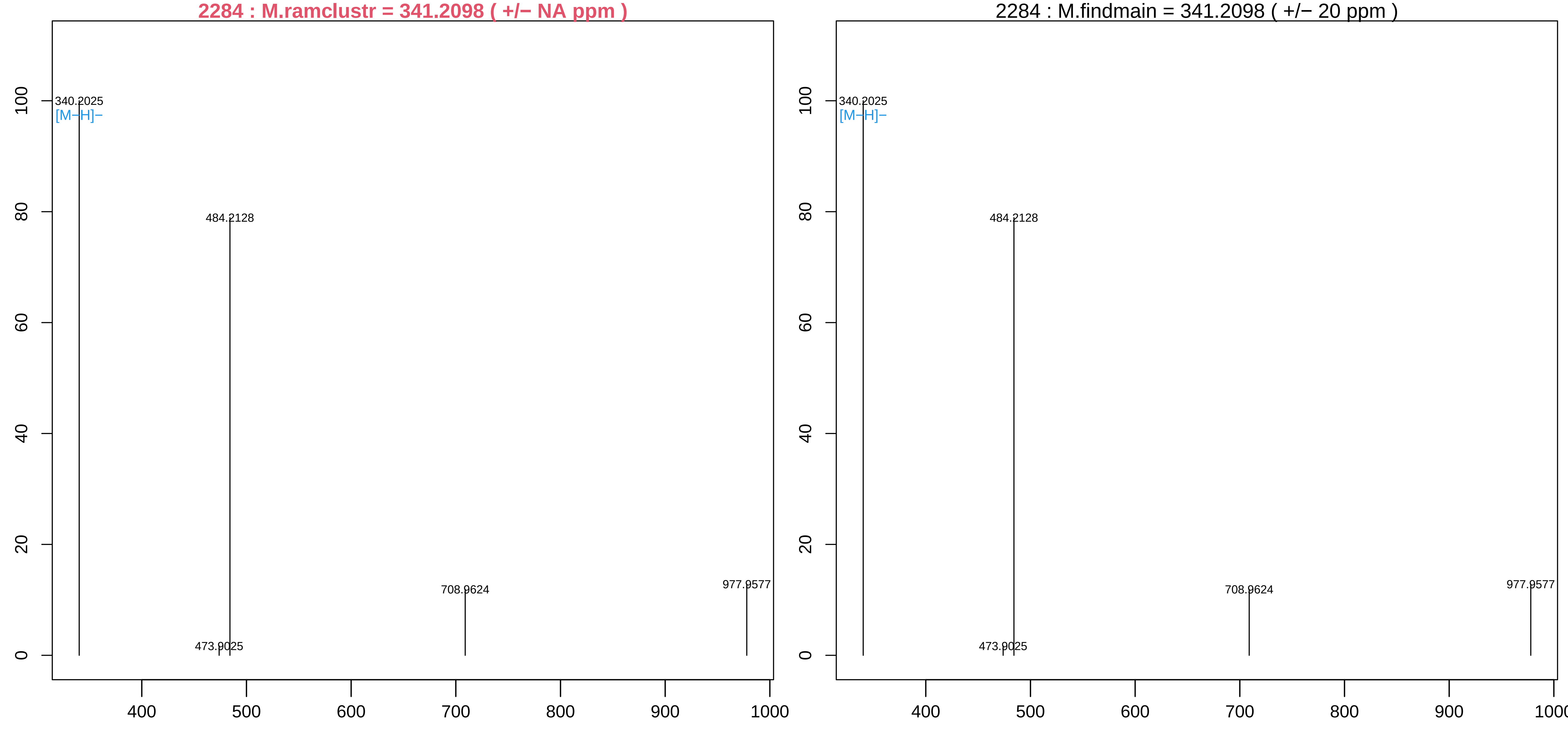
2284 : M.ramclustr = 341.2098 (+/− NA ppm)
340.2025
[M−H]−
484.2128
473.9025
708.9624
977.9577
0
20
40
60
80
100
400
500
600
700
800
900
1000
2284 : M.findmain = 341.2098 (+/− 20 ppm)
340.2025
[M−H]−
484.2128
473.9025
708.9624
977.9577
0
20
40
60
80
100
400
500
600
700
800
900
1000

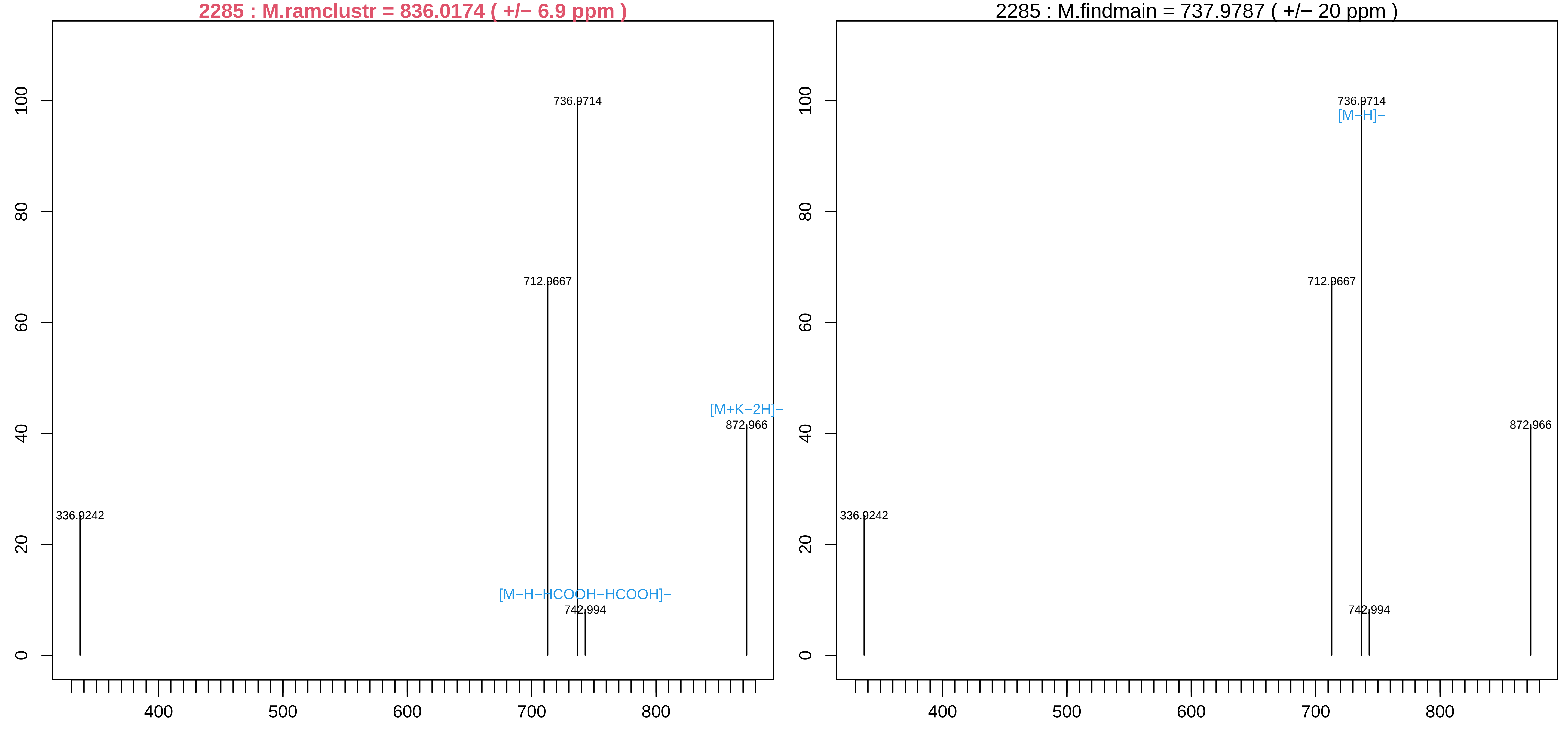
2285 : M.ramclustr = 836.0174 (+/− 6.9 ppm)
736.9714
712.9667
[M+K−2H]−
872.966
336.9242
[M−H−HCOOH−HCOOH]−
742.994
2285 : M.findmain = 737.9787 (+/− 20 ppm)
736.9714
[M−H]−
712.9667
872.966
336.9242
742.994
0
20
40
60
80
100
400
500
600
700
800

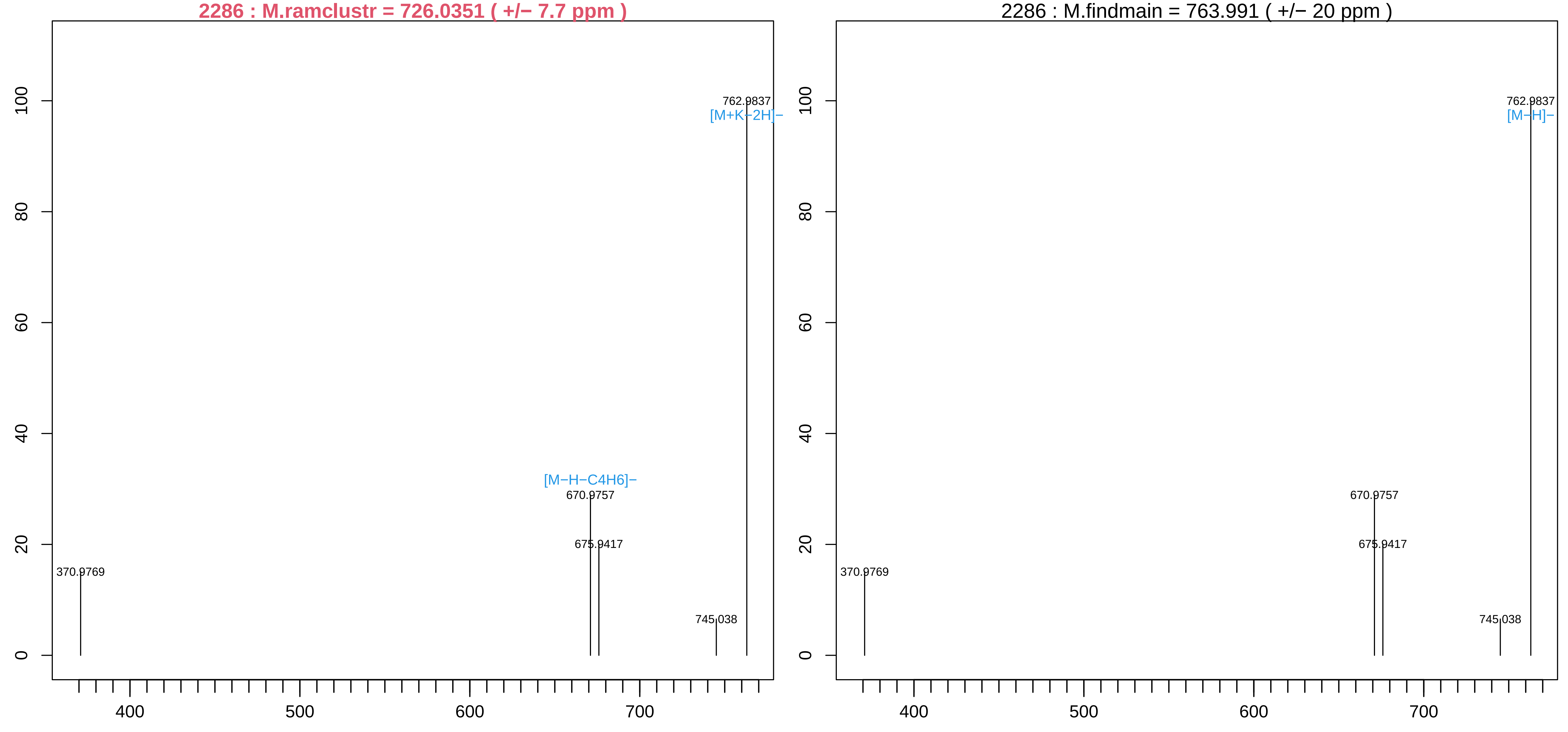

2286 : M.ramclustr = 726.0351 (+/− 7.7 ppm)
762.9837
[M+K−2H]−
[M−H−C4H6]−
670.9757
675.9417
370.9769
745.038
2286 : M.findmain = 763.991 (+/− 20 ppm)
762.9837
[M−H]−
670.9757
675.9417
370.9769
745.038

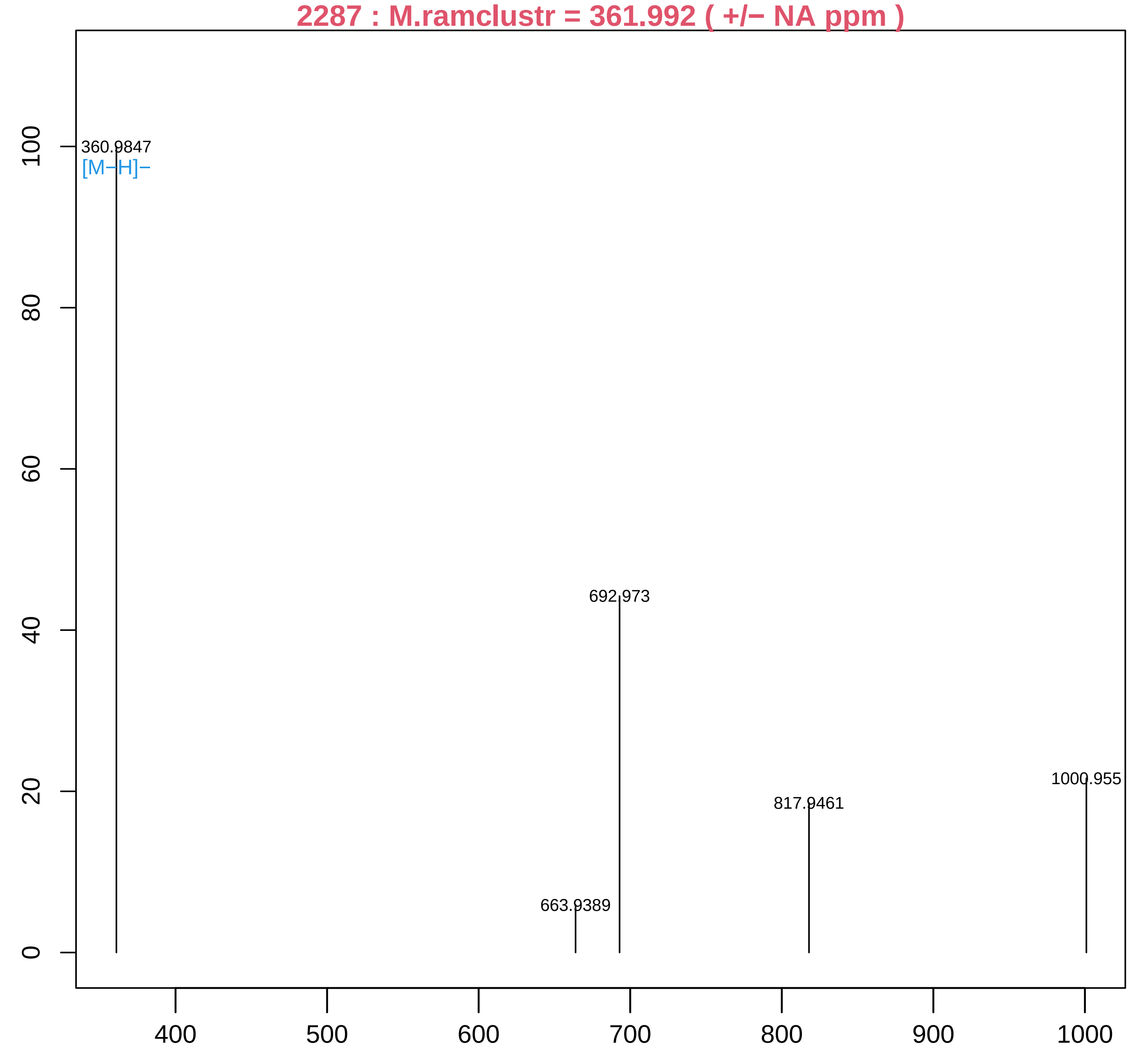
2287 : M.ramclustr = 361.992 (+/− NA ppm)
360.9847
[M−H]−
692.973
663.9389
817.9461
1000.955
0
20
40
60
80
100
400
500
600
700
800
900
1000
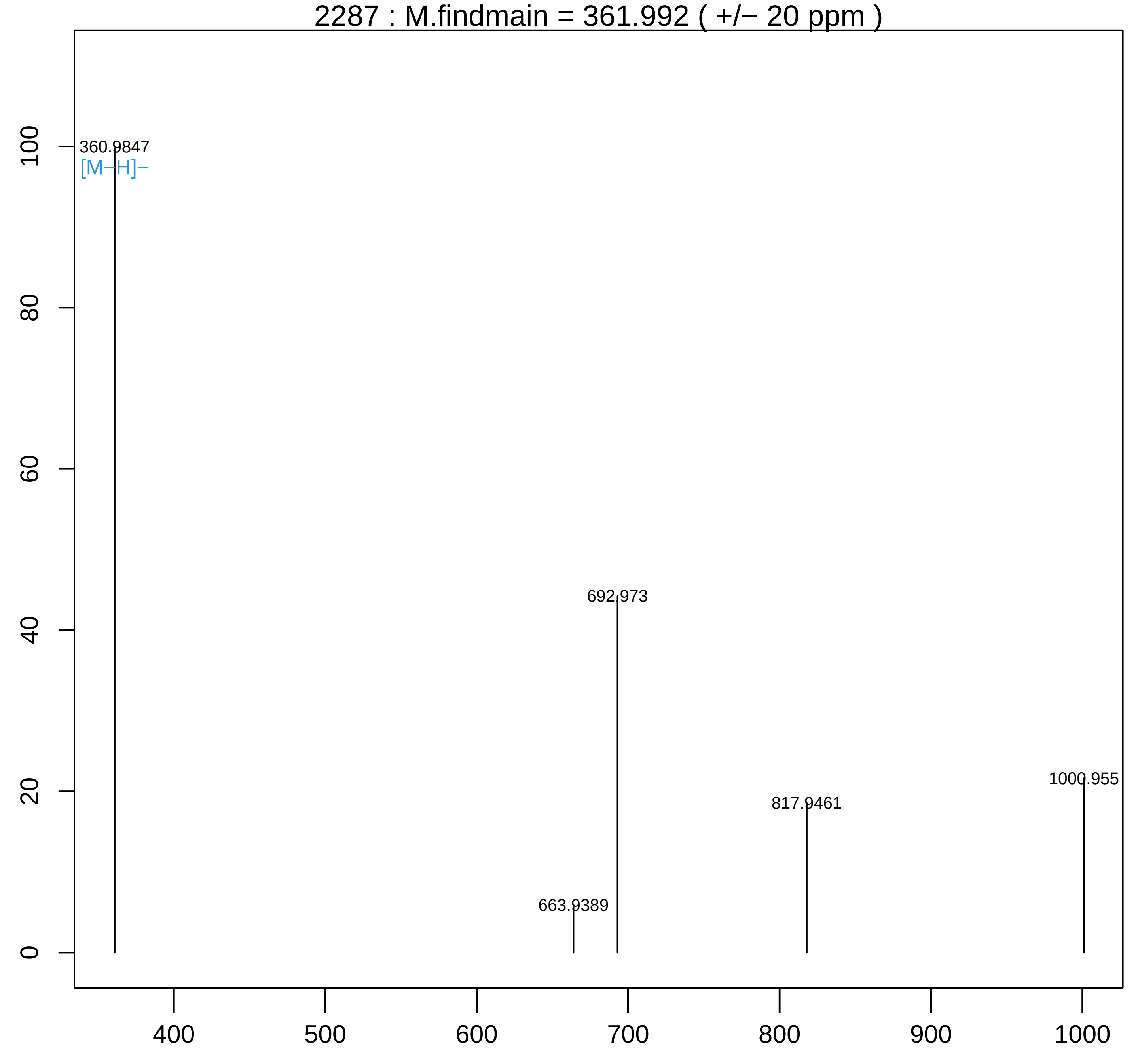
2287 : M.findmain = 361.992 (+/− 20 ppm)
360.9847
[M−H]−
692.973
663.9389
817.9461
1000.955
0
20
40
60
80
100
400
500
600
700
800
900
1000

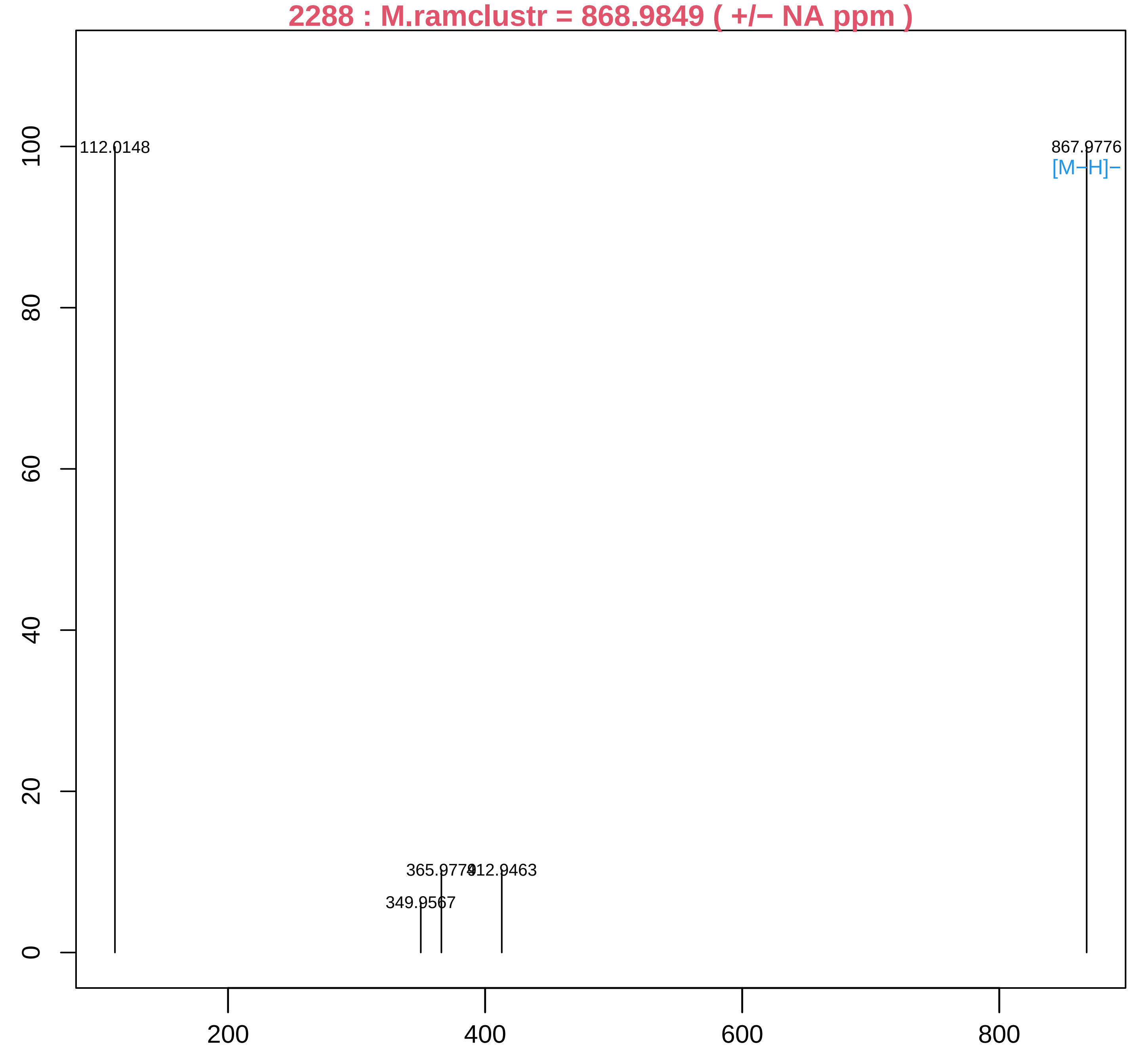
2288 : M.ramclustr = 868.9849 (+/− NA ppm)
112.0148
867.9776
[M−H]−
365.9779
412.9463
349.9567
0
20
40
60
80
100
200
400
600
800

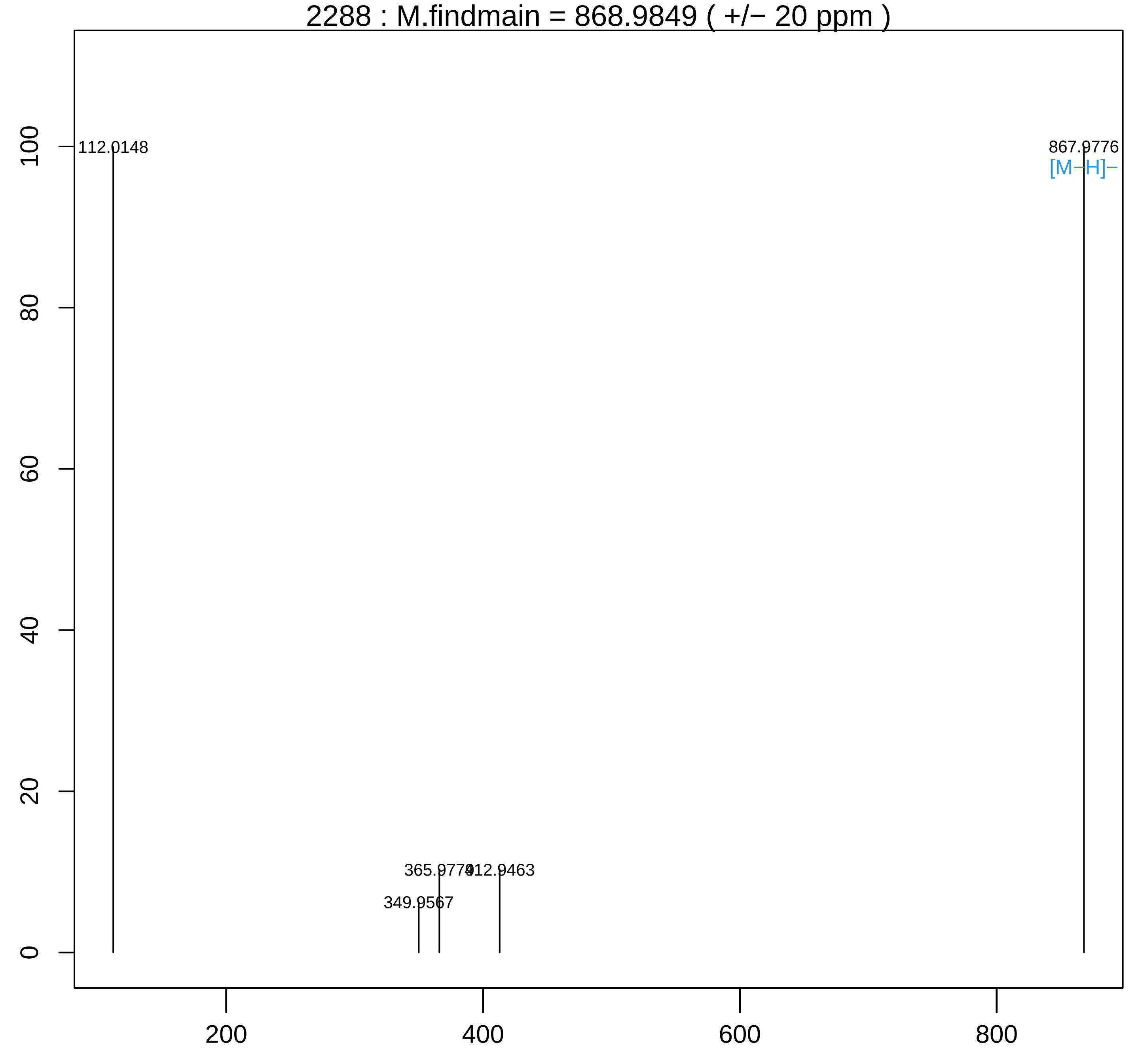
2288 : M.findmain = 868.9849 (+/− 20 ppm)
112.0148
867.9776
[M−H]−
365.9779
412.9463
349.9567
0
20
40
60
80
100
200
400
600
800

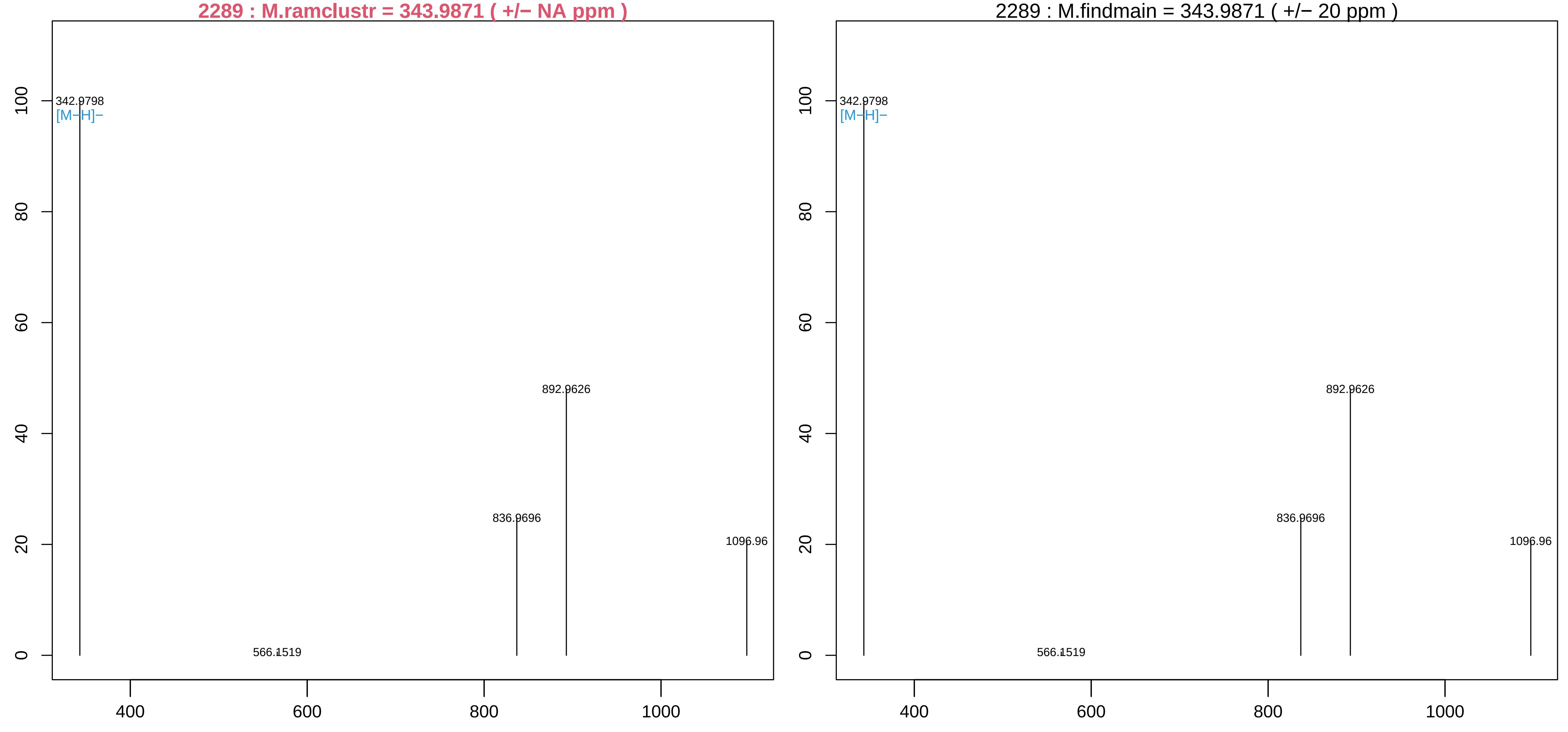
2289 : M.ramclustr = 343.9871 (+/− NA ppm)
342.9798
[M−H]−
892.9626
836.9696
1096.96
566.1519
0
20
40
60
80
100
400
600
800
1000
2289 : M.findmain = 343.9871 (+/− 20 ppm)
342.9798
[M−H]−
892.9626
836.9696
1096.96
566.1519
0
20
40
60
80
100
400
600
800
1000

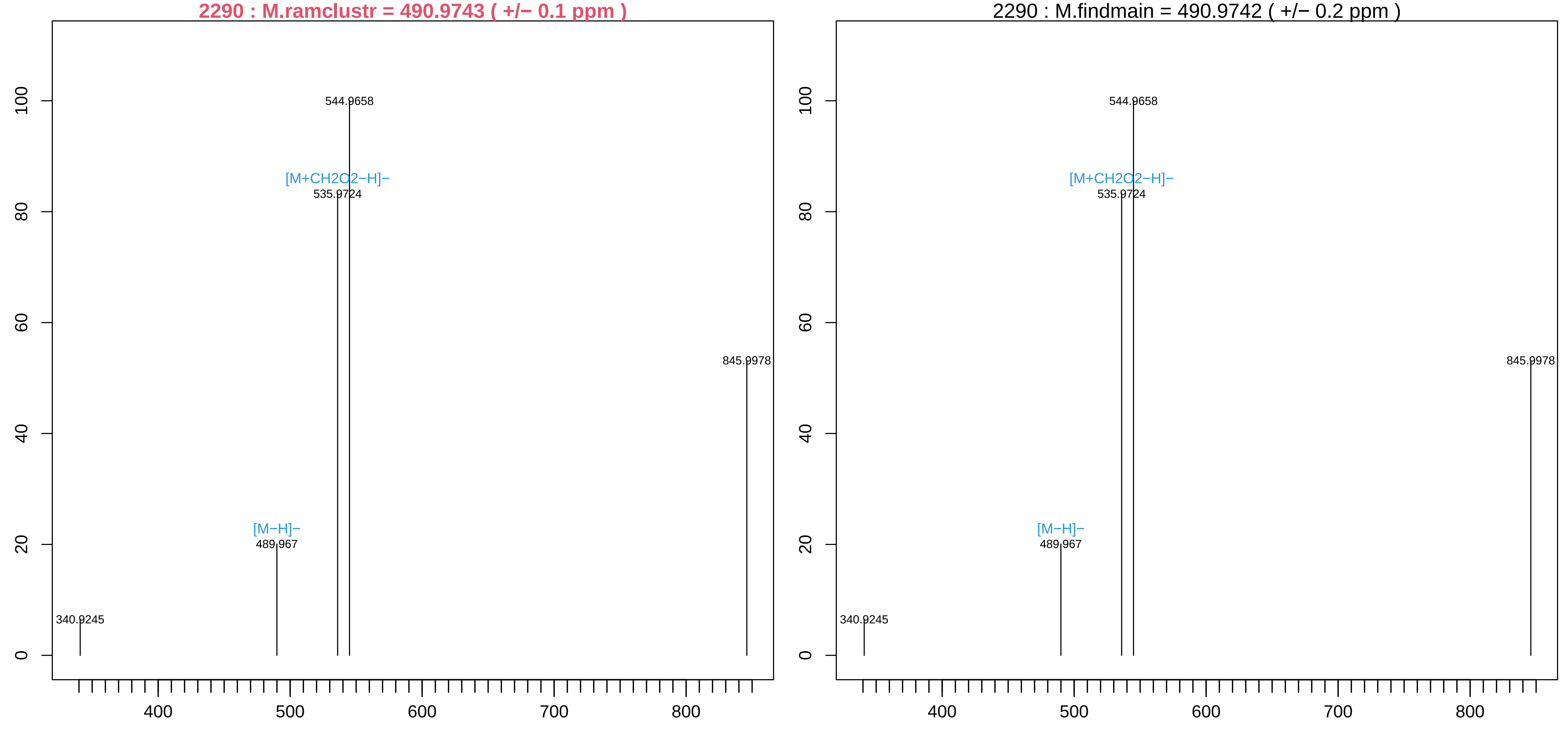
2290 : M.ramclustr = 490.9743 (+/− 0.1 ppm)
544.9658
[M+CH2O2−H]−
535.9724
845.9978
[M−H]−
489.967
340.9245
0
20
40
60
80
100
400
500
600
700
800
2290 : M.findmain = 490.9742 (+/− 0.2 ppm)
544.9658
[M+CH2O2−H]−
535.9724
845.9978
[M−H]−
489.967
340.9245
0
20
40
60
80
100
400
500
600
700
800

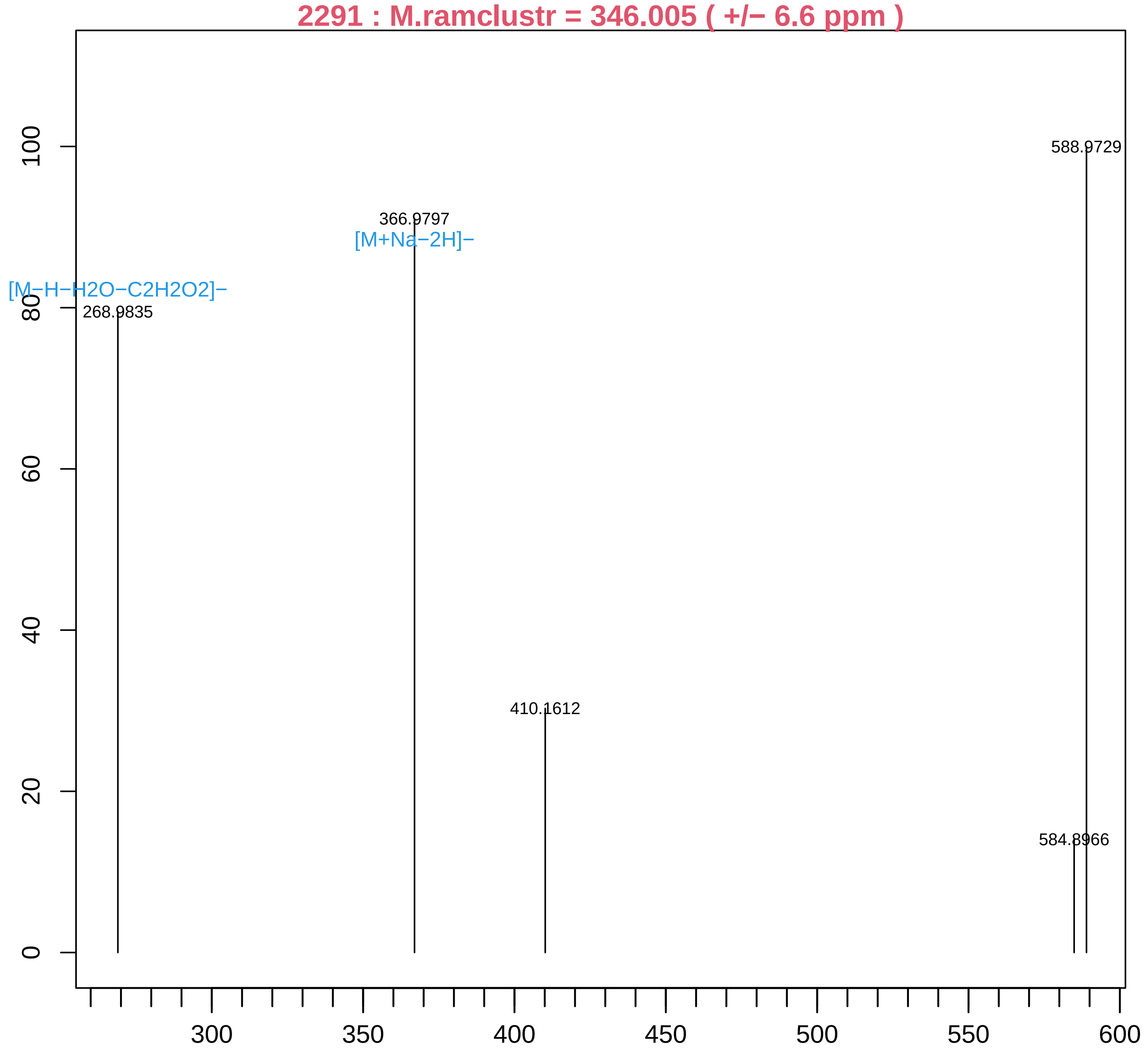

2291 : M.ramclustr = 346.005 (+/− 6.6 ppm)
588.9729
366.9797
[M+Na−2H]−
[M−H−H2O−C2H2O2]−
268.9835
410.1612
584.8966
0
20
40
60
80
100
300
350
400
450
500
550
600

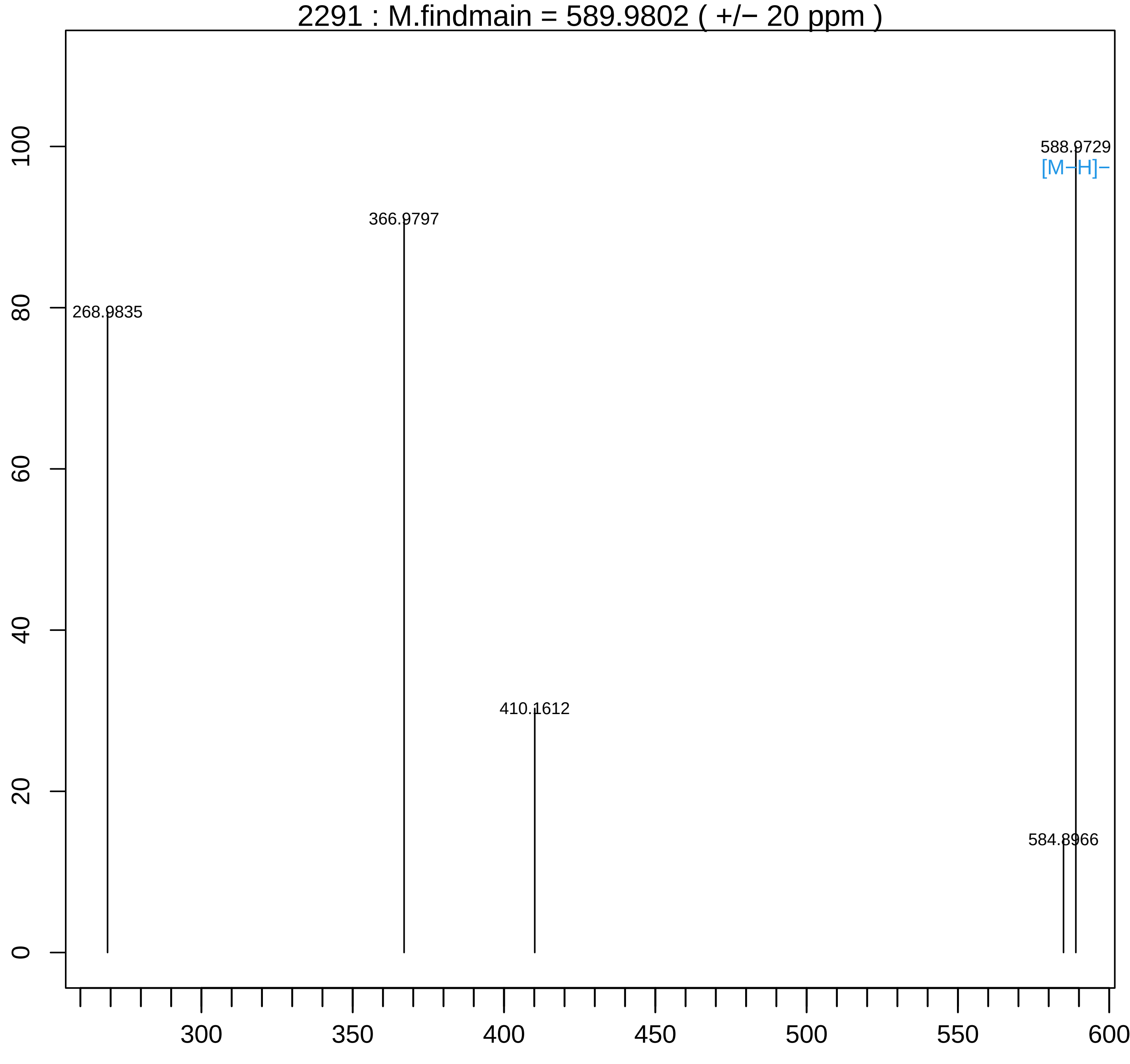

2291 : M.findmain = 589.9802 (+/− 20 ppm)
588.9729
[M−H]−
366.9797
268.9835
410.1612
584.8966
0
20
40
60
80
100
300
350
400
450
500
550
600

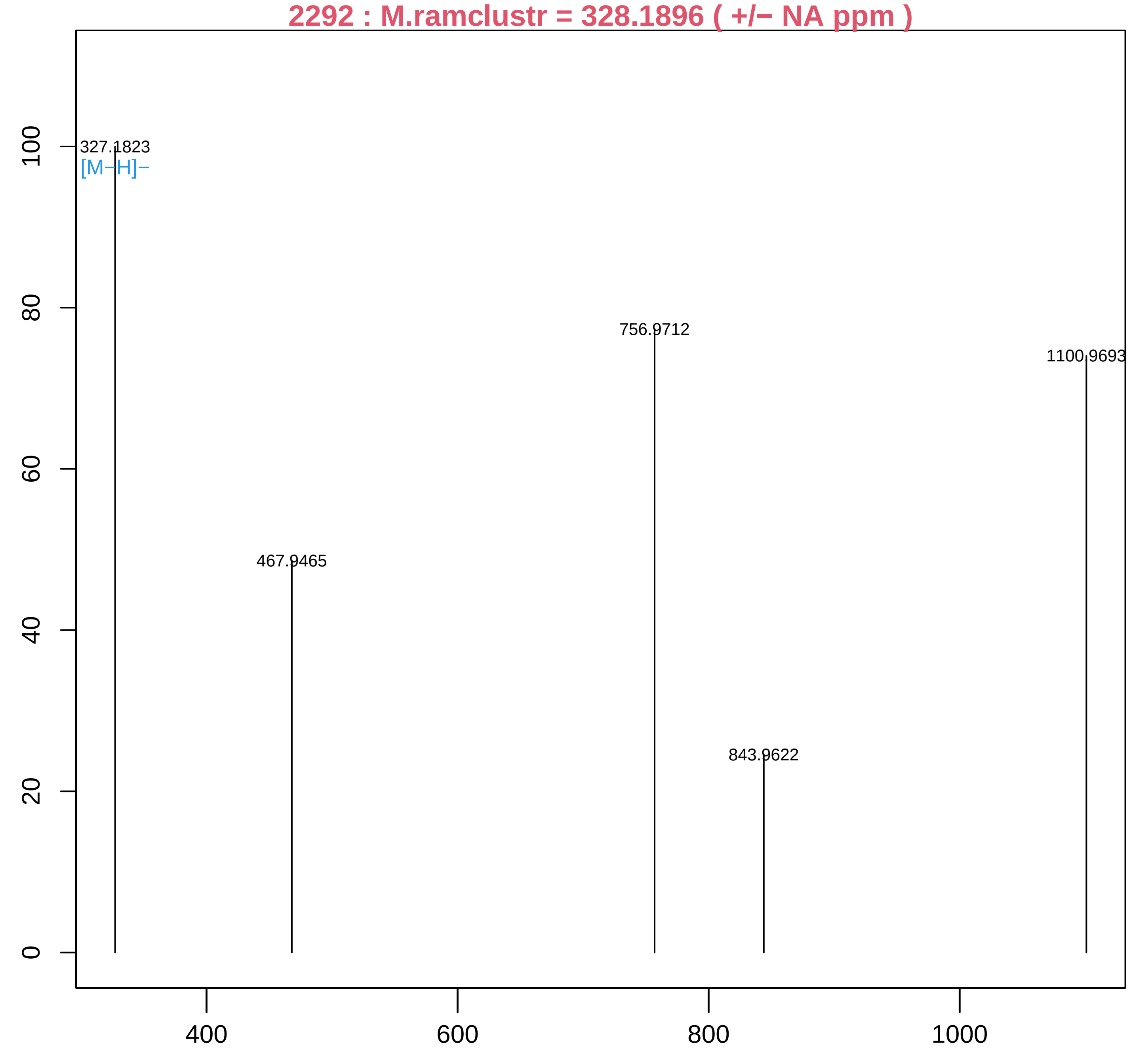
2292 : M.ramclustr = 328.1896 (+/− NA ppm)
327.1823
[M−H]−
467.9465
756.9712
843.9622
1100.9693
0
20
40
60
80
100
400
600
800
1000

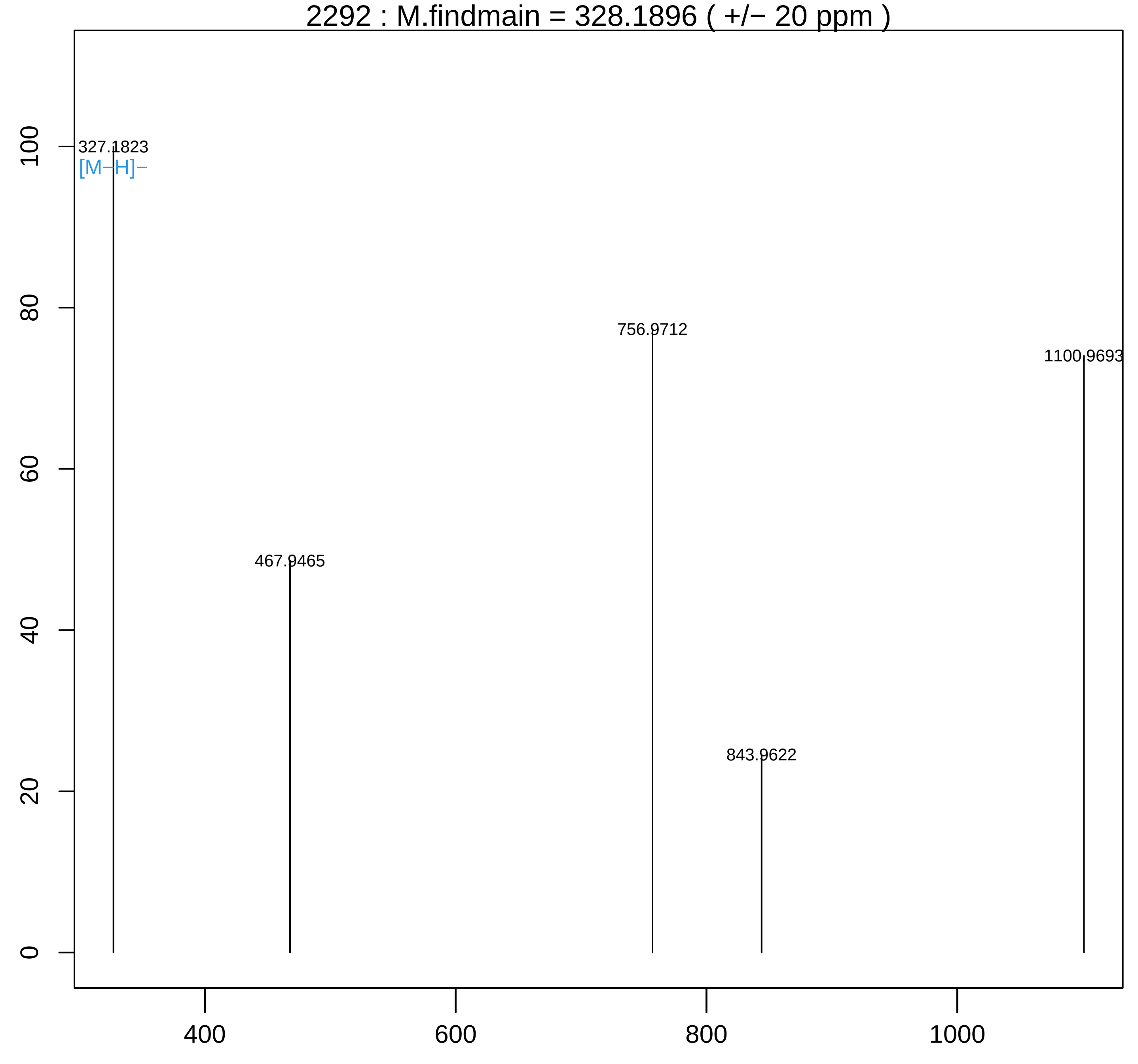
2292 : M.findmain = 328.1896 (+/− 20 ppm)
327.1823
[M−H]−
467.9465
756.9712
843.9622
1100.9693
0
20
40
60
80
100
400
600
800
1000

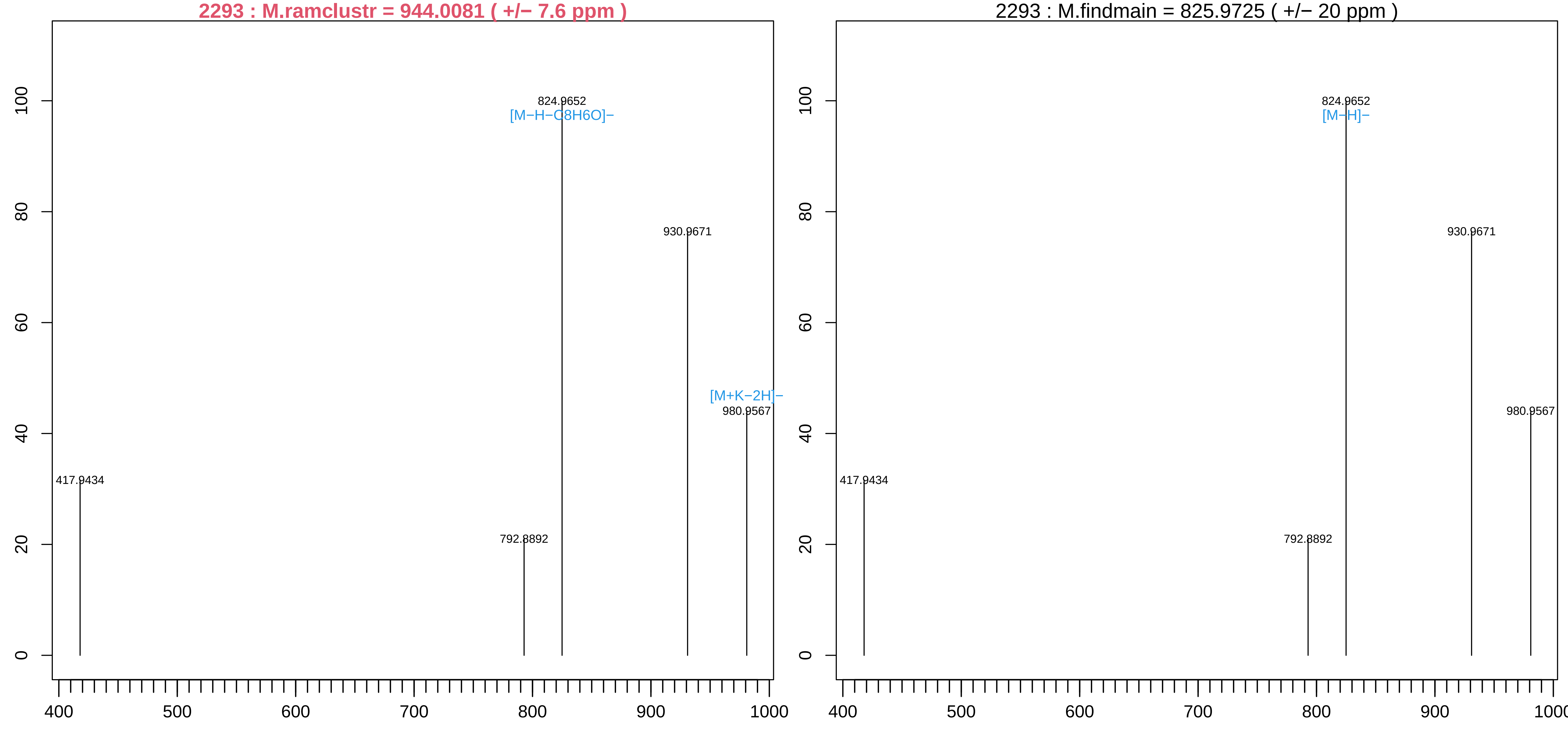

2293 : M.ramclustr = 944.0081 (+/− 7.6 ppm)
824.9652
[M−H−C8H6O]−
930.9671
[M+K−2H]−
980.9567
417.9434
792.8892
2293 : M.findmain = 825.9725 (+/− 20 ppm)
824.9652
[M−H]−
930.9671
980.9567
417.9434
792.8892
0
20
40
60
80
100
400
500
600
700
800
900
1000

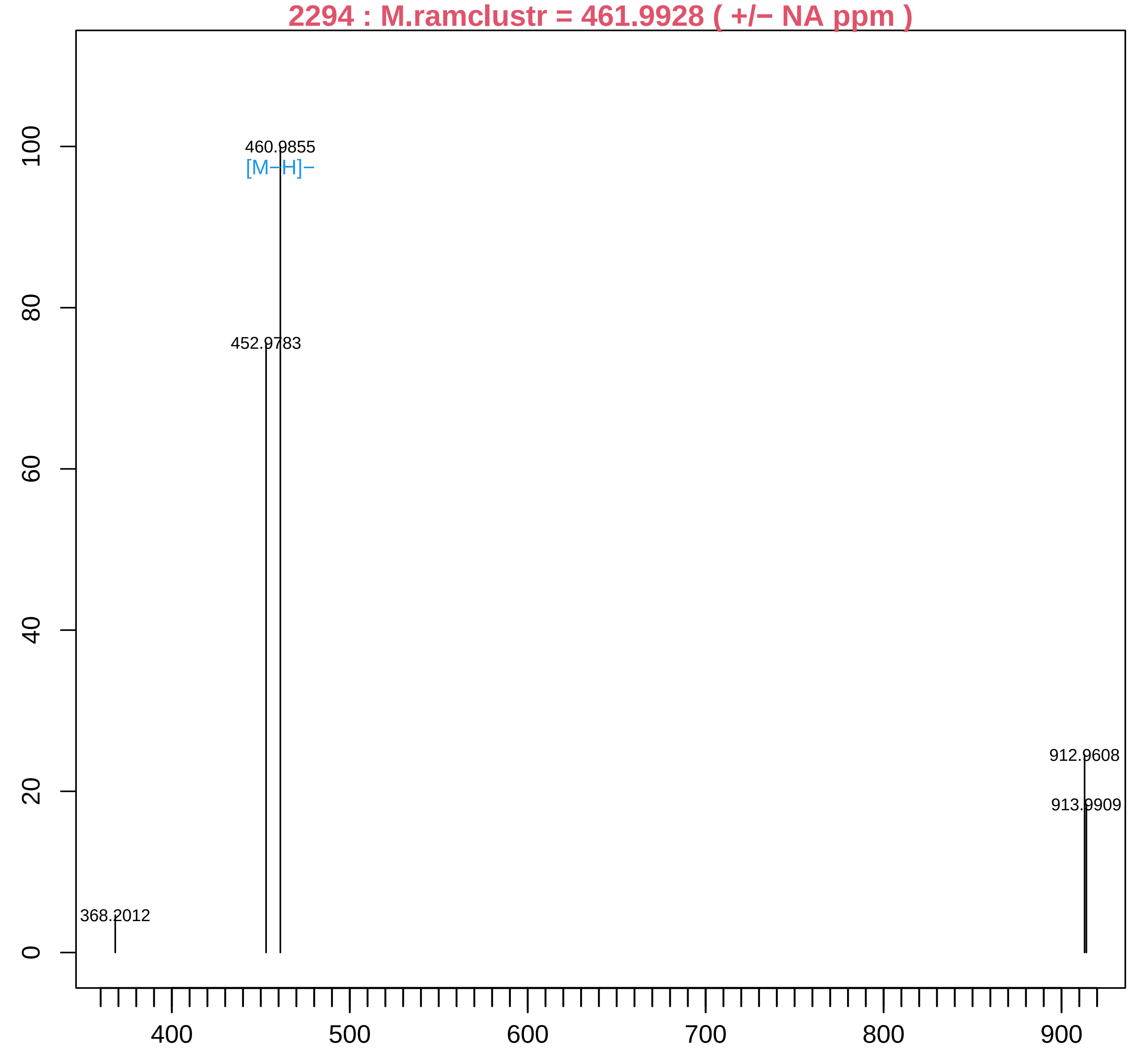

2294 : M.ramclustr = 461.9928 (+/− NA ppm)
460.9855
[M−H]−
452.9783
912.9608
913.9909
368.2012
0
20
40
60
80
100
400
500
600
700
800
900

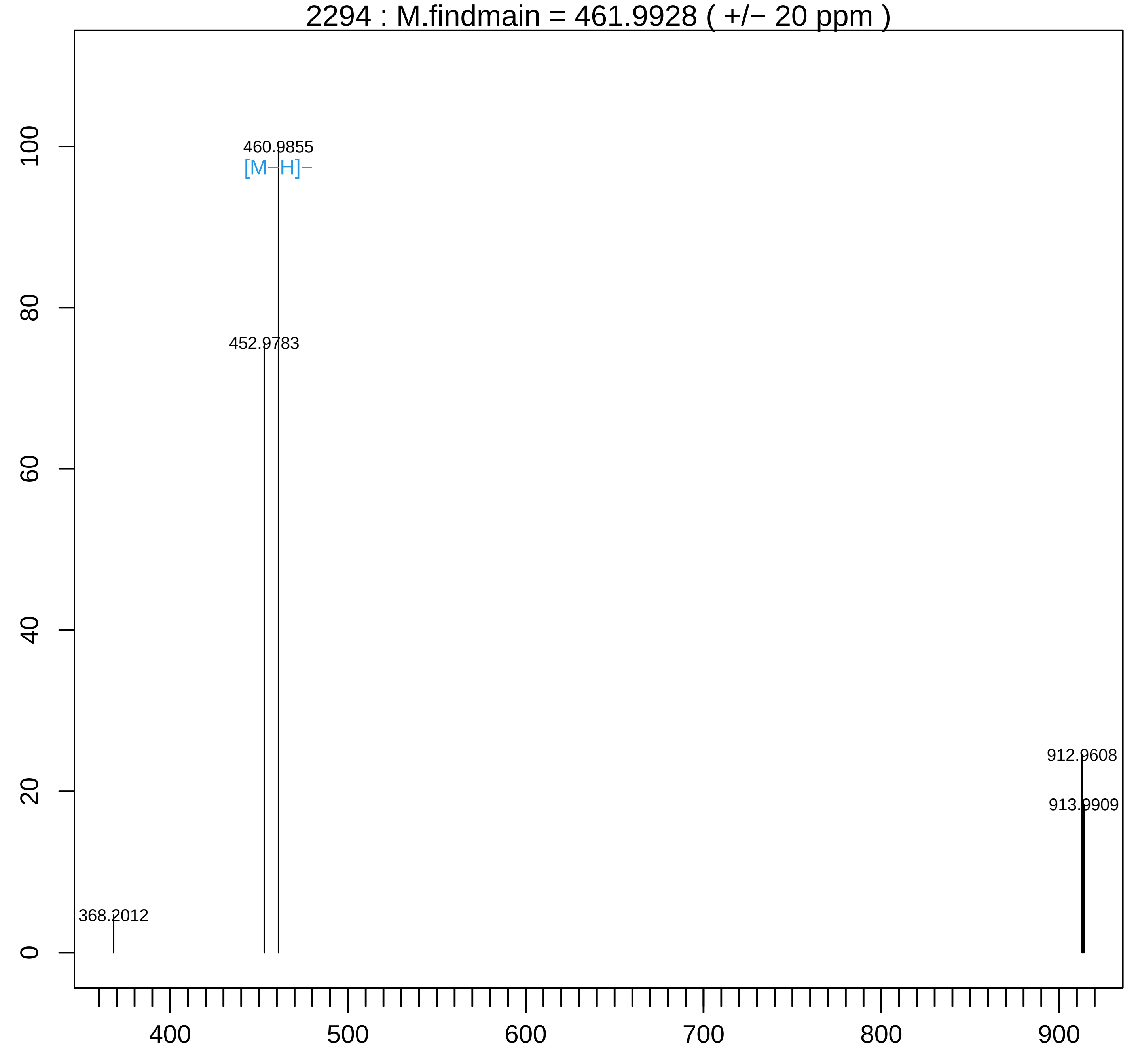

2294 : M.findmain = 461.9928 (+/− 20 ppm)
460.9855
[M−H]−
452.9783
912.9608
913.9909
368.2012
0
20
40
60
80
100
400
500
600
700
800
900

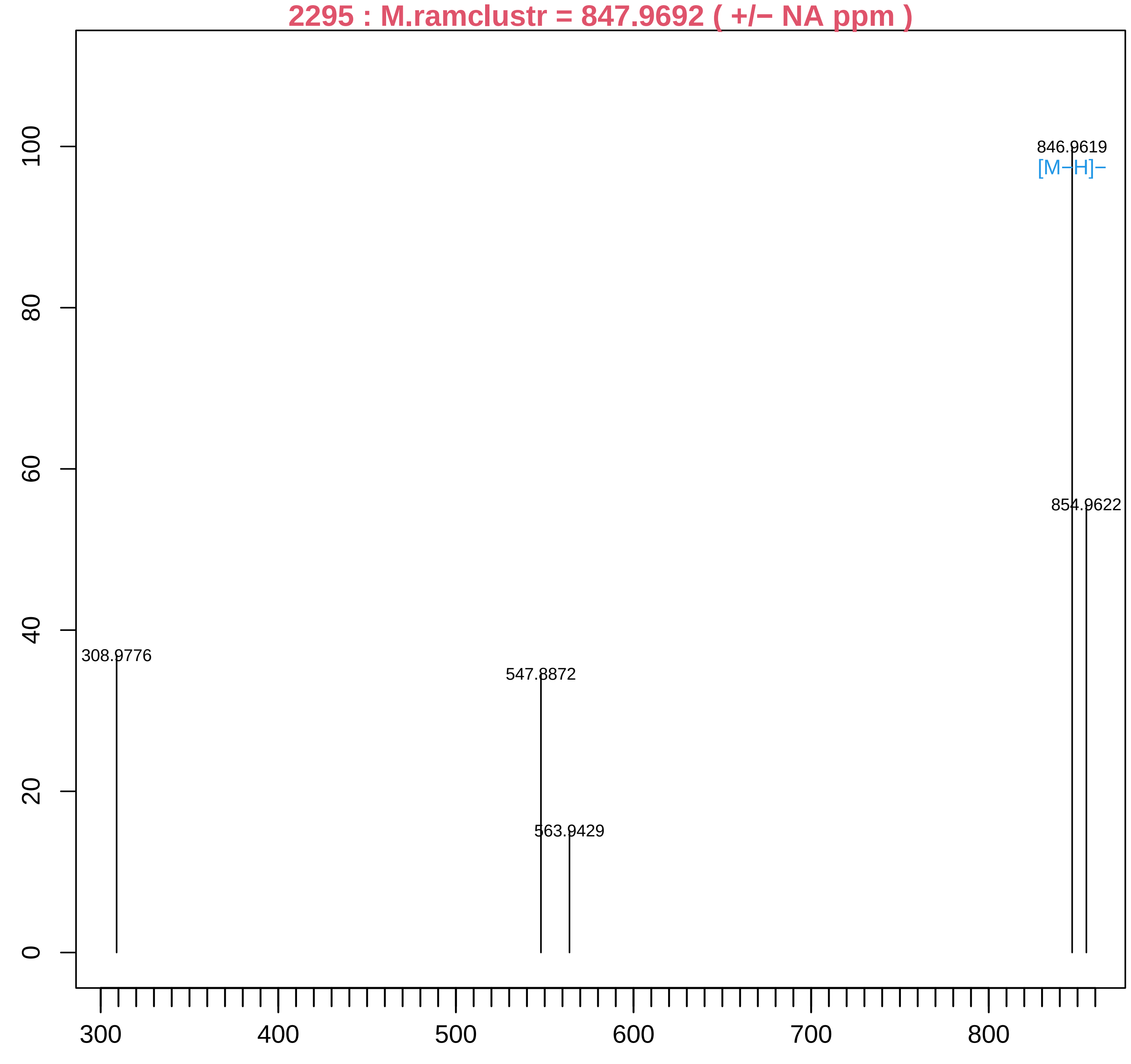
2295 : M.ramclustr = 847.9692 (+/− NA ppm)
846.9619
[M−H]−
854.9622
308.9776
547.8872
563.9429
0
20
40
60
80
100
300
400
500
600
700
800

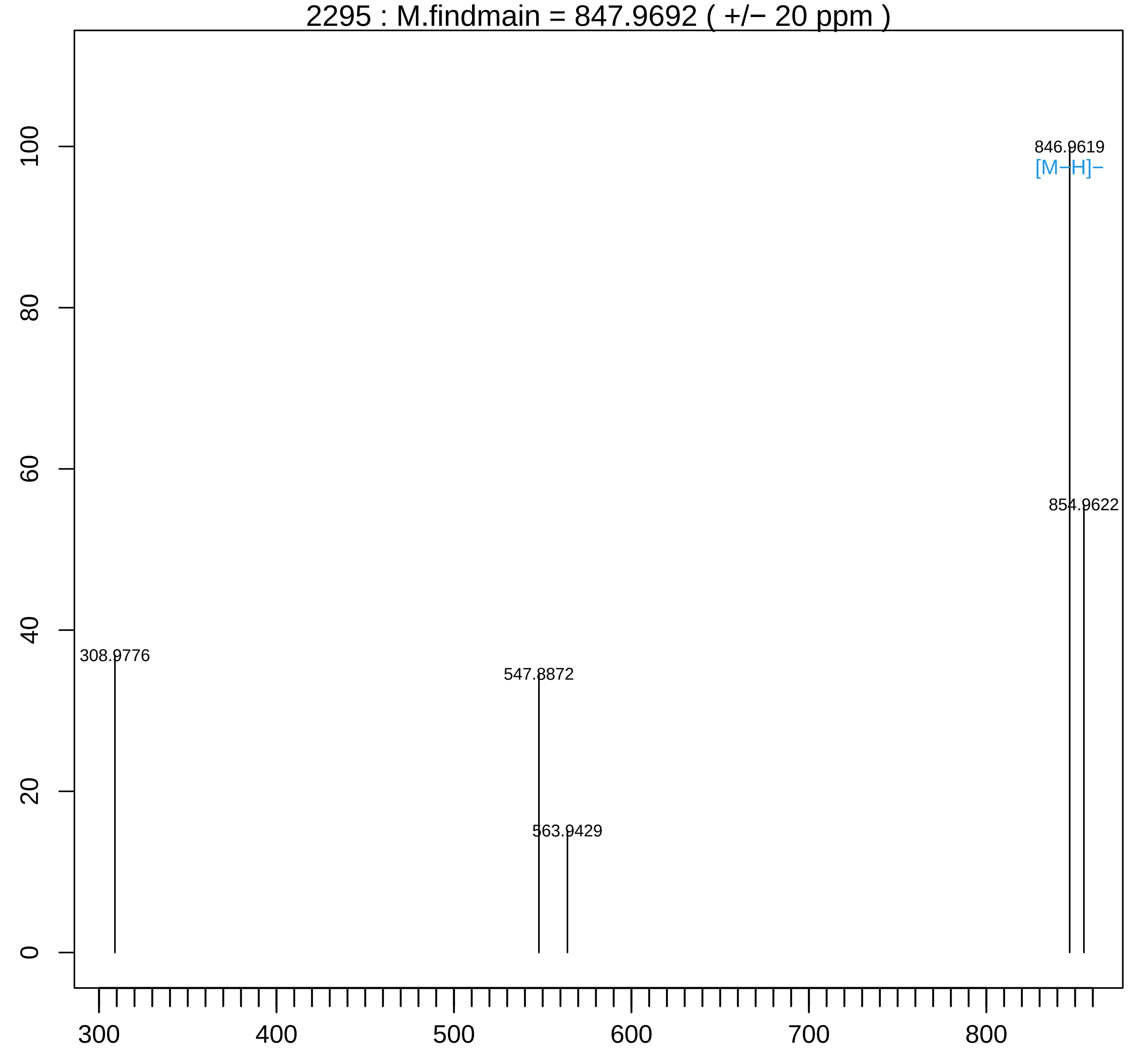
2295 : M.findmain = 847.9692 (+/− 20 ppm)
846.9619
[M−H]−
854.9622
308.9776
547.8872
563.9429
0
20
40
60
80
100
300
400
500
600
700
800

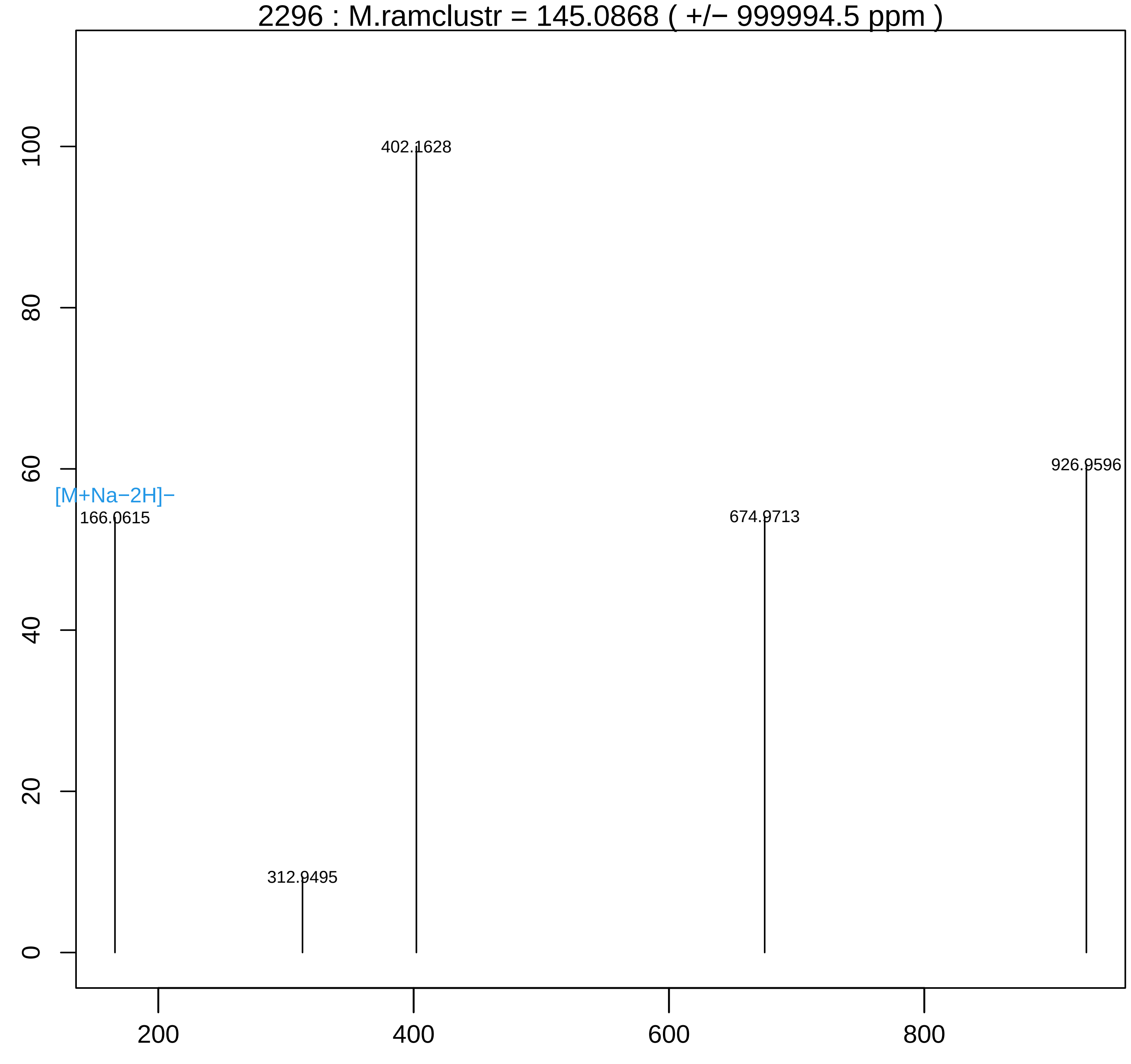
2296 : M.ramclustr = 145.0868 (+/− 999994.5 ppm)
402.1628
926.9596
[M+Na−2H]−
166.0615
674.9713
312.9495
0
20
40
60
80
100
200
400
600
800

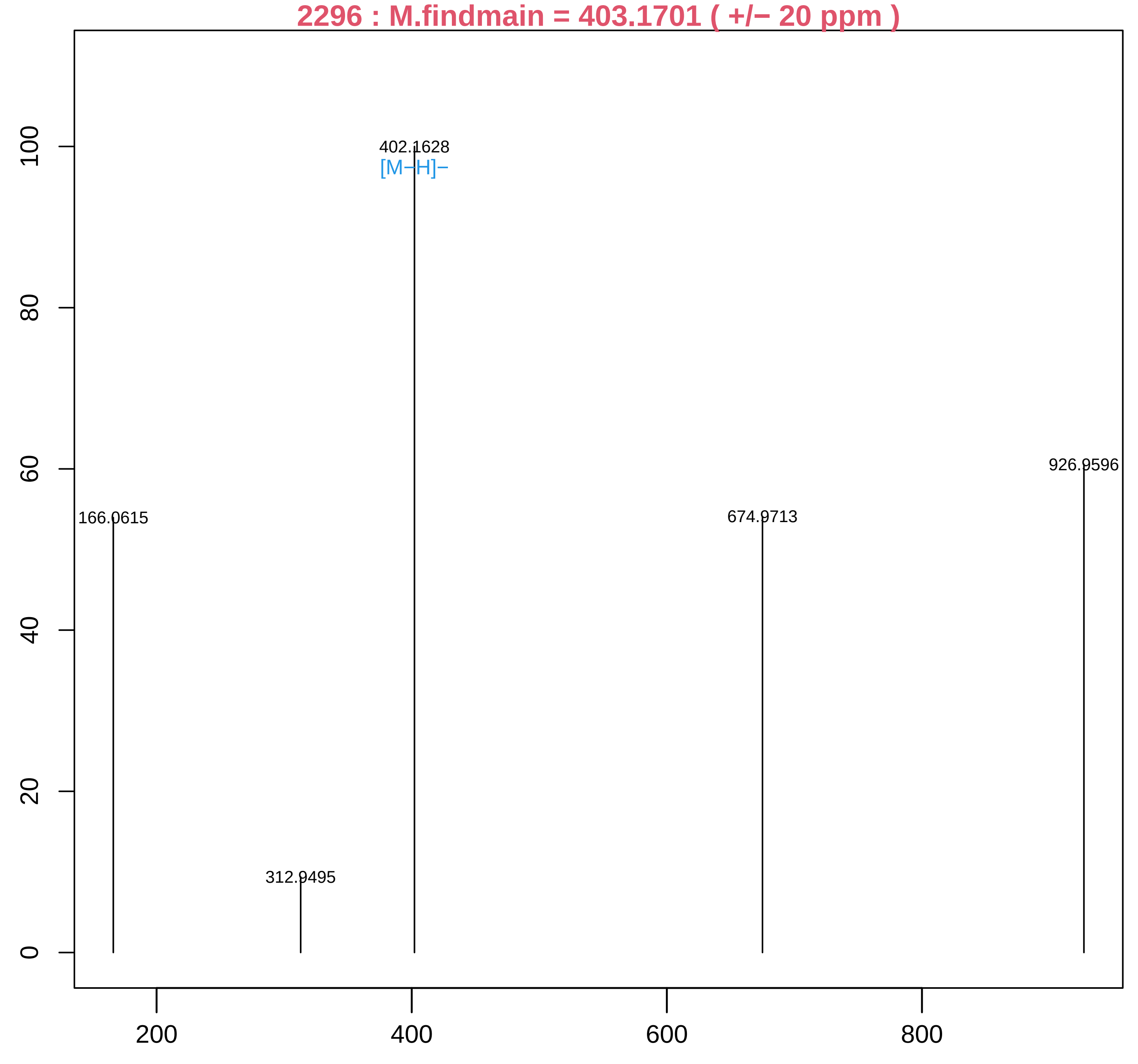
2296 : M.findmain = 403.1701 (+/− 20 ppm)
402.1628
[M−H]−
926.9596
166.0615
674.9713
312.9495
0
20
40
60
80
100
200
400
600
800

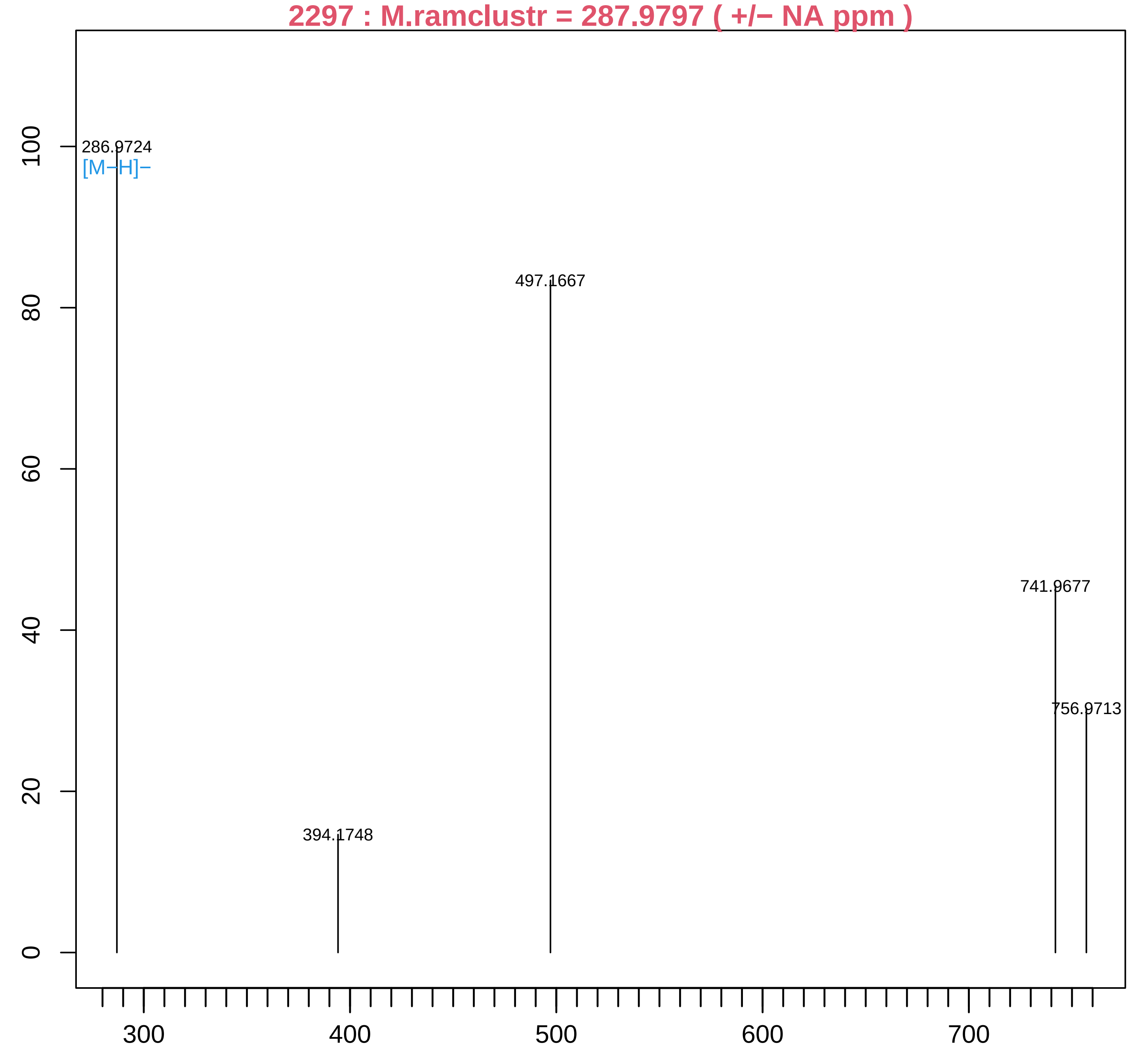
2297 : M.ramclustr = 287.9797 (+/− NA ppm)
286.9724
[M−H]−
497.1667
741.9677
756.9713
394.1748
0
20
40
60
80
100
300
400
500
600
700

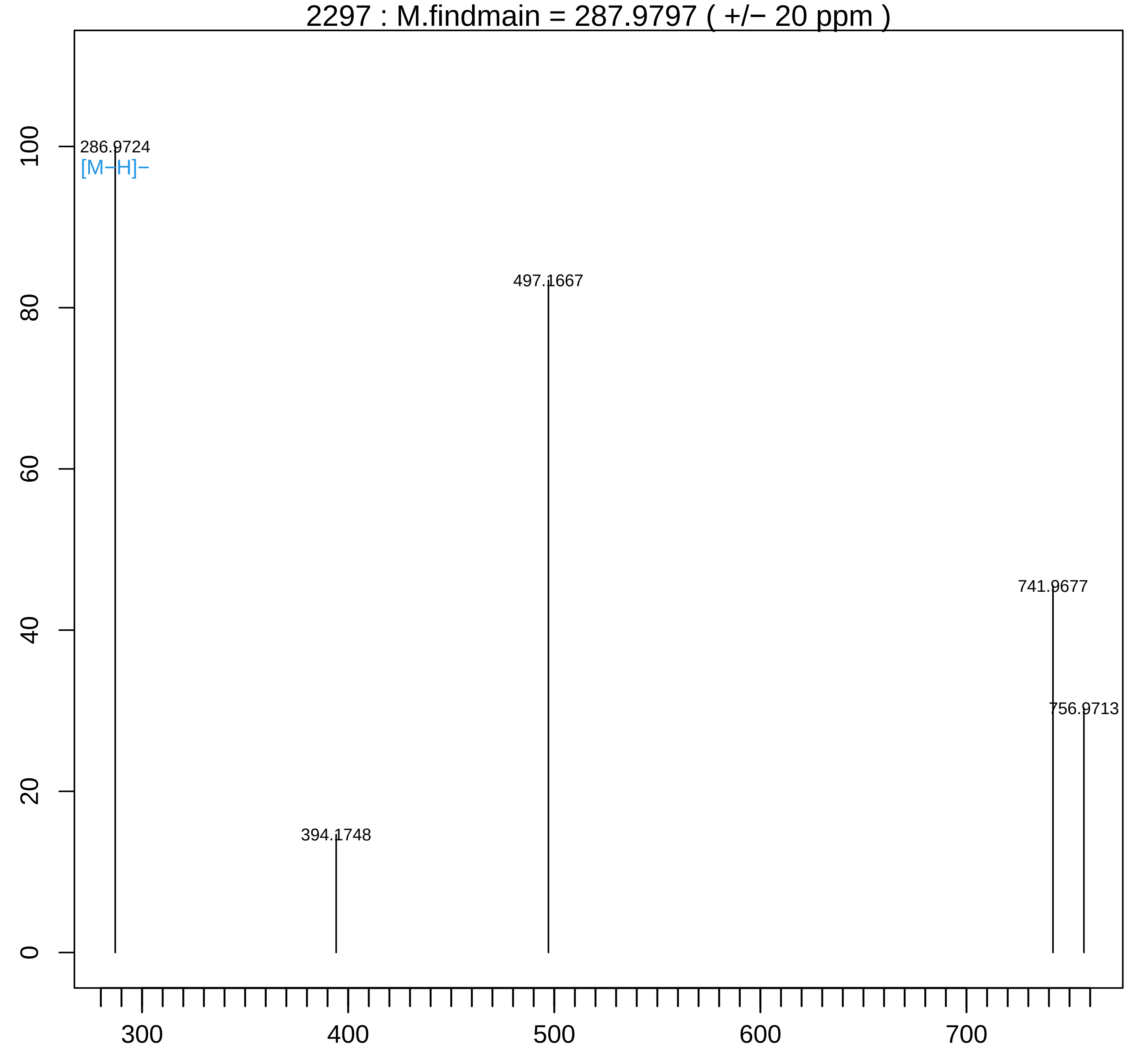
2297 : M.findmain = 287.9797 (+/− 20 ppm)
286.9724
[M−H]−
497.1667
741.9677
756.9713
394.1748
0
20
40
60
80
100
300
400
500
600
700

2298 : M.ramclustr = 214.0225 (+/− NA ppm)

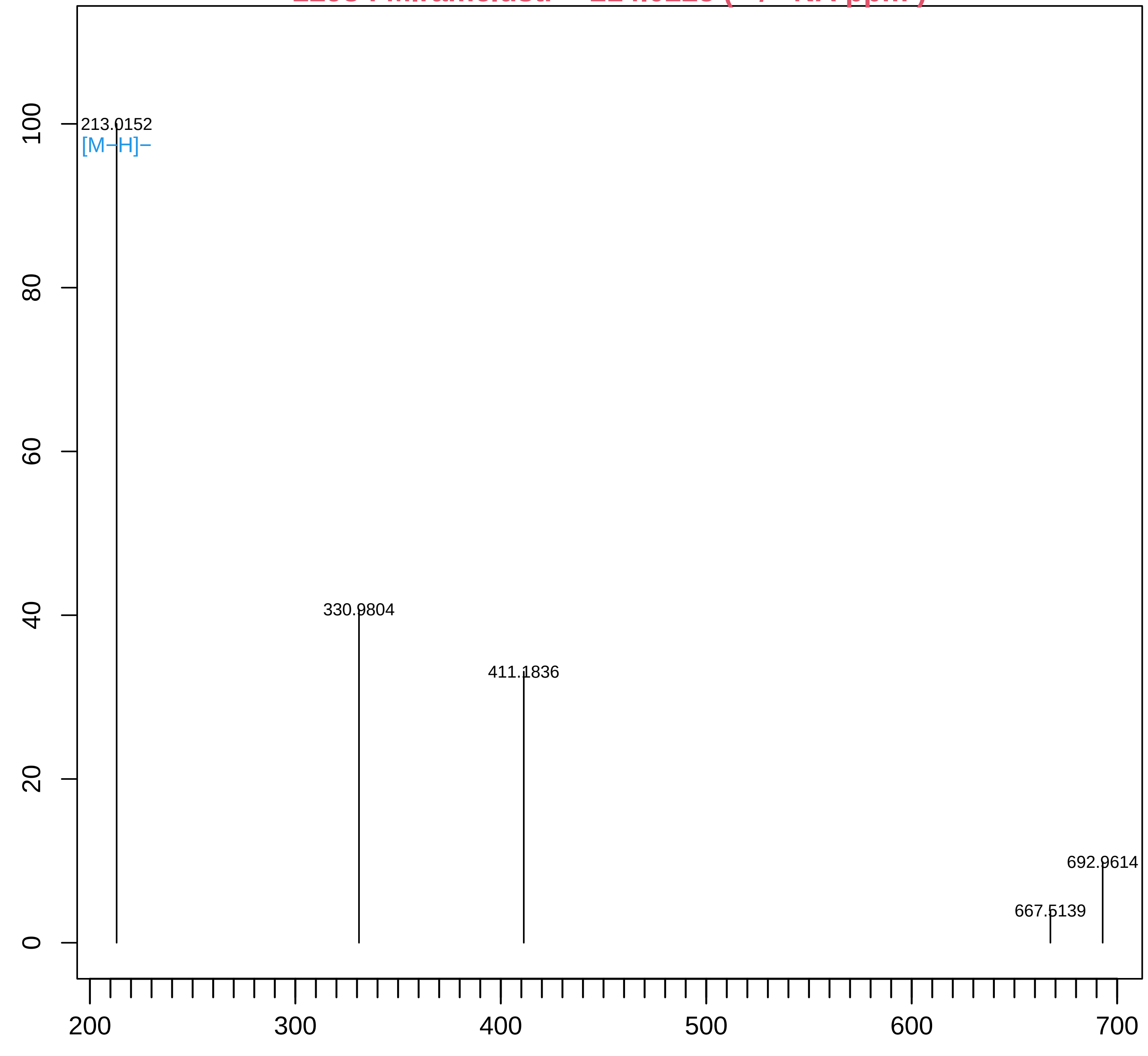

2298 : M.findmain = 214.0225 (+/− 20 ppm)

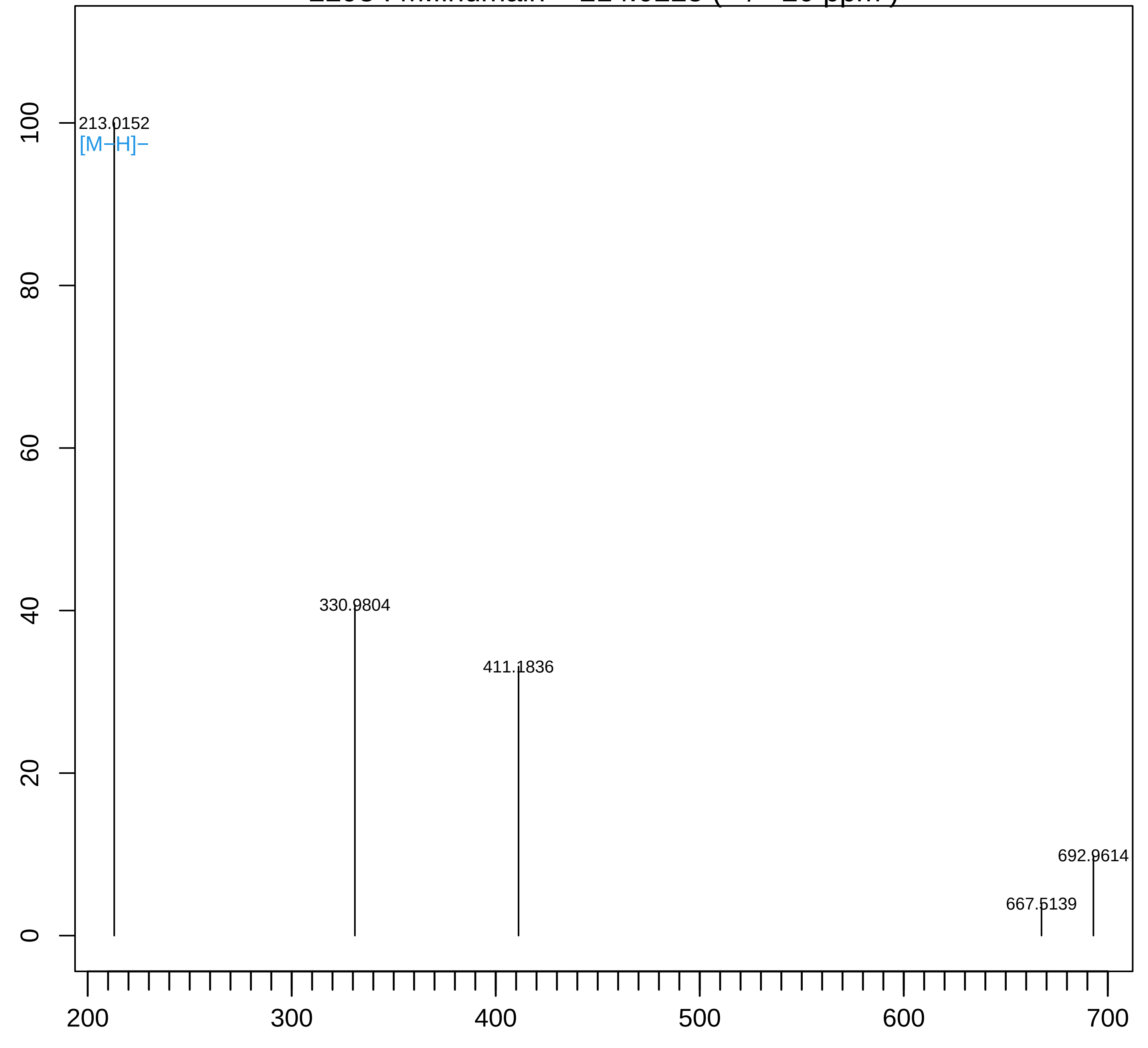

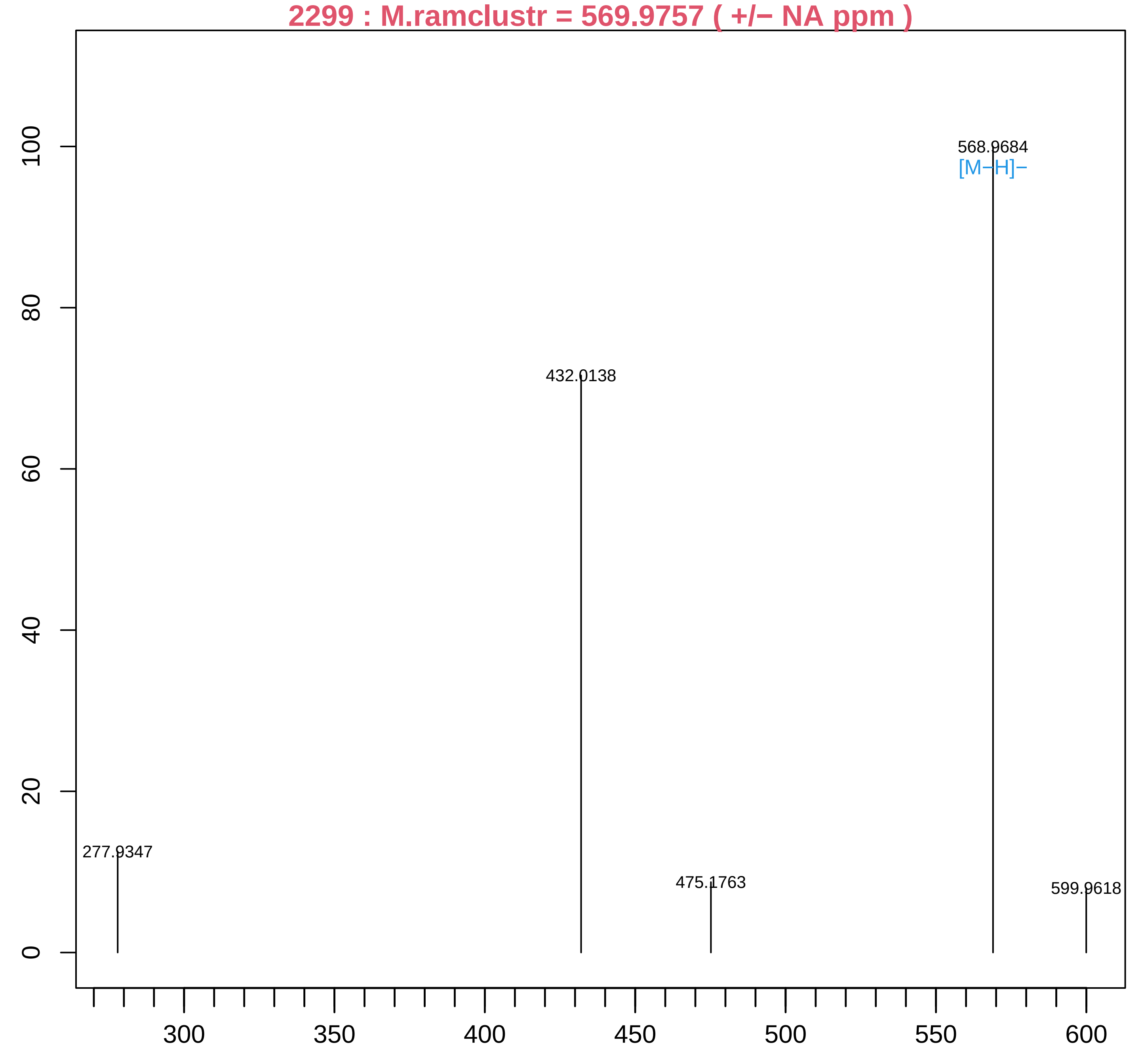
2299 : M.ramclustr = 569.9757 (+/− NA ppm)
568.9684
[M−H]−
432.0138
277.9347
475.1763
599.9618
0
20
40
60
80
100
300
350
400
450
500
550
600

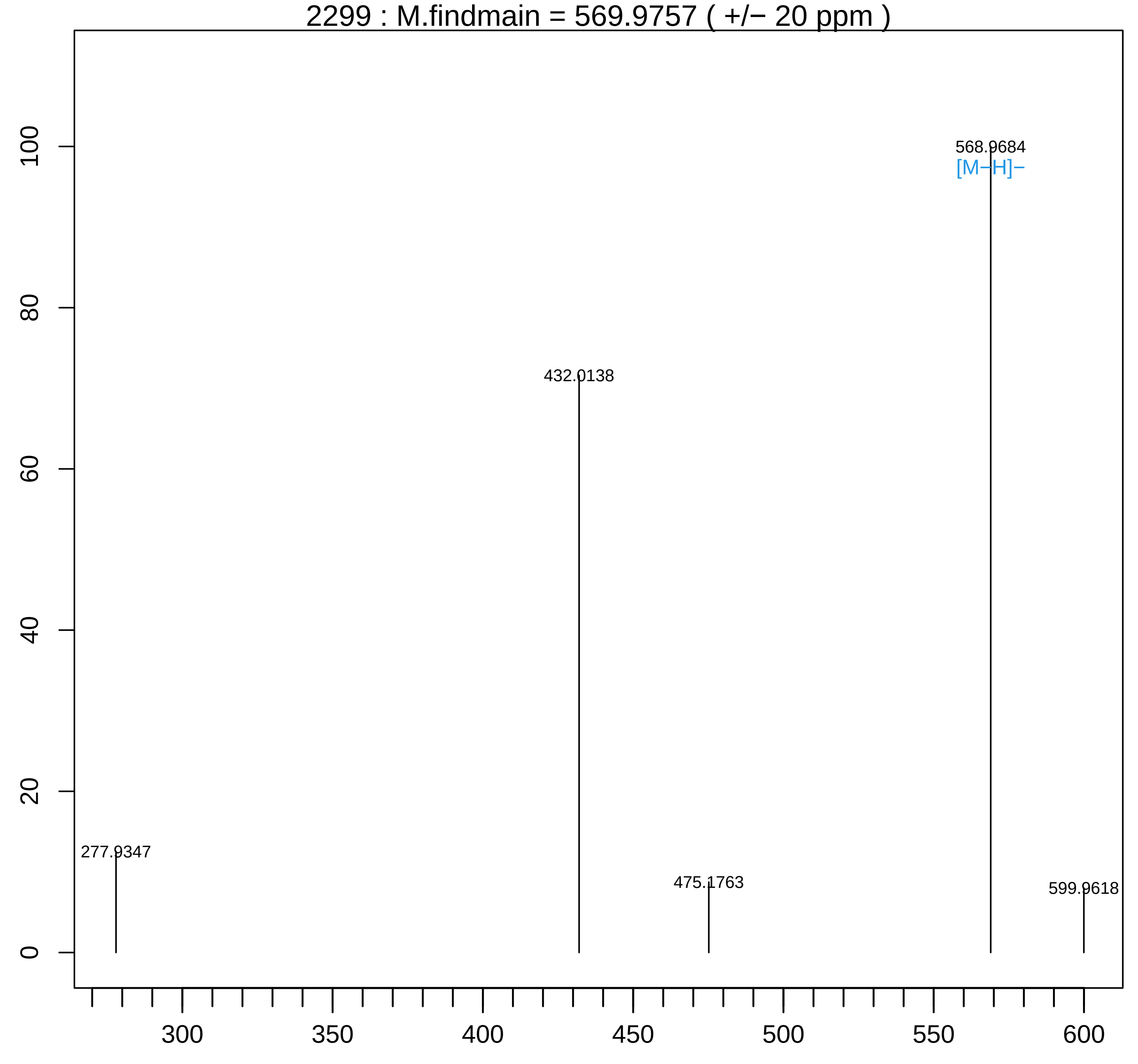
2299 : M.findmain = 569.9757 (+/− 20 ppm)
568.9684
[M−H]−
432.0138
277.9347
475.1763
599.9618
0
20
40
60
80
100
300
350
400
450
500
550
600

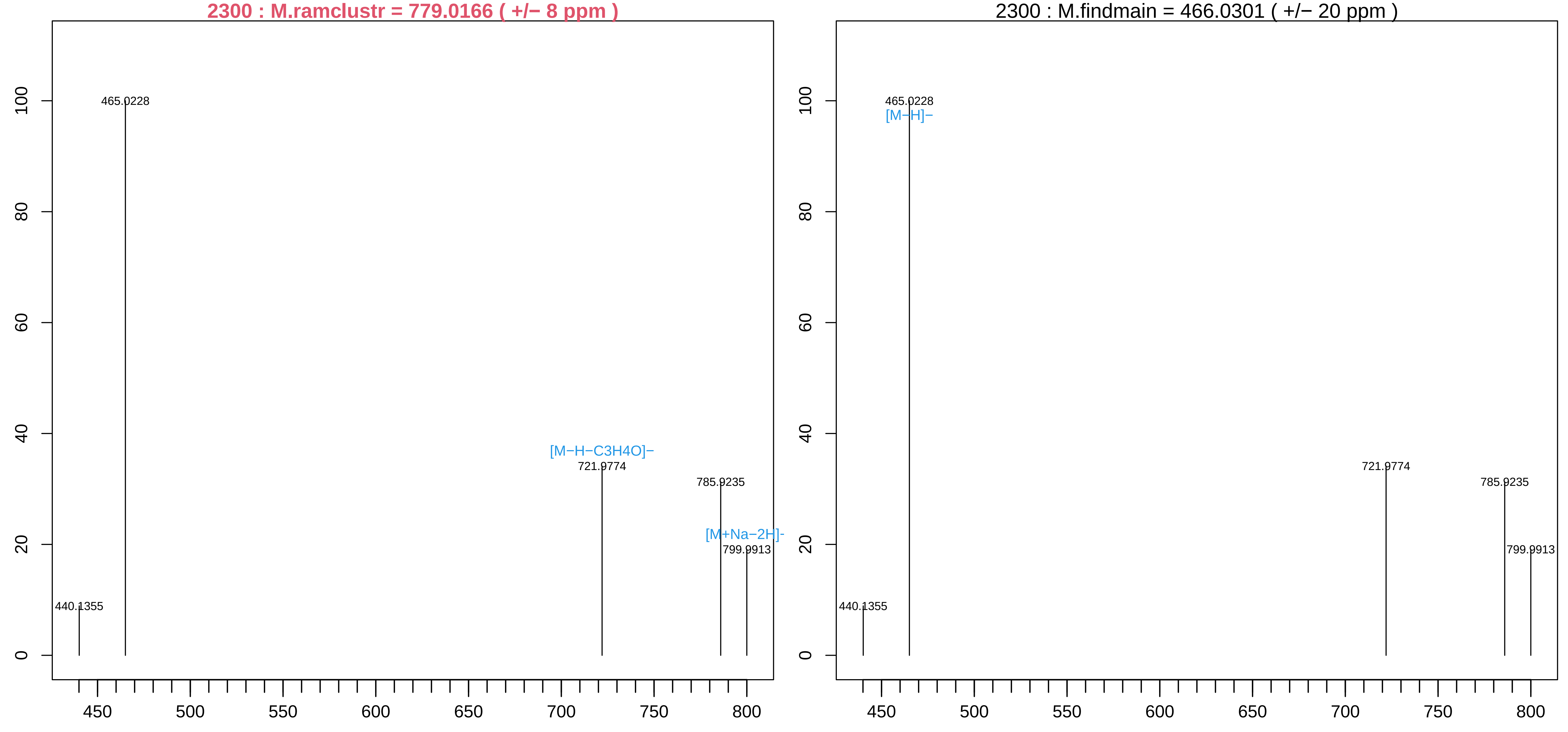

2300 : M.ramclustr = 779.0166 (+/− 8 ppm)
465.0228
[M−H−C3H4O]−
721.9774
785.9235
[M+Na−2H]-
799.9913
440.1355
2300 : M.findmain = 466.0301 (+/− 20 ppm)
465.0228
[M−H]−
721.9774
785.9235
799.9913
440.1355
0
20
40
60
80
100
450
500
550
600
650
700
750
800

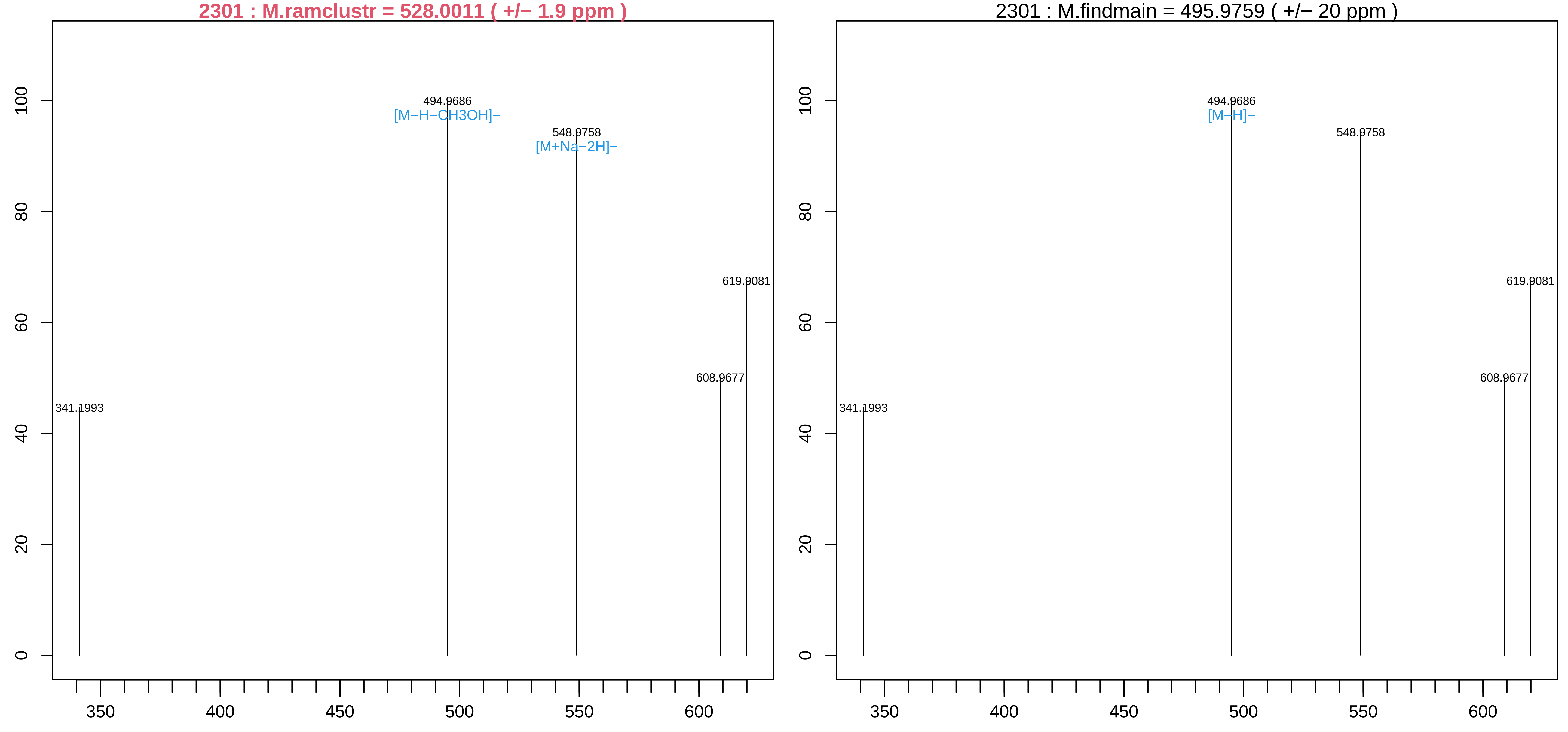
2301 : M.ramclustr = 528.0011 (+/− 1.9 ppm)
494.9686
[M−H−CH3OH]−
548.9758
[M+Na−2H]−
619.9081
608.9677
341.1993
0
20
40
60
80
100
350
400
450
500
550
600
2301 : M.findmain = 495.9759 (+/− 20 ppm)
494.9686
[M−H]−
548.9758
619.9081
608.9677
341.1993
0
20
40
60
80
100
350
400
450
500
550
600

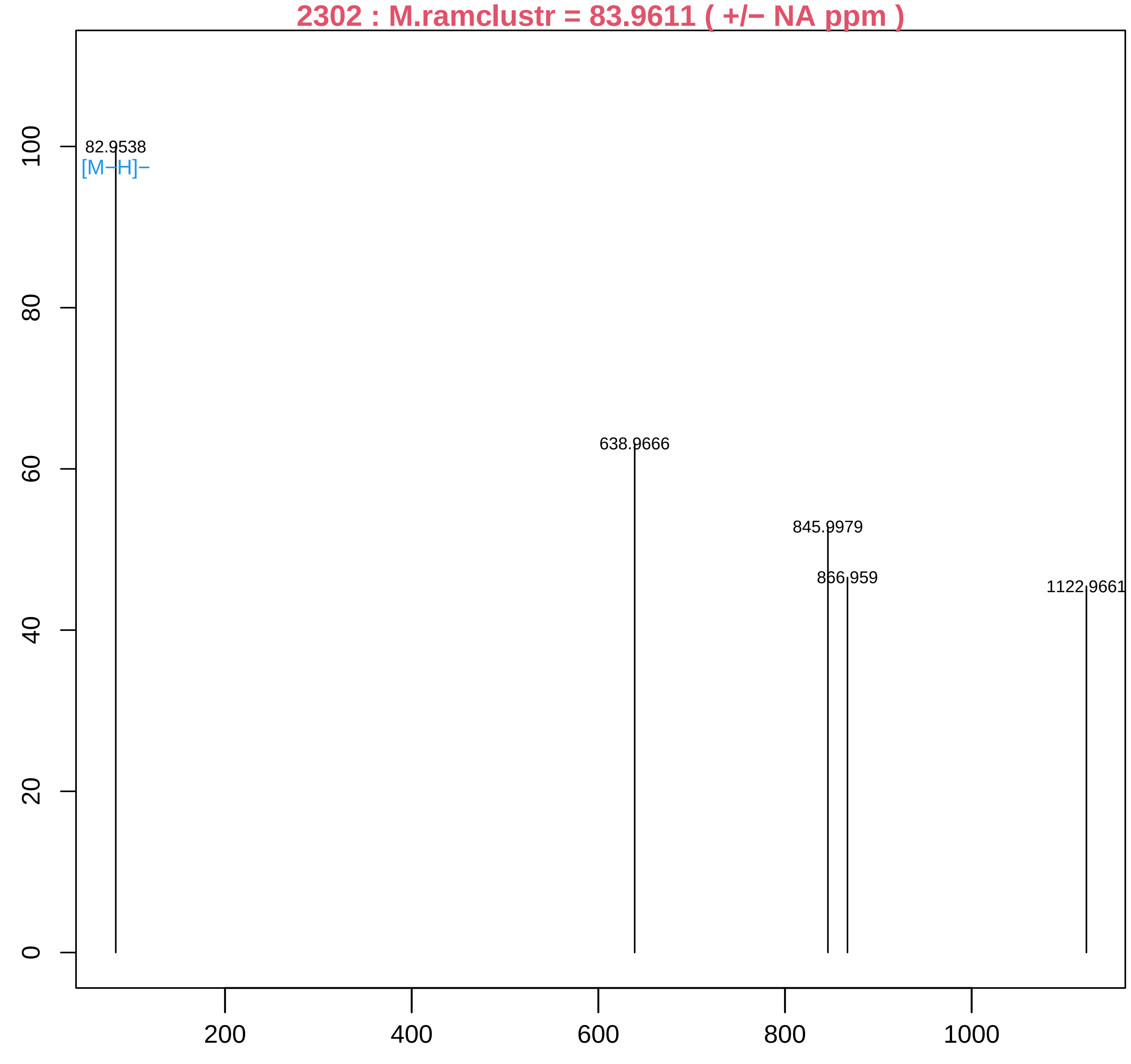
2302 : M.ramclustr = 83.9611 (+/− NA ppm)
82.9538
[M−H]−
638.9666
845.9979
866.959
1122.9661
0
20
40
60
80
100
200
400
600
800
1000

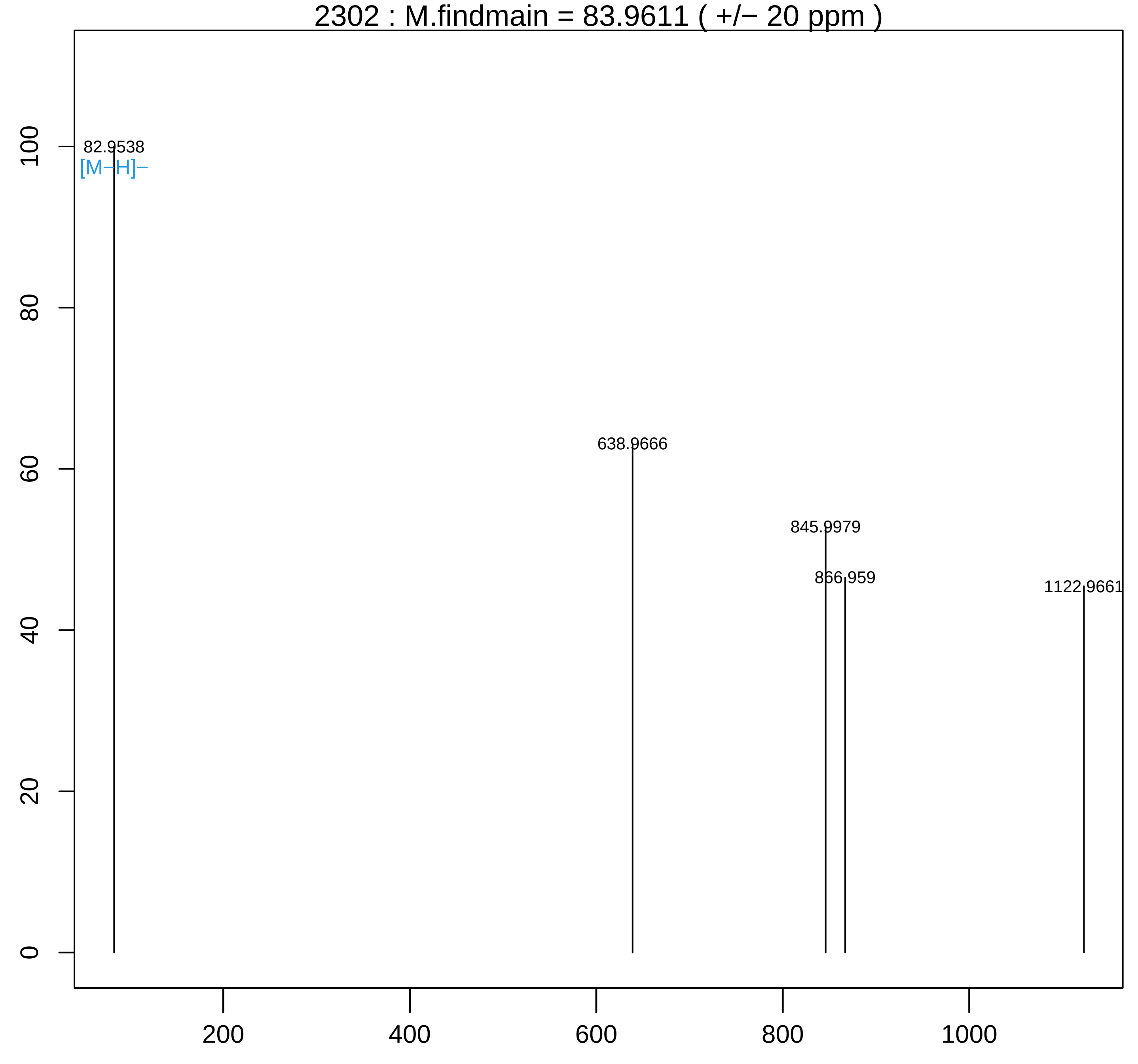
2302 : M.findmain = 83.9611 (+/− 20 ppm)
82.9538
[M−H]−
638.9666
845.9979
866.959
1122.9661
0
20
40
60
80
100
200
400
600
800
1000

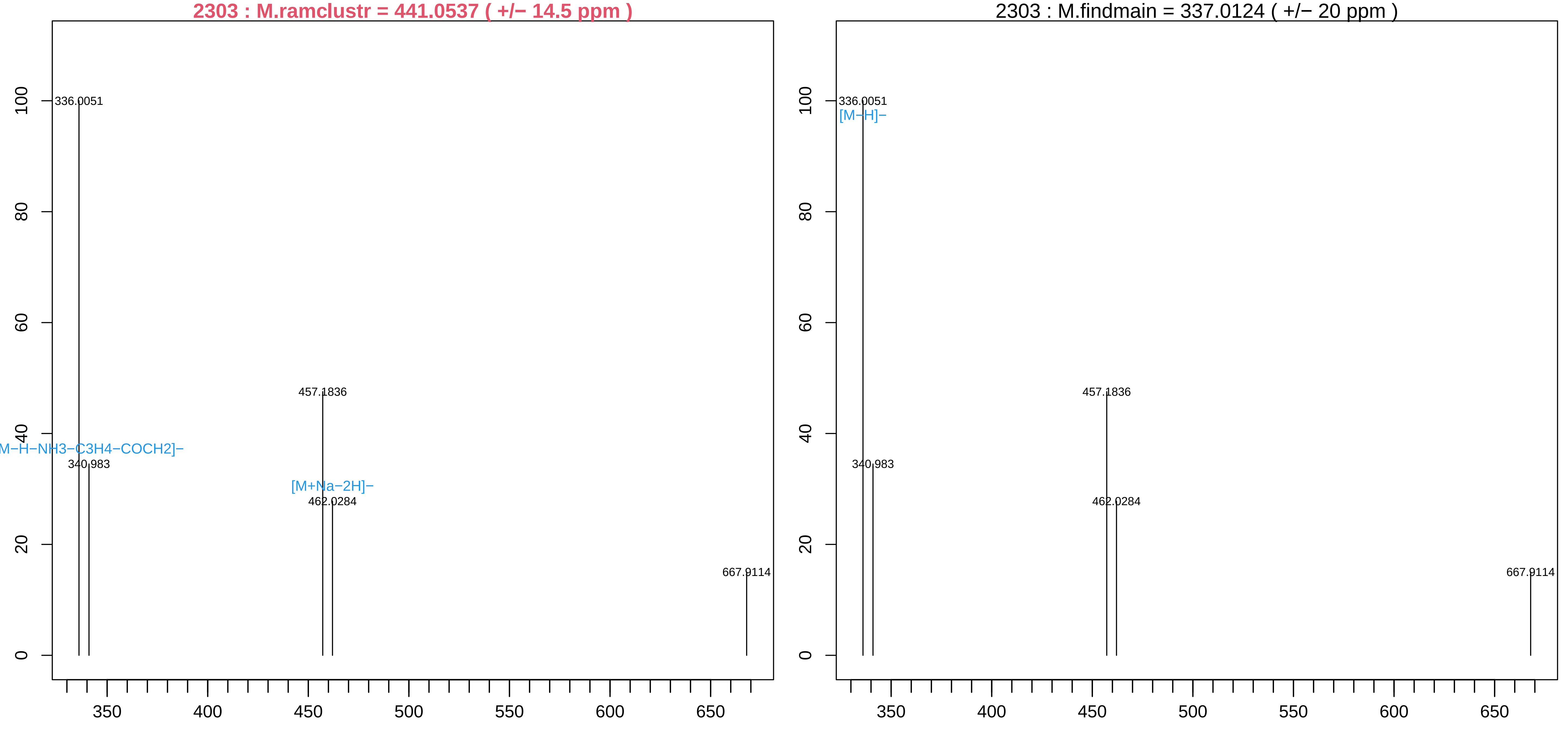
2303 : M.ramclustr = 441.0537 (+/− 14.5 ppm)
336.0051
[M−H−NH3−C3H4−COCH2]−
340.983
457.1836
[M+Na−2H]−
462.0284
667.9114
2303 : M.findmain = 337.0124 (+/− 20 ppm)
336.0051
[M−H]−
340.983
457.1836
462.0284
667.9114

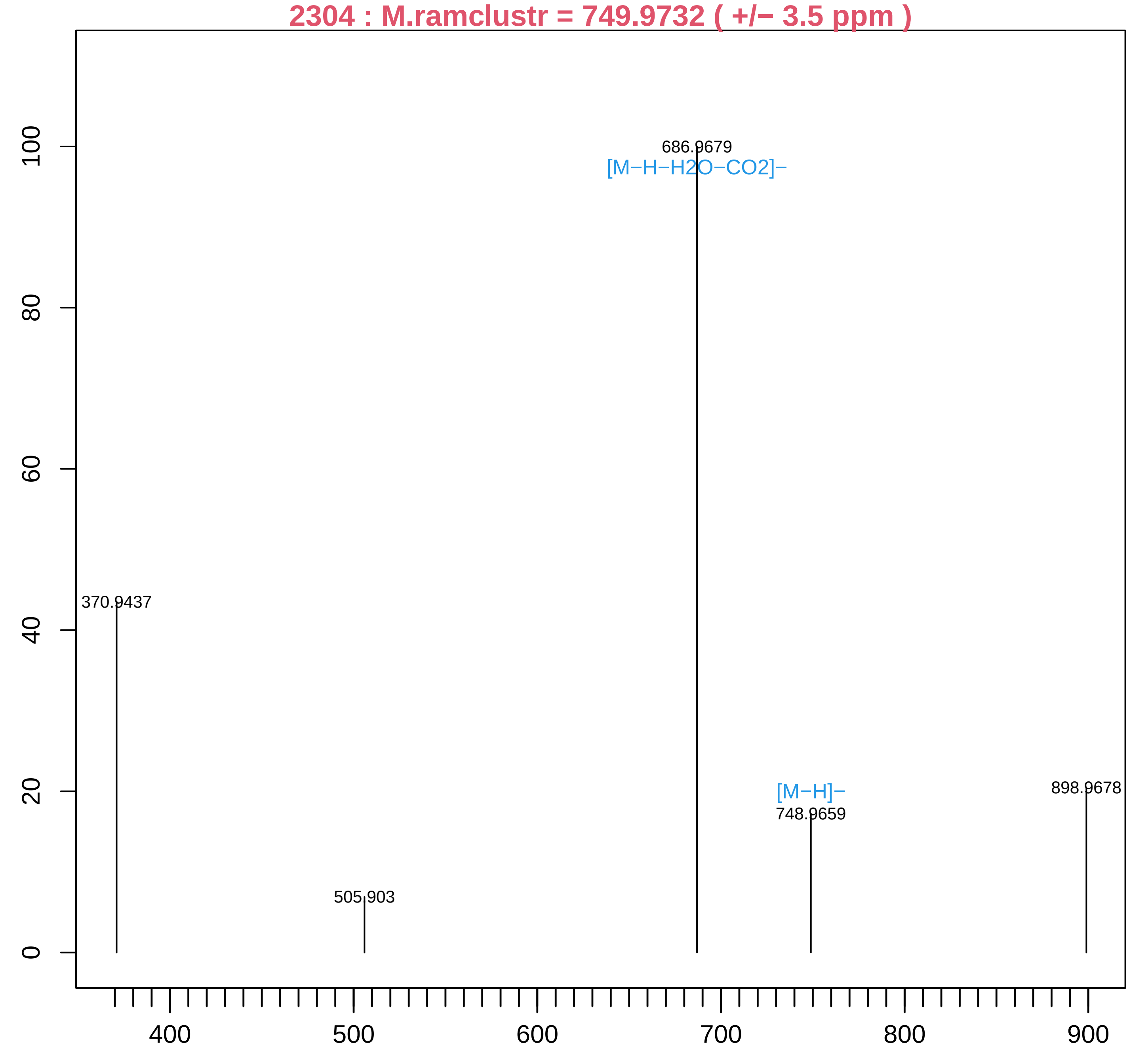

2304 : M.ramclustr = 749.9732 (+/− 3.5 ppm)
686.9679
[M−H−H2O−CO2]−
370.9437
505.903
[M−H]−
748.9659
898.9678
0
20
40
60
80
100
400
500
600
700
800
900

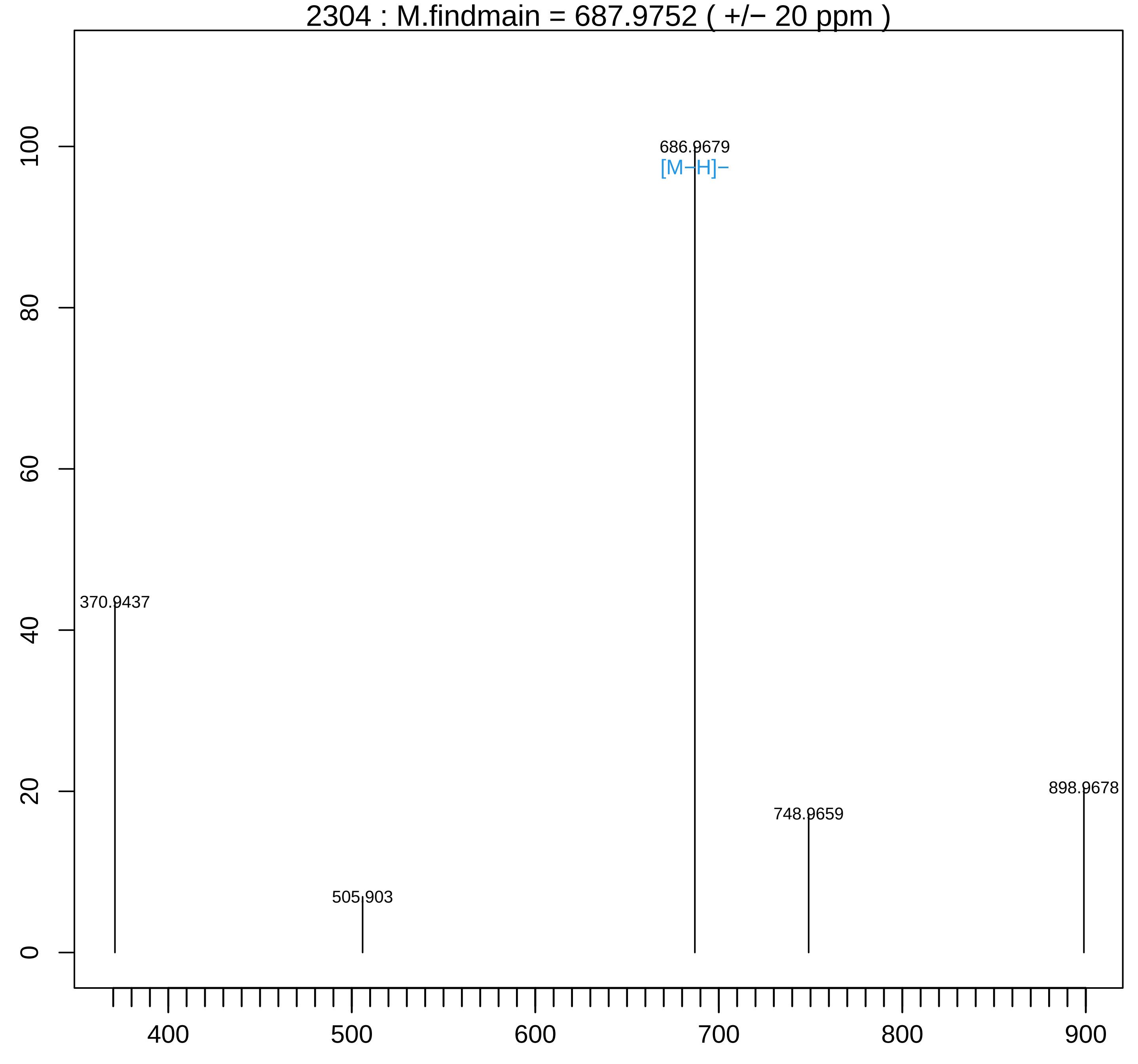

2304 : M.findmain = 687.9752 (+/− 20 ppm)
686.9679
[M−H]−
370.9437
505.903
748.9659
898.9678
0
20
40
60
80
100
400
500
600
700
800
900

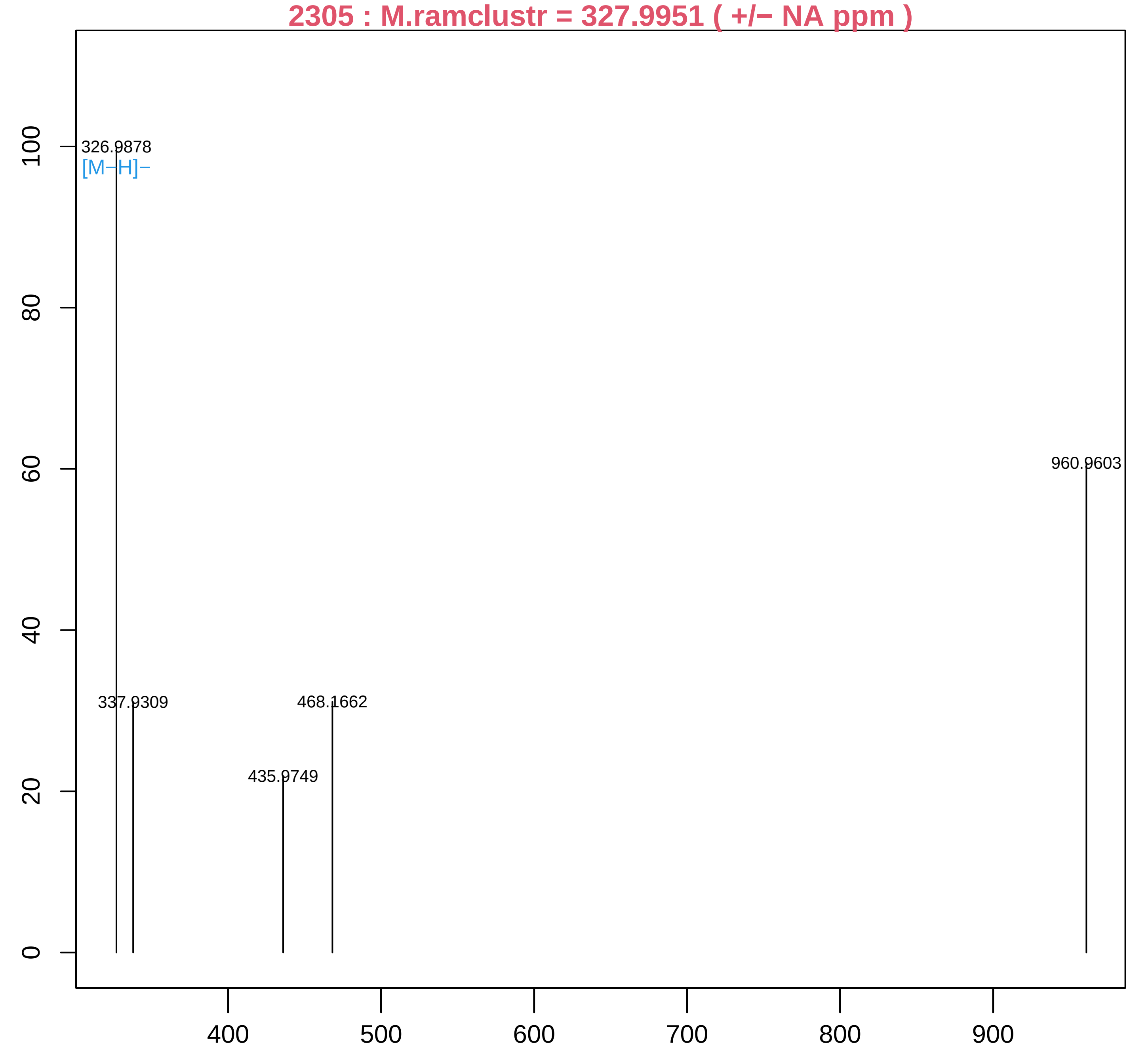
2305 : M.ramclustr = 327.9951 (+/− NA ppm)
326.9878
[M−H]−
337.9309
435.9749
468.1662
960.9603
0
20
40
60
80
100
400
500
600
700
800
900

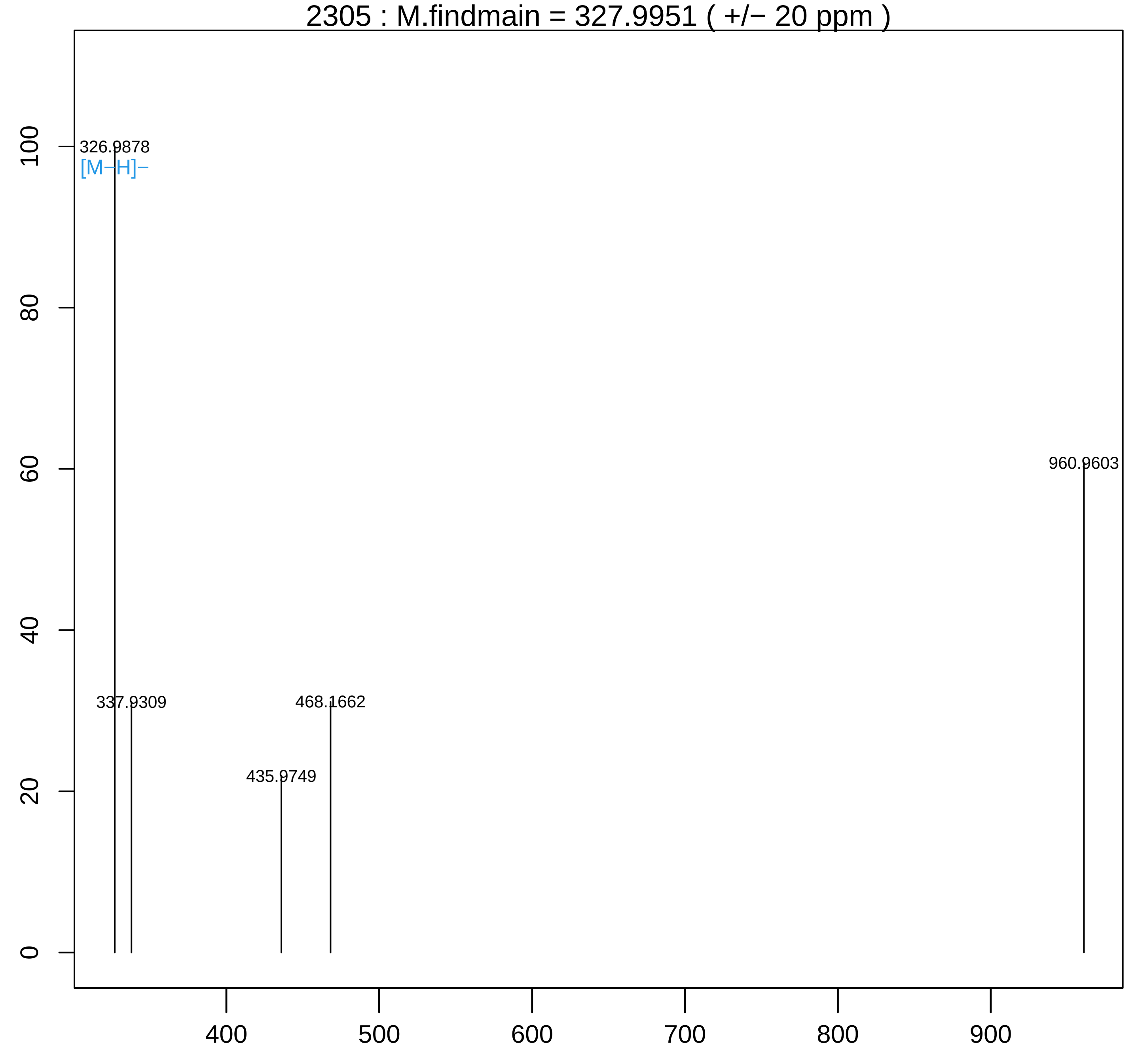
2305 : M.findmain = 327.9951 (+/− 20 ppm)
326.9878
[M−H]−
337.9309
435.9749
468.1662
960.9603
0
20
40
60
80
100
400
500
600
700
800
900

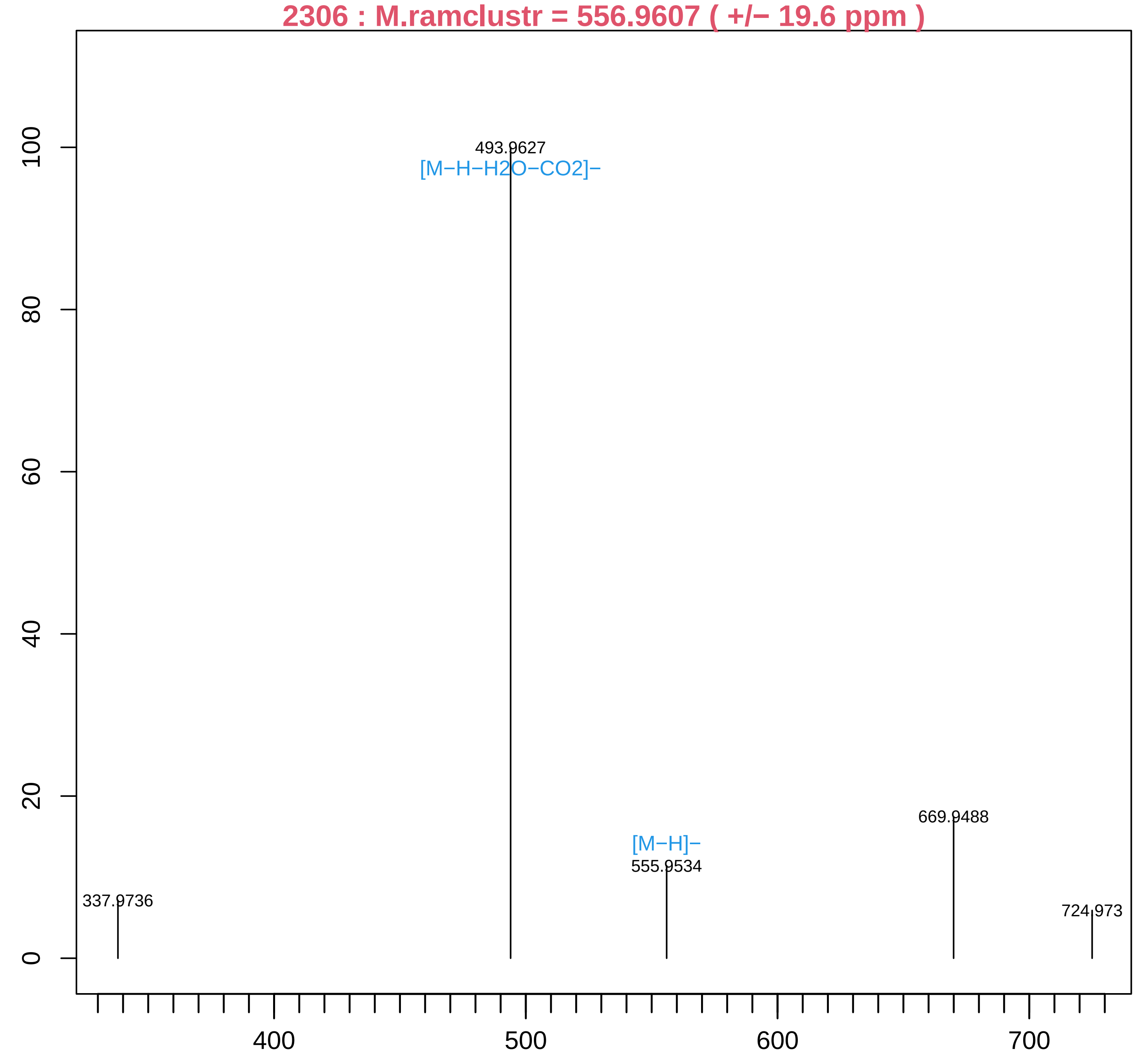
2306 : M.ramclustr = 556.9607 (+/− 19.6 ppm)
493.9627
[M−H−H2O−CO2]−
[M−H]−
555.9534
669.9488
337.9736
724.973
0
20
40
60
80
100
400
500
600
700

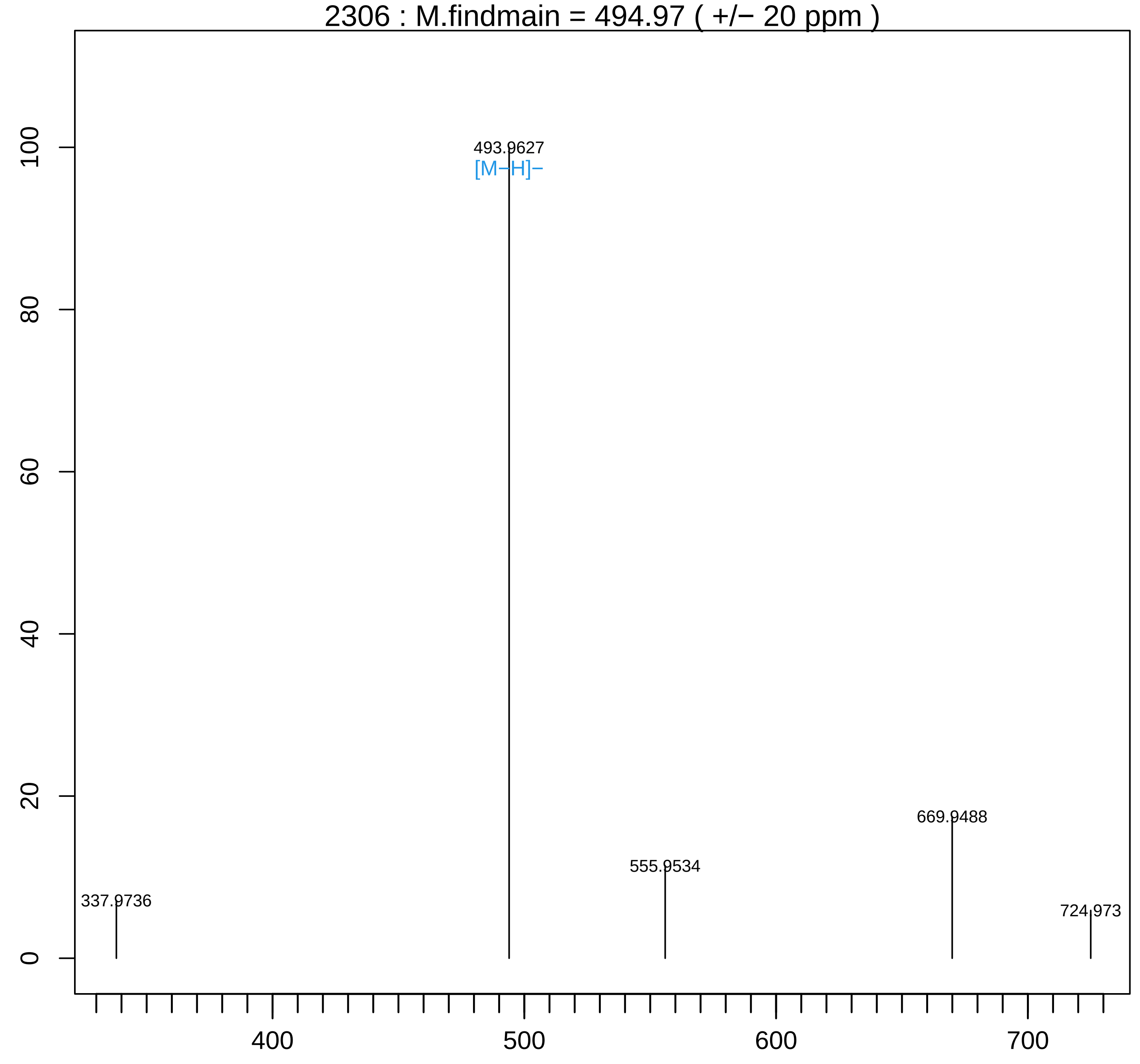
2306 : M.findmain = 494.97 (+/− 20 ppm)
493.9627
[M−H]−
555.9534
669.9488
337.9736
724.973
0
20
40
60
80
100
400
500
600
700

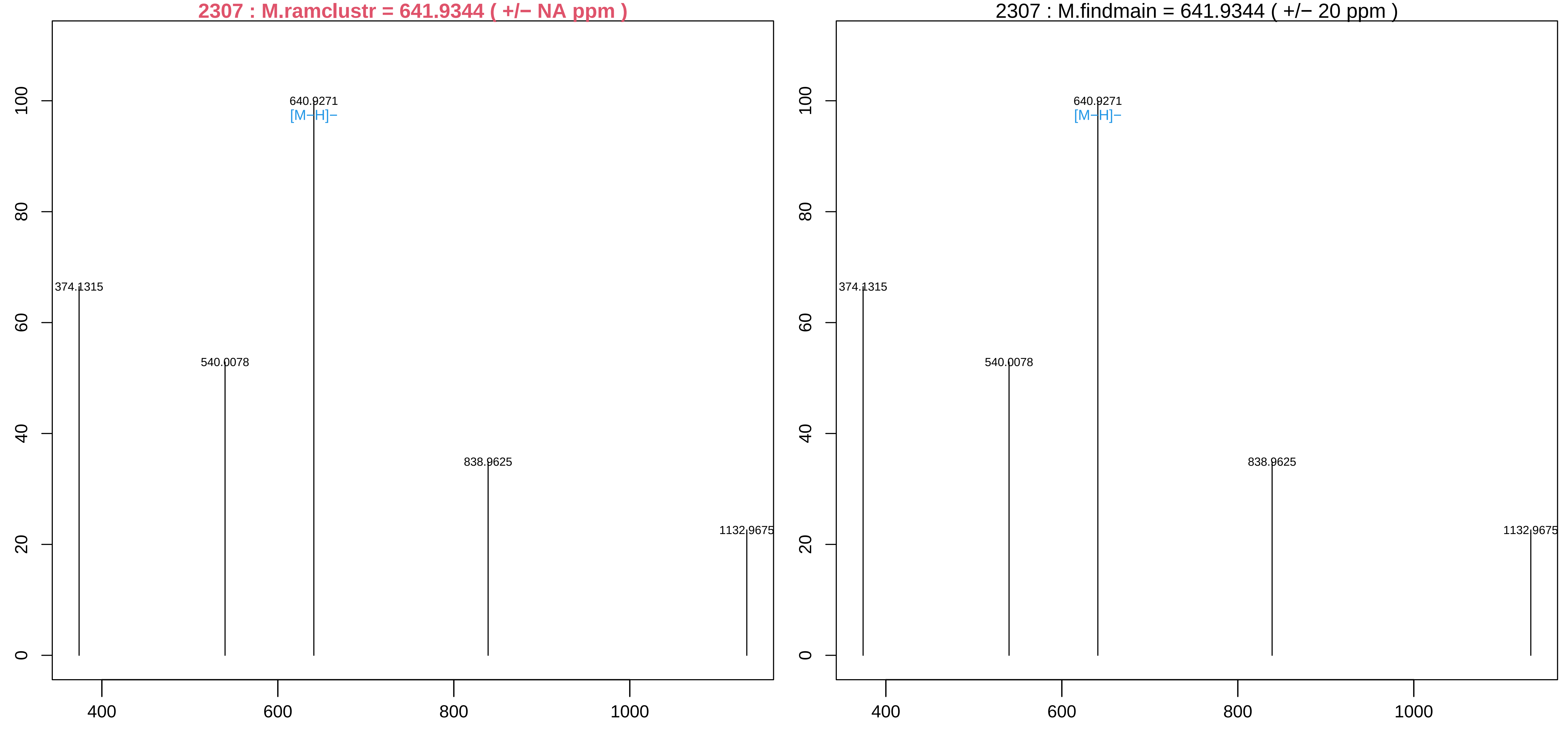
2307 : M.ramclustr = 641.9344 (+/− NA ppm)
374.1315
540.0078
640.9271
[M−H]−
838.9625
1132.9675
2307 : M.findmain = 641.9344 (+/− 20 ppm)
374.1315
540.0078
640.9271
[M−H]−
838.9625
1132.9675
0
20
40
60
80
100
400
600
800
1000

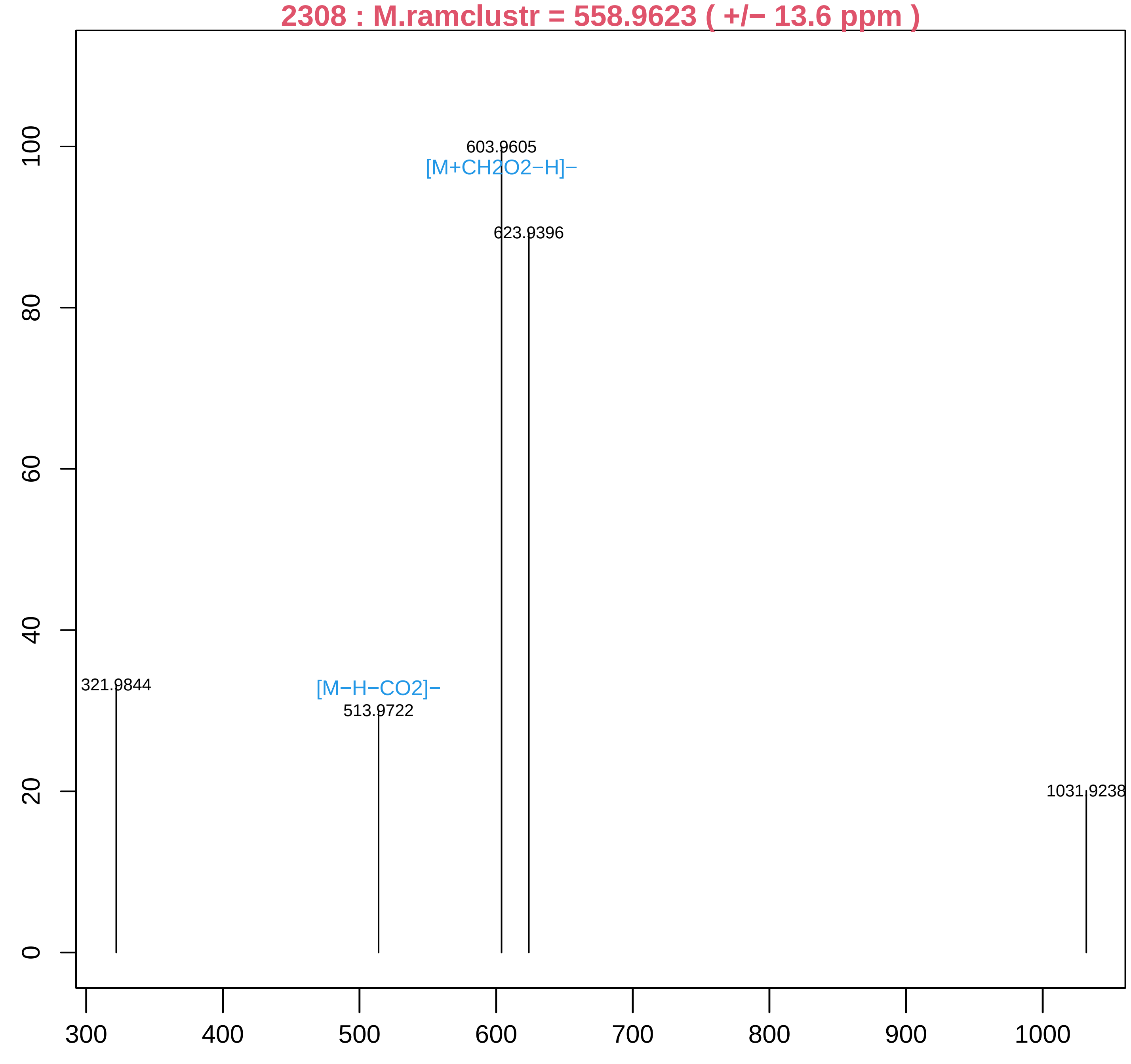

2308 : M.ramclustr = 558.9623 (+/− 13.6 ppm)
603.9605
[M+CH2O2−H]−
623.9396
321.9844
[M−H−CO2]−
513.9722
1031.9238

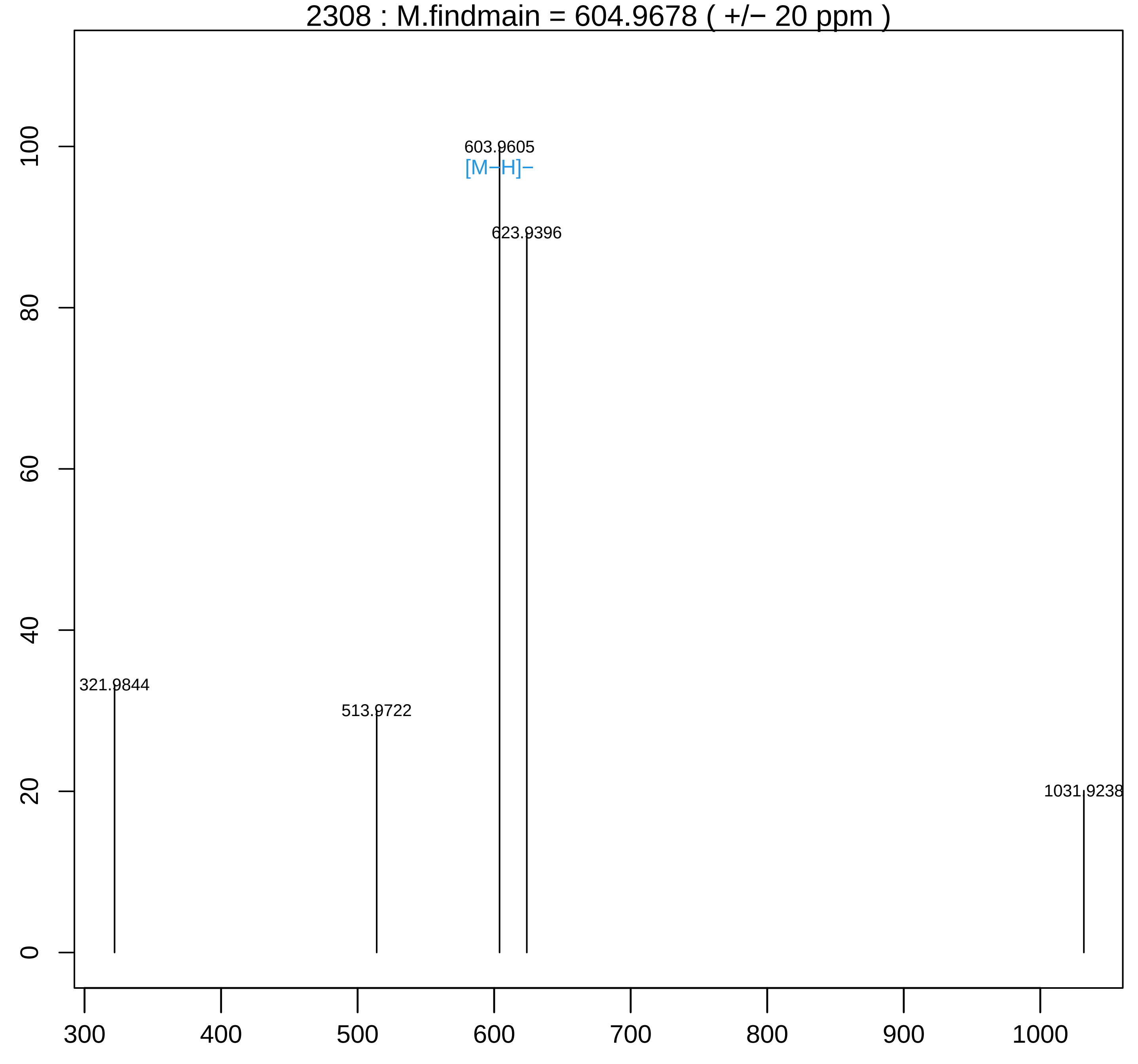

2308 : M.findmain = 604.9678 (+/− 20 ppm)
603.9605
[M−H]−
623.9396
321.9844
513.9722
1031.9238

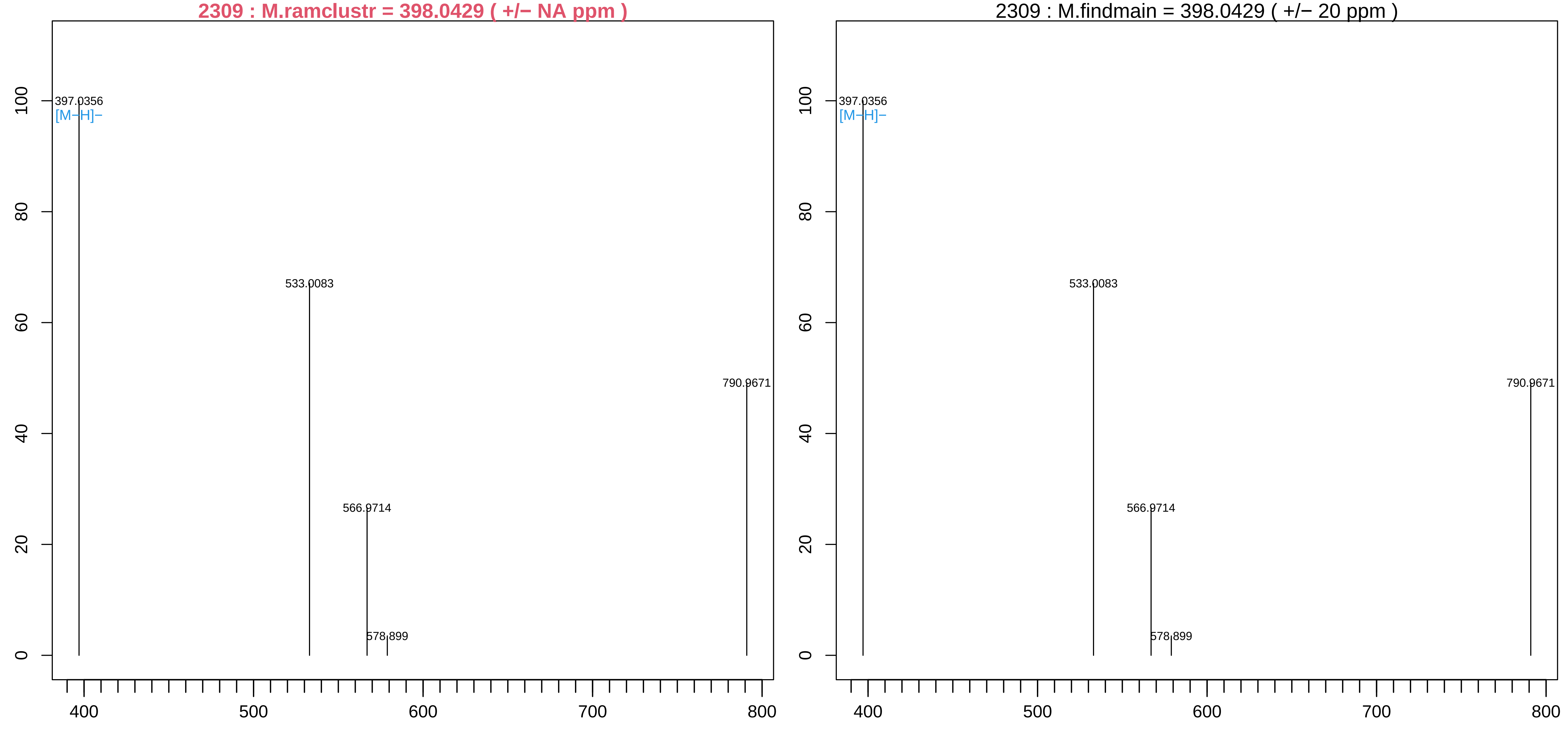
2309 : M.ramclustr = 398.0429 (+/− NA ppm)
397.0356
[M−H]−
533.0083
566.9714
578.899
790.9671
0
20
40
60
80
100
400
500
600
700
800
2309 : M.findmain = 398.0429 (+/− 20 ppm)
397.0356
[M−H]−
533.0083
566.9714
578.899
790.9671

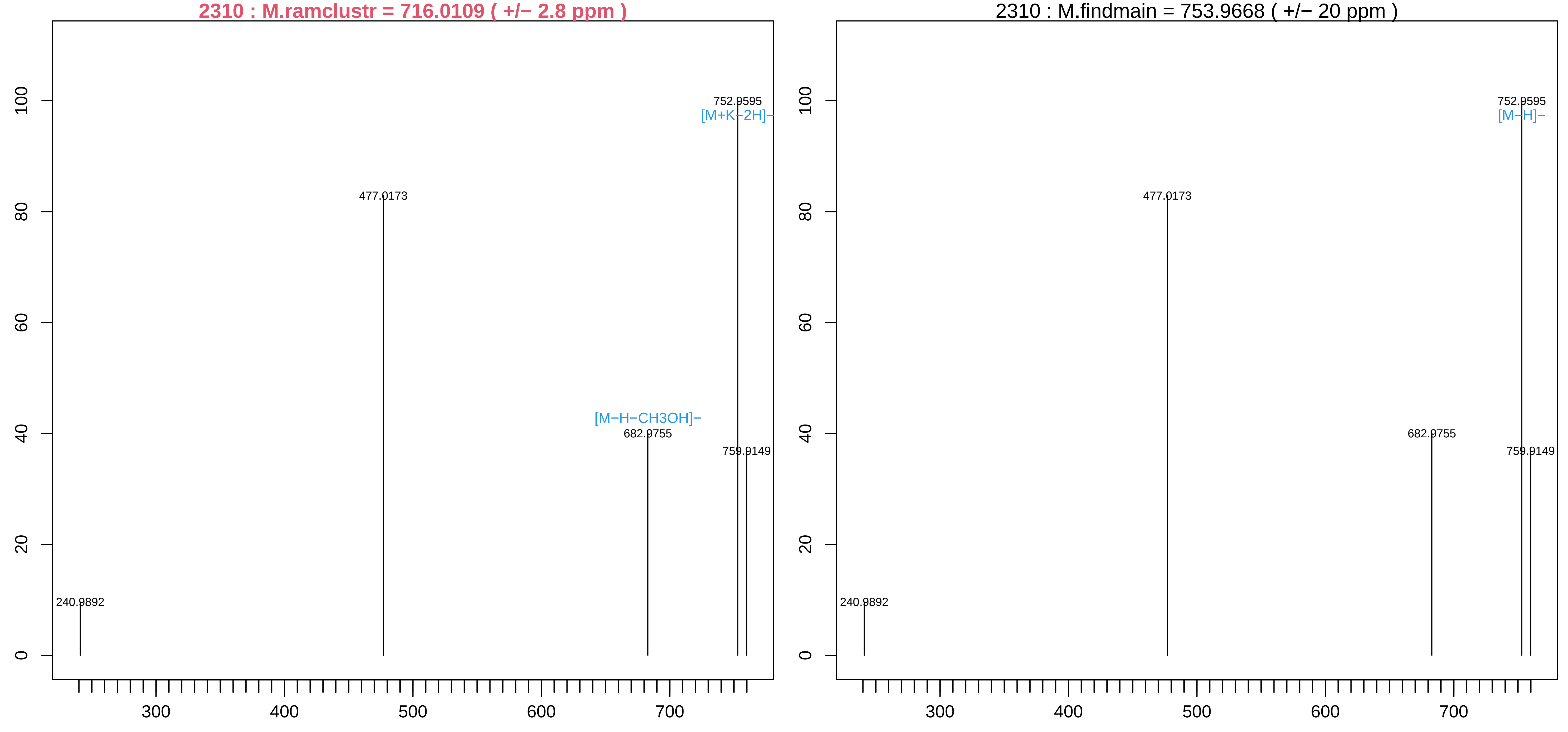
2310 : M.ramclustr = 716.0109 (+/− 2.8 ppm)
752.9595
[M+K−2H]−
477.0173
[M−H−CH3OH]−
682.9755
759.9149
240.9892
0
20
40
60
80
100
300
400
500
600
700
2310 : M.findmain = 753.9668 (+/− 20 ppm)
752.9595
[M−H]−
477.0173
682.9755
759.9149
240.9892

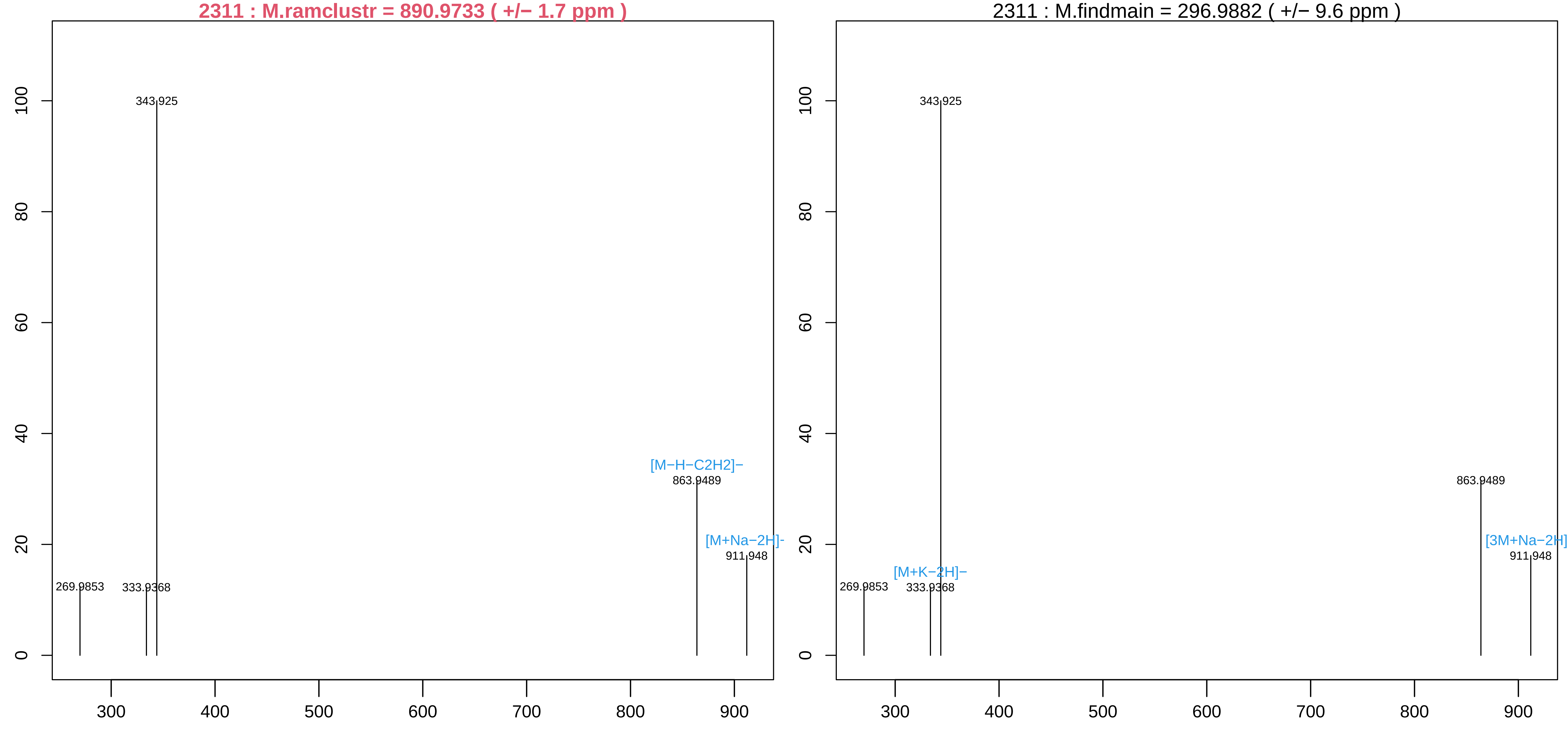

2311 : M.ramclustr = 890.9733 (+/− 1.7 ppm)
343.925
[M−H−C2H2]−
863.9489
[M+Na−2H]-
911.948
269.9853
333.9368
0
20
40
60
80
100
300
400
500
600
700
800
900
2311 : M.findmain = 296.9882 (+/− 9.6 ppm)
343.925
863.9489
[3M+Na−2H]
911.948
[M+K−2H]−
269.9853
333.9368
0
20
40
60
80
100
300
400
500
600
700
800
900

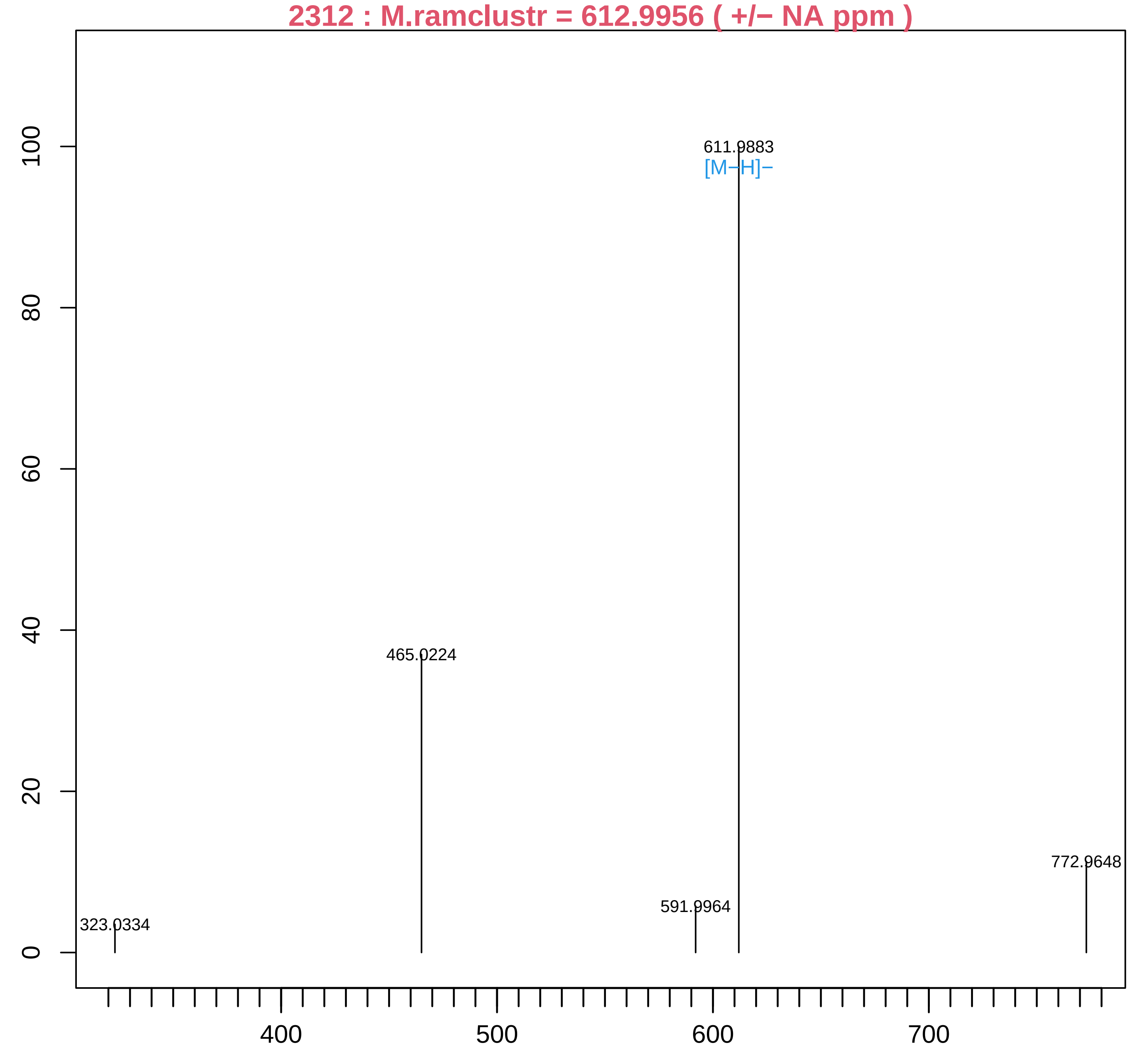
2312 : M.ramclustr = 612.9956 (+/− NA ppm)
611.9883
[M−H]−
465.0224
591.9964
772.9648
323.0334
0
20
40
60
80
100
400
500
600
700

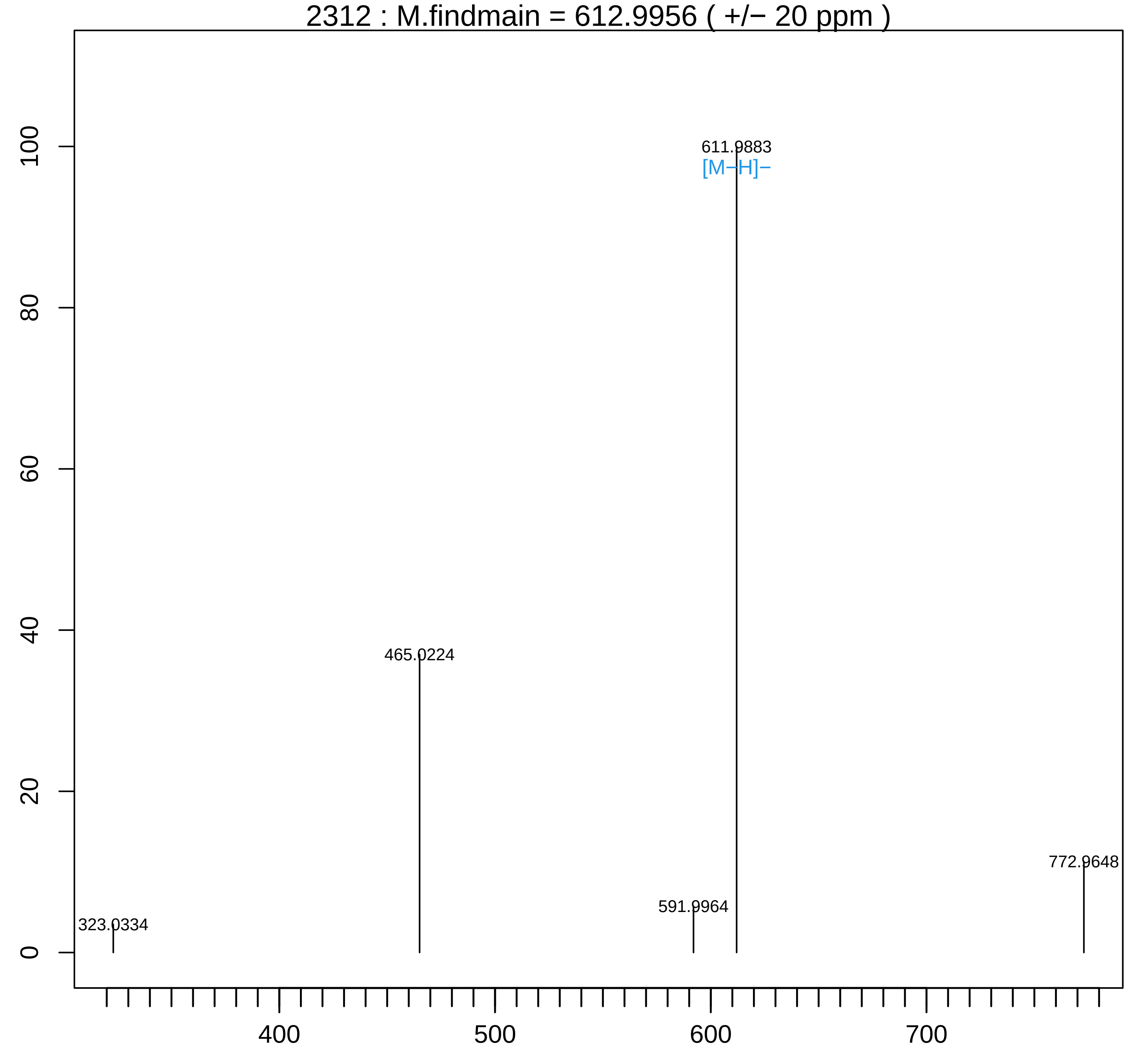
2312 : M.findmain = 612.9956 (+/− 20 ppm)
611.9883
[M−H]−
465.0224
591.9964
772.9648
323.0334
0
20
40
60
80
100
400
500
600
700

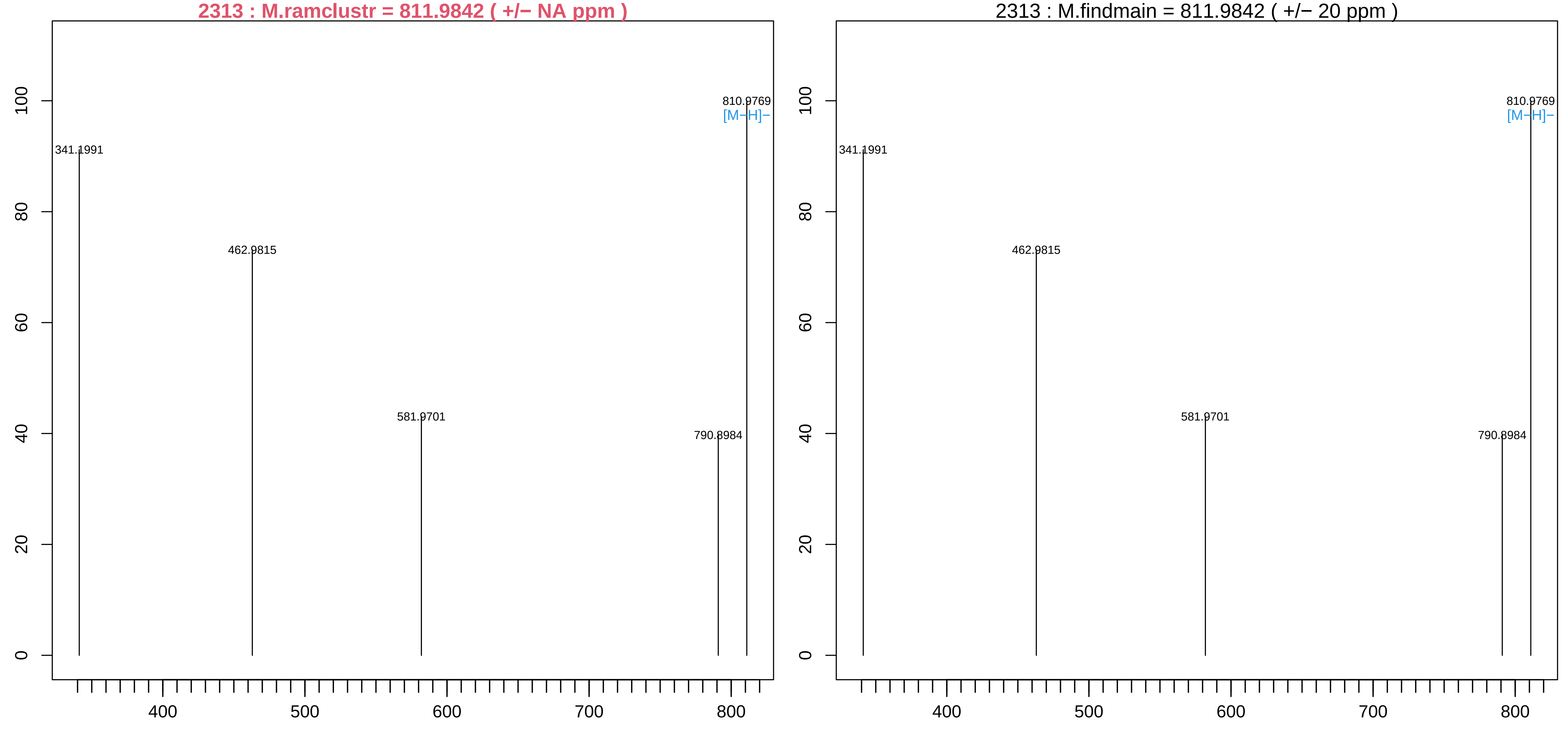

2313 : M.ramclustr = 811.9842 (+/− NA ppm)
2313 : M.findmain = 811.9842 (+/− 20 ppm)
810.9769
[M−H]−
341.1991
462.9815
581.9701
790.8984
0
20
40
60
80
100
400
500
600
700
800

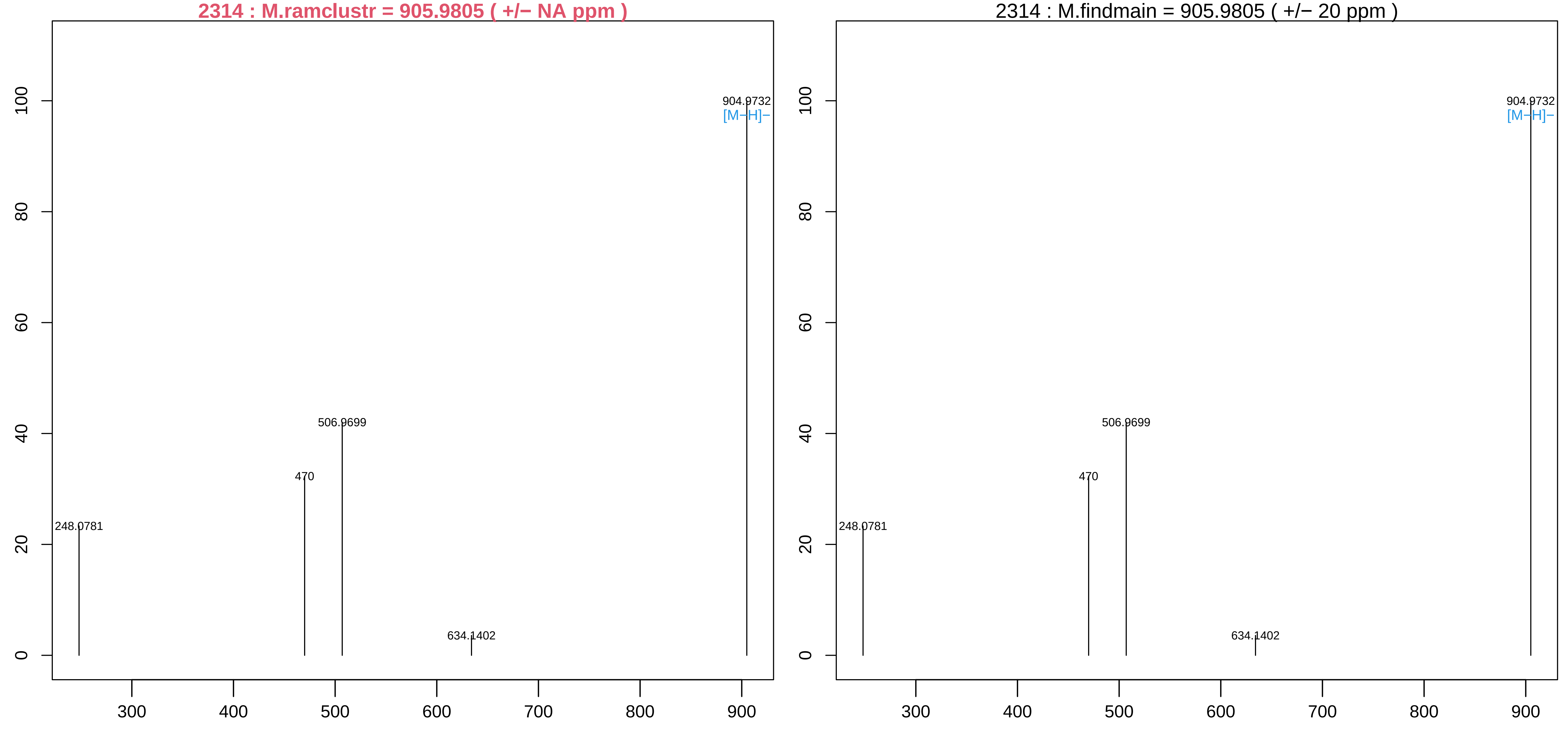

2314 : M.ramclustr = 905.9805 (+/− NA ppm)
2314 : M.findmain = 905.9805 (+/− 20 ppm)
904.9732
[M−H]−
506.9699
470
248.0781
634.1402
0
20
40
60
80
100
300
400
500
600
700
800
900

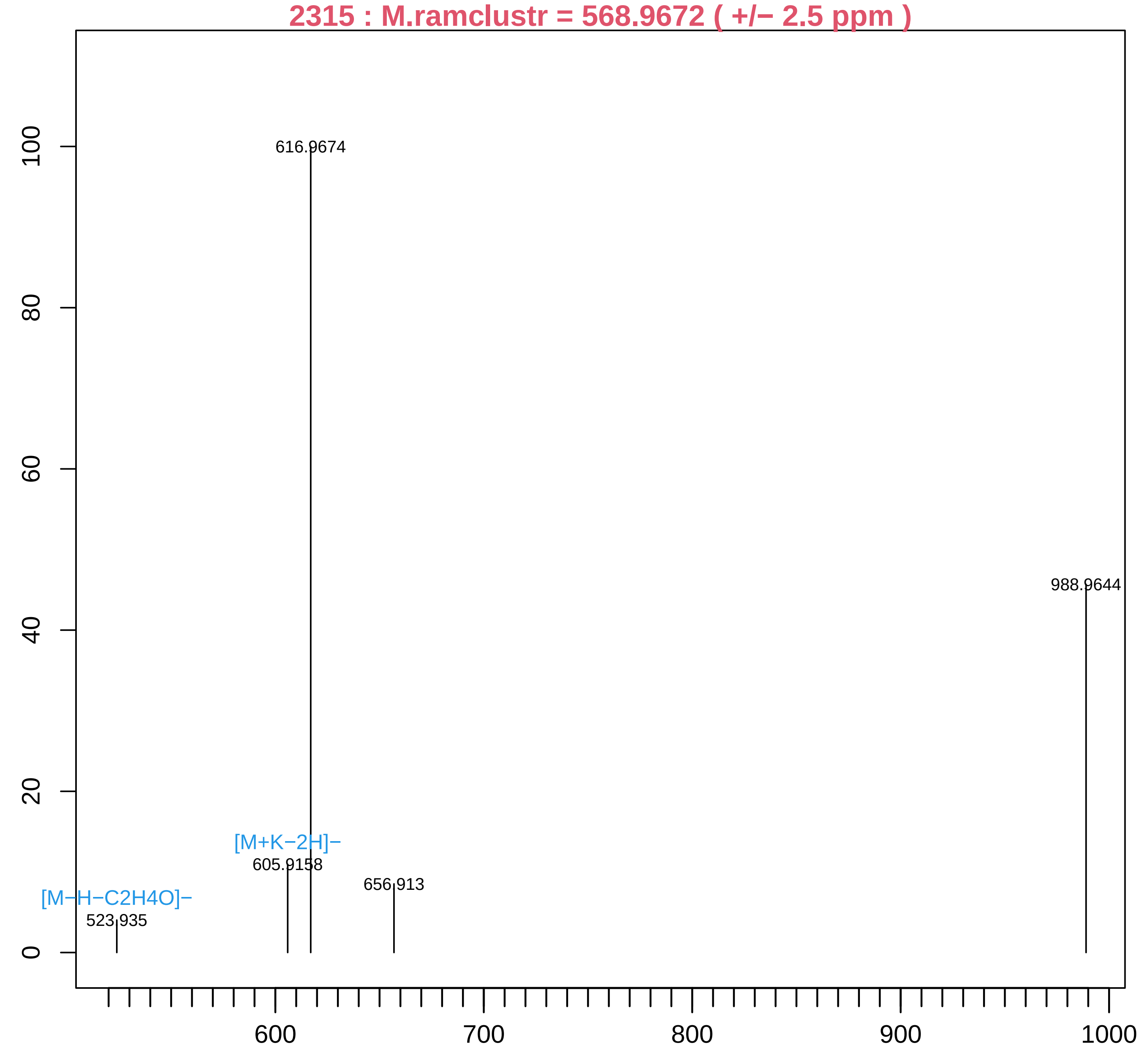
2315 : M.ramclustr = 568.9672 (+/− 2.5 ppm)
616.9674
988.9644
[M+K−2H]−
605.9158
656.913
[M−H−C2H4O]−
523.935
0
20
40
60
80
100
600
700
800
900
1000

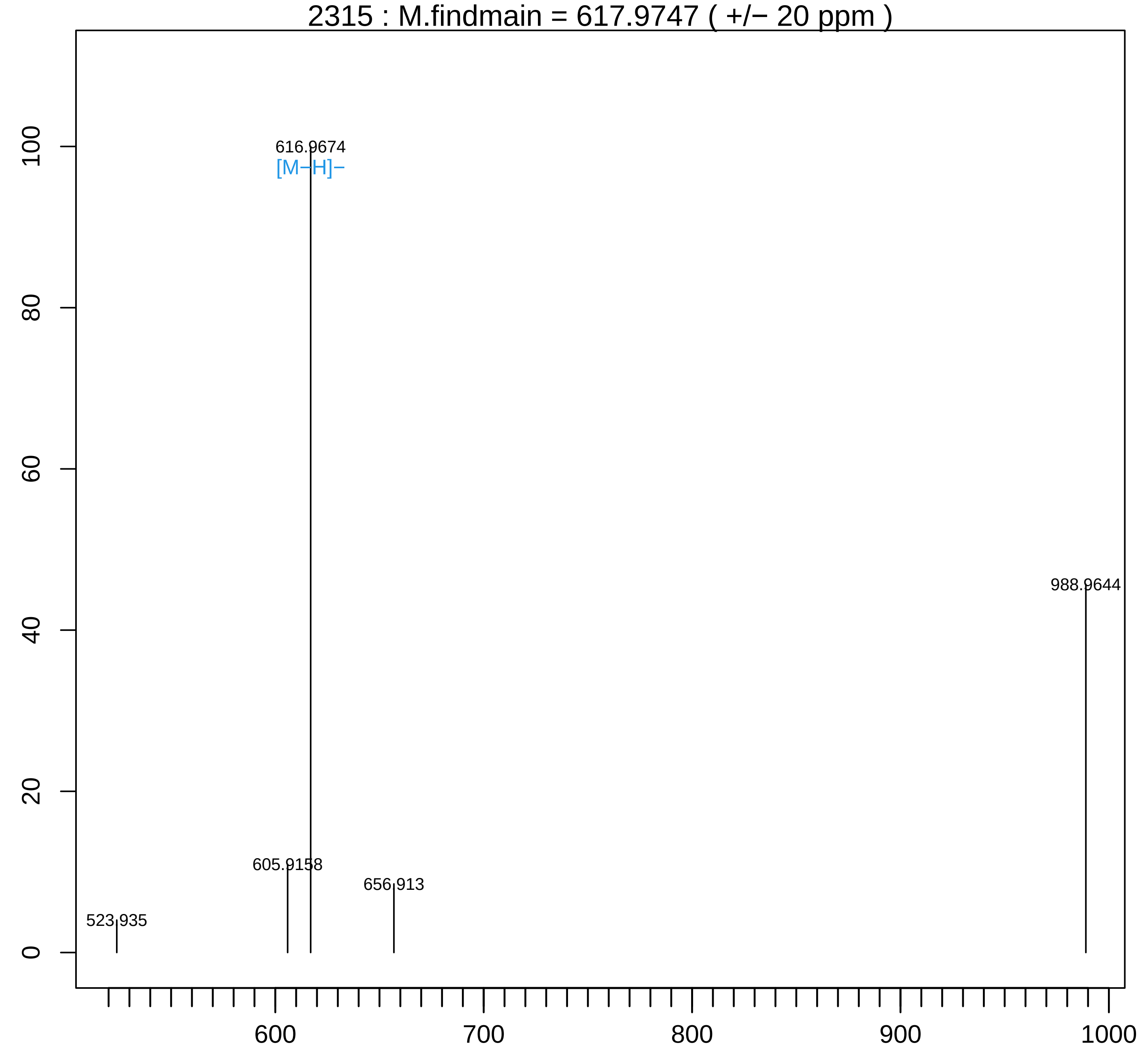
2315 : M.findmain = 617.9747 (+/− 20 ppm)
616.9674
[M−H]−
988.9644
605.9158
656.913
523.935
0
20
40
60
80
100
600
700
800
900
1000

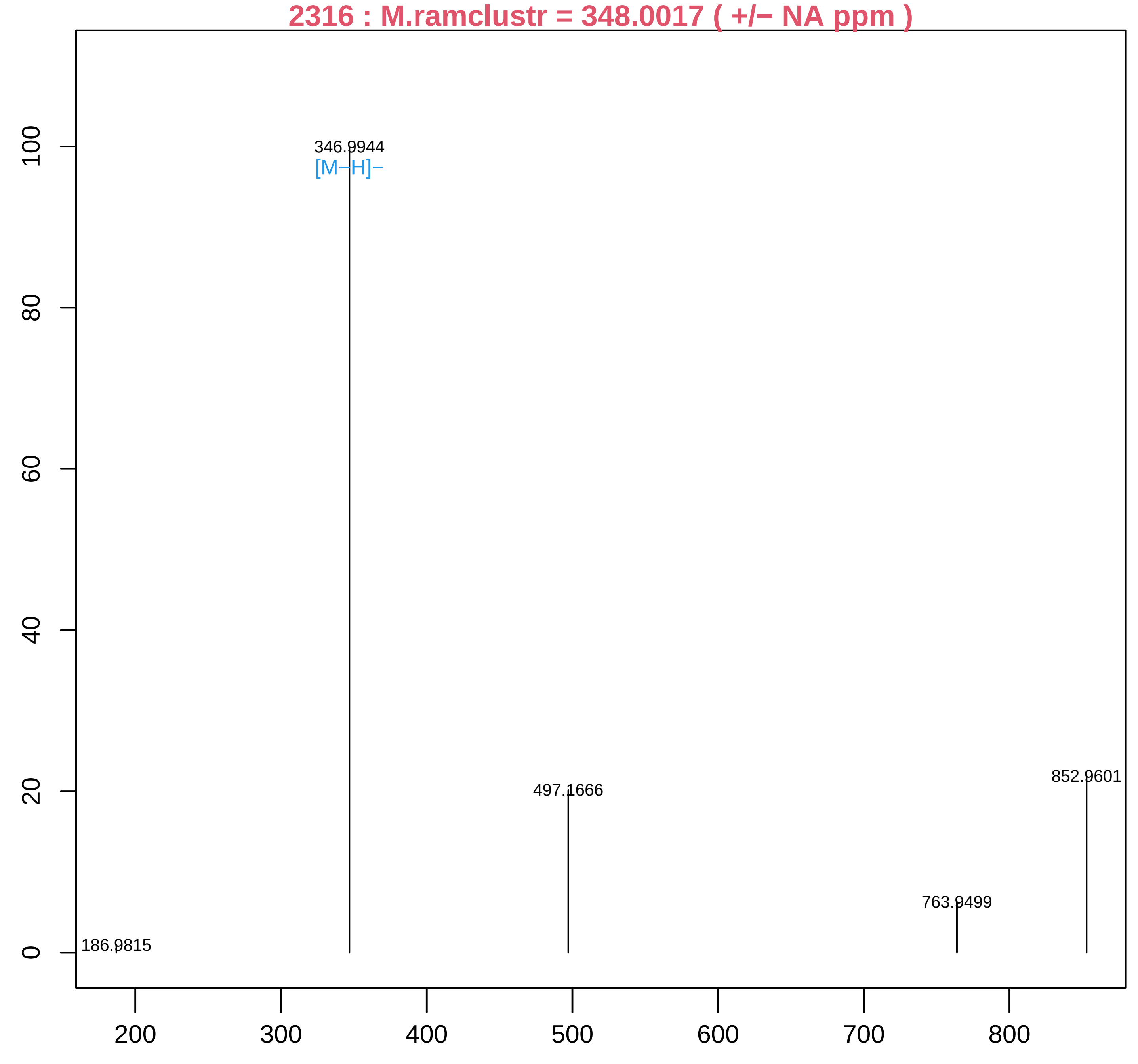

2316 : M.ramclustr = 348.0017 (+/− NA ppm)
346.9944
[M−H]−
497.1666
852.9601
763.9499
186.9815
0
20
40
60
80
100
200
300
400
500
600
700
800

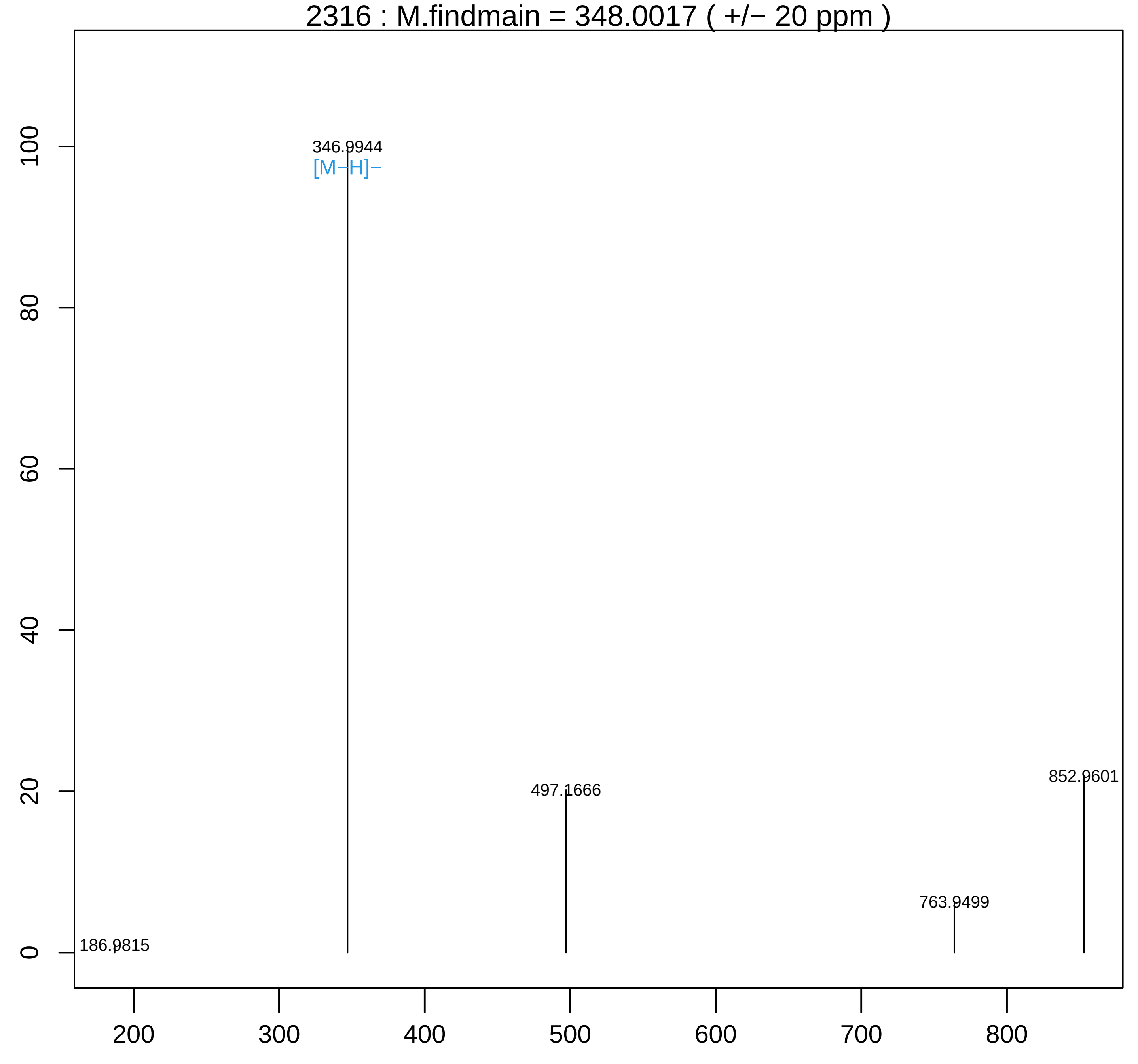

2316 : M.findmain = 348.0017 (+/− 20 ppm)
346.9944
[M−H]−
497.1666
852.9601
763.9499
186.9815
0
20
40
60
80
100
200
300
400
500
600
700
800

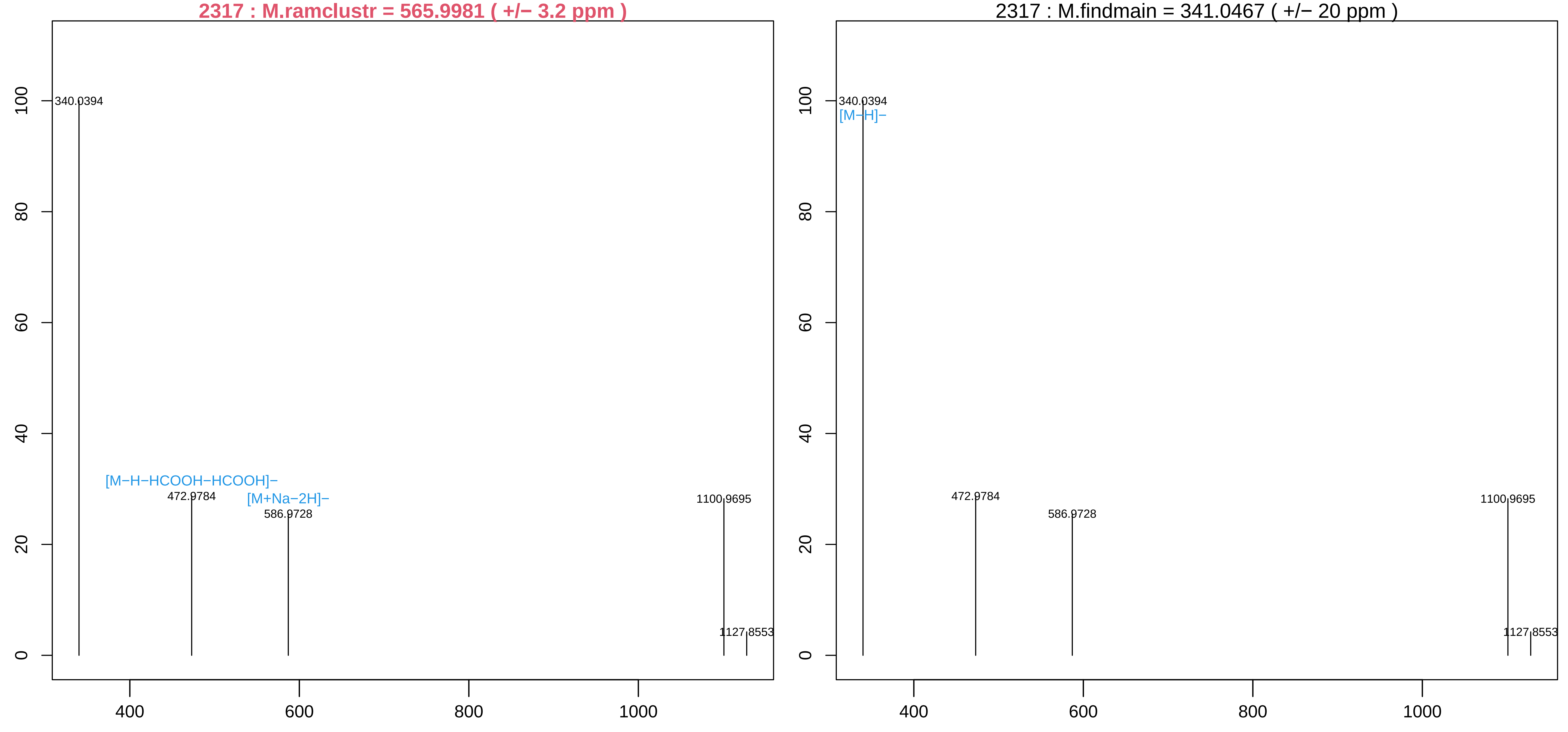
2317 : M.ramclustr = 565.9981 (+/− 3.2 ppm)
340.0394
[M−H−HCOOH−HCOOH]−
472.9784
[M+Na−2H]−
586.9728
1100.9695
1127.8553
0
20
40
60
80
100
400
600
800
1000
2317 : M.findmain = 341.0467 (+/− 20 ppm)
340.0394
[M−H]−
472.9784
586.9728
1100.9695
1127.8553

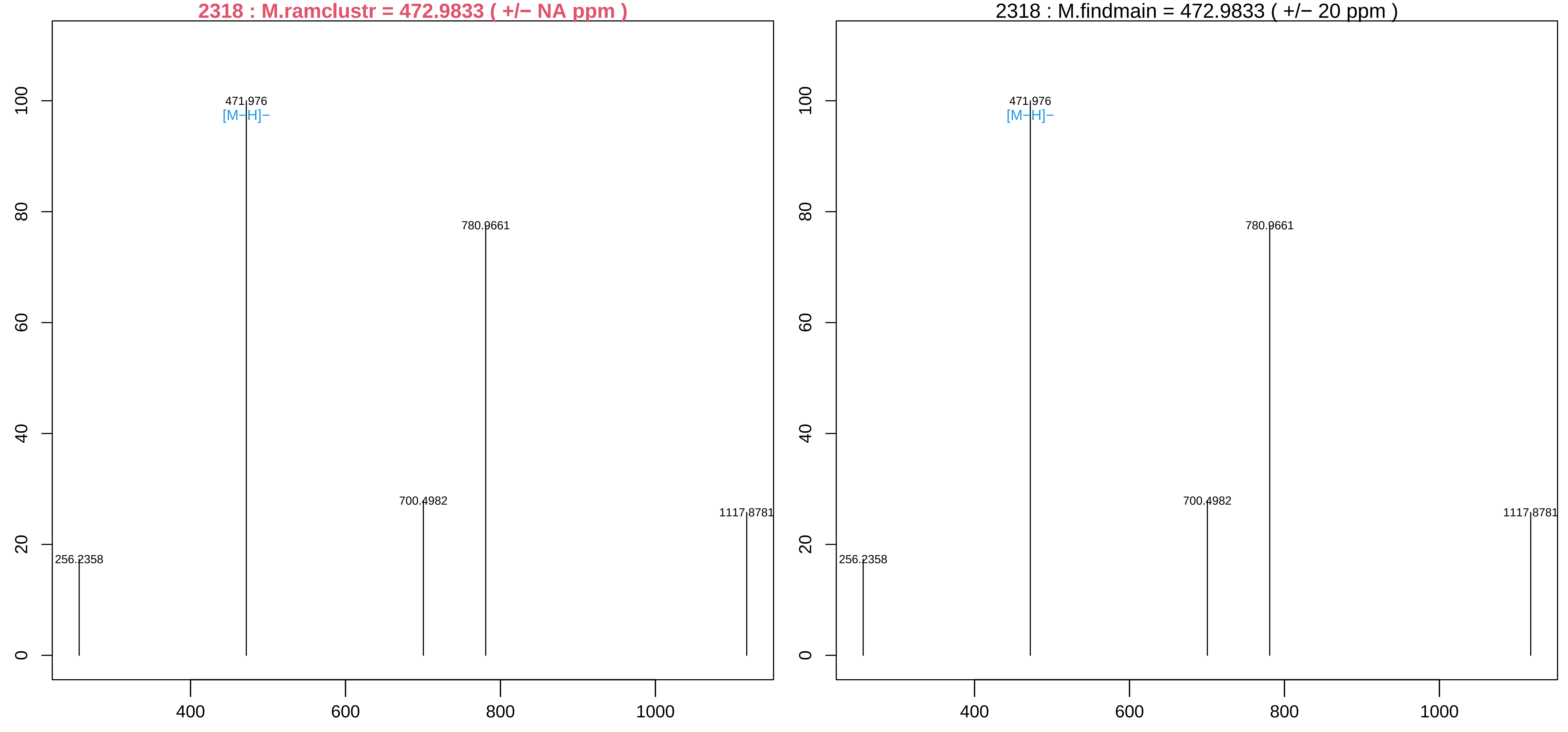

2318 : M.ramclustr = 472.9833 (+/− NA ppm)
2318 : M.findmain = 472.9833 (+/− 20 ppm)
471.976
[M−H]−
780.9661
700.4982
1117.8781
256.2358
0
20
40
60
80
100
400
600
800
1000

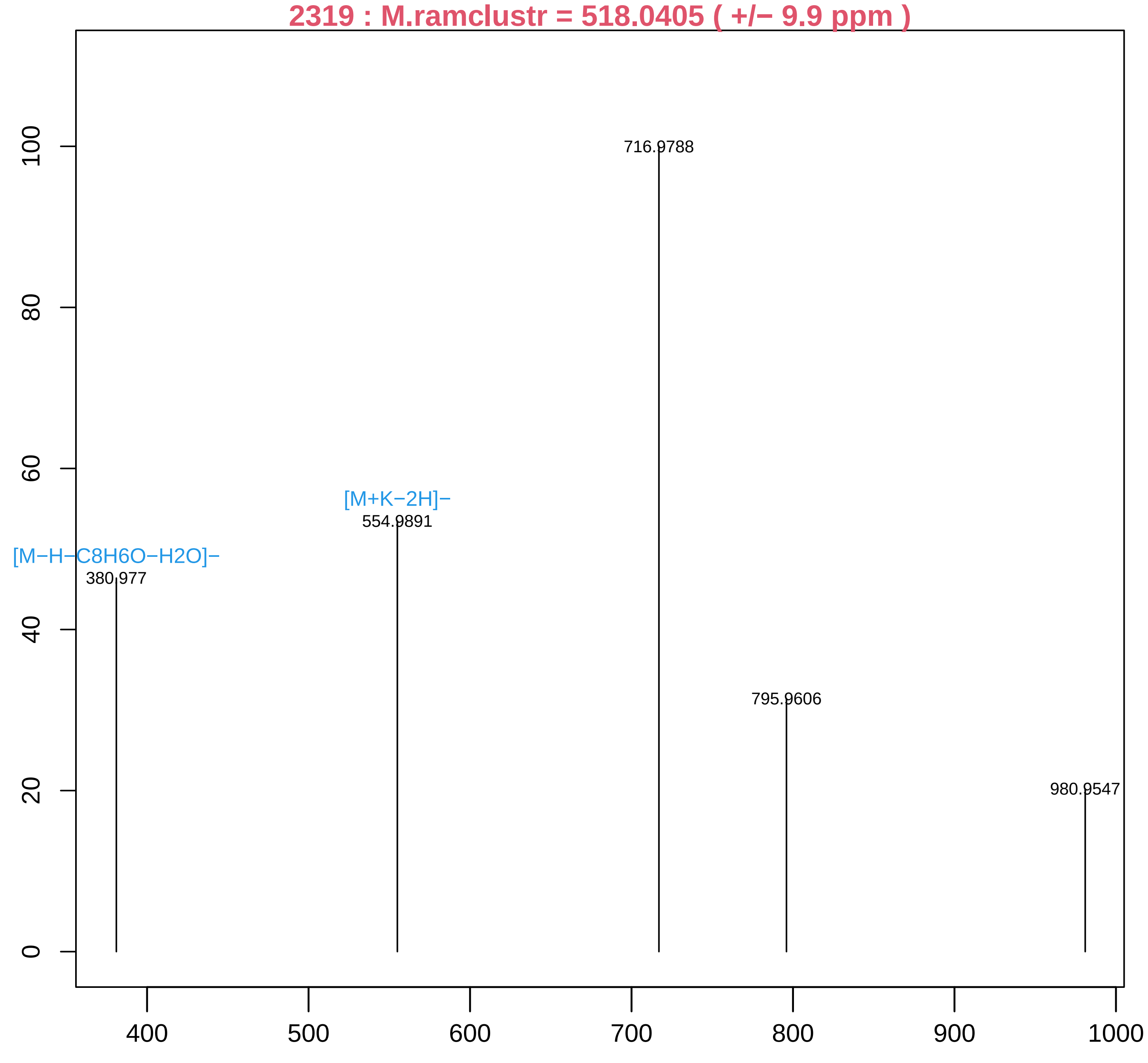

2319 : M.ramclustr = 518.0405 (+/− 9.9 ppm)
716.9788
[M+K−2H]−
554.9891
[M−H−C8H6O−H2O]−
380.977
795.9606
980.9547
0
20
40
60
80
100
400
500
600
700
800
900
1000

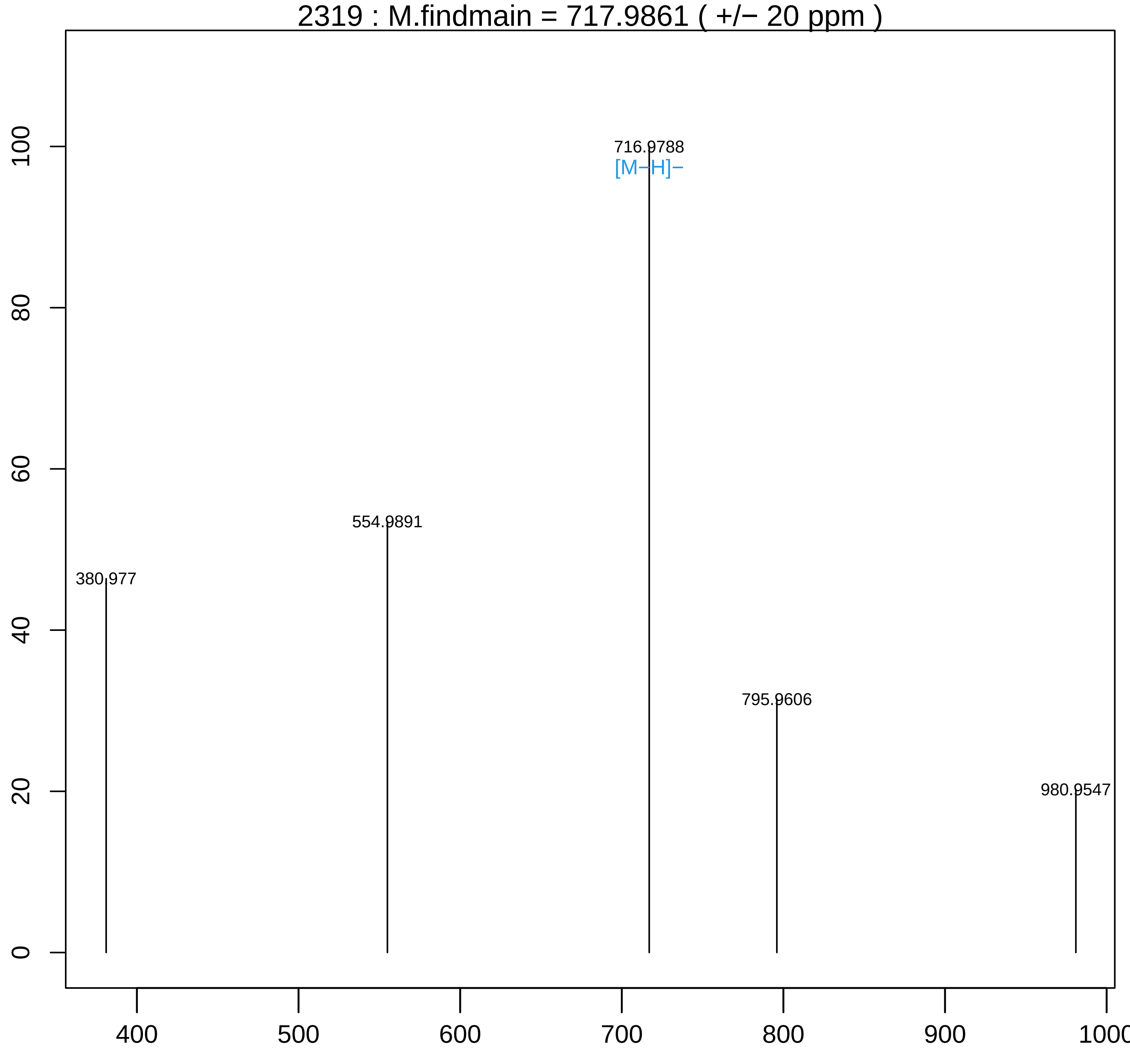

2319 : M.findmain = 717.9861 (+/− 20 ppm)
716.9788
[M−H]−
554.9891
380.977
795.9606
980.9547
0
20
40
60
80
100
400
500
600
700
800
900
1000

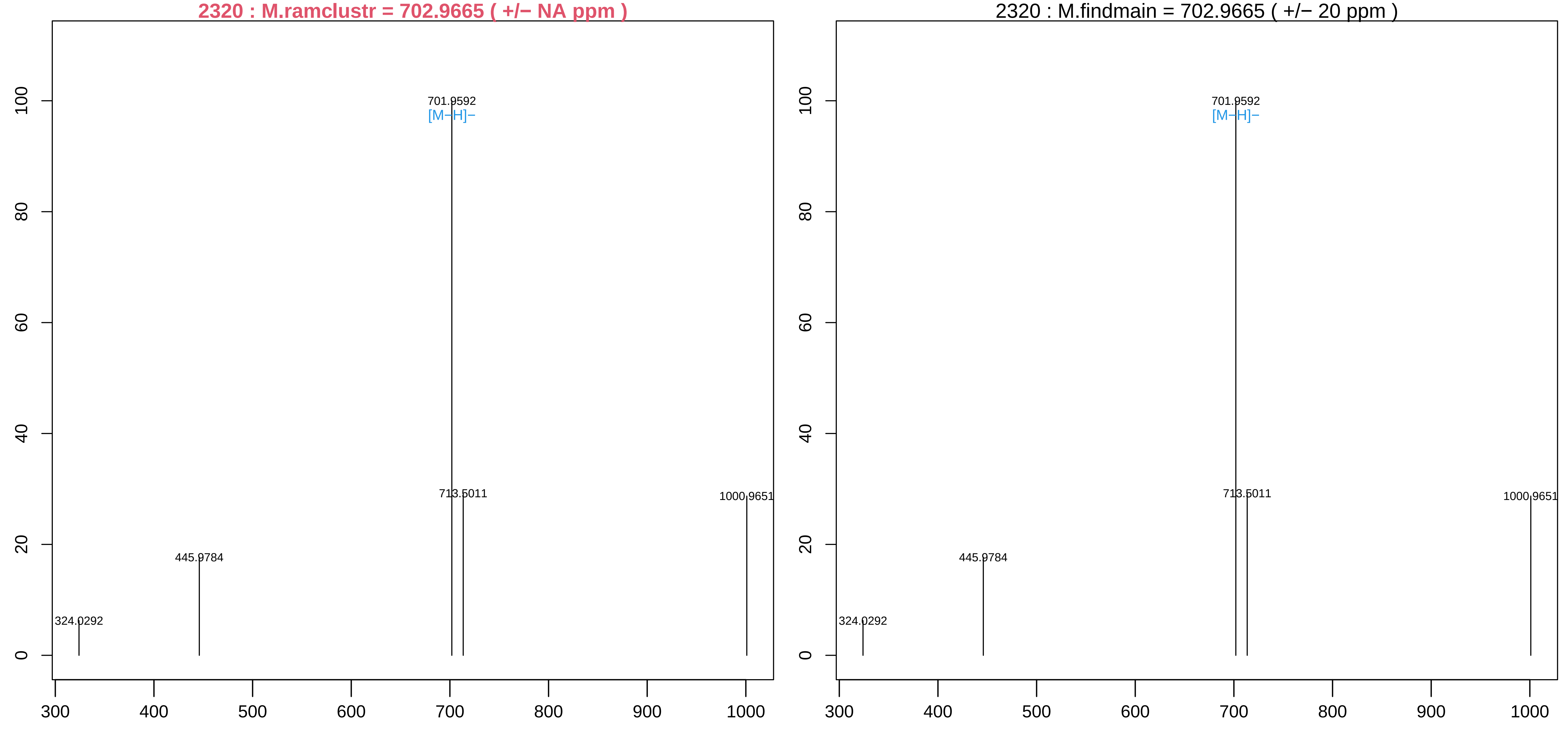
2320 : M.ramclustr = 702.9665 (+/− NA ppm)
2320 : M.findmain = 702.9665 (+/− 20 ppm)
701.9592
[M−H]−
713.5011
1000.9651
445.9784
324.0292
0
20
40
60
80
100
300
400
500
600
700
800
900
1000

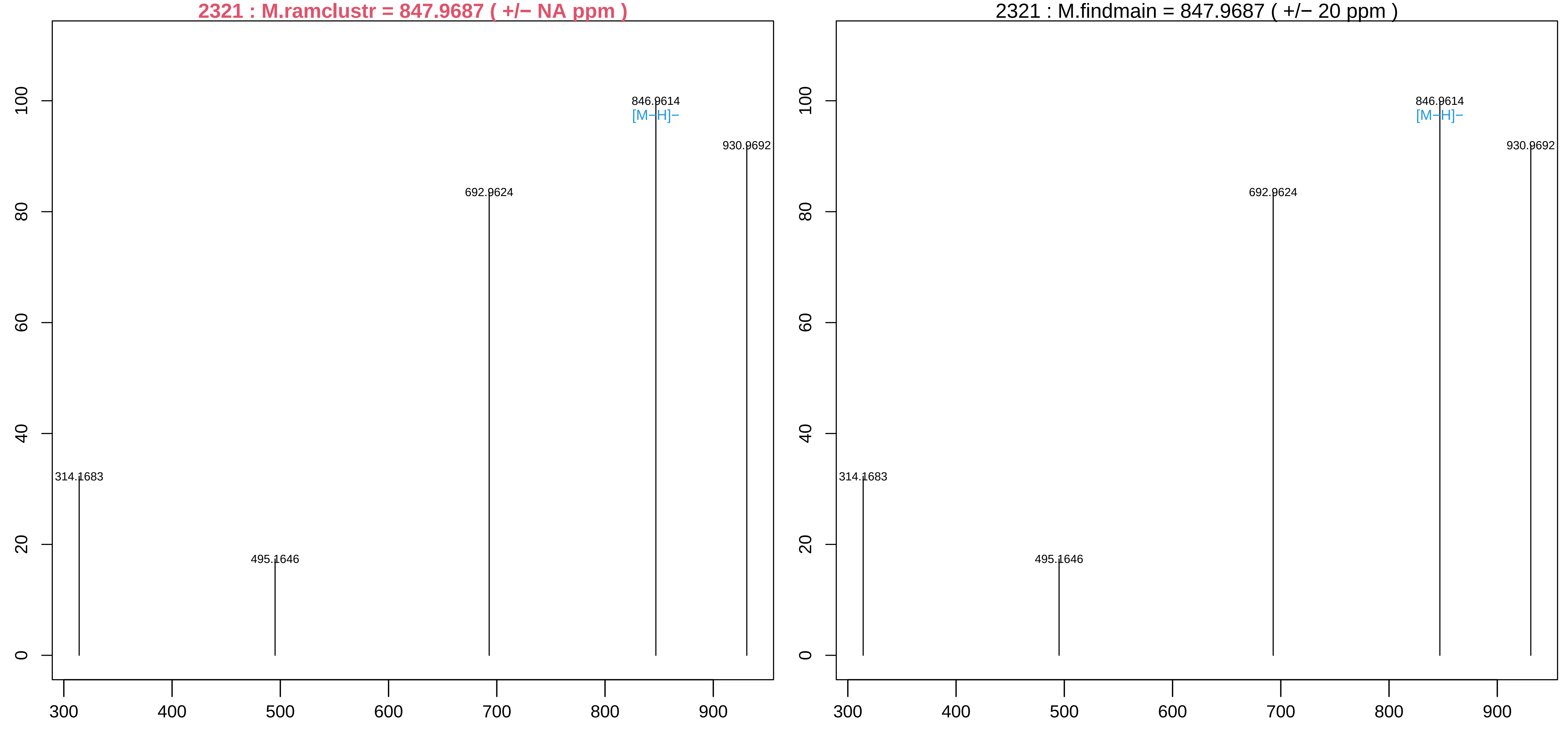
2321 : M.ramclustr = 847.9687 (+/− NA ppm)
2321 : M.findmain = 847.9687 (+/− 20 ppm)
314.1683
495.1646
692.9624
846.9614
[M−H]−
930.9692

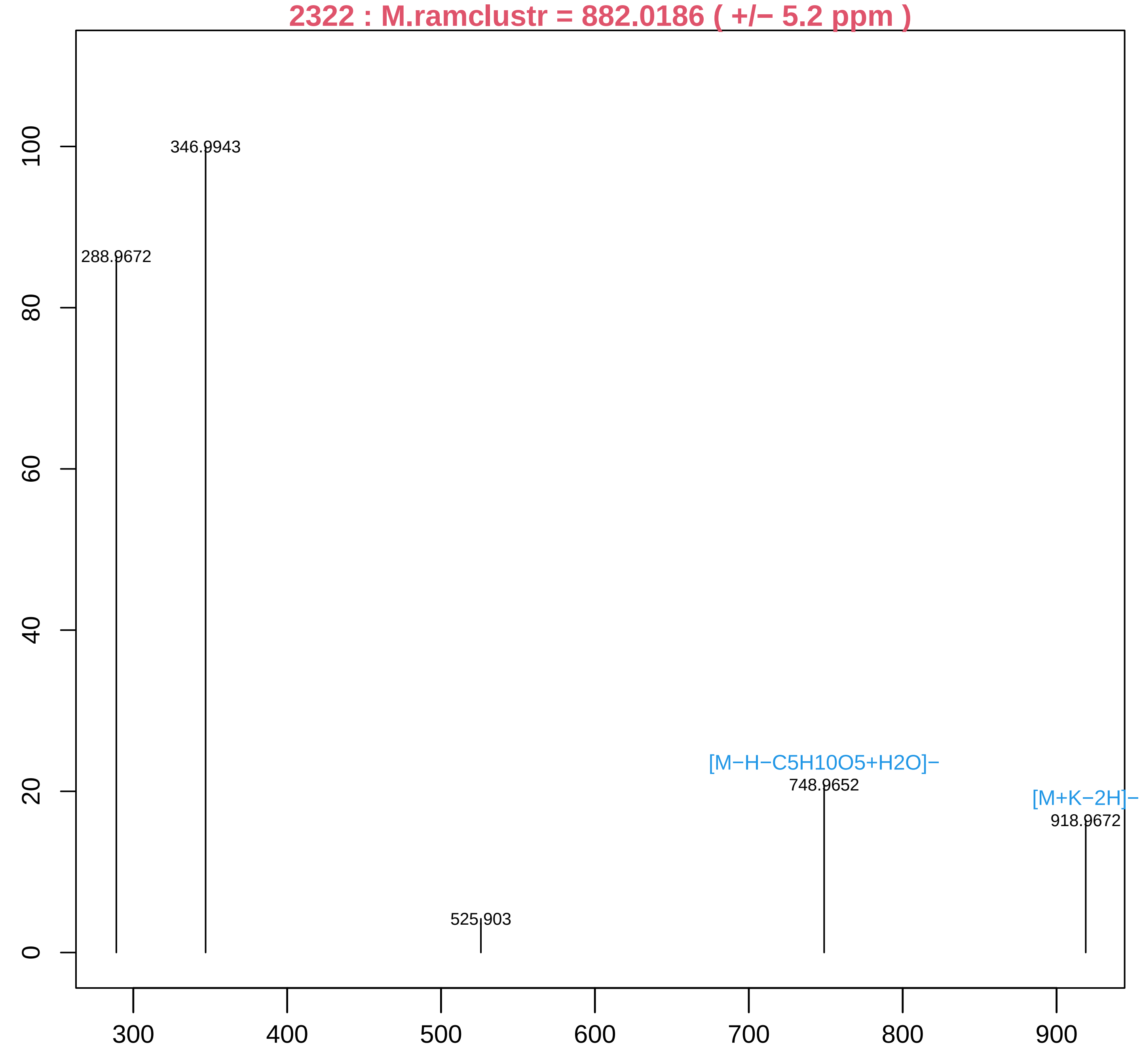
2322 : M.ramclustr = 882.0186 (+/− 5.2 ppm)
346.9943
288.9672
525.903
[M−H−C5H10O5+H2O]−
748.9652
[M+K−2H]−
918.9672
0
20
40
60
80
100
300
400
500
600
700
800
900

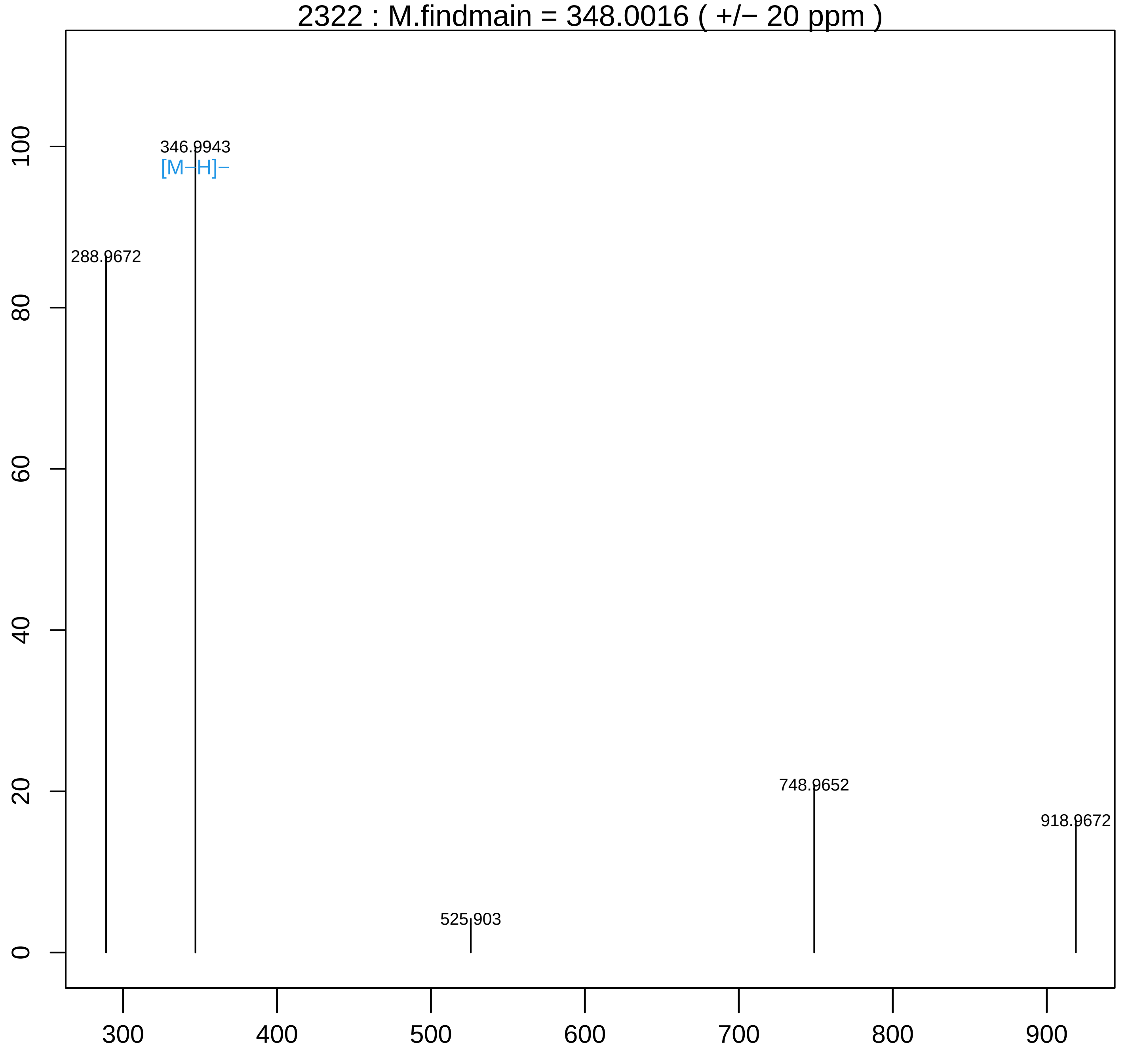
2322 : M.findmain = 348.0016 (+/− 20 ppm)
346.9943
[M−H]−
288.9672
525.903
748.9652
918.9672
0
20
40
60
80
100
300
400
500
600
700
800
900

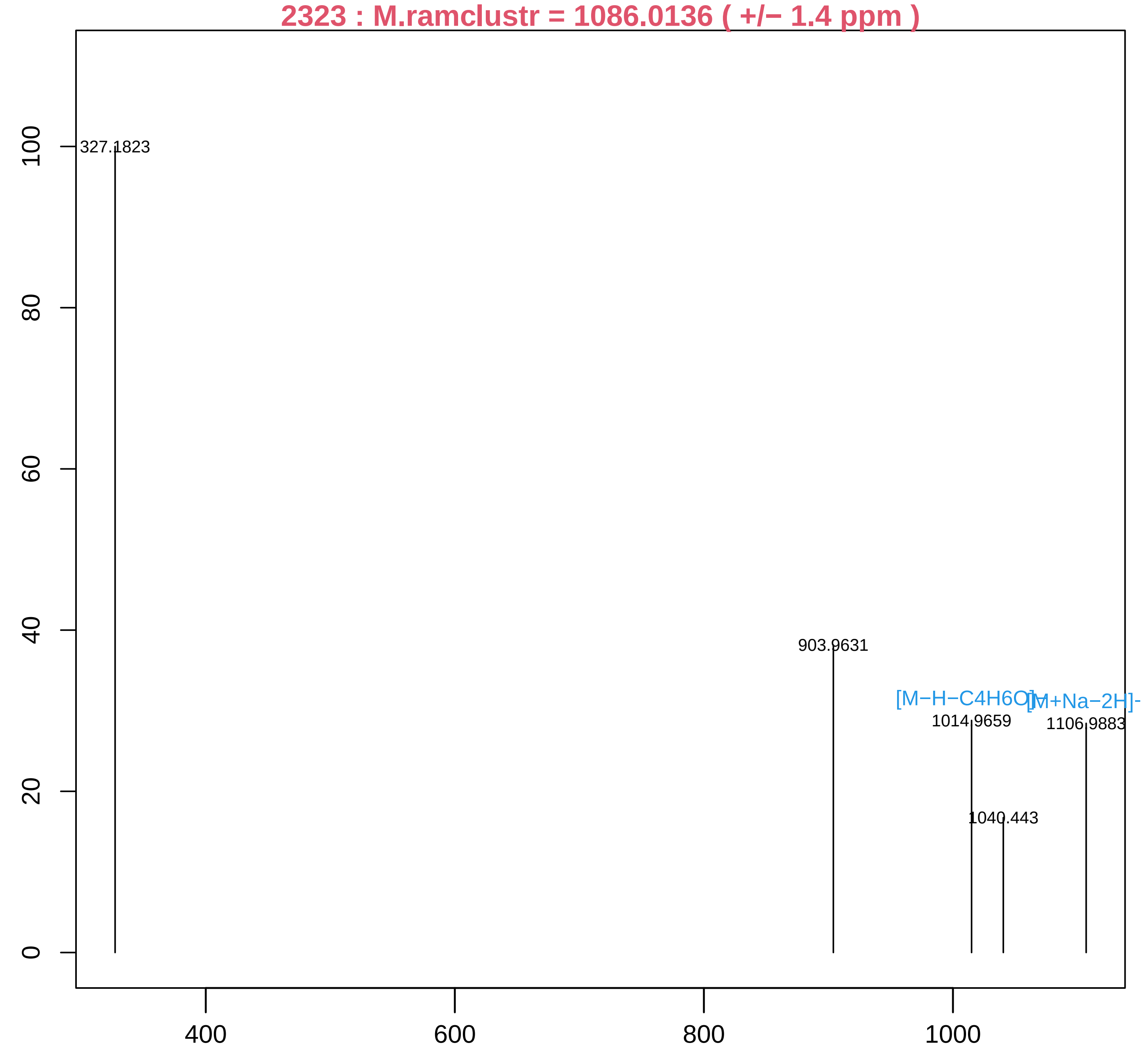
2323 : M.ramclustr = 1086.0136 (+/− 1.4 ppm)
327.1823
903.9631
[M−H−C4H6O]−
[M+Na−2H]-
1014.9659
1106.9883
1040.443
0
20
40
60
80
100
400
600
800
1000

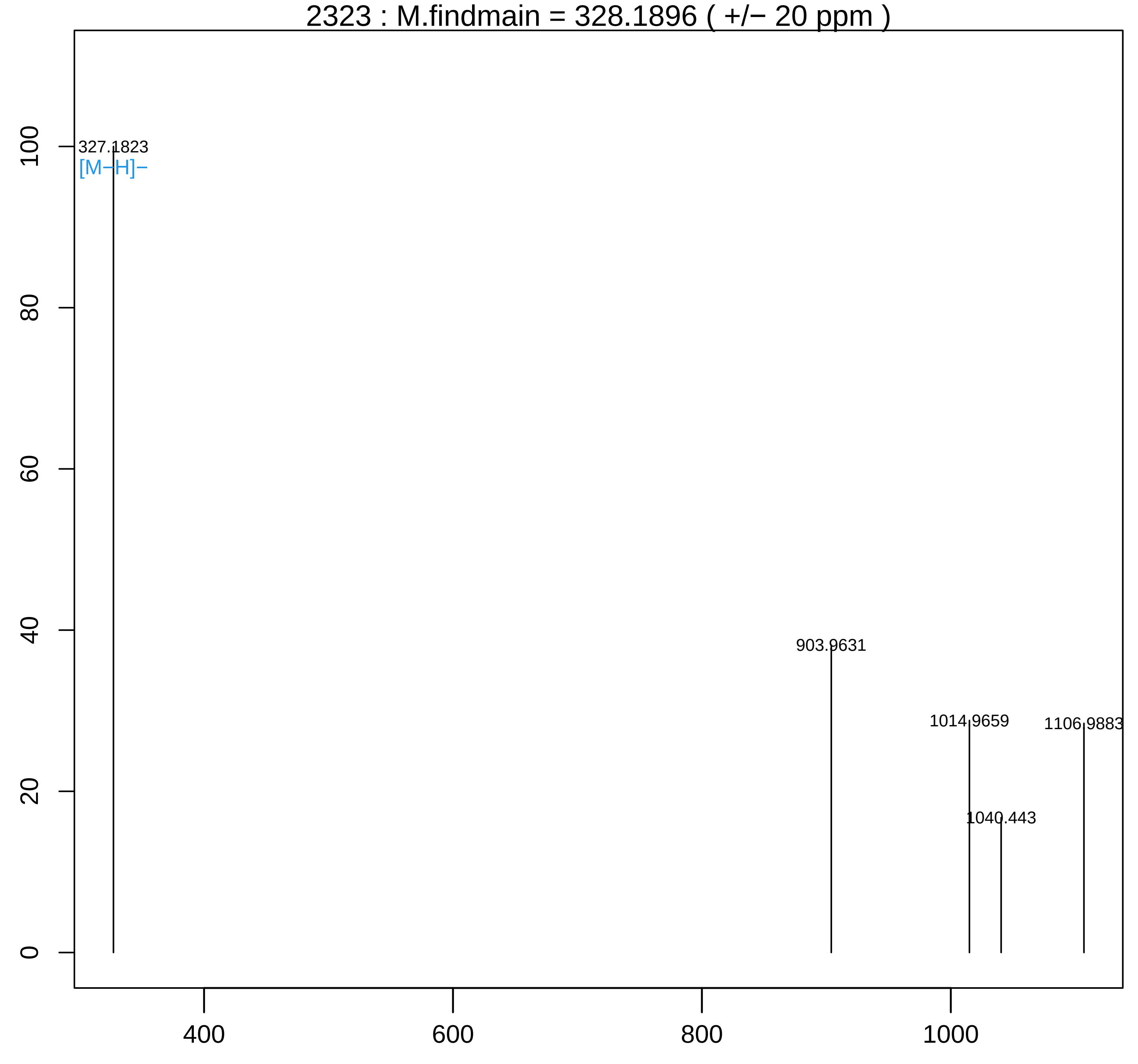
2323 : M.findmain = 328.1896 (+/− 20 ppm)
327.1823
[M−H]−
903.9631
1014.9659
1106.9883
1040.443
0
20
40
60
80
100
400
600
800
1000

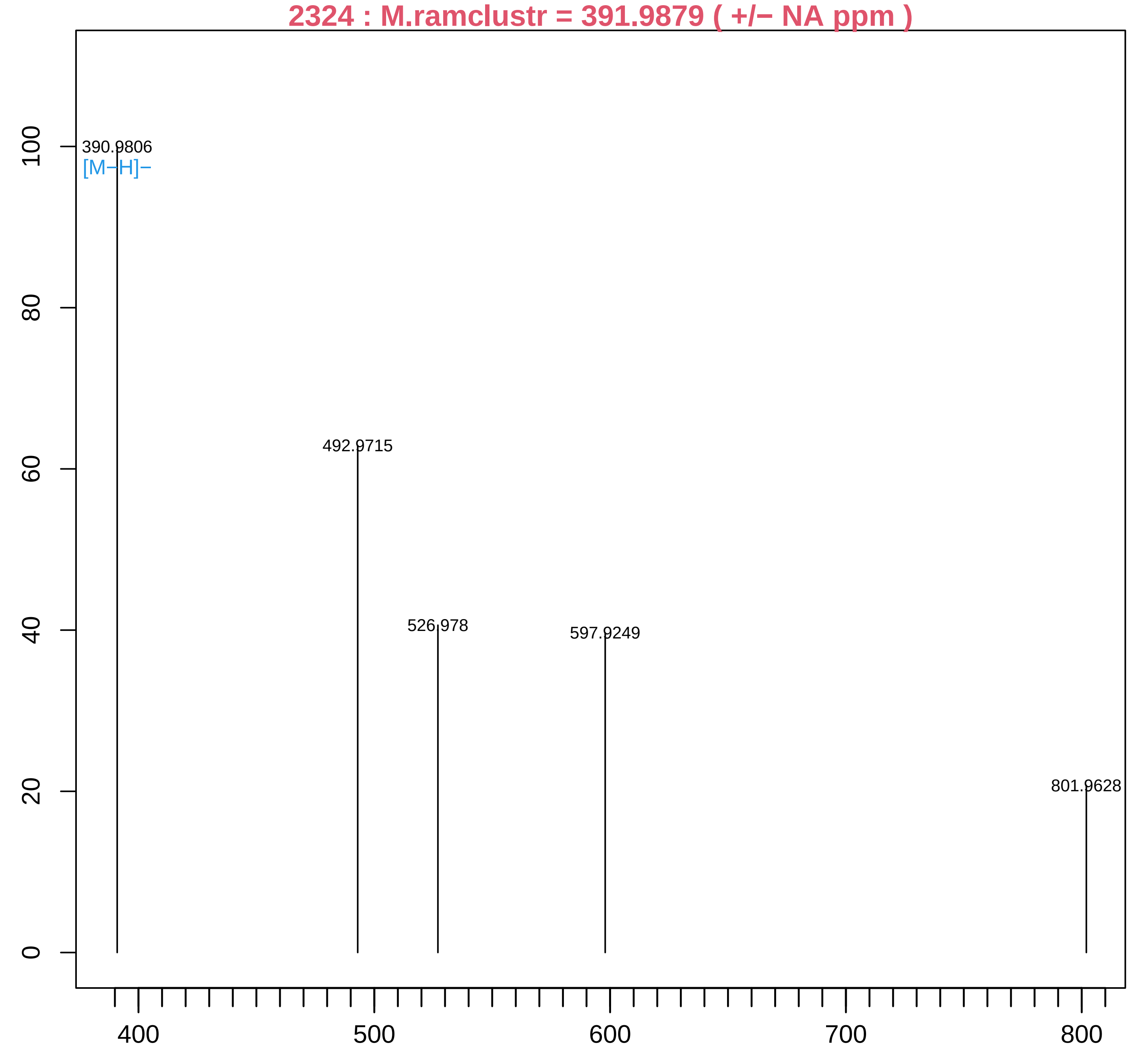
2324 : M.ramclustr = 391.9879 (+/− NA ppm)
390.9806
[M−H]−
492.9715
526.978
597.9249
801.9628
0
20
40
60
80
100
400
500
600
700
800

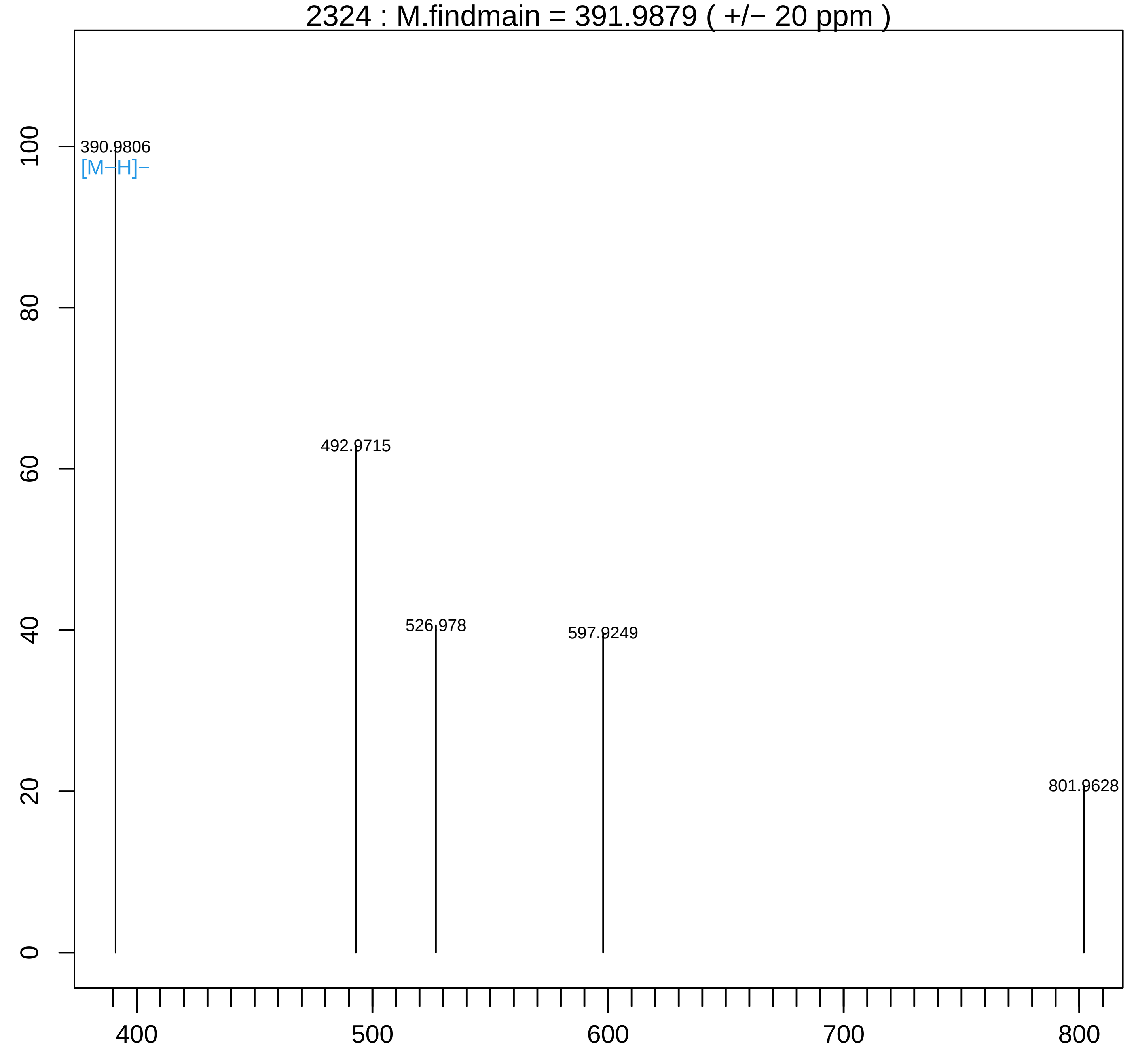
2324 : M.findmain = 391.9879 (+/− 20 ppm)
390.9806
[M−H]−
492.9715
526.978
597.9249
801.9628
0
20
40
60
80
100
400
500
600
700
800

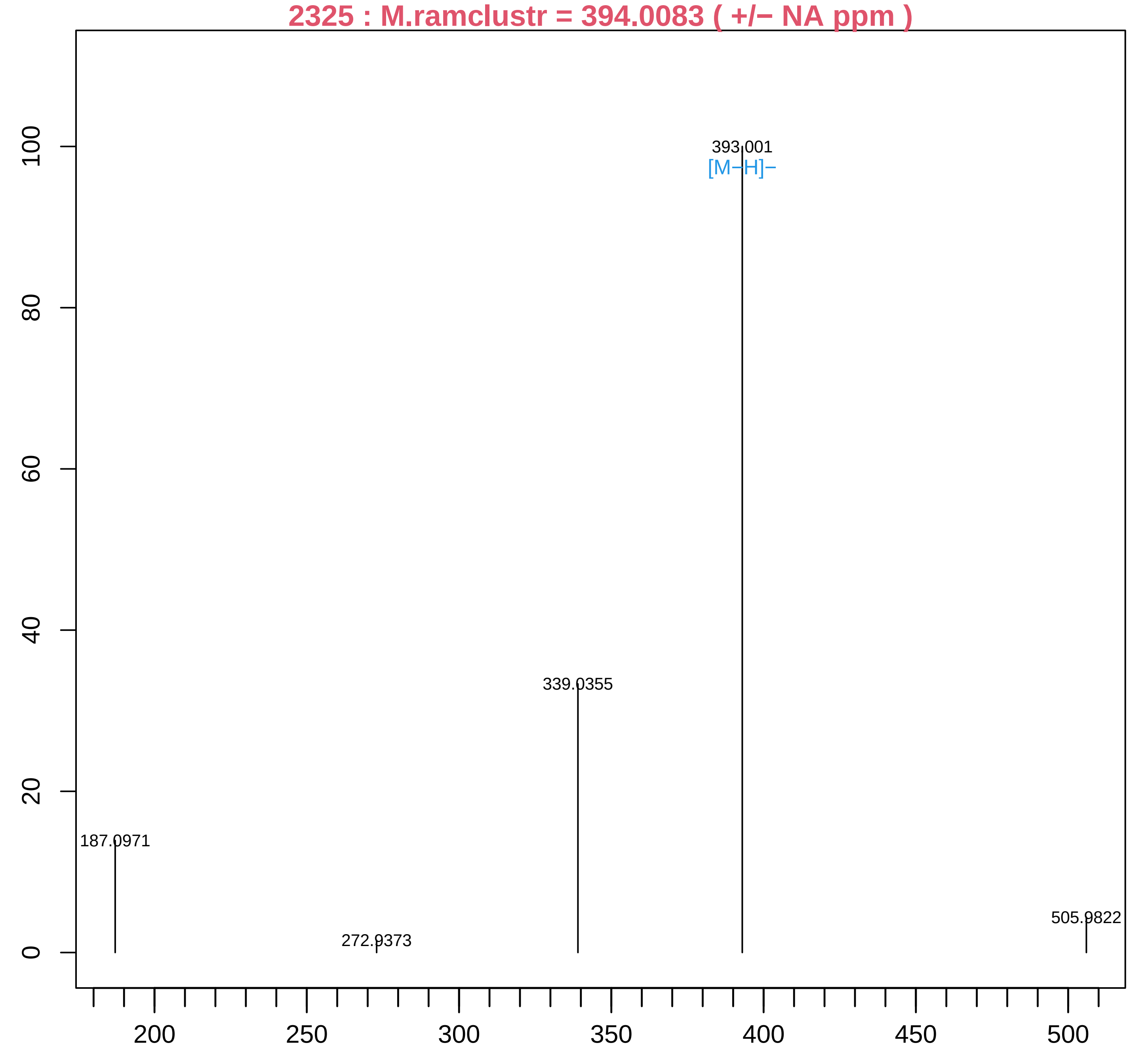

2325 : M.ramclustr = 394.0083 (+/− NA ppm)
393.001
[M−H]−
339.0355
187.0971
272.9373
505.9822
0
20
40
60
80
100
200
250
300
350
400
450
500

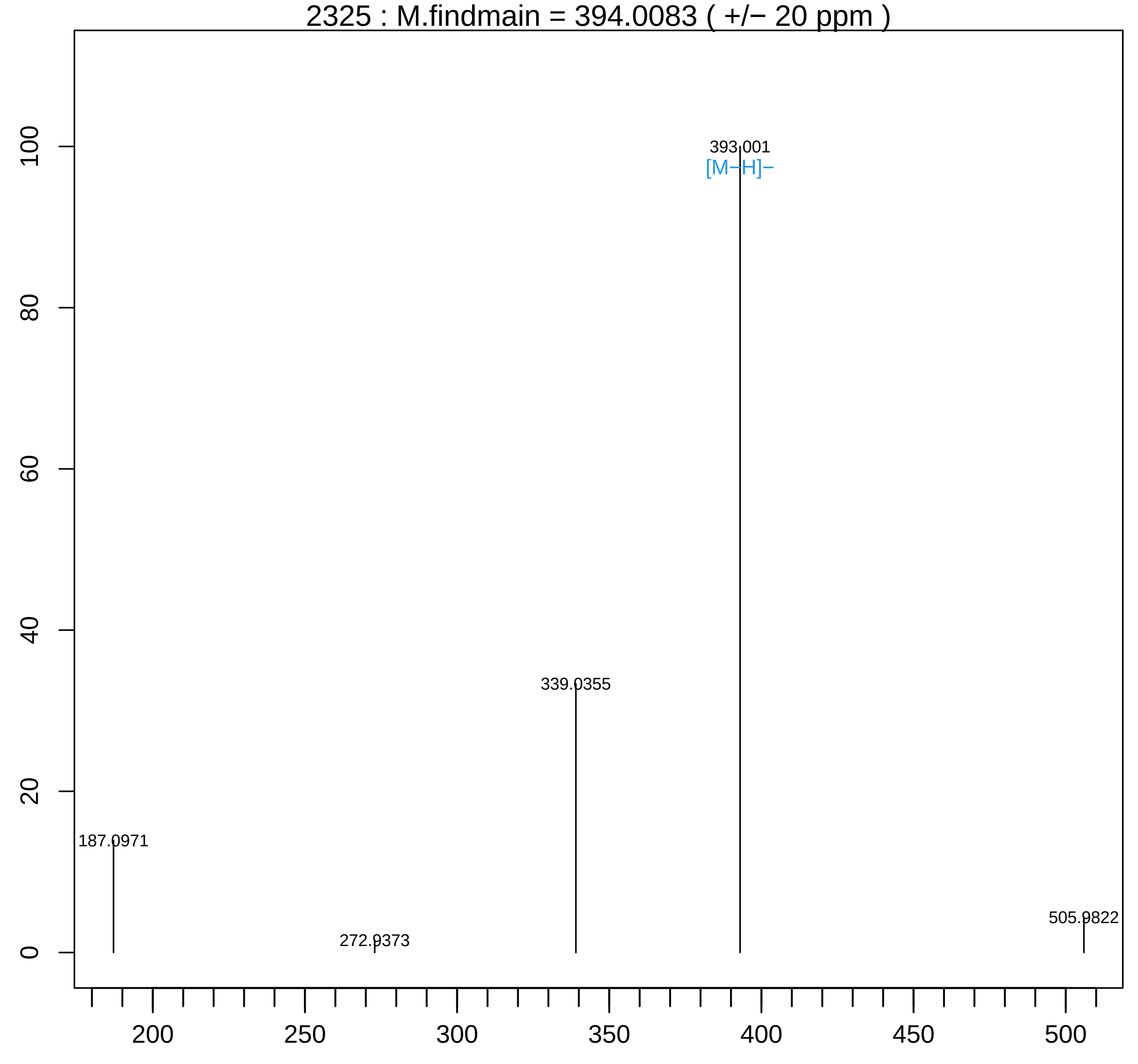

2325 : M.findmain = 394.0083 (+/− 20 ppm)
393.001
[M−H]−
339.0355
187.0971
272.9373
505.9822
0
20
40
60
80
100
200
250
300
350
400
450
500

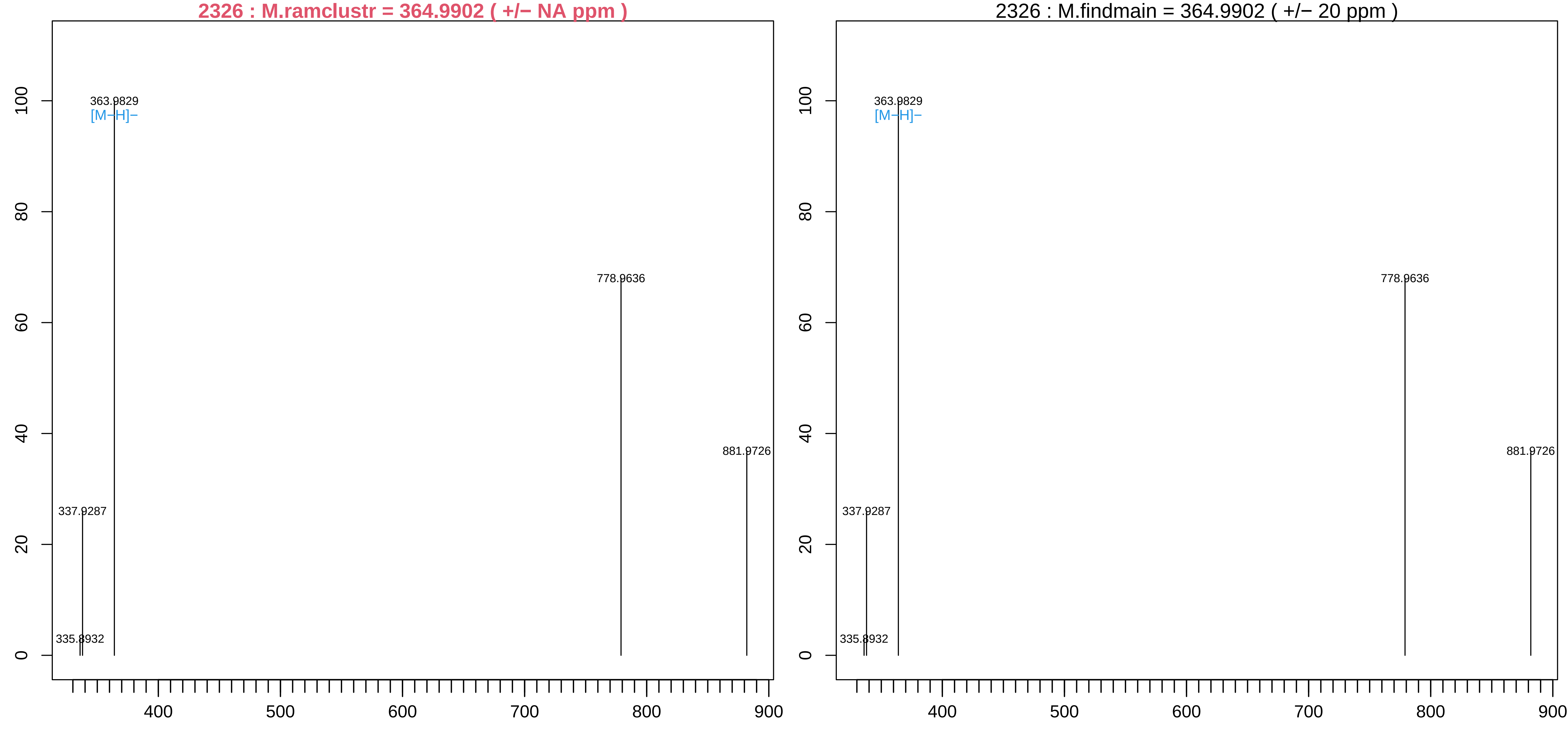
2326 : M.ramclustr = 364.9902 (+/− NA ppm)
2326 : M.findmain = 364.9902 (+/− 20 ppm)
363.9829
[M−H]−
778.9636
881.9726
337.9287
335.8932
0
20
40
60
80
100
400
500
600
700
800
900

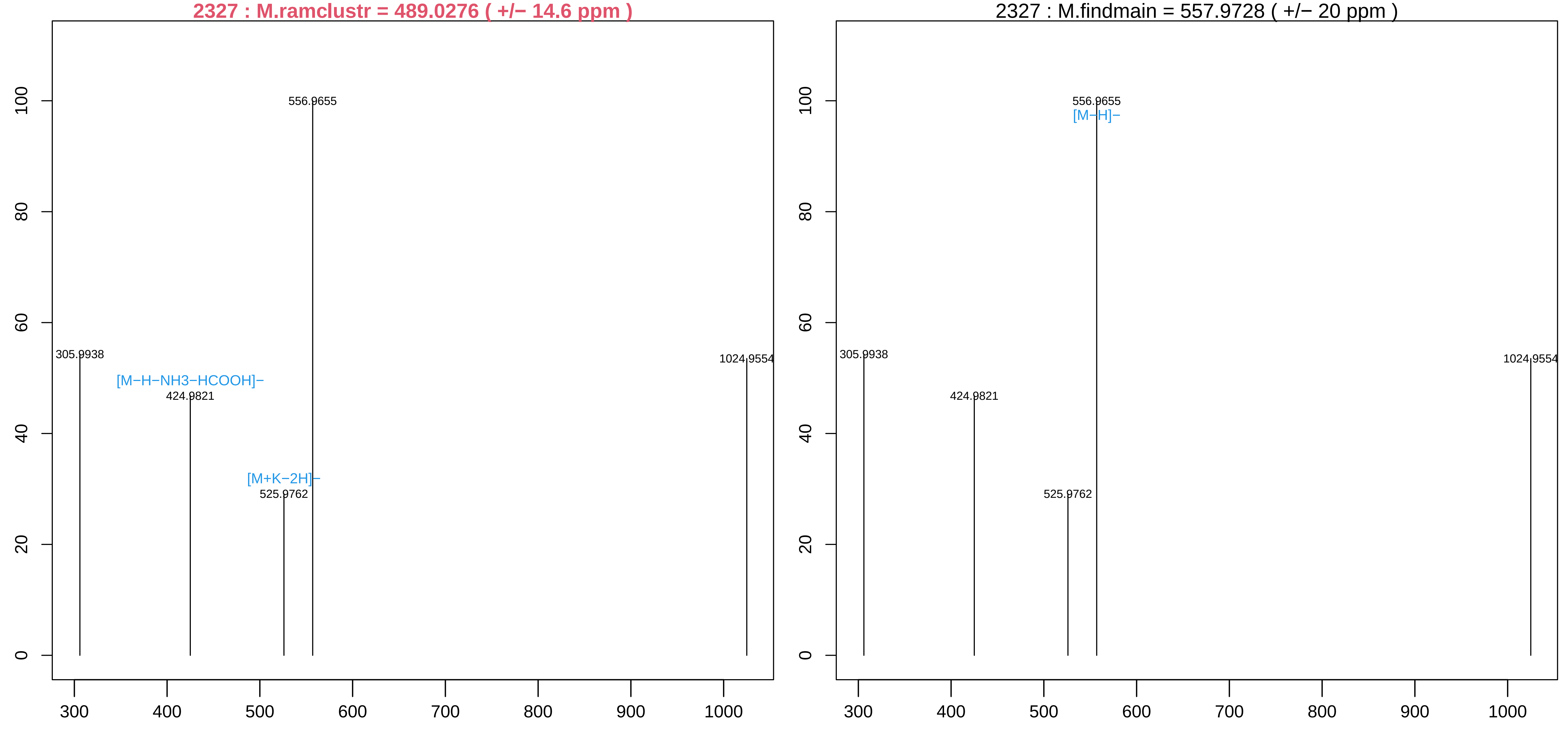
2327 : M.ramclustr = 489.0276 (+/− 14.6 ppm)
556.9655
305.9938
1024.9554
[M−H−NH3−HCOOH]−
424.9821
[M+K−2H]−
525.9762
2327 : M.findmain = 557.9728 (+/− 20 ppm)
556.9655
[M−H]−
305.9938
1024.9554
424.9821
525.9762
0
20
40
60
80
100
300
400
500
600
700
800
900
1000

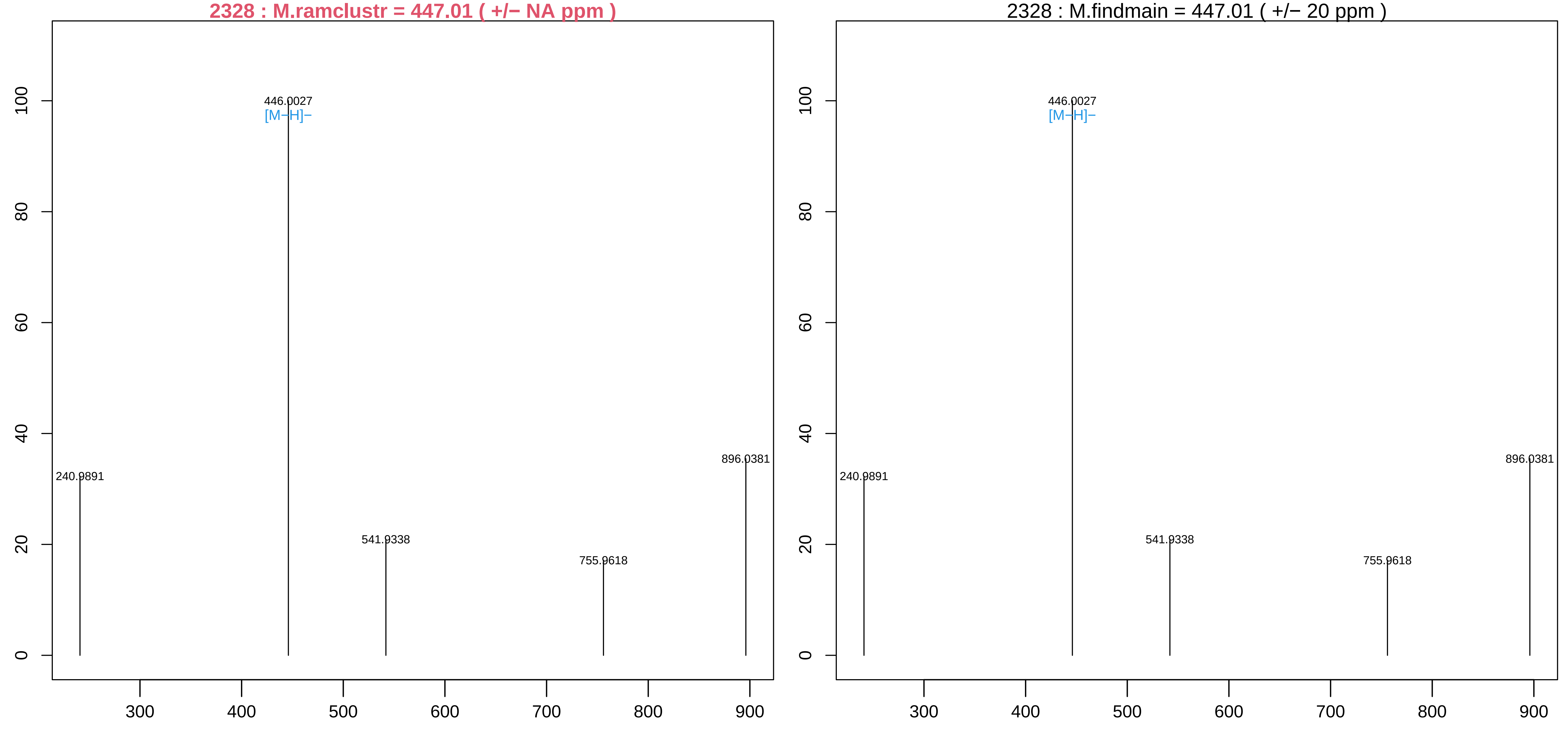

2328 : M.ramclustr = 447.01 (+/− NA ppm)
446.0027
[M−H]−
240.9891
541.9338
755.9618
896.0381
2328 : M.findmain = 447.01 (+/− 20 ppm)
446.0027
[M−H]−
240.9891
541.9338
755.9618
896.0381

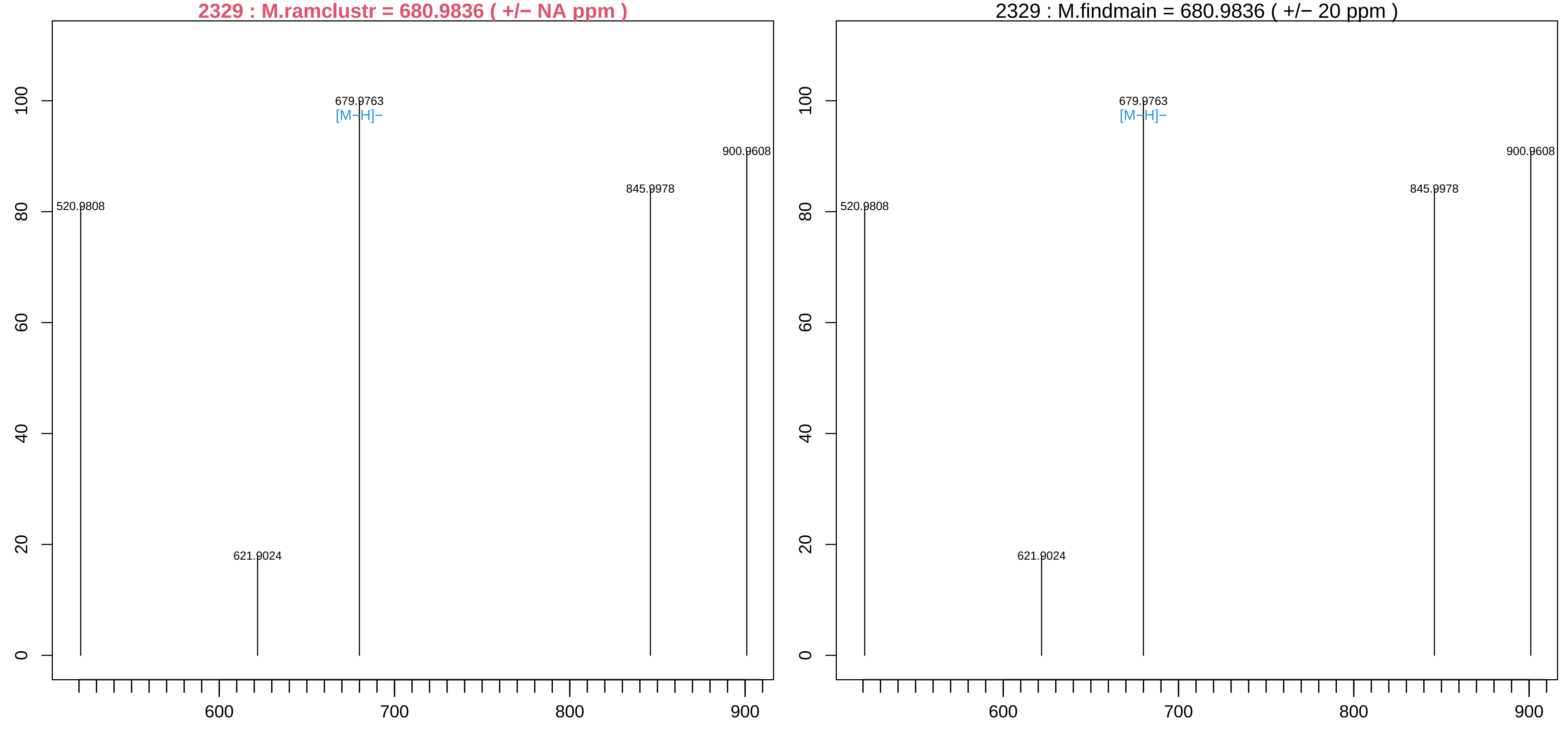

2329 : M.ramclustr = 680.9836 (+/− NA ppm)
520.9808
621.9024
679.9763
[M−H]−
845.9978
900.9608
0
20
40
60
80
100
600
700
800
900
2329 : M.findmain = 680.9836 (+/− 20 ppm)
520.9808
621.9024
679.9763
[M−H]−
845.9978
900.9608
0
20
40
60
80
100
600
700
800
900

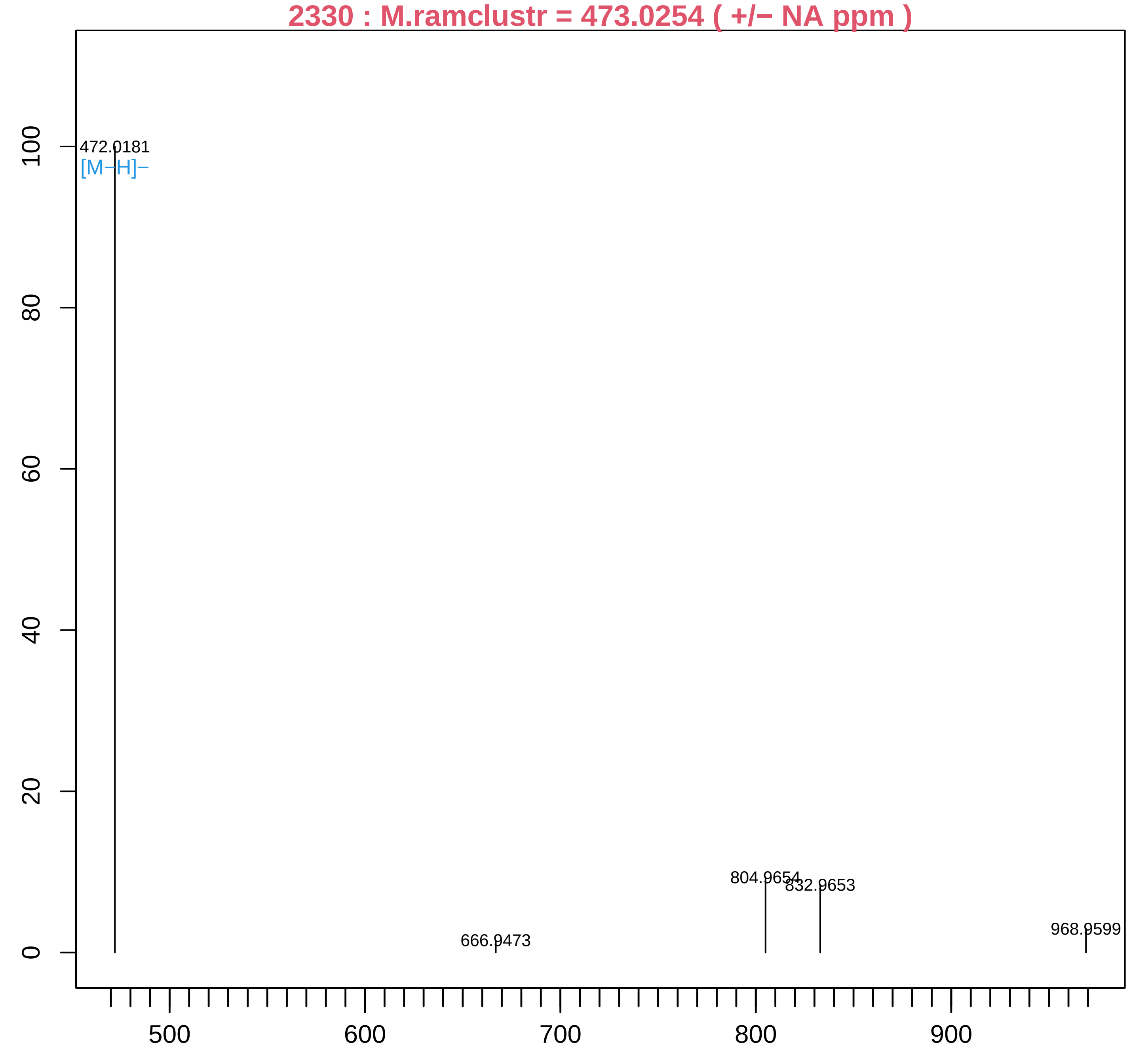

2330 : M.ramclustr = 473.0254 (+/− NA ppm)
472.0181
[M−H]−
666.9473
804.9654
832.9653
968.9599
0
20
40
60
80
100
500
600
700
800
900

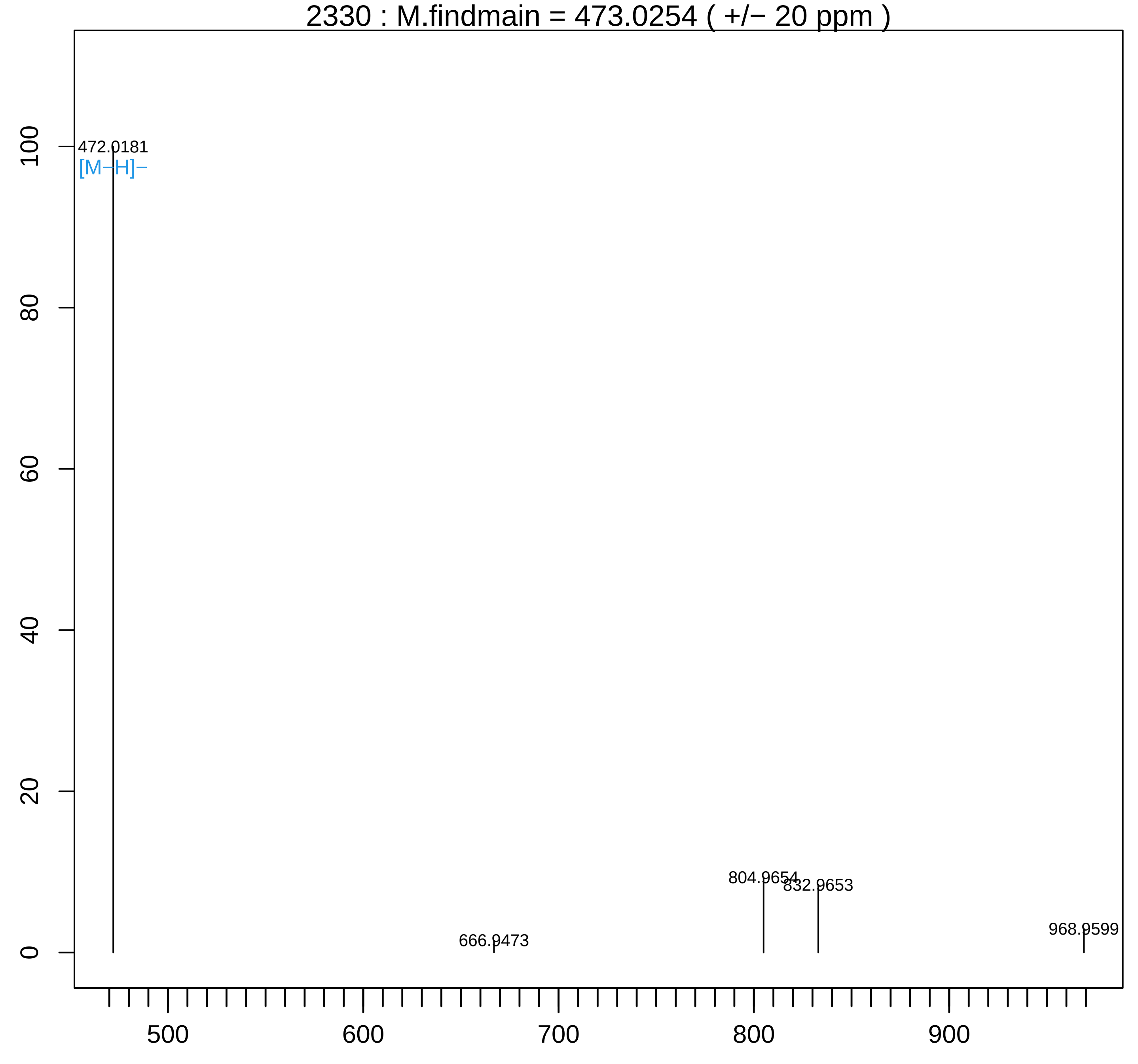

2330 : M.findmain = 473.0254 (+/− 20 ppm)
472.0181
[M−H]−
666.9473
804.9654
832.9653
968.9599
0
20
40
60
80
100
500
600
700
800
900

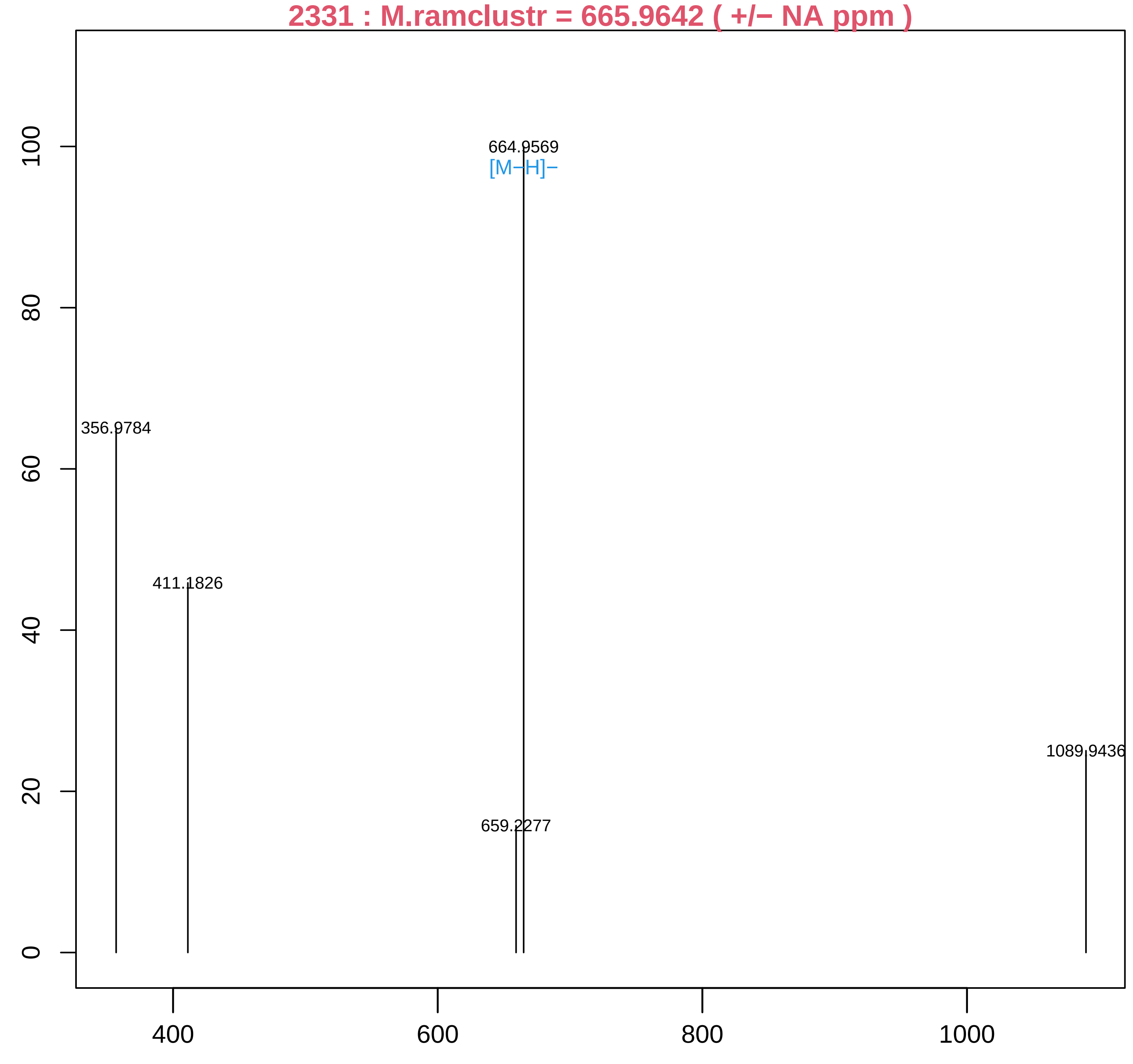
2331 : M.ramclustr = 665.9642 (+/− NA ppm)
356.9784
411.1826
664.9569
[M−H]−
659.2277
1089.9436
0
20
40
60
80
100
400
600
800
1000

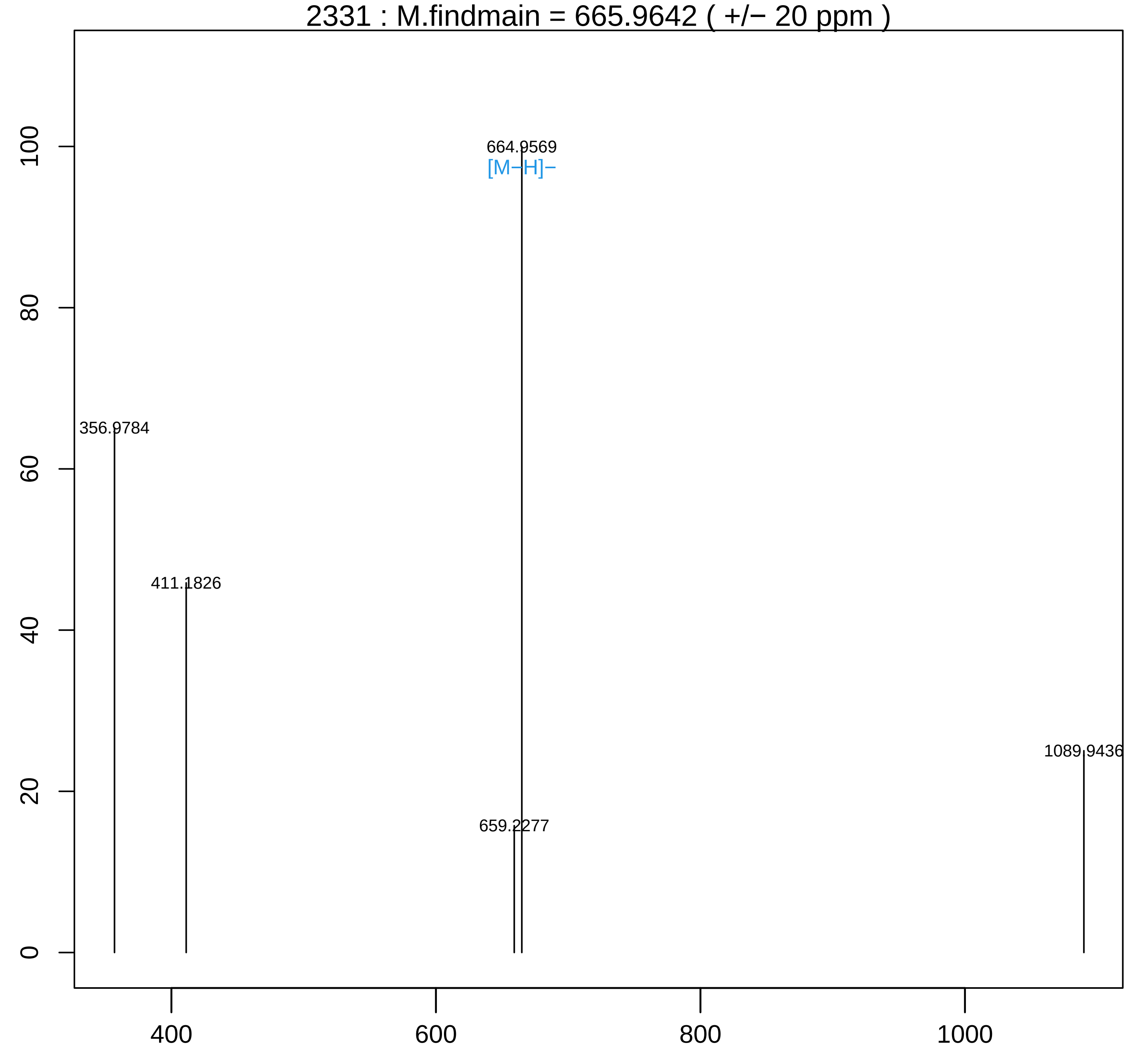
2331 : M.findmain = 665.9642 (+/− 20 ppm)
356.9784
411.1826
664.9569
[M−H]−
659.2277
1089.9436
0
20
40
60
80
100
400
600
800
1000

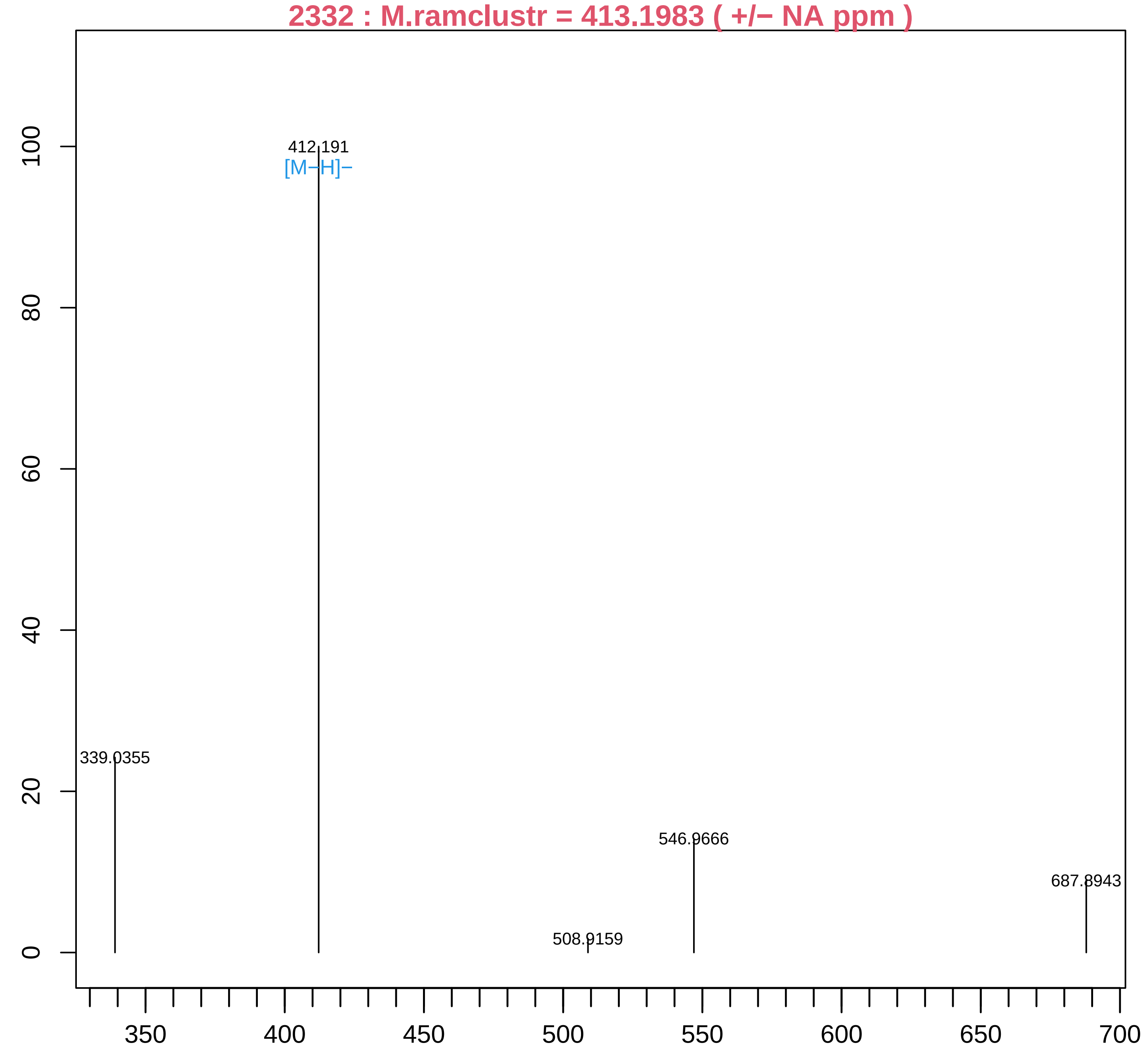
2332 : M.ramclustr = 413.1983 (+/− NA ppm)
412.191
[M−H]−
339.0355
546.9666
687.8943
508.9159
0
20
40
60
80
100
350
400
450
500
550
600
650
700

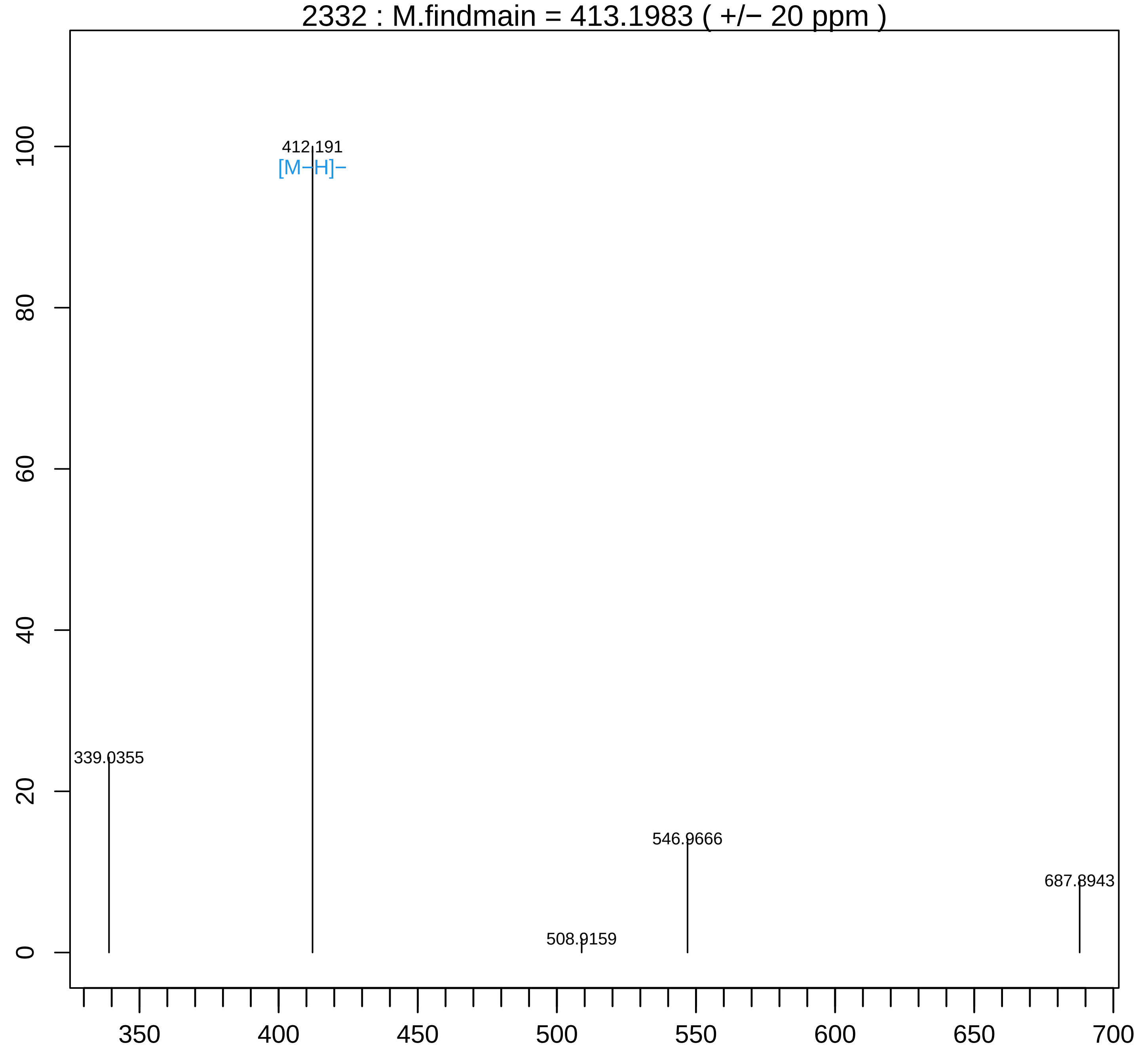
2332 : M.findmain = 413.1983 (+/− 20 ppm)
412.191
[M−H]−
339.0355
546.9666
687.8943
508.9159
0
20
40
60
80
100
350
400
450
500
550
600
650
700

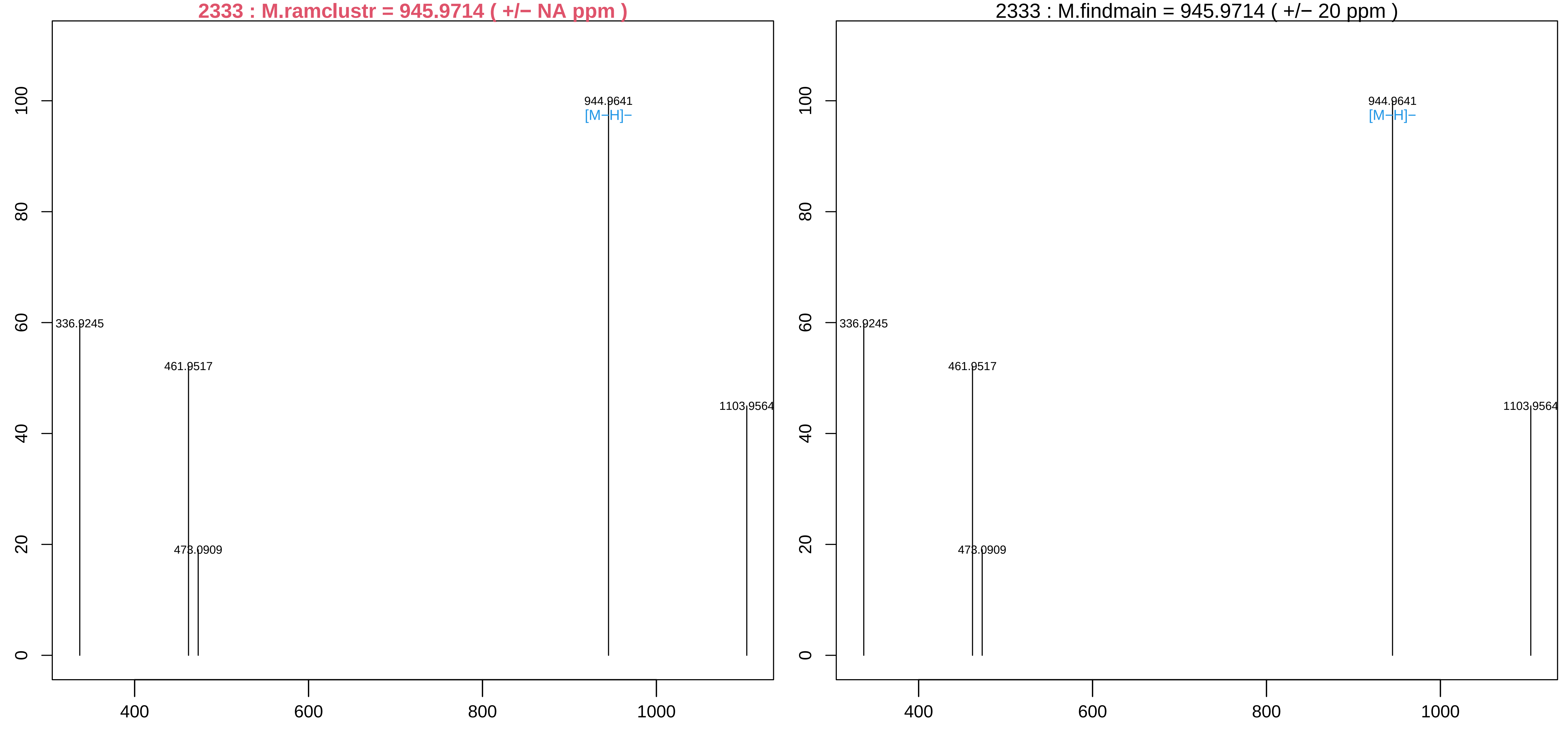
2333 : M.ramclustr = 945.9714 (+/− NA ppm)
2333 : M.findmain = 945.9714 (+/− 20 ppm)
944.9641
[M−H]−
336.9245
461.9517
473.0909
1103.9564
0
20
40
60
80
100
400
600
800
1000

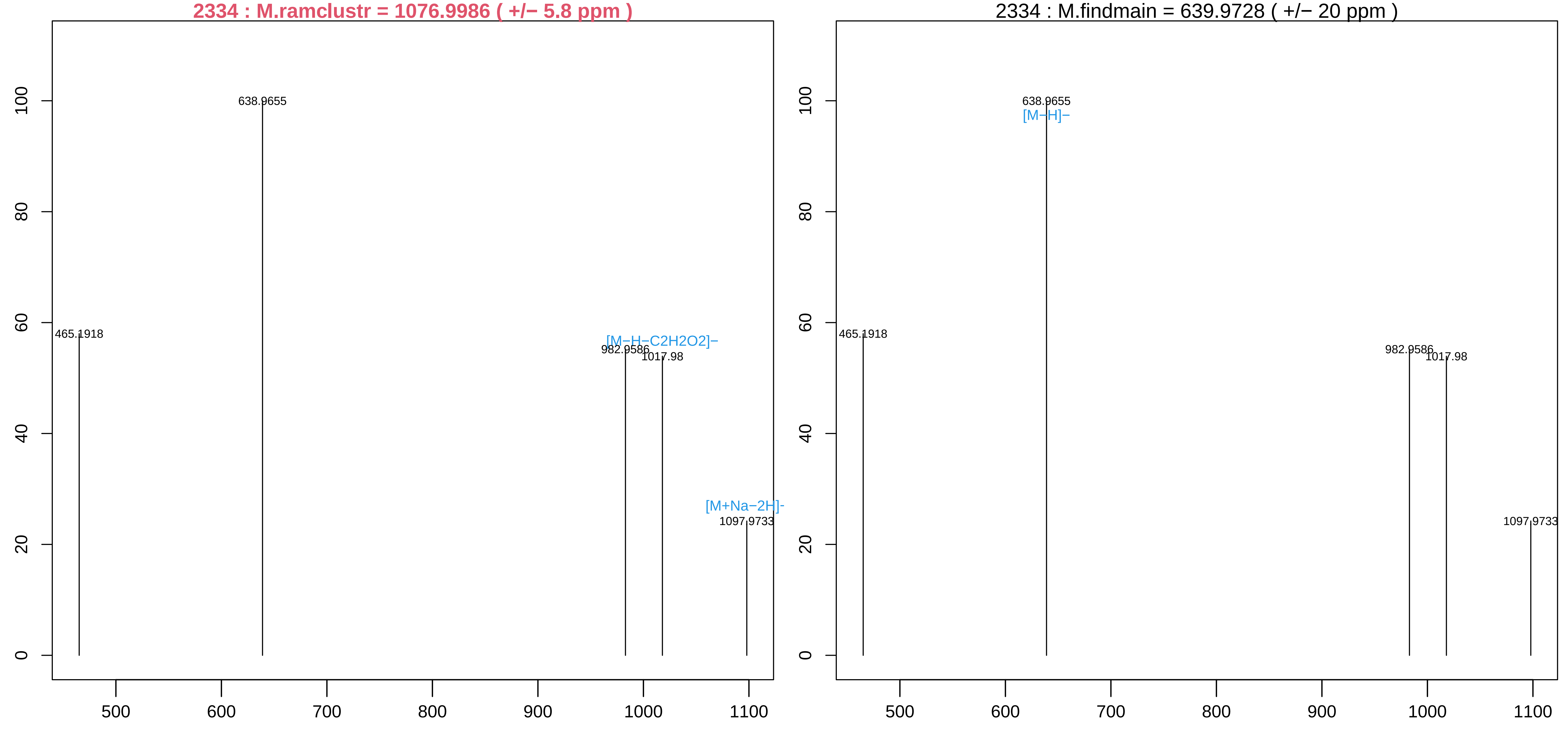
2334 : M.ramclustr = 1076.9986 (+/− 5.8 ppm)
638.9655
465.1918
[M−H−C2H2O2]−
982.9586
1017.98
[M+Na−2H]-
1097.9733
0
20
40
60
80
100
500
600
700
800
900
1000
1100
2334 : M.findmain = 639.9728 (+/− 20 ppm)
638.9655
[M−H]−
465.1918
982.9586
1017.98
1097.9733
0
20
40
60
80
100
500
600
700
800
900
1000
1100

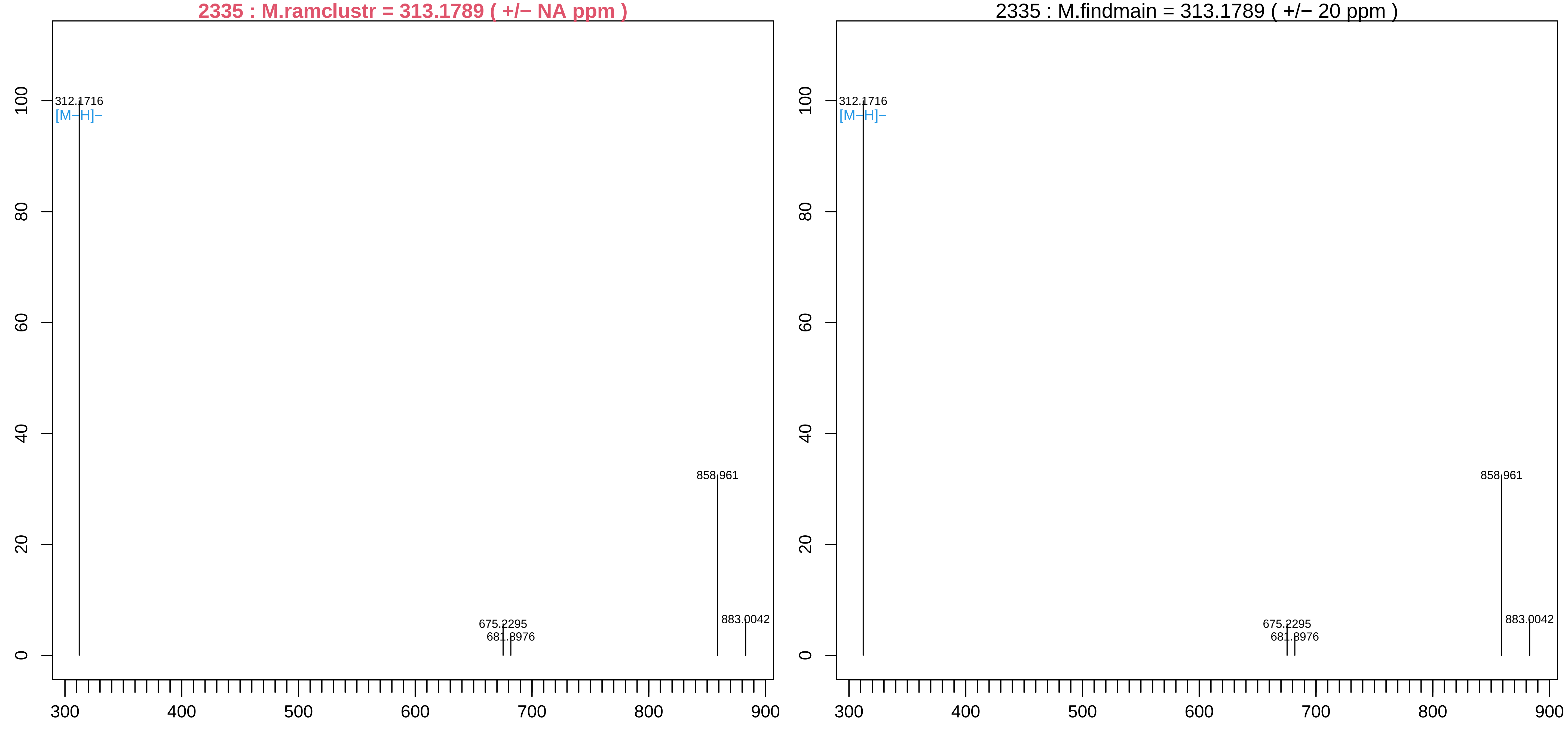
2335 : M.ramclustr = 313.1789 (+/− NA ppm)
312.1716
[M−H]−
675.2295
681.8976
858.961
883.0042
2335 : M.findmain = 313.1789 (+/− 20 ppm)
312.1716
[M−H]−
675.2295
681.8976
858.961
883.0042

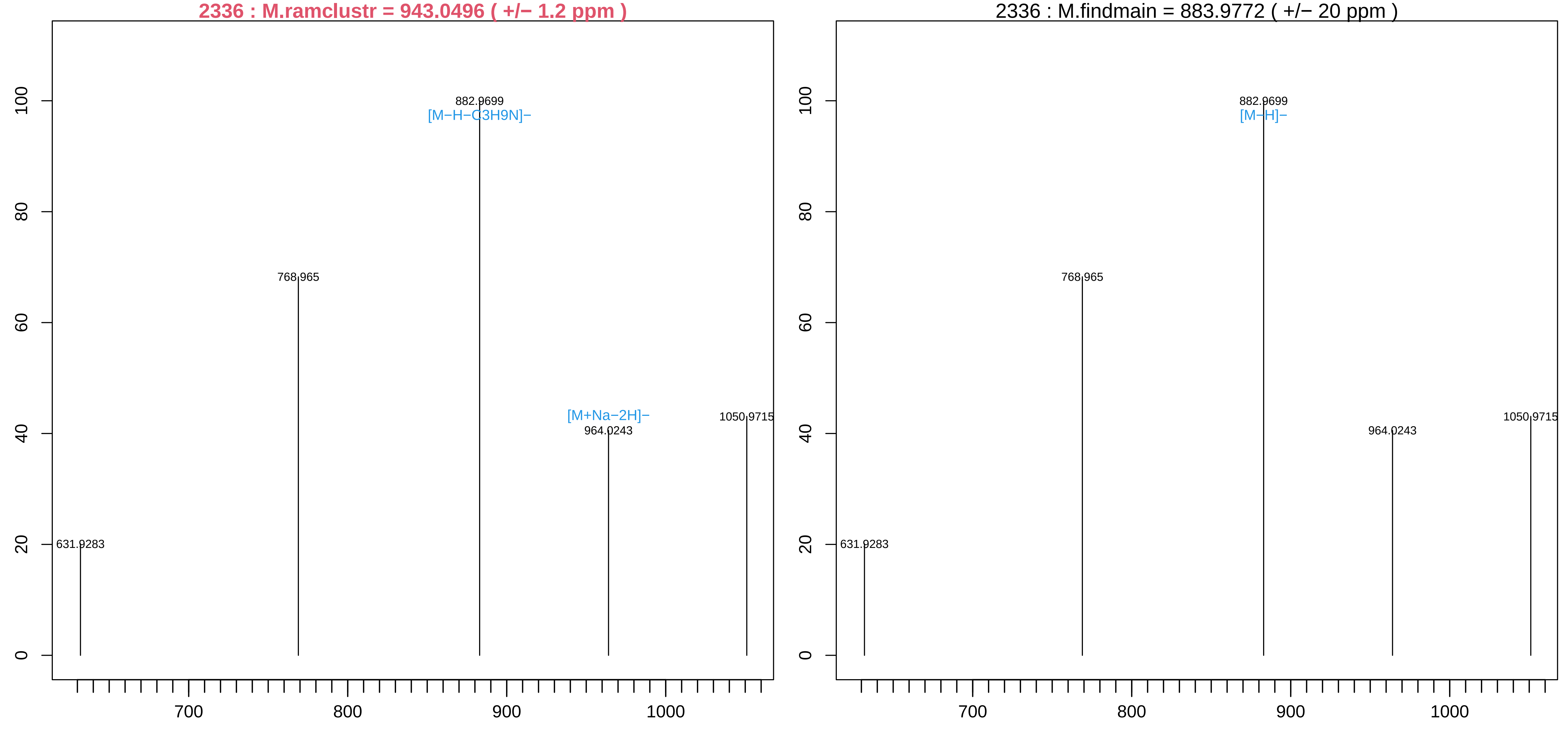
2336 : M.ramclustr = 943.0496 (+/− 1.2 ppm)
882.9699
[M−H−C3H9N]−
768.965
[M+Na−2H]−
964.0243
1050.9715
631.9283
2336 : M.findmain = 883.9772 (+/− 20 ppm)
882.9699
[M−H]−
768.965
964.0243
1050.9715
631.9283

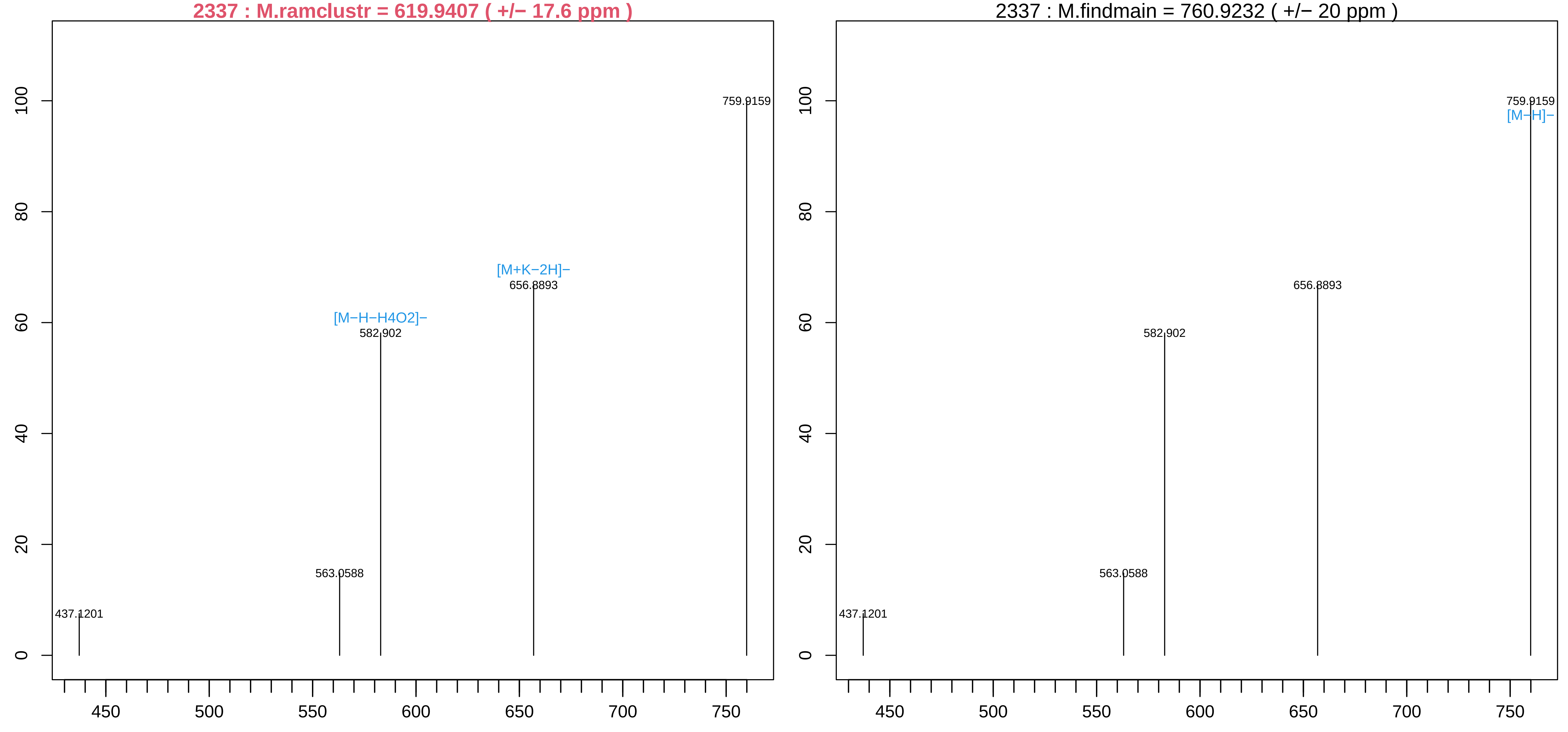

2337 : M.ramclustr = 619.9407 (+/− 17.6 ppm)
759.9159
[M+K−2H]−
656.8893
[M−H−H4O2]−
582.902
563.0588
437.1201
2337 : M.findmain = 760.9232 (+/− 20 ppm)
759.9159
[M−H]−
656.8893
582.902
563.0588
437.1201

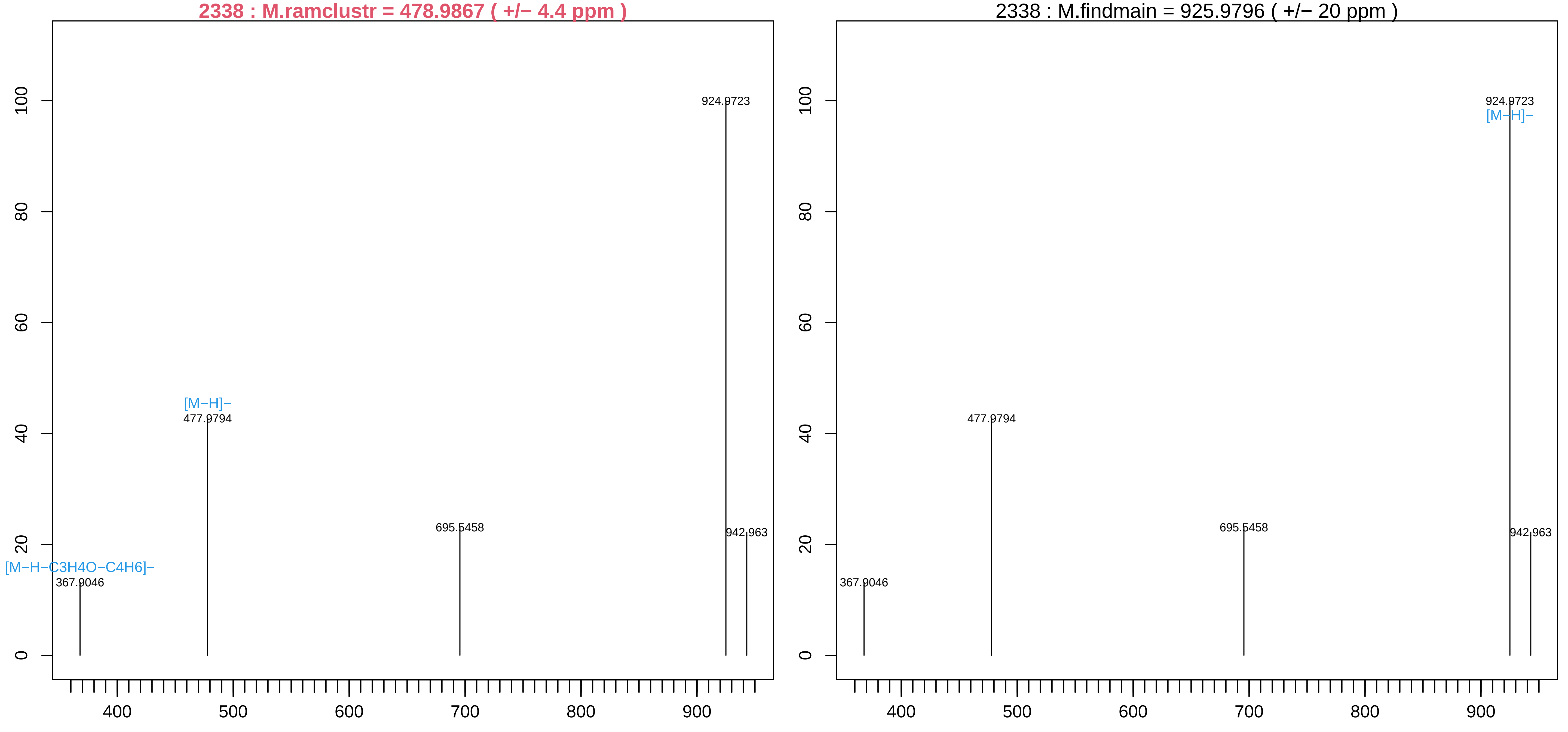

2338 : M.ramclustr = 478.9867 (+/− 4.4 ppm)
924.9723
[M−H]−
477.9794
695.5458
942.963
[M−H−C3H4O−C4H6]−
367.9046
2338 : M.findmain = 925.9796 (+/− 20 ppm)
924.9723
[M−H]−
477.9794
695.5458
942.963
367.9046

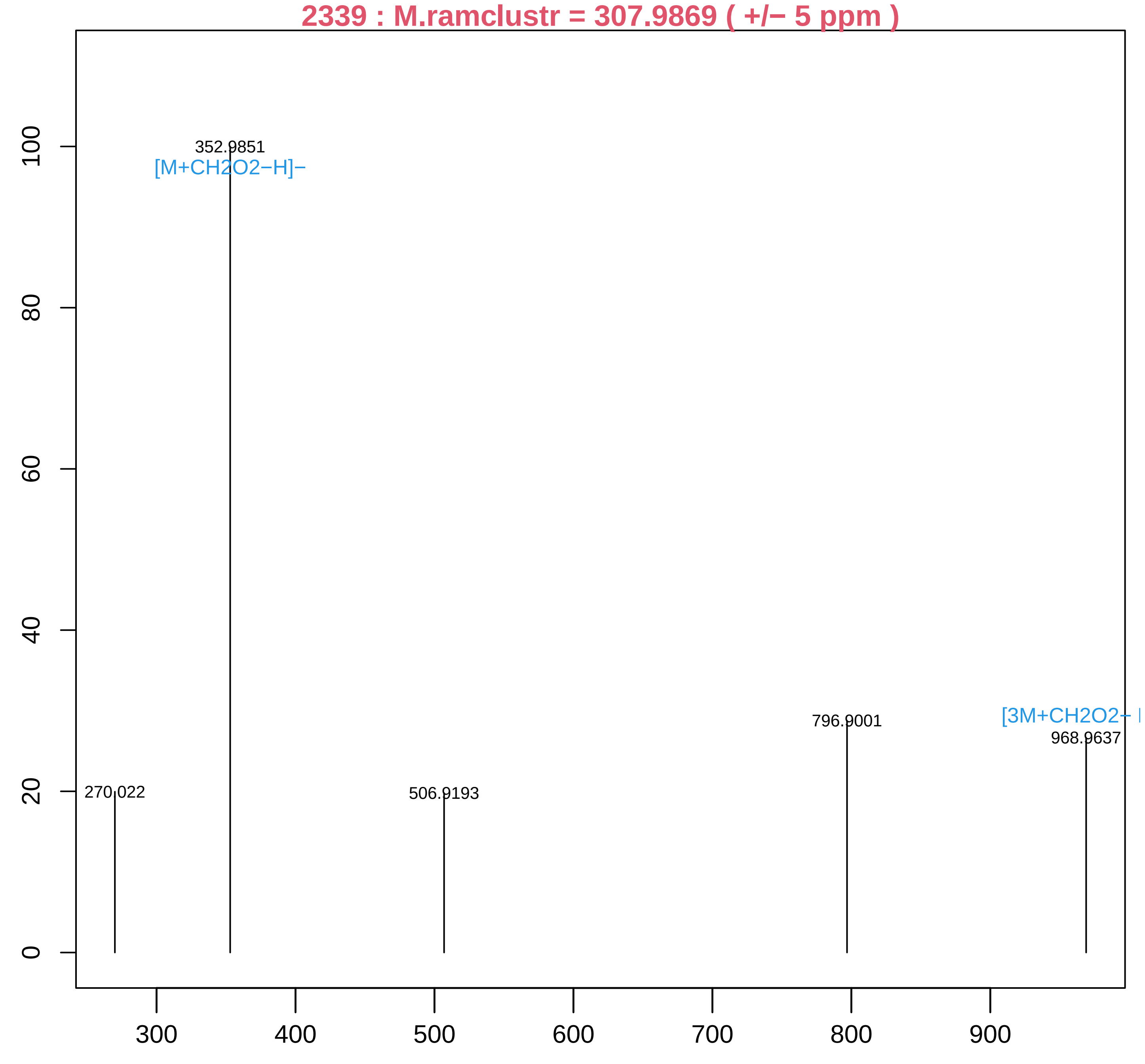

2339 : M.ramclustr = 307.9869 (+/− 5 ppm)
352.9851
[M+CH2O2−H]−
796.9001
[3M+CH2O2−
968.9637
270.022
506.9193
0
20
40
60
80
100
300
400
500
600
700
800
900

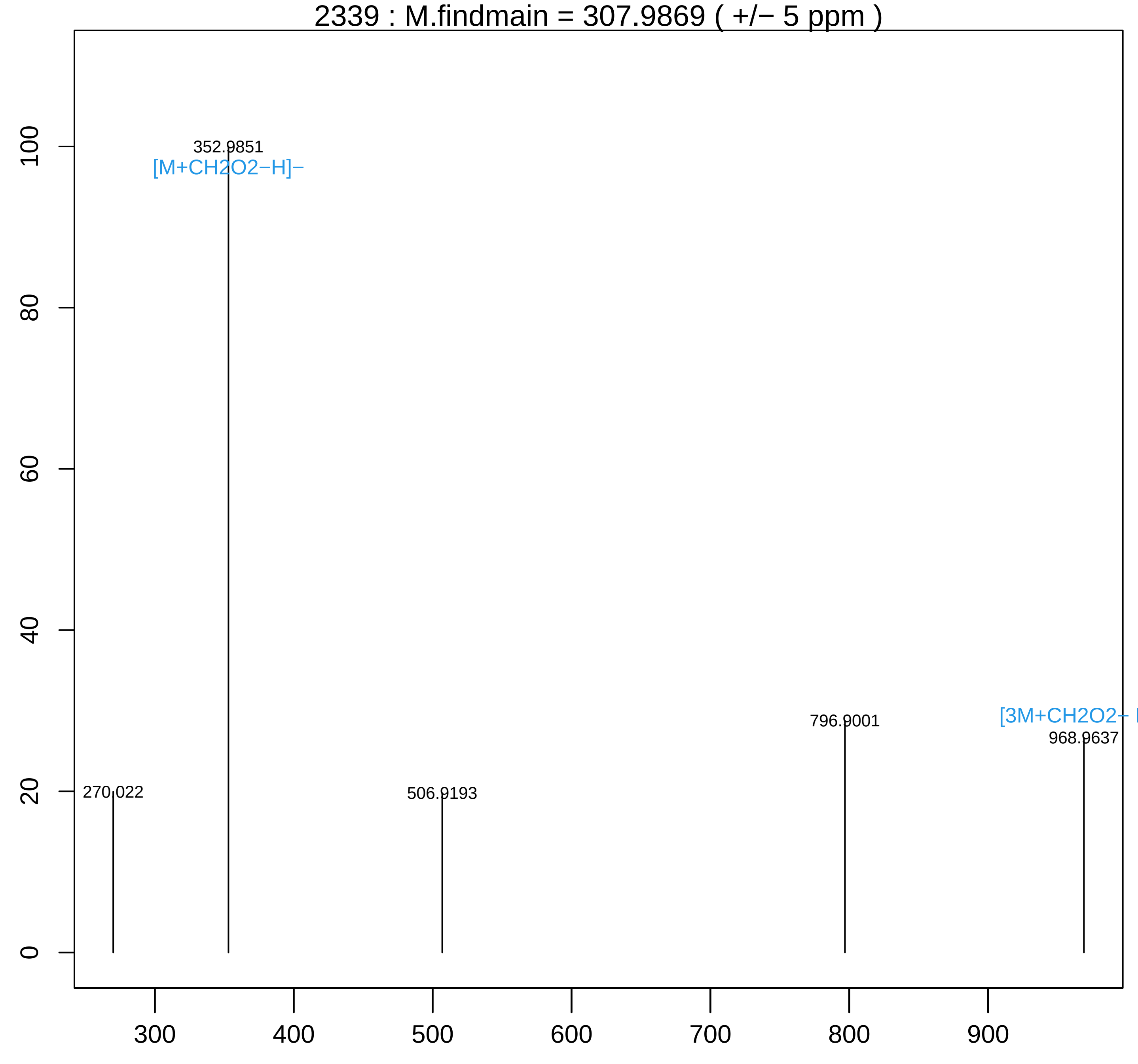

2339 : M.findmain = 307.9869 (+/− 5 ppm)
352.9851
[M+CH2O2−H]−
796.9001
[3M+CH2O2−
968.9637
270.022
506.9193
0
20
40
60
80
100
300
400
500
600
700
800
900

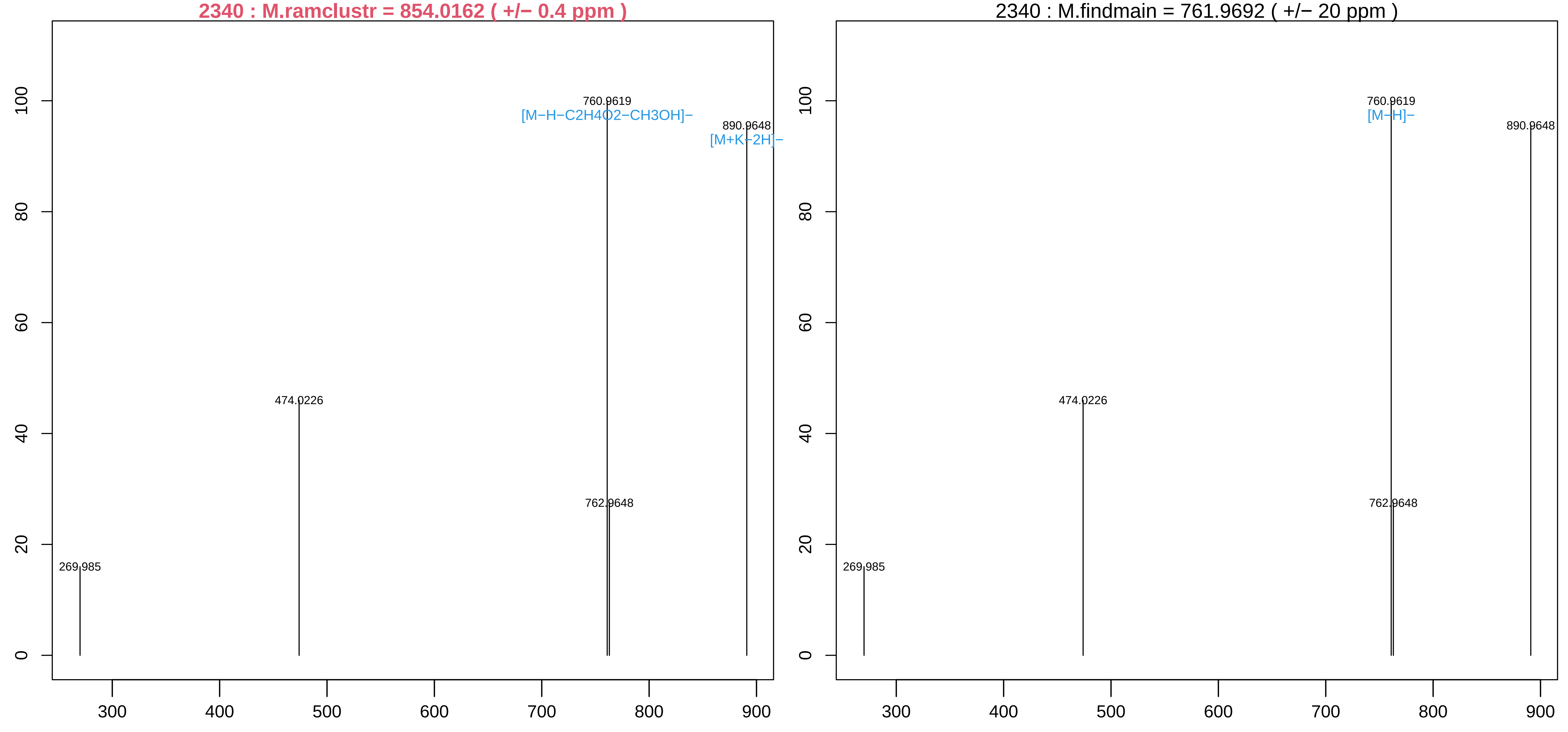
2340 : M.ramclustr = 854.0162 (+/− 0.4 ppm)
760.9619
[M−H−C2H4O2−CH3OH]−
890.9648
[M+K−2H]−
474.0226
762.9648
269.985
0
20
40
60
80
100
300
400
500
600
700
800
900
2340 : M.findmain = 761.9692 (+/− 20 ppm)
760.9619
[M−H]−
890.9648
474.0226
762.9648
269.985

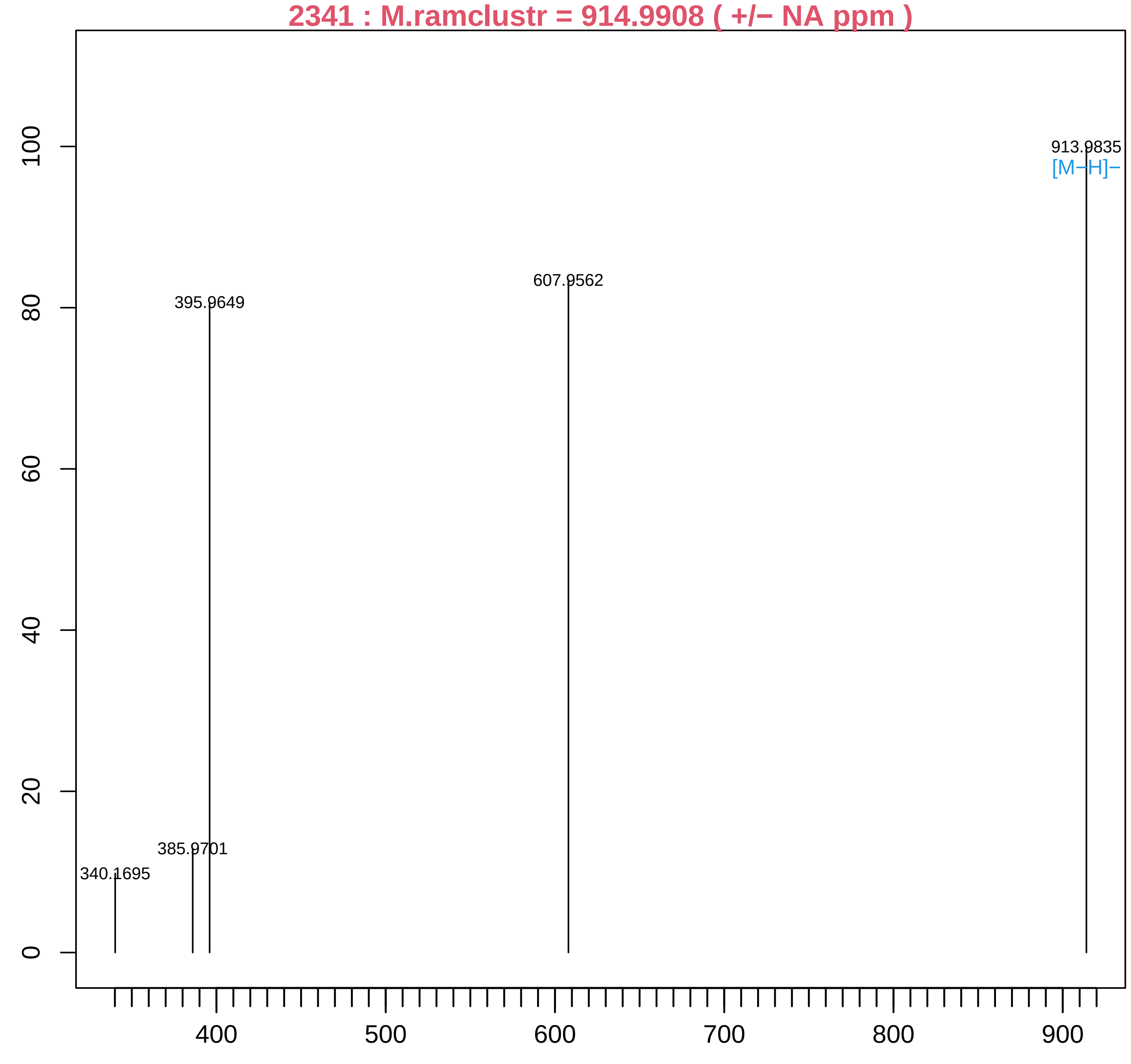
2341 : M.ramclustr = 914.9908 (+/− NA ppm)
913.9835
[M−H]−
607.9562
395.9649
385.9701
340.1695
0
20
40
60
80
100
400
500
600
700
800
900

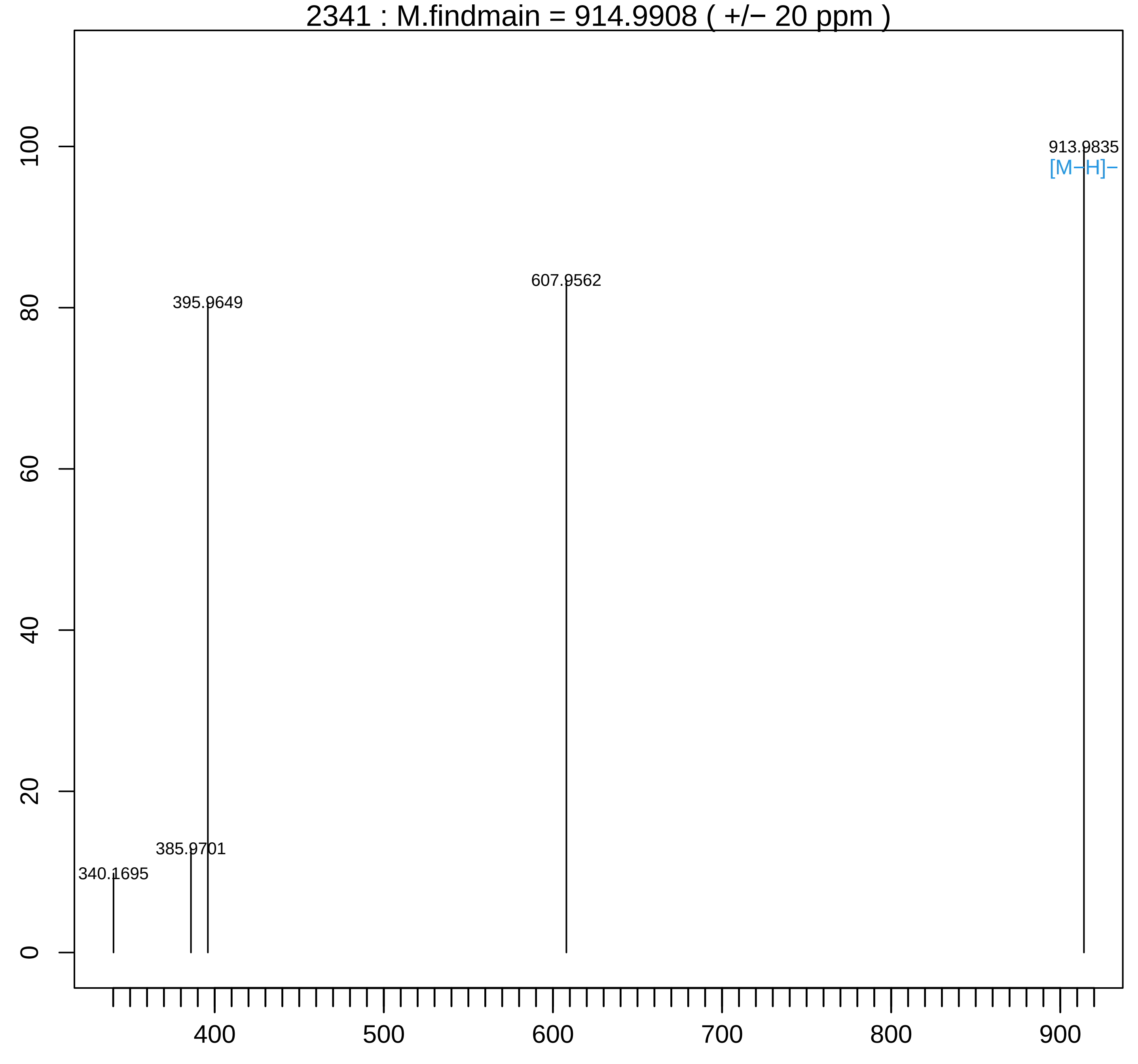
2341 : M.findmain = 914.9908 (+/− 20 ppm)
913.9835
[M−H]−
607.9562
395.9649
385.9701
340.1695
0
20
40
60
80
100
400
500
600
700
800
900

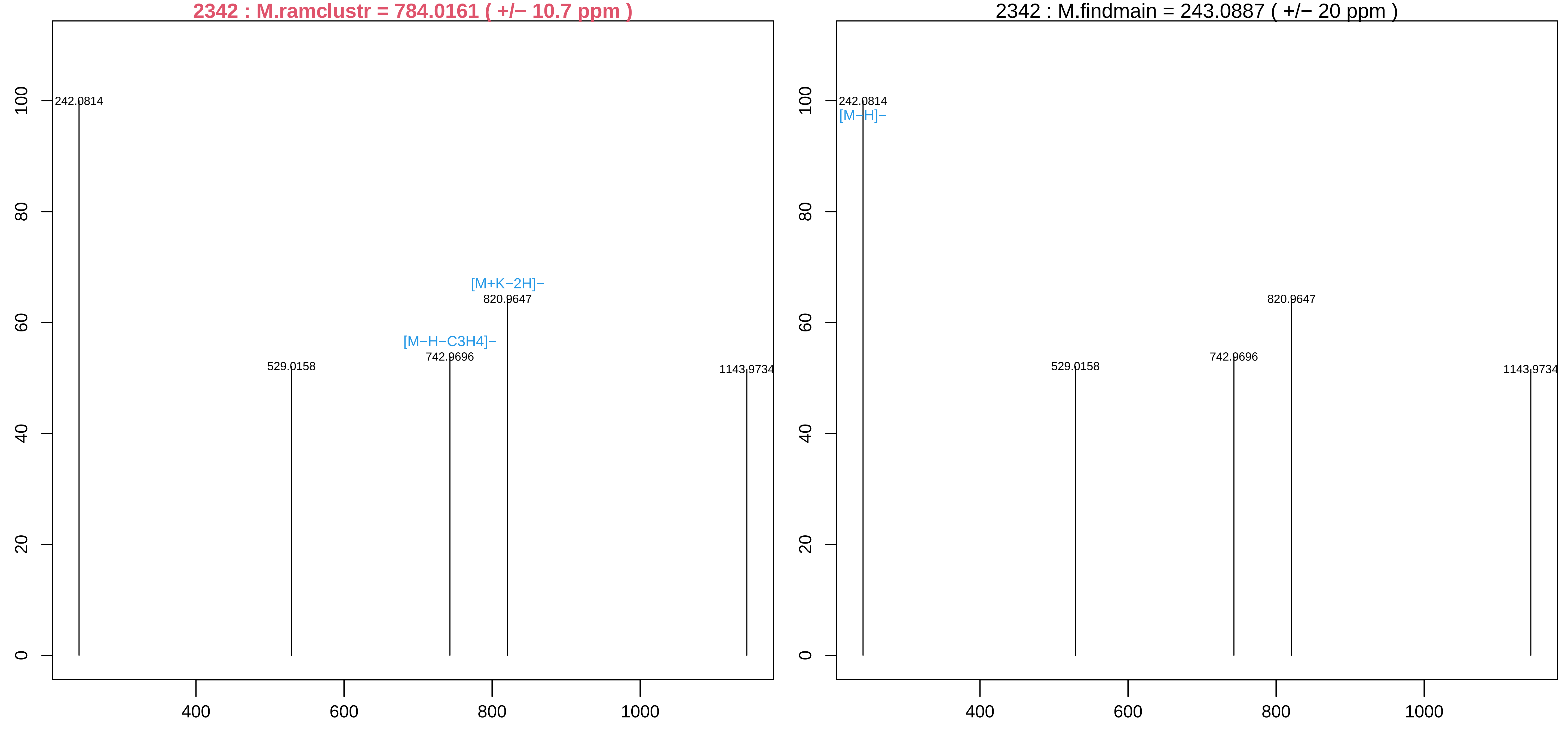
2342 : M.ramclustr = 784.0161 (+/− 10.7 ppm)
242.0814
529.0158
[M−H−C3H4]−
742.9696
[M+K−2H]−
820.9647
1143.9734
0
20
40
60
80
100
400
600
800
1000
2342 : M.findmain = 243.0887 (+/− 20 ppm)
242.0814
[M−H]−
529.0158
742.9696
820.9647
1143.9734
0
20
40
60
80
100
400
600
800
1000

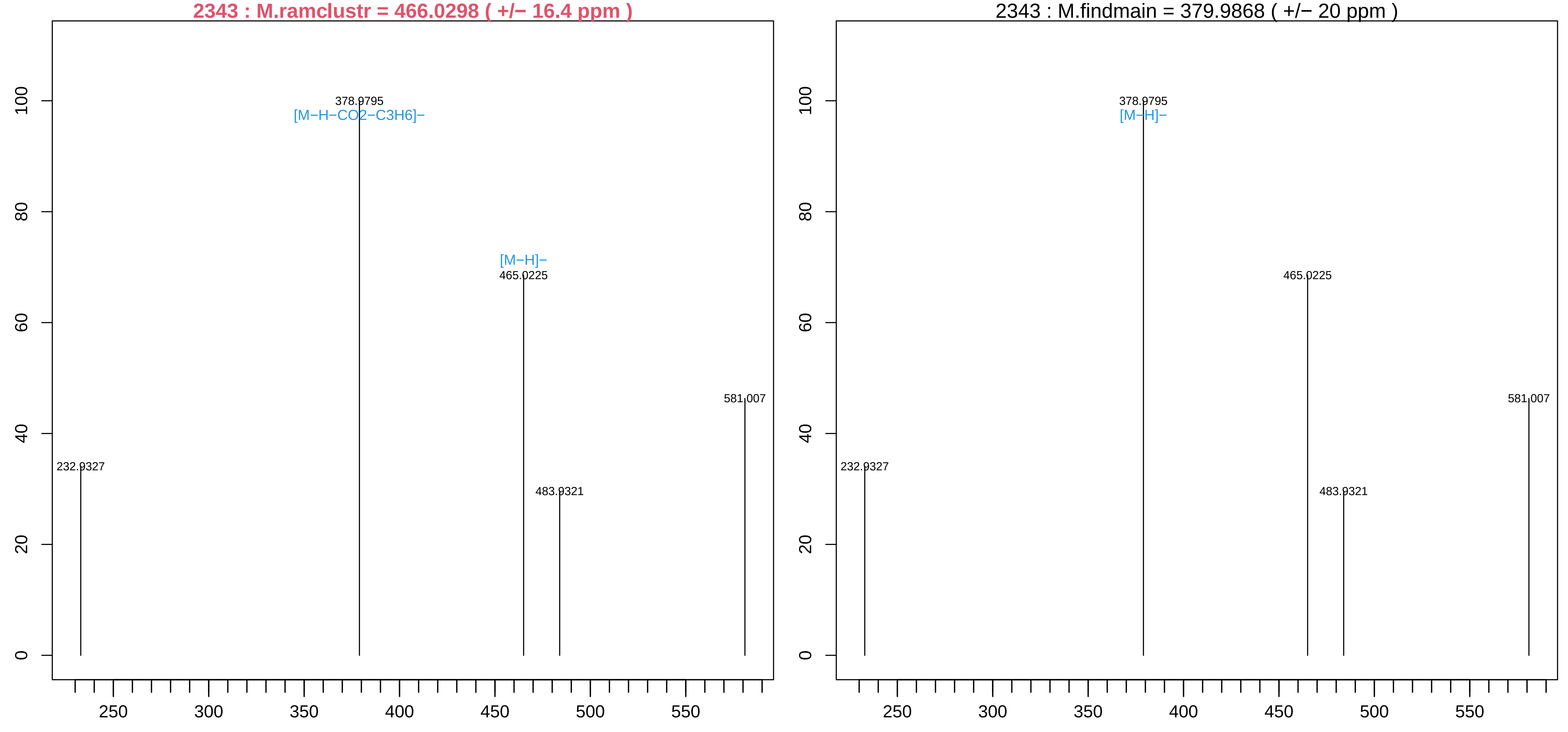
2343 : M.ramclustr = 466.0298 (+/− 16.4 ppm)
378.9795
[M−H−CO2−C3H6]−
[M−H]−
465.0225
581.007
232.9327
483.9321
2343 : M.findmain = 379.9868 (+/− 20 ppm)
378.9795
[M−H]−
465.0225
581.007
232.9327
483.9321

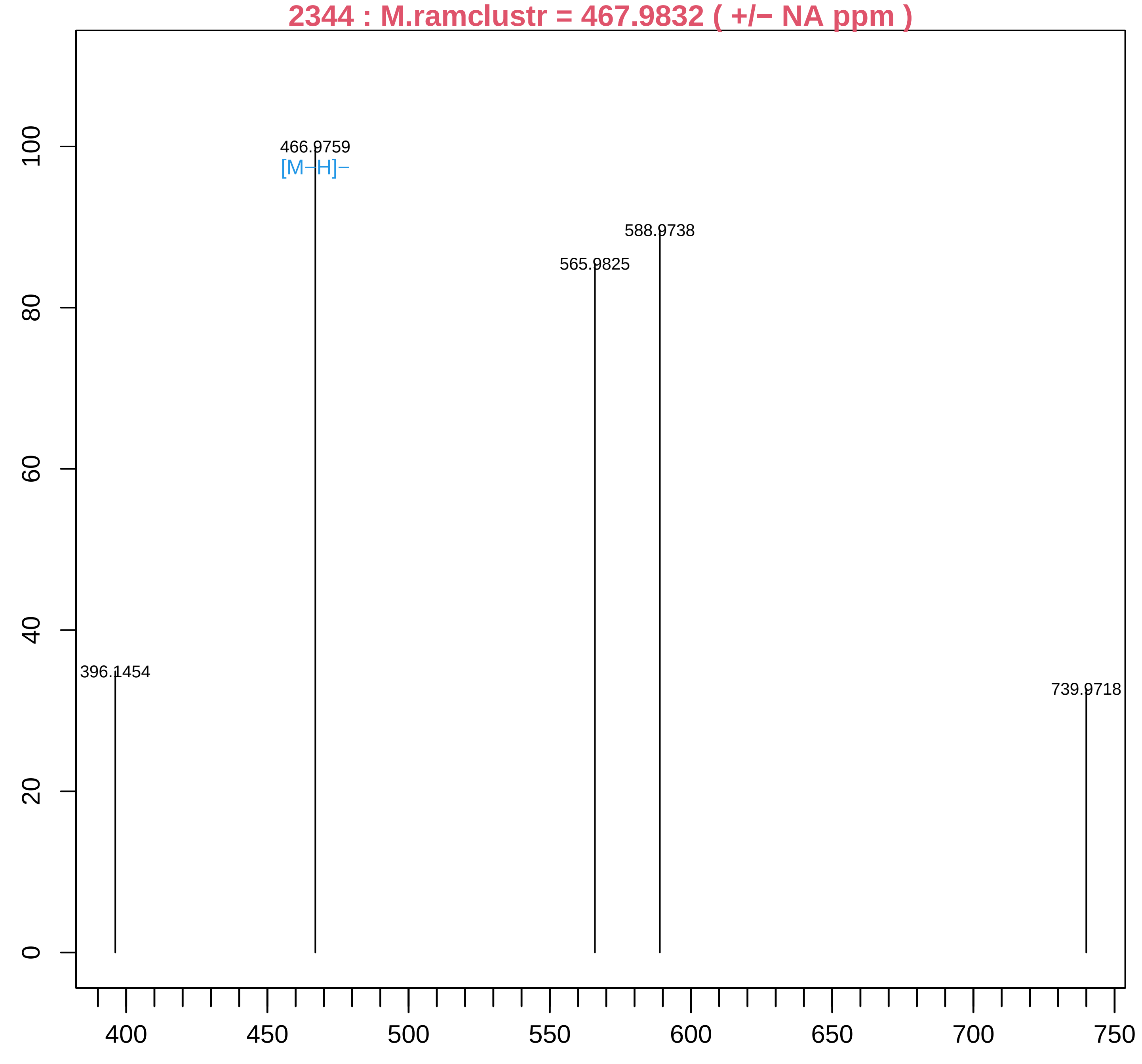

2344 : M.ramclustr = 467.9832 (+/− NA ppm)
466.9759
[M−H]−
588.9738
565.9825
396.1454
739.9718
0
20
40
60
80
100
400
450
500
550
600
650
700
750

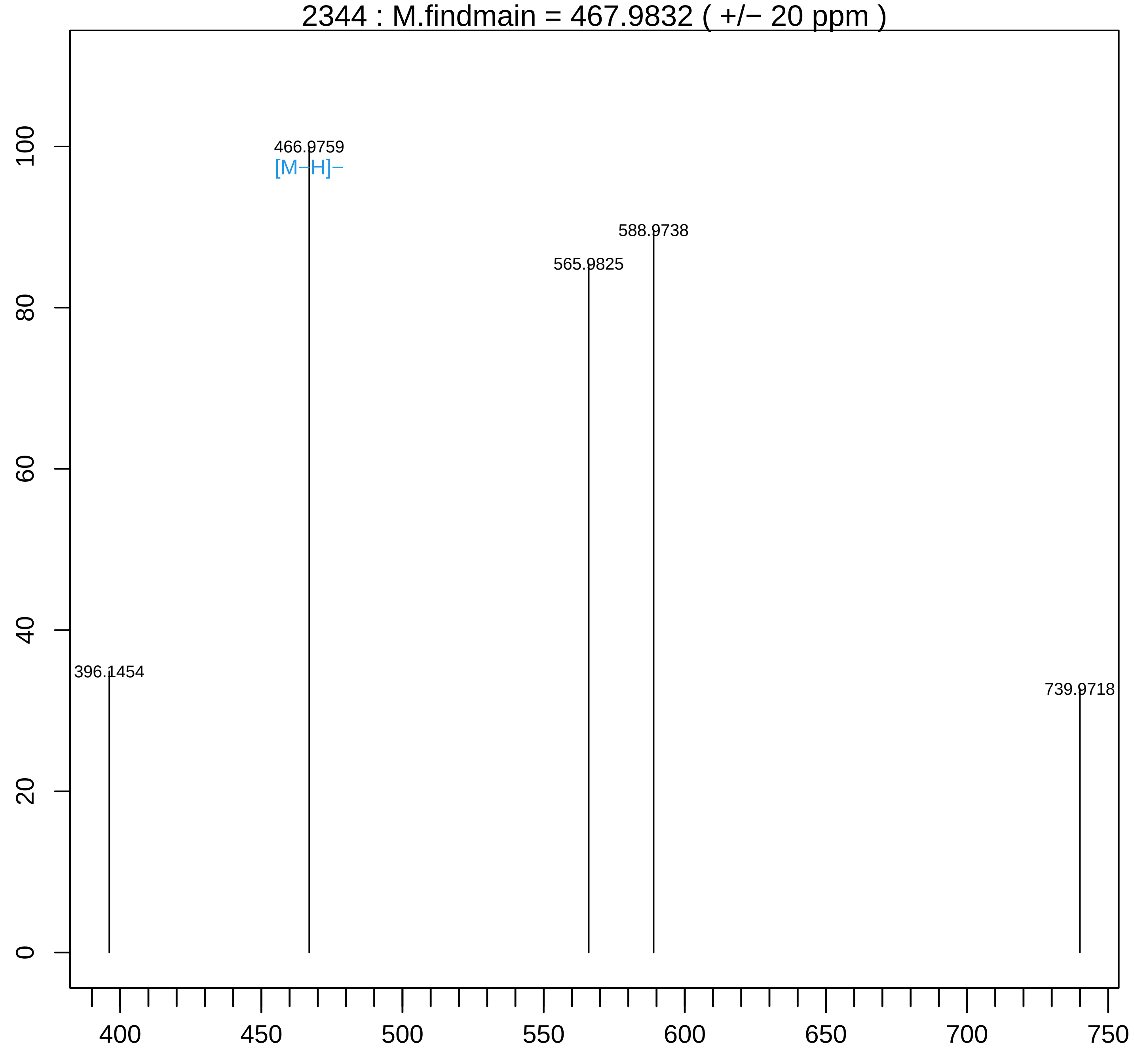

2344 : M.findmain = 467.9832 (+/− 20 ppm)
466.9759
[M−H]−
588.9738
565.9825
396.1454
739.9718
0
20
40
60
80
100
400
450
500
550
600
650
700
750

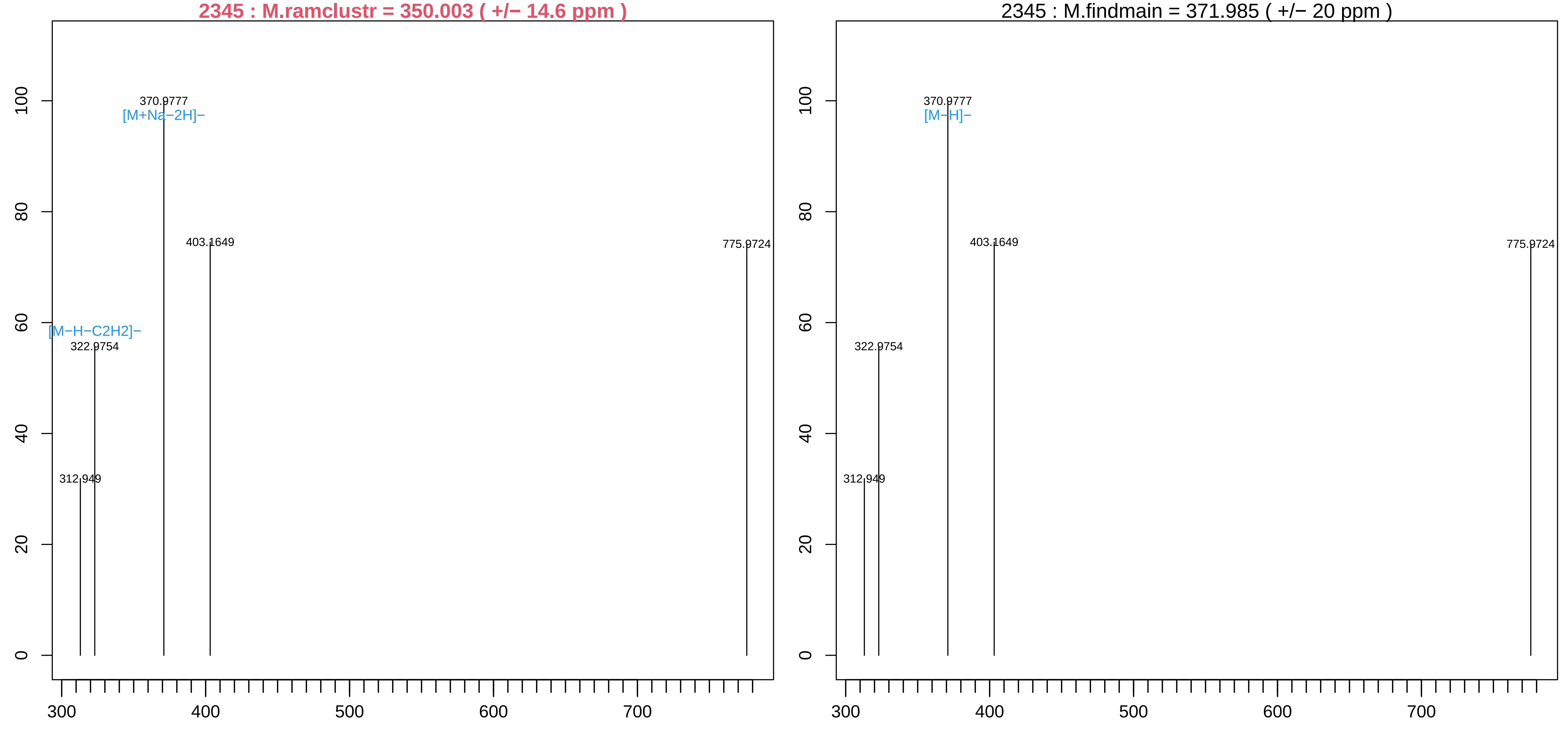

2345 : M.ramclustr = 350.003 (+/− 14.6 ppm)
370.9777
[M+Na−2H]−
403.1649
775.9724
[M−H−C2H2]−
322.9754
312.949
2345 : M.findmain = 371.985 (+/− 20 ppm)
370.9777
[M−H]−
403.1649
775.9724
322.9754
312.949
0
20
40
60
80
100
300
400
500
600
700

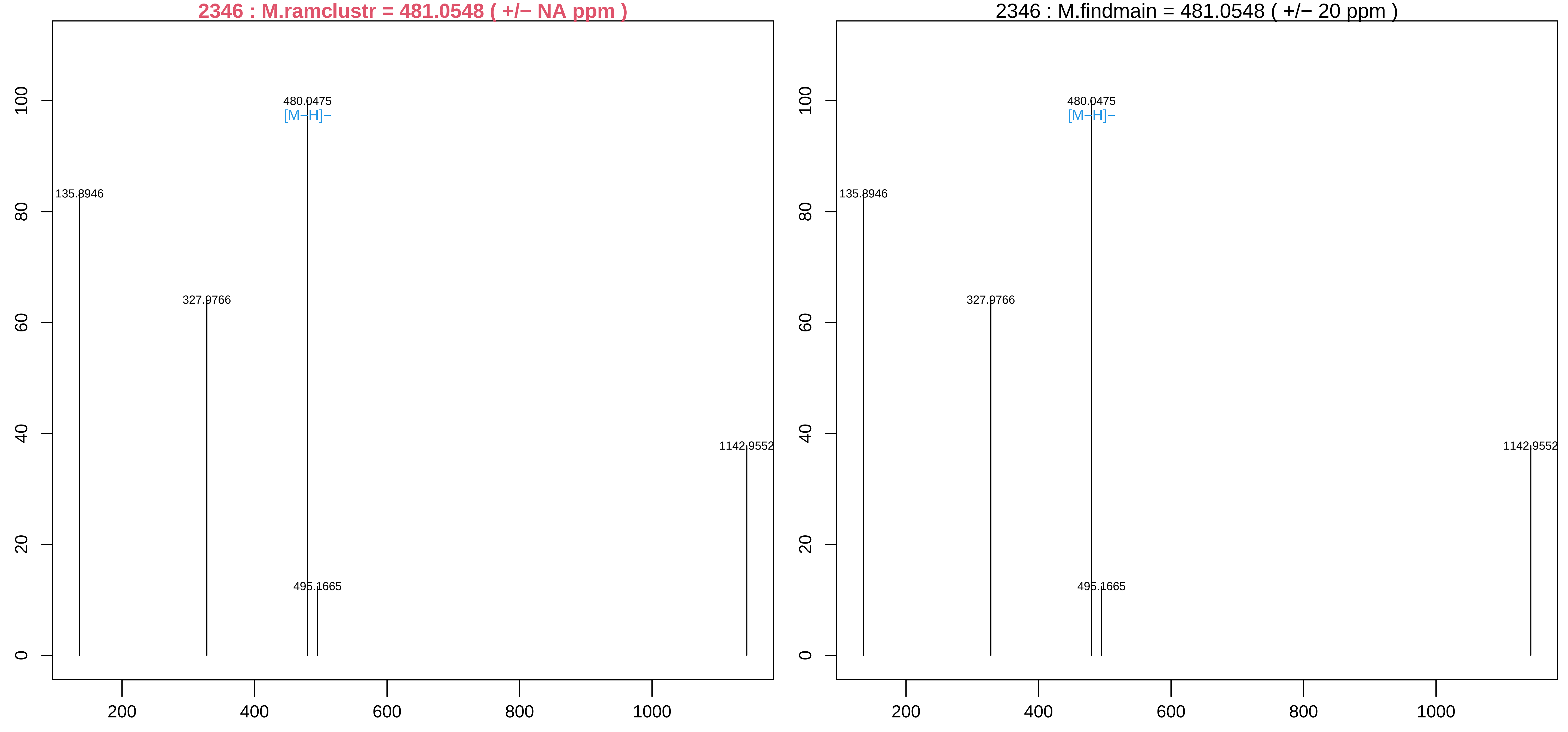
2346 : M.ramclustr = 481.0548 (+/− NA ppm)
2346 : M.findmain = 481.0548 (+/− 20 ppm)
135.8946
327.9766
480.0475
[M−H]−
495.1665
1142.9552
0
20
40
60
80
100
200
400
600
800
1000

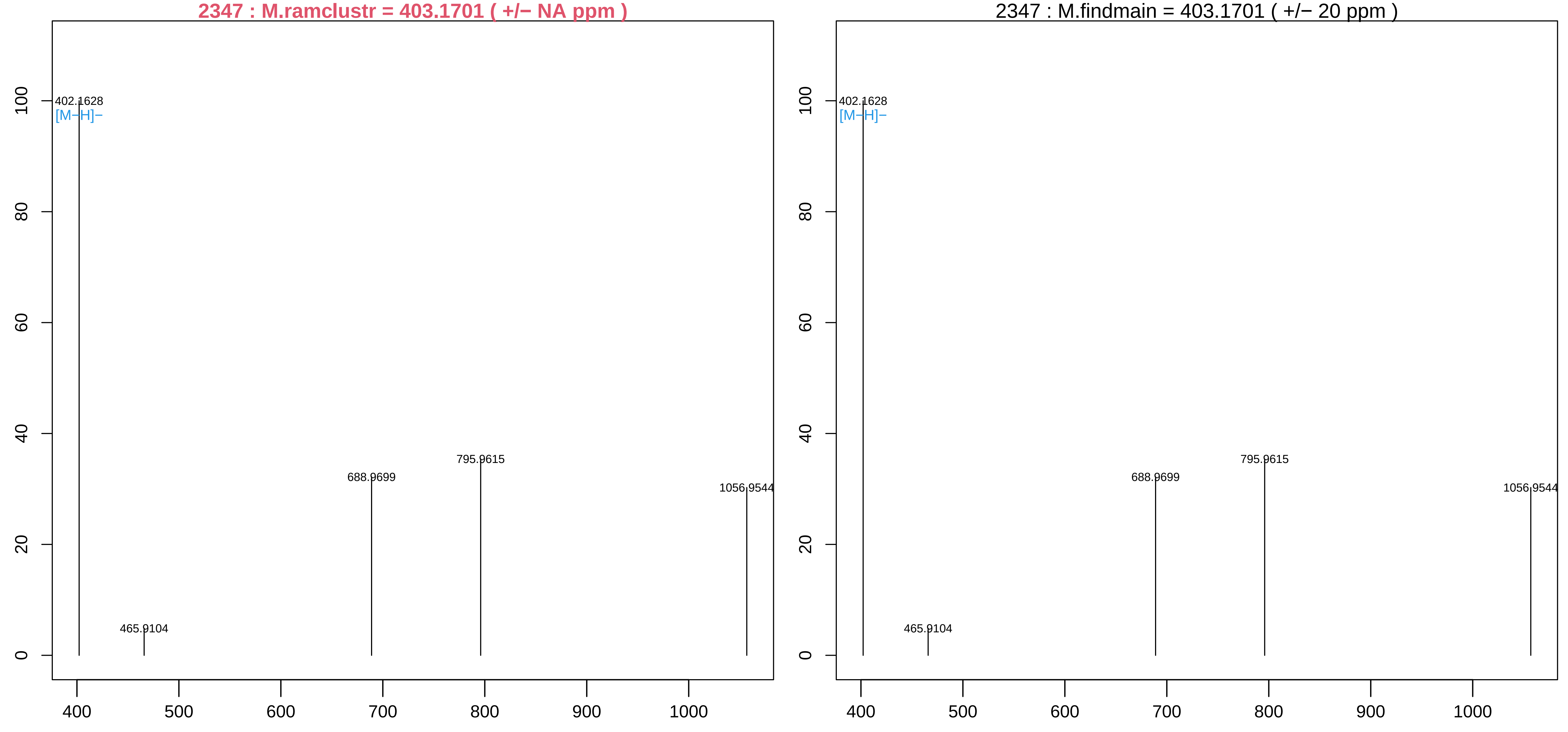
2347 : M.ramclustr = 403.1701 (+/− NA ppm)
402.1628
[M−H]−
465.9104
688.9699
795.9615
1056.9544
2347 : M.findmain = 403.1701 (+/− 20 ppm)
402.1628
[M−H]−
465.9104
688.9699
795.9615
1056.9544

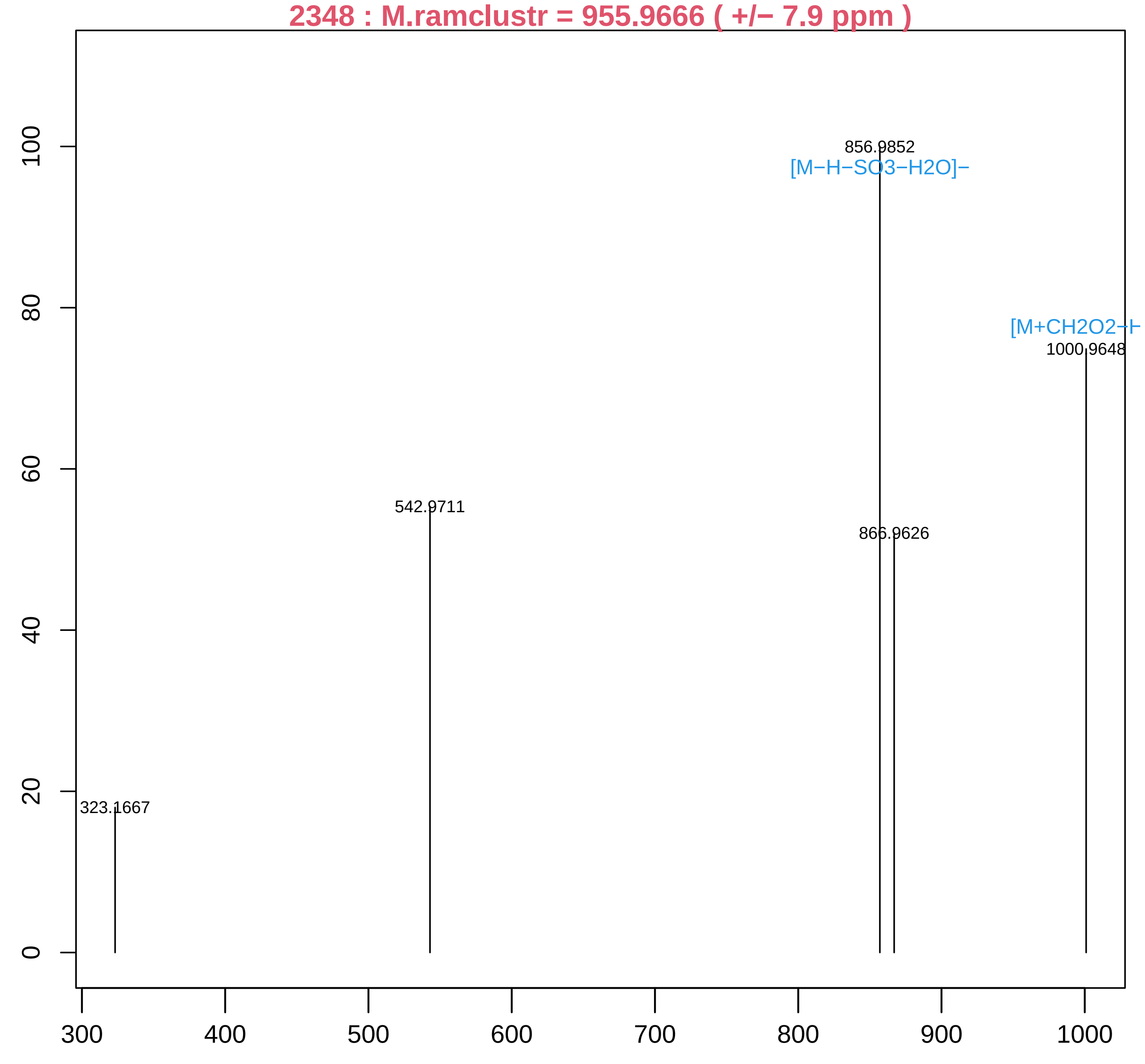
2348 : M.ramclustr = 955.9666 (+/− 7.9 ppm)
856.9852
[M−H−SO3−H2O]−
[M+CH2O2−H
1000.9648
542.9711
866.9626
323.1667
0
20
40
60
80
100
300
400
500
600
700
800
900
1000

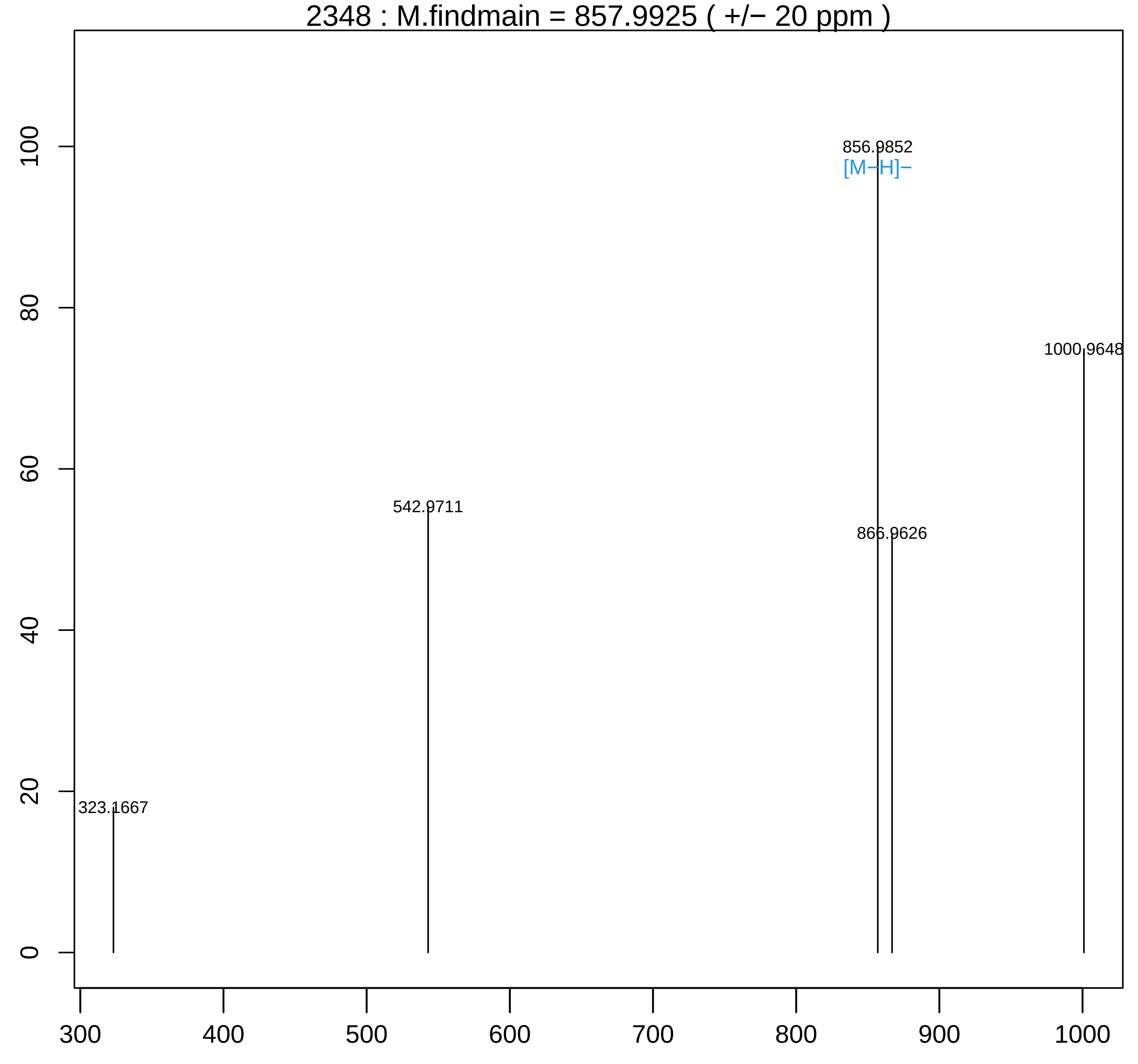
2348 : M.findmain = 857.9925 (+/− 20 ppm)
856.9852
[M−H]−
1000.9648
542.9711
866.9626
323.1667
0
20
40
60
80
100
300
400
500
600
700
800
900
1000

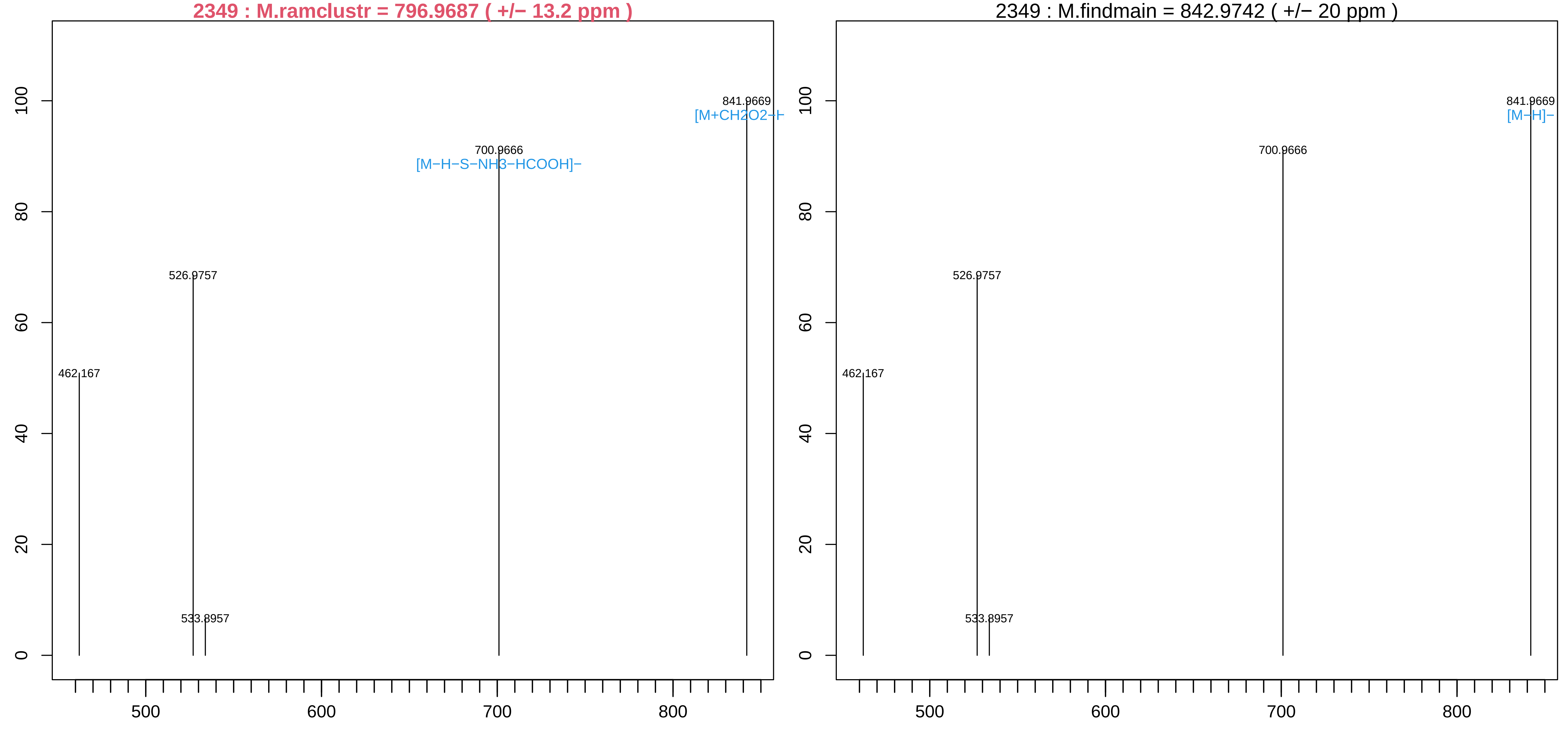

2349 : M.ramclustr = 796.9687 (+/− 13.2 ppm)
841.9669
[M+CH2O2-H
700.9666
[M−H−S−NH3−HCOOH]−
526.9757
462.167
533.8957
2349 : M.findmain = 842.9742 (+/− 20 ppm)
841.9669
[M−H]−
700.9666
526.9757
462.167
533.8957
0
20
40
60
80
100
500
600
700
800

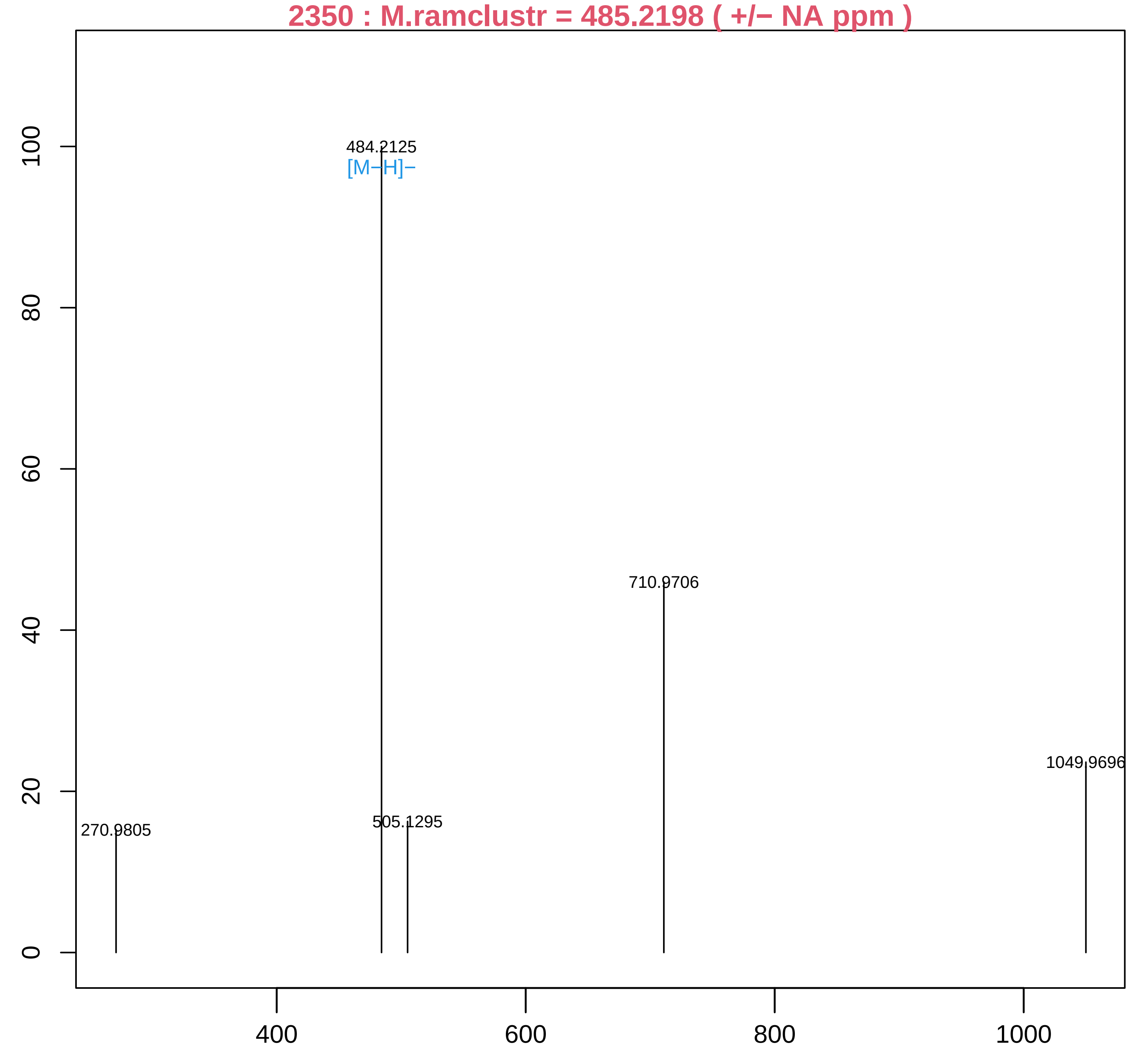
2350 : M.ramclustr = 485.2198 (+/− NA ppm)
484.2125
[M−H]−
710.9706
1049.9696
505.1295
270.9805
0
20
40
60
80
100
400
600
800
1000

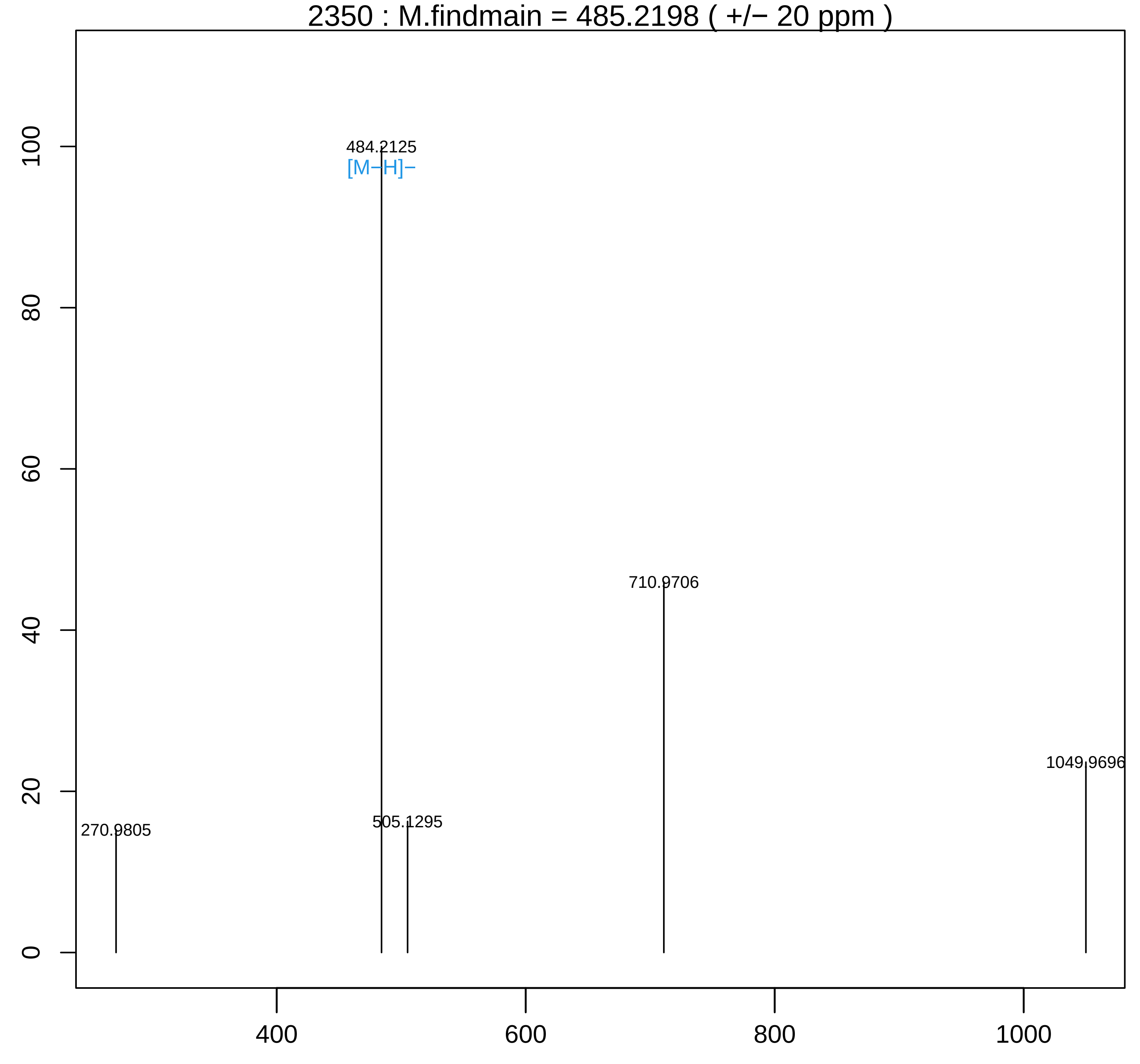
2350 : M.findmain = 485.2198 (+/− 20 ppm)
484.2125
[M−H]−
710.9706
1049.9696
505.1295
270.9805
0
20
40
60
80
100
400
600
800
1000

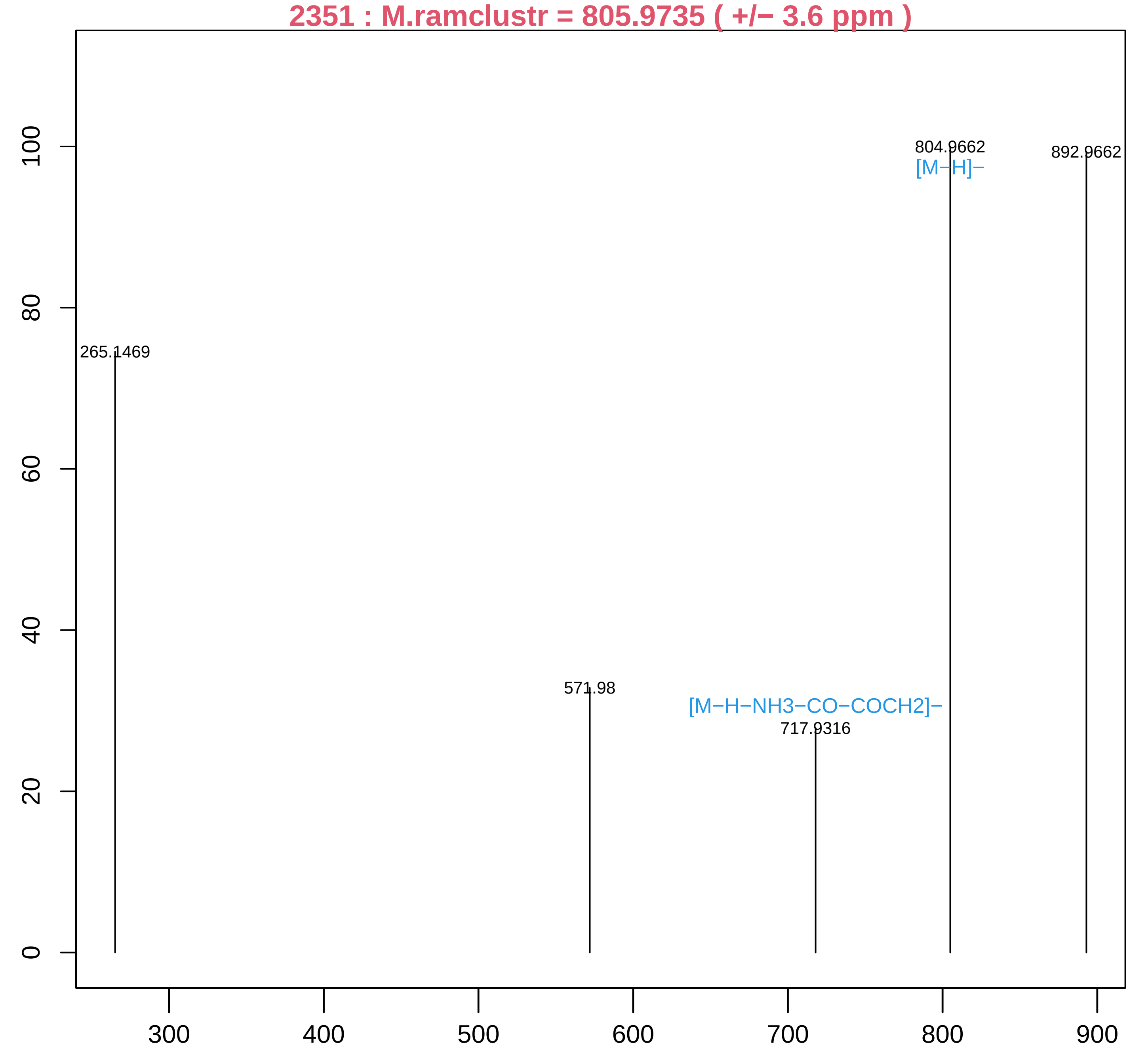
2351 : M.ramclustr = 805.9735 (+/− 3.6 ppm)
265.1469
571.98
[M−H−NH3−CO−COCH2]−
717.9316
804.9662
[M−H]−
892.9662
0
20
40
60
80
100
300
400
500
600
700
800
900

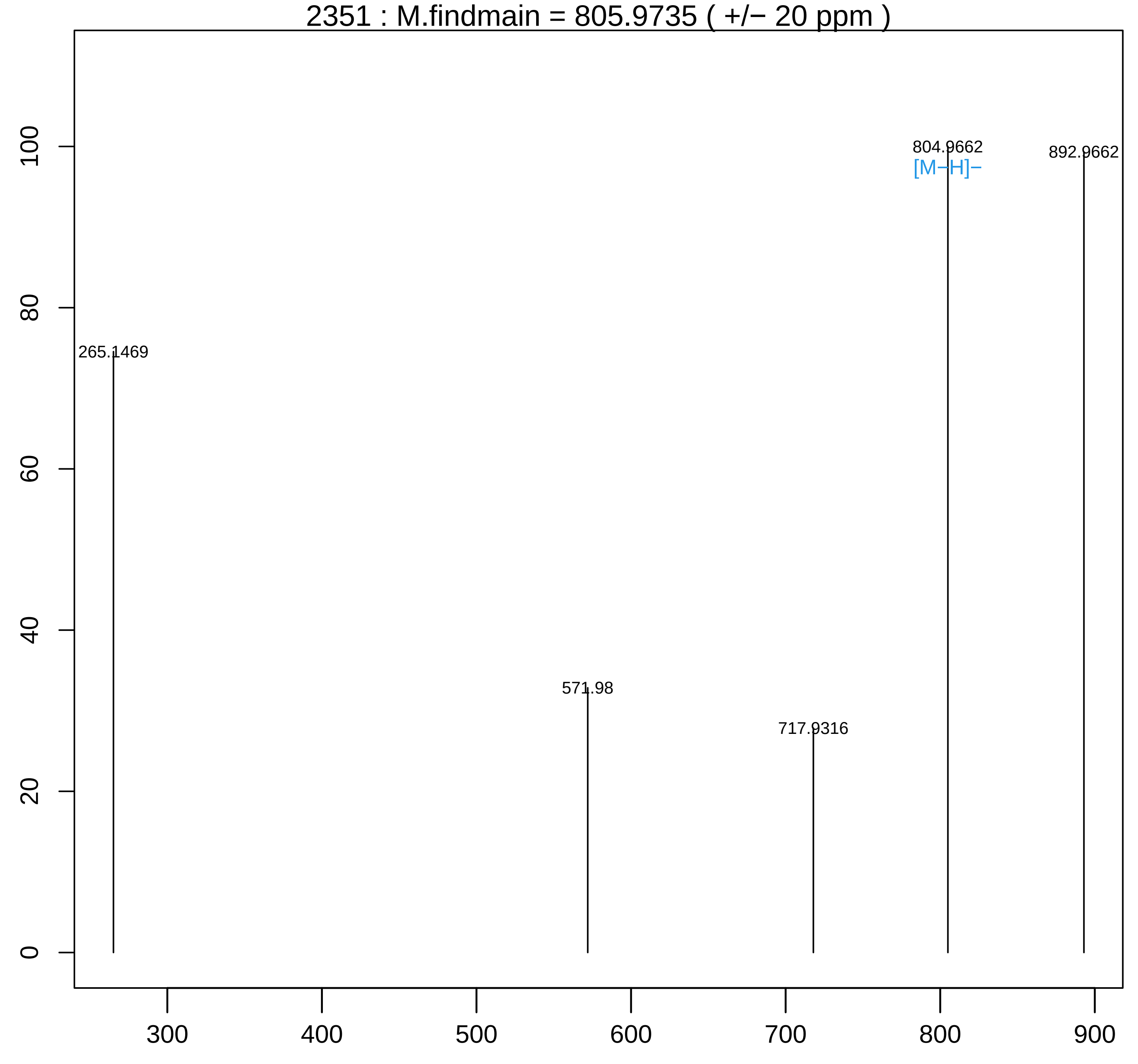
2351 : M.findmain = 805.9735 (+/− 20 ppm)
265.1469
571.98
717.9316
804.9662
[M−H]−
892.9662
0
20
40
60
80
100
300
400
500
600
700
800
900

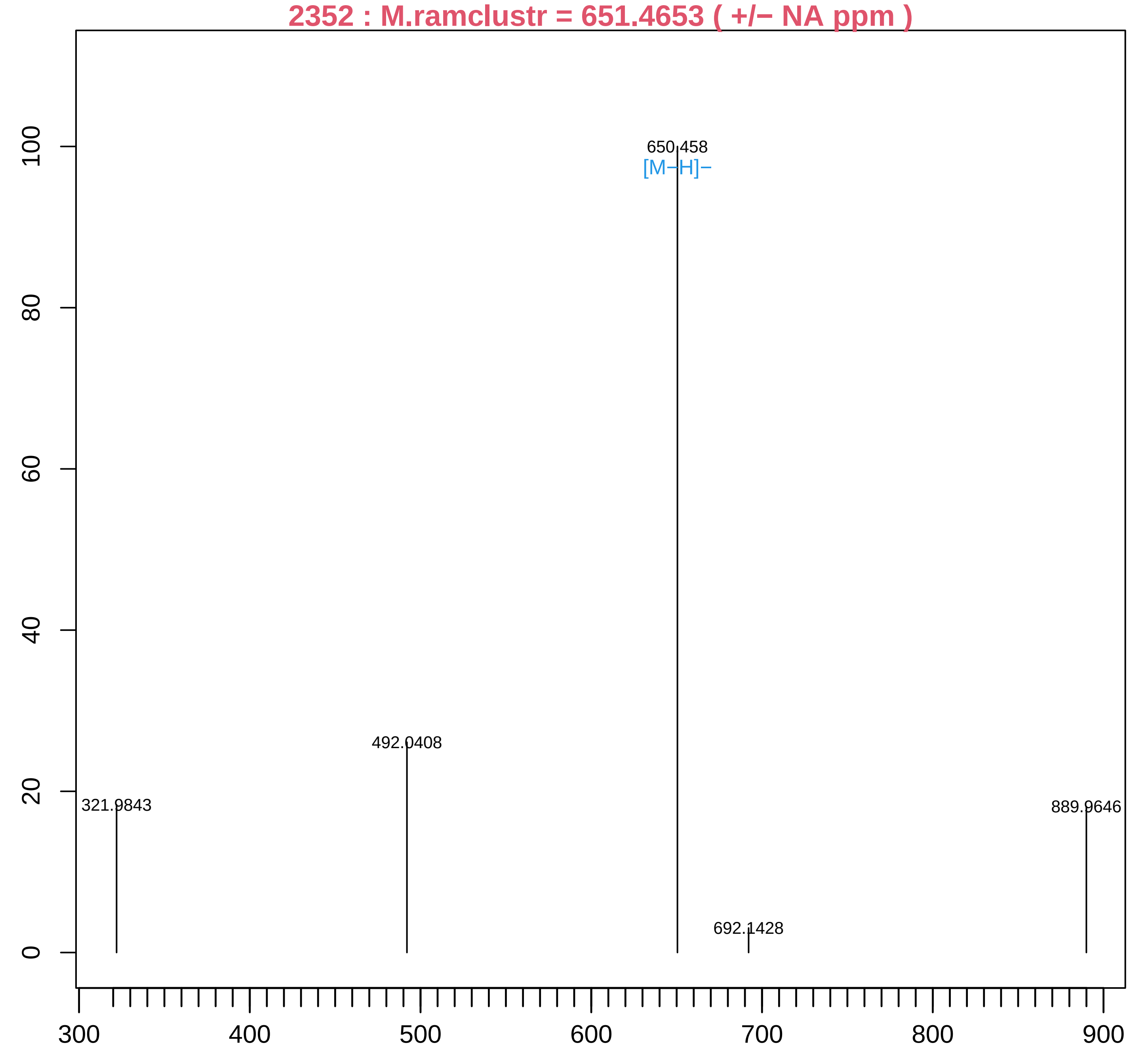
2352 : M.ramclustr = 651.4653 (+/− NA ppm)
650.458
[M−H]−
492.0408
321.9843
889.9646
692.1428

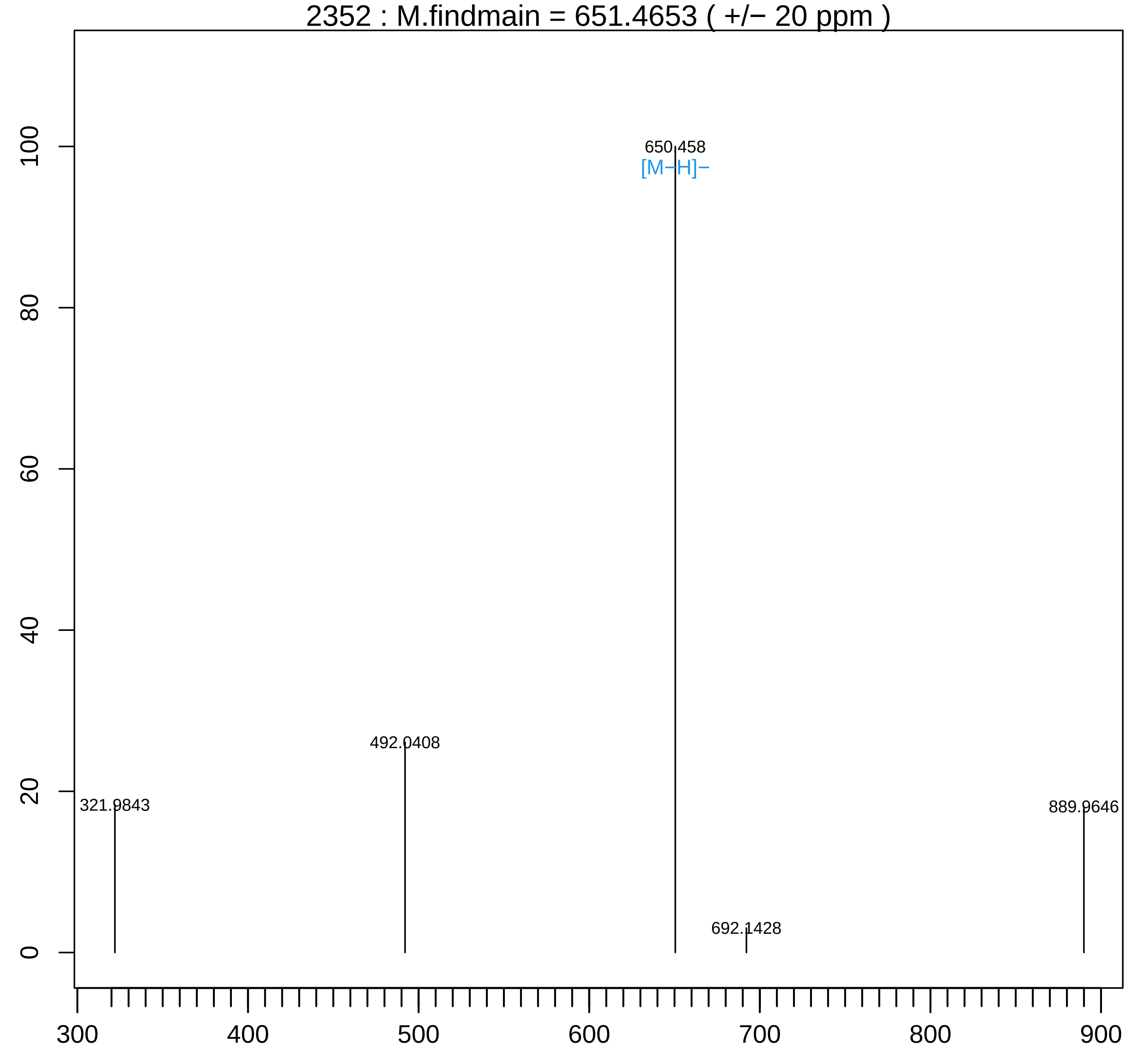
2352 : M.findmain = 651.4653 (+/− 20 ppm)
650.458
[M−H]−
492.0408
321.9843
889.9646
692.1428

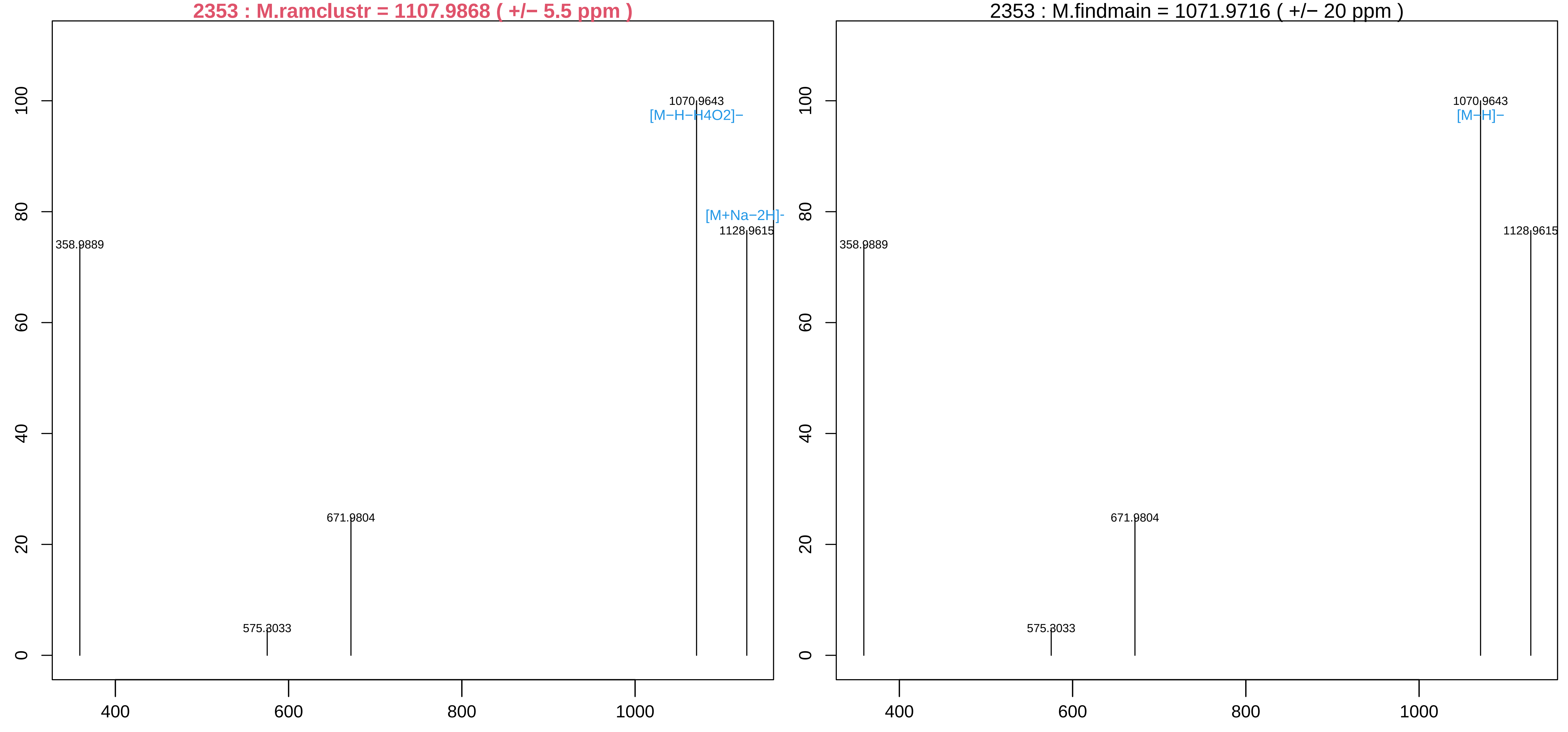

2353 : M.ramclustr = 1107.9868 (+/− 5.5 ppm)
1070.9643
[M−H−H4O2]−
[M+Na−2H]-
1128.9615
358.9889
671.9804
575.3033
0
20
40
60
80
100
400
600
800
1000
2353 : M.findmain = 1071.9716 (+/− 20 ppm)
1070.9643
[M−H]−
1128.9615
358.9889
671.9804
575.3033
0
20
40
60
80
100
400
600
800
1000

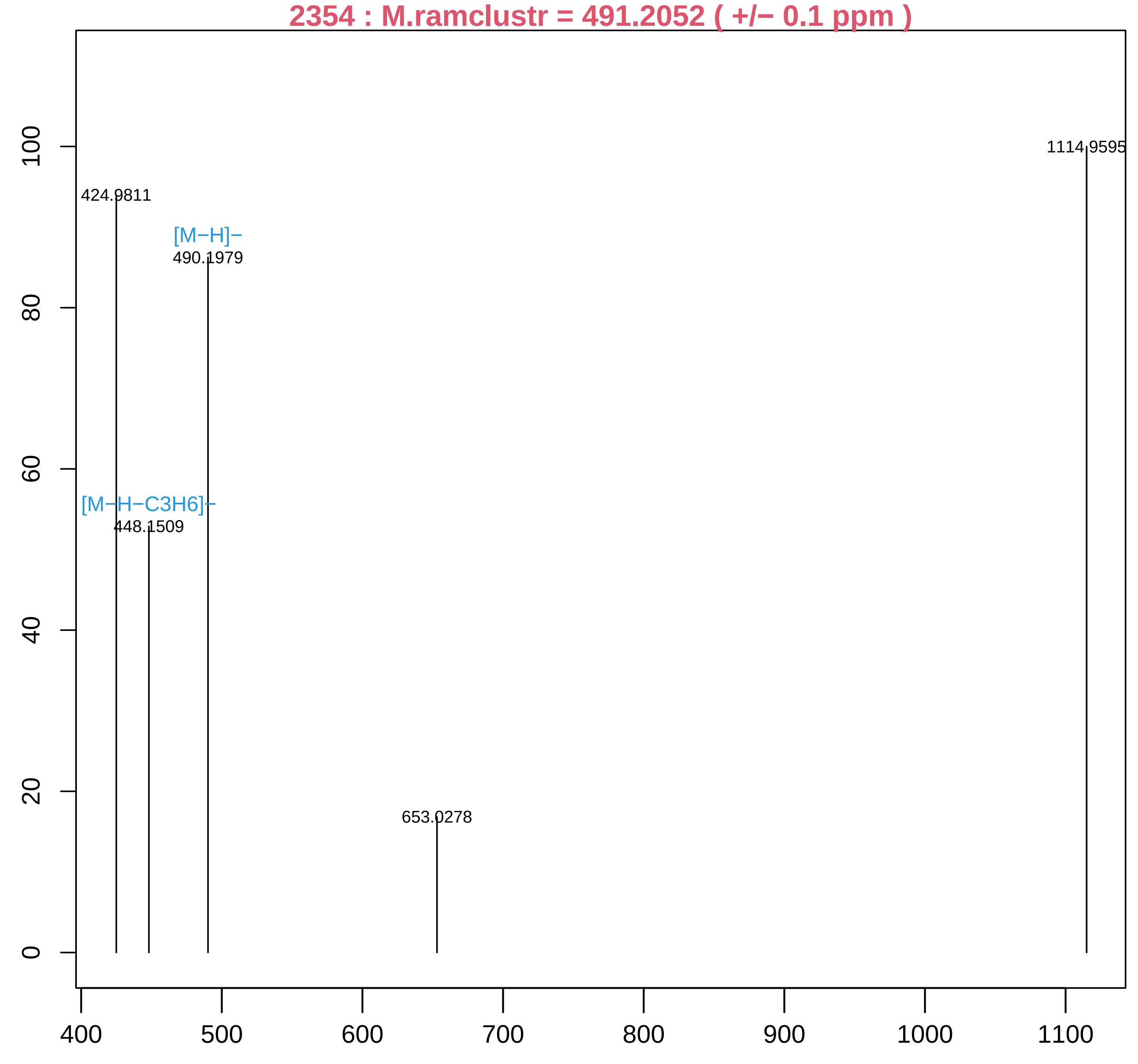
2354 : M.ramclustr = 491.2052 (+/− 0.1 ppm)
1114.9595
424.9811
[M−H]−
490.1979
[M−H−C3H6]−
448.1509
653.0278
0
20
40
60
80
100
400
500
600
700
800
900
1000
1100

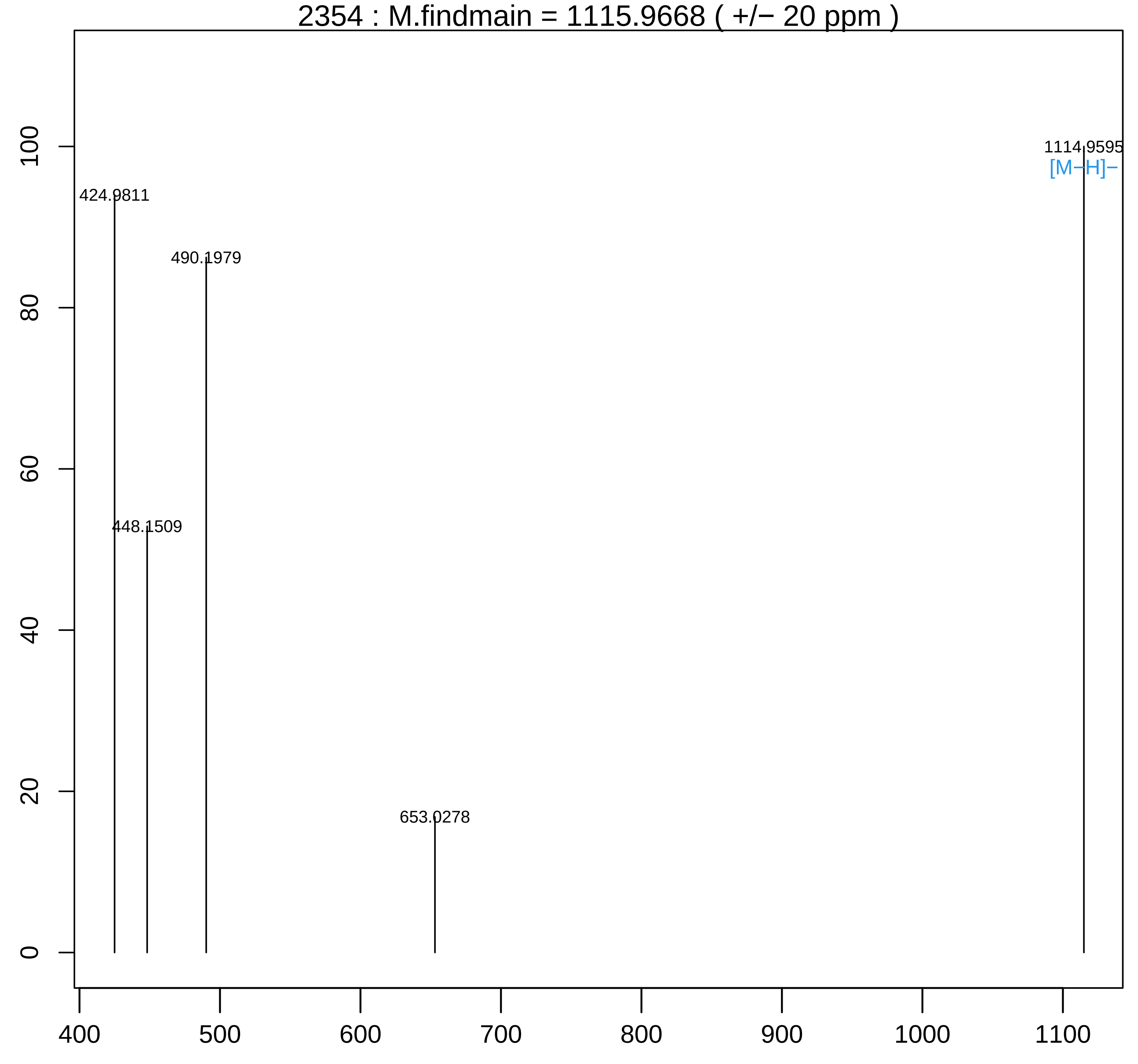
2354 : M.findmain = 1115.9668 (+/− 20 ppm)
1114.9595
[M−H]−
424.9811
490.1979
448.1509
653.0278
0
20
40
60
80
100
400
500
600
700
800
900
1000
1100

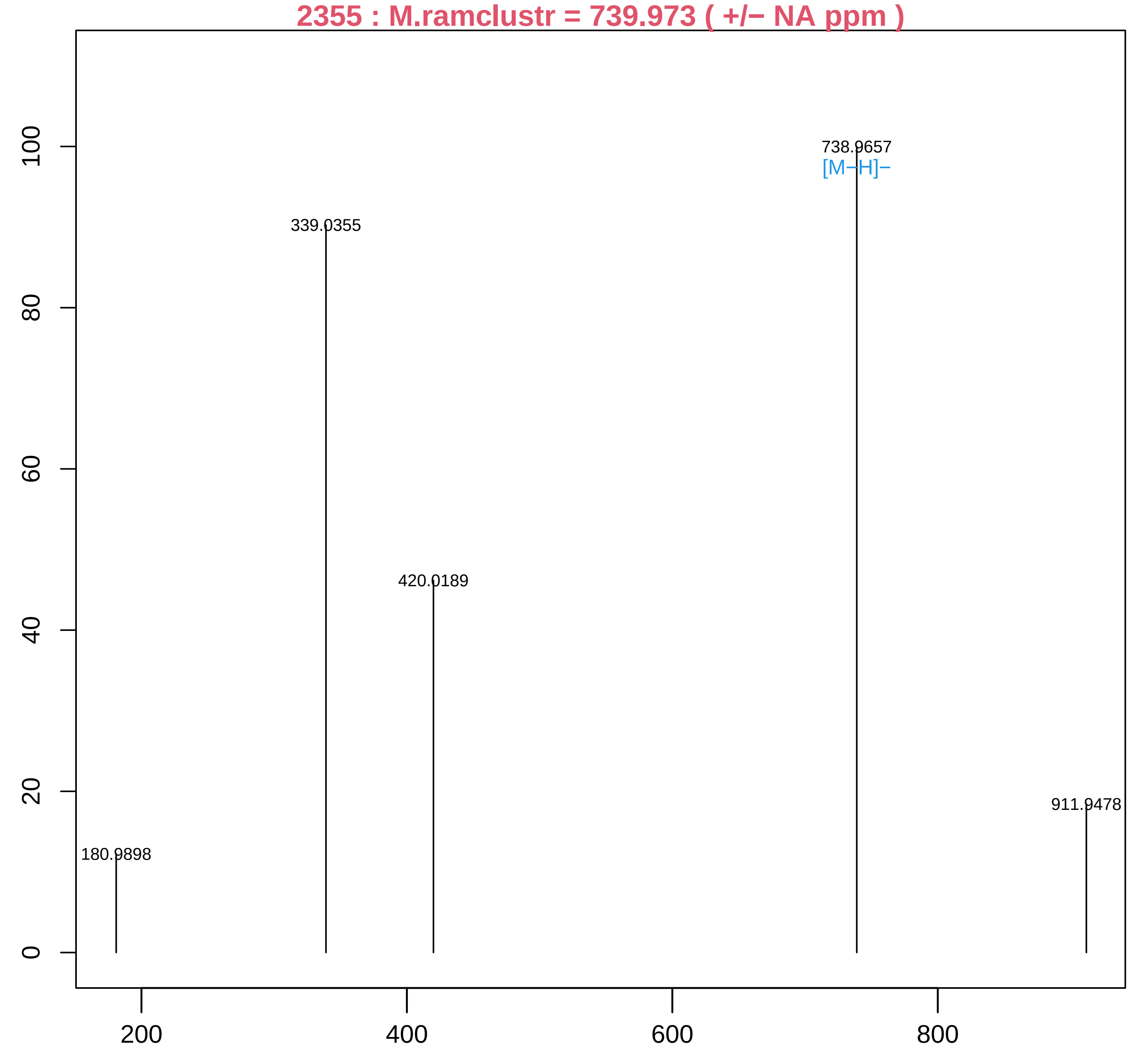
2355 : M.ramclustr = 739.973 (+/− NA ppm)
180.9898
339.0355
420.0189
738.9657
[M−H]−
911.9478
0
20
40
60
80
100
200
400
600
800

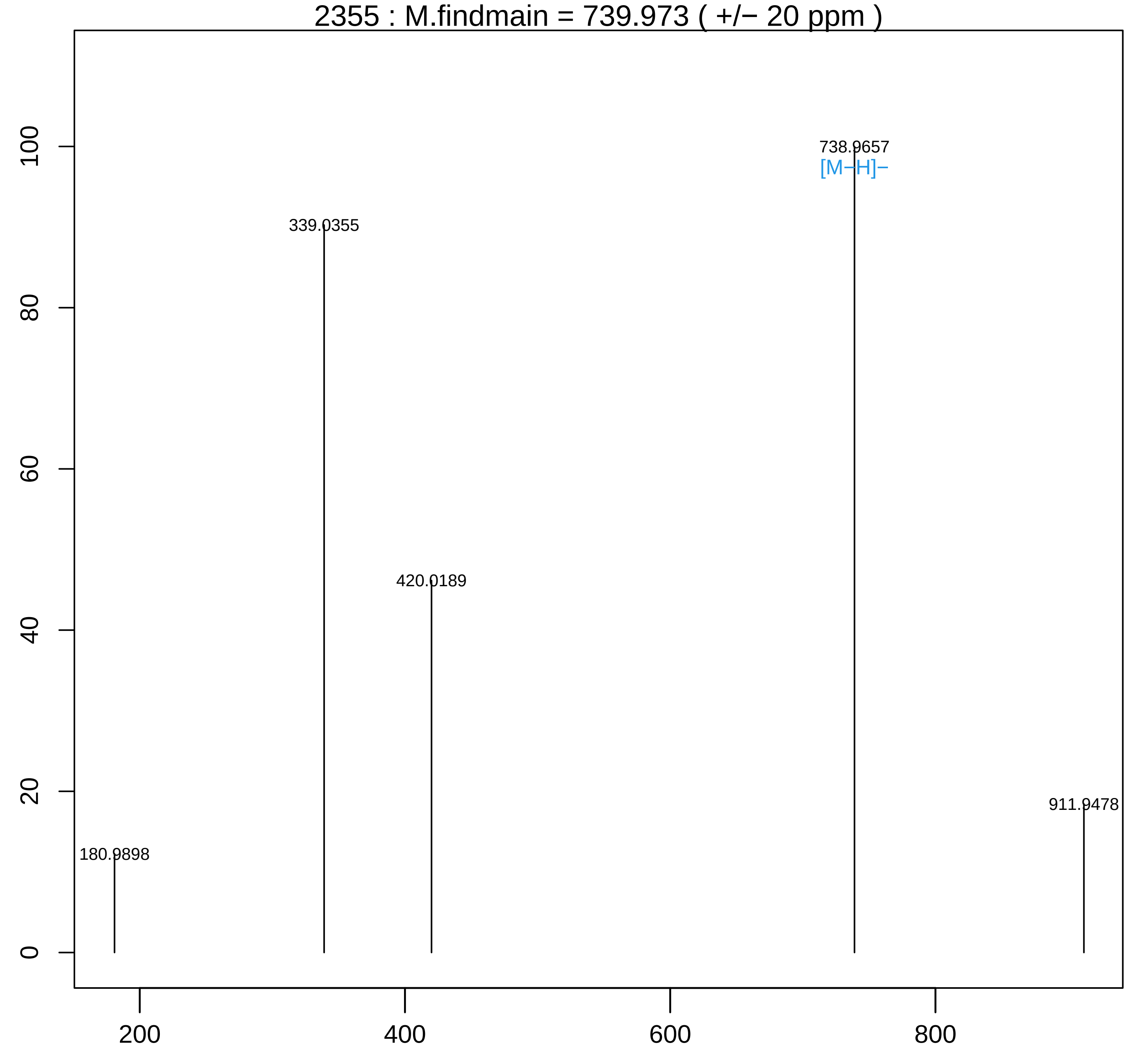
2355 : M.findmain = 739.973 (+/− 20 ppm)
180.9898
339.0355
420.0189
738.9657
[M−H]−
911.9478
0
20
40
60
80
100
200
400
600
800

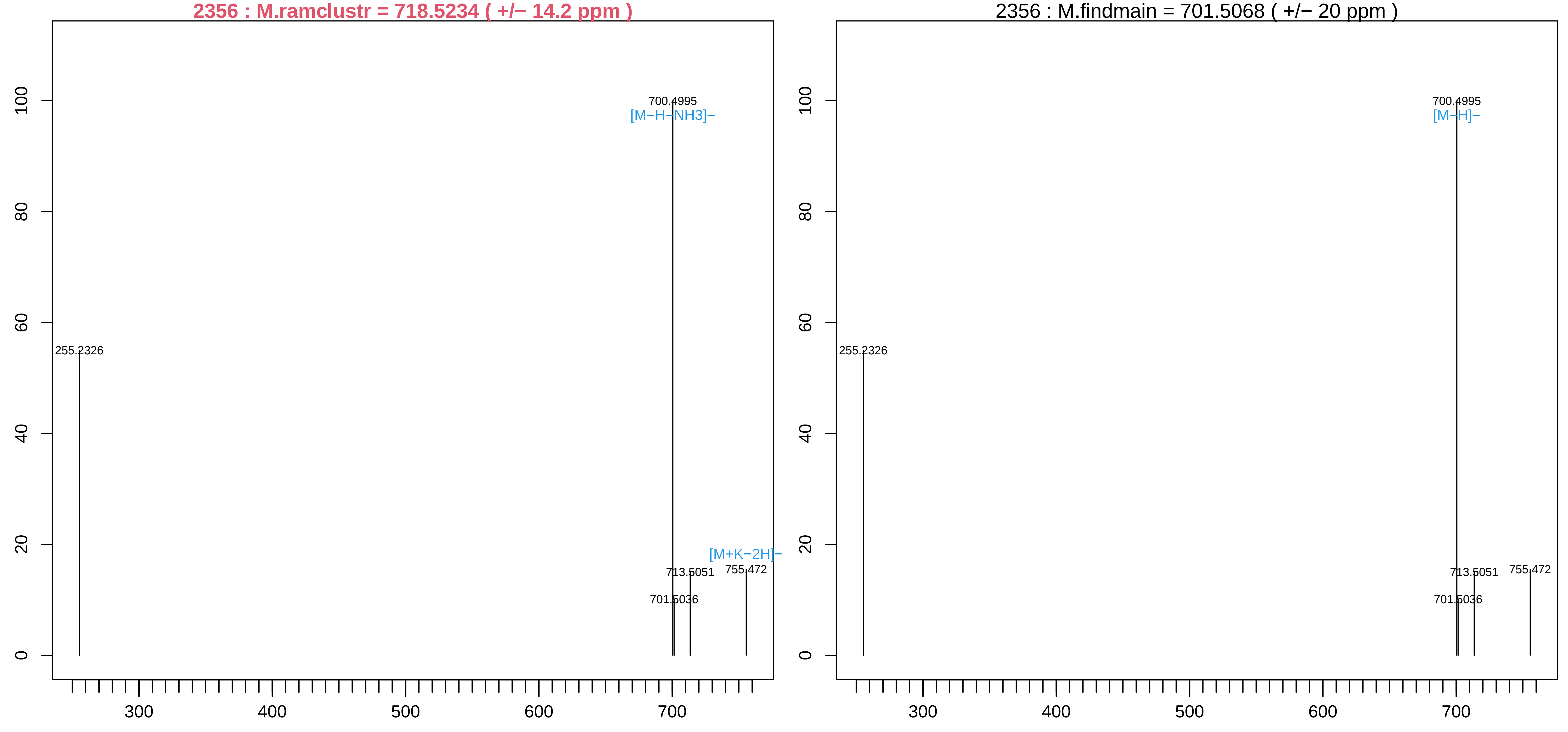

2356 : M.ramclustr = 718.5234 (+/− 14.2 ppm)
700.4995
[M−H−NH3]−
255.2326
[M+K−2H]−
713.5051
755.472
701.5036
2356 : M.findmain = 701.5068 (+/− 20 ppm)
700.4995
[M−H]−
255.2326
713.5051
755.472
701.5036
0
20
40
60
80
100
300
400
500
600
700

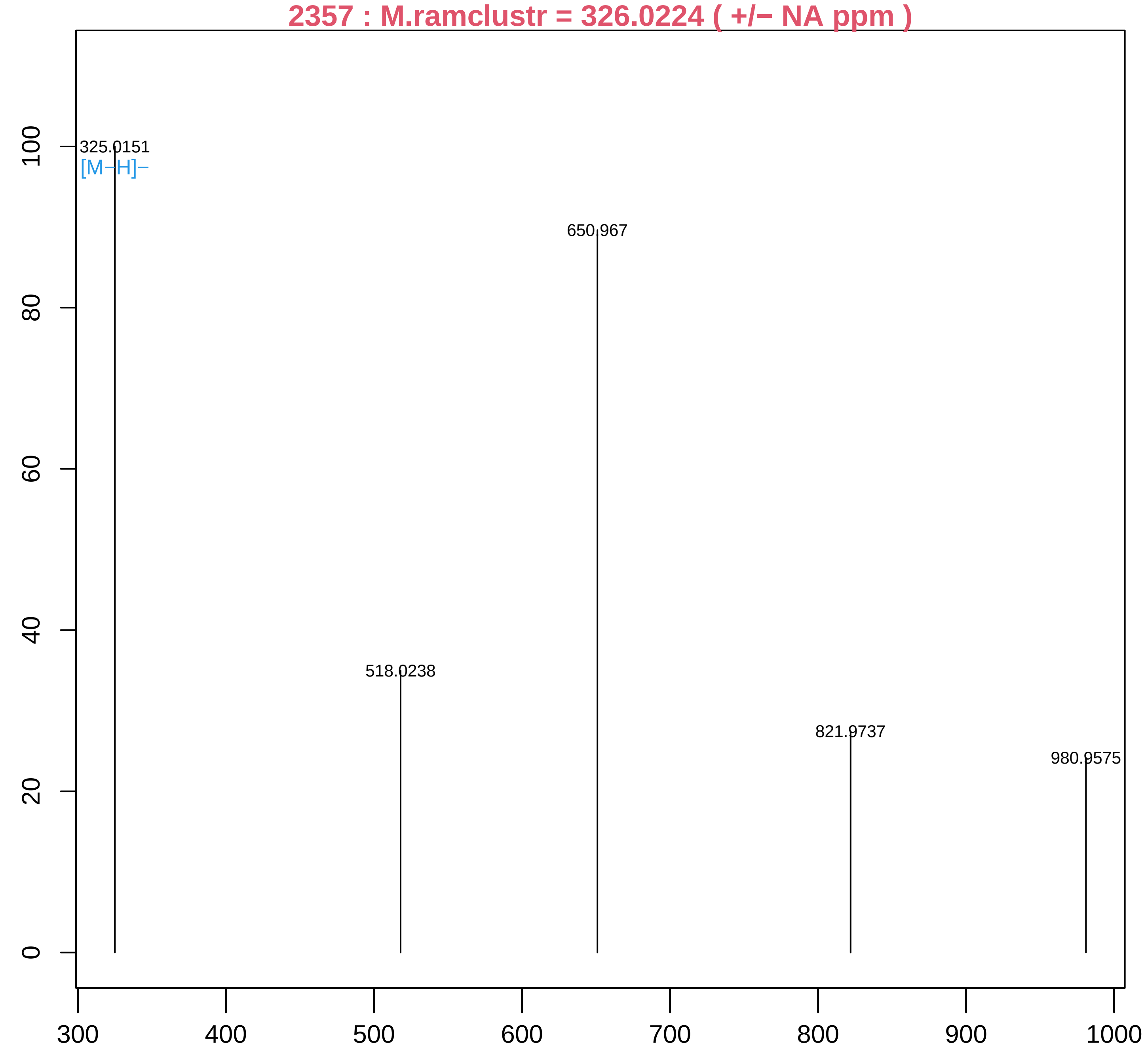

2357 : M.ramclustr = 326.0224 (+/− NA ppm)
325.0151
[M−H]−
650.967
518.0238
821.9737
980.9575
0
20
40
60
80
100
300
400
500
600
700
800
900
1000

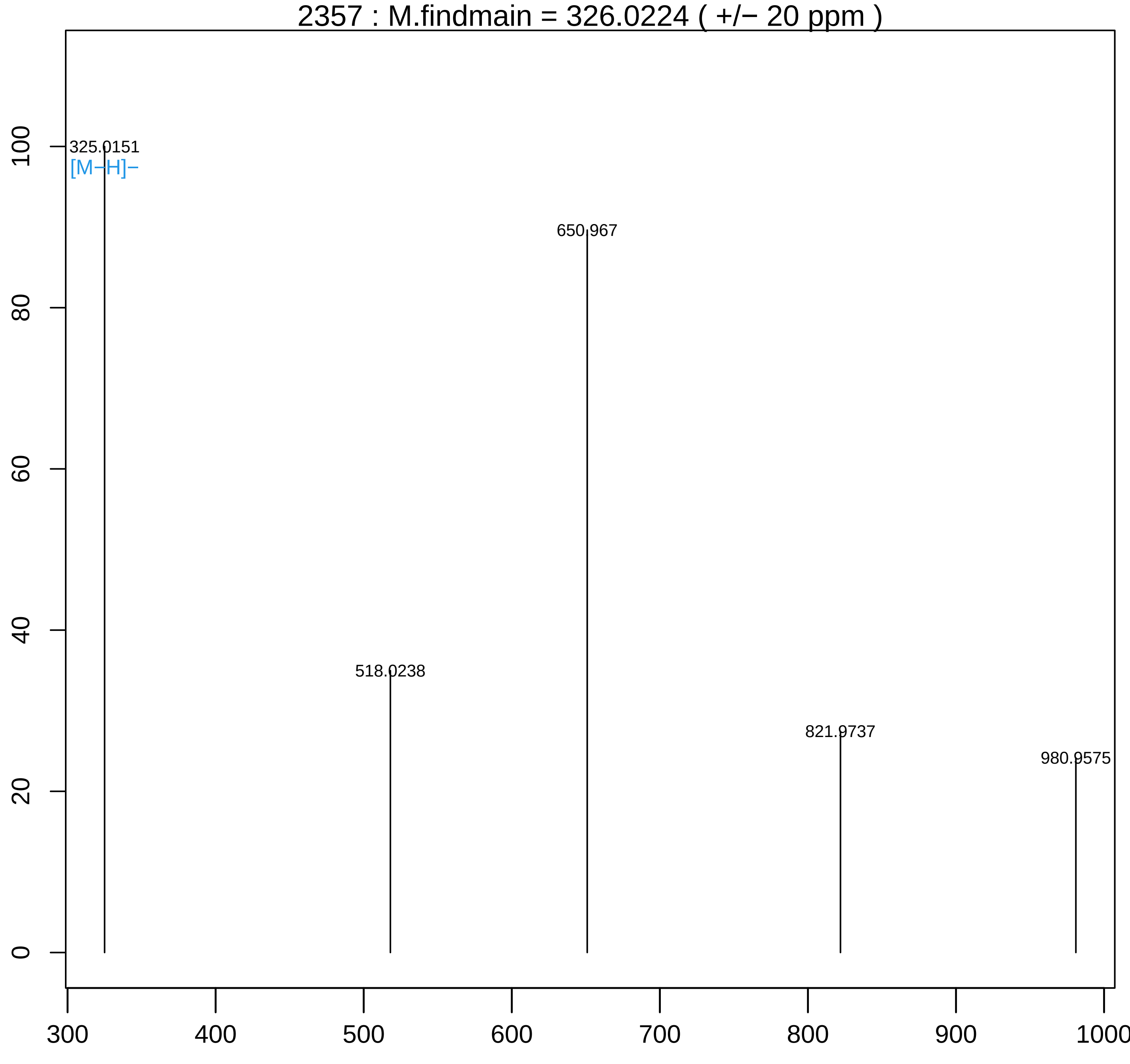

2357 : M.findmain = 326.0224 (+/− 20 ppm)
325.0151
[M−H]−
650.967
518.0238
821.9737
980.9575
0
20
40
60
80
100
300
400
500
600
700
800
900
1000

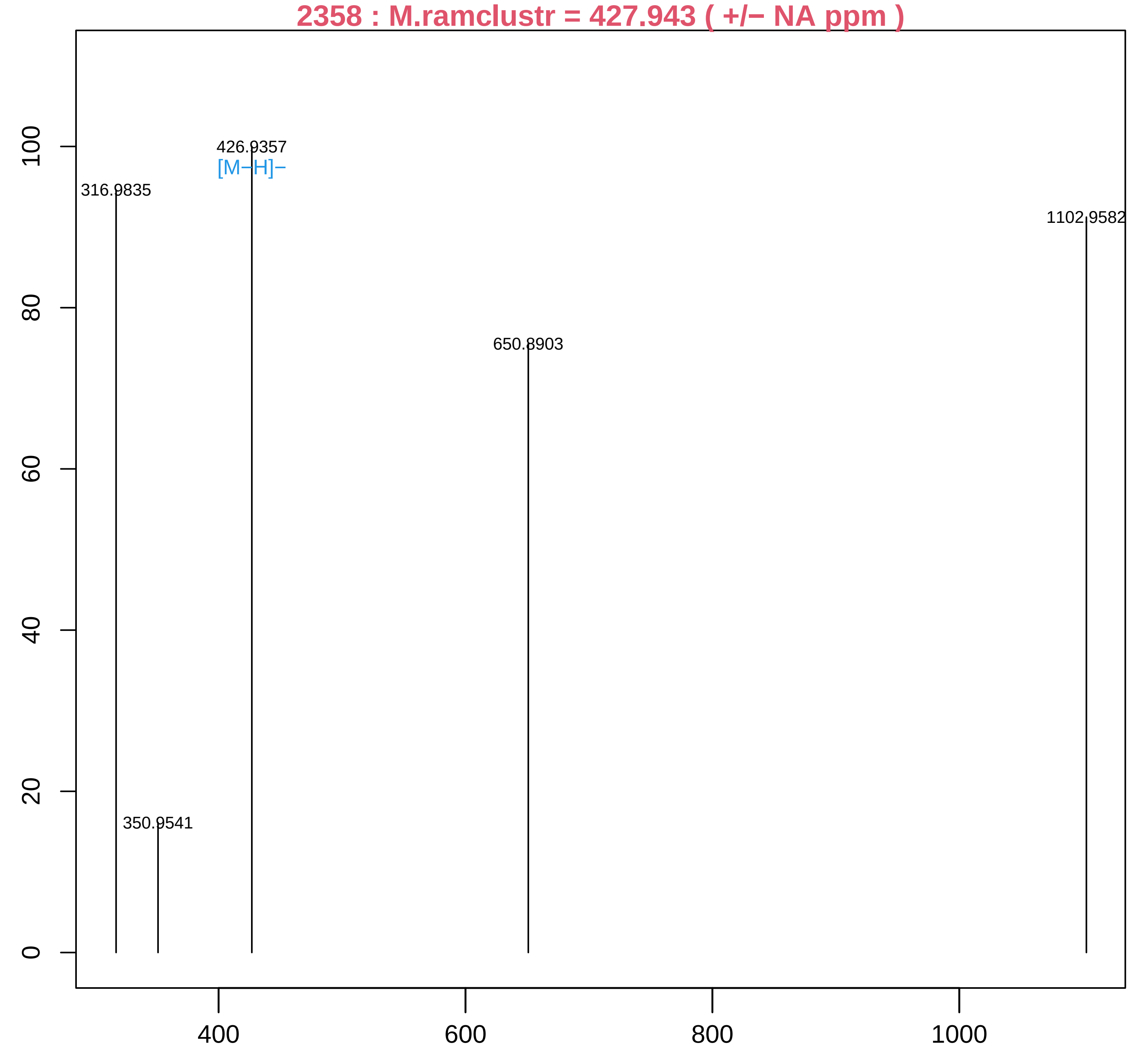

2358 : M.ramclustr = 427.943 (+/− NA ppm)
316.9835
350.9541
426.9357
[M−H]−
650.8903
1102.9582
0
20
40
60
80
100
400
600
800
1000

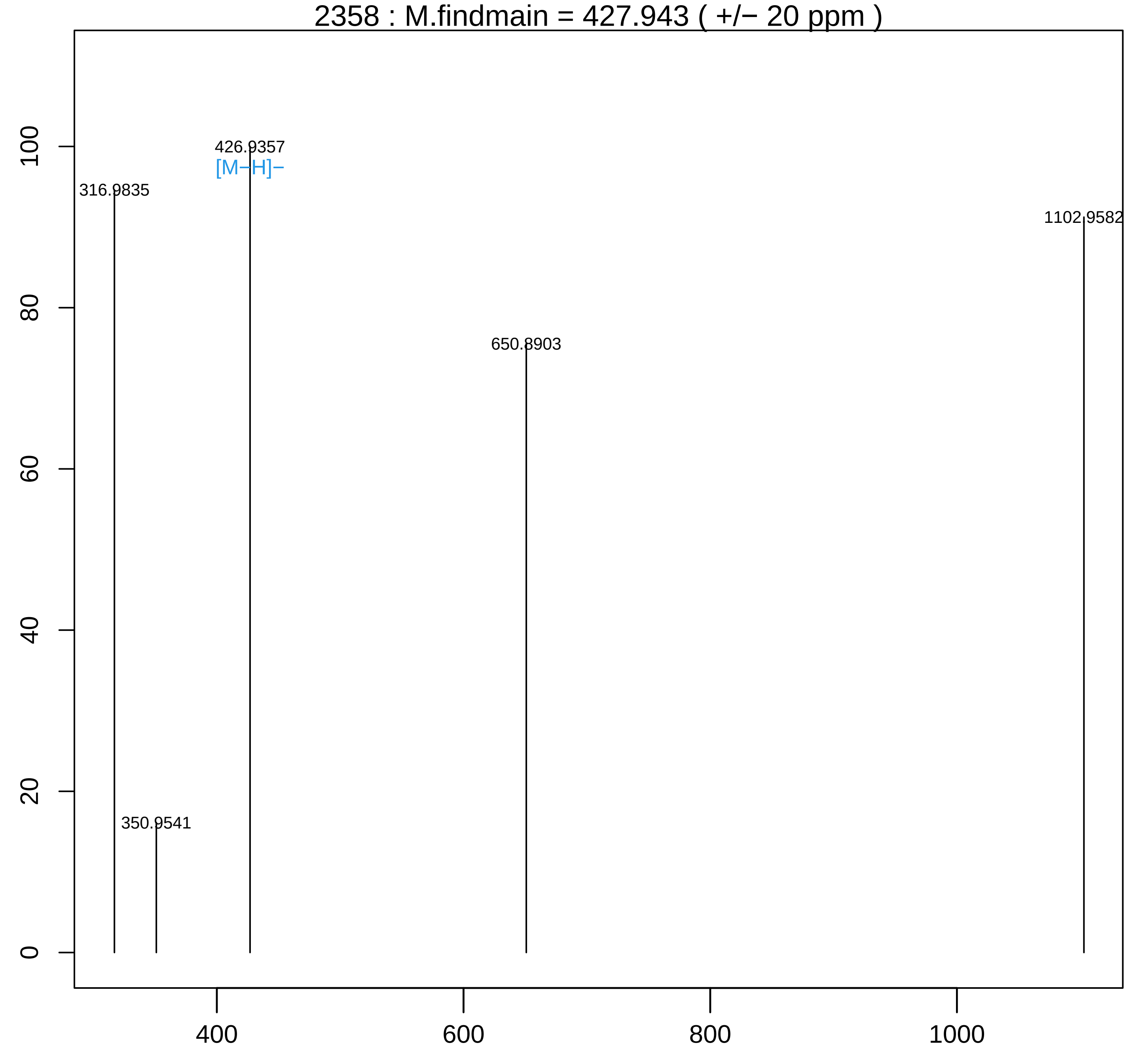

2358 : M.findmain = 427.943 (+/− 20 ppm)
316.9835
350.9541
426.9357
[M−H]−
650.8903
1102.9582
0
20
40
60
80
100
400
600
800
1000

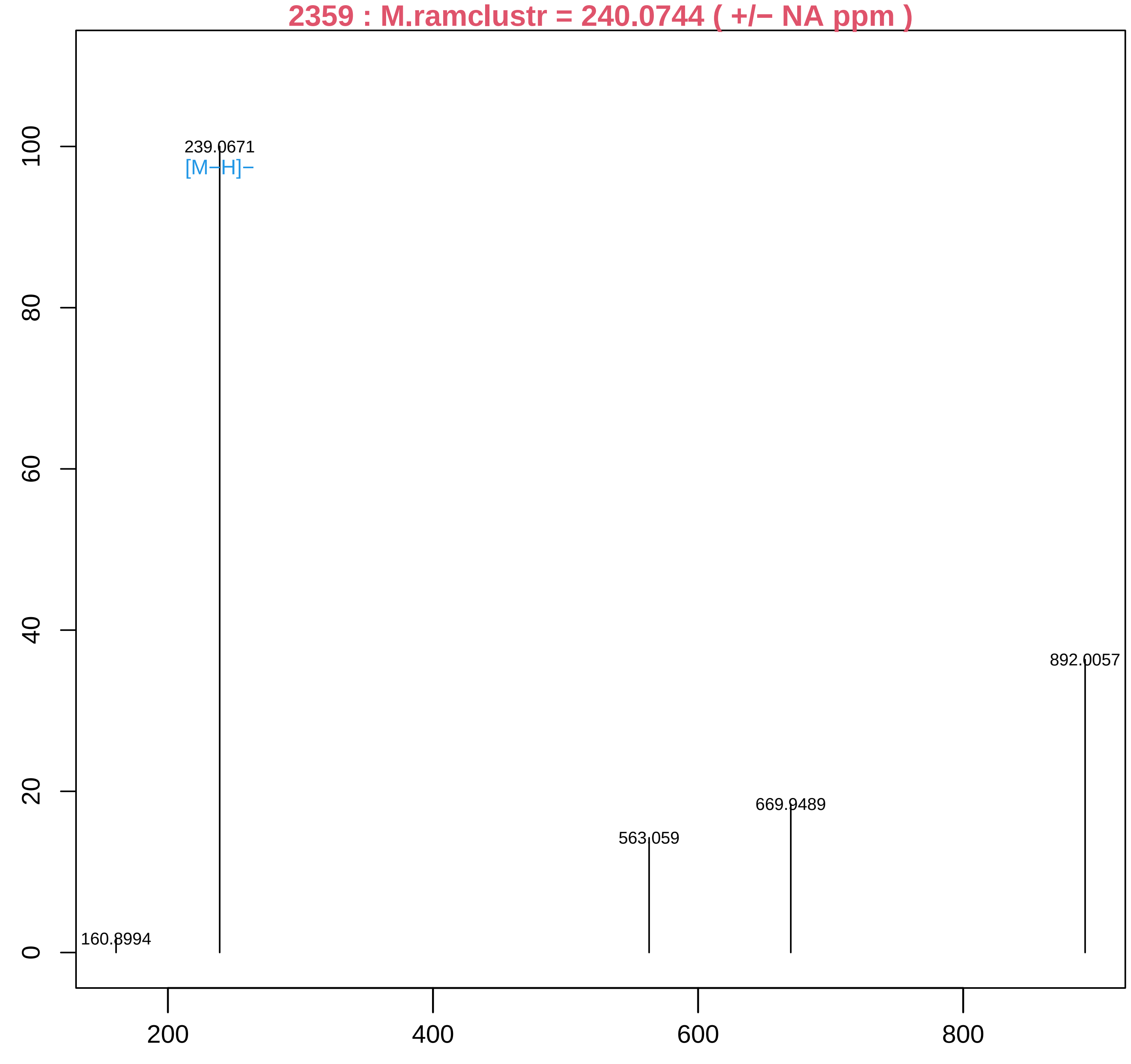

2359 : M.ramclustr = 240.0744 (+/− NA ppm)
239.0671
[M−H]−
892.0057
669.9489
563.059
160.8994
0
20
40
60
80
100
200
400
600
800

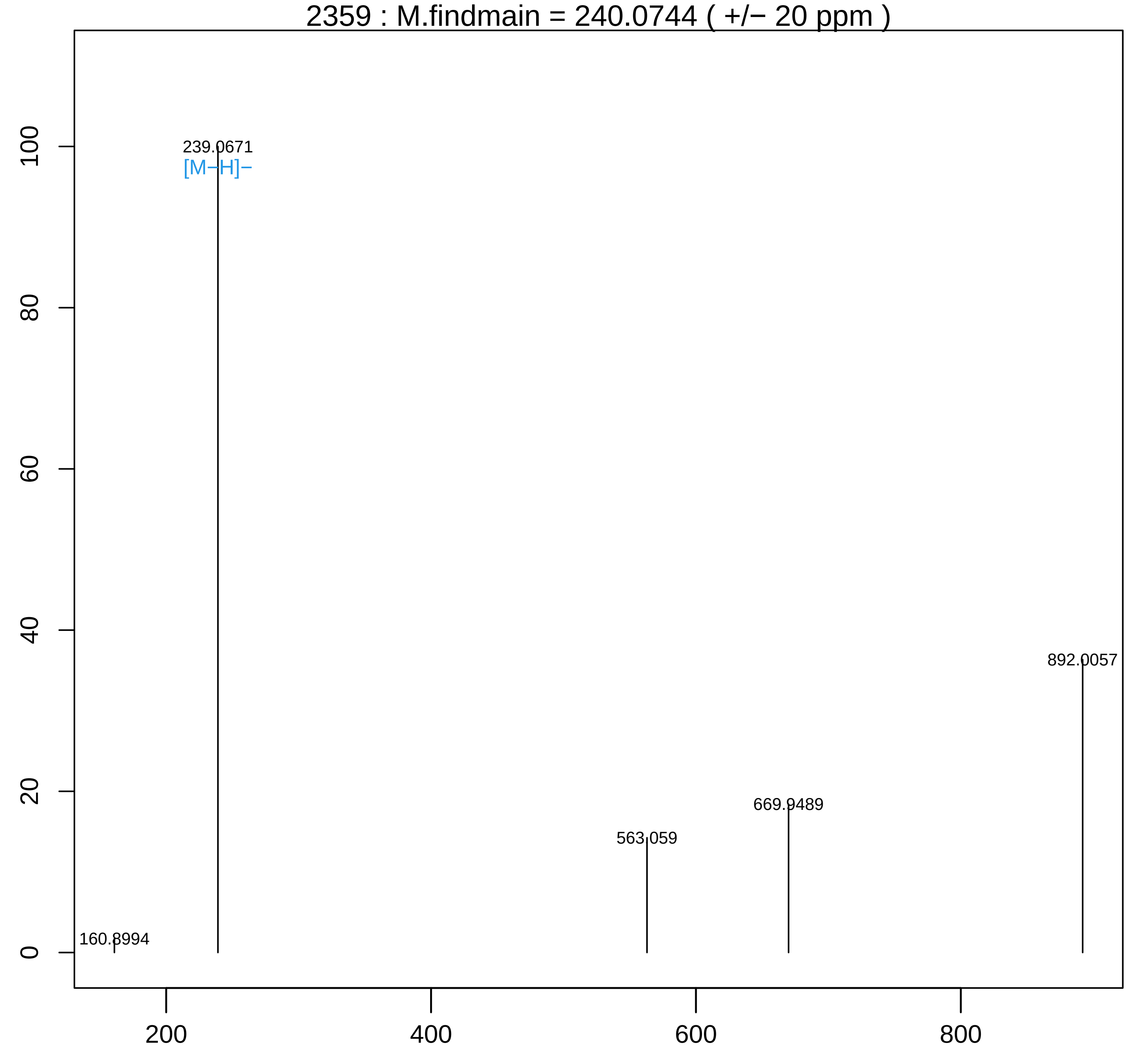

2359 : M.findmain = 240.0744 (+/− 20 ppm)
239.0671
[M−H]−
892.0057
669.9489
563.059
160.8994
0
20
40
60
80
100
200
400
600
800

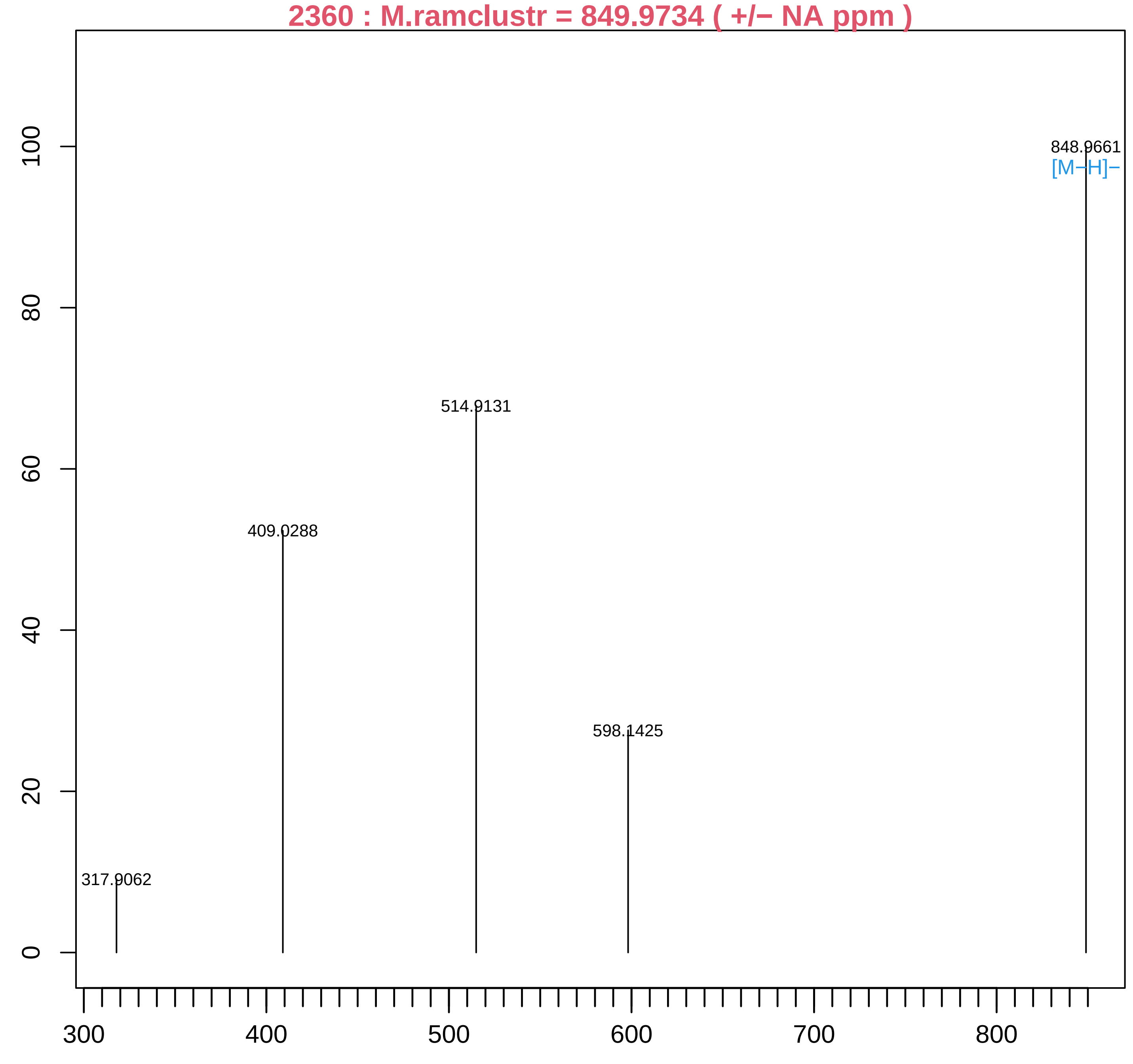
2360 : M.ramclustr = 849.9734 (+/− NA ppm)
848.9661
[M−H]−
514.9131
409.0288
598.1425
317.9062
0
20
40
60
80
100
300
400
500
600
700
800

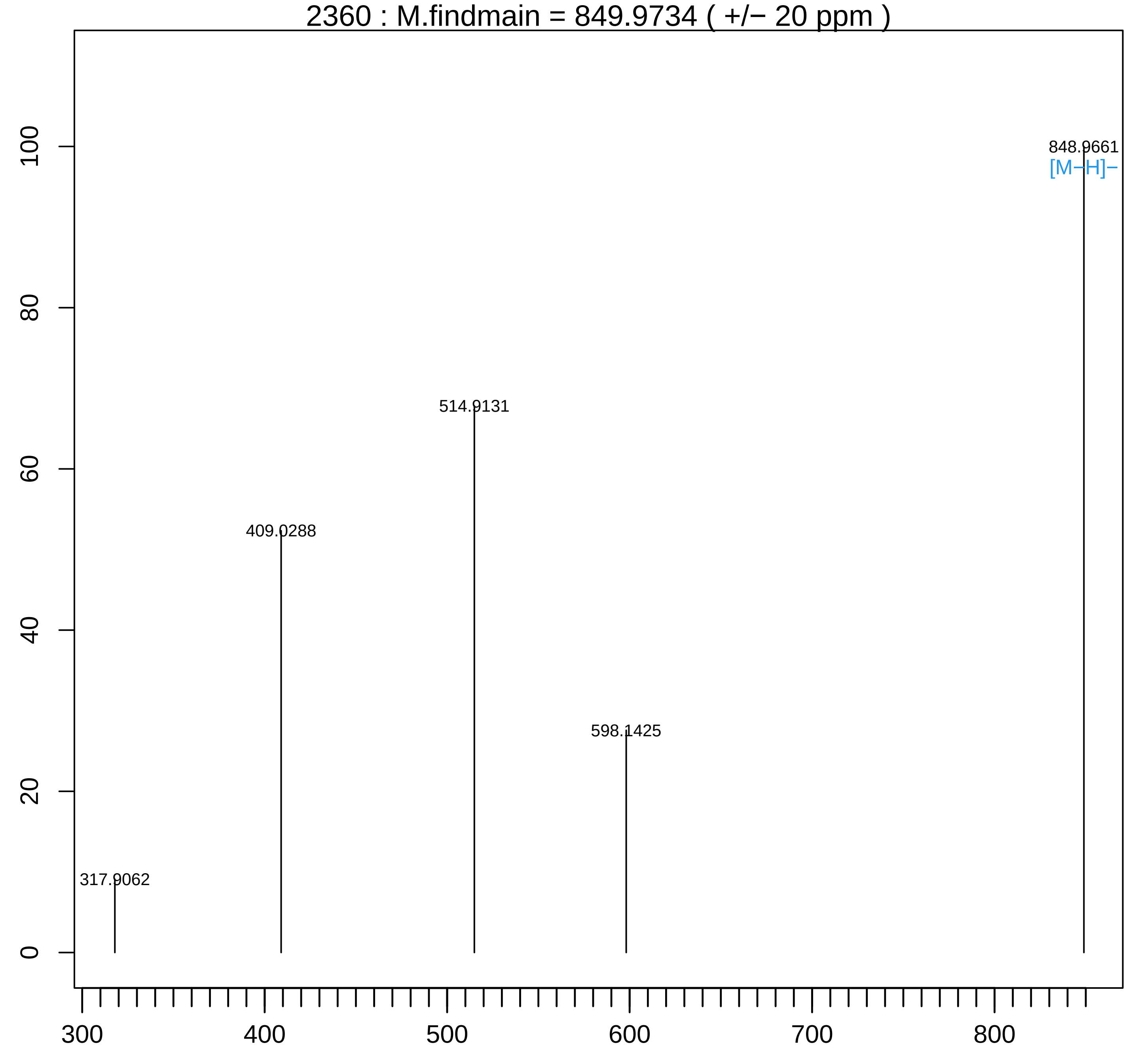
2360 : M.findmain = 849.9734 (+/− 20 ppm)
848.9661
[M−H]−
514.9131
409.0288
598.1425
317.9062
0
20
40
60
80
100
300
400
500
600
700
800

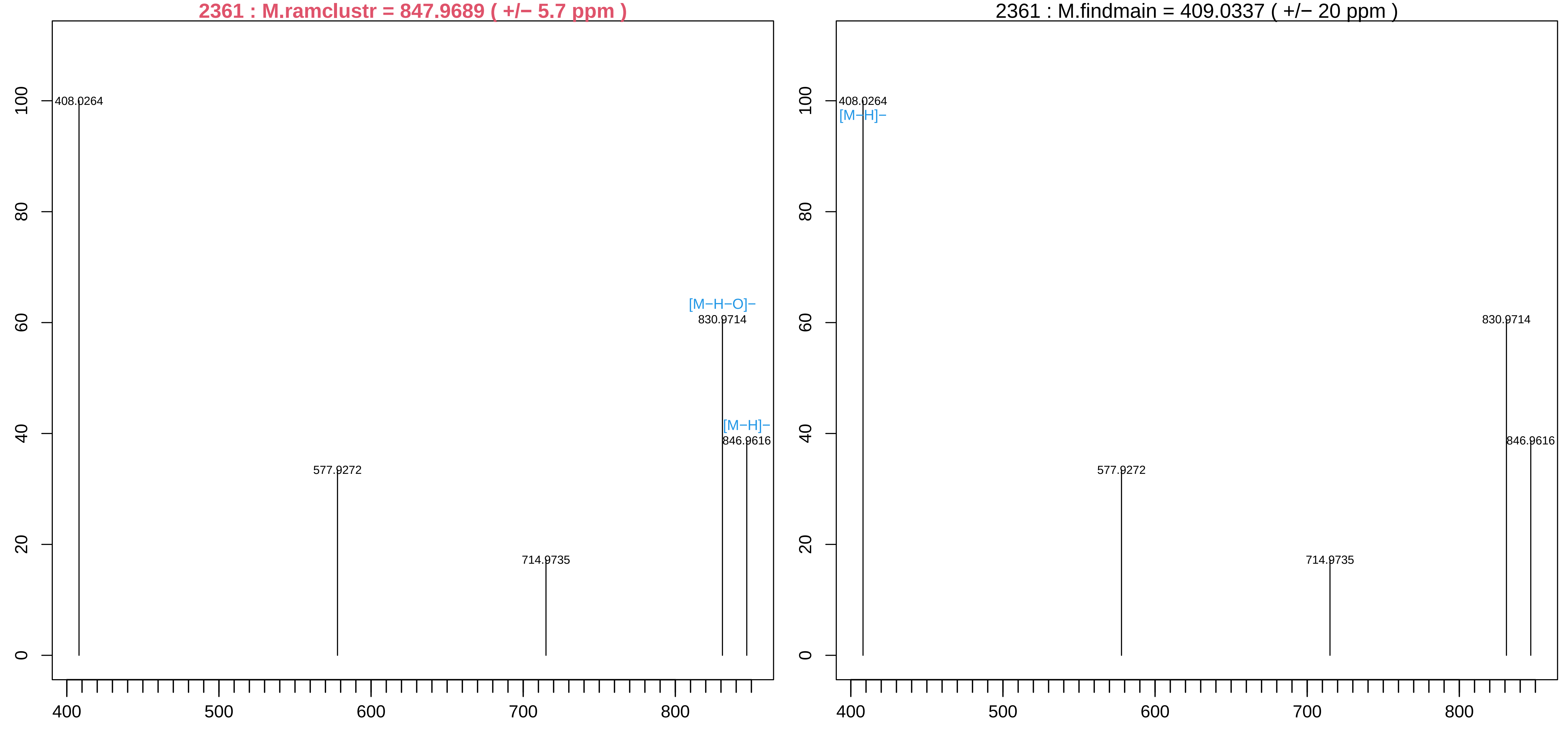
2361 : M.ramclustr = 847.9689 (+/− 5.7 ppm)
408.0264
577.9272
714.9735
[M−H−O]−
830.9714
[M−H]−
846.9616
2361 : M.findmain = 409.0337 (+/− 20 ppm)
408.0264
[M−H]−
577.9272
714.9735
830.9714
846.9616
0
20
40
60
80
100
400
500
600
700
800

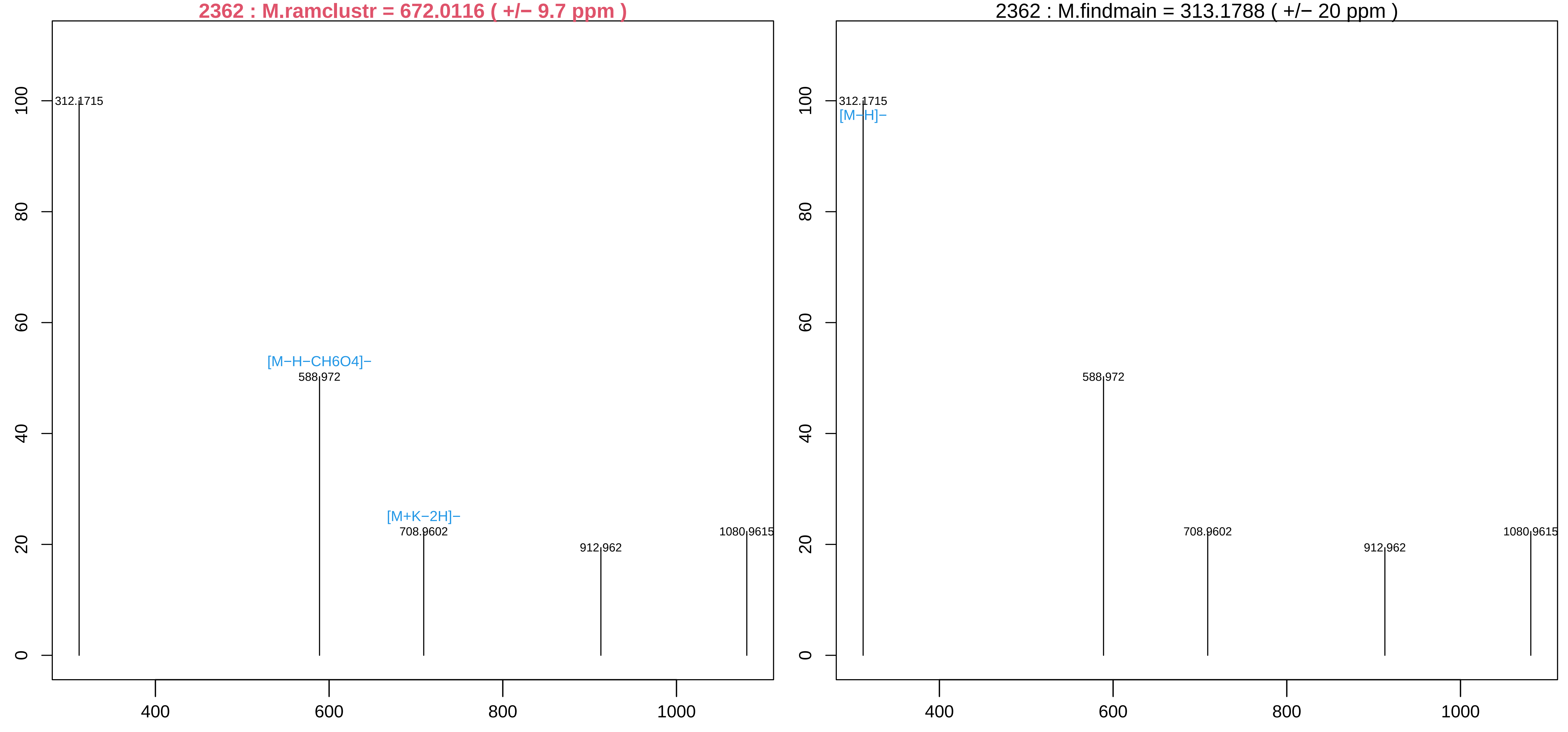
2362 : M.ramclustr = 672.0116 (+/− 9.7 ppm)
312.1715
[M−H−CH6O4]−
588.972
[M+K−2H]−
708.9602
912.962
1080.9615
2362 : M.findmain = 313.1788 (+/− 20 ppm)
312.1715
[M−H]−
588.972
708.9602
912.962
1080.9615

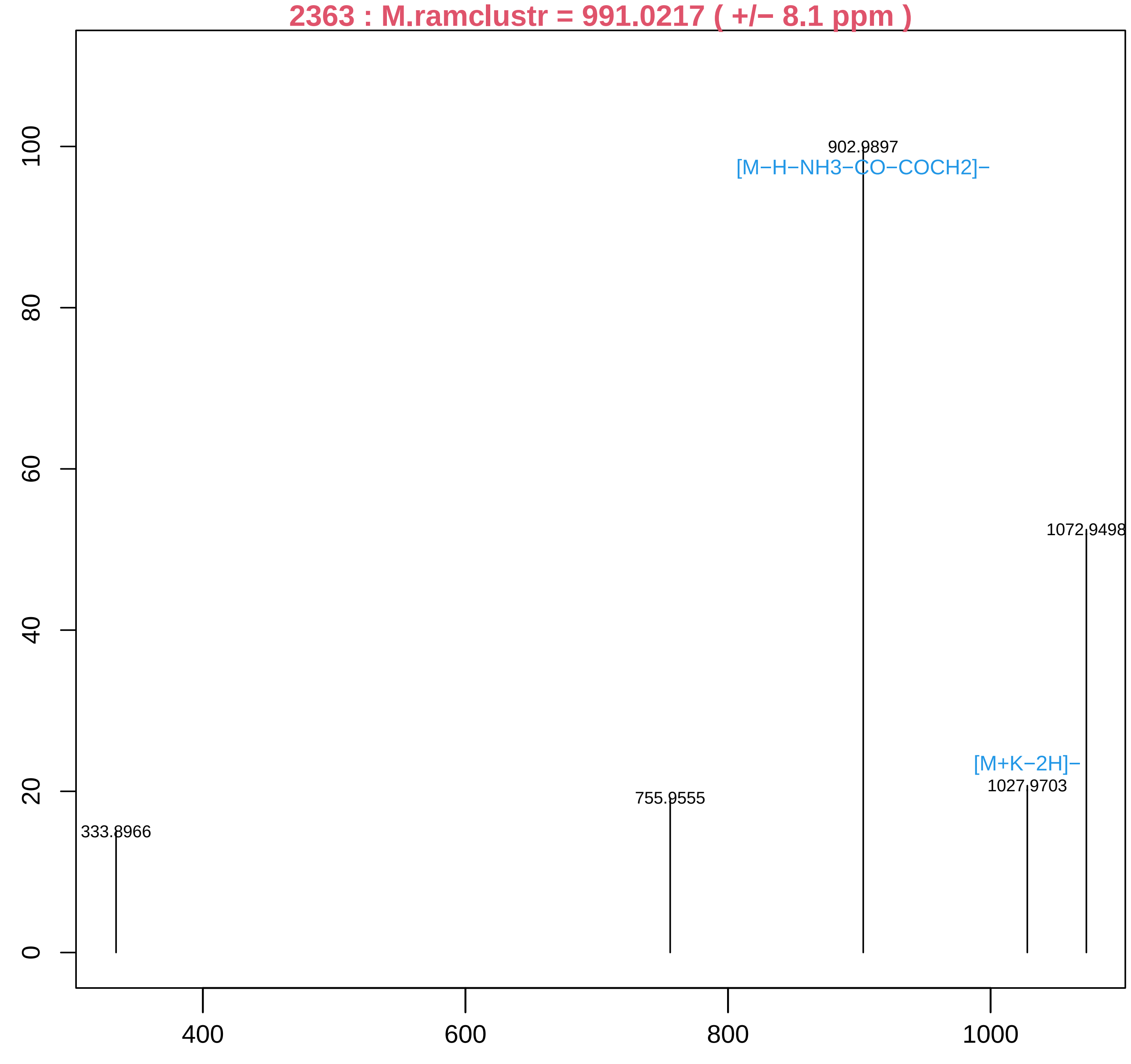
2363 : M.ramclustr = 991.0217 (+/− 8.1 ppm)
902.9897
[M−H−NH3−CO−COCH2]−
1072.9498
[M+K−2H]−
1027.9703
755.9555
333.8966
0
20
40
60
80
100
400
600
800
1000

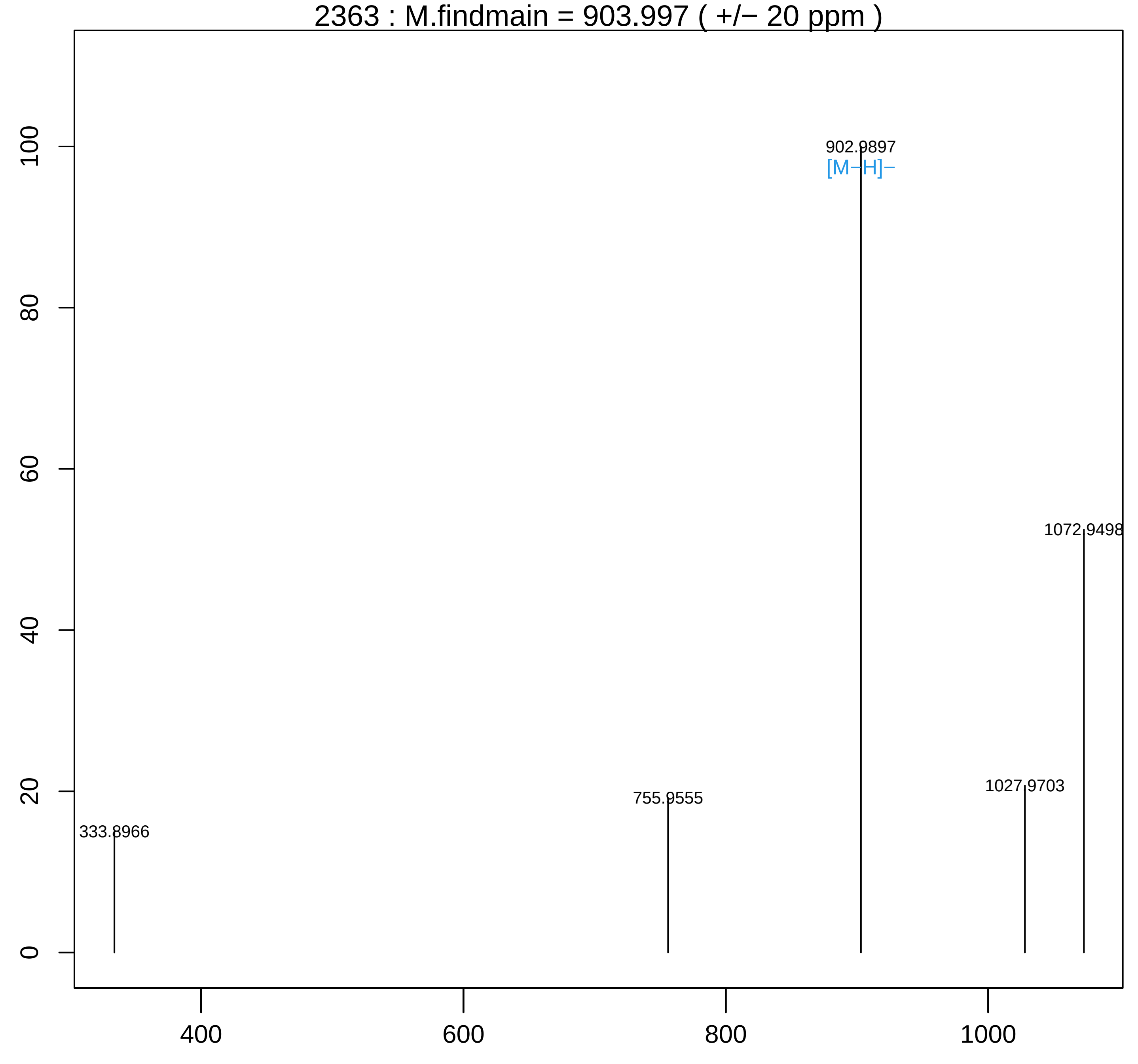
2363 : M.findmain = 903.997 (+/− 20 ppm)
902.9897
[M−H]−
1072.9498
1027.9703
755.9555
333.8966
0
20
40
60
80
100
400
600
800
1000

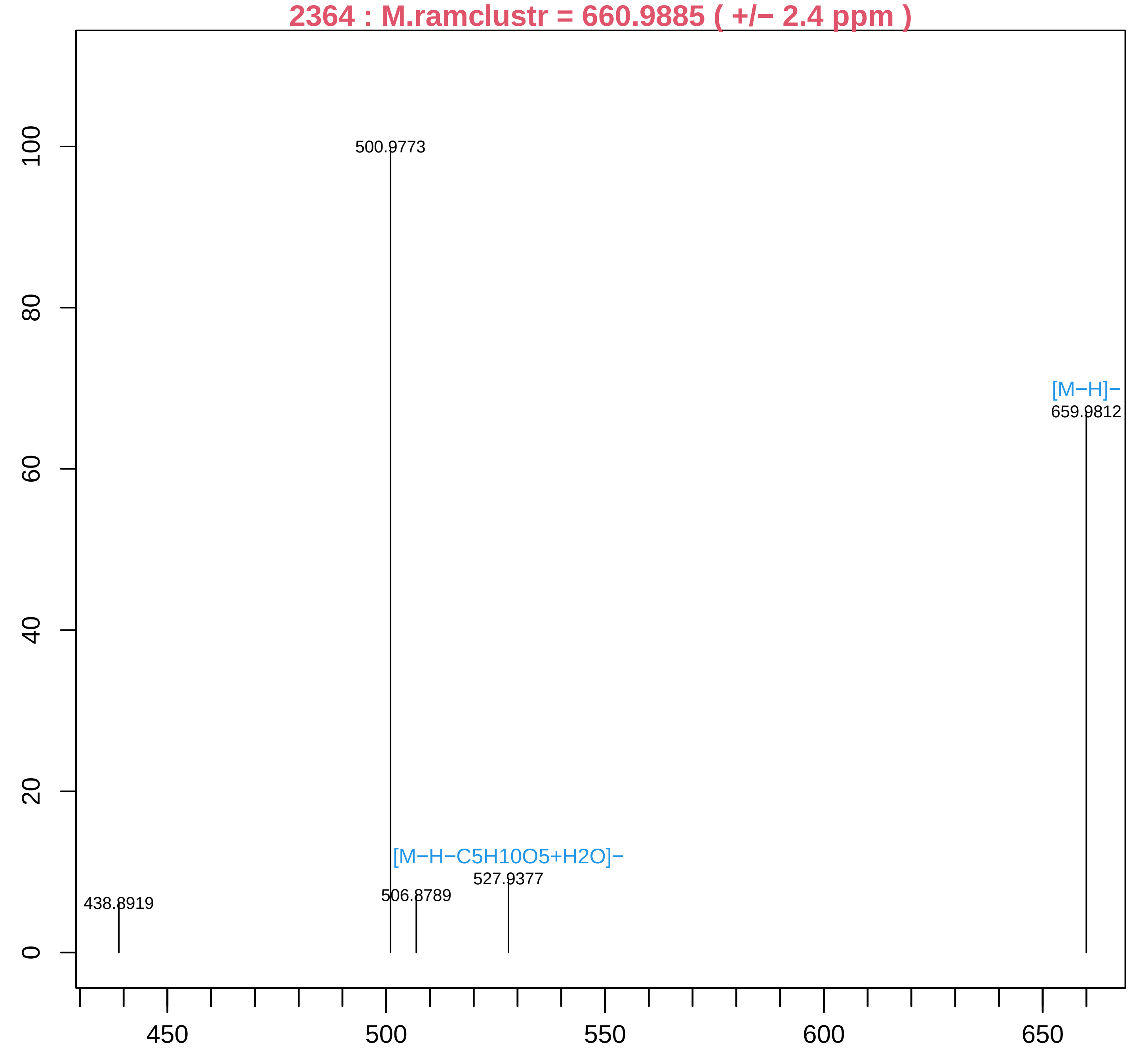

2364 : M.ramclustr = 660.9885 (+/− 2.4 ppm)
500.9773
[M−H]−
659.9812
[M−H−C5H10O5+H2O]−
527.9377
438.8919
506.8789
0
20
40
60
80
100
450
500
550
600
650

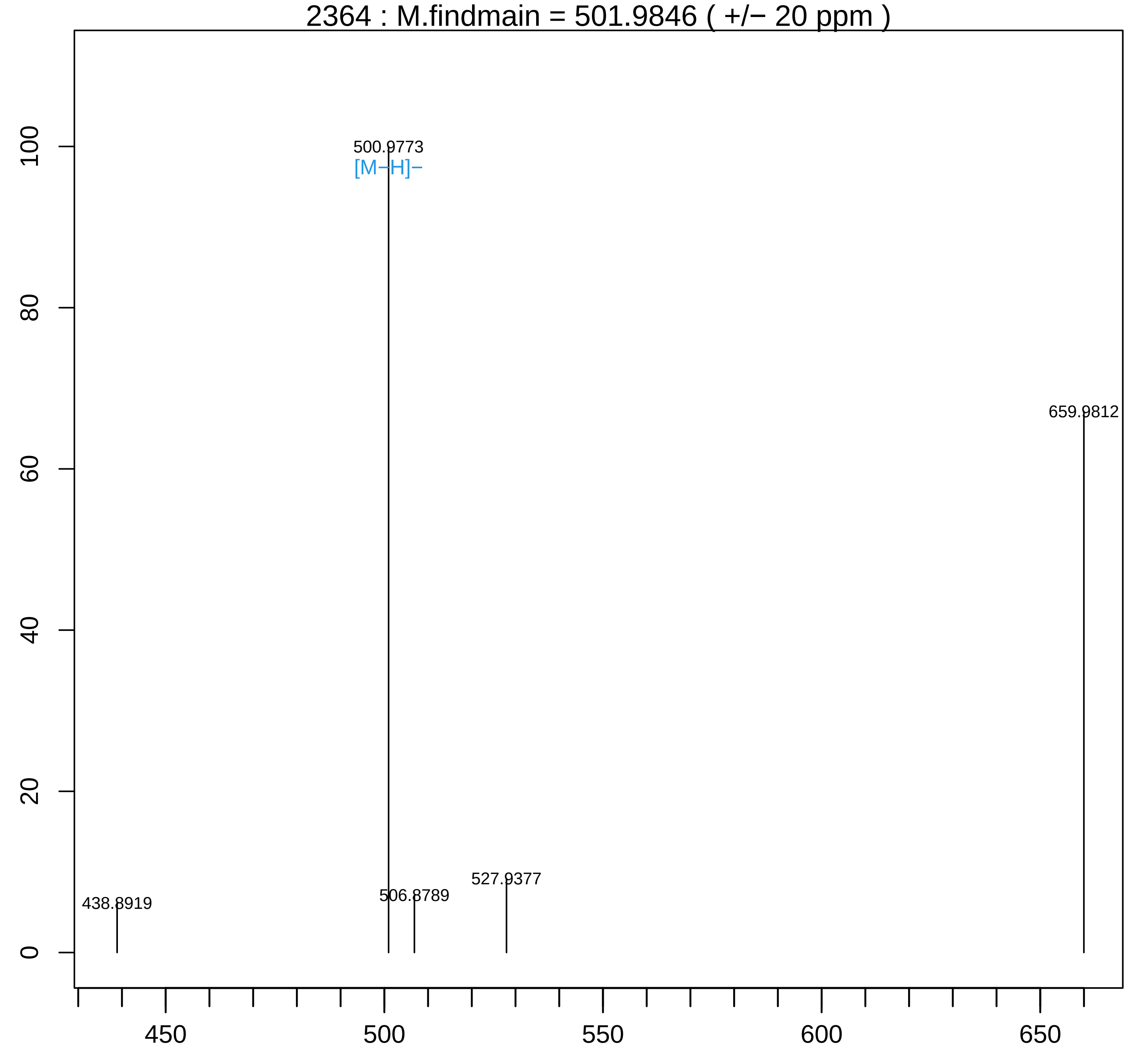

2364 : M.findmain = 501.9846 (+/− 20 ppm)
500.9773
[M−H]−
659.9812
527.9377
438.8919
506.8789
0
20
40
60
80
100
450
500
550
600
650

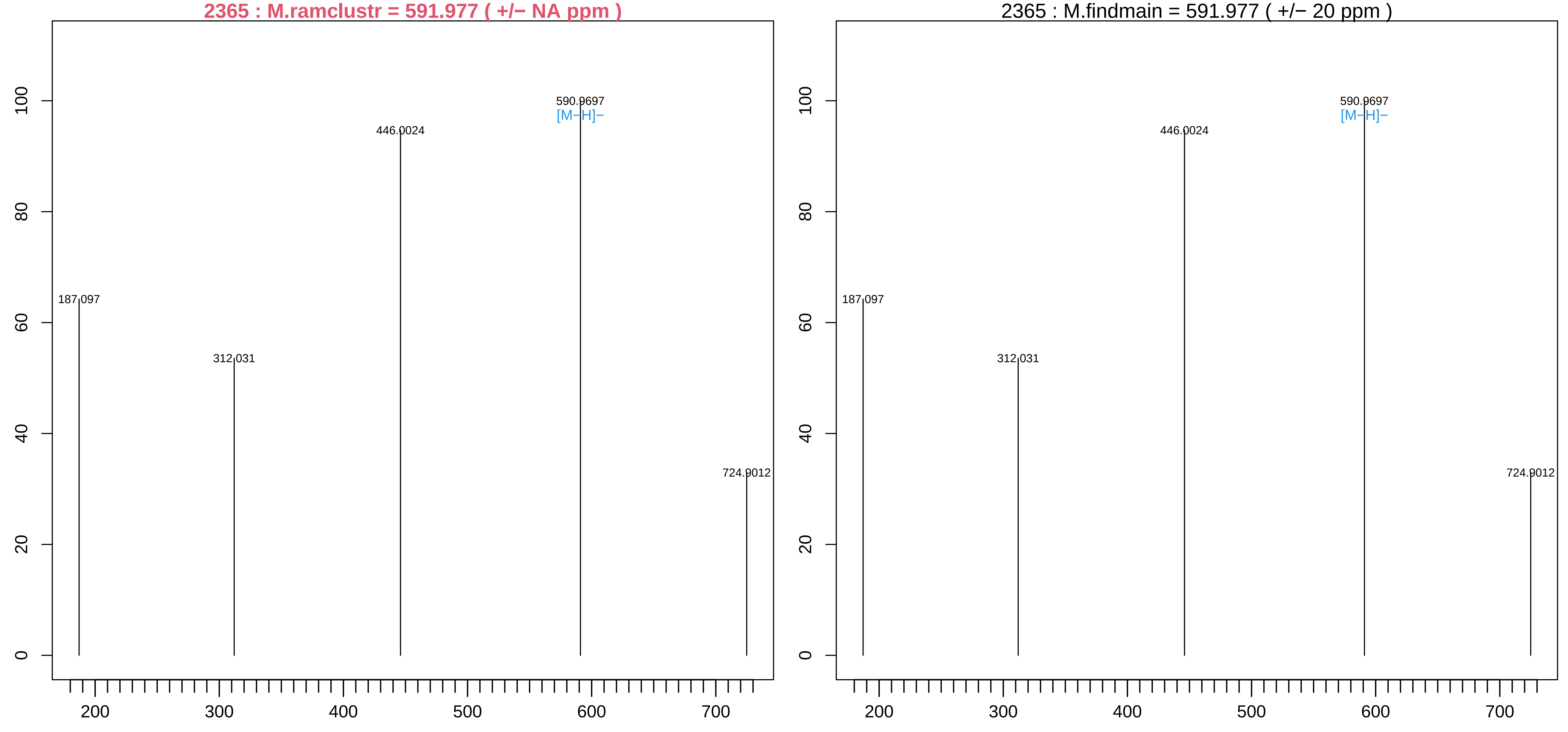

2365 : M.ramclustr = 591.977 (+/− NA ppm)
2365 : M.findmain = 591.977 (+/− 20 ppm)
187.097
312.031
446.0024
590.9697
[M−H]−
724.9012
0
20
40
60
80
100
200
300
400
500
600
700

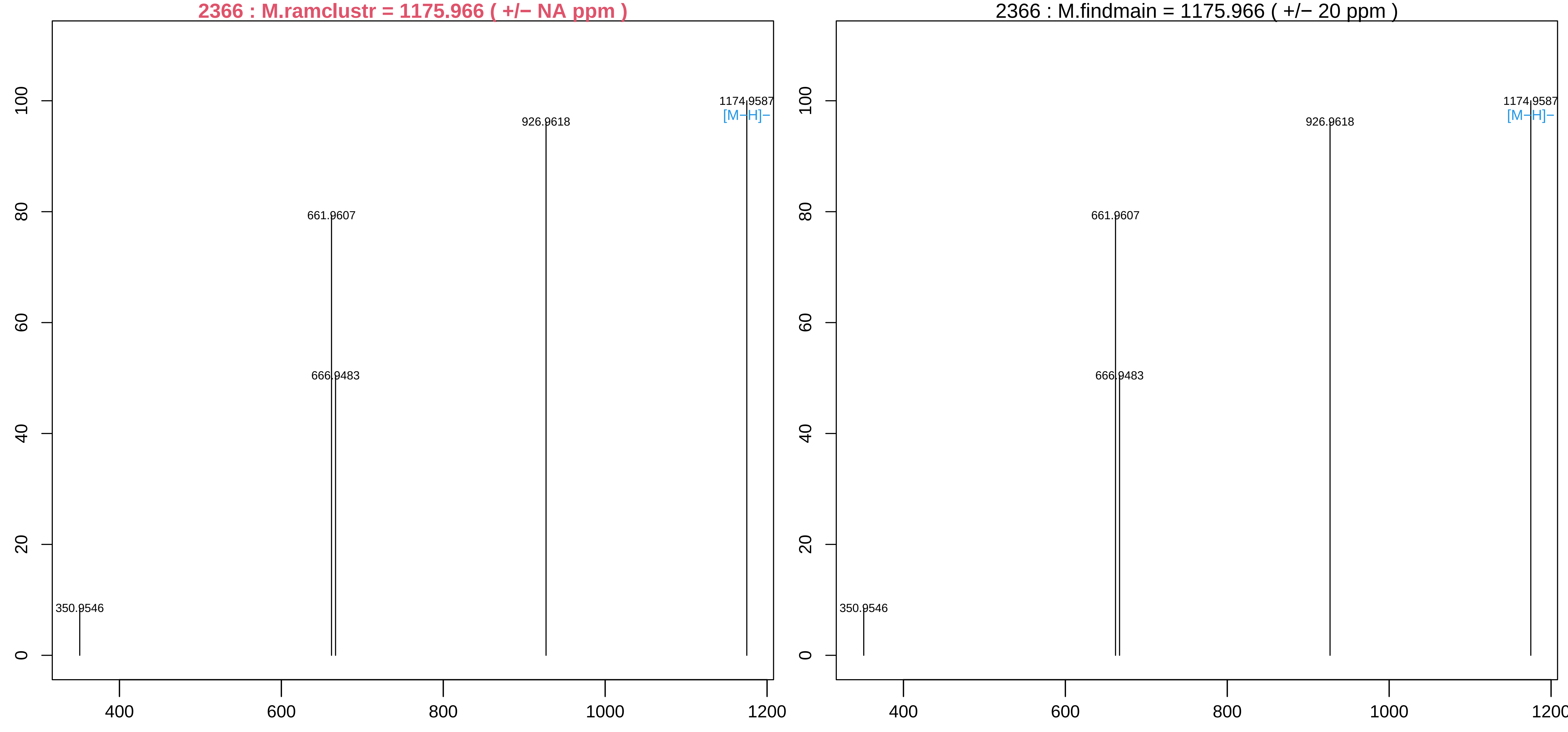

2366 : M.ramclustr = 1175.966 (+/− NA ppm)
1174.9587
[M−H]−
926.9618
661.9607
666.9483
350.9546
2366 : M.findmain = 1175.966 (+/− 20 ppm)
1174.9587
[M−H]−
926.9618
661.9607
666.9483
350.9546
0
20
40
60
80
100
400
600
800
1000
1200

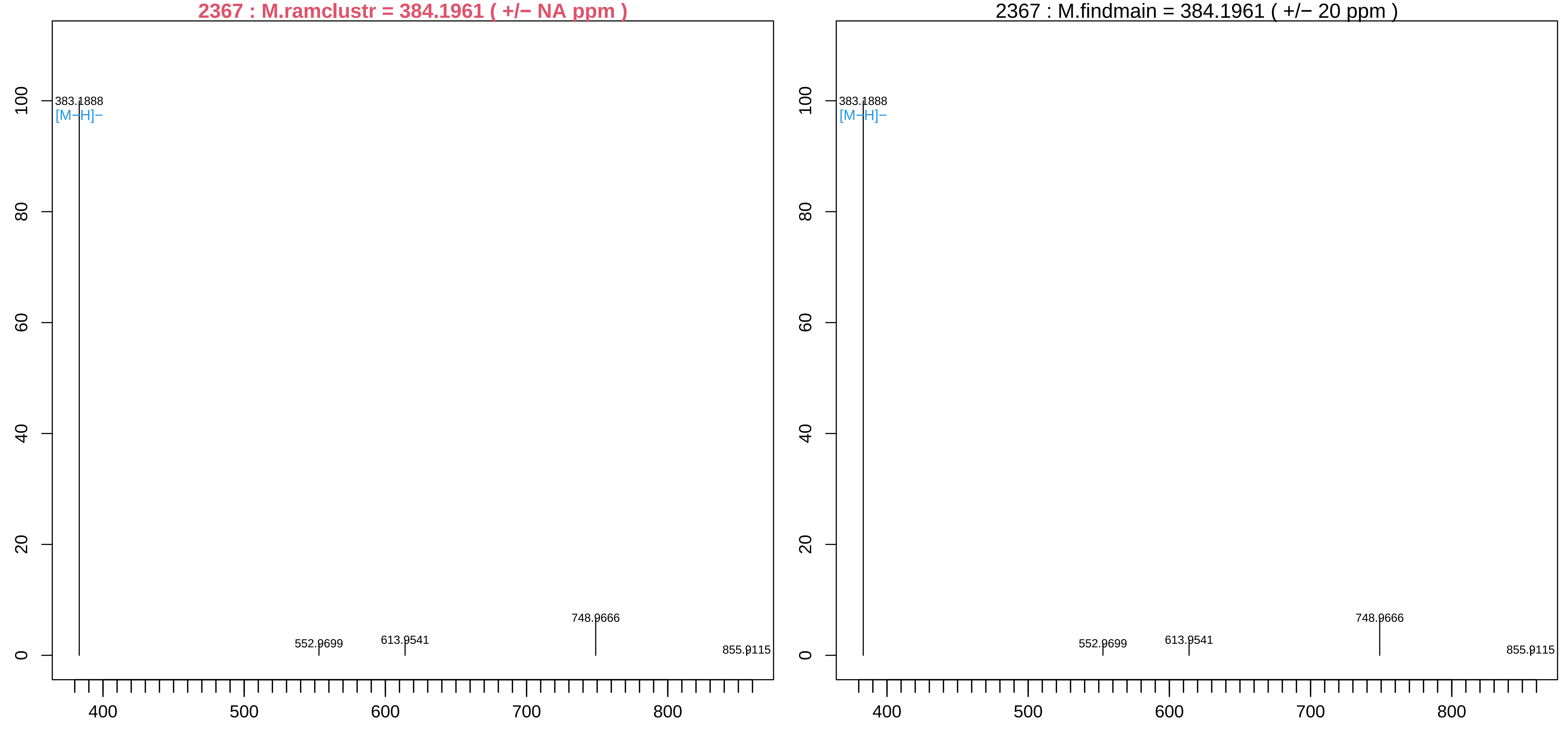

2367 : M.ramclustr = 384.1961 (+/− NA ppm)
383.1888
[M−H]−
552.9699
613.9541
748.9666
855.9115
2367 : M.findmain = 384.1961 (+/− 20 ppm)
383.1888
[M−H]−
552.9699
613.9541
748.9666
855.9115
0
20
40
60
80
100
400
500
600
700
800

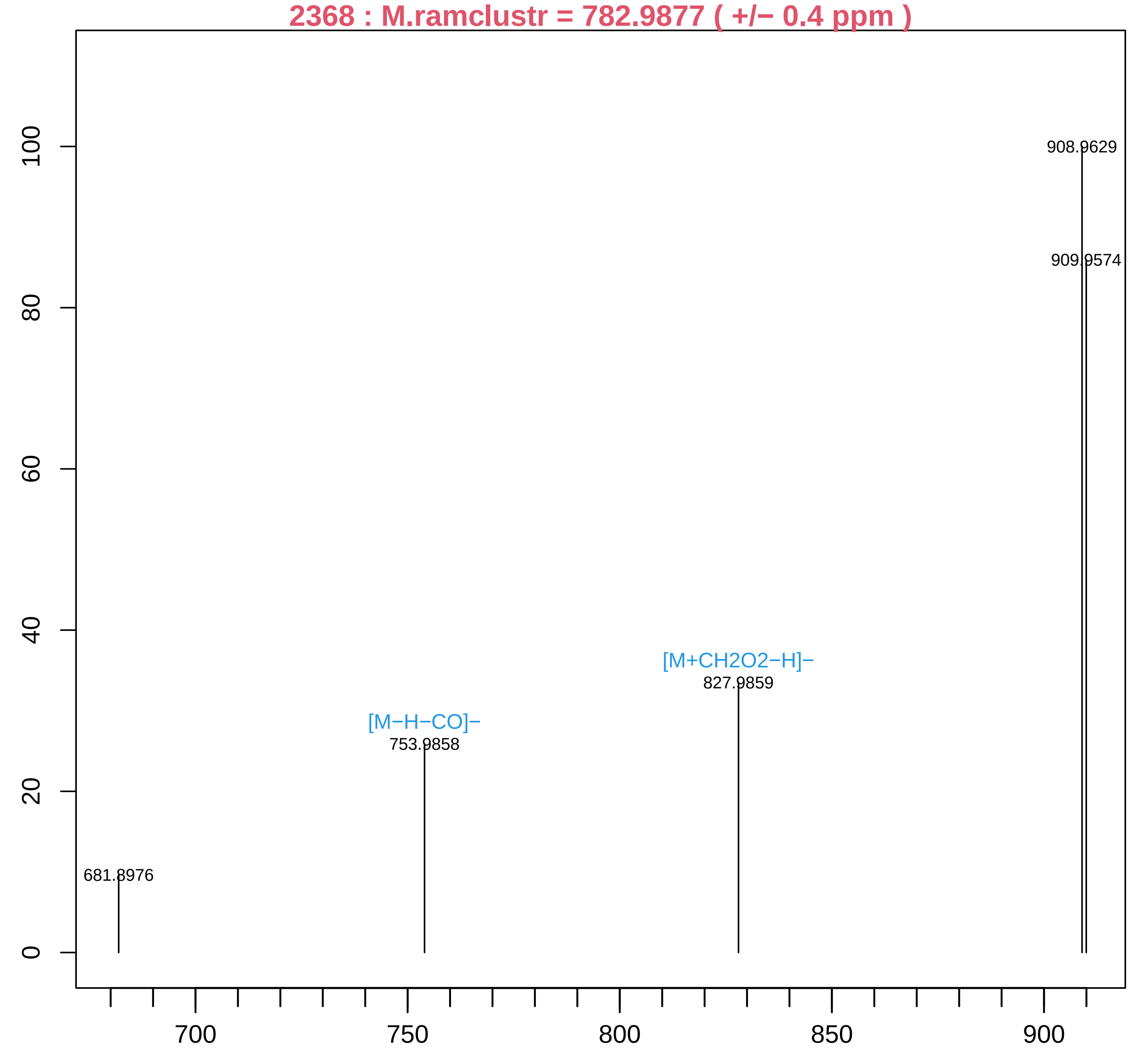
2368 : M.ramclustr = 782.9877 (+/− 0.4 ppm)
908.9629
909.9574
[M+CH2O2−H]−
827.9859
[M−H−CO]−
753.9858
681.8976
0
20
40
60
80
100
700
750
800
850
900

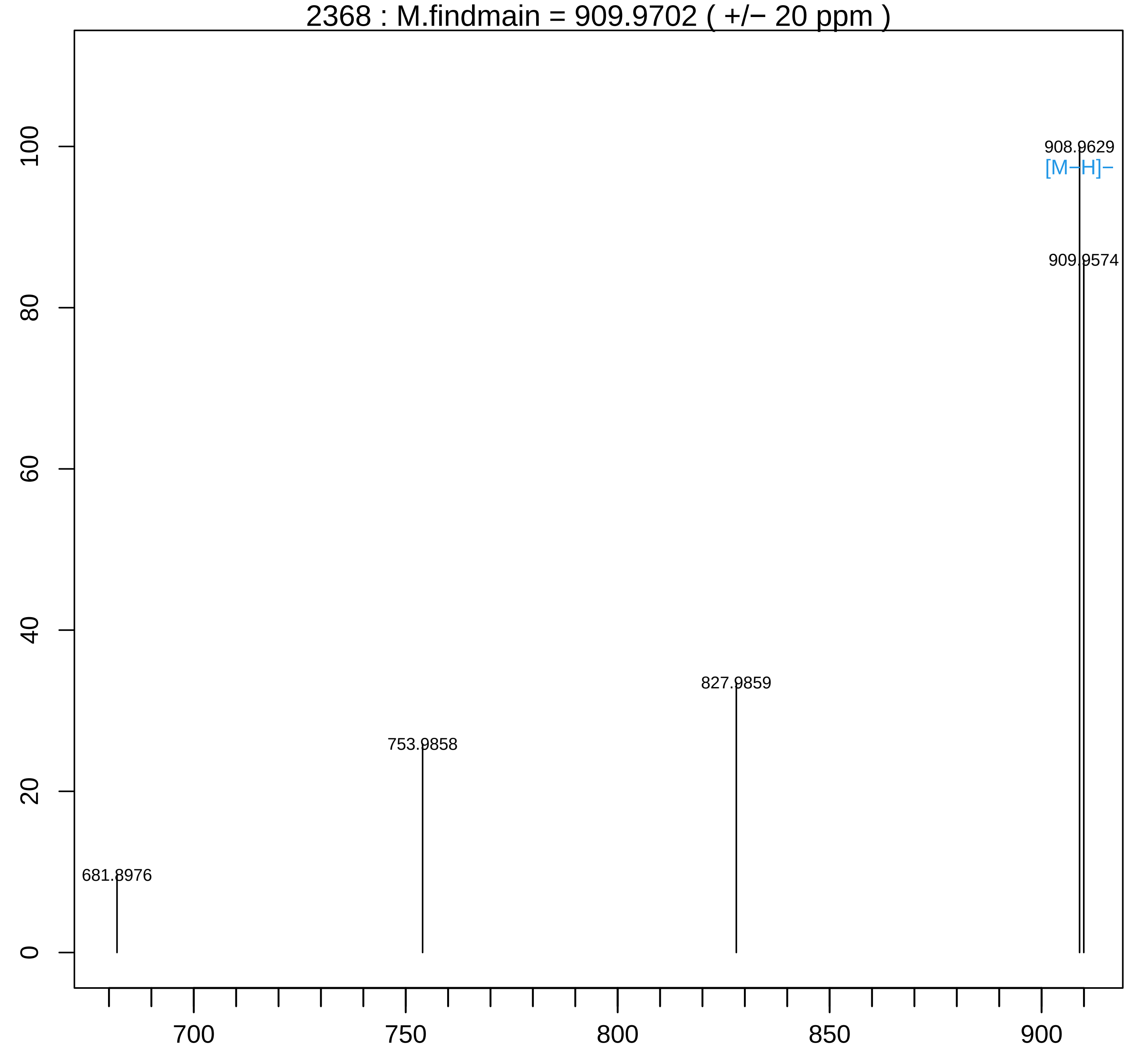
2368 : M.findmain = 909.9702 (+/− 20 ppm)
908.9629
[M−H]−
909.9574
827.9859
753.9858
681.8976
0
20
40
60
80
100
700
750
800
850
900

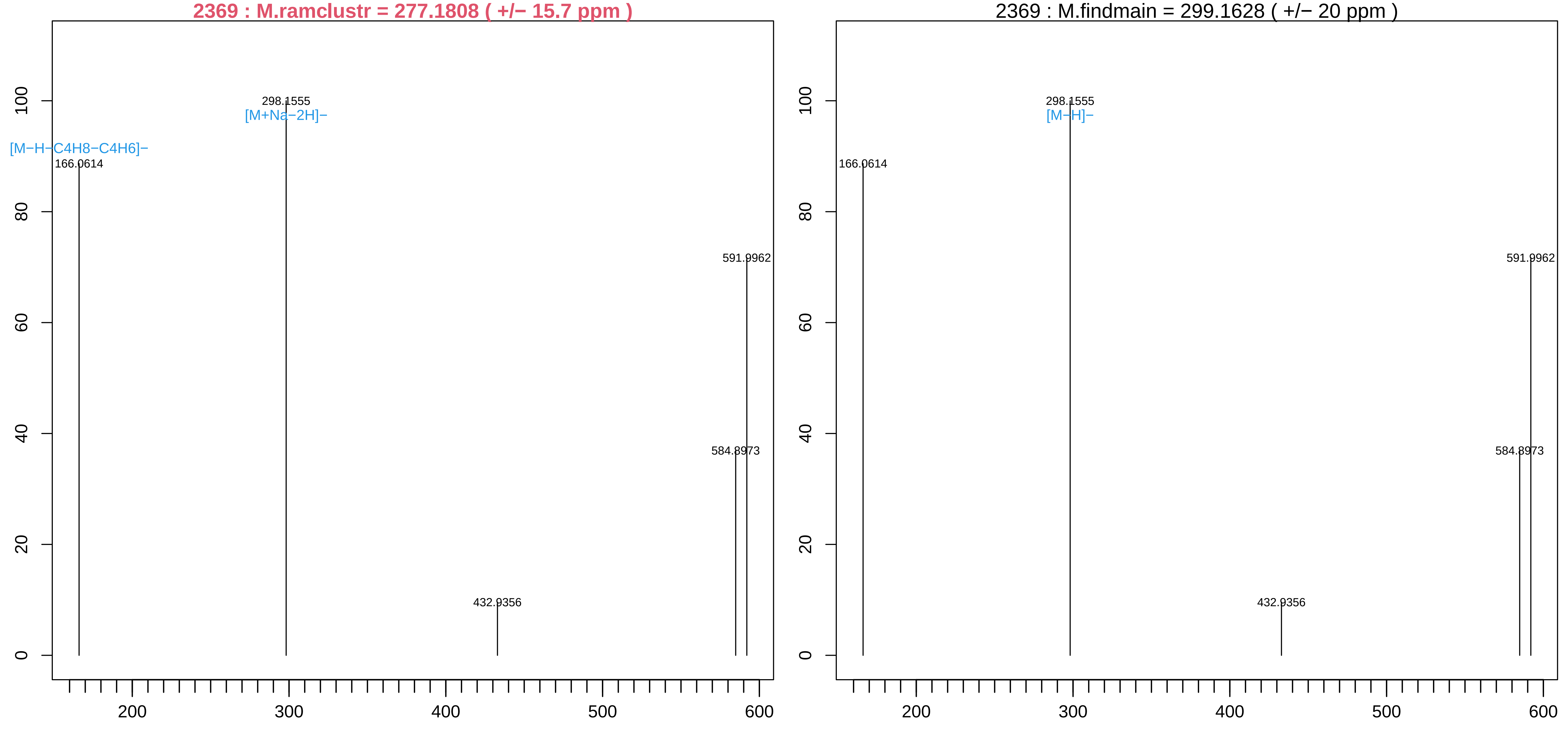
2369 : M.ramclustr = 277.1808 (+/− 15.7 ppm)
[M−H−C4H8−C4H6]−
166.0614
298.1555
[M+Na−2H]−
432.9356
584.8973
591.9962
0
20
40
60
80
100
200
300
400
500
600
2369 : M.findmain = 299.1628 (+/− 20 ppm)
166.0614
298.1555
[M−H]−
432.9356
584.8973
591.9962

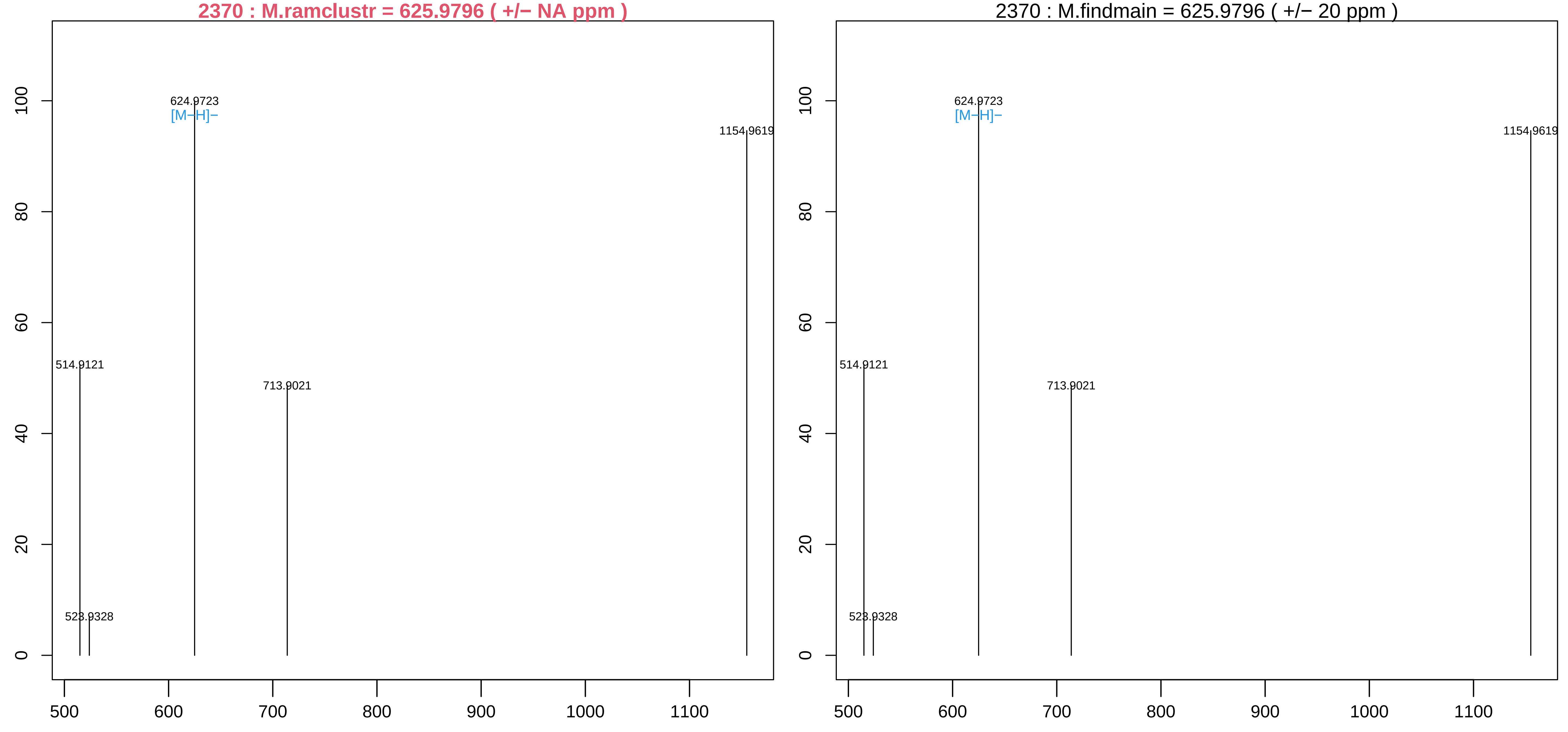
2370 : M.ramclustr = 625.9796 (+/− NA ppm)
624.9723
[M−H]−
1154.9619
514.9121
713.9021
523.9328
0
20
40
60
80
100
500
600
700
800
900
1000
1100
2370 : M.findmain = 625.9796 (+/− 20 ppm)
624.9723
[M−H]−
1154.9619
514.9121
713.9021
523.9328
0
20
40
60
80
100
500
600
700
800
900
1000
1100

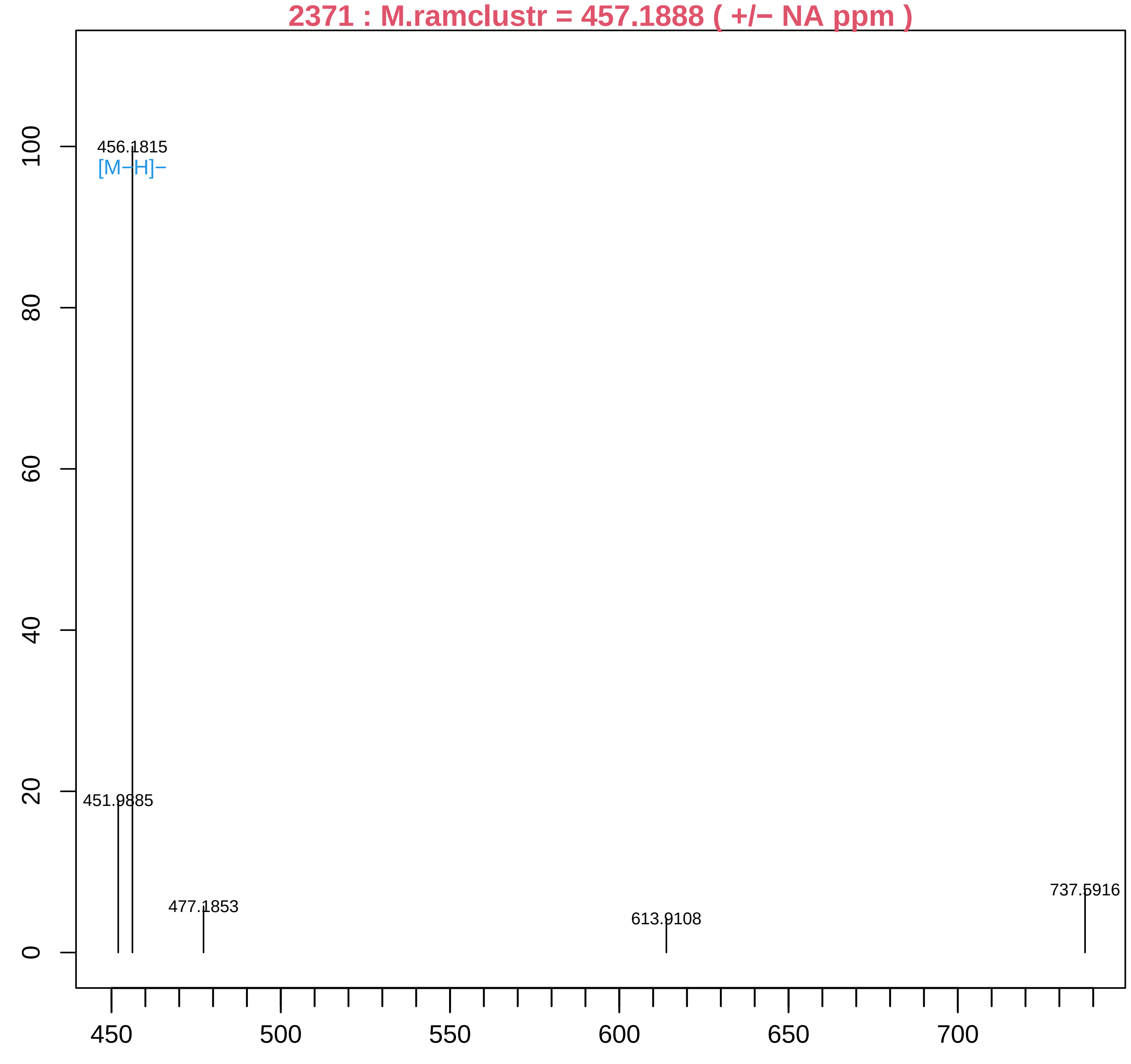
2371 : M.ramclustr = 457.1888 (+/− NA ppm)
456.1815
[M−H]−
451.9885
477.1853
613.9108
737.5916
0
20
40
60
80
100
450
500
550
600
650
700

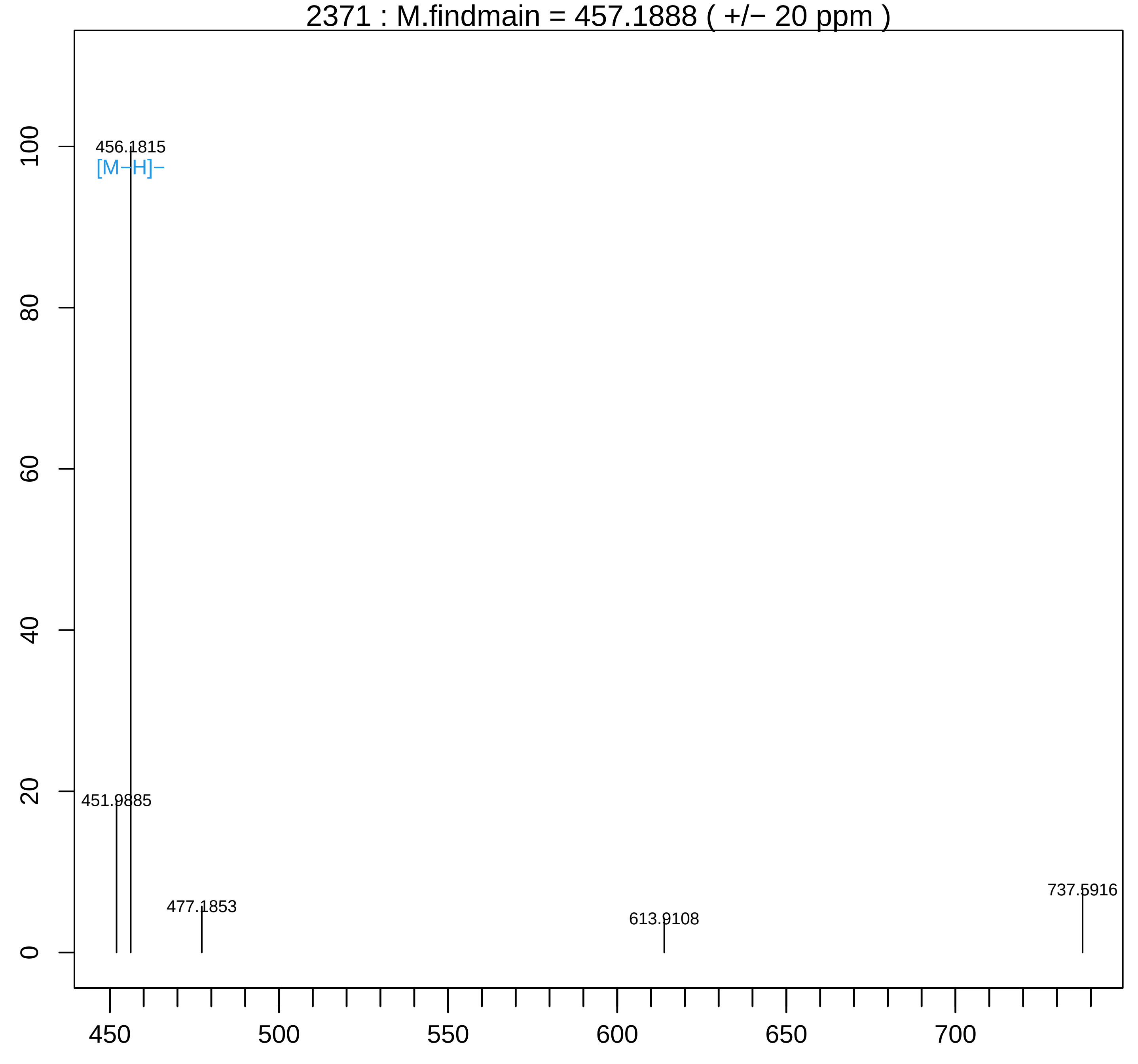
2371 : M.findmain = 457.1888 (+/− 20 ppm)
456.1815
[M−H]−
451.9885
477.1853
613.9108
737.5916
0
20
40
60
80
100
450
500
550
600
650
700

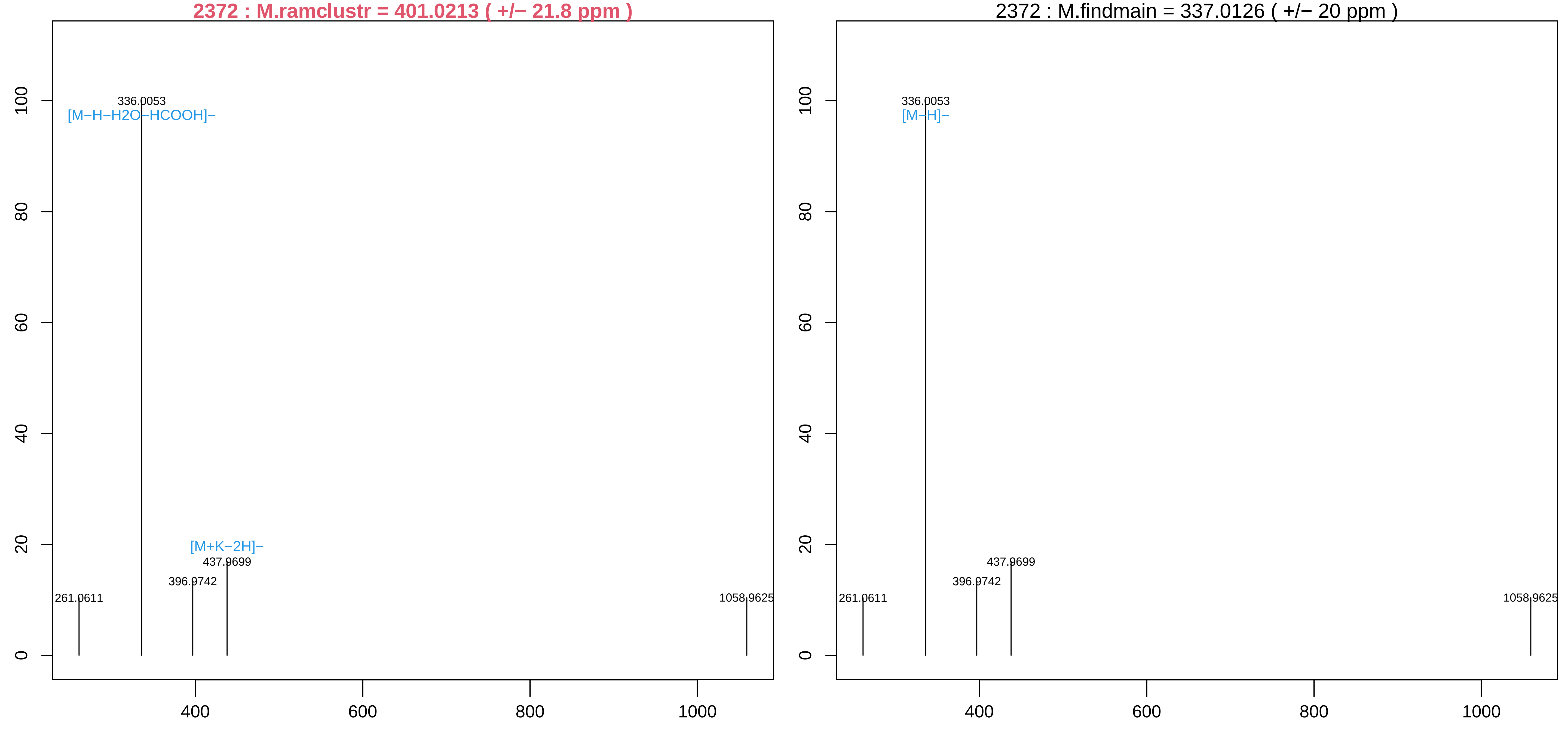
2372 : M.ramclustr = 401.0213 (+/− 21.8 ppm)
336.0053
[M−H−H2O−HCOOH]−
[M+K−2H]−
437.9699
396.9742
261.0611
1058.9625
2372 : M.findmain = 337.0126 (+/− 20 ppm)
336.0053
[M−H]−
437.9699
396.9742
261.0611
1058.9625
0
20
40
60
80
100
400
600
800
1000

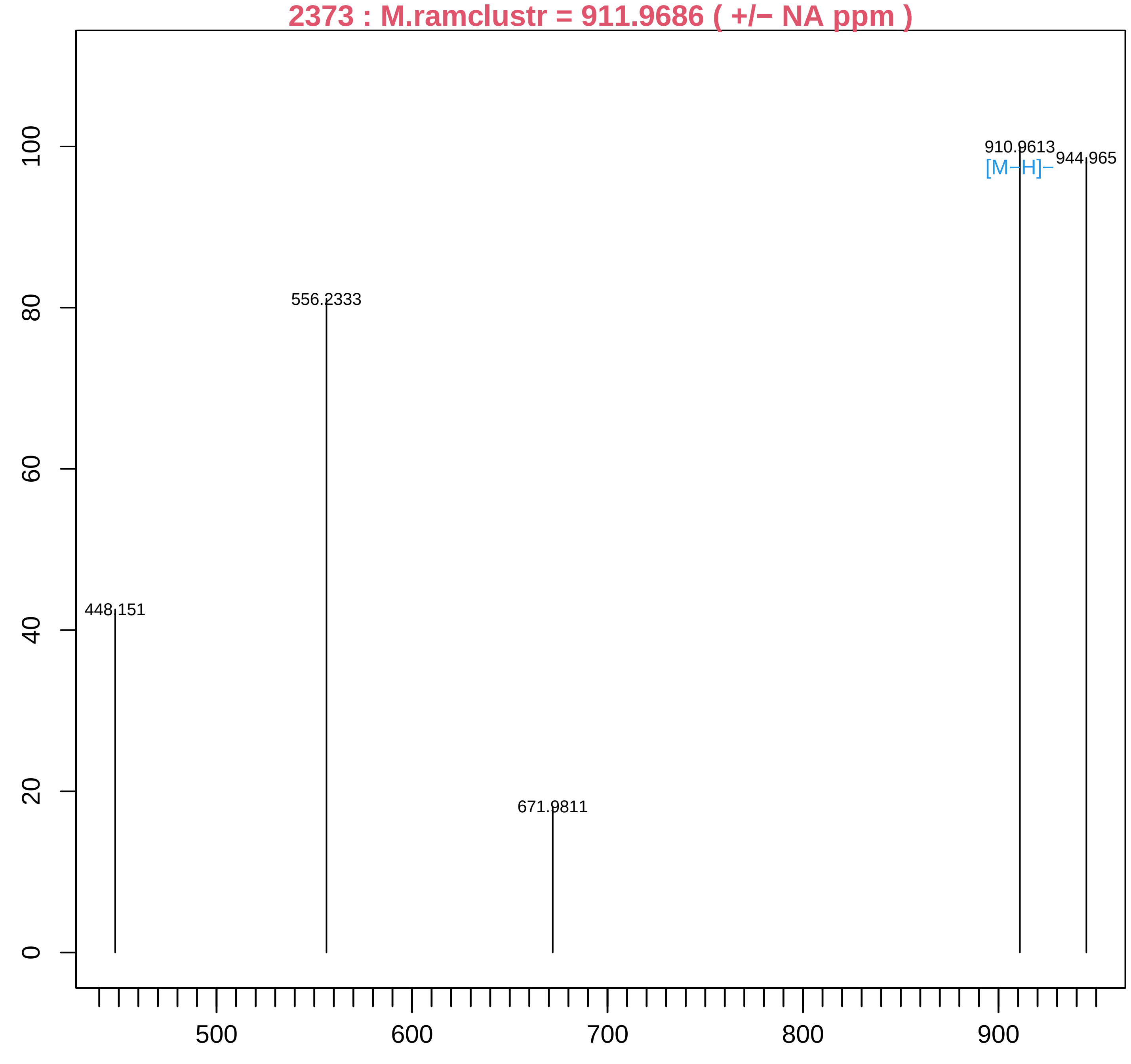
2373 : M.ramclustr = 911.9686 (+/− NA ppm)
910.9613
[M−H]−
944.965
556.2333
448.151
671.9811
0
20
40
60
80
100
500
600
700
800
900

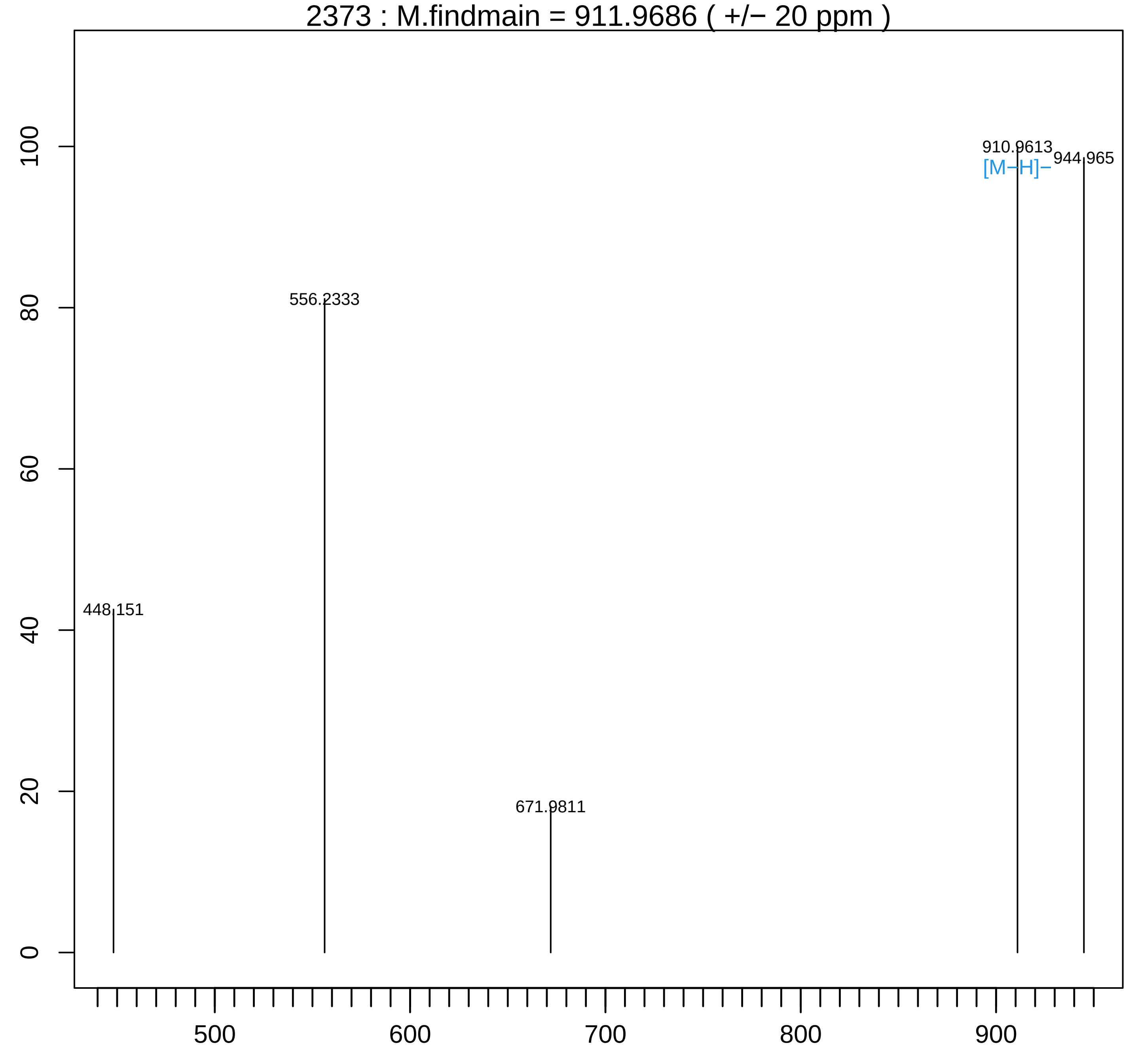
2373 : M.findmain = 911.9686 (+/− 20 ppm)
910.9613
[M−H]−
944.965
556.2333
448.151
671.9811
0
20
40
60
80
100
500
600
700
800
900

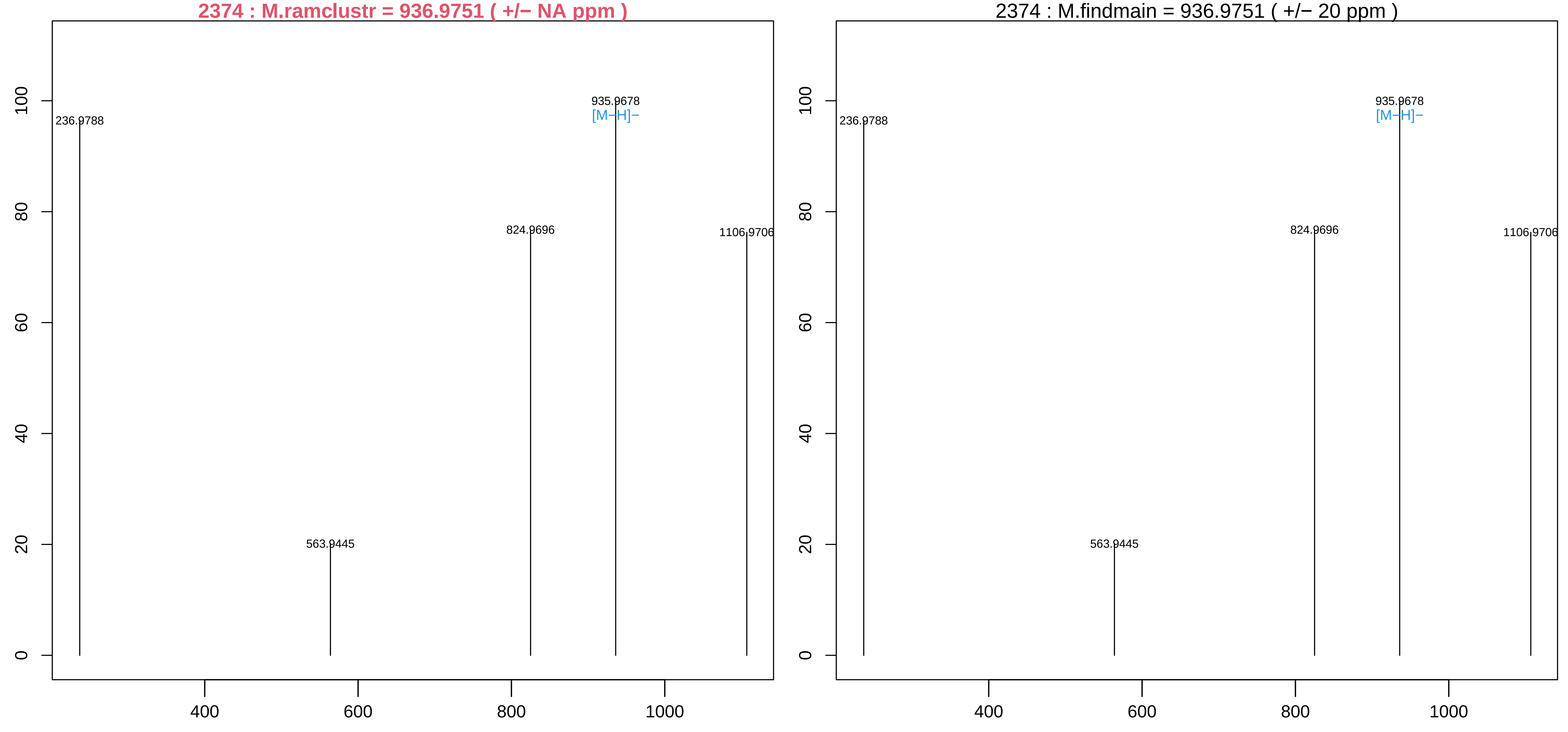
2374 : M.ramclustr = 936.9751 (+/− NA ppm)
236.9788
563.9445
824.9696
935.9678
[M−H]−
1106.9706
0
20
40
60
80
100
400
600
800
1000
2374 : M.findmain = 936.9751 (+/− 20 ppm)
236.9788
563.9445
824.9696
935.9678
[M−H]−
1106.9706
0
20
40
60
80
100
400
600
800
1000

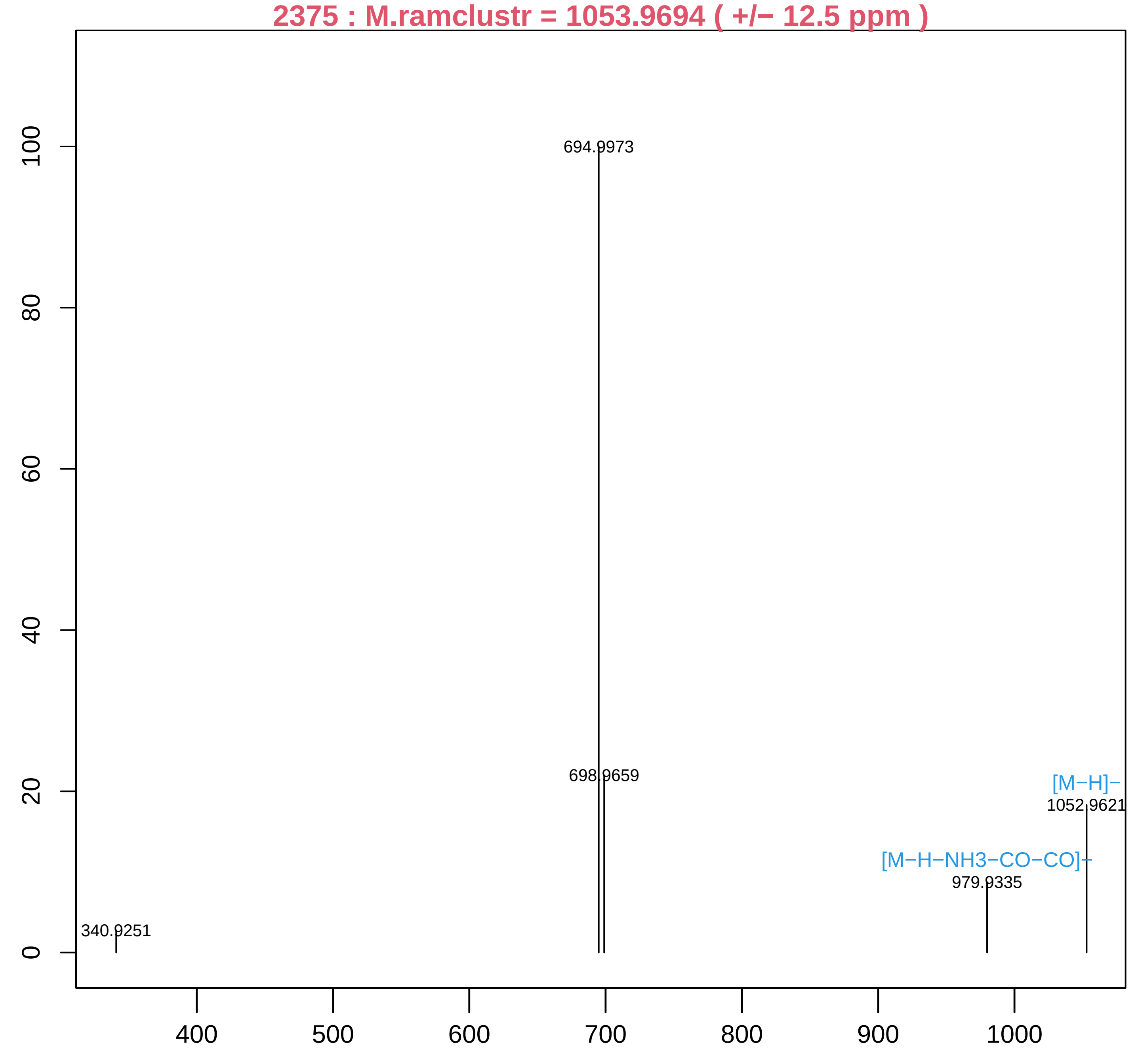

2375 : M.ramclustr = 1053.9694 (+/− 12.5 ppm)
694.9973
698.9659
[M−H]−
1052.9621
[M−H−NH3−CO−CO]−
979.9335
340.9251
0
20
40
60
80
100
400
500
600
700
800
900
1000

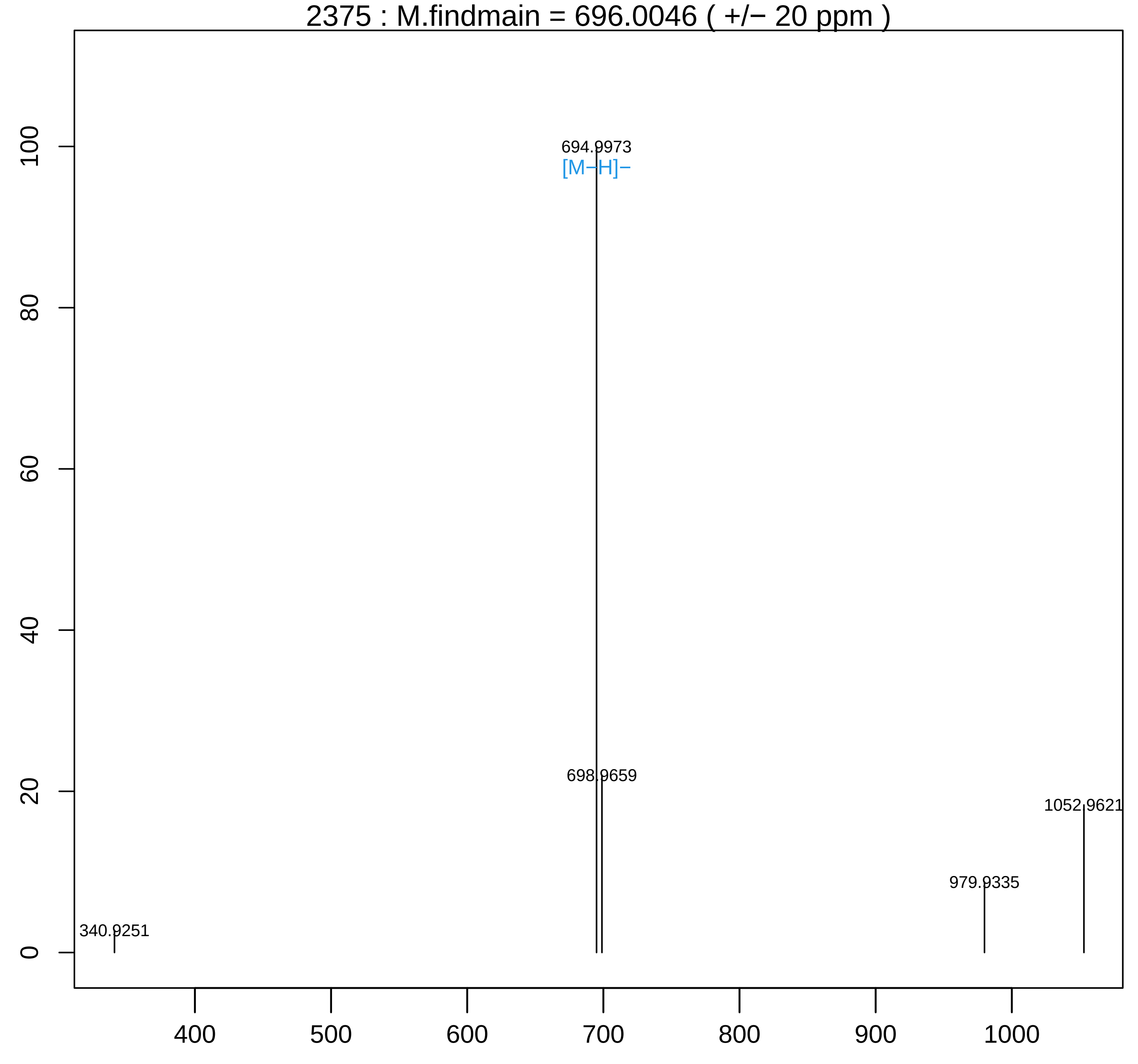

2375 : M.findmain = 696.0046 (+/− 20 ppm)
694.9973
[M−H]−
698.9659
1052.9621
979.9335
340.9251
0
20
40
60
80
100
400
500
600
700
800
900
1000

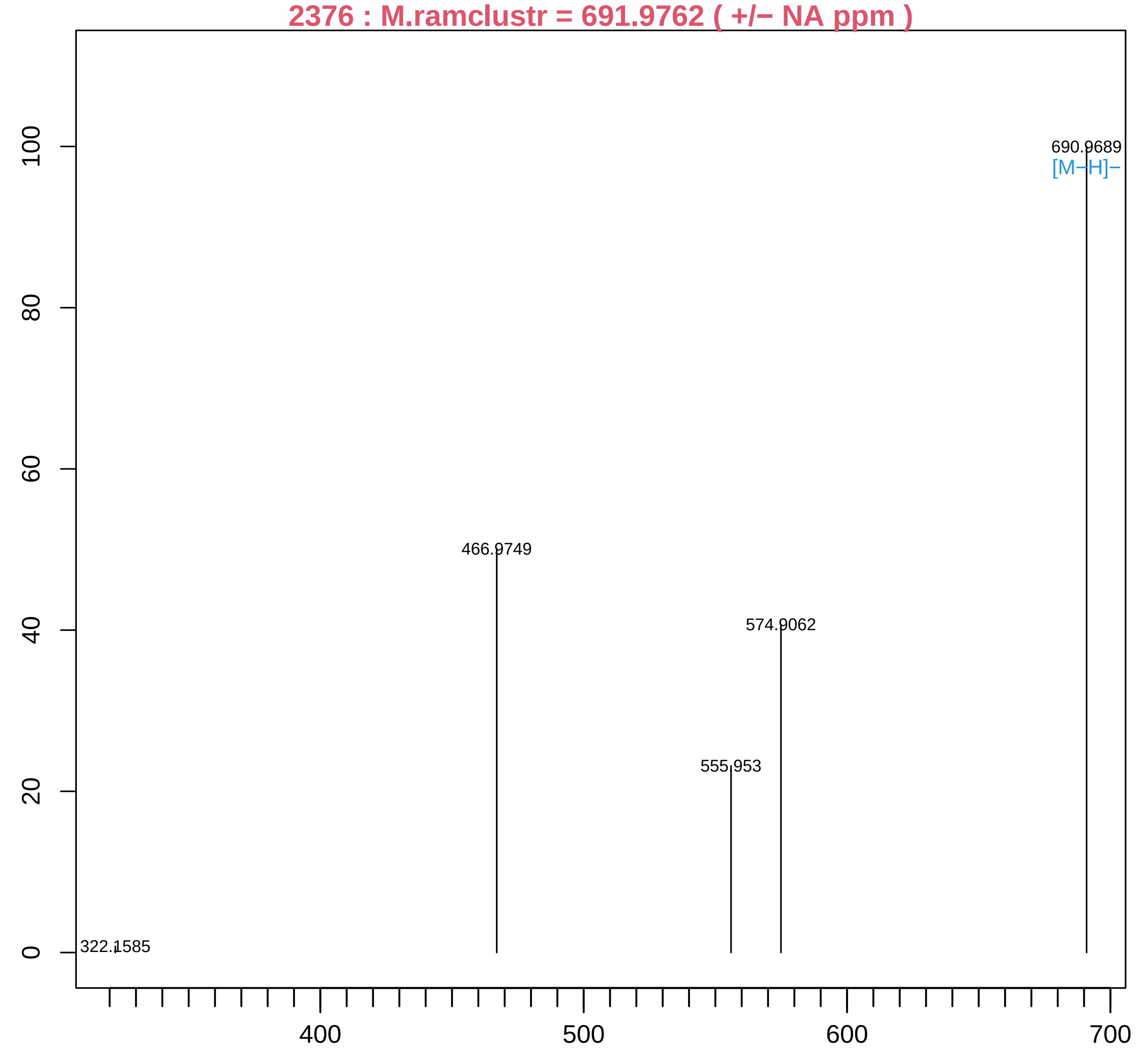

2376 : M.ramclustr = 691.9762 (+/− NA ppm)
690.9689
[M−H]−
466.9749
574.9062
555.953
322.1585
0
20
40
60
80
100
400
500
600
700

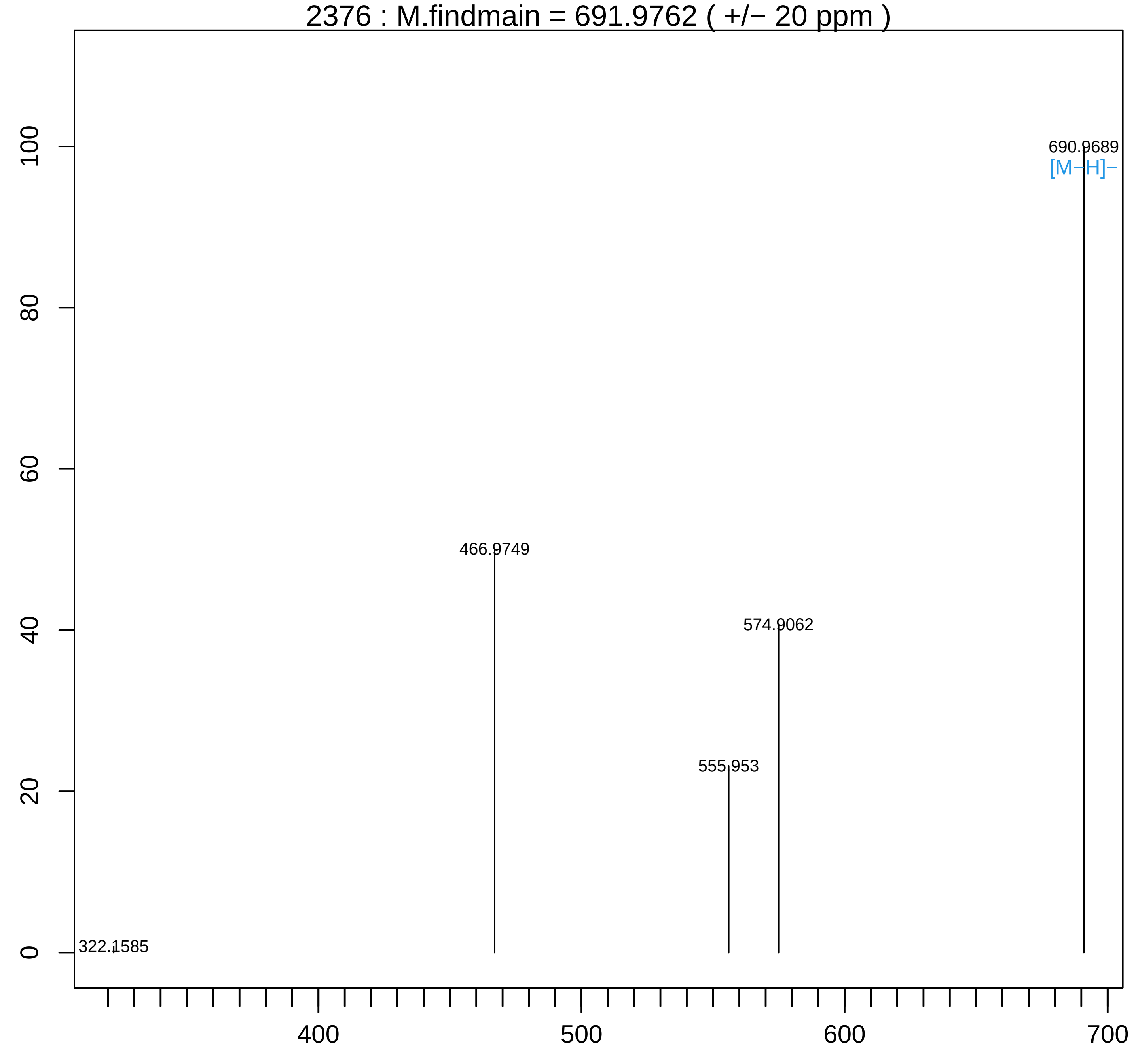

2376 : M.findmain = 691.9762 (+/− 20 ppm)
690.9689
[M−H]−
466.9749
574.9062
555.953
322.1585
0
20
40
60
80
100
400
500
600
700

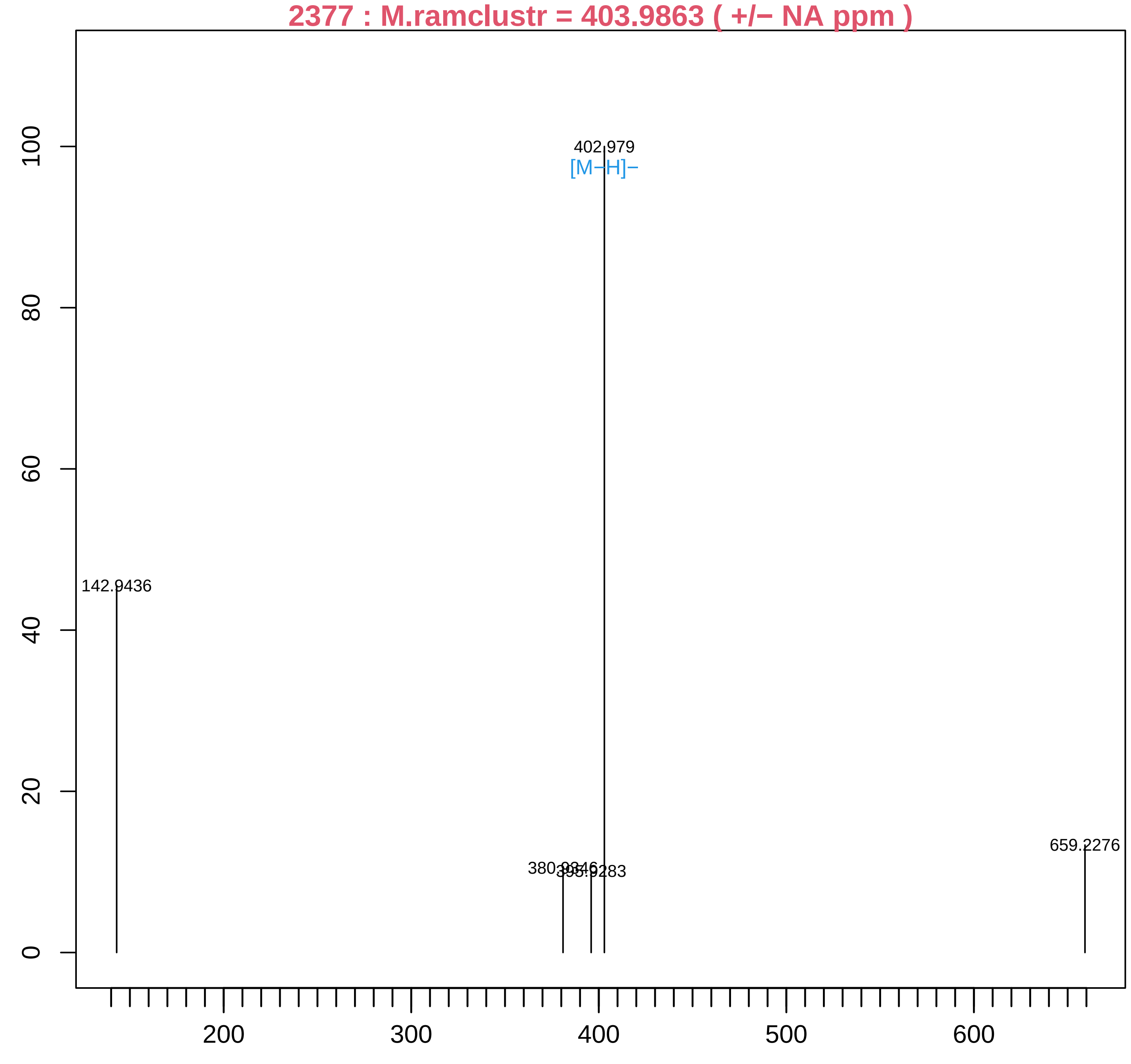

2377 : M.ramclustr = 403.9863 (+/− NA ppm)
402.979
[M−H]−
142.9436
380.9846
395.9283
659.2276
0
20
40
60
80
100
200
300
400
500
600

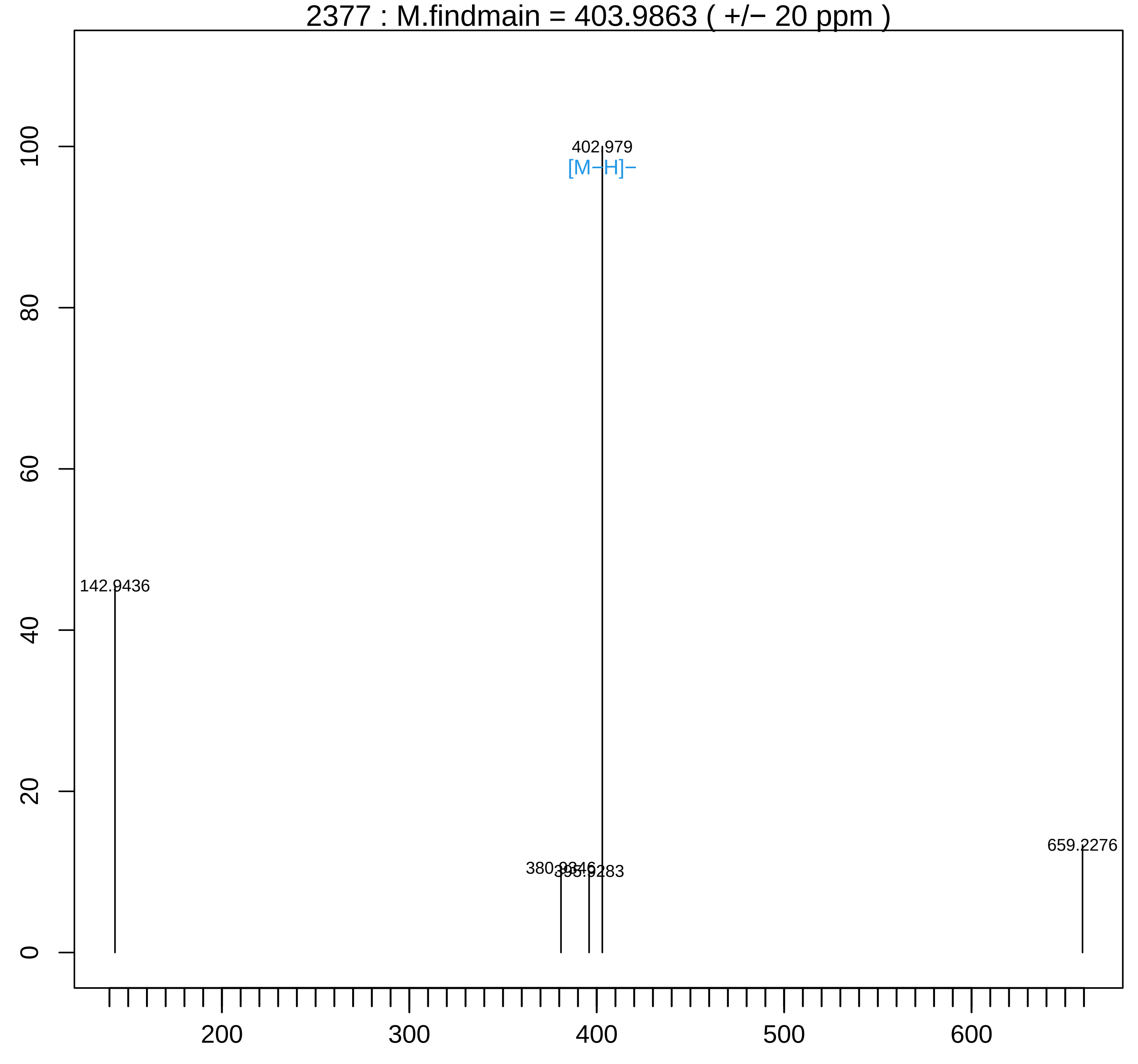

2377 : M.findmain = 403.9863 (+/− 20 ppm)
402.979
[M−H]−
142.9436
380.9846
395.9283
659.2276
0
20
40
60
80
100
200
300
400
500
600

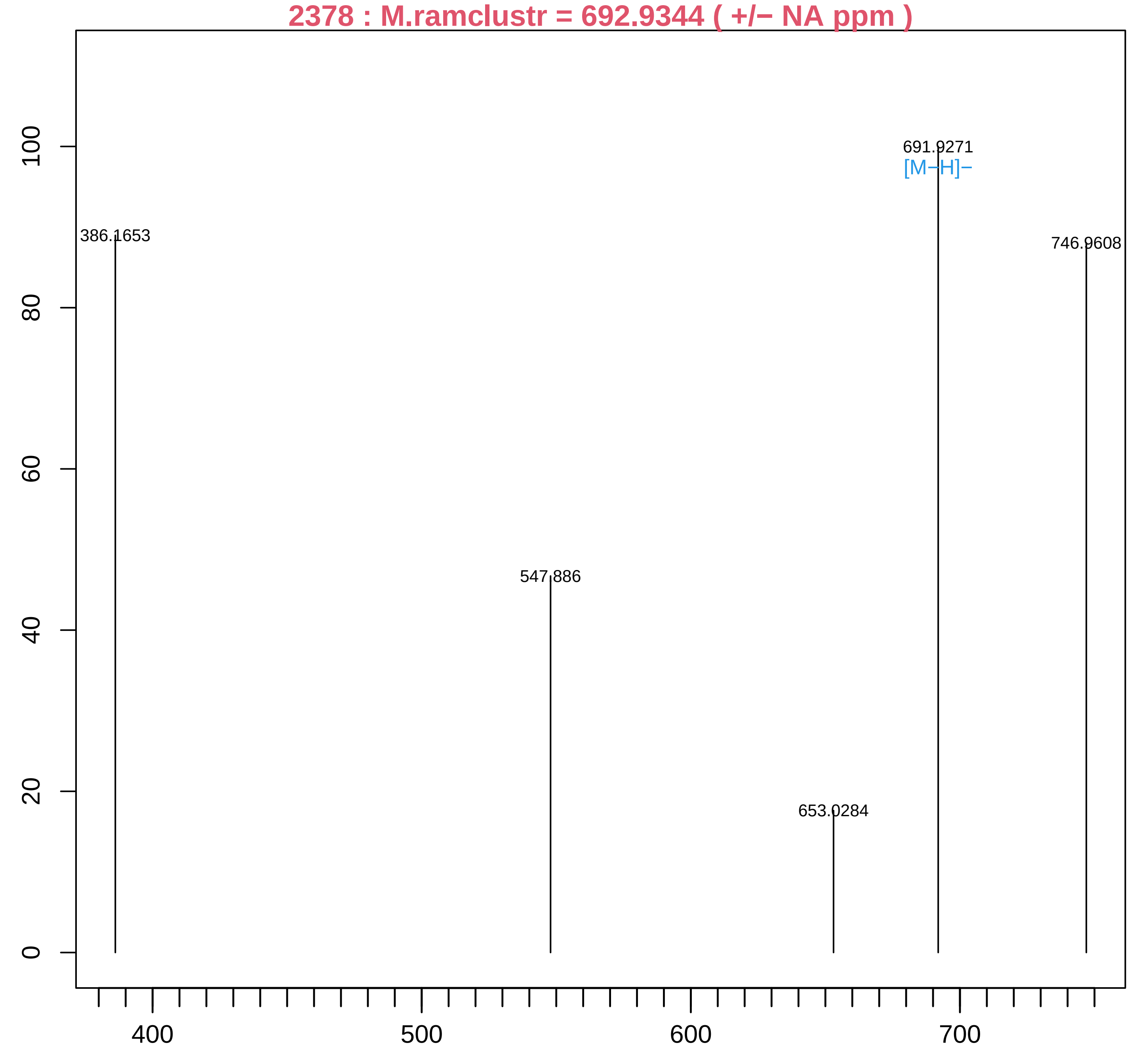
2378 : M.ramclustr = 692.9344 (+/− NA ppm)
386.1653
547.886
653.0284
691.9271
[M−H]−
746.9608
0
20
40
60
80
100
400
500
600
700

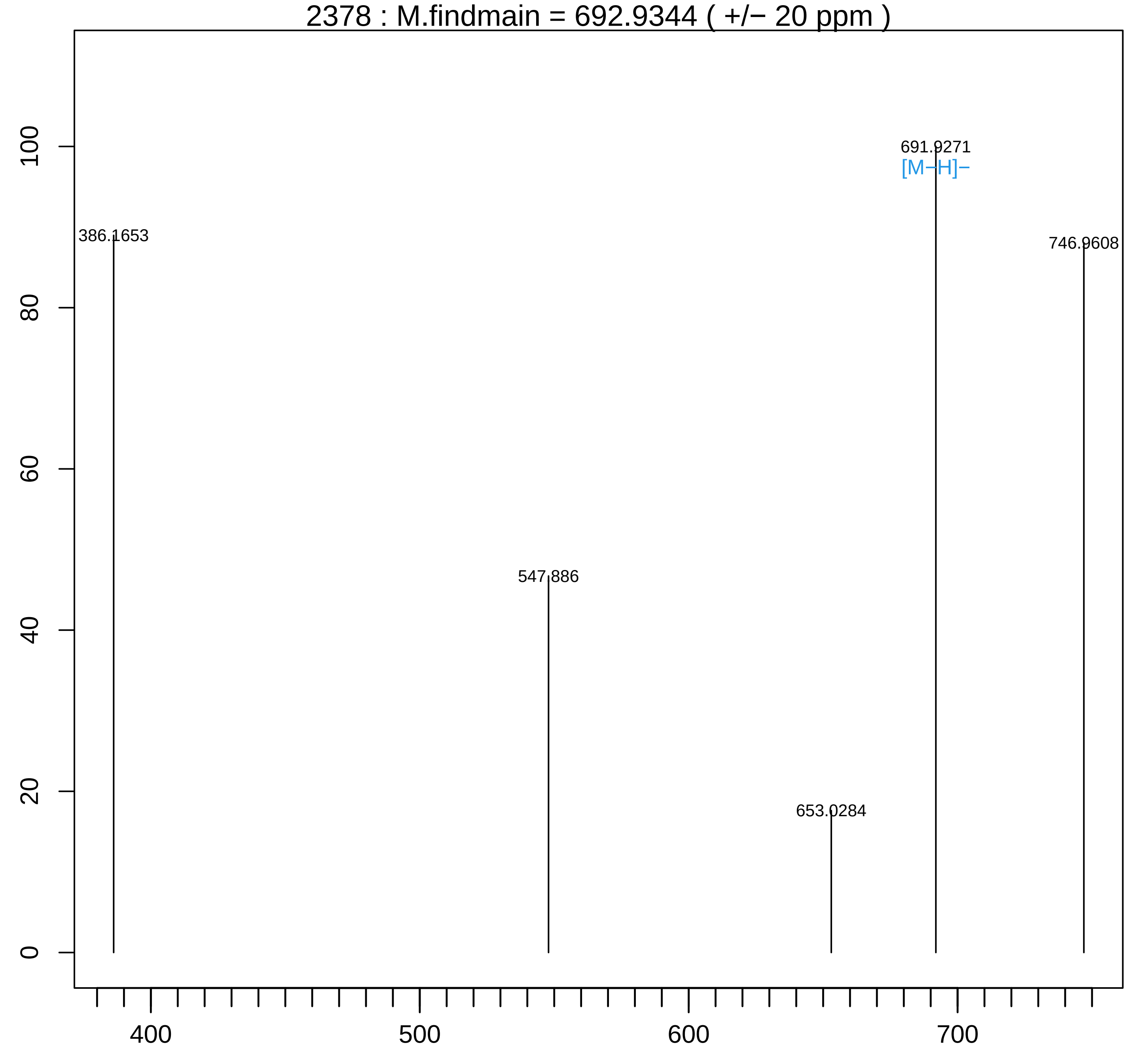
2378 : M.findmain = 692.9344 (+/− 20 ppm)
386.1653
547.886
653.0284
691.9271
[M−H]−
746.9608
0
20
40
60
80
100
400
500
600
700

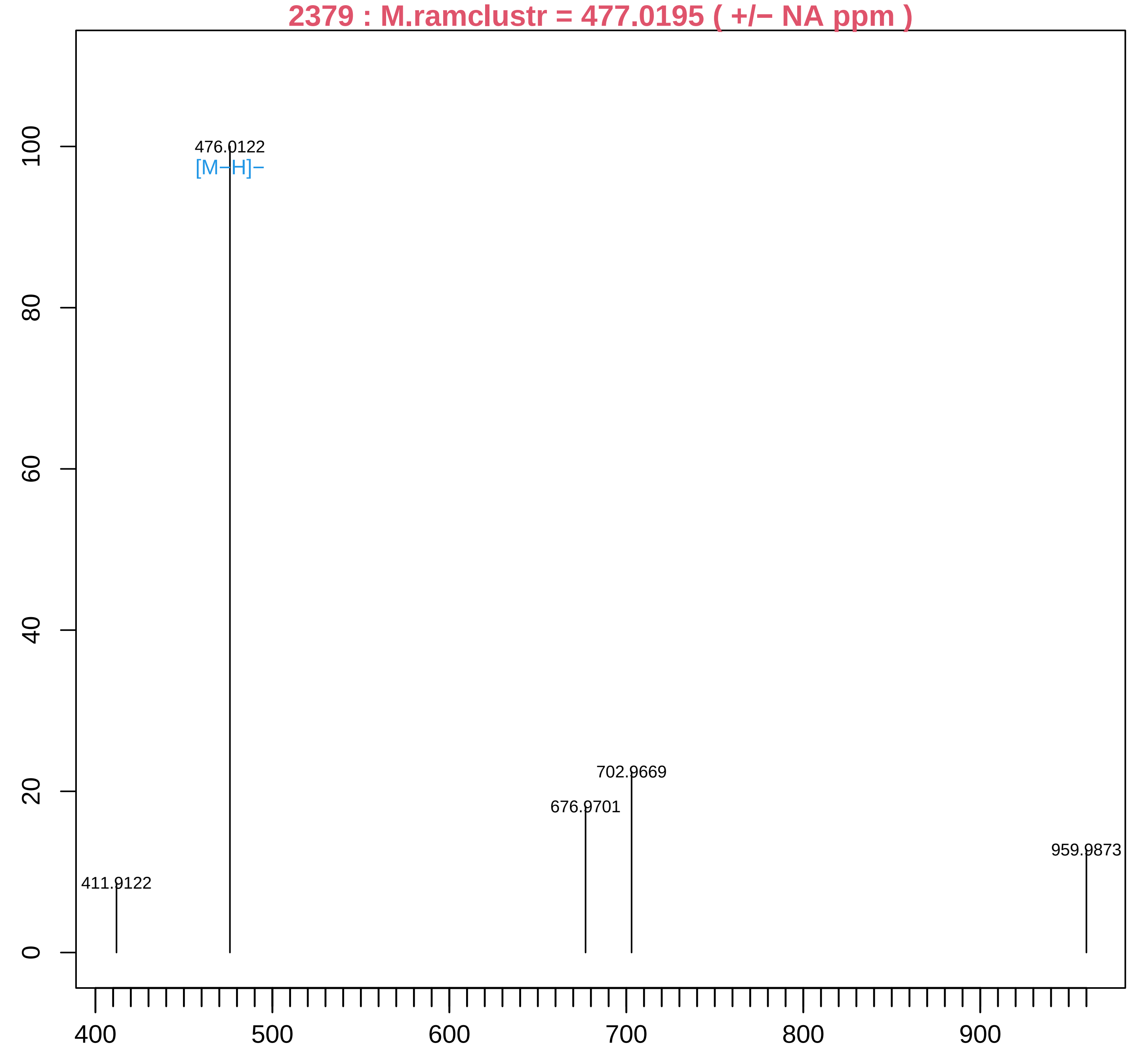
2379 : M.ramclustr = 477.0195 (+/− NA ppm)
476.0122
[M−H]−
702.9669
676.9701
959.9873
411.9122
0
20
40
60
80
100
400
500
600
700
800
900

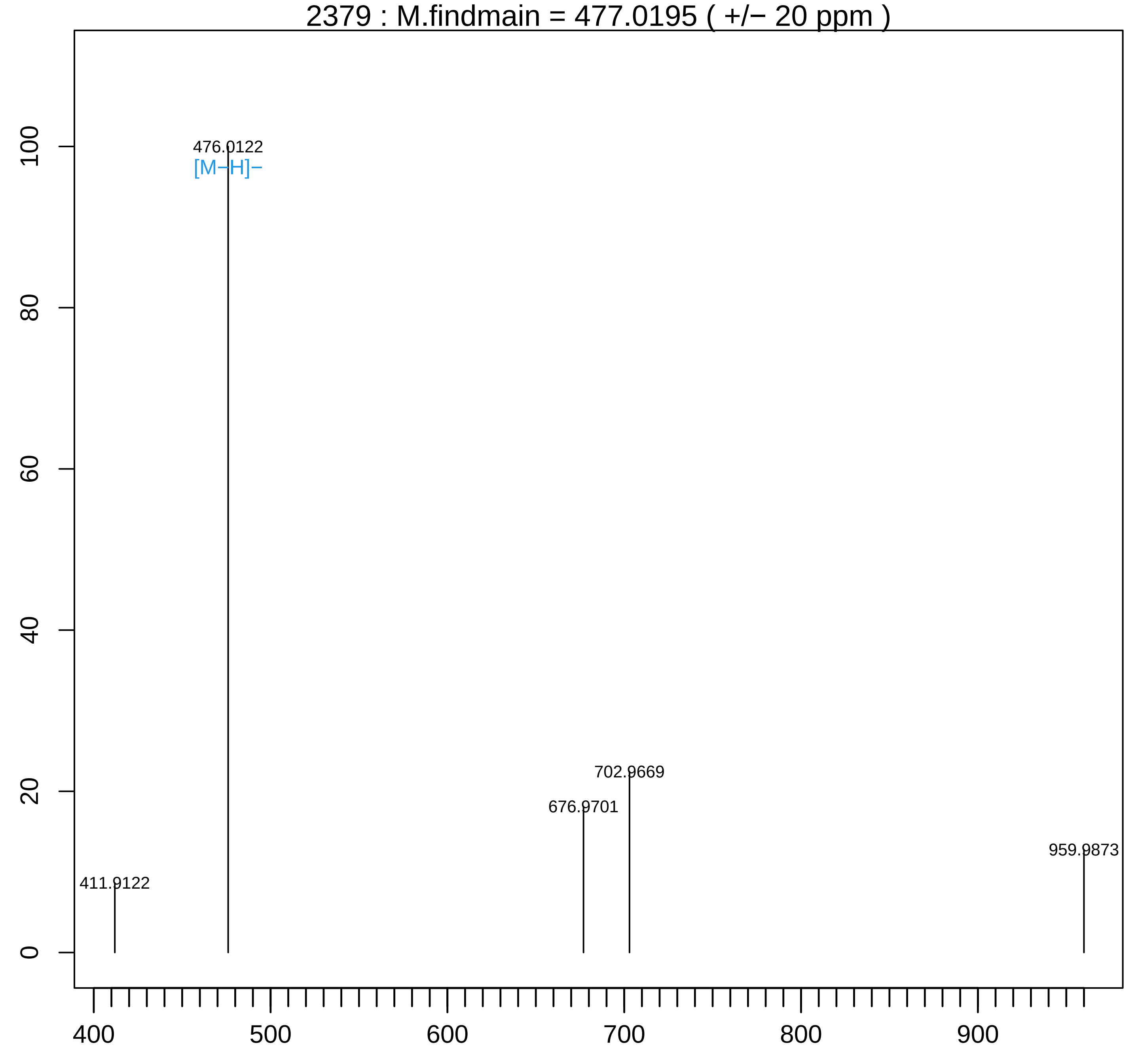
2379 : M.findmain = 477.0195 (+/− 20 ppm)
476.0122
[M−H]−
702.9669
676.9701
959.9873
411.9122
0
20
40
60
80
100
400
500
600
700
800
900

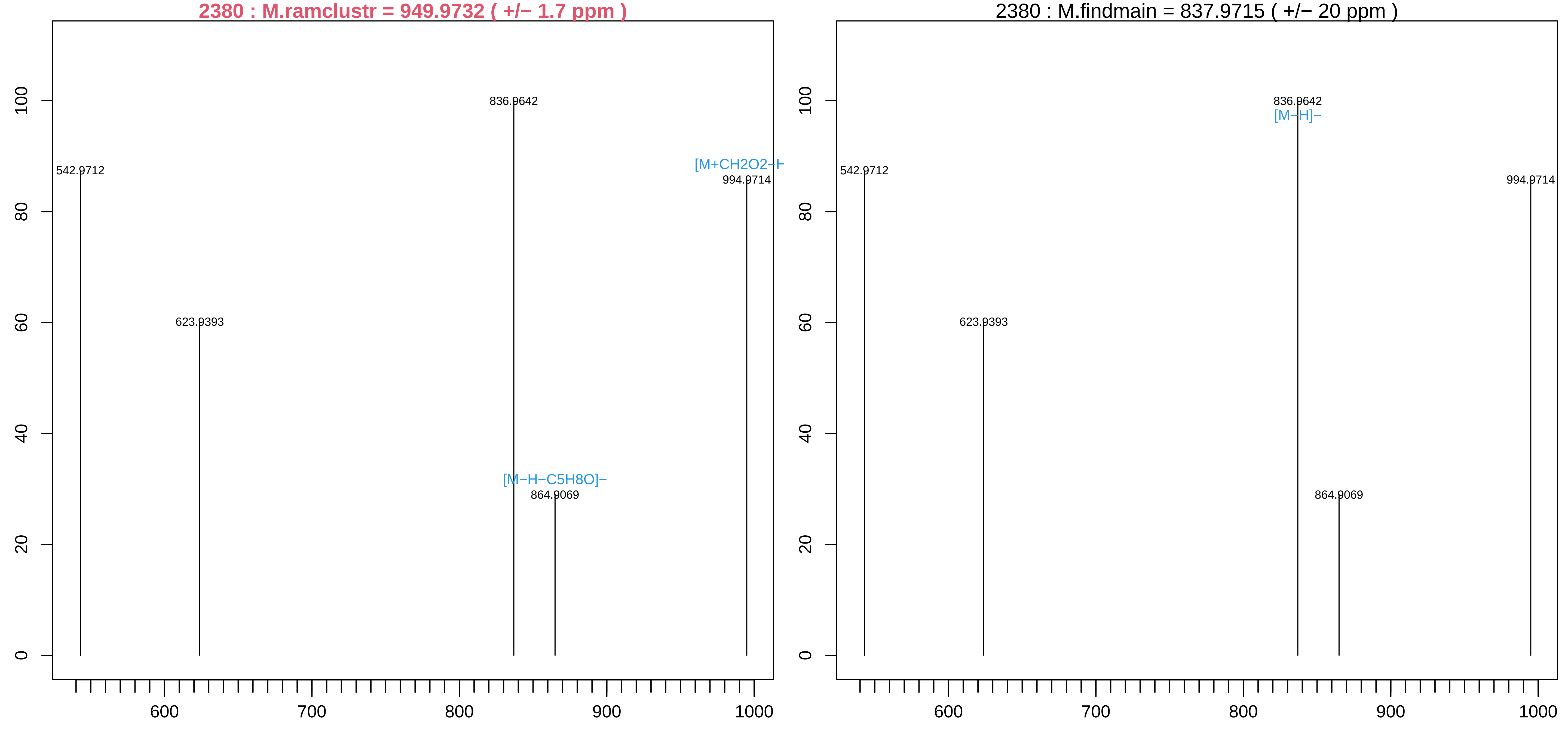
2380 : M.ramclustr = 949.9732 (+/− 1.7 ppm)
542.9712
623.9393
836.9642
[M−H−C5H8O]−
864.9069
[M+CH2O2−H
994.9714
2380 : M.findmain = 837.9715 (+/− 20 ppm)
542.9712
623.9393
836.9642
[M−H]−
864.9069
994.9714
0
20
40
60
80
100
600
700
800
900
1000

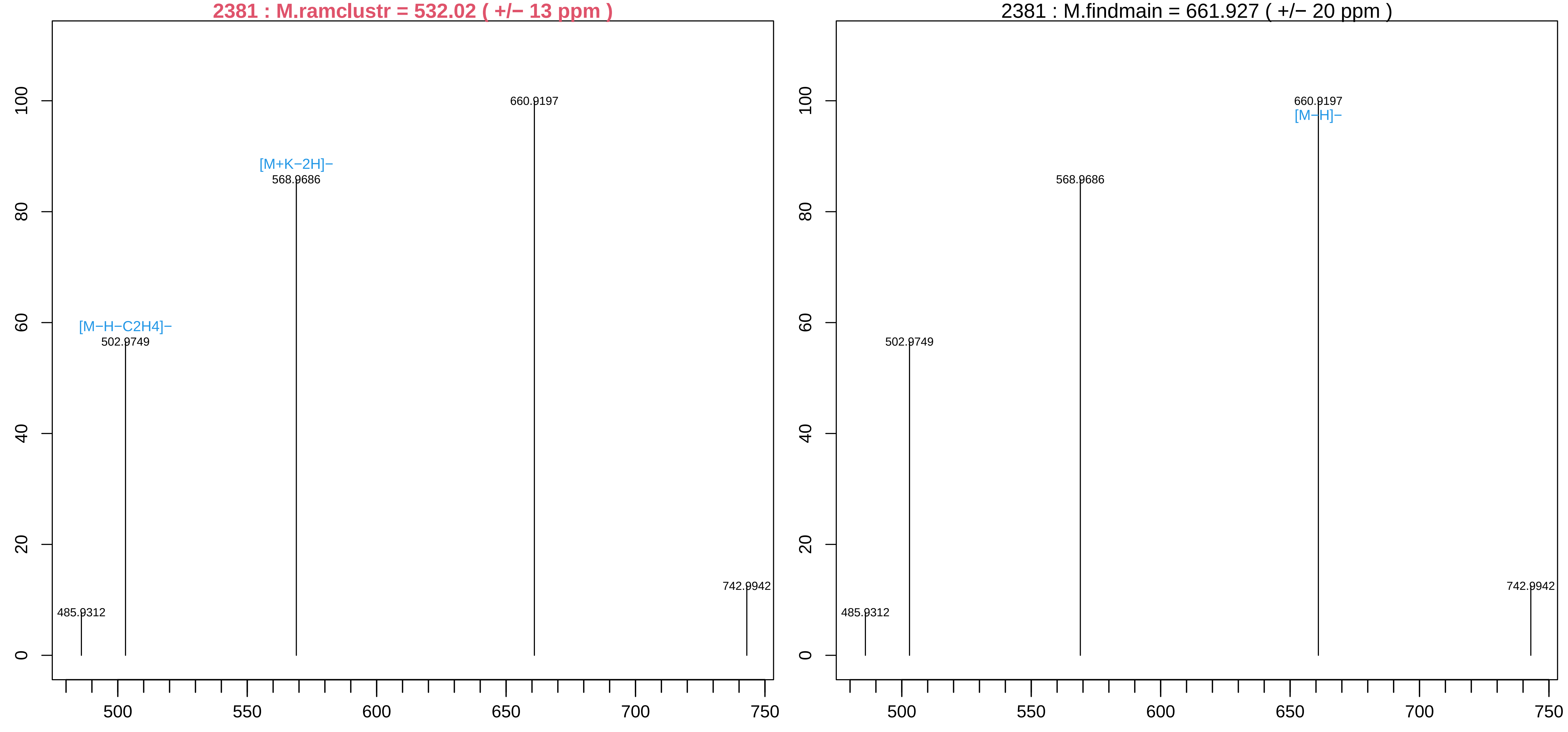
2381 : M.ramclustr = 532.02 (+/− 13 ppm)
[M−H−C2H4]−
502.9749
[M+K−2H]−
568.9686
660.9197
485.9312
742.9942
2381 : M.findmain = 661.927 (+/− 20 ppm)
660.9197
[M−H]−
568.9686
502.9749
485.9312
742.9942

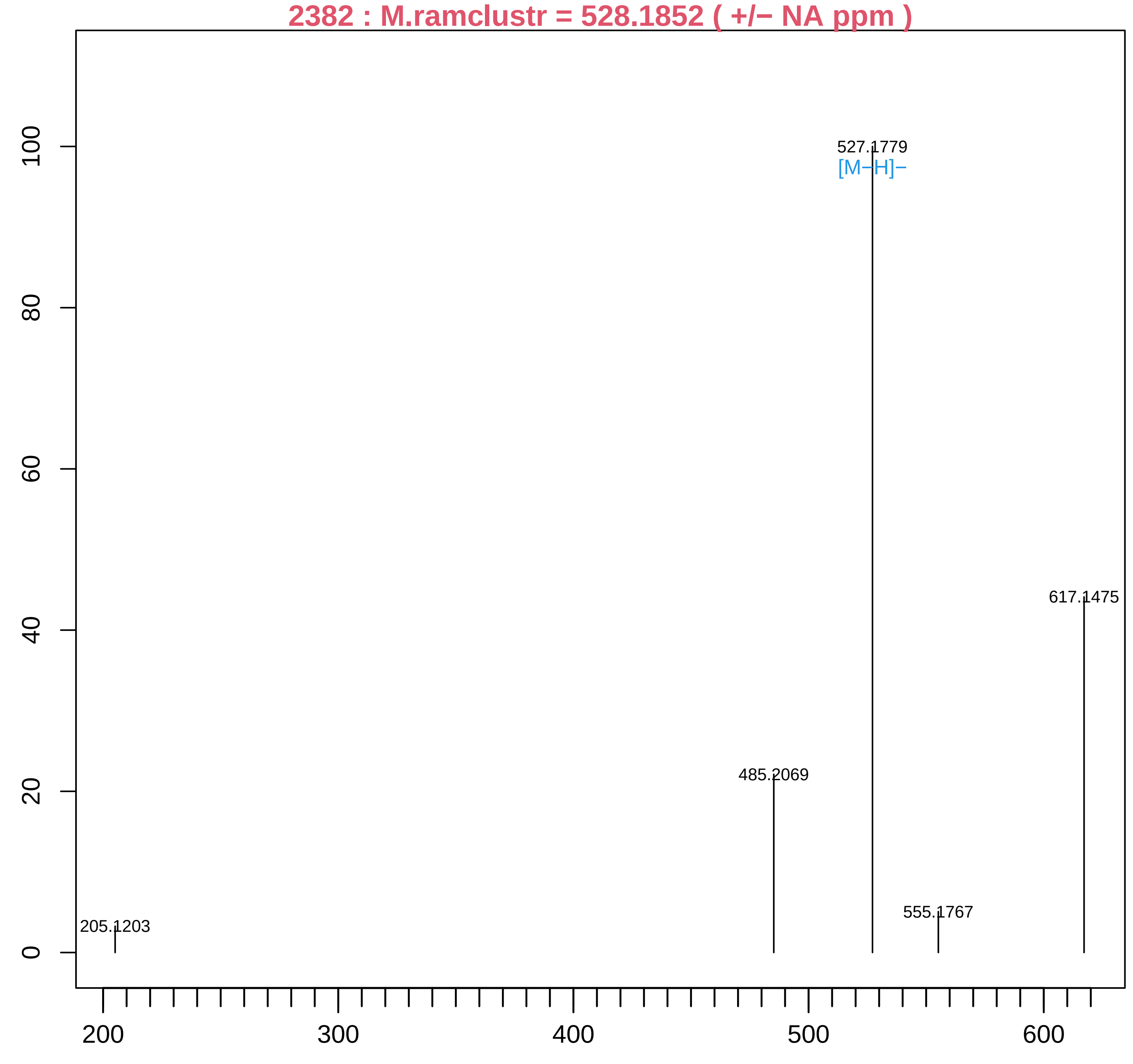

2382 : M.ramclustr = 528.1852 (+/− NA ppm)
527.1779
[M−H]−
617.1475
485.2069
555.1767
205.1203
0
20
40
60
80
100
200
300
400
500
600

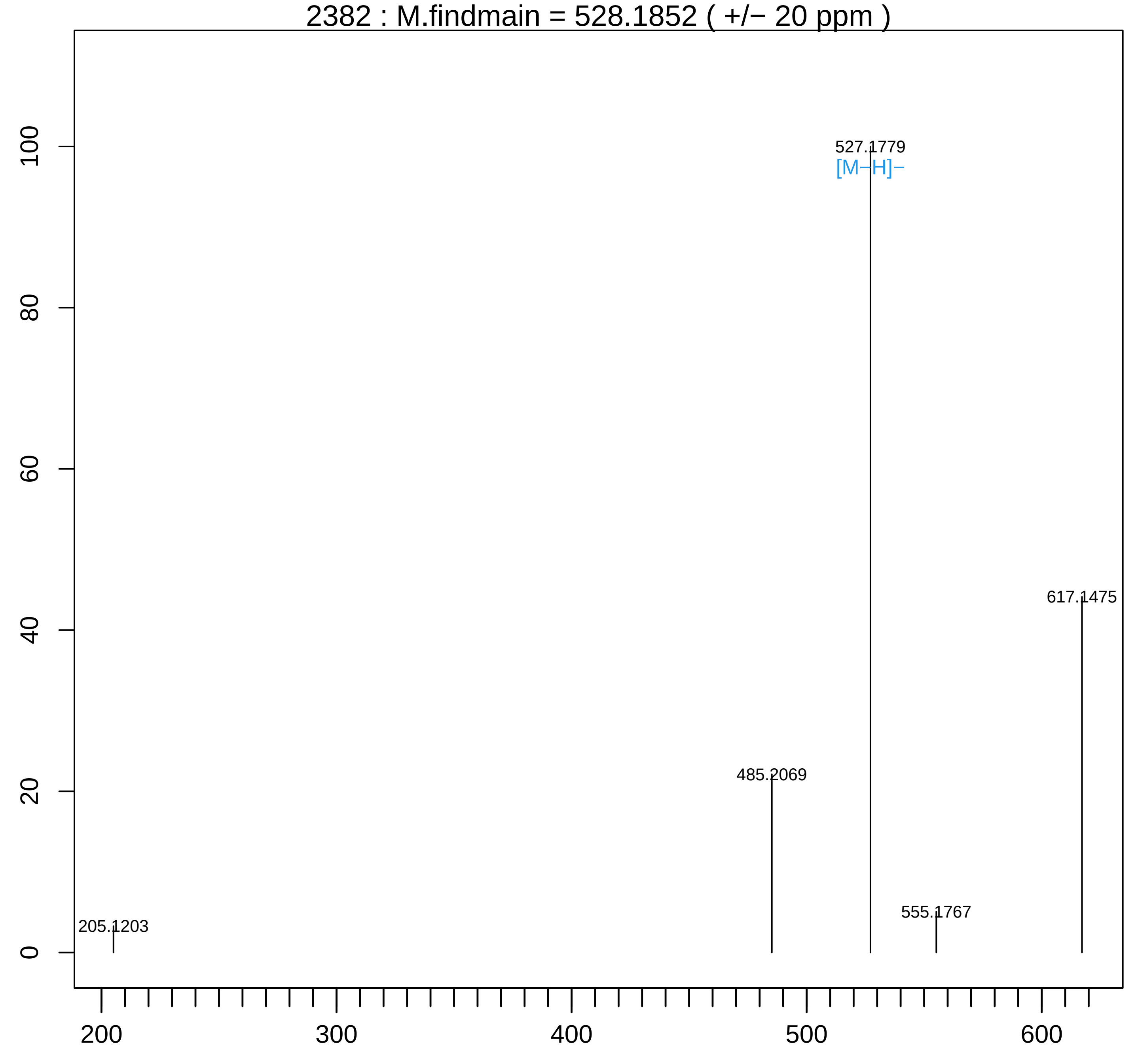

2382 : M.findmain = 528.1852 (+/− 20 ppm)
527.1779
[M−H]−
617.1475
485.2069
555.1767
205.1203
0
20
40
60
80
100
200
300
400
500
600

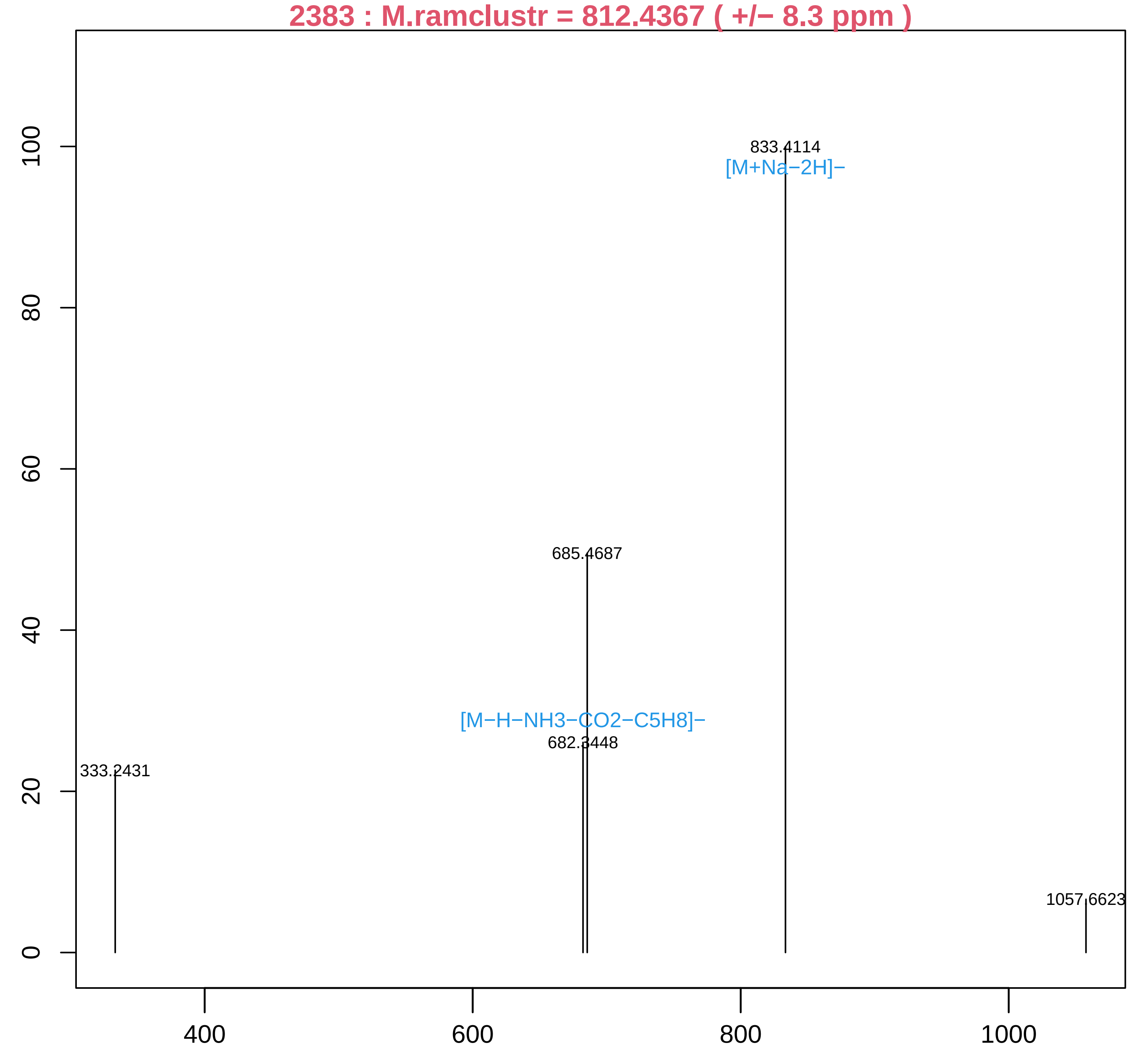
2383 : M.ramclustr = 812.4367 (+/− 8.3 ppm)
833.4114
[M+Na−2H]−
685.4687
[M−H−NH3−CO2−C5H8]−
682.3448
333.2431
1057.6623
0
20
40
60
80
100
400
600
800
1000

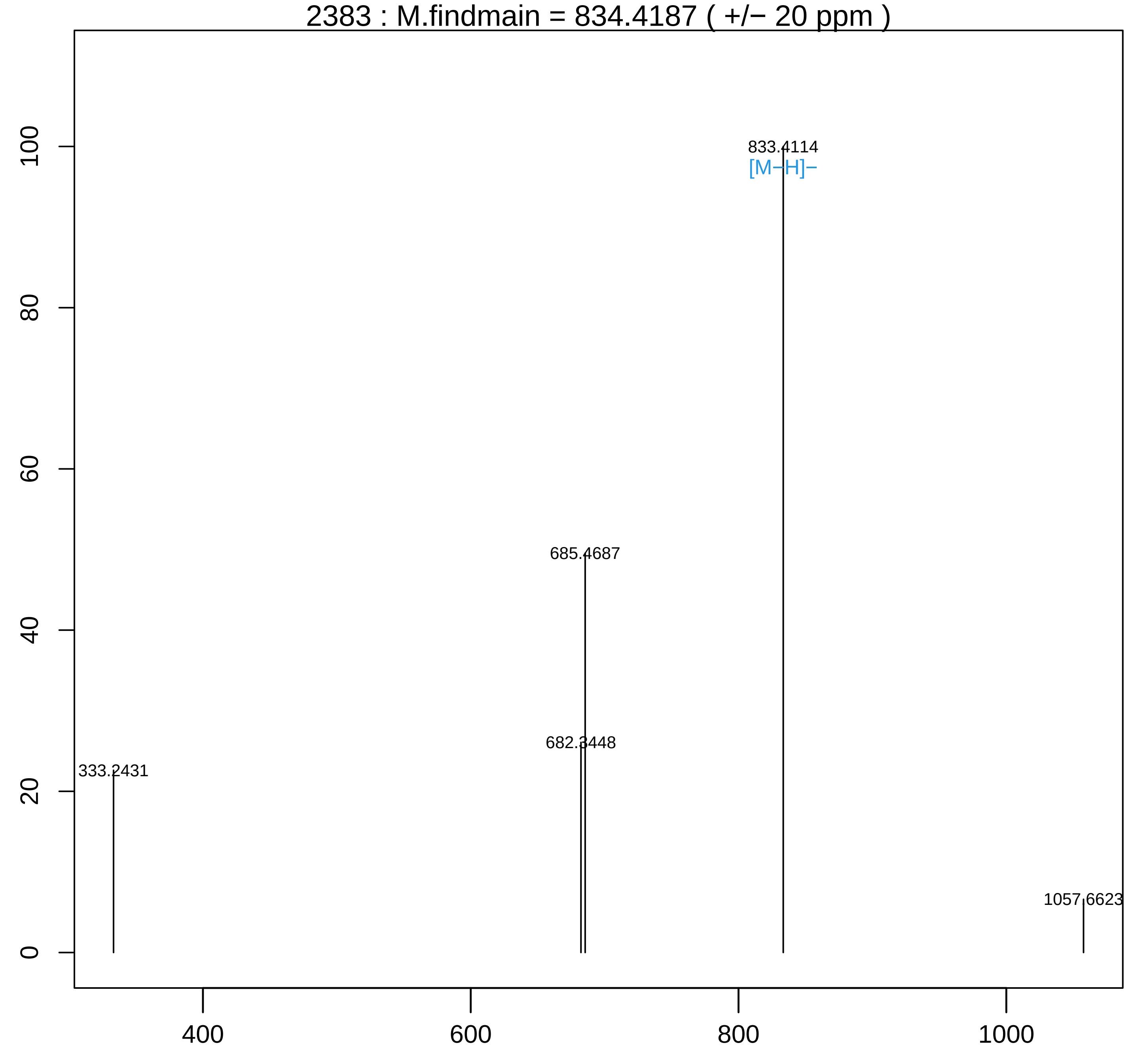
2383 : M.findmain = 834.4187 (+/− 20 ppm)
833.4114
[M−H]−
685.4687
682.3448
333.2431
1057.6623
0
20
40
60
80
100
400
600
800
1000

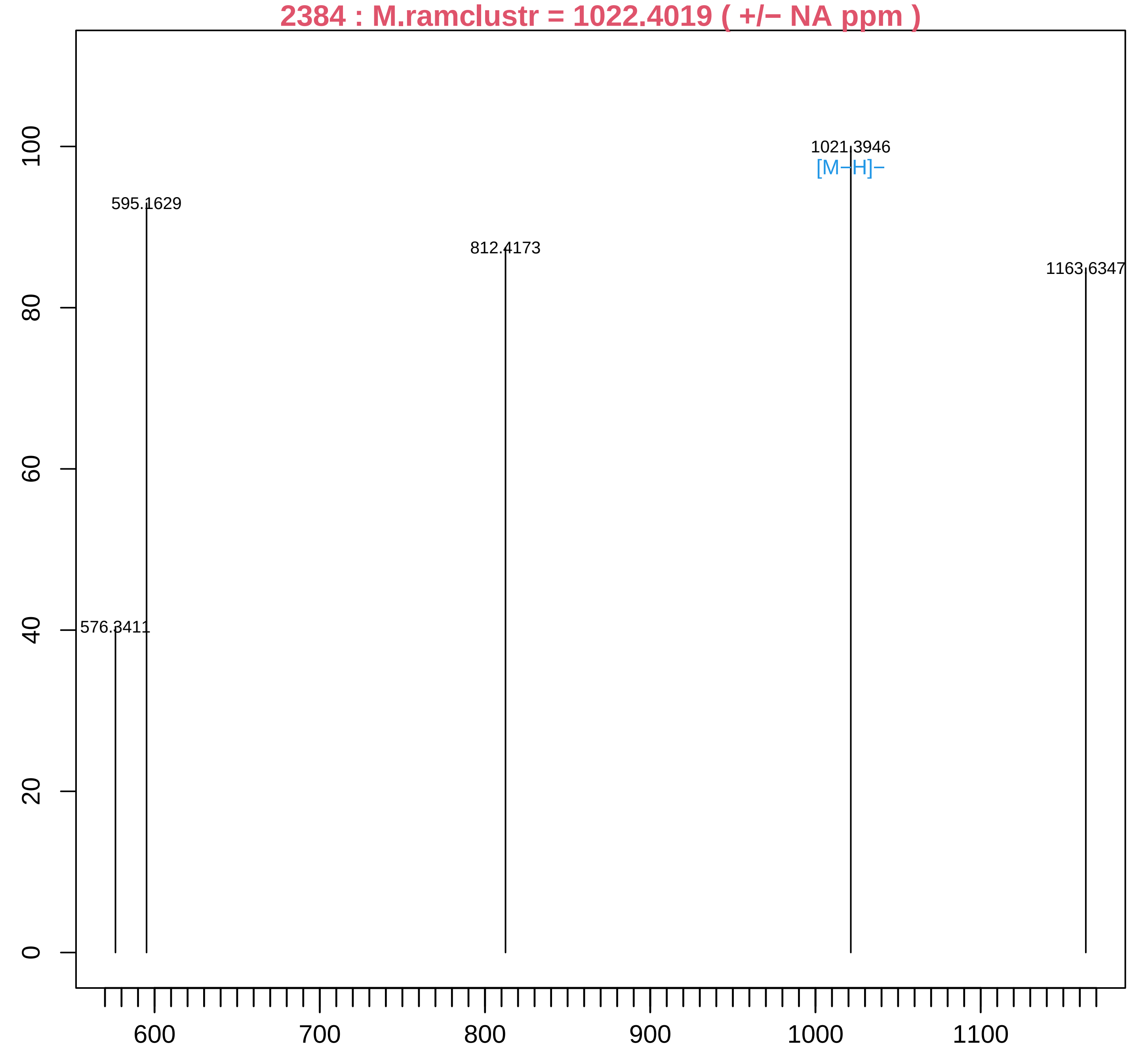
2384 : M.ramclustr = 1022.4019 (+/− NA ppm)
1021.3946
[M−H]−
595.1629
812.4173
1163.6347
576.3411
0
20
40
60
80
100
600
700
800
900
1000
1100

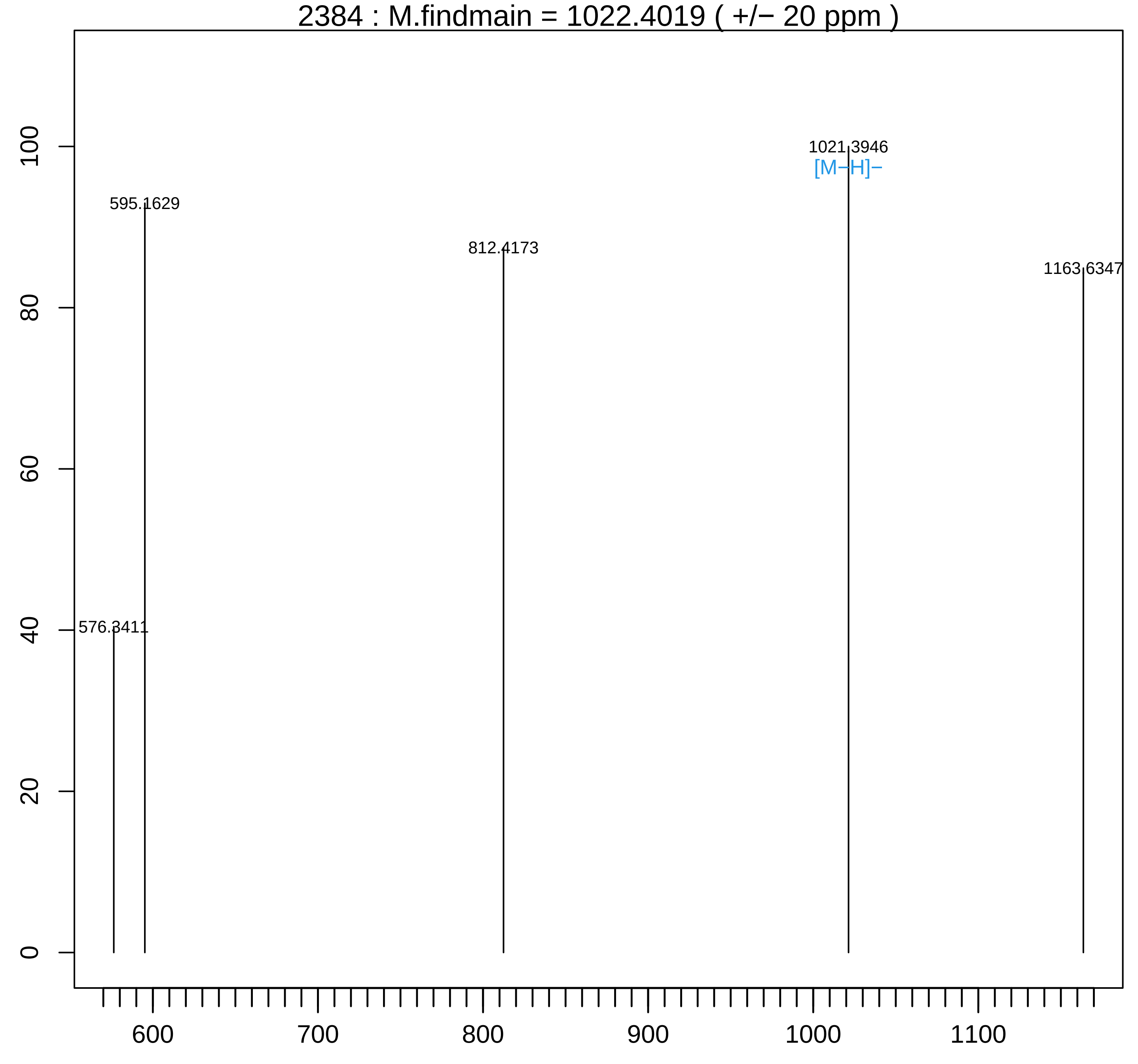
2384 : M.findmain = 1022.4019 (+/− 20 ppm)
1021.3946
[M−H]−
595.1629
812.4173
1163.6347
576.3411
0
20
40
60
80
100
600
700
800
900
1000
1100

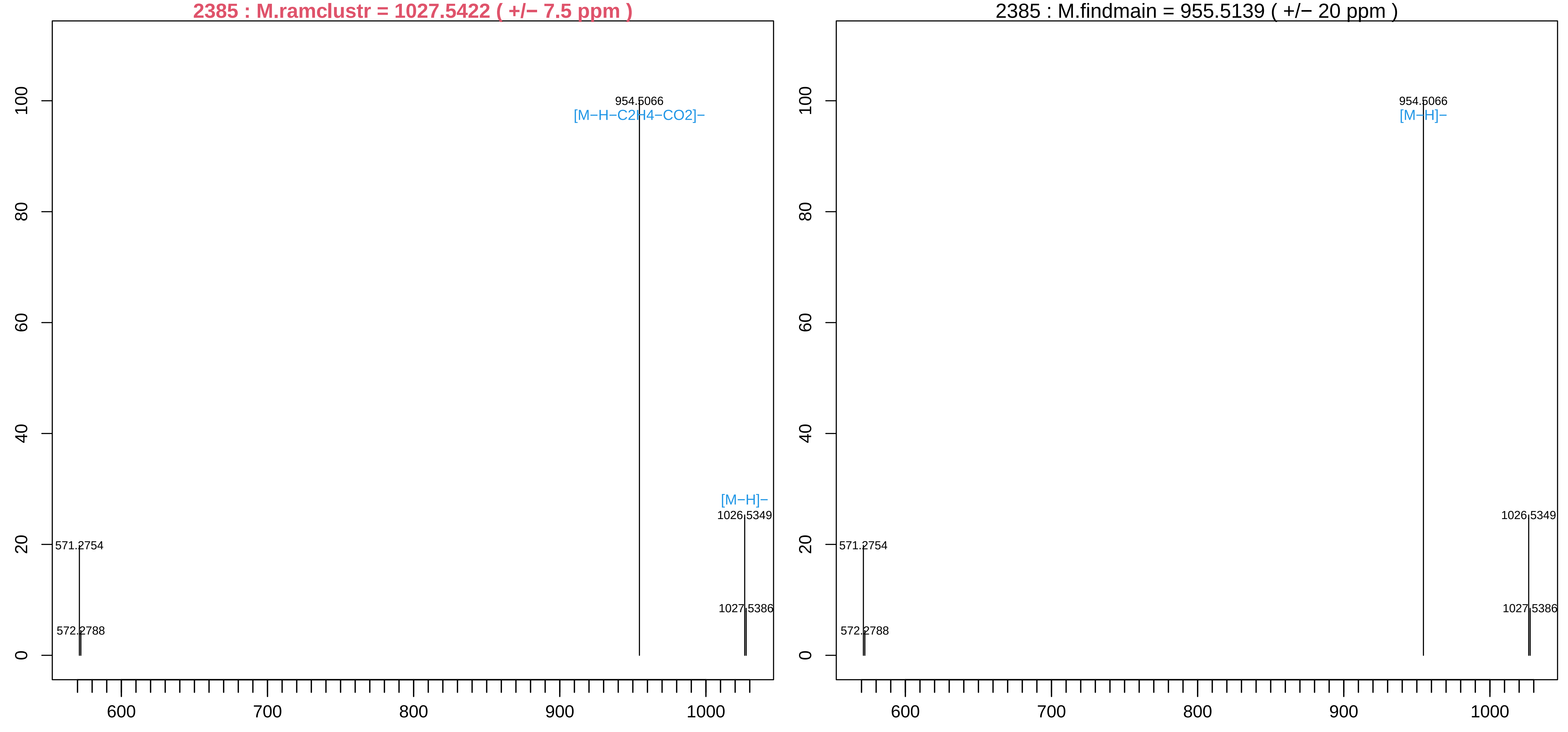

2385 : M.ramclustr = 1027.5422 (+/− 7.5 ppm)
954.5066
[M−H−C2H4−CO2]−
[M−H]−
1026.5349
571.2754
1027.5386
572.2788
2385 : M.findmain = 955.5139 (+/− 20 ppm)
954.5066
[M−H]−
1026.5349
571.2754
1027.5386
572.2788
0
20
40
60
80
100
600
700
800
900
1000

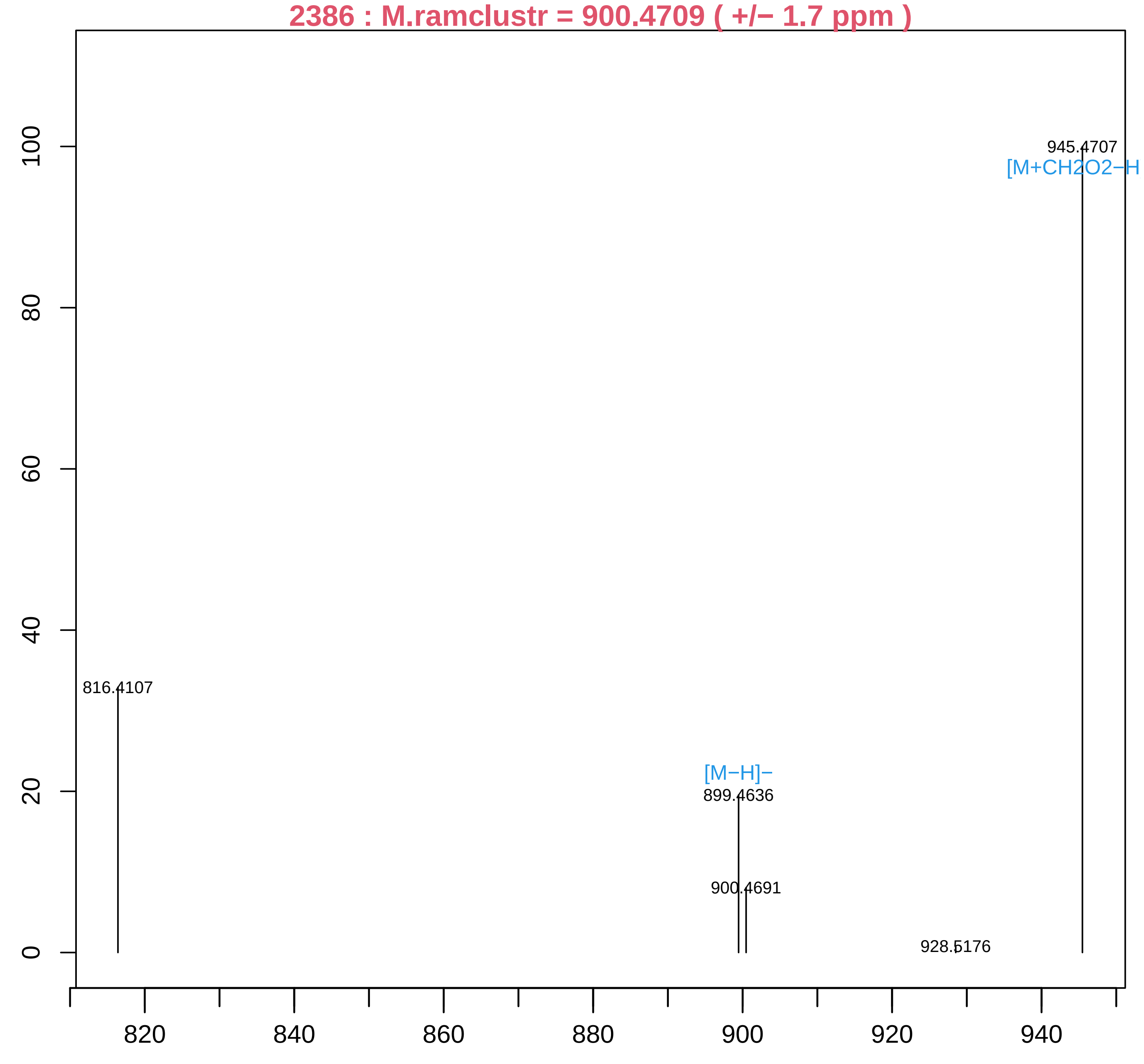
2386 : M.ramclustr = 900.4709 (+/− 1.7 ppm)
945.4707
[M+CH2O2−H
816.4107
[M−H]−
899.4636
900.4691
928.5176
0
20
40
60
80
100
820
840
860
880
900
920
940
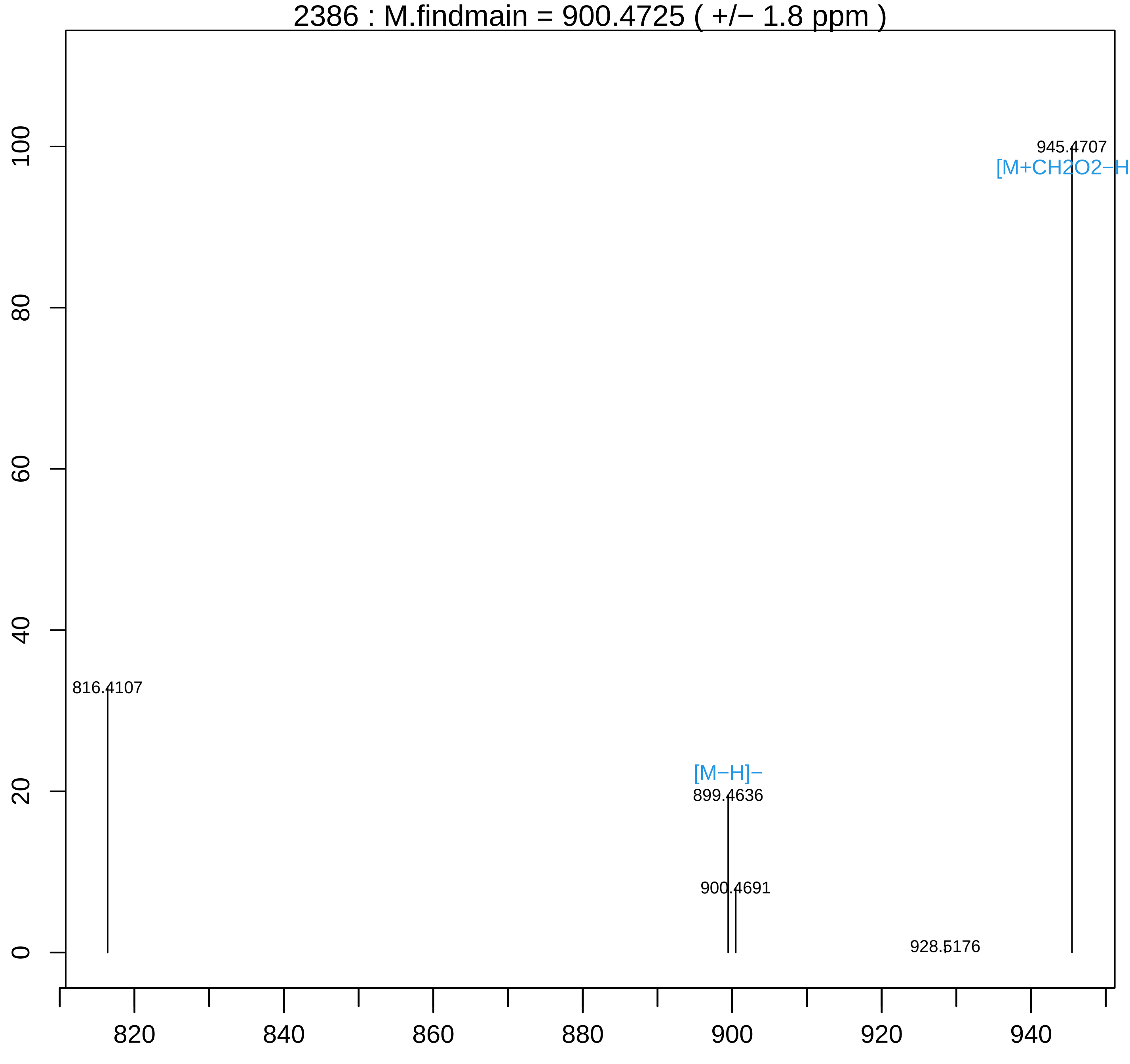
2386 : M.findmain = 900.4725 (+/− 1.8 ppm)
945.4707
[M+CH2O2−H
816.4107
[M−H]−
899.4636
900.4691
928.5176
0
20
40
60
80
100
820
840
860
880
900
920
940

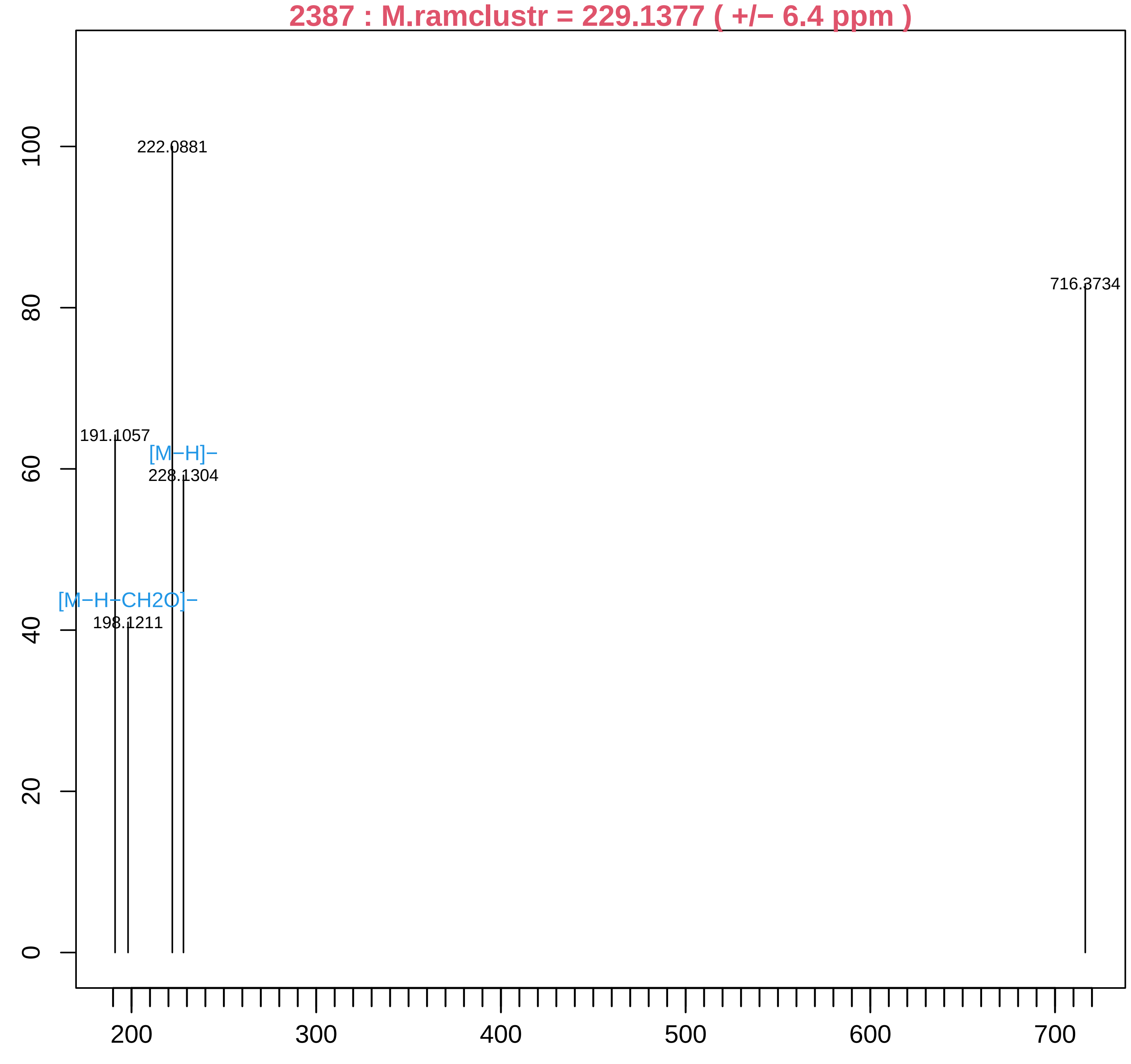
2387 : M.ramclustr = 229.1377 (+/− 6.4 ppm)
222.0881
716.3734
191.1057
[M−H]−
228.1304
[M−H−CH2O]−
198.1211
0
20
40
60
80
100
200
300
400
500
600
700

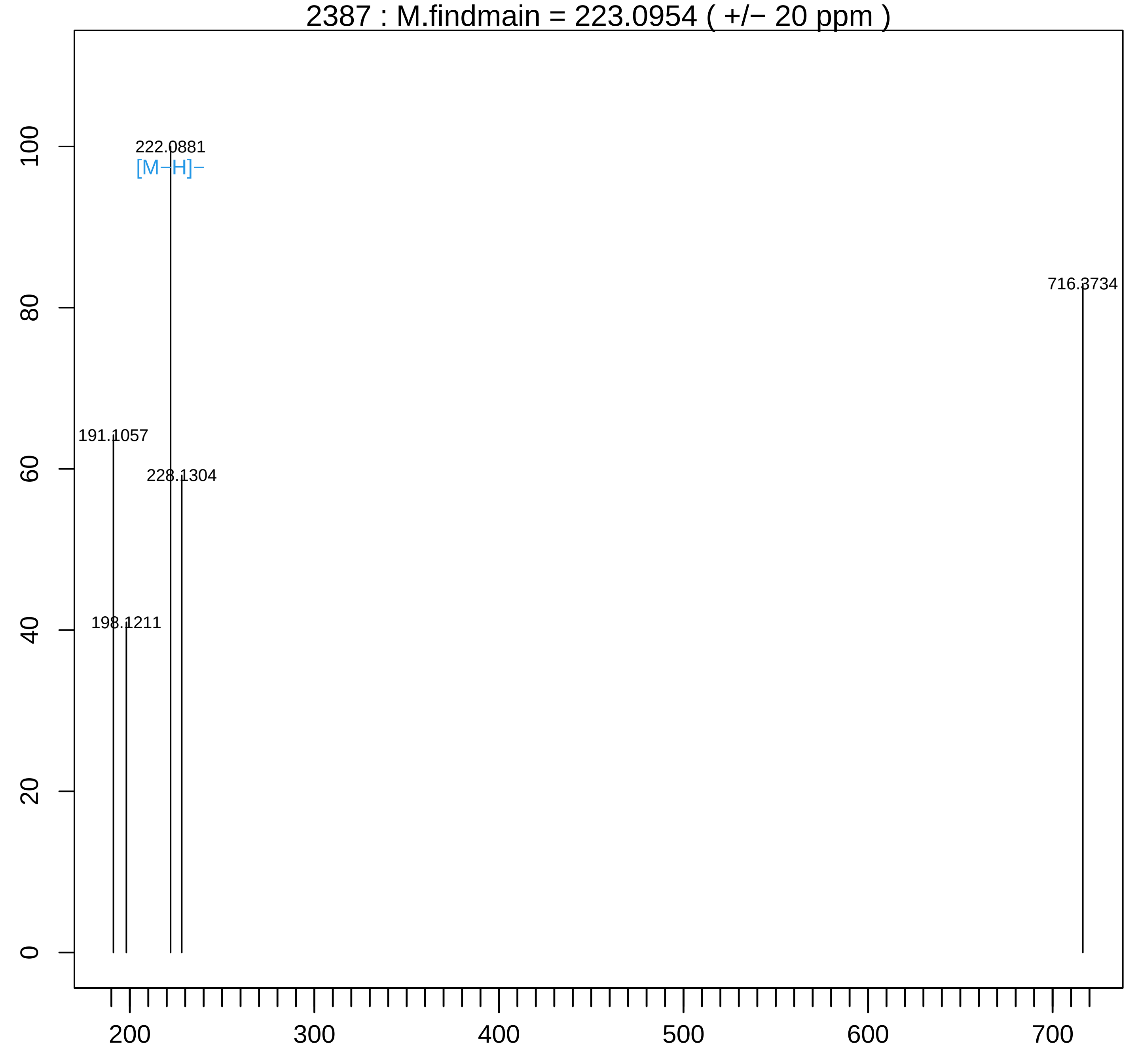
2387 : M.findmain = 223.0954 (+/− 20 ppm)
222.0881
[M−H]−
716.3734
191.1057
228.1304
198.1211
0
20
40
60
80
100
200
300
400
500
600
700

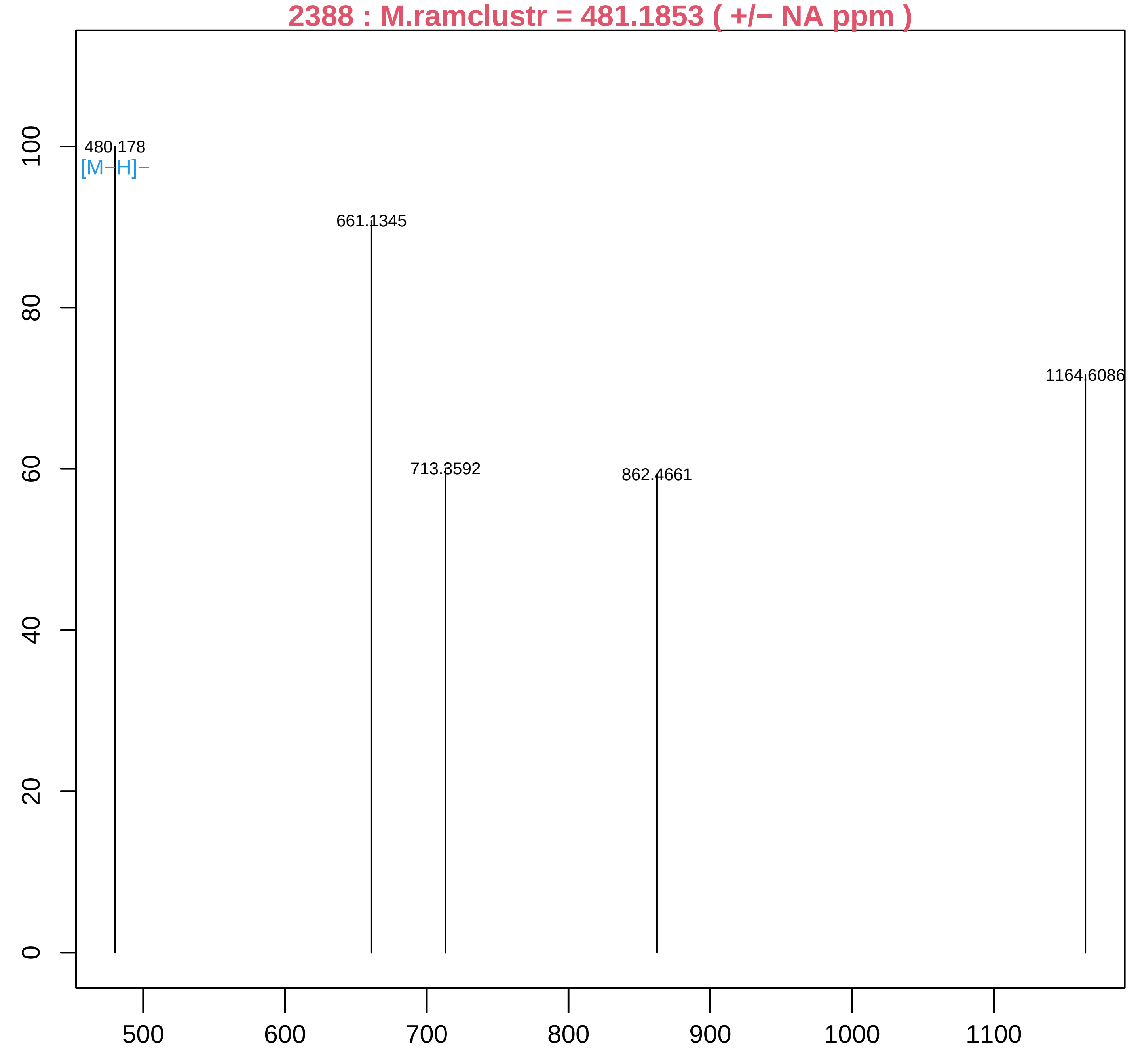
2388 : M.ramclustr = 481.1853 (+/− NA ppm)
480.178
[M−H]−
661.1345
713.3592
862.4661
1164.6086
0
20
40
60
80
100
500
600
700
800
900
1000
1100

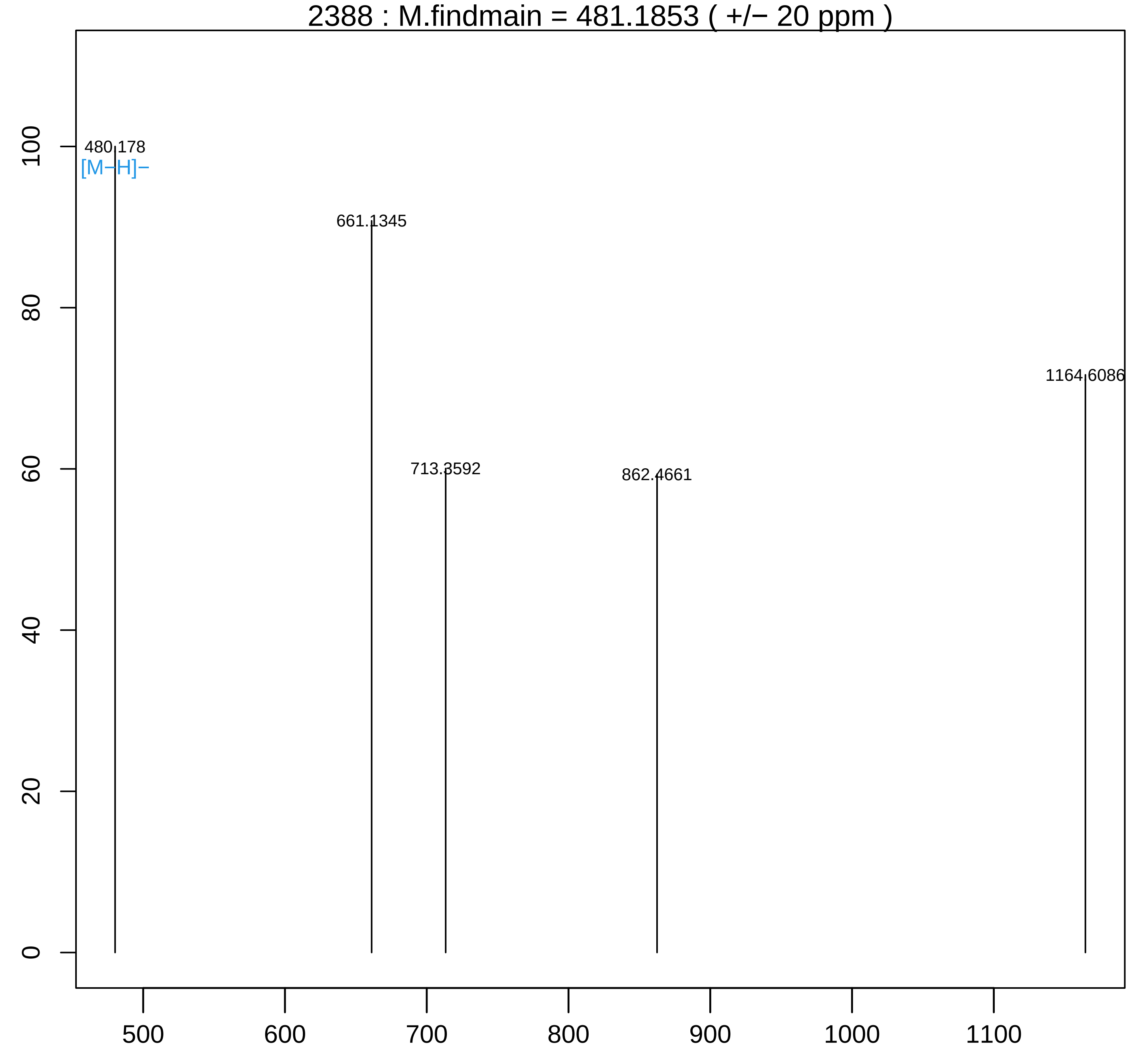
2388 : M.findmain = 481.1853 (+/− 20 ppm)
480.178
[M−H]−
661.1345
713.3592
862.4661
1164.6086
0
20
40
60
80
100
500
600
700
800
900
1000
1100

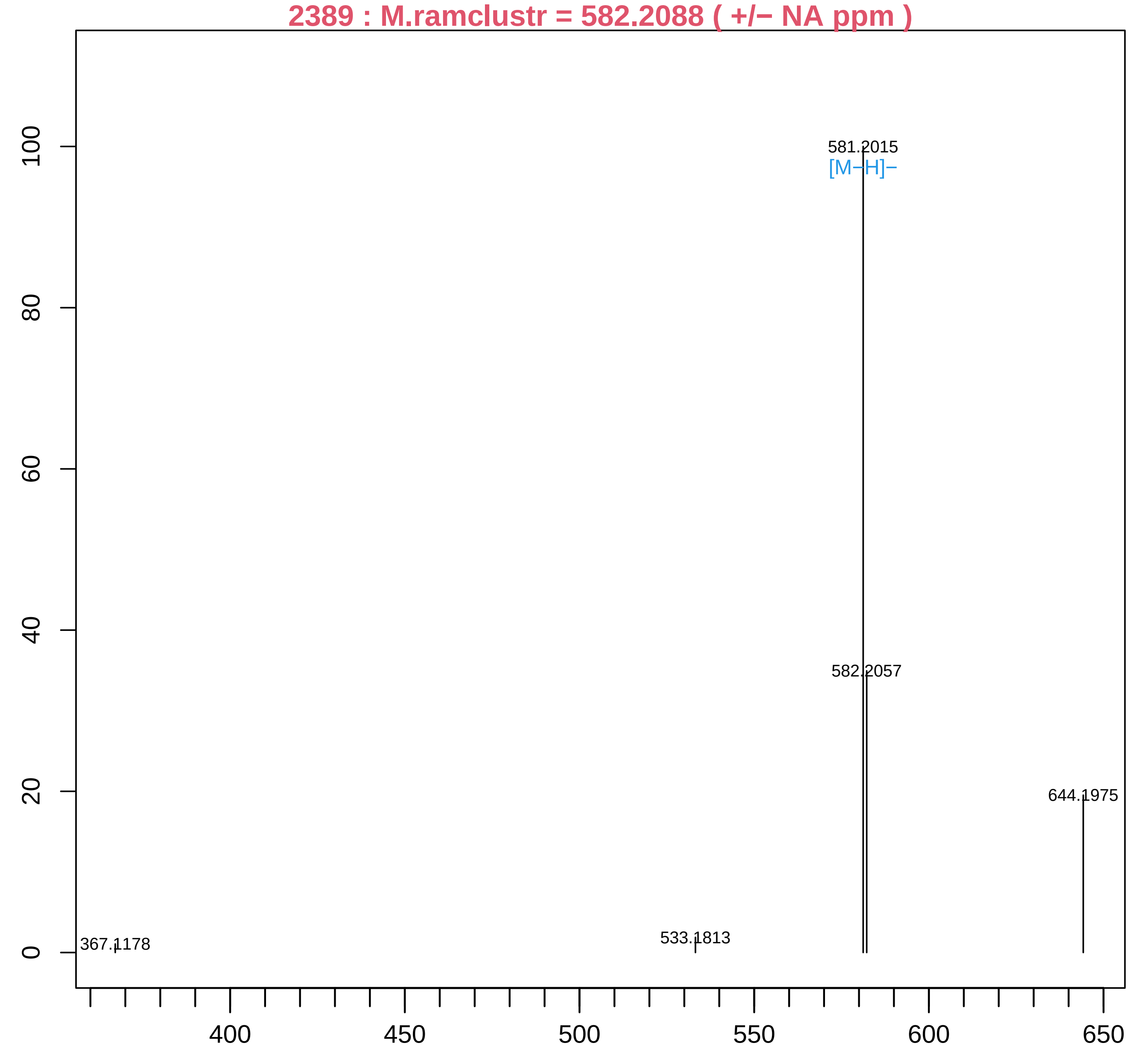
2389 : M.ramclustr = 582.2088 (+/− NA ppm)
581.2015
[M−H]−
582.2057
644.1975
533.1813
367.1178
0
20
40
60
80
100
400
450
500
550
600
650
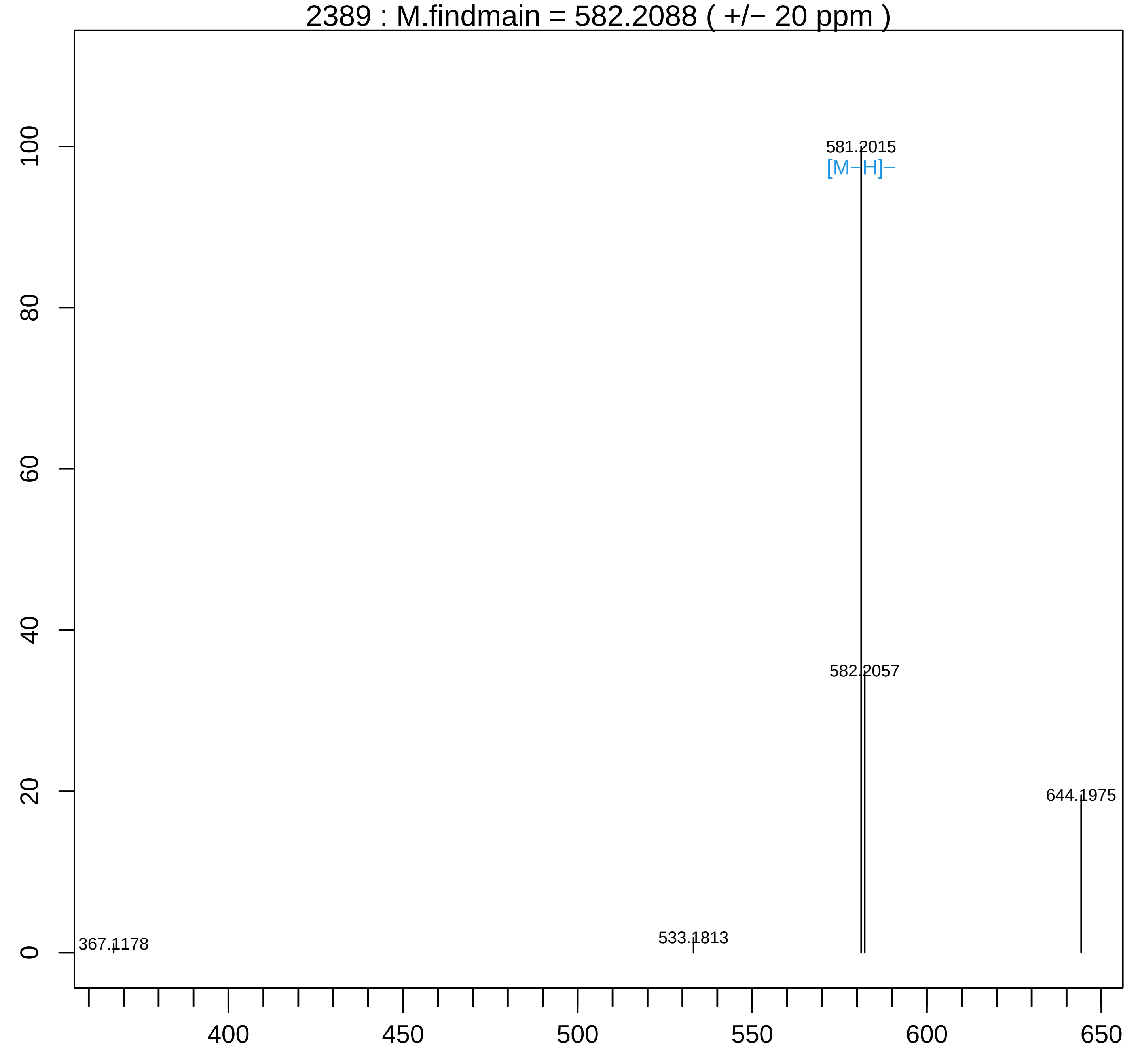
2389 : M.findmain = 582.2088 (+/− 20 ppm)
581.2015
[M−H]−
582.2057
644.1975
533.1813
367.1178
0
20
40
60
80
100
400
450
500
550
600
650

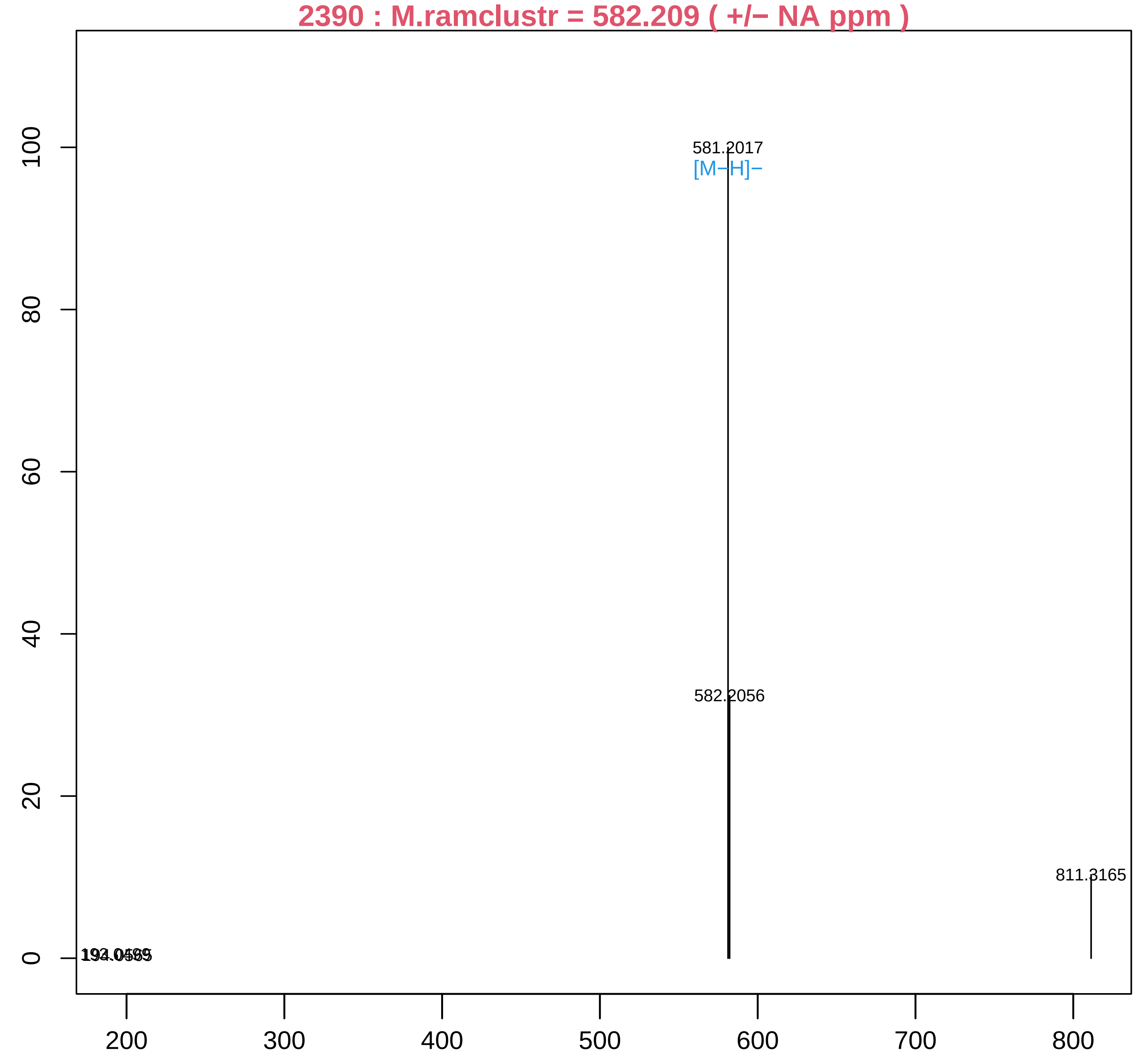
2390 : M.ramclustr = 582.209 (+/− NA ppm)
581.2017
[M−H]−
582.2056
811.3165
0
20
40
60
80
100
200
300
400
500
600
700
800

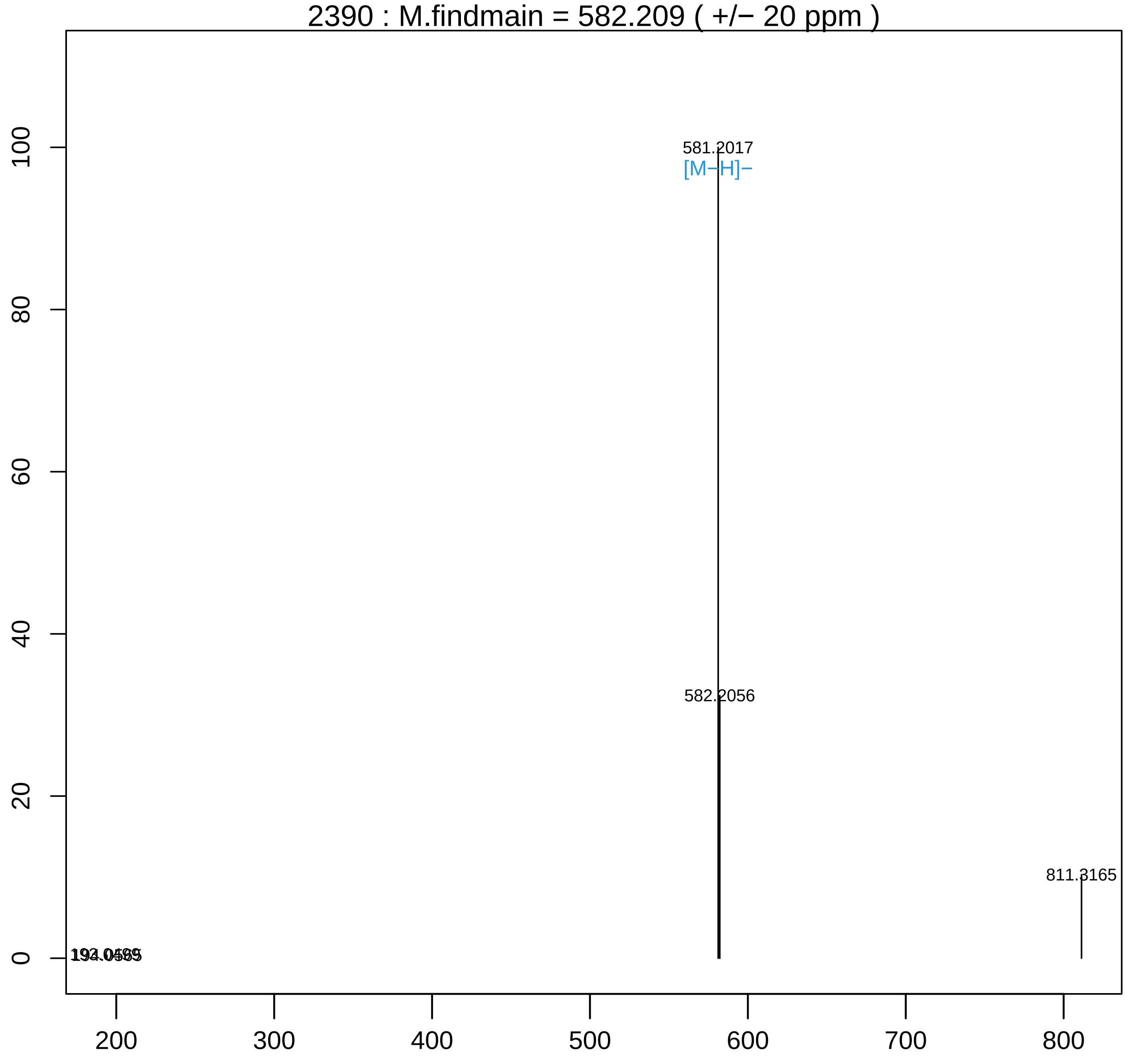
2390 : M.findmain = 582.209 (+/− 20 ppm)
581.2017
[M−H]−
582.2056
811.3165
0
20
40
60
80
100
200
300
400
500
600
700
800

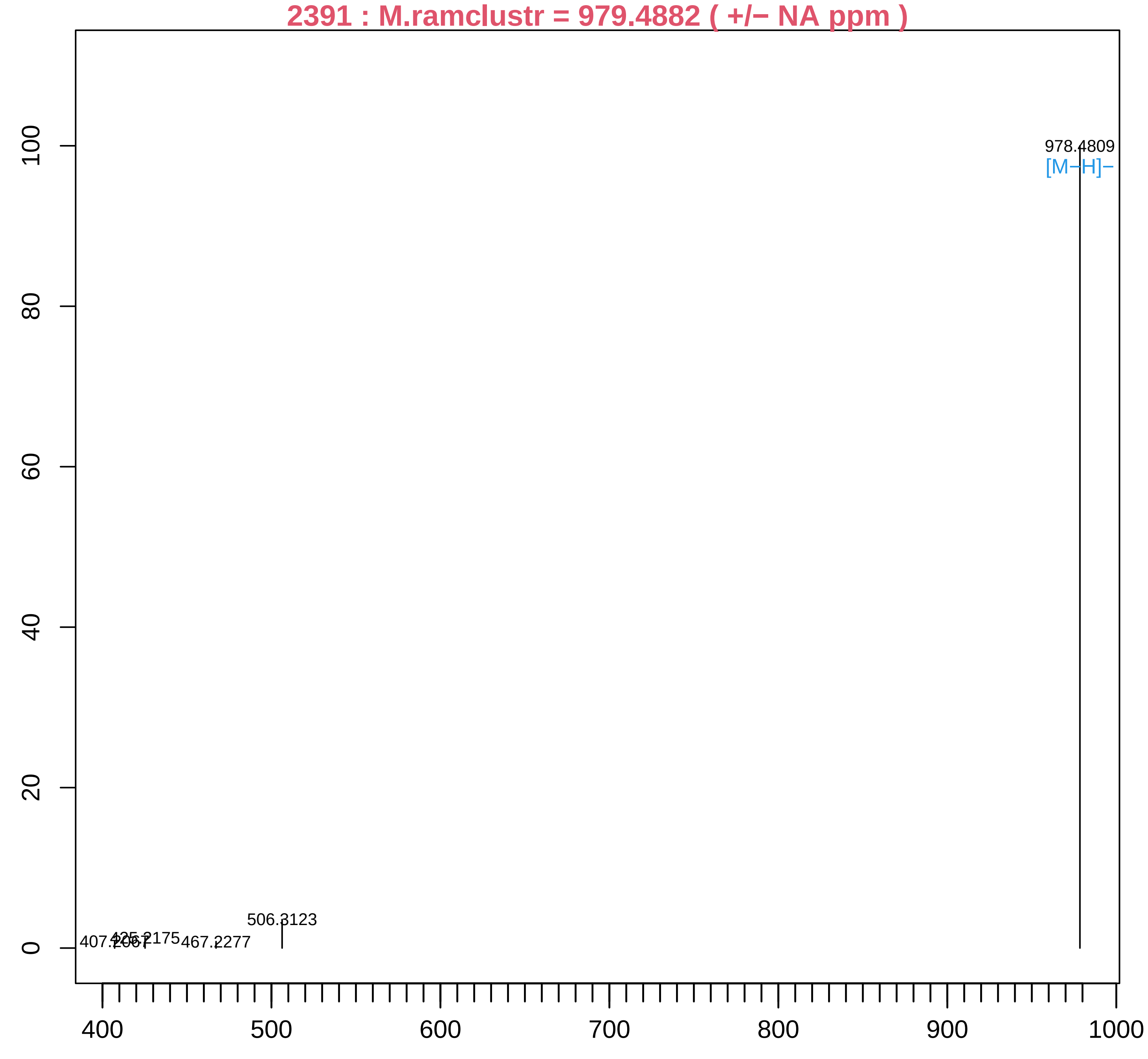
2391 : M.ramclustr = 979.4882 (+/− NA ppm)
978.4809
[M−H]−
506.3123
407.2067
425.2175
467.2277
0
20
40
60
80
100
400
500
600
700
800
900
1000

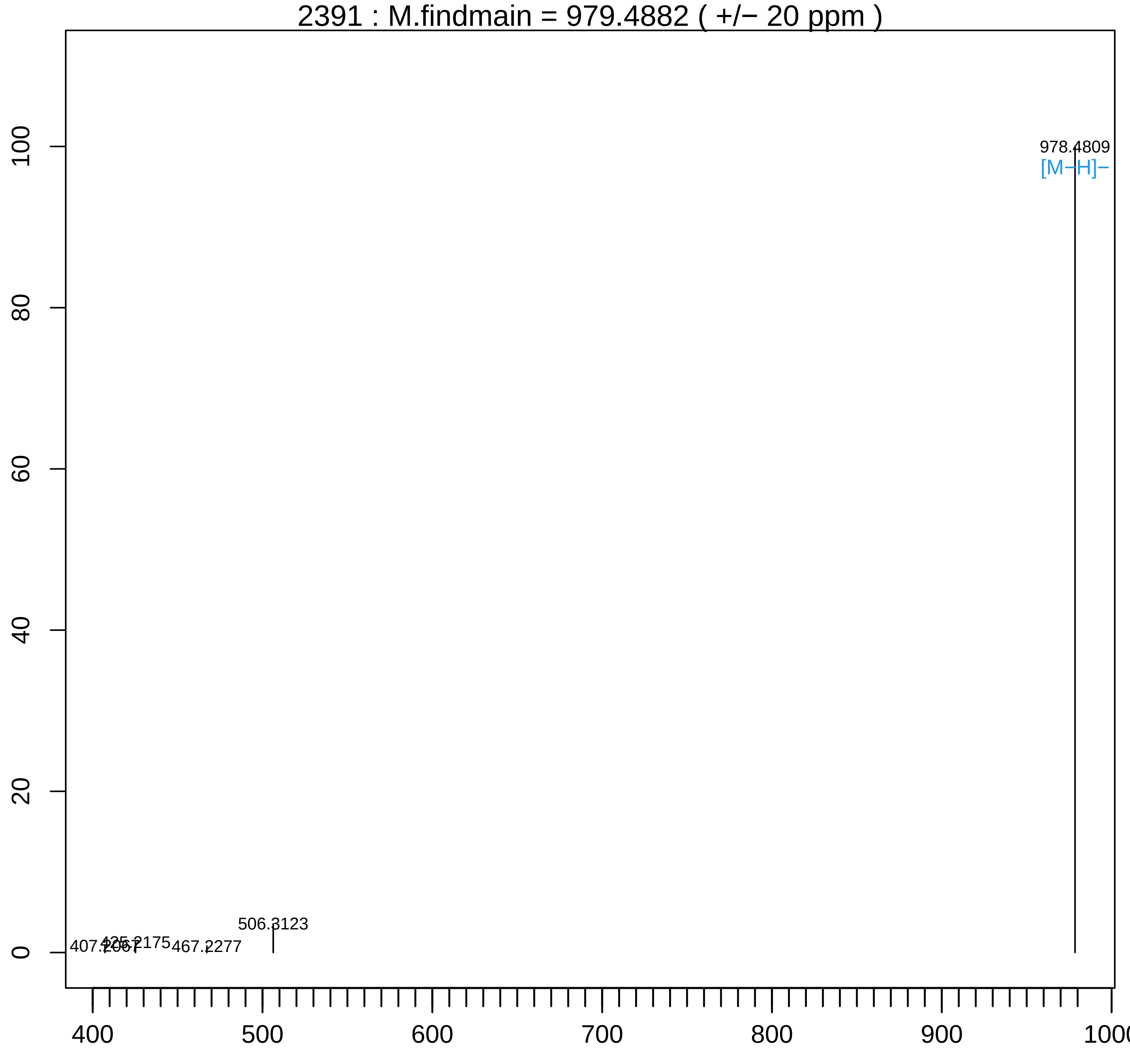
2391 : M.findmain = 979.4882 (+/− 20 ppm)
978.4809
[M−H]−
506.3123
407.2067
425.2175
467.2277
0
20
40
60
80
100
400
500
600
700
800
900
1000

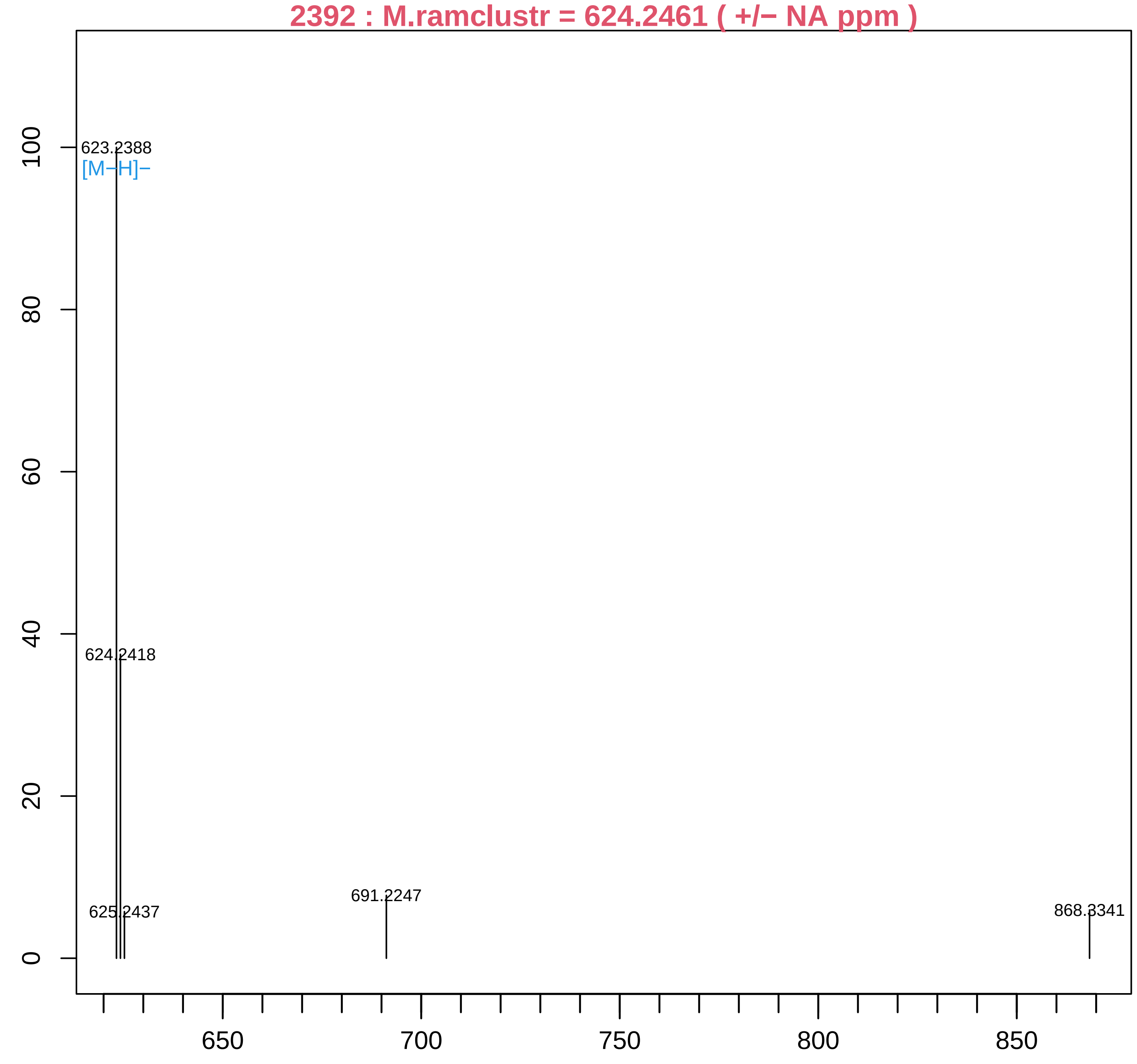
2392 : M.ramclustr = 624.2461 (+/− NA ppm)
623.2388
[M−H]−
624.2418
625.2437
691.2247
868.3341
0
20
40
60
80
100
650
700
750
800
850

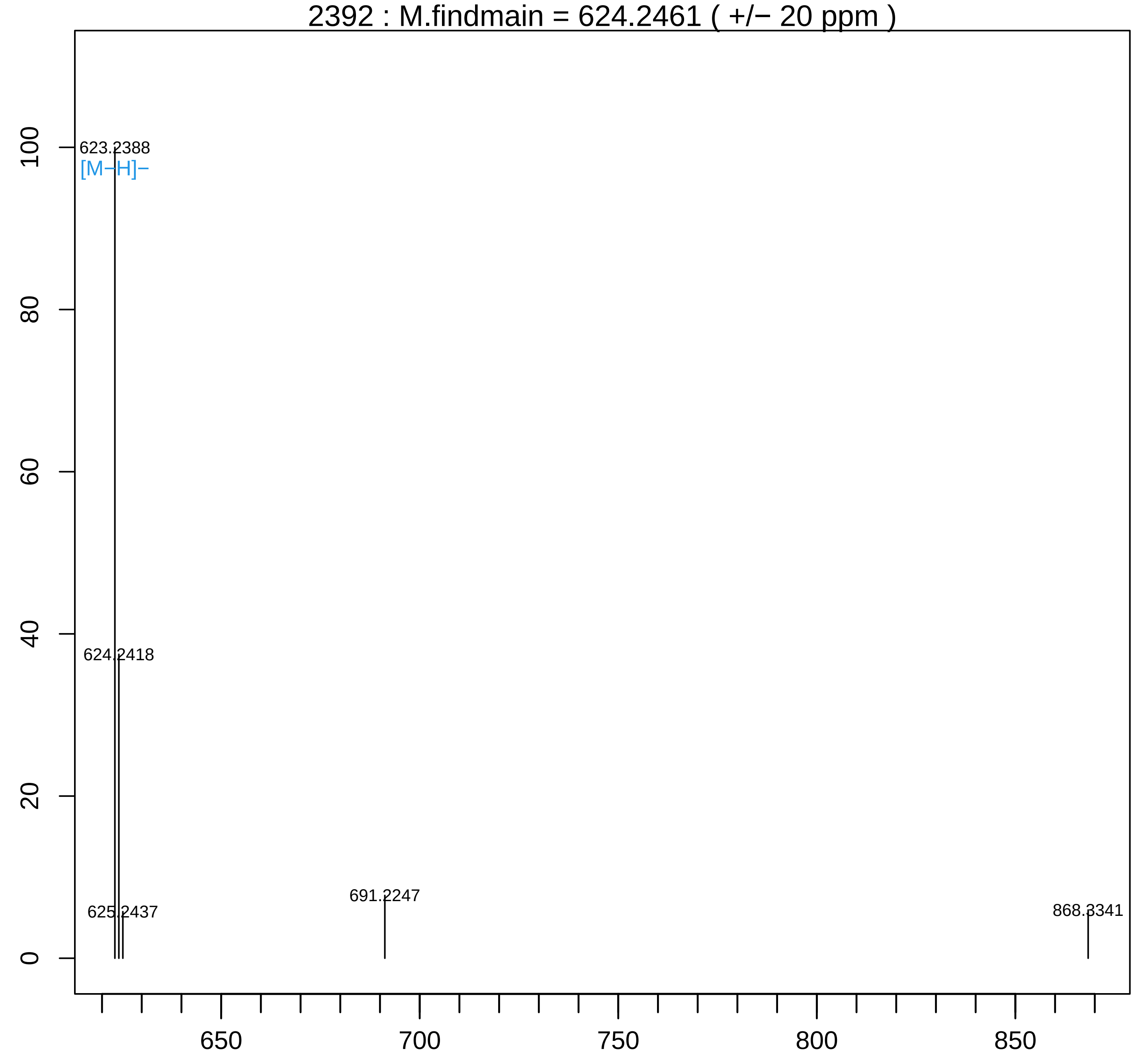
2392 : M.findmain = 624.2461 (+/− 20 ppm)
623.2388
[M−H]−
624.2418
625.2437
691.2247
868.3341
0
20
40
60
80
100
650
700
750
800
850

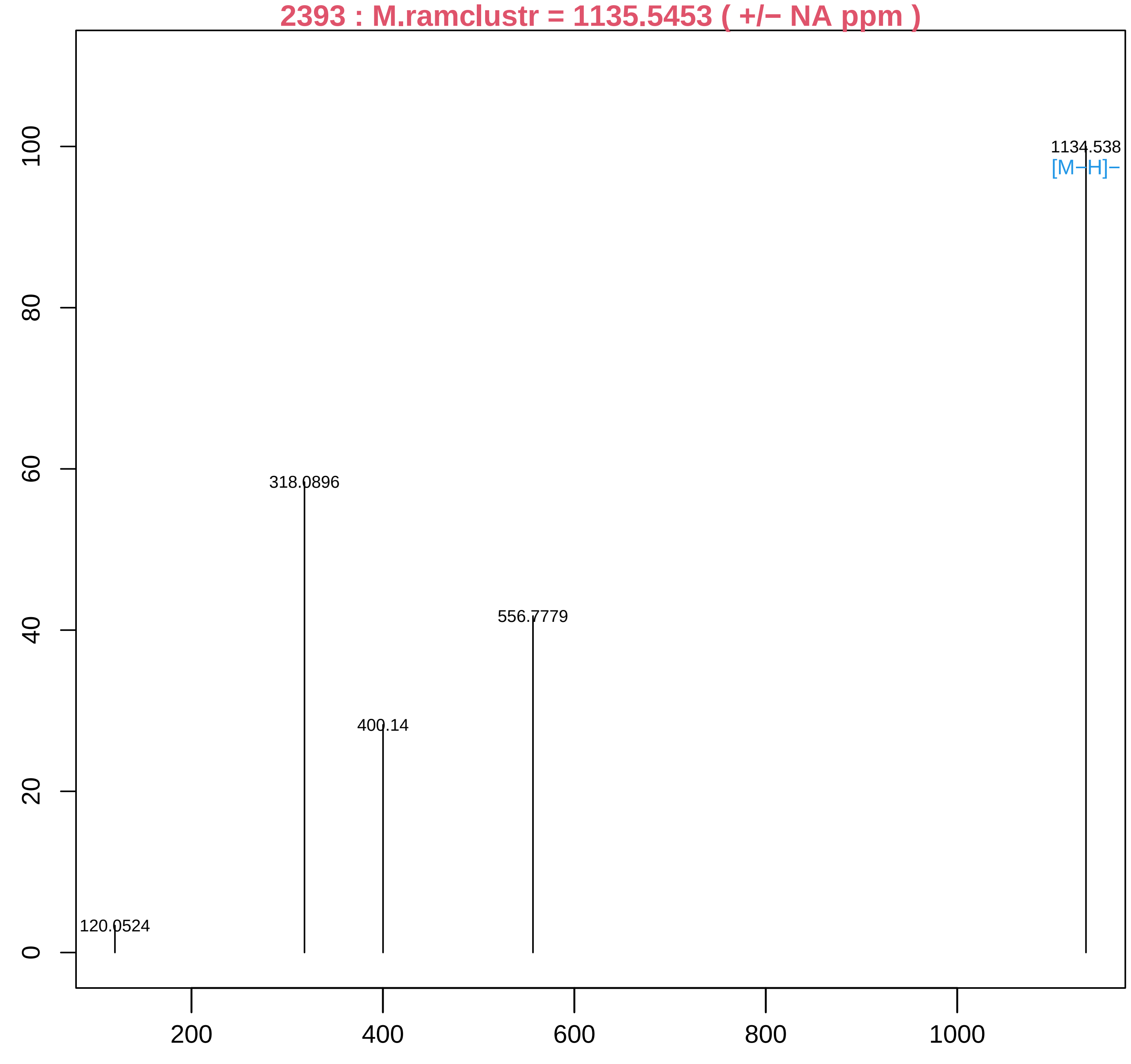

2393 : M.ramclustr = 1135.5453 (+/− NA ppm)
1134.538
[M−H]−
318.0896
556.7779
400.14
120.0524
0
20
40
60
80
100
200
400
600
800
1000

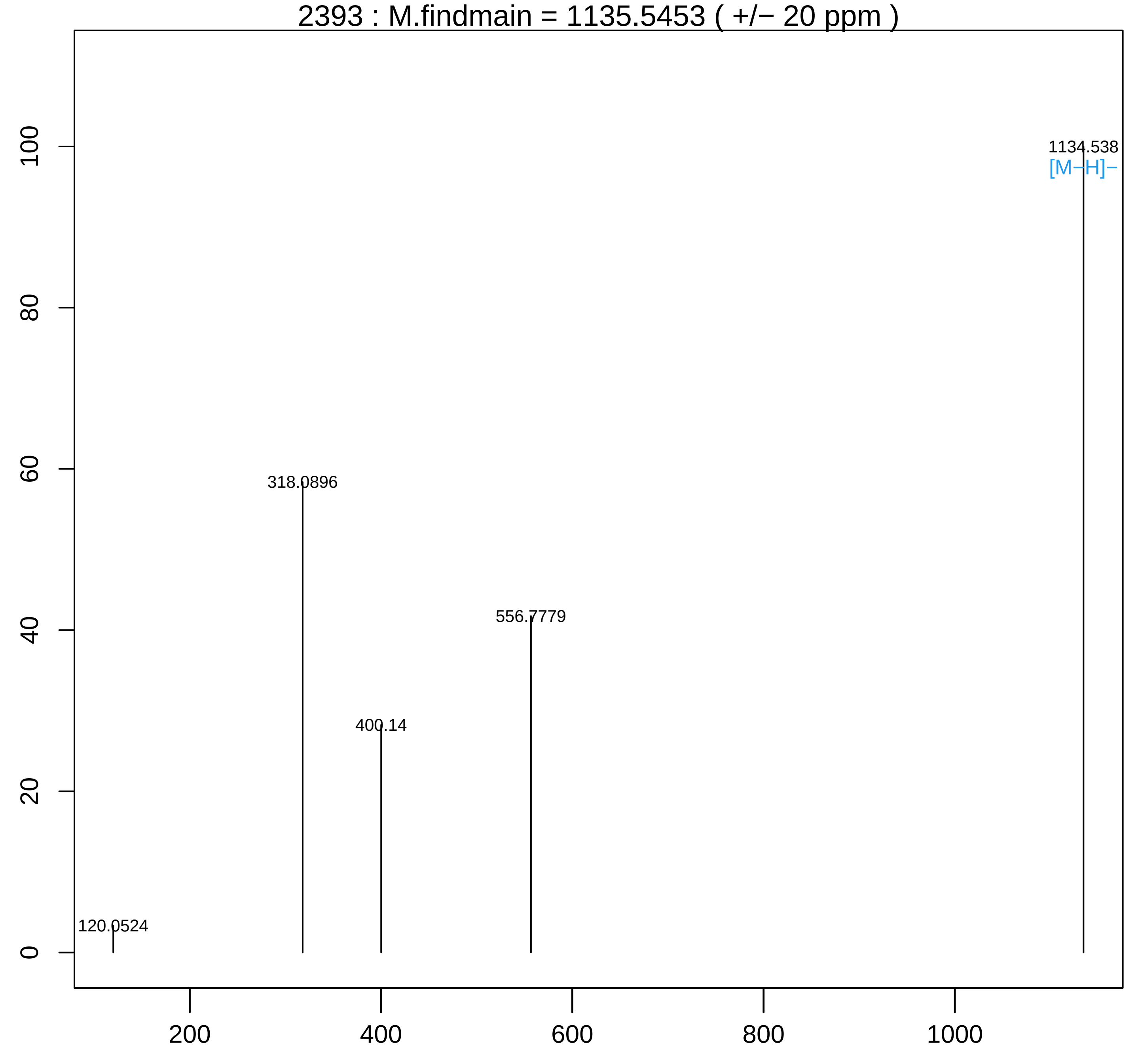

2393 : M.findmain = 1135.5453 (+/− 20 ppm)
1134.538
[M−H]−
318.0896
556.7779
400.14
120.0524
0
20
40
60
80
100
200
400
600
800
1000

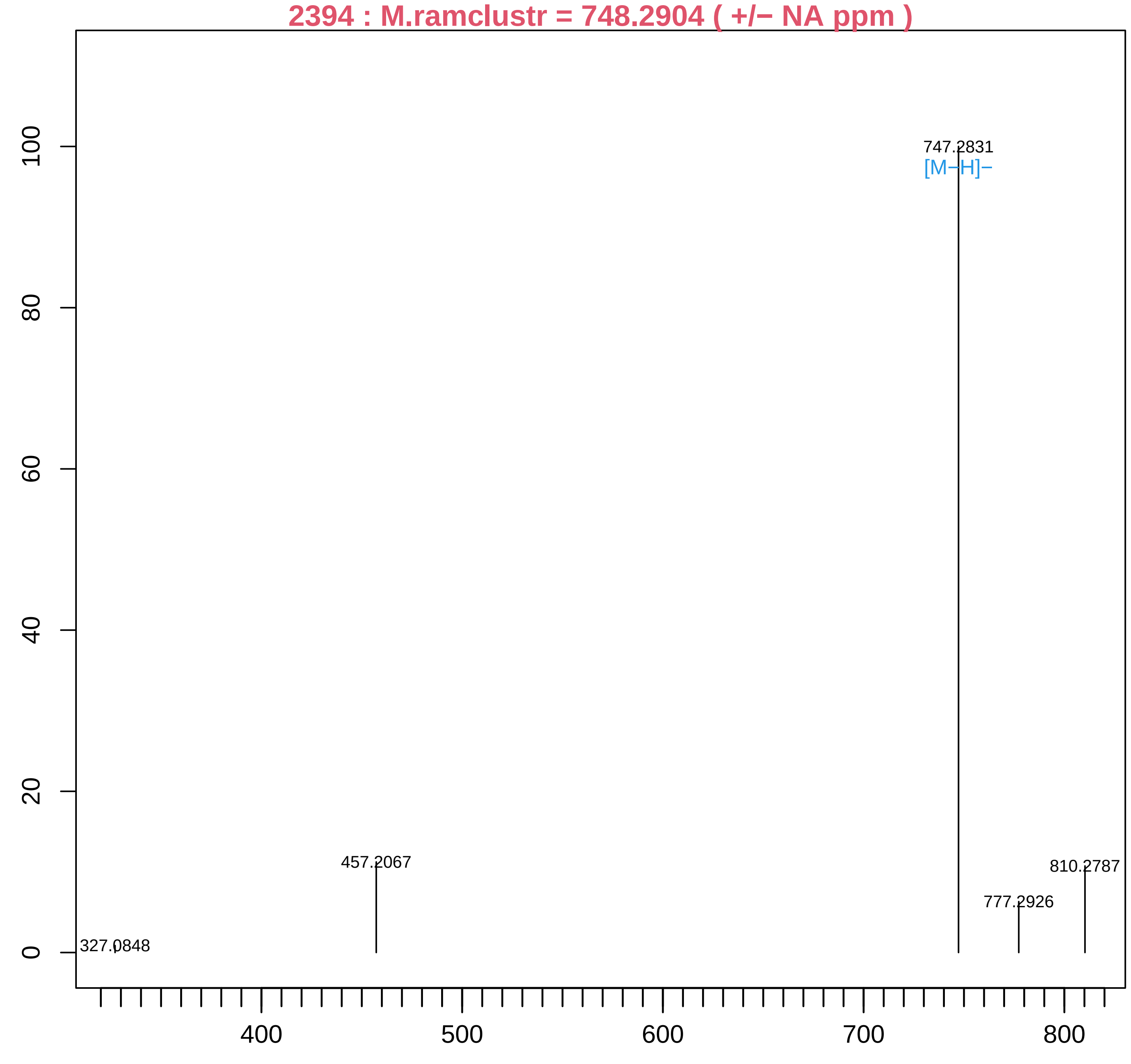

2394 : M.ramclustr = 748.2904 (+/− NA ppm)
747.2831
[M−H]−
457.2067
810.2787
777.2926
327.0848
0
20
40
60
80
100
400
500
600
700
800

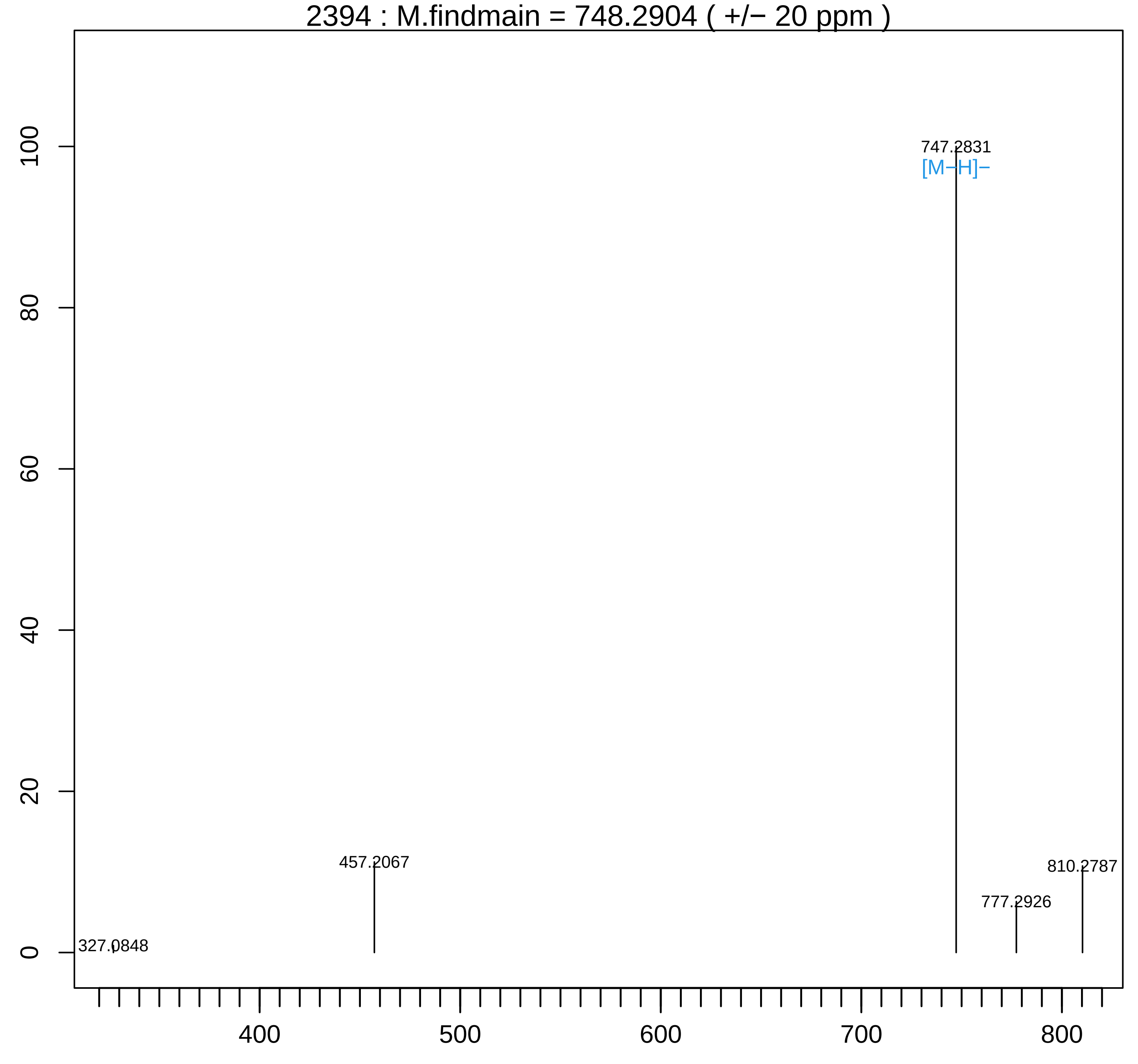

2394 : M.findmain = 748.2904 (+/− 20 ppm)
747.2831
[M−H]−
457.2067
810.2787
777.2926
327.0848
0
20
40
60
80
100
400
500
600
700
800

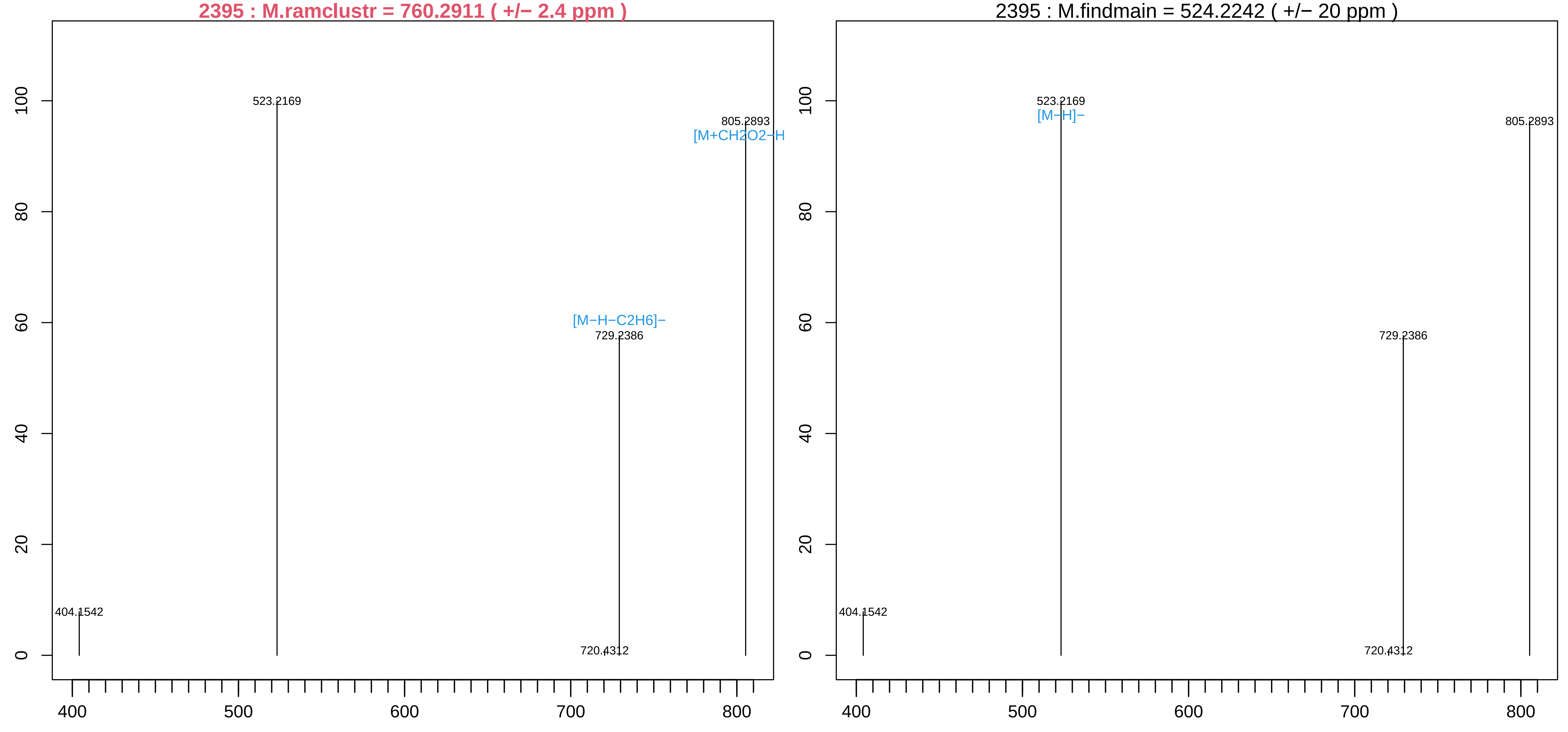

2395 : M.ramclustr = 760.2911 (+/− 2.4 ppm)
523.2169
805.2893
[M+CH2O2−H
[M−H−C2H6]−
729.2386
404.1542
720.4312
2395 : M.findmain = 524.2242 (+/− 20 ppm)
523.2169
[M−H]−
805.2893
729.2386
404.1542
720.4312
0
20
40
60
80
100
400
500
600
700
800

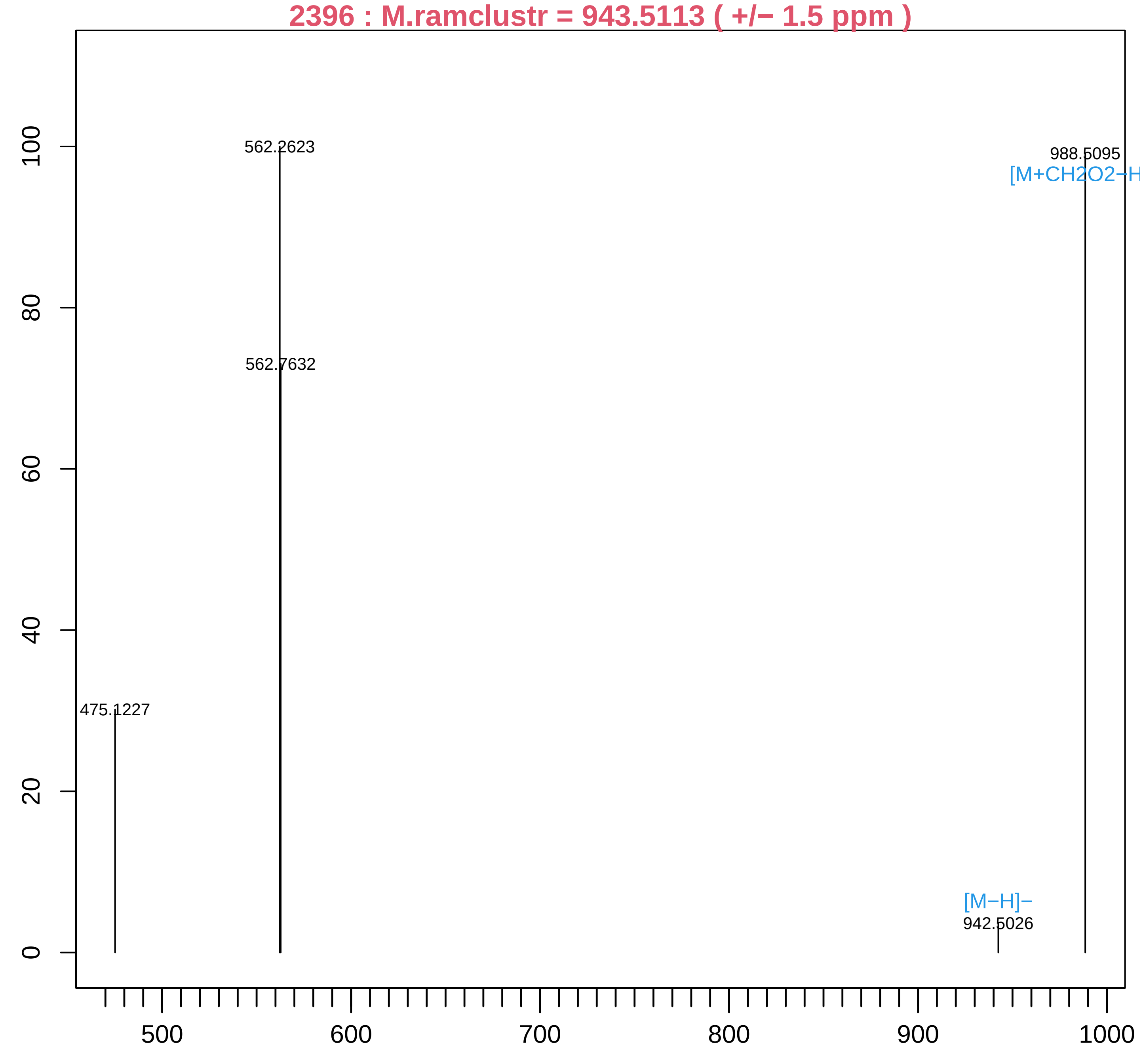
2396 : M.ramclustr = 943.5113 (+/− 1.5 ppm)
562.2623
562.7632
475.1227
988.5095
[M+CH2O2−H
[M−H]−
942.5026
0
20
40
60
80
100
500
600
700
800
900
1000

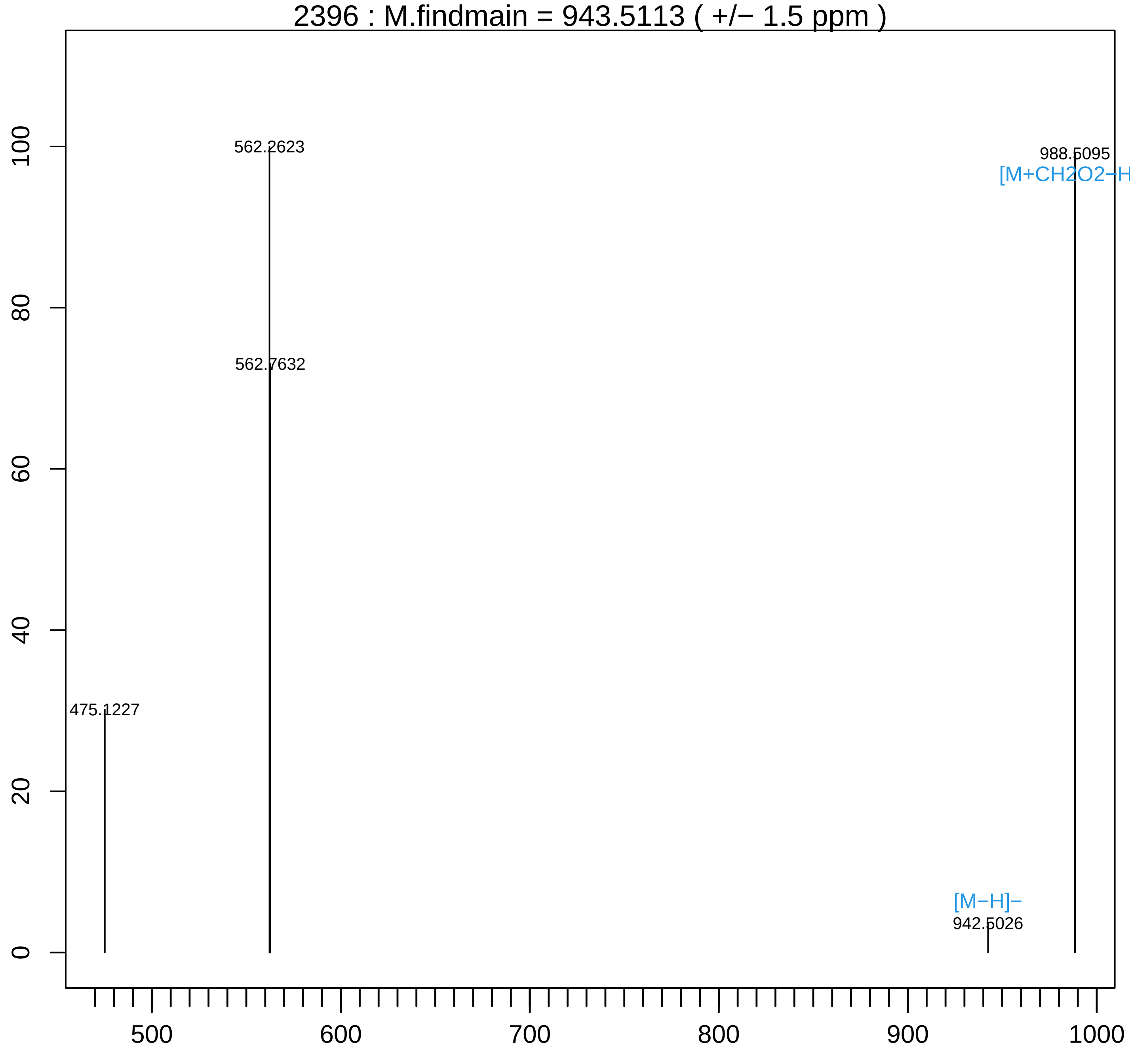
2396 : M.findmain = 943.5113 (+/− 1.5 ppm)
562.2623
562.7632
475.1227
988.5095
[M+CH2O2−H
[M−H]−
942.5026
0
20
40
60
80
100
500
600
700
800
900
1000

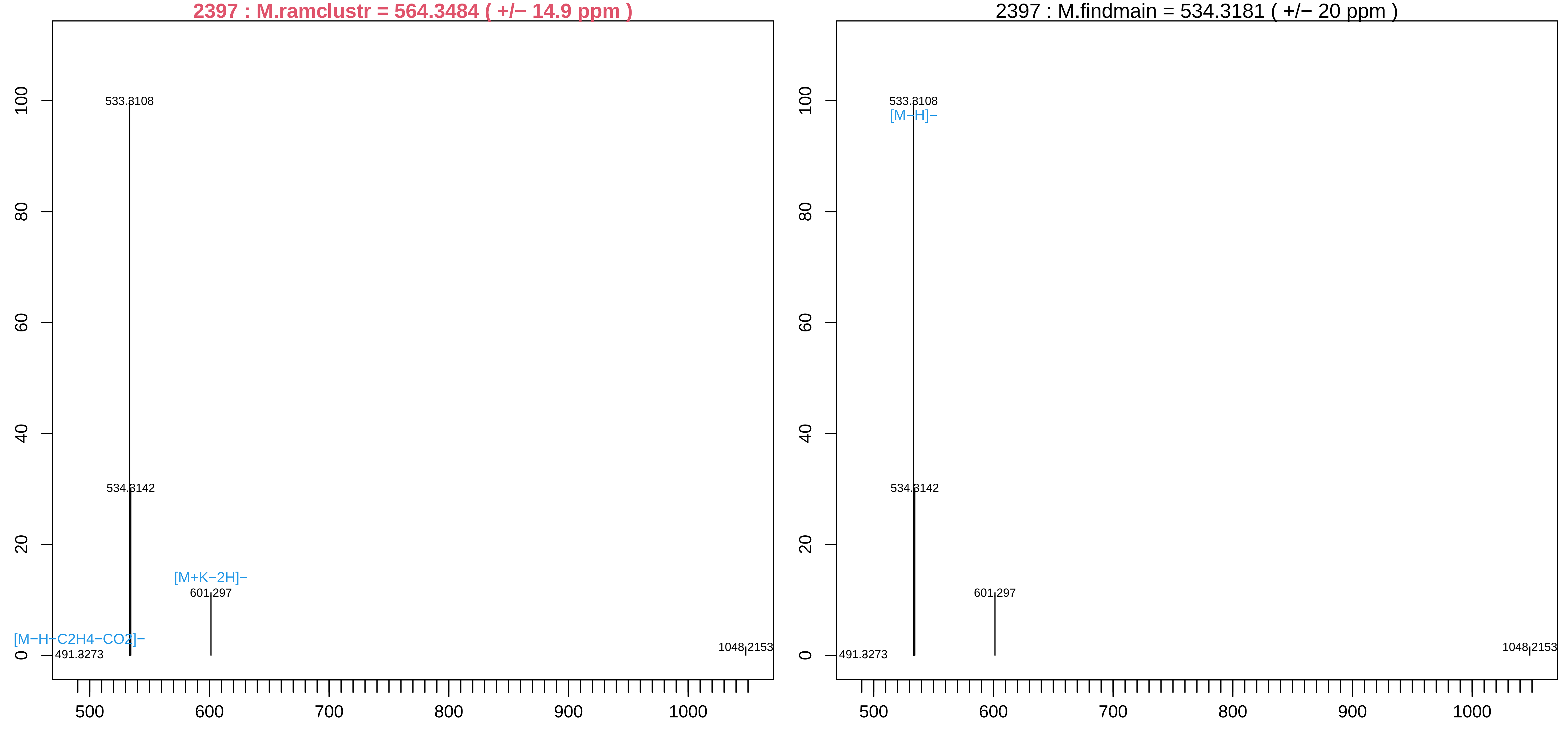

2397 : M.ramclustr = 564.3484 (+/− 14.9 ppm)
533.3108
534.3142
[M+K−2H]−
601.297
[M−H−C2H4−CO2]−
491.3273
1048.2153
0
20
40
60
80
100
500
600
700
800
900
1000
2397 : M.findmain = 534.3181 (+/− 20 ppm)
533.3108
[M−H]−
534.3142
601.297
491.3273
1048.2153

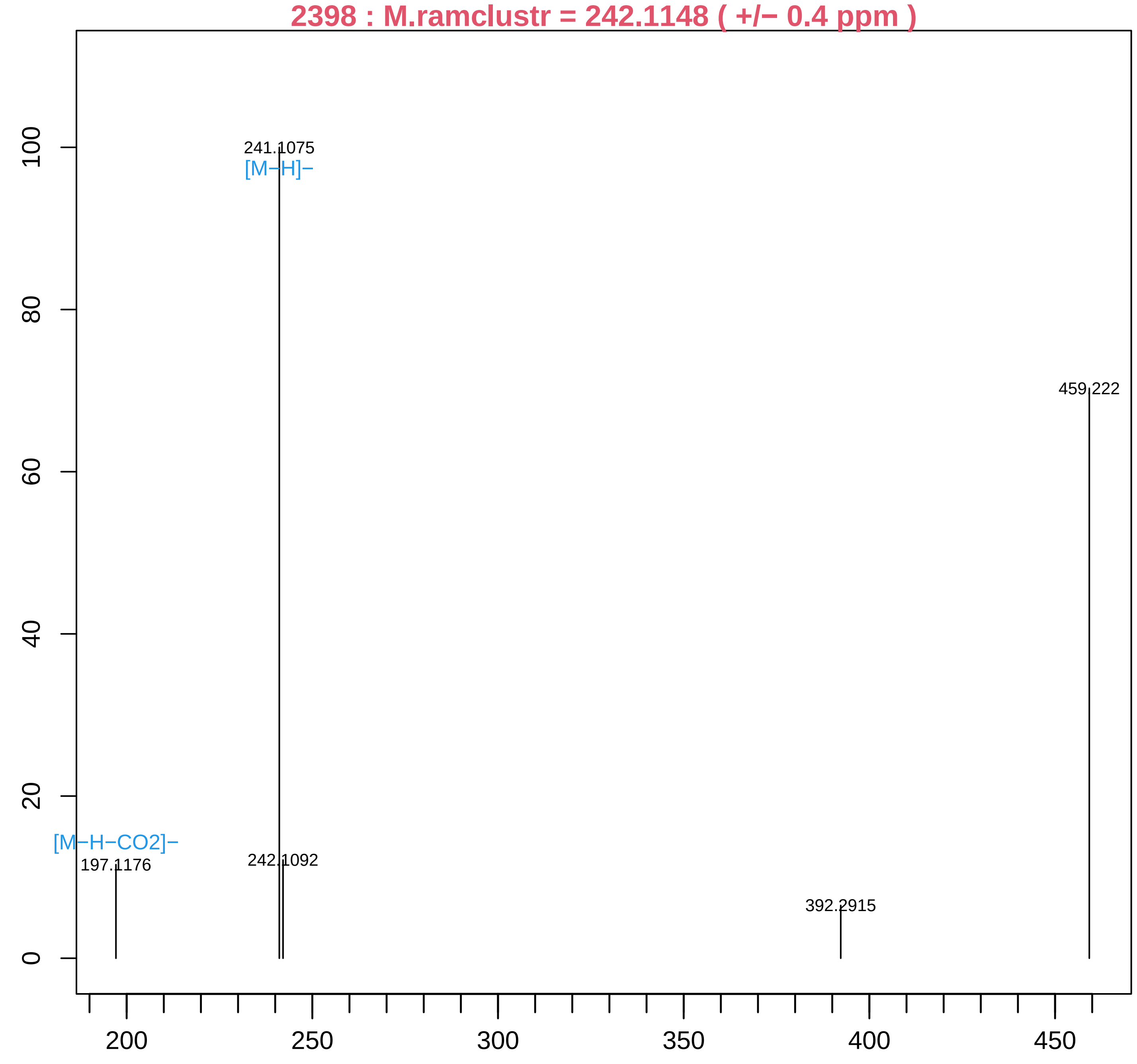
2398 : M.ramclustr = 242.1148 (+/− 0.4 ppm)
241.1075
[M−H]−
459.222
[M−H−CO2]−
197.1176
242.1092
392.2915
0
20
40
60
80
100
200
250
300
350
400
450

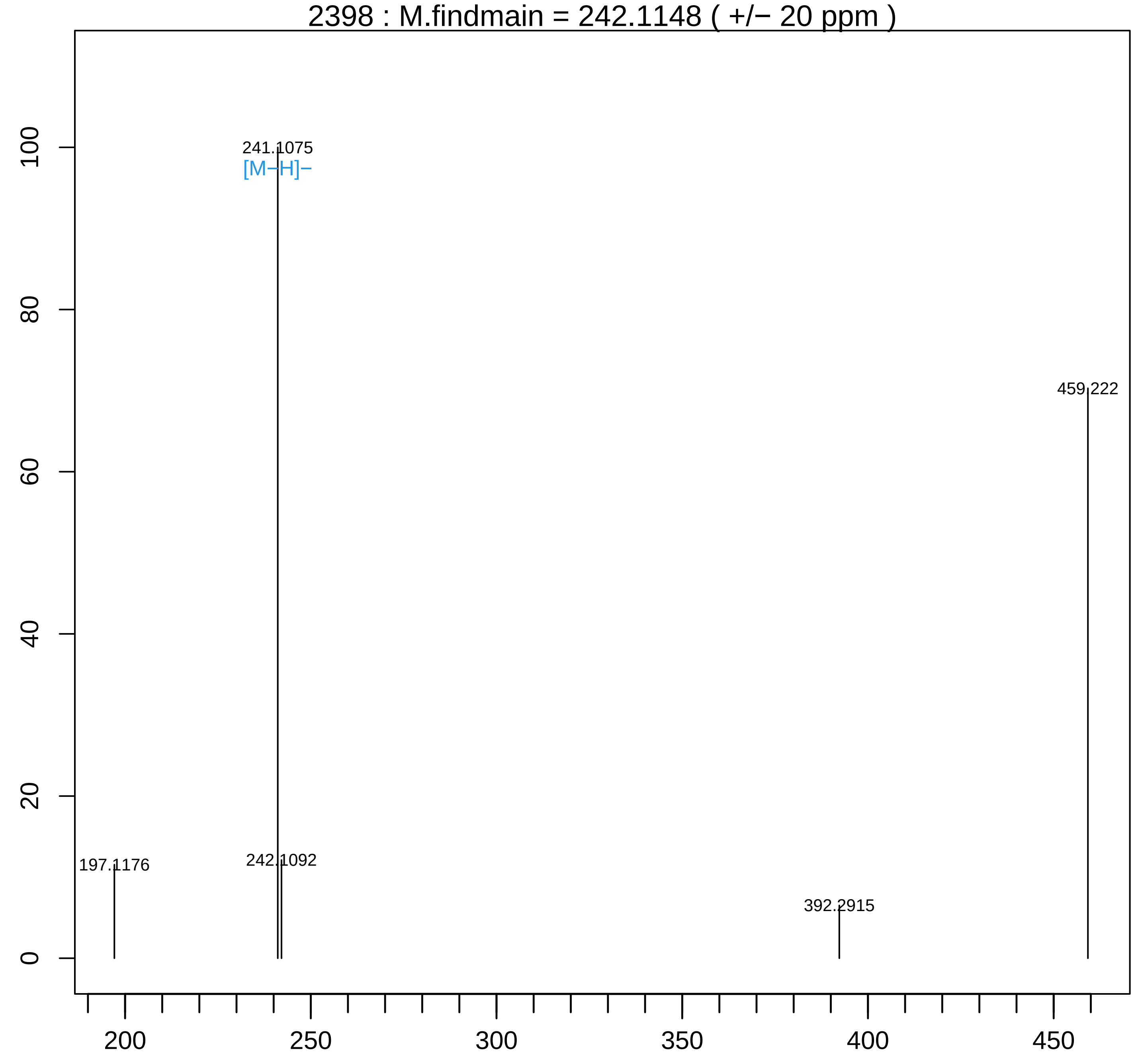
2398 : M.findmain = 242.1148 (+/− 20 ppm)
241.1075
[M−H]−
459.222
197.1176
242.1092
392.2915
0
20
40
60
80
100
200
250
300
350
400
450

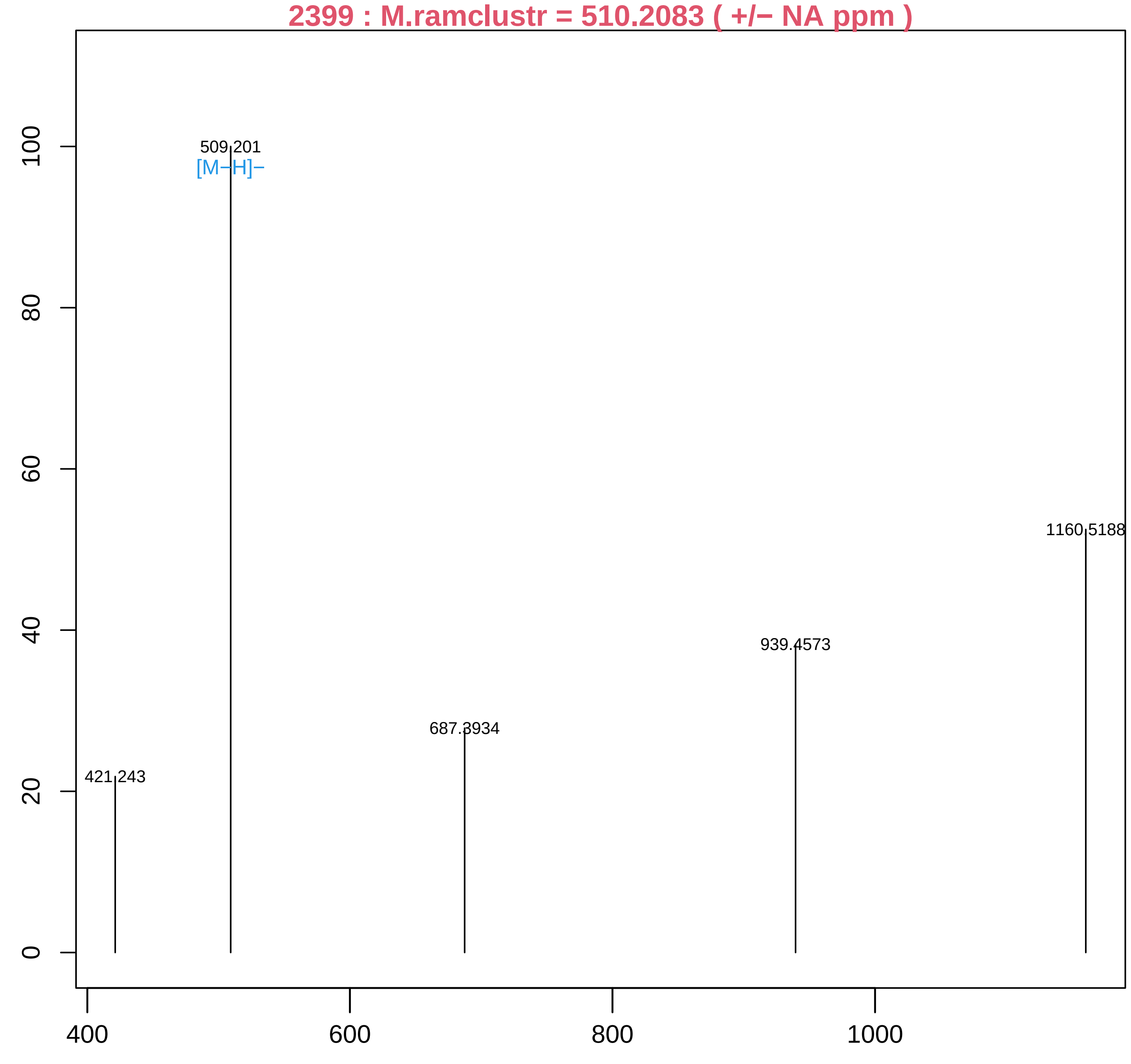

2399 : M.ramclustr = 510.2083 (+/− NA ppm)
509.201
[M−H]−
421.243
687.3934
939.4573
1160.5188
0
20
40
60
80
100
400
600
800
1000

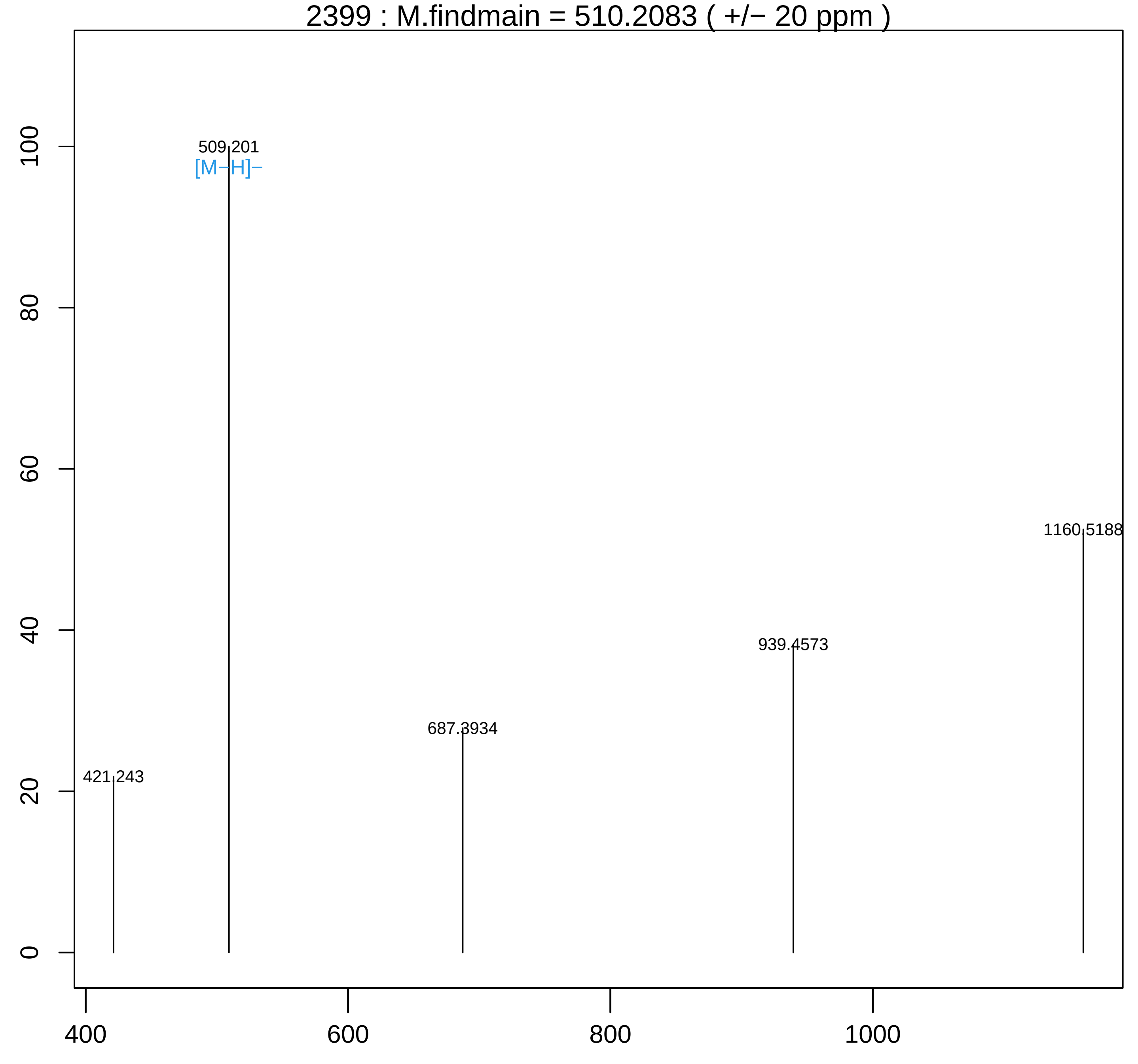

2399 : M.findmain = 510.2083 (+/− 20 ppm)
509.201
[M−H]−
421.243
687.3934
939.4573
1160.5188
0
20
40
60
80
100
400
600
800
1000

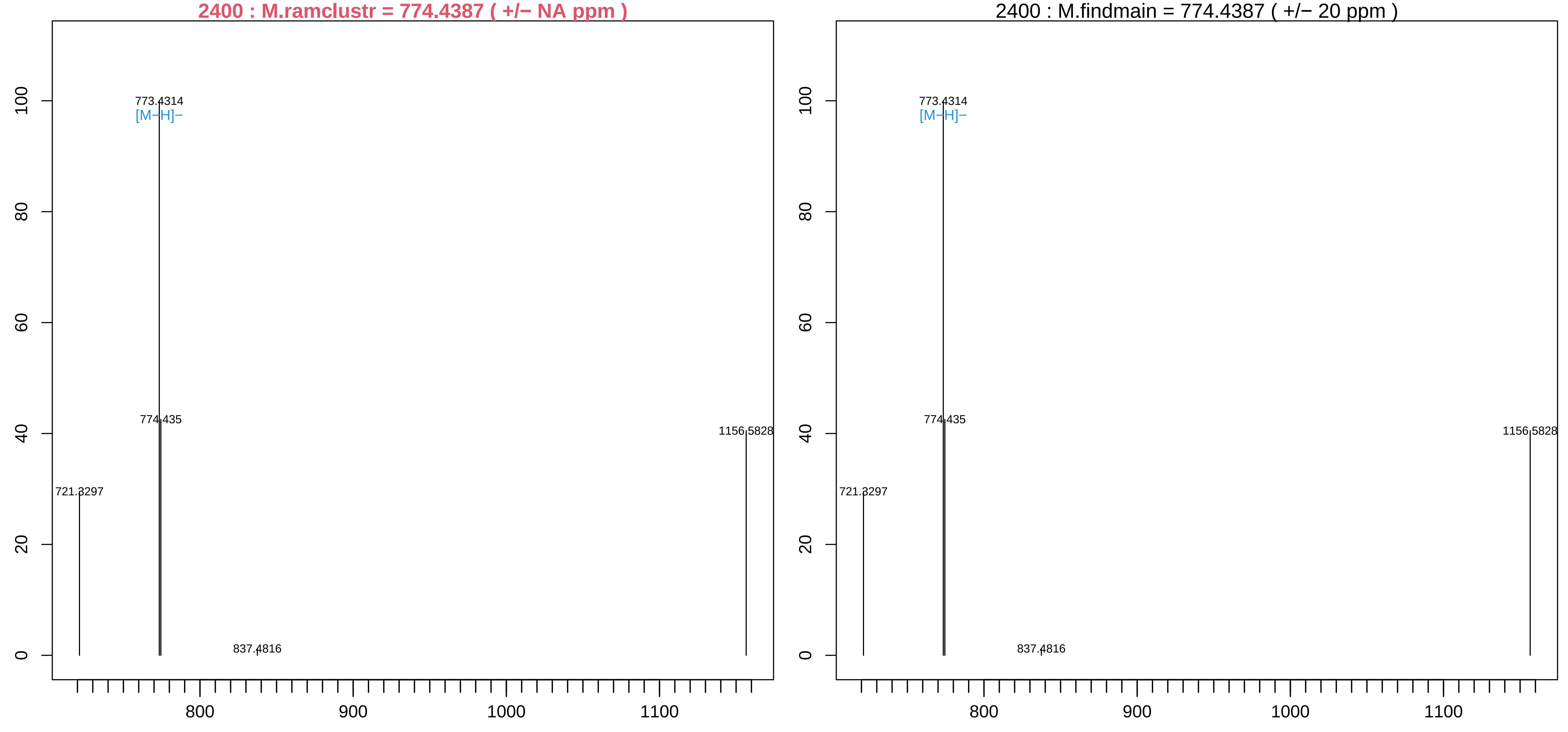
2400 : M.ramclustr = 774.4387 (+/− NA ppm)
773.4314
[M−H]−
774.435
1156.5828
721.3297
837.4816
0
20
40
60
80
100
800
900
1000
1100
2400 : M.findmain = 774.4387 (+/− 20 ppm)
773.4314
[M−H]−
774.435
1156.5828
721.3297
837.4816
0
20
40
60
80
100
800
900
1000
1100

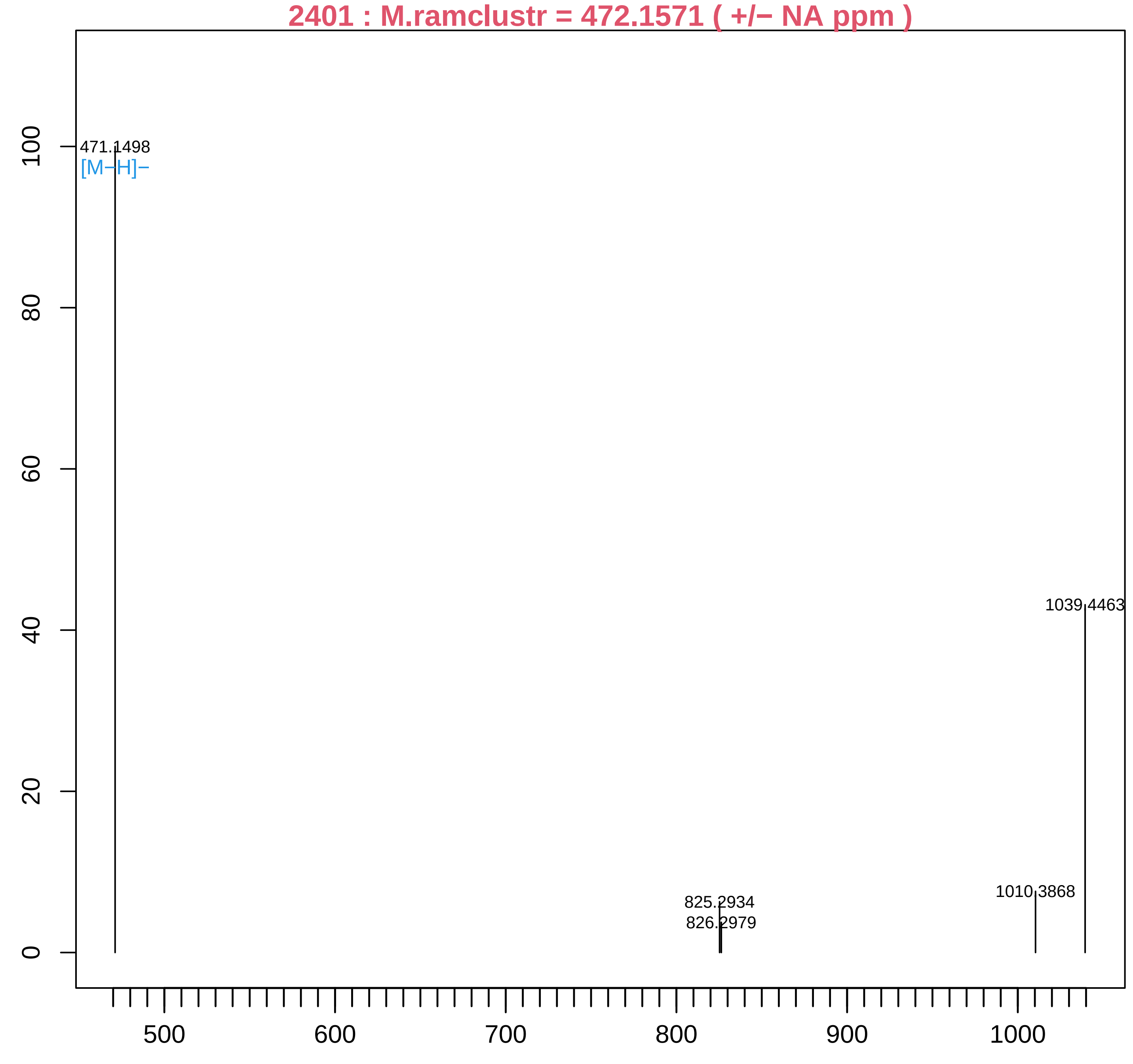
2401 : M.ramclustr = 472.1571 (+/− NA ppm)
471.1498
[M−H]−
825.2934
826.2979
1010.3868
1039.4463
0
20
40
60
80
100
500
600
700
800
900
1000

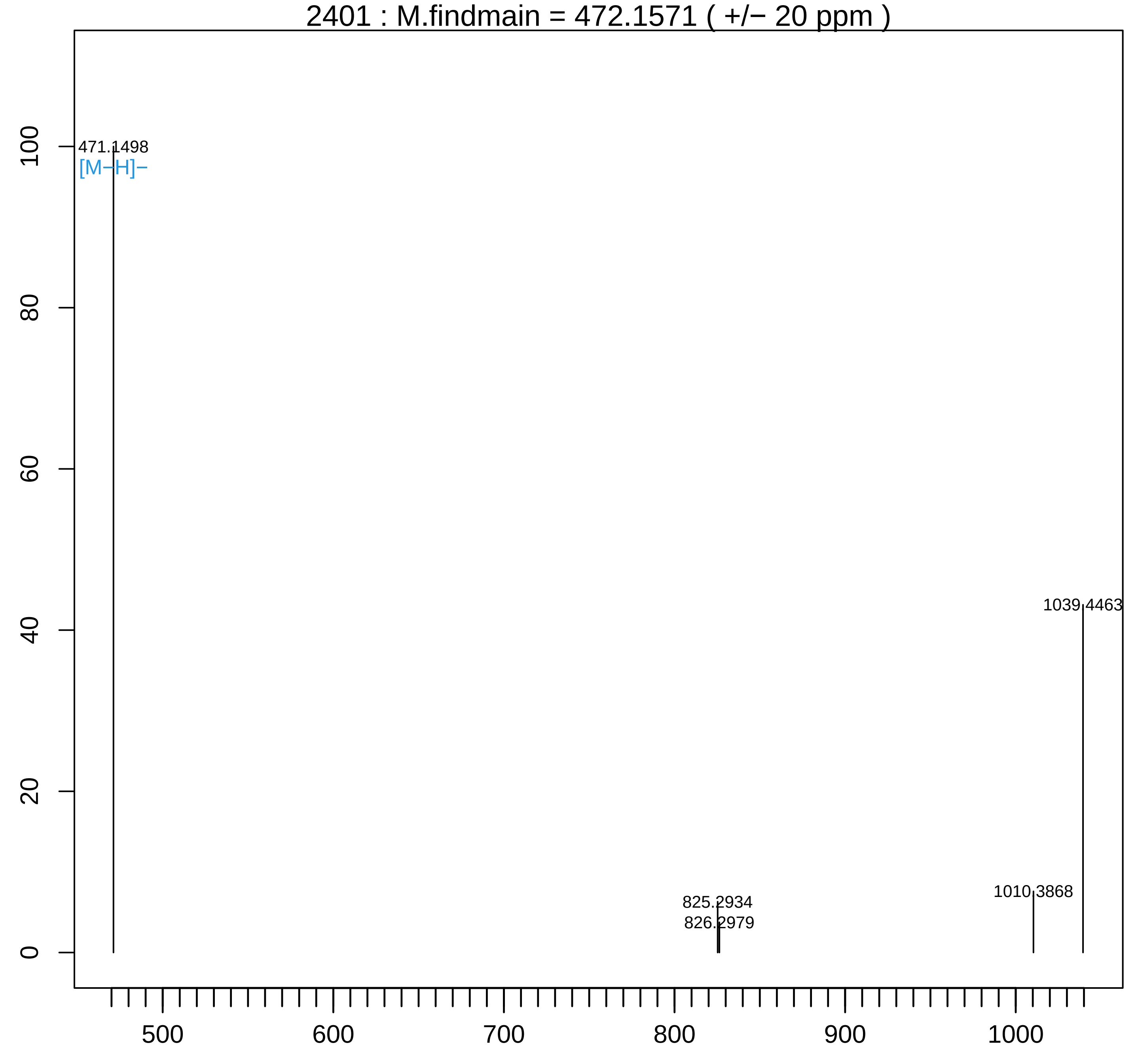
2401 : M.findmain = 472.1571 (+/− 20 ppm)
471.1498
[M−H]−
825.2934
826.2979
1010.3868
1039.4463
0
20
40
60
80
100
500
600
700
800
900
1000

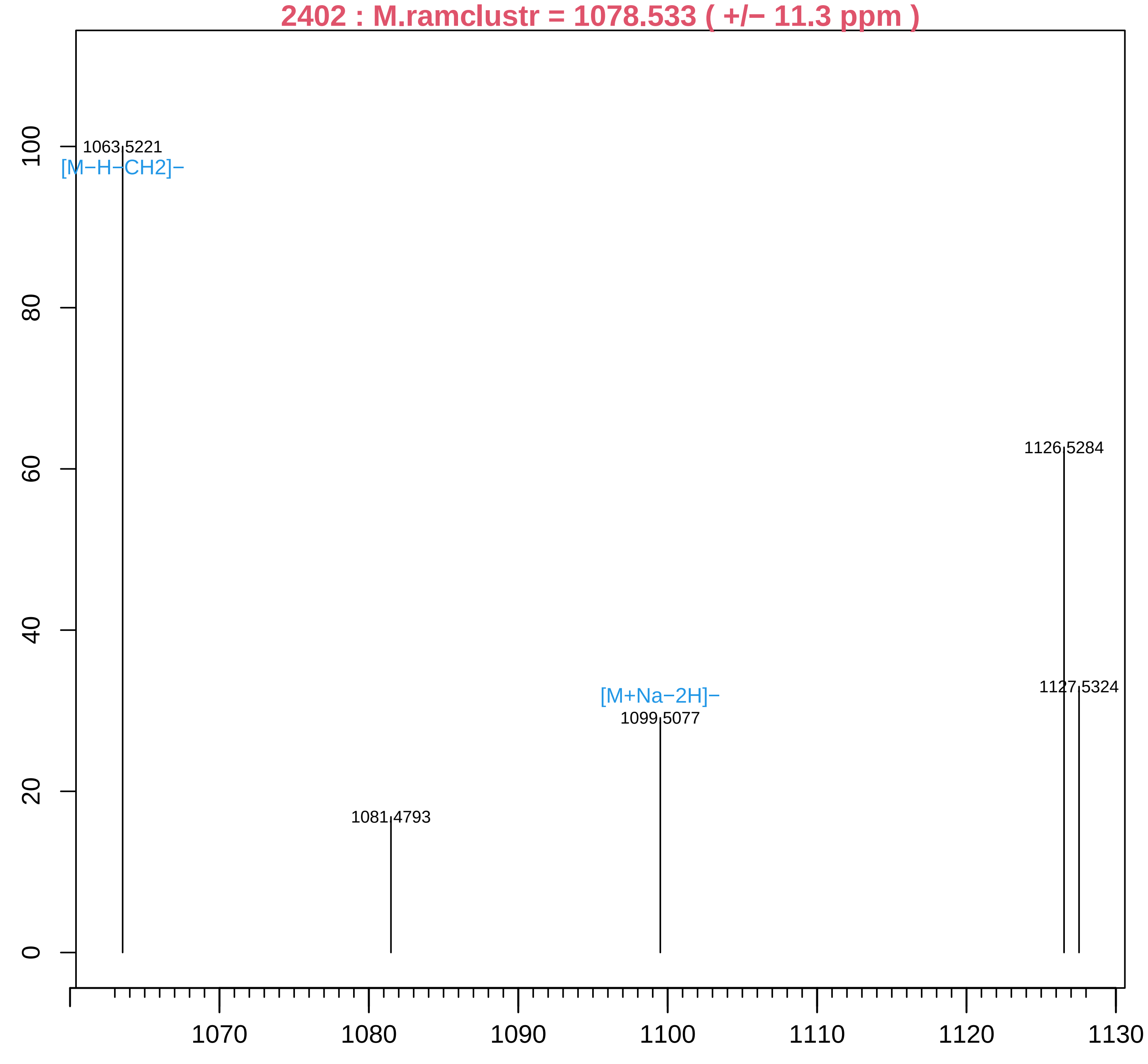
2402 : M.ramclustr = 1078.533 (+/− 11.3 ppm)
1063.5221
[M−H−CH2]−
1081.4793
[M+Na−2H]−
1099.5077
1126.5284
1127.5324
0
20
40
60
80
100
1070
1080
1090
1100
1110
1120
1130

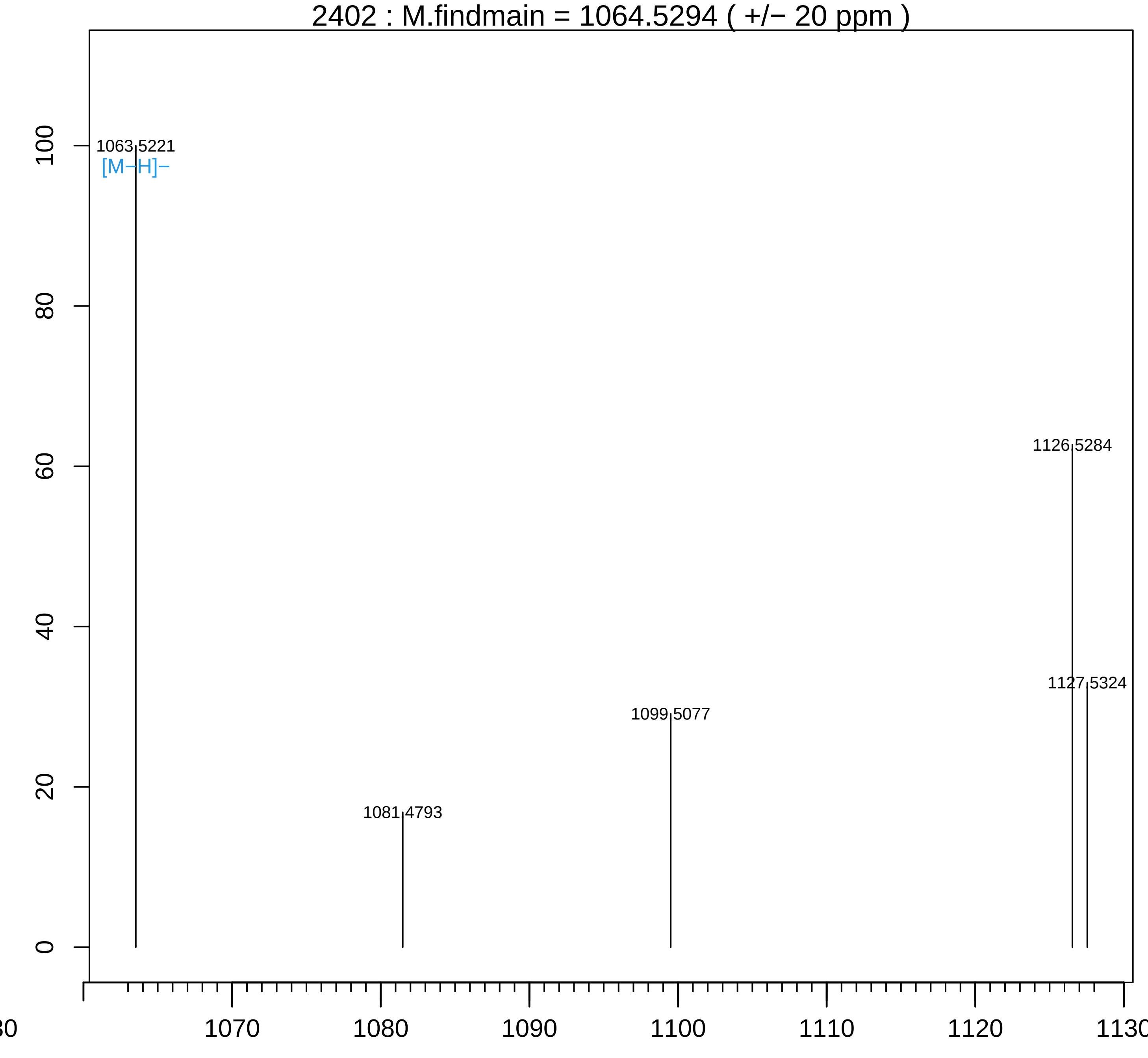
2402 : M.findmain = 1064.5294 (+/− 20 ppm)
1063.5221
[M−H]−
1081.4793
1099.5077
1126.5284
1127.5324
0
20
40
60
80
100
1070
1080
1090
1100
1110
1120
1130

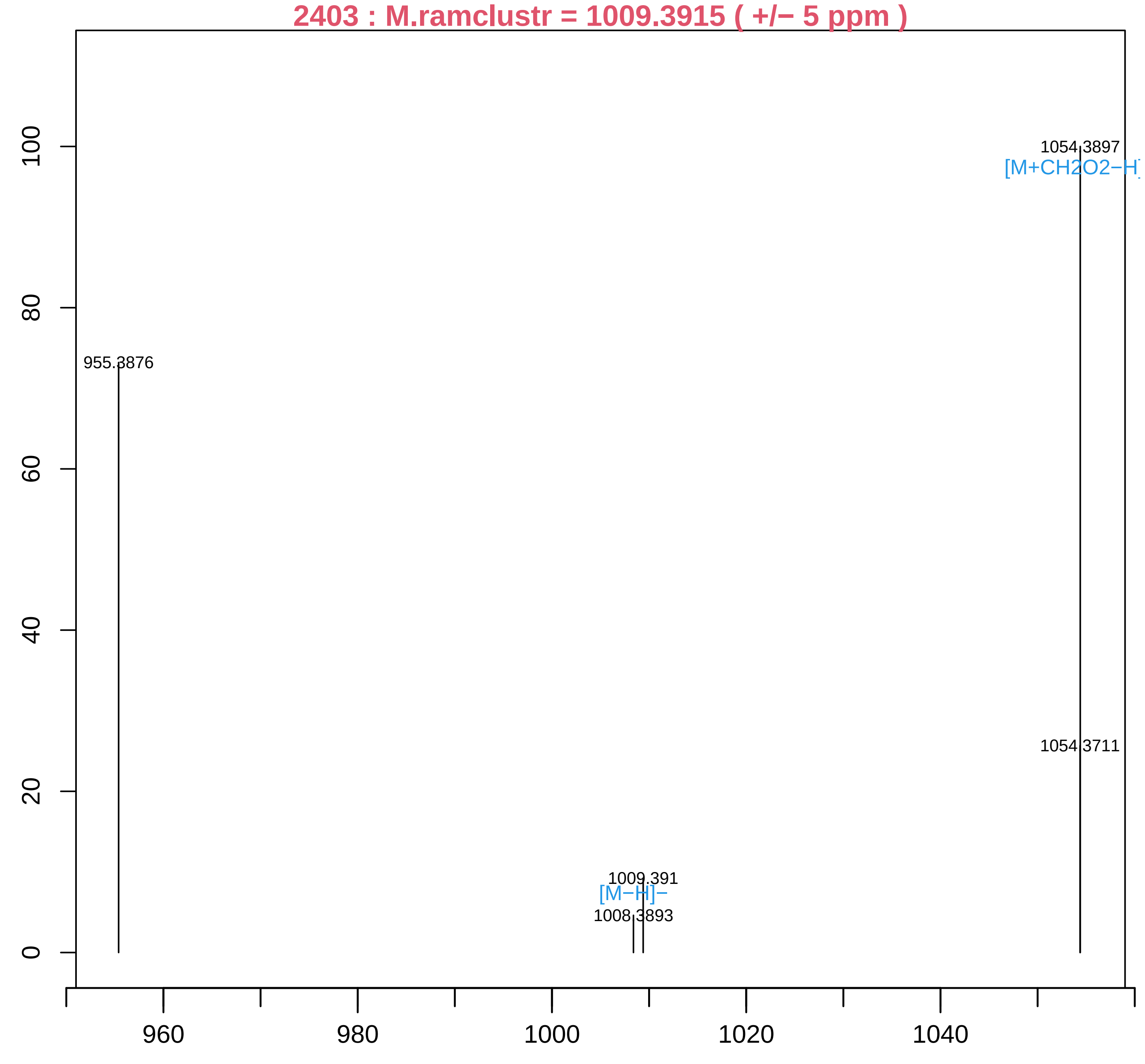
2403 : M.ramclustr = 1009.3915 (+/− 5 ppm)
1054.3897
[M+CH2O2−H]
955.3876
1054.3711
1009.391
[M−H]−
1008.3893
0
20
40
60
80
100
960
980
1000
1020
1040

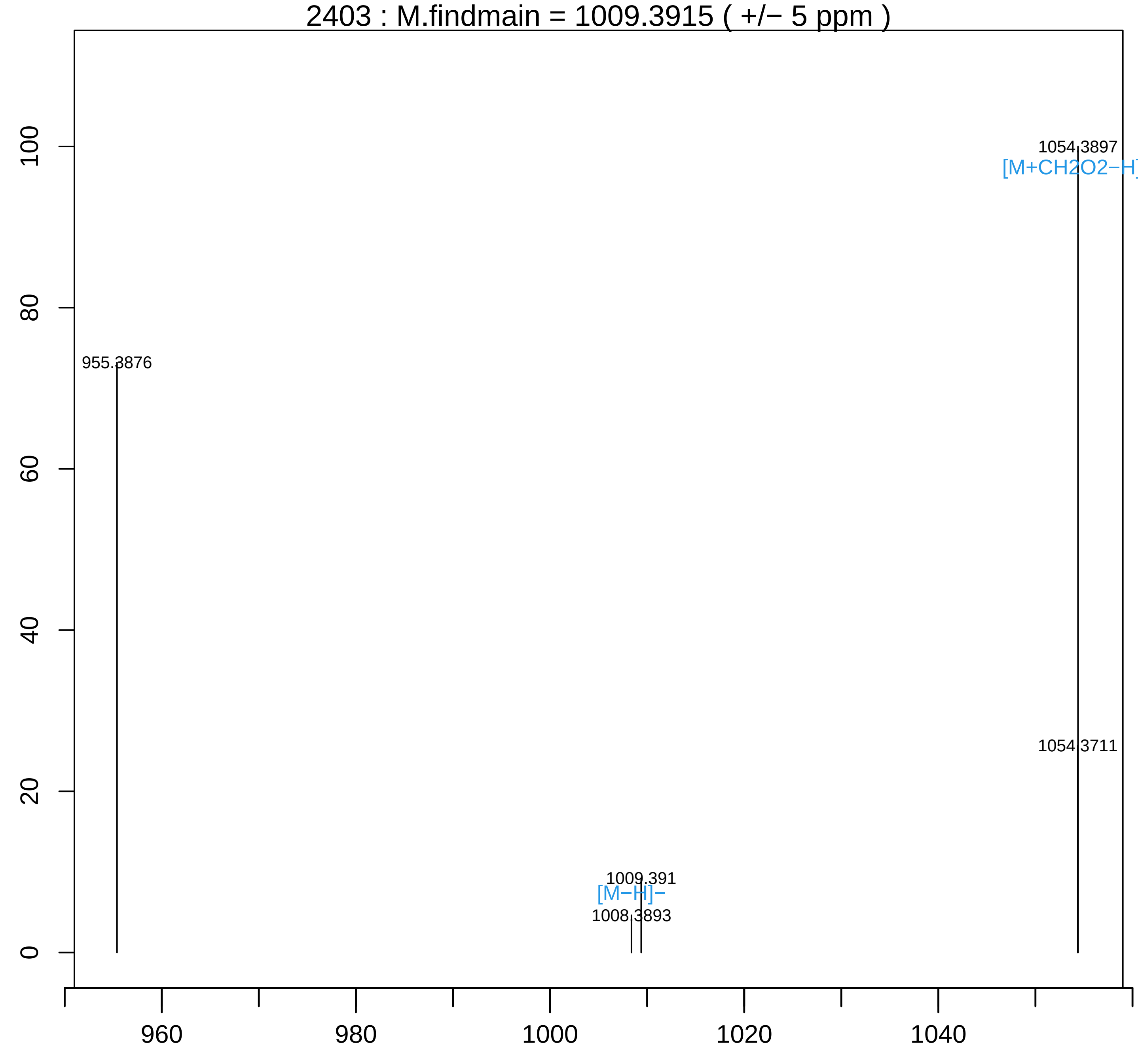
2403 : M.findmain = 1009.3915 (+/− 5 ppm)
1054.3897
[M+CH2O2−H]
955.3876
1054.3711
1009.391
[M−H]−
1008.3893
0
20
40
60
80
100
960
980
1000
1020
1040

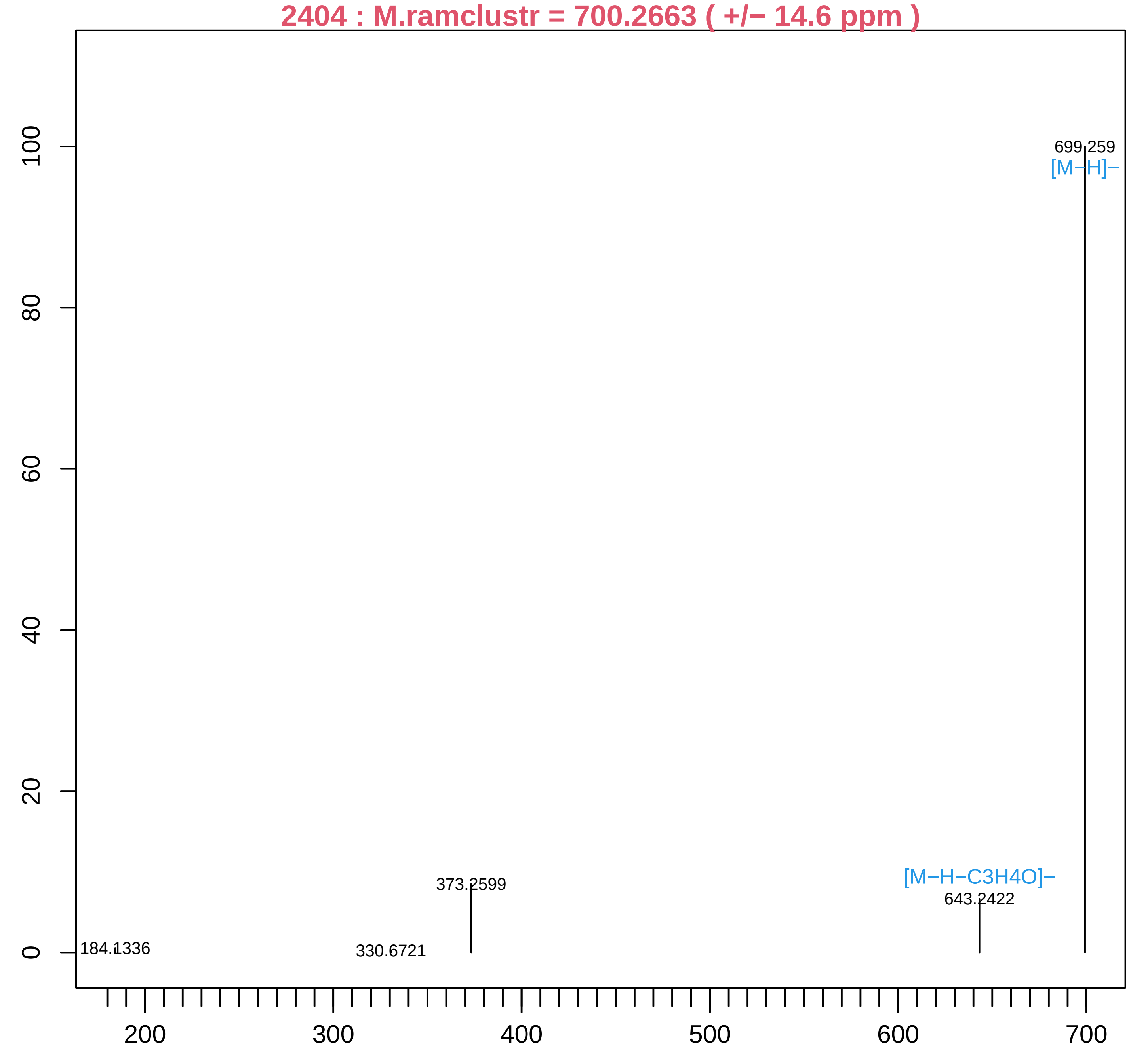
2404 : M.ramclustr = 700.2663 (+/− 14.6 ppm)
699.259
[M−H]−
373.2599
[M−H−C3H4O]−
643.2422
184.1336
330.6721
0
20
40
60
80
100
200
300
400
500
600
700

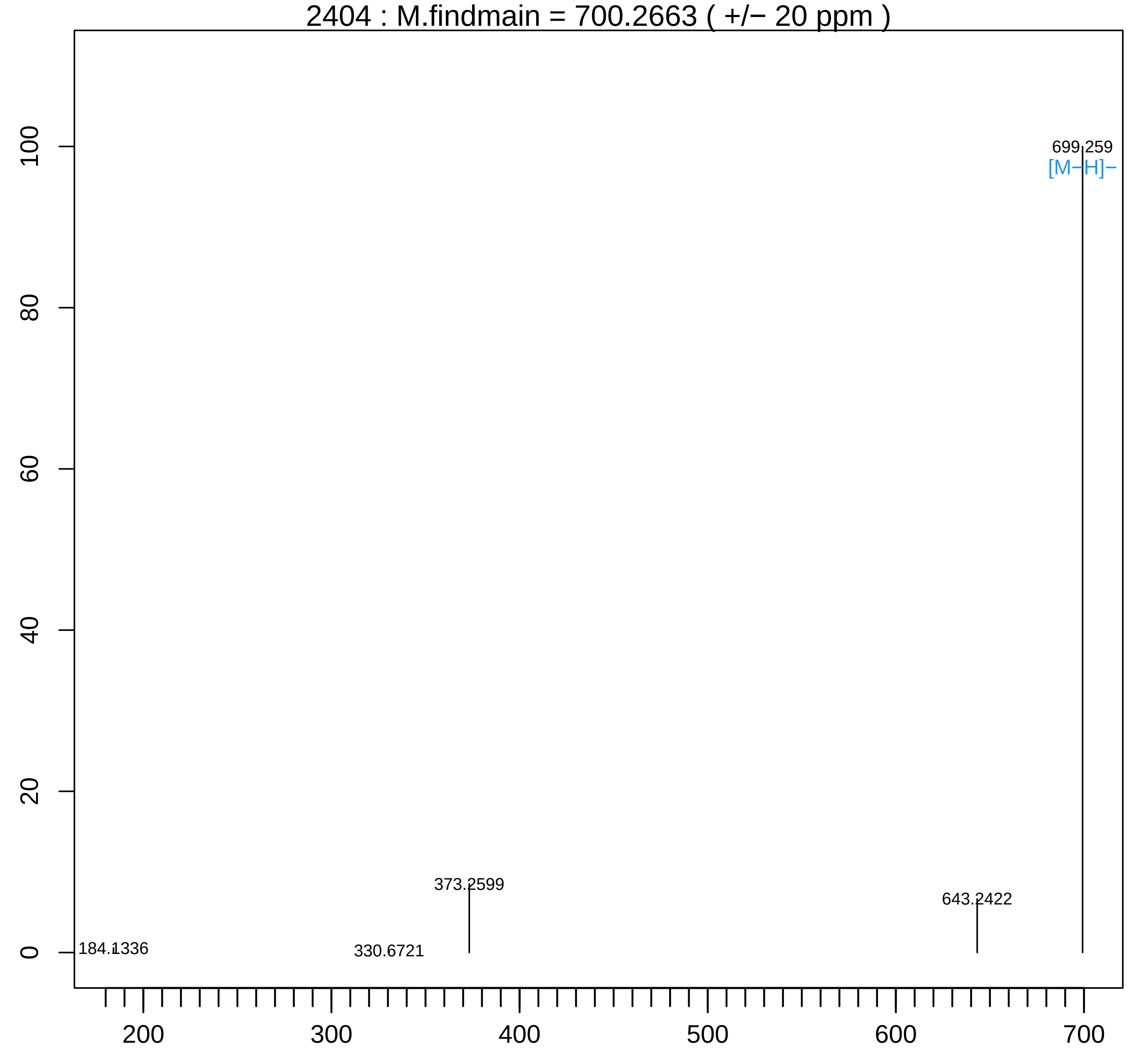
2404 : M.findmain = 700.2663 (+/− 20 ppm)
699.259
[M−H]−
373.2599
643.2422
184.1336
330.6721
0
20
40
60
80
100
200
300
400
500
600
700

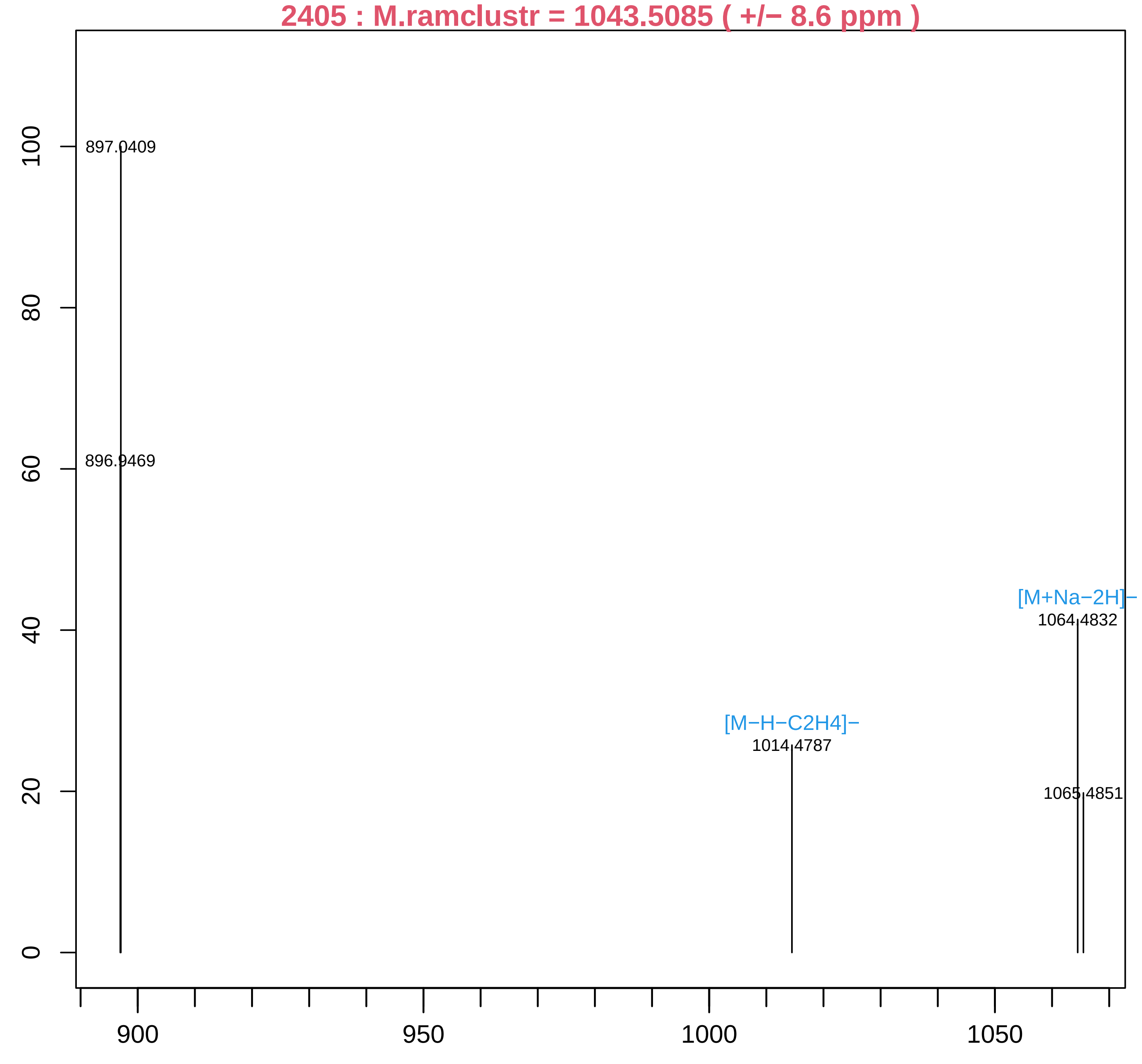
2405 : M.ramclustr = 1043.5085 (+/− 8.6 ppm)
897.0409
896.9469
[M+Na−2H]−
1064.4832
[M−H−C2H4]−
1014.4787
1065.4851
0
20
40
60
80
100
900
950
1000
1050

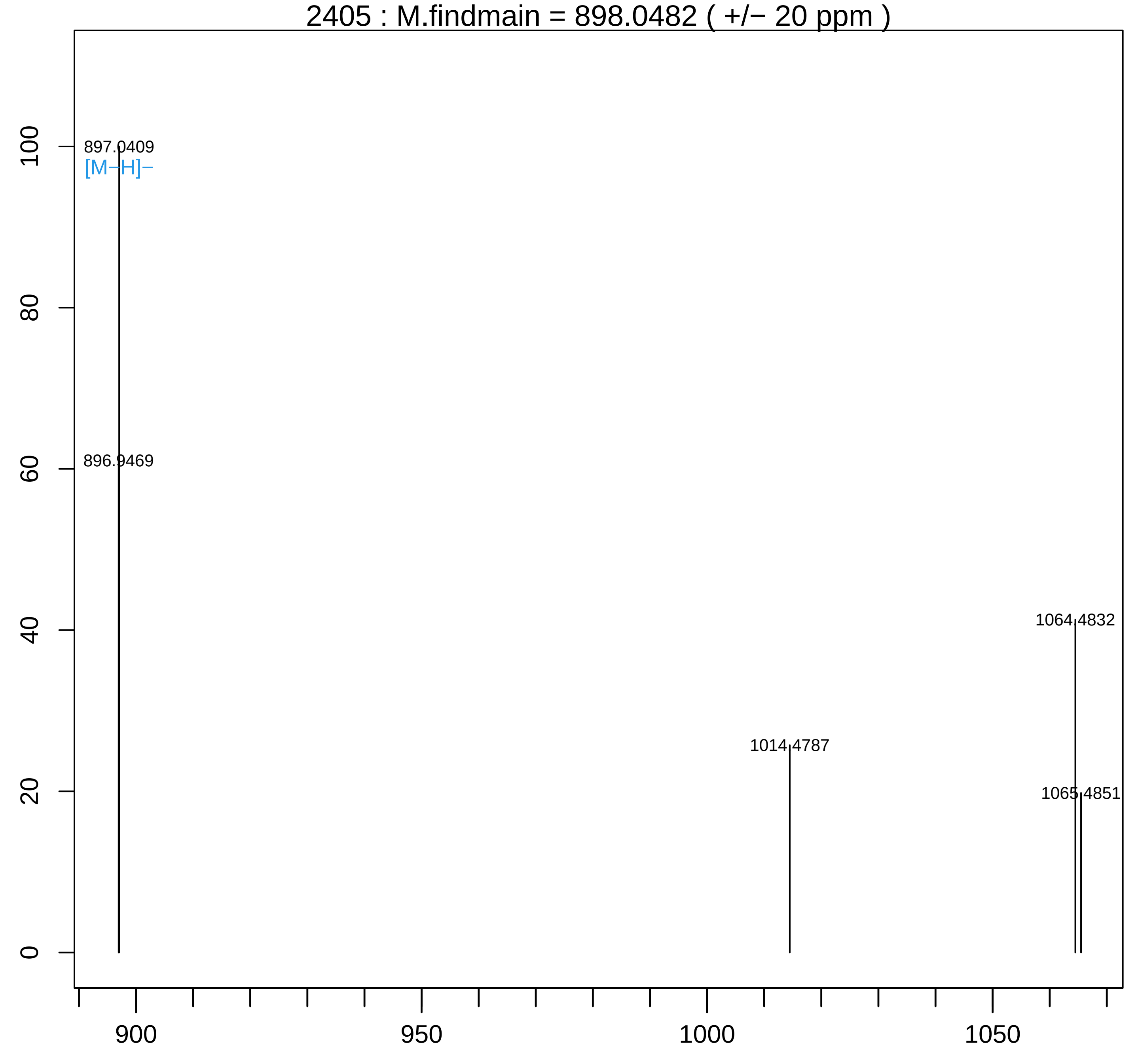
2405 : M.findmain = 898.0482 (+/− 20 ppm)
897.0409
[M−H]−
896.9469
1064.4832
1014.4787
1065.4851
0
20
40
60
80
100
900
950
1000
1050

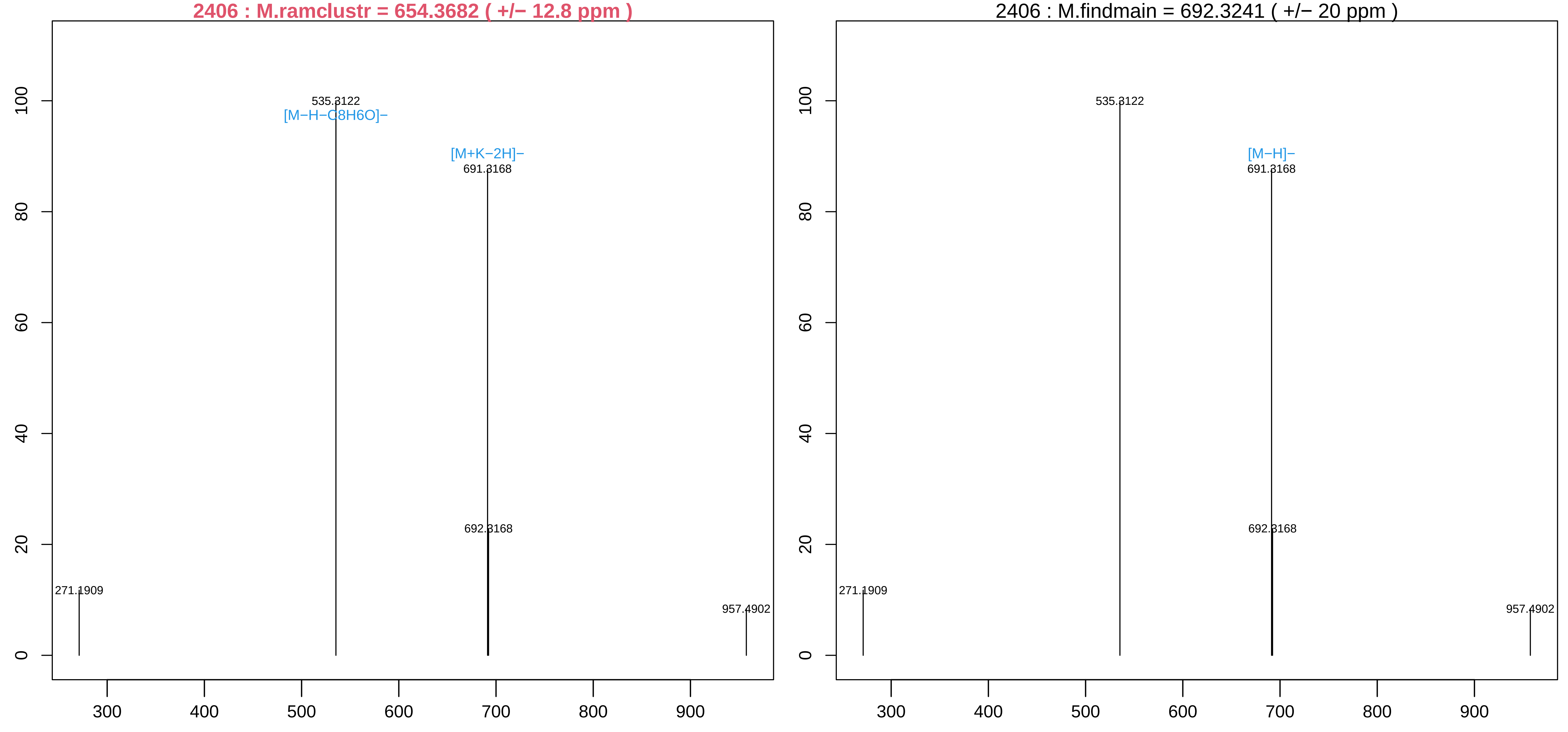
2406 : M.ramclustr = 654.3682 (+/− 12.8 ppm)
535.3122
[M−H−C8H6O]−
[M+K−2H]−
691.3168
692.3168
271.1909
957.4902
0
20
40
60
80
100
300
400
500
600
700
800
900
2406 : M.findmain = 692.3241 (+/− 20 ppm)
535.3122
[M−H]−
691.3168
692.3168
271.1909
957.4902
0
20
40
60
80
100
300
400
500
600
700
800
900

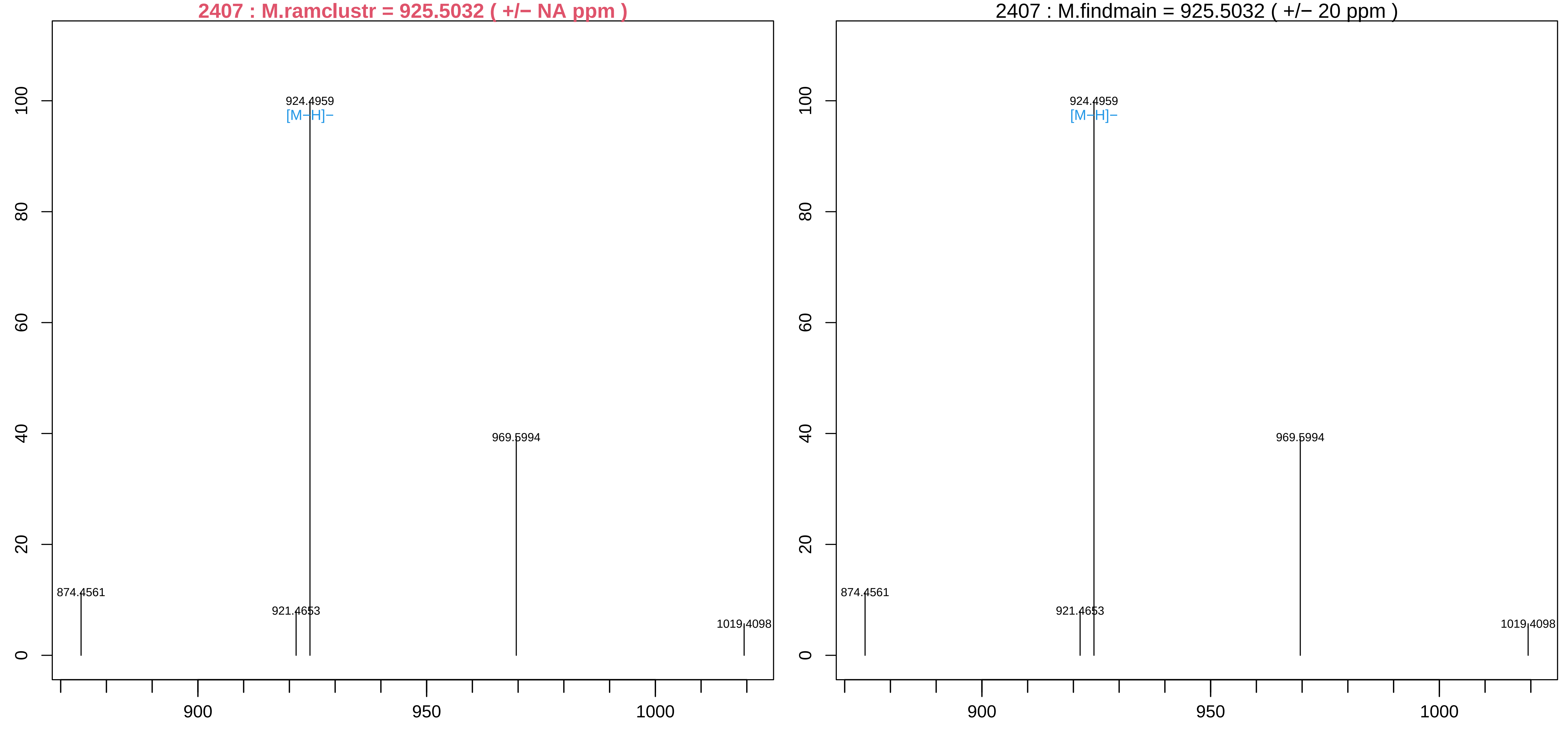

2407 : M.ramclustr = 925.5032 (+/− NA ppm)
924.4959
[M−H]−
969.5994
874.4561
921.4653
1019.4098
0
20
40
60
80
100
900
950
1000
2407 : M.findmain = 925.5032 (+/− 20 ppm)
924.4959
[M−H]−
969.5994
874.4561
921.4653
1019.4098
0
20
40
60
80
100
900
950
1000

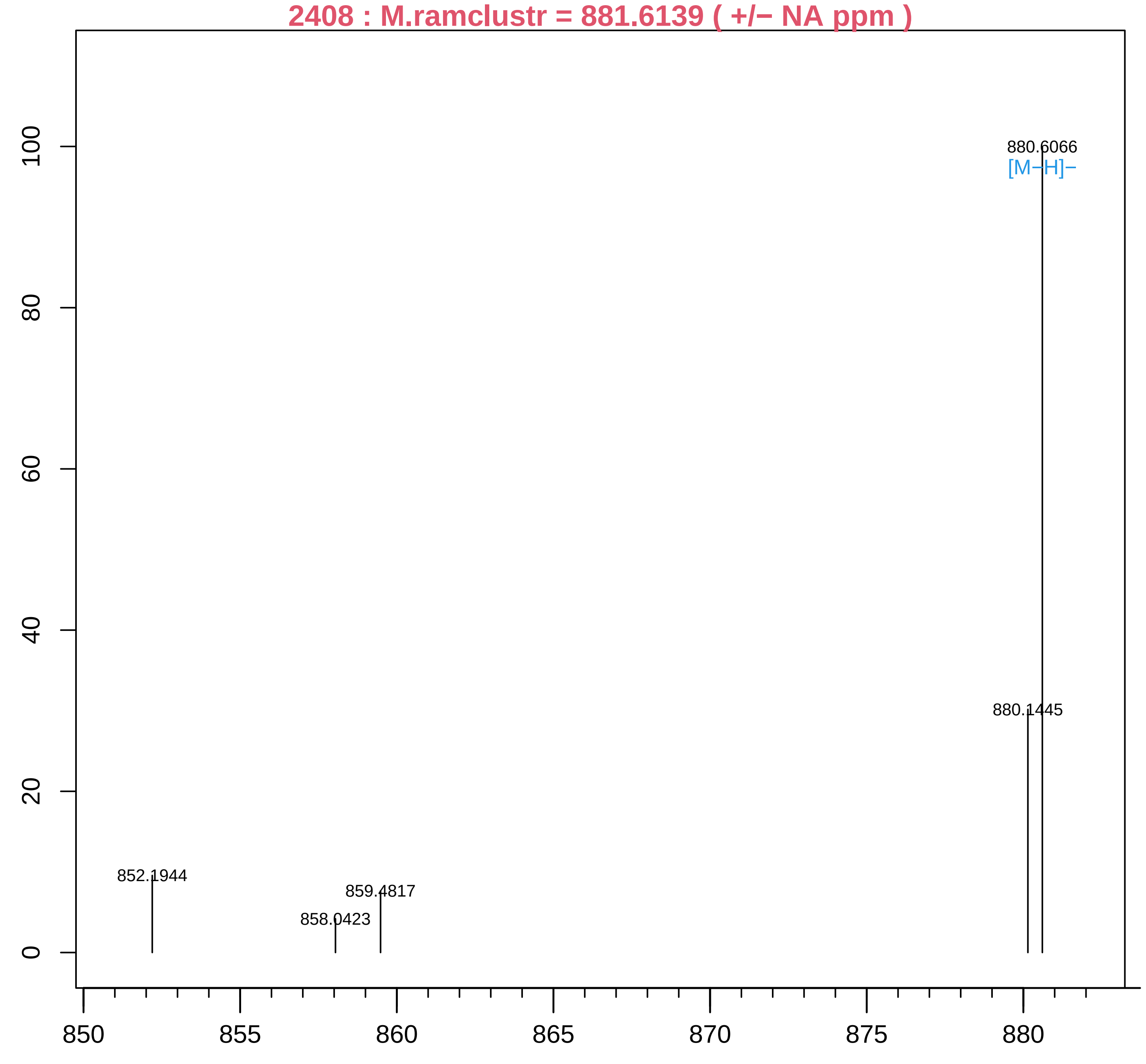
2408 : M.ramclustr = 881.6139 (+/− NA ppm)
880.6066
[M−H]−
880.1445
852.1944
859.4817
858.0423
0
20
40
60
80
100
850
855
860
865
870
875
880

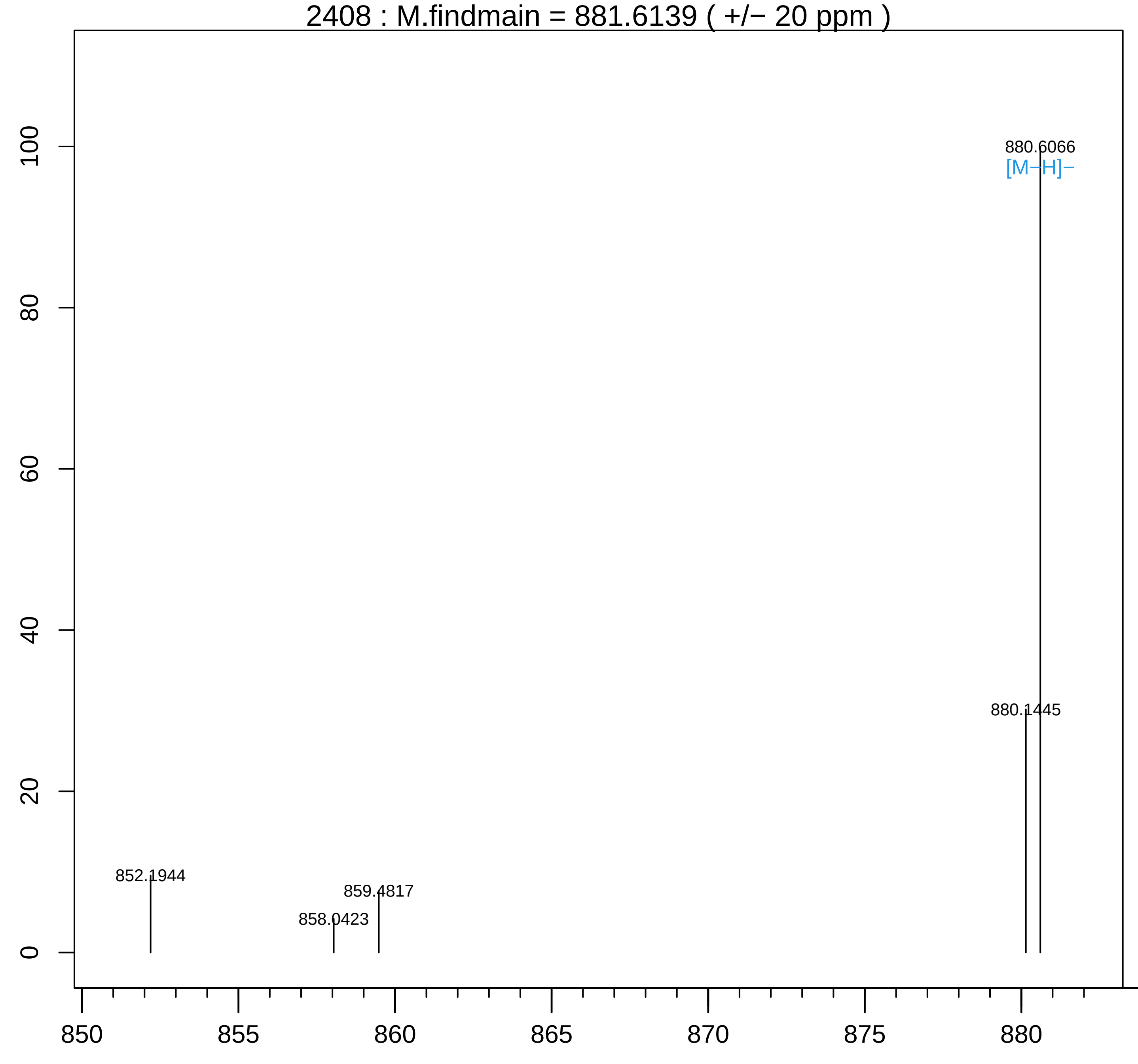
2408 : M.findmain = 881.6139 (+/− 20 ppm)
880.6066
[M−H]−
880.1445
852.1944
859.4817
858.0423
0
20
40
60
80
100
850
855
860
865
870
875
880

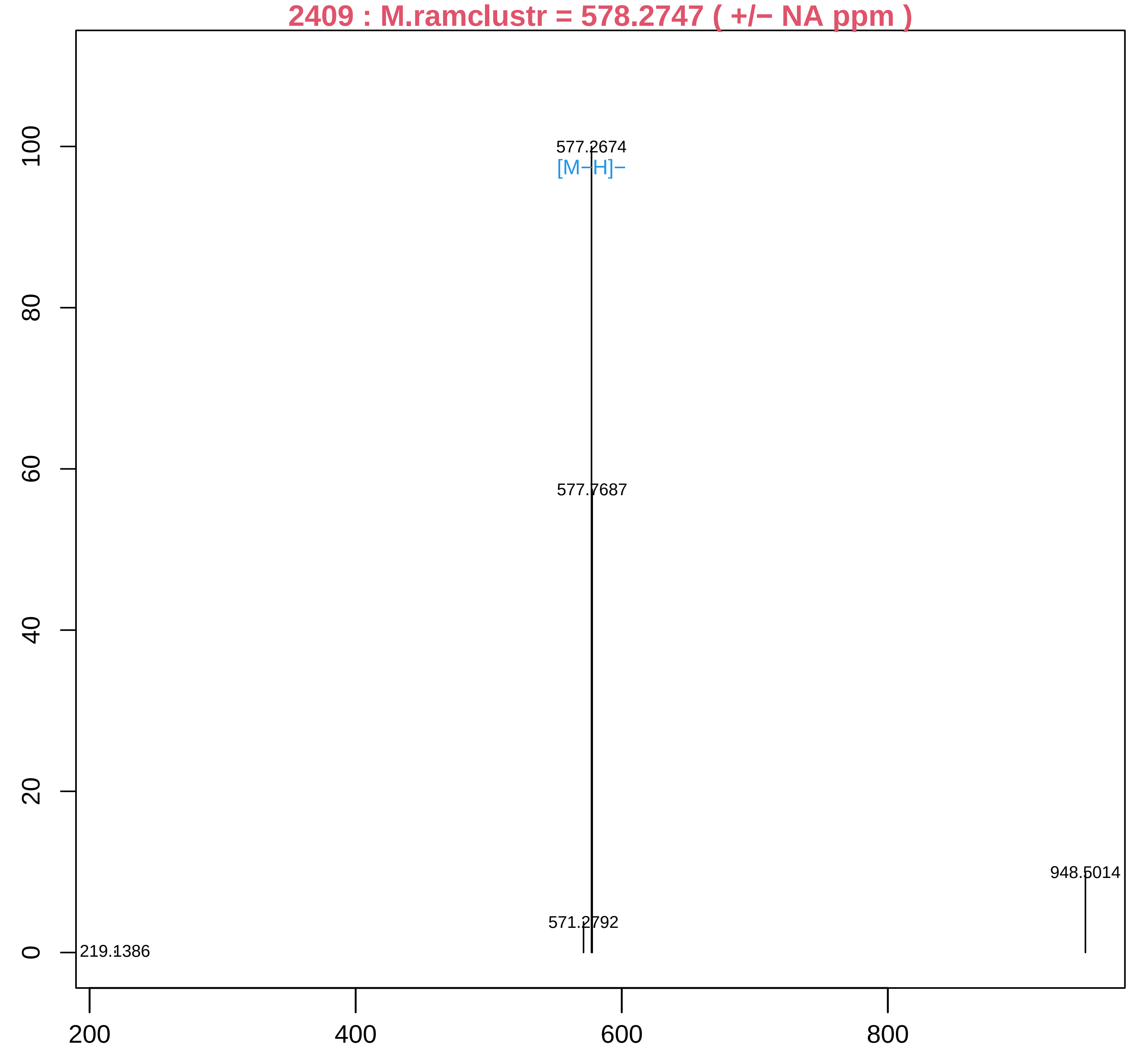

2409 : M.ramclustr = 578.2747 (+/− NA ppm)
577.2674
[M−H]−
577.7687
948.5014
571.2792
219.1386
0
20
40
60
80
100
200
400
600
800

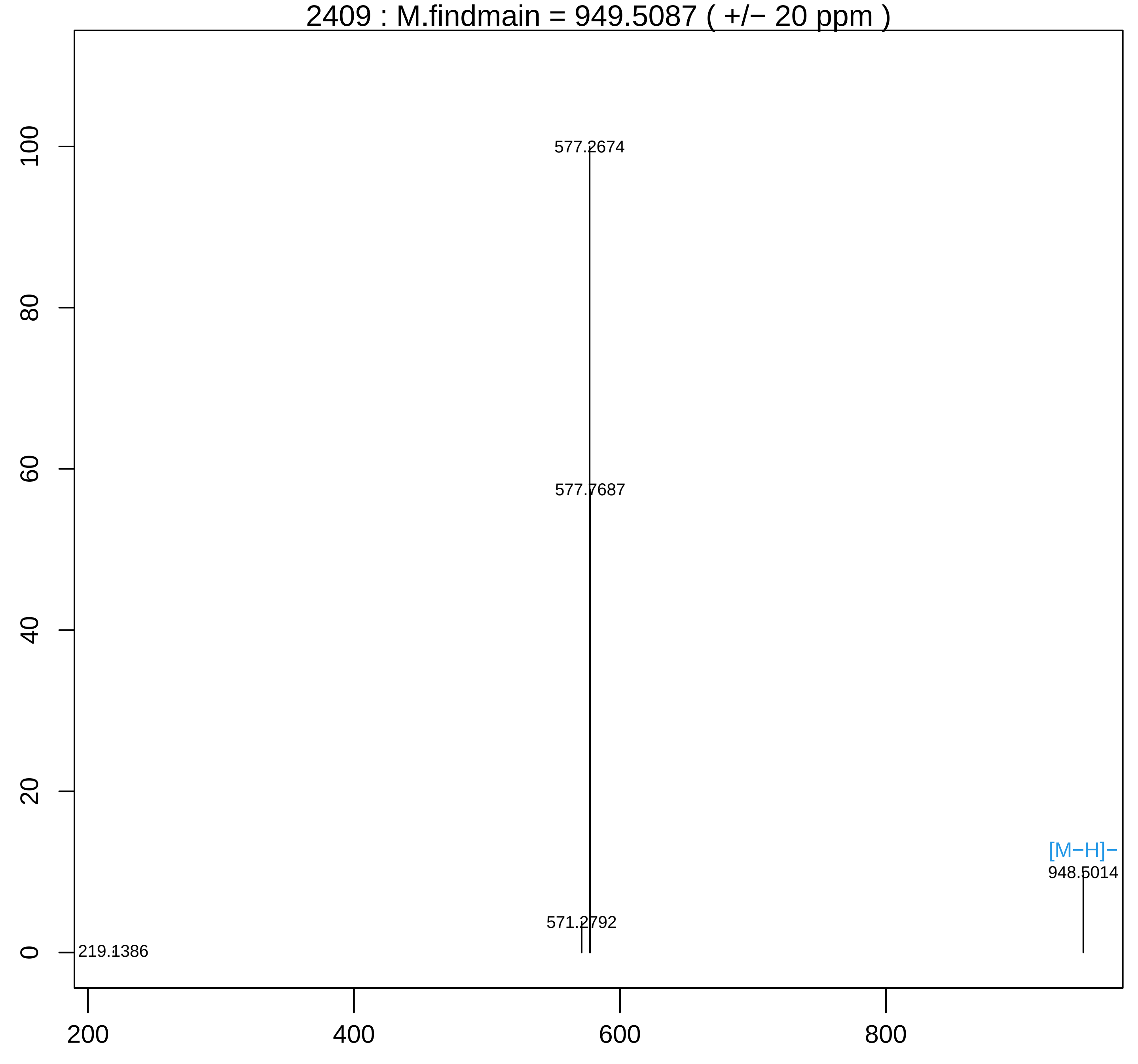

2409 : M.findmain = 949.5087 (+/− 20 ppm)
577.2674
577.7687
[M−H]−
948.5014
571.2792
219.1386
0
20
40
60
80
100
200
400
600
800

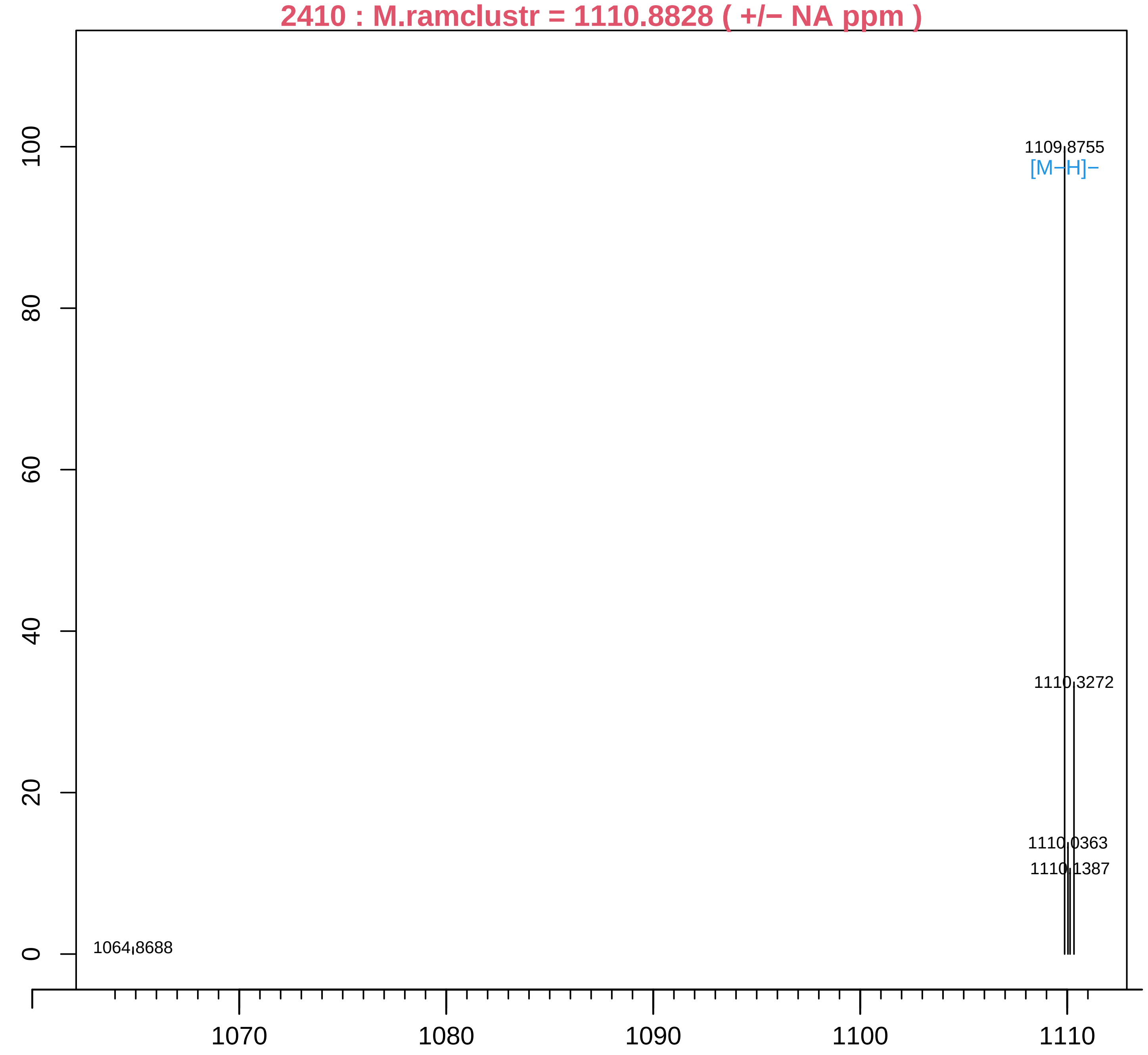
2410 : M.ramclustr = 1110.8828 (+/− NA ppm)
1109.8755
[M−H]−
1110.3272
1110.0363
1110.1387
1064.8688
0
20
40
60
80
100
1070
1080
1090
1100
1110

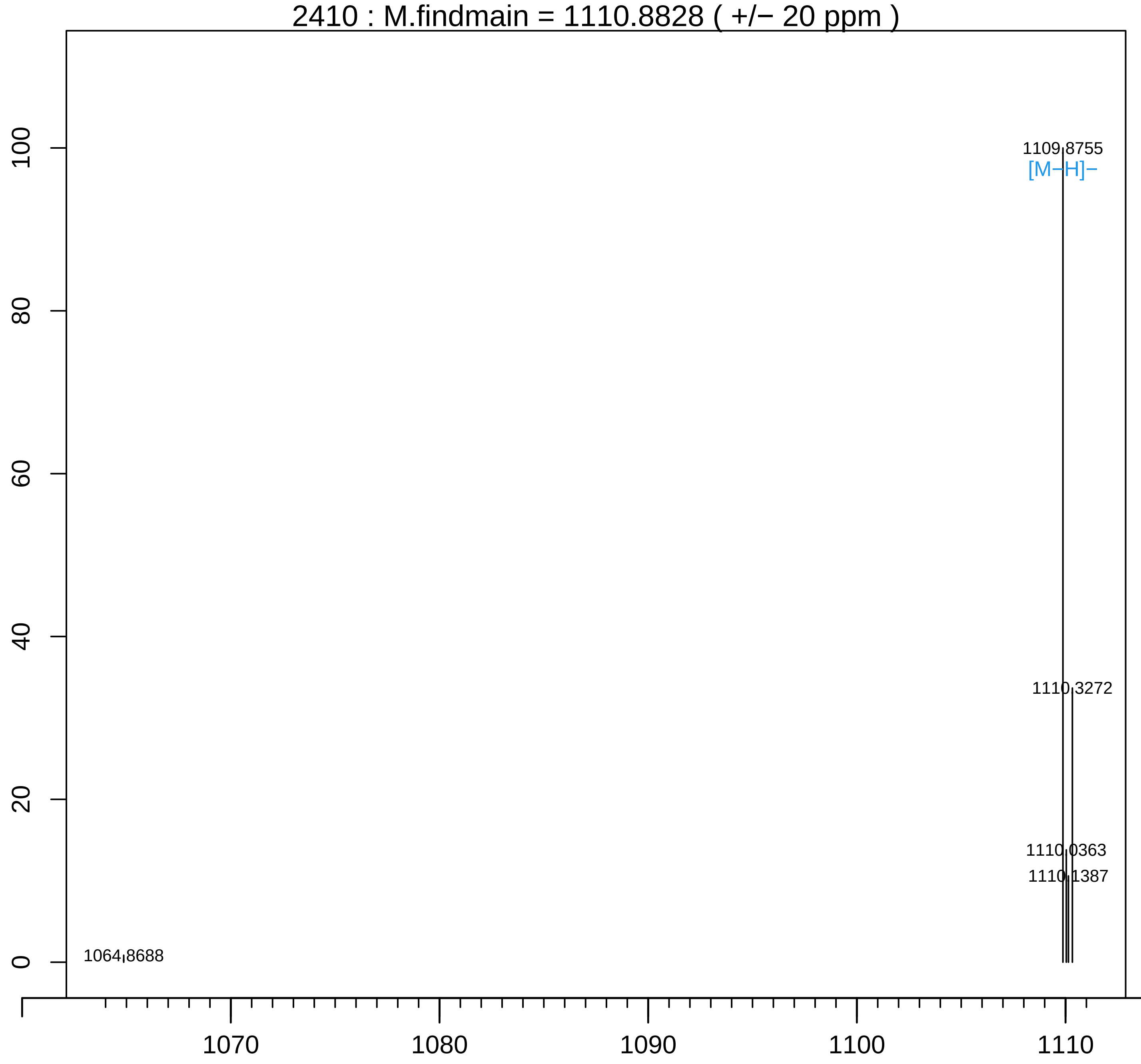
2410 : M.findmain = 1110.8828 (+/− 20 ppm)
1109.8755
[M−H]−
1110.3272
1110.0363
1110.1387
1064.8688
0
20
40
60
80
100
1070
1080
1090
1100
1110

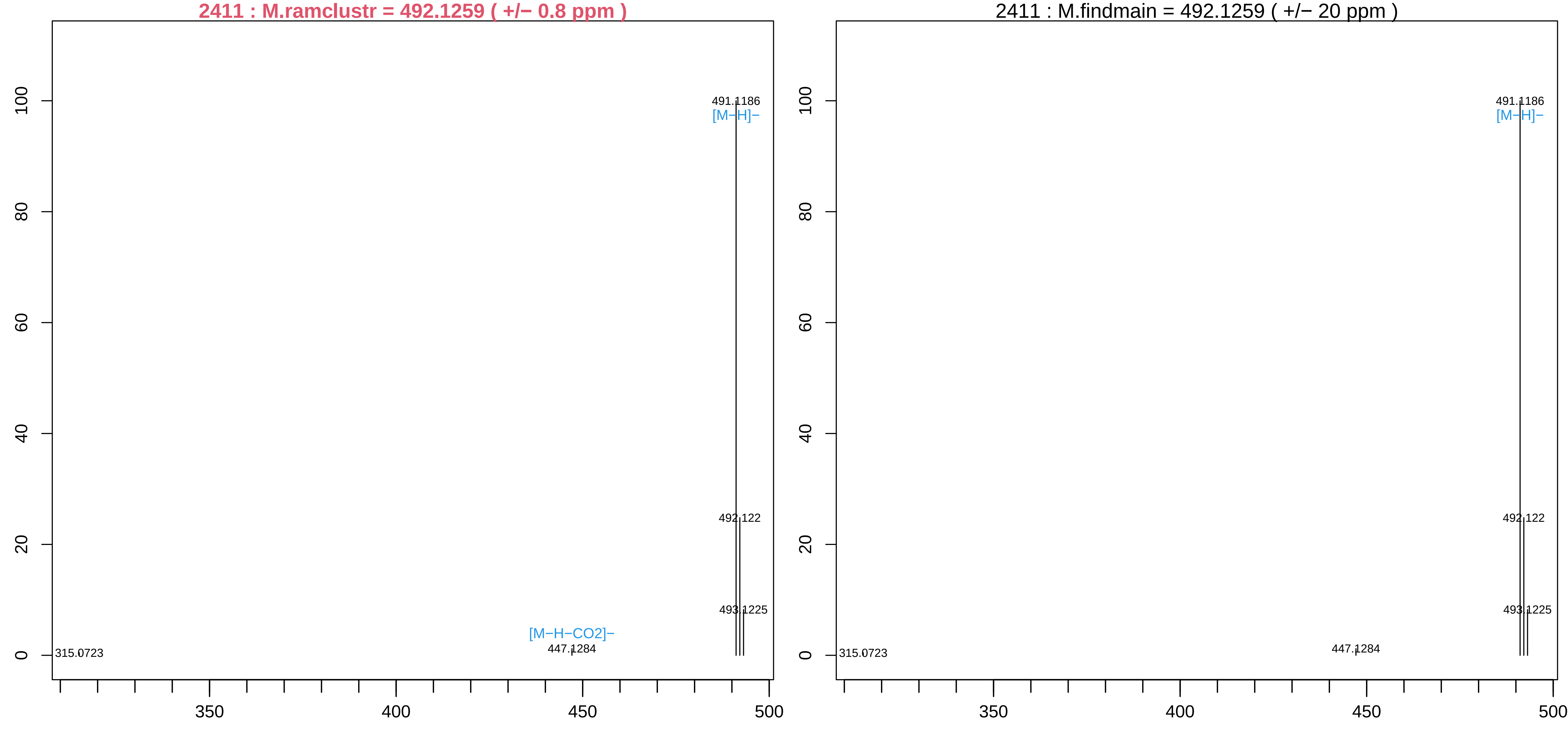
2411 : M.ramclustr = 492.1259 (+/− 0.8 ppm)
491.1186
[M−H]−
492.122
493.1225
[M−H−CO2]−
447.1284
315.0723
0
20
40
60
80
100
350
400
450
500
2411 : M.findmain = 492.1259 (+/− 20 ppm)
491.1186
[M−H]−
492.122
493.1225
447.1284
315.0723
0
20
40
60
80
100
350
400
450
500

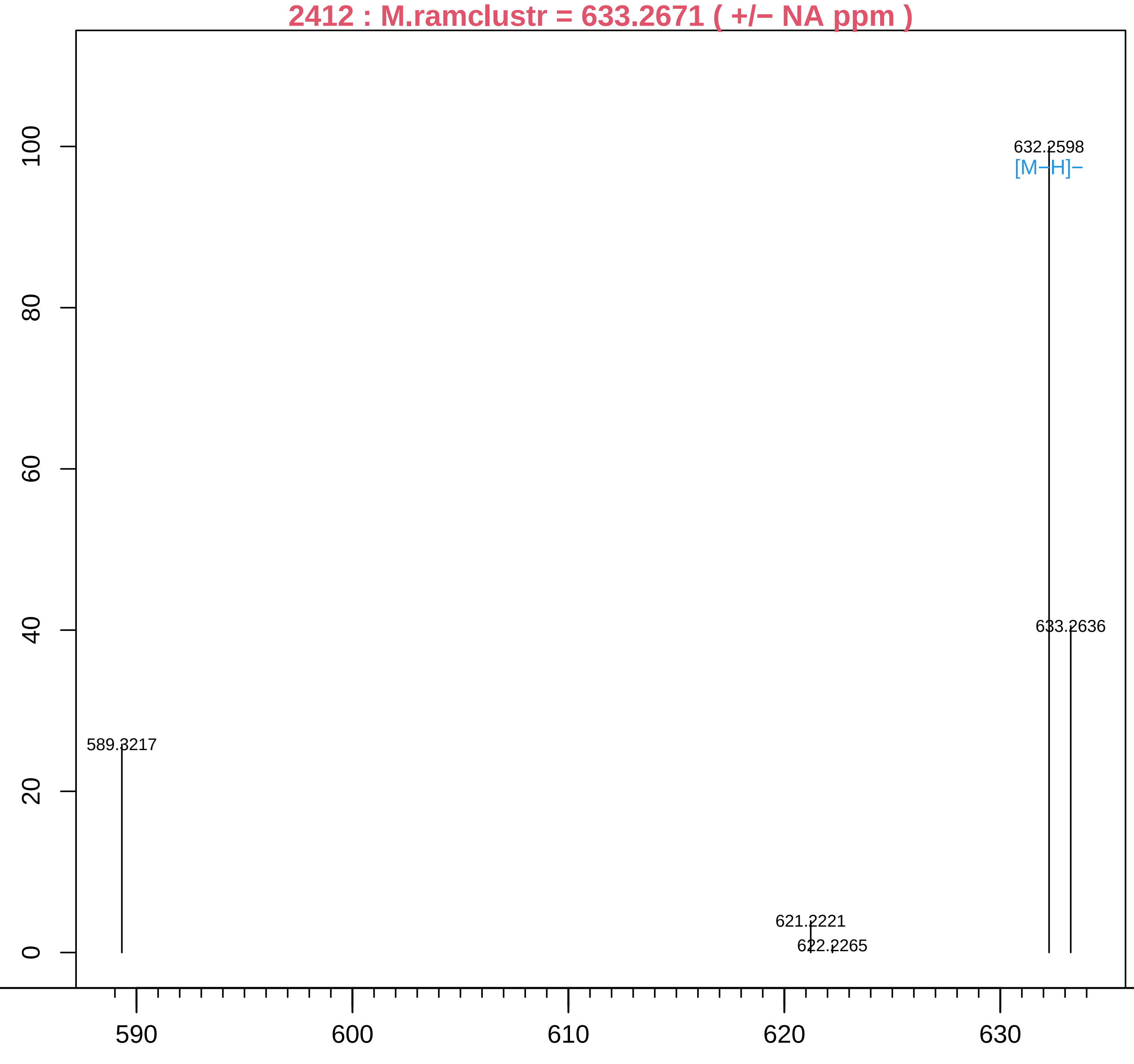
2412 : M.ramclustr = 633.2671 (+/− NA ppm)
632.2598
[M−H]−
633.2636
589.3217
621.2221
622.2265
0
20
40
60
80
100
590
600
610
620
630

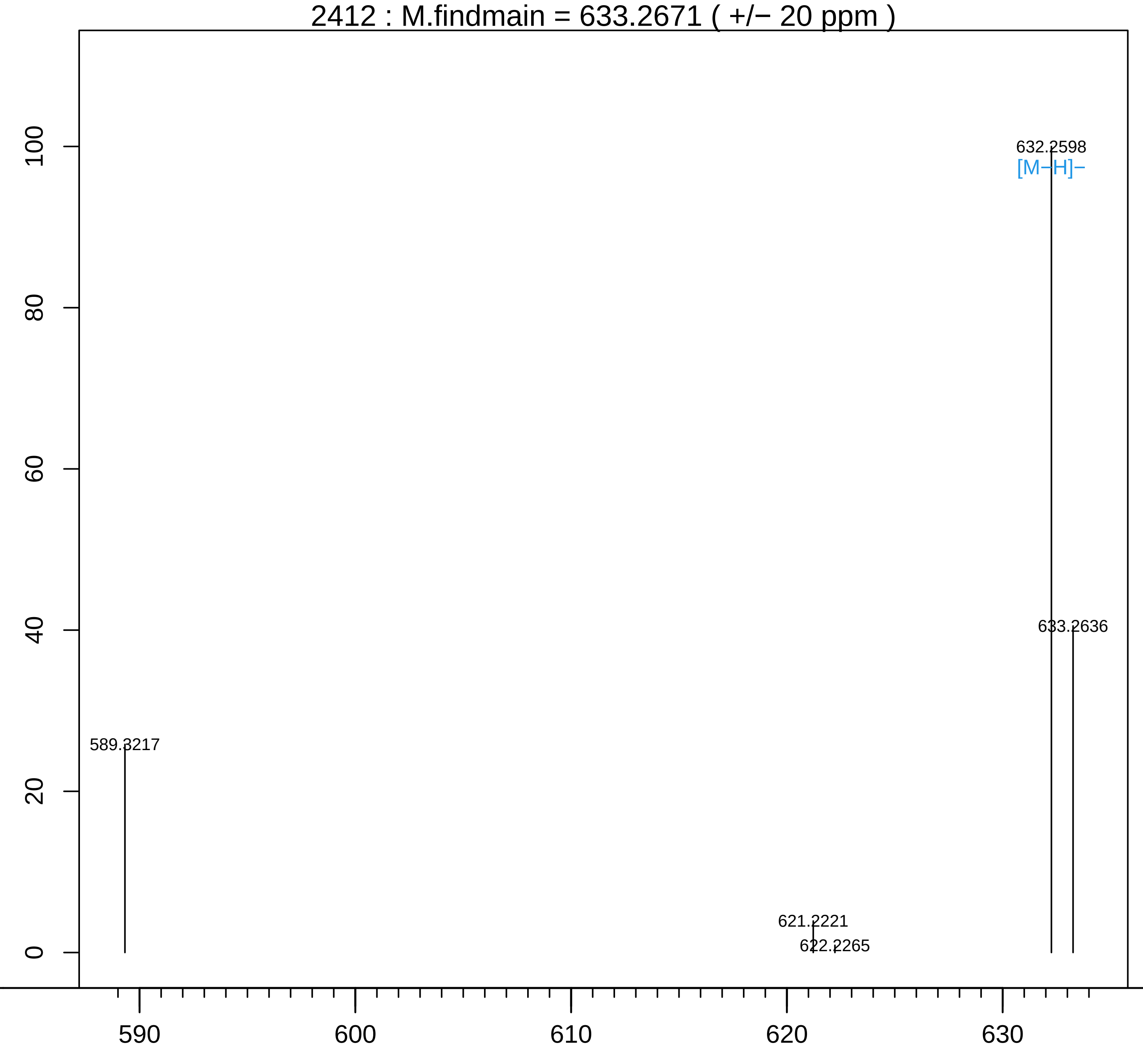
2412 : M.findmain = 633.2671 (+/− 20 ppm)
632.2598
[M−H]−
633.2636
589.3217
621.2221
622.2265
0
20
40
60
80
100
590
600
610
620
630

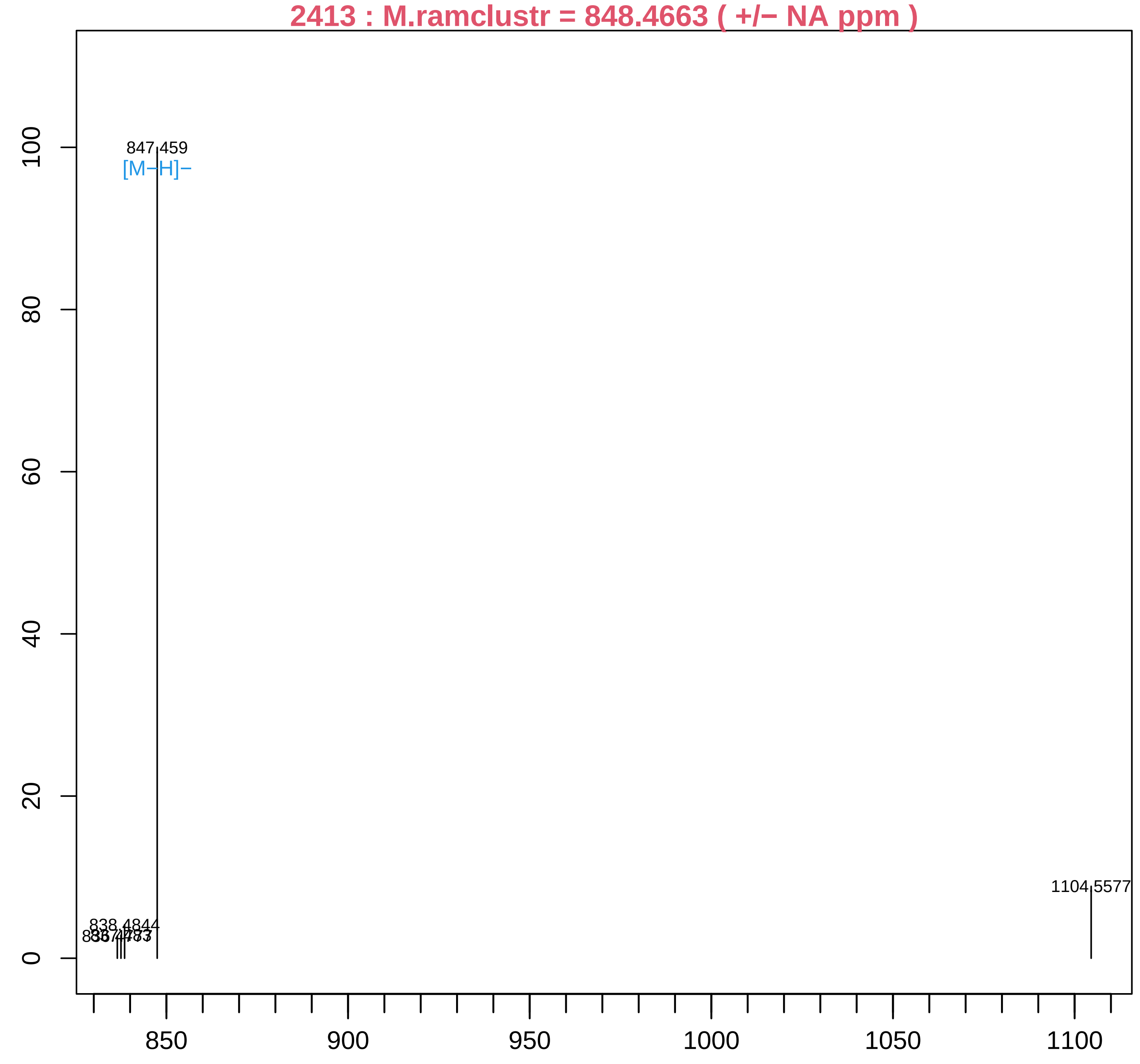
2413 : M.ramclustr = 848.4663 (+/− NA ppm)
847.459
[M−H]−
838.4844
1104.5577
0
20
40
60
80
100
850
900
950
1000
1050
1100

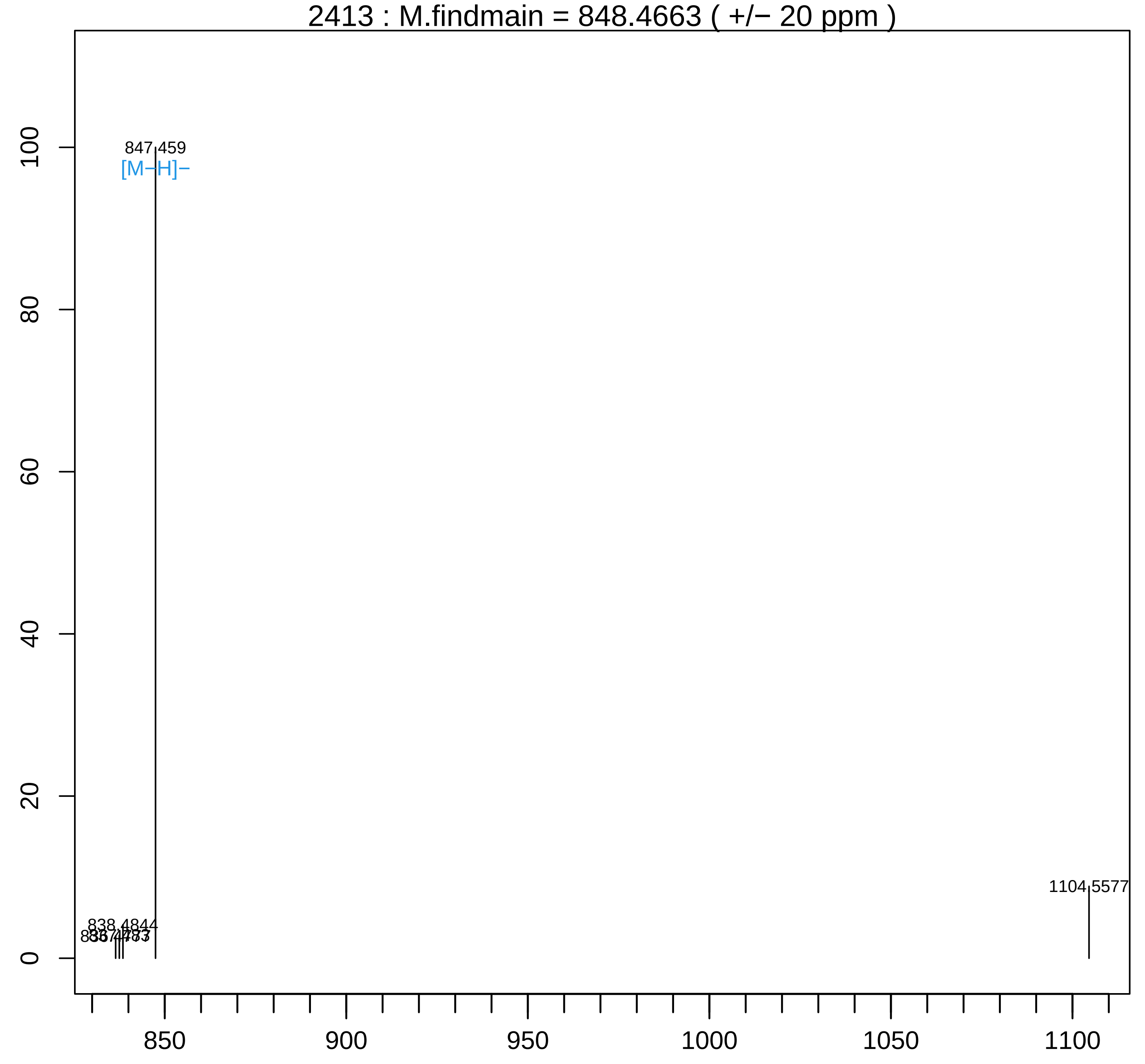
2413 : M.findmain = 848.4663 (+/− 20 ppm)
847.459
[M−H]−
838.4844
1104.5577
0
20
40
60
80
100
850
900
950
1000
1050
1100

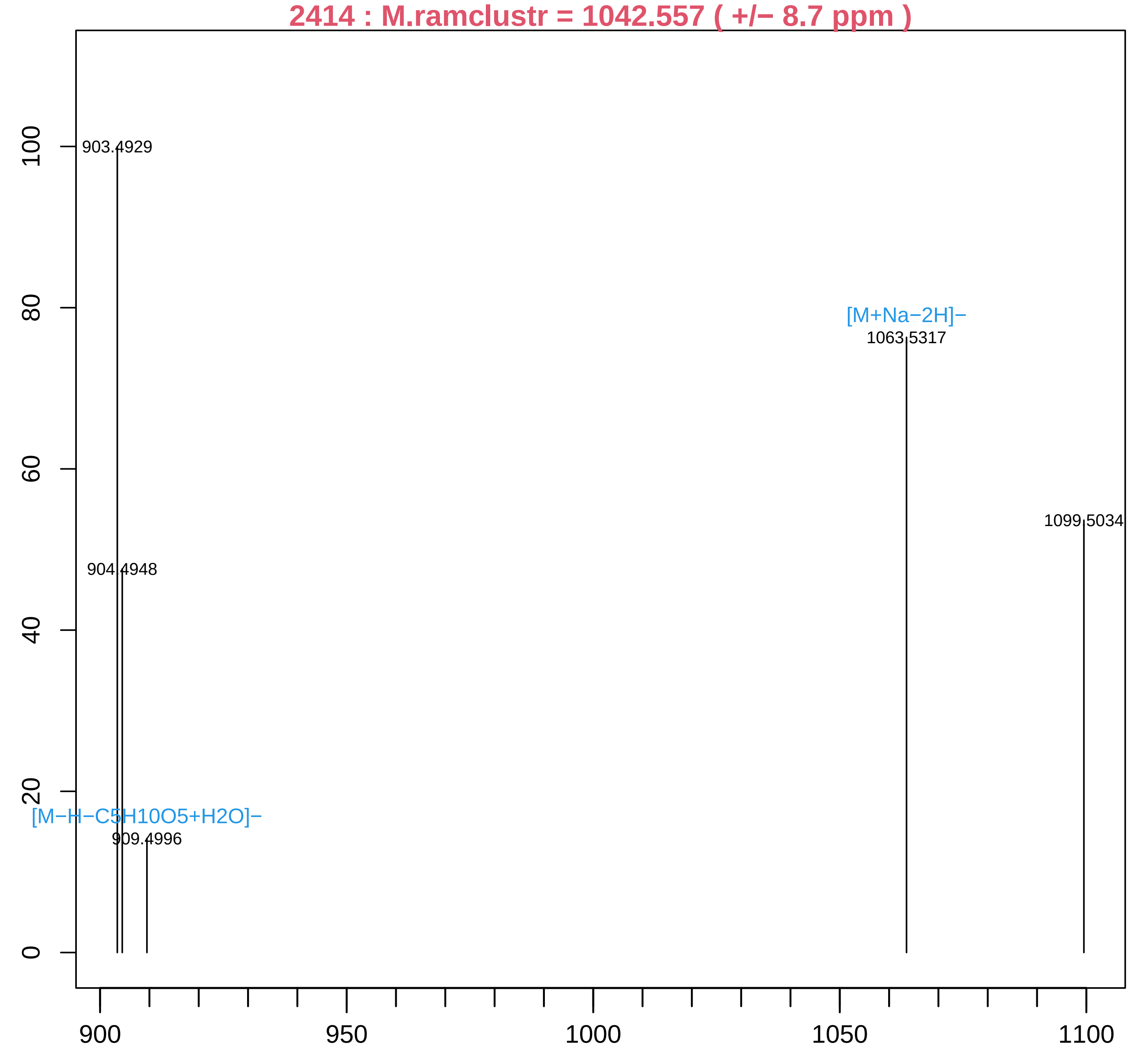
2414 : M.ramclustr = 1042.557 (+/− 8.7 ppm)
903.4929
904.4948
909.4996
[M−H−C5H10O5+H2O]−
[M+Na−2H]−
1063.5317
1099.5034
0
20
40
60
80
100
900
950
1000
1050
1100

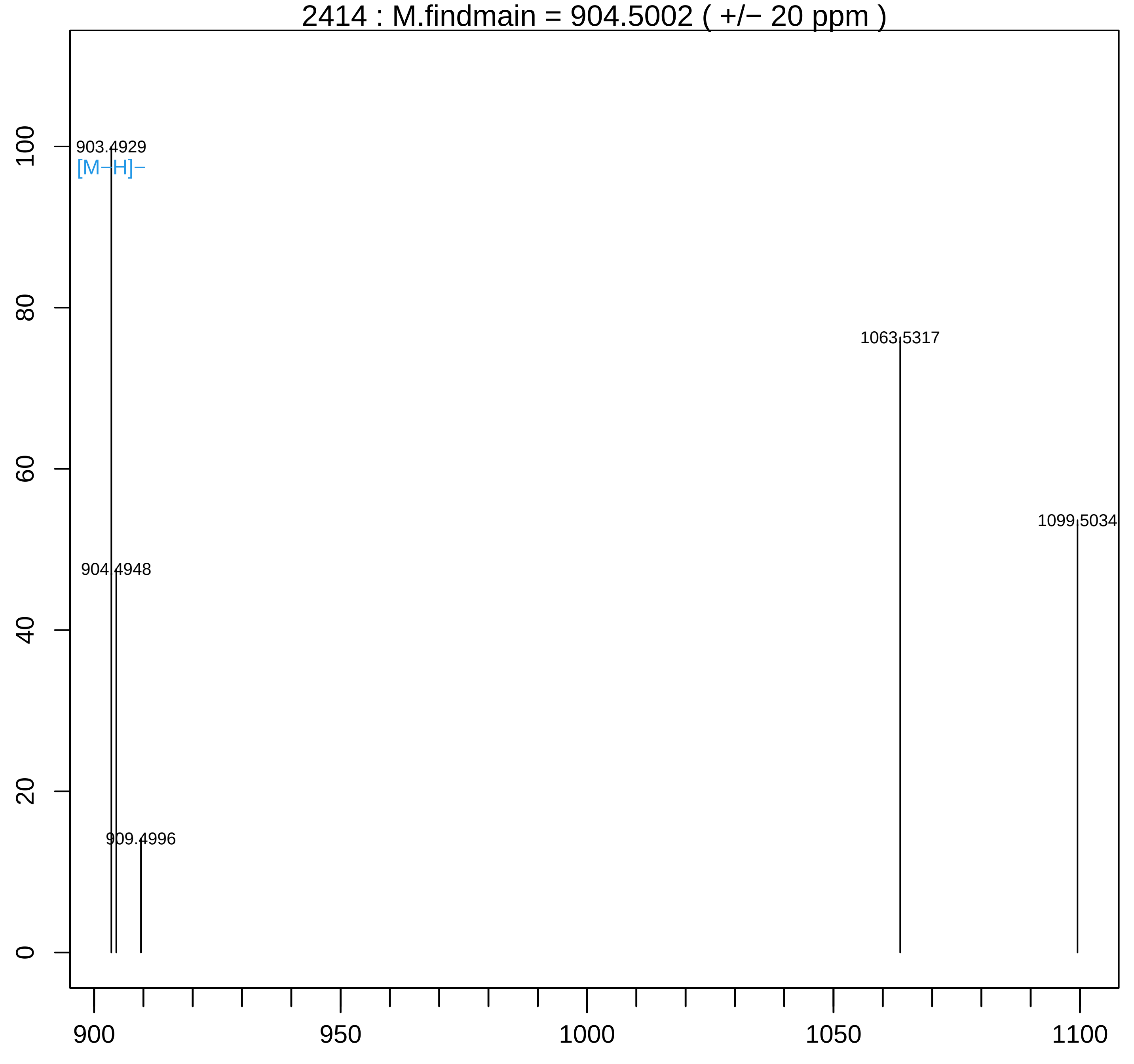
2414 : M.findmain = 904.5002 (+/− 20 ppm)
903.4929
[M−H]−
904.4948
909.4996
1063.5317
1099.5034
0
20
40
60
80
100
900
950
1000
1050
1100

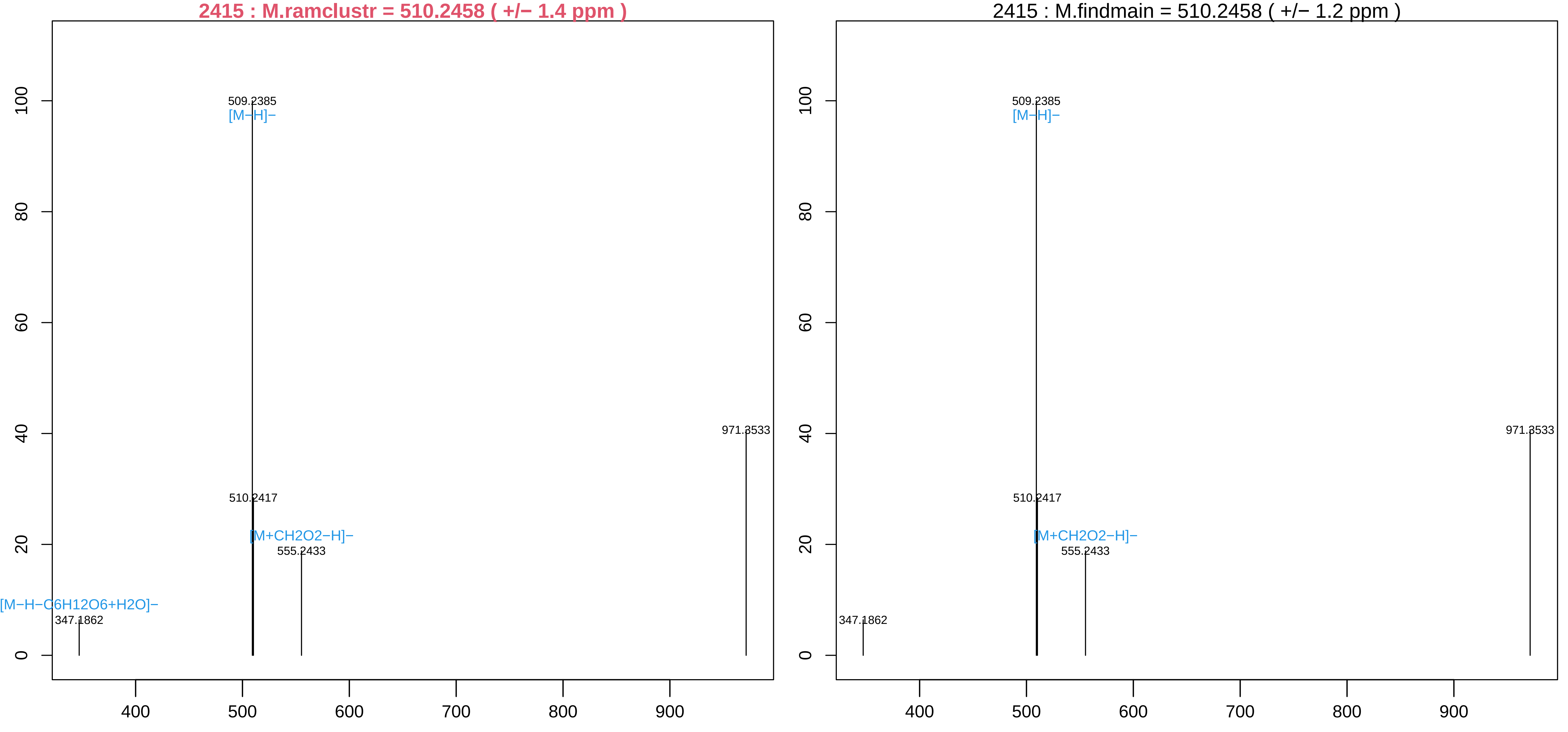
2415 : M.ramclustr = 510.2458 (+/− 1.4 ppm)
509.2385
[M−H]−
971.3533
510.2417
[M+CH2O2−H]−
555.2433
[M−H−C6H12O6+H2O]−
347.1862
0
20
40
60
80
100
400
500
600
700
800
900
2415 : M.findmain = 510.2458 (+/− 1.2 ppm)
509.2385
[M−H]−
971.3533
510.2417
[M+CH2O2−H]−
555.2433
347.1862
0
20
40
60
80
100
400
500
600
700
800
900

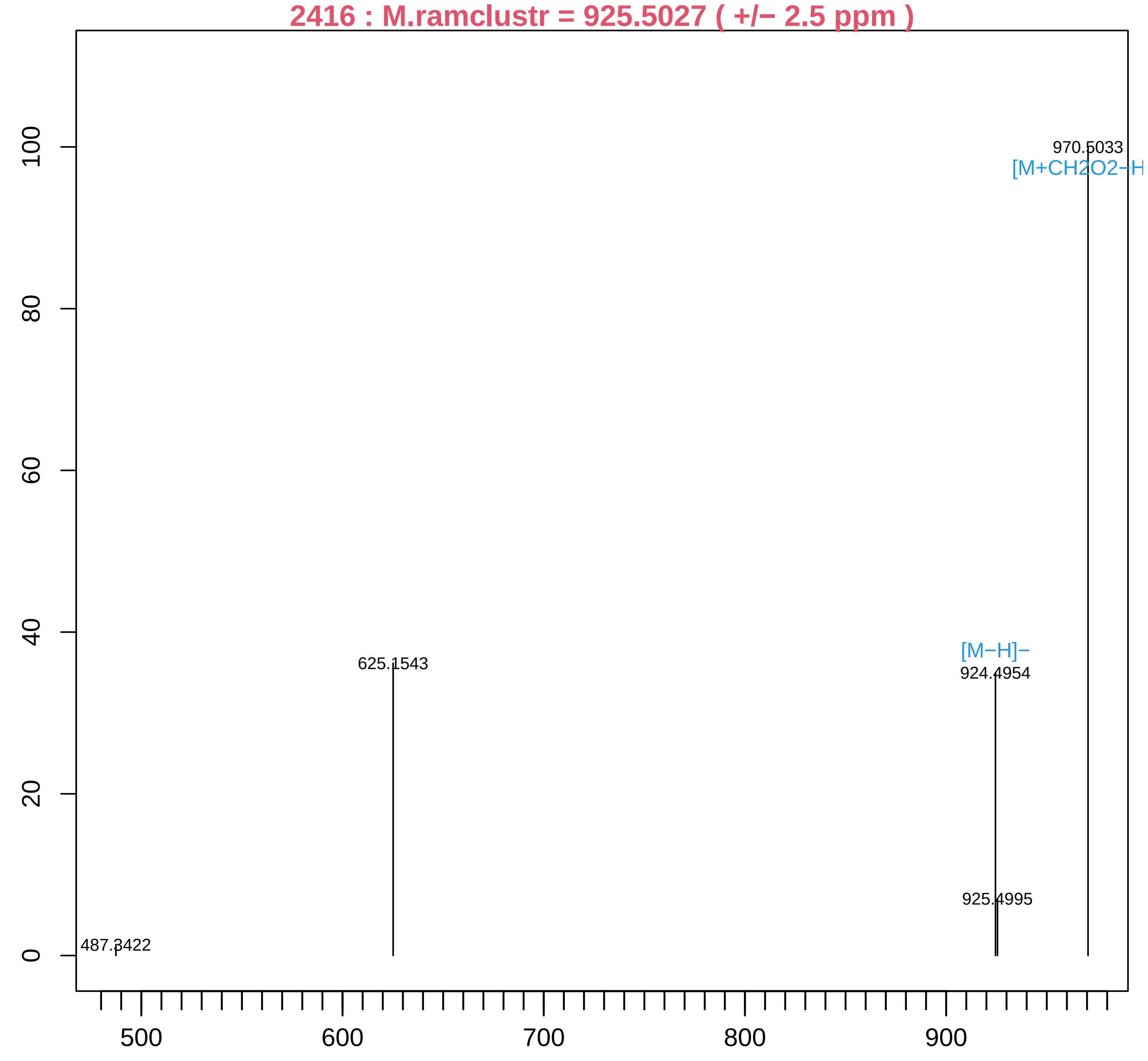
2416 : M.ramclustr = 925.5027 (+/− 2.5 ppm)
970.5033
[M+CH2O2−H
[M−H]−
625.1543
924.4954
925.4995
487.3422
0
20
40
60
80
100
500
600
700
800
900

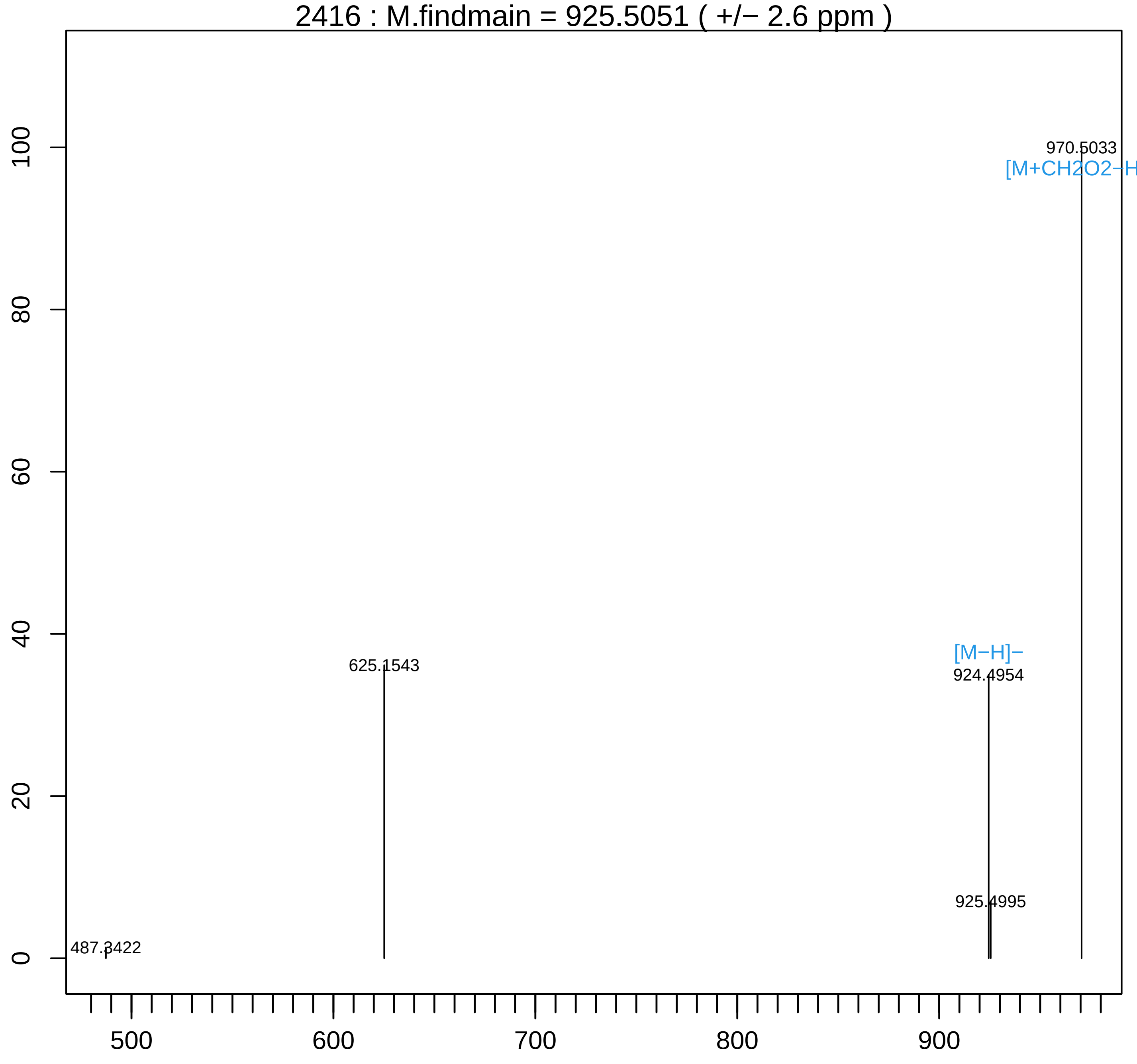
2416 : M.findmain = 925.5051 (+/− 2.6 ppm)
970.5033
[M+CH2O2−H
[M−H]−
625.1543
924.4954
925.4995
487.3422
0
20
40
60
80
100
500
600
700
800
900

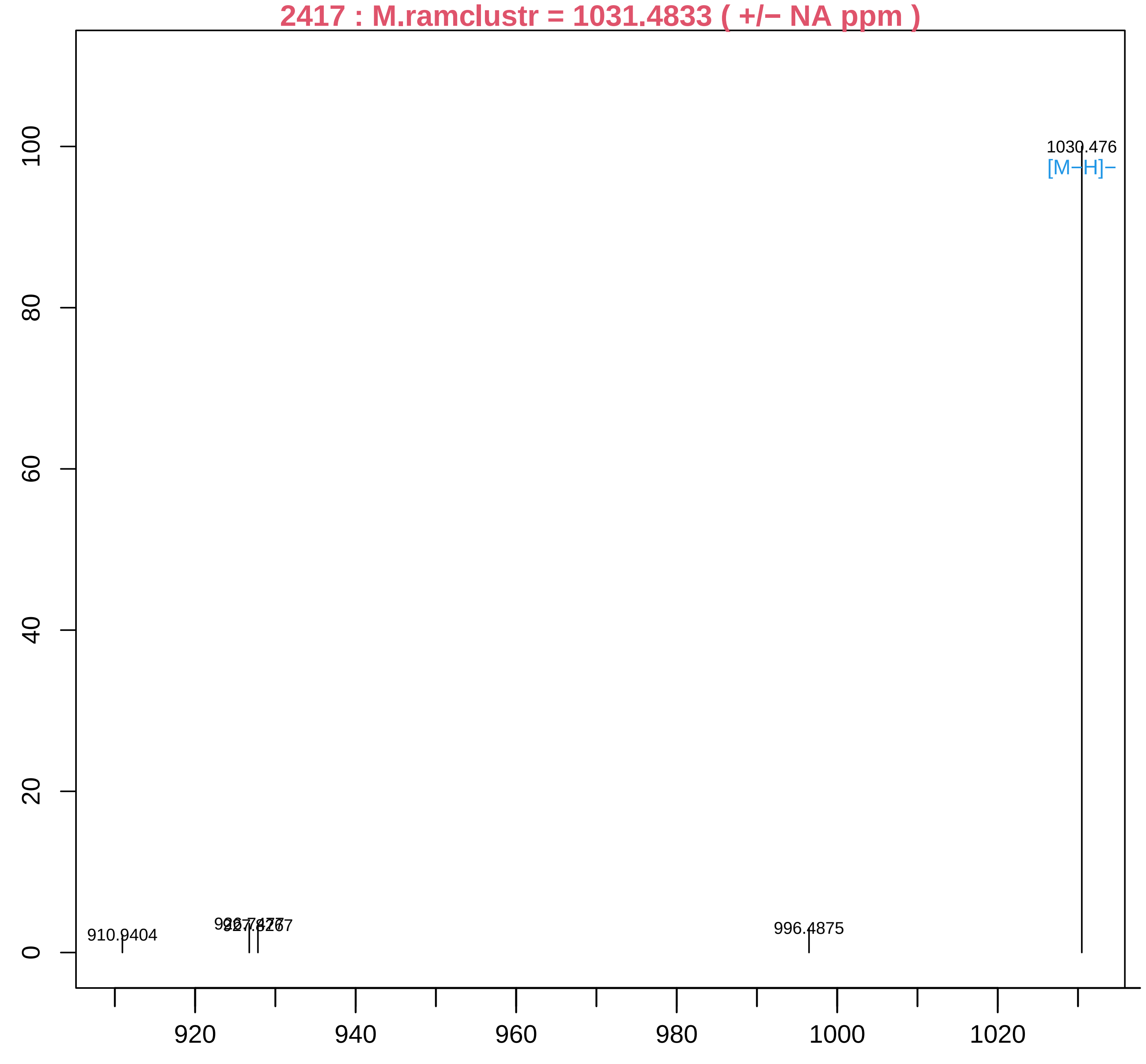
2417 : M.ramclustr = 1031.4833 (+/− NA ppm)
1030.476
[M−H]−
910.9404
996.4875
0
20
40
60
80
100
920
940
960
980
1000
1020

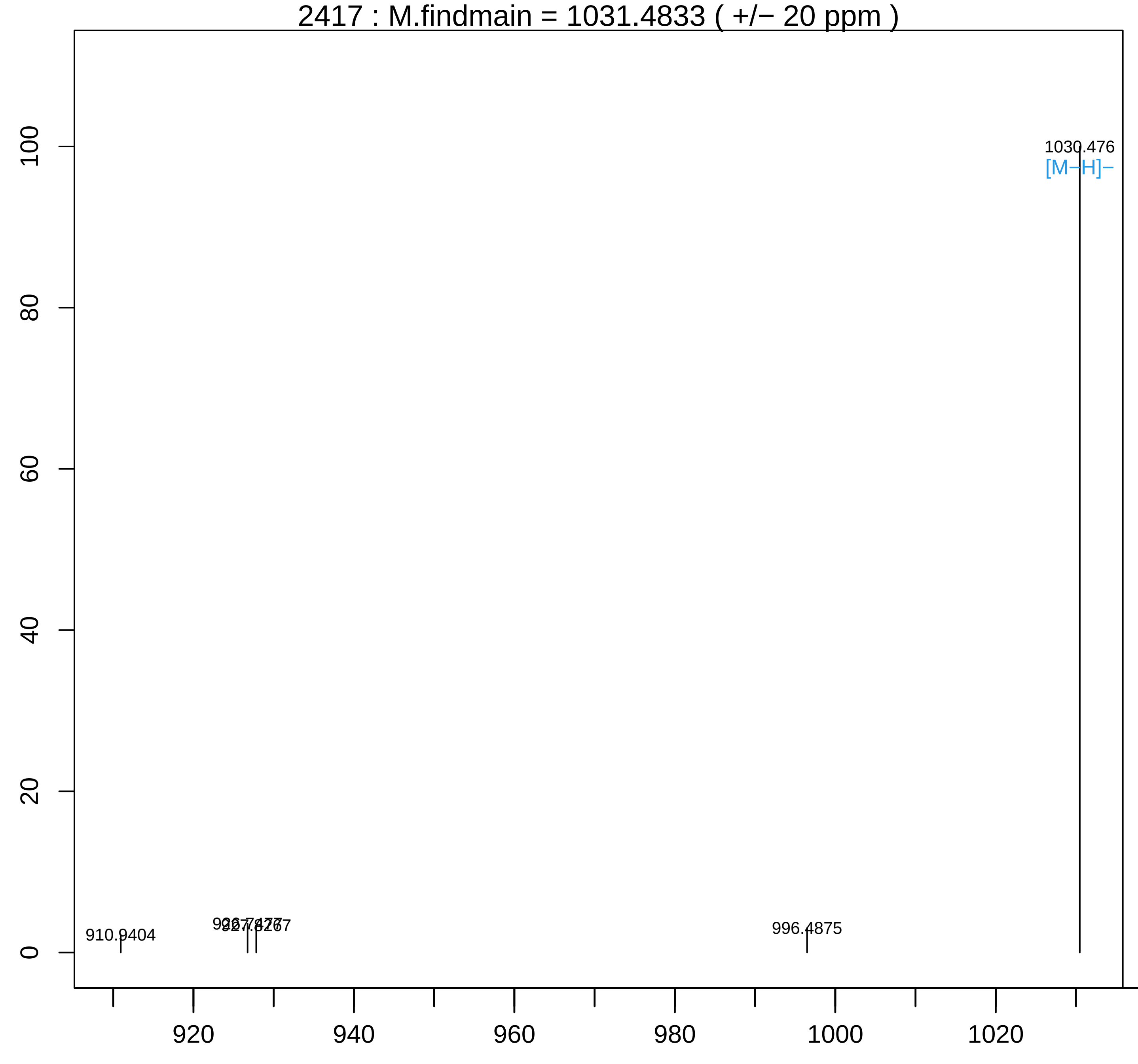
2417 : M.findmain = 1031.4833 (+/− 20 ppm)
1030.476
[M−H]−
910.9404
996.4875
0
20
40
60
80
100
920
940
960
980
1000
1020

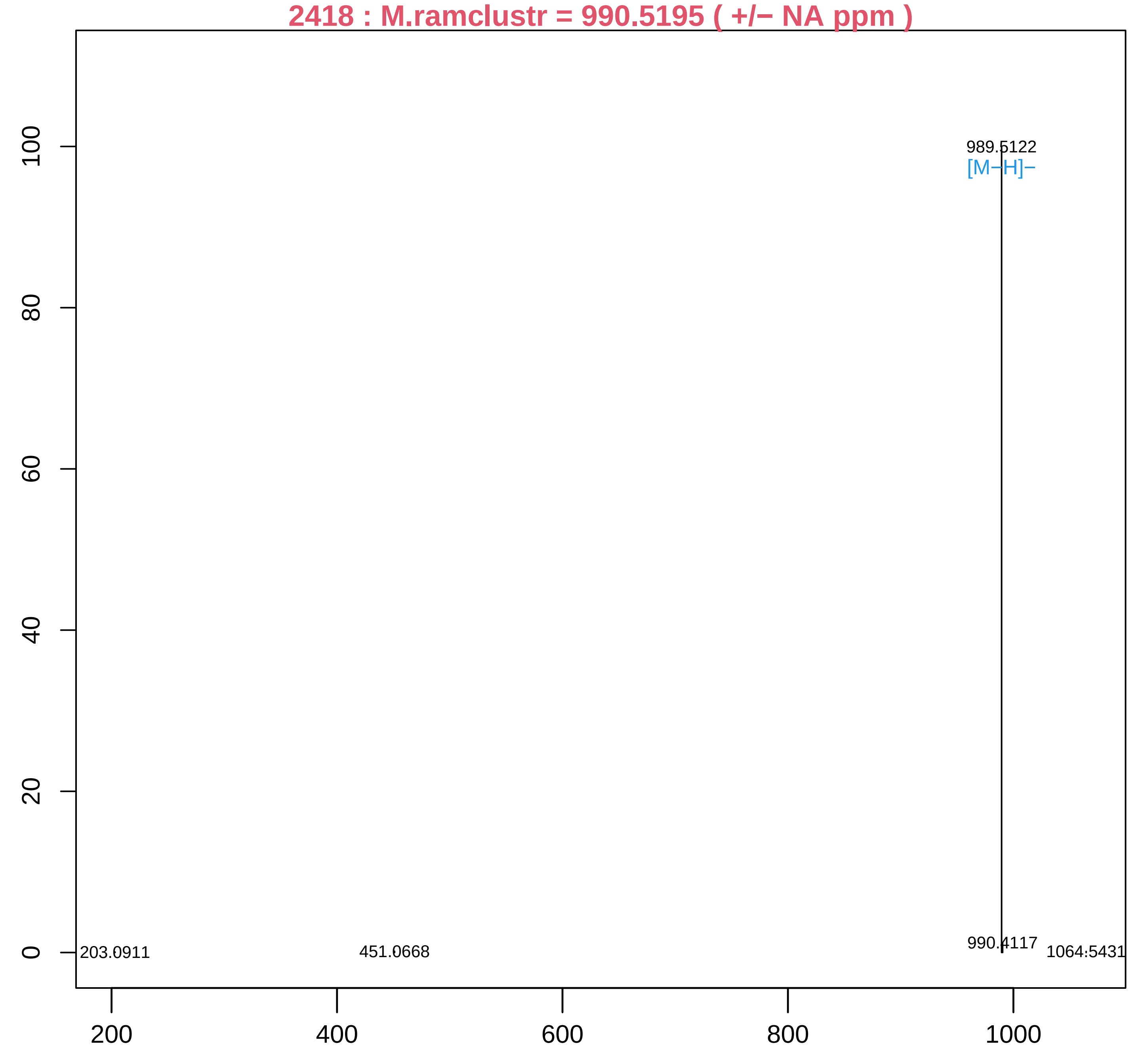
2418 : M.ramclustr = 990.5195 (+/− NA ppm)
989.5122
[M−H]−
990.4117
203.0911
451.0668
1064.5431
0
20
40
60
80
100
200
400
600
800
1000

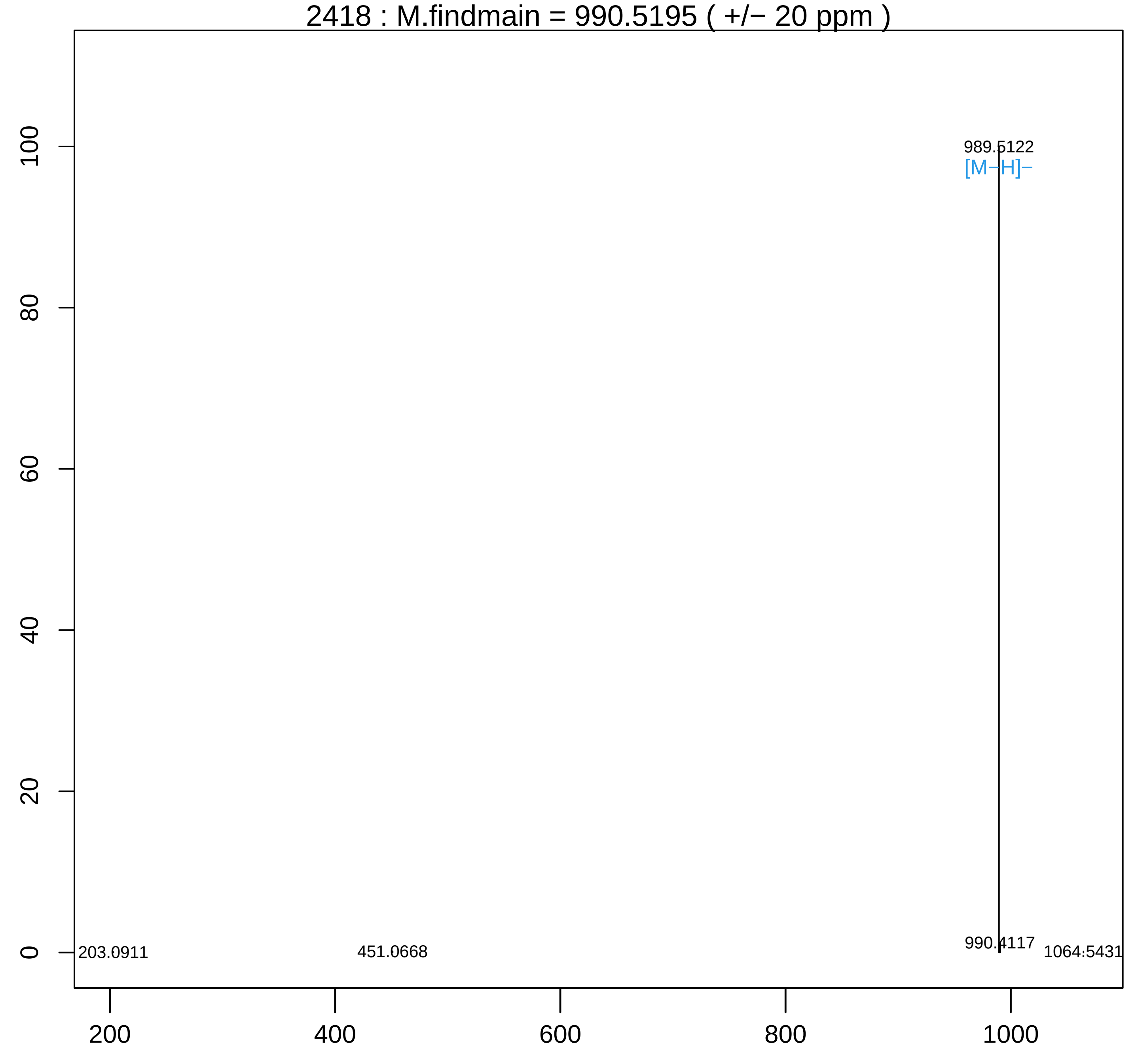
2418 : M.findmain = 990.5195 (+/− 20 ppm)
989.5122
[M−H]−
990.4117
203.0911
451.0668
1064.5431
0
20
40
60
80
100
200
400
600
800
1000

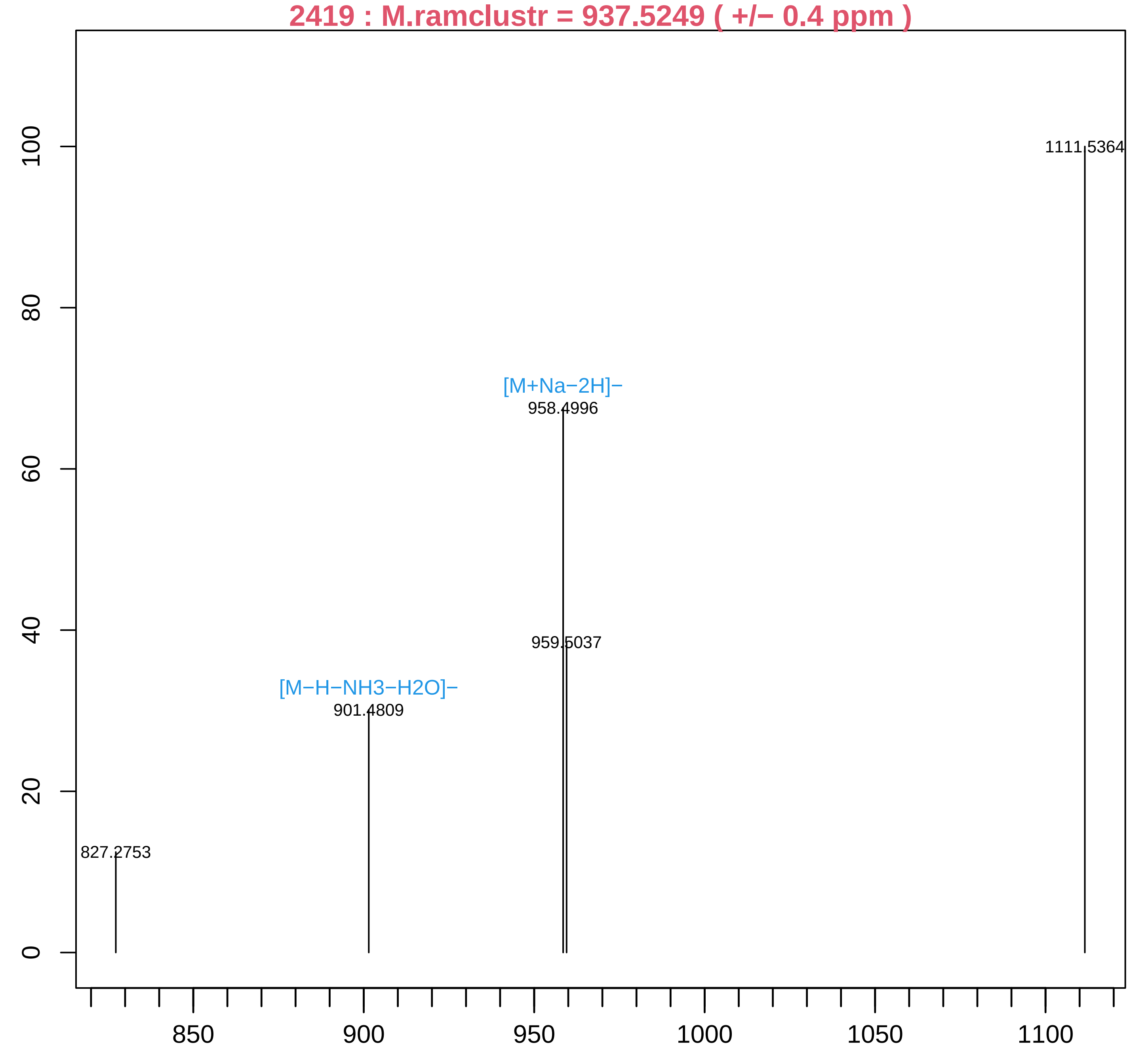

2419 : M.ramclustr = 937.5249 (+/− 0.4 ppm)
[M+Na−2H]−
958.4996
959.5037
[M−H−NH3−H2O]−
901.4809
827.2753
1111.5364
0
20
40
60
80
100
850
900
950
1000
1050
1100

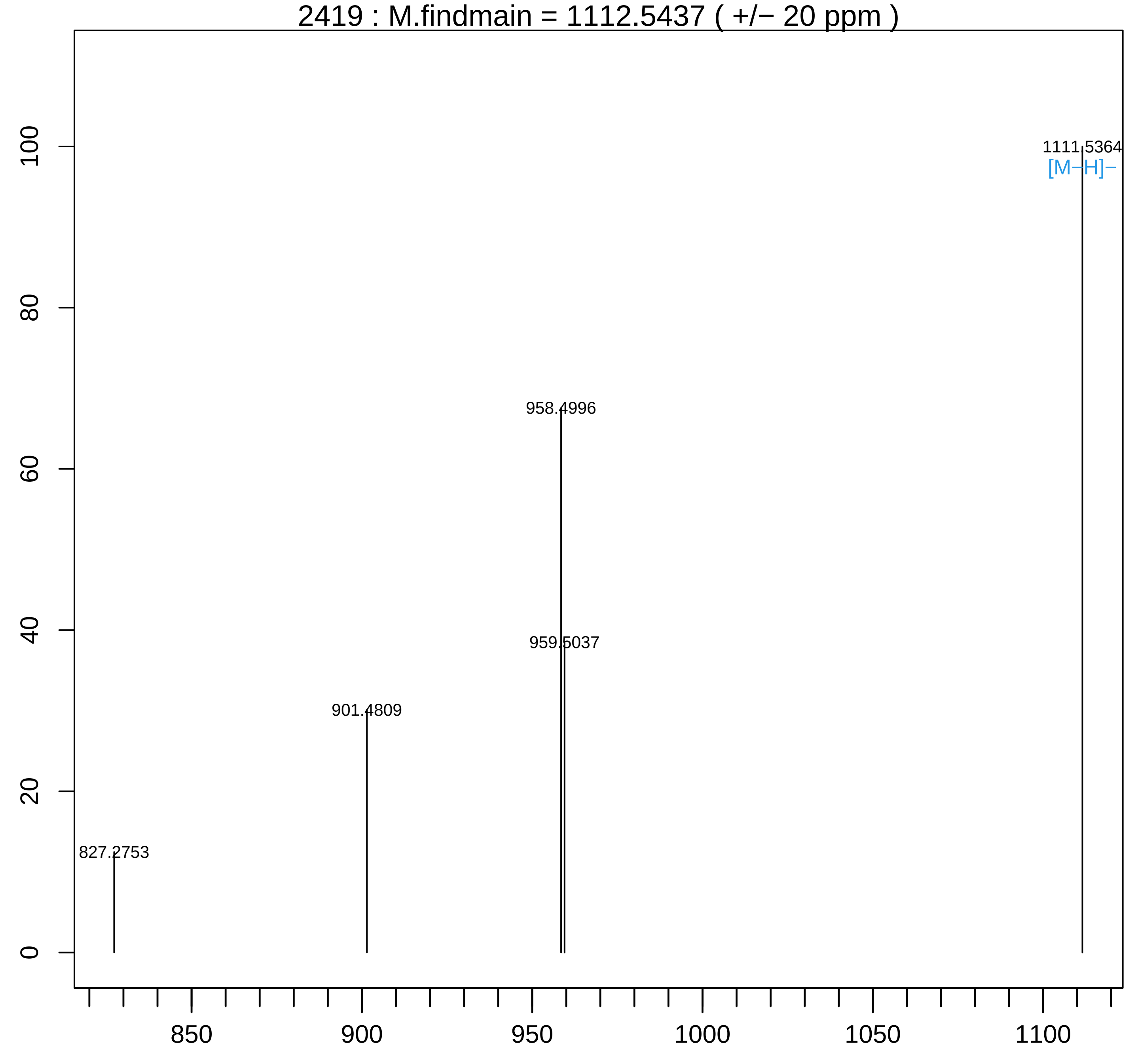

2419 : M.findmain = 1112.5437 (+/− 20 ppm)
1111.5364
[M−H]−
958.4996
959.5037
901.4809
827.2753
0
20
40
60
80
100
850
900
950
1000
1050
1100

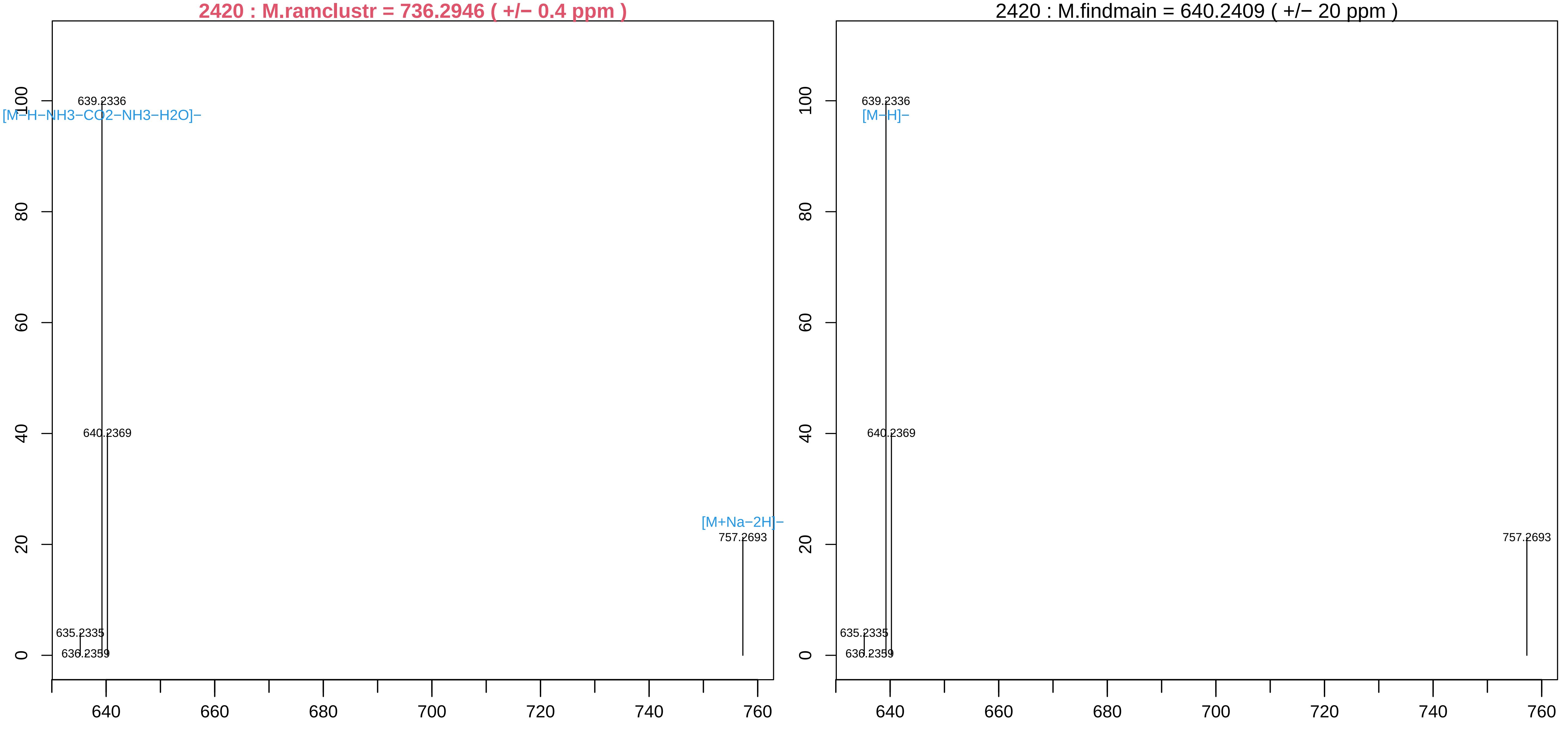
2420 : M.ramclustr = 736.2946 (+/− 0.4 ppm)
639.2336
[M−H−NH3−CO2−NH3−H2O]−
640.2369
635.2335
636.2359
[M+Na−2H]−
757.2693
2420 : M.findmain = 640.2409 (+/− 20 ppm)
639.2336
[M−H]−
640.2369
635.2335
636.2359
757.2693
0
20
40
60
80
100
640
660
680
700
720
740
760

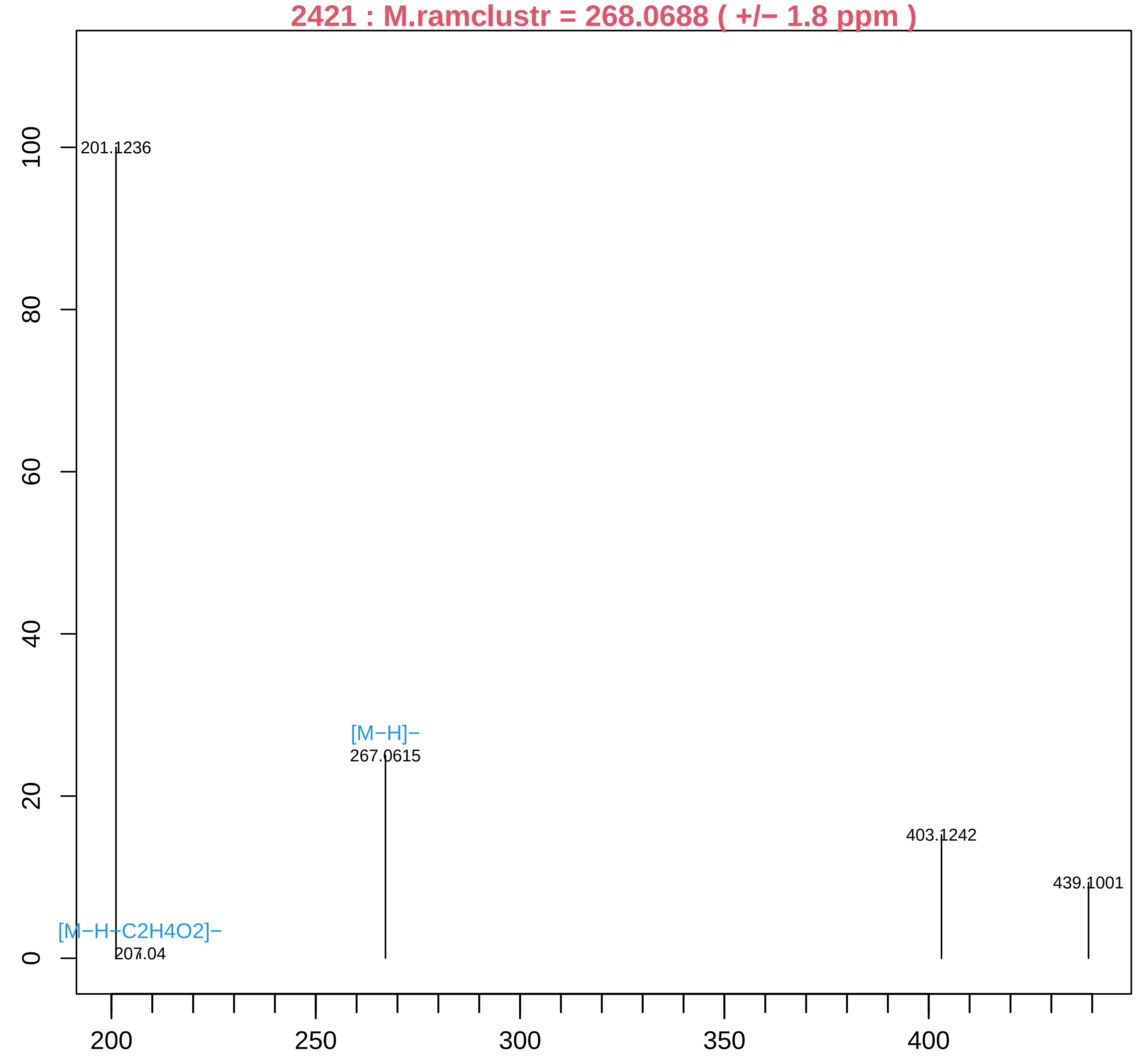
2421 : M.ramclustr = 268.0688 (+/− 1.8 ppm)
201.1236
[M−H]−
267.0615
403.1242
439.1001
[M−H−C2H4O2]−
207.04
0
20
40
60
80
100
200
250
300
350
400

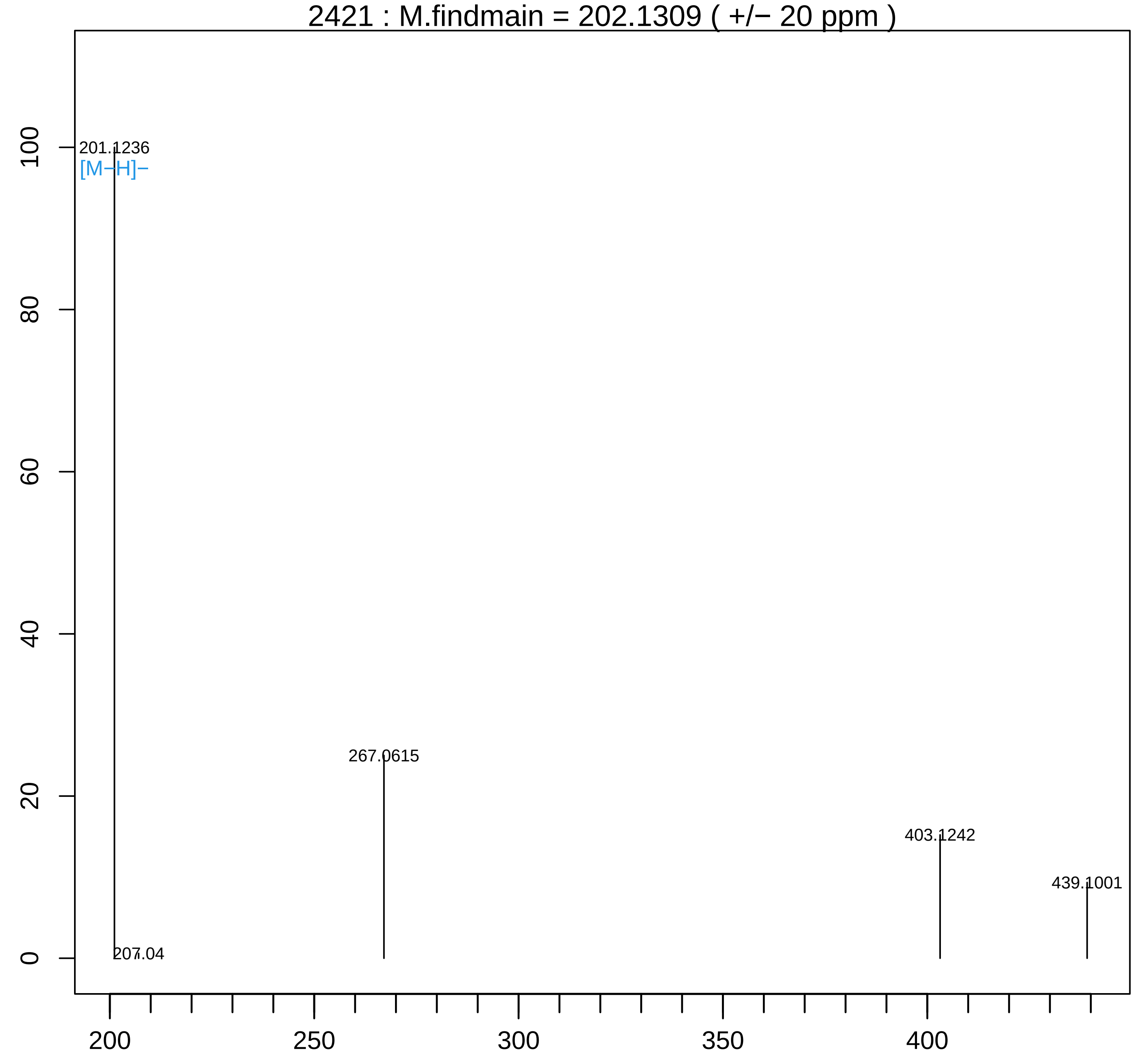
2421 : M.findmain = 202.1309 (+/− 20 ppm)
201.1236
[M−H]−
267.0615
403.1242
439.1001
207.04
0
20
40
60
80
100
200
250
300
350
400

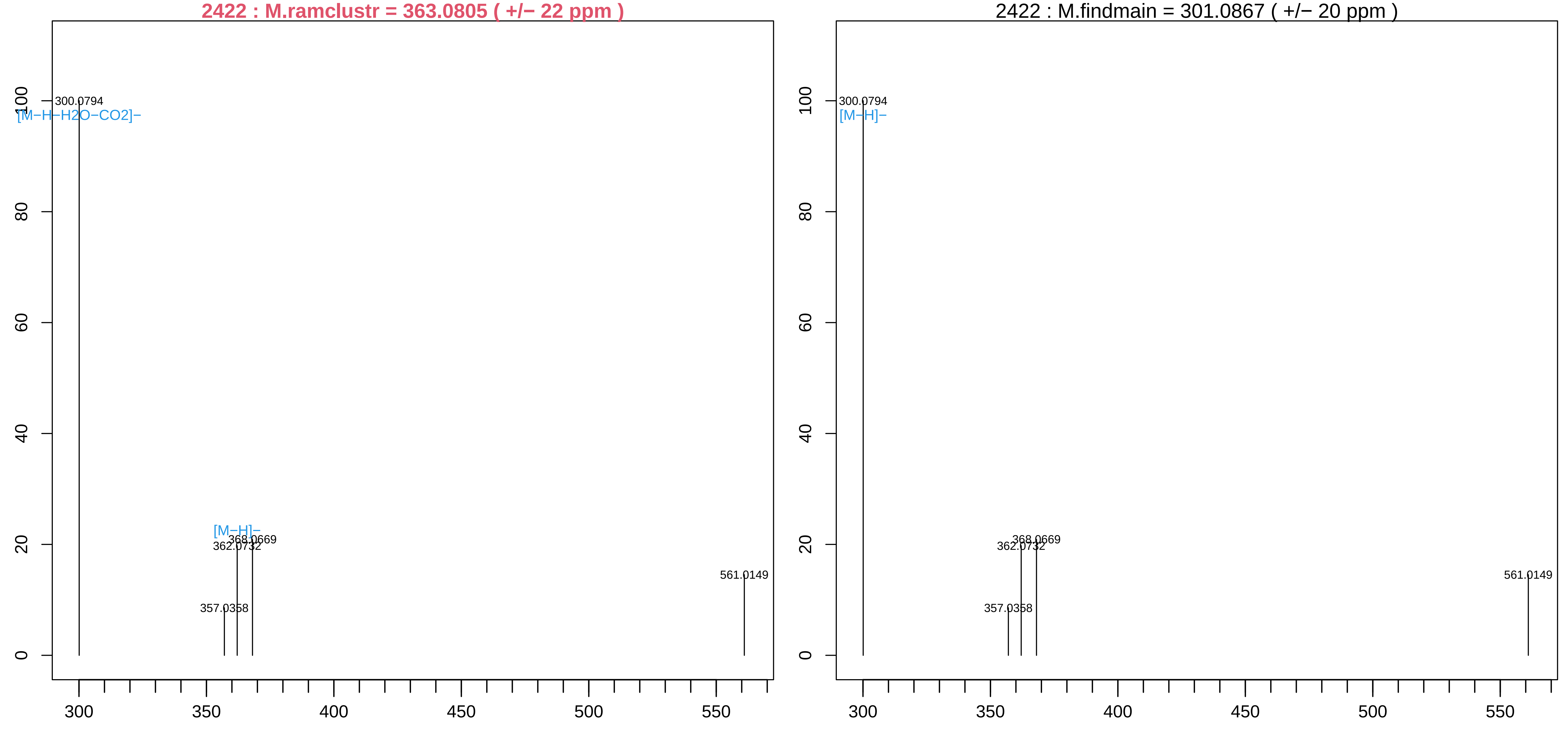

2422 : M.ramclustr = 363.0805 (+/− 22 ppm)
300.0794
[M−H−H2O−CO2]−
[M−H]−
368.0669
362.0732
357.0358
561.0149
2422 : M.findmain = 301.0867 (+/− 20 ppm)
300.0794
[M−H]−
368.0669
362.0732
357.0358
561.0149

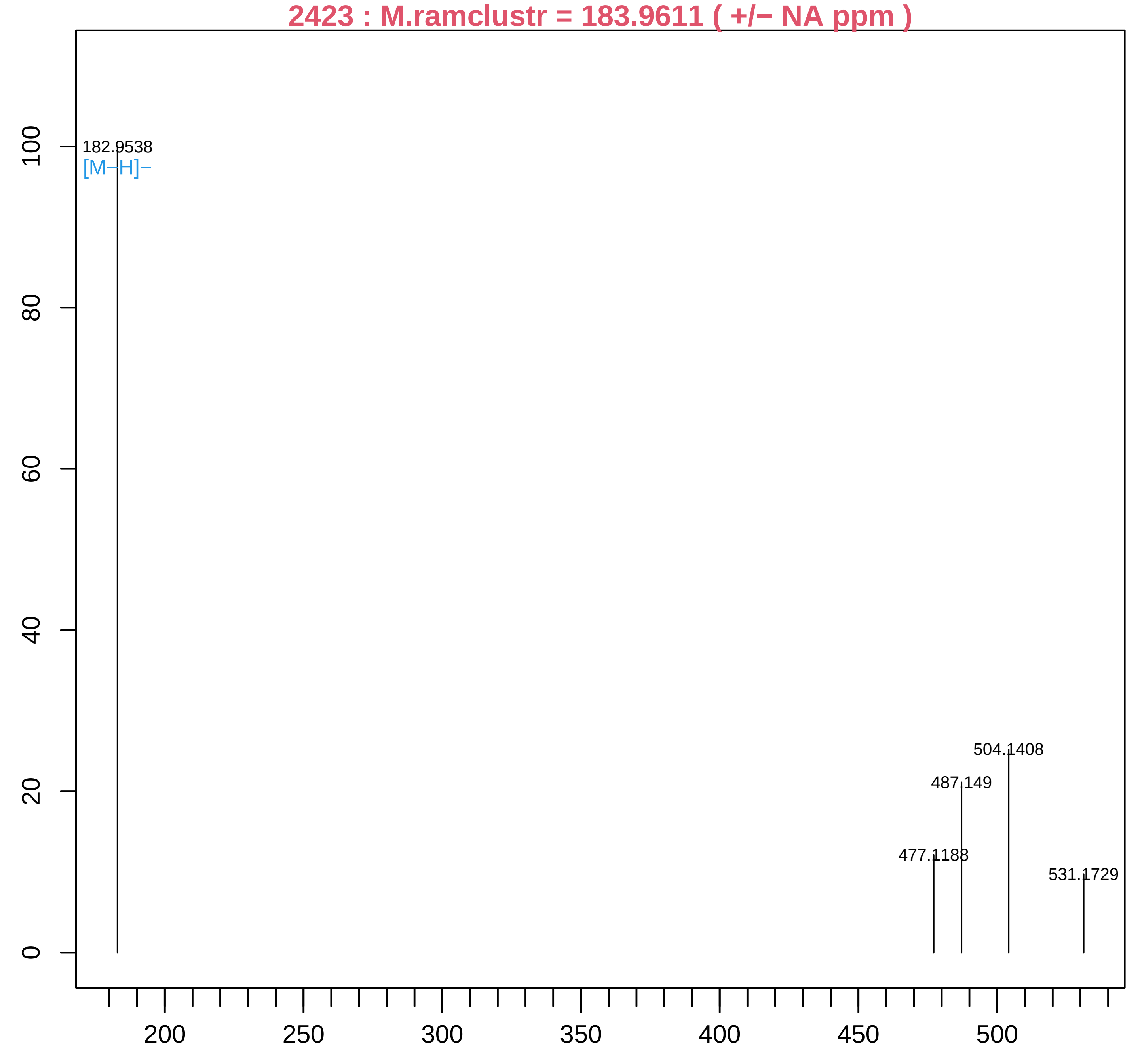
2423 : M.ramclustr = 183.9611 (+/− NA ppm)
182.9538
[M−H]−
504.1408
487.149
477.1188
531.1729
0
20
40
60
80
100
200
250
300
350
400
450
500

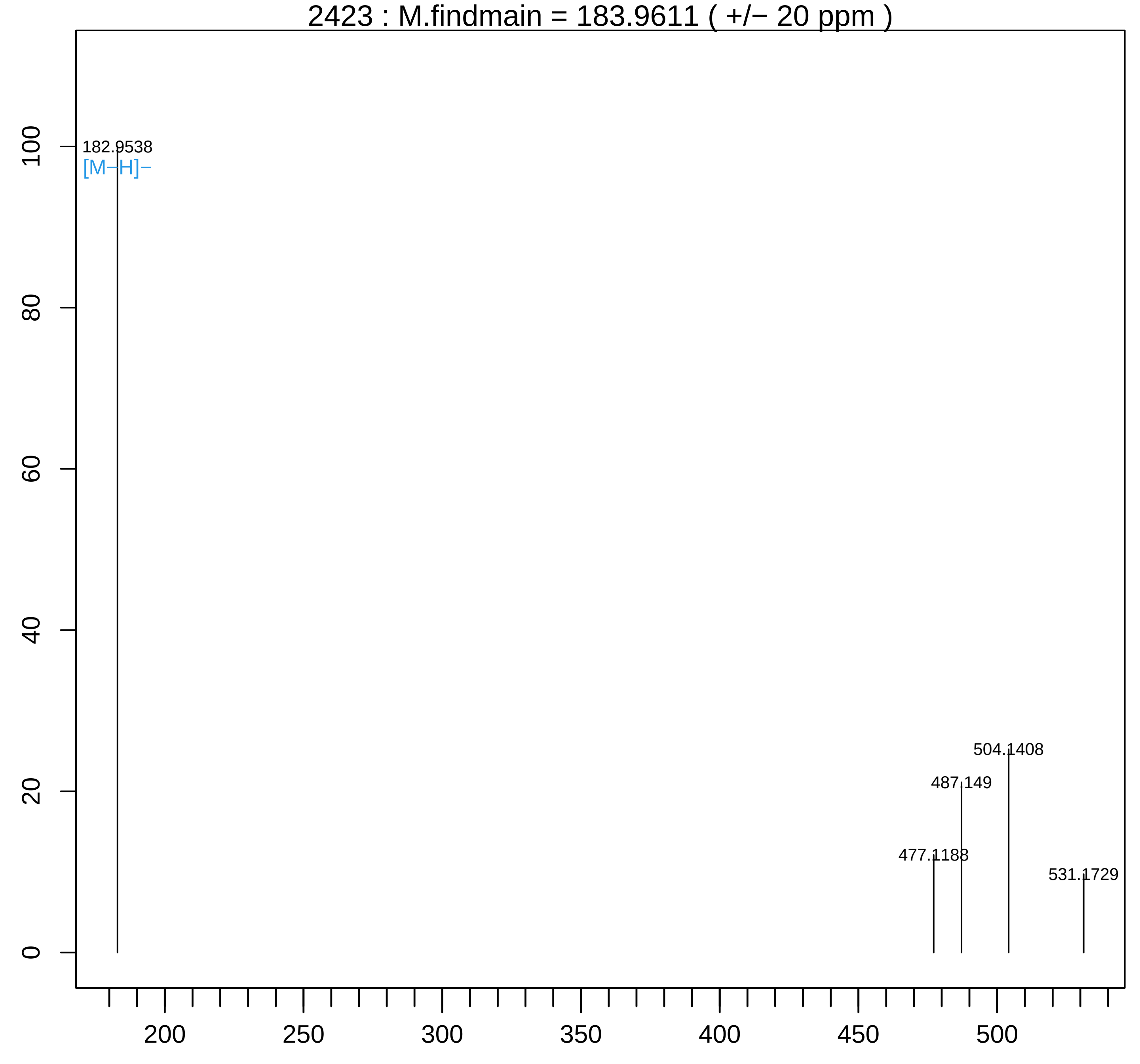
2423 : M.findmain = 183.9611 (+/− 20 ppm)
182.9538
[M−H]−
504.1408
487.149
477.1188
531.1729
0
20
40
60
80
100
200
250
300
350
400
450
500

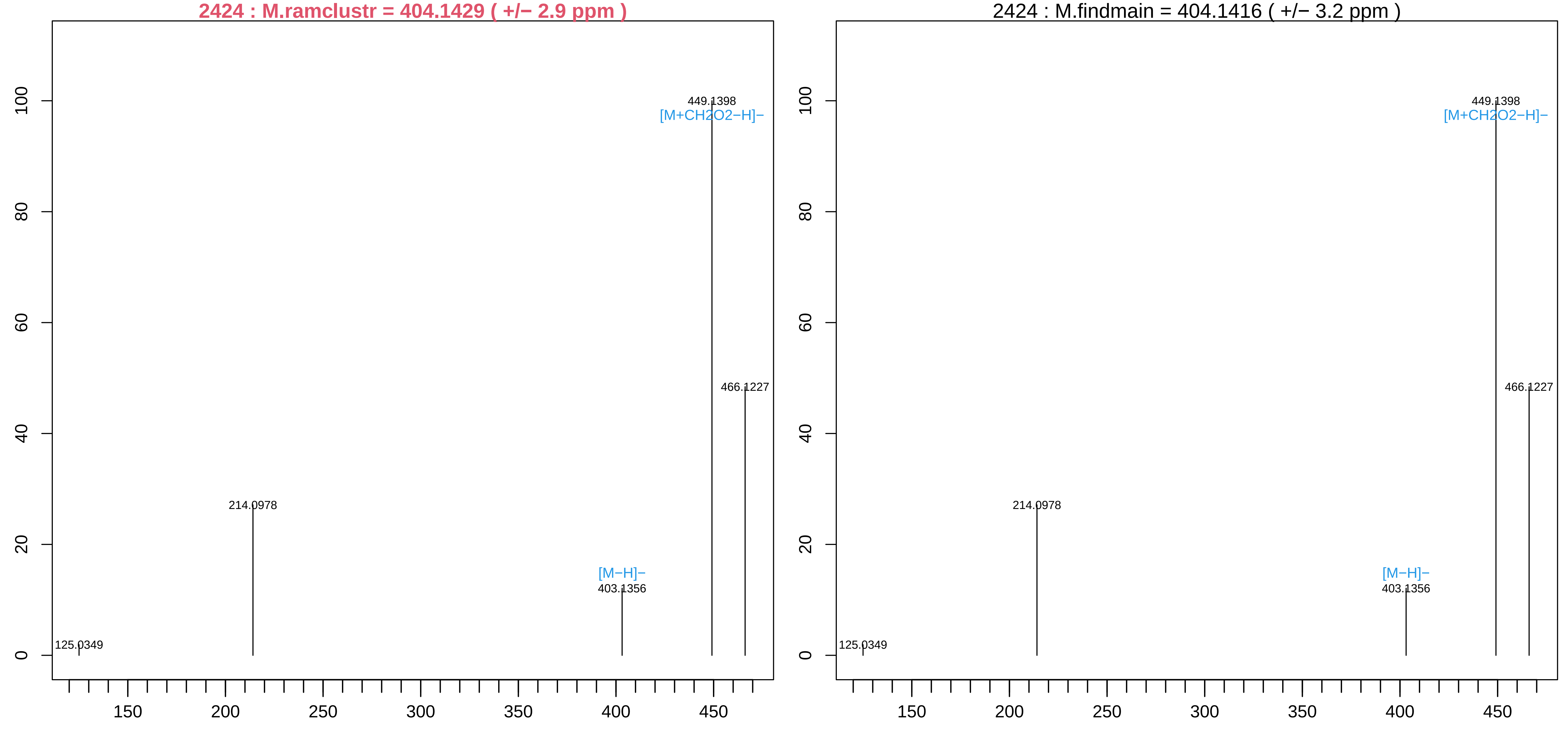
2424 : M.ramclustr = 404.1429 (+/− 2.9 ppm)
2424 : M.findmain = 404.1416 (+/− 3.2 ppm)
449.1398
[M+CH2O2−H]−
466.1227
214.0978
[M−H]−
403.1356
125.0349
0
20
40
60
80
100
150
200
250
300
350
400
450

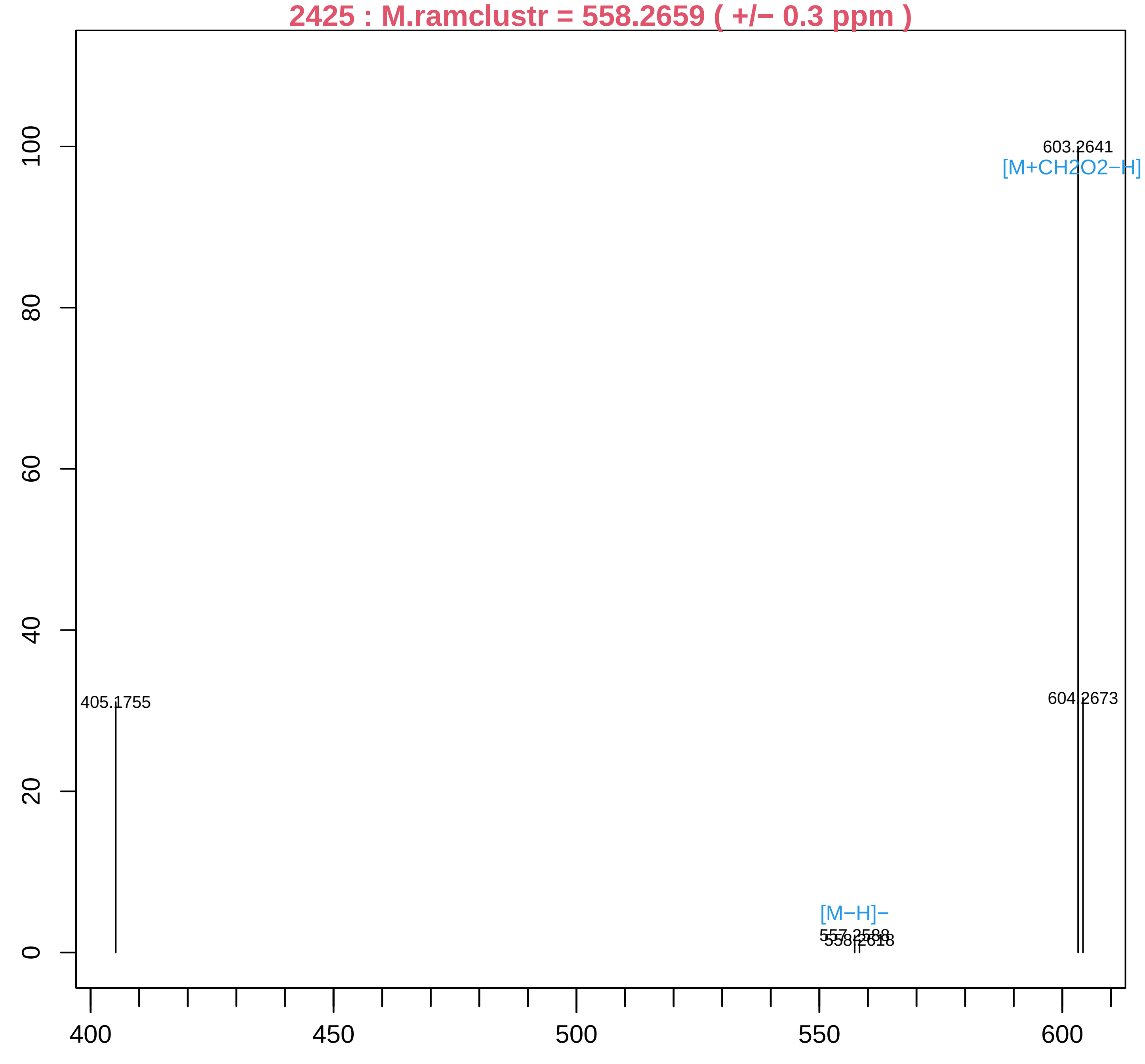

2425 : M.ramclustr = 558.2659 (+/− 0.3 ppm)
603.2641
[M+CH2O2−H]
405.1755
604.2673
[M−H]−
557.2588
558.2618
0
20
40
60
80
100
400
450
500
550
600

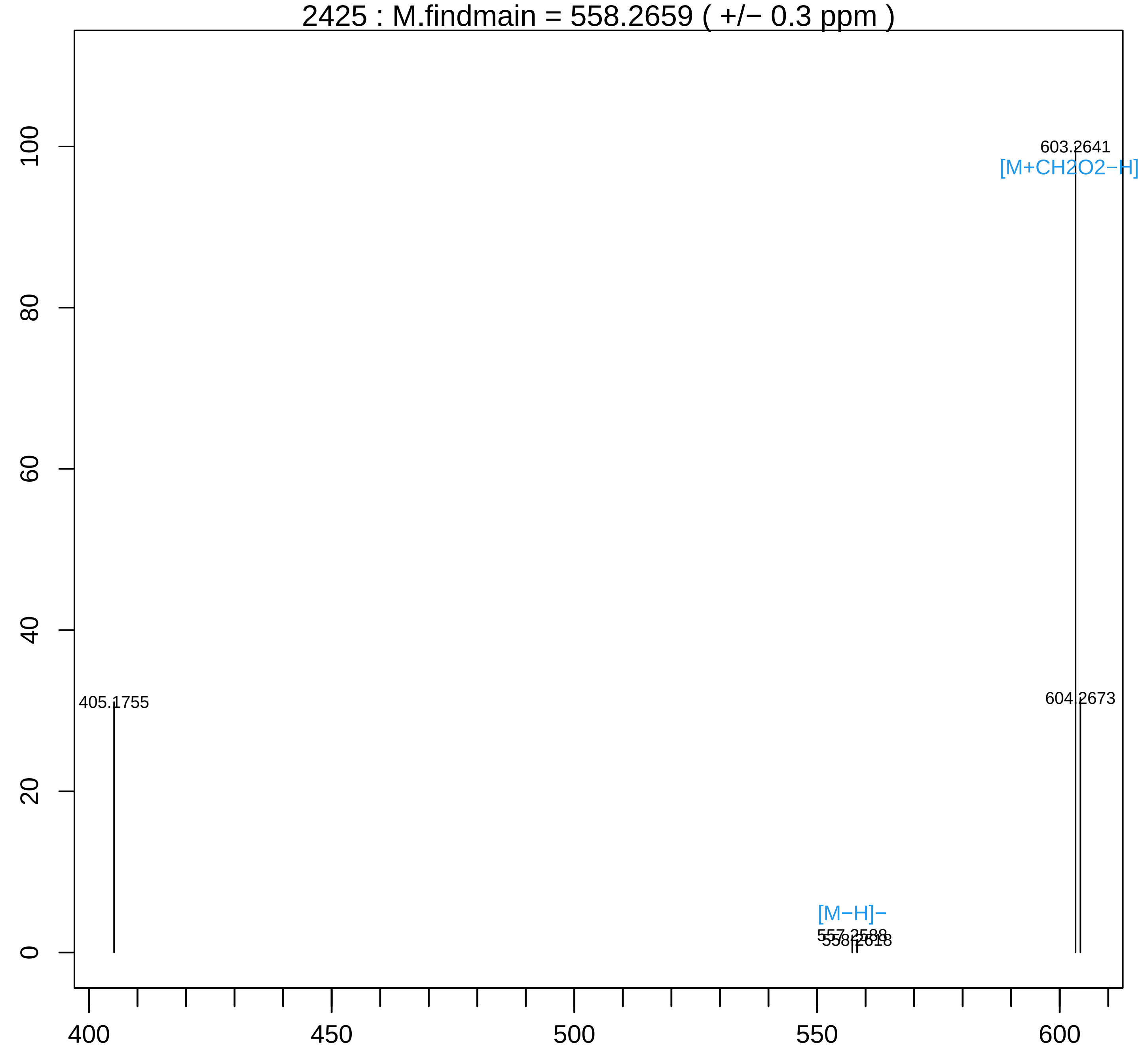

2425 : M.findmain = 558.2659 (+/− 0.3 ppm)
603.2641
[M+CH2O2−H]
405.1755
604.2673
[M−H]−
557.2588
558.2618
0
20
40
60
80
100
400
450
500
550
600

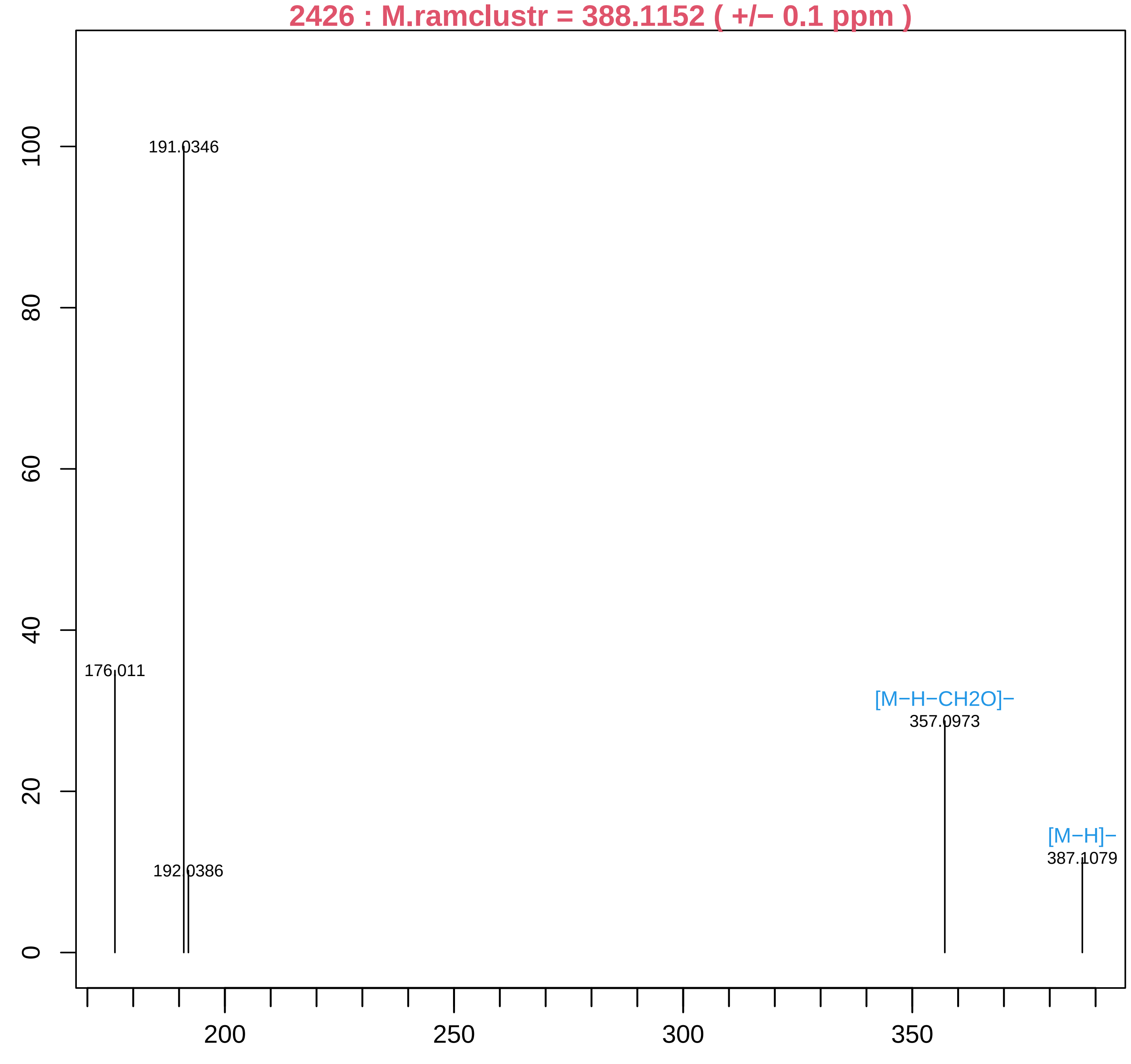
2426 : M.ramclustr = 388.1152 (+/− 0.1 ppm)
191.0346
176.011
192.0386
[M−H−CH2O]−
357.0973
[M−H]−
387.1079
0
20
40
60
80
100
200
250
300
350

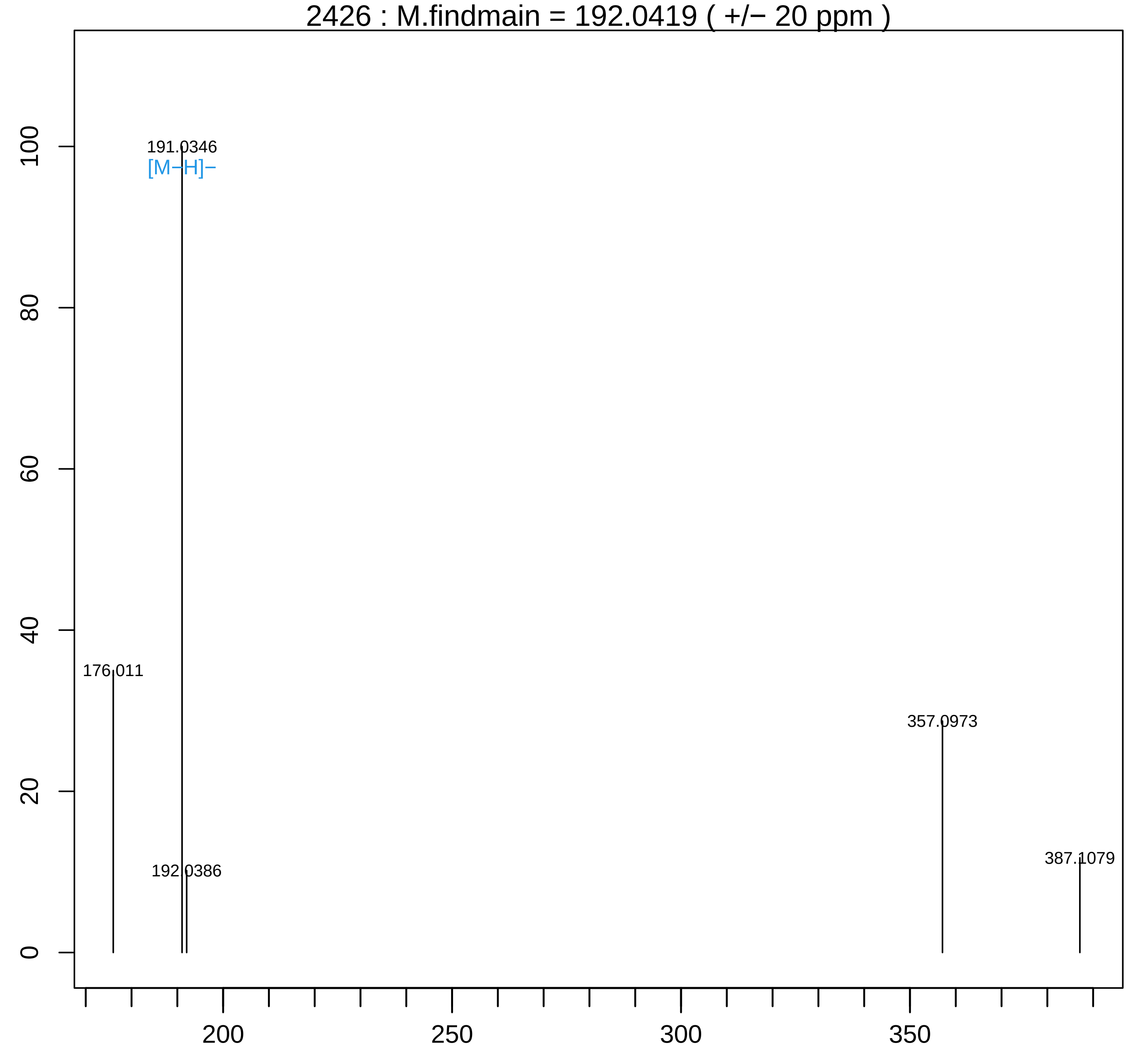
2426 : M.findmain = 192.0419 (+/− 20 ppm)
191.0346
[M−H]−
176.011
192.0386
357.0973
387.1079
0
20
40
60
80
100
200
250
300
350

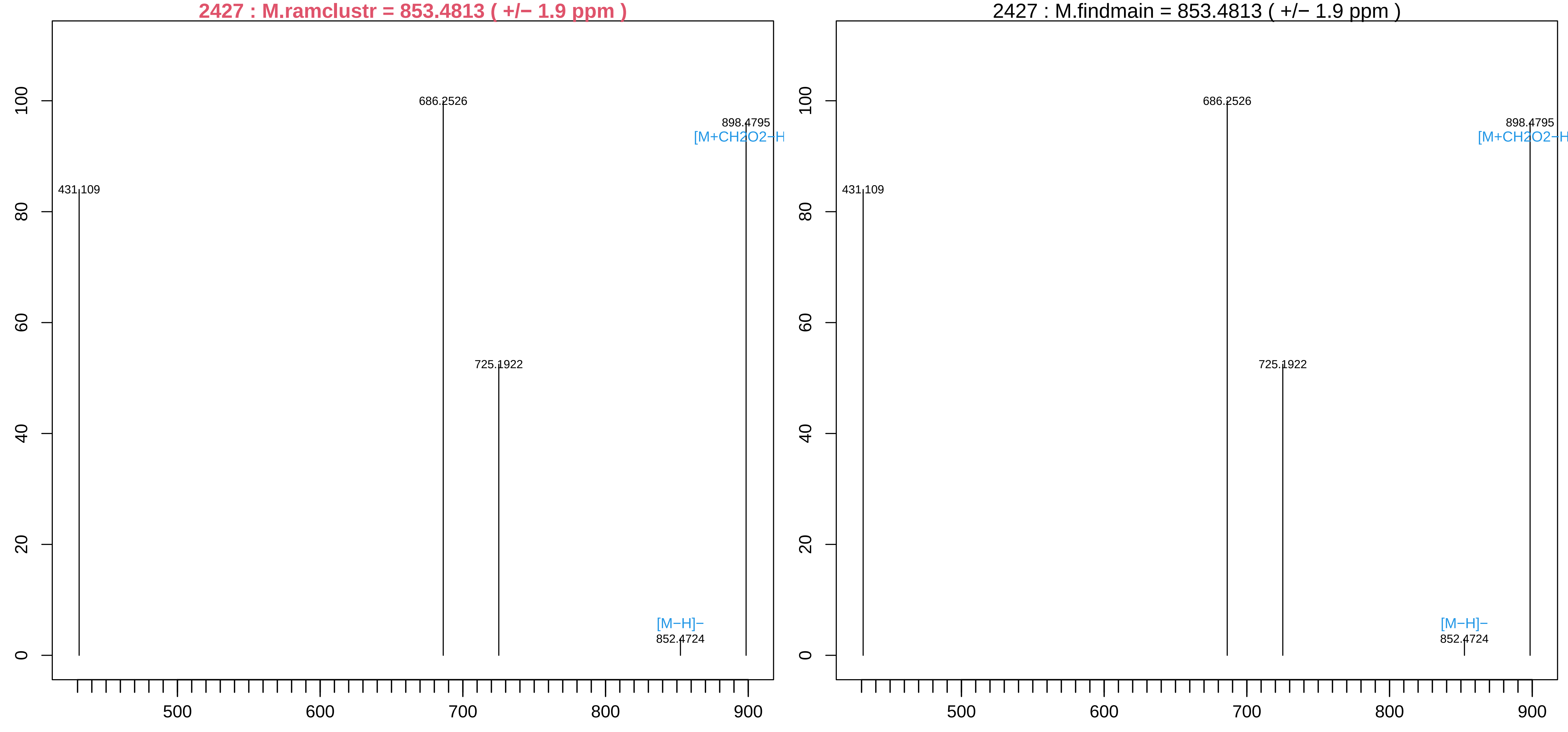

2427 : M.ramclustr = 853.4813 (+/− 1.9 ppm)
2427 : M.findmain = 853.4813 (+/− 1.9 ppm)
431.109
686.2526
725.1922
898.4795
[M+CH2O2−H
[M−H]−
852.4724
0
20
40
60
80
100
500
600
700
800
900

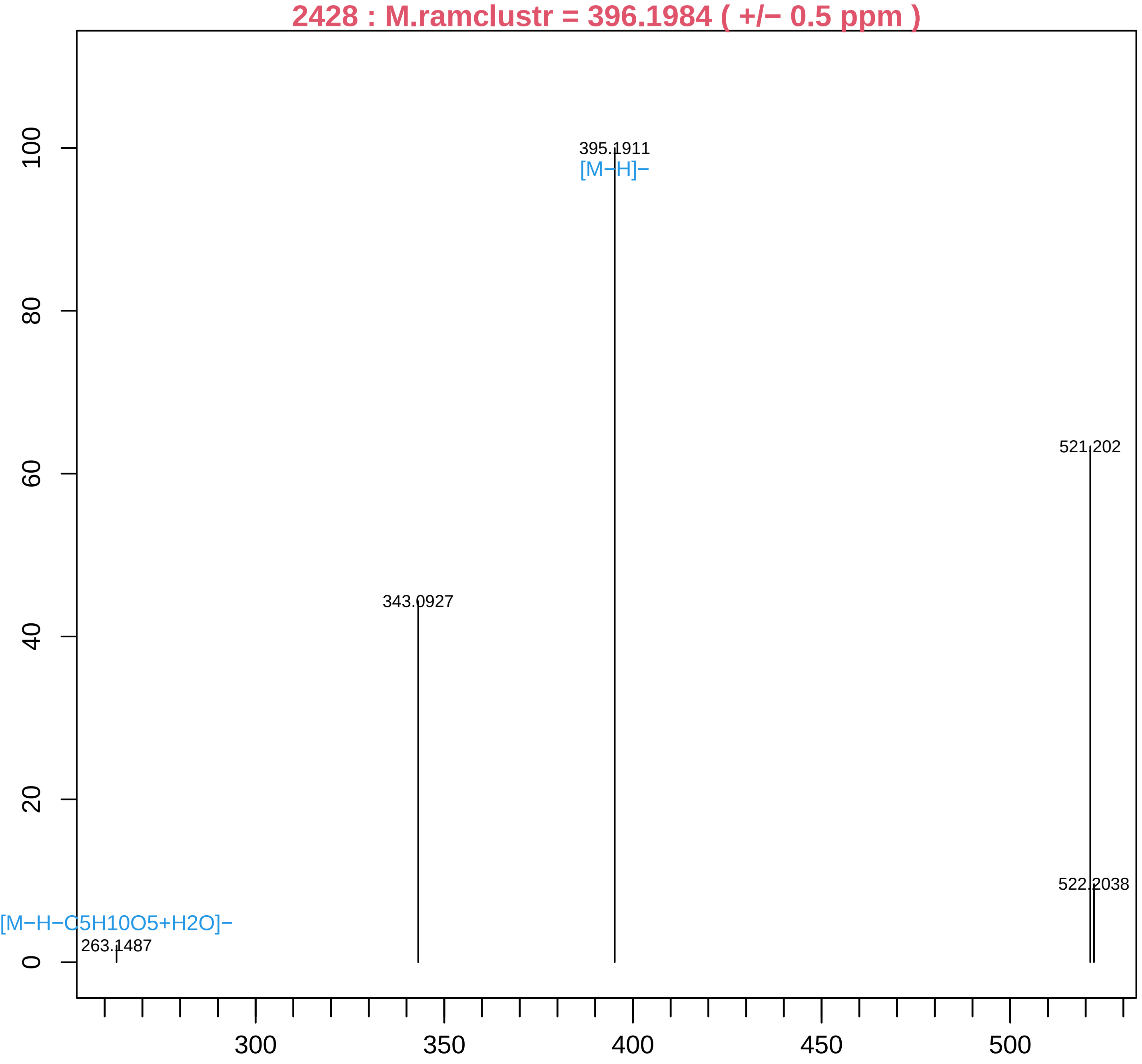

2428 : M.ramclustr = 396.1984 (+/− 0.5 ppm)
395.1911
[M−H]−
521.202
343.0927
522.2038
[M−H−C5H10O5+H2O]−
263.1487
0
20
40
60
80
100
300
350
400
450
500

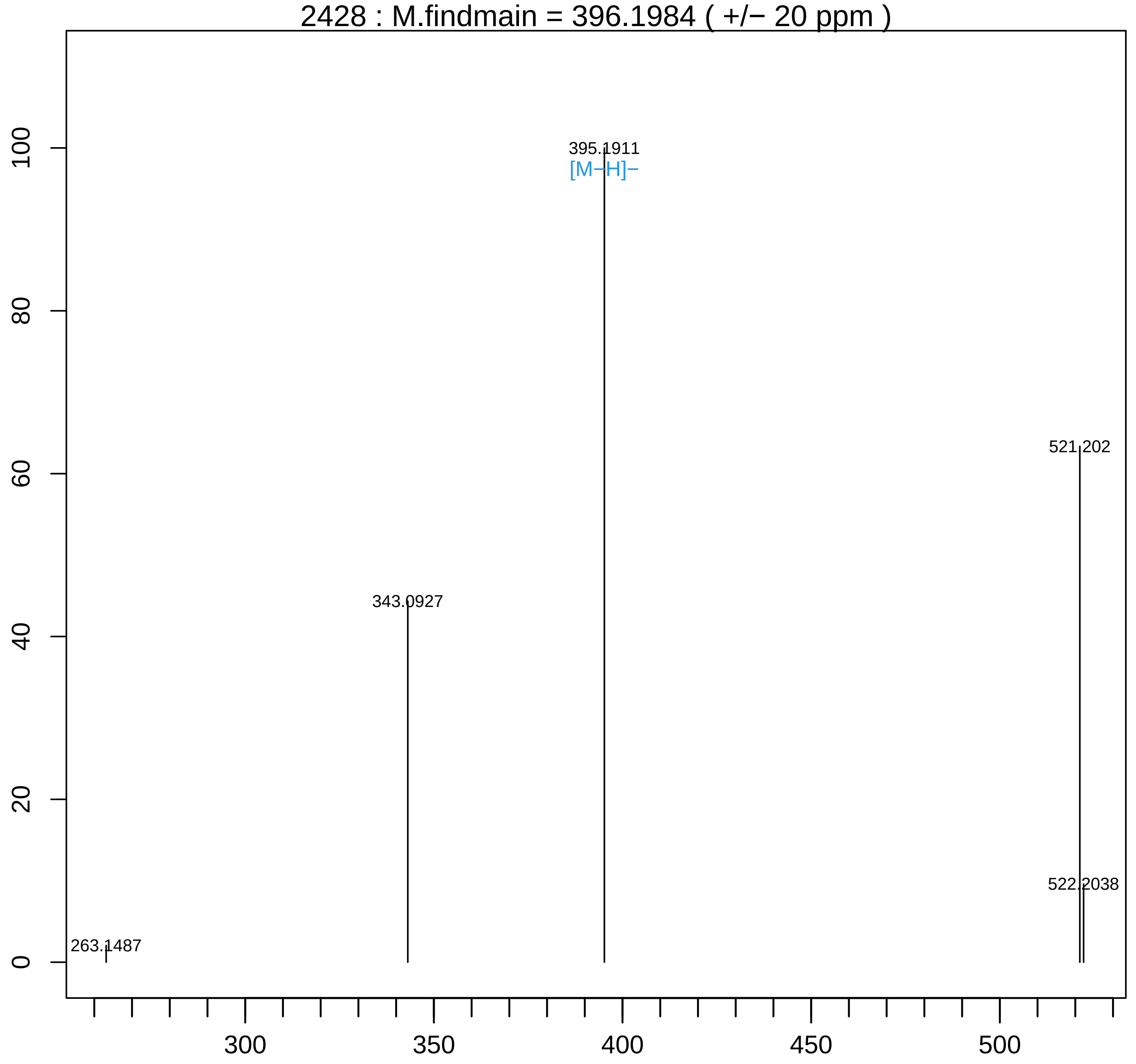

2428 : M.findmain = 396.1984 (+/− 20 ppm)
395.1911
[M−H]−
521.202
343.0927
522.2038
263.1487
0
20
40
60
80
100
300
350
400
450
500

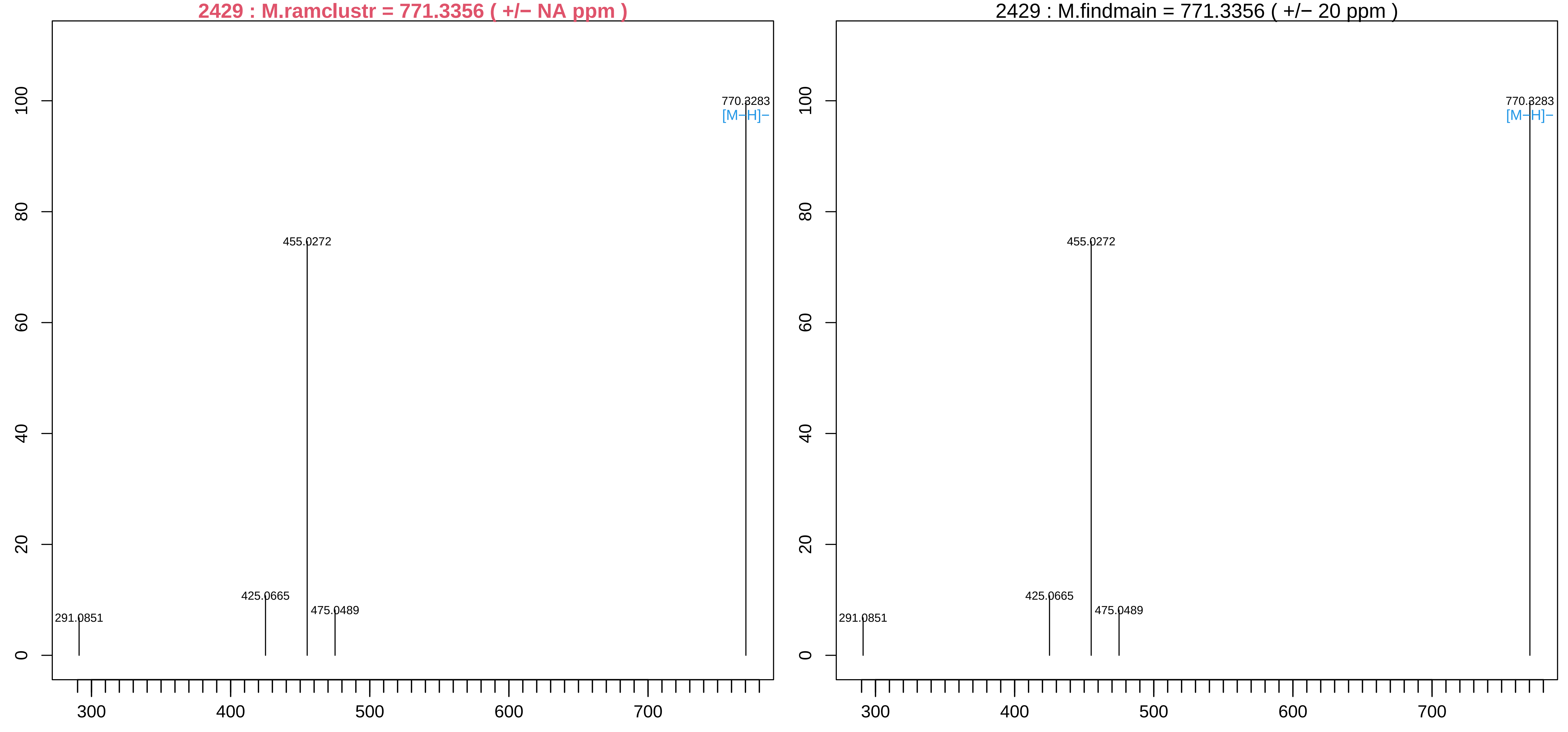

2429 : M.ramclustr = 771.3356 (+/− NA ppm)
770.3283
[M−H]−
455.0272
425.0665
475.0489
291.0851
0
20
40
60
80
100
300
400
500
600
700
2429 : M.findmain = 771.3356 (+/− 20 ppm)
770.3283
[M−H]−
455.0272
425.0665
475.0489
291.0851
0
20
40
60
80
100
300
400
500
600
700

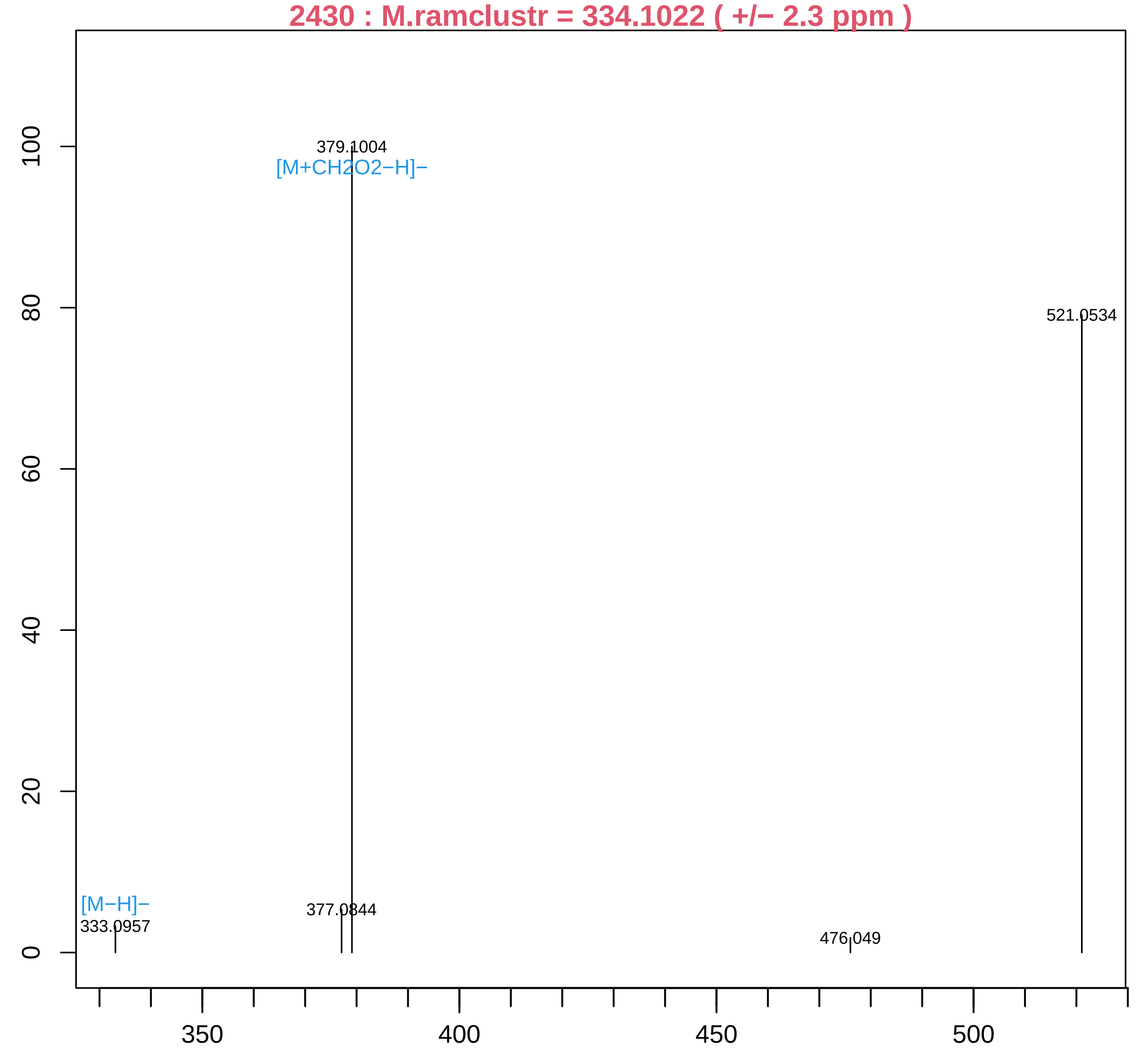
2430 : M.ramclustr = 334.1022 (+/− 2.3 ppm)
379.1004
[M+CH2O2−H]−
521.0534
[M−H]−
333.0957
377.0844
476.049
0
20
40
60
80
100
350
400
450
500

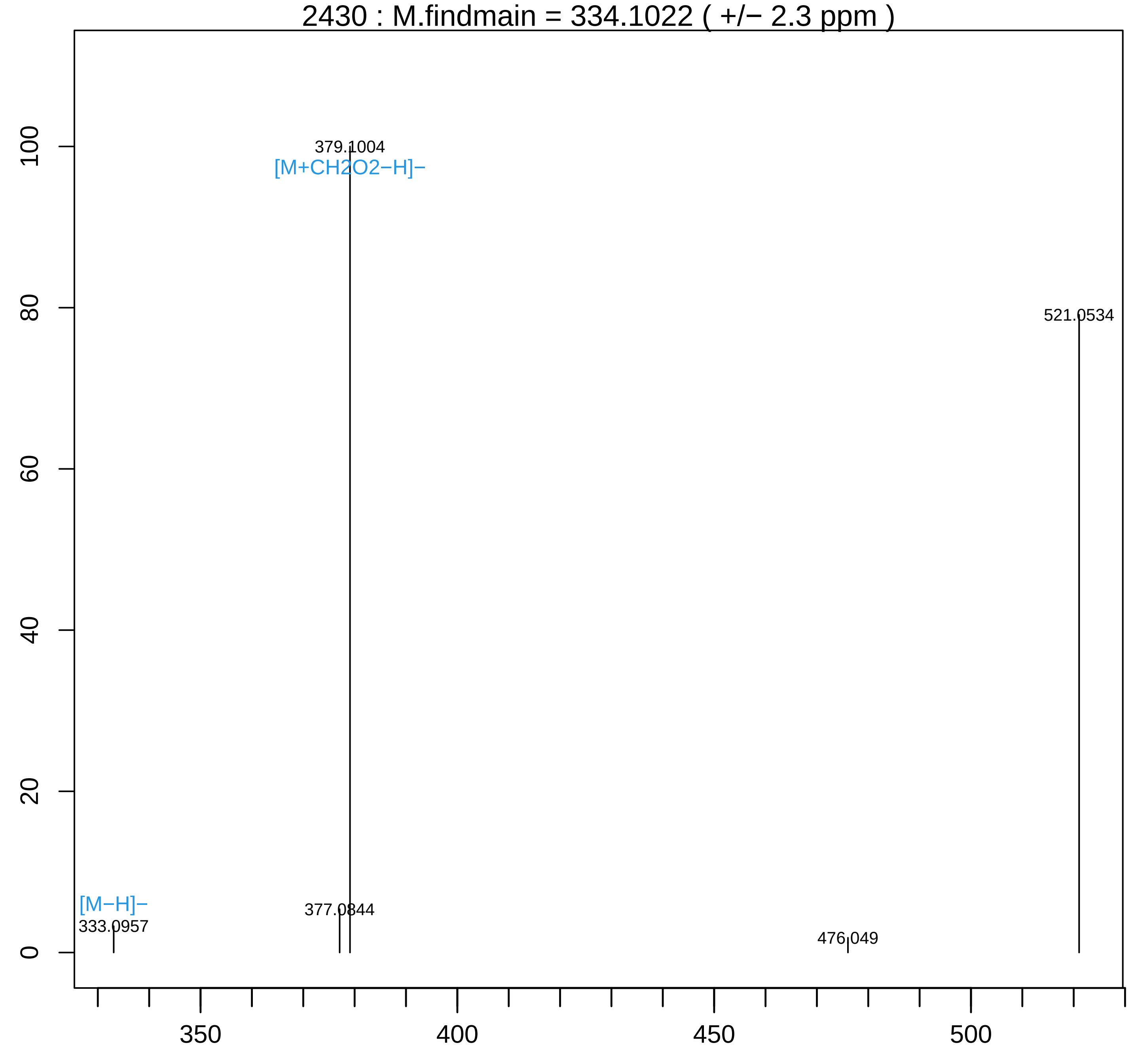
2430 : M.findmain = 334.1022 (+/− 2.3 ppm)
379.1004
[M+CH2O2−H]−
521.0534
[M−H]−
333.0957
377.0844
476.049
0
20
40
60
80
100
350
400
450
500

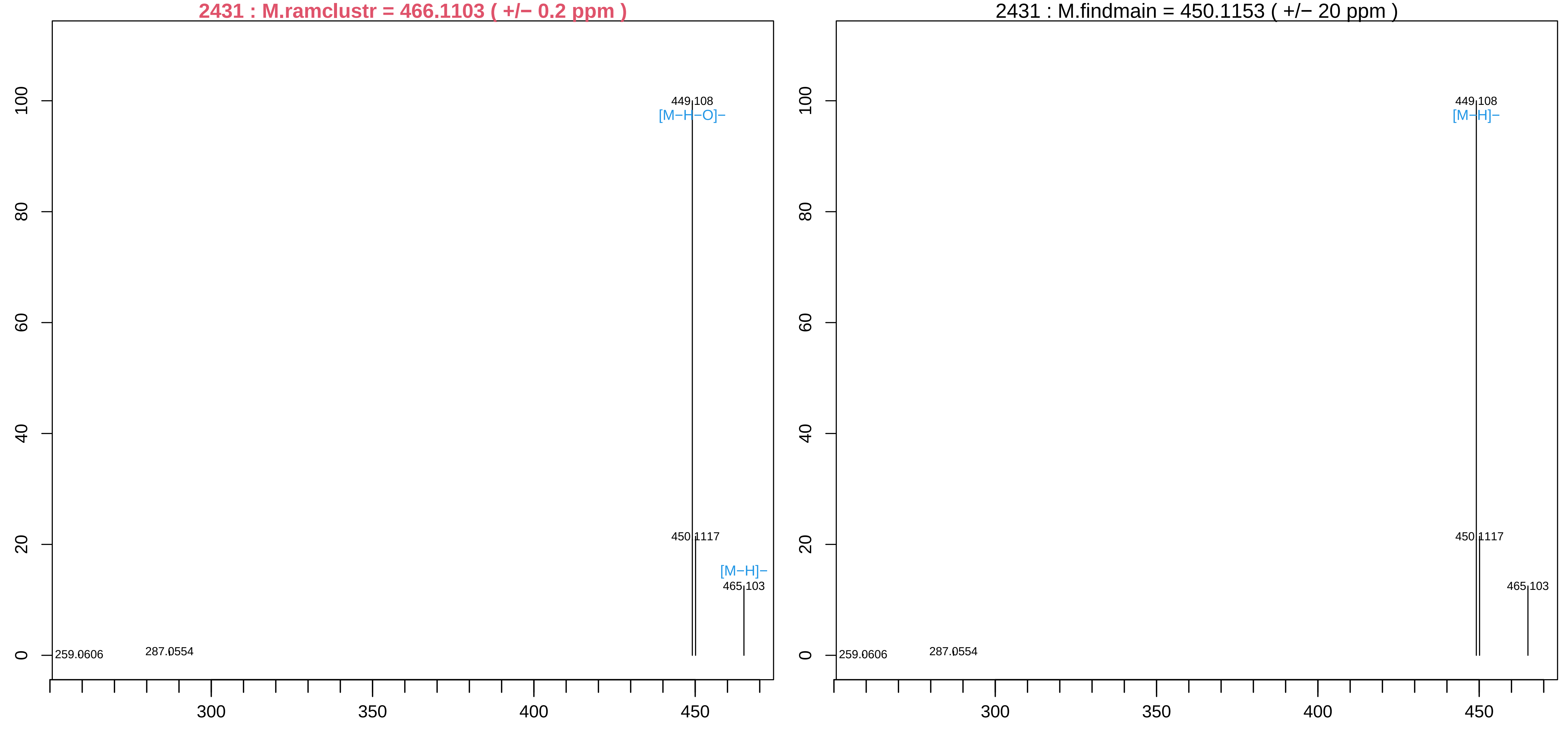
2431 : M.ramclustr = 466.1103 (+/− 0.2 ppm)
449.108
[M−H−O]−
450.1117
[M−H]−
465.103
259.0606
287.0554
2431 : M.findmain = 450.1153 (+/− 20 ppm)
449.108
[M−H]−
450.1117
465.103
259.0606
287.0554
0
20
40
60
80
100
300
350
400
450

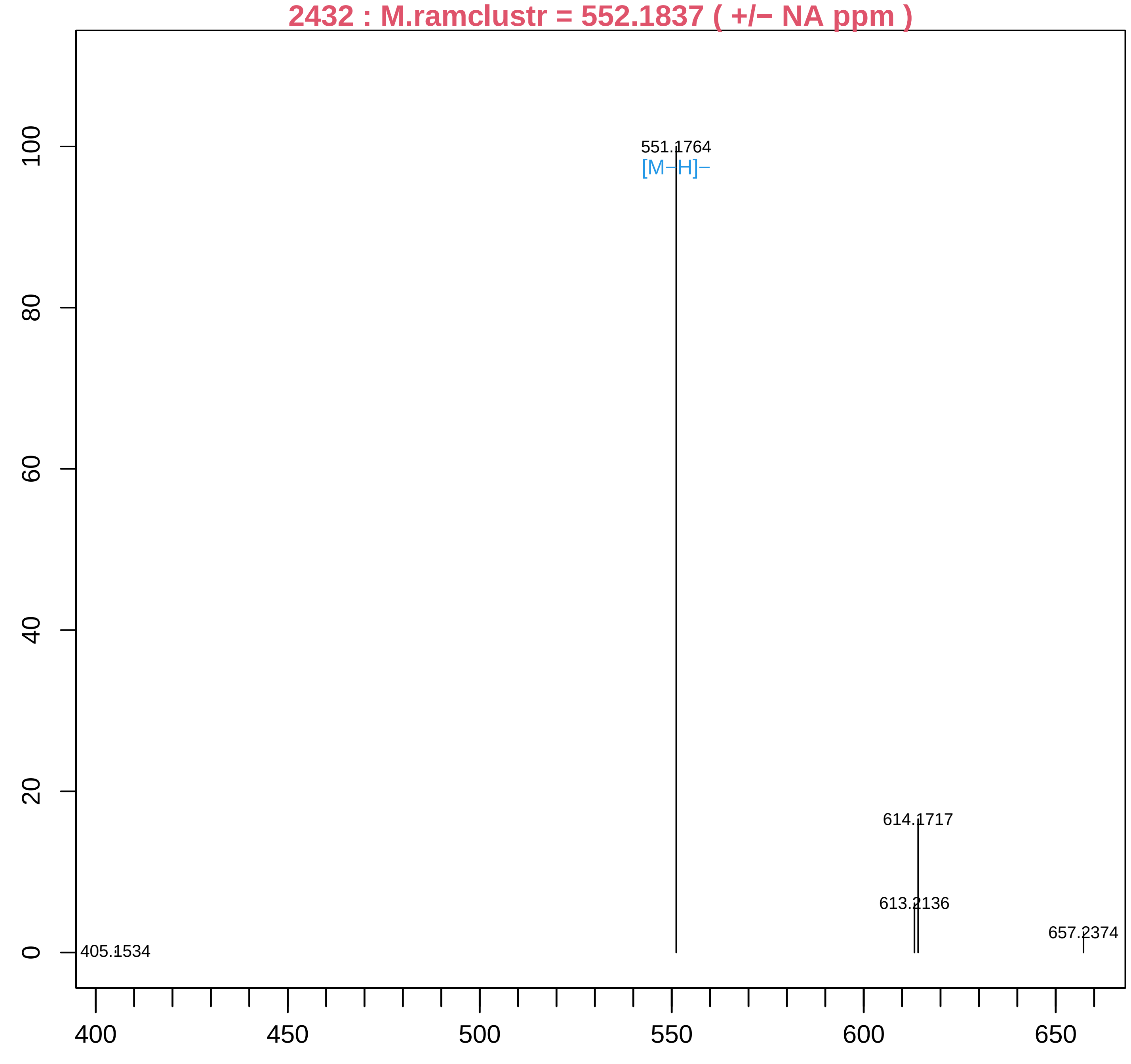

2432 : M.ramclustr = 552.1837 (+/− NA ppm)
551.1764
[M−H]−
614.1717
613.2136
657.2374
405.1534
0
20
40
60
80
100
400
450
500
550
600
650

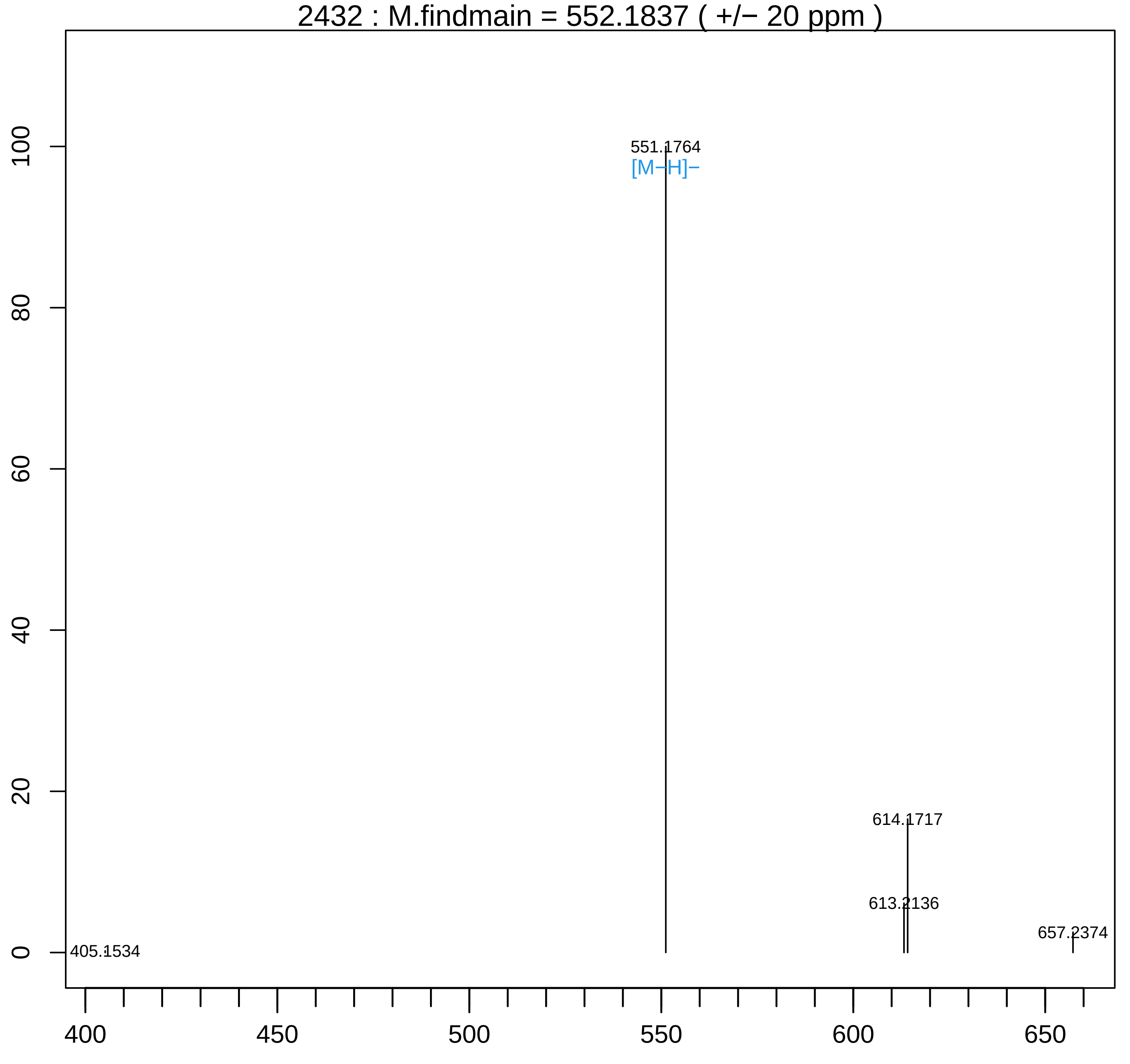

2432 : M.findmain = 552.1837 (+/− 20 ppm)
551.1764
[M−H]−
614.1717
613.2136
657.2374
405.1534
0
20
40
60
80
100
400
450
500
550
600
650

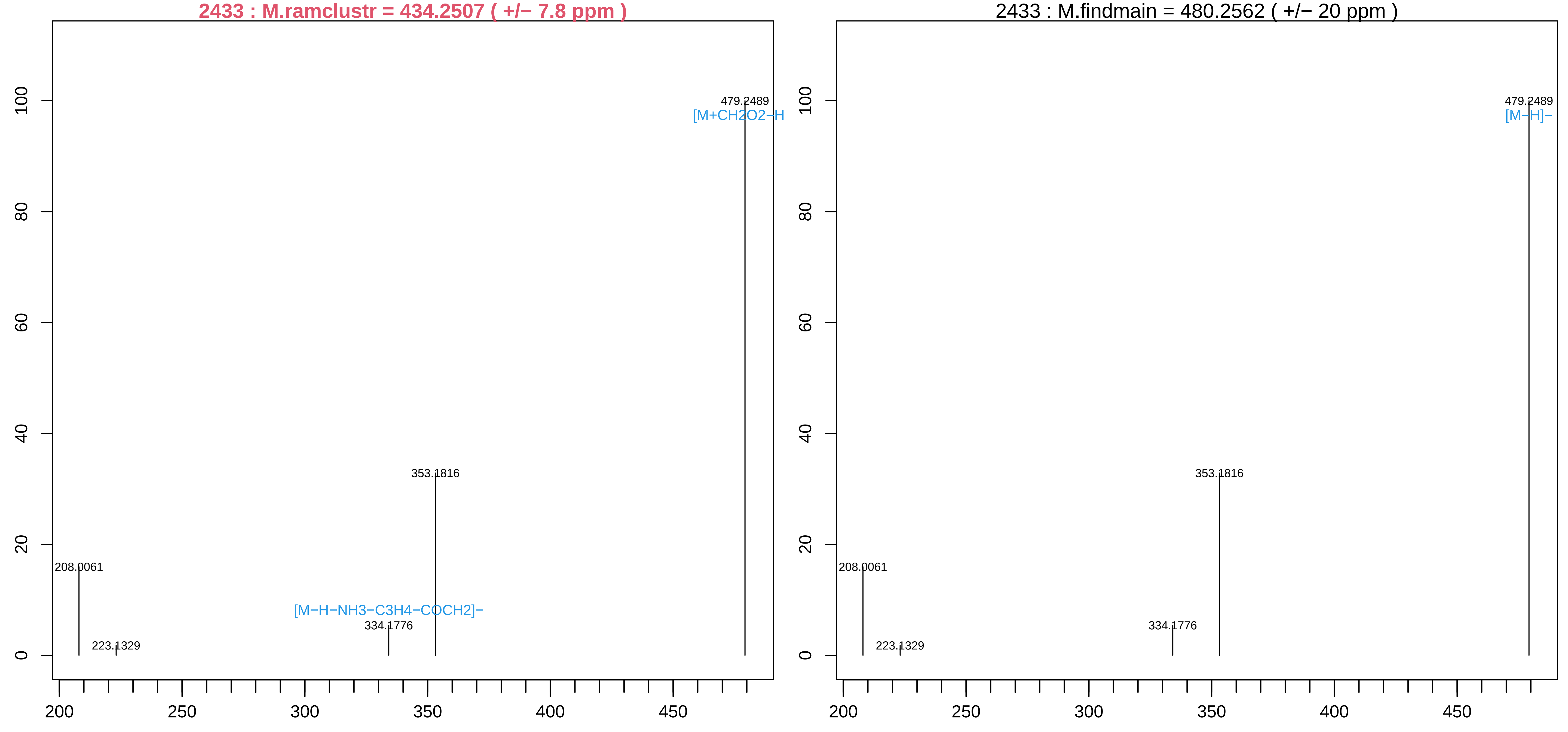
2433 : M.ramclustr = 434.2507 (+/− 7.8 ppm)
479.2489
[M+CH2O2−H
353.1816
208.0061
[M−H−NH3−C3H4−COCH2]−
334.1776
223.1329
2433 : M.findmain = 480.2562 (+/− 20 ppm)
479.2489
[M−H]−
353.1816
208.0061
334.1776
223.1329
0
20
40
60
80
100
200
250
300
350
400
450

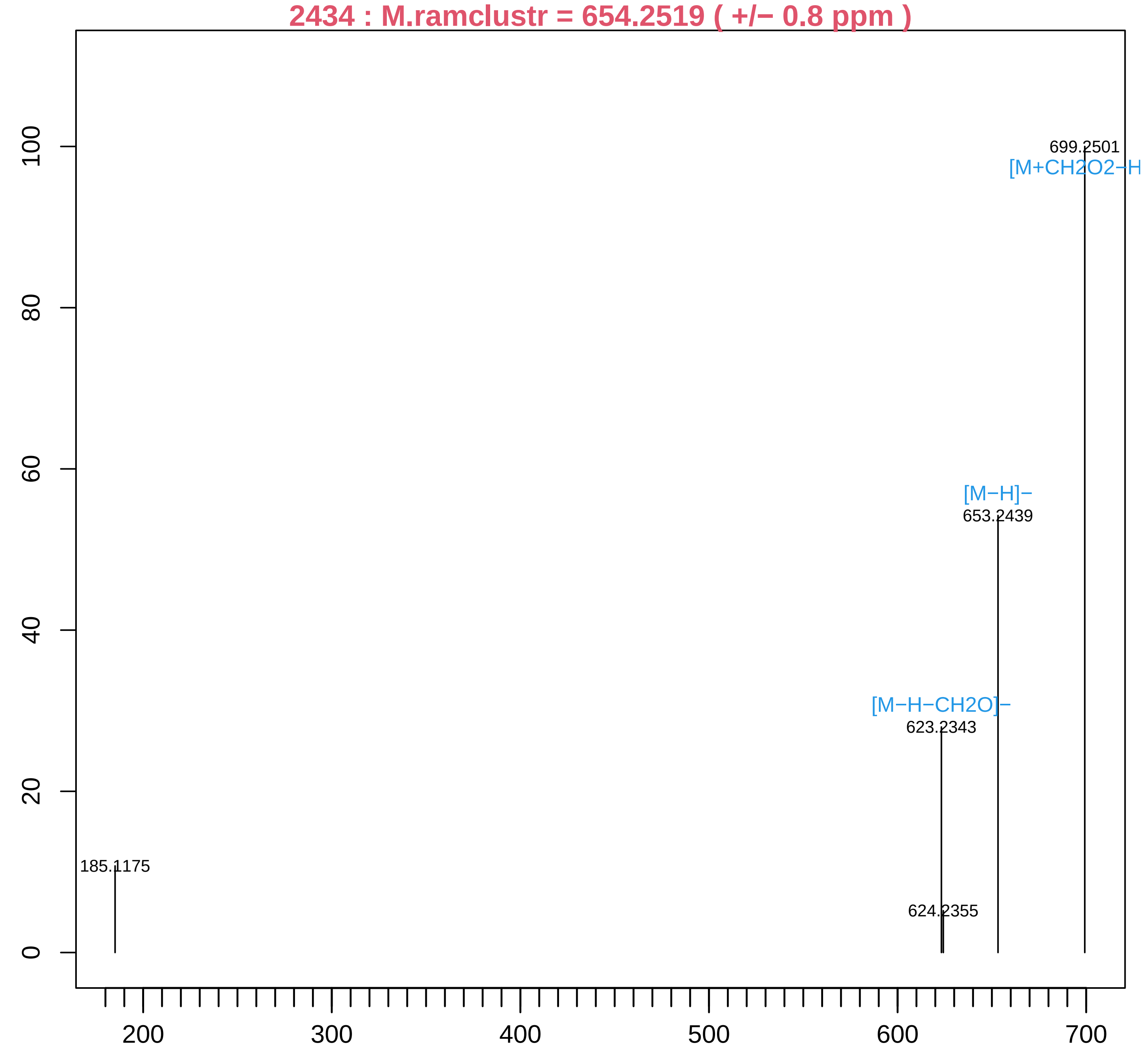

2434 : M.ramclustr = 654.2519 (+/− 0.8 ppm)
699.2501
[M+CH2O2−H
[M−H]−
653.2439
[M−H−CH2O]−
623.2343
624.2355
185.1175
0
20
40
60
80
100
200
300
400
500
600
700

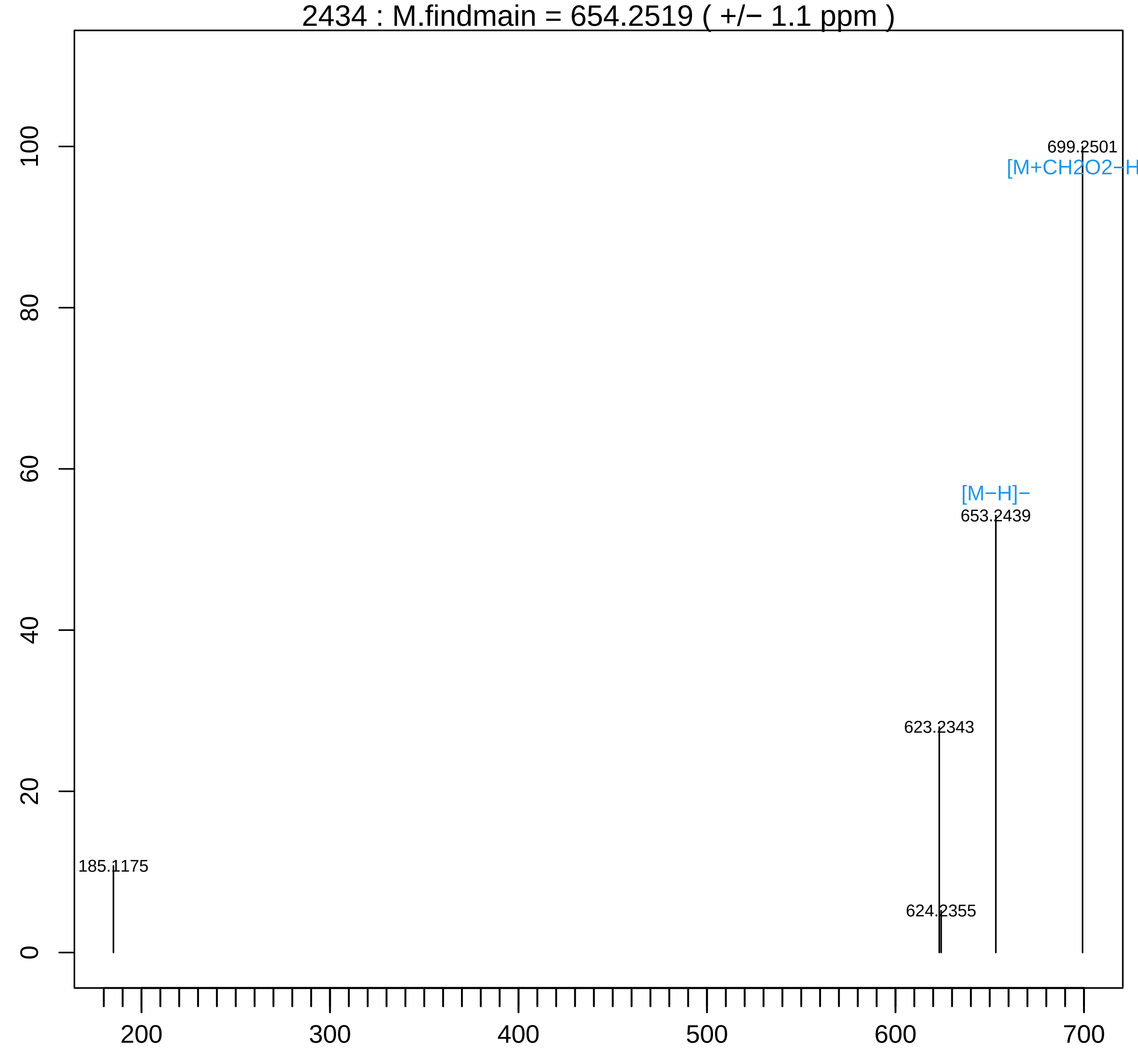

2434 : M.findmain = 654.2519 (+/− 1.1 ppm)
699.2501
[M+CH2O2−H
[M−H]−
653.2439
623.2343
624.2355
185.1175
0
20
40
60
80
100
200
300
400
500
600
700

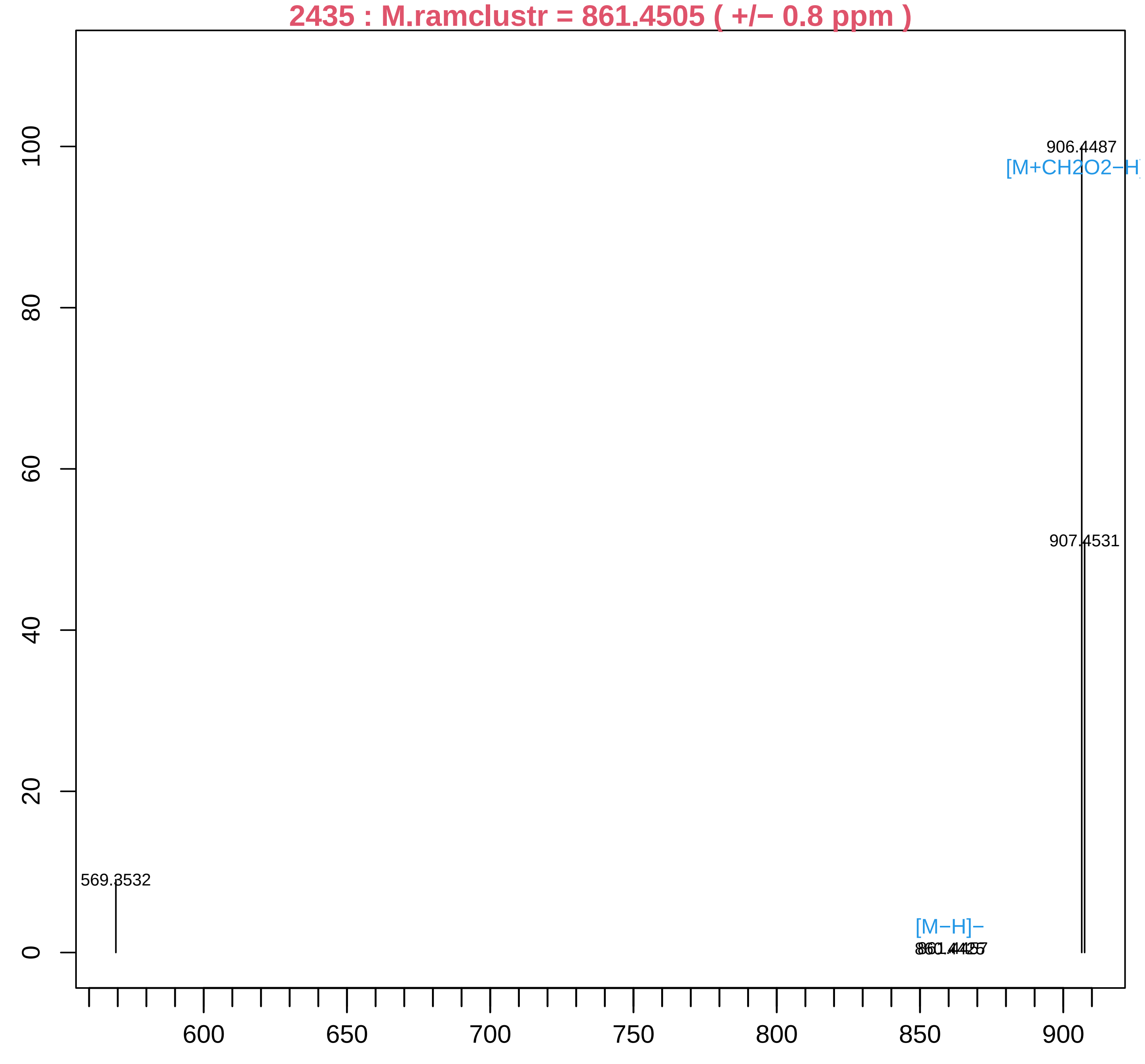
2435 : M.ramclustr = 861.4505 (+/− 0.8 ppm)
906.4487
[M+CH2O2−H
907.4531
569.3532
[M−H]−
100
80
60
40
20
0
600
650
700
750
800
850
900

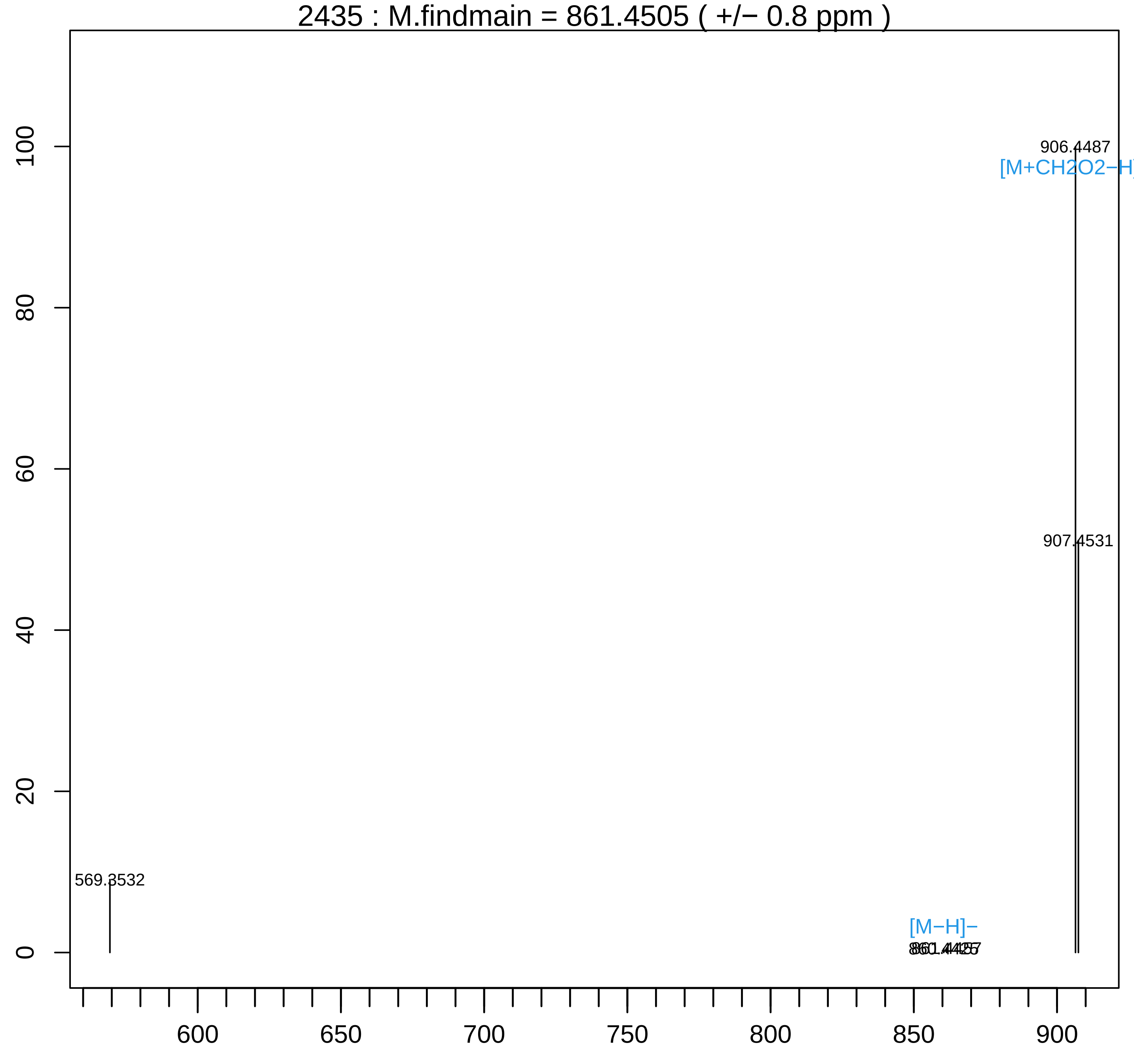
2435 : M.findmain = 861.4505 (+/− 0.8 ppm)
906.4487
[M+CH2O2−H
907.4531
569.3532
[M−H]−
100
80
60
40
20
0
600
650
700
750
800
850
900

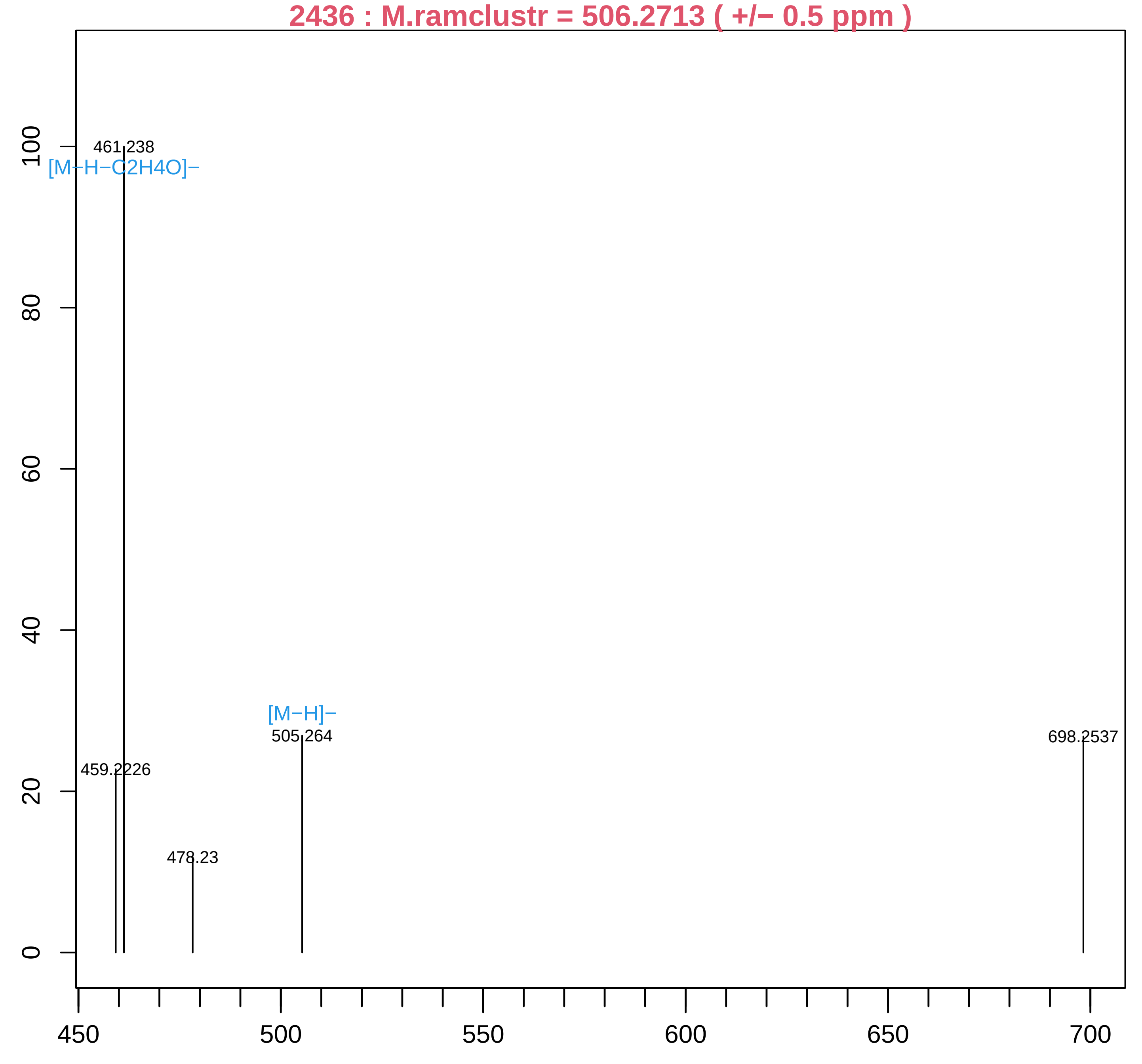
2436 : M.ramclustr = 506.2713 (+/− 0.5 ppm)
461.238
[M−H−C2H4O]−
[M−H]−
505.264
698.2537
459.2226
478.23
0
20
40
60
80
100
450
500
550
600
650
700

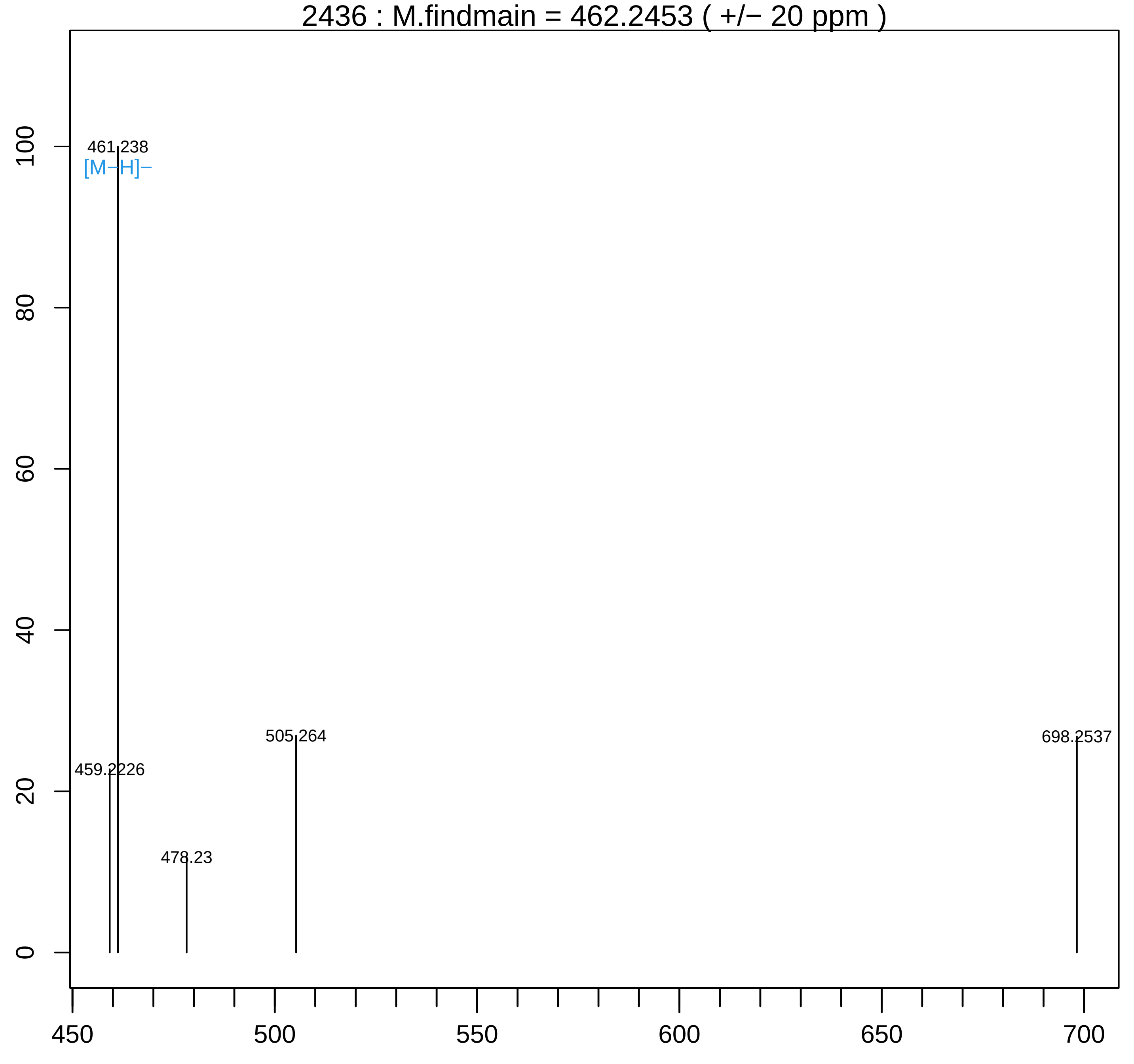
2436 : M.findmain = 462.2453 (+/− 20 ppm)
461.238
[M−H]−
505.264
698.2537
459.2226
478.23
0
20
40
60
80
100
450
500
550
600
650
700

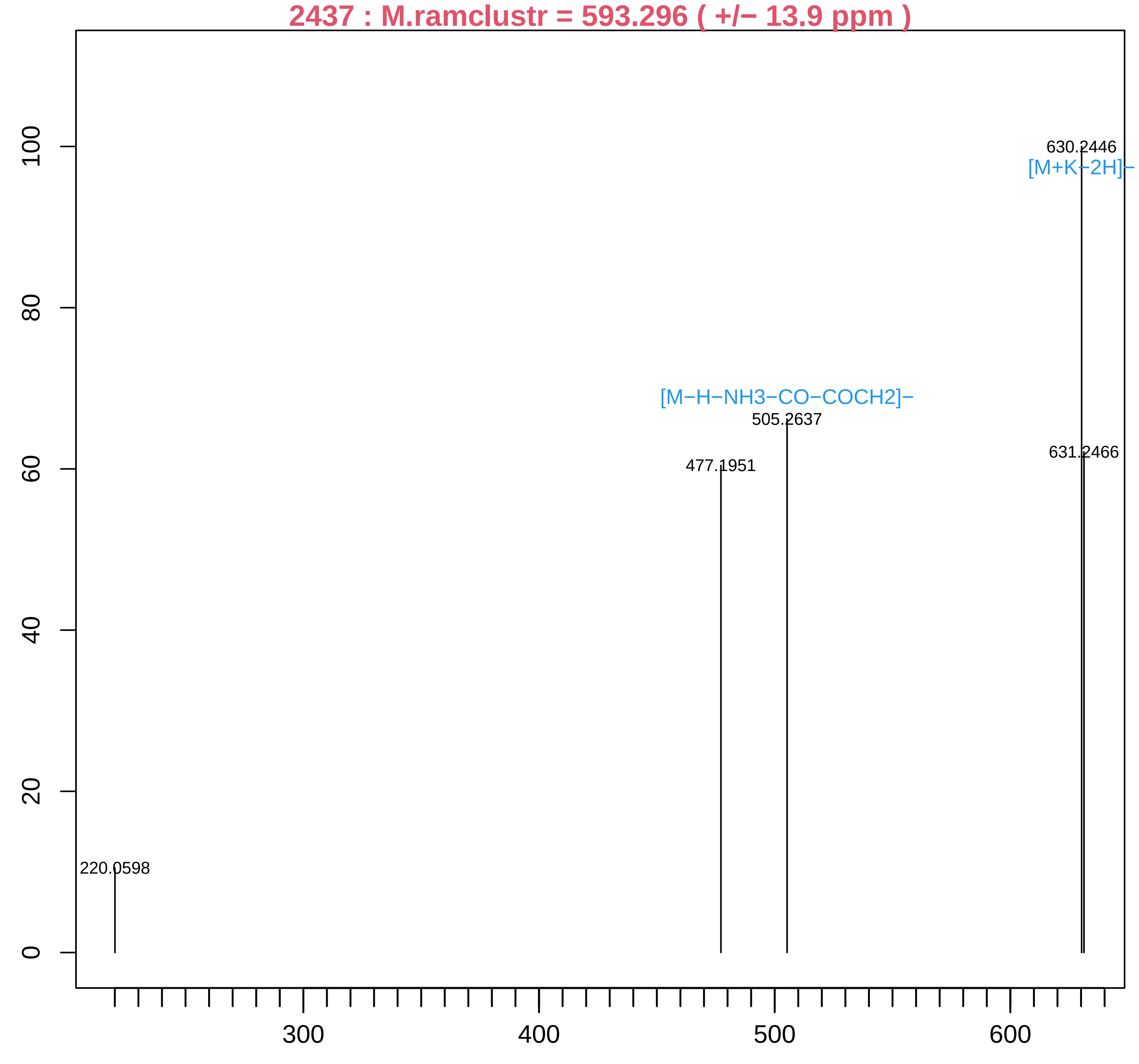
2437 : M.ramclustr = 593.296 (+/− 13.9 ppm)
630.2446
[M+K−2H]−
[M−H−NH3−CO−COCH2]−
505.2637
477.1951
631.2466
220.0598
0
20
40
60
80
100
300
400
500
600
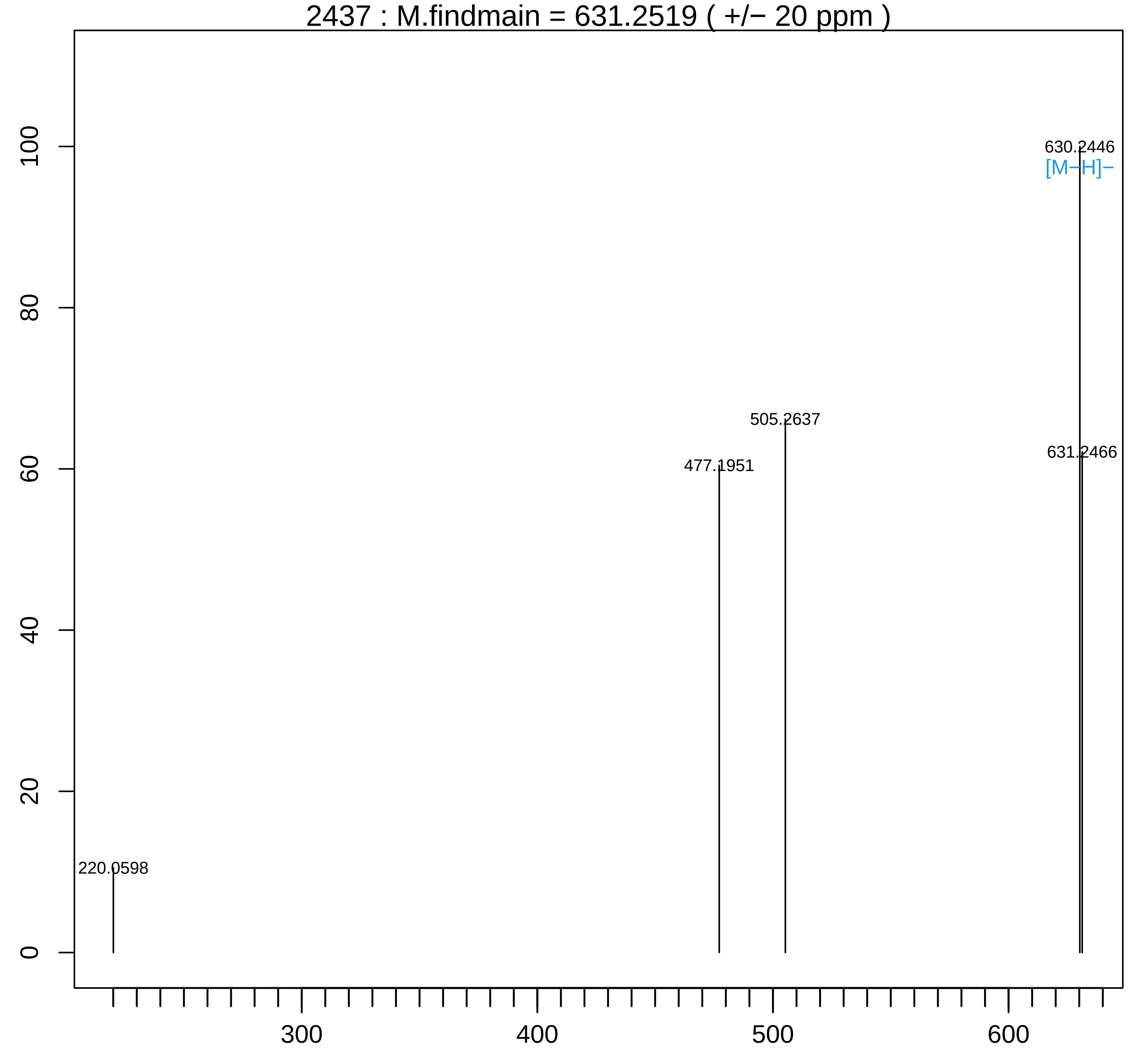
2437 : M.findmain = 631.2519 (+/− 20 ppm)
630.2446
[M−H]−
505.2637
477.1951
631.2466
220.0598
0
20
40
60
80
100
300
400
500
600

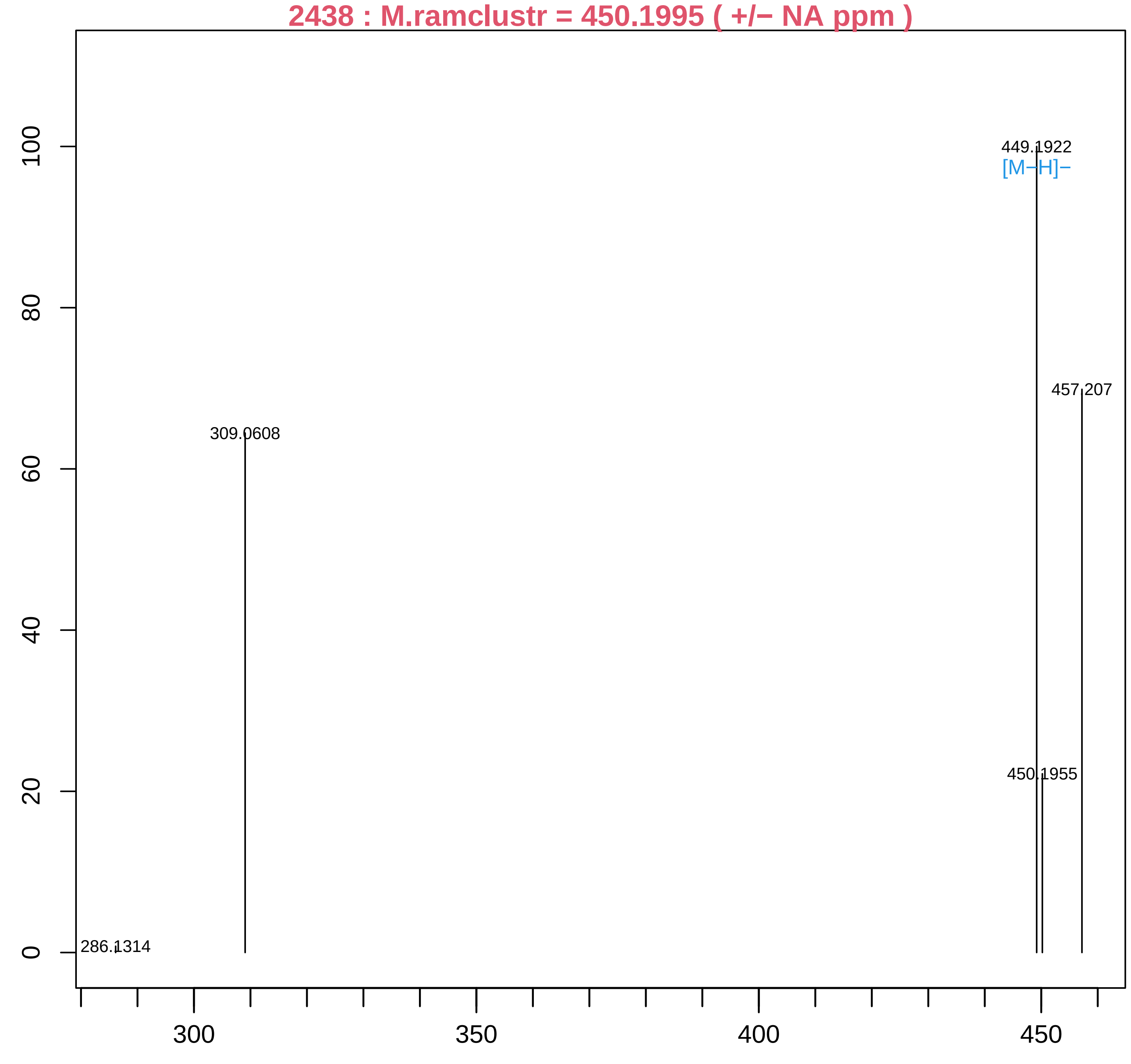
2438 : M.ramclustr = 450.1995 (+/− NA ppm)
449.1922
[M−H]−
457.207
309.0608
450.1955
286.1314
0
20
40
60
80
100
300
350
400
450

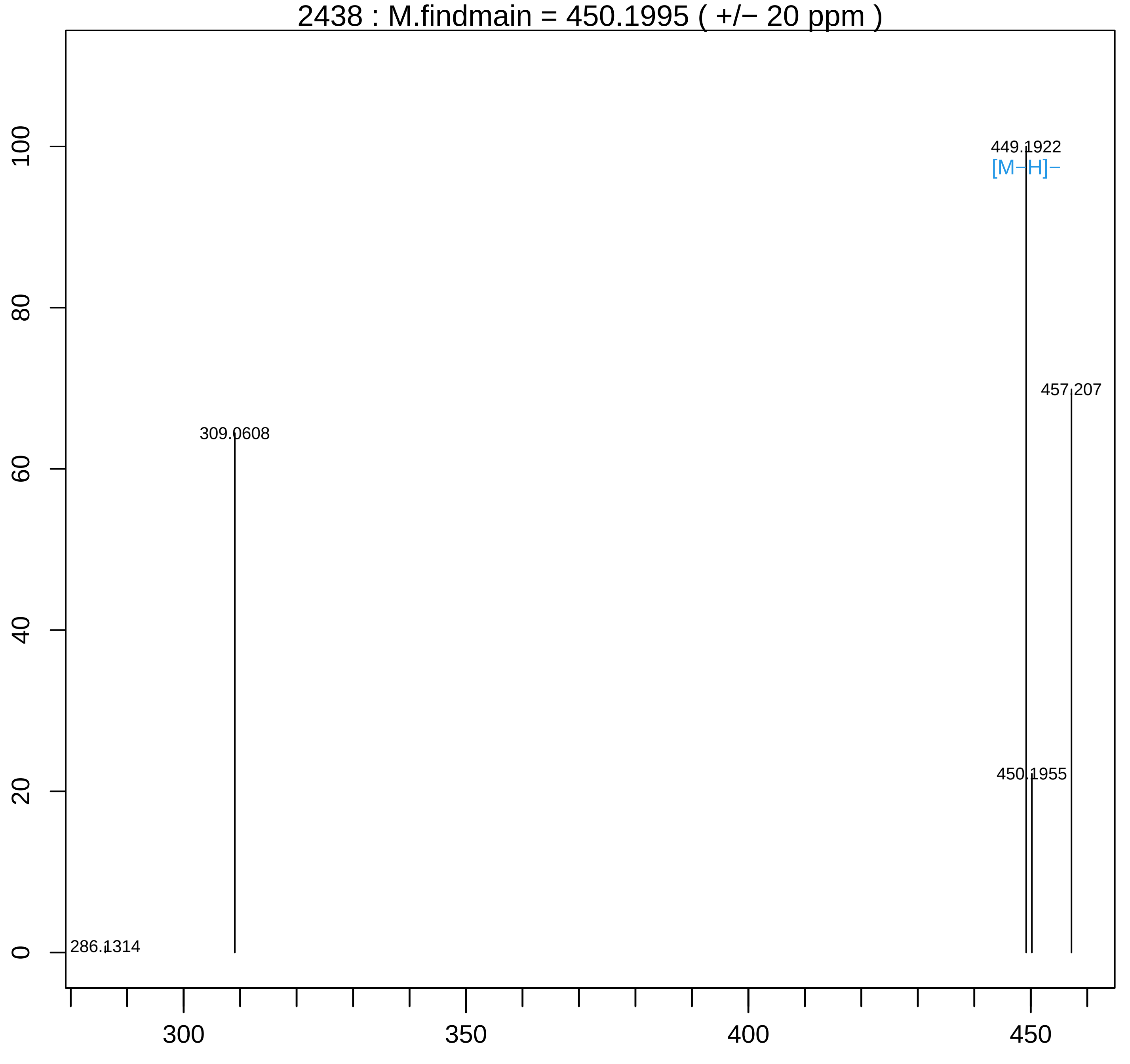
2438 : M.findmain = 450.1995 (+/− 20 ppm)
449.1922
[M−H]−
457.207
309.0608
450.1955
286.1314
0
20
40
60
80
100
300
350
400
450

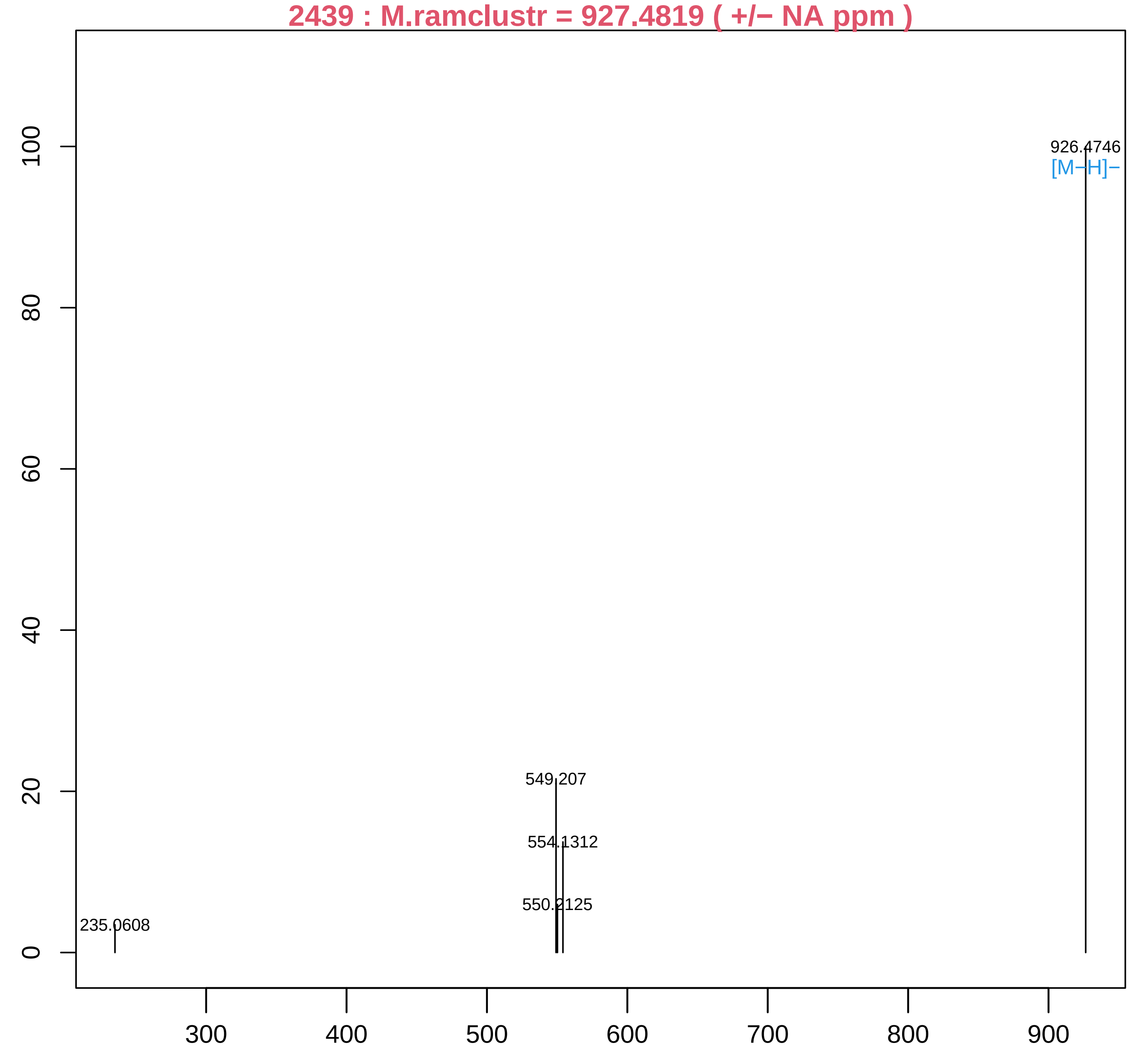

2439 : M.ramclustr = 927.4819 (+/− NA ppm)
926.4746
[M−H]−
549.207
554.1312
550.2125
235.0608
0
20
40
60
80
100
300
400
500
600
700
800
900

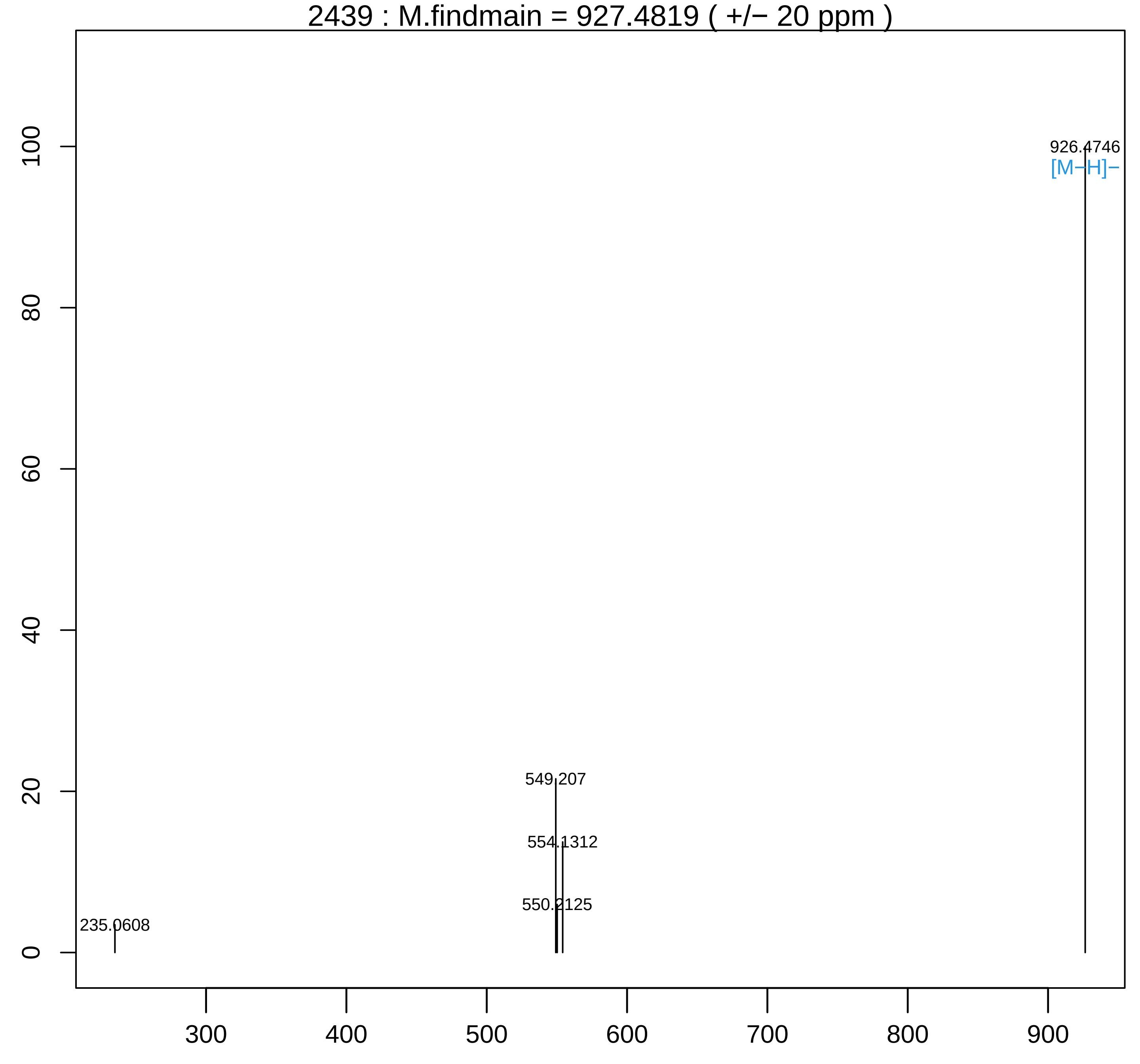

2439 : M.findmain = 927.4819 (+/− 20 ppm)
926.4746
[M−H]−
549.207
554.1312
550.2125
235.0608
0
20
40
60
80
100
300
400
500
600
700
800
900

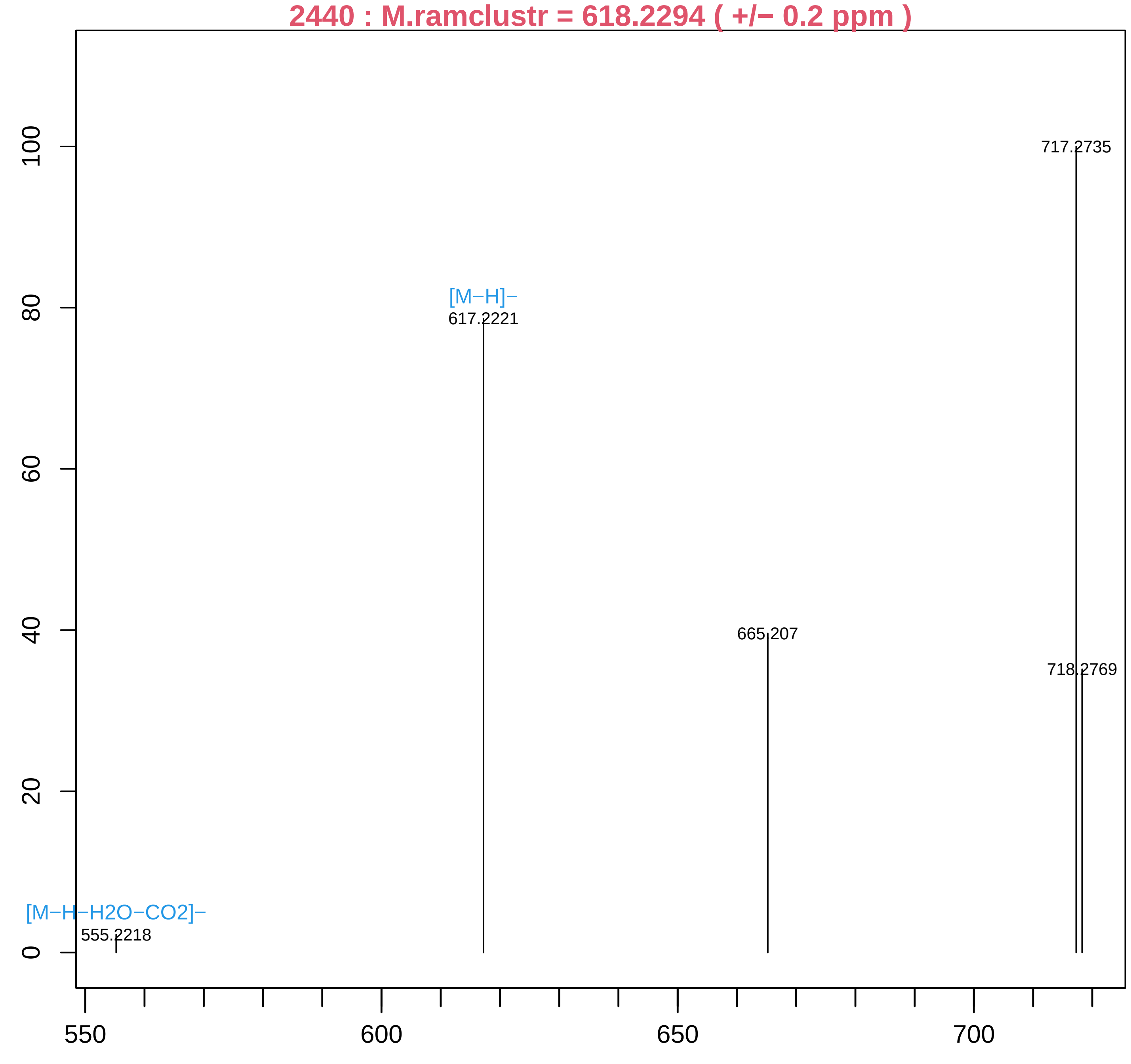
2440 : M.ramclustr = 618.2294 (+/− 0.2 ppm)
[M−H]−
617.2221
717.2735
665.207
718.2769
[M−H−H2O−CO2]−
555.2218
0
20
40
60
80
100
550
600
650
700

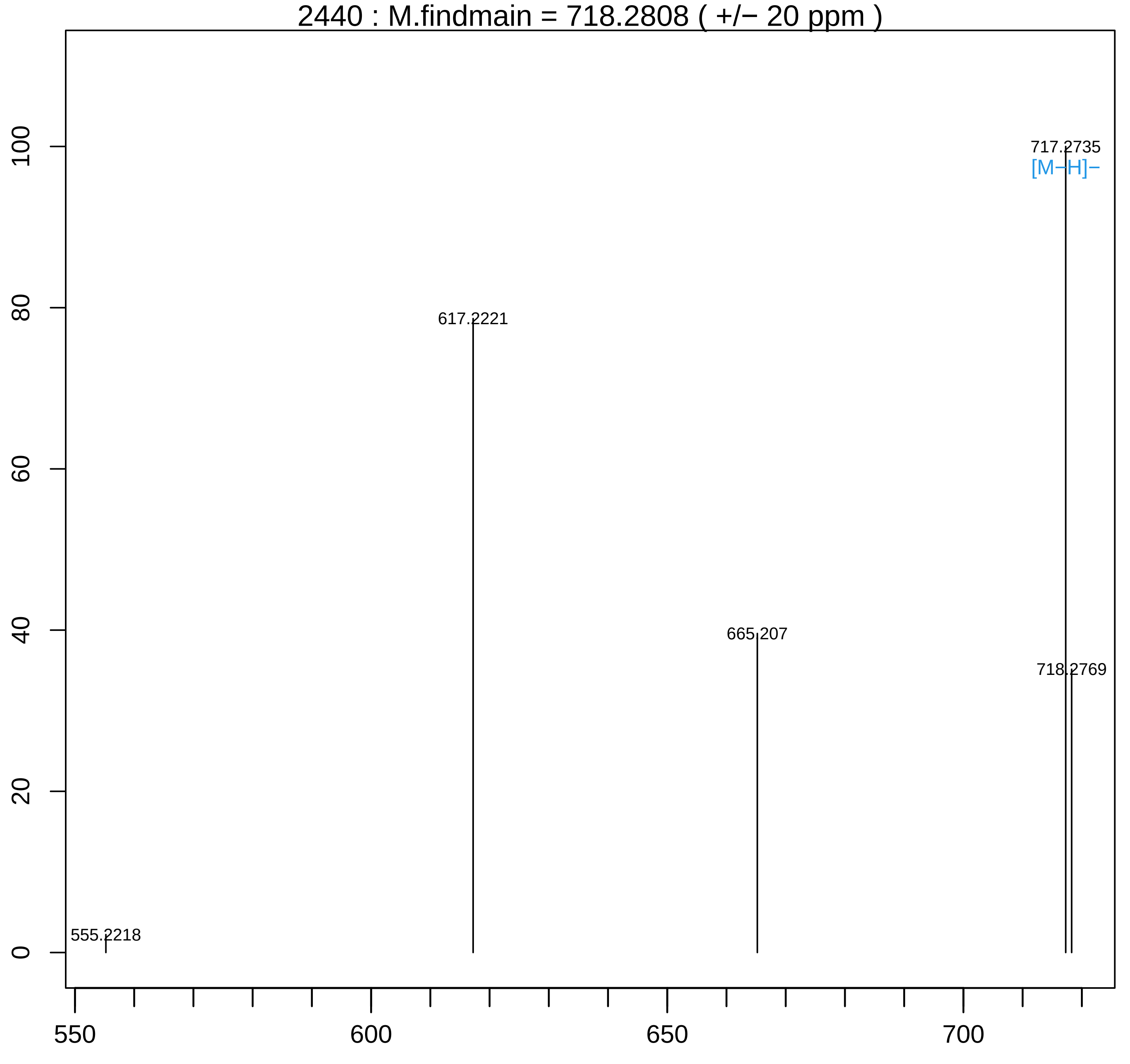
2440 : M.findmain = 718.2808 (+/− 20 ppm)
717.2735
[M−H]−
617.2221
665.207
718.2769
555.2218
0
20
40
60
80
100
550
600
650
700

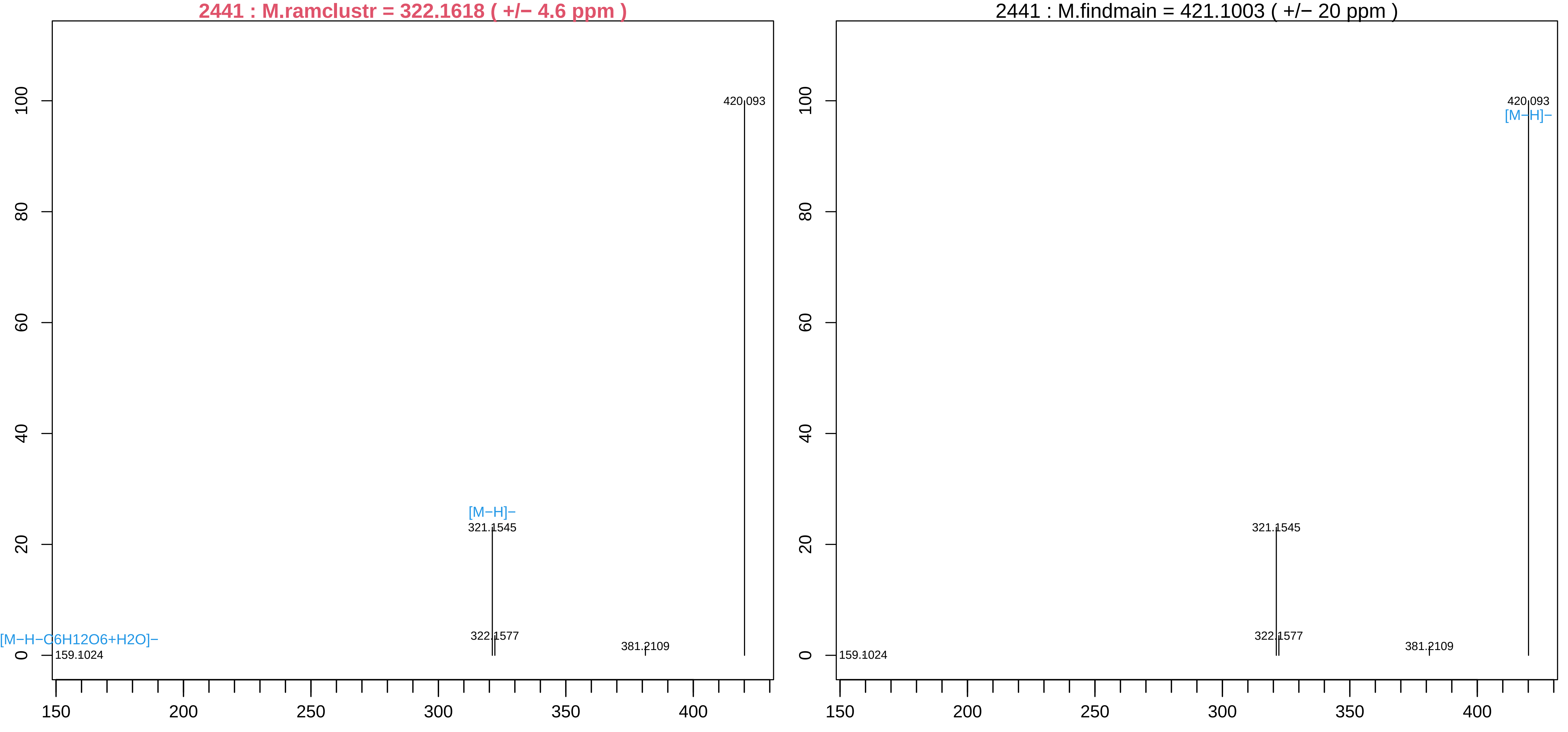
2441 : M.ramclustr = 322.1618 (+/− 4.6 ppm)
[M−H]−
321.1545
420.093
322.1577
381.2109
[M−H−C6H12O6+H2O]−
159.1024
2441 : M.findmain = 421.1003 (+/− 20 ppm)
420.093
[M−H]−
321.1545
322.1577
381.2109
159.1024
0
20
40
60
80
100
150
200
250
300
350
400

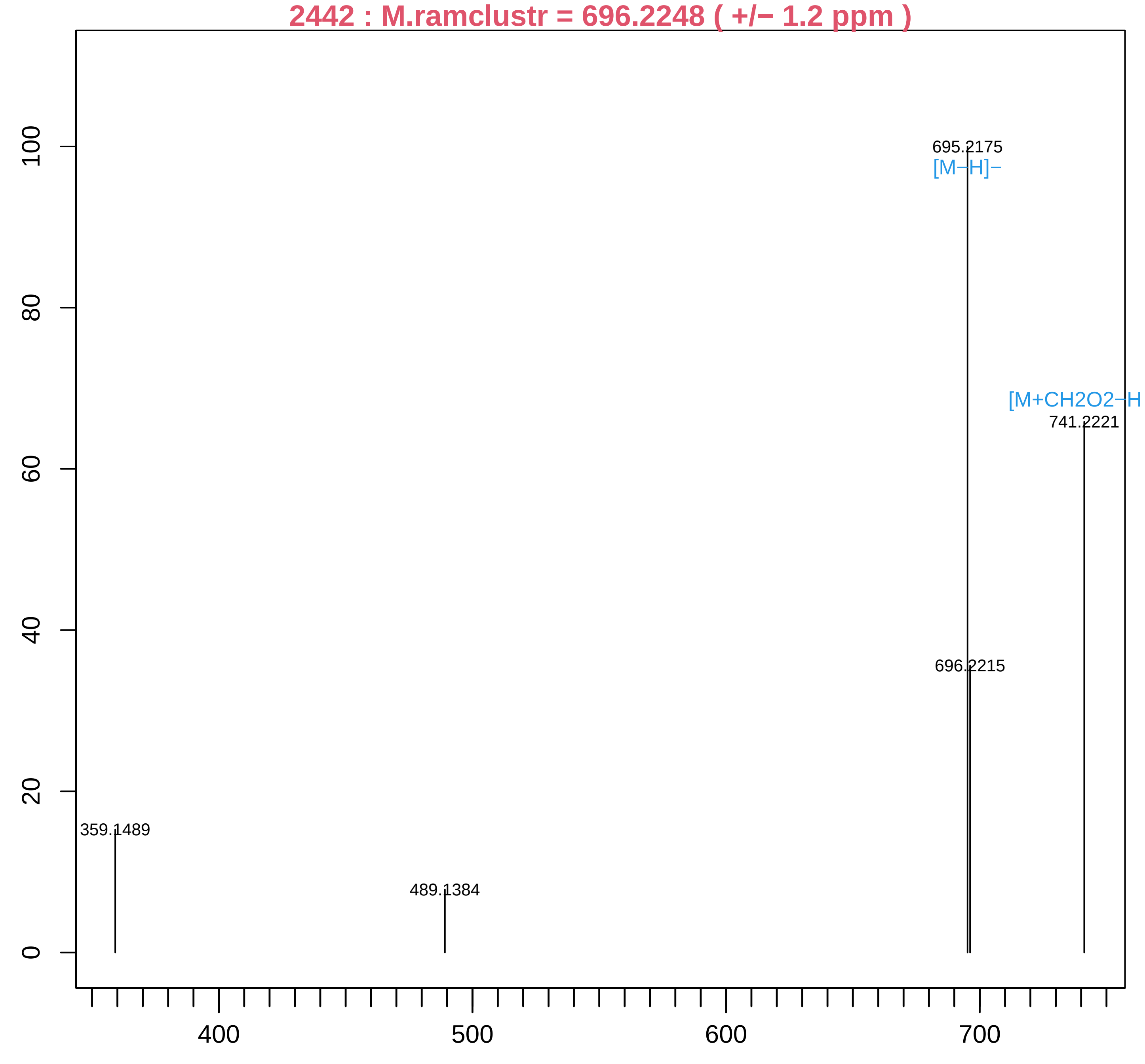
2442 : M.ramclustr = 696.2248 (+/− 1.2 ppm)
695.2175
[M−H]−
[M+CH2O2−H
741.2221
696.2215
359.1489
489.1384
0
20
40
60
80
100
400
500
600
700

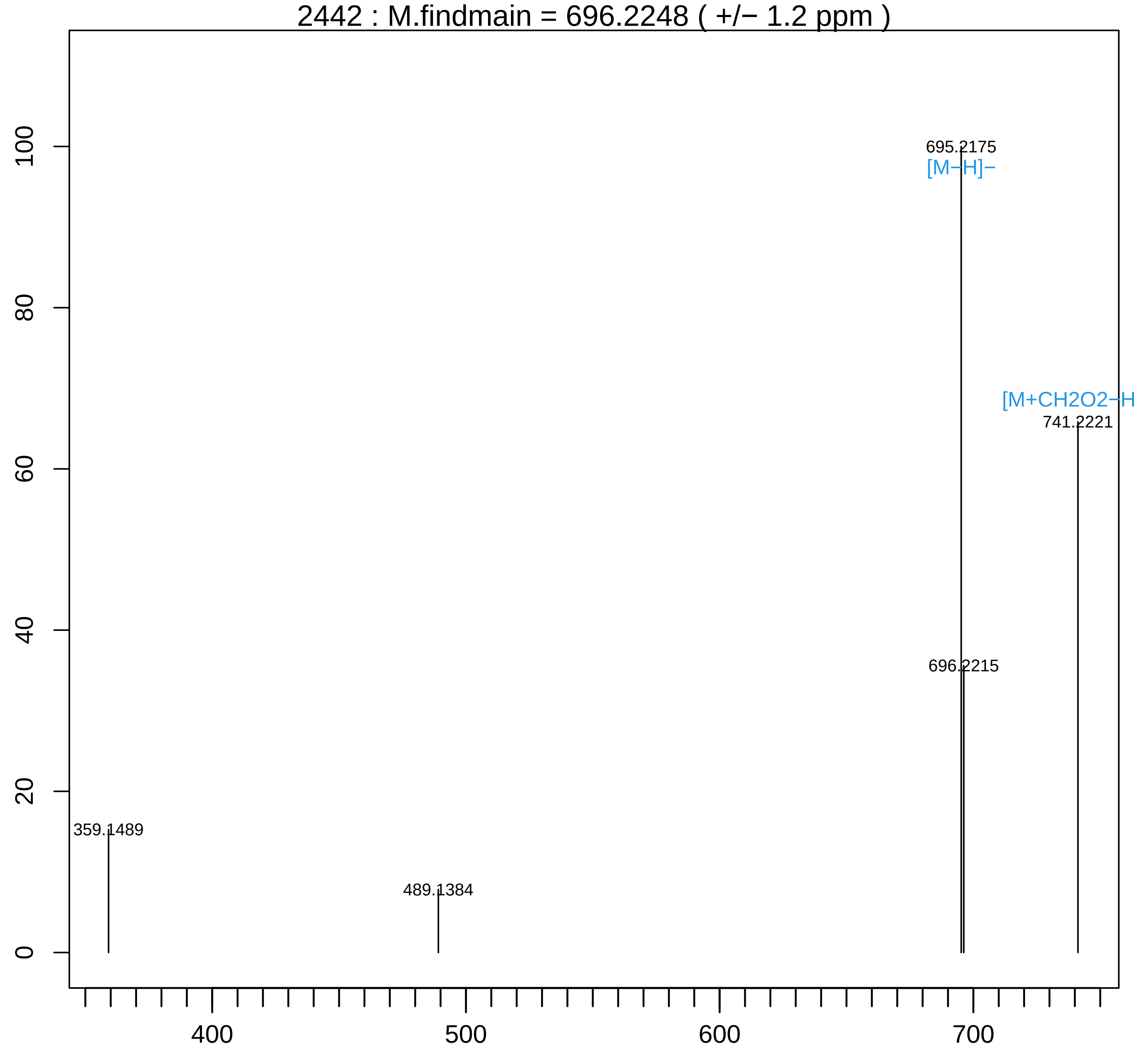
2442 : M.findmain = 696.2248 (+/− 1.2 ppm)
695.2175
[M−H]−
[M+CH2O2−H
741.2221
696.2215
359.1489
489.1384
0
20
40
60
80
100
400
500
600
700

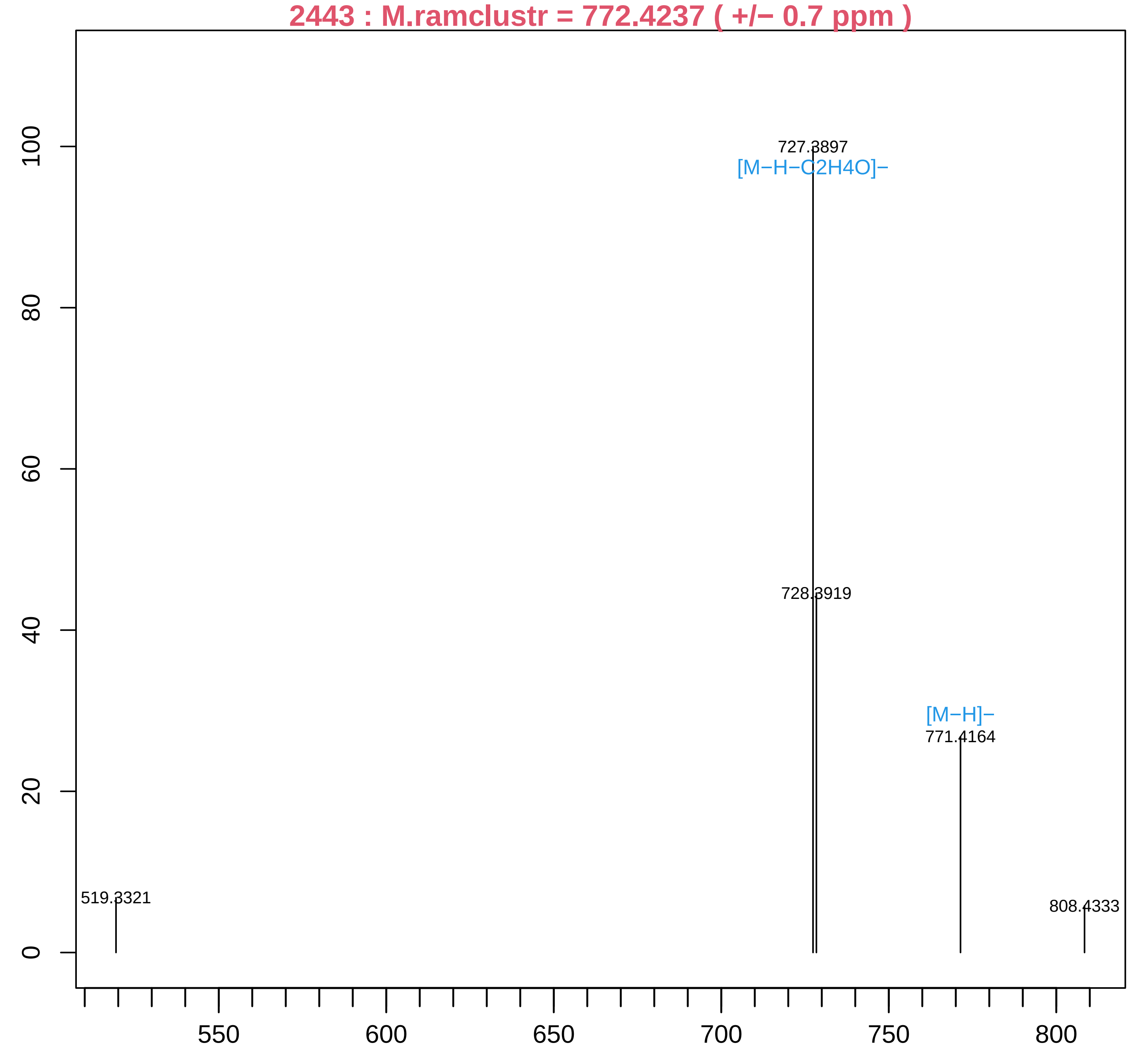

2443 : M.ramclustr = 772.4237 (+/− 0.7 ppm)
727.3897
[M−H−C2H4O]−
728.3919
[M−H]−
771.4164
519.3321
808.4333
0
20
40
60
80
100
550
600
650
700
750
800

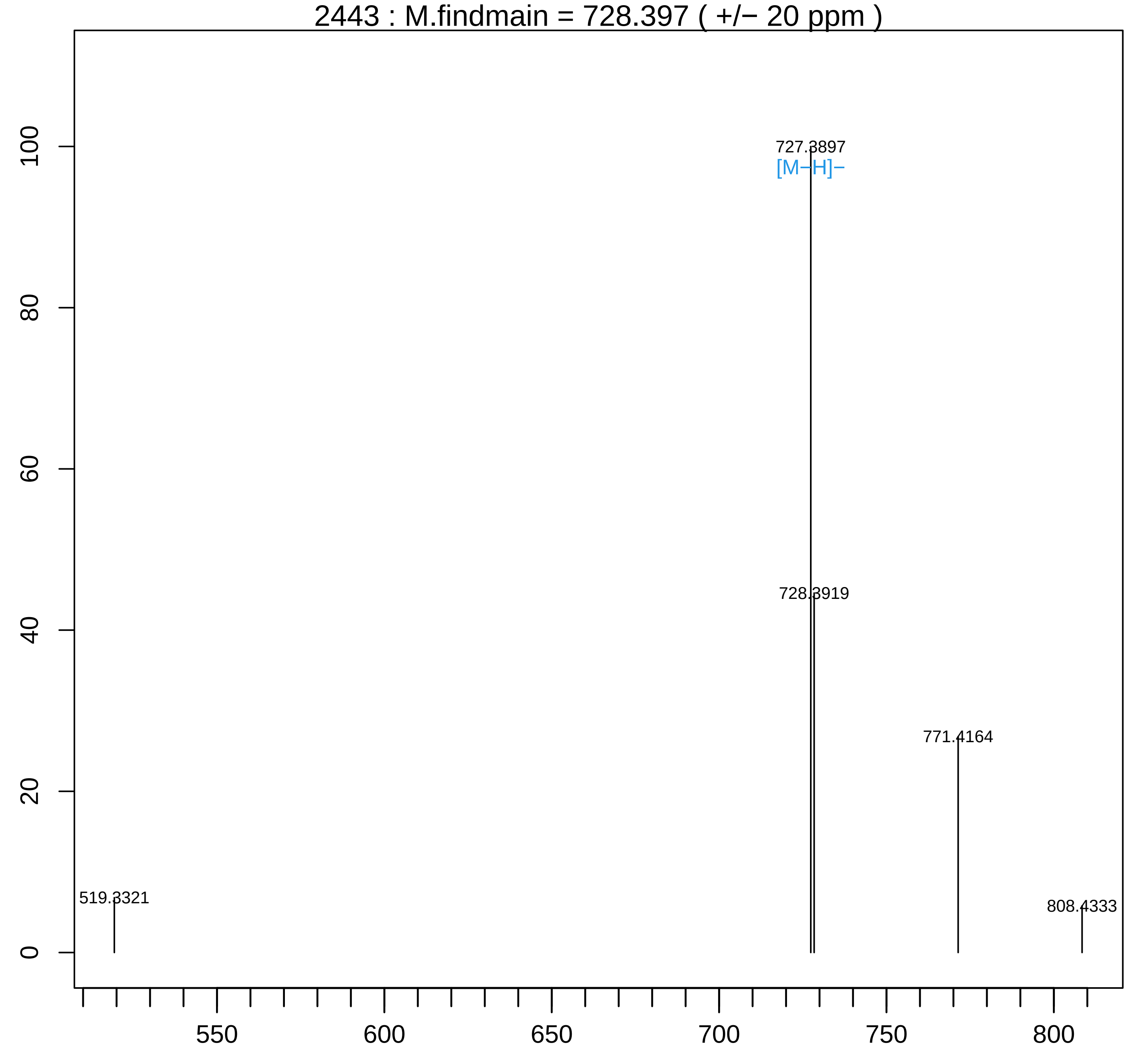

2443 : M.findmain = 728.397 (+/− 20 ppm)
727.3897
[M−H]−
728.3919
771.4164
519.3321
808.4333
0
20
40
60
80
100
550
600
650
700
750
800

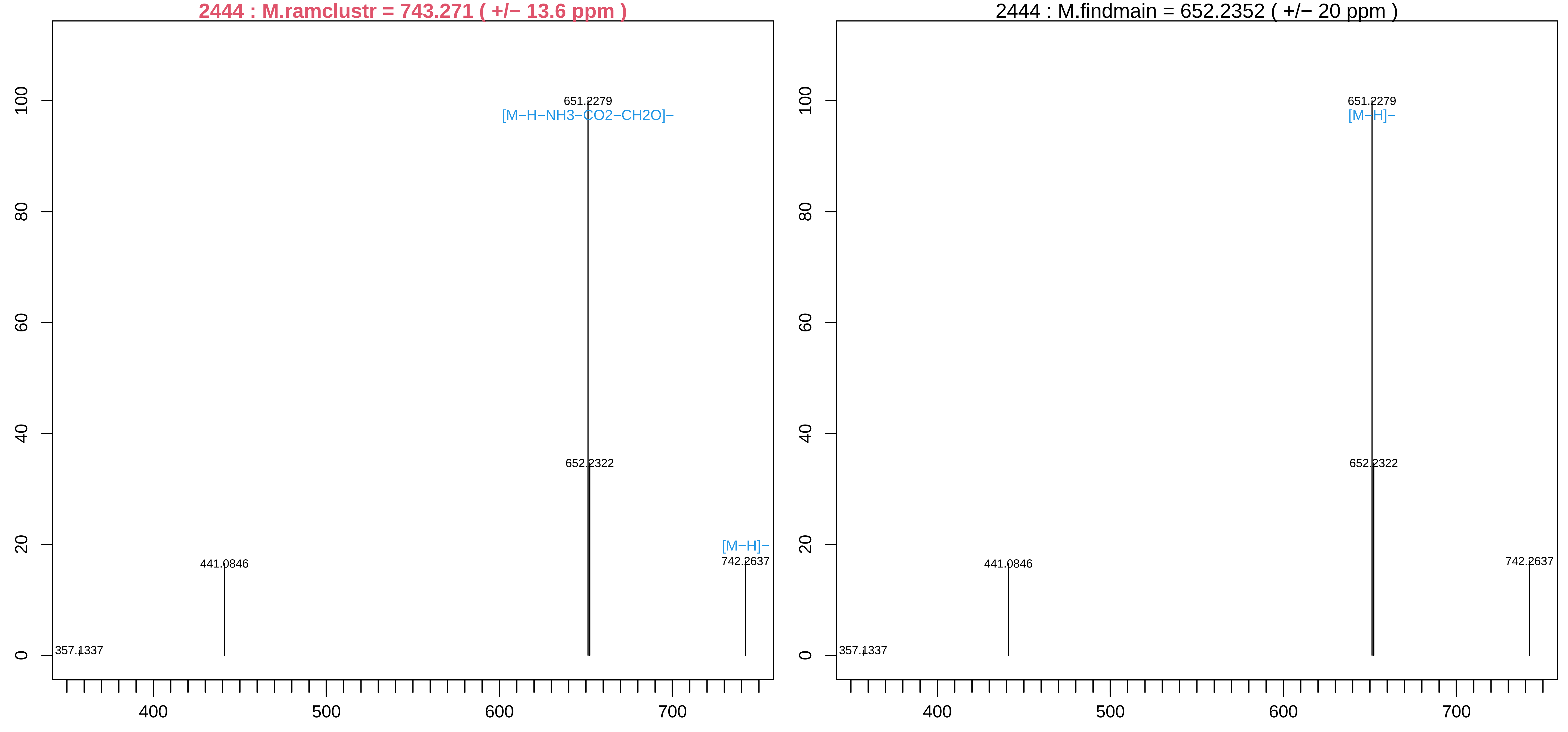

2444 : M.ramclustr = 743.271 (+/− 13.6 ppm)
651.2279
[M−H−NH3−CO2−CH2O]−
652.2322
441.0846
[M−H]−
742.2637
357.1337
2444 : M.findmain = 652.2352 (+/− 20 ppm)
651.2279
[M−H]−
652.2322
441.0846
742.2637
357.1337
0
20
40
60
80
100
400
500
600
700

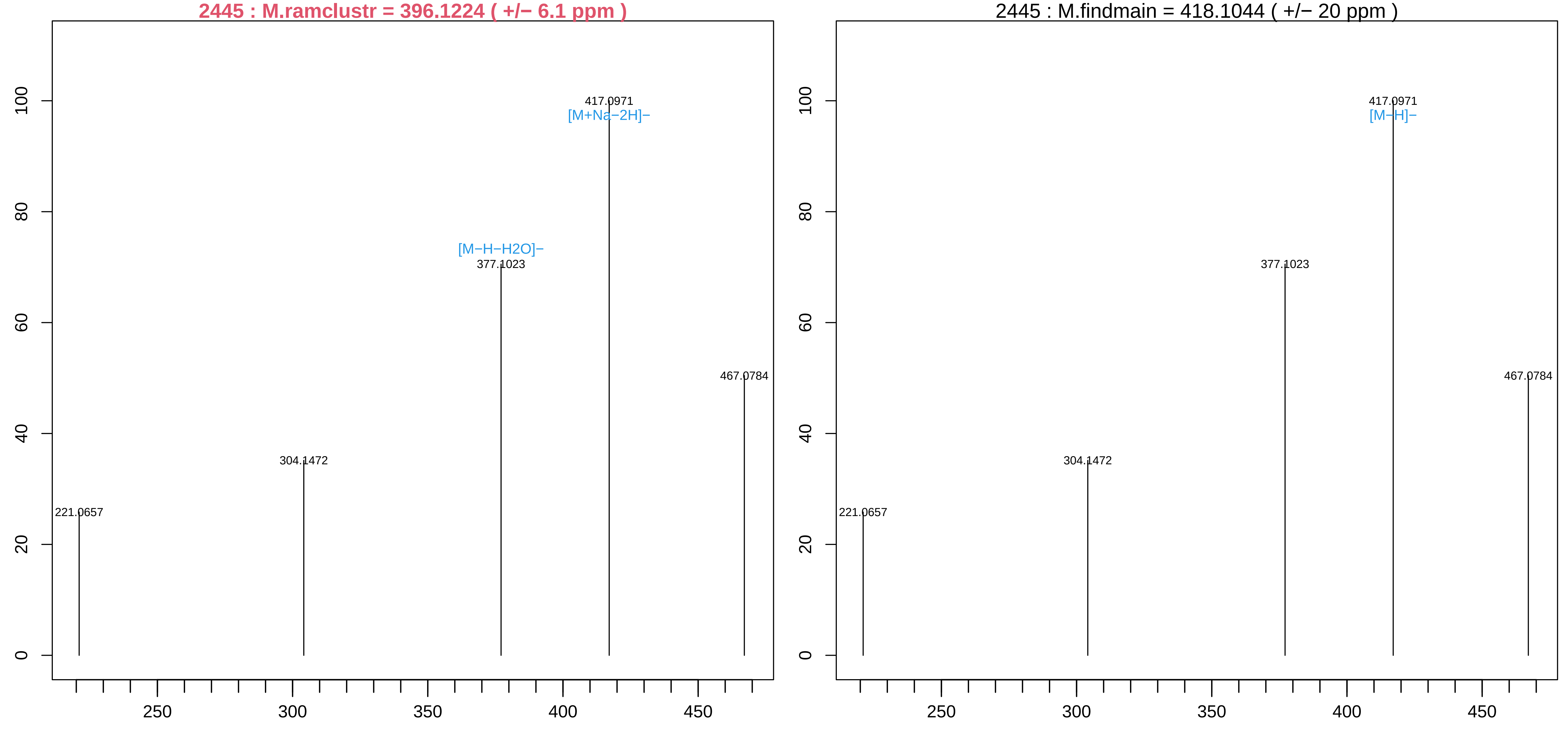
2445 : M.ramclustr = 396.1224 (+/− 6.1 ppm)
417.0971
[M+Na−2H]−
[M−H−H2O]−
377.1023
467.0784
304.1472
221.0657
0
20
40
60
80
100
250
300
350
400
450
2445 : M.findmain = 418.1044 (+/− 20 ppm)
417.0971
[M−H]−
377.1023
467.0784
304.1472
221.0657
0
20
40
60
80
100
250
300
350
400
450

2446 : M.ramclustr = 1126.5401 (+/− NA ppm)

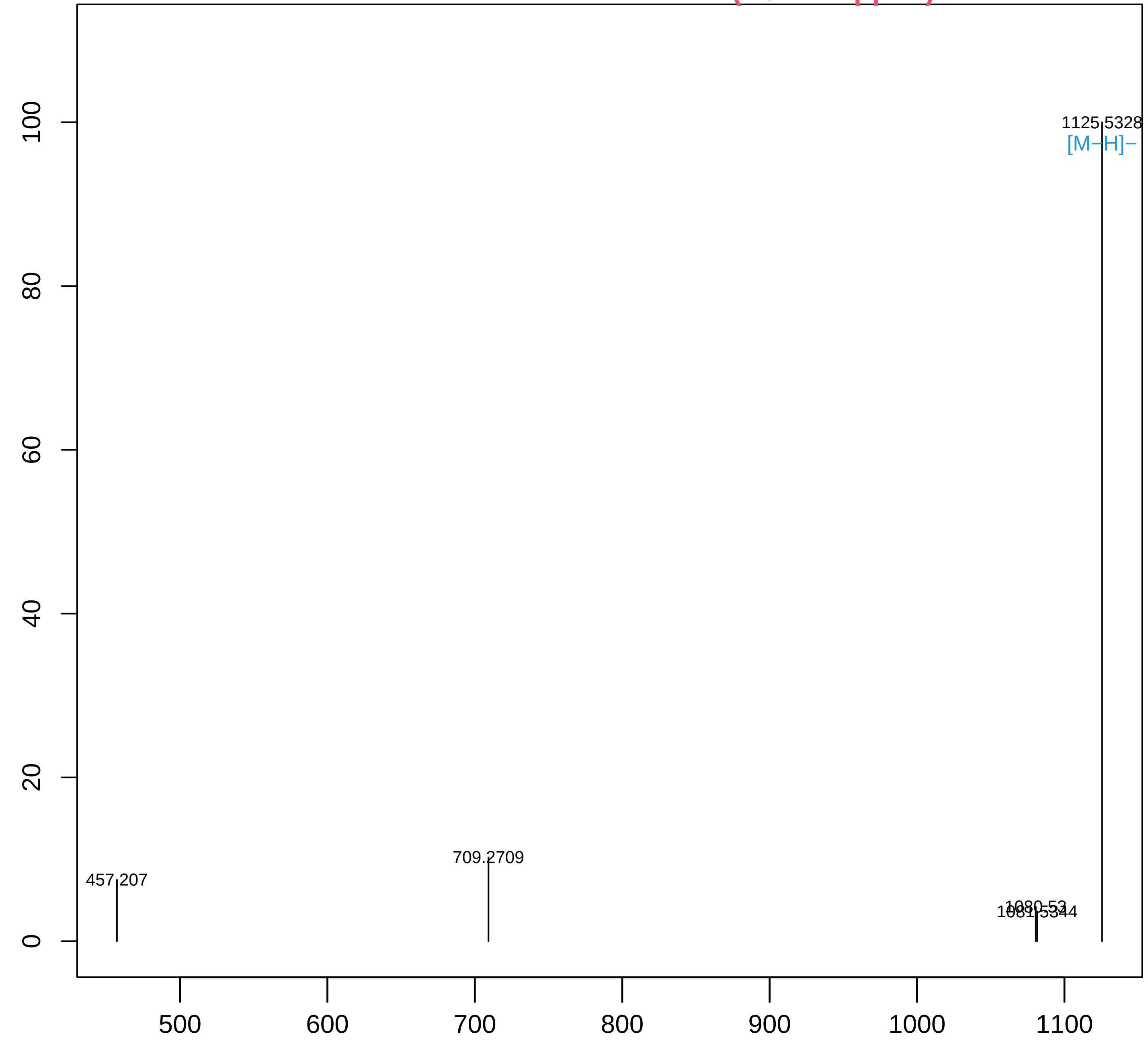

2446 : M.findmain = 1126.5401 (+/− 20 ppm)

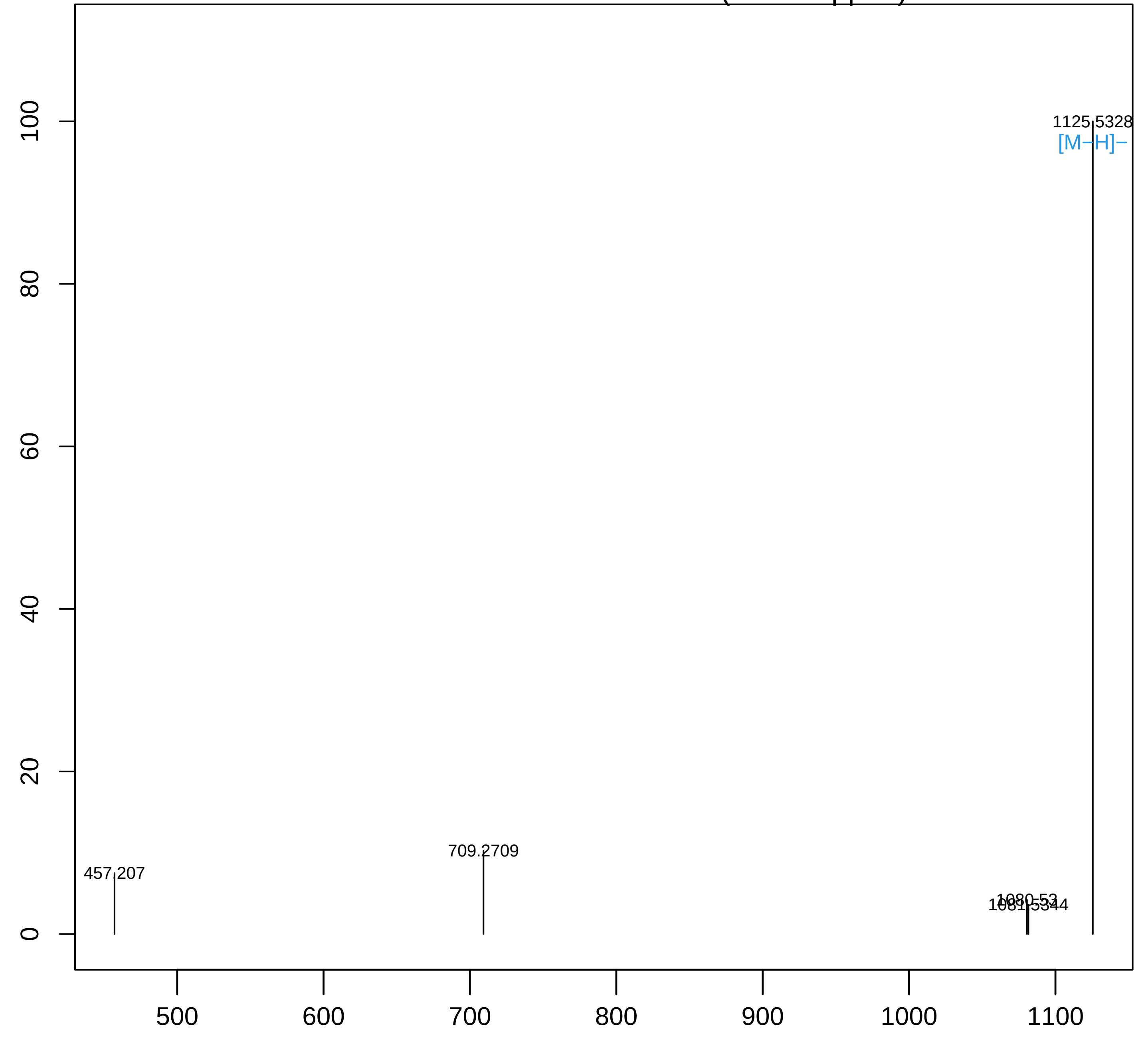

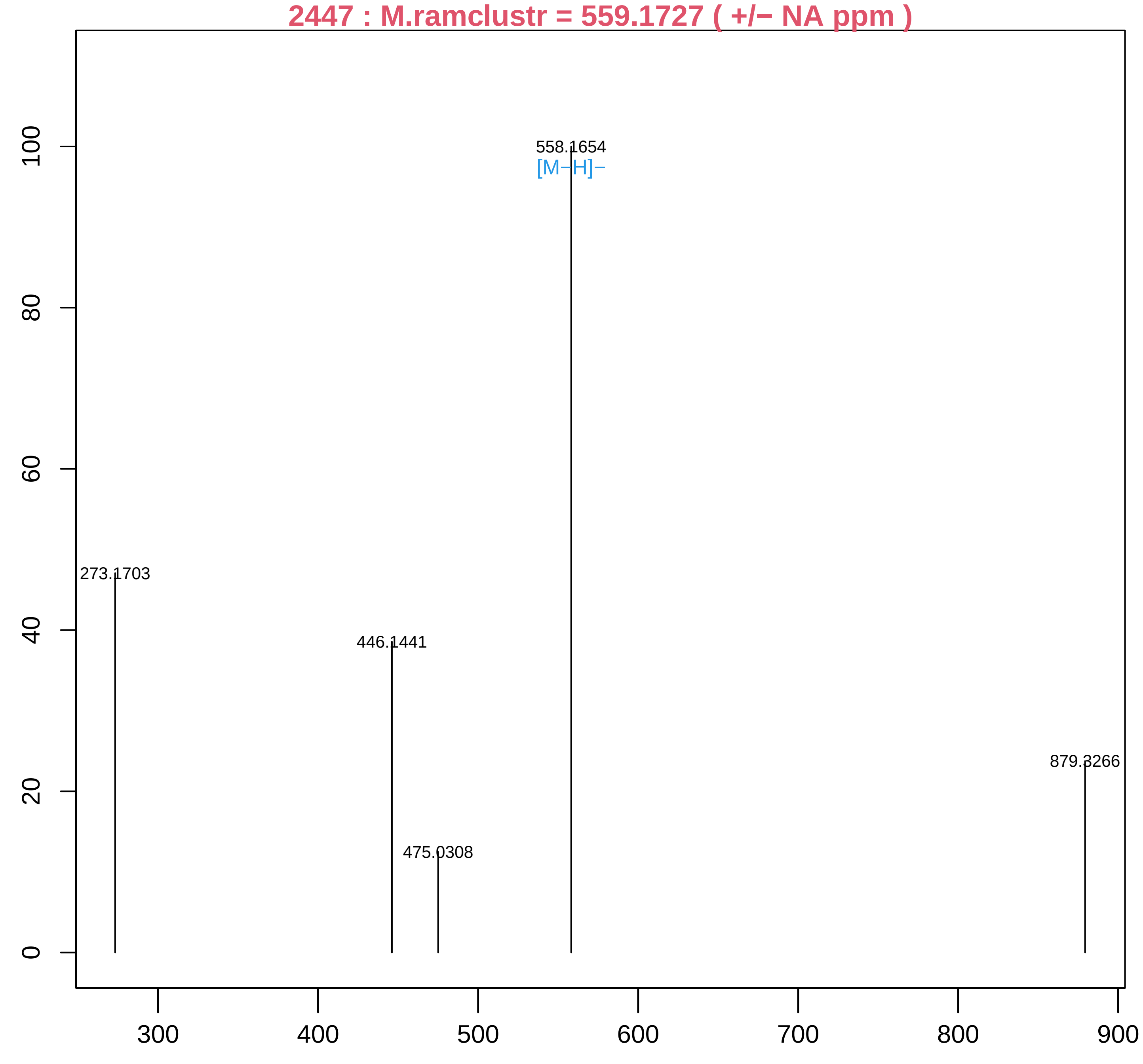
2447 : M.ramclustr = 559.1727 (+/− NA ppm)
558.1654
[M−H]−
273.1703
446.1441
475.0308
879.3266
0
20
40
60
80
100
300
400
500
600
700
800
900

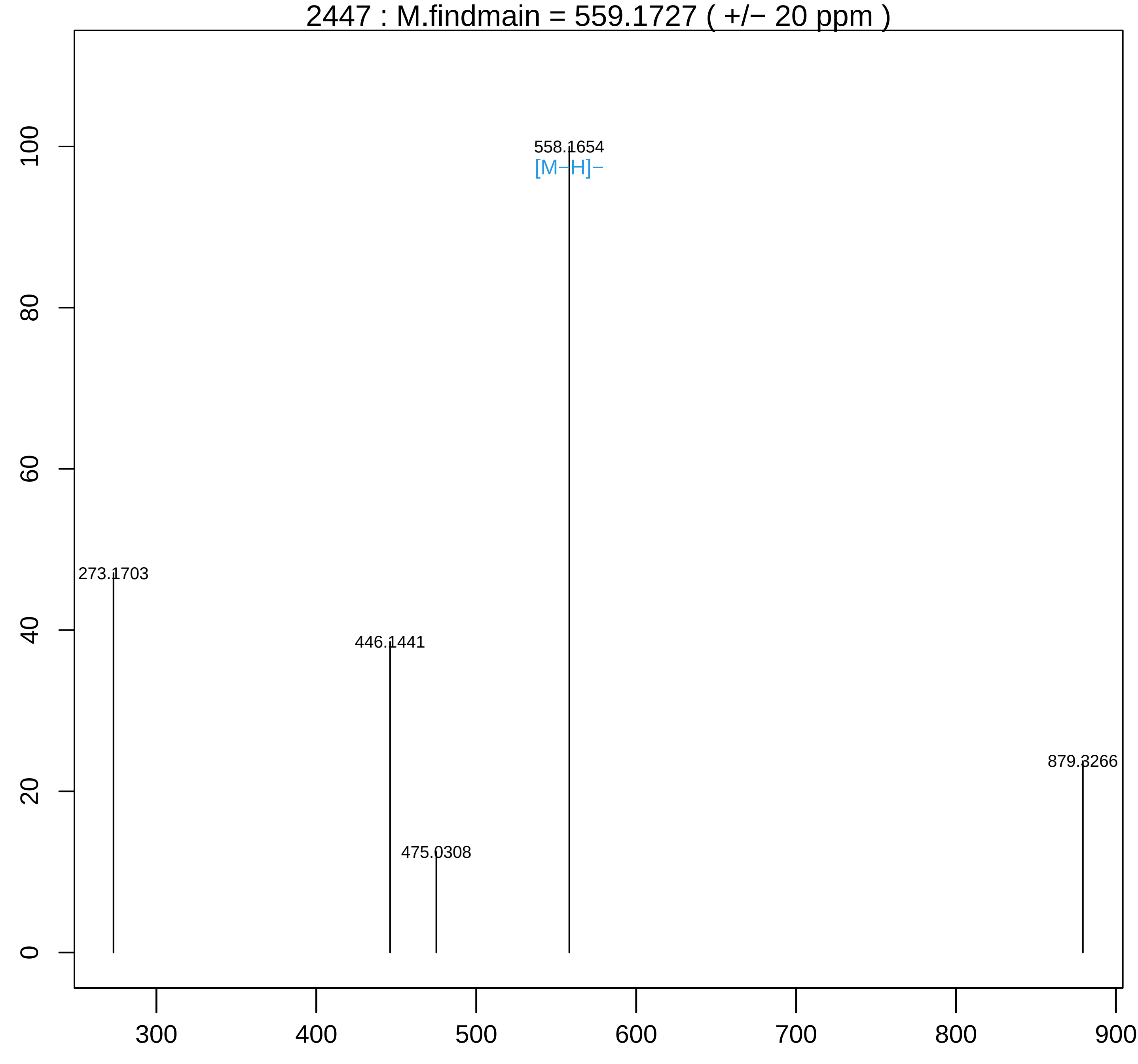
2447 : M.findmain = 559.1727 (+/− 20 ppm)
558.1654
[M−H]−
273.1703
446.1441
475.0308
879.3266
0
20
40
60
80
100
300
400
500
600
700
800
900

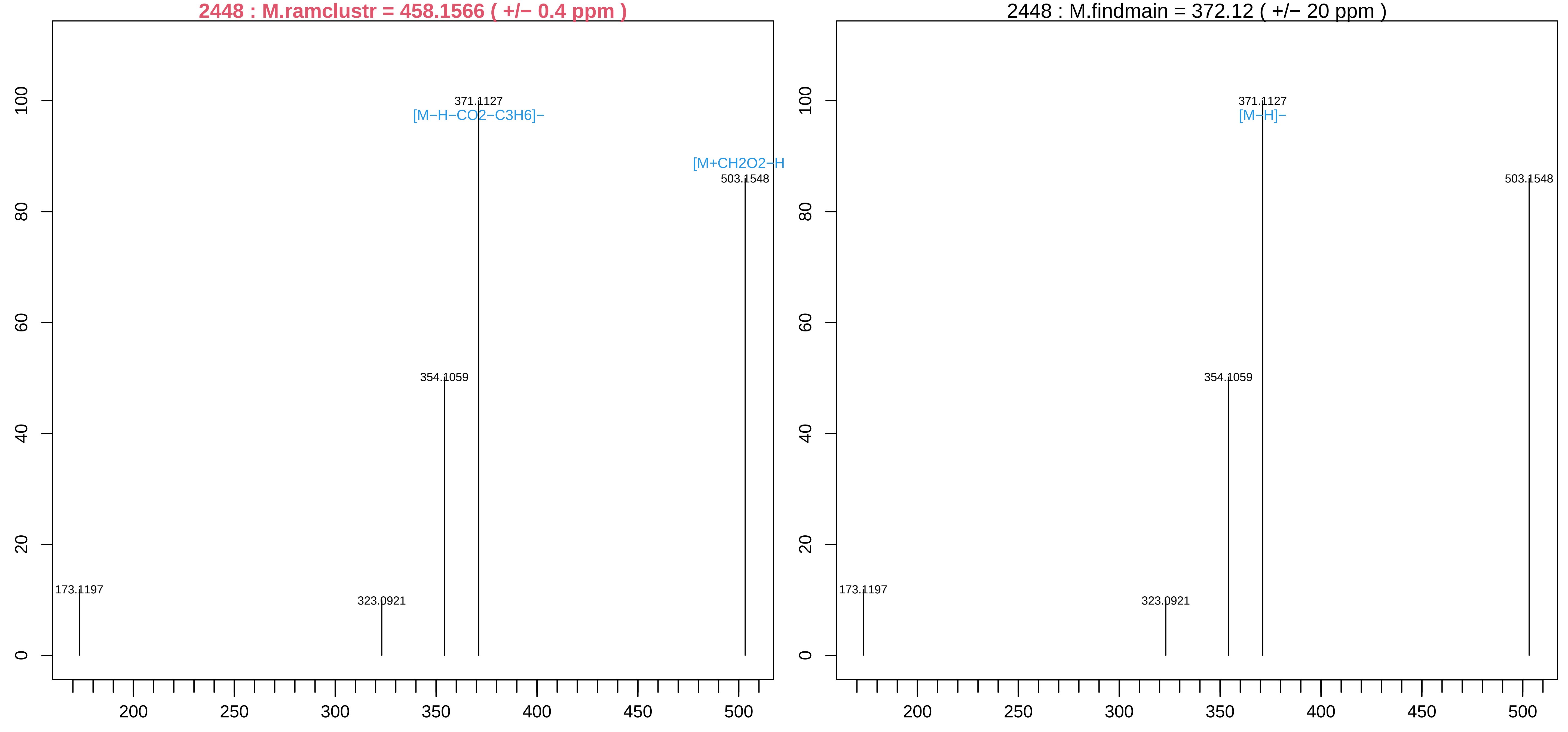

2448 : M.ramclustr = 458.1566 (+/− 0.4 ppm)
371.1127
[M−H−CO2−C3H6]−
[M+CH2O2−H
503.1548
354.1059
173.1197
323.0921
0
20
40
60
80
100
200
250
300
350
400
450
500
2448 : M.findmain = 372.12 (+/− 20 ppm)
371.1127
[M−H]−
503.1548
354.1059
173.1197
323.0921

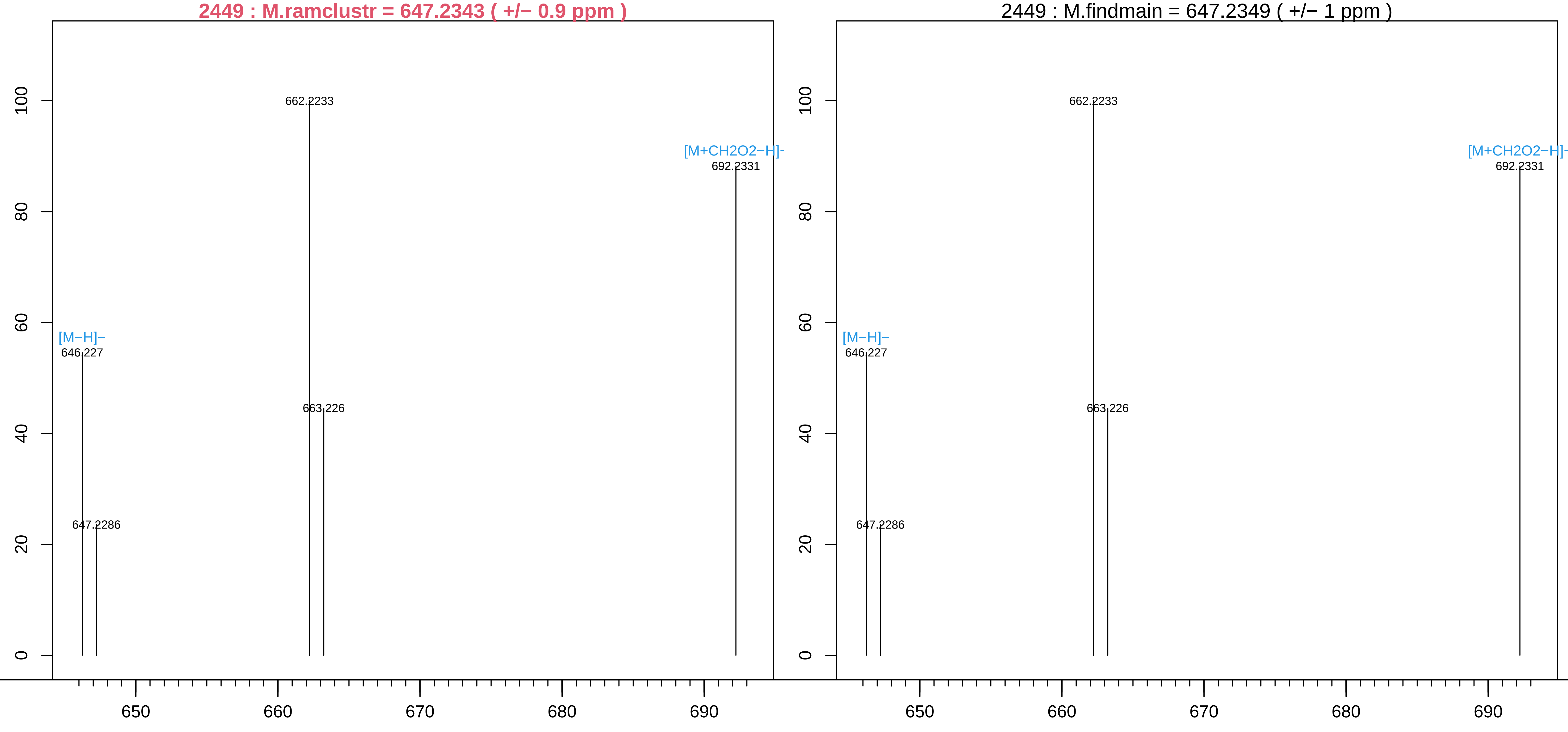
2449 : M.ramclustr = 647.2343 (+/− 0.9 ppm)
2449 : M.findmain = 647.2349 (+/− 1 ppm)
[M−H]−
646.227
647.2286
662.2233
663.226
[M+CH2O2−H]-
692.2331
0
20
40
60
80
100
650
660
670
680
690

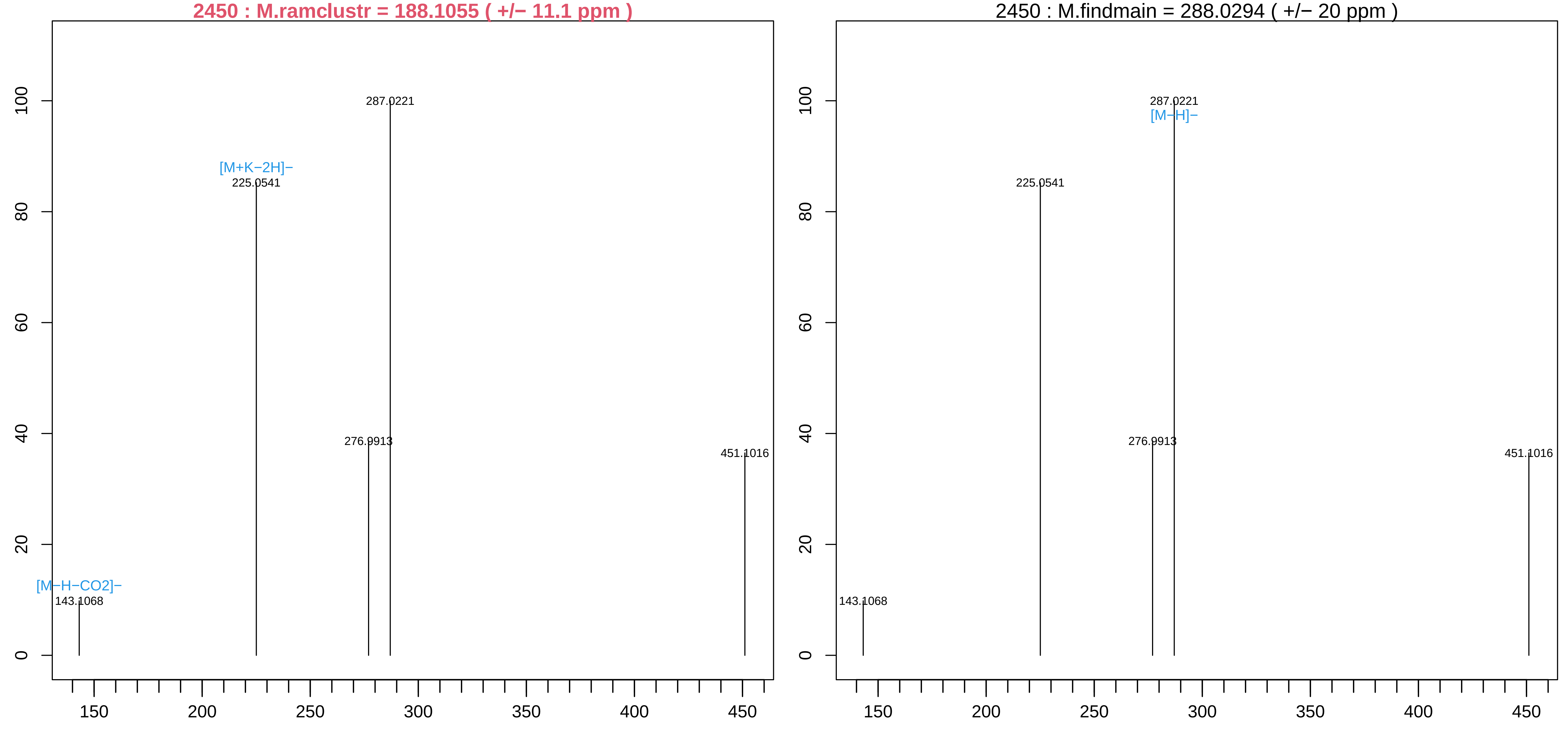
2450 : M.ramclustr = 188.1055 (+/− 11.1 ppm)
[M−H−CO2]−
143.1068
[M+K−2H]−
225.0541
276.9913
287.0221
451.1016
2450 : M.findmain = 288.0294 (+/− 20 ppm)
143.1068
225.0541
276.9913
287.0221
[M−H]−
451.1016
0
20
40
60
80
100
150
200
250
300
350
400
450

2451 : M.ramclustr = 588.1136 (+/− 0.4 ppm)

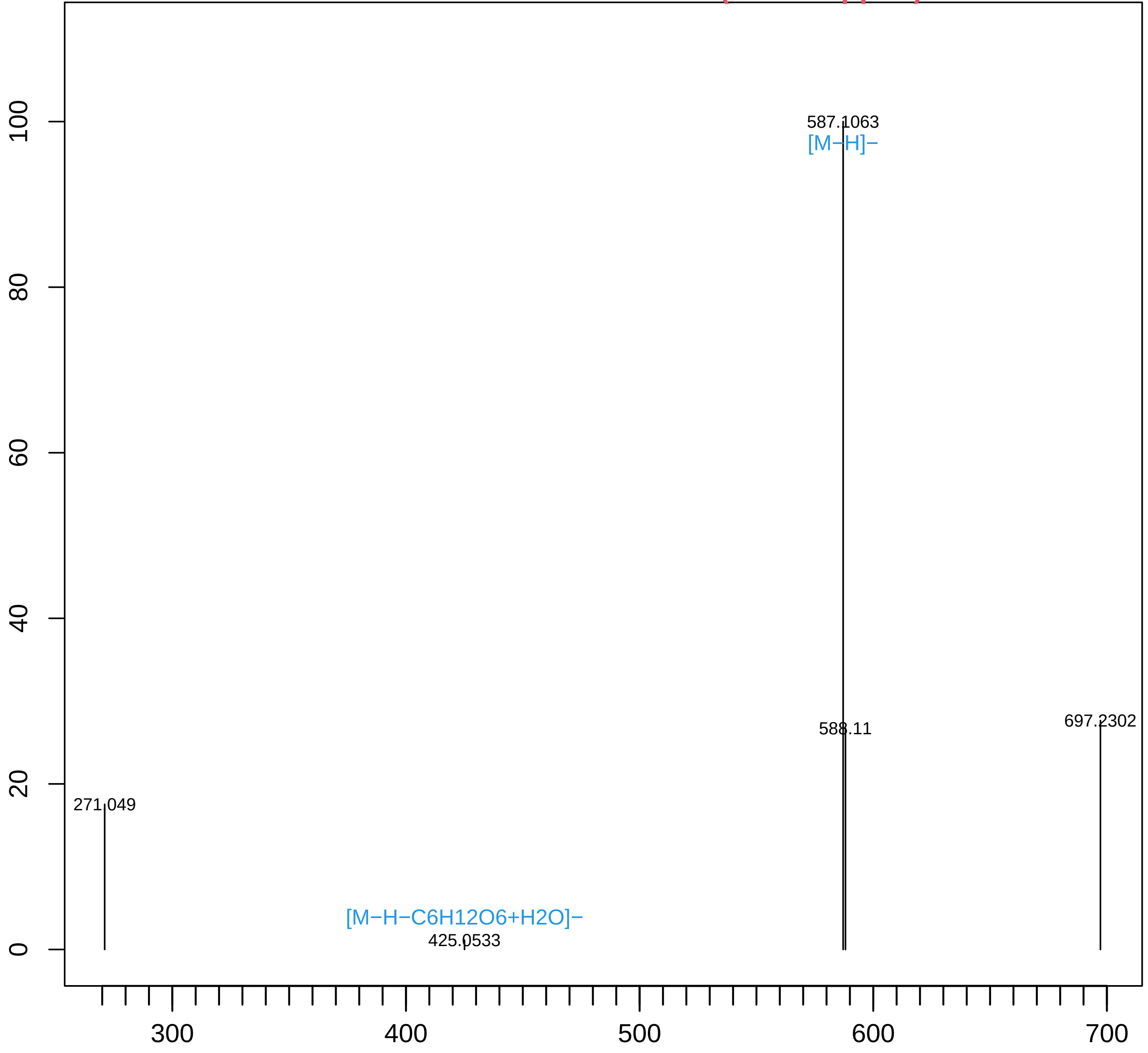

2451 : M.findmain = 588.1136 (+/− 20 ppm)

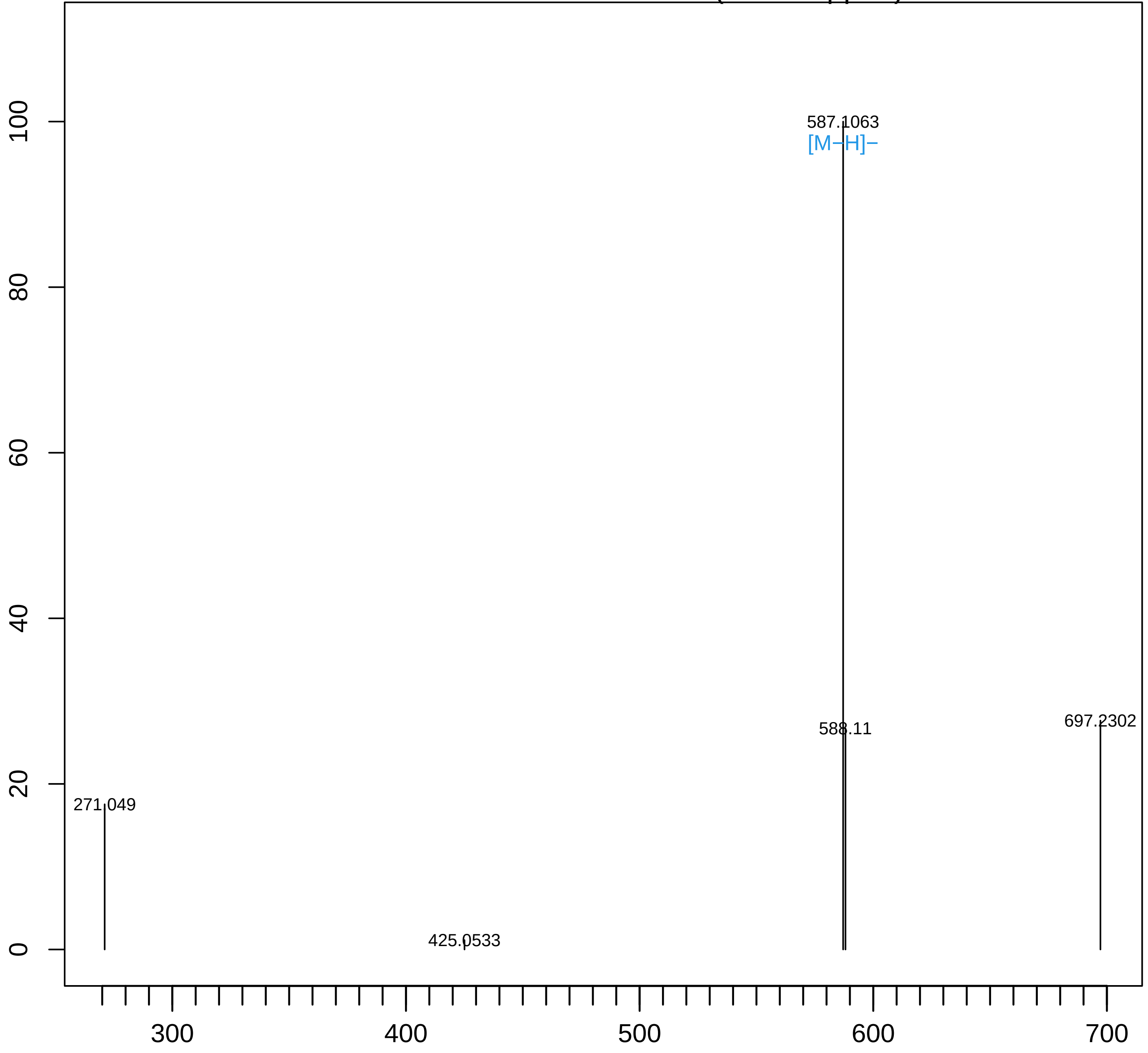

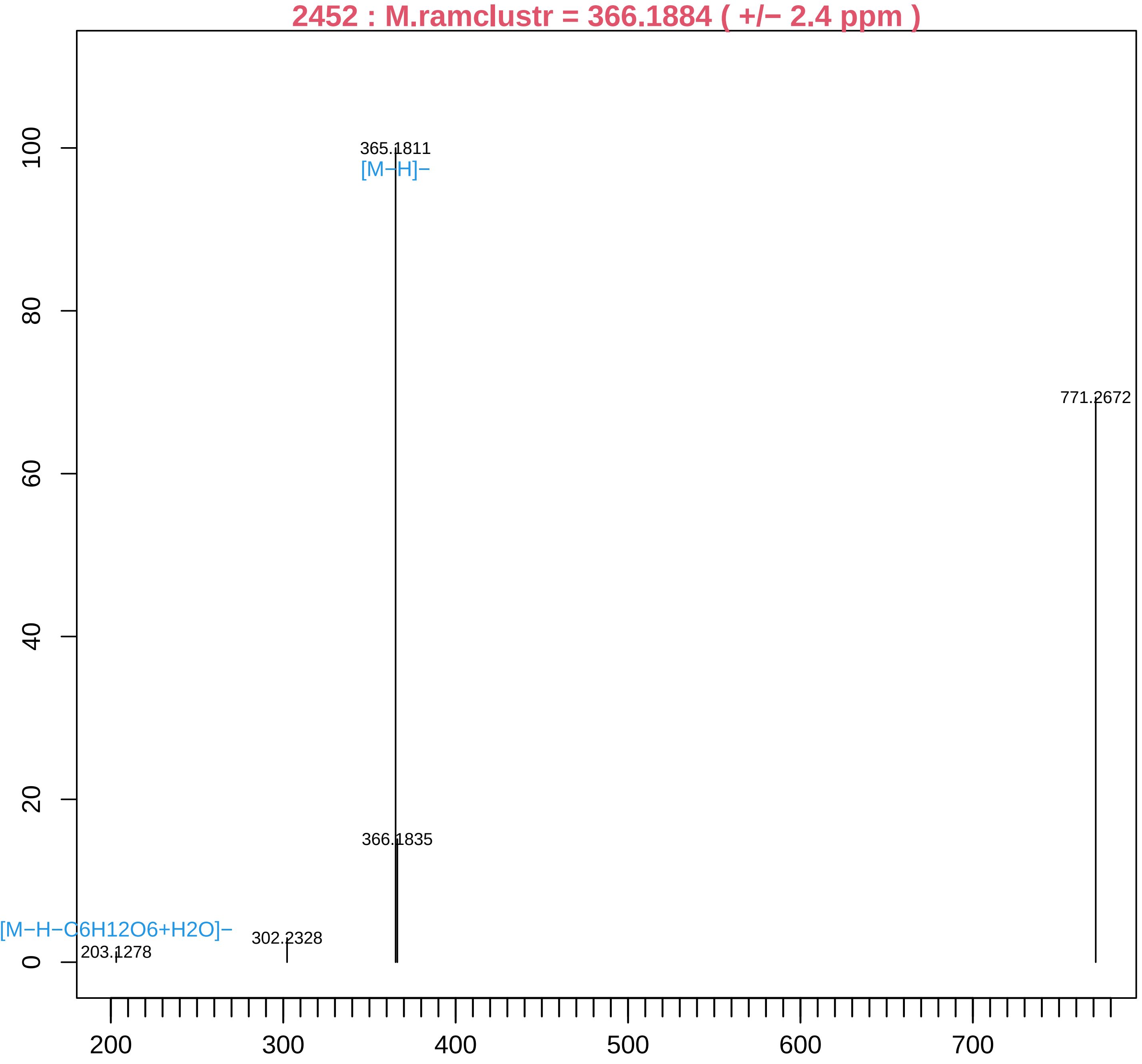
2452 : M.ramclustr = 366.1884 (+/− 2.4 ppm)
365.1811
[M−H]−
771.2672
366.1835
[M−H−C6H12O6+H2O]−
203.1278
302.2328
0
20
40
60
80
100
200
300
400
500
600
700

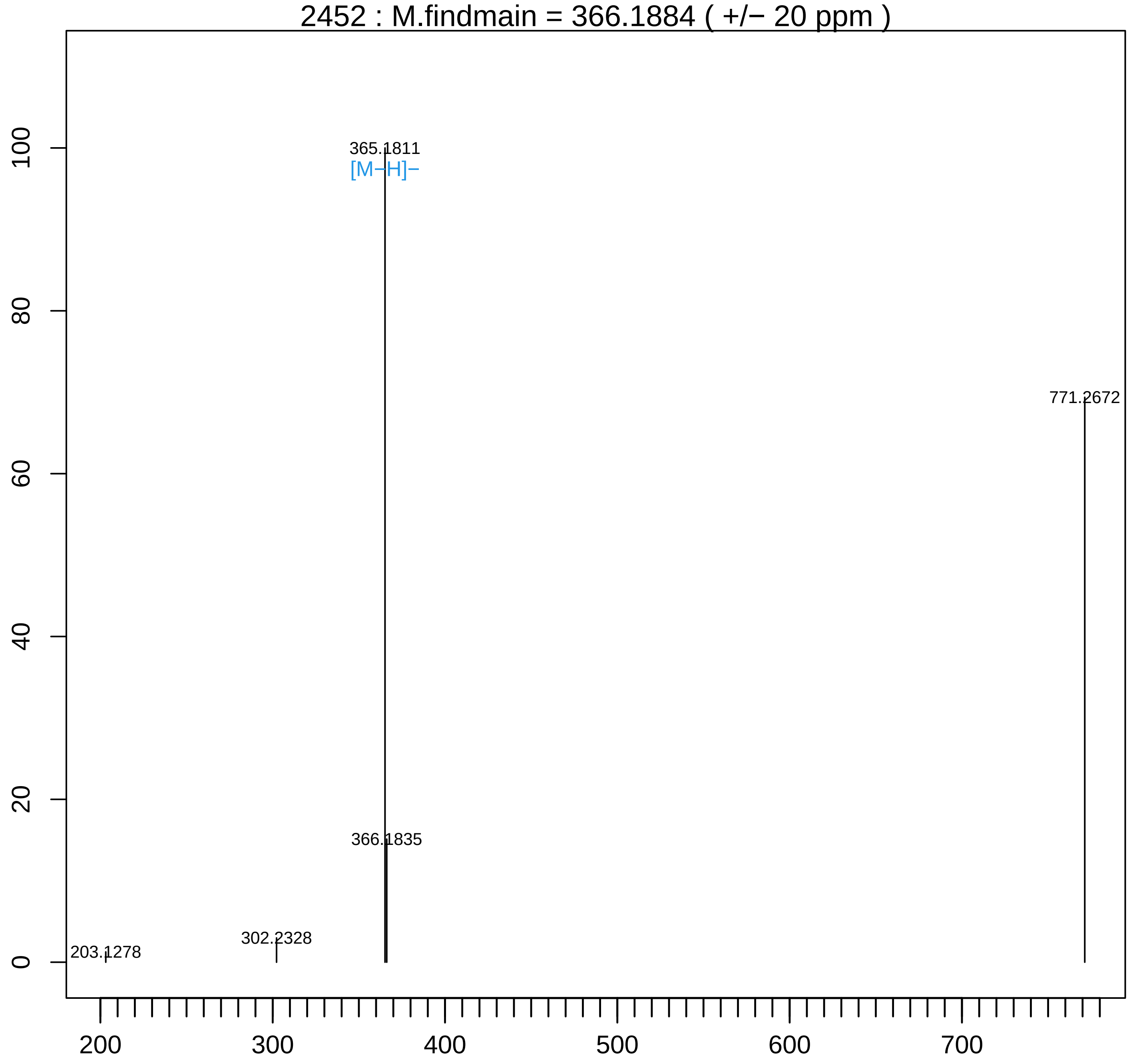
2452 : M.findmain = 366.1884 (+/− 20 ppm)
365.1811
[M−H]−
771.2672
366.1835
203.1278
302.2328
0
20
40
60
80
100
200
300
400
500
600
700

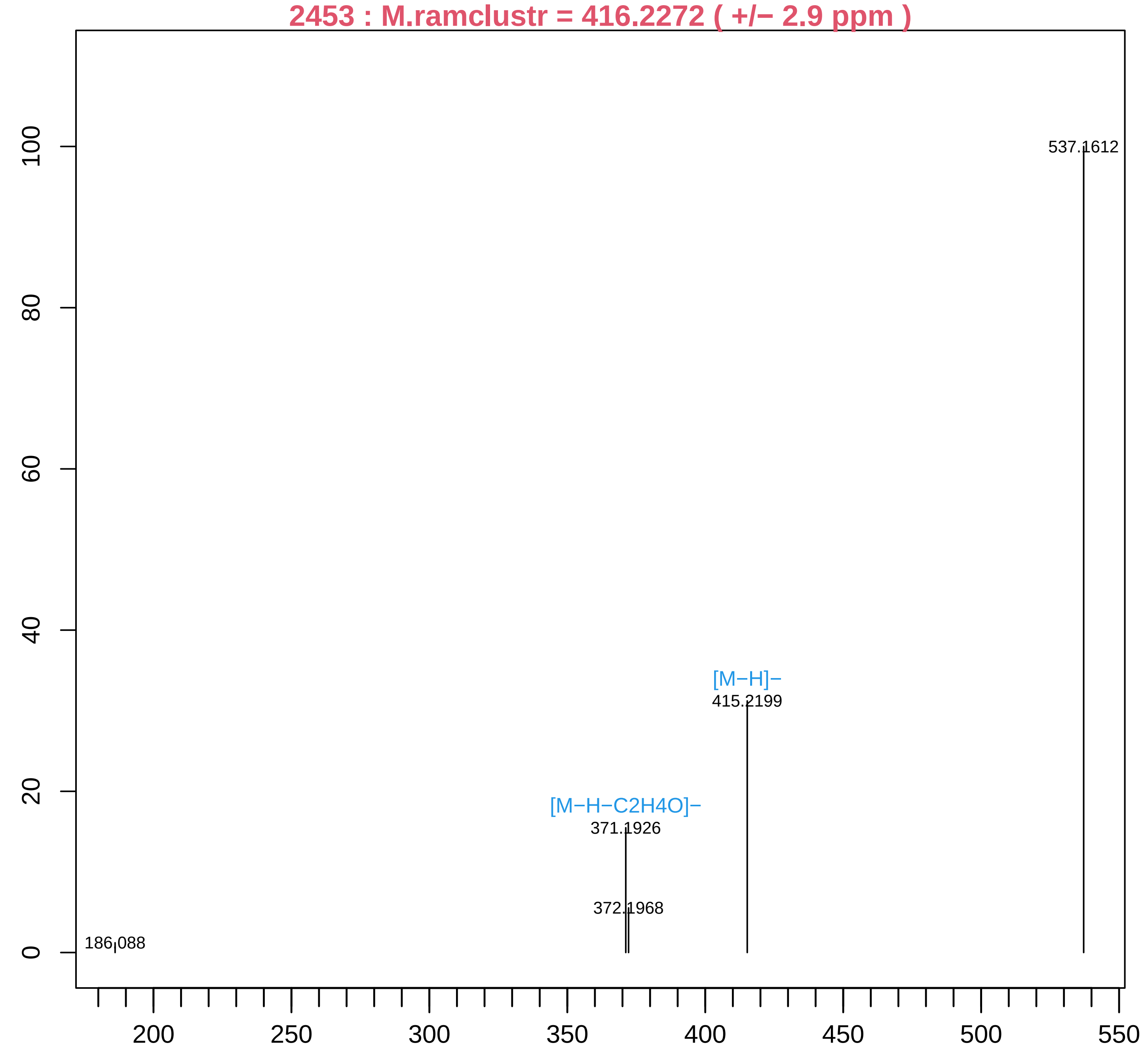
2453 : M.ramclustr = 416.2272 (+/− 2.9 ppm)
537.1612
[M−H]−
415.2199
[M−H−C2H4O]−
371.1926
372.1968
186.088
0
20
40
60
80
100
200
250
300
350
400
450
500
550

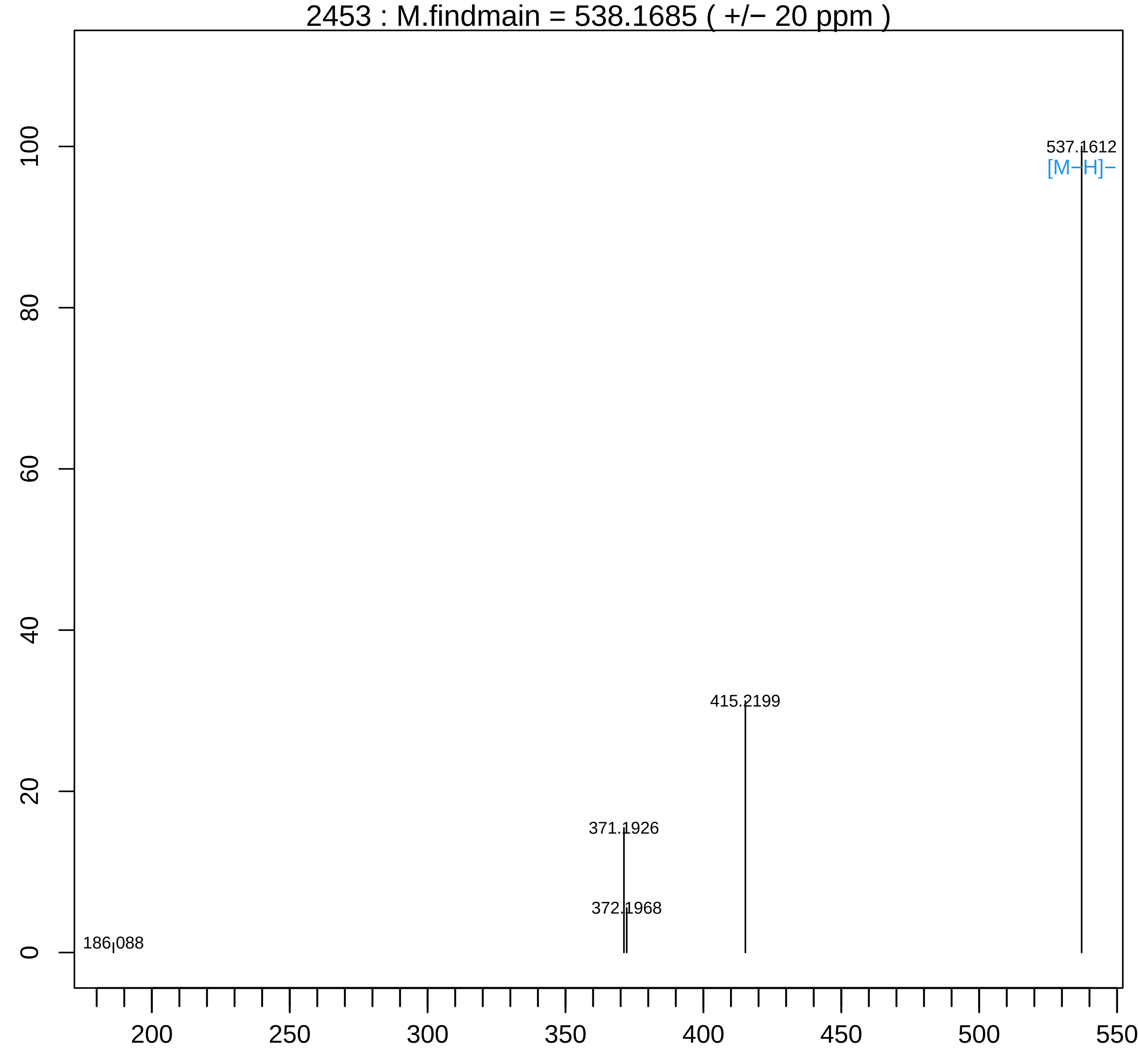
2453 : M.findmain = 538.1685 (+/− 20 ppm)
537.1612
[M−H]−
415.2199
371.1926
372.1968
186.088
0
20
40
60
80
100
200
250
300
350
400
450
500
550

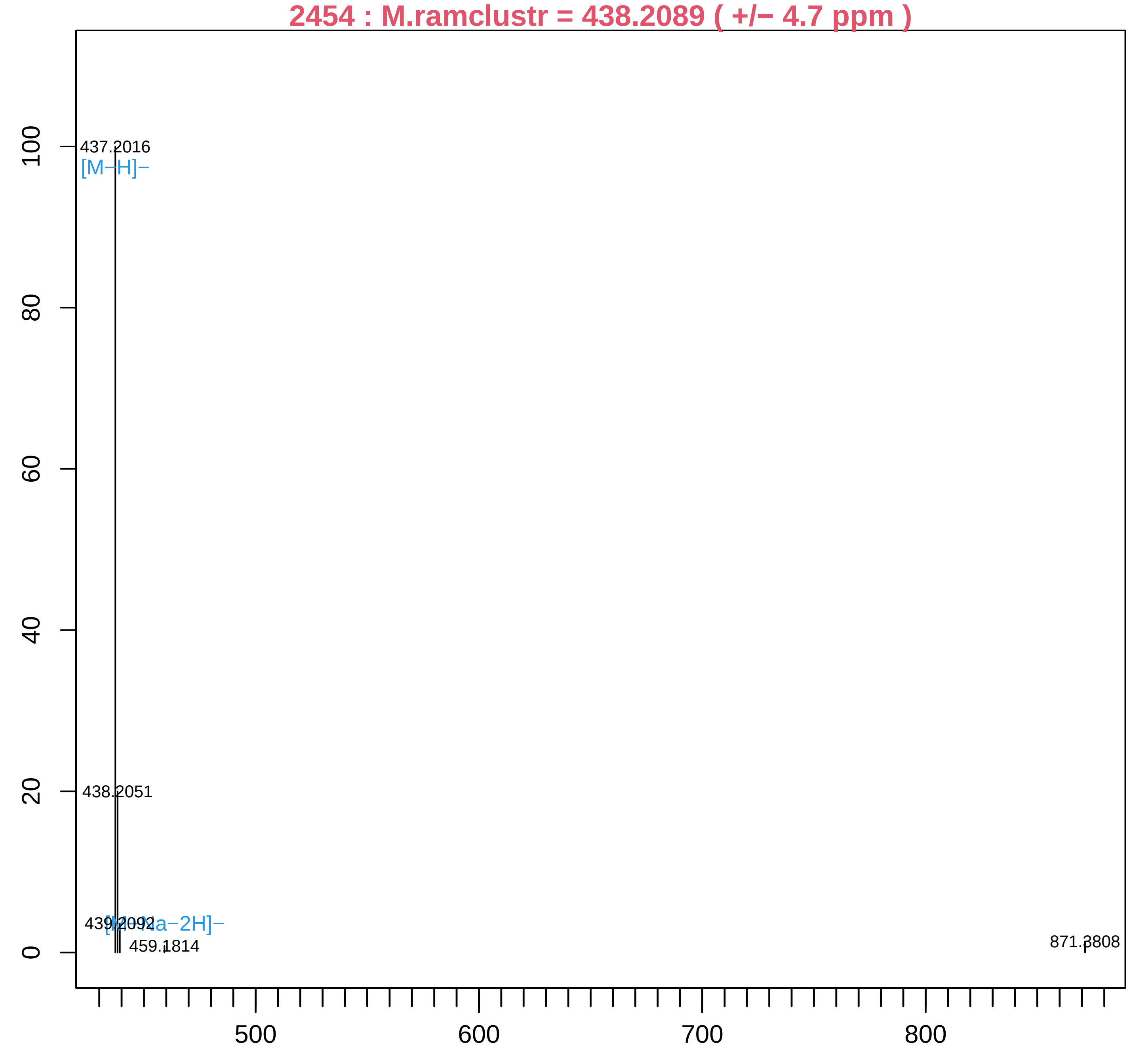
2454 : M.ramclustr = 438.2089 (+/− 4.7 ppm)
437.2016
[M−H]−
438.2051
439.2092
[M+Na−2H]−
459.1814
871.3808
0
20
40
60
80
100
500
600
700
800

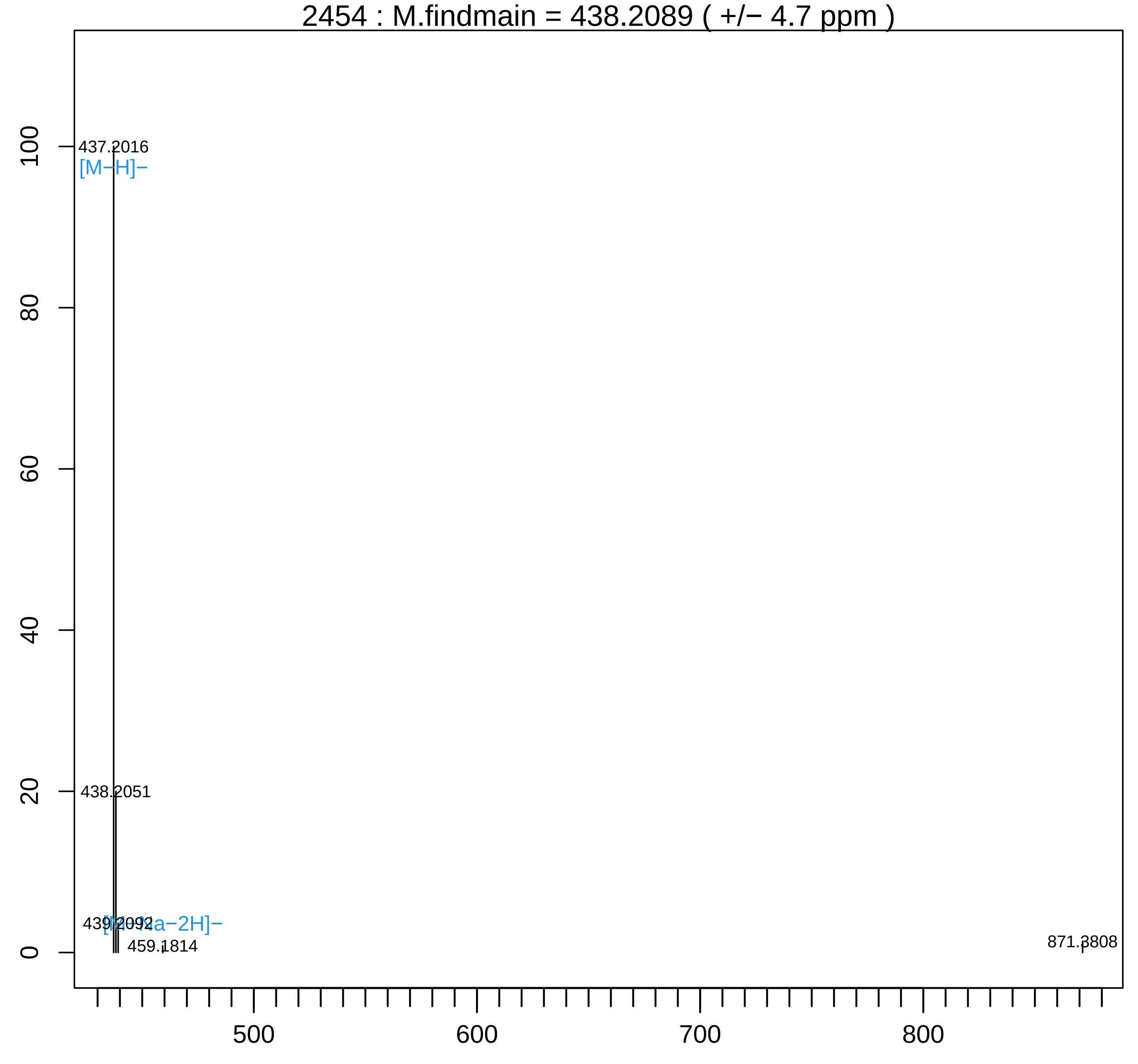
2454 : M.findmain = 438.2089 (+/− 4.7 ppm)
437.2016
[M−H]−
438.2051
439.2092
[M+Na−2H]−
459.1814
871.3808
0
20
40
60
80
100
500
600
700
800

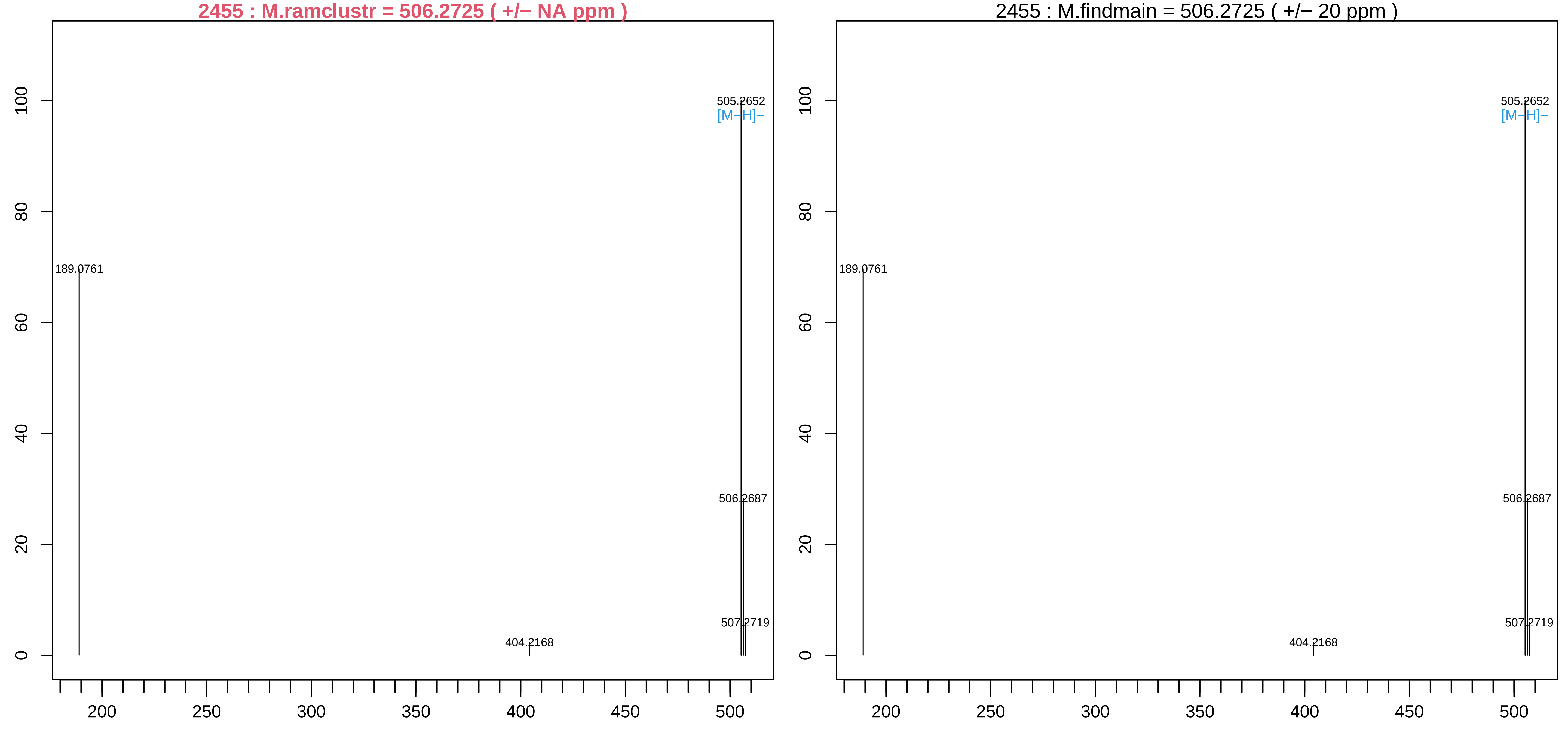
2455 : M.ramclustr = 506.2725 (+/− NA ppm)
2455 : M.findmain = 506.2725 (+/− 20 ppm)
505.2652
[M−H]−
189.0761
506.2687
507.2719
404.2168
0
20
40
60
80
100
200
250
300
350
400
450
500

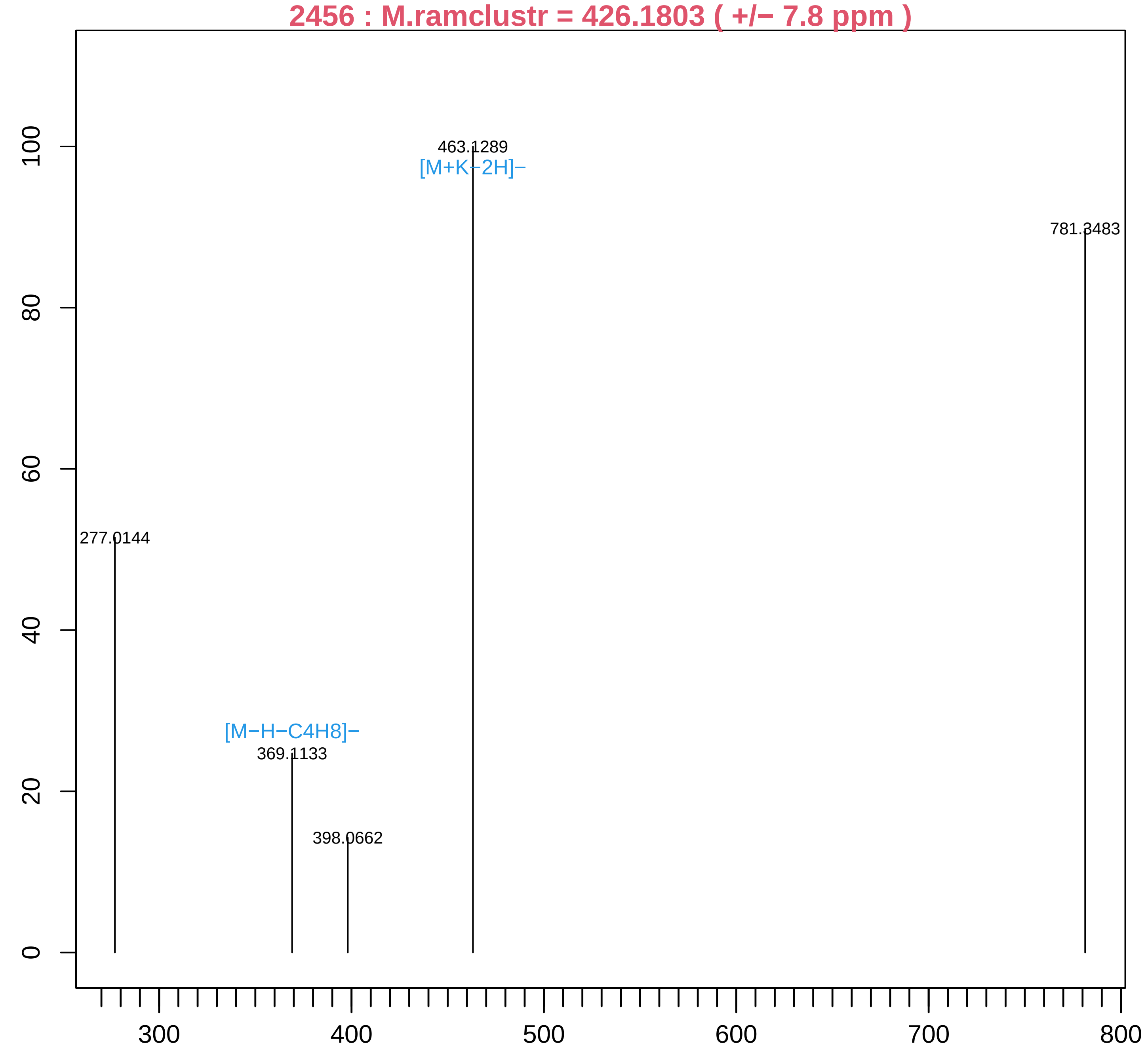
2456 : M.ramclustr = 426.1803 (+/− 7.8 ppm)
463.1289
[M+K−2H]−
781.3483
277.0144
[M−H−C4H8]−
369.1133
398.0662
0
20
40
60
80
100
300
400
500
600
700
800

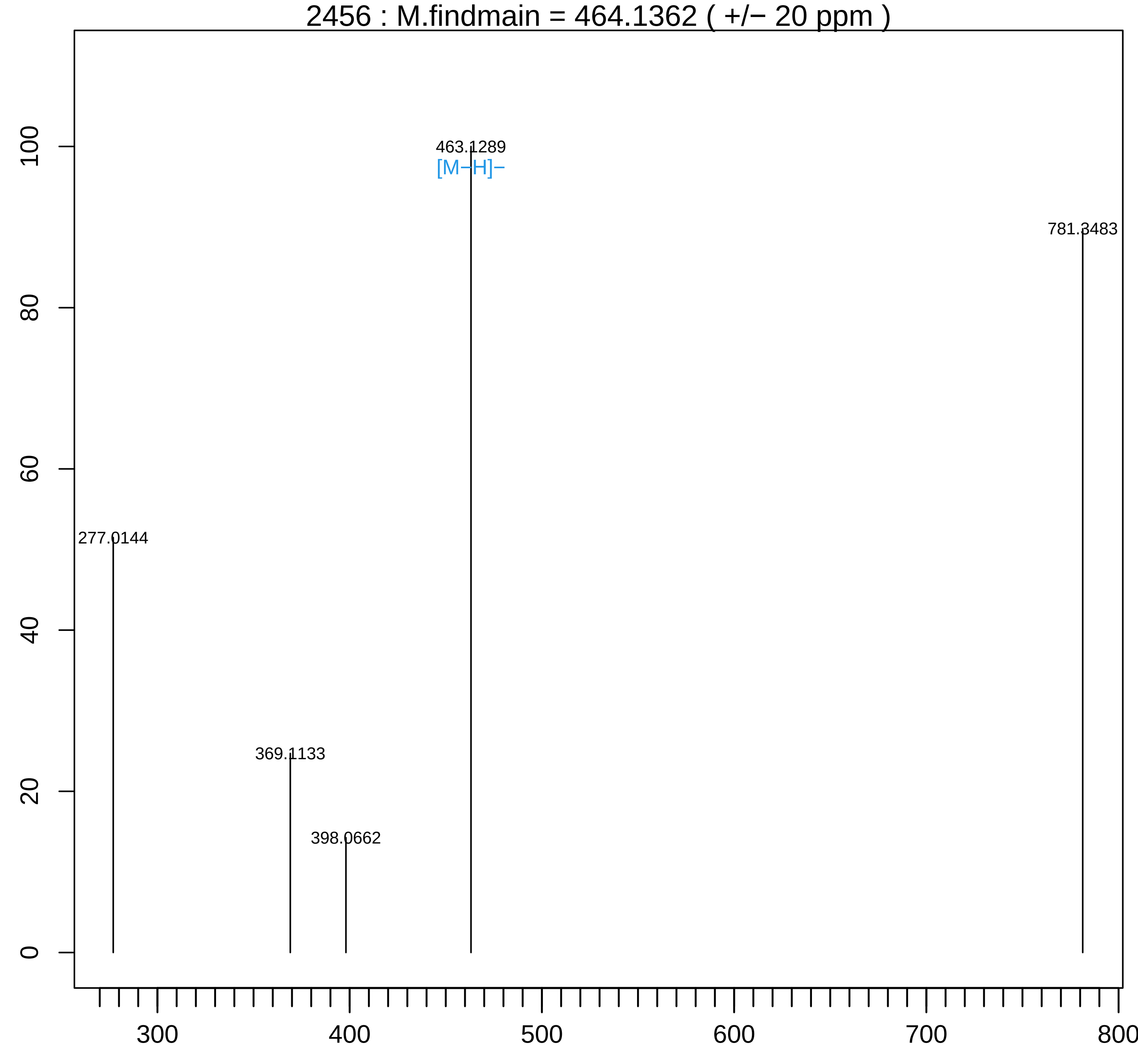
2456 : M.findmain = 464.1362 (+/− 20 ppm)
463.1289
[M−H]−
781.3483
277.0144
369.1133
398.0662
0
20
40
60
80
100
300
400
500
600
700
800

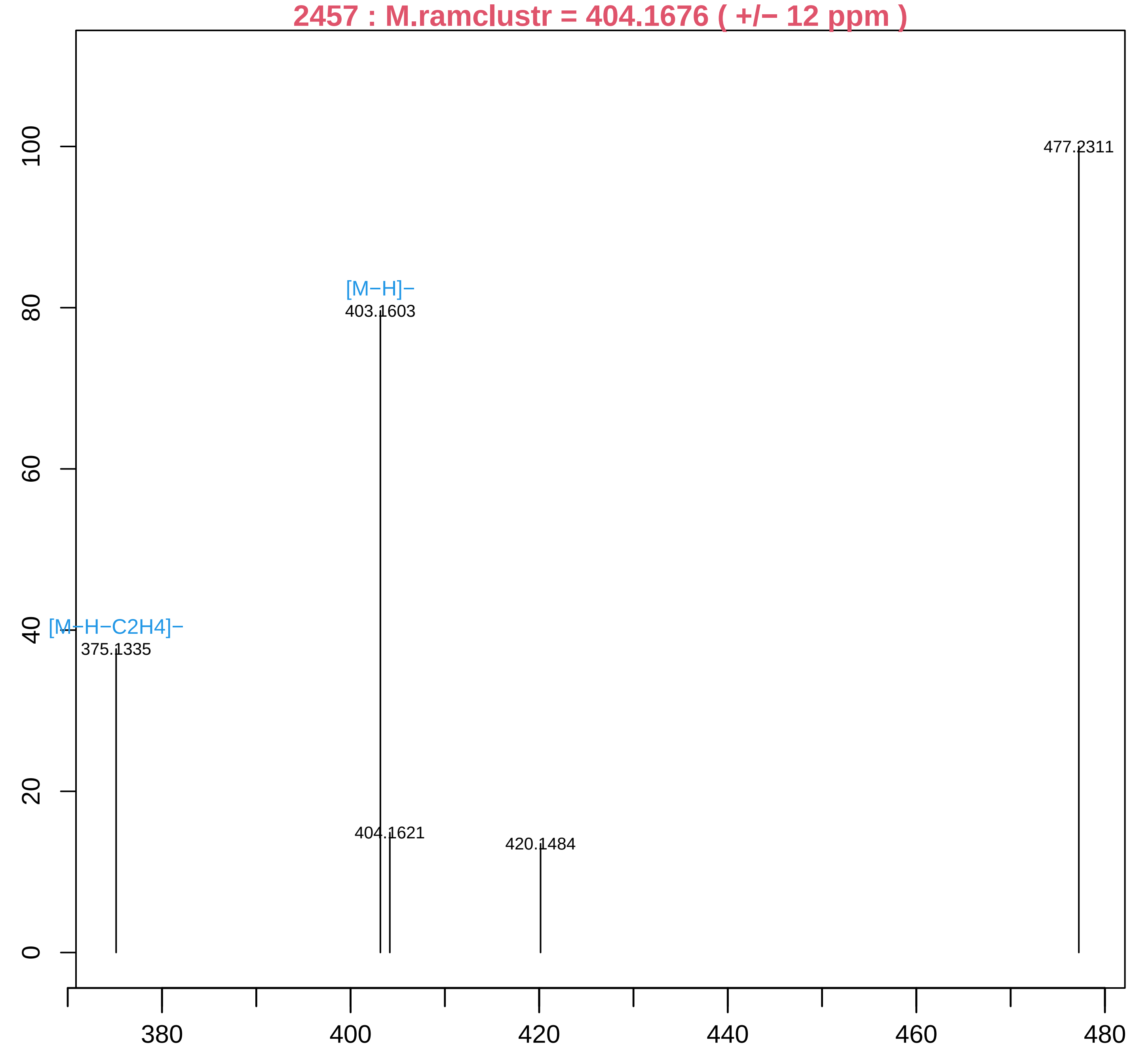

2457 : M.ramclustr = 404.1676 (+/− 12 ppm)
477.2311
[M−H]−
403.1603
[M−H−C2H4]−
375.1335
404.1621
420.1484
0
20
40
60
80
100
380
400
420
440
460
480

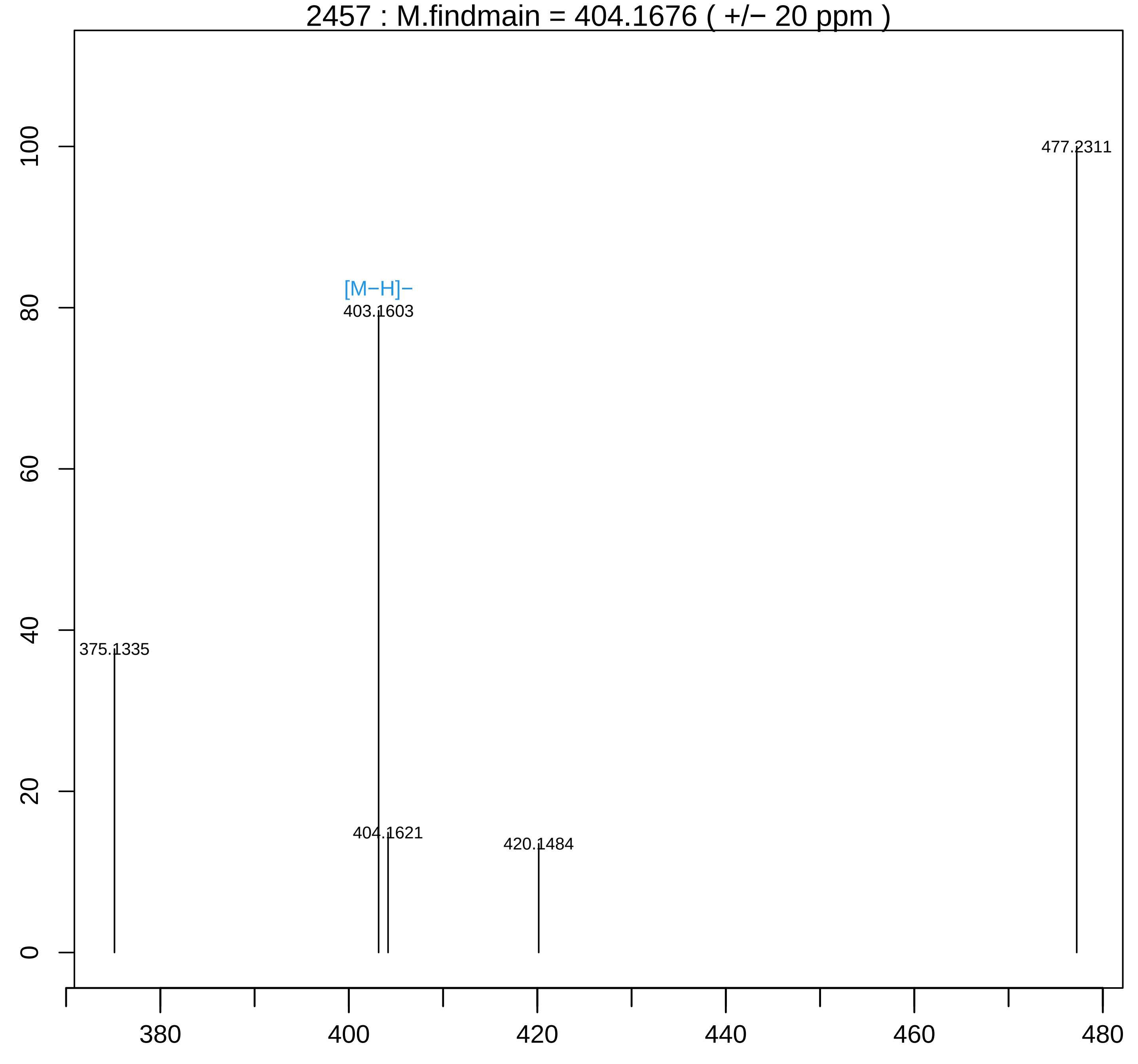

2457 : M.findmain = 404.1676 (+/− 20 ppm)
477.2311
[M−H]−
403.1603
375.1335
404.1621
420.1484
0
20
40
60
80
100
380
400
420
440
460
480

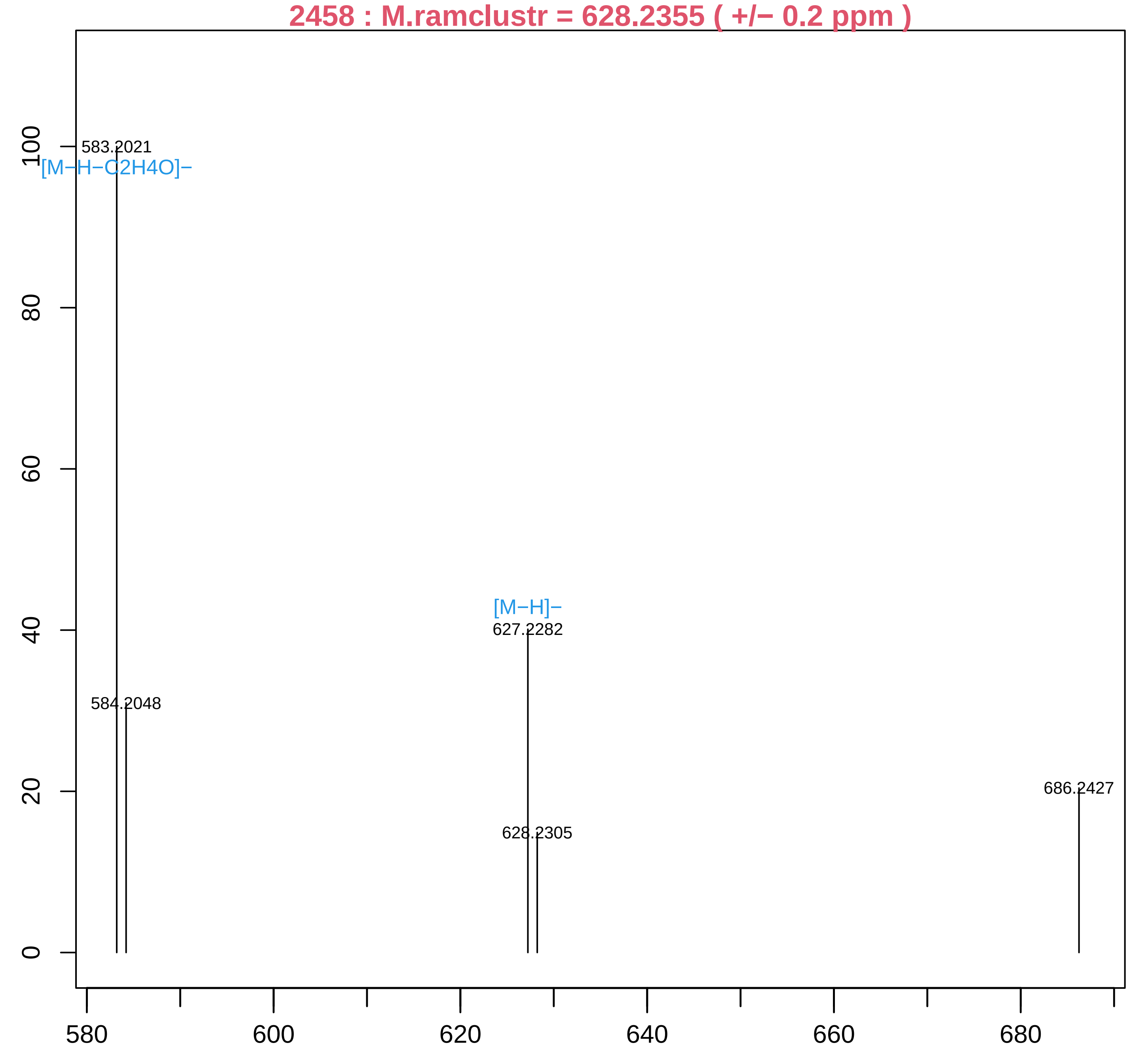

2458 : M.ramclustr = 628.2355 (+/− 0.2 ppm)
583.2021
[M−H−C2H4O]−
584.2048
[M−H]−
627.2282
628.2305
686.2427
0
20
40
60
80
100
580
600
620
640
660
680

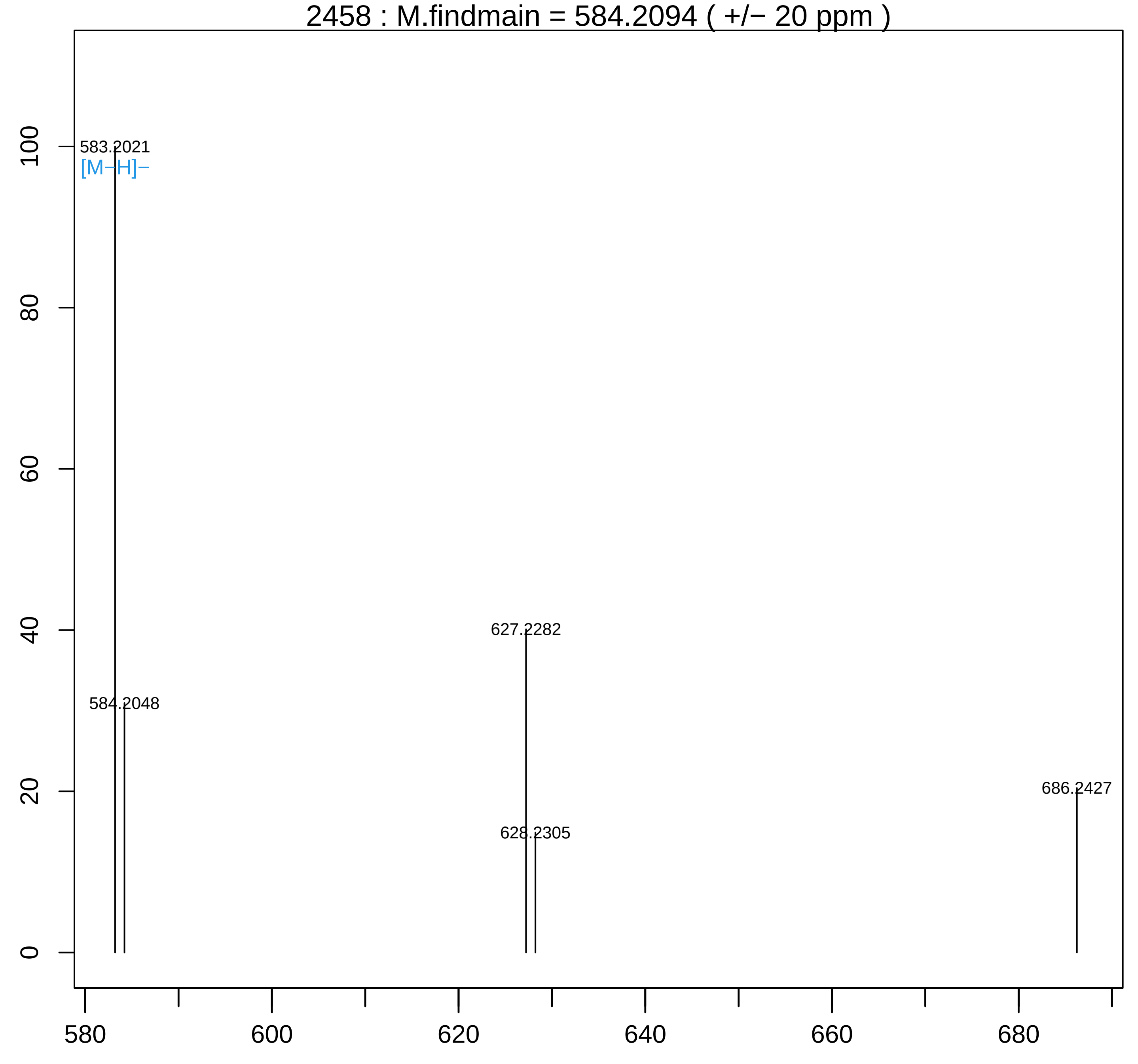

2458 : M.findmain = 584.2094 (+/− 20 ppm)
583.2021
[M−H]−
584.2048
627.2282
628.2305
686.2427
0
20
40
60
80
100
580
600
620
640
660
680

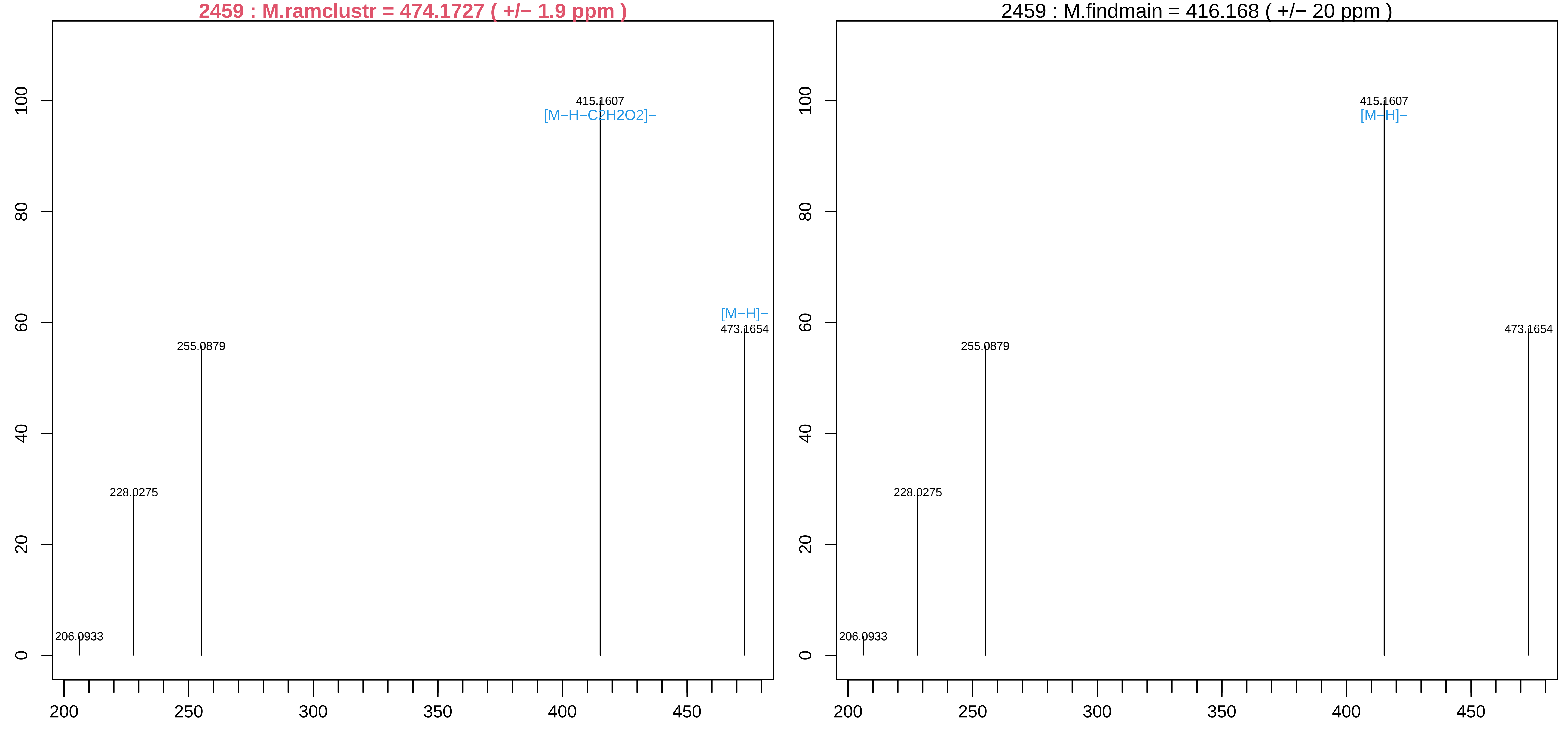
2459 : M.ramclustr = 474.1727 (+/− 1.9 ppm)
415.1607
[M−H−C2H2O2]−
[M−H]−
473.1654
255.0879
228.0275
206.0933
2459 : M.findmain = 416.168 (+/− 20 ppm)
415.1607
[M−H]−
473.1654
255.0879
228.0275
206.0933

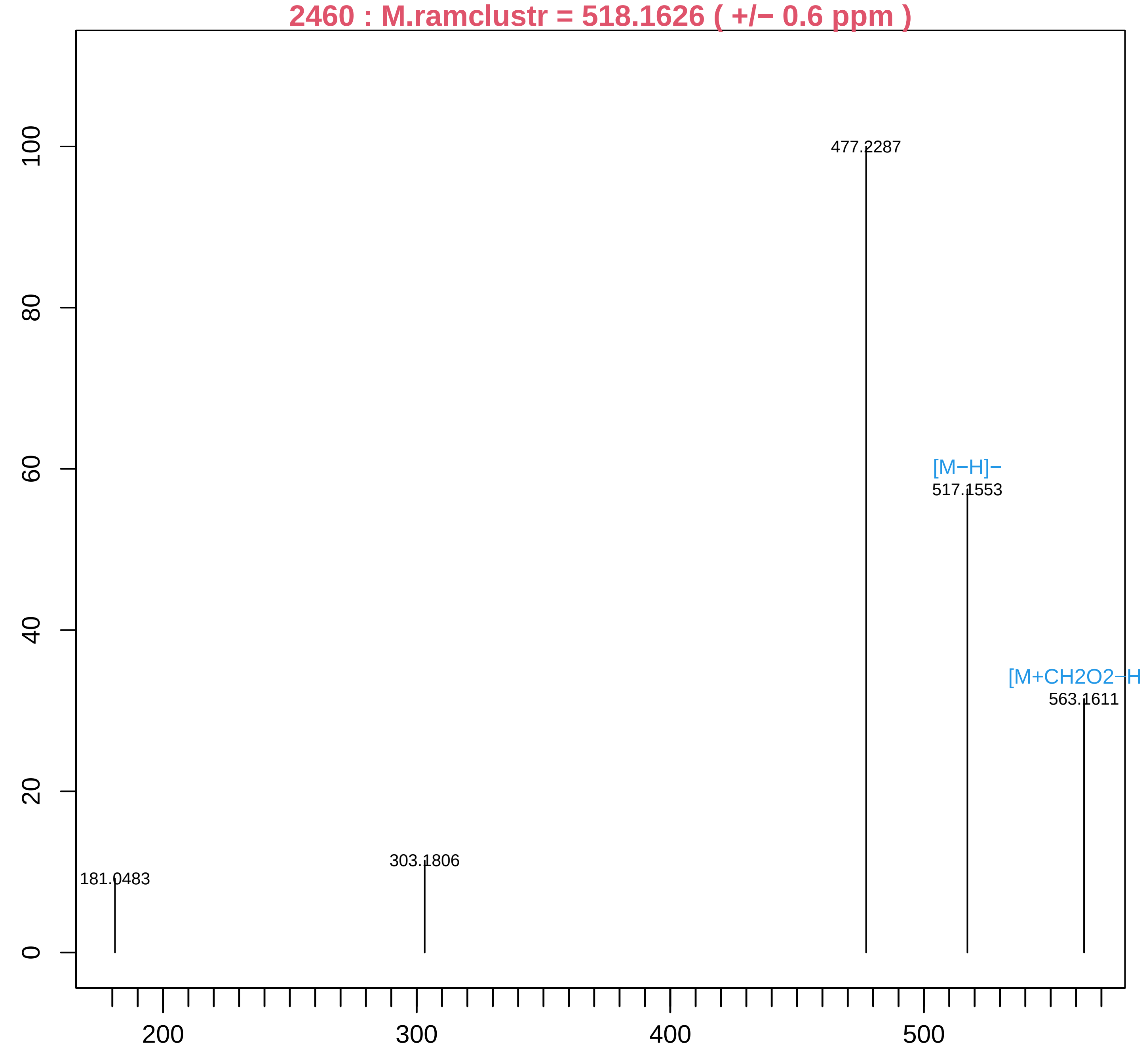

2460 : M.ramclustr = 518.1626 (+/− 0.6 ppm)
477.2287
[M−H]−
517.1553
[M+CH2O2−H
563.1611
303.1806
181.0483
0
20
40
60
80
100
200
300
400
500

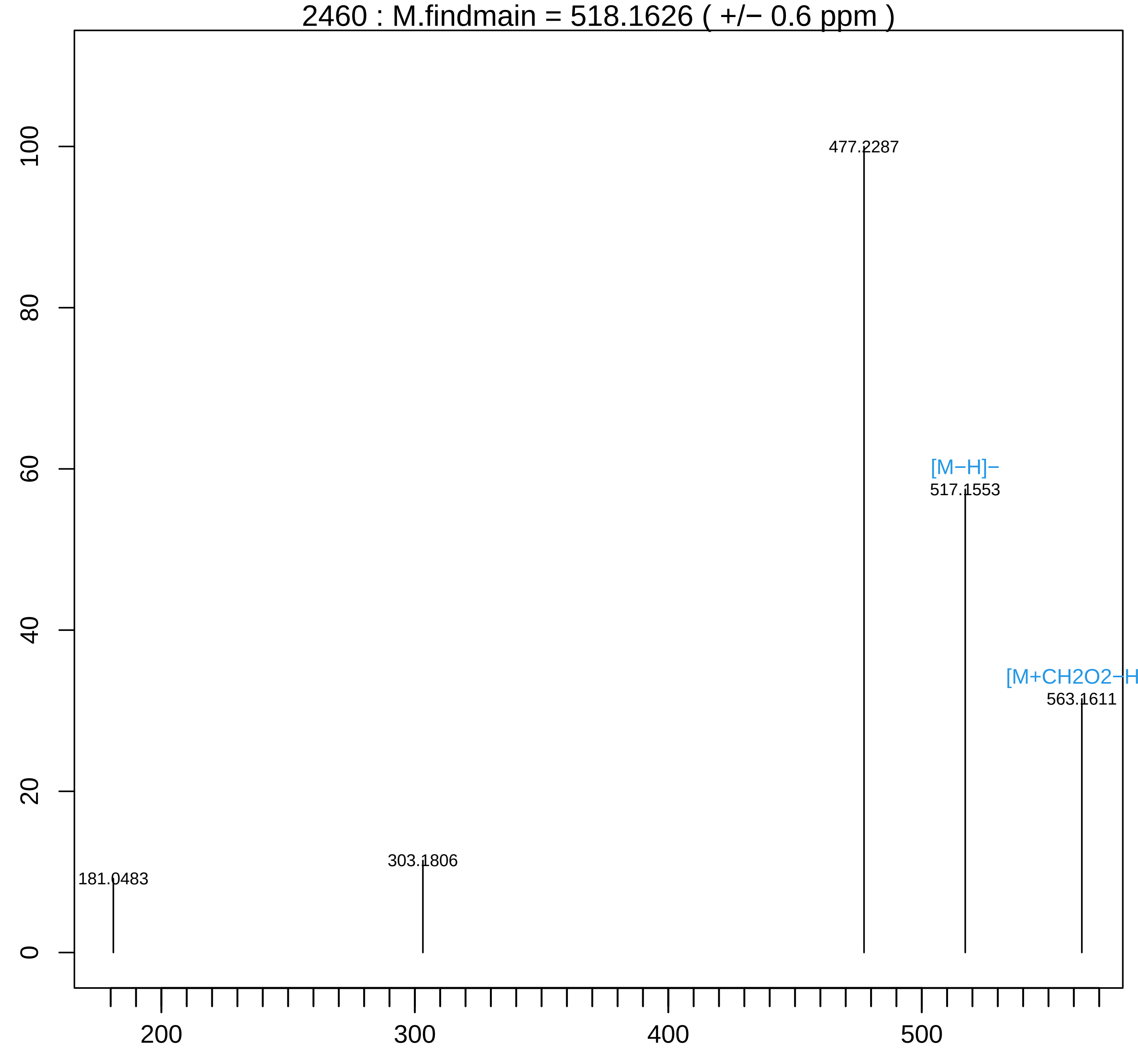

2460 : M.findmain = 518.1626 (+/− 0.6 ppm)
477.2287
[M−H]−
517.1553
[M+CH2O2−H
563.1611
303.1806
181.0483
0
20
40
60
80
100
200
300
400
500

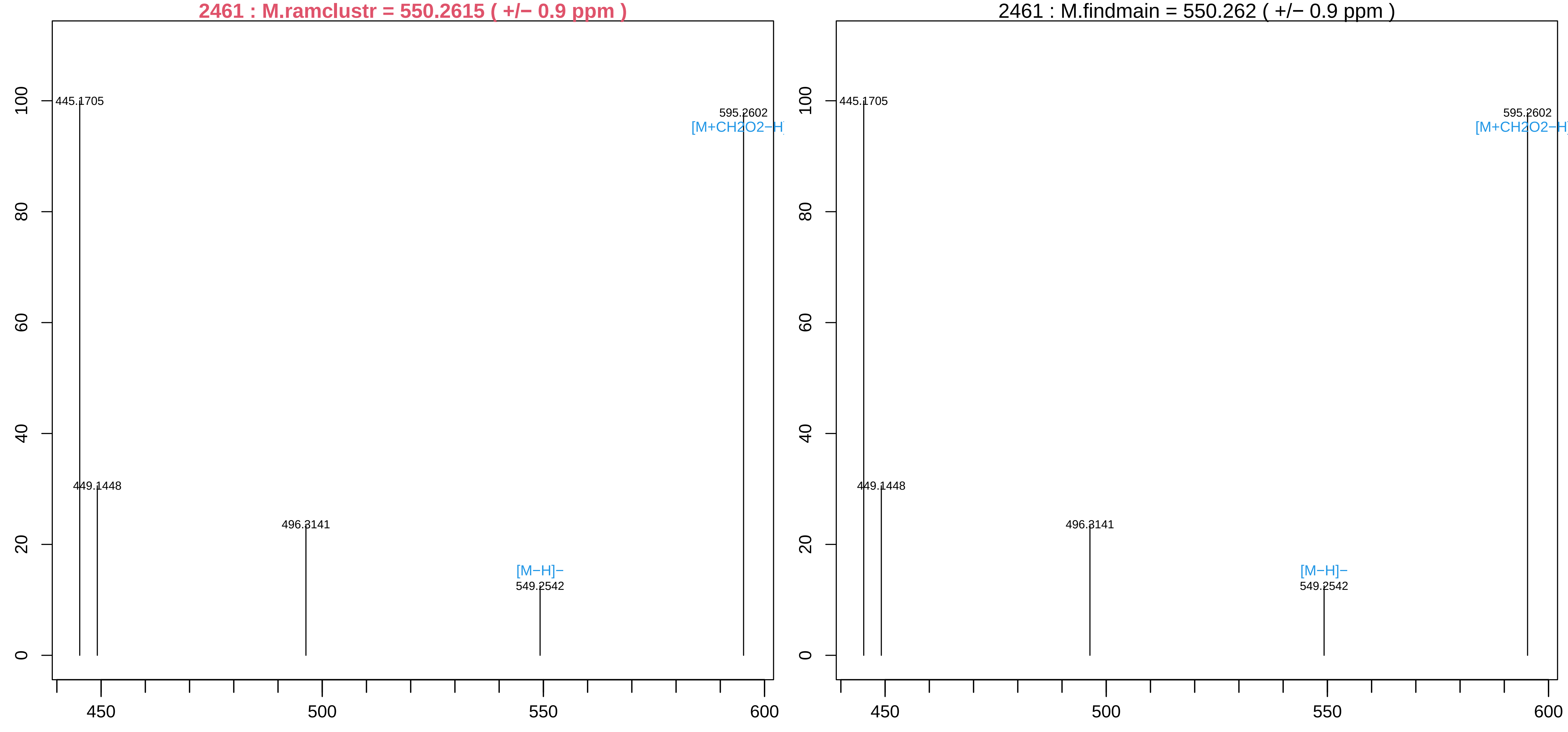
2461 : M.ramclustr = 550.2615 (+/− 0.9 ppm)
445.1705
595.2602
[M+CH2O2−H]
449.1448
496.3141
[M−H]−
549.2542
0
20
40
60
80
100
450
500
550
600
2461 : M.findmain = 550.262 (+/− 0.9 ppm)
445.1705
595.2602
[M+CH2O2−H]
449.1448
496.3141
[M−H]−
549.2542
0
20
40
60
80
100
450
500
550
600

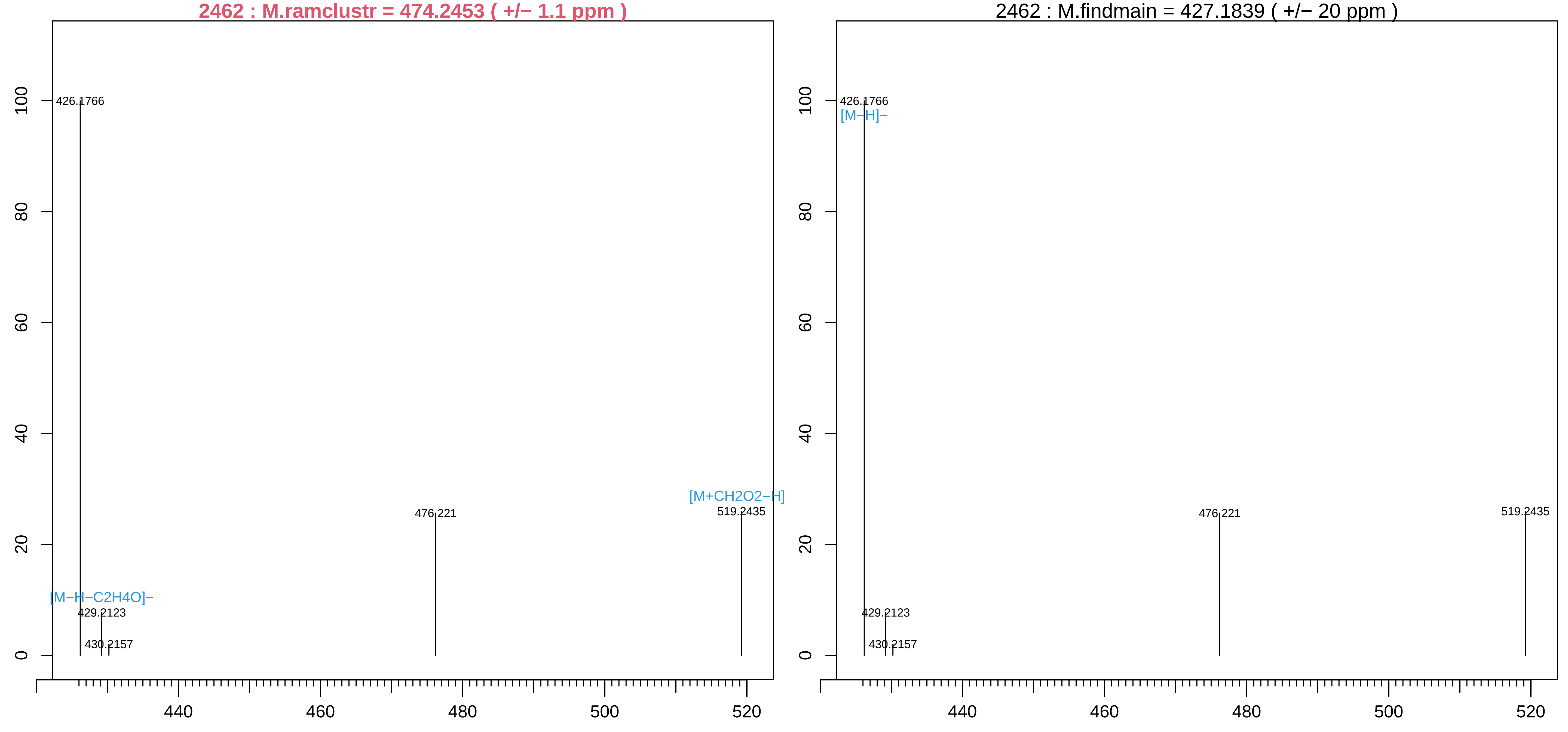

2462 : M.ramclustr = 474.2453 (+/− 1.1 ppm)
426.1766
[M−H−C2H4O]−
429.2123
430.2157
476.221
[M+CH2O2−H]
519.2435
2462 : M.findmain = 427.1839 (+/− 20 ppm)
426.1766
[M−H]−
429.2123
430.2157
476.221
519.2435
0
20
40
60
80
100
440
460
480
500
520

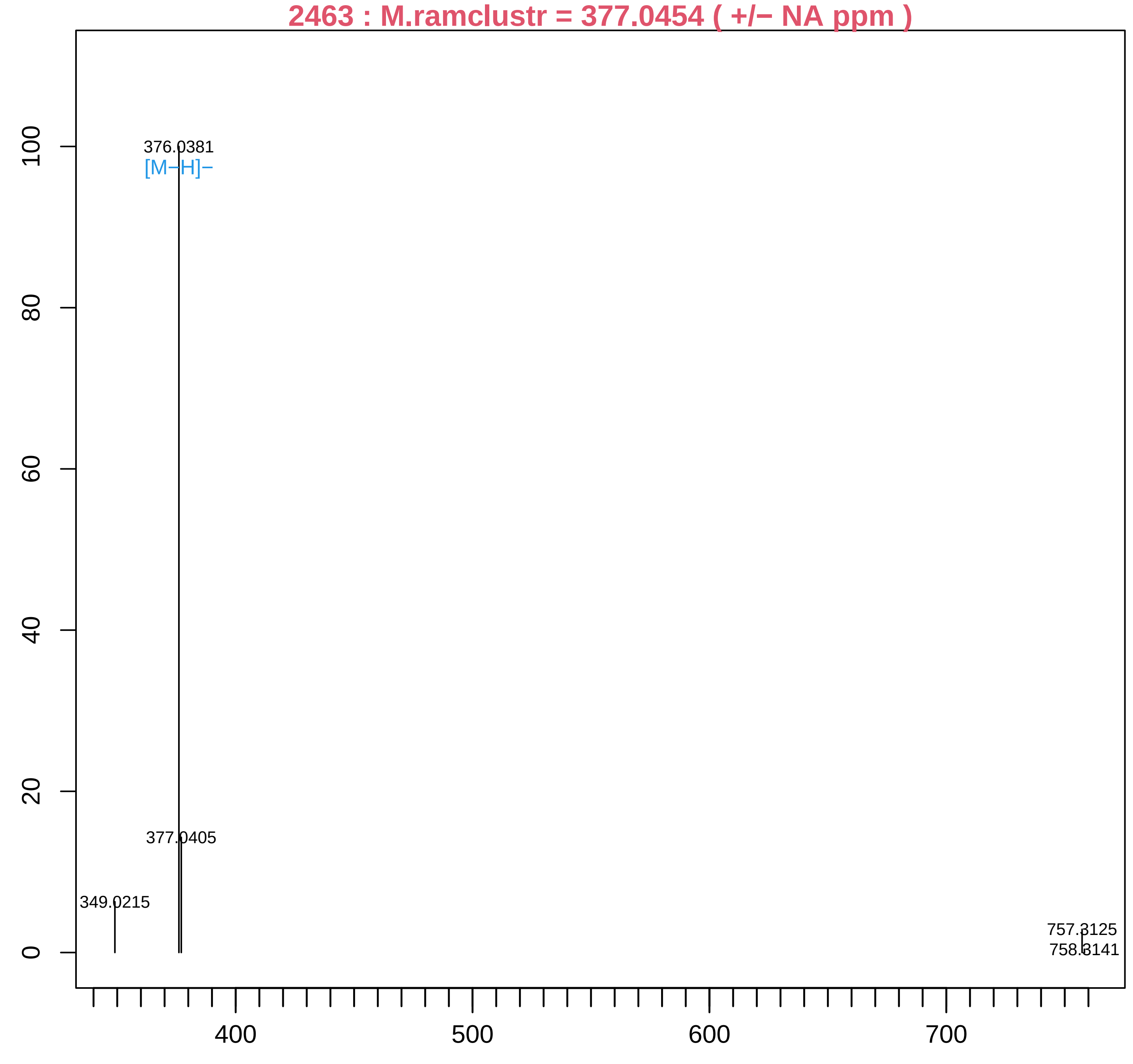
2463 : M.ramclustr = 377.0454 (+/− NA ppm)
376.0381
[M−H]−
377.0405
349.0215
757.3125
758.3141
0
20
40
60
80
100
400
500
600
700

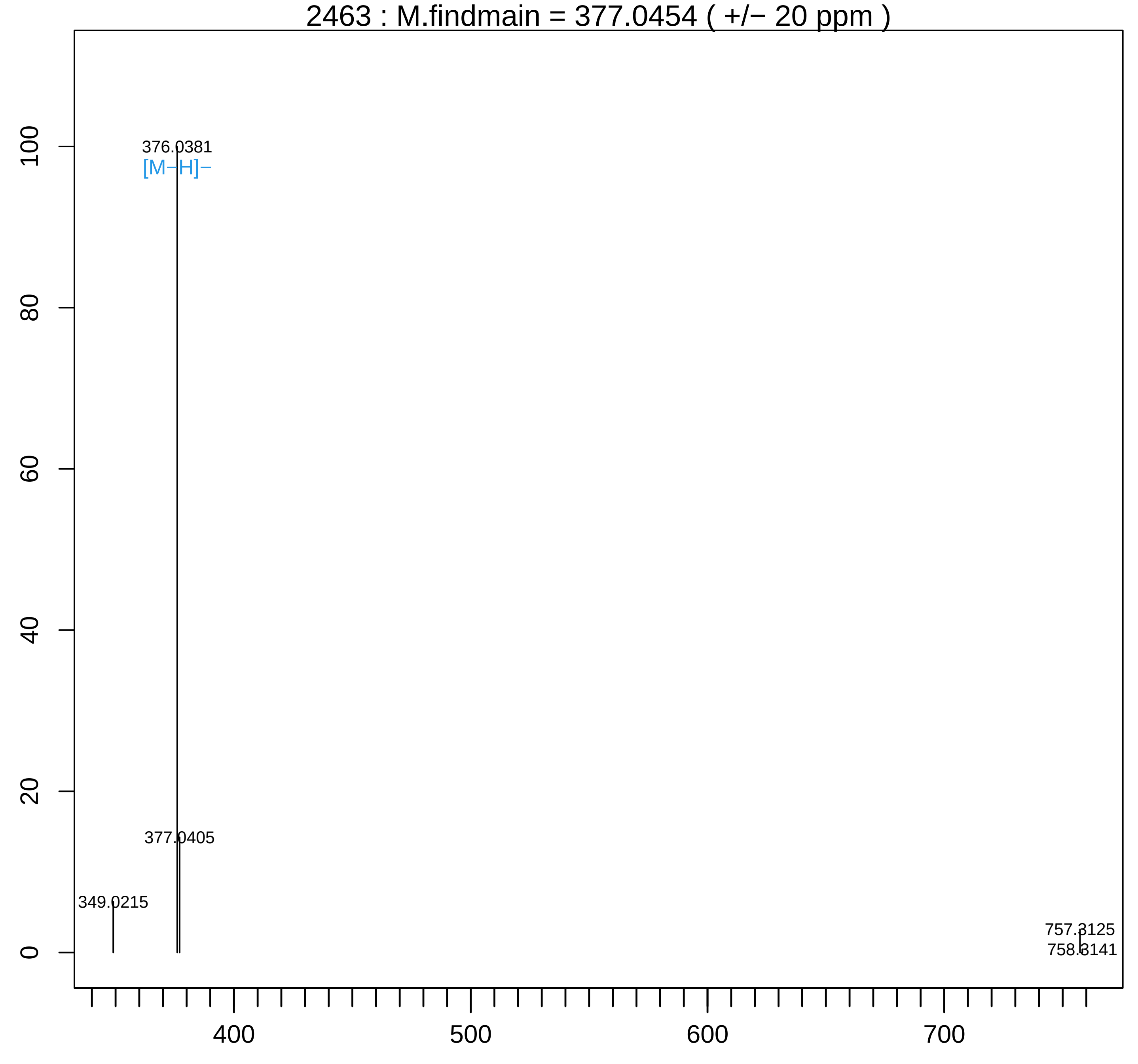
2463 : M.findmain = 377.0454 (+/− 20 ppm)
376.0381
[M−H]−
377.0405
349.0215
757.3125
758.3141
0
20
40
60
80
100
400
500
600
700

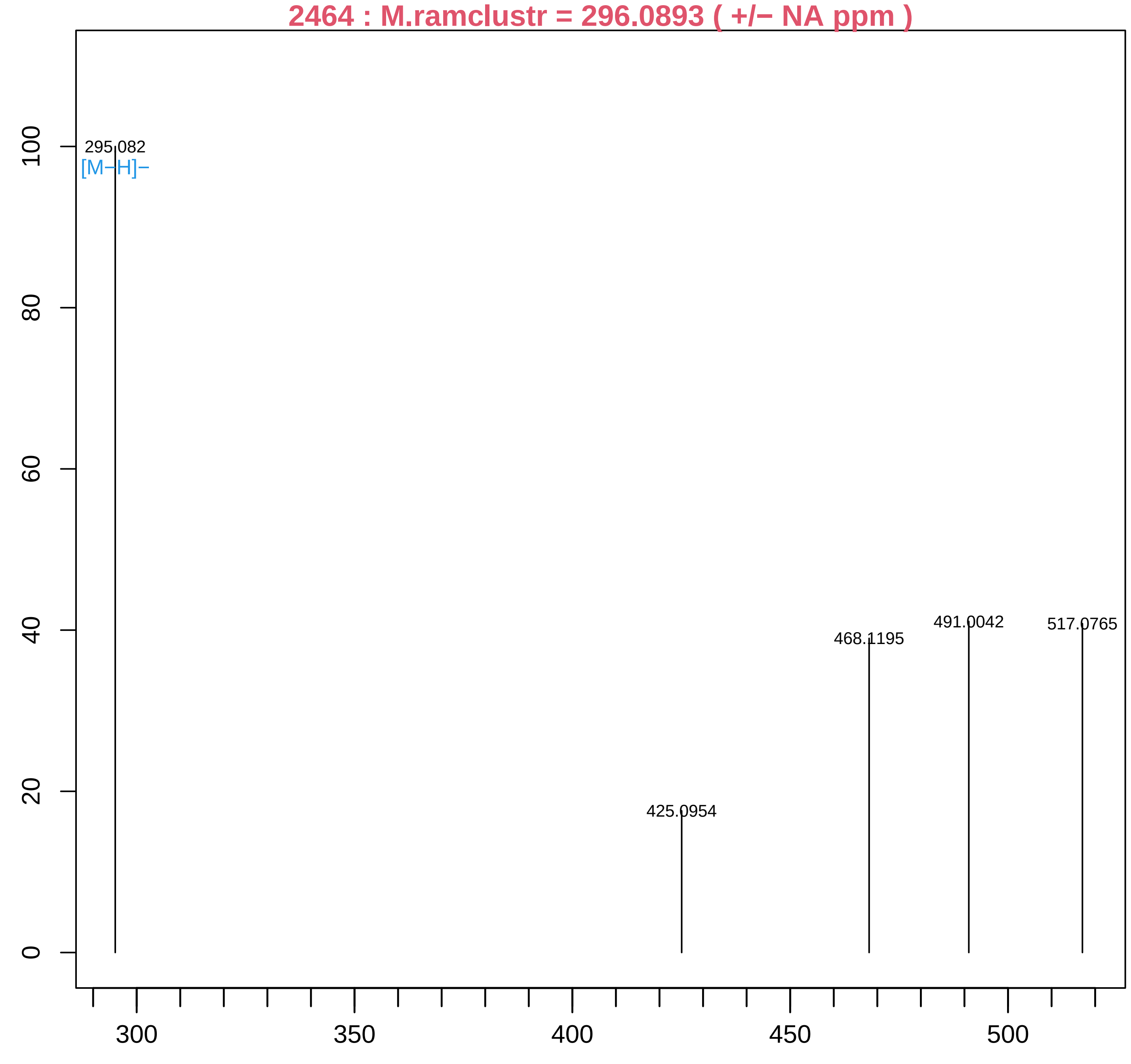
2464 : M.ramclustr = 296.0893 (+/− NA ppm)
295.082
[M−H]−
425.0954
468.1195
491.0042
517.0765
0
20
40
60
80
100
300
350
400
450
500

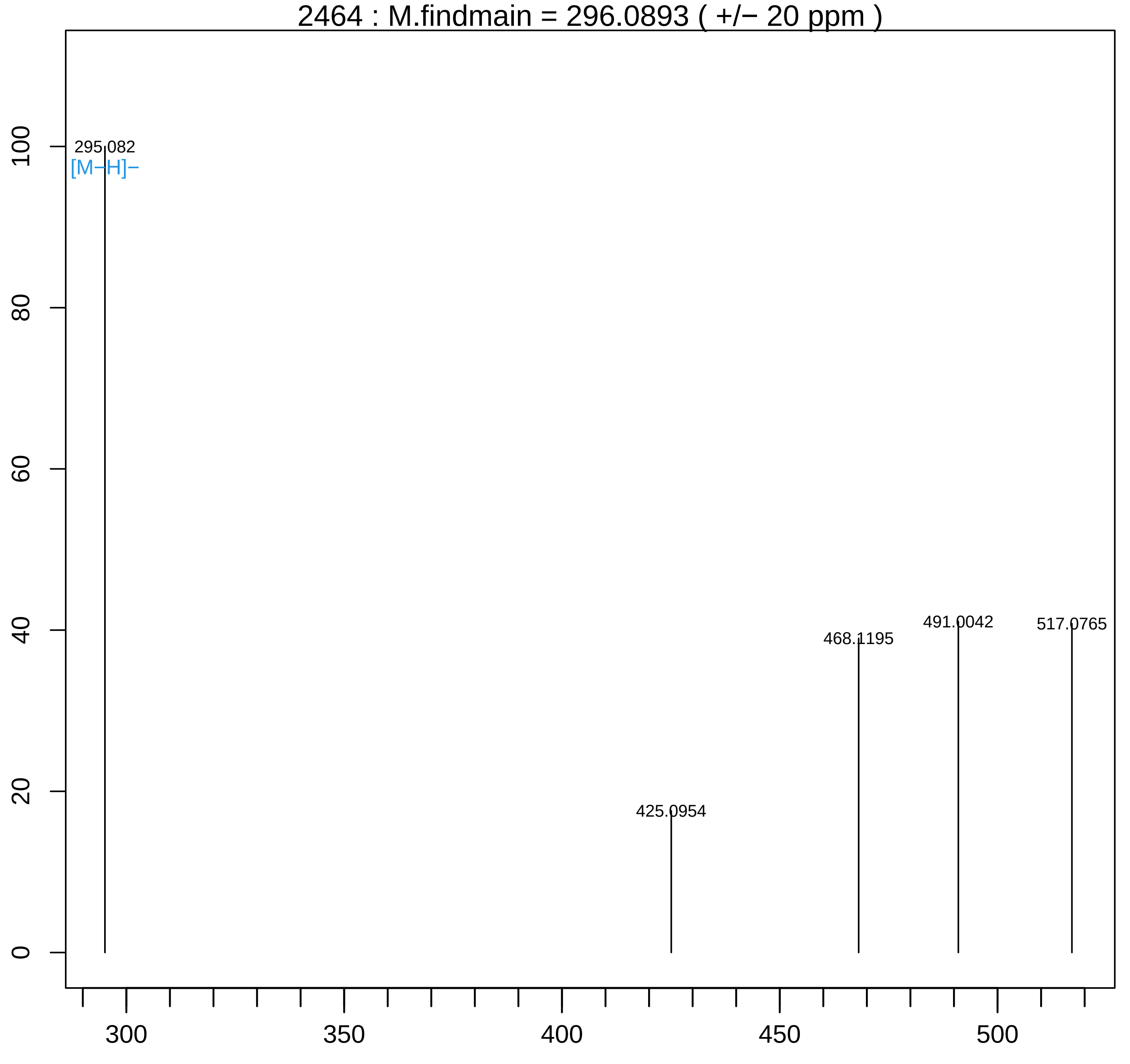
2464 : M.findmain = 296.0893 (+/− 20 ppm)
295.082
[M−H]−
425.0954
468.1195
491.0042
517.0765
0
20
40
60
80
100
300
350
400
450
500

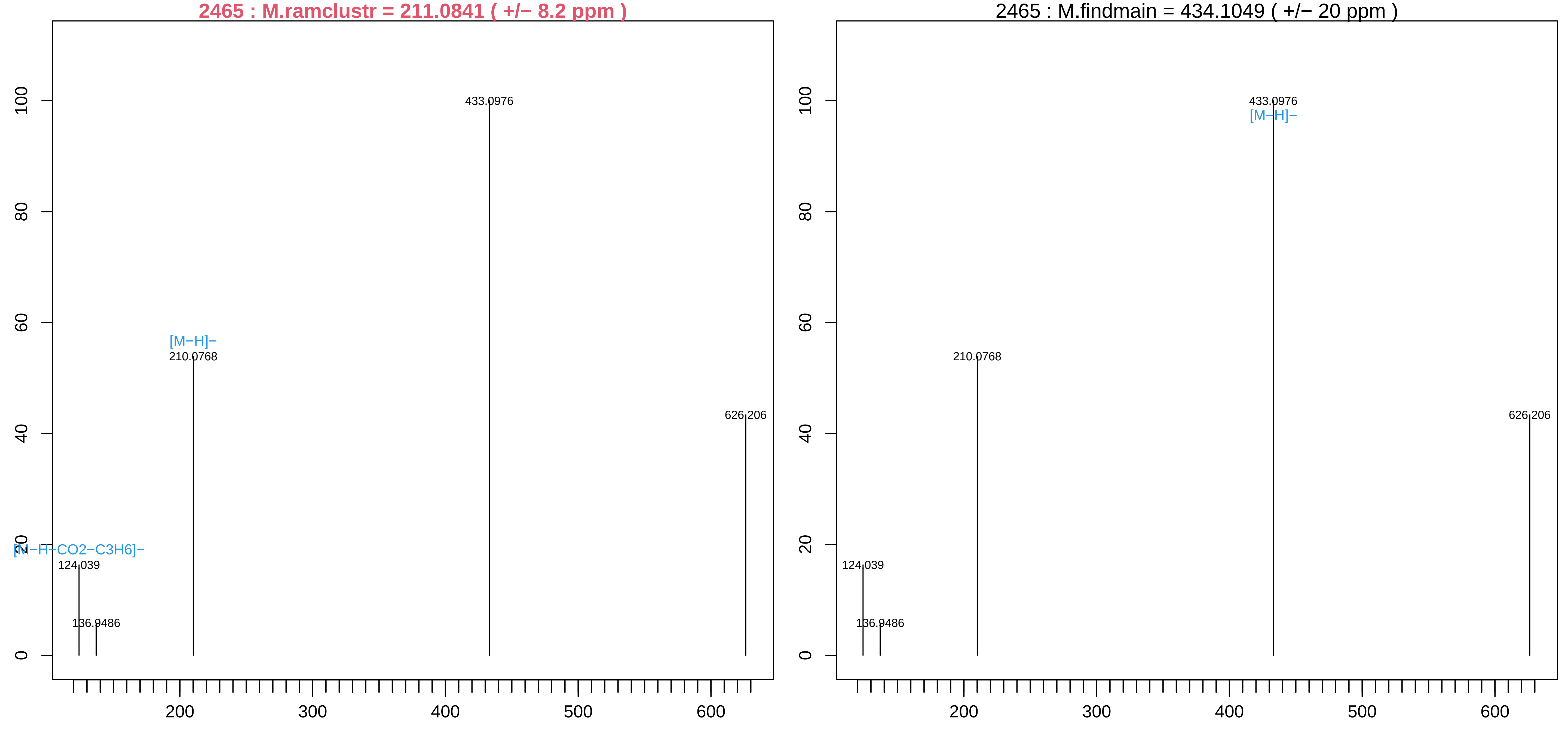
2465 : M.ramclustr = 211.0841 (+/− 8.2 ppm)
[M−H]−
210.0768
433.0976
626.206
[M−H−CO2−C3H6]−
124.039
136.9486
2465 : M.findmain = 434.1049 (+/− 20 ppm)
433.0976
[M−H]−
210.0768
626.206
124.039
136.9486
0
20
40
60
80
100
200
300
400
500
600

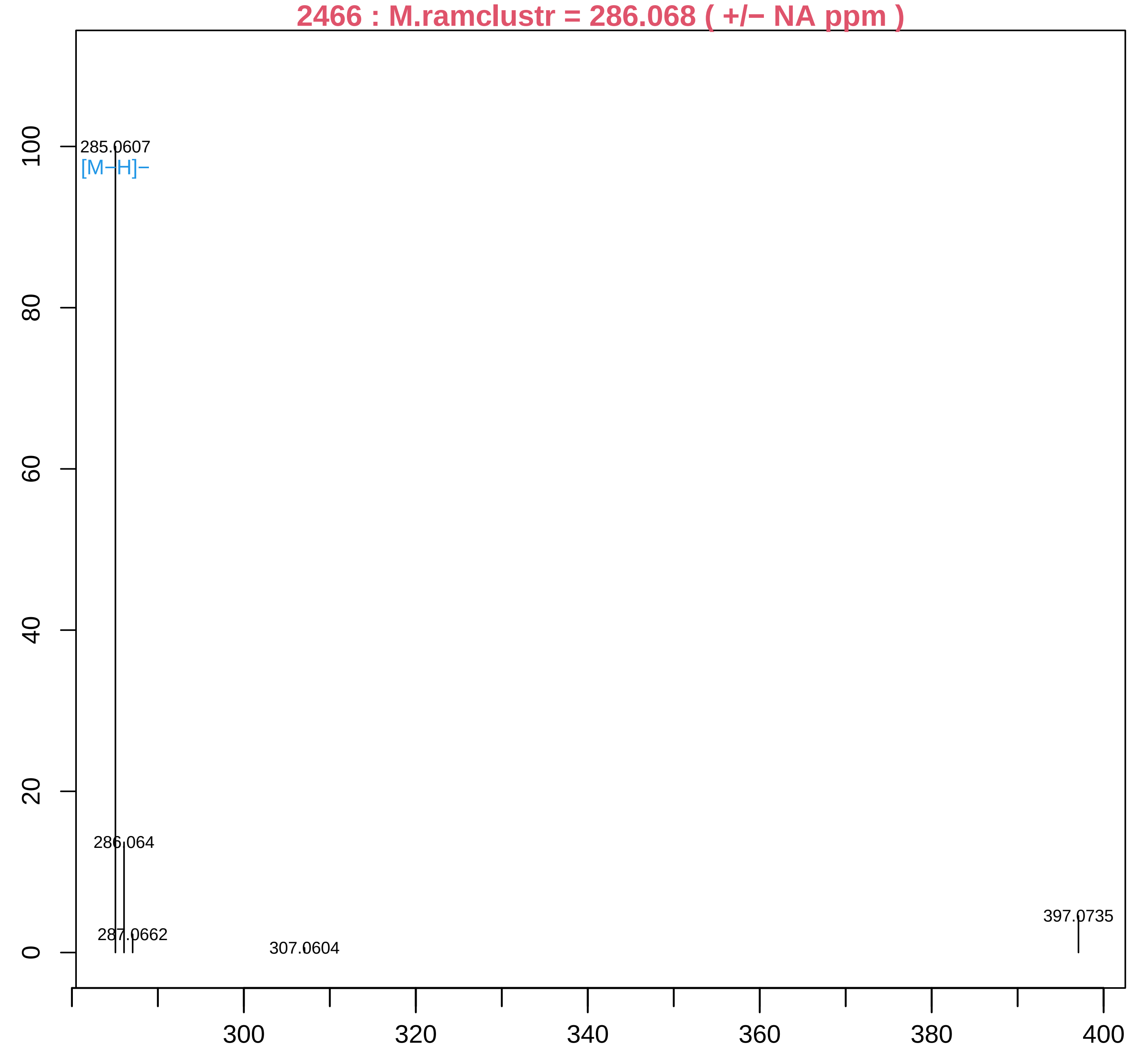

2466 : M.ramclustr = 286.068 (+/− NA ppm)
285.0607
[M−H]−
286.064
287.0662
307.0604
397.0735
0
20
40
60
80
100
300
320
340
360
380
400

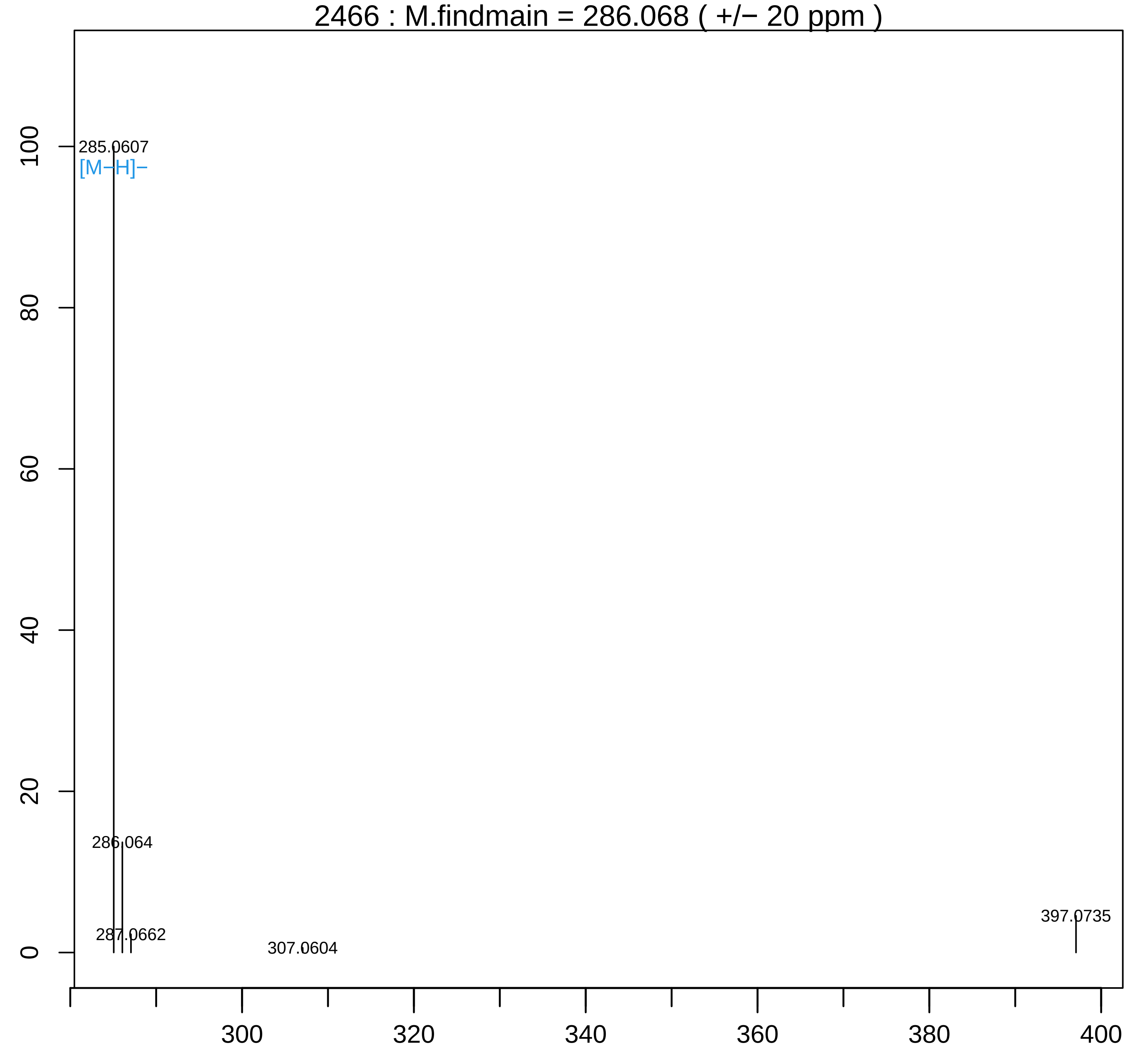

2466 : M.findmain = 286.068 (+/− 20 ppm)
285.0607
[M−H]−
286.064
287.0662
307.0604
397.0735
0
20
40
60
80
100
300
320
340
360
380
400

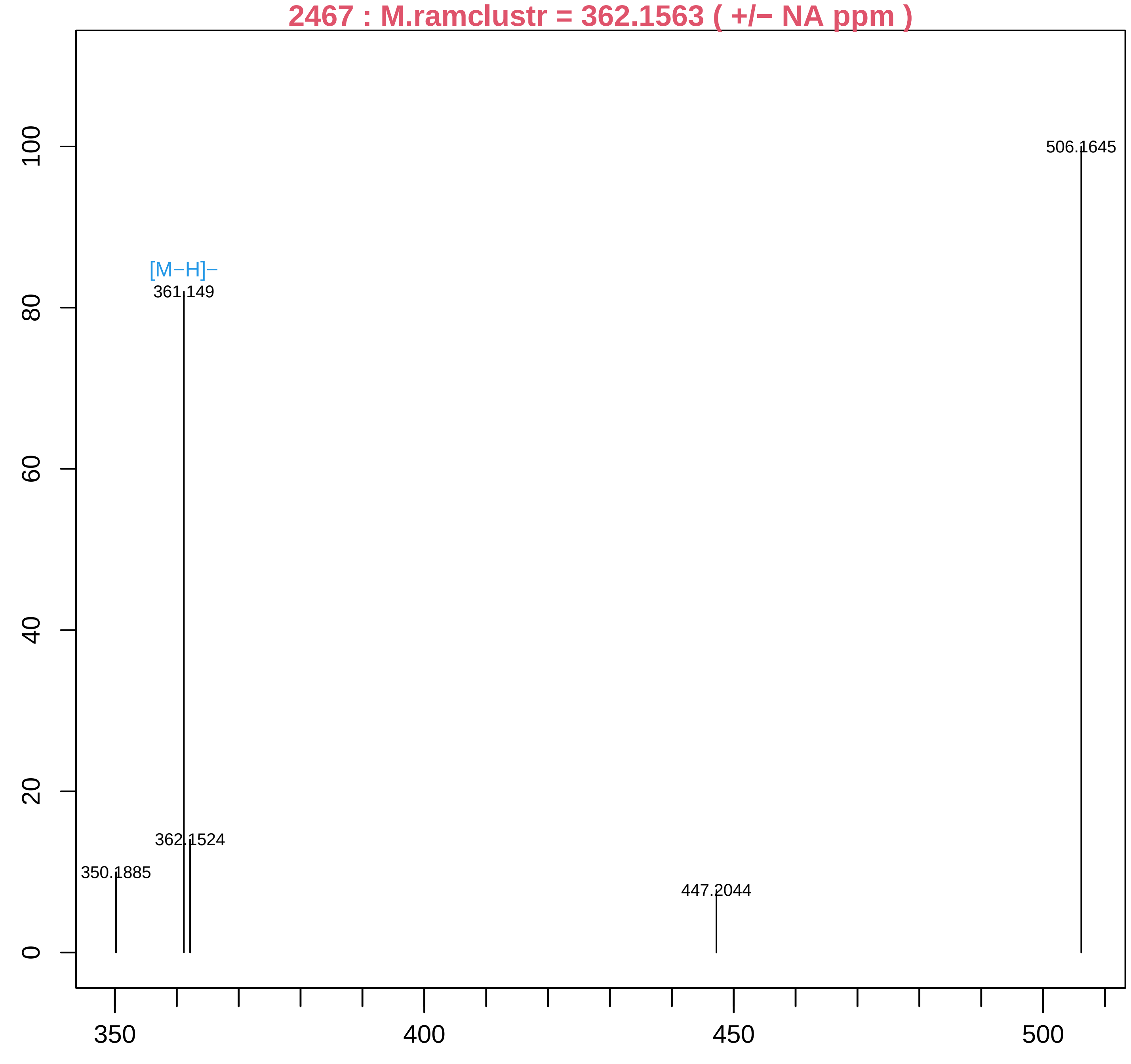
2467 : M.ramclustr = 362.1563 (+/− NA ppm)
506.1645
[M−H]−
361.149
362.1524
350.1885
447.2044
0
20
40
60
80
100
350
400
450
500

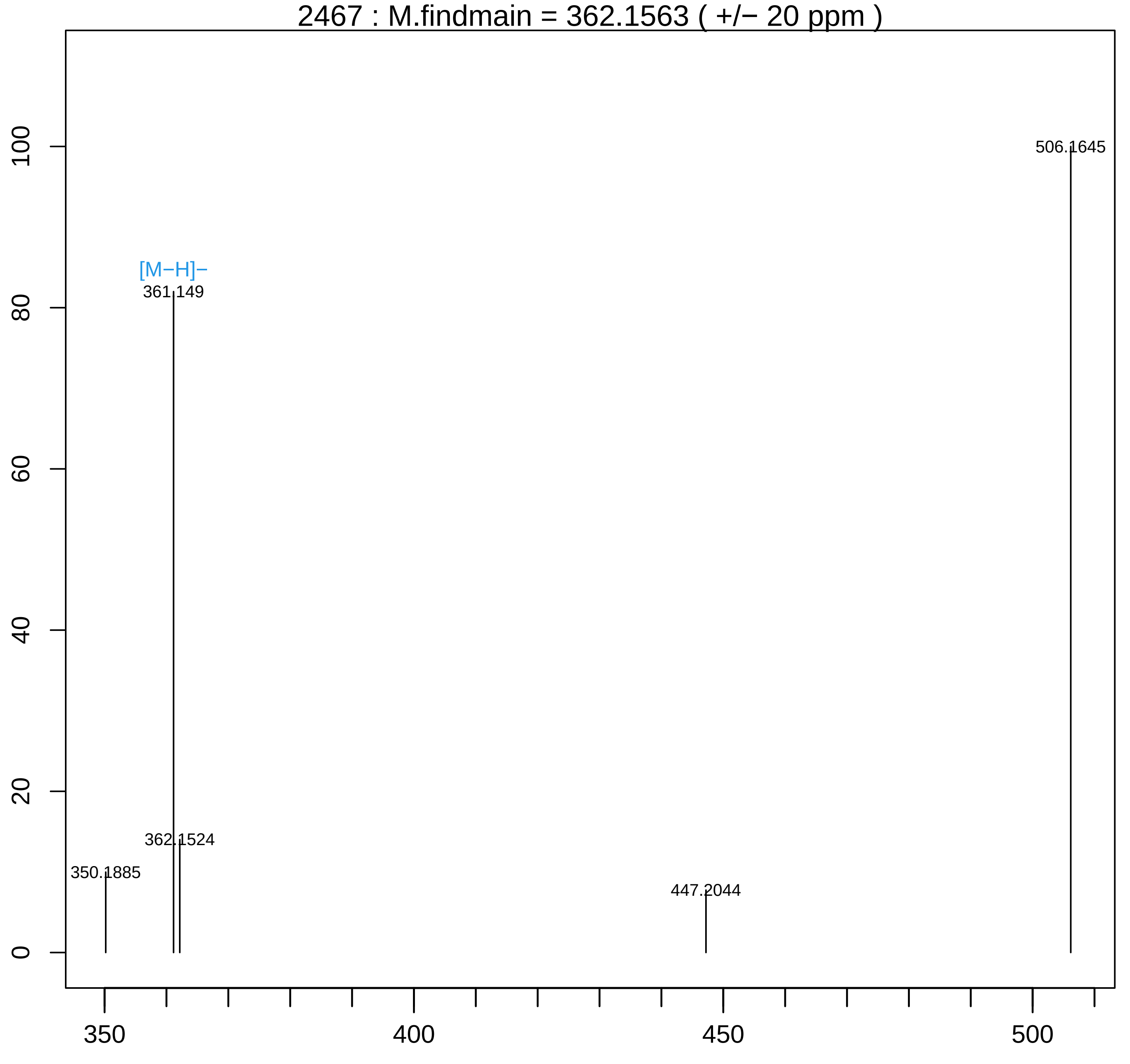
2467 : M.findmain = 362.1563 (+/− 20 ppm)
506.1645
[M−H]−
361.149
362.1524
350.1885
447.2044
0
20
40
60
80
100
350
400
450
500

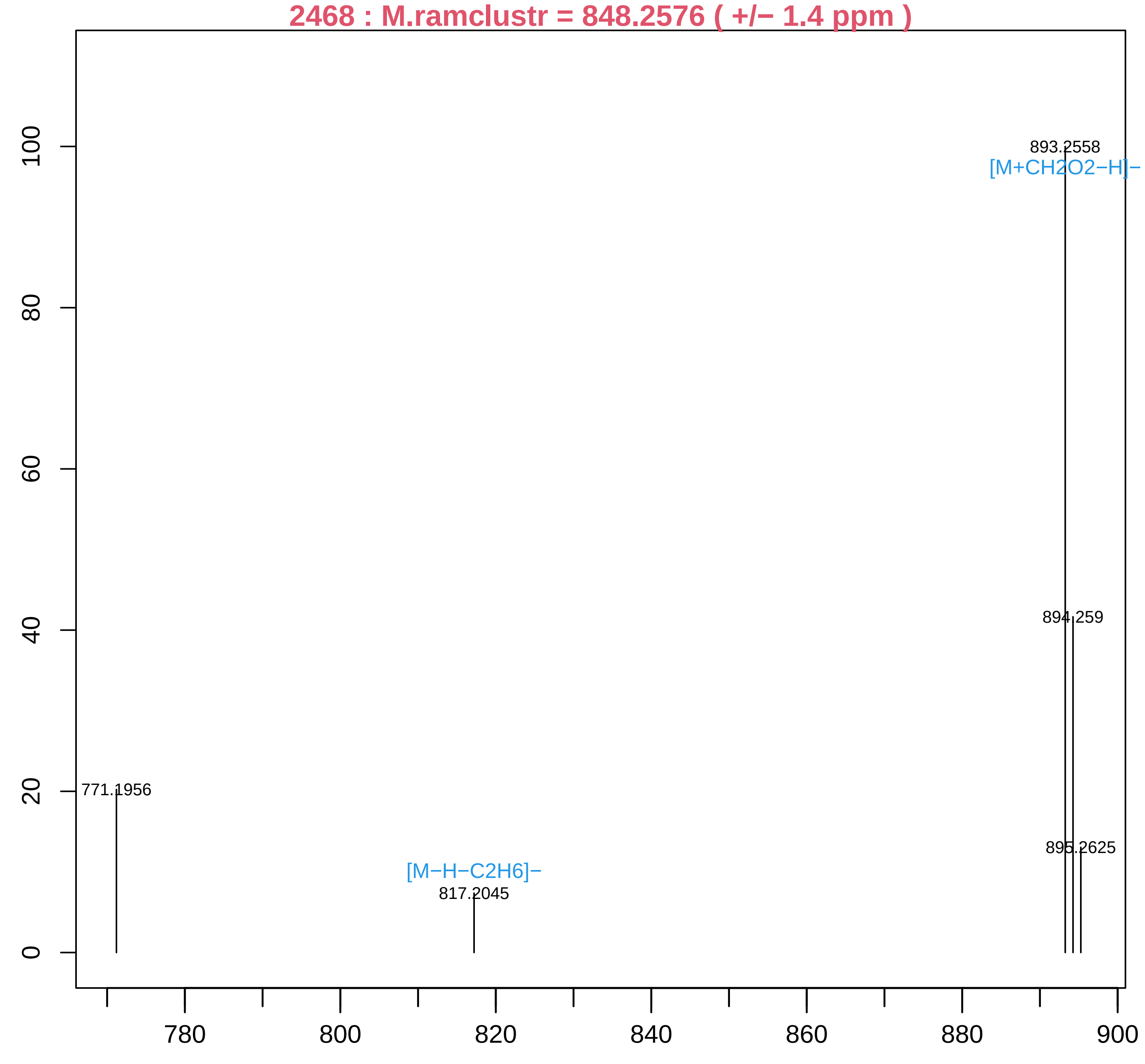

2468 : M.ramclustr = 848.2576 (+/− 1.4 ppm)
893.2558
[M+CH2O2−H]−
894.259
895.2625
771.1956
[M−H−C2H6]−
817.2045
0
20
40
60
80
100
780
800
820
840
860
880
900

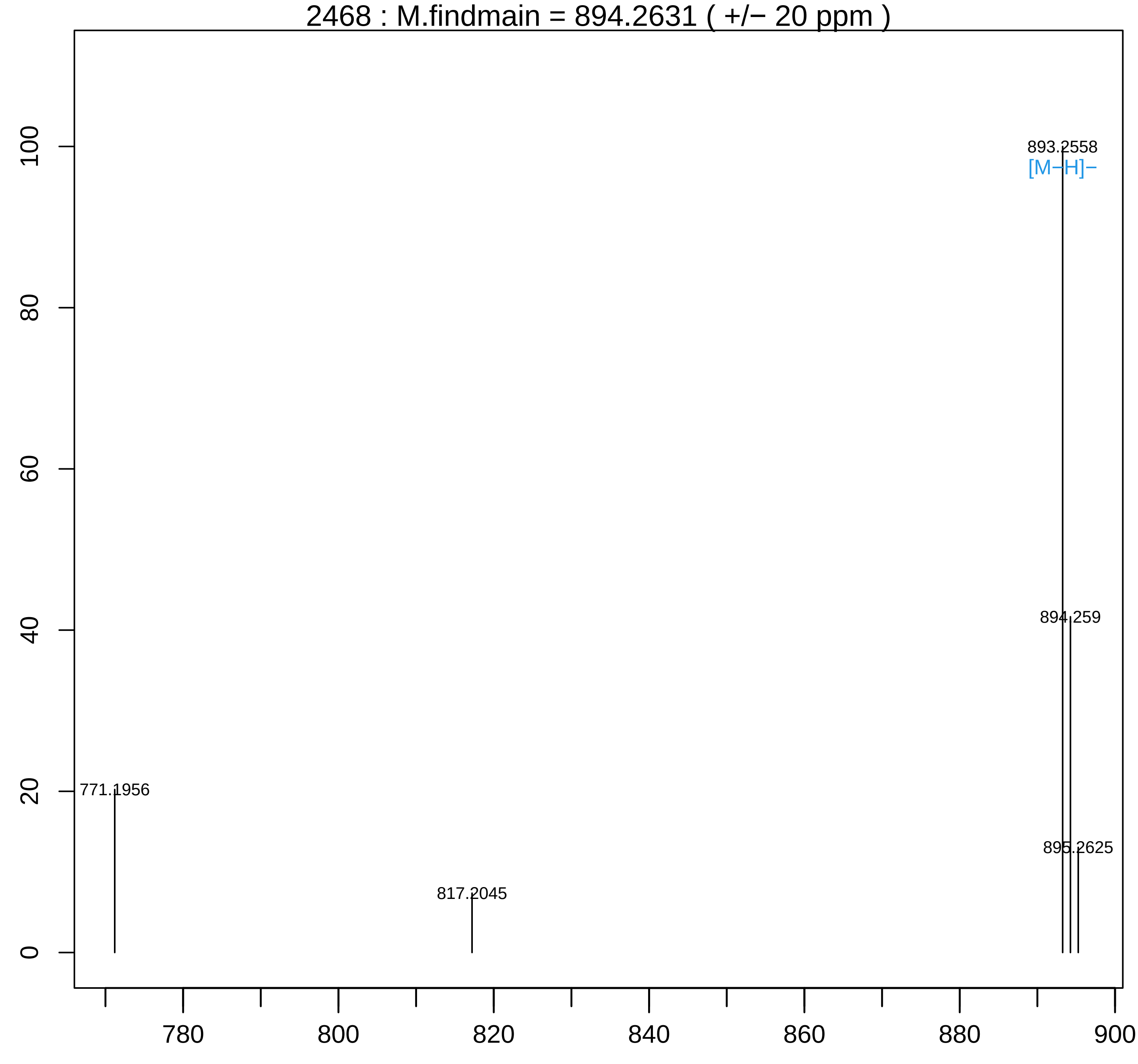

2468 : M.findmain = 894.2631 (+/− 20 ppm)
893.2558
[M−H]−
894.259
895.2625
771.1956
817.2045
0
20
40
60
80
100
780
800
820
840
860
880
900

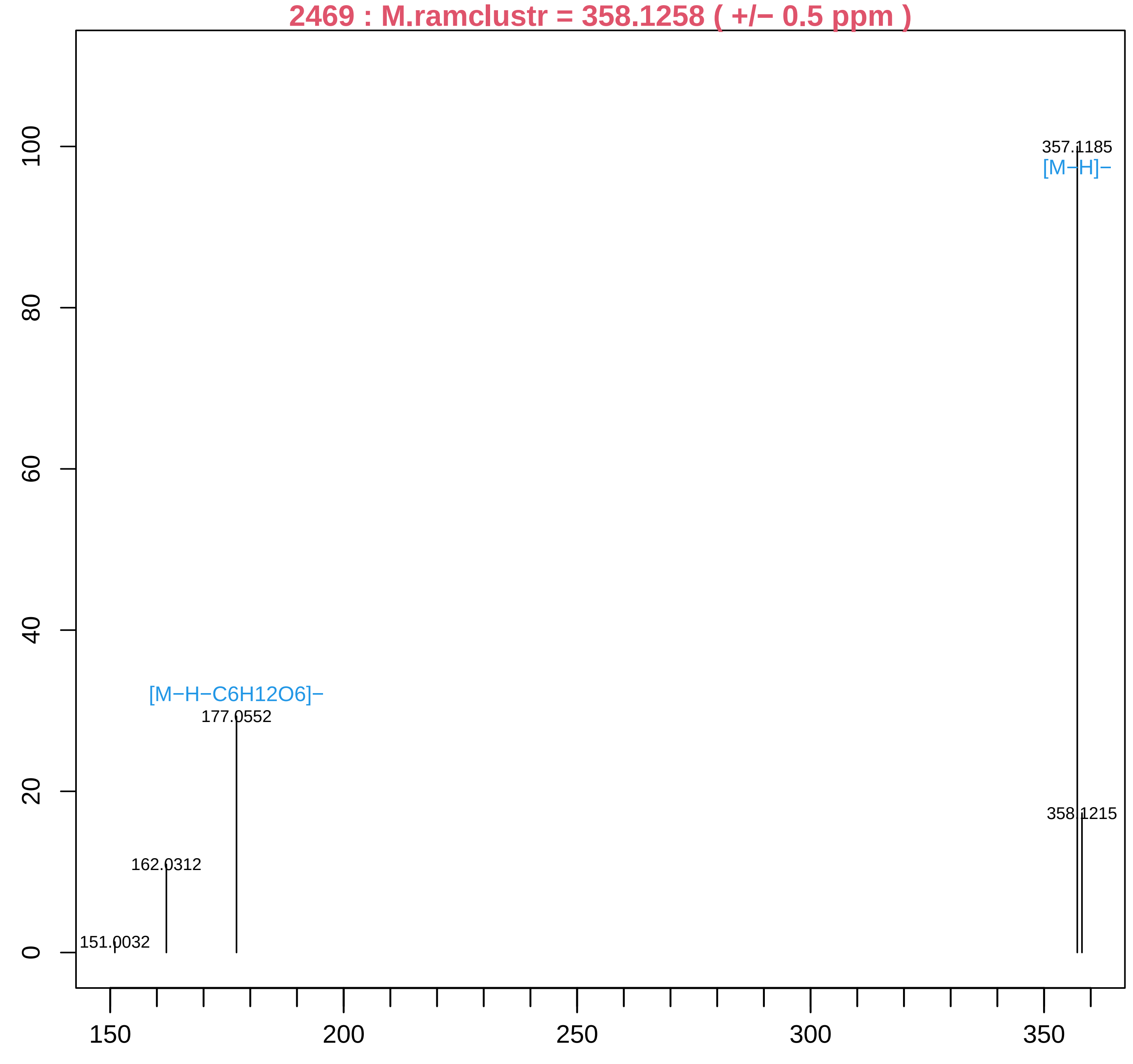

2469 : M.ramclustr = 358.1258 (+/− 0.5 ppm)
357.1185
[M−H]−
[M−H−C6H12O6]−
177.0552
358.1215
162.0312
151.0032
0
20
40
60
80
100
150
200
250
300
350

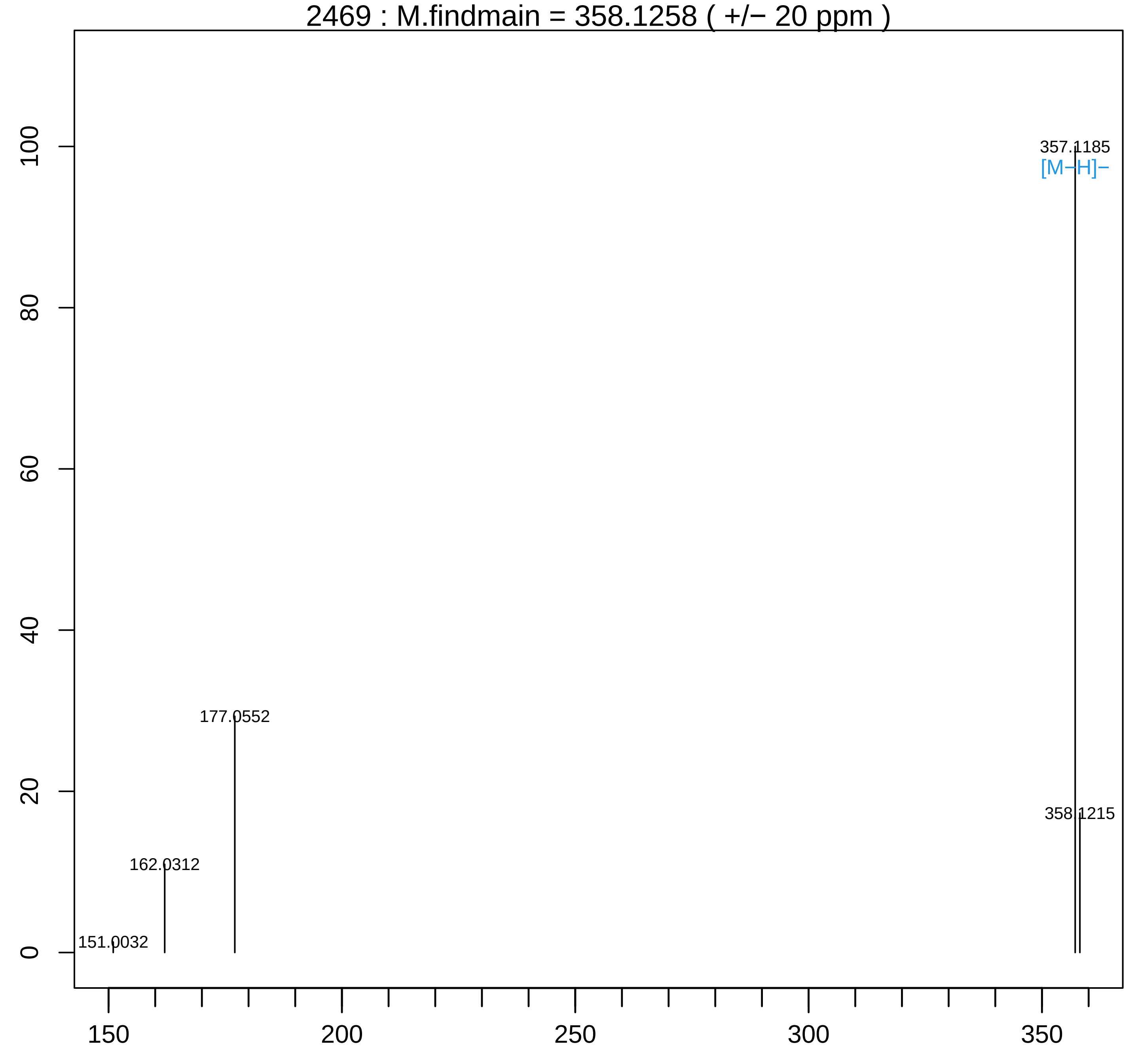

2469 : M.findmain = 358.1258 (+/− 20 ppm)
357.1185
[M−H]−
177.0552
358.1215
162.0312
151.0032
0
20
40
60
80
100
150
200
250
300
350

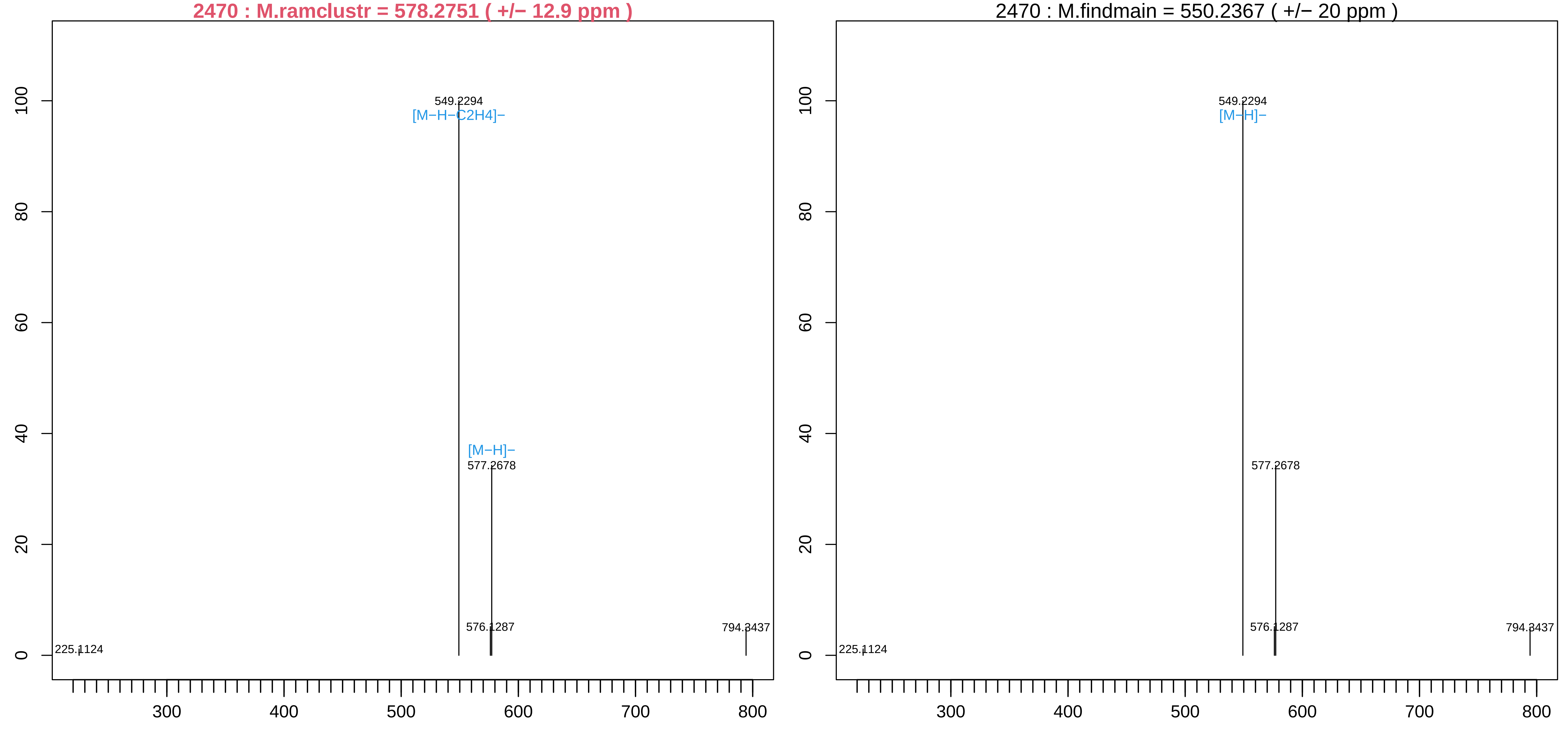

2470 : M.ramclustr = 578.2751 (+/− 12.9 ppm)
549.2294
[M−H−C2H4]−
[M−H]−
577.2678
576.1287
794.3437
225.1124
0
20
40
60
80
100
300
400
500
600
700
800
2470 : M.findmain = 550.2367 (+/− 20 ppm)
549.2294
[M−H]−
577.2678
576.1287
794.3437
225.1124

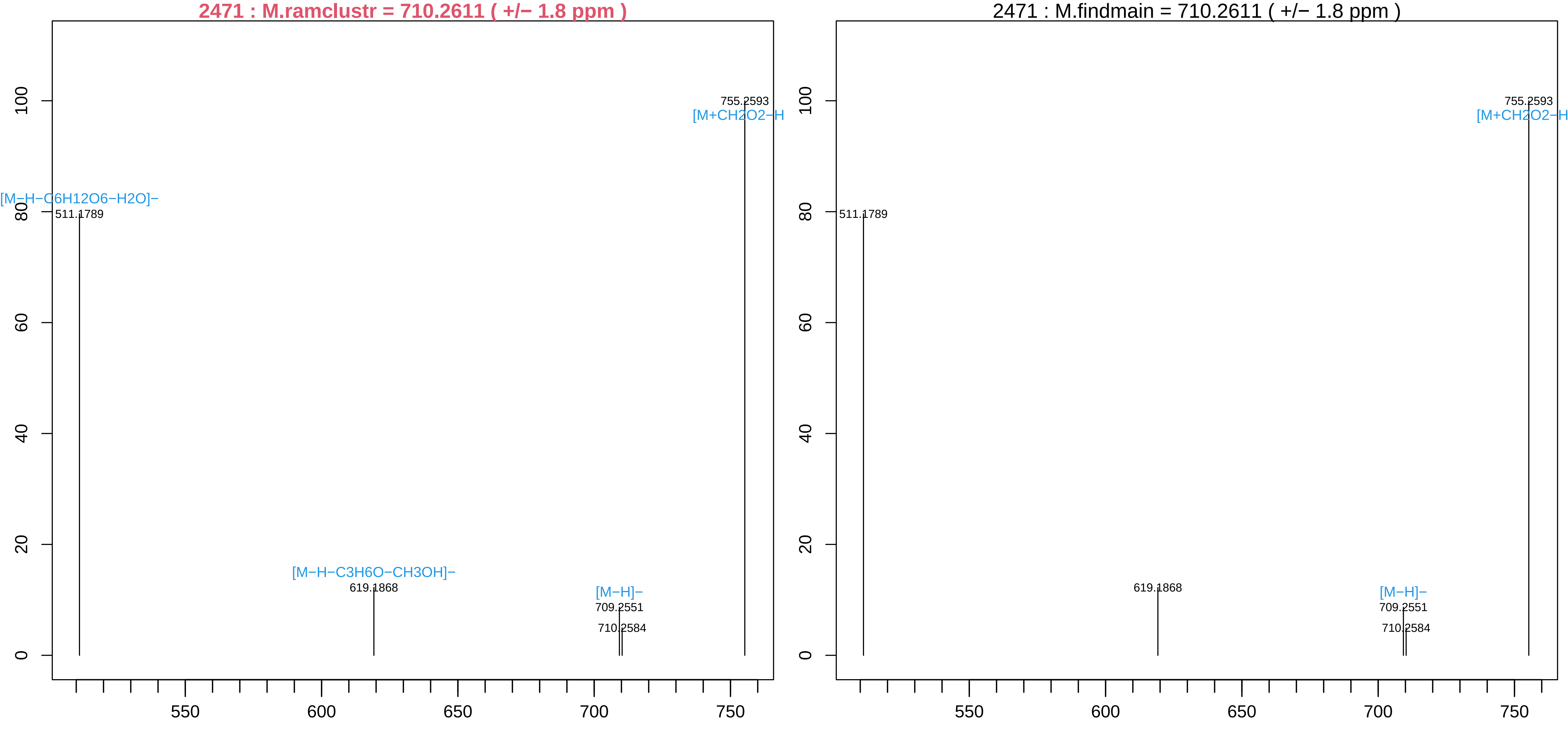

2471 : M.ramclustr = 710.2611 (+/− 1.8 ppm)
2471 : M.findmain = 710.2611 (+/− 1.8 ppm)
[M−H−C6H12O6−H2O]−
511.1789
[M−H−C3H6O−CH3OH]−
619.1868
[M−H]−
709.2551
710.2584
755.2593
[M+CH2O2−H
0
20
40
60
80
100
550
600
650
700
750

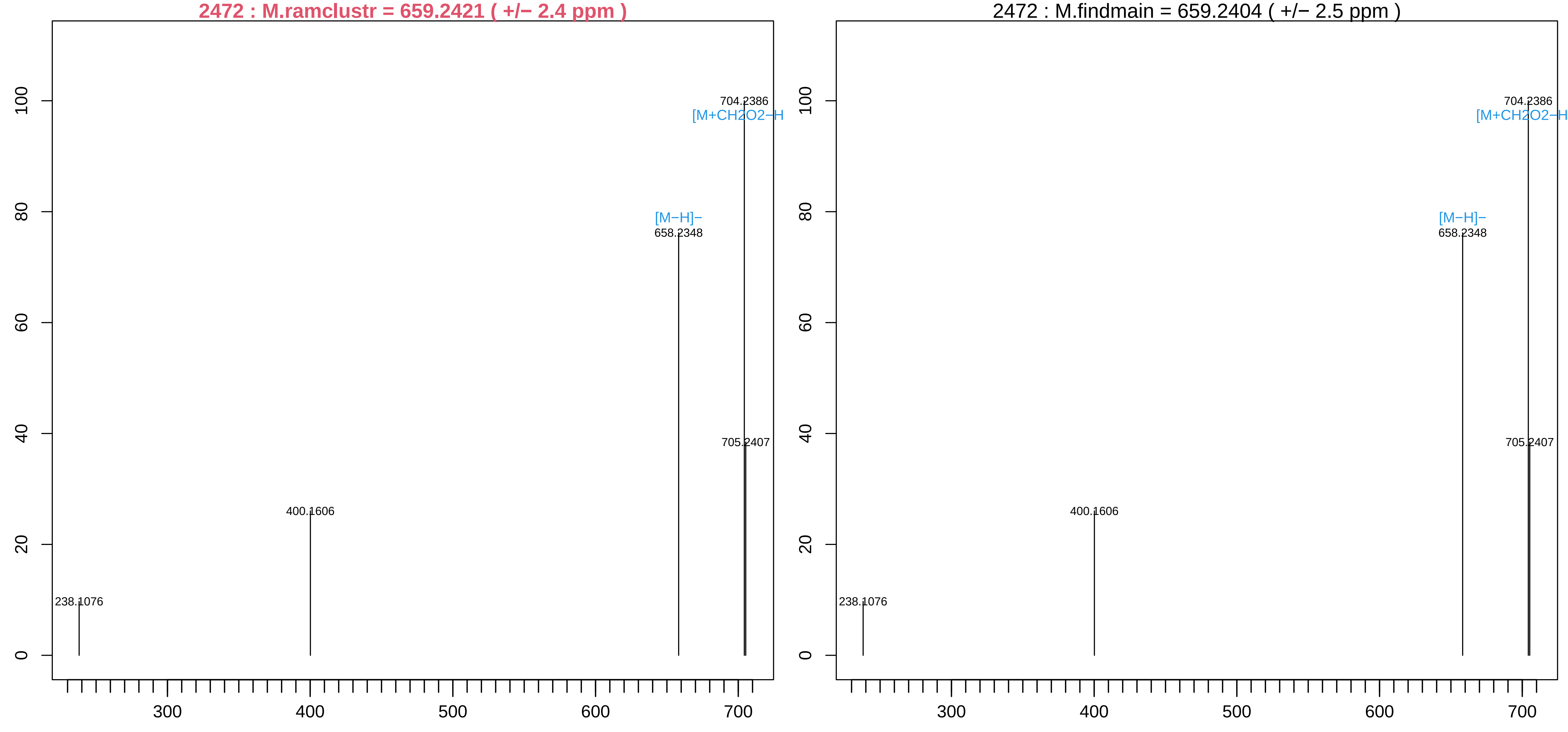

2472 : M.ramclustr = 659.2421 (+/− 2.4 ppm)
704.2386
[M+CH2O2−H
[M−H]−
658.2348
705.2407
400.1606
238.1076
0
20
40
60
80
100
300
400
500
600
700
2472 : M.findmain = 659.2404 (+/− 2.5 ppm)
704.2386
[M+CH2O2−H
[M−H]−
658.2348
705.2407
400.1606
238.1076
0
20
40
60
80
100
300
400
500
600
700

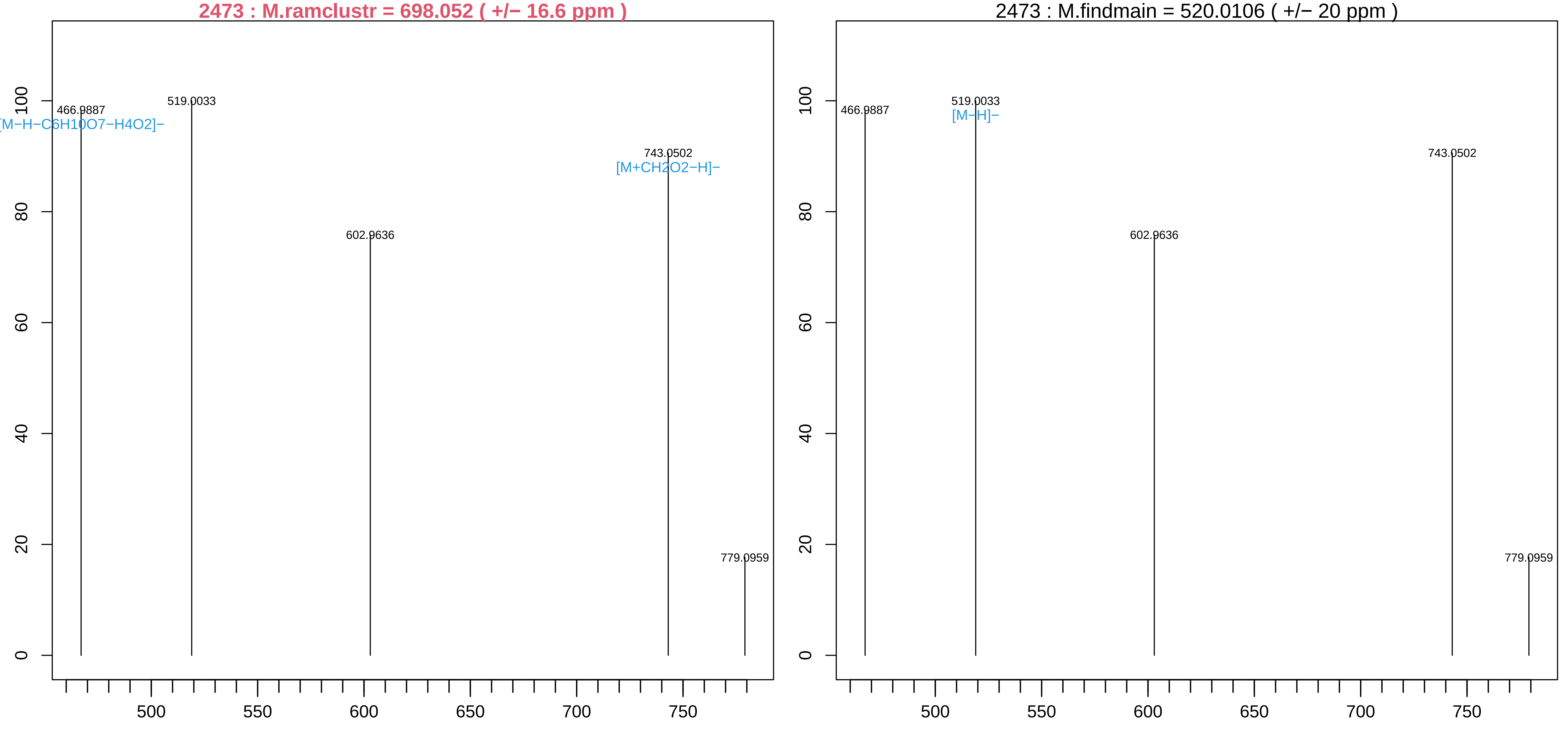
2473 : M.ramclustr = 698.052 (+/− 16.6 ppm)
466.9887
[M−H−C6H10O7−H4O2]−
519.0033
602.9636
743.0502
[M+CH2O2−H]−
779.0959
2473 : M.findmain = 520.0106 (+/− 20 ppm)
466.9887
519.0033
[M−H]−
602.9636
743.0502
779.0959
0
20
40
60
80
100
500
550
600
650
700
750

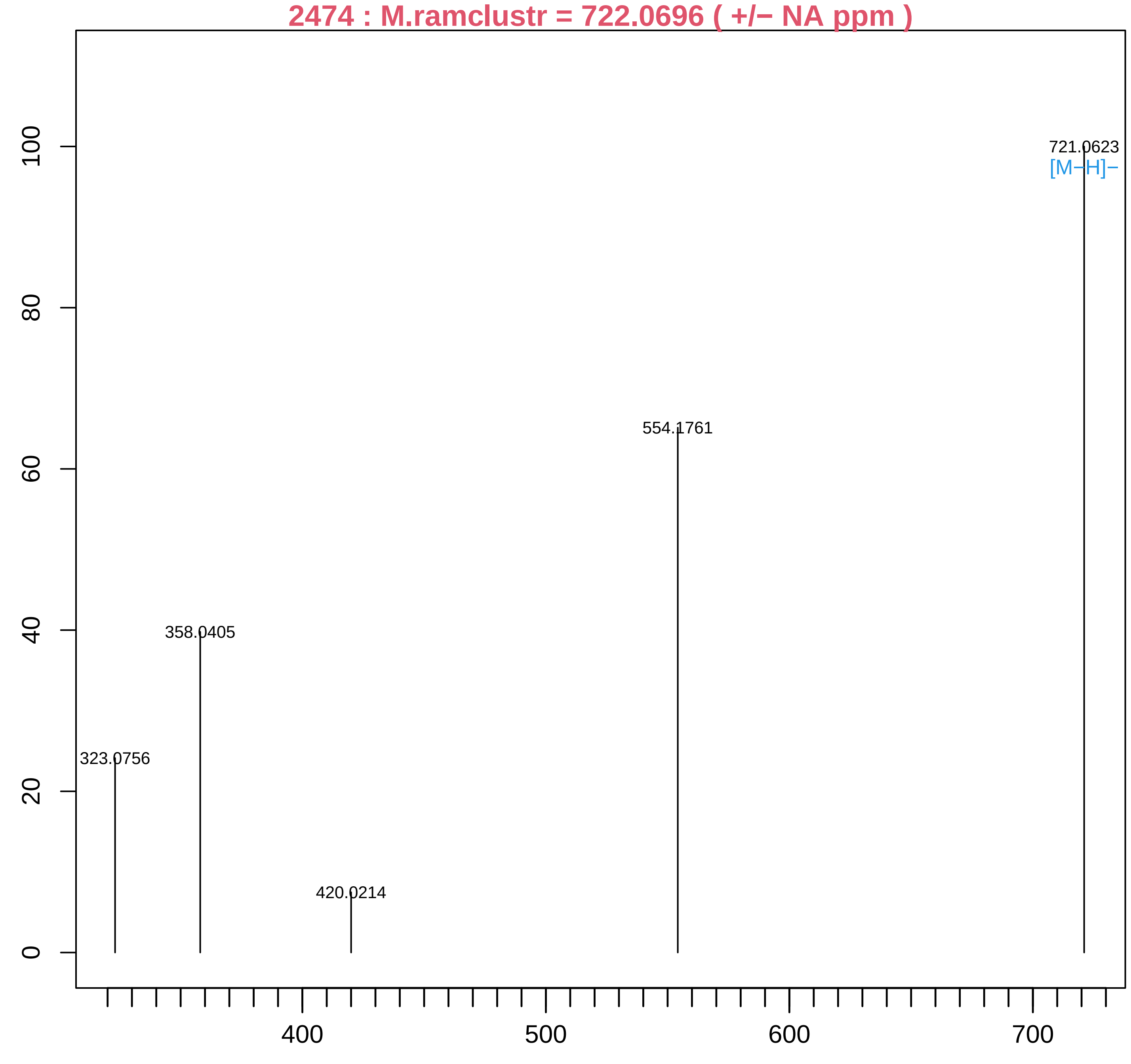
2474 : M.ramclustr = 722.0696 (+/− NA ppm)
721.0623
[M−H]−
554.1761
358.0405
323.0756
420.0214
0
20
40
60
80
100
400
500
600
700

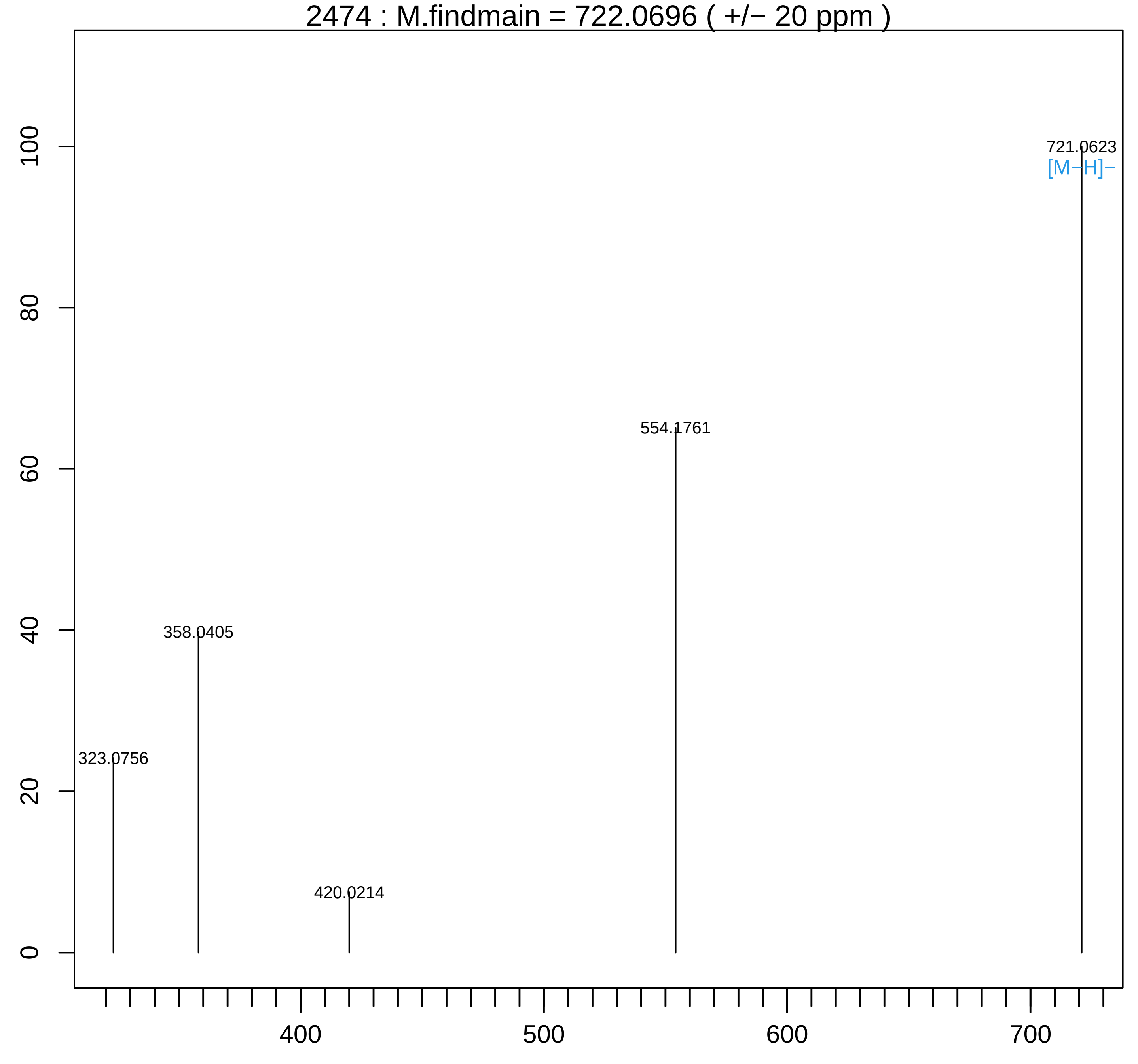
2474 : M.findmain = 722.0696 (+/− 20 ppm)
721.0623
[M−H]−
554.1761
358.0405
323.0756
420.0214
0
20
40
60
80
100
400
500
600
700

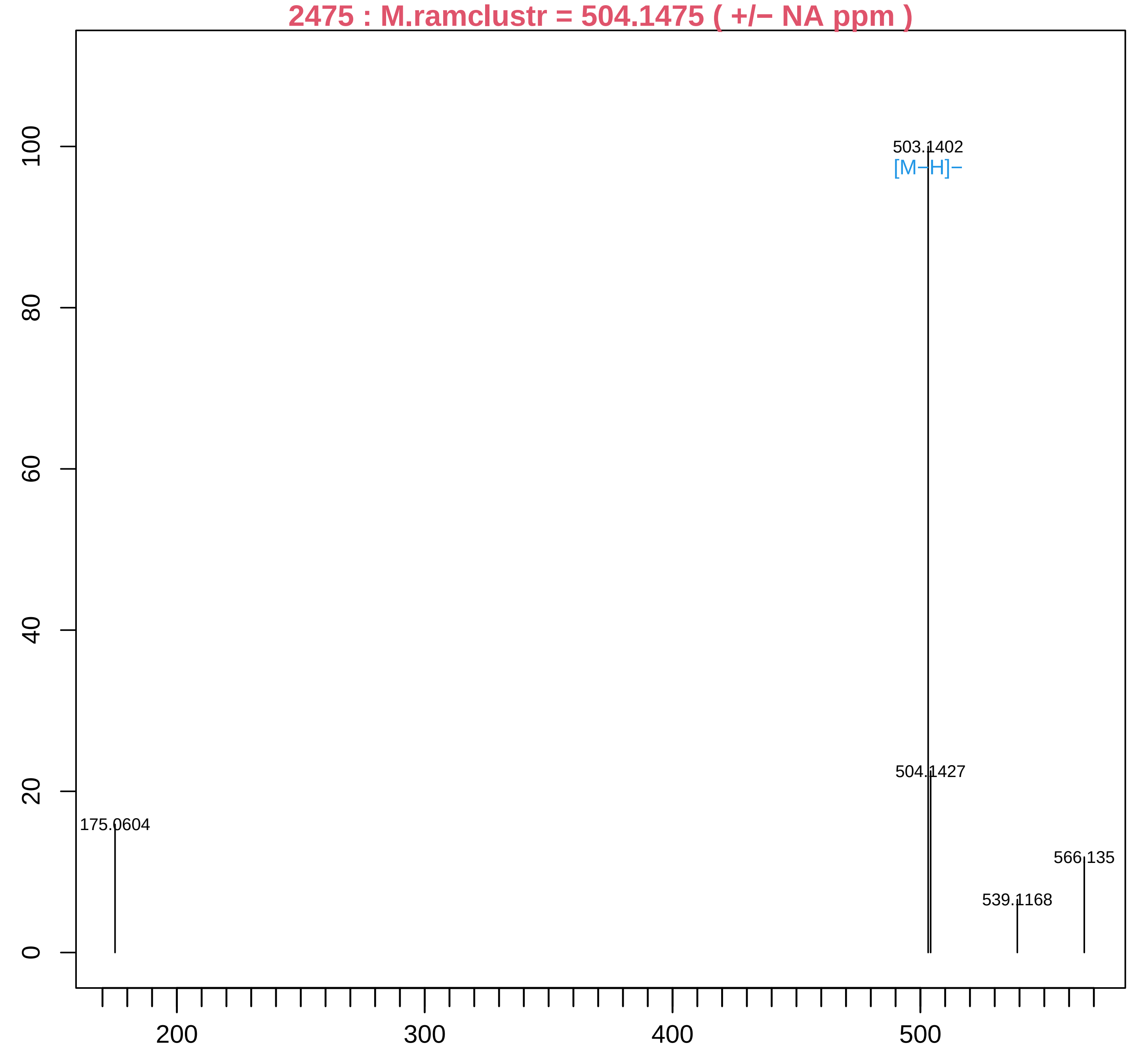

2475 : M.ramclustr = 504.1475 (+/− NA ppm)
0
20
40
60
80
100
200
300
400
500
175.0604
503.1402
[M−H]−
504.1427
539.1168
566.135

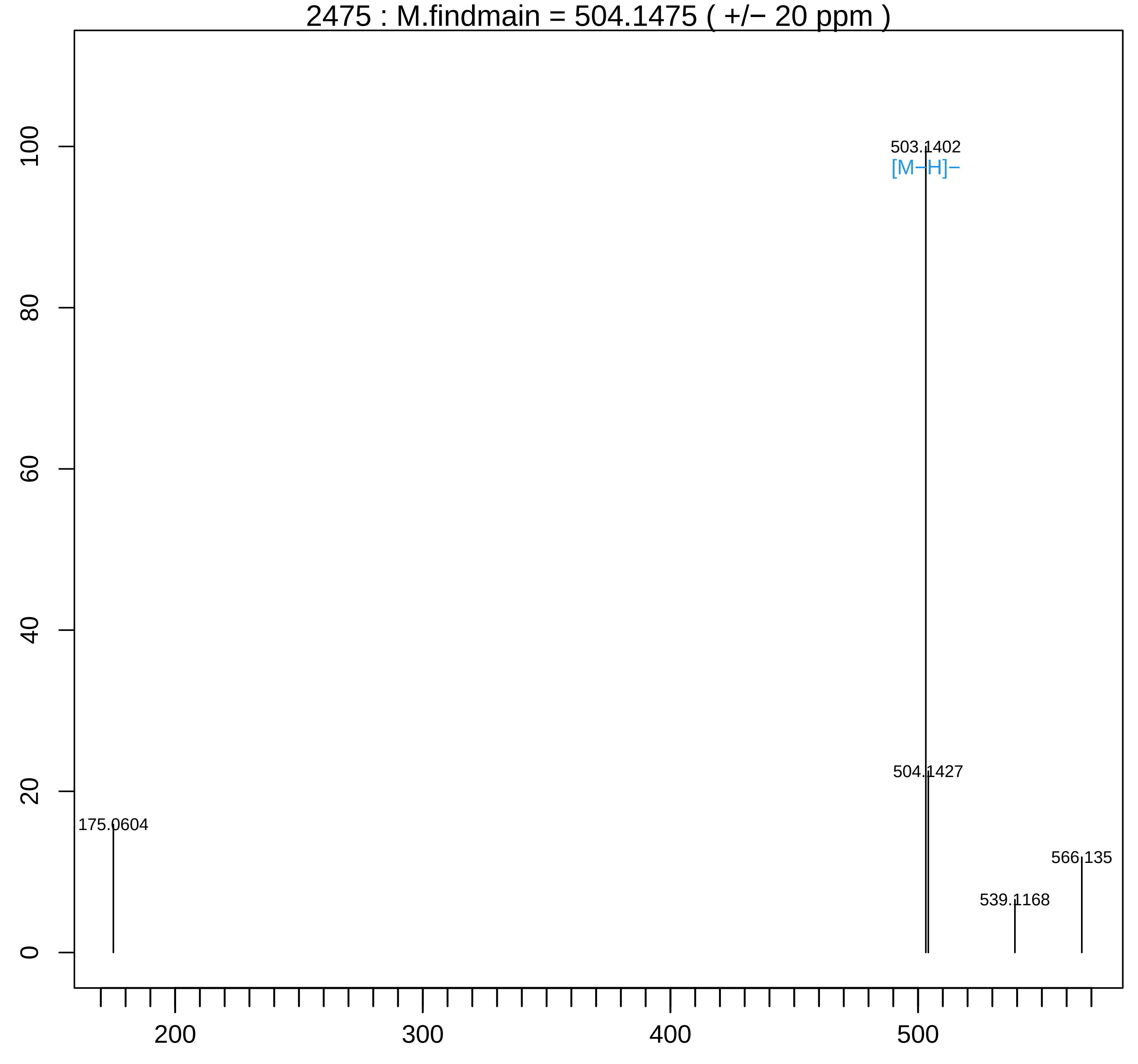

2475 : M.findmain = 504.1475 (+/− 20 ppm)
0
20
40
60
80
100
200
300
400
500
175.0604
503.1402
[M−H]−
504.1427
539.1168
566.135

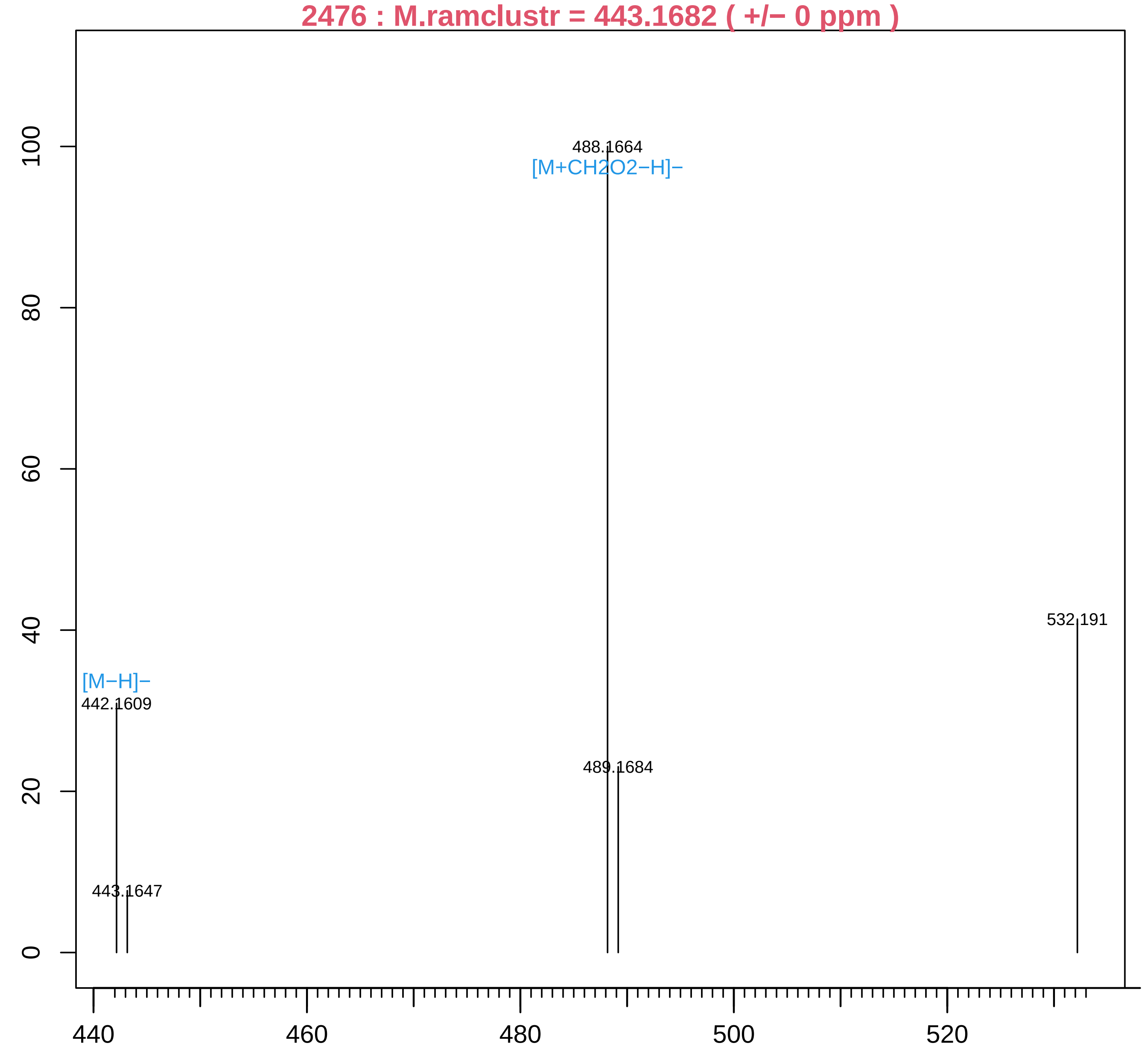
2476 : M.ramclustr = 443.1682 (+/− 0 ppm)
488.1664
[M+CH2O2−H]−
532.191
[M−H]−
442.1609
489.1684
443.1647
0
20
40
60
80
100
440
460
480
500
520

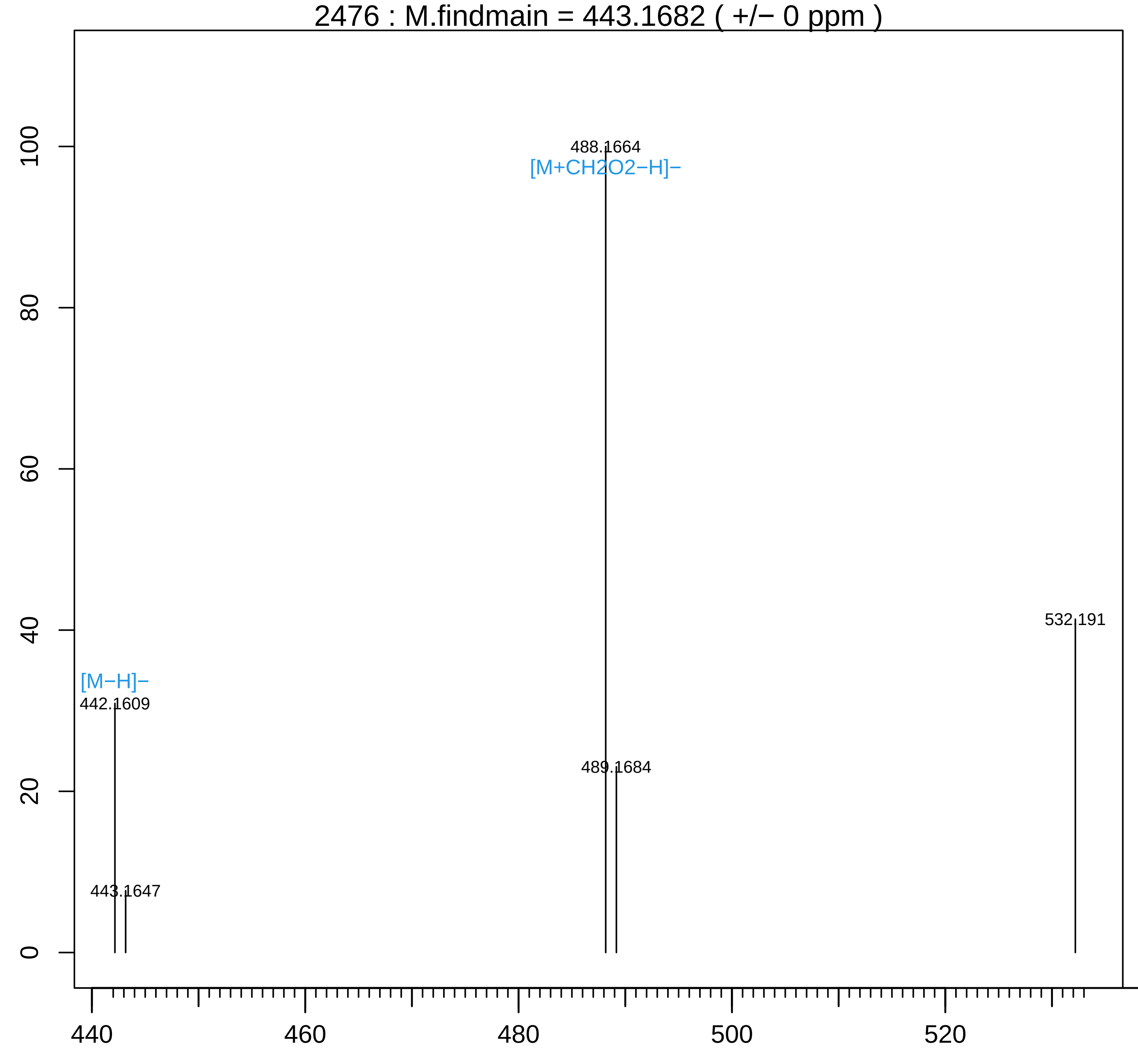
2476 : M.findmain = 443.1682 (+/− 0 ppm)
488.1664
[M+CH2O2−H]−
532.191
[M−H]−
442.1609
489.1684
443.1647
0
20
40
60
80
100
440
460
480
500
520

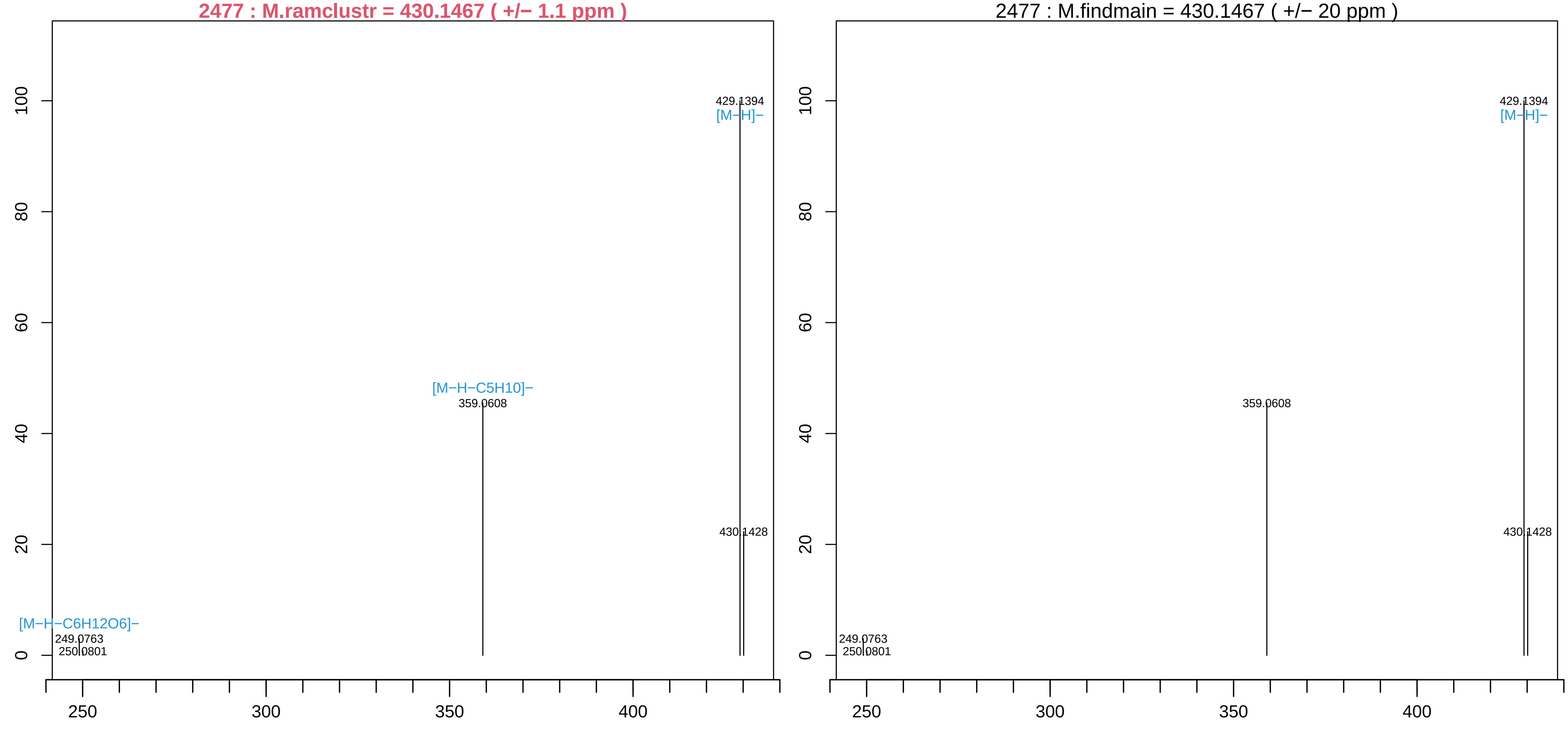
2477 : M.ramclustr = 430.1467 (+/− 1.1 ppm)
429.1394
[M−H]−
[M−H−C5H10]−
359.0608
430.1428
[M−H−C6H12O6]−
249.0763
250.0801
0
20
40
60
80
100
250
300
350
400
2477 : M.findmain = 430.1467 (+/− 20 ppm)
429.1394
[M−H]−
359.0608
430.1428
249.0763
250.0801

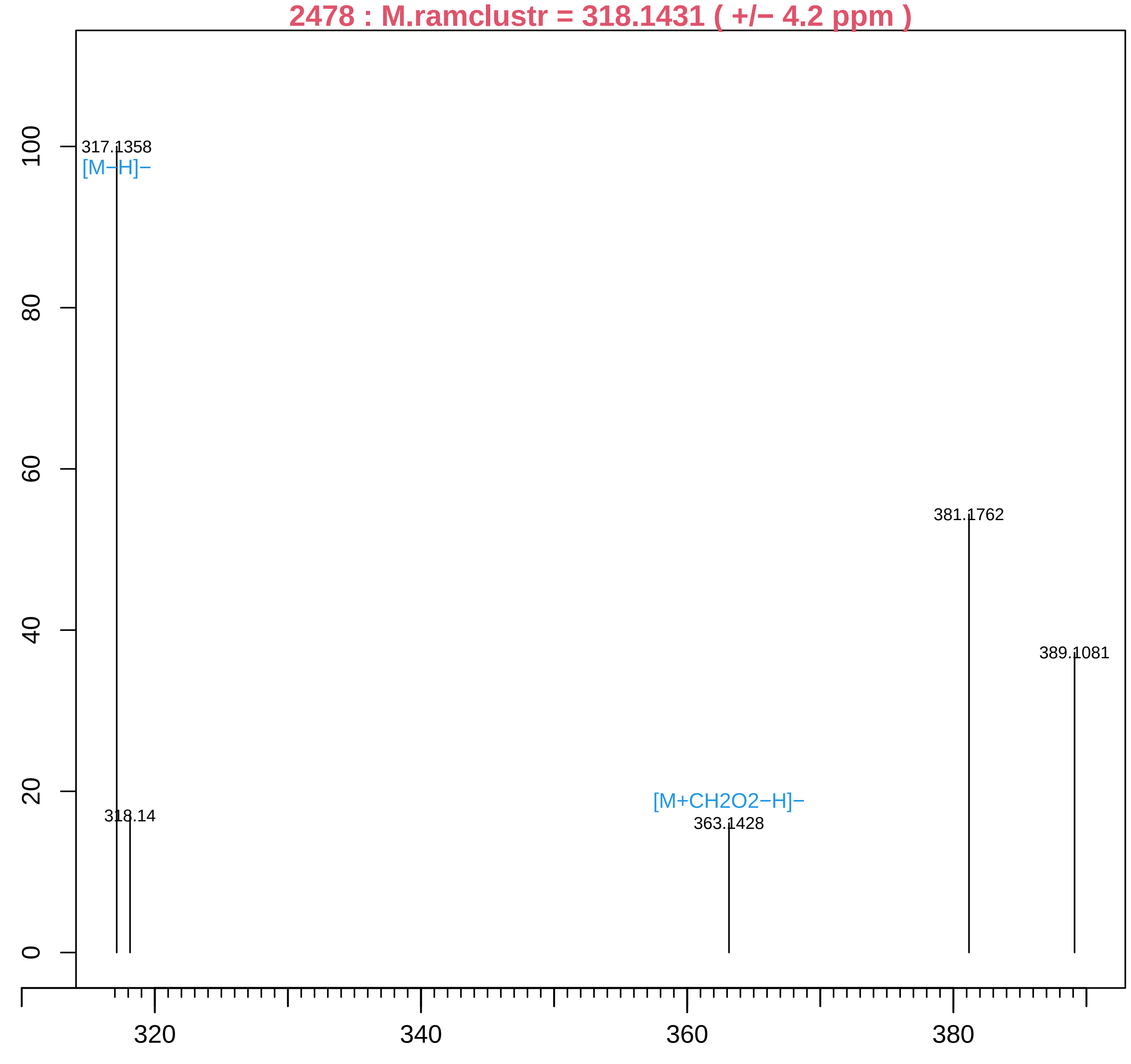
2478 : M.ramclustr = 318.1431 (+/− 4.2 ppm)
317.1358
[M−H]−
318.14
[M+CH2O2−H]−
363.1428
381.1762
389.1081
0
20
40
60
80
100
320
340
360
380

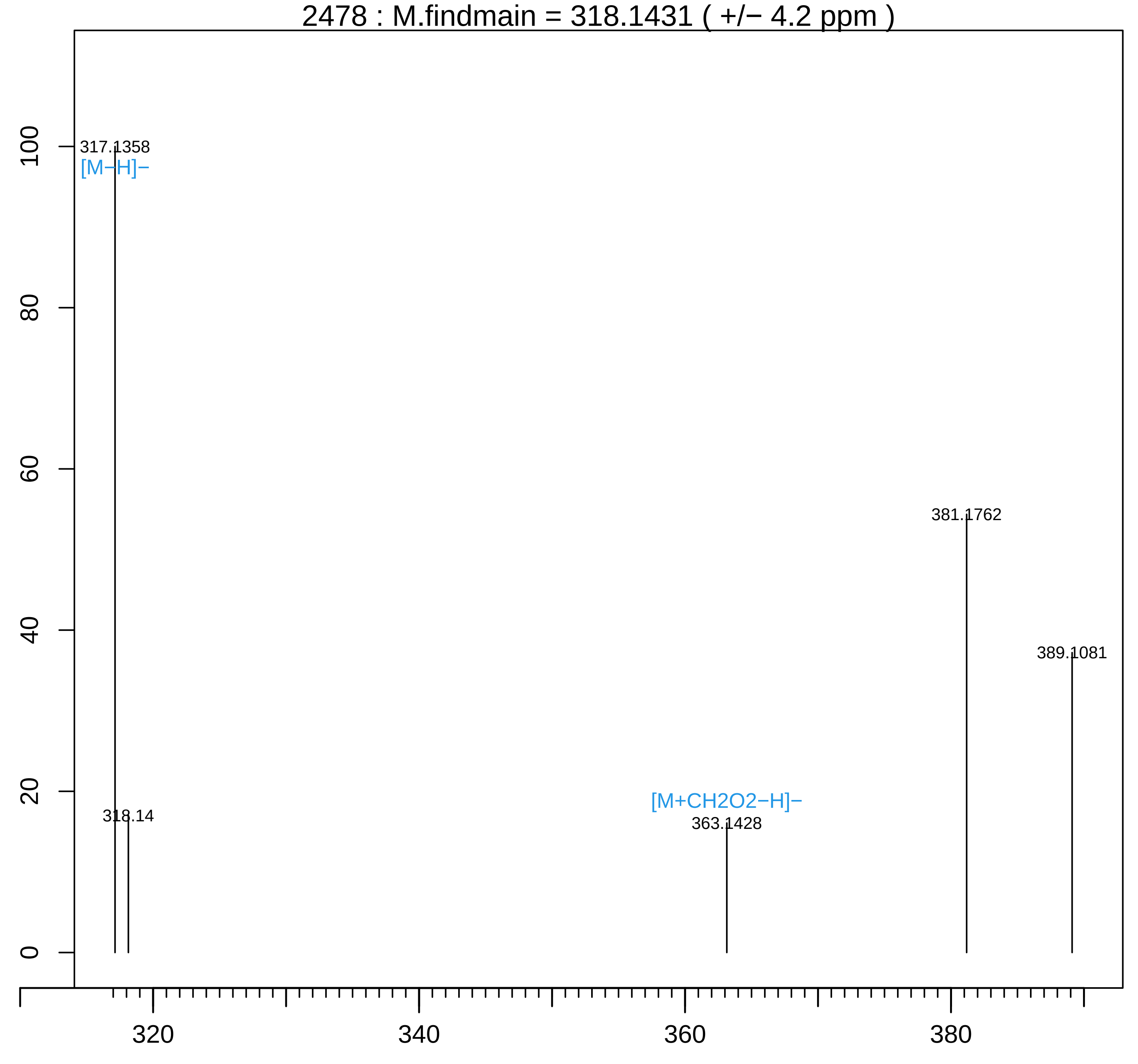
2478 : M.findmain = 318.1431 (+/− 4.2 ppm)
317.1358
[M−H]−
318.14
[M+CH2O2−H]−
363.1428
381.1762
389.1081
0
20
40
60
80
100
320
340
360
380

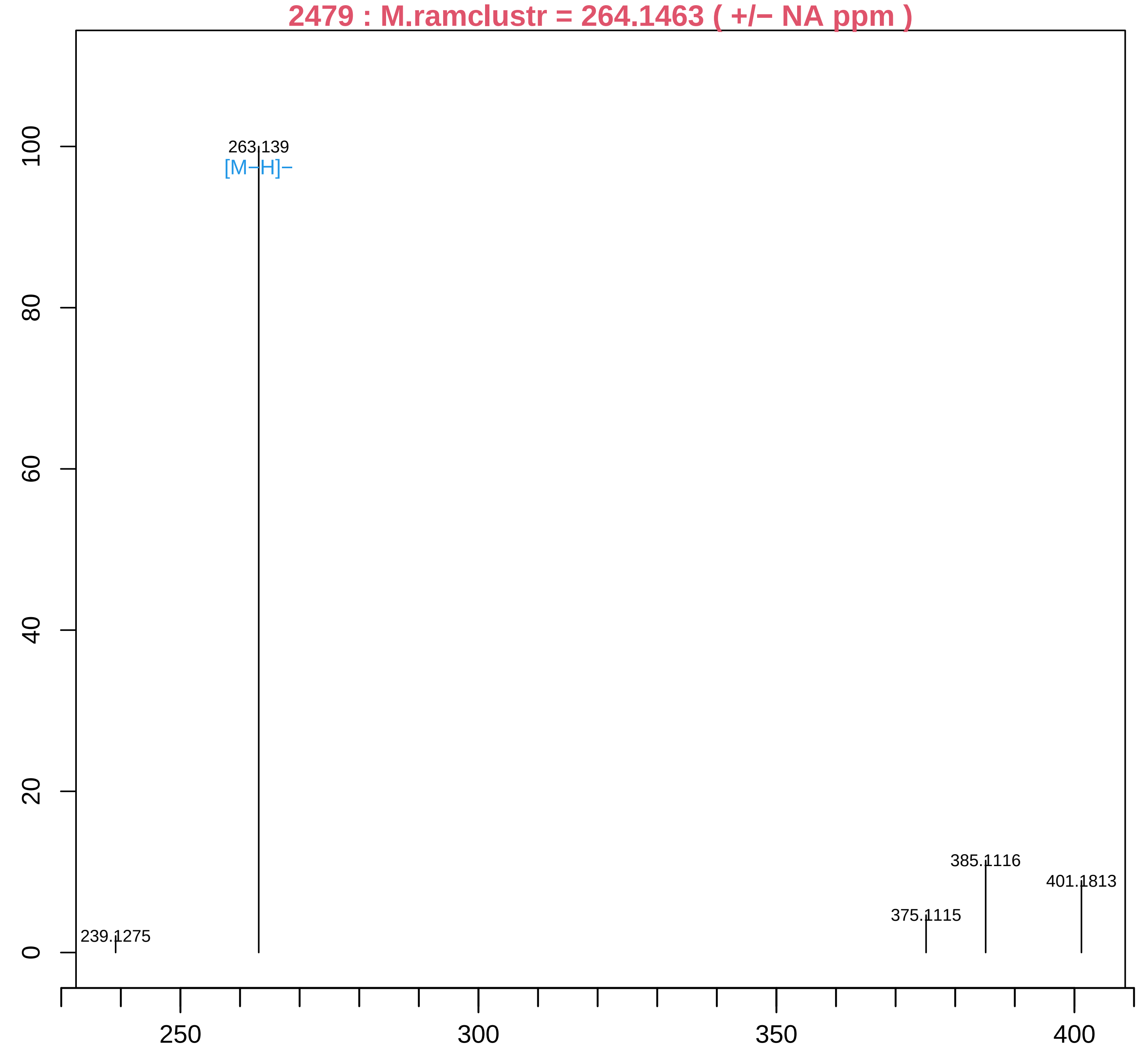
2479 : M.ramclustr = 264.1463 (+/− NA ppm)
263.139
[M−H]−
239.1275
375.1115
385.1116
401.1813
0
20
40
60
80
100
250
300
350
400

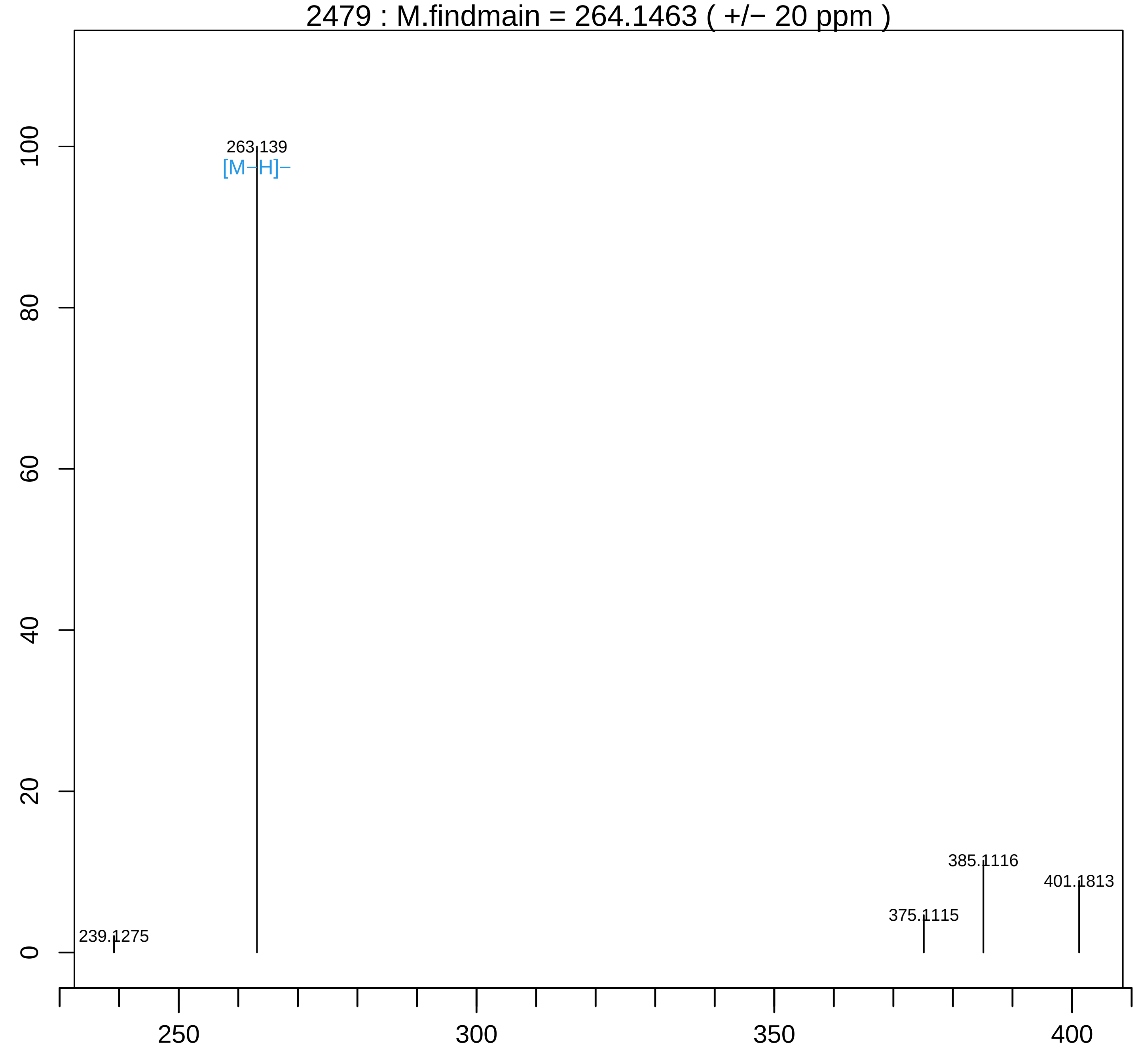
2479 : M.findmain = 264.1463 (+/− 20 ppm)
263.139
[M−H]−
239.1275
375.1115
385.1116
401.1813
0
20
40
60
80
100
250
300
350
400

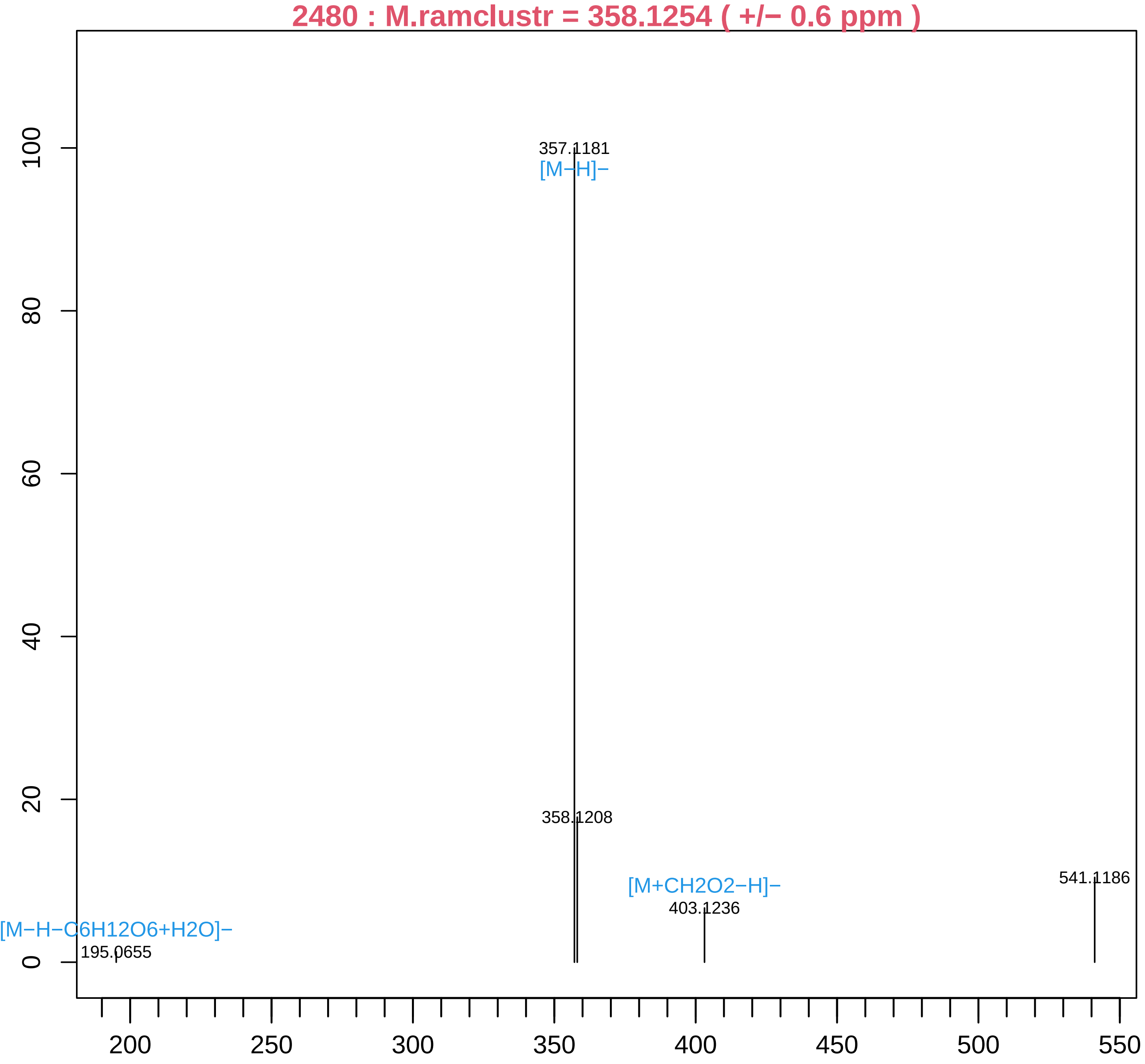

2480 : M.ramclustr = 358.1254 (+/− 0.6 ppm)
357.1181
[M−H]−
358.1208
[M+CH2O2−H]−
403.1236
541.1186
[M−H−C6H12O6+H2O]−
195.0655
0
20
40
60
80
100
200
250
300
350
400
450
500
550

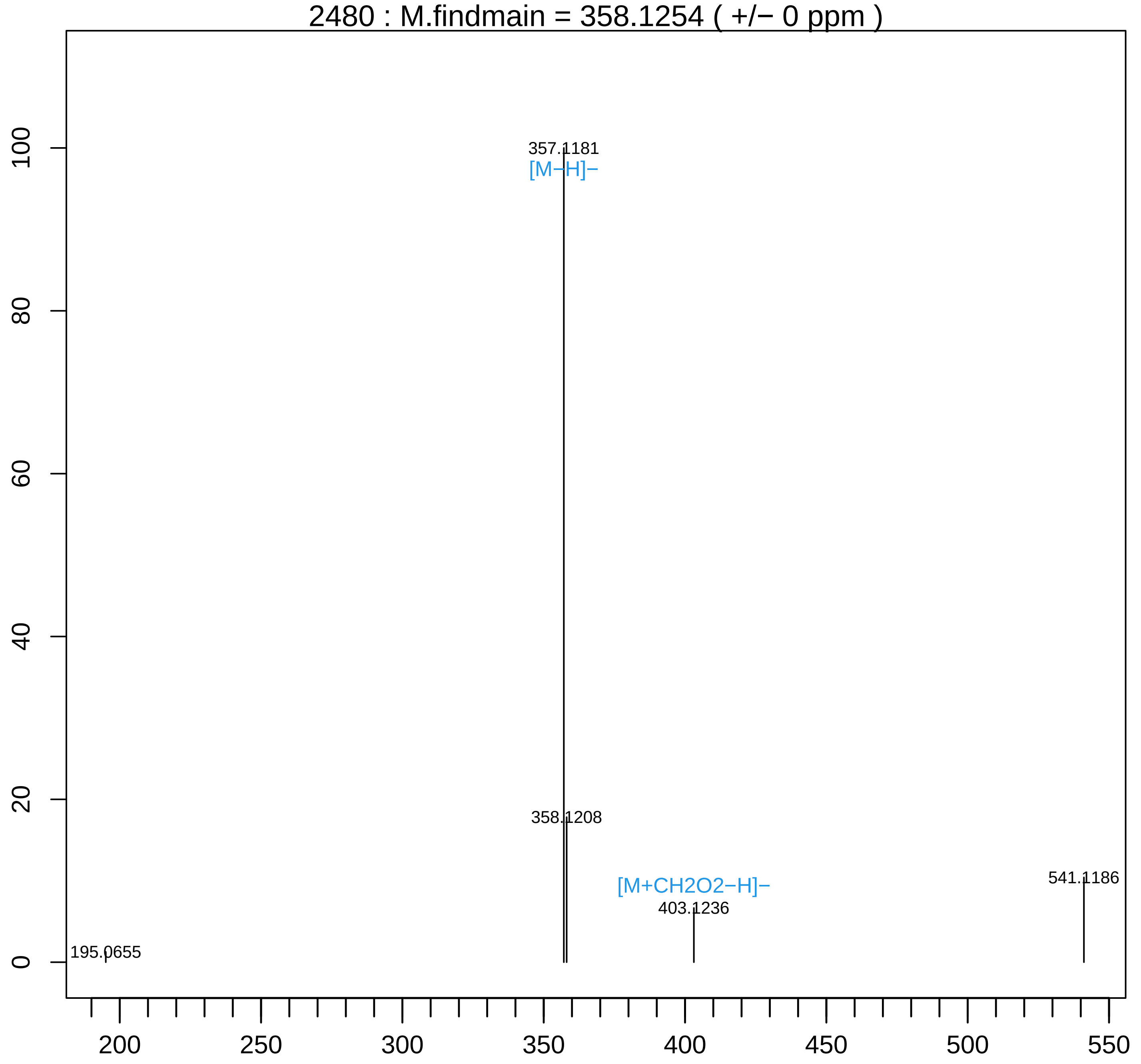

2480 : M.findmain = 358.1254 (+/− 0 ppm)
357.1181
[M−H]−
358.1208
[M+CH2O2−H]−
403.1236
541.1186
195.0655
0
20
40
60
80
100
200
250
300
350
400
450
500
550

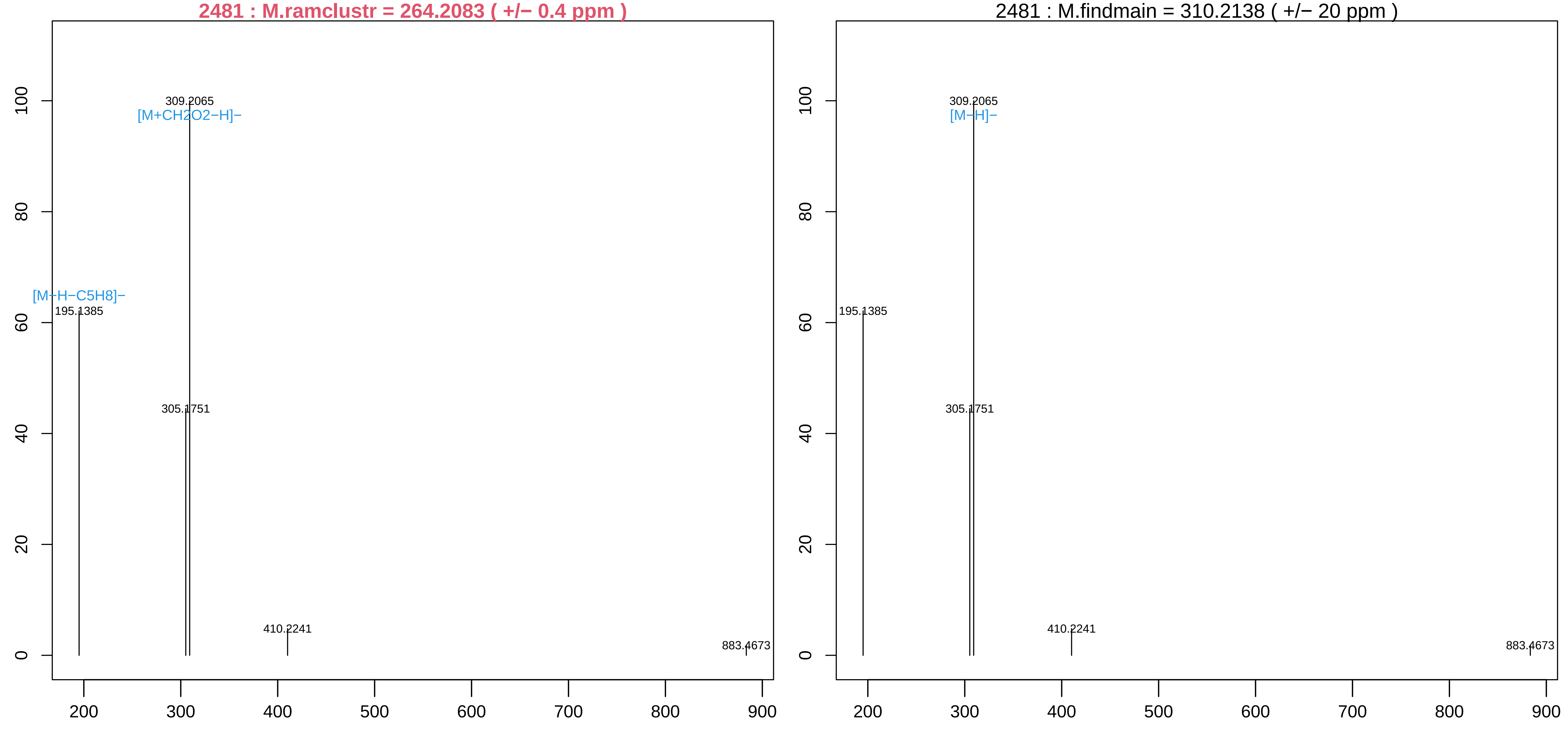

2481 : M.ramclustr = 264.2083 (+/− 0.4 ppm)
309.2065
[M+CH2O2−H]−
[M−H−C5H8]−
195.1385
305.1751
410.2241
883.4673
0
20
40
60
80
100
200
300
400
500
600
700
800
900
2481 : M.findmain = 310.2138 (+/− 20 ppm)
309.2065
[M−H]−
195.1385
305.1751
410.2241
883.4673
0
20
40
60
80
100
200
300
400
500
600
700
800
900

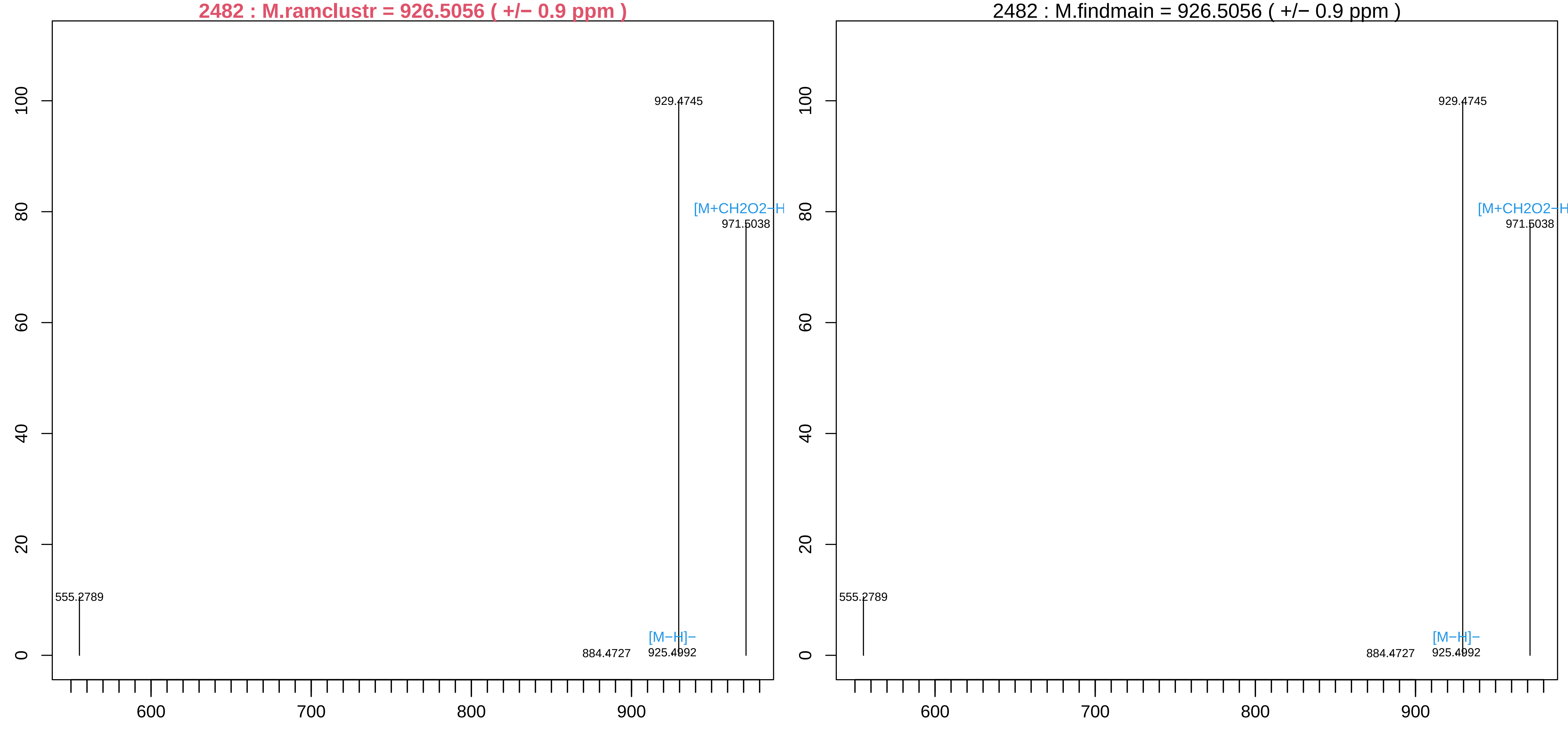
2482 : M.ramclustr = 926.5056 (+/− 0.9 ppm)
929.4745
[M+CH2O2−H
971.5038
555.2789
[M−H]−
884.4727
925.4992
2482 : M.findmain = 926.5056 (+/− 0.9 ppm)
929.4745
[M+CH2O2−H
971.5038
555.2789
[M−H]−
884.4727
925.4992

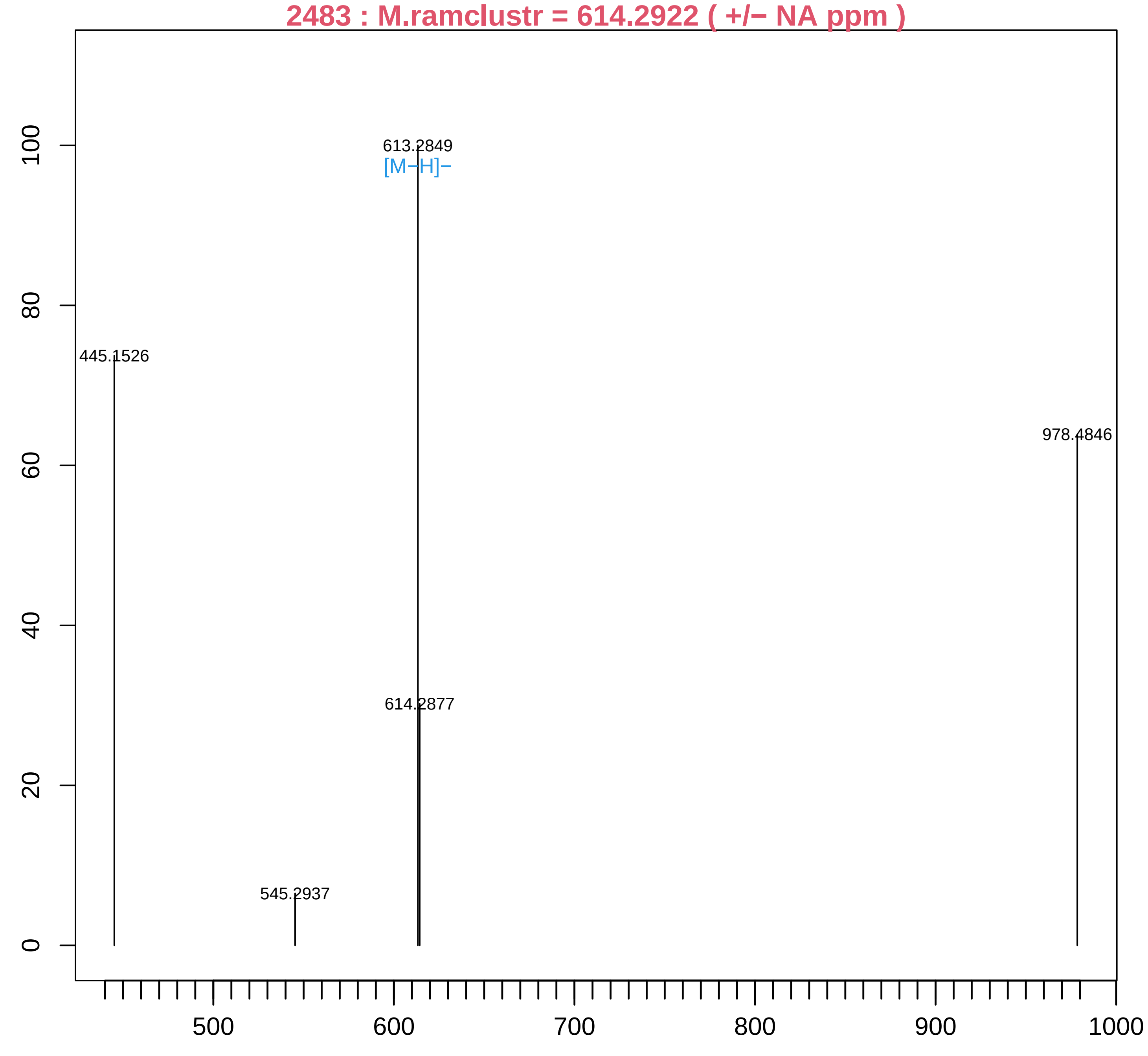
2483 : M.ramclustr = 614.2922 (+/− NA ppm)
613.2849
[M−H]−
445.1526
978.4846
614.2877
545.2937
0
20
40
60
80
100
500
600
700
800
900
1000

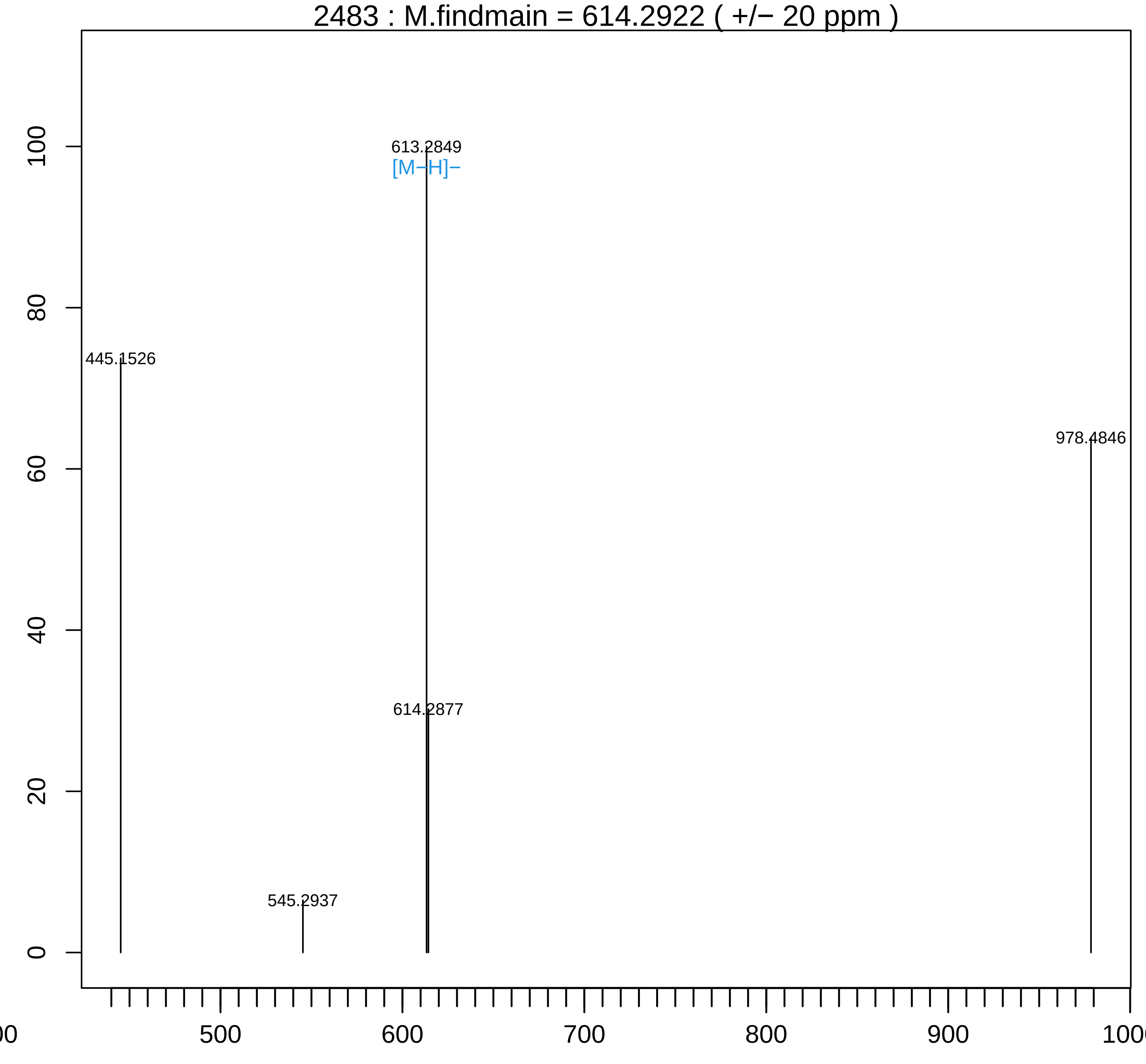
2483 : M.findmain = 614.2922 (+/− 20 ppm)
613.2849
[M−H]−
445.1526
978.4846
614.2877
545.2937
0
20
40
60
80
100
500
600
700
800
900

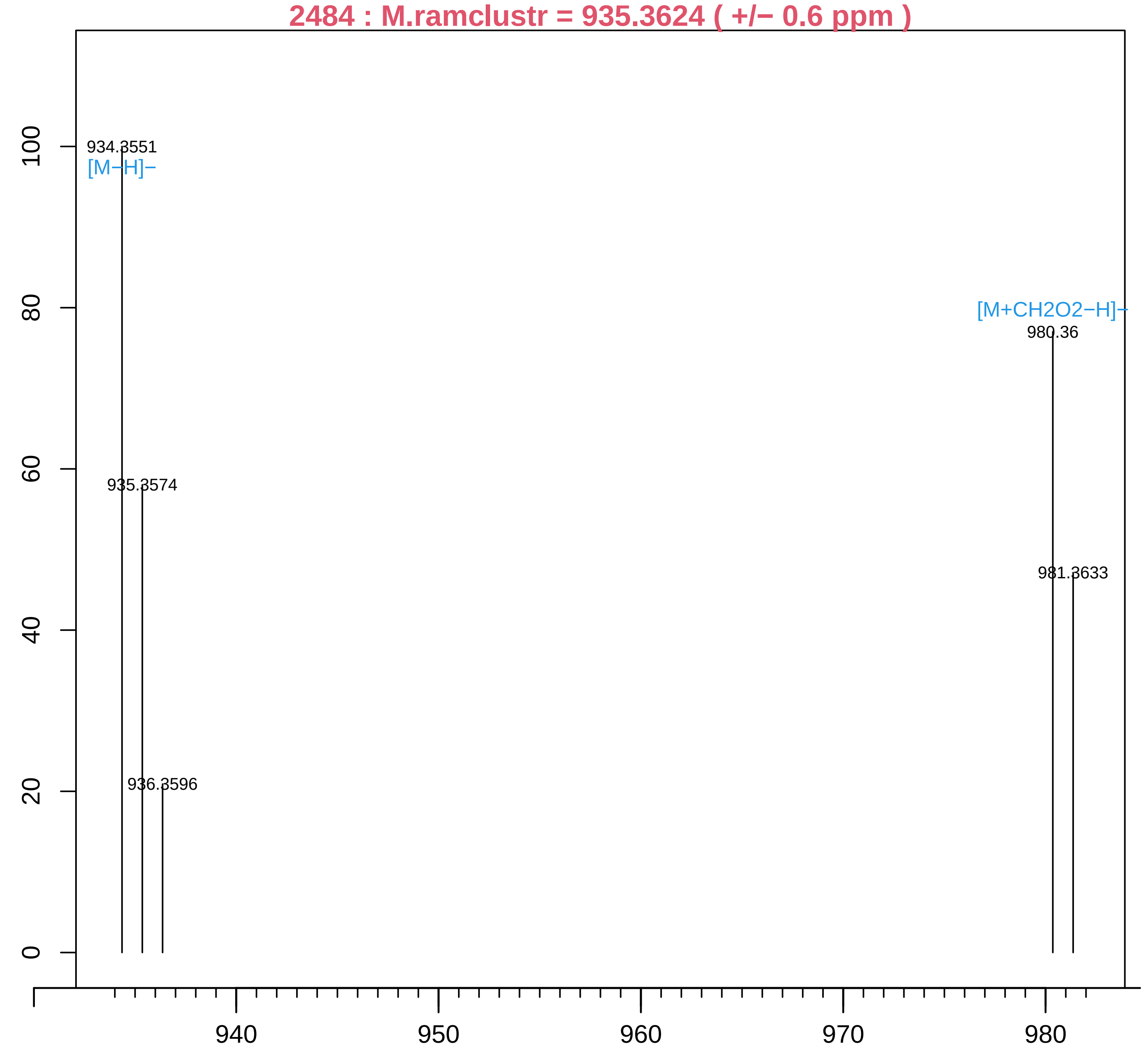
2484 : M.ramclustr = 935.3624 (+/− 0.6 ppm)
934.3551
[M−H]−
935.3574
936.3596
[M+CH2O2−H]−
980.36
981.3633
0
20
40
60
80
100
940
950
960
970
980

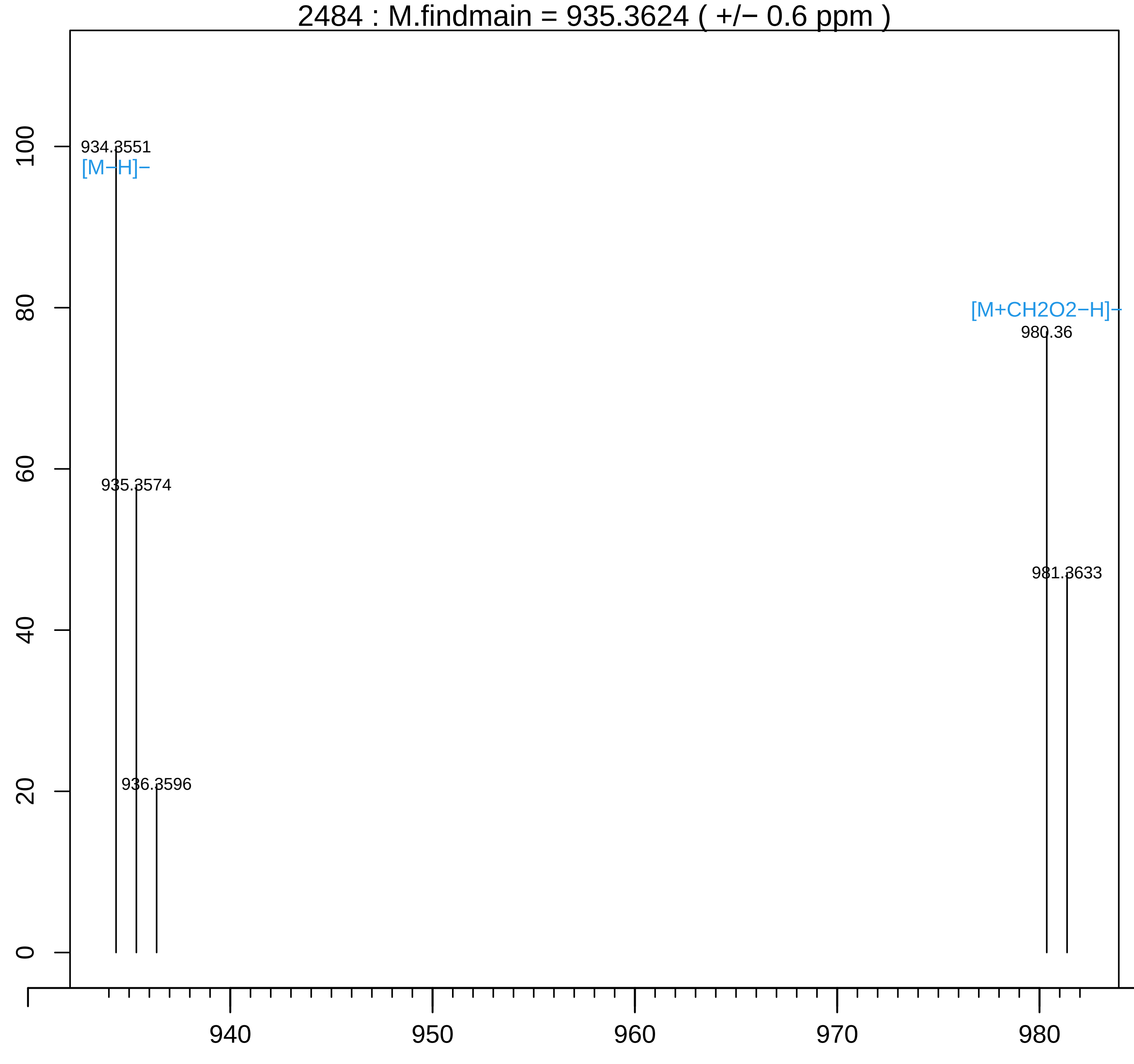
2484 : M.findmain = 935.3624 (+/− 0.6 ppm)
934.3551
[M−H]−
935.3574
936.3596
[M+CH2O2−H]−
980.36
981.3633
0
20
40
60
80
100
940
950
960
970
980

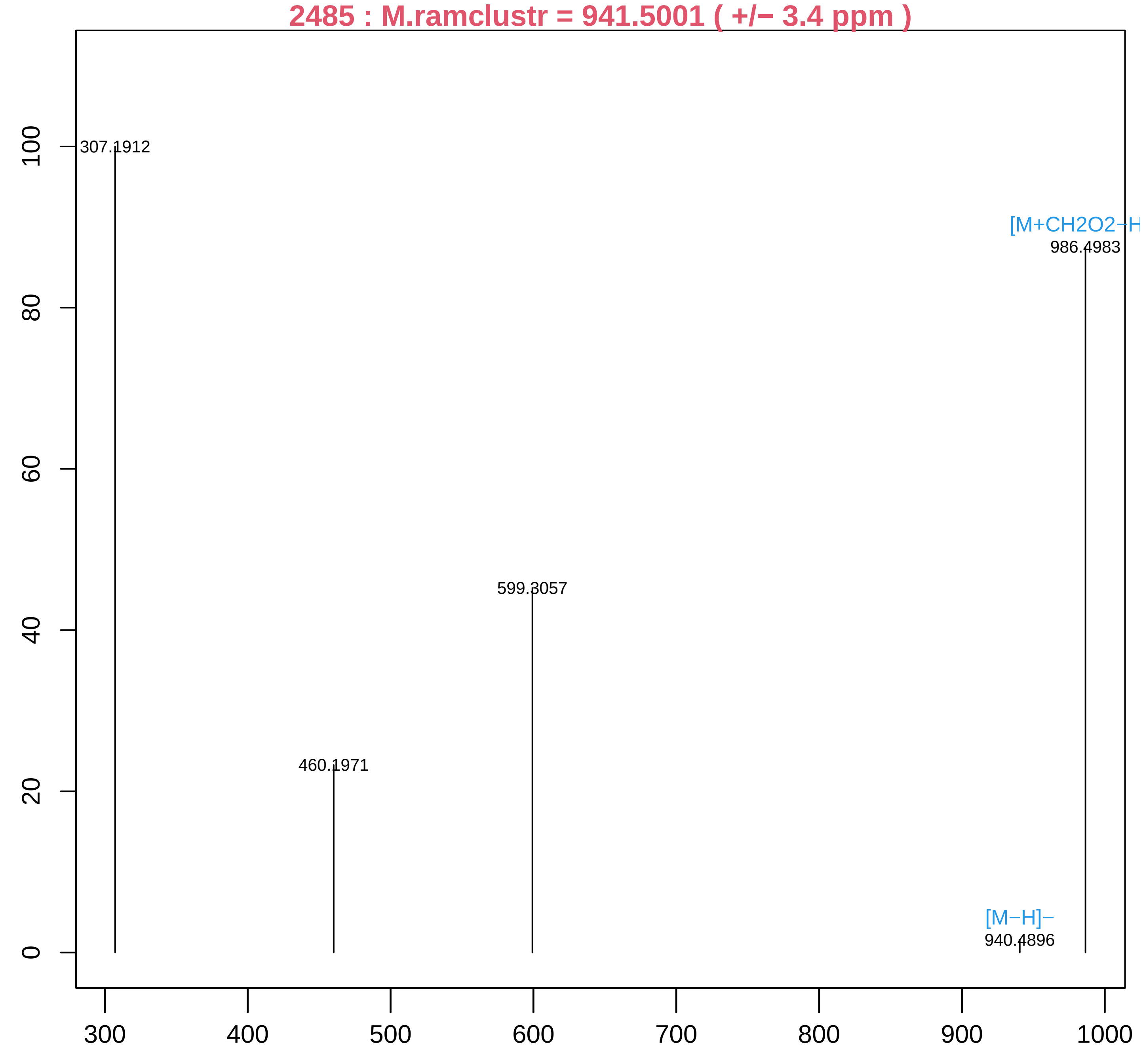
2485 : M.ramclustr = 941.5001 (+/− 3.4 ppm)
307.1912
460.1971
599.3057
[M+CH2O2−H
986.4983
[M−H]−
940.4896
0
20
40
60
80
100
300
400
500
600
700
800
900
1000

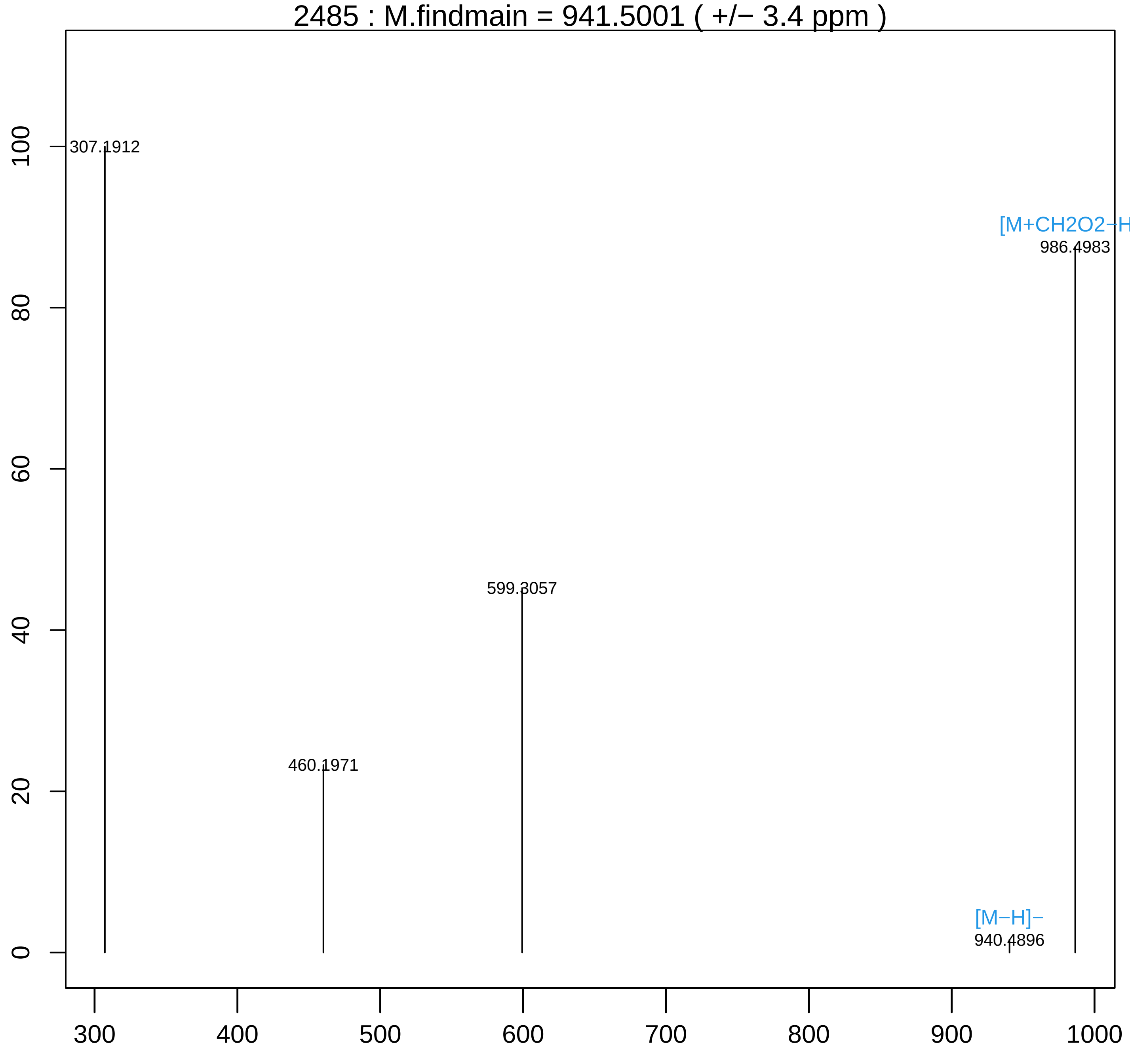
2485 : M.findmain = 941.5001 (+/− 3.4 ppm)
307.1912
460.1971
599.3057
[M+CH2O2−H
986.4983
[M−H]−
940.4896
0
20
40
60
80
100
300
400
500
600
700
800
900
1000

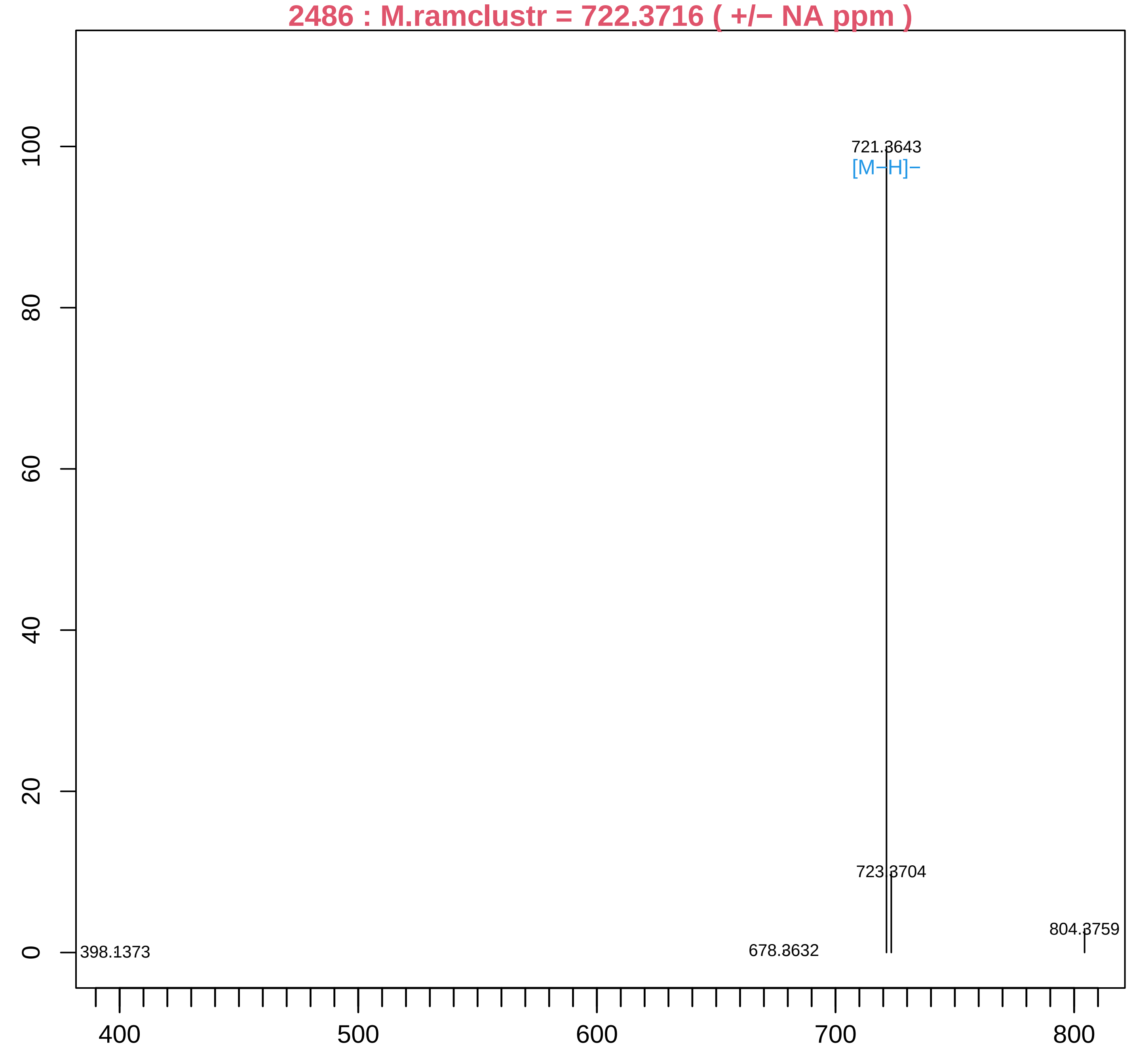
2486 : M.ramclustr = 722.3716 (+/− NA ppm)
721.3643
[M−H]−
723.3704
678.3632
804.3759
398.1373
0
20
40
60
80
100
400
500
600
700
800

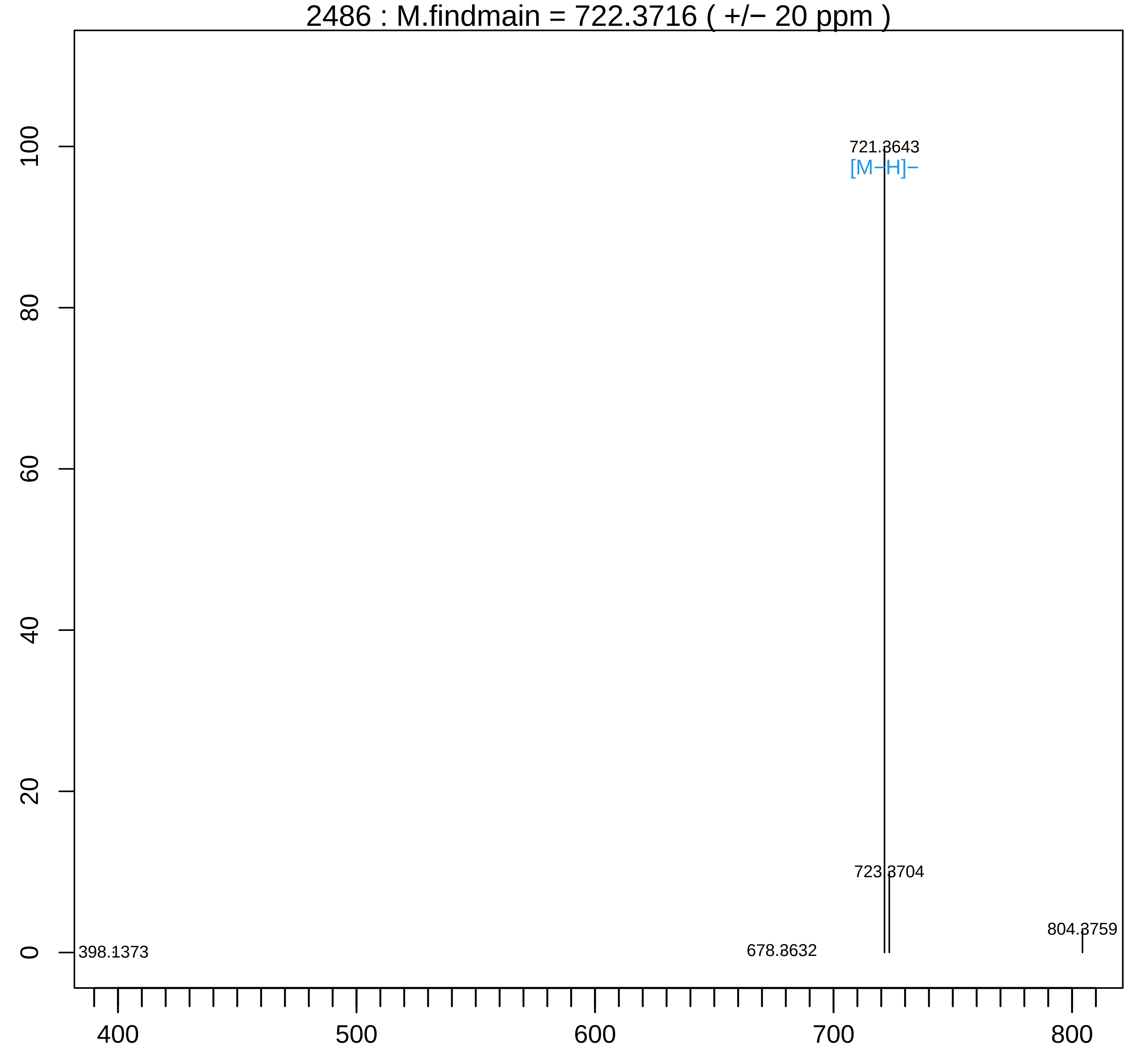
2486 : M.findmain = 722.3716 (+/− 20 ppm)
721.3643
[M−H]−
723.3704
678.3632
804.3759
398.1373
0
20
40
60
80
100
400
500
600
700
800

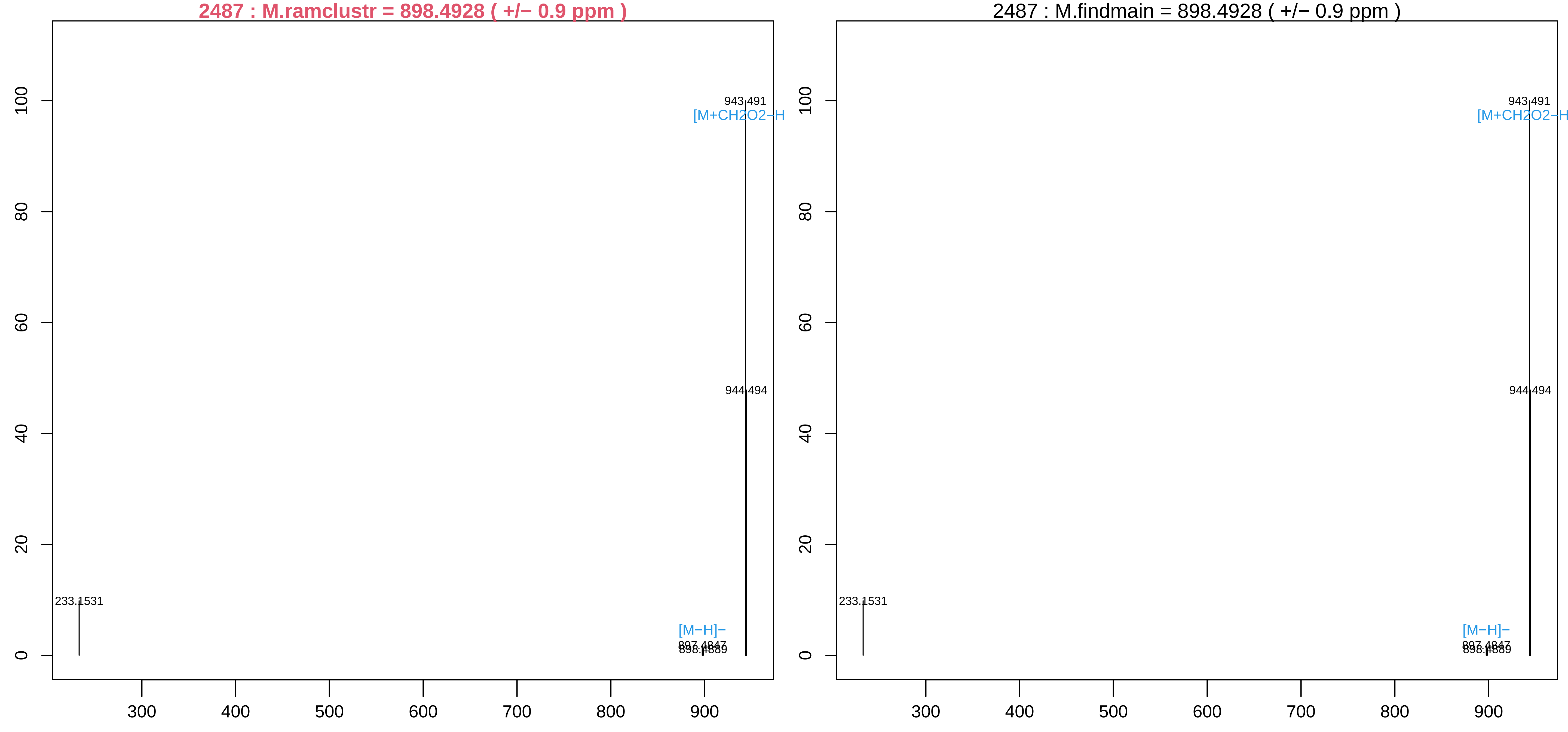
2487 : M.ramclustr = 898.4928 (+/− 0.9 ppm)
943.491
[M+CH2O2−H
944.494
233.1531
[M−H]−
897.4847
898.4889
0
20
40
60
80
100
300
400
500
600
700
800
900
2487 : M.findmain = 898.4928 (+/− 0.9 ppm)
943.491
[M+CH2O2−H
944.494
233.1531
[M−H]−
897.4847
898.4889

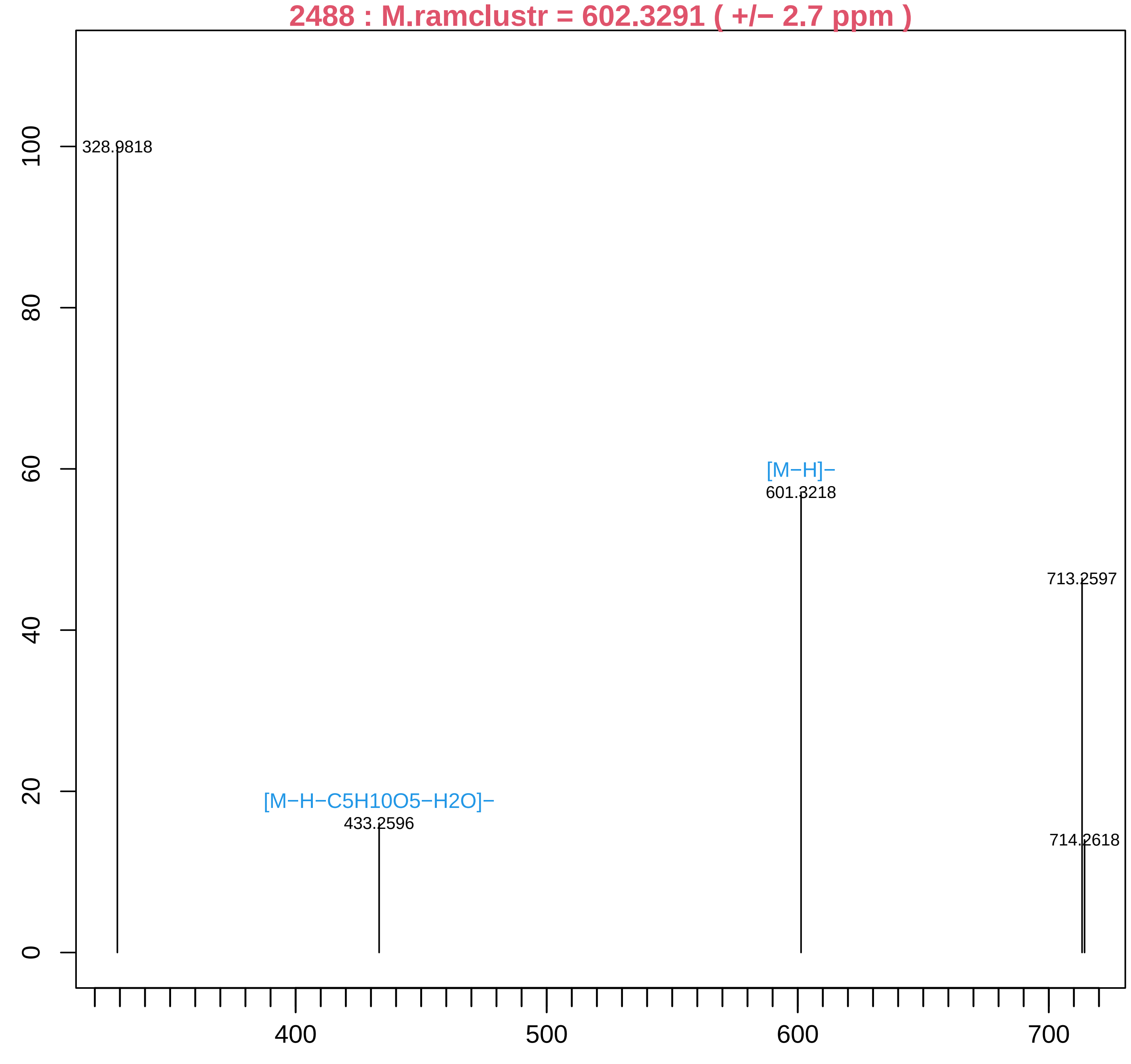
2488 : M.ramclustr = 602.3291 (+/− 2.7 ppm)
328.9818
[M−H−C5H10O5−H2O]−
433.2596
[M−H]−
601.3218
713.2597
714.2618
0
20
40
60
80
100
400
500
600
700

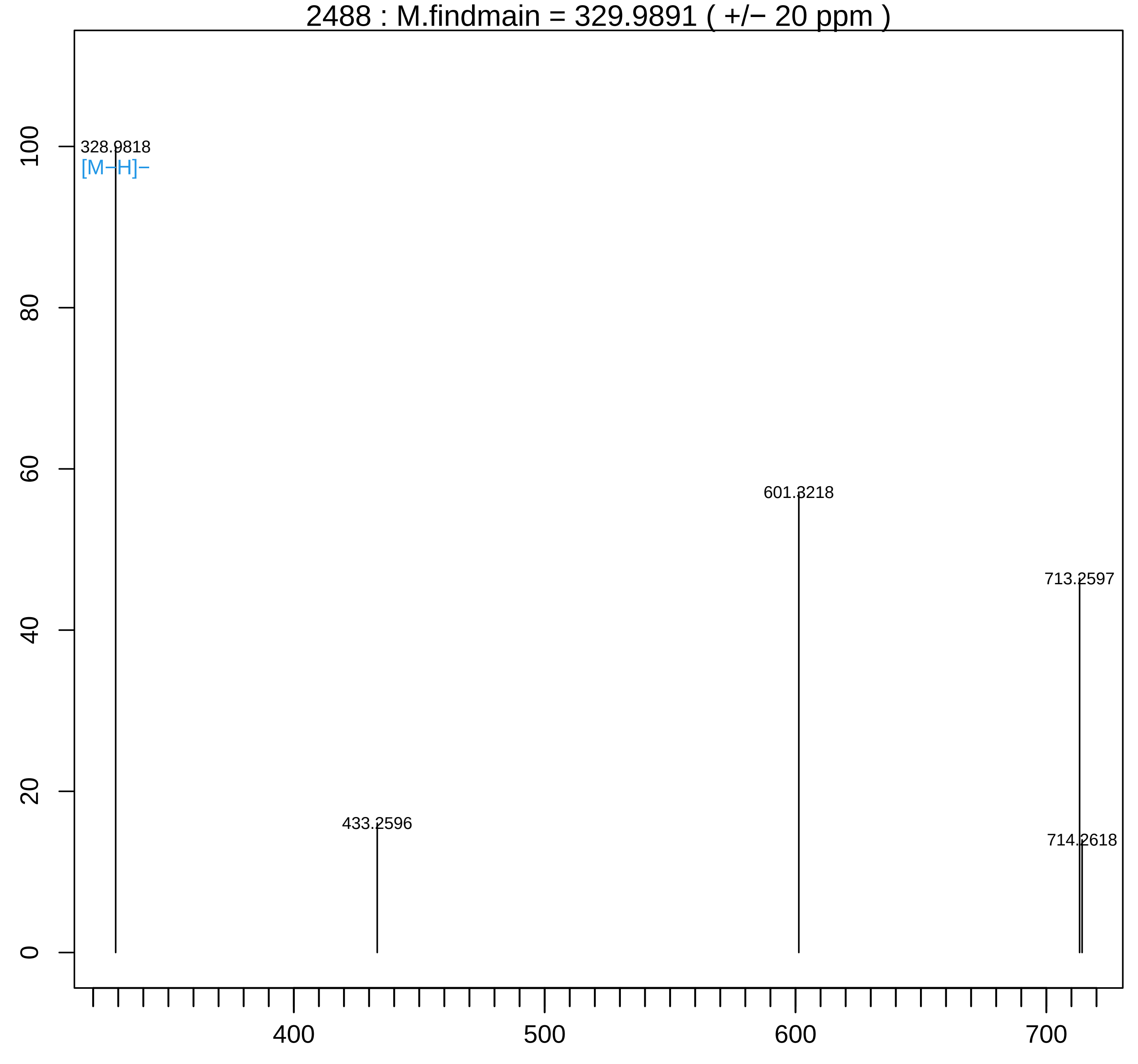
2488 : M.findmain = 329.9891 (+/− 20 ppm)
328.9818
[M−H]−
433.2596
601.3218
713.2597
714.2618
0
20
40
60
80
100
400
500
600
700

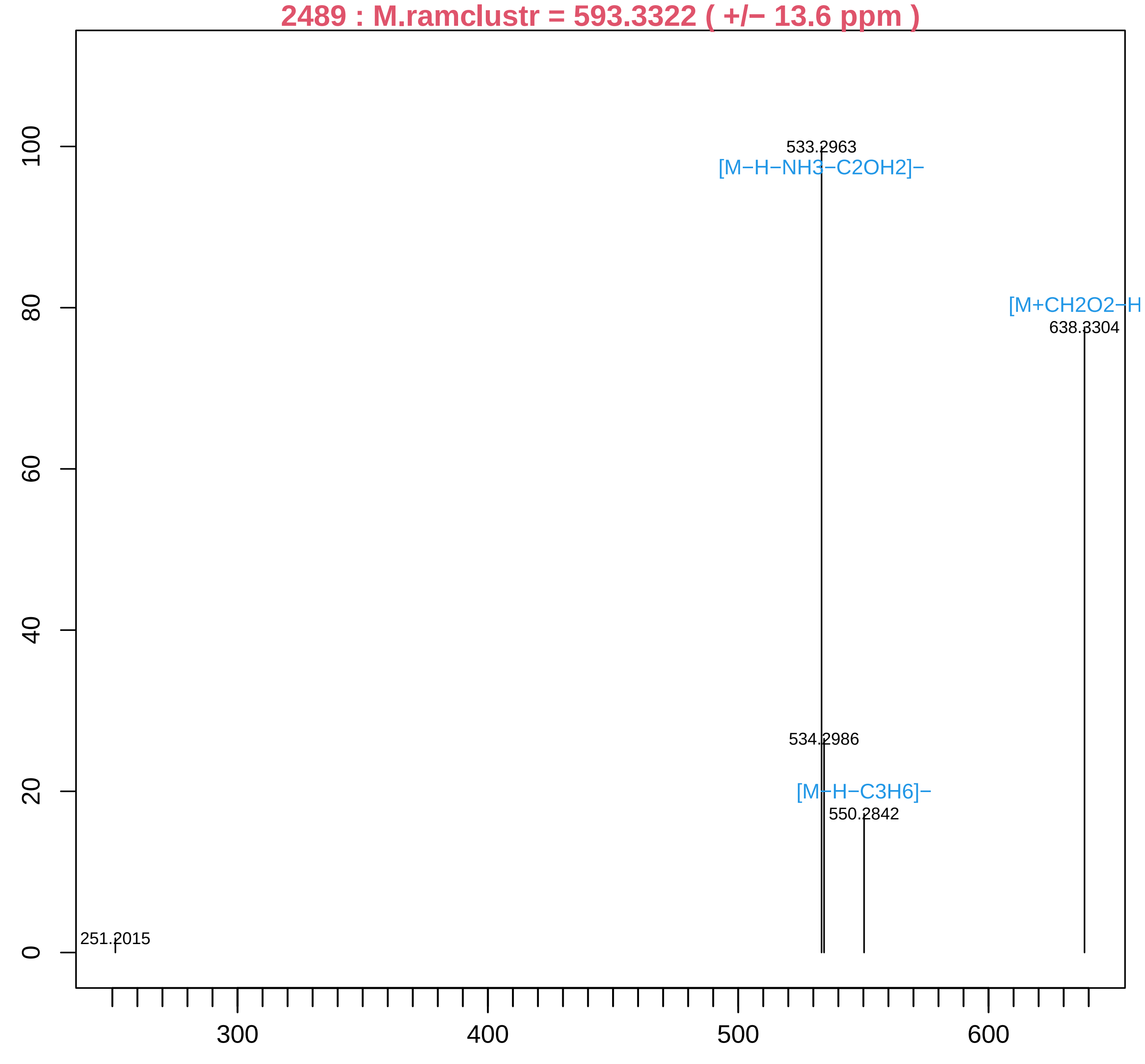
2489 : M.ramclustr = 593.3322 (+/− 13.6 ppm)
533.2963
[M−H−NH3−C2OH2]−
[M+CH2O2−H
638.3304
534.2986
[M−H−C3H6]−
550.2842
251.2015
0
20
40
60
80
100
300
400
500
600

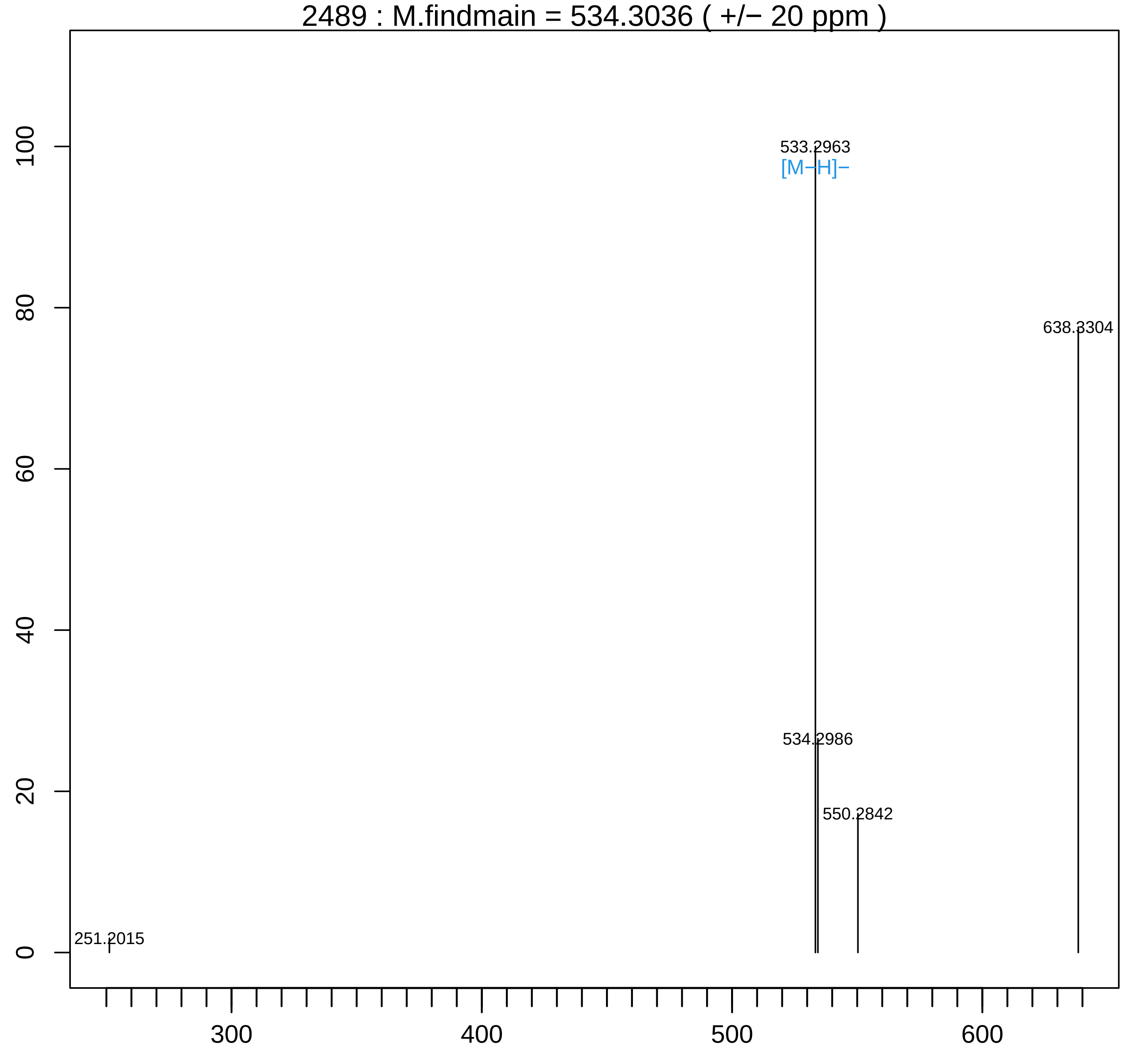
2489 : M.findmain = 534.3036 (+/− 20 ppm)
533.2963
[M−H]−
638.3304
534.2986
550.2842
251.2015
0
20
40
60
80
100
300
400
500
600

2490 : M.ramclustr = 475.2689 (+/− NA ppm)

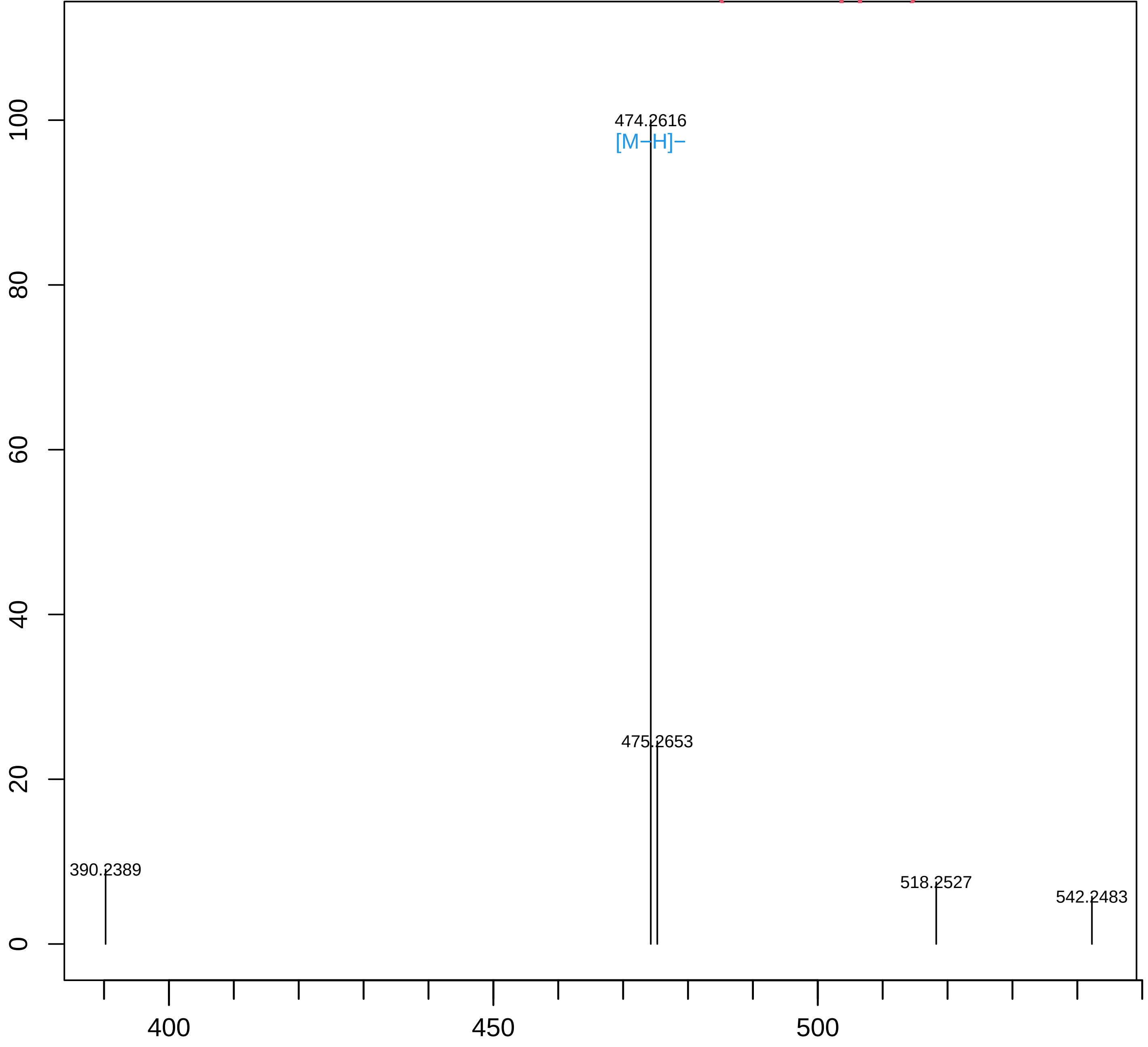

2490 : M.findmain = 475.2689 (+/− 20 ppm)

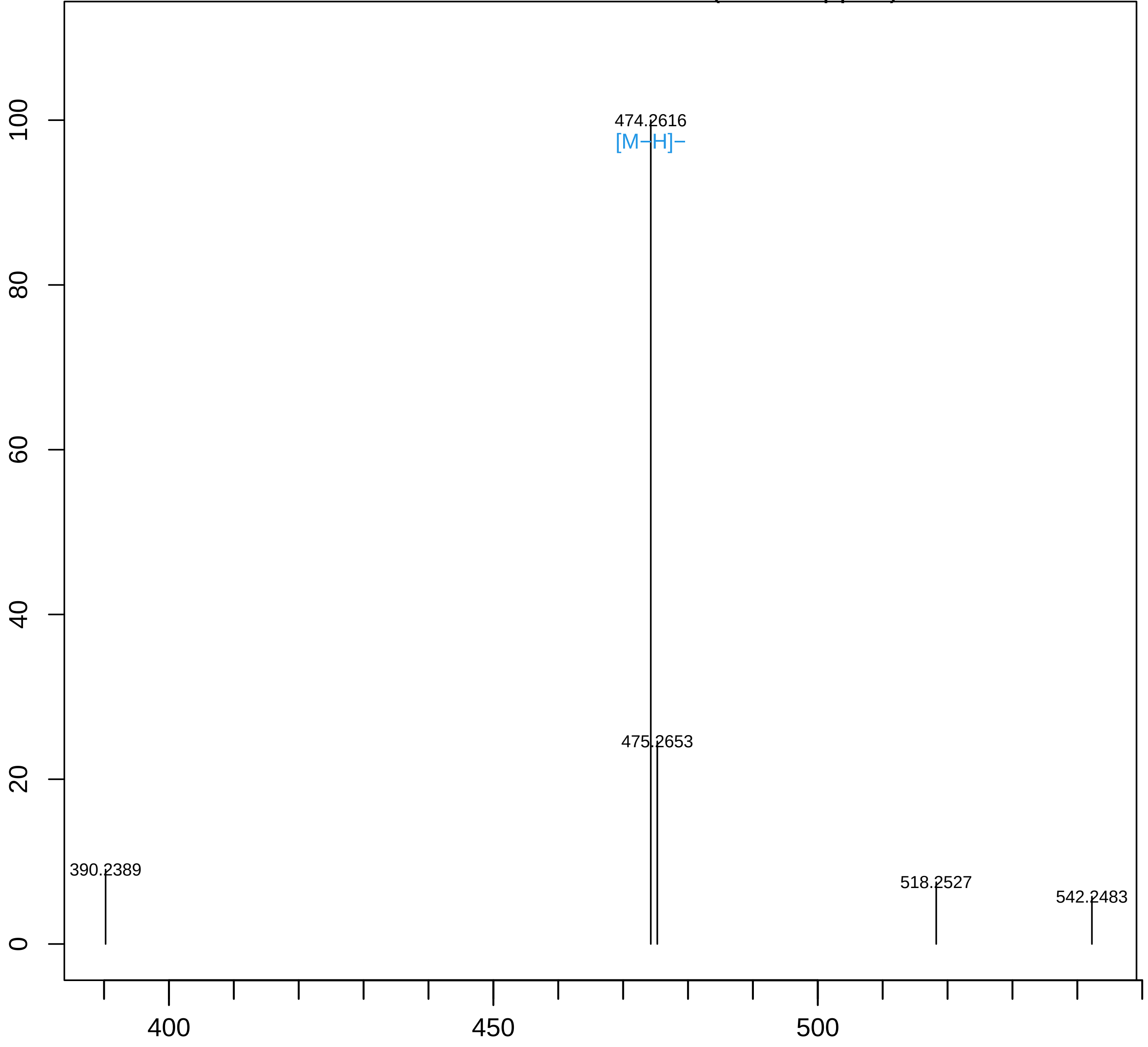

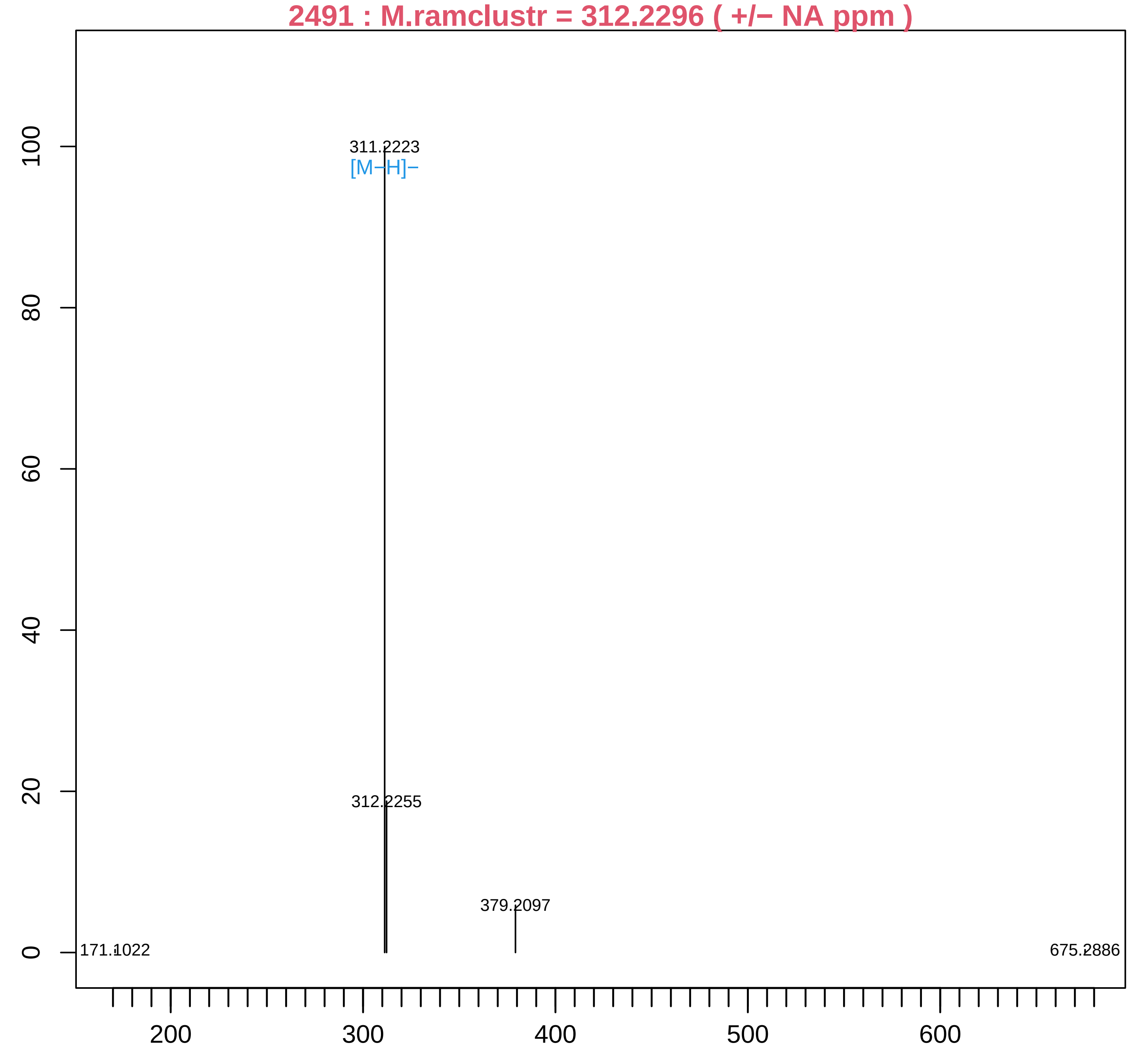
2491 : M.ramclustr = 312.2296 (+/− NA ppm)
311.2223
[M−H]−
312.2255
379.2097
171.1022
675.2886
0
20
40
60
80
100
200
300
400
500
600
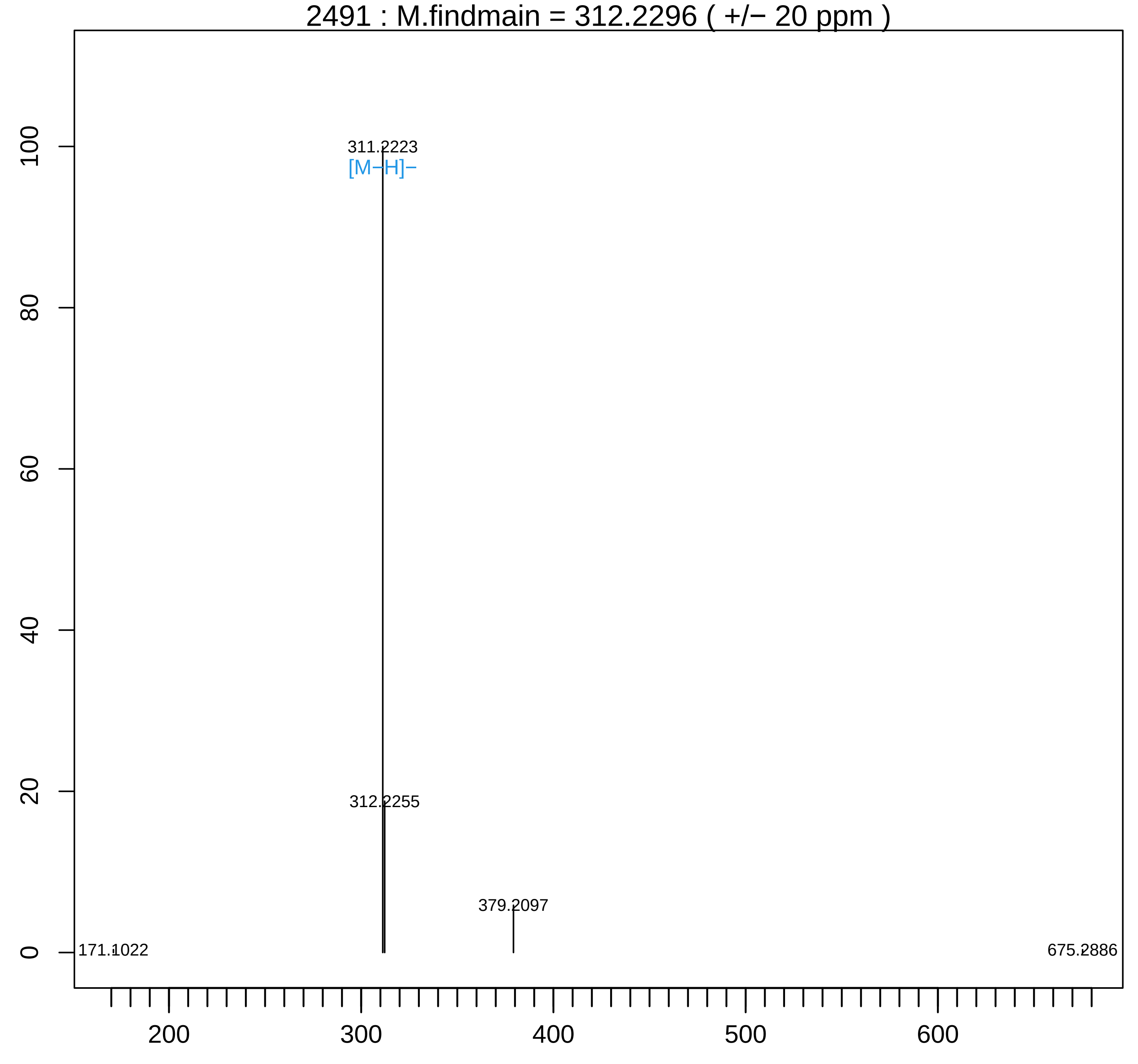
2491 : M.findmain = 312.2296 (+/− 20 ppm)
311.2223
[M−H]−
312.2255
379.2097
171.1022
675.2886
0
20
40
60
80
100
200
300
400
500
600

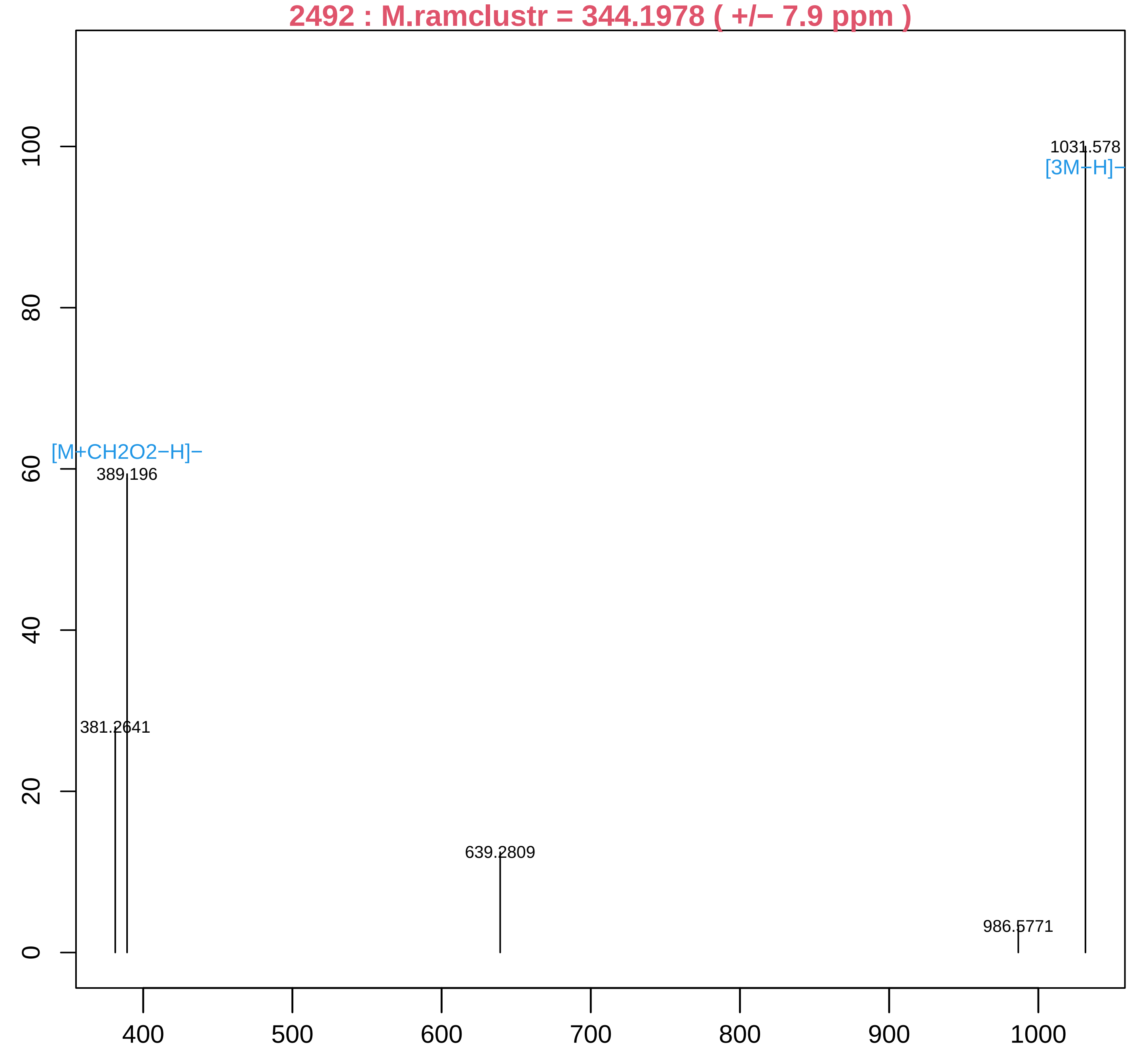
2492 : M.ramclustr = 344.1978 (+/− 7.9 ppm)
1031.578
[3M−H]−
[M+CH2O2−H]−
389.196
381.2641
639.2809
986.5771

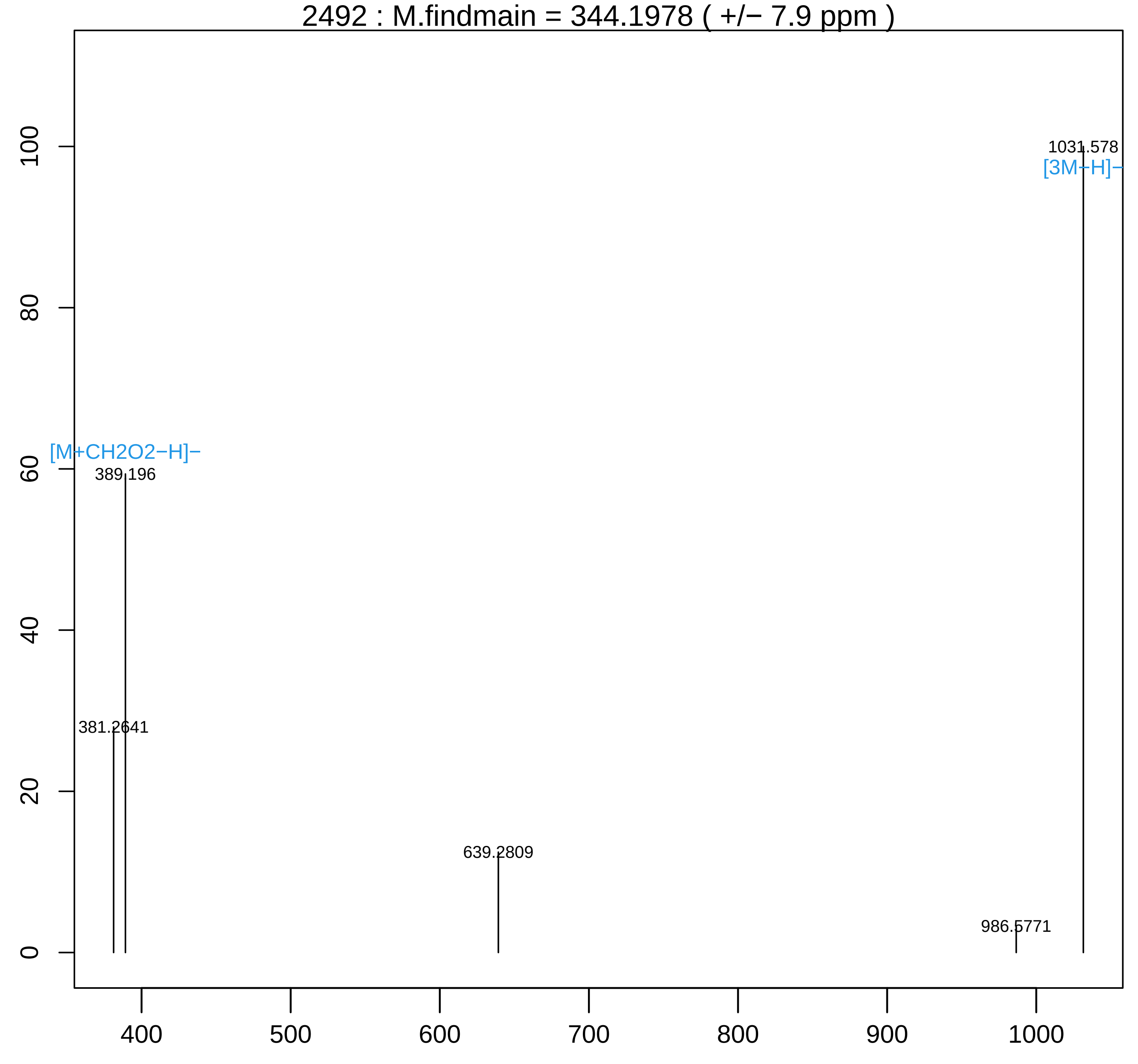
2492 : M.findmain = 344.1978 (+/− 7.9 ppm)
1031.578
[3M−H]−
[M+CH2O2−H]−
389.196
381.2641
639.2809
986.5771

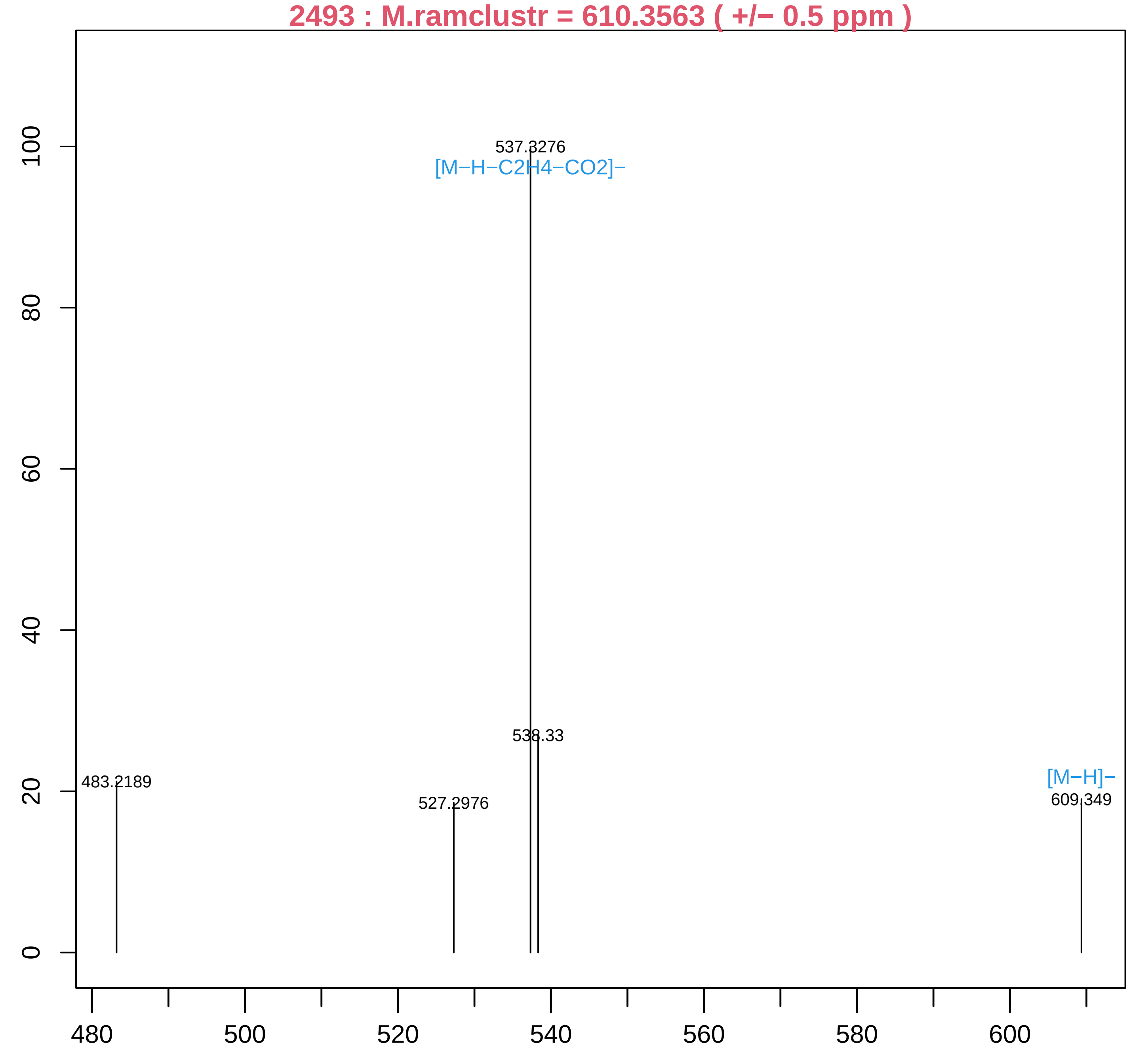

2493 : M.ramclustr = 610.3563 (+/− 0.5 ppm)
537.3276
[M−H−C2H4−CO2]−
538.33
483.2189
527.2976
[M−H]−
609.349
0
20
40
60
80
100
480
500
520
540
560
580
600

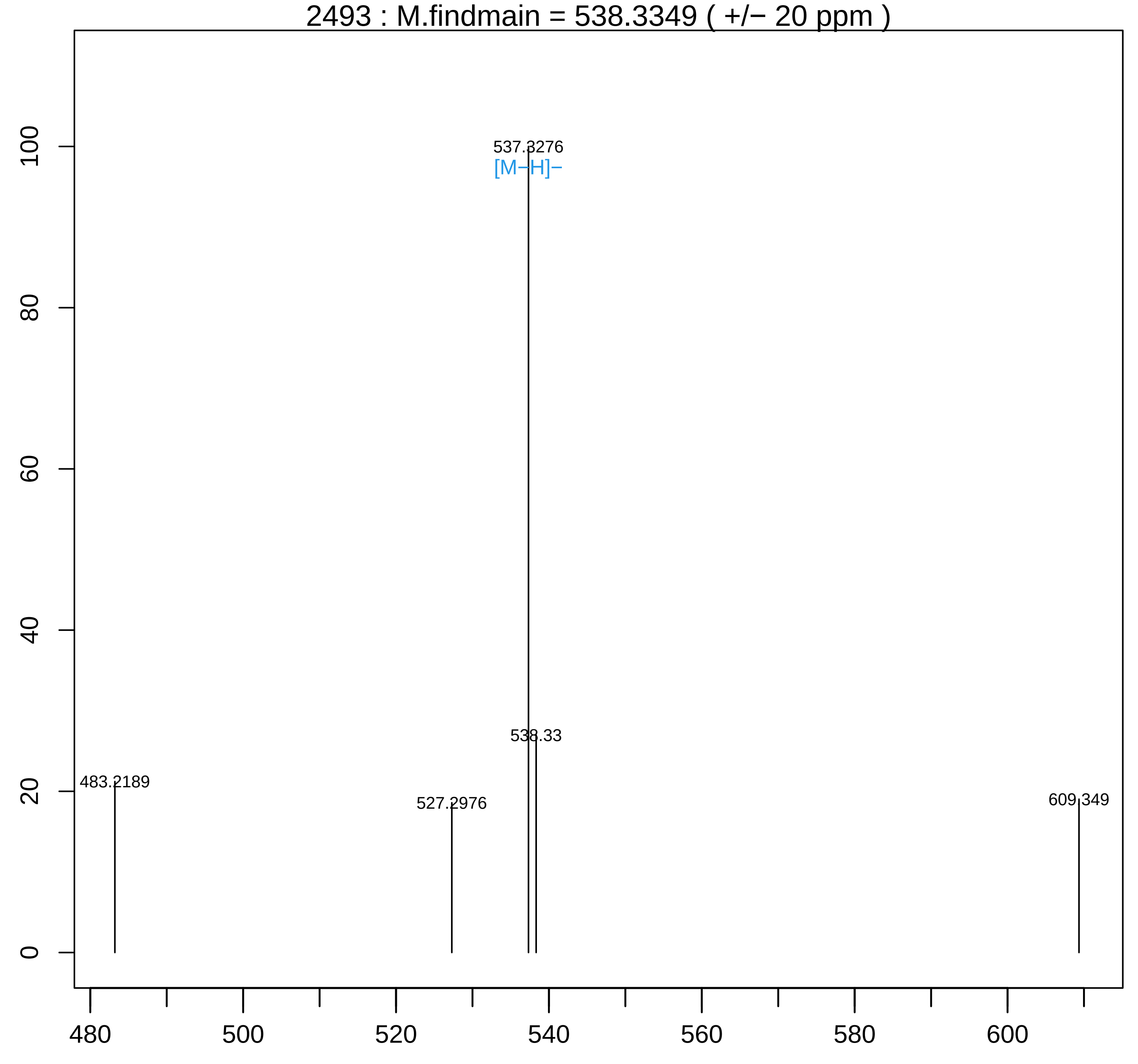

2493 : M.findmain = 538.3349 (+/− 20 ppm)
537.3276
[M−H]−
538.33
483.2189
527.2976
609.349
0
20
40
60
80
100
480
500
520
540
560
580
600

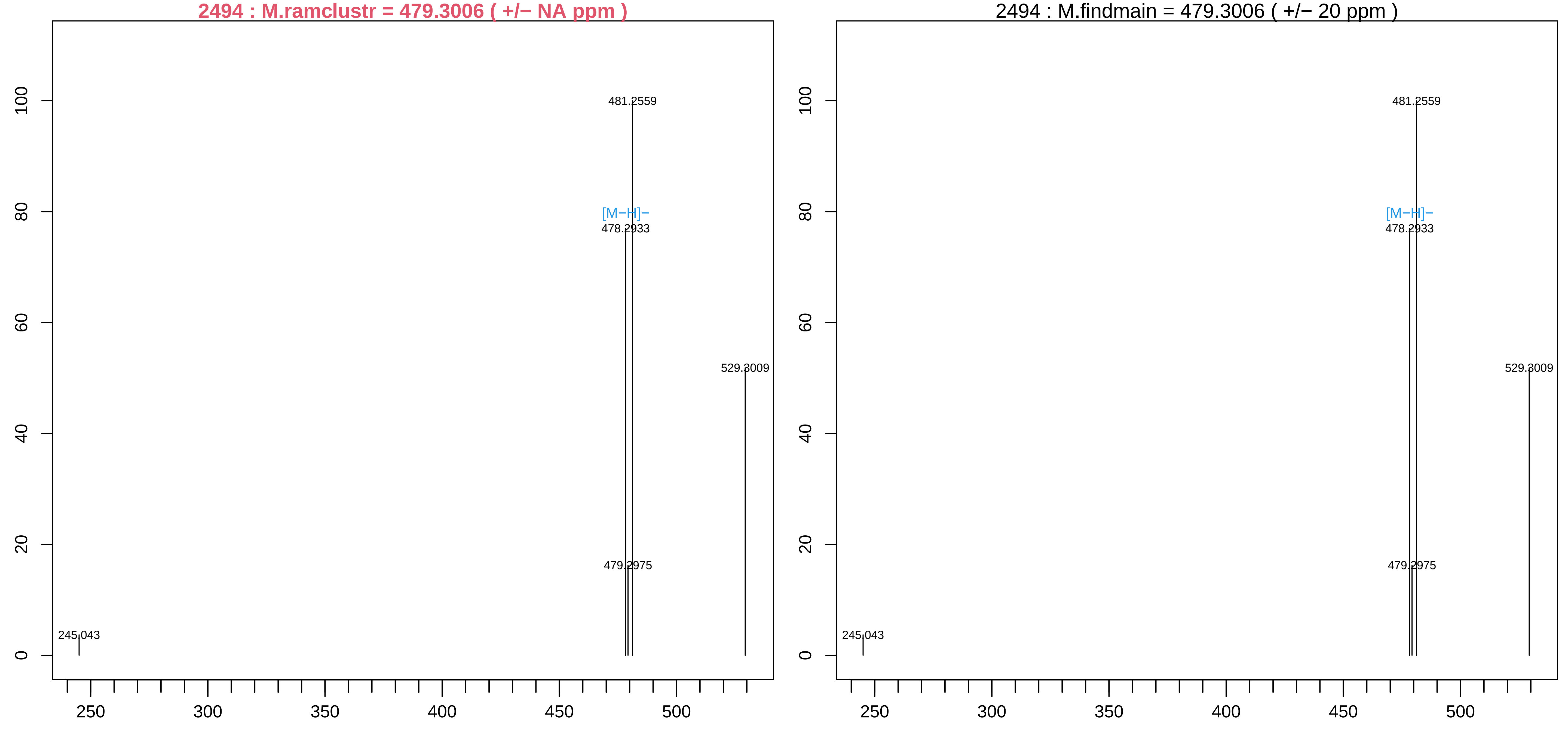
2494 : M.ramclustr = 479.3006 (+/− NA ppm)
481.2559
[M−H]−
478.2933
529.3009
479.2975
245.043
2494 : M.findmain = 479.3006 (+/− 20 ppm)
481.2559
[M−H]−
478.2933
529.3009
479.2975
245.043

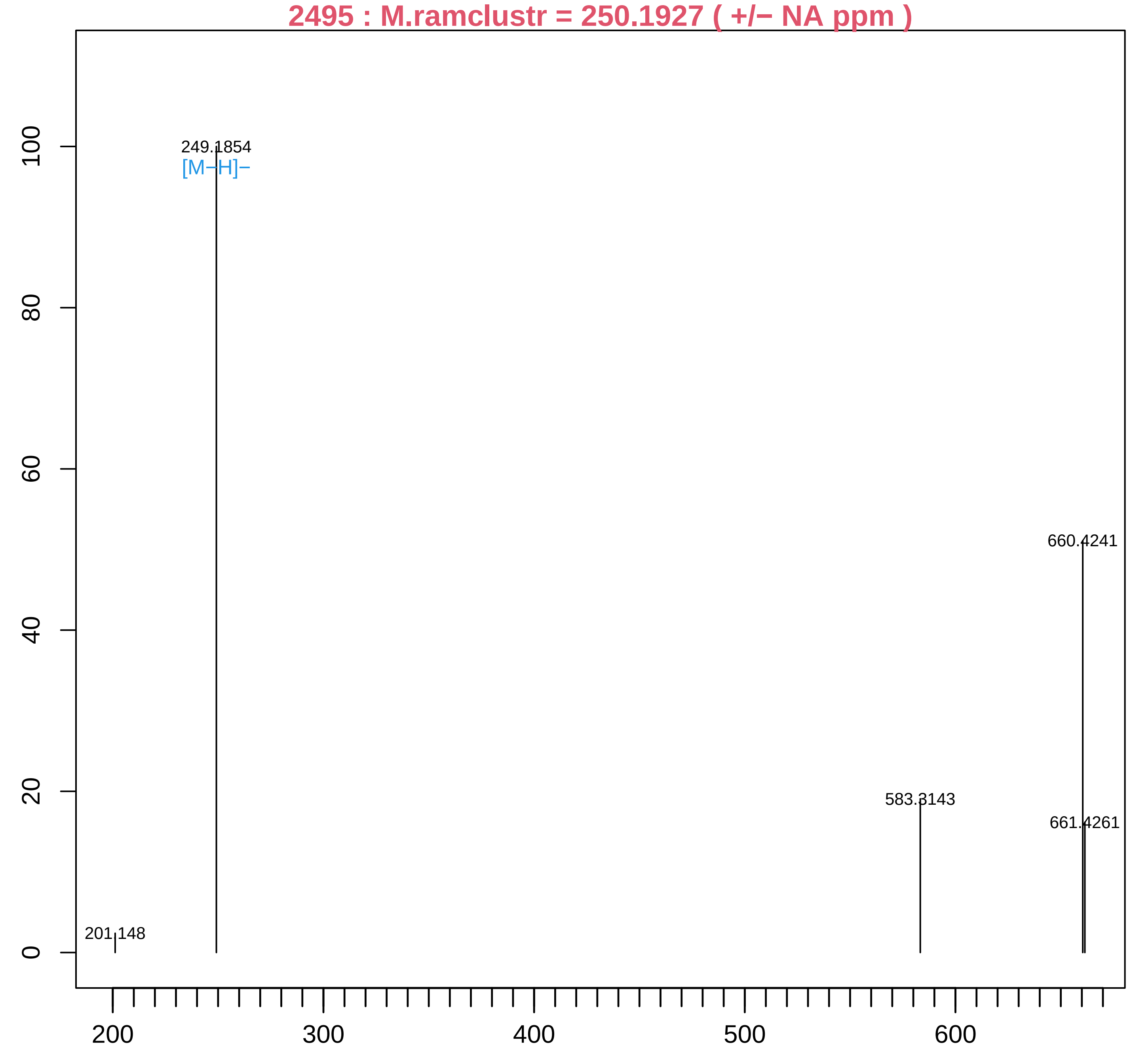
2495 : M.ramclustr = 250.1927 (+/− NA ppm)
249.1854
[M−H]−
660.4241
583.3143
661.4261
201.148
0
20
40
60
80
100
200
300
400
500
600

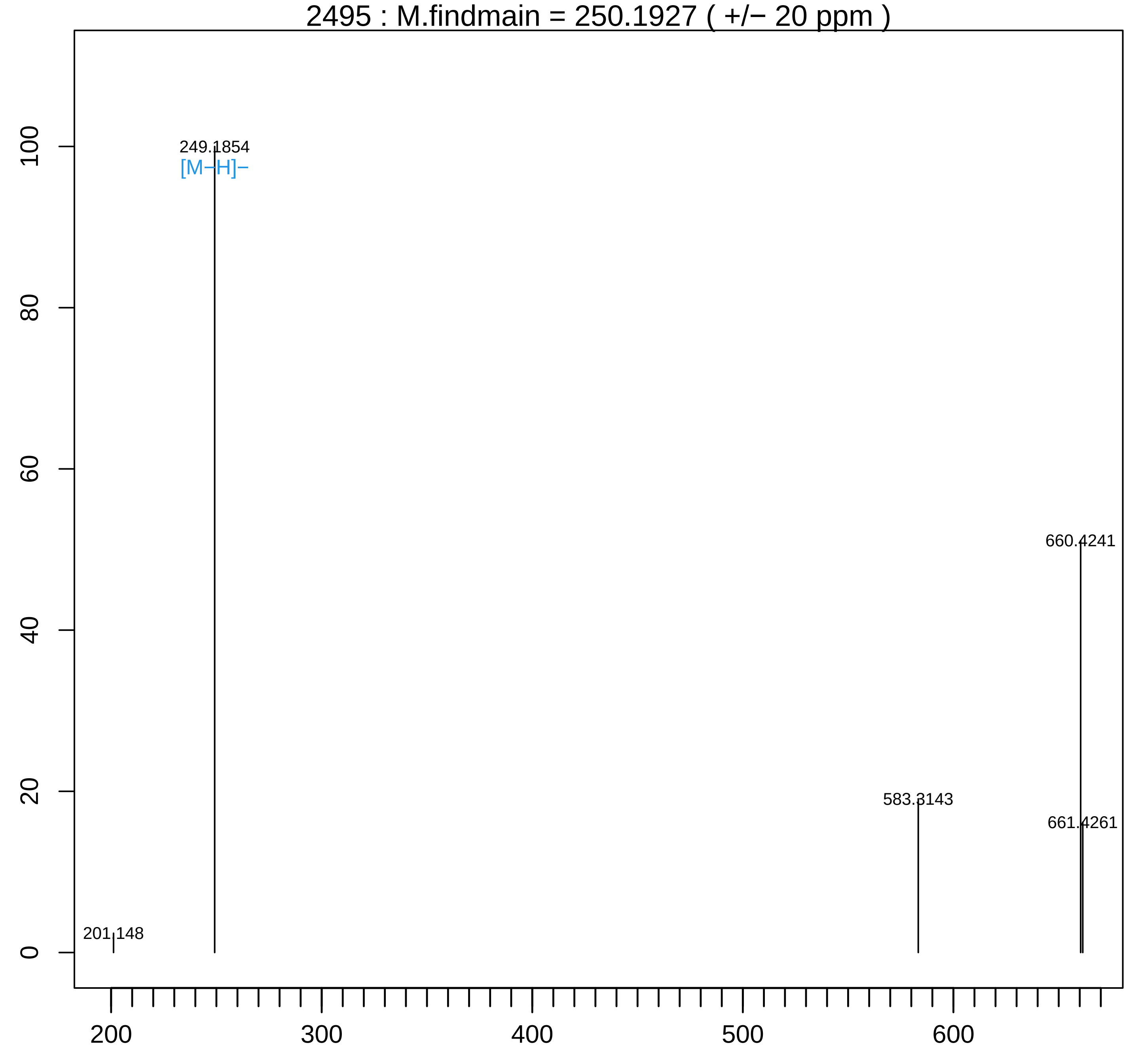
2495 : M.findmain = 250.1927 (+/− 20 ppm)
249.1854
[M−H]−
660.4241
583.3143
661.4261
201.148
0
20
40
60
80
100
200
300
400
500
600

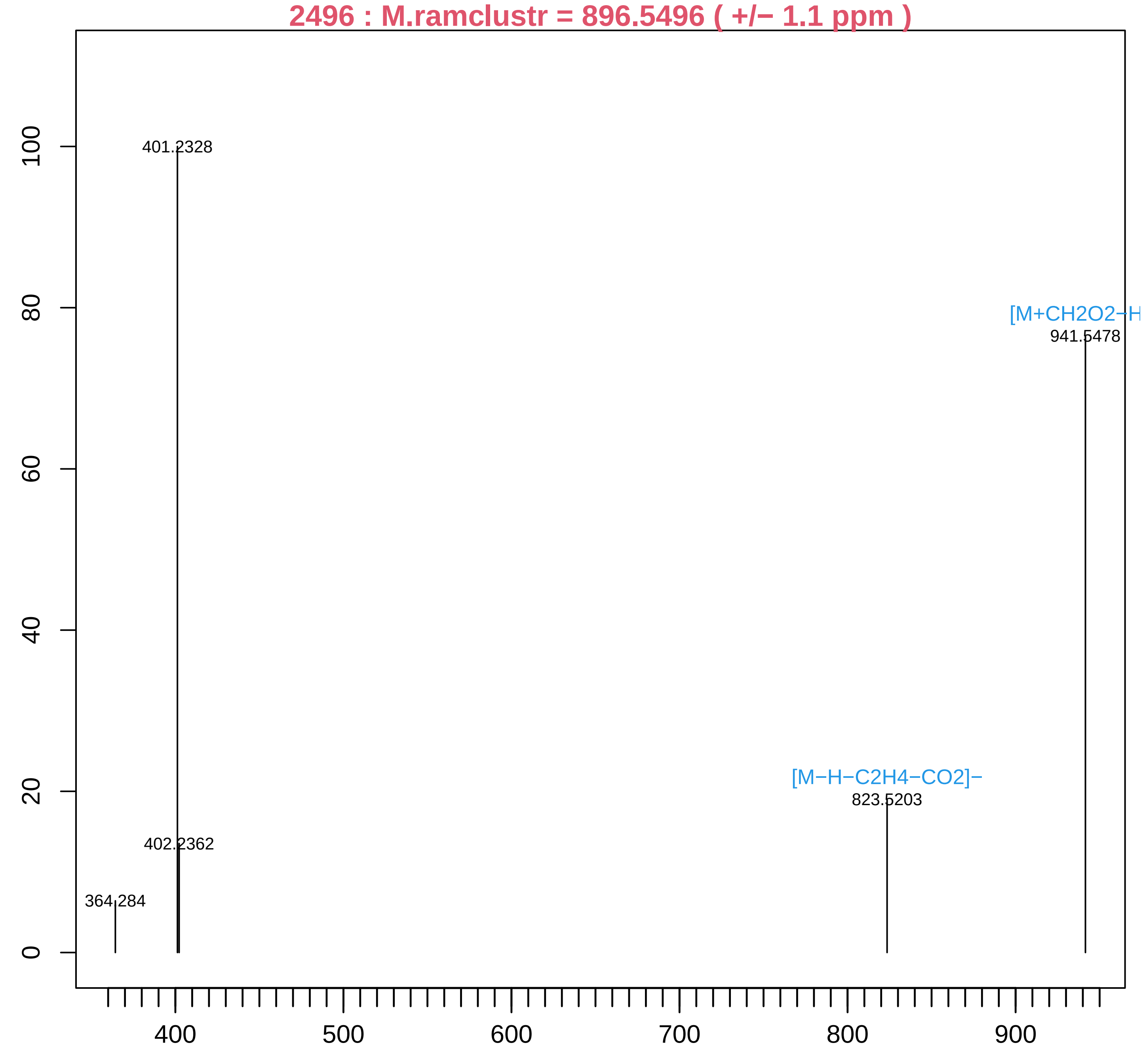
2496 : M.ramclustr = 896.5496 (+/− 1.1 ppm)
401.2328
[M+CH2O2−H
941.5478
[M−H−C2H4−CO2]−
823.5203
402.2362
364.284
0
20
40
60
80
100
400
500
600
700
800
900

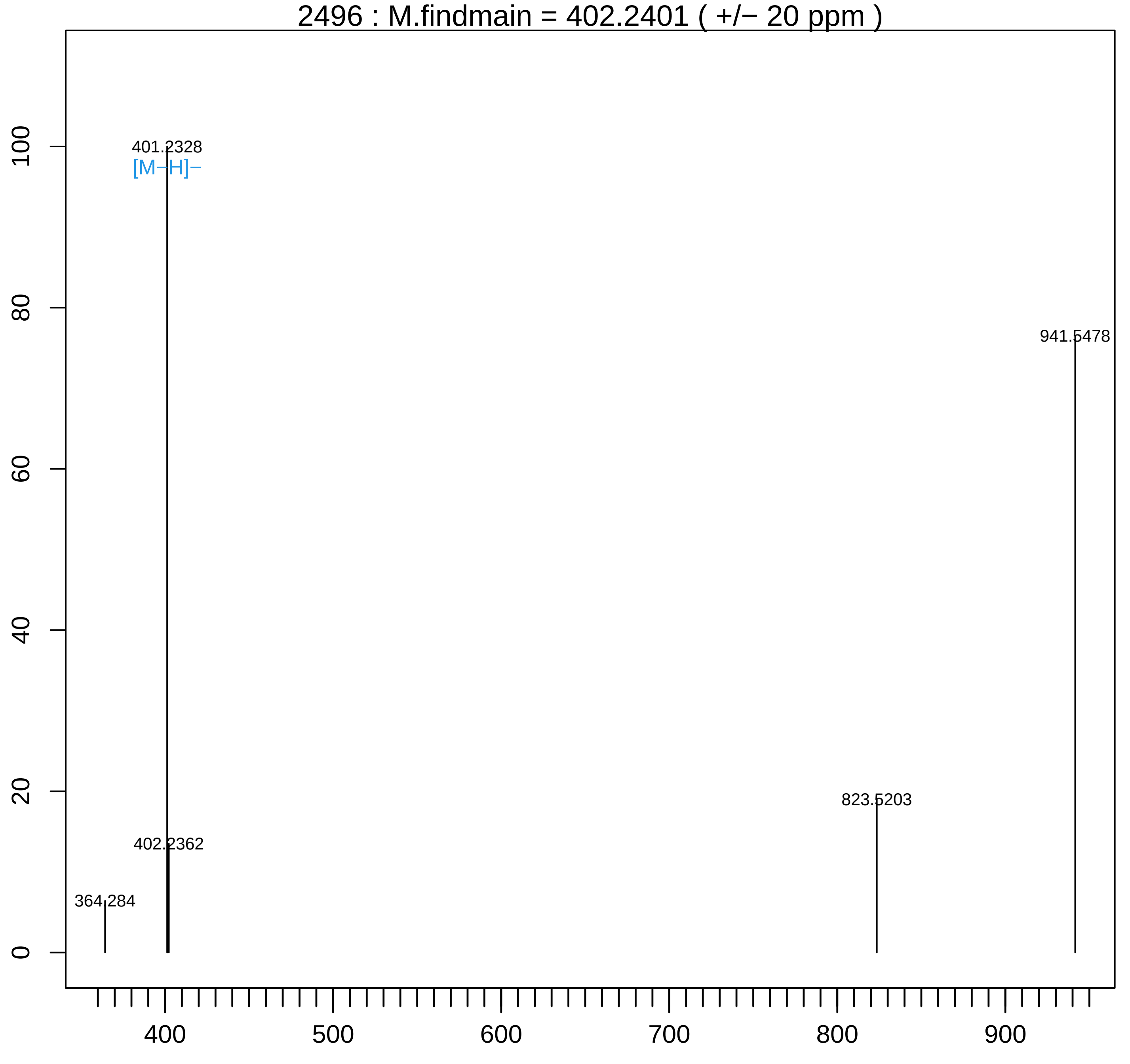
2496 : M.findmain = 402.2401 (+/− 20 ppm)
401.2328
[M−H]−
941.5478
823.5203
402.2362
364.284
0
20
40
60
80
100
400
500
600
700
800
900

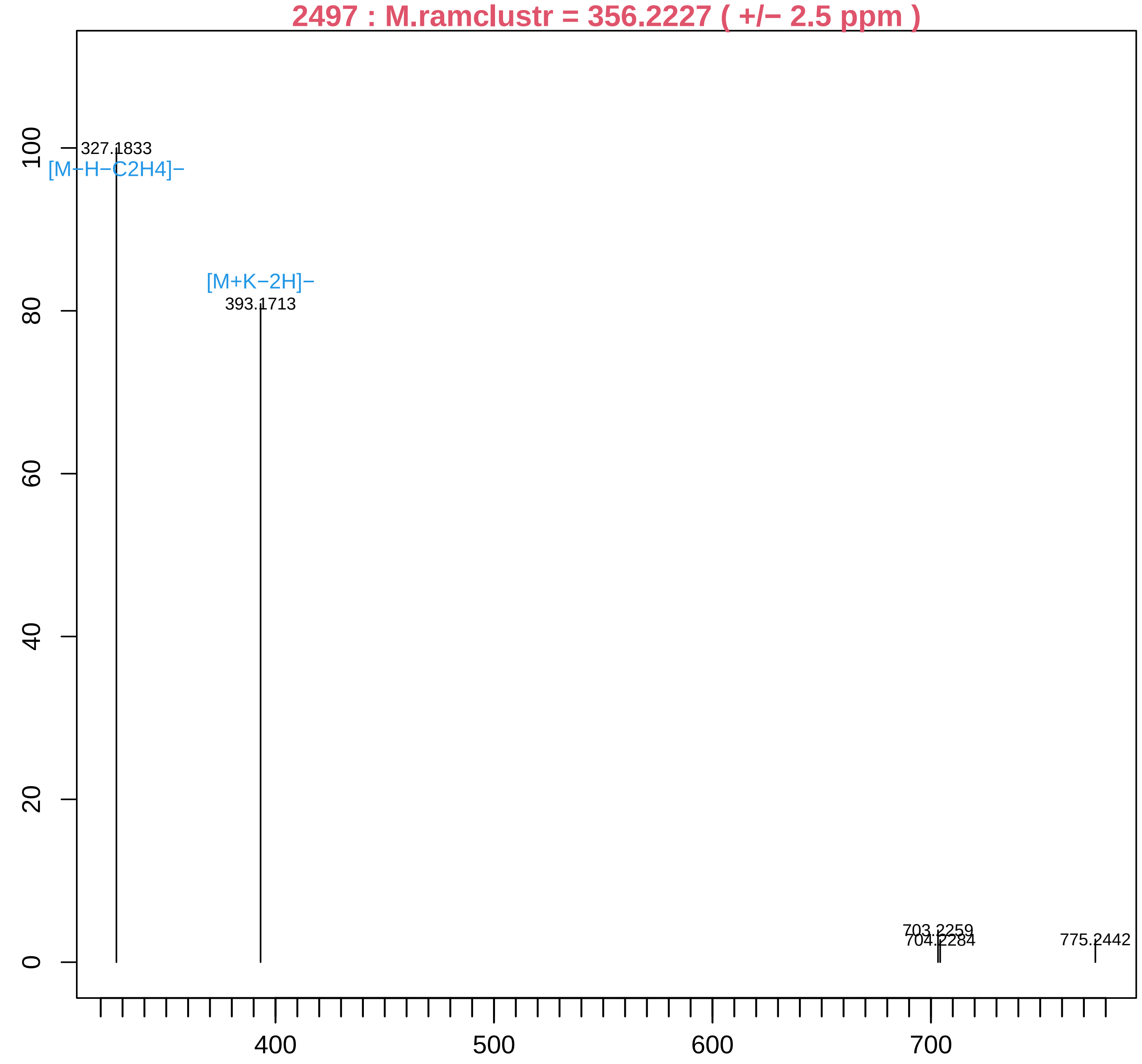

2497 : M.ramclustr = 356.2227 (+/− 2.5 ppm)
327.1833
[M−H−C2H4]−
[M+K−2H]−
393.1713
703.2259
704.2284
775.2442
0
20
40
60
80
100
400
500
600
700

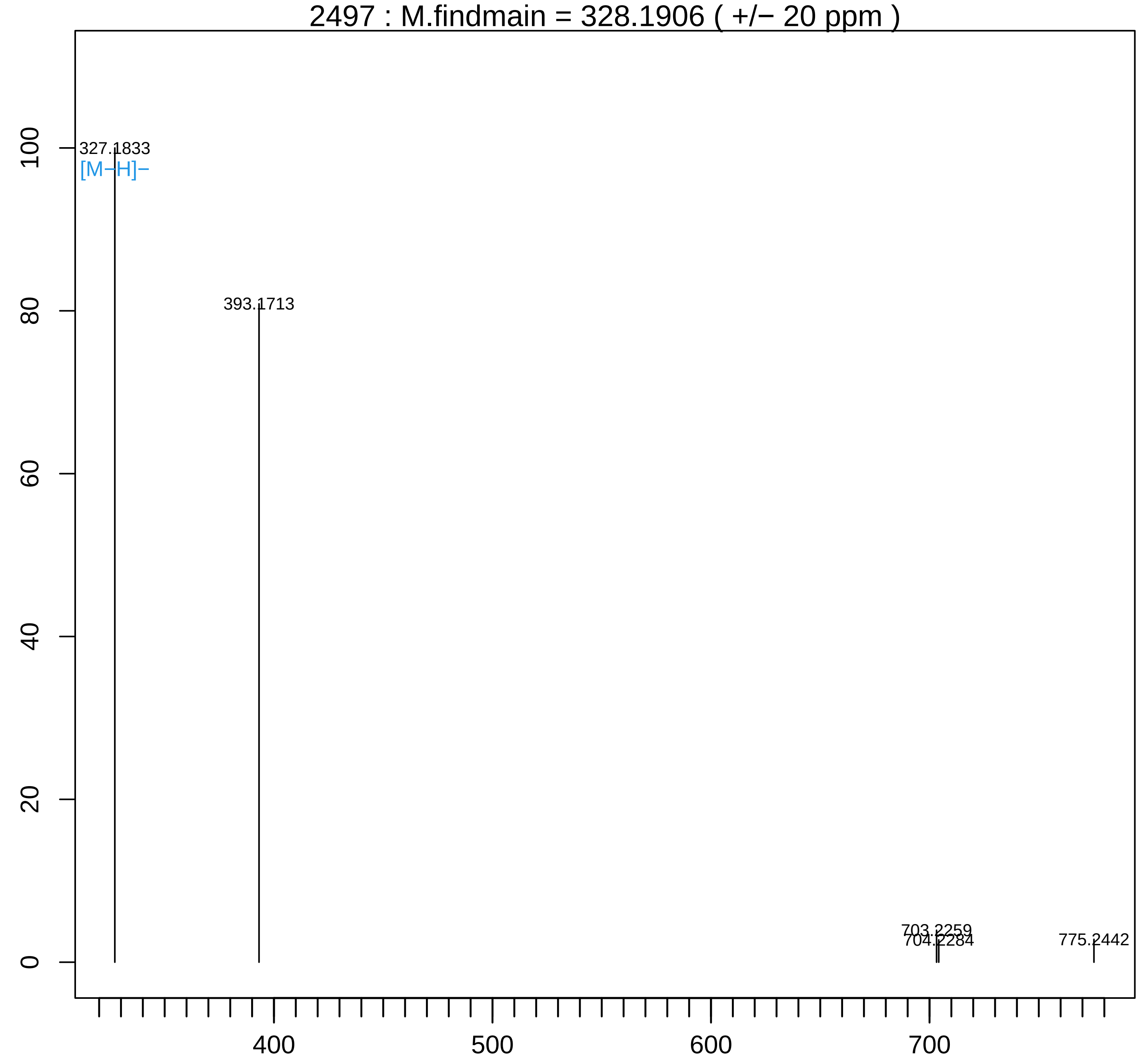

2497 : M.findmain = 328.1906 (+/− 20 ppm)
327.1833
[M−H]−
393.1713
703.2259
704.2284
775.2442
0
20
40
60
80
100
400
500
600
700

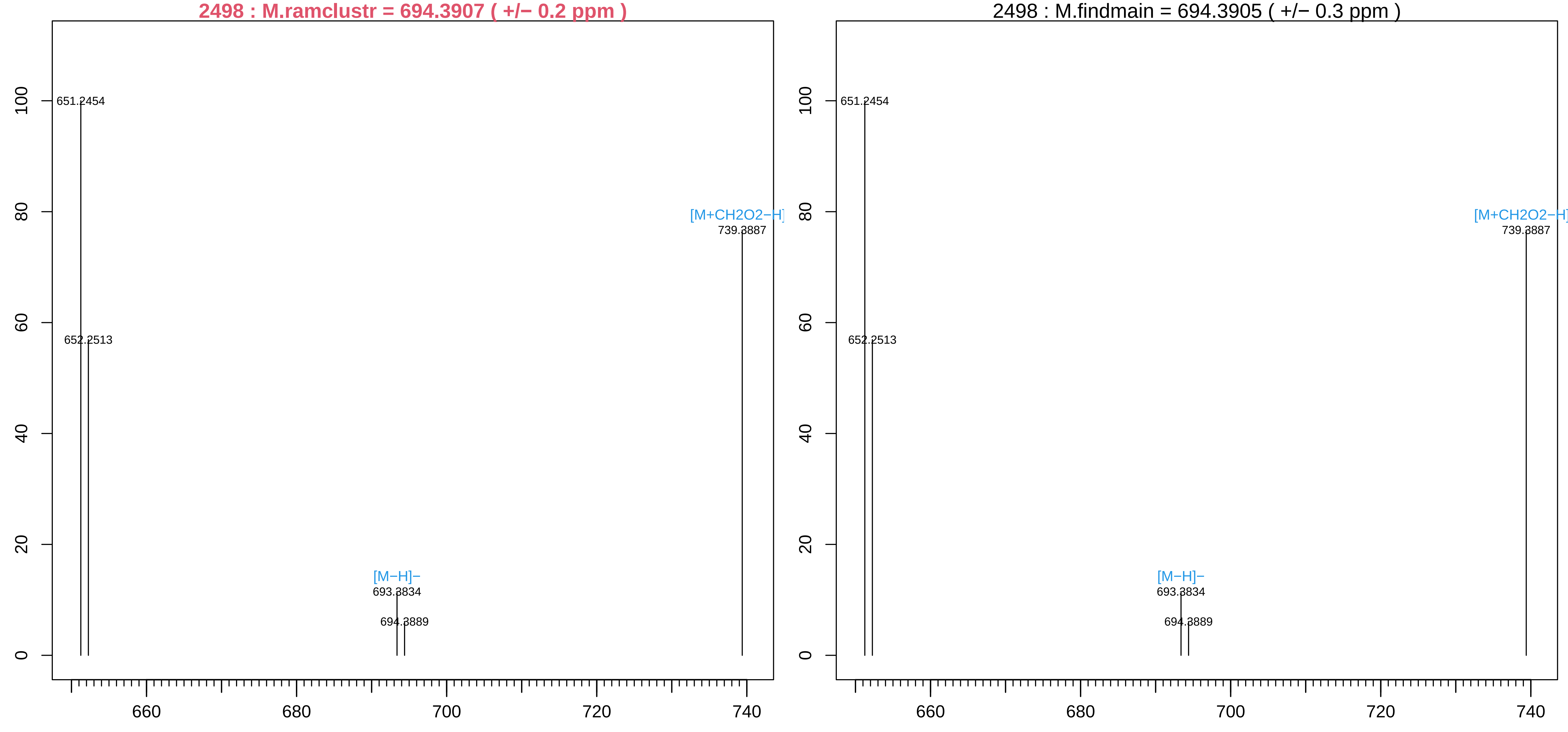
2498 : M.ramclustr = 694.3907 (+/− 0.2 ppm)
651.2454
652.2513
[M−H]−
693.3834
694.3889
[M+CH2O2−H]
739.3887
2498 : M.findmain = 694.3905 (+/− 0.3 ppm)
651.2454
652.2513
[M−H]−
693.3834
694.3889
[M+CH2O2−H]
739.3887

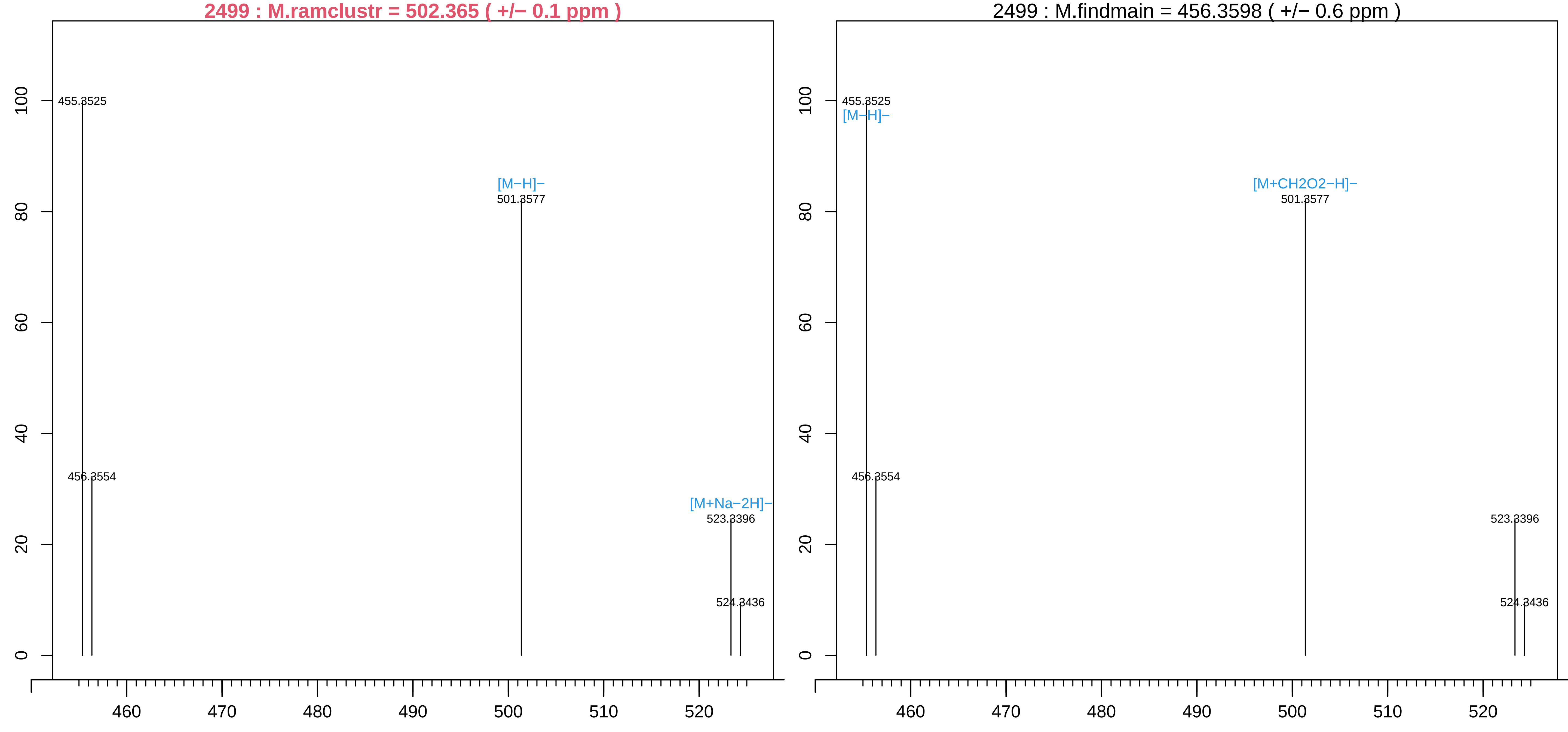
2499 : M.ramclustr = 502.365 (+/− 0.1 ppm)
455.3525
456.3554
[M−H]−
501.3577
[M+Na−2H]−
523.3396
524.3436
0
20
40
60
80
100
460
470
480
490
500
510
520
2499 : M.findmain = 456.3598 (+/− 0.6 ppm)
455.3525
[M−H]−
456.3554
[M+CH2O2−H]−
501.3577
523.3396
524.3436

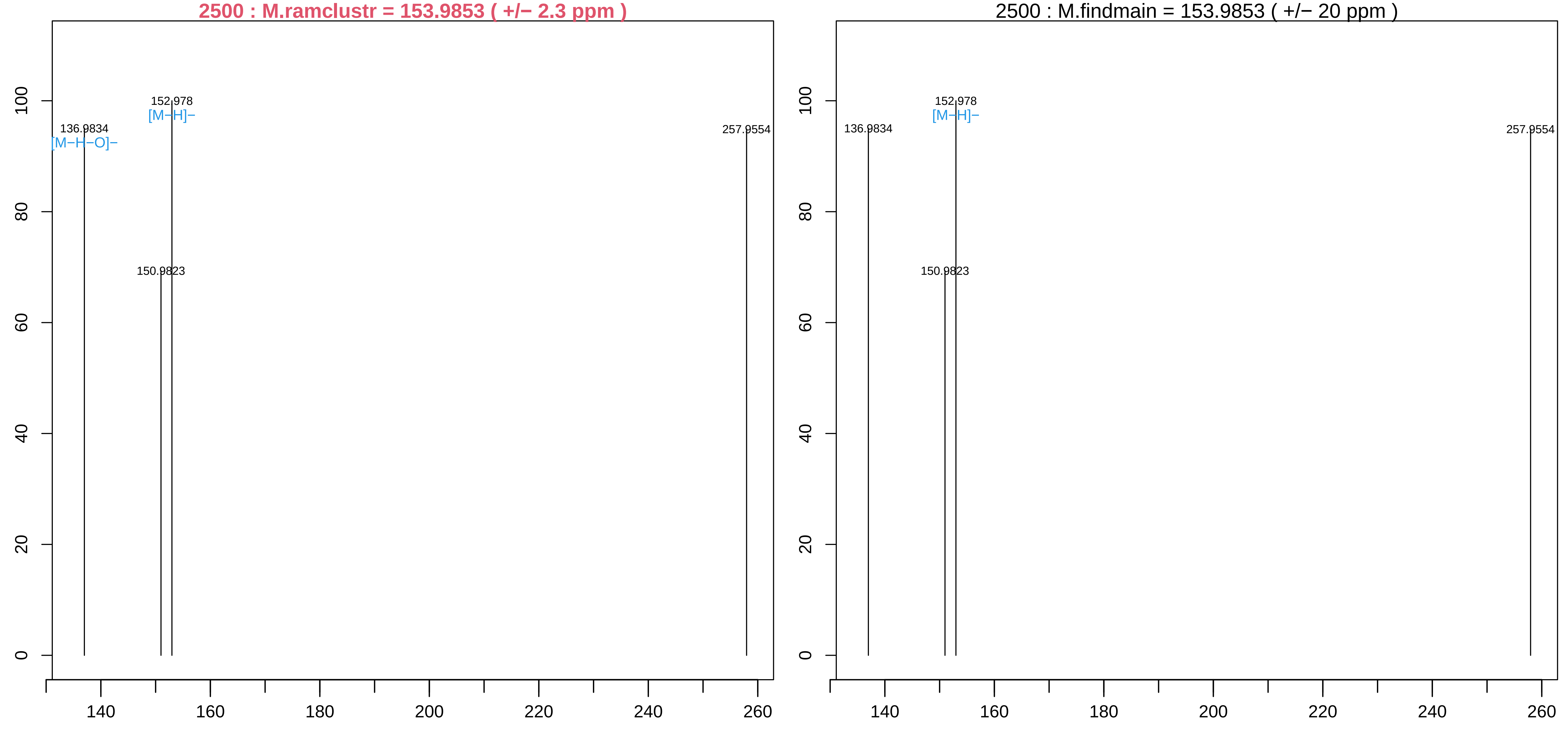
2500 : M.ramclustr = 153.9853 (+/− 2.3 ppm)
136.9834
[M−H−O]−
152.978
[M−H]−
150.9823
257.9554
2500 : M.findmain = 153.9853 (+/− 20 ppm)
136.9834
152.978
[M−H]−
150.9823
257.9554
0
20
40
60
80
100
140
160
180
200
220
240
260

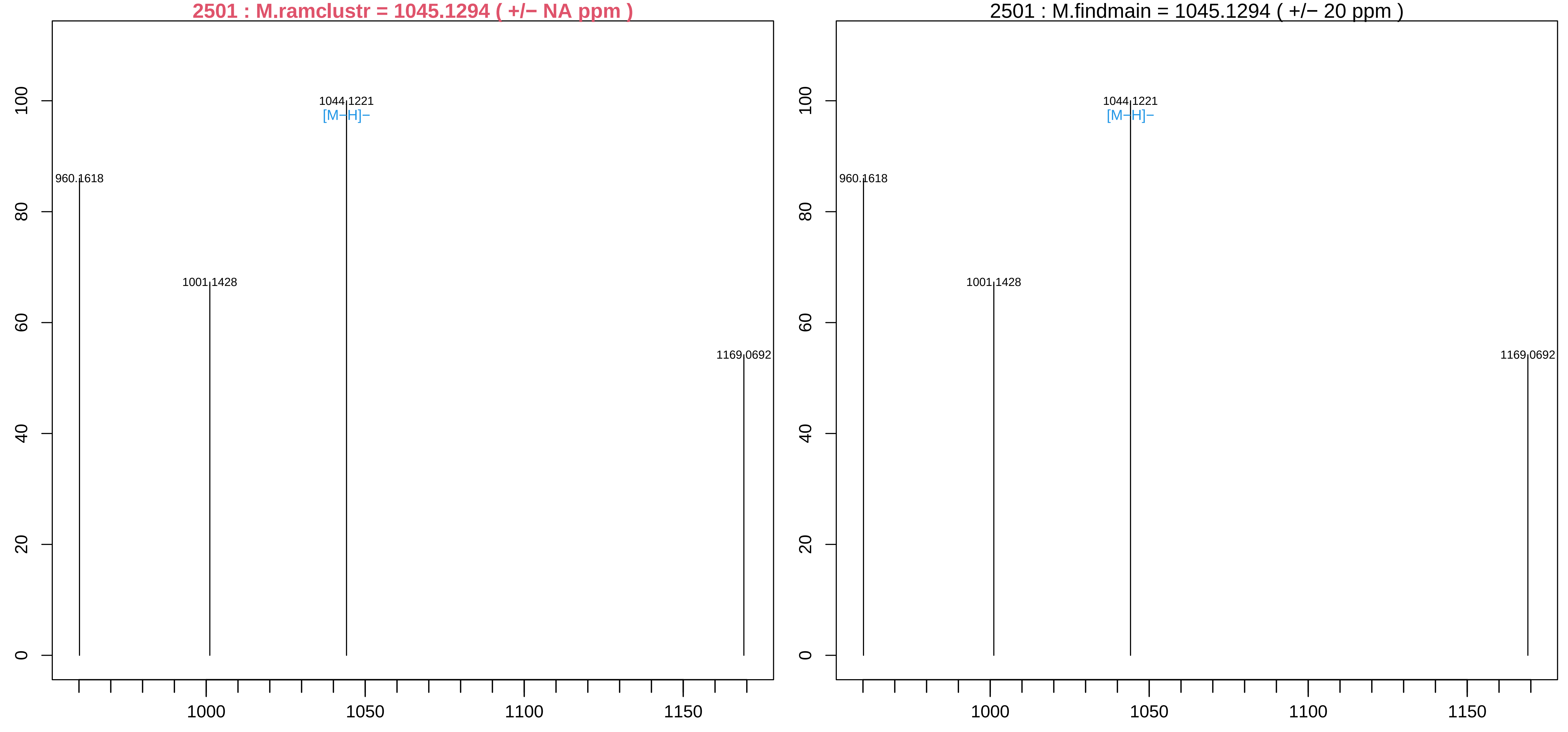
2501 : M.ramclustr = 1045.1294 (+/− NA ppm)
2501 : M.findmain = 1045.1294 (+/− 20 ppm)
960.1618
1001.1428
1044.1221
[M−H]−
1169.0692
0
20
40
60
80
100
1000
1050
1100
1150

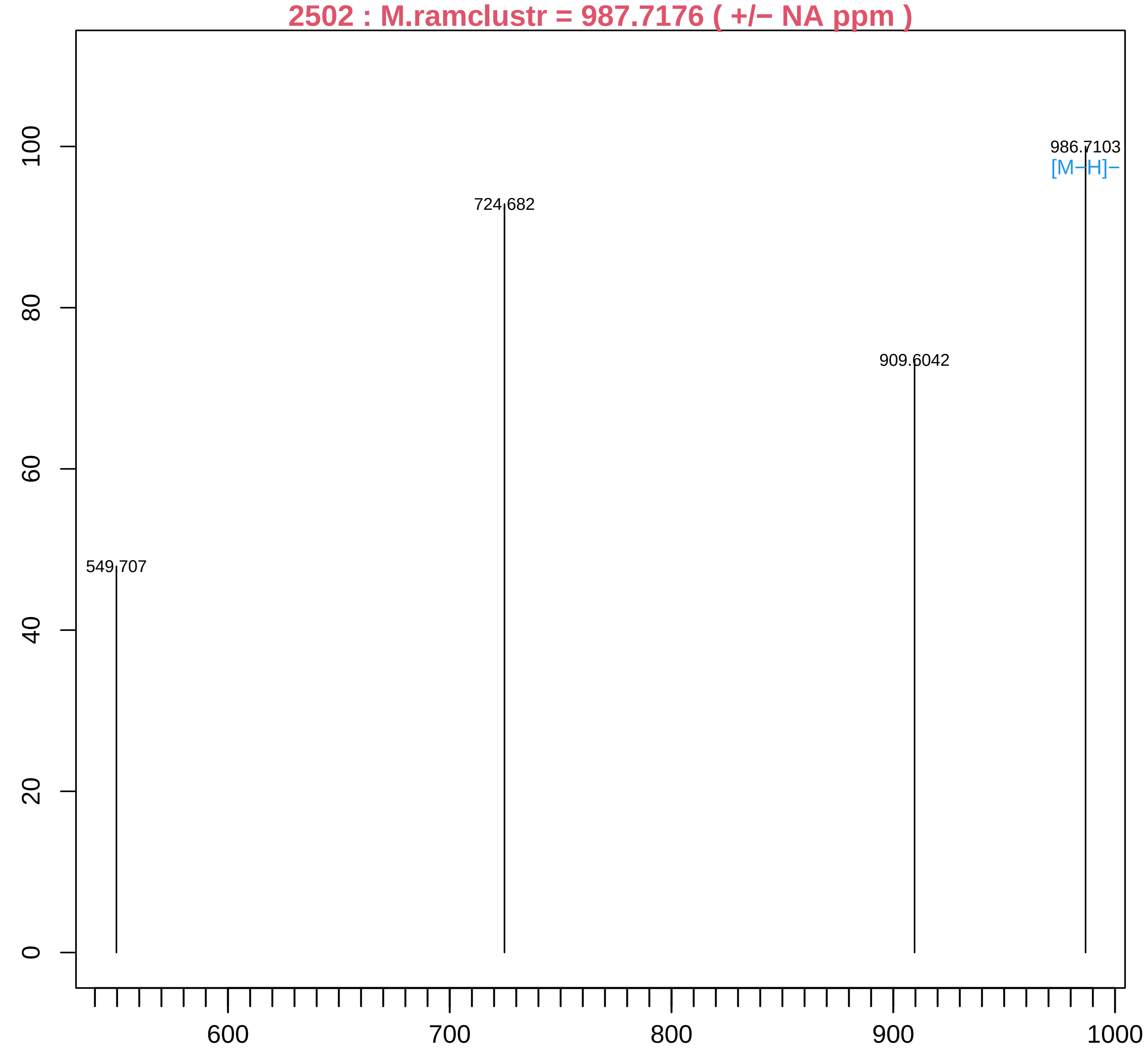

2502 : M.ramclustr = 987.7176 (+/− NA ppm)
986.7103
[M−H]−
724.682
909.6042
549.707

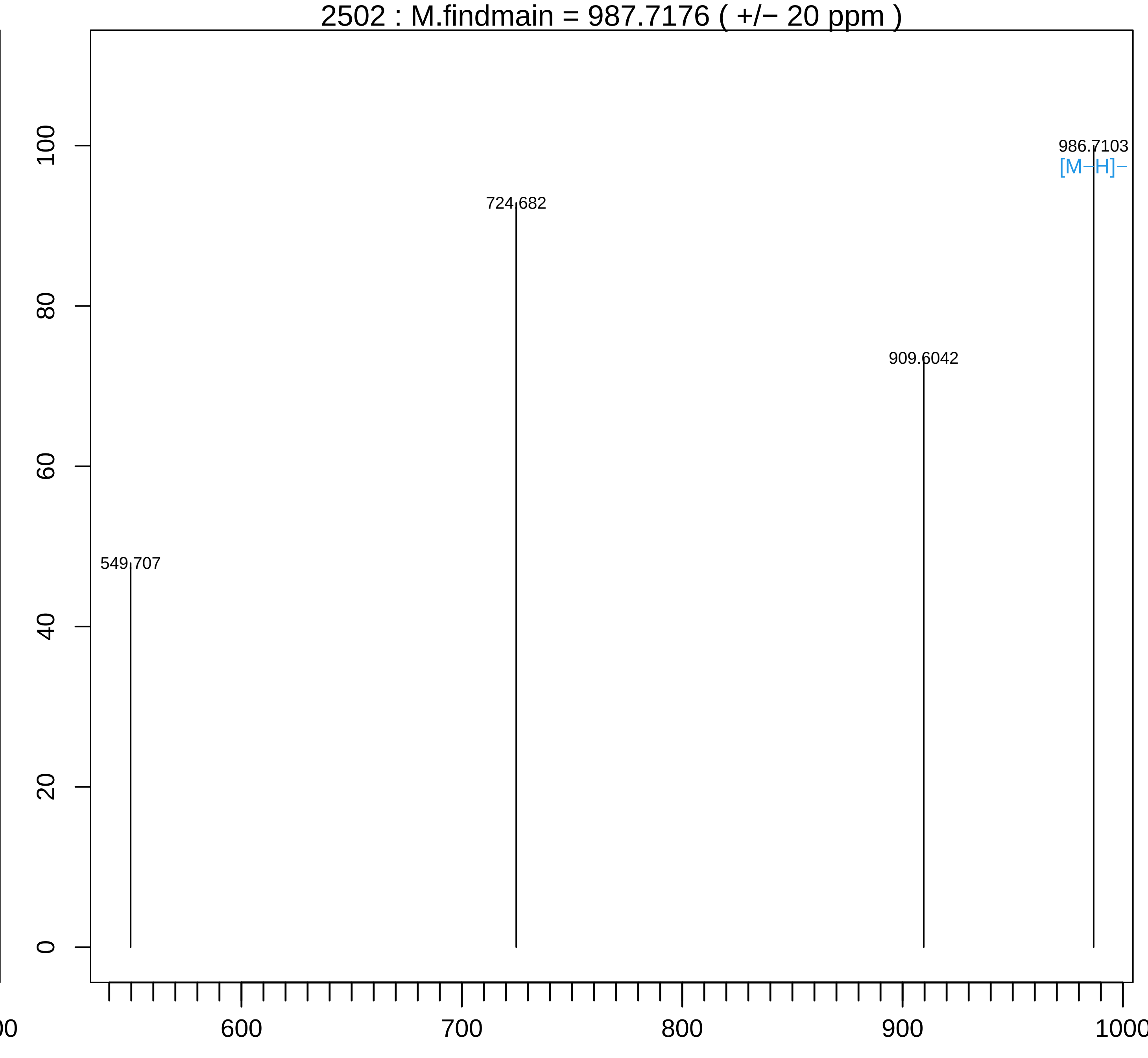

2502 : M.findmain = 987.7176 (+/− 20 ppm)
986.7103
[M−H]−
724.682
909.6042
549.707

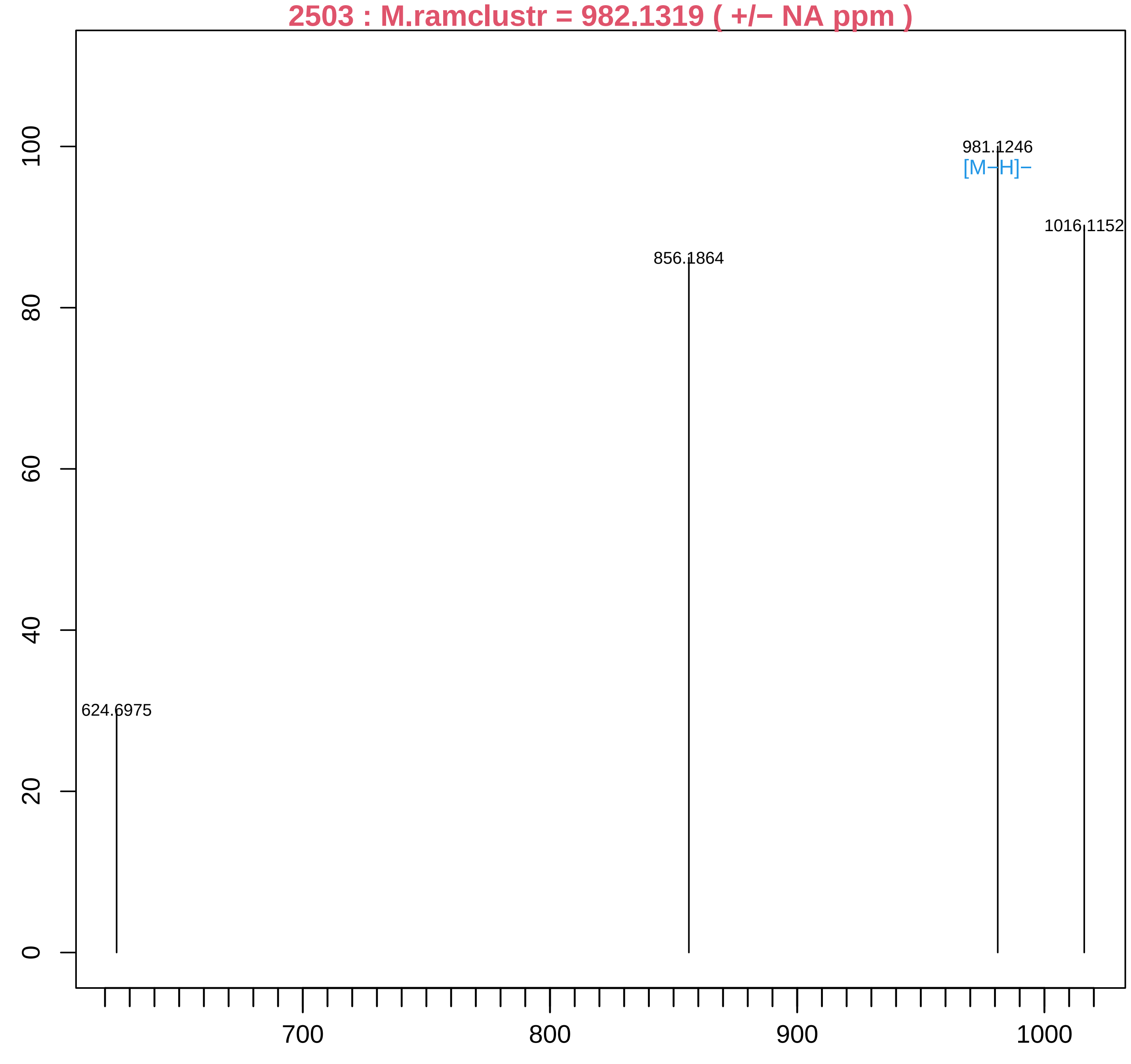

2503 : M.ramclustr = 982.1319 (+/− NA ppm)
981.1246
[M−H]−
1016.1152
856.1864
624.6975
0
20
40
60
80
100
700
800
900
1000

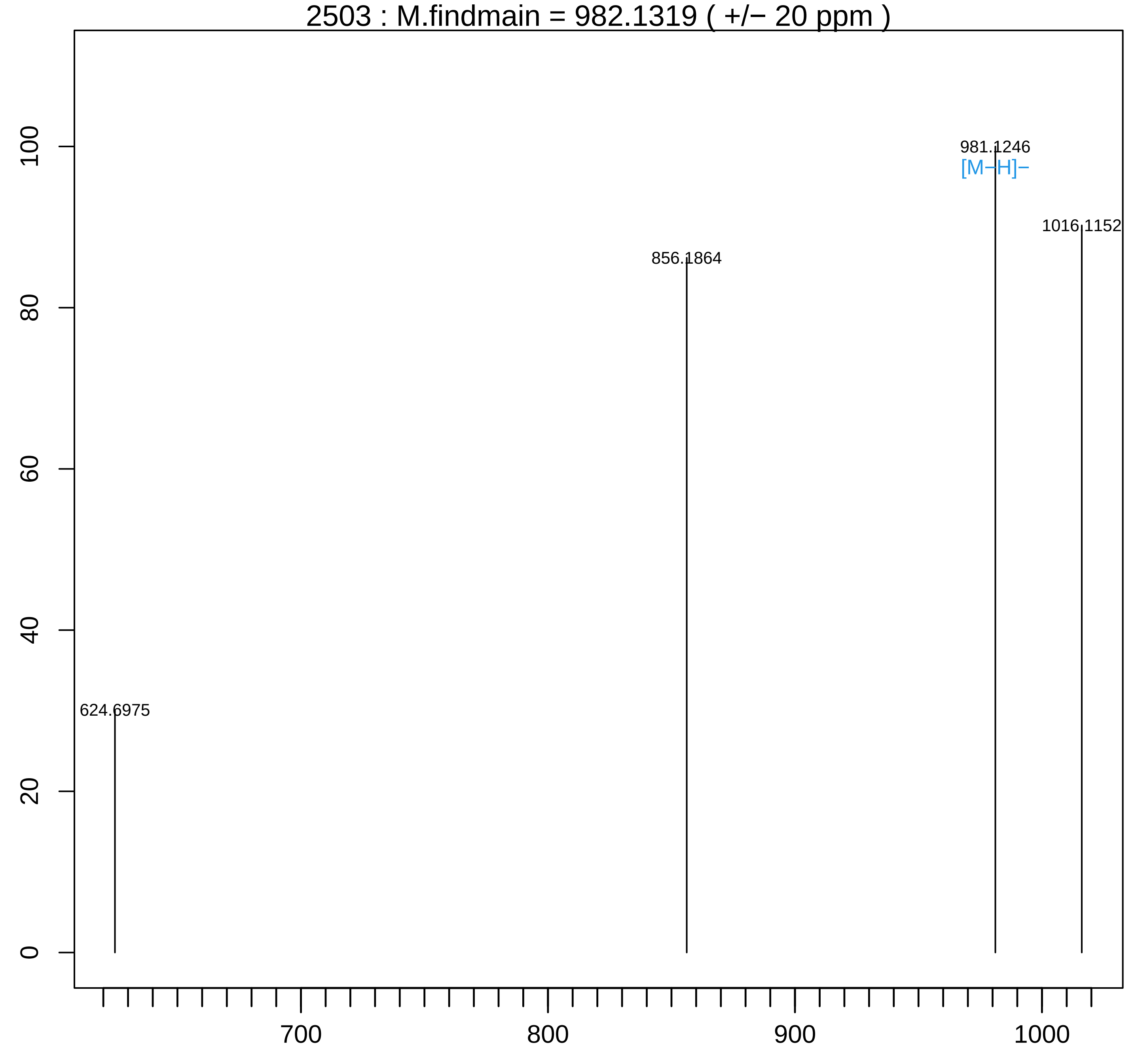

2503 : M.findmain = 982.1319 (+/− 20 ppm)
981.1246
[M−H]−
1016.1152
856.1864
624.6975
0
20
40
60
80
100
700
800
900
1000

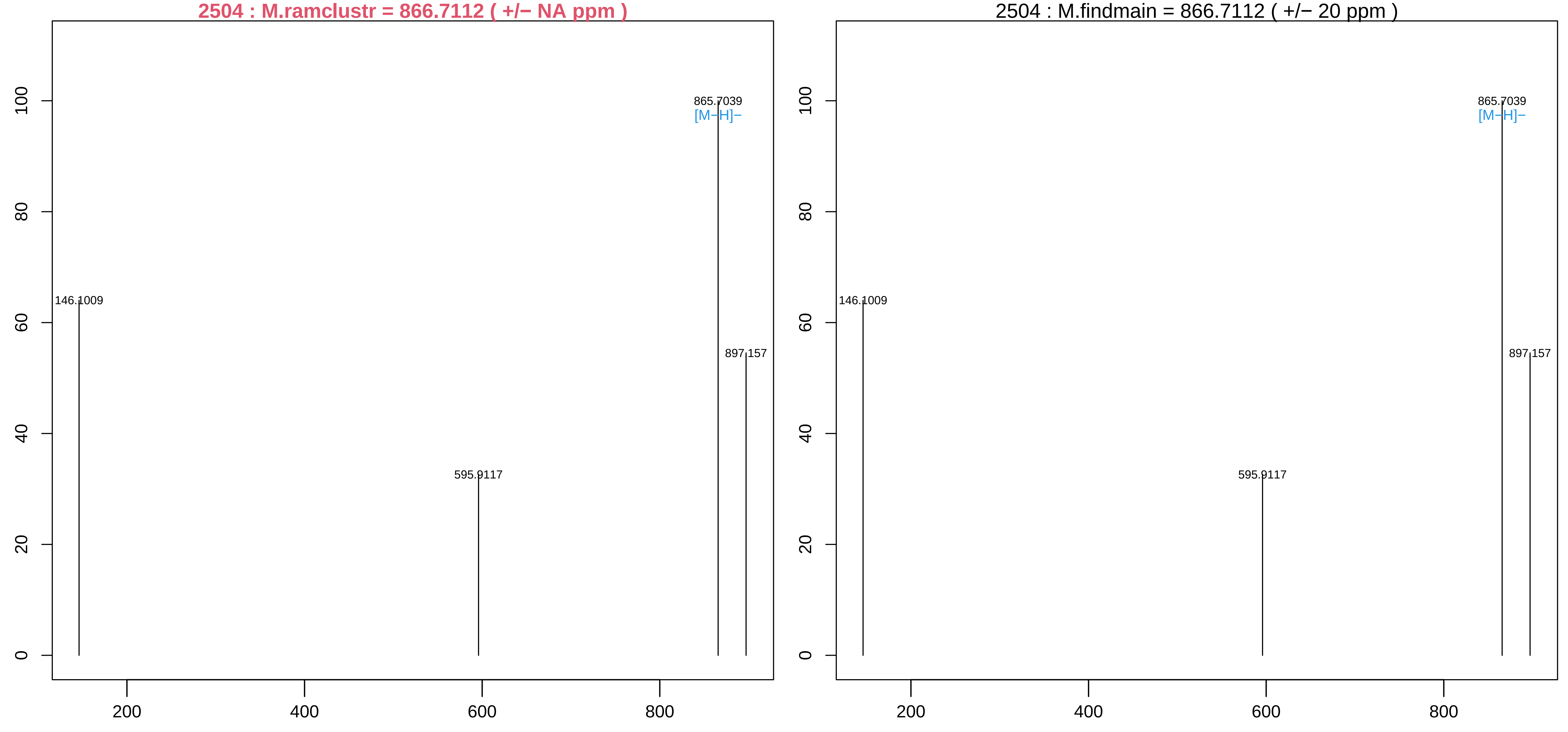

2504 : M.ramclustr = 866.7112 (+/− NA ppm)
2504 : M.findmain = 866.7112 (+/− 20 ppm)
146.1009
595.9117
865.7039
[M−H]−
897.157
0
20
40
60
80
100
200
400
600
800

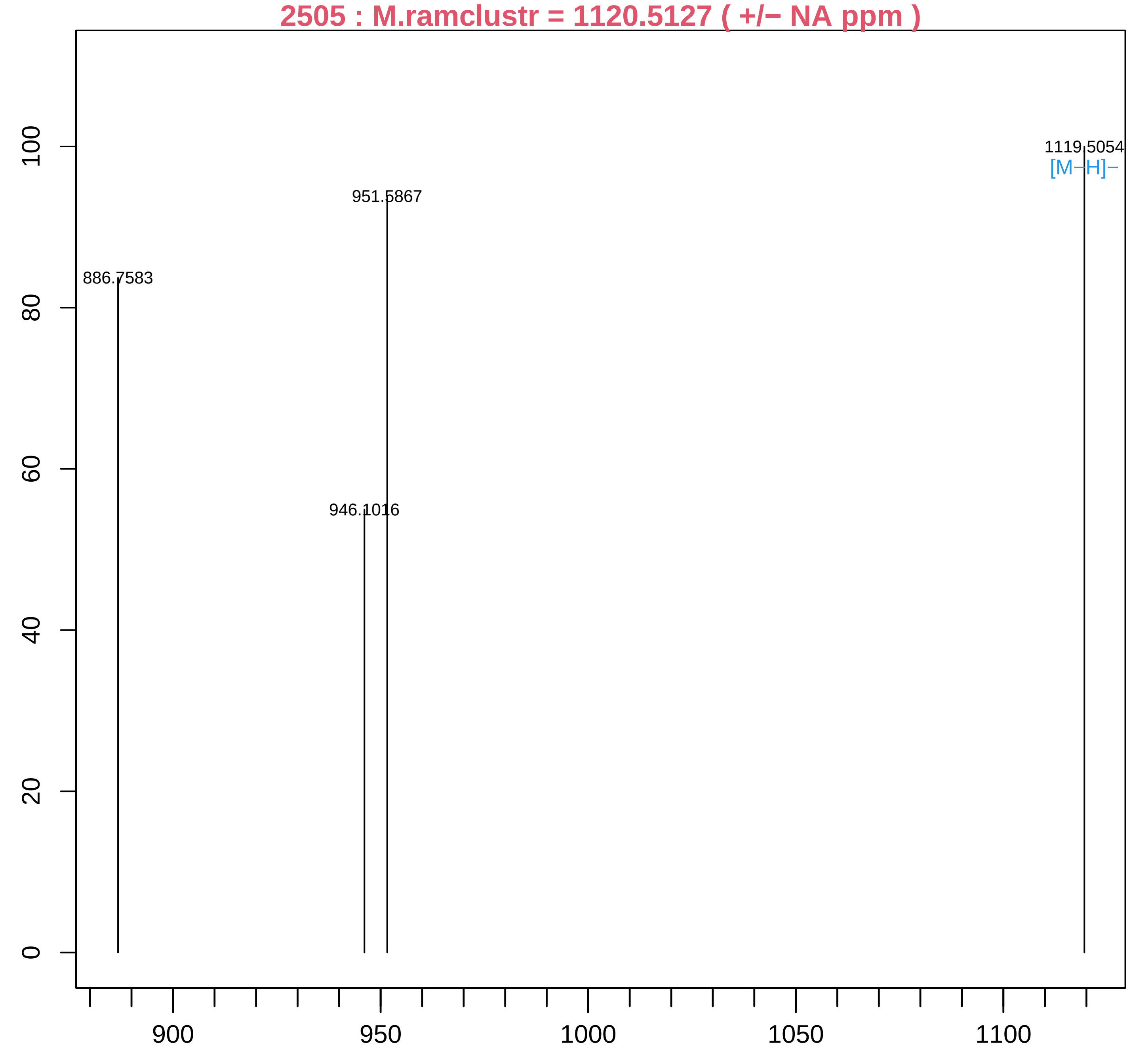
2505 : M.ramclustr = 1120.5127 (+/− NA ppm)
1119.5054
[M−H]−
951.5867
886.7583
946.1016
0
20
40
60
80
100
900
950
1000
1050
1100

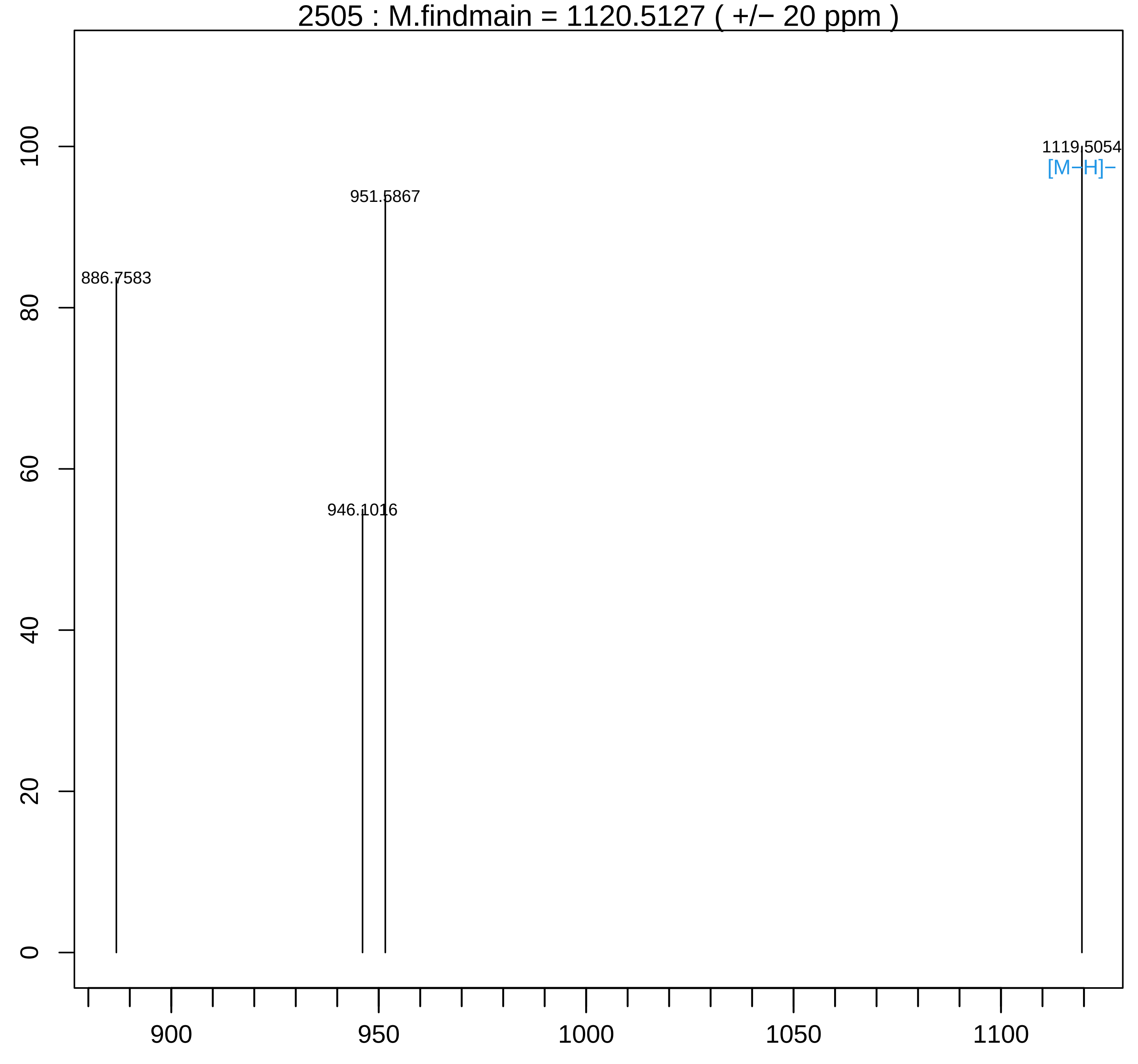
2505 : M.findmain = 1120.5127 (+/− 20 ppm)
1119.5054
[M−H]−
951.5867
886.7583
946.1016
0
20
40
60
80
100
900
950
1000
1050
1100

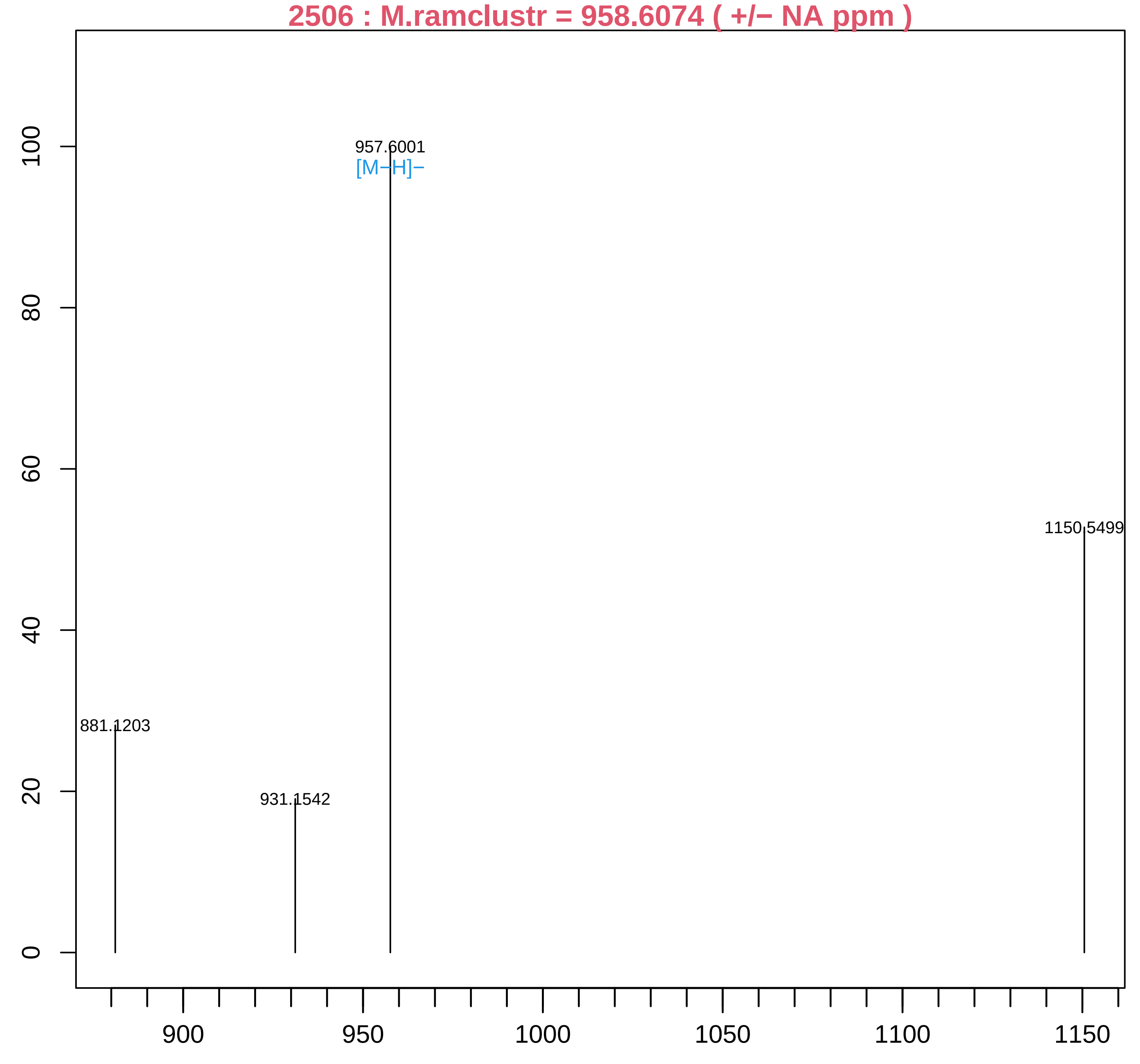
2506 : M.ramclustr = 958.6074 (+/− NA ppm)
957.6001
[M−H]−
1150.5499
881.1203
931.1542
0
20
40
60
80
100
900
950
1000
1050
1100
1150

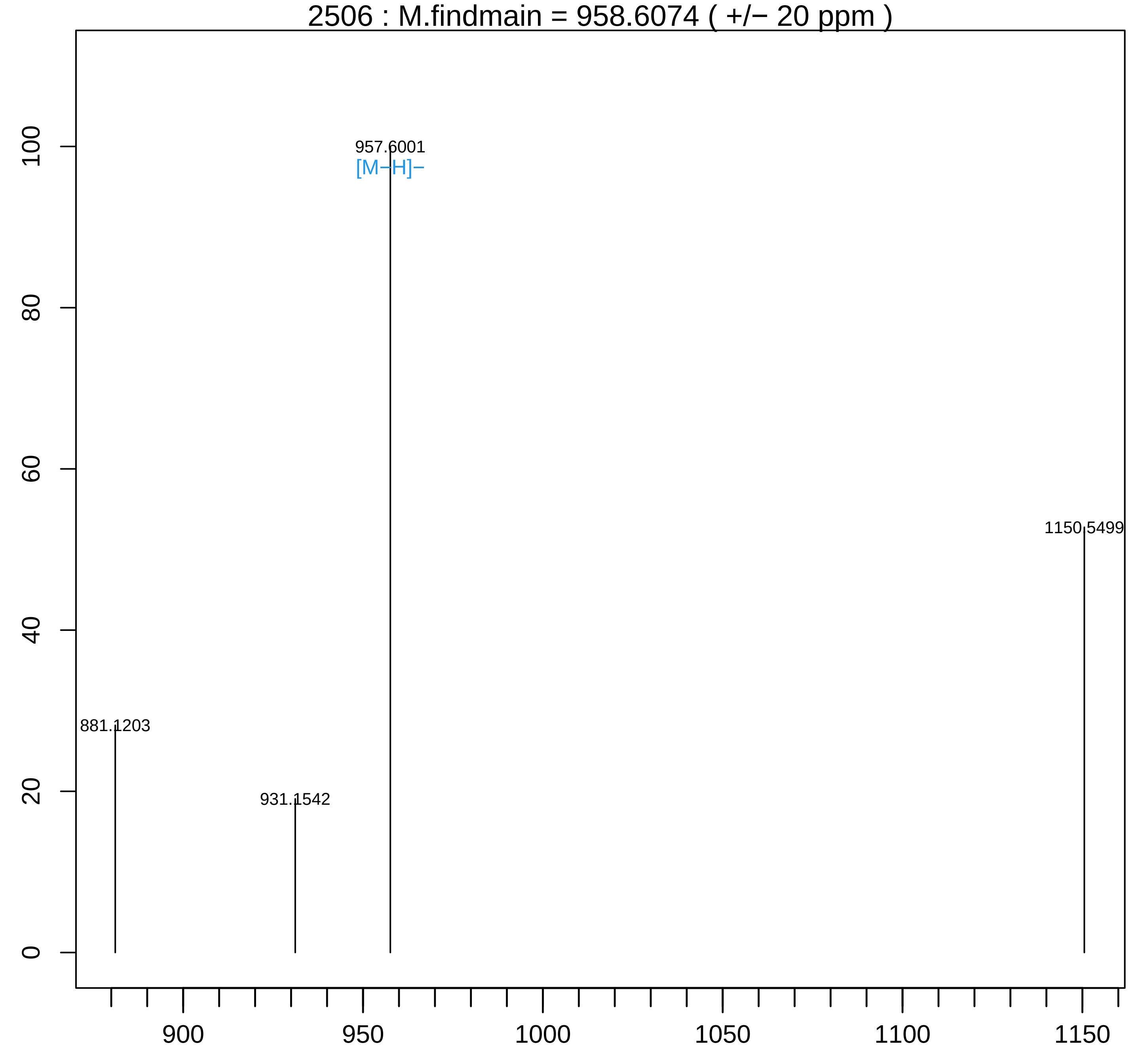
2506 : M.findmain = 958.6074 (+/− 20 ppm)
957.6001
[M−H]−
1150.5499
881.1203
931.1542
0
20
40
60
80
100
900
950
1000
1050
1100
1150

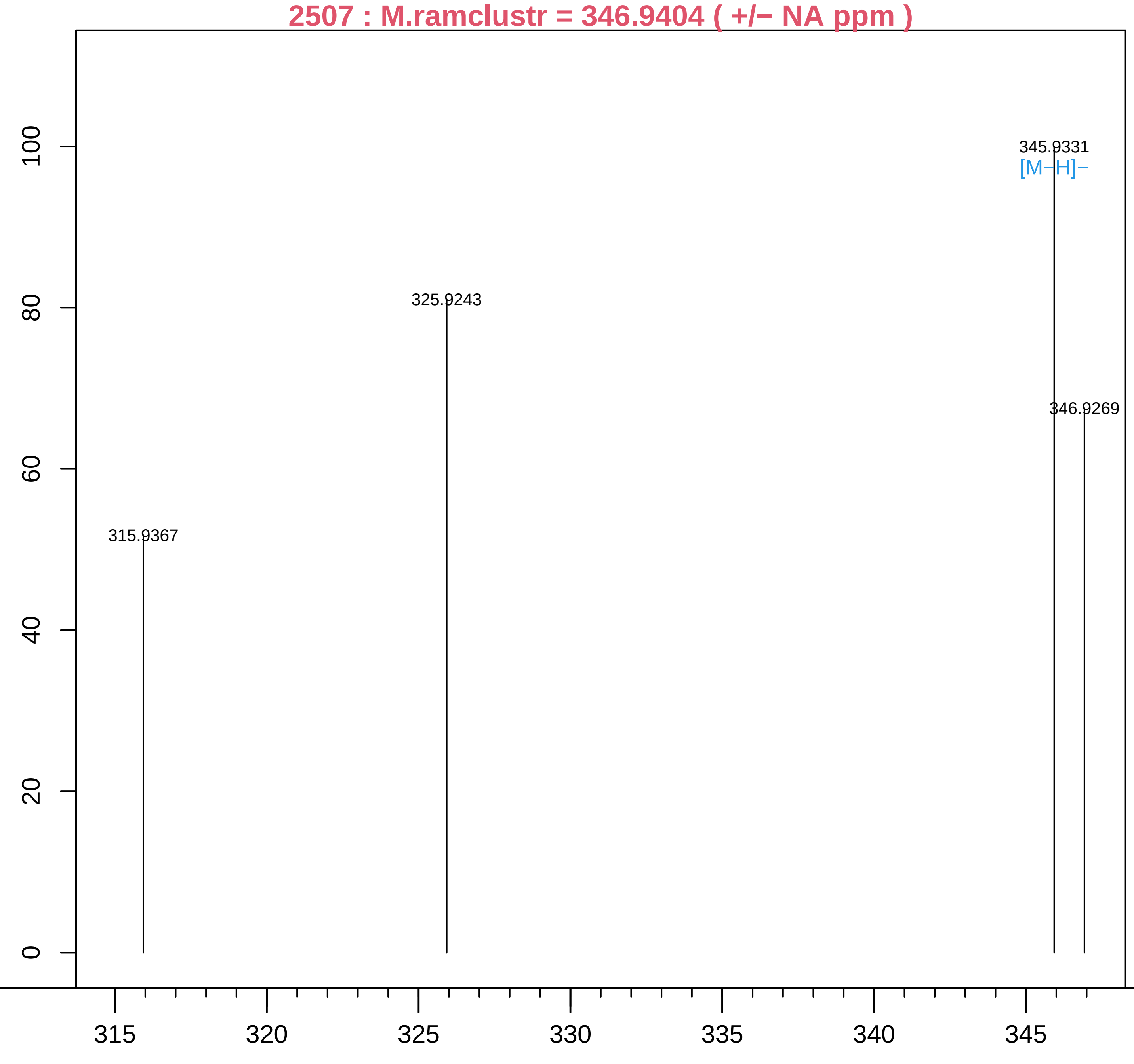
2507 : M.ramclustr = 346.9404 (+/− NA ppm)
315.9367
325.9243
345.9331
[M−H]−
346.9269
0
20
40
60
80
100
315
320
325
330
335
340
345

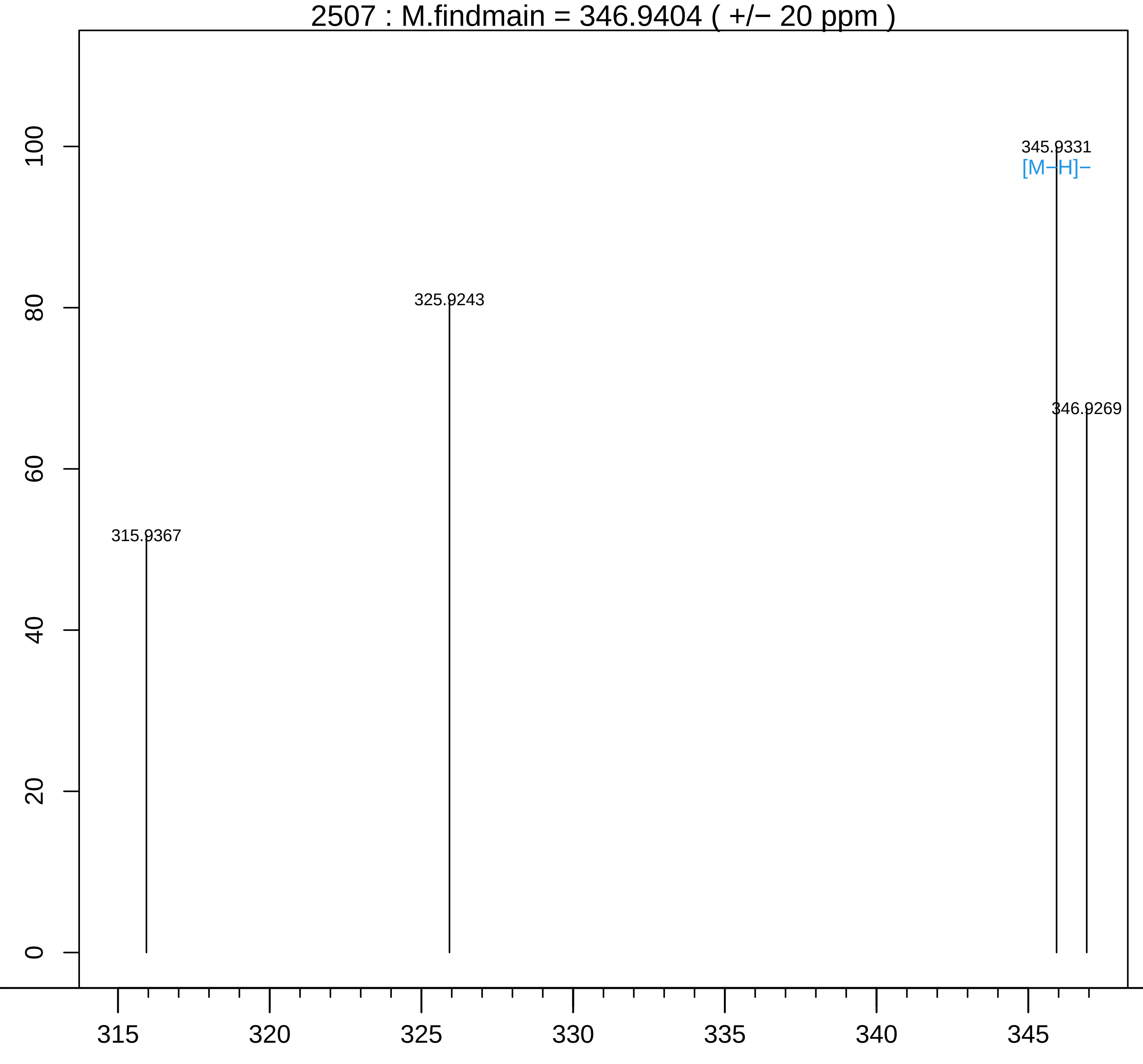
2507 : M.findmain = 346.9404 (+/− 20 ppm)
315.9367
325.9243
345.9331
[M−H]−
346.9269
0
20
40
60
80
100
315
320
325
330
335
340
345

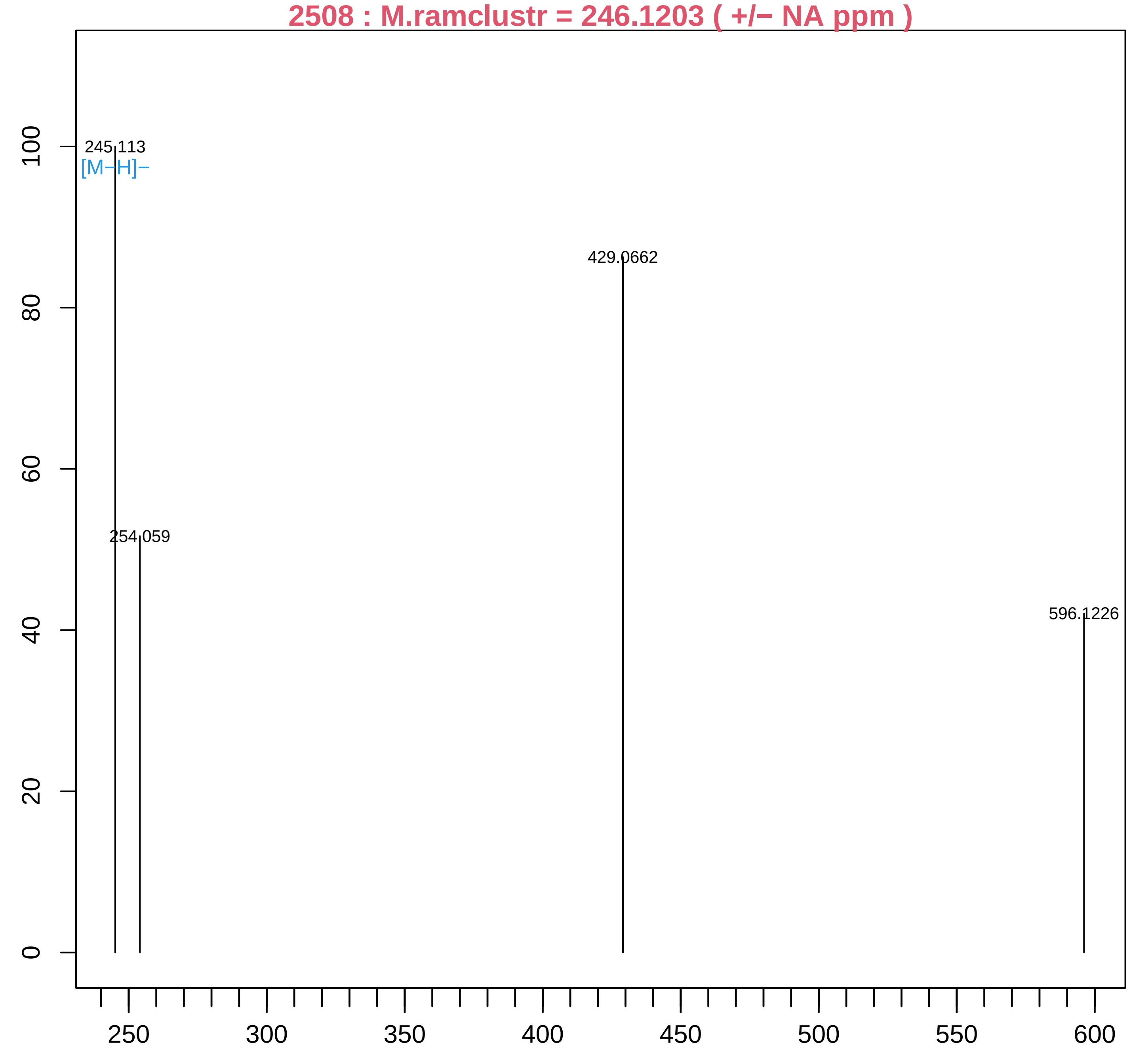

2508 : M.ramclustr = 246.1203 (+/− NA ppm)
245.113
[M−H]−
254.059
429.0662
596.1226
0
20
40
60
80
100
250
300
350
400
450
500
550
600

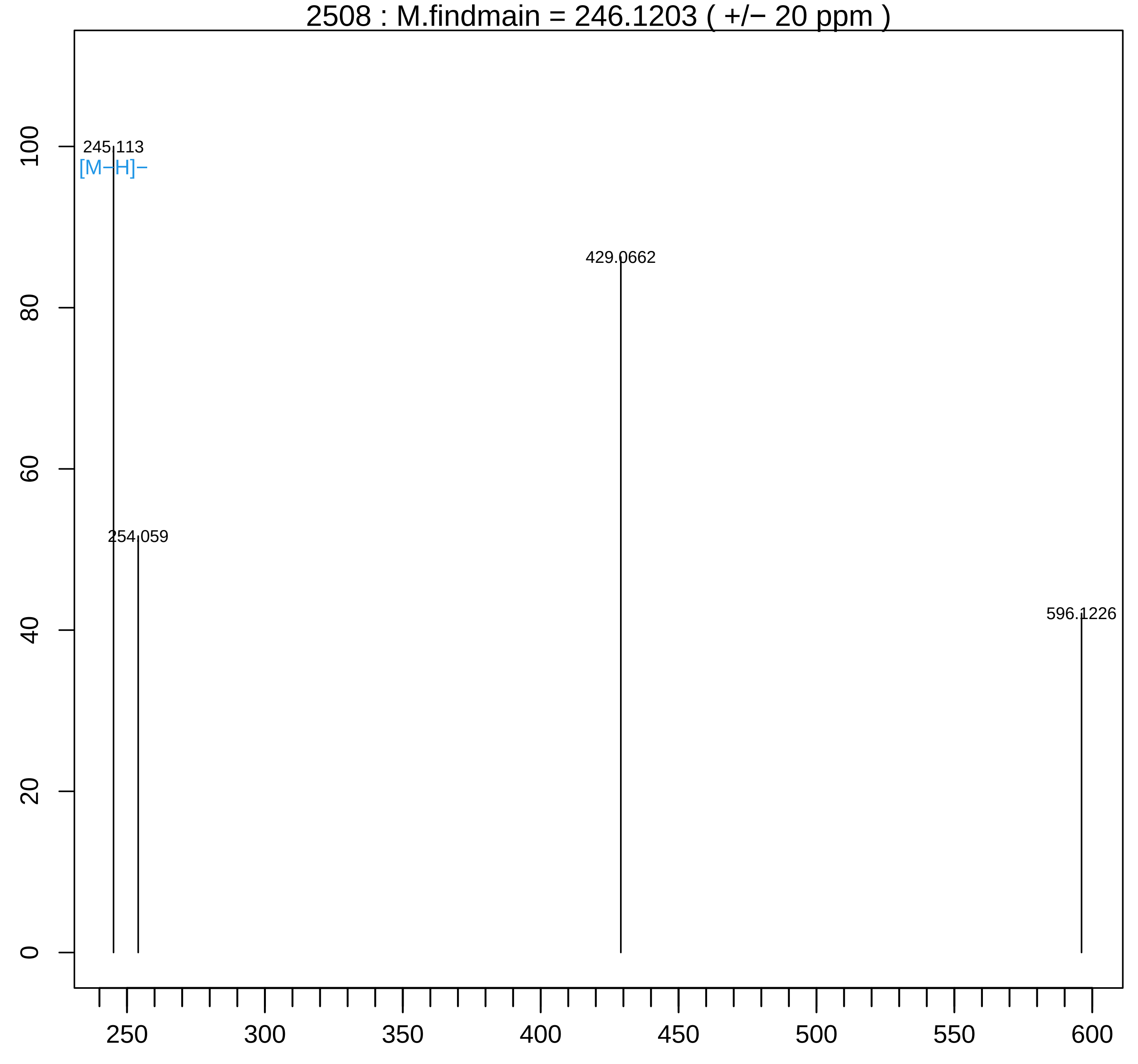

2508 : M.findmain = 246.1203 (+/− 20 ppm)
245.113
[M−H]−
254.059
429.0662
596.1226
0
20
40
60
80
100
250
300
350
400
450
500
550
600

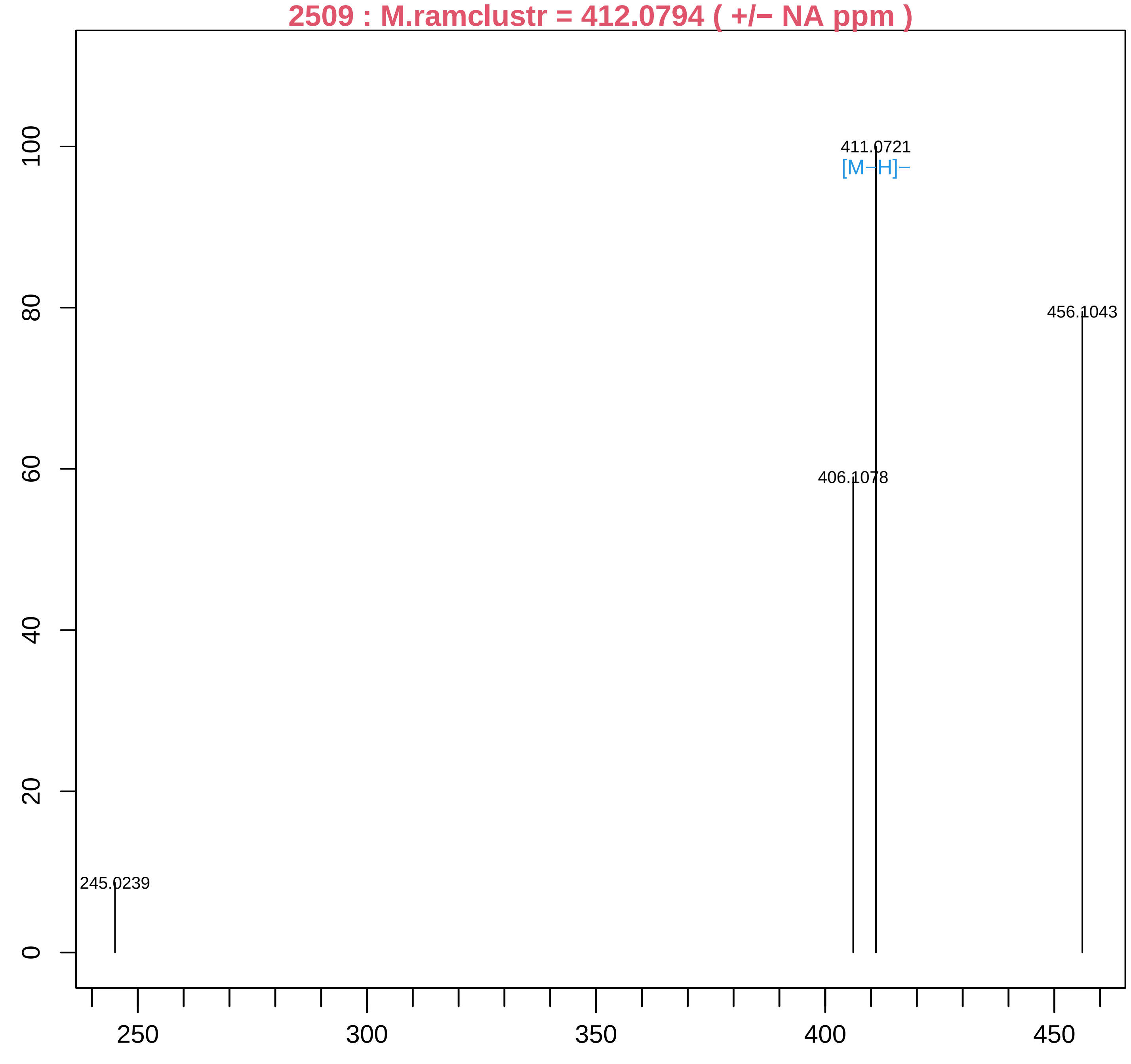
2509 : M.ramclustr = 412.0794 (+/− NA ppm)
411.0721
[M−H]−
456.1043
406.1078
245.0239
0
20
40
60
80
100
250
300
350
400
450

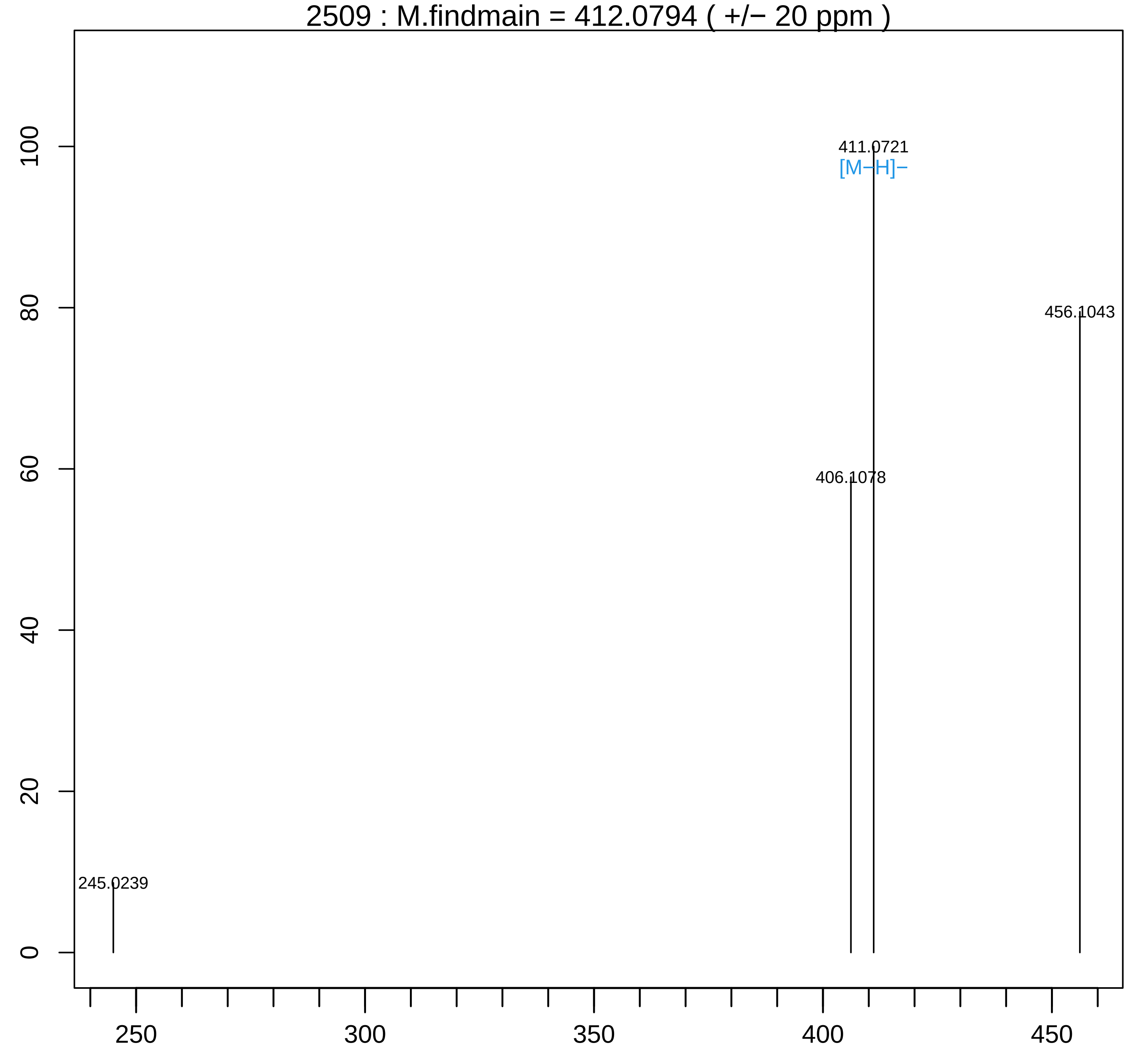
2509 : M.findmain = 412.0794 (+/− 20 ppm)
411.0721
[M−H]−
456.1043
406.1078
245.0239
0
20
40
60
80
100
250
300
350
400
450

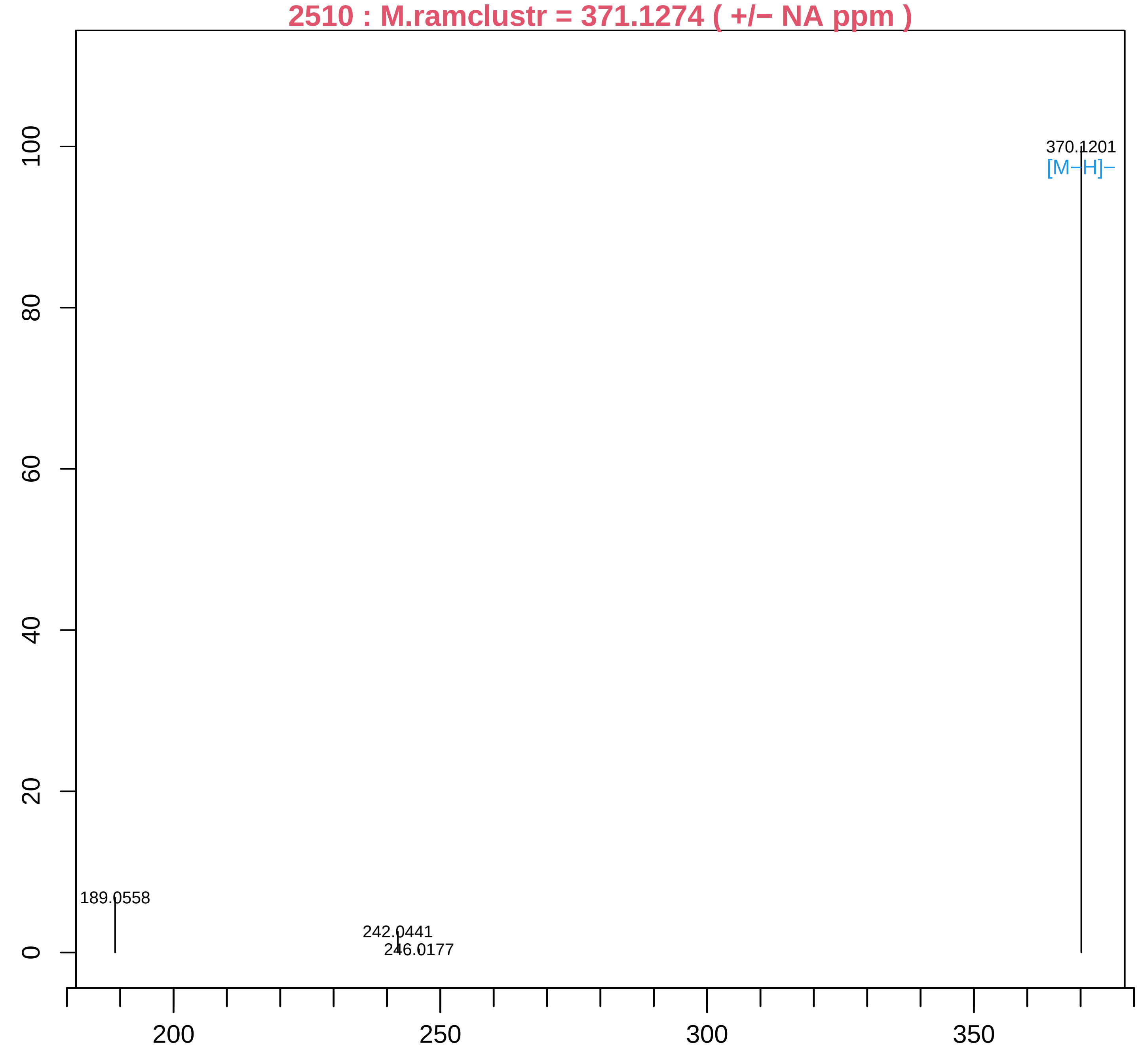
2510 : M.ramclustr = 371.1274 (+/− NA ppm)
370.1201
[M−H]−
189.0558
242.0441
246.0177
0
20
40
60
80
100
200
250
300
350

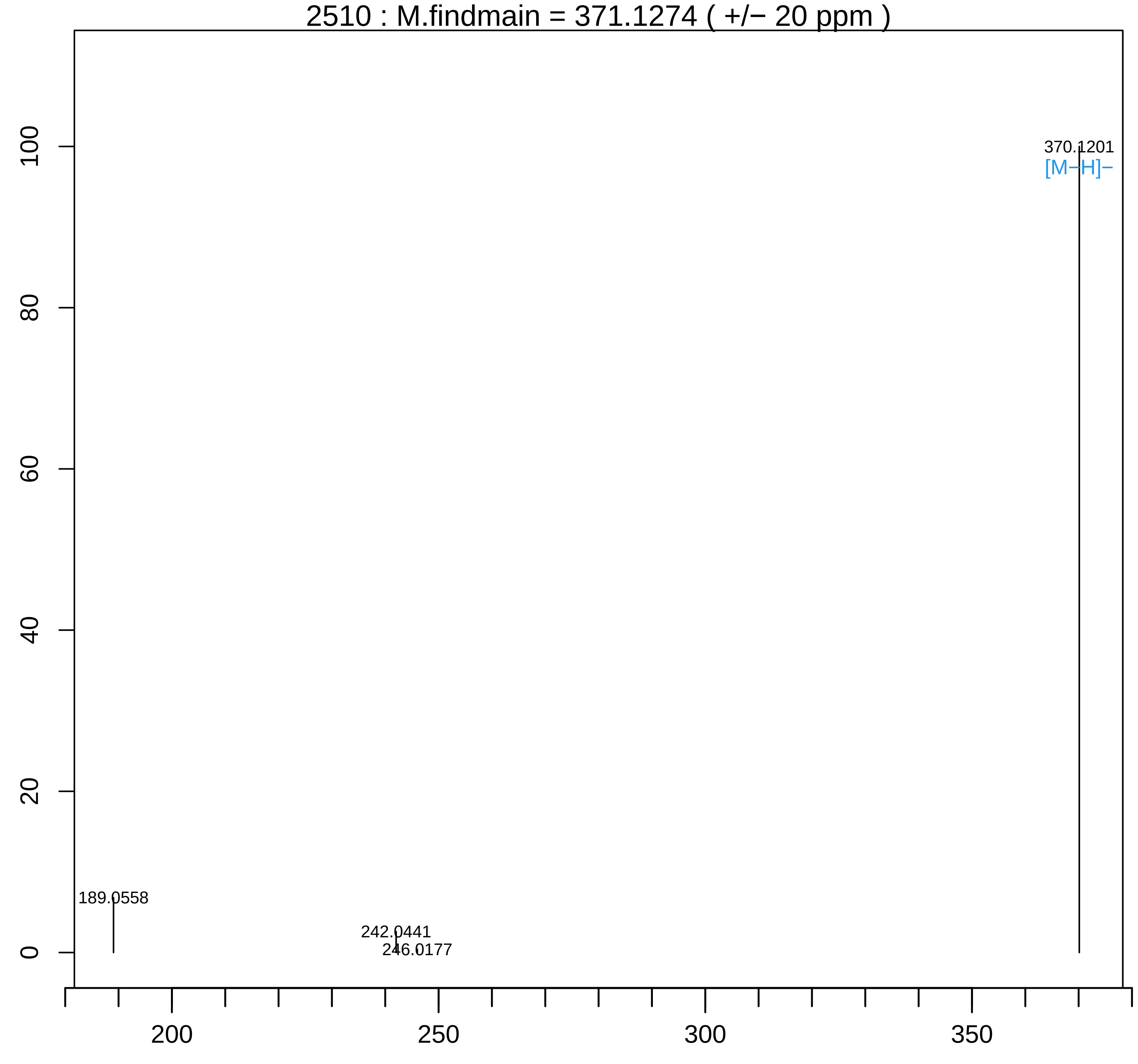
2510 : M.findmain = 371.1274 (+/− 20 ppm)
370.1201
[M−H]−
189.0558
242.0441
246.0177
0
20
40
60
80
100
200
250
300
350

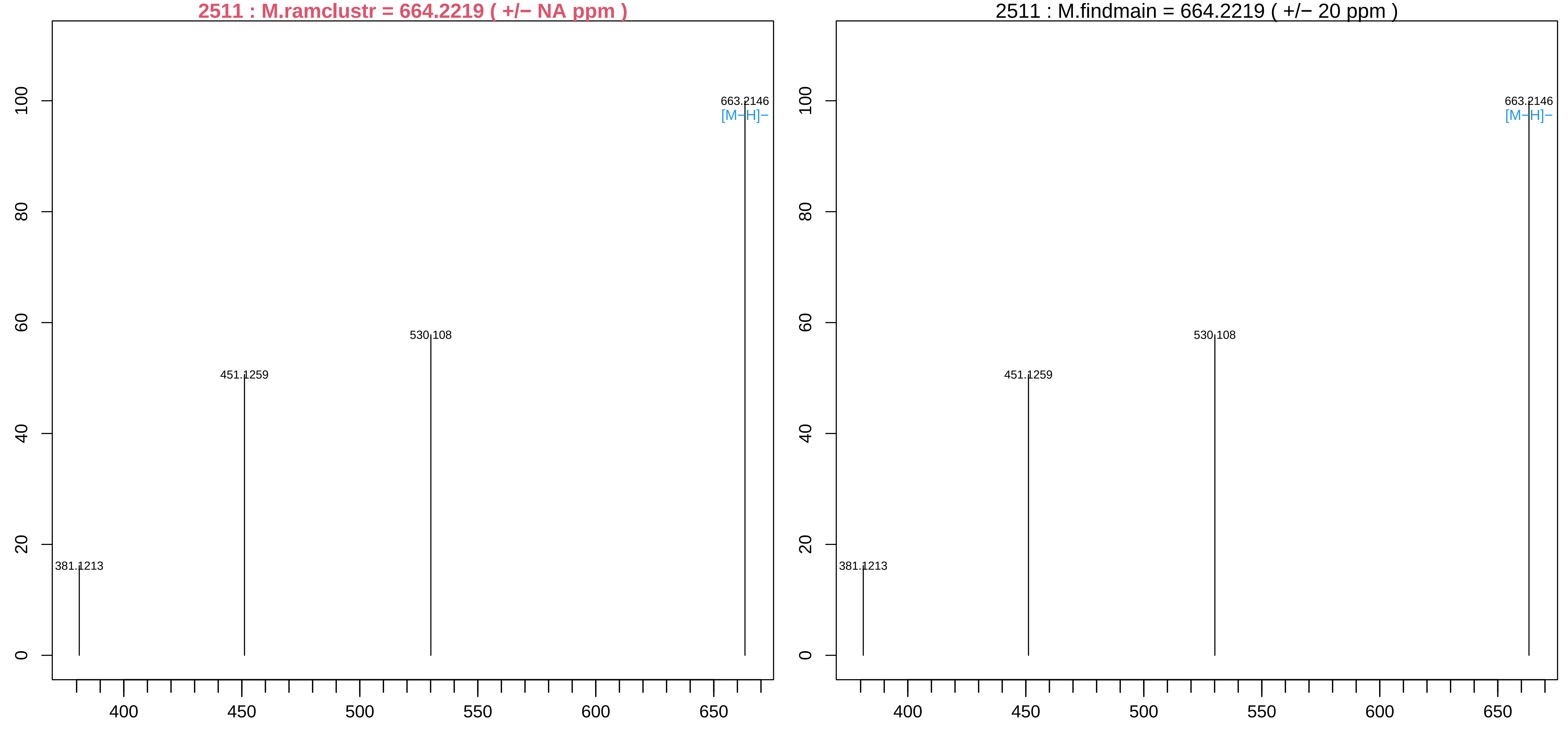
2511 : M.ramclustr = 664.2219 (+/− NA ppm)
663.2146
[M−H]−
530.108
451.1259
381.1213
0
20
40
60
80
100
400
450
500
550
600
650
2511 : M.findmain = 664.2219 (+/− 20 ppm)
663.2146
[M−H]−
530.108
451.1259
381.1213
0
20
40
60
80
100
400
450
500
550
600
650

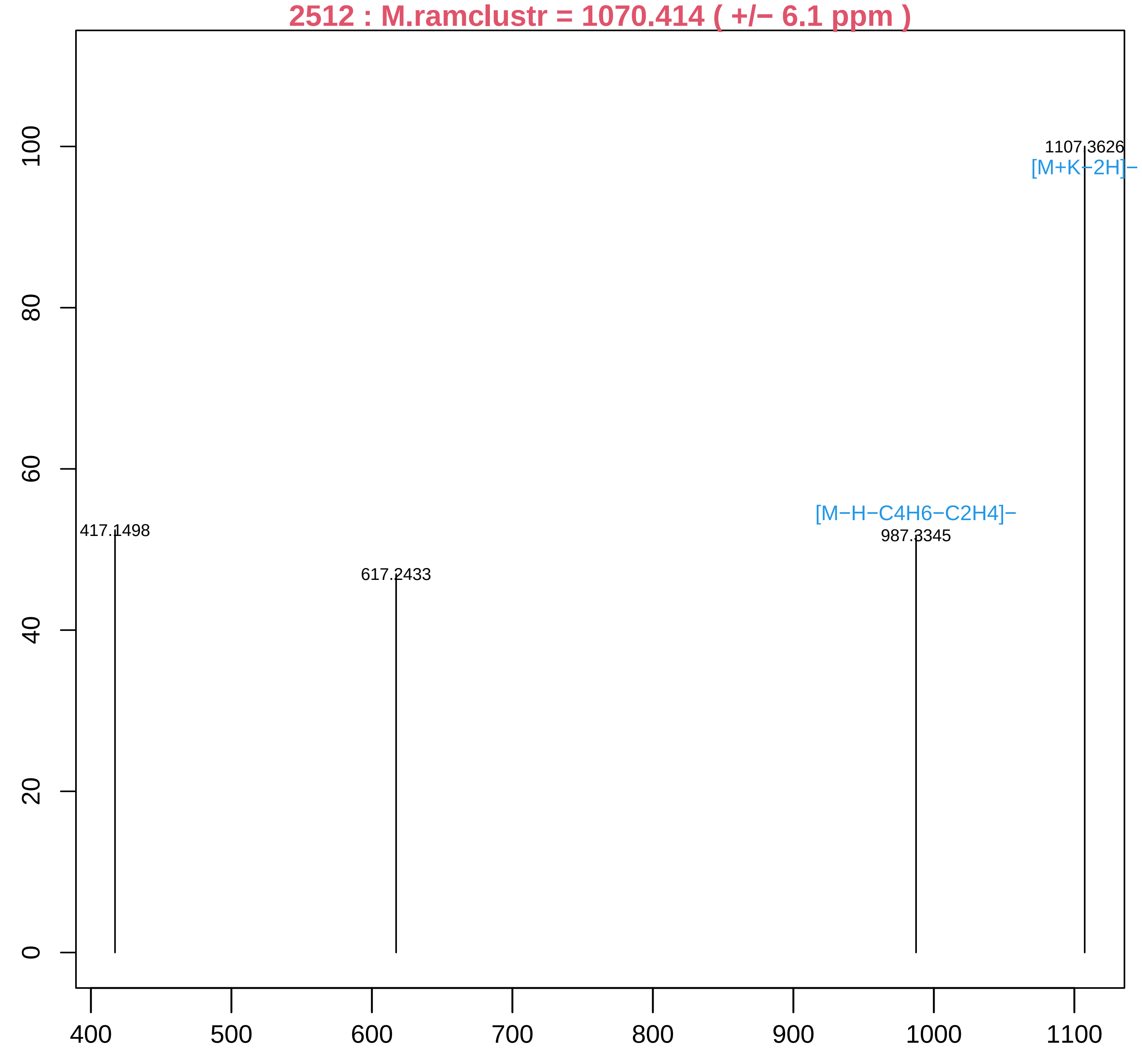
2512 : M.ramclustr = 1070.414 (+/− 6.1 ppm)
1107.3626
[M+K−2H]−
[M−H−C4H6−C2H4]−
987.3345
417.1498
617.2433
0
20
40
60
80
100
400
500
600
700
800
900
1000
1100

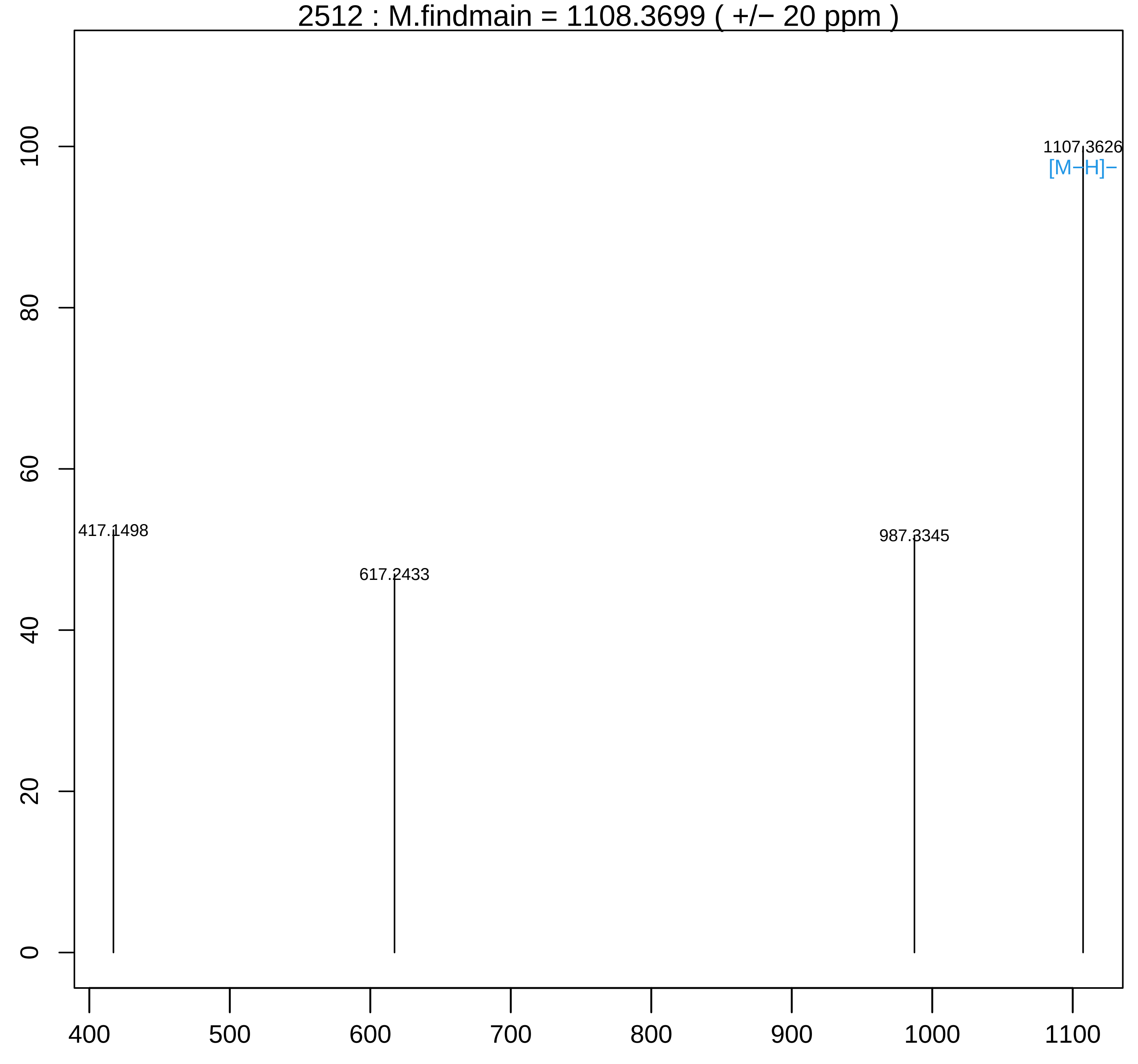
2512 : M.findmain = 1108.3699 (+/− 20 ppm)
1107.3626
[M−H]−
417.1498
617.2433
987.3345
0
20
40
60
80
100
400
500
600
700
800
900
1000
1100

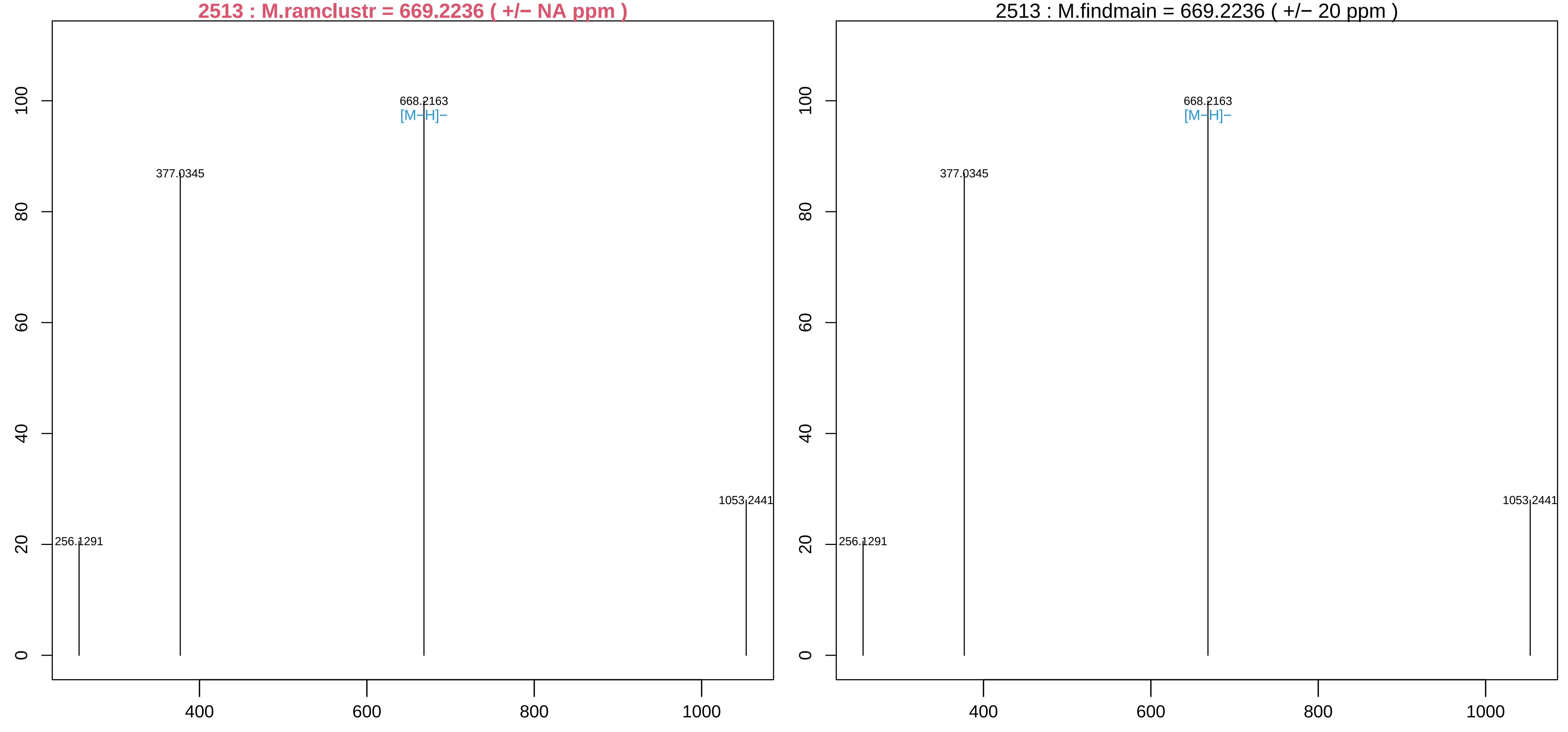
2513 : M.ramclustr = 669.2236 (+/− NA ppm)
668.2163
[M−H]−
377.0345
1053.2441
256.1291
0
20
40
60
80
100
400
600
800
1000
2513 : M.findmain = 669.2236 (+/− 20 ppm)
668.2163
[M−H]−
377.0345
1053.2441
256.1291
0
20
40
60
80
100
400
600
800
1000

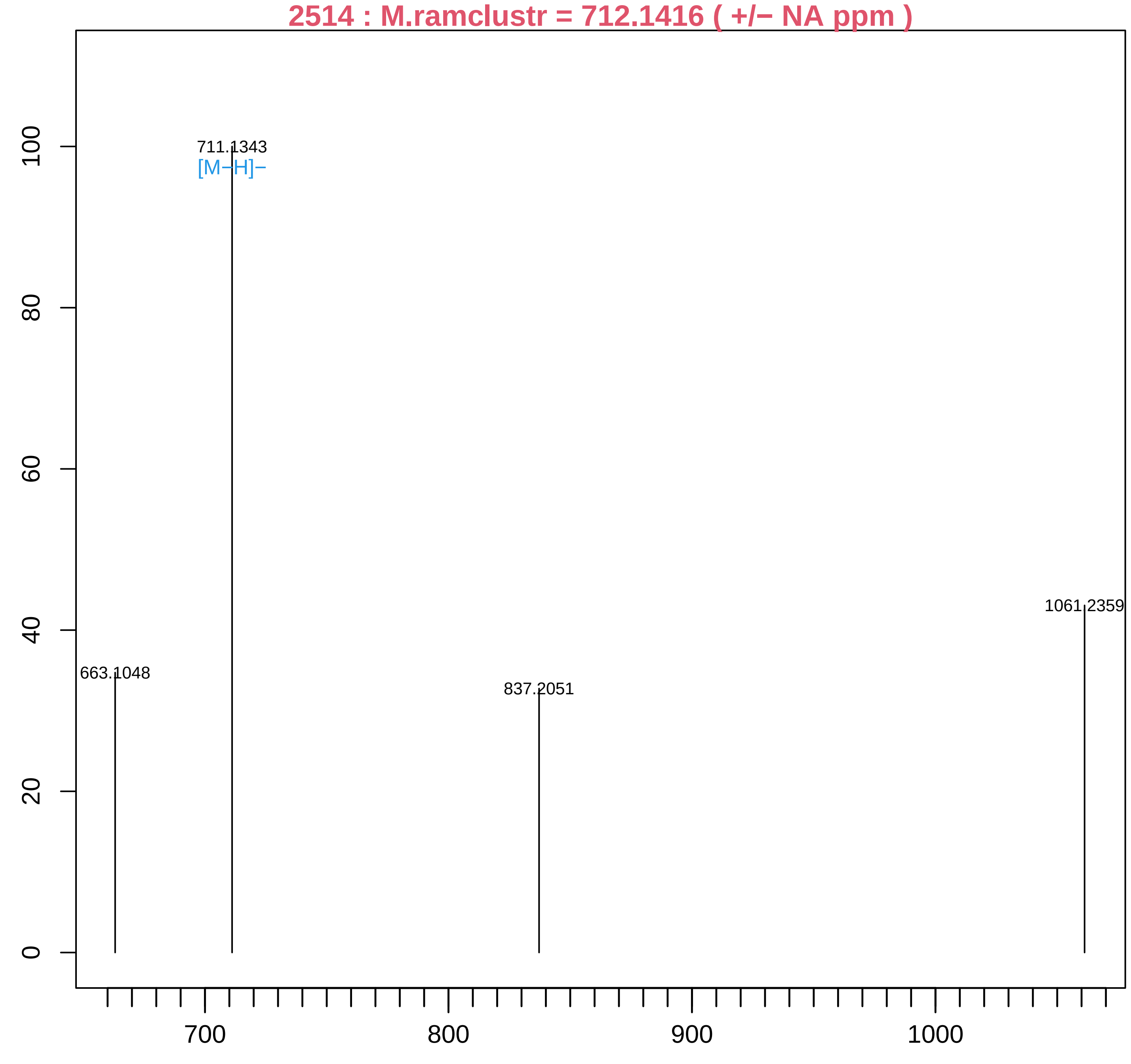
2514 : M.ramclustr = 712.1416 (+/− NA ppm)
711.1343
[M−H]−
663.1048
837.2051
1061.2359
0
20
40
60
80
100
700
800
900
1000

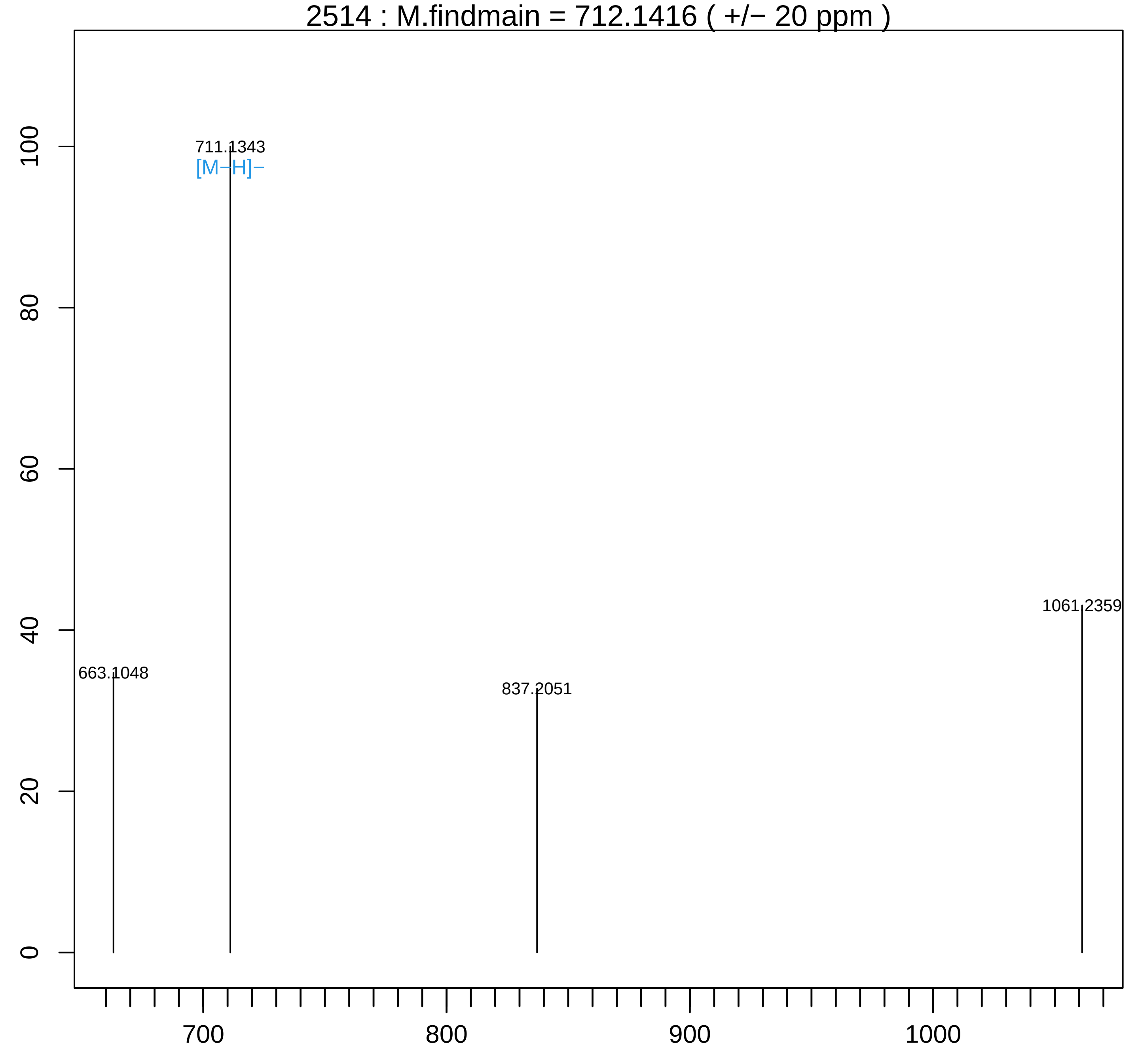
2514 : M.findmain = 712.1416 (+/− 20 ppm)
711.1343
[M−H]−
663.1048
837.2051
1061.2359
0
20
40
60
80
100
700
800
900
1000

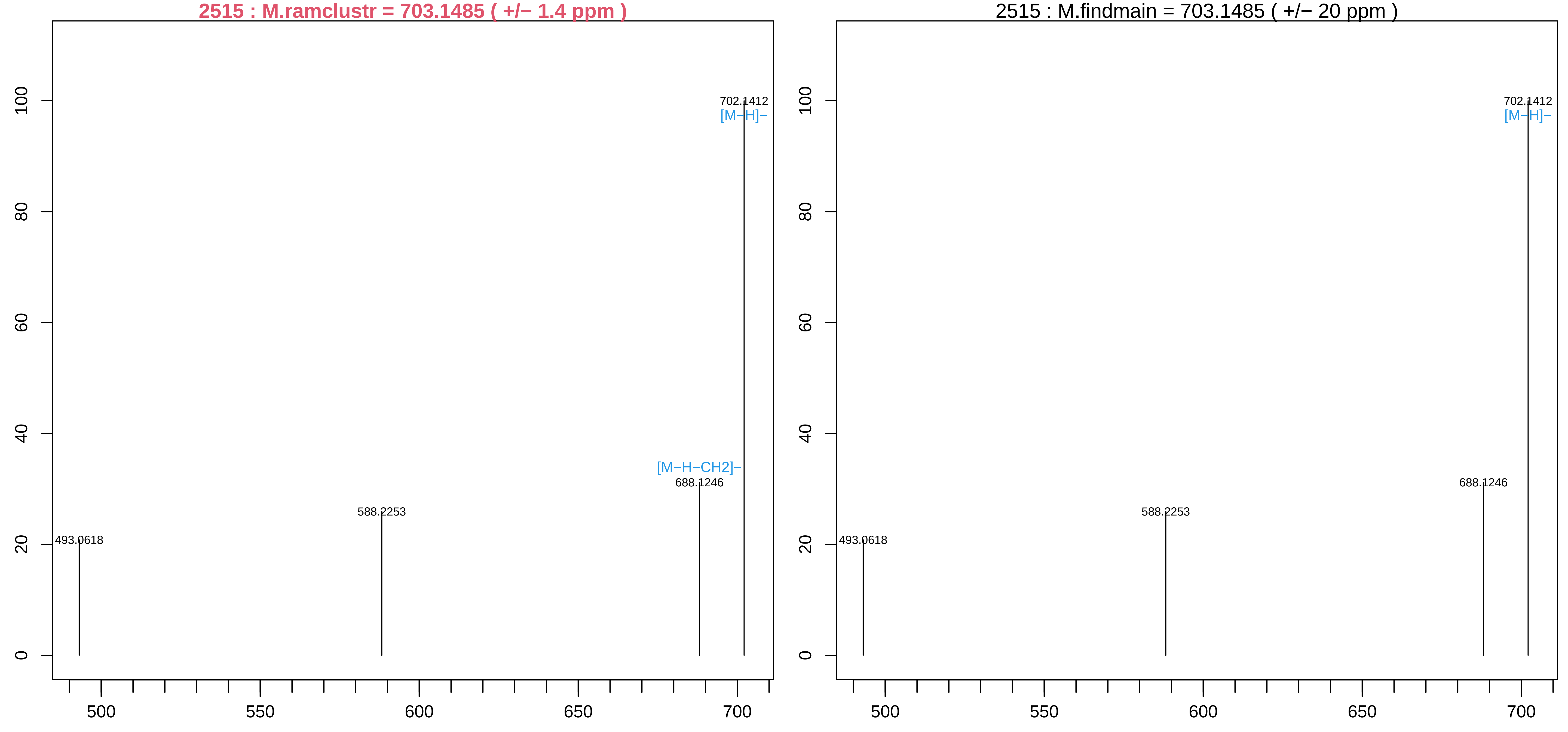
2515 : M.ramclustr = 703.1485 (+/− 1.4 ppm)
702.1412
[M−H]−
[M−H−CH2]−
688.1246
588.2253
493.0618
2515 : M.findmain = 703.1485 (+/− 20 ppm)
702.1412
[M−H]−
688.1246
588.2253
493.0618

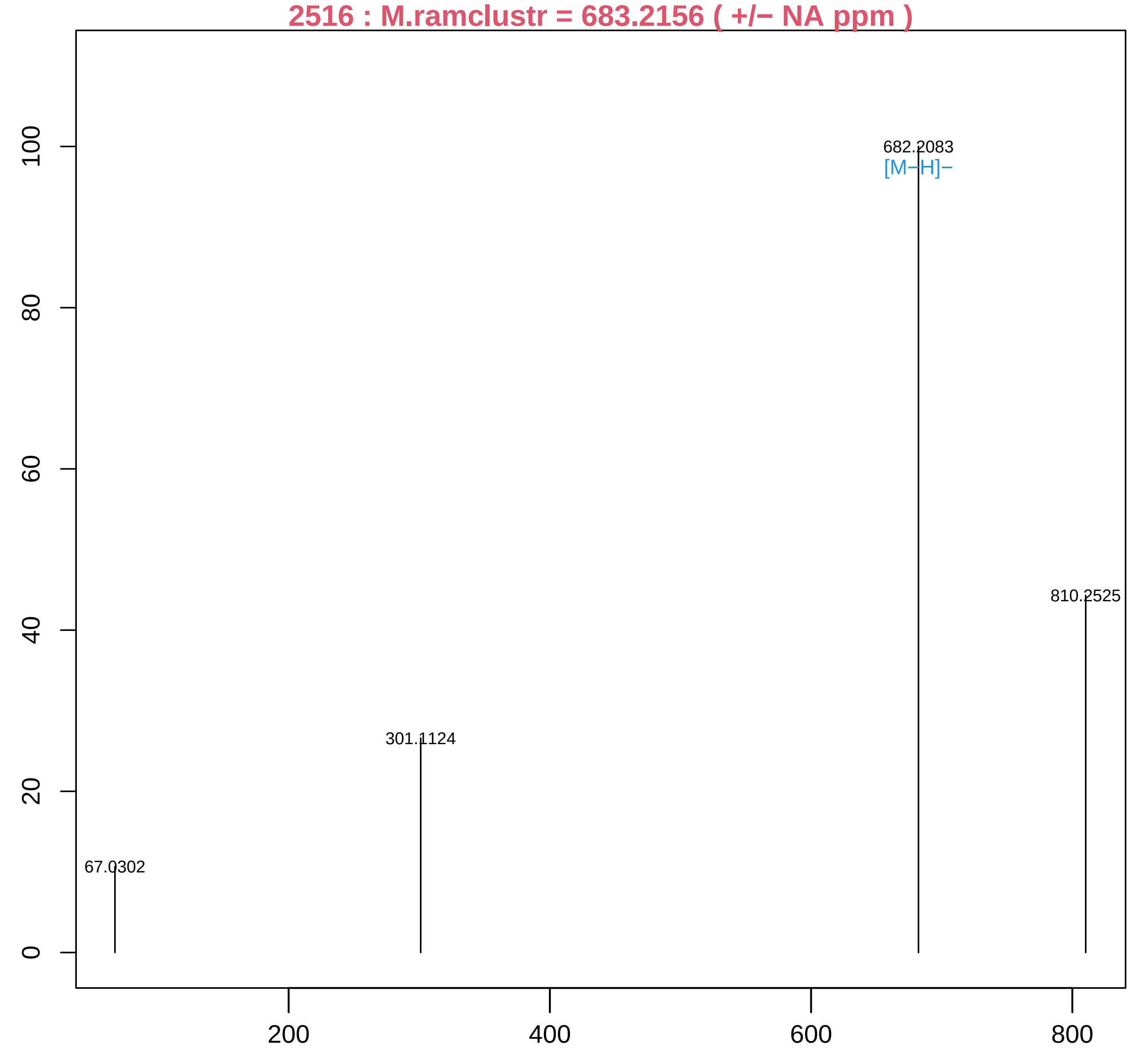
2516 : M.ramclustr = 683.2156 (+/− NA ppm)
682.2083
[M−H]−
810.2525
301.1124
67.0302
0
20
40
60
80
100
200
400
600
800
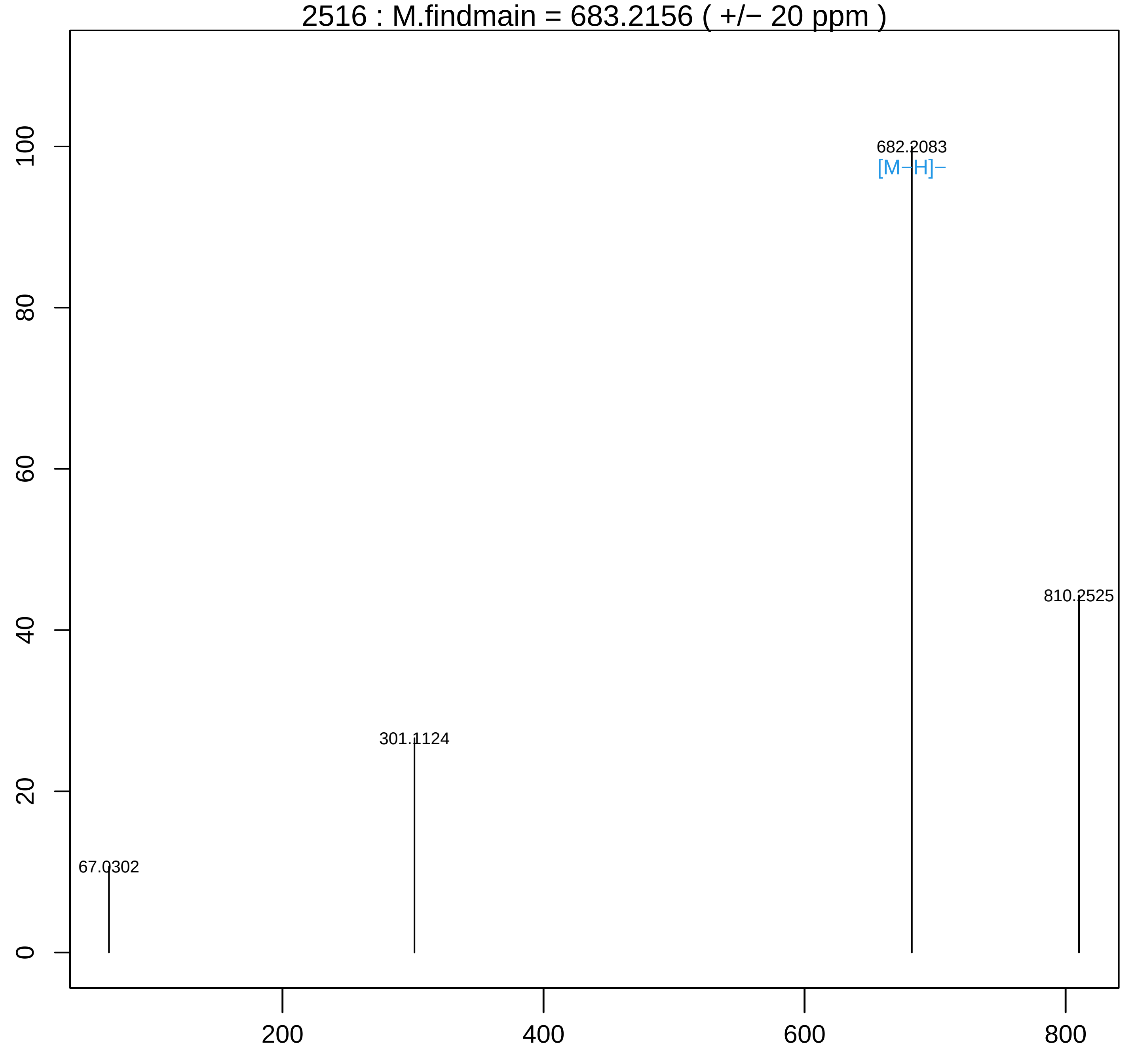
2516 : M.findmain = 683.2156 (+/− 20 ppm)
682.2083
[M−H]−
810.2525
301.1124
67.0302
0
20
40
60
80
100
200
400
600
800

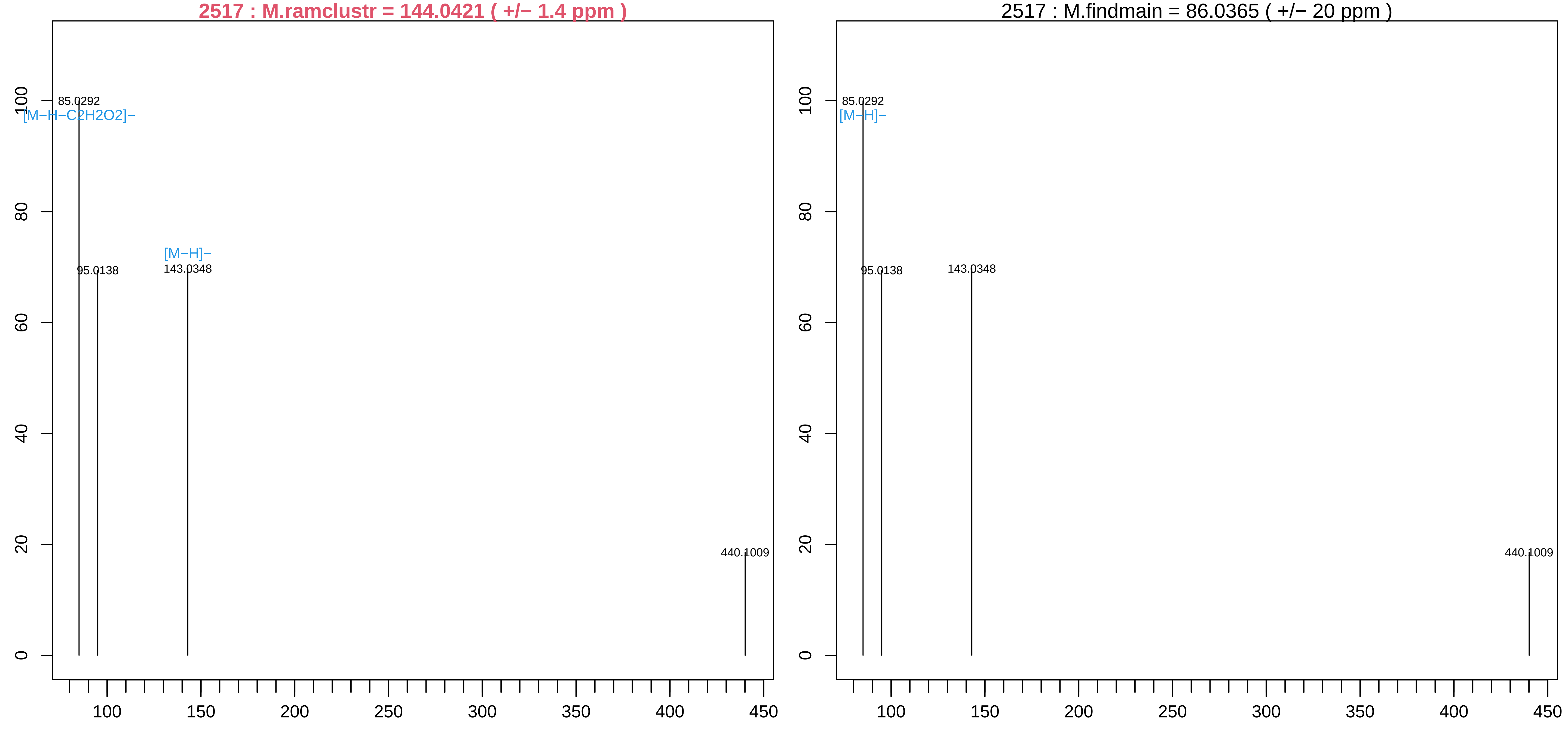
2517 : M.ramclustr = 144.0421 (+/− 1.4 ppm)
85.0292
[M−H−C2H2O2]−
95.0138
[M−H]−
143.0348
440.1009
2517 : M.findmain = 86.0365 (+/− 20 ppm)
85.0292
[M−H]−
95.0138
143.0348
440.1009
0
20
40
60
80
100
100
150
200
250
300
350
400
450

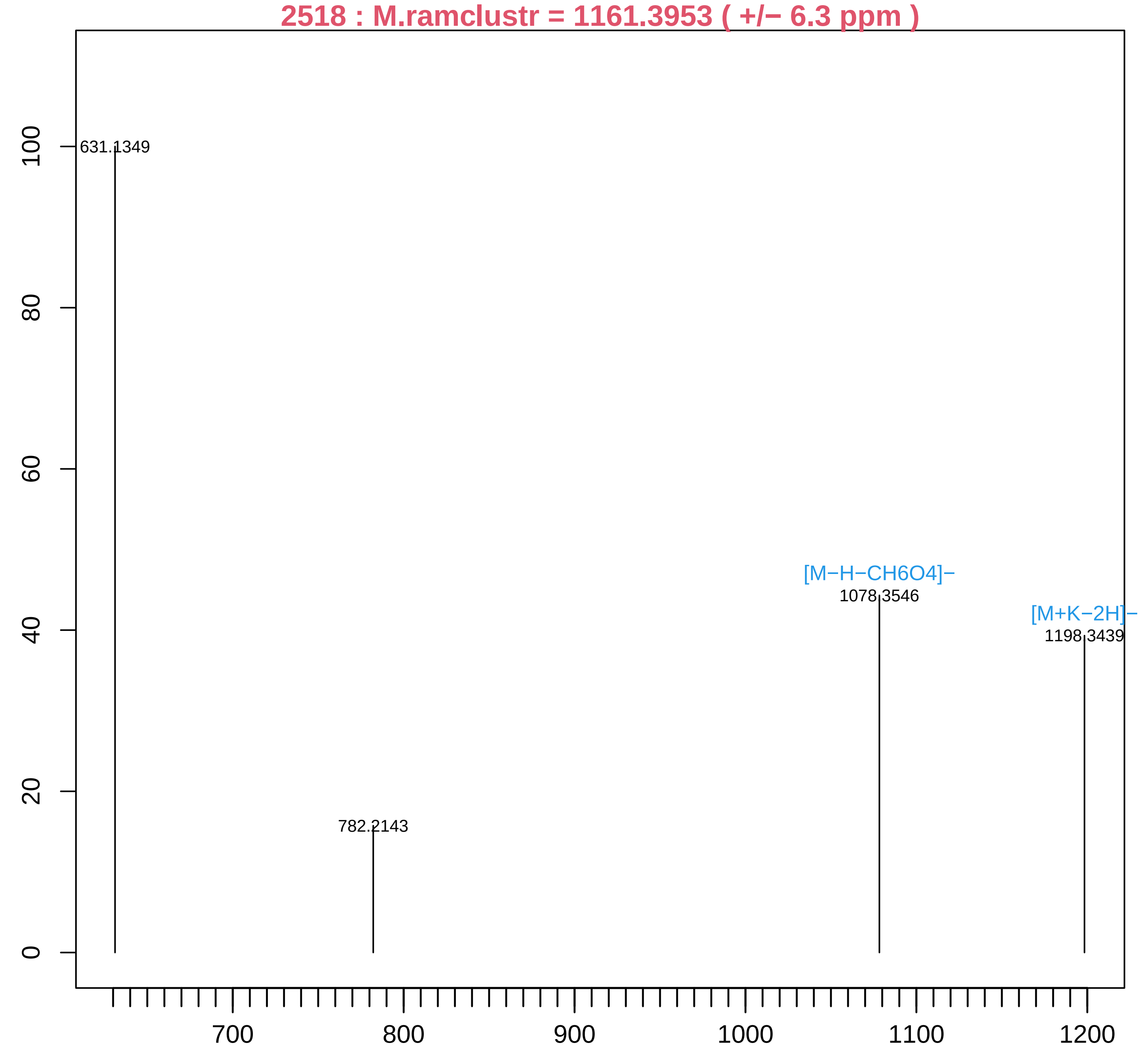
2518 : M.ramclustr = 1161.3953 (+/− 6.3 ppm)
631.1349
782.2143
[M−H−CH6O4]−
1078.3546
[M+K−2H]−
1198.3439
0
20
40
60
80
100
700
800
900
1000
1100
1200

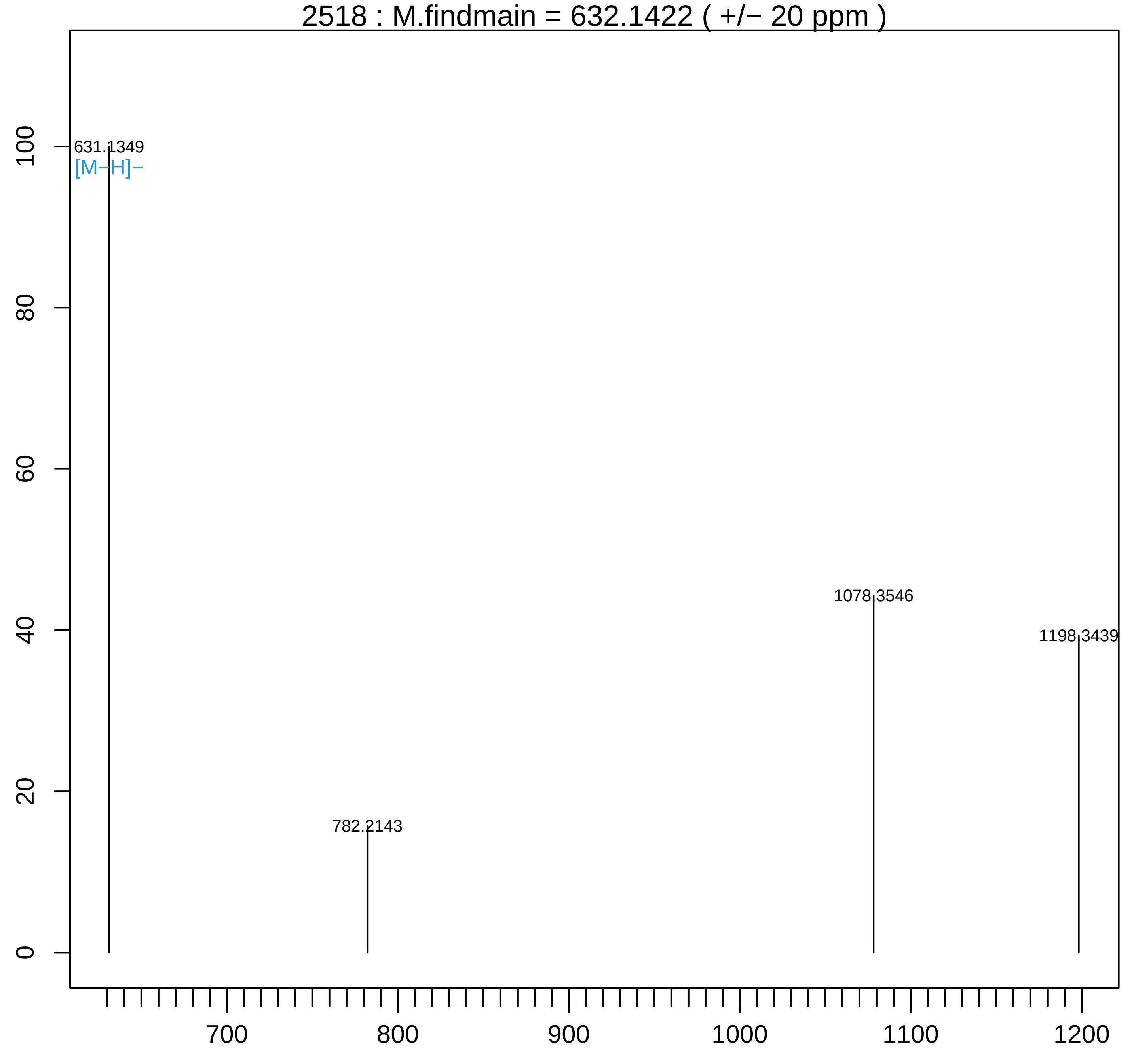
2518 : M.findmain = 632.1422 (+/− 20 ppm)
631.1349
[M−H]−
782.2143
1078.3546
1198.3439
0
20
40
60
80
100
700
800
900
1000
1100
1200

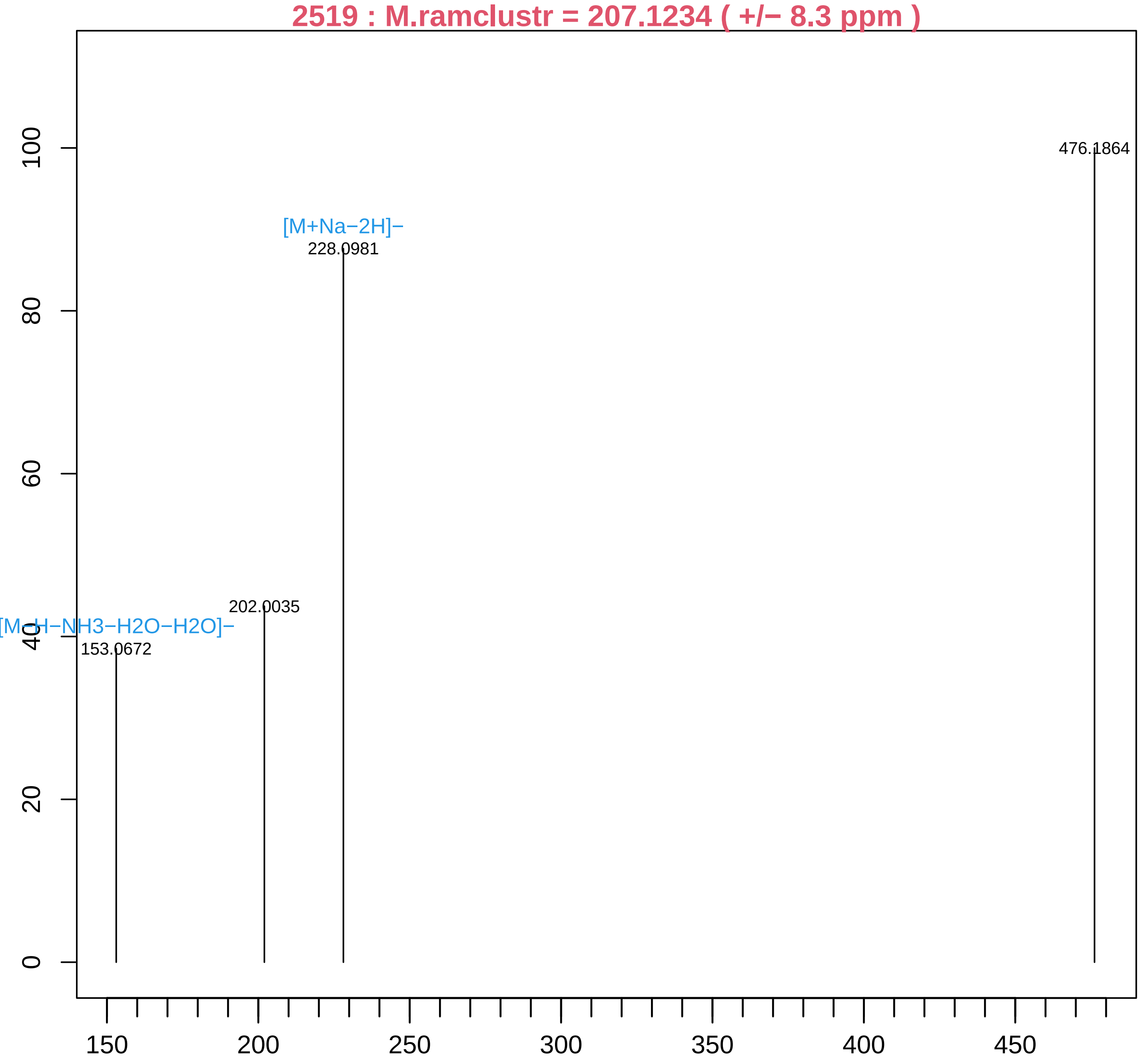
2519 : M.ramclustr = 207.1234 (+/− 8.3 ppm)
476.1864
[M+Na−2H]−
228.0981
202.0035
[M−H−NH3−H2O−H2O]−
153.0672
0
20
40
60
80
100
150
200
250
300
350
400
450

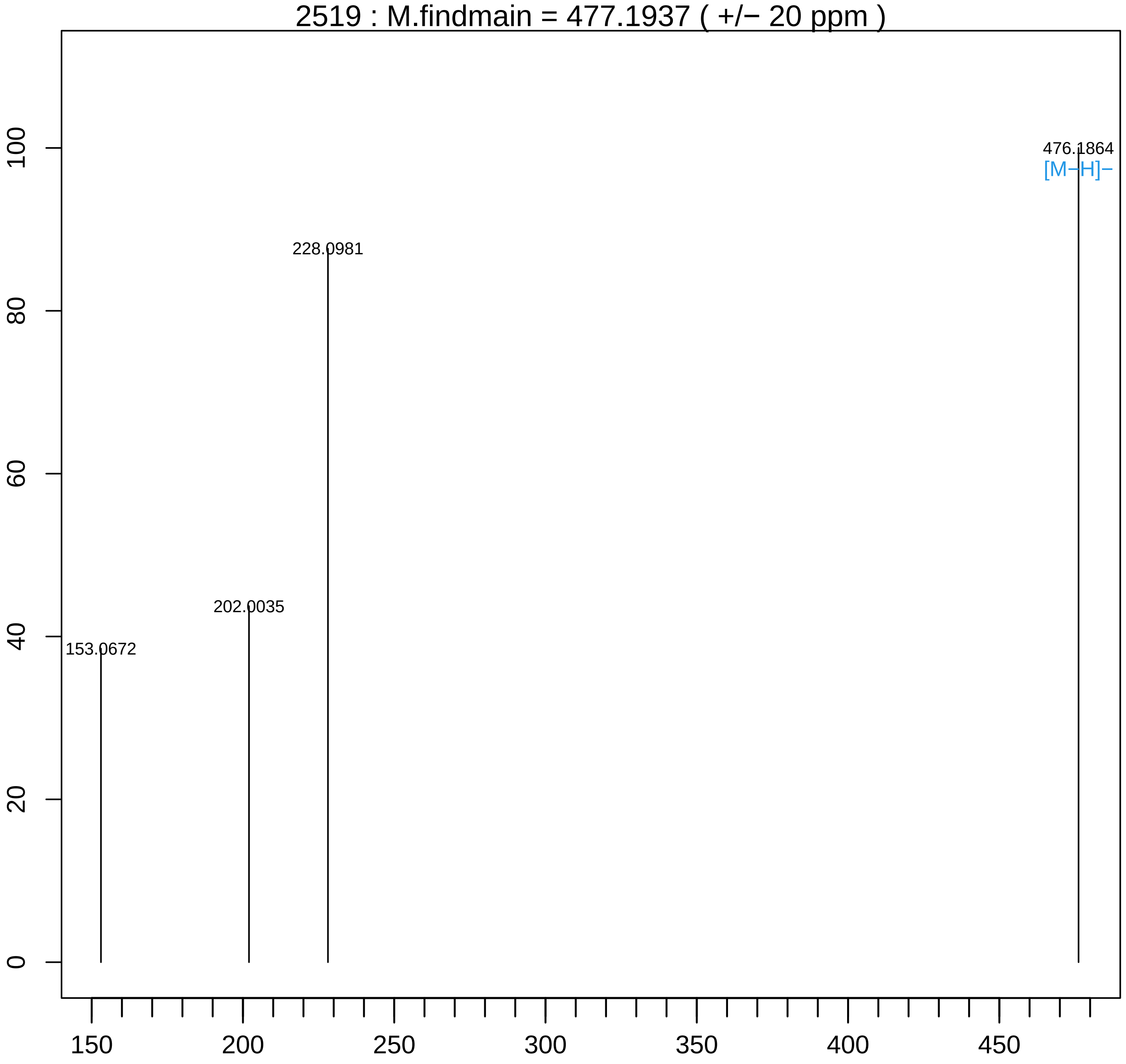
2519 : M.findmain = 477.1937 (+/− 20 ppm)
476.1864
[M−H]−
228.0981
202.0035
153.0672
0
20
40
60
80
100
150
200
250
300
350
400
450

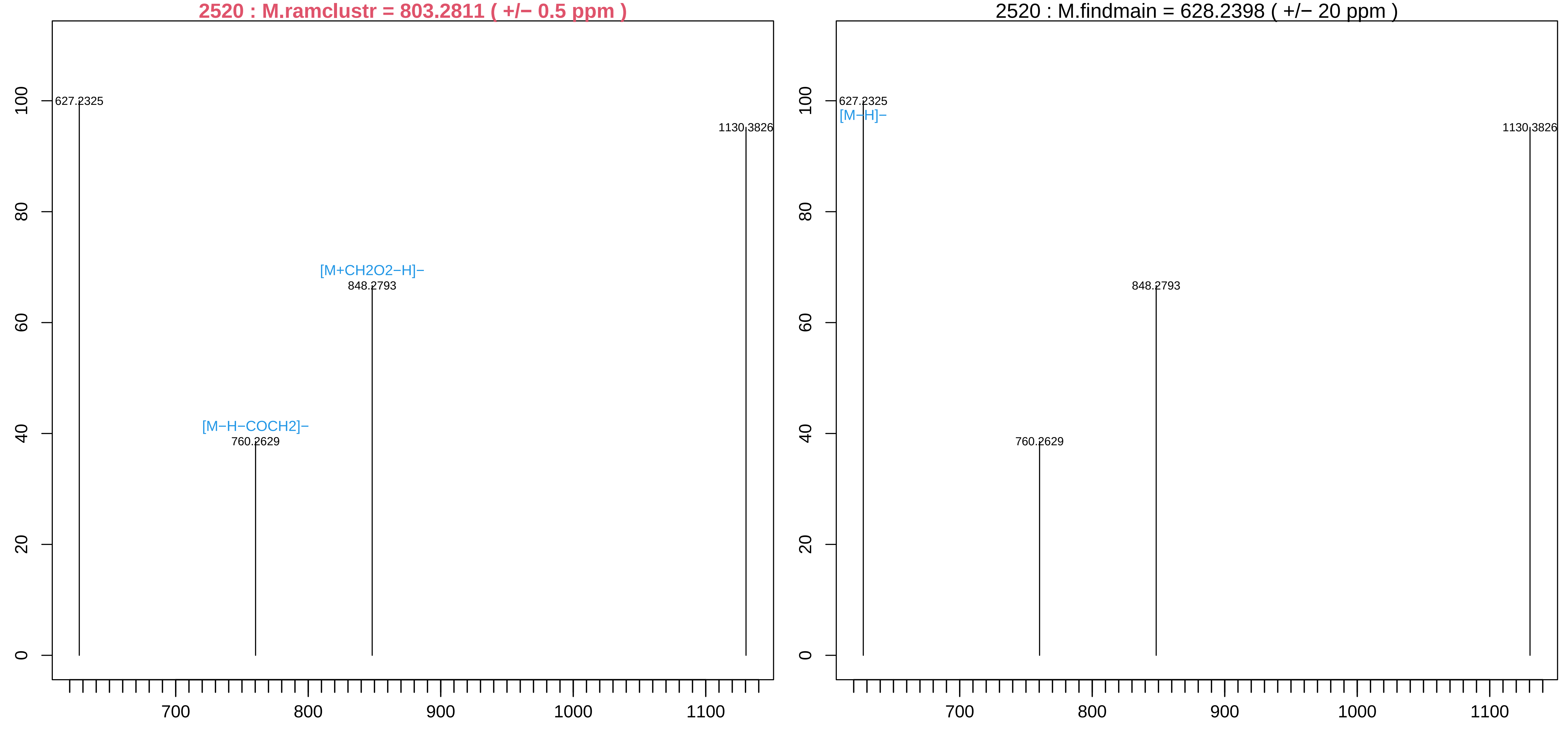

2520 : M.ramclustr = 803.2811 (+/− 0.5 ppm)
627.2325
[M−H−COCH2]−
760.2629
[M+CH2O2−H]−
848.2793
1130.3826
2520 : M.findmain = 628.2398 (+/− 20 ppm)
627.2325
[M−H]−
760.2629
848.2793
1130.3826
0
20
40
60
80
100
700
800
900
1000
1100

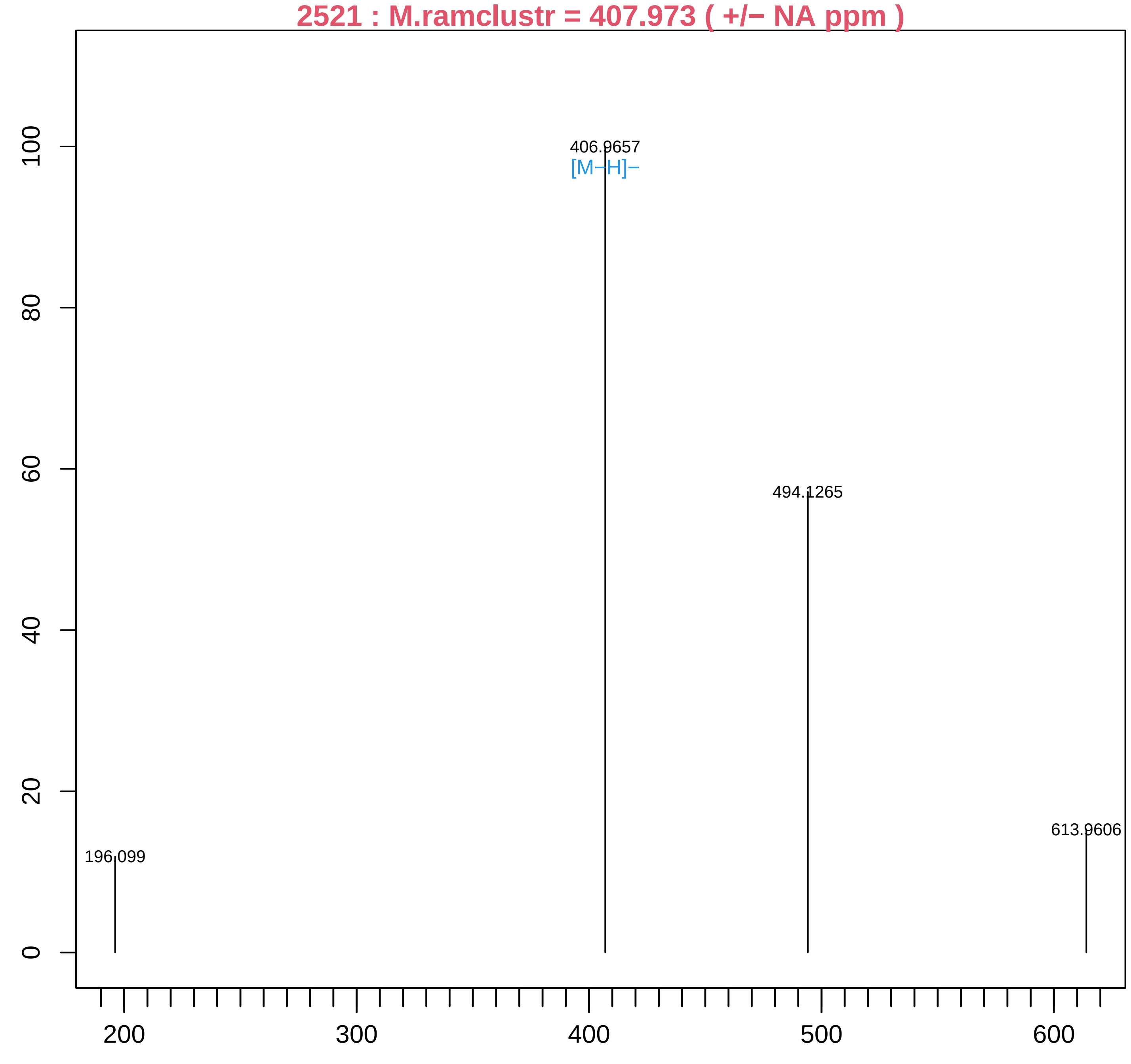
2521 : M.ramclustr = 407.973 (+/− NA ppm)
406.9657
[M−H]−
494.1265
613.9606
196.099
0
20
40
60
80
100
200
300
400
500
600

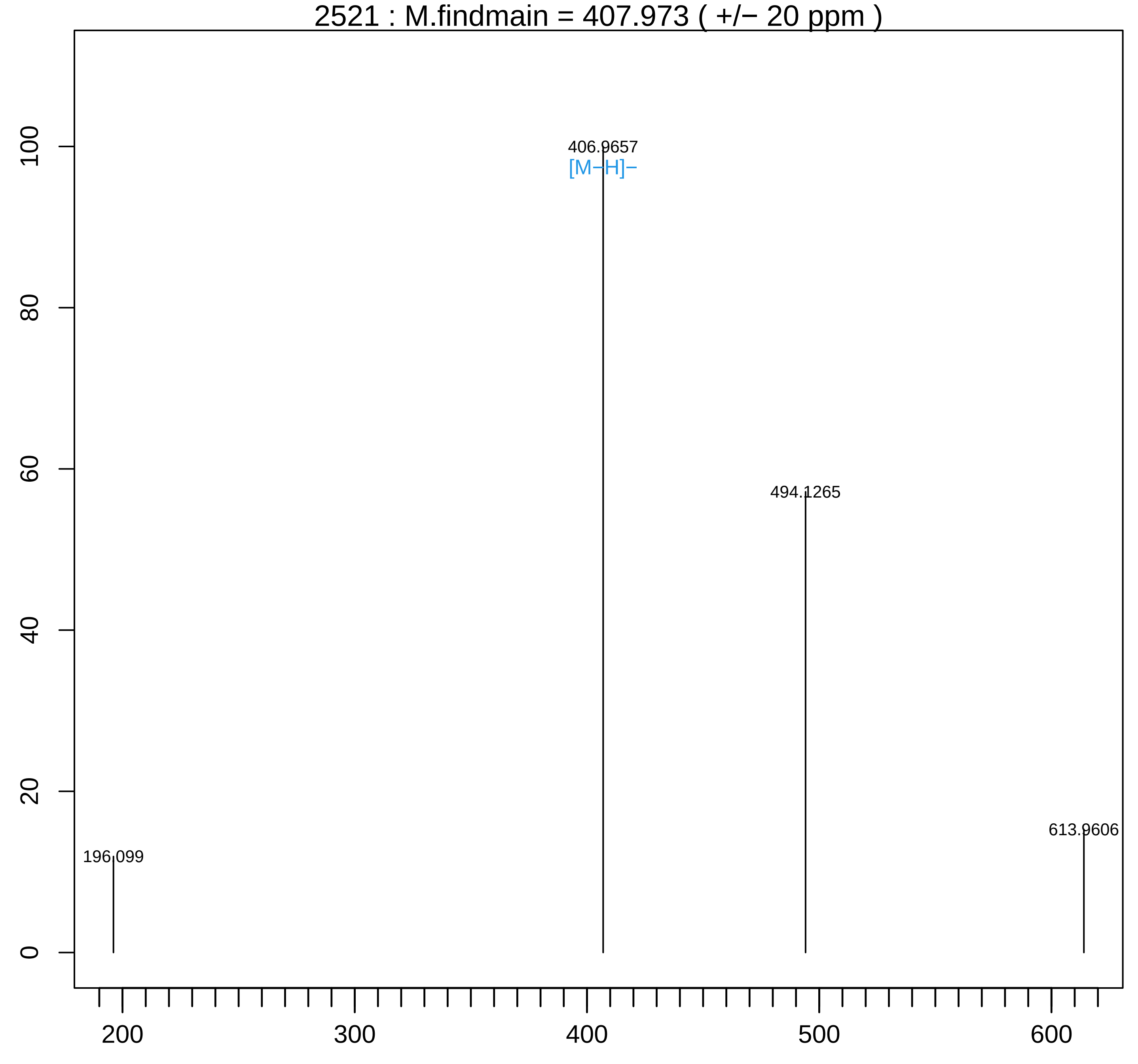
2521 : M.findmain = 407.973 (+/− 20 ppm)
406.9657
[M−H]−
494.1265
613.9606
196.099
0
20
40
60
80
100
200
300
400
500
600

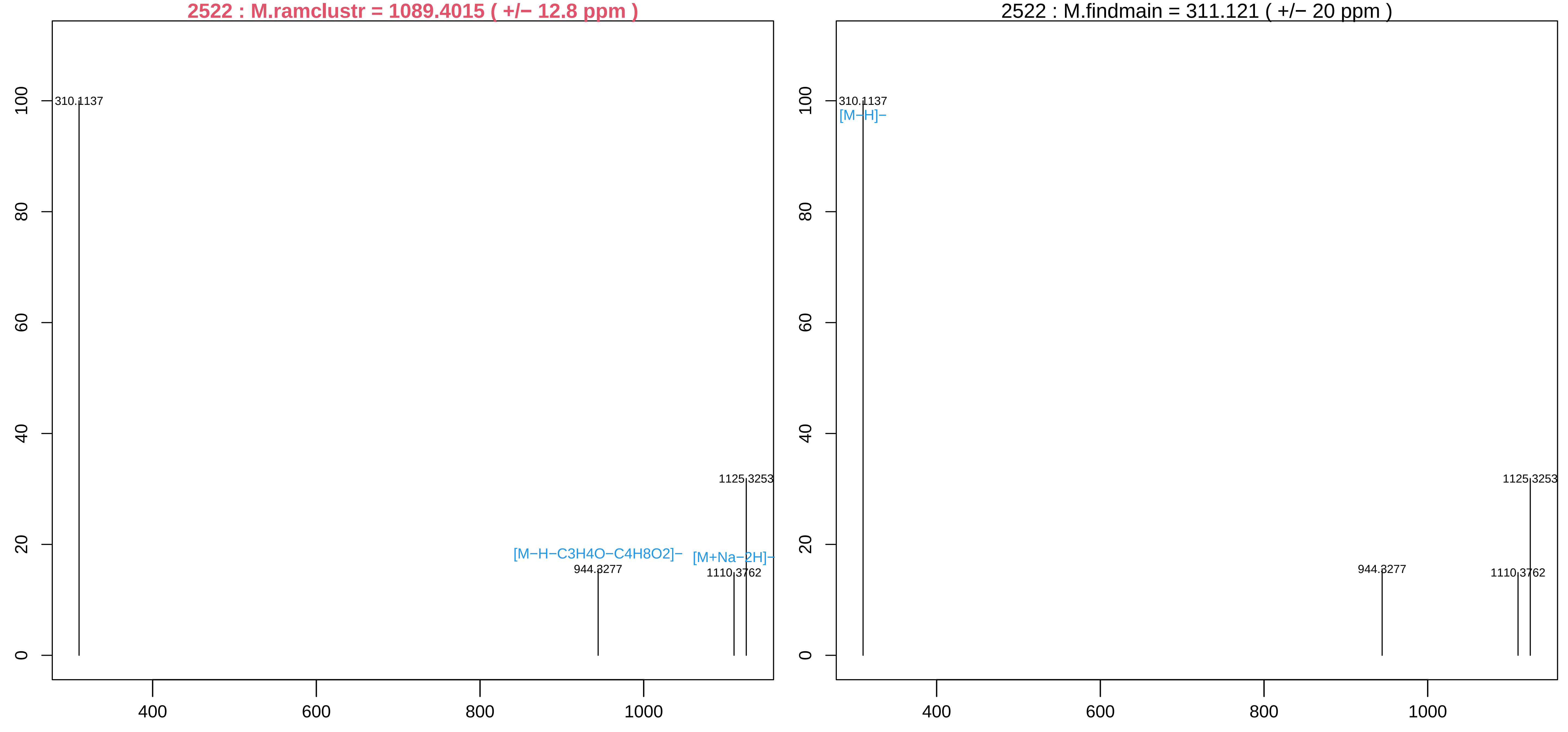

2522 : M.ramclustr = 1089.4015 (+/− 12.8 ppm)
310.1137
[M−H−C3H4O−C4H8O2]−
944.3277
[M+Na−2H]−
1110.3762
1125.3253
0
20
40
60
80
100
400
600
800
1000
2522 : M.findmain = 311.121 (+/− 20 ppm)
310.1137
[M−H]−
944.3277
1110.3762
1125.3253
0
20
40
60
80
100
400
600
800
1000

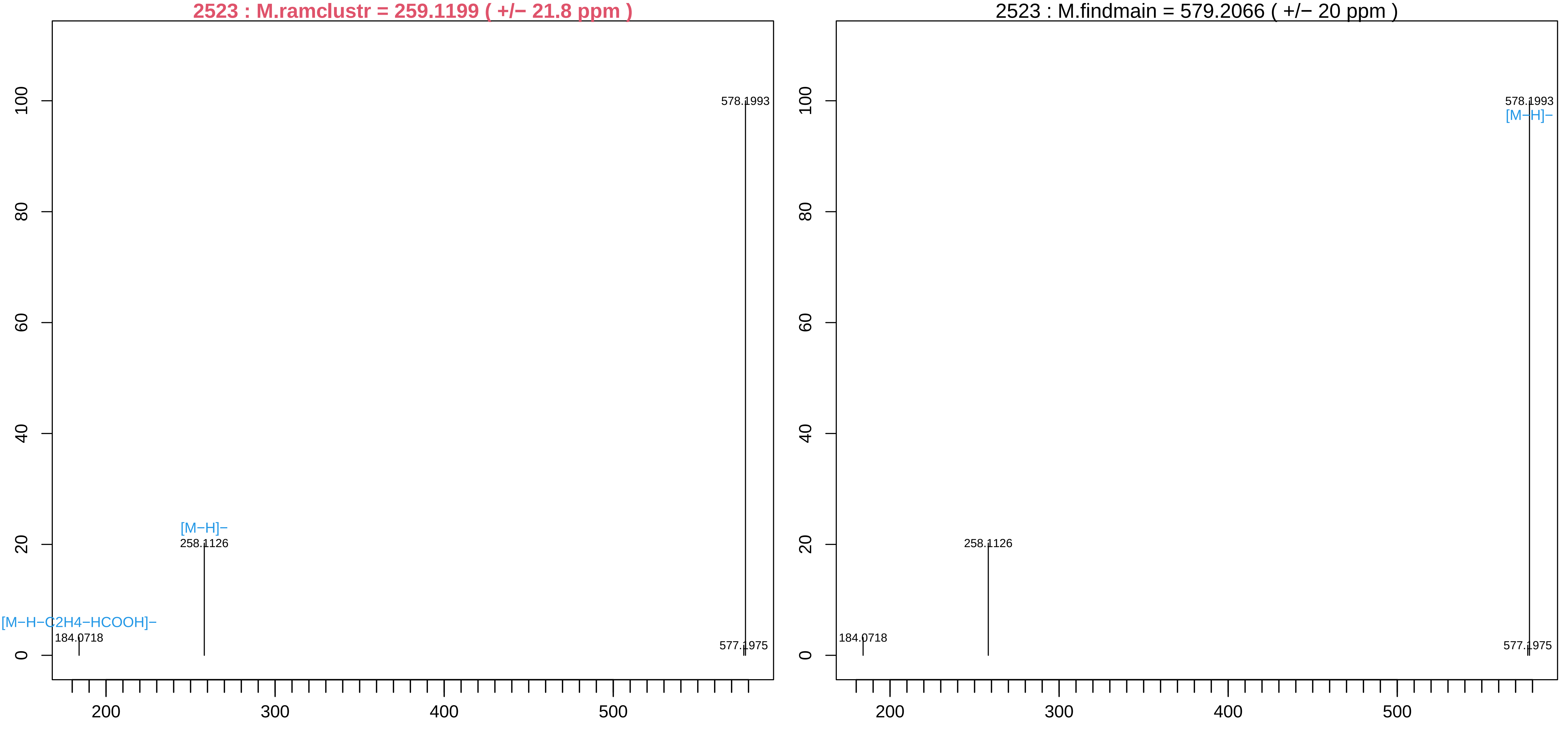

2523 : M.ramclustr = 259.1199 (+/− 21.8 ppm)
578.1993
[M−H]−
258.1126
[M−H−C2H4−HCOOH]−
184.0718
577.1975
2523 : M.findmain = 579.2066 (+/− 20 ppm)
578.1993
[M−H]−
258.1126
184.0718
577.1975

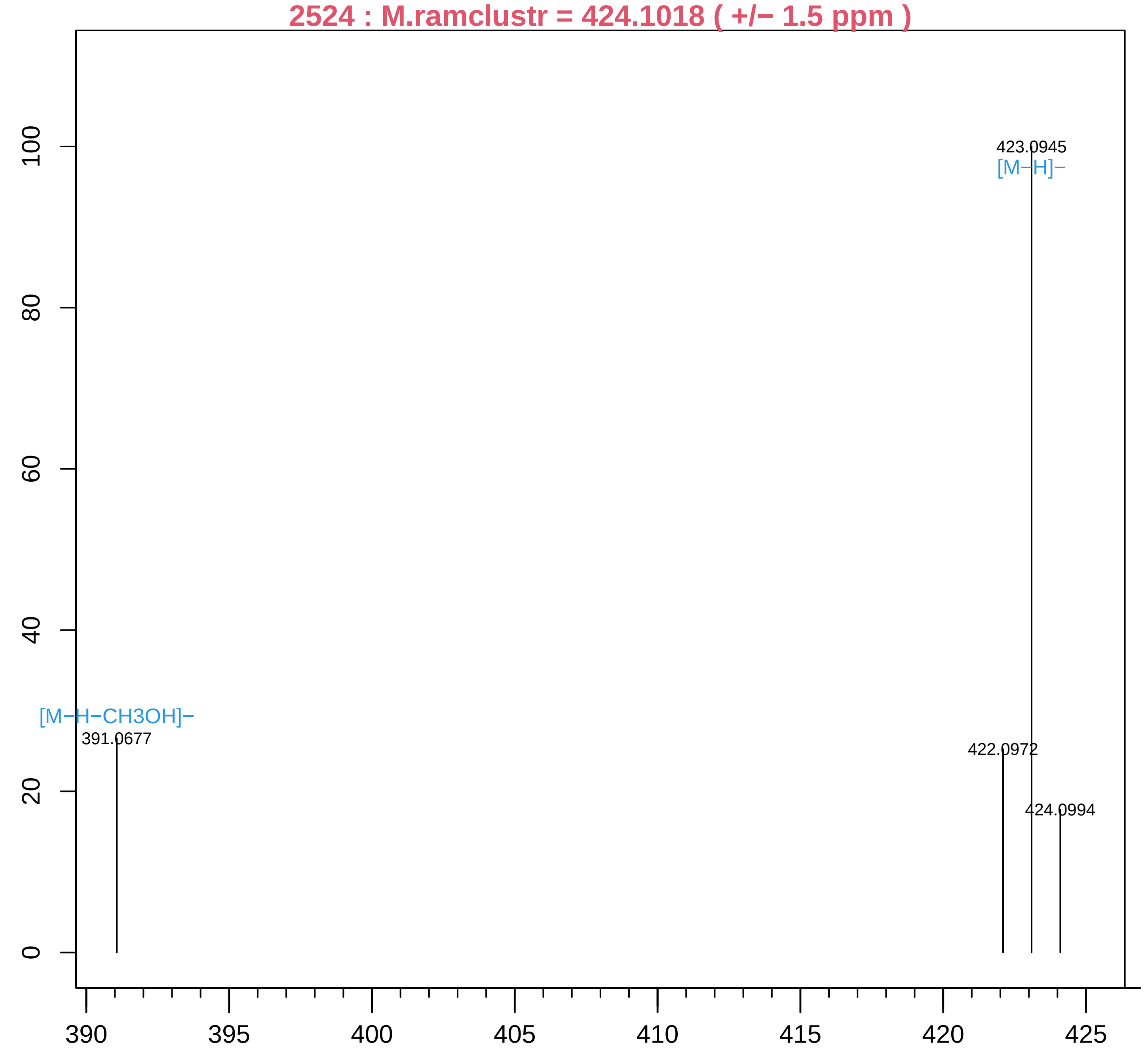

2524 : M.ramclustr = 424.1018 (+/− 1.5 ppm)
423.0945
[M−H]−
[M−H−CH3OH]−
391.0677
422.0972
424.0994

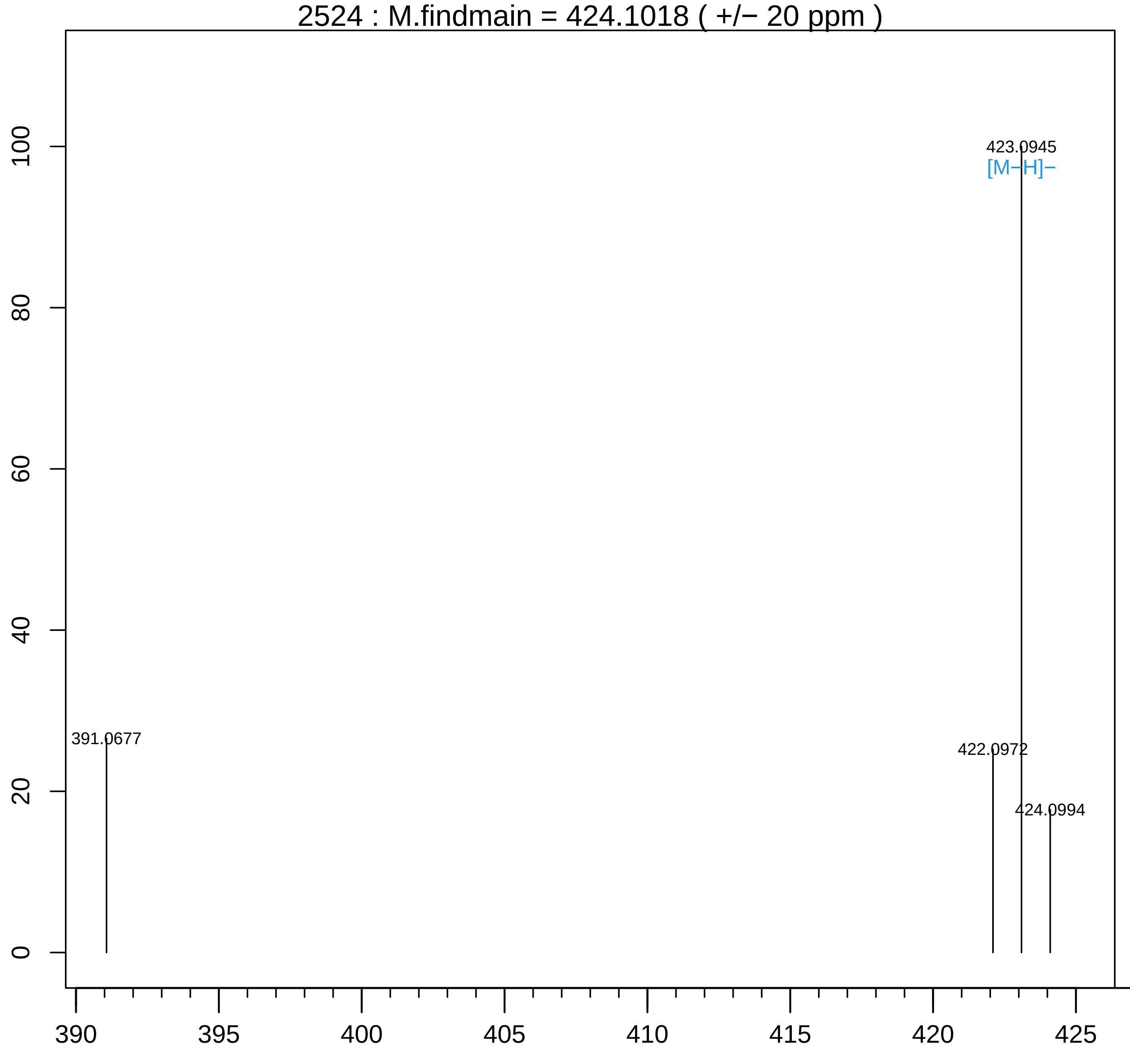

2524 : M.findmain = 424.1018 (+/− 20 ppm)
423.0945
[M−H]−
391.0677
422.0972
424.0994

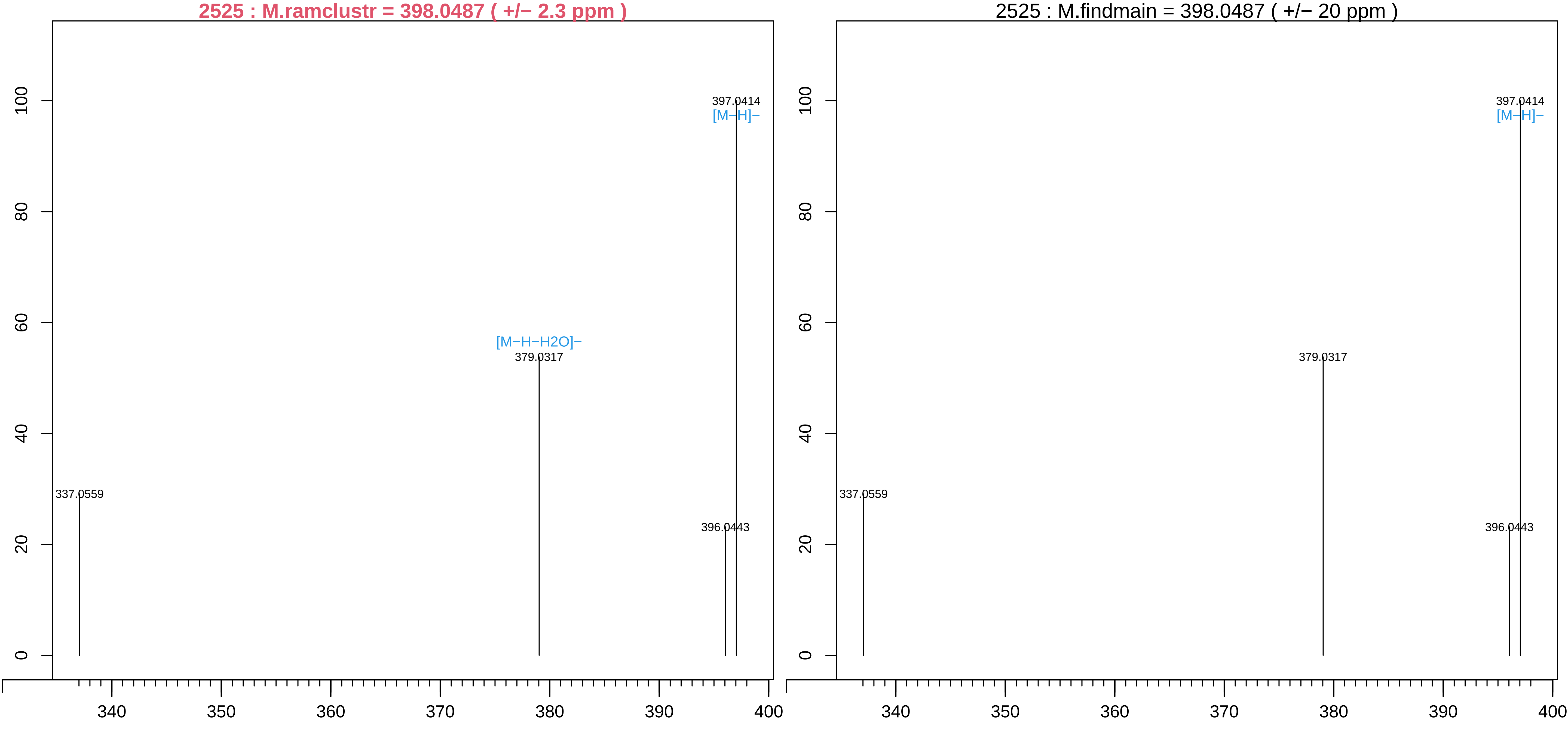

2525 : M.ramclustr = 398.0487 (+/− 2.3 ppm)
397.0414
[M−H]−
[M−H−H2O]−
379.0317
337.0559
396.0443
2525 : M.findmain = 398.0487 (+/− 20 ppm)
397.0414
[M−H]−
379.0317
337.0559
396.0443
0
20
40
60
80
100
340
350
360
370
380
390
400

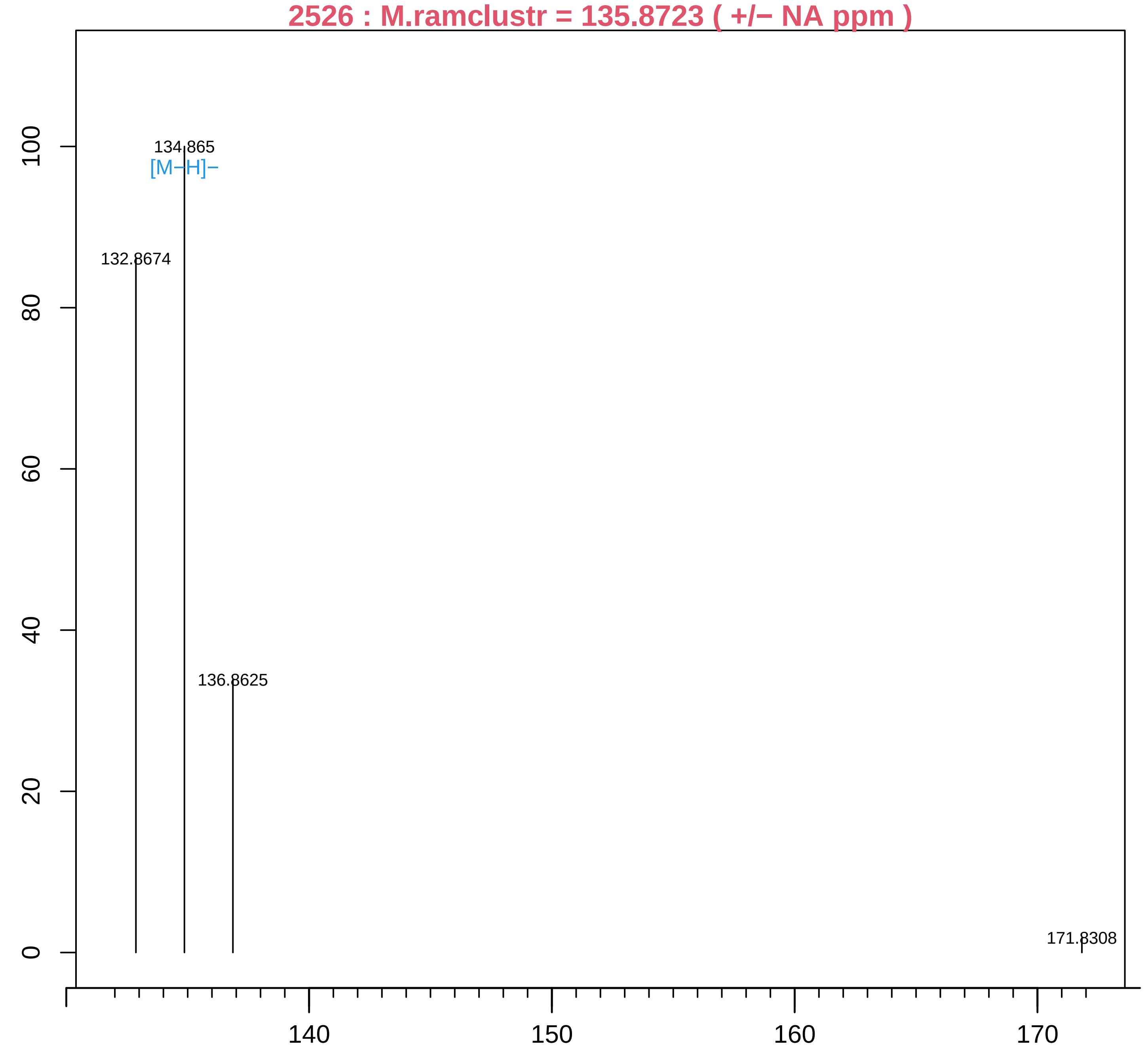

2526 : M.ramclustr = 135.8723 (+/− NA ppm)
134.865
[M−H]−
132.8674
136.8625
171.8308
0
20
40
60
80
100
140
150
160
170

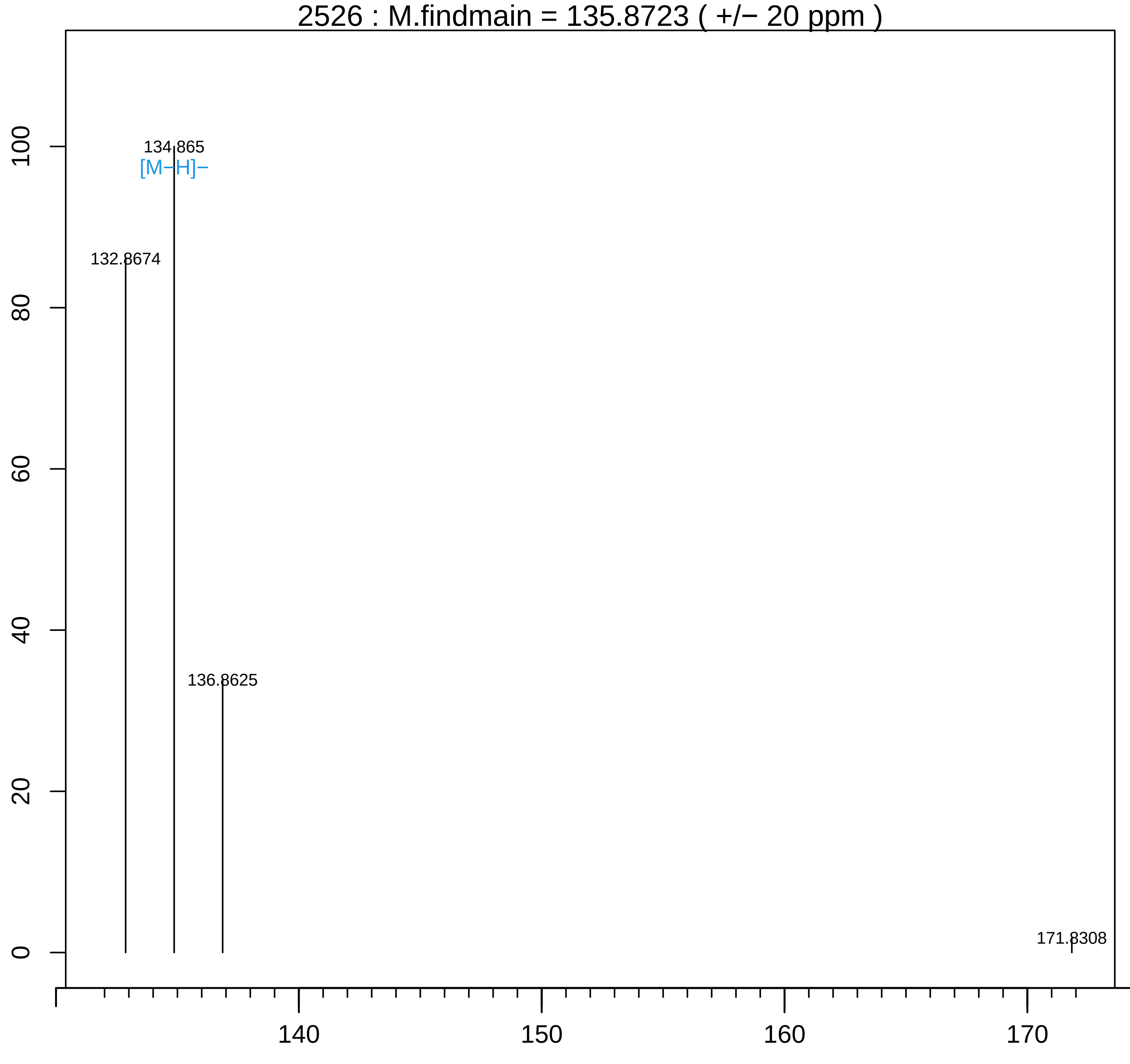

2526 : M.findmain = 135.8723 (+/− 20 ppm)
134.865
[M−H]−
132.8674
136.8625
171.8308
0
20
40
60
80
100
140
150
160
170

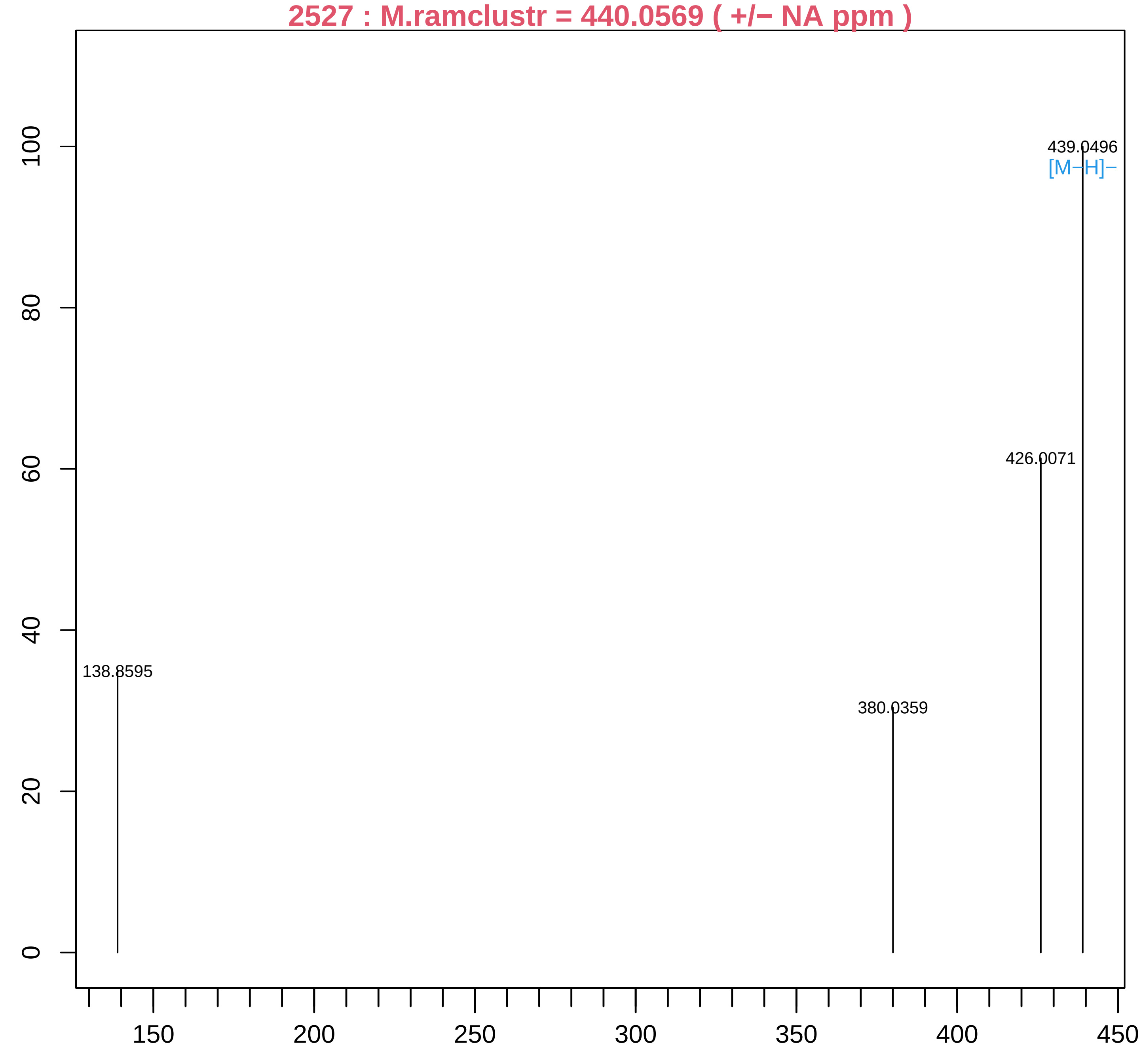
2527 : M.ramclustr = 440.0569 (+/− NA ppm)
439.0496
[M−H]−
426.0071
380.0359
138.8595
0
20
40
60
80
100
150
200
250
300
350
400
450

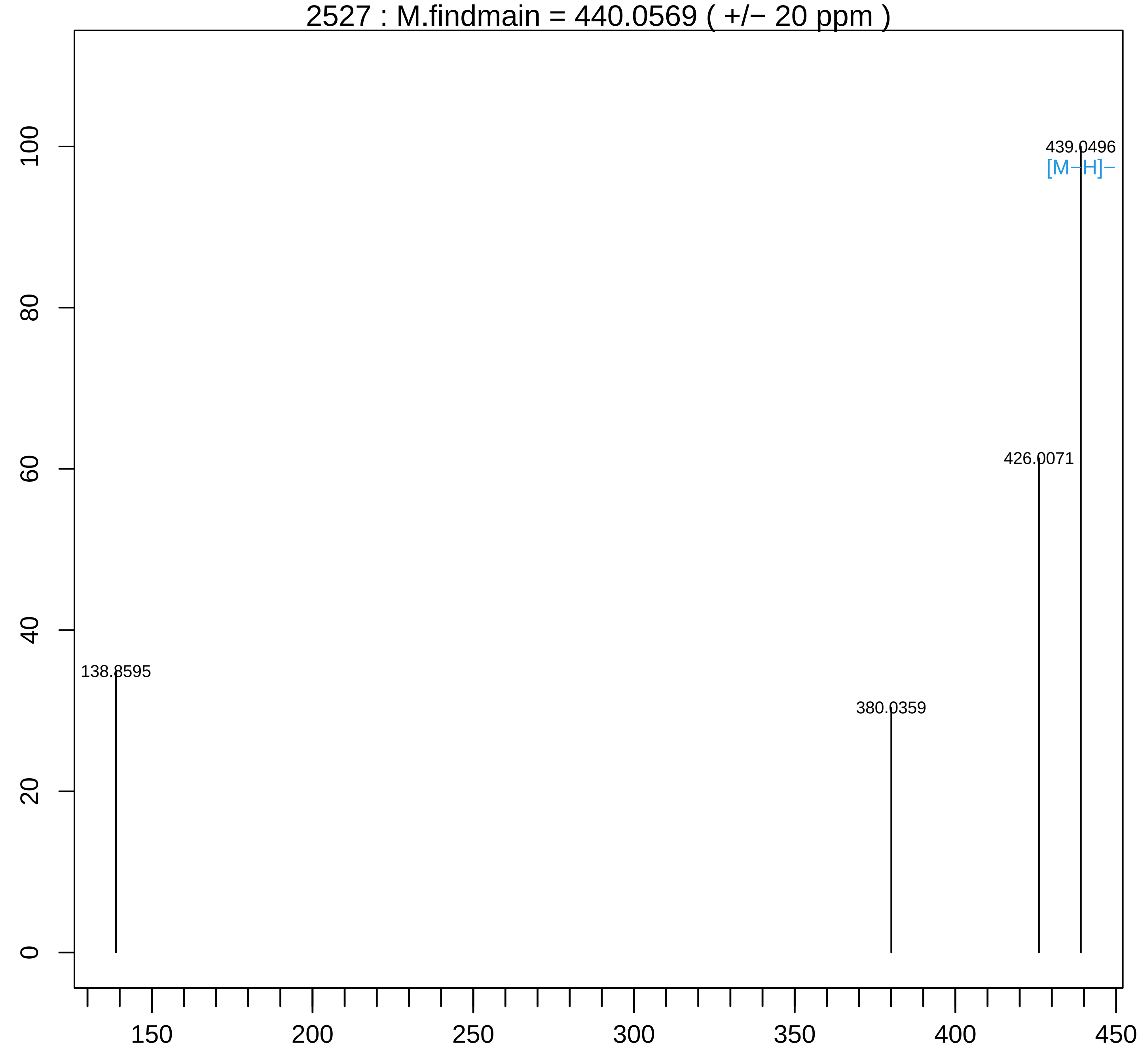
2527 : M.findmain = 440.0569 (+/− 20 ppm)
439.0496
[M−H]−
426.0071
380.0359
138.8595
0
20
40
60
80
100
150
200
250
300
350
400
450

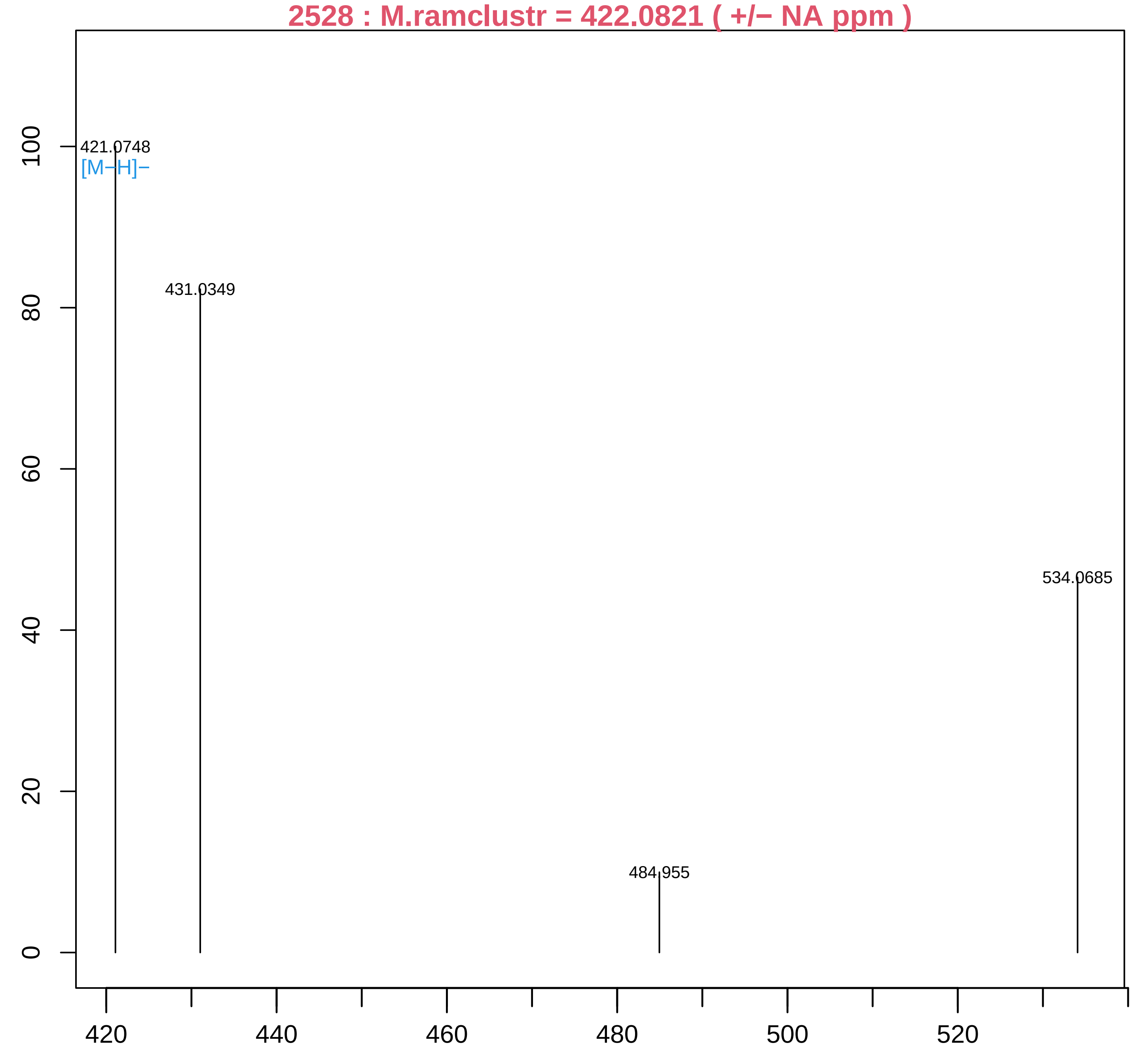

2528 : M.ramclustr = 422.0821 (+/− NA ppm)
421.0748
[M−H]−
431.0349
534.0685
484.955
0
20
40
60
80
100
420
440
460
480
500
520

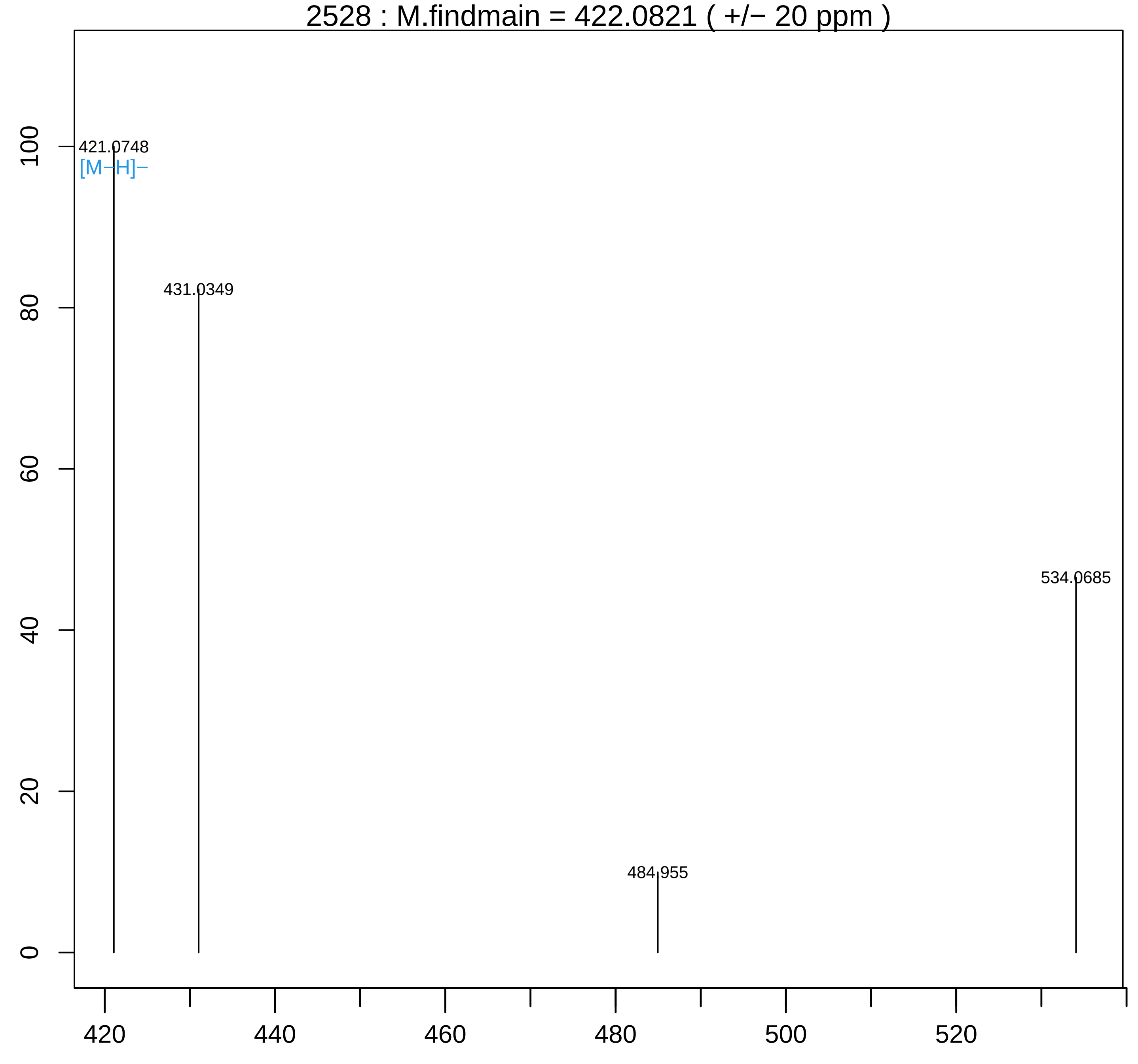

2528 : M.findmain = 422.0821 (+/− 20 ppm)
421.0748
[M−H]−
431.0349
534.0685
484.955
0
20
40
60
80
100
420
440
460
480
500
520

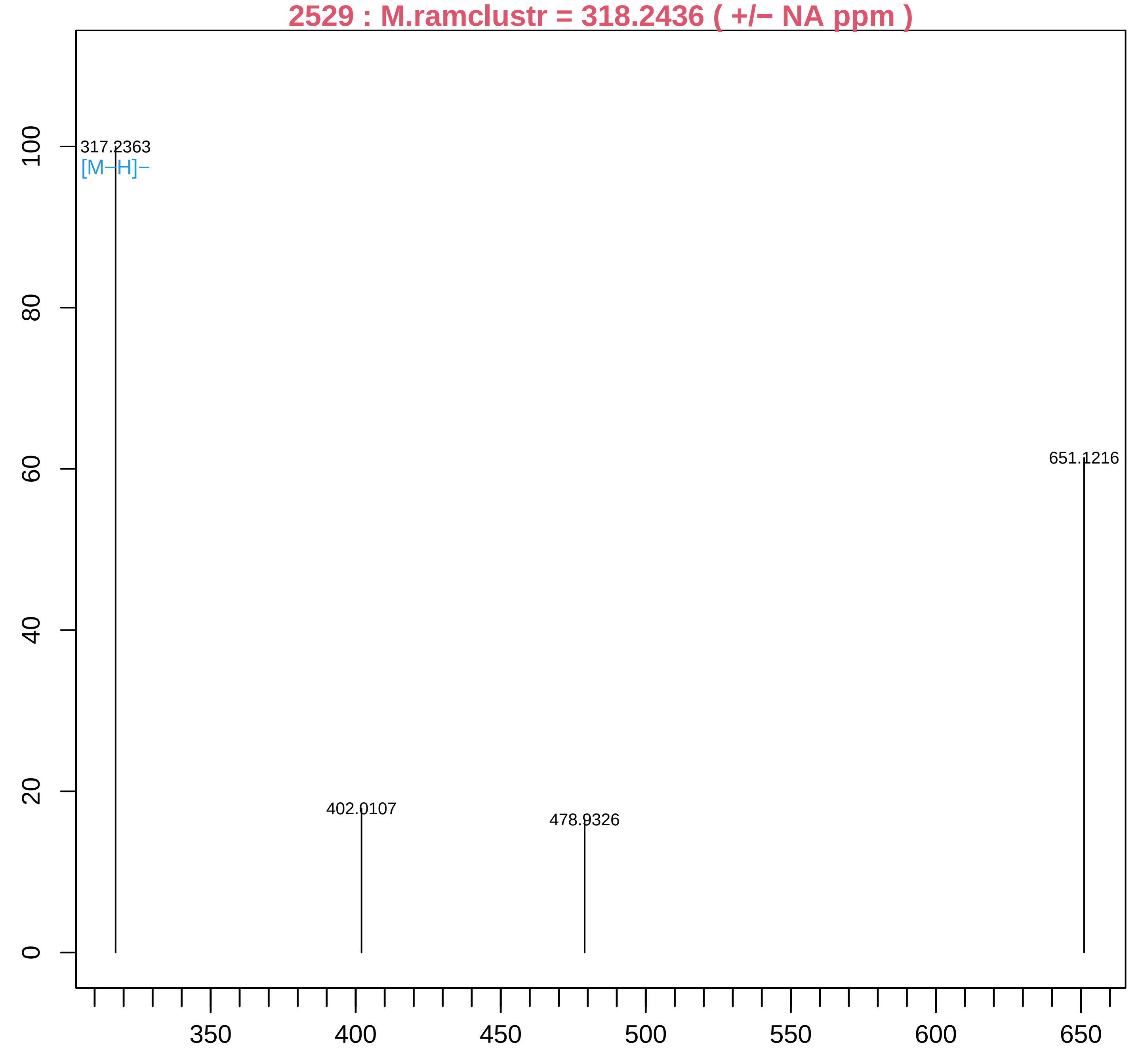

2529 : M.ramclustr = 318.2436 (+/− NA ppm)
317.2363
[M−H]−
402.0107
478.9326
651.1216
0
20
40
60
80
100
350
400
450
500
550
600
650

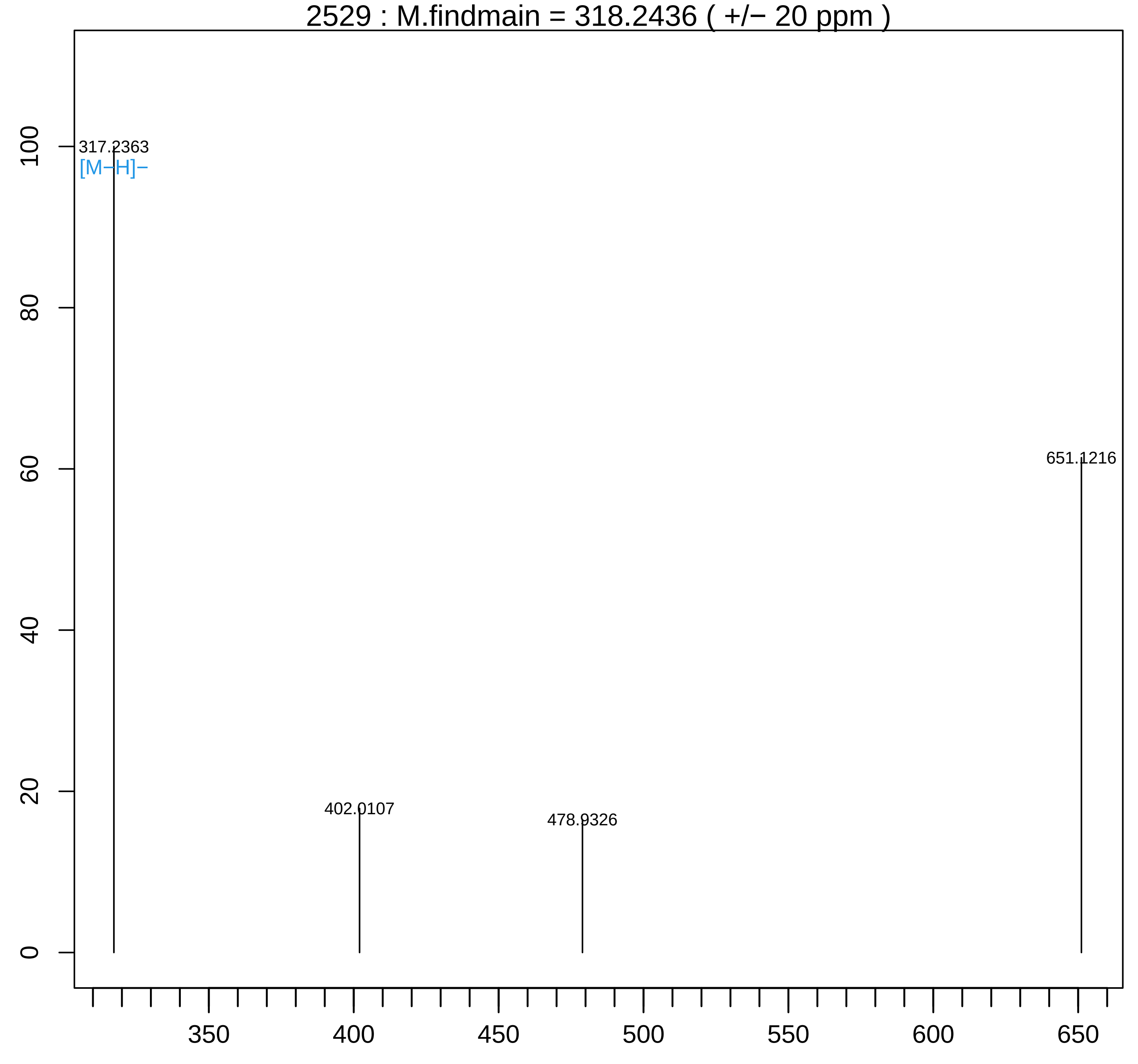

2529 : M.findmain = 318.2436 (+/− 20 ppm)
317.2363
[M−H]−
402.0107
478.9326
651.1216
0
20
40
60
80
100
350
400
450
500
550
600
650

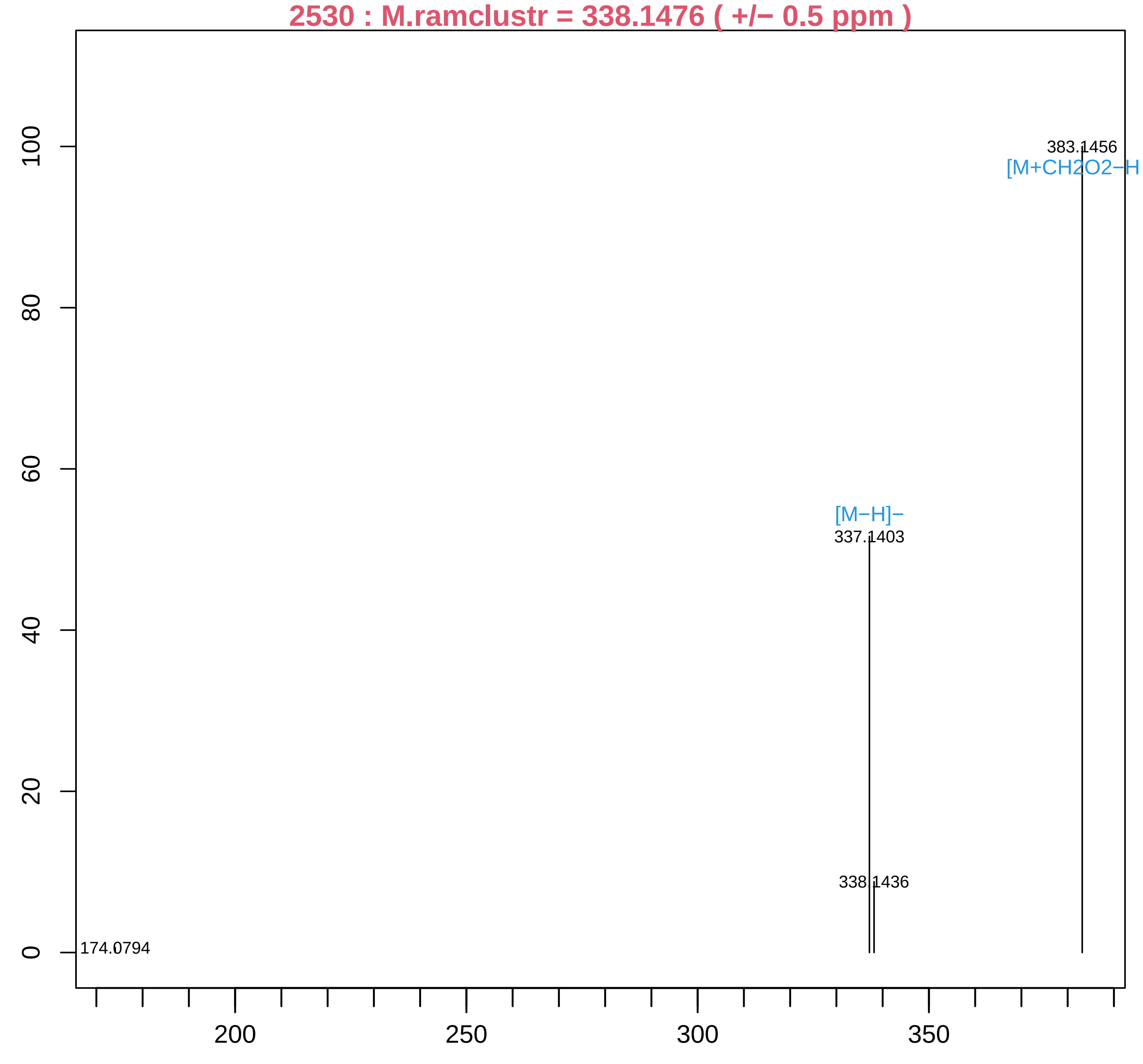
2530 : M.ramclustr = 338.1476 (+/− 0.5 ppm)
383.1456
[M+CH2O2−H
[M−H]−
337.1403
338.1436
174.0794
0
20
40
60
80
100
200
250
300
350

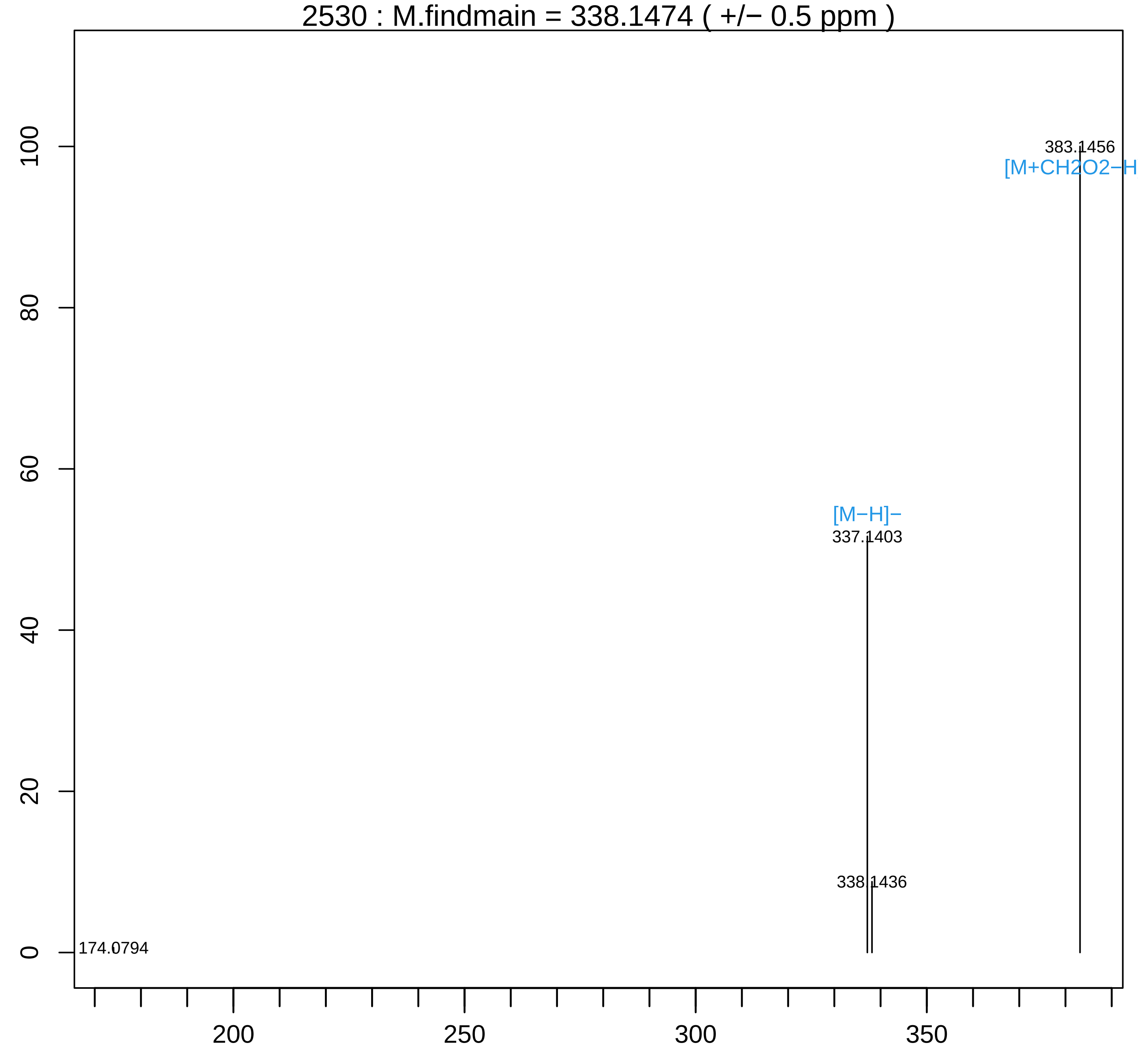
2530 : M.findmain = 338.1474 (+/− 0.5 ppm)
383.1456
[M+CH2O2−H
[M−H]−
337.1403
338.1436
174.0794
0
20
40
60
80
100
200
250
300
350

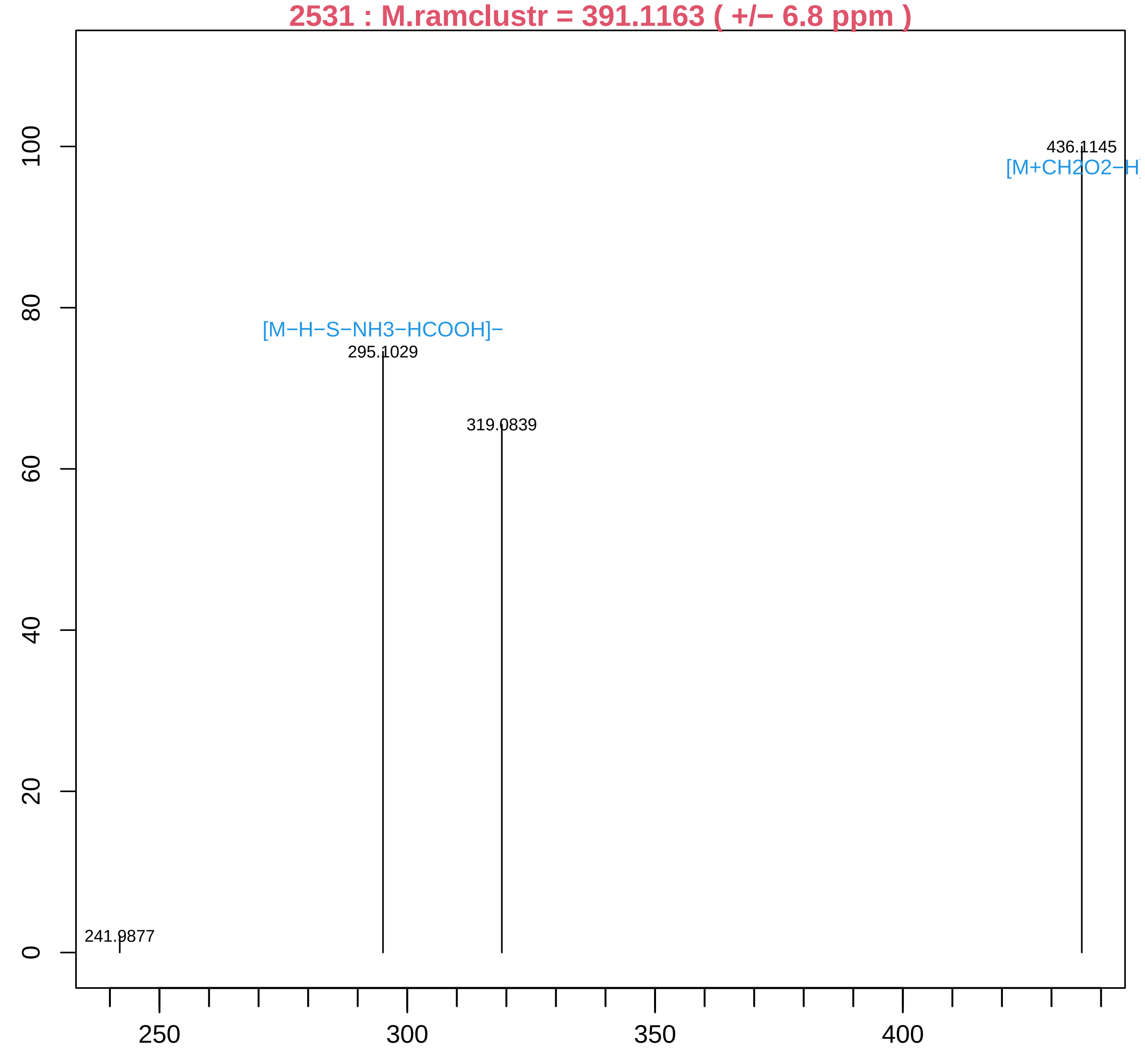
2531 : M.ramclustr = 391.1163 (+/− 6.8 ppm)
436.1145
[M+CH2O2−H
[M−H−S−NH3−HCOOH]−
295.1029
319.0839
241.9877
0
20
40
60
80
100
250
300
350
400

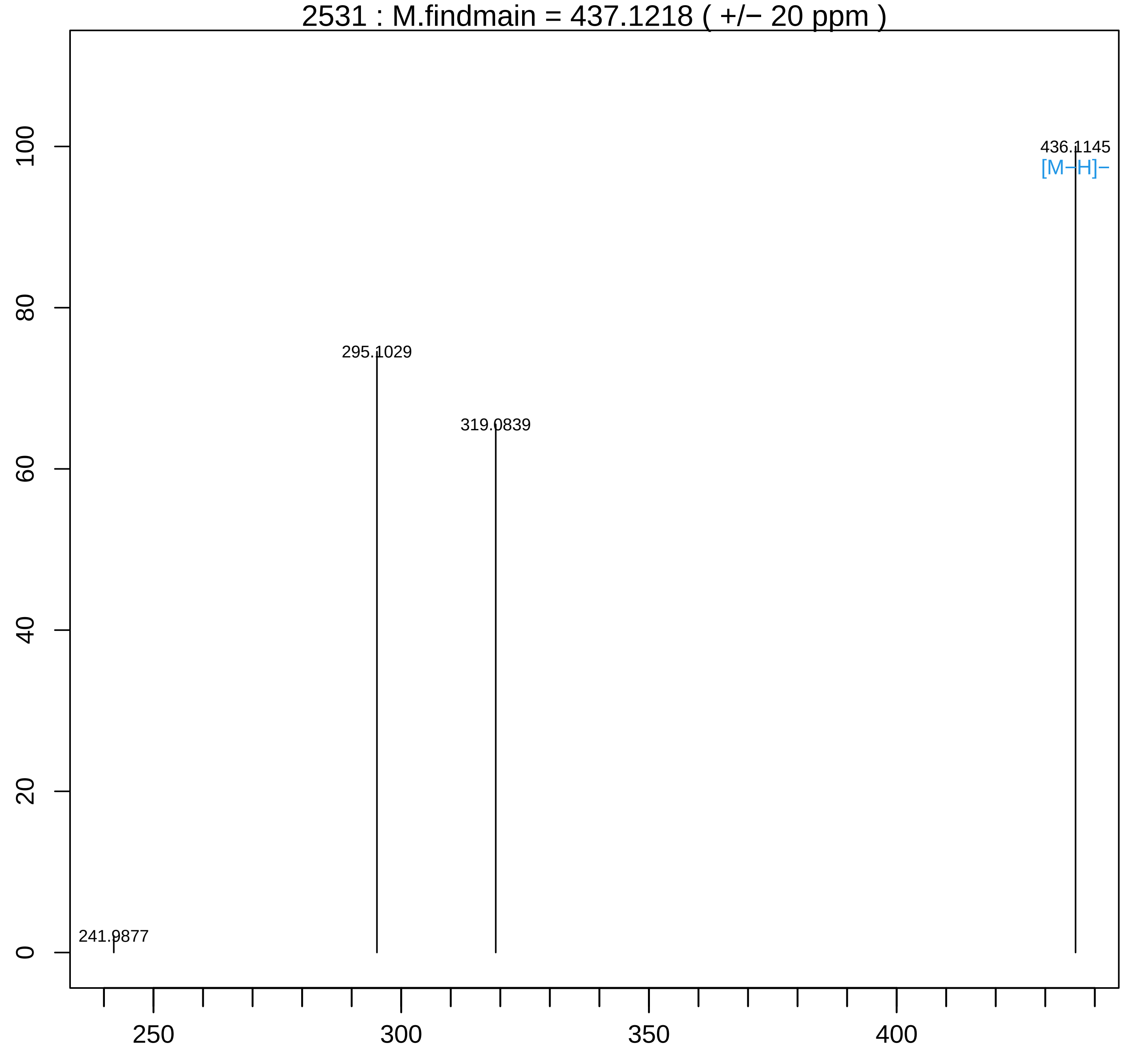
2531 : M.findmain = 437.1218 (+/− 20 ppm)
436.1145
[M−H]−
295.1029
319.0839
241.9877
0
20
40
60
80
100
250
300
350
400

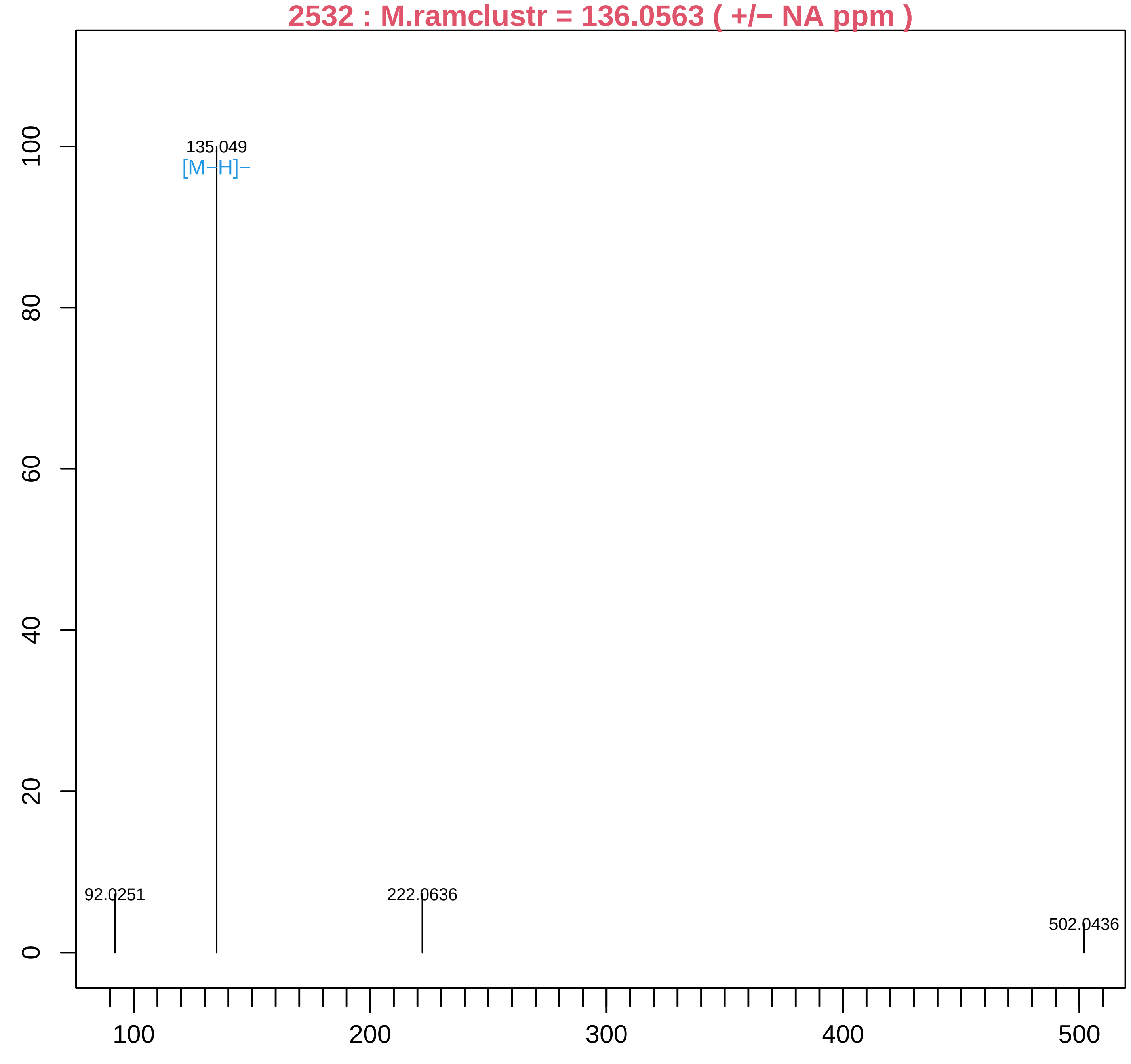
2532 : M.ramclustr = 136.0563 (+/− NA ppm)
135.049
[M−H]−
92.0251
222.0636
502.0436
0
20
40
60
80
100
100
200
300
400
500

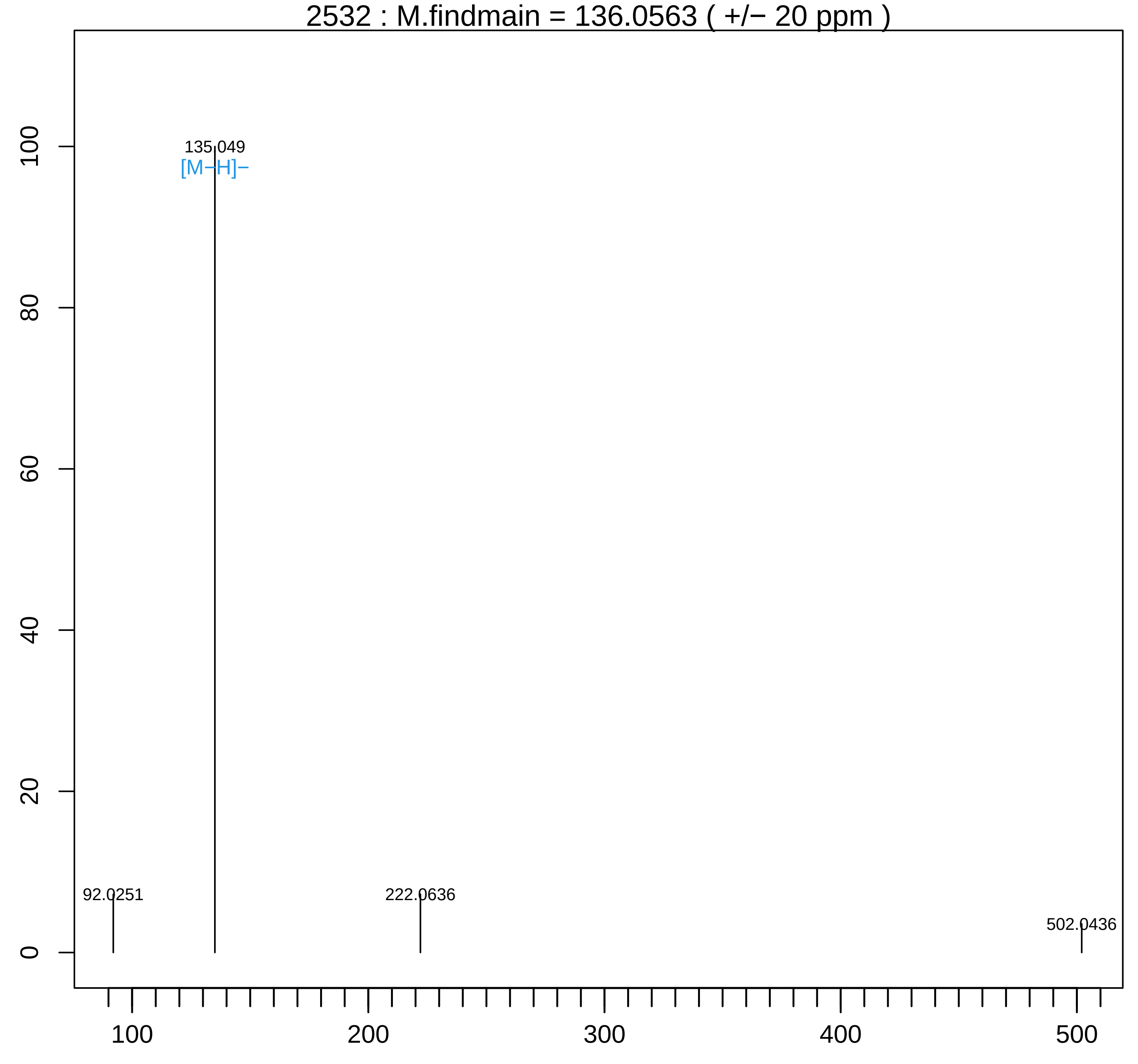
2532 : M.findmain = 136.0563 (+/− 20 ppm)
135.049
[M−H]−
92.0251
222.0636
502.0436
0
20
40
60
80
100
100
200
300
400
500

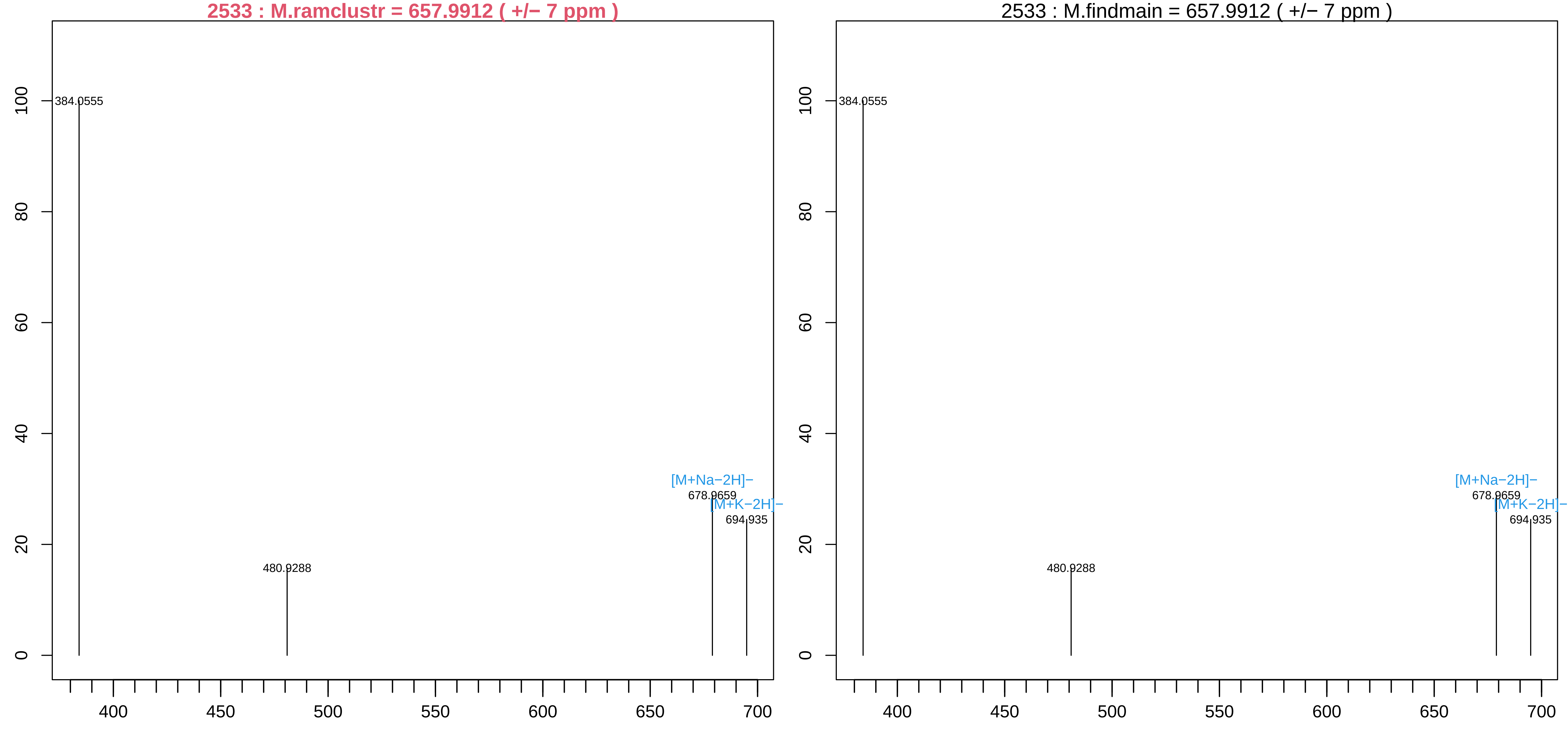

2533 : M.ramclustr = 657.9912 (+/− 7 ppm)
384.0555
480.9288
[M+Na−2H]−
678.9659
[M+K−2H]−
694.935
2533 : M.findmain = 657.9912 (+/− 7 ppm)
384.0555
480.9288
[M+Na−2H]−
678.9659
[M+K−2H]−
694.935

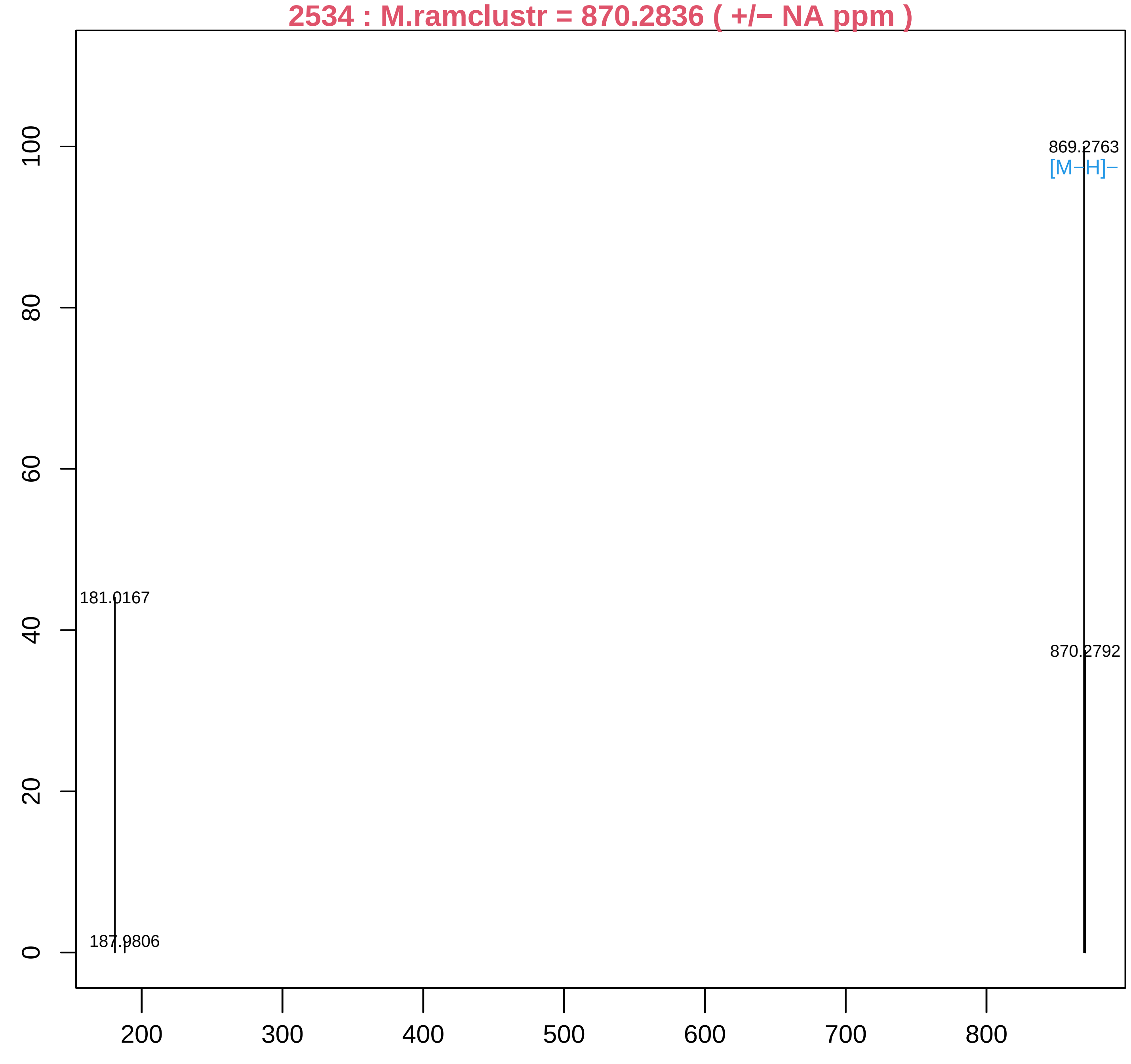
2534 : M.ramclustr = 870.2836 (+/− NA ppm)
869.2763
[M−H]−
181.0167
870.2792
187.9806
0
20
40
60
80
100
200
300
400
500
600
700
800

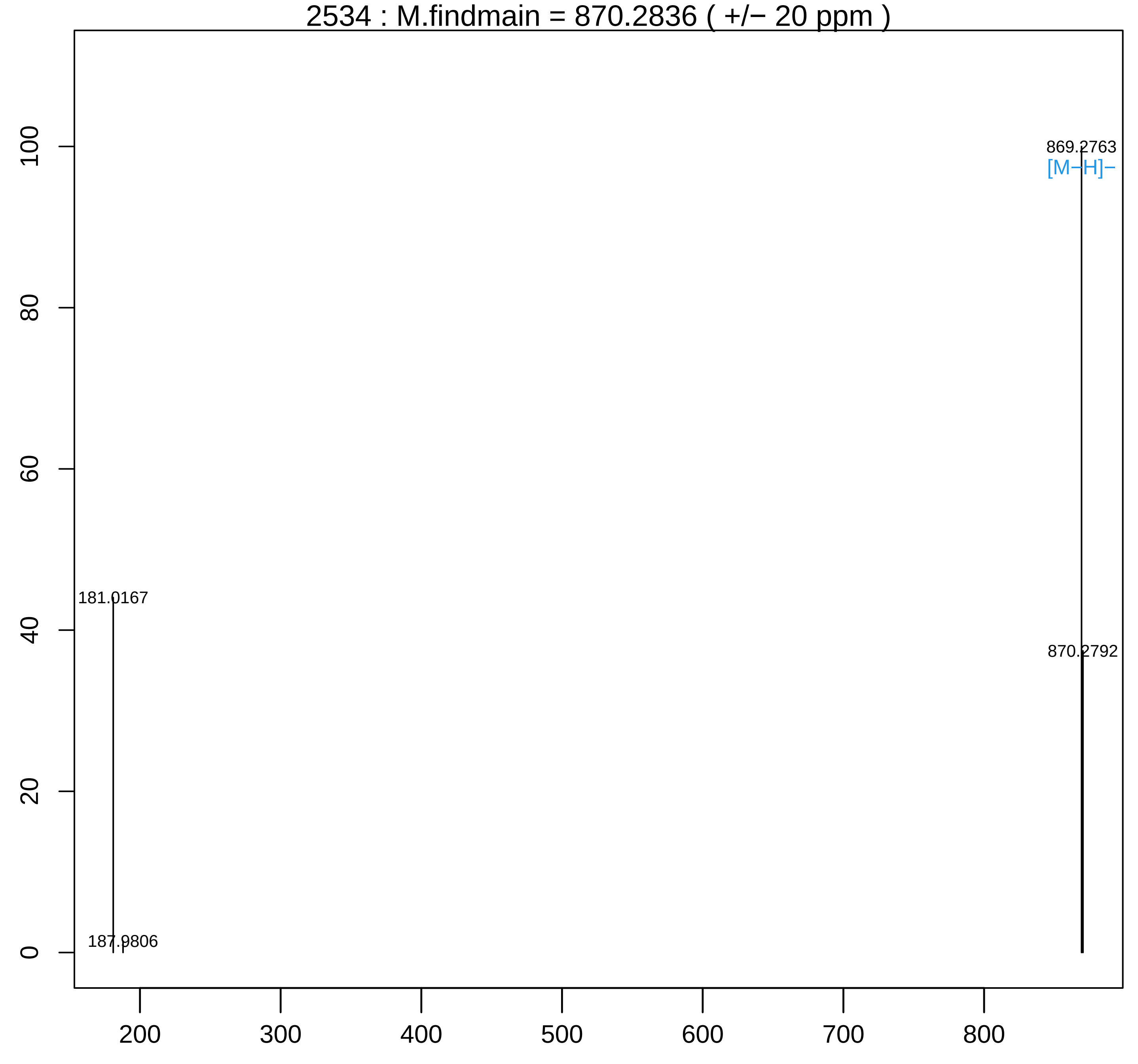
2534 : M.findmain = 870.2836 (+/− 20 ppm)
869.2763
[M−H]−
181.0167
870.2792
187.9806
0
20
40
60
80
100
200
300
400
500
600
700
800

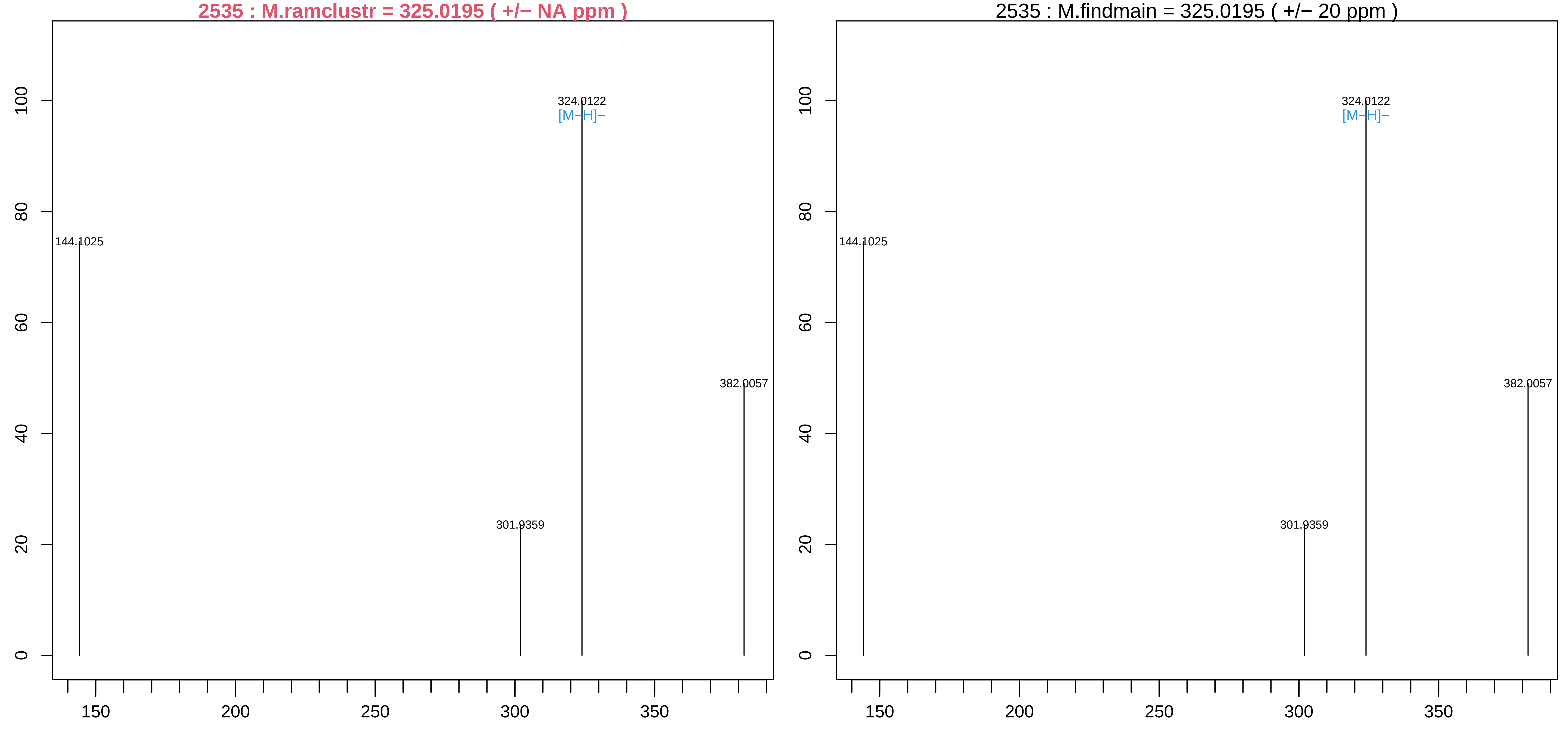
2535 : M.ramclustr = 325.0195 (+/− NA ppm)
2535 : M.findmain = 325.0195 (+/− 20 ppm)
144.1025
301.9359
324.0122
[M−H]−
382.0057
0
20
40
60
80
100
150
200
250
300
350

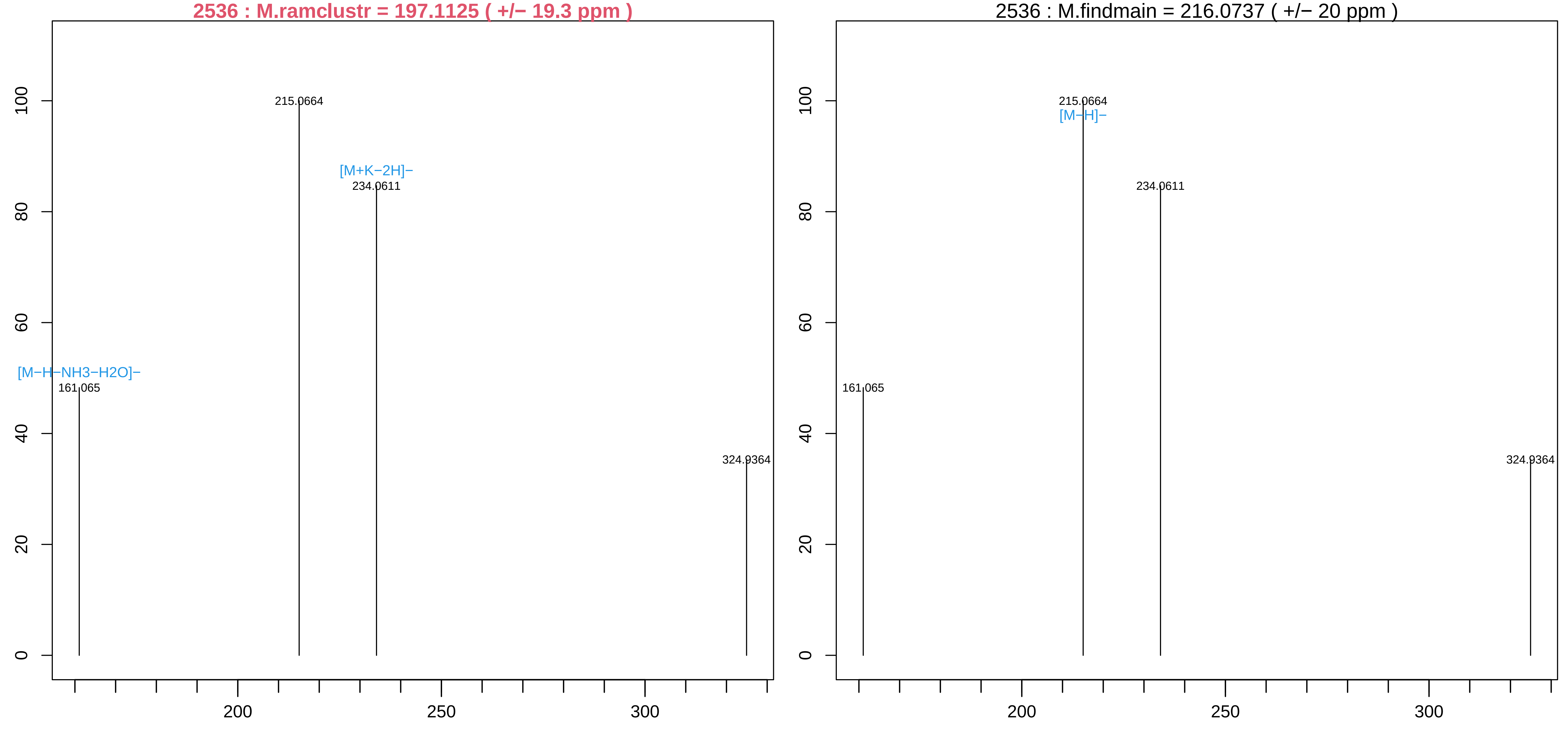
2536 : M.ramclustr = 197.1125 (+/− 19.3 ppm)
215.0664
[M+K−2H]−
234.0611
[M−H−NH3−H2O]−
161.065
324.9364
0
20
40
60
80
100
200
250
300
2536 : M.findmain = 216.0737 (+/− 20 ppm)
215.0664
[M−H]−
234.0611
161.065
324.9364
0
20
40
60
80
100
200
250
300

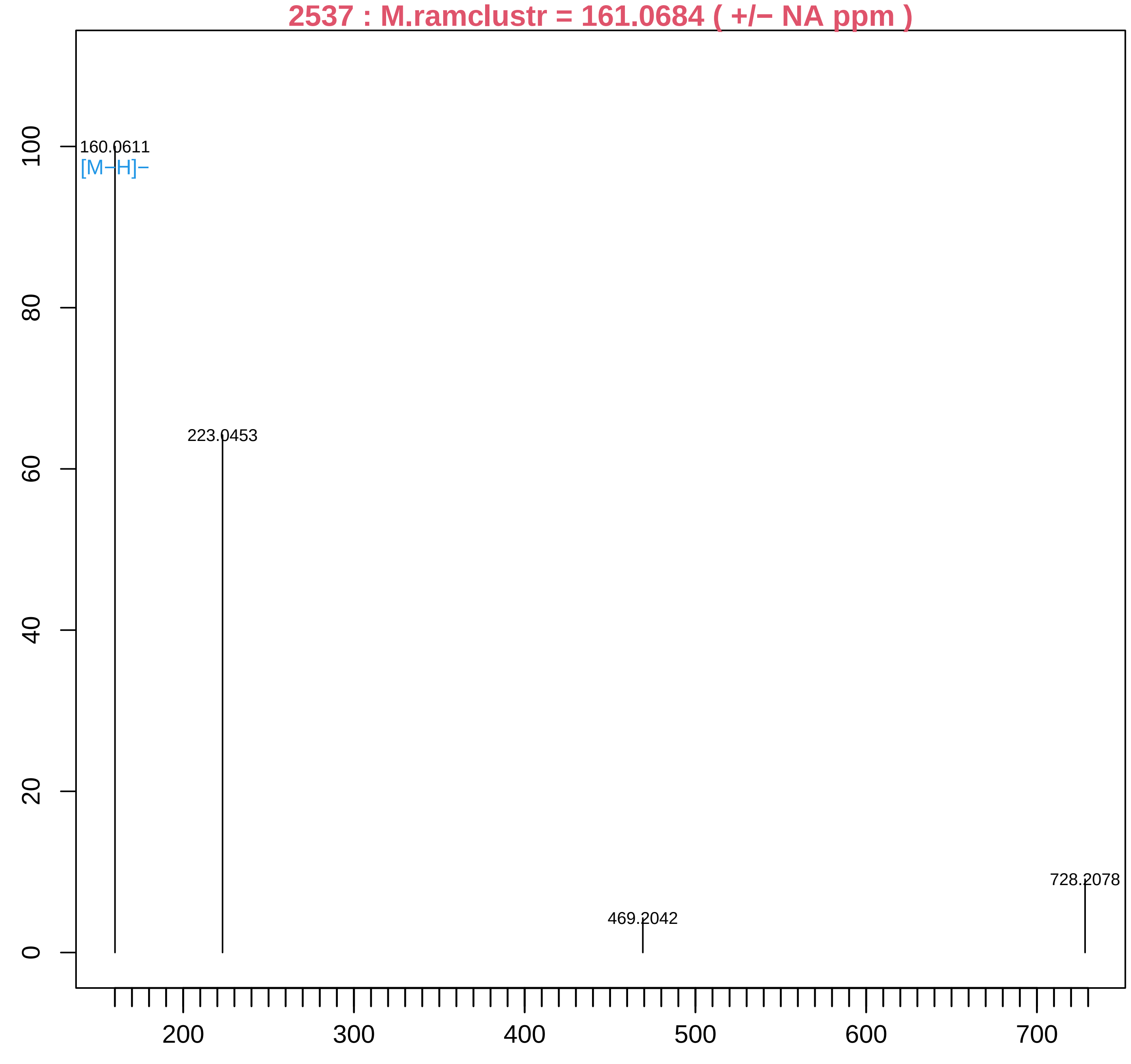
2537 : M.ramclustr = 161.0684 (+/− NA ppm)
160.0611
[M−H]−
223.0453
469.2042
728.2078
0
20
40
60
80
100
200
300
400
500
600
700
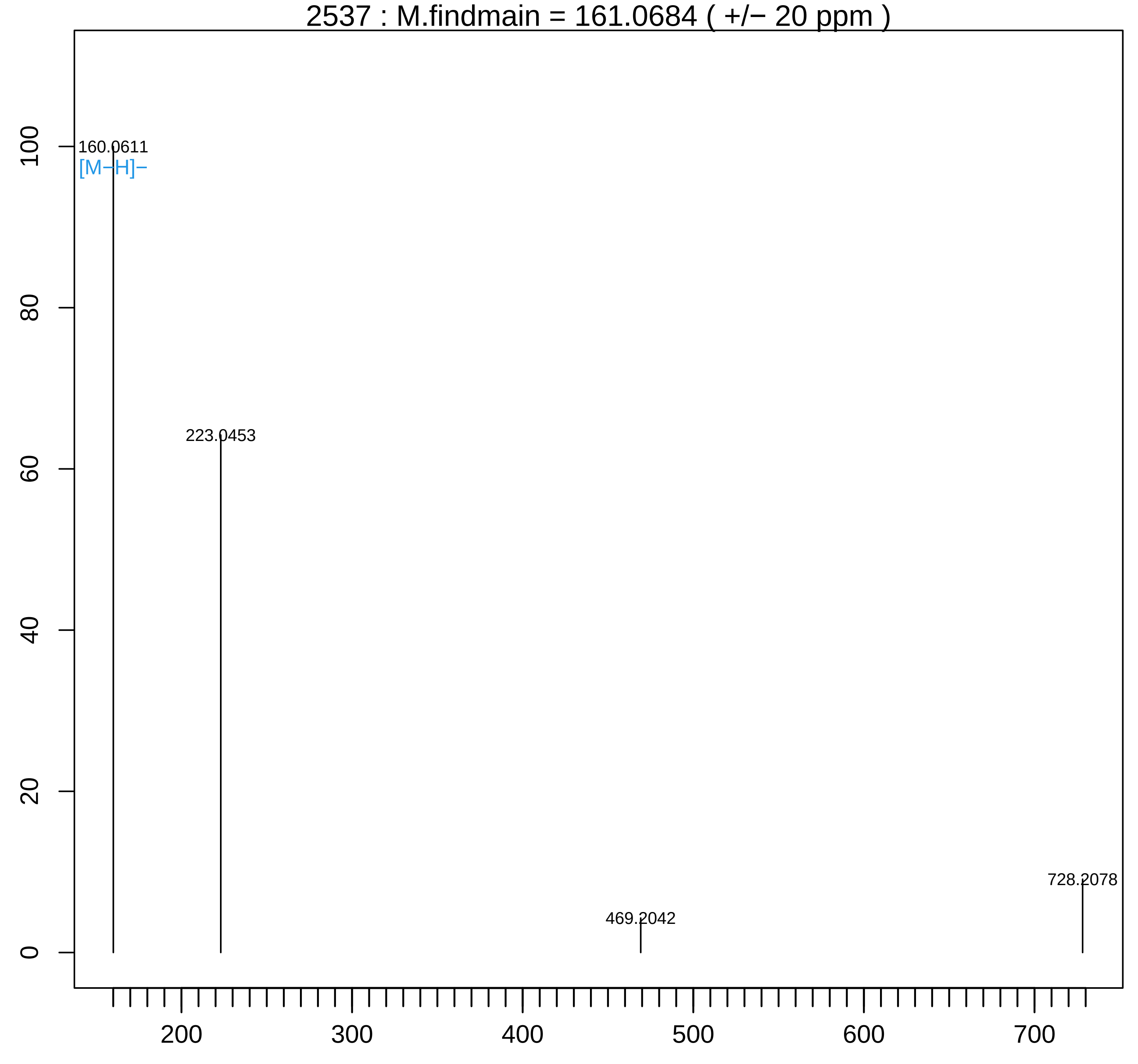
2537 : M.findmain = 161.0684 (+/− 20 ppm)
160.0611
[M−H]−
223.0453
469.2042
728.2078
0
20
40
60
80
100
200
300
400
500
600
700

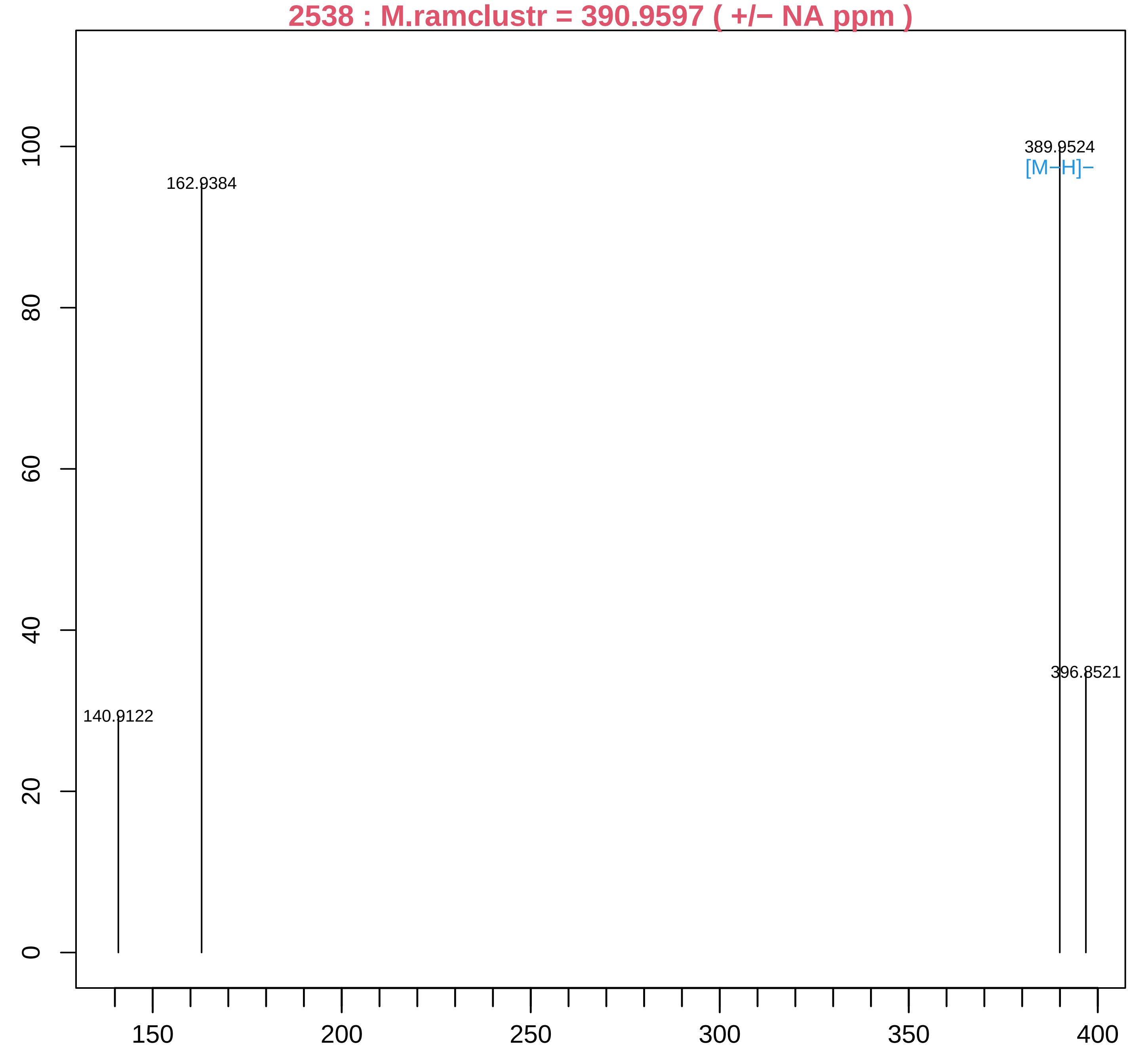
2538 : M.ramclustr = 390.9597 (+/− NA ppm)
140.9122
162.9384
389.9524
[M−H]−
396.8521
0
20
40
60
80
100
150
200
250
300
350
400

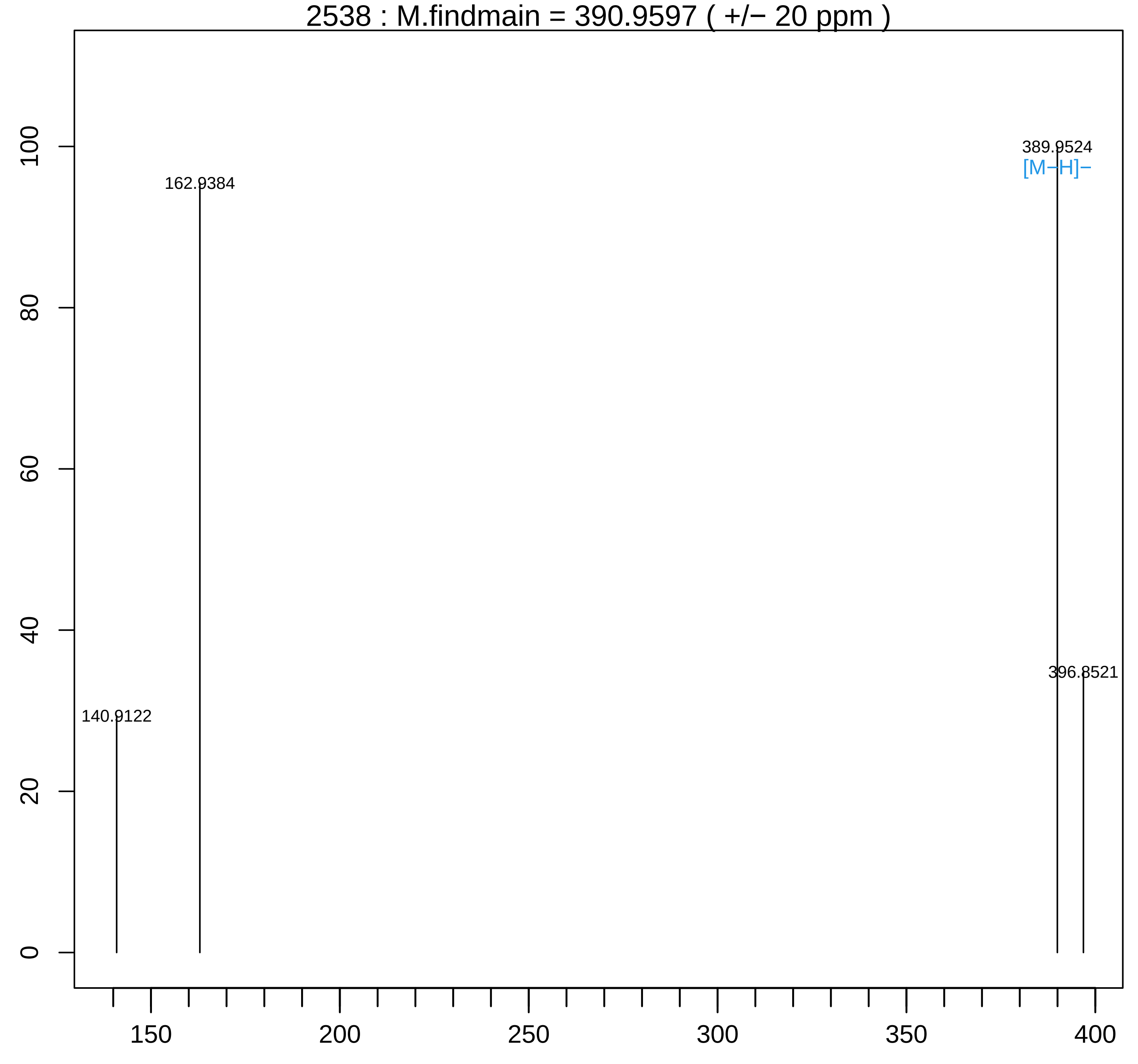
2538 : M.findmain = 390.9597 (+/− 20 ppm)
140.9122
162.9384
389.9524
[M−H]−
396.8521
0
20
40
60
80
100
150
200
250
300
350
400

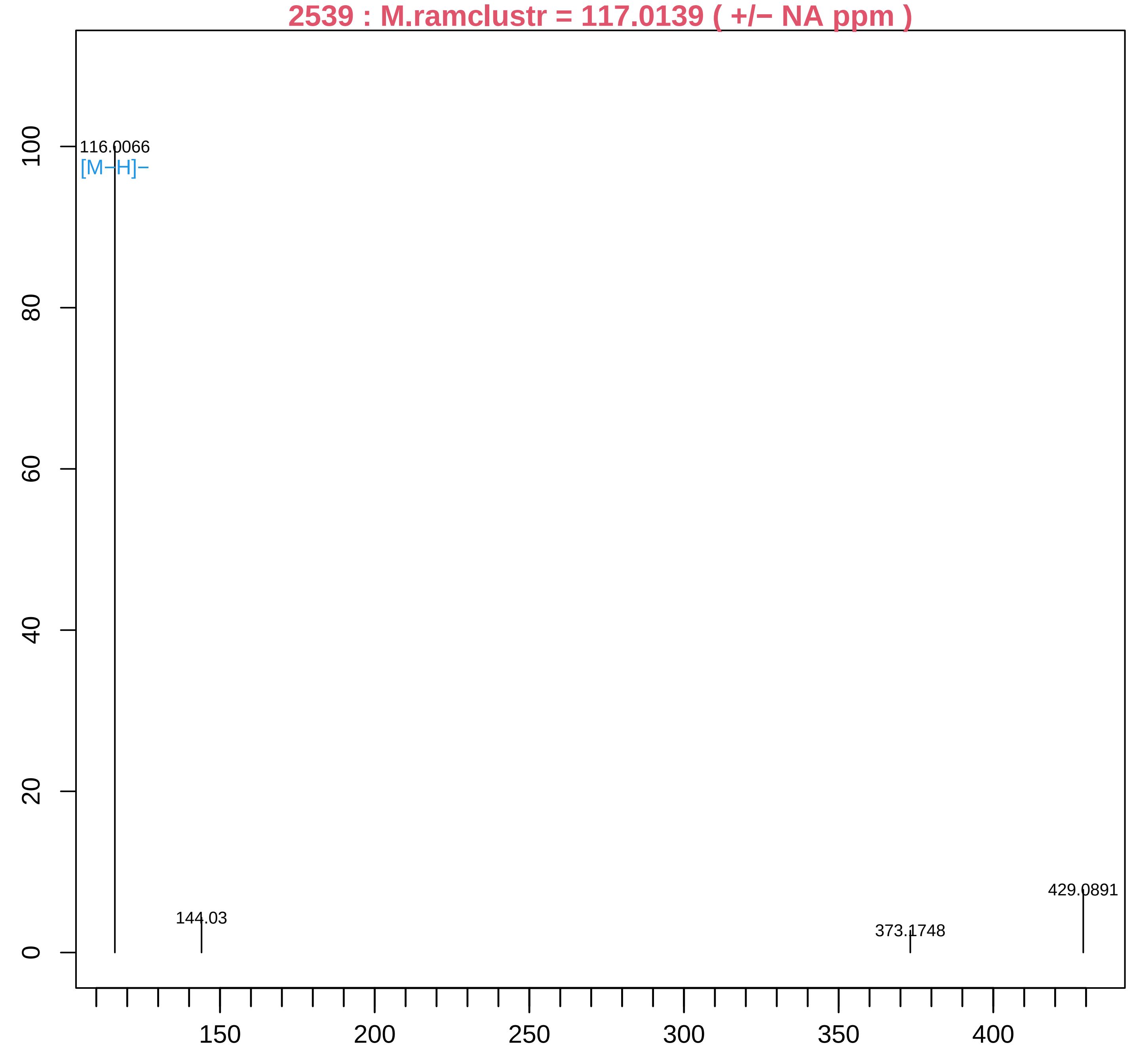

2539 : M.ramclustr = 117.0139 (+/− NA ppm)
116.0066
[M−H]−
144.03
373.1748
429.0891
0
20
40
60
80
100
150
200
250
300
350
400

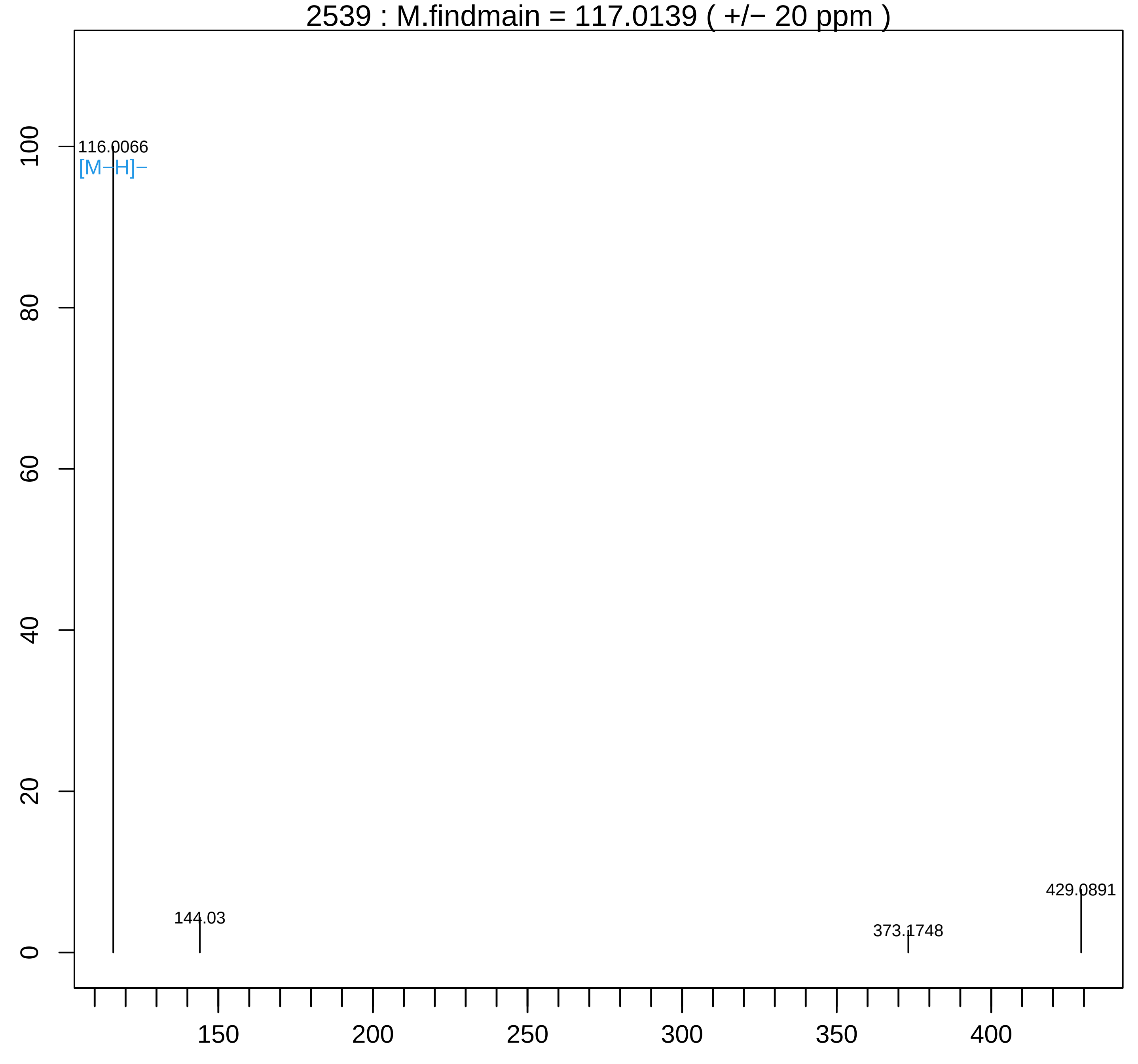

2539 : M.findmain = 117.0139 (+/− 20 ppm)
116.0066
[M−H]−
144.03
373.1748
429.0891
0
20
40
60
80
100
150
200
250
300
350
400

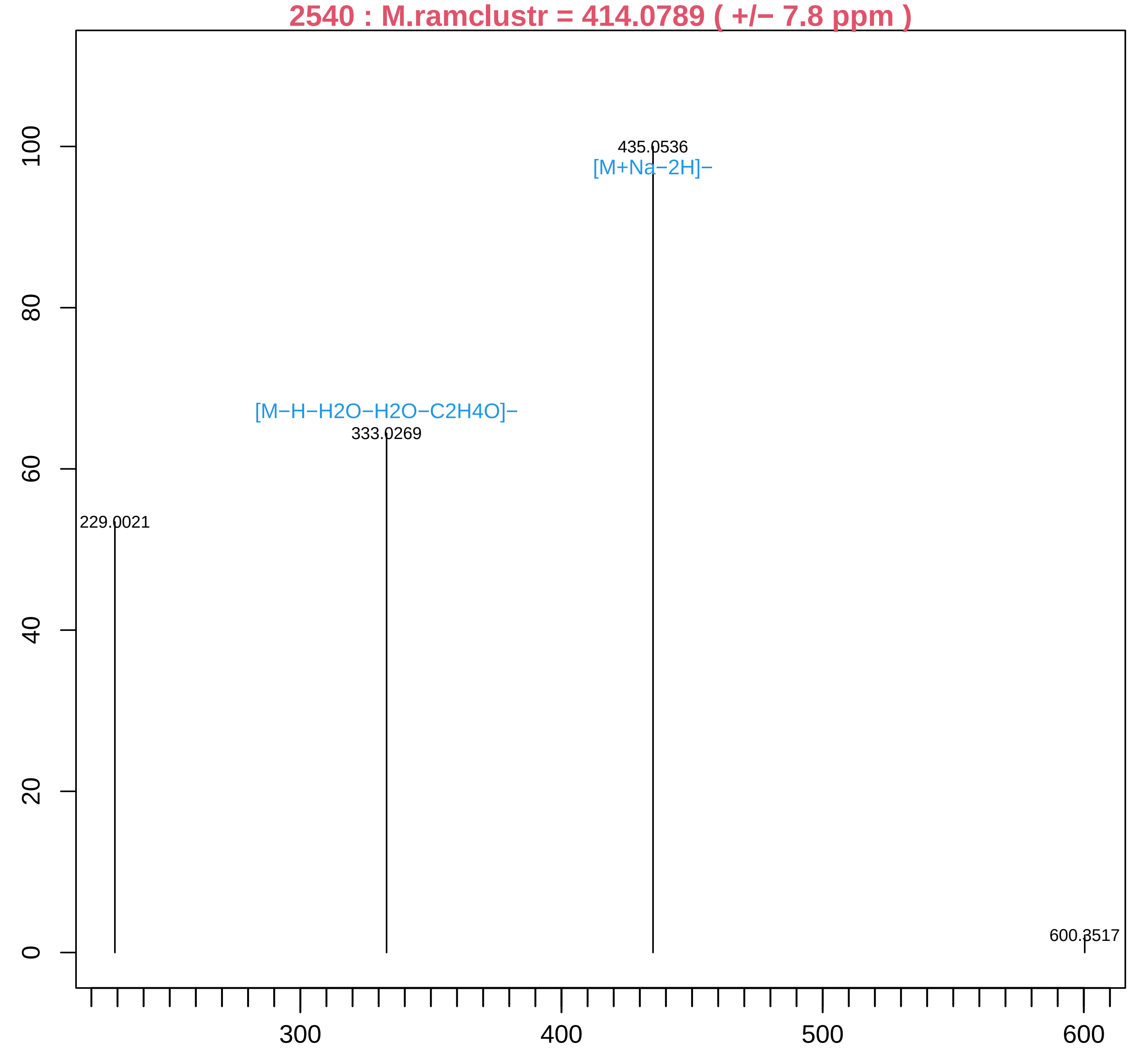
2540 : M.ramclustr = 414.0789 (+/− 7.8 ppm)
435.0536
[M+Na−2H]−
[M−H−H2O−H2O−C2H4O]−
333.0269
229.0021
600.3517
0
20
40
60
80
100
300
400
500
600

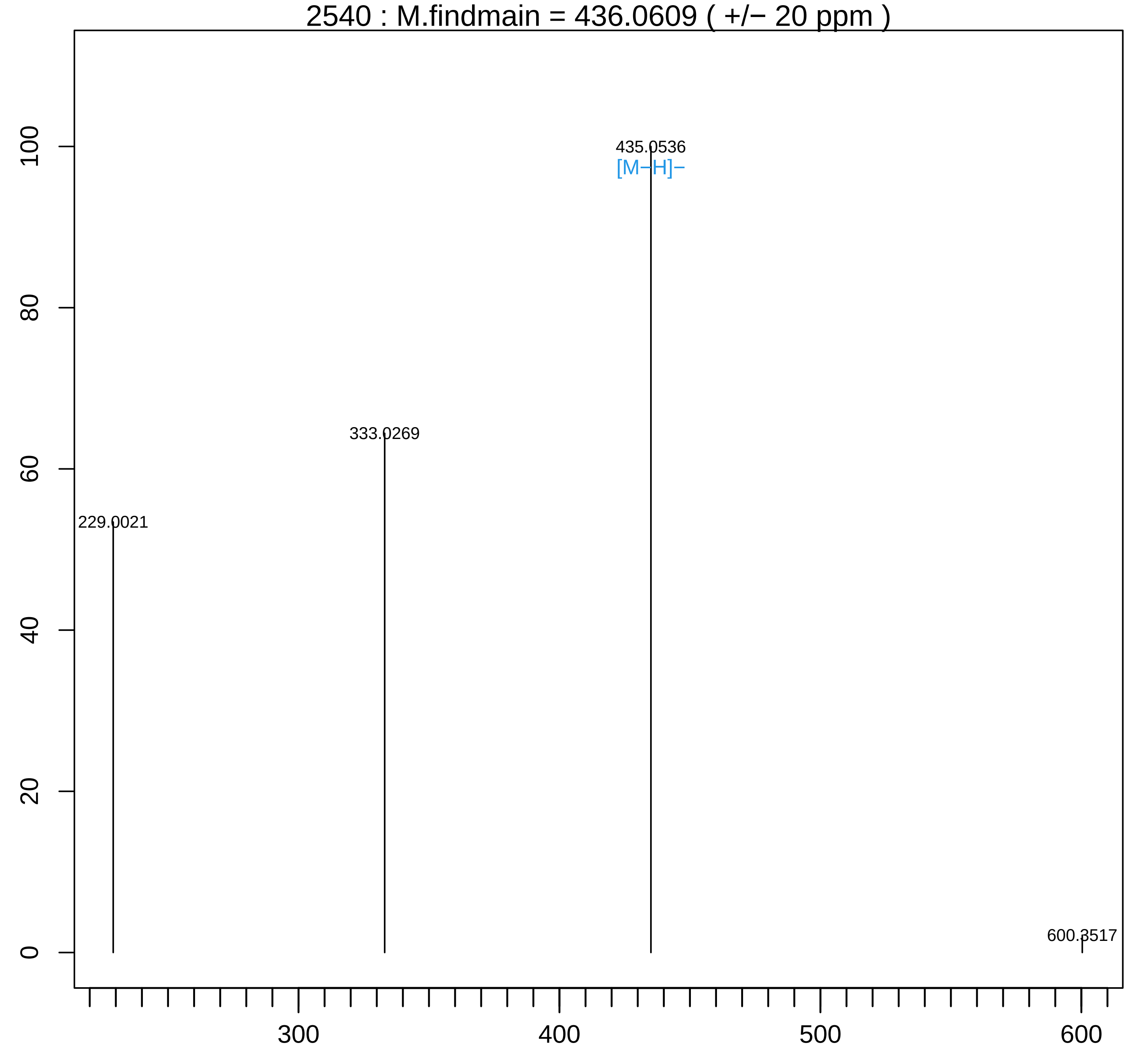
2540 : M.findmain = 436.0609 (+/− 20 ppm)
435.0536
[M−H]−
333.0269
229.0021
600.3517
0
20
40
60
80
100
300
400
500
600

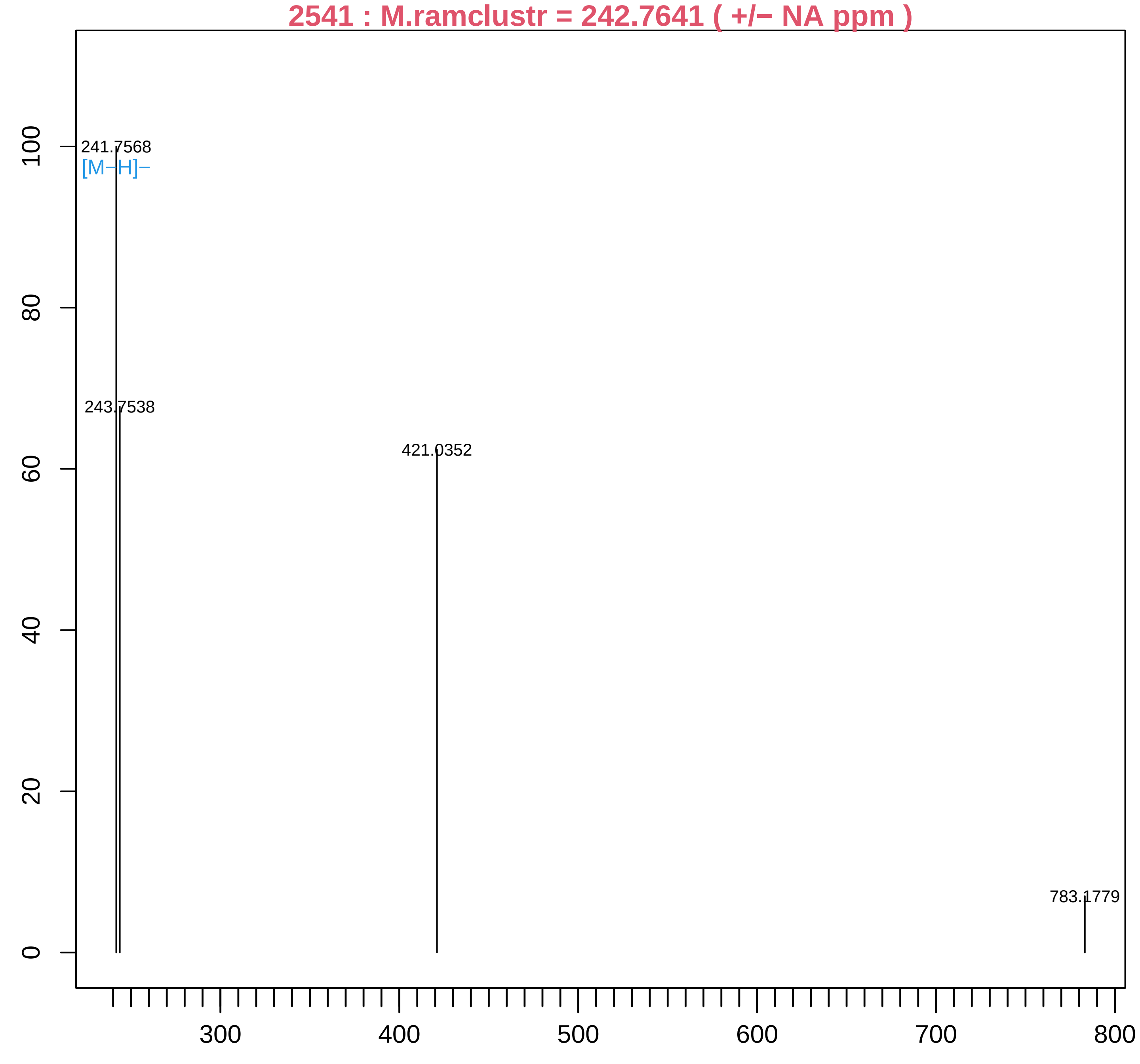

2541 : M.ramclustr = 242.7641 (+/− NA ppm)
241.7568
[M−H]−
243.7538
421.0352
783.1779
0
20
40
60
80
100
300
400
500
600
700
800

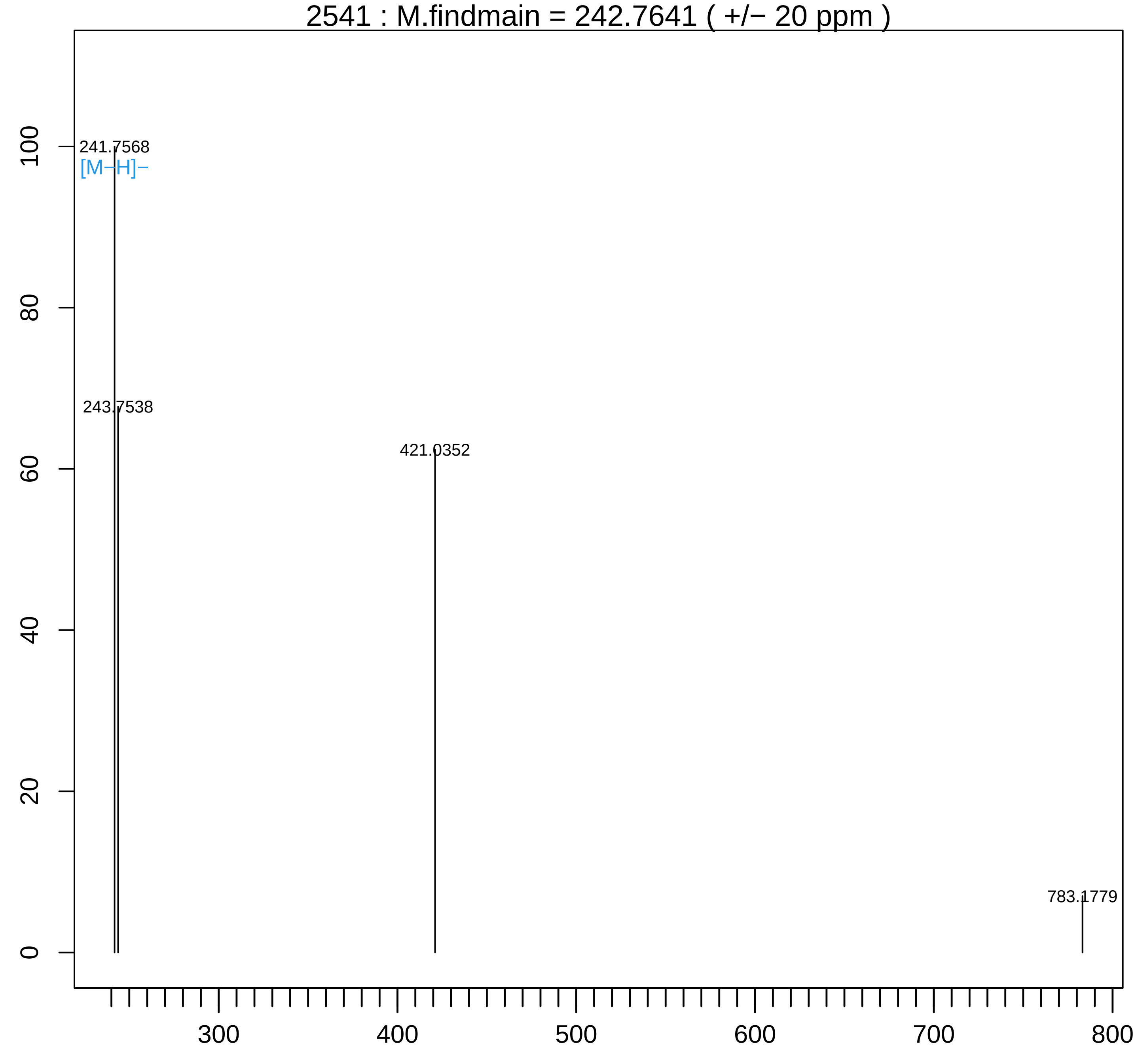

2541 : M.findmain = 242.7641 (+/− 20 ppm)
241.7568
[M−H]−
243.7538
421.0352
783.1779
0
20
40
60
80
100
300
400
500
600
700
800

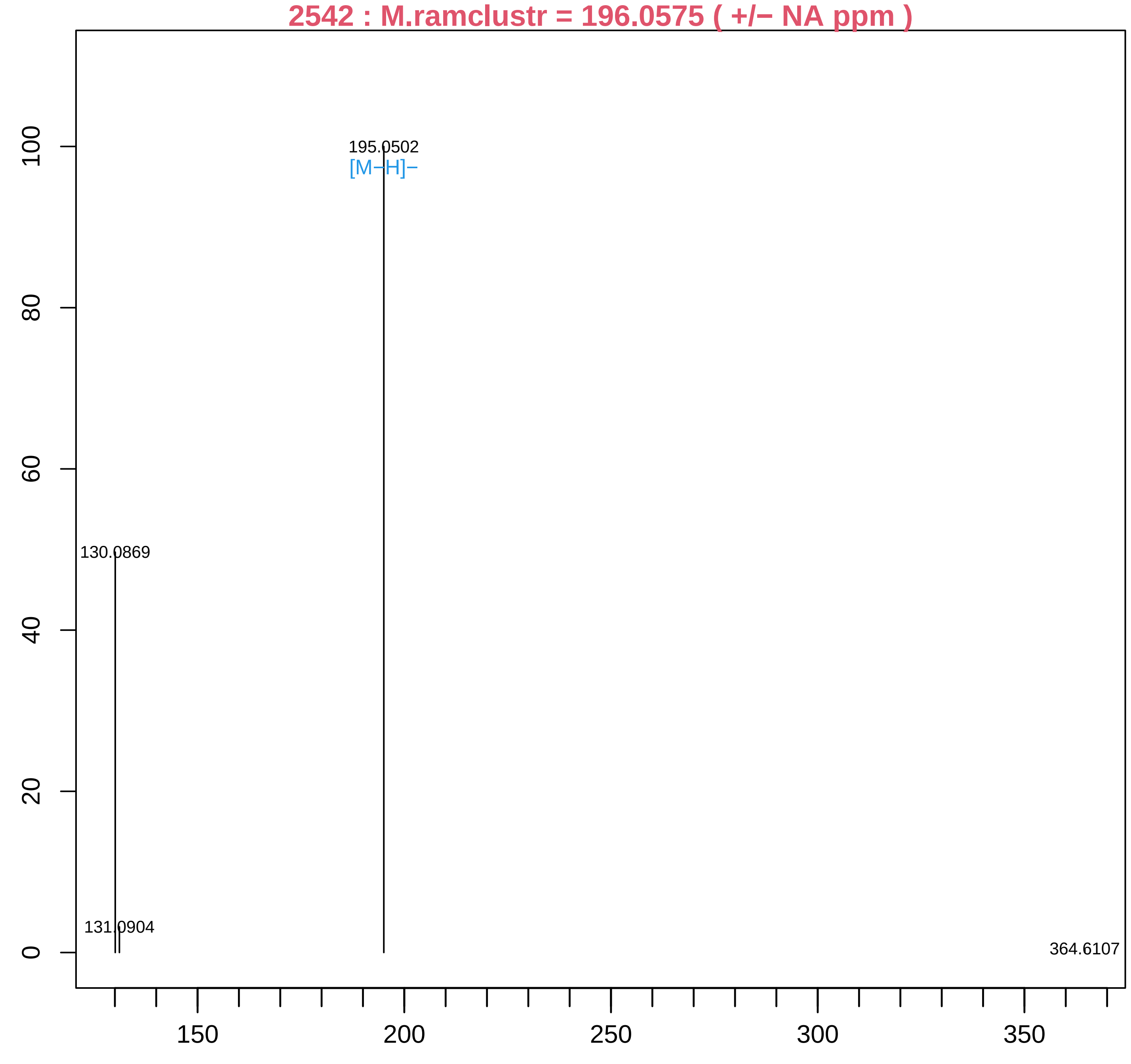
2542 : M.ramclustr = 196.0575 (+/− NA ppm)
195.0502
[M−H]−
130.0869
131.0904
364.6107
0
20
40
60
80
100
150
200
250
300
350

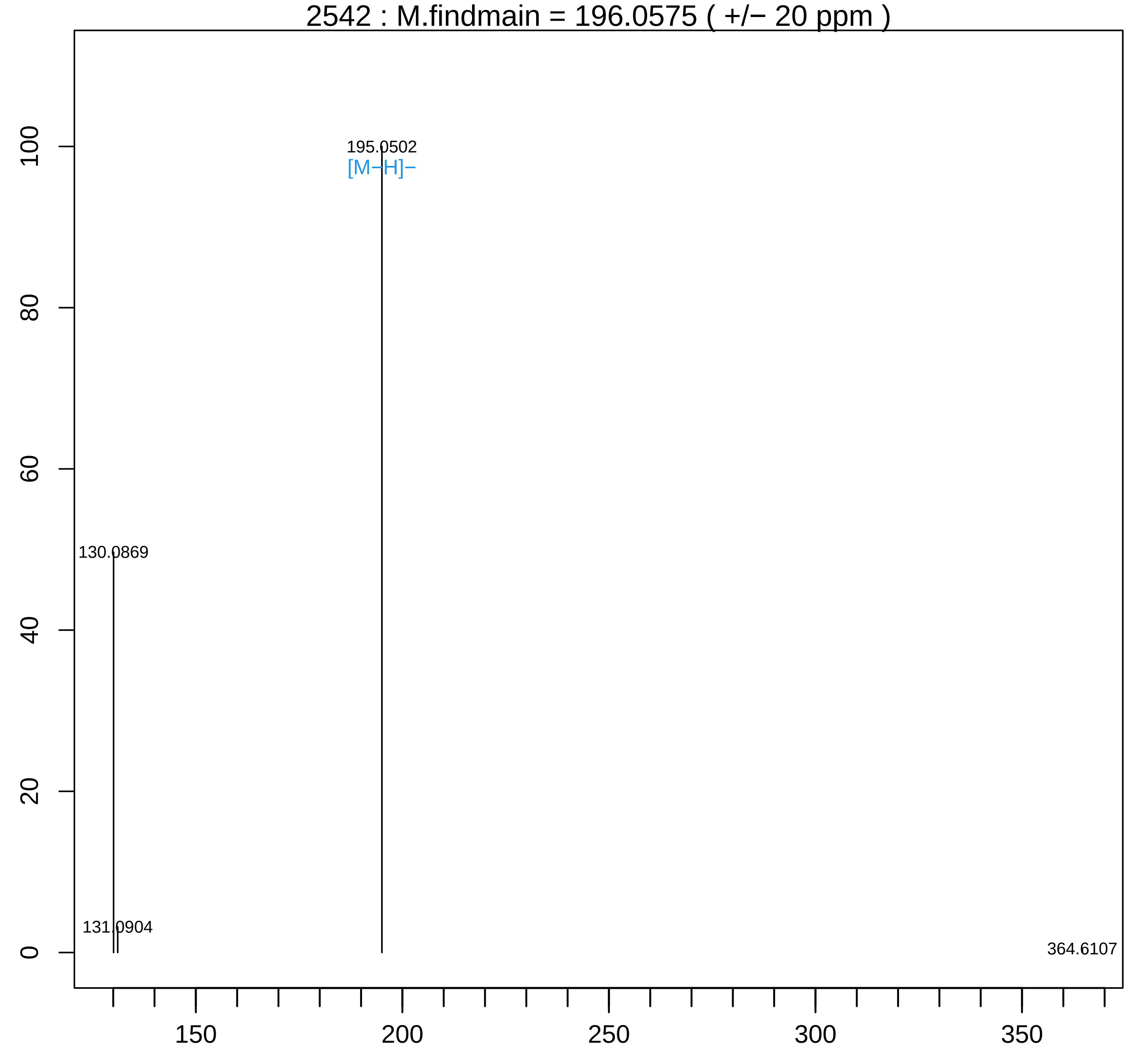
2542 : M.findmain = 196.0575 (+/− 20 ppm)
195.0502
[M−H]−
130.0869
131.0904
364.6107
0
20
40
60
80
100
150
200
250
300
350

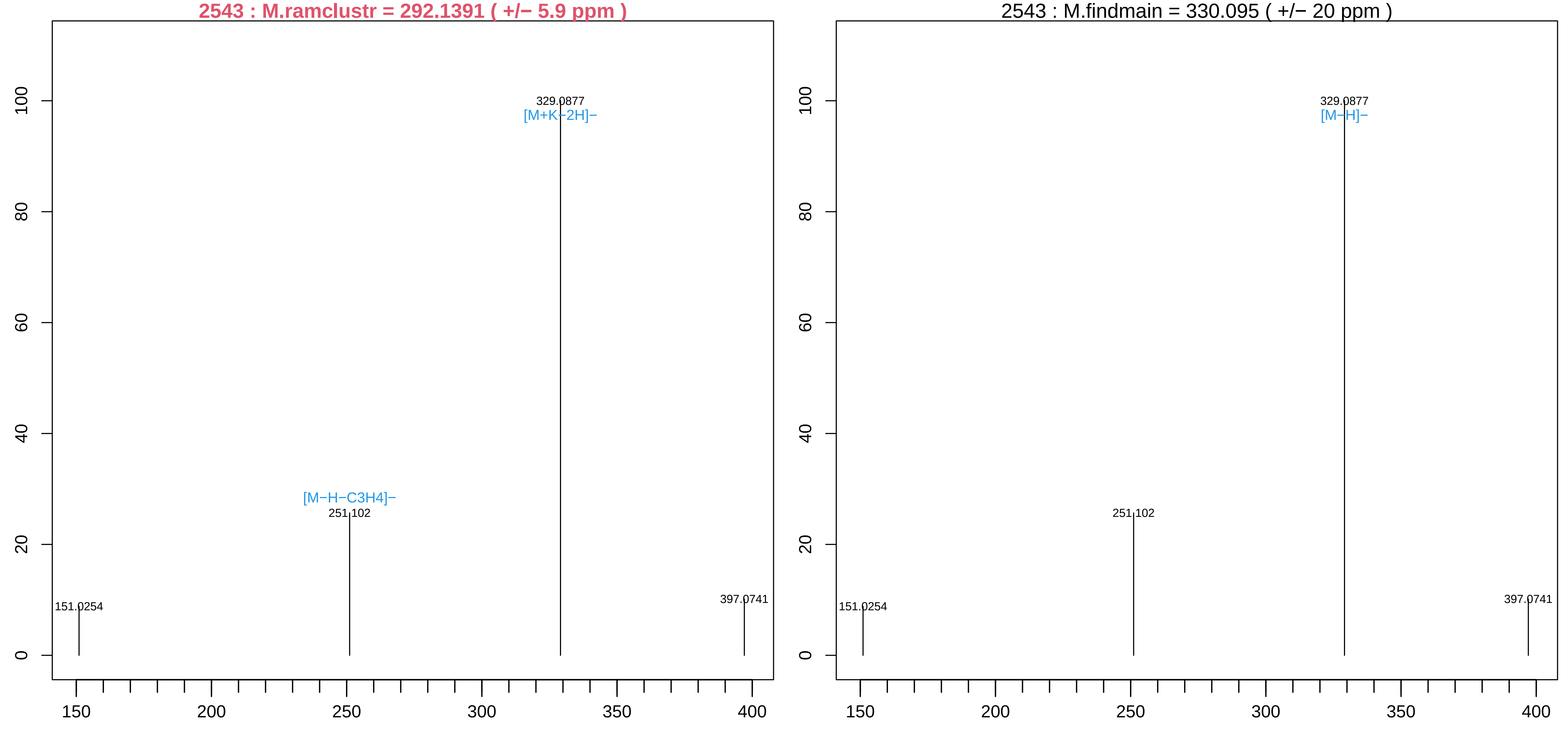

2543 : M.ramclustr = 292.1391 (+/− 5.9 ppm)
329.0877
[M+K−2H]−
[M−H−C3H4]−
251.102
151.0254
397.0741
2543 : M.findmain = 330.095 (+/− 20 ppm)
329.0877
[M−H]−
251.102
151.0254
397.0741

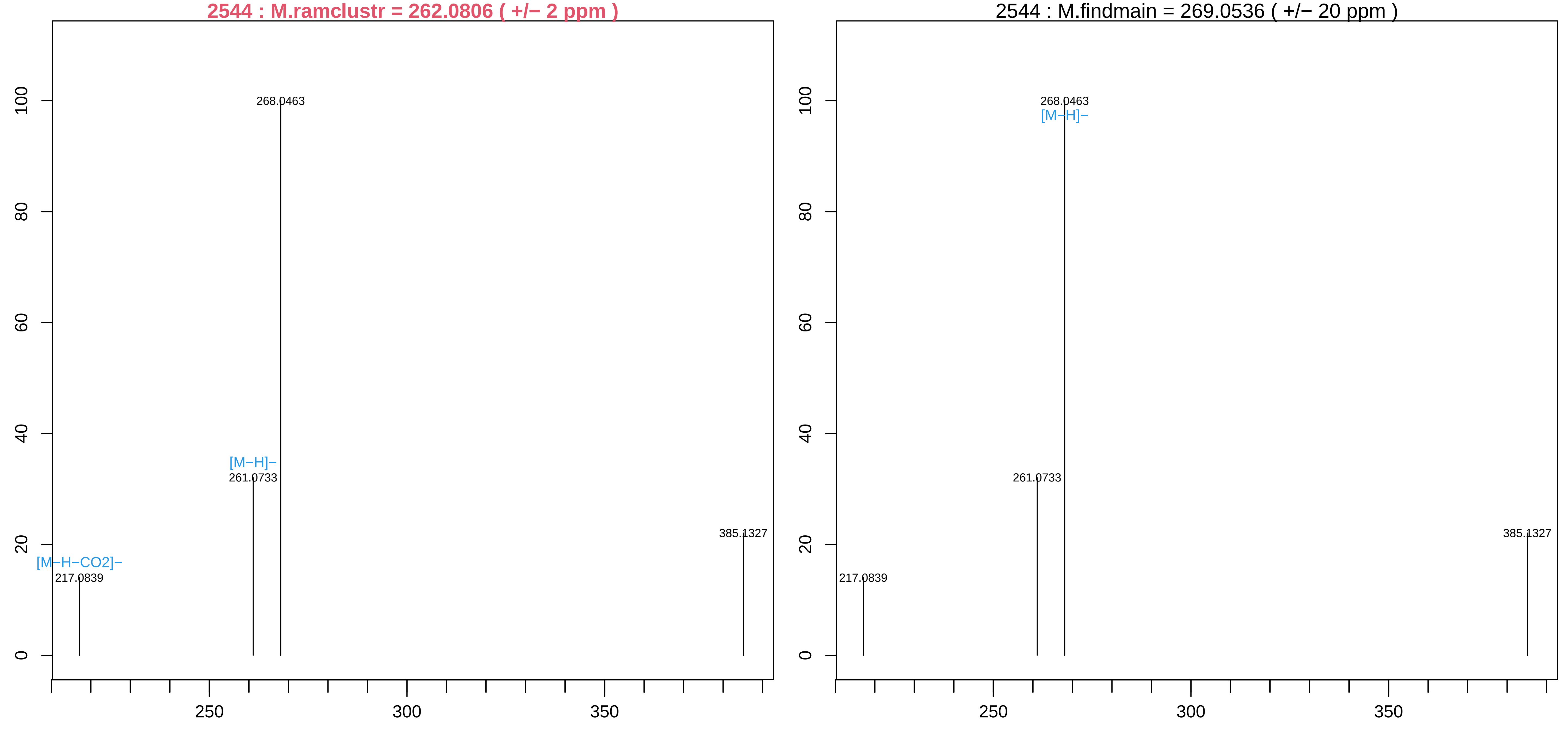
2544 : M.ramclustr = 262.0806 (+/− 2 ppm)
268.0463
[M−H]−
261.0733
385.1327
[M−H−CO2]−
217.0839
2544 : M.findmain = 269.0536 (+/− 20 ppm)
268.0463
[M−H]−
261.0733
385.1327
217.0839
0
20
40
60
80
100
250
300
350

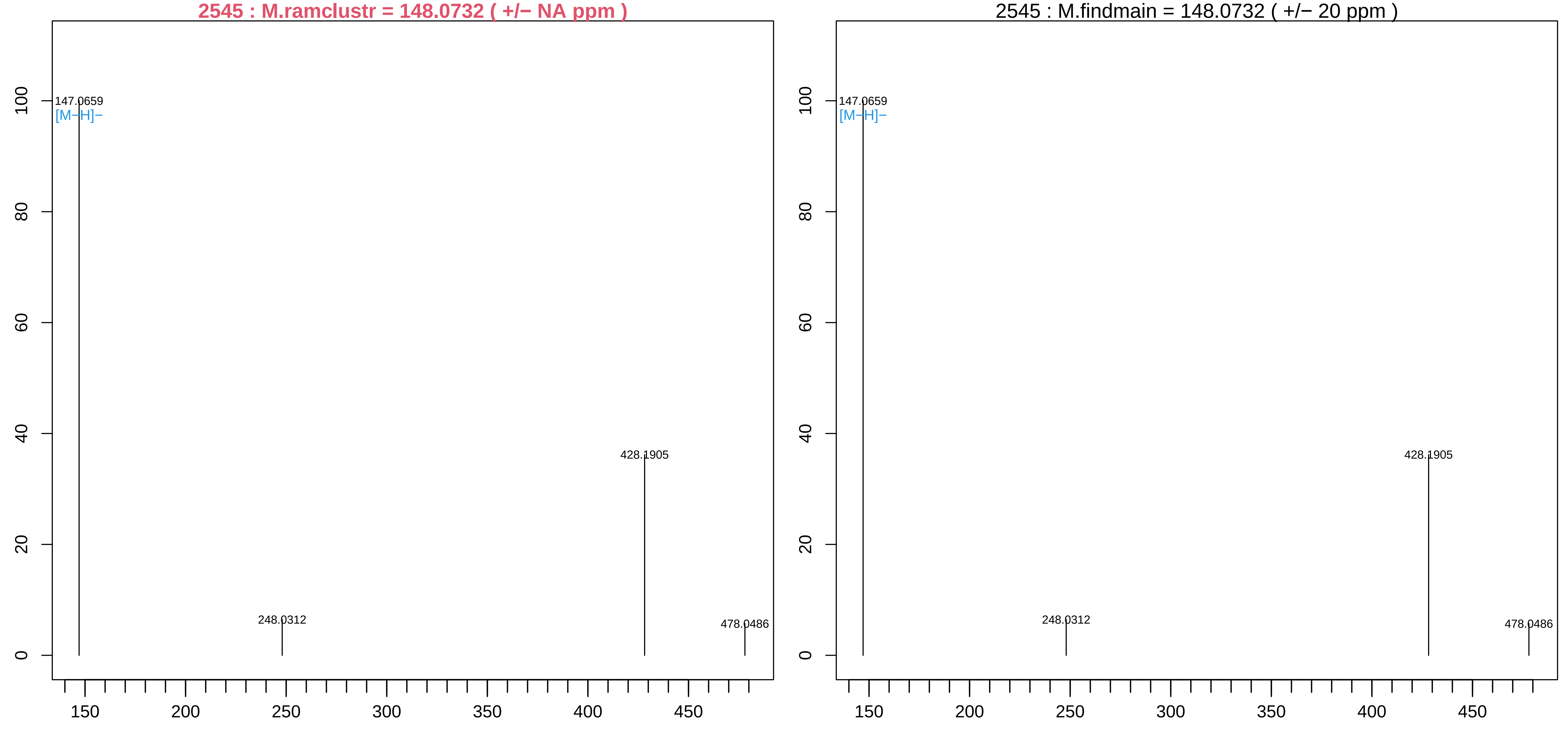
2545 : M.ramclustr = 148.0732 (+/− NA ppm)
2545 : M.findmain = 148.0732 (+/− 20 ppm)
147.0659
[M−H]−
248.0312
428.1905
478.0486
0
20
40
60
80
100
150
200
250
300
350
400
450

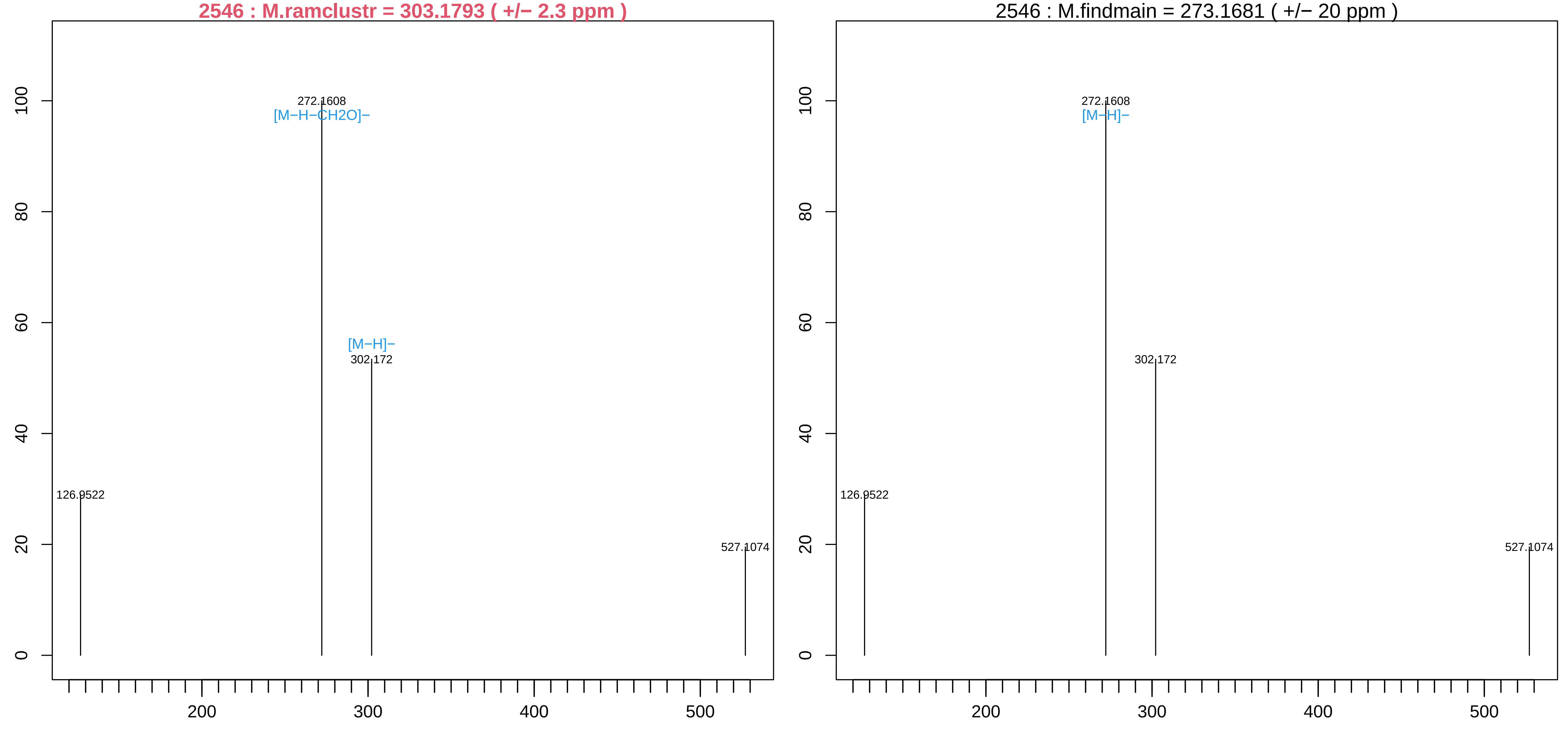

2546 : M.ramclustr = 303.1793 (+/− 2.3 ppm)
272.1608
[M−H−CH2O]−
[M−H]−
302.172
126.9522
527.1074
0
20
40
60
80
100
200
300
400
500
2546 : M.findmain = 273.1681 (+/− 20 ppm)
272.1608
[M−H]−
302.172
126.9522
527.1074
0
20
40
60
80
100
200
300
400
500

2547 : M.ramclustr = 286.0791 (+/− 29.9 ppm)

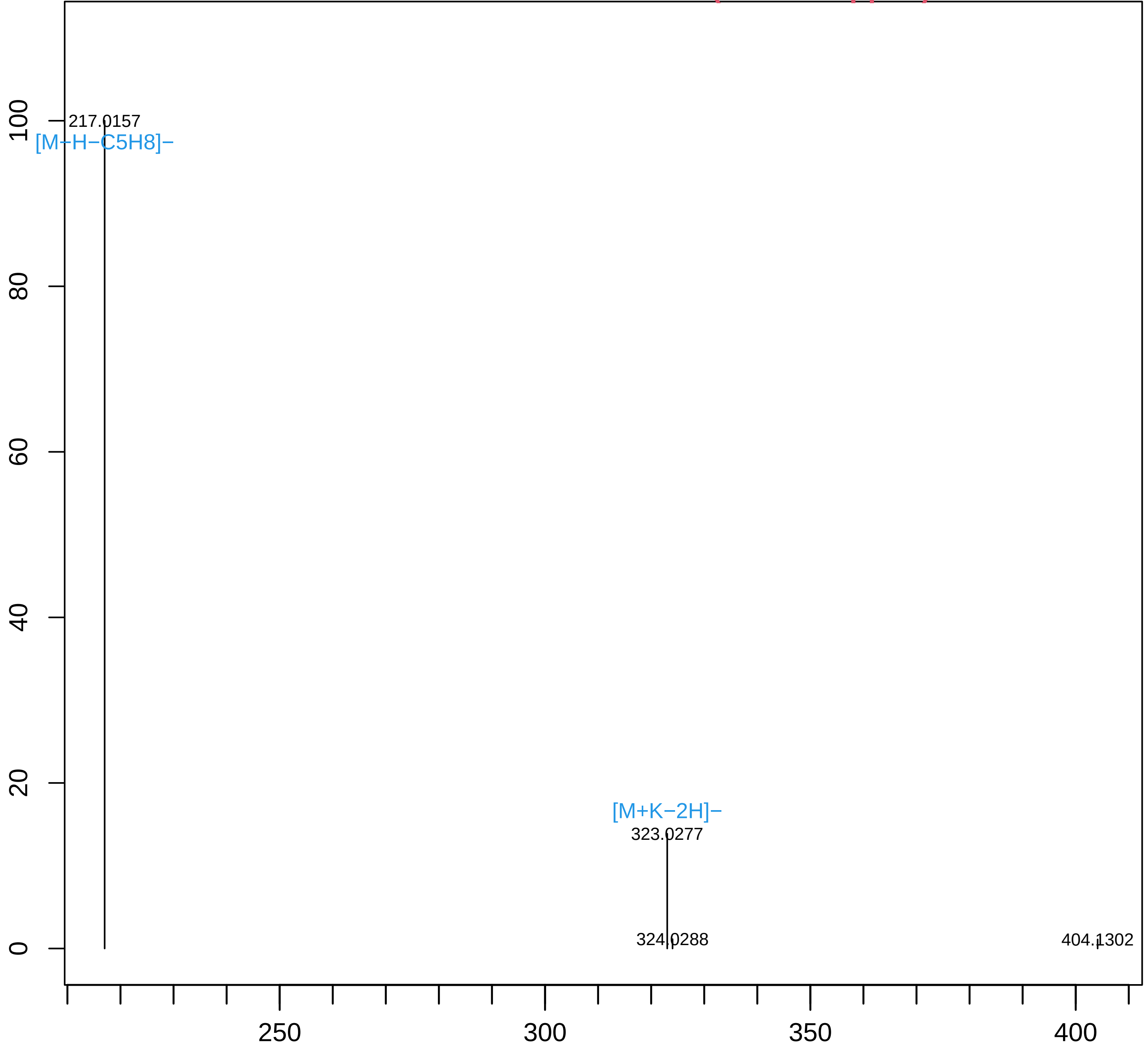

2547 : M.findmain = 218.023 (+/− 20 ppm)

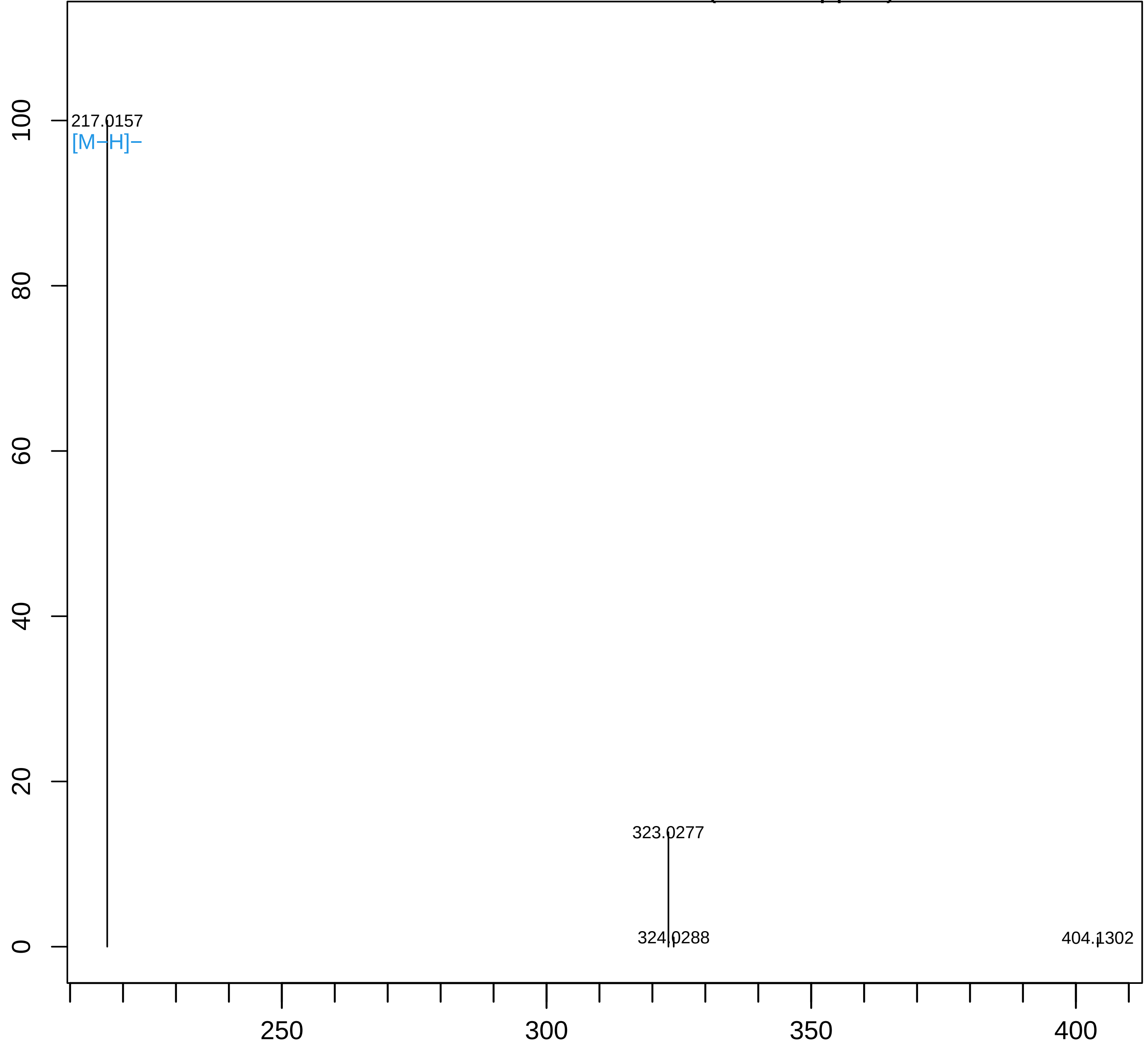

2548 : M.ramclustr = 274.1286 (+/− 27.7 ppm)

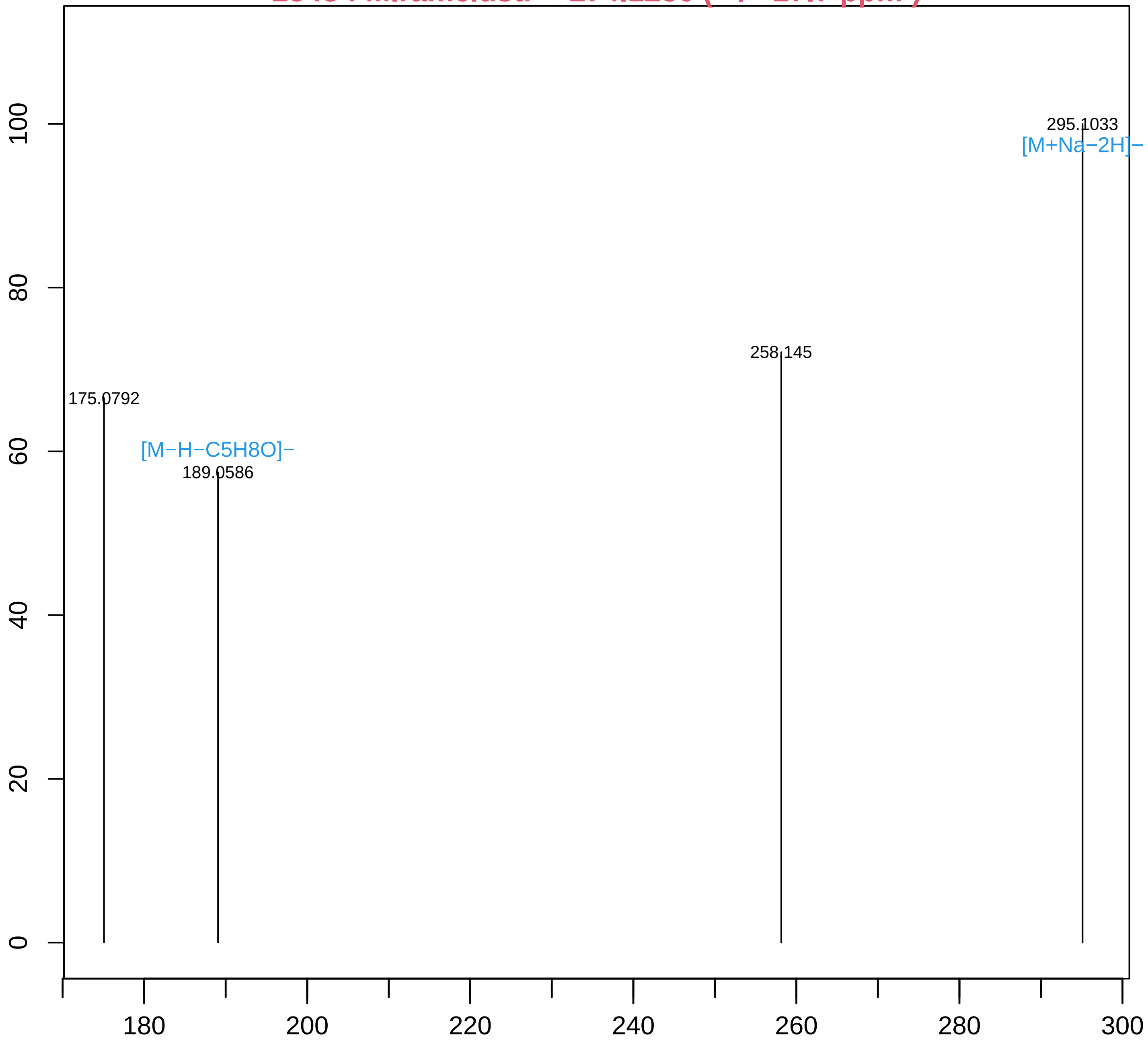

2548 : M.findmain = 296.1106 (+/− 20 ppm)

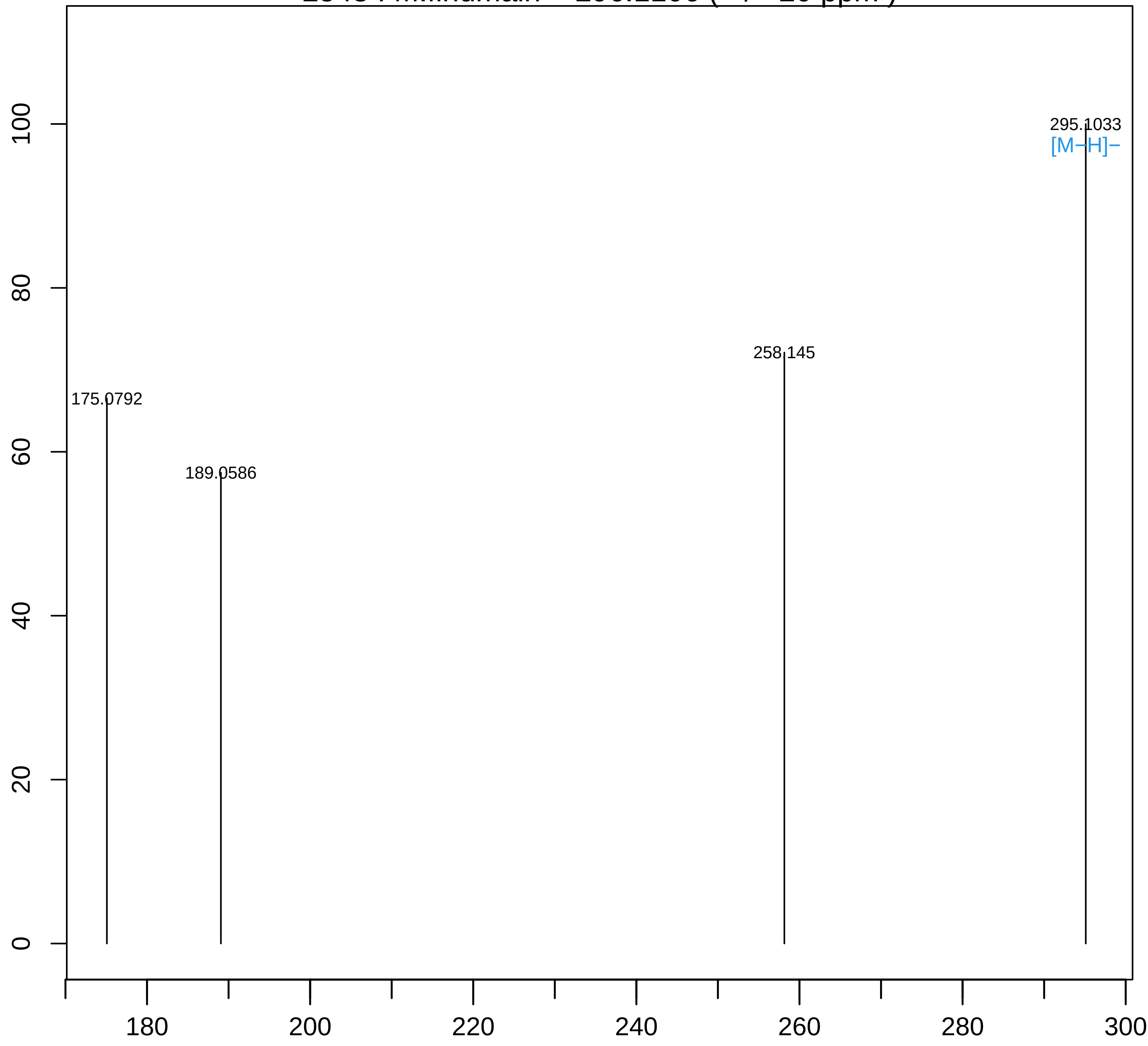

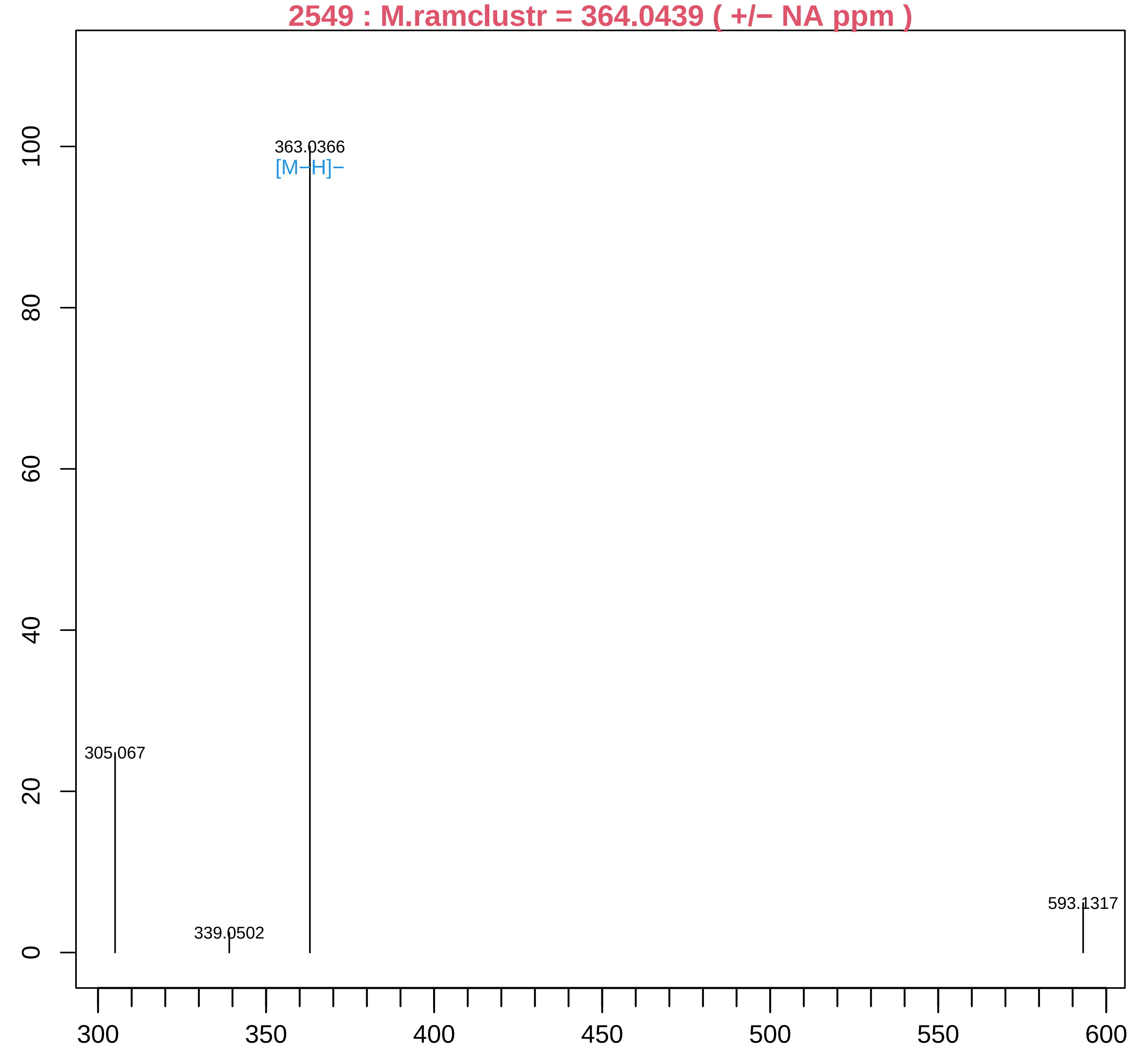
2549 : M.ramclustr = 364.0439 (+/− NA ppm)
363.0366
[M−H]−
305.067
339.0502
593.1317
0
20
40
60
80
100
300
350
400
450
500
550
600

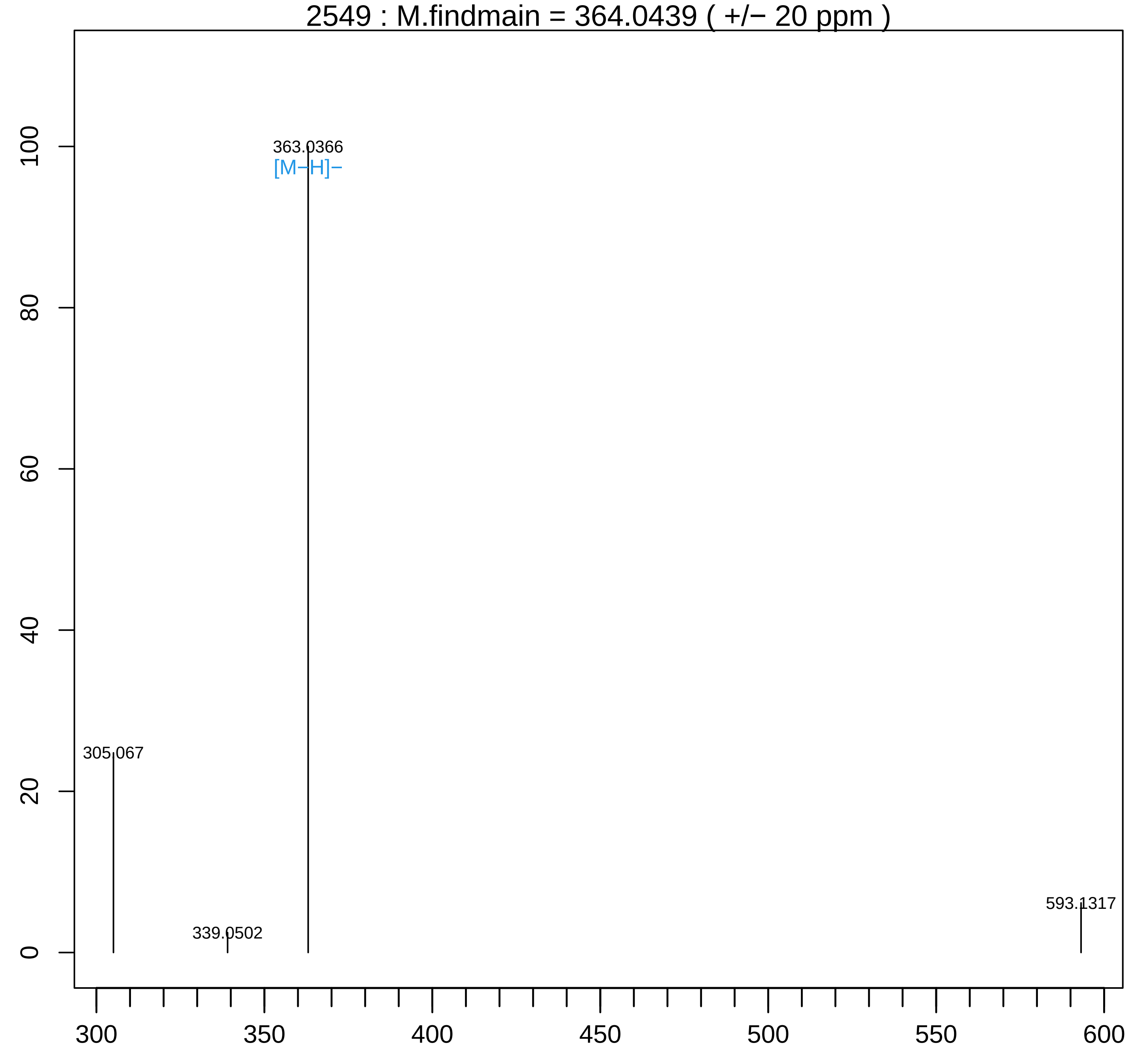
2549 : M.findmain = 364.0439 (+/− 20 ppm)
363.0366
[M−H]−
305.067
339.0502
593.1317
0
20
40
60
80
100
300
350
400
450
500
550
600

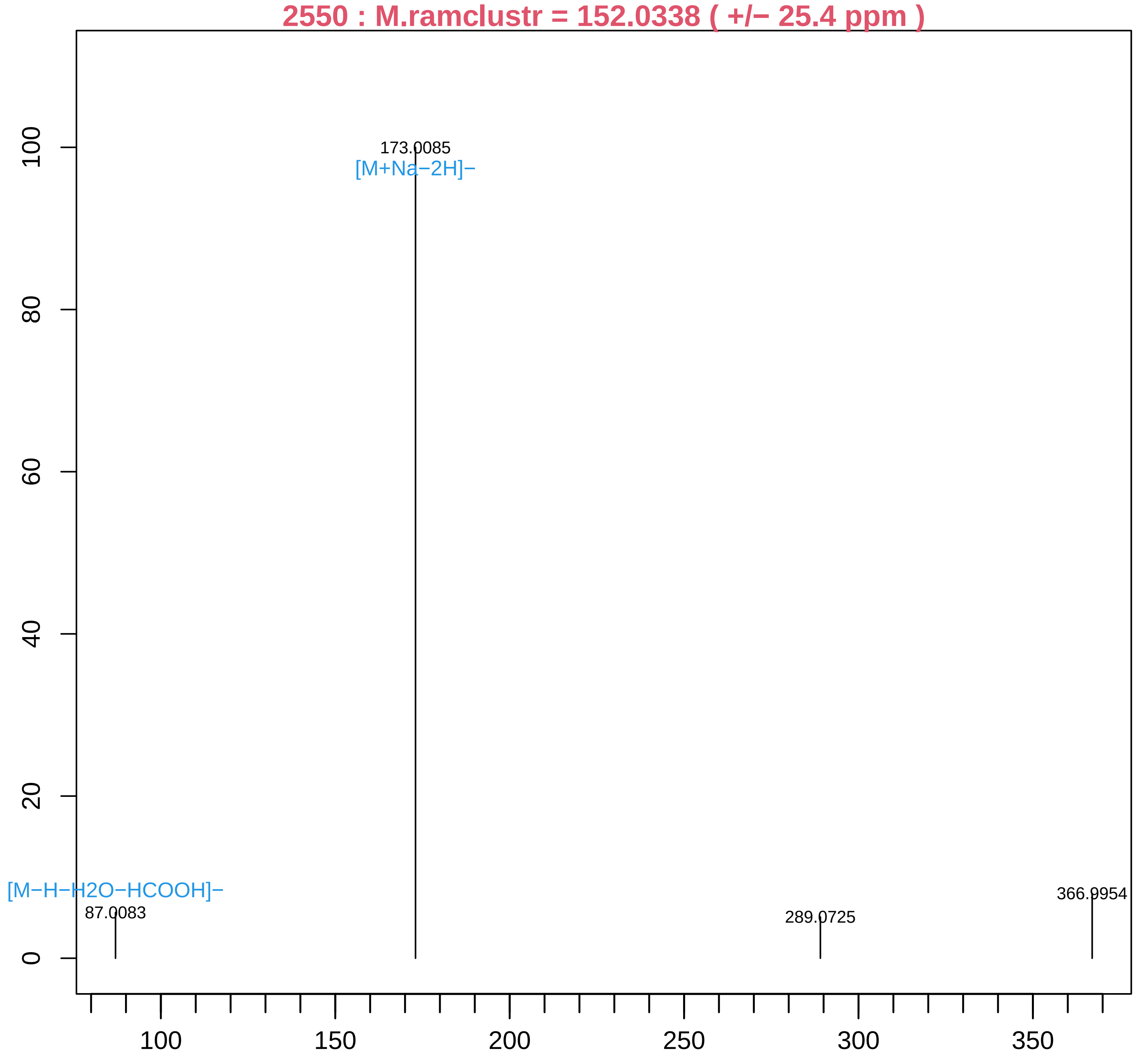

2550 : M.ramclustr = 152.0338 (+/− 25.4 ppm)
173.0085
[M+Na−2H]−
[M−H−H2O−HCOOH]−
87.0083
289.0725
366.9954
0
20
40
60
80
100
100
150
200
250
300
350

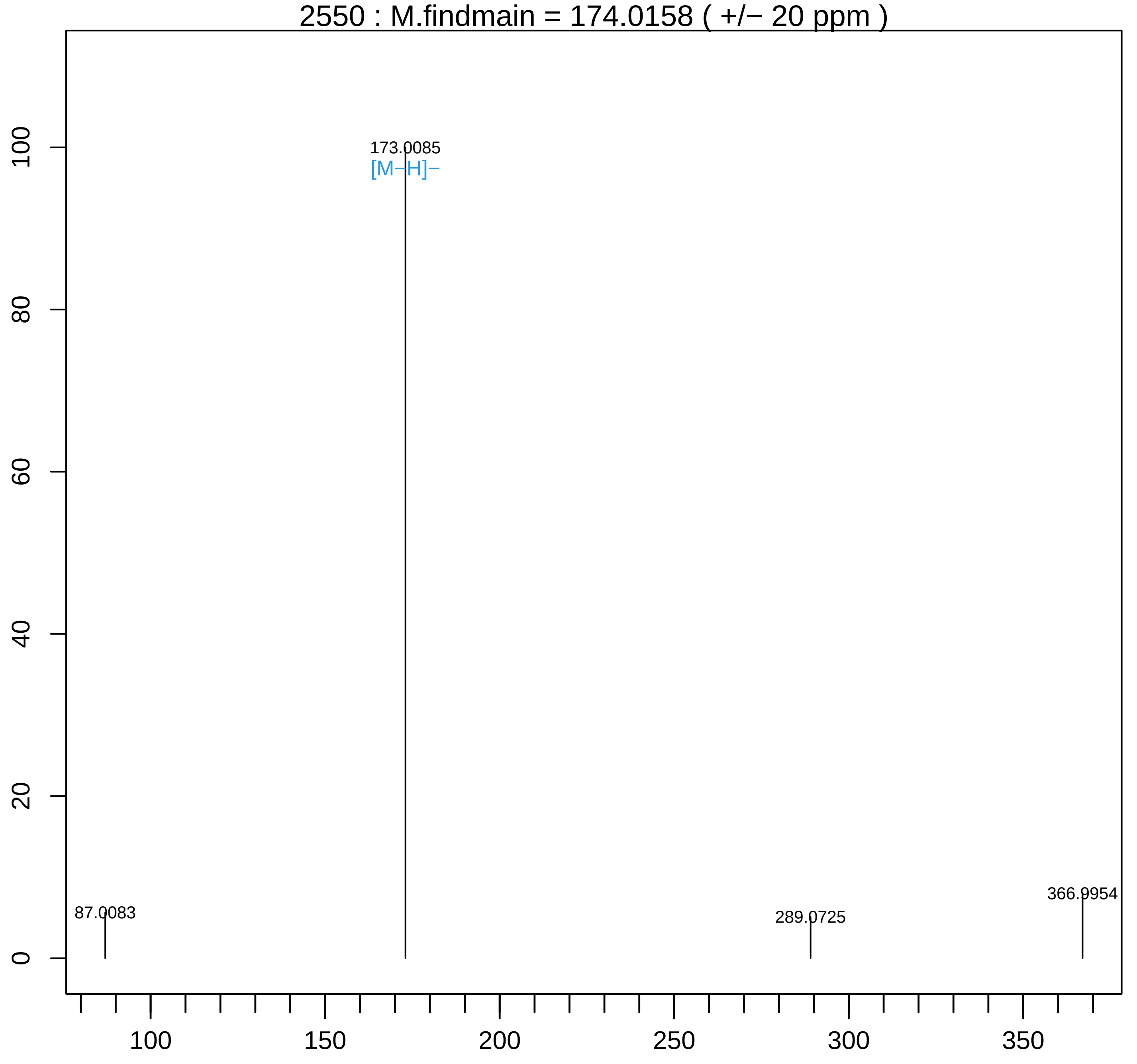

2550 : M.findmain = 174.0158 (+/− 20 ppm)
173.0085
[M−H]−
87.0083
289.0725
366.9954
0
20
40
60
80
100
100
150
200
250
300
350

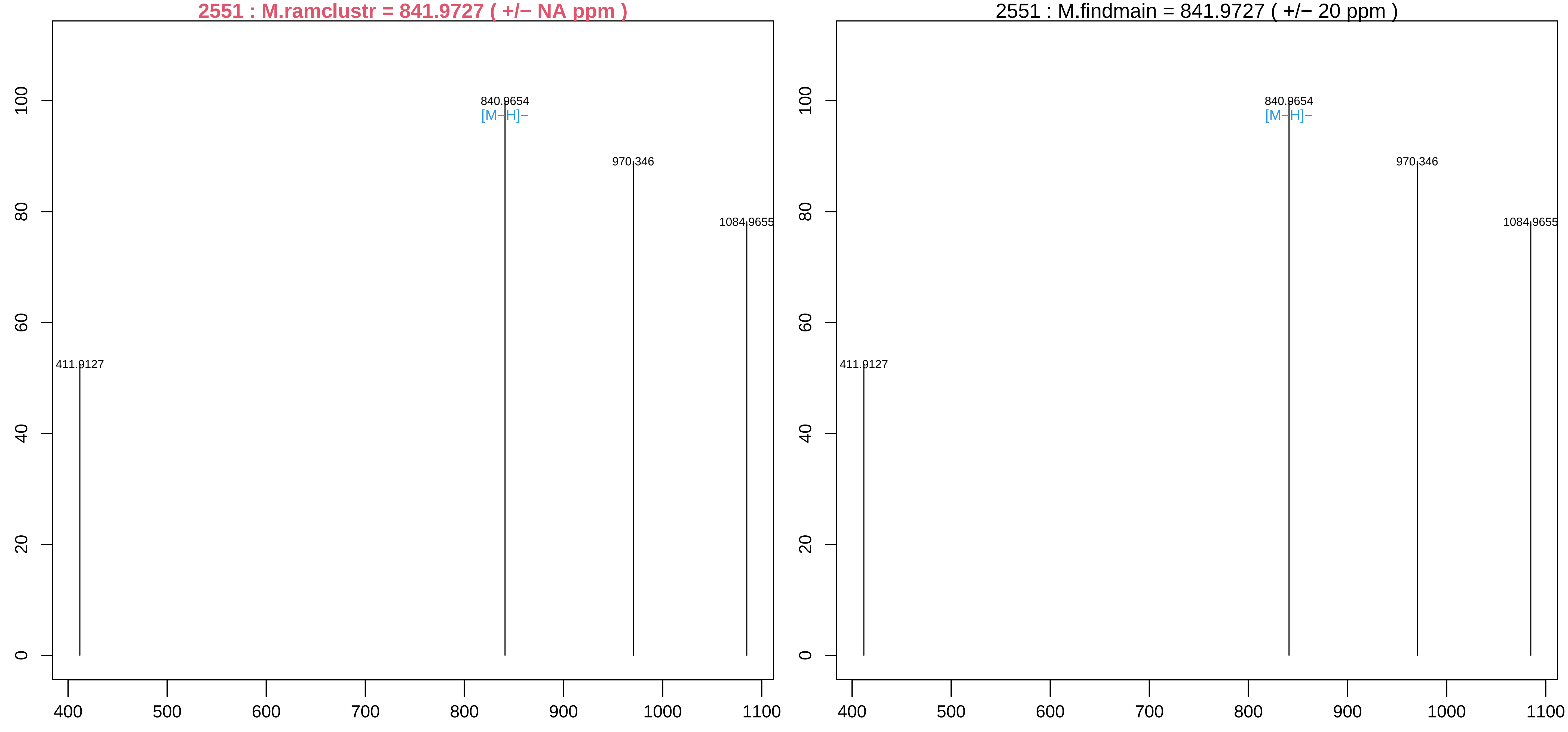

2551 : M.ramclustr = 841.9727 (+/− NA ppm)
840.9654
[M−H]−
970.346
1084.9655
411.9127
2551 : M.findmain = 841.9727 (+/− 20 ppm)
840.9654
[M−H]−
970.346
1084.9655
411.9127

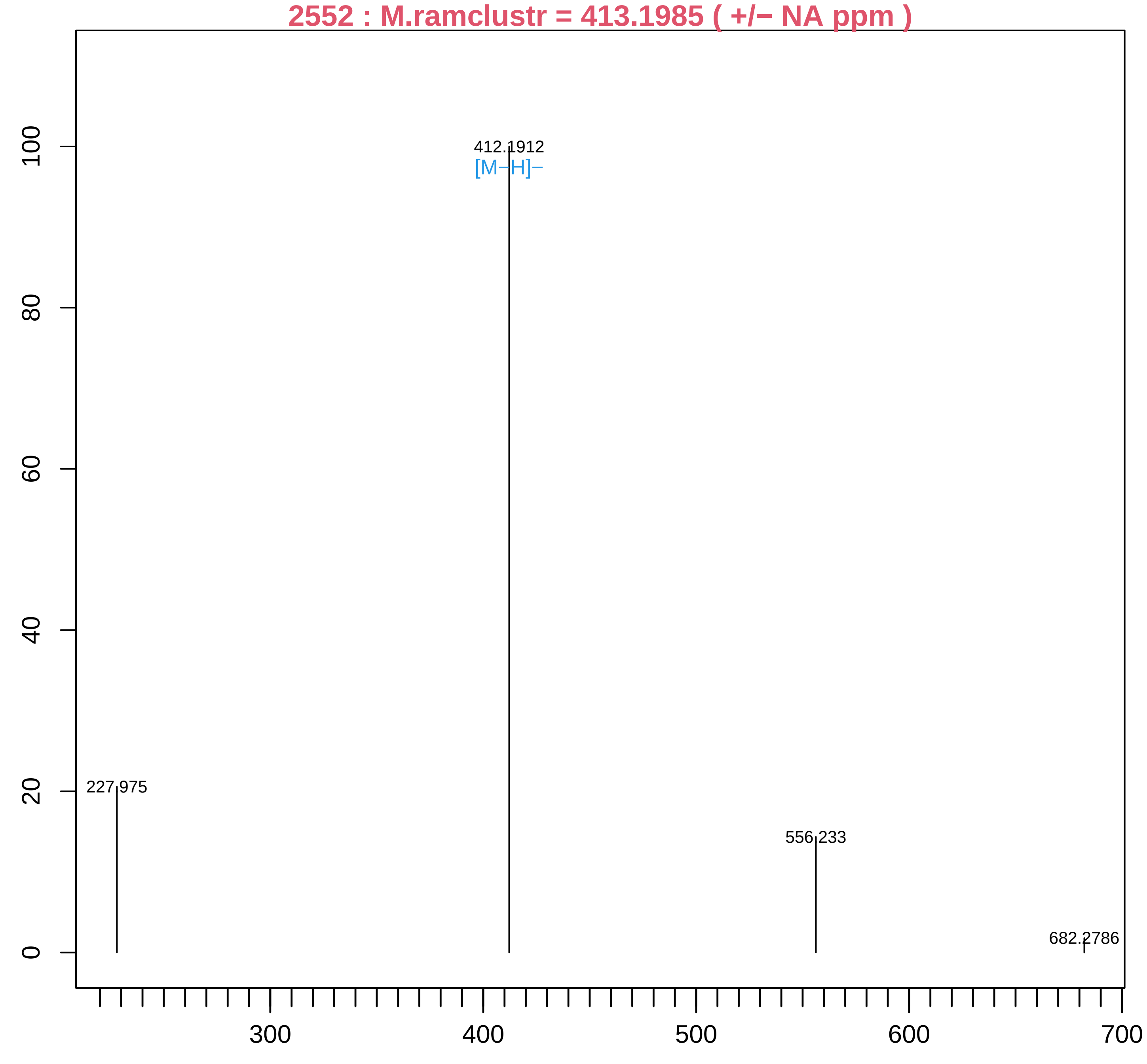
2552 : M.ramclustr = 413.1985 (+/− NA ppm)
412.1912
[M−H]−
227.975
556.233
682.2786
0
20
40
60
80
100
300
400
500
600
700

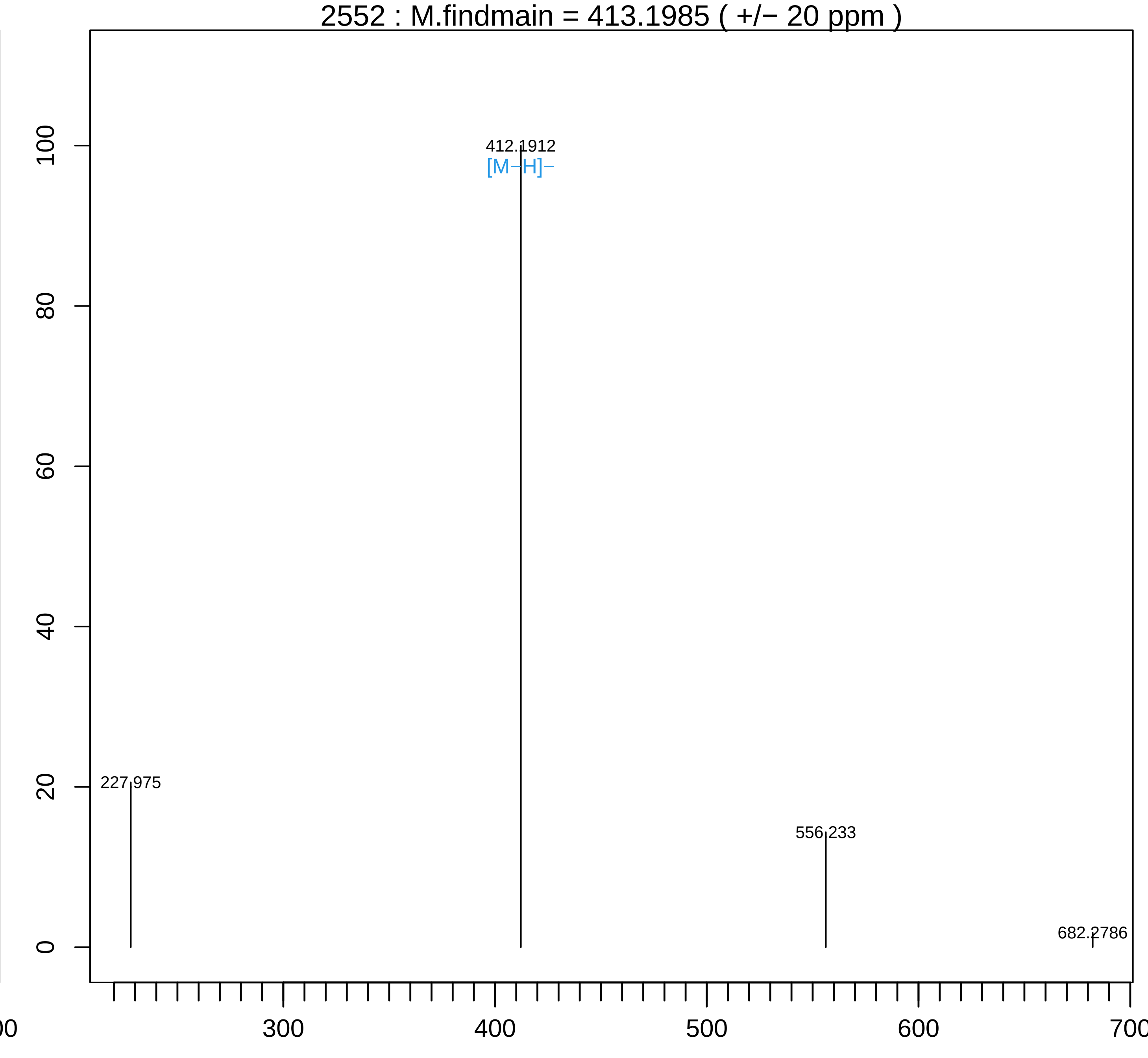
2552 : M.findmain = 413.1985 (+/− 20 ppm)
412.1912
[M−H]−
227.975
556.233
682.2786
0
20
40
60
80
100
300
400
500
600
700

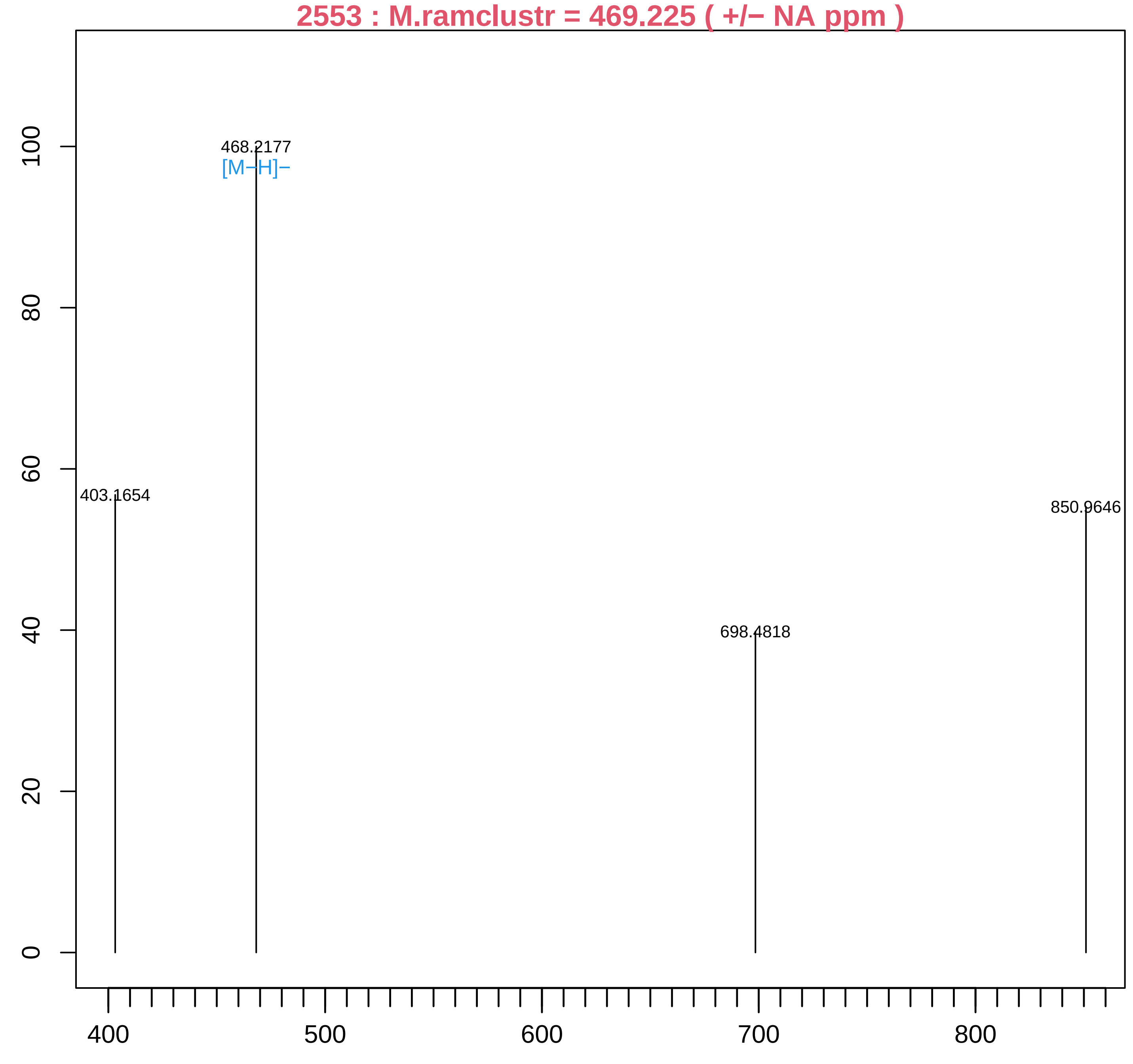
2553 : M.ramclustr = 469.225 (+/− NA ppm)
468.2177
[M−H]−
403.1654
850.9646
698.4818
0
20
40
60
80
100
400
500
600
700
800

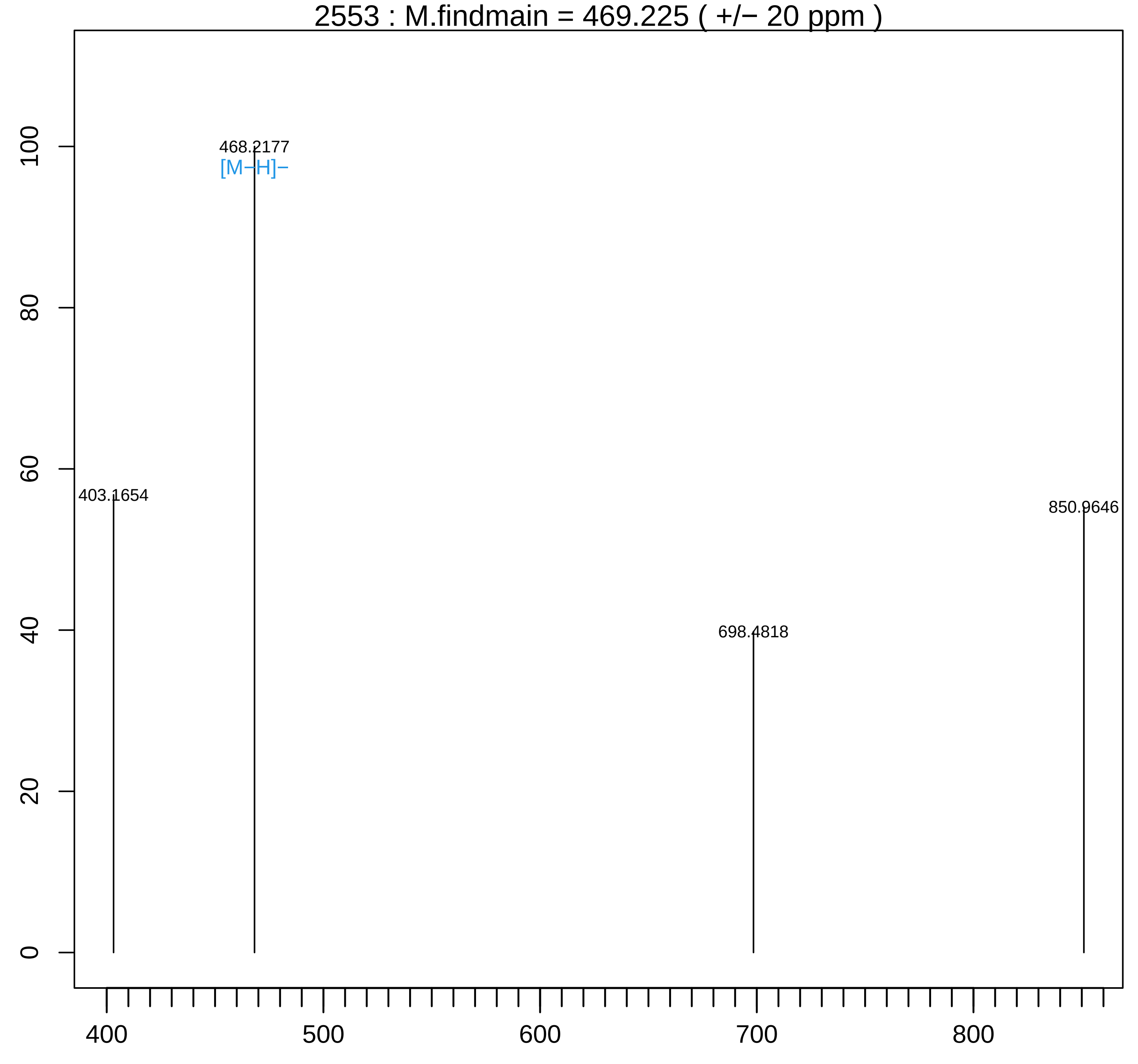
2553 : M.findmain = 469.225 (+/− 20 ppm)
468.2177
[M−H]−
403.1654
850.9646
698.4818
0
20
40
60
80
100
400
500
600
700
800

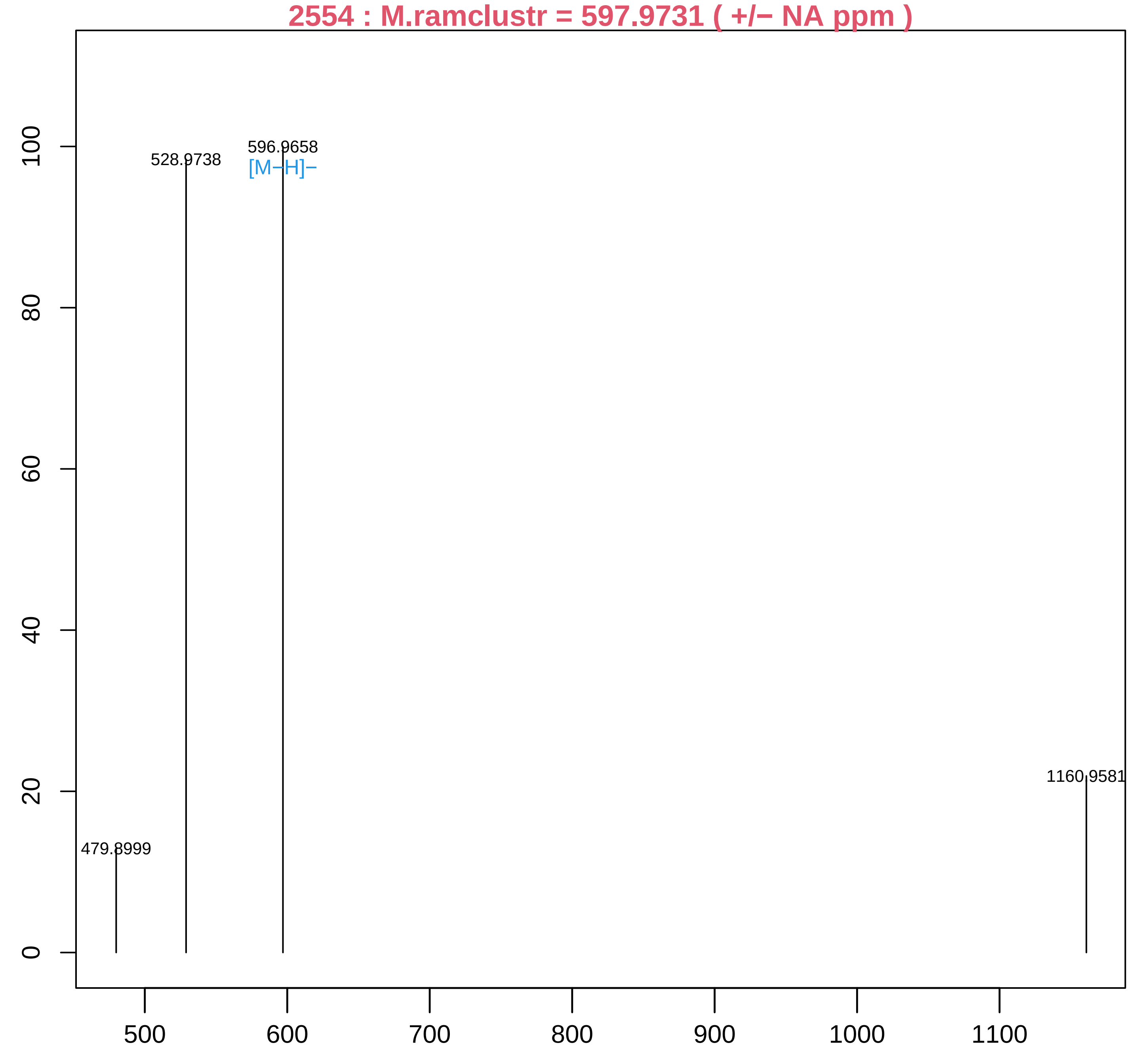

2554 : M.ramclustr = 597.9731 (+/− NA ppm)
528.9738
596.9658
[M−H]−
1160.9581
479.8999
0
20
40
60
80
100
500
600
700
800
900
1000
1100

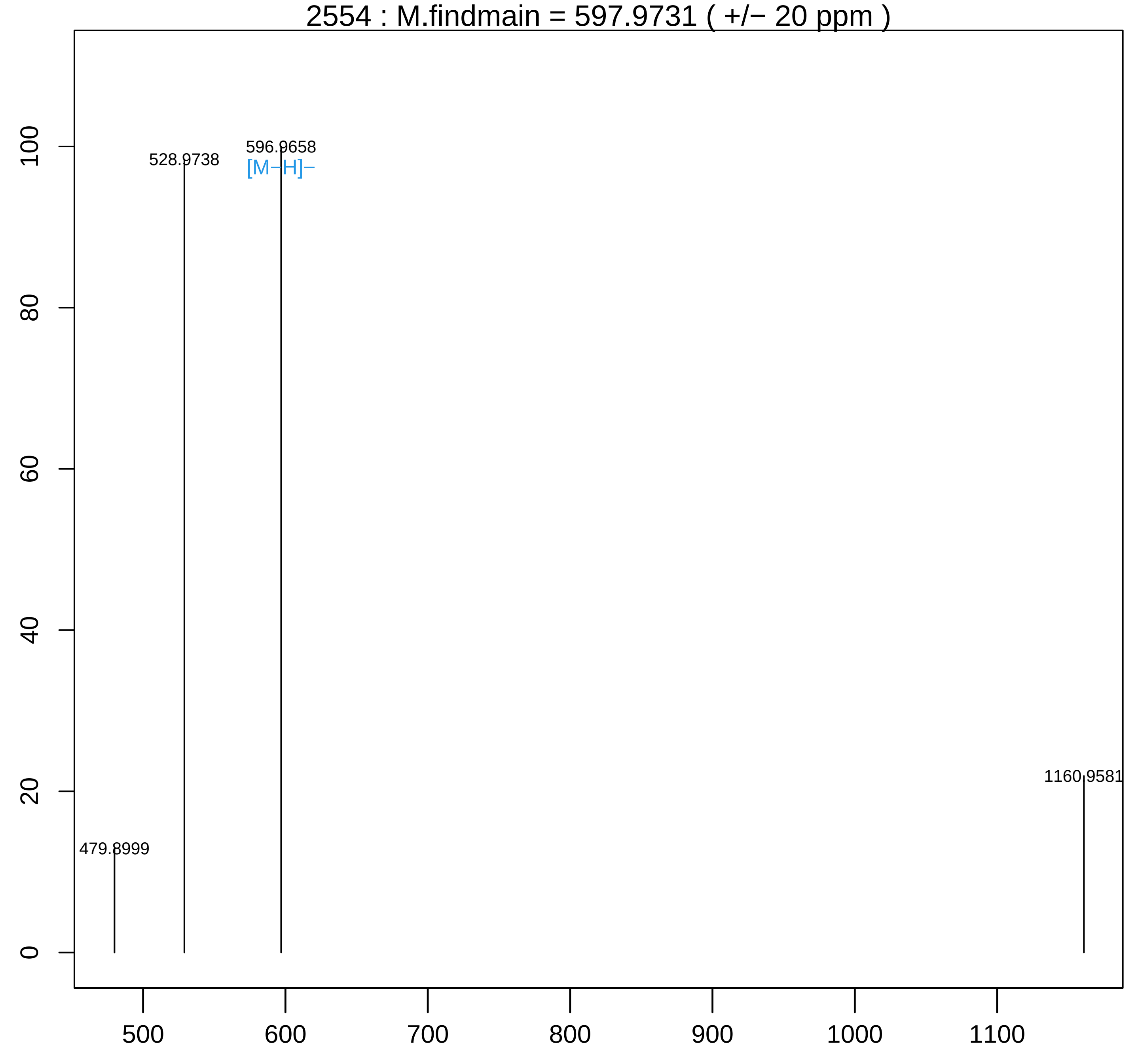

2554 : M.findmain = 597.9731 (+/− 20 ppm)
528.9738
596.9658
[M−H]−
1160.9581
479.8999
0
20
40
60
80
100
500
600
700
800
900
1000
1100

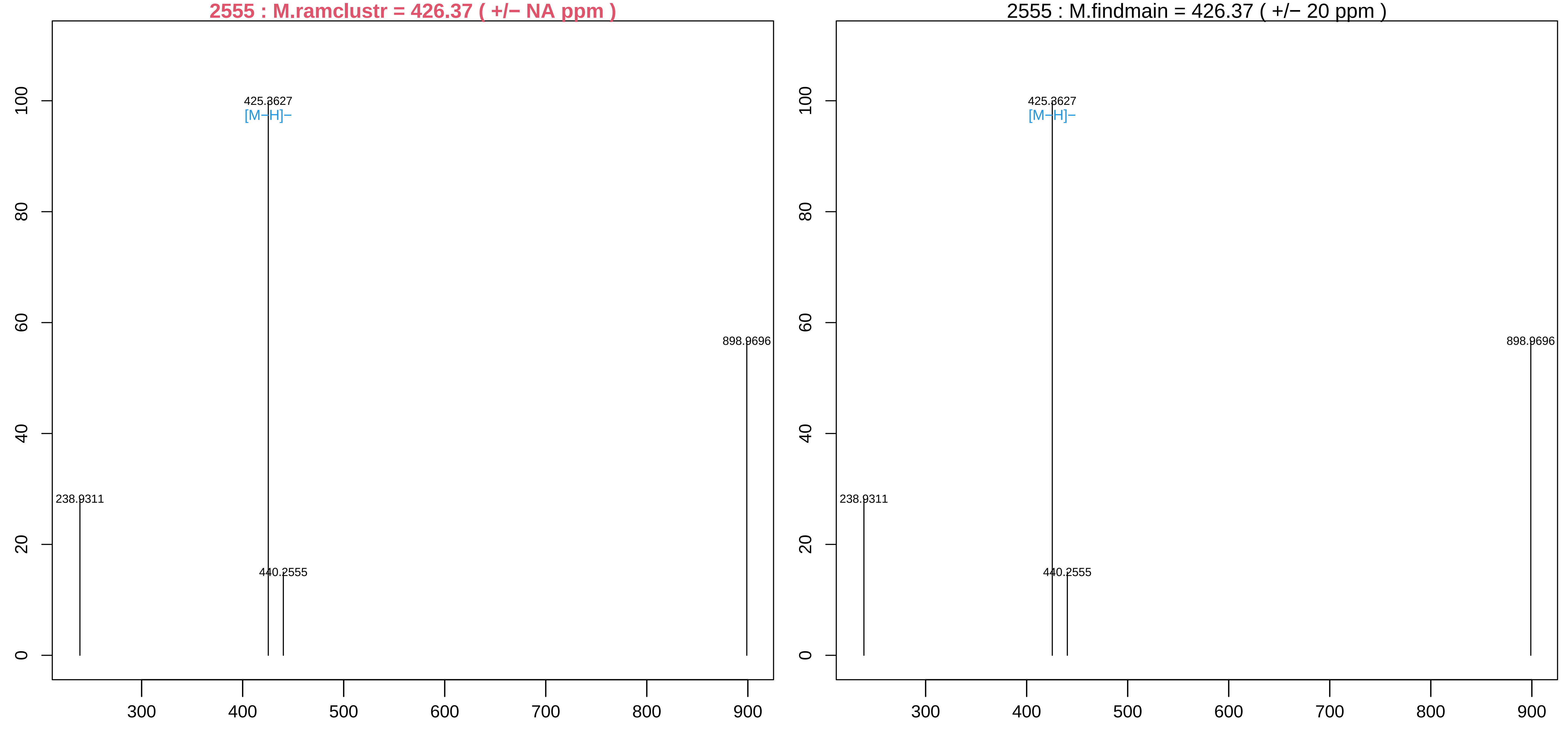

2555 : M.ramclustr = 426.37 (+/− NA ppm)
425.3627
[M−H]−
898.9696
238.9311
440.2555
2555 : M.findmain = 426.37 (+/− 20 ppm)
425.3627
[M−H]−
898.9696
238.9311
440.2555

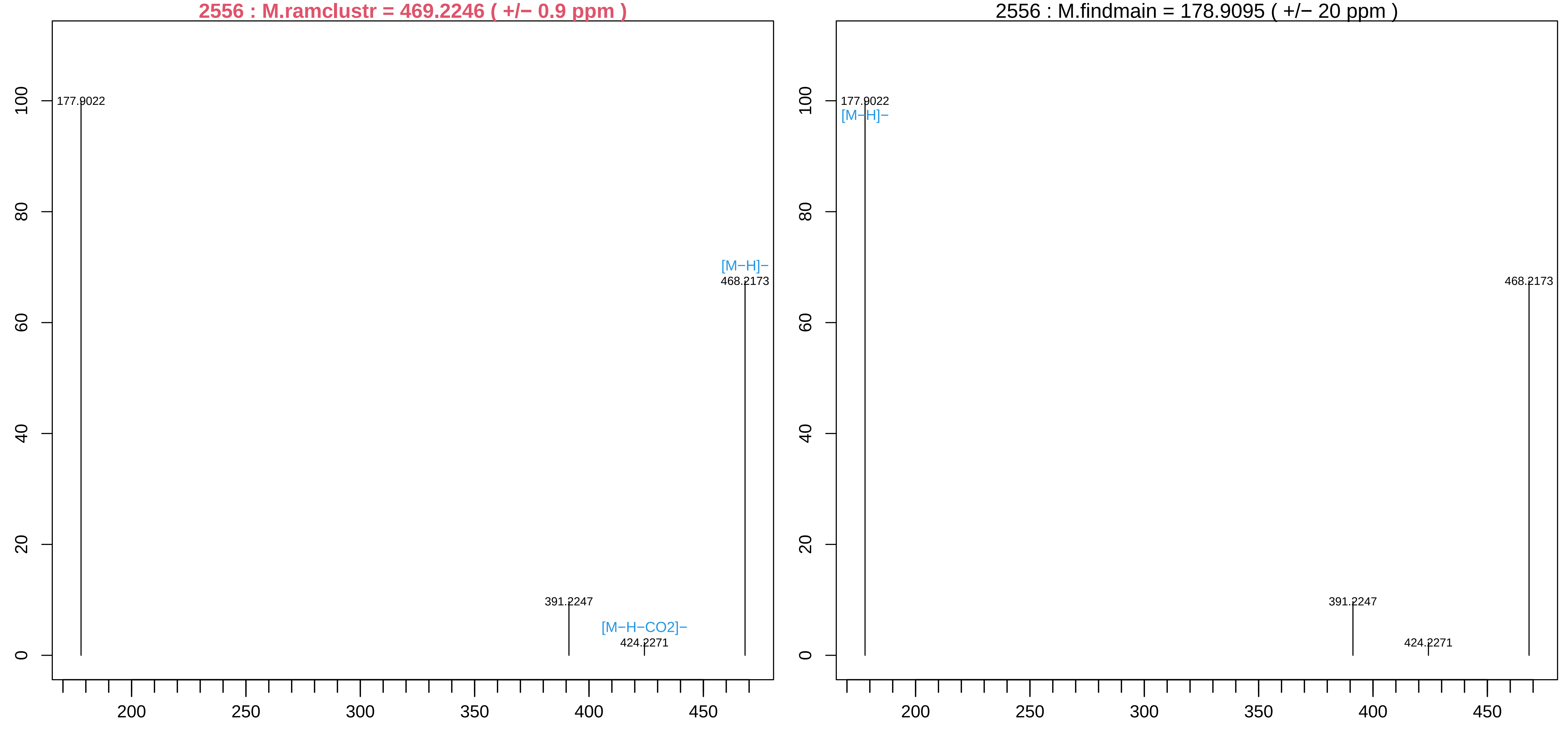
2556 : M.ramclustr = 469.2246 (+/− 0.9 ppm)
177.9022
[M−H]−
468.2173
391.2247
[M−H−CO2]−
424.2271
0
20
40
60
80
100
200
250
300
350
400
450
2556 : M.findmain = 178.9095 (+/− 20 ppm)
177.9022
[M−H]−
468.2173
391.2247
424.2271
0
20
40
60
80
100
200
250
300
350
400
450

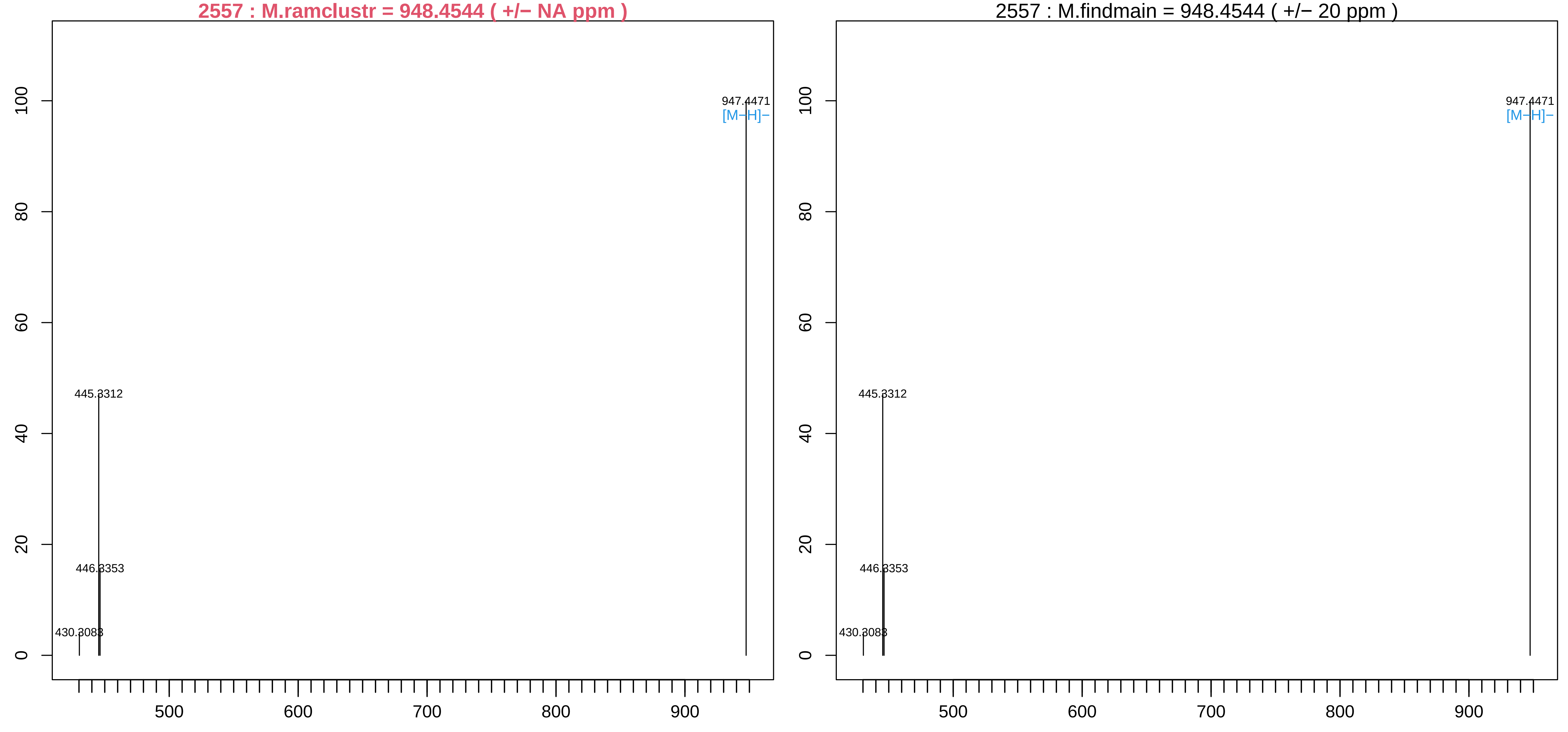
2557 : M.ramclustr = 948.4544 (+/− NA ppm)
947.4471
[M−H]−
445.3312
446.3353
430.3083
2557 : M.findmain = 948.4544 (+/− 20 ppm)
947.4471
[M−H]−
445.3312
446.3353
430.3083

2558 : M.ramclustr = 485.2196 (+/− NA ppm)

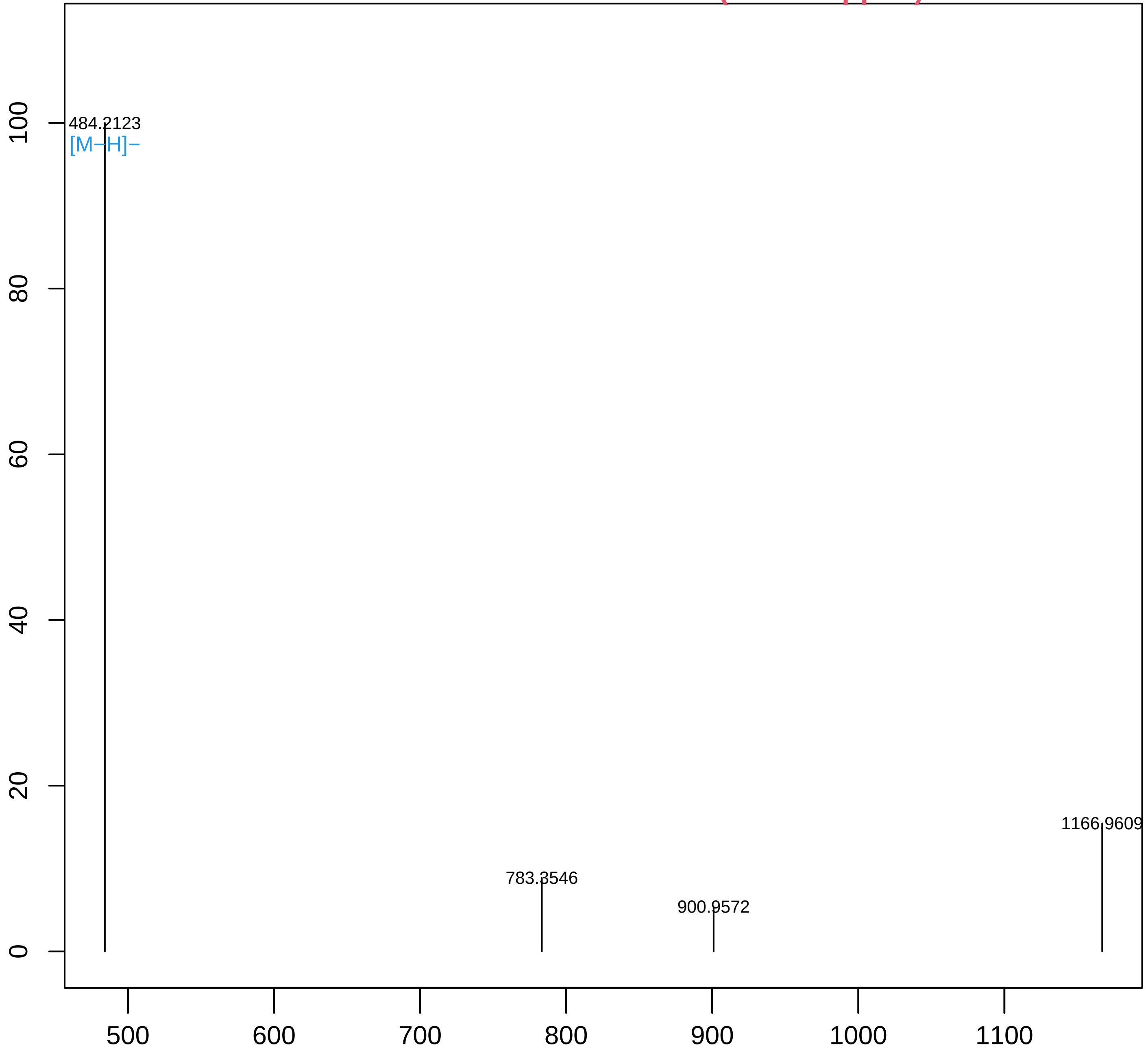

2558 : M.findmain = 485.2196 (+/− 20 ppm)

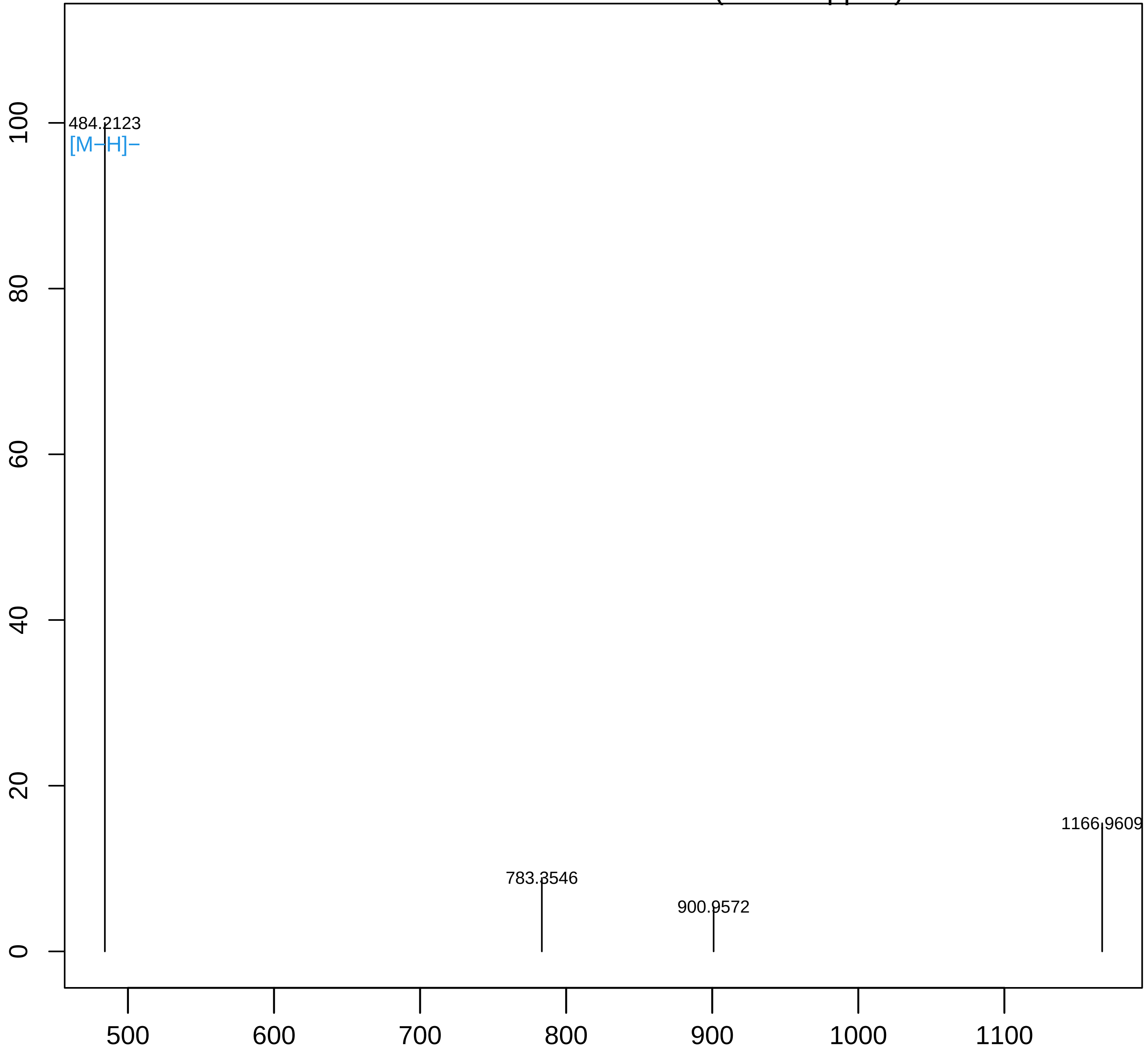

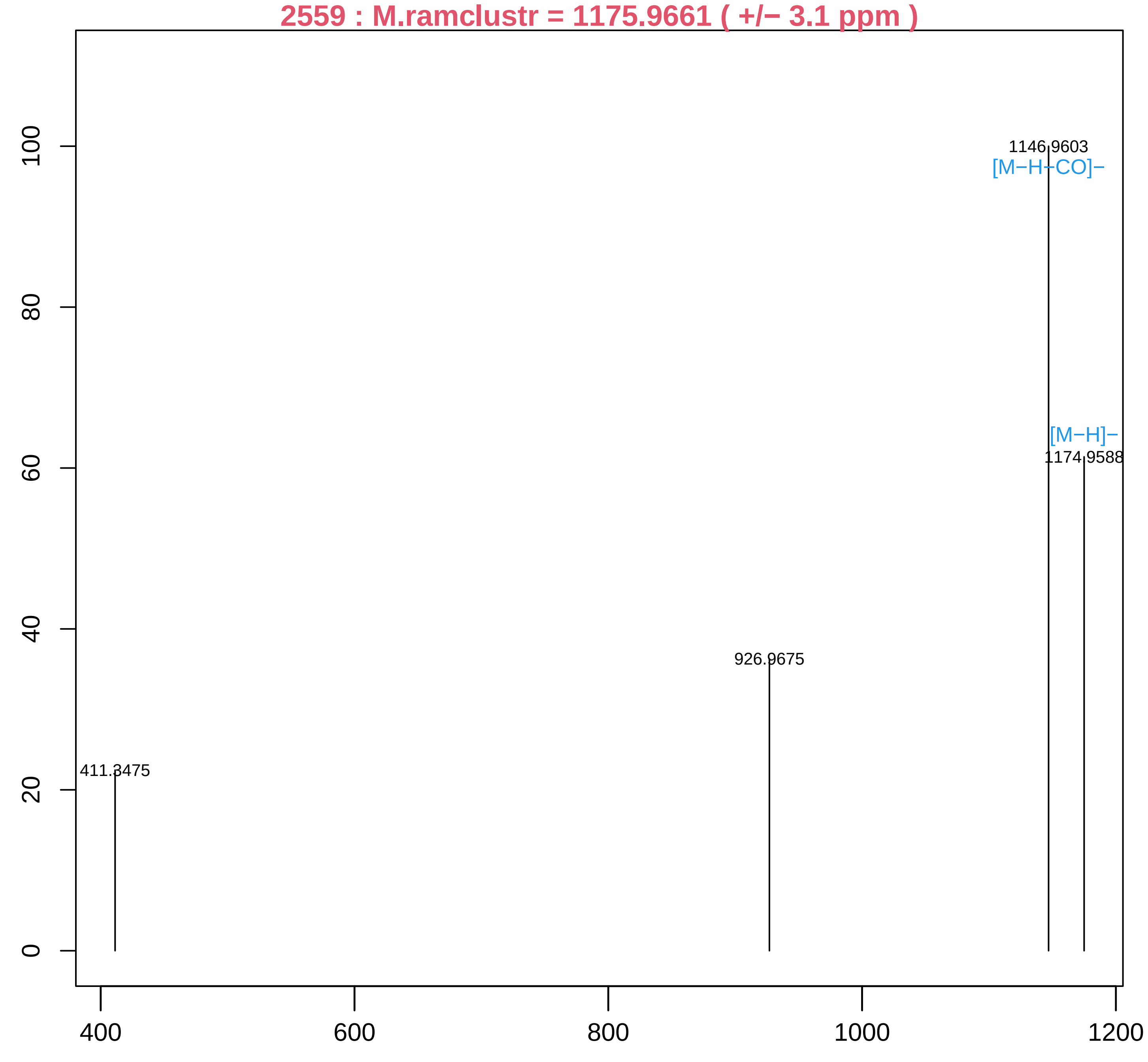

2559 : M.ramclustr = 1175.9661 (+/− 3.1 ppm)
1146.9603
[M−H−CO]−
[M−H]−
1174.9588
926.9675
411.3475
0
20
40
60
80
100
400
600
800
1000
1200

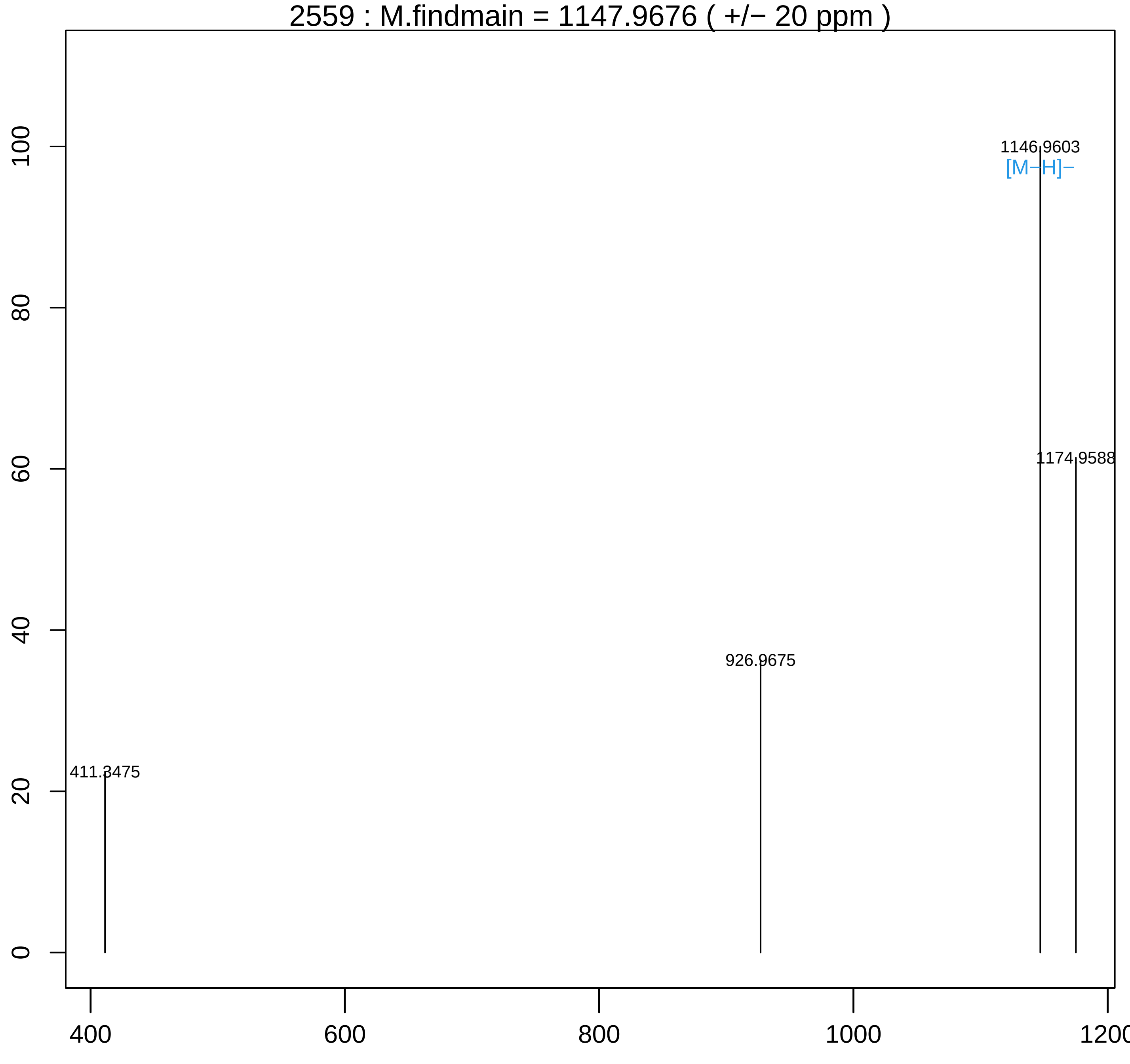

2559 : M.findmain = 1147.9676 (+/− 20 ppm)
1146.9603
[M−H]−
1174.9588
926.9675
411.3475
0
20
40
60
80
100
400
600
800
1000
1200

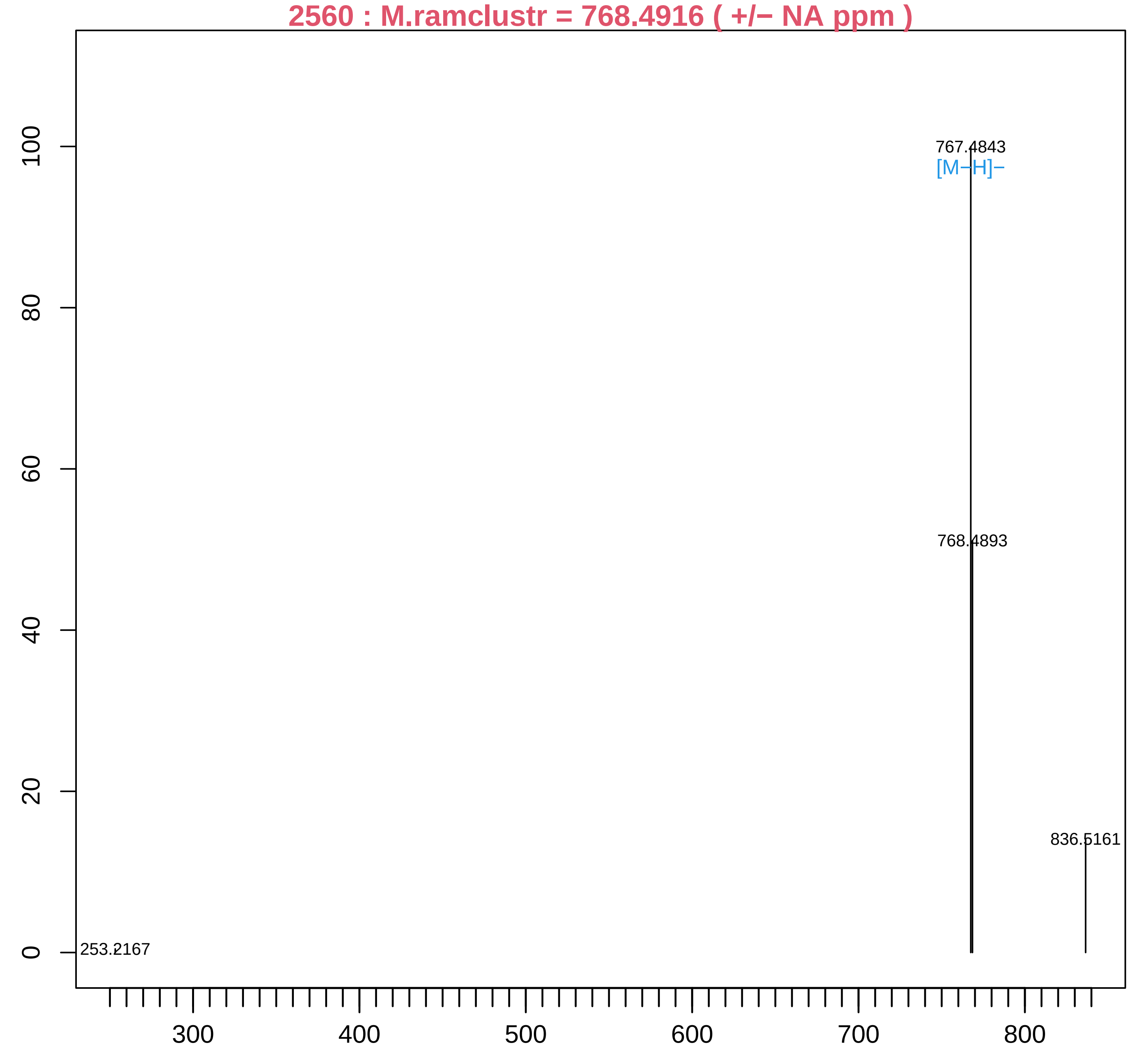
2560 : M.ramclustr = 768.4916 (+/− NA ppm)
767.4843
[M−H]−
768.4893
836.5161
253.2167
0
20
40
60
80
100
300
400
500
600
700
800

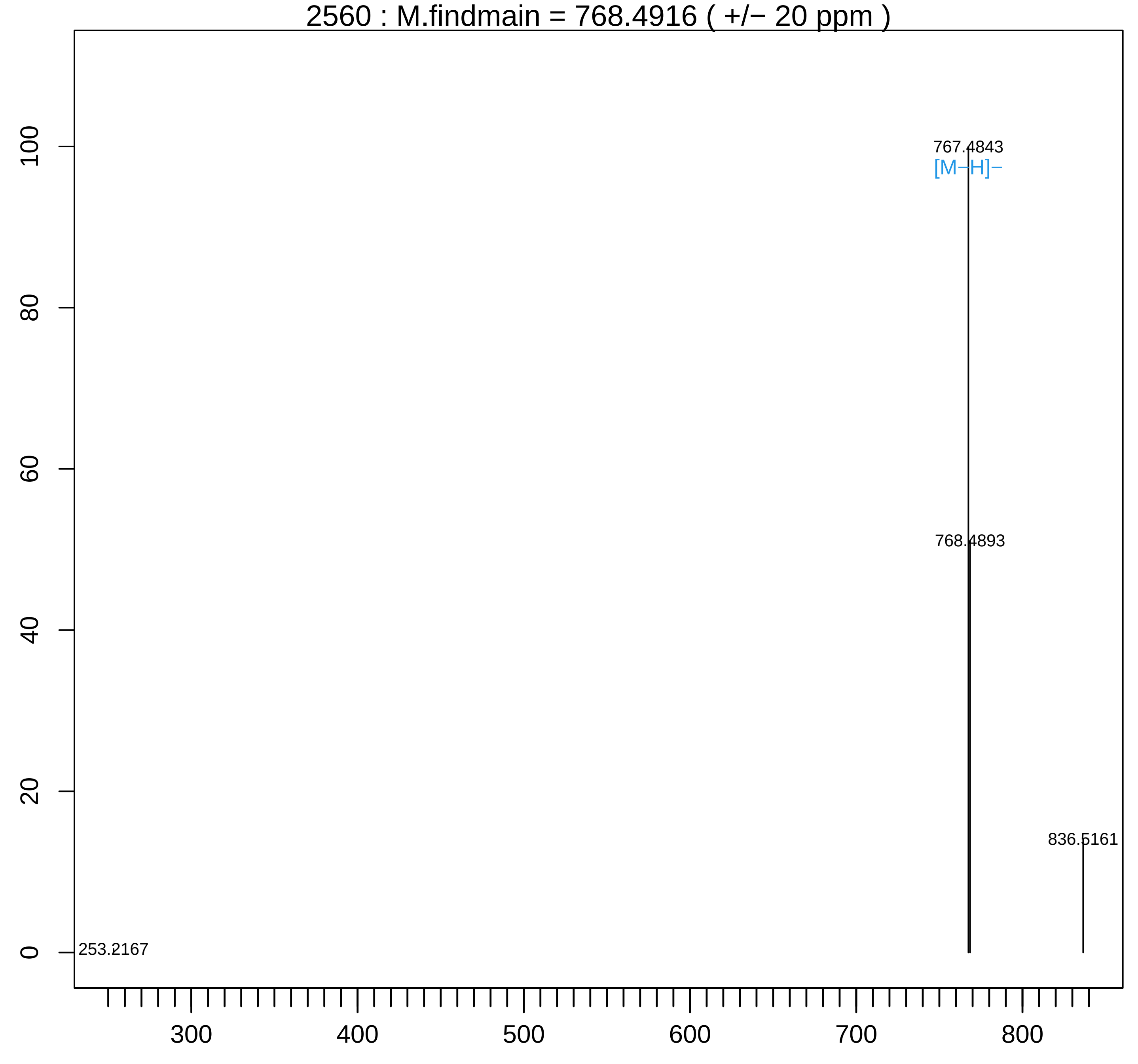
2560 : M.findmain = 768.4916 (+/− 20 ppm)
767.4843
[M−H]−
768.4893
836.5161
253.2167
0
20
40
60
80
100
300
400
500
600
700
800

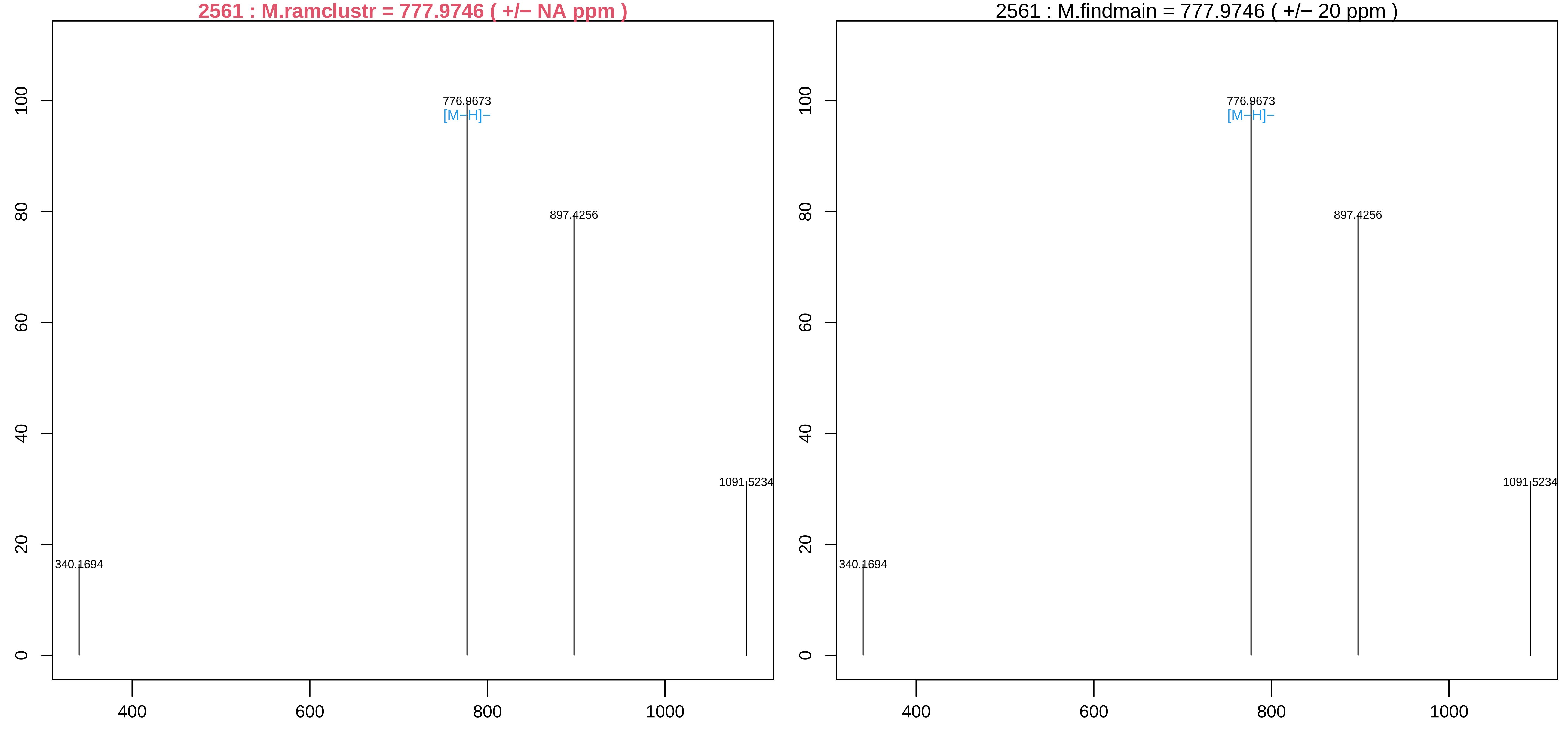
2561 : M.ramclustr = 777.9746 (+/− NA ppm)
2561 : M.findmain = 777.9746 (+/− 20 ppm)
776.9673
[M−H]−
897.4256
1091.5234
340.1694
0
20
40
60
80
100
400
600
800
1000

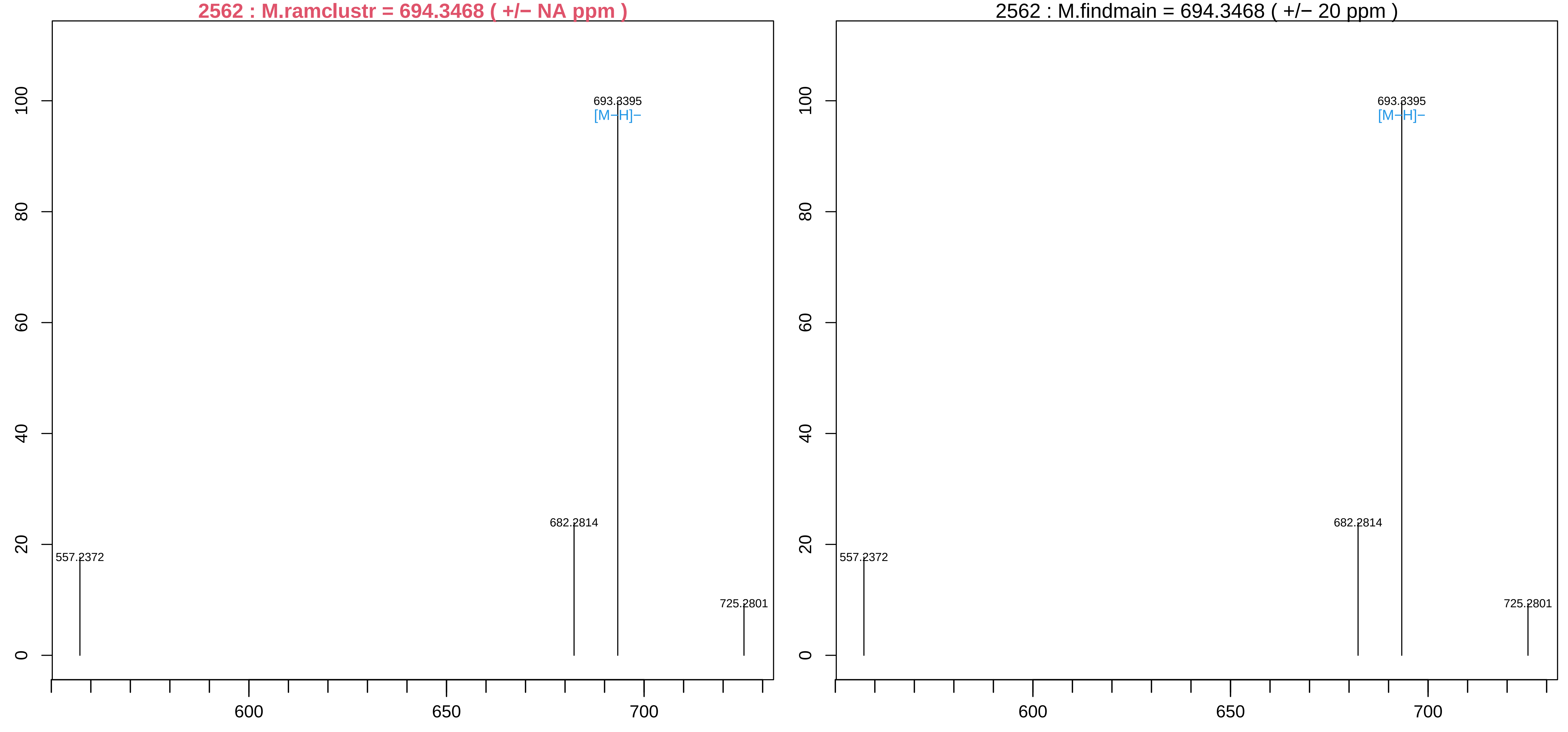

2562 : M.ramclustr = 694.3468 (+/− NA ppm)
693.3395
[M−H]−
682.2814
557.2372
725.2801
0
20
40
60
80
100
600
650
700
2562 : M.findmain = 694.3468 (+/− 20 ppm)
693.3395
[M−H]−
682.2814
557.2372
725.2801
0
20
40
60
80
100
600
650
700

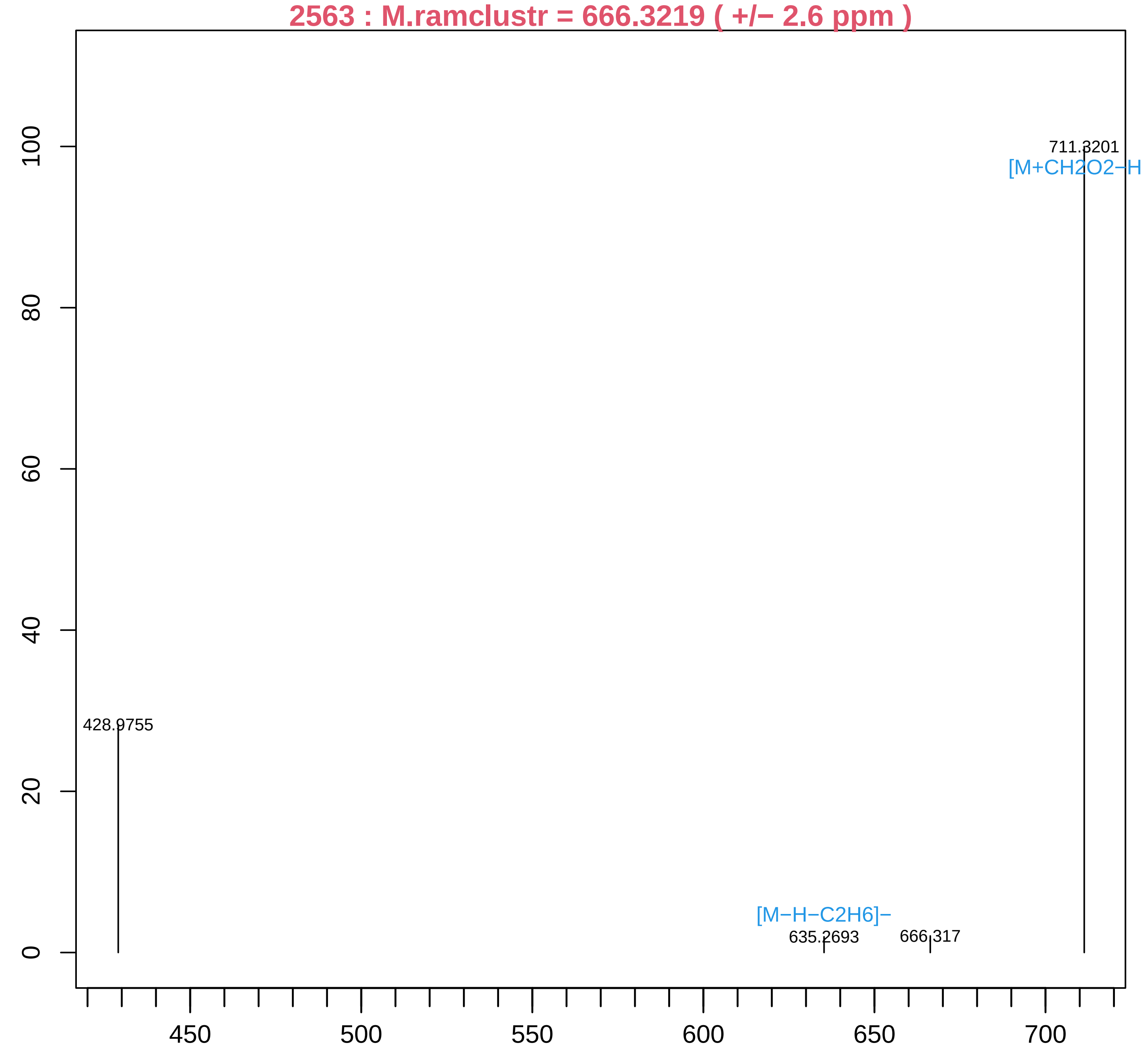
2563 : M.ramclustr = 666.3219 (+/− 2.6 ppm)
711.3201
[M+CH2O2−H
428.9755
[M−H−C2H6]−
635.2693
666.317
0
20
40
60
80
100
450
500
550
600
650
700

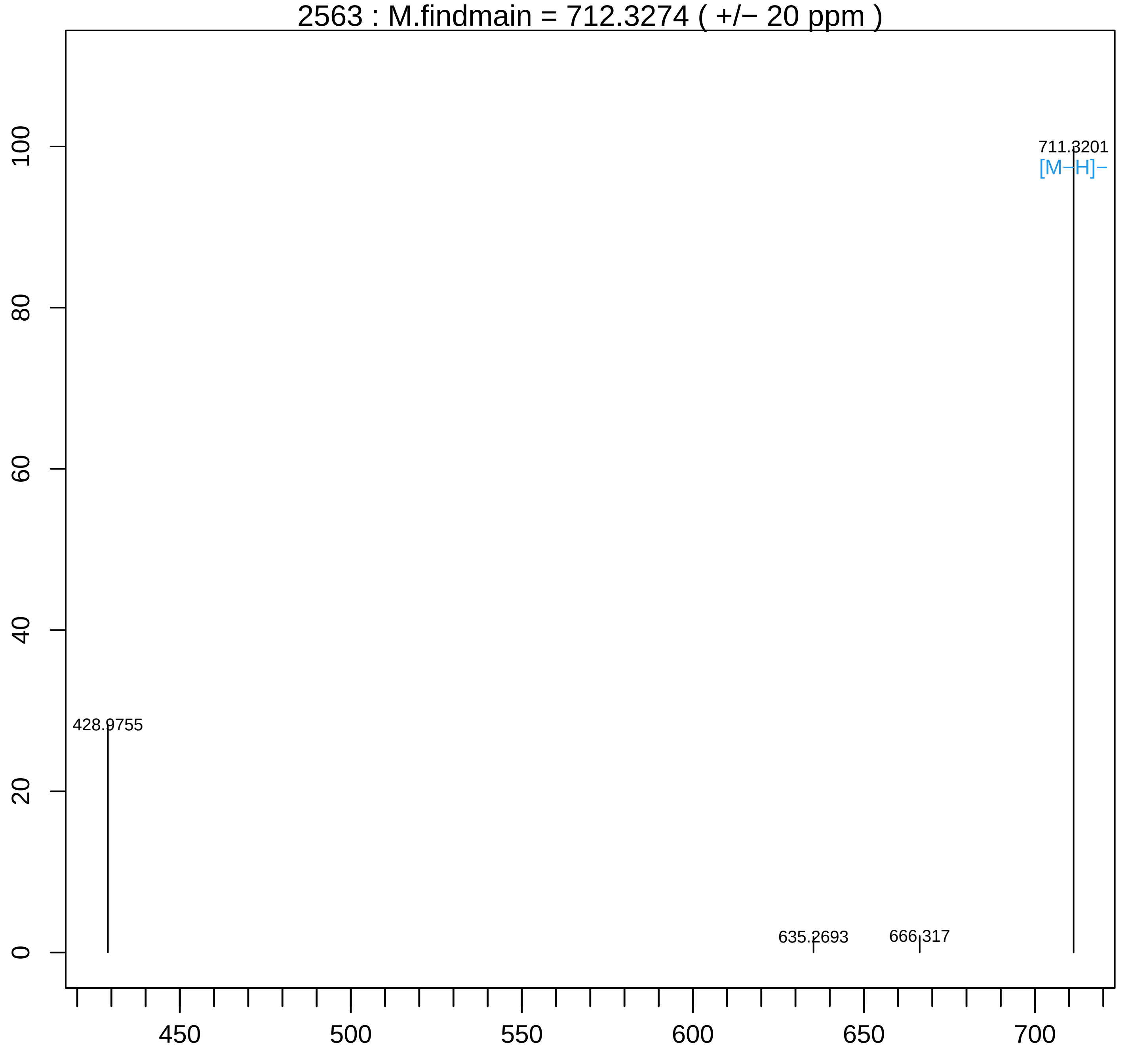
2563 : M.findmain = 712.3274 (+/− 20 ppm)
711.3201
[M−H]−
428.9755
635.2693
666.317
0
20
40
60
80
100
450
500
550
600
650
700

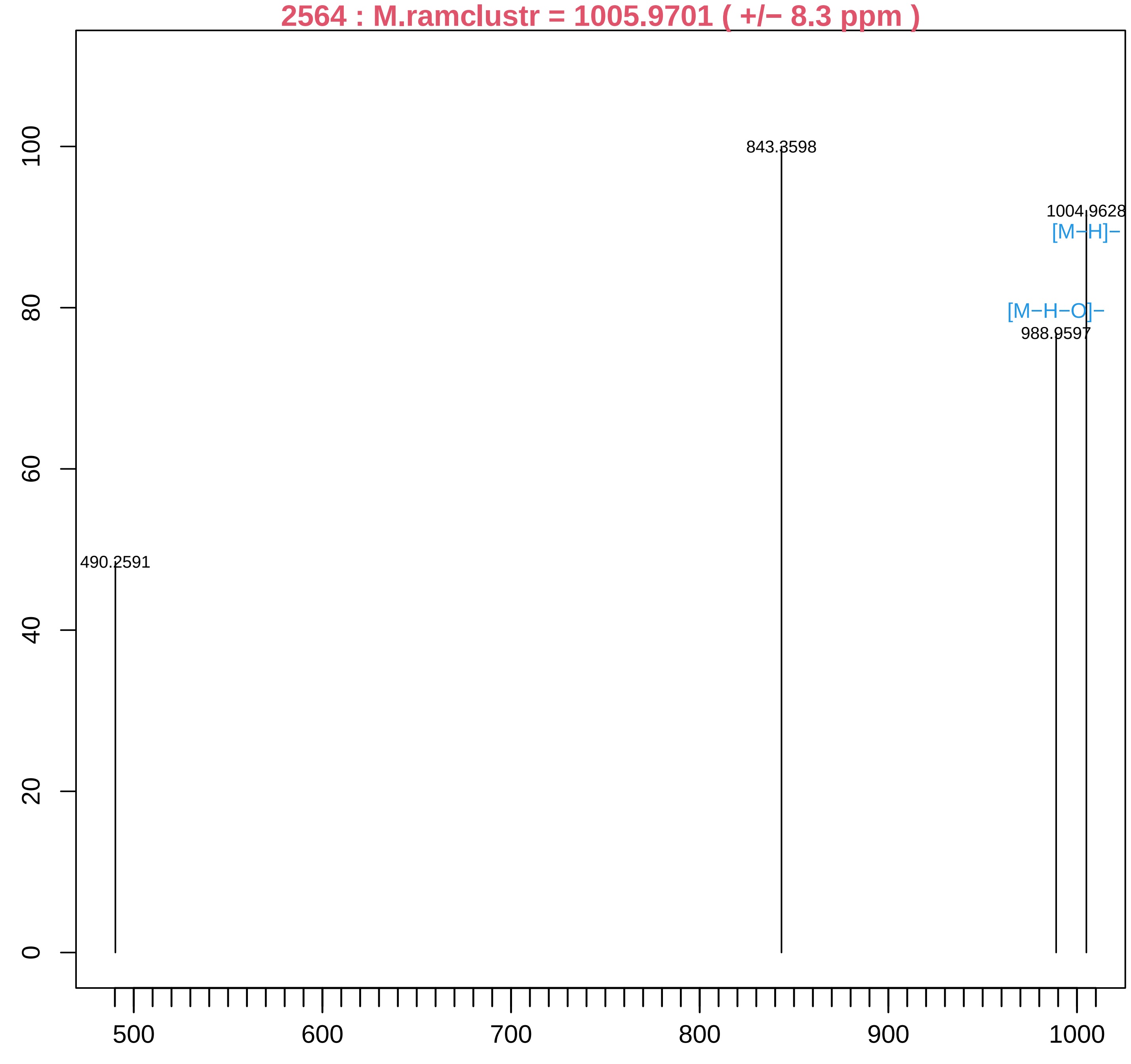

2564 : M.ramclustr = 1005.9701 (+/− 8.3 ppm)
843.3598
1004.9628
[M−H]−
[M−H−O]−
988.9597
490.2591
0
20
40
60
80
100
500
600
700
800
900
1000

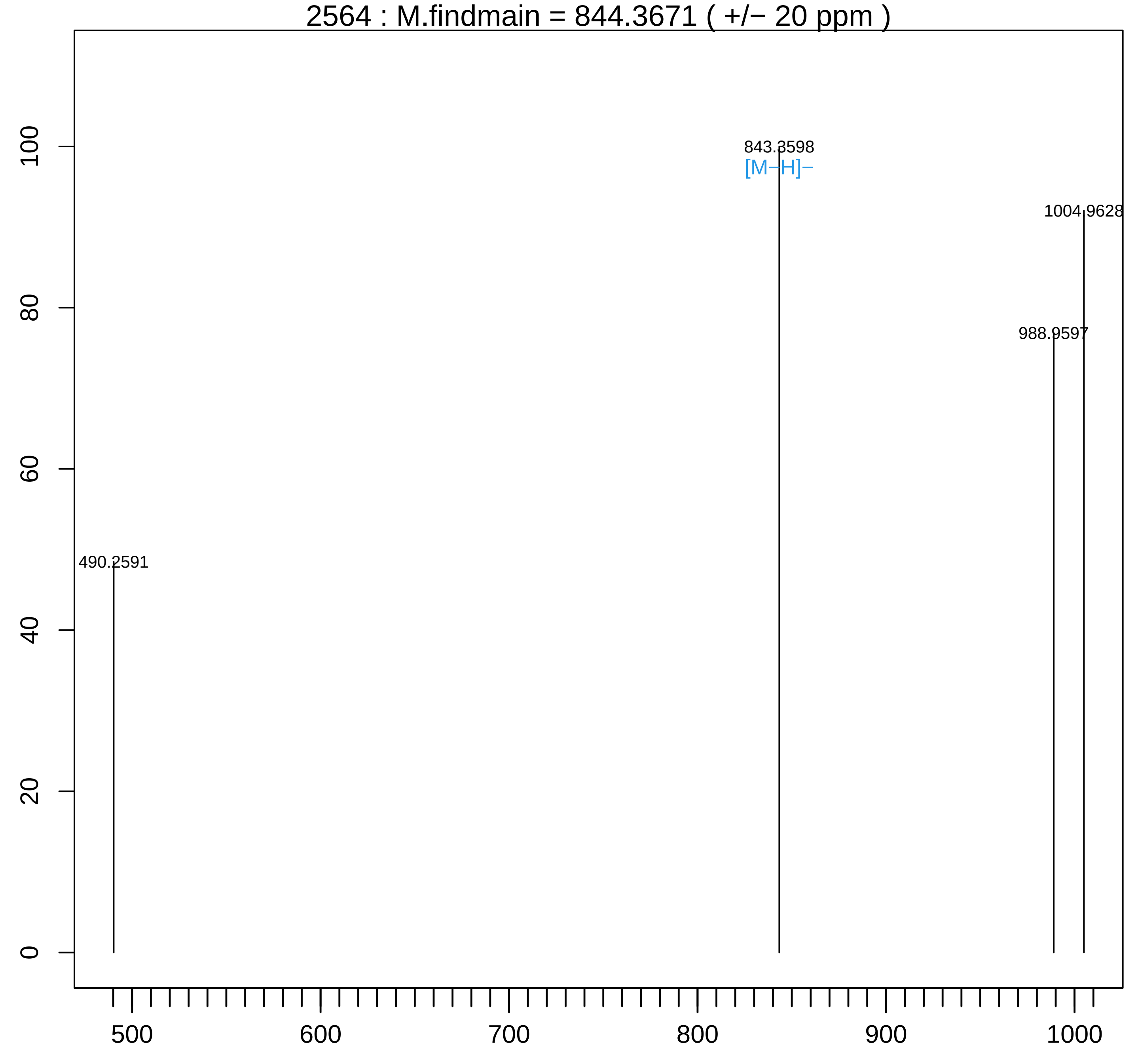

2564 : M.findmain = 844.3671 (+/− 20 ppm)
843.3598
[M−H]−
1004.9628
988.9597
490.2591
0
20
40
60
80
100
500
600
700
800
900
1000

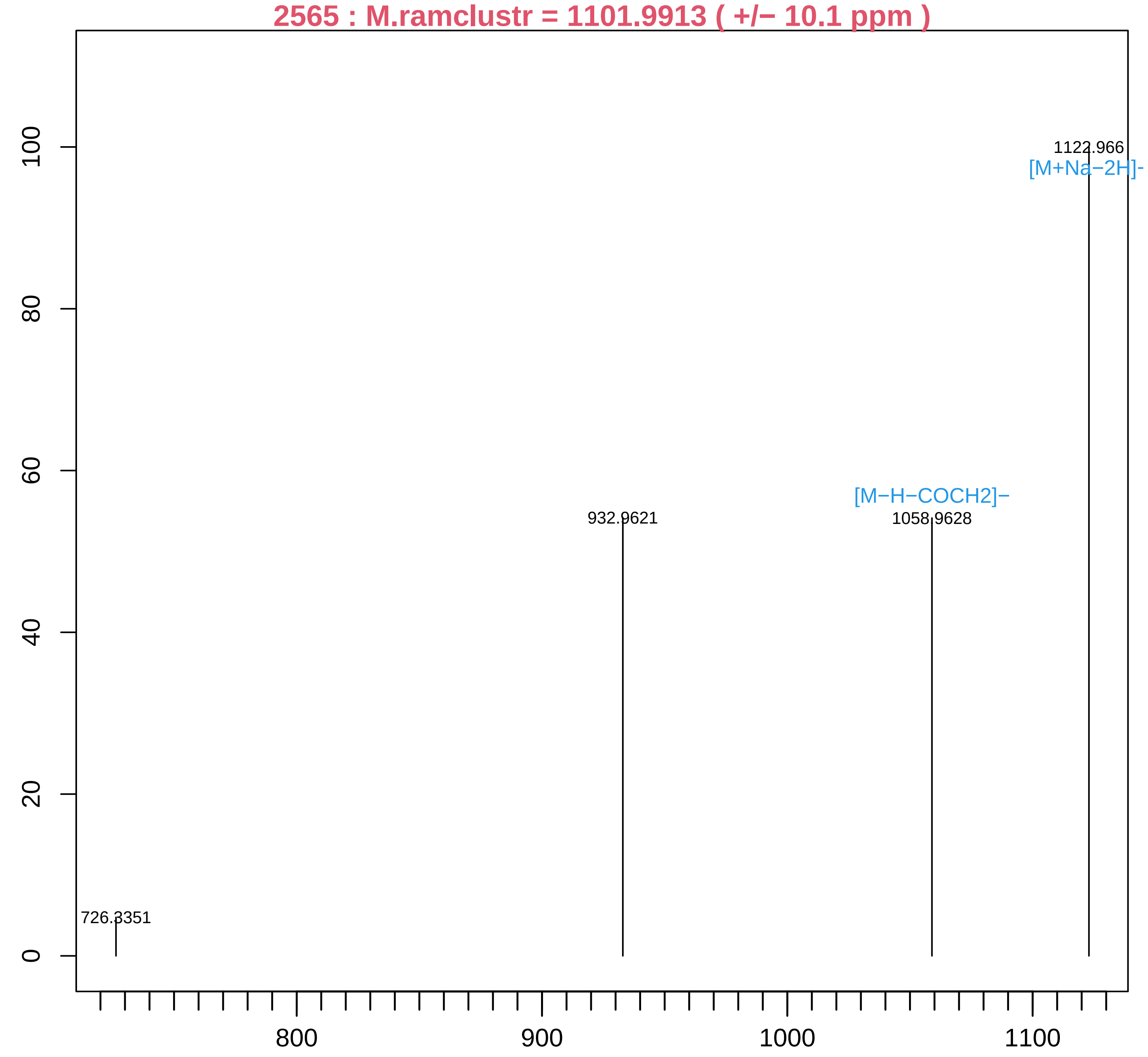
2565 : M.ramclustr = 1101.9913 (+/− 10.1 ppm)
1122.966
[M+Na−2H]-
[M−H−COCH2]−
932.9621
1058.9628
726.3351
0
20
40
60
80
100
800
900
1000
1100

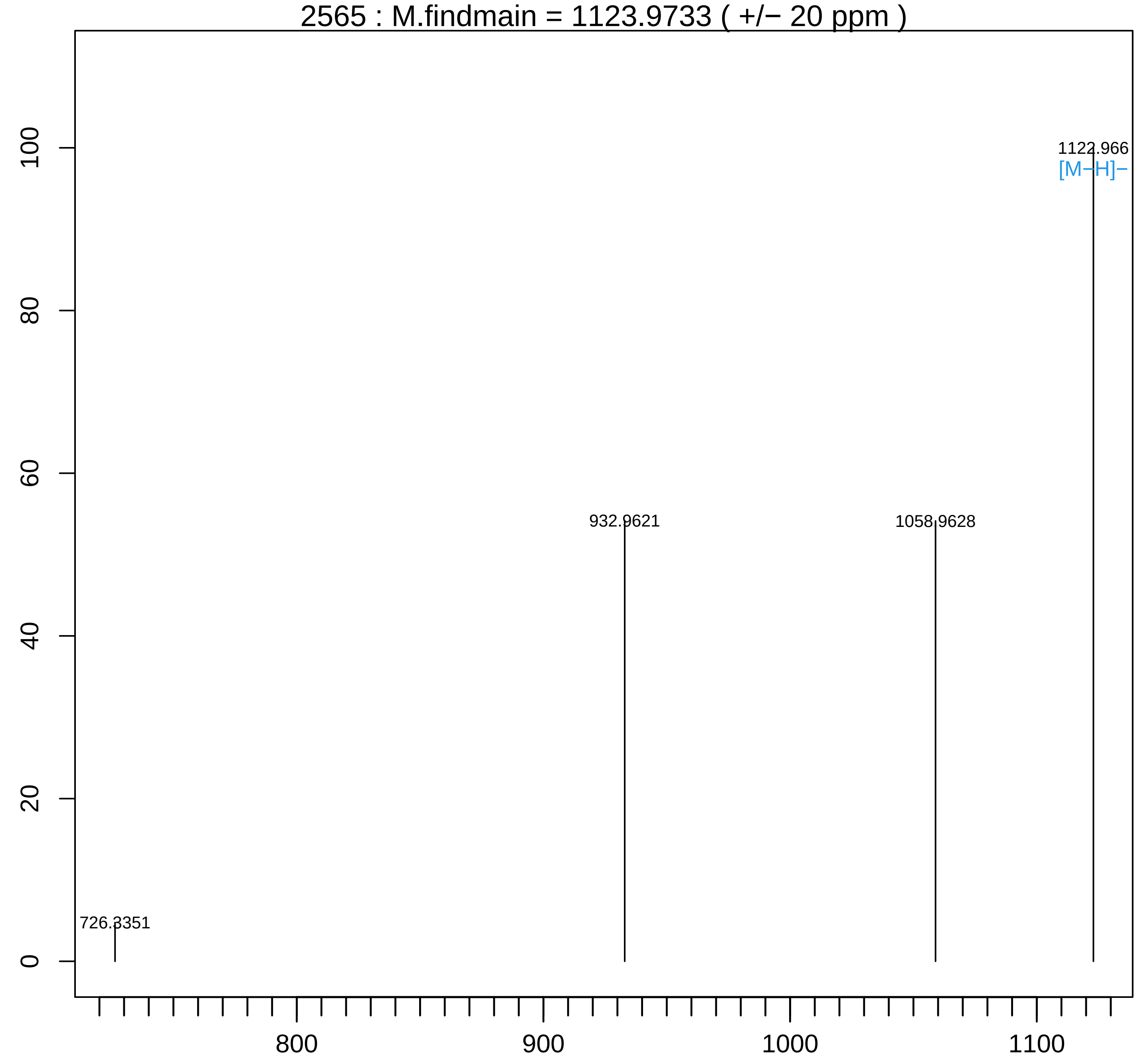
2565 : M.findmain = 1123.9733 (+/− 20 ppm)
1122.966
[M−H]−
932.9621
1058.9628
726.3351
0
20
40
60
80
100
800
900
1000
1100

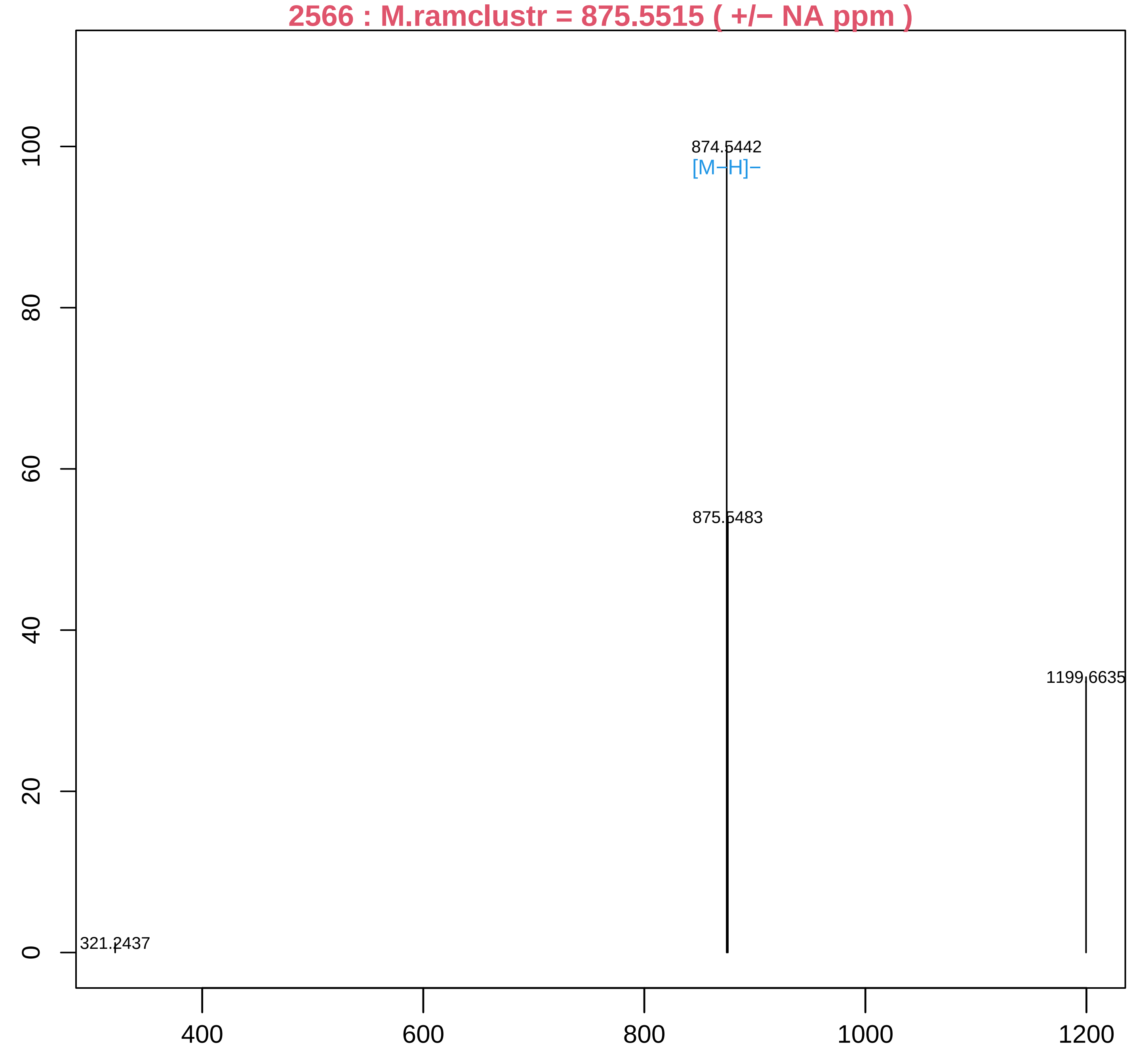

2566 : M.ramclustr = 875.5515 (+/− NA ppm)
874.5442
[M−H]−
875.5483
1199.6635
321.2437
0
20
40
60
80
100
400
600
800
1000
1200

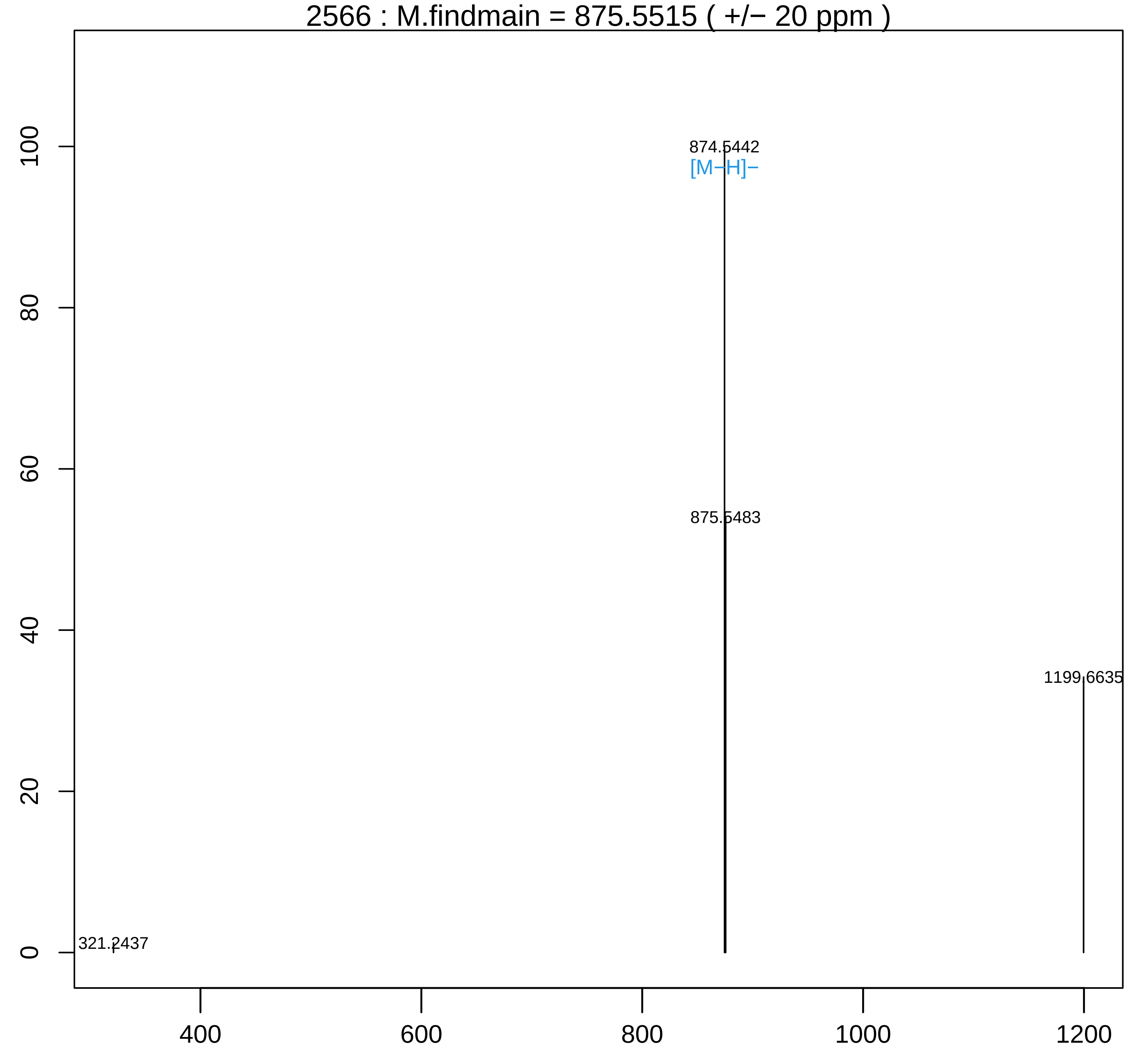

2566 : M.findmain = 875.5515 (+/− 20 ppm)
874.5442
[M−H]−
875.5483
1199.6635
321.2437
0
20
40
60
80
100
400
600
800
1000
1200

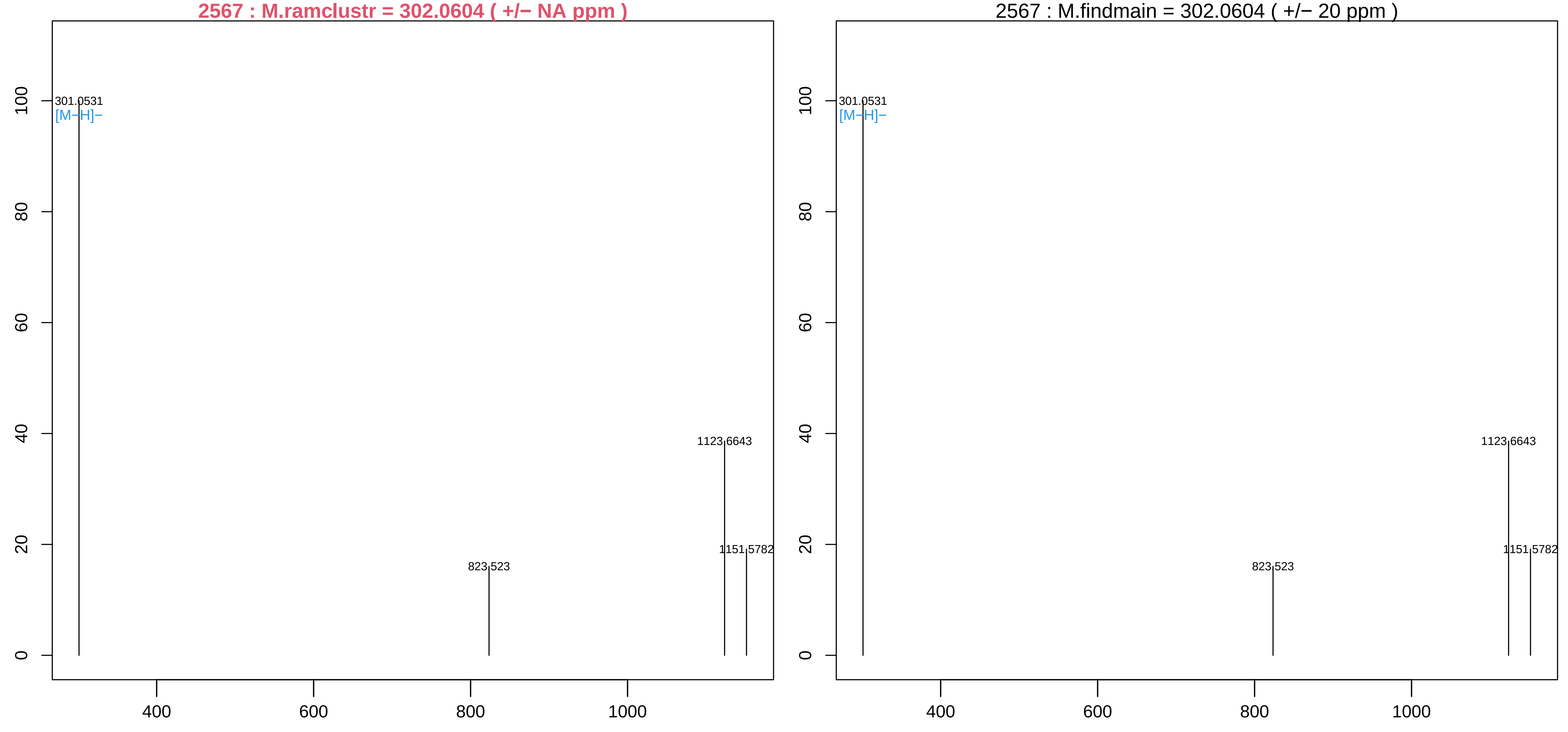

2567 : M.ramclustr = 302.0604 (+/− NA ppm)
301.0531
[M−H]−
1123.6643
1151.5782
823.523
2567 : M.findmain = 302.0604 (+/− 20 ppm)
301.0531
[M−H]−
1123.6643
1151.5782
823.523
0
20
40
60
80
100
400
600
800
1000

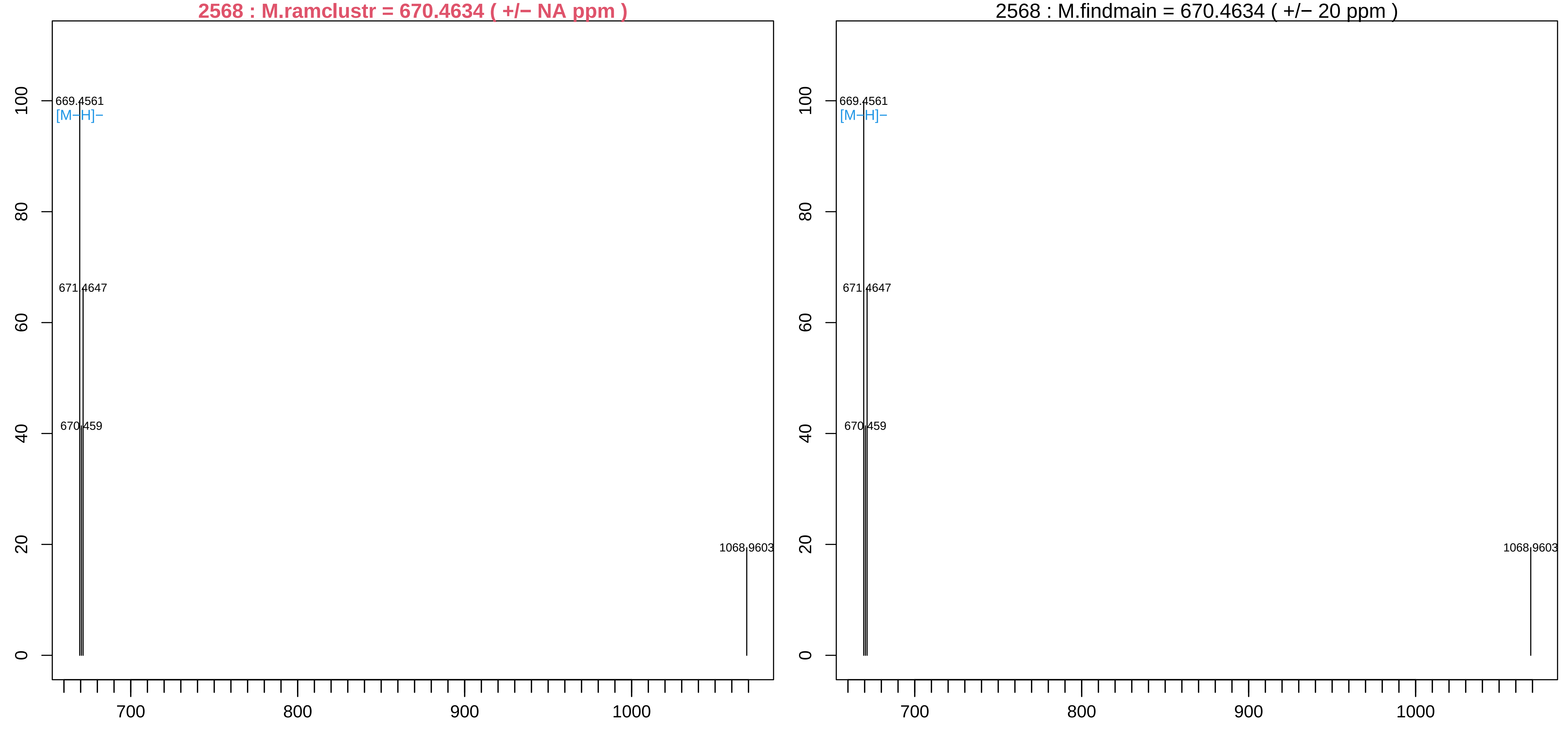
2568 : M.ramclustr = 670.4634 (+/− NA ppm)
669.4561
[M−H]−
671.4647
670.459
1068.9603
0
20
40
60
80
100
700
800
900
1000
2568 : M.findmain = 670.4634 (+/− 20 ppm)
669.4561
[M−H]−
671.4647
670.459
1068.9603
0
20
40
60
80
100
700
800
900
1000

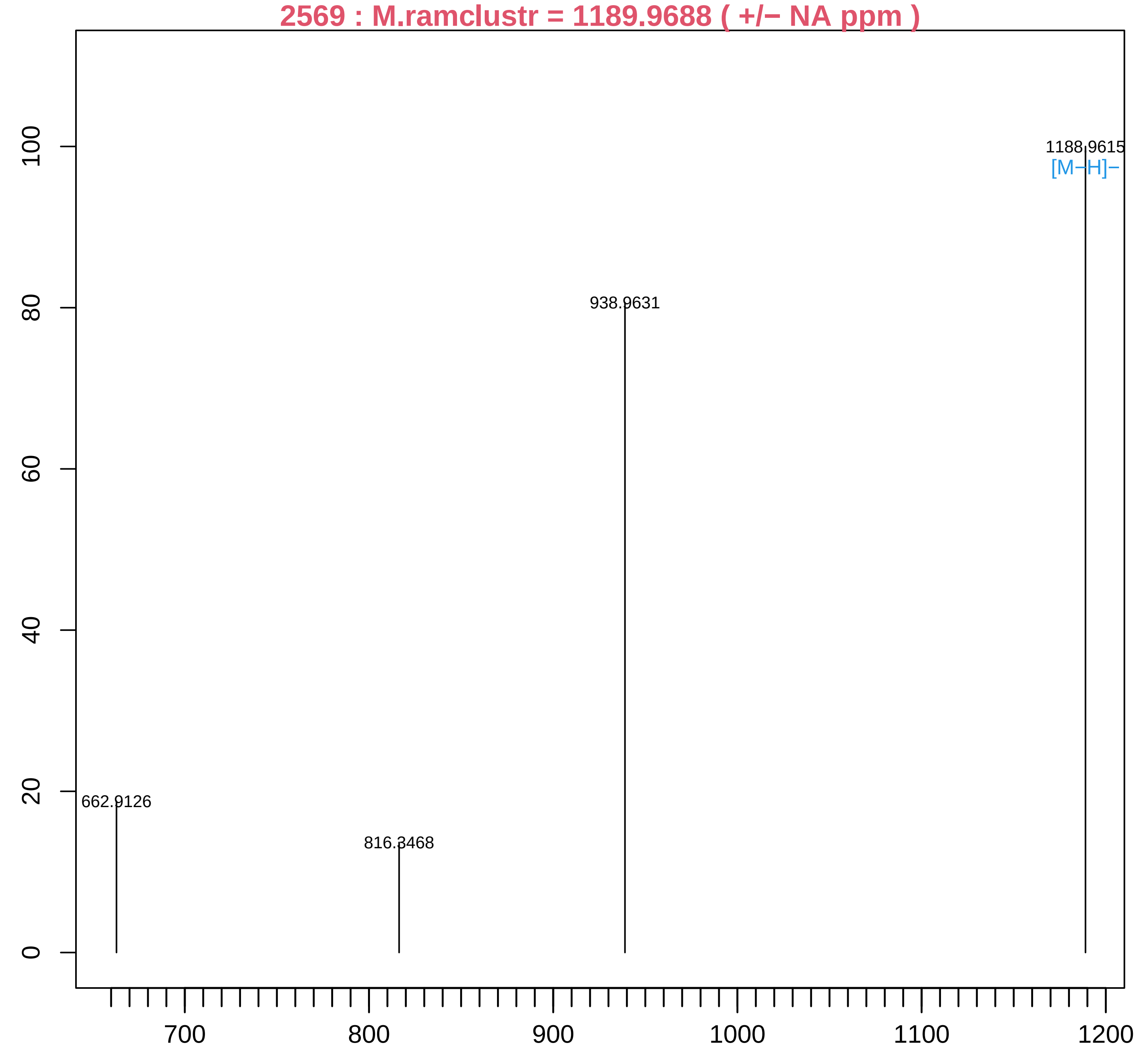
2569 : M.ramclustr = 1189.9688 (+/− NA ppm)
1188.9615
[M−H]−
938.9631
662.9126
816.3468
0
20
40
60
80
100
700
800
900
1000
1100
1200

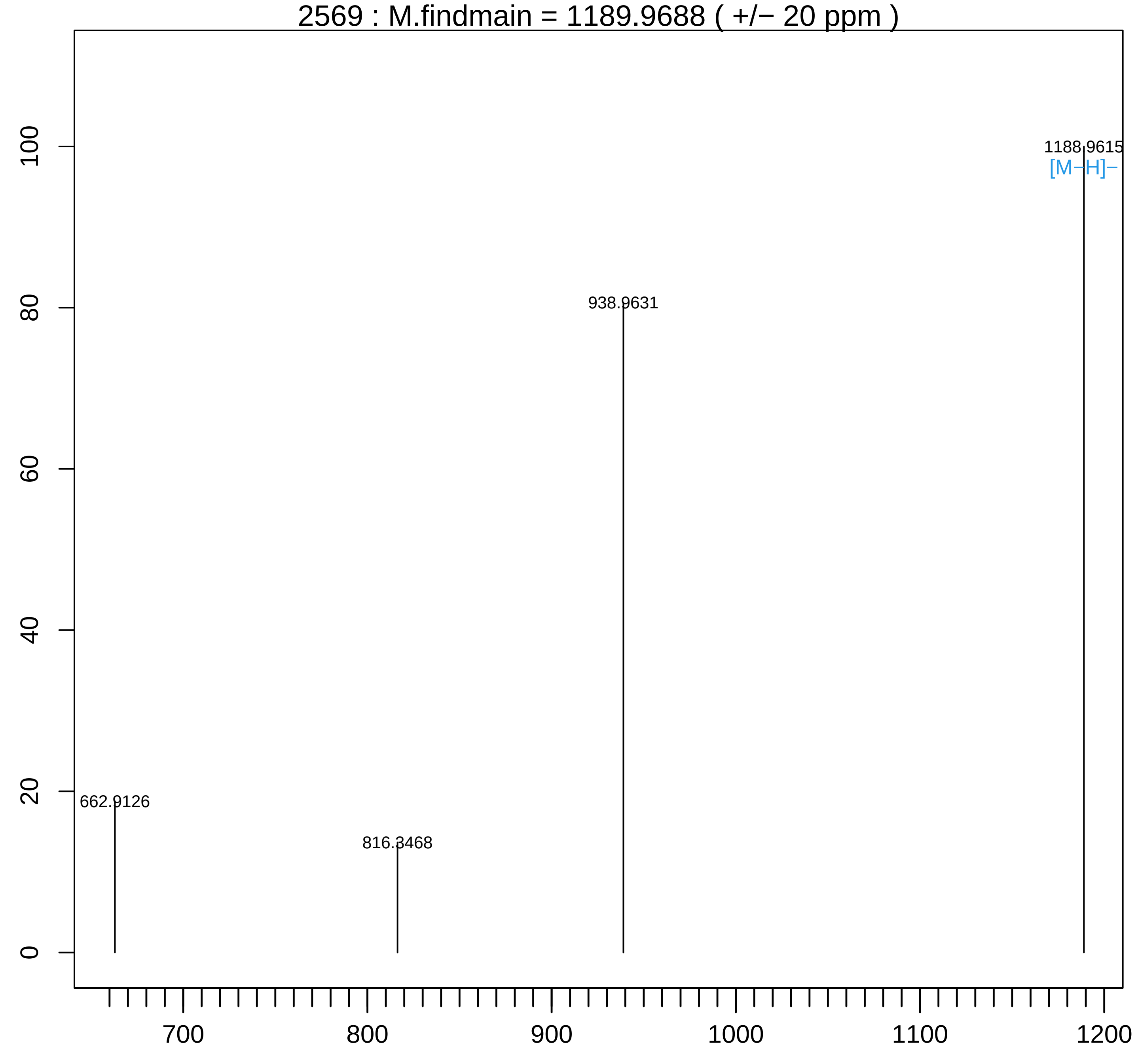
2569 : M.findmain = 1189.9688 (+/− 20 ppm)
1188.9615
[M−H]−
938.9631
662.9126
816.3468
0
20
40
60
80
100
700
800
900
1000
1100
1200

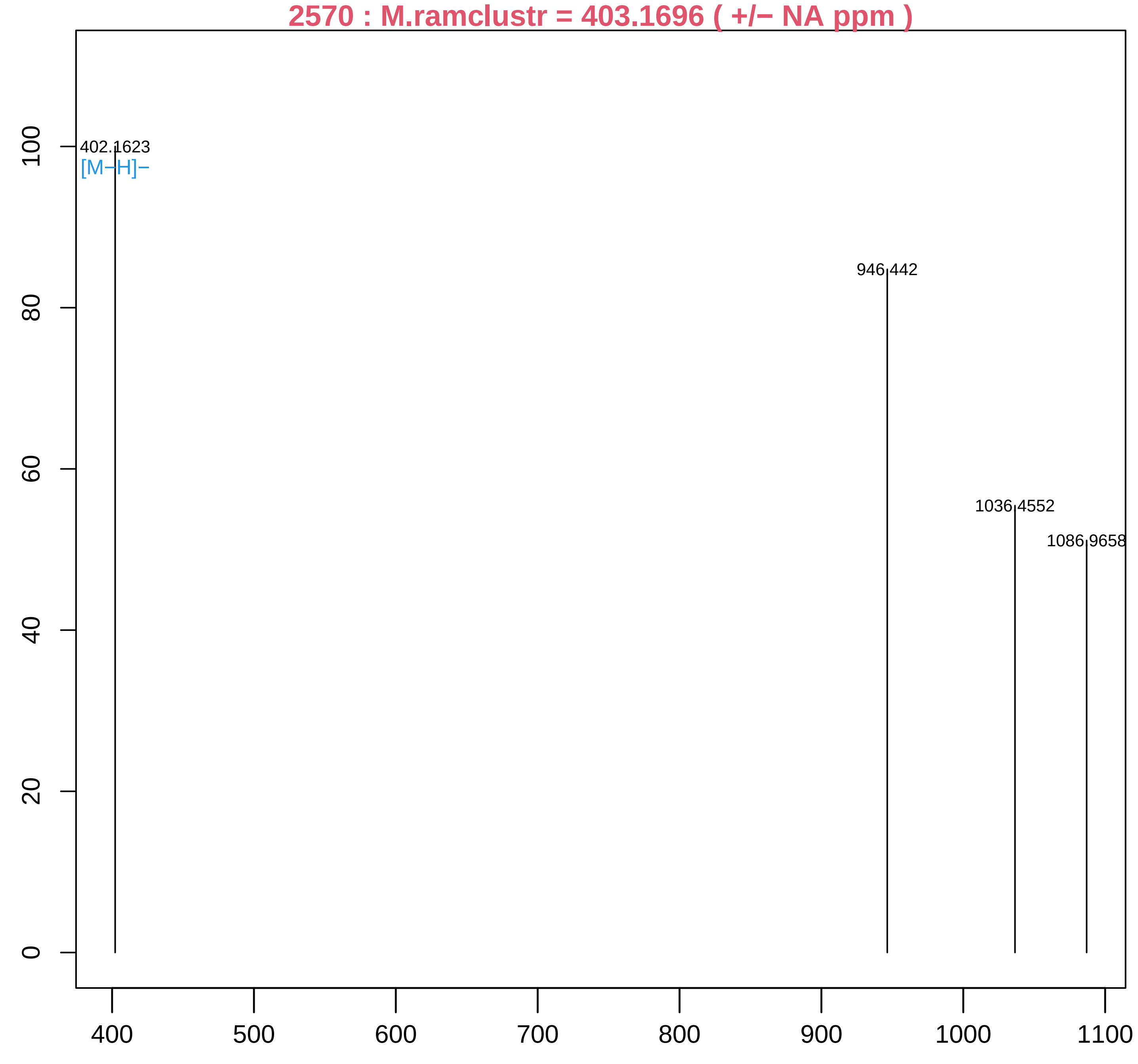

2570 : M.ramclustr = 403.1696 (+/− NA ppm)
402.1623
[M−H]−
946.442
1036.4552
1086.9658

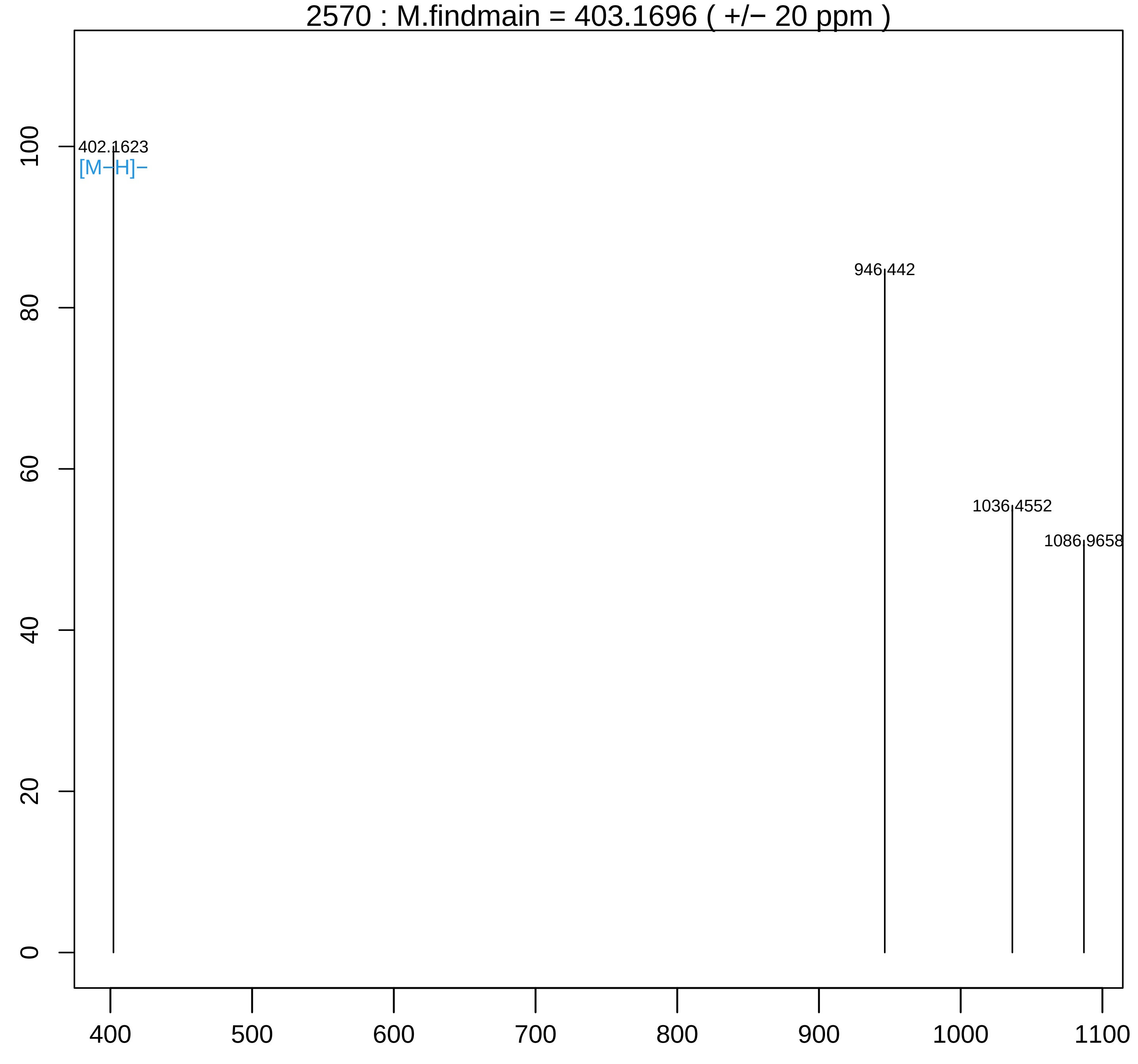

2570 : M.findmain = 403.1696 (+/− 20 ppm)
402.1623
[M−H]−
946.442
1036.4552
1086.9658

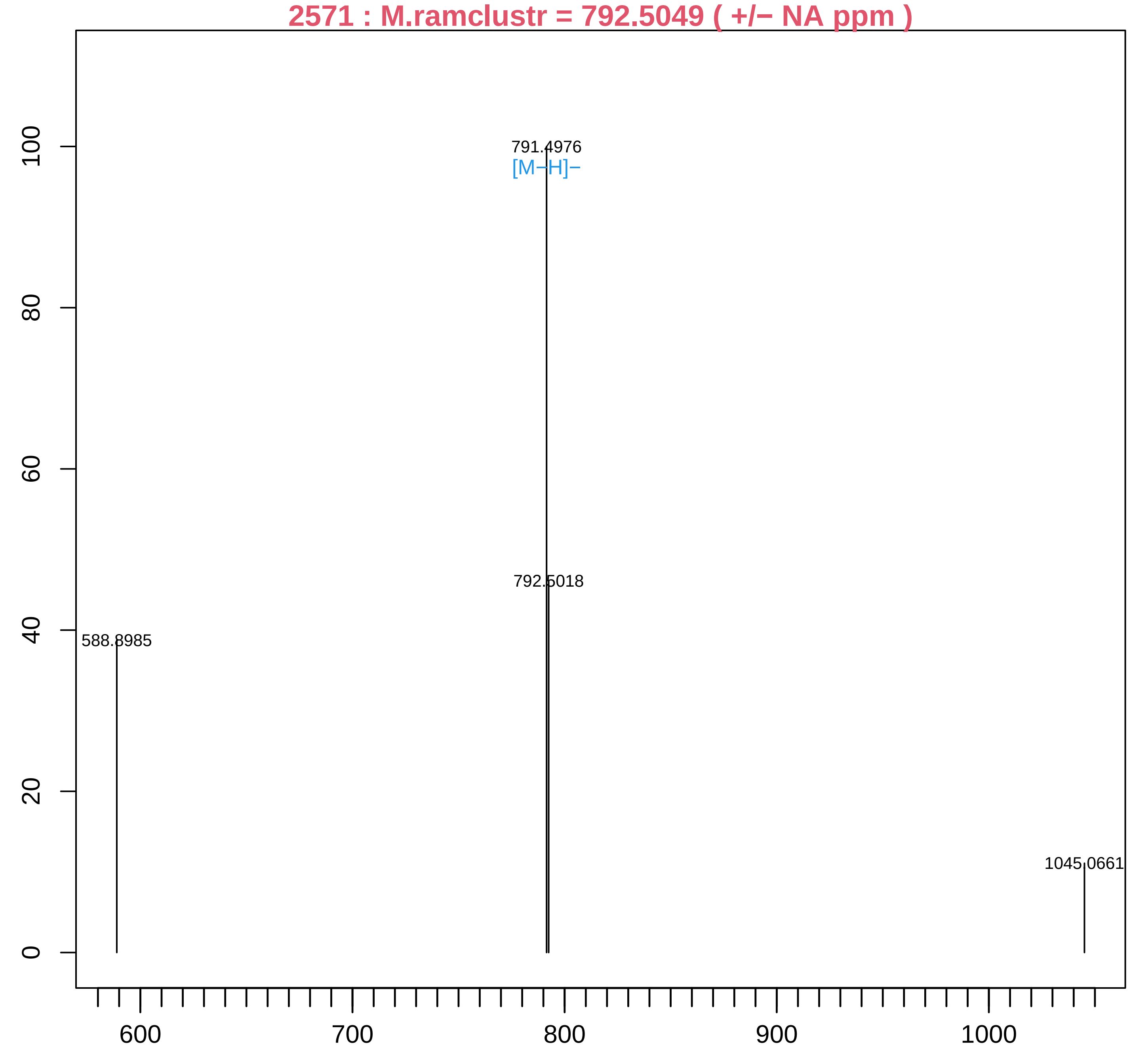
2571 : M.ramclustr = 792.5049 (+/− NA ppm)
791.4976
[M−H]−
792.5018
588.8985
1045.0661
0
20
40
60
80
100
600
700
800
900
1000

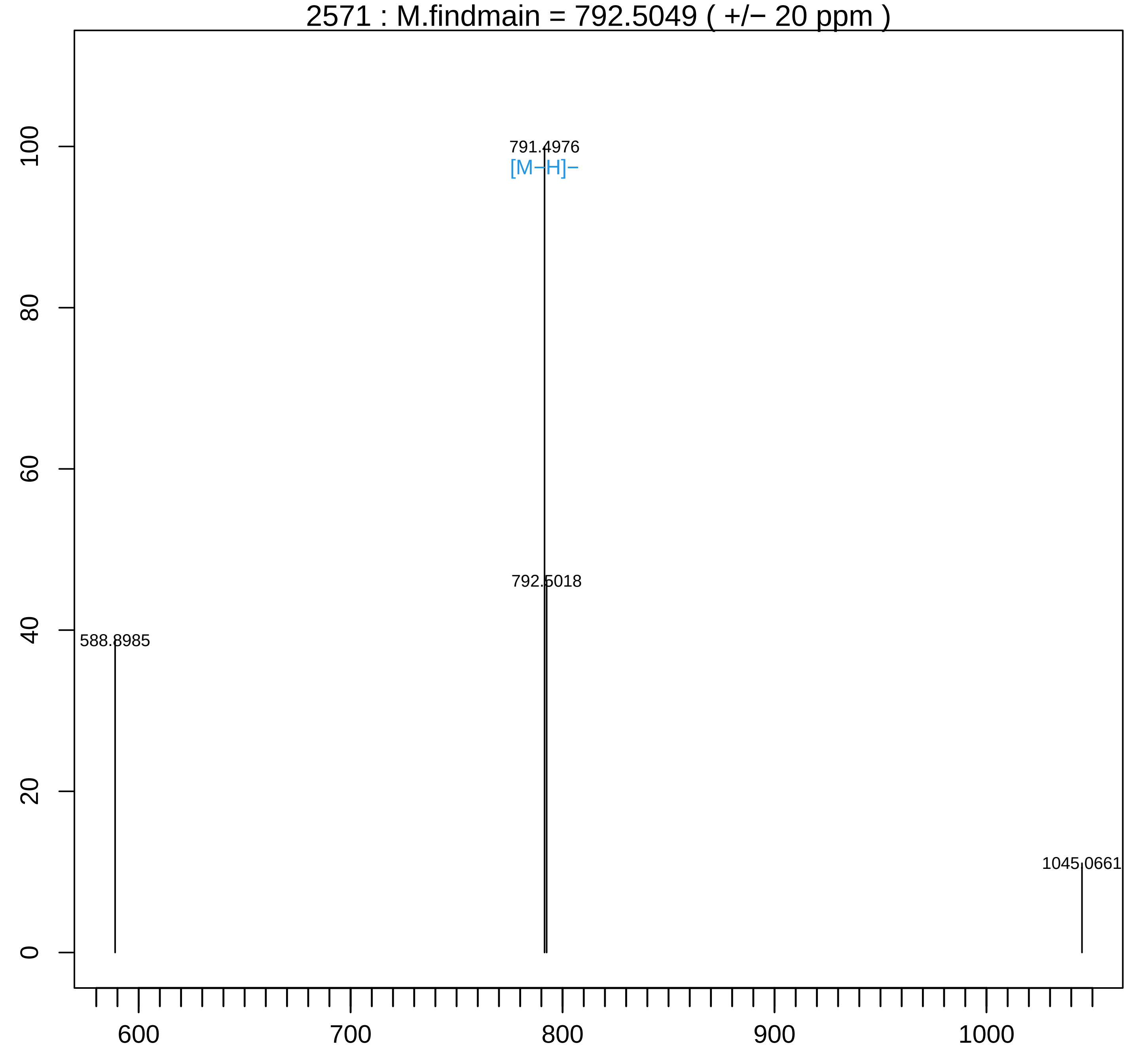
2571 : M.findmain = 792.5049 (+/− 20 ppm)
791.4976
[M−H]−
792.5018
588.8985
1045.0661
0
20
40
60
80
100
600
700
800
900
1000

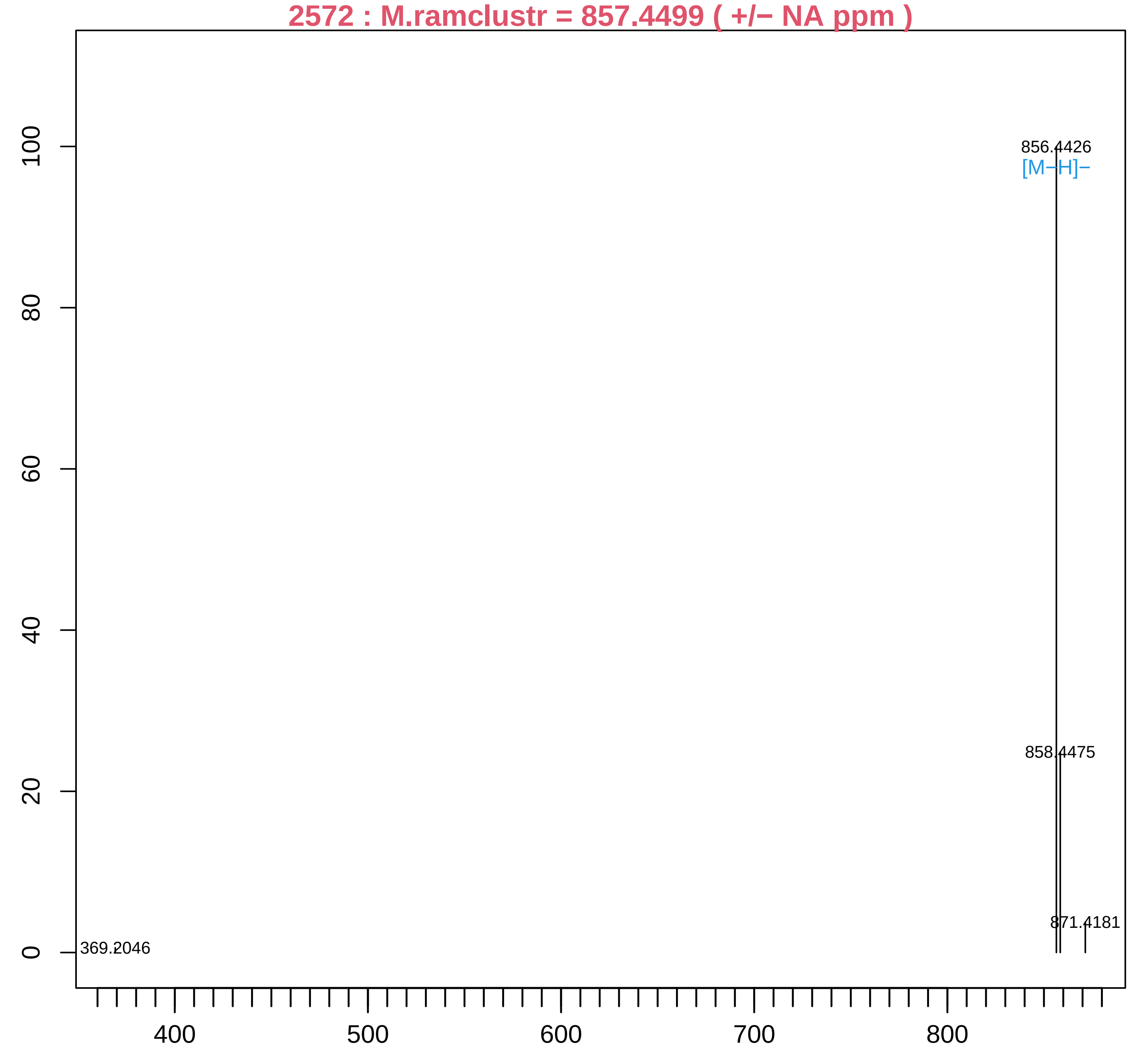

2572 : M.ramclustr = 857.4499 (+/− NA ppm)
856.4426
[M−H]−
858.4475
871.4181
369.2046
0
20
40
60
80
100
400
500
600
700
800

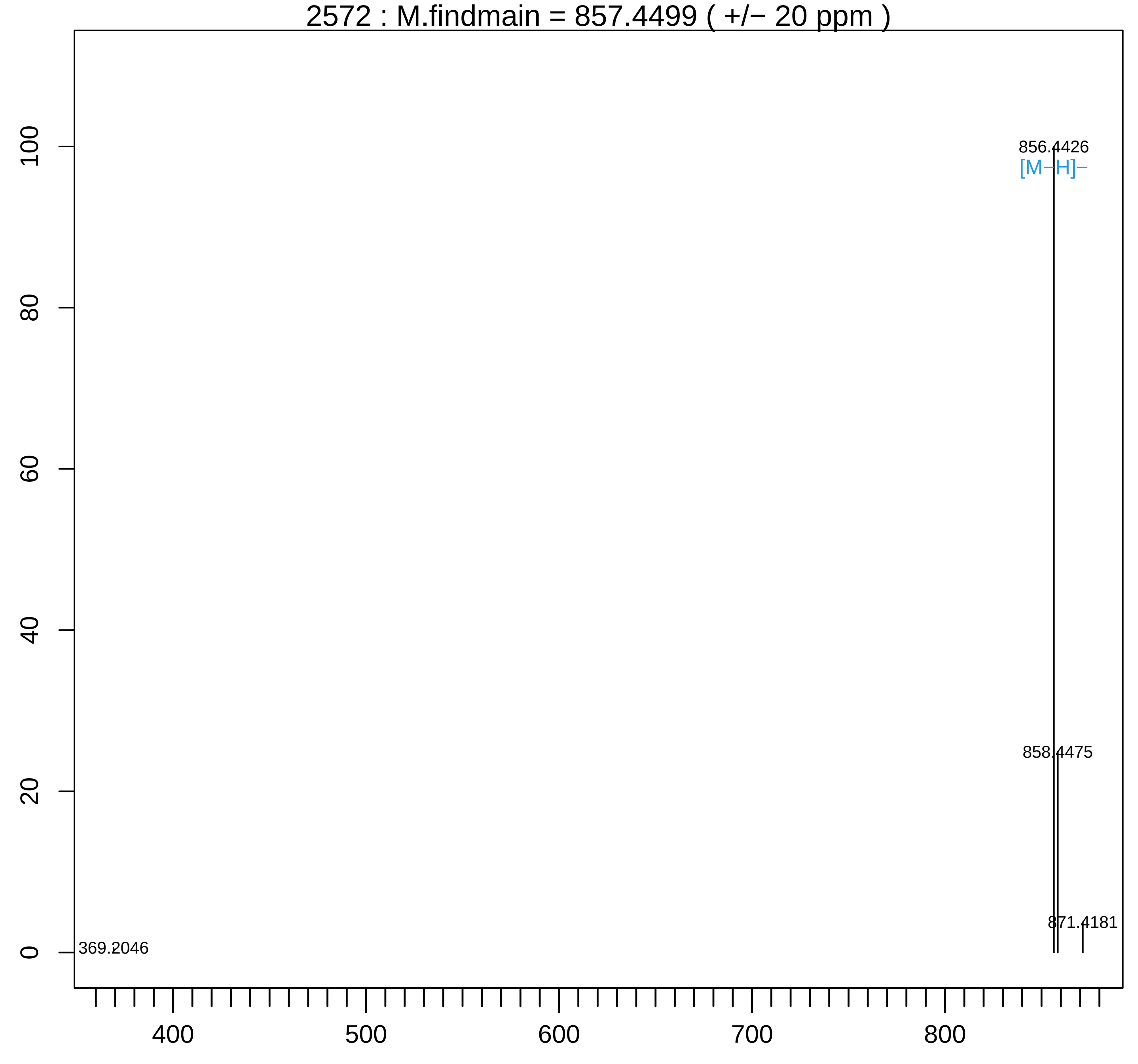

2572 : M.findmain = 857.4499 (+/− 20 ppm)
856.4426
[M−H]−
858.4475
871.4181
369.2046
0
20
40
60
80
100
400
500
600
700
800

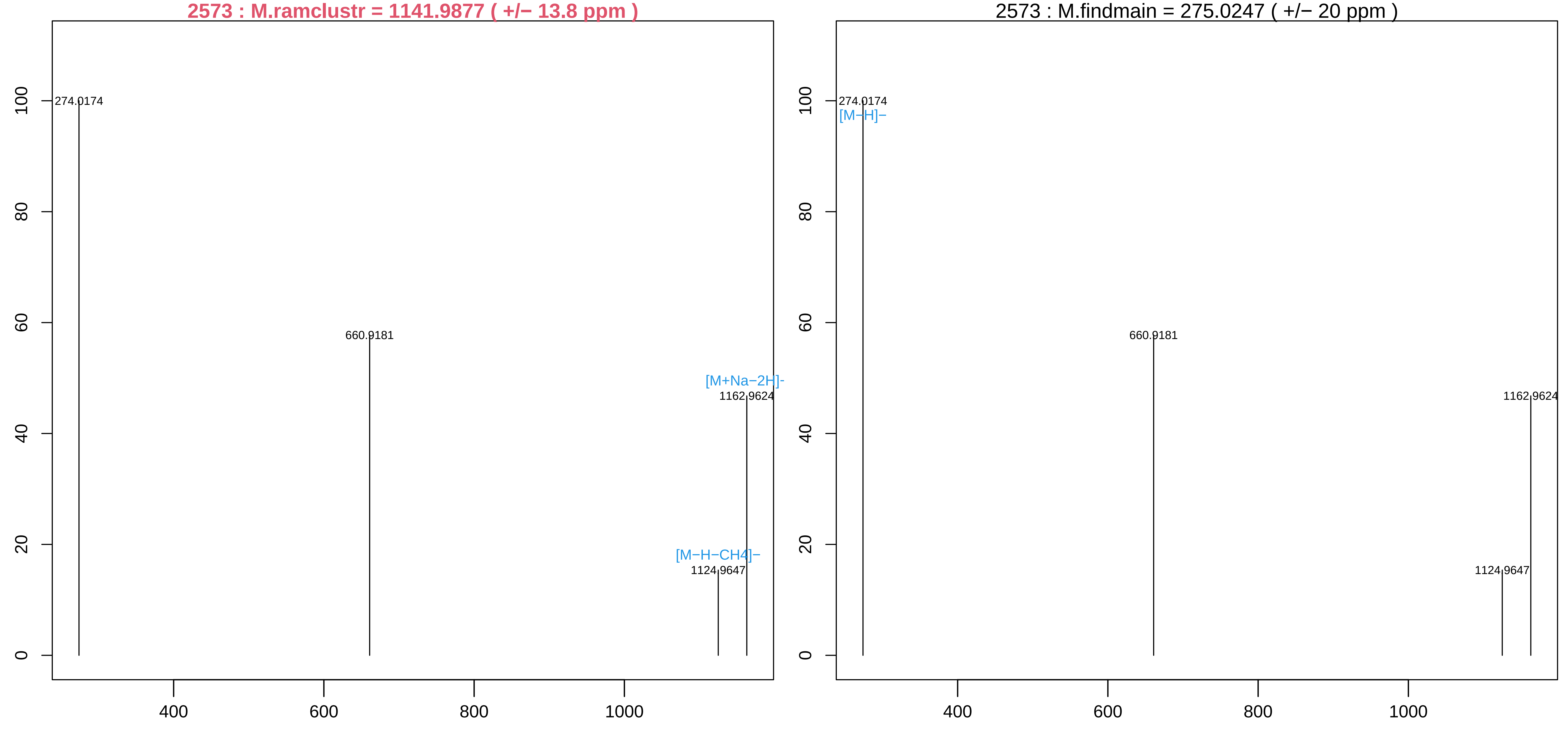

2573 : M.ramclustr = 1141.9877 (+/− 13.8 ppm)
274.0174
660.9181
[M+Na−2H]-
1162.9624
[M−H−CH4]−
1124.9647
0
20
40
60
80
100
400
600
800
1000
2573 : M.findmain = 275.0247 (+/− 20 ppm)
274.0174
[M−H]−
660.9181
1162.9624
1124.9647
0
20
40
60
80
100
400
600
800
1000

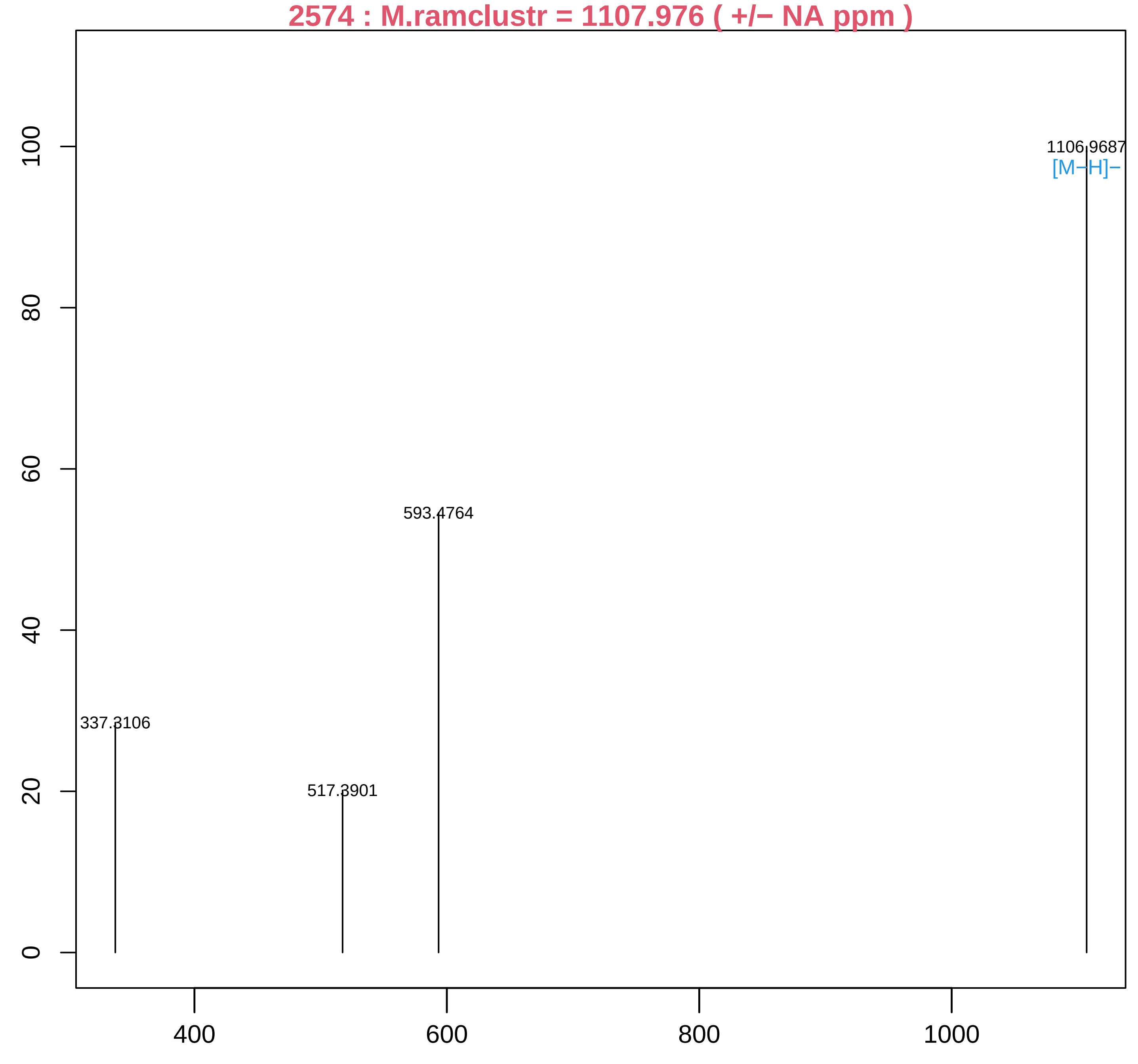
2574 : M.ramclustr = 1107.976 (+/− NA ppm)
1106.9687
[M−H]−
593.4764
337.3106
517.3901
0
20
40
60
80
100
400
600
800
1000
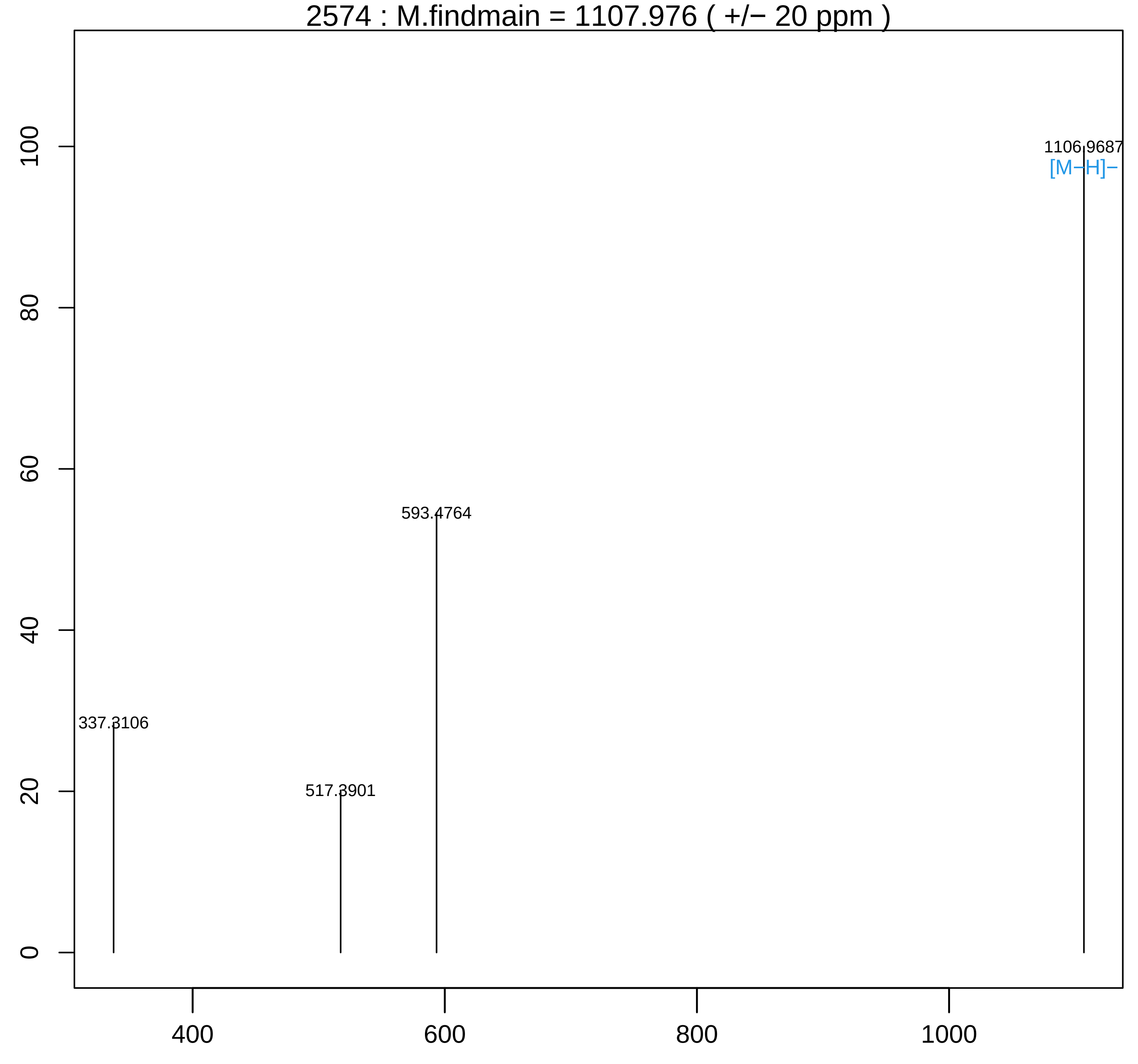
2574 : M.findmain = 1107.976 (+/− 20 ppm)
1106.9687
[M−H]−
593.4764
337.3106
517.3901
0
20
40
60
80
100
400
600
800
1000

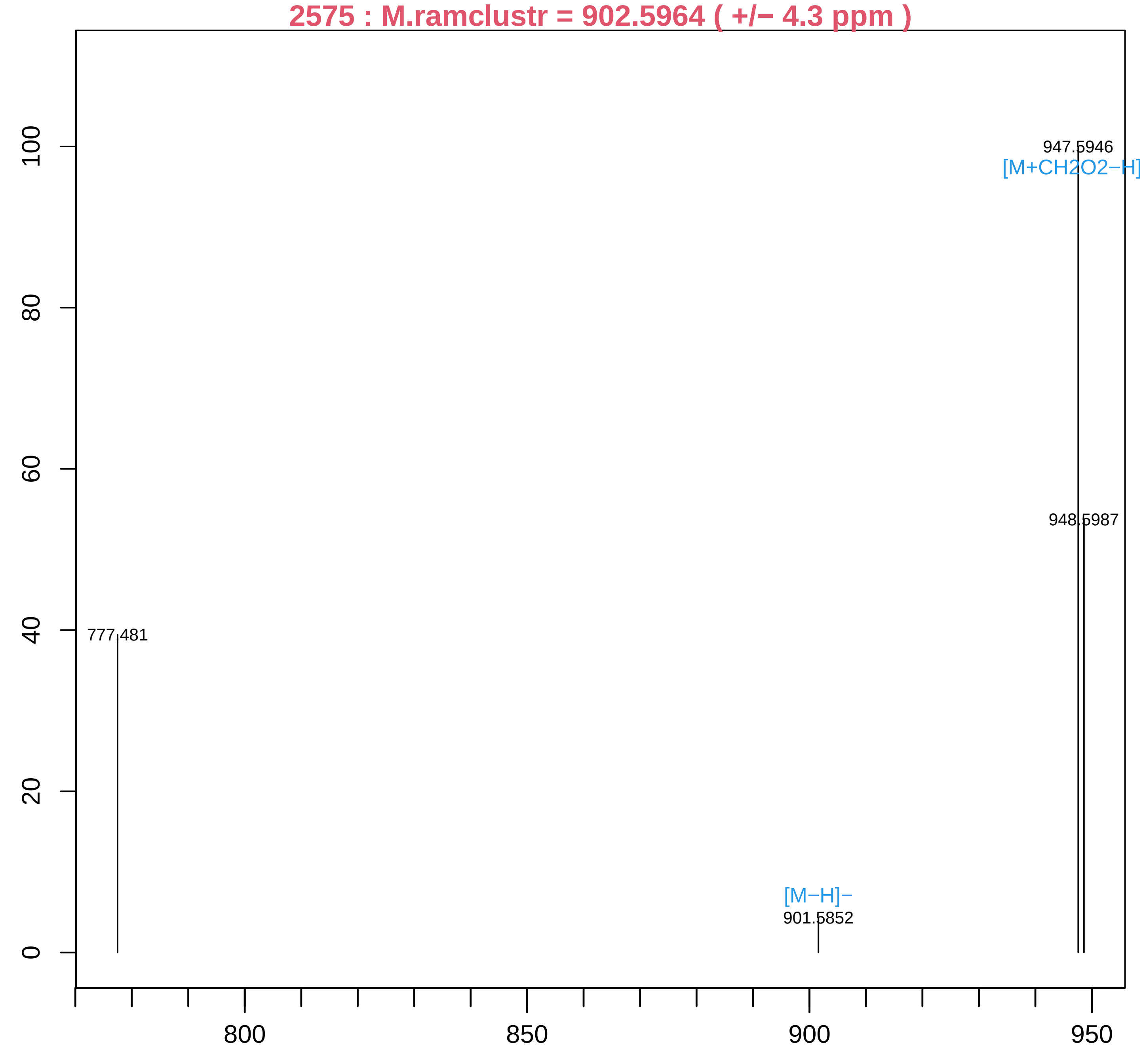
2575 : M.ramclustr = 902.5964 (+/− 4.3 ppm)
947.5946
[M+CH2O2−H]
948.5987
777.481
[M−H]−
901.5852
0
20
40
60
80
100
800
850
900
950

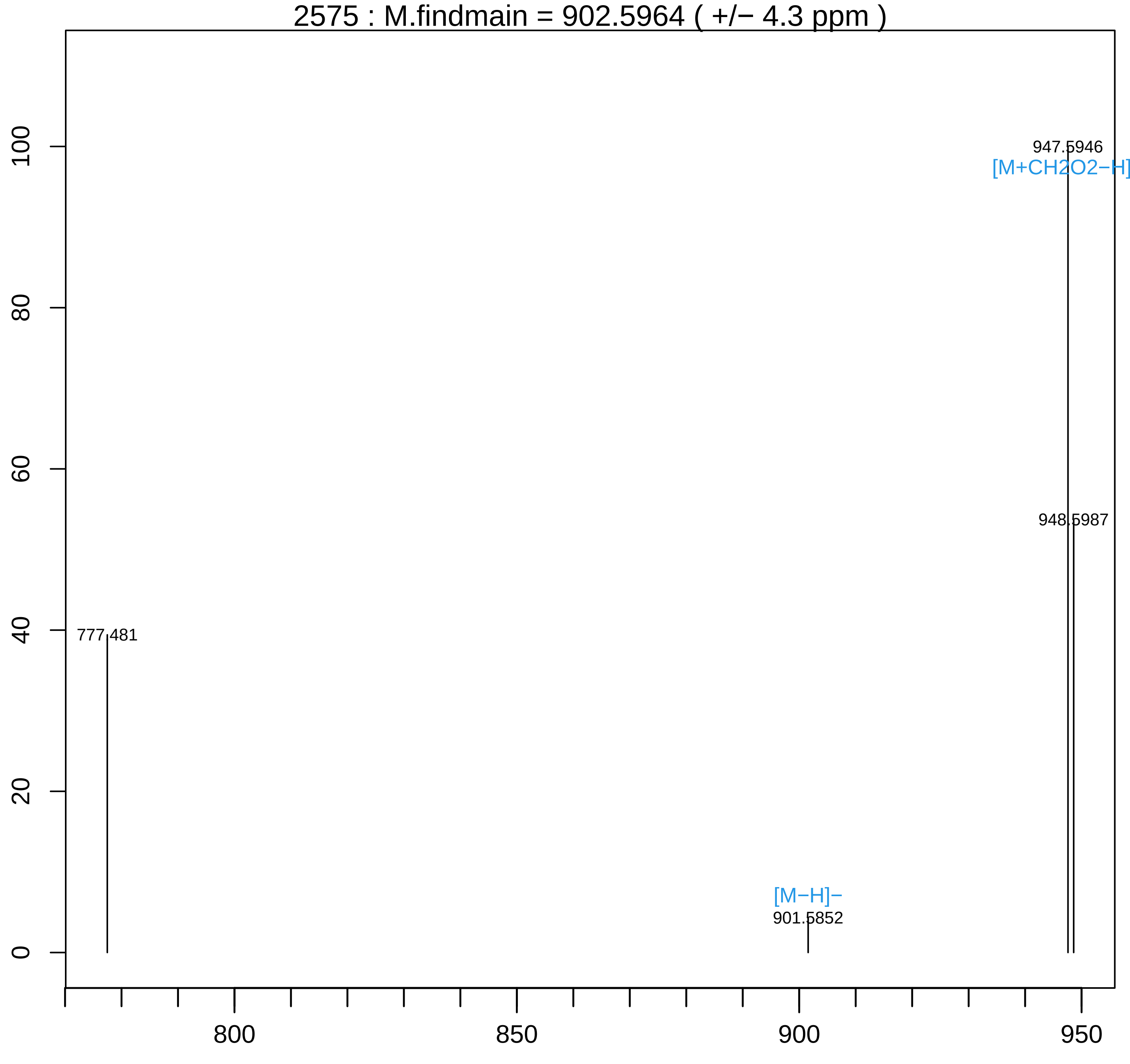
2575 : M.findmain = 902.5964 (+/− 4.3 ppm)
947.5946
[M+CH2O2−H]
948.5987
777.481
[M−H]−
901.5852
0
20
40
60
80
100
800
850
900
950

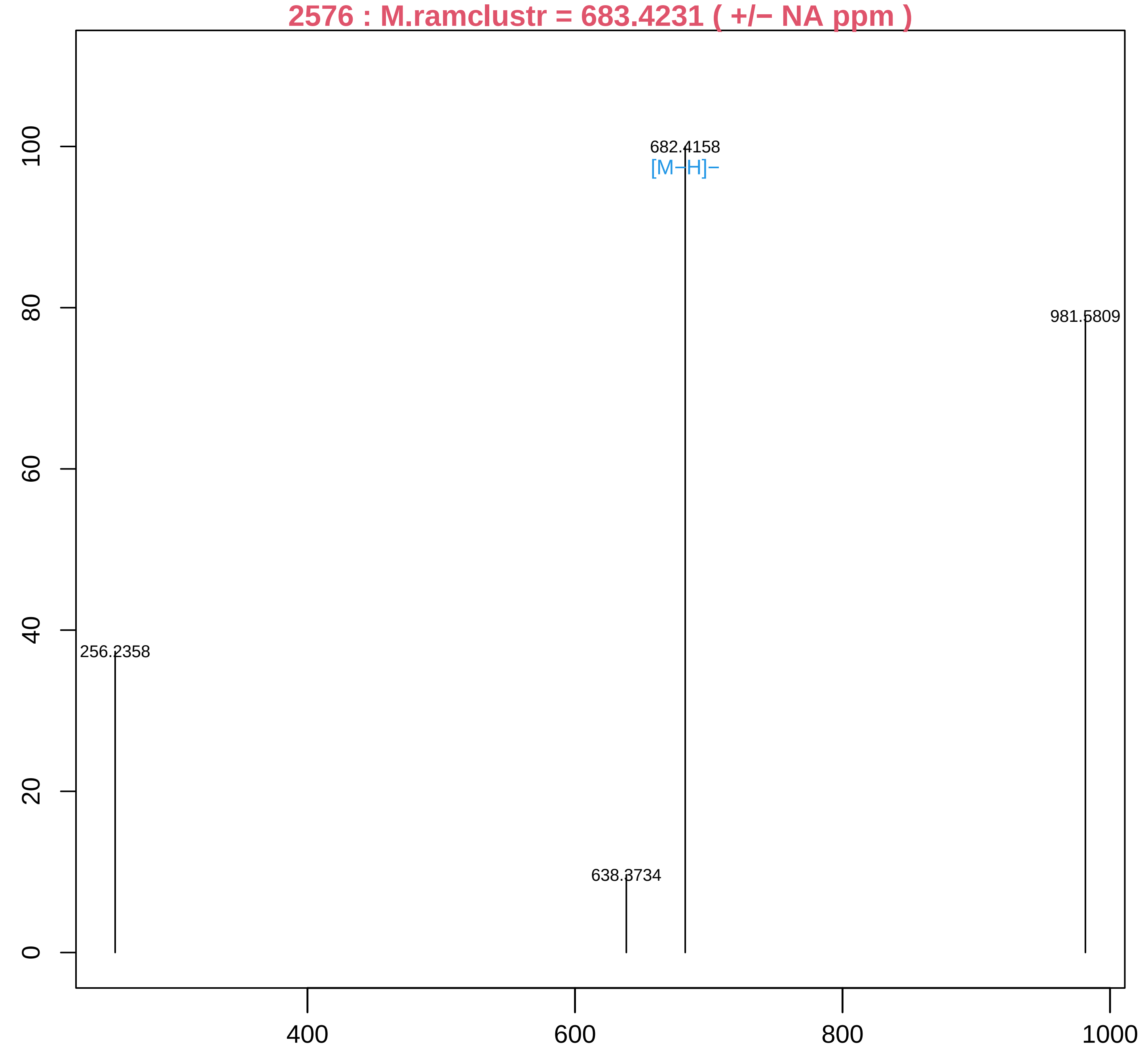

2576 : M.ramclustr = 683.4231 (+/− NA ppm)
682.4158
[M−H]−
981.5809
256.2358
638.3734
0
20
40
60
80
100
400
600
800
1000

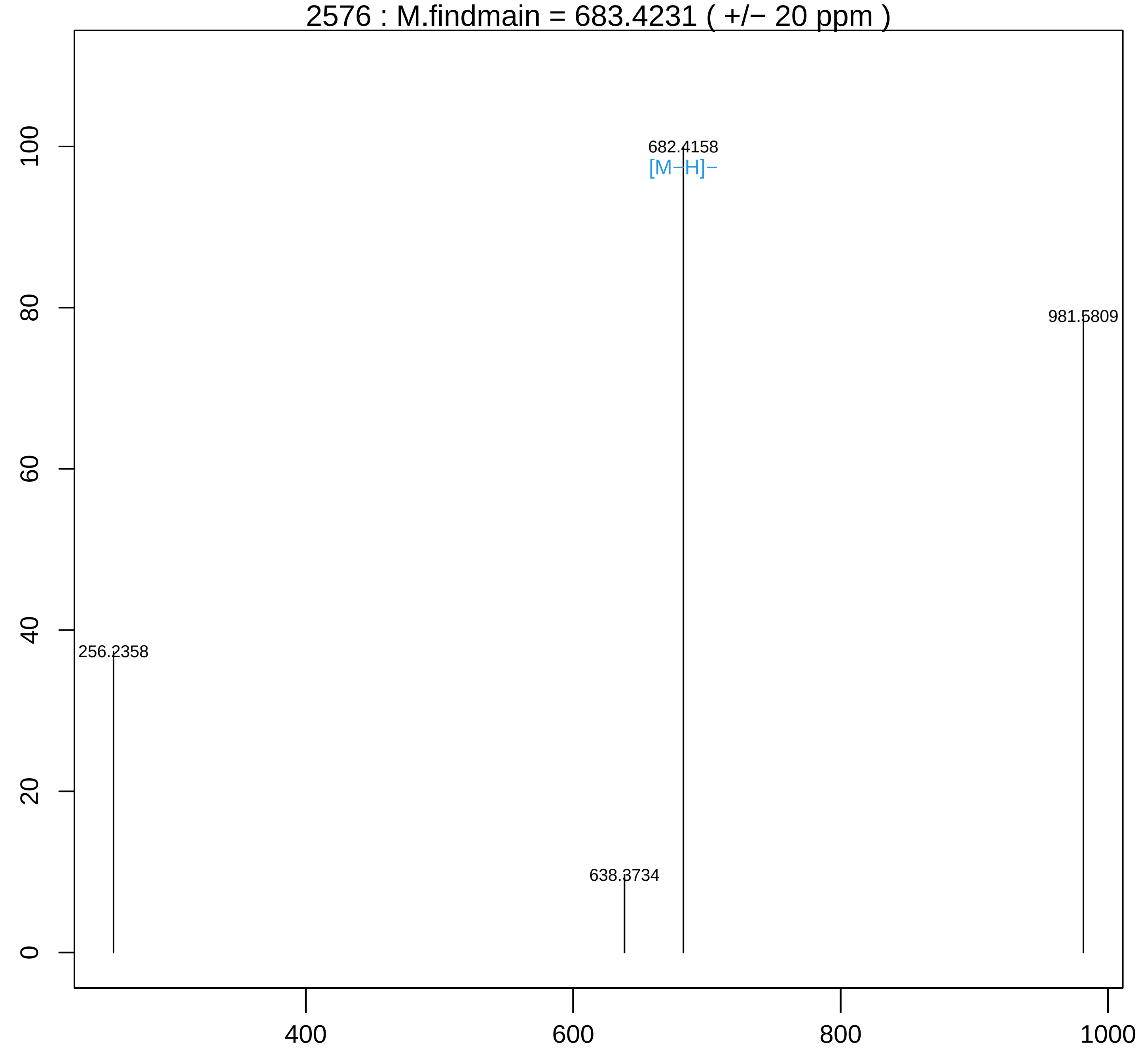

2576 : M.findmain = 683.4231 (+/− 20 ppm)
682.4158
[M−H]−
981.5809
256.2358
638.3734
0
20
40
60
80
100
400
600
800
1000

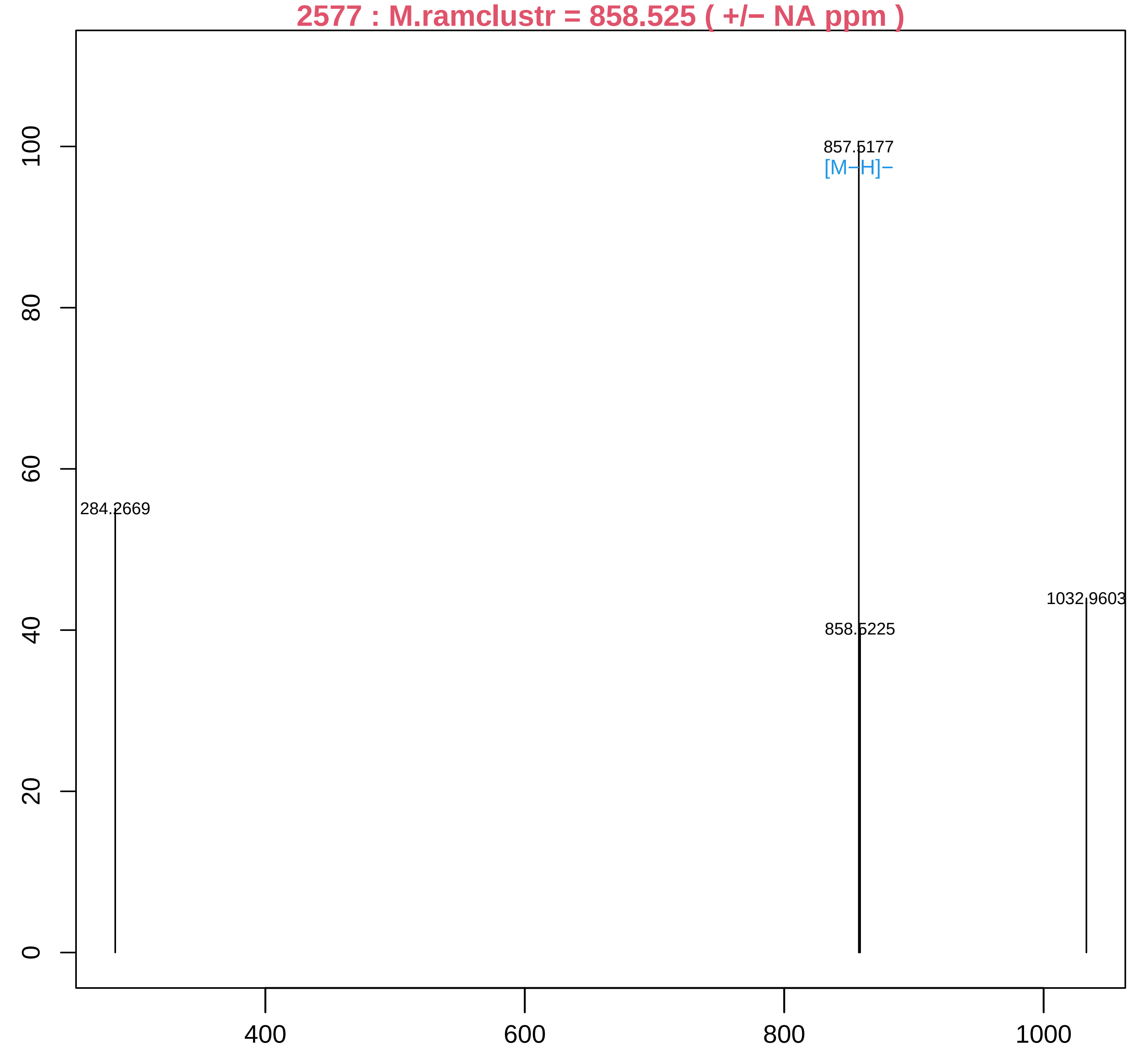

2577 : M.ramclustr = 858.525 (+/− NA ppm)
857.5177
[M−H]−
284.2669
1032.9603
858.5225
0
20
40
60
80
100
400
600
800
1000

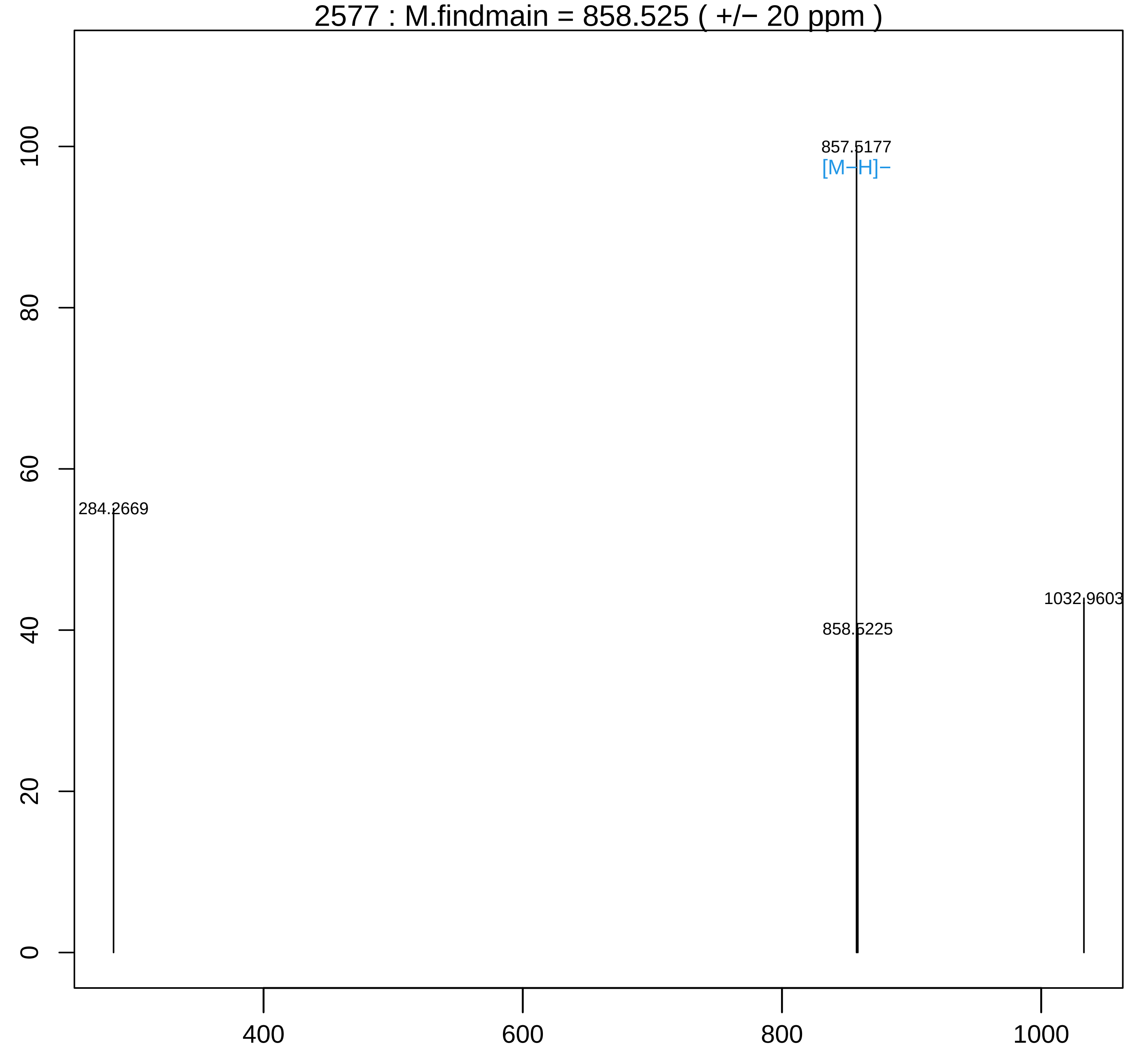

2577 : M.findmain = 858.525 (+/− 20 ppm)
857.5177
[M−H]−
284.2669
1032.9603
858.5225
0
20
40
60
80
100
400
600
800
1000

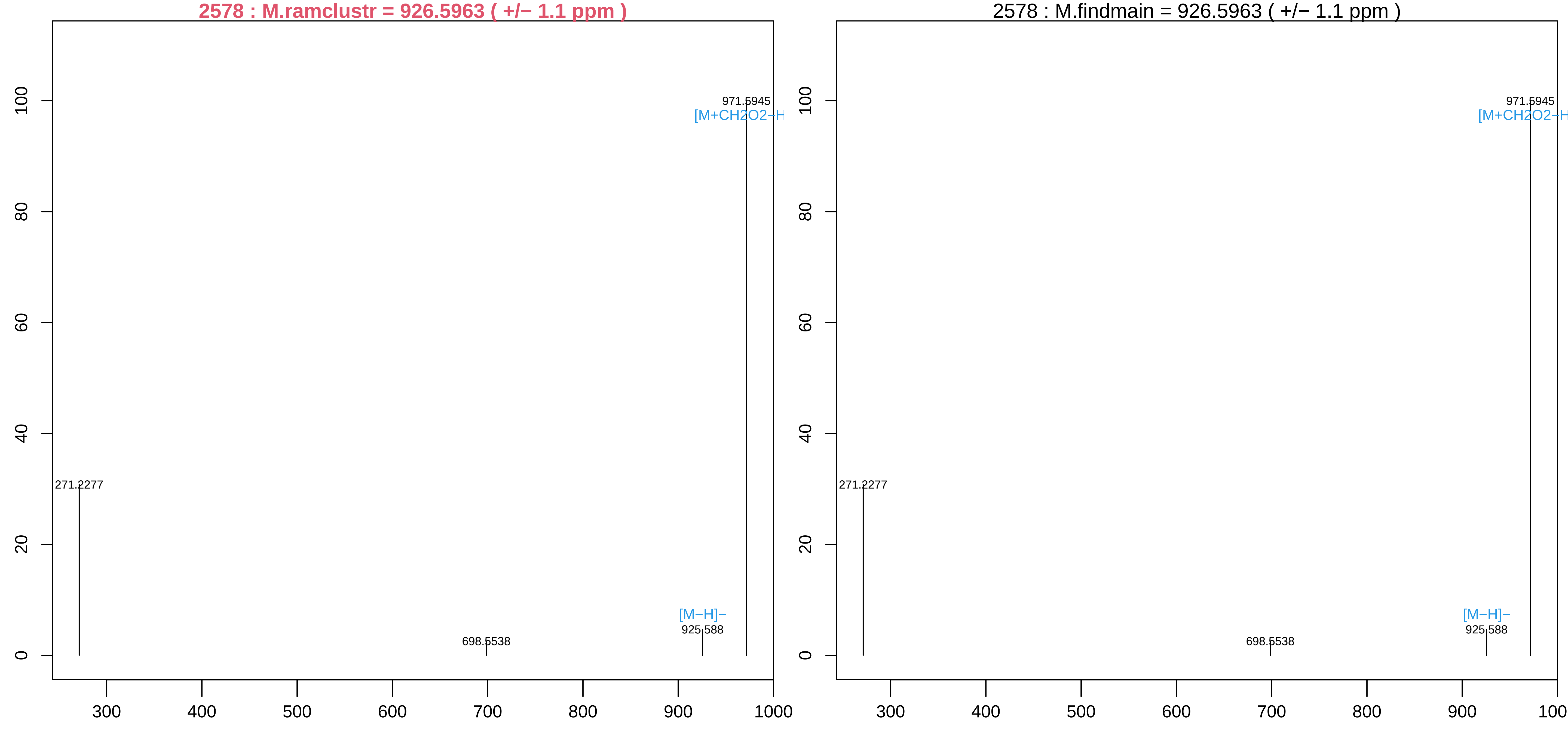
2578 : M.ramclustr = 926.5963 (+/− 1.1 ppm)
971.5945
[M+CH2O2−H
271.2277
698.5538
[M−H]−
925.588
0
20
40
60
80
100
300
400
500
600
700
800
900
1000
2578 : M.findmain = 926.5963 (+/− 1.1 ppm)
971.5945
[M+CH2O2−H
271.2277
698.5538
[M−H]−
925.588
0
20
40
60
80
100
300
400
500
600
700
800
900
100

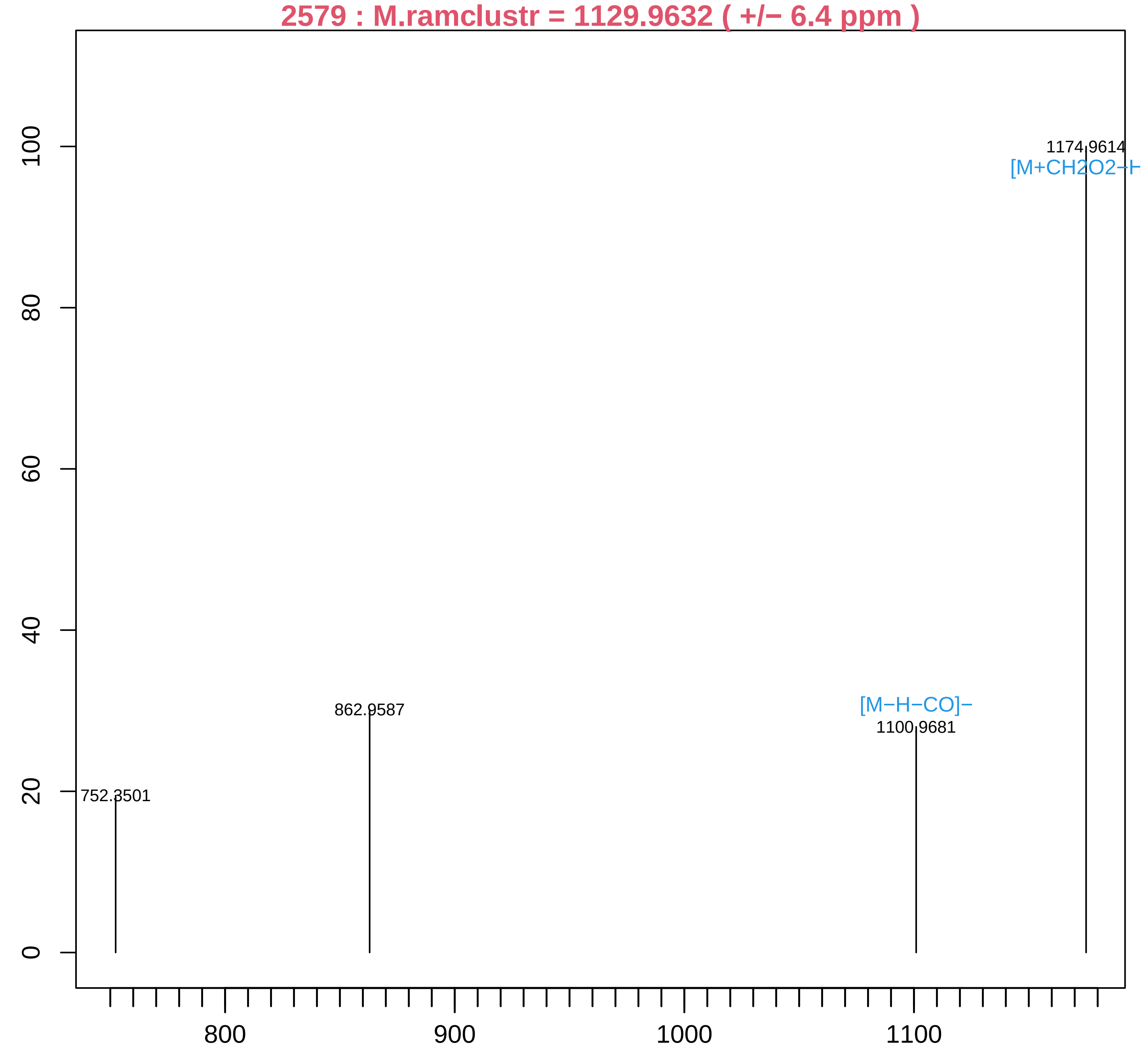

2579 : M.ramclustr = 1129.9632 (+/− 6.4 ppm)
1174.9614
[M+CH2O2−H
862.9587
[M−H−CO]−
1100.9681
752.3501
0
20
40
60
80
100
800
900
1000
1100

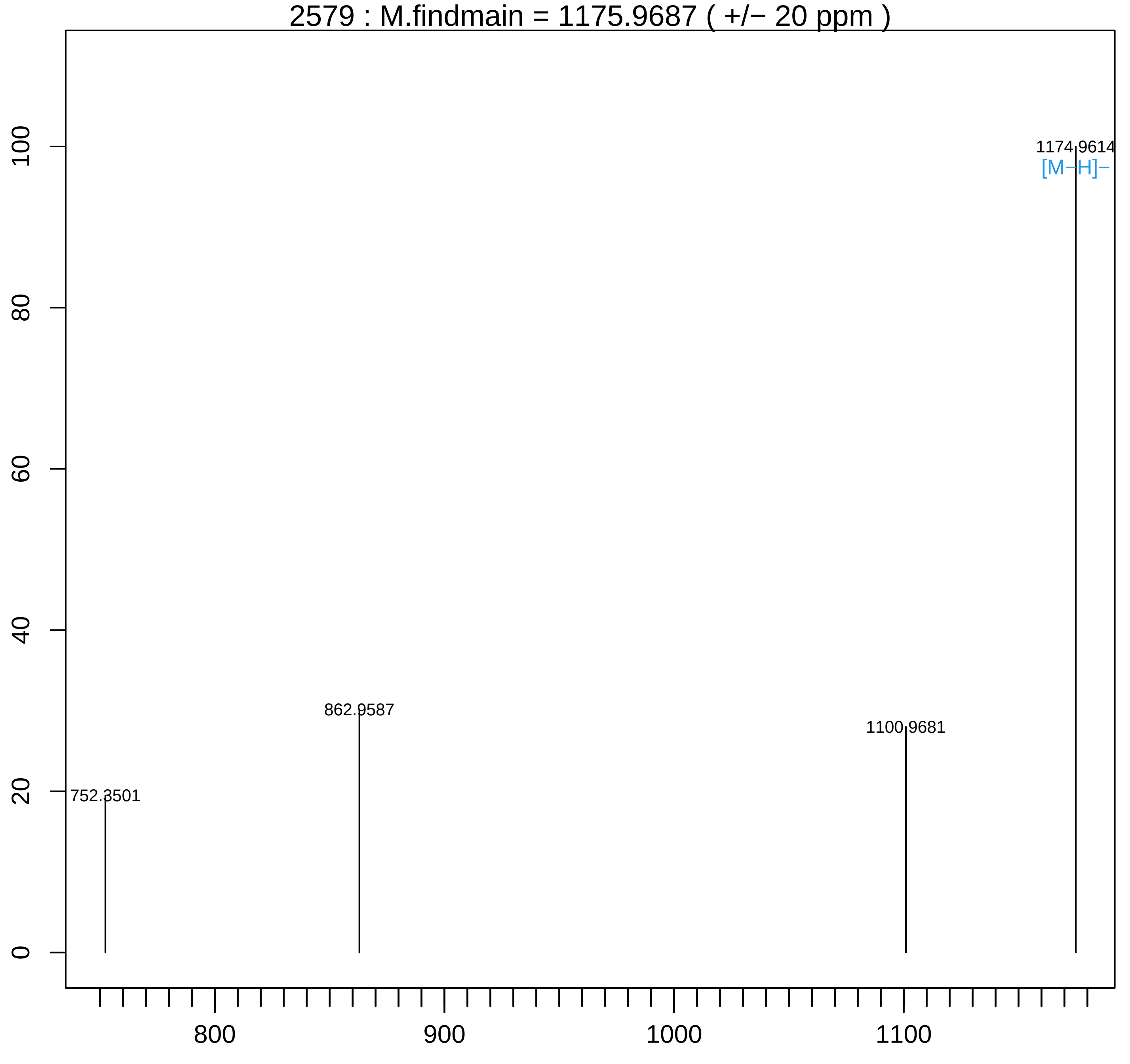

2579 : M.findmain = 1175.9687 (+/− 20 ppm)
1174.9614
[M−H]−
862.9587
1100.9681
752.3501
0
20
40
60
80
100
800
900
1000
1100

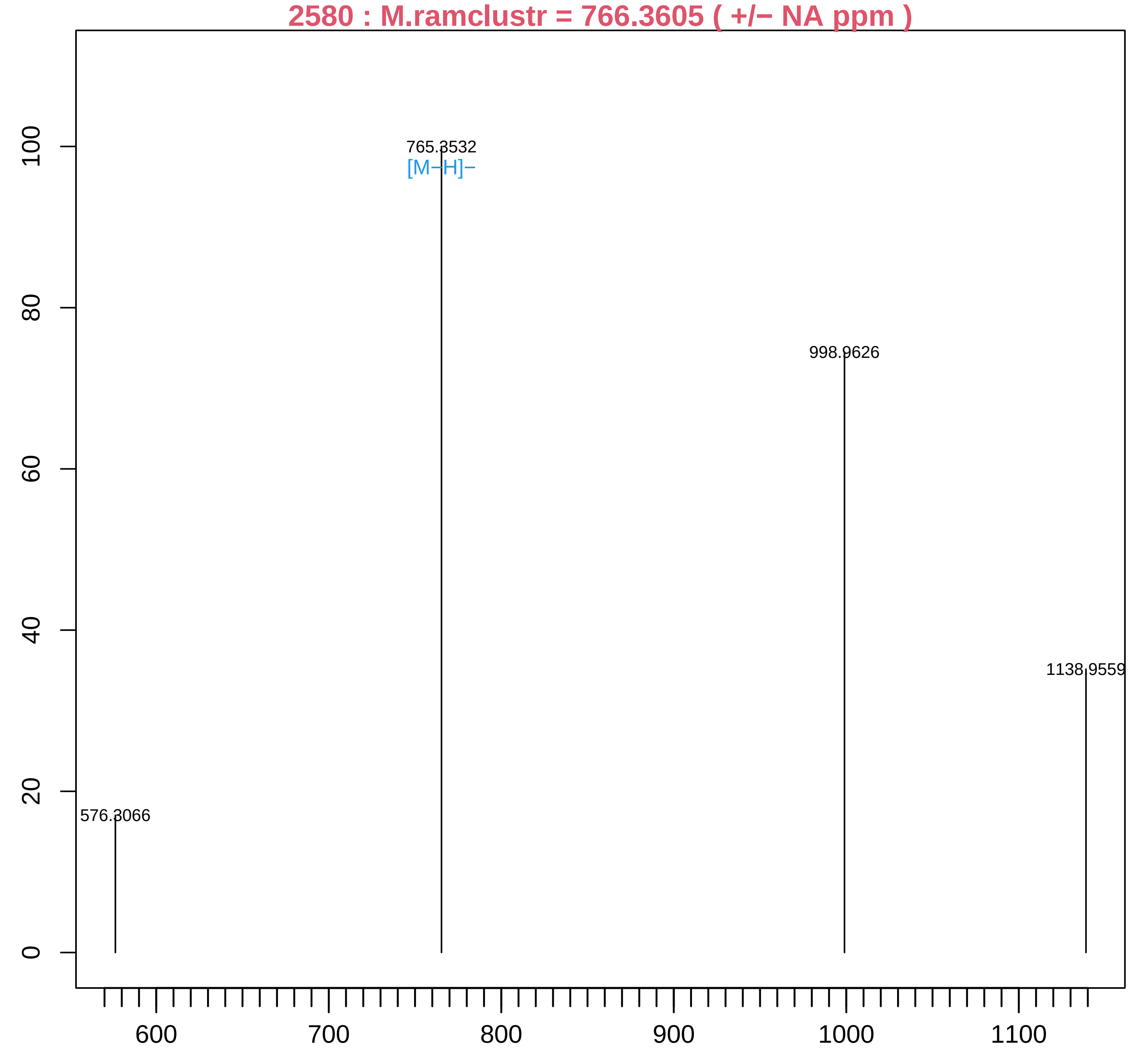

2580 : M.ramclustr = 766.3605 (+/− NA ppm)
765.3532
[M−H]−
998.9626
1138.9559
576.3066
0
20
40
60
80
100
600
700
800
900
1000
1100

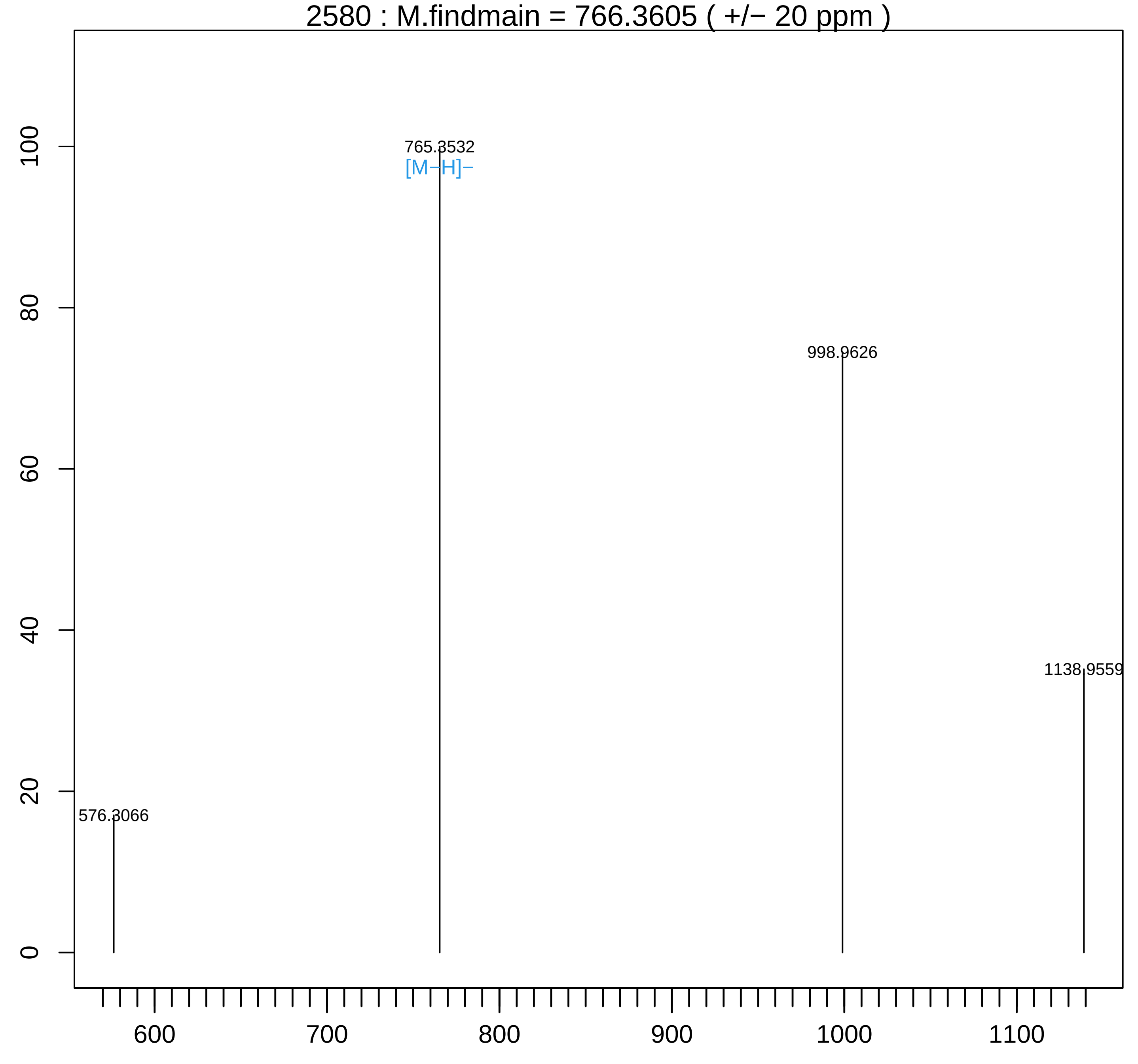

2580 : M.findmain = 766.3605 (+/− 20 ppm)
765.3532
[M−H]−
998.9626
1138.9559
576.3066
0
20
40
60
80
100
600
700
800
900
1000
1100

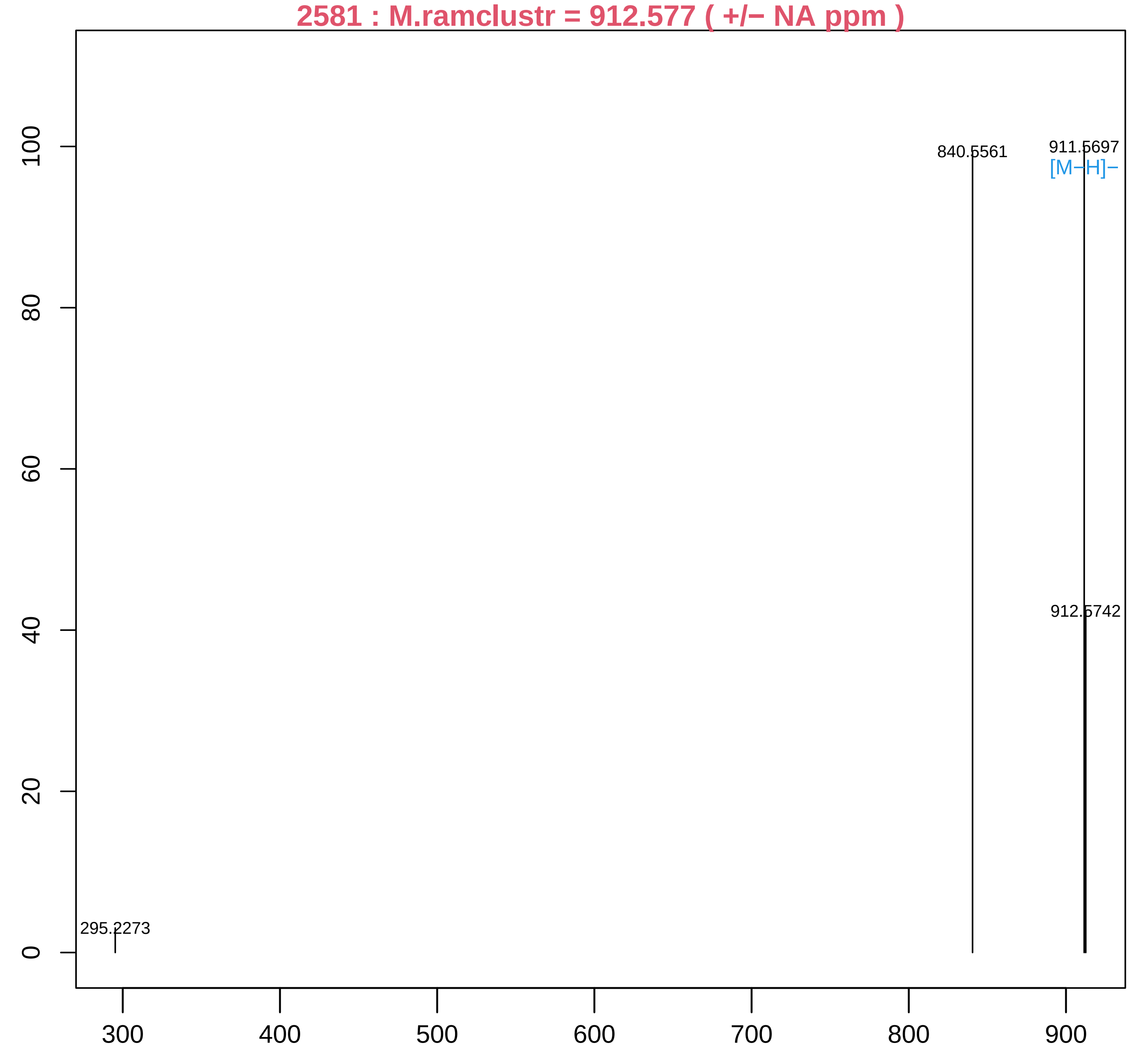
2581 : M.ramclustr = 912.577 (+/− NA ppm)
840.5561
911.5697
[M−H]−
912.5742
295.2273
0
20
40
60
80
100
300
400
500
600
700
800
900

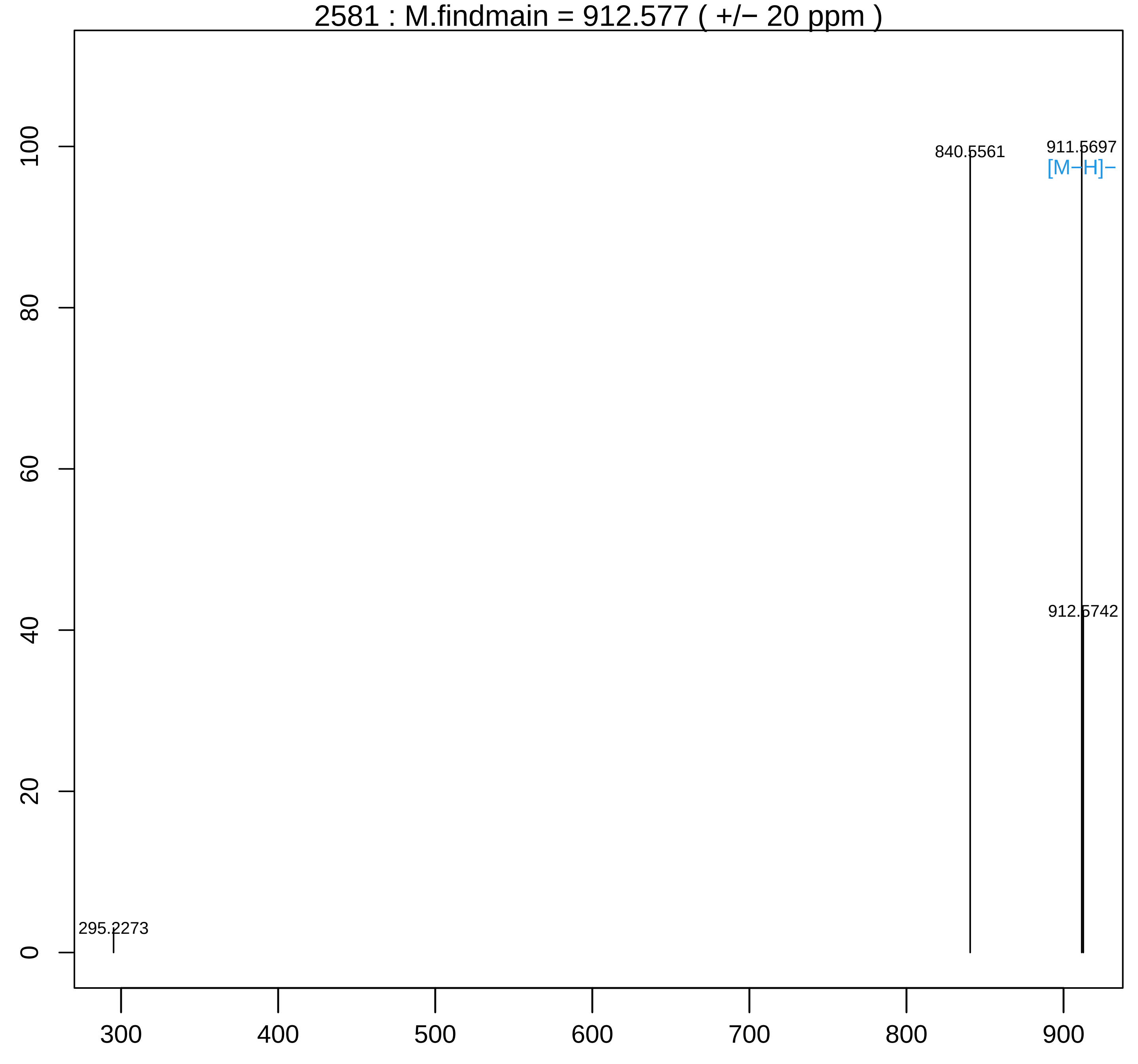
2581 : M.findmain = 912.577 (+/− 20 ppm)
840.5561
911.5697
[M−H]−
912.5742
295.2273
0
20
40
60
80
100
300
400
500
600
700
800
900

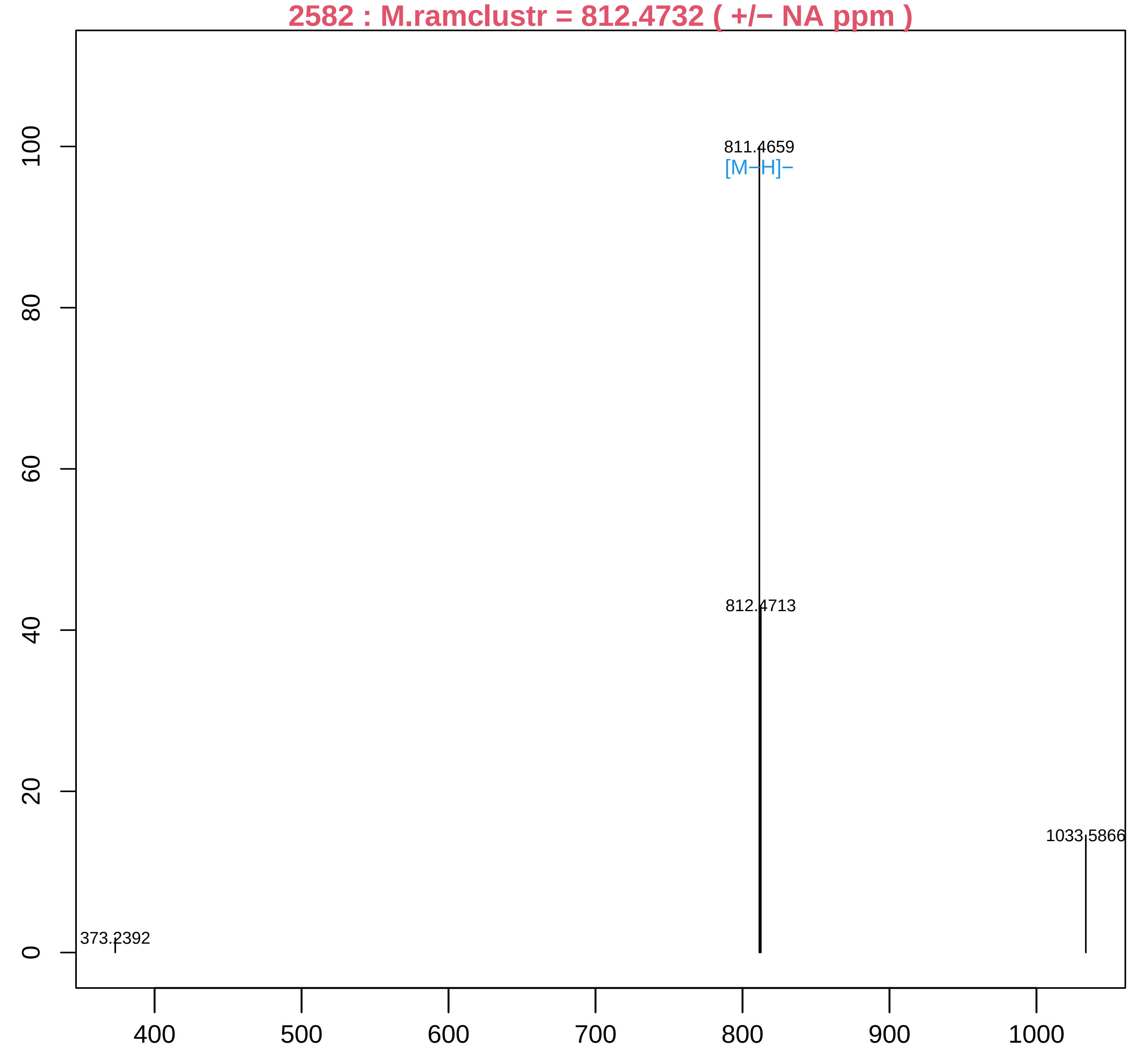
2582 : M.ramclustr = 812.4732 (+/− NA ppm)
811.4659
[M−H]−
812.4713
1033.5866
373.2392
0
20
40
60
80
100
400
500
600
700
800
900
1000

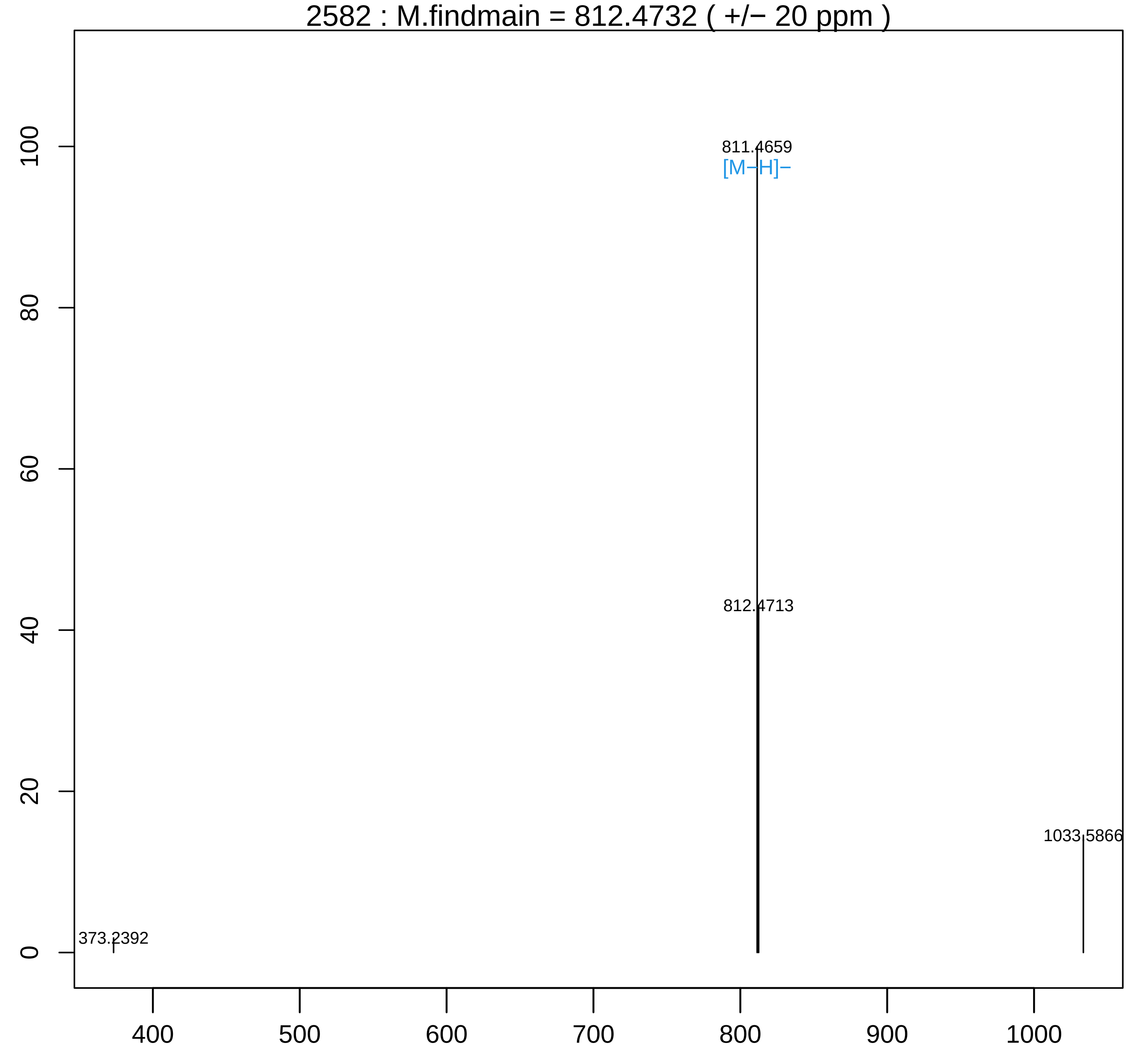
2582 : M.findmain = 812.4732 (+/− 20 ppm)
811.4659
[M−H]−
812.4713
1033.5866
373.2392
0
20
40
60
80
100
400
500
600
700
800
900
1000

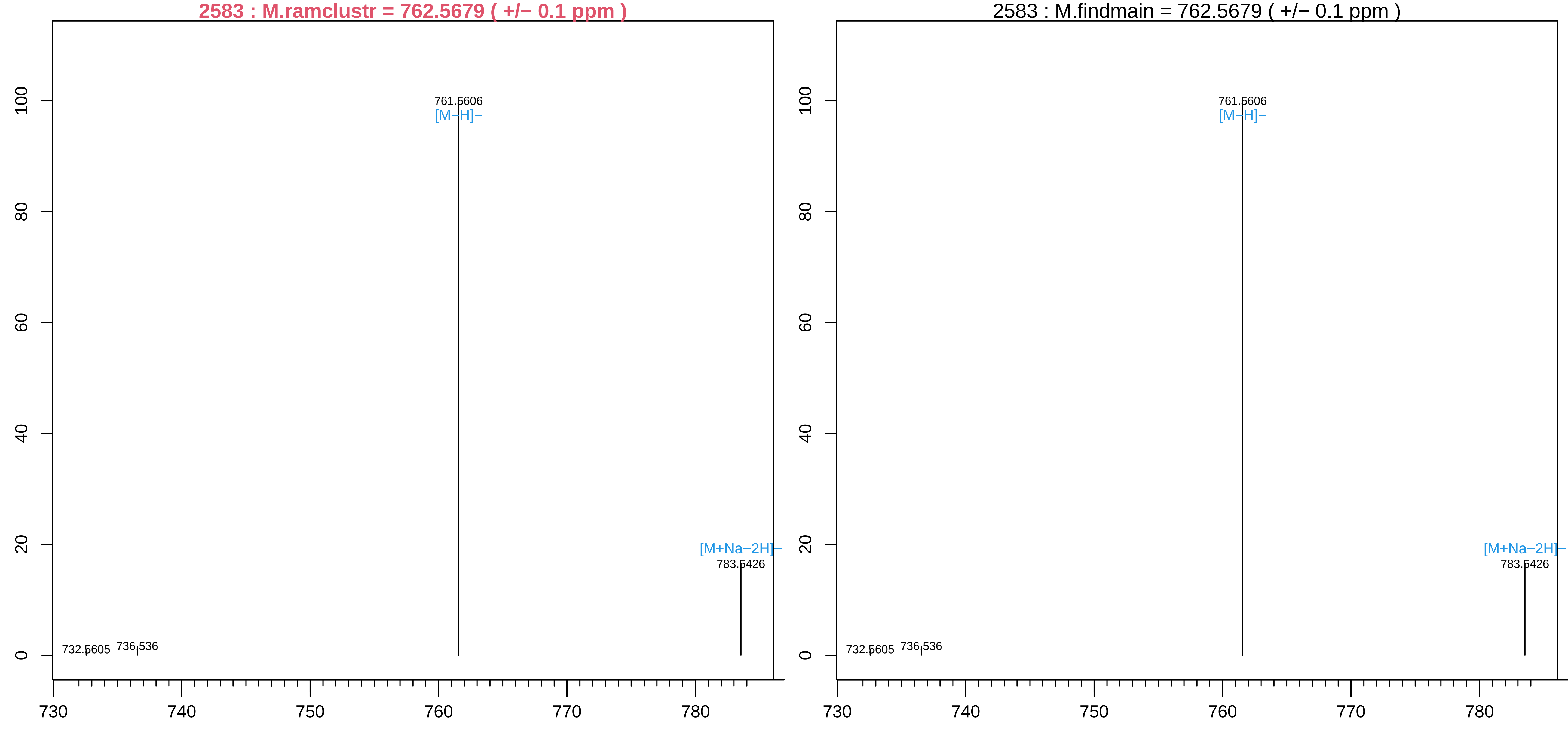
2583 : M.ramclustr = 762.5679 (+/− 0.1 ppm)
761.5606
[M−H]−
[M+Na−2H]−
783.5426
732.5605
736.536
2583 : M.findmain = 762.5679 (+/− 0.1 ppm)
761.5606
[M−H]−
[M+Na−2H]−
783.5426
732.5605
736.536

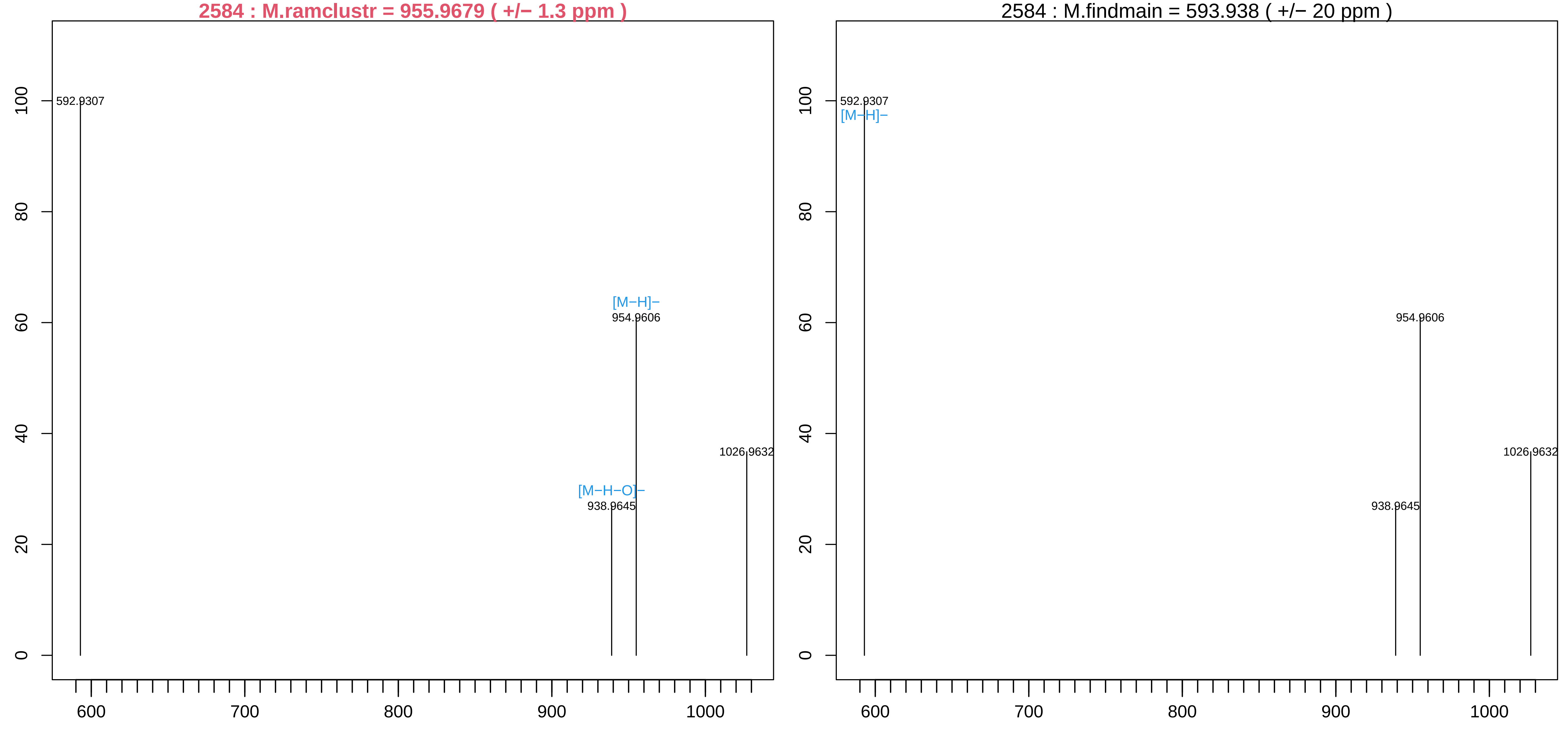

2584 : M.ramclustr = 955.9679 (+/− 1.3 ppm)
592.9307
[M−H]−
954.9606
1026.9632
[M−H−O]−
938.9645
0
20
40
60
80
100
600
700
800
900
1000
2584 : M.findmain = 593.938 (+/− 20 ppm)
592.9307
[M−H]−
954.9606
1026.9632
938.9645
0
20
40
60
80
100
600
700
800
900
1000

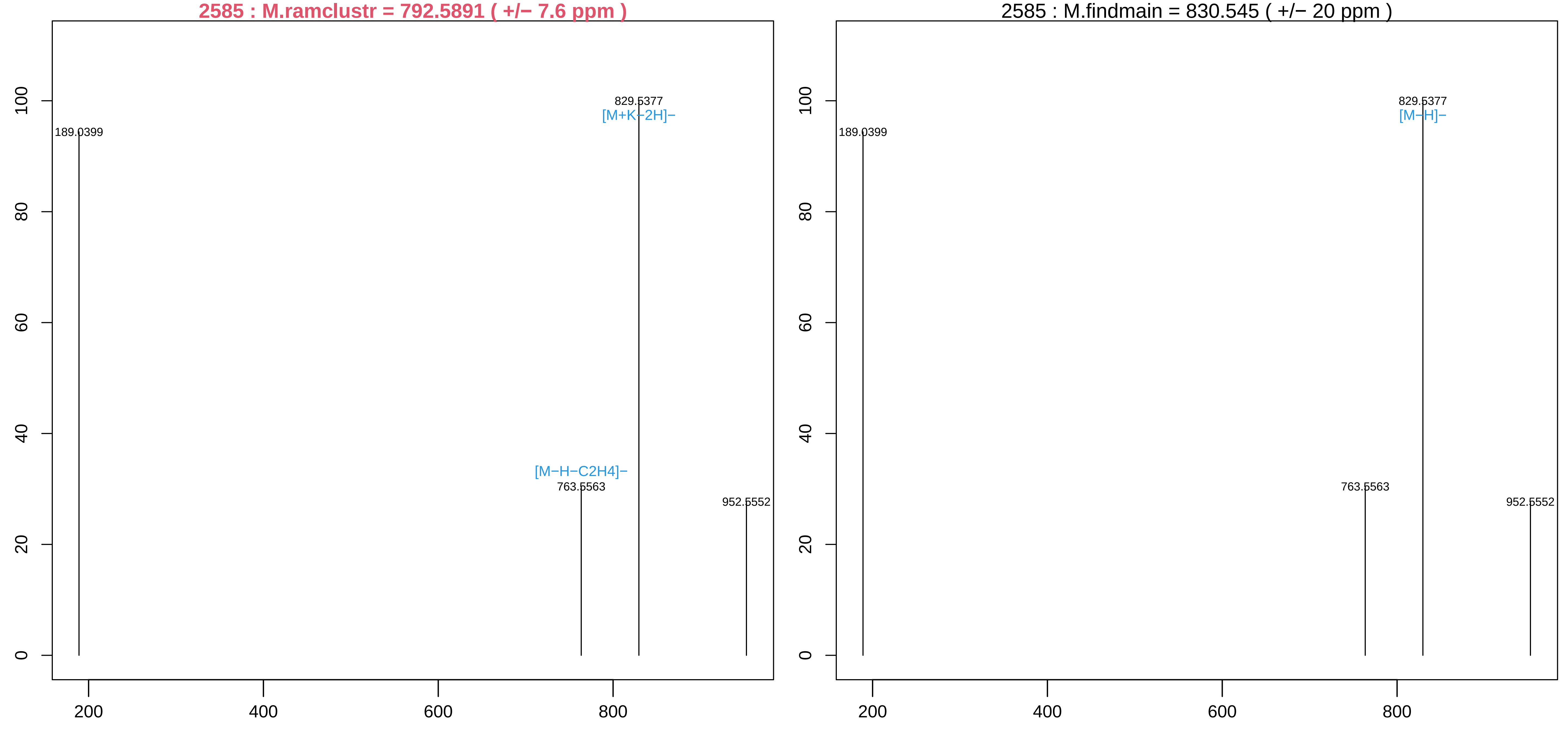
2585 : M.ramclustr = 792.5891 (+/− 7.6 ppm)
189.0399
829.5377
[M+K−2H]−
[M−H−C2H4]−
763.5563
952.5552
0
20
40
60
80
100
200
400
600
800
2585 : M.findmain = 830.545 (+/− 20 ppm)
189.0399
829.5377
[M−H]−
763.5563
952.5552
0
20
40
60
80
100
200
400
600
800

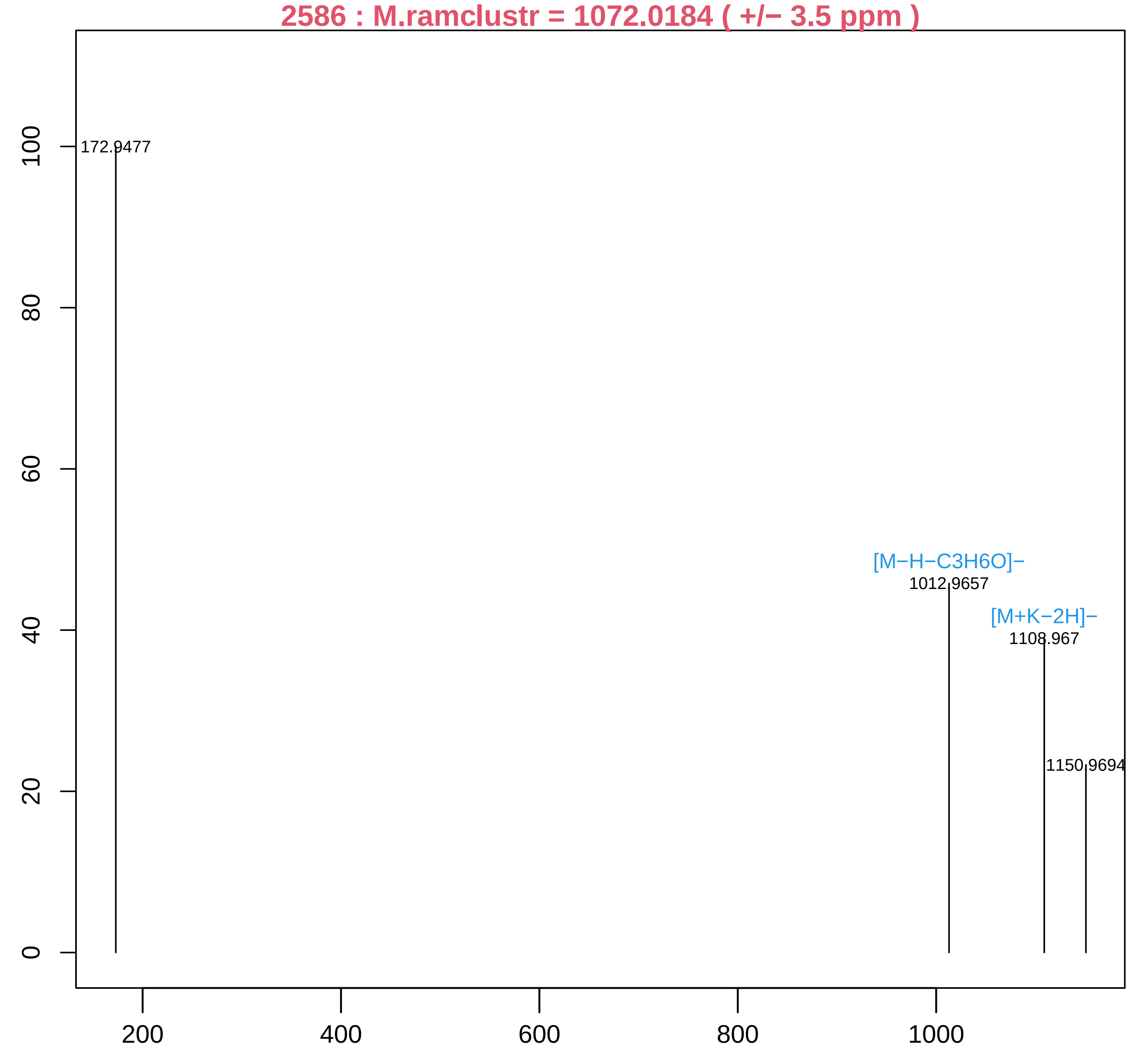

2586 : M.ramclustr = 1072.0184 (+/− 3.5 ppm)
172.9477
[M−H−C3H6O]−
1012.9657
[M+K−2H]−
1108.967
1150.9694
0
20
40
60
80
100
200
400
600
800
1000

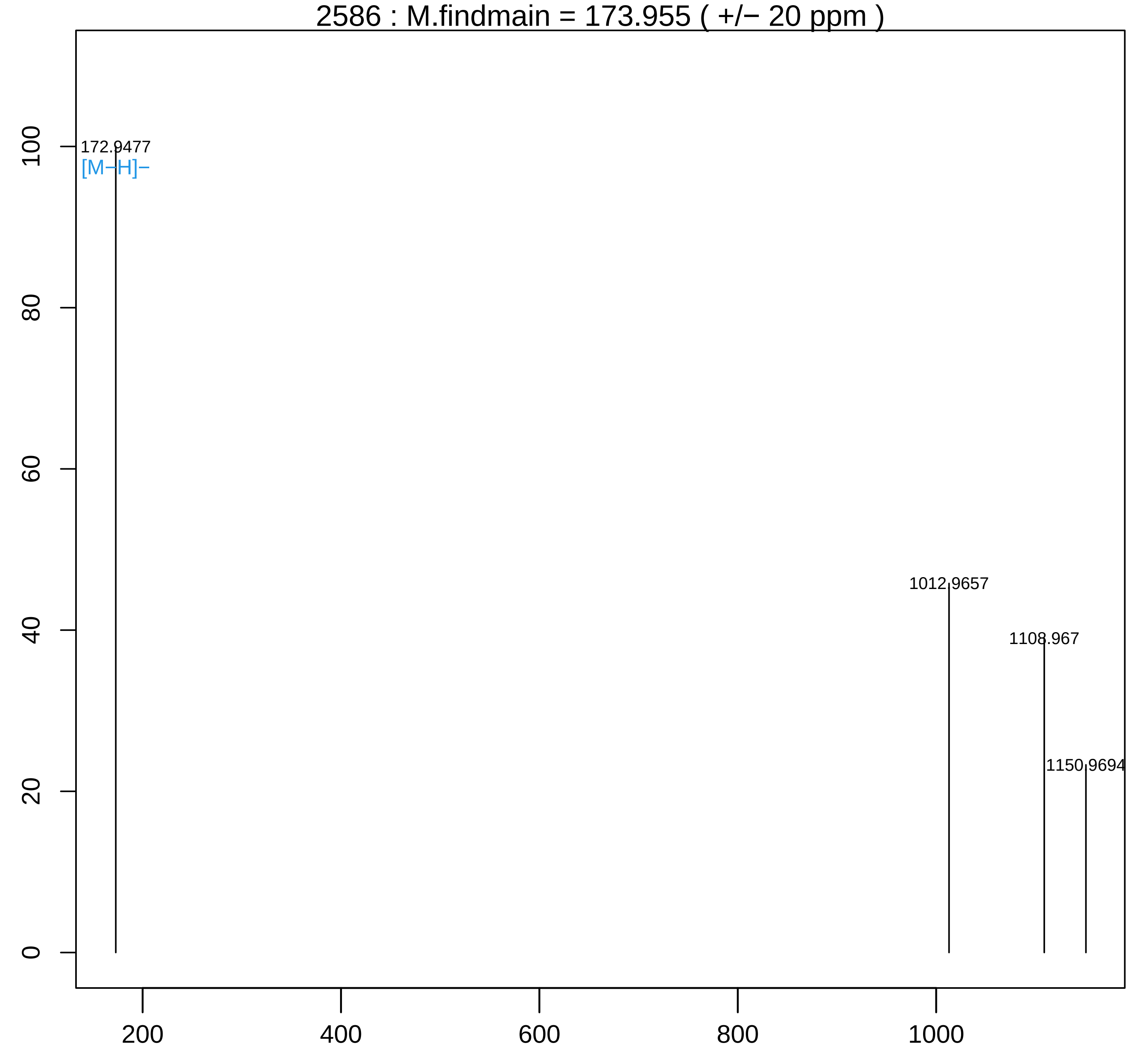

2586 : M.findmain = 173.955 (+/− 20 ppm)
172.9477
[M−H]−
1012.9657
1108.967
1150.9694
0
20
40
60
80
100
200
400
600
800
1000

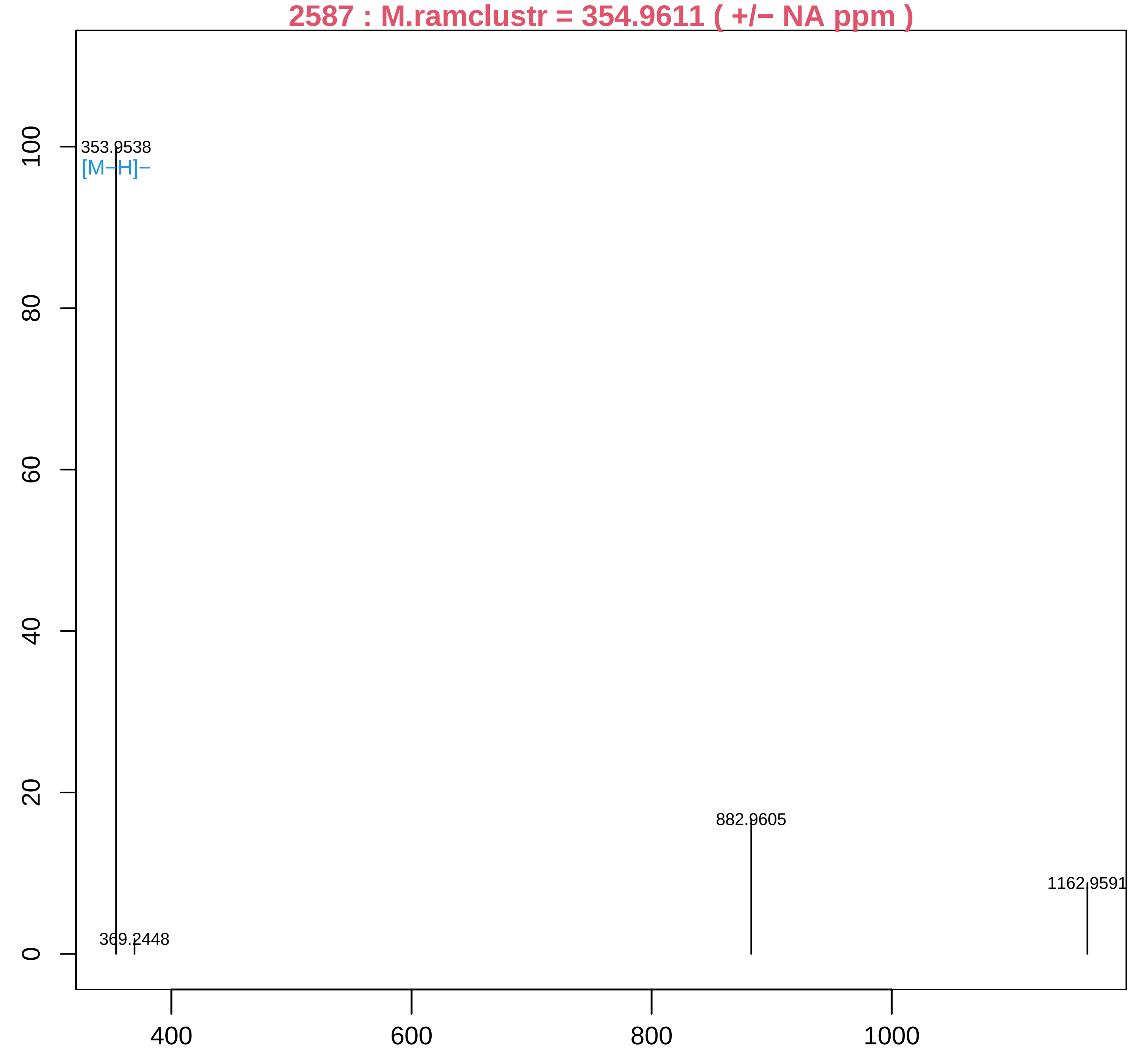
2587 : M.ramclustr = 354.9611 (+/− NA ppm)
353.9538
[M−H]−
369.2448
882.9605
1162.9591
0
20
40
60
80
100
400
600
800
1000

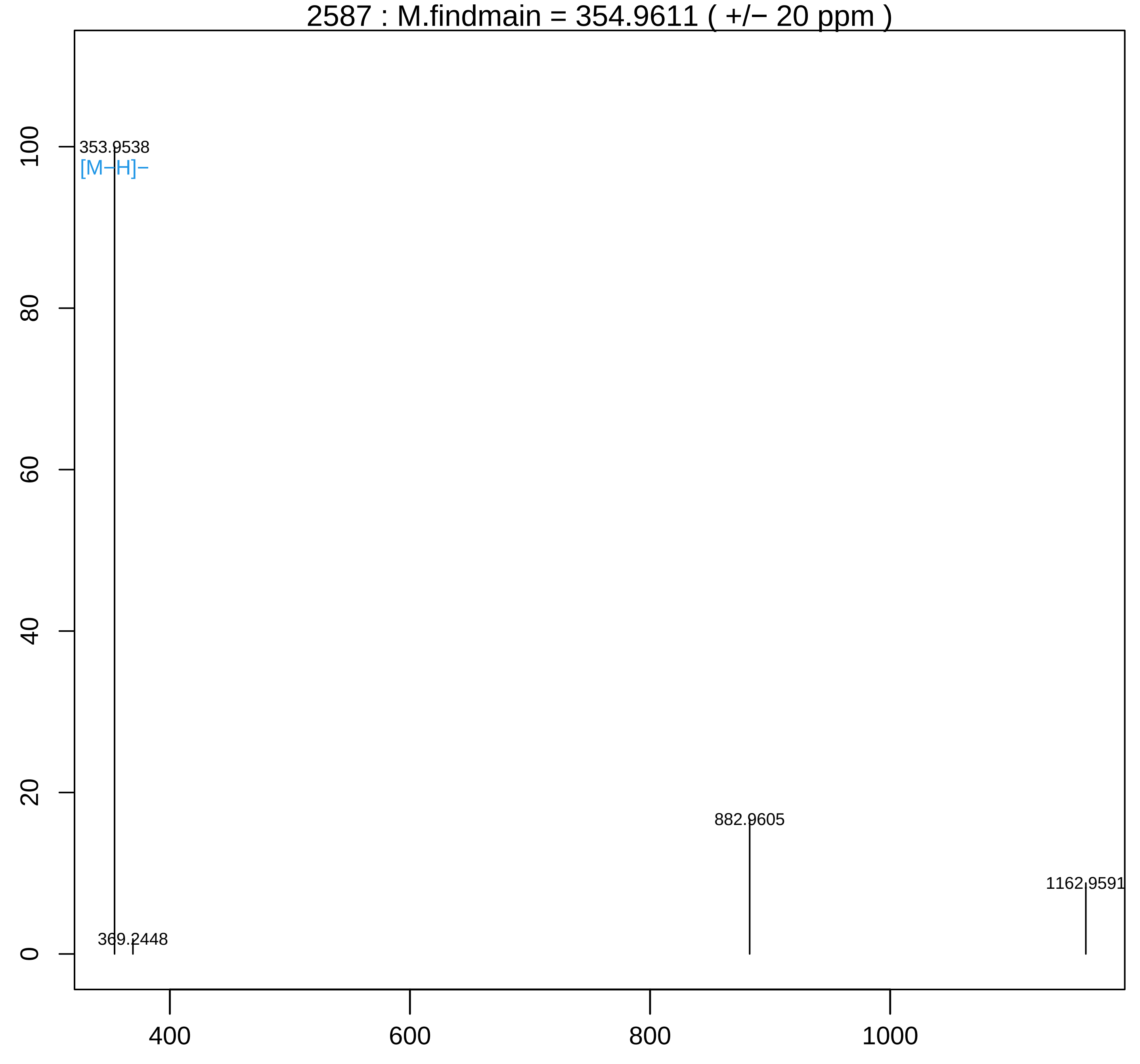
2587 : M.findmain = 354.9611 (+/− 20 ppm)
353.9538
[M−H]−
369.2448
882.9605
1162.9591
0
20
40
60
80
100
400
600
800
1000

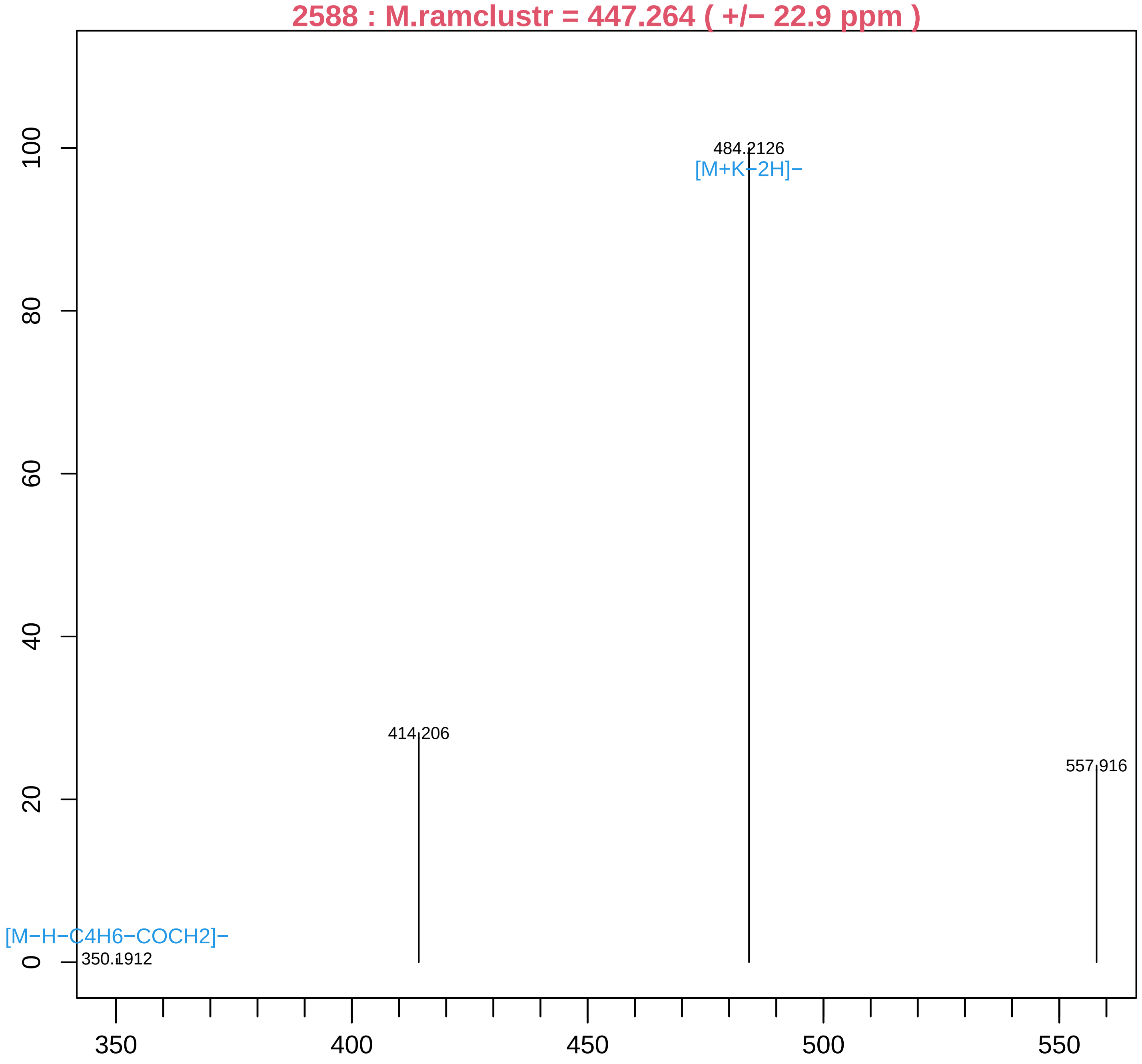
2588 : M.ramclustr = 447.264 (+/− 22.9 ppm)
484.2126
[M+K−2H]−
414.206
557.916
[M−H−C4H6−COCH2]−
350.1912
0
20
40
60
80
100
350
400
450
500
550

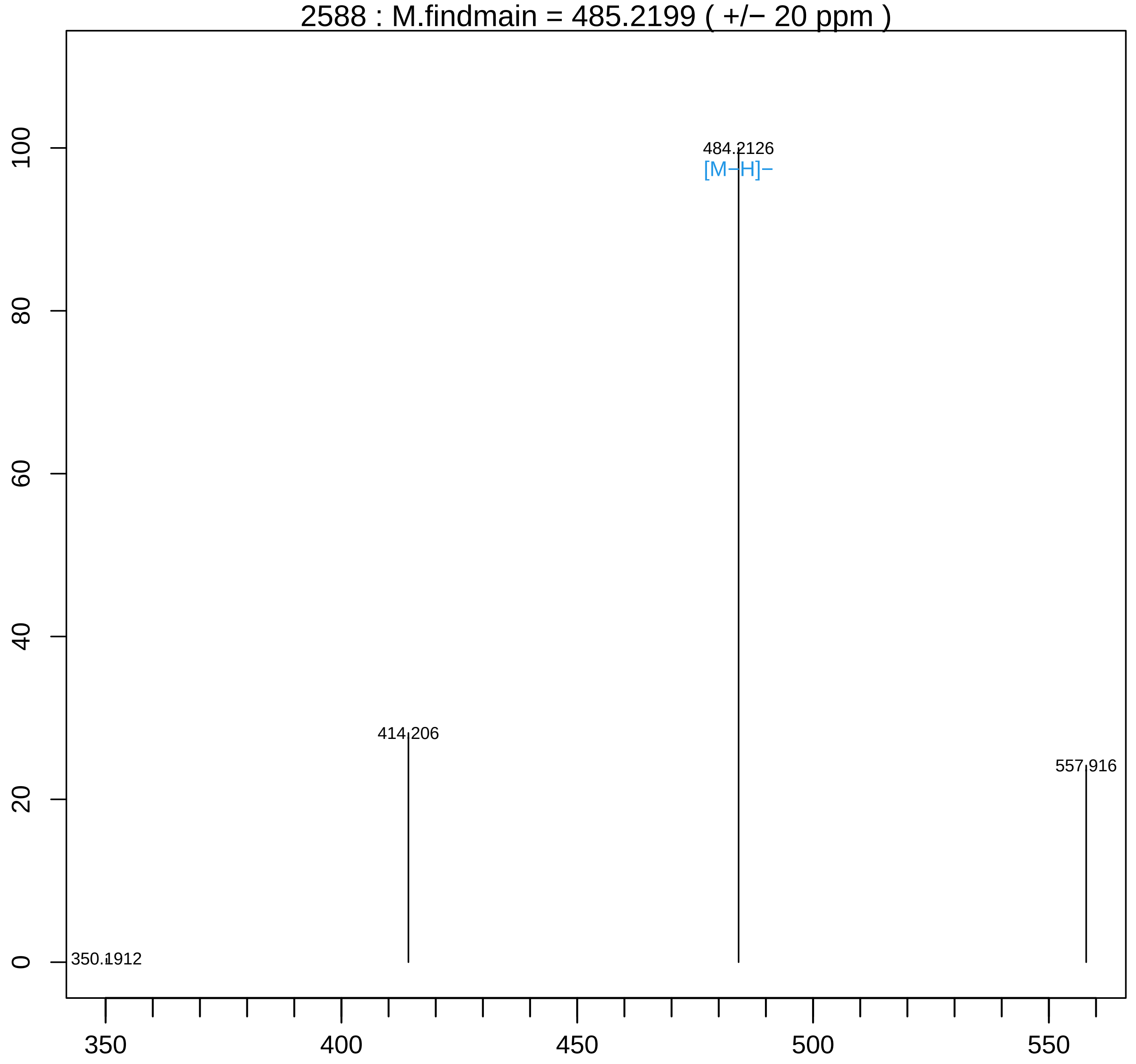
2588 : M.findmain = 485.2199 (+/− 20 ppm)
484.2126
[M−H]−
414.206
557.916
350.1912
0
20
40
60
80
100
350
400
450
500
550

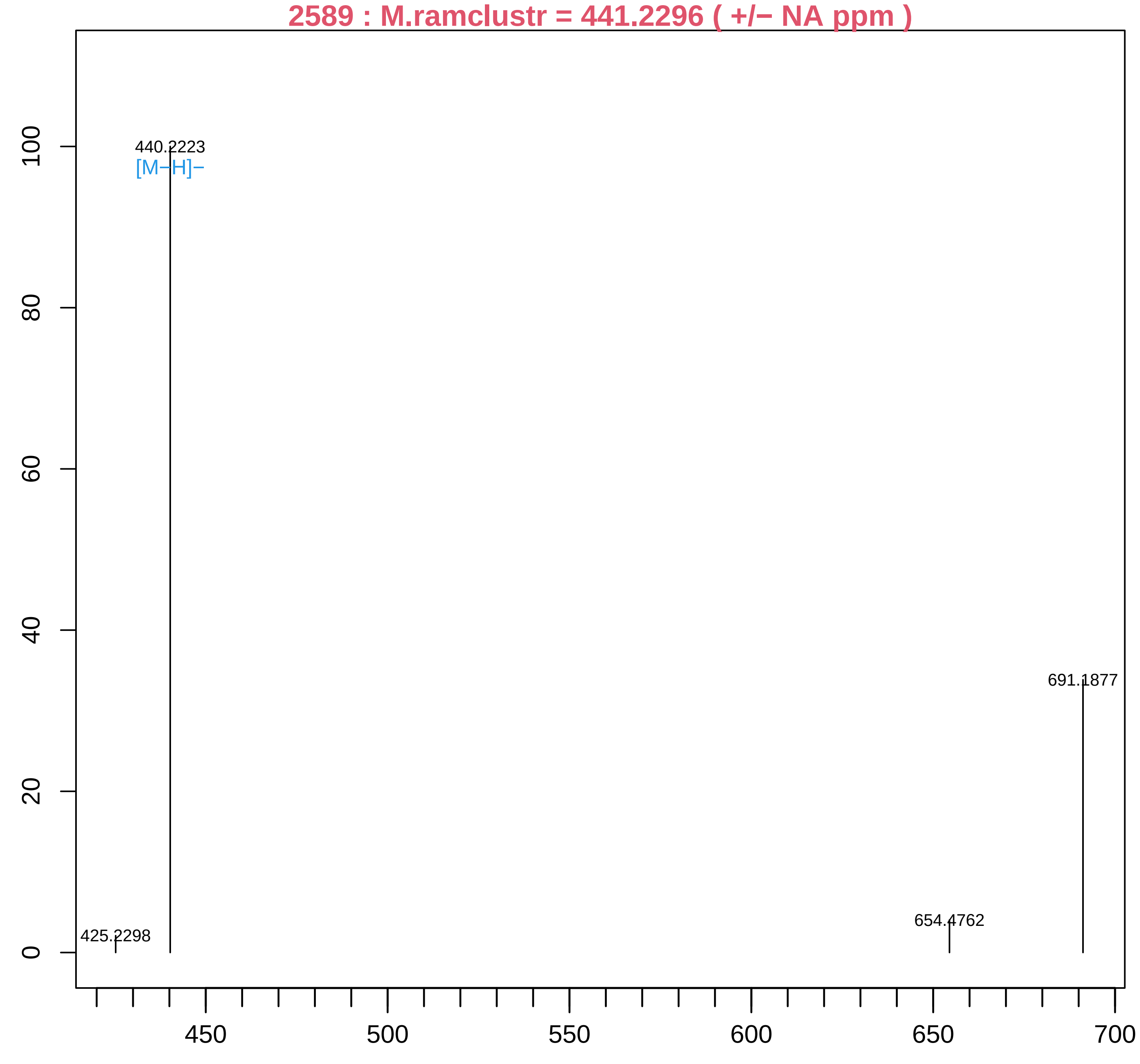

2589 : M.ramclustr = 441.2296 (+/− NA ppm)
440.2223
[M−H]−
691.1877
654.4762
425.2298
0
20
40
60
80
100
450
500
550
600
650
700

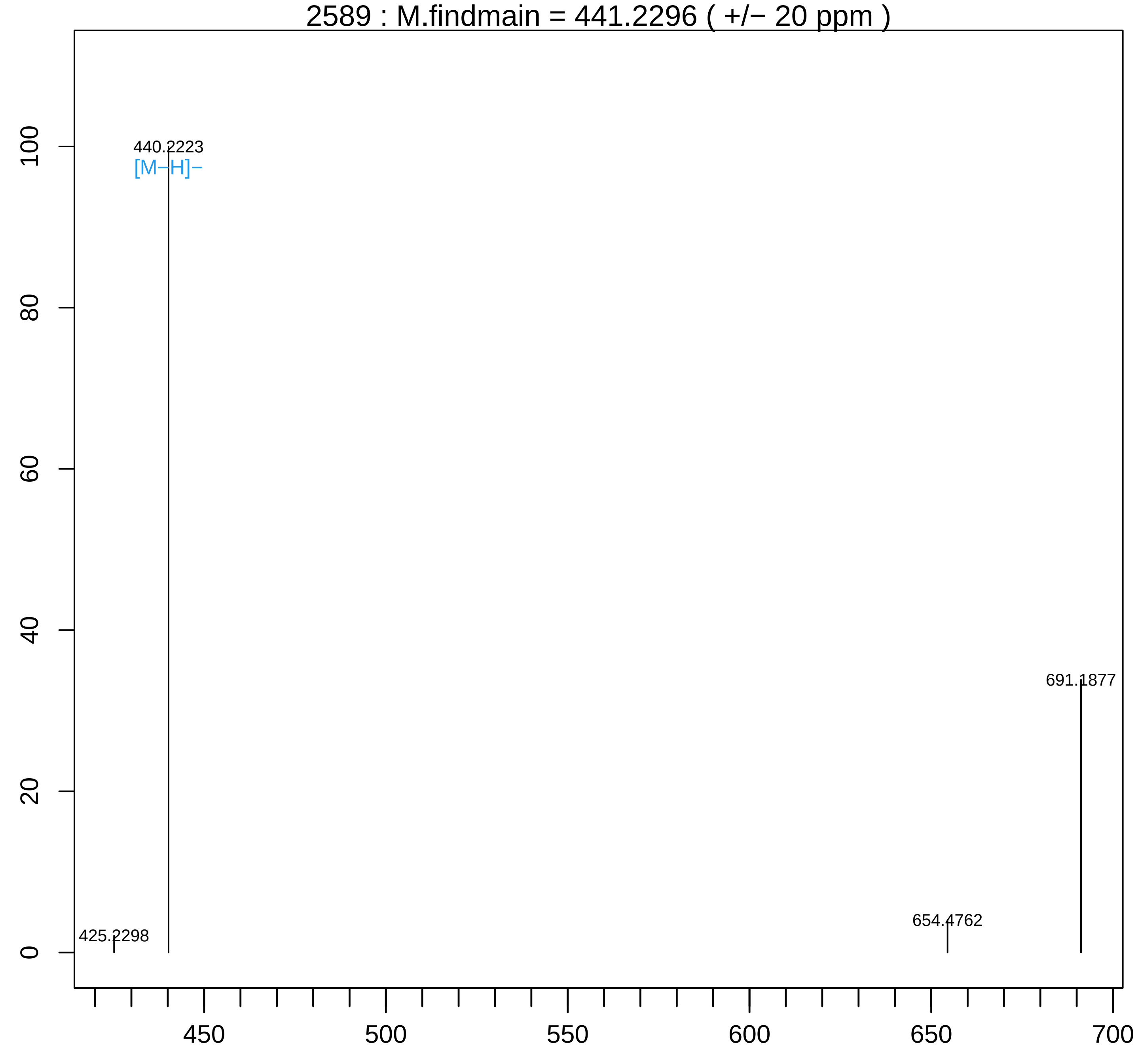

2589 : M.findmain = 441.2296 (+/− 20 ppm)
440.2223
[M−H]−
691.1877
654.4762
425.2298
0
20
40
60
80
100
450
500
550
600
650
700

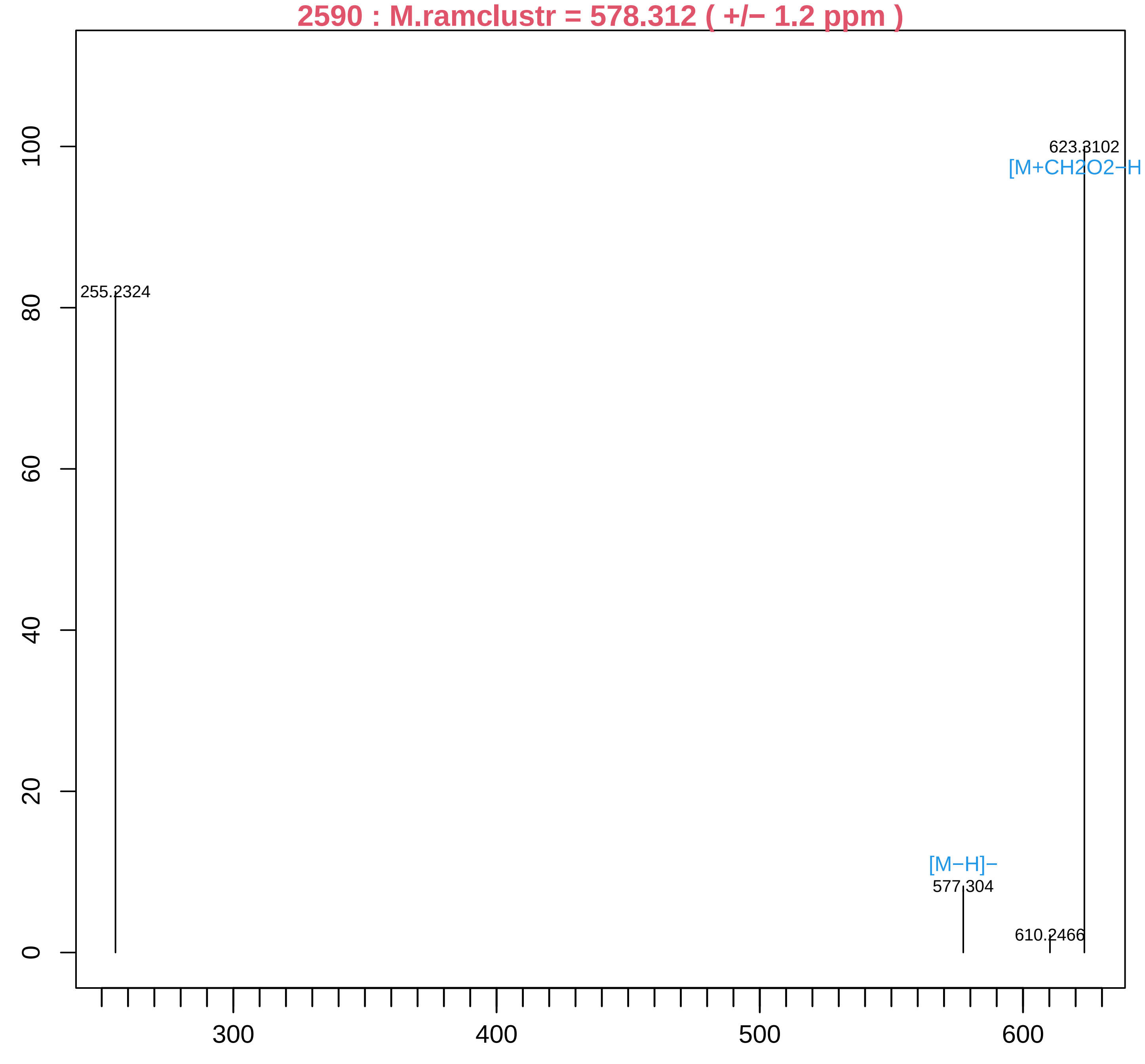

2590 : M.ramclustr = 578.312 (+/− 1.2 ppm)
623.3102
[M+CH2O2−H
255.2324
[M−H]−
577.304
610.2466
0
20
40
60
80
100
300
400
500
600

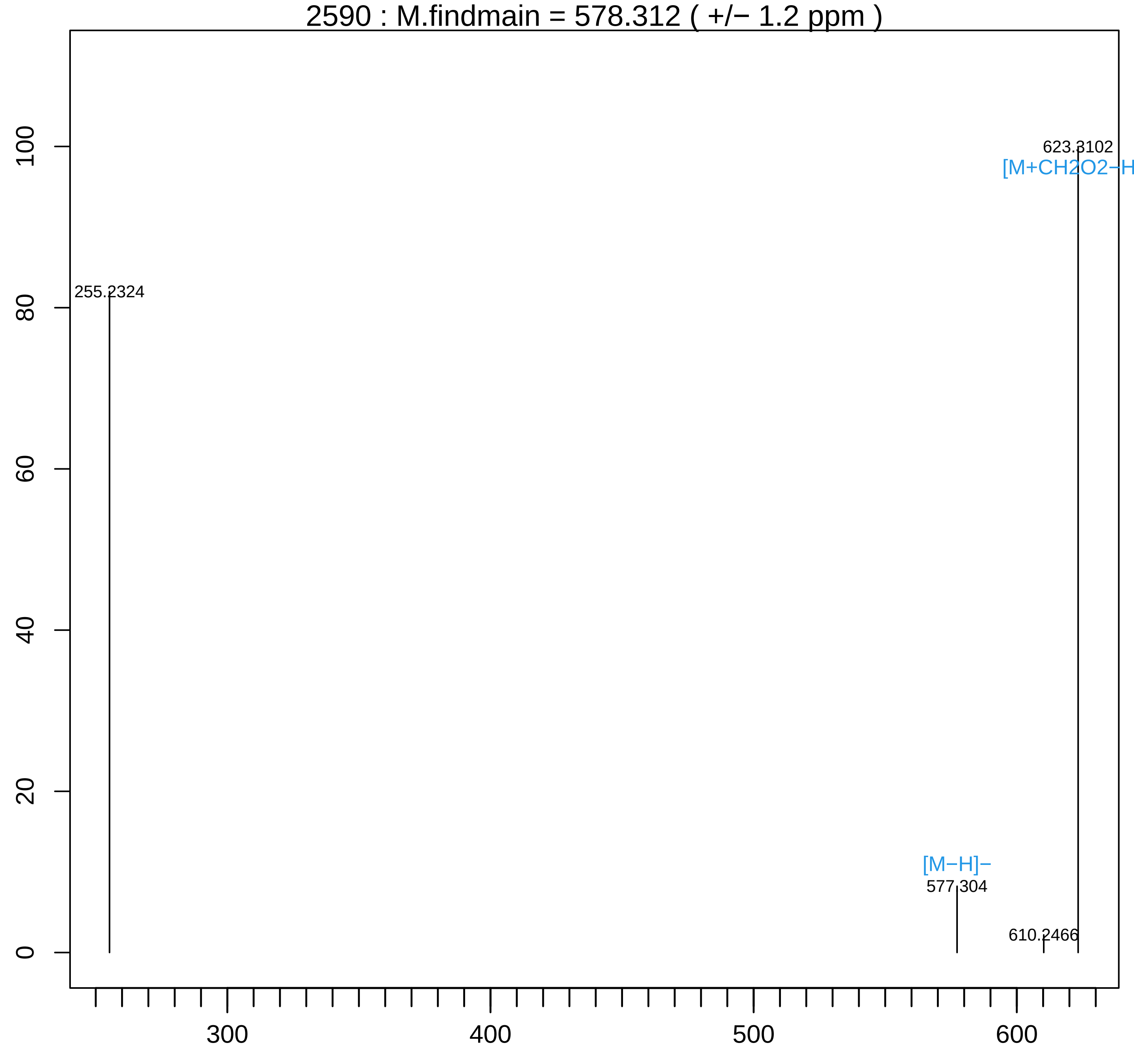

2590 : M.findmain = 578.312 (+/− 1.2 ppm)
623.3102
[M+CH2O2−H
255.2324
[M−H]−
577.304
610.2466
0
20
40
60
80
100
300
400
500
600

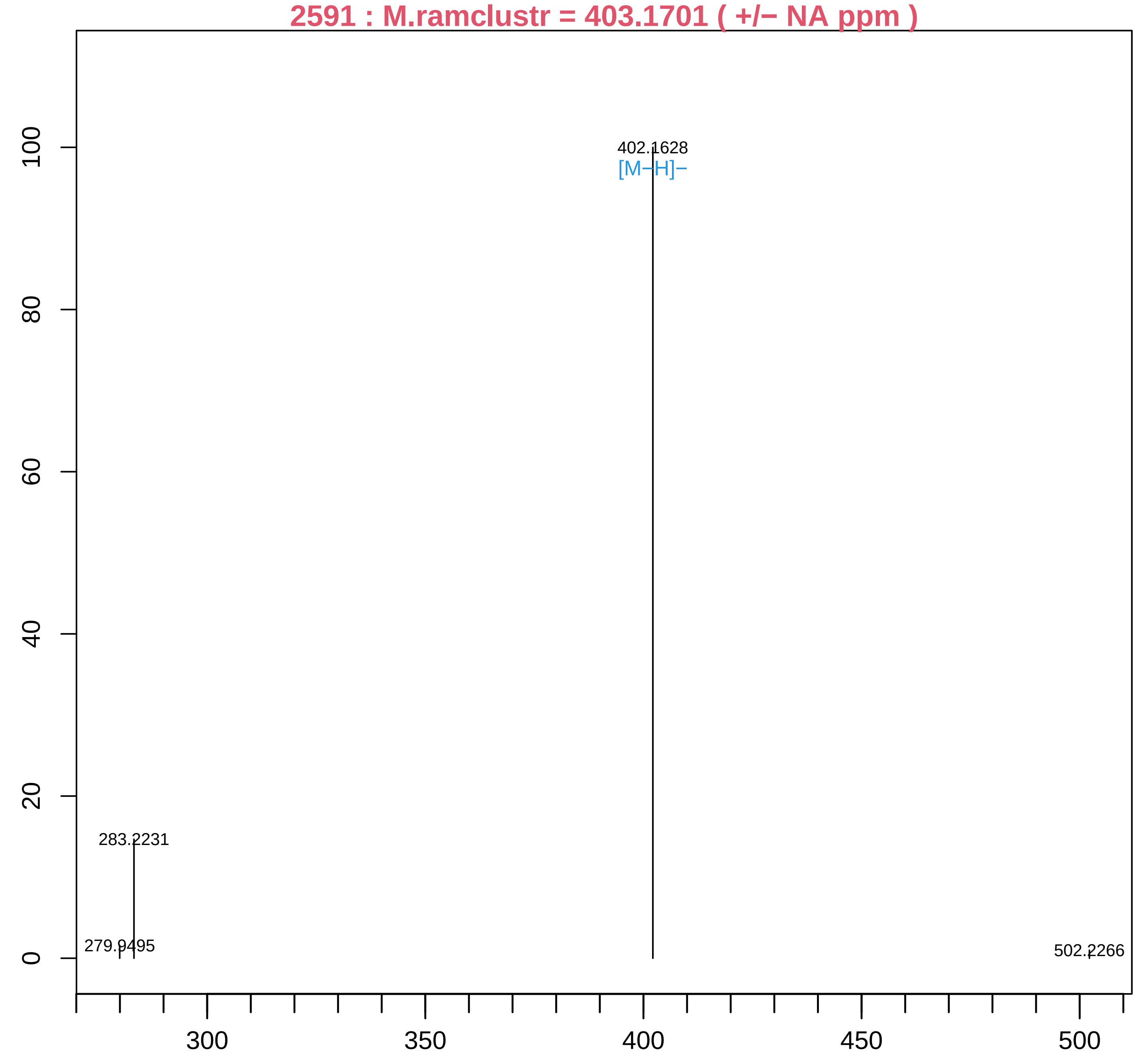
2591 : M.ramclustr = 403.1701 (+/− NA ppm)
402.1628
[M−H]−
283.2231
279.9495
502.2266
0
20
40
60
80
100
300
350
400
450
500

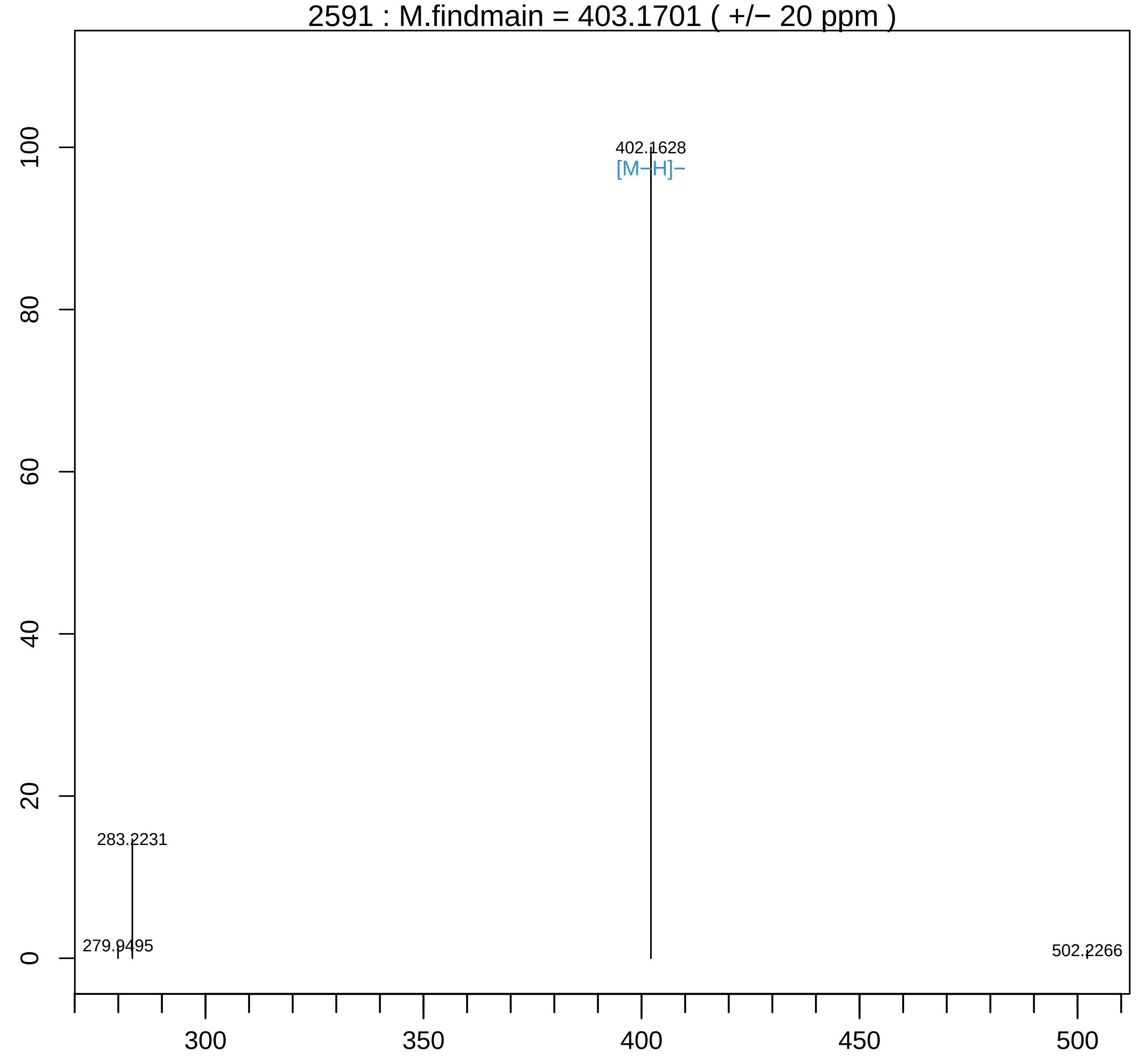
2591 : M.findmain = 403.1701 (+/− 20 ppm)
402.1628
[M−H]−
283.2231
279.9495
502.2266
0
20
40
60
80
100
300
350
400
450
500

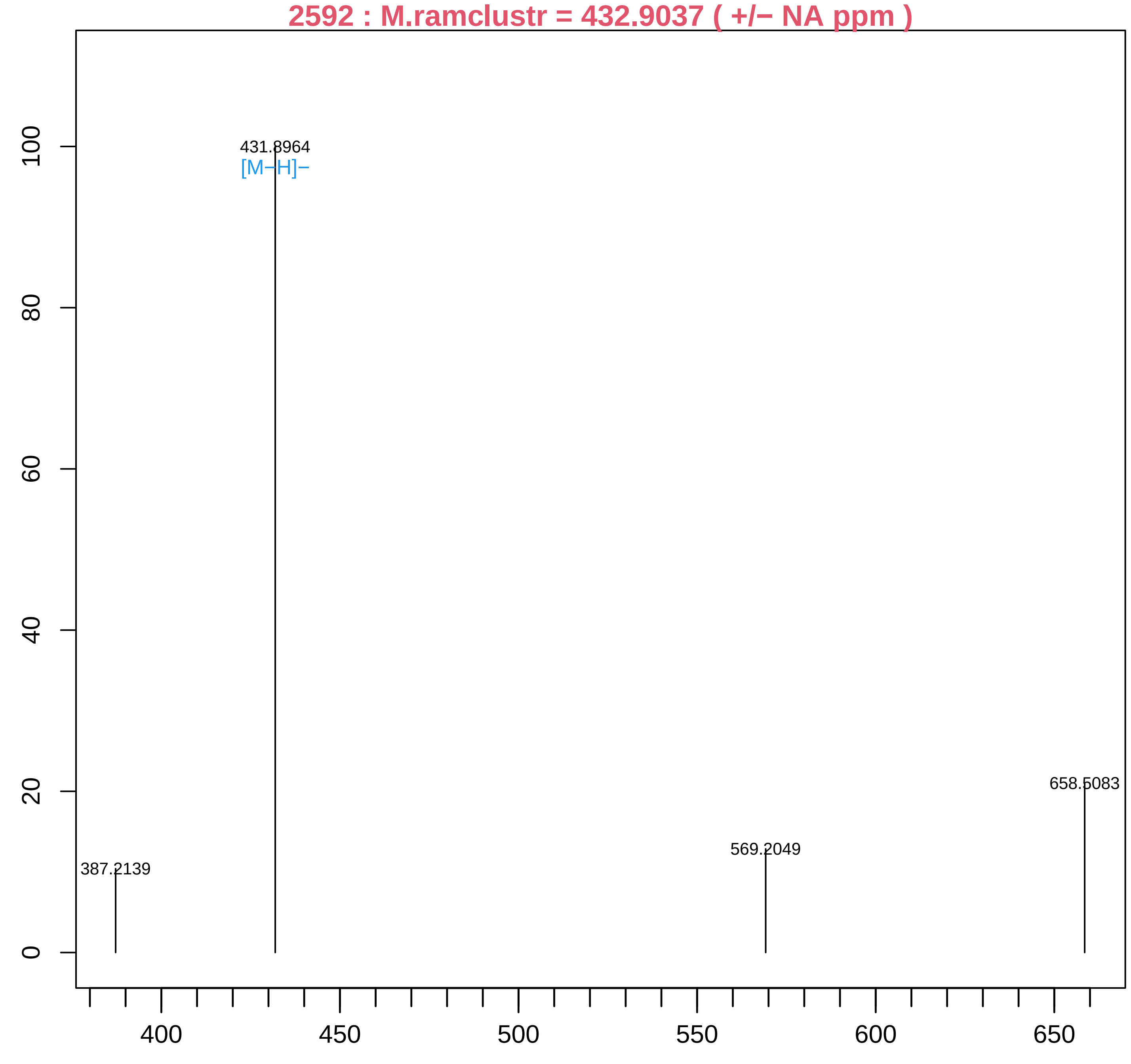
2592 : M.ramclustr = 432.9037 (+/− NA ppm)
431.8964
[M−H]−
387.2139
569.2049
658.5083
0
20
40
60
80
100
400
450
500
550
600
650

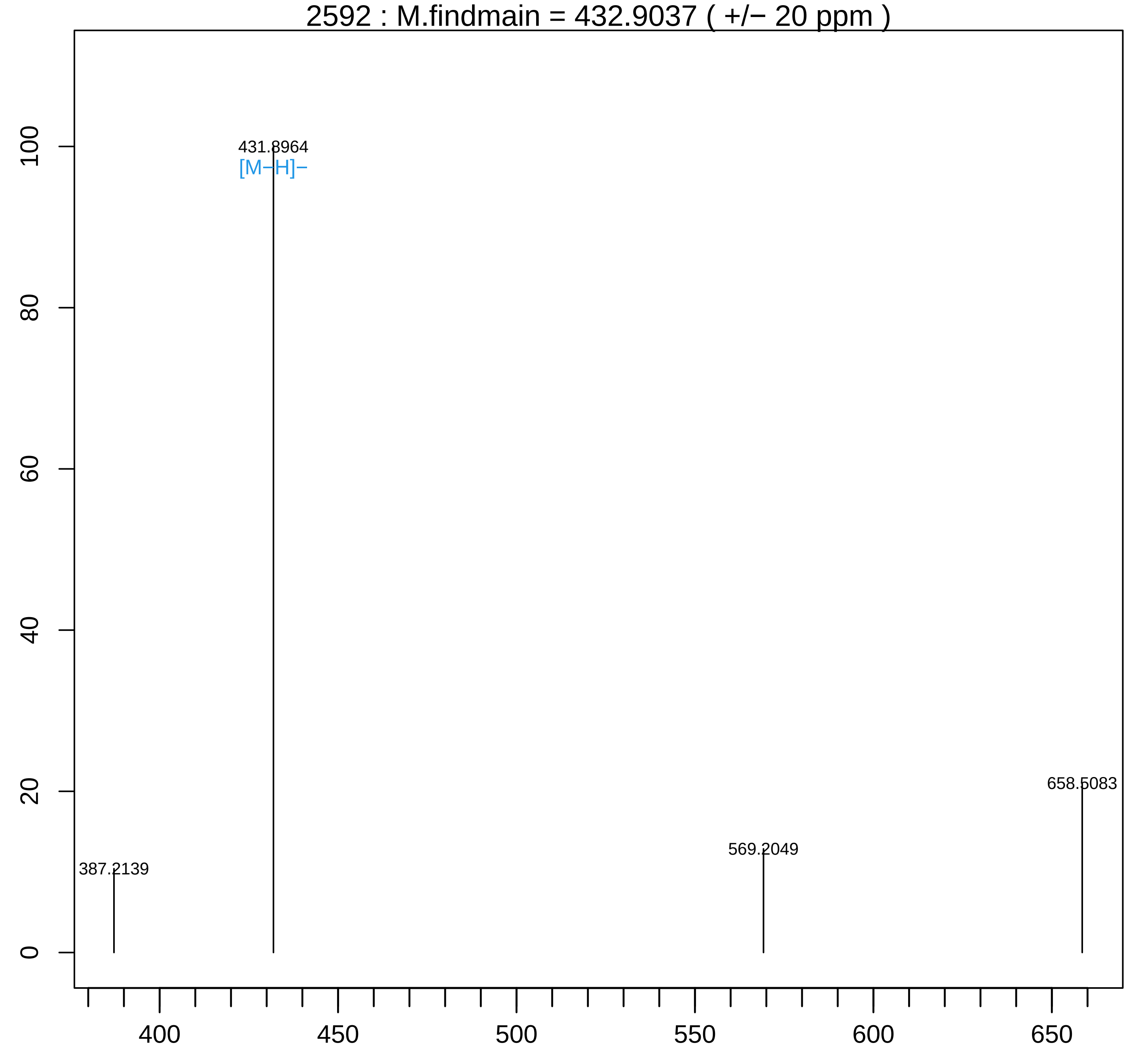
2592 : M.findmain = 432.9037 (+/− 20 ppm)
431.8964
[M−H]−
387.2139
569.2049
658.5083
0
20
40
60
80
100
400
450
500
550
600
650

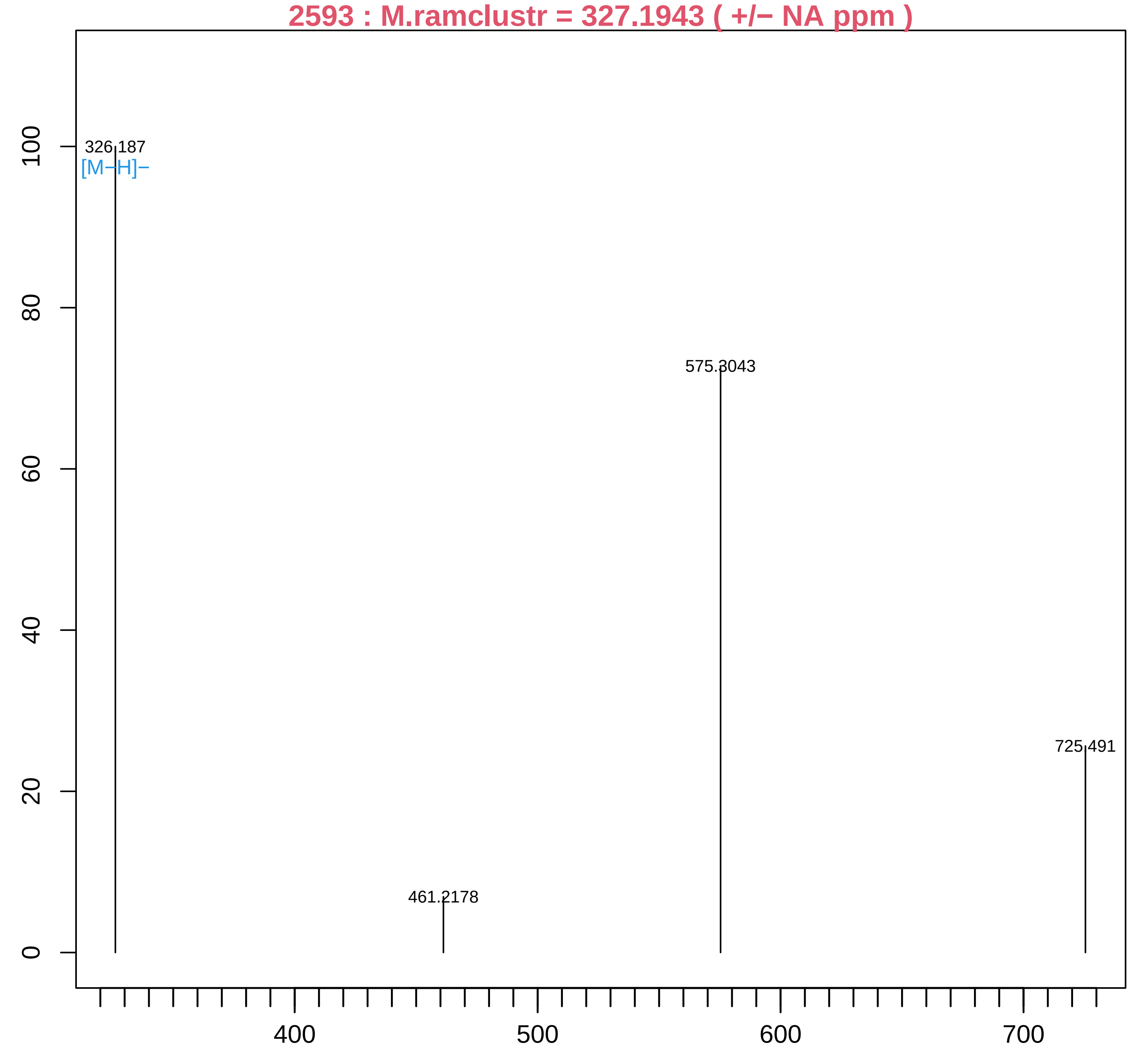
2593 : M.ramclustr = 327.1943 (+/− NA ppm)
326.187
[M−H]−
575.3043
461.2178
725.491
0
20
40
60
80
100
400
500
600
700
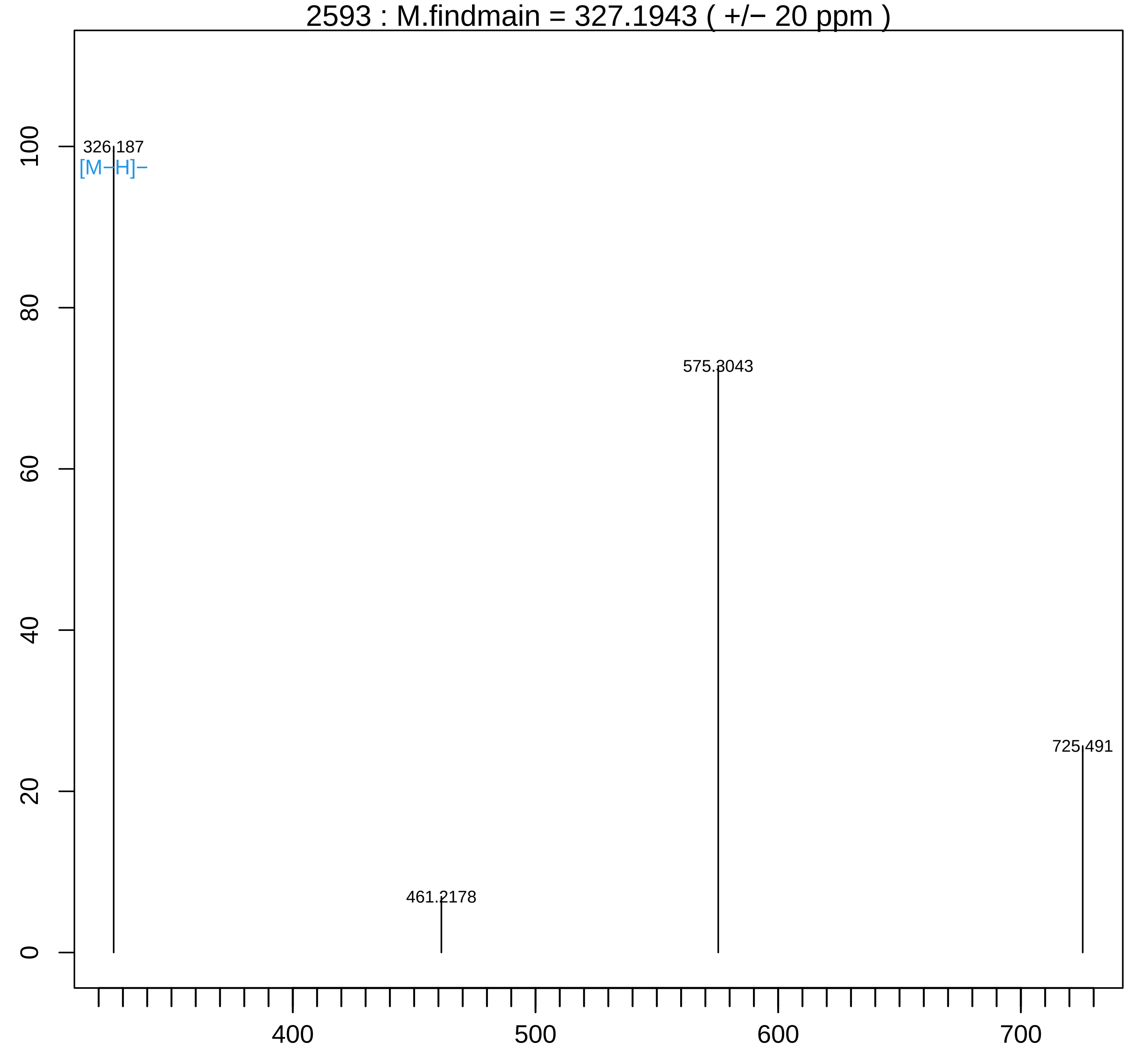
2593 : M.findmain = 327.1943 (+/− 20 ppm)
326.187
[M−H]−
575.3043
461.2178
725.491
0
20
40
60
80
100
400
500
600
700

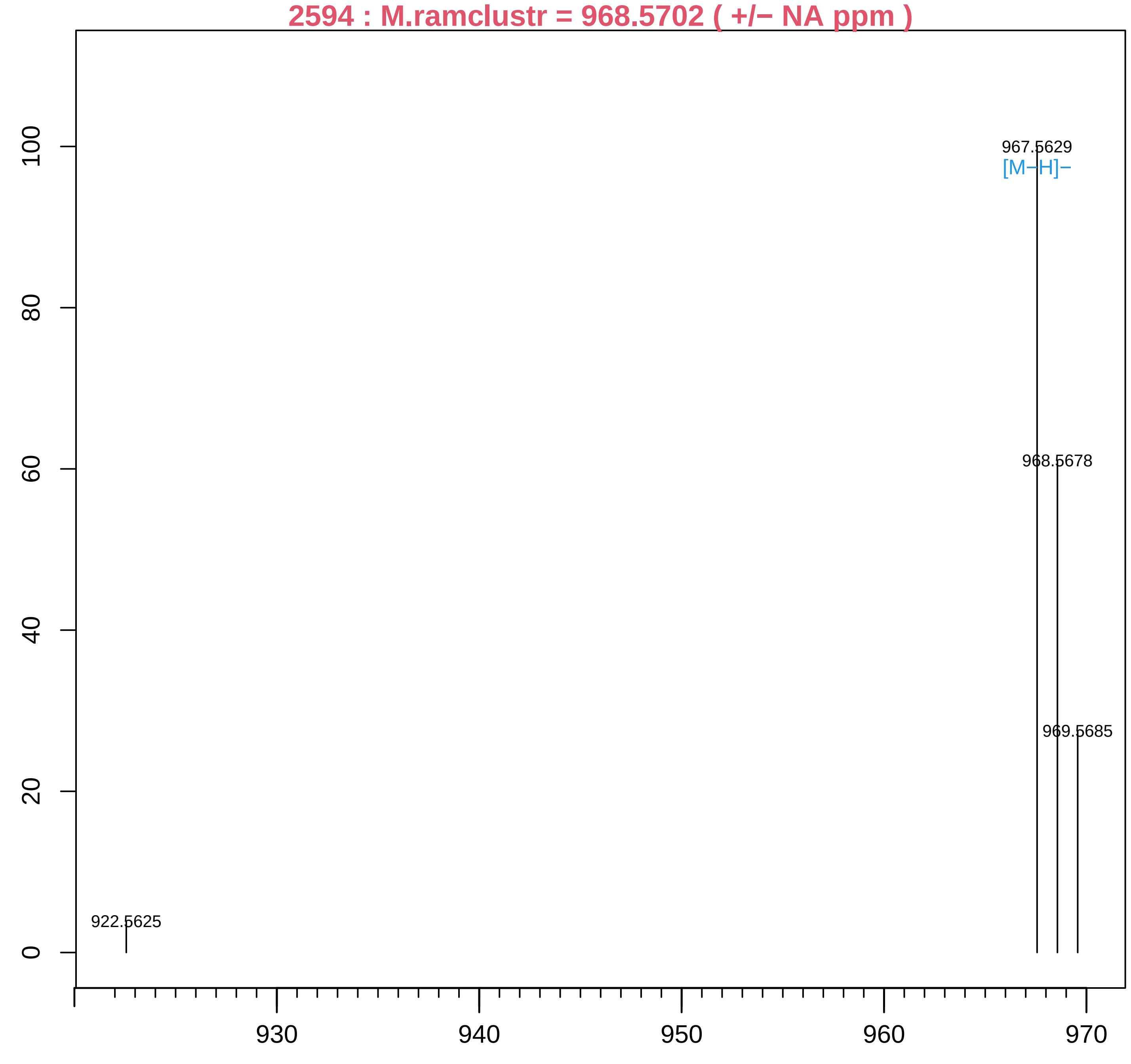
2594 : M.ramclustr = 968.5702 (+/− NA ppm)
967.5629
[M−H]−
968.5678
969.5685
922.5625
0
20
40
60
80
100
930
940
950
960
970

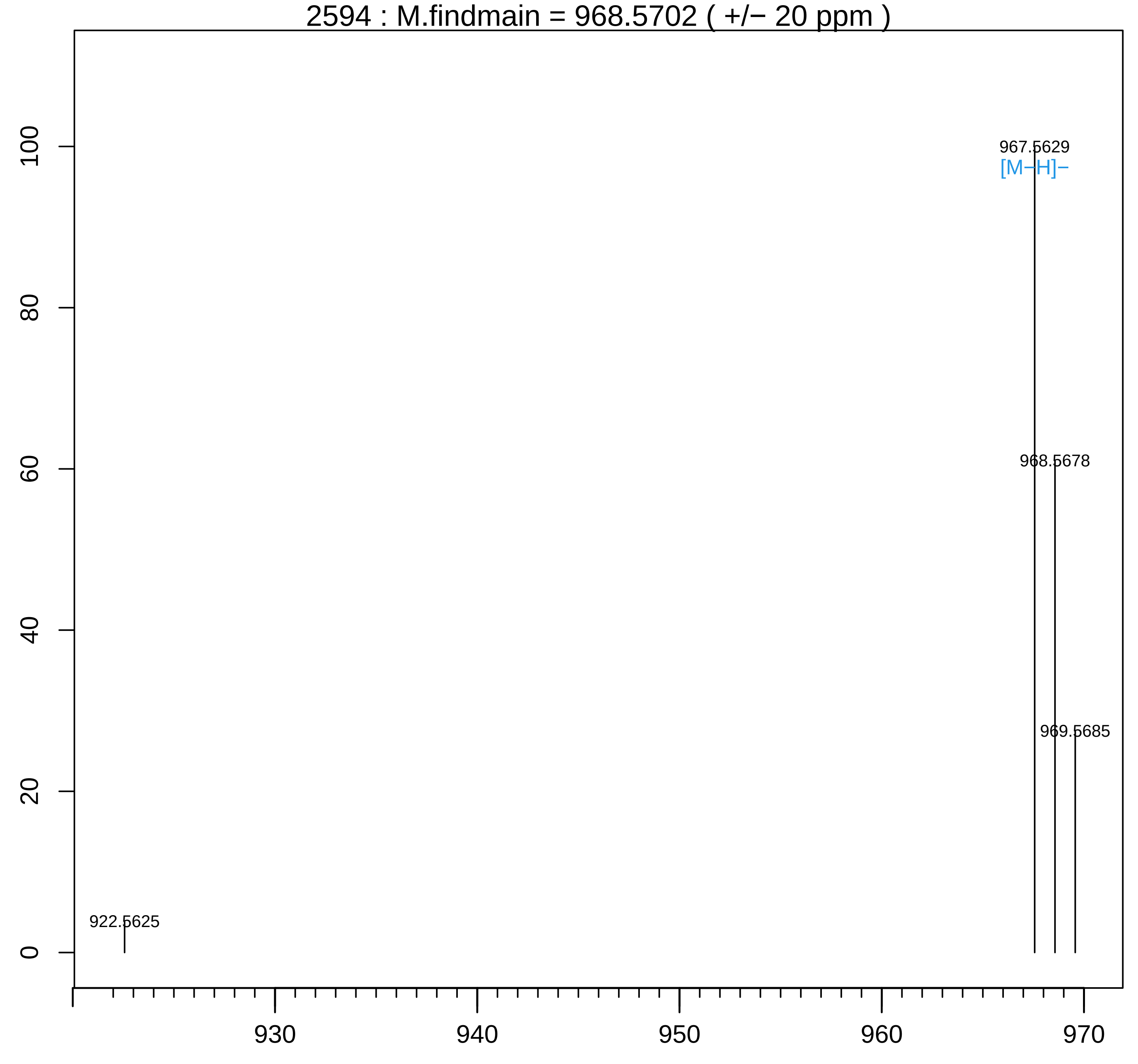
2594 : M.findmain = 968.5702 (+/− 20 ppm)
967.5629
[M−H]−
968.5678
969.5685
922.5625
0
20
40
60
80
100
930
940
950
960
970

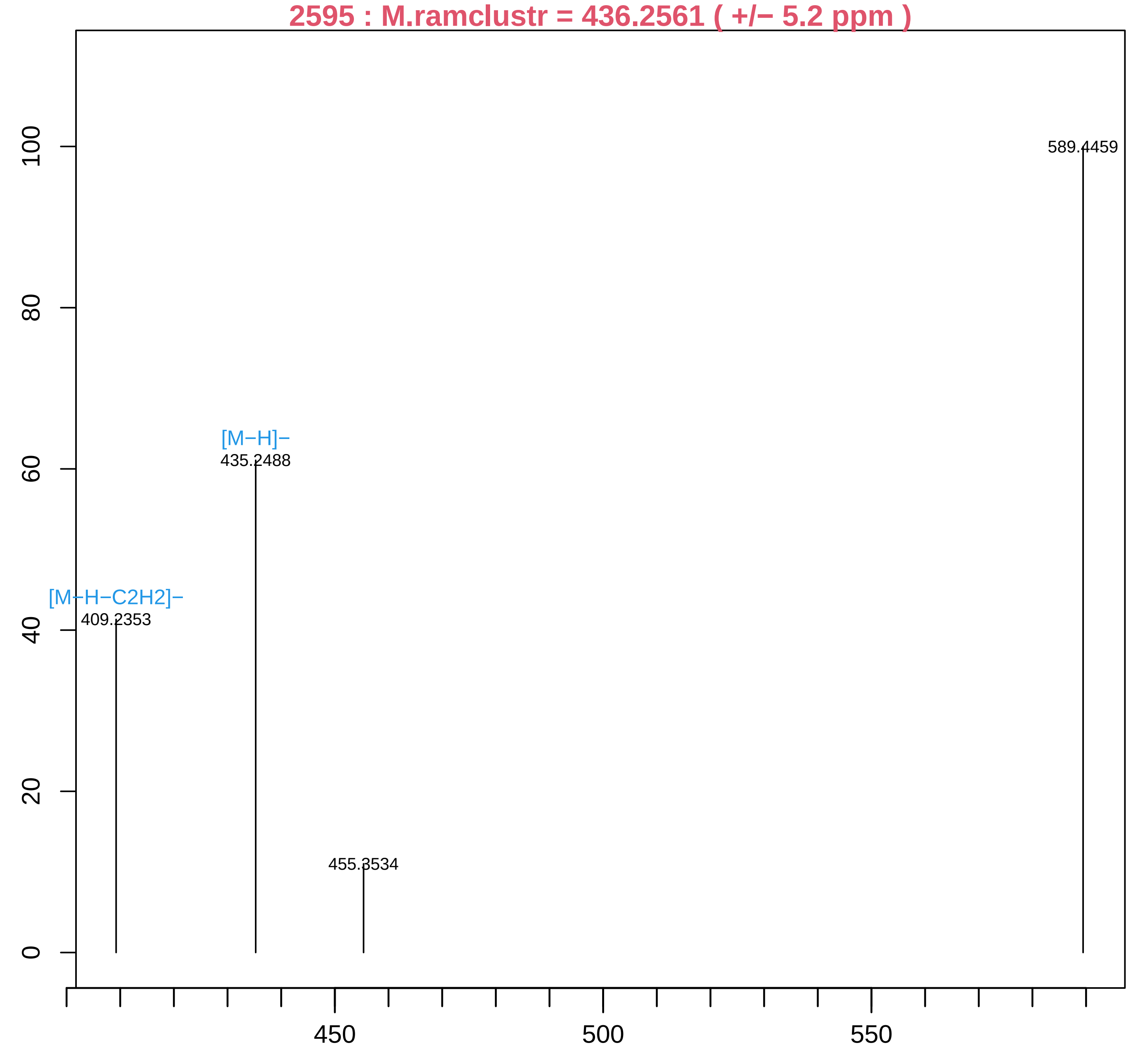

2595 : M.ramclustr = 436.2561 (+/− 5.2 ppm)
589.4459
[M−H]−
435.2488
[M−H−C2H2]−
409.2353
455.3534
0
20
40
60
80
100
450
500
550

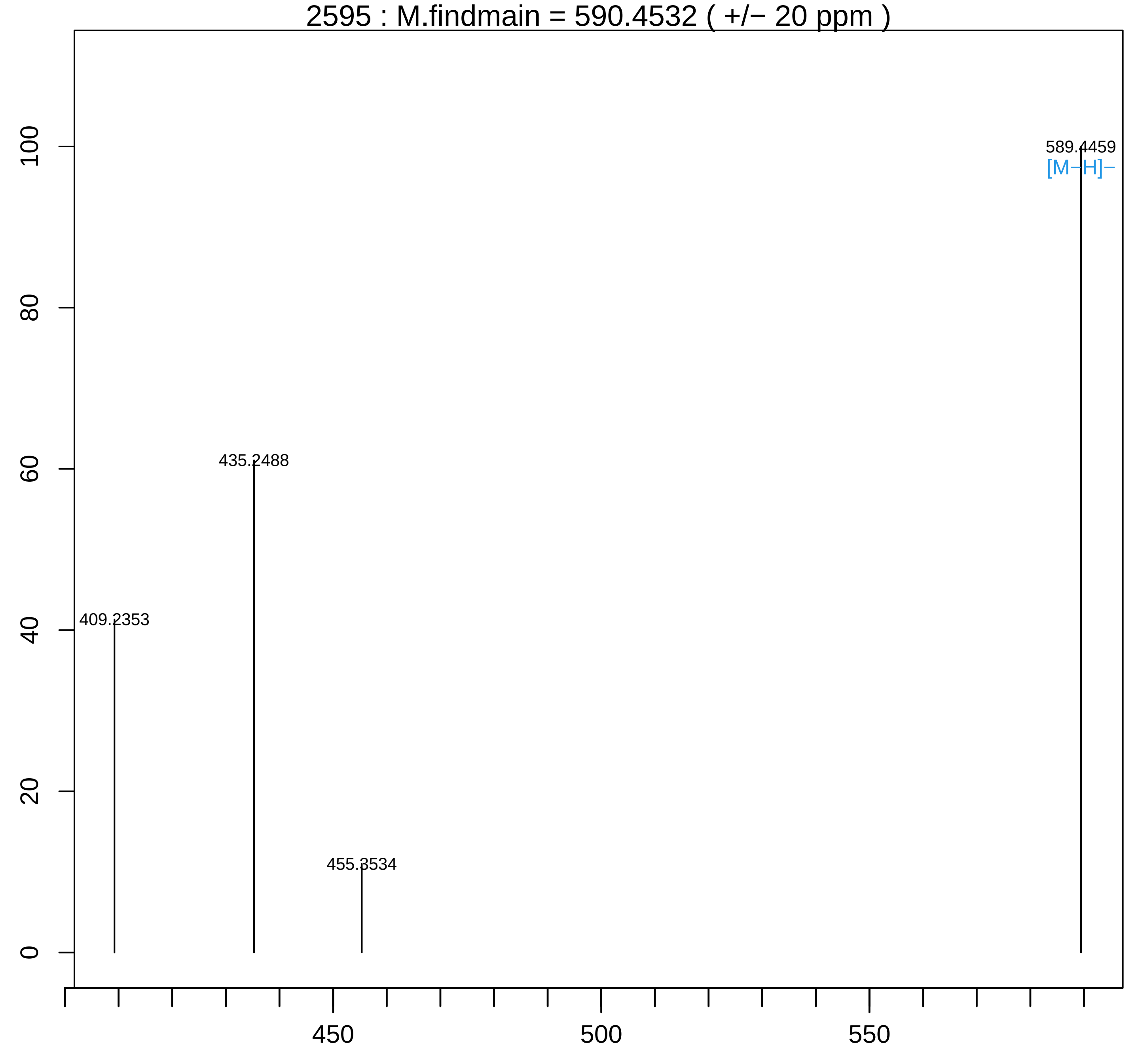

2595 : M.findmain = 590.4532 (+/− 20 ppm)
589.4459
[M−H]−
435.2488
409.2353
455.3534
0
20
40
60
80
100
450
500
550

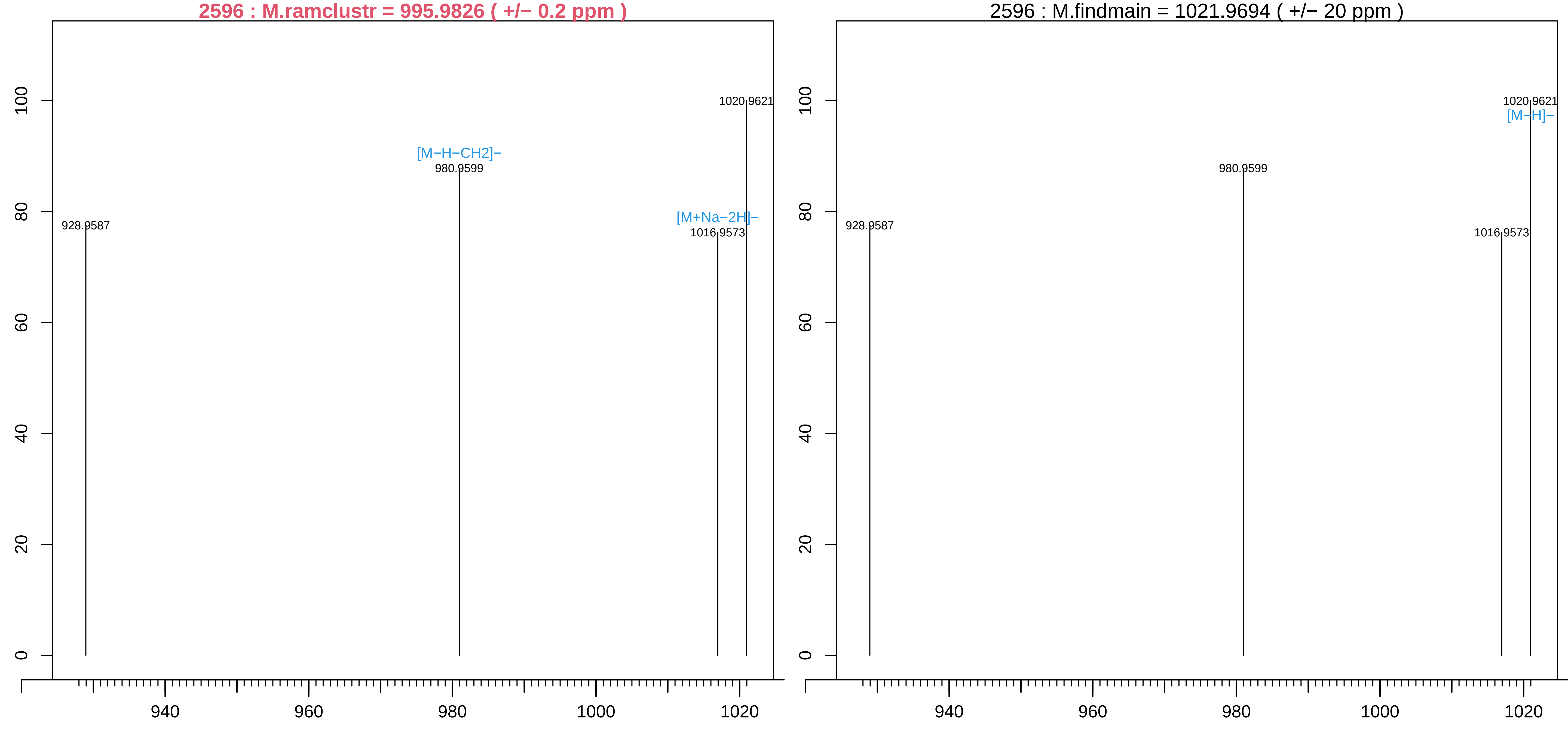
2596 : M.ramclustr = 995.9826 (+/− 0.2 ppm)
[M−H−CH2]−
980.9599
928.9587
[M+Na−2H]−
1016.9573
1020.9621
2596 : M.findmain = 1021.9694 (+/− 20 ppm)
1020.9621
[M−H]−
980.9599
928.9587
1016.9573
0
20
40
60
80
100
940
960
980
1000
1020

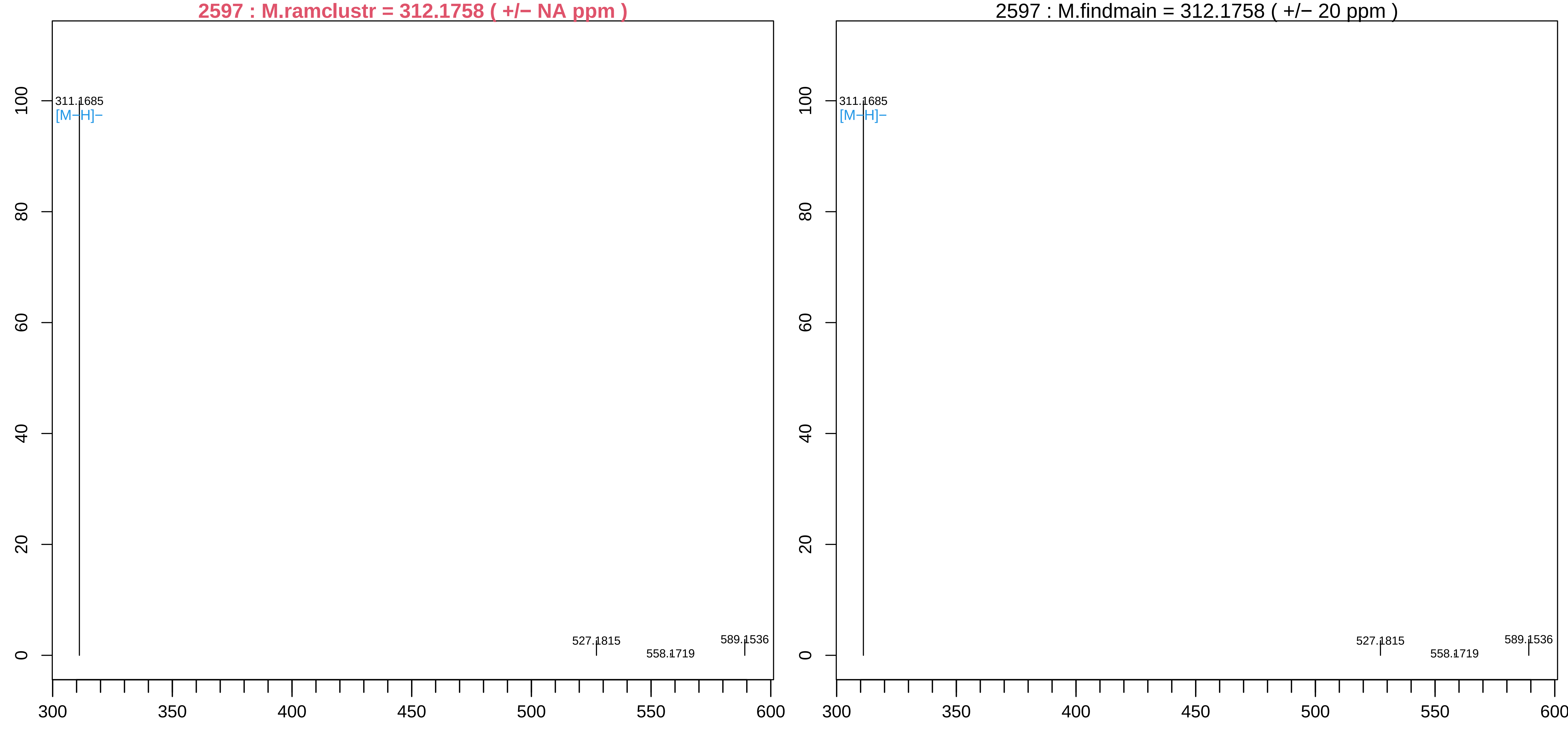
2597 : M.ramclustr = 312.1758 (+/− NA ppm)
311.1685
[M−H]−
527.1815
558.1719
589.1536
2597 : M.findmain = 312.1758 (+/− 20 ppm)
311.1685
[M−H]−
527.1815
558.1719
589.1536
0
20
40
60
80
100
300
350
400
450
500
550
600

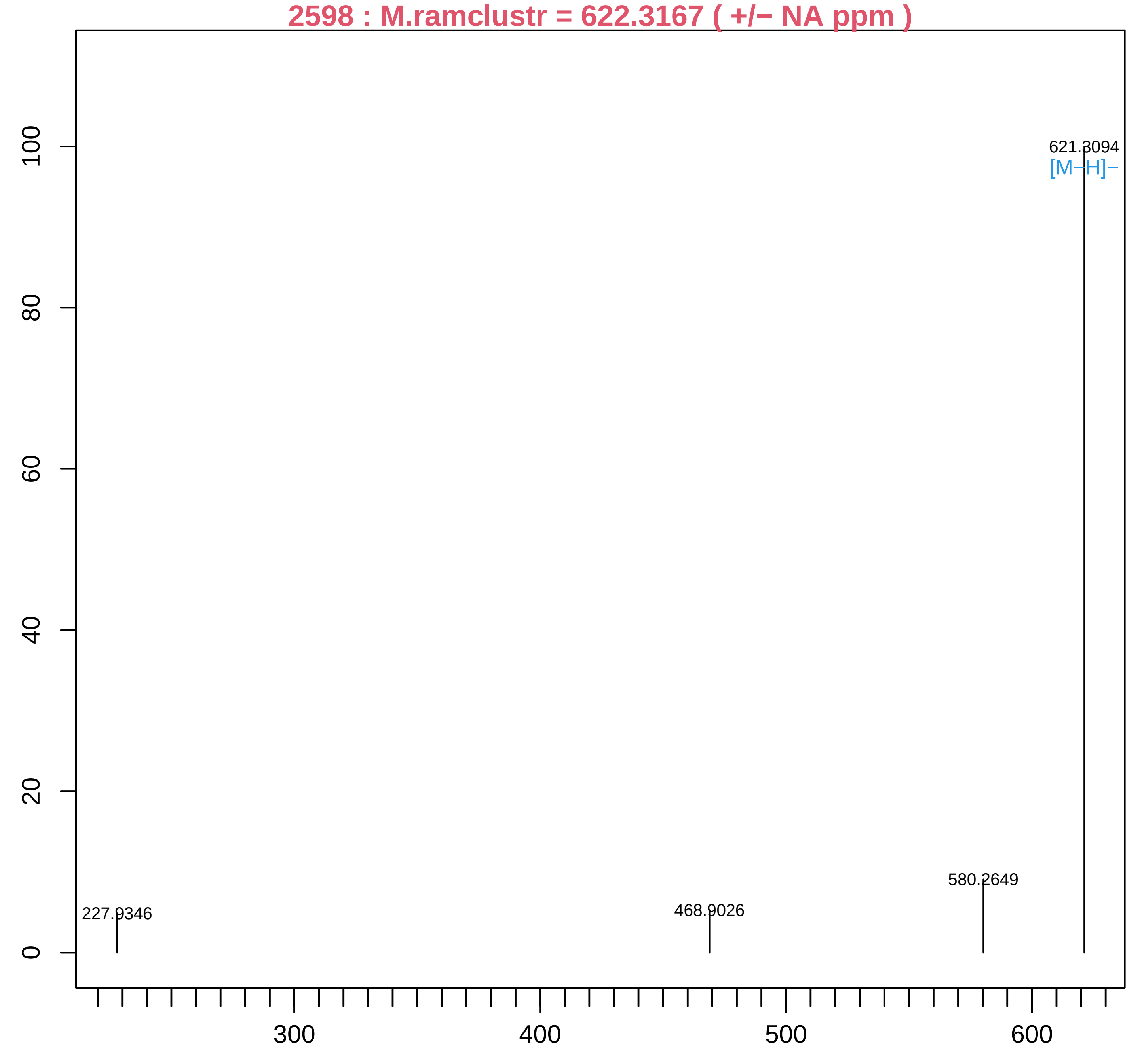
2598 : M.ramclustr = 622.3167 (+/− NA ppm)
621.3094
[M−H]−
580.2649
468.9026
227.9346
0
20
40
60
80
100
300
400
500
600

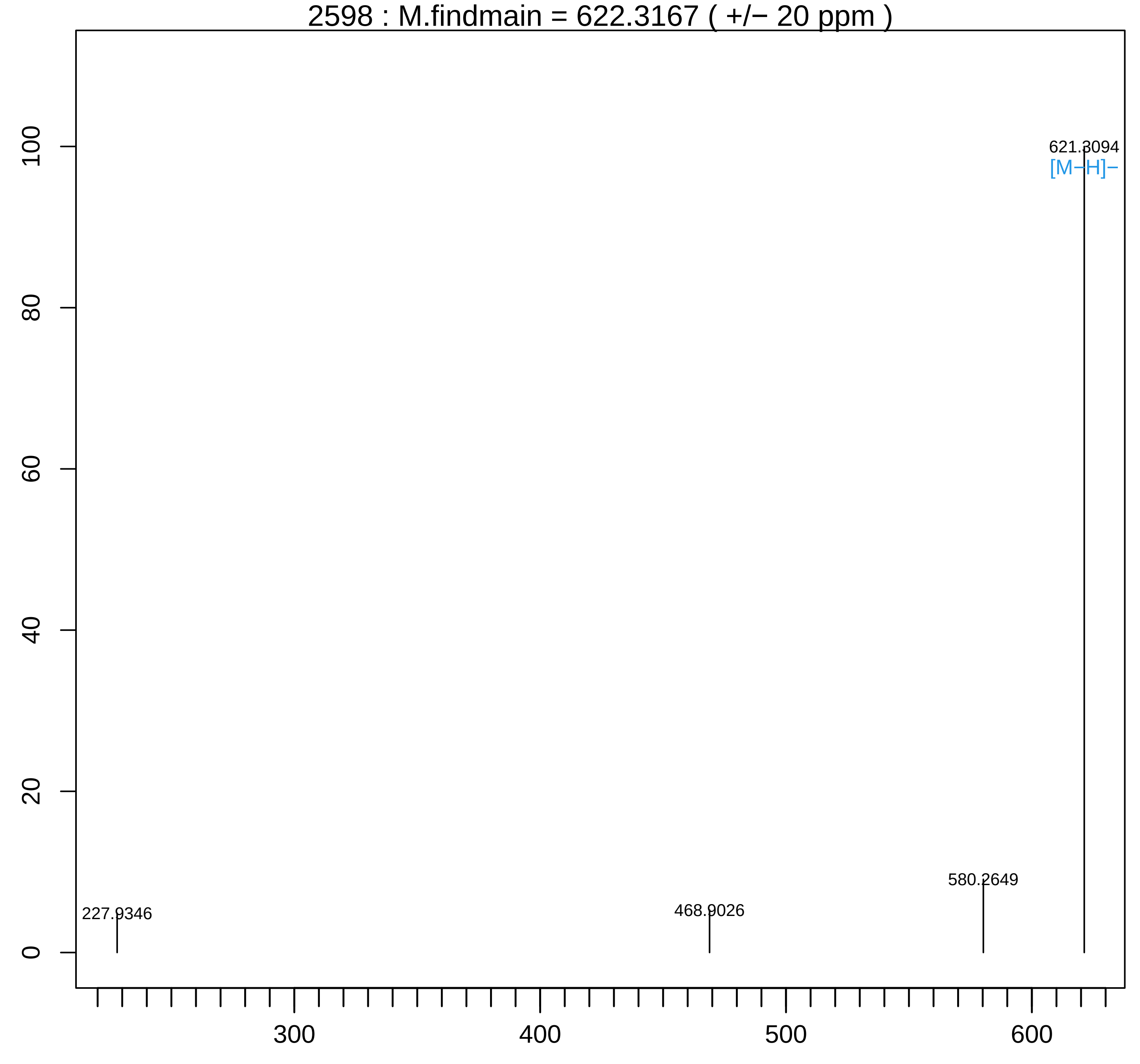
2598 : M.findmain = 622.3167 (+/− 20 ppm)
621.3094
[M−H]−
580.2649
468.9026
227.9346
0
20
40
60
80
100
300
400
500
600

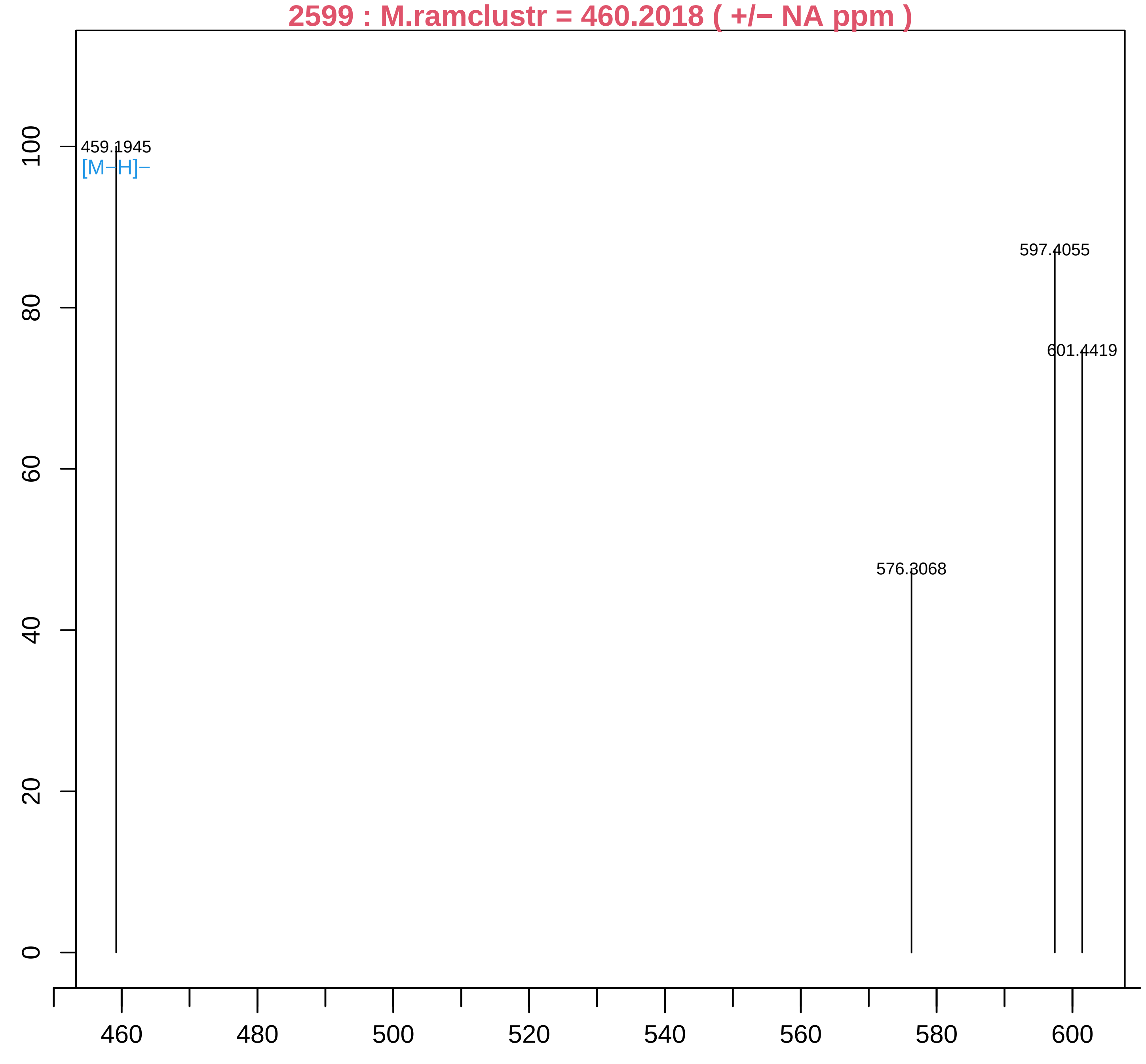
2599 : M.ramclustr = 460.2018 (+/− NA ppm)
459.1945
[M−H]−
597.4055
601.4419
576.3068
0
20
40
60
80
100
460
480
500
520
540
560
580
600
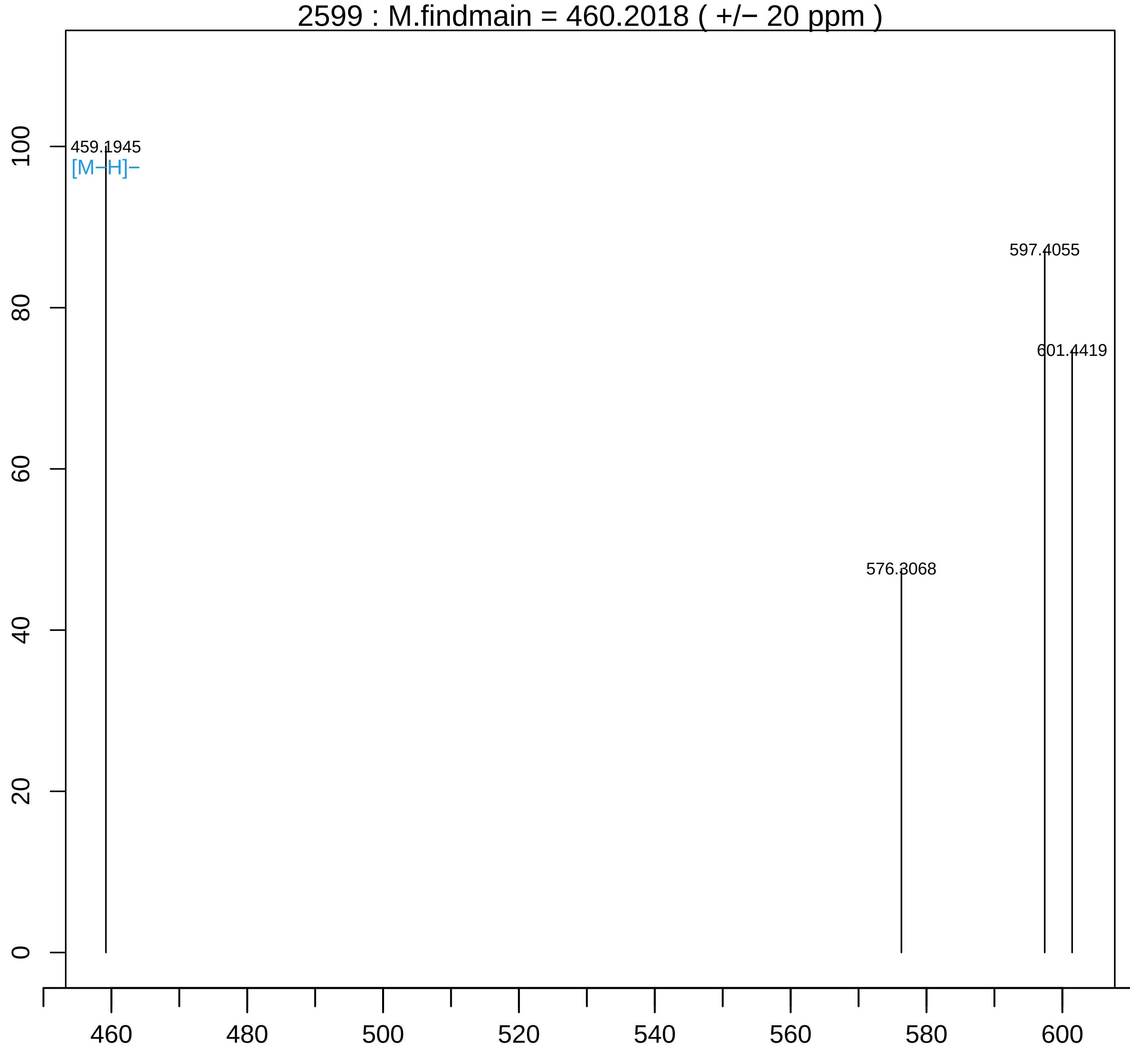
2599 : M.findmain = 460.2018 (+/− 20 ppm)
459.1945
[M−H]−
597.4055
601.4419
576.3068
0
20
40
60
80
100
460
480
500
520
540
560
580
600

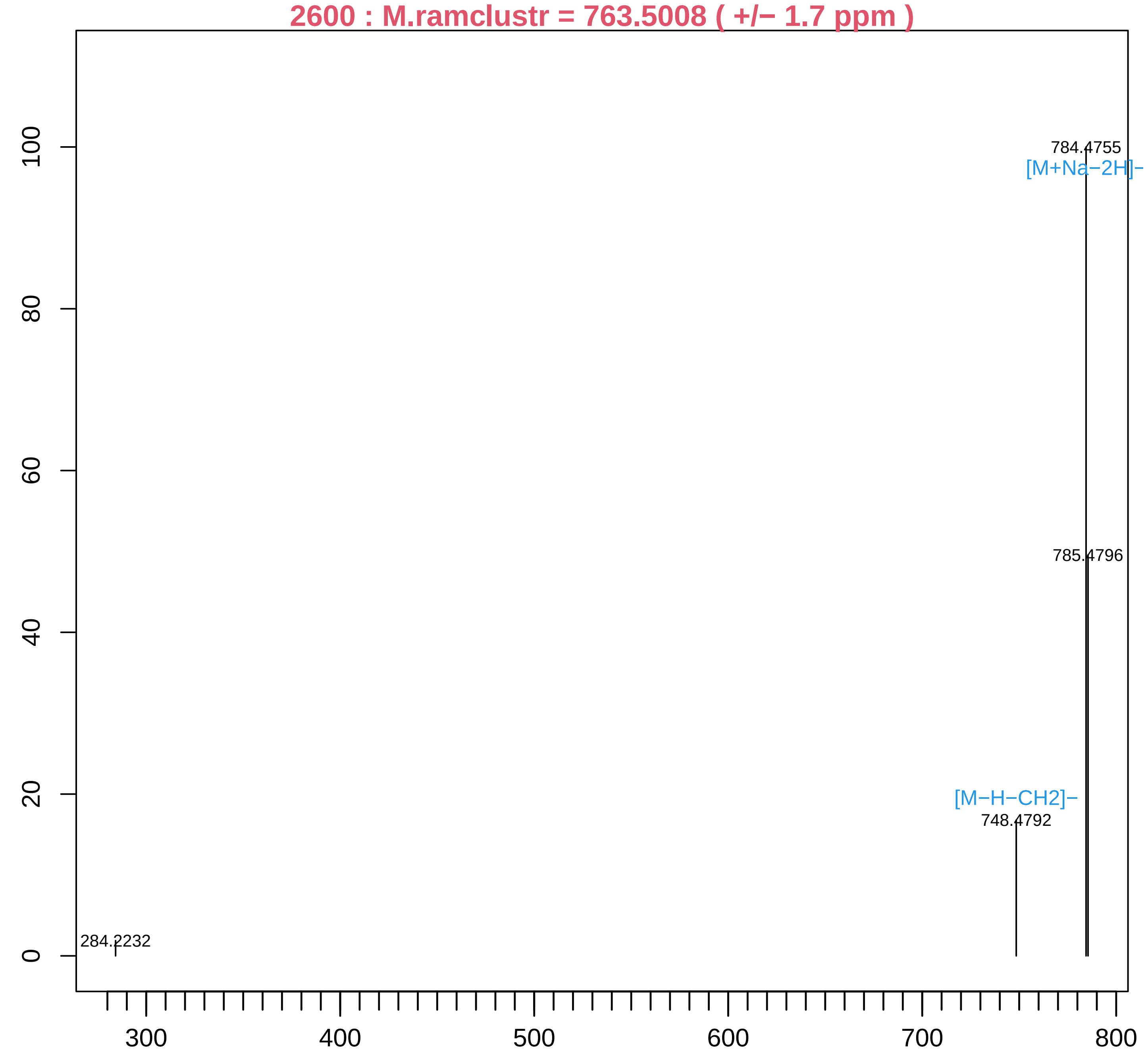
2600 : M.ramclustr = 763.5008 (+/− 1.7 ppm)
784.4755
[M+Na−2H]−
785.4796
[M−H−CH2]−
748.4792
284.2232
0
20
40
60
80
100
300
400
500
600
700
800

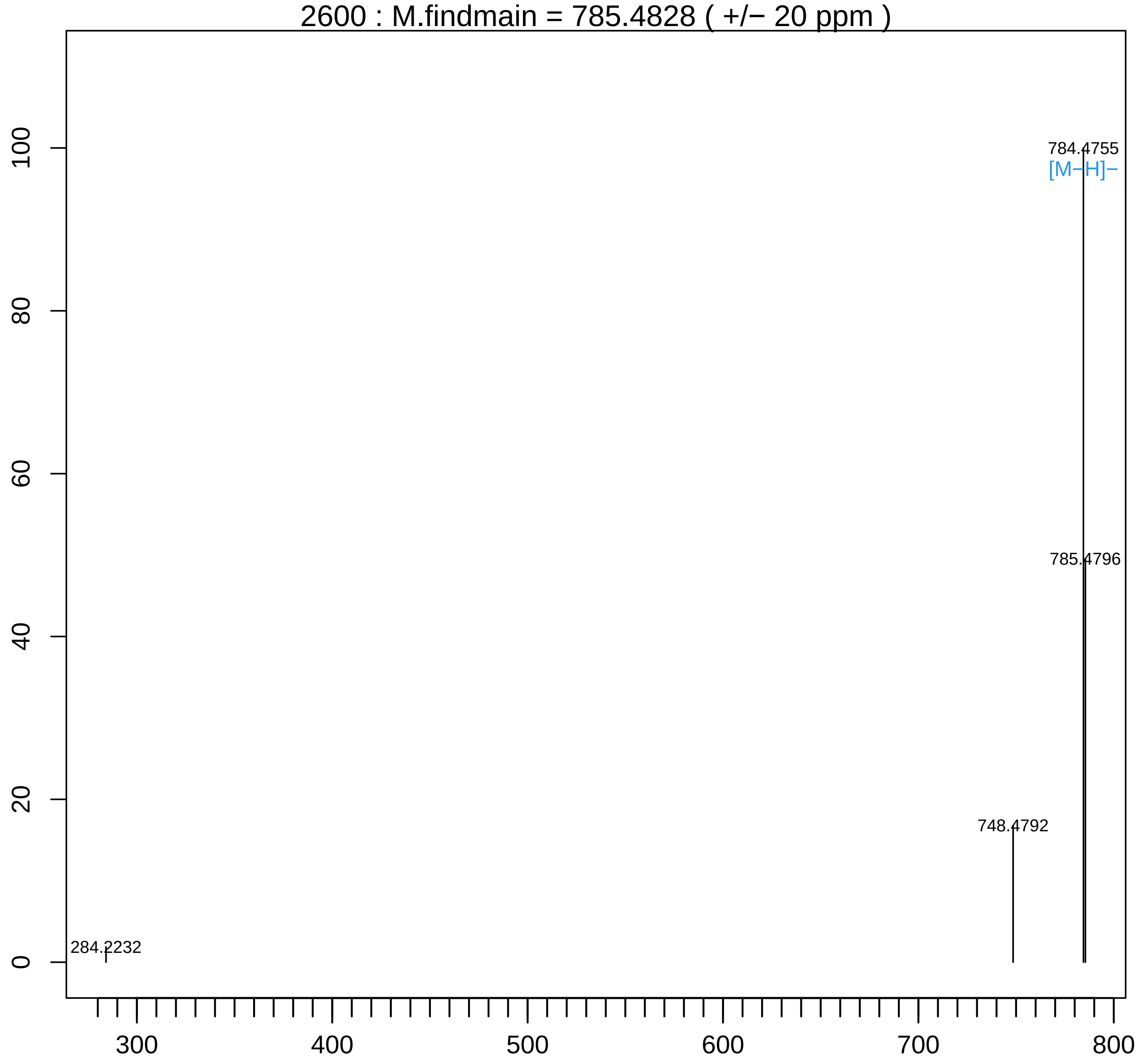
2600 : M.findmain = 785.4828 (+/− 20 ppm)
784.4755
[M−H]−
785.4796
748.4792
284.2232
0
20
40
60
80
100
300
400
500
600
700
800

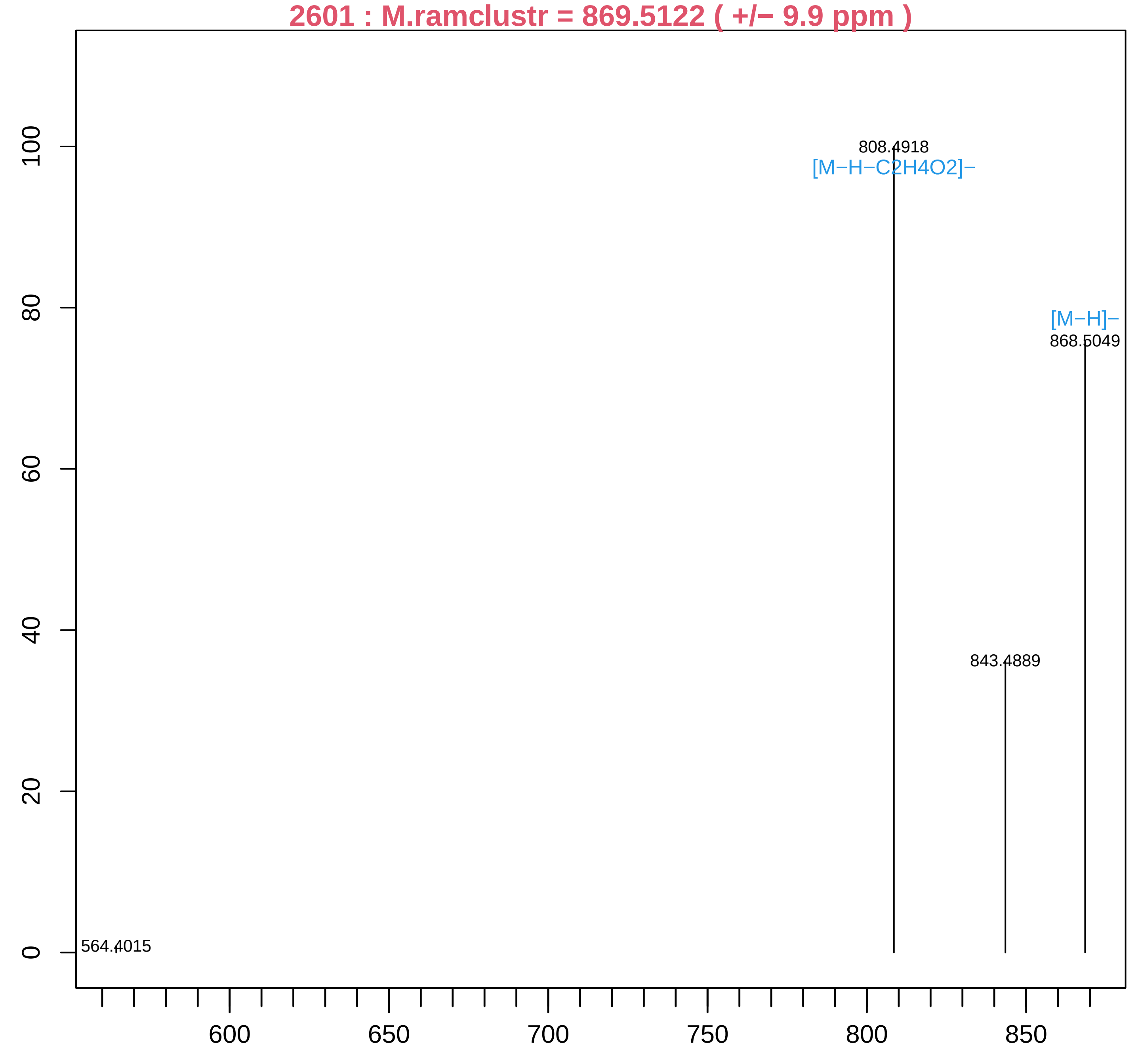

2601 : M.ramclustr = 869.5122 (+/− 9.9 ppm)
808.4918
[M−H−C2H4O2]−
[M−H]−
868.5049
843.4889
564.4015

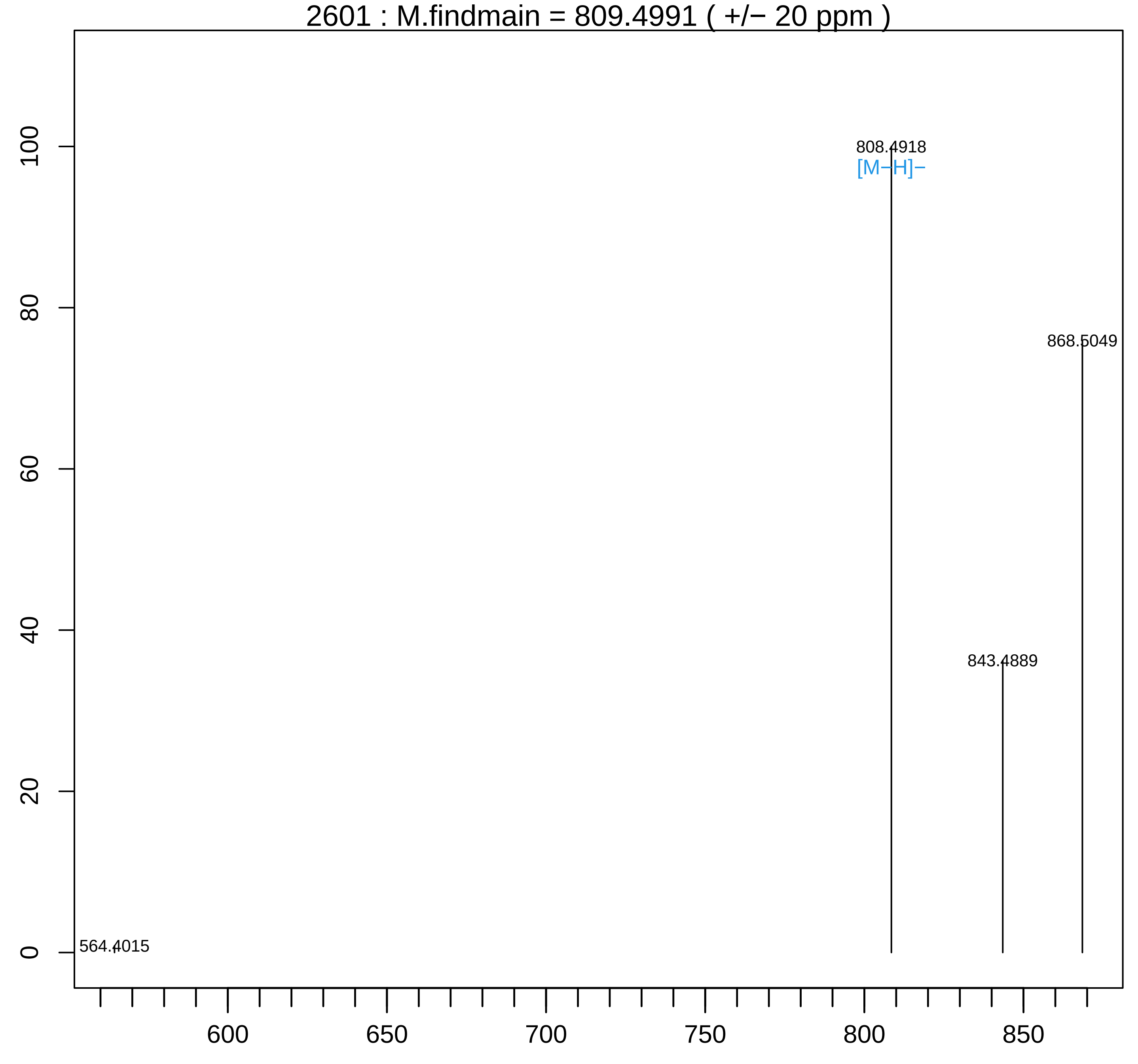

2601 : M.findmain = 809.4991 (+/− 20 ppm)
808.4918
[M−H]−
868.5049
843.4889
564.4015

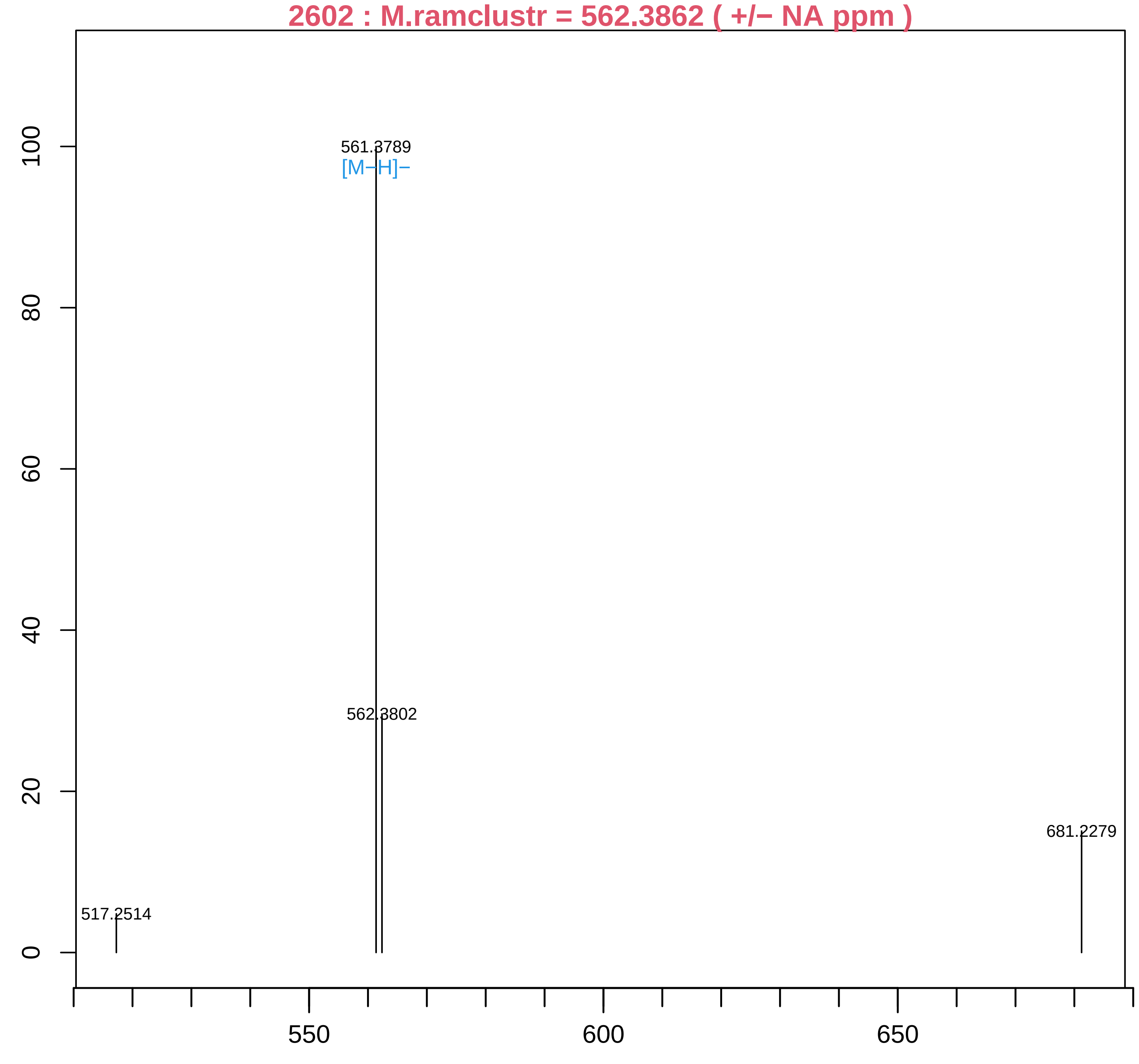
2602 : M.ramclustr = 562.3862 (+/− NA ppm)
561.3789
[M−H]−
562.3802
681.2279
517.2514
0
20
40
60
80
100
550
600
650

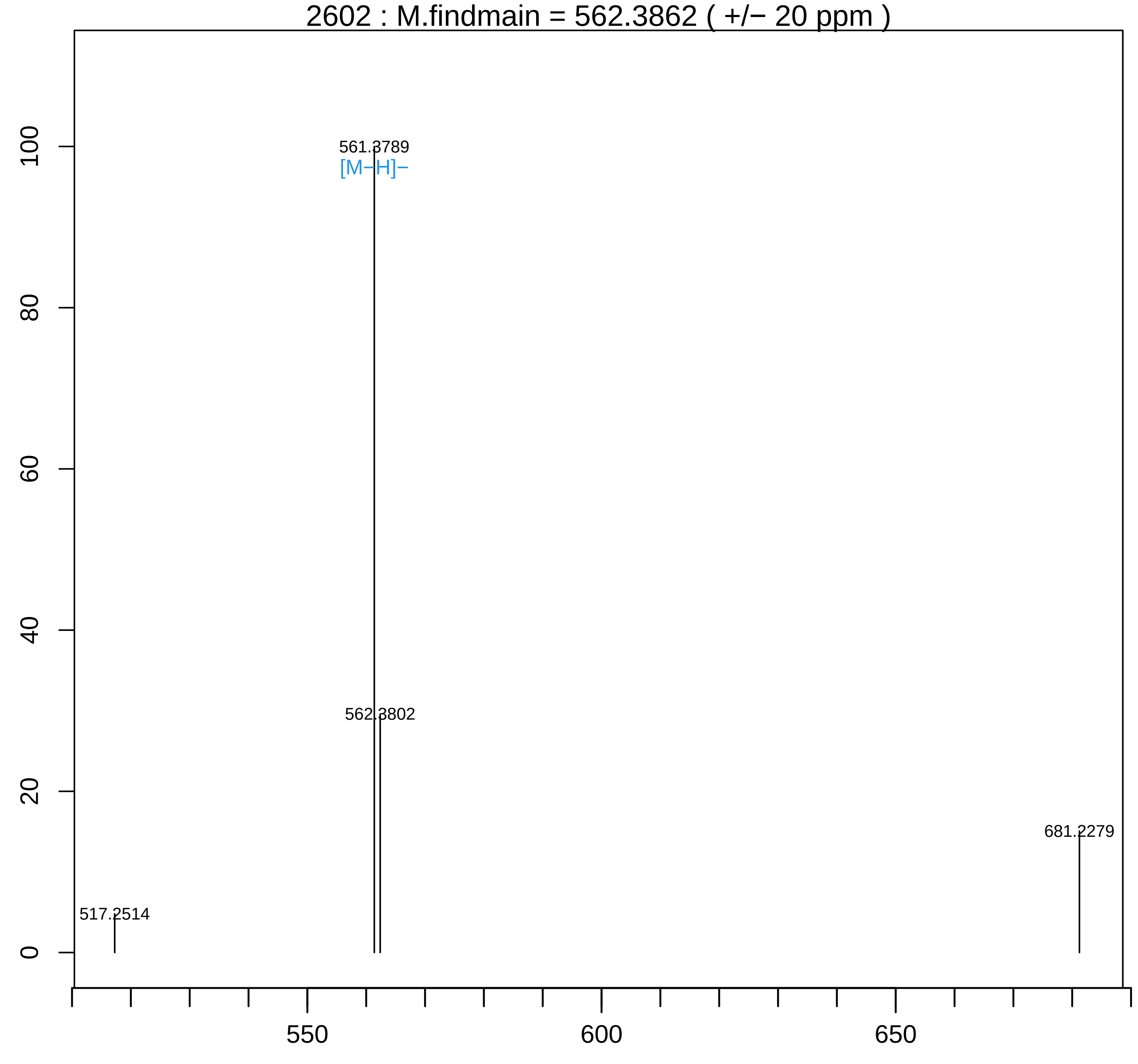
2602 : M.findmain = 562.3862 (+/− 20 ppm)
561.3789
[M−H]−
562.3802
681.2279
517.2514
0
20
40
60
80
100
550
600
650

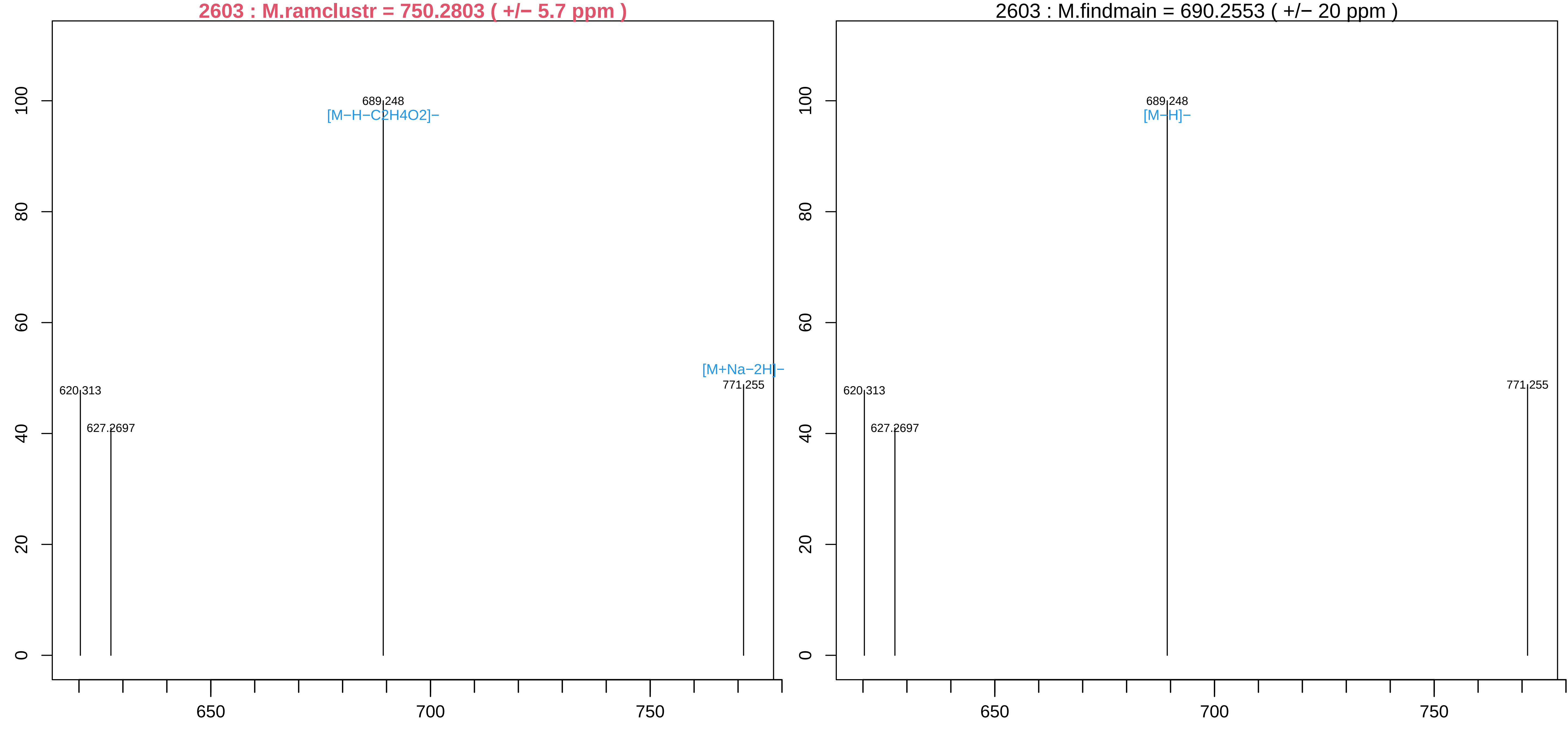

2603 : M.ramclustr = 750.2803 (+/− 5.7 ppm)
689.248
[M−H−C2H4O2]−
[M+Na−2H]−
771.255
620.313
627.2697
2603 : M.findmain = 690.2553 (+/− 20 ppm)
689.248
[M−H]−
620.313
627.2697
771.255
0
20
40
60
80
100
650
700
750

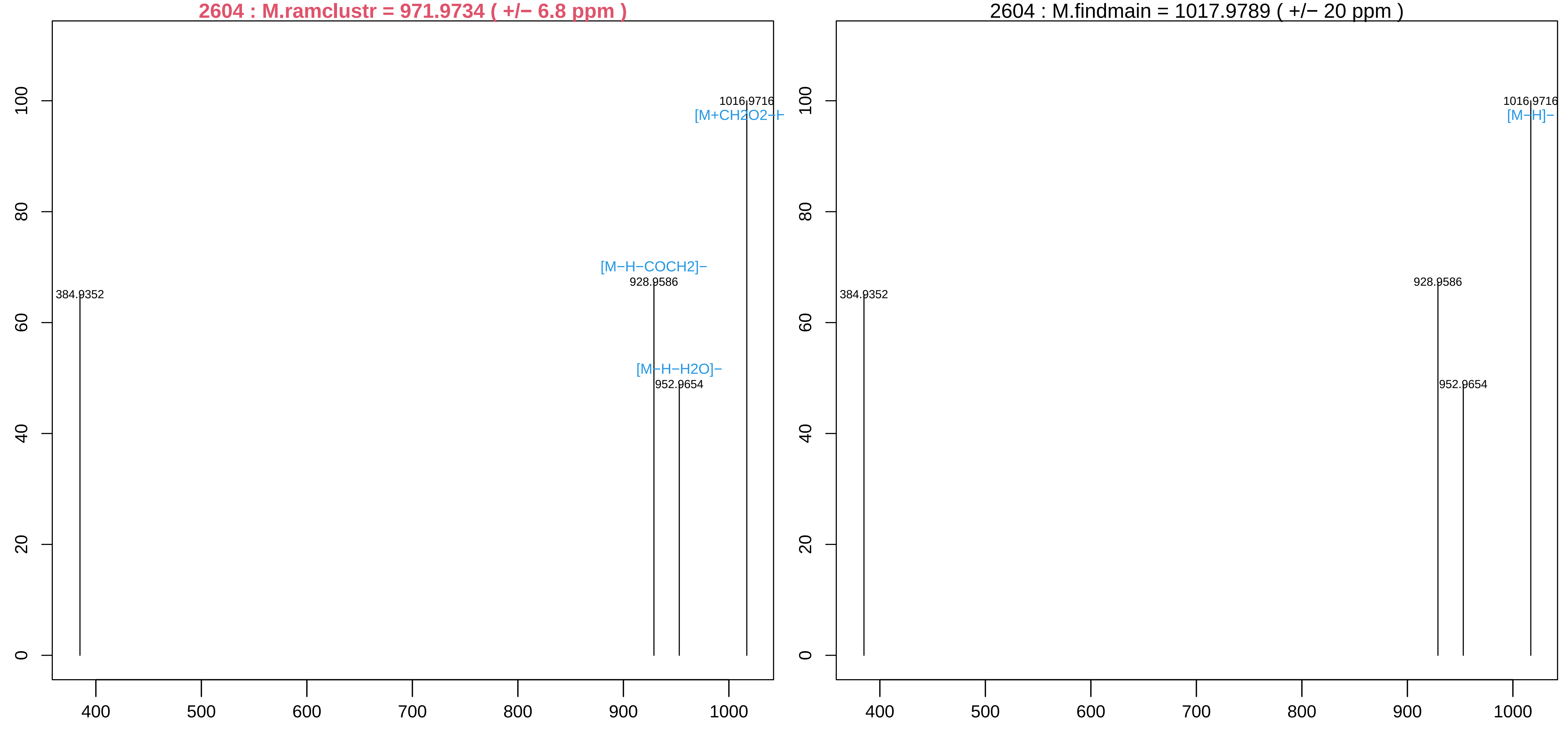

2604 : M.ramclustr = 971.9734 (+/− 6.8 ppm)
1016.9716
[M+CH2O2-H
[M−H−COCH2]−
928.9586
384.9352
[M−H−H2O]−
952.9654
2604 : M.findmain = 1017.9789 (+/− 20 ppm)
1016.9716
[M−H]−
928.9586
384.9352
952.9654
0
20
40
60
80
100
400
500
600
700
800
900
1000

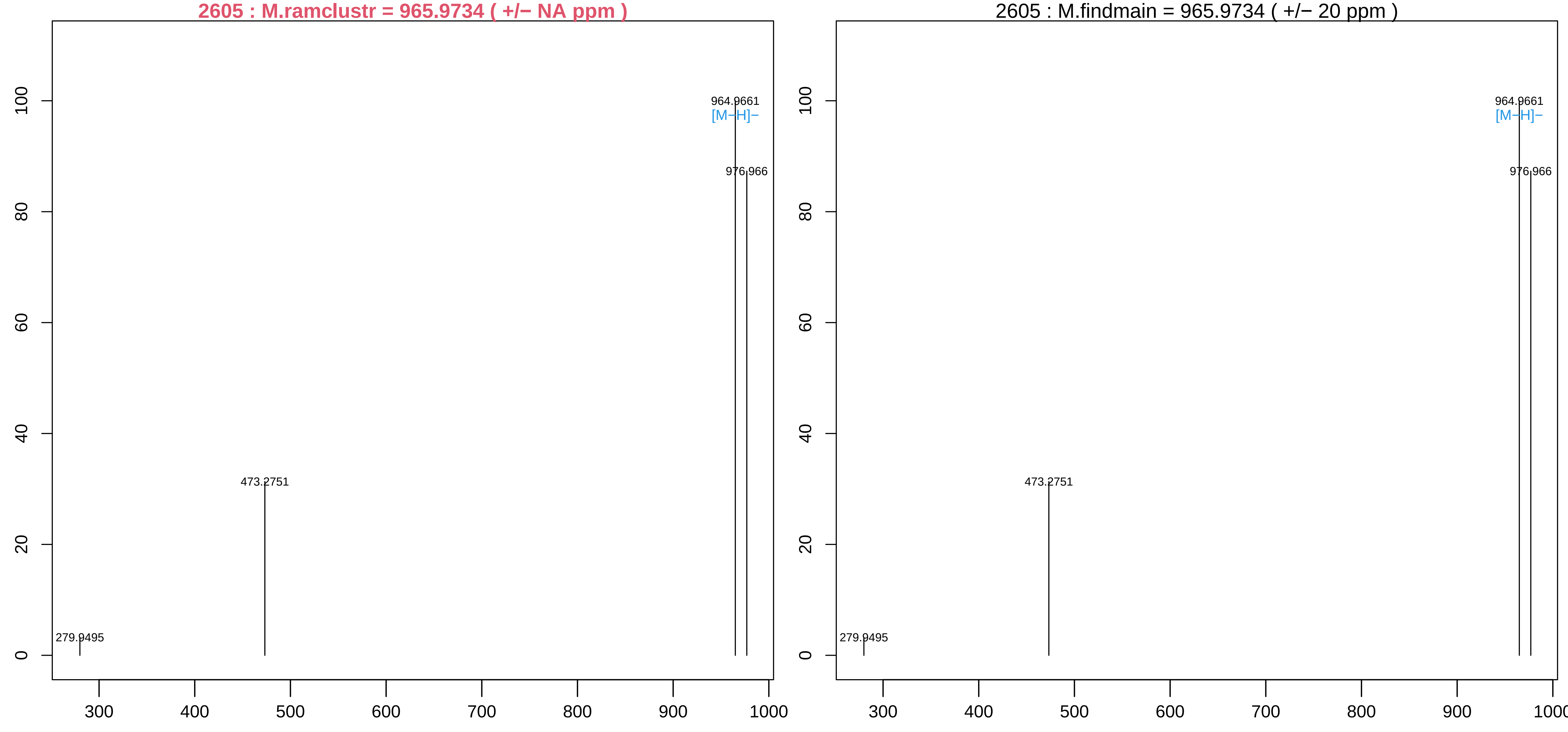
2605 : M.ramclustr = 965.9734 (+/− NA ppm)
964.9661
[M−H]−
976.966
473.2751
279.9495
0
20
40
60
80
100
300
400
500
600
700
800
900
1000
2605 : M.findmain = 965.9734 (+/− 20 ppm)
964.9661
[M−H]−
976.966
473.2751
279.9495

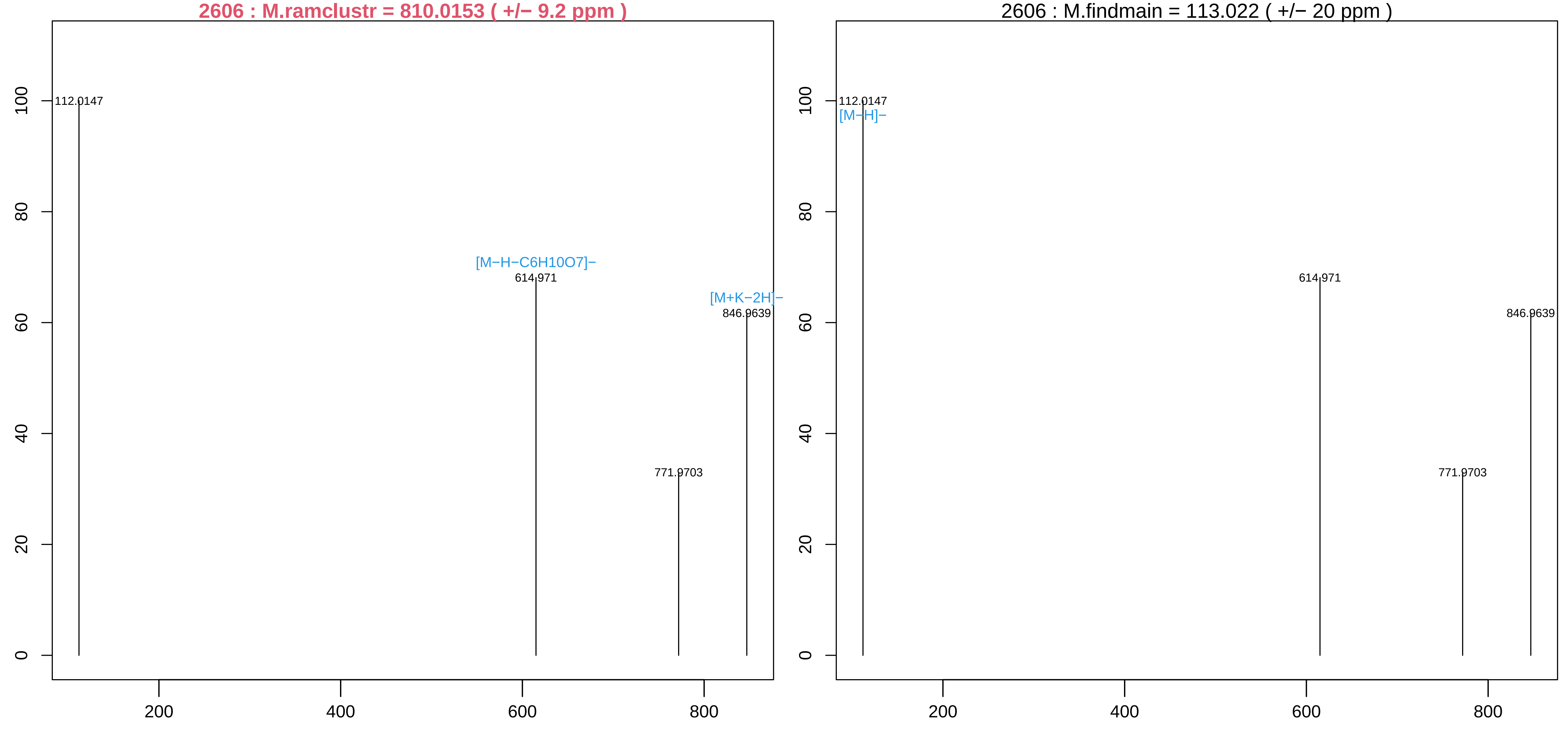
2606 : M.ramclustr = 810.0153 (+/− 9.2 ppm)
112.0147
[M−H−C6H10O7]−
614.971
[M+K−2H]−
846.9639
771.9703
2606 : M.findmain = 113.022 (+/− 20 ppm)
112.0147
[M−H]−
614.971
846.9639
771.9703

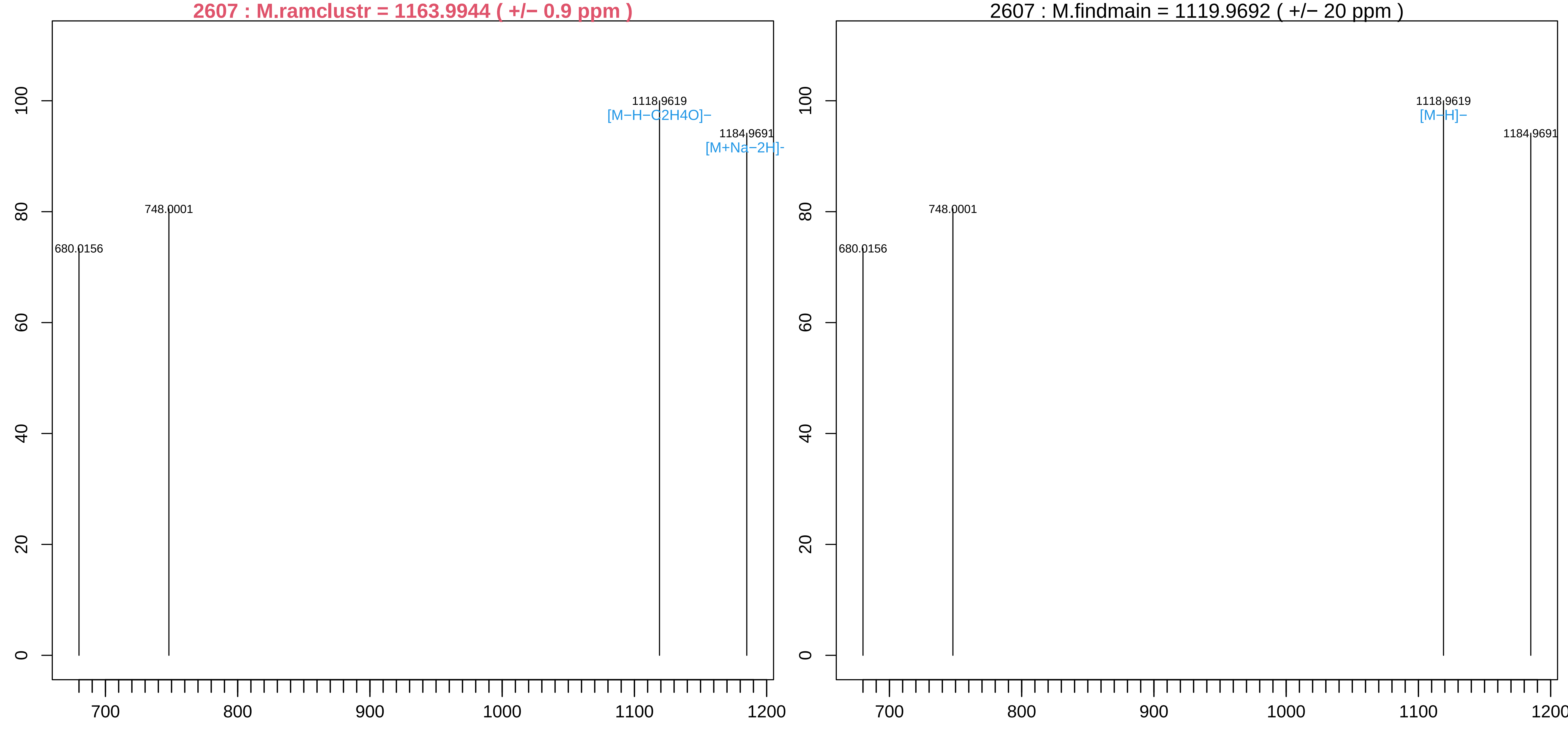

2607 : M.ramclustr = 1163.9944 (+/− 0.9 ppm)
680.0156
748.0001
1118.9619
[M−H−C2H4O]−
1184.9691
[M+Na−2H]-
0
20
40
60
80
100
700
800
900
1000
1100
1200
2607 : M.findmain = 1119.9692 (+/− 20 ppm)
680.0156
748.0001
1118.9619
[M−H]−
1184.9691

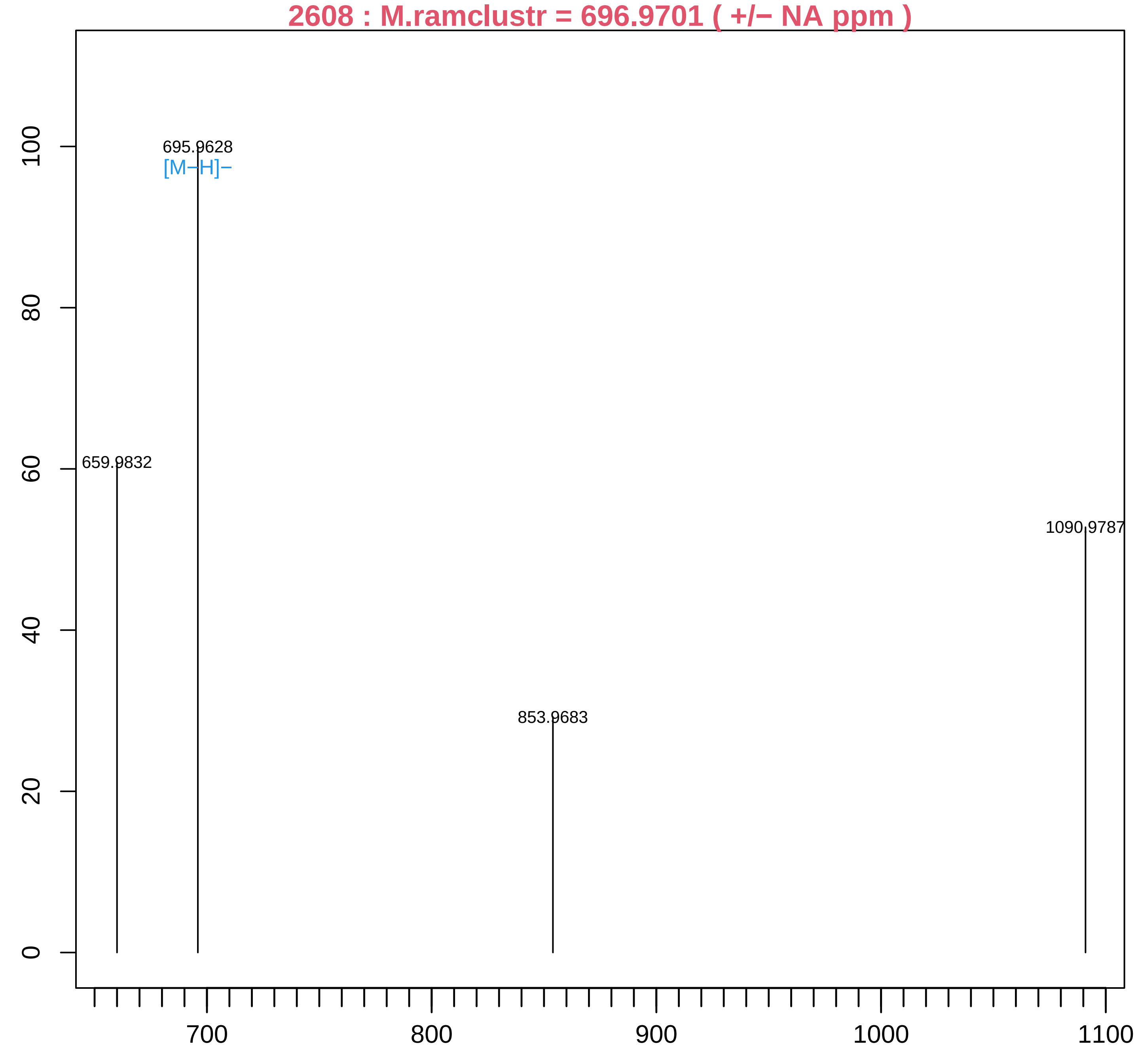

2608 : M.ramclustr = 696.9701 (+/− NA ppm)
695.9628
[M−H]−
659.9832
853.9683
1090.9787
0
20
40
60
80
100
700
800
900
1000
1100

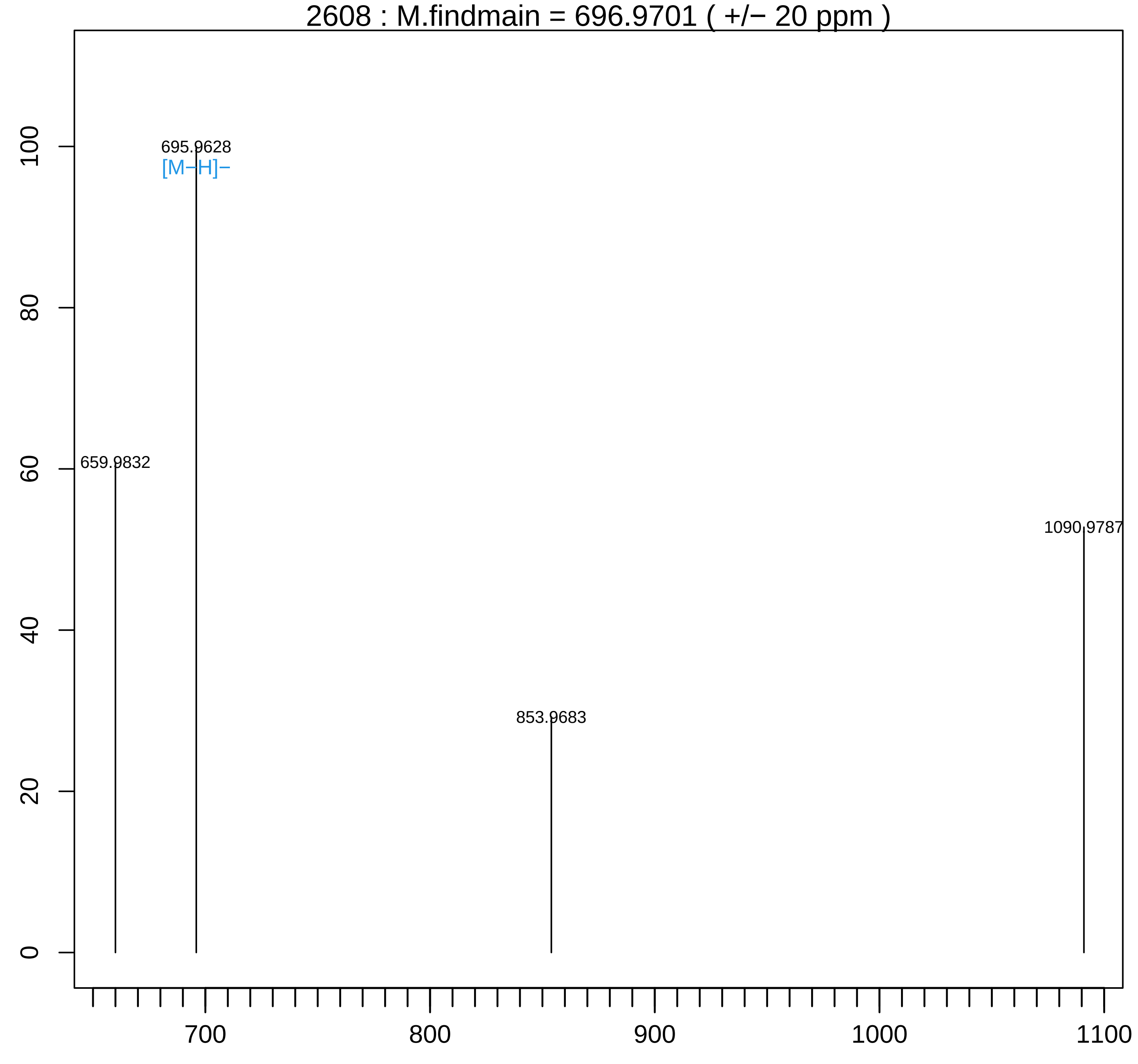

2608 : M.findmain = 696.9701 (+/− 20 ppm)
695.9628
[M−H]−
659.9832
853.9683
1090.9787
0
20
40
60
80
100
700
800
900
1000
1100

2609 : M.ramclustr = 591.9782 (+/− NA ppm)

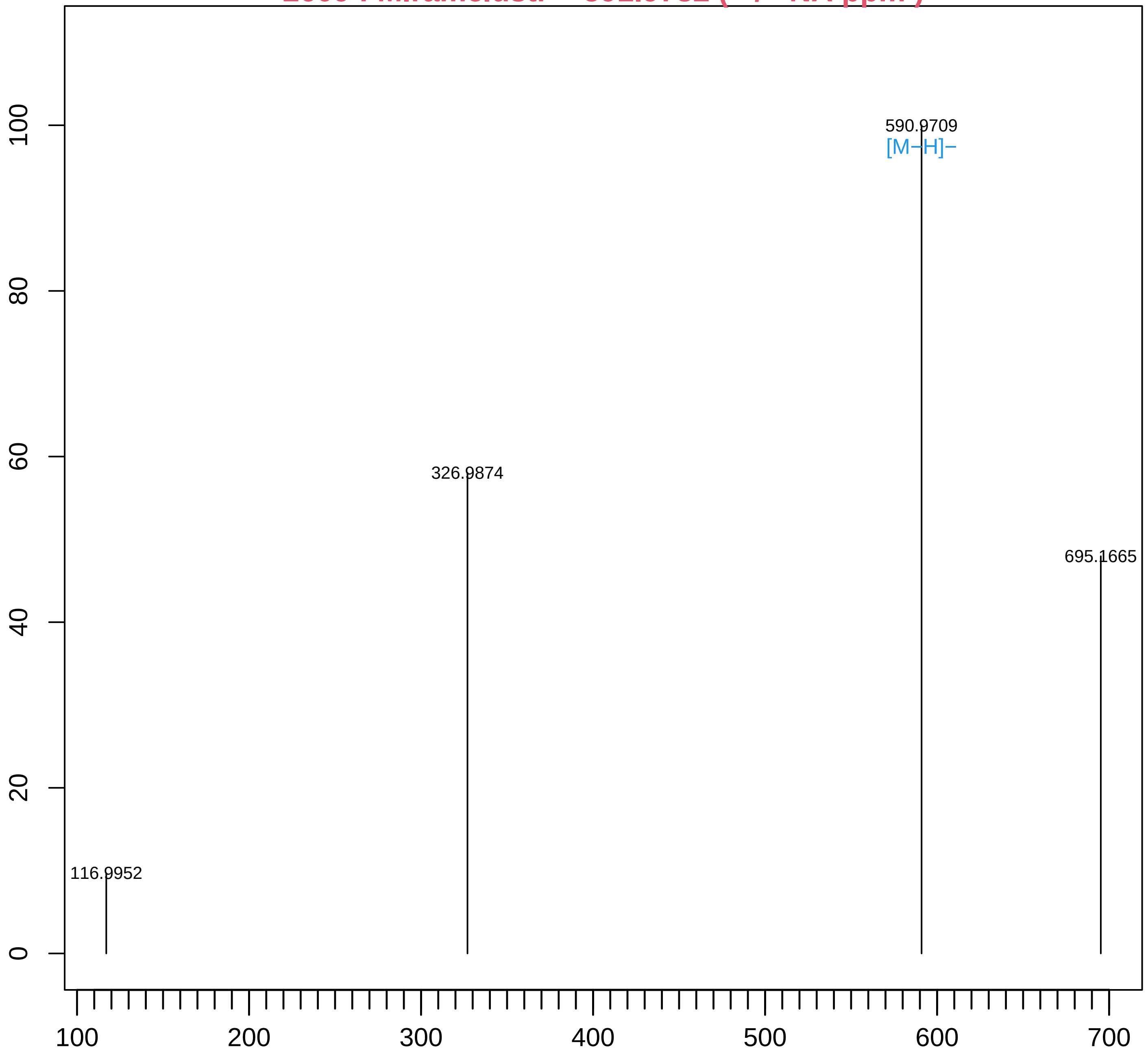

2609 : M.findmain = 591.9782 (+/− 20 ppm)

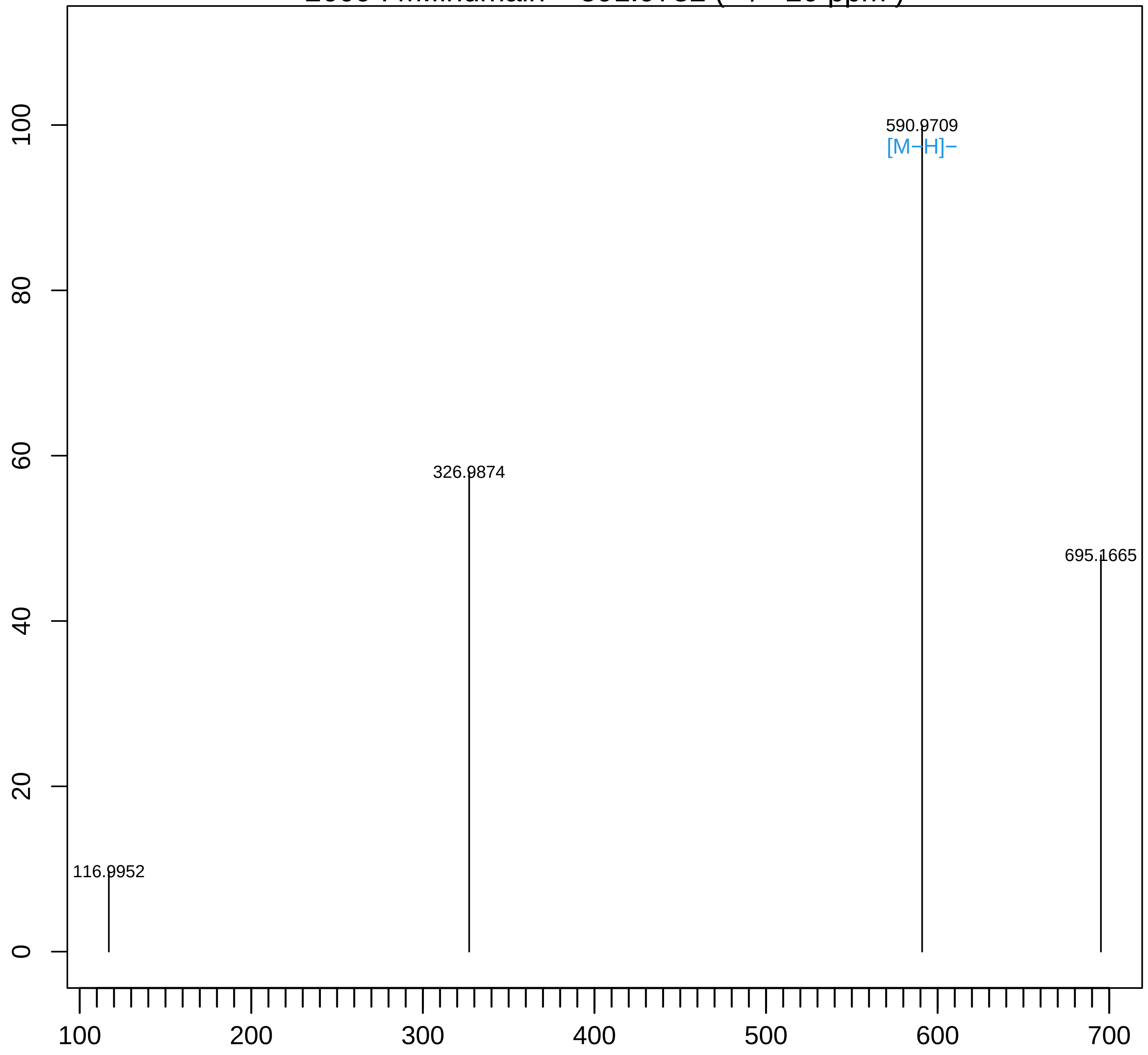

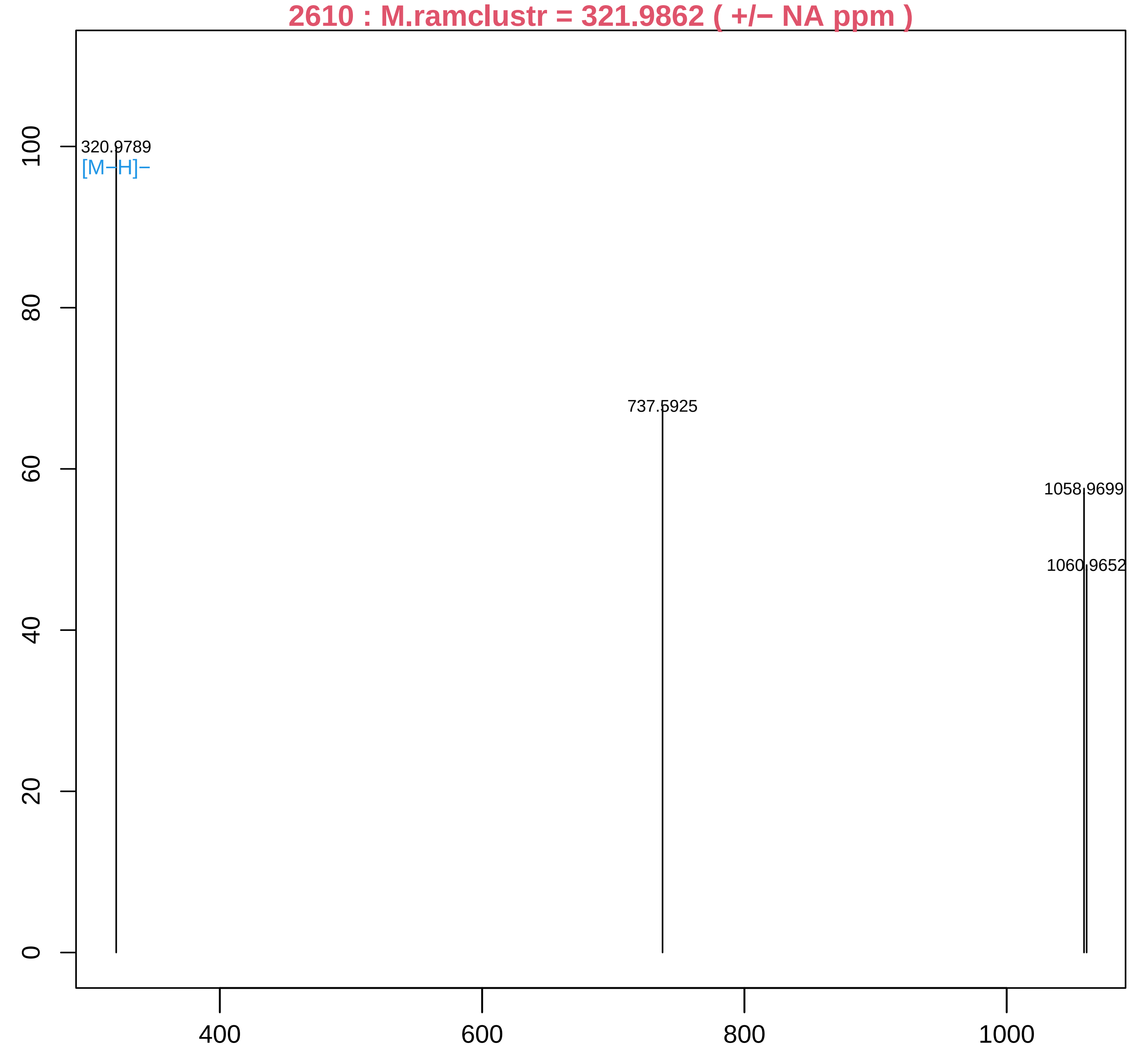

2610 : M.ramclustr = 321.9862 (+/− NA ppm)
320.9789
[M−H]−
737.5925
1058.9699
1060.9652
0
20
40
60
80
100
400
600
800
1000

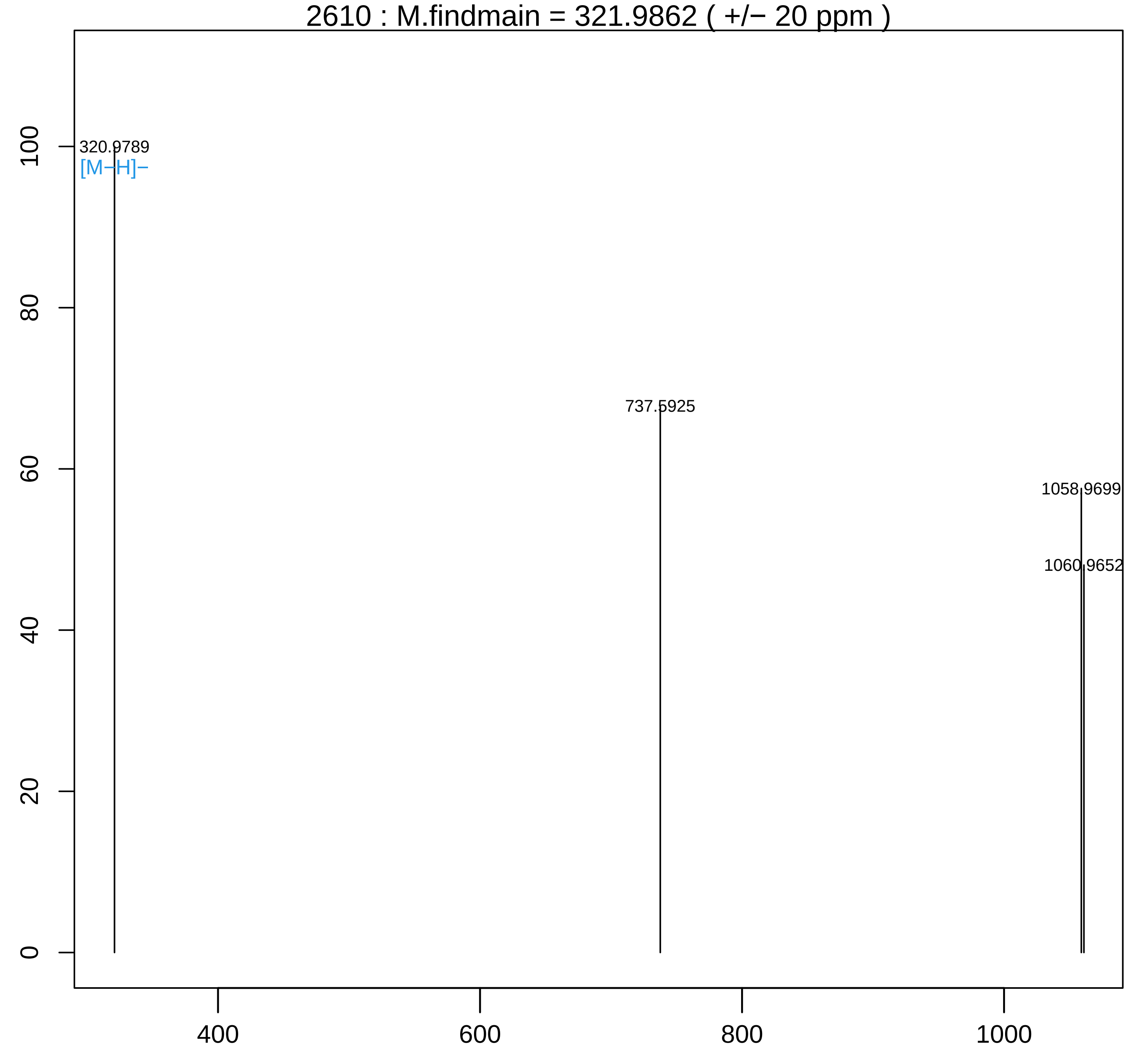

2610 : M.findmain = 321.9862 (+/− 20 ppm)
320.9789
[M−H]−
737.5925
1058.9699
1060.9652
0
20
40
60
80
100
400
600
800
1000

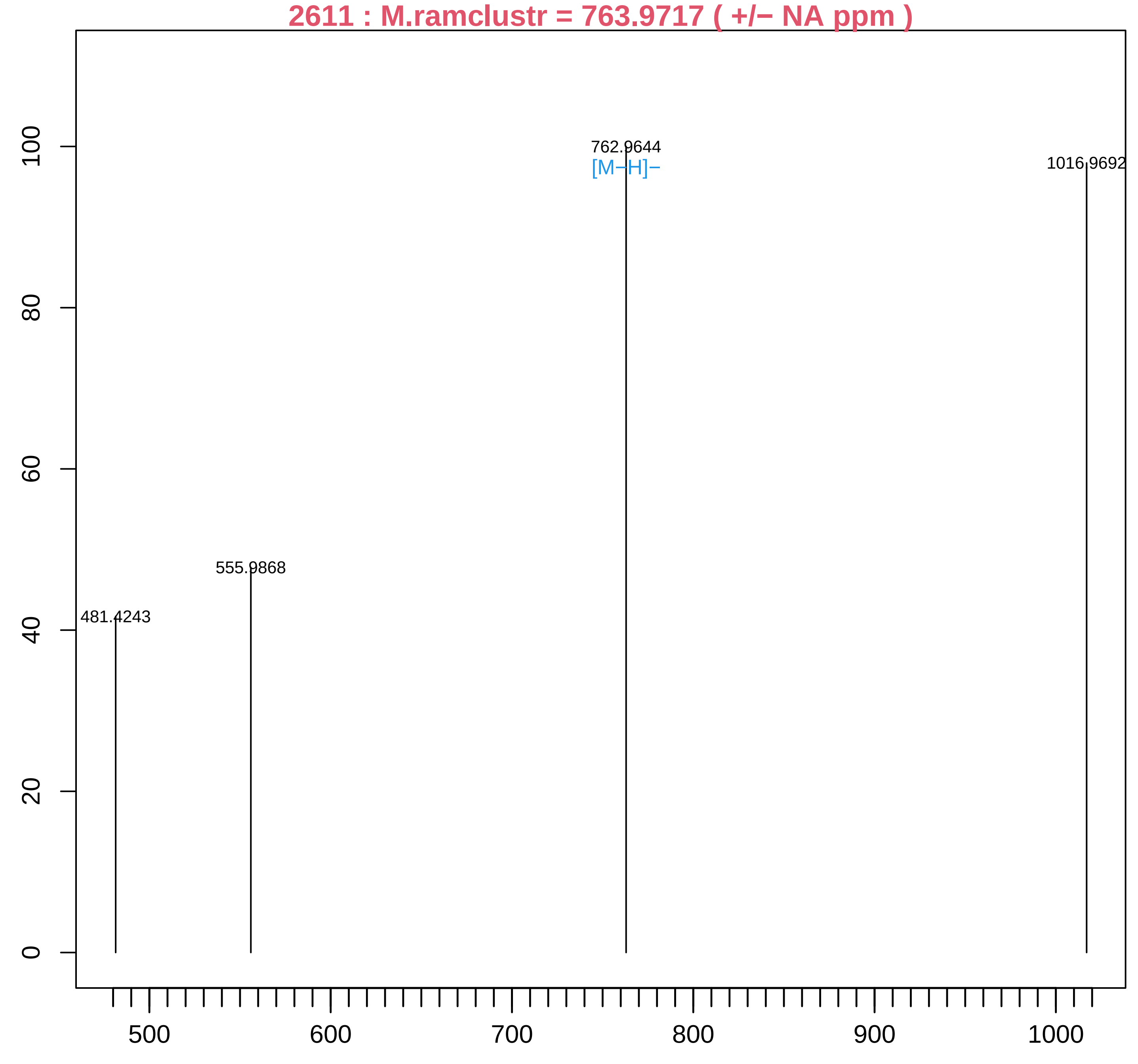

2611 : M.ramclustr = 763.9717 (+/− NA ppm)
762.9644
[M−H]−
1016.9692
555.9868
481.4243
0
20
40
60
80
100
500
600
700
800
900
1000

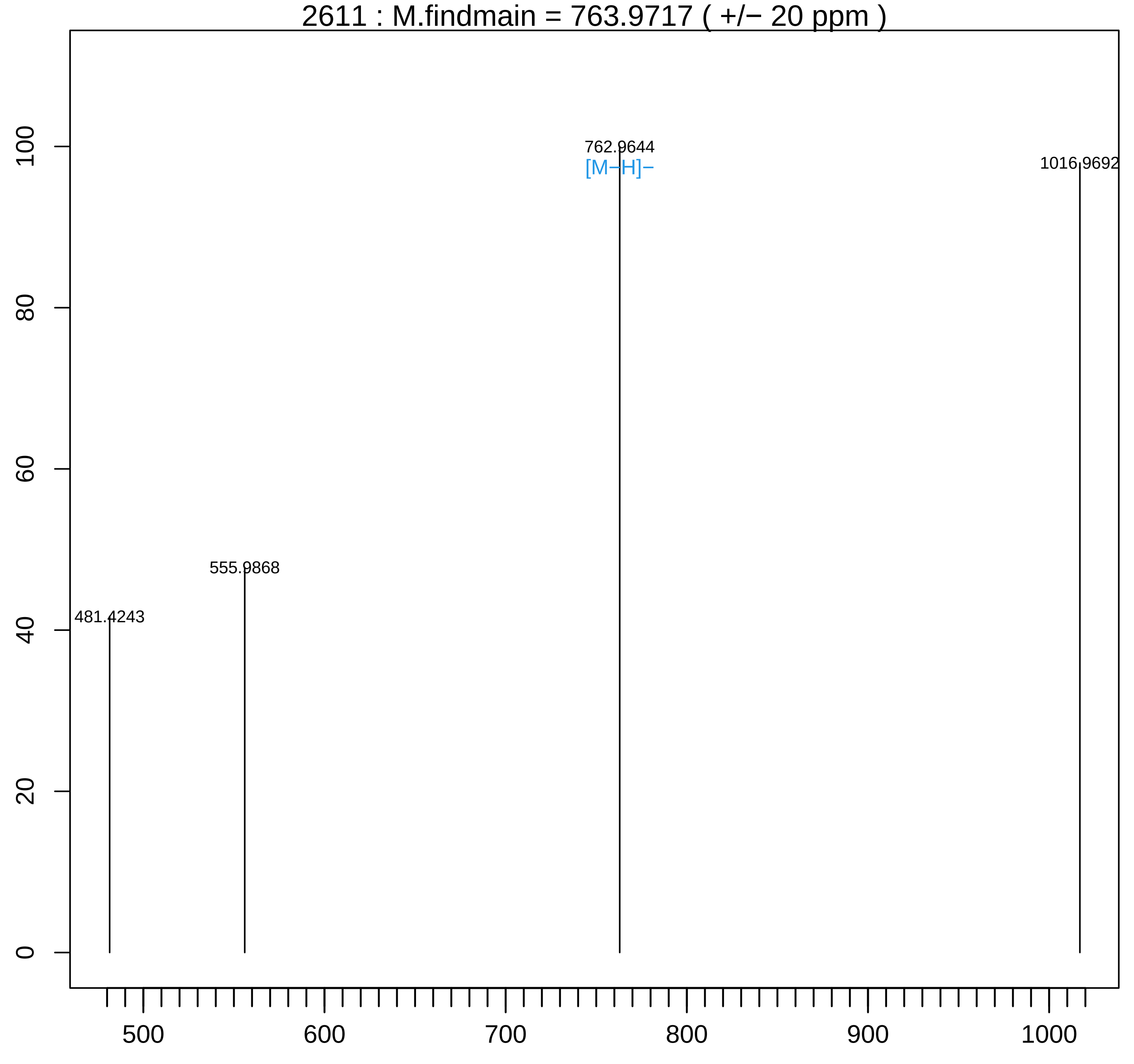

2611 : M.findmain = 763.9717 (+/− 20 ppm)
762.9644
[M−H]−
1016.9692
555.9868
481.4243
0
20
40
60
80
100
500
600
700
800
900
1000

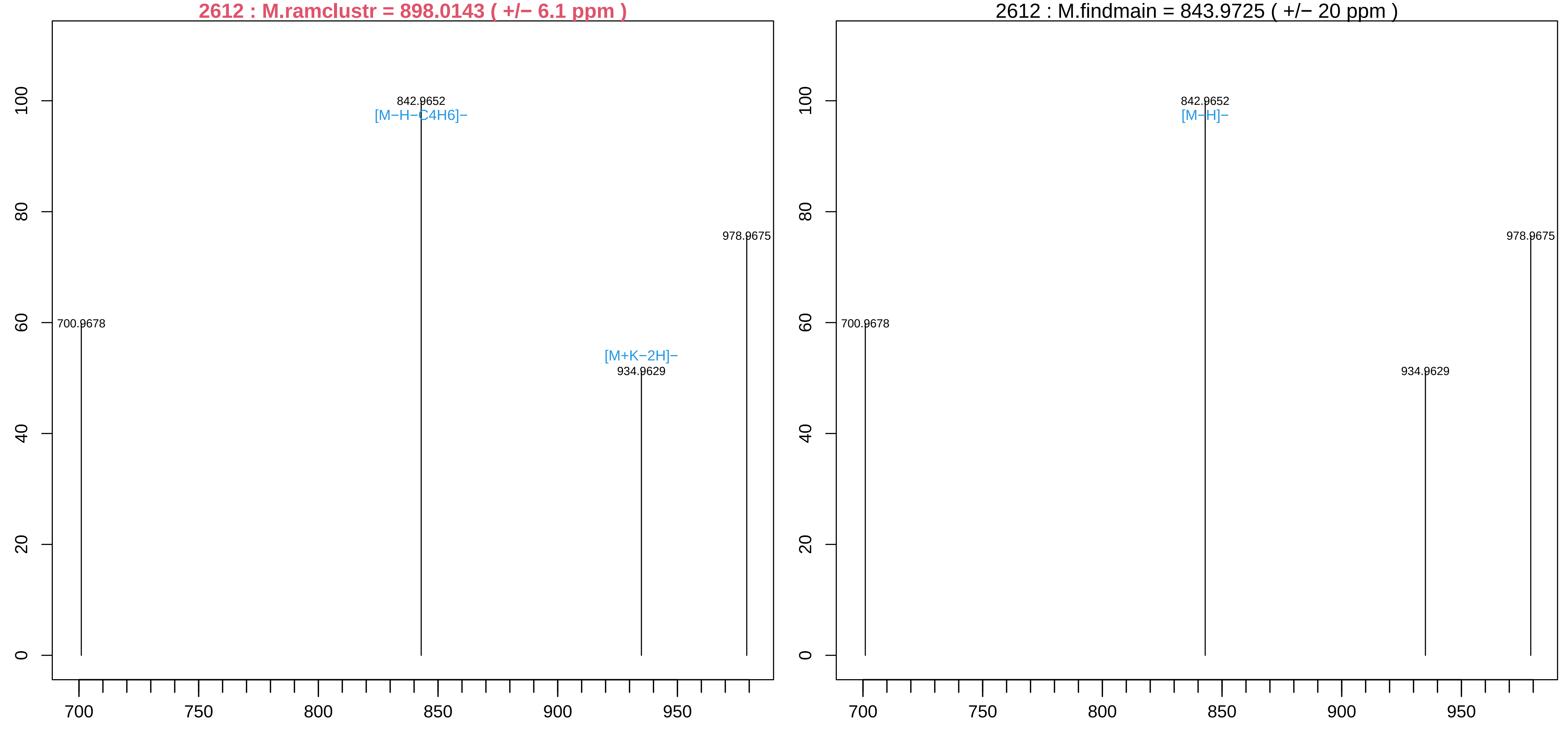
2612 : M.ramclustr = 898.0143 (+/− 6.1 ppm)
842.9652
[M−H−C4H6]−
978.9675
700.9678
[M+K−2H]−
934.9629
0
20
40
60
80
100
700
750
800
850
900
950
2612 : M.findmain = 843.9725 (+/− 20 ppm)
842.9652
[M−H]−
978.9675
700.9678
934.9629
0
20
40
60
80
100
700
750
800
850
900
950

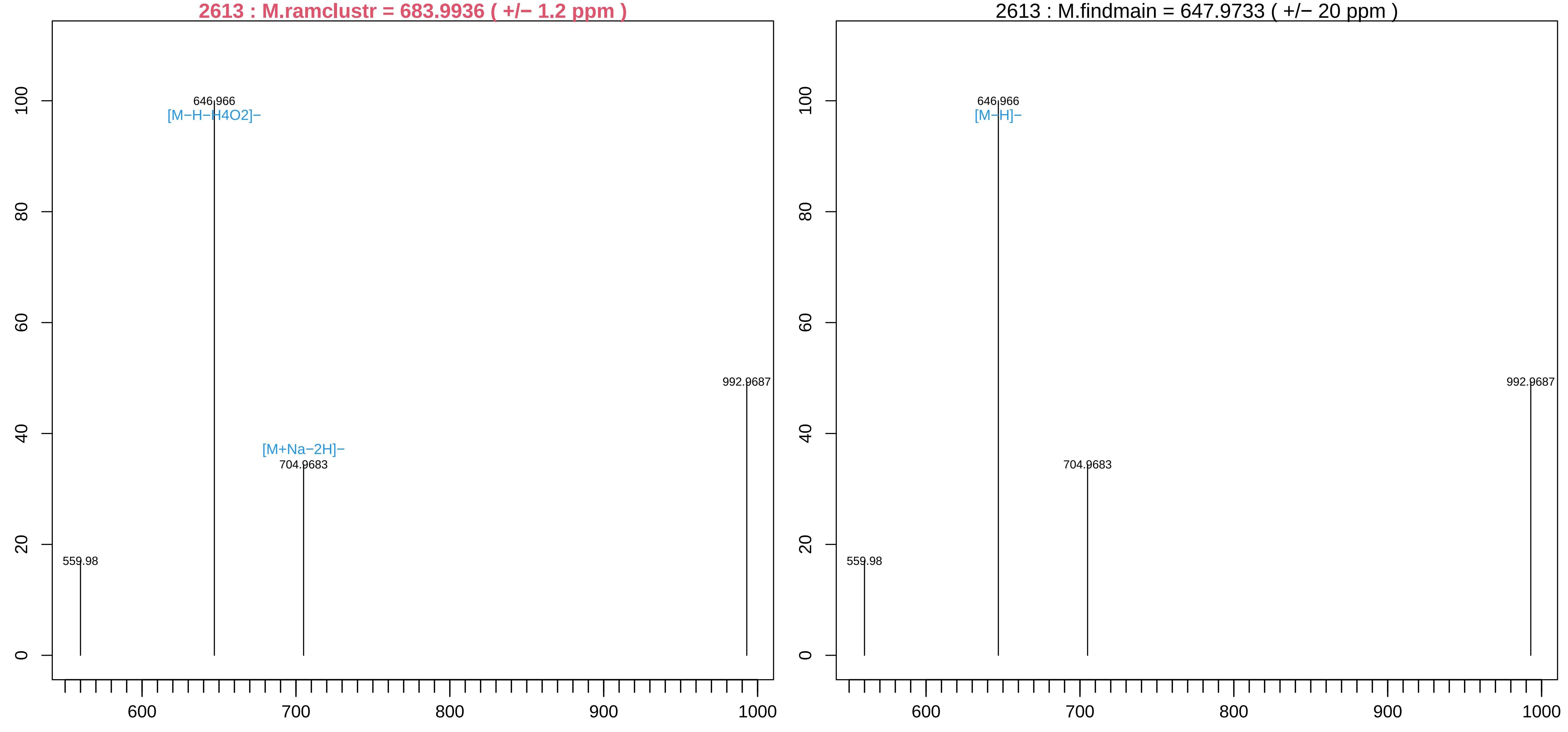

2613 : M.ramclustr = 683.9936 (+/− 1.2 ppm)
646.966
[M−H−H4O2]−
[M+Na−2H]−
704.9683
992.9687
559.98
0
20
40
60
80
100
600
700
800
900
1000
2613 : M.findmain = 647.9733 (+/− 20 ppm)
646.966
[M−H]−
704.9683
992.9687
559.98

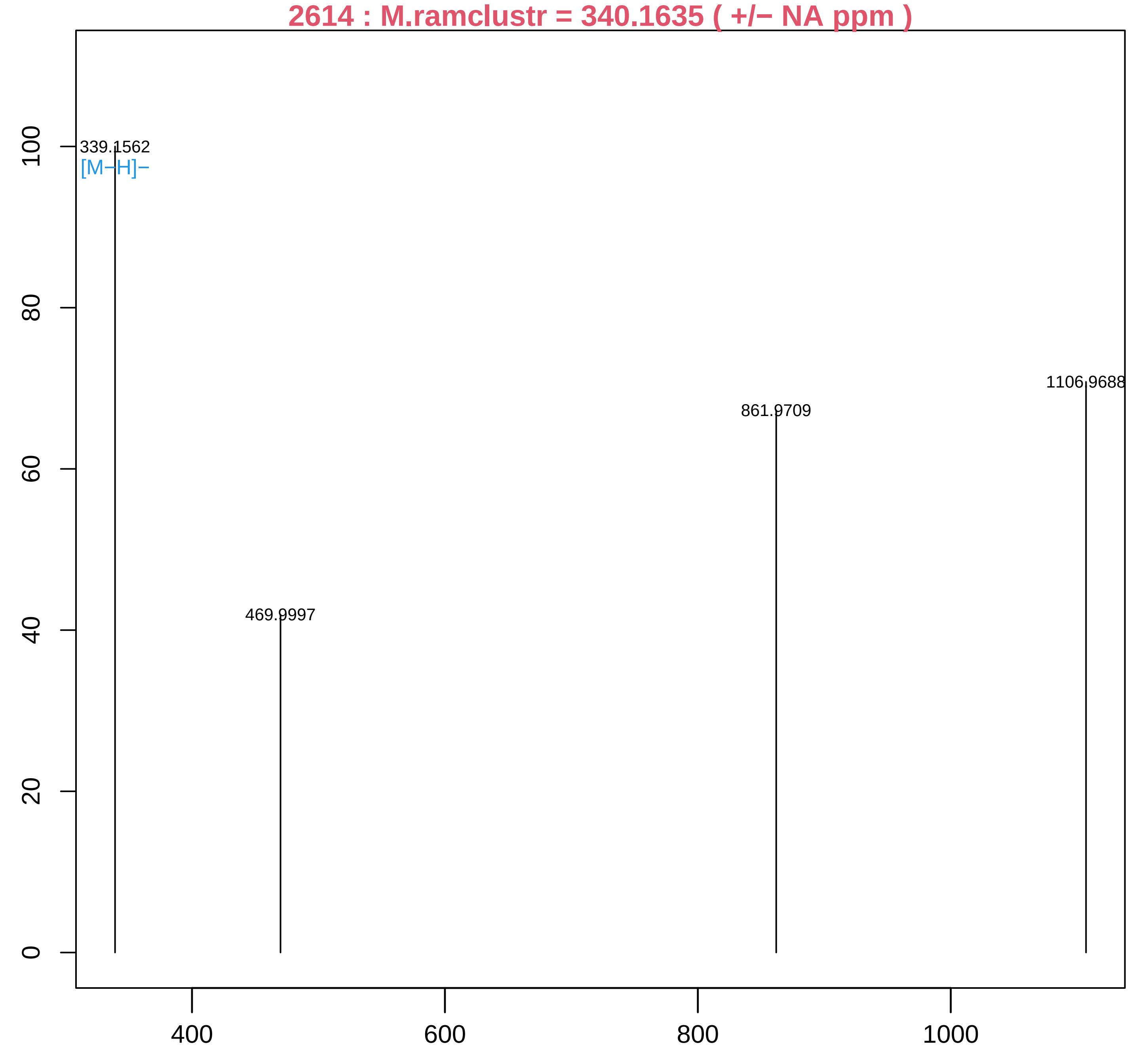

2614 : M.ramclustr = 340.1635 (+/− NA ppm)
339.1562
[M−H]−
469.9997
861.9709
1106.9688
0
20
40
60
80
100
400
600
800
1000

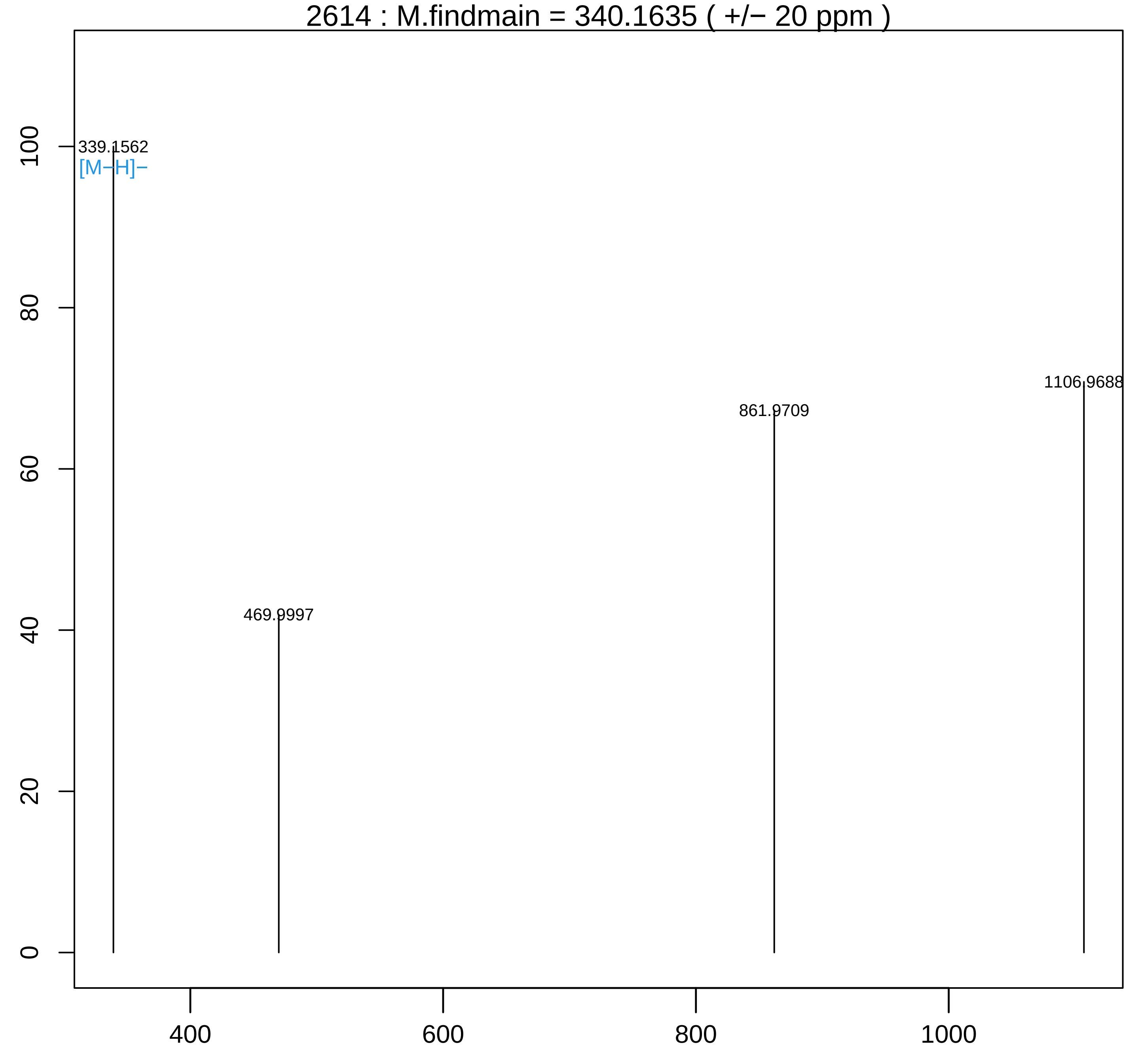

2614 : M.findmain = 340.1635 (+/− 20 ppm)
339.1562
[M−H]−
469.9997
861.9709
1106.9688
0
20
40
60
80
100
400
600
800
1000

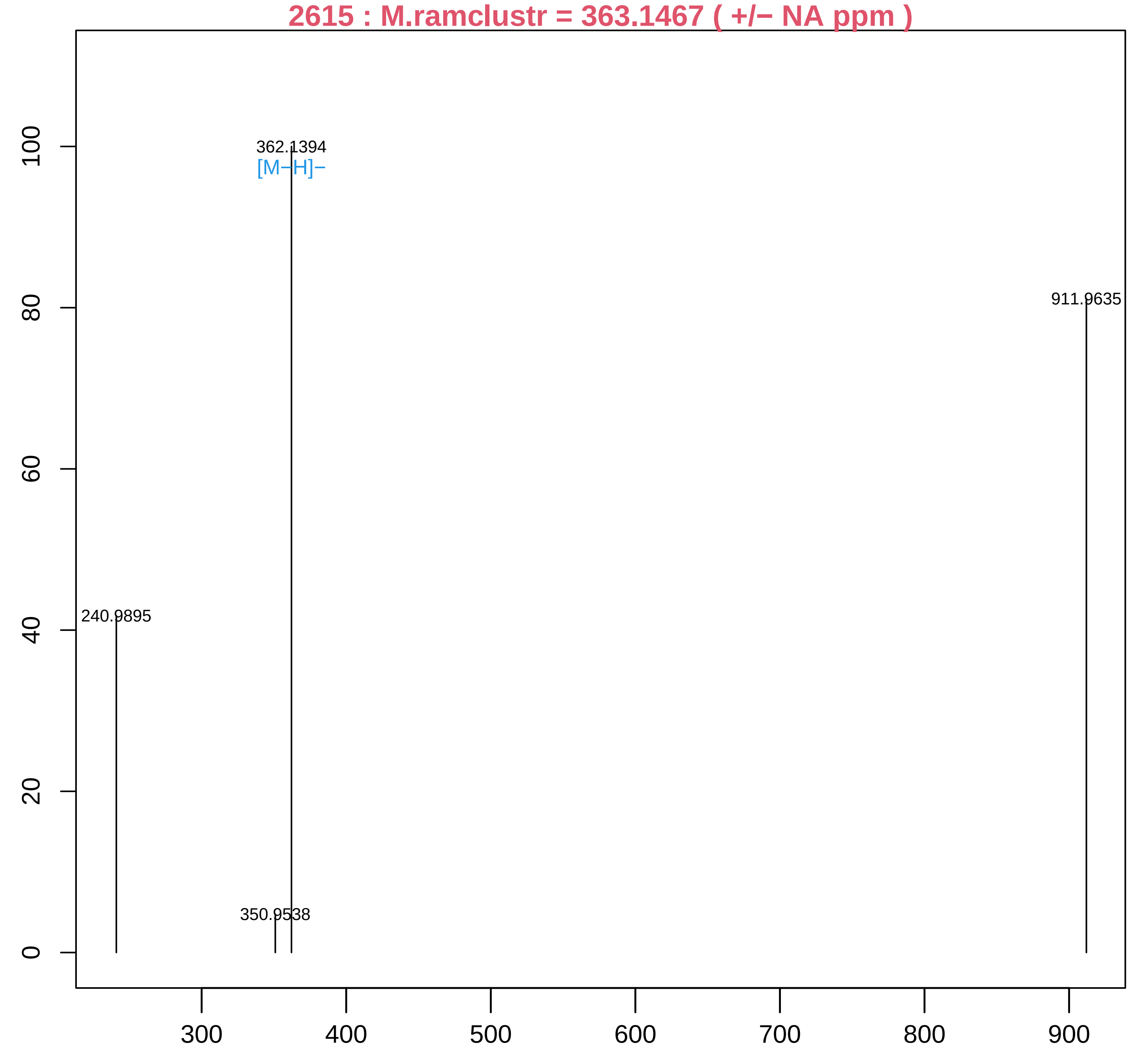
2615 : M.ramclustr = 363.1467 (+/− NA ppm)
362.1394
[M−H]−
911.9635
240.9895
350.9538
0
20
40
60
80
100
300
400
500
600
700
800
900

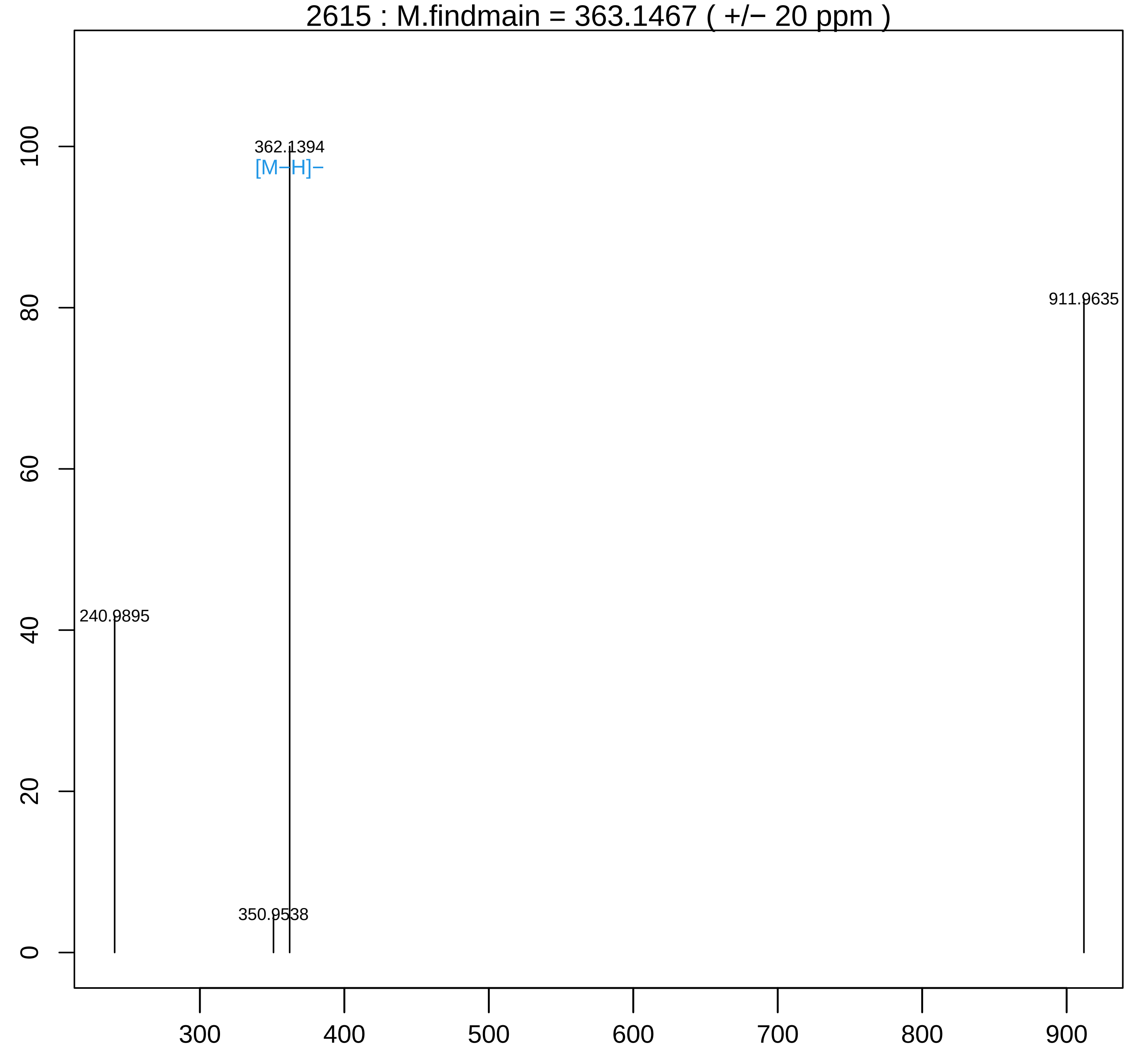
2615 : M.findmain = 363.1467 (+/− 20 ppm)
362.1394
[M−H]−
911.9635
240.9895
350.9538
0
20
40
60
80
100
300
400
500
600
700
800
900

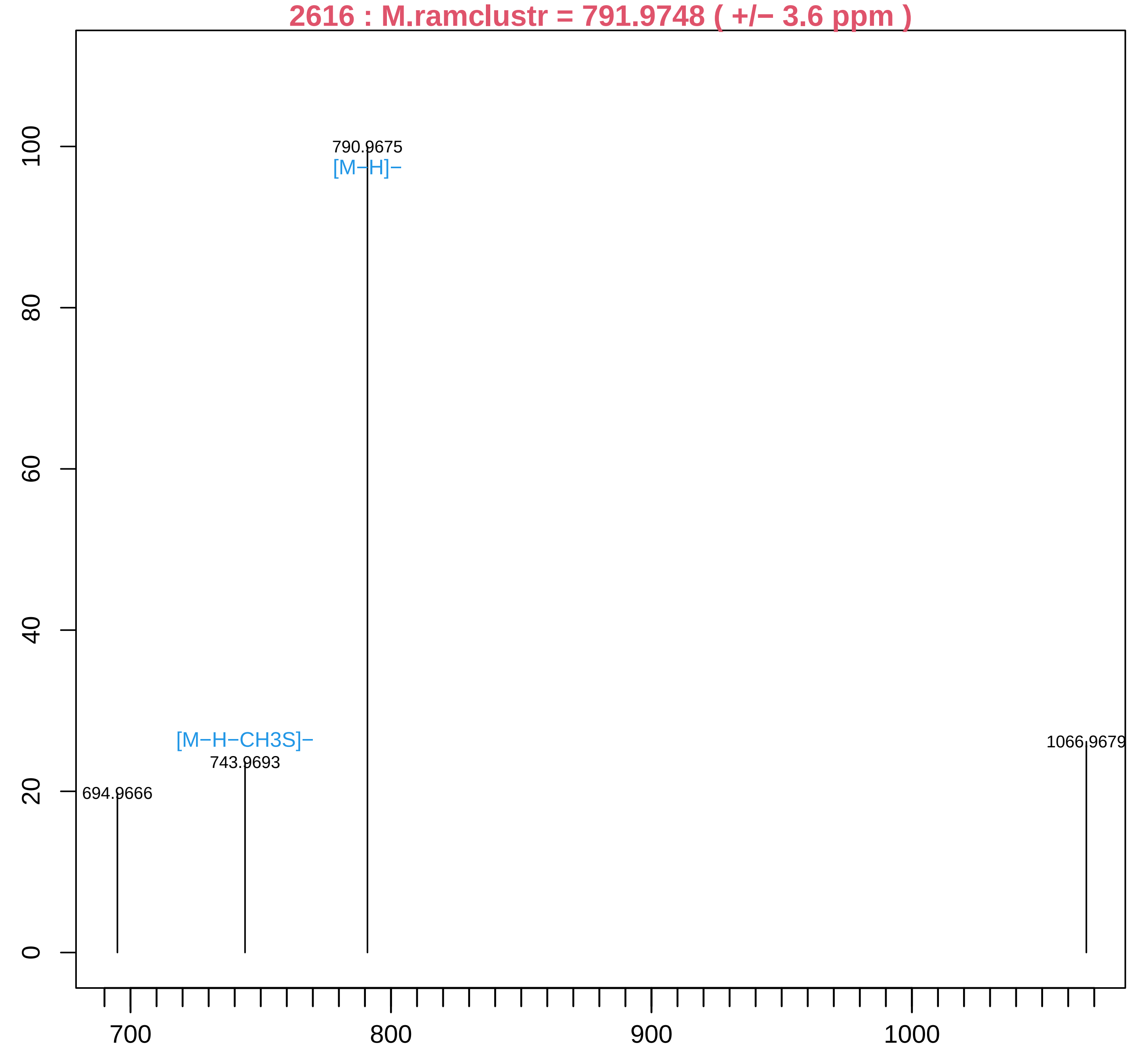

2616 : M.ramclustr = 791.9748 (+/− 3.6 ppm)
790.9675
[M−H]−
[M−H−CH3S]−
743.9693
694.9666
1066.9679
0
20
40
60
80
100
700
800
900
1000

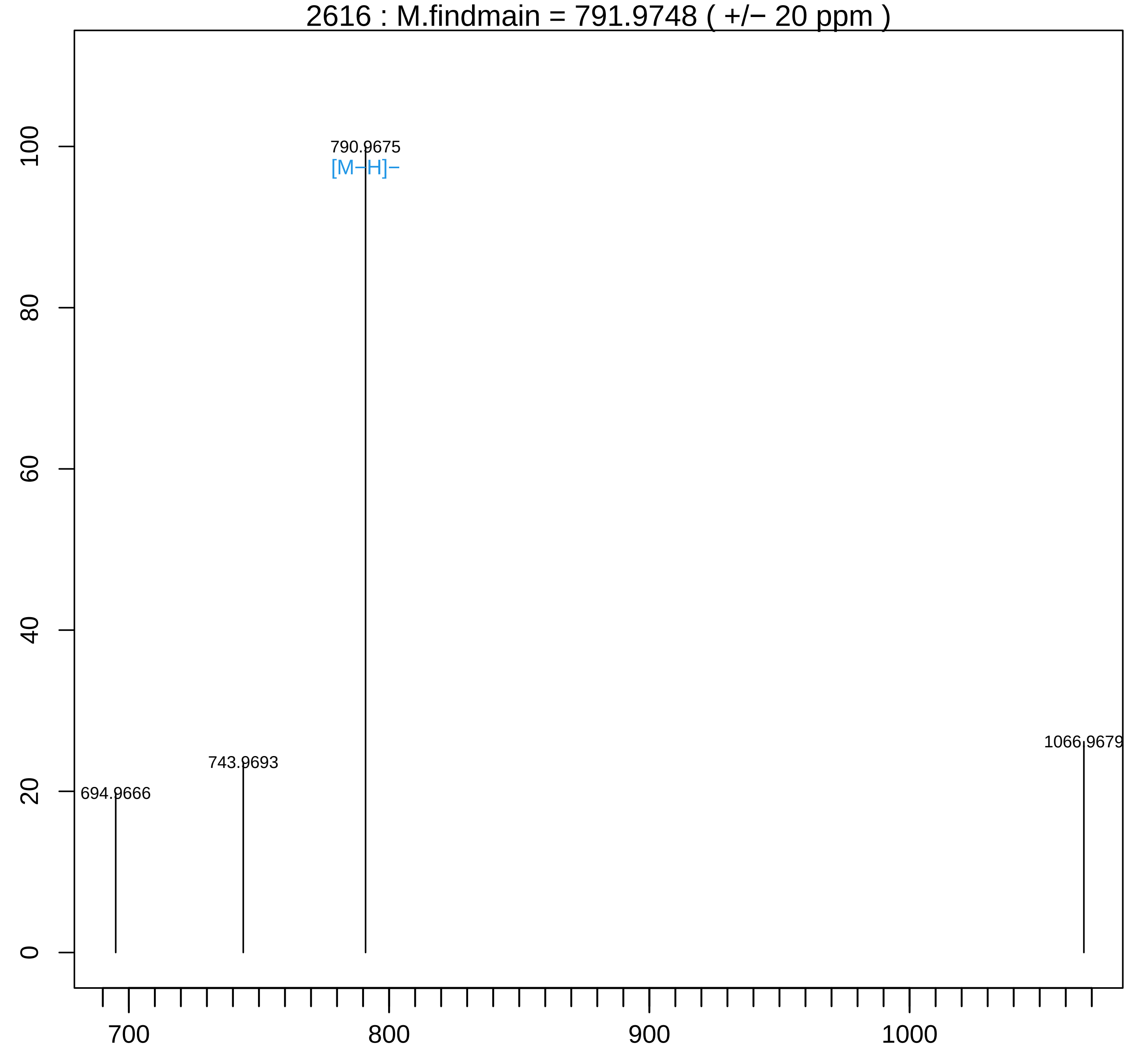

2616 : M.findmain = 791.9748 (+/− 20 ppm)
790.9675
[M−H]−
743.9693
694.9666
1066.9679
0
20
40
60
80
100
700
800
900
1000

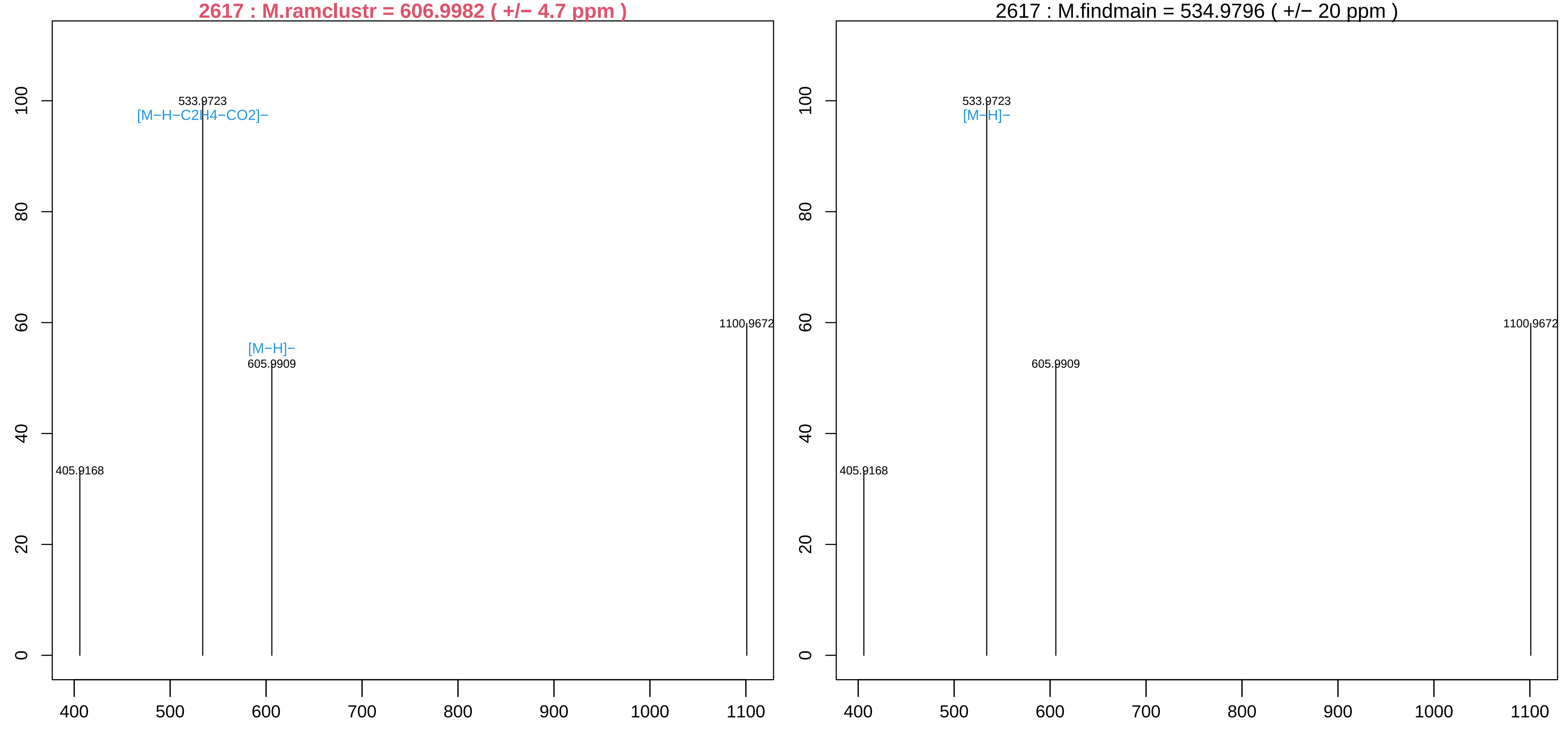
2617 : M.ramclustr = 606.9982 (+/− 4.7 ppm)
533.9723
[M−H−C2H4−CO2]−
[M−H]−
605.9909
405.9168
1100.9672
2617 : M.findmain = 534.9796 (+/− 20 ppm)
533.9723
[M−H]−
605.9909
405.9168
1100.9672
0
20
40
60
80
100
400
500
600
700
800
900
1000
1100

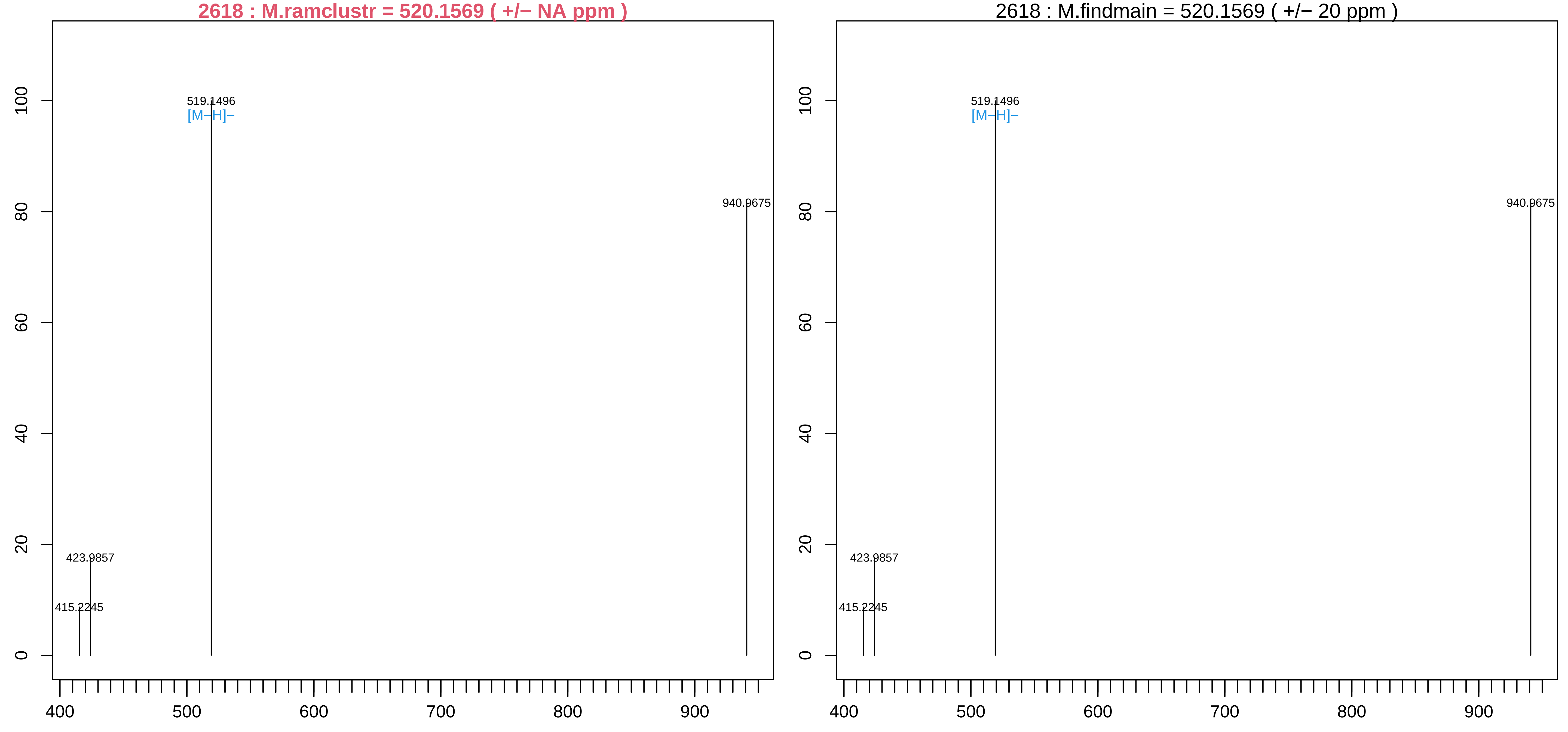
2618 : M.ramclustr = 520.1569 (+/− NA ppm)
519.1496
[M−H]−
940.9675
423.9857
415.2245
0
20
40
60
80
100
400
500
600
700
800
900
2618 : M.findmain = 520.1569 (+/− 20 ppm)
519.1496
[M−H]−
940.9675
423.9857
415.2245
0
20
40
60
80
100
400
500
600
700
800
900

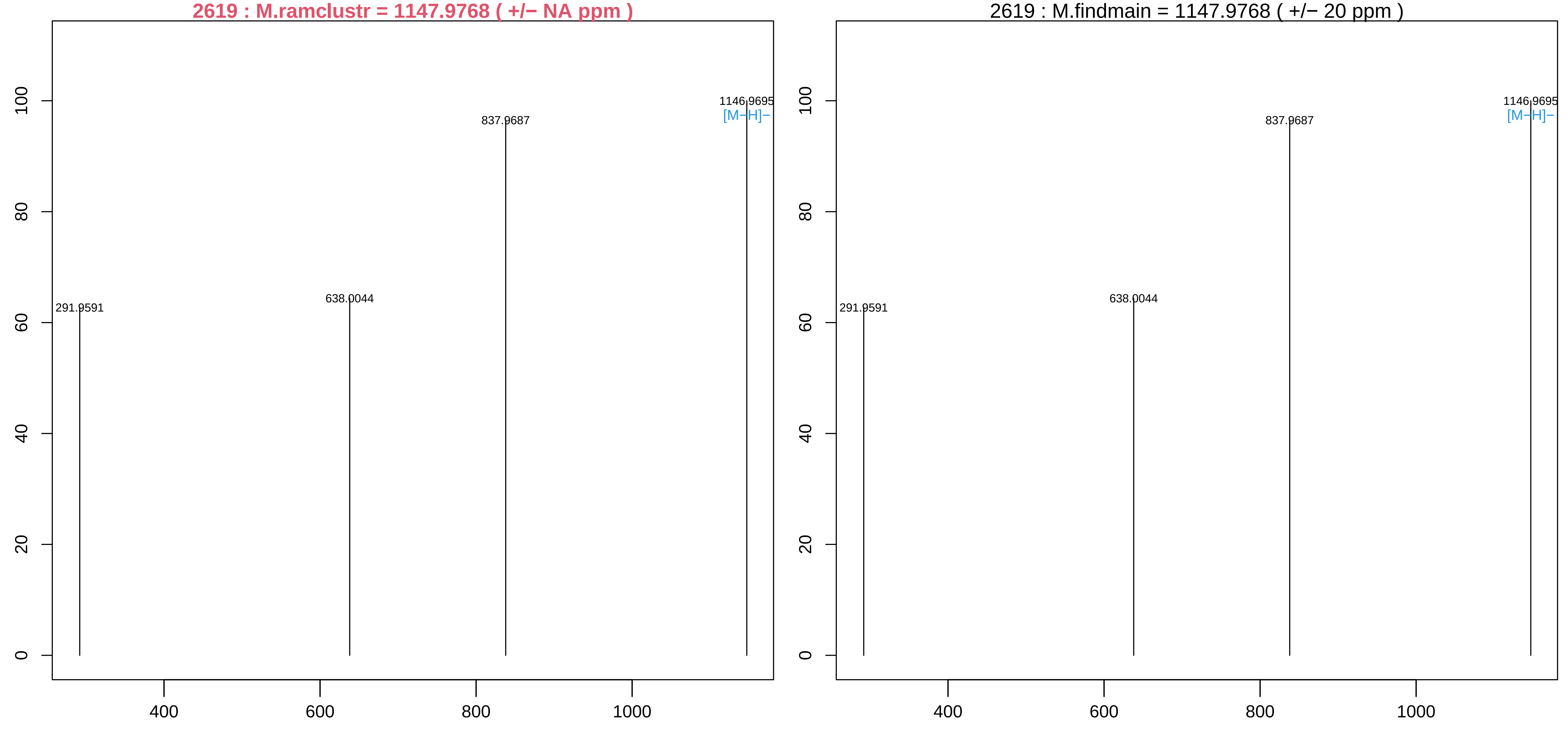
2619 : M.ramclustr = 1147.9768 (+/− NA ppm)
2619 : M.findmain = 1147.9768 (+/− 20 ppm)
291.9591
638.0044
837.9687
1146.9695
[M−H]−
0
20
40
60
80
100
400
600
800
1000

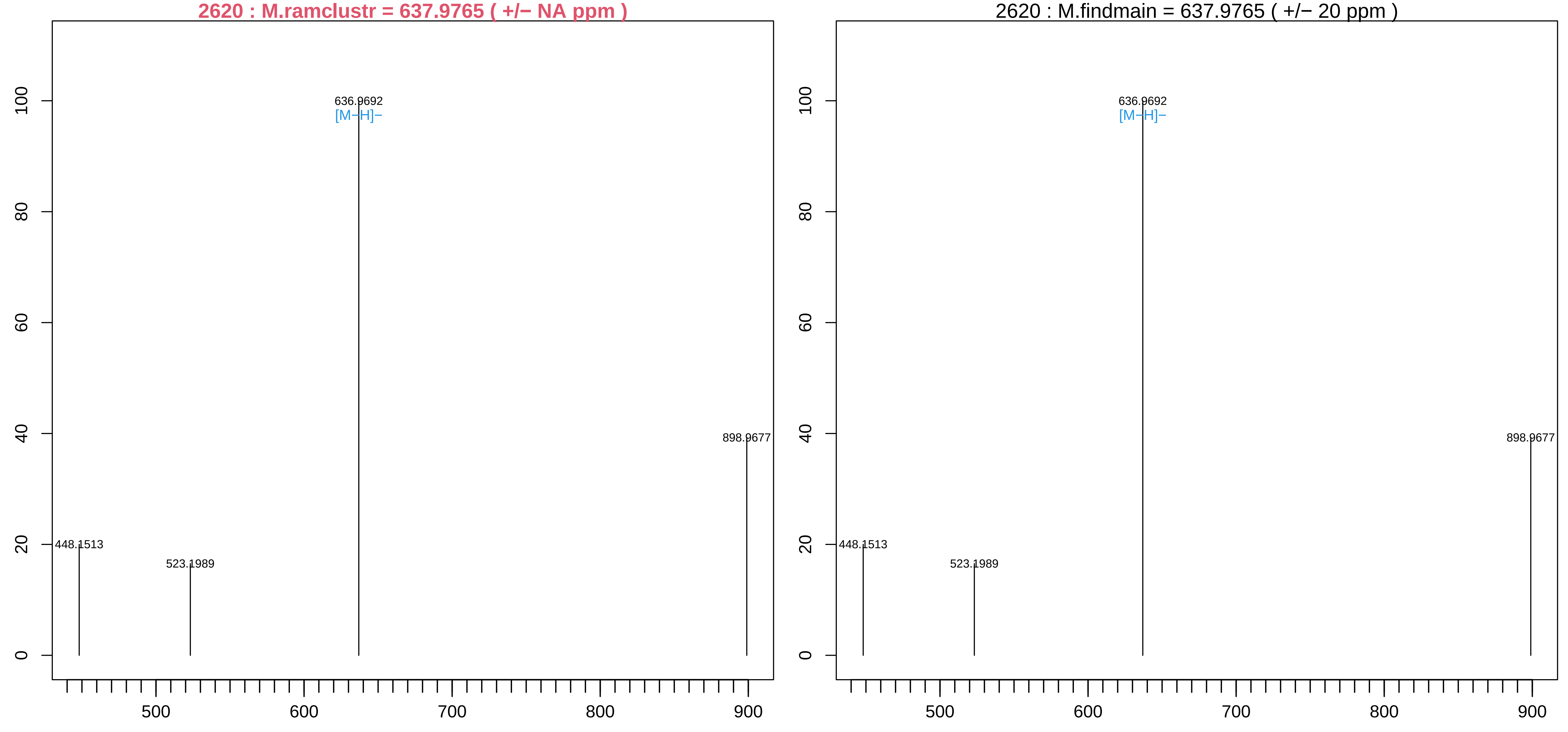
2620 : M.ramclustr = 637.9765 (+/− NA ppm)
636.9692
[M−H]−
898.9677
448.1513
523.1989
0
20
40
60
80
100
500
600
700
800
900
2620 : M.findmain = 637.9765 (+/− 20 ppm)
636.9692
[M−H]−
898.9677
448.1513
523.1989
0
20
40
60
80
100
500
600
700
800
900

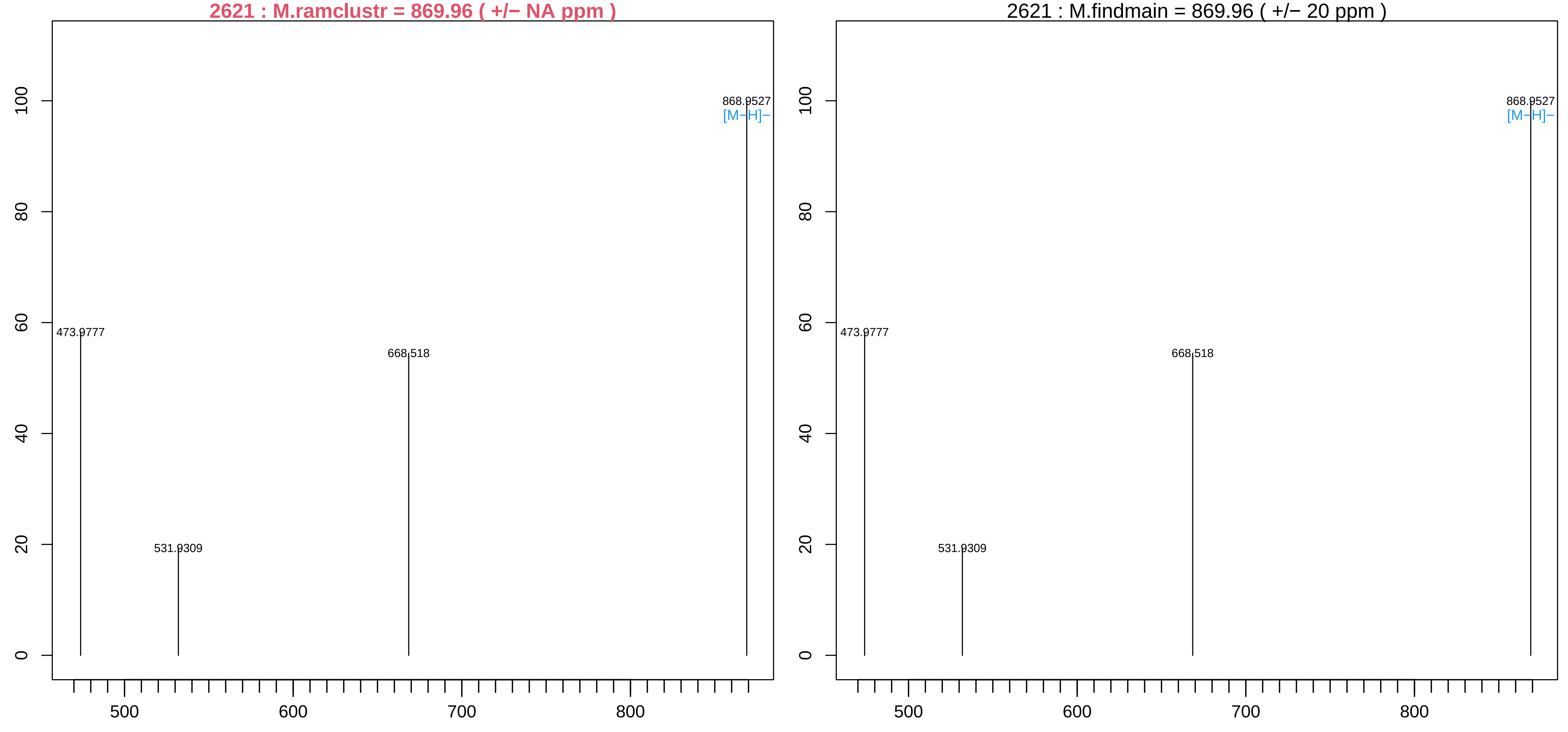
2621 : M.ramclustr = 869.96 (+/− NA ppm)
868.9527
[M−H]−
473.9777
668.518
531.9309
0
20
40
60
80
100
500
600
700
800
2621 : M.findmain = 869.96 (+/− 20 ppm)
868.9527
[M−H]−
473.9777
668.518
531.9309

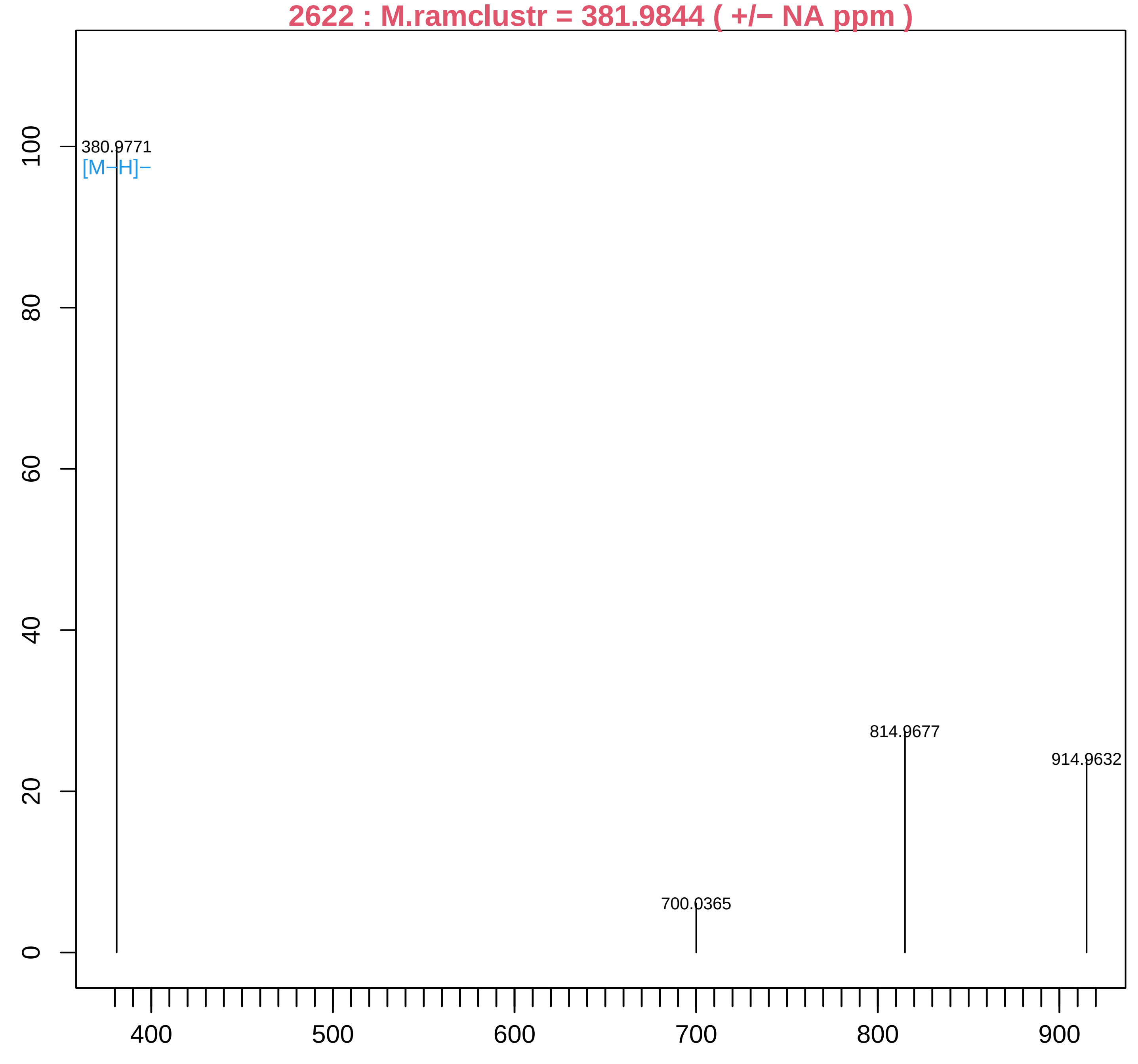
2622 : M.ramclustr = 381.9844 (+/− NA ppm)
380.9771
[M−H]−
700.0365
814.9677
914.9632
0
20
40
60
80
100
400
500
600
700
800
900

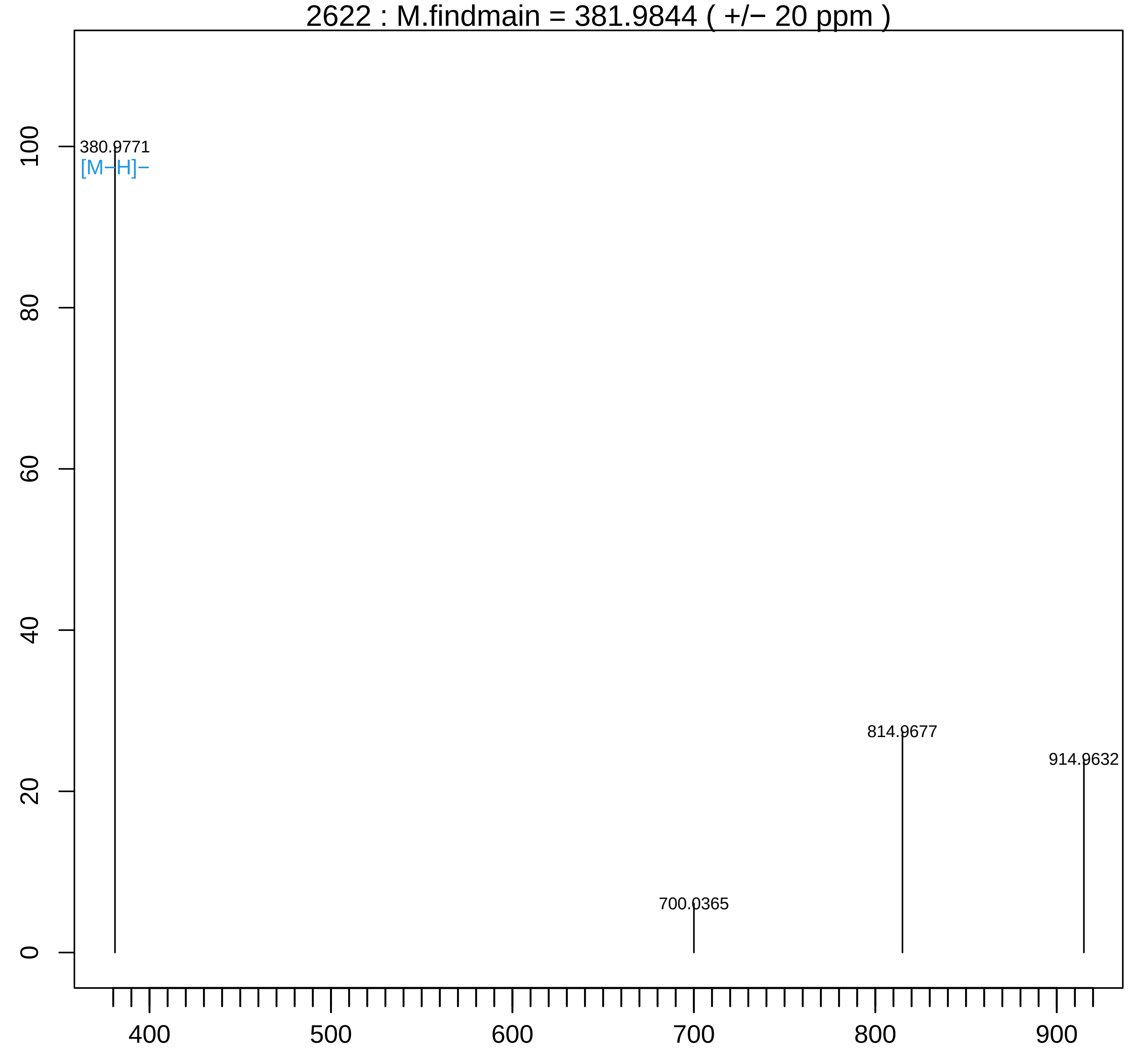
2622 : M.findmain = 381.9844 (+/− 20 ppm)
380.9771
[M−H]−
700.0365
814.9677
914.9632
0
20
40
60
80
100
400
500
600
700
800
900

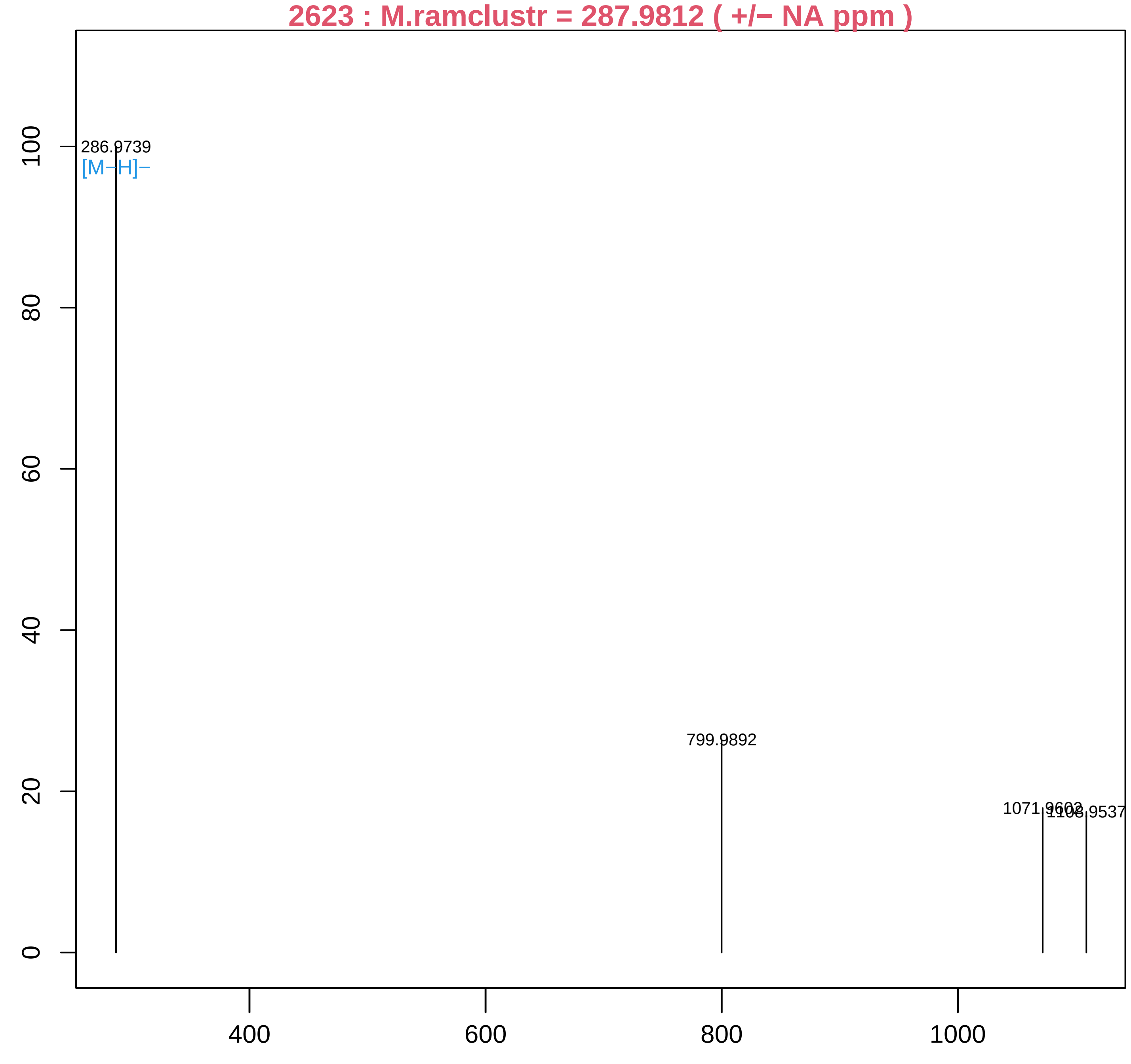
2623 : M.ramclustr = 287.9812 (+/− NA ppm)
286.9739
[M−H]−
799.9892
1071.9602
1106.9537
0
20
40
60
80
100
400
600
800
1000

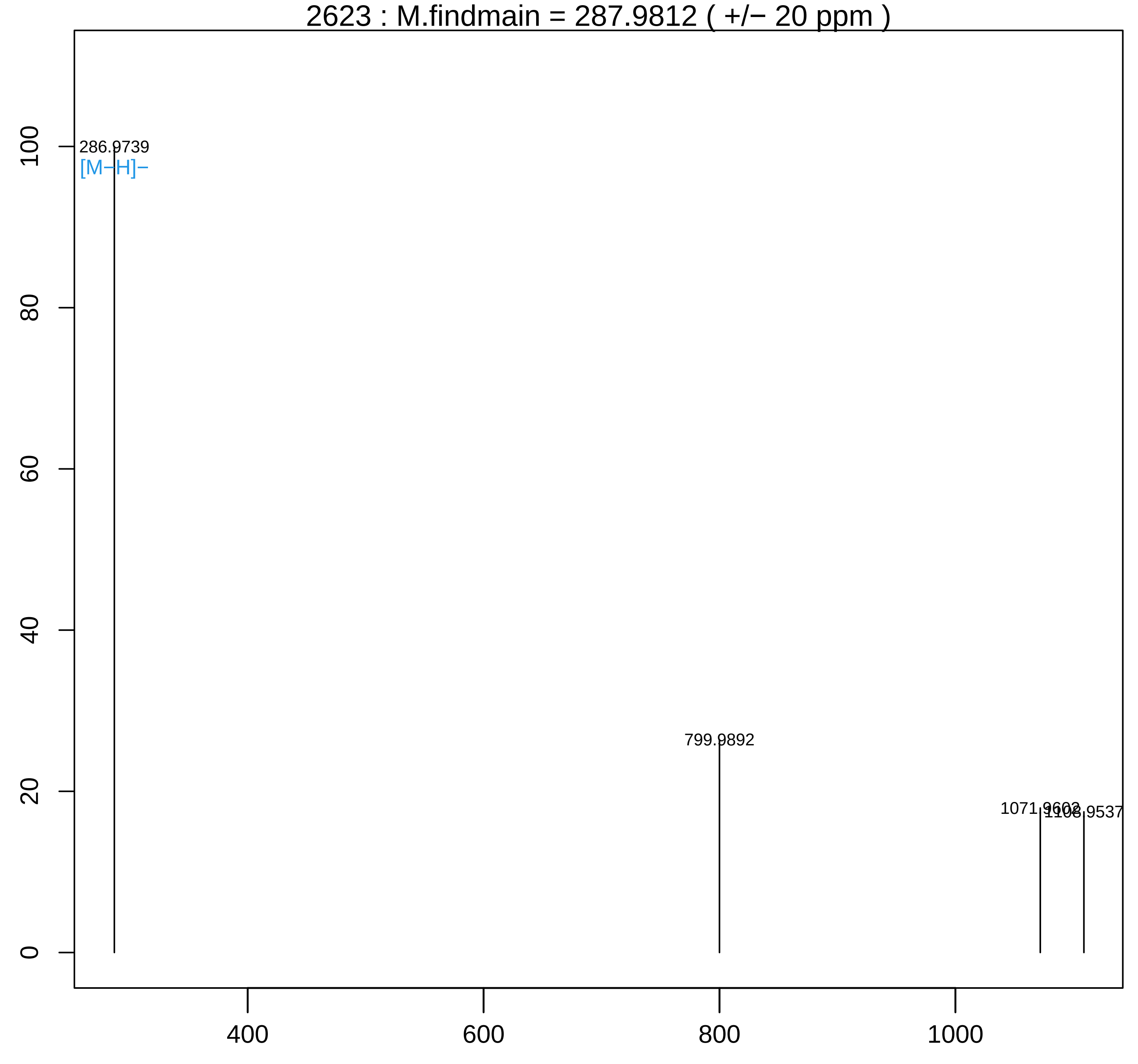
2623 : M.findmain = 287.9812 (+/− 20 ppm)
286.9739
[M−H]−
799.9892
1071.9602
1106.9537
0
20
40
60
80
100
400
600
800
1000

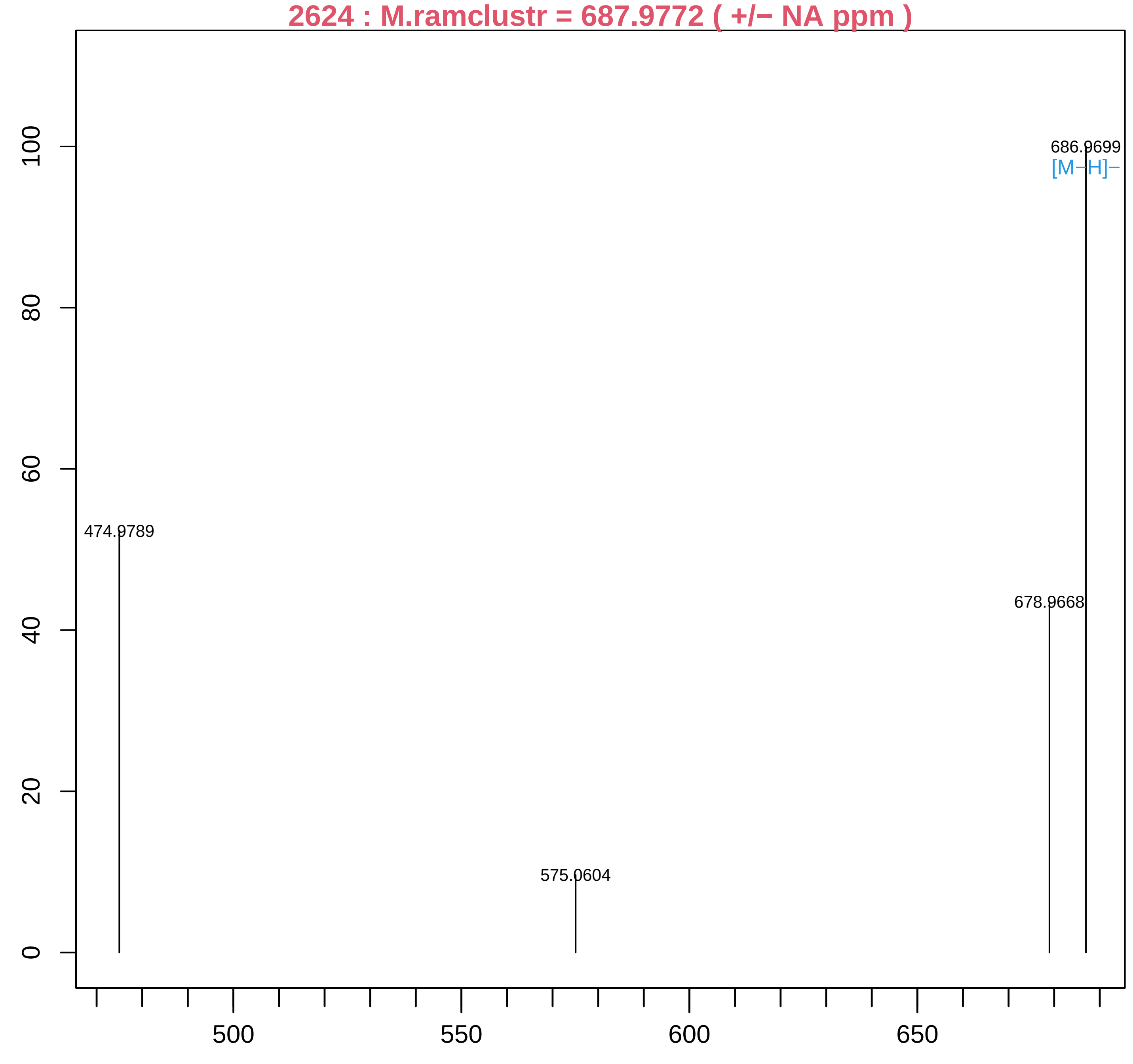
2624 : M.ramclustr = 687.9772 (+/− NA ppm)
686.9699
[M−H]−
474.9789
678.9668
575.0604
0
20
40
60
80
100
500
550
600
650

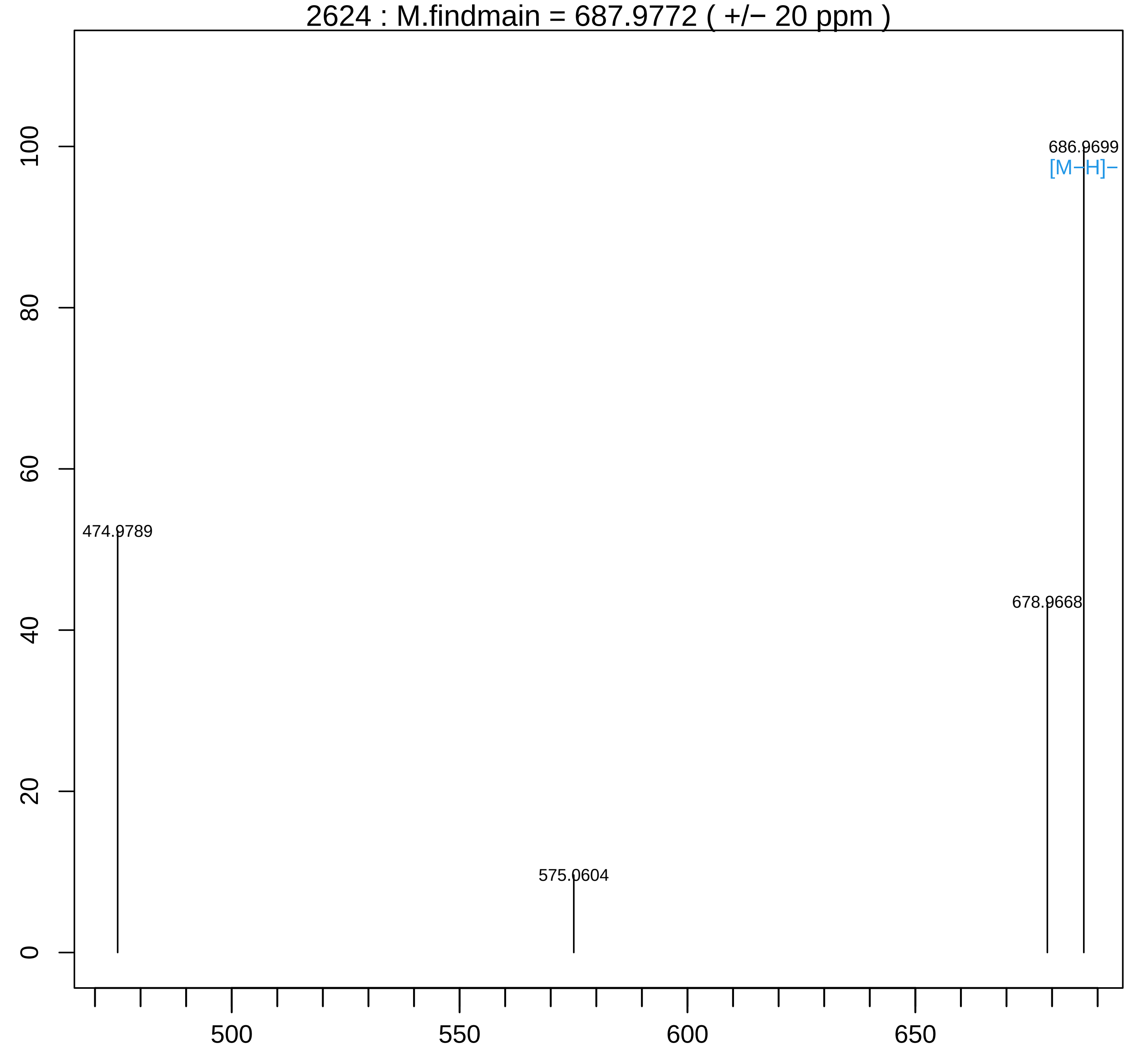
2624 : M.findmain = 687.9772 (+/− 20 ppm)
686.9699
[M−H]−
474.9789
678.9668
575.0604
0
20
40
60
80
100
500
550
600
650

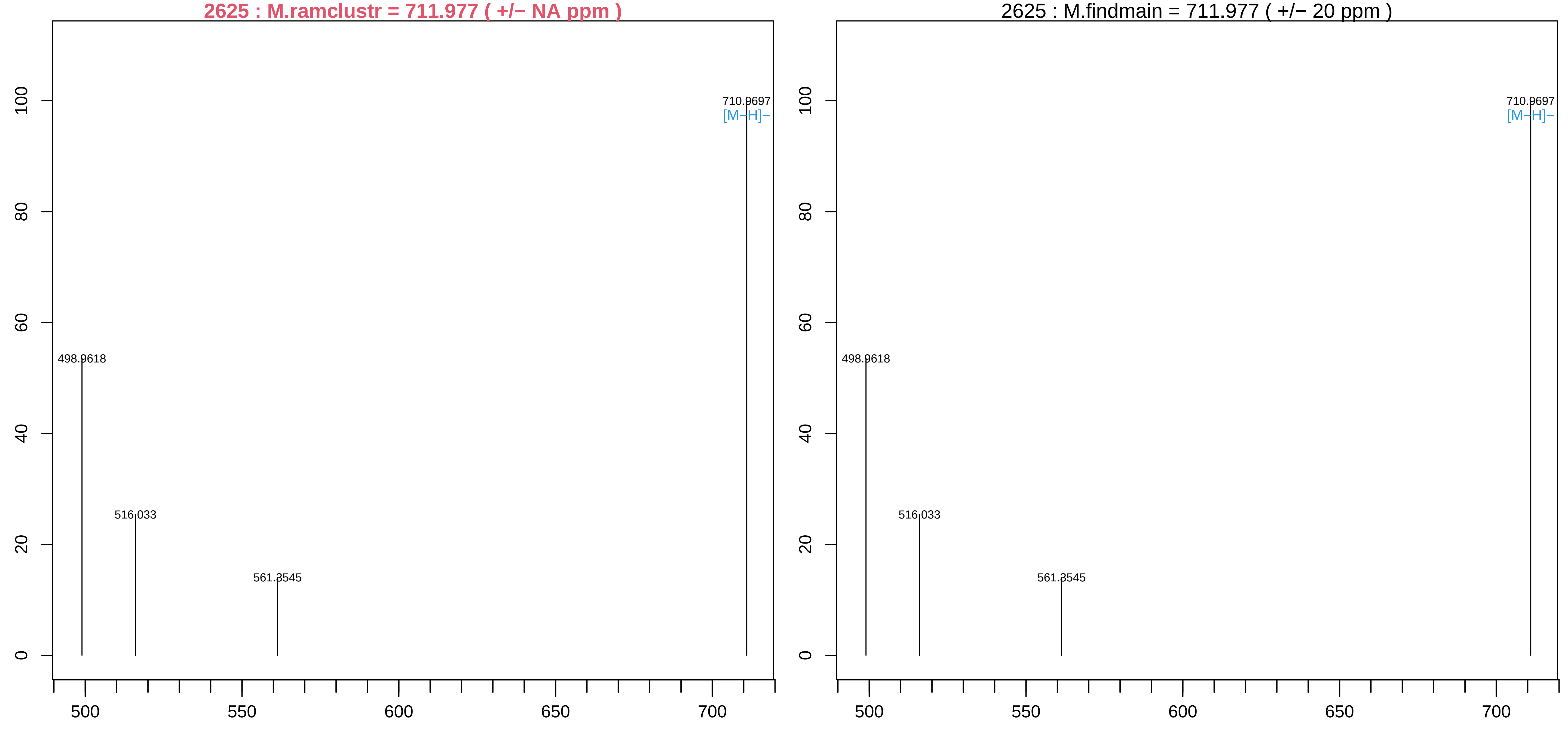
2625 : M.ramclustr = 711.977 (+/− NA ppm)
710.9697
[M−H]−
498.9618
516.033
561.3545
2625 : M.findmain = 711.977 (+/− 20 ppm)
710.9697
[M−H]−
498.9618
516.033
561.3545

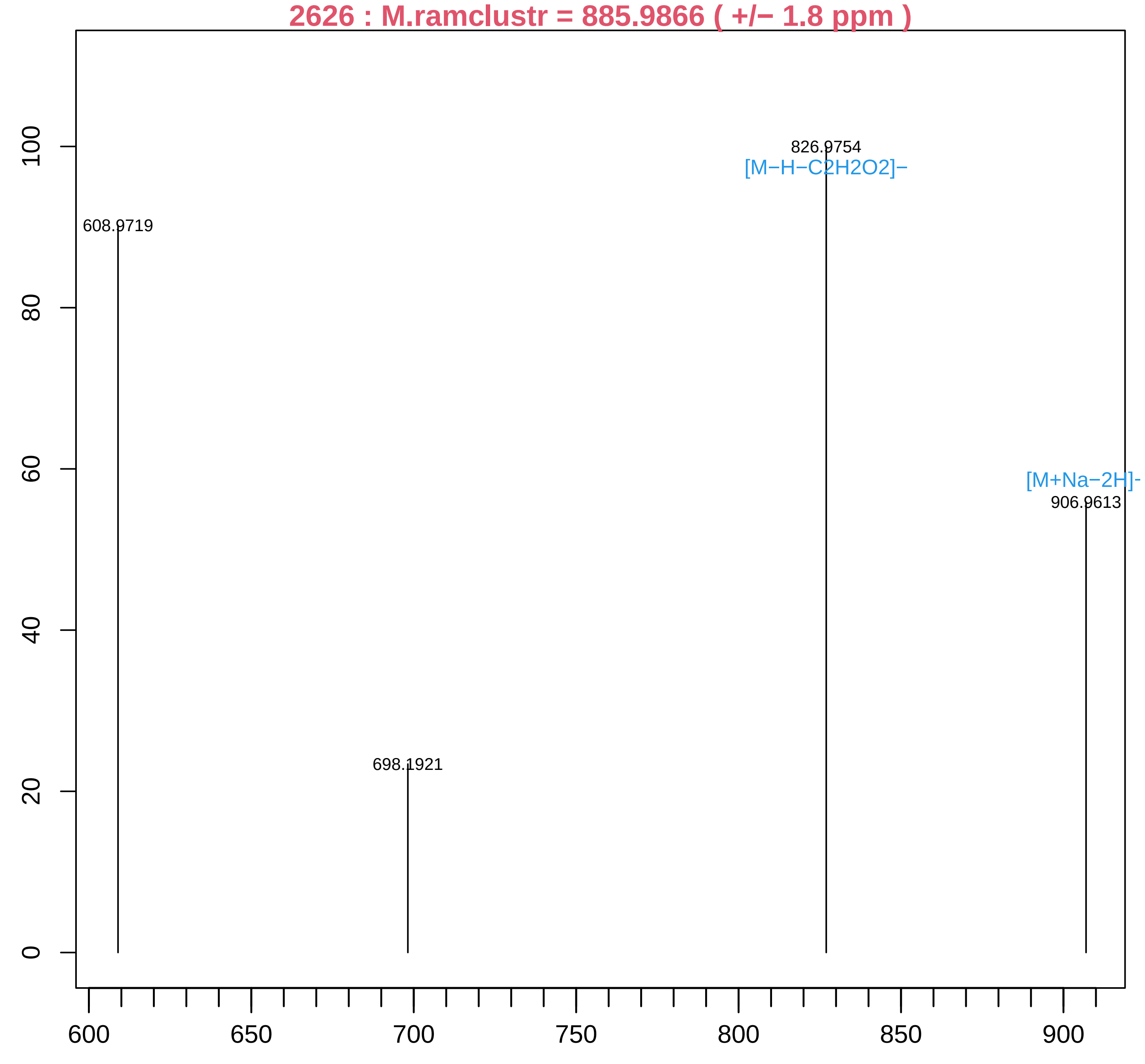
2626 : M.ramclustr = 885.9866 (+/− 1.8 ppm)
608.9719
698.1921
826.9754
[M−H−C2H2O2]−
[M+Na−2H]-
906.9613
0
20
40
60
80
100
600
650
700
750
800
850
900

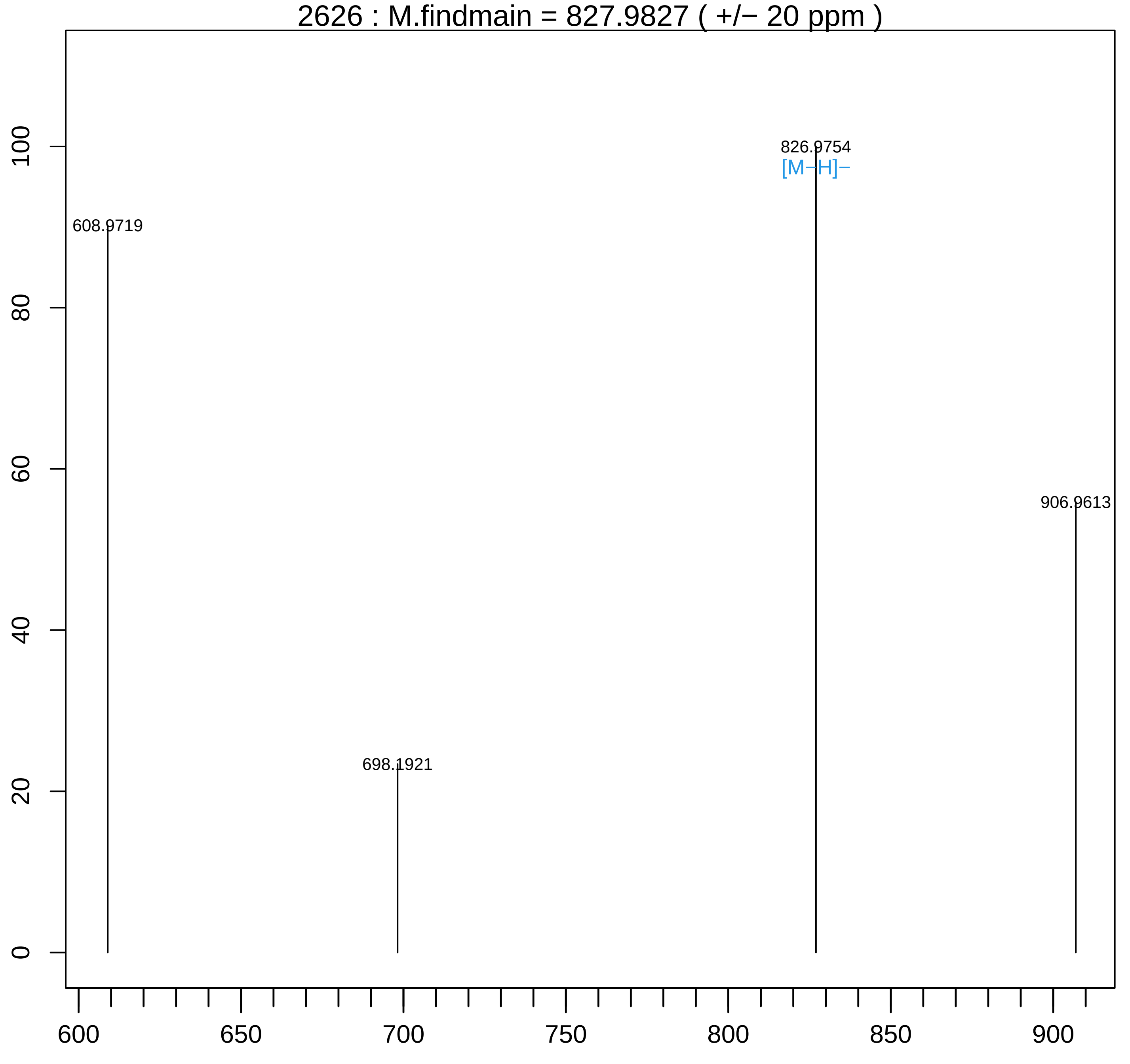
2626 : M.findmain = 827.9827 (+/− 20 ppm)
608.9719
698.1921
826.9754
[M−H]−
906.9613
0
20
40
60
80
100
600
650
700
750
800
850
900

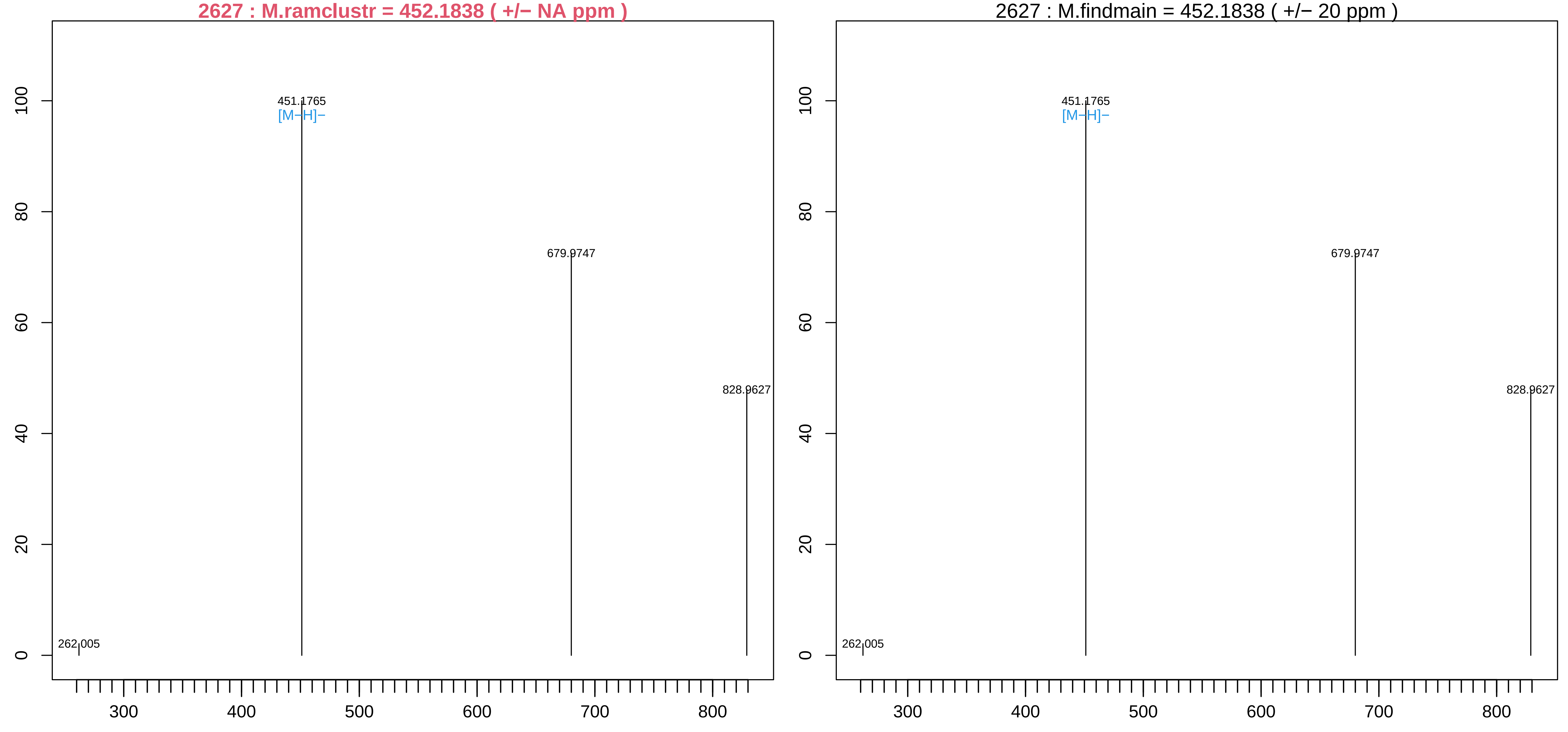
2627 : M.ramclustr = 452.1838 (+/− NA ppm)
2627 : M.findmain = 452.1838 (+/− 20 ppm)
451.1765
[M−H]−
679.9747
828.9627
262.005
0
20
40
60
80
100
300
400
500
600
700
800

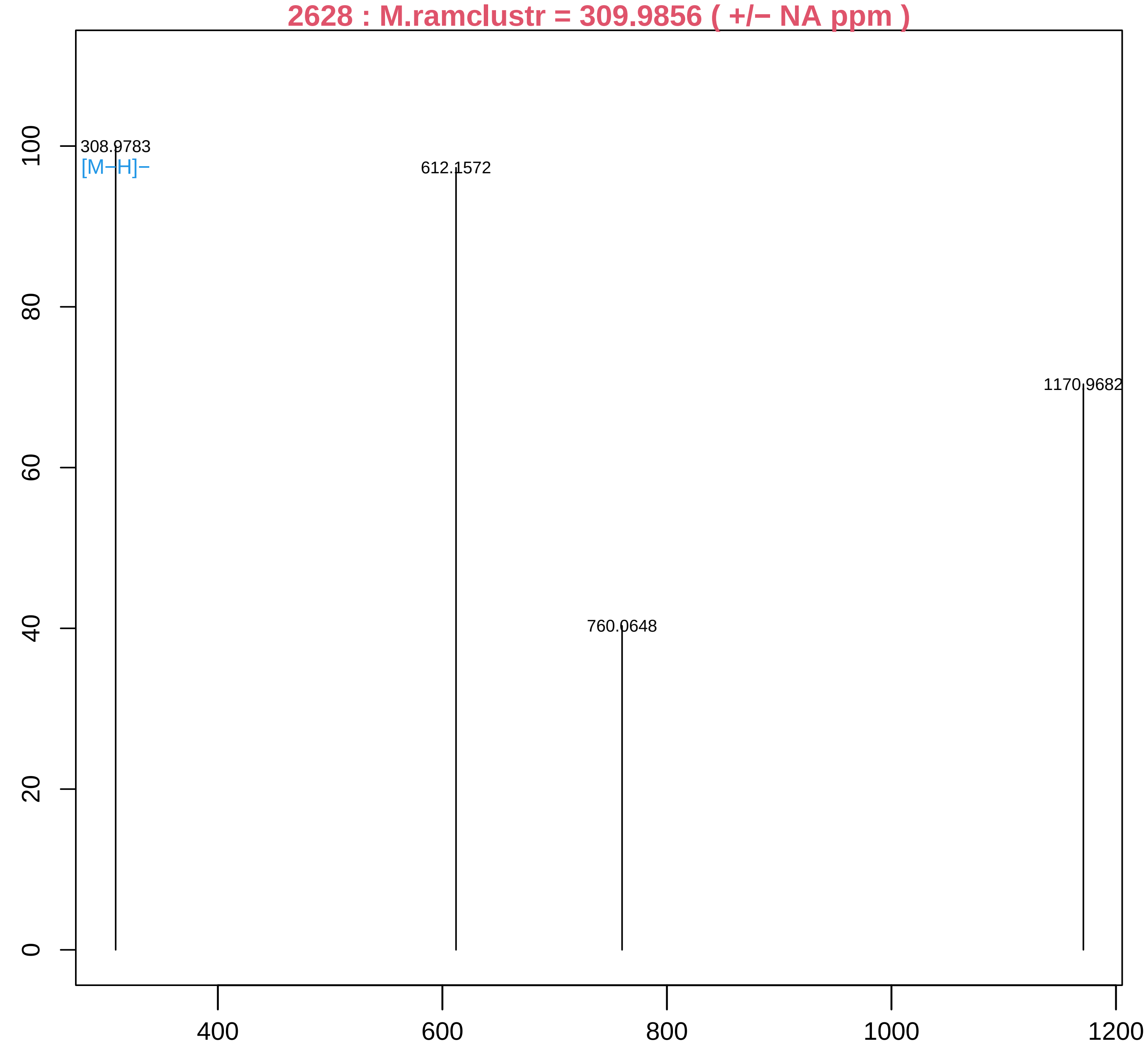

2628 : M.ramclustr = 309.9856 (+/− NA ppm)
308.9783
[M−H]−
612.1572
760.0648
1170.9682
0
20
40
60
80
100
400
600
800
1000
1200

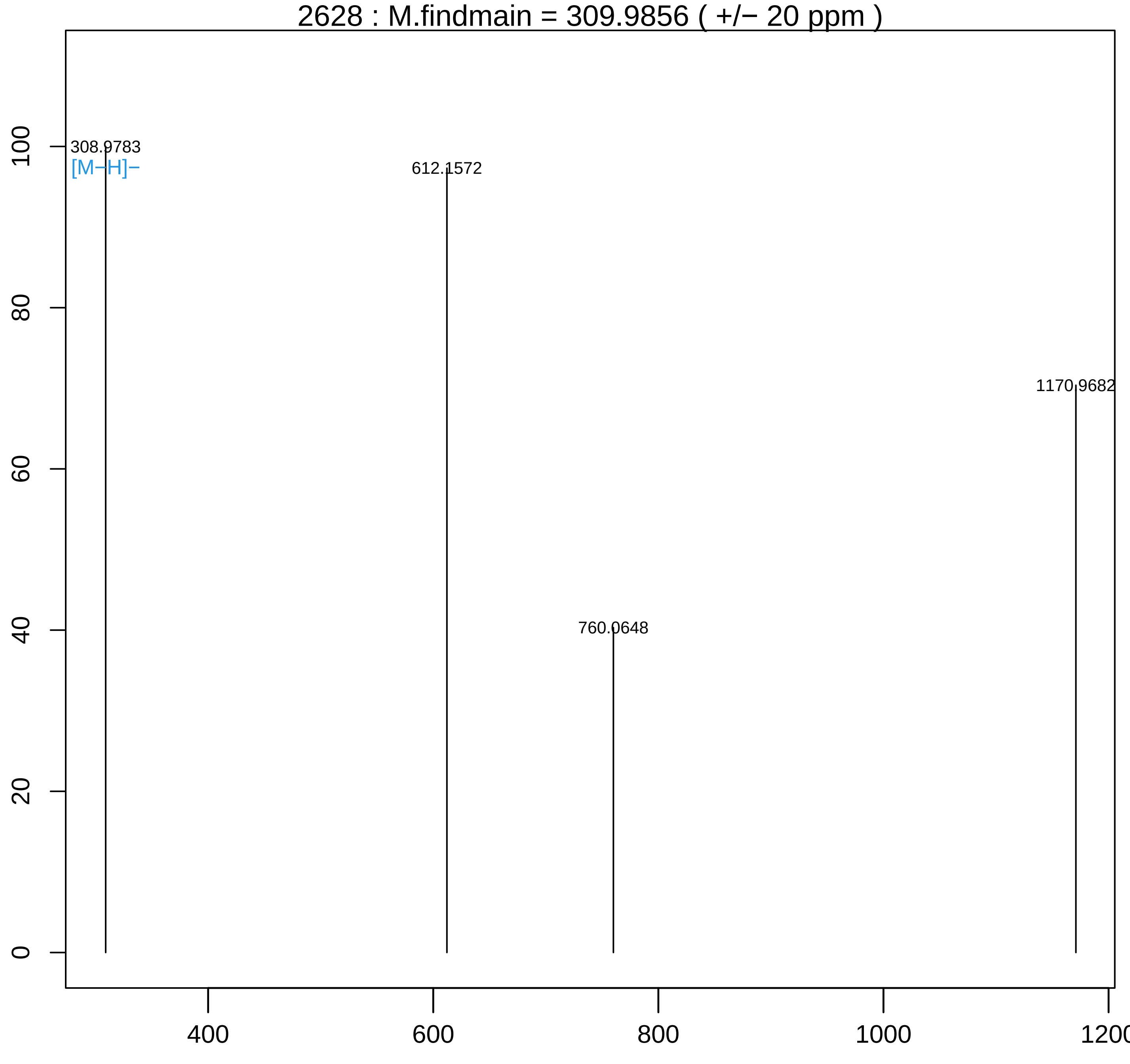

2628 : M.findmain = 309.9856 (+/− 20 ppm)
308.9783
[M−H]−
612.1572
760.0648
1170.9682
0
20
40
60
80
100
400
600
800
1000
1200

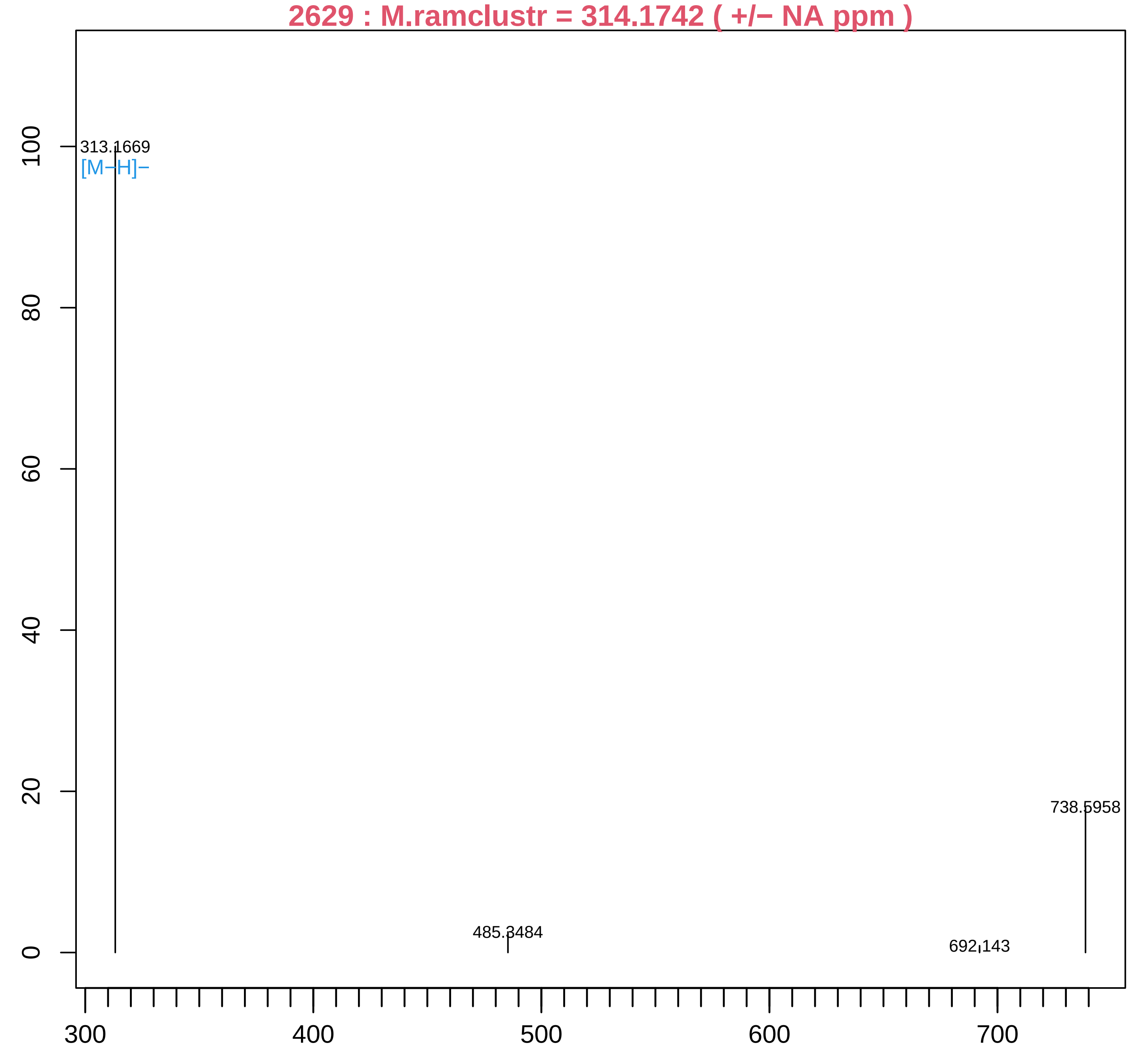
2629 : M.ramclustr = 314.1742 (+/− NA ppm)
313.1669
[M−H]−
485.3484
692.143
738.5958
0
20
40
60
80
100
300
400
500
600
700

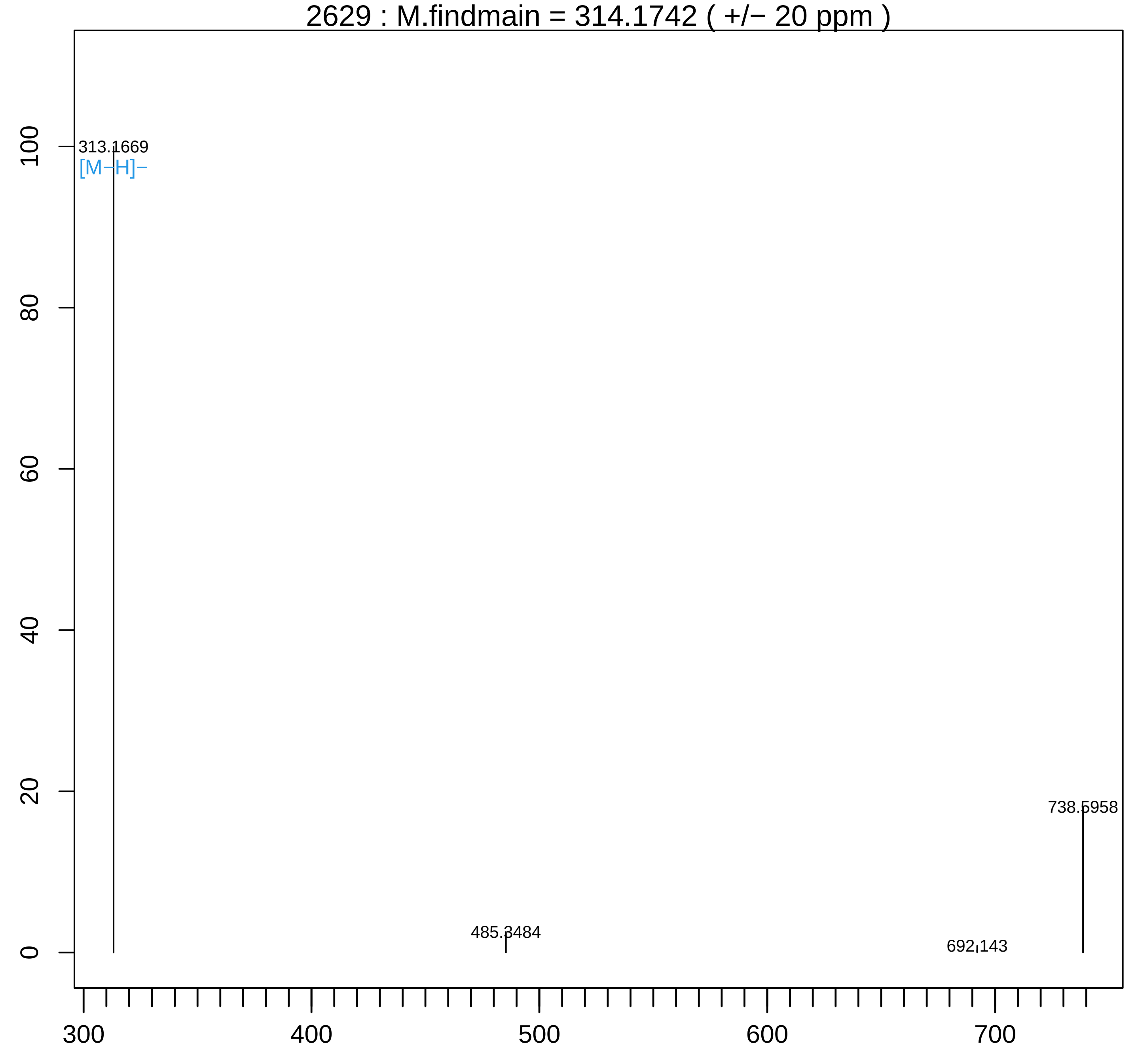
2629 : M.findmain = 314.1742 (+/− 20 ppm)
313.1669
[M−H]−
485.3484
692.143
738.5958
0
20
40
60
80
100
300
400
500
600
700

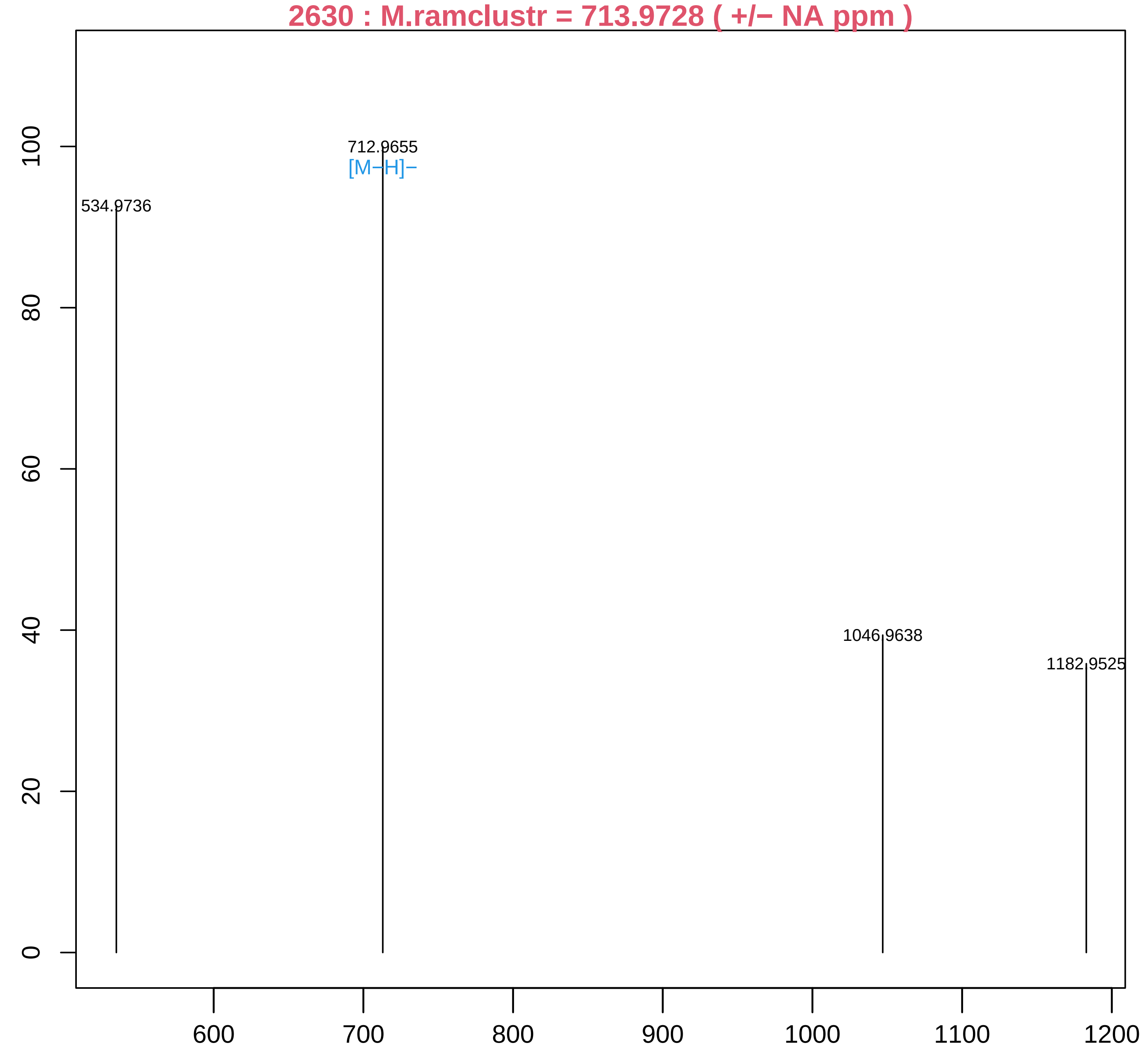
2630 : M.ramclustr = 713.9728 (+/− NA ppm)
712.9655
[M−H]−
534.9736
1046.9638
1182.9525
0
20
40
60
80
100
600
700
800
900
1000
1100
1200
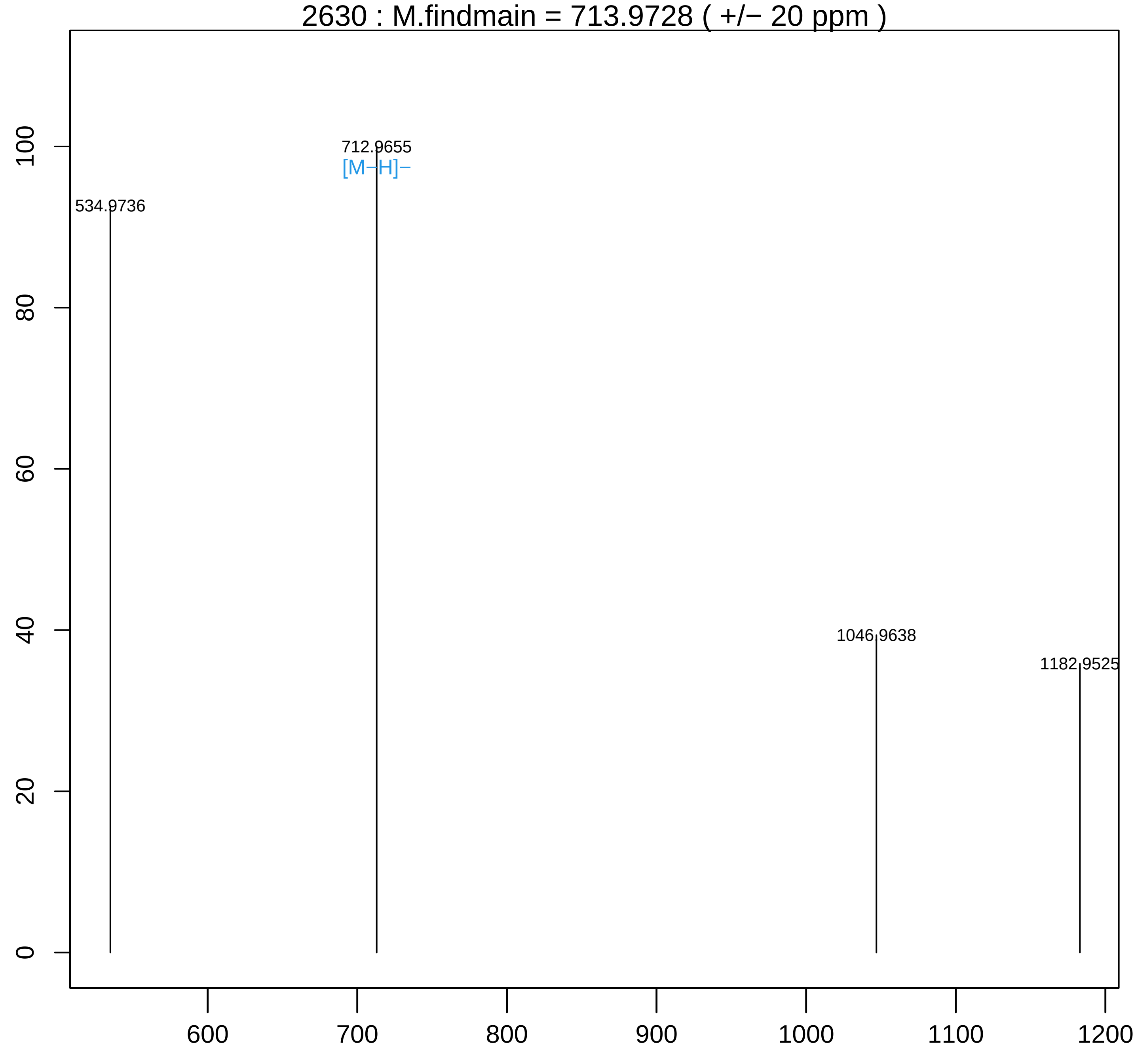
2630 : M.findmain = 713.9728 (+/− 20 ppm)
712.9655
[M−H]−
534.9736
1046.9638
1182.9525
0
20
40
60
80
100
600
700
800
900
1000
1100
1200

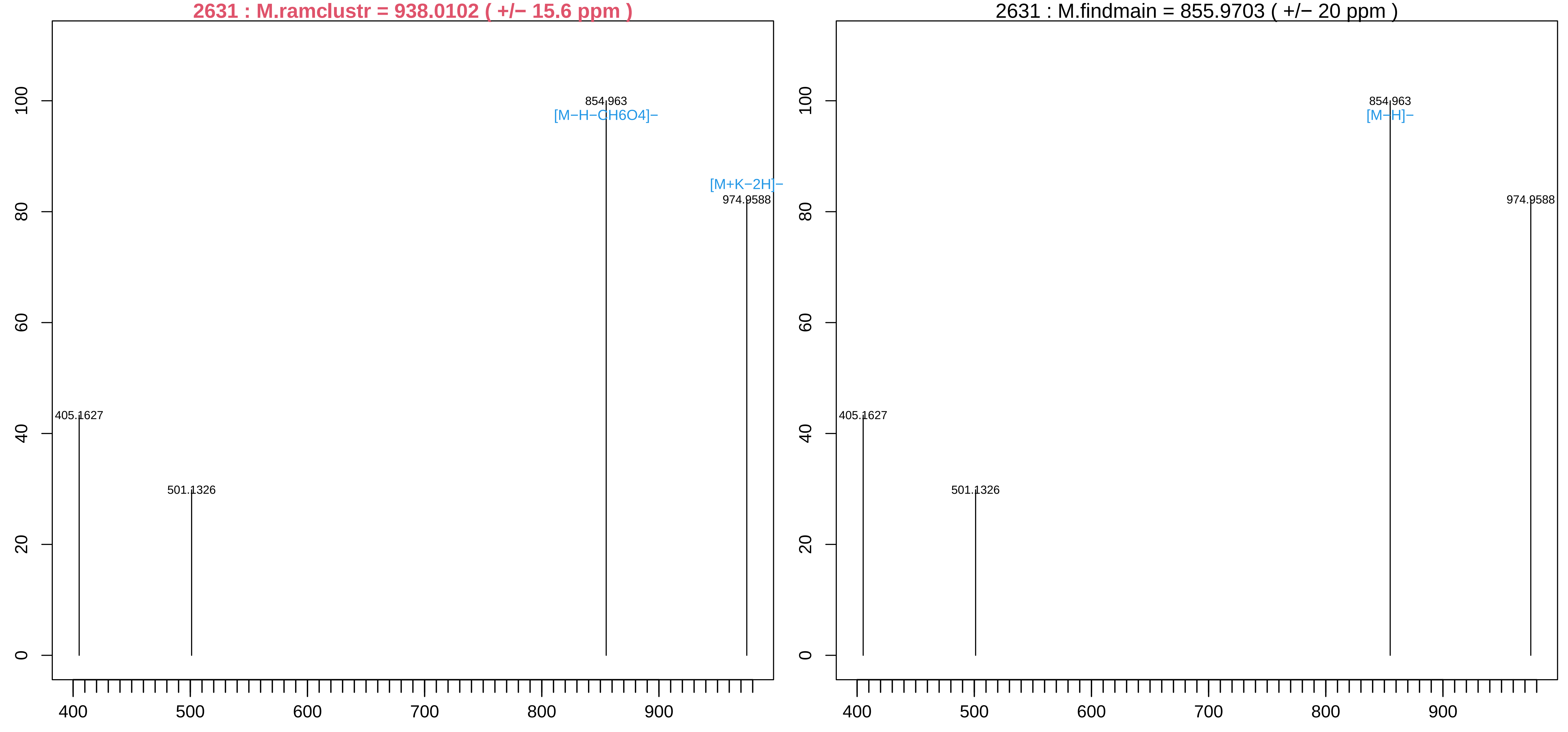
2631 : M.ramclustr = 938.0102 (+/− 15.6 ppm)
854.963
[M−H−CH6O4]−
[M+K−2H]−
974.9588
405.1627
501.1326
2631 : M.findmain = 855.9703 (+/− 20 ppm)
854.963
[M−H]−
974.9588
405.1627
501.1326
0
20
40
60
80
100
400
500
600
700
800
900

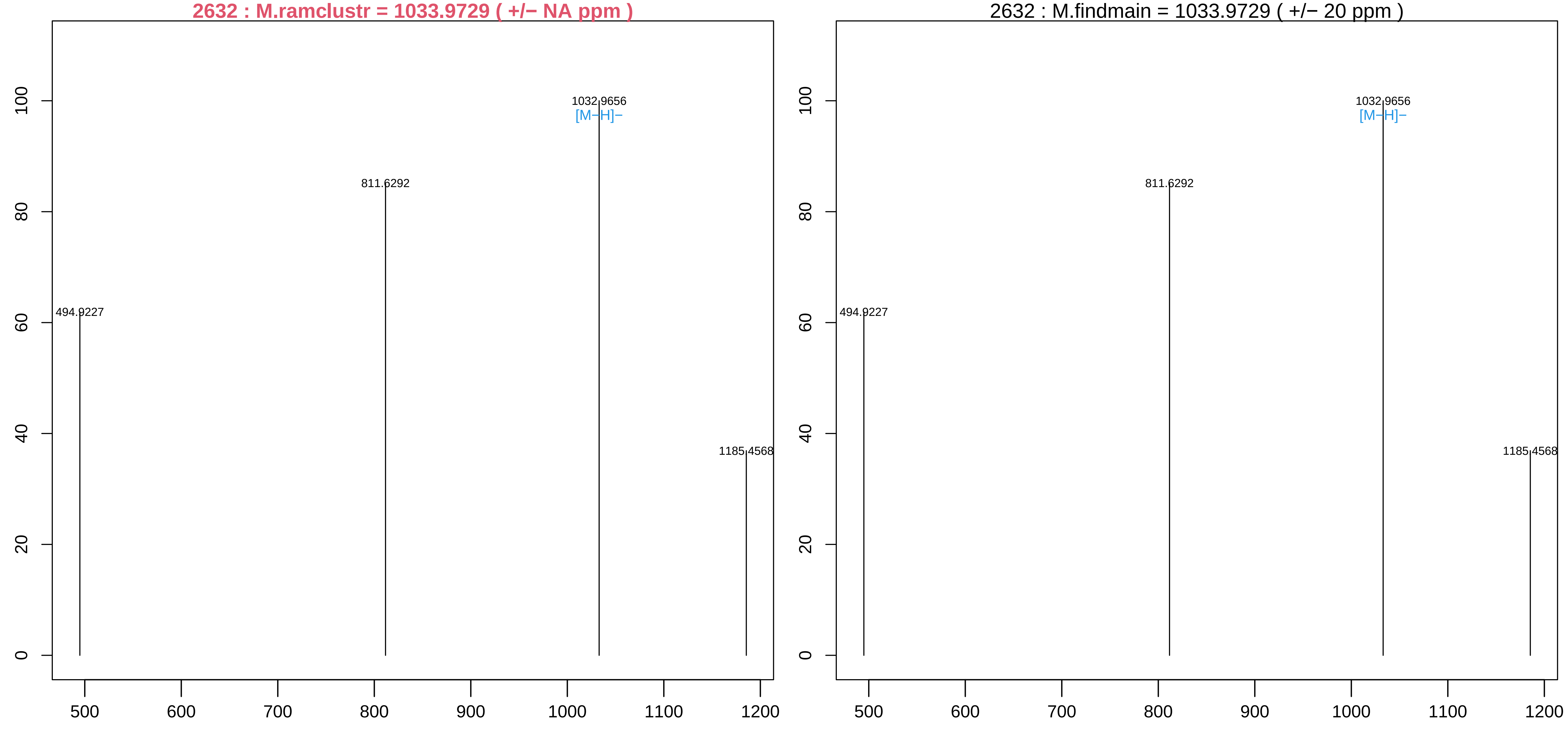

2632 : M.ramclustr = 1033.9729 (+/− NA ppm)
494.9227
811.6292
1032.9656
[M−H]−
1185.4568
2632 : M.findmain = 1033.9729 (+/− 20 ppm)
494.9227
811.6292
1032.9656
[M−H]−
1185.4568

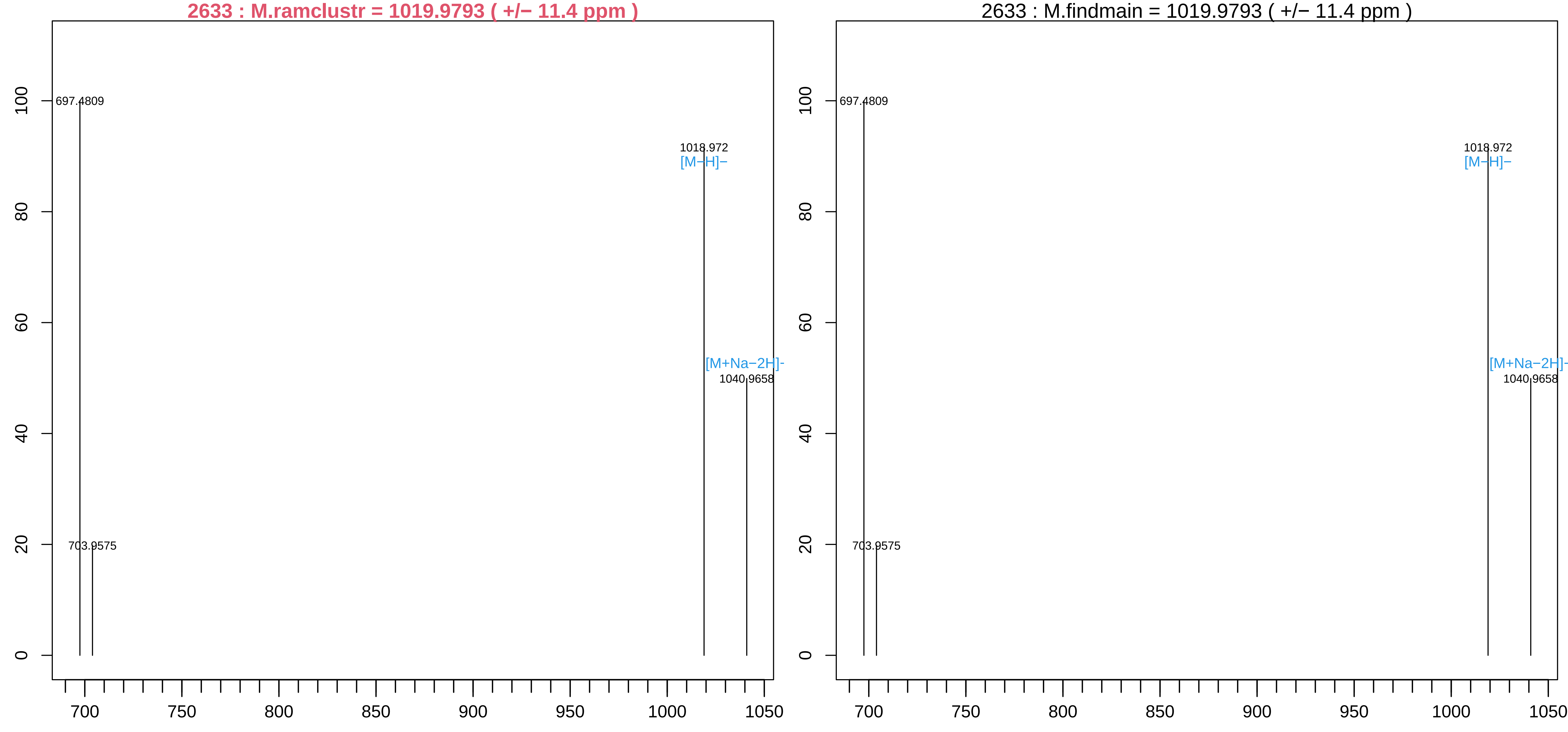
2633 : M.ramclustr = 1019.9793 (+/− 11.4 ppm)
697.4809
703.9575
1018.972
[M−H]−
[M+Na−2H]-
1040.9658
0
20
40
60
80
100
700
750
800
850
900
950
1000
1050
2633 : M.findmain = 1019.9793 (+/− 11.4 ppm)
697.4809
703.9575
1018.972
[M−H]−
[M+Na−2H]-
1040.9658

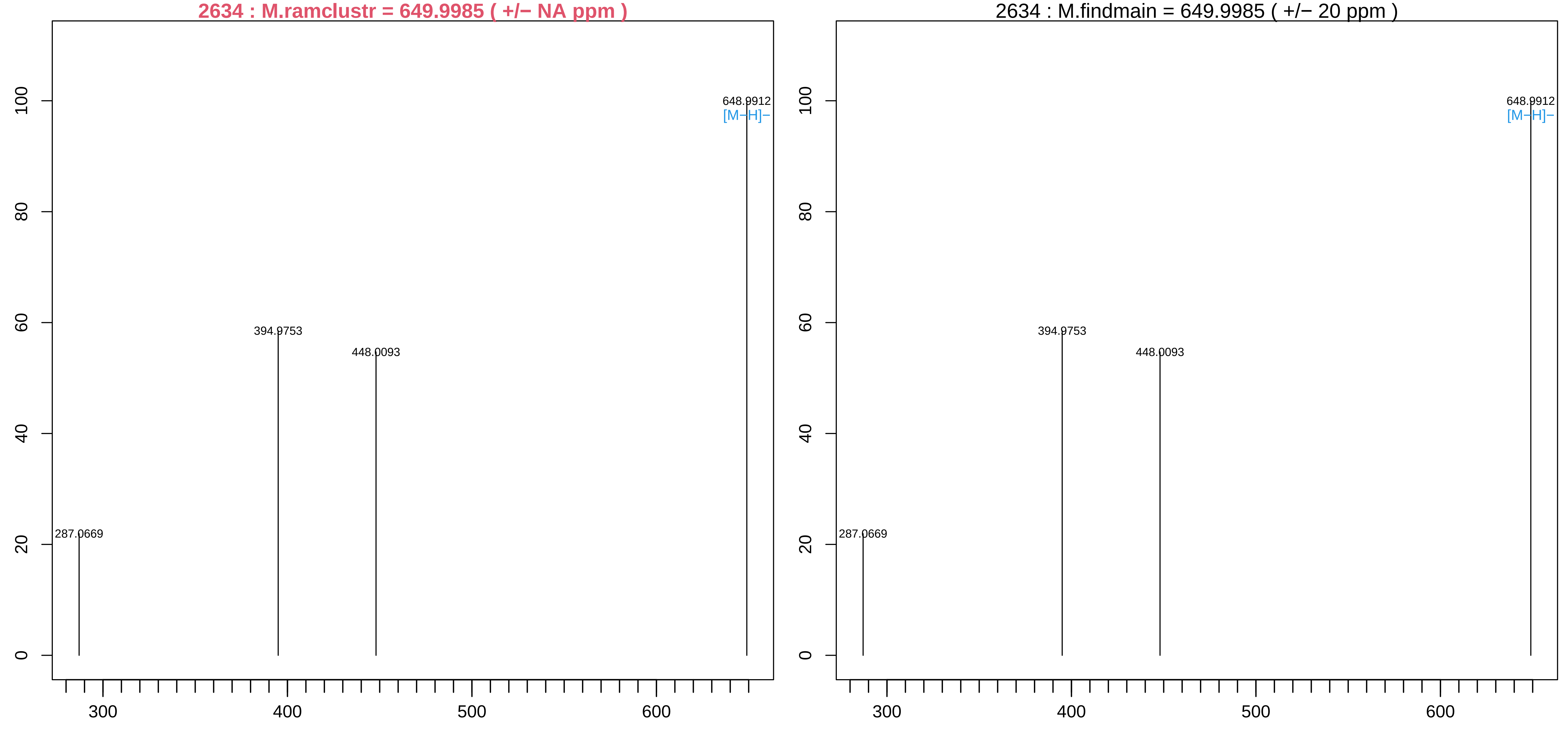

2634 : M.ramclustr = 649.9985 (+/− NA ppm)
2634 : M.findmain = 649.9985 (+/− 20 ppm)
648.9912
[M−H]−
394.9753
448.0093
287.0669
0
20
40
60
80
100
300
400
500
600

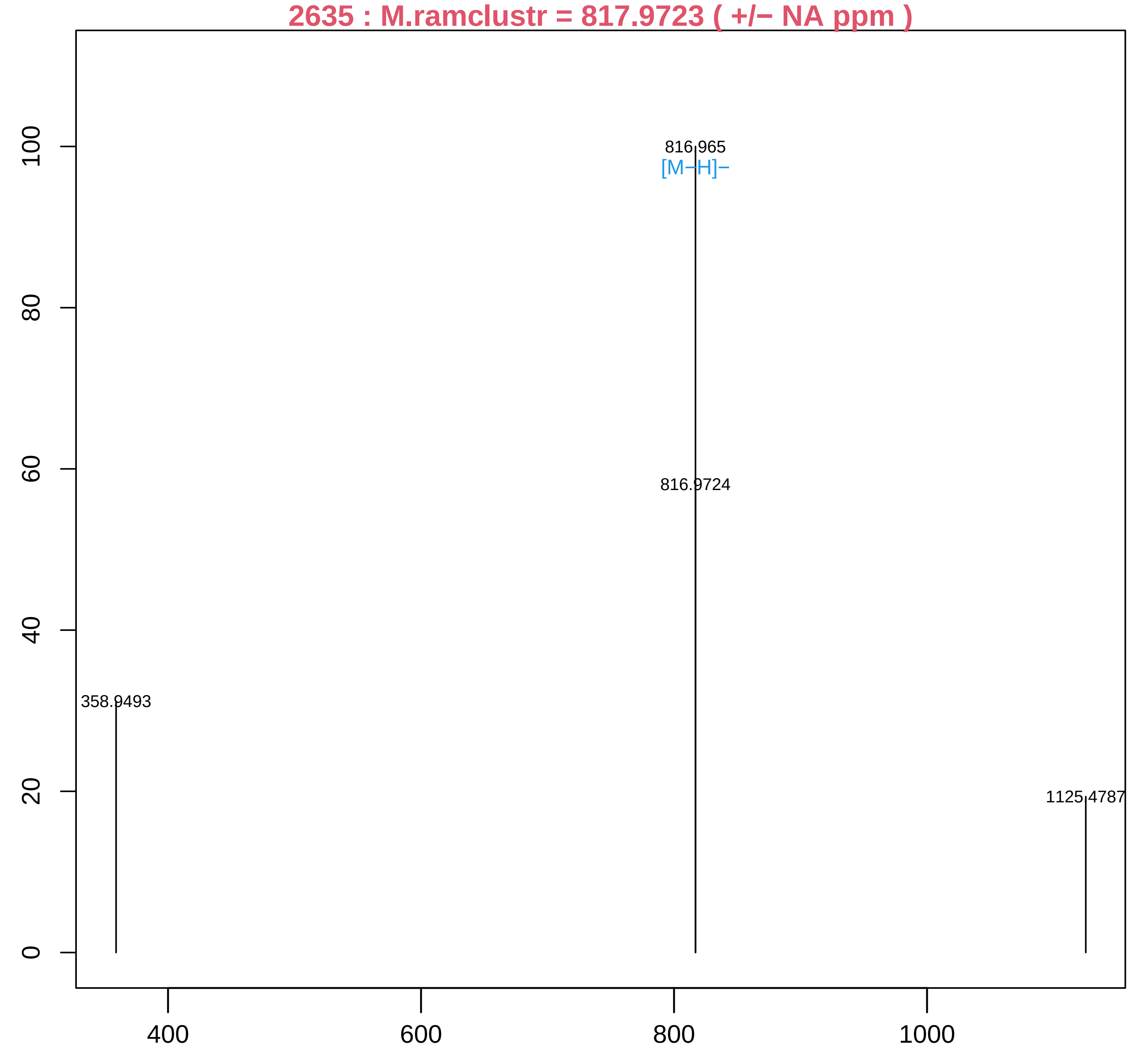

2635 : M.ramclustr = 817.9723 (+/− NA ppm)
816.965
[M−H]−
816.9724
358.9493
1125.4787
0
20
40
60
80
100
400
600
800
1000

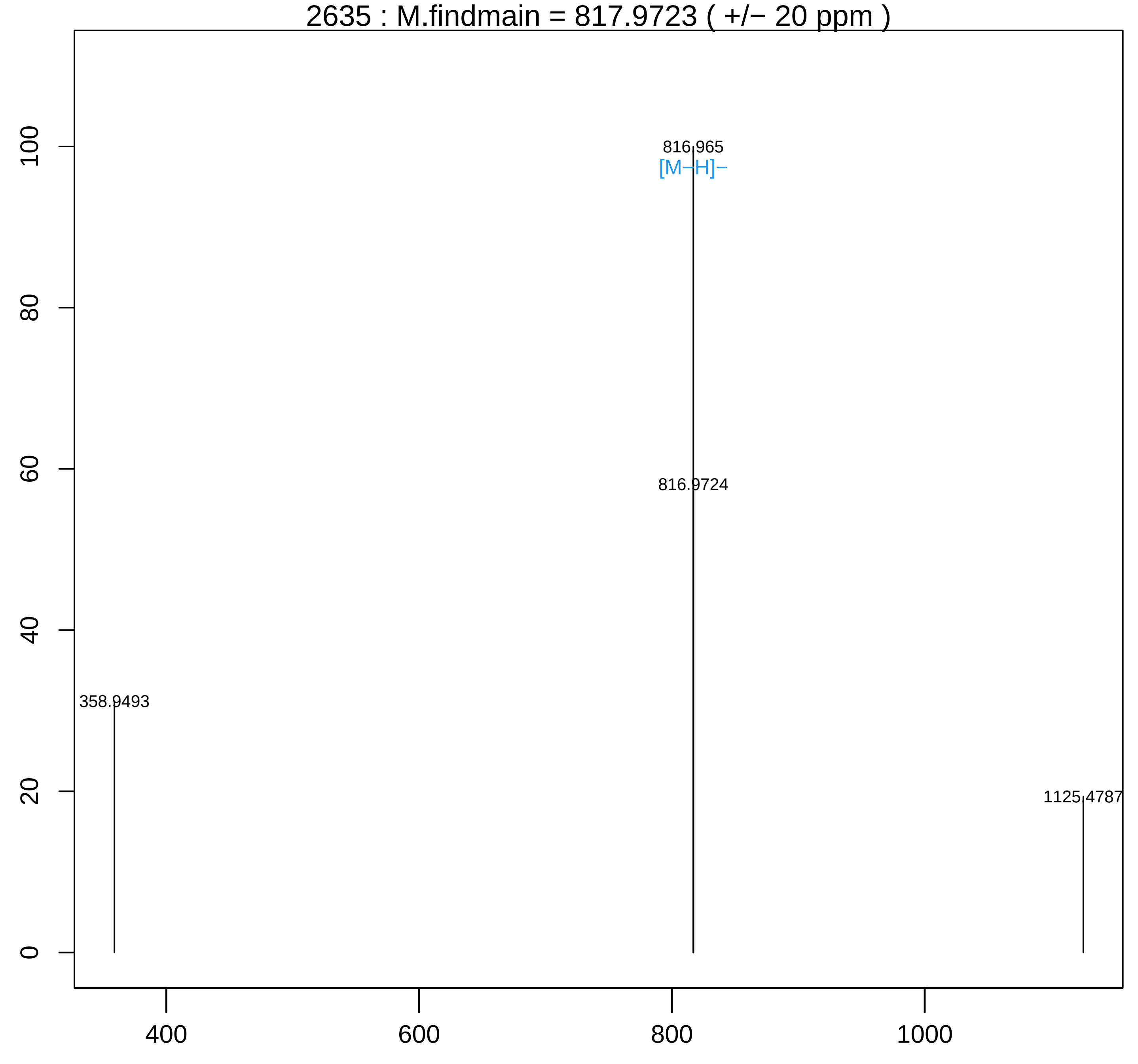

2635 : M.findmain = 817.9723 (+/− 20 ppm)
816.965
[M−H]−
816.9724
358.9493
1125.4787
0
20
40
60
80
100
400
600
800
1000

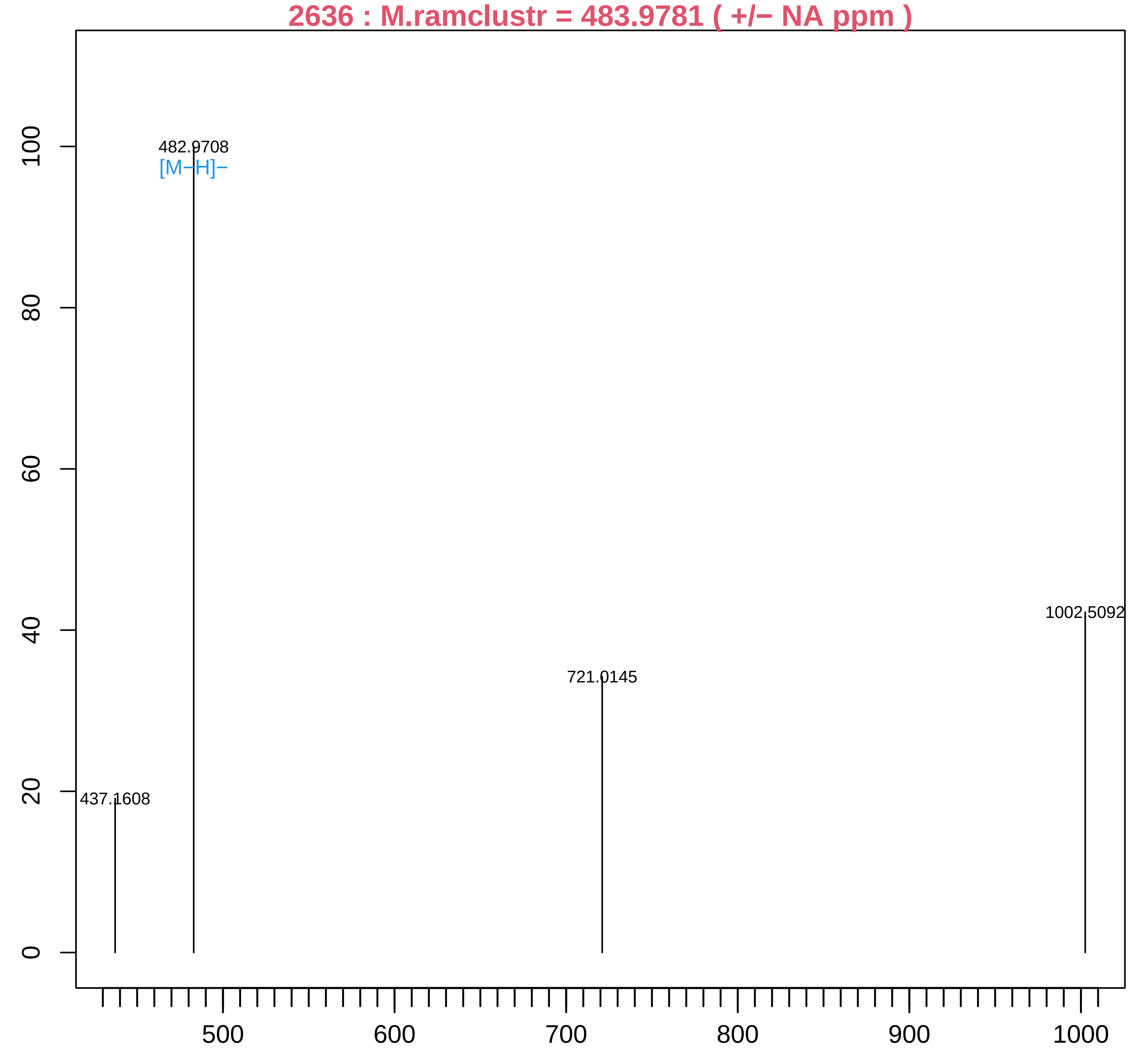
2636 : M.ramclustr = 483.9781 (+/− NA ppm)
482.9708
[M−H]−
437.1608
721.0145
1002.5092
0
20
40
60
80
100
500
600
700
800
900
1000

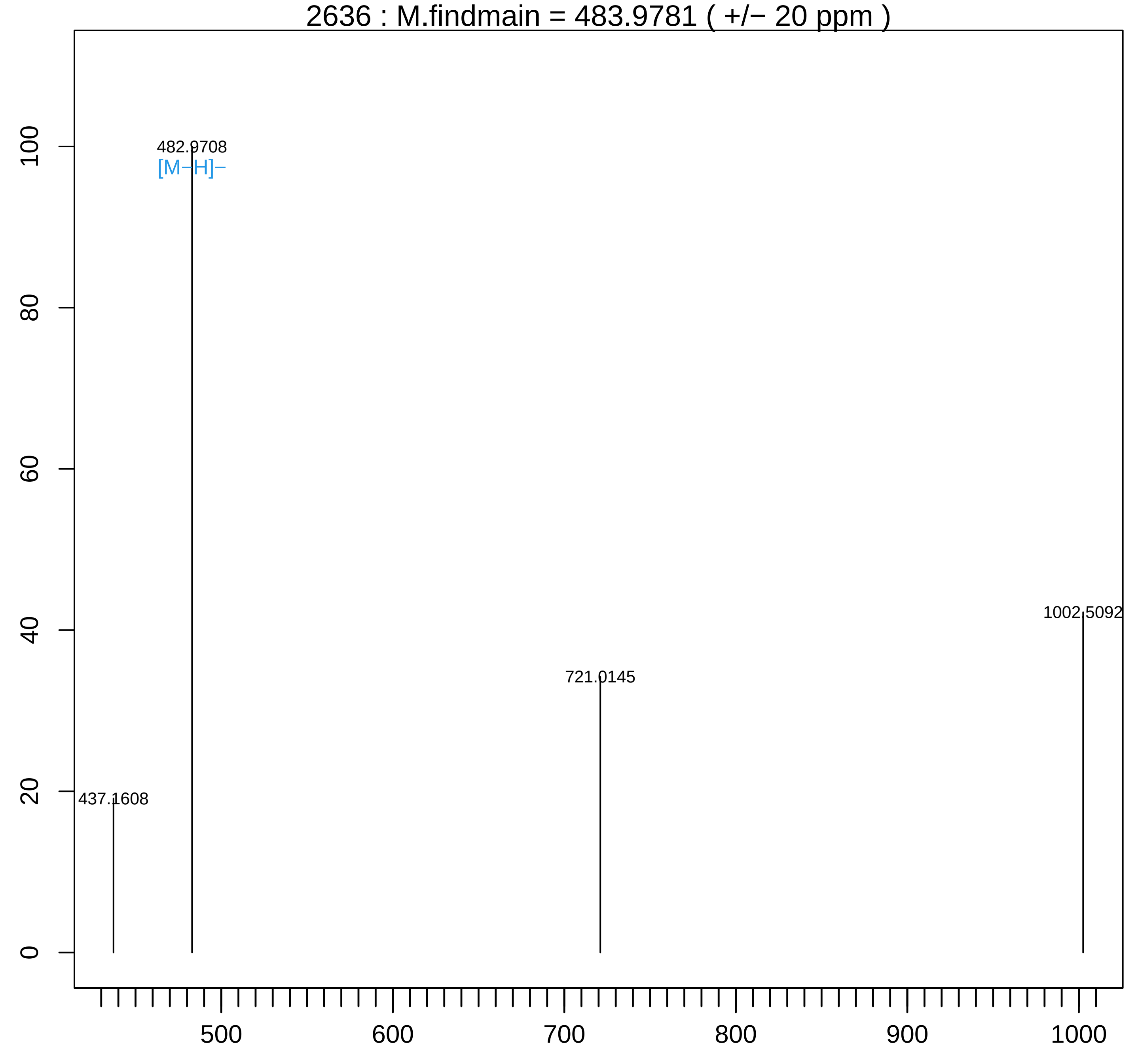
2636 : M.findmain = 483.9781 (+/− 20 ppm)
482.9708
[M−H]−
437.1608
721.0145
1002.5092
0
20
40
60
80
100
500
600
700
800
900
1000

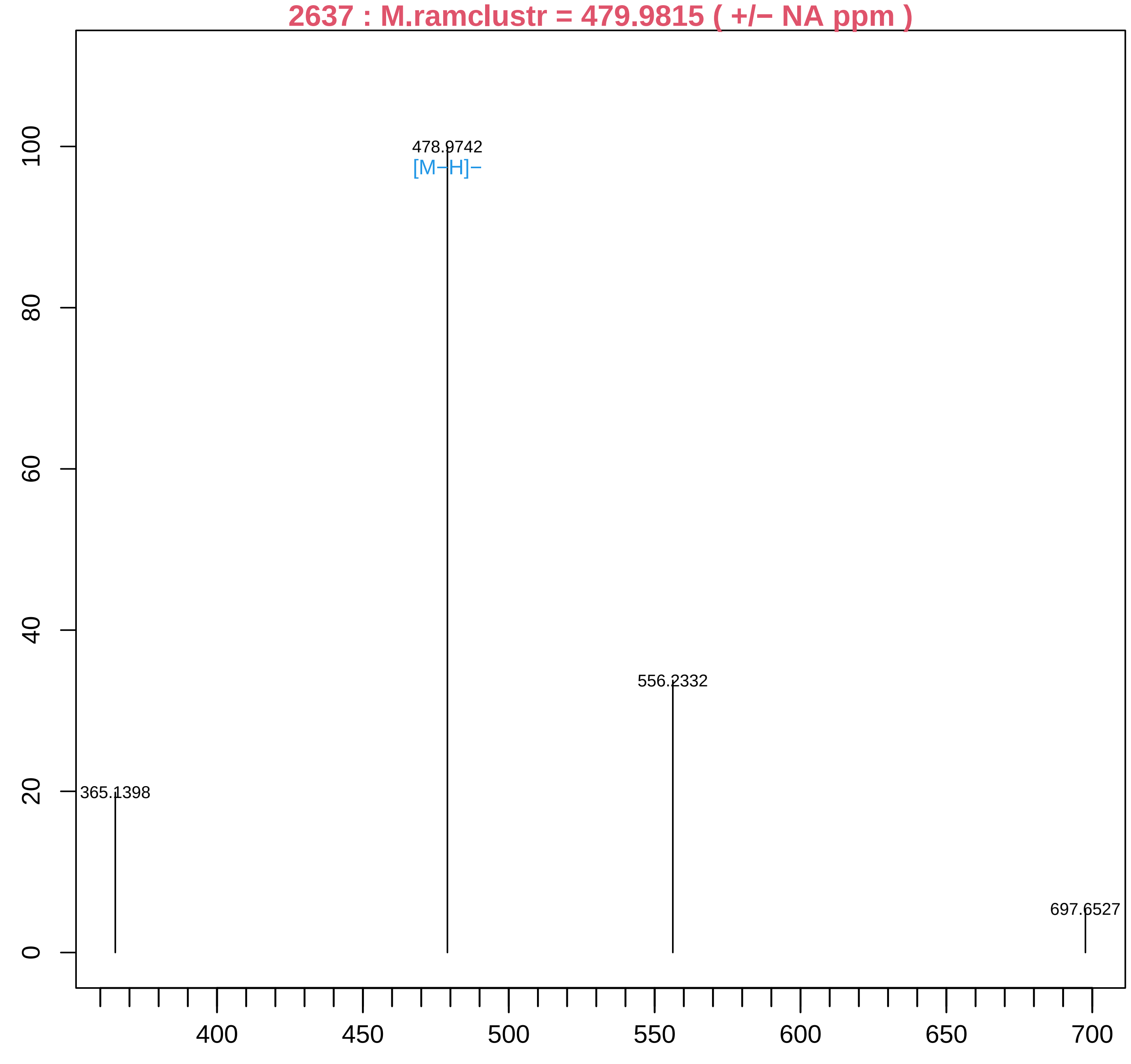
2637 : M.ramclustr = 479.9815 (+/− NA ppm)
478.9742
[M−H]−
556.2332
365.1398
697.6527
0
20
40
60
80
100
400
450
500
550
600
650
700

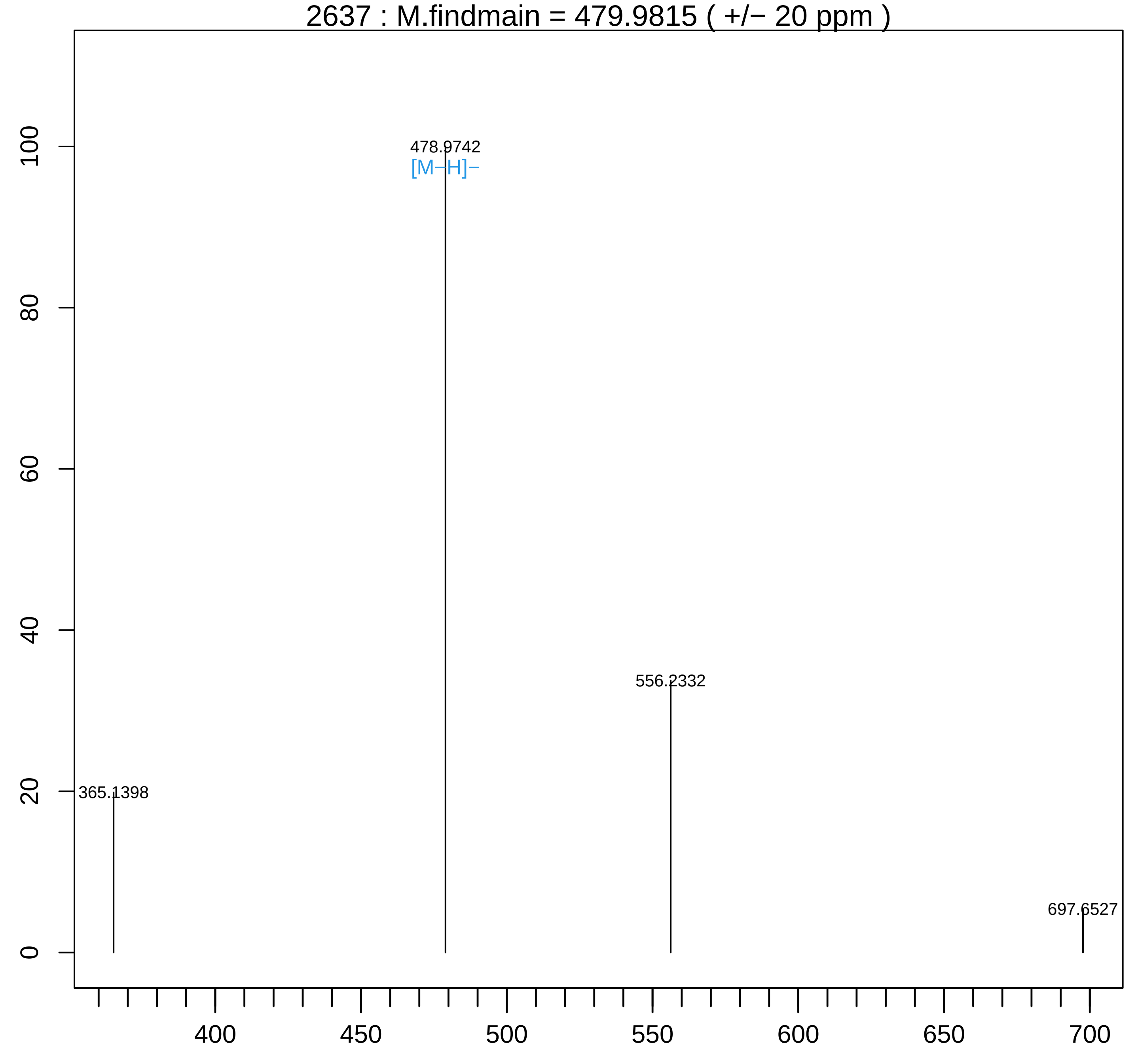
2637 : M.findmain = 479.9815 (+/− 20 ppm)
478.9742
[M−H]−
556.2332
365.1398
697.6527
0
20
40
60
80
100
400
450
500
550
600
650
700

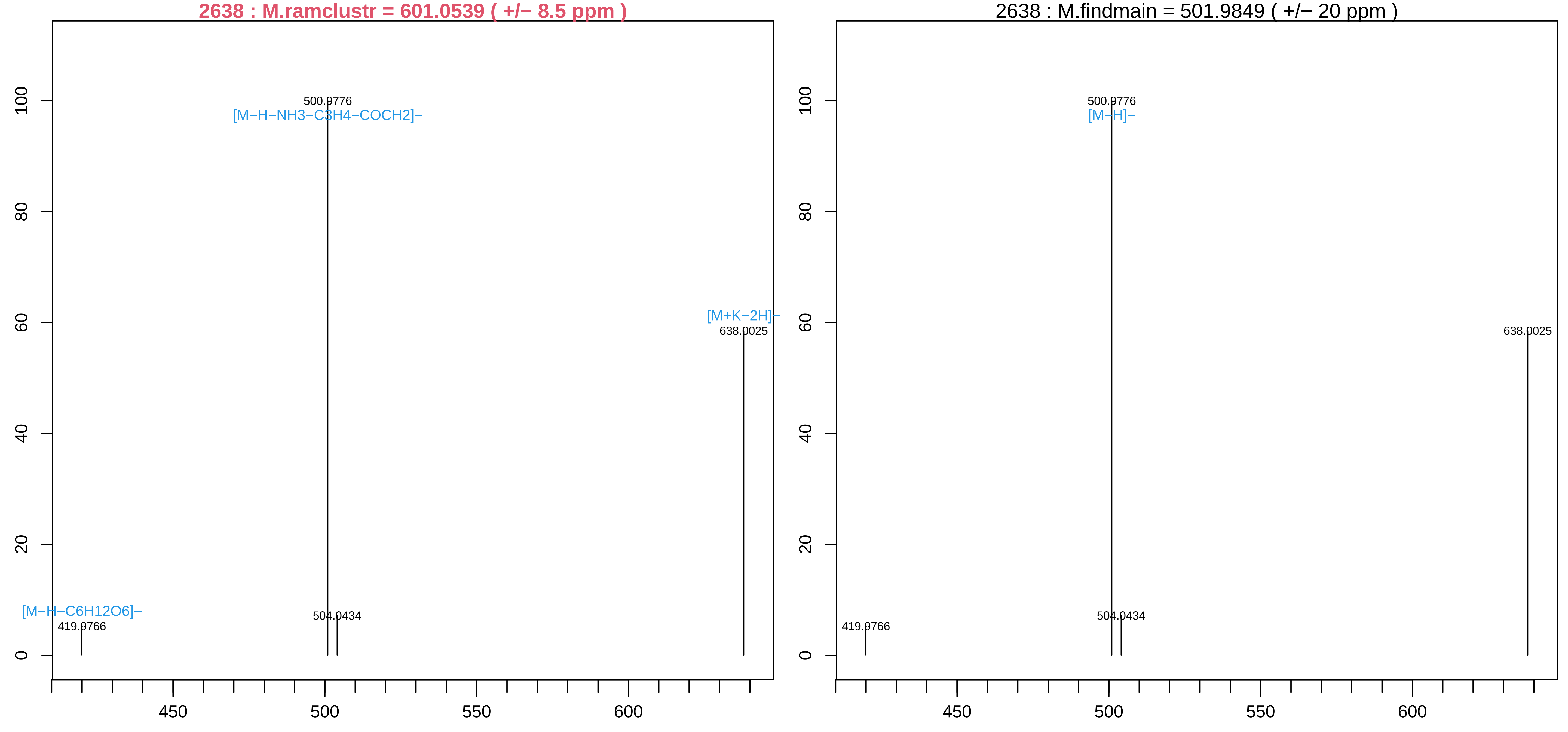

2638 : M.ramclustr = 601.0539 (+/− 8.5 ppm)
500.9776
[M−H−NH3−C3H4−COCH2]−
[M+K−2H]−
638.0025
[M−H−C6H12O6]−
419.9766
504.0434
2638 : M.findmain = 501.9849 (+/− 20 ppm)
500.9776
[M−H]−
638.0025
419.9766
504.0434
0
20
40
60
80
100
450
500
550
600

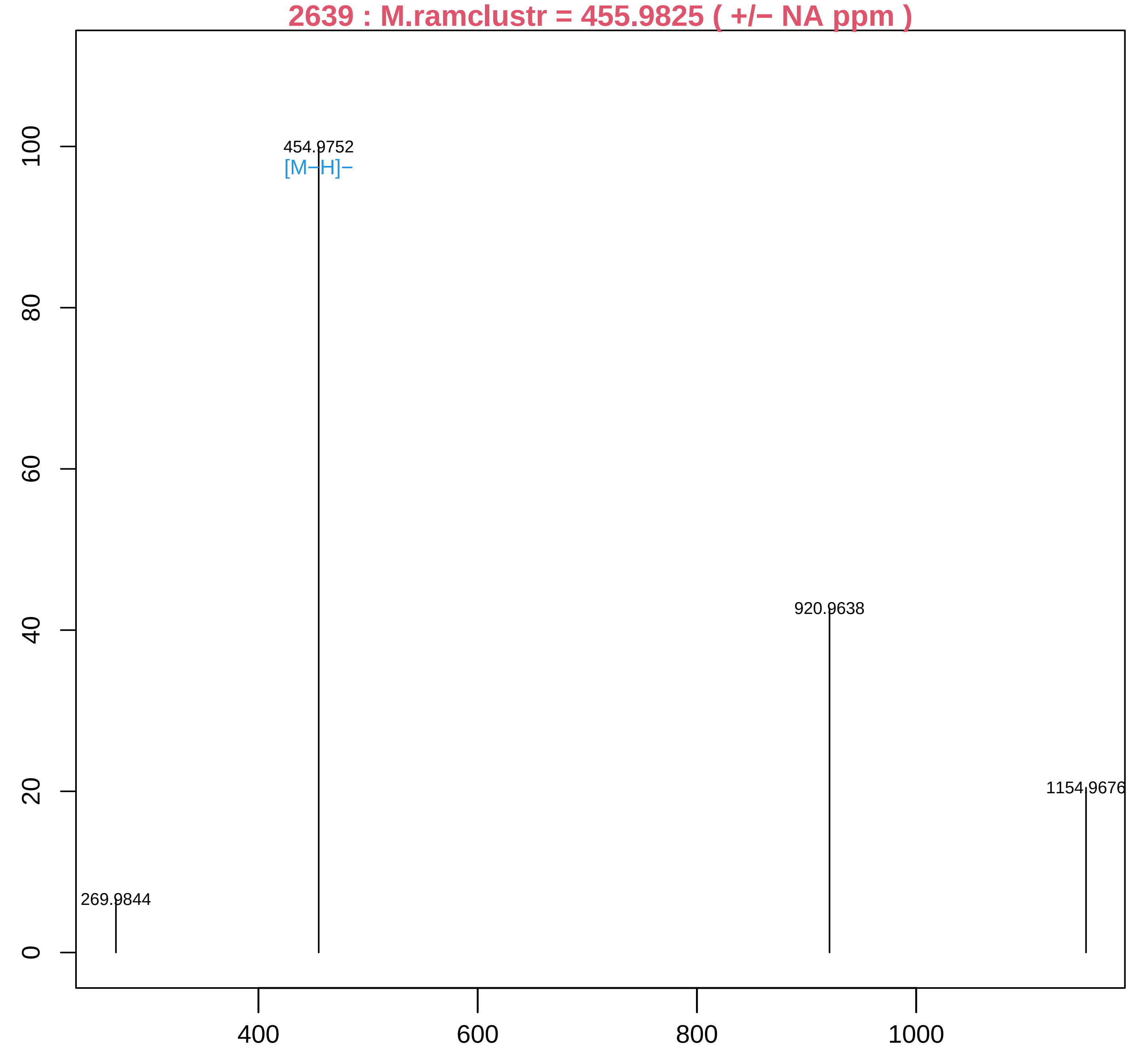
2639 : M.ramclustr = 455.9825 (+/− NA ppm)
454.9752
[M−H]−
920.9638
1154.9676
269.9844
0
20
40
60
80
100
400
600
800
1000
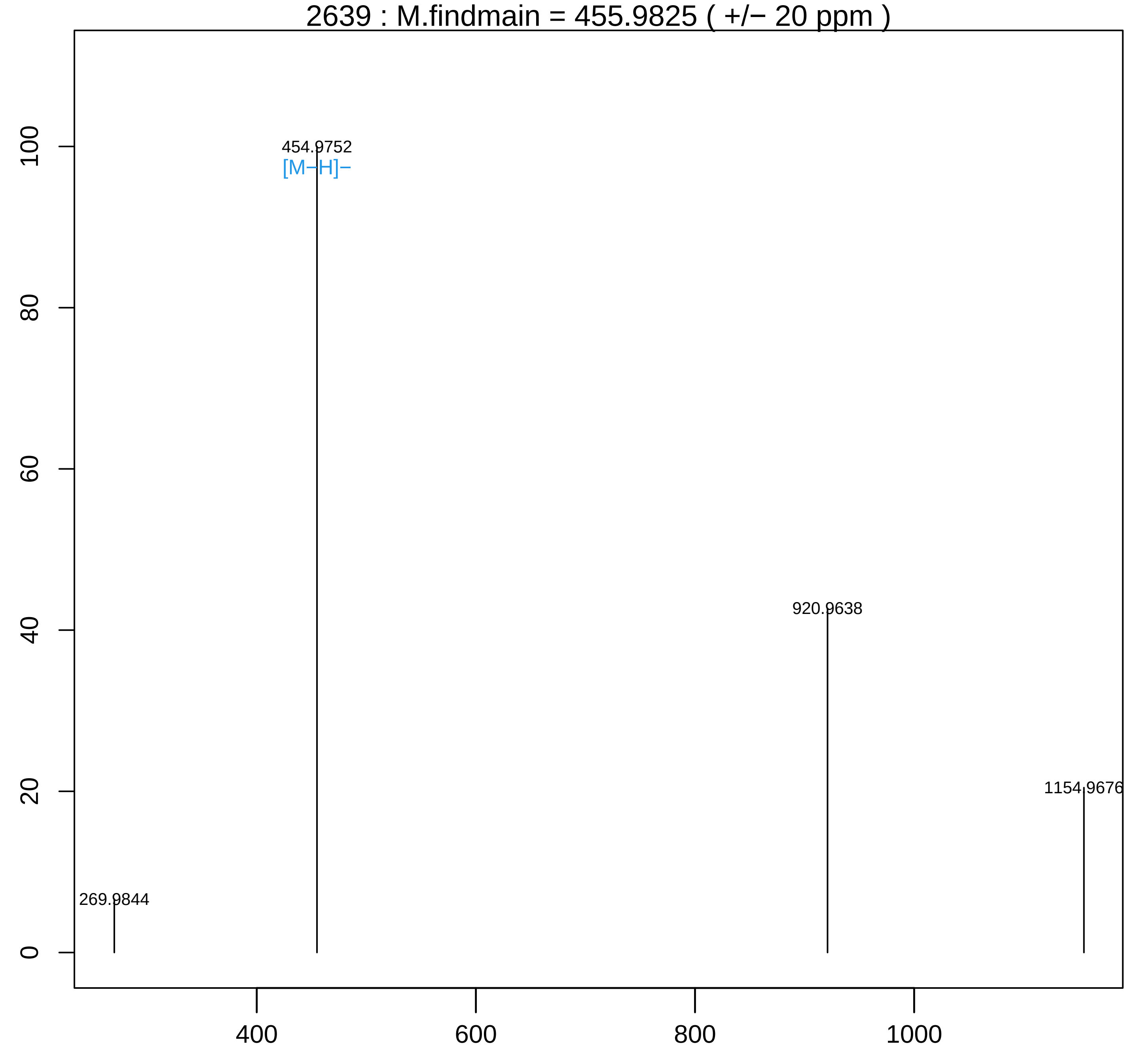
2639 : M.findmain = 455.9825 (+/− 20 ppm)
454.9752
[M−H]−
920.9638
1154.9676
269.9844
0
20
40
60
80
100
400
600
800
1000

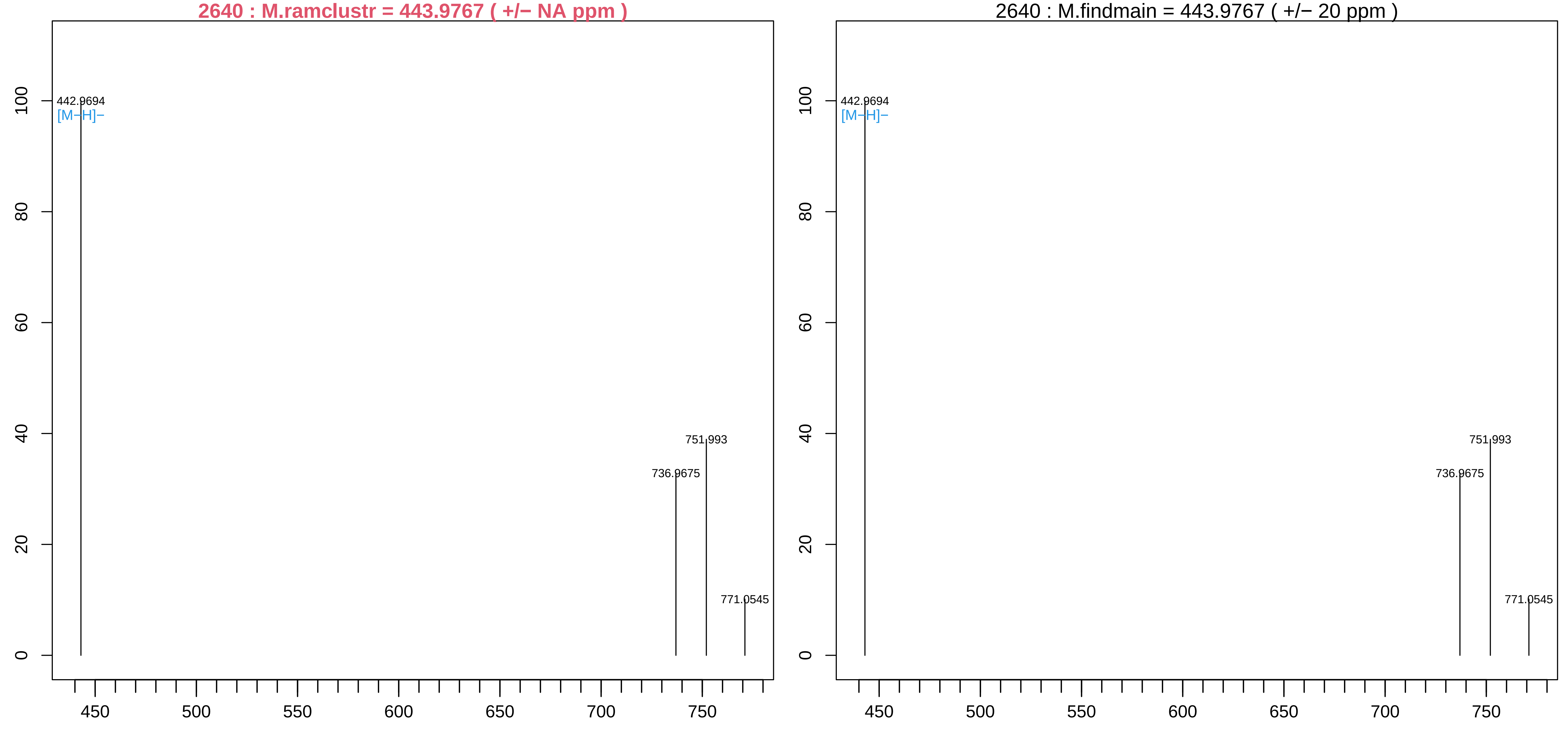
2640 : M.ramclustr = 443.9767 (+/− NA ppm)
442.9694
[M−H]−
751.993
736.9675
771.0545
2640 : M.findmain = 443.9767 (+/− 20 ppm)
442.9694
[M−H]−
751.993
736.9675
771.0545
0
20
40
60
80
100
450
500
550
600
650
700
750

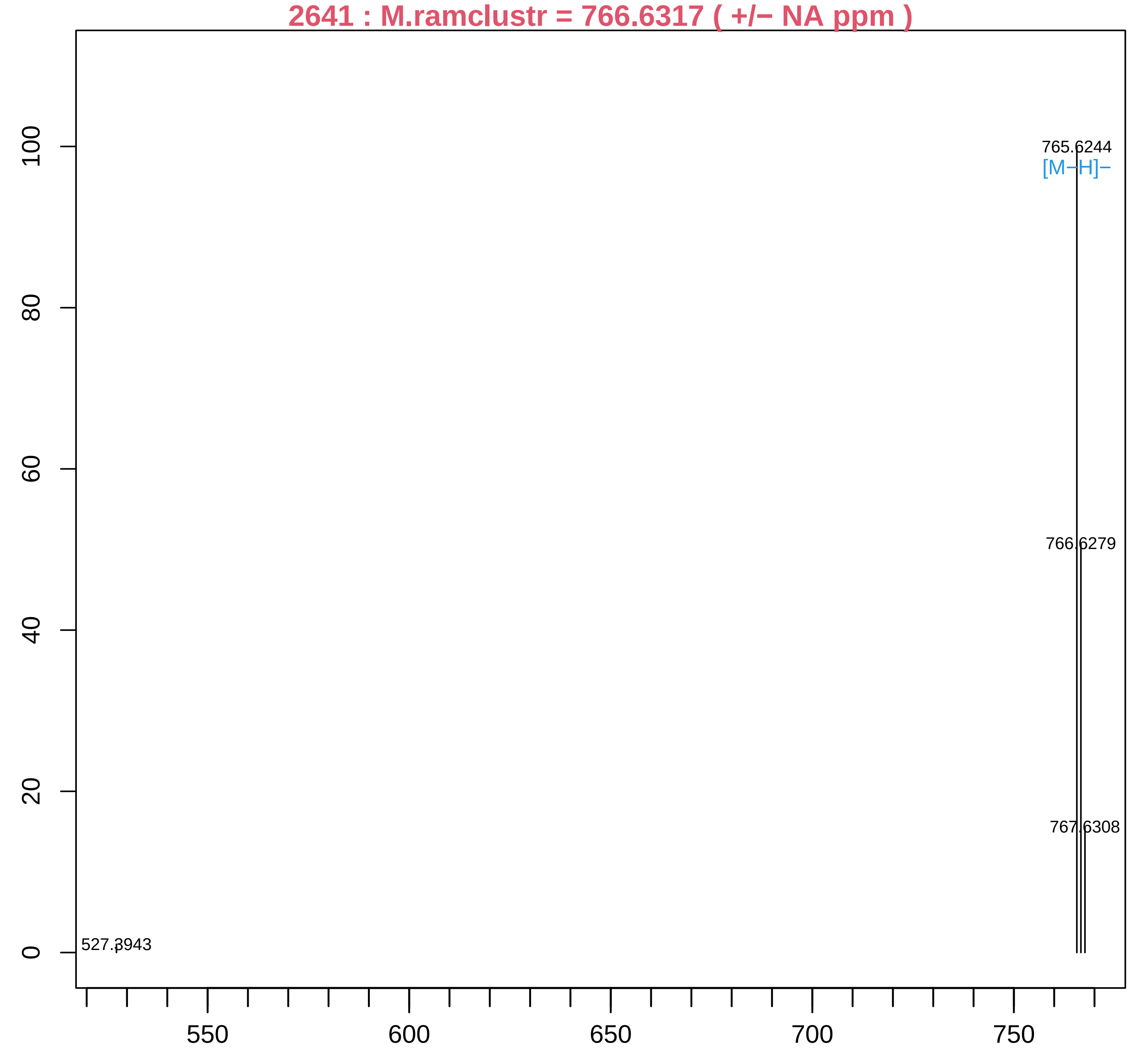
2641 : M.ramclustr = 766.6317 (+/− NA ppm)
765.6244
[M−H]−
766.6279
767.6308
527.3943
0
20
40
60
80
100
550
600
650
700
750

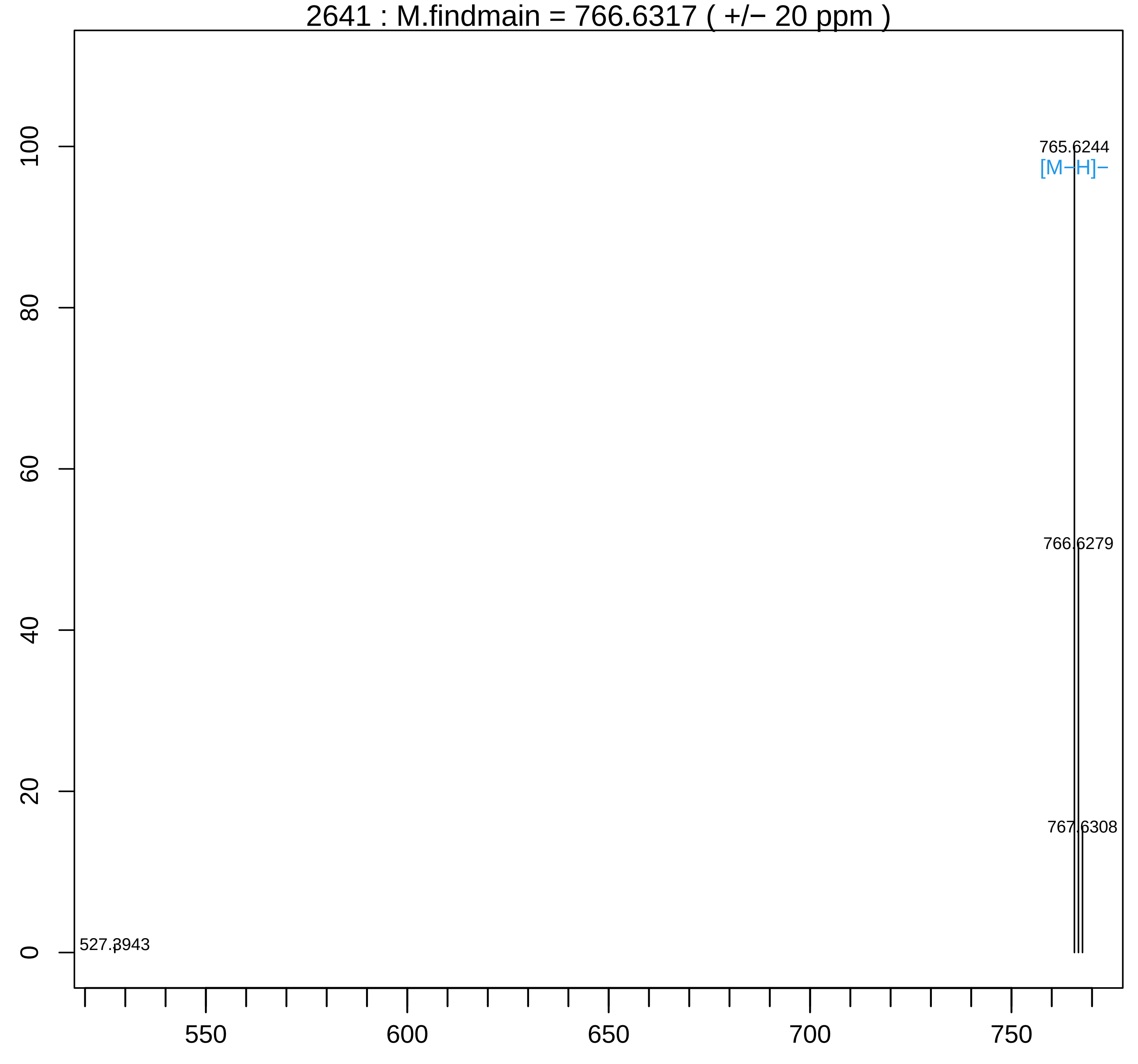
2641 : M.findmain = 766.6317 (+/− 20 ppm)
765.6244
[M−H]−
766.6279
767.6308
527.3943
0
20
40
60
80
100
550
600
650
700
750

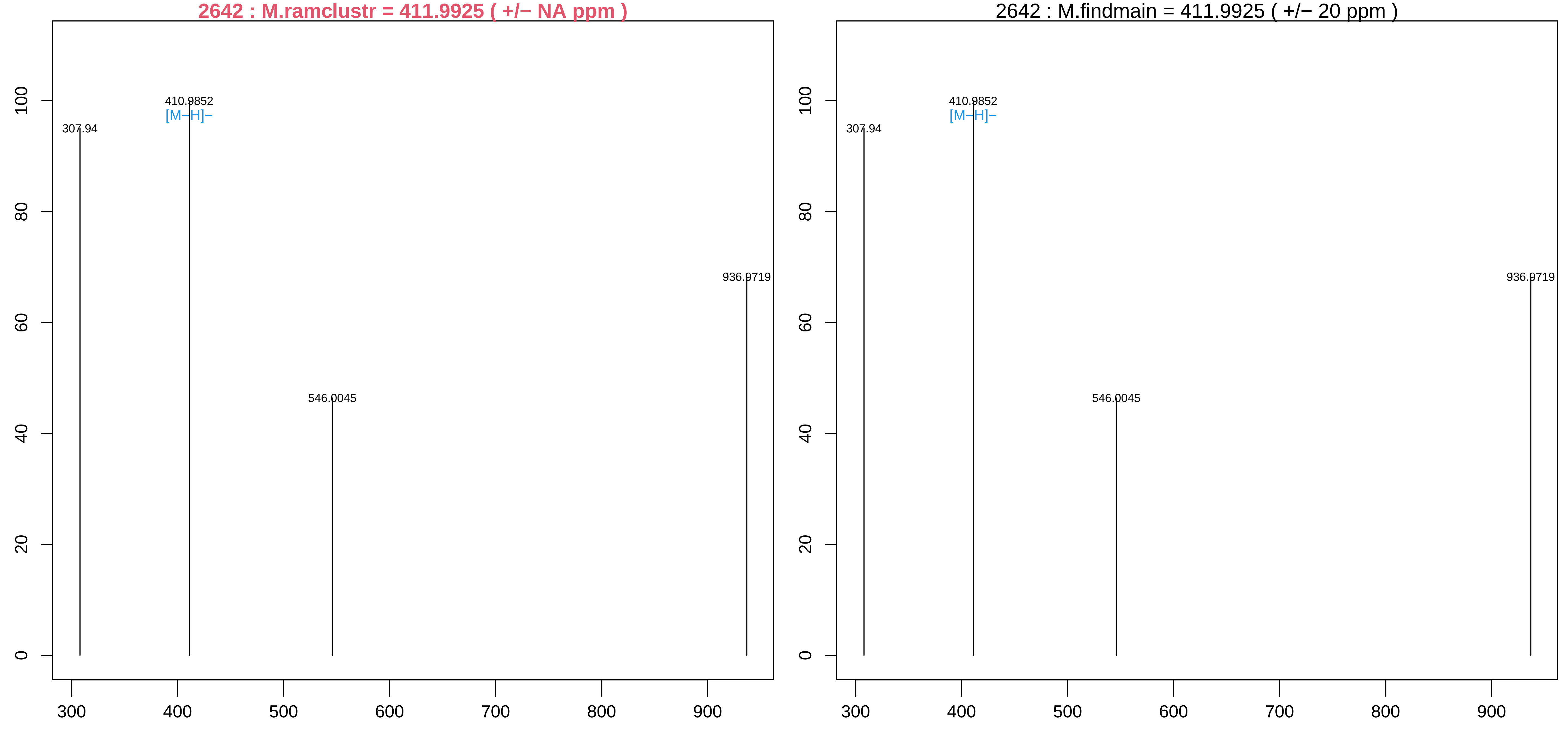
2642 : M.ramclustr = 411.9925 (+/− NA ppm)
307.94
410.9852
[M−H]−
546.0045
936.9719
2642 : M.findmain = 411.9925 (+/− 20 ppm)
307.94
410.9852
[M−H]−
546.0045
936.9719
0
20
40
60
80
100
300
400
500
600
700
800
900

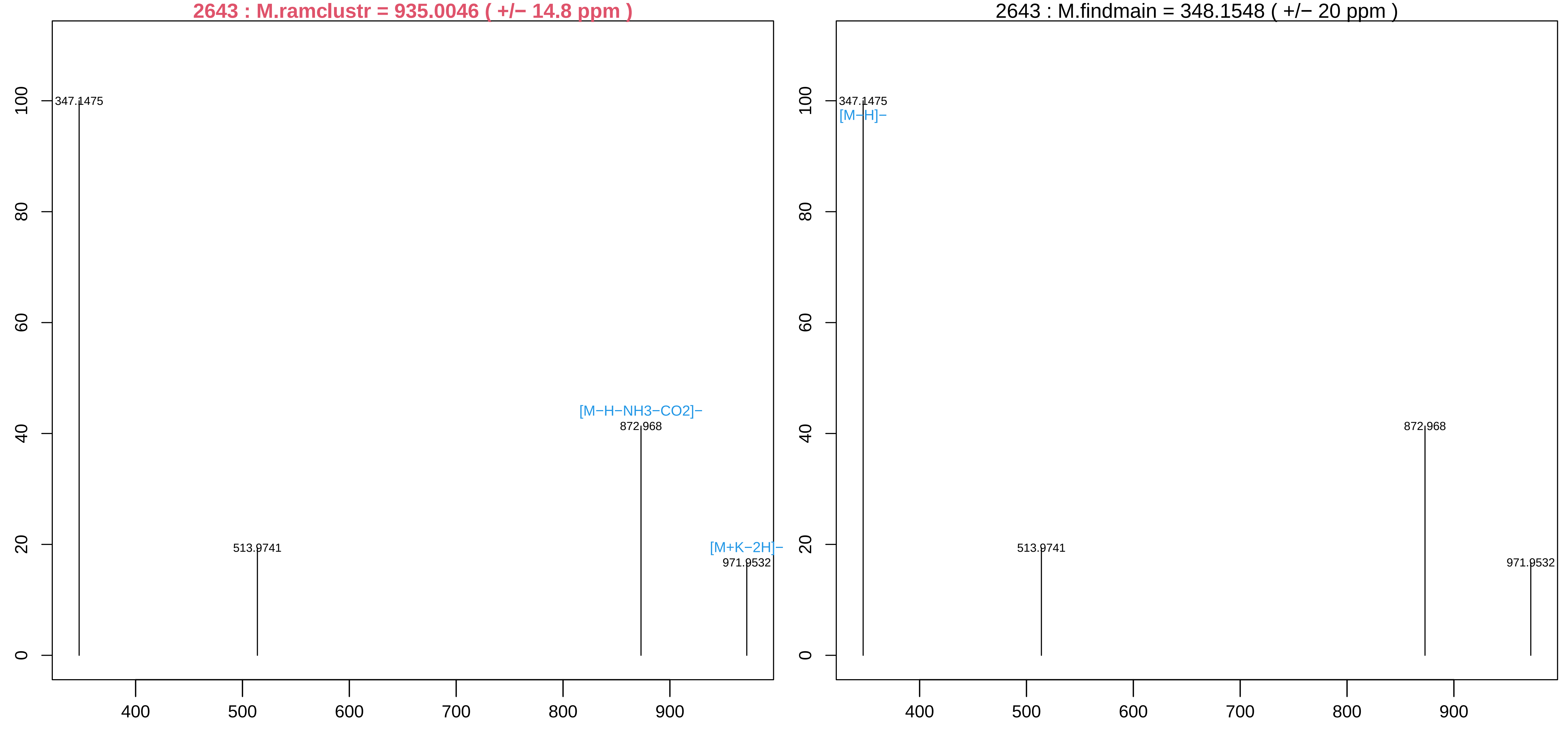
2643 : M.ramclustr = 935.0046 (+/− 14.8 ppm)
347.1475
513.9741
[M−H−NH3−CO2]−
872.968
[M+K−2H]−
971.9532
2643 : M.findmain = 348.1548 (+/− 20 ppm)
347.1475
[M−H]−
513.9741
872.968
971.9532
0
20
40
60
80
100
400
500
600
700
800
900

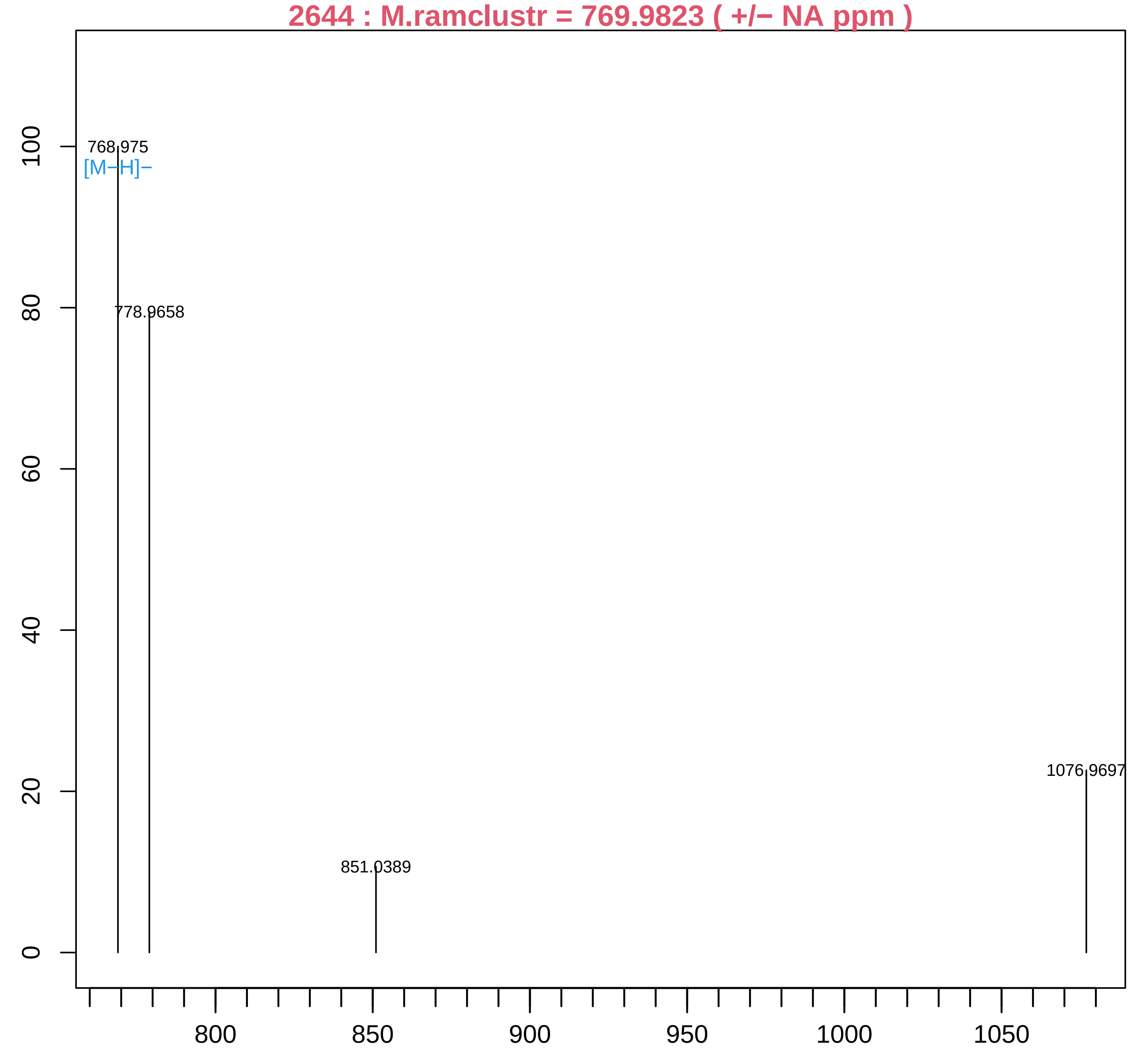
2644 : M.ramclustr = 769.9823 (+/− NA ppm)
768.975
[M−H]−
778.9658
851.0389
1076.9697
0
20
40
60
80
100
800
850
900
950
1000
1050

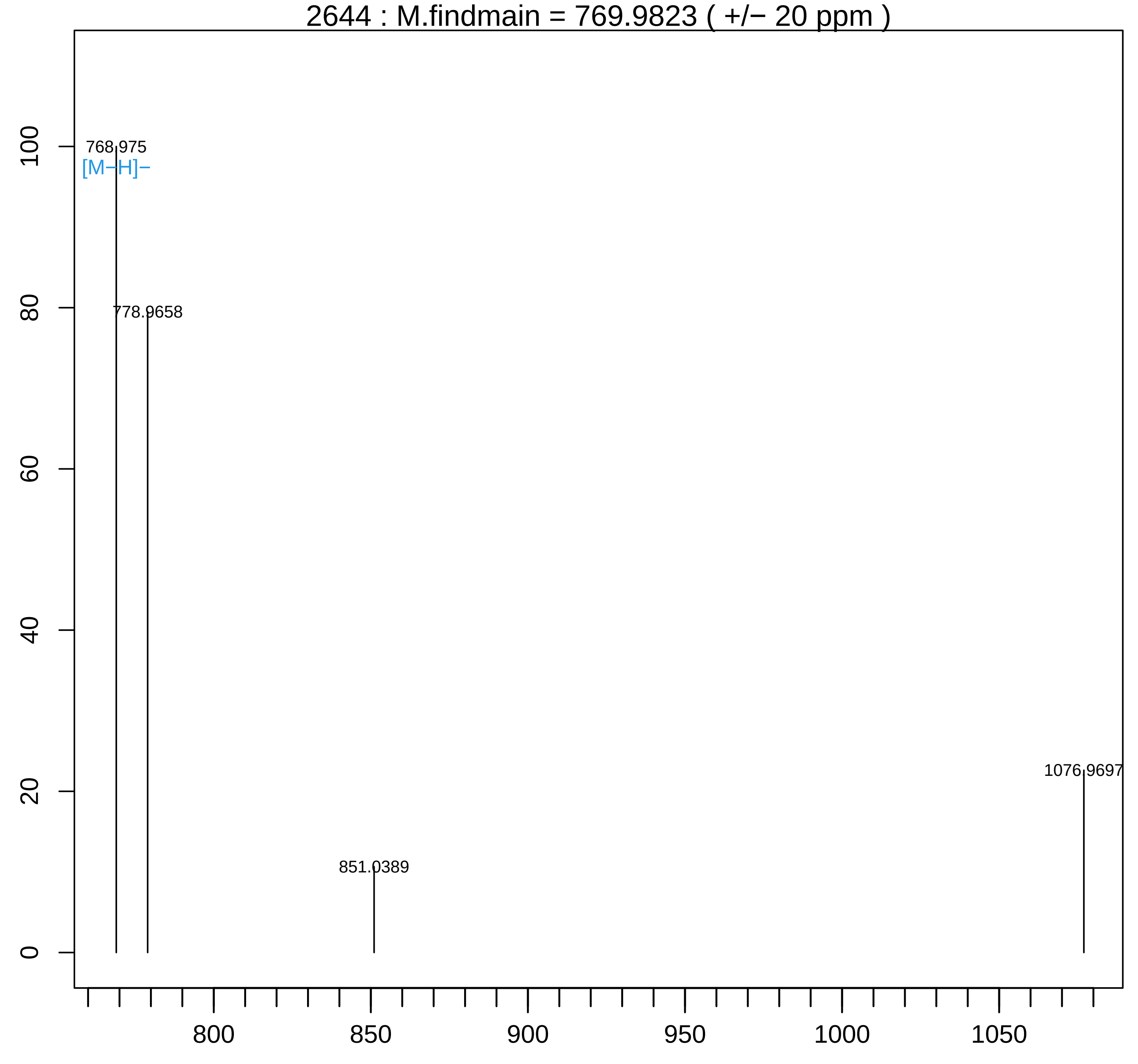
2644 : M.findmain = 769.9823 (+/− 20 ppm)
768.975
[M−H]−
778.9658
851.0389
1076.9697
0
20
40
60
80
100
800
850
900
950
1000
1050

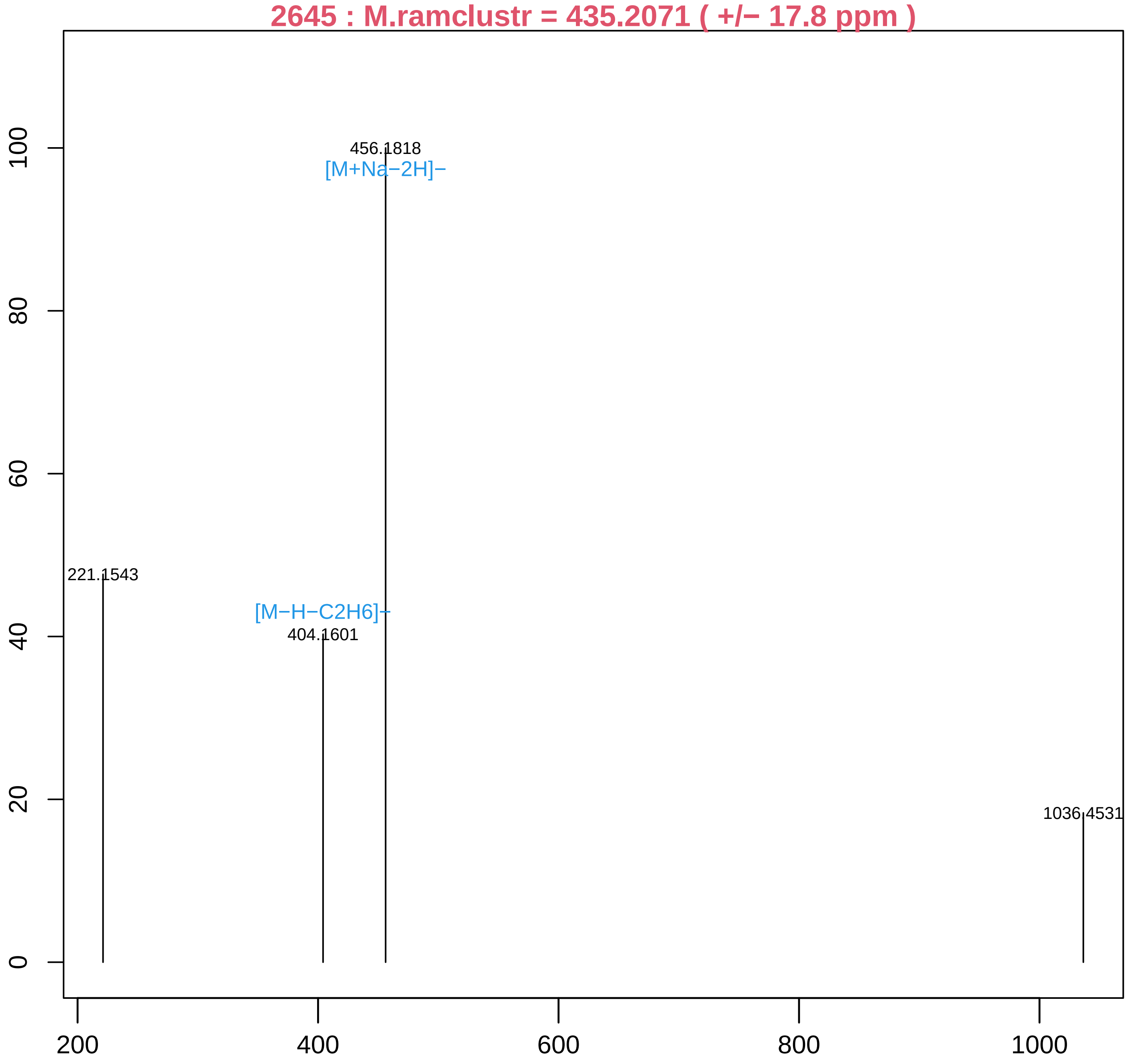

2645 : M.ramclustr = 435.2071 (+/− 17.8 ppm)
456.1818
[M+Na−2H]−
221.1543
[M−H−C2H6]−
404.1601
1036.4531
0
20
40
60
80
100
200
400
600
800
1000

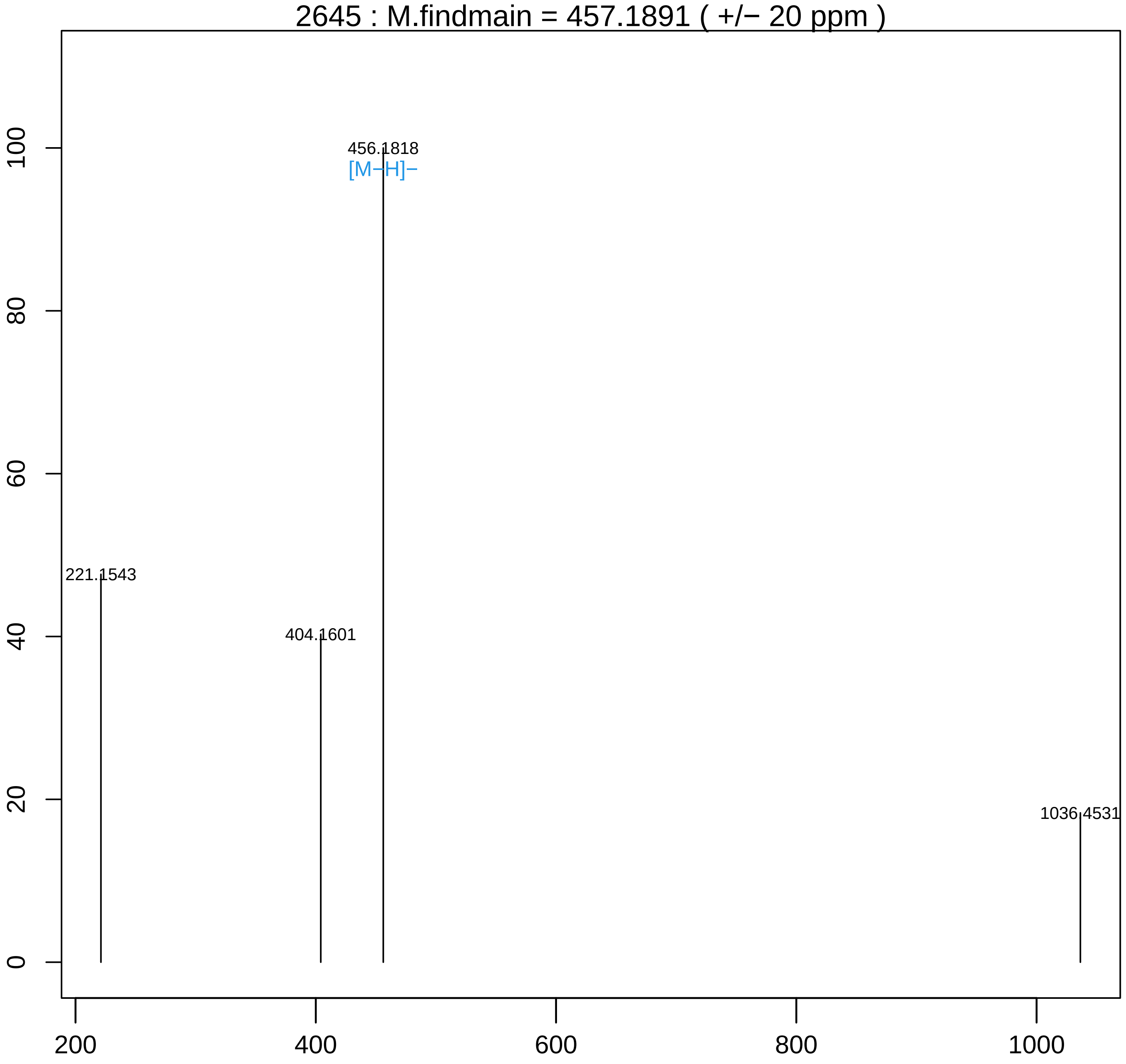

2645 : M.findmain = 457.1891 (+/− 20 ppm)
456.1818
[M−H]−
221.1543
404.1601
1036.4531
0
20
40
60
80
100
200
400
600
800
1000

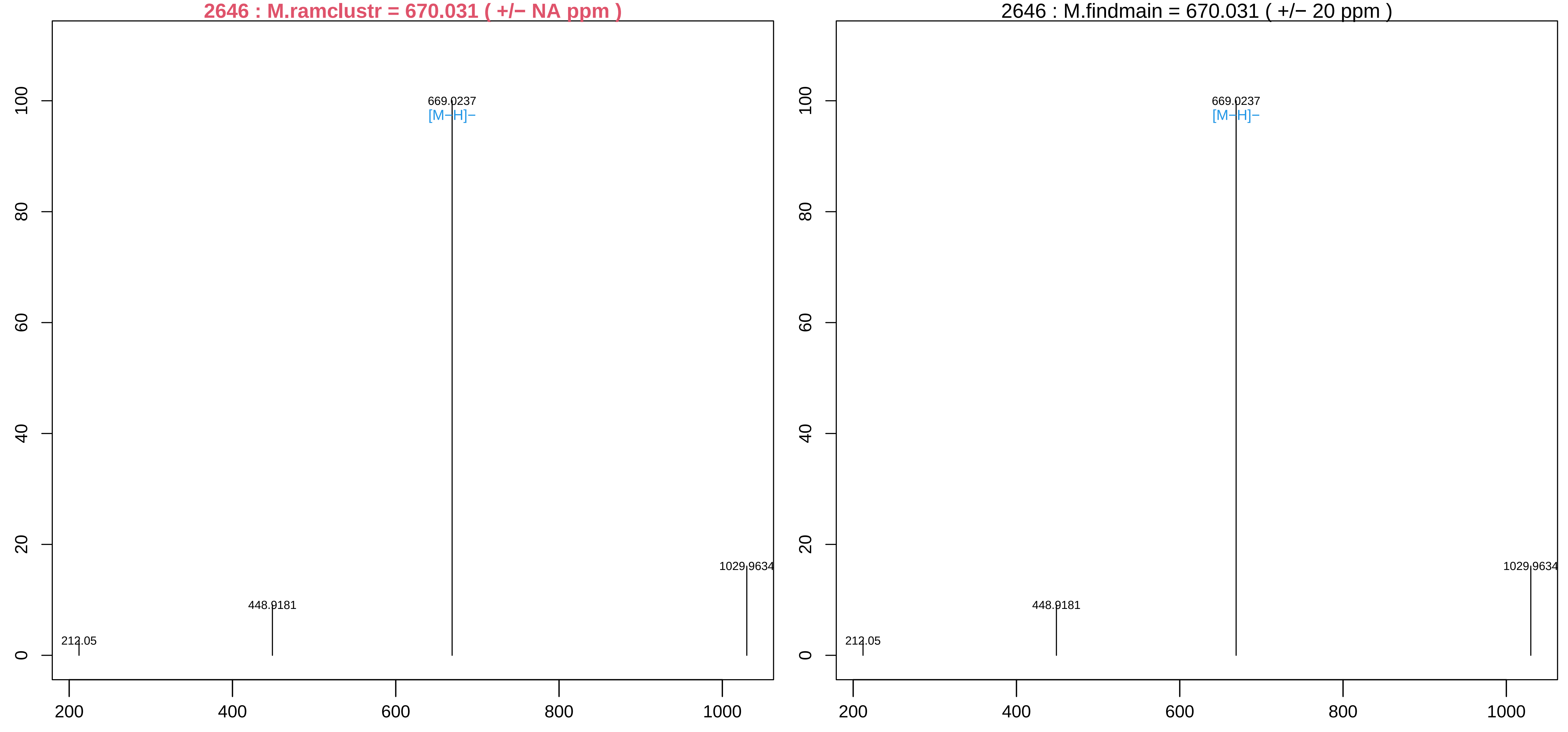
2646 : M.ramclustr = 670.031 (+/− NA ppm)
2646 : M.findmain = 670.031 (+/− 20 ppm)
669.0237
[M−H]−
1029.9634
448.9181
212.05
0
20
40
60
80
100
200
400
600
800
1000

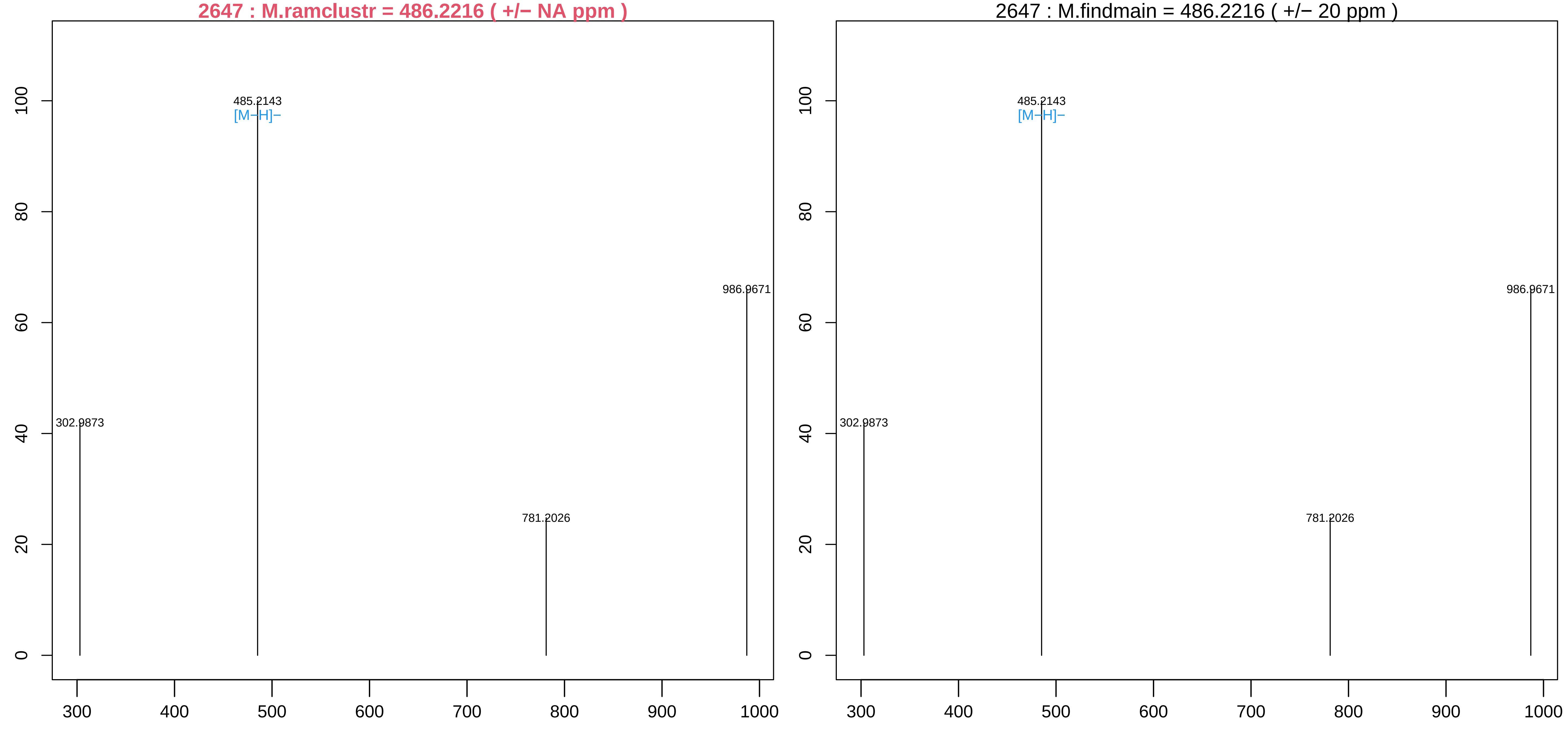
2647 : M.ramclustr = 486.2216 (+/− NA ppm)
485.2143
[M−H]−
986.9671
302.9873
781.2026
2647 : M.findmain = 486.2216 (+/− 20 ppm)
485.2143
[M−H]−
986.9671
302.9873
781.2026
0
20
40
60
80
100
300
400
500
600
700
800
900
1000

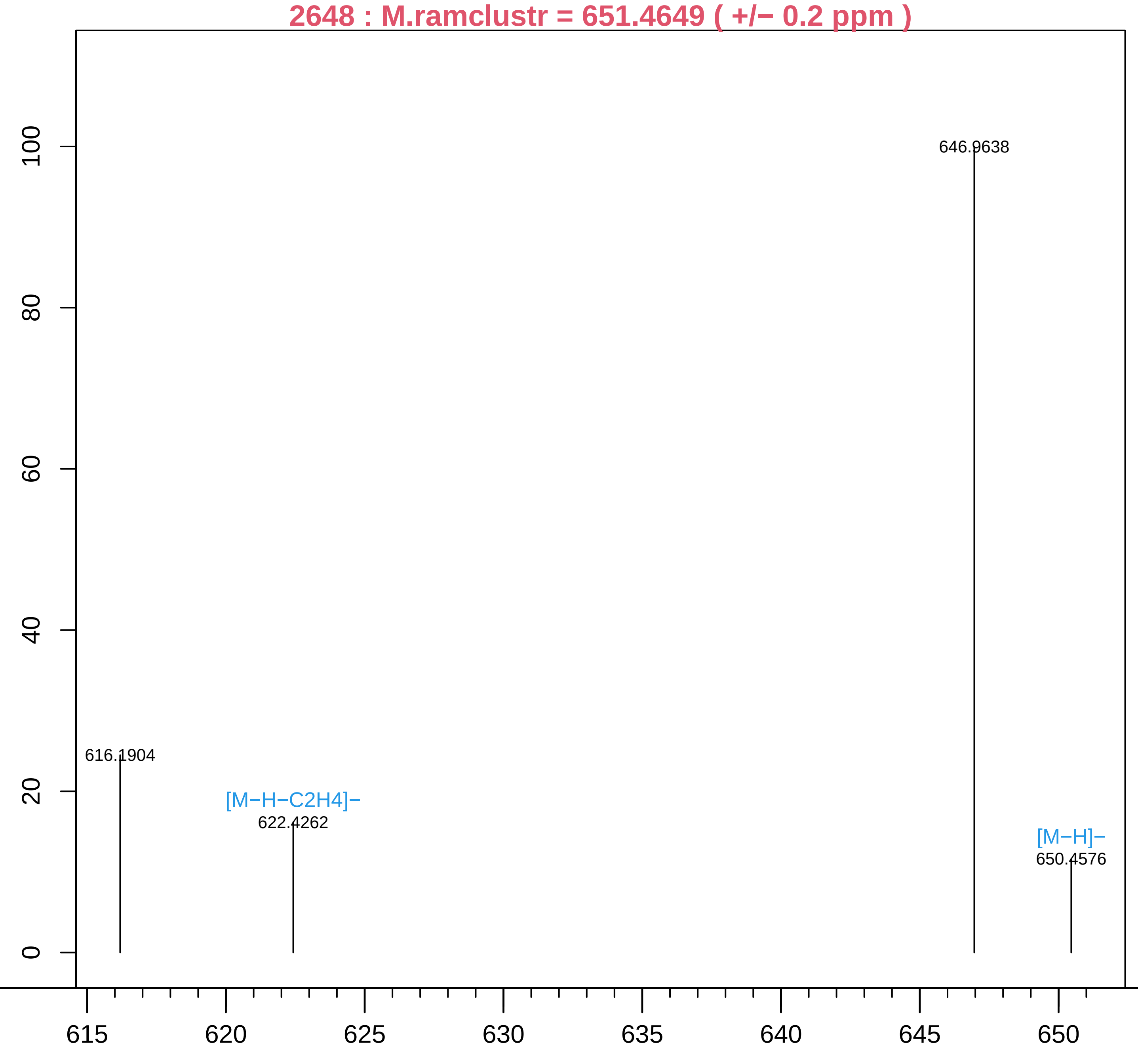
2648 : M.ramclustr = 651.4649 (+/− 0.2 ppm)
646.9638
616.1904
[M−H−C2H4]−
622.4262
[M−H]−
650.4576
0
20
40
60
80
100
615
620
625
630
635
640
645
650

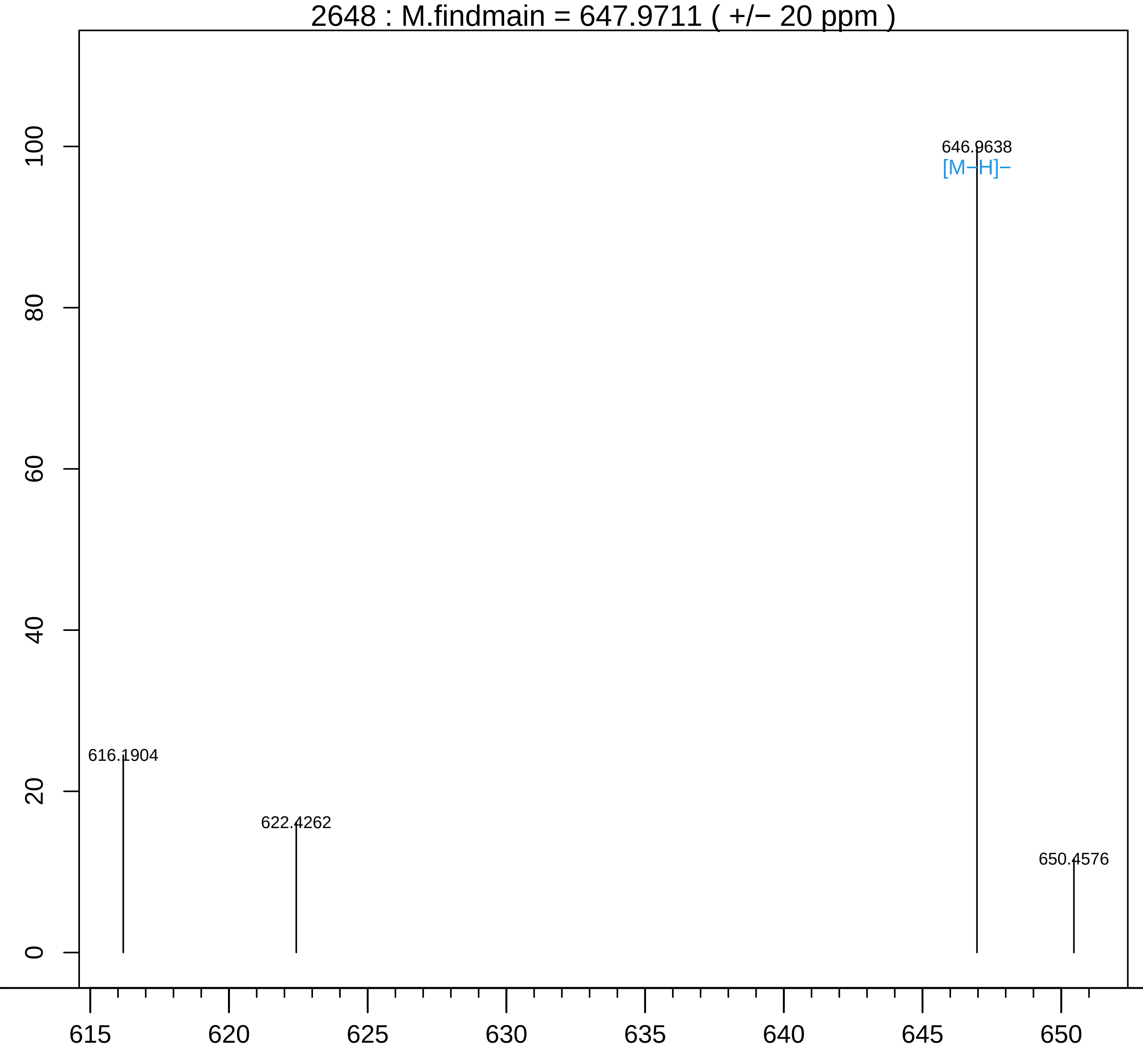
2648 : M.findmain = 647.9711 (+/− 20 ppm)
646.9638
[M−H]−
616.1904
622.4262
650.4576
0
20
40
60
80
100
615
620
625
630
635
640
645
650

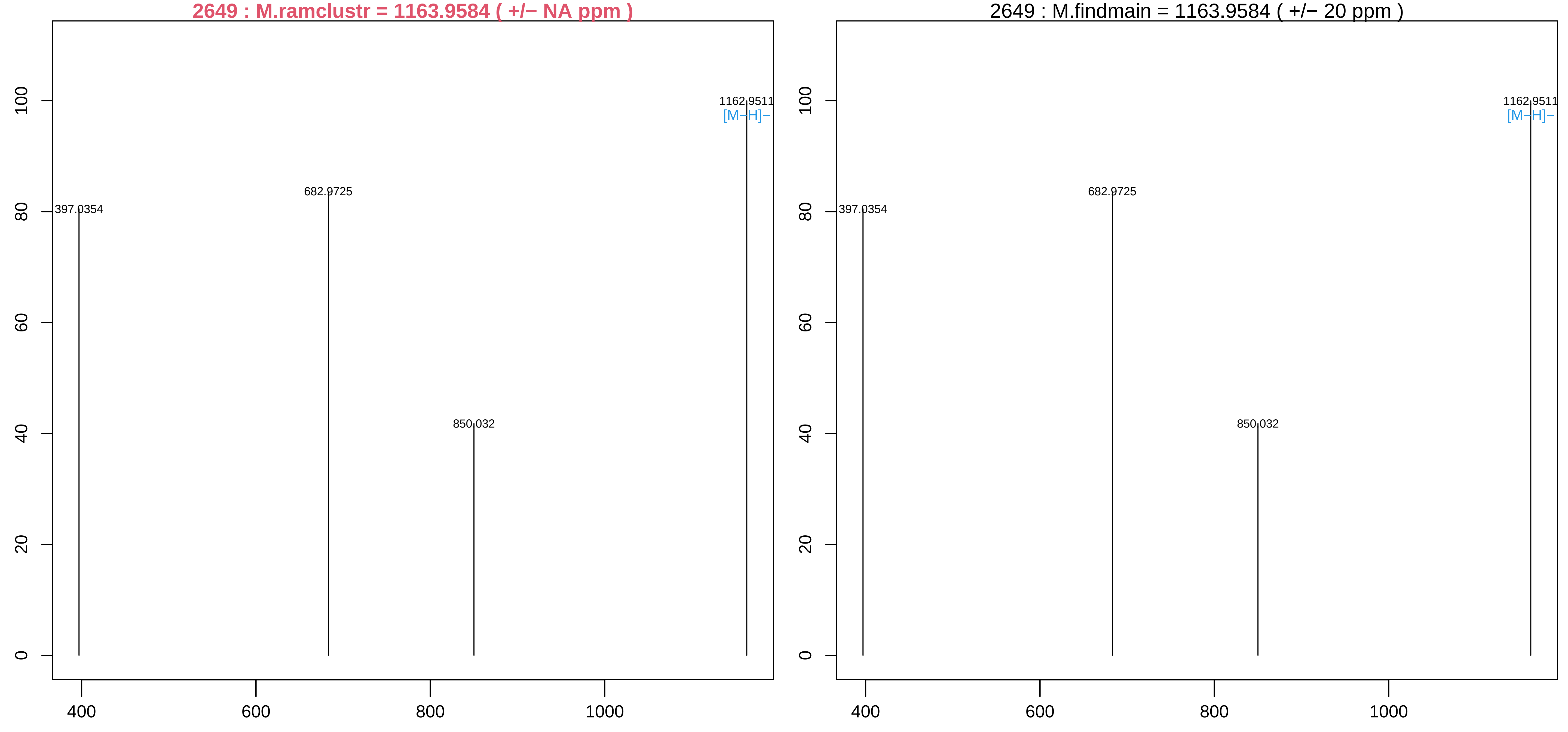

2649 : M.ramclustr = 1163.9584 (+/− NA ppm)
2649 : M.findmain = 1163.9584 (+/− 20 ppm)
1162.9511
[M−H]−
682.9725
397.0354
850.032
0
20
40
60
80
100
400
600
800
1000

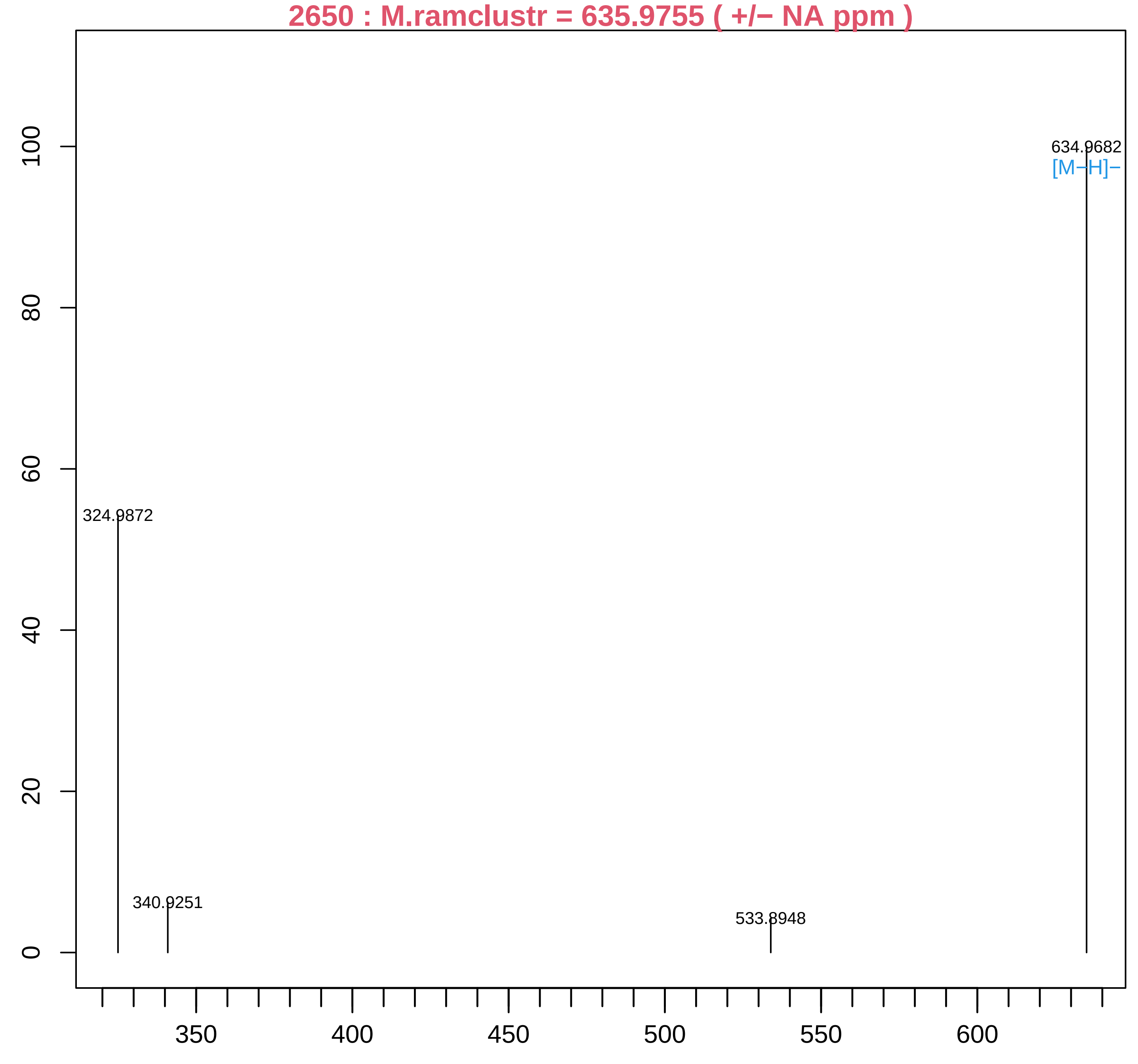
2650 : M.ramclustr = 635.9755 (+/− NA ppm)
634.9682
[M−H]−
324.9872
340.9251
533.8948
0
20
40
60
80
100
350
400
450
500
550
600

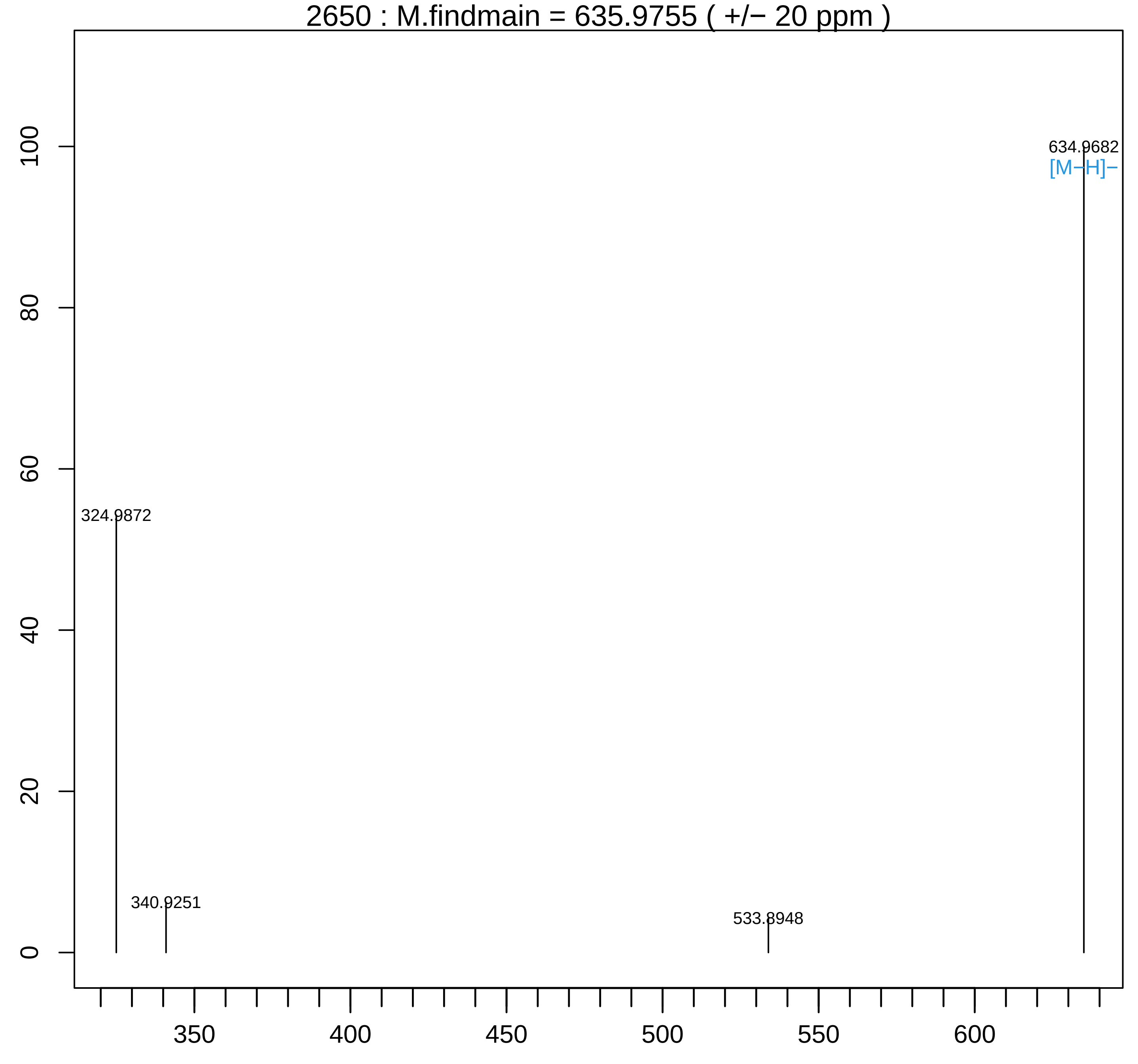
2650 : M.findmain = 635.9755 (+/− 20 ppm)
634.9682
[M−H]−
324.9872
340.9251
533.8948
0
20
40
60
80
100
350
400
450
500
550
600

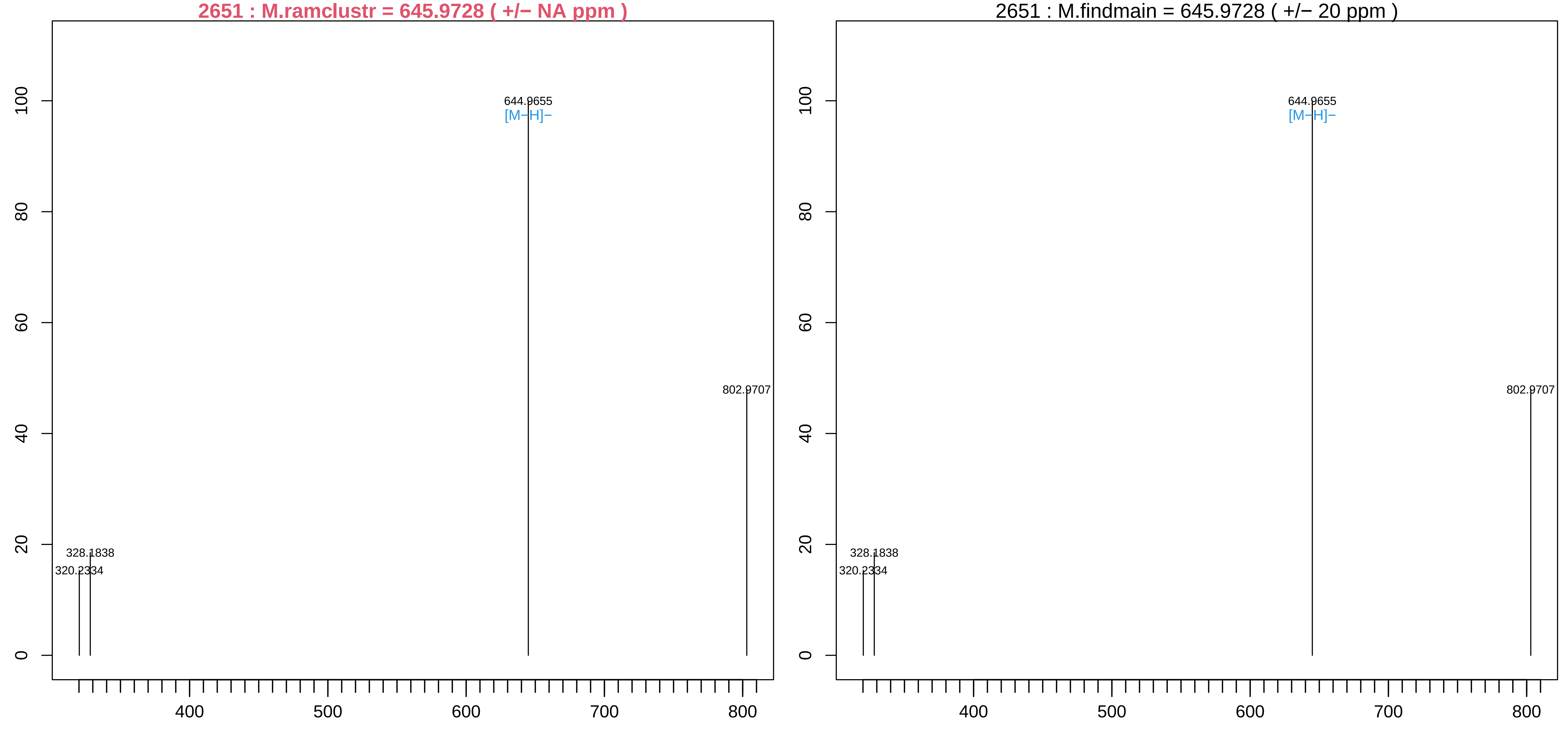
2651 : M.ramclustr = 645.9728 (+/− NA ppm)
2651 : M.findmain = 645.9728 (+/− 20 ppm)
644.9655
[M−H]−
802.9707
328.1838
320.2334
0
20
40
60
80
100
400
500
600
700
800

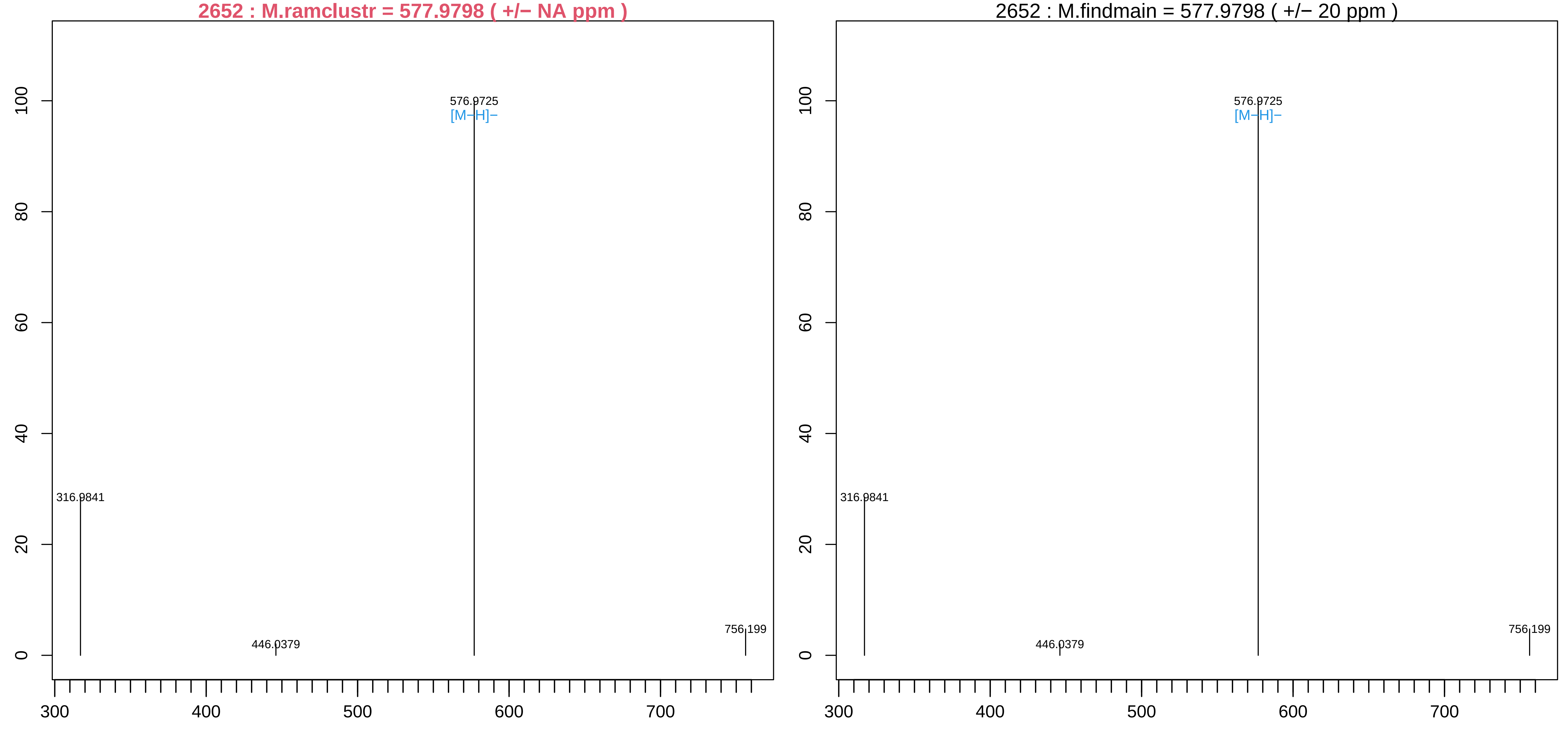

2652 : M.ramclustr = 577.9798 (+/− NA ppm)
2652 : M.findmain = 577.9798 (+/− 20 ppm)
576.9725
[M−H]−
316.9841
446.0379
756.199
0
20
40
60
80
100
300
400
500
600
700

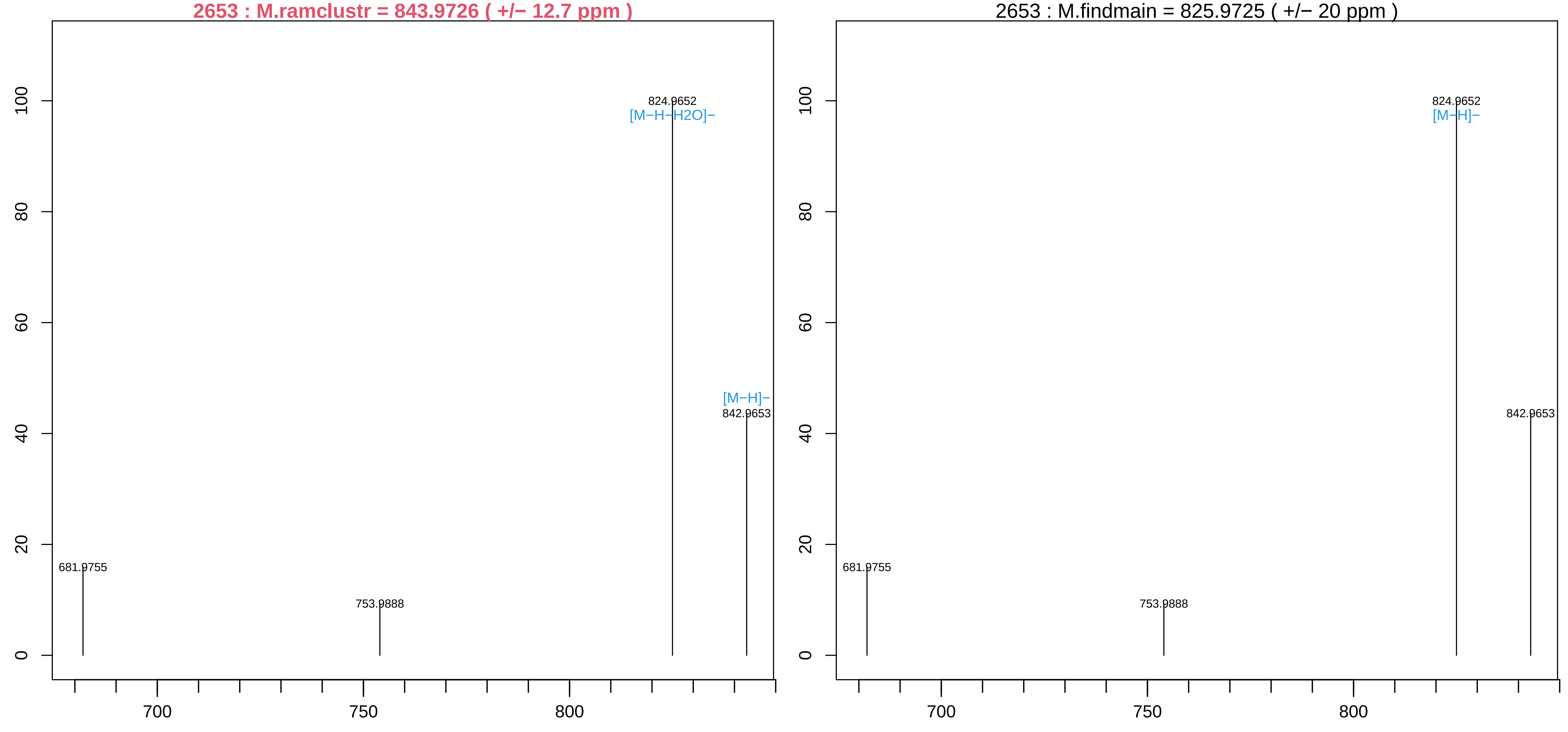
2653 : M.ramclustr = 843.9726 (+/− 12.7 ppm)
824.9652
[M−H−H2O]−
[M−H]−
842.9653
681.9755
753.9888
0
20
40
60
80
100
700
750
800
2653 : M.findmain = 825.9725 (+/− 20 ppm)
824.9652
[M−H]−
842.9653
681.9755
753.9888
0
20
40
60
80
100
700
750
800

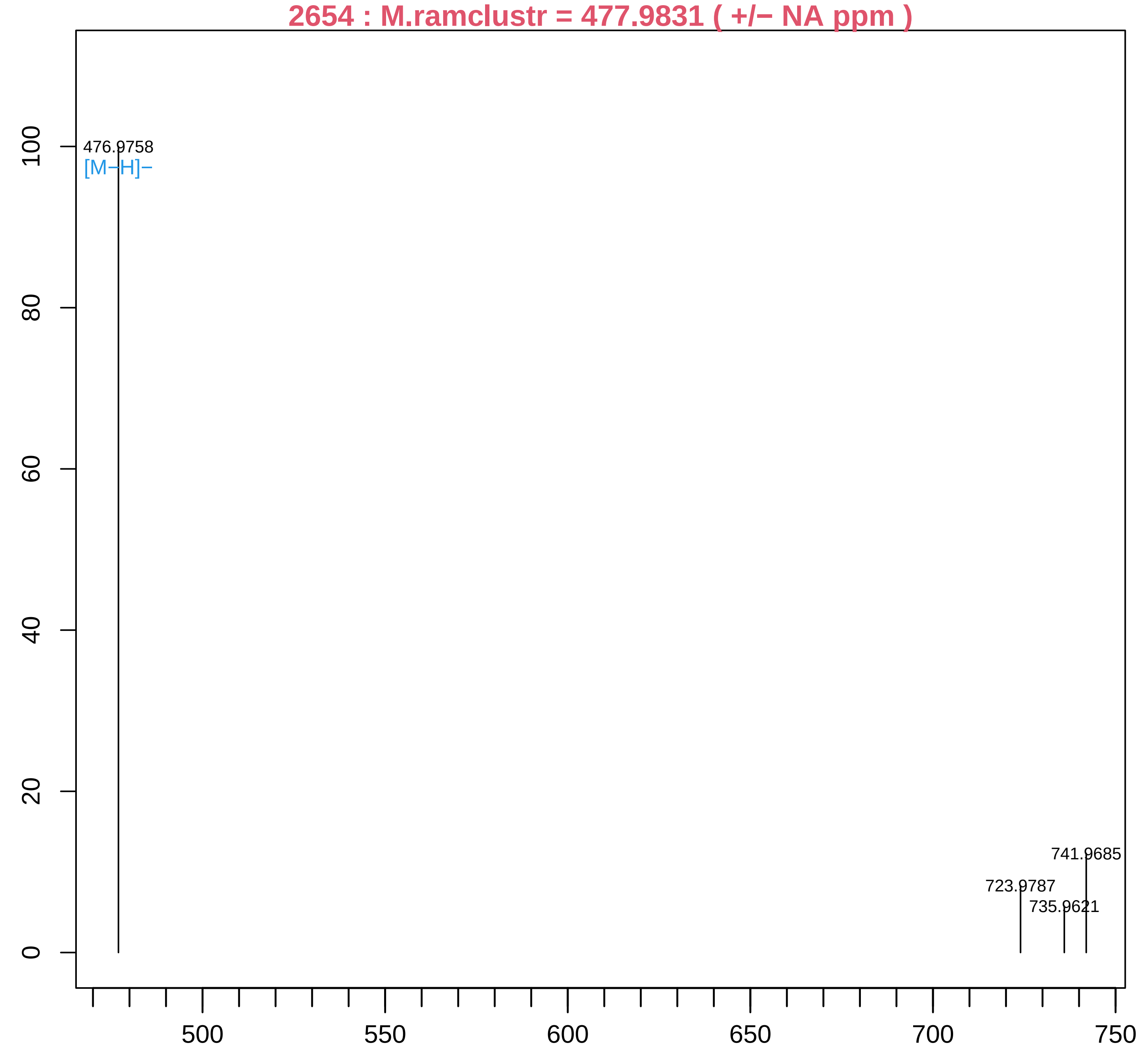

2654 : M.ramclustr = 477.9831 (+/− NA ppm)
476.9758
[M−H]−
723.9787
735.9621
741.9685
0
20
40
60
80
100
500
550
600
650
700
750

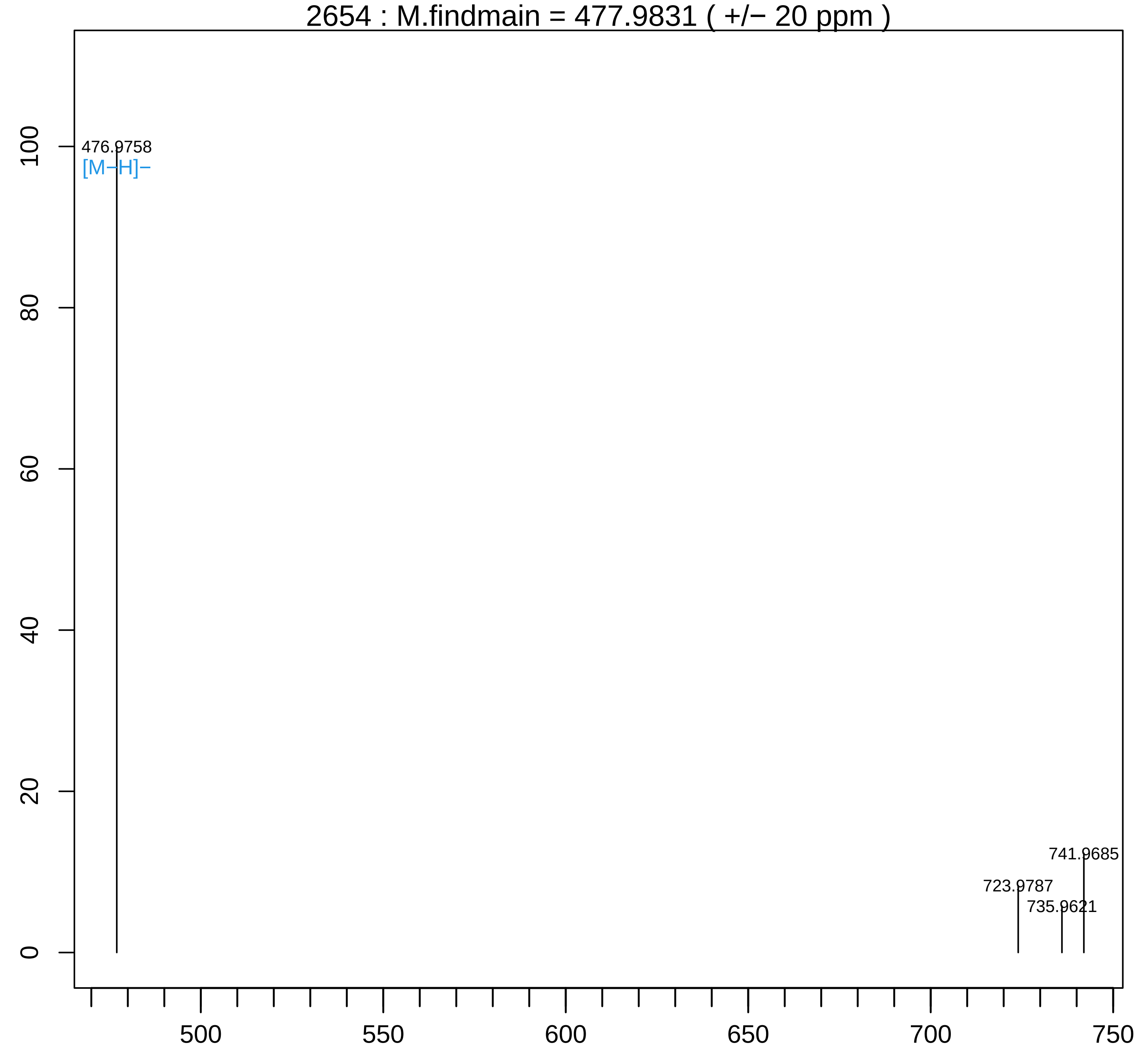

2654 : M.findmain = 477.9831 (+/− 20 ppm)
476.9758
[M−H]−
723.9787
735.9621
741.9685
0
20
40
60
80
100
500
550
600
650
700
750

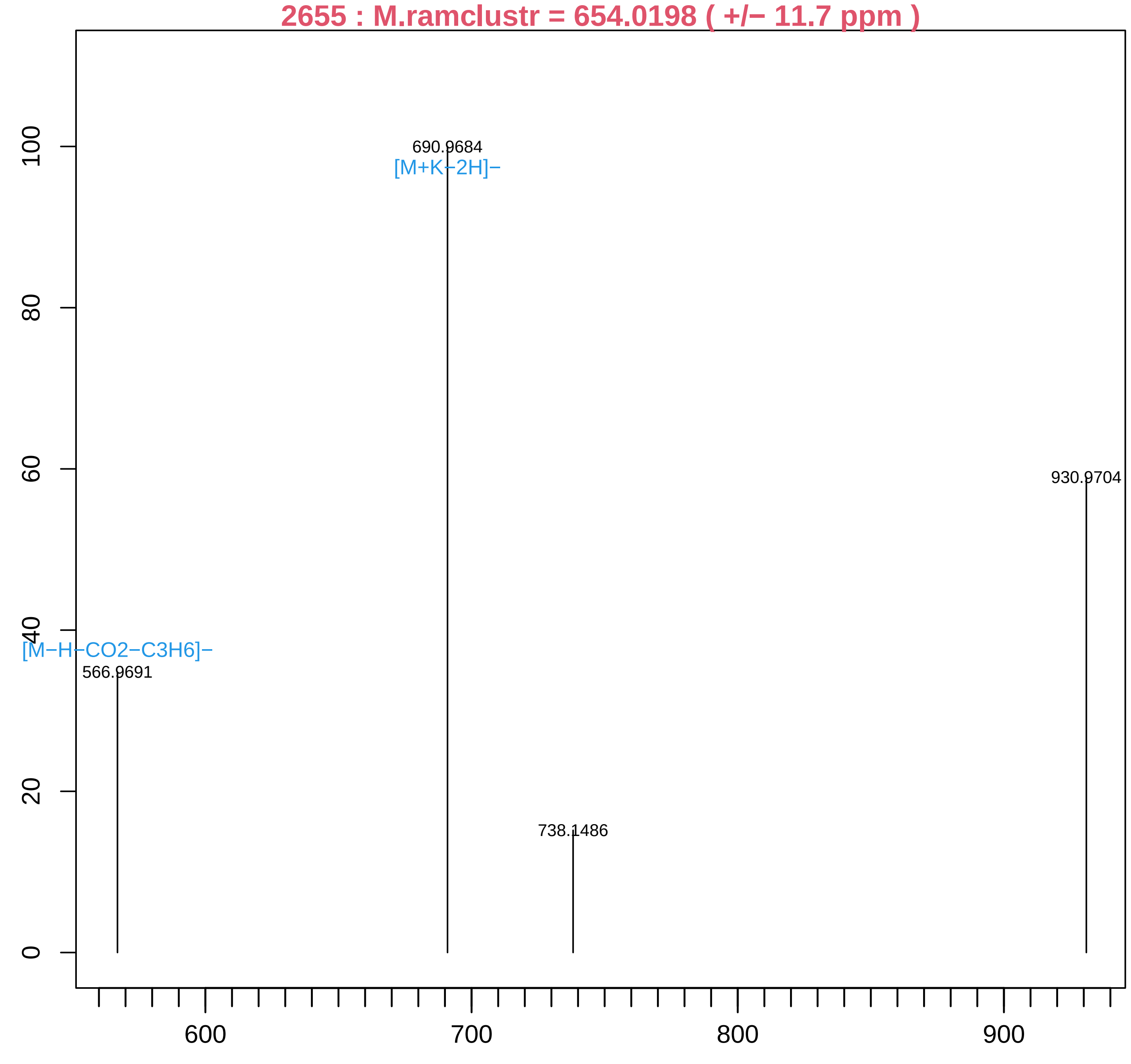
2655 : M.ramclustr = 654.0198 (+/− 11.7 ppm)
690.9684
[M+K−2H]−
930.9704
[M−H−CO2−C3H6]−
566.9691
738.1486
0
20
40
60
80
100
600
700
800
900

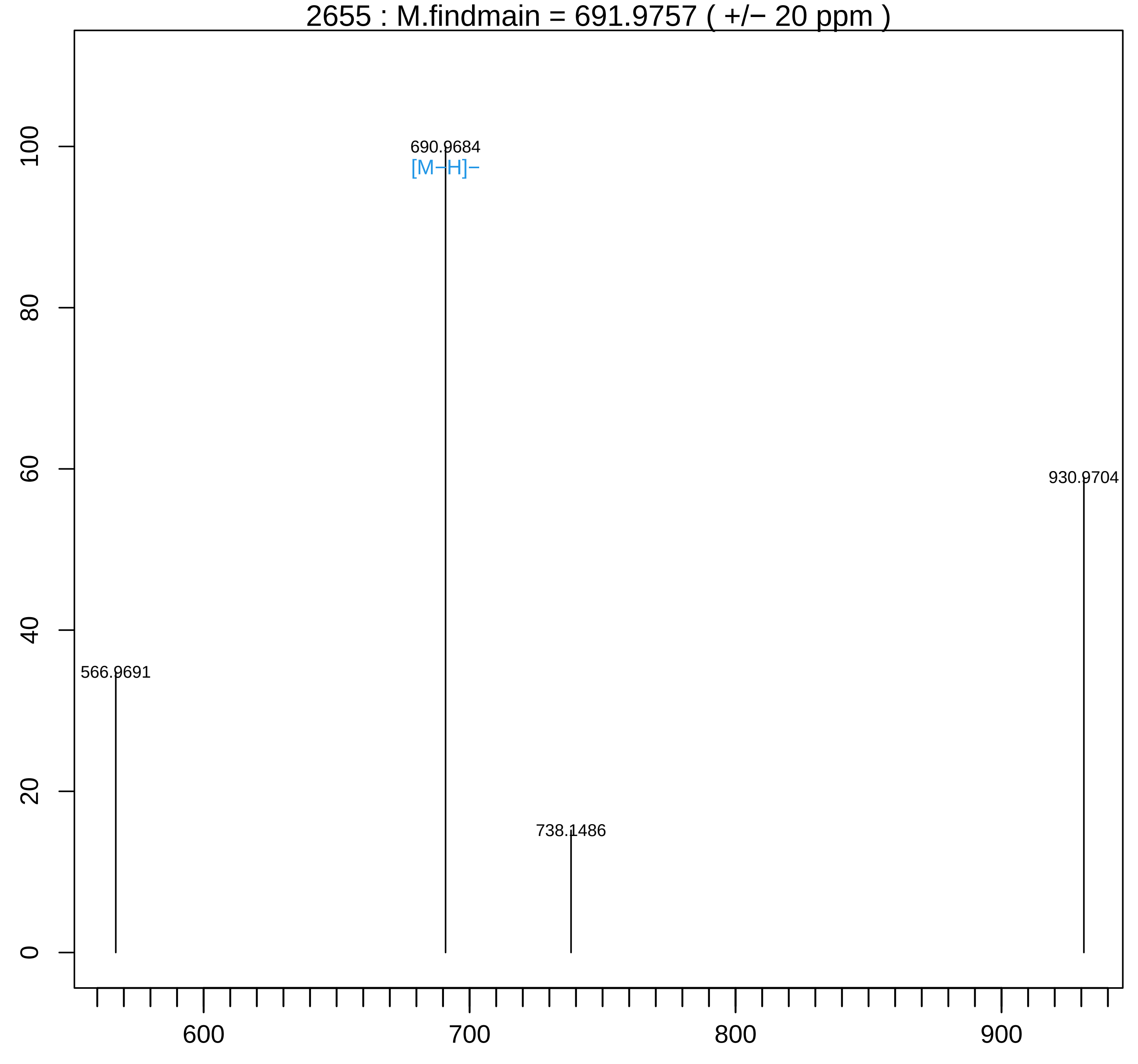
2655 : M.findmain = 691.9757 (+/− 20 ppm)
690.9684
[M−H]−
930.9704
566.9691
738.1486
0
20
40
60
80
100
600
700
800
900

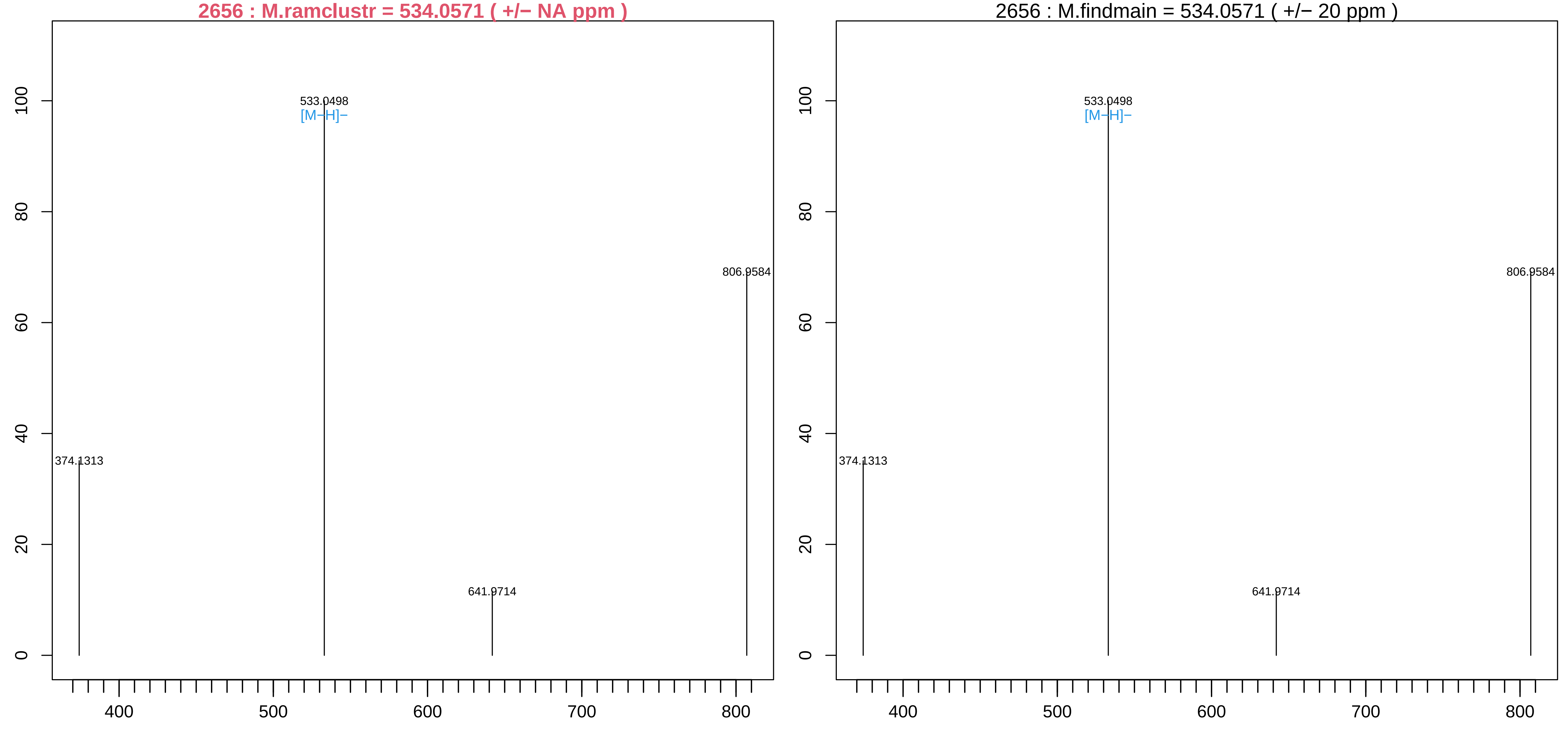

2656 : M.ramclustr = 534.0571 (+/− NA ppm)
533.0498
[M−H]−
806.9584
374.1313
641.9714
0
20
40
60
80
100
400
500
600
700
800
2656 : M.findmain = 534.0571 (+/− 20 ppm)
533.0498
[M−H]−
806.9584
374.1313
641.9714
0
20
40
60
80
100
400
500
600
700
800

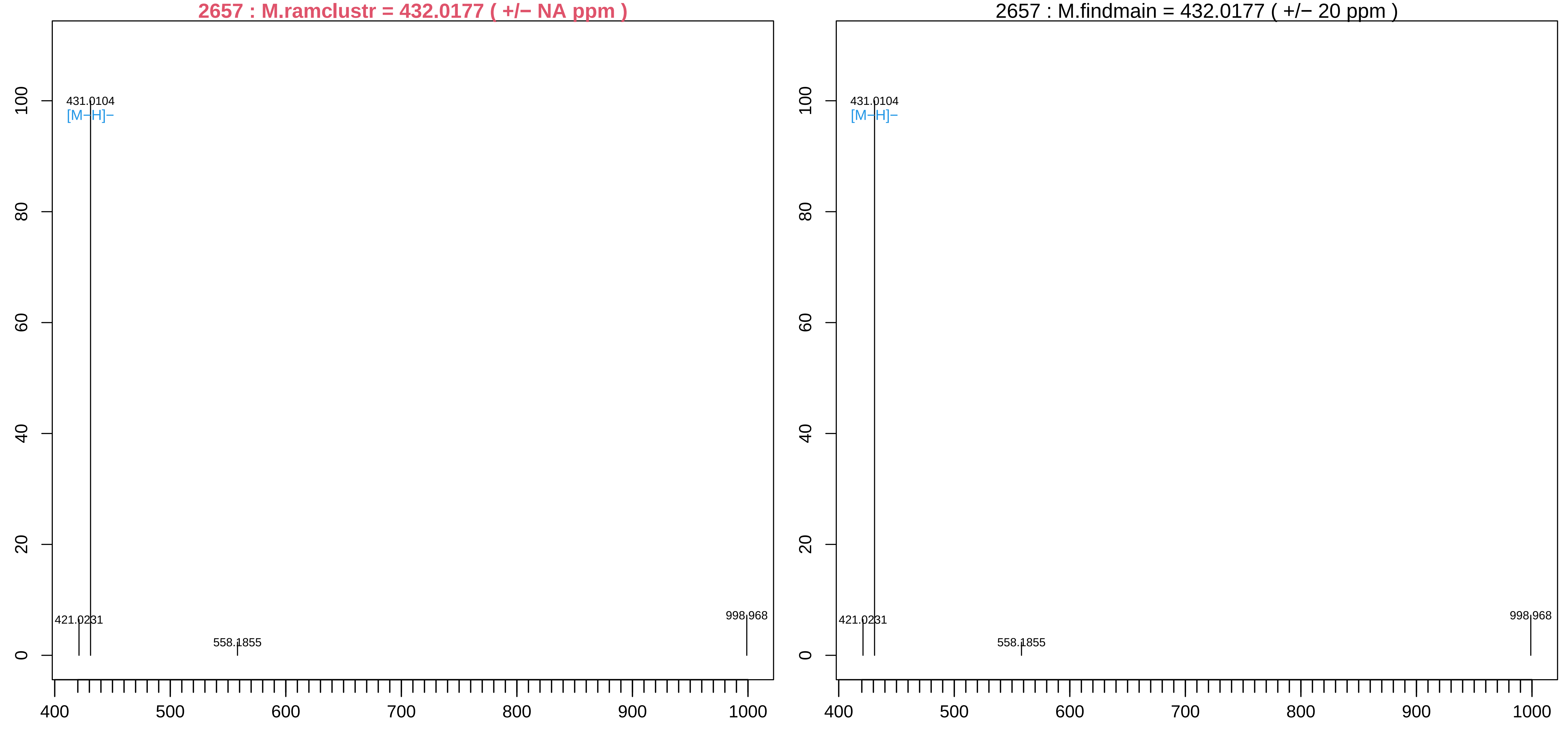
2657 : M.ramclustr = 432.0177 (+/− NA ppm)
431.0104
[M−H]−
421.0231
558.1855
998.968
2657 : M.findmain = 432.0177 (+/− 20 ppm)
431.0104
[M−H]−
421.0231
558.1855
998.968
0
20
40
60
80
100
400
500
600
700
800
900
1000

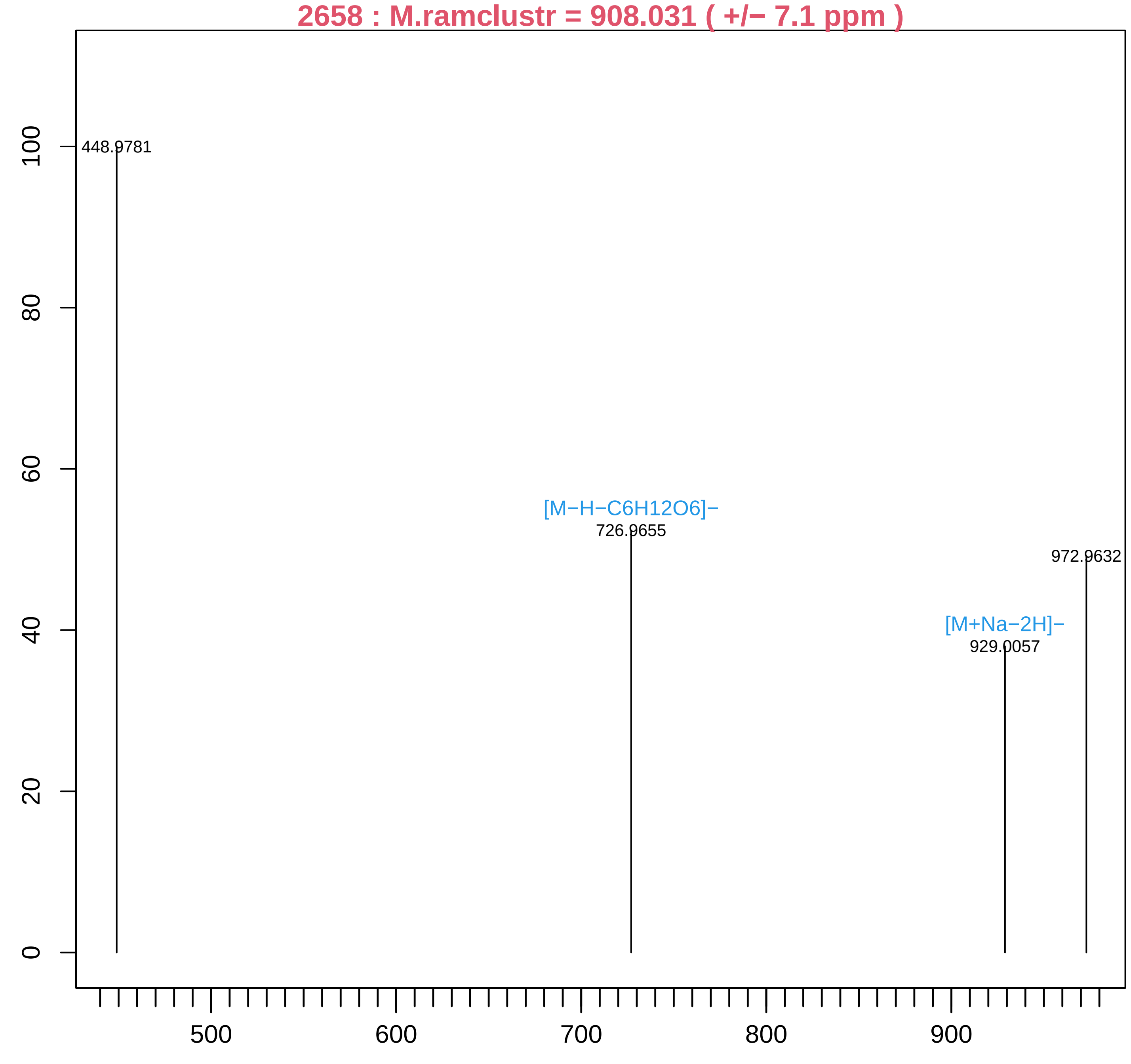
2658 : M.ramclustr = 908.031 (+/− 7.1 ppm)
448.9781
[M−H−C6H12O6]−
726.9655
972.9632
[M+Na−2H]−
929.0057
0
20
40
60
80
100
500
600
700
800
900

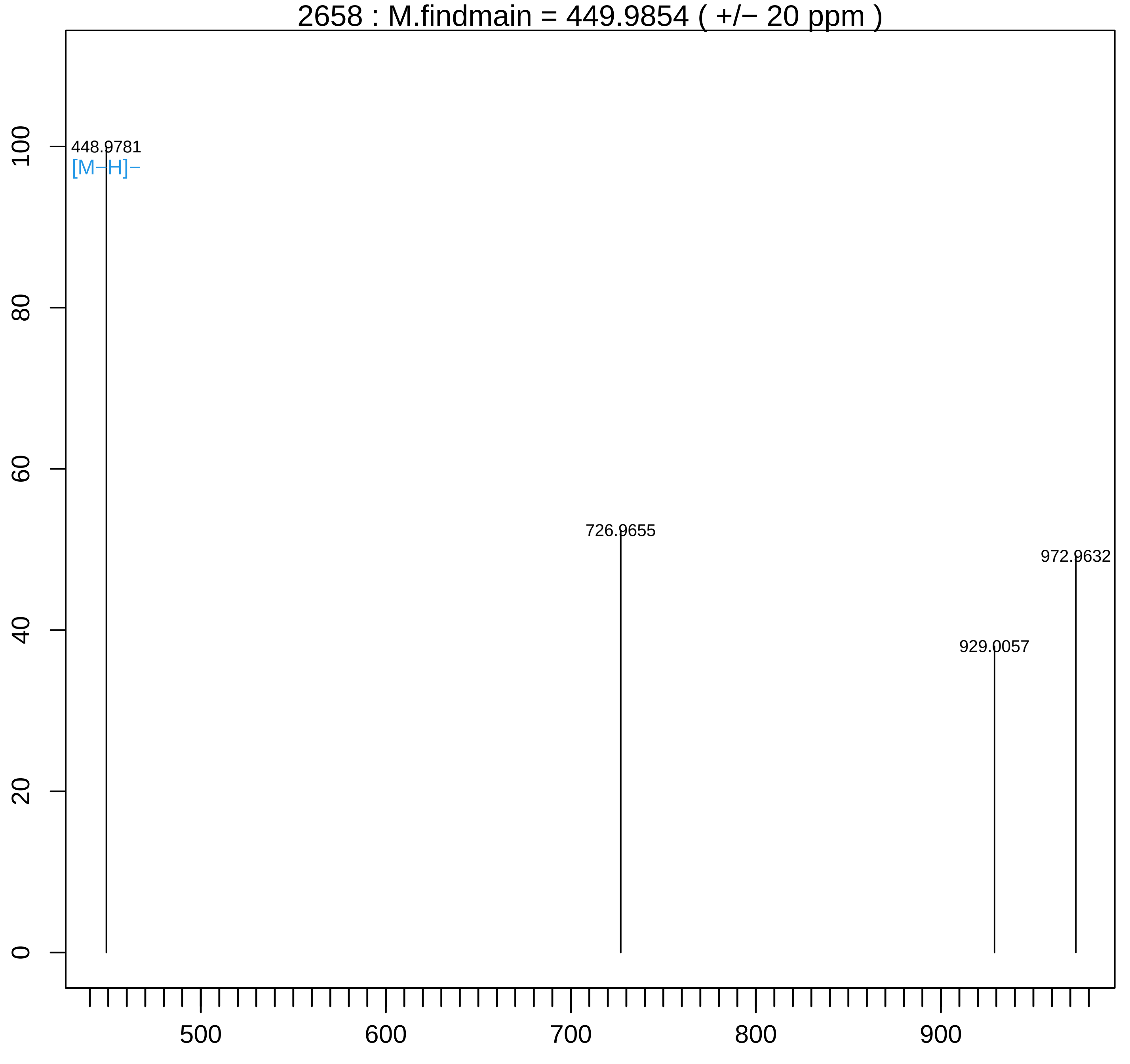
2658 : M.findmain = 449.9854 (+/− 20 ppm)
448.9781
[M−H]−
726.9655
972.9632
929.0057
0
20
40
60
80
100
500
600
700
800
900

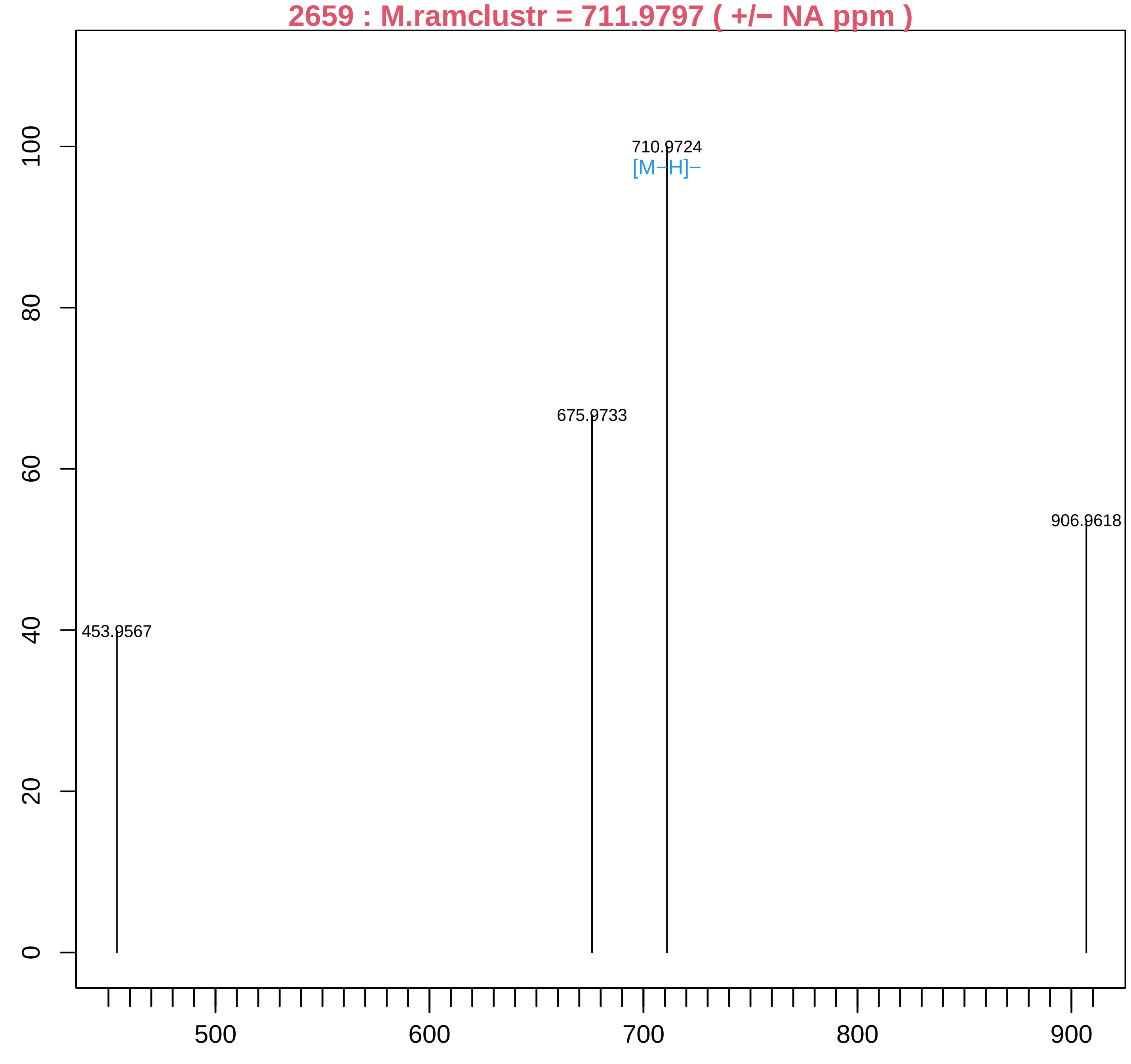
2659 : M.ramclustr = 711.9797 (+/− NA ppm)
710.9724
[M−H]−
675.9733
906.9618
453.9567
0
20
40
60
80
100
500
600
700
800
900

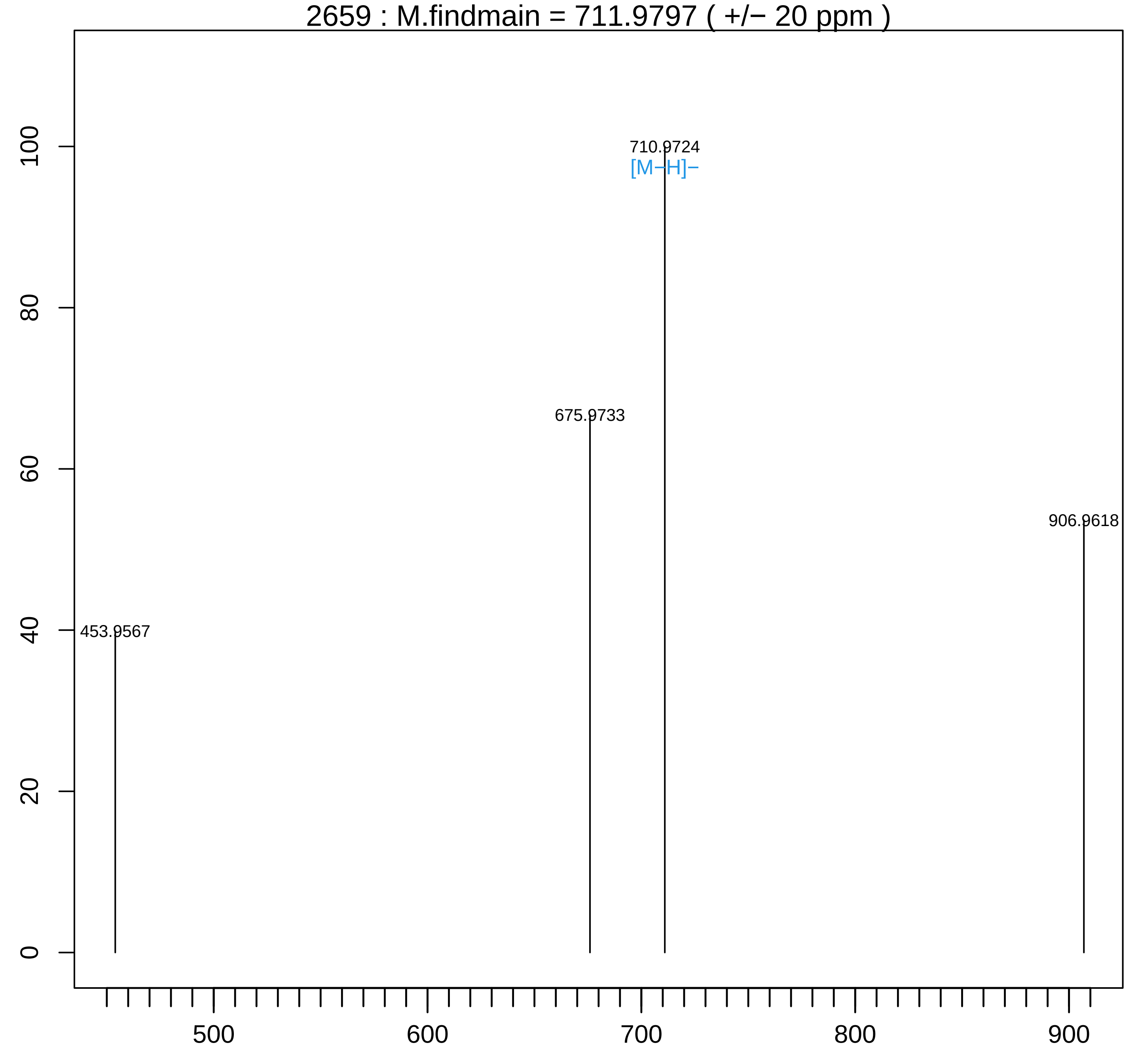
2659 : M.findmain = 711.9797 (+/− 20 ppm)
710.9724
[M−H]−
675.9733
906.9618
453.9567
0
20
40
60
80
100
500
600
700
800
900

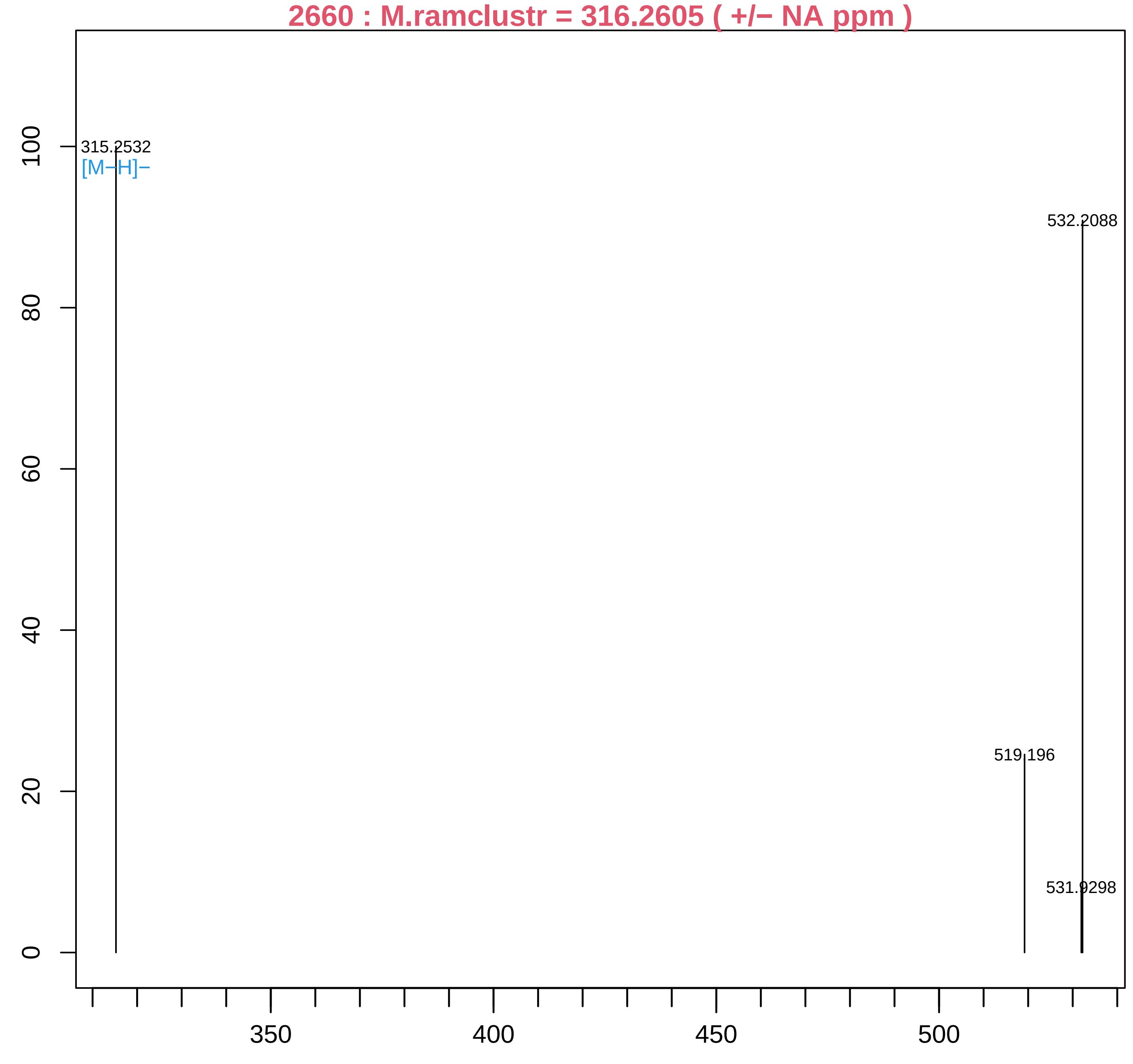

2660 : M.ramclustr = 316.2605 (+/− NA ppm)
315.2532
[M−H]−
532.2088
519.196
531.9298
0
20
40
60
80
100
350
400
450
500

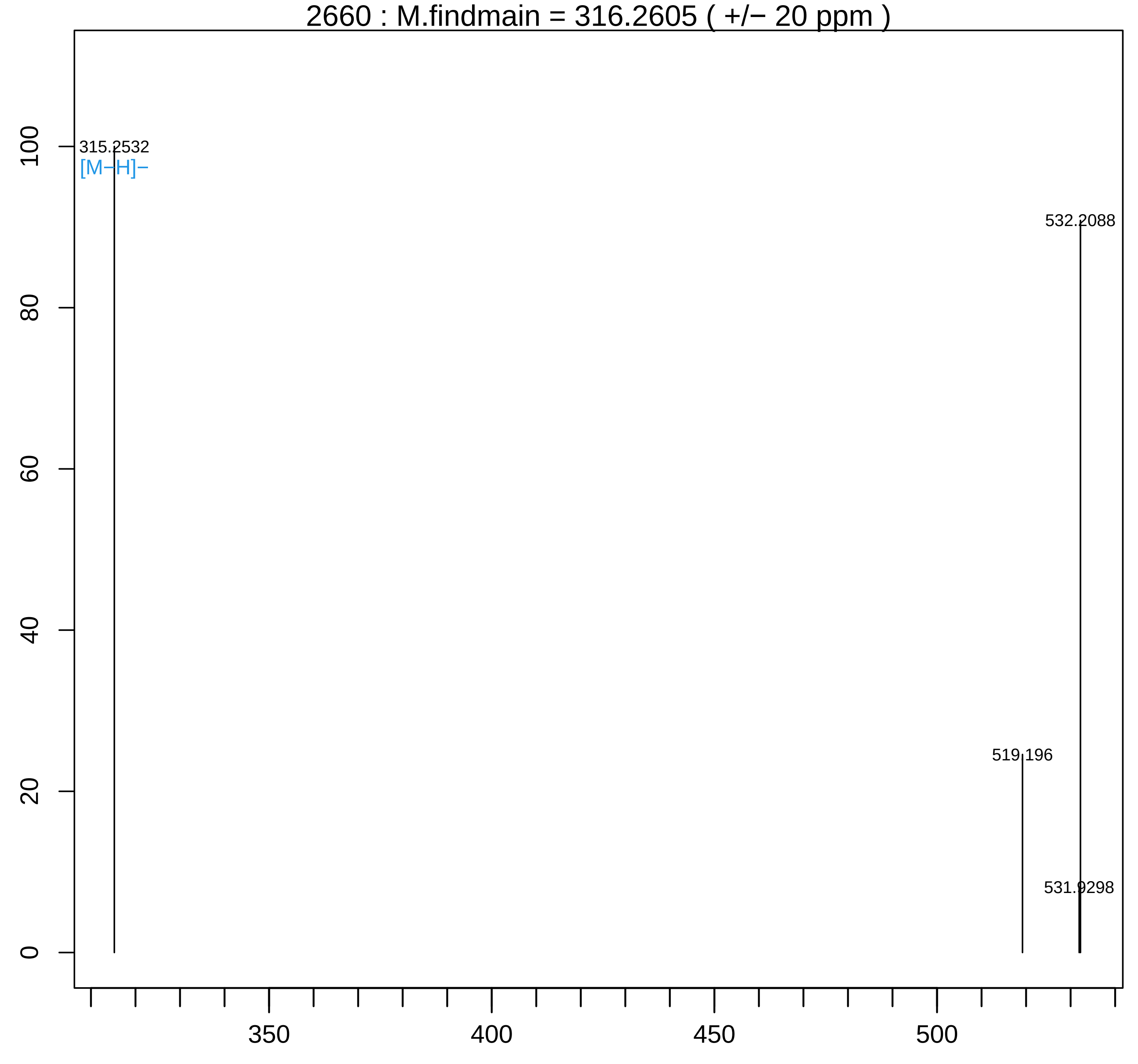

2660 : M.findmain = 316.2605 (+/− 20 ppm)
315.2532
[M−H]−
532.2088
519.196
531.9298
0
20
40
60
80
100
350
400
450
500

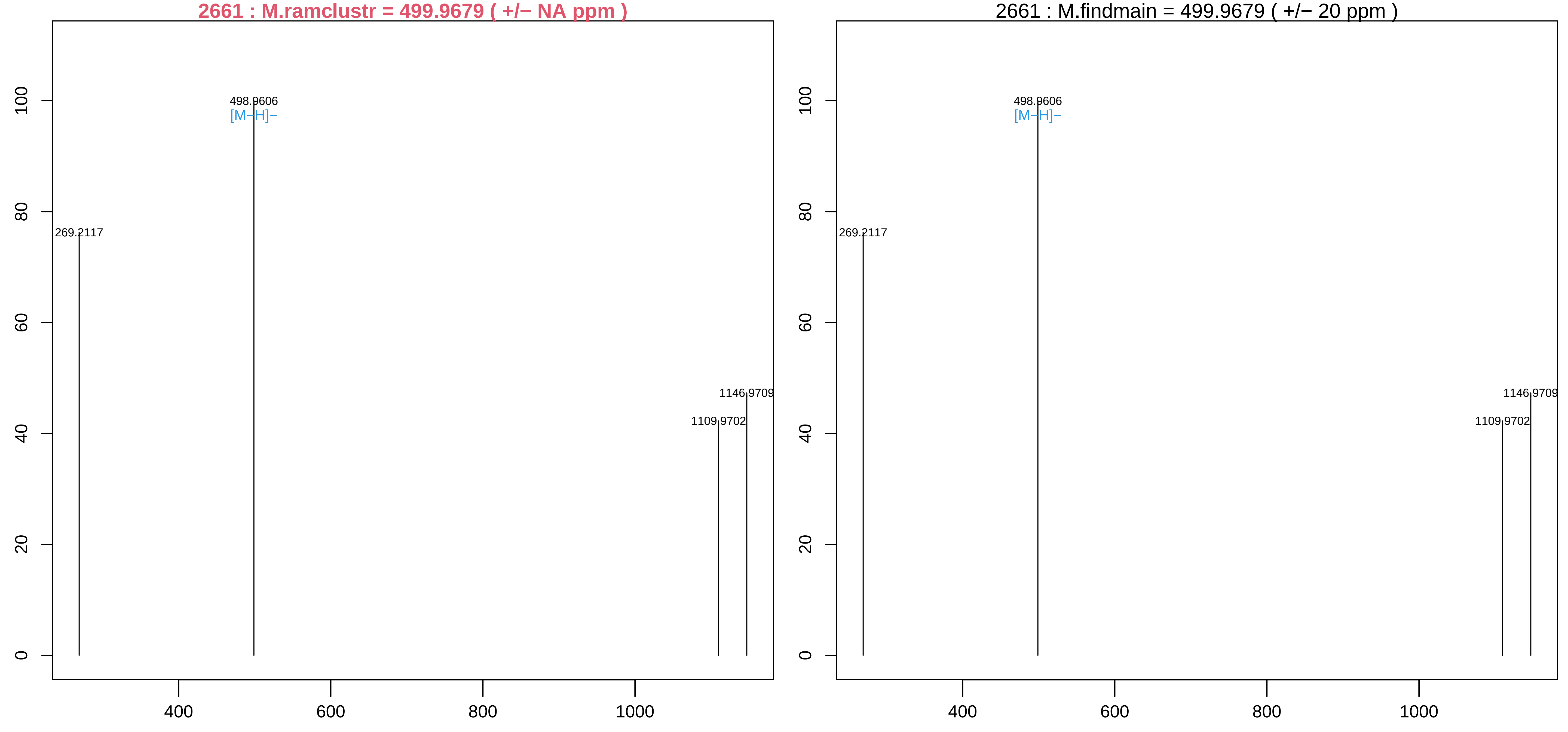
2661 : M.ramclustr = 499.9679 (+/− NA ppm)
498.9606
[M−H]−
269.2117
1146.9709
1109.9702
0
20
40
60
80
100
400
600
800
1000
2661 : M.findmain = 499.9679 (+/− 20 ppm)
498.9606
[M−H]−
269.2117
1146.9709
1109.9702
0
20
40
60
80
100
400
600
800
1000

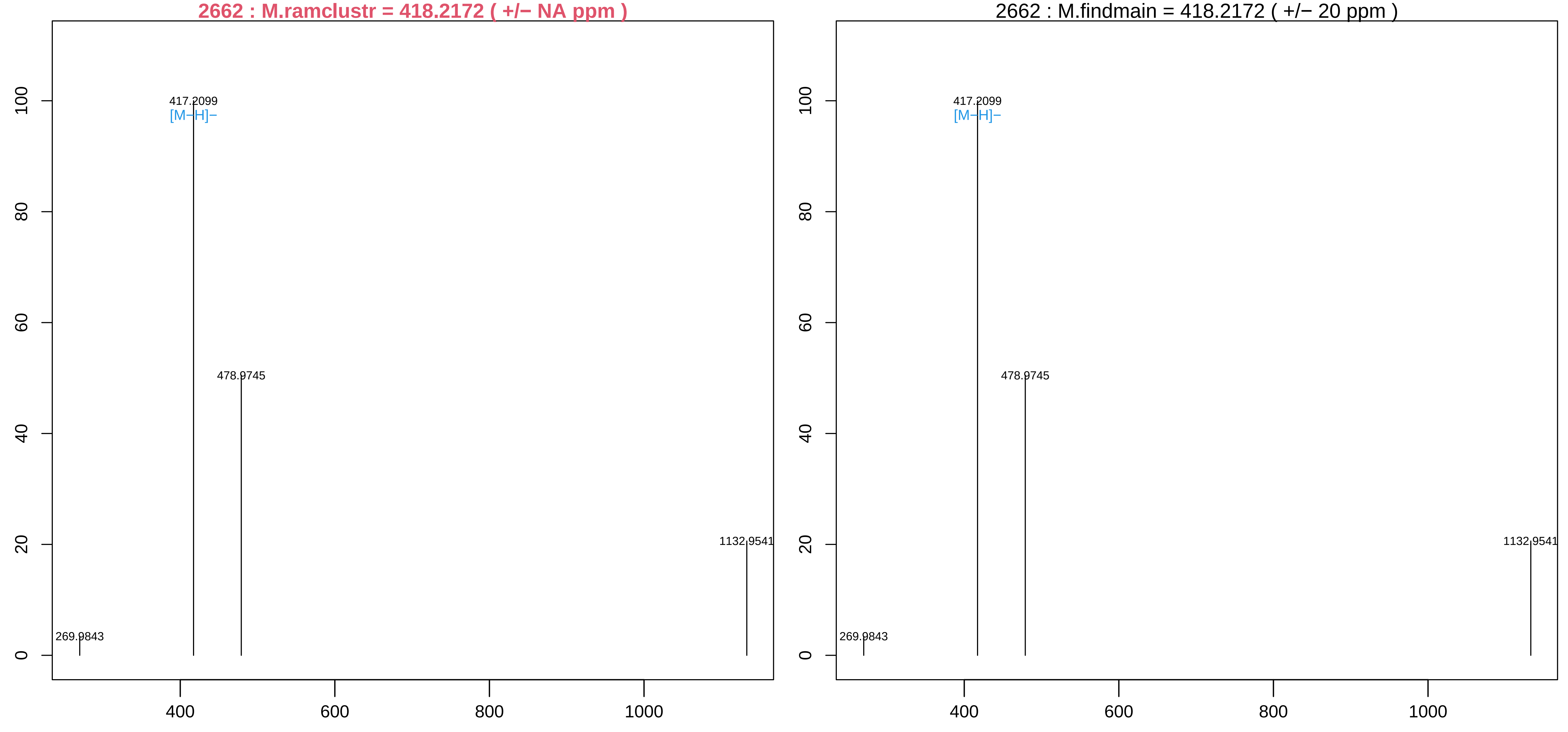
2662 : M.ramclustr = 418.2172 (+/− NA ppm)
2662 : M.findmain = 418.2172 (+/− 20 ppm)
417.2099
[M−H]−
478.9745
1132.9541
269.9843
0
20
40
60
80
100
400
600
800
1000

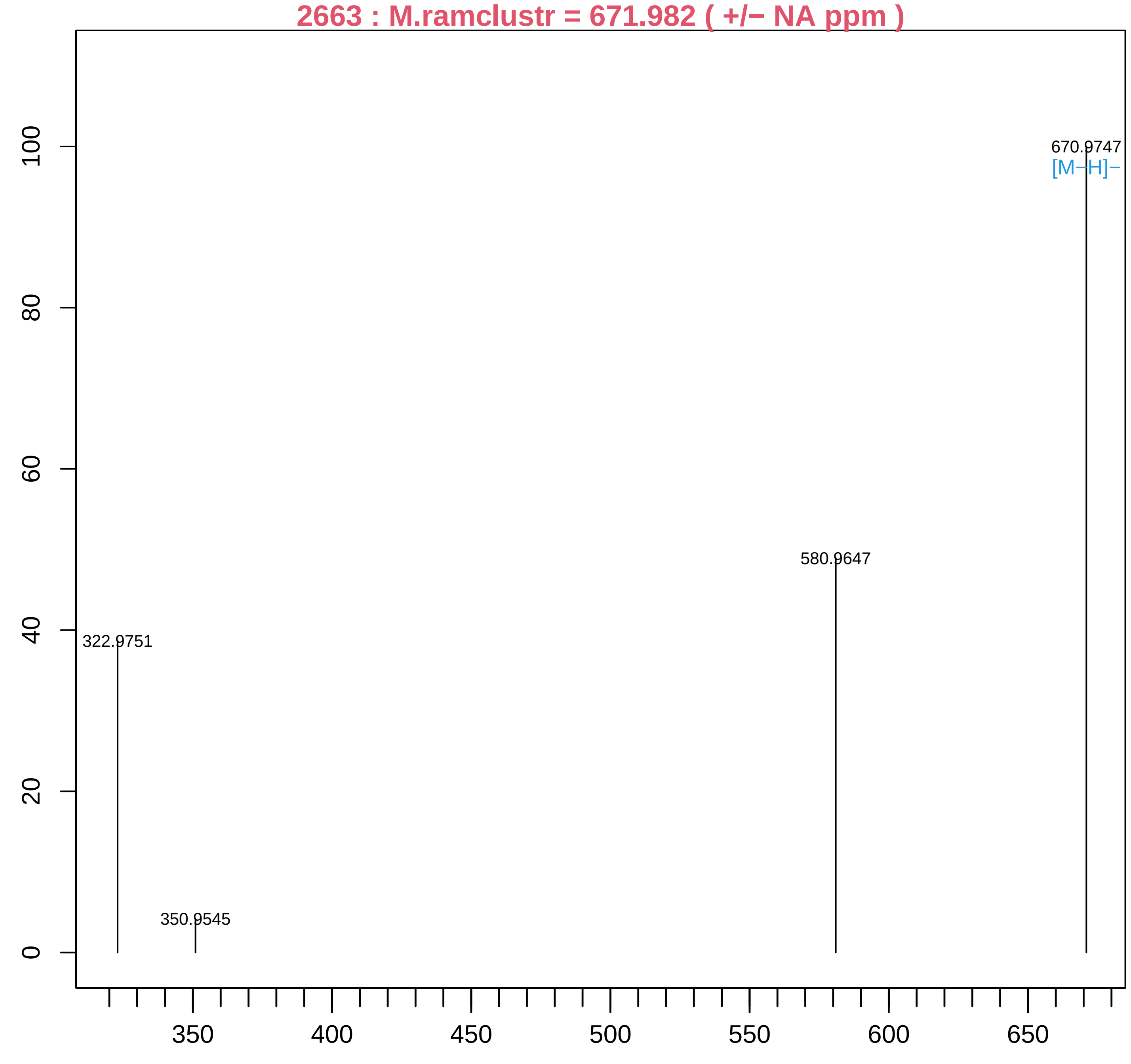

2663 : M.ramclustr = 671.982 (+/− NA ppm)
670.9747
[M−H]−
580.9647
322.9751
350.9545
0
20
40
60
80
100
350
400
450
500
550
600
650

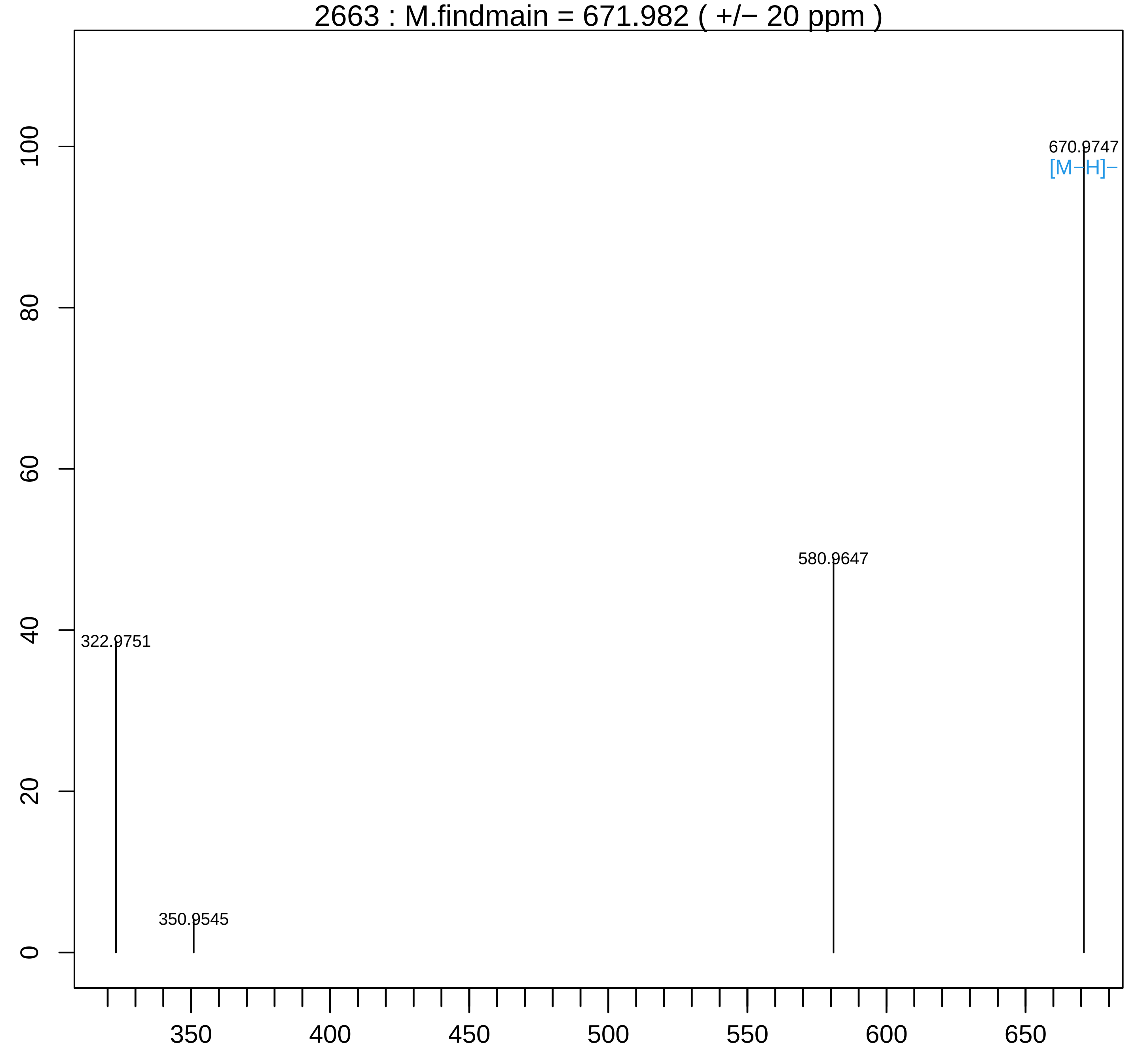

2663 : M.findmain = 671.982 (+/− 20 ppm)
670.9747
[M−H]−
580.9647
322.9751
350.9545
0
20
40
60
80
100
350
400
450
500
550
600
650

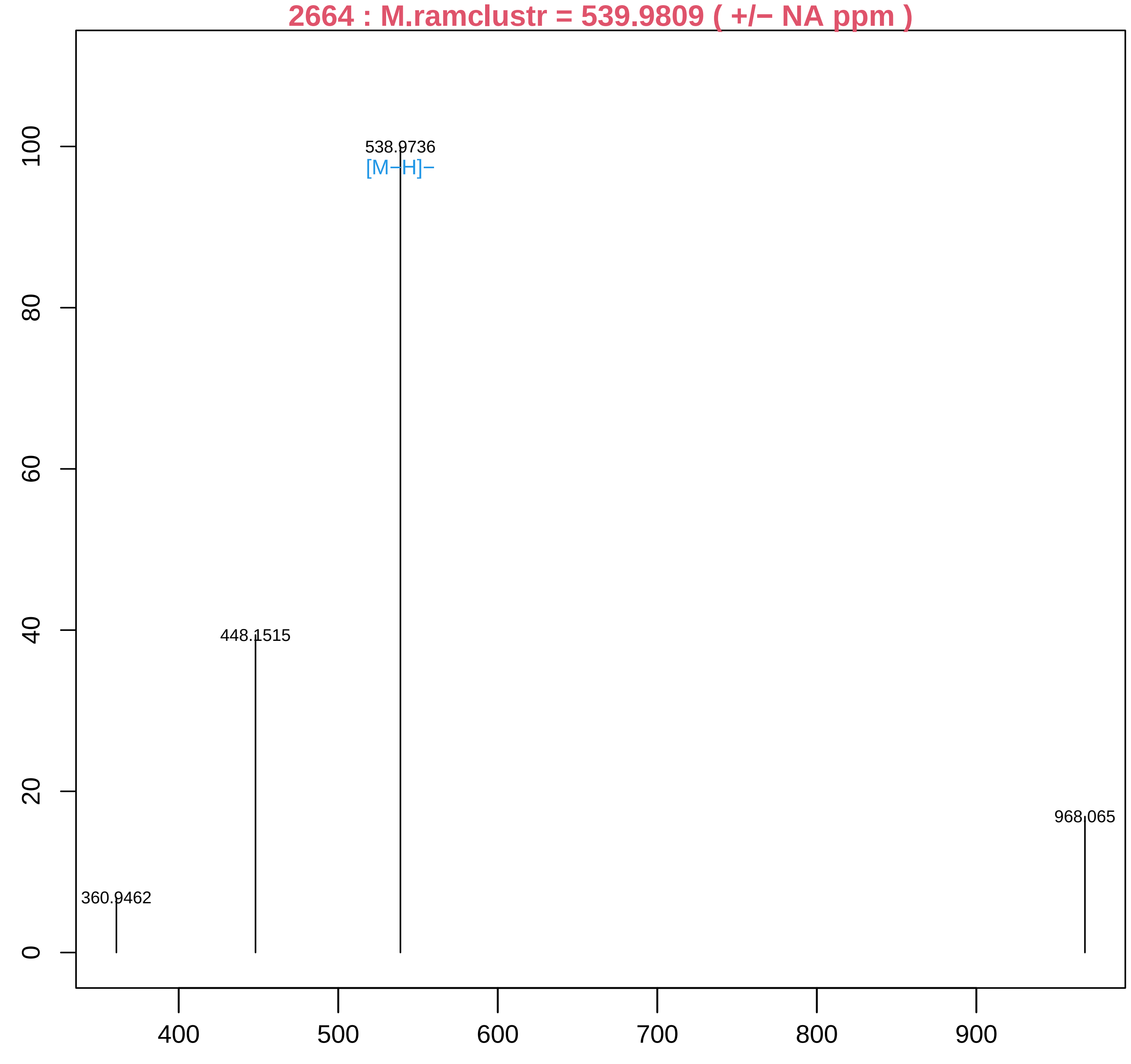
2664 : M.ramclustr = 539.9809 (+/− NA ppm)
538.9736
[M−H]−
448.1515
360.9462
968.065
0
20
40
60
80
100
400
500
600
700
800
900

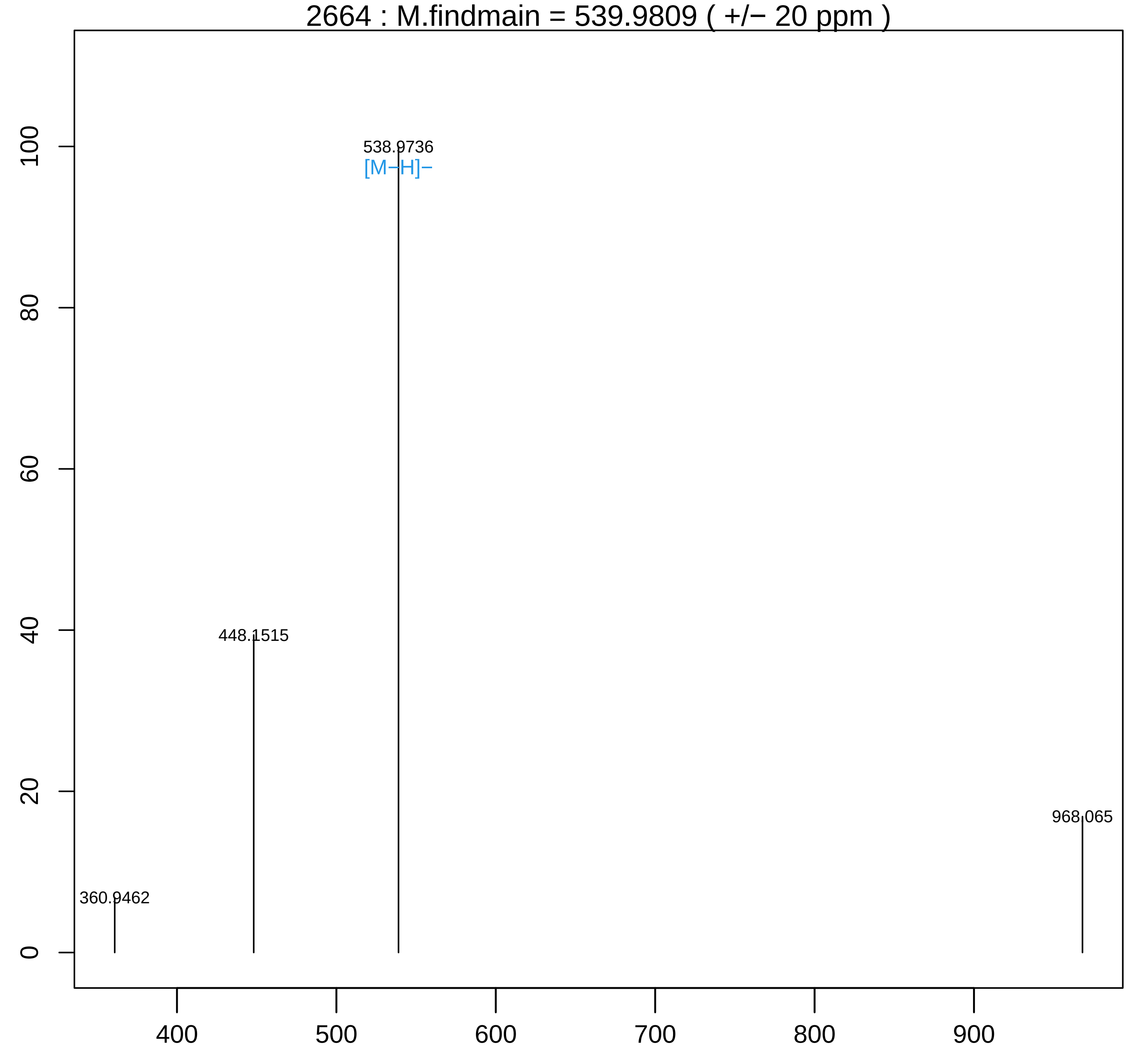
2664 : M.findmain = 539.9809 (+/− 20 ppm)
538.9736
[M−H]−
448.1515
360.9462
968.065
0
20
40
60
80
100
400
500
600
700
800
900

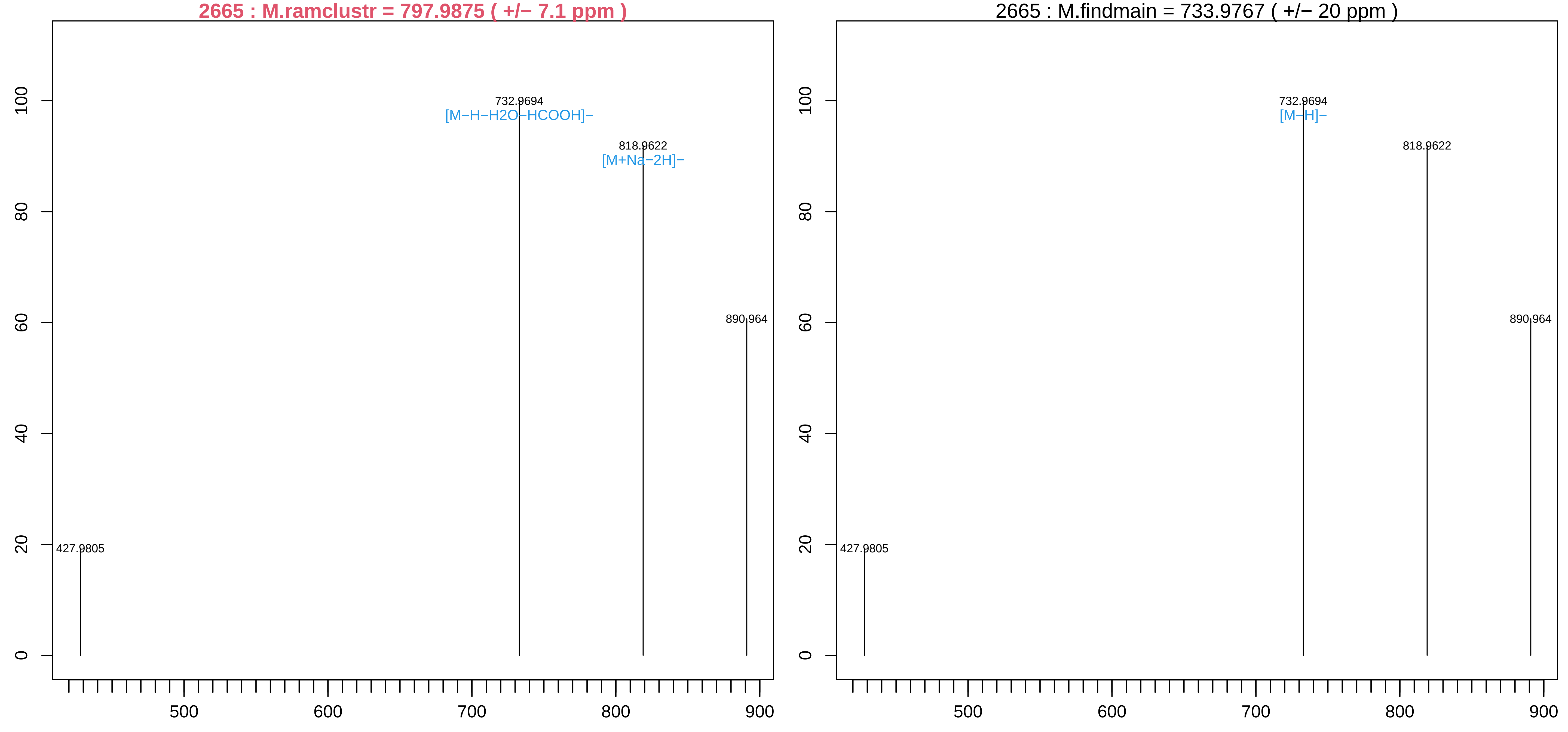
2665 : M.ramclustr = 797.9875 (+/− 7.1 ppm)
732.9694
[M−H−H2O−HCOOH]−
818.9622
[M+Na−2H]−
890.964
427.9805
2665 : M.findmain = 733.9767 (+/− 20 ppm)
732.9694
[M−H]−
818.9622
890.964
427.9805

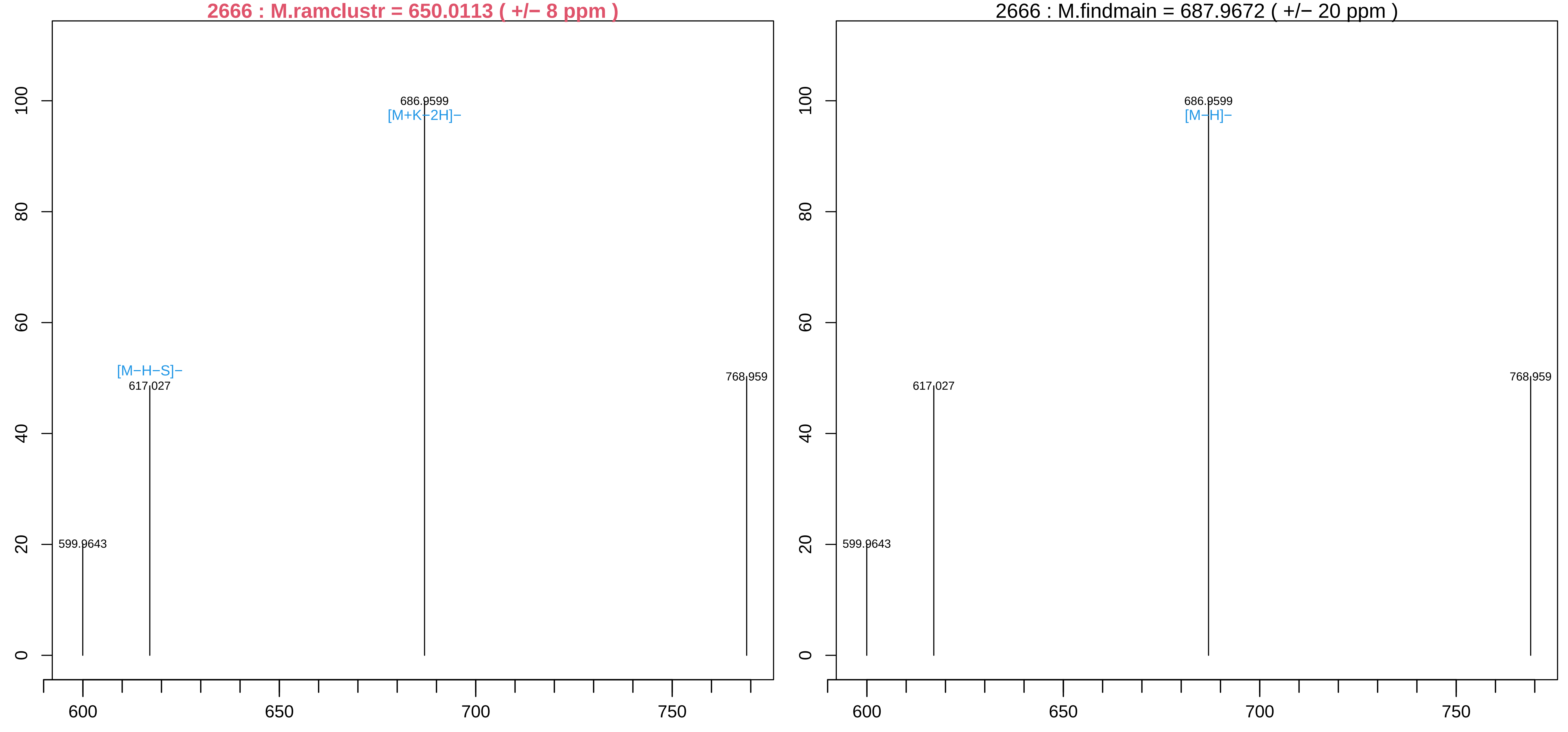
2666 : M.ramclustr = 650.0113 (+/− 8 ppm)
686.9599
[M+K−2H]−
[M−H−S]−
617.027
599.9643
768.959
2666 : M.findmain = 687.9672 (+/− 20 ppm)
686.9599
[M−H]−
617.027
599.9643
768.959

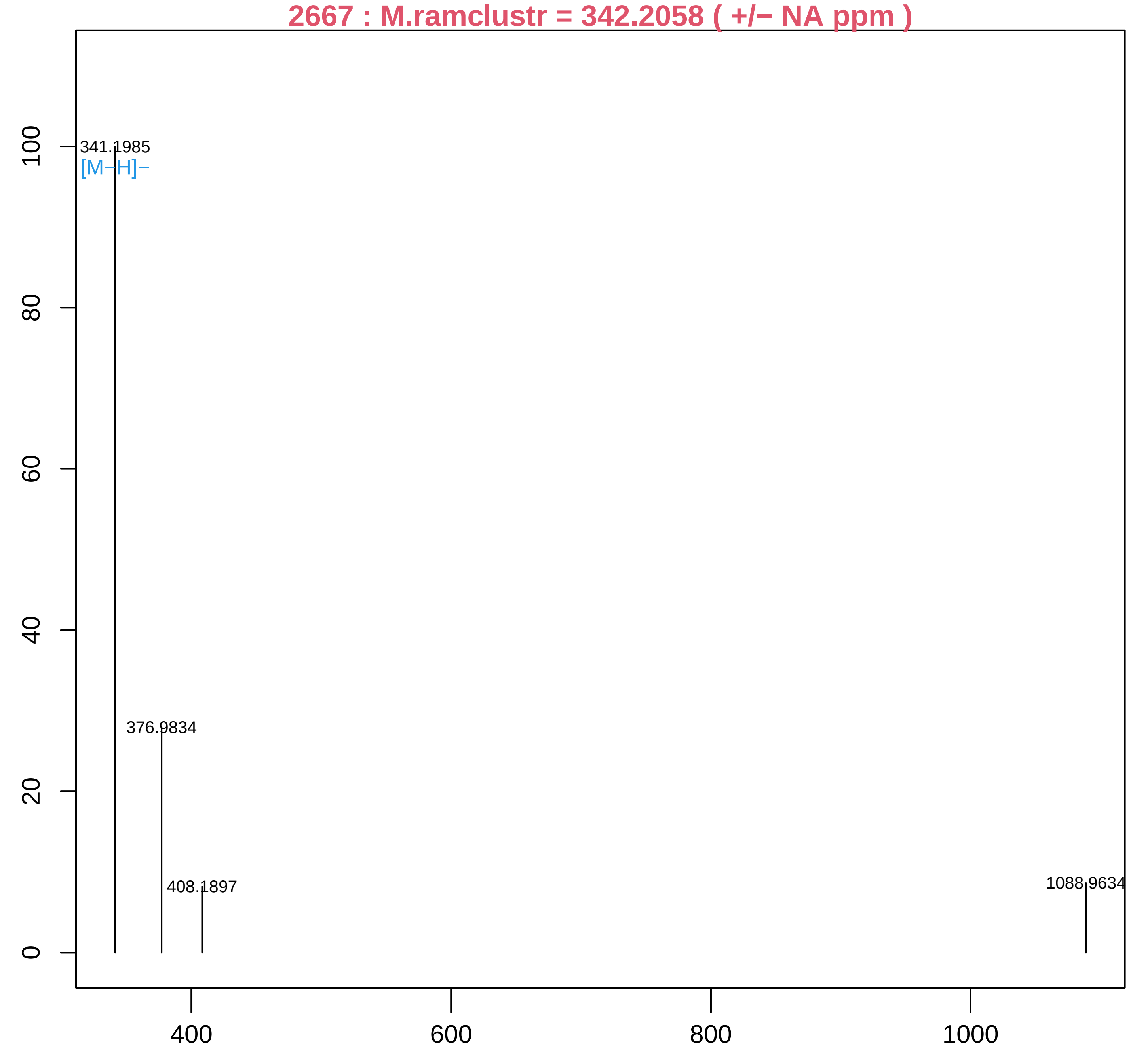
2667 : M.ramclustr = 342.2058 (+/− NA ppm)
341.1985
[M−H]−
376.9834
408.1897
1088.9634
0
20
40
60
80
100
400
600
800
1000

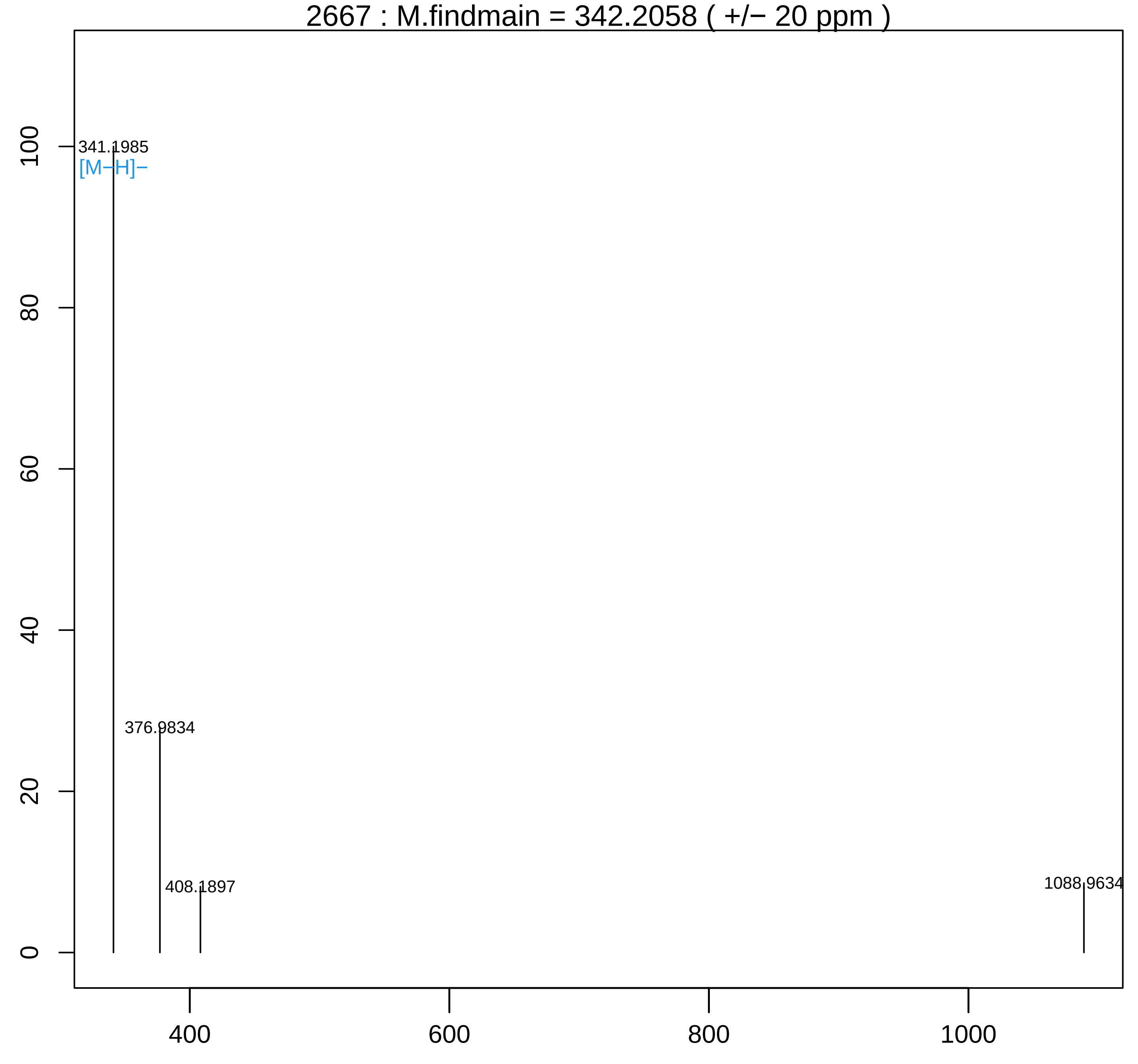
2667 : M.findmain = 342.2058 (+/− 20 ppm)
341.1985
[M−H]−
376.9834
408.1897
1088.9634
0
20
40
60
80
100
400
600
800
1000

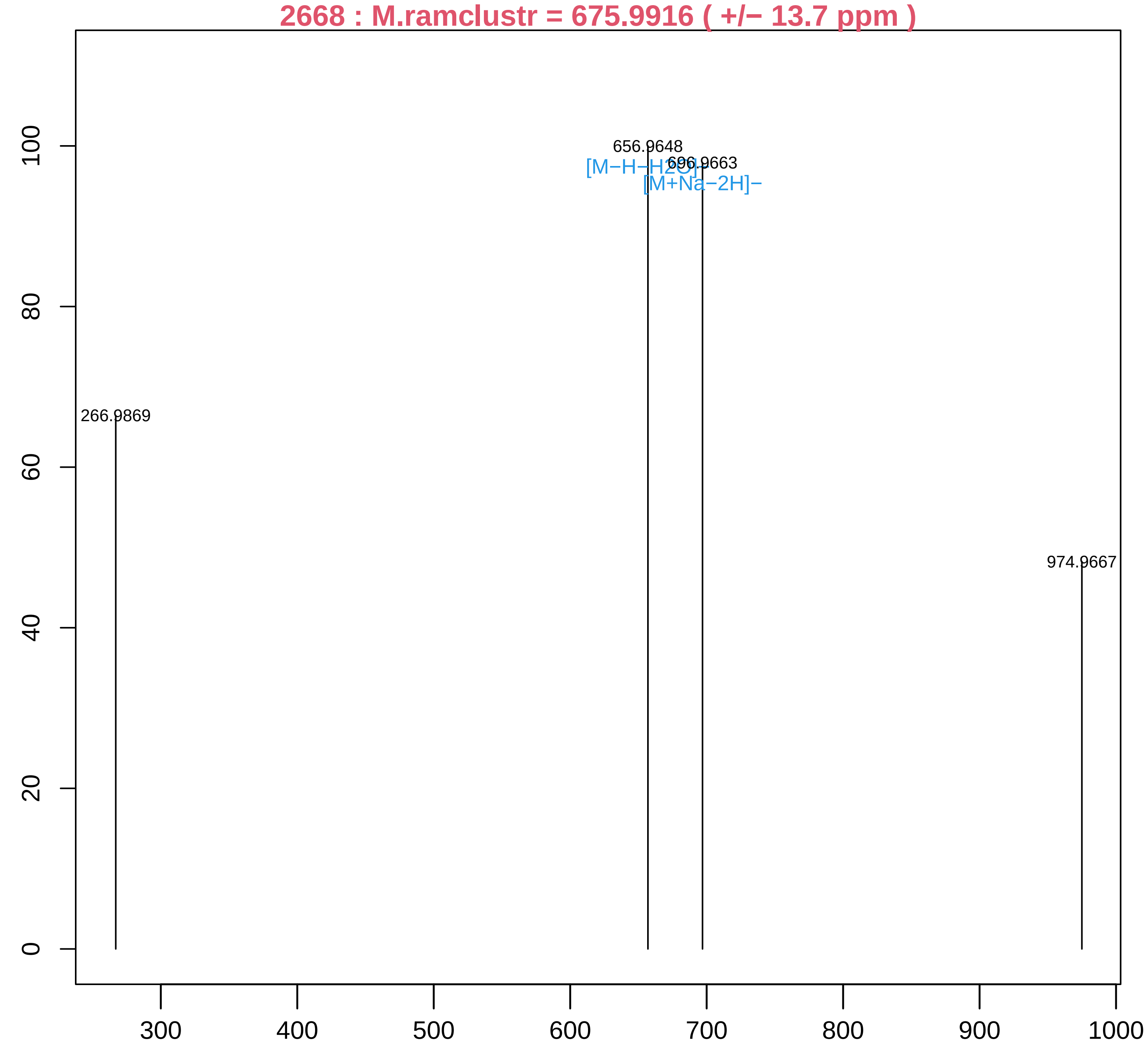
2668 : M.ramclustr = 675.9916 (+/− 13.7 ppm)
656.9648
[M−H−H2O]−
696.9663
[M+Na−2H]−
266.9869
974.9667
0
20
40
60
80
100
300
400
500
600
700
800
900
1000

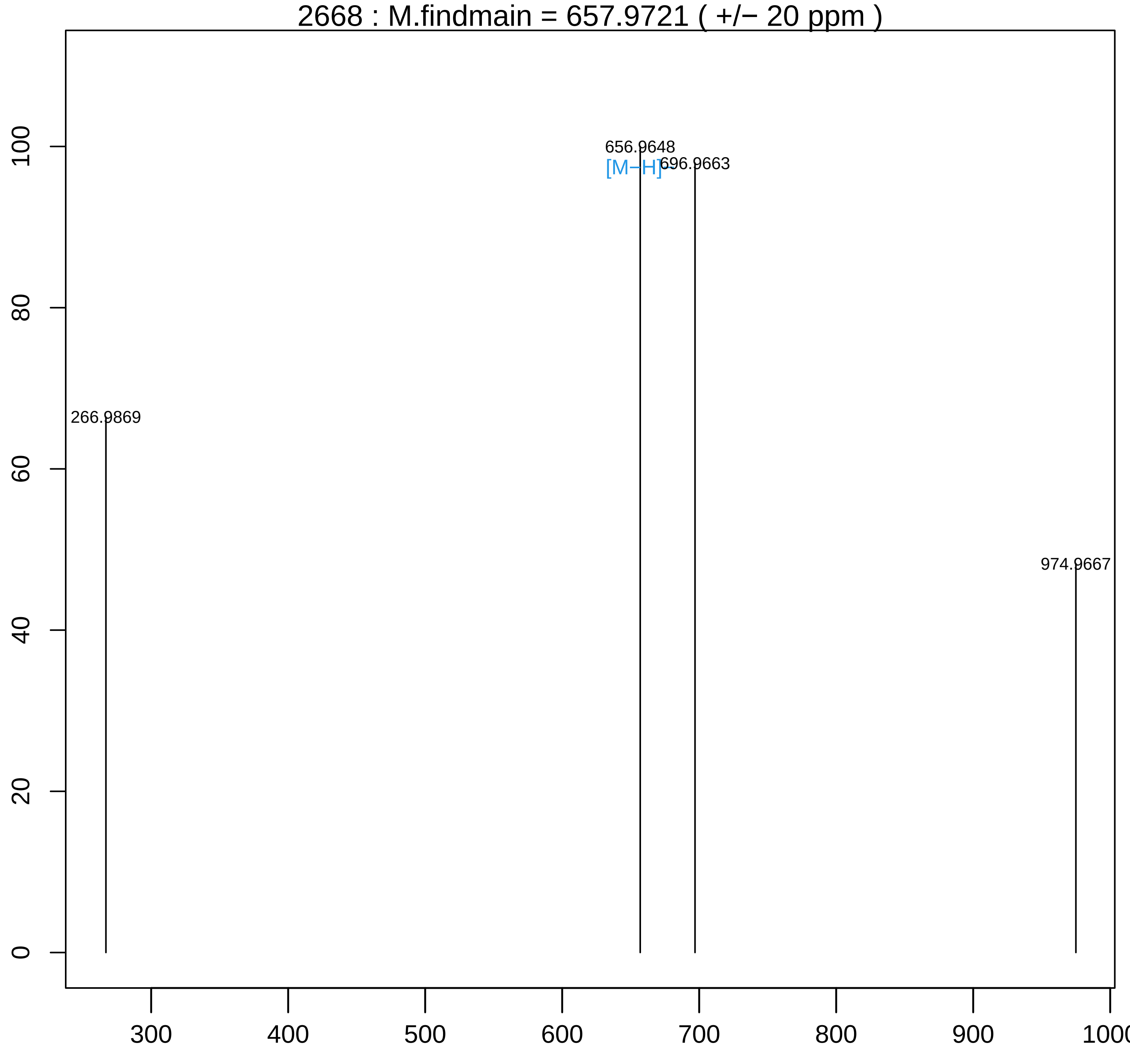
2668 : M.findmain = 657.9721 (+/− 20 ppm)
656.9648
[M−H]−
696.9663
266.9869
974.9667
0
20
40
60
80
100
300
400
500
600
700
800
900
1000

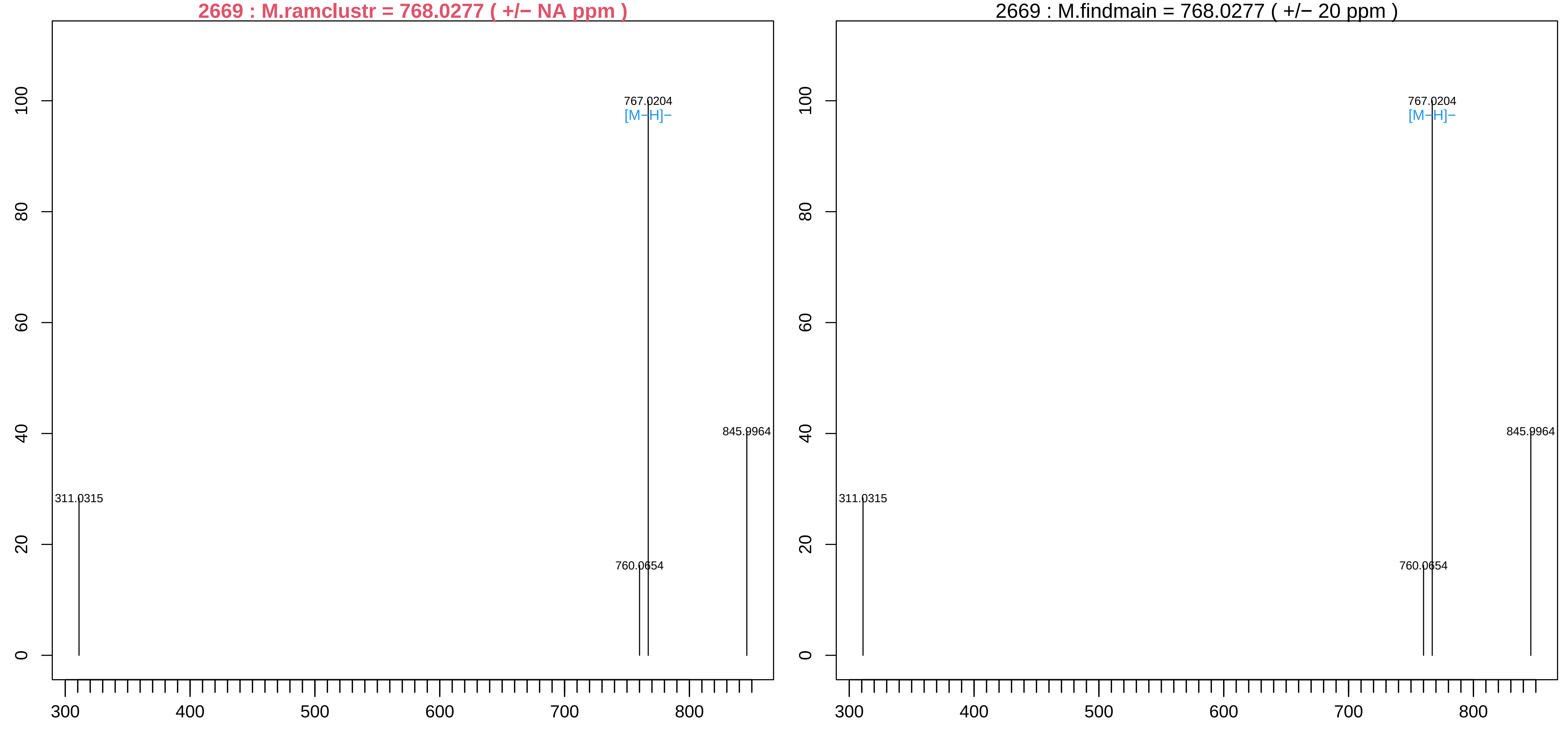
2669 : M.ramclustr = 768.0277 (+/− NA ppm)
767.0204
[M−H]−
845.9964
311.0315
760.0654
2669 : M.findmain = 768.0277 (+/− 20 ppm)
767.0204
[M−H]−
845.9964
311.0315
760.0654
0
20
40
60
80
100
300
400
500
600
700
800

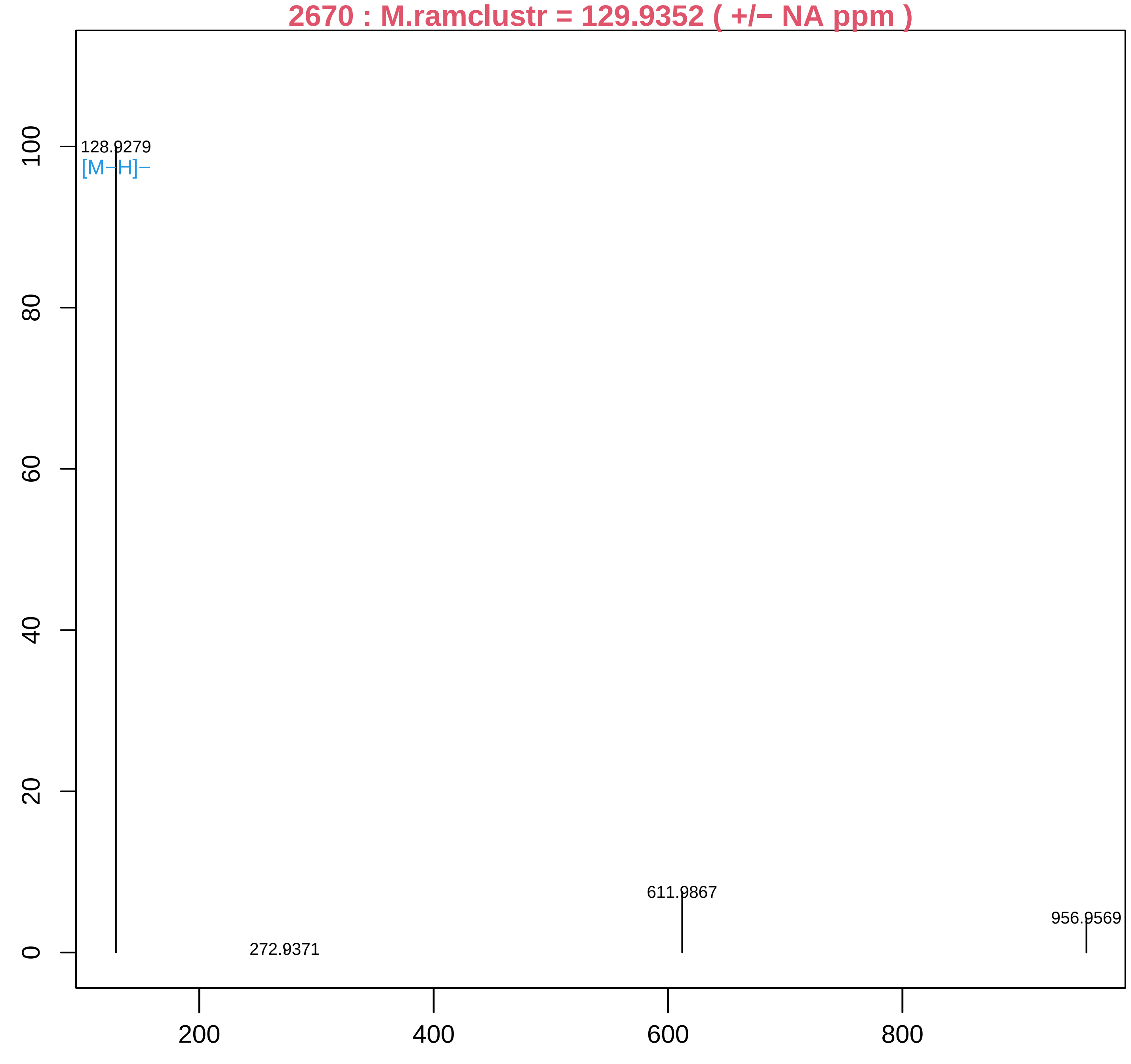

2670 : M.ramclustr = 129.9352 (+/− NA ppm)
128.9279
[M−H]−
272.9371
611.9867
956.9569
0
20
40
60
80
100
200
400
600
800

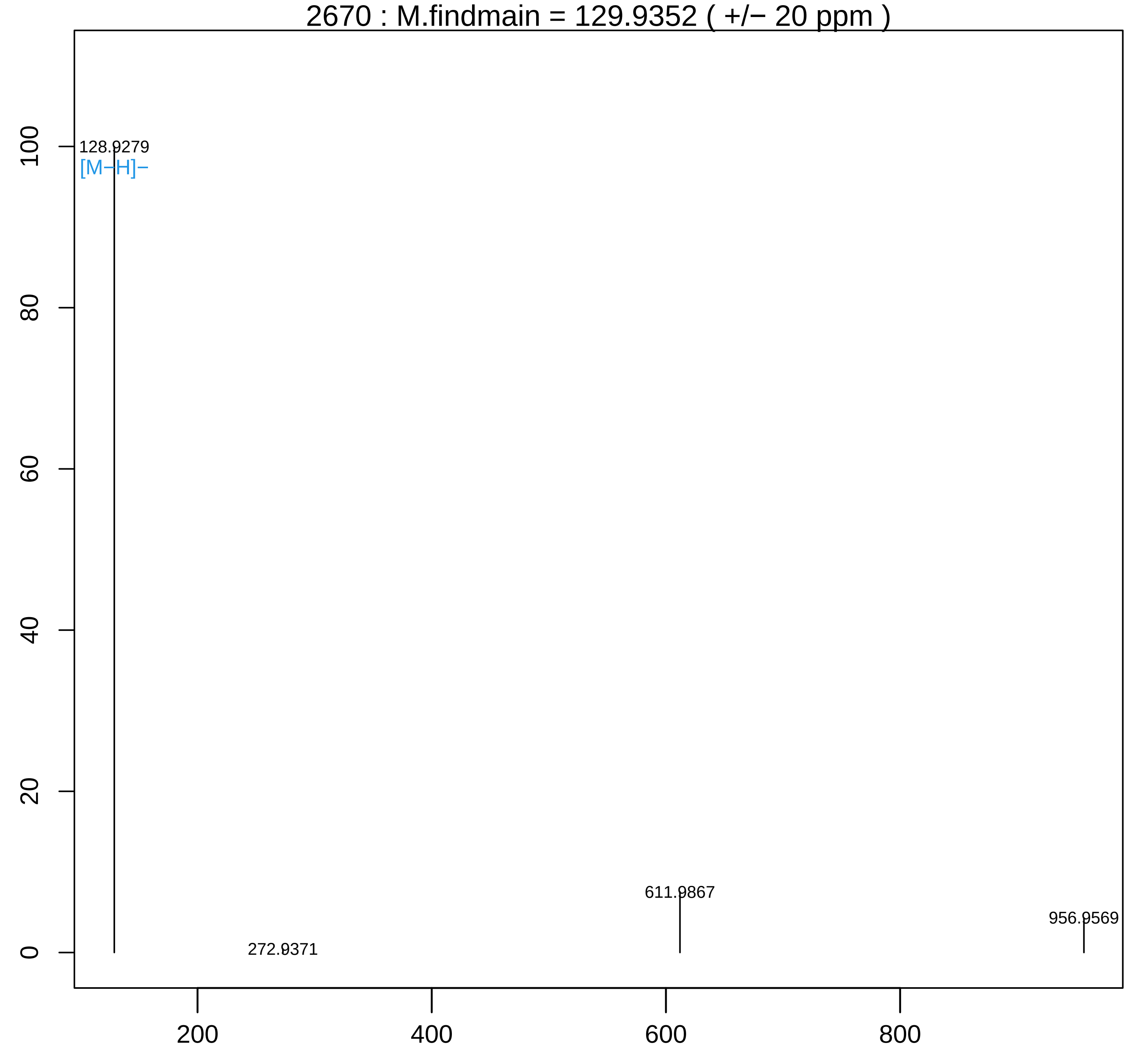

2670 : M.findmain = 129.9352 (+/− 20 ppm)
128.9279
[M−H]−
272.9371
611.9867
956.9569
0
20
40
60
80
100
200
400
600
800

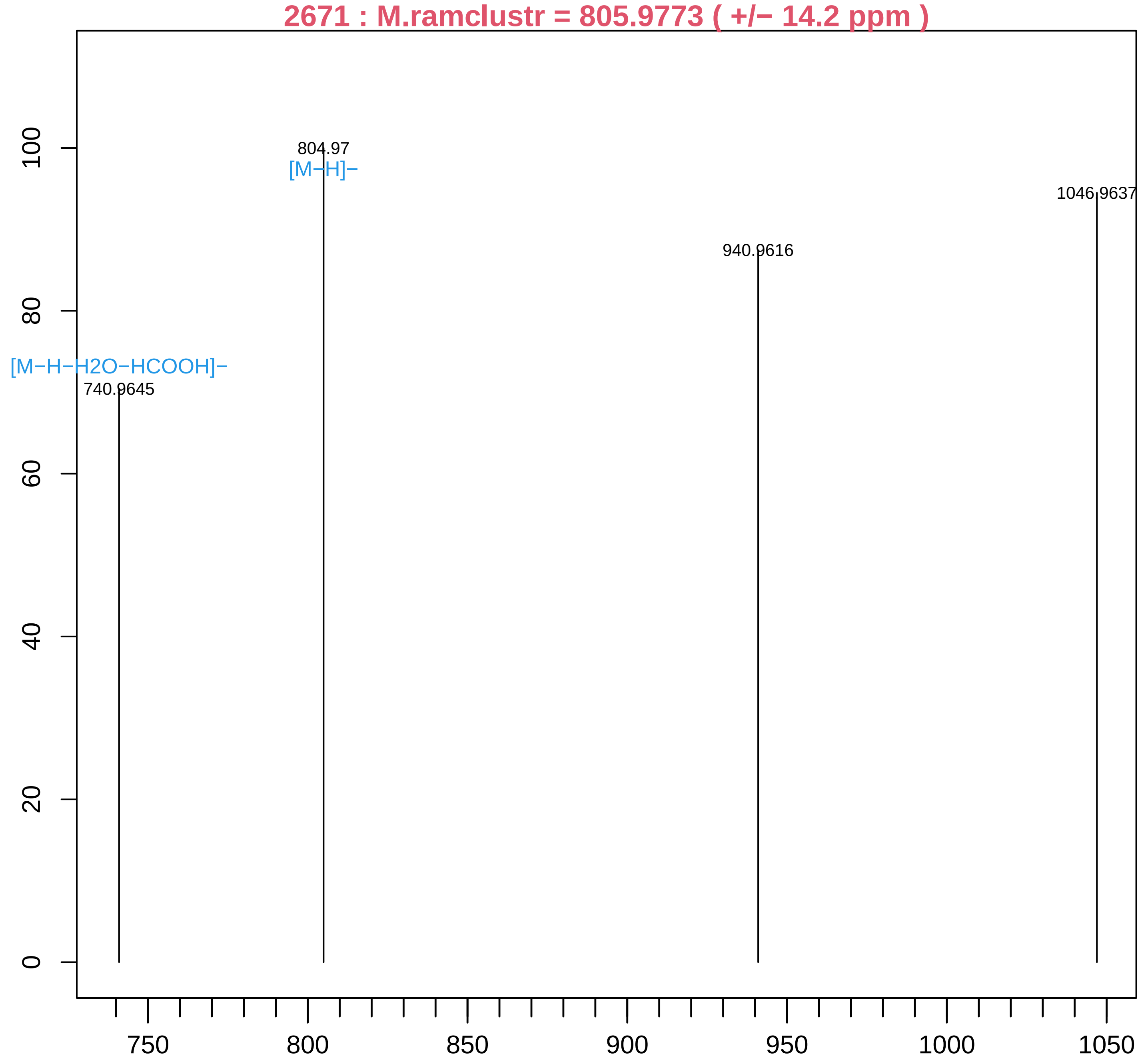
2671 : M.ramclustr = 805.9773 (+/− 14.2 ppm)
804.97
[M−H]−
1046.9637
940.9616
[M−H−H2O−HCOOH]−
740.9645
0
20
40
60
80
100
750
800
850
900
950
1000
1050

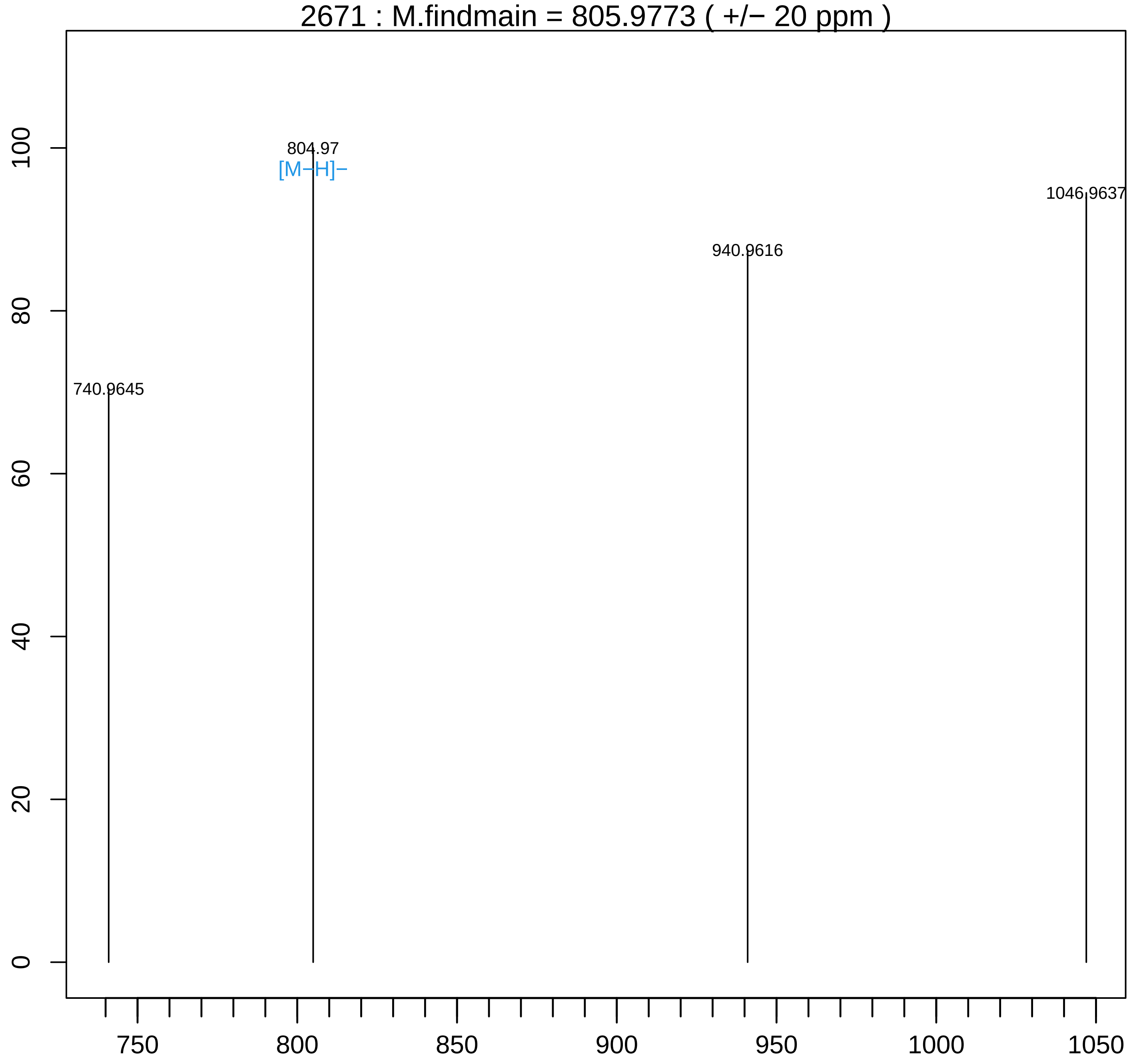
2671 : M.findmain = 805.9773 (+/− 20 ppm)
804.97
[M−H]−
1046.9637
940.9616
740.9645
0
20
40
60
80
100
750
800
850
900
950
1000
1050

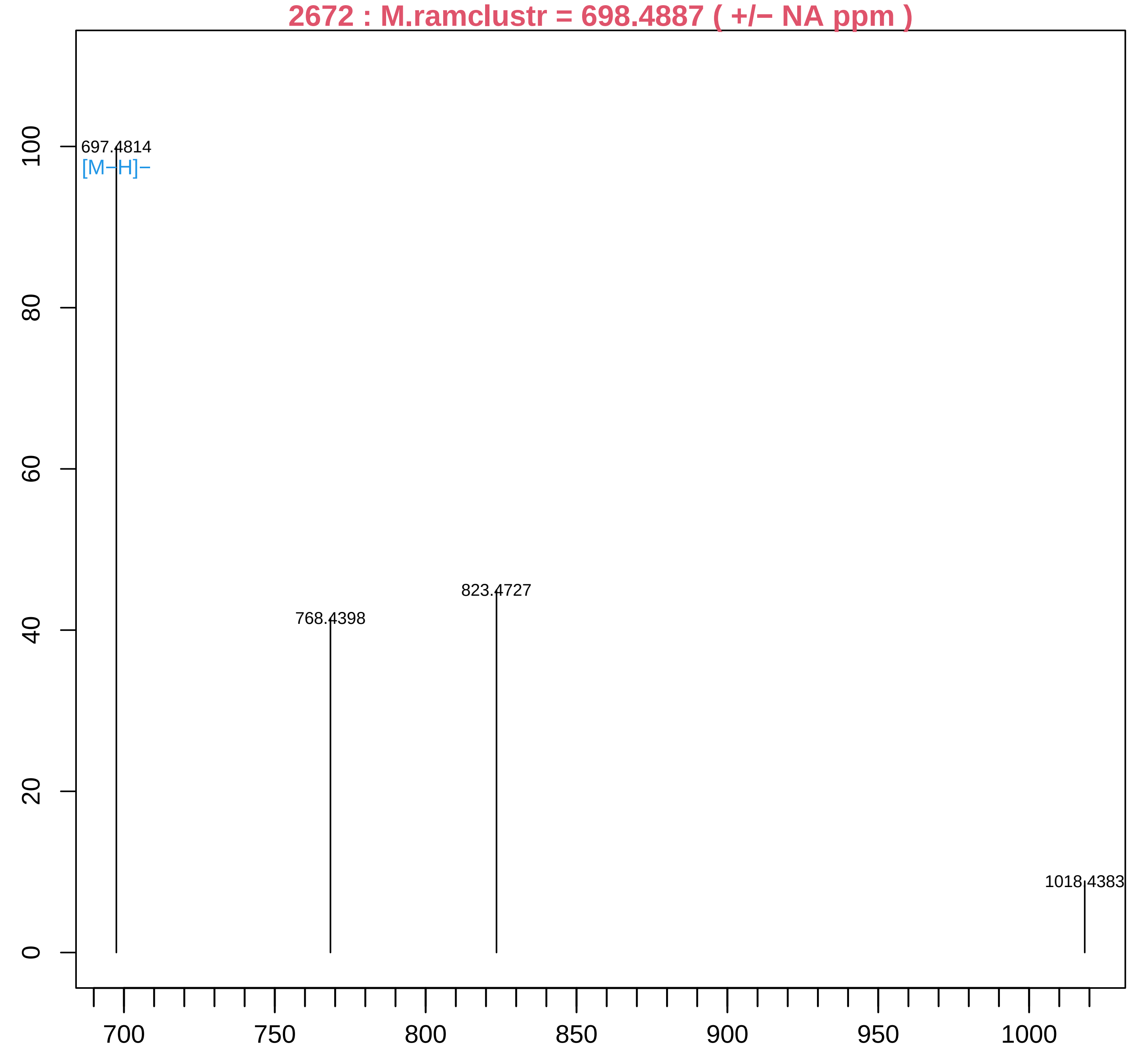
2672 : M.ramclustr = 698.4887 (+/− NA ppm)
697.4814
[M−H]−
768.4398
823.4727
1018.4383
0
20
40
60
80
100
700
750
800
850
900
950
1000

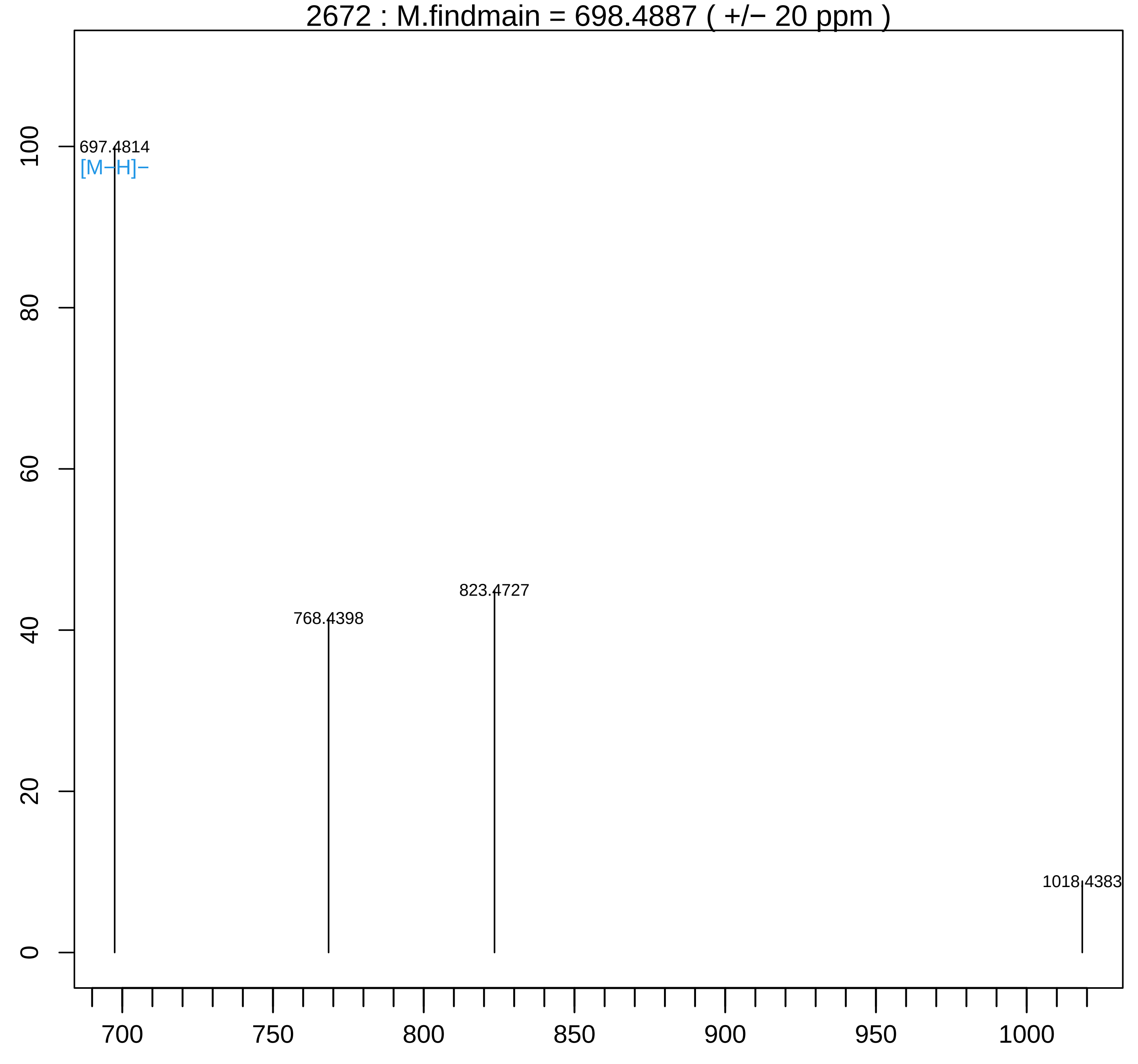
2672 : M.findmain = 698.4887 (+/− 20 ppm)
697.4814
[M−H]−
768.4398
823.4727
1018.4383
0
20
40
60
80
100
700
750
800
850
900
950
1000

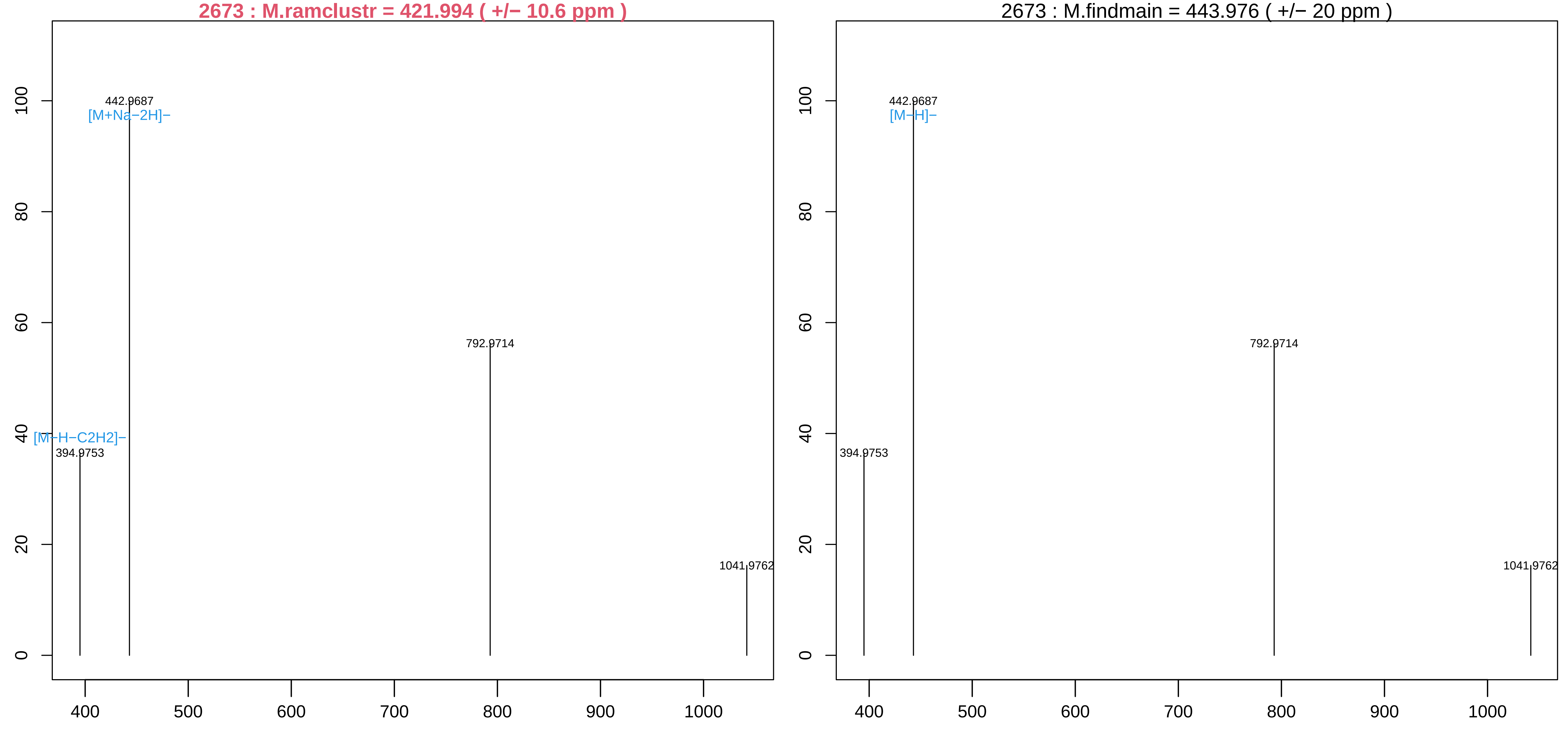
2673 : M.ramclustr = 421.994 (+/− 10.6 ppm)
442.9687
[M+Na−2H]−
792.9714
[M−H−C2H2]−
394.9753
1041.9762
2673 : M.findmain = 443.976 (+/− 20 ppm)
442.9687
[M−H]−
792.9714
394.9753
1041.9762

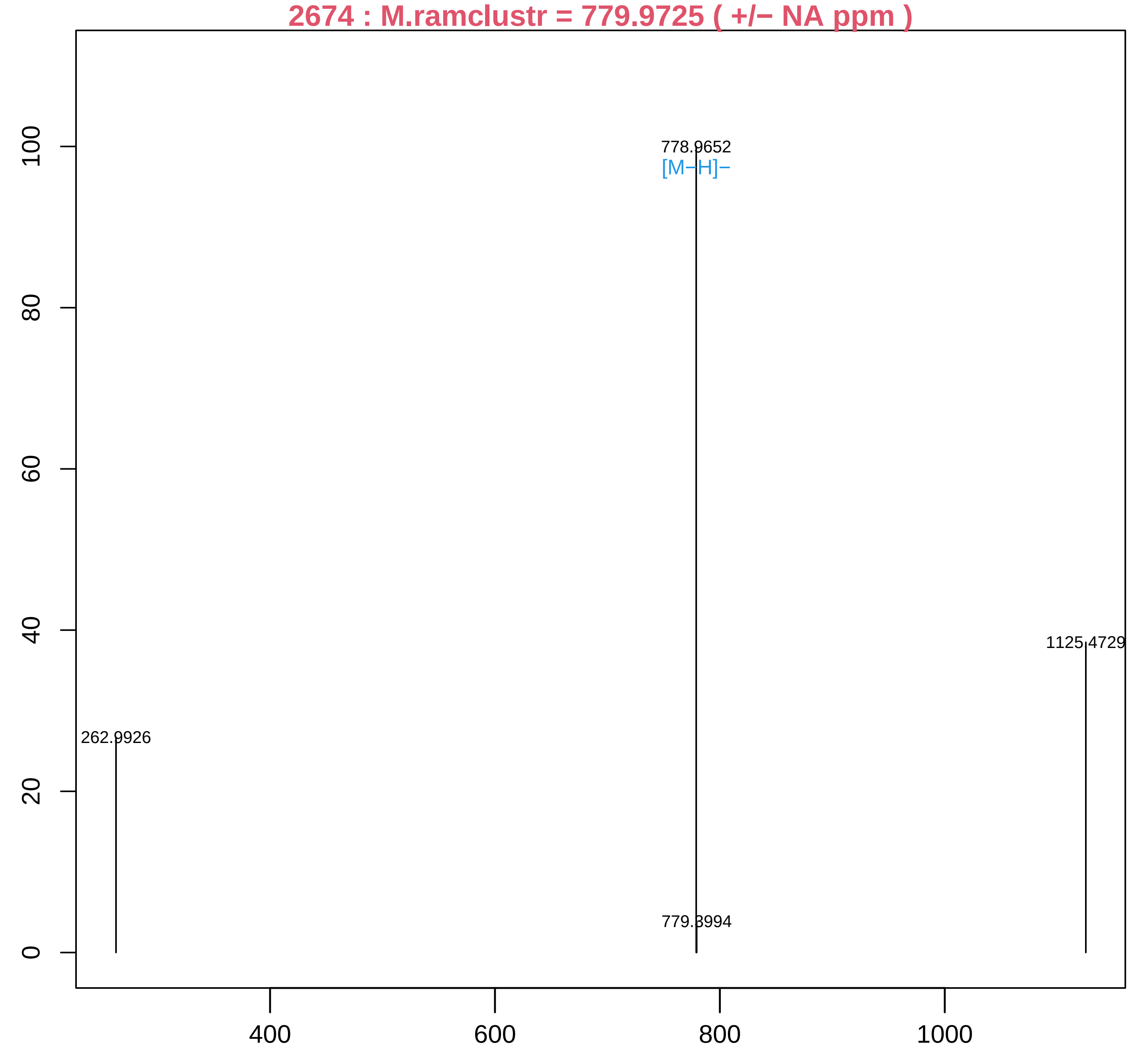

2674 : M.ramclustr = 779.9725 (+/− NA ppm)
778.9652
[M−H]−
1125.4729
262.9926
779.3994
0
20
40
60
80
100
400
600
800
1000

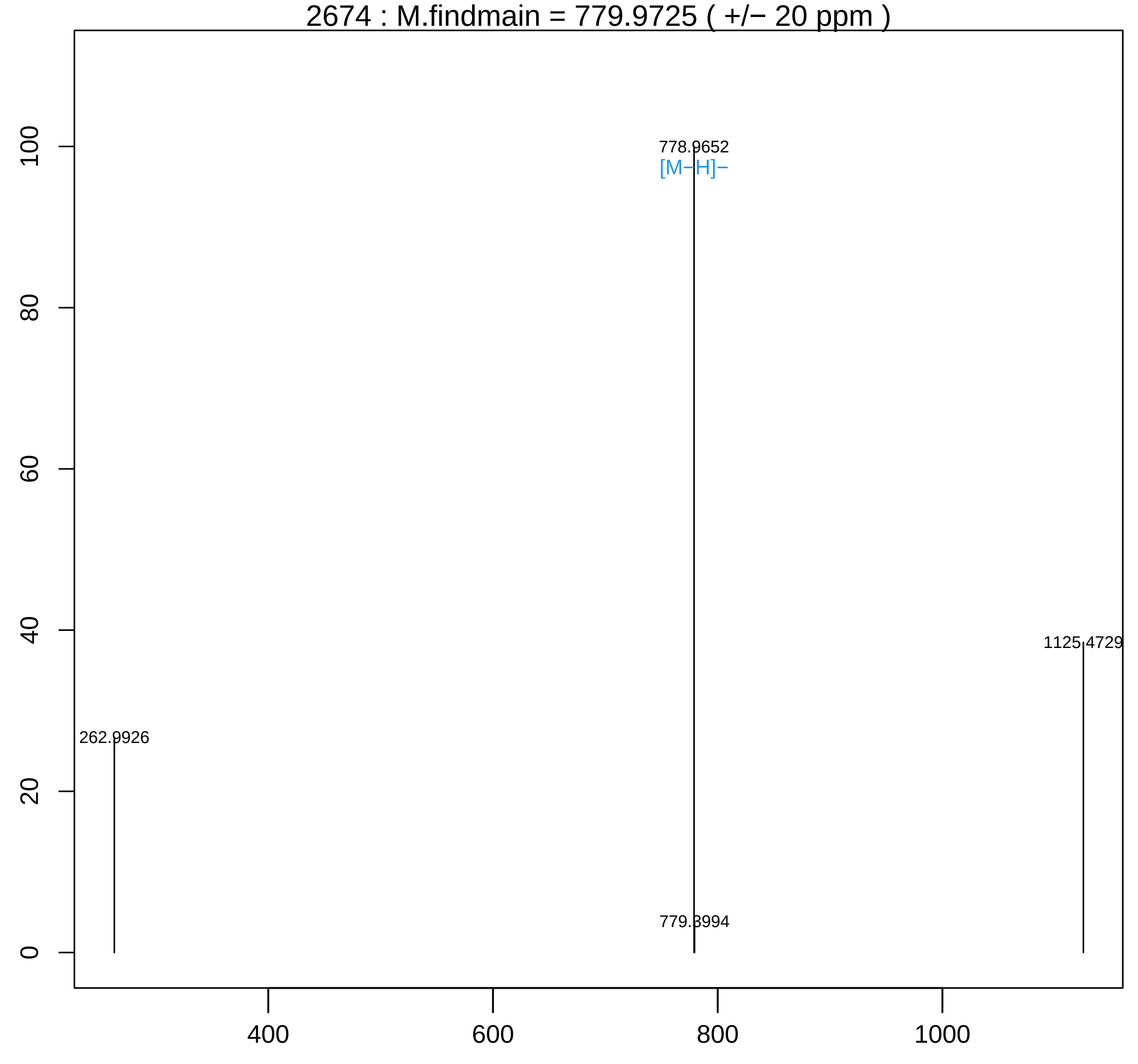

2674 : M.findmain = 779.9725 (+/− 20 ppm)
778.9652
[M−H]−
1125.4729
262.9926
779.3994
0
20
40
60
80
100
400
600
800
1000

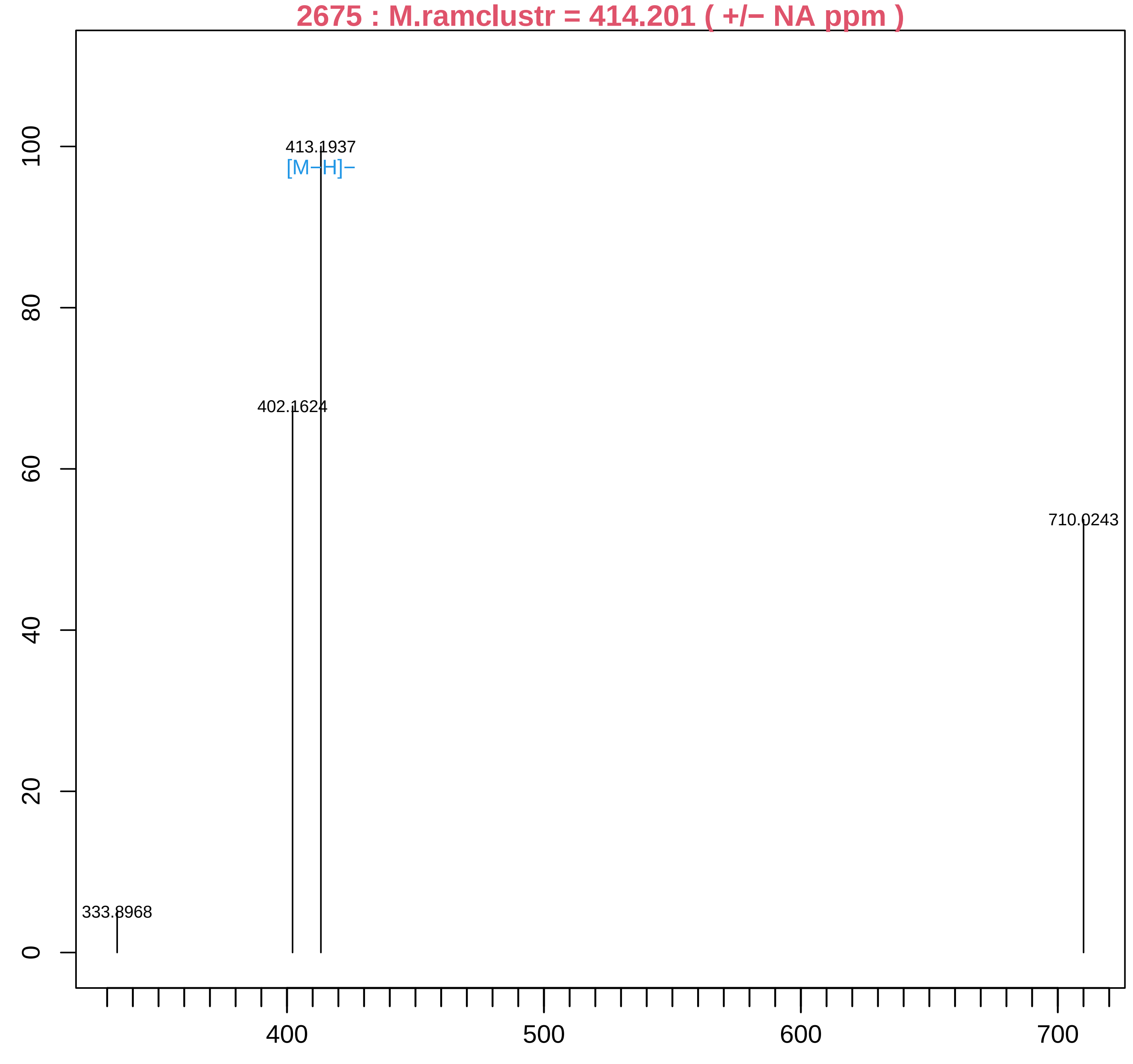

2675 : M.ramclustr = 414.201 (+/− NA ppm)
413.1937
[M−H]−
402.1624
710.0243
333.8968
0
20
40
60
80
100
400
500
600
700

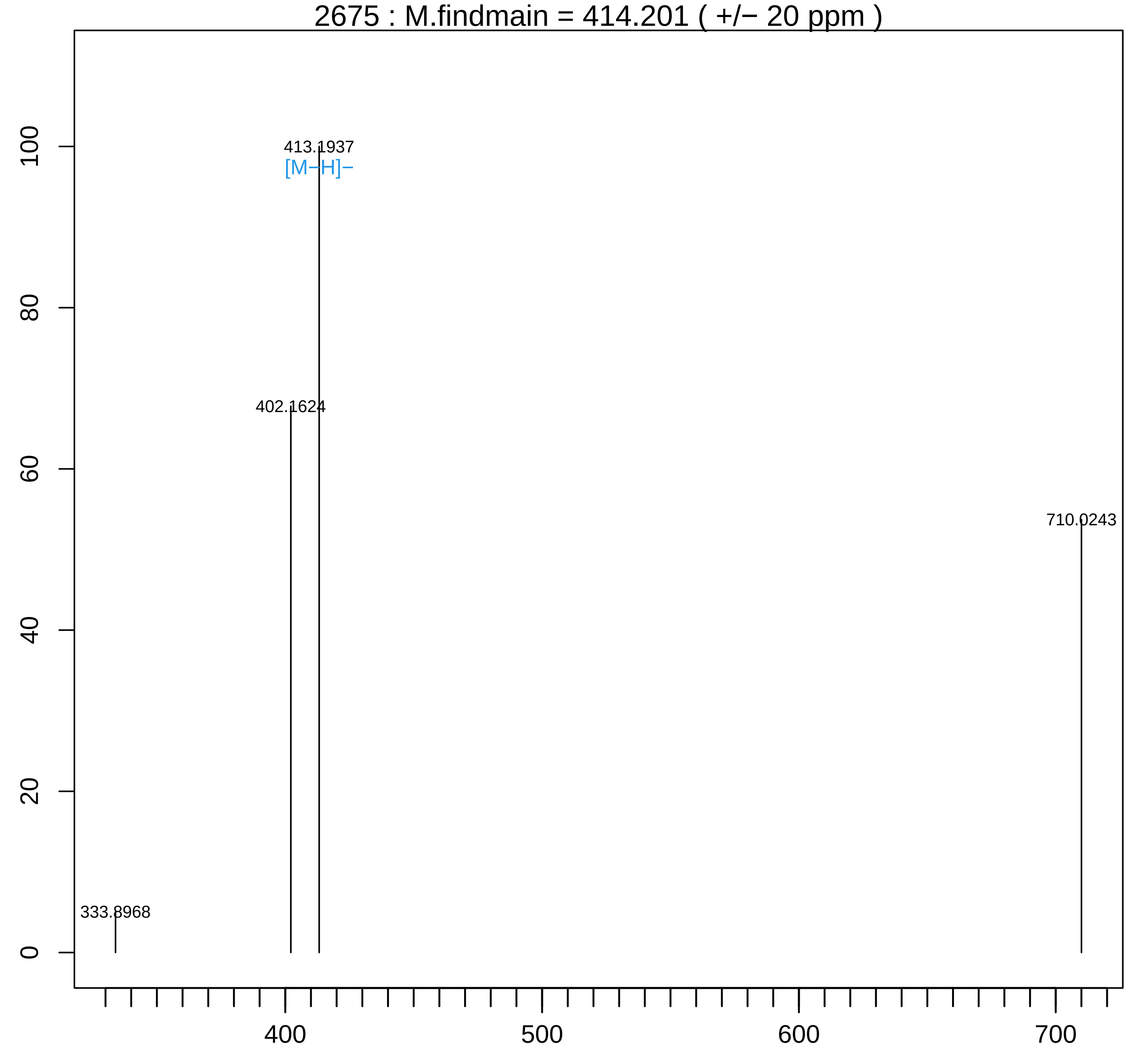

2675 : M.findmain = 414.201 (+/− 20 ppm)
413.1937
[M−H]−
402.1624
710.0243
333.8968
0
20
40
60
80
100
400
500
600
700

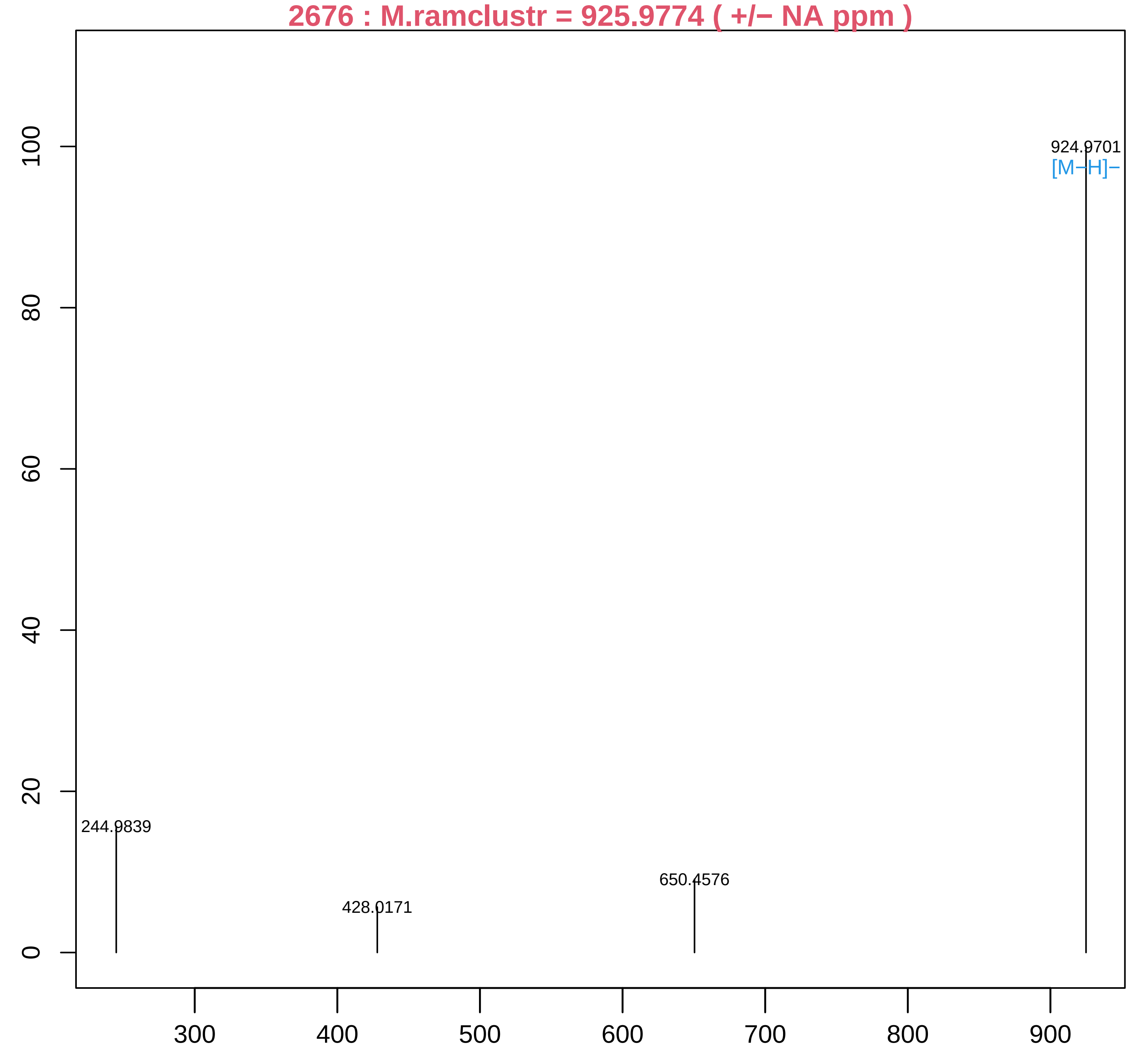
2676 : M.ramclustr = 925.9774 (+/− NA ppm)
924.9701
[M−H]−
244.9839
428.0171
650.4576
0
20
40
60
80
100
300
400
500
600
700
800
900

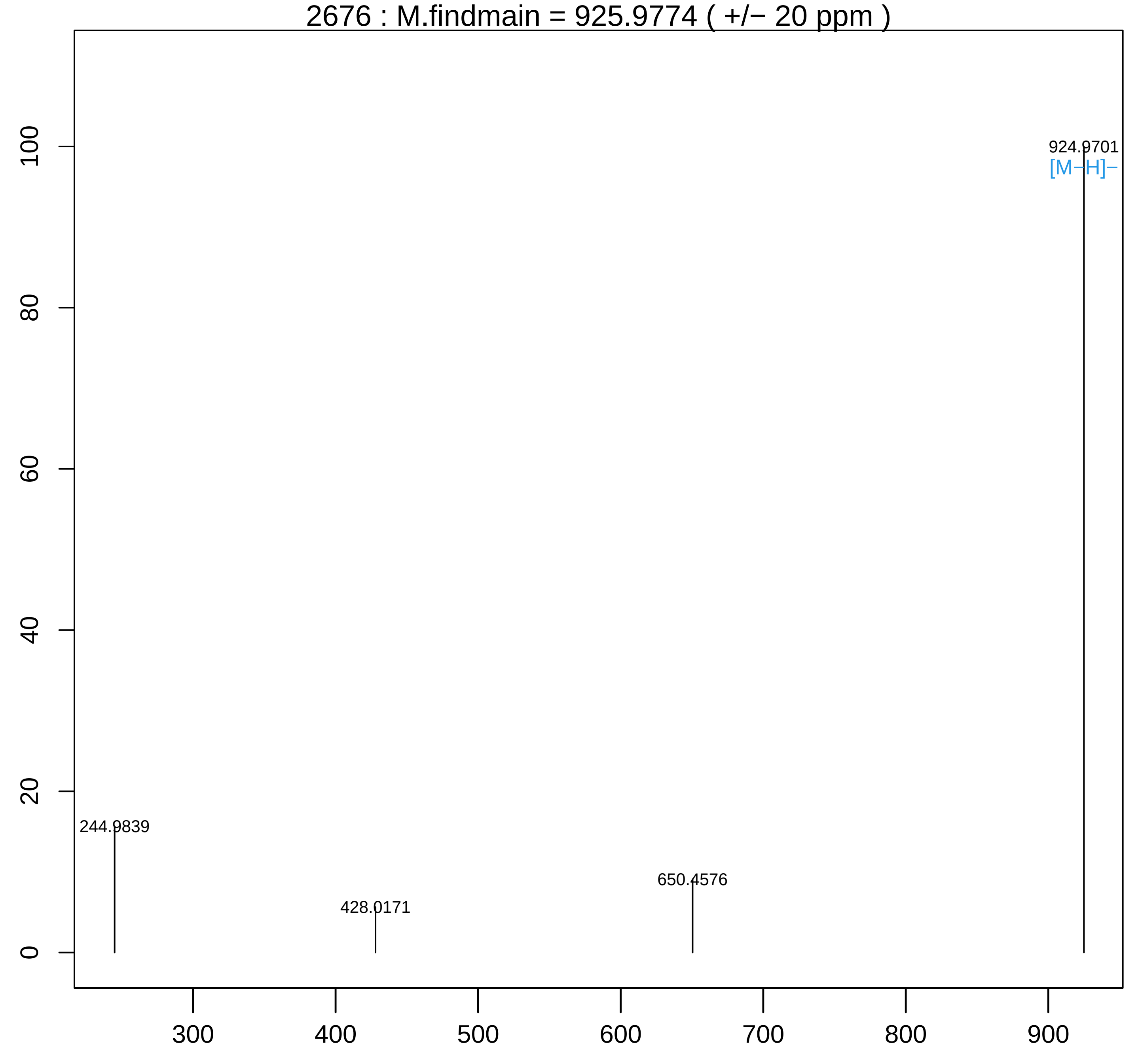
2676 : M.findmain = 925.9774 (+/− 20 ppm)
924.9701
[M−H]−
244.9839
428.0171
650.4576
0
20
40
60
80
100
300
400
500
600
700
800
900

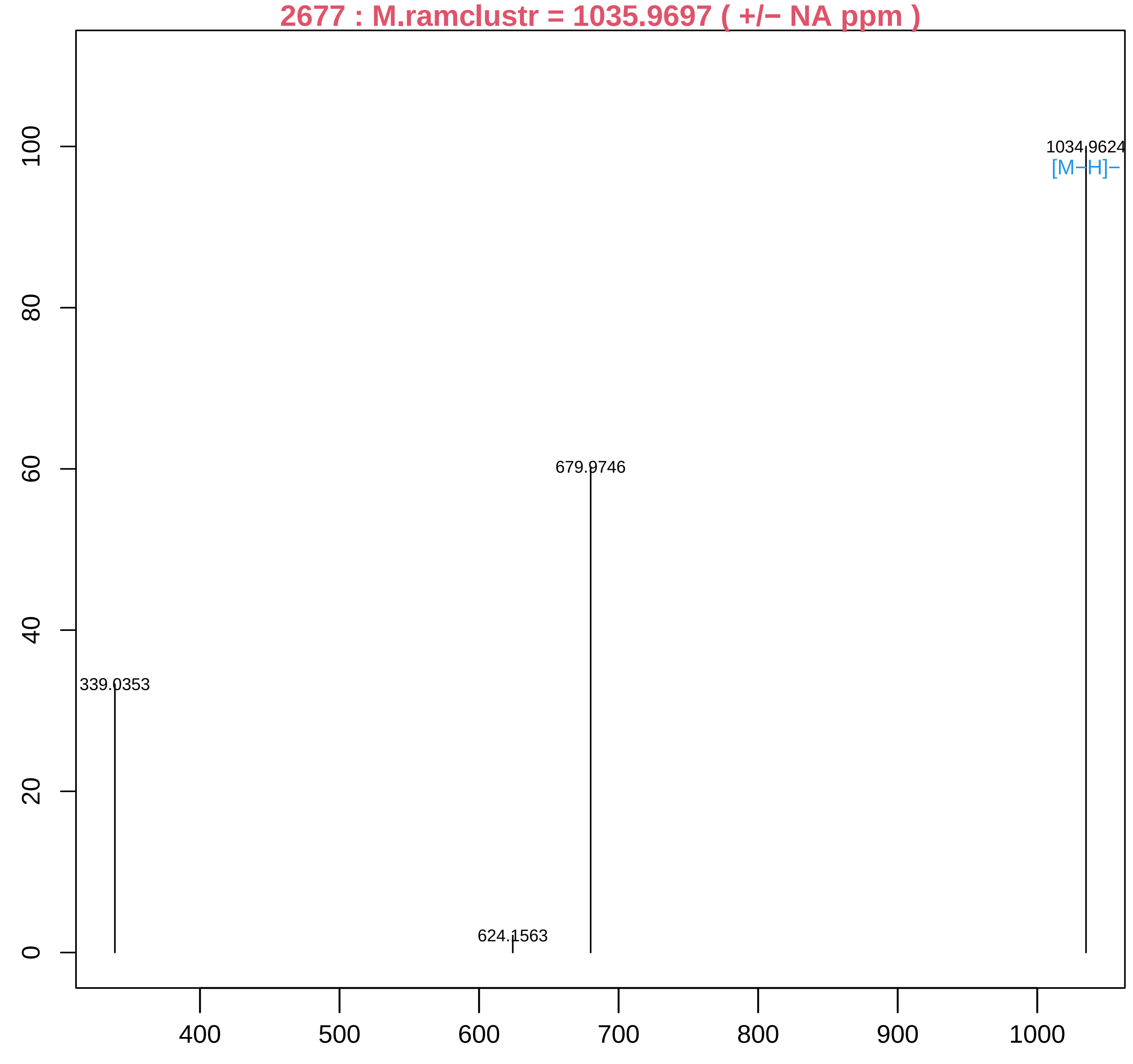
2677 : M.ramclustr = 1035.9697 (+/− NA ppm)
1034.9624
[M−H]−
679.9746
339.0353
624.1563
0
20
40
60
80
100
400
500
600
700
800
900
1000

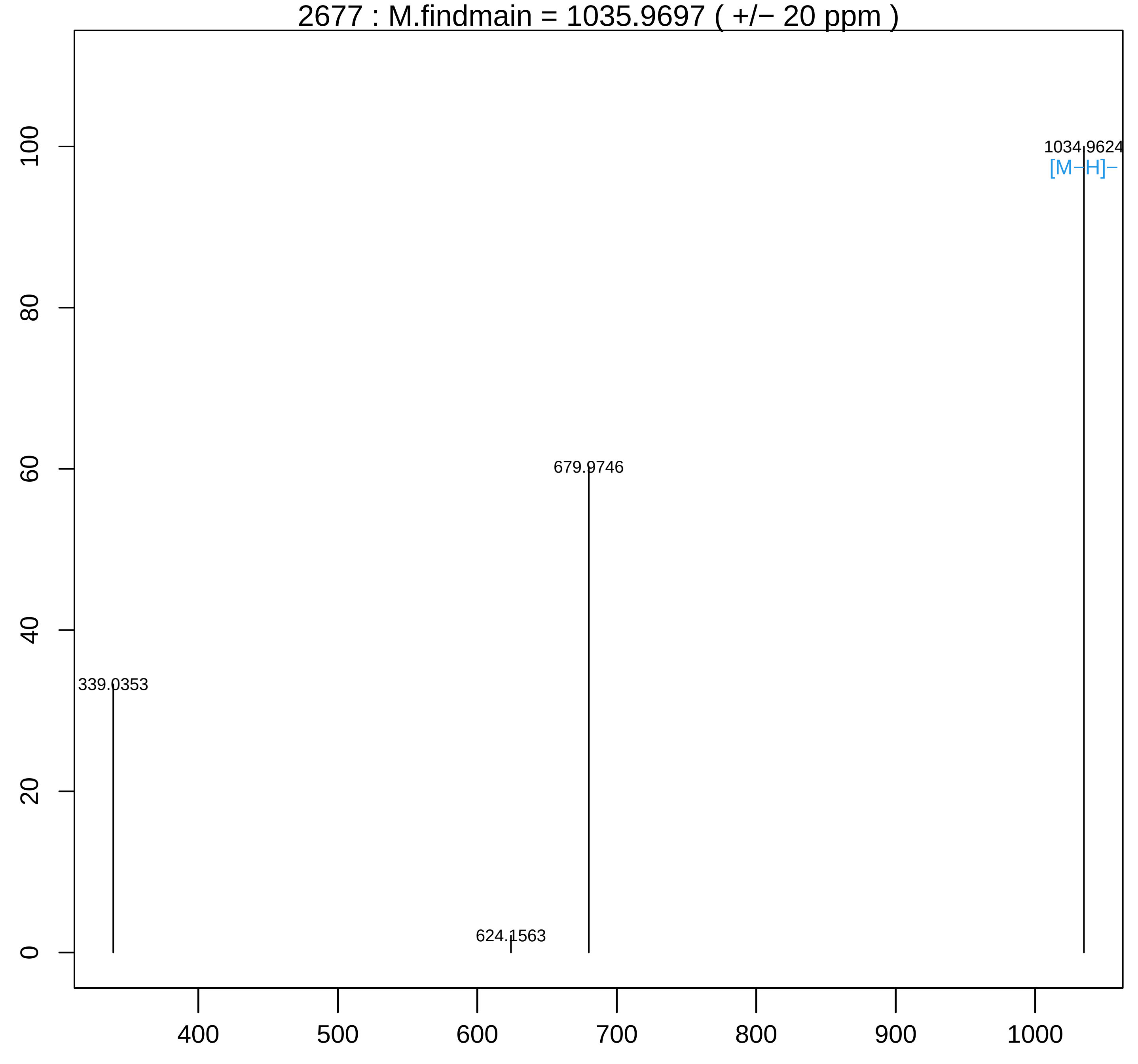
2677 : M.findmain = 1035.9697 (+/− 20 ppm)
1034.9624
[M−H]−
679.9746
339.0353
624.1563
0
20
40
60
80
100
400
500
600
700
800
900
1000

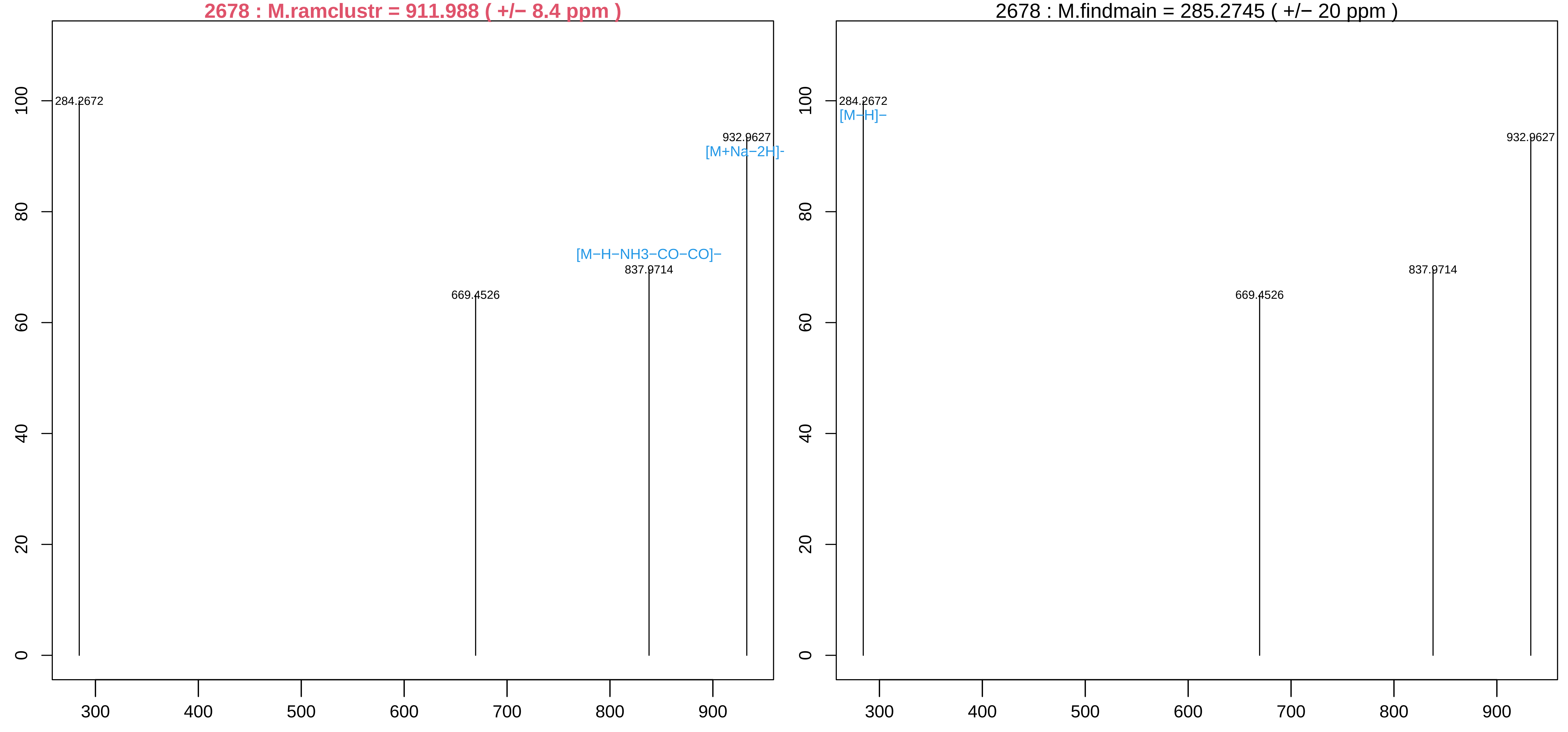
2678 : M.ramclustr = 911.988 (+/− 8.4 ppm)
284.2672
932.9627
[M+Na−2H]-
[M−H−NH3−CO−CO]−
837.9714
669.4526
0
20
40
60
80
100
300
400
500
600
700
800
900
2678 : M.findmain = 285.2745 (+/− 20 ppm)
284.2672
[M−H]−
932.9627
837.9714
669.4526

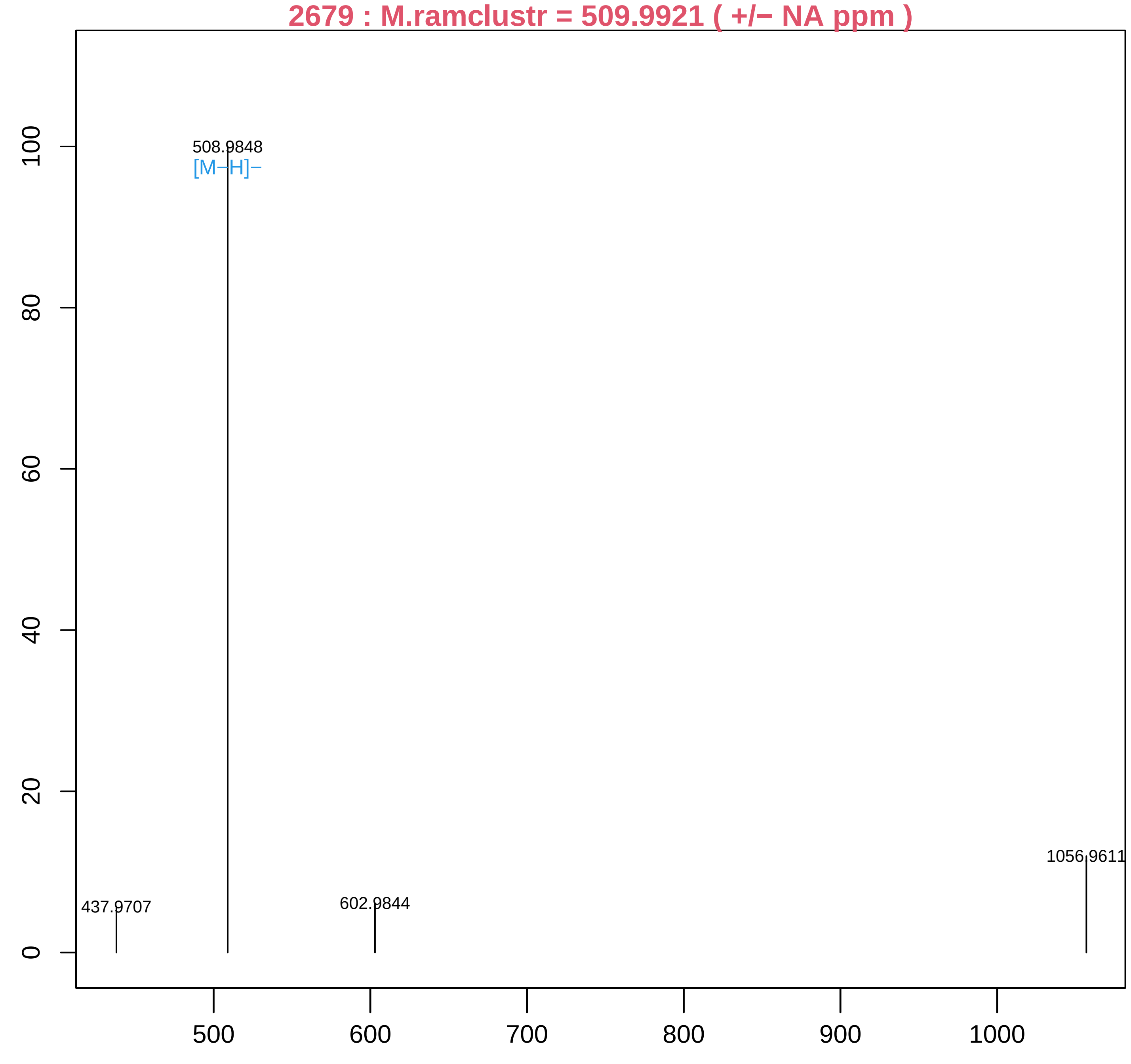
2679 : M.ramclustr = 509.9921 (+/− NA ppm)
508.9848
[M−H]−
437.9707
602.9844
1056.9611
0
20
40
60
80
100
500
600
700
800
900
1000

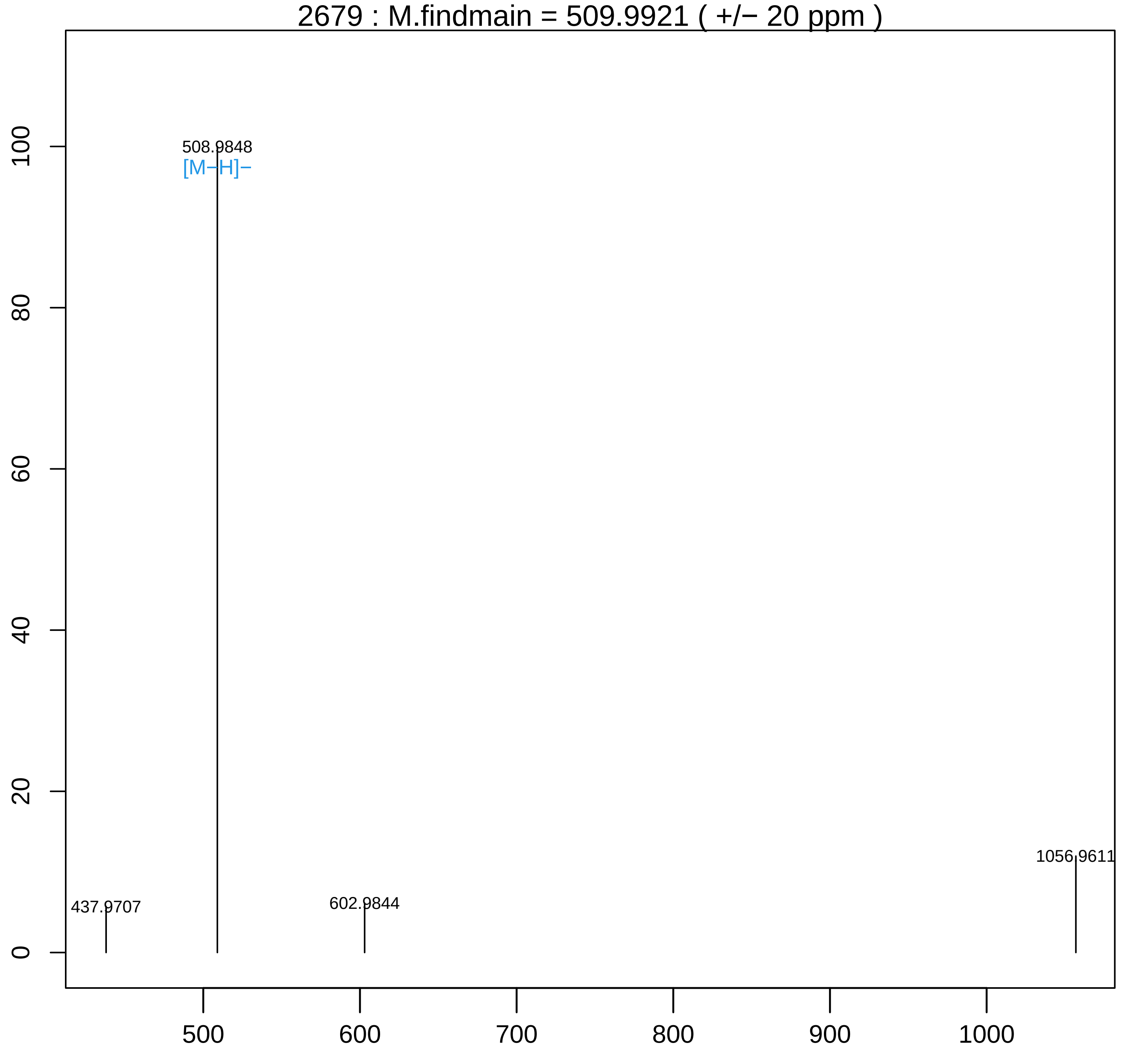
2679 : M.findmain = 509.9921 (+/− 20 ppm)
508.9848
[M−H]−
437.9707
602.9844
1056.9611
0
20
40
60
80
100
500
600
700
800
900
1000

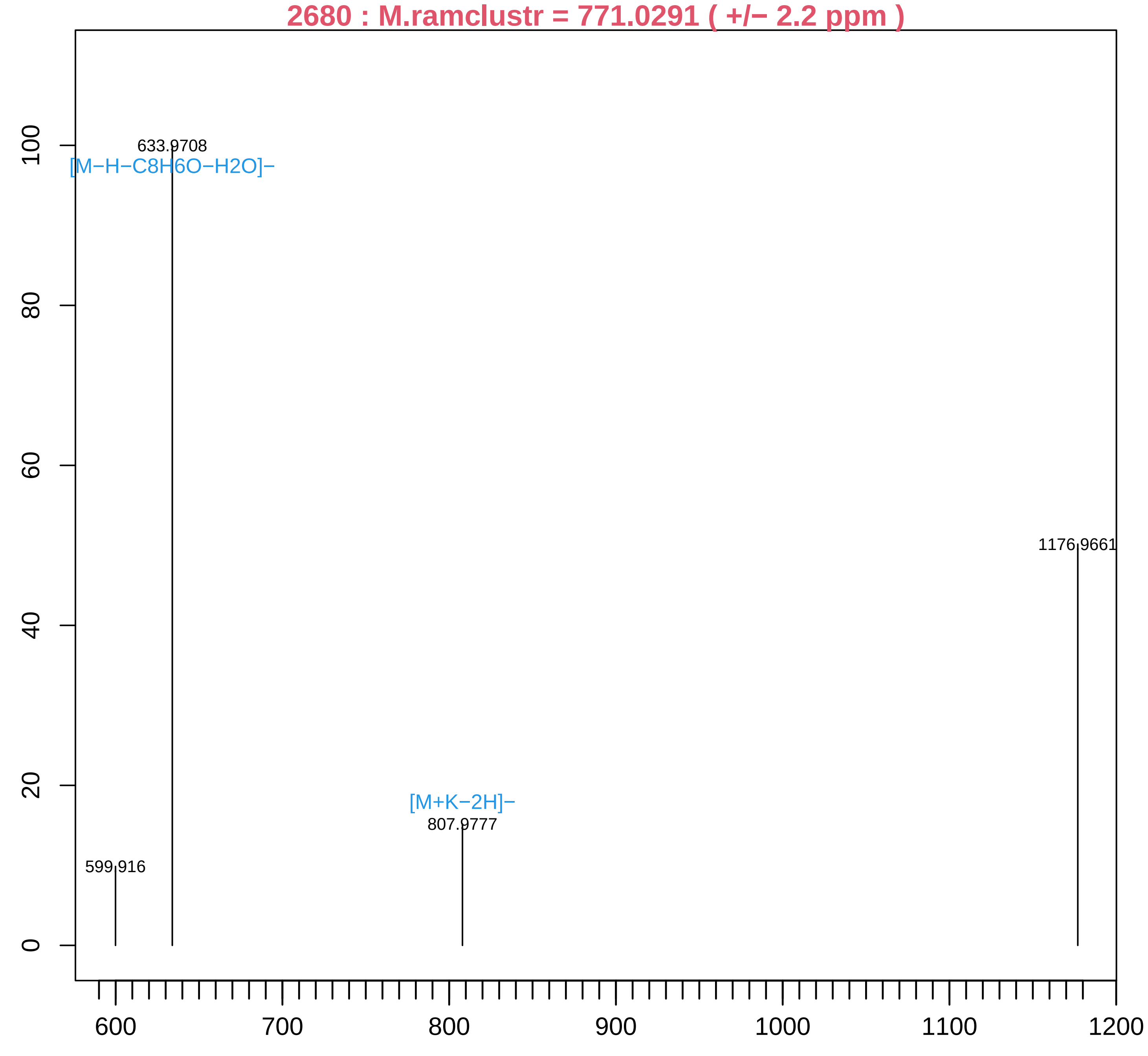
2680 : M.ramclustr = 771.0291 (+/− 2.2 ppm)
633.9708
[M−H−C8H6O−H2O]−
[M+K−2H]−
807.9777
599.916
1176.9661
0
20
40
60
80
100
600
700
800
900
1000
1100
1200

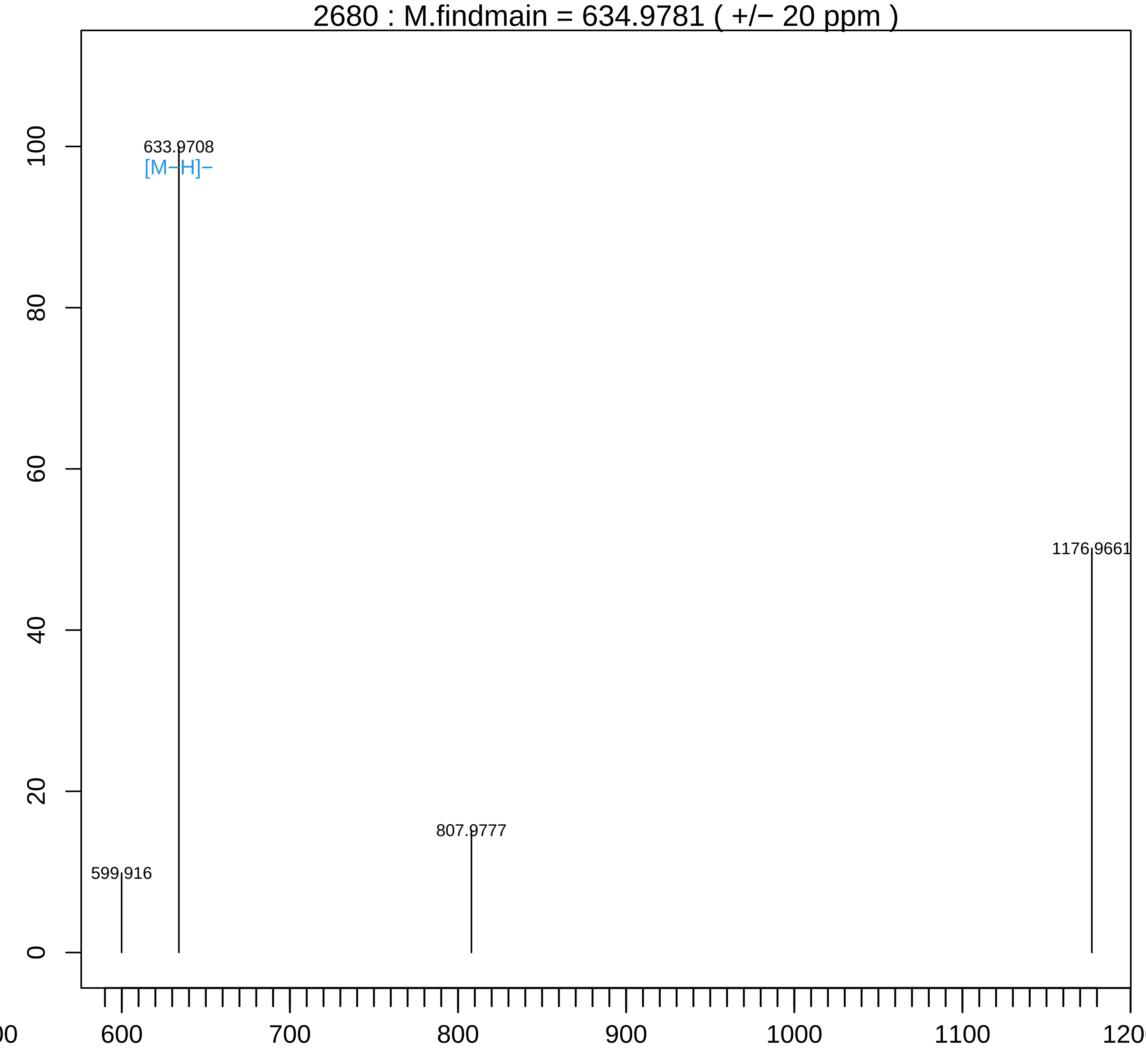
2680 : M.findmain = 634.9781 (+/− 20 ppm)
633.9708
[M−H]−
807.9777
599.916
1176.9661
0
20
40
60
80
100
600
700
800
900
1000
1100
1200

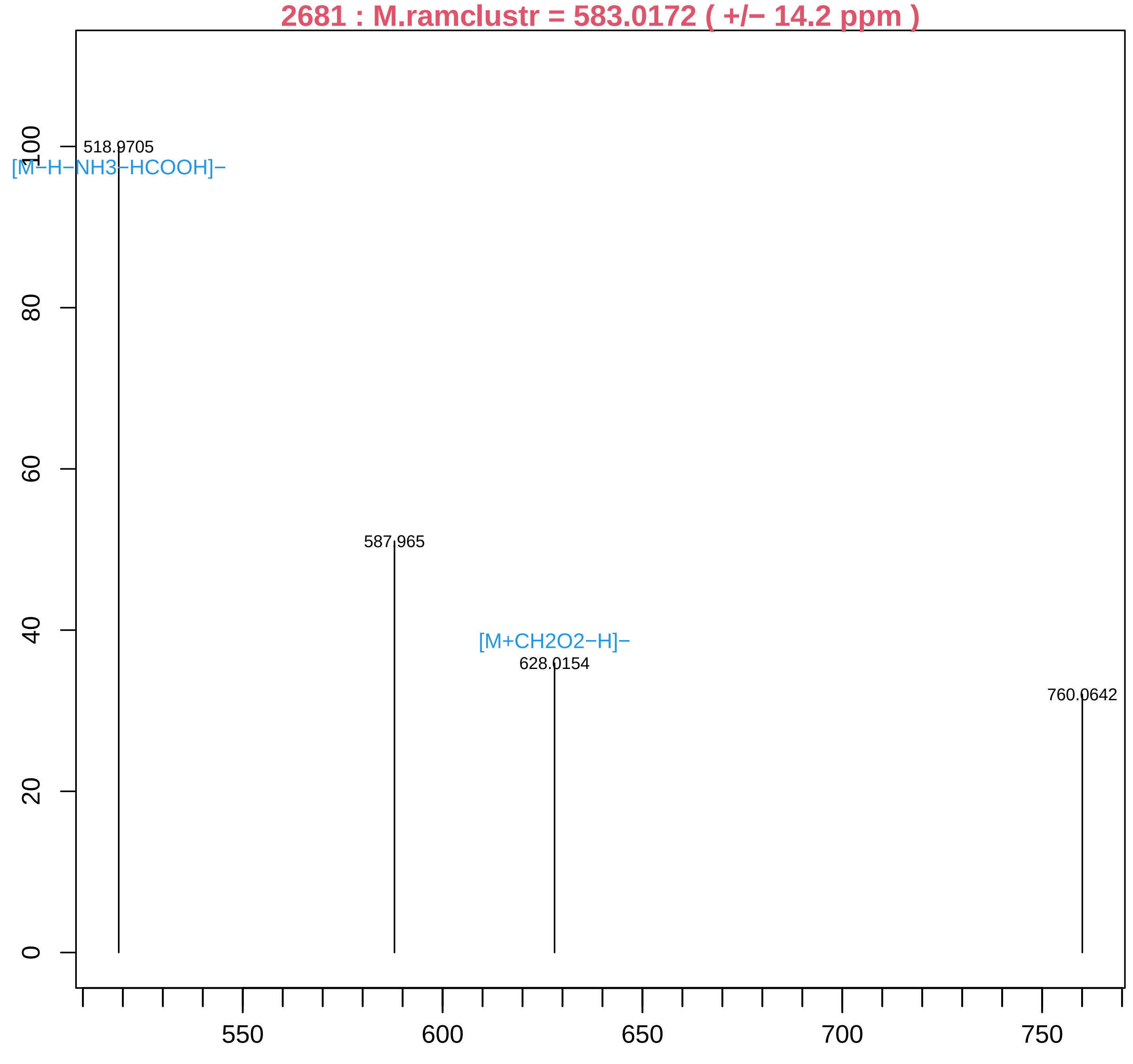
2681 : M.ramclustr = 583.0172 (+/− 14.2 ppm)
518.9705
[M−H−NH3−HCOOH]−
587.965
[M+CH2O2−H]−
628.0154
760.0642
0
20
40
60
80
100
550
600
650
700
750

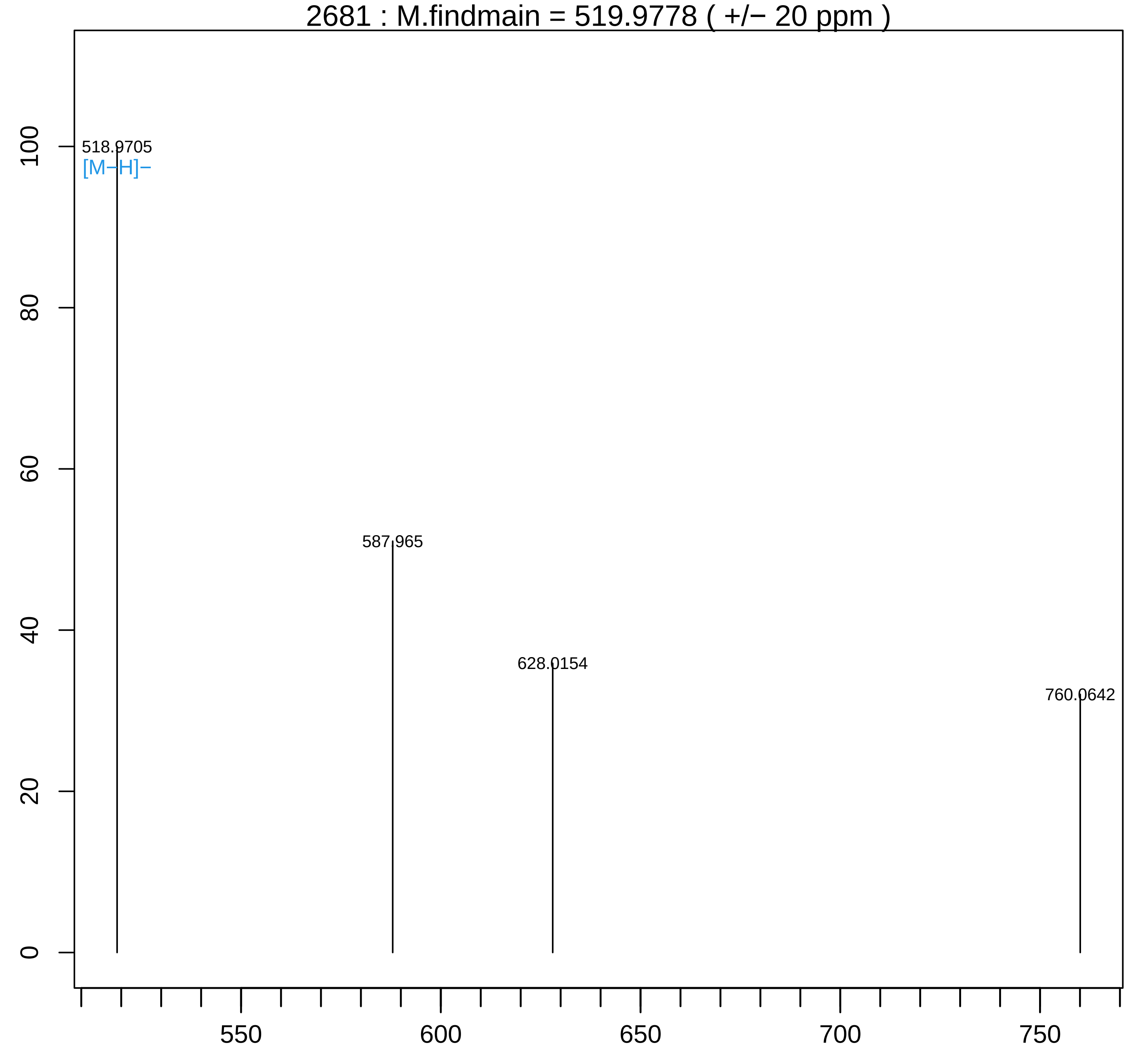
2681 : M.findmain = 519.9778 (+/− 20 ppm)
518.9705
[M−H]−
587.965
628.0154
760.0642
0
20
40
60
80
100
550
600
650
700
750

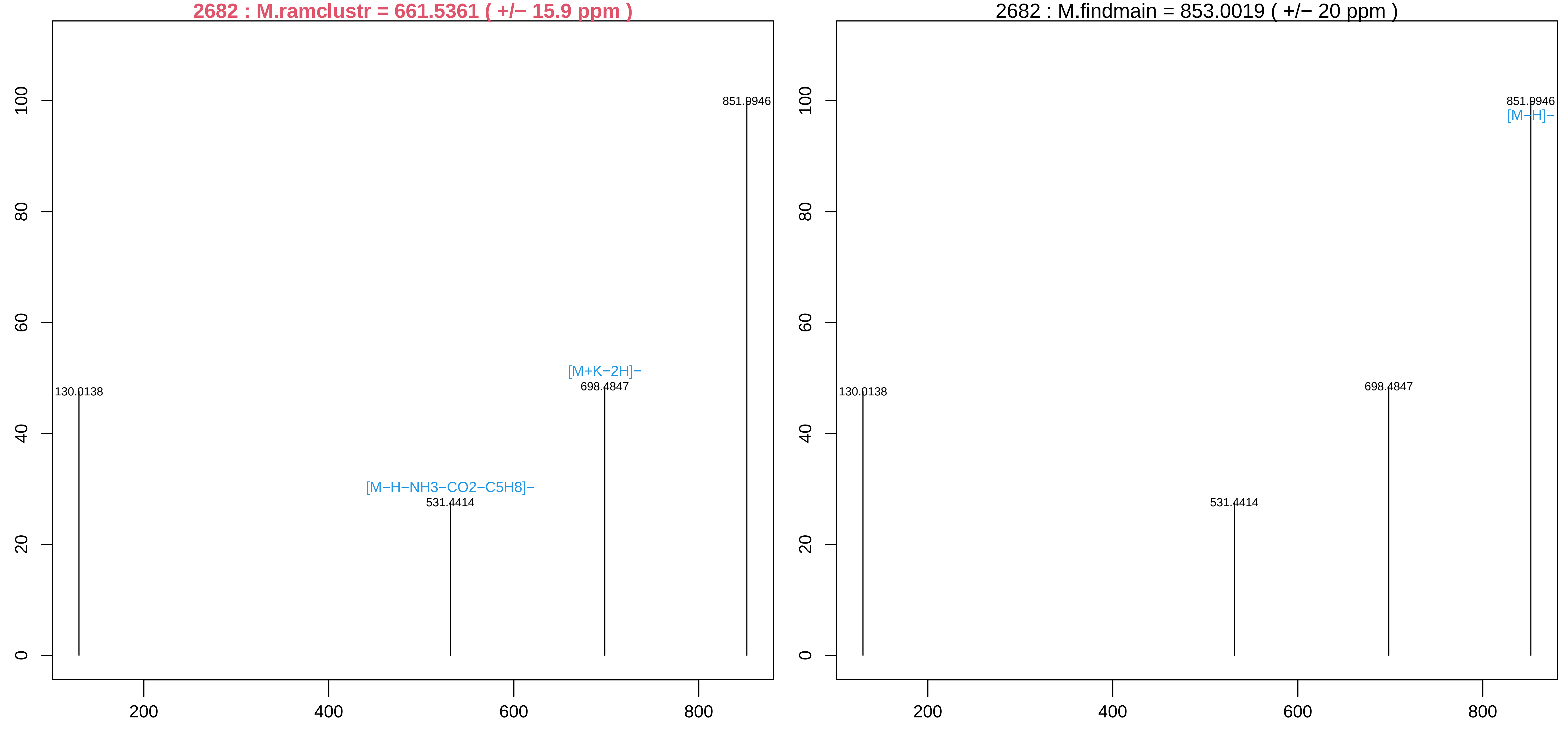
2682 : M.ramclustr = 661.5361 (+/− 15.9 ppm)
851.9946
[M+K−2H]−
698.4847
130.0138
[M−H−NH3−CO2−C5H8]−
531.4414
2682 : M.findmain = 853.0019 (+/− 20 ppm)
851.9946
[M−H]−
130.0138
698.4847
531.4414
0
20
40
60
80
100
200
400
600
800

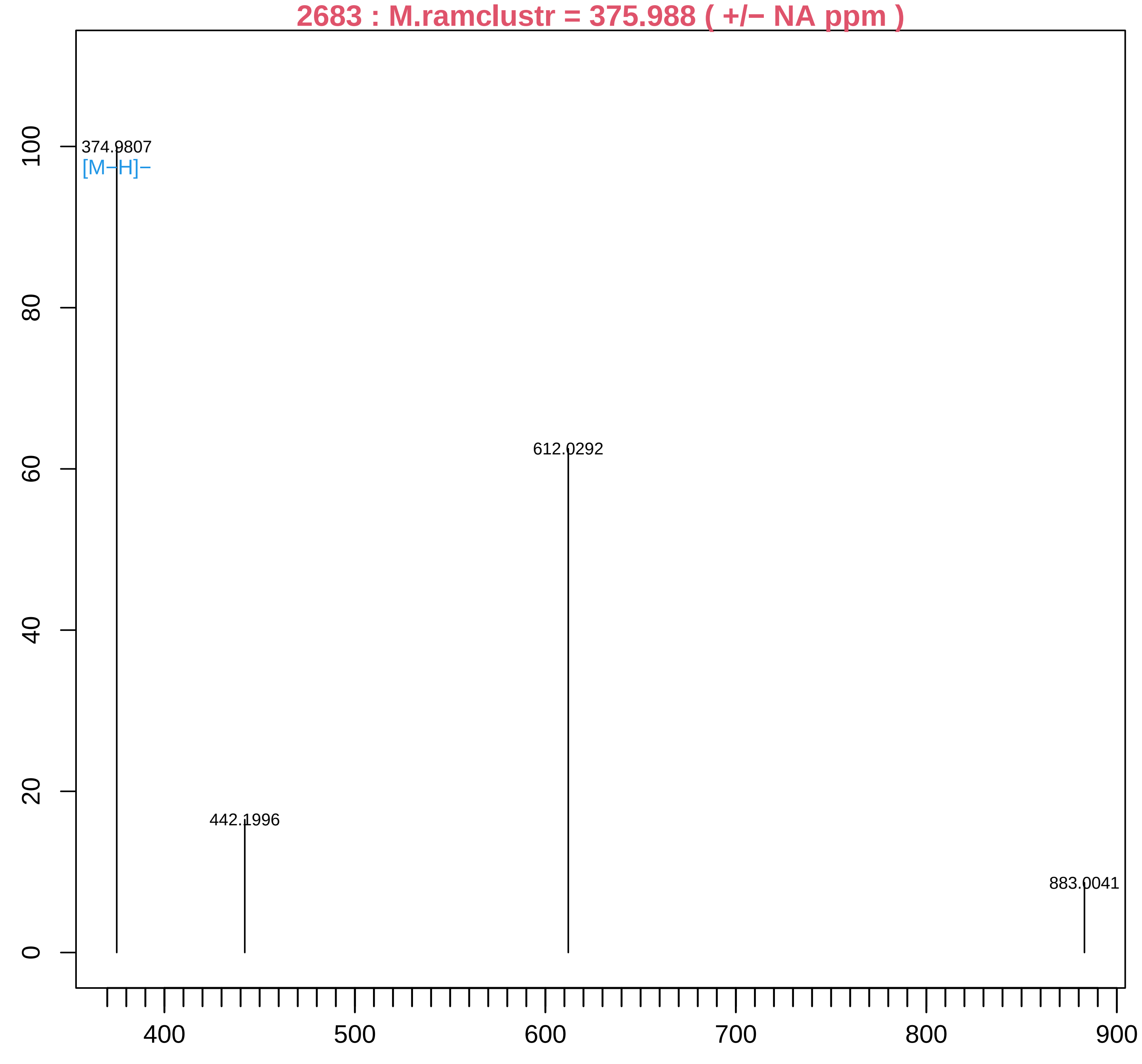

2683 : M.ramclustr = 375.988 (+/− NA ppm)
374.9807
[M−H]−
442.1996
612.0292
883.0041
0
20
40
60
80
100
400
500
600
700
800
900

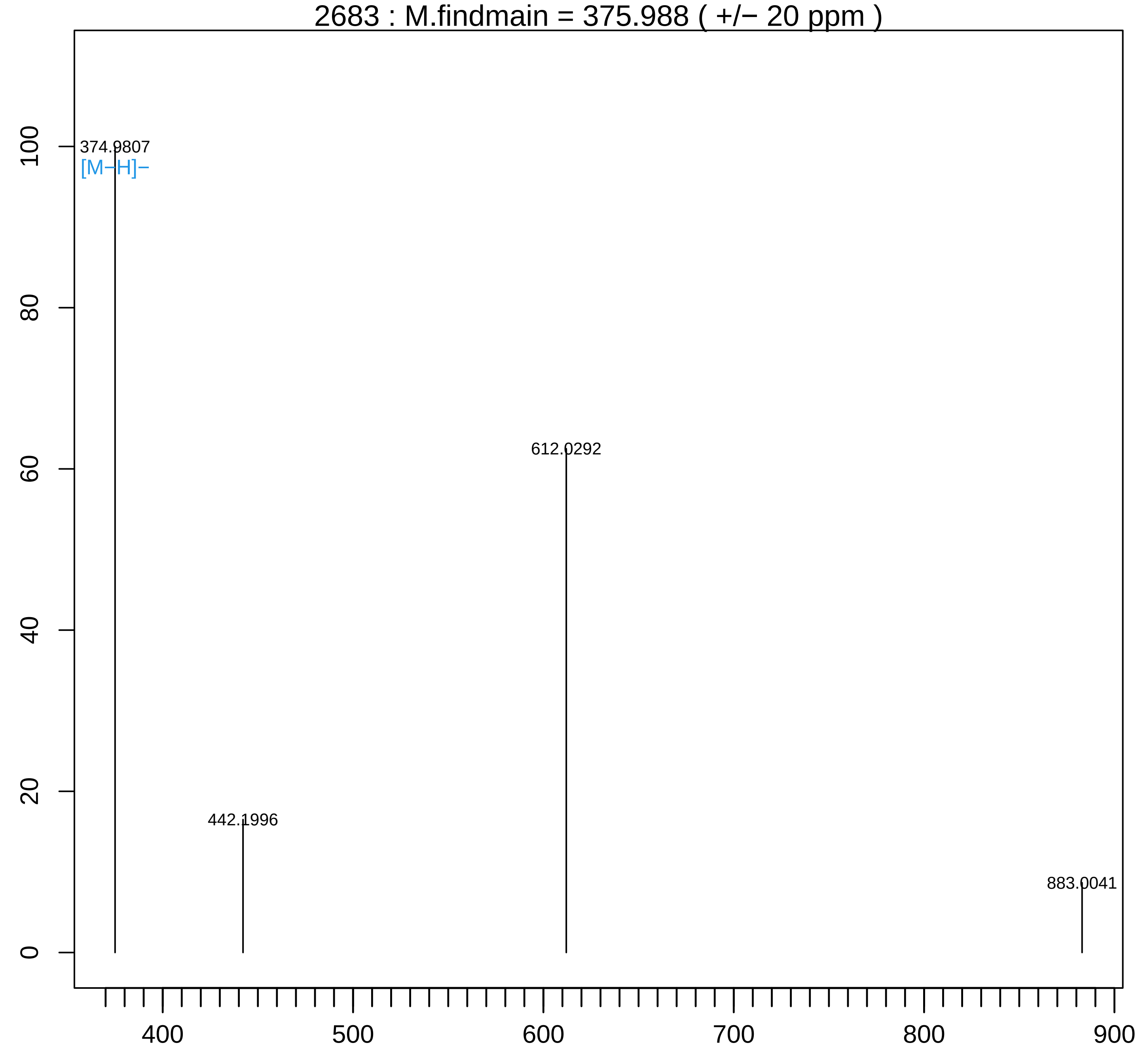

2683 : M.findmain = 375.988 (+/− 20 ppm)
374.9807
[M−H]−
442.1996
612.0292
883.0041
0
20
40
60
80
100
400
500
600
700
800
900

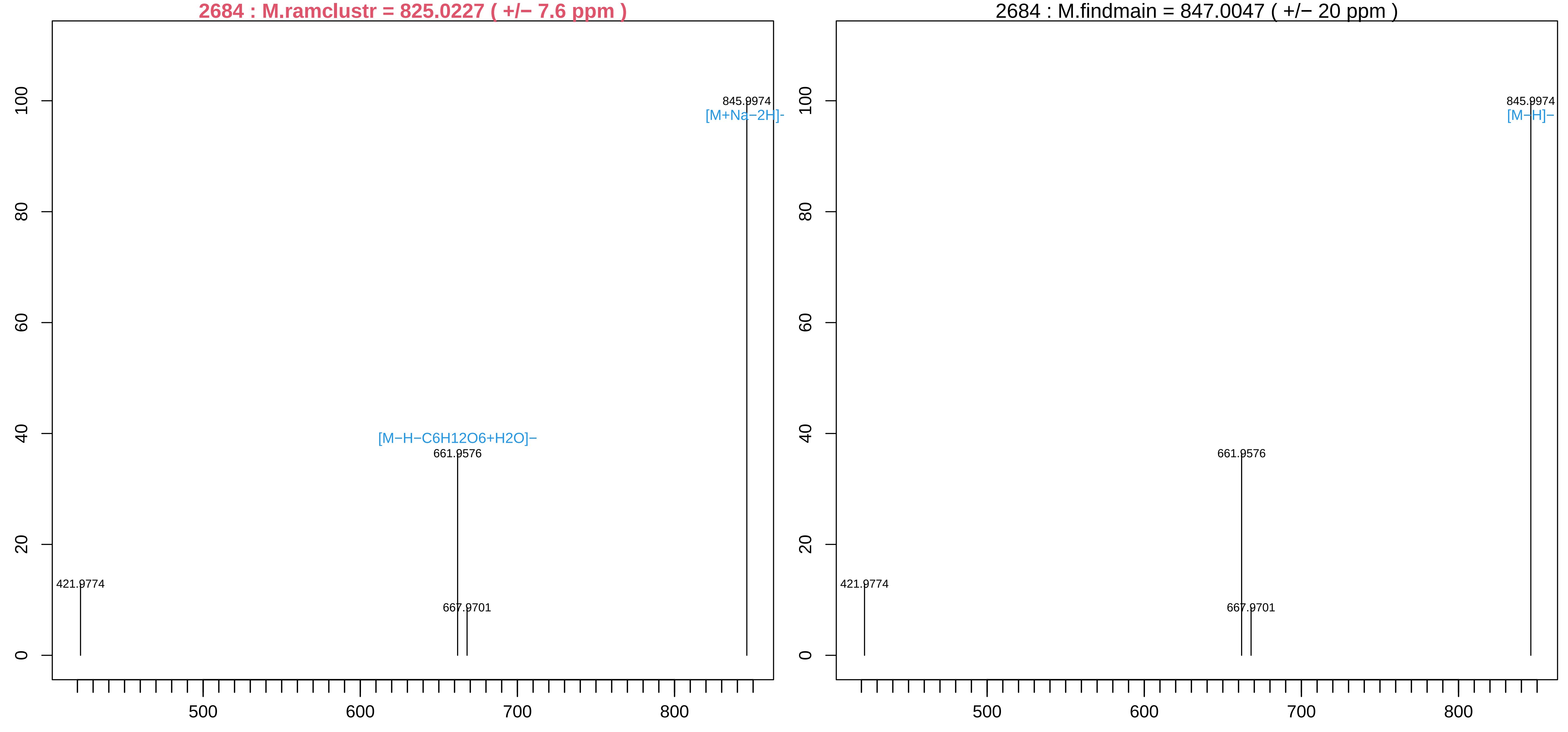
2684 : M.ramclustr = 825.0227 (+/− 7.6 ppm)
845.9974
[M+Na−2H]-
[M−H−C6H12O6+H2O]−
661.9576
421.9774
667.9701
0
20
40
60
80
100
500
600
700
800
2684 : M.findmain = 847.0047 (+/− 20 ppm)
845.9974
[M−H]−
661.9576
421.9774
667.9701
0
20
40
60
80
100
500
600
700
800

2685 : M.ramclustr = 1053.9831 (+/− 14.1 ppm)

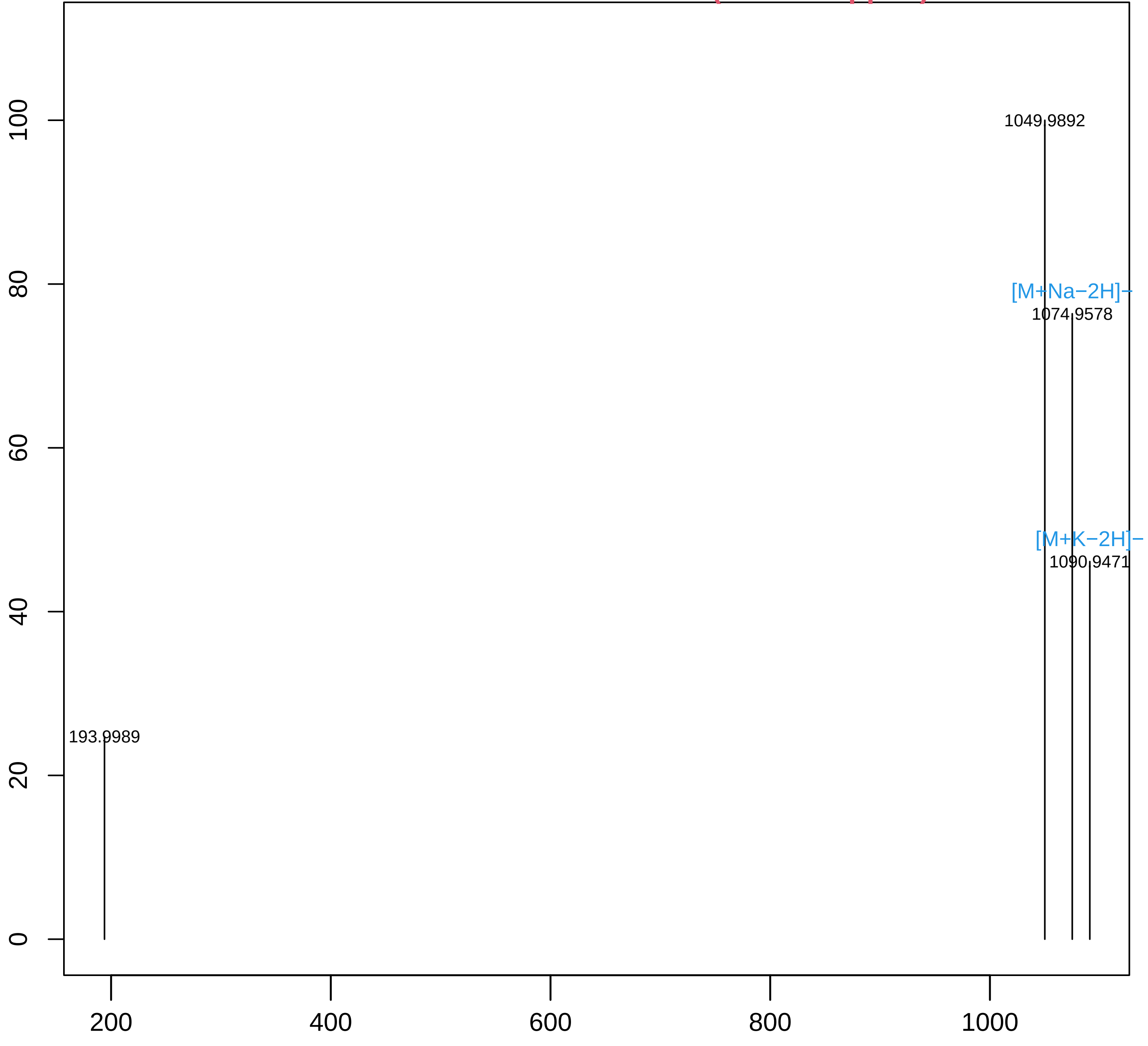

2685 : M.findmain = 1053.9831 (+/− 14.1 ppm)

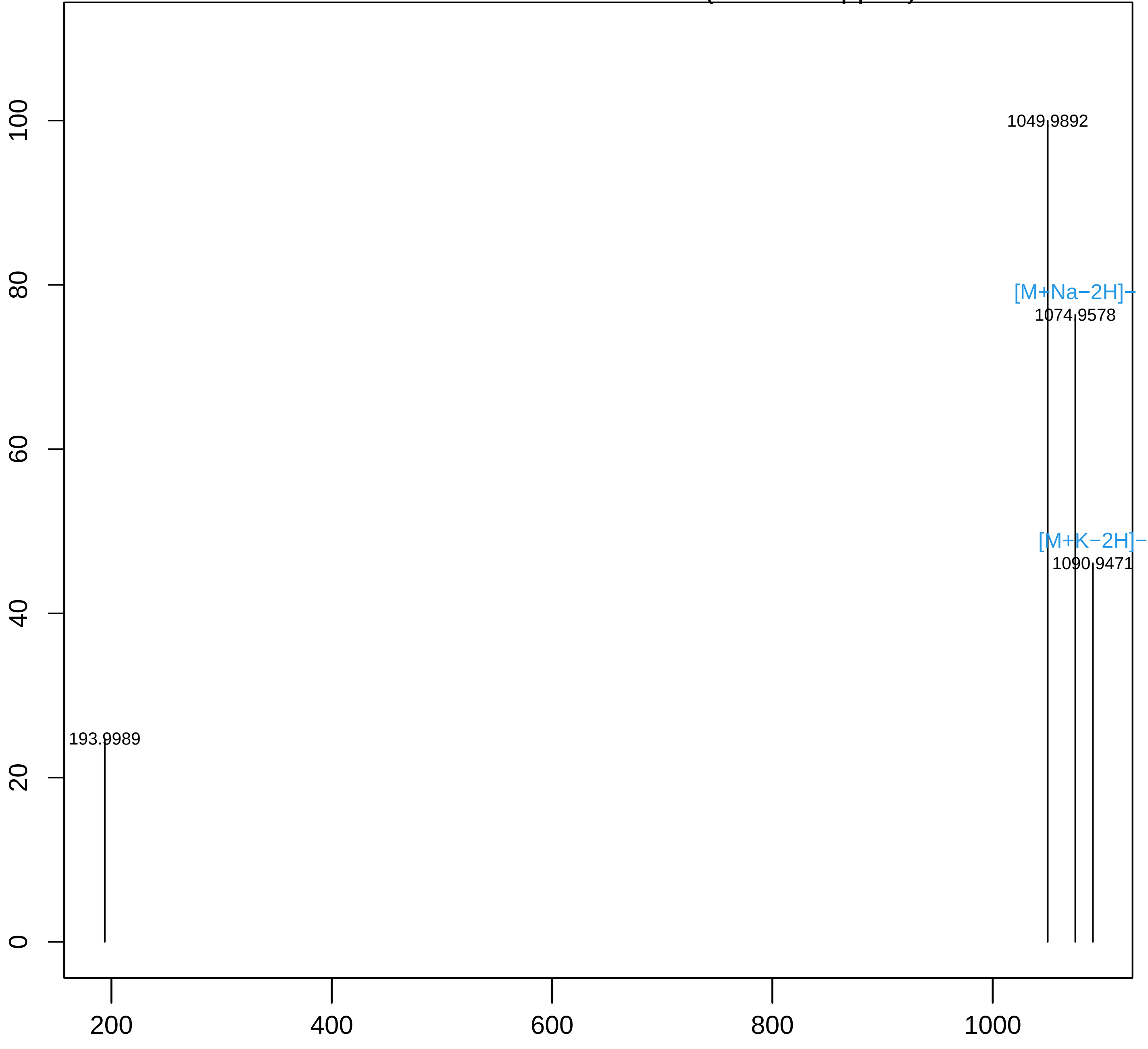

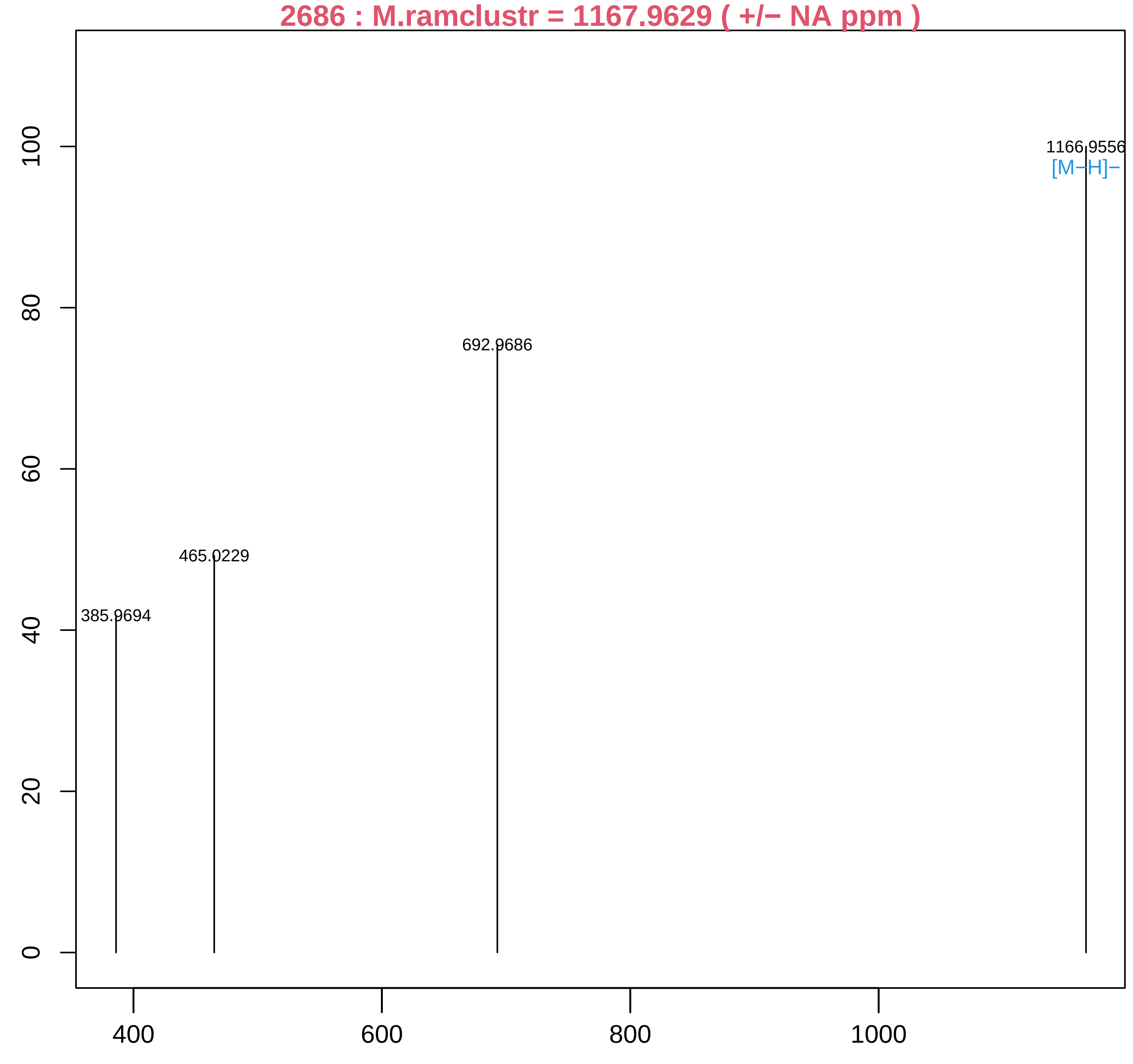
2686 : M.ramclustr = 1167.9629 (+/− NA ppm)
1166.9556
[M−H]−
692.9686
465.0229
385.9694
0
20
40
60
80
100
400
600
800
1000

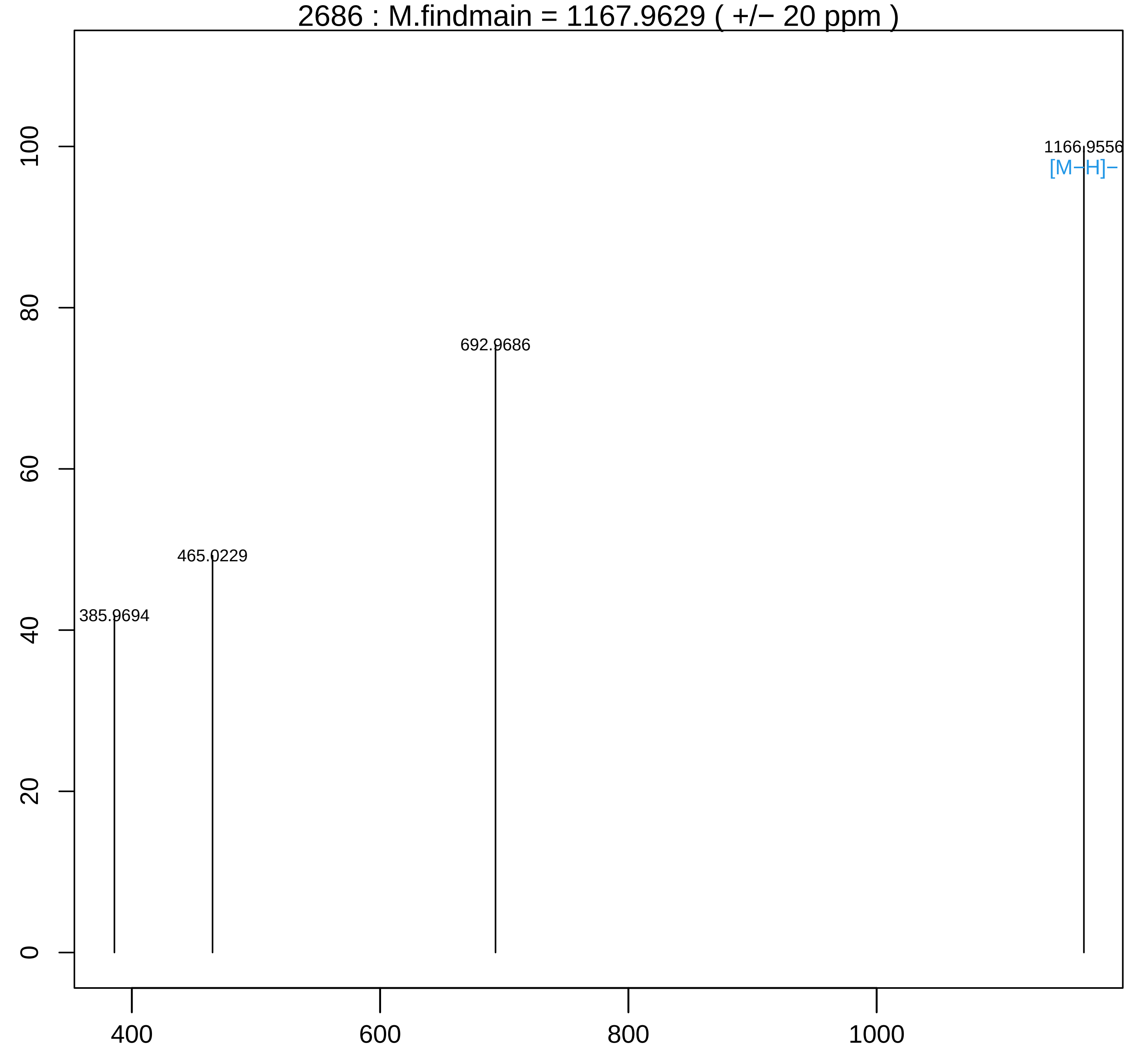
2686 : M.findmain = 1167.9629 (+/− 20 ppm)
1166.9556
[M−H]−
692.9686
465.0229
385.9694
0
20
40
60
80
100
400
600
800
1000

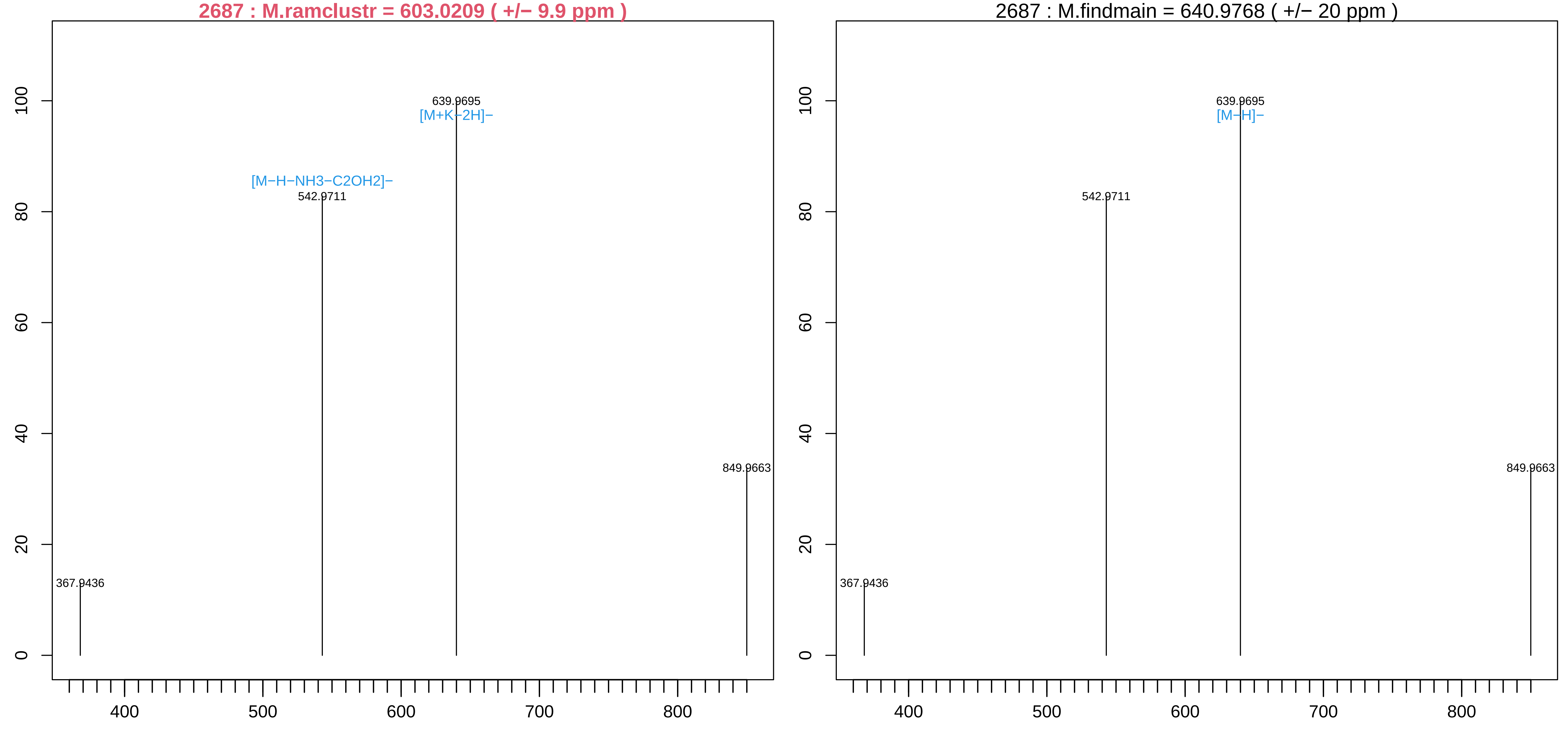
2687 : M.ramclustr = 603.0209 (+/− 9.9 ppm)
639.9695
[M+K−2H]−
[M−H−NH3−C2OH2]−
542.9711
849.9663
367.9436
2687 : M.findmain = 640.9768 (+/− 20 ppm)
639.9695
[M−H]−
542.9711
849.9663
367.9436

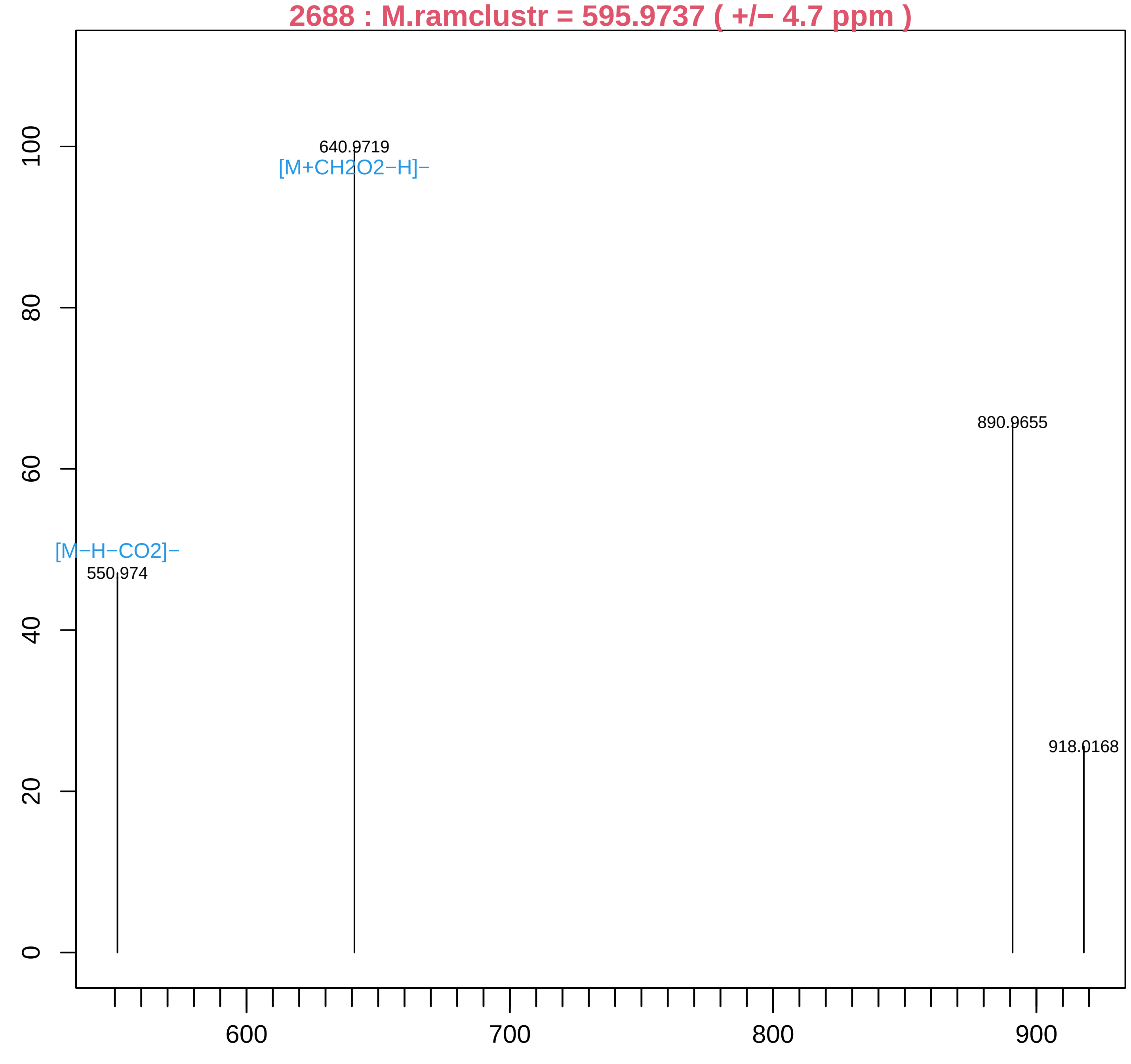
2688 : M.ramclustr = 595.9737 (+/− 4.7 ppm)
640.9719
[M+CH2O2−H]−
890.9655
[M−H−CO2]−
550.974
918.0168
0
20
40
60
80
100
600
700
800
900

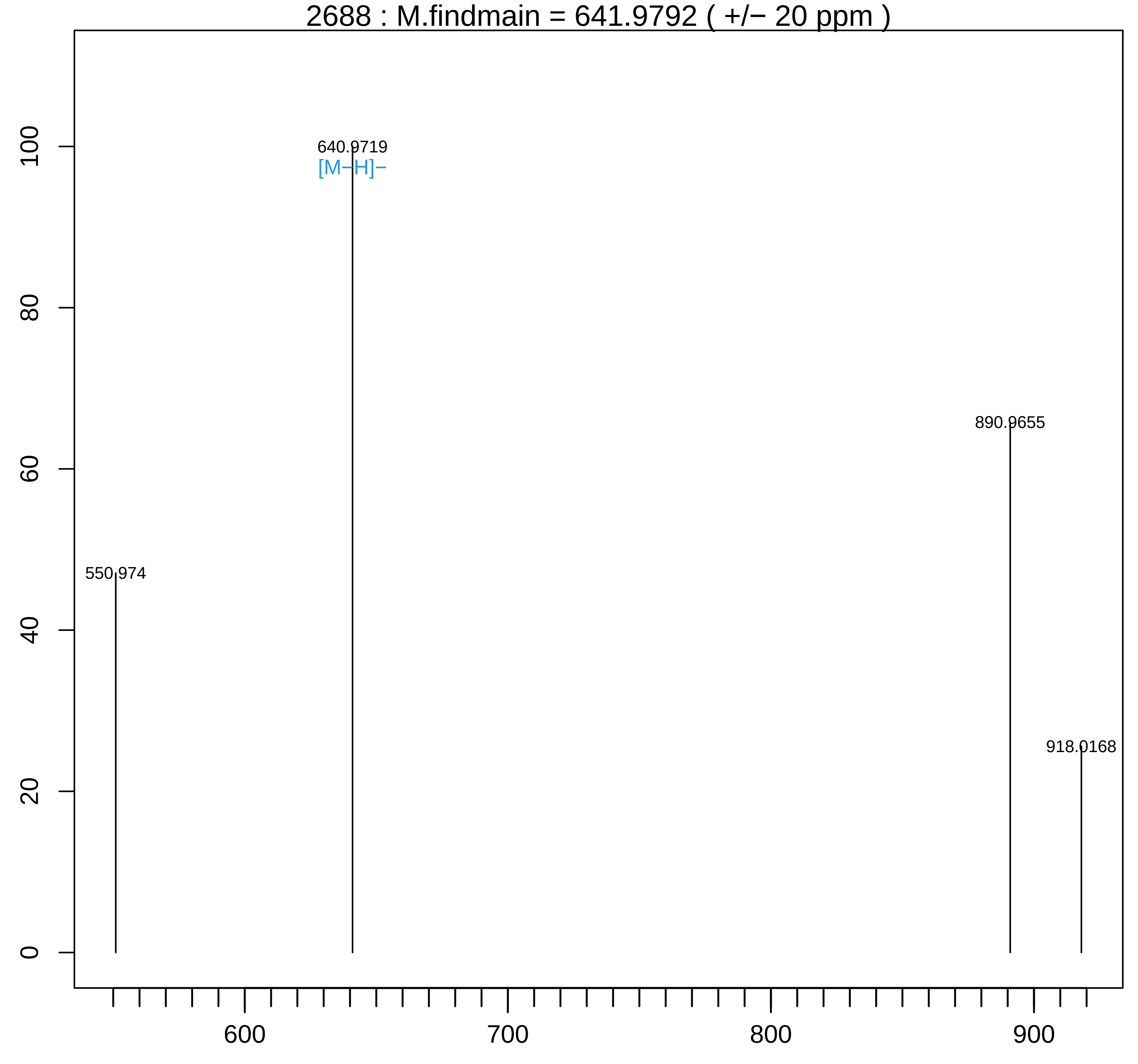
2688 : M.findmain = 641.9792 (+/− 20 ppm)
640.9719
[M−H]−
890.9655
550.974
918.0168
0
20
40
60
80
100
600
700
800
900

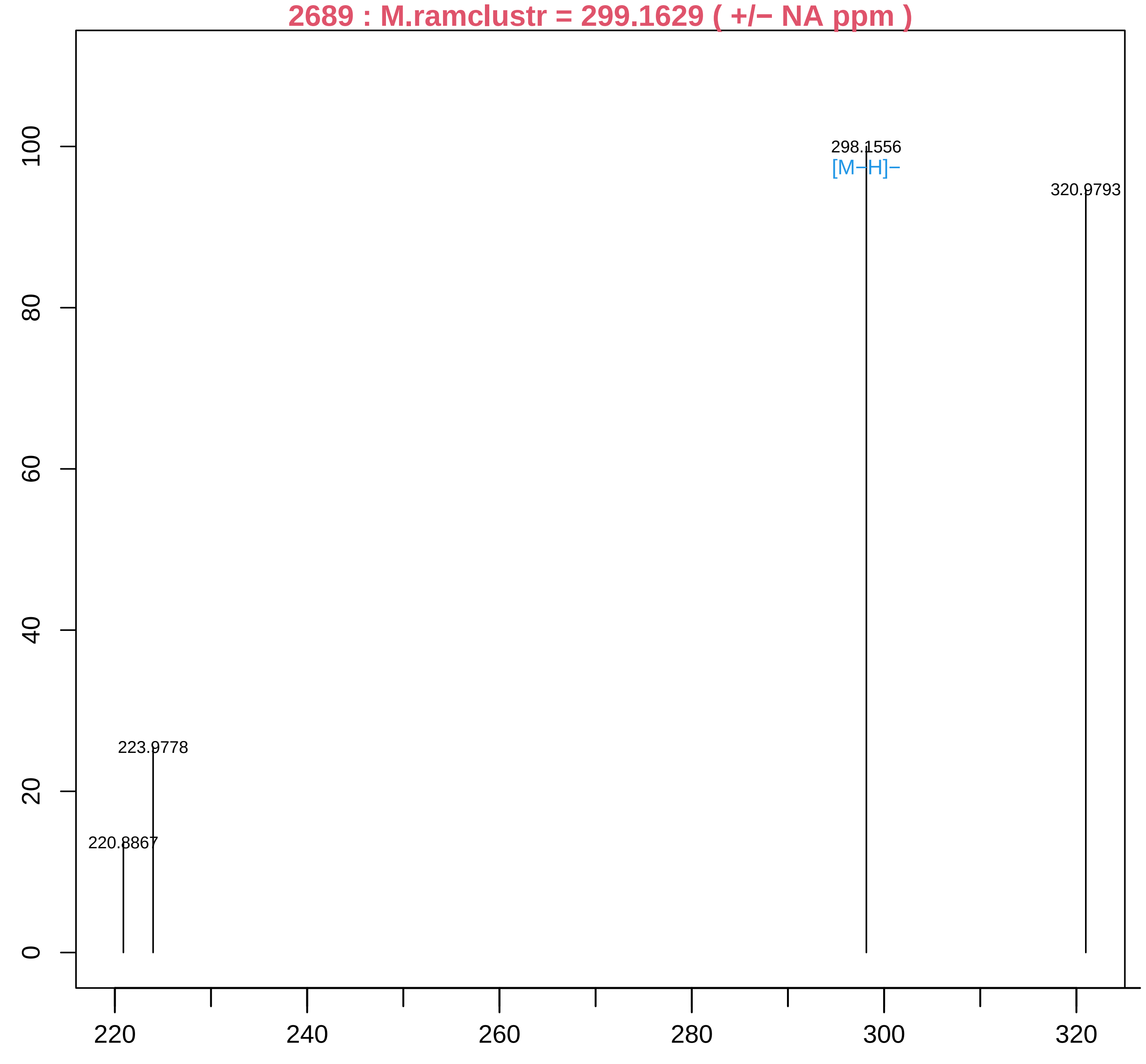

2689 : M.ramclustr = 299.1629 (+/− NA ppm)
298.1556
[M−H]−
320.9793
223.9778
220.8867
0
20
40
60
80
100
220
240
260
280
300
320

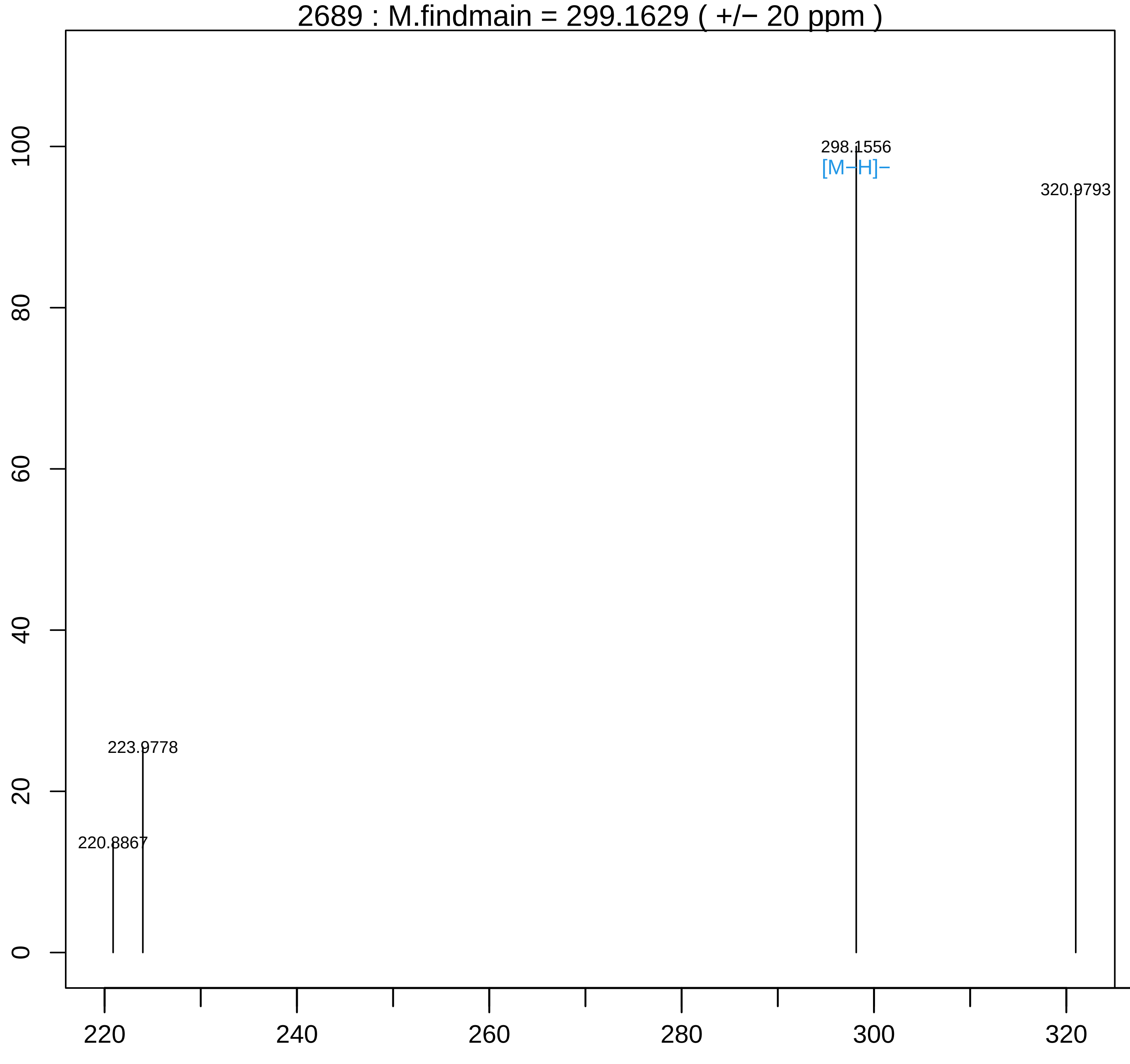

2689 : M.findmain = 299.1629 (+/− 20 ppm)
298.1556
[M−H]−
320.9793
223.9778
220.8867
0
20
40
60
80
100
220
240
260
280
300
320

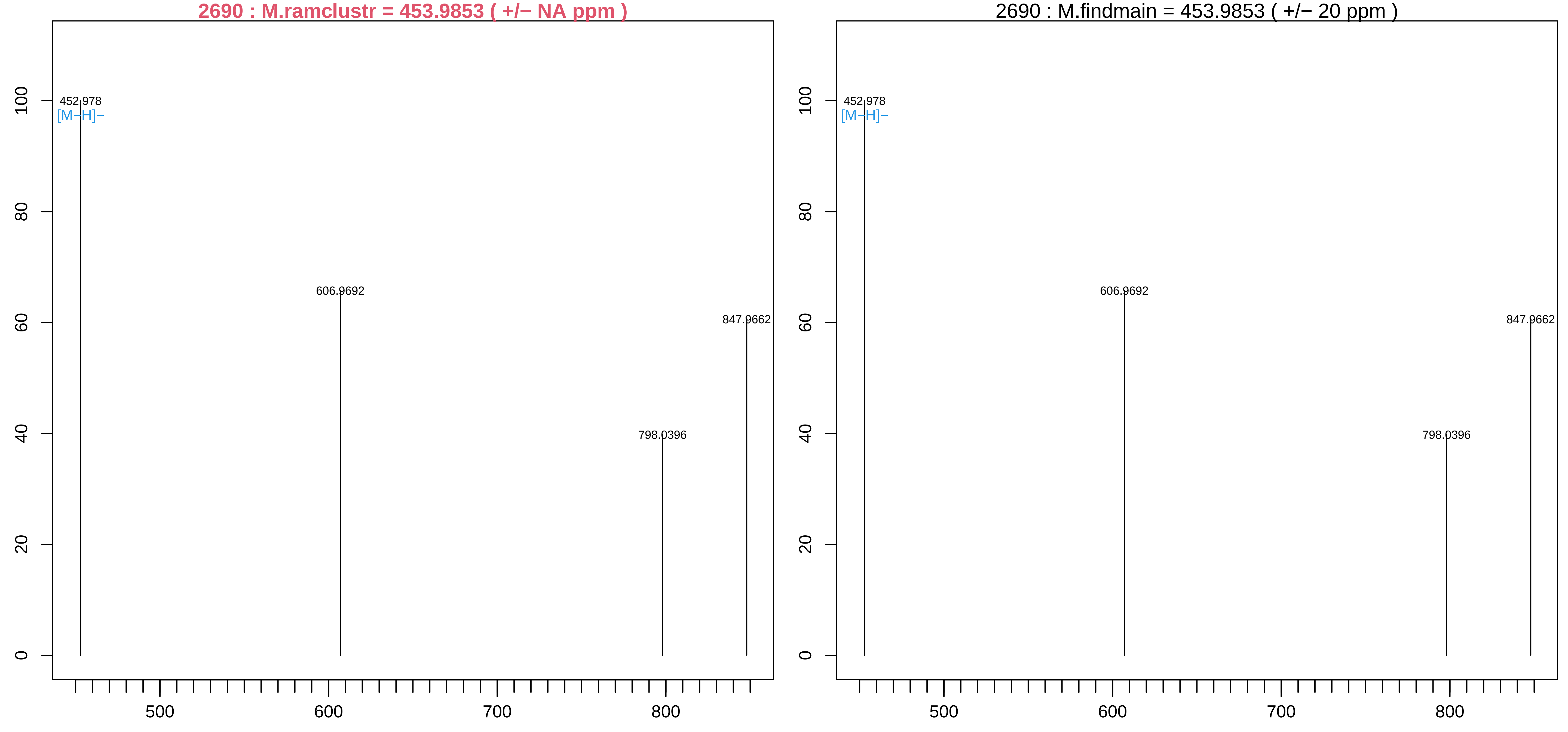

2690 : M.ramclustr = 453.9853 (+/− NA ppm)
2690 : M.findmain = 453.9853 (+/− 20 ppm)
452.978
[M−H]−
606.9692
798.0396
847.9662
0
20
40
60
80
100
500
600
700
800

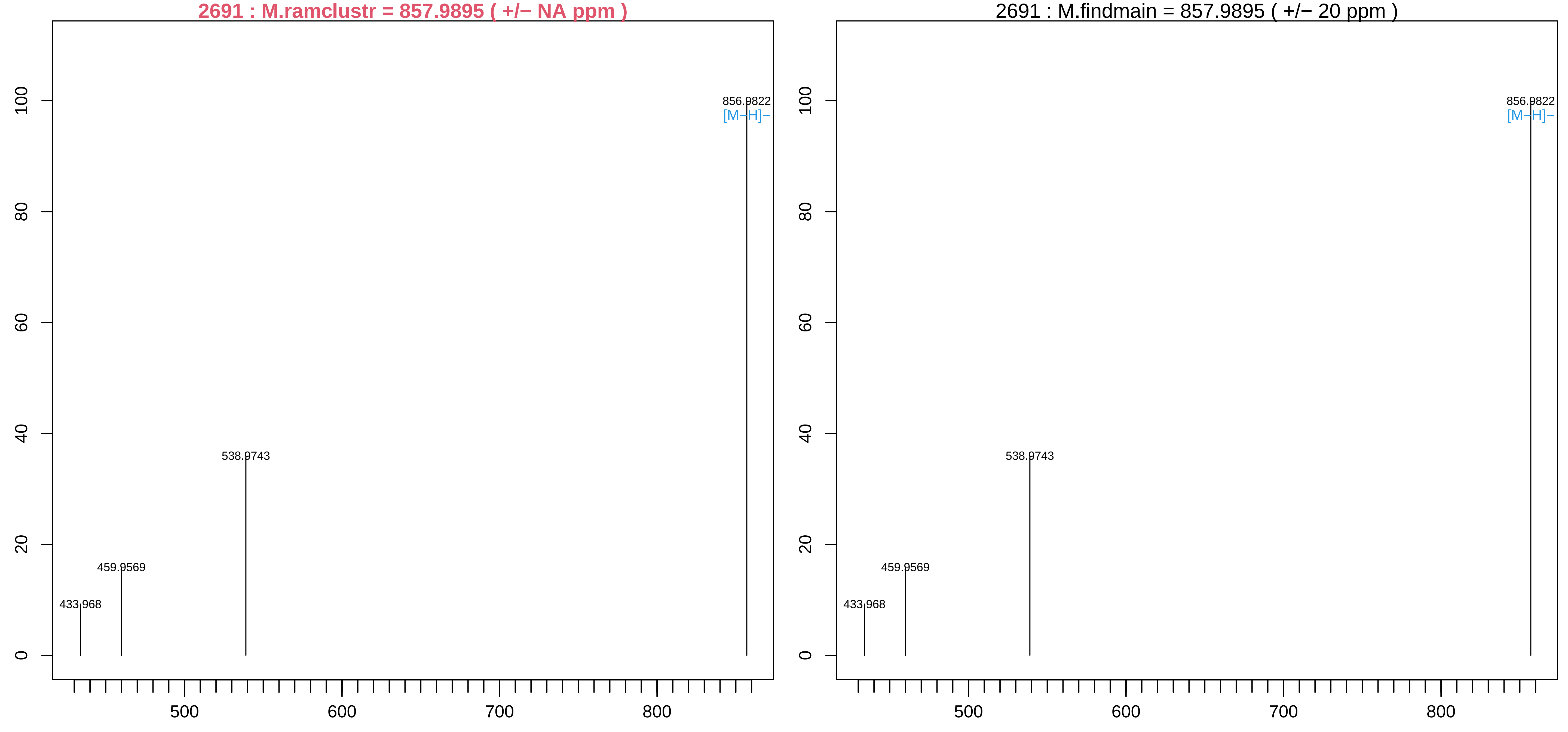
2691 : M.ramclustr = 857.9895 (+/− NA ppm)
856.9822
[M−H]−
538.9743
459.9569
433.968
2691 : M.findmain = 857.9895 (+/− 20 ppm)
856.9822
[M−H]−
538.9743
459.9569
433.968
0
20
40
60
80
100
500
600
700
800

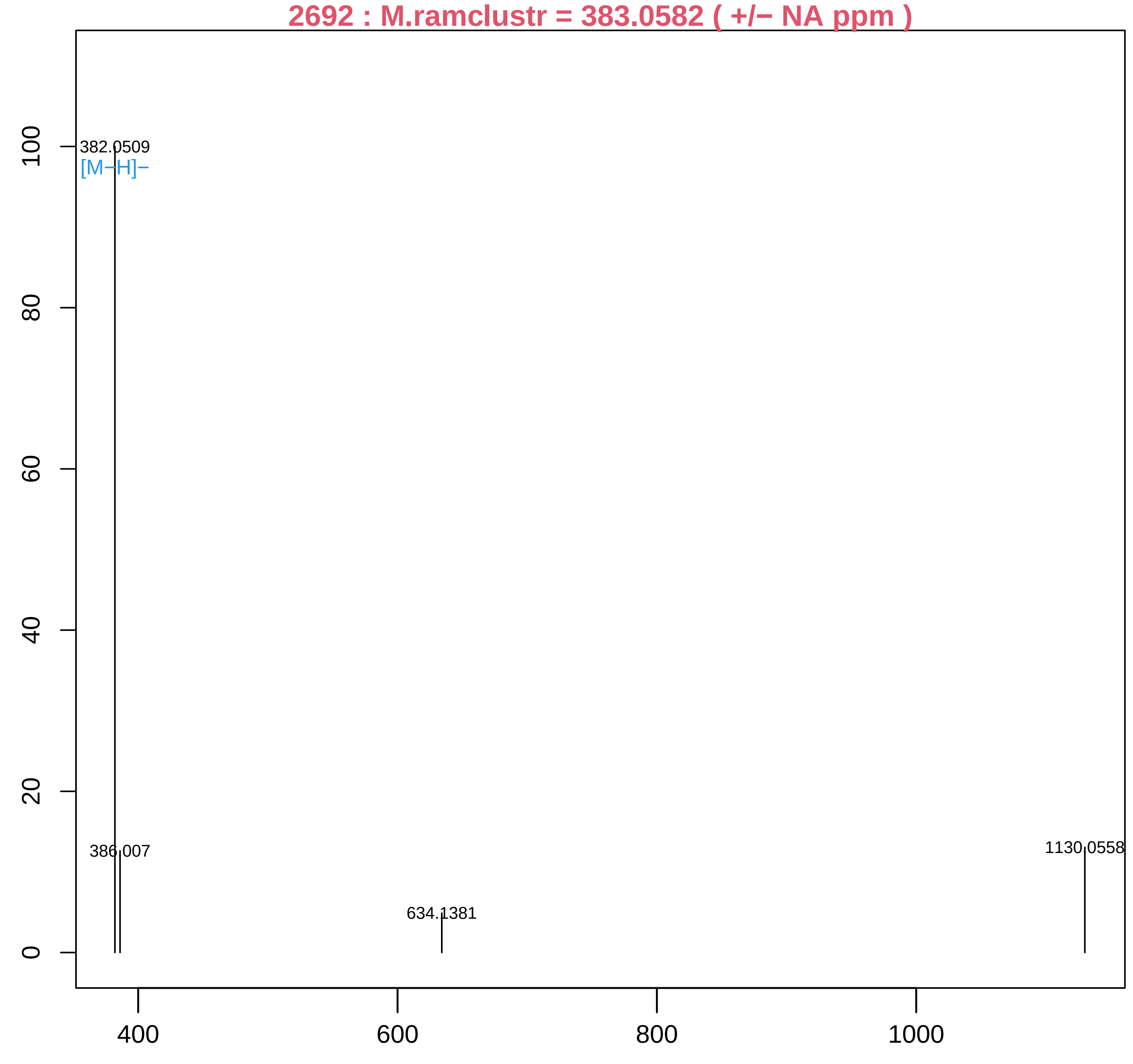

2692 : M.ramclustr = 383.0582 (+/− NA ppm)
382.0509
[M−H]−
386.007
634.1381
1130.0558
0
20
40
60
80
100
400
600
800
1000

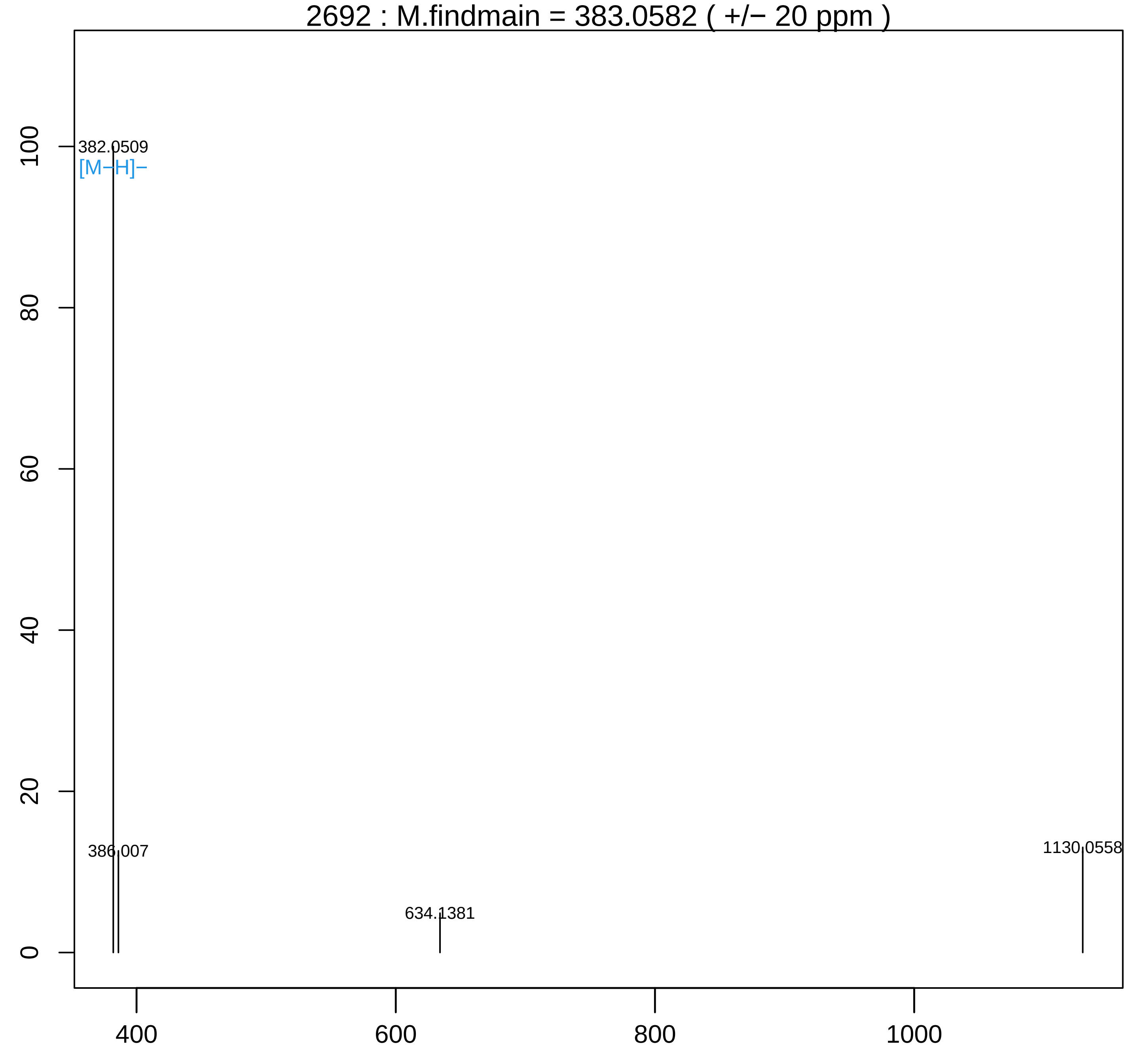

2692 : M.findmain = 383.0582 (+/− 20 ppm)
382.0509
[M−H]−
386.007
634.1381
1130.0558
0
20
40
60
80
100
400
600
800
1000

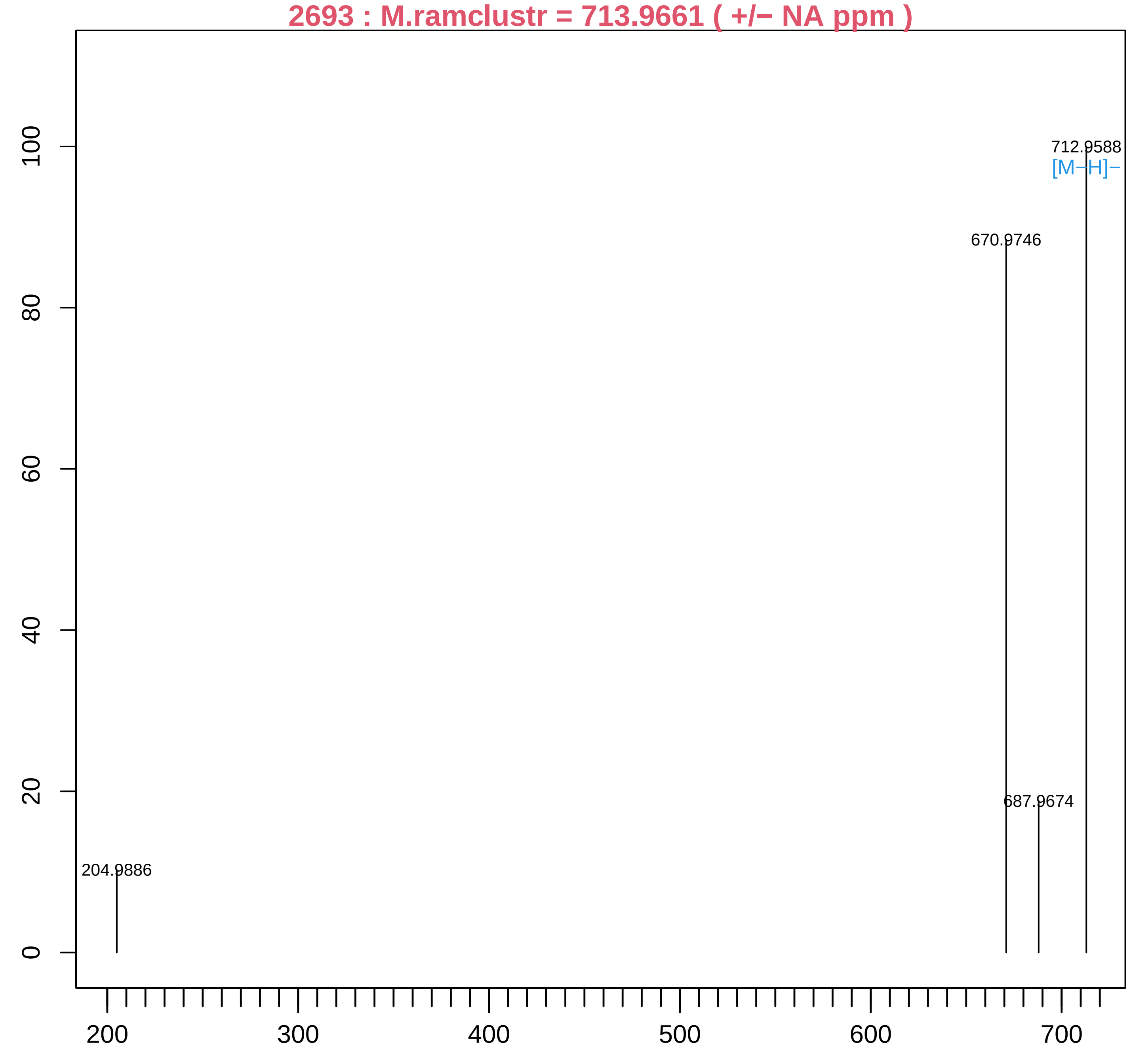
2693 : M.ramclustr = 713.9661 (+/− NA ppm)
712.9588
[M−H]−
670.9746
687.9674
204.9886
0
20
40
60
80
100
200
300
400
500
600
700

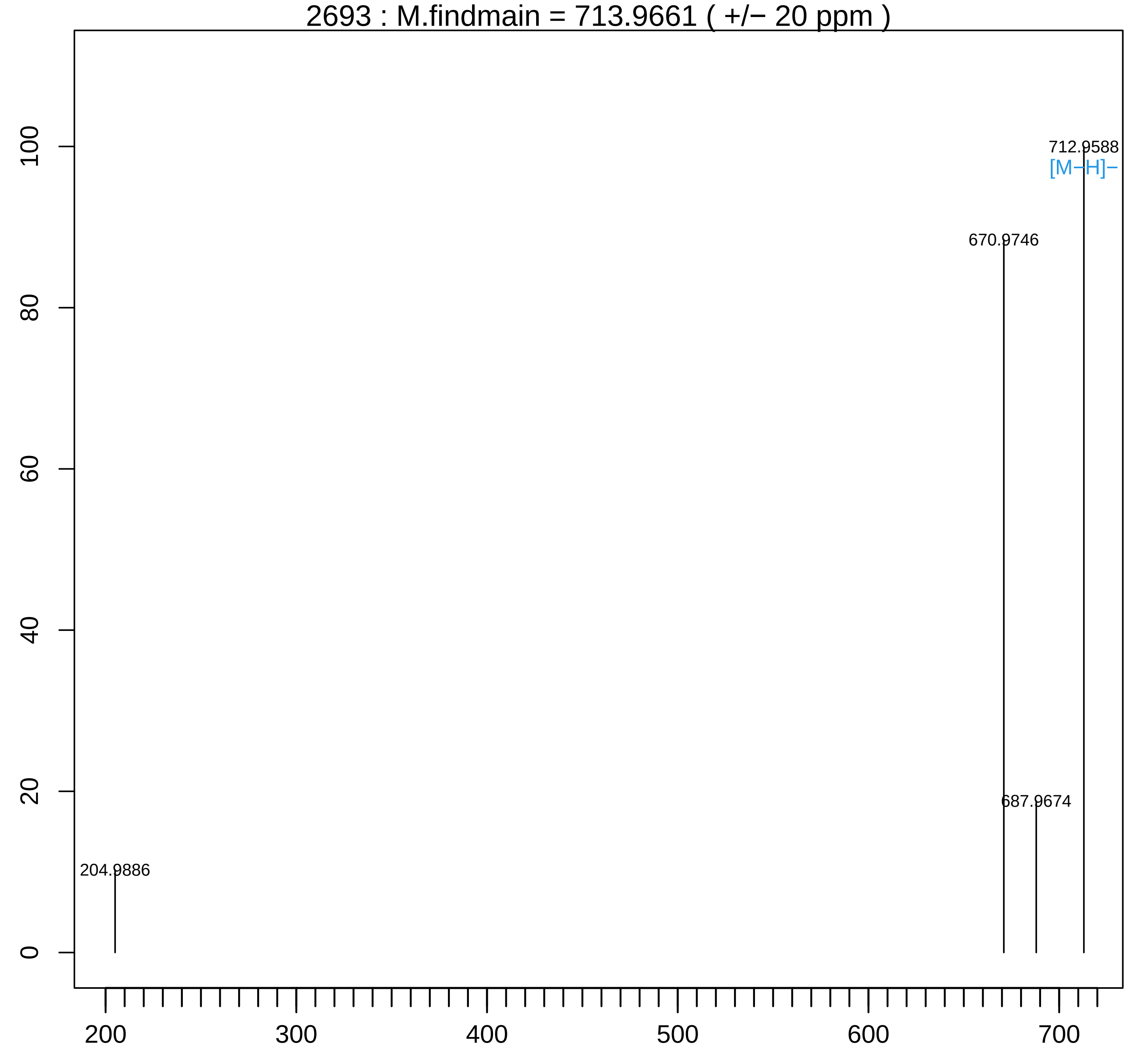
2693 : M.findmain = 713.9661 (+/− 20 ppm)
712.9588
[M−H]−
670.9746
687.9674
204.9886
0
20
40
60
80
100
200
300
400
500
600
700

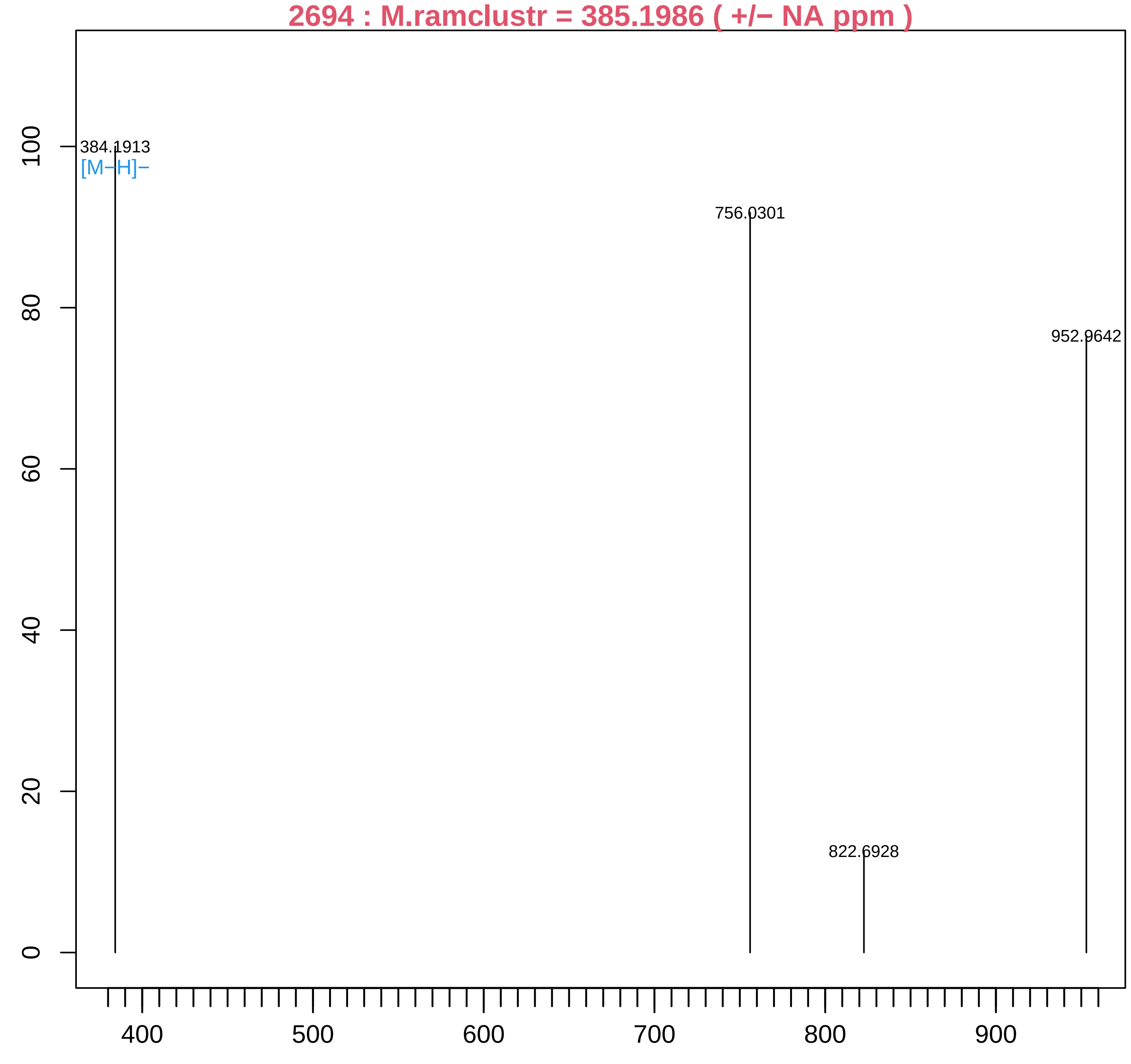

2694 : M.ramclustr = 385.1986 (+/− NA ppm)
384.1913
[M−H]−
756.0301
952.9642
822.6928
0
20
40
60
80
100
400
500
600
700
800
900

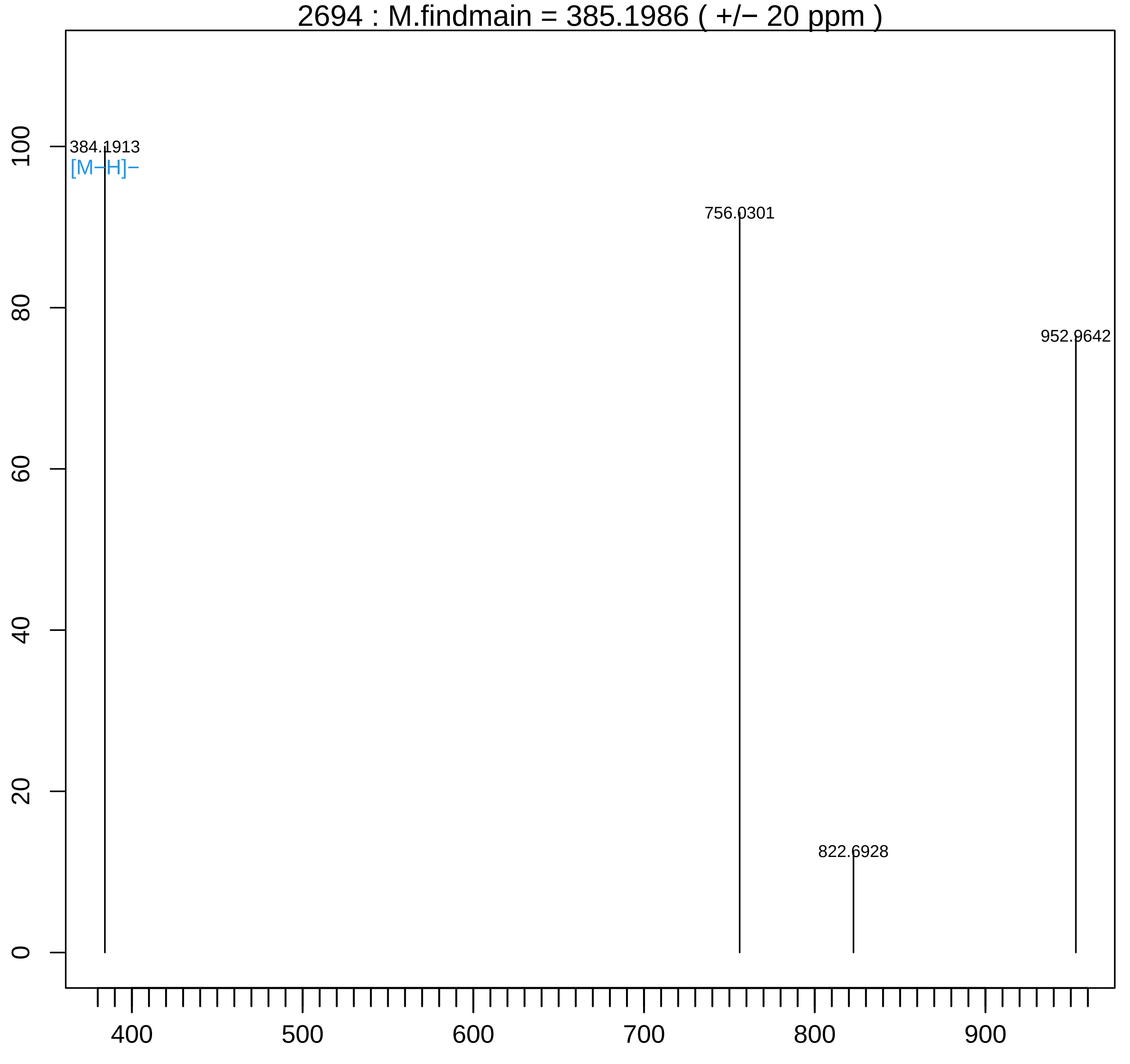

2694 : M.findmain = 385.1986 (+/− 20 ppm)
384.1913
[M−H]−
756.0301
952.9642
822.6928
0
20
40
60
80
100
400
500
600
700
800
900

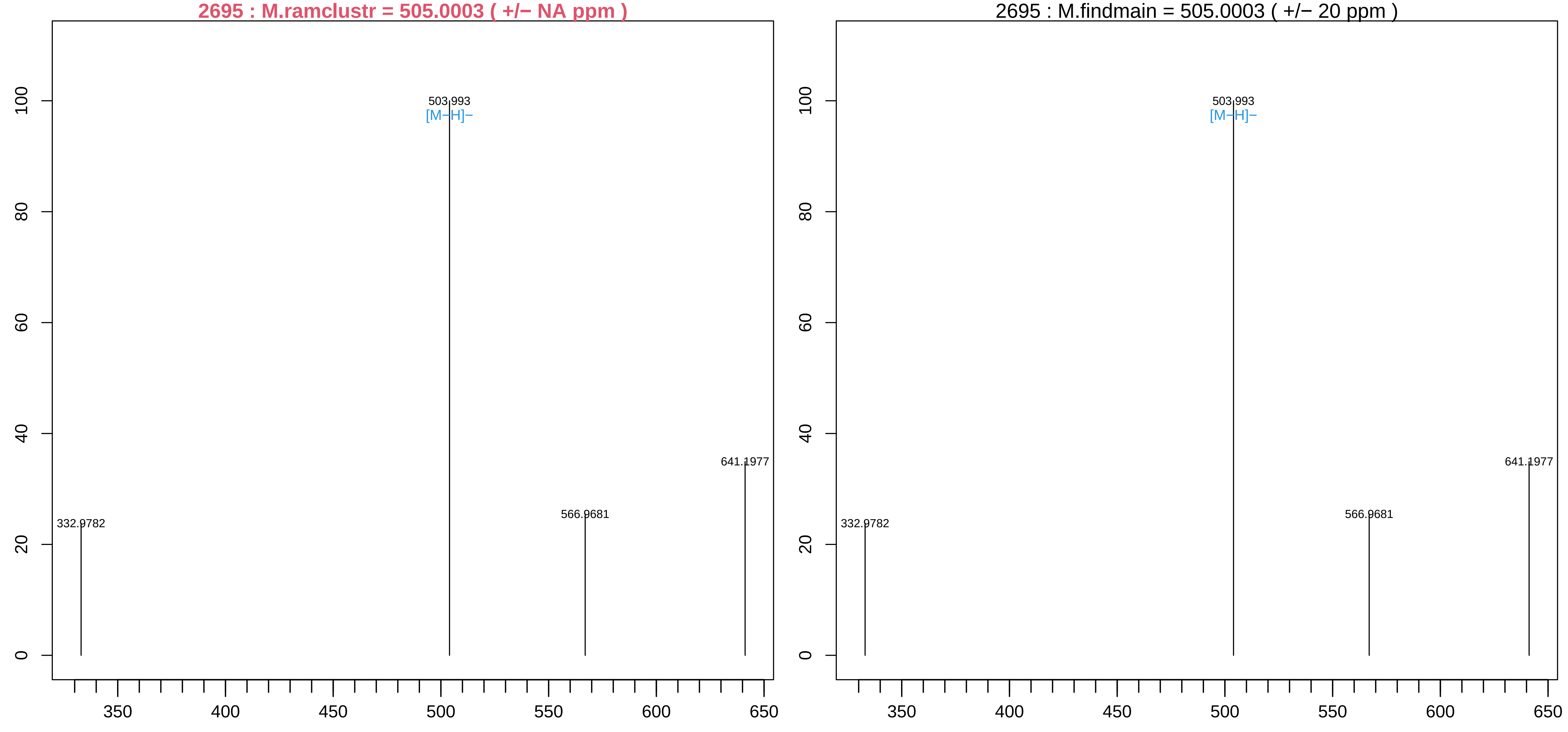
2695 : M.ramclustr = 505.0003 (+/− NA ppm)
2695 : M.findmain = 505.0003 (+/− 20 ppm)
503.993
[M−H]−
332.9782
566.9681
641.1977
0
20
40
60
80
100
350
400
450
500
550
600
650

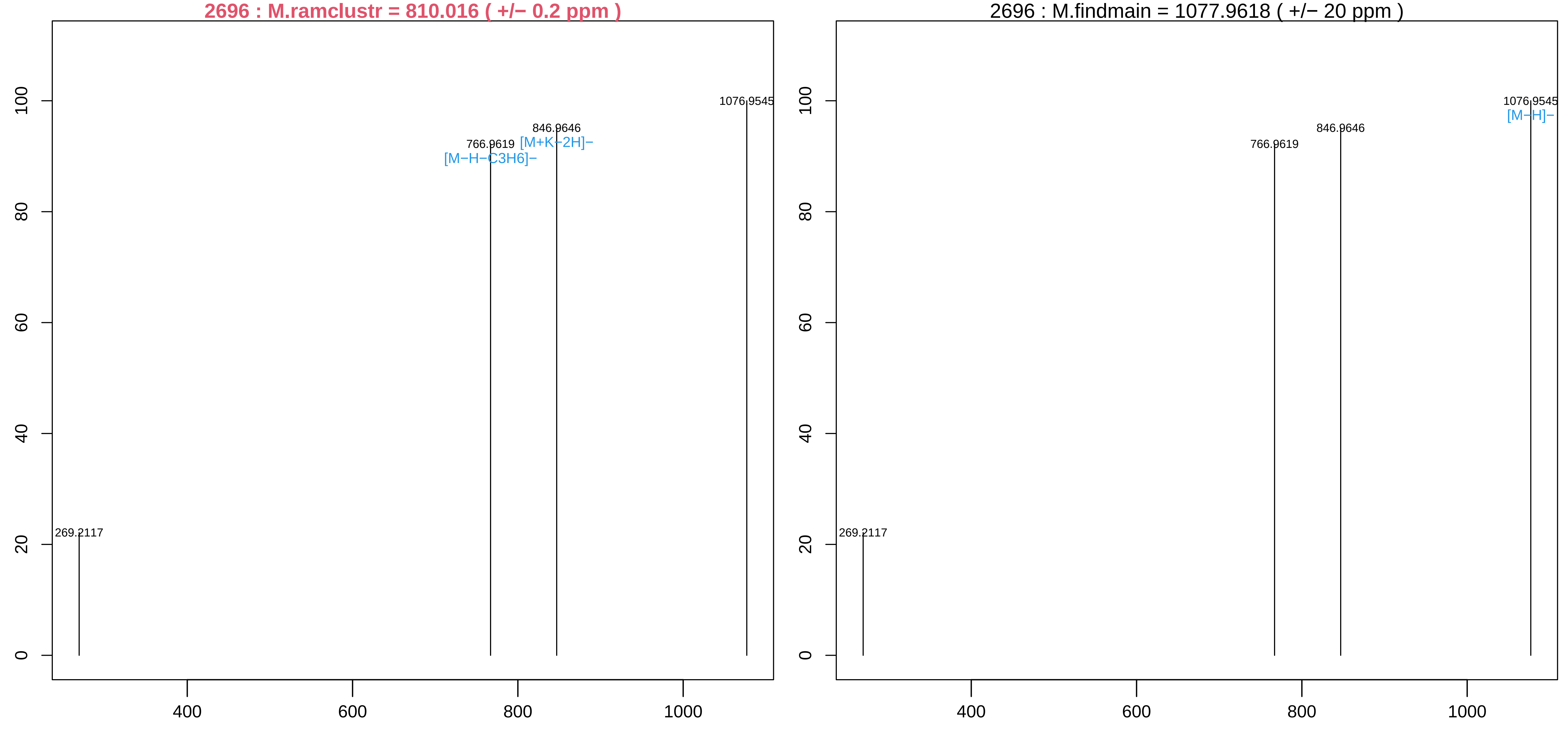

2696 : M.ramclustr = 810.016 (+/− 0.2 ppm)
2696 : M.findmain = 1077.9618 (+/− 20 ppm)
0
20
40
60
80
100
400
600
800
1000
269.2117
766.9619
[M−H−C3H6]−
846.9646
[M+K−2H]−
1076.9545
[M−H]−

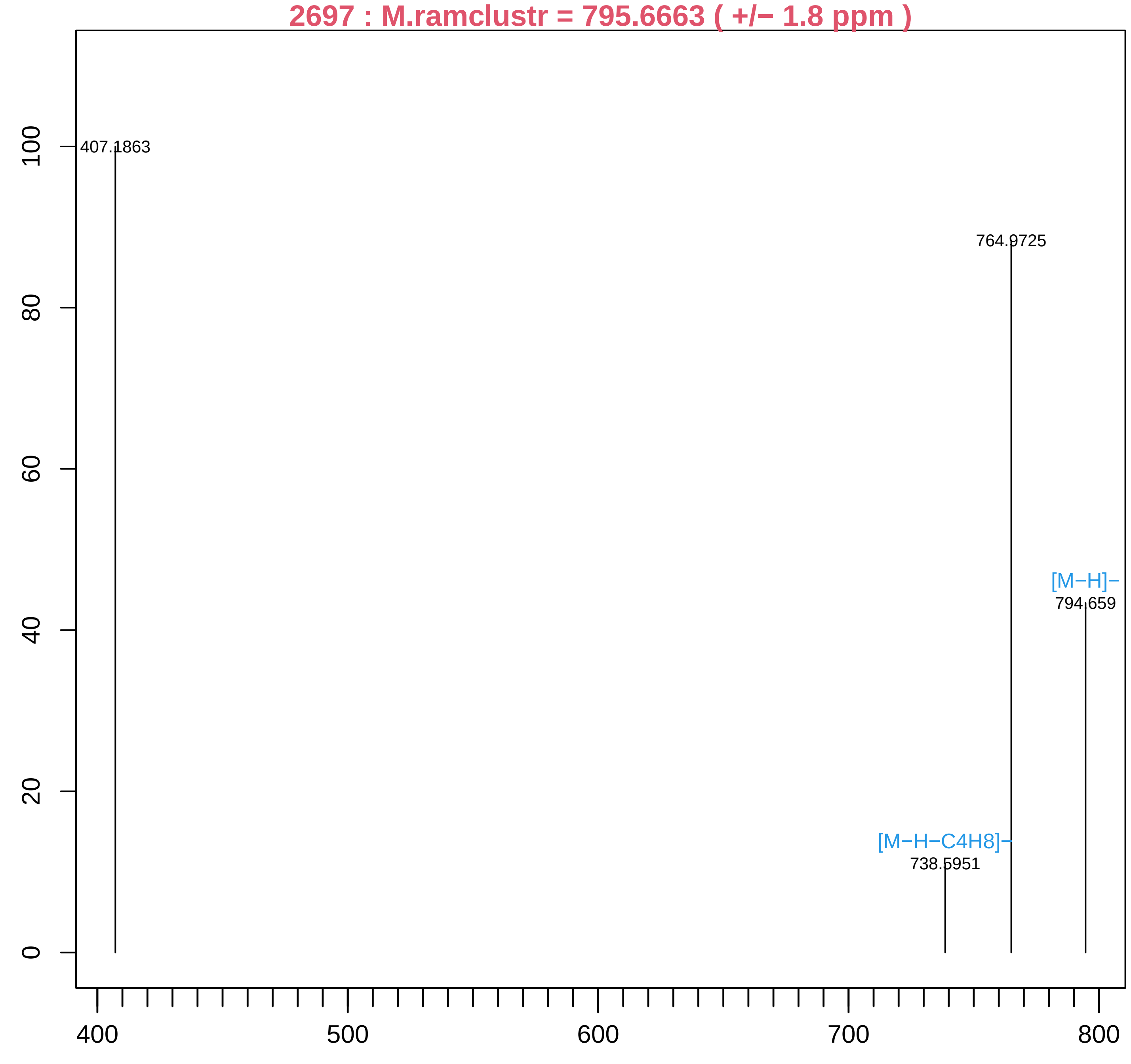

2697 : M.ramclustr = 795.6663 (+/− 1.8 ppm)
407.1863
764.9725
[M−H]−
794.659
[M−H−C4H8]−
738.5951
0
20
40
60
80
100
400
500
600
700
800

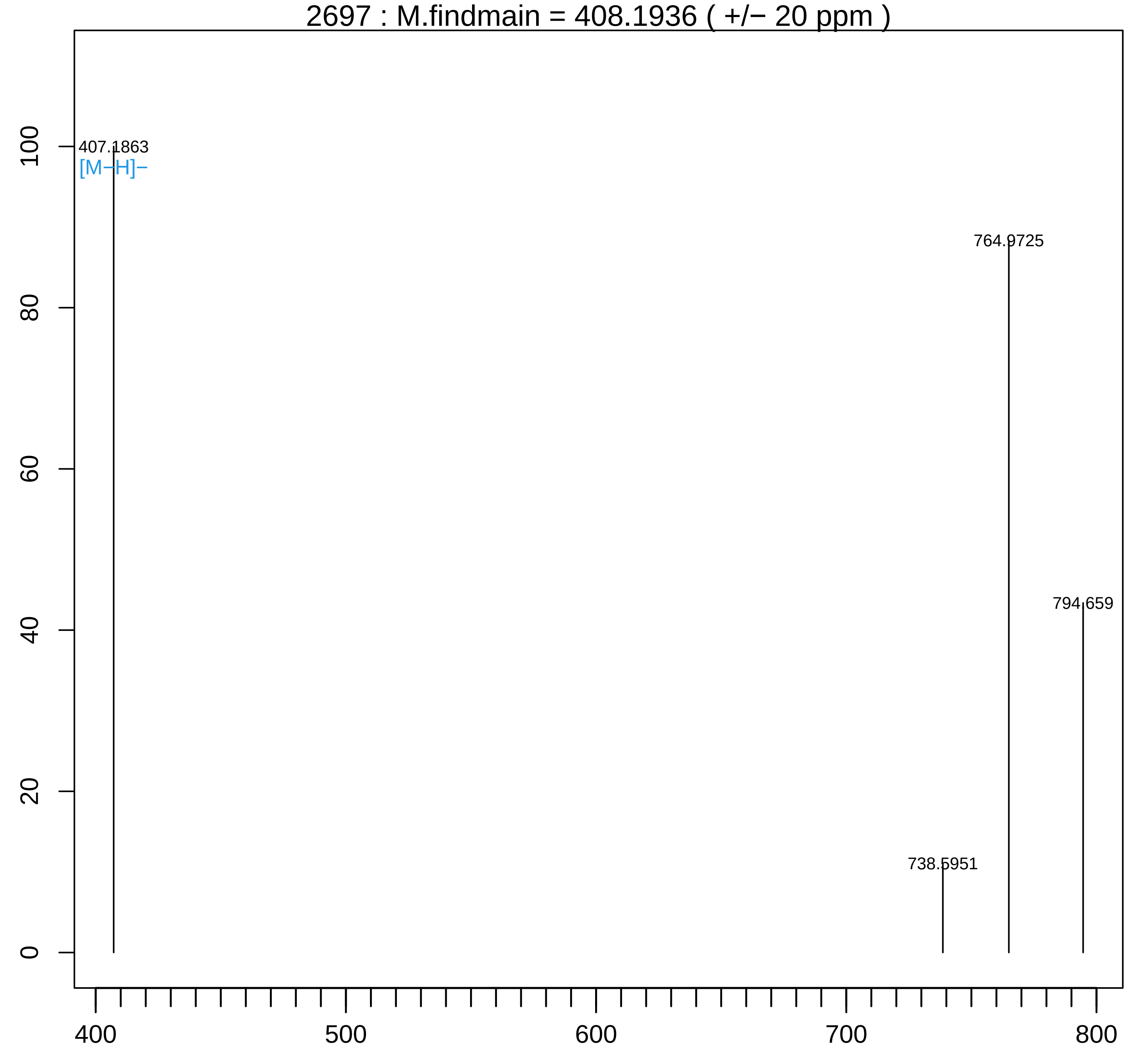

2697 : M.findmain = 408.1936 (+/− 20 ppm)
407.1863
[M−H]−
764.9725
794.659
738.5951
0
20
40
60
80
100
400
500
600
700
800

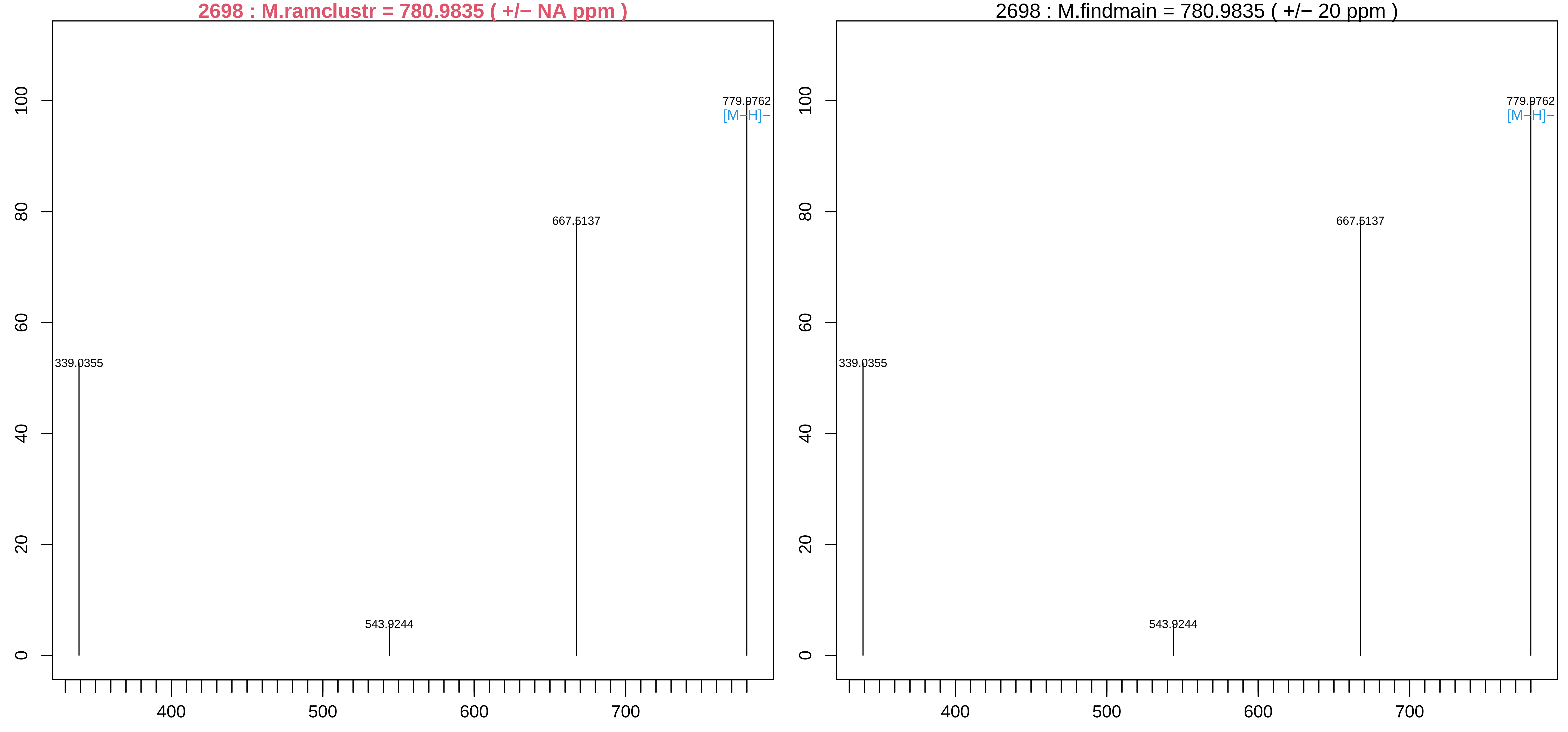
2698 : M.ramclustr = 780.9835 (+/− NA ppm)
779.9762
[M−H]−
667.5137
339.0355
543.9244
2698 : M.findmain = 780.9835 (+/− 20 ppm)
779.9762
[M−H]−
667.5137
339.0355
543.9244
0
20
40
60
80
100
400
500
600
700

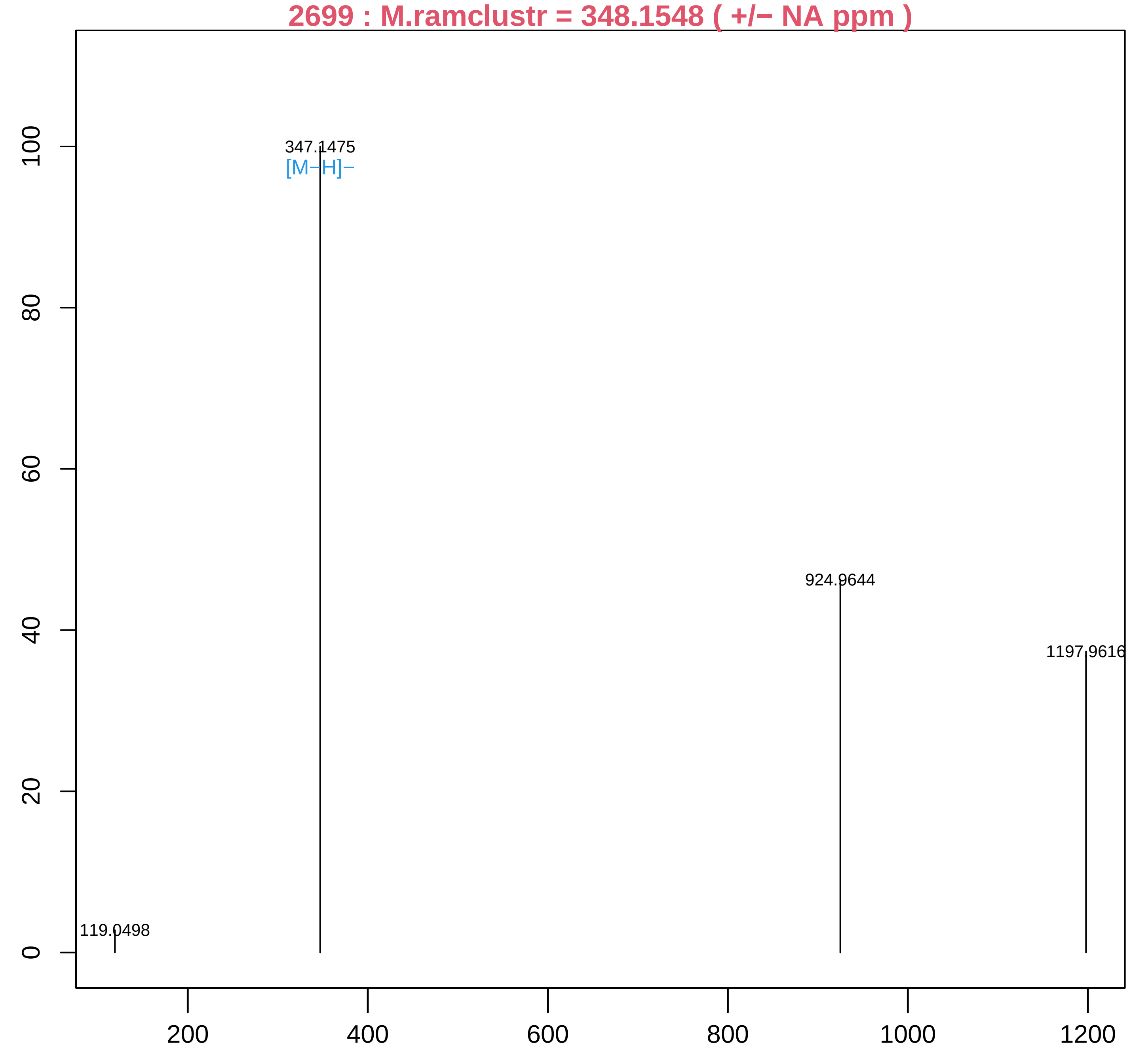

2699 : M.ramclustr = 348.1548 (+/− NA ppm)
347.1475
[M−H]−
924.9644
1197.9616
119.0498

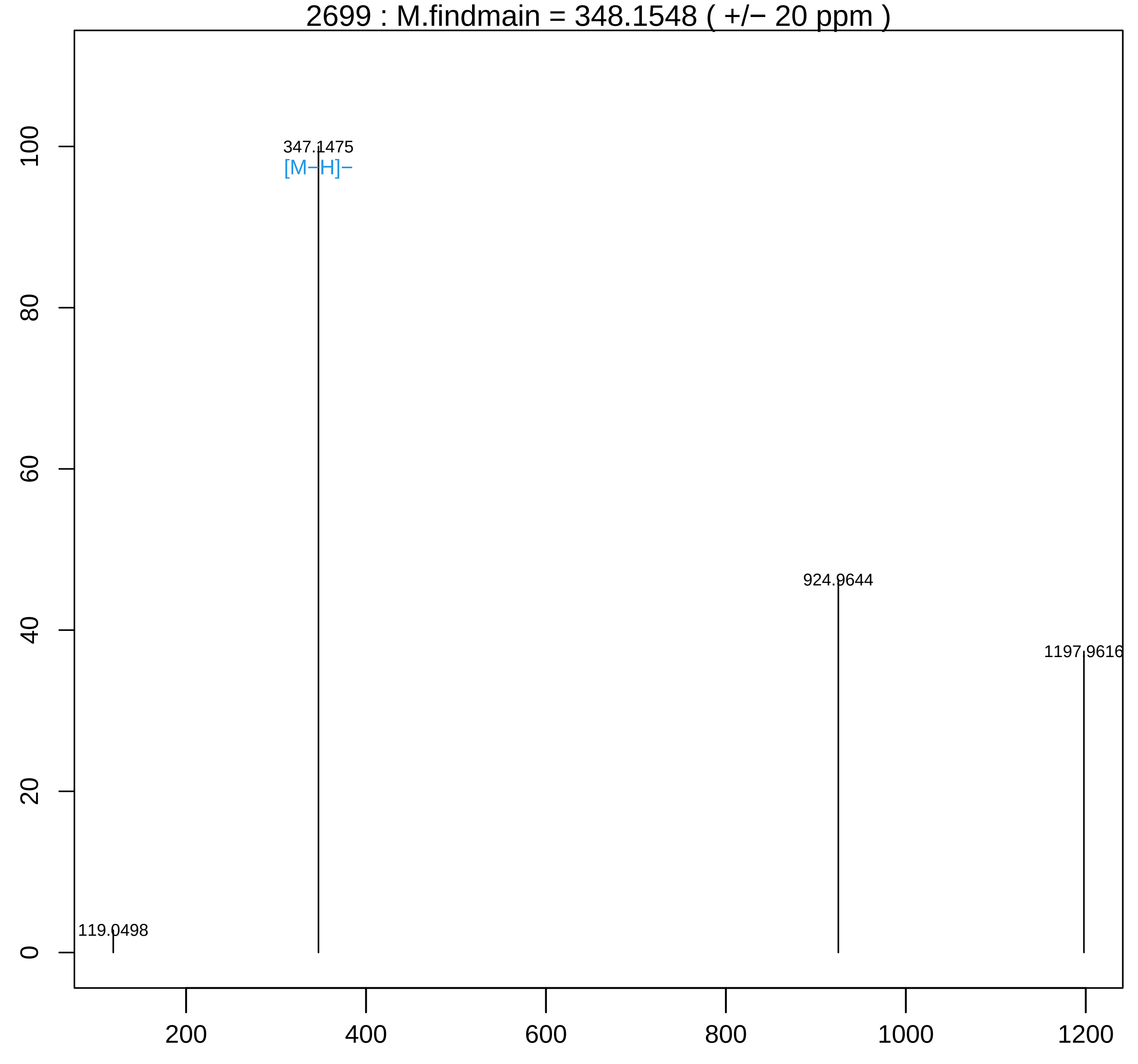

2699 : M.findmain = 348.1548 (+/− 20 ppm)
347.1475
[M−H]−
924.9644
1197.9616
119.0498

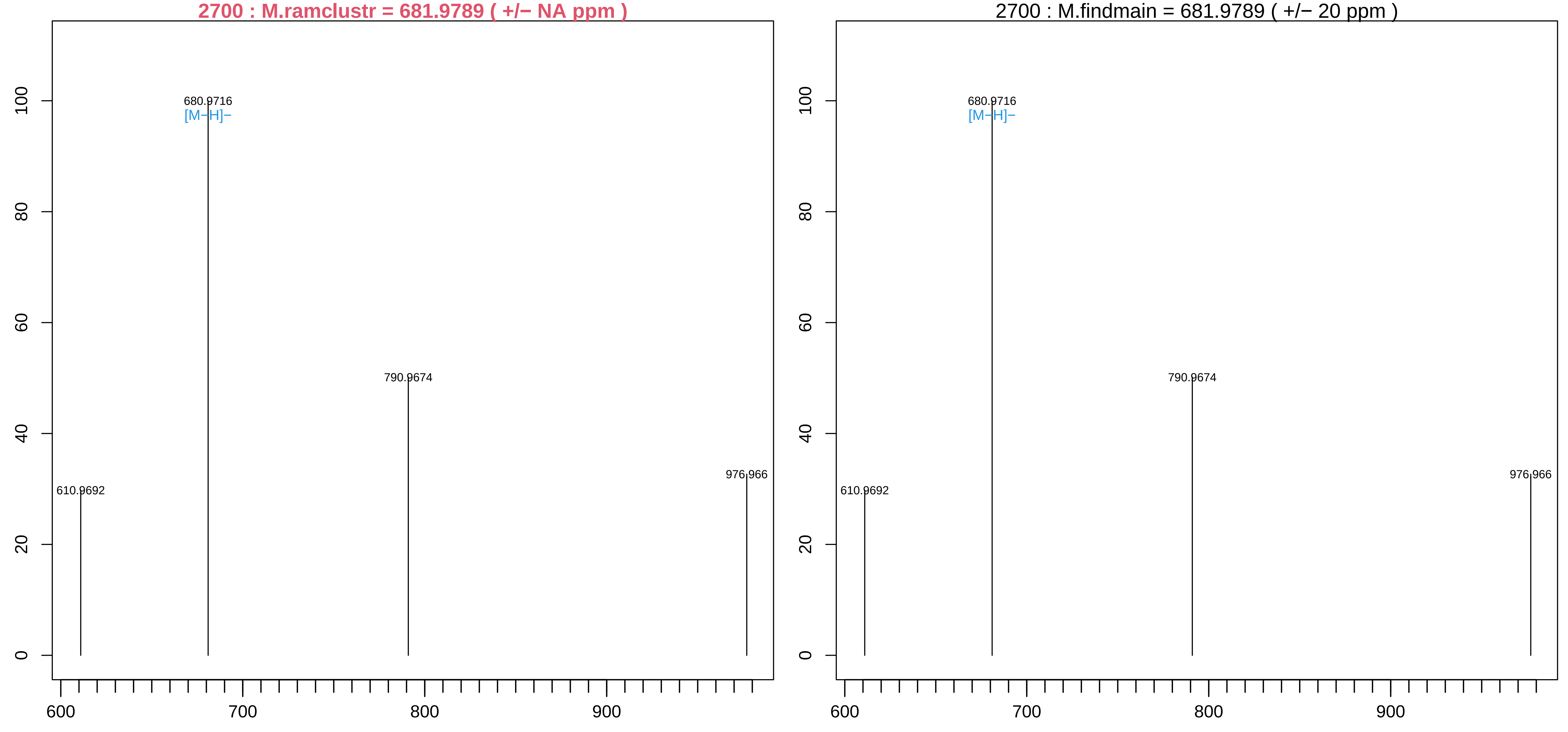
2700 : M.ramclustr = 681.9789 (+/− NA ppm)
680.9716
[M−H]−
790.9674
976.966
610.9692
2700 : M.findmain = 681.9789 (+/− 20 ppm)
680.9716
[M−H]−
790.9674
976.966
610.9692
0
20
40
60
80
100
600
700
800
900

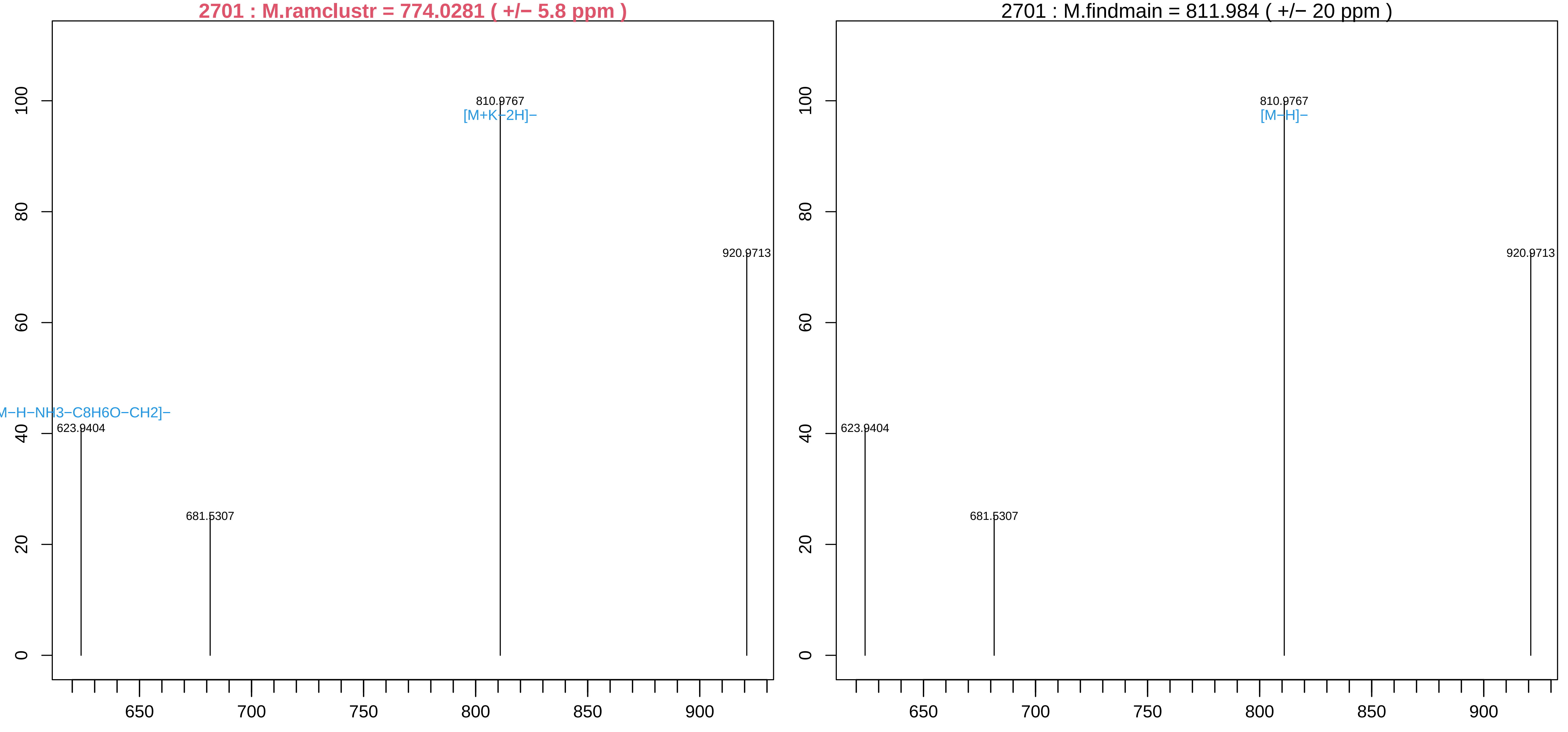
2701 : M.ramclustr = 774.0281 (+/− 5.8 ppm)
810.9767
[M+K−2H]−
920.9713
M−H−NH3−C8H6O−CH2]−
623.9404
681.5307
0
20
40
60
80
100
650
700
750
800
850
900
2701 : M.findmain = 811.984 (+/− 20 ppm)
810.9767
[M−H]−
920.9713
623.9404
681.5307
0
20
40
60
80
100
650
700
750
800
850
900

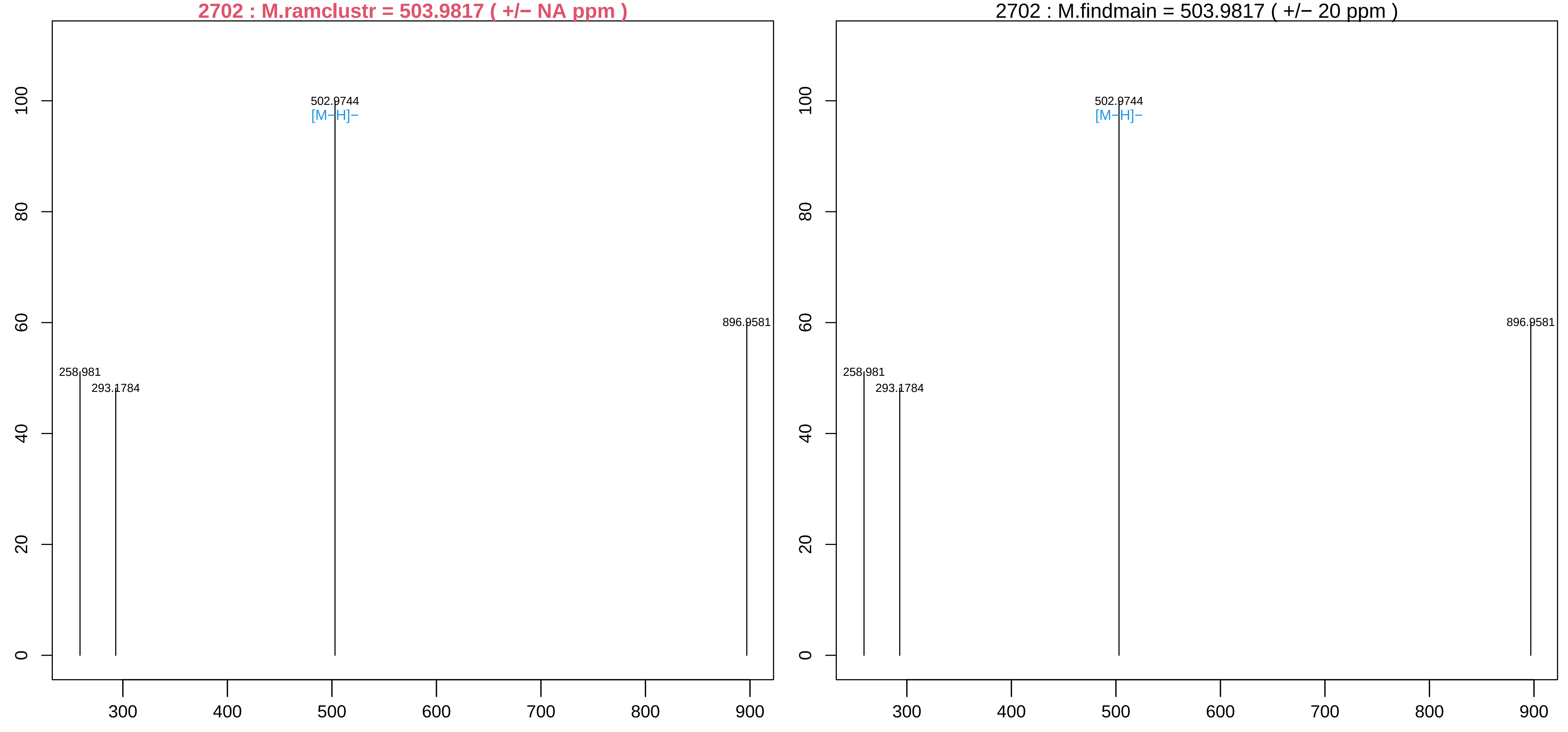
2702 : M.ramclustr = 503.9817 (+/− NA ppm)
2702 : M.findmain = 503.9817 (+/− 20 ppm)
502.9744
[M−H]−
896.9581
258.981
293.1784
0
20
40
60
80
100
300
400
500
600
700
800
900

2703 : M.ramclustr = 1082.0147 (+/− 9.8 ppm)

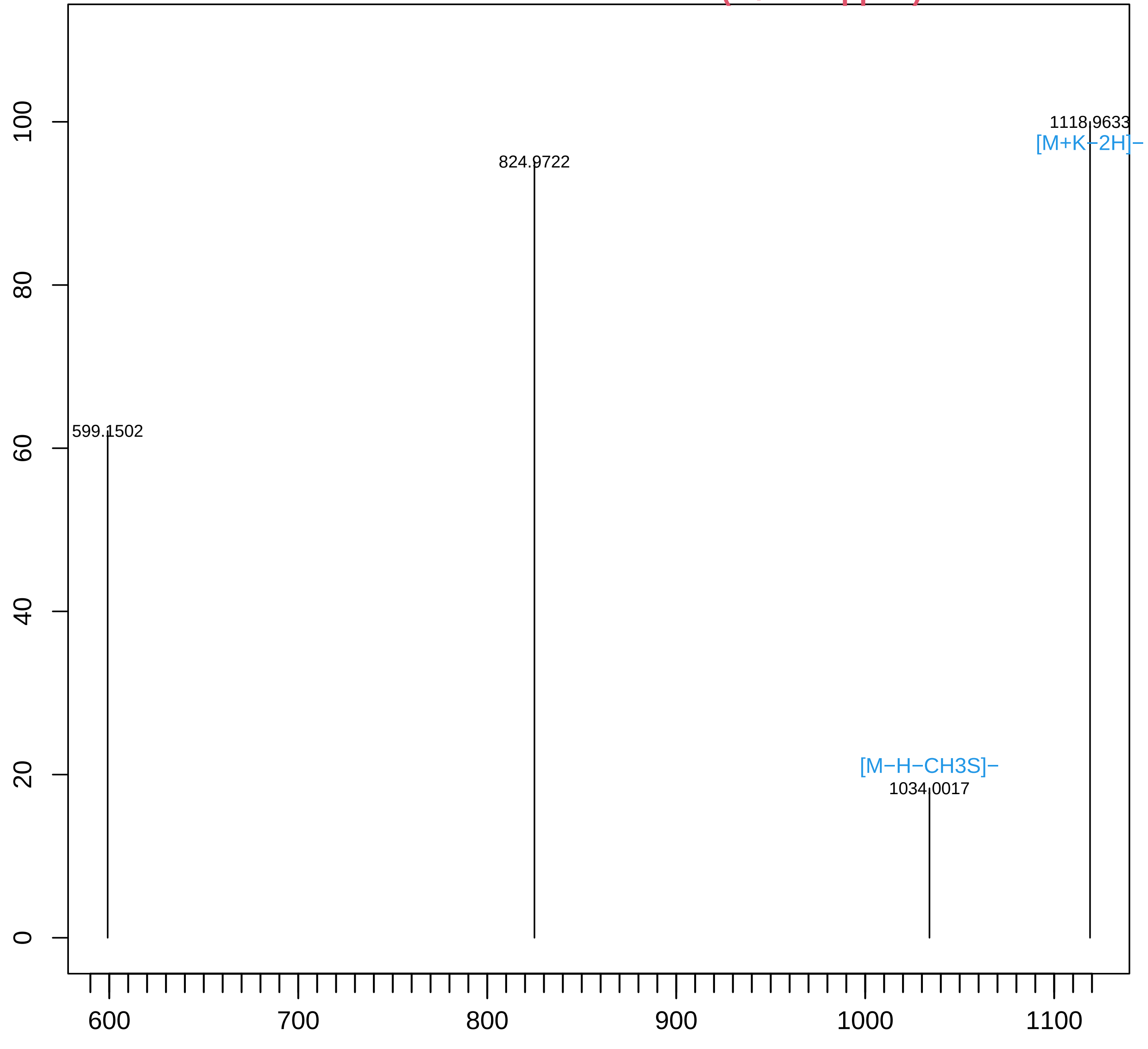

2703 : M.findmain = 1119.9706 (+/− 20 ppm)

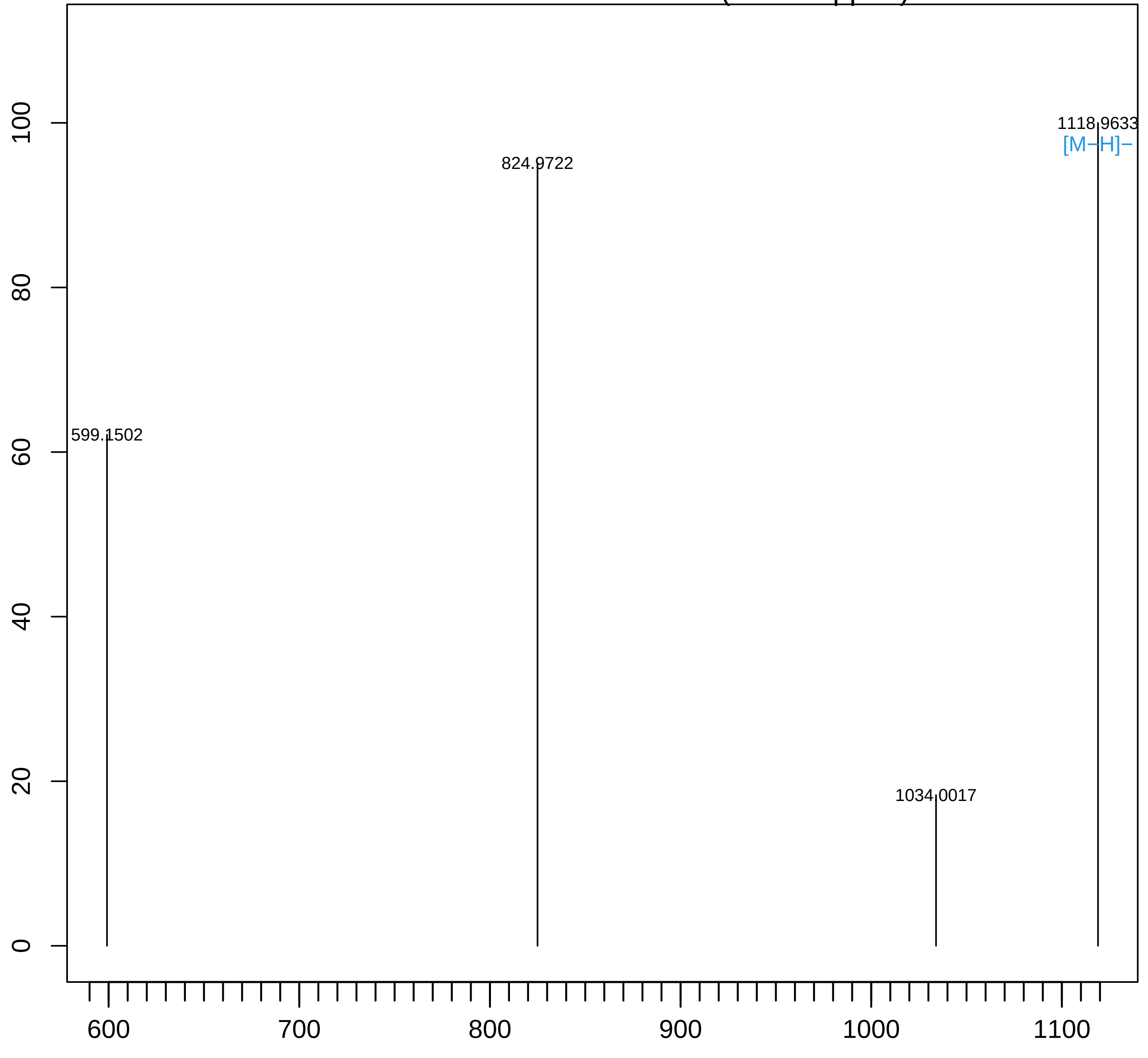

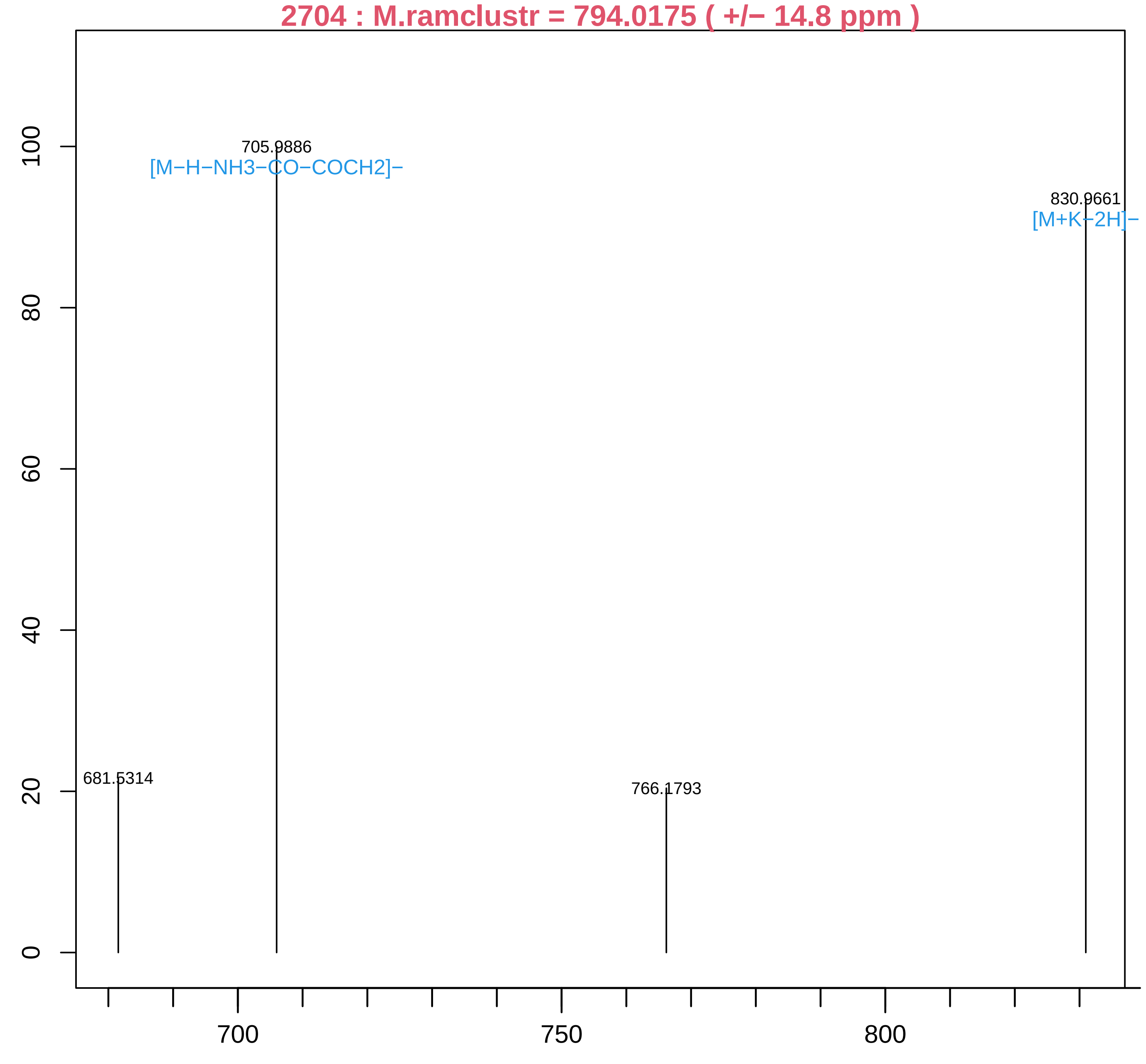
2704 : M.ramclustr = 794.0175 (+/− 14.8 ppm)
705.9886
[M−H−NH3−CO−COCH2]−
830.9661
[M+K−2H]−
681.5314
766.1793
0
20
40
60
80
100
700
750
800

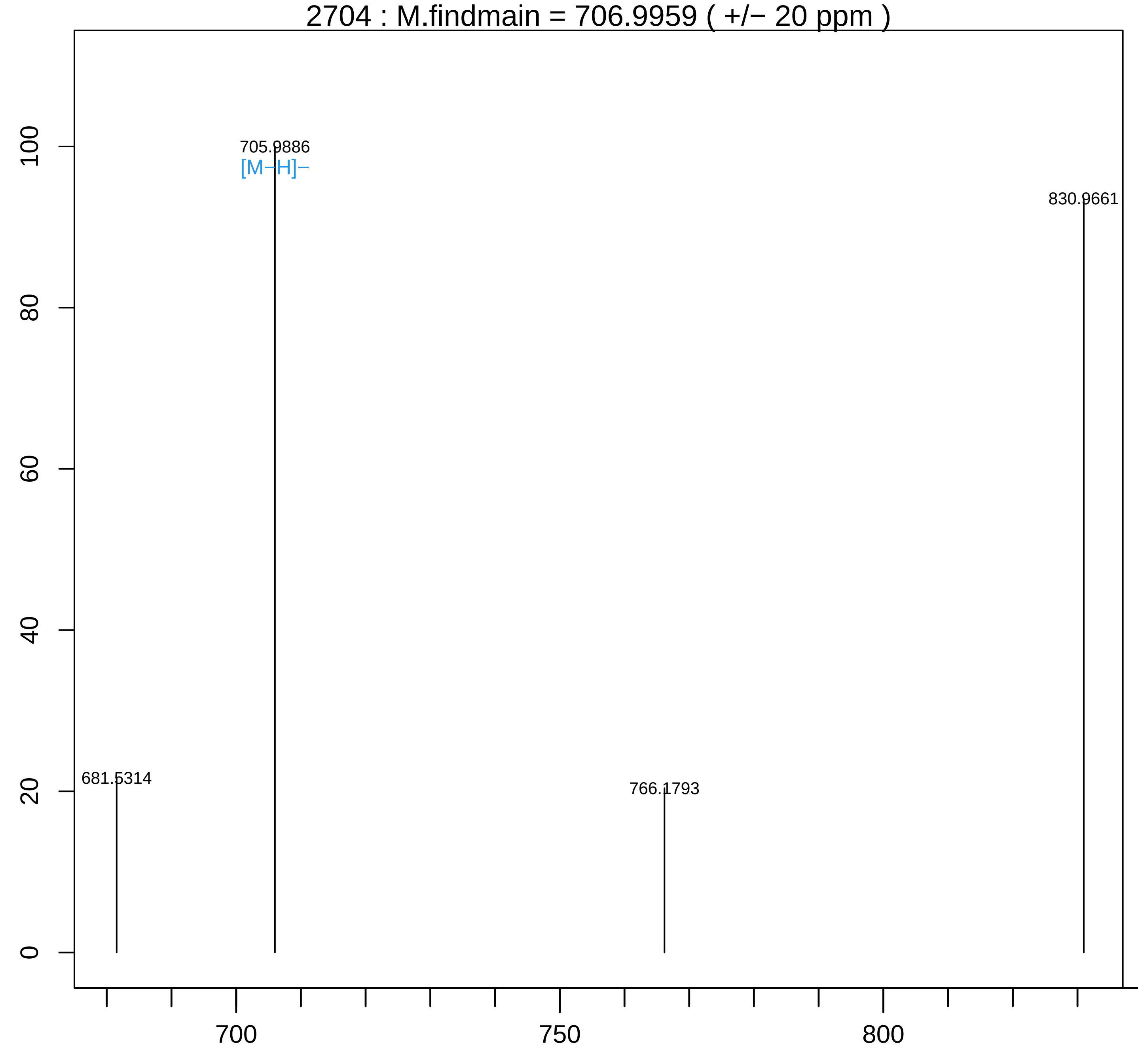
2704 : M.findmain = 706.9959 (+/− 20 ppm)
705.9886
[M−H]−
830.9661
681.5314
766.1793
0
20
40
60
80
100
700
750
800

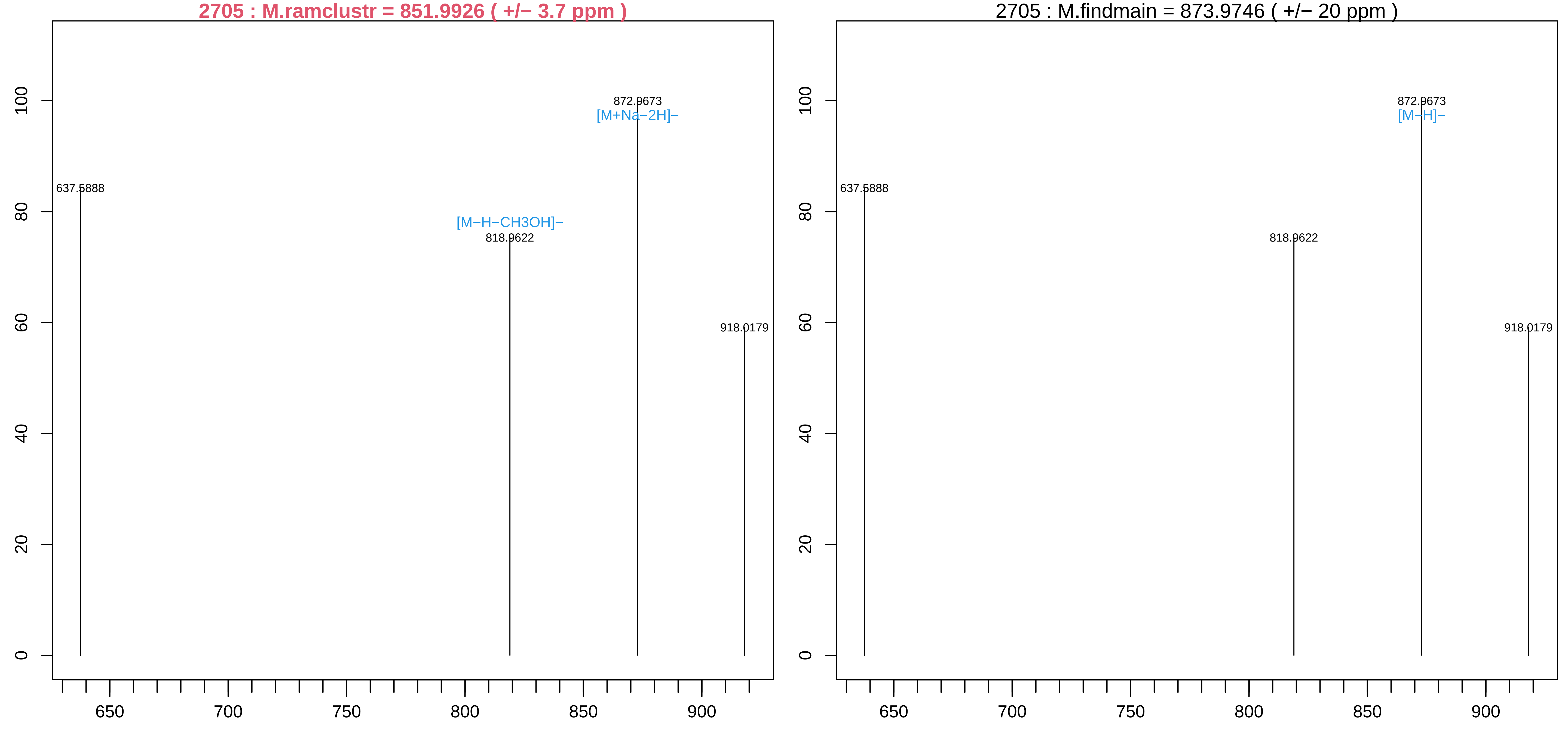

2705 : M.ramclustr = 851.9926 (+/− 3.7 ppm)
637.5888
[M−H−CH3OH]−
818.9622
872.9673
[M+Na−2H]−
918.0179
0
20
40
60
80
100
650
700
750
800
850
900
2705 : M.findmain = 873.9746 (+/− 20 ppm)
637.5888
818.9622
872.9673
[M−H]−
918.0179
0
20
40
60
80
100
650
700
750
800
850
900

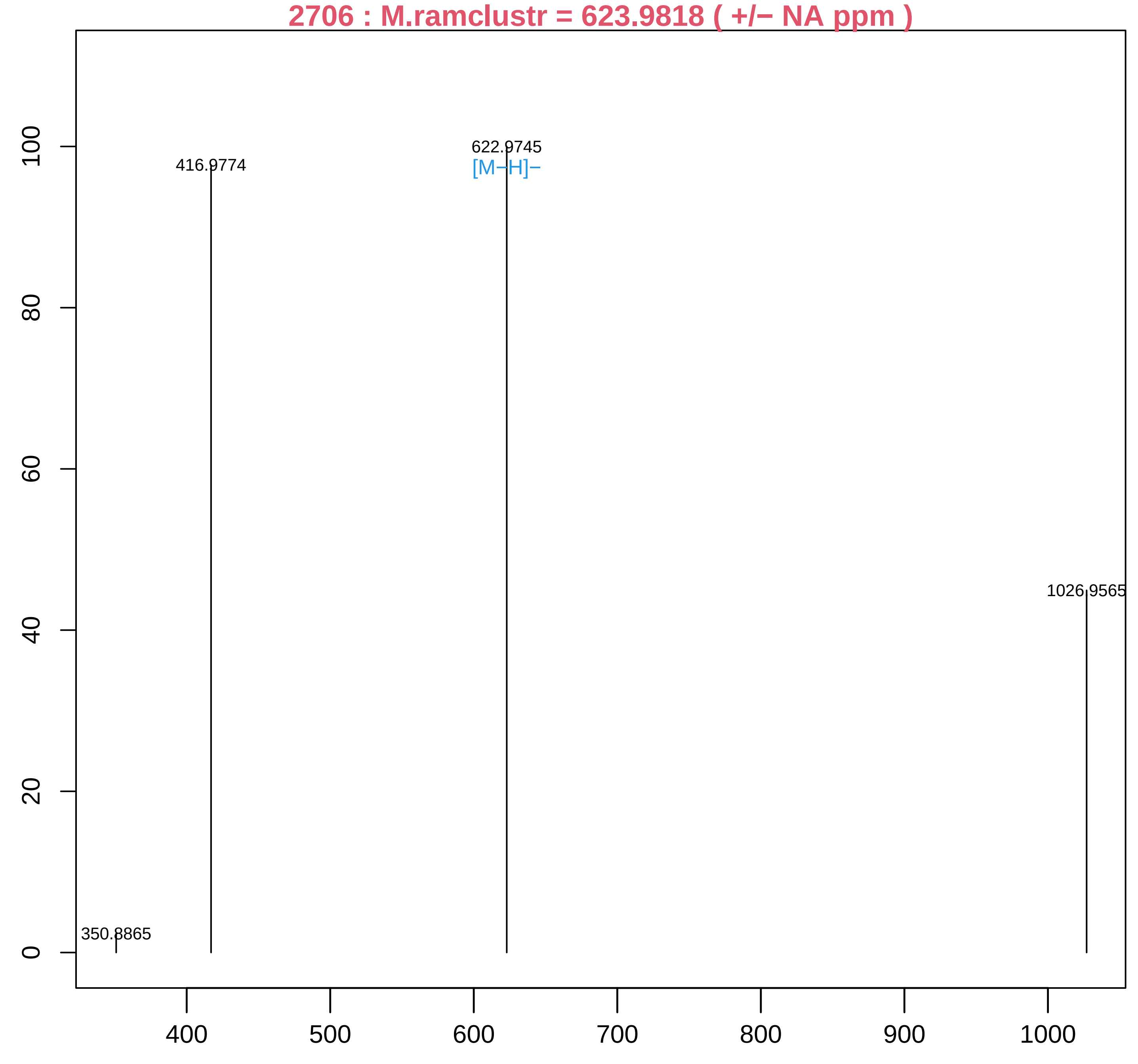
2706 : M.ramclustr = 623.9818 (+/− NA ppm)
416.9774
622.9745
[M−H]−
1026.9565
350.8865
0
20
40
60
80
100
400
500
600
700
800
900
1000

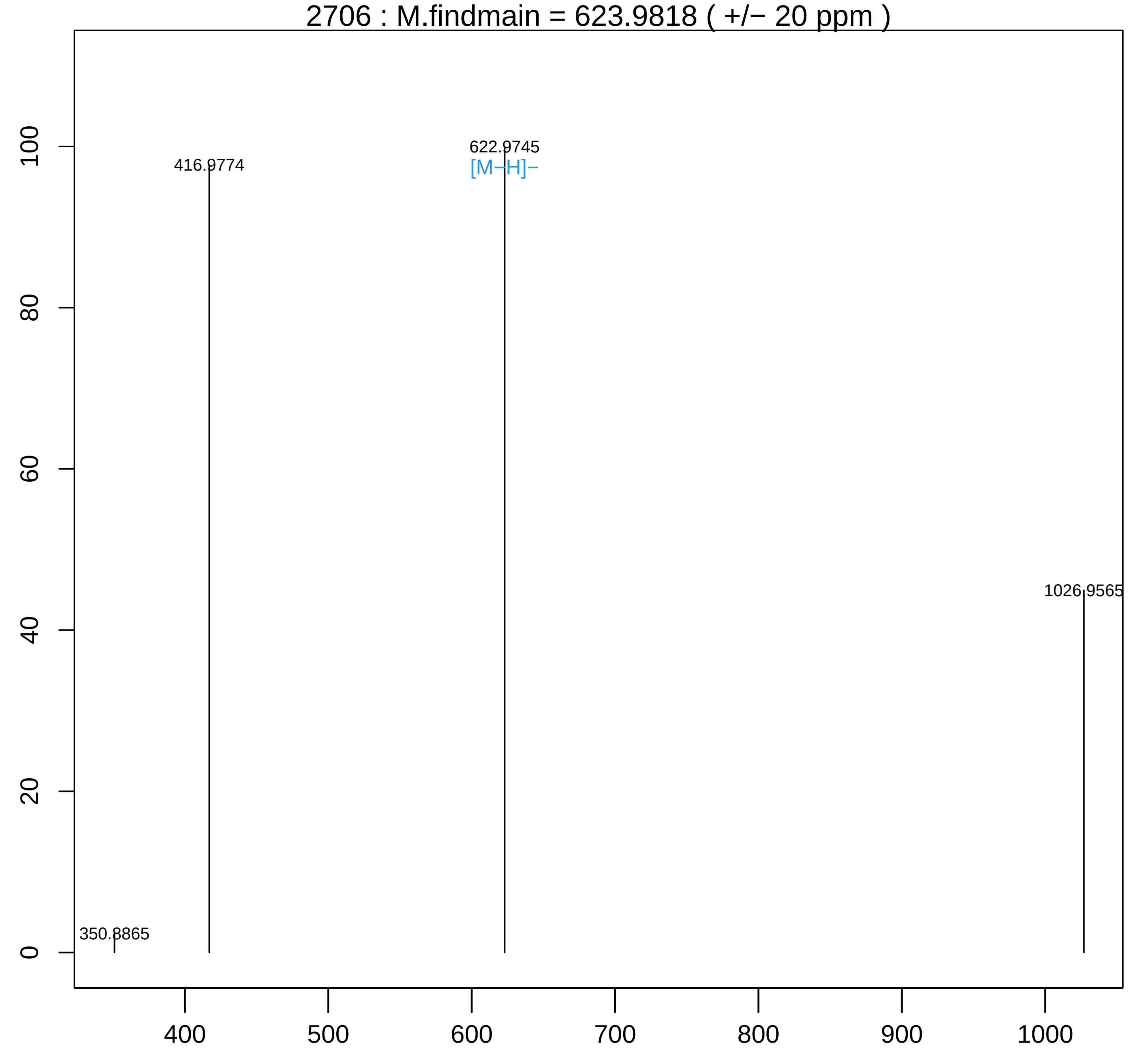
2706 : M.findmain = 623.9818 (+/− 20 ppm)
416.9774
622.9745
[M−H]−
1026.9565
350.8865
0
20
40
60
80
100
400
500
600
700
800
900
1000

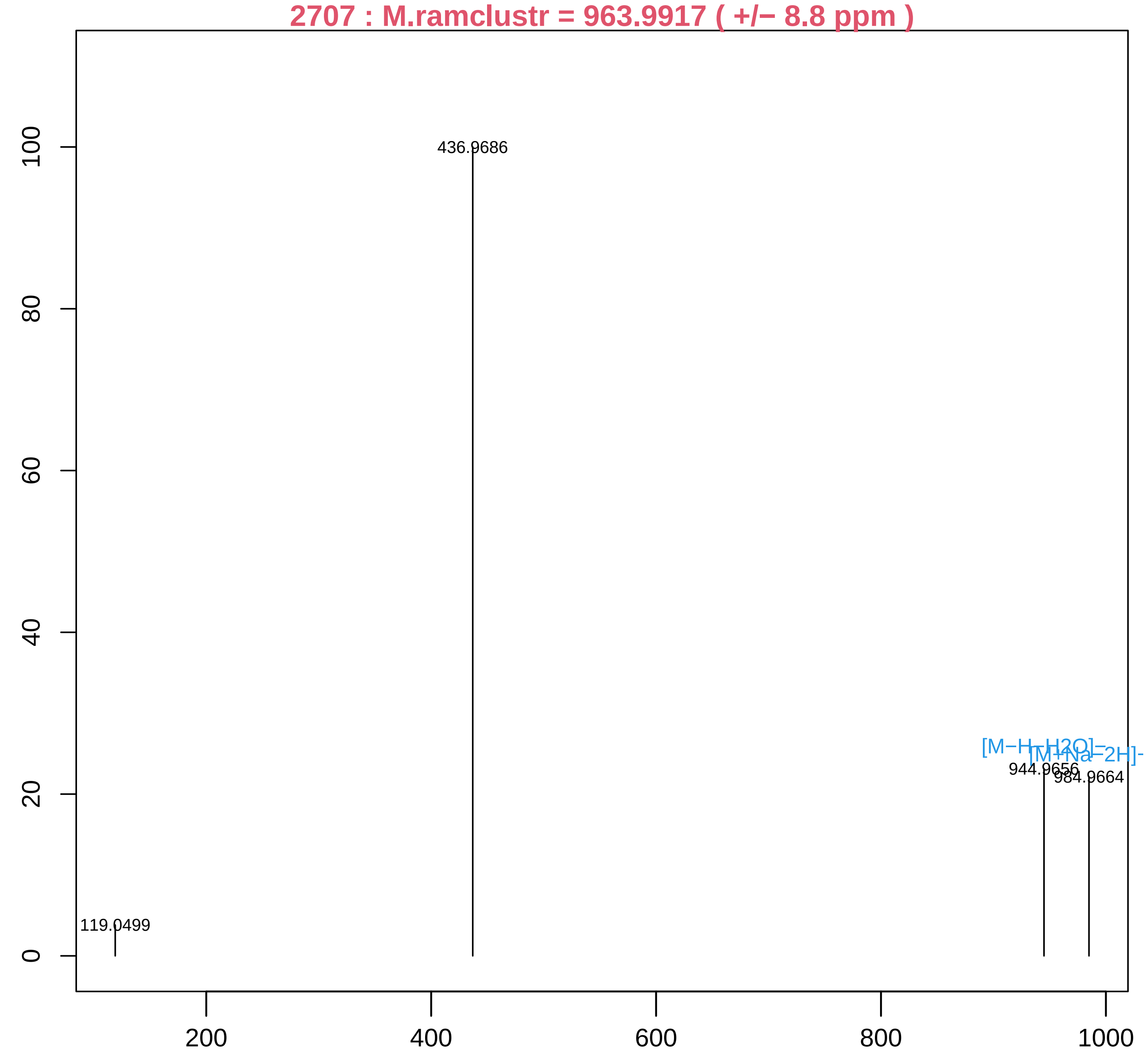
2707 : M.ramclustr = 963.9917 (+/− 8.8 ppm)
436.9686
[M−H−H2O]−
[M+Na−2H]-
944.9656
984.9664
119.0499
0
20
40
60
80
100
200
400
600
800
1000

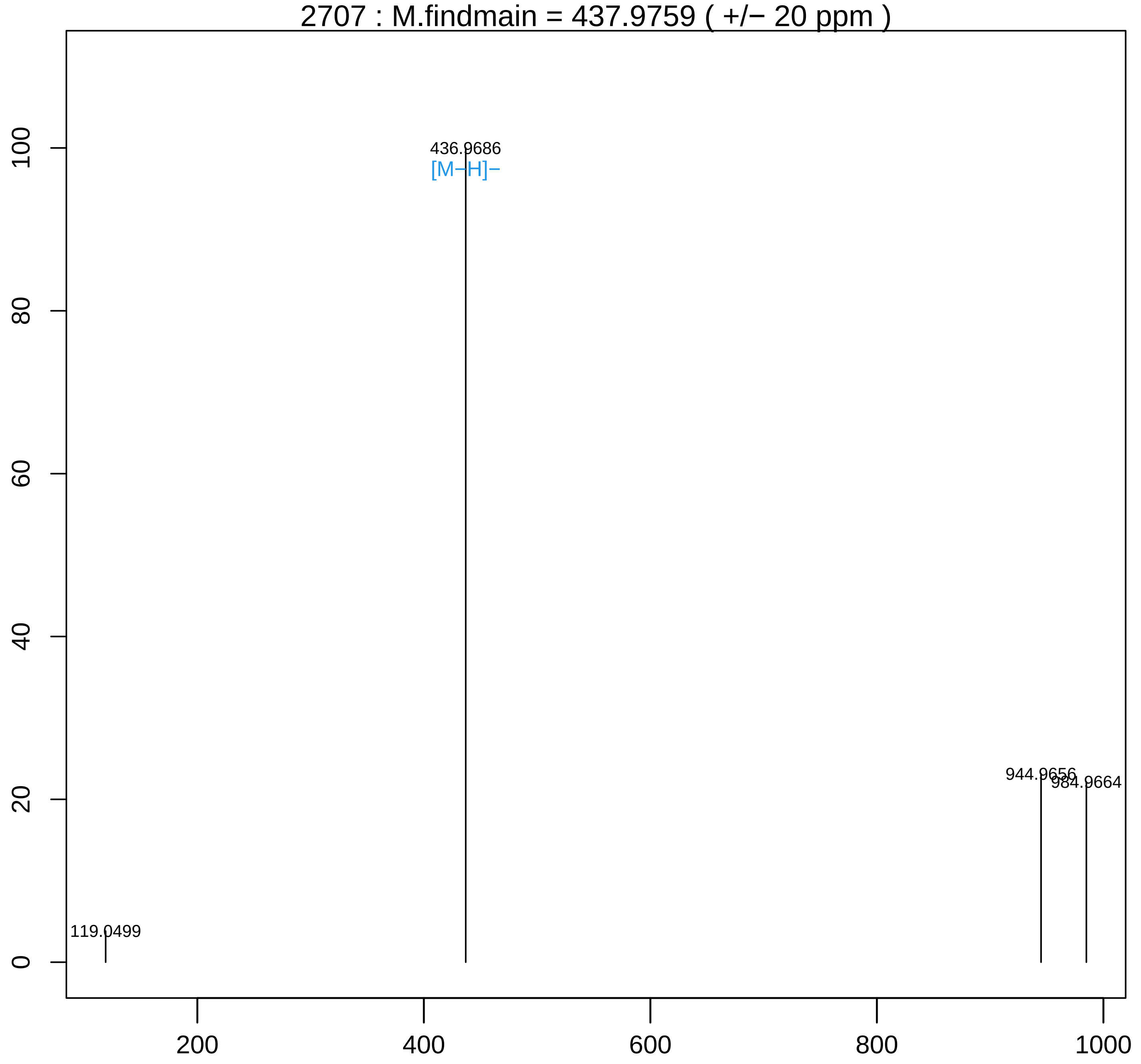
2707 : M.findmain = 437.9759 (+/− 20 ppm)
436.9686
[M−H]−
944.9656
984.9664
119.0499
0
20
40
60
80
100
200
400
600
800
1000

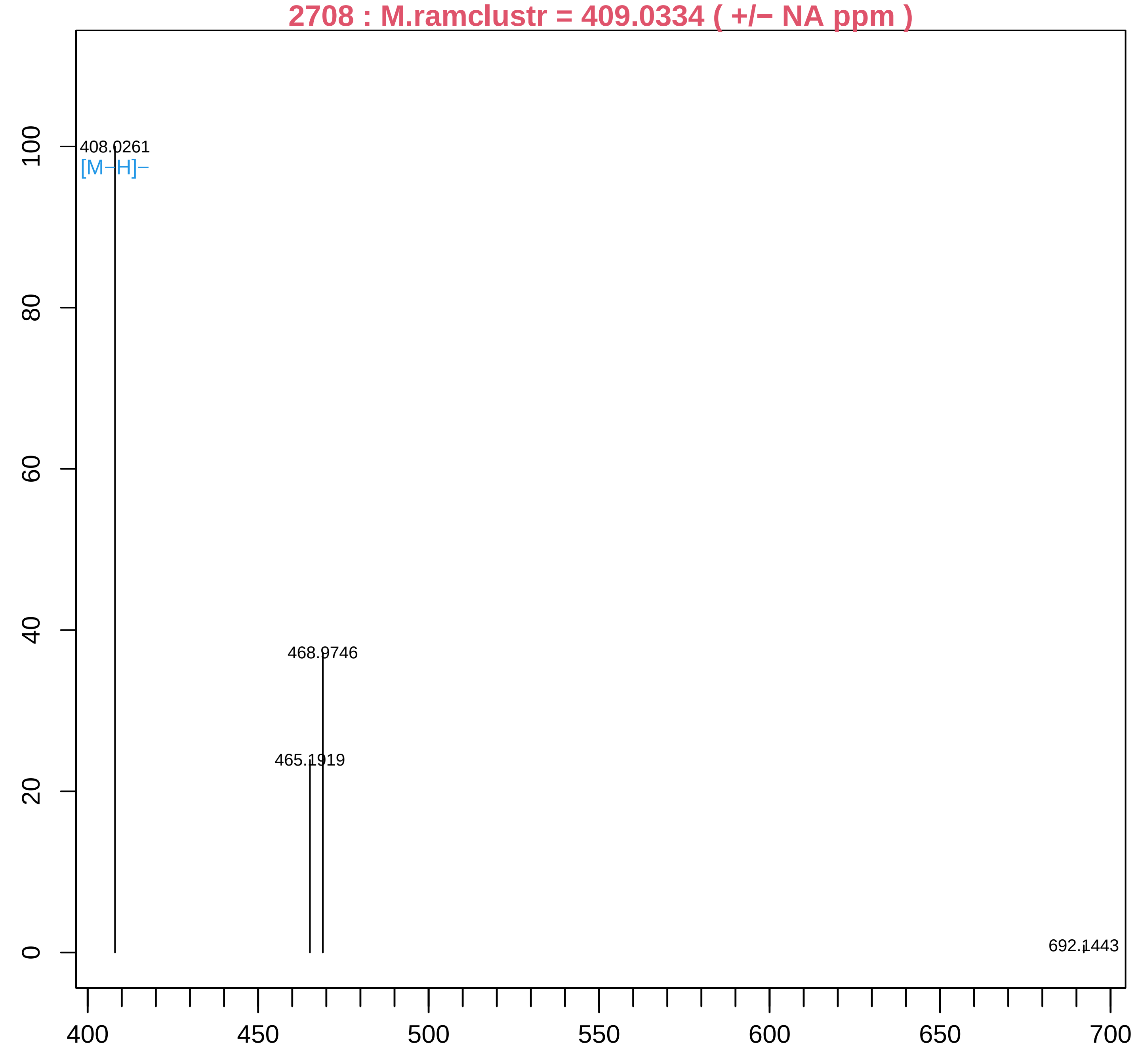
2708 : M.ramclustr = 409.0334 (+/− NA ppm)
408.0261
[M−H]−
468.9746
465.1919
692.1443
0
20
40
60
80
100
400
450
500
550
600
650
700

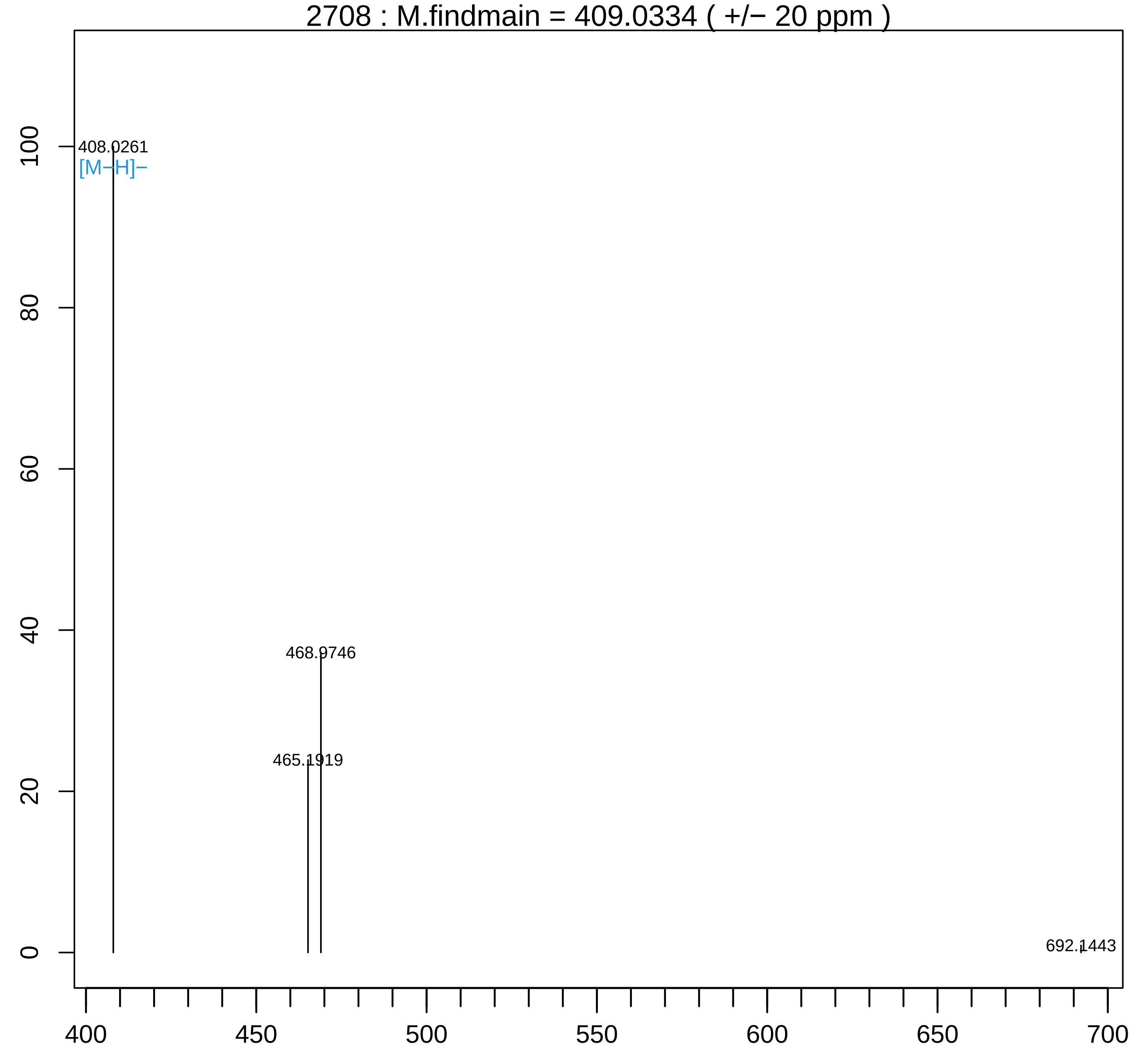
2708 : M.findmain = 409.0334 (+/− 20 ppm)
408.0261
[M−H]−
468.9746
465.1919
692.1443
0
20
40
60
80
100
400
450
500
550
600
650
700

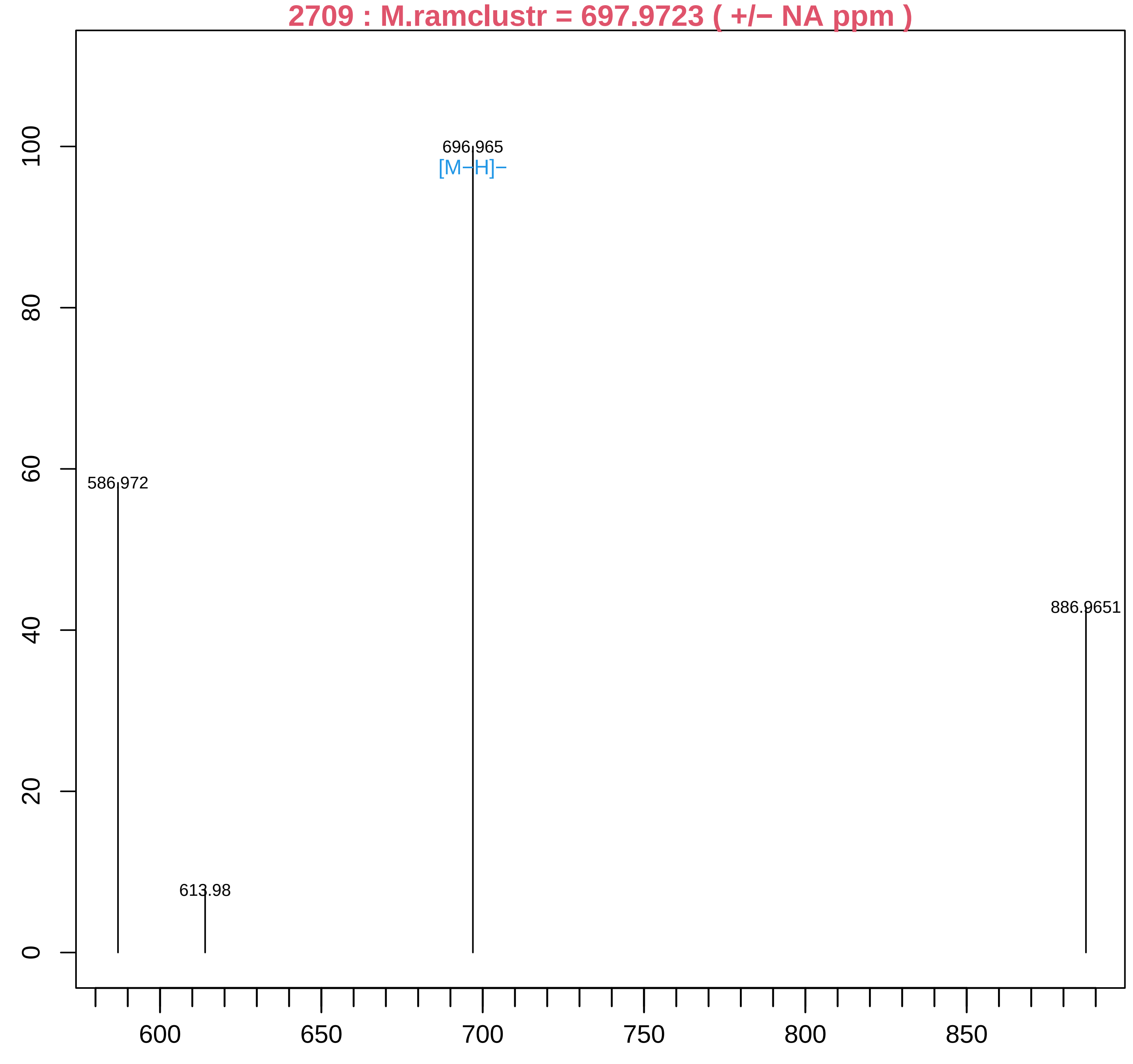

2709 : M.ramclustr = 697.9723 (+/− NA ppm)
696.965
[M−H]−
586.972
613.98
886.9651
0
20
40
60
80
100
600
650
700
750
800
850

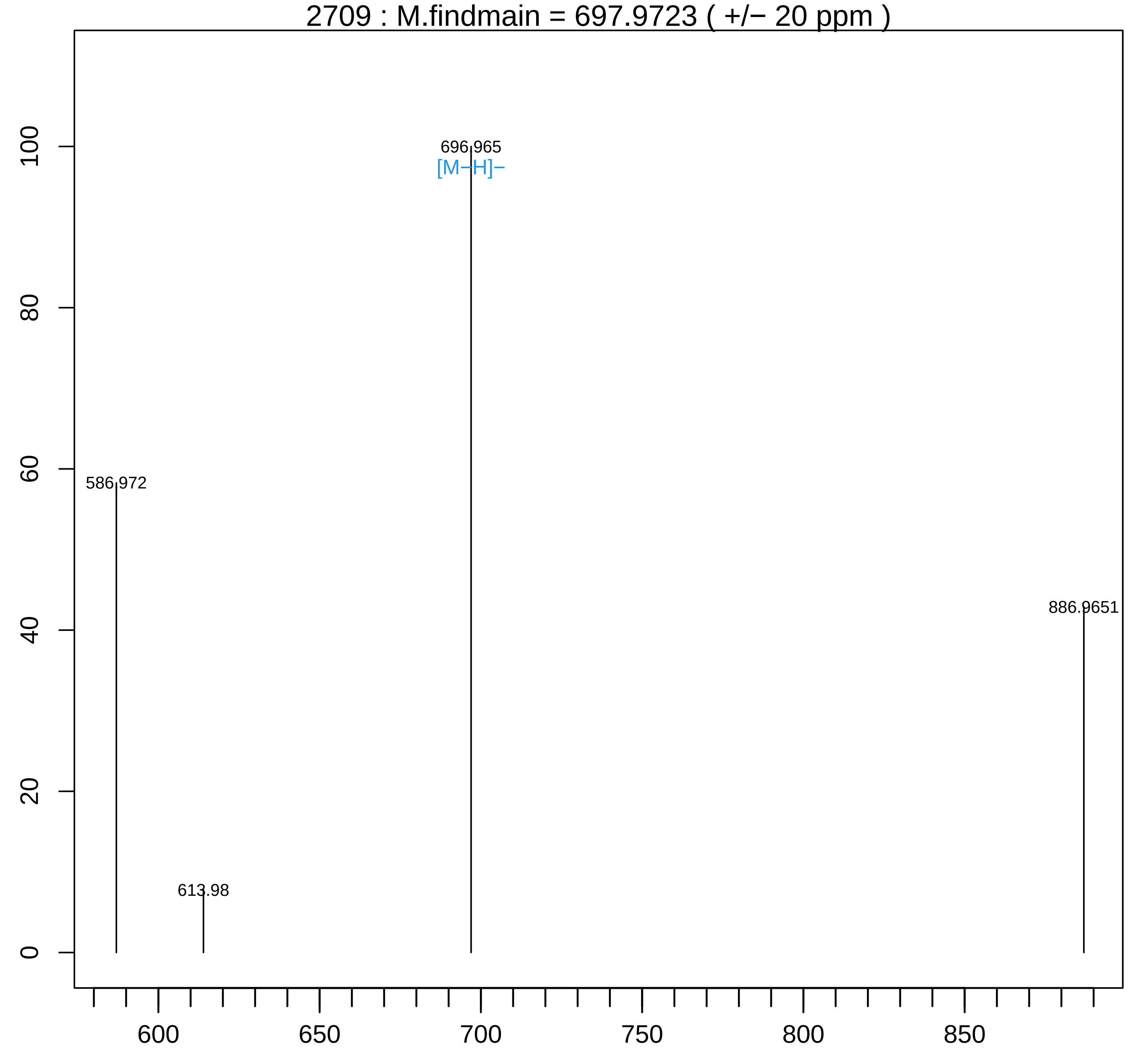

2709 : M.findmain = 697.9723 (+/− 20 ppm)
696.965
[M−H]−
586.972
613.98
886.9651
0
20
40
60
80
100
600
650
700
750
800
850

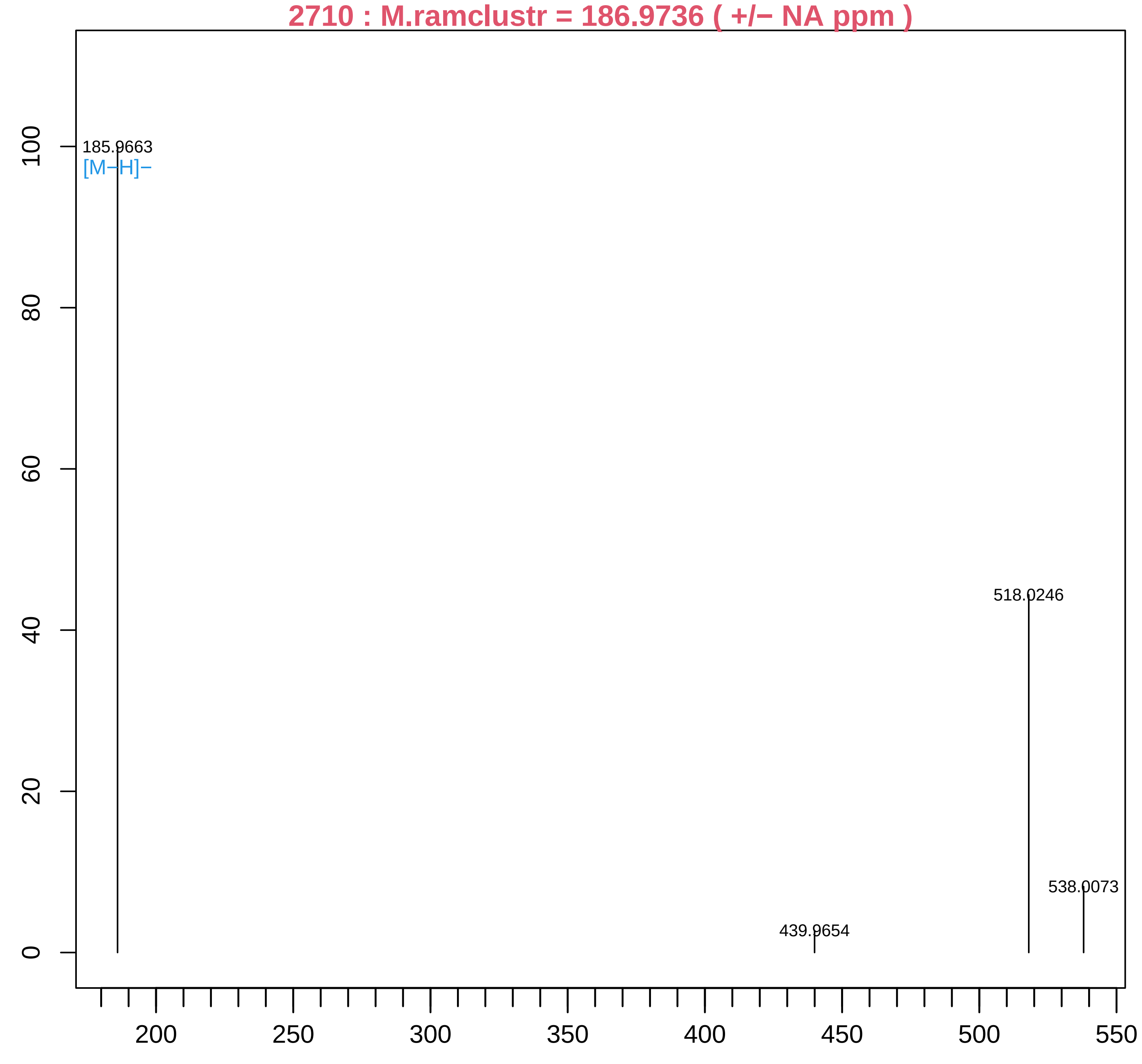
2710 : M.ramclustr = 186.9736 (+/− NA ppm)
185.9663
[M−H]−
518.0246
538.0073
439.9654
0
20
40
60
80
100
200
250
300
350
400
450
500
550

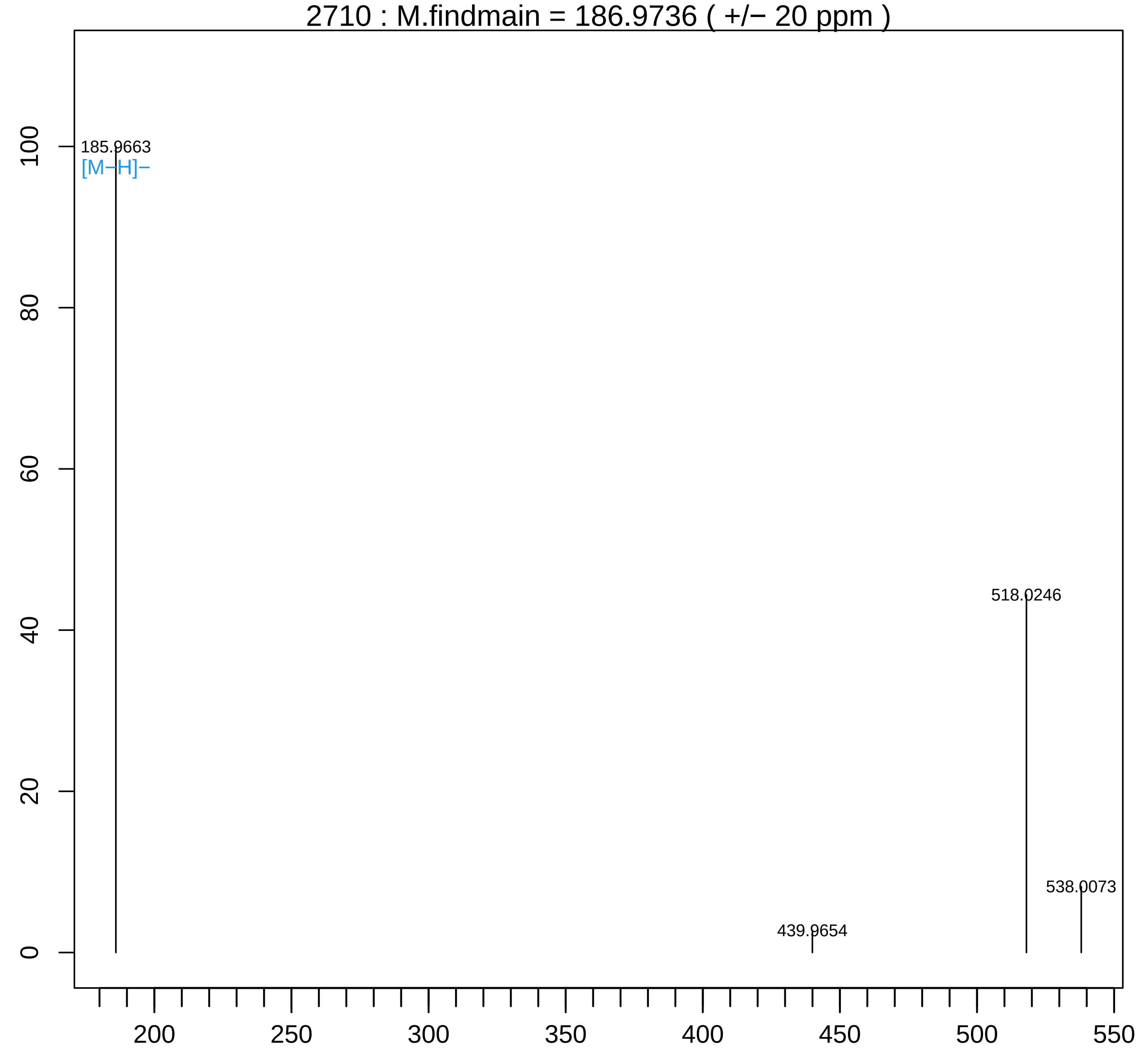
2710 : M.findmain = 186.9736 (+/− 20 ppm)
185.9663
[M−H]−
518.0246
538.0073
439.9654
0
20
40
60
80
100
200
250
300
350
400
450
500
550

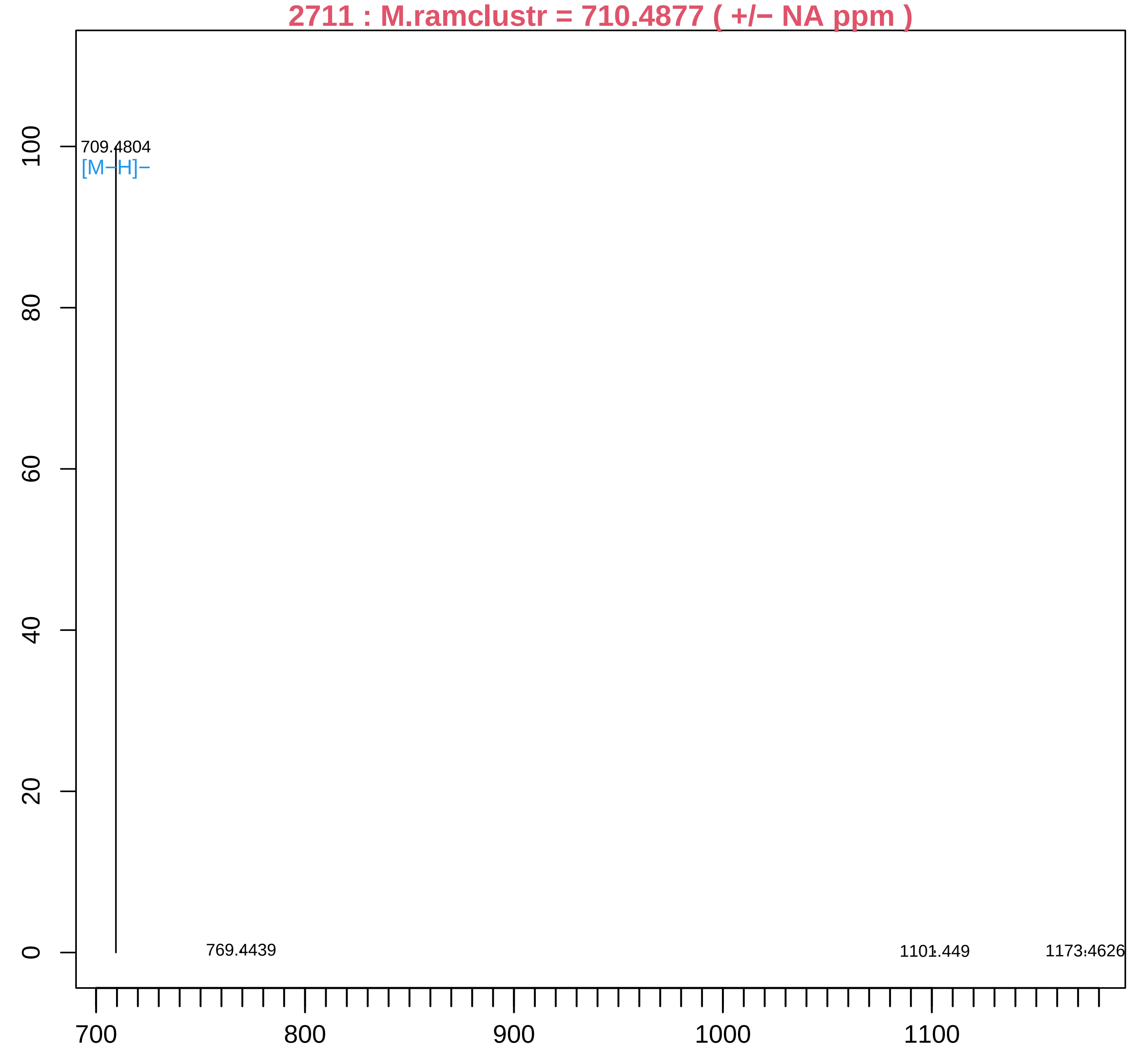

2711 : M.ramclustr = 710.4877 (+/− NA ppm)
709.4804
[M−H]−
769.4439
1101.449
1173.4626

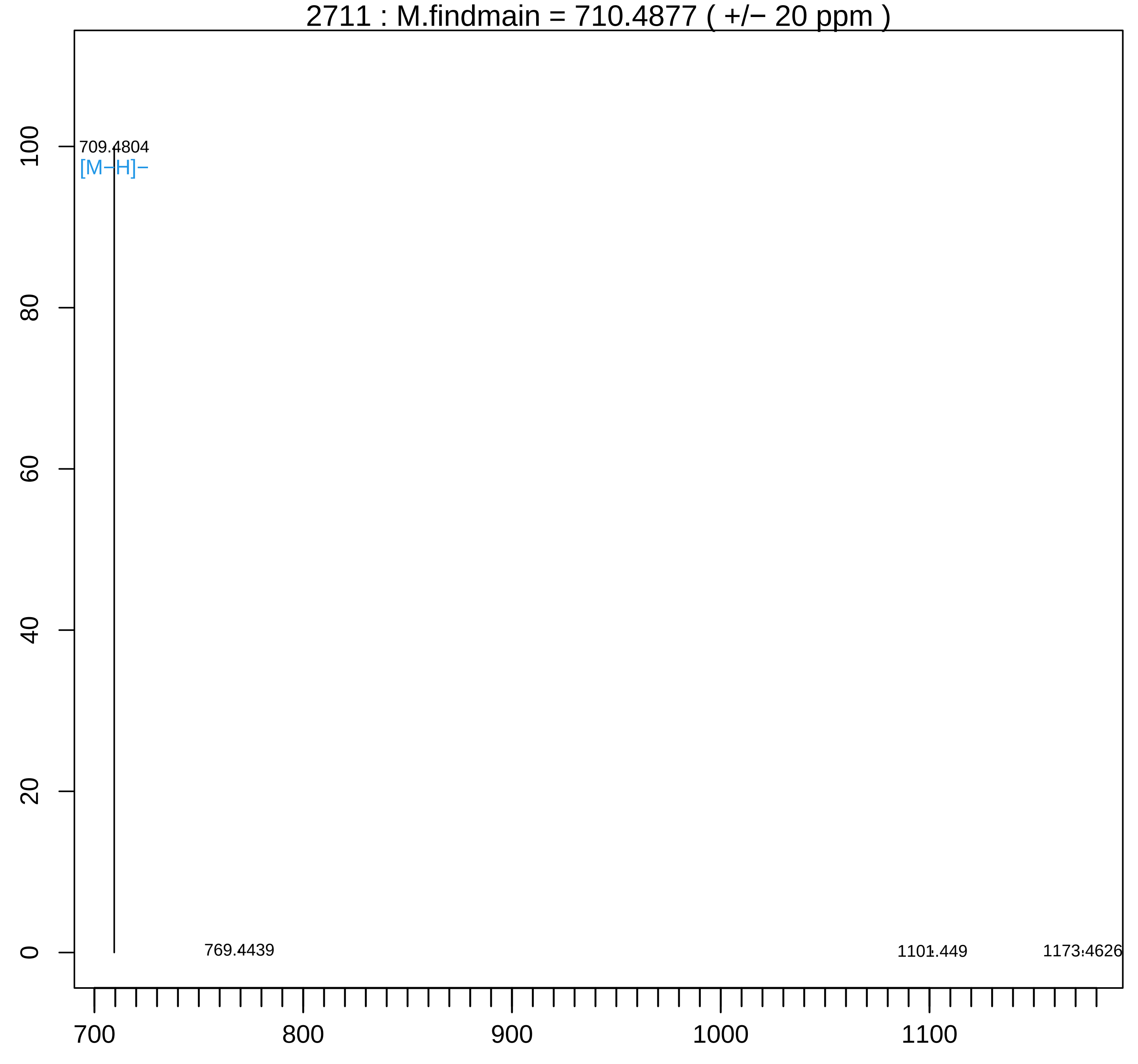

2711 : M.findmain = 710.4877 (+/− 20 ppm)
709.4804
[M−H]−
769.4439
1101.449
1173.4626

2712 : M.ramclustr = 689.0503 (+/− NA ppm)

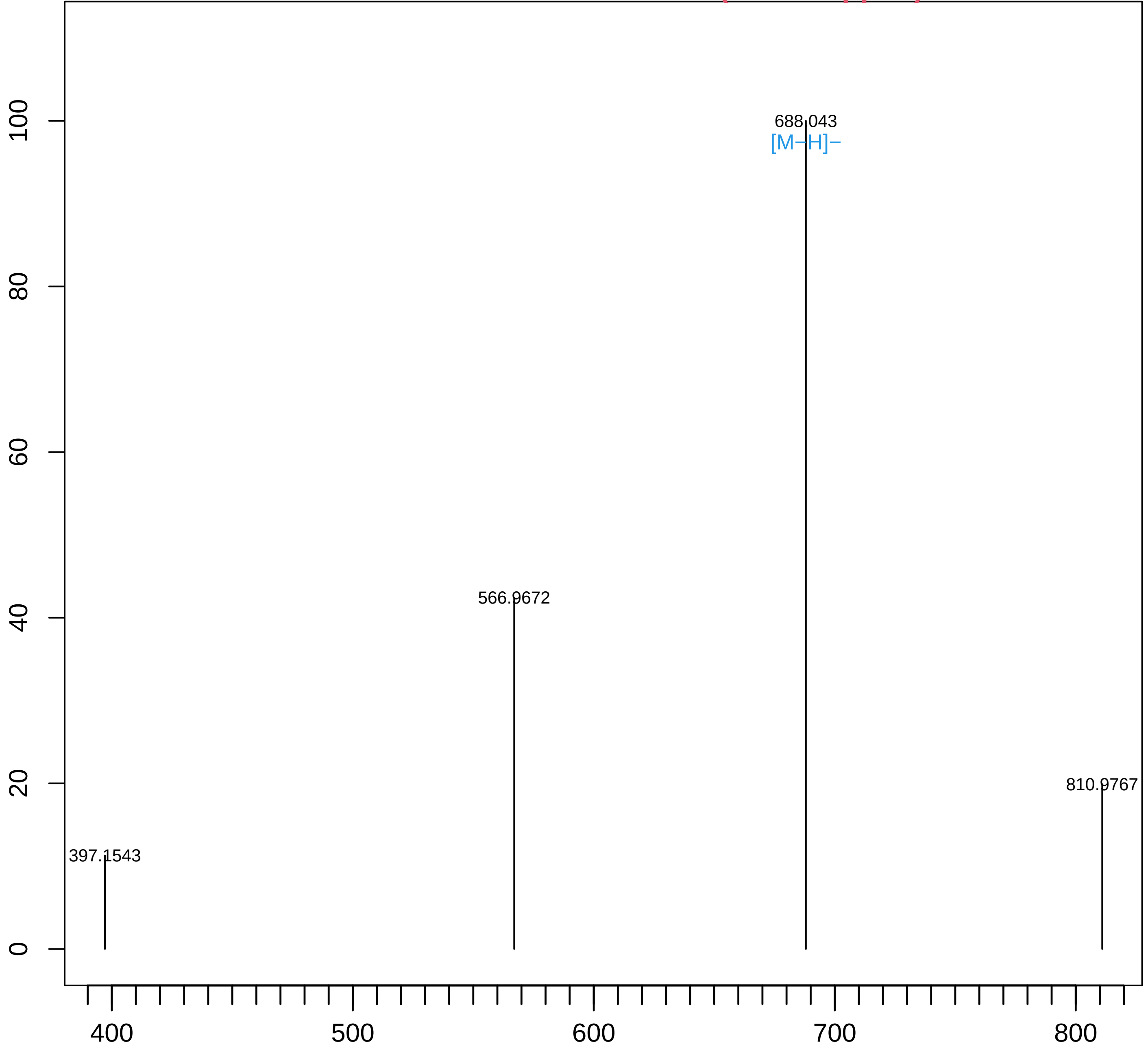

2712 : M.findmain = 689.0503 (+/− 20 ppm)

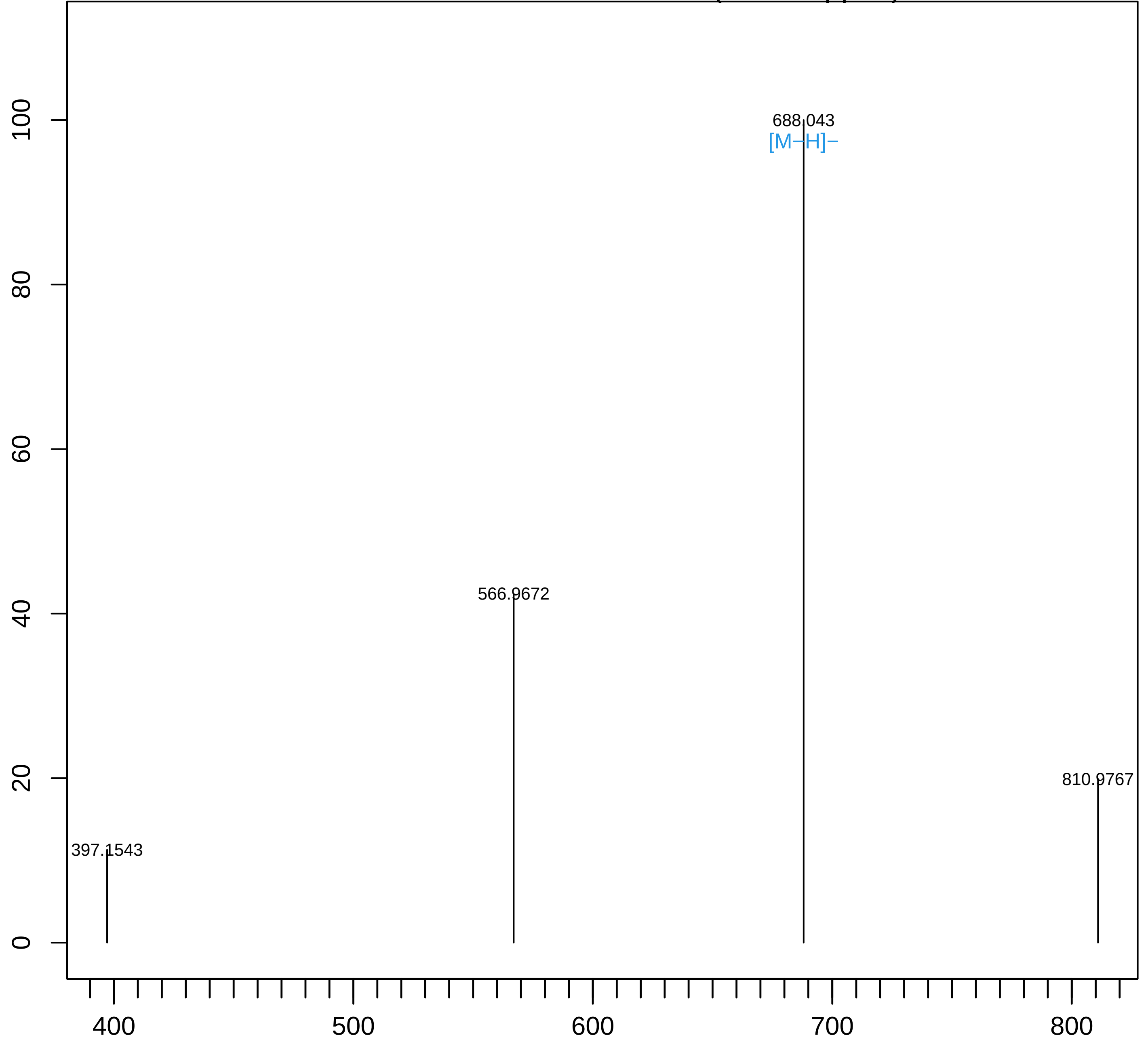

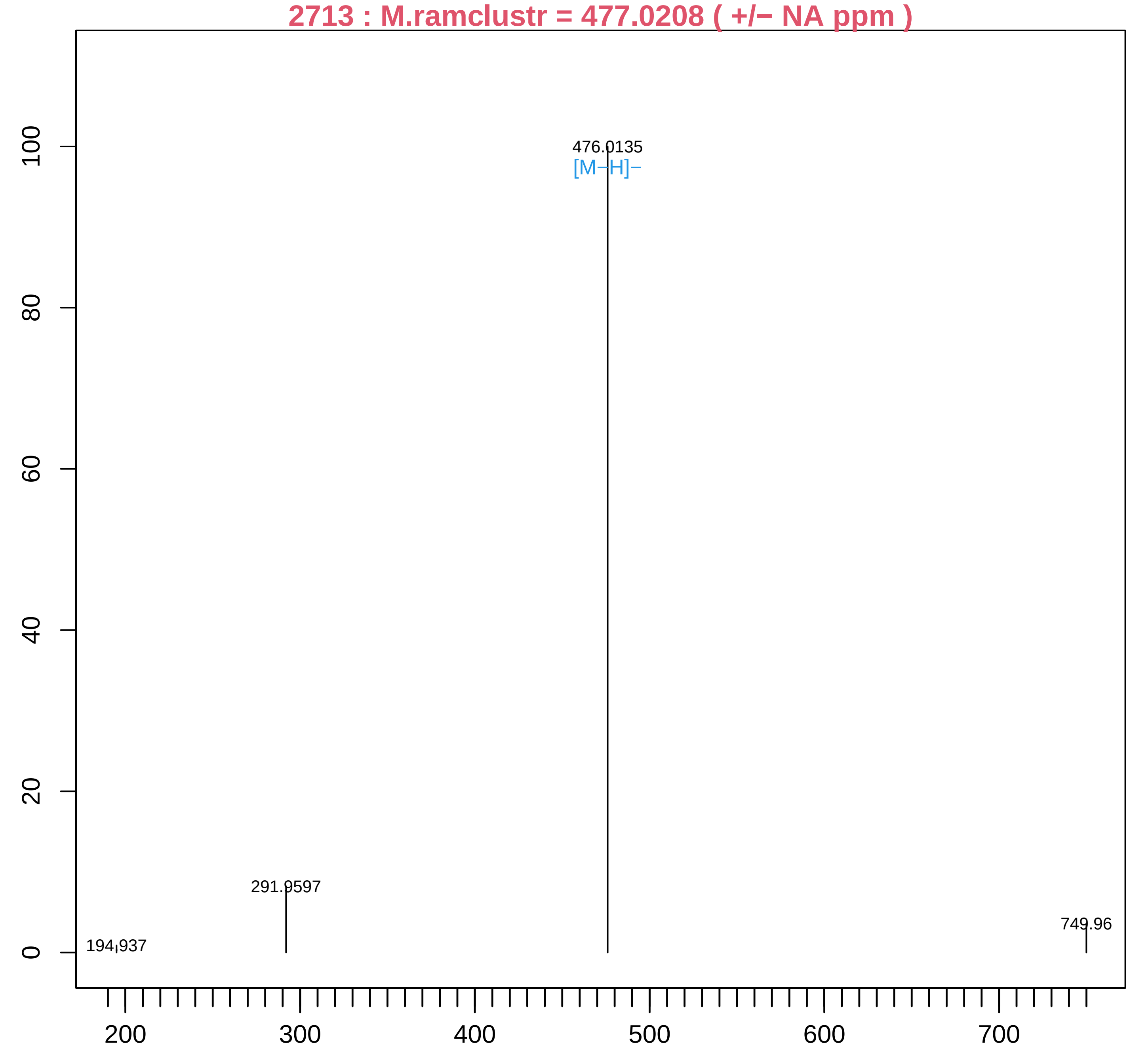
2713 : M.ramclustr = 477.0208 (+/− NA ppm)
476.0135
[M−H]−
291.9597
194.937
749.96
0
20
40
60
80
100
200
300
400
500
600
700

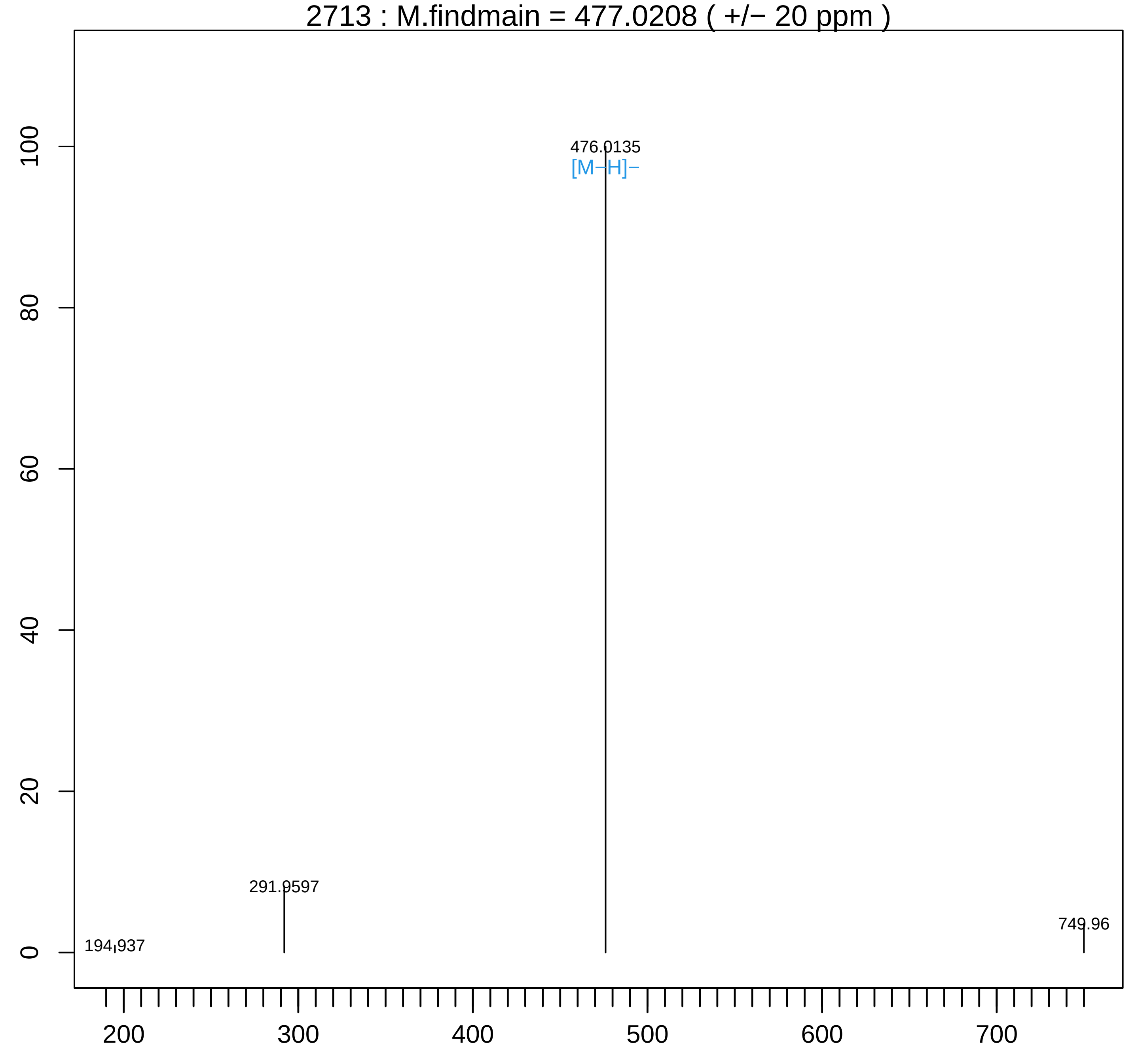
2713 : M.findmain = 477.0208 (+/− 20 ppm)
476.0135
[M−H]−
291.9597
194.937
749.96
0
20
40
60
80
100
200
300
400
500
600
700

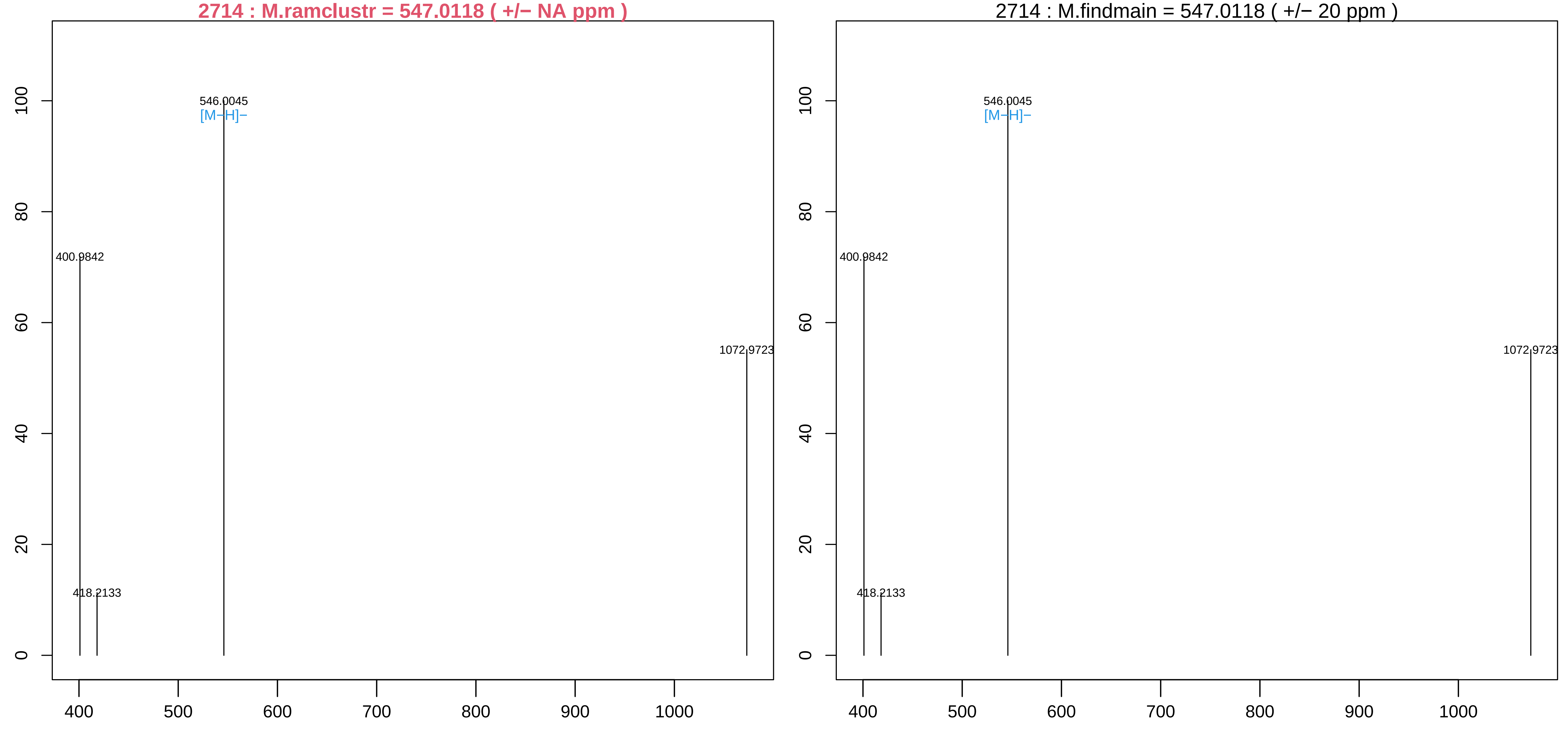
2714 : M.ramclustr = 547.0118 (+/− NA ppm)
546.0045
[M−H]−
400.9842
418.2133
1072.9723
2714 : M.findmain = 547.0118 (+/− 20 ppm)
546.0045
[M−H]−
400.9842
418.2133
1072.9723

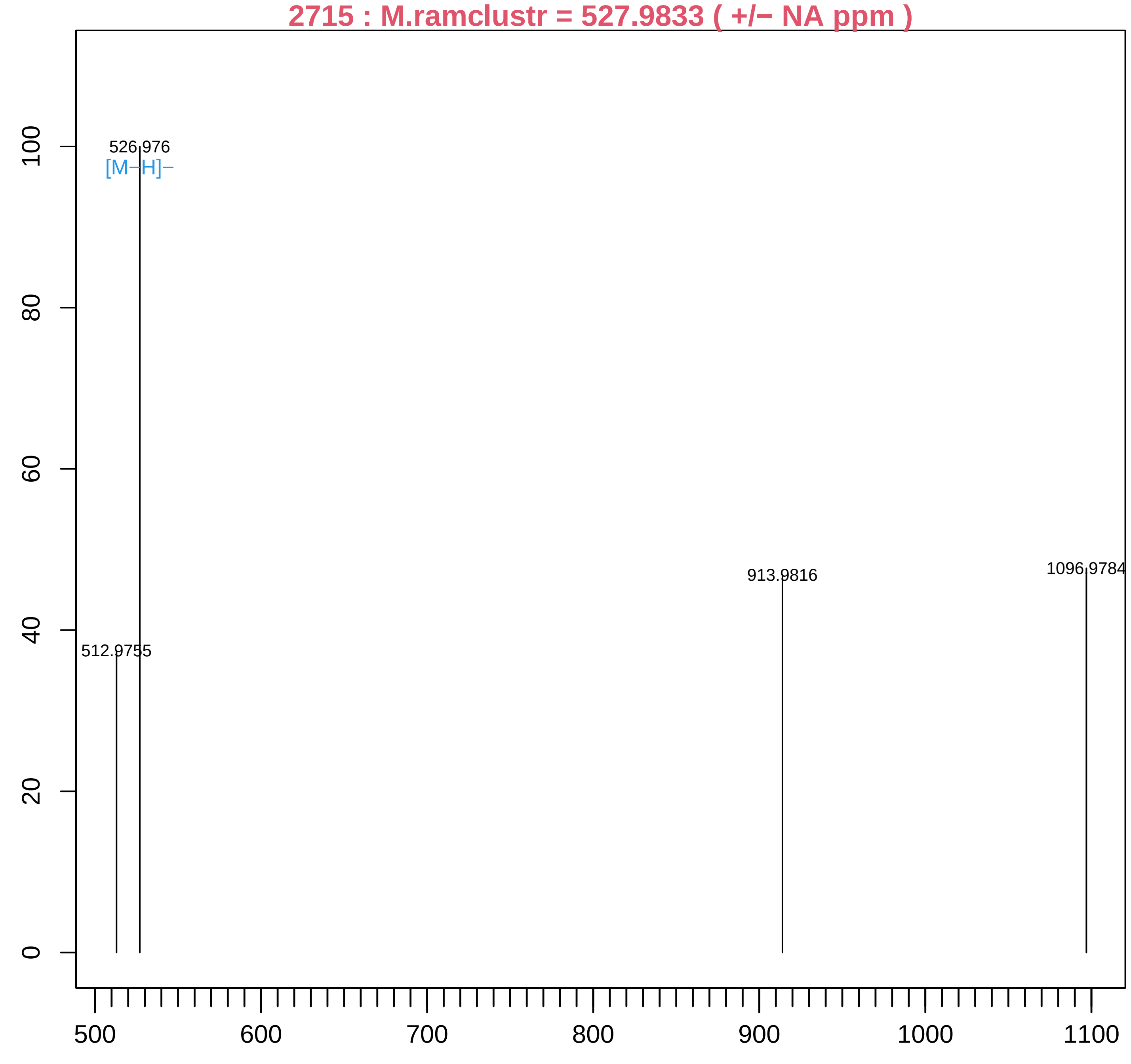
2715 : M.ramclustr = 527.9833 (+/− NA ppm)
526.976
[M−H]−
512.9755
913.9816
1096.9784
0
20
40
60
80
100
500
600
700
800
900
1000
1100

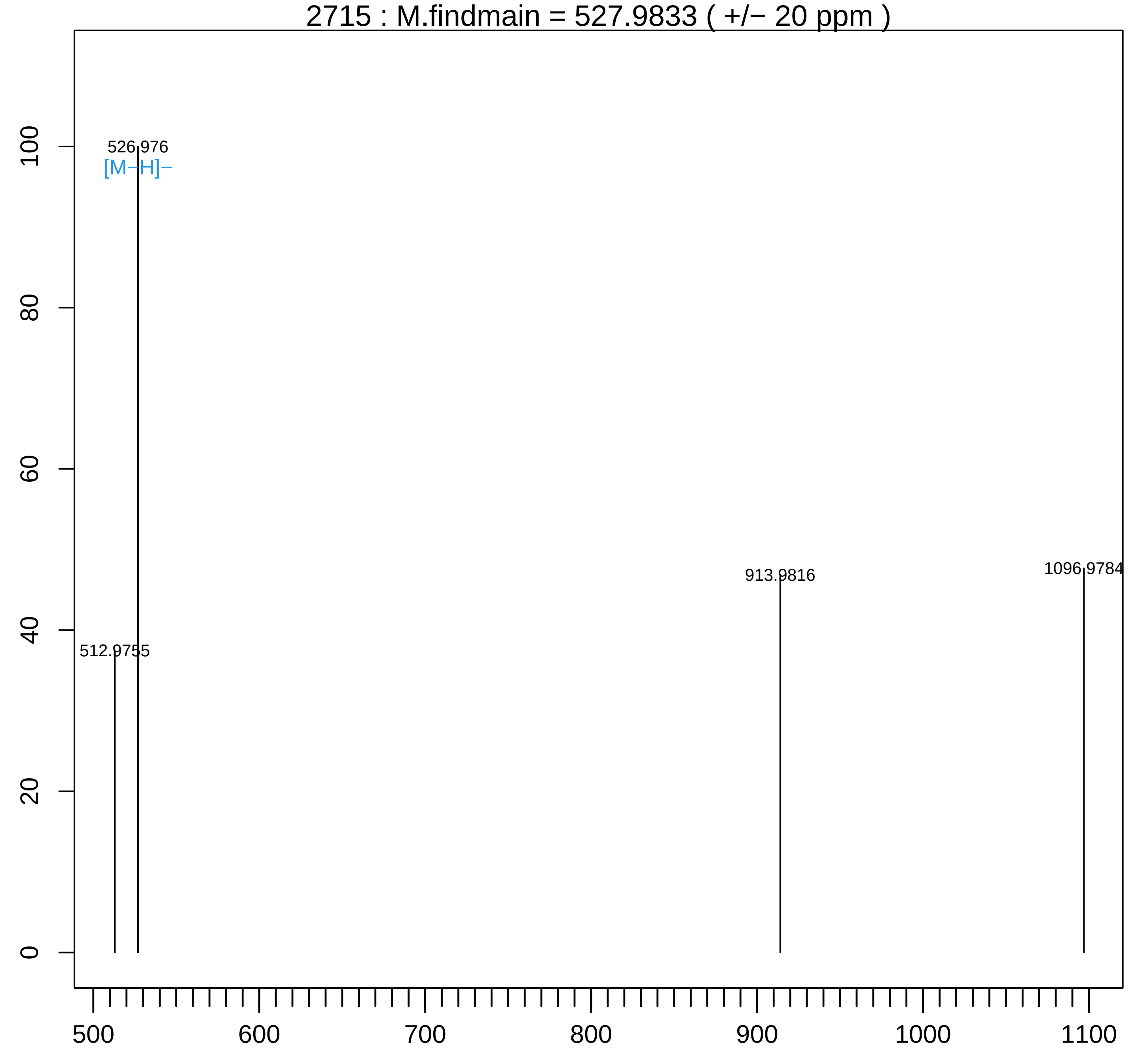
2715 : M.findmain = 527.9833 (+/− 20 ppm)
526.976
[M−H]−
512.9755
913.9816
1096.9784
0
20
40
60
80
100
500
600
700
800
900
1000
1100

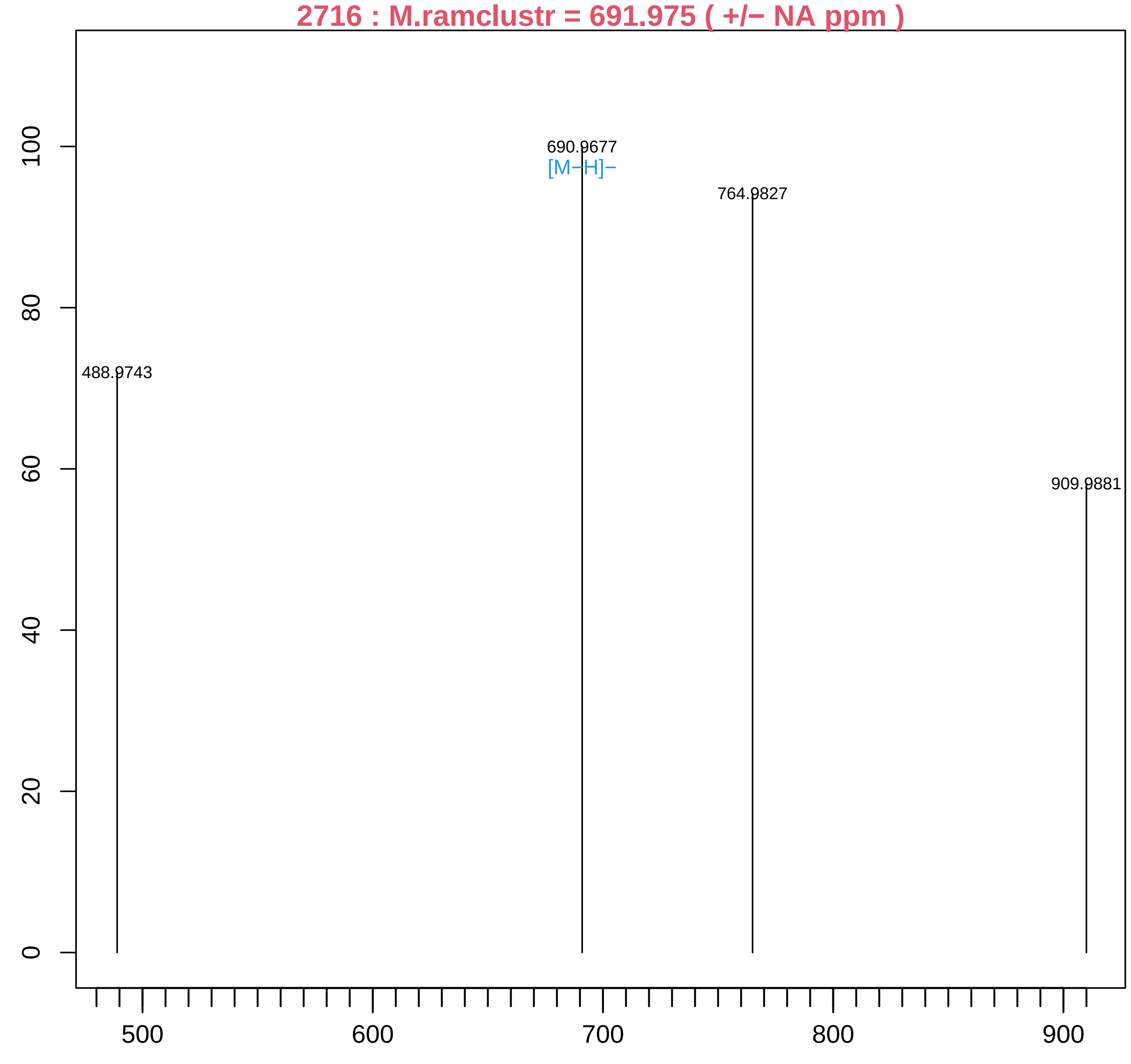
2716 : M.ramclustr = 691.975 (+/− NA ppm)
488.9743
690.9677
[M−H]−
764.9827
909.9881
0
20
40
60
80
100
500
600
700
800
900

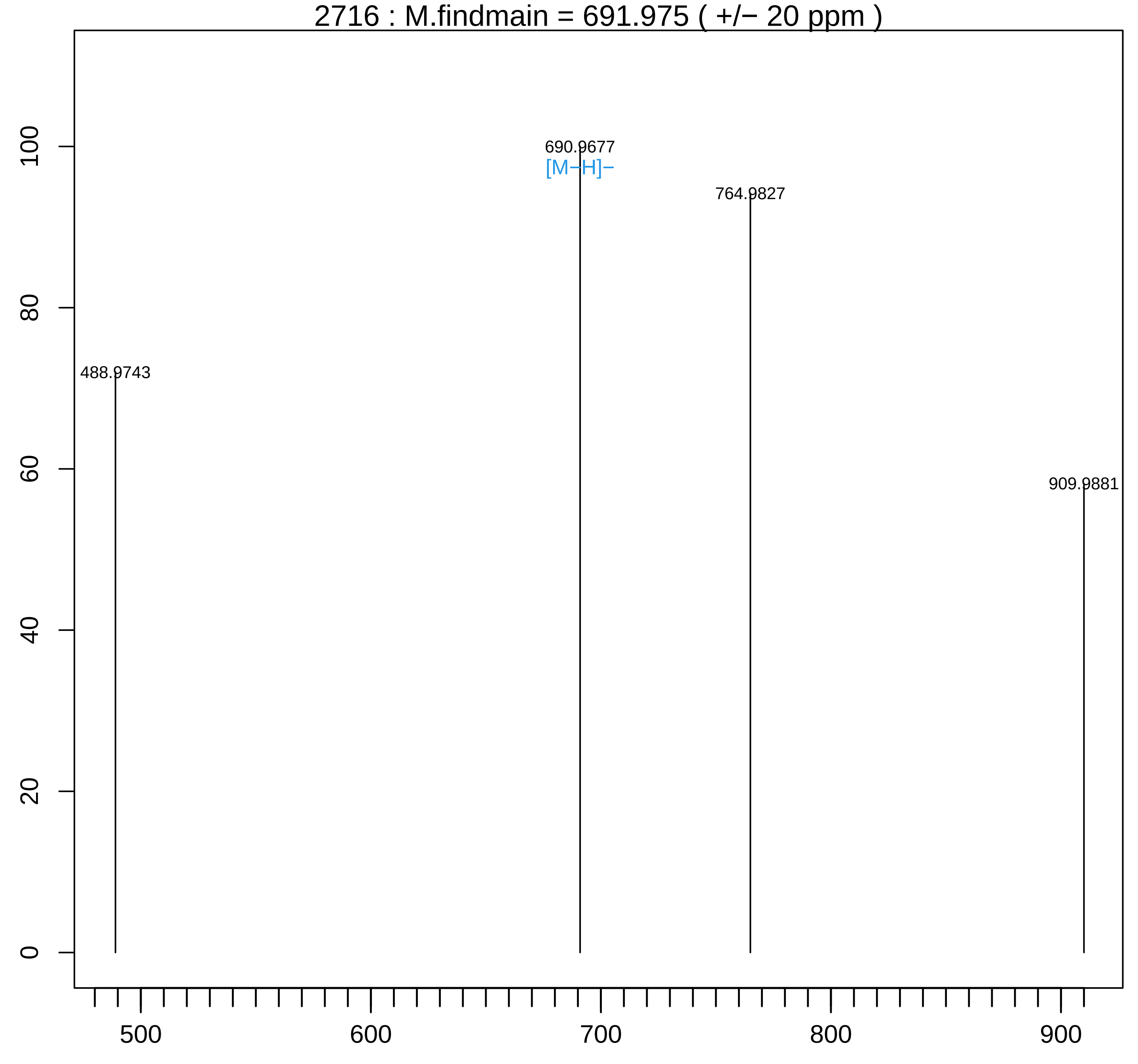
2716 : M.findmain = 691.975 (+/− 20 ppm)
488.9743
690.9677
[M−H]−
764.9827
909.9881
0
20
40
60
80
100
500
600
700
800
900

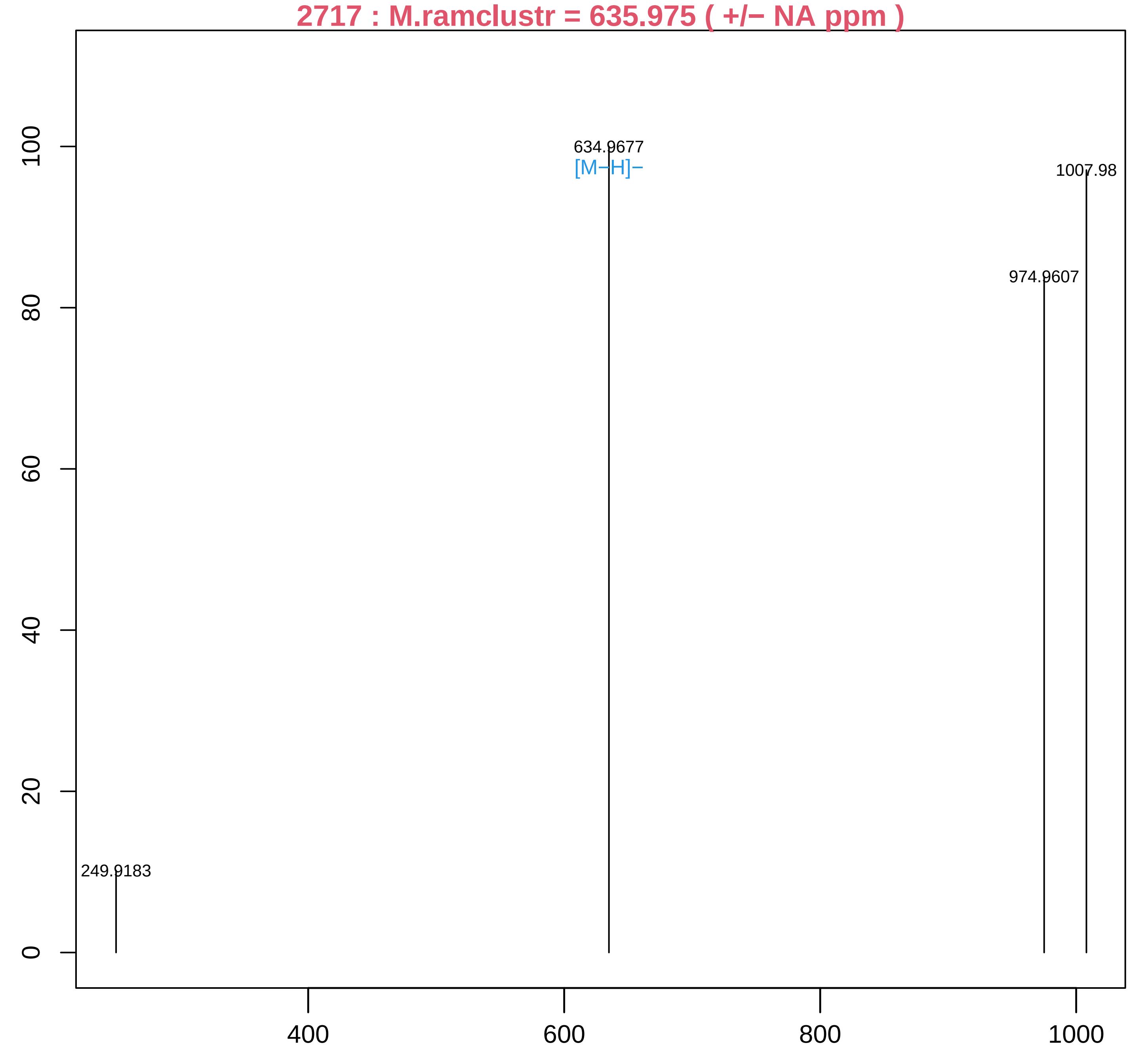
2717 : M.ramclustr = 635.975 (+/− NA ppm)
634.9677
[M−H]−
1007.98
974.9607
249.9183
0
20
40
60
80
100
400
600
800
1000

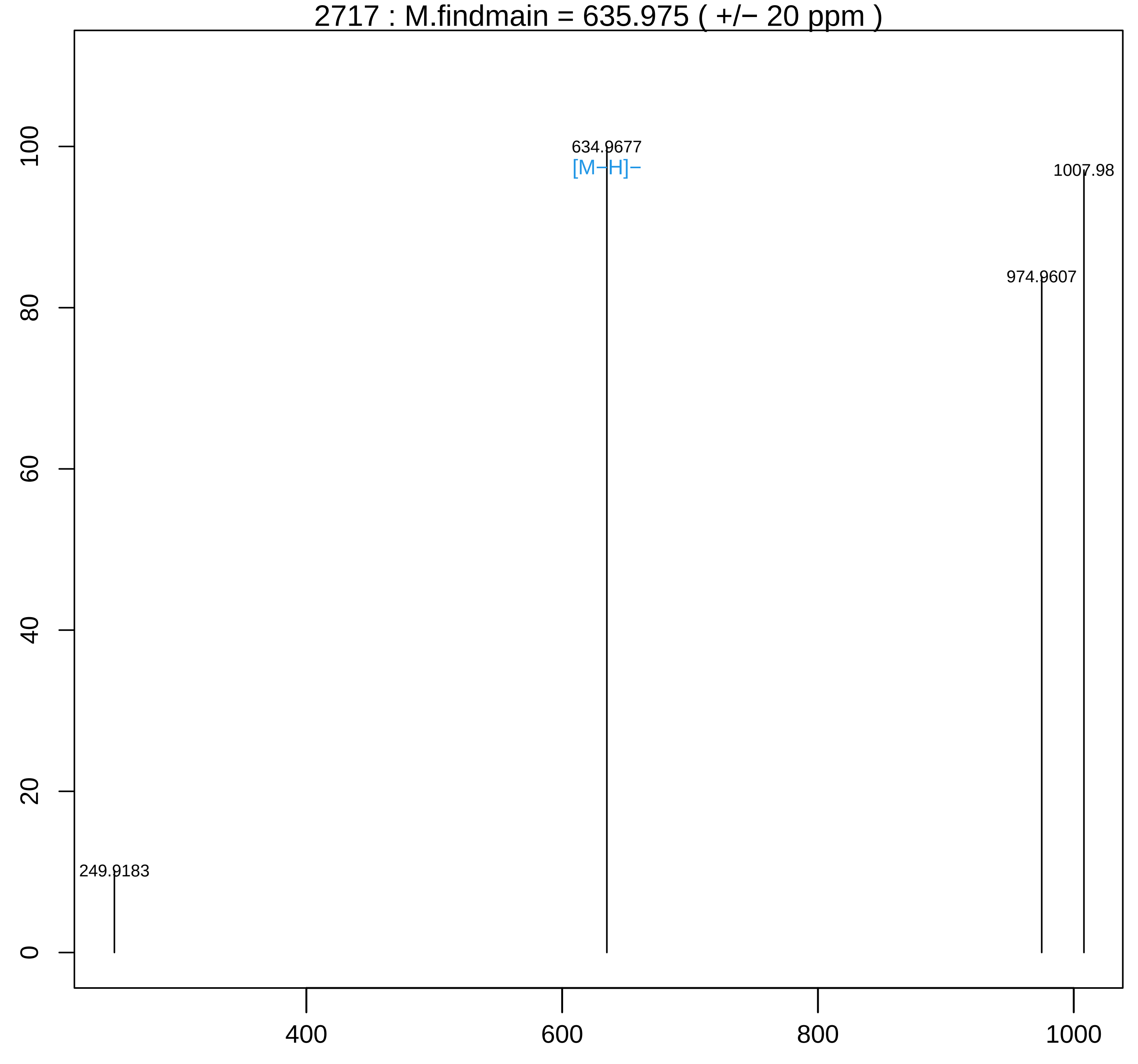
2717 : M.findmain = 635.975 (+/− 20 ppm)
634.9677
[M−H]−
1007.98
974.9607
249.9183
0
20
40
60
80
100
400
600
800
1000

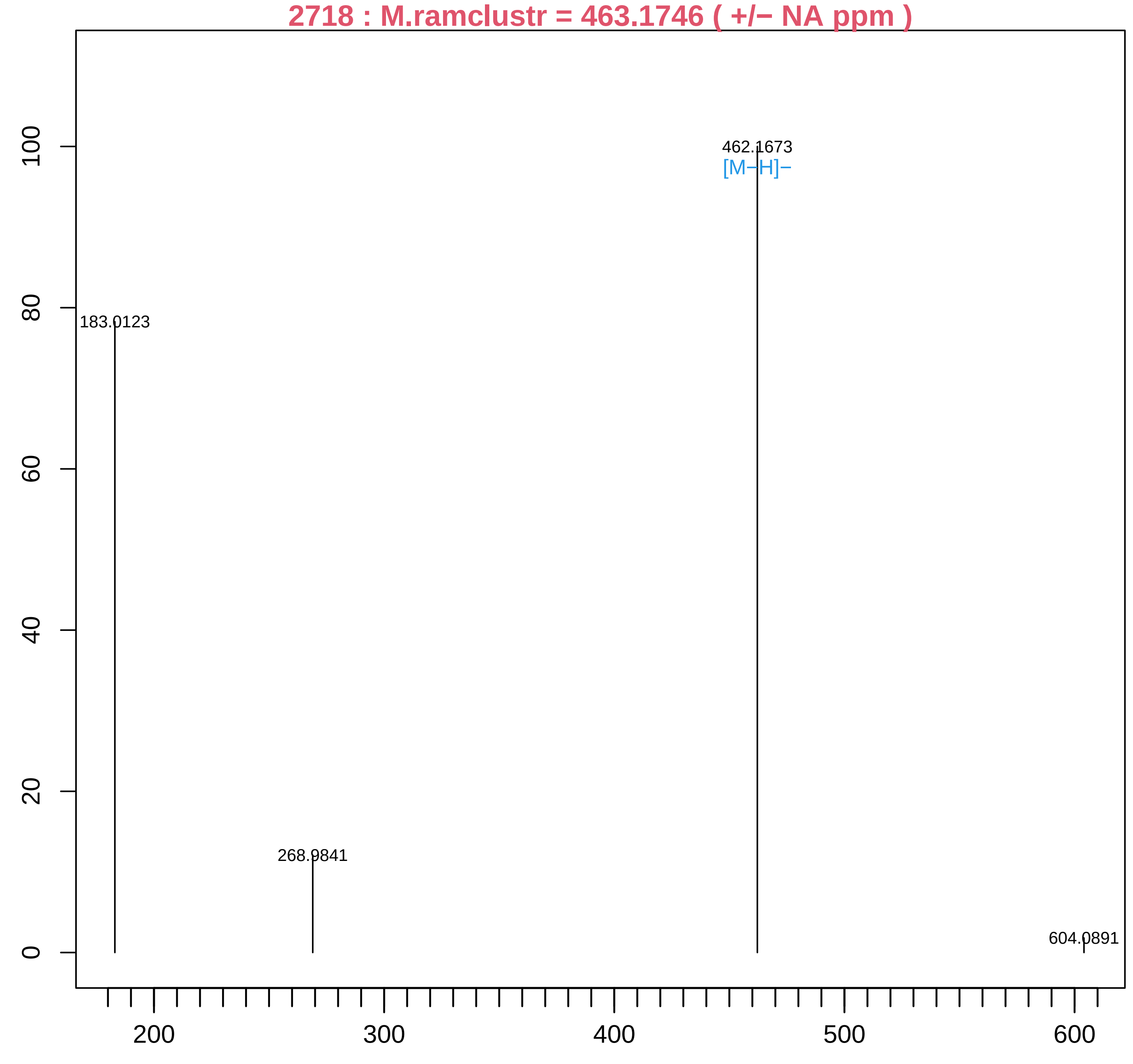

2718 : M.ramclustr = 463.1746 (+/− NA ppm)
183.0123
268.9841
462.1673
[M−H]−
604.0891
0
20
40
60
80
100
200
300
400
500
600

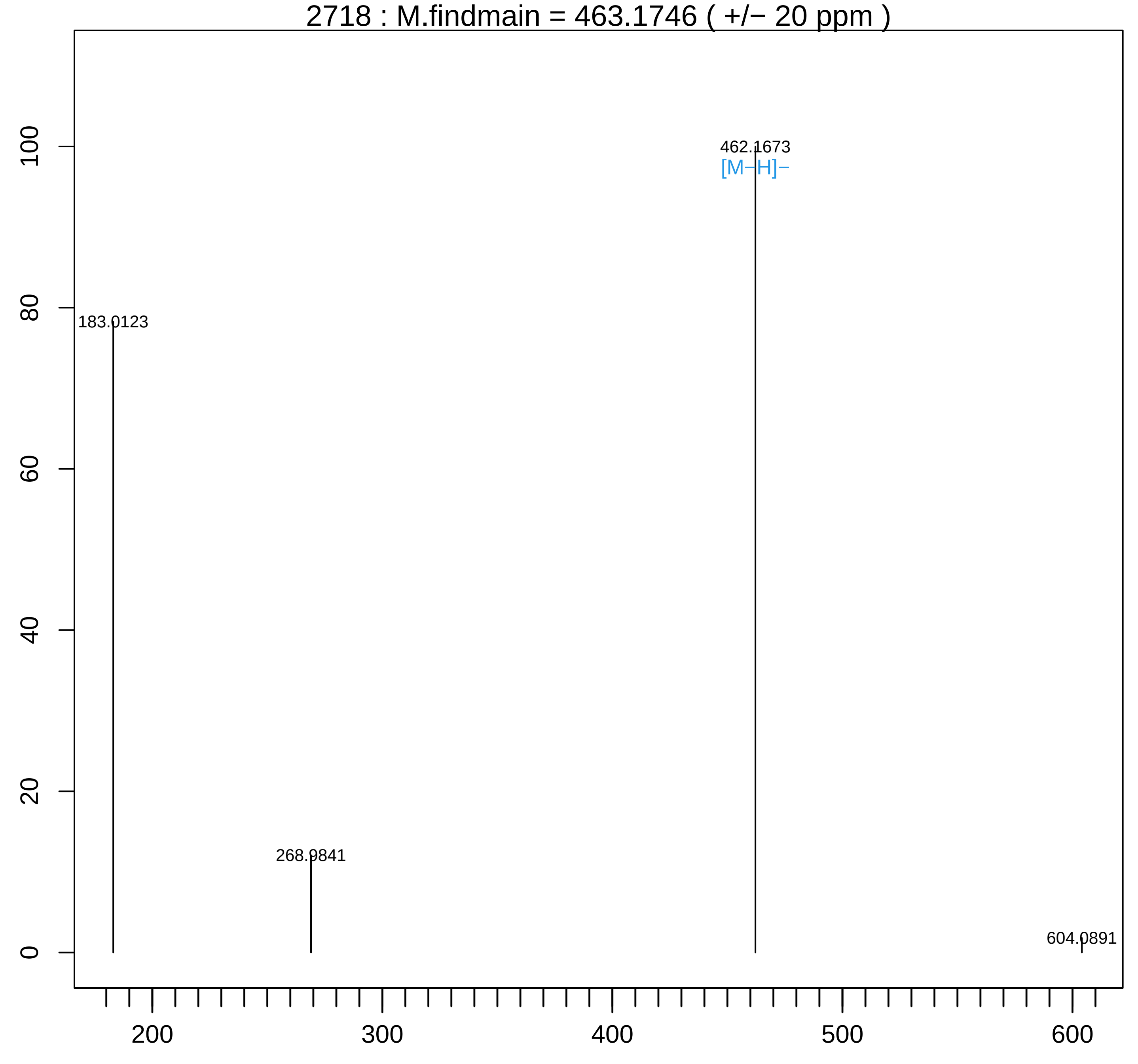

2718 : M.findmain = 463.1746 (+/− 20 ppm)
183.0123
268.9841
462.1673
[M−H]−
604.0891
0
20
40
60
80
100
200
300
400
500
600

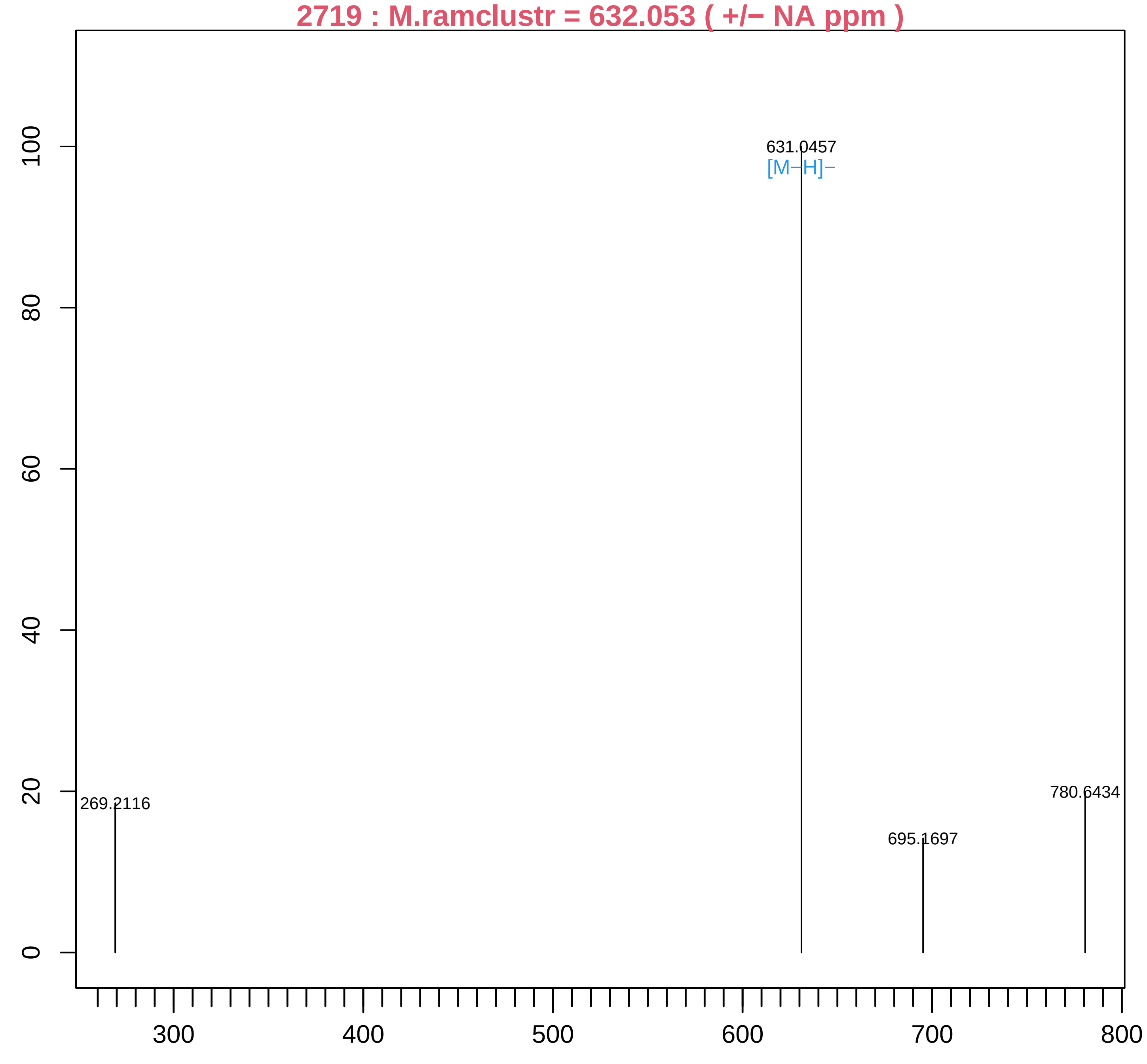
2719 : M.ramclustr = 632.053 (+/− NA ppm)
631.0457
[M−H]−
269.2116
695.1697
780.6434
0
20
40
60
80
100
300
400
500
600
700
800

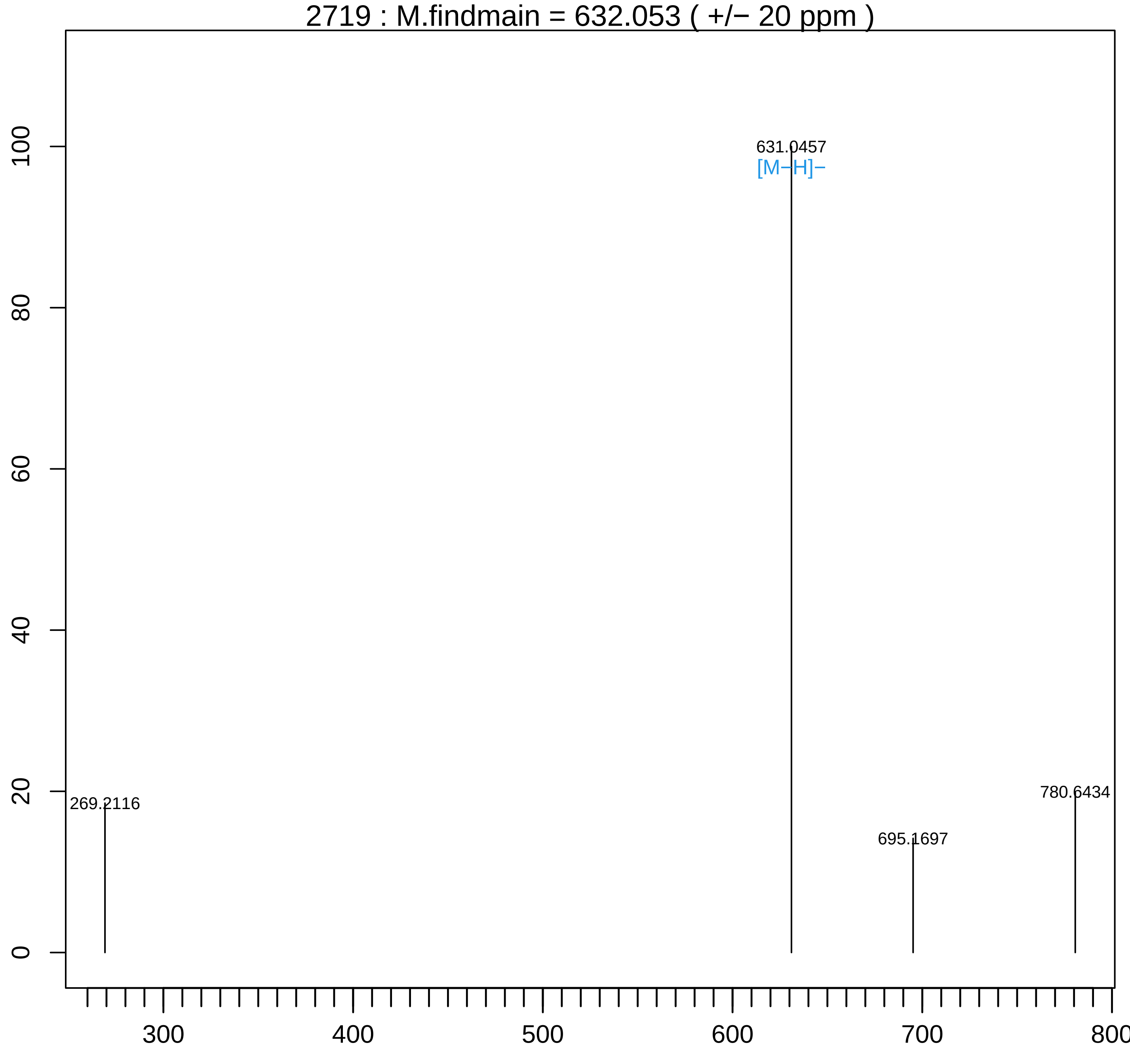
2719 : M.findmain = 632.053 (+/− 20 ppm)
631.0457
[M−H]−
269.2116
695.1697
780.6434
0
20
40
60
80
100
300
400
500
600
700
800

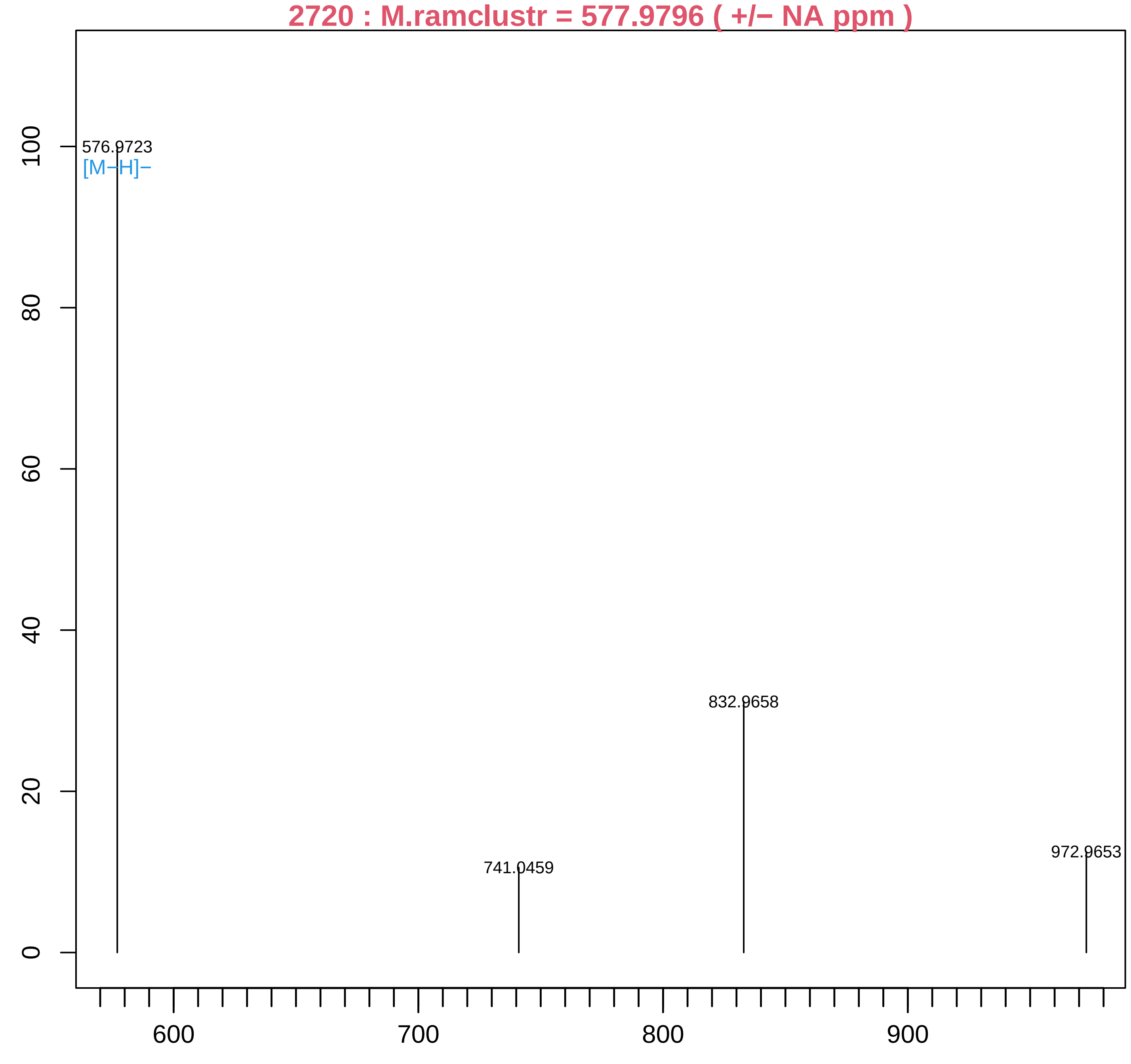
2720 : M.ramclustr = 577.9796 (+/− NA ppm)
576.9723
[M−H]−
741.0459
832.9658
972.9653
0
20
40
60
80
100
600
700
800
900

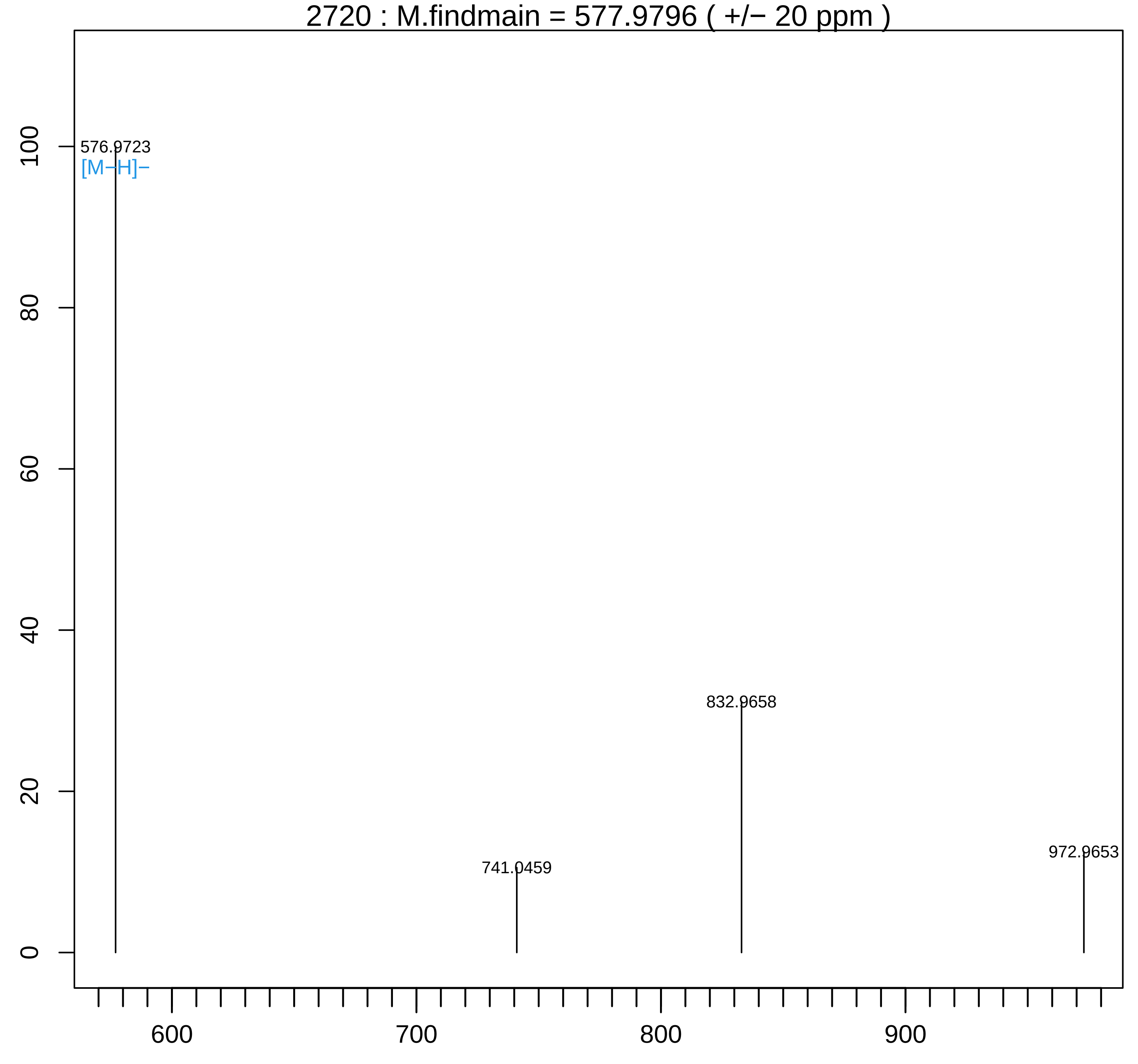
2720 : M.findmain = 577.9796 (+/− 20 ppm)
576.9723
[M−H]−
741.0459
832.9658
972.9653
0
20
40
60
80
100
600
700
800
900

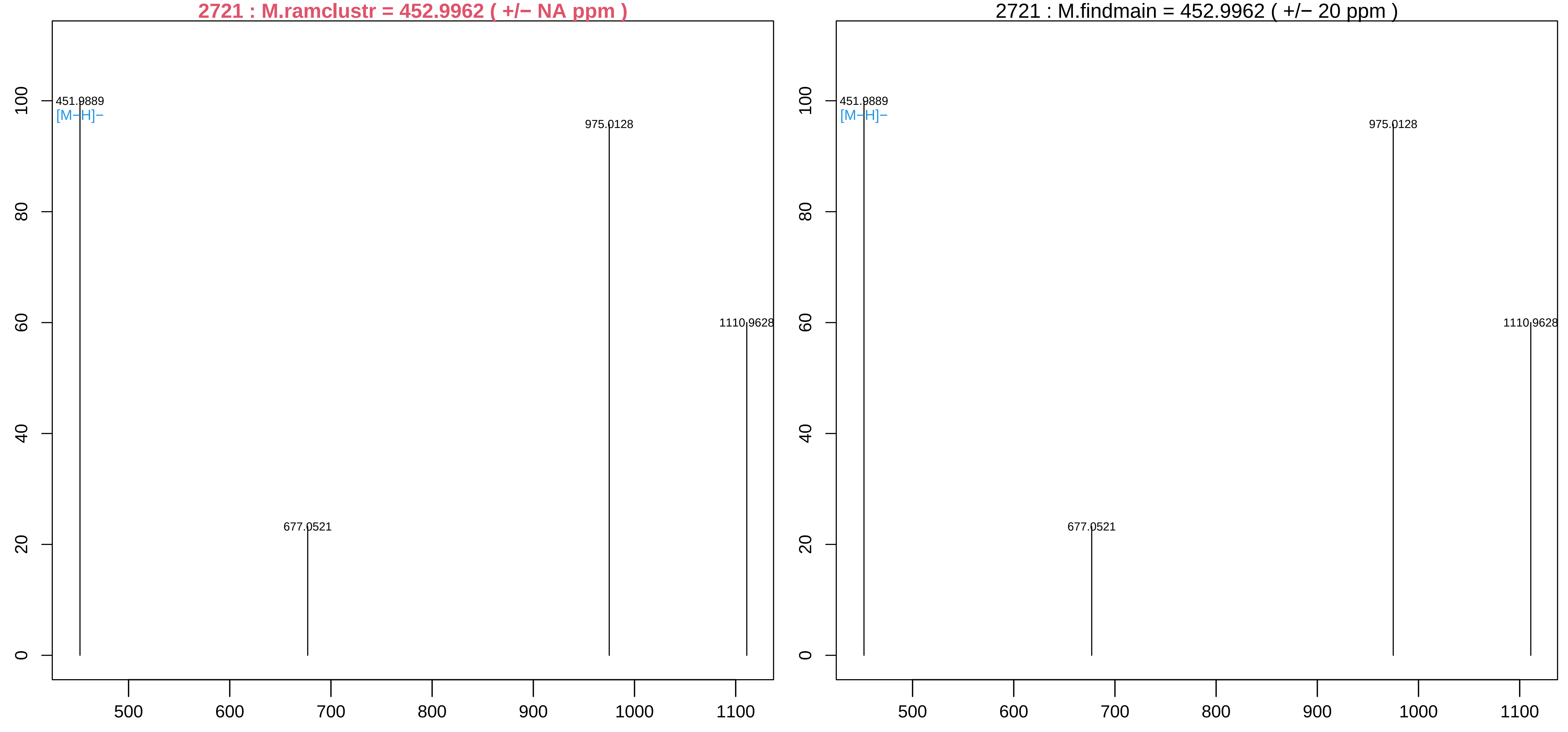
2721 : M.ramclustr = 452.9962 (+/− NA ppm)
451.9889
[M−H]−
975.0128
677.0521
1110.9628
2721 : M.findmain = 452.9962 (+/− 20 ppm)
451.9889
[M−H]−
975.0128
677.0521
1110.9628

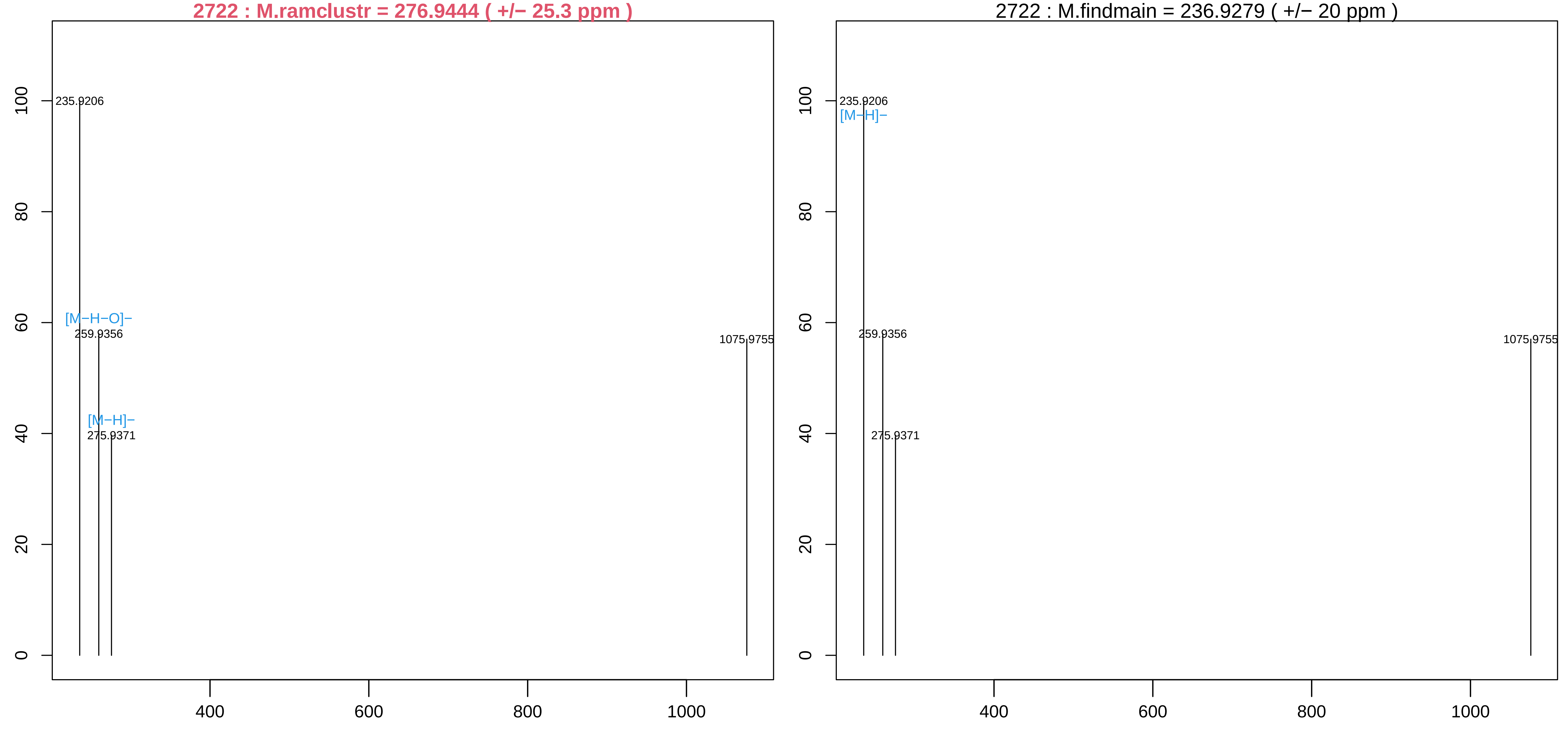
2722 : M.ramclustr = 276.9444 (+/− 25.3 ppm)
235.9206
[M−H−O]−
259.9356
[M−H]−
275.9371
1075.9755
0
20
40
60
80
100
400
600
800
1000
2722 : M.findmain = 236.9279 (+/− 20 ppm)
235.9206
[M−H]−
259.9356
275.9371
1075.9755
0
20
40
60
80
100
400
600
800
1000

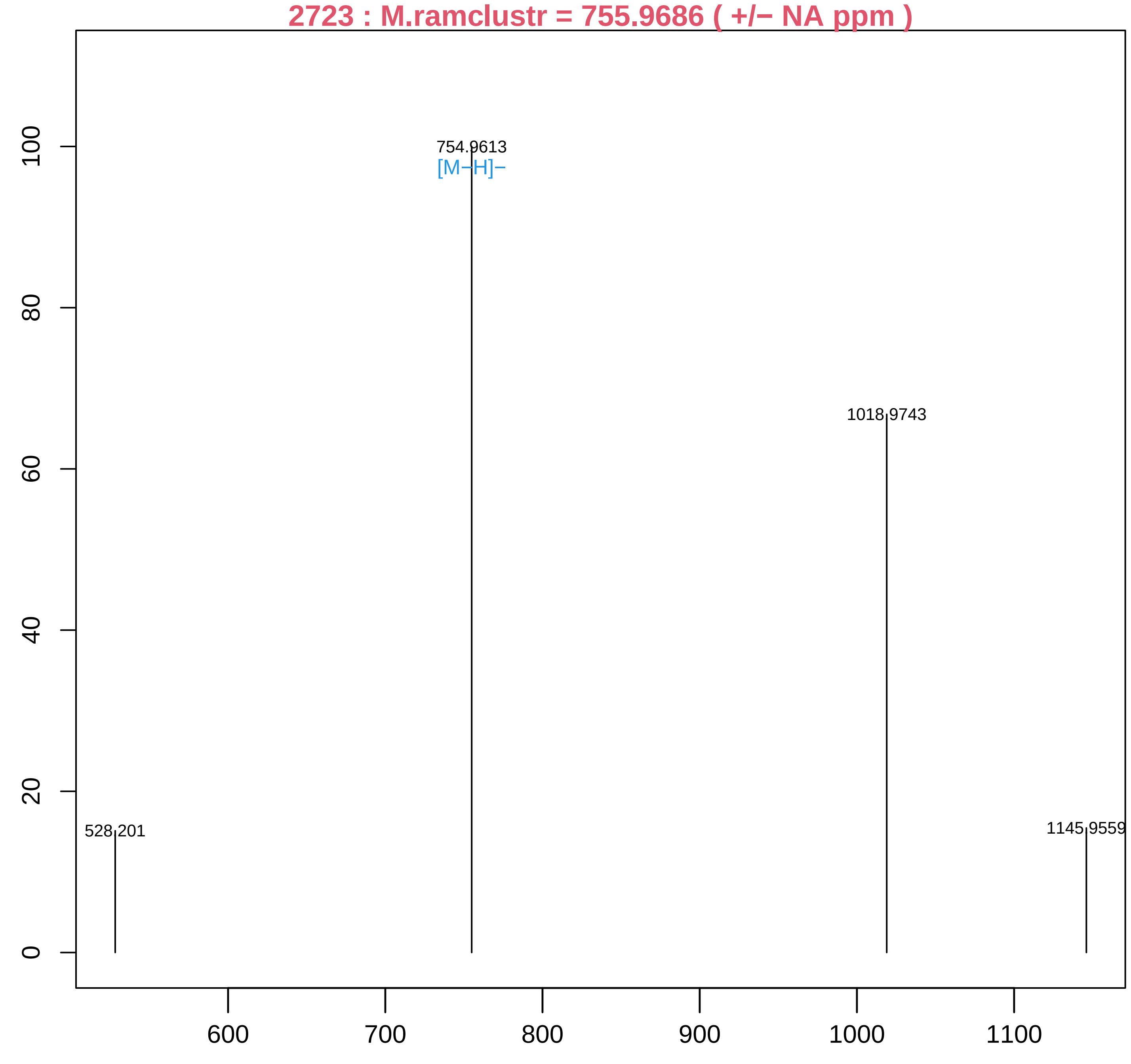

2723 : M.ramclustr = 755.9686 (+/− NA ppm)
754.9613
[M−H]−
1018.9743
528.201
1145.9559
0
20
40
60
80
100
600
700
800
900
1000
1100

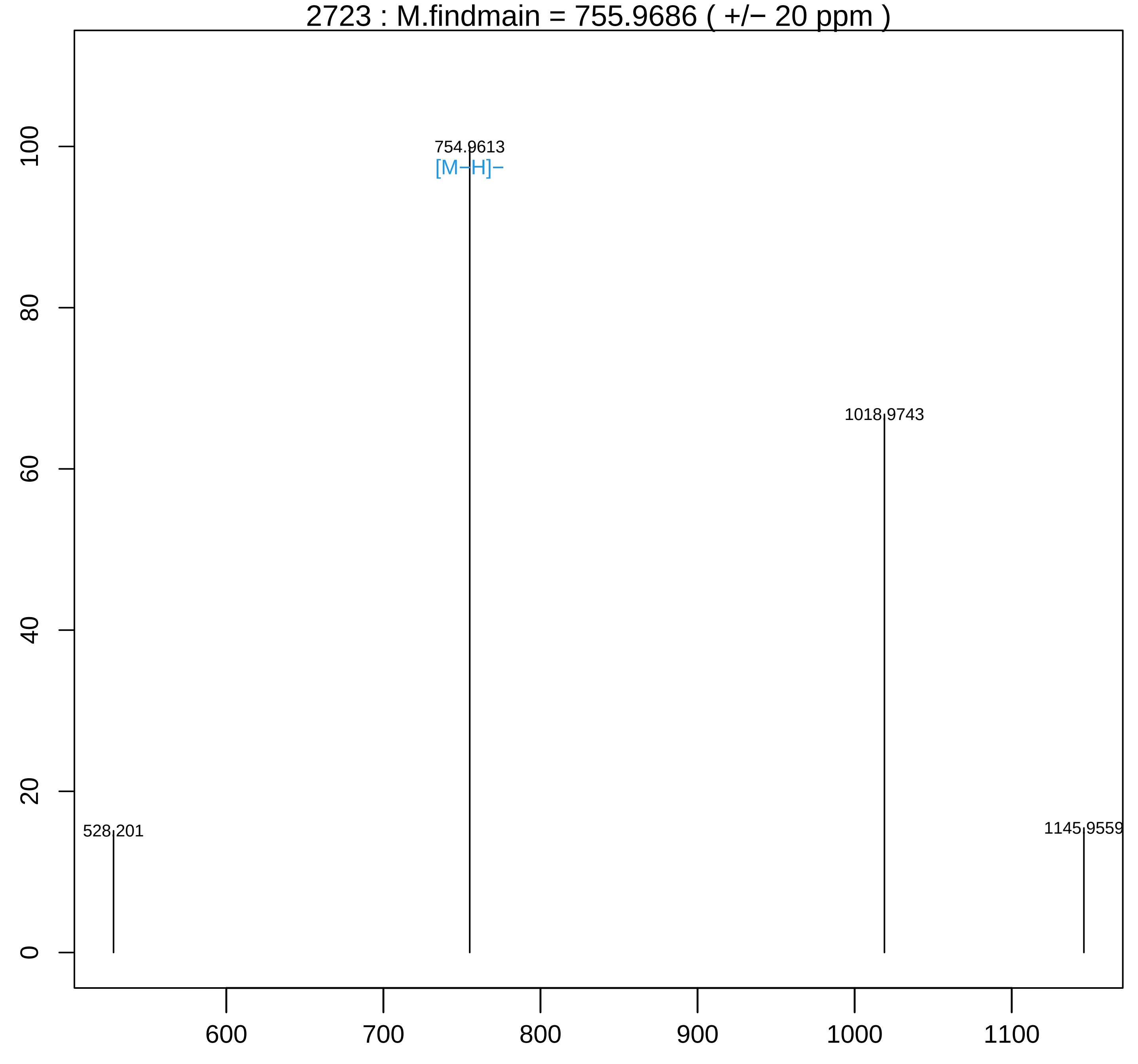

2723 : M.findmain = 755.9686 (+/− 20 ppm)
754.9613
[M−H]−
1018.9743
528.201
1145.9559
0
20
40
60
80
100
600
700
800
900
1000
1100

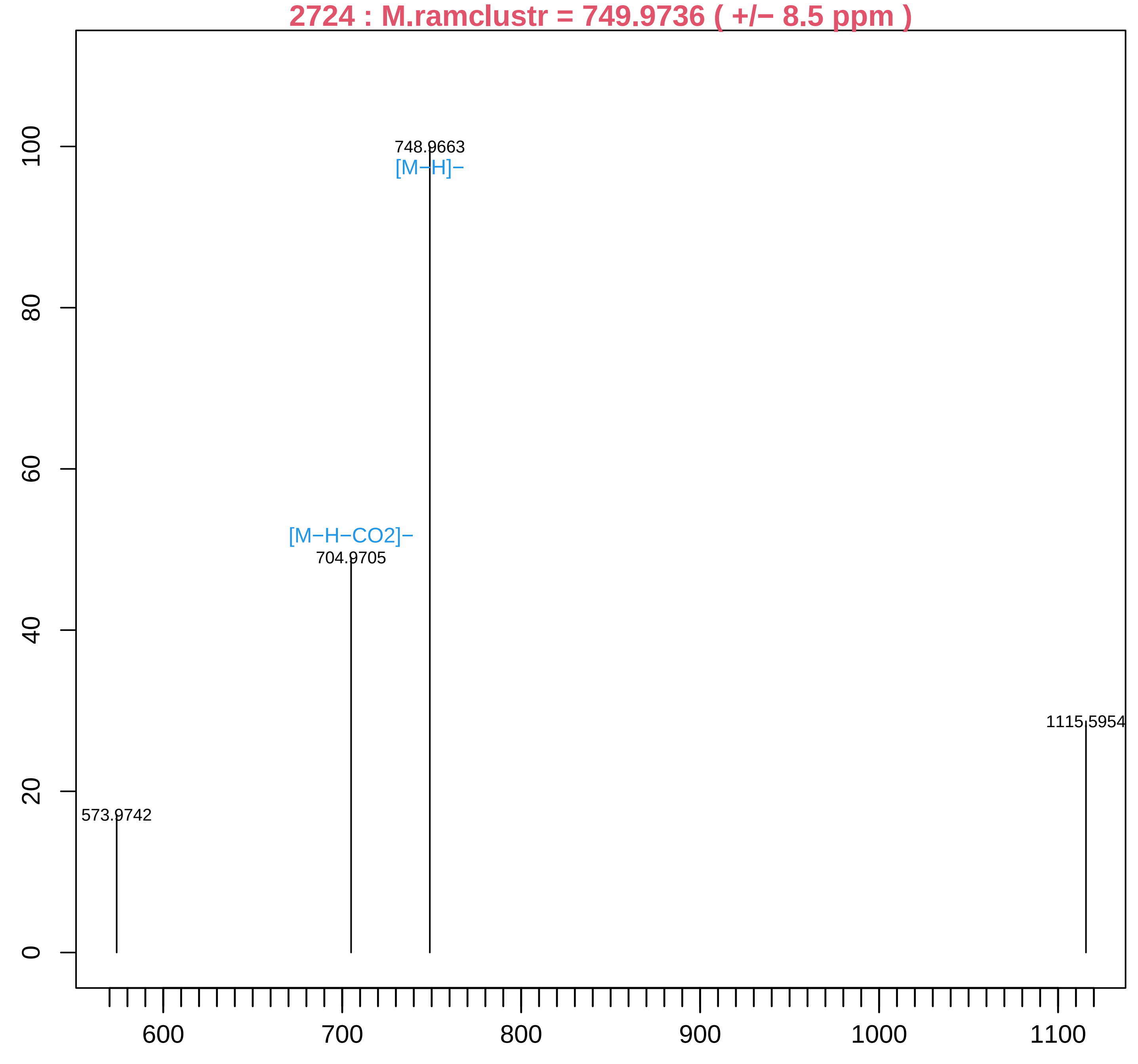
2724 : M.ramclustr = 749.9736 (+/− 8.5 ppm)
748.9663
[M−H]−
[M−H−CO2]−
704.9705
1115.5954
573.9742
0
20
40
60
80
100
600
700
800
900
1000
1100

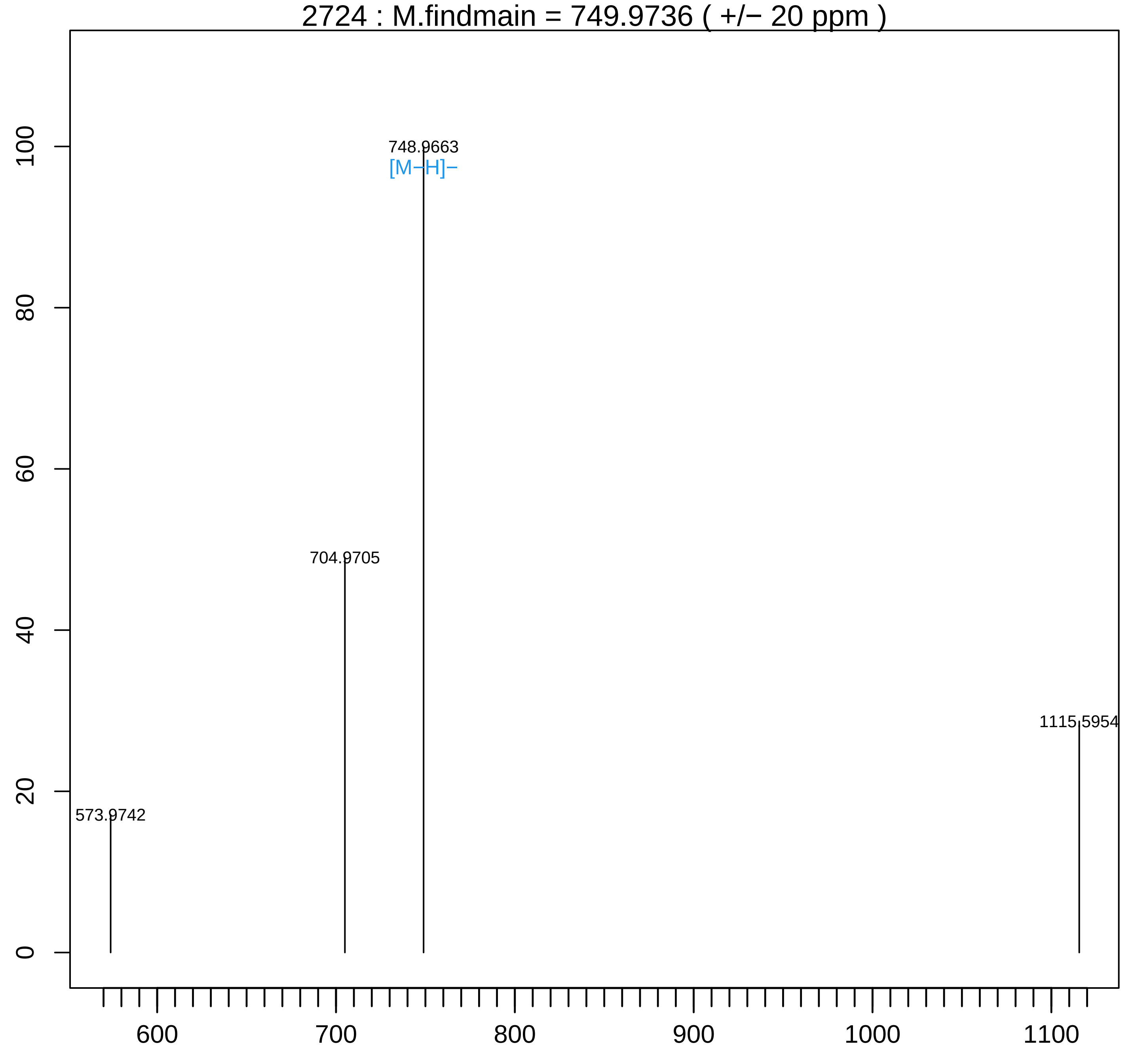
2724 : M.findmain = 749.9736 (+/− 20 ppm)
748.9663
[M−H]−
704.9705
1115.5954
573.9742
0
20
40
60
80
100
600
700
800
900
1000
1100

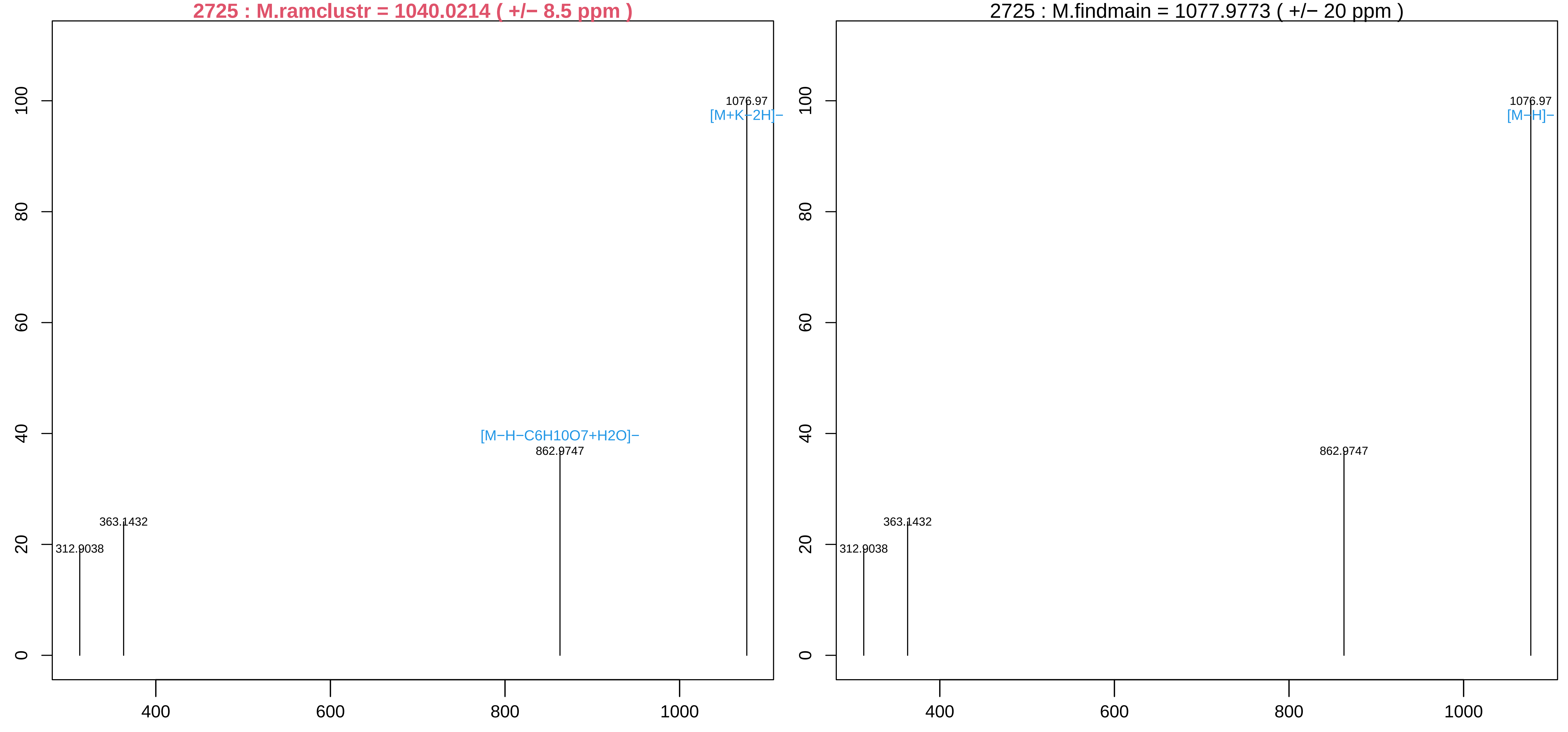
2725 : M.ramclustr = 1040.0214 (+/− 8.5 ppm)
1076.97
[M+K−2H]−
[M−H−C6H10O7+H2O]−
862.9747
363.1432
312.9038
2725 : M.findmain = 1077.9773 (+/− 20 ppm)
1076.97
[M−H]−
862.9747
363.1432
312.9038
0
20
40
60
80
100
400
600
800
1000

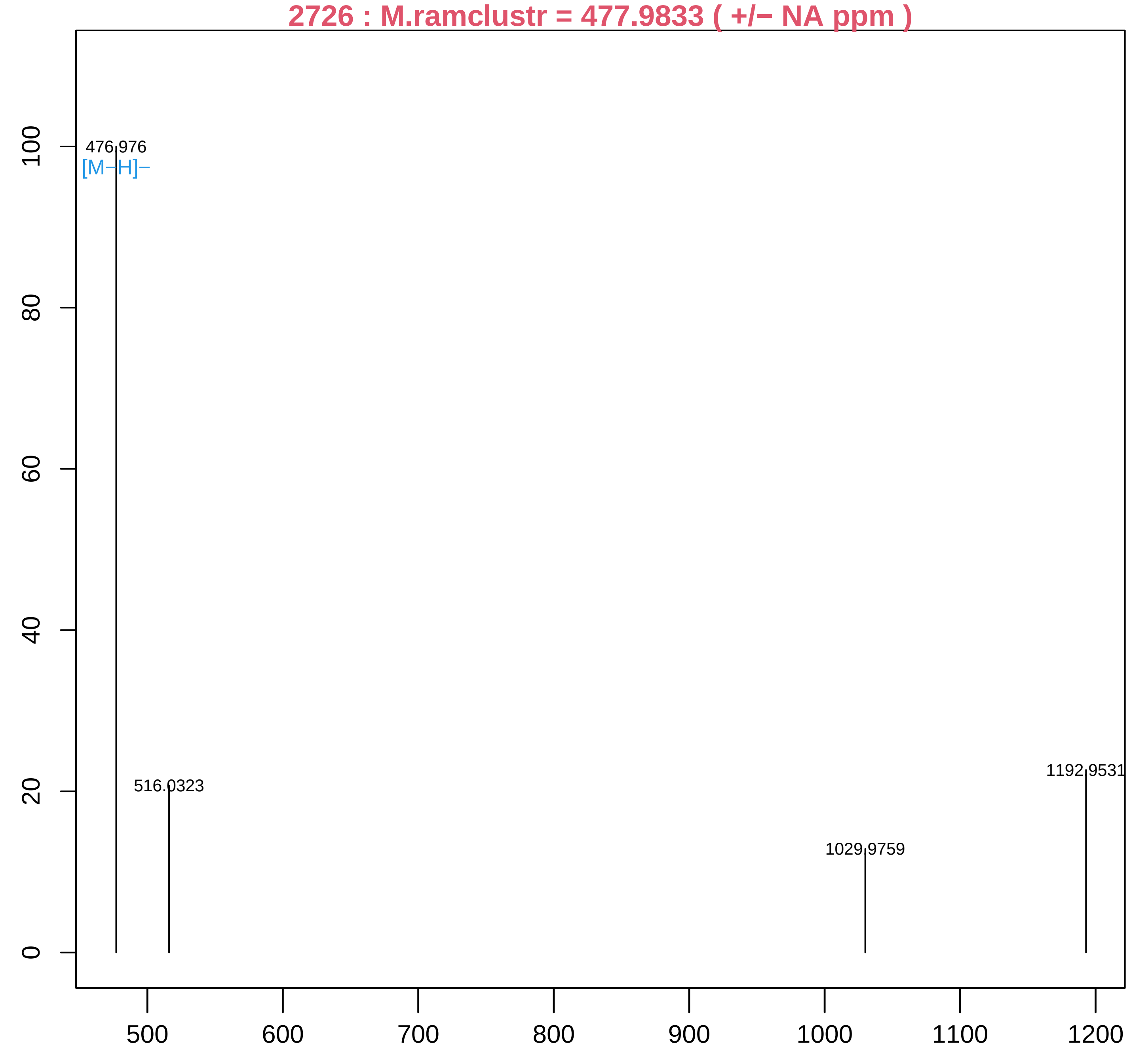

2726 : M.ramclustr = 477.9833 (+/− NA ppm)
476.976
[M−H]−
516.0323
1029.9759
1192.9531
0
20
40
60
80
100
500
600
700
800
900
1000
1100
1200

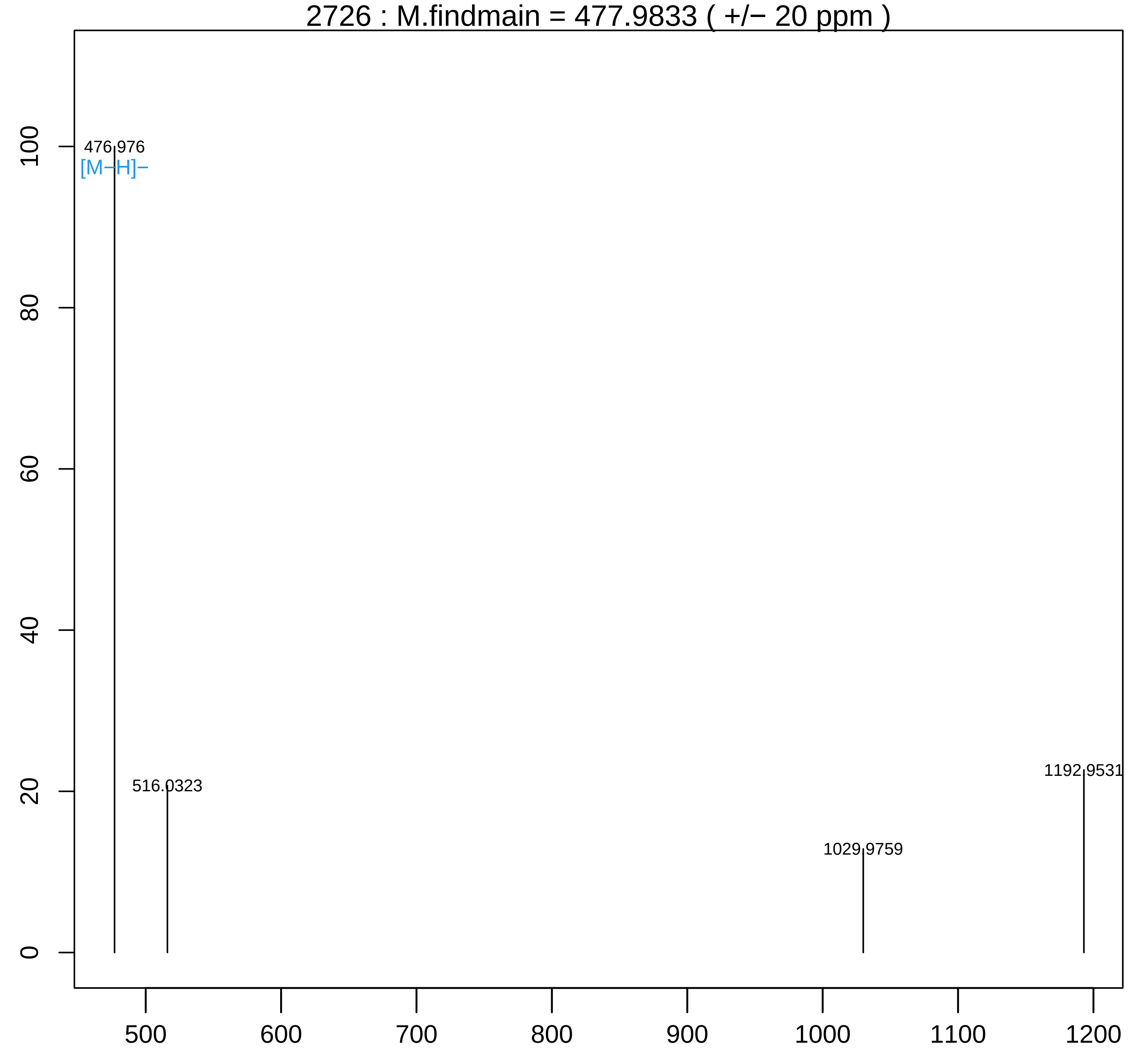

2726 : M.findmain = 477.9833 (+/− 20 ppm)
476.976
[M−H]−
516.0323
1029.9759
1192.9531
0
20
40
60
80
100
500
600
700
800
900
1000
1100
1200

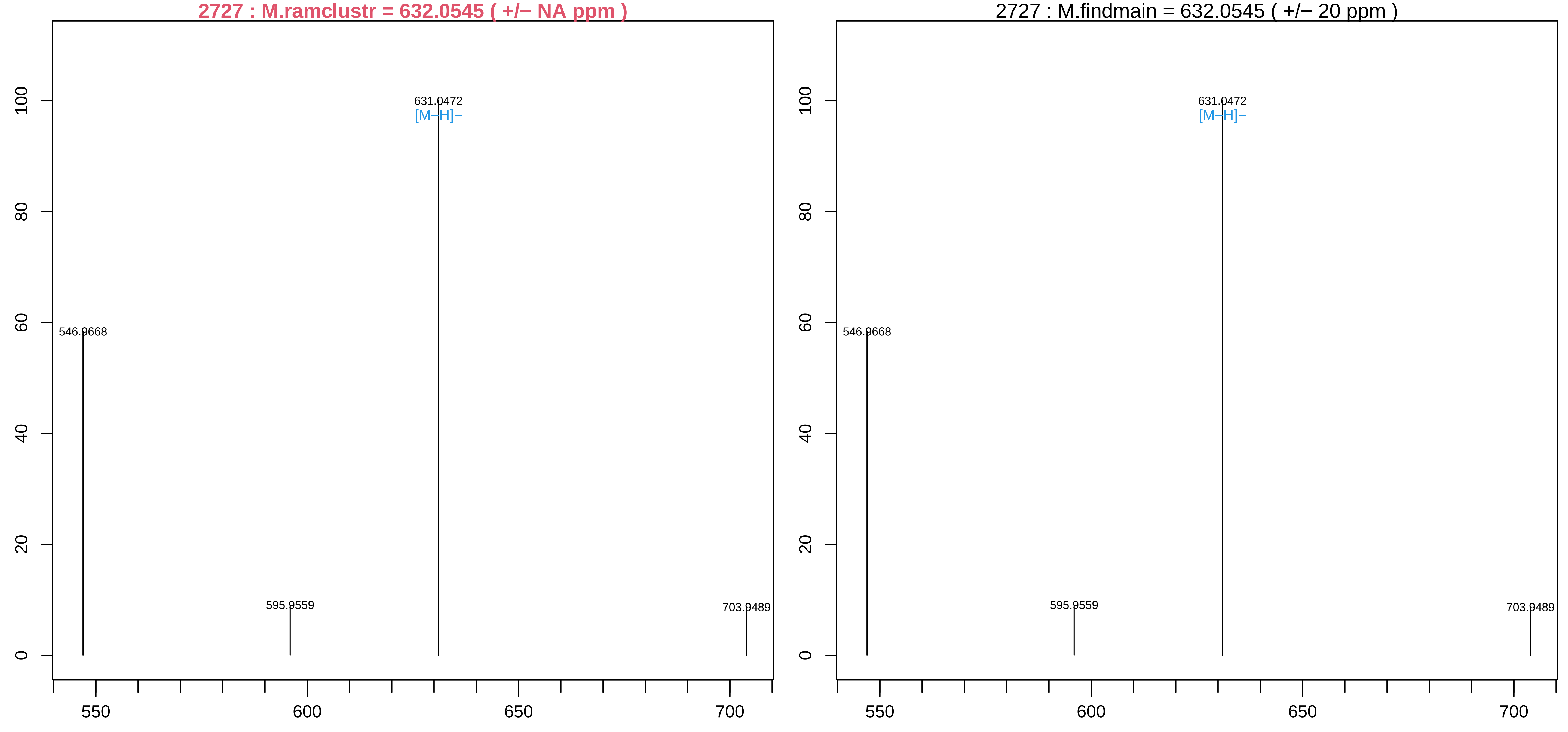

2727 : M.ramclustr = 632.0545 (+/− NA ppm)
631.0472
[M−H]−
546.9668
595.9559
703.9489
0
20
40
60
80
100
550
600
650
700
2727 : M.findmain = 632.0545 (+/− 20 ppm)
631.0472
[M−H]−
546.9668
595.9559
703.9489
0
20
40
60
80
100
550
600
650
700

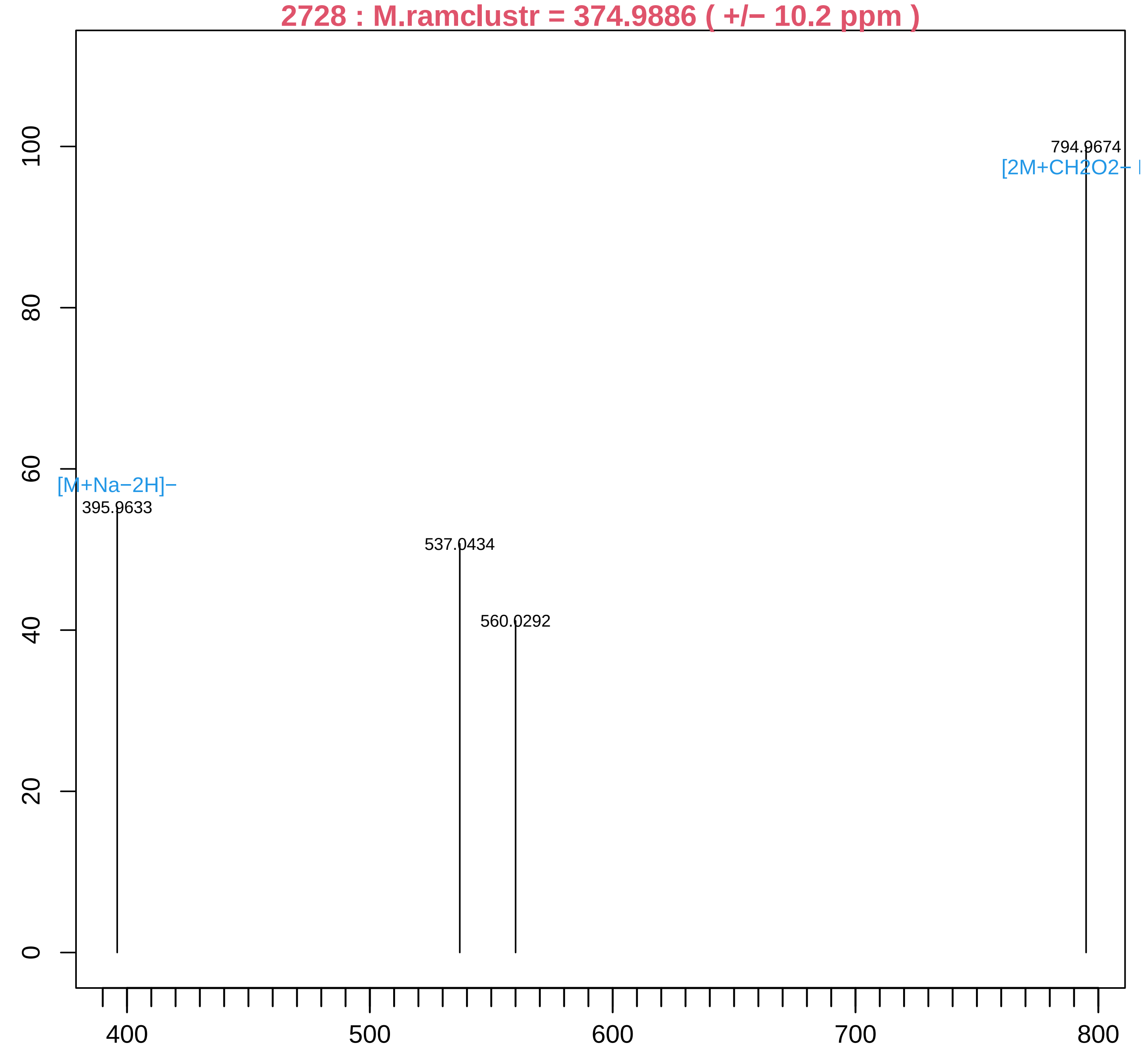
2728 : M.ramclustr = 374.9886 (+/− 10.2 ppm)
794.9674
[2M+CH2O2−
[M+Na−2H]−
395.9633
537.0434
560.0292
0
20
40
60
80
100
400
500
600
700
800

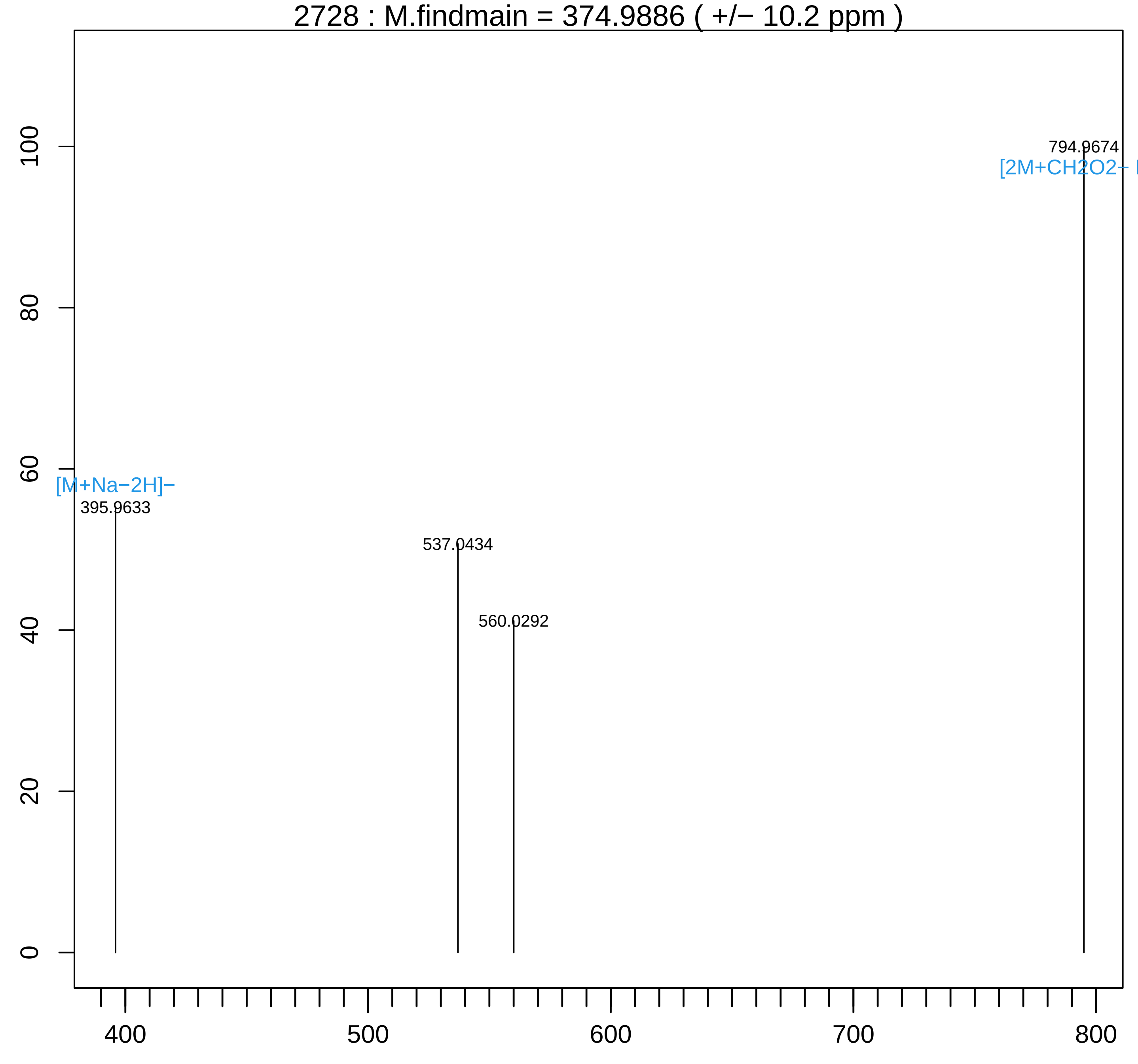
2728 : M.findmain = 374.9886 (+/− 10.2 ppm)
794.9674
[2M+CH2O2−
[M+Na−2H]−
395.9633
537.0434
560.0292
0
20
40
60
80
100
400
500
600
700
800

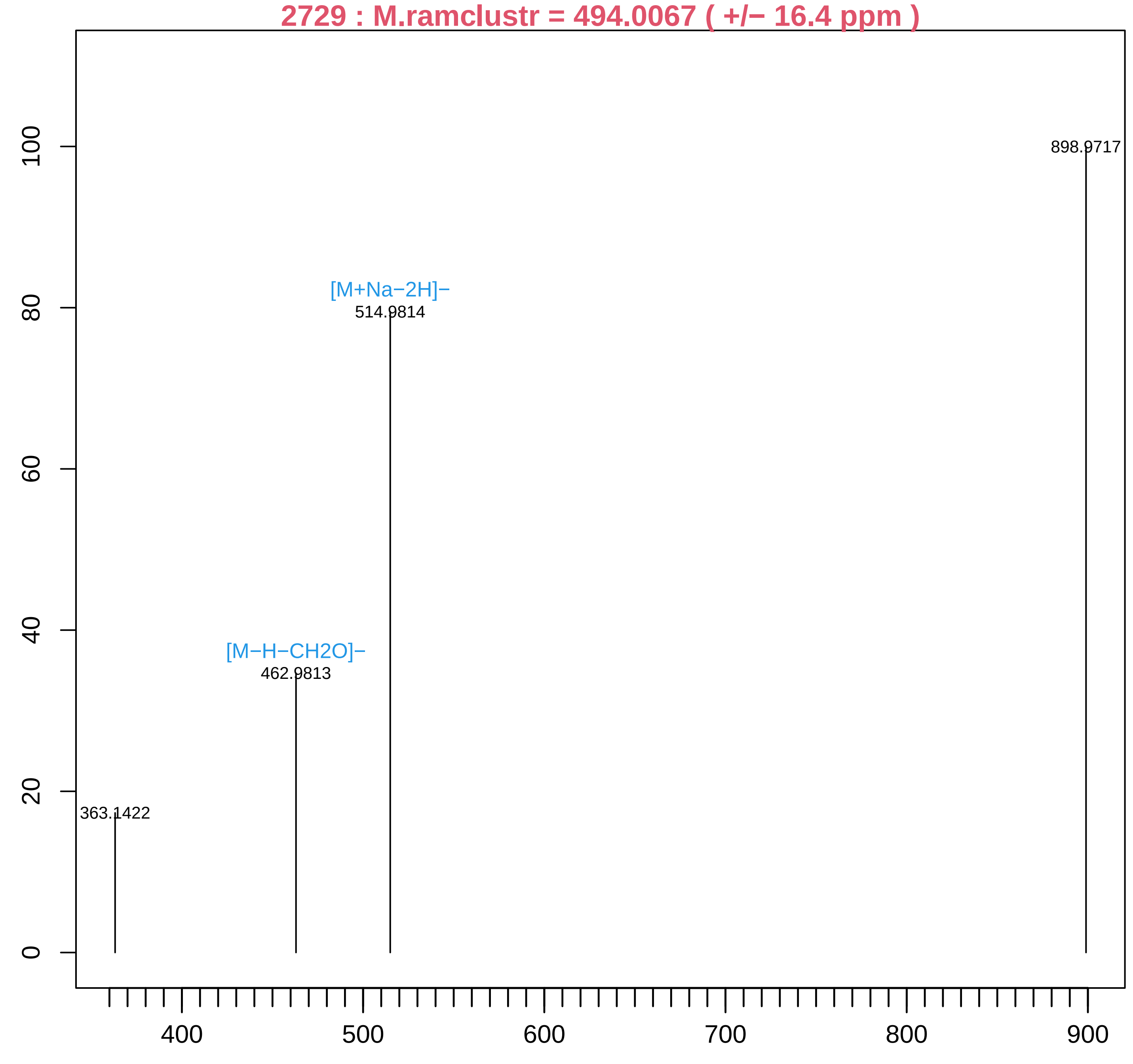

2729 : M.ramclustr = 494.0067 (+/− 16.4 ppm)
[M+Na−2H]−
514.9814
898.9717
[M−H−CH2O]−
462.9813
363.1422

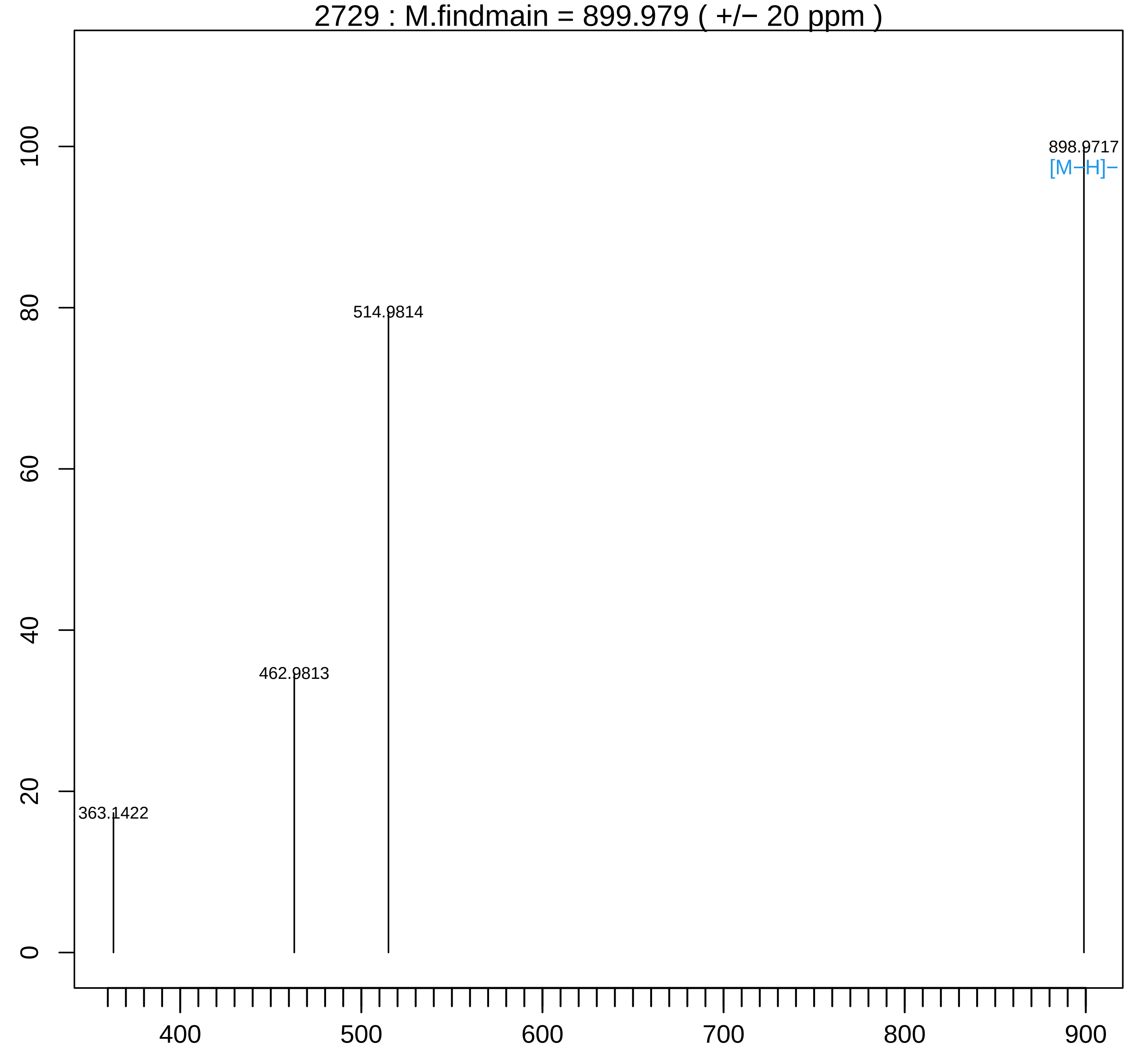

2729 : M.findmain = 899.979 (+/− 20 ppm)
898.9717
[M−H]−
514.9814
462.9813
363.1422

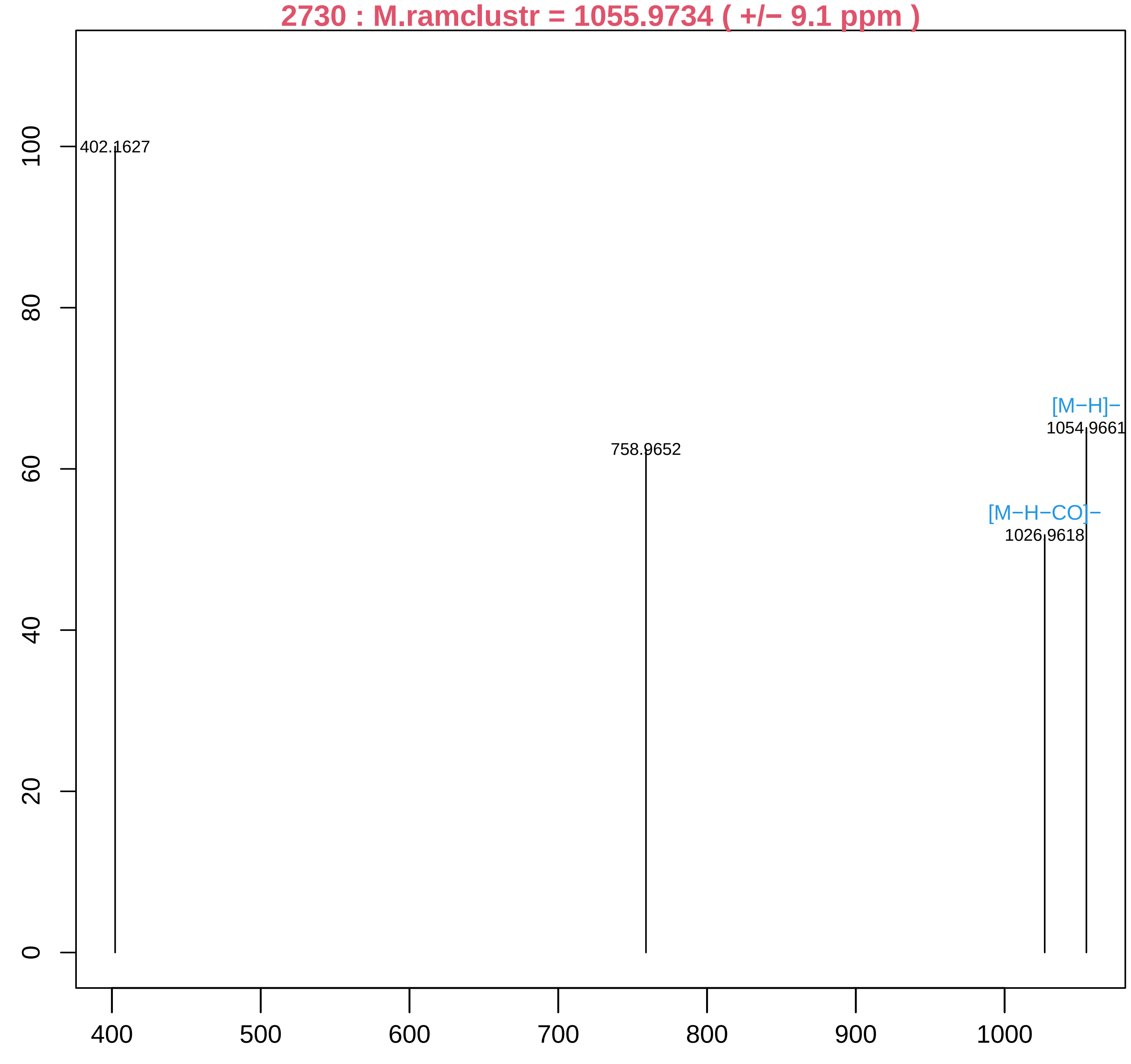

2730 : M.ramclustr = 1055.9734 (+/− 9.1 ppm)
402.1627
758.9652
[M−H]−
1054.9661
[M−H−CO]−
1026.9618
0
20
40
60
80
100
400
500
600
700
800
900
1000

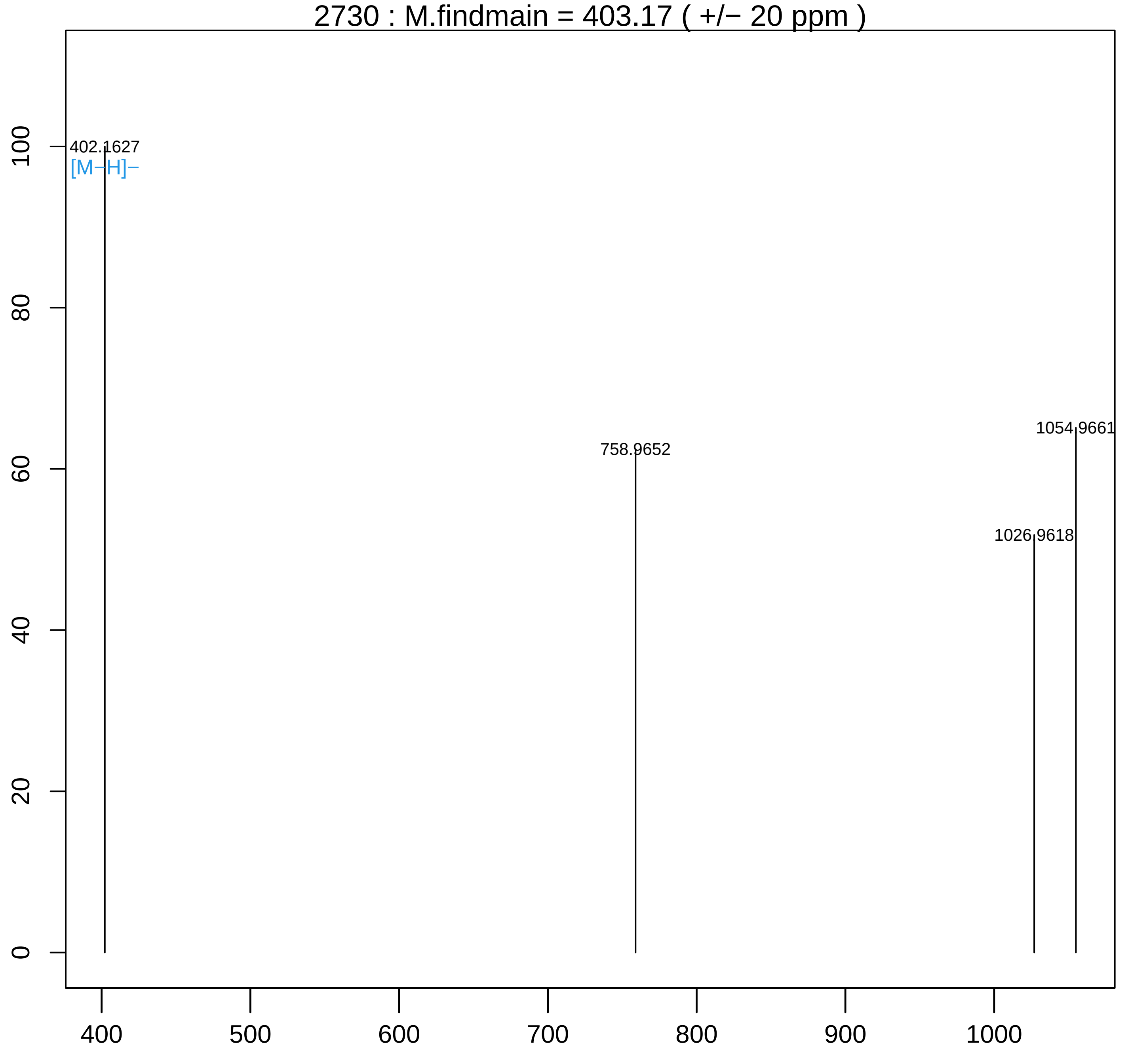

2730 : M.findmain = 403.17 (+/− 20 ppm)
402.1627
[M−H]−
758.9652
1054.9661
1026.9618
0
20
40
60
80
100
400
500
600
700
800
900
1000

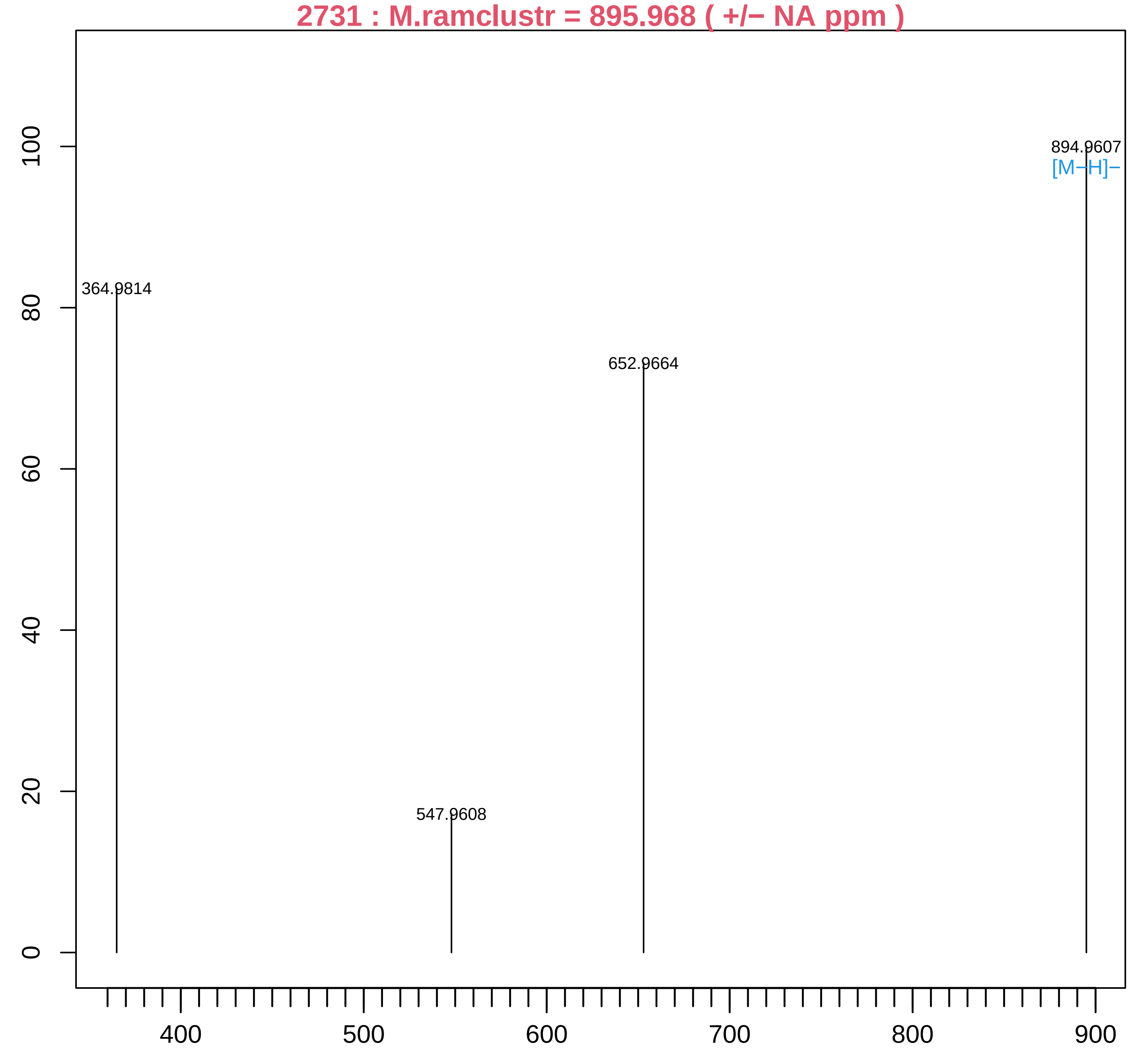
2731 : M.ramclustr = 895.968 (+/− NA ppm)
894.9607
[M−H]−
364.9814
652.9664
547.9608
0
20
40
60
80
100
400
500
600
700
800
900

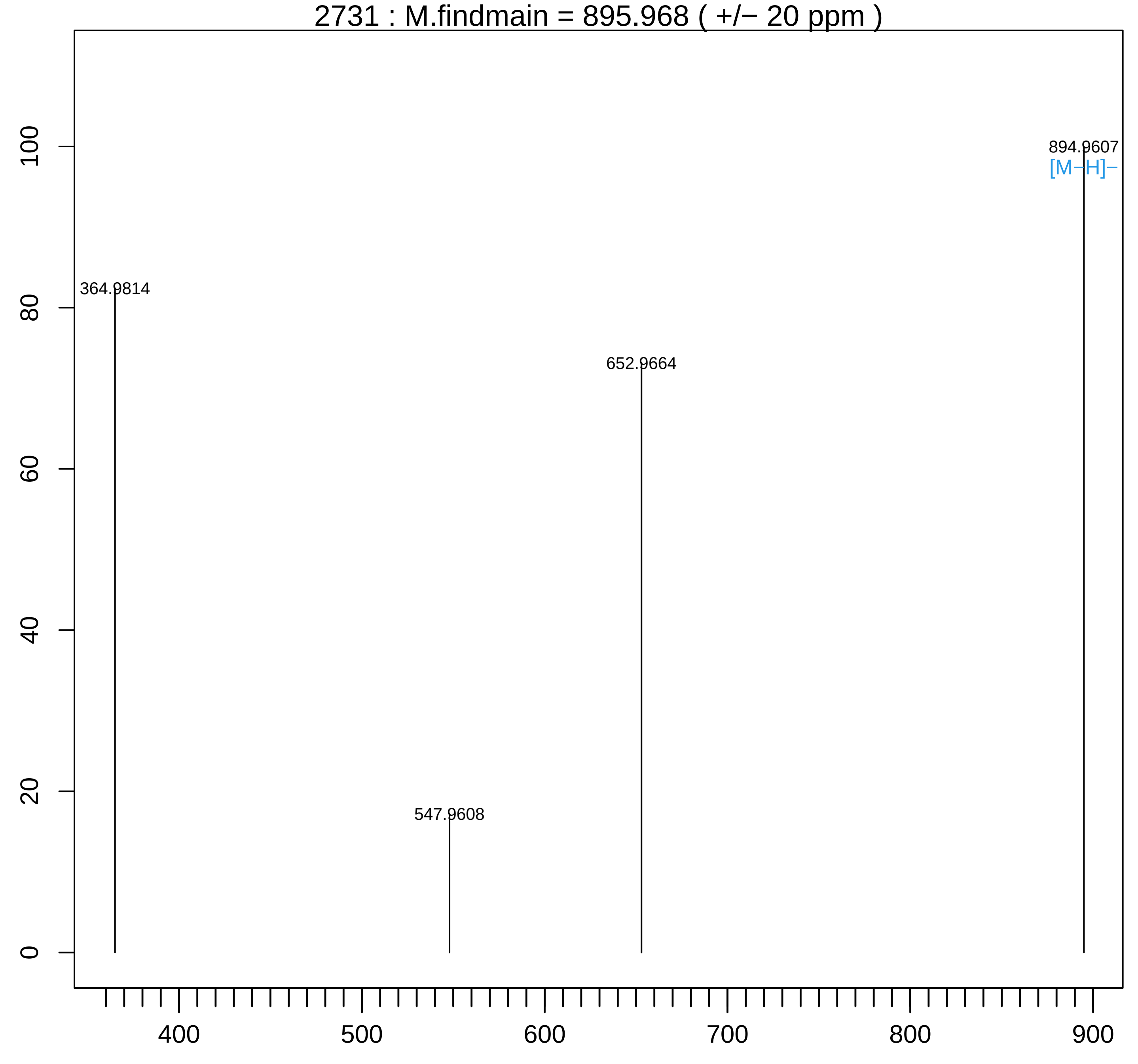
2731 : M.findmain = 895.968 (+/− 20 ppm)
894.9607
[M−H]−
364.9814
652.9664
547.9608
0
20
40
60
80
100
400
500
600
700
800
900

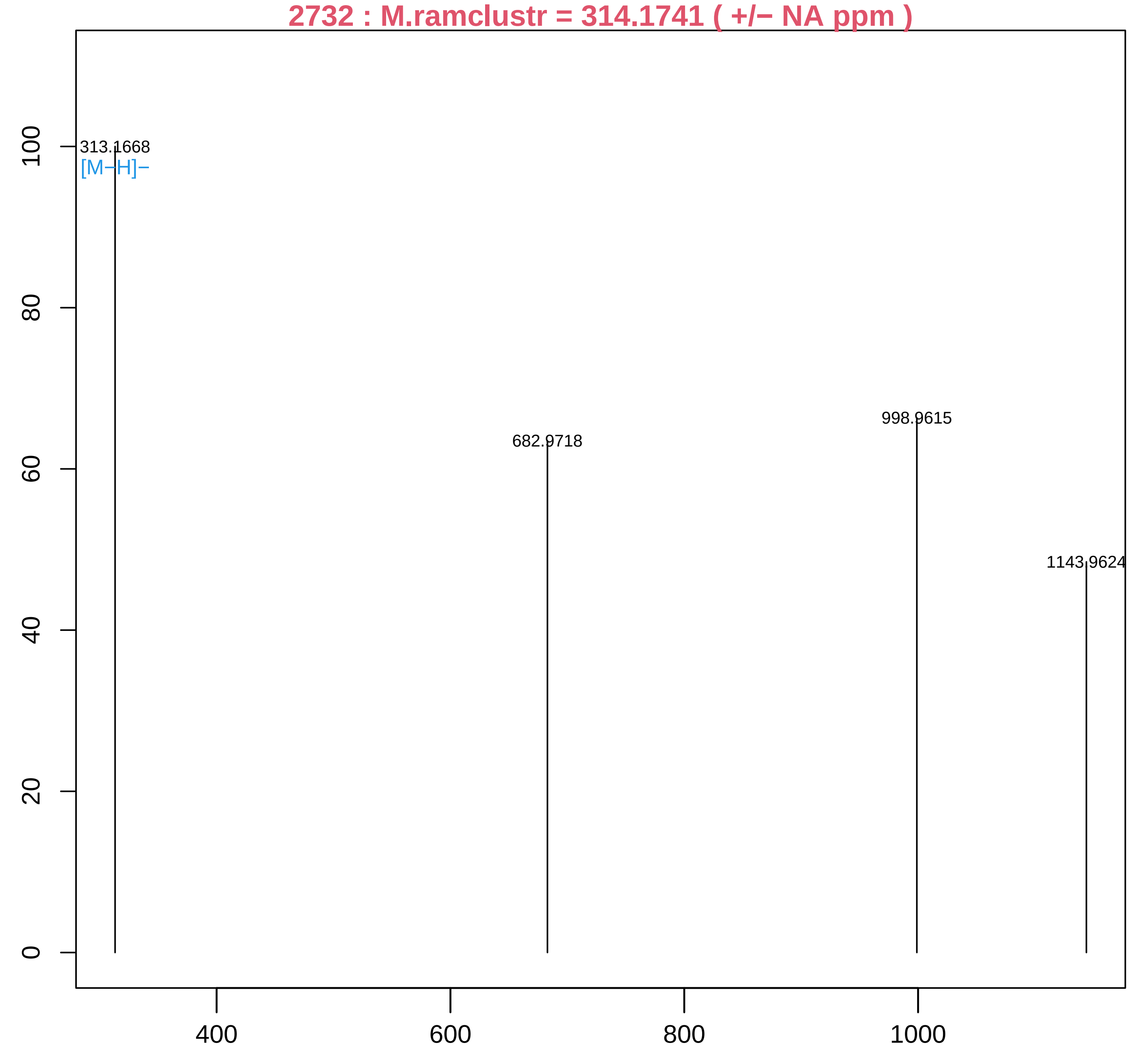
2732 : M.ramclustr = 314.1741 (+/− NA ppm)
313.1668
[M−H]−
682.9718
998.9615
1143.9624
0
20
40
60
80
100
400
600
800
1000

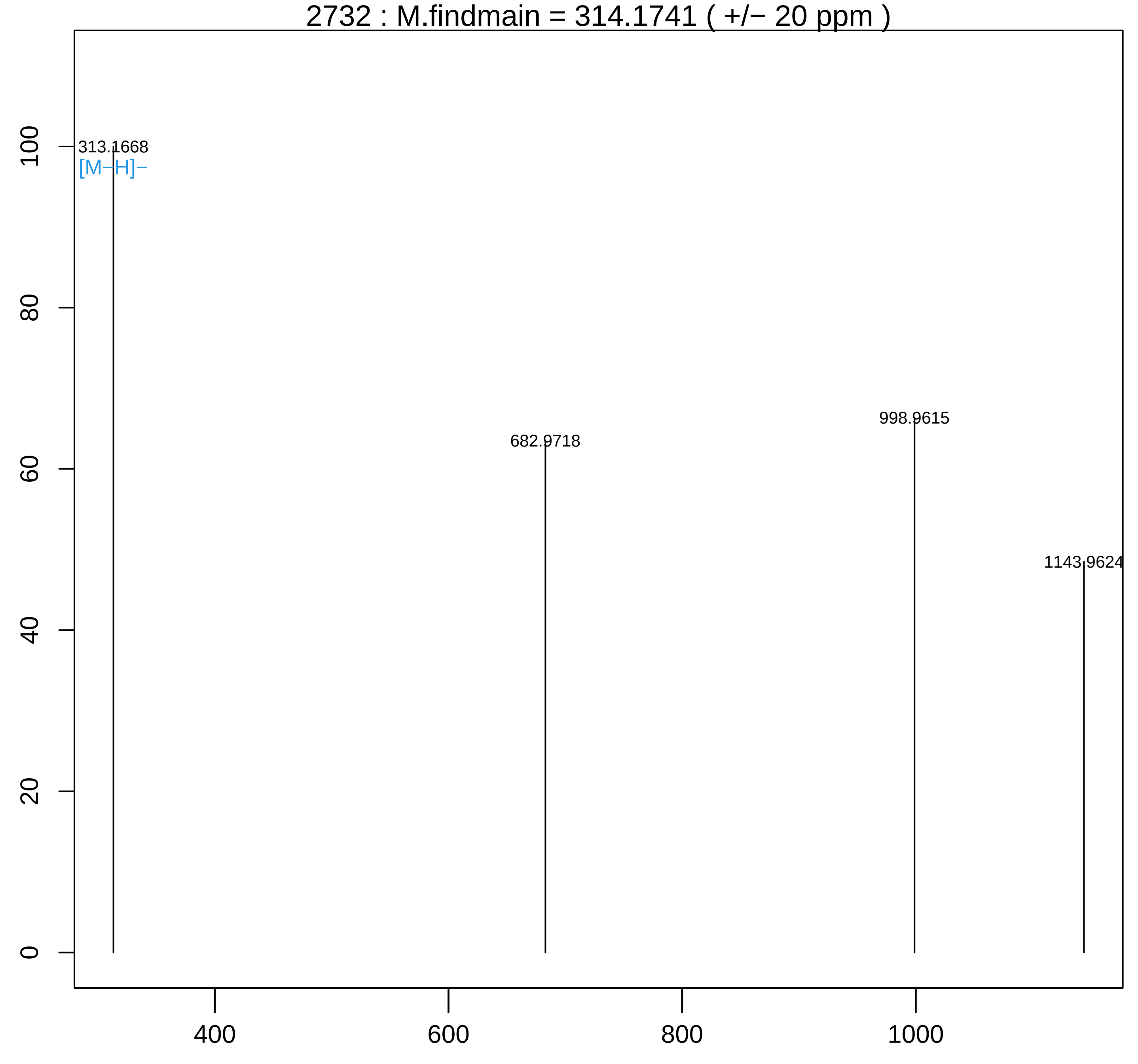
2732 : M.findmain = 314.1741 (+/− 20 ppm)
313.1668
[M−H]−
682.9718
998.9615
1143.9624
0
20
40
60
80
100
400
600
800
1000

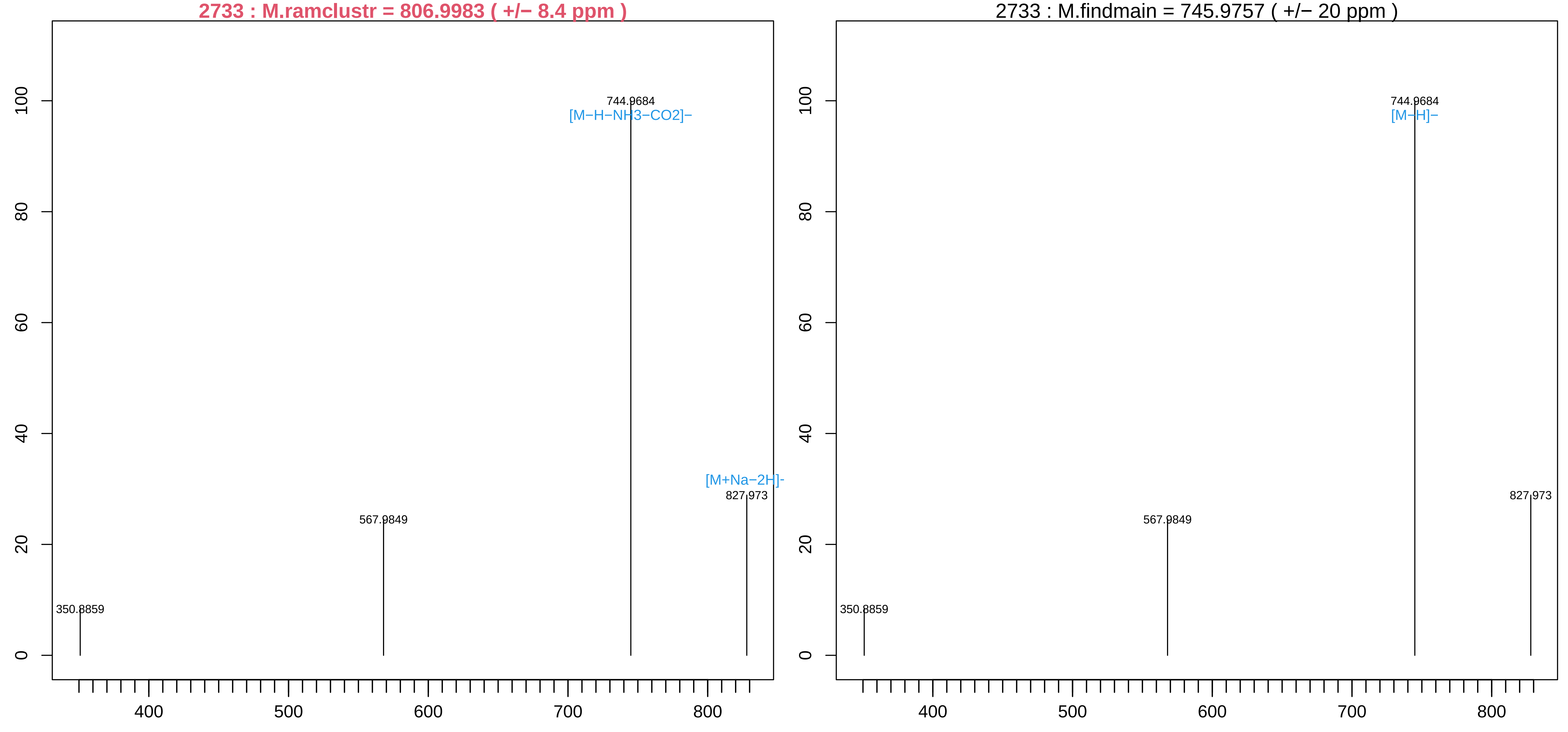
2733 : M.ramclustr = 806.9983 (+/− 8.4 ppm)
744.9684
[M−H−NH3−CO2]−
[M+Na−2H]-
827.973
567.9849
350.8859
2733 : M.findmain = 745.9757 (+/− 20 ppm)
744.9684
[M−H]−
827.973
567.9849
350.8859

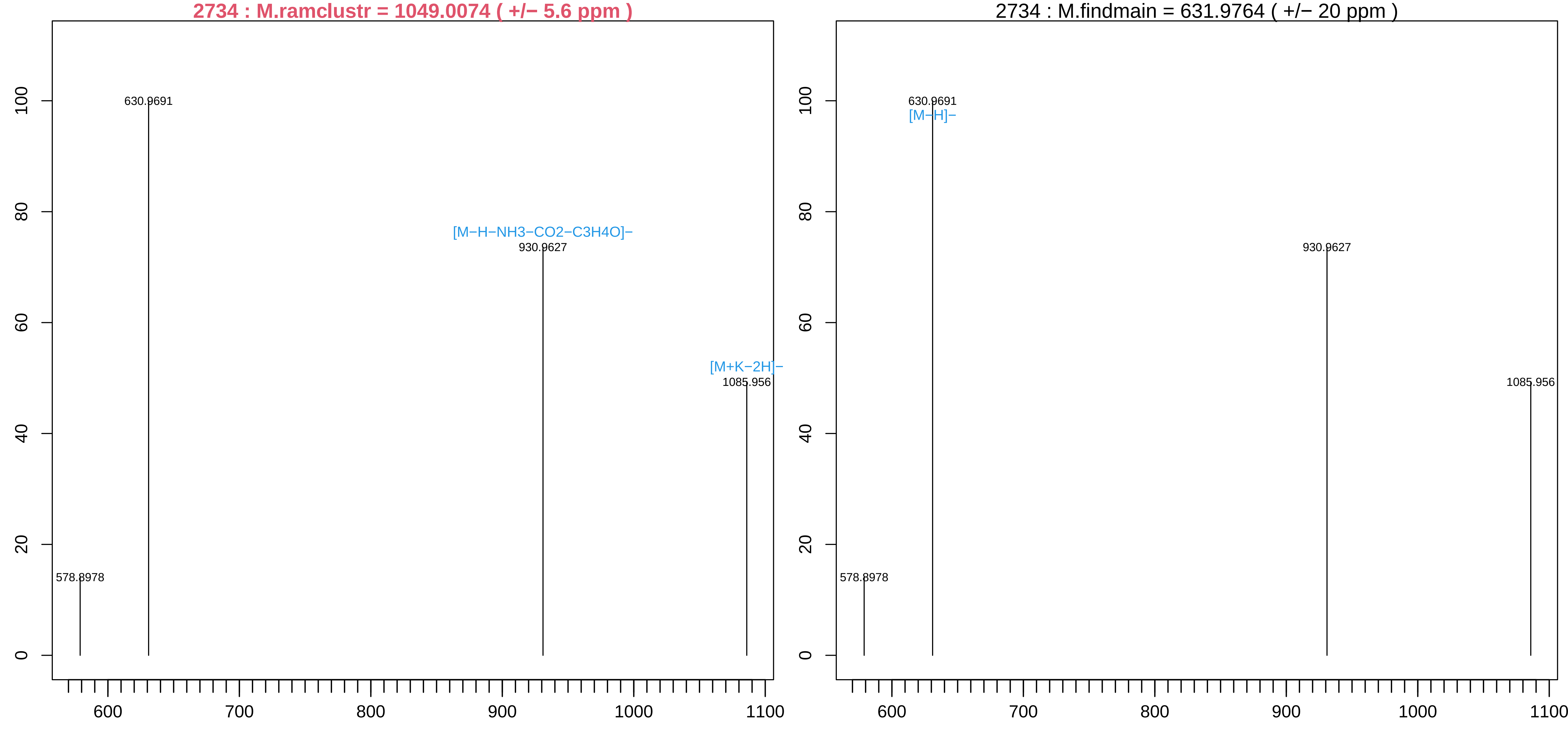
2734 : M.ramclustr = 1049.0074 (+/− 5.6 ppm)
630.9691
[M−H−NH3−CO2−C3H4O]−
930.9627
[M+K−2H]−
1085.956
578.8978
0
20
40
60
80
100
600
700
800
900
1000
1100
2734 : M.findmain = 631.9764 (+/− 20 ppm)
630.9691
[M−H]−
930.9627
1085.956
578.8978
0
20
40
60
80
100
600
700
800
900
1000
1100

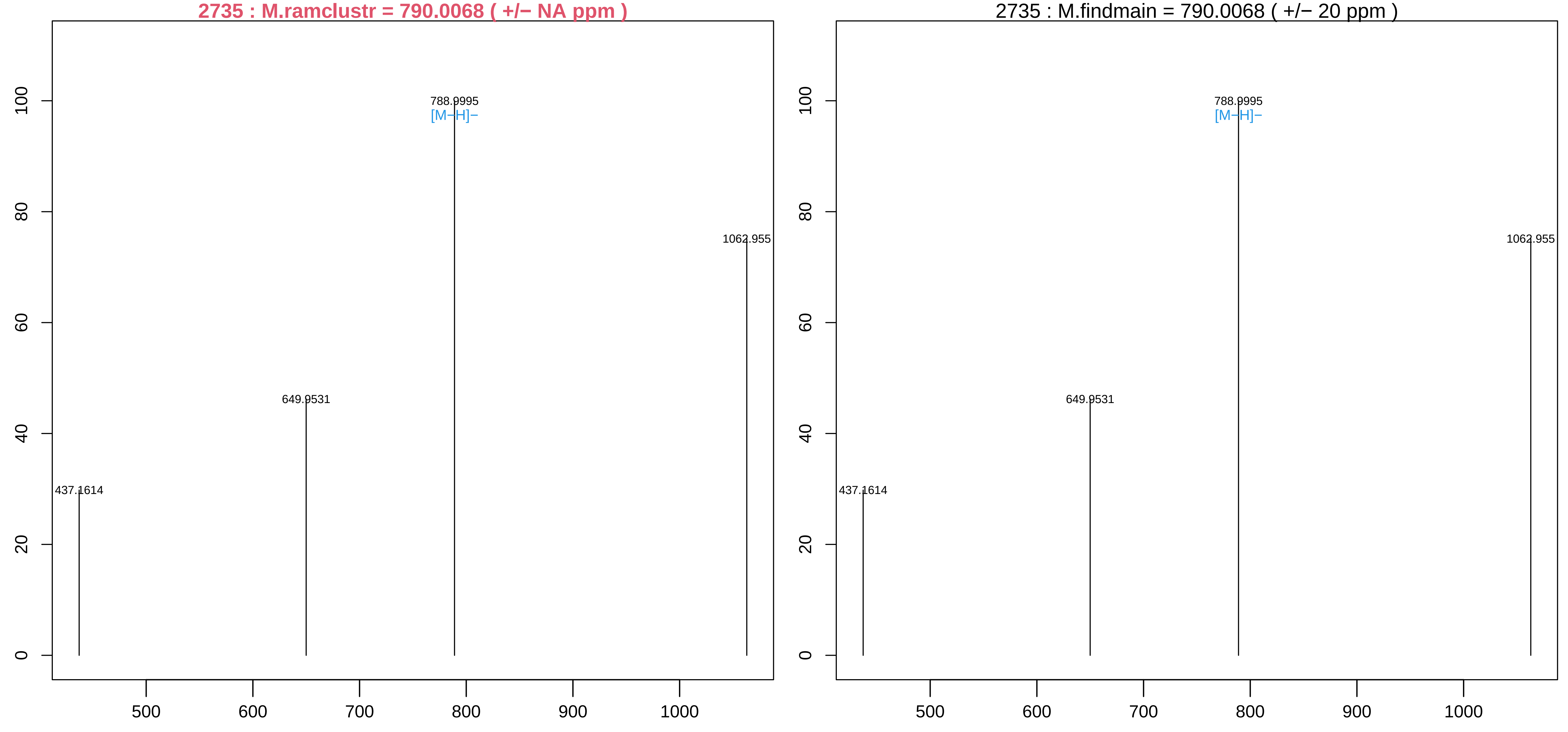
2735 : M.ramclustr = 790.0068 (+/− NA ppm)
2735 : M.findmain = 790.0068 (+/− 20 ppm)
788.9995
[M−H]−
1062.955
649.9531
437.1614
0
20
40
60
80
100
500
600
700
800
900
1000

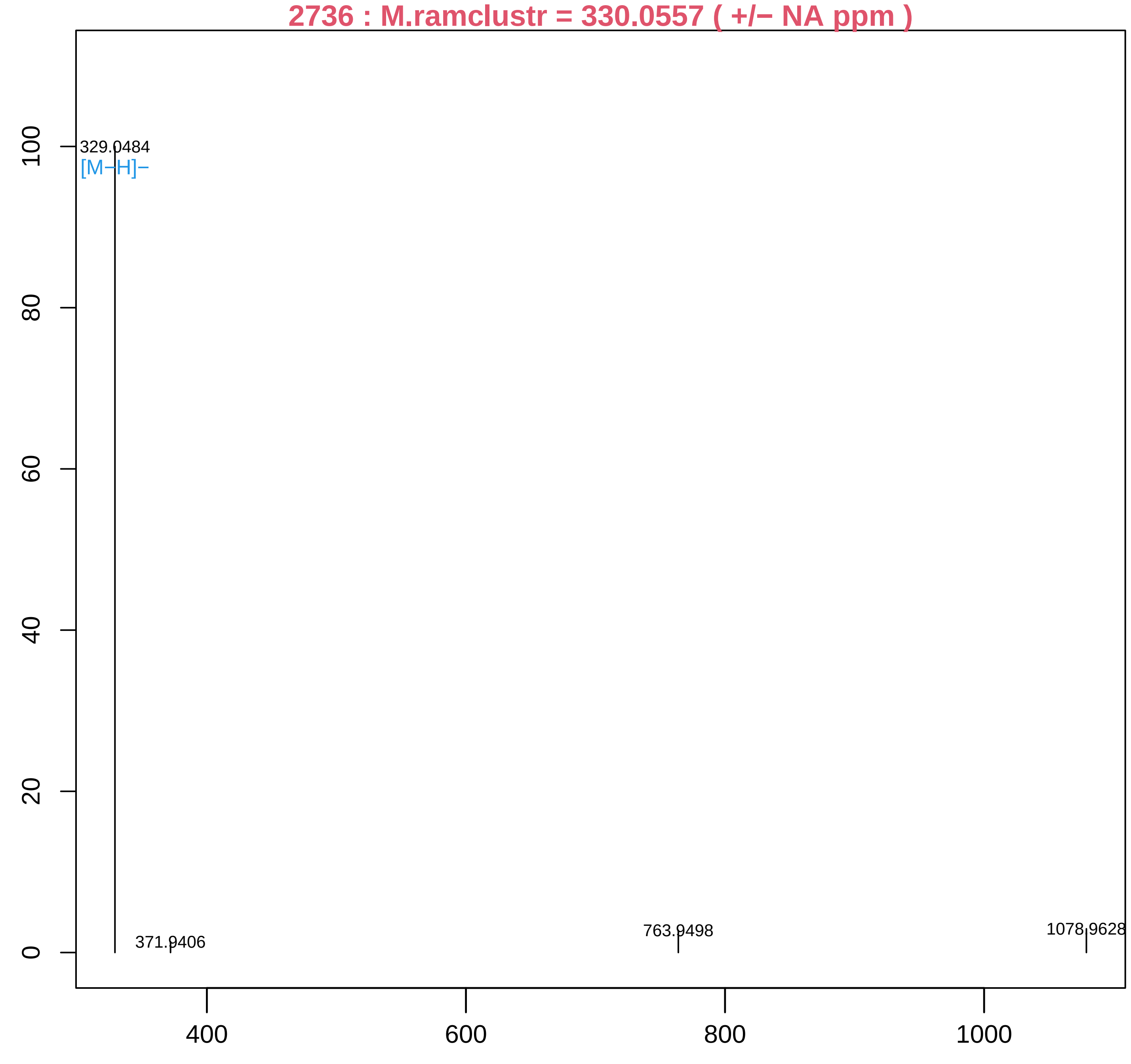
2736 : M.ramclustr = 330.0557 (+/− NA ppm)
329.0484
[M−H]−
371.9406
763.9498
1078.9628
0
20
40
60
80
100
400
600
800
1000

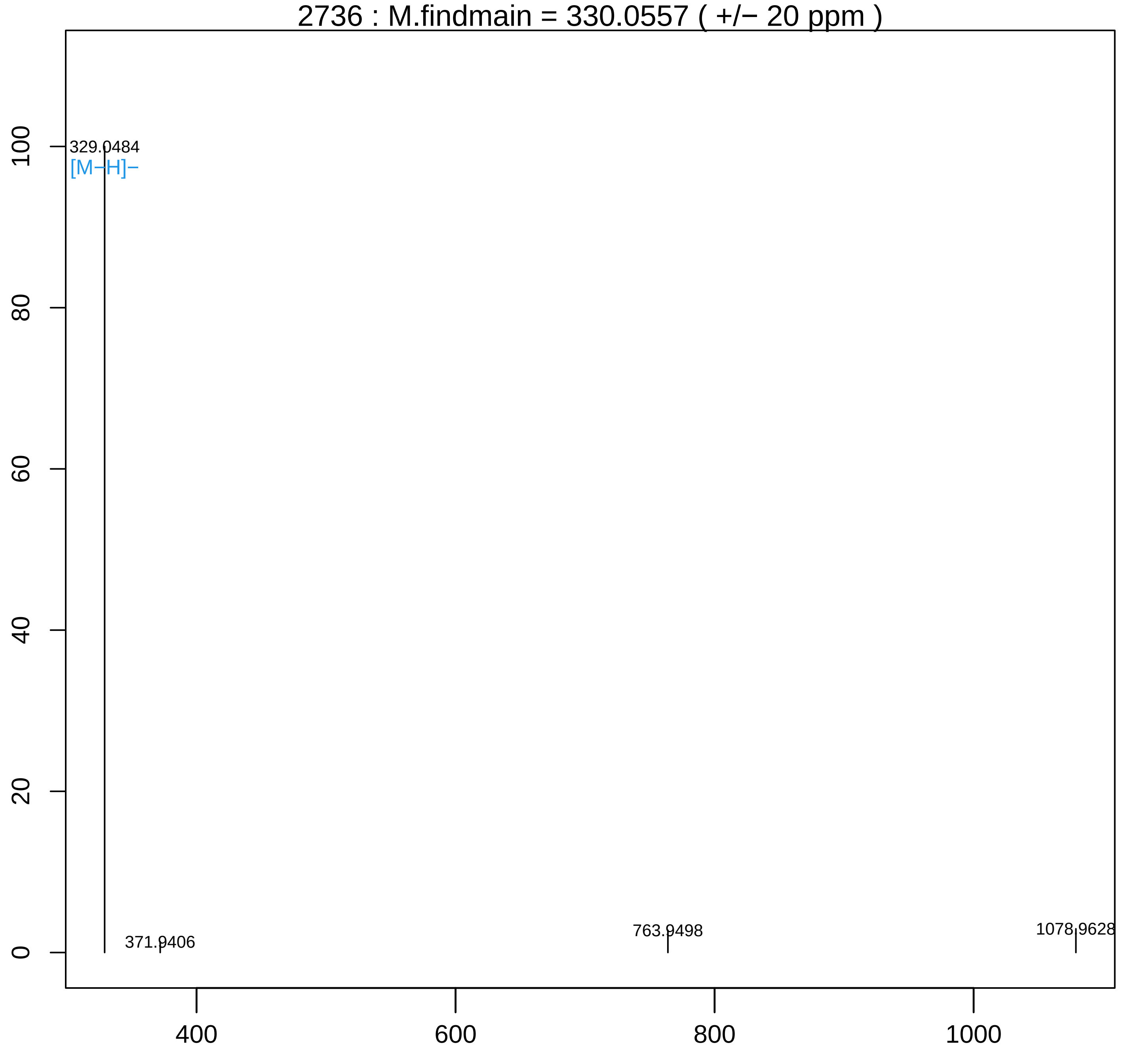
2736 : M.findmain = 330.0557 (+/− 20 ppm)
329.0484
[M−H]−
371.9406
763.9498
1078.9628
0
20
40
60
80
100
400
600
800
1000

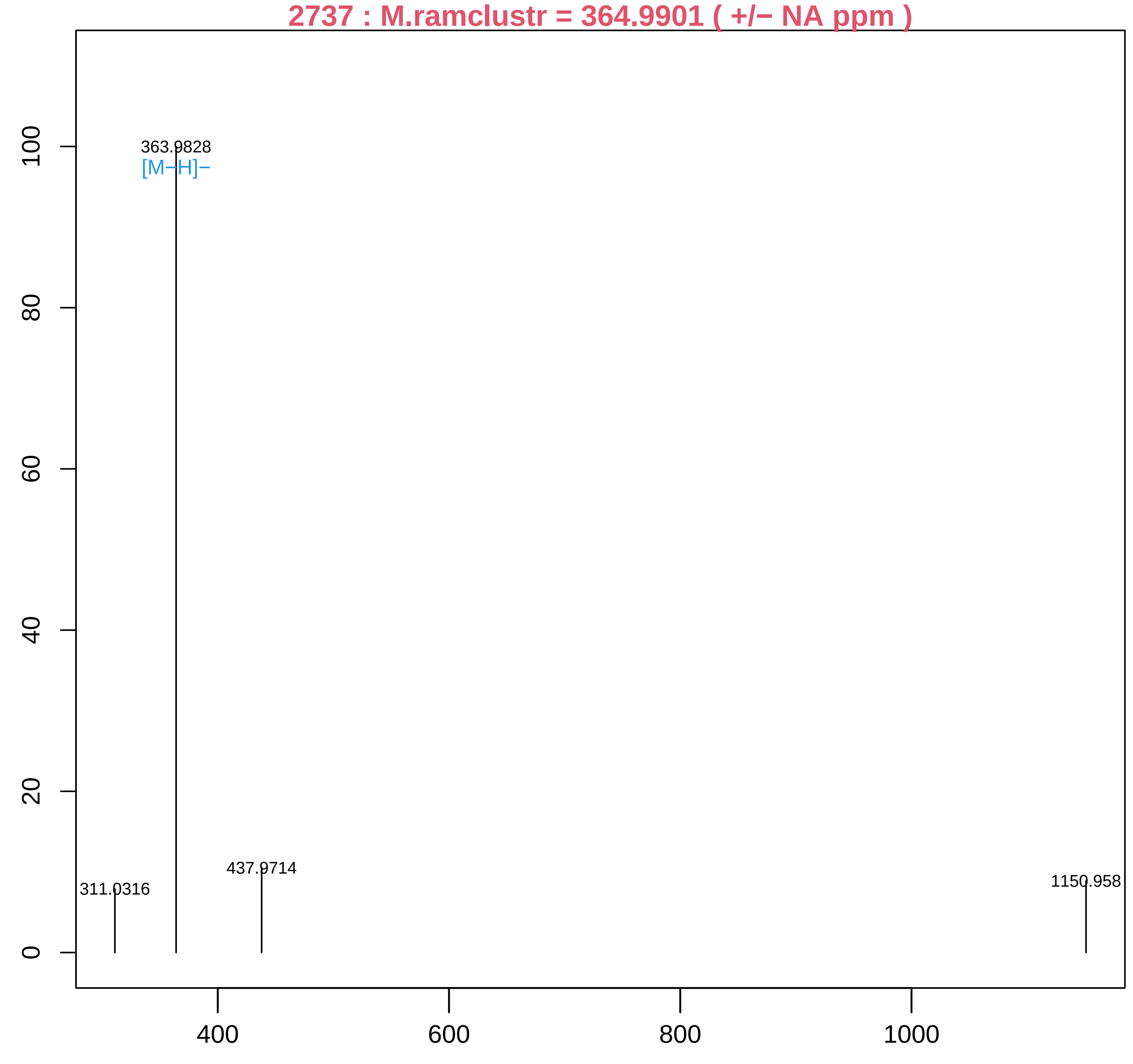
2737 : M.ramclustr = 364.9901 (+/− NA ppm)
363.9828
[M−H]−
311.0316
437.9714
1150.958
0
20
40
60
80
100
400
600
800
1000

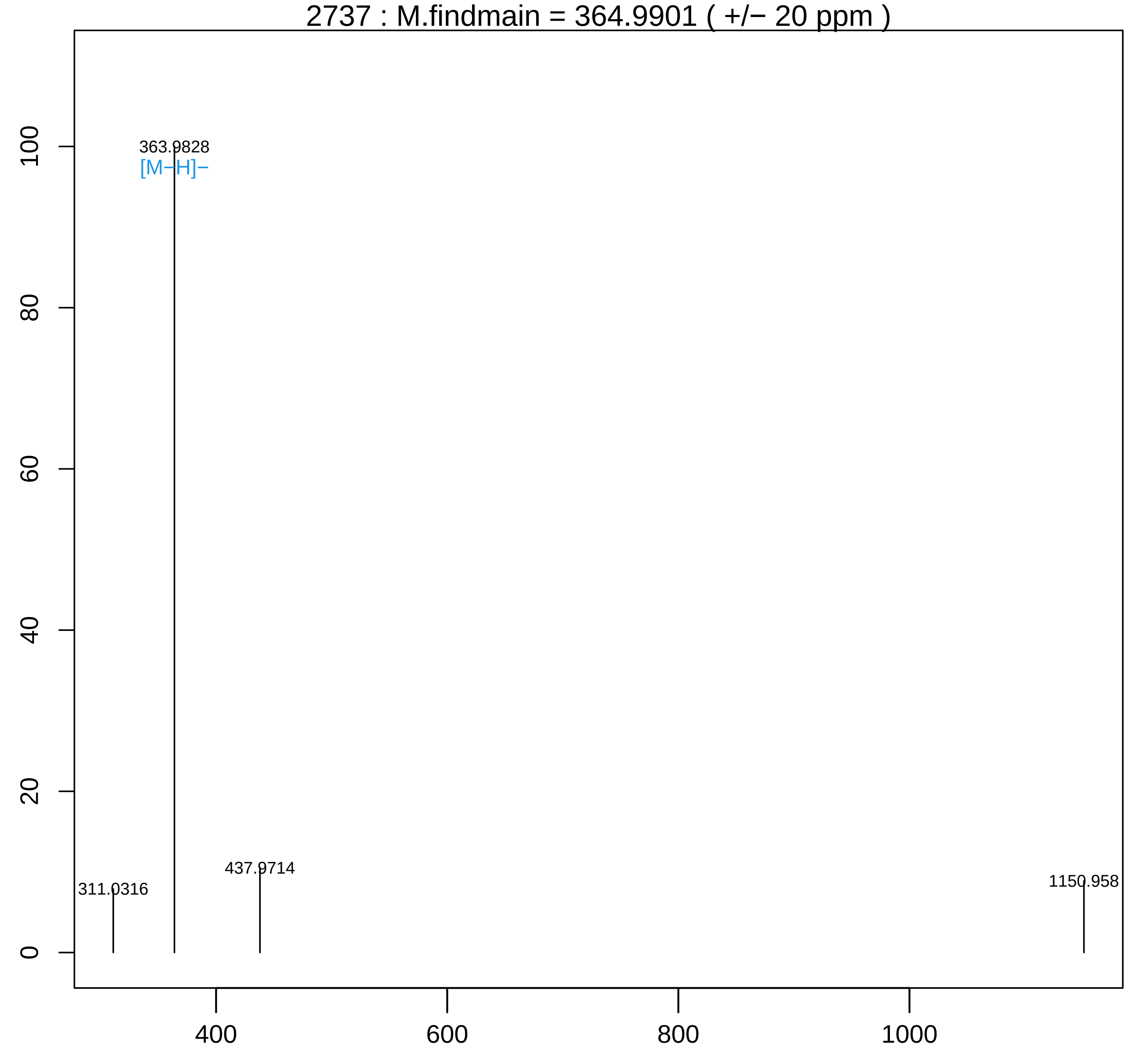
2737 : M.findmain = 364.9901 (+/− 20 ppm)
363.9828
[M−H]−
311.0316
437.9714
1150.958
0
20
40
60
80
100
400
600
800
1000

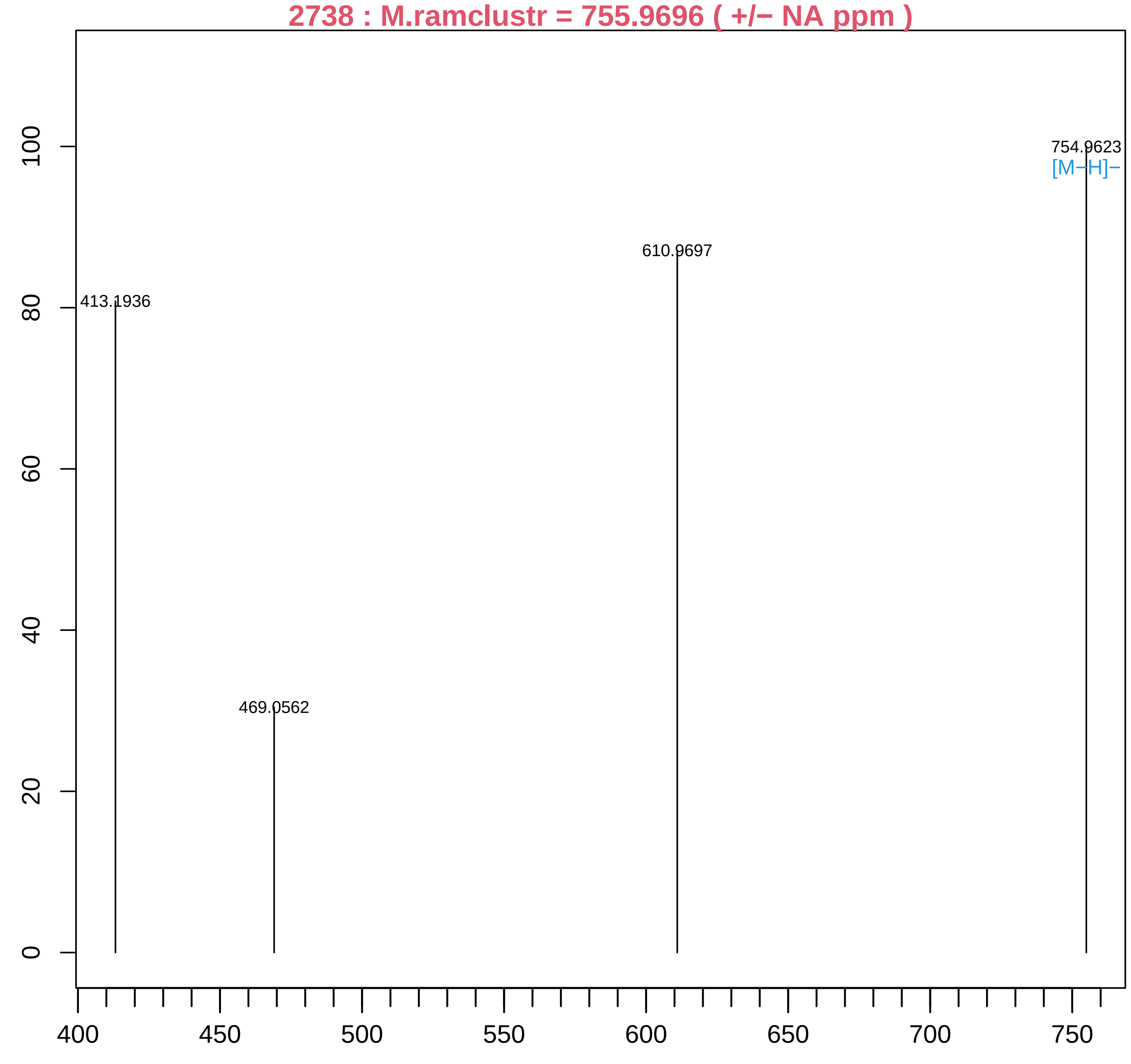
2738 : M.ramclustr = 755.9696 (+/− NA ppm)
754.9623
[M−H]−
610.9697
413.1936
469.0562
0
20
40
60
80
100
400
450
500
550
600
650
700
750

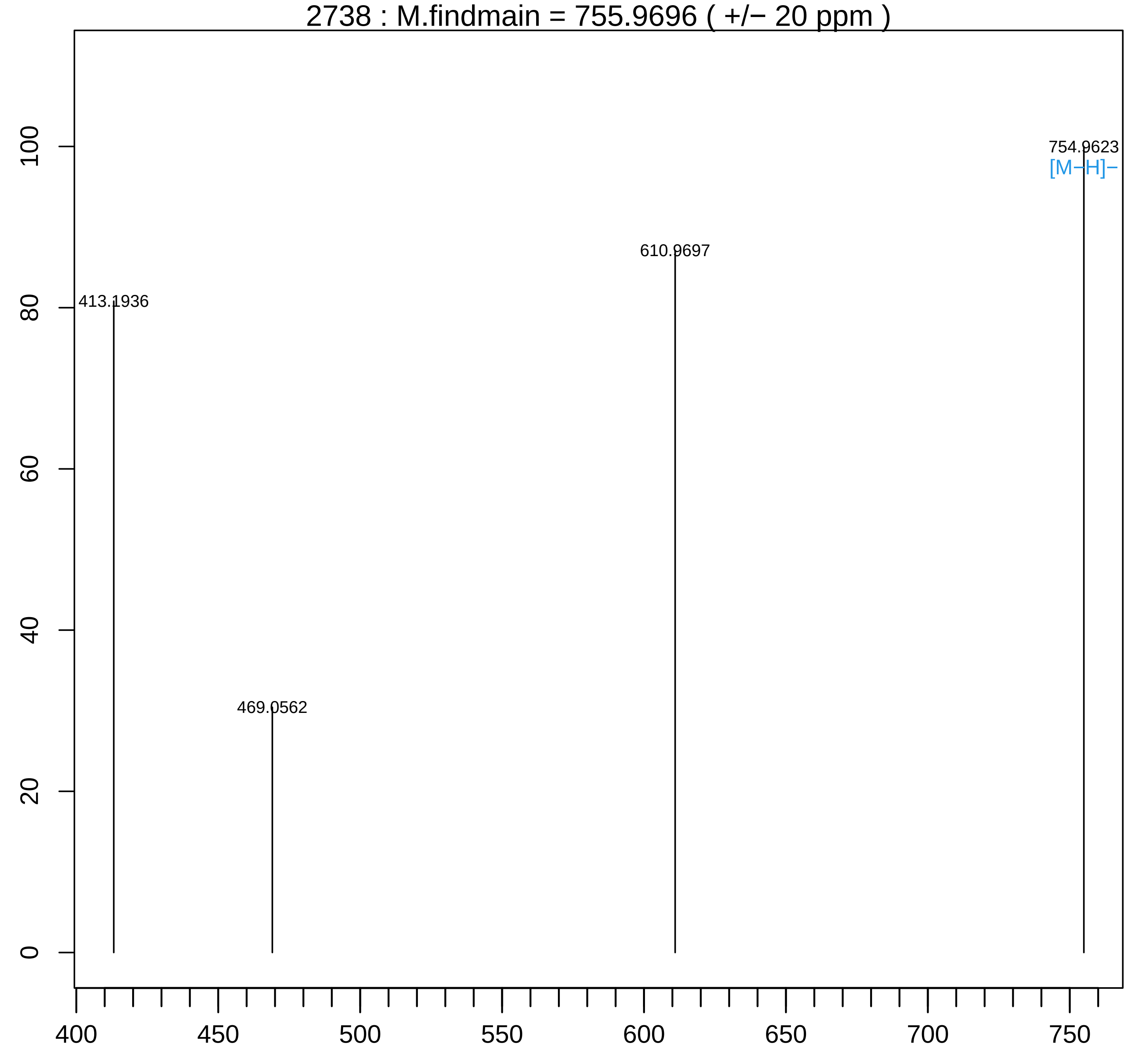
2738 : M.findmain = 755.9696 (+/− 20 ppm)
754.9623
[M−H]−
610.9697
413.1936
469.0562
0
20
40
60
80
100
400
450
500
550
600
650
700
750

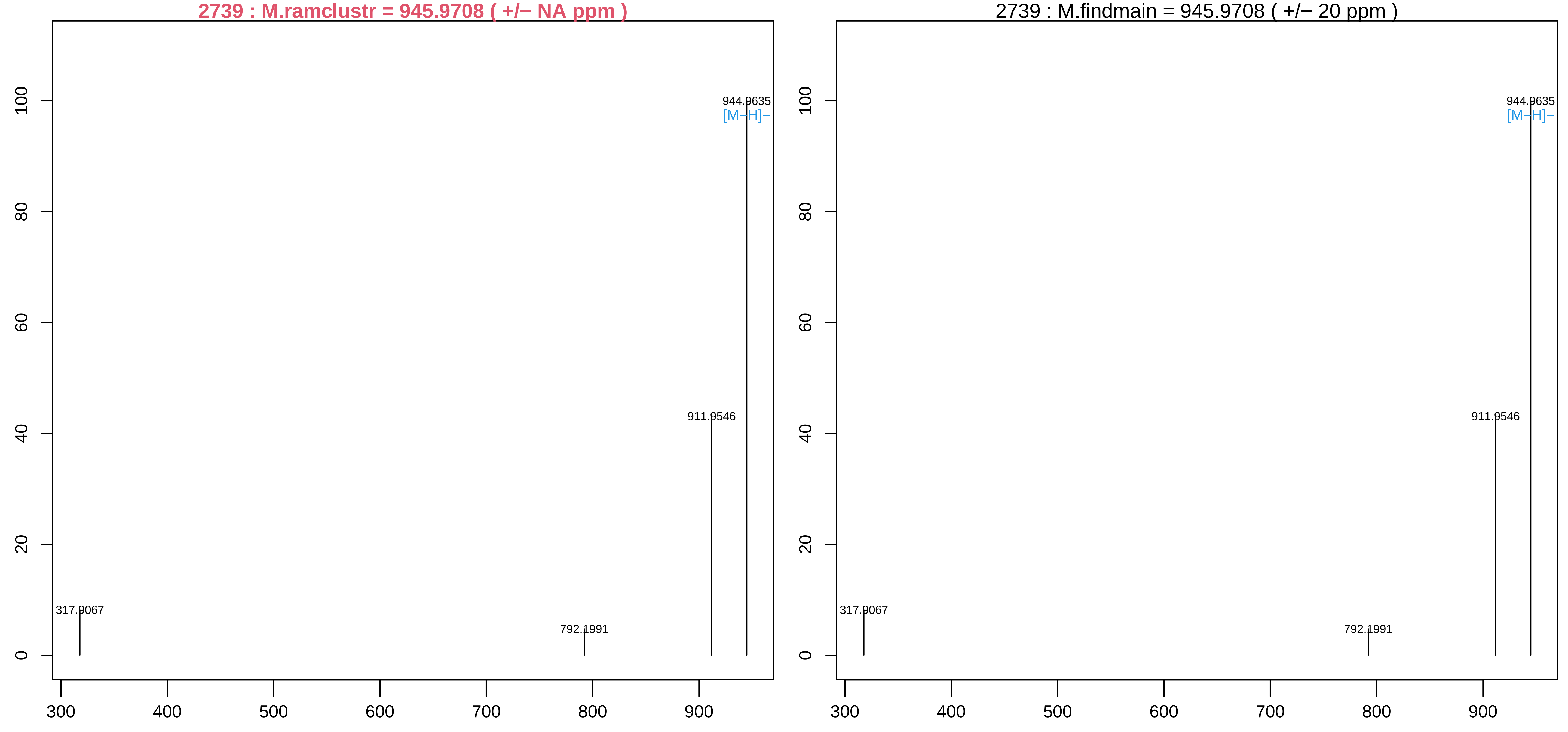

2739 : M.ramclustr = 945.9708 (+/− NA ppm)
944.9635
[M−H]−
911.9546
792.1991
317.9067
2739 : M.findmain = 945.9708 (+/− 20 ppm)
944.9635
[M−H]−
911.9546
792.1991
317.9067

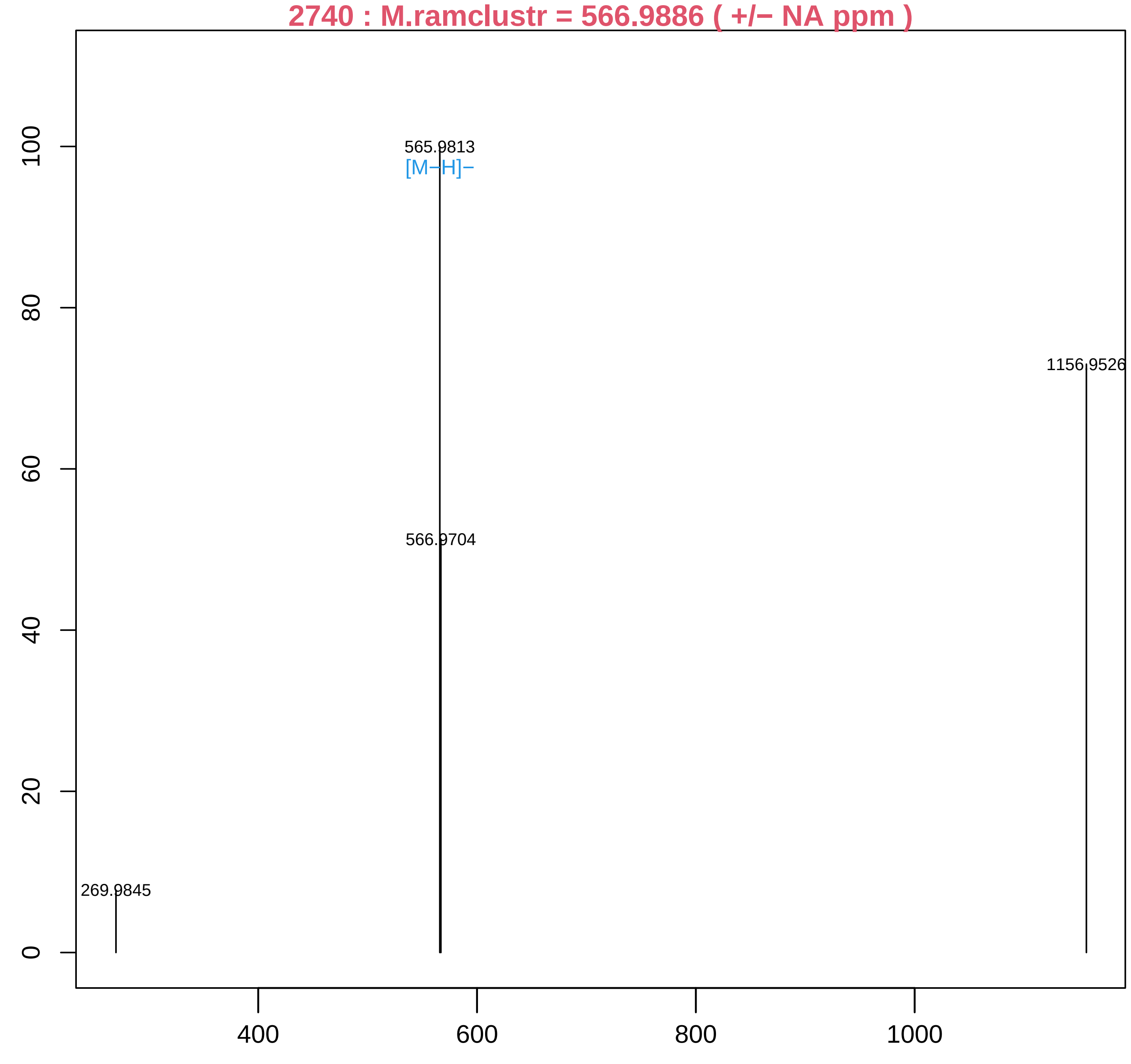
2740 : M.ramclustr = 566.9886 (+/− NA ppm)
565.9813
[M−H]−
566.9704
1156.9526
269.9845
0
20
40
60
80
100
400
600
800
1000

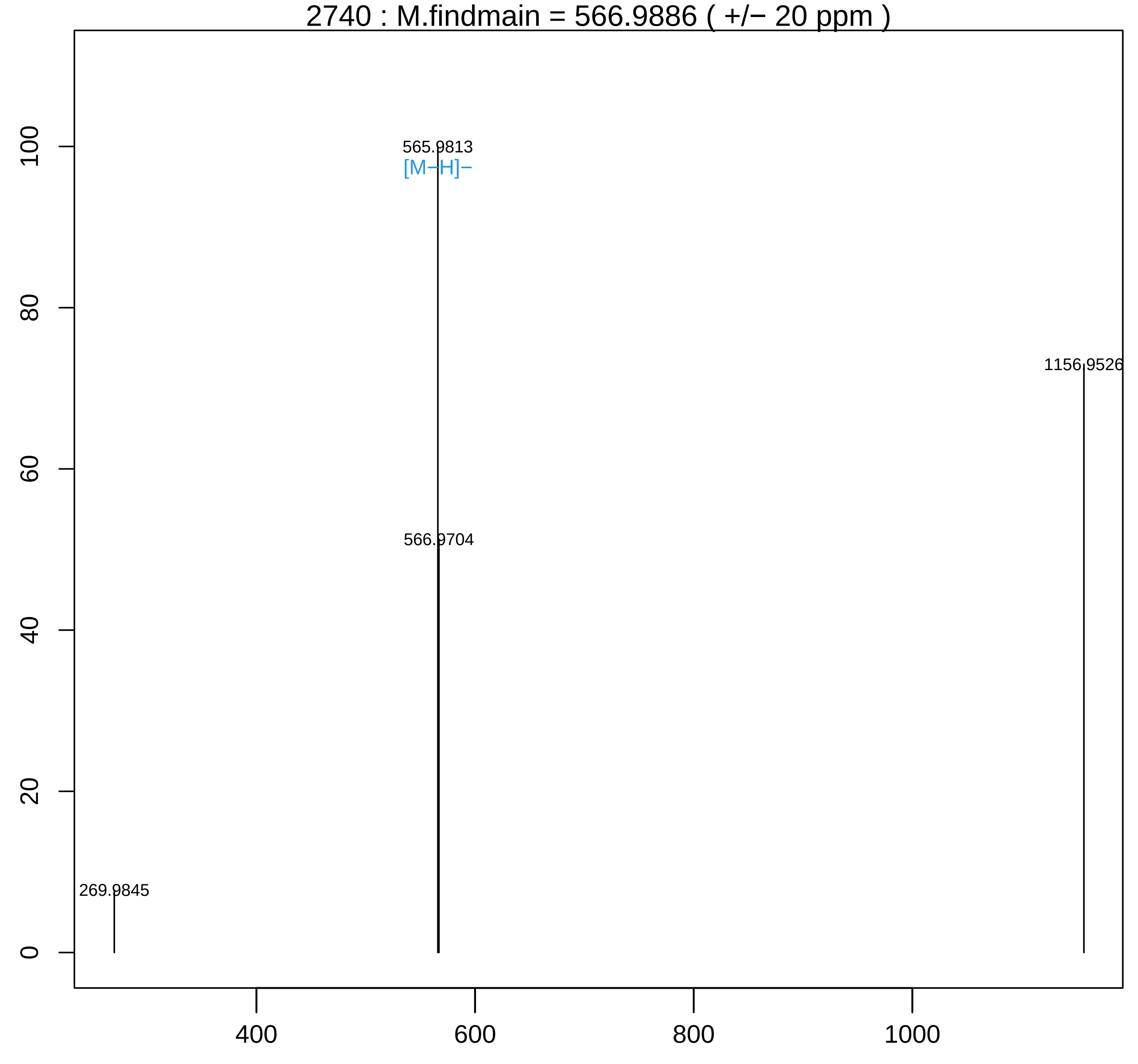
2740 : M.findmain = 566.9886 (+/− 20 ppm)
565.9813
[M−H]−
566.9704
1156.9526
269.9845
0
20
40
60
80
100
400
600
800
1000

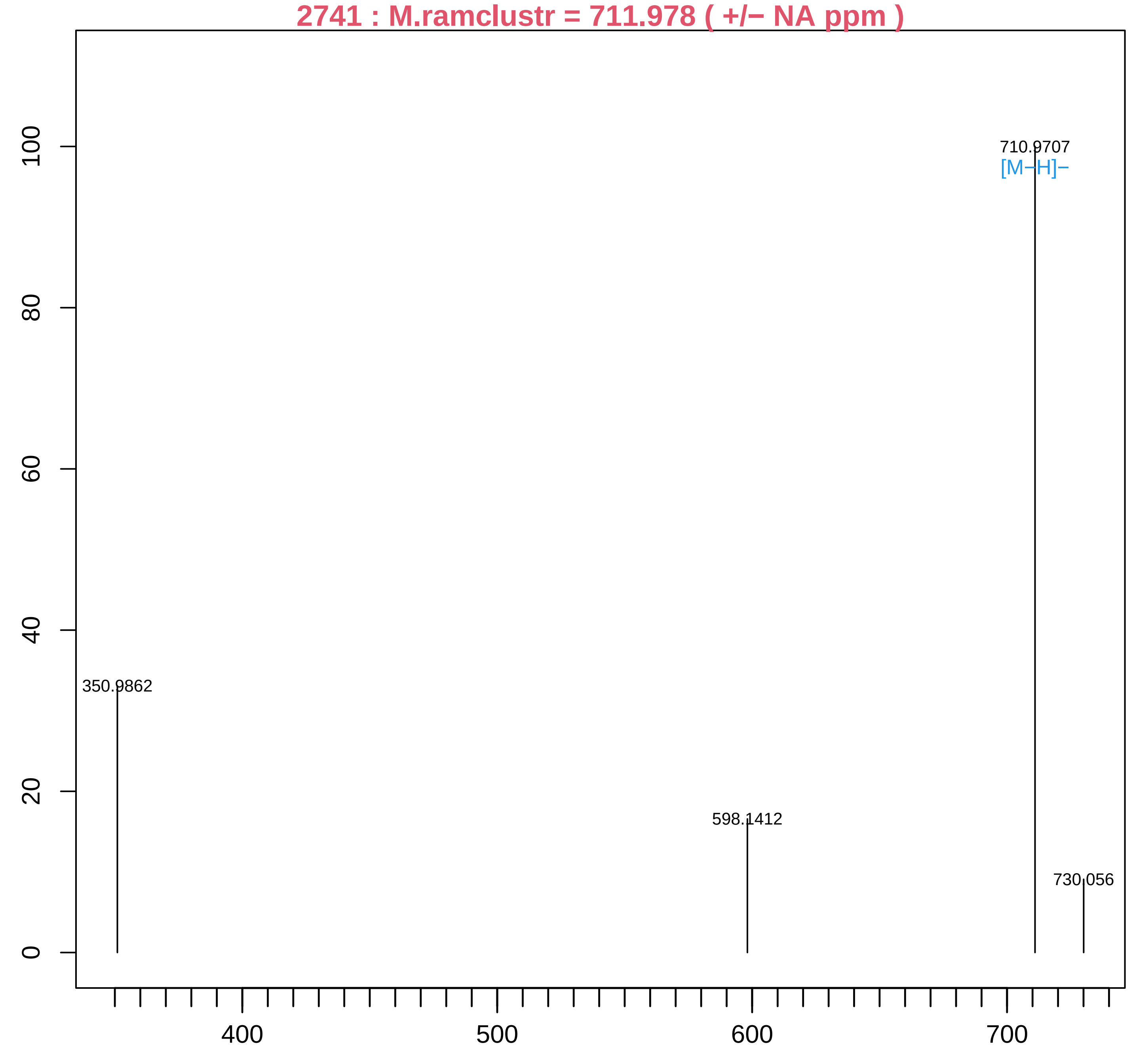
2741 : M.ramclustr = 711.978 (+/− NA ppm)
710.9707
[M−H]−
350.9862
598.1412
730.056
0
20
40
60
80
100
400
500
600
700

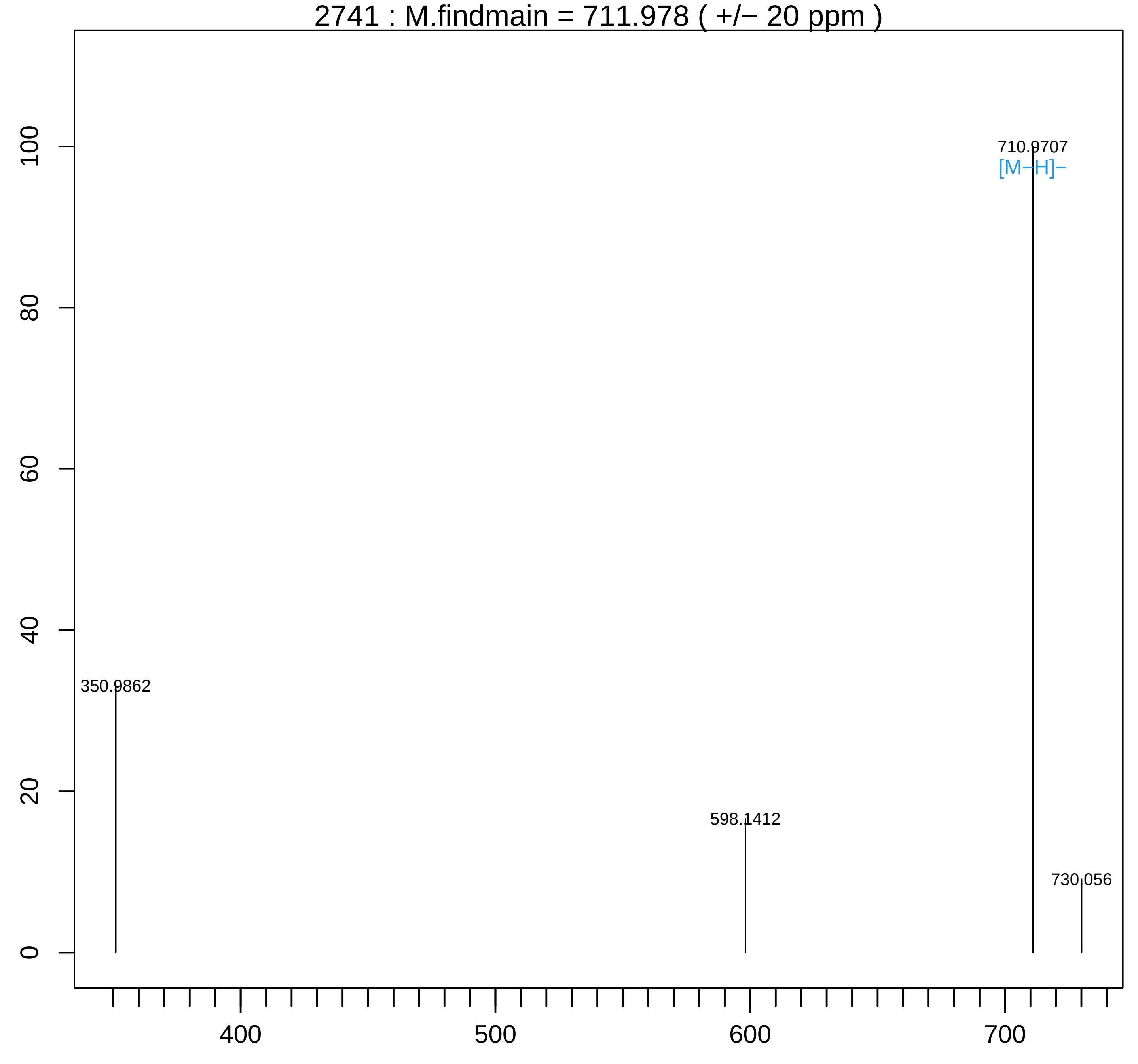
2741 : M.findmain = 711.978 (+/− 20 ppm)
710.9707
[M−H]−
350.9862
598.1412
730.056
0
20
40
60
80
100
400
500
600
700

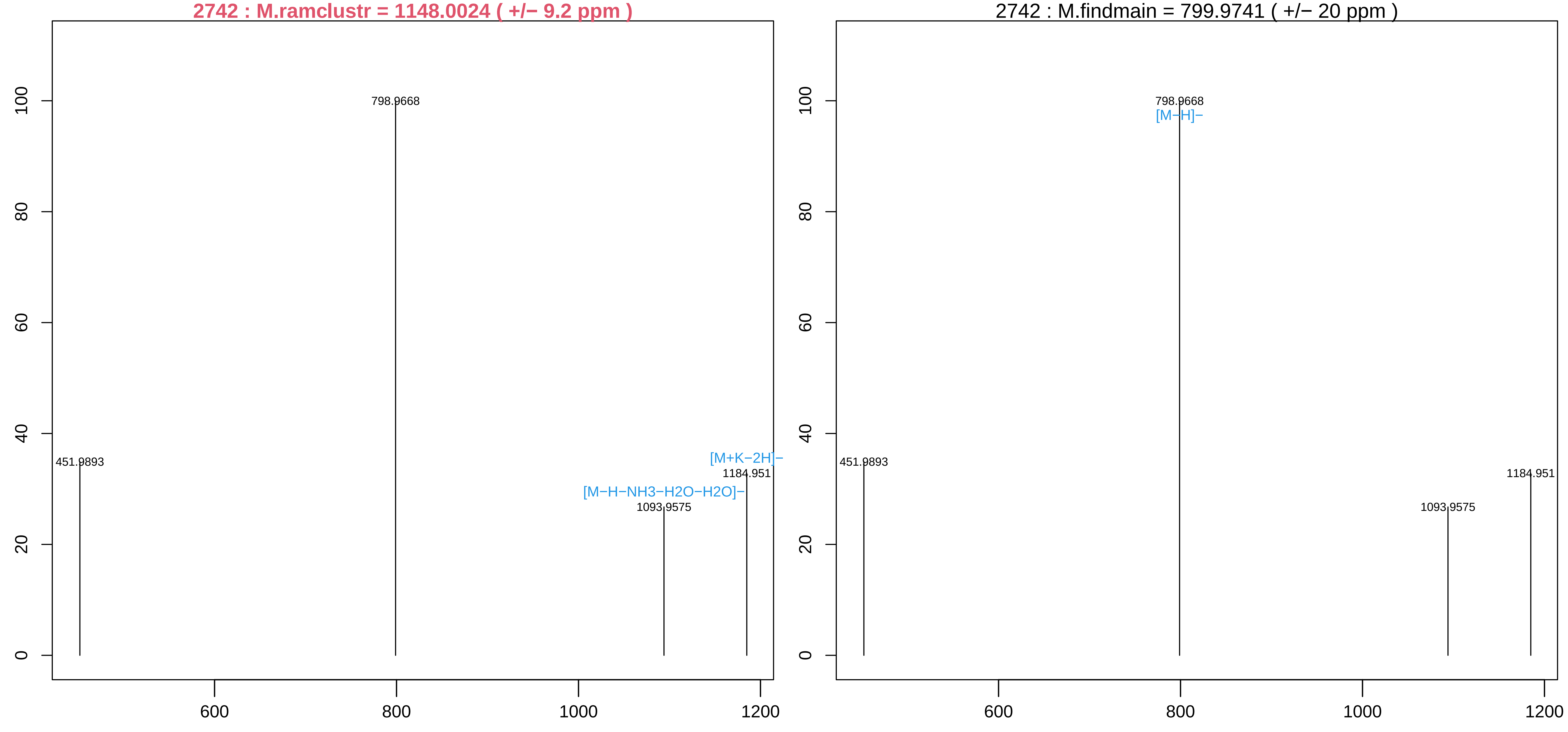

2742 : M.ramclustr = 1148.0024 (+/− 9.2 ppm)
798.9668
451.9893
[M+K−2H]−
1184.951
[M−H−NH3−H2O−H2O]−
1093.9575
0
20
40
60
80
100
600
800
1000
1200
2742 : M.findmain = 799.9741 (+/− 20 ppm)
798.9668
[M−H]−
451.9893
1184.951
1093.9575
0
20
40
60
80
100
600
800
1000
1200

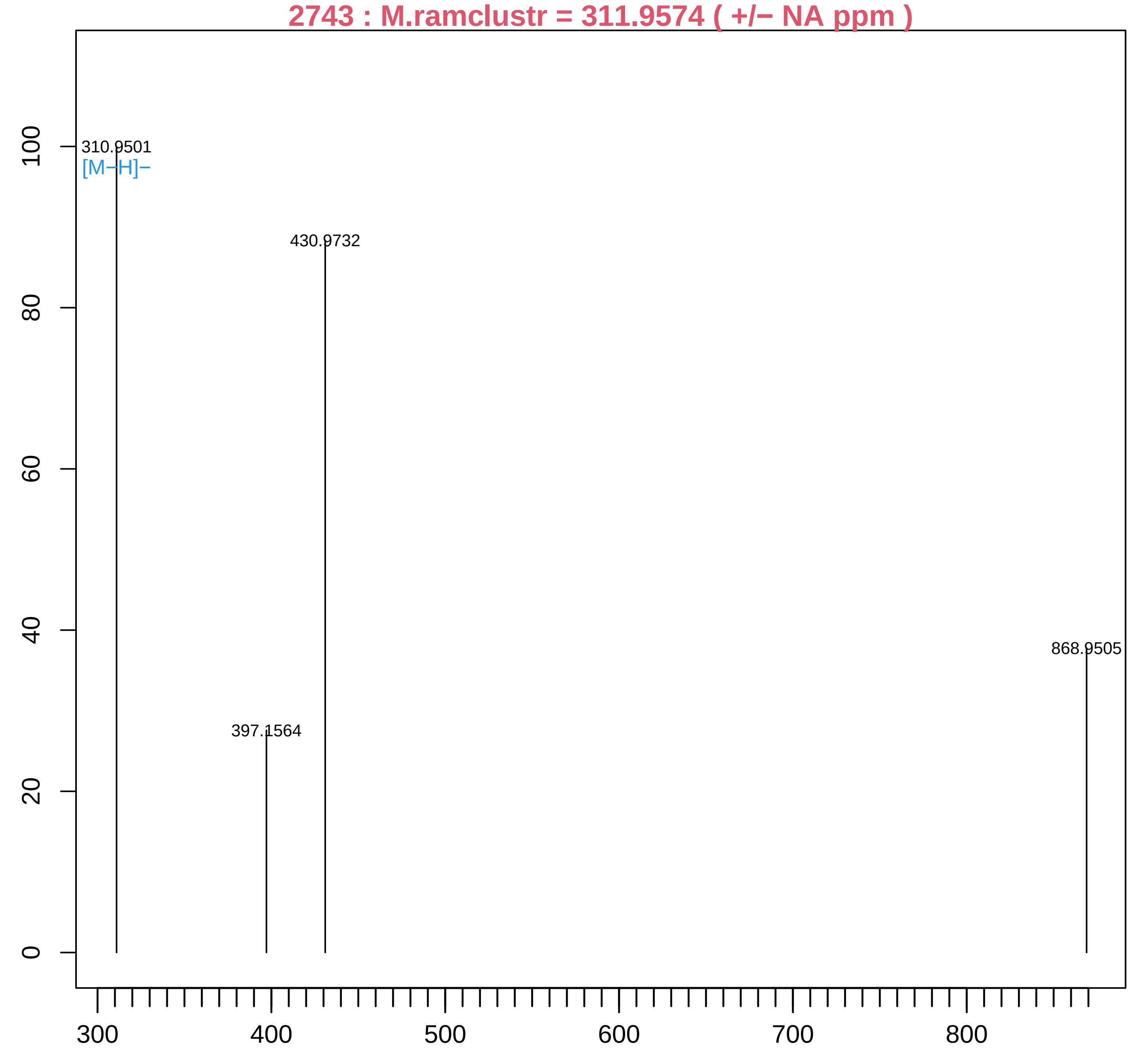
2743 : M.ramclustr = 311.9574 (+/− NA ppm)
310.9501
[M−H]−
430.9732
397.1564
868.9505
0
20
40
60
80
100
300
400
500
600
700
800

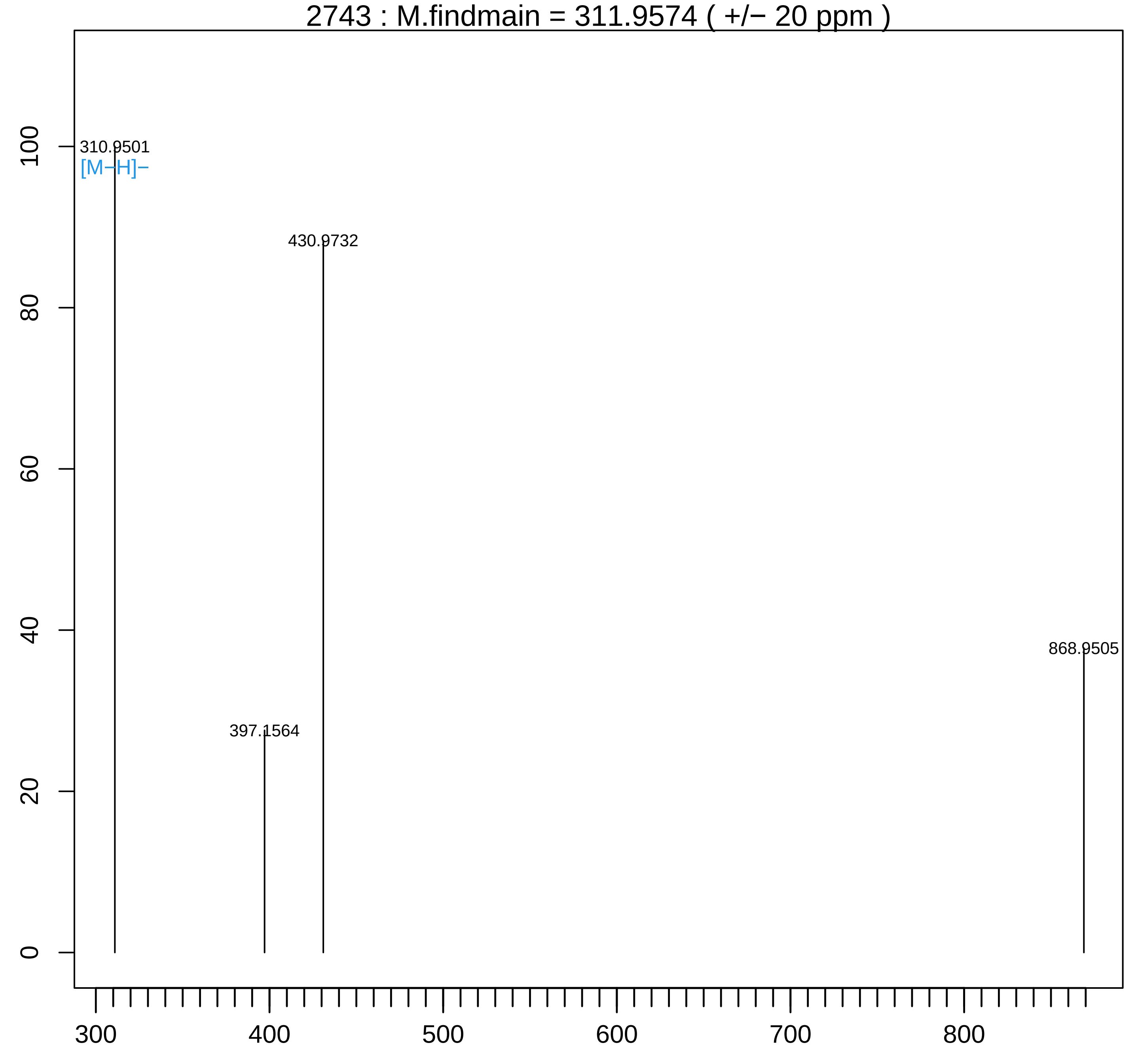
2743 : M.findmain = 311.9574 (+/− 20 ppm)
310.9501
[M−H]−
430.9732
397.1564
868.9505
0
20
40
60
80
100
300
400
500
600
700
800

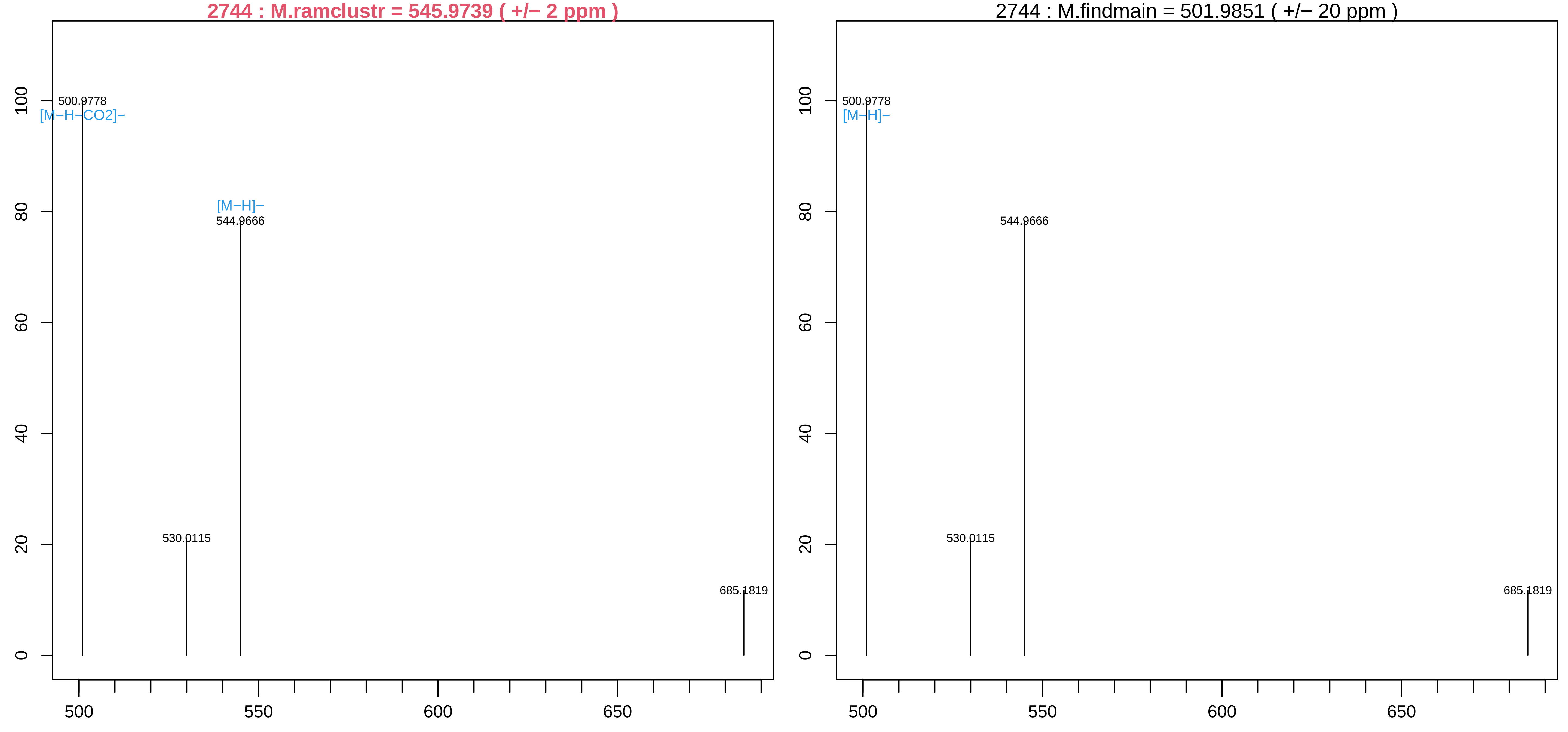

2744 : M.ramclustr = 545.9739 (+/− 2 ppm)
500.9778
[M−H−CO2]−
[M−H]−
544.9666
530.0115
685.1819
2744 : M.findmain = 501.9851 (+/− 20 ppm)
500.9778
[M−H]−
544.9666
530.0115
685.1819
0
20
40
60
80
100
500
550
600
650

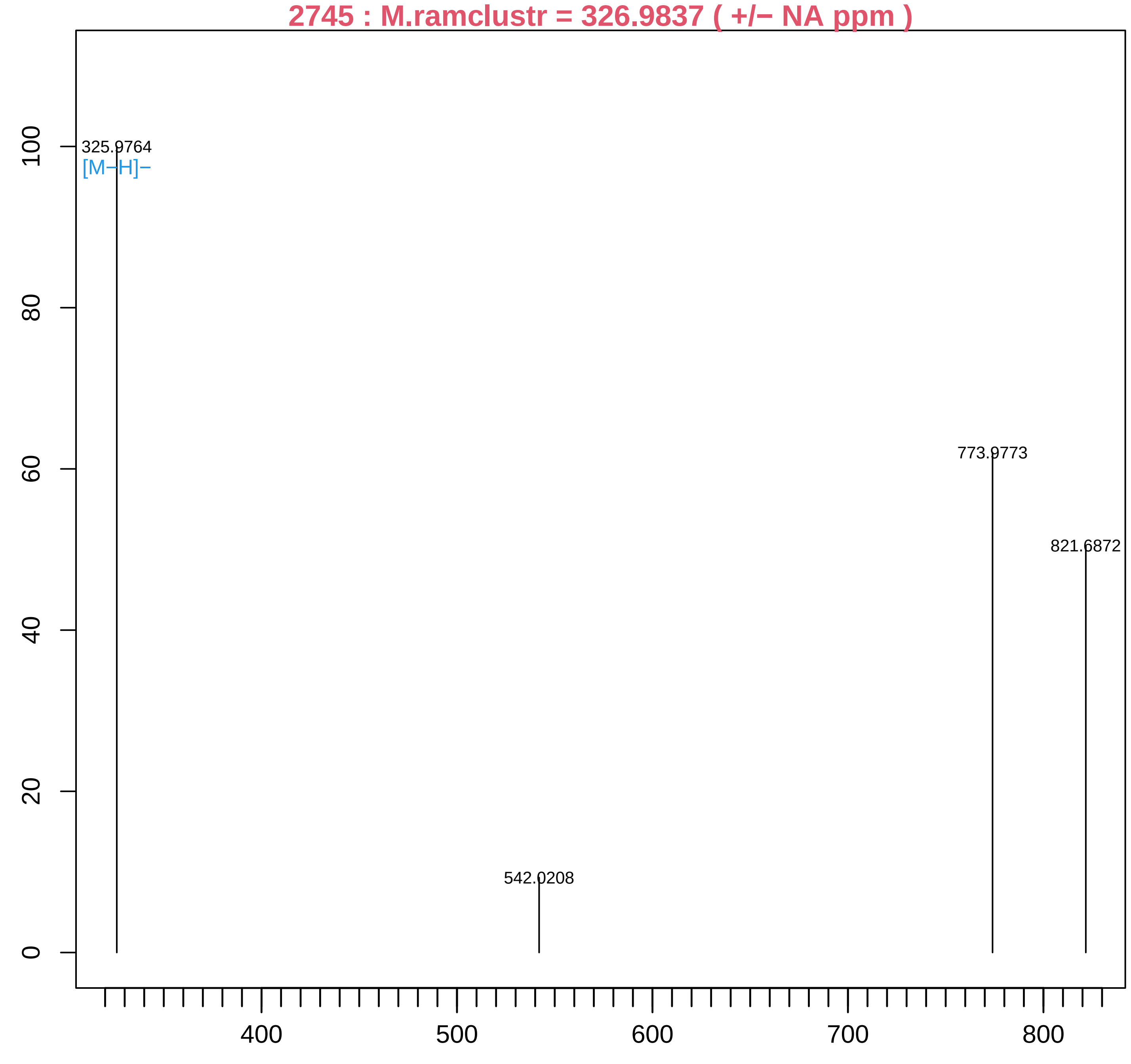

2745 : M.ramclustr = 326.9837 (+/− NA ppm)
325.9764
[M−H]−
542.0208
773.9773
821.6872
0
20
40
60
80
100
400
500
600
700
800

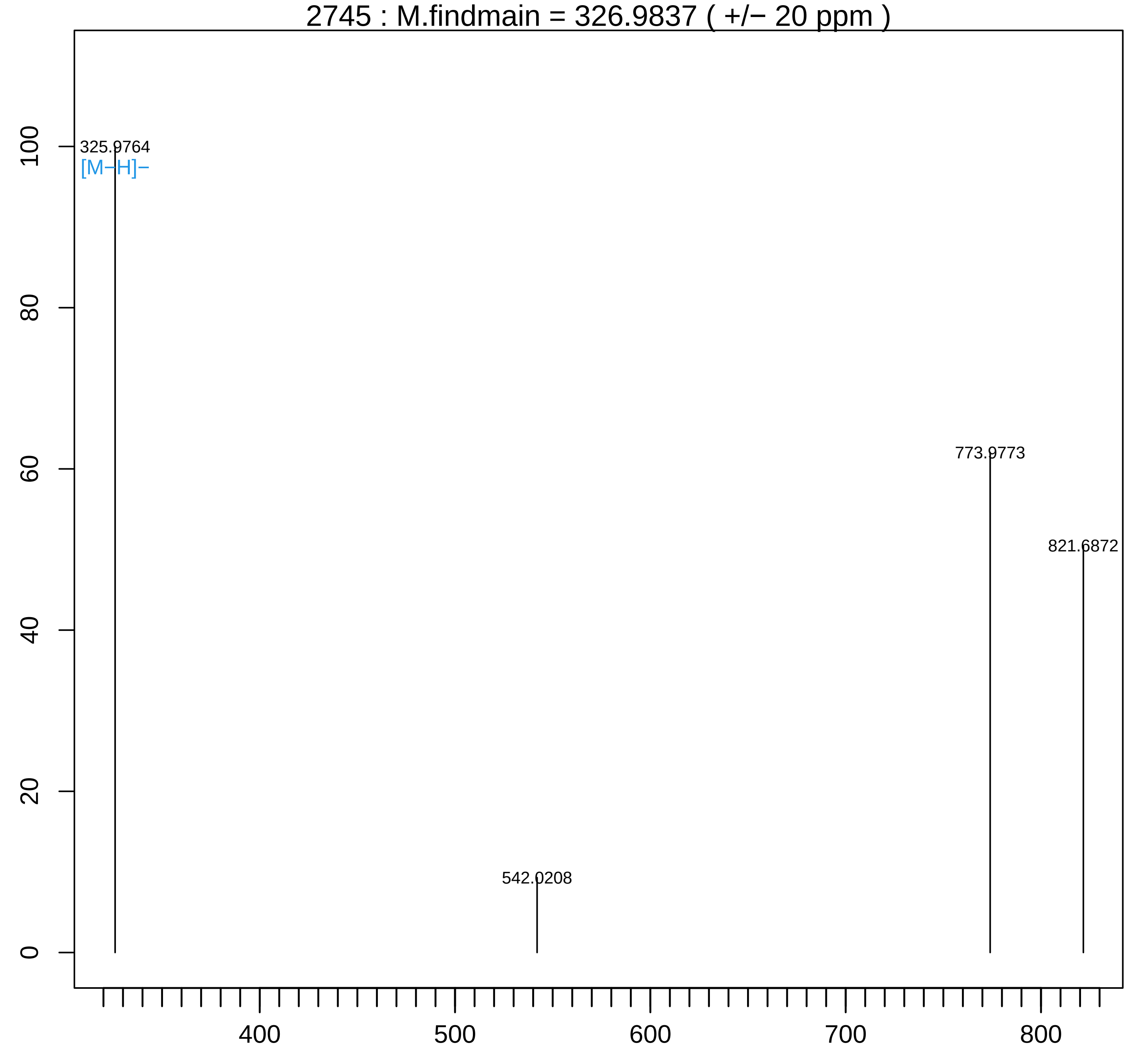

2745 : M.findmain = 326.9837 (+/− 20 ppm)
325.9764
[M−H]−
542.0208
773.9773
821.6872
0
20
40
60
80
100
400
500
600
700
800

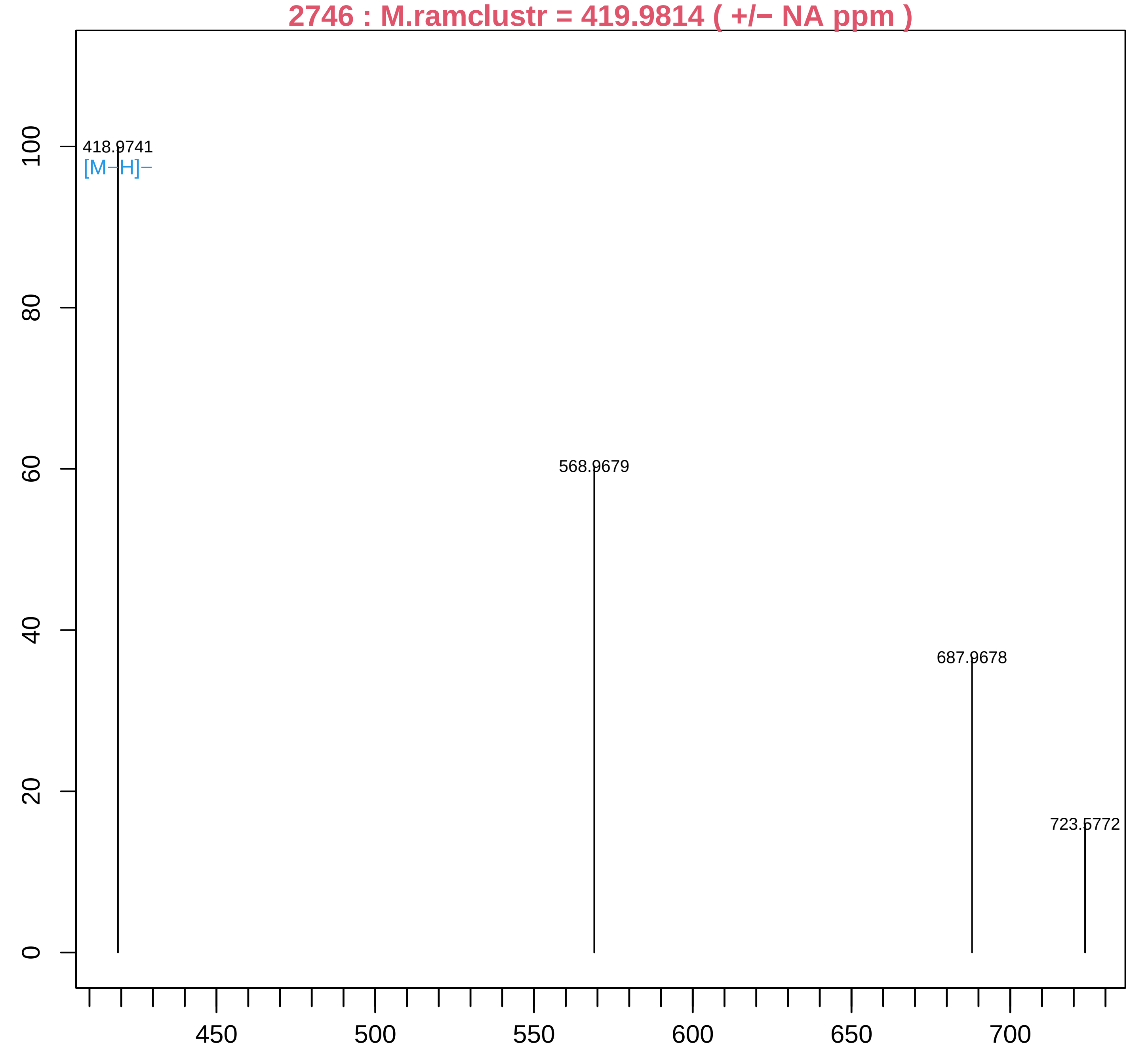
2746 : M.ramclustr = 419.9814 (+/− NA ppm)
418.9741
[M−H]−
568.9679
687.9678
723.5772
0
20
40
60
80
100
450
500
550
600
650
700

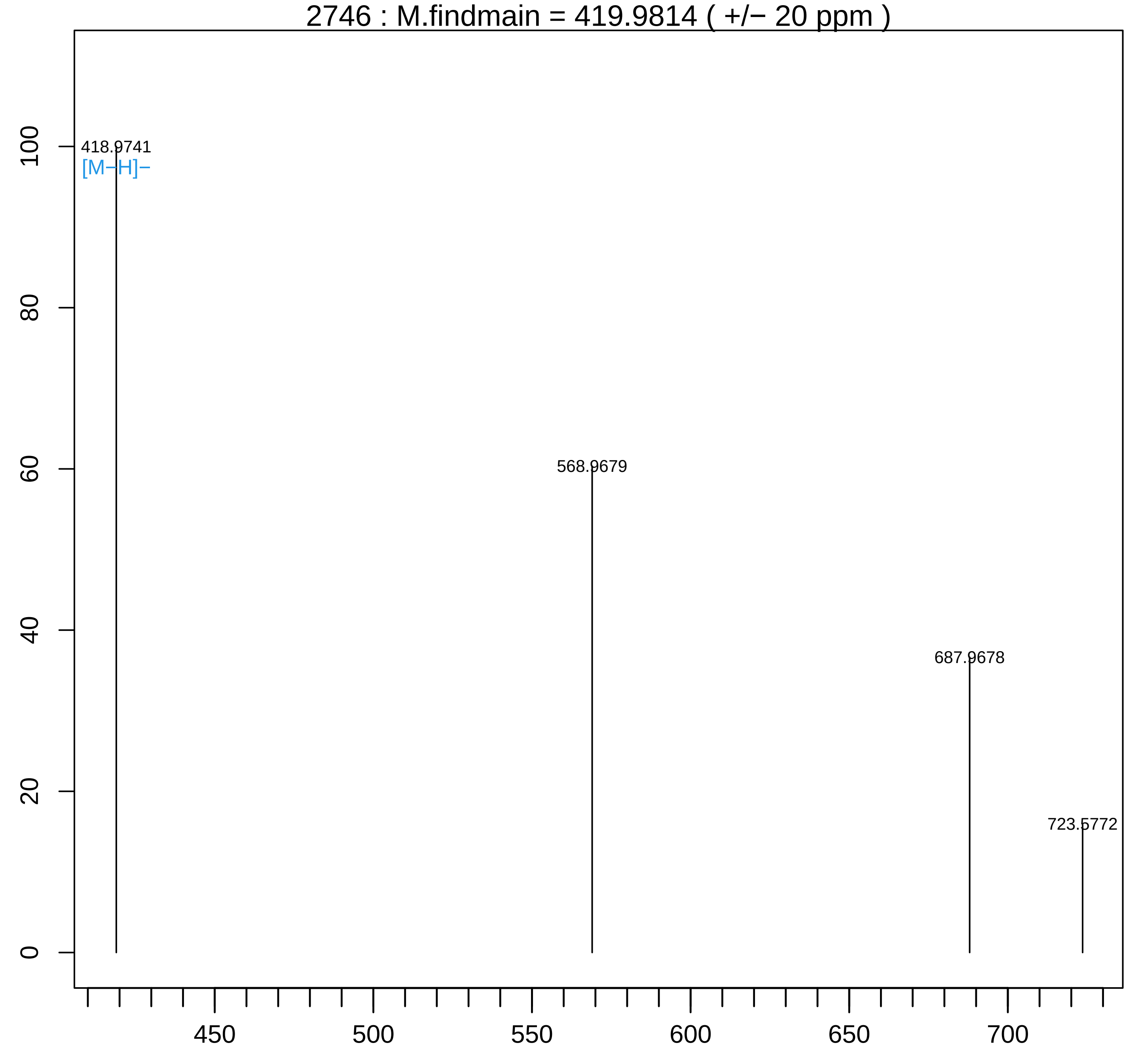
2746 : M.findmain = 419.9814 (+/− 20 ppm)
418.9741
[M−H]−
568.9679
687.9678
723.5772
0
20
40
60
80
100
450
500
550
600
650
700

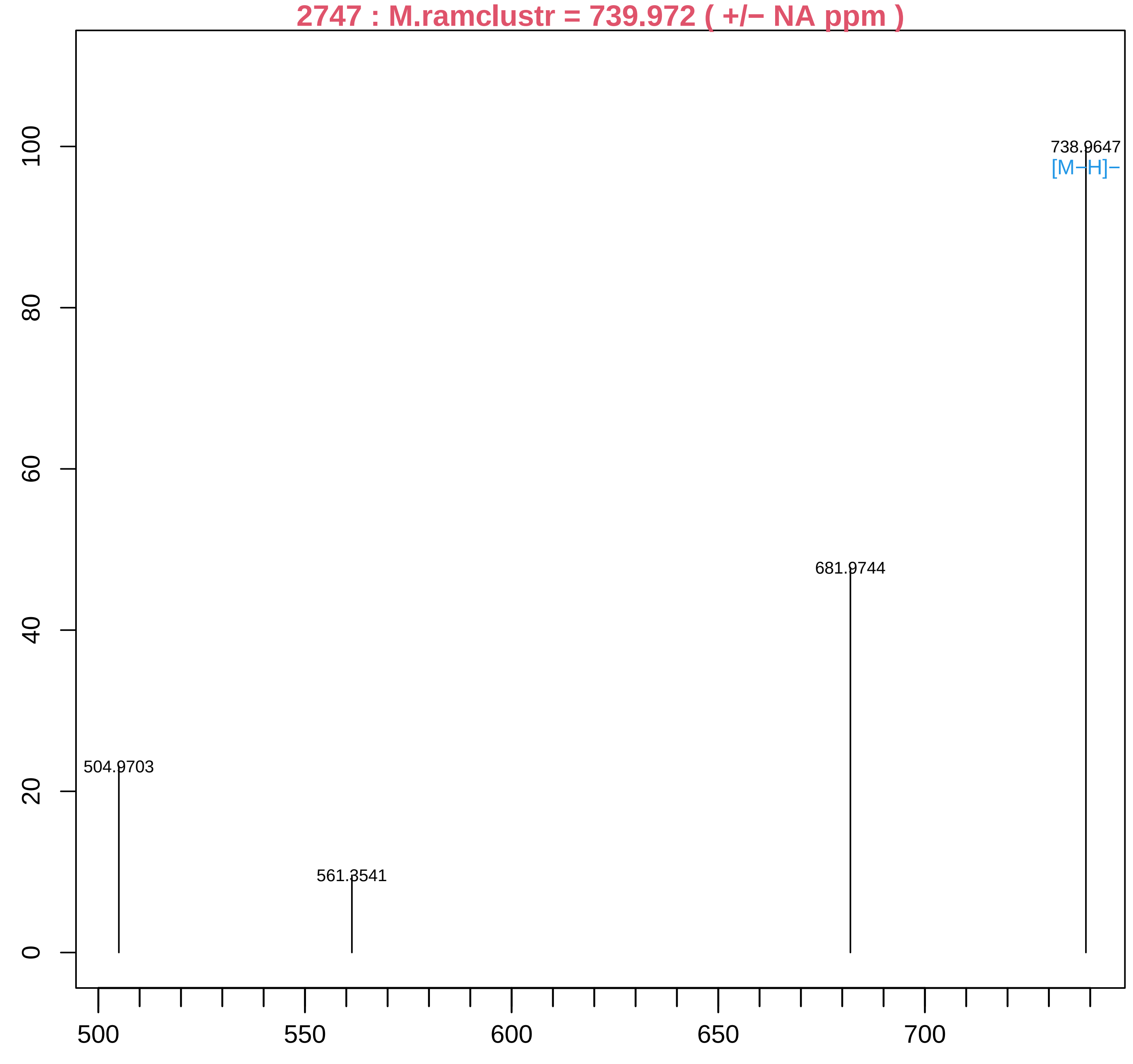
2747 : M.ramclustr = 739.972 (+/− NA ppm)
738.9647
[M−H]−
681.9744
504.9703
561.3541
0
20
40
60
80
100
500
550
600
650
700

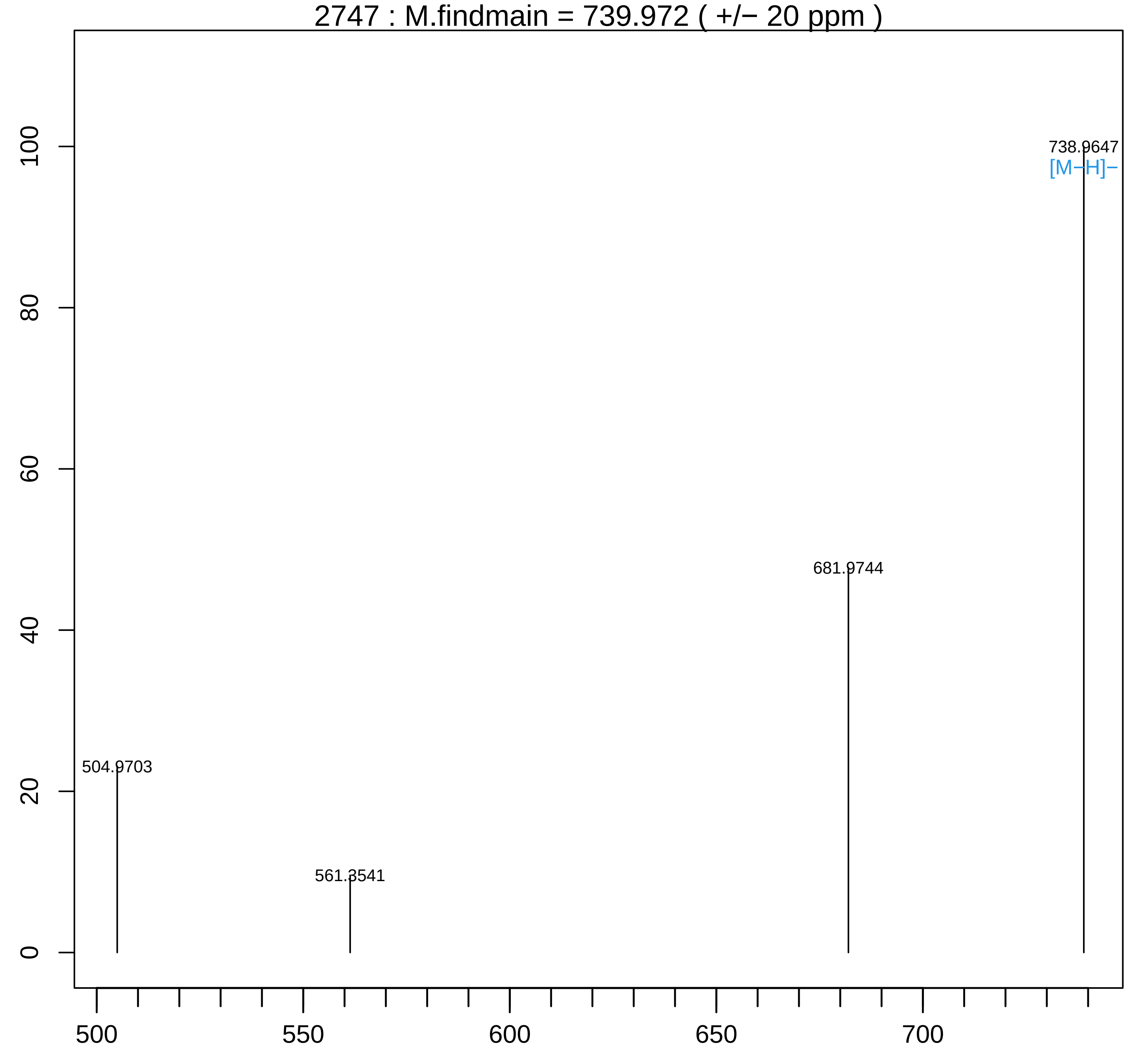
2747 : M.findmain = 739.972 (+/− 20 ppm)
738.9647
[M−H]−
681.9744
504.9703
561.3541
0
20
40
60
80
100
500
550
600
650
700

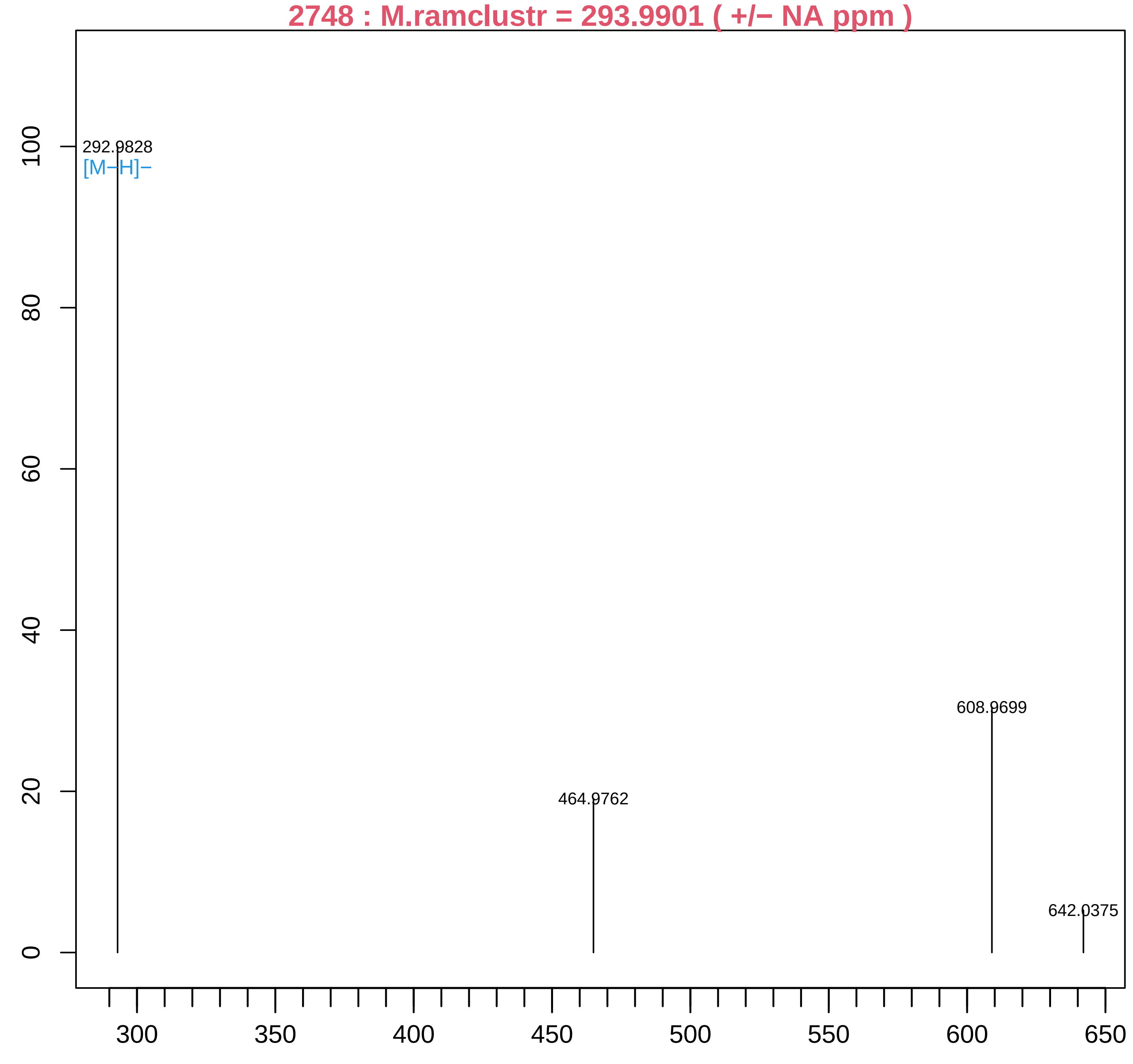

2748 : M.ramclustr = 293.9901 (+/− NA ppm)
292.9828
[M−H]−
464.9762
608.9699
642.0375
0
20
40
60
80
100
300
350
400
450
500
550
600
650

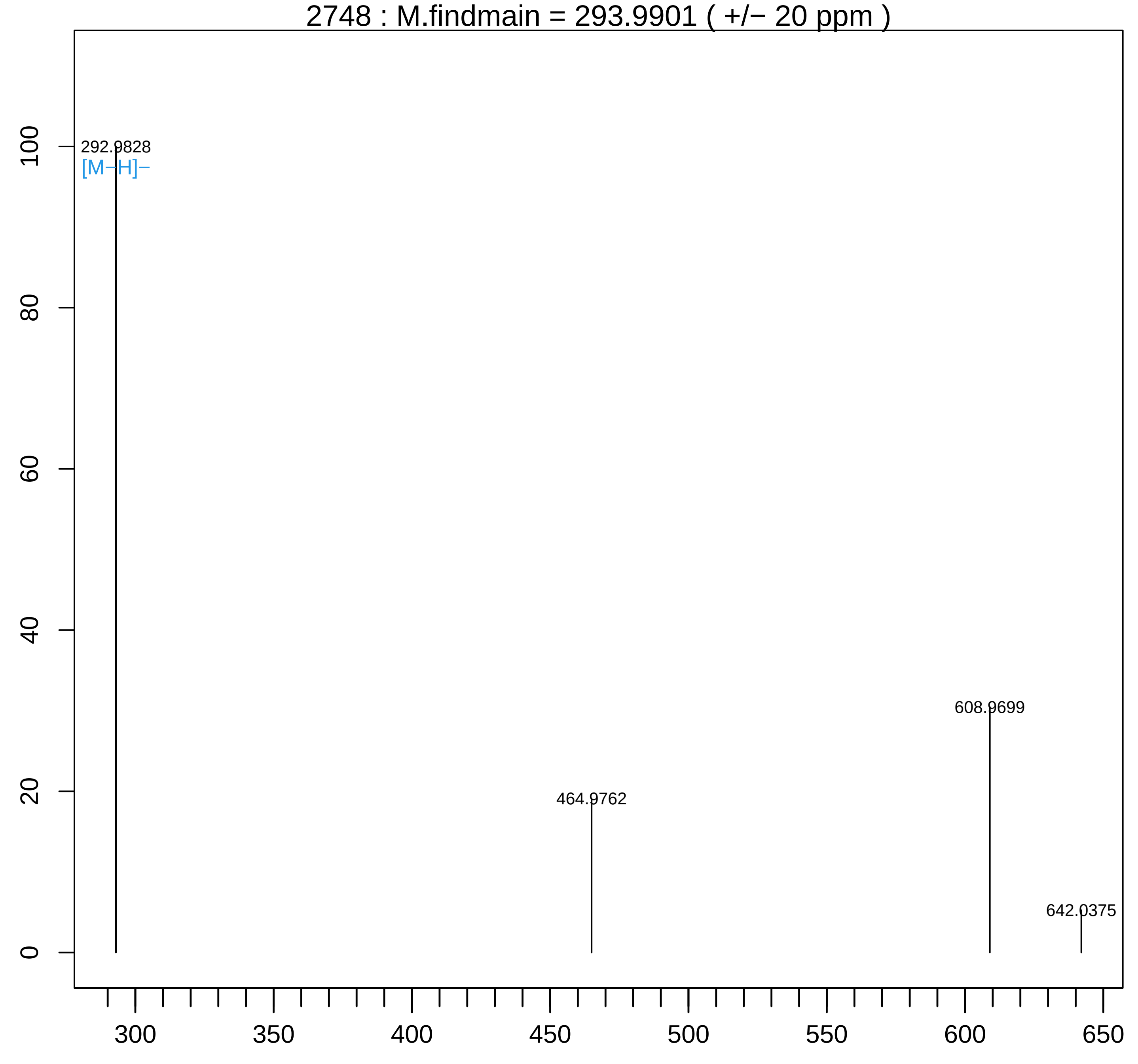

2748 : M.findmain = 293.9901 (+/− 20 ppm)
292.9828
[M−H]−
464.9762
608.9699
642.0375
0
20
40
60
80
100
300
350
400
450
500
550
600
650

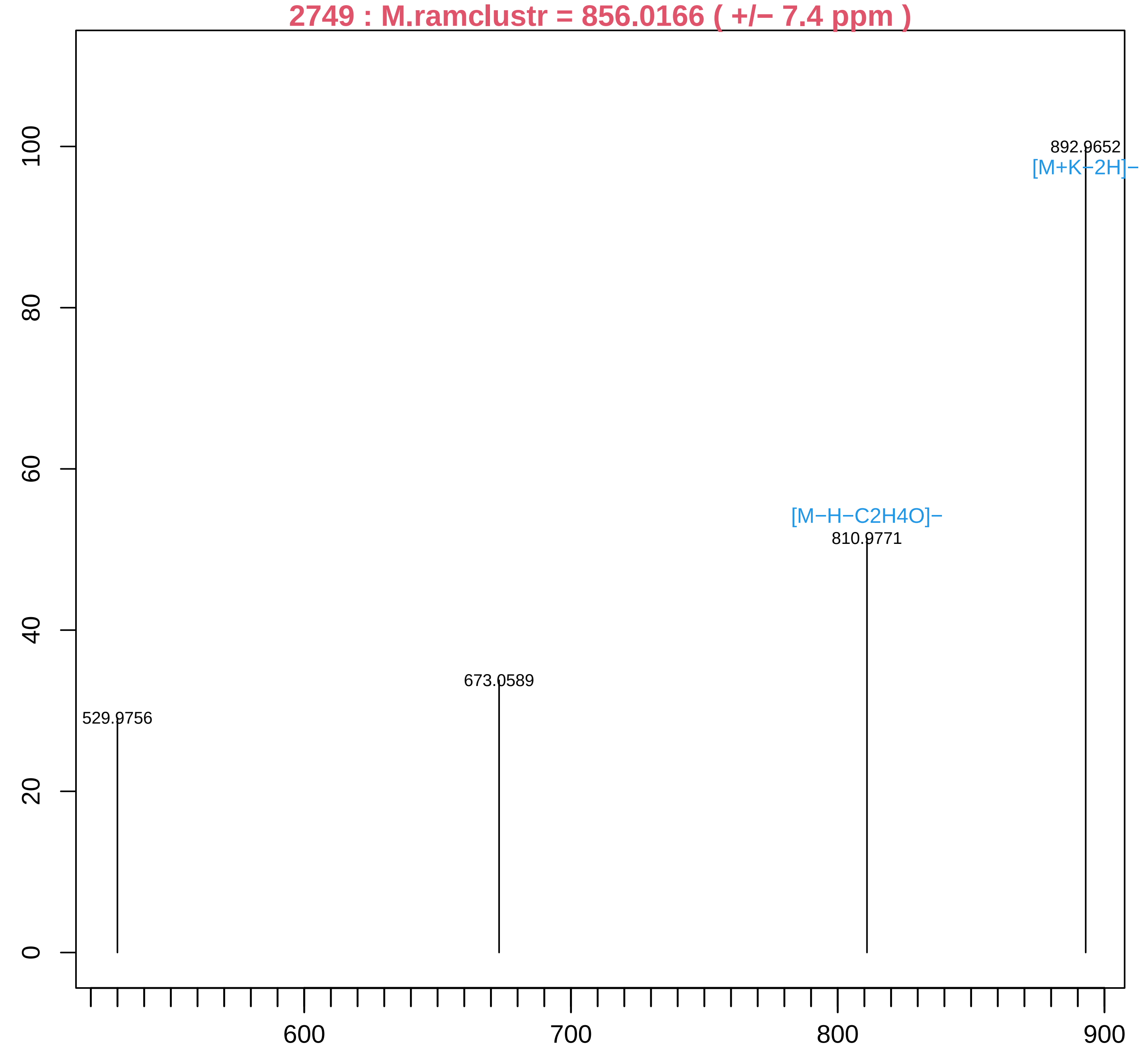
2749 : M.ramclustr = 856.0166 (+/− 7.4 ppm)
892.9652
[M+K−2H]−
[M−H−C2H4O]−
810.9771
673.0589
529.9756
0
20
40
60
80
100
600
700
800
900

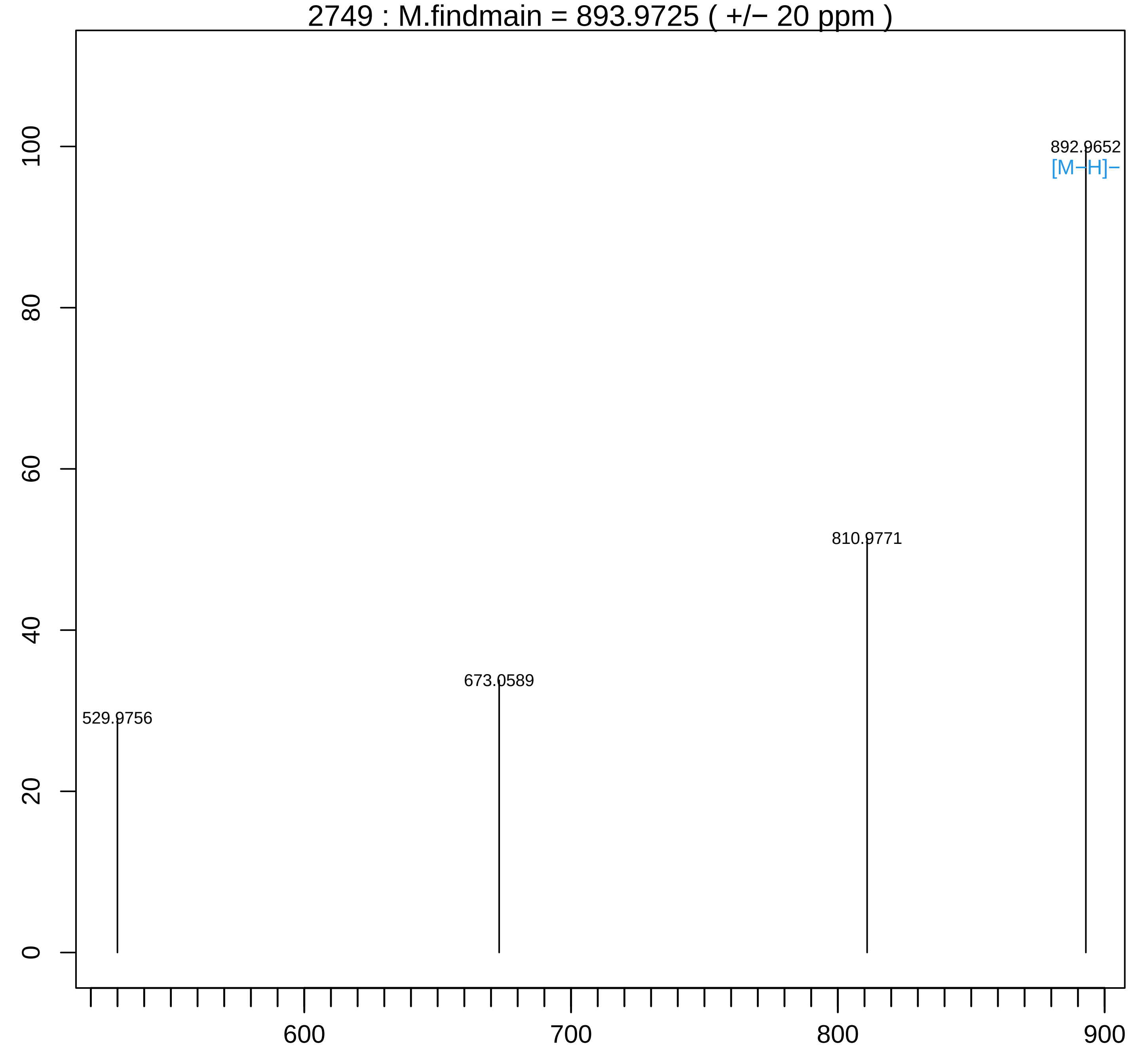
2749 : M.findmain = 893.9725 (+/− 20 ppm)
892.9652
[M−H]−
810.9771
673.0589
529.9756
0
20
40
60
80
100
600
700
800
900

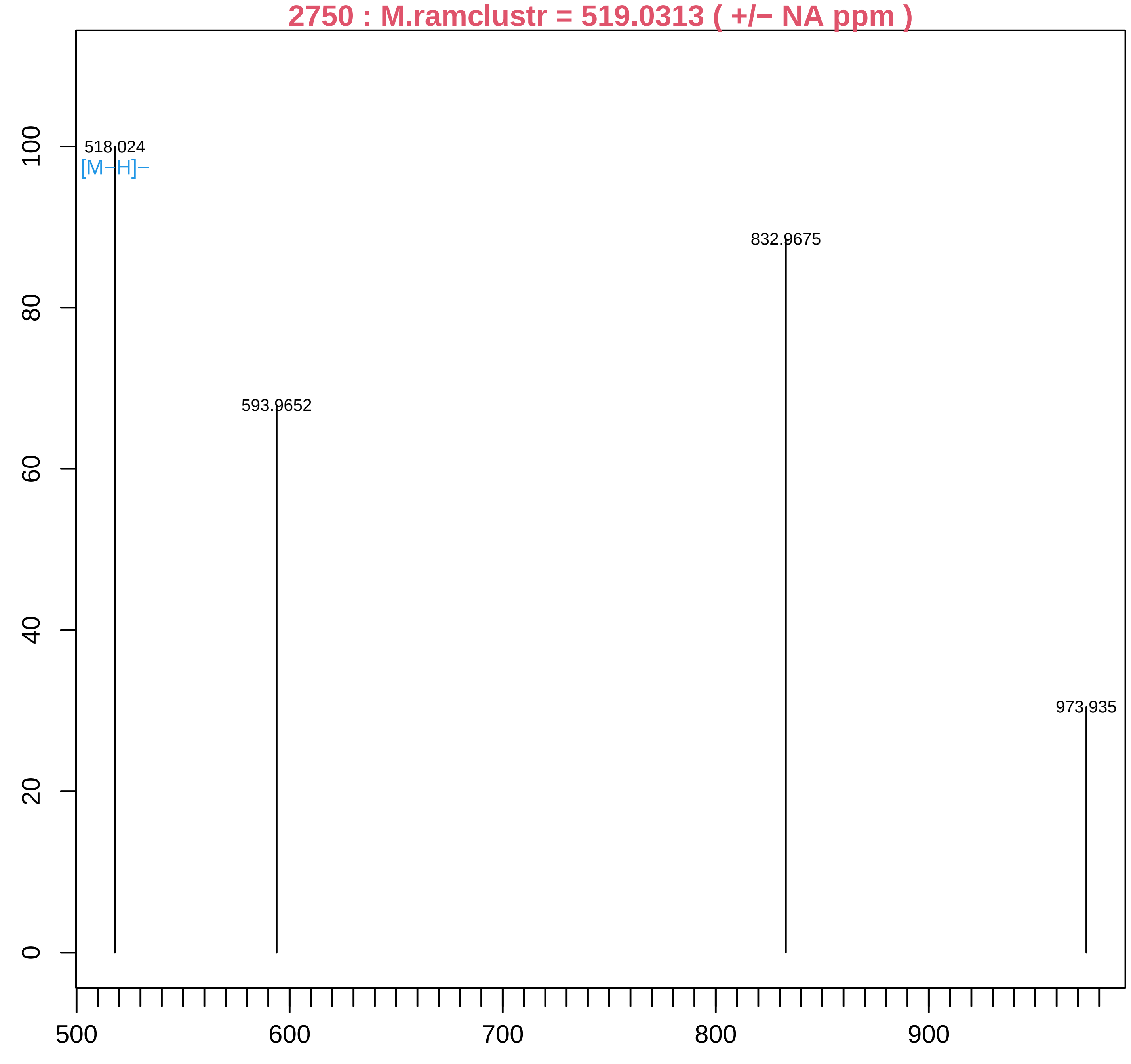
2750 : M.ramclustr = 519.0313 (+/− NA ppm)
518.024
[M−H]−
593.9652
832.9675
973.935
0
20
40
60
80
100
500
600
700
800
900

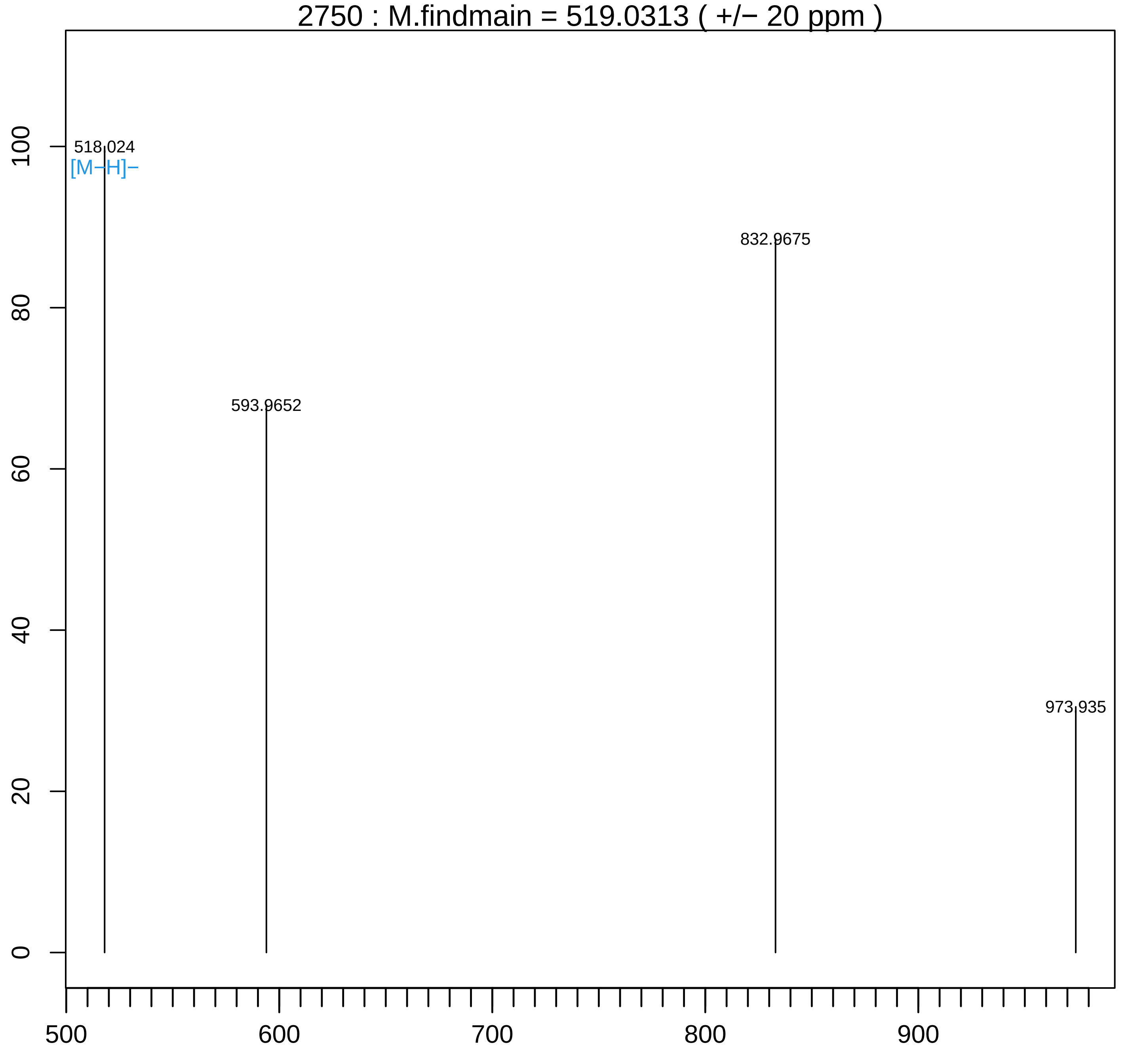
2750 : M.findmain = 519.0313 (+/− 20 ppm)
518.024
[M−H]−
593.9652
832.9675
973.935
0
20
40
60
80
100
500
600
700
800
900

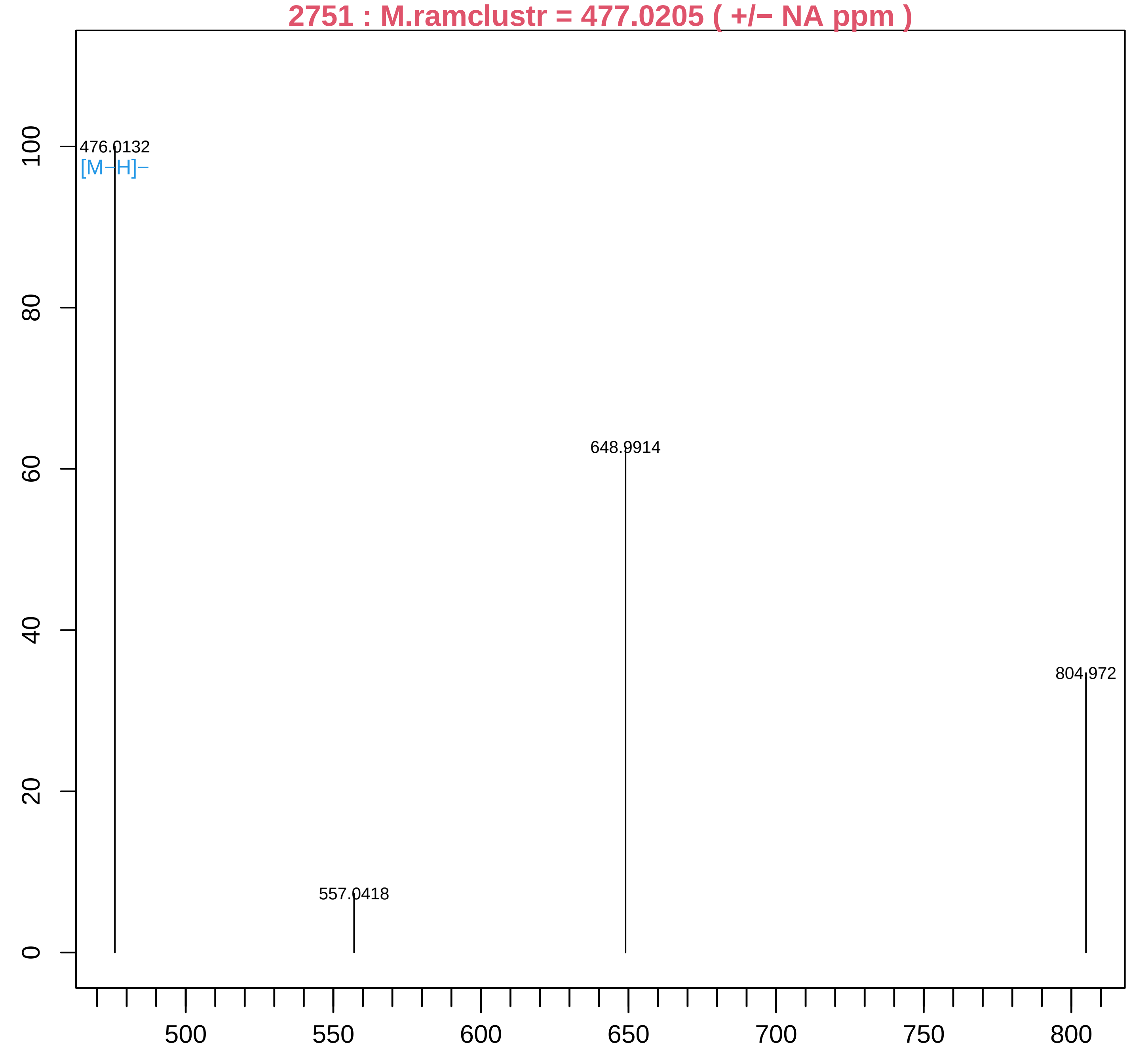

2751 : M.ramclustr = 477.0205 (+/− NA ppm)
476.0132
[M−H]−
557.0418
648.9914
804.972
0
20
40
60
80
100
500
550
600
650
700
750
800

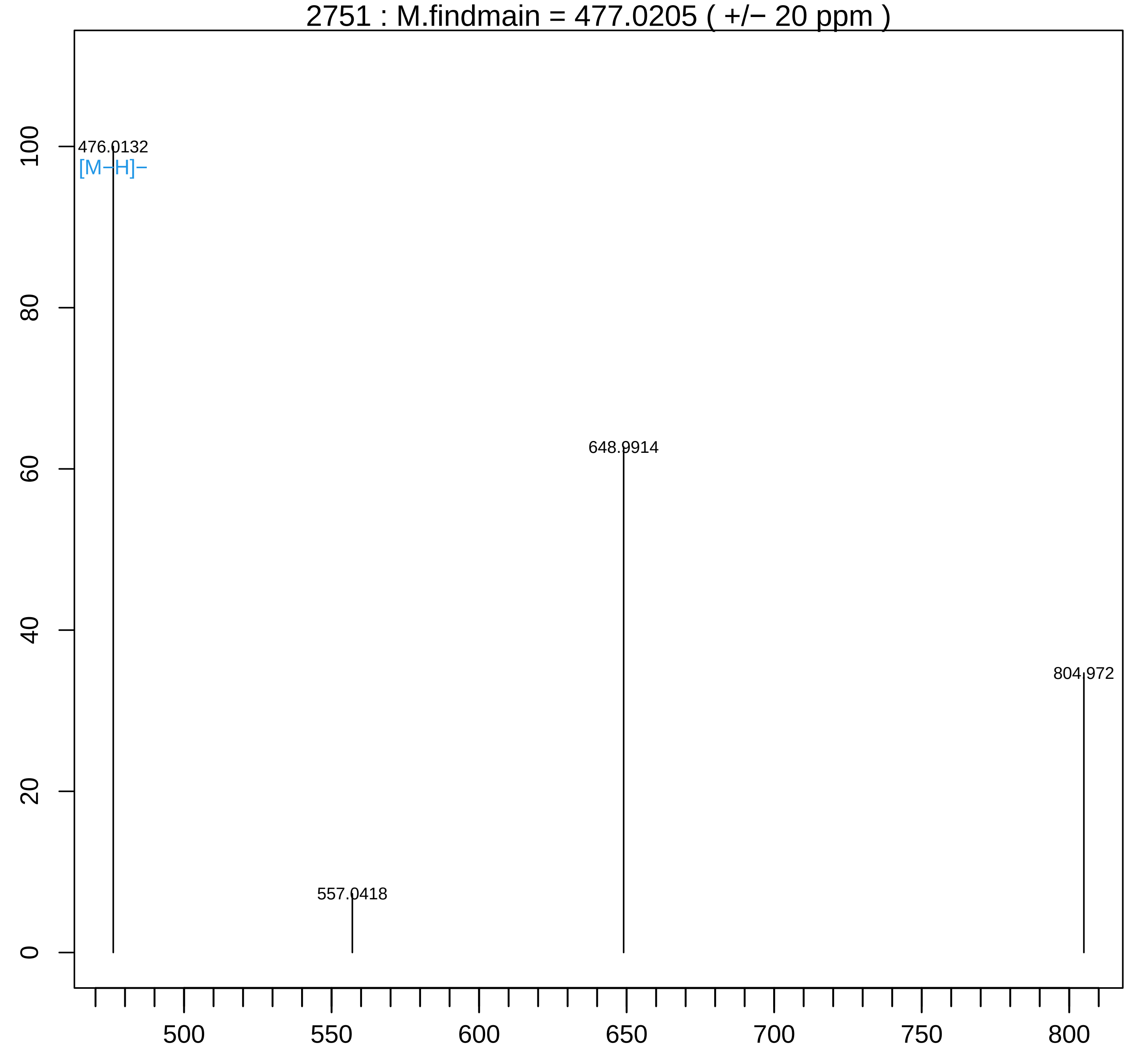

2751 : M.findmain = 477.0205 (+/− 20 ppm)
476.0132
[M−H]−
557.0418
648.9914
804.972
0
20
40
60
80
100
500
550
600
650
700
750
800

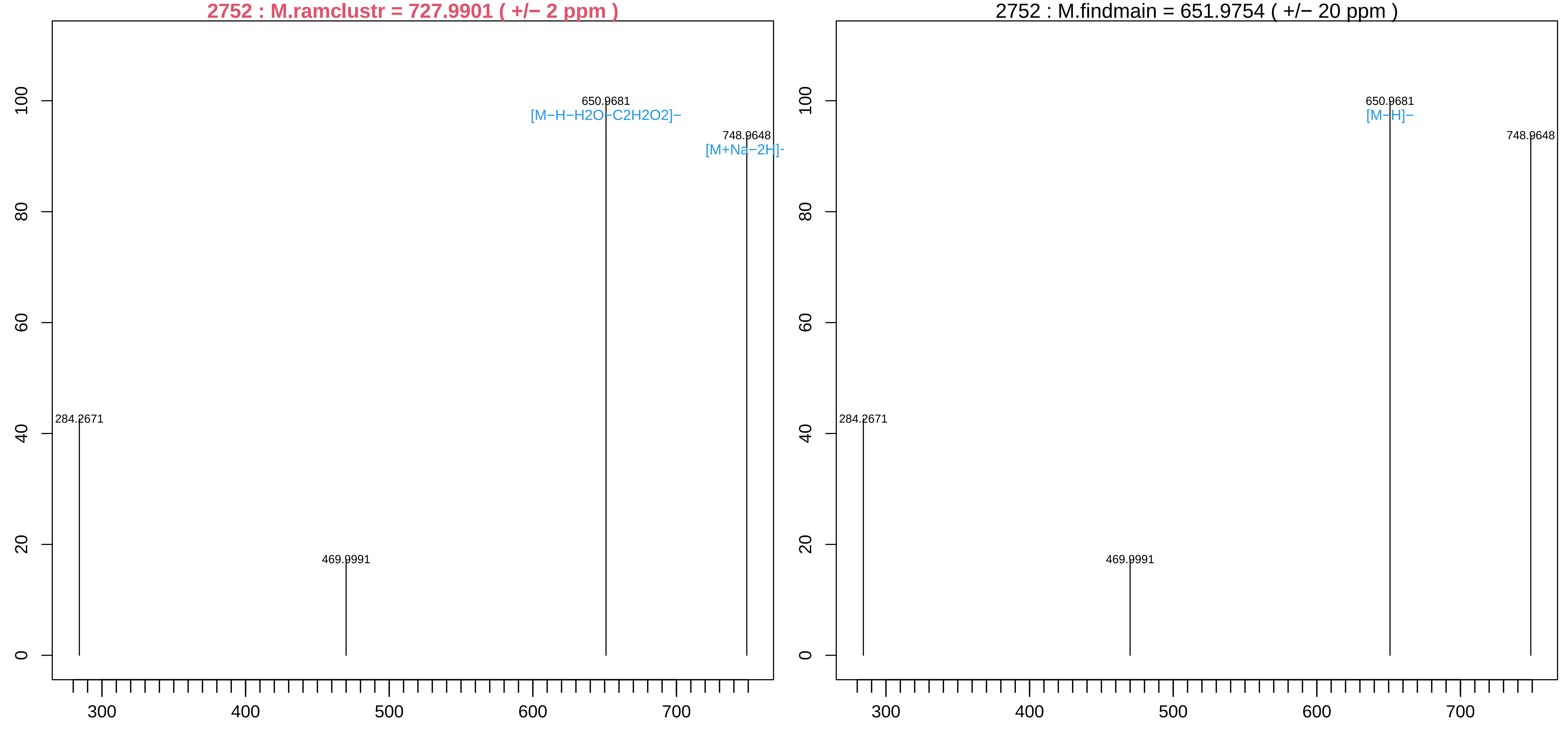
2752 : M.ramclustr = 727.9901 (+/− 2 ppm)
650.9681
[M−H−H2O−C2H2O2]−
748.9648
[M+Na−2H]-
284.2671
469.9991
2752 : M.findmain = 651.9754 (+/− 20 ppm)
650.9681
[M−H]−
748.9648
284.2671
469.9991

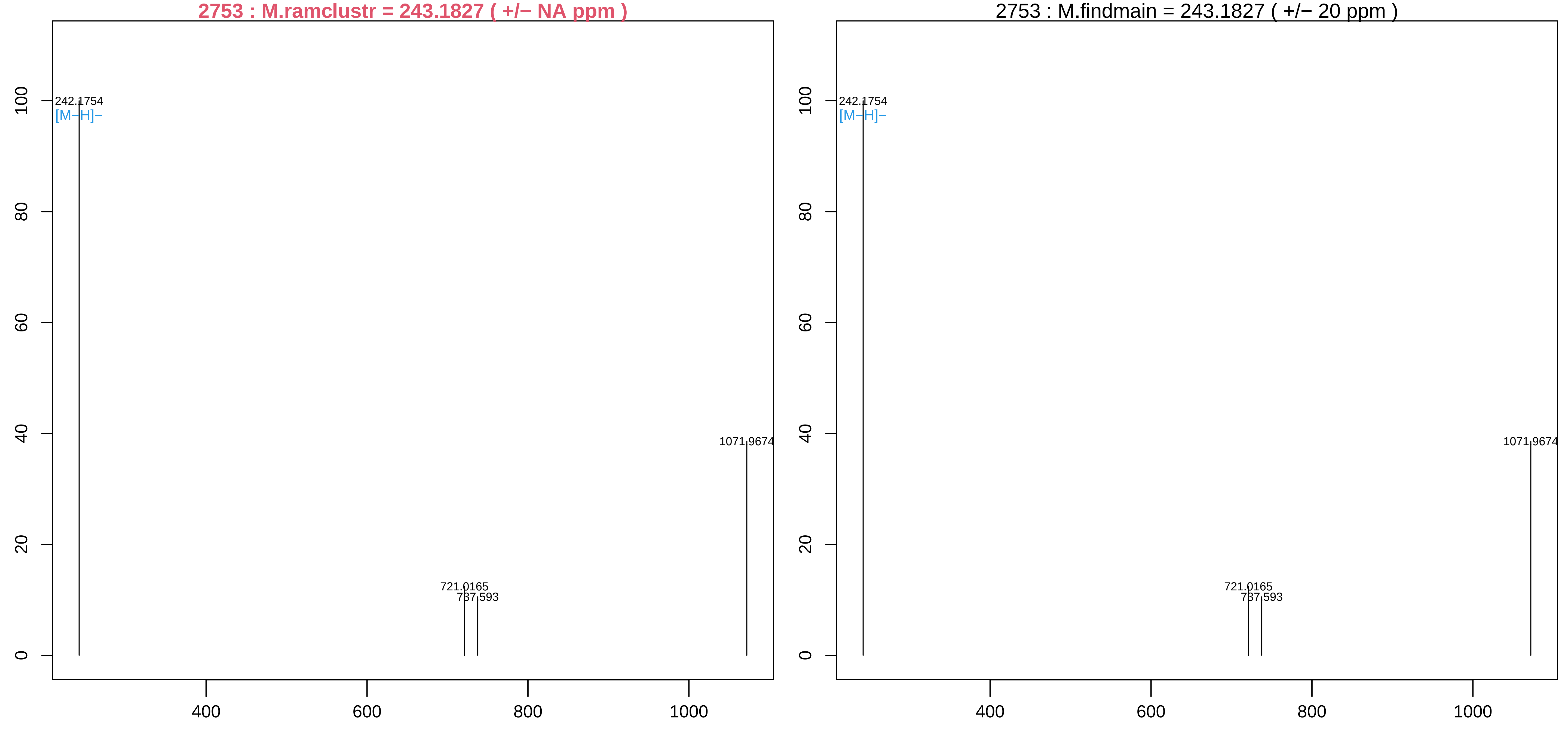

2753 : M.ramclustr = 243.1827 (+/− NA ppm)
242.1754
[M−H]−
721.0165
737.593
1071.9674
0
20
40
60
80
100
400
600
800
1000
2753 : M.findmain = 243.1827 (+/− 20 ppm)
242.1754
[M−H]−
721.0165
737.593
1071.9674
0
20
40
60
80
100
400
600
800
1000

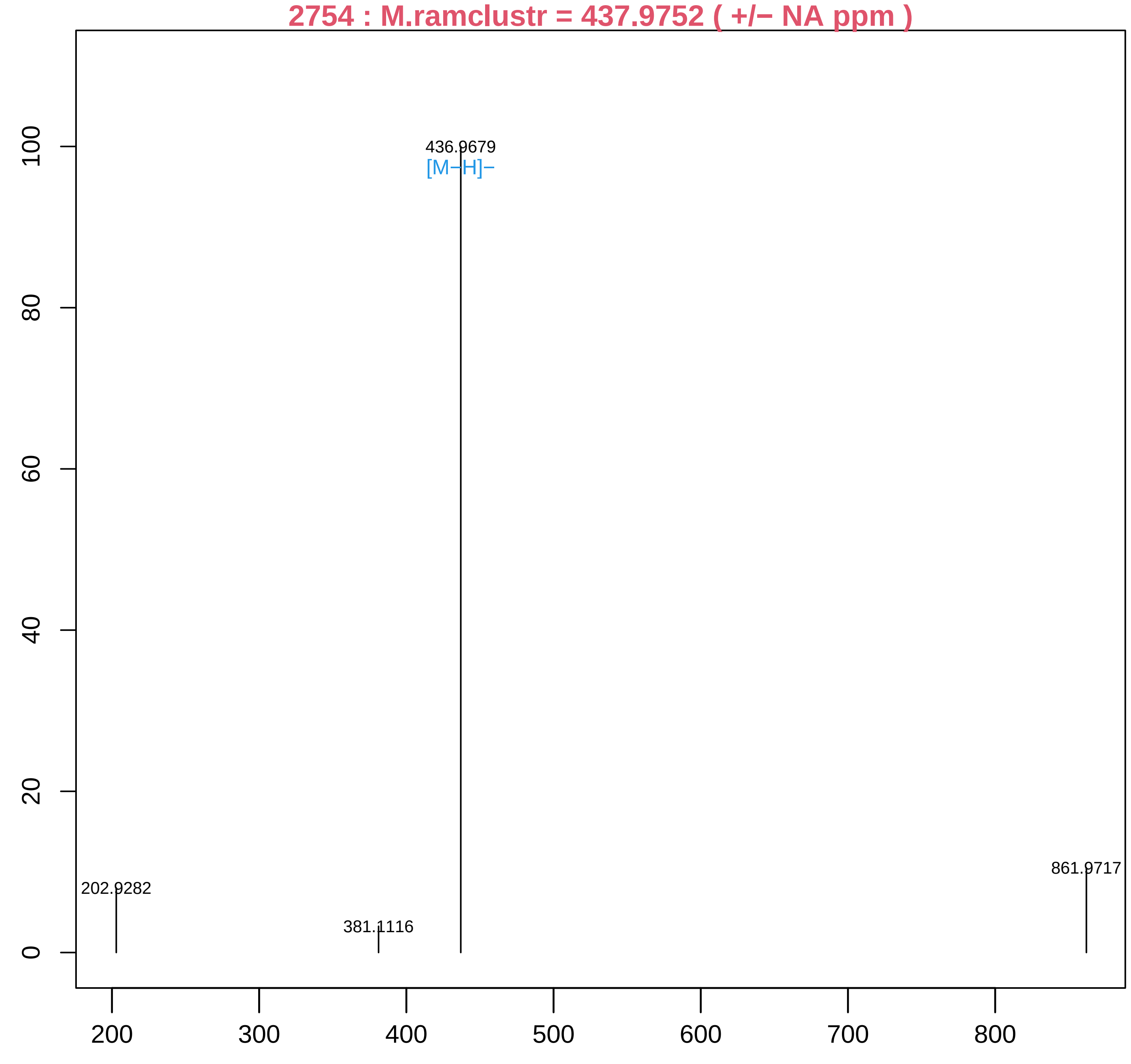
2754 : M.ramclustr = 437.9752 (+/− NA ppm)
436.9679
[M−H]−
202.9282
381.1116
861.9717
0
20
40
60
80
100
200
300
400
500
600
700
800

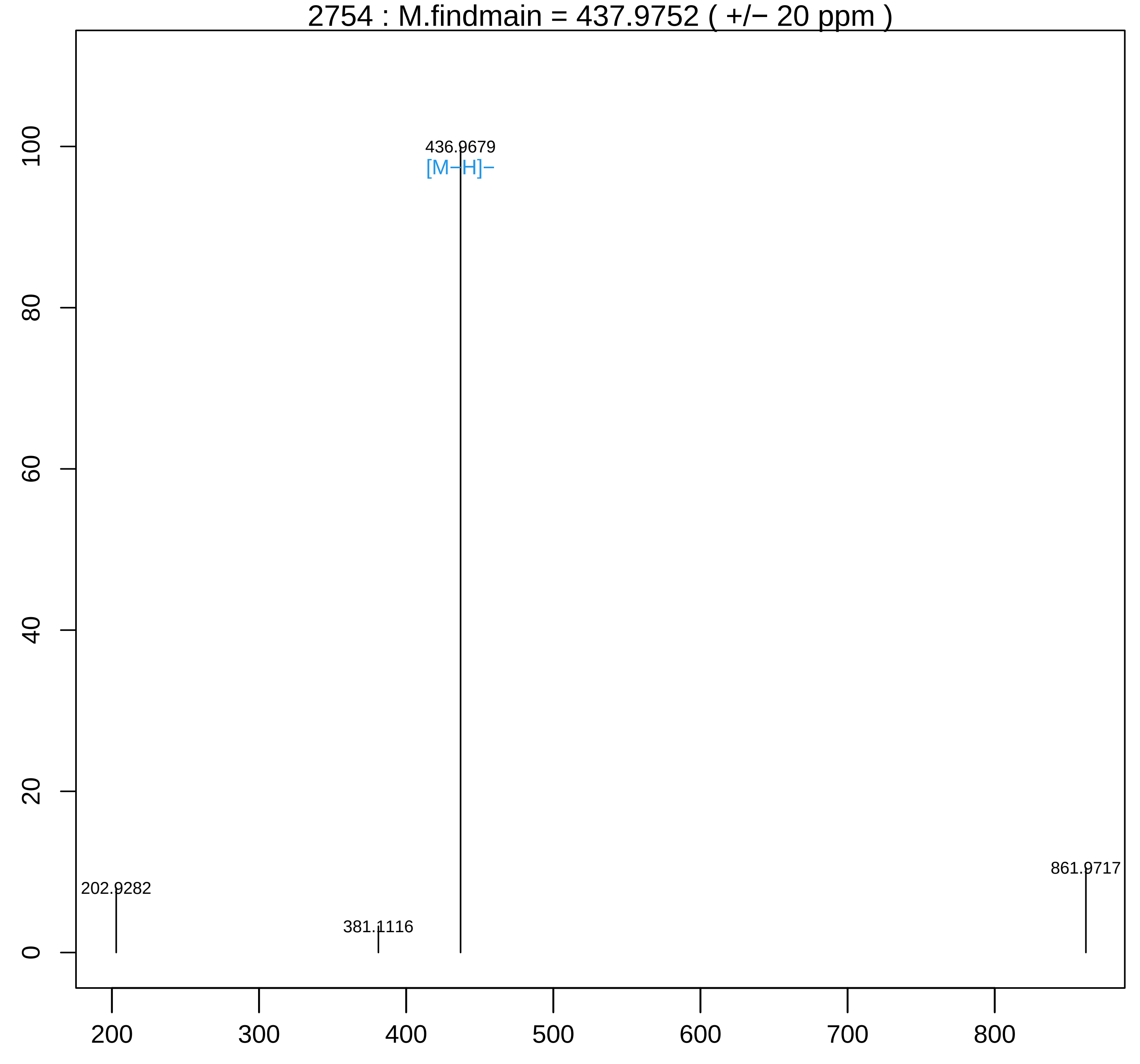
2754 : M.findmain = 437.9752 (+/− 20 ppm)
436.9679
[M−H]−
202.9282
381.1116
861.9717
0
20
40
60
80
100
200
300
400
500
600
700
800

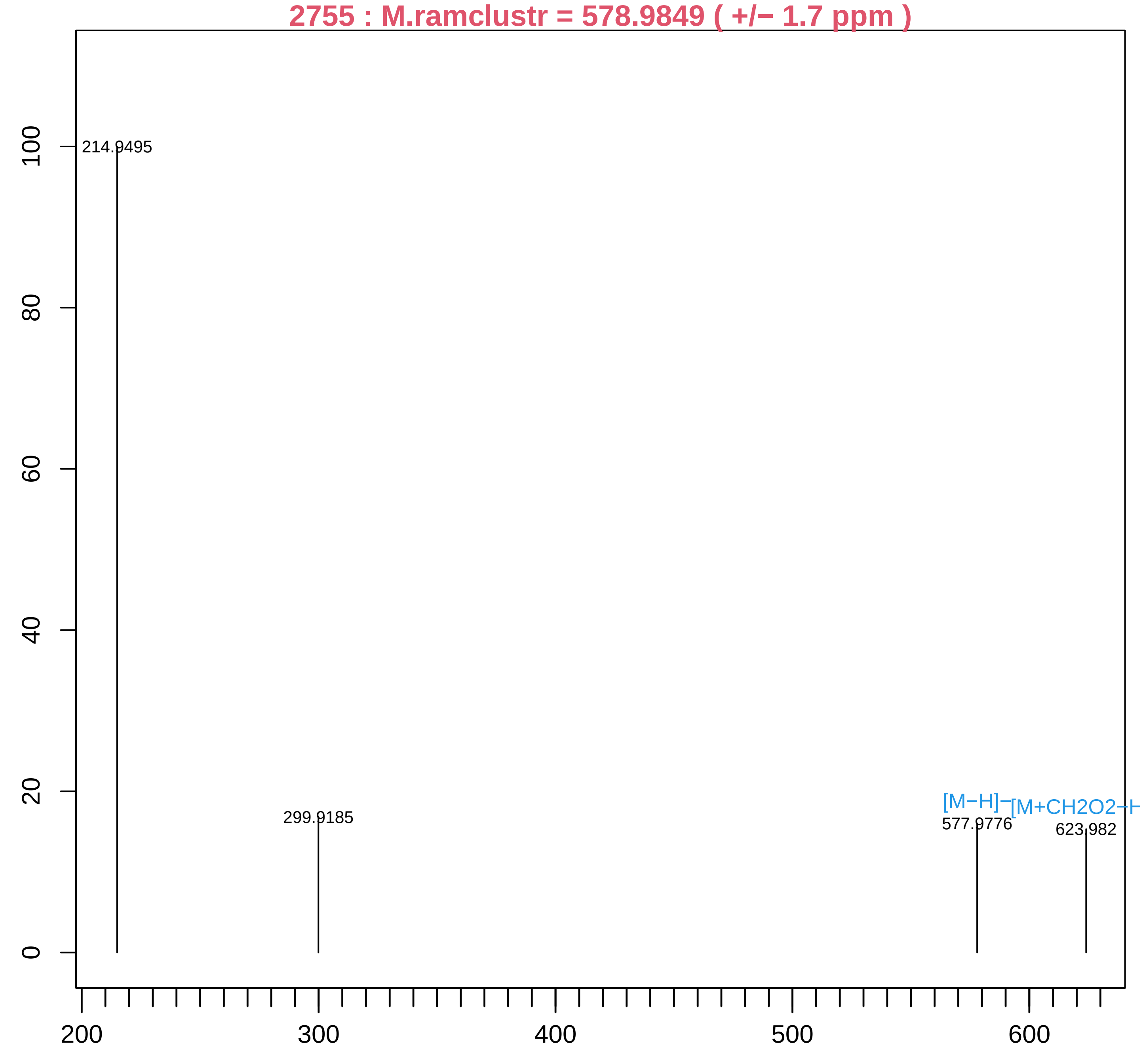
2755 : M.ramclustr = 578.9849 (+/− 1.7 ppm)
214.9495
299.9185
[M−H]−
577.9776
[M+CH2O2−H
623.982
0
20
40
60
80
100
200
300
400
500
600

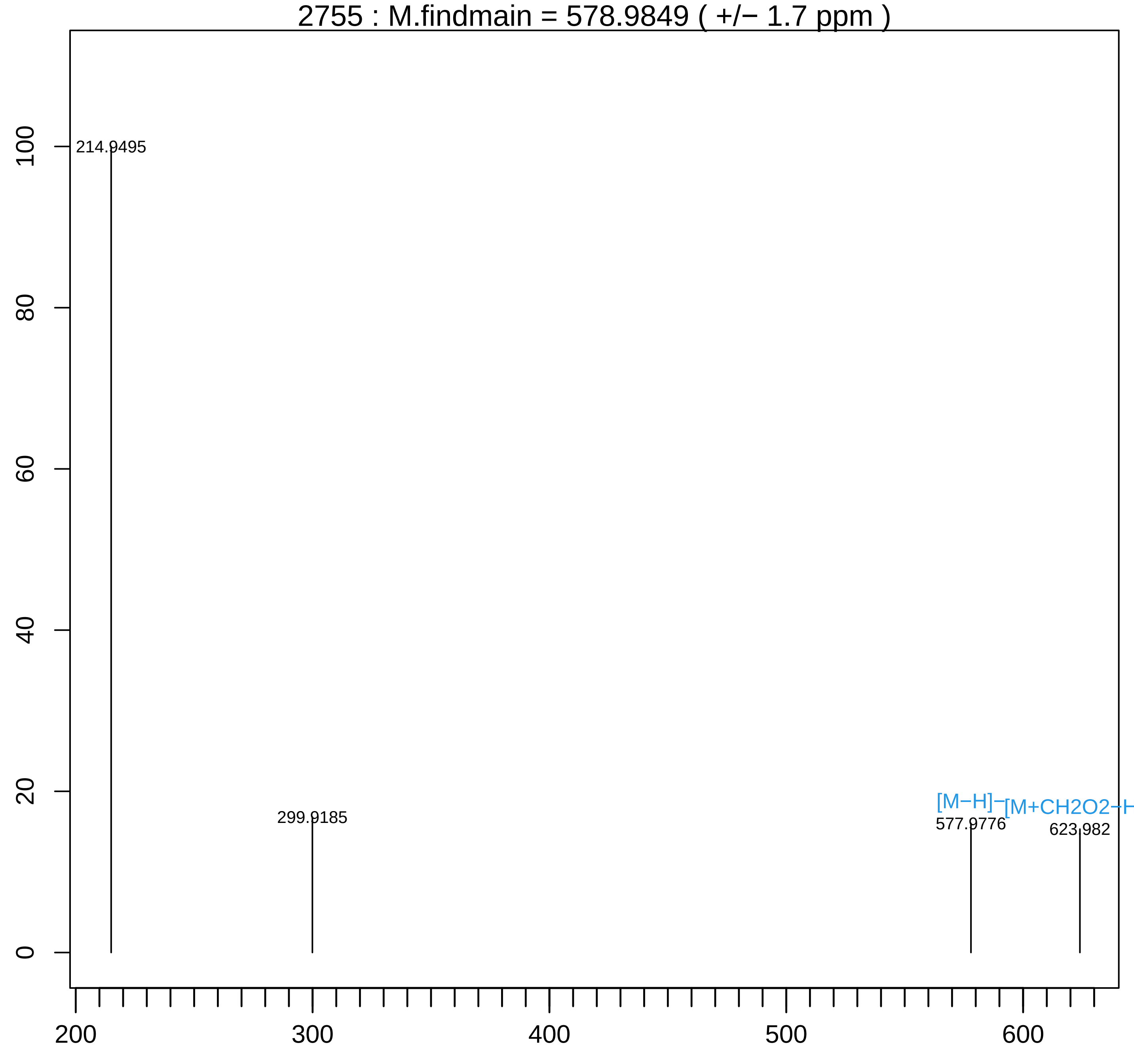
2755 : M.findmain = 578.9849 (+/− 1.7 ppm)
214.9495
299.9185
[M−H]−
577.9776
[M+CH2O2−H
623.982
0
20
40
60
80
100
200
300
400
500
600

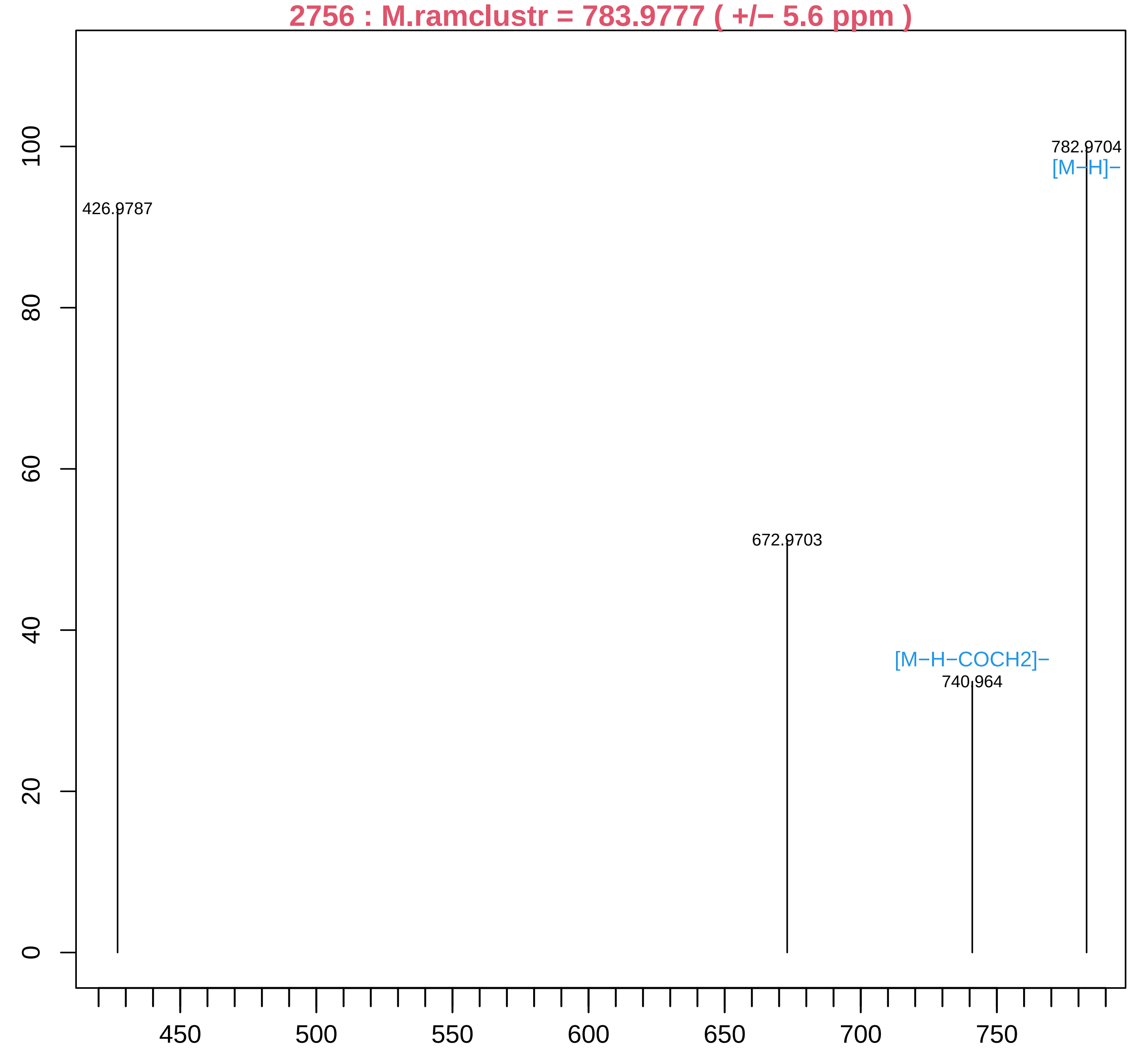
2756 : M.ramclustr = 783.9777 (+/− 5.6 ppm)
100
80
60
40
20
0
450
500
550
600
650
700
750
782.9704
[M−H]−
426.9787
672.9703
[M−H−COCH2]−
740.964

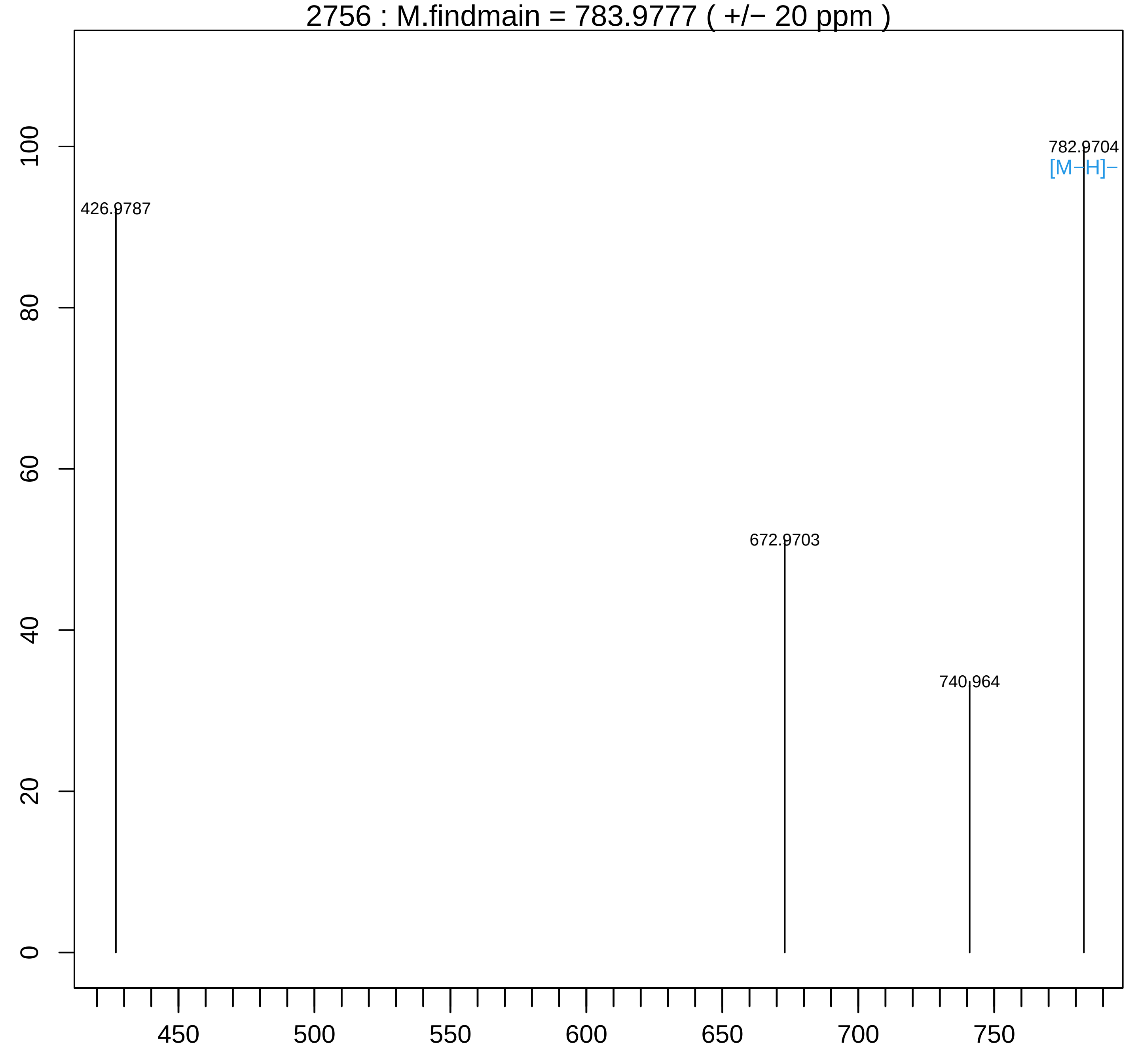
2756 : M.findmain = 783.9777 (+/− 20 ppm)
100
80
60
40
20
0
450
500
550
600
650
700
750
782.9704
[M−H]−
426.9787
672.9703
740.964

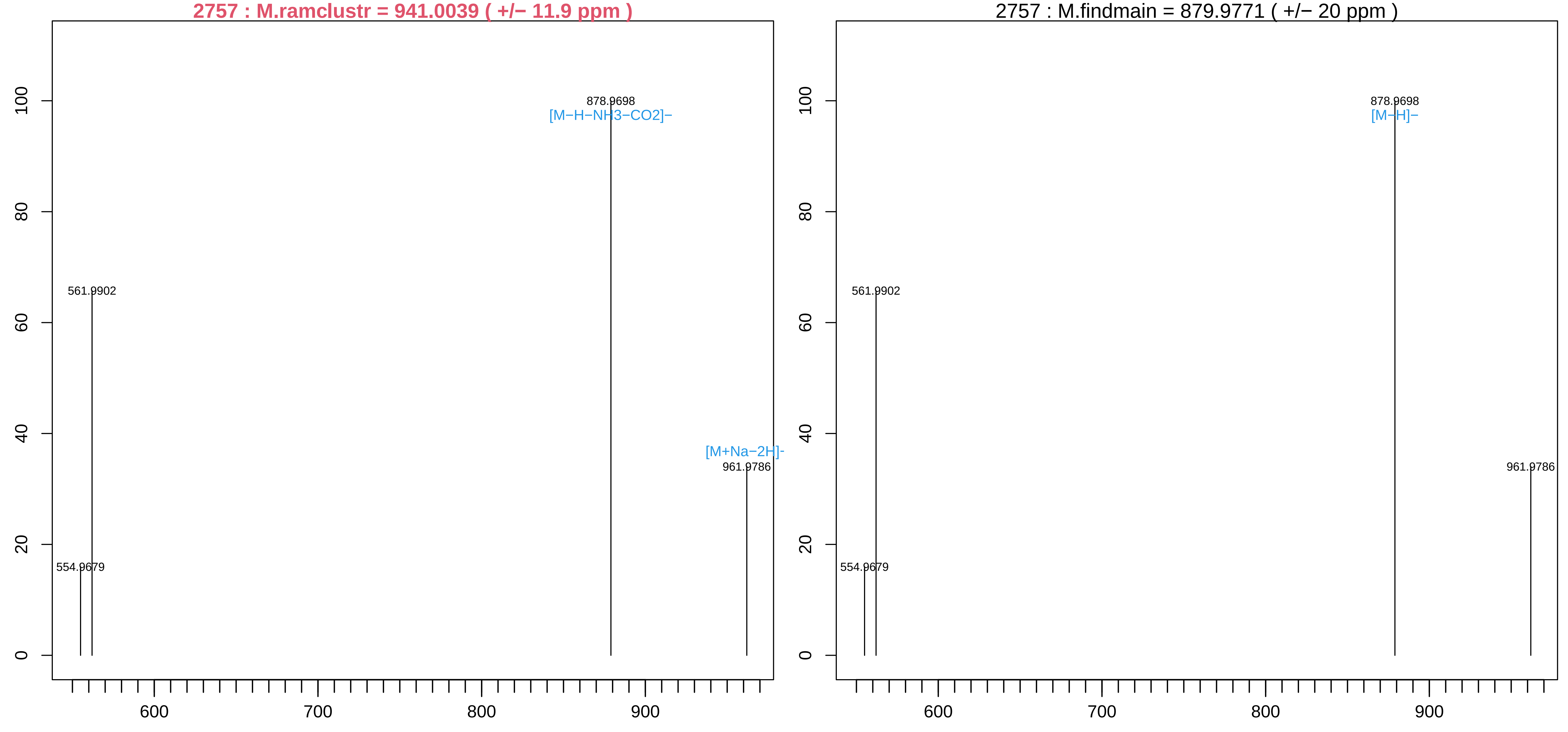

2757 : M.ramclustr = 941.0039 (+/− 11.9 ppm)
878.9698
[M−H−NH3−CO2]−
561.9902
[M+Na−2H]-
961.9786
554.9679
0
20
40
60
80
100
600
700
800
900
2757 : M.findmain = 879.9771 (+/− 20 ppm)
878.9698
[M−H]−
561.9902
961.9786
554.9679
0
20
40
60
80
100
600
700
800
900

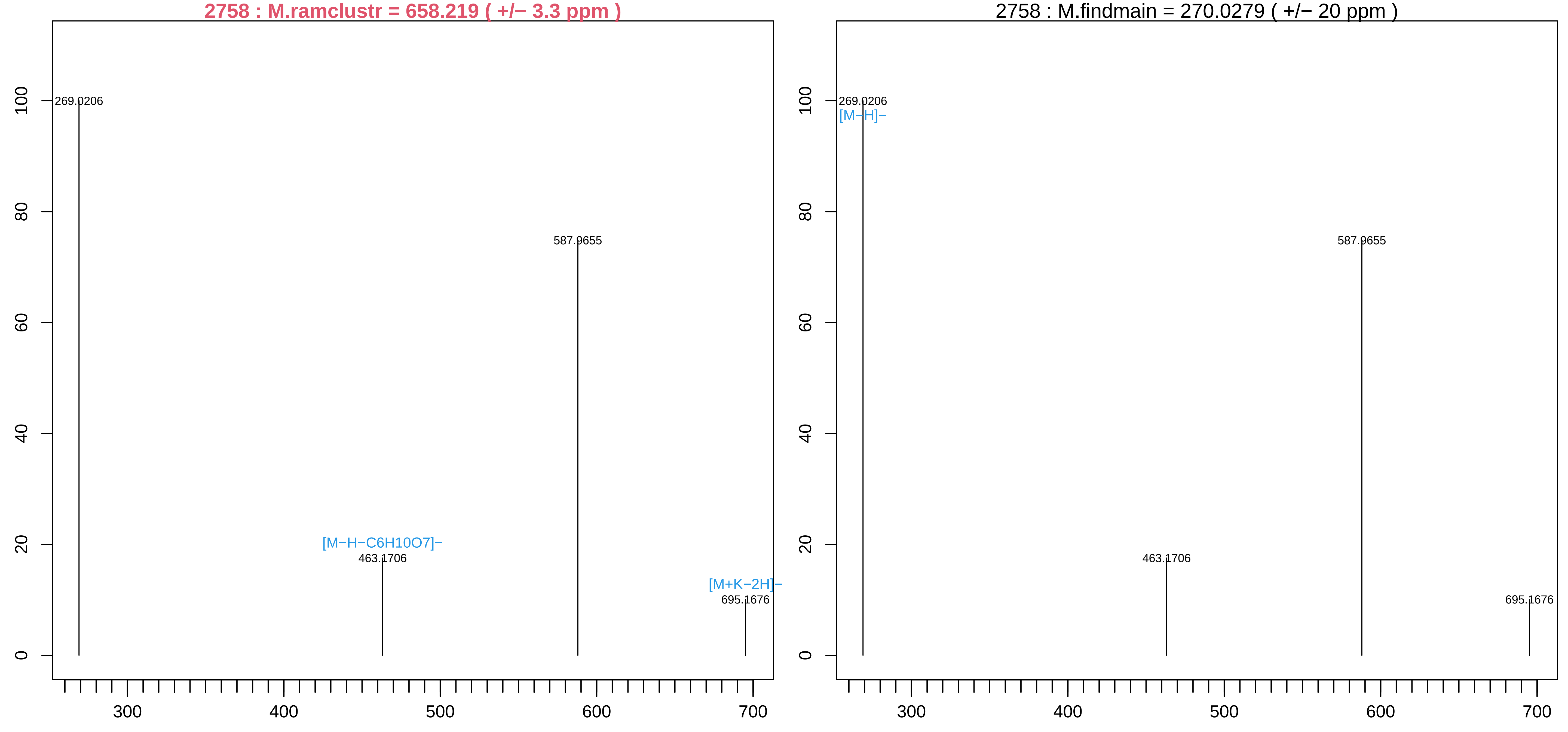

2758 : M.ramclustr = 658.219 (+/− 3.3 ppm)
269.0206
[M−H−C6H10O7]−
463.1706
587.9655
[M+K−2H]−
695.1676
2758 : M.findmain = 270.0279 (+/− 20 ppm)
269.0206
[M−H]−
463.1706
587.9655
695.1676
0
20
40
60
80
100
300
400
500
600
700

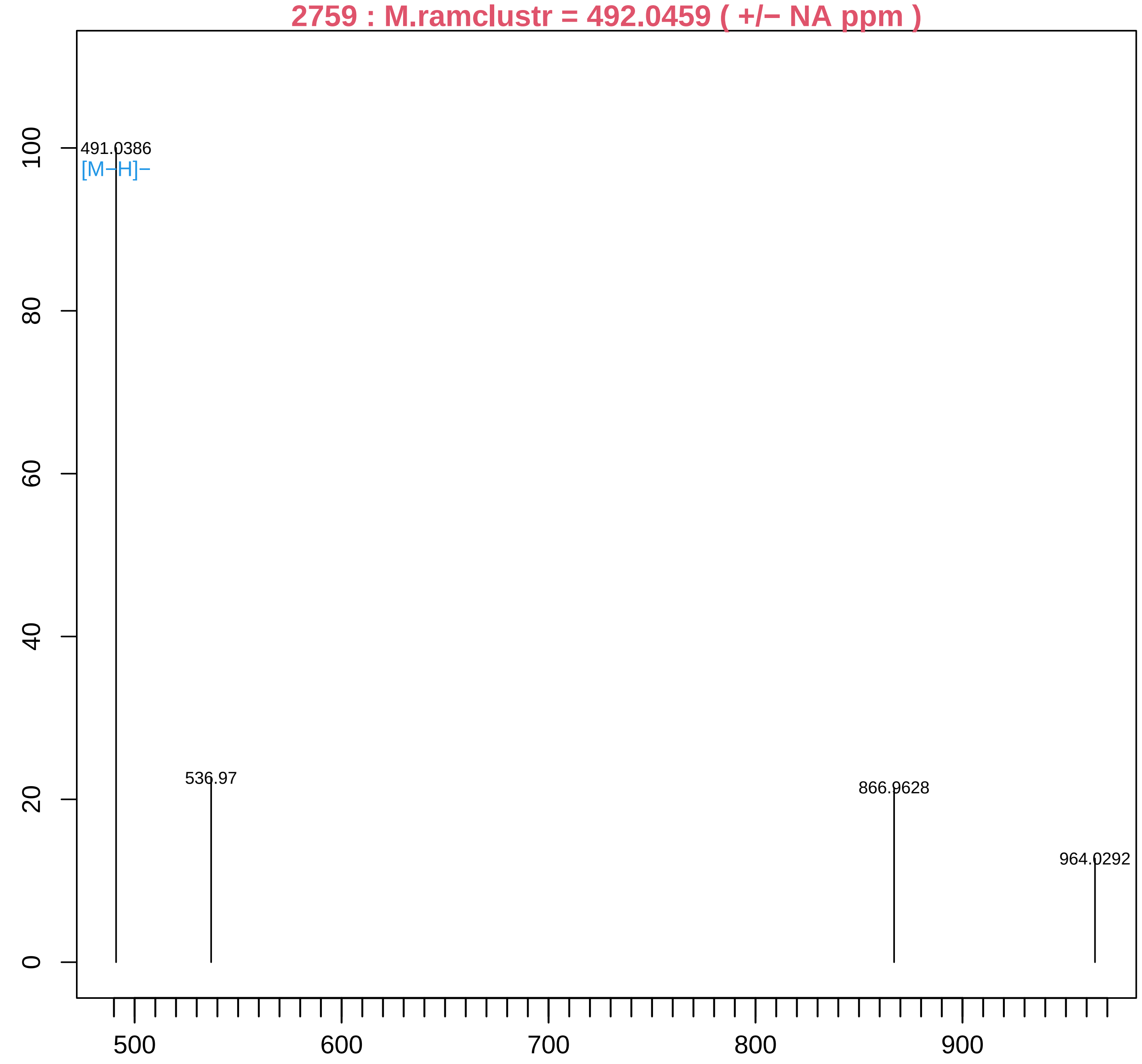
2759 : M.ramclustr = 492.0459 (+/− NA ppm)
491.0386
[M−H]−
536.97
866.9628
964.0292
0
20
40
60
80
100
500
600
700
800
900

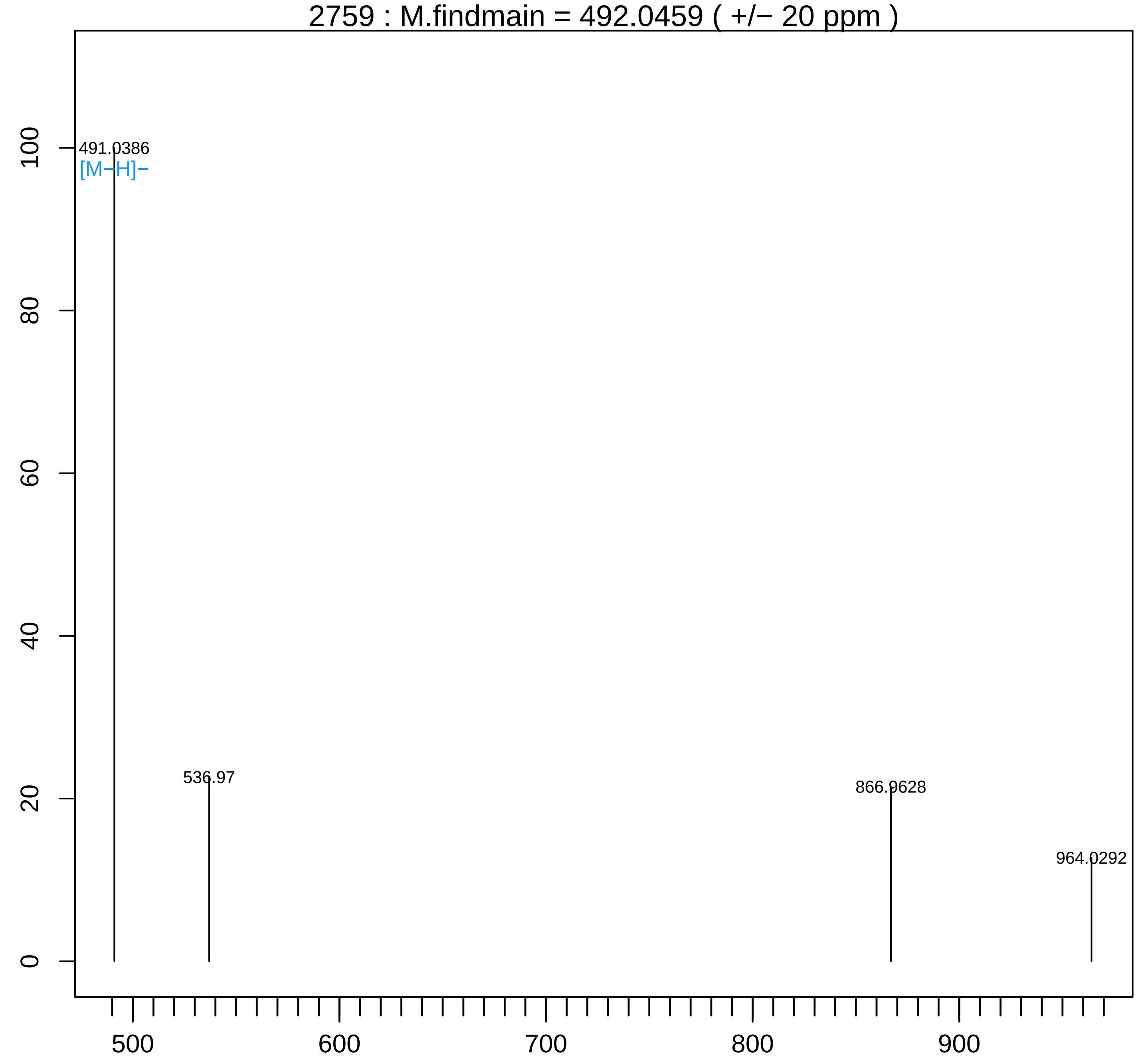
2759 : M.findmain = 492.0459 (+/− 20 ppm)
491.0386
[M−H]−
536.97
866.9628
964.0292
0
20
40
60
80
100
500
600
700
800
900

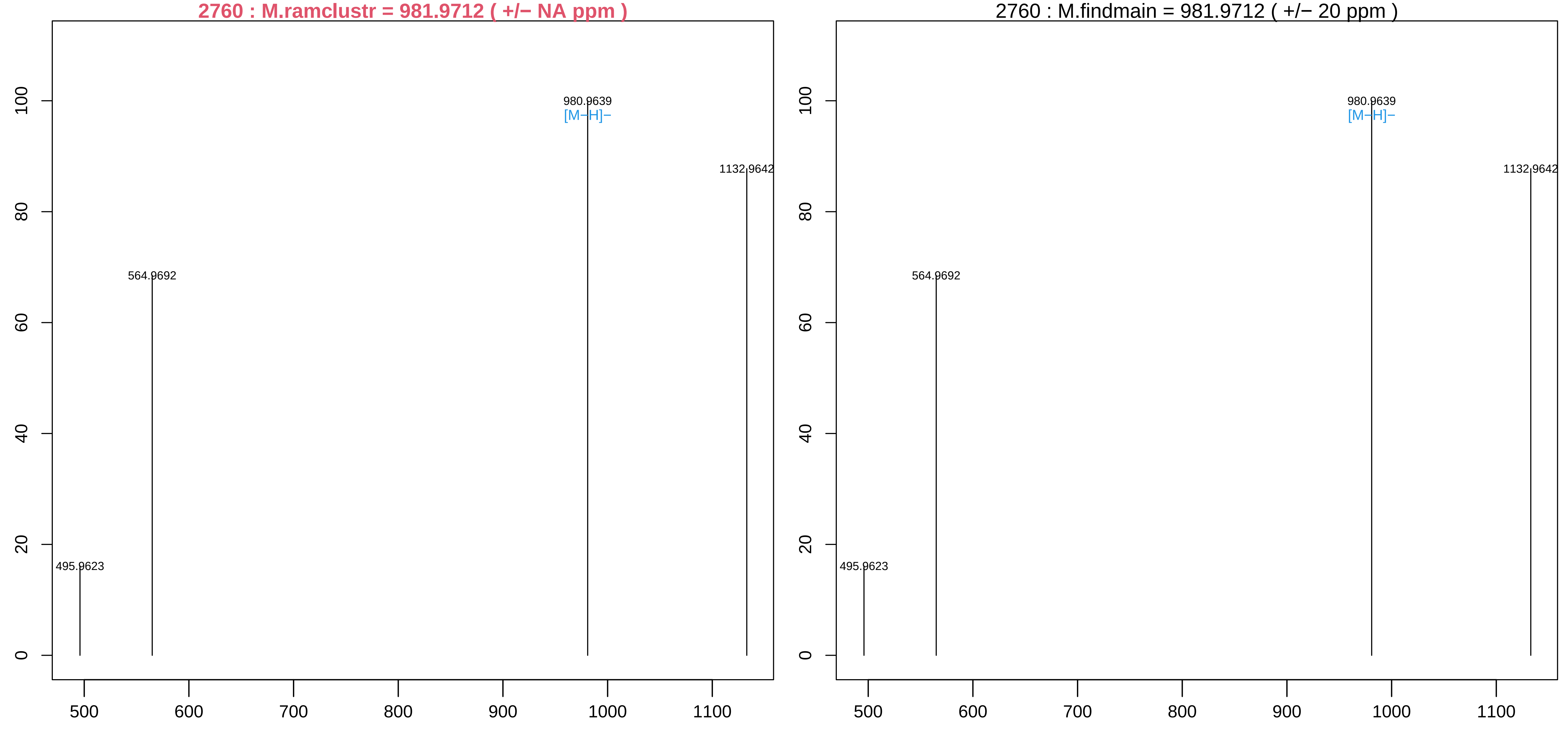
2760 : M.ramclustr = 981.9712 (+/− NA ppm)
2760 : M.findmain = 981.9712 (+/− 20 ppm)
980.9639
[M−H]−
1132.9642
564.9692
495.9623
0
20
40
60
80
100
500
600
700
800
900
1000
1100

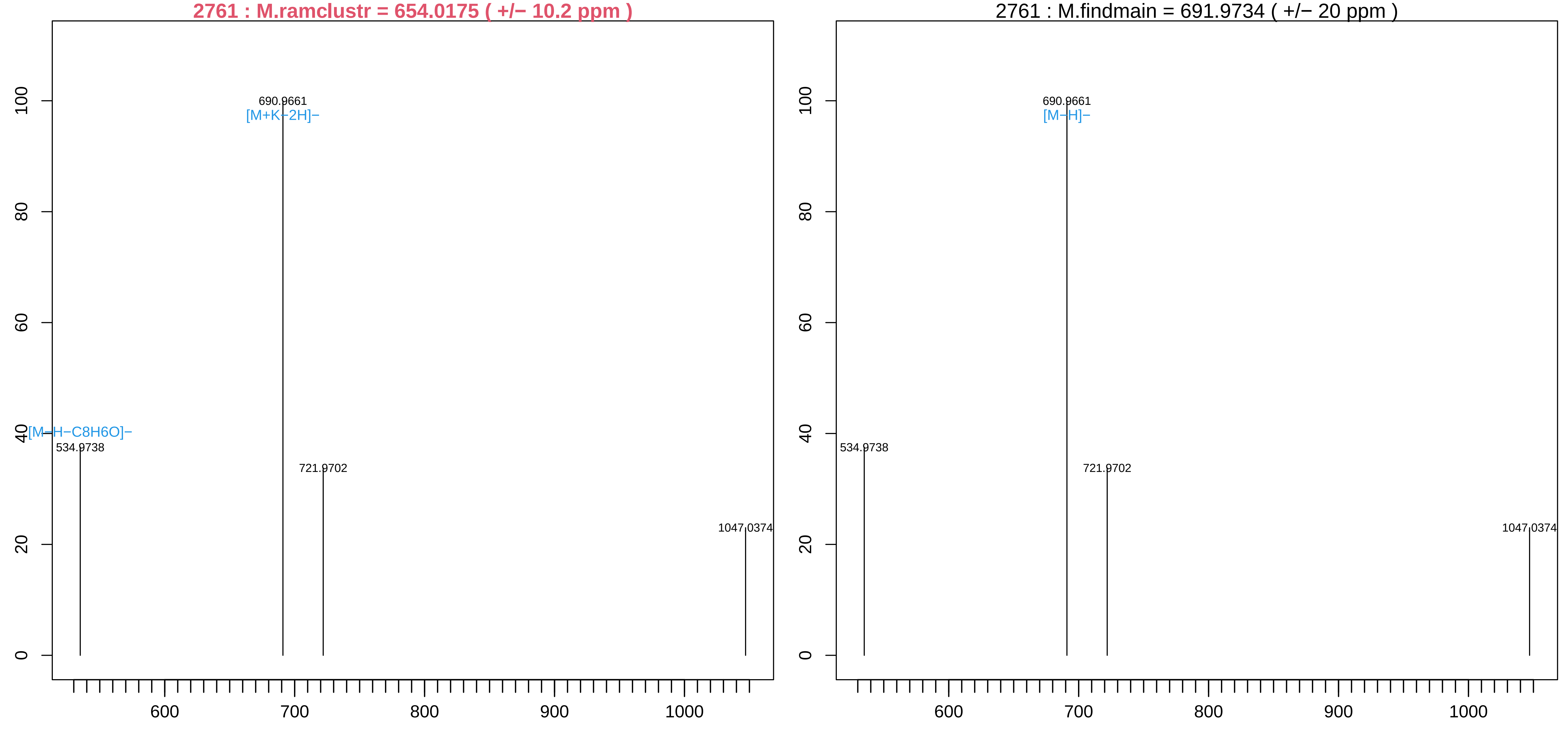
2761 : M.ramclustr = 654.0175 (+/− 10.2 ppm)
690.9661
[M+K−2H]−
[M−H−C8H6O]−
534.9738
721.9702
1047.0374
0
20
40
60
80
100
600
700
800
900
1000
2761 : M.findmain = 691.9734 (+/− 20 ppm)
690.9661
[M−H]−
534.9738
721.9702
1047.0374

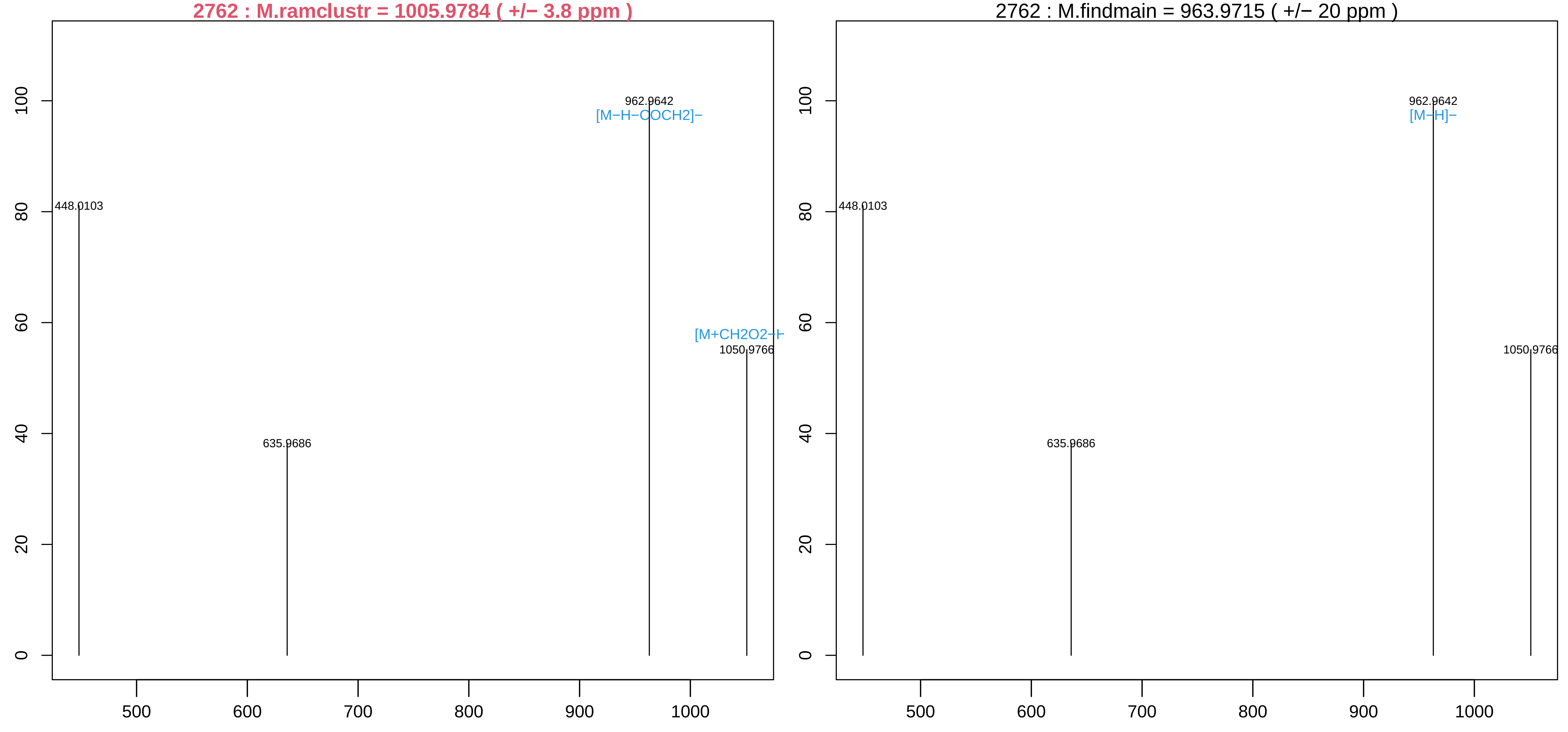
2762 : M.ramclustr = 1005.9784 (+/− 3.8 ppm)
962.9642
[M−H−COCH2]−
448.0103
635.9686
[M+CH2O2−H
1050.9766
2762 : M.findmain = 963.9715 (+/− 20 ppm)
962.9642
[M−H]−
448.0103
635.9686
1050.9766
0
20
40
60
80
100
500
600
700
800
900
1000

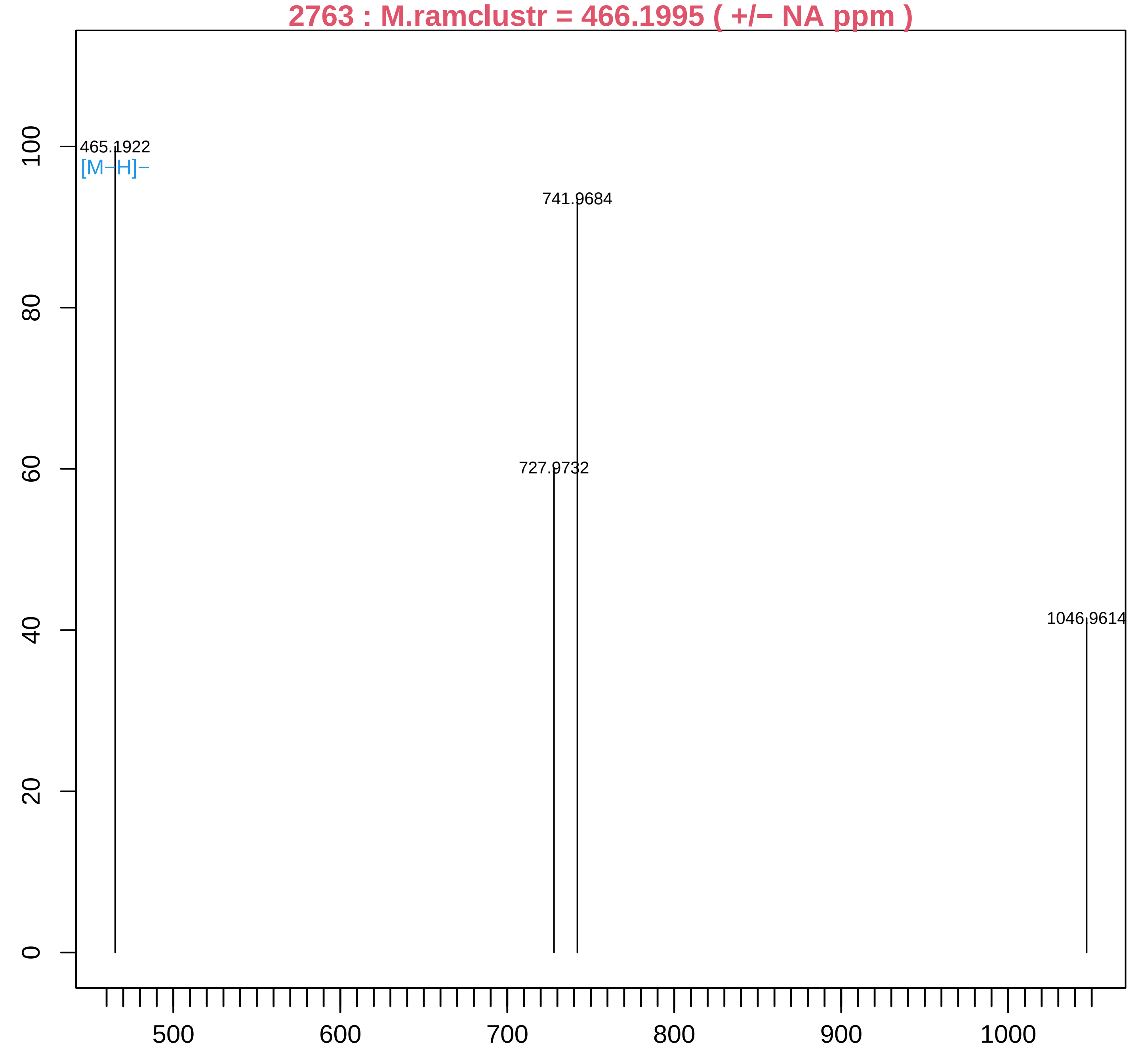
2763 : M.ramclustr = 466.1995 (+/− NA ppm)
465.1922
[M−H]−
741.9684
727.9732
1046.9614
0
20
40
60
80
100
500
600
700
800
900
1000

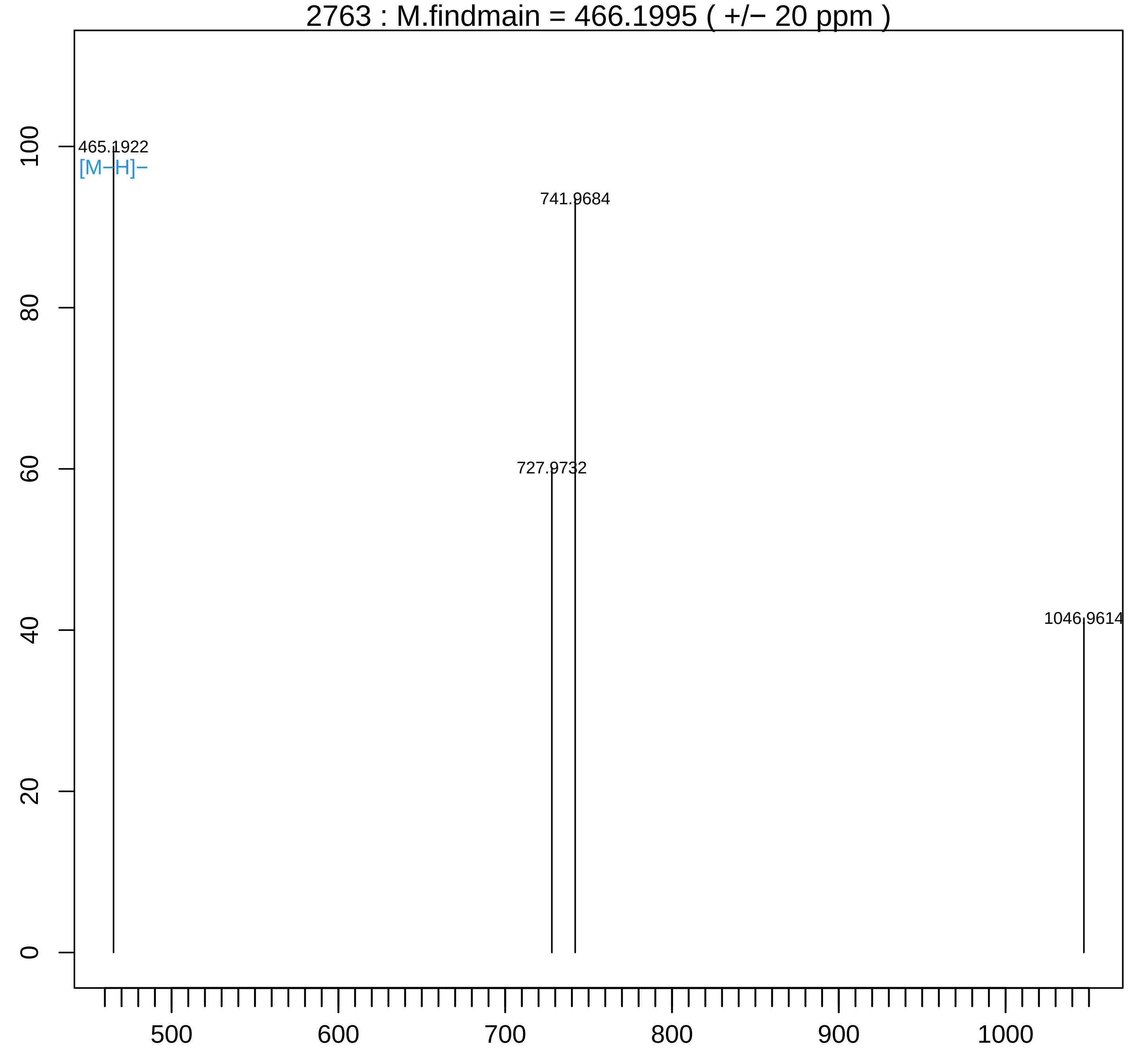
2763 : M.findmain = 466.1995 (+/− 20 ppm)
465.1922
[M−H]−
741.9684
727.9732
1046.9614
0
20
40
60
80
100
500
600
700
800
900
1000

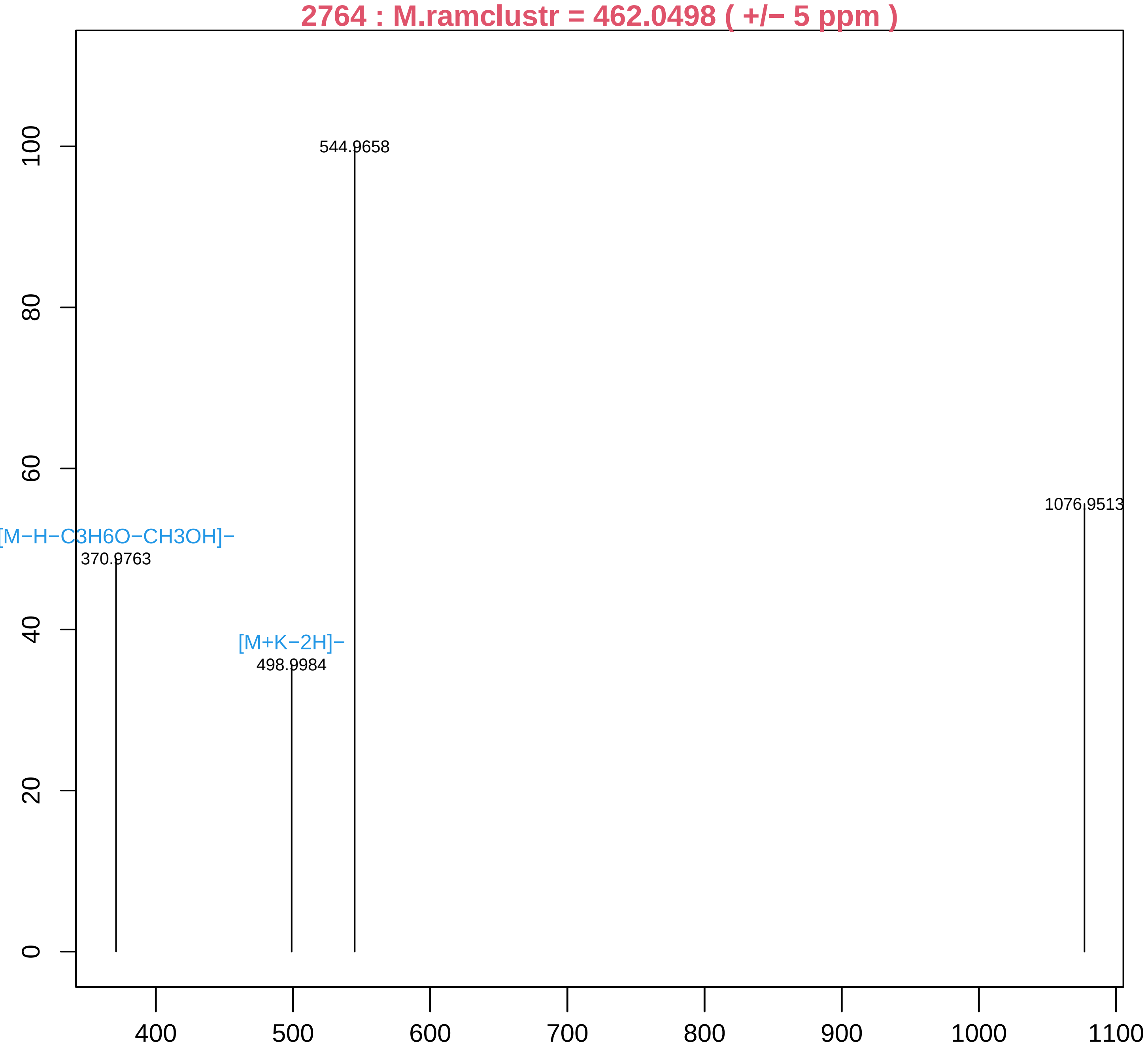

2764 : M.ramclustr = 462.0498 (+/− 5 ppm)
544.9658
1076.9513
[M−H−C3H6O−CH3OH]−
370.9763
[M+K−2H]−
498.9984
0
20
40
60
80
100
400
500
600
700
800
900
1000
1100

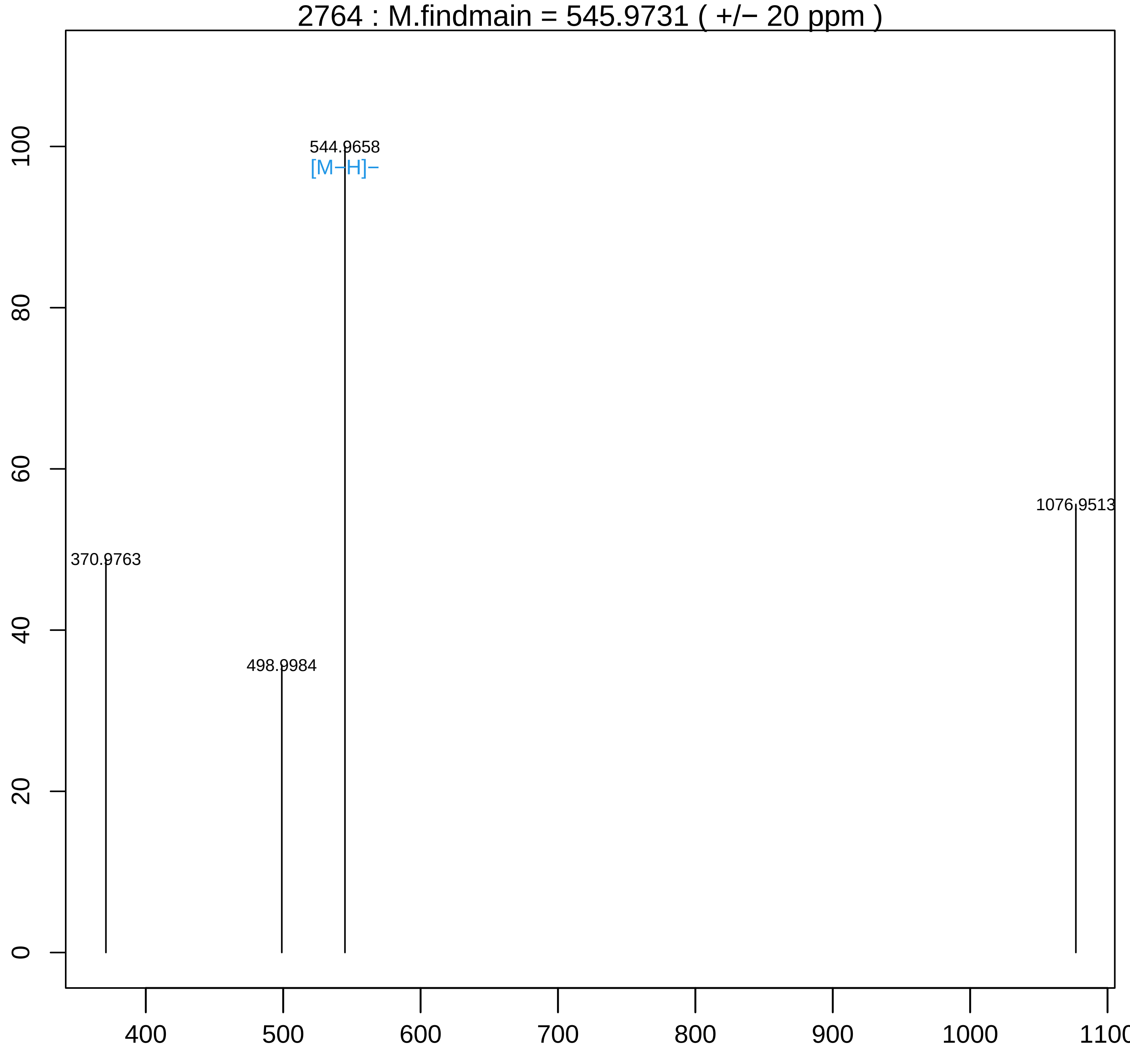

2764 : M.findmain = 545.9731 (+/− 20 ppm)
544.9658
[M−H]−
1076.9513
370.9763
498.9984
0
20
40
60
80
100
400
500
600
700
800
900
1000
1100

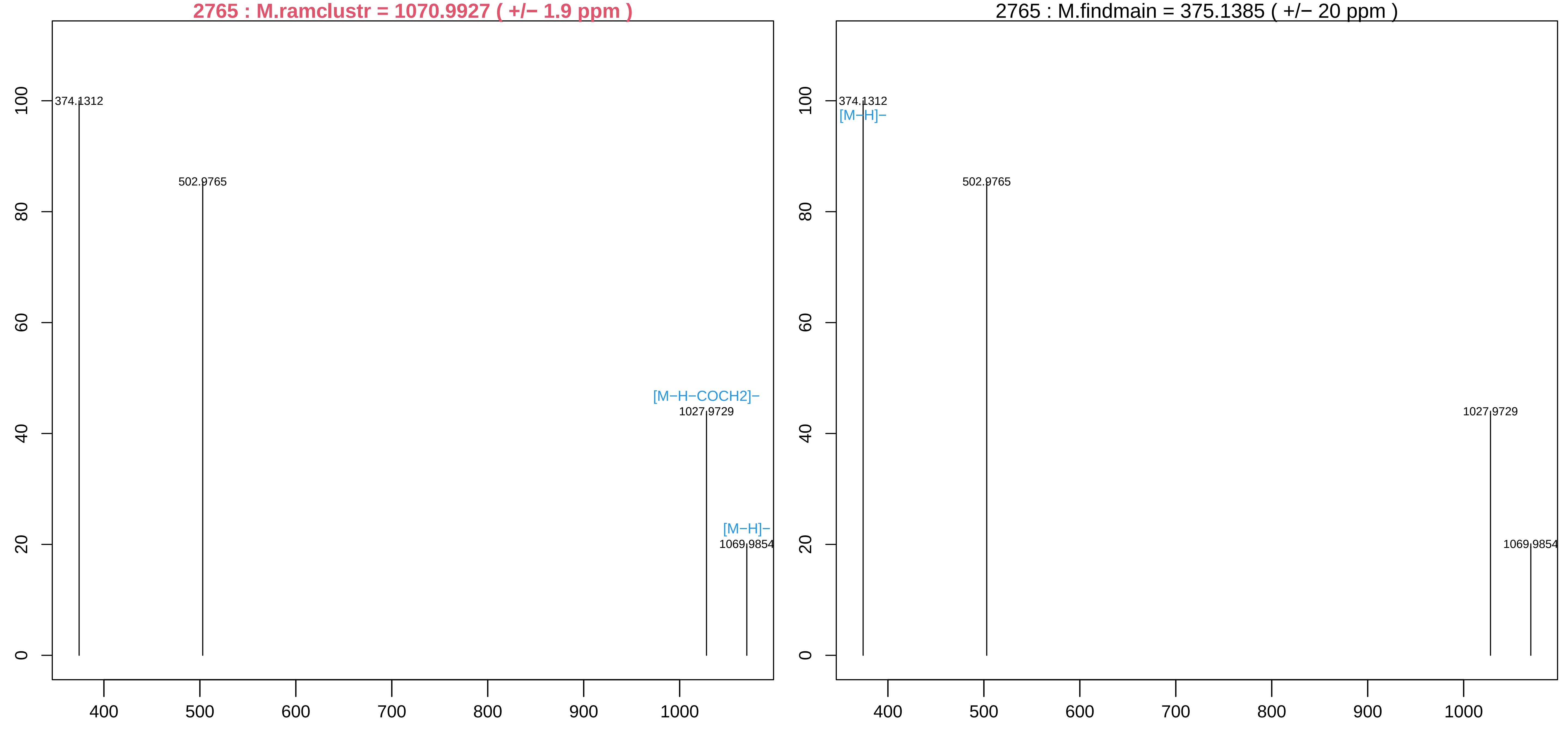
2765 : M.ramclustr = 1070.9927 (+/− 1.9 ppm)
374.1312
502.9765
[M−H−COCH2]−
1027.9729
[M−H]−
1069.9854
2765 : M.findmain = 375.1385 (+/− 20 ppm)
374.1312
[M−H]−
502.9765
1027.9729
1069.9854

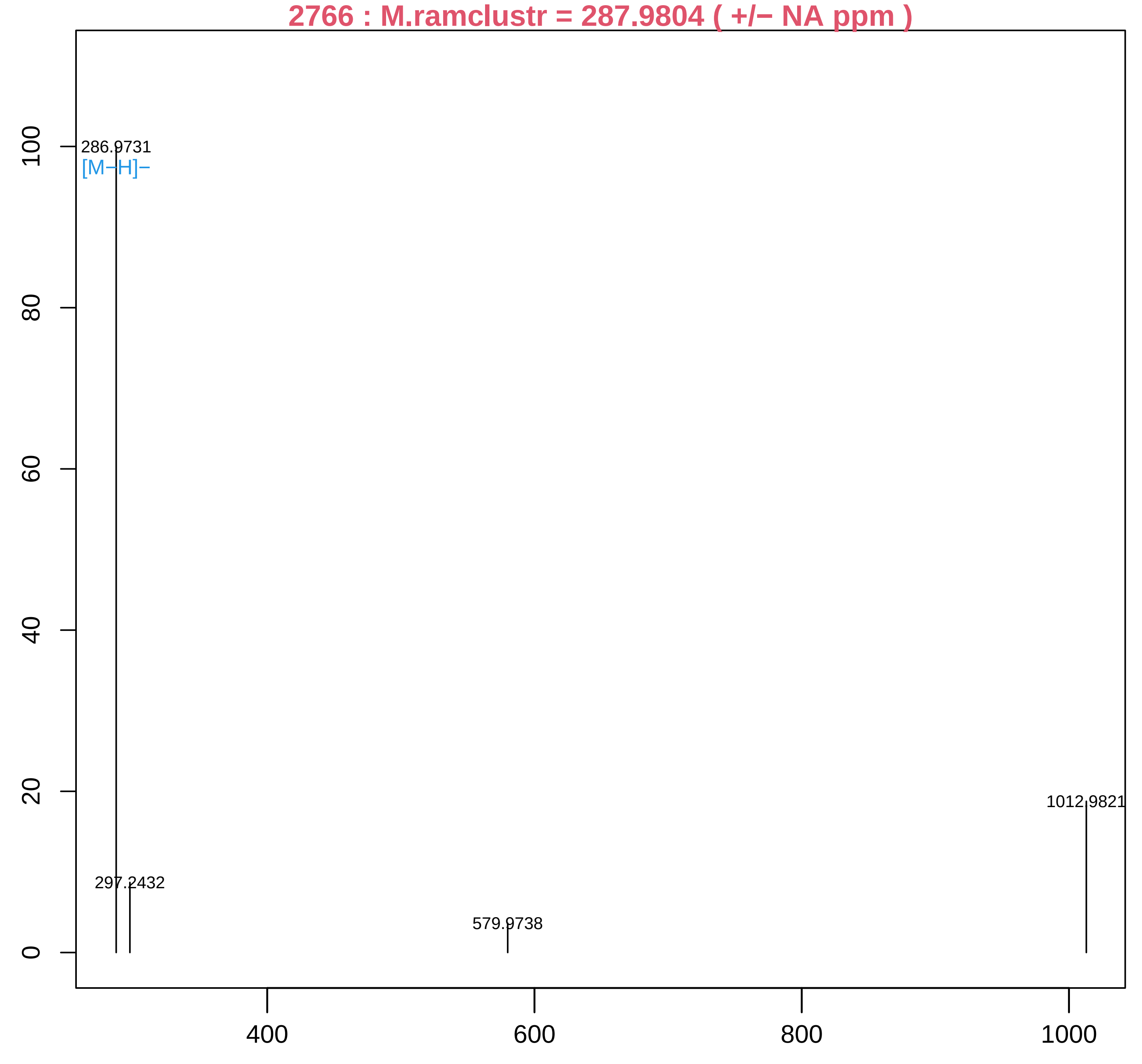
2766 : M.ramclustr = 287.9804 (+/− NA ppm)
286.9731
[M−H]−
297.2432
579.9738
1012.9821
0
20
40
60
80
100
400
600
800
1000
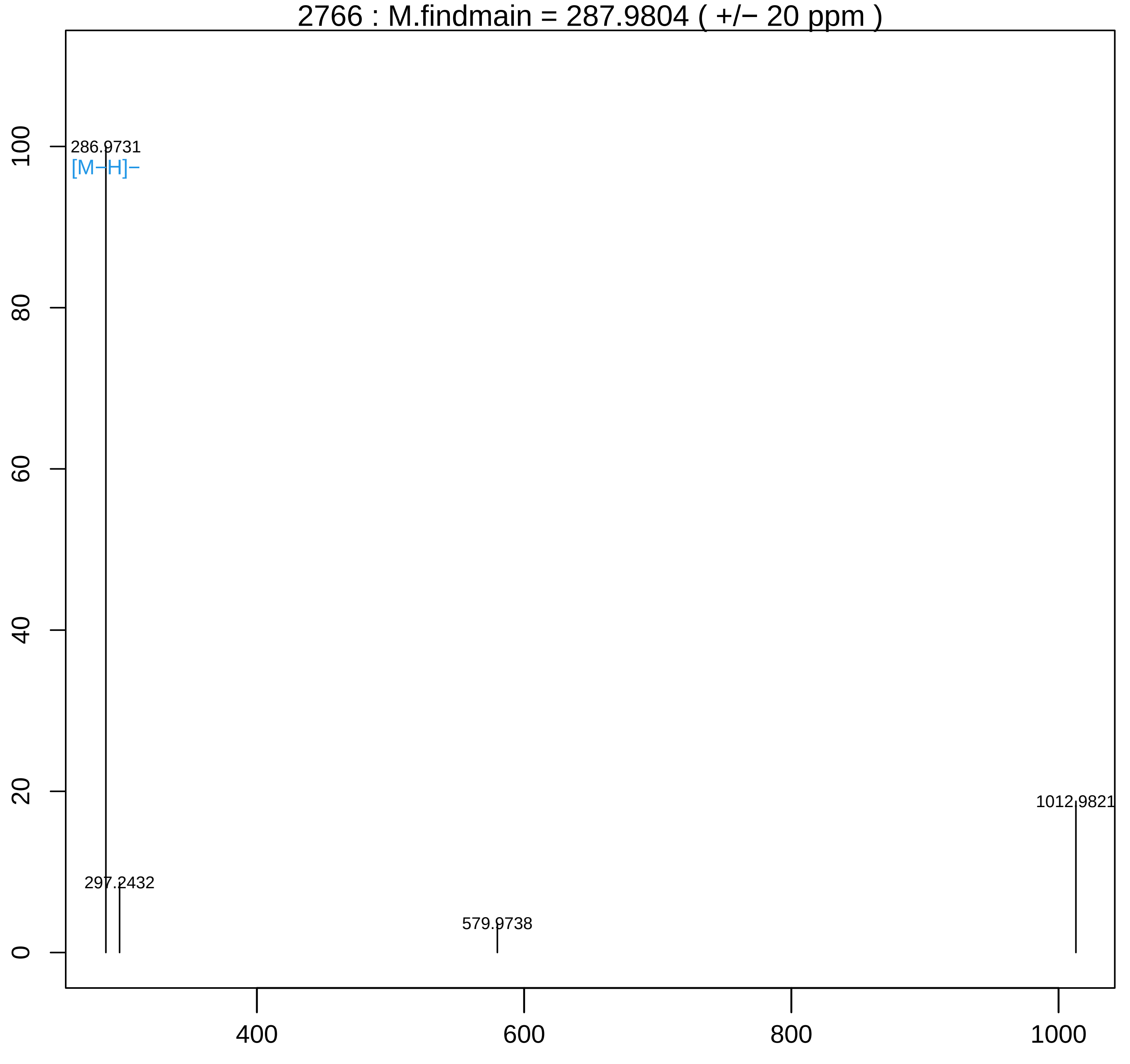
2766 : M.findmain = 287.9804 (+/− 20 ppm)
286.9731
[M−H]−
297.2432
579.9738
1012.9821
0
20
40
60
80
100
400
600
800
1000

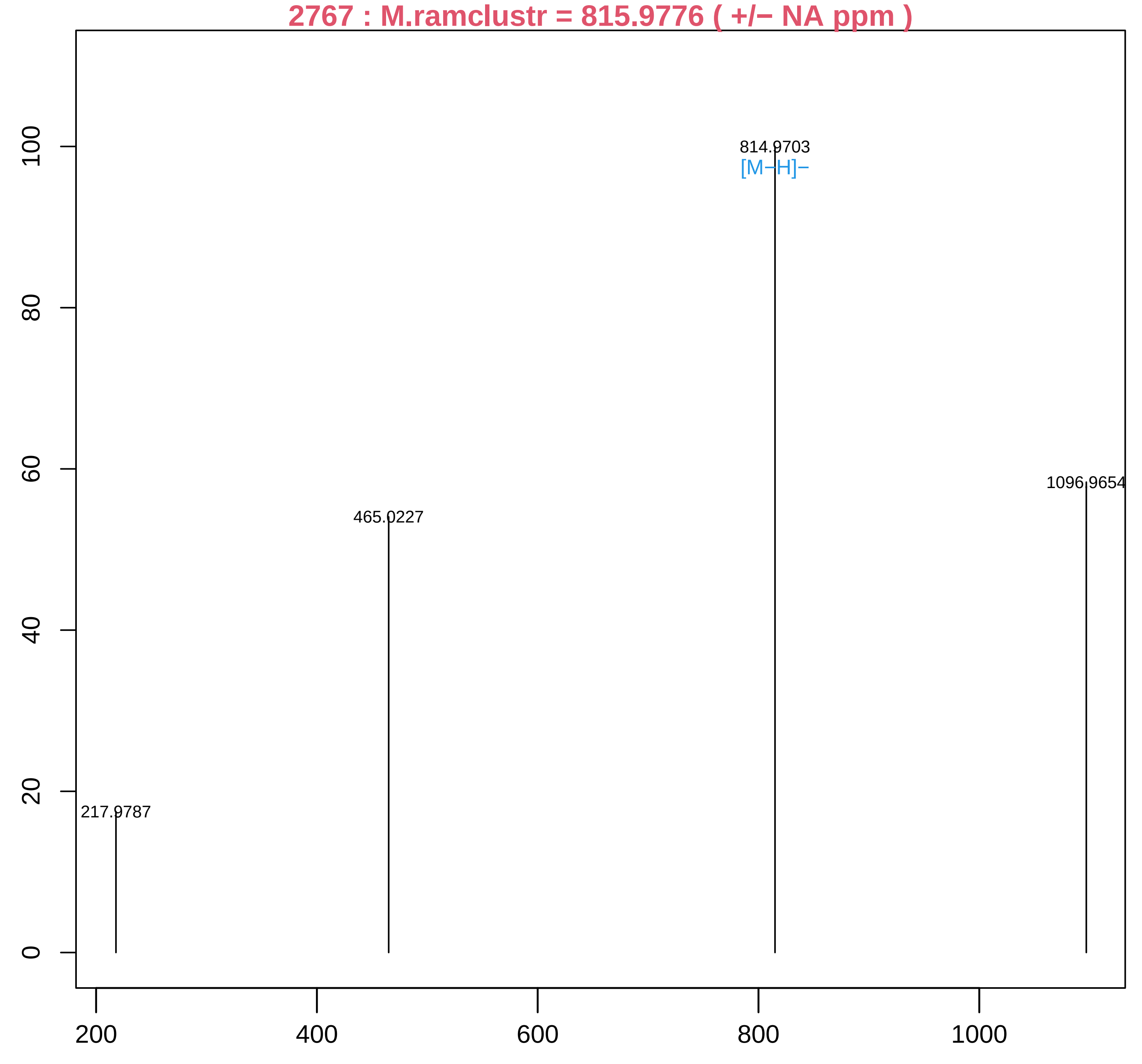
2767 : M.ramclustr = 815.9776 (+/− NA ppm)
814.9703
[M−H]−
465.0227
1096.9654
217.9787
0
20
40
60
80
100
200
400
600
800
1000

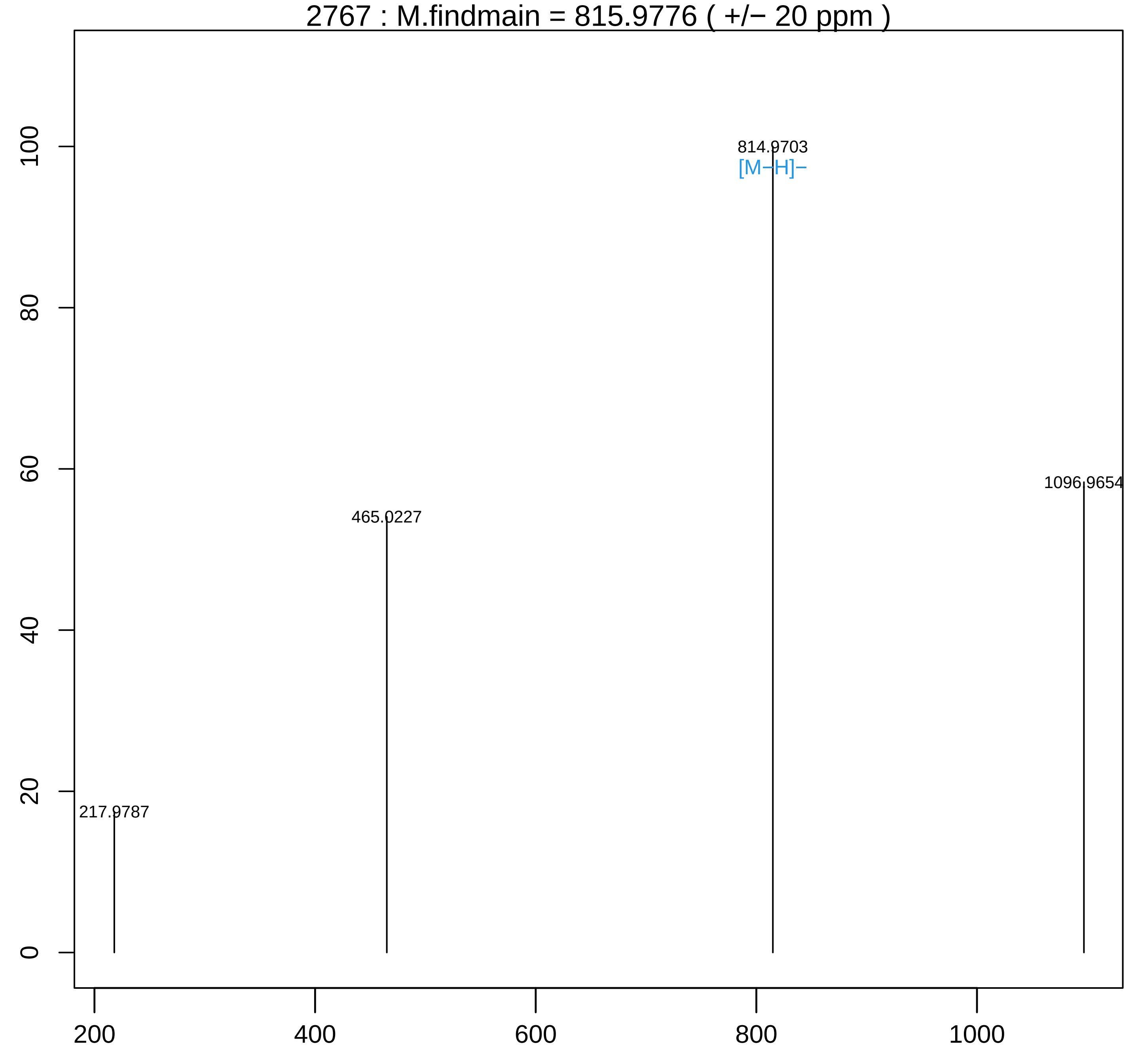
2767 : M.findmain = 815.9776 (+/− 20 ppm)
814.9703
[M−H]−
465.0227
1096.9654
217.9787
0
20
40
60
80
100
200
400
600
800
1000

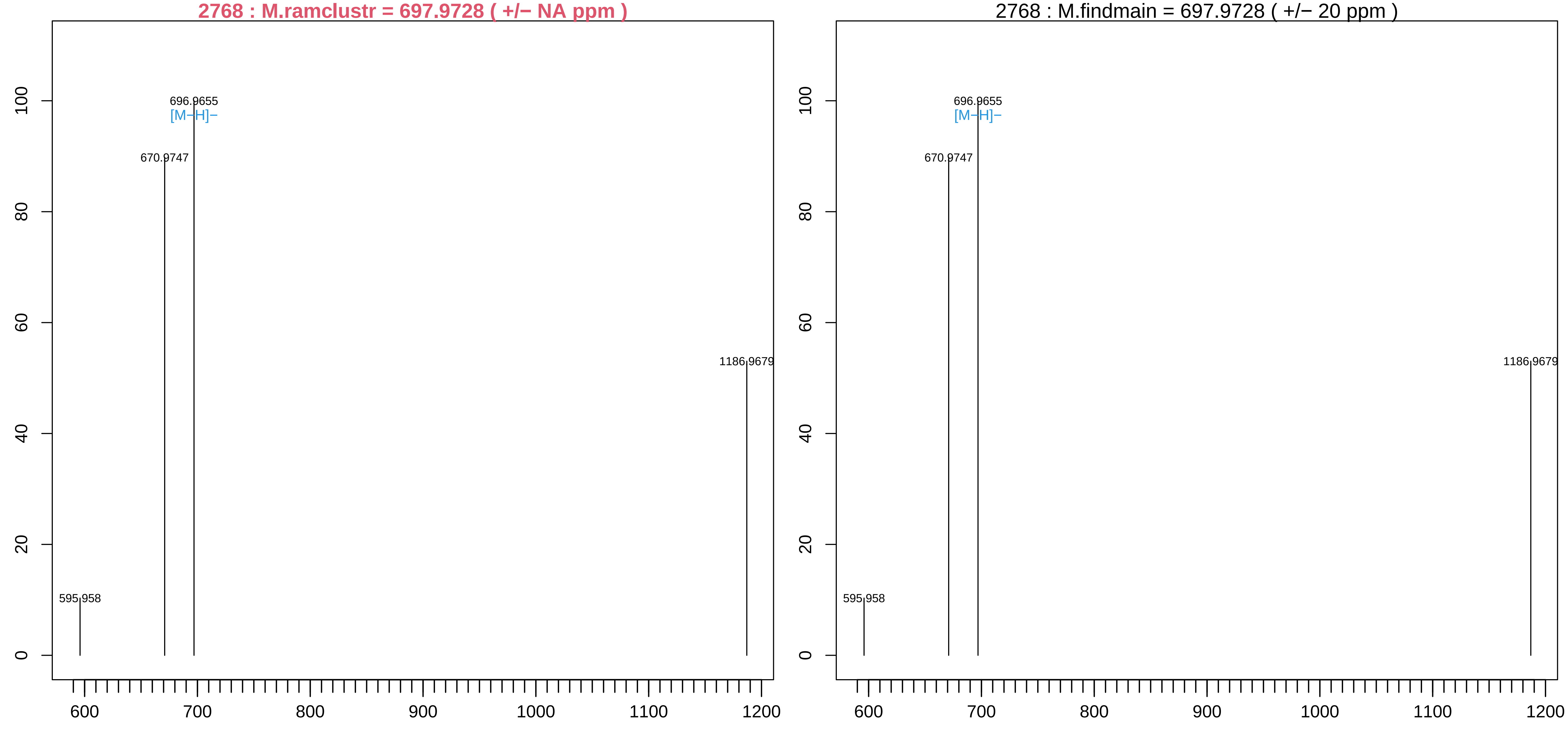
2768 : M.ramclustr = 697.9728 (+/− NA ppm)
696.9655
[M−H]−
670.9747
1186.9679
595.958
2768 : M.findmain = 697.9728 (+/− 20 ppm)
696.9655
[M−H]−
670.9747
1186.9679
595.958
0
20
40
60
80
100
600
700
800
900
1000
1100
1200

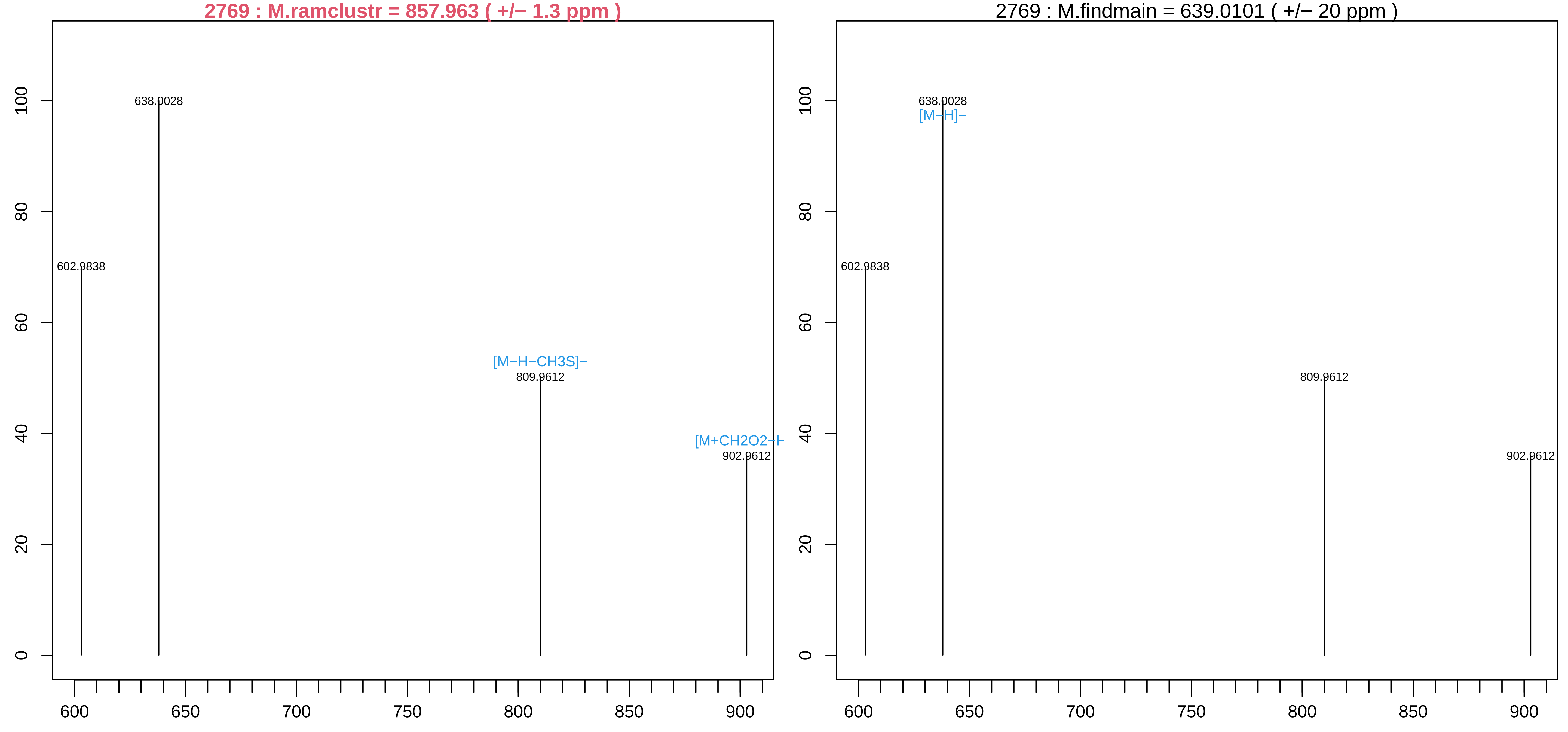
2769 : M.ramclustr = 857.963 (+/− 1.3 ppm)
602.9838
638.0028
[M−H−CH3S]−
809.9612
[M+CH2O2−H
902.9612
2769 : M.findmain = 639.0101 (+/− 20 ppm)
602.9838
638.0028
[M−H]−
809.9612
902.9612
0
20
40
60
80
100
600
650
700
750
800
850
900

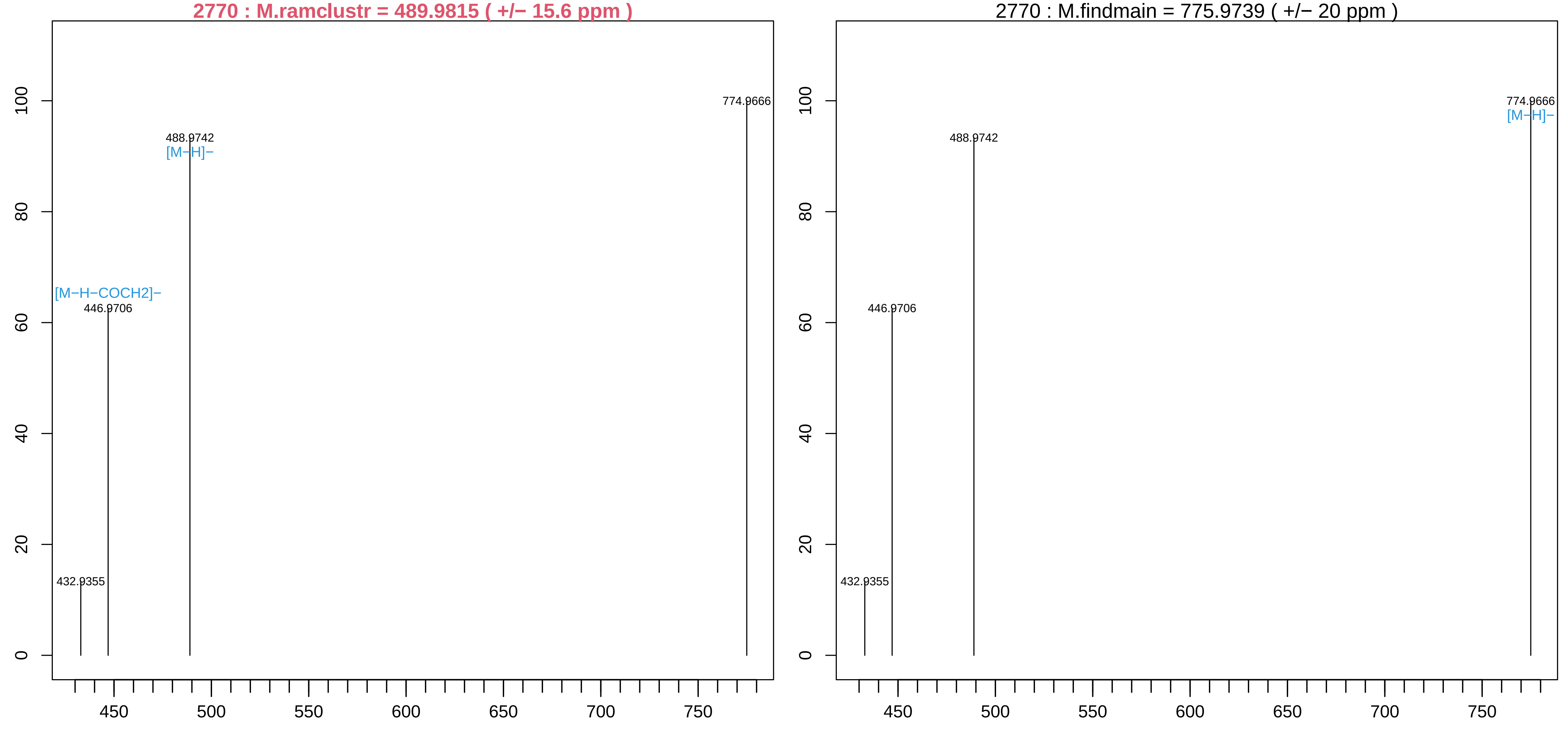

2770 : M.ramclustr = 489.9815 (+/− 15.6 ppm)
774.9666
488.9742
[M−H]−
[M−H−COCH2]−
446.9706
432.9355
2770 : M.findmain = 775.9739 (+/− 20 ppm)
774.9666
[M−H]−
488.9742
446.9706
432.9355
0
20
40
60
80
100
450
500
550
600
650
700
750

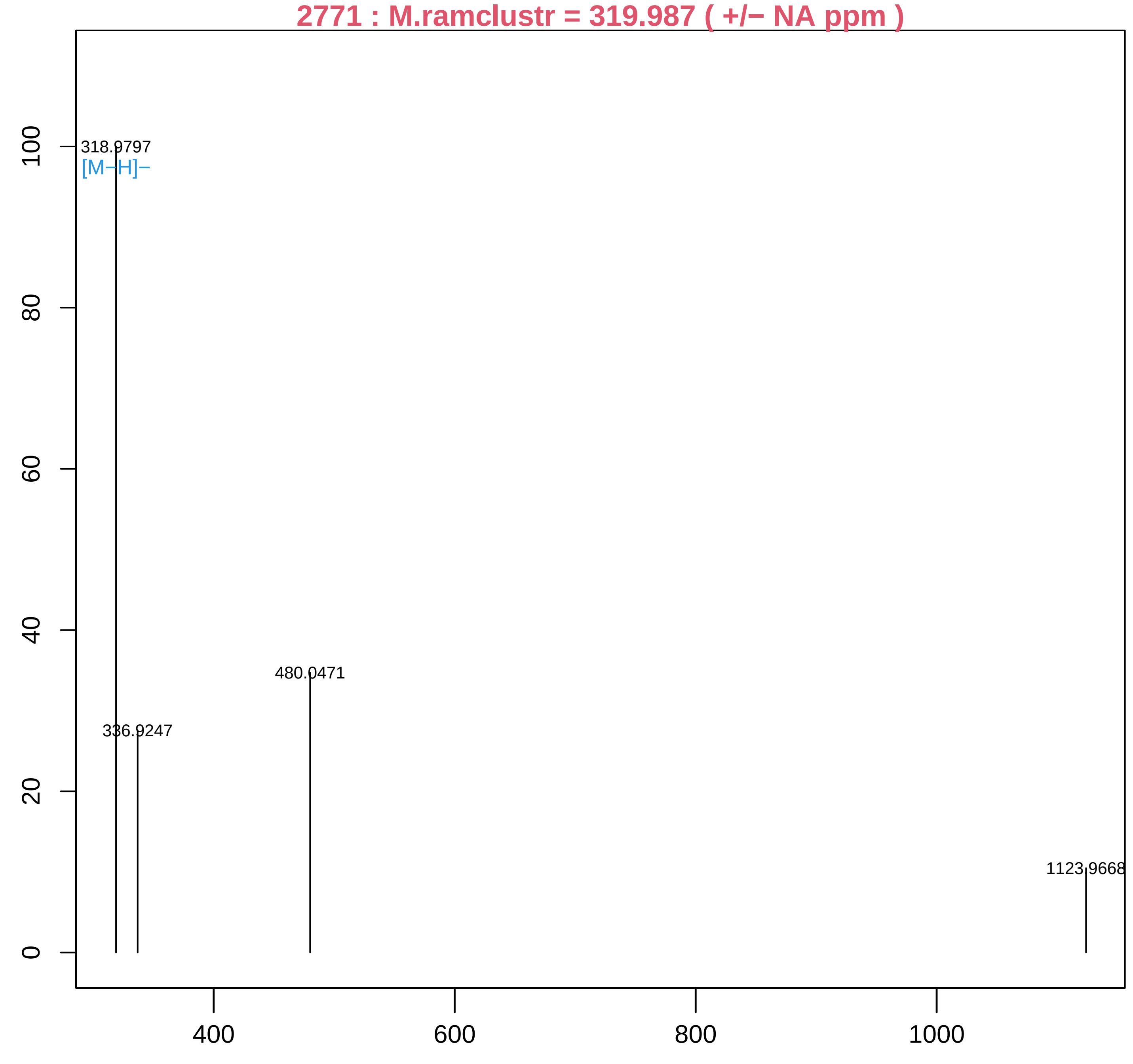
2771 : M.ramclustr = 319.987 (+/− NA ppm)
318.9797
[M−H]−
336.9247
480.0471
1123.9668
0
20
40
60
80
100
400
600
800
1000

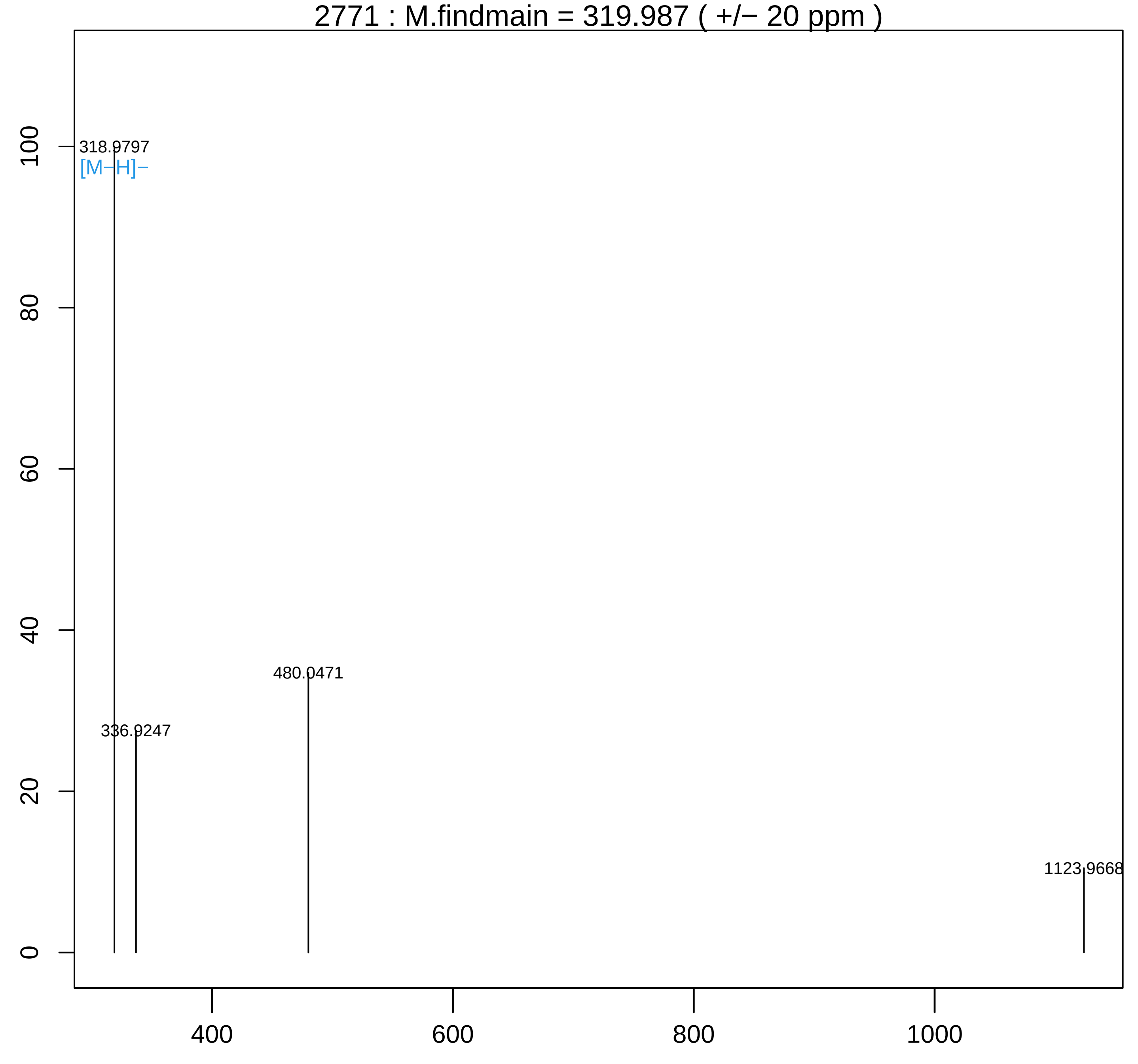
2771 : M.findmain = 319.987 (+/− 20 ppm)
318.9797
[M−H]−
336.9247
480.0471
1123.9668
0
20
40
60
80
100
400
600
800
1000

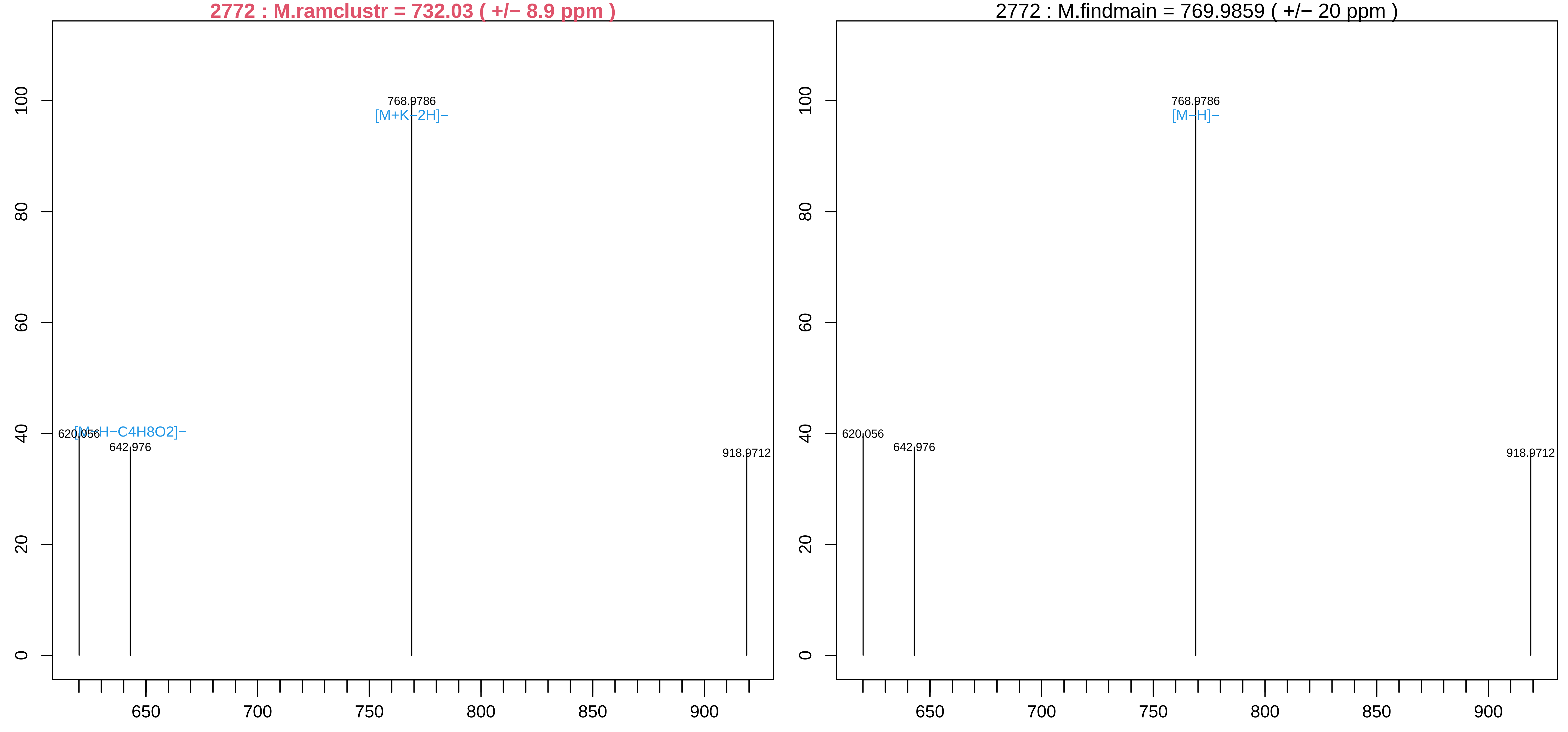
2772 : M.ramclustr = 732.03 (+/− 8.9 ppm)
768.9786
[M+K−2H]−
620.056
[M−H−C4H8O2]−
642.976
918.9712
2772 : M.findmain = 769.9859 (+/− 20 ppm)
768.9786
[M−H]−
620.056
642.976
918.9712
0
20
40
60
80
100
650
700
750
800
850
900

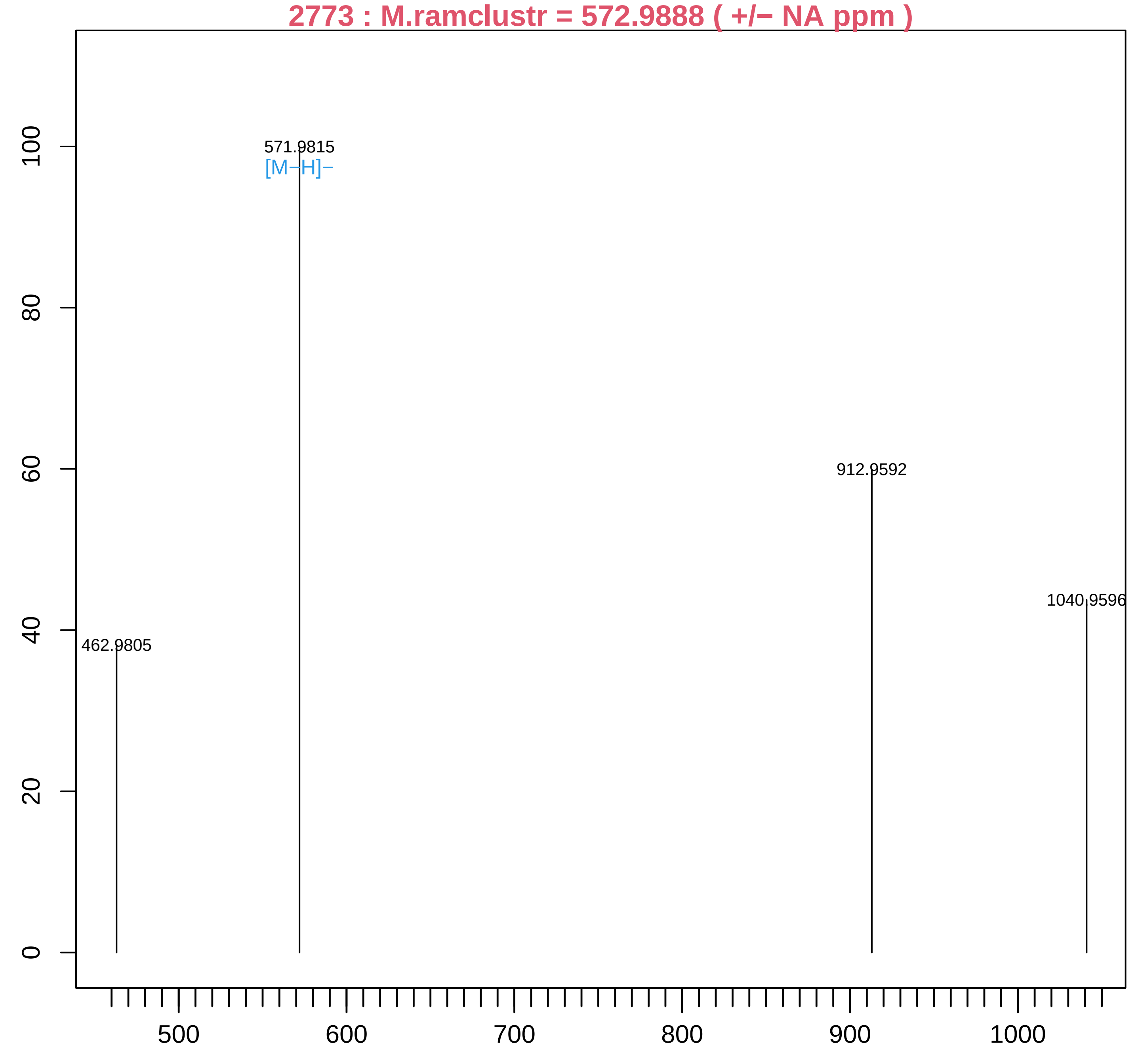
2773 : M.ramclustr = 572.9888 (+/− NA ppm)
571.9815
[M−H]−
912.9592
1040.9596
462.9805
0
20
40
60
80
100
500
600
700
800
900
1000

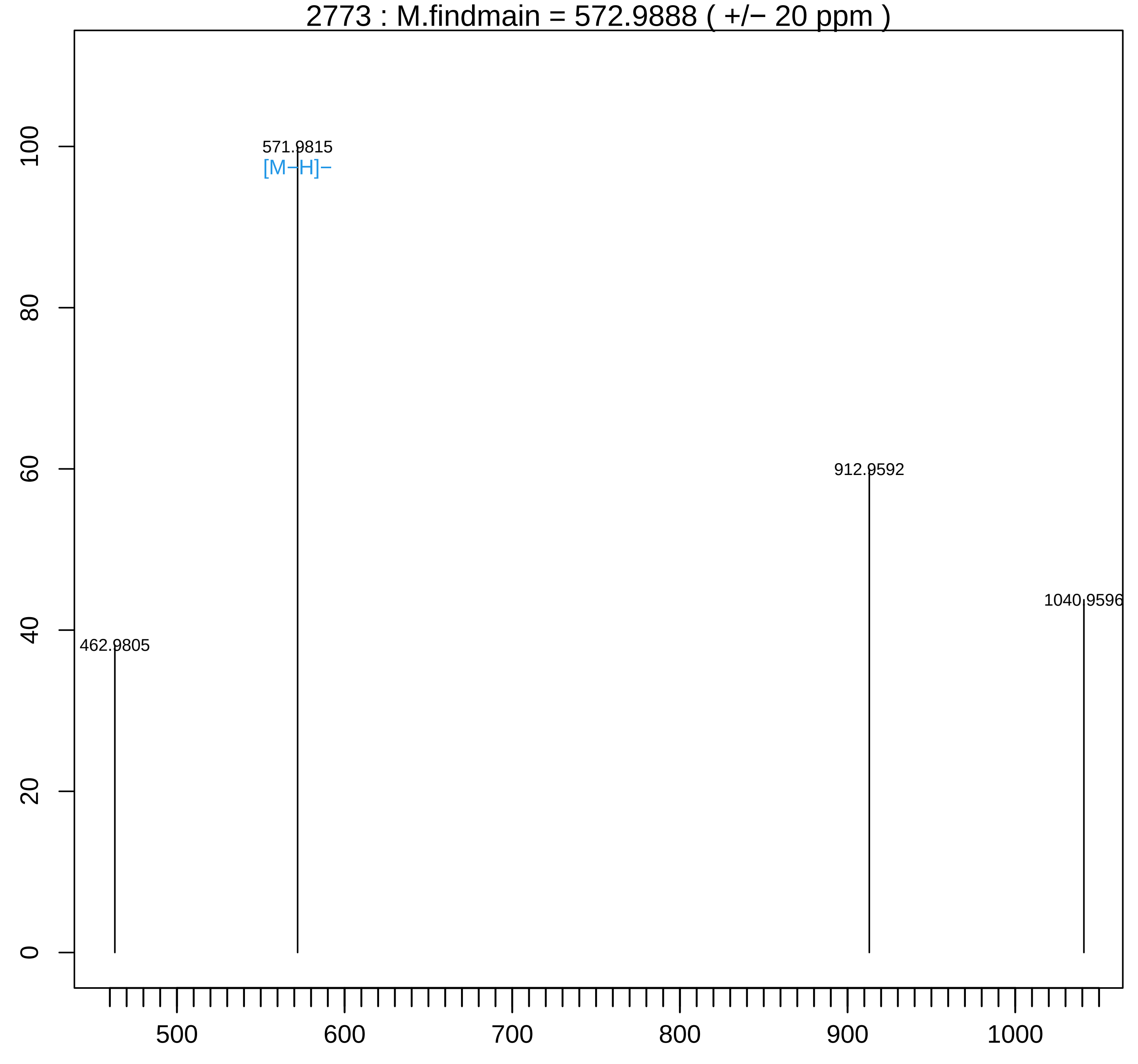
2773 : M.findmain = 572.9888 (+/− 20 ppm)
571.9815
[M−H]−
912.9592
1040.9596
462.9805
0
20
40
60
80
100
500
600
700
800
900
1000

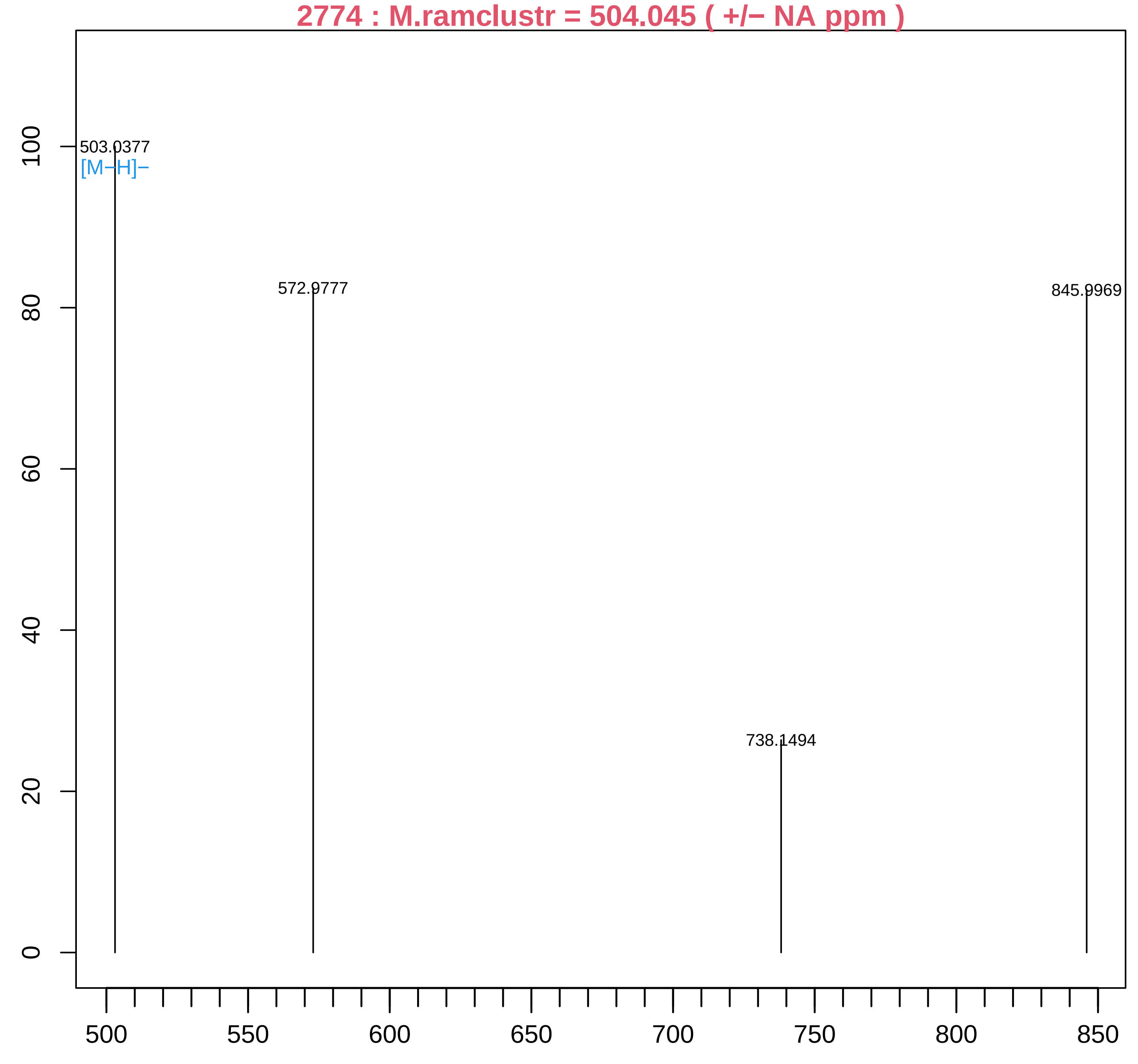

2774 : M.ramclustr = 504.045 (+/− NA ppm)
503.0377
[M−H]−
572.9777
845.9969
738.1494

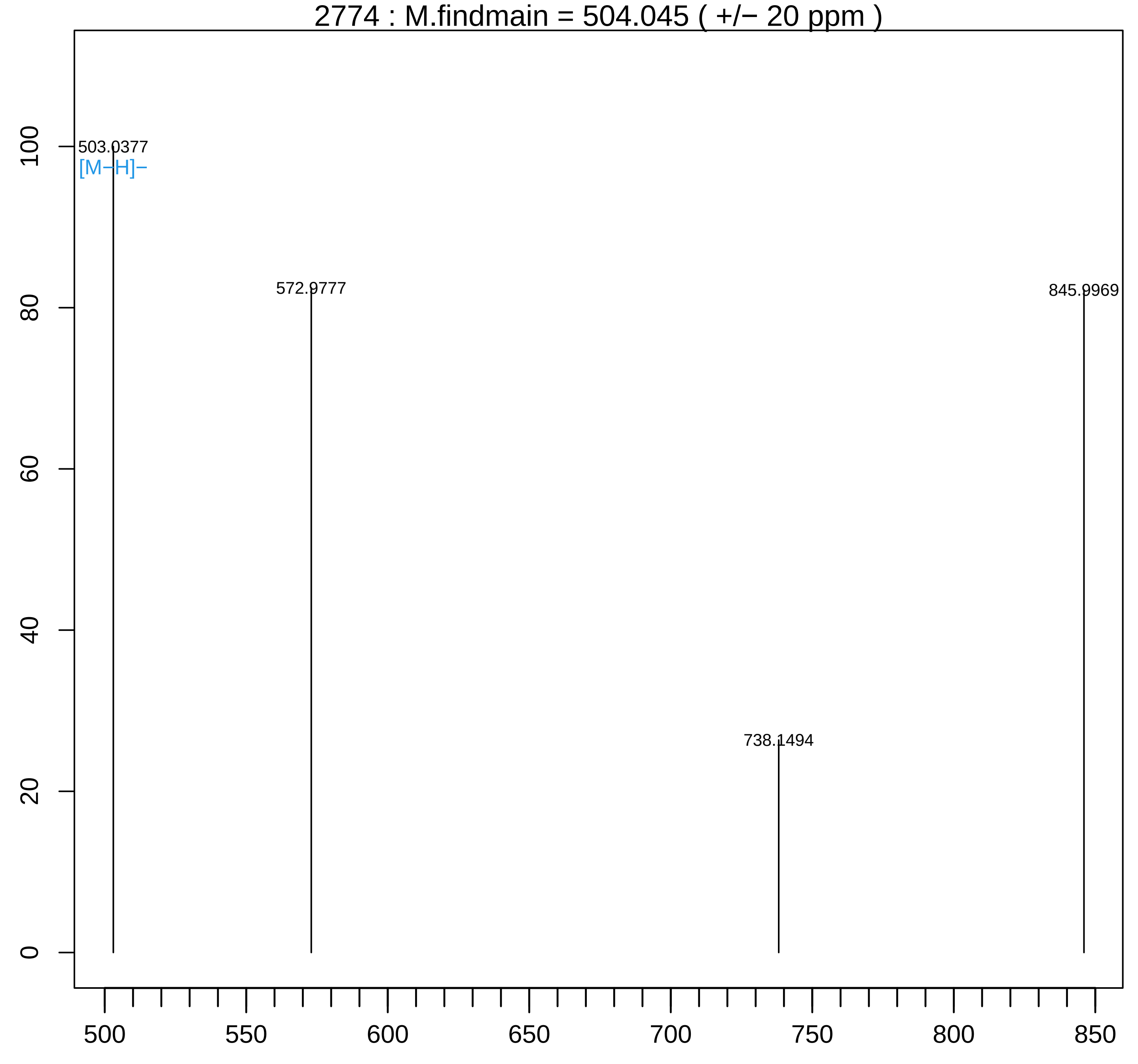

2774 : M.findmain = 504.045 (+/− 20 ppm)
503.0377
[M−H]−
572.9777
845.9969
738.1494

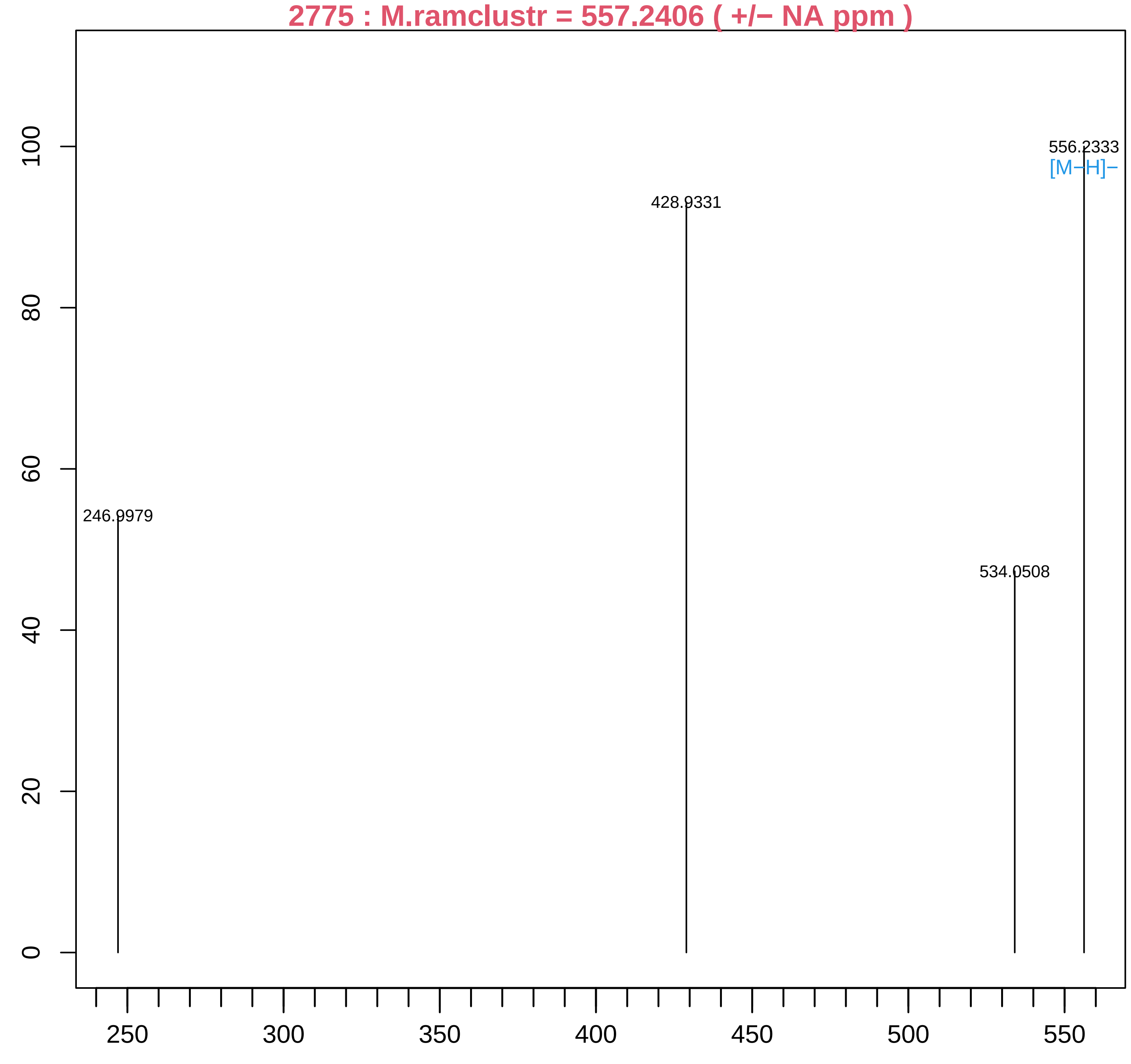
2775 : M.ramclustr = 557.2406 (+/− NA ppm)
556.2333
[M−H]−
428.9331
246.9979
534.0508
0
20
40
60
80
100
250
300
350
400
450
500
550

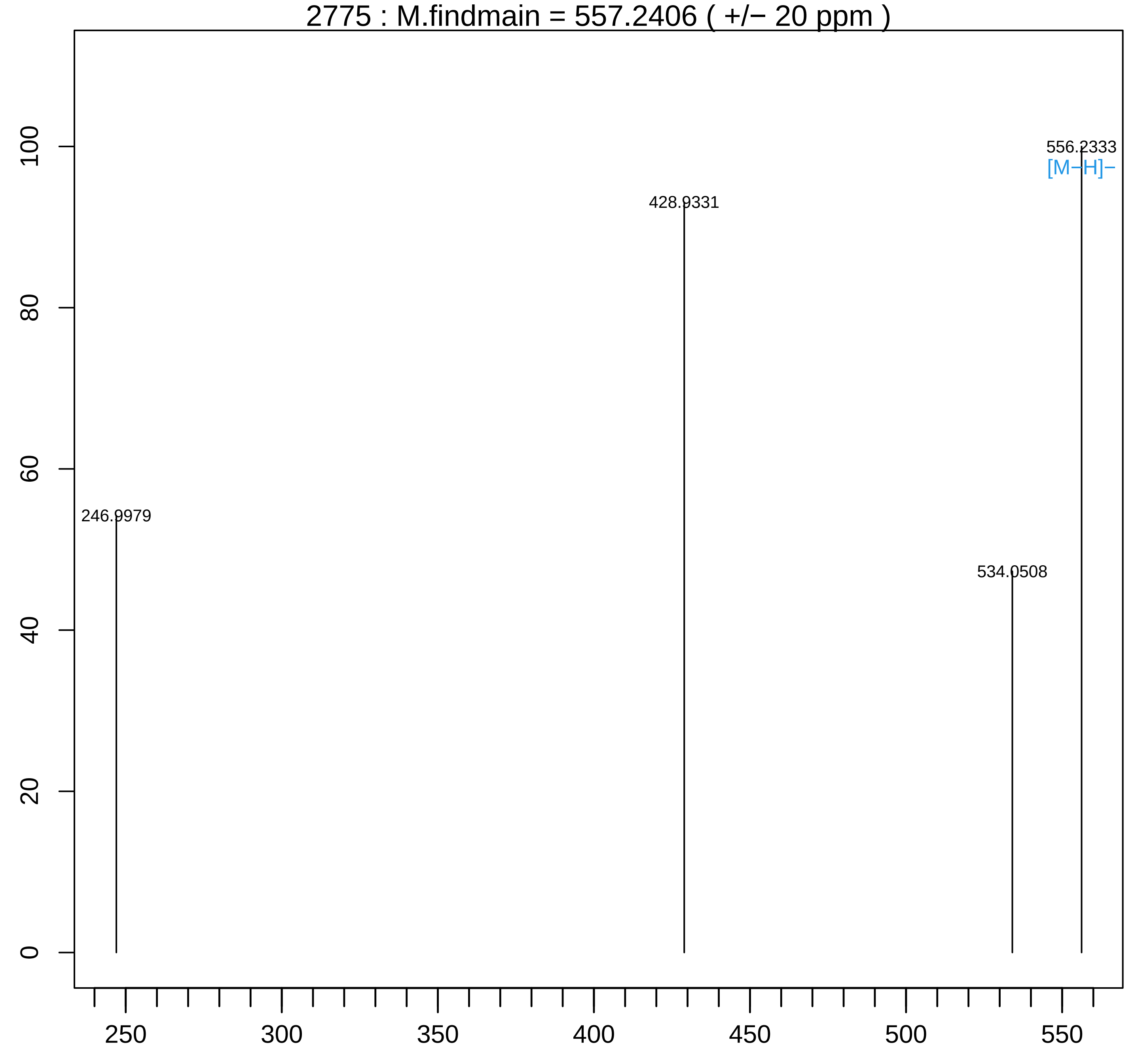
2775 : M.findmain = 557.2406 (+/− 20 ppm)
556.2333
[M−H]−
428.9331
246.9979
534.0508
0
20
40
60
80
100
250
300
350
400
450
500
550

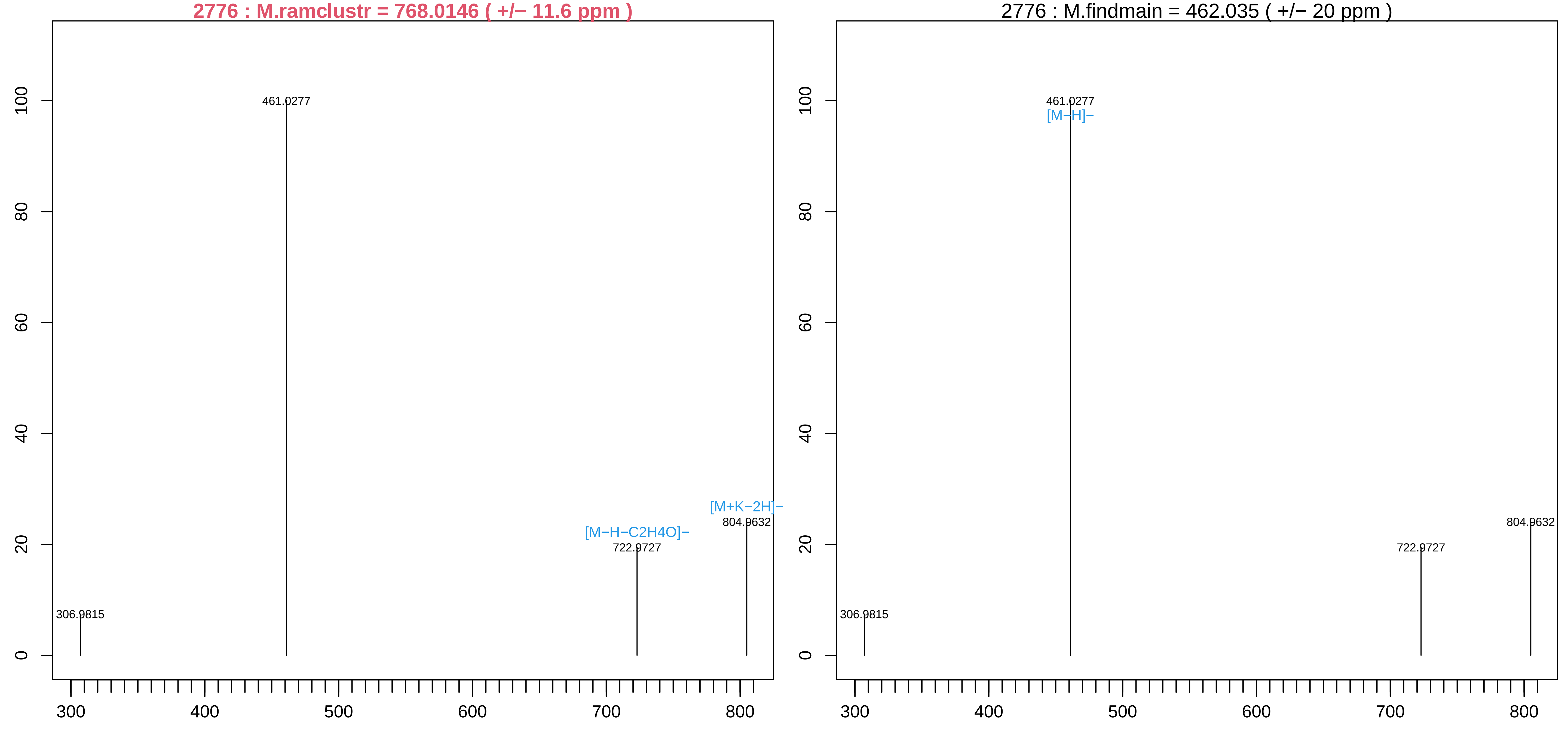
2776 : M.ramclustr = 768.0146 (+/− 11.6 ppm)
461.0277
[M−H−C2H4O]−
722.9727
[M+K−2H]−
804.9632
306.9815
0
20
40
60
80
100
300
400
500
600
700
800
2776 : M.findmain = 462.035 (+/− 20 ppm)
461.0277
[M−H]−
722.9727
804.9632
306.9815

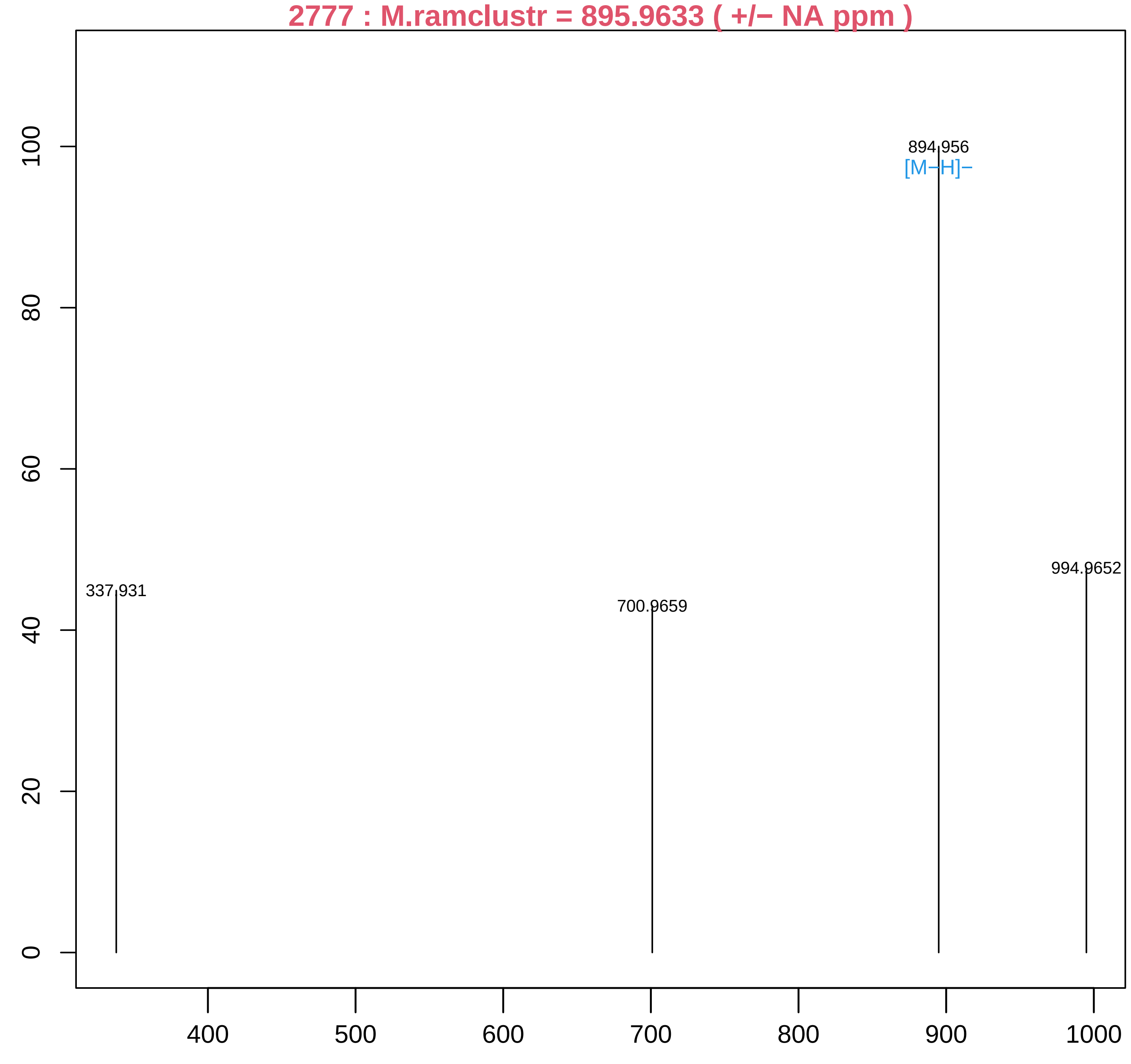

2777 : M.ramclustr = 895.9633 (+/− NA ppm)
894.956
[M−H]−
337.931
700.9659
994.9652

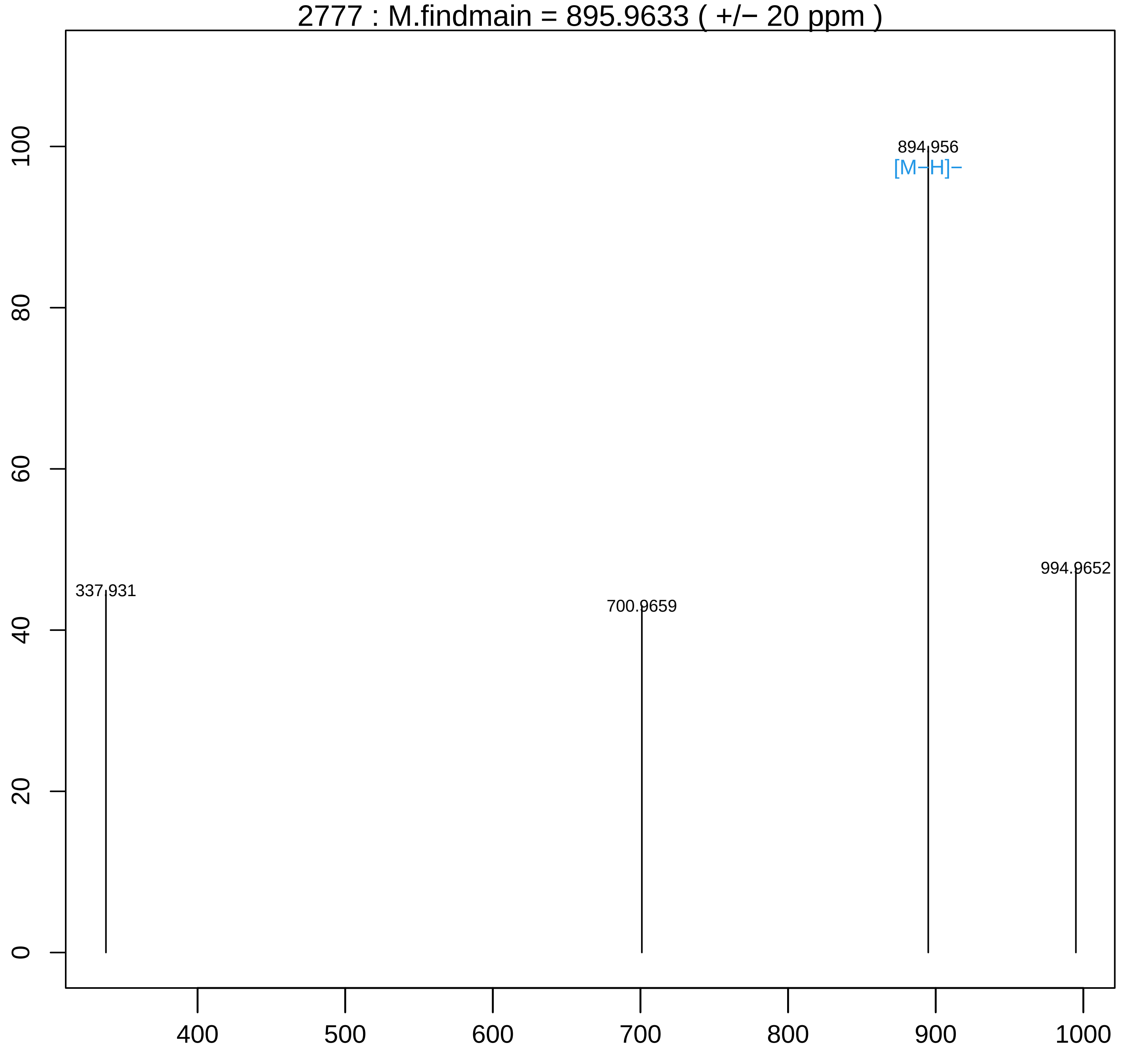

2777 : M.findmain = 895.9633 (+/− 20 ppm)
894.956
[M−H]−
337.931
700.9659
994.9652

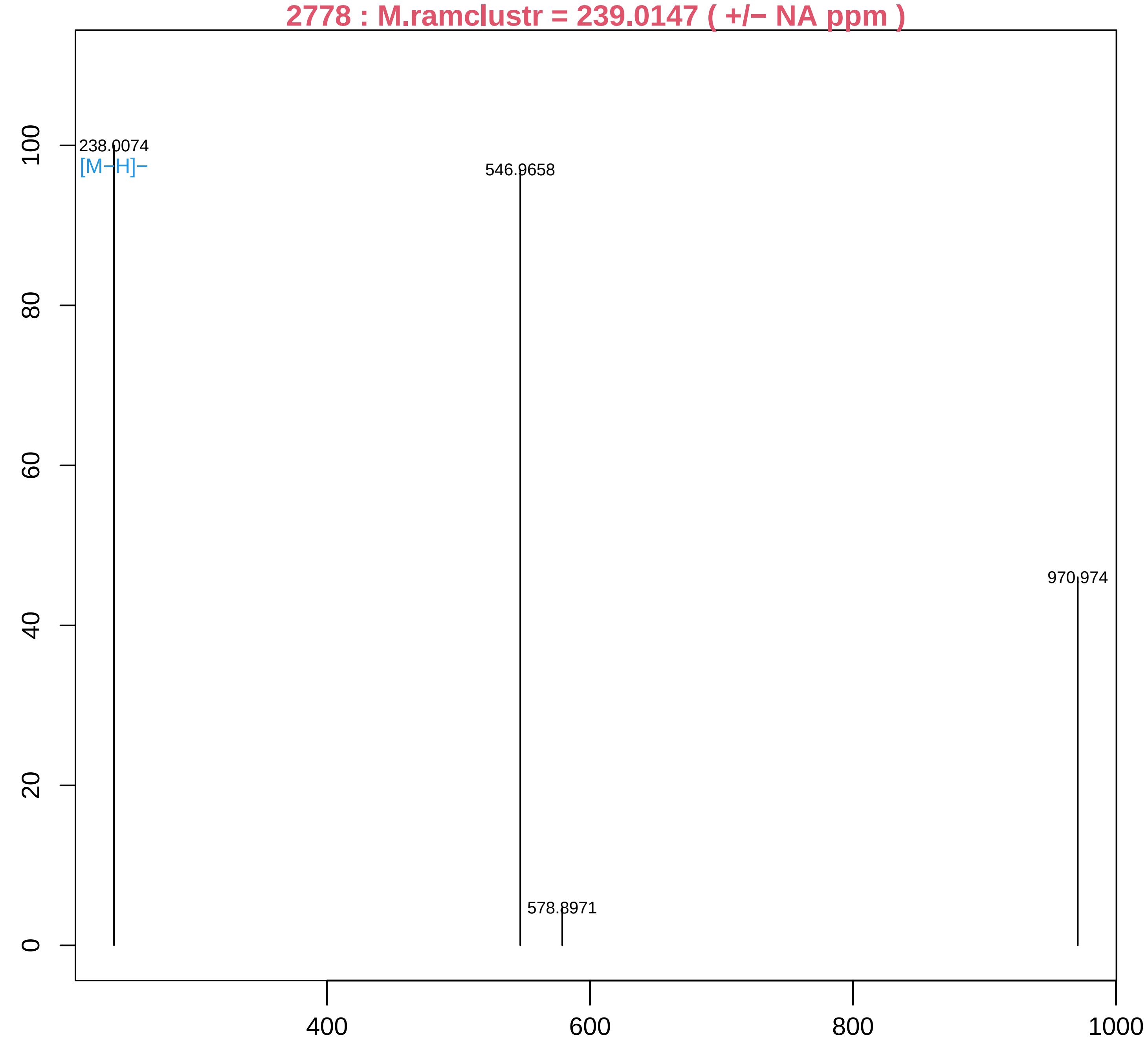
2778 : M.ramclustr = 239.0147 (+/− NA ppm)
238.0074
[M−H]−
546.9658
970.974
578.8971
0
20
40
60
80
100
400
600
800
1000

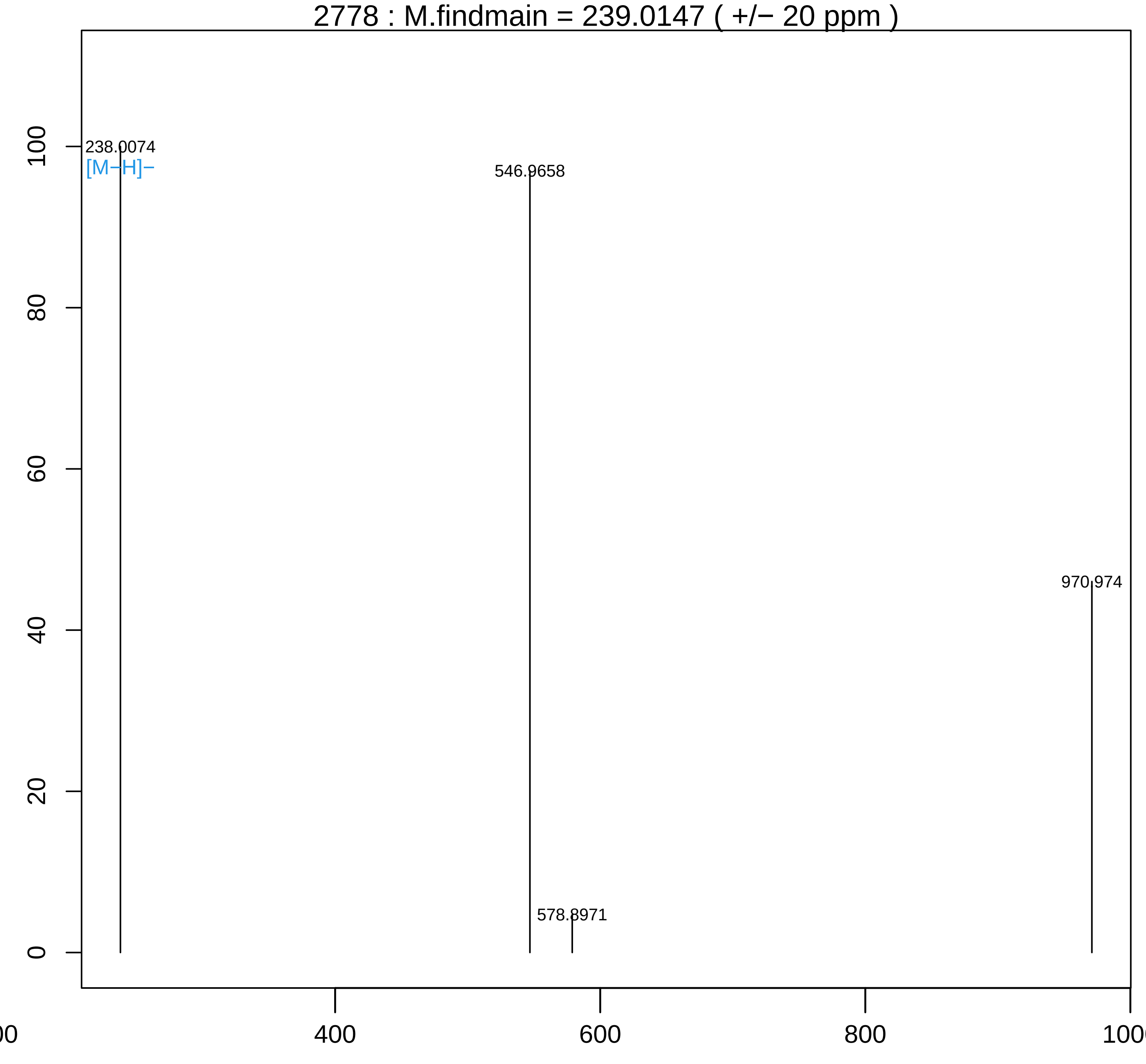
2778 : M.findmain = 239.0147 (+/− 20 ppm)
238.0074
[M−H]−
546.9658
970.974
578.8971
0
20
40
60
80
100
400
600
800
1000

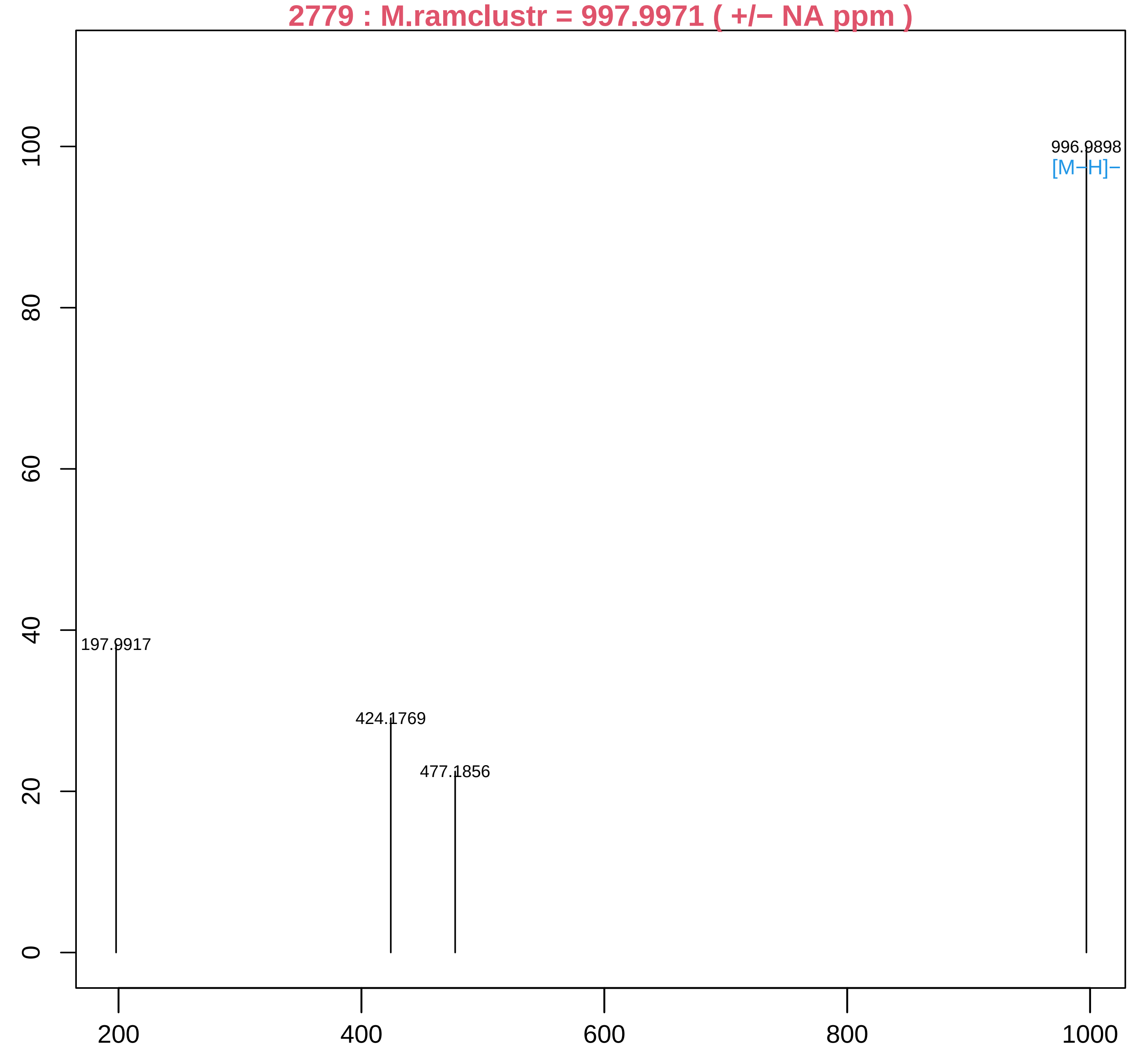

2779 : M.ramclustr = 997.9971 (+/− NA ppm)
996.9898
[M−H]−
197.9917
424.1769
477.1856
0
20
40
60
80
100
200
400
600
800
1000

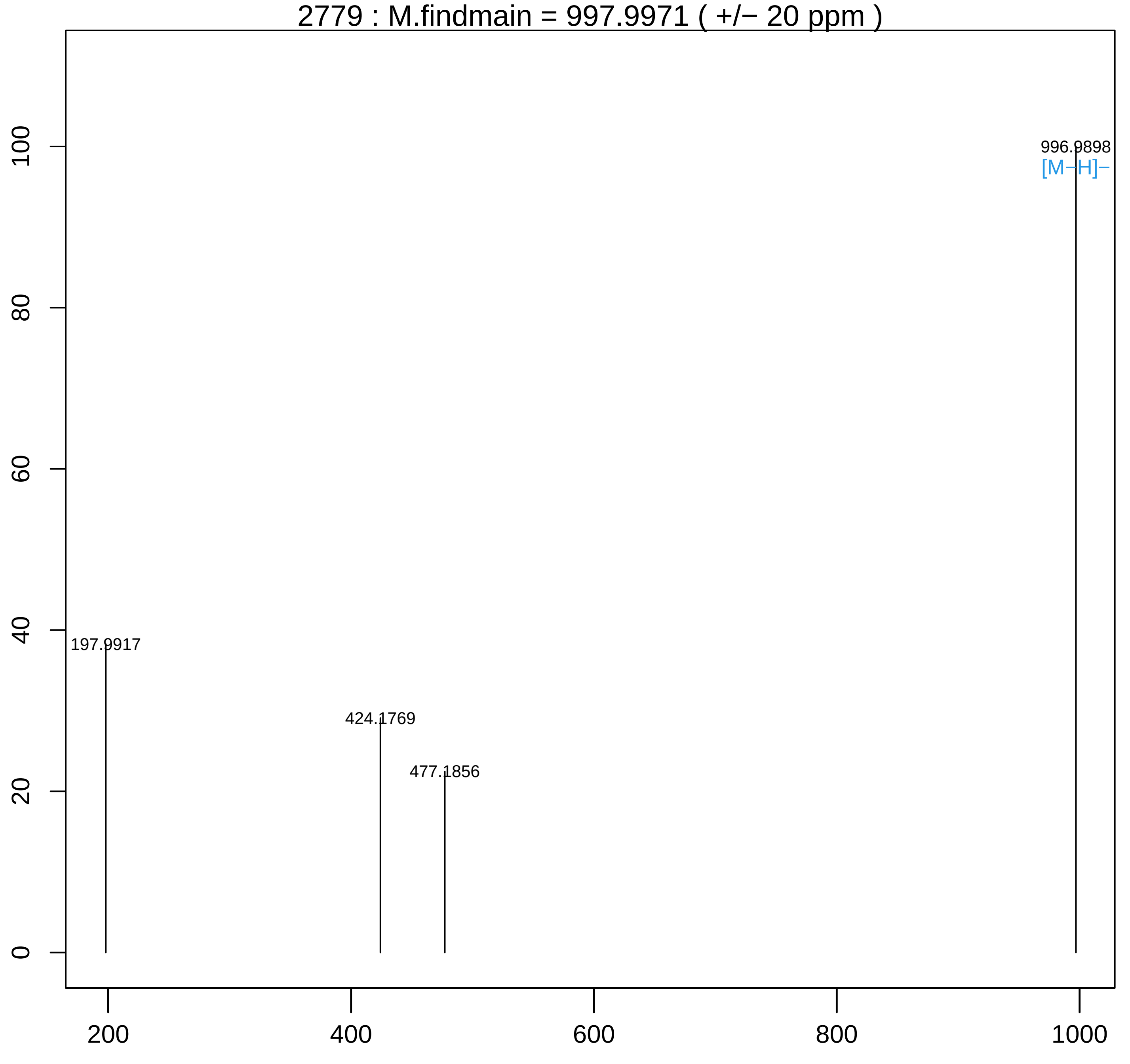

2779 : M.findmain = 997.9971 (+/− 20 ppm)
996.9898
[M−H]−
197.9917
424.1769
477.1856
0
20
40
60
80
100
200
400
600
800
1000

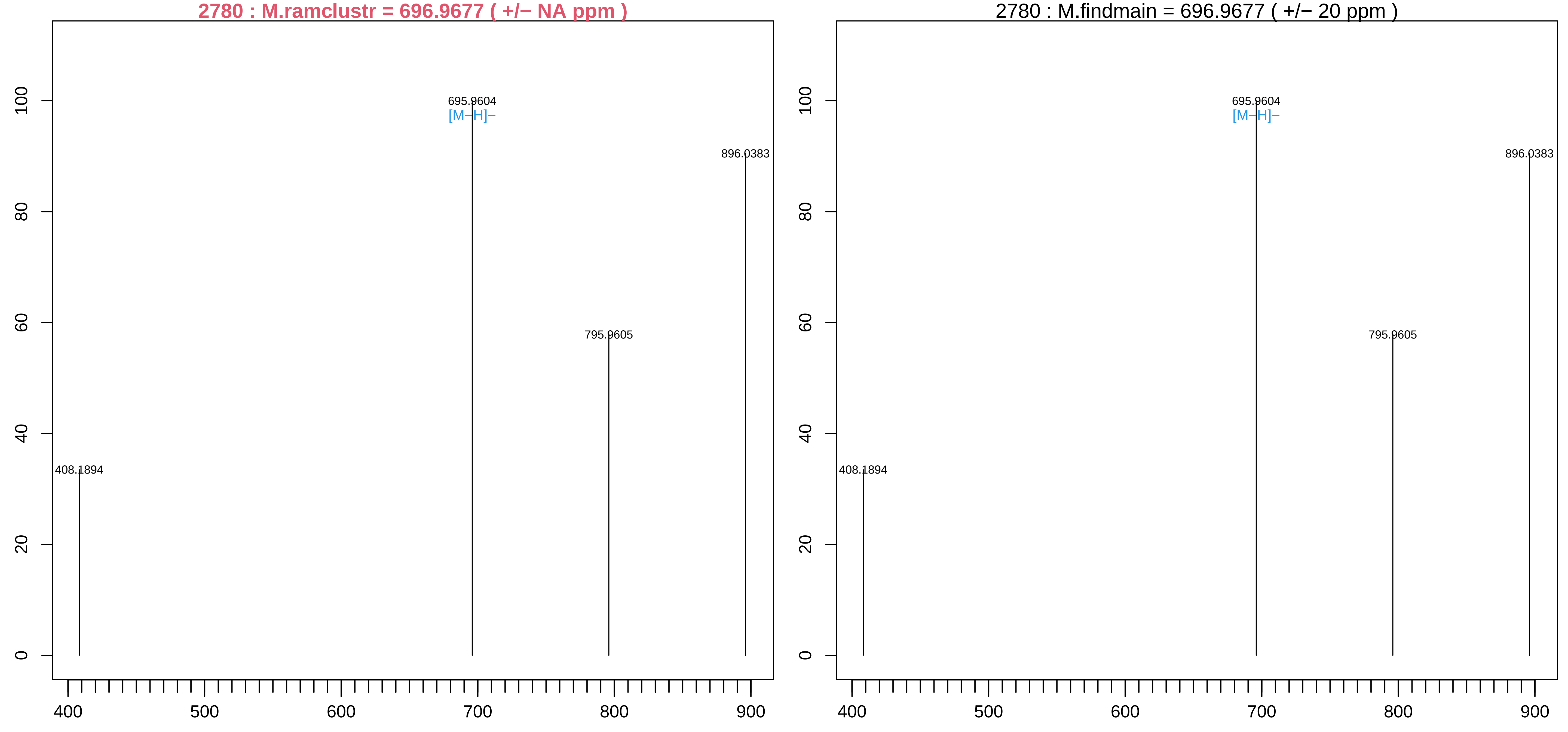
2780 : M.ramclustr = 696.9677 (+/− NA ppm)
695.9604
[M−H]−
896.0383
795.9605
408.1894
2780 : M.findmain = 696.9677 (+/− 20 ppm)
695.9604
[M−H]−
896.0383
795.9605
408.1894

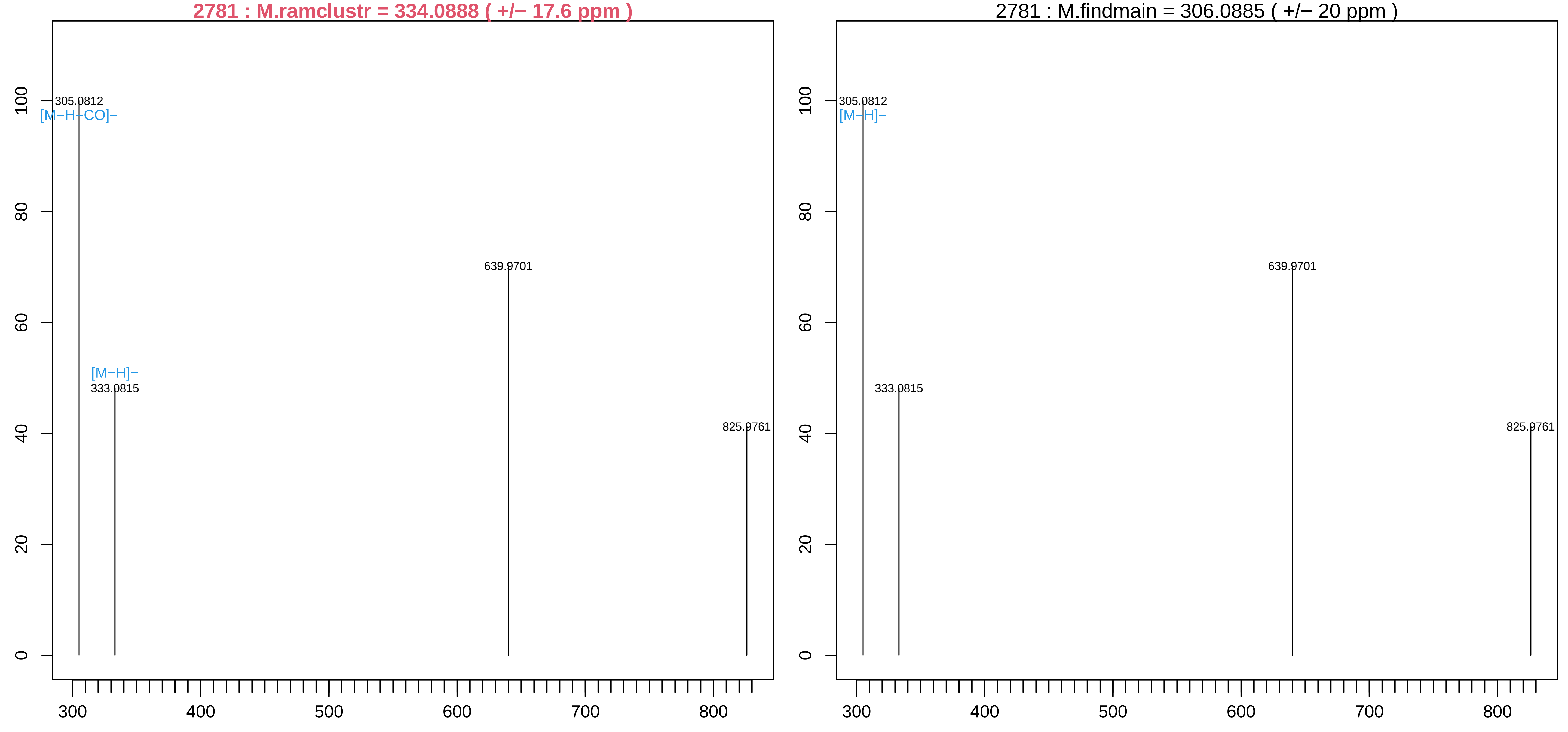
2781 : M.ramclustr = 334.0888 (+/− 17.6 ppm)
305.0812
[M−H−CO]−
[M−H]−
333.0815
639.9701
825.9761
2781 : M.findmain = 306.0885 (+/− 20 ppm)
305.0812
[M−H]−
333.0815
639.9701
825.9761

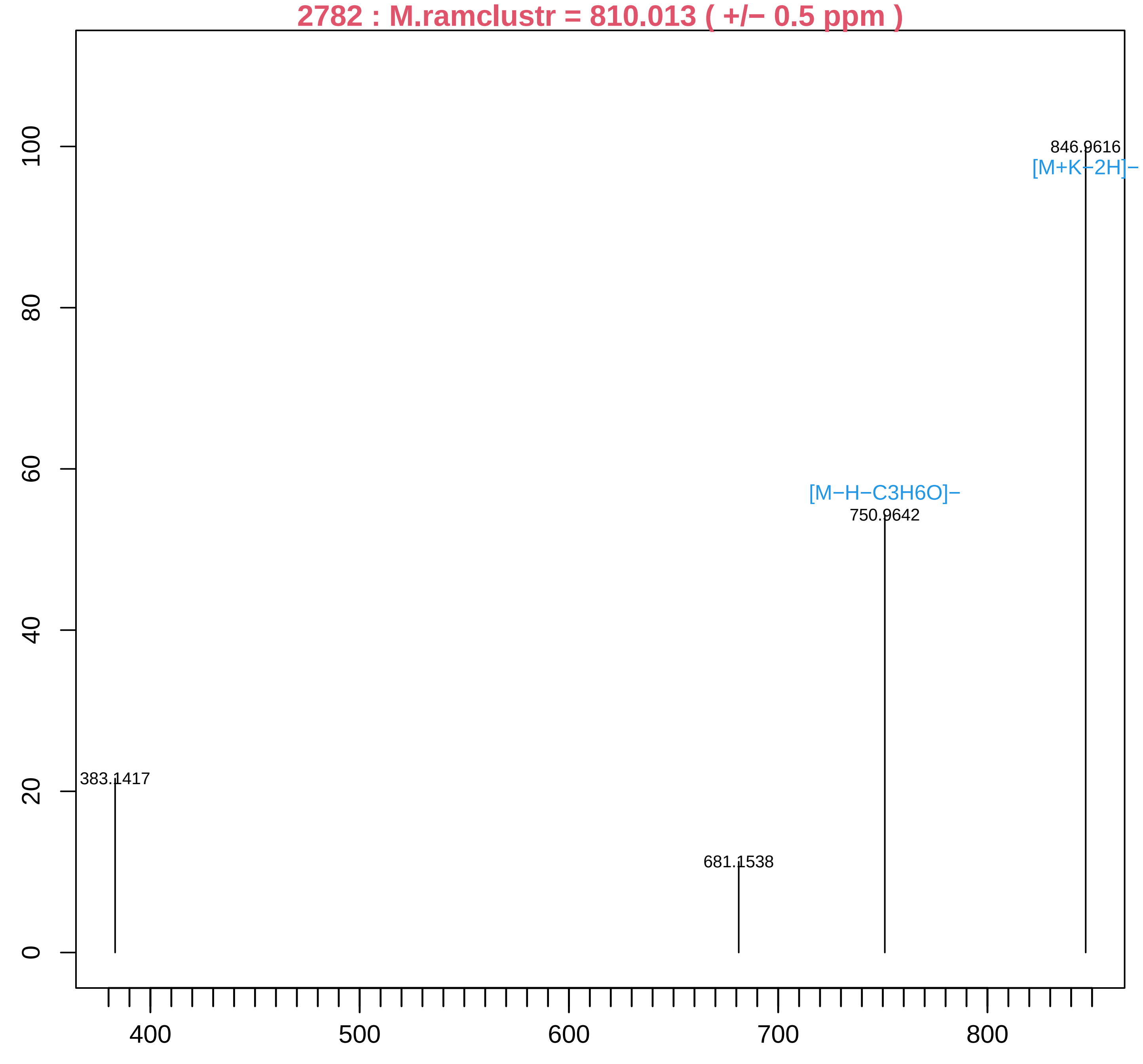
2782 : M.ramclustr = 810.013 (+/− 0.5 ppm)
846.9616
[M+K−2H]−
[M−H−C3H6O]−
750.9642
383.1417
681.1538
0
20
40
60
80
100
400
500
600
700
800

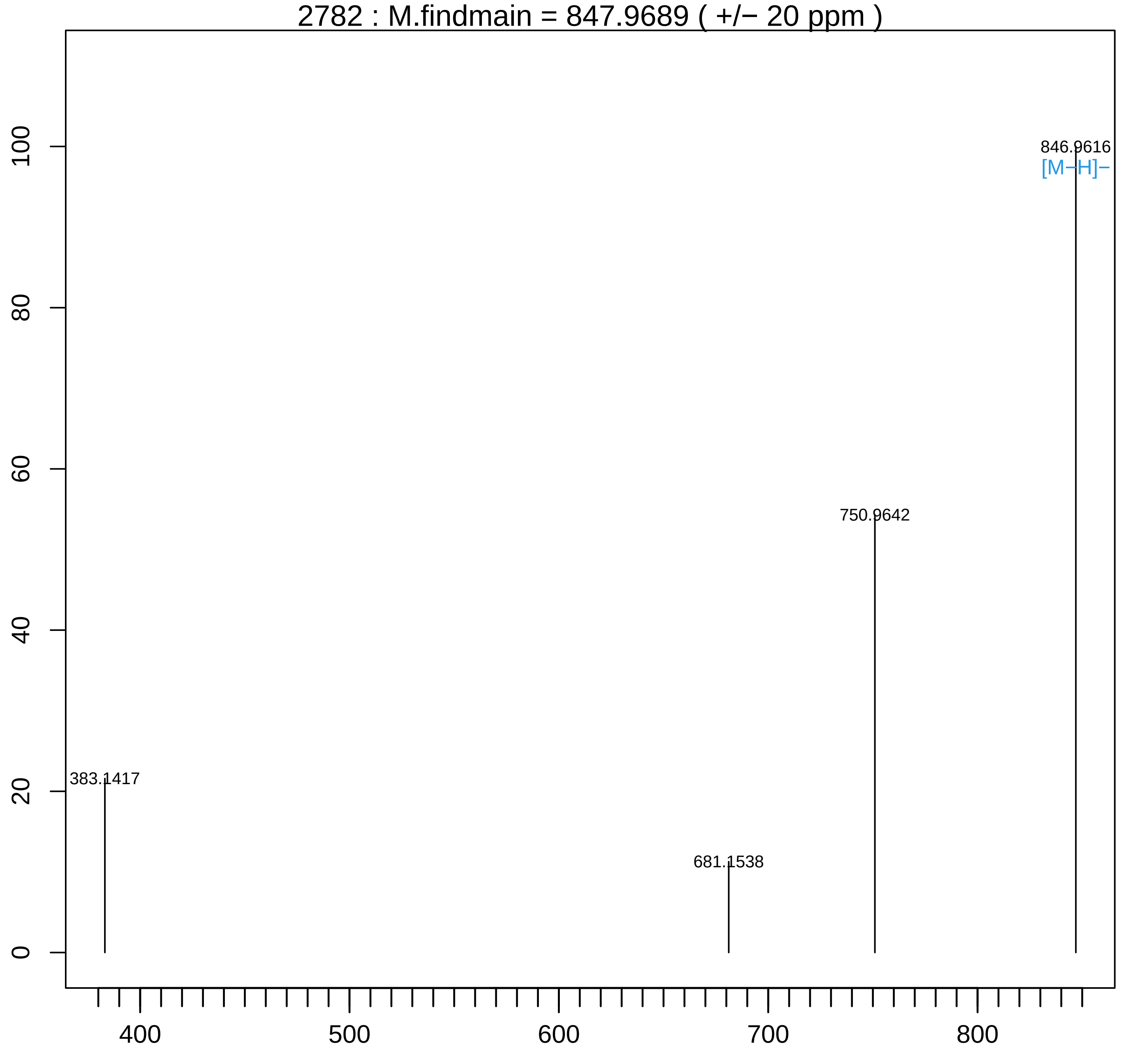
2782 : M.findmain = 847.9689 (+/− 20 ppm)
846.9616
[M−H]−
750.9642
383.1417
681.1538
0
20
40
60
80
100
400
500
600
700
800

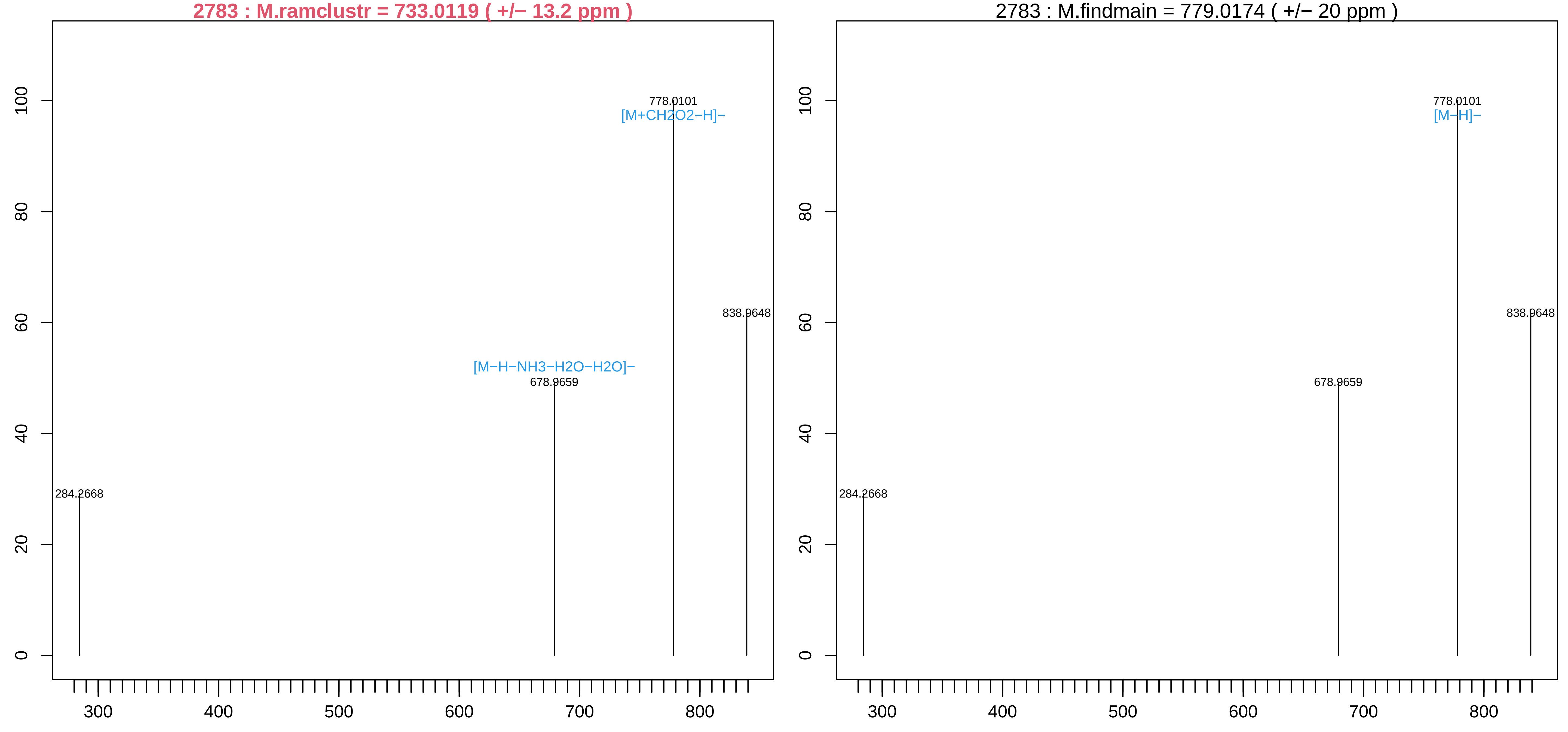

2783 : M.ramclustr = 733.0119 (+/− 13.2 ppm)
778.0101
[M+CH2O2−H]−
838.9648
[M−H−NH3−H2O−H2O]−
678.9659
284.2668
0
20
40
60
80
100
300
400
500
600
700
800
2783 : M.findmain = 779.0174 (+/− 20 ppm)
778.0101
[M−H]−
838.9648
678.9659
284.2668

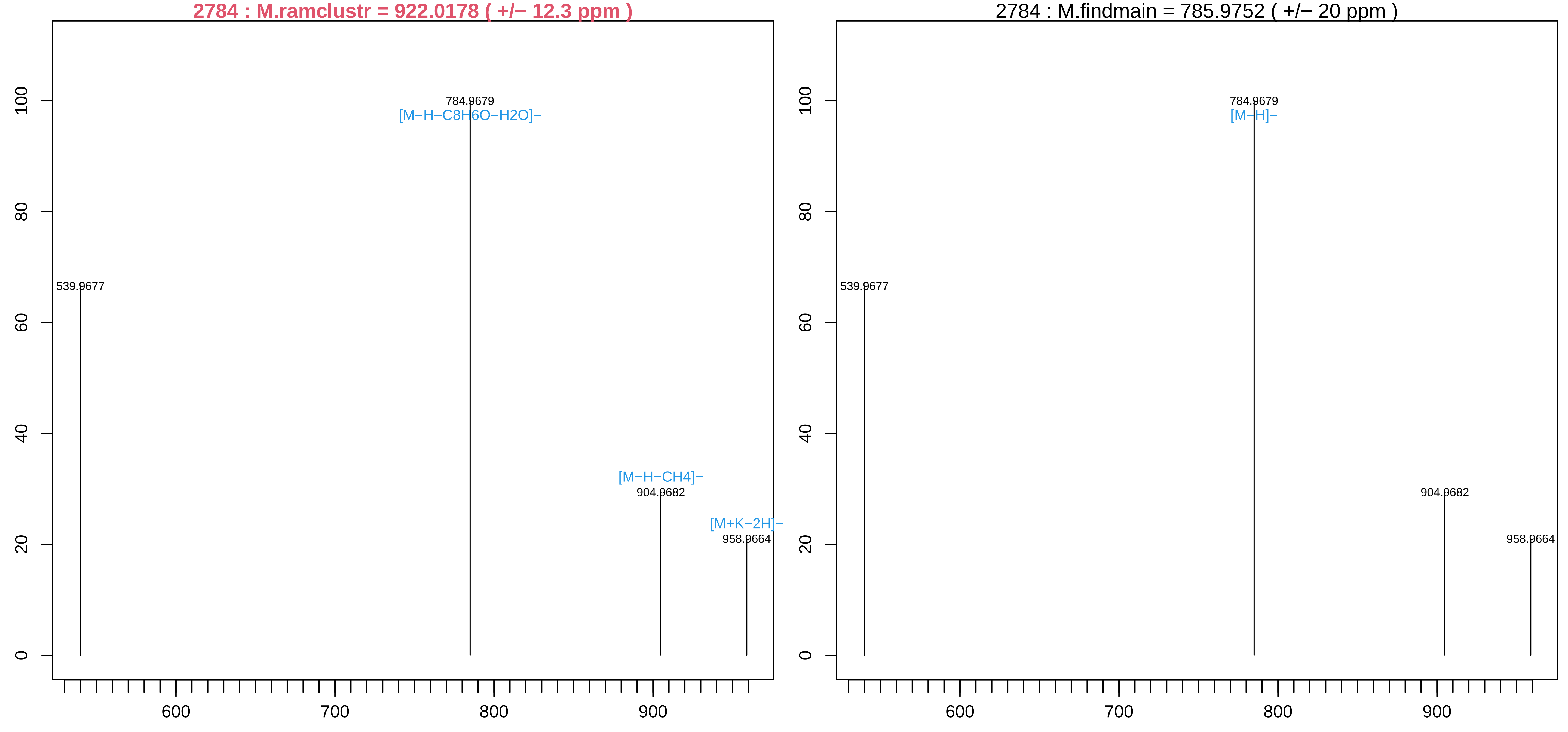

2784 : M.ramclustr = 922.0178 (+/− 12.3 ppm)
784.9679
[M−H−C8H6O−H2O]−
539.9677
[M−H−CH4]−
904.9682
[M+K−2H]−
958.9664
2784 : M.findmain = 785.9752 (+/− 20 ppm)
784.9679
[M−H]−
539.9677
904.9682
958.9664
0
20
40
60
80
100
600
700
800
900

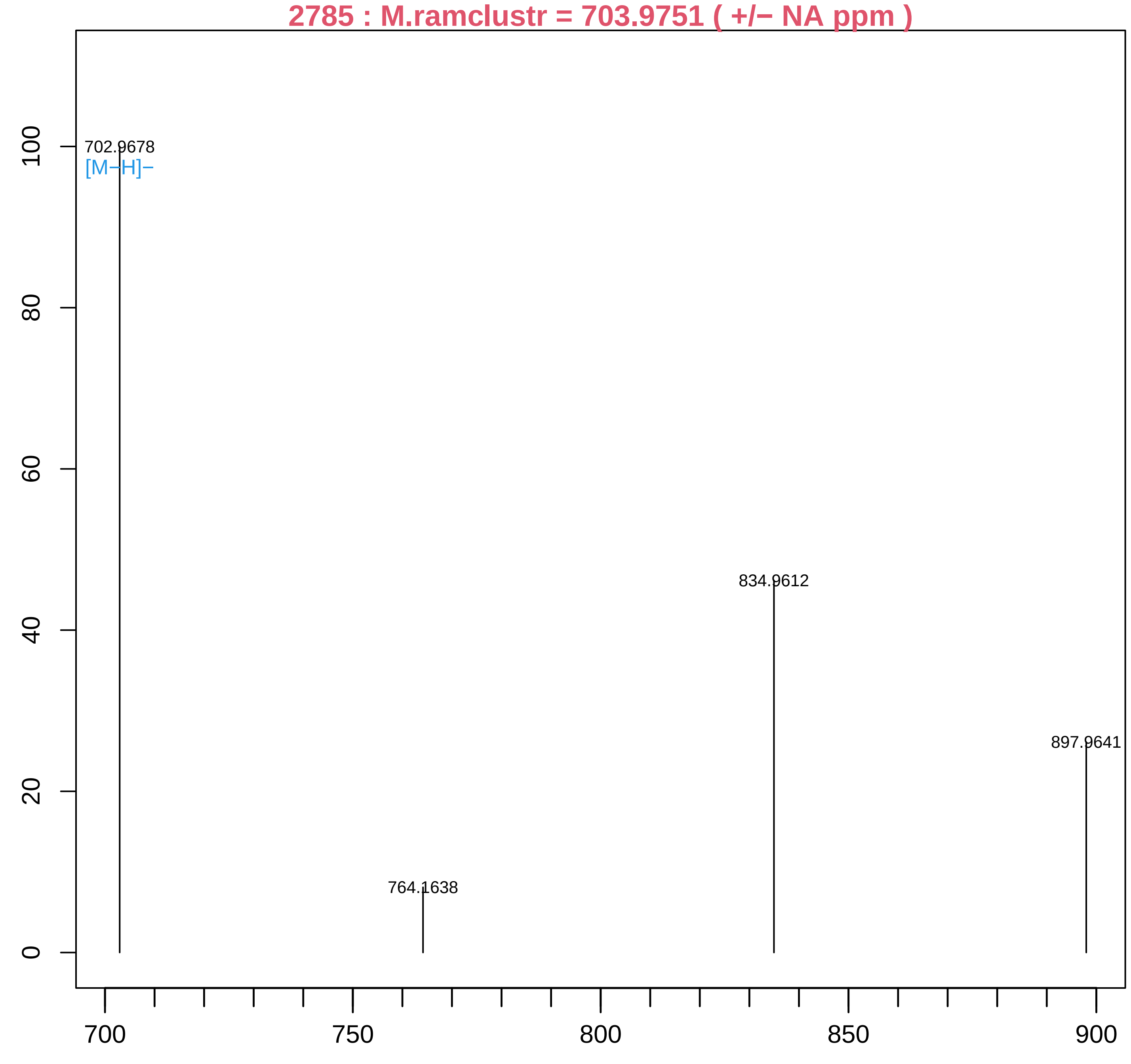
2785 : M.ramclustr = 703.9751 (+/− NA ppm)
702.9678
[M−H]−
764.1638
834.9612
897.9641
0
20
40
60
80
100
700
750
800
850
900

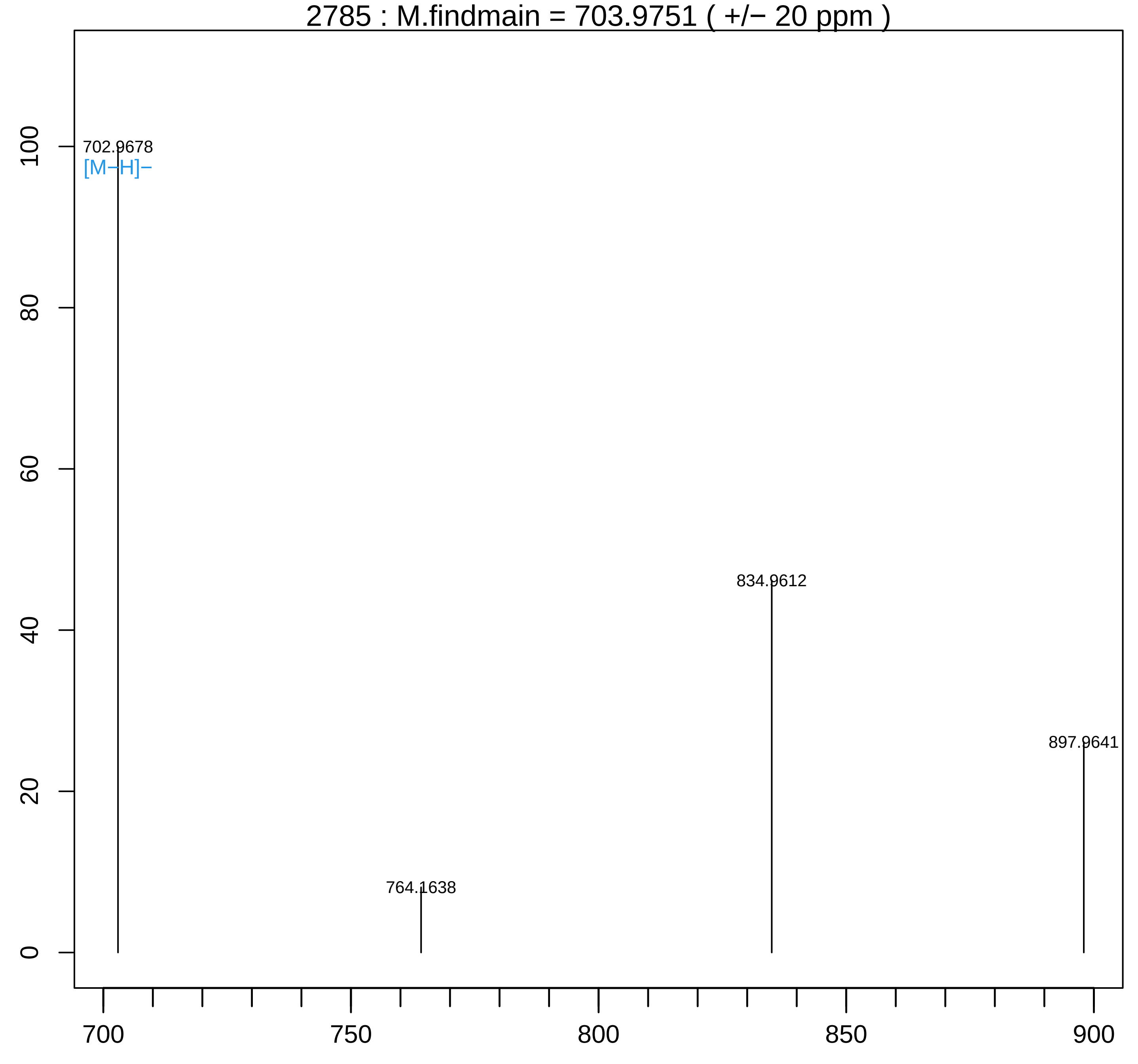
2785 : M.findmain = 703.9751 (+/− 20 ppm)
702.9678
[M−H]−
764.1638
834.9612
897.9641
0
20
40
60
80
100
700
750
800
850
900

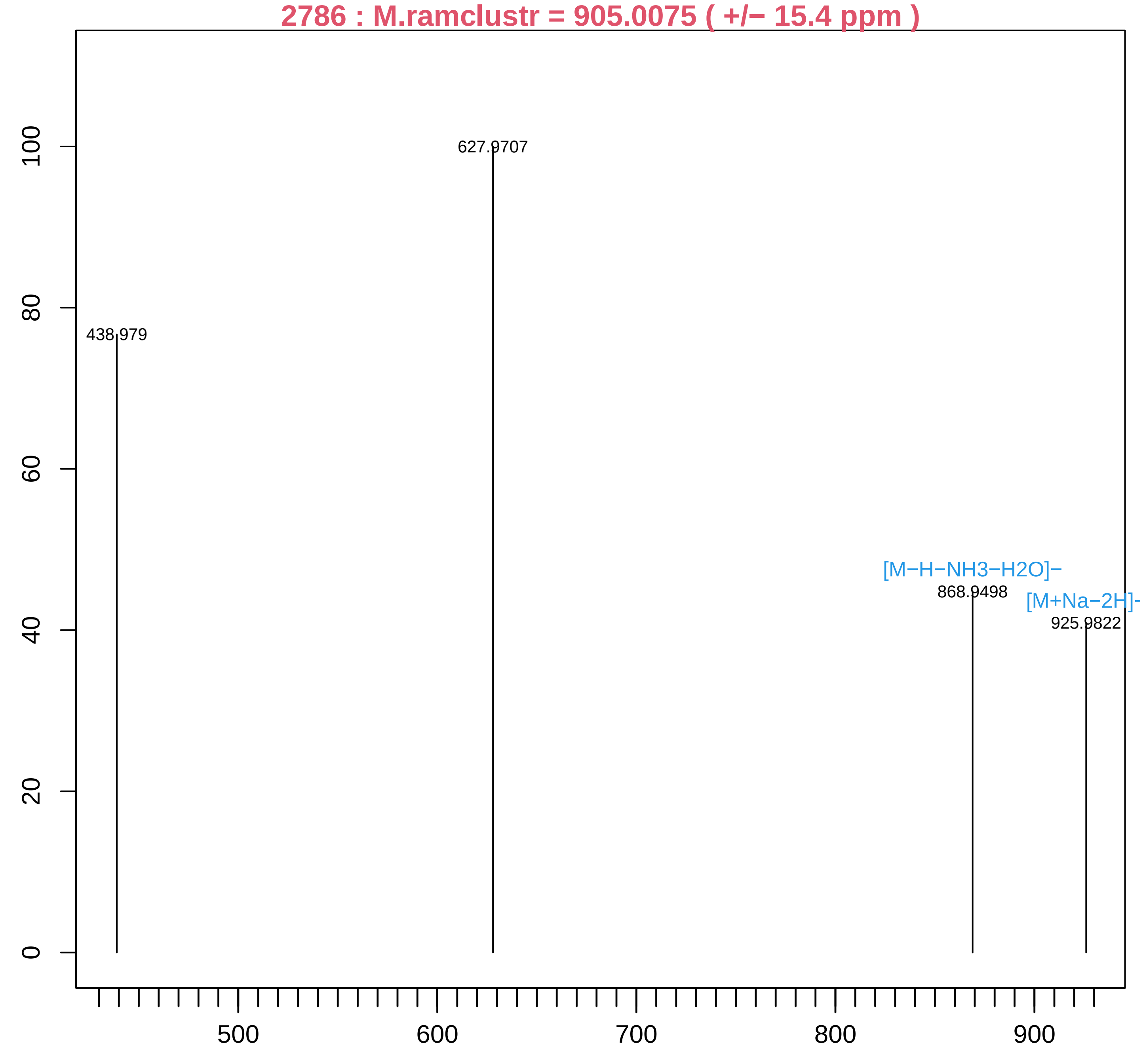
2786 : M.ramclustr = 905.0075 (+/− 15.4 ppm)
627.9707
438.979
[M−H−NH3−H2O]−
868.9498
[M+Na−2H]-
925.9822
0
20
40
60
80
100
500
600
700
800
900

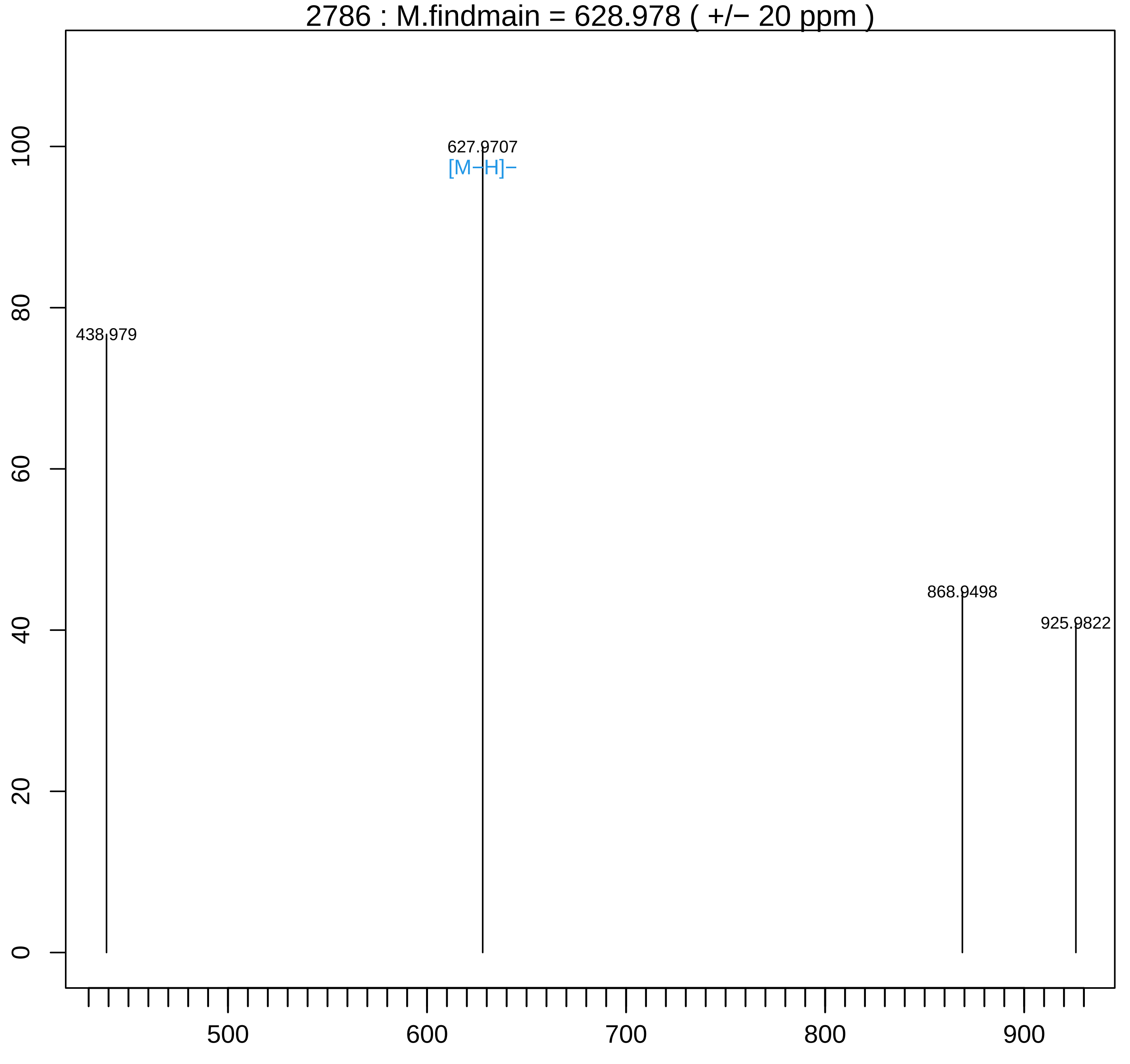
2786 : M.findmain = 628.978 (+/− 20 ppm)
627.9707
[M−H]−
438.979
868.9498
925.9822
0
20
40
60
80
100
500
600
700
800
900

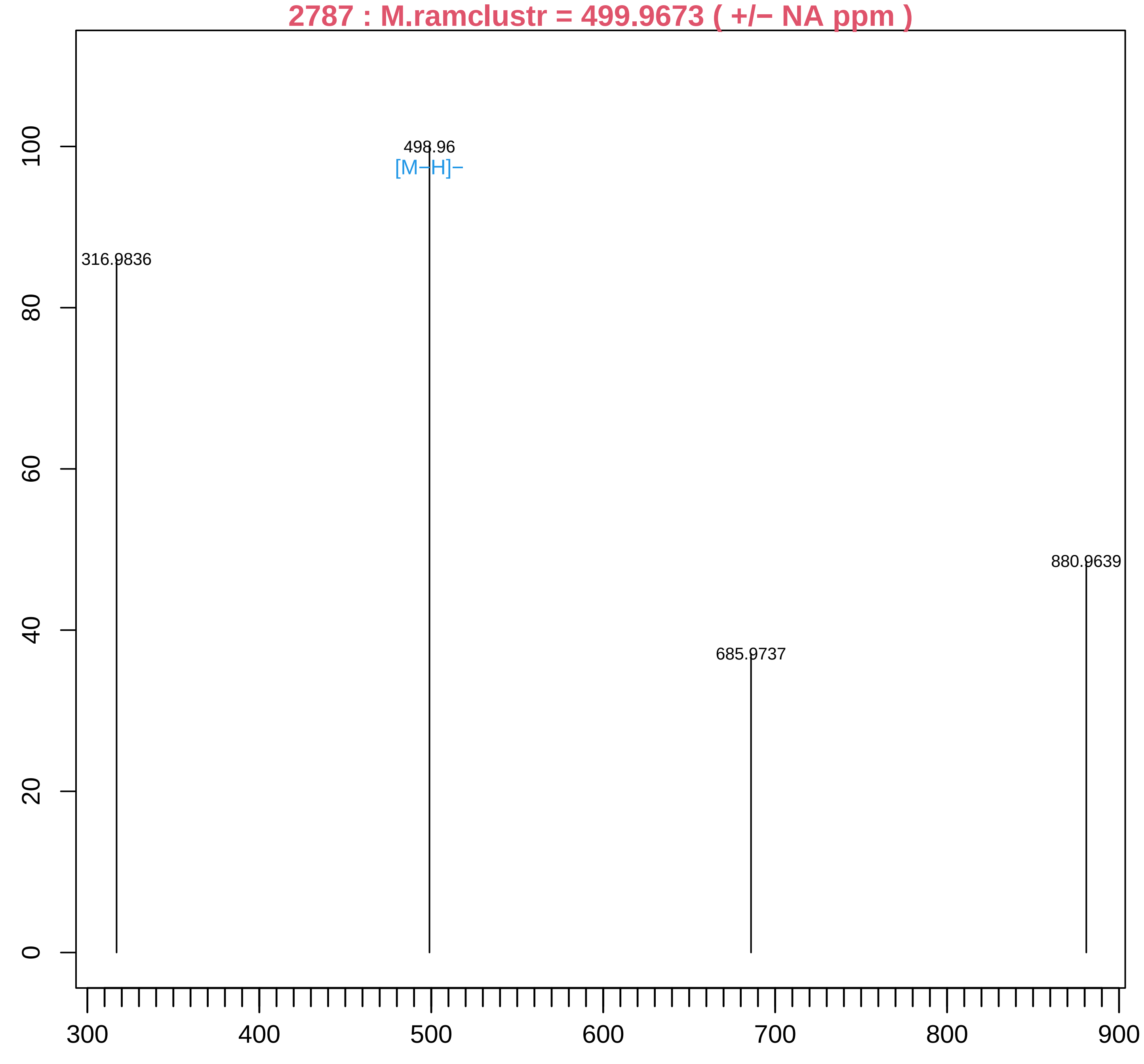
2787 : M.ramclustr = 499.9673 (+/− NA ppm)
316.9836
498.96
[M−H]−
685.9737
880.9639
0
20
40
60
80
100
300
400
500
600
700
800
900

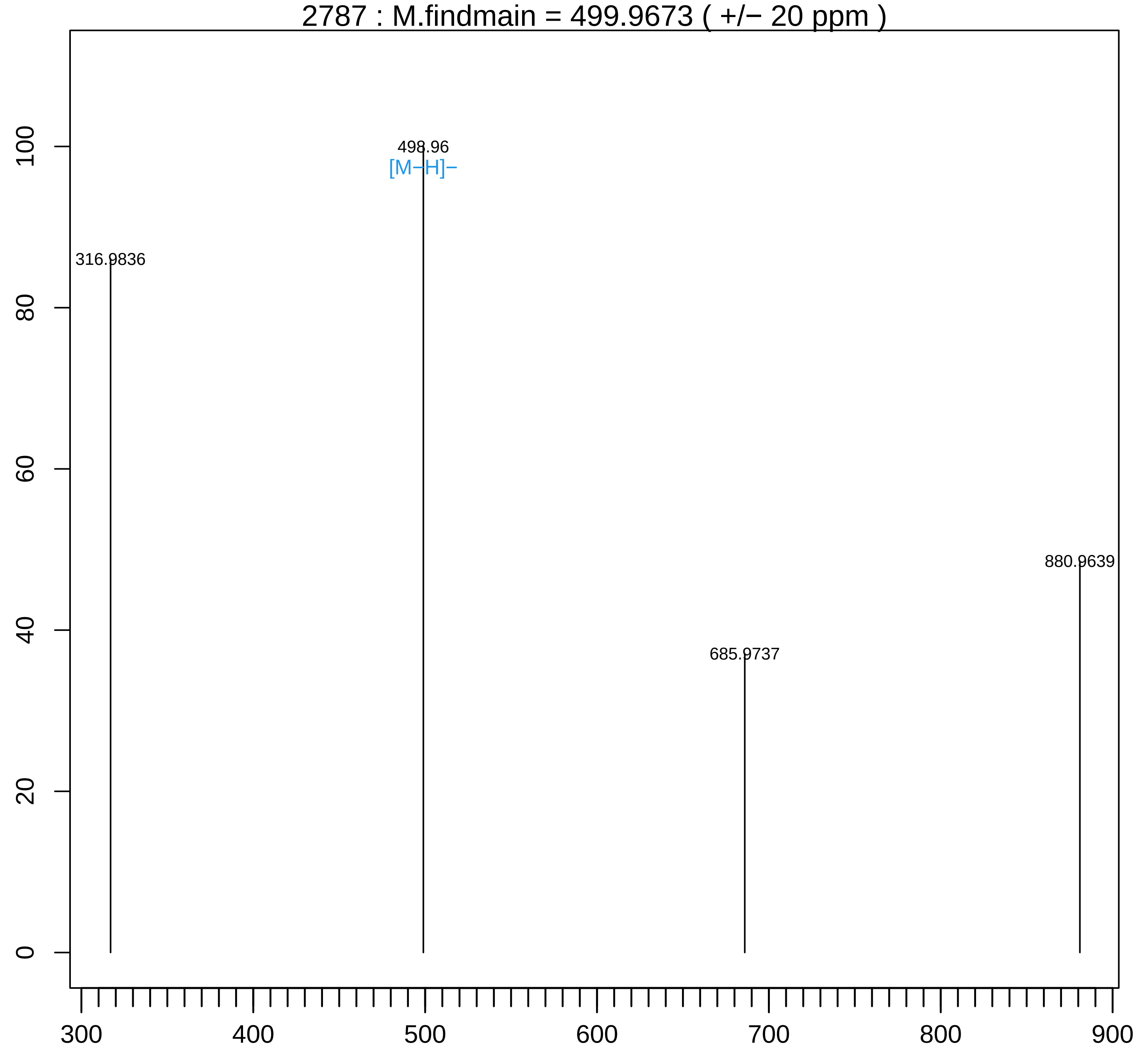
2787 : M.findmain = 499.9673 (+/− 20 ppm)
316.9836
498.96
[M−H]−
685.9737
880.9639
0
20
40
60
80
100
300
400
500
600
700
800
900

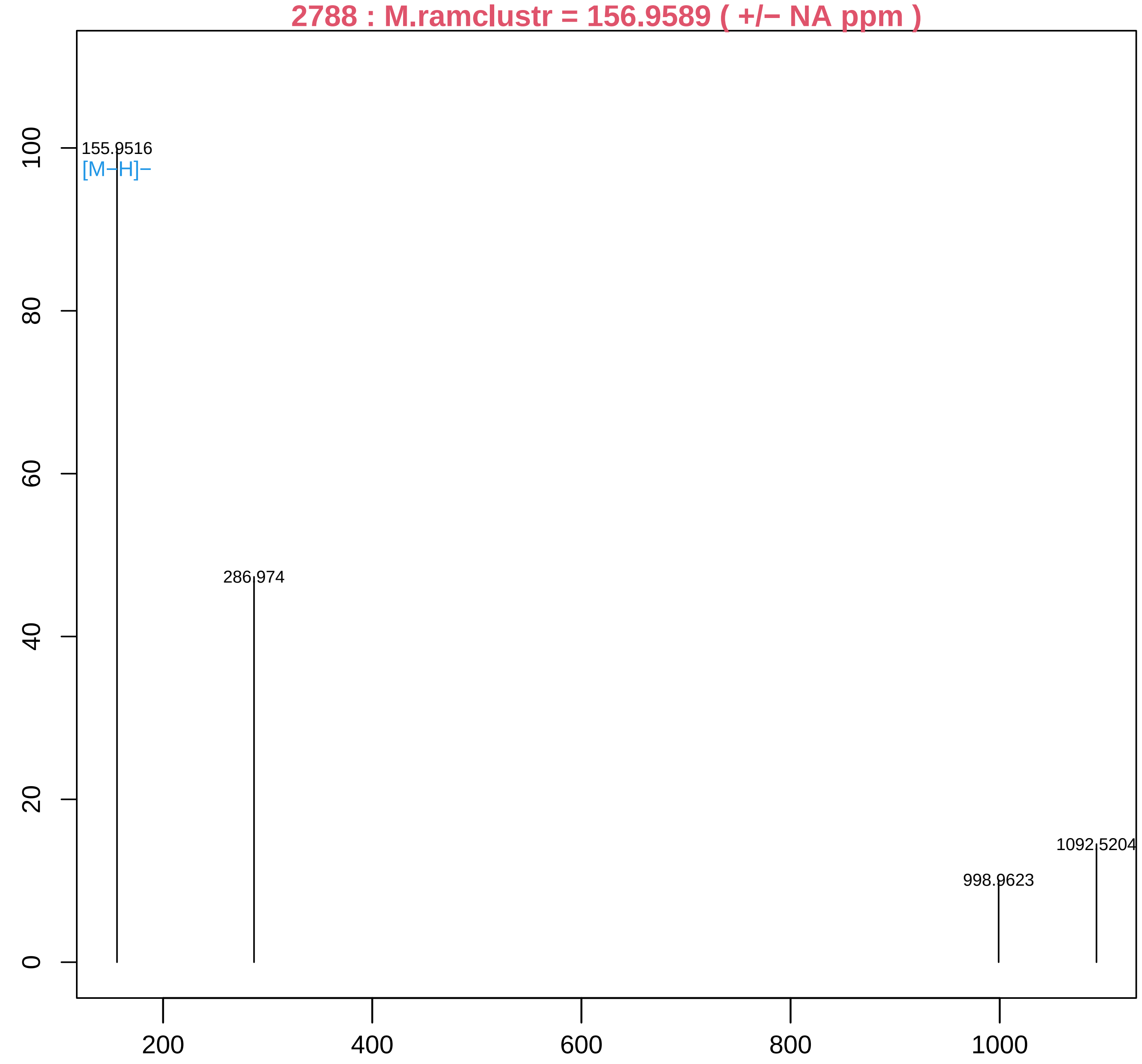
2788 : M.ramclustr = 156.9589 (+/− NA ppm)
155.9516
[M−H]−
286.974
998.9623
1092.5204
0
20
40
60
80
100
200
400
600
800
1000
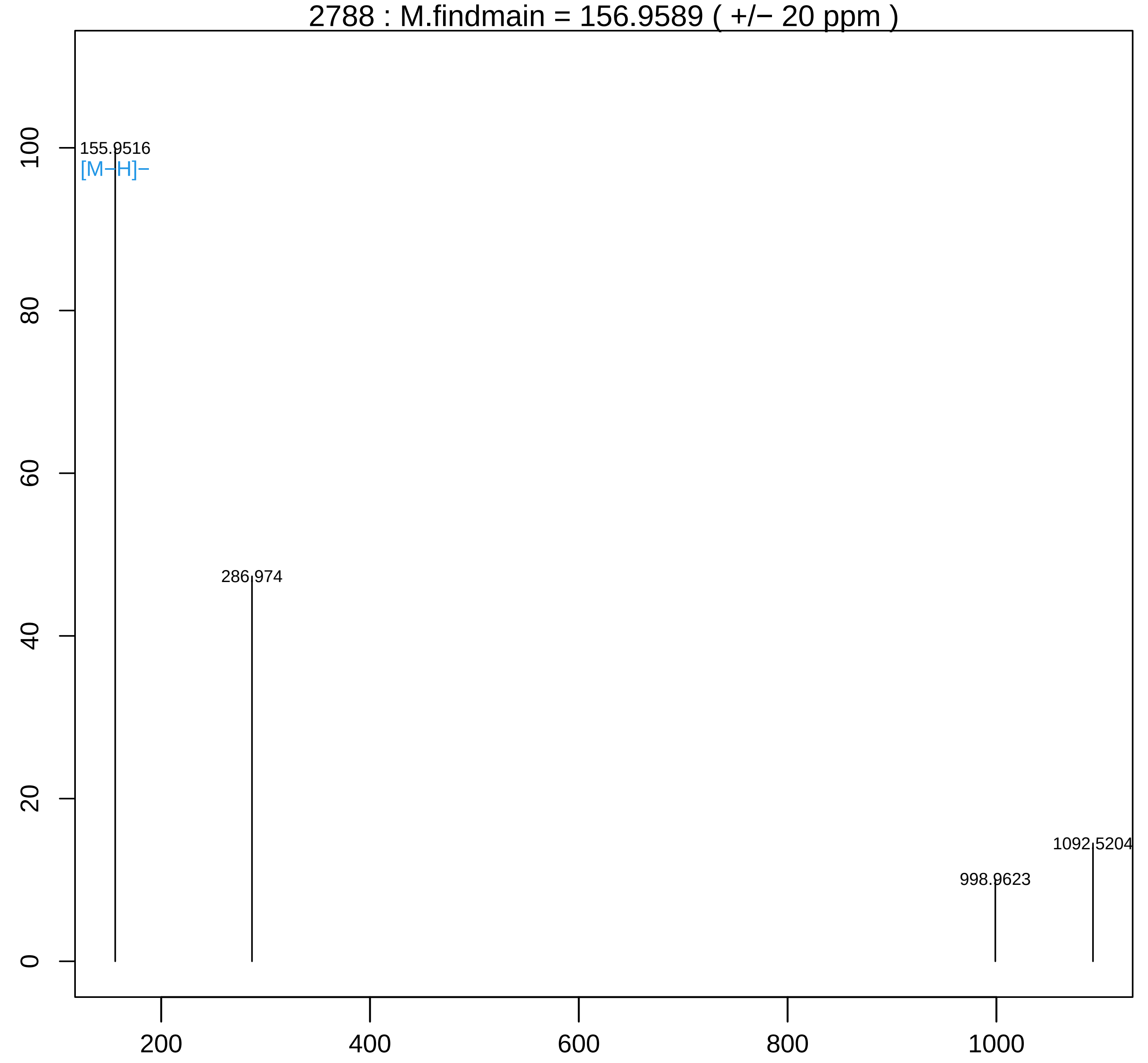
2788 : M.findmain = 156.9589 (+/− 20 ppm)
155.9516
[M−H]−
286.974
998.9623
1092.5204
0
20
40
60
80
100
200
400
600
800
1000

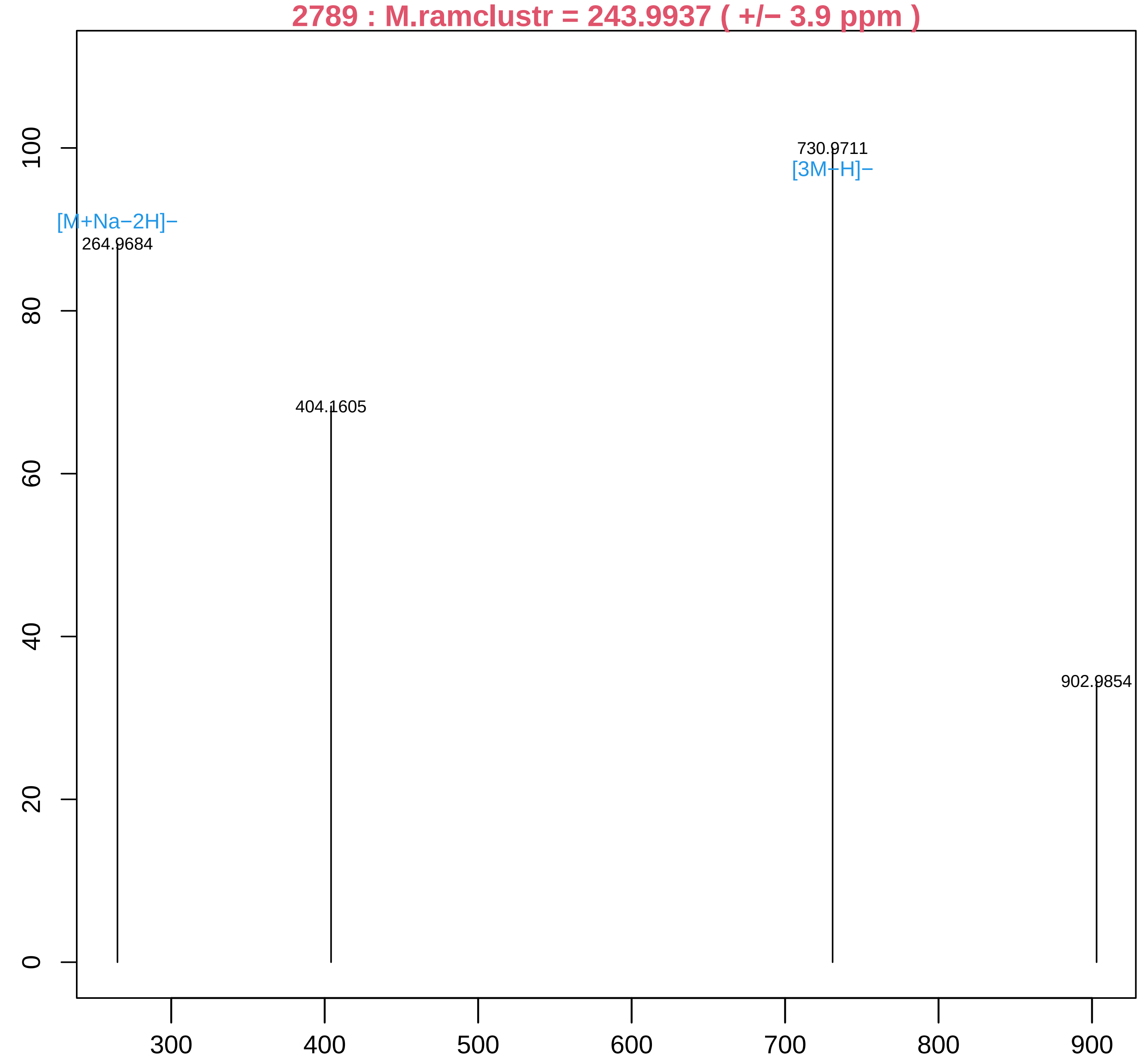

2789 : M.ramclustr = 243.9937 (+/− 3.9 ppm)
[M+Na−2H]−
264.9684
404.1605
730.9711
[3M−H]−
902.9854

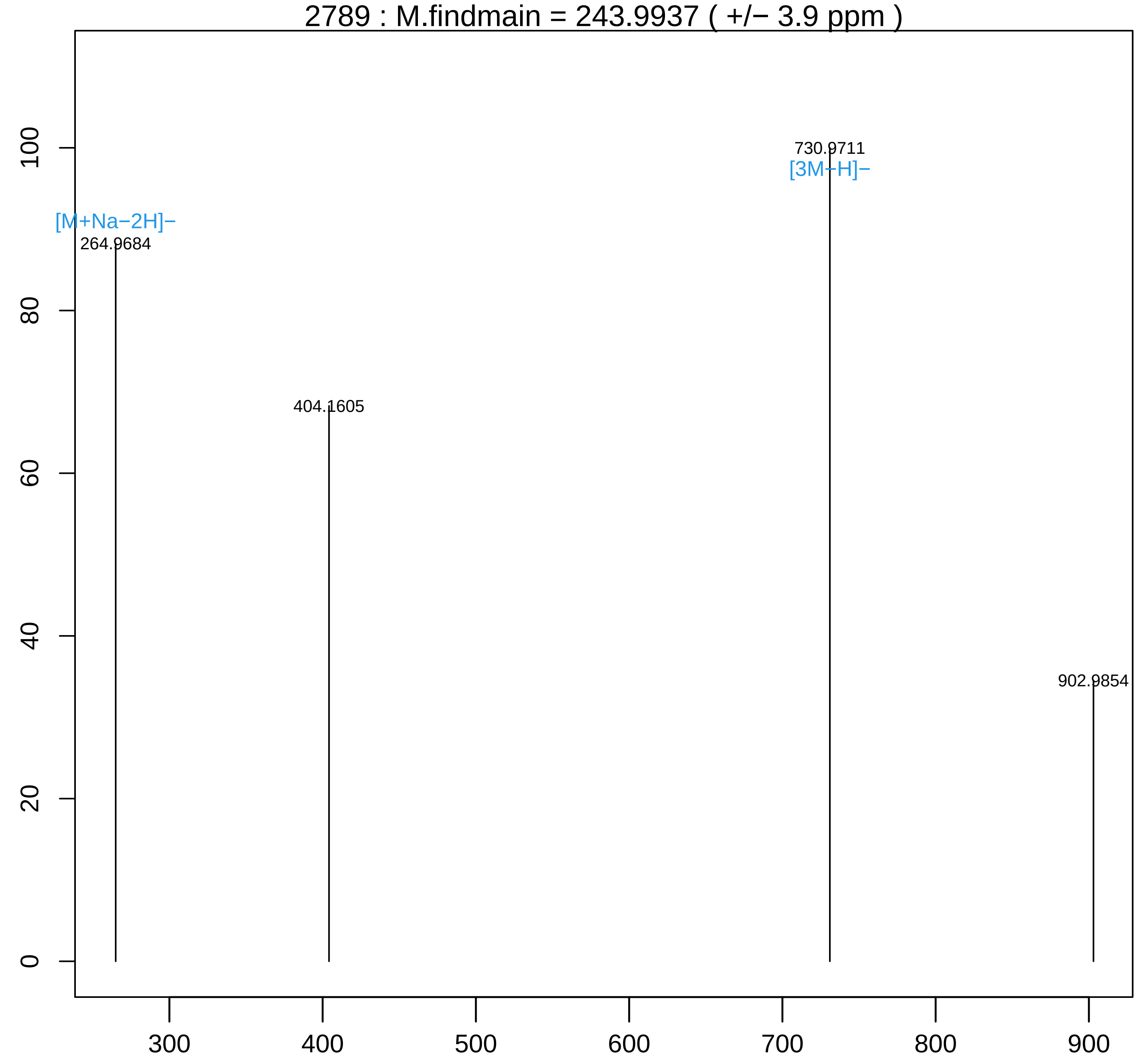

2789 : M.findmain = 243.9937 (+/− 3.9 ppm)
[M+Na−2H]−
264.9684
404.1605
730.9711
[3M−H]−
902.9854

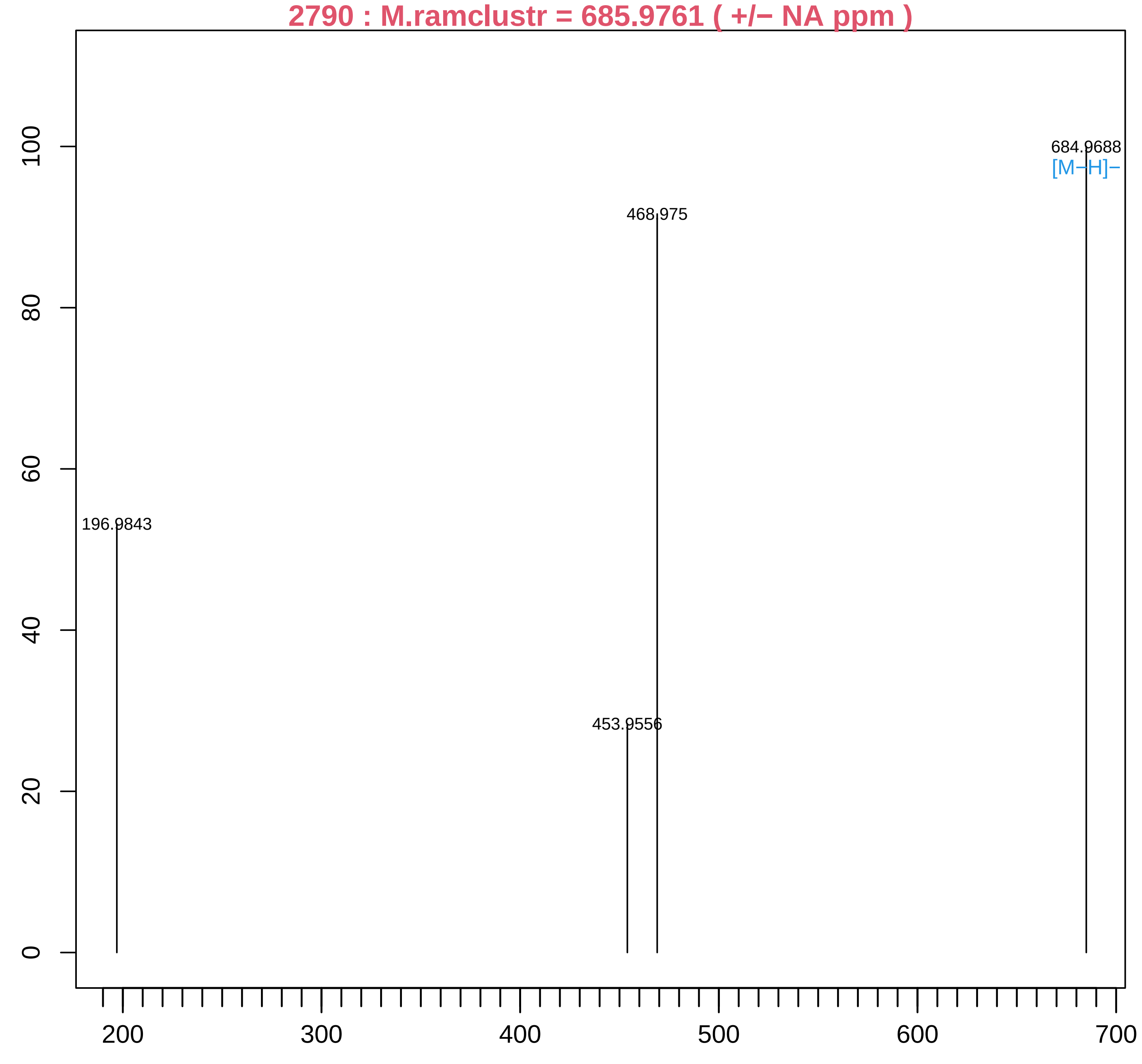
2790 : M.ramclustr = 685.9761 (+/− NA ppm)
684.9688
[M−H]−
468.975
196.9843
453.9556

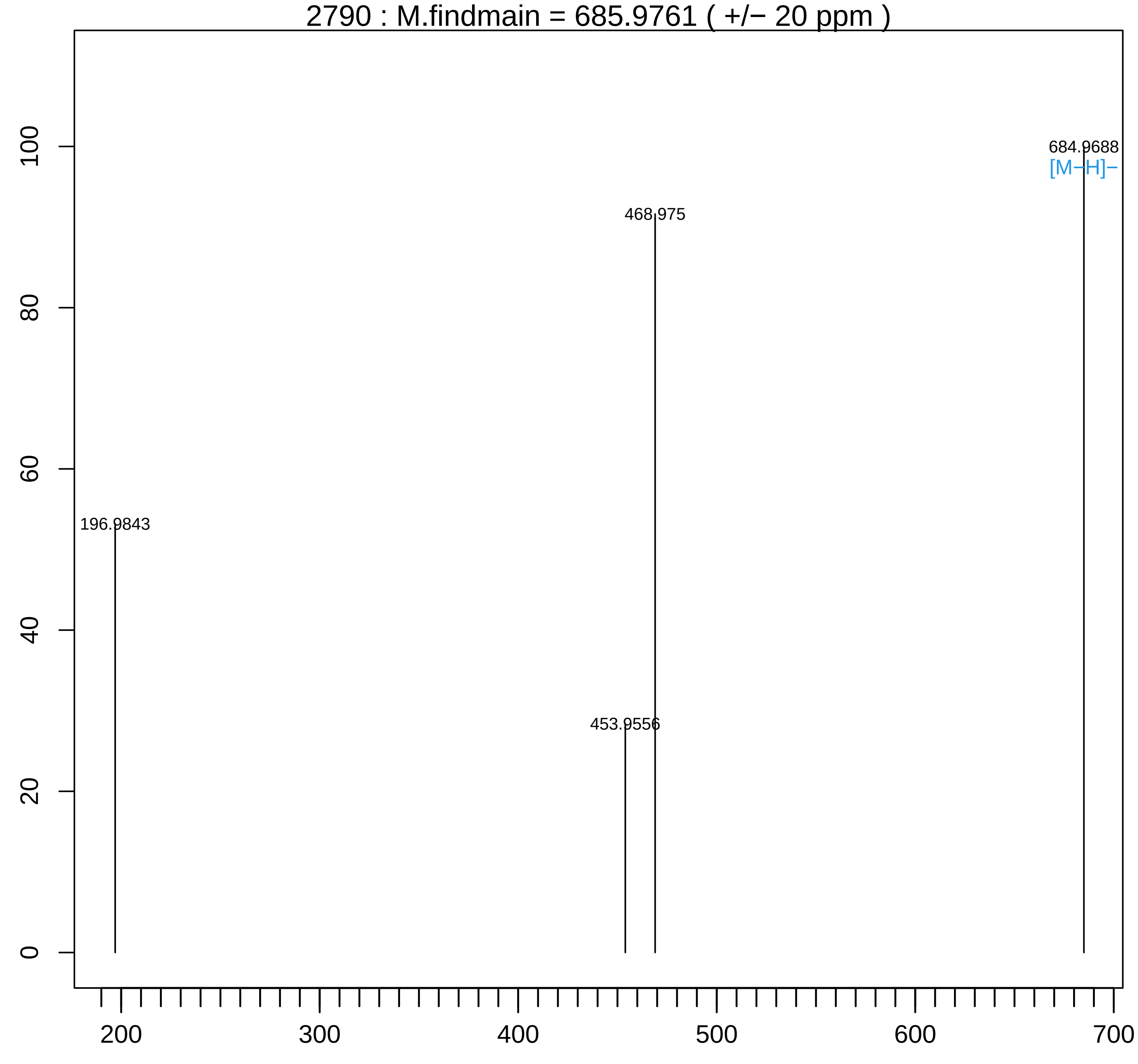
2790 : M.findmain = 685.9761 (+/− 20 ppm)
684.9688
[M−H]−
468.975
196.9843
453.9556

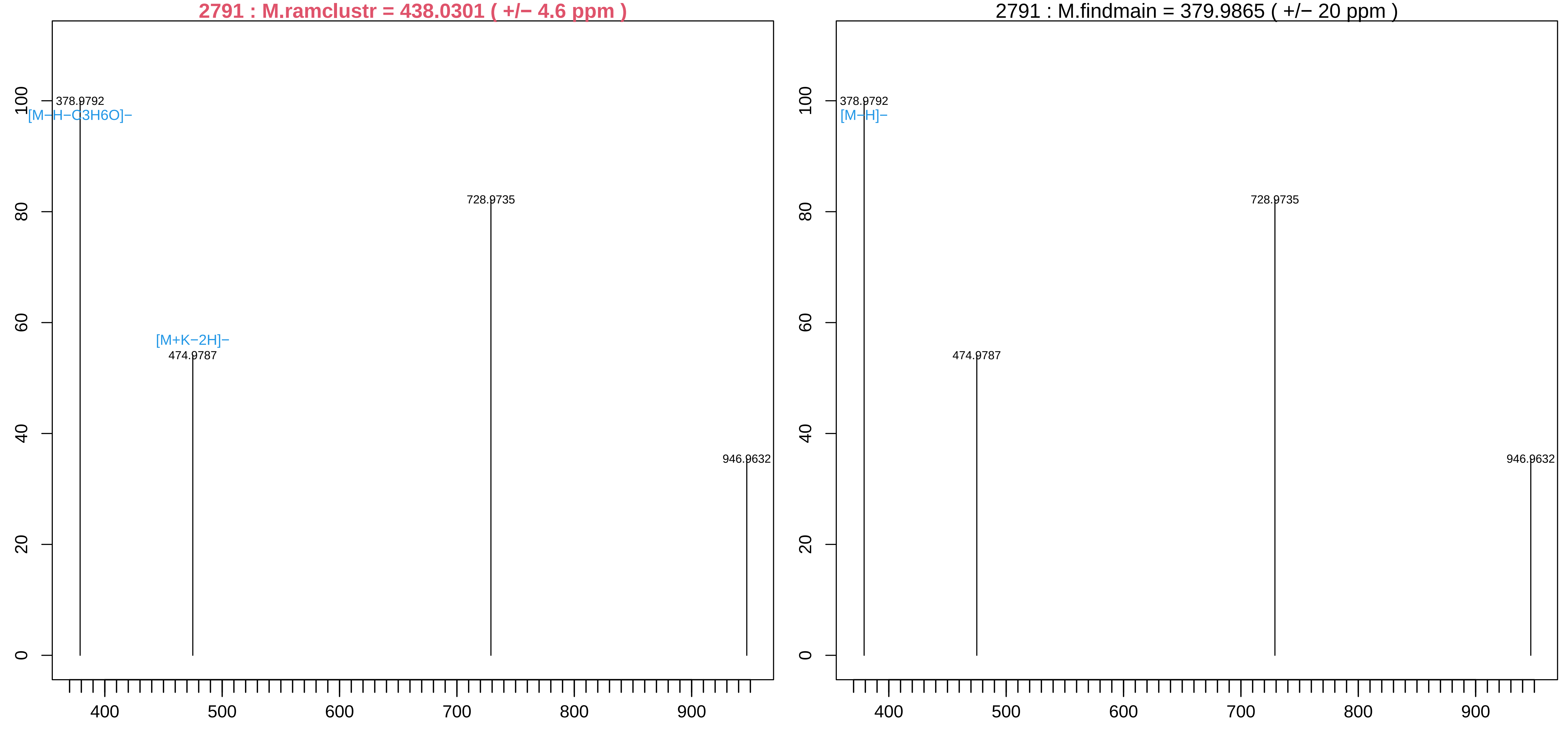
2791 : M.ramclustr = 438.0301 (+/− 4.6 ppm)
378.9792
[M−H−C3H6O]−
[M+K−2H]−
474.9787
728.9735
946.9632
2791 : M.findmain = 379.9865 (+/− 20 ppm)
378.9792
[M−H]−
474.9787
728.9735
946.9632
0
20
40
60
80
100
400
500
600
700
800
900

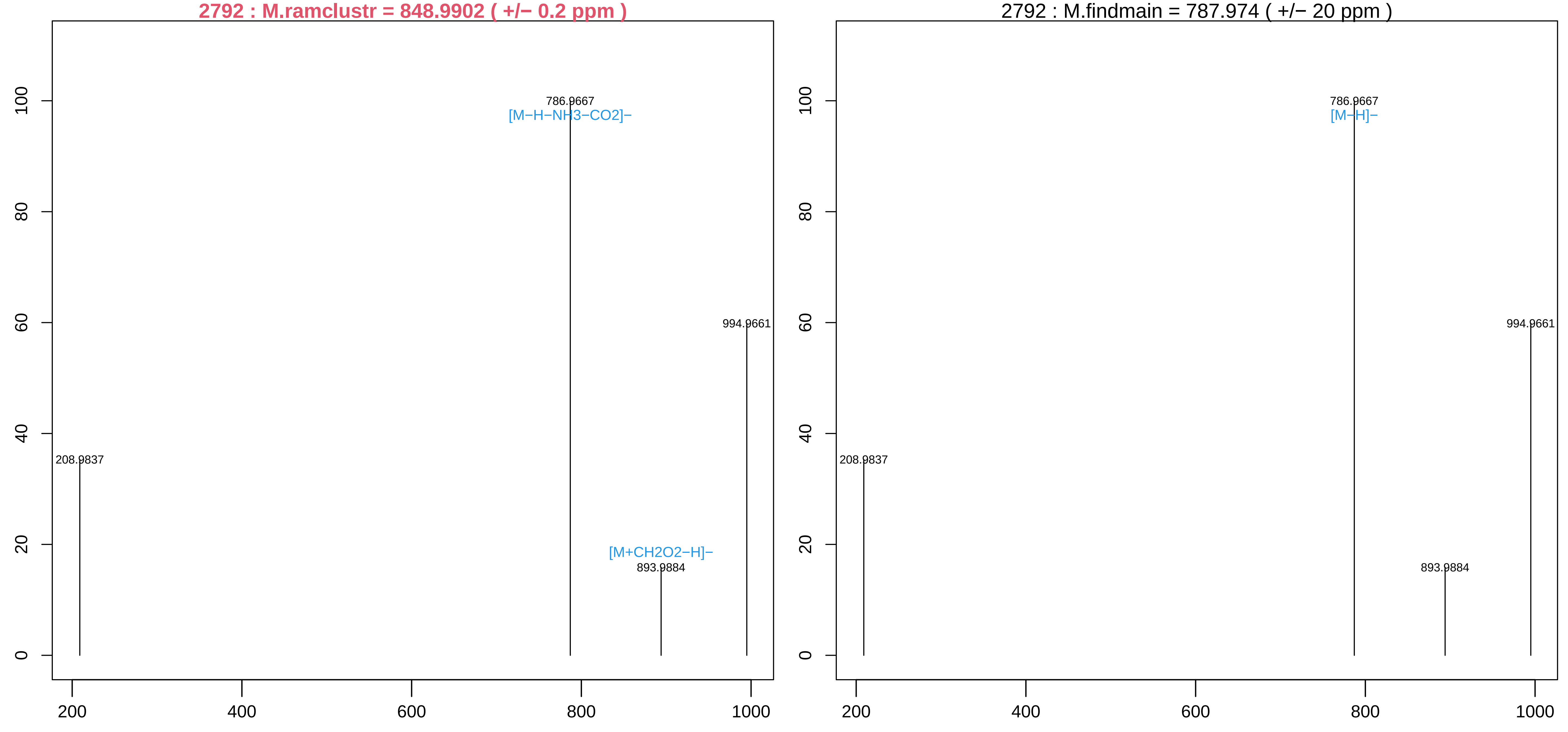

2792 : M.ramclustr = 848.9902 (+/− 0.2 ppm)
786.9667
[M−H−NH3−CO2]−
994.9661
208.9837
[M+CH2O2−H]−
893.9884
2792 : M.findmain = 787.974 (+/− 20 ppm)
786.9667
[M−H]−
994.9661
208.9837
893.9884
0
20
40
60
80
100
200
400
600
800
1000

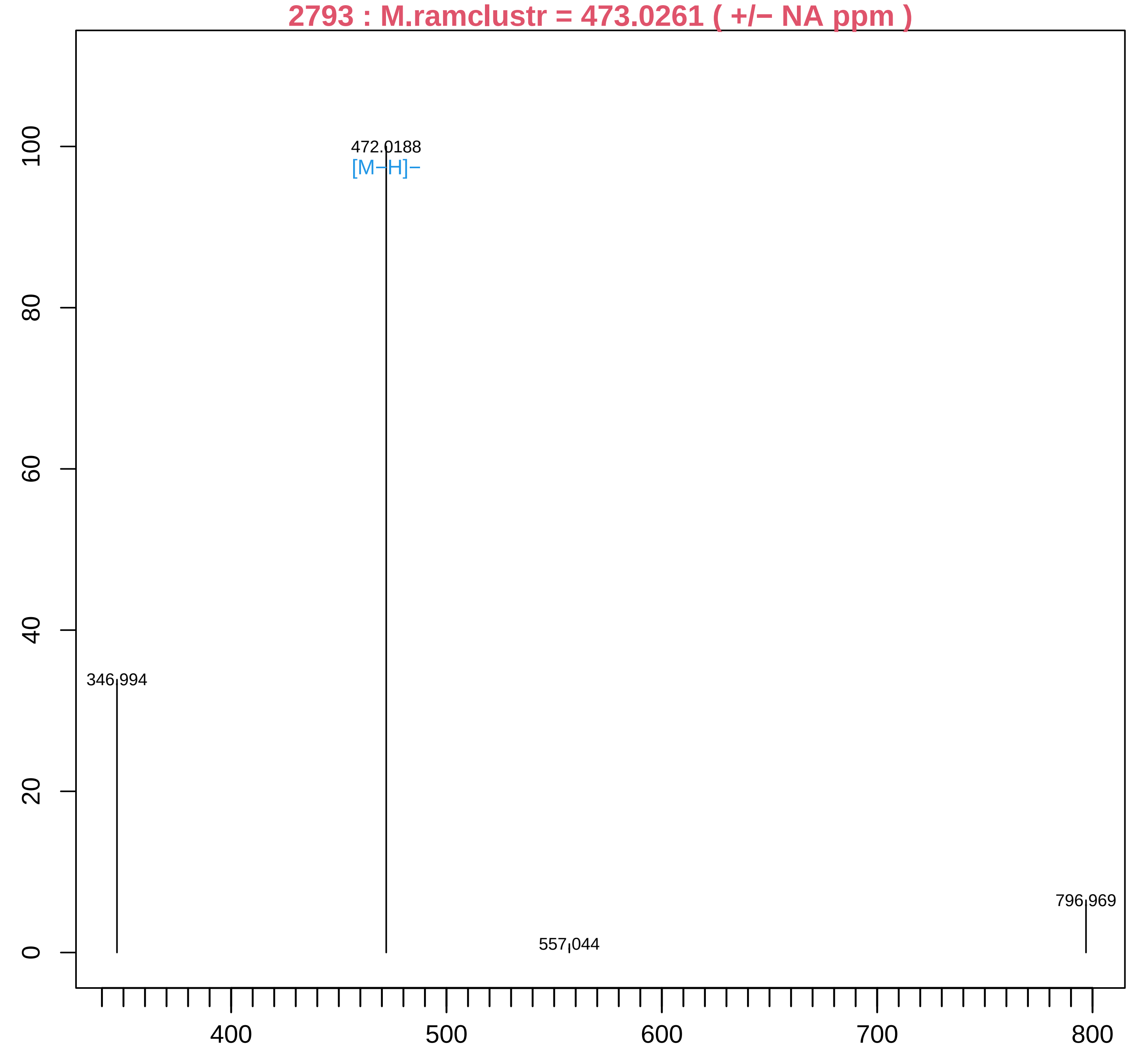

2793 : M.ramclustr = 473.0261 (+/− NA ppm)
472.0188
[M−H]−
346.994
557.044
796.969
0
20
40
60
80
100
400
500
600
700
800

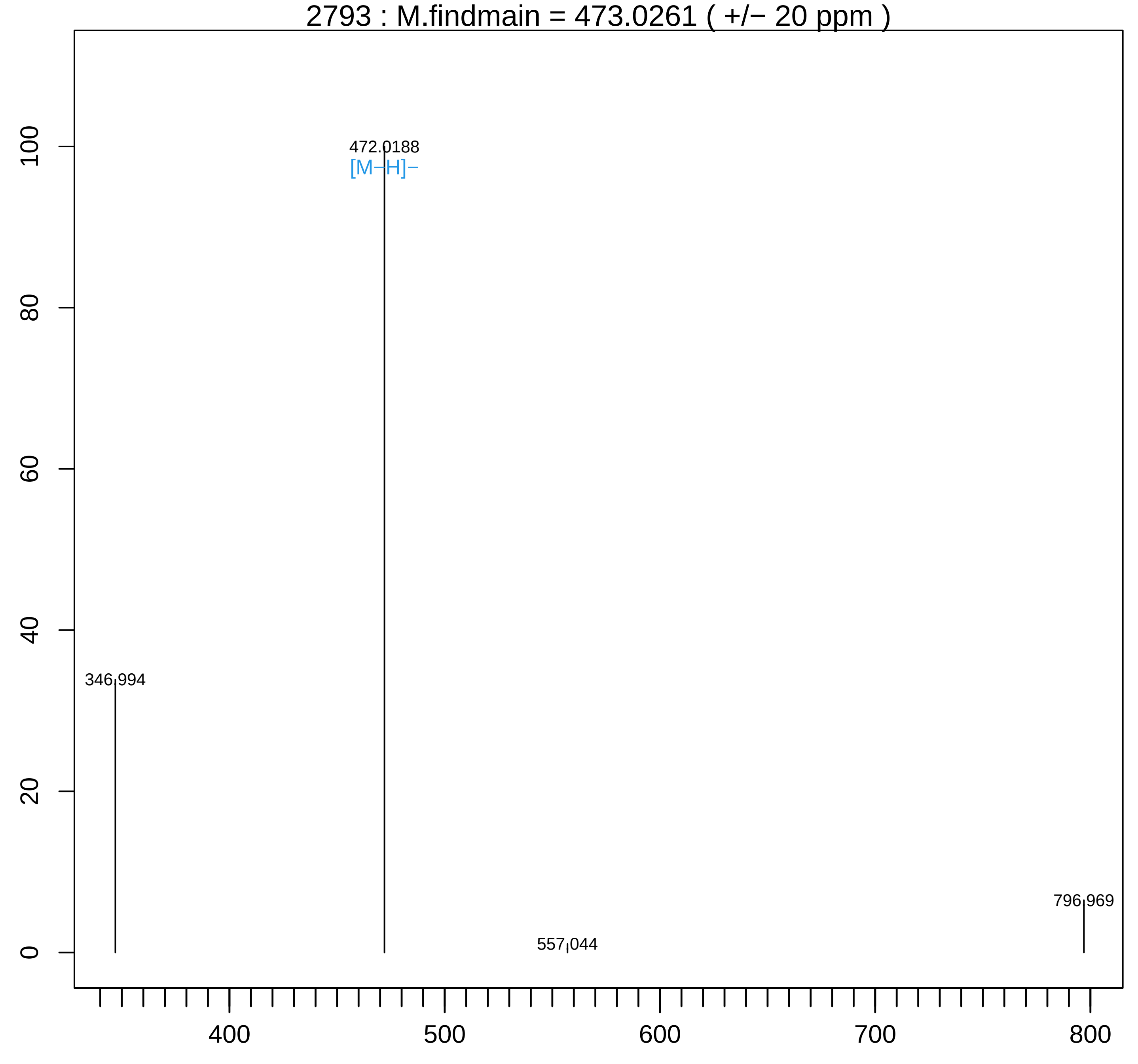

2793 : M.findmain = 473.0261 (+/− 20 ppm)
472.0188
[M−H]−
346.994
557.044
796.969
0
20
40
60
80
100
400
500
600
700
800

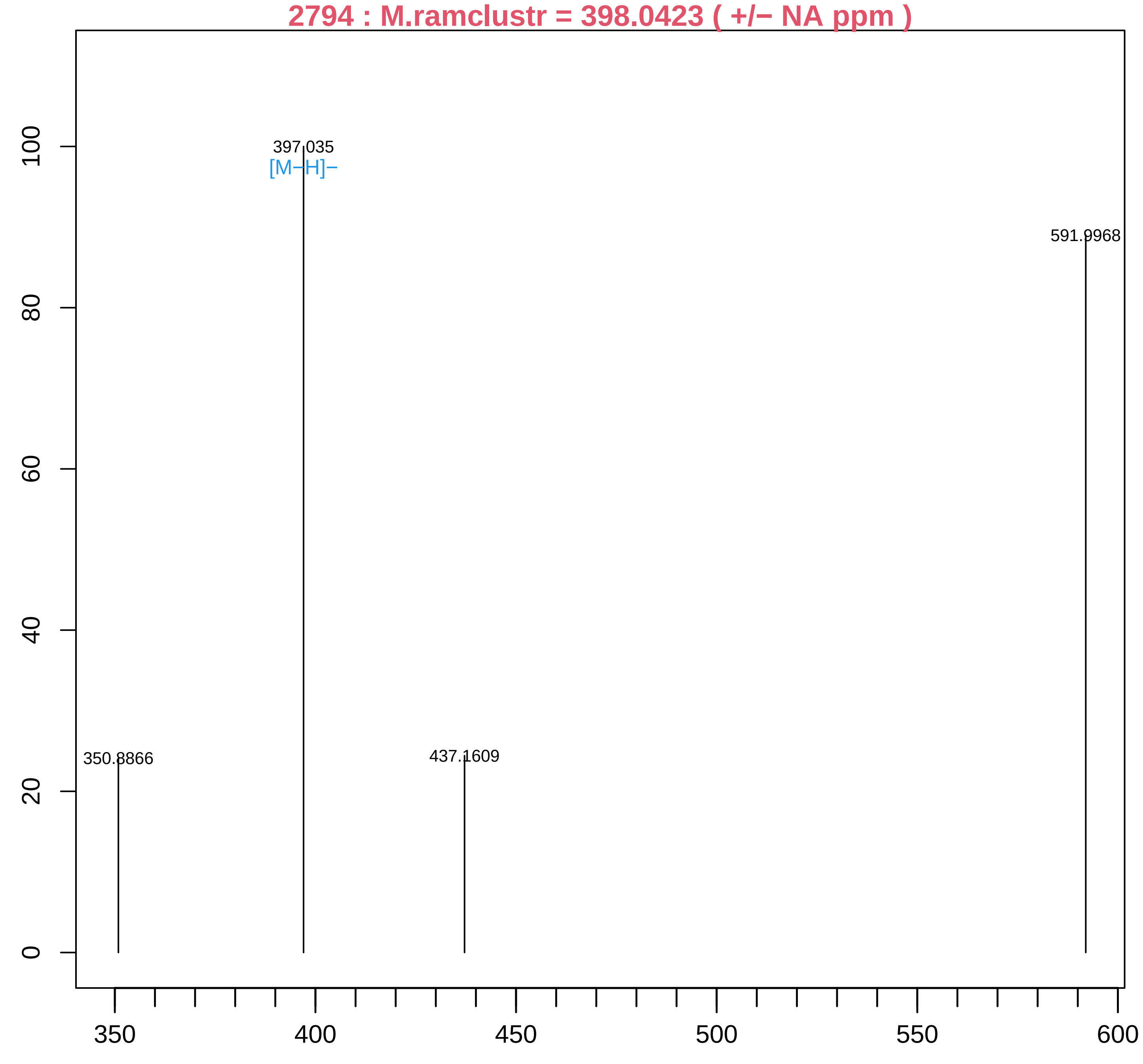
2794 : M.ramclustr = 398.0423 (+/− NA ppm)
397.035
[M−H]−
591.9968
350.8866
437.1609
0
20
40
60
80
100
350
400
450
500
550
600
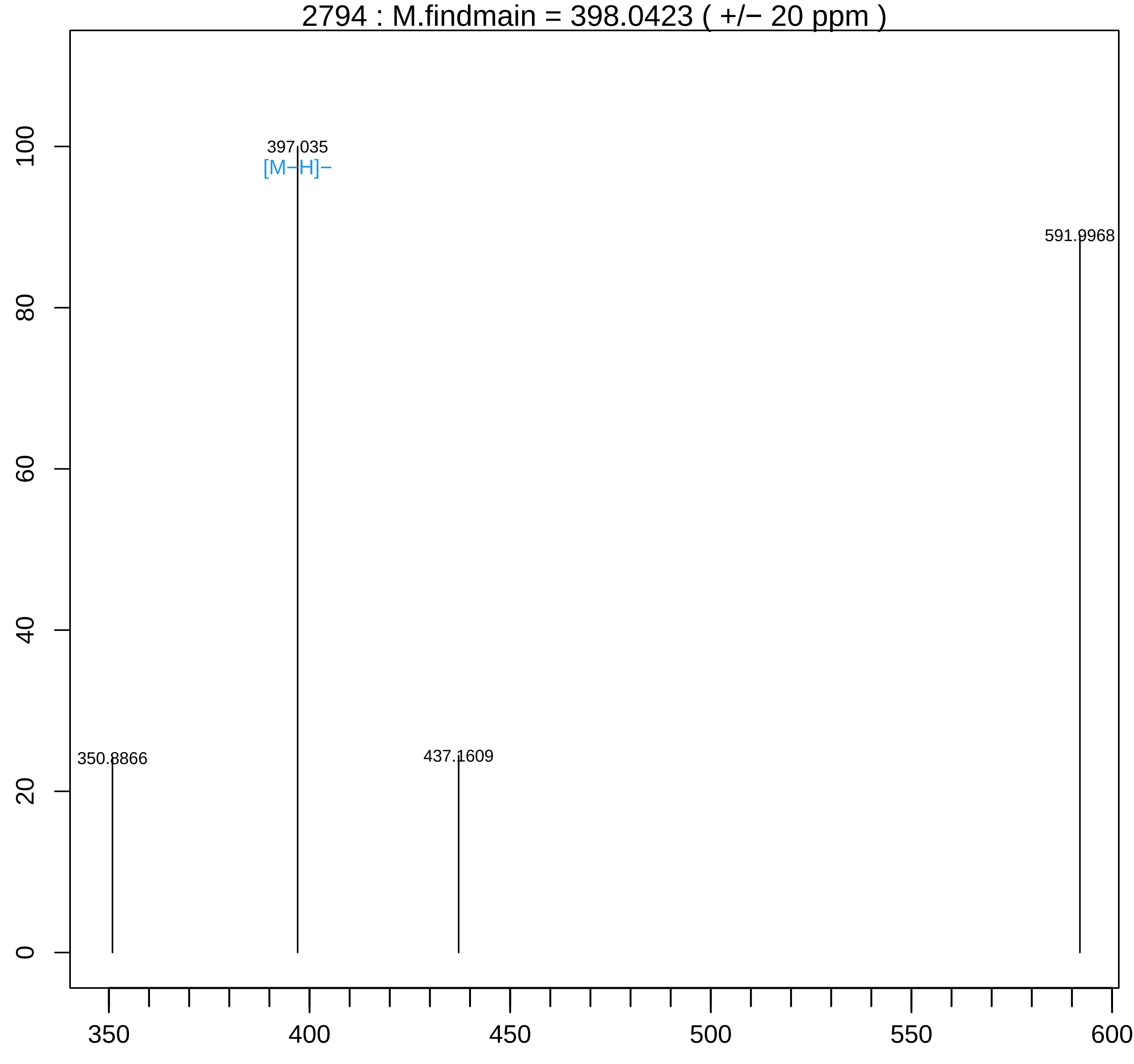
2794 : M.findmain = 398.0423 (+/− 20 ppm)
397.035
[M−H]−
591.9968
350.8866
437.1609
0
20
40
60
80
100
350
400
450
500
550
600

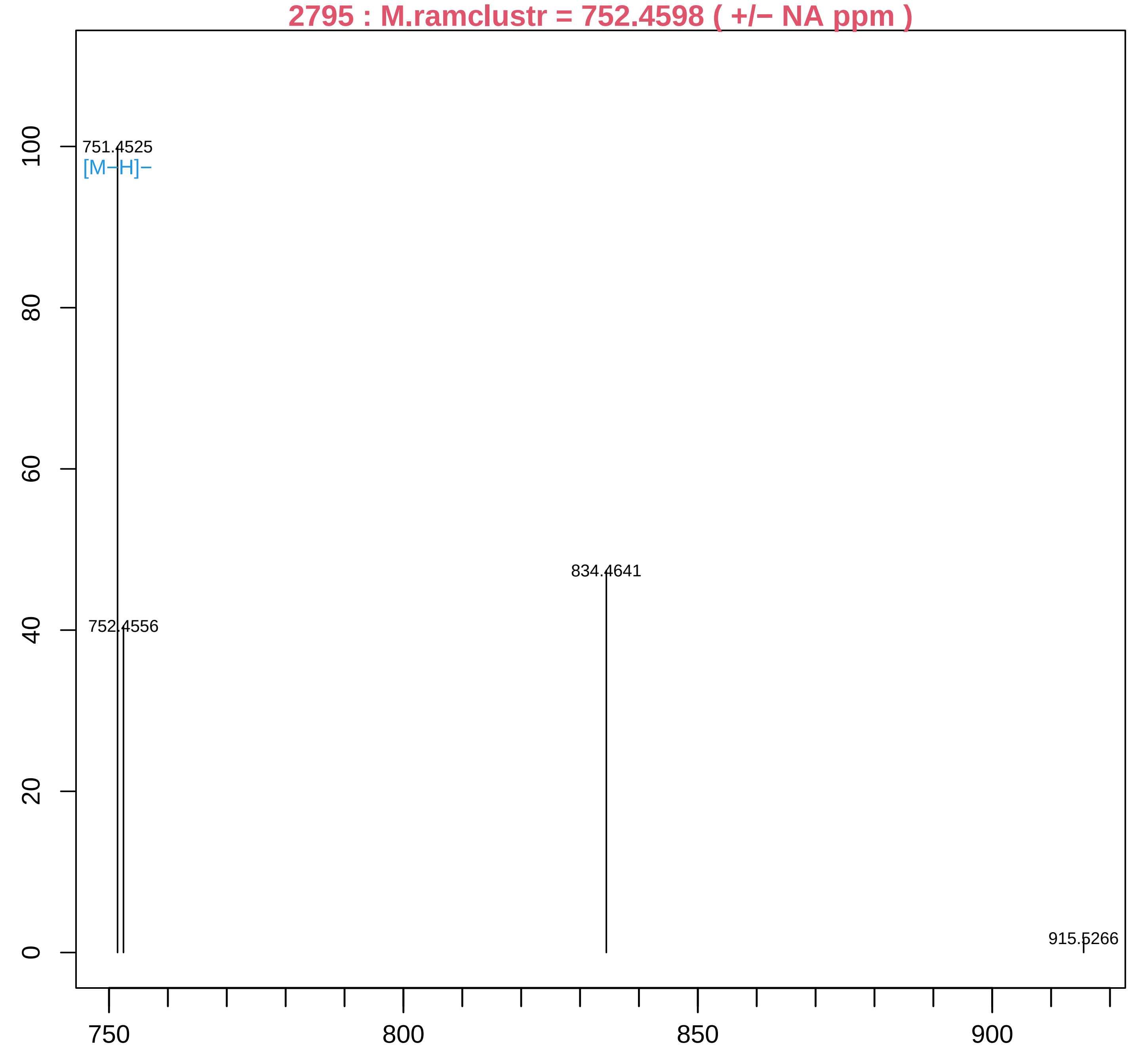

2795 : M.ramclustr = 752.4598 (+/− NA ppm)
751.4525
[M−H]−
752.4556
834.4641
915.5266
0
20
40
60
80
100
750
800
850
900

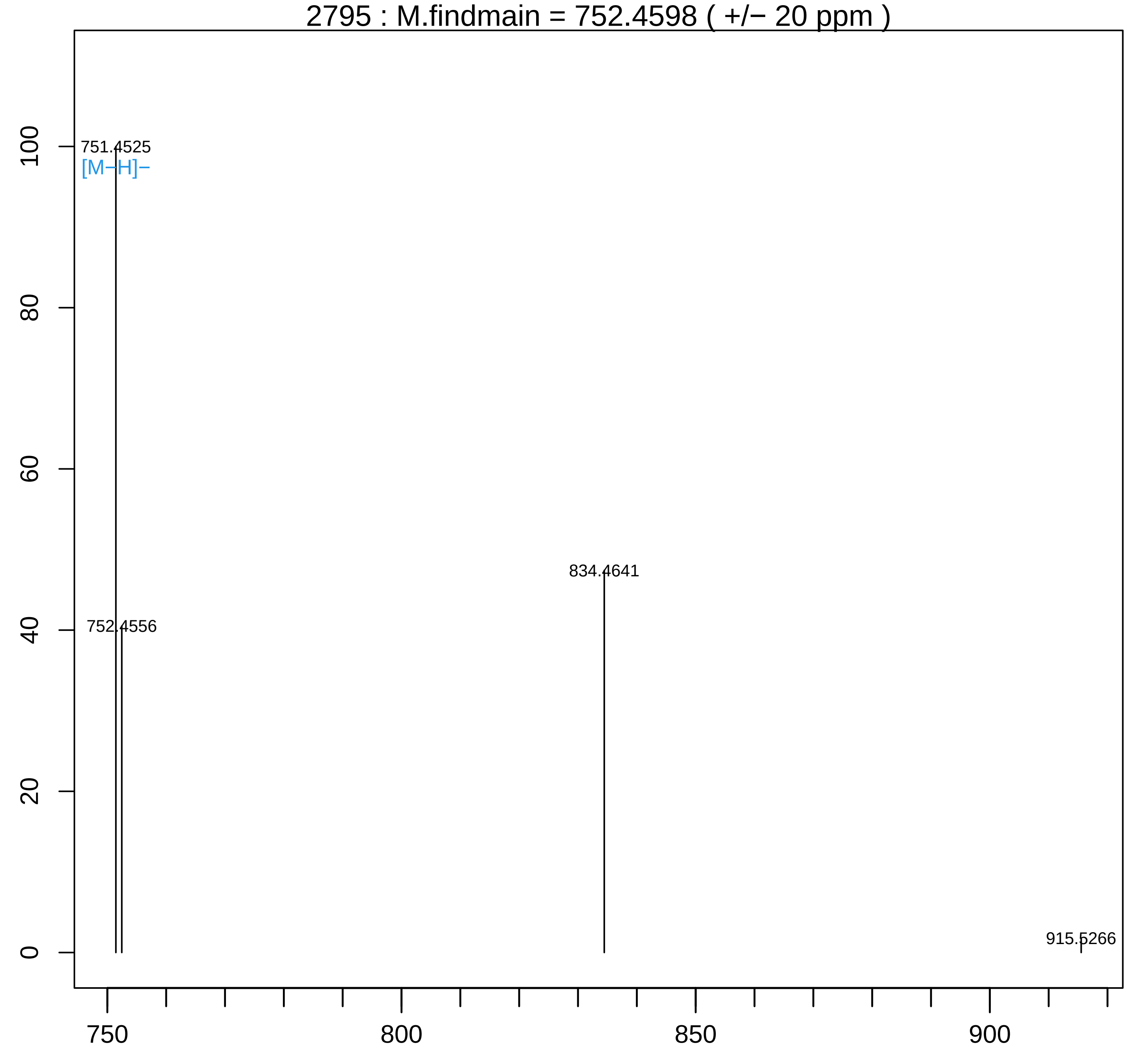

2795 : M.findmain = 752.4598 (+/− 20 ppm)
751.4525
[M−H]−
752.4556
834.4641
915.5266
0
20
40
60
80
100
750
800
850
900

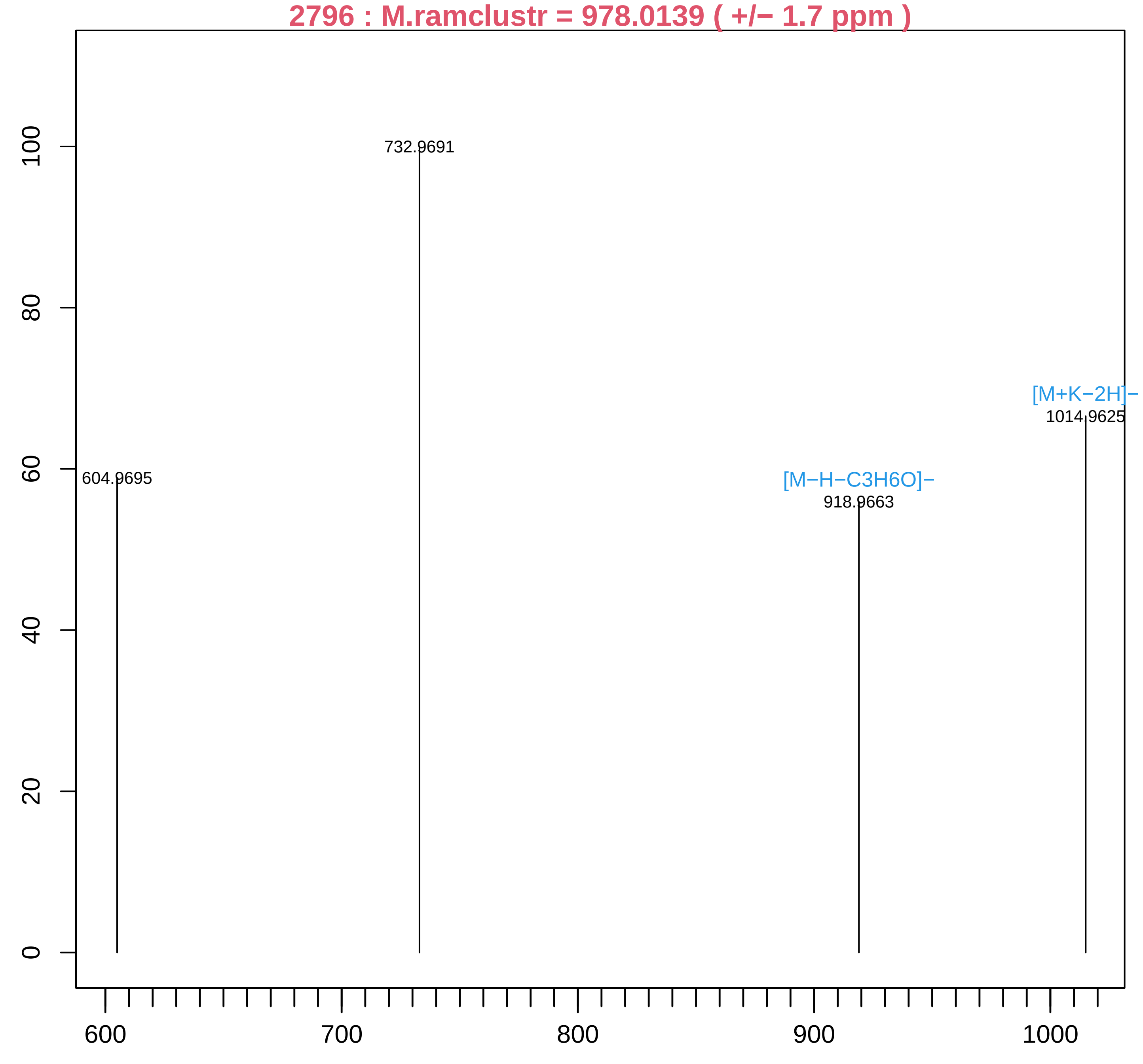
2796 : M.ramclustr = 978.0139 (+/− 1.7 ppm)
732.9691
604.9695
[M−H−C3H6O]−
918.9663
[M+K−2H]−
1014.9625
0
20
40
60
80
100
600
700
800
900
1000

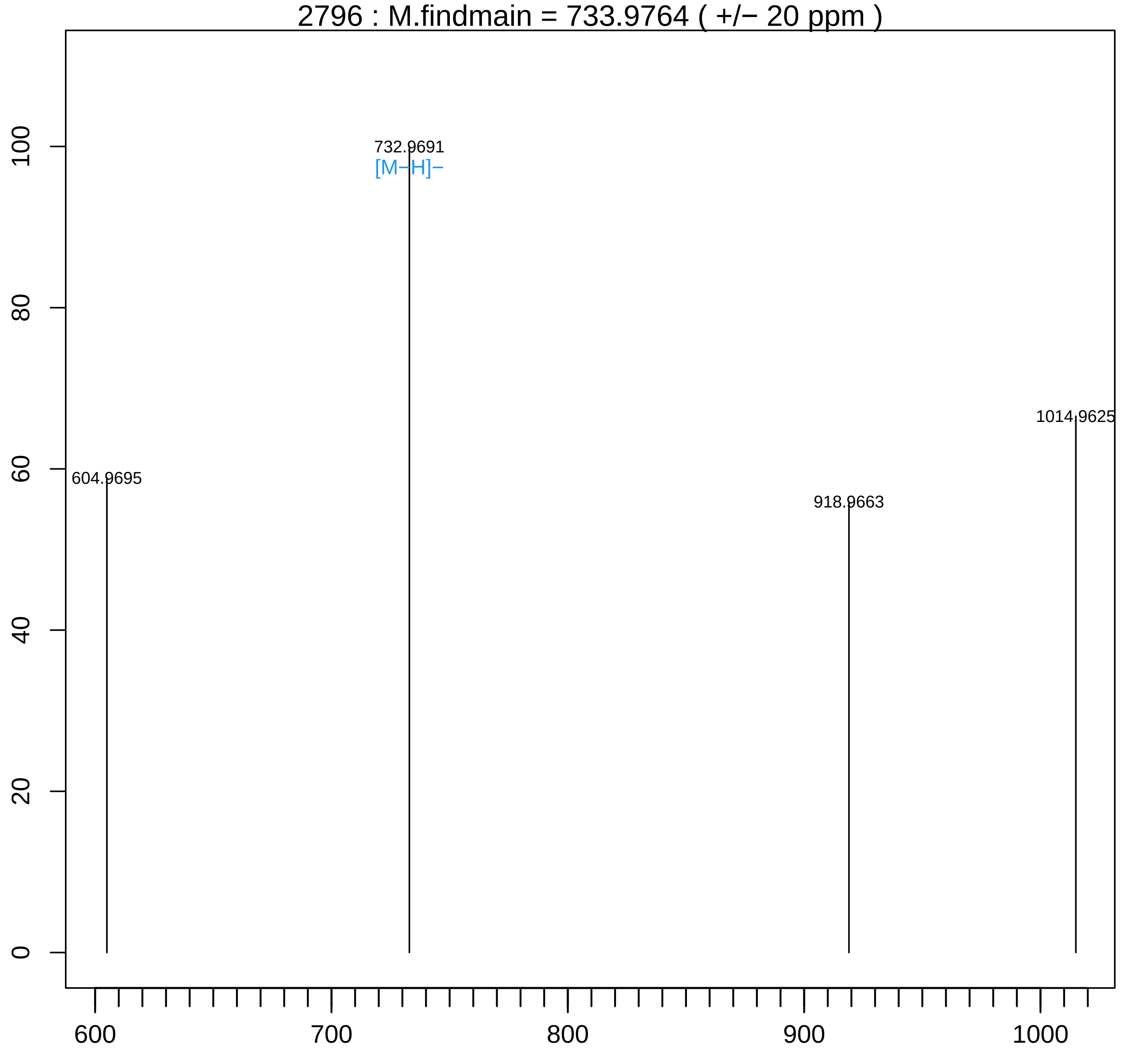
2796 : M.findmain = 733.9764 (+/− 20 ppm)
732.9691
[M−H]−
604.9695
918.9663
1014.9625
0
20
40
60
80
100
600
700
800
900
1000

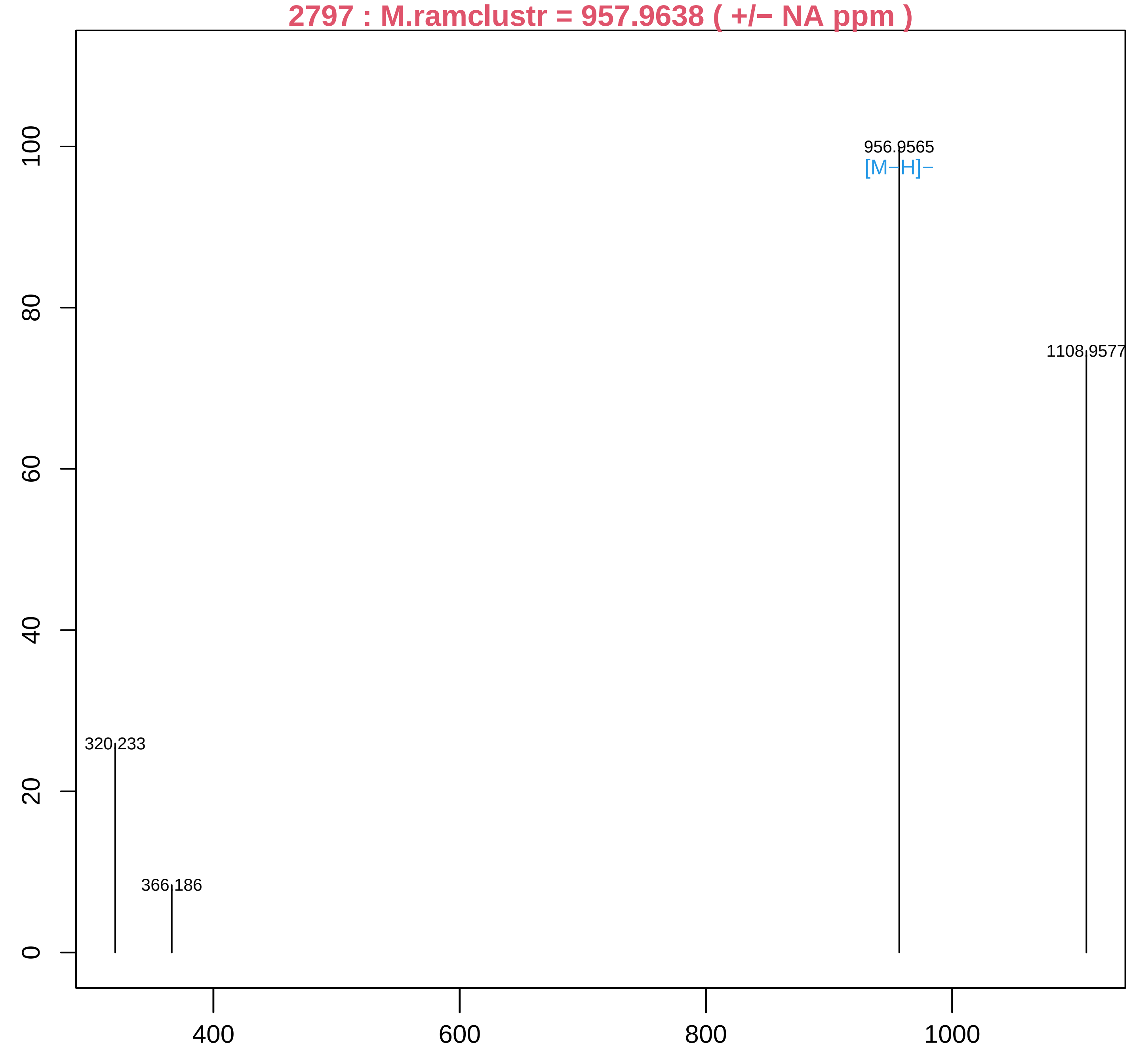
2797 : M.ramclustr = 957.9638 (+/− NA ppm)
956.9565
[M−H]−
1108.9577
320.233
366.186
0
20
40
60
80
100
400
600
800
1000

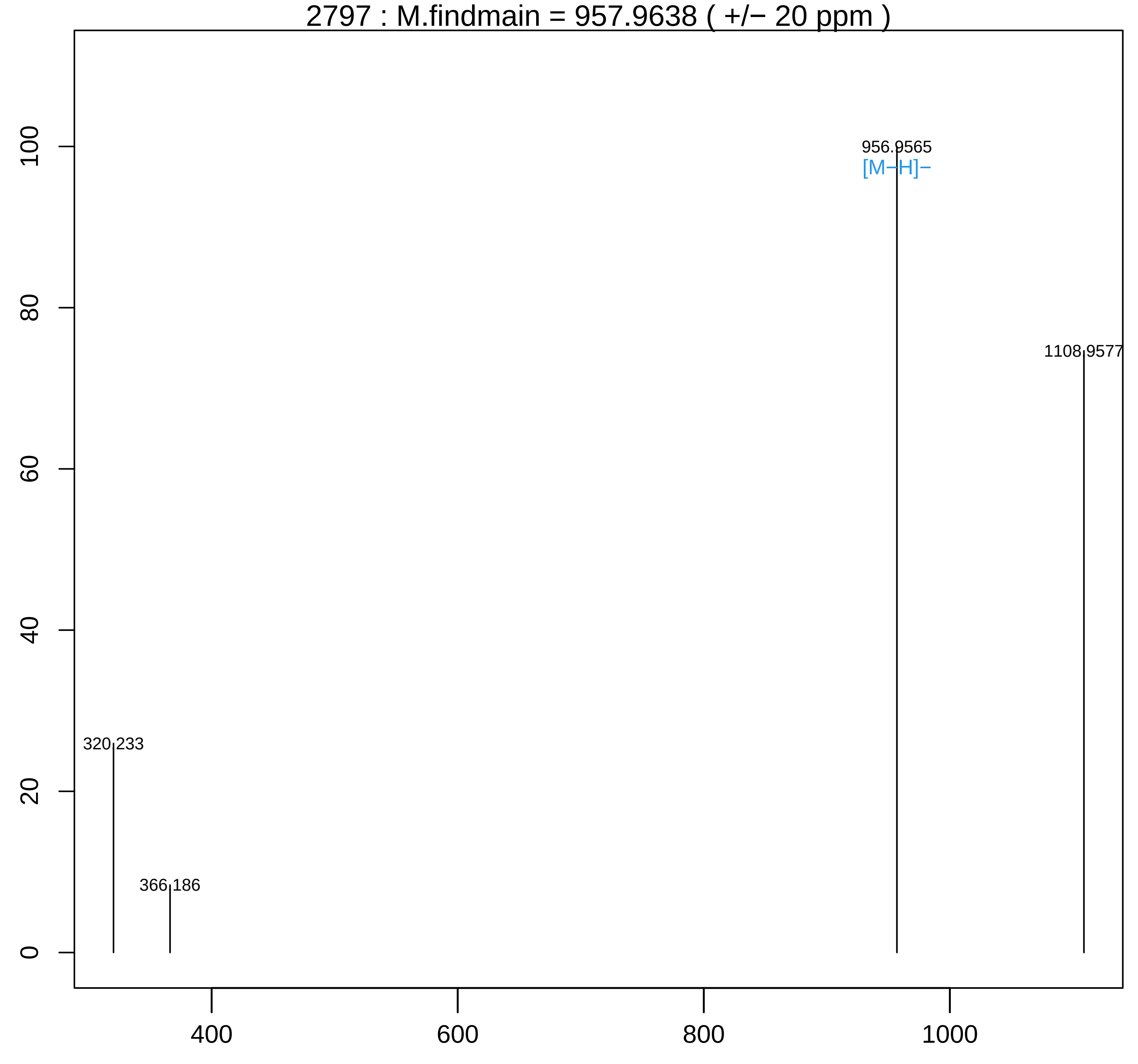
2797 : M.findmain = 957.9638 (+/− 20 ppm)
956.9565
[M−H]−
1108.9577
320.233
366.186
0
20
40
60
80
100
400
600
800
1000

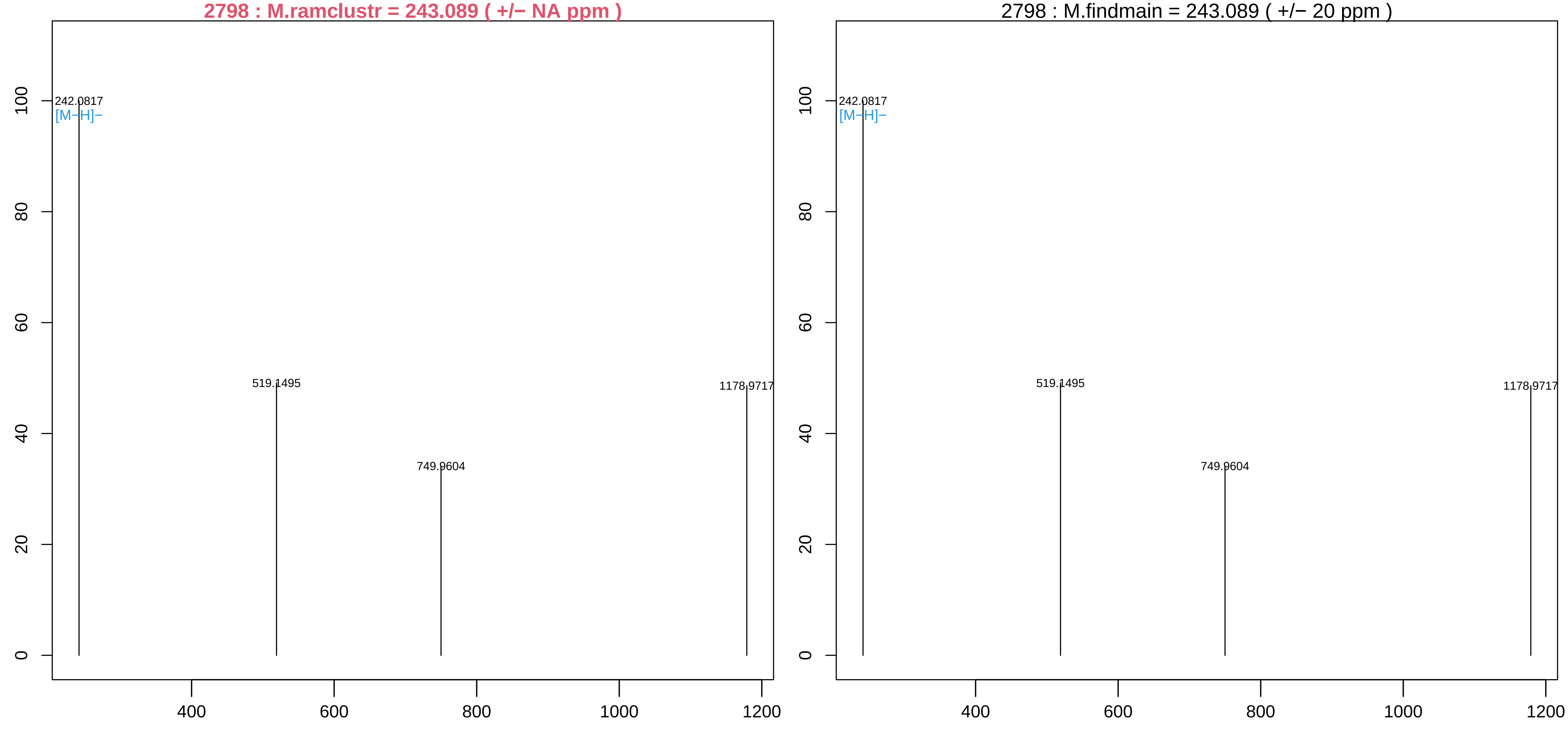
2798 : M.ramclustr = 243.089 (+/− NA ppm)
242.0817
[M−H]−
519.1495
749.9604
1178.9717
0
20
40
60
80
100
400
600
800
1000
1200
2798 : M.findmain = 243.089 (+/− 20 ppm)
242.0817
[M−H]−
519.1495
749.9604
1178.9717
0
20
40
60
80
100
400
600
800
1000
1200

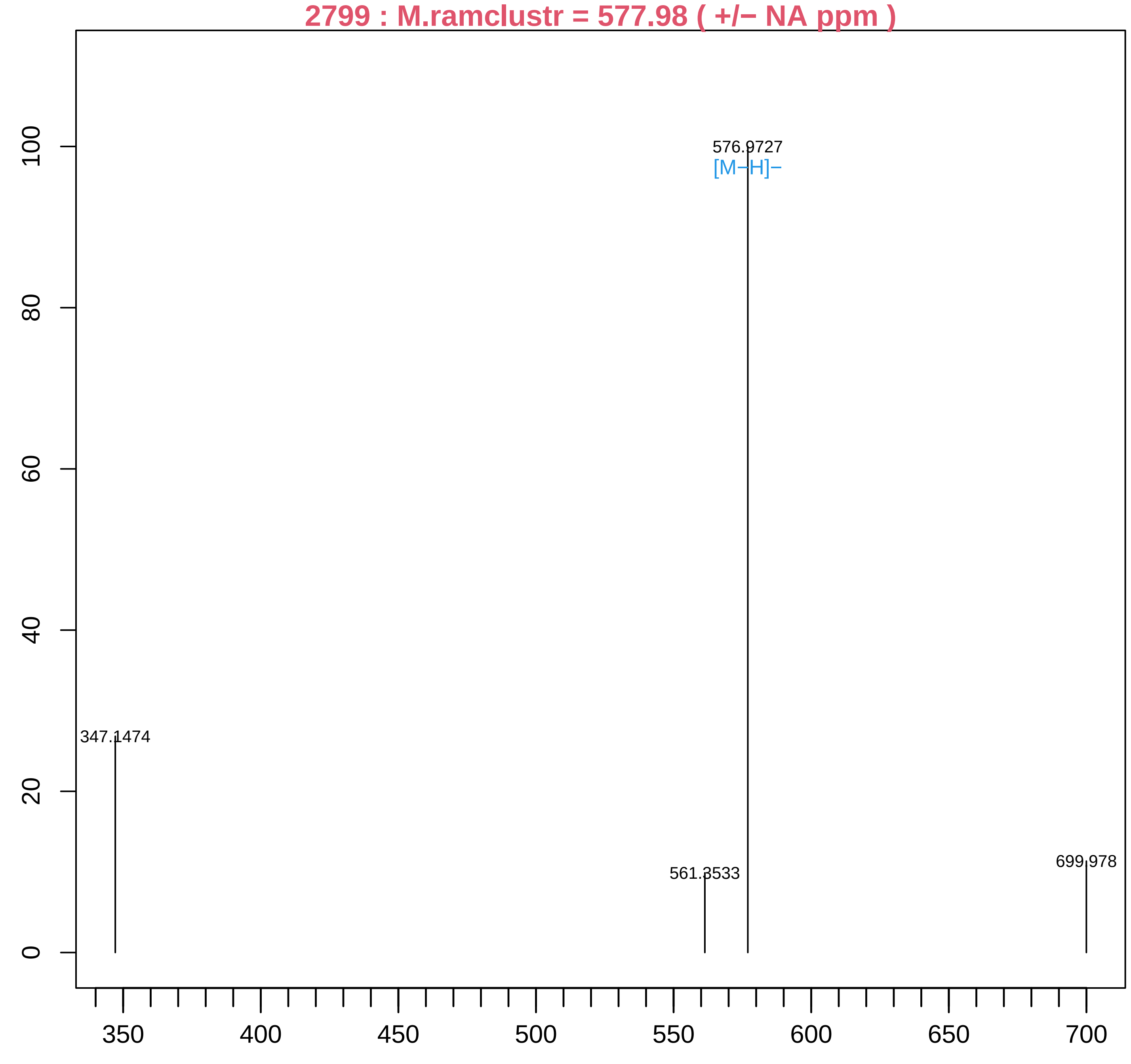

2799 : M.ramclustr = 577.98 (+/− NA ppm)
576.9727
[M−H]−
347.1474
561.3533
699.978

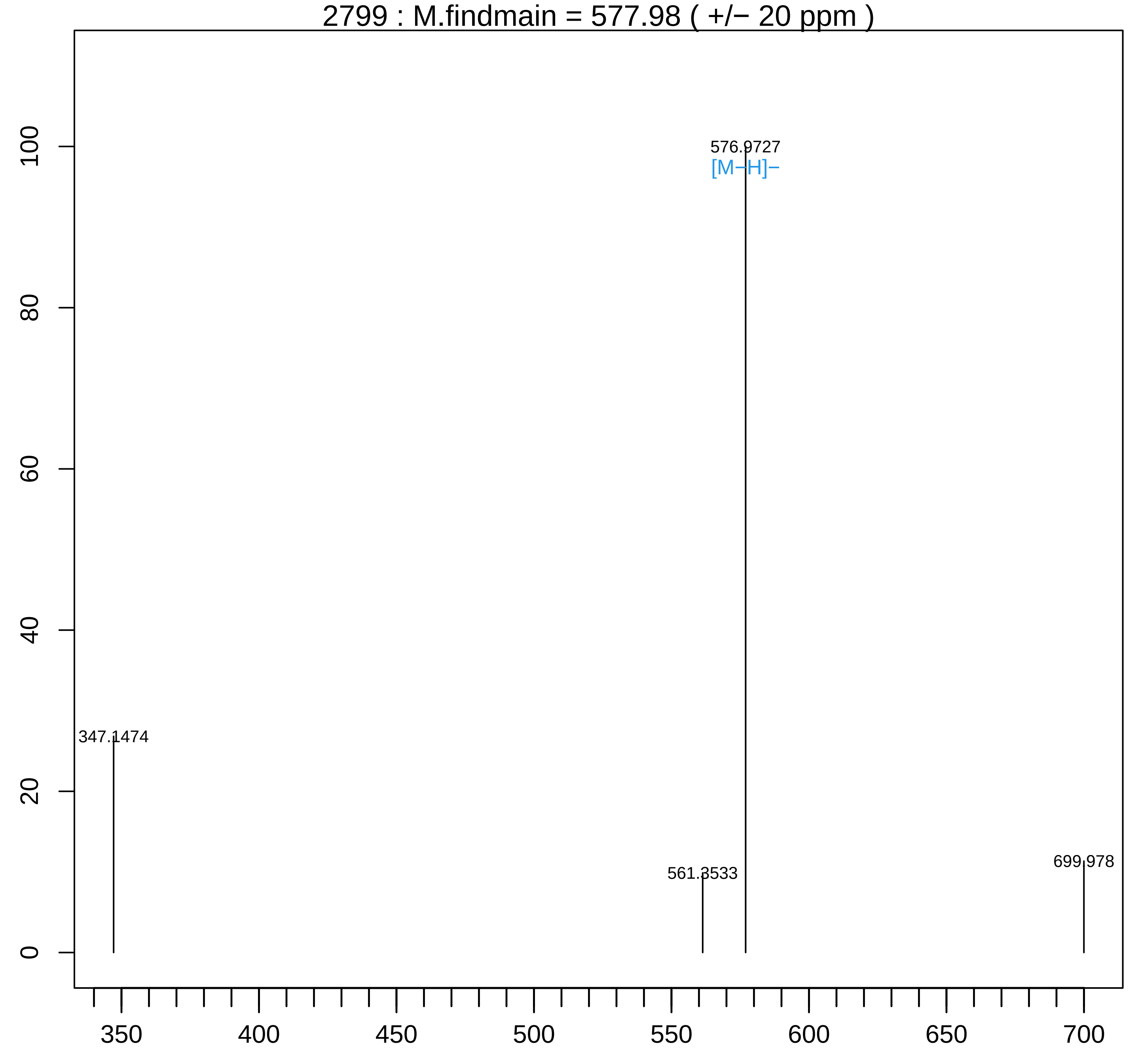

2799 : M.findmain = 577.98 (+/− 20 ppm)
576.9727
[M−H]−
347.1474
561.3533
699.978

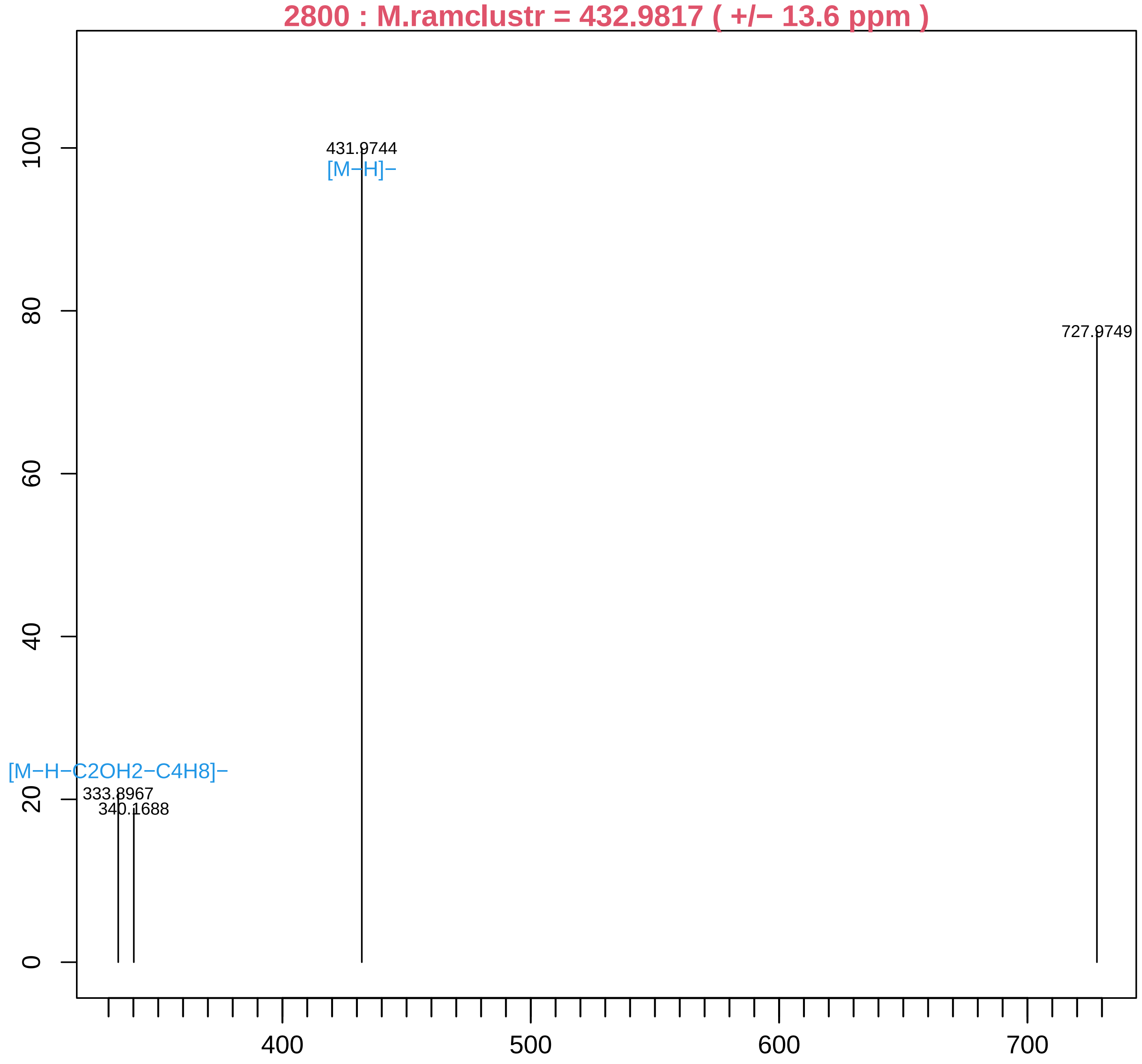
2800 : M.ramclustr = 432.9817 (+/− 13.6 ppm)
431.9744
[M−H]−
727.9749
[M−H−C2OH2−C4H8]−
333.8967
340.1688
0
20
40
60
80
100
400
500
600
700

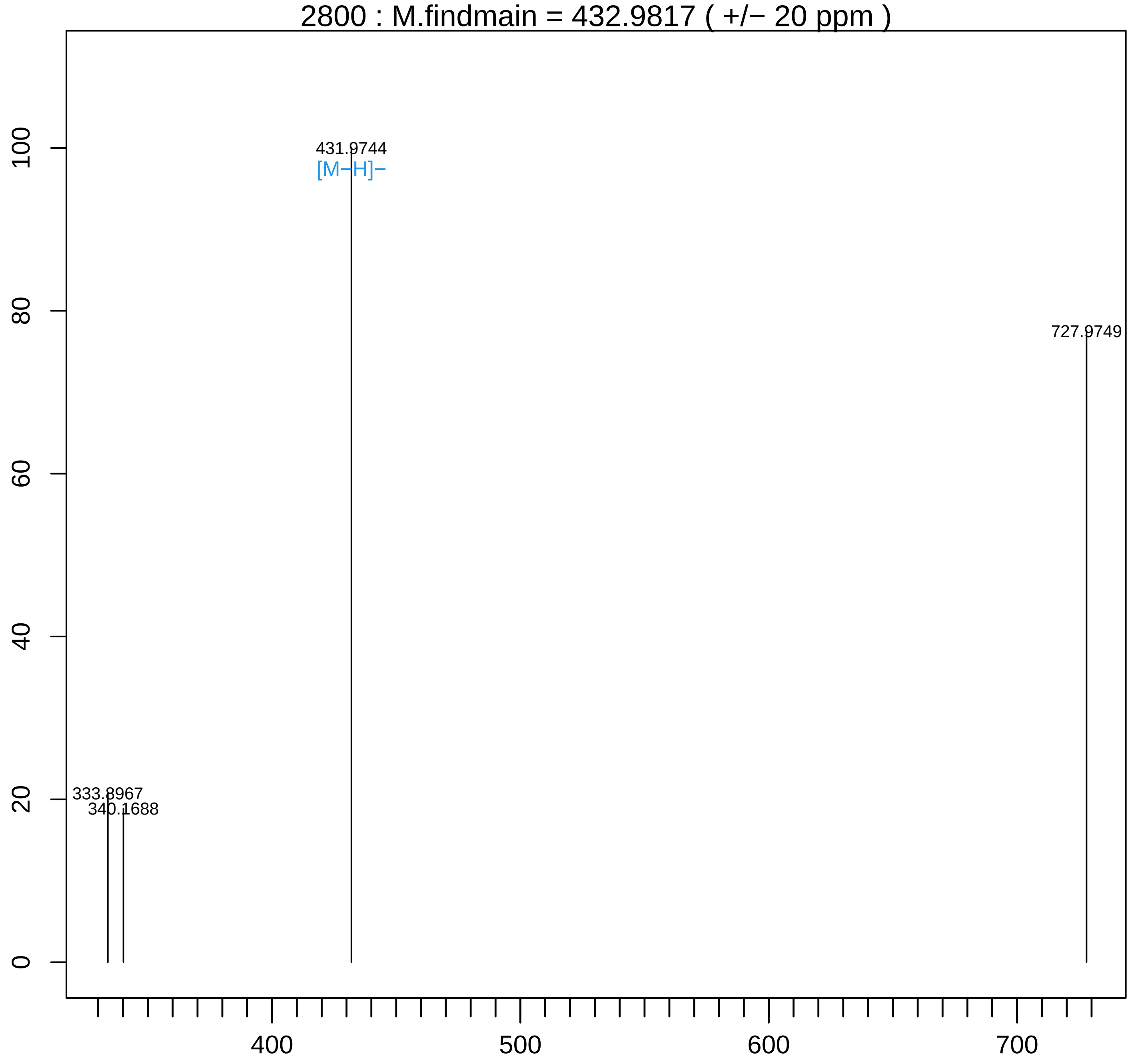
2800 : M.findmain = 432.9817 (+/− 20 ppm)
431.9744
[M−H]−
727.9749
333.8967
340.1688
0
20
40
60
80
100
400
500
600
700

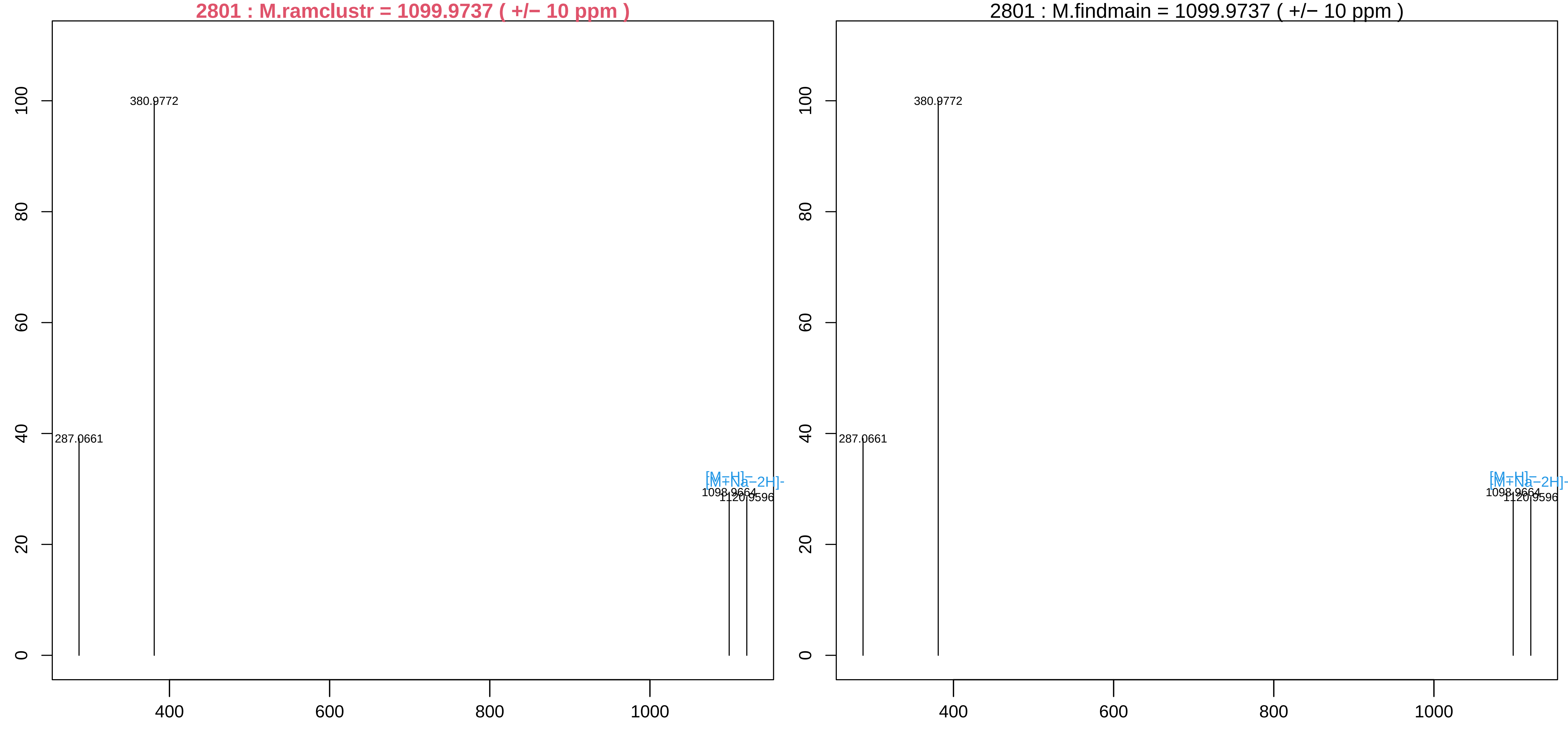
2801 : M.ramclustr = 1099.9737 (+/− 10 ppm)
380.9772
287.0661
[M−H]−
[M+Na−2H]-
1098.9664
1120.9596
0
20
40
60
80
100
400
600
800
1000
2801 : M.findmain = 1099.9737 (+/− 10 ppm)
380.9772
287.0661
[M−H]−
[M+Na−2H]-
1098.9664
1120.9596
0
20
40
60
80
100
400
600
800
1000

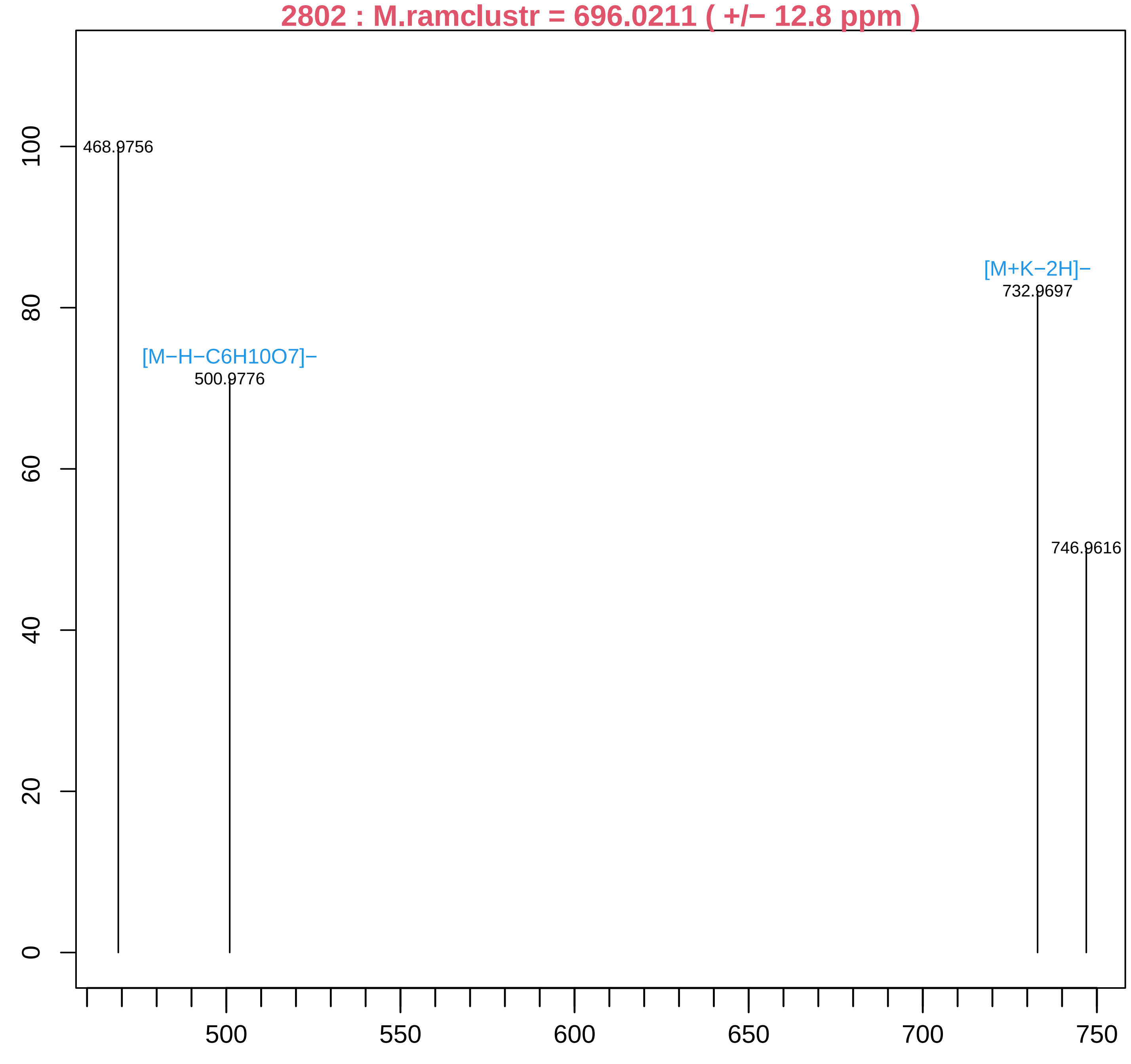

2802 : M.ramclustr = 696.0211 (+/− 12.8 ppm)
468.9756
[M−H−C6H10O7]−
500.9776
[M+K−2H]−
732.9697
746.9616
0
20
40
60
80
100
500
550
600
650
700
750

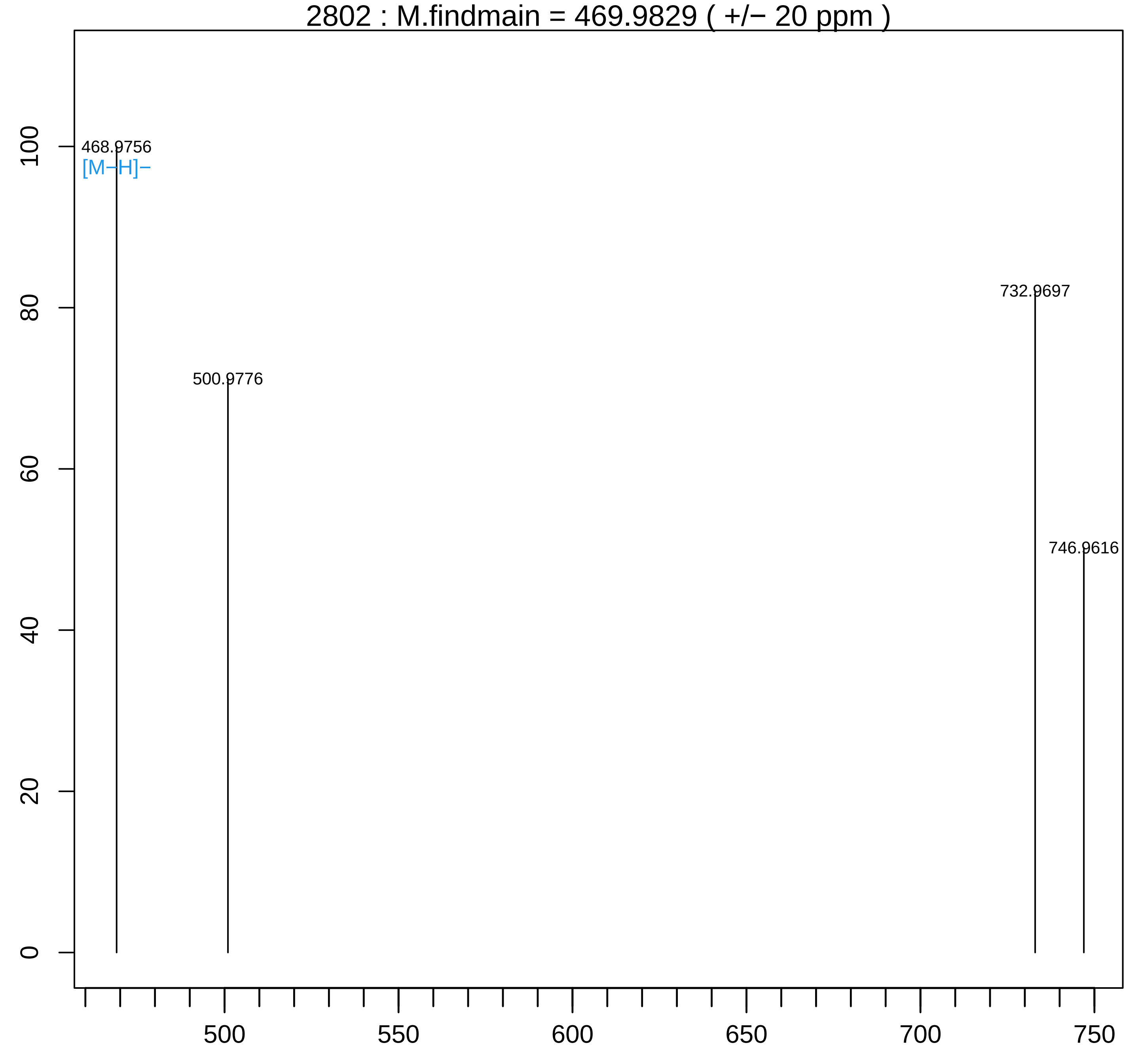

2802 : M.findmain = 469.9829 (+/− 20 ppm)
468.9756
[M−H]−
500.9776
732.9697
746.9616
0
20
40
60
80
100
500
550
600
650
700
750

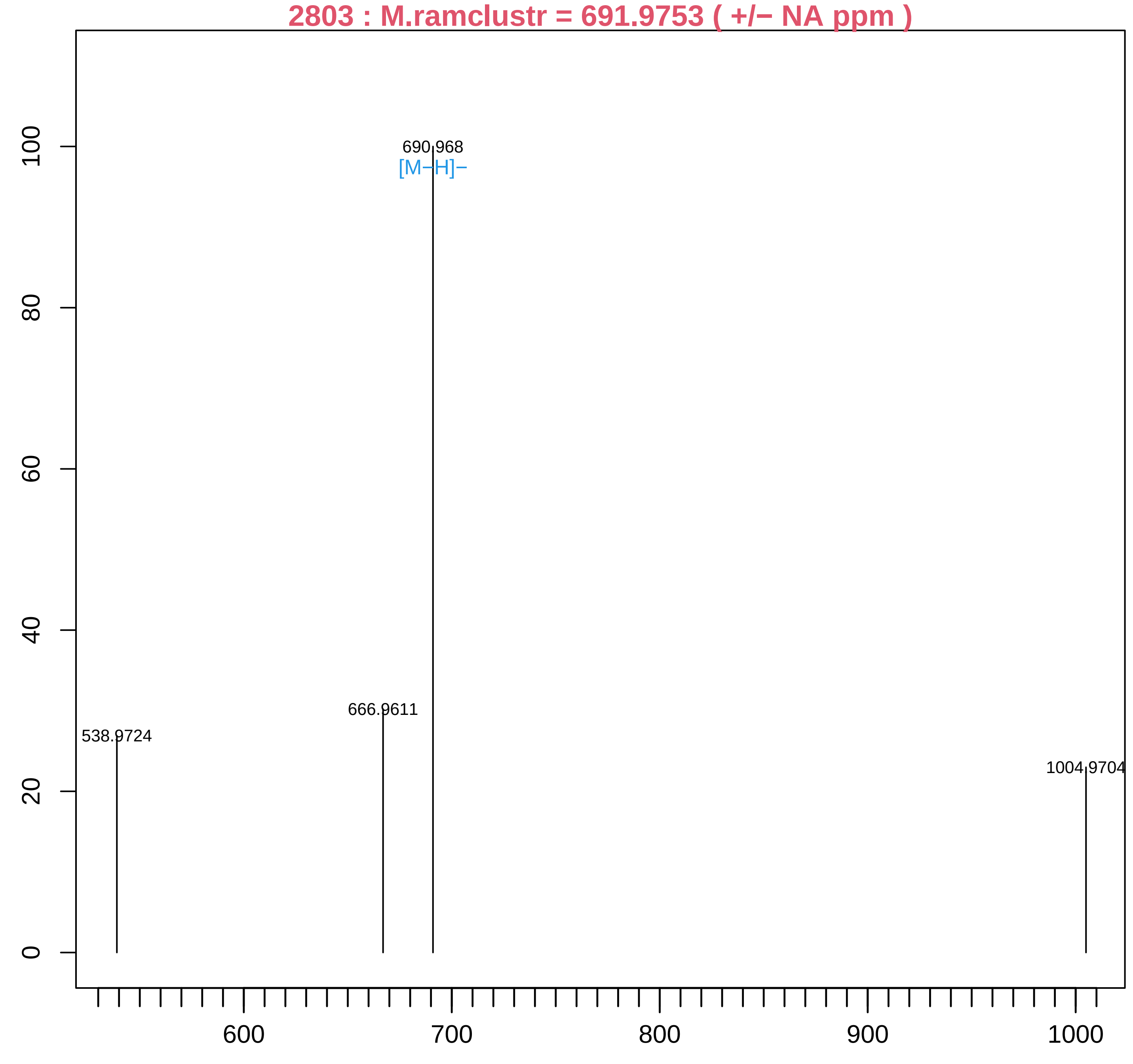
2803 : M.ramclustr = 691.9753 (+/− NA ppm)
690.968
[M−H]−
666.9611
538.9724
1004.9704
0
20
40
60
80
100
600
700
800
900
1000

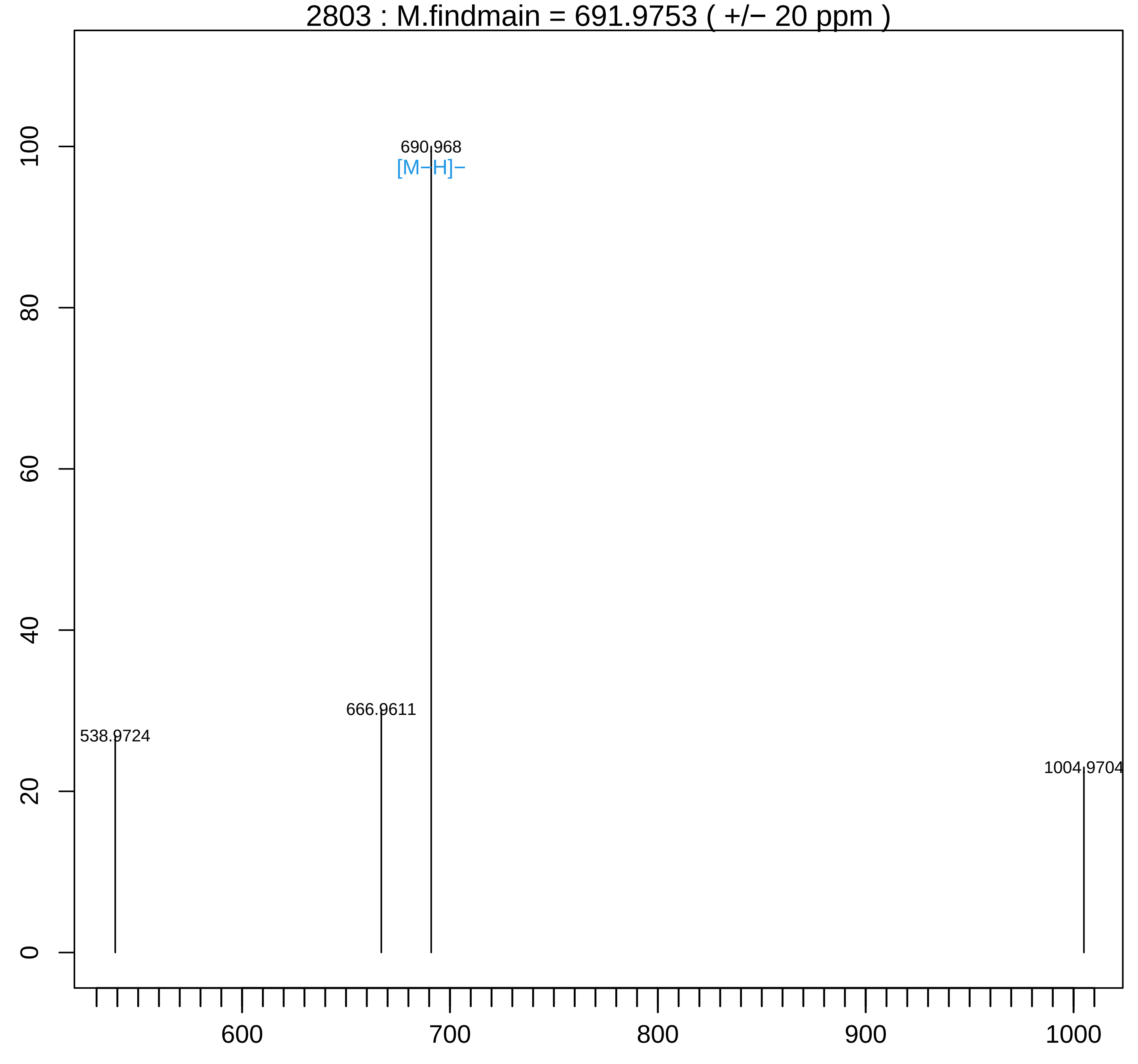
2803 : M.findmain = 691.9753 (+/− 20 ppm)
690.968
[M−H]−
666.9611
538.9724
1004.9704
0
20
40
60
80
100
600
700
800
900
1000

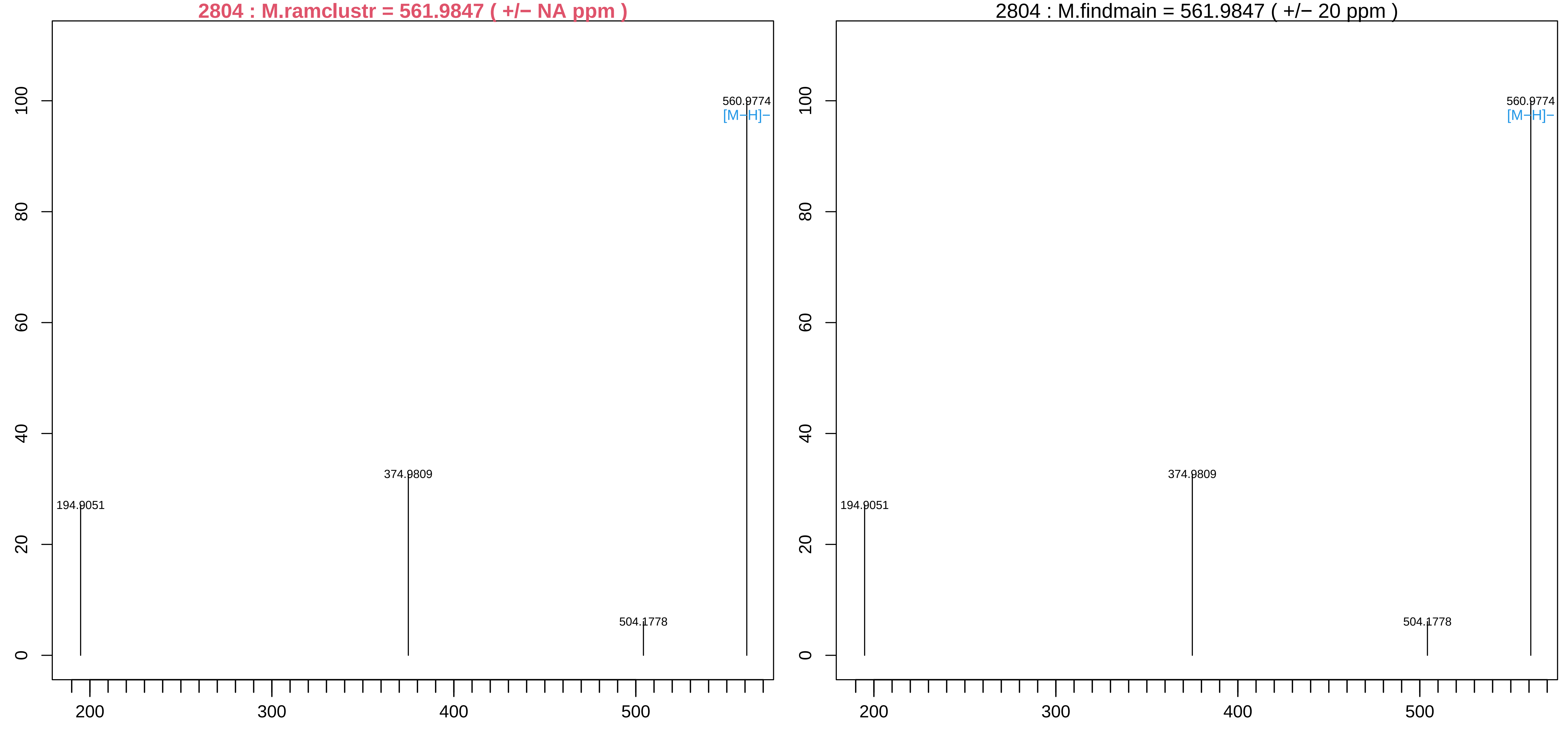
2804 : M.ramclustr = 561.9847 (+/− NA ppm)
2804 : M.findmain = 561.9847 (+/− 20 ppm)
560.9774
[M−H]−
374.9809
194.9051
504.1778
0
20
40
60
80
100
200
300
400
500

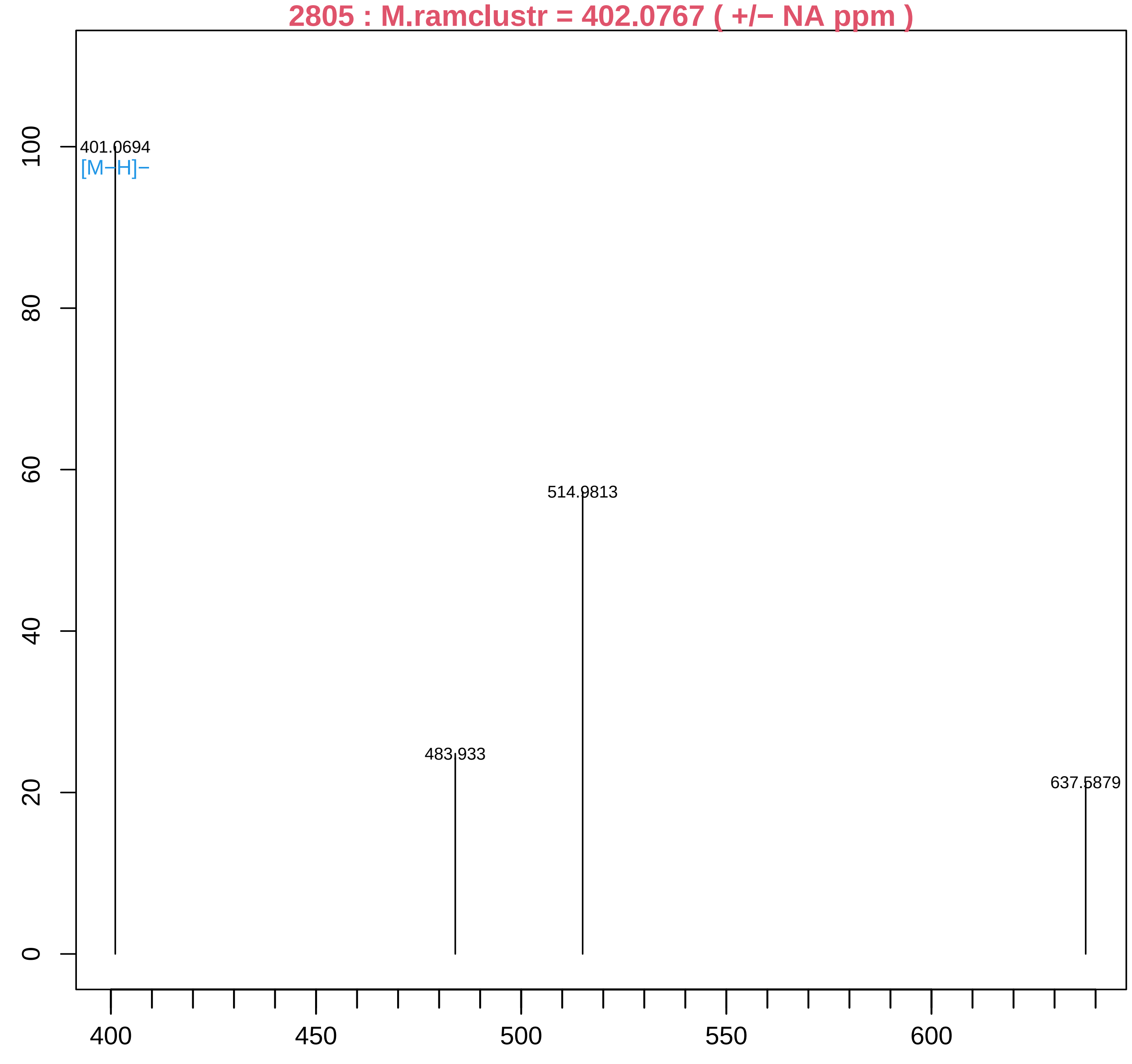
2805 : M.ramclustr = 402.0767 (+/− NA ppm)
401.0694
[M−H]−
514.9813
483.933
637.5879
0
20
40
60
80
100
400
450
500
550
600

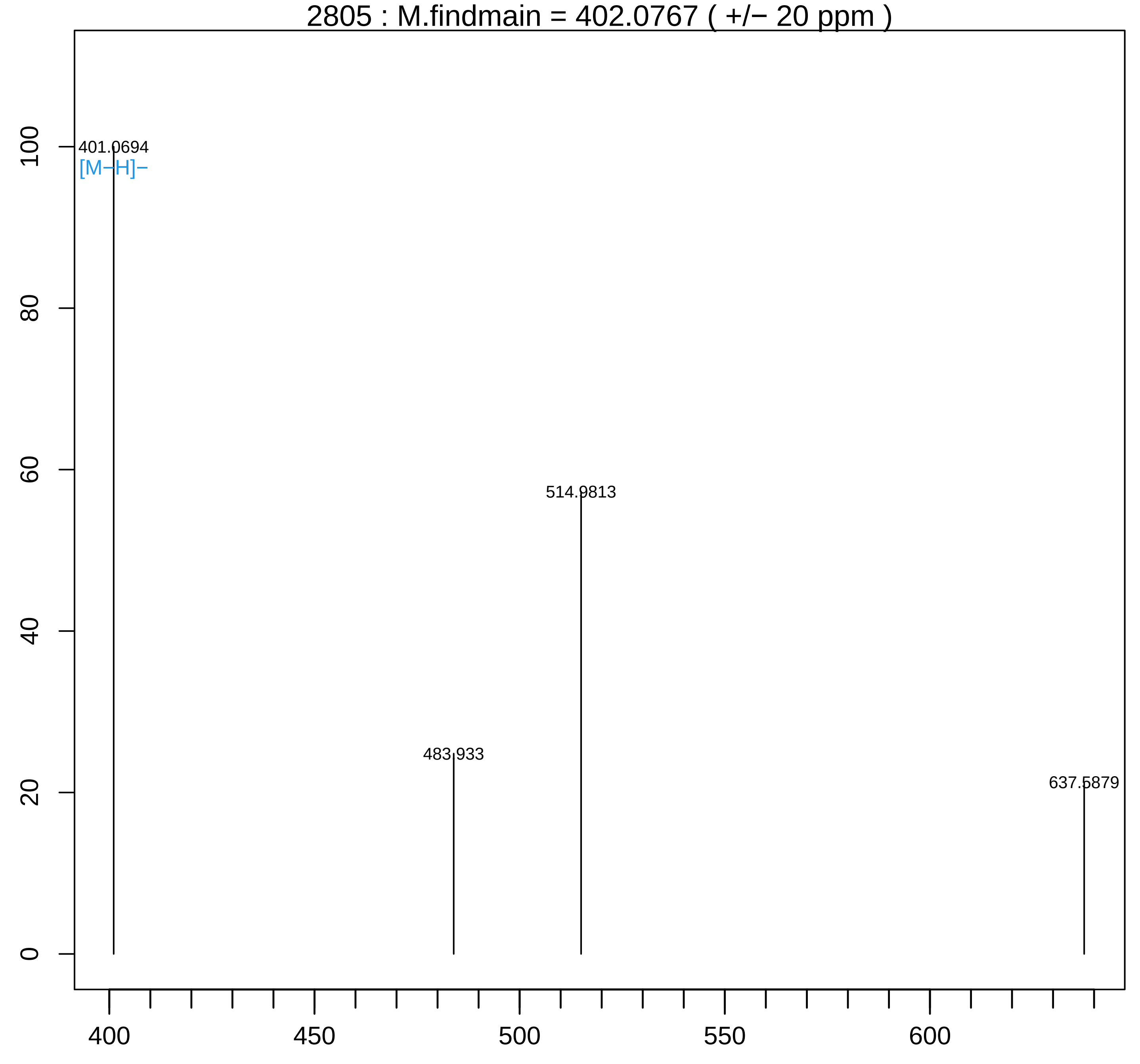
2805 : M.findmain = 402.0767 (+/− 20 ppm)
401.0694
[M−H]−
514.9813
483.933
637.5879
0
20
40
60
80
100
400
450
500
550
600

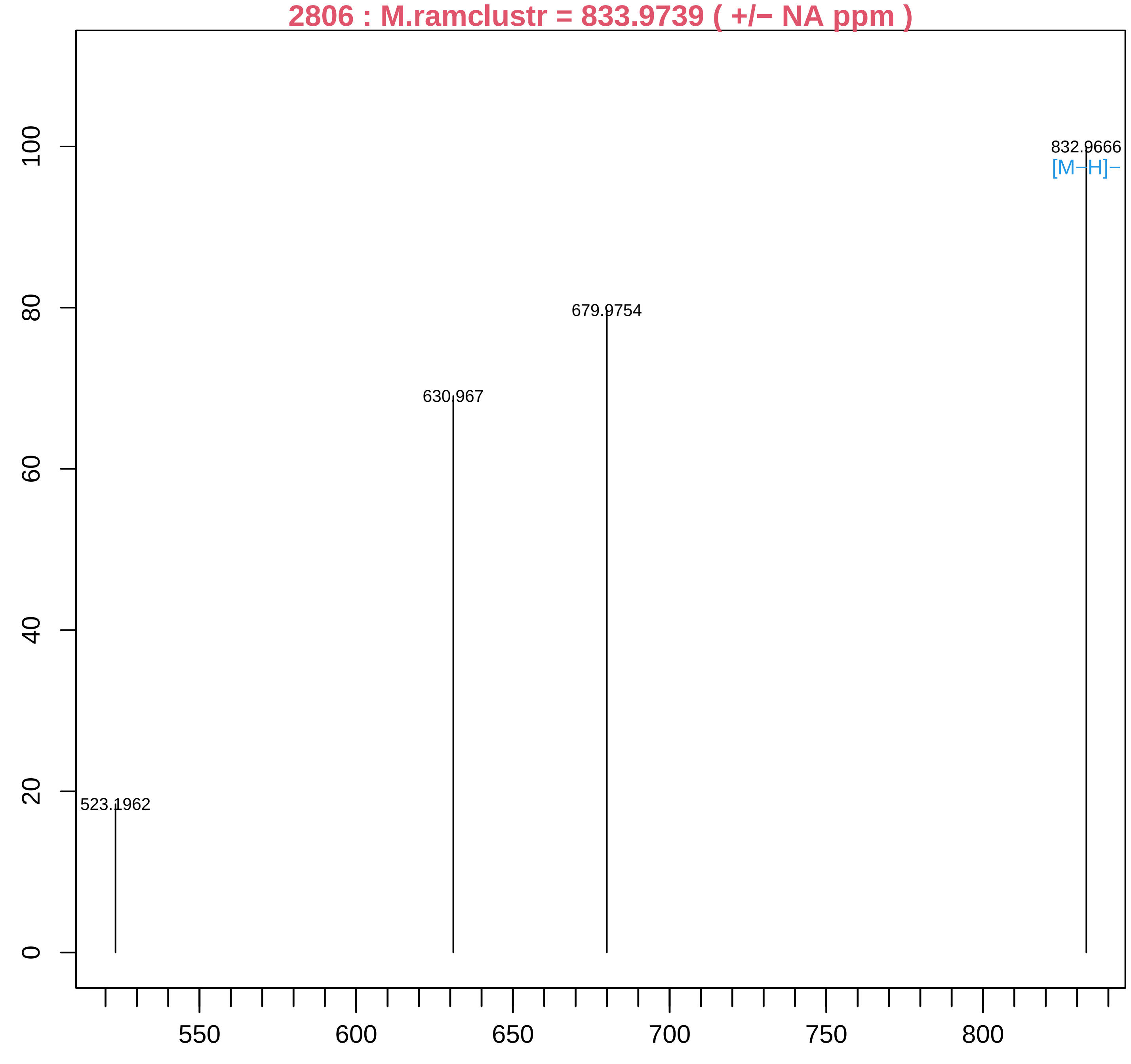

2806 : M.ramclustr = 833.9739 (+/− NA ppm)
832.9666
[M−H]−
679.9754
630.967
523.1962

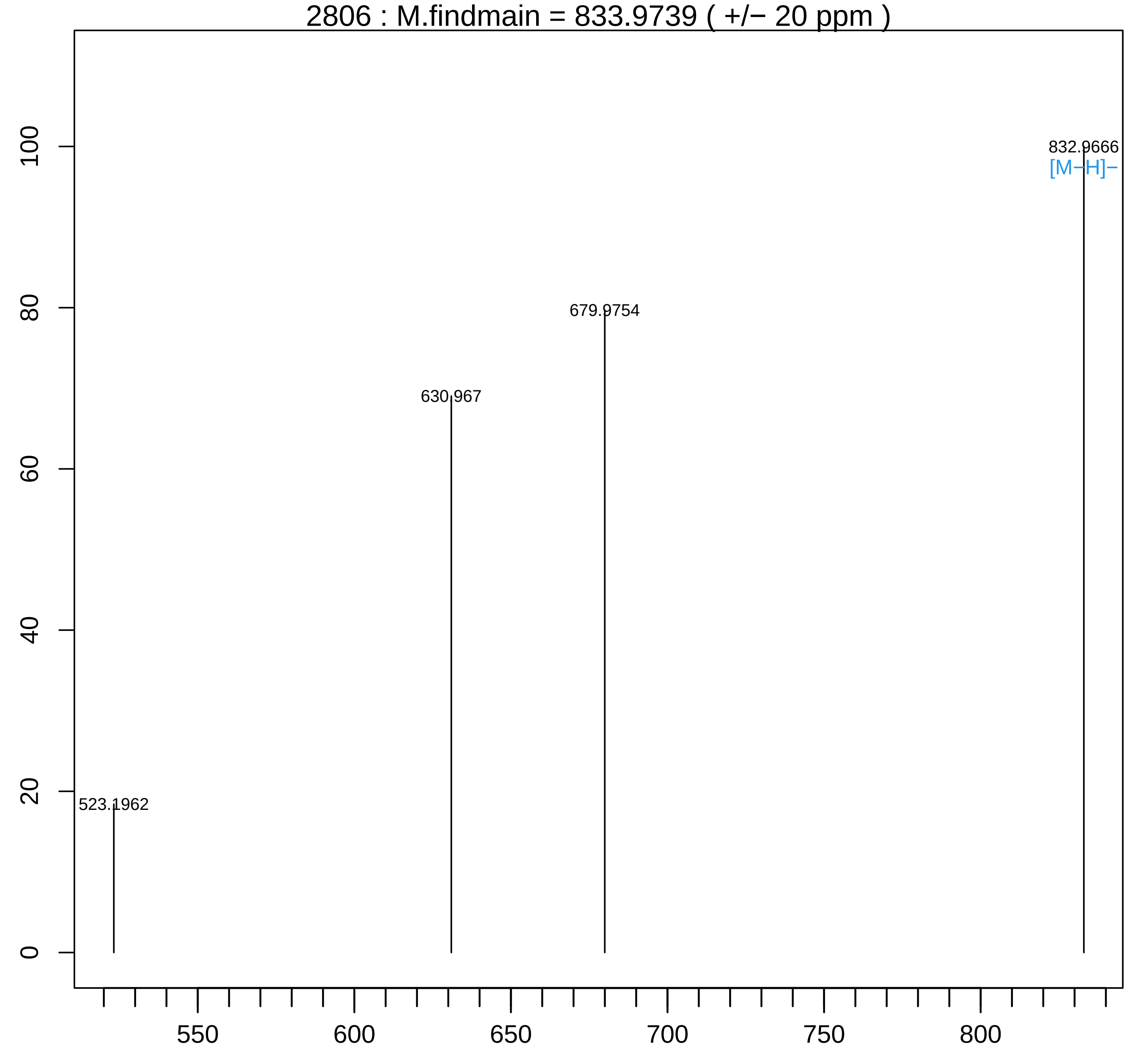

2806 : M.findmain = 833.9739 (+/− 20 ppm)
832.9666
[M−H]−
679.9754
630.967
523.1962

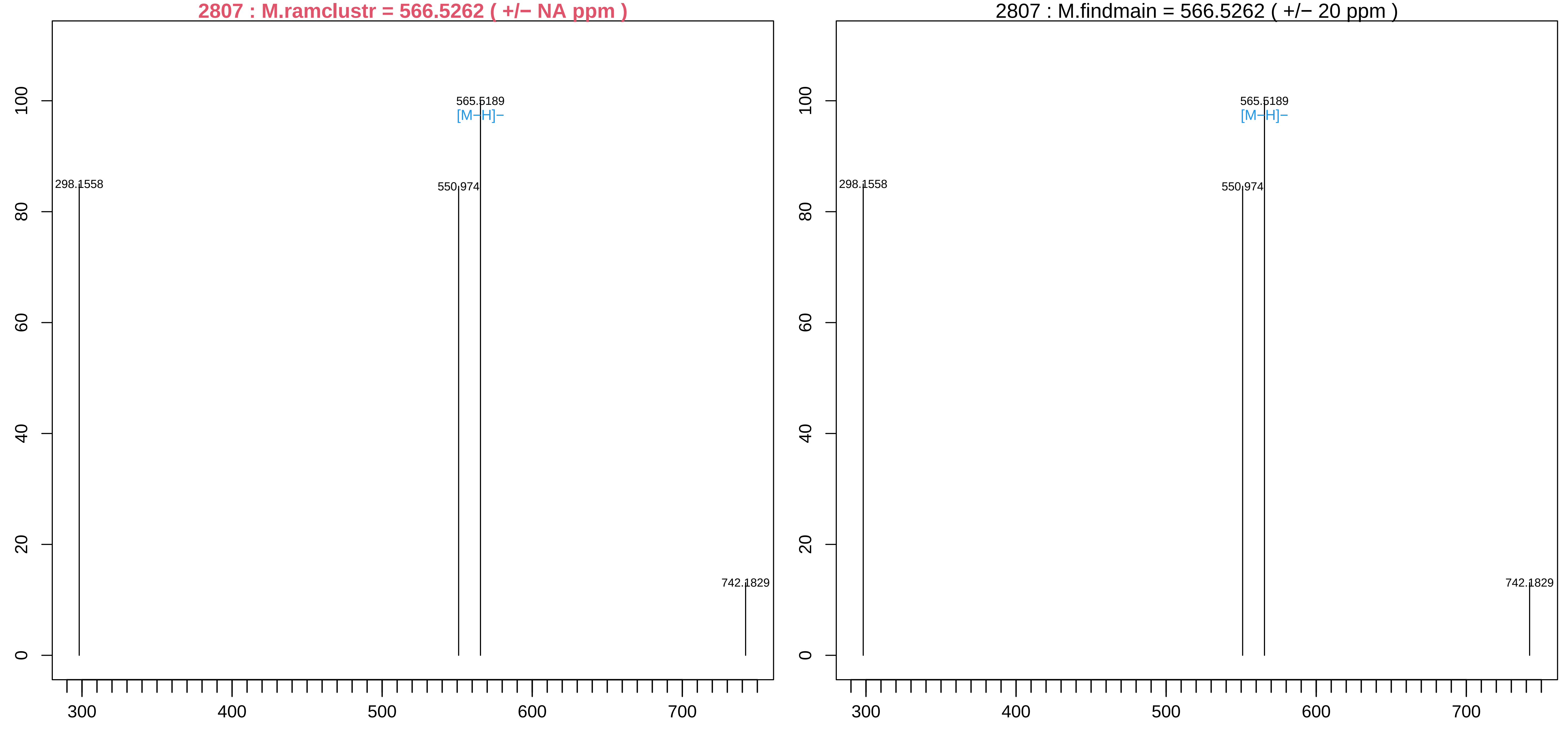
2807 : M.ramclustr = 566.5262 (+/− NA ppm)
298.1558
550.974
565.5189
[M−H]−
742.1829
2807 : M.findmain = 566.5262 (+/− 20 ppm)
298.1558
550.974
565.5189
[M−H]−
742.1829
0
20
40
60
80
100
300
400
500
600
700

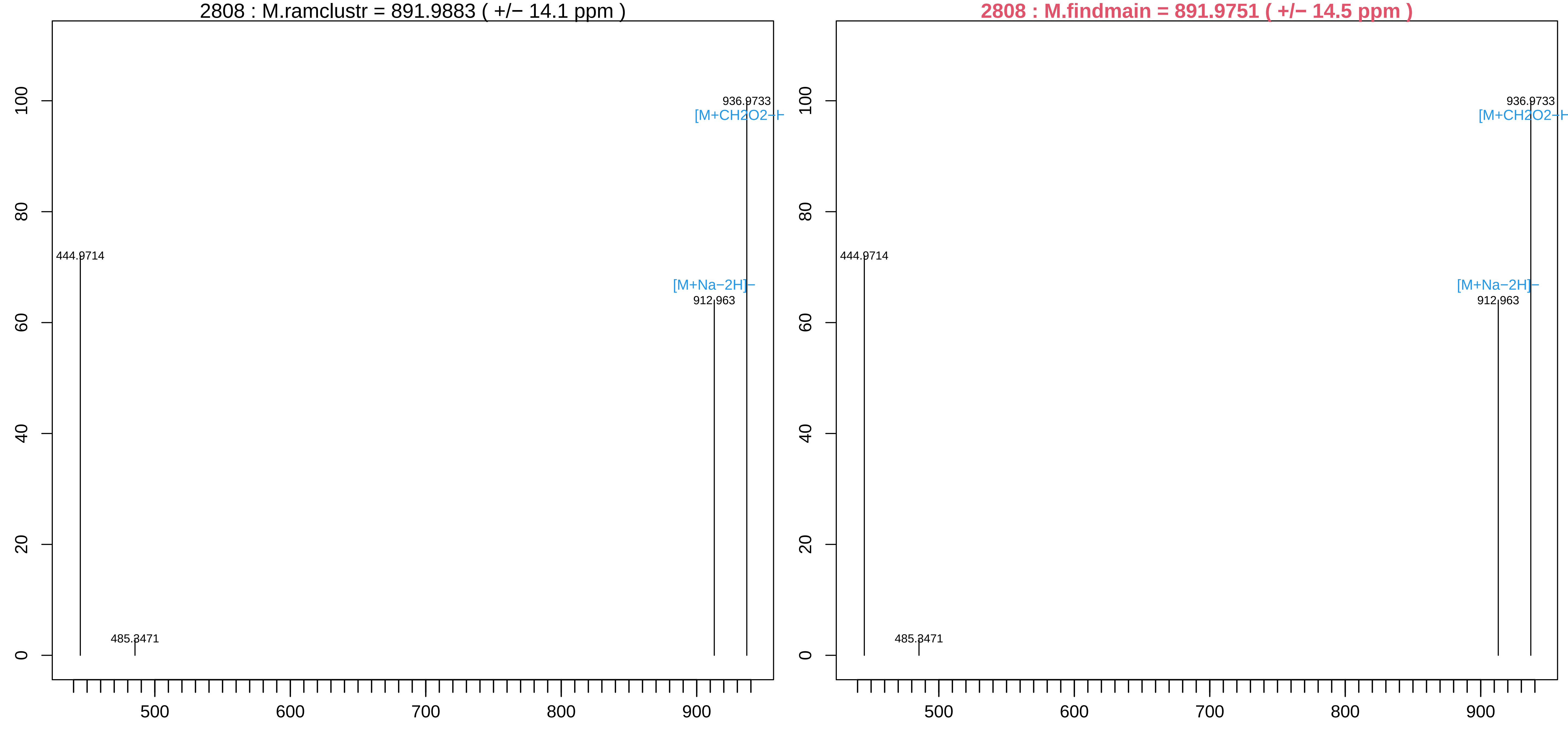

2808 : M.ramclustr = 891.9883 (+/− 14.1 ppm)
2808 : M.findmain = 891.9751 (+/− 14.5 ppm)
936.9733
[M+CH2O2−H
444.9714
[M+Na−2H]−
912.963
485.3471
0
20
40
60
80
100
500
600
700
800
900

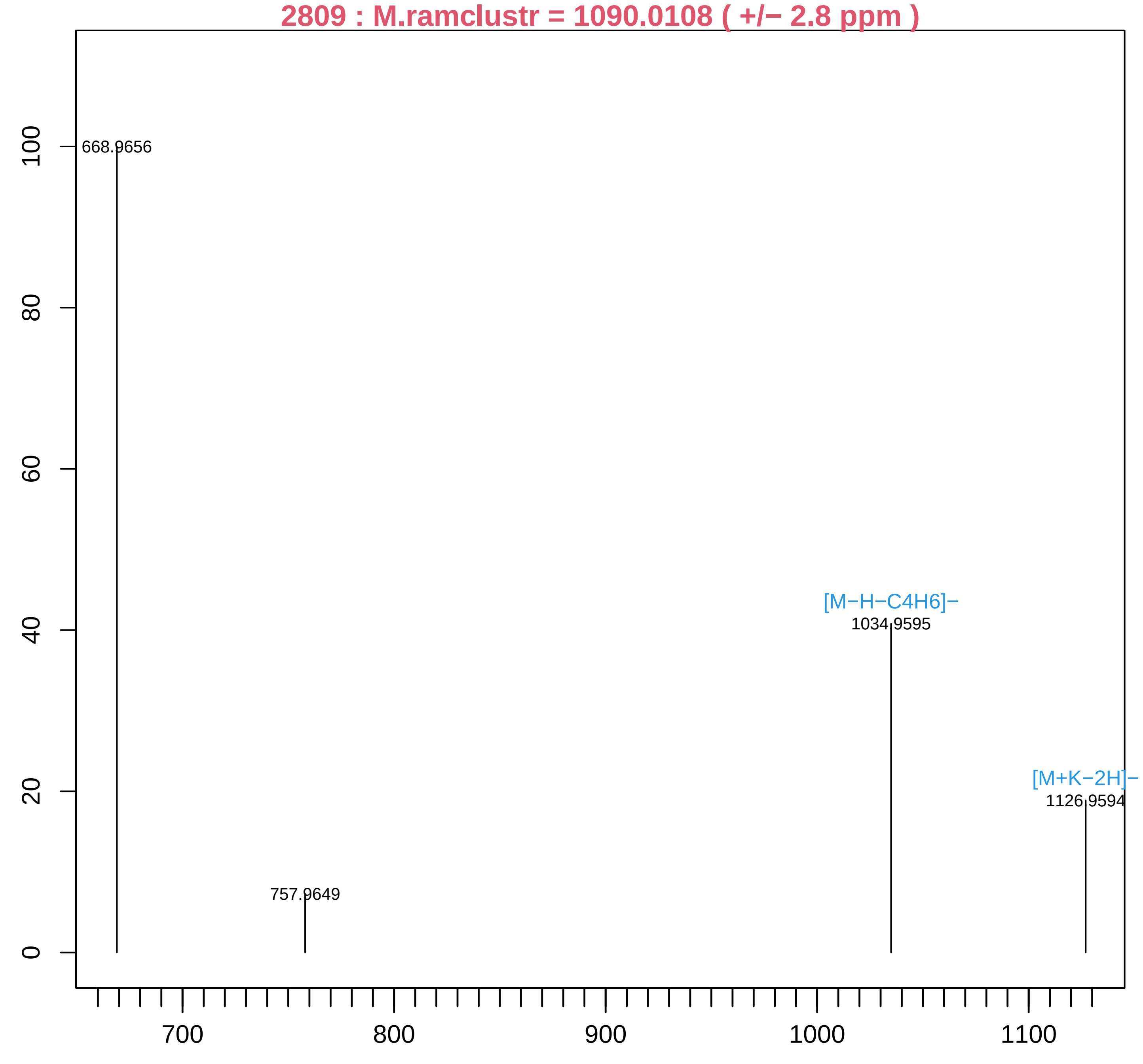

2809 : M.ramclustr = 1090.0108 (+/− 2.8 ppm)
668.9656
757.9649
[M−H−C4H6]−
1034.9595
[M+K−2H]−
1126.9594
0
20
40
60
80
100
700
800
900
1000
1100

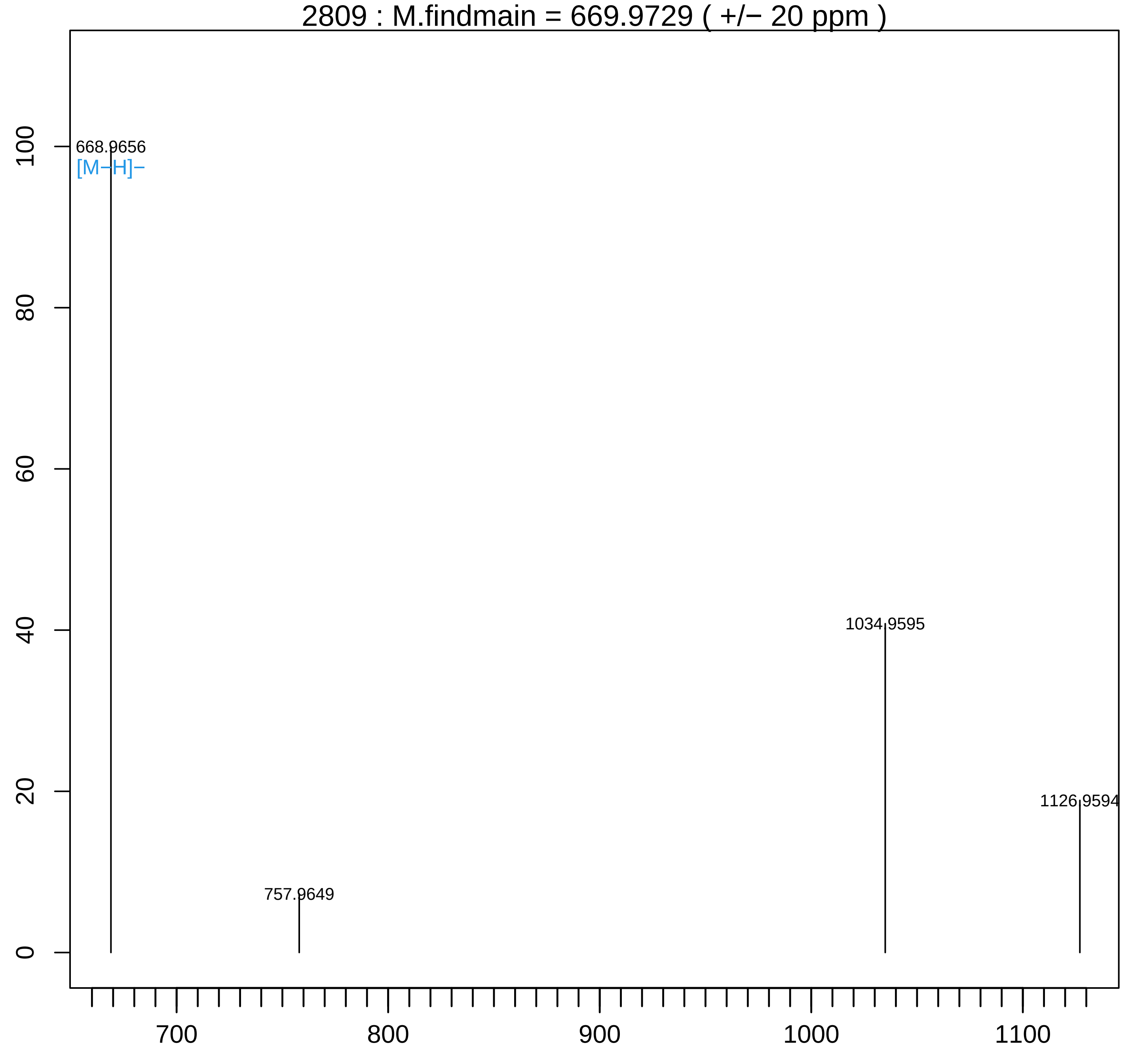

2809 : M.findmain = 669.9729 (+/− 20 ppm)
668.9656
[M−H]−
757.9649
1034.9595
1126.9594
0
20
40
60
80
100
700
800
900
1000
1100

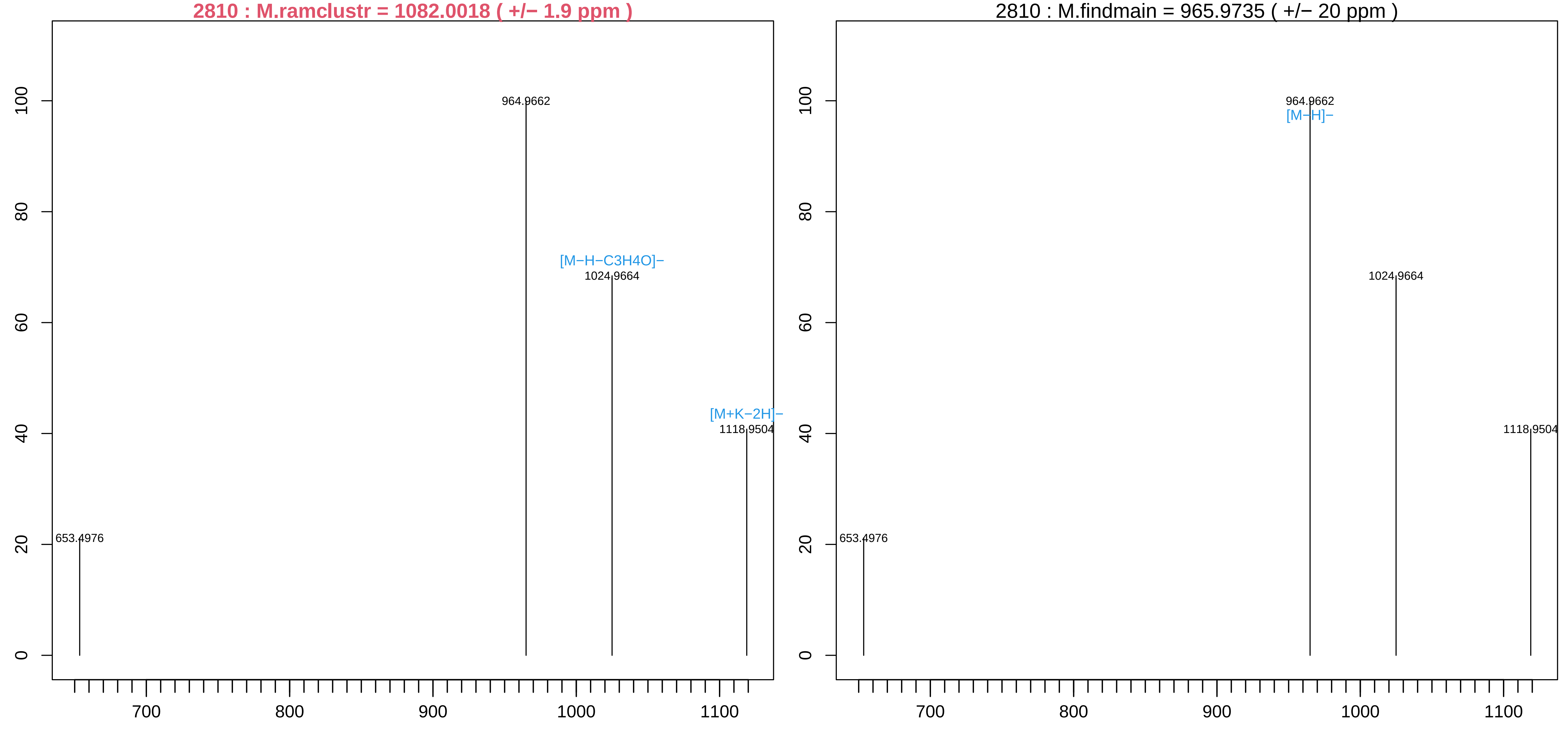
2810 : M.ramclustr = 1082.0018 (+/− 1.9 ppm)
964.9662
[M−H−C3H4O]−
1024.9664
[M+K−2H]−
1118.9504
653.4976
2810 : M.findmain = 965.9735 (+/− 20 ppm)
964.9662
[M−H]−
1024.9664
1118.9504
653.4976
0
20
40
60
80
100
700
800
900
1000
1100

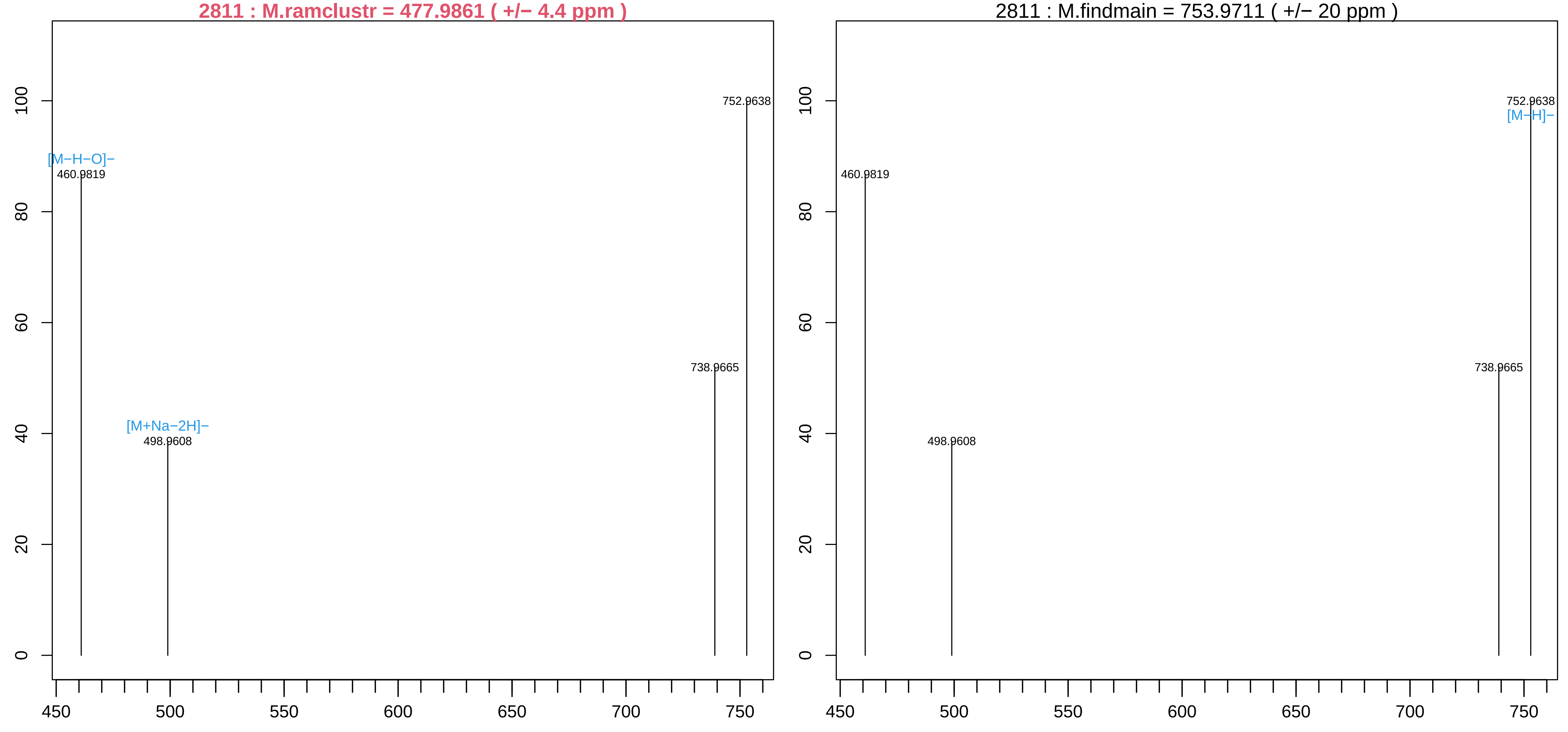

2811 : M.ramclustr = 477.9861 (+/− 4.4 ppm)
[M−H−O]−
460.9819
[M+Na−2H]−
498.9608
738.9665
752.9638
2811 : M.findmain = 753.9711 (+/− 20 ppm)
460.9819
498.9608
738.9665
752.9638
[M−H]−

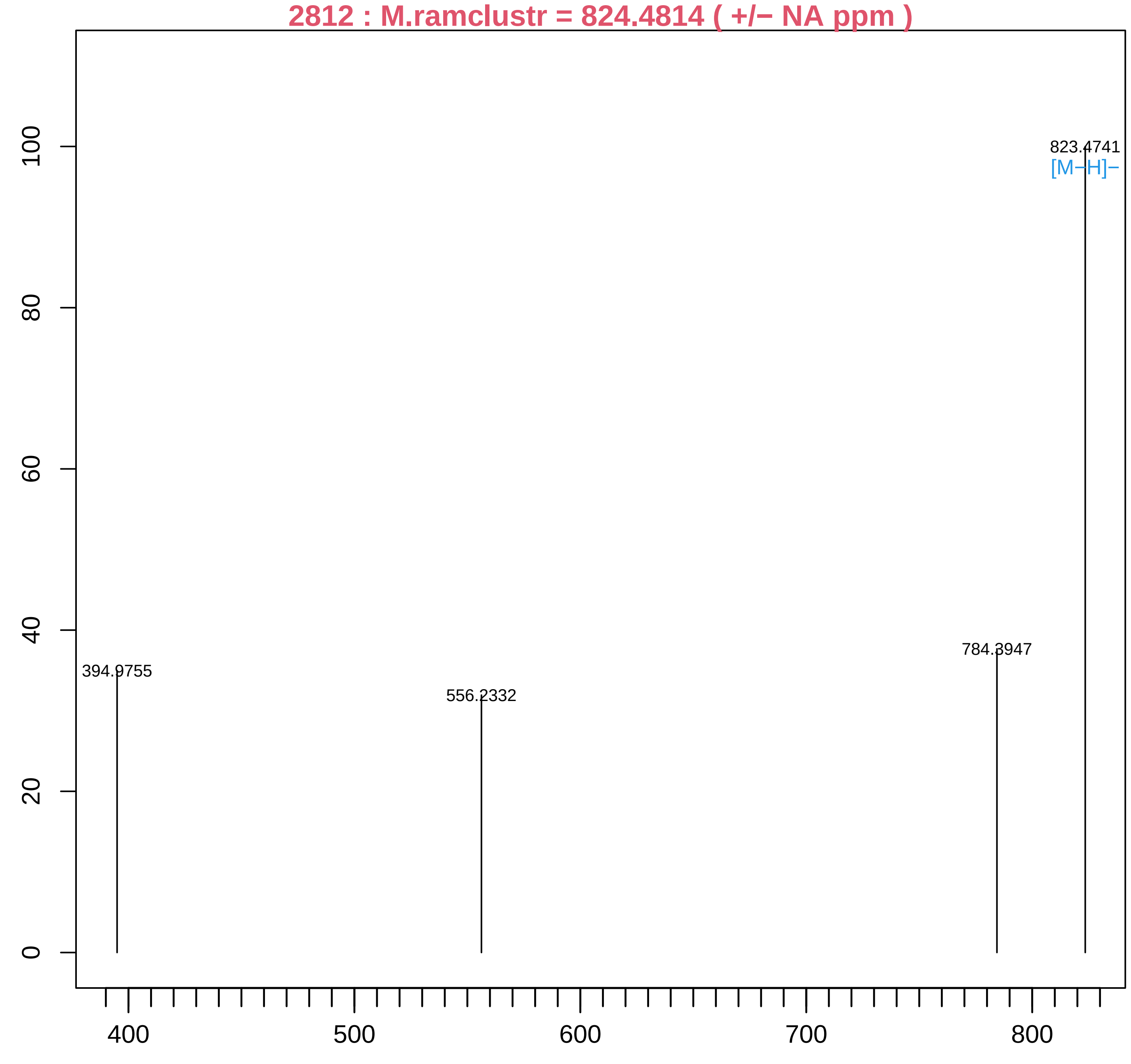
2812 : M.ramclustr = 824.4814 (+/− NA ppm)
823.4741
[M−H]−
784.3947
556.2332
394.9755
0
20
40
60
80
100
400
500
600
700
800

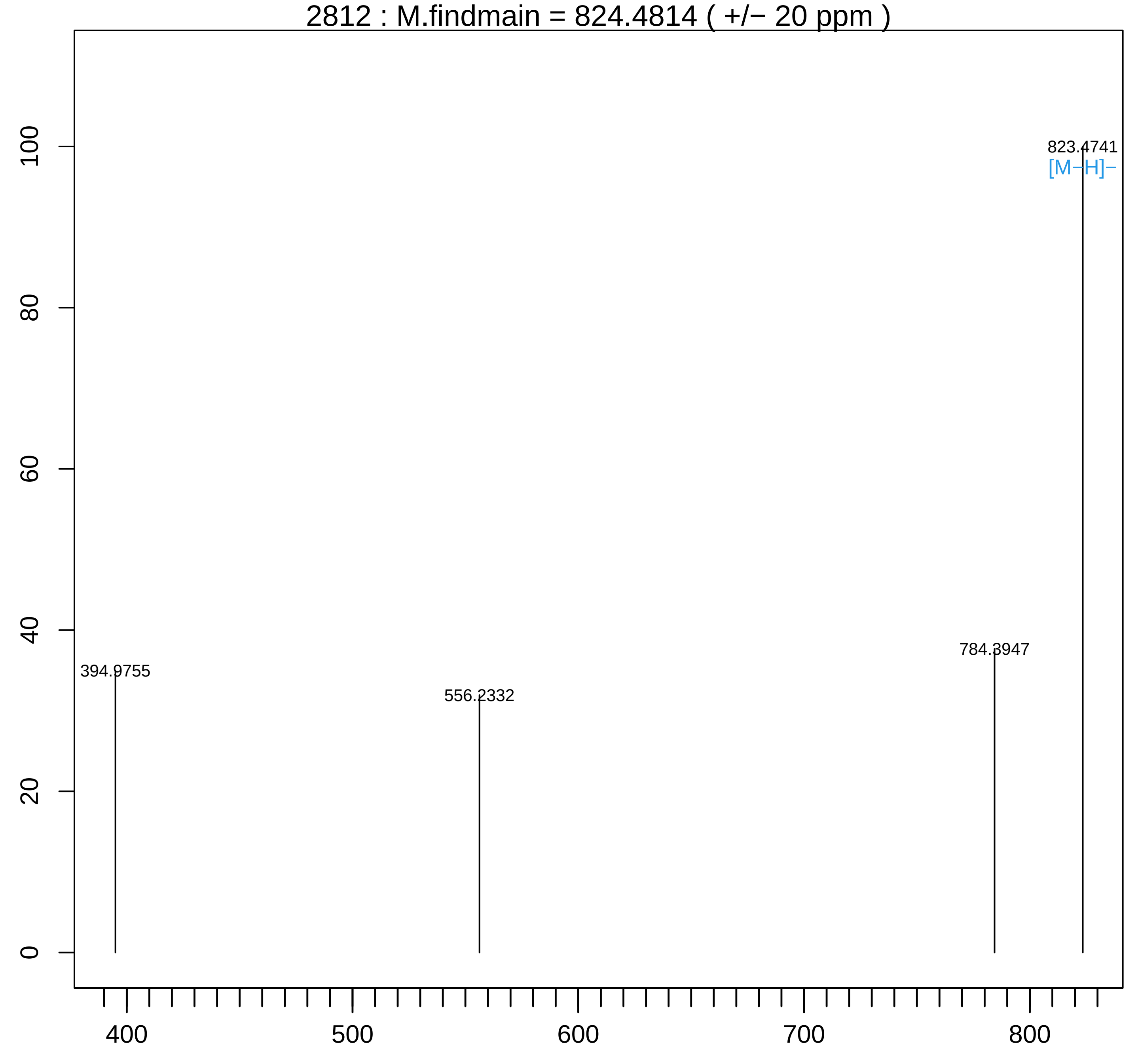
2812 : M.findmain = 824.4814 (+/− 20 ppm)
823.4741
[M−H]−
784.3947
556.2332
394.9755
0
20
40
60
80
100
400
500
600
700
800

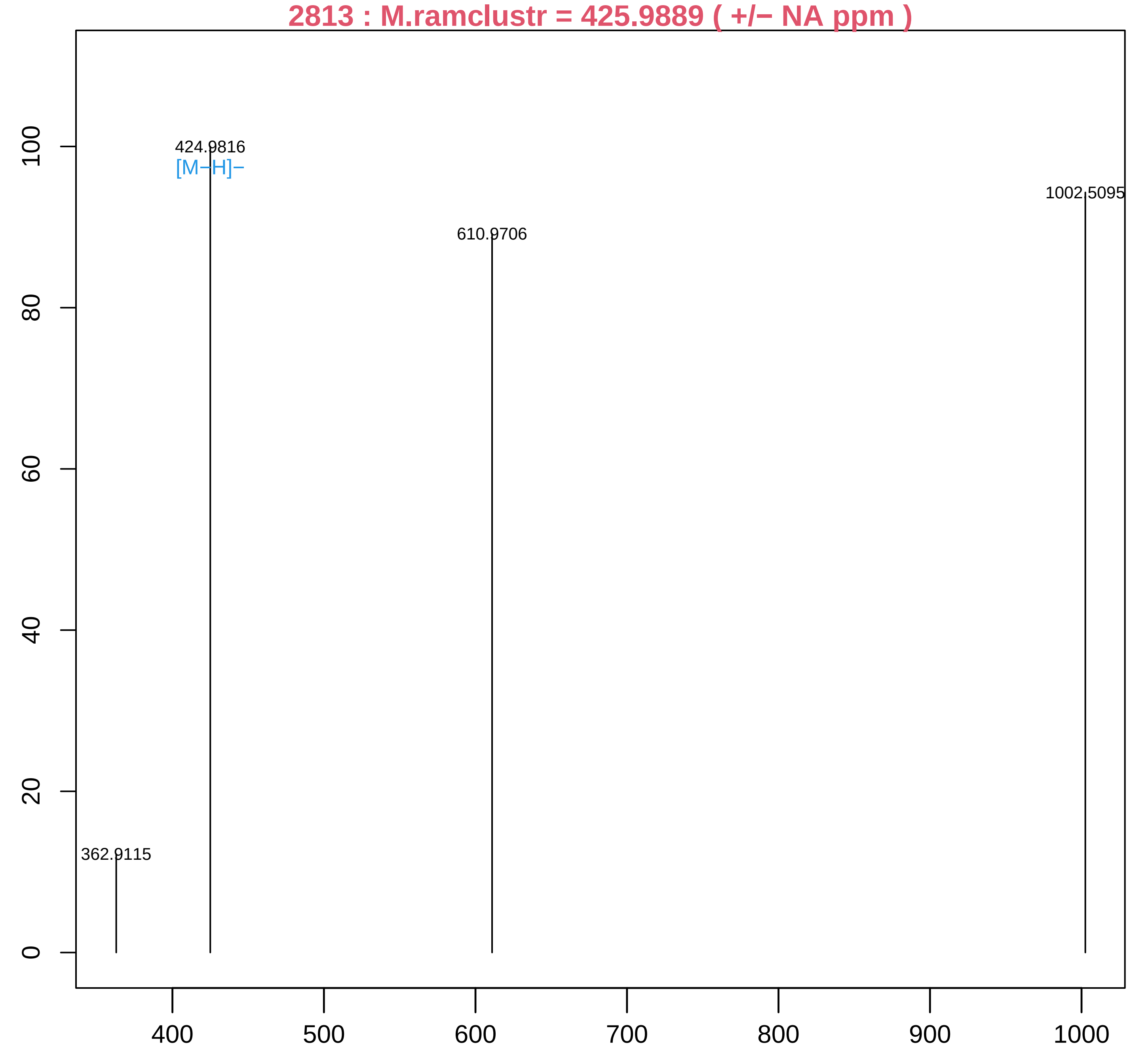
2813 : M.ramclustr = 425.9889 (+/− NA ppm)
424.9816
[M−H]−
1002.5095
610.9706
362.9115
0
20
40
60
80
100
400
500
600
700
800
900
1000

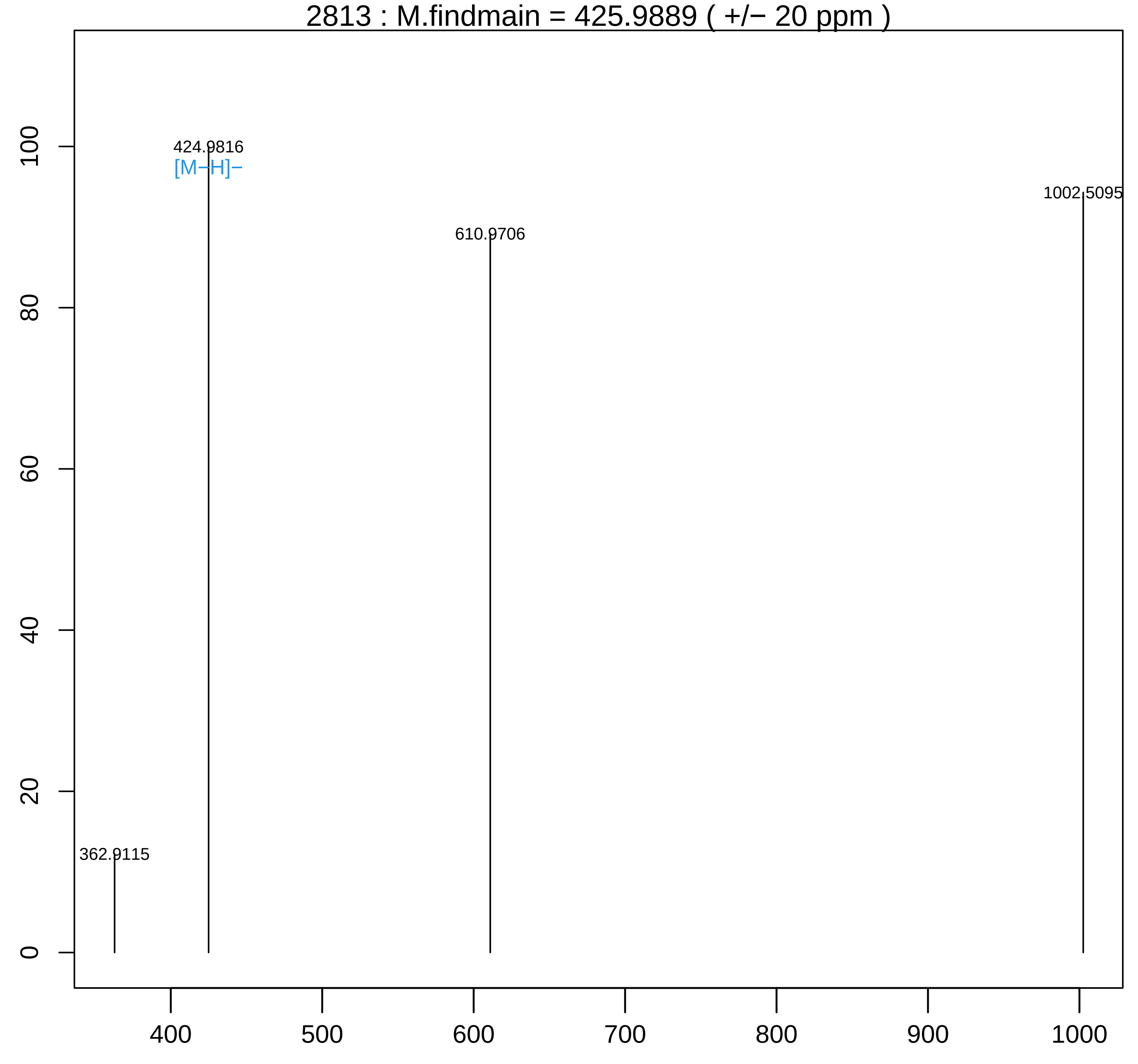
2813 : M.findmain = 425.9889 (+/− 20 ppm)
424.9816
[M−H]−
1002.5095
610.9706
362.9115
0
20
40
60
80
100
400
500
600
700
800
900
1000

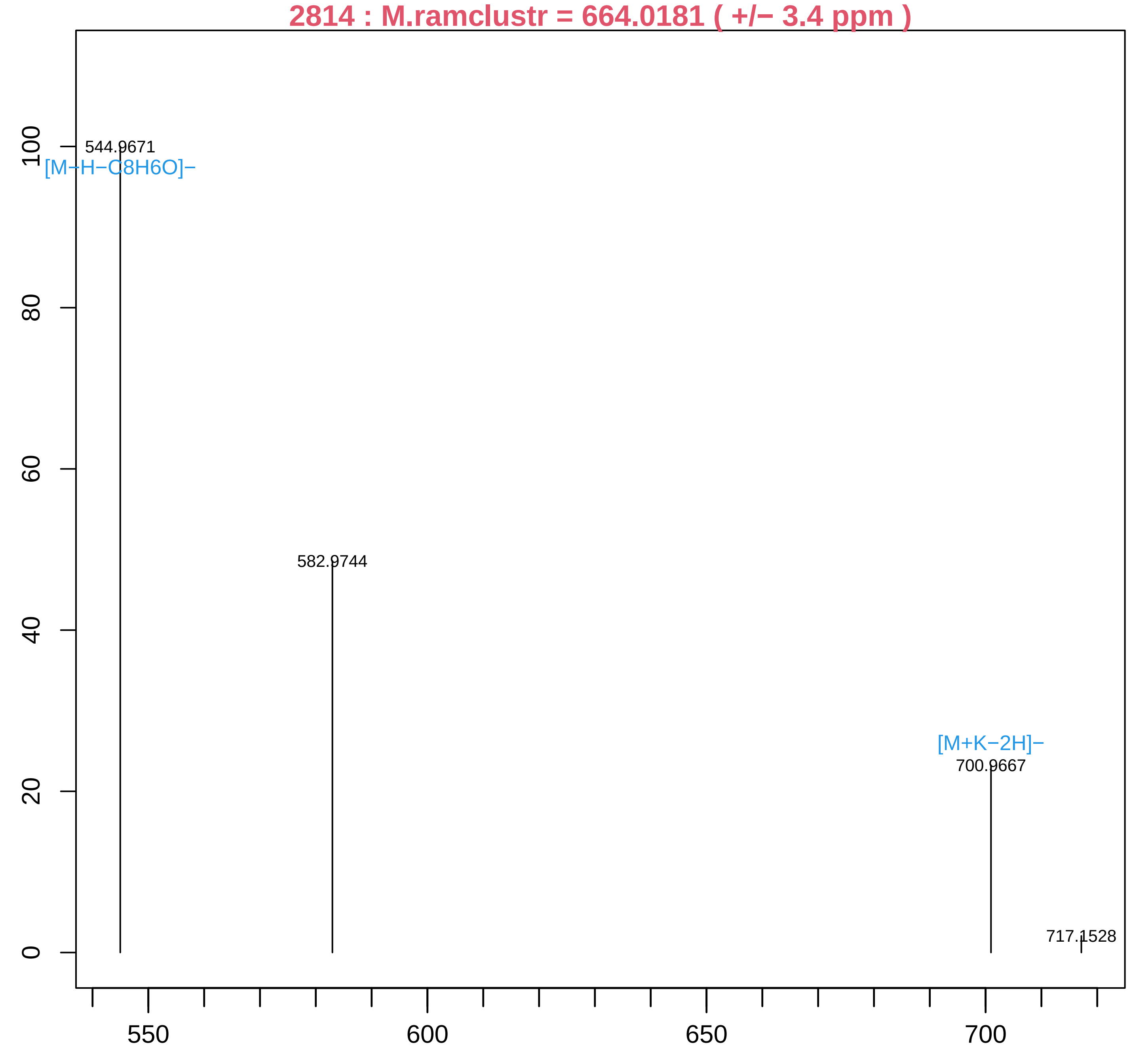
2814 : M.ramclustr = 664.0181 (+/− 3.4 ppm)
544.9671
[M−H−C8H6O]−
582.9744
[M+K−2H]−
700.9667
717.1528
0
20
40
60
80
100
550
600
650
700

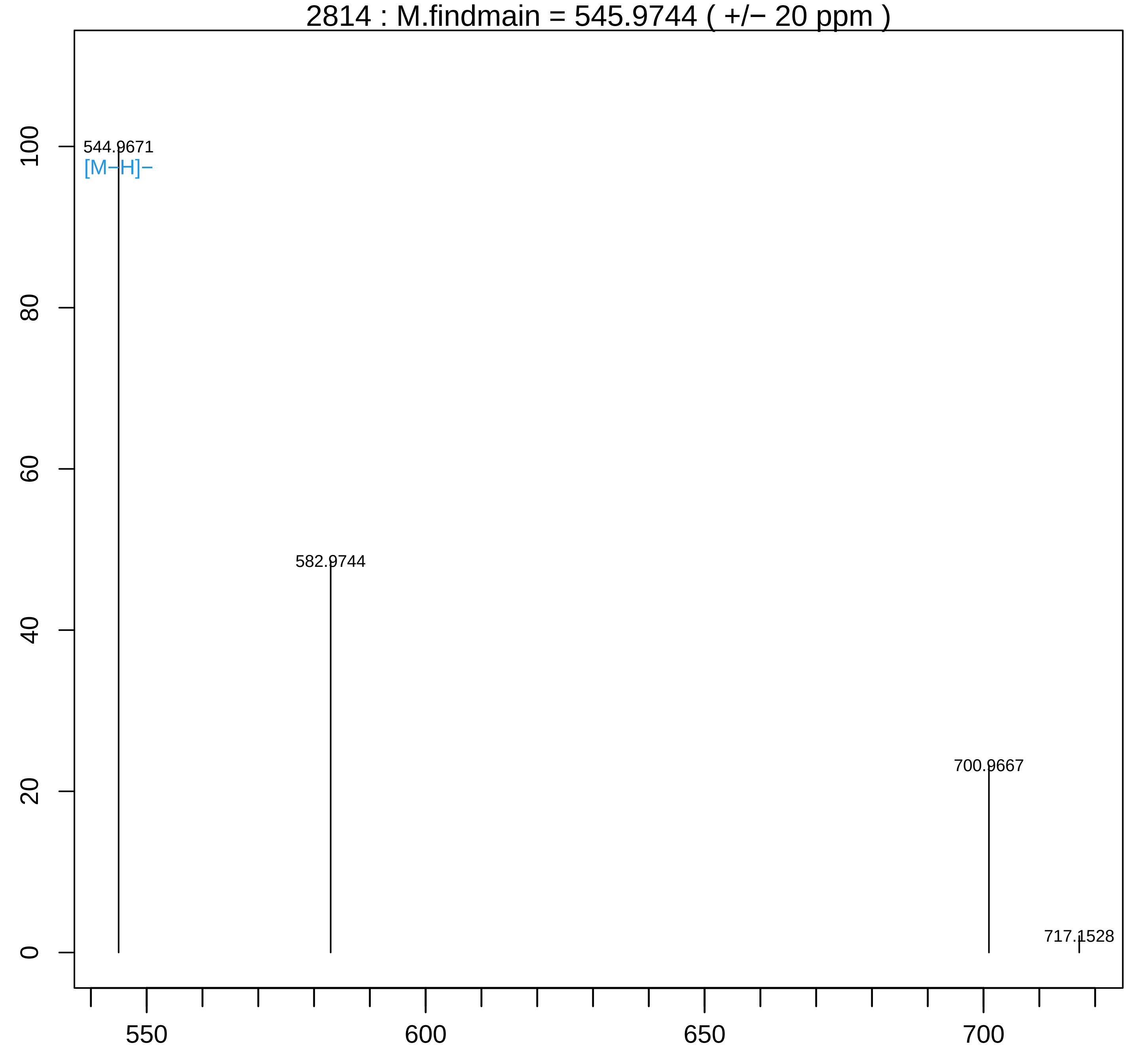
2814 : M.findmain = 545.9744 (+/− 20 ppm)
544.9671
[M−H]−
582.9744
700.9667
717.1528
0
20
40
60
80
100
550
600
650
700

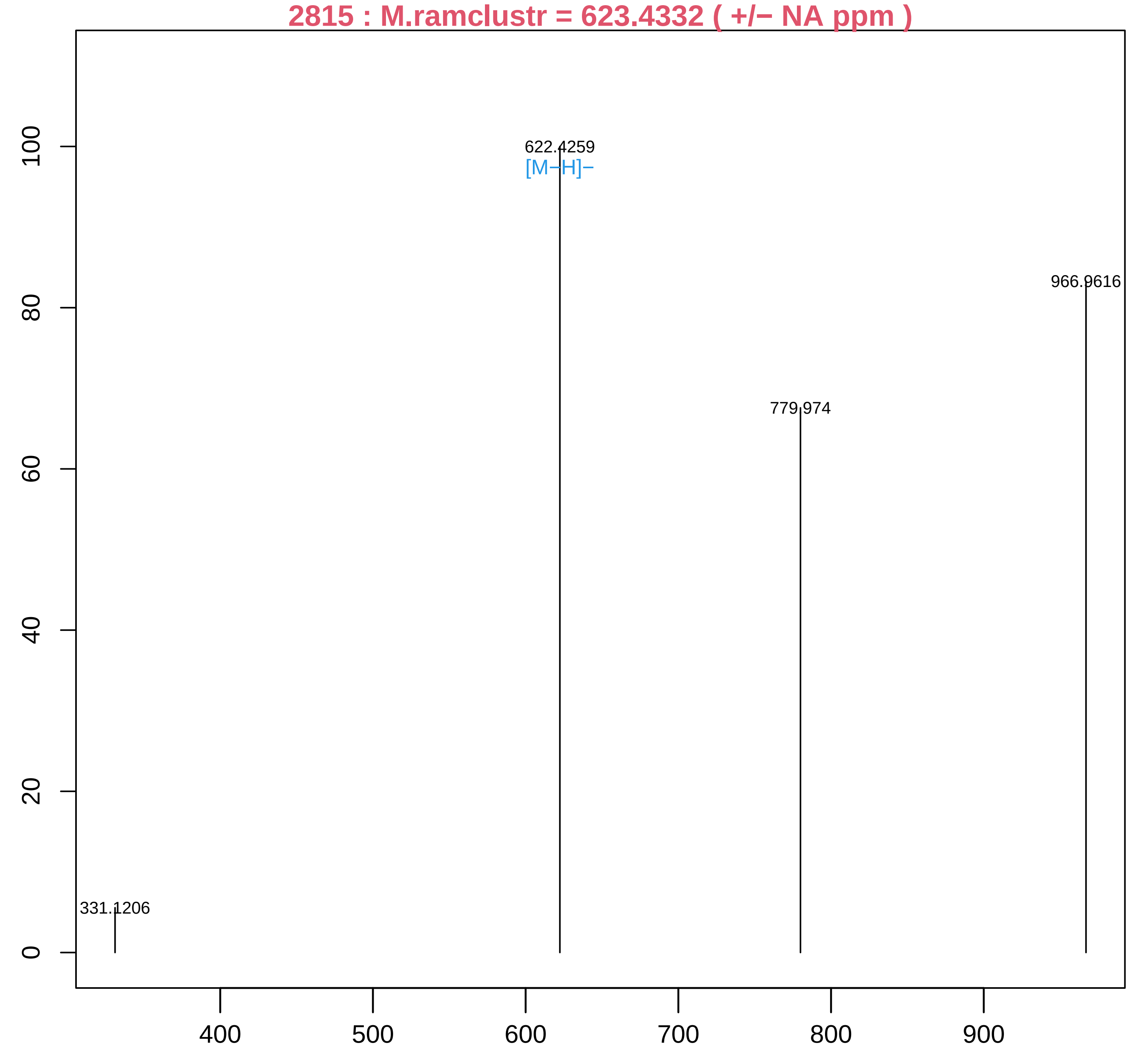
2815 : M.ramclustr = 623.4332 (+/− NA ppm)
622.4259
[M−H]−
966.9616
779.974
331.1206
0
20
40
60
80
100
400
500
600
700
800
900

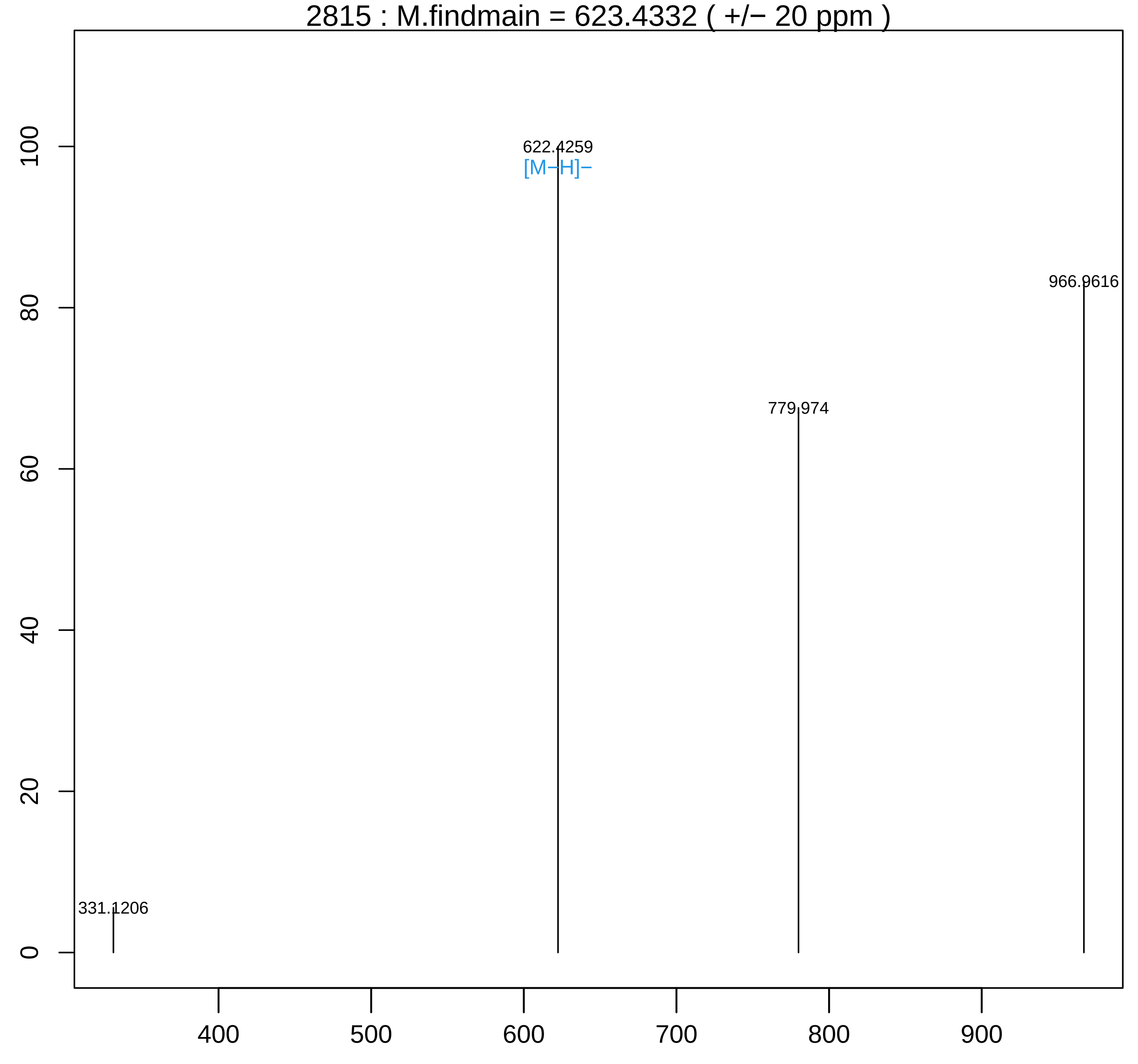
2815 : M.findmain = 623.4332 (+/− 20 ppm)
622.4259
[M−H]−
966.9616
779.974
331.1206
0
20
40
60
80
100
400
500
600
700
800
900

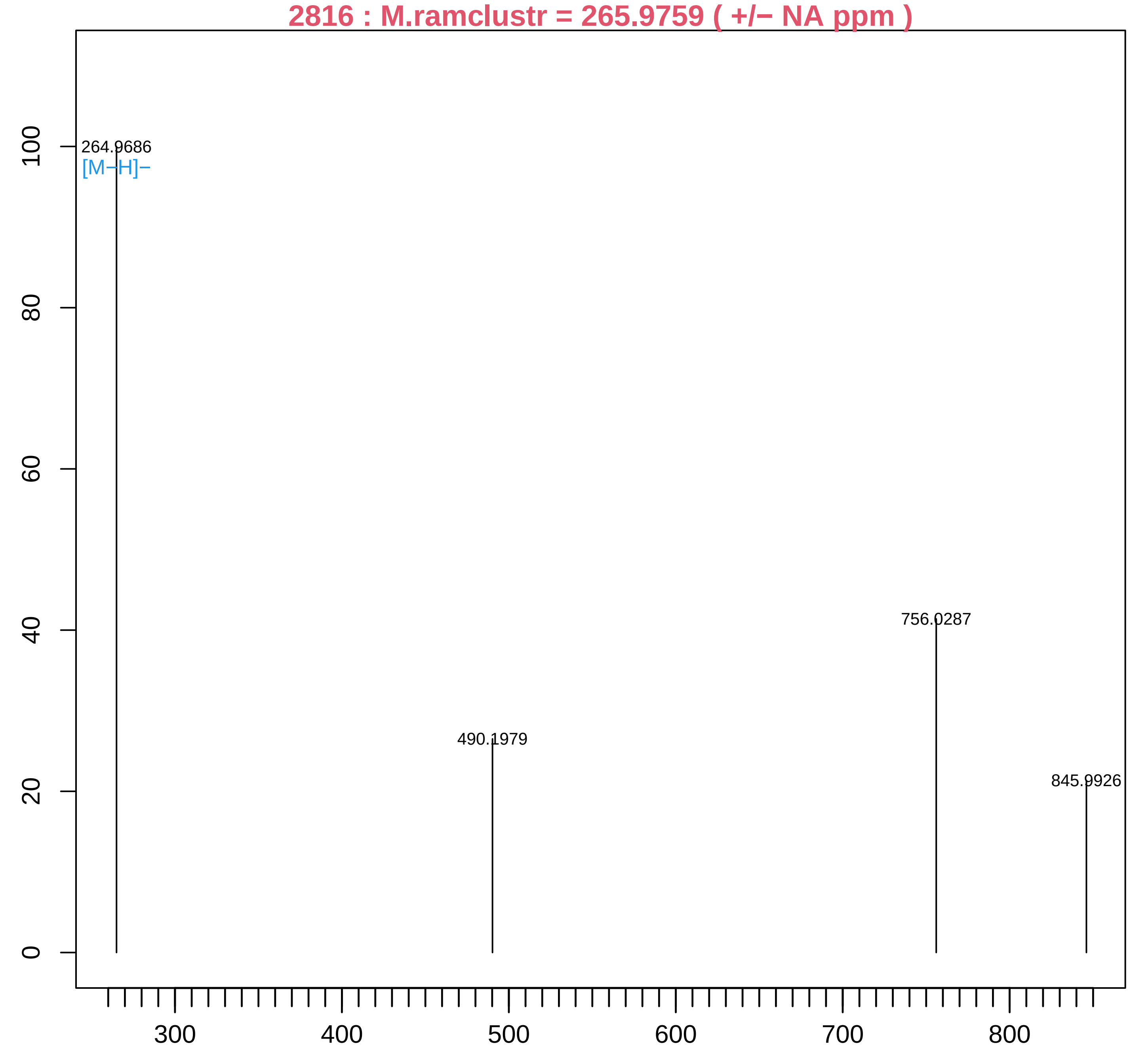
2816 : M.ramclustr = 265.9759 (+/− NA ppm)
264.9686
[M−H]−
490.1979
756.0287
845.9926
0
20
40
60
80
100
300
400
500
600
700
800

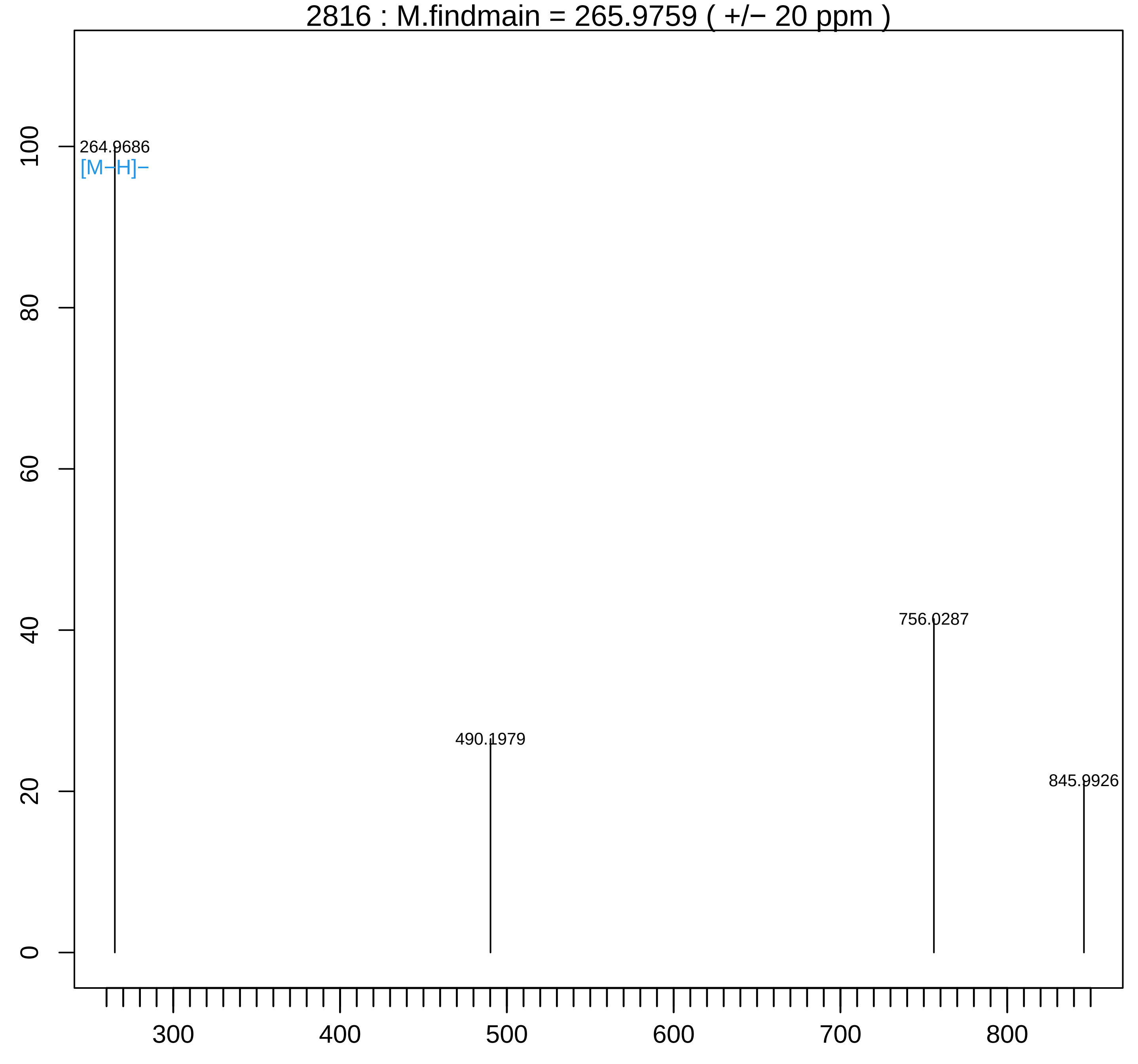
2816 : M.findmain = 265.9759 (+/− 20 ppm)
264.9686
[M−H]−
490.1979
756.0287
845.9926
0
20
40
60
80
100
300
400
500
600
700
800

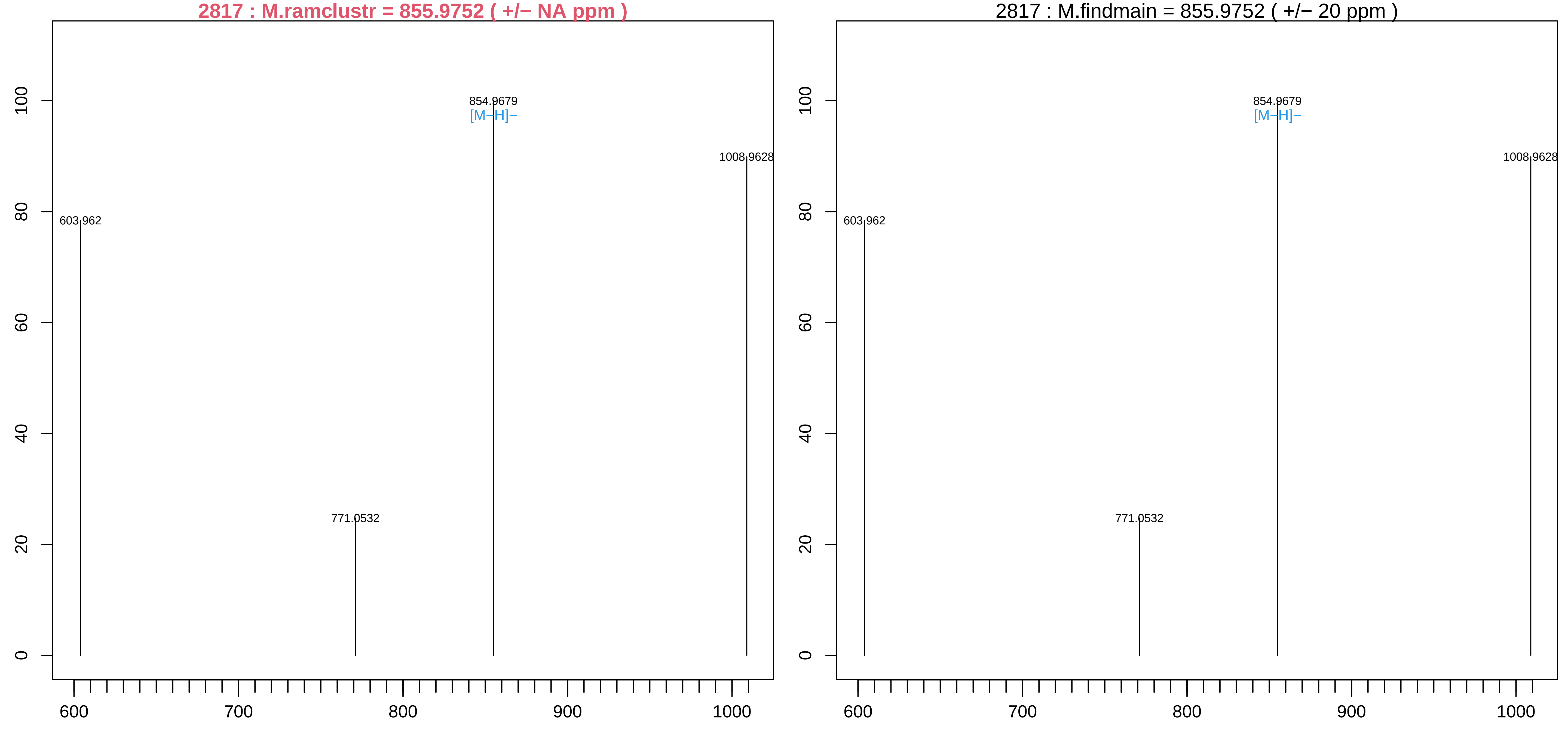
2817 : M.ramclustr = 855.9752 (+/− NA ppm)
603.962
771.0532
854.9679
[M−H]−
1008.9628
0
20
40
60
80
100
600
700
800
900
1000
2817 : M.findmain = 855.9752 (+/− 20 ppm)
603.962
771.0532
854.9679
[M−H]−
1008.9628
0
20
40
60
80
100
600
700
800
900
1000

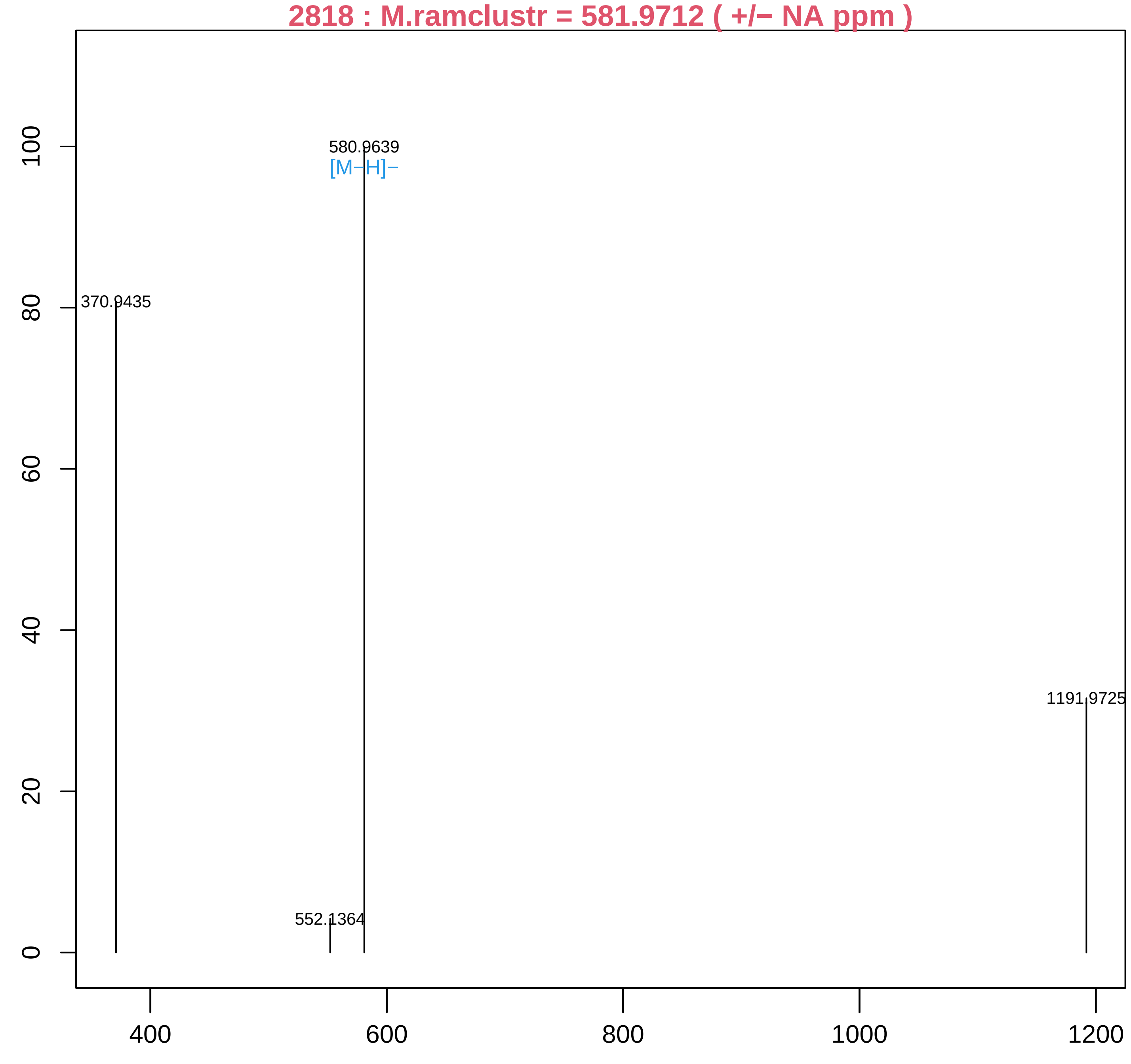
2818 : M.ramclustr = 581.9712 (+/− NA ppm)
580.9639
[M−H]−
370.9435
1191.9725
552.1364
0
20
40
60
80
100
400
600
800
1000
1200

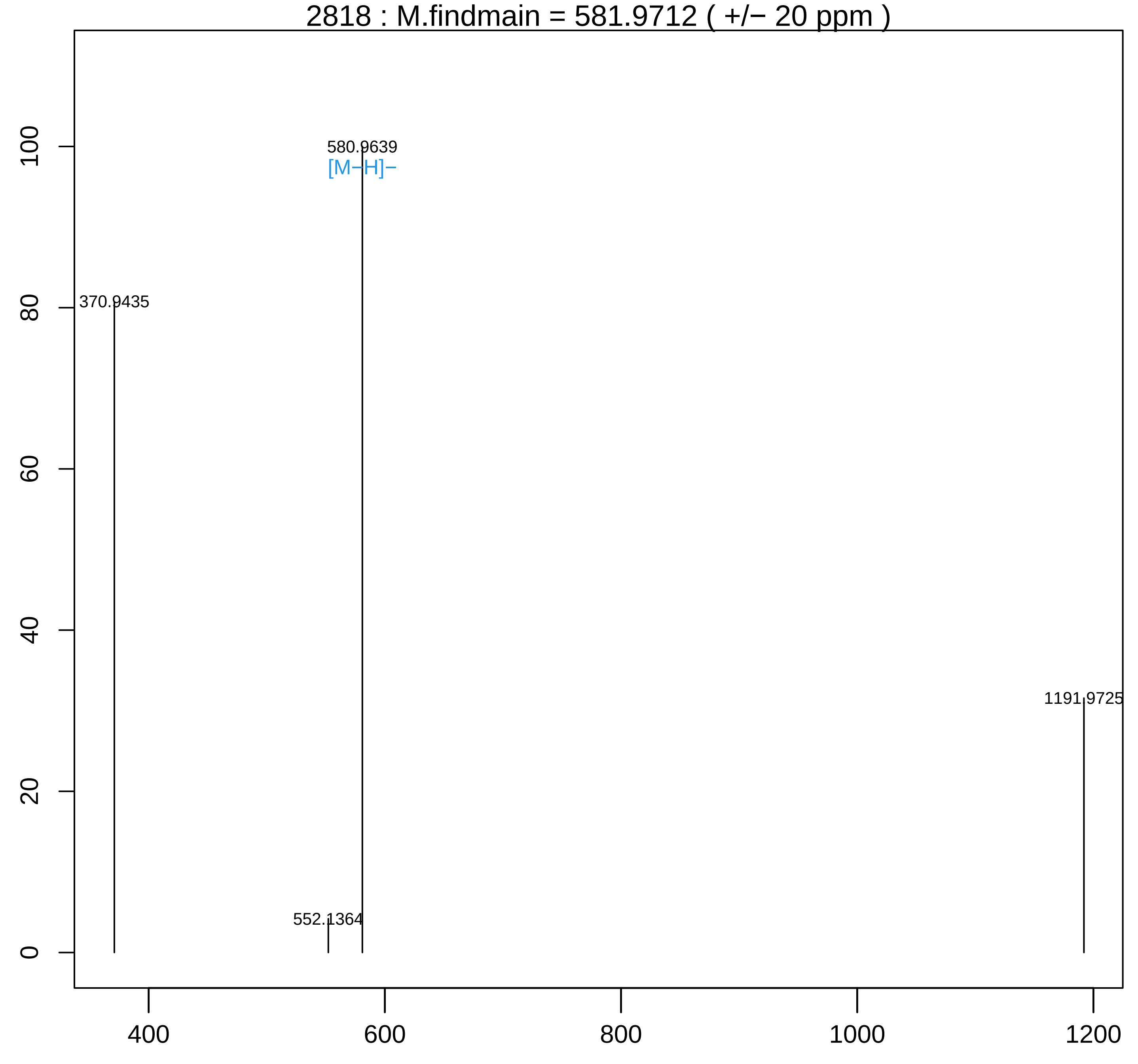
2818 : M.findmain = 581.9712 (+/− 20 ppm)
580.9639
[M−H]−
370.9435
1191.9725
552.1364
0
20
40
60
80
100
400
600
800
1000
1200

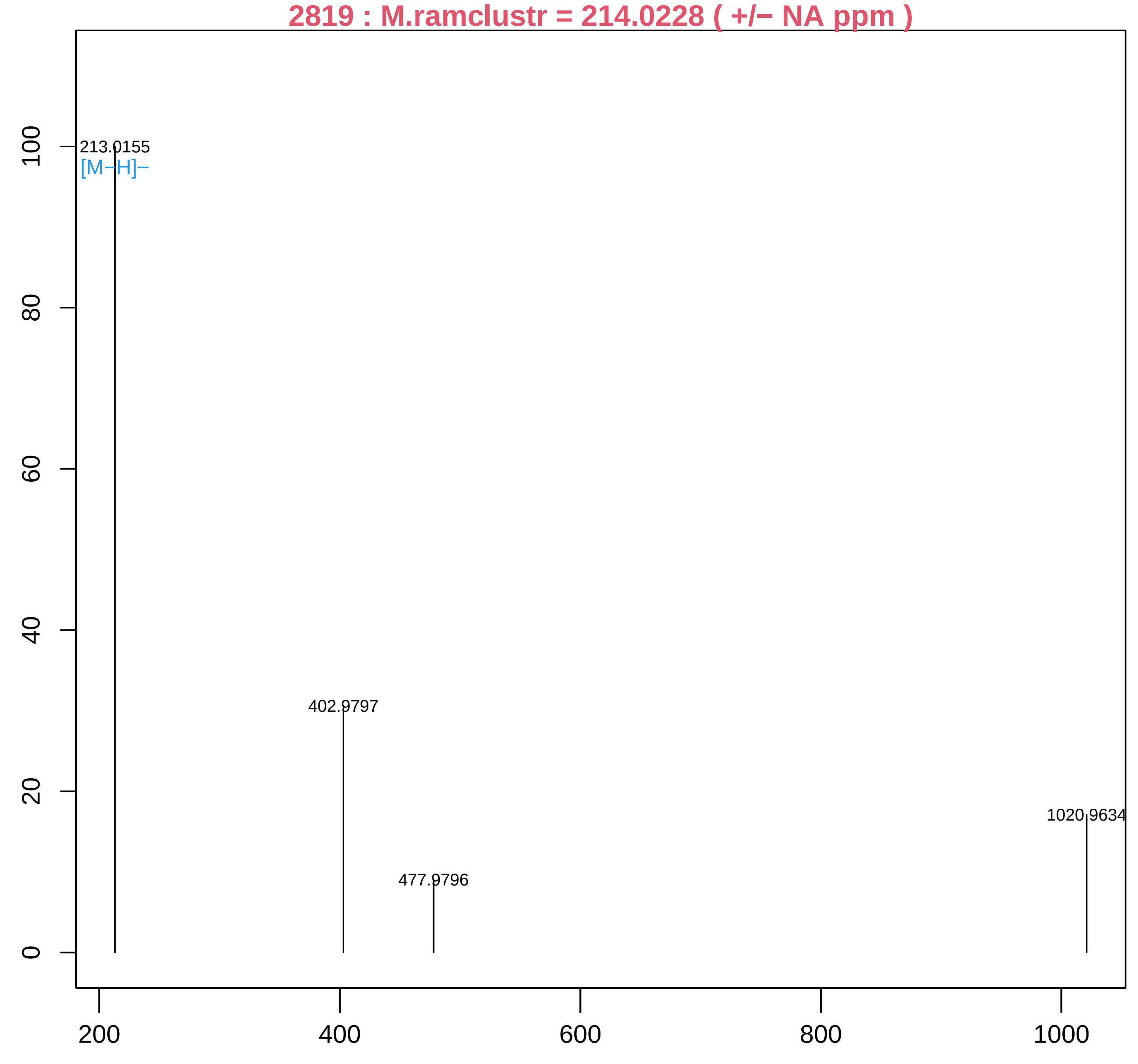
2819 : M.ramclustr = 214.0228 (+/− NA ppm)
213.0155
[M−H]−
402.9797
477.9796
1020.9634
0
20
40
60
80
100
200
400
600
800
1000

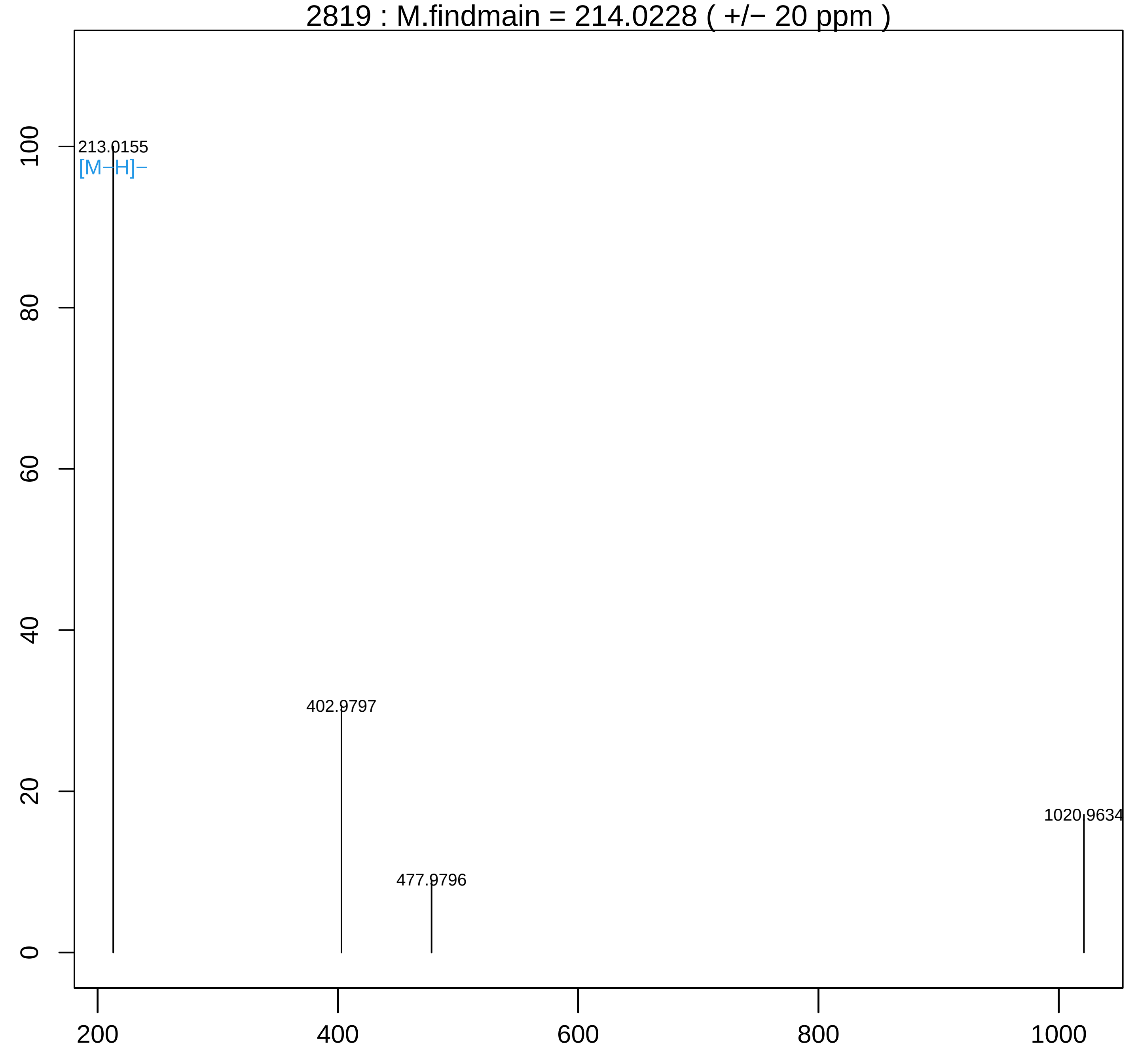
2819 : M.findmain = 214.0228 (+/− 20 ppm)
213.0155
[M−H]−
402.9797
477.9796
1020.9634
0
20
40
60
80
100
200
400
600
800
1000

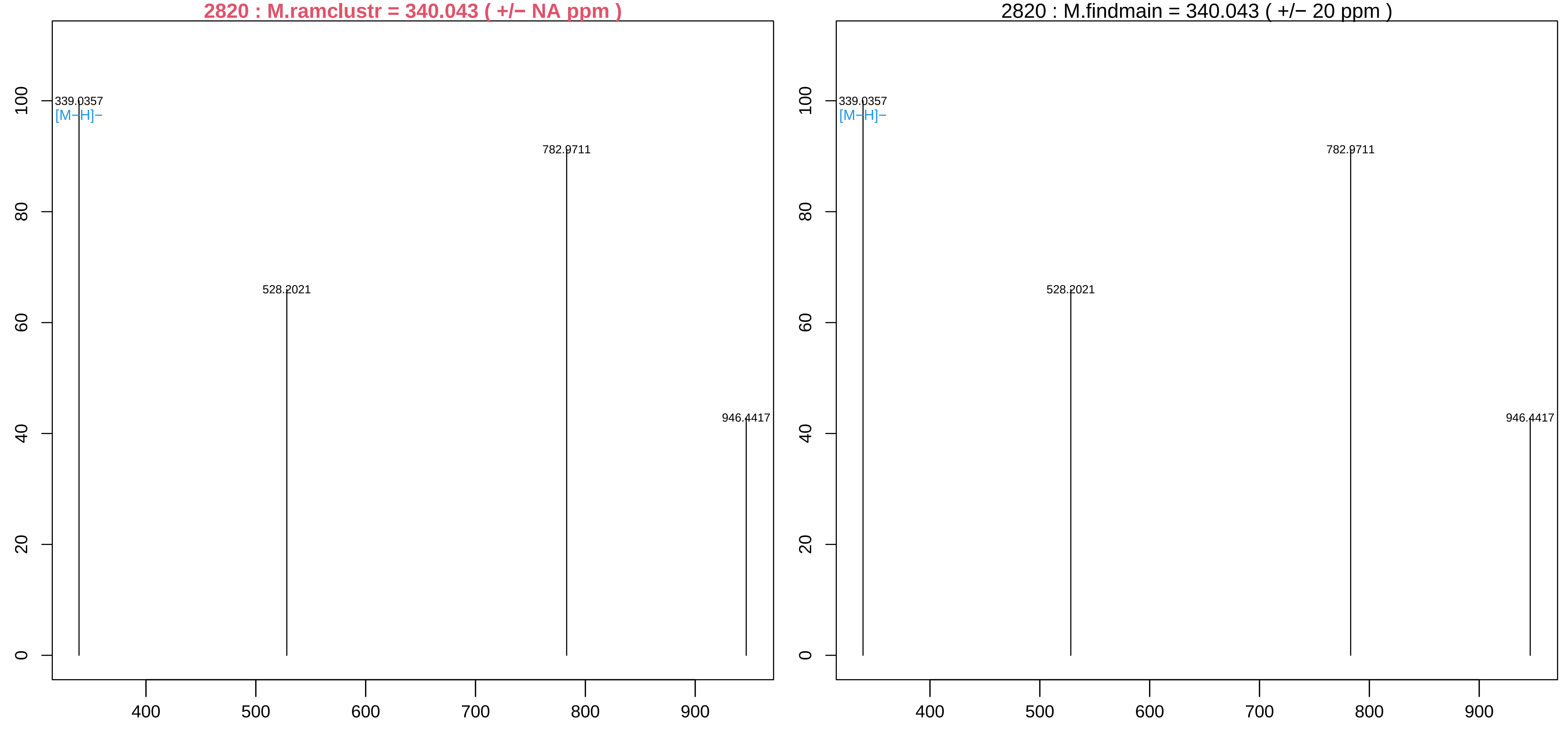

2820 : M.ramclustr = 340.043 (+/− NA ppm)
339.0357
[M−H]−
528.2021
782.9711
946.4417
0
20
40
60
80
100
400
500
600
700
800
900
2820 : M.findmain = 340.043 (+/− 20 ppm)
339.0357
[M−H]−
528.2021
782.9711
946.4417
0
20
40
60
80
100
400
500
600
700
800
900

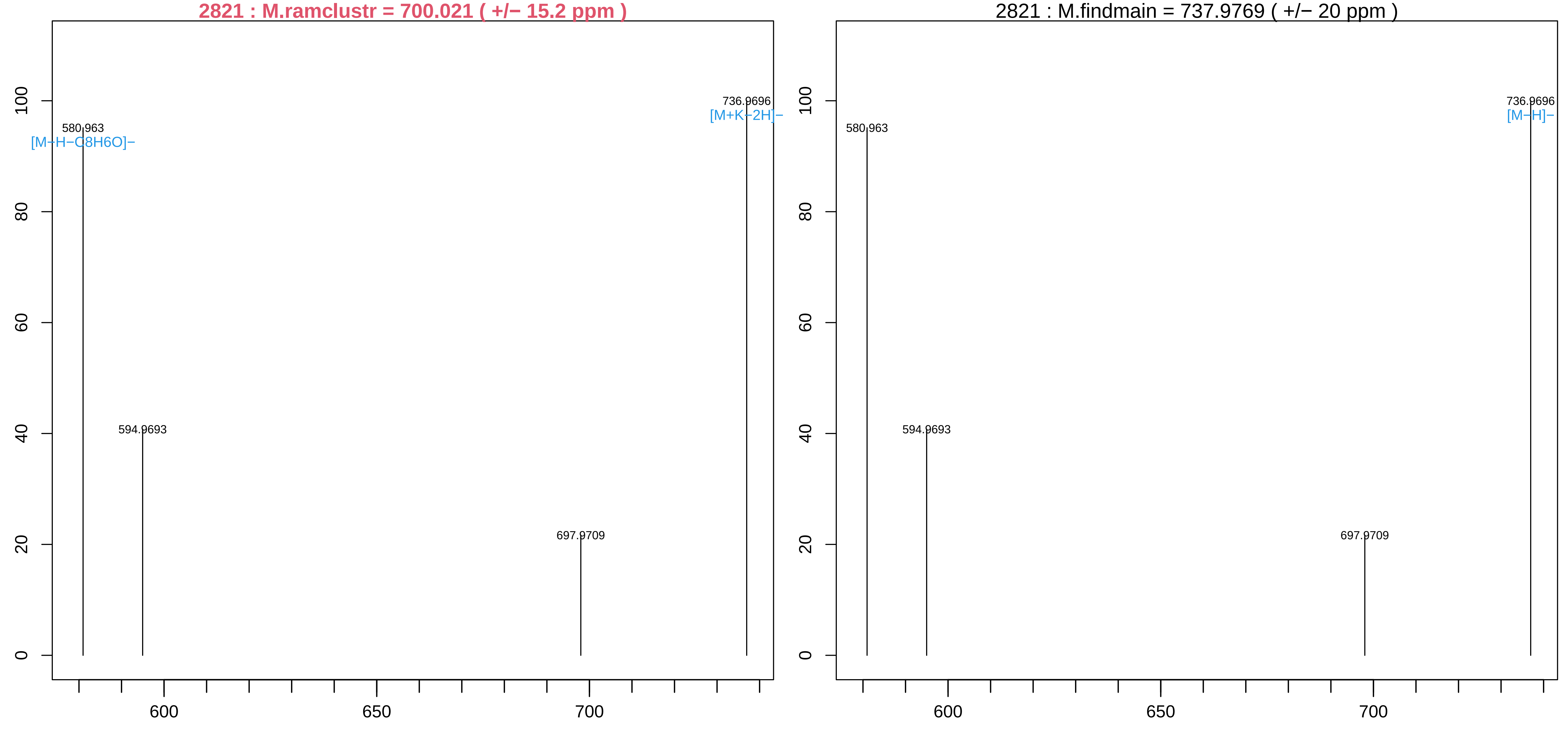

2821 : M.ramclustr = 700.021 (+/− 15.2 ppm)
580.963
[M−H−C8H6O]−
594.9693
697.9709
736.9696
[M+K−2H]−
0
20
40
60
80
100
600
650
700
2821 : M.findmain = 737.9769 (+/− 20 ppm)
580.963
594.9693
697.9709
736.9696
[M−H]−
0
20
40
60
80
100
600
650
700

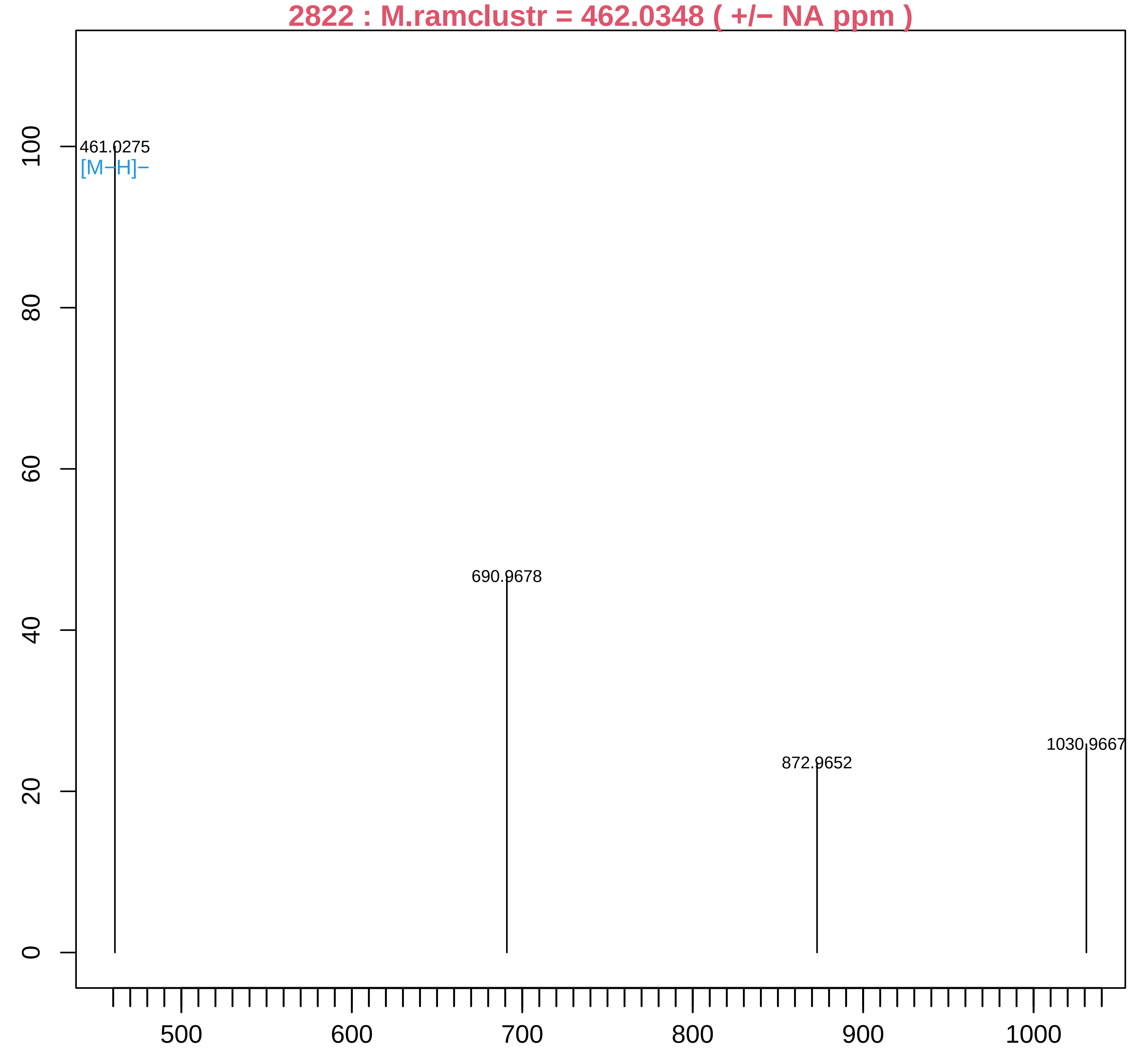

2822 : M.ramclustr = 462.0348 (+/− NA ppm)
461.0275
[M−H]−
690.9678
872.9652
1030.9667
0
20
40
60
80
100
500
600
700
800
900
1000

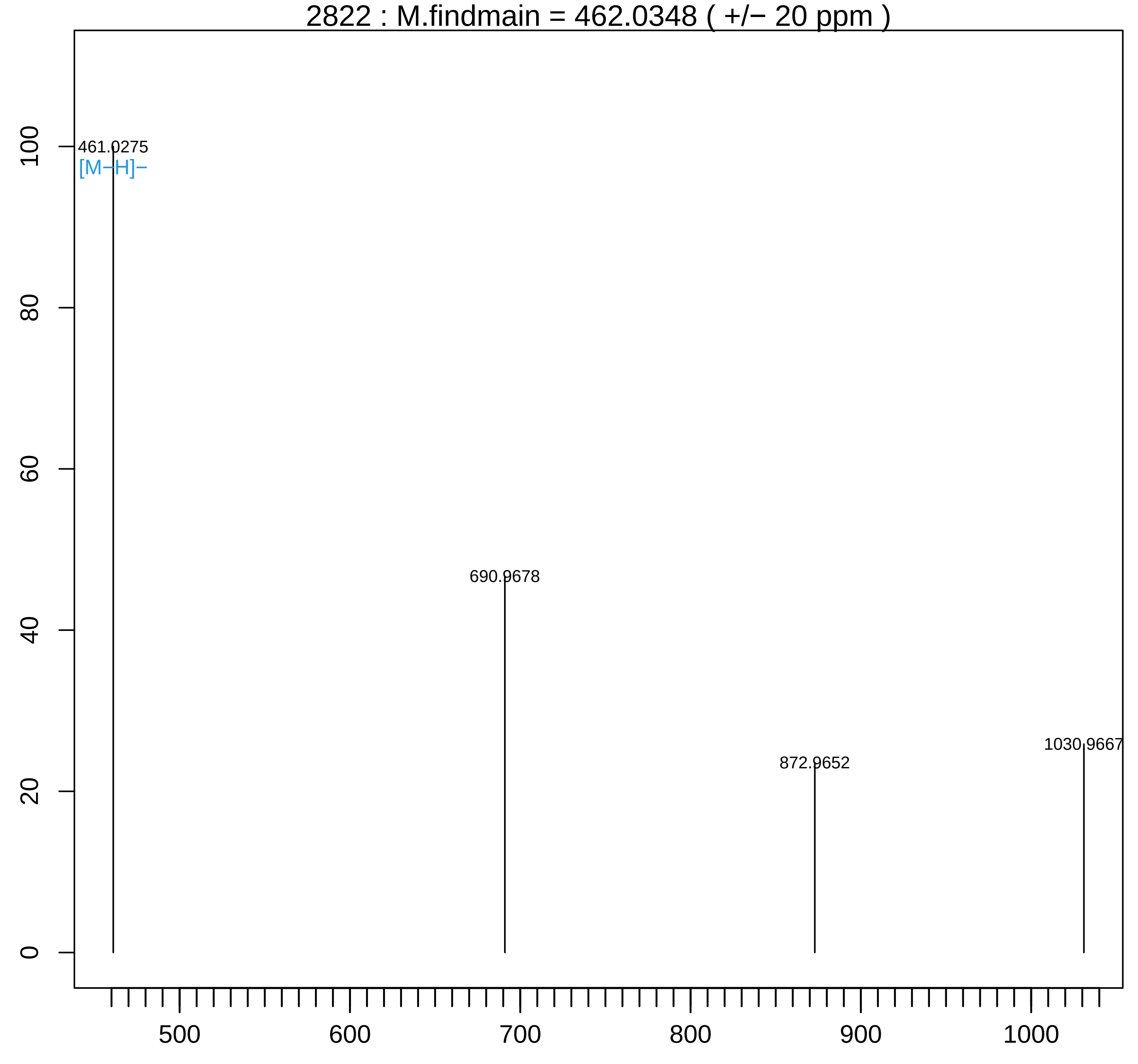

2822 : M.findmain = 462.0348 (+/− 20 ppm)
461.0275
[M−H]−
690.9678
872.9652
1030.9667
0
20
40
60
80
100
500
600
700
800
900
1000

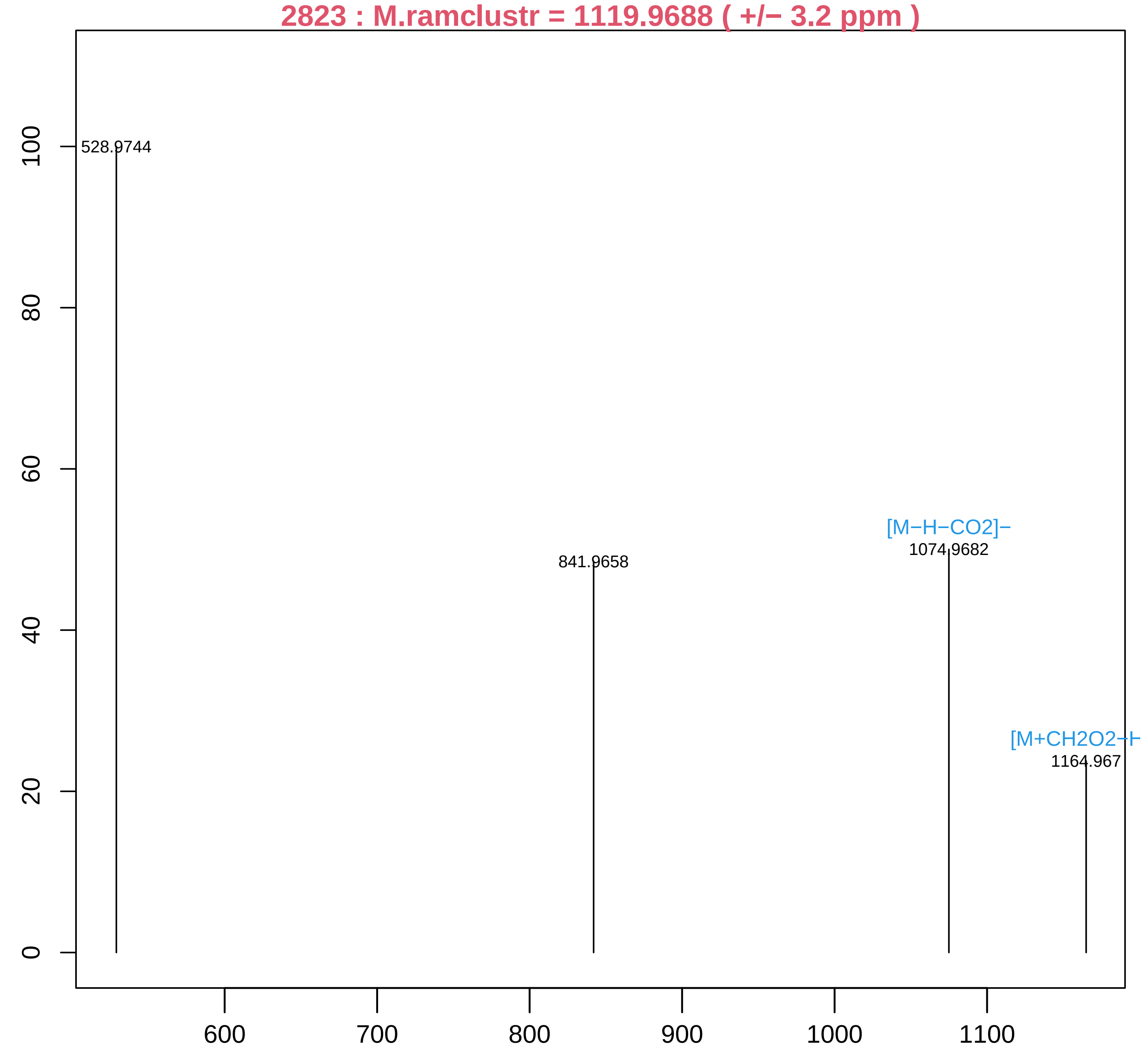

2823 : M.ramclustr = 1119.9688 (+/− 3.2 ppm)
528.9744
841.9658
[M−H−CO2]−
1074.9682
[M+CH2O2−H
1164.967
0
20
40
60
80
100
600
700
800
900
1000
1100

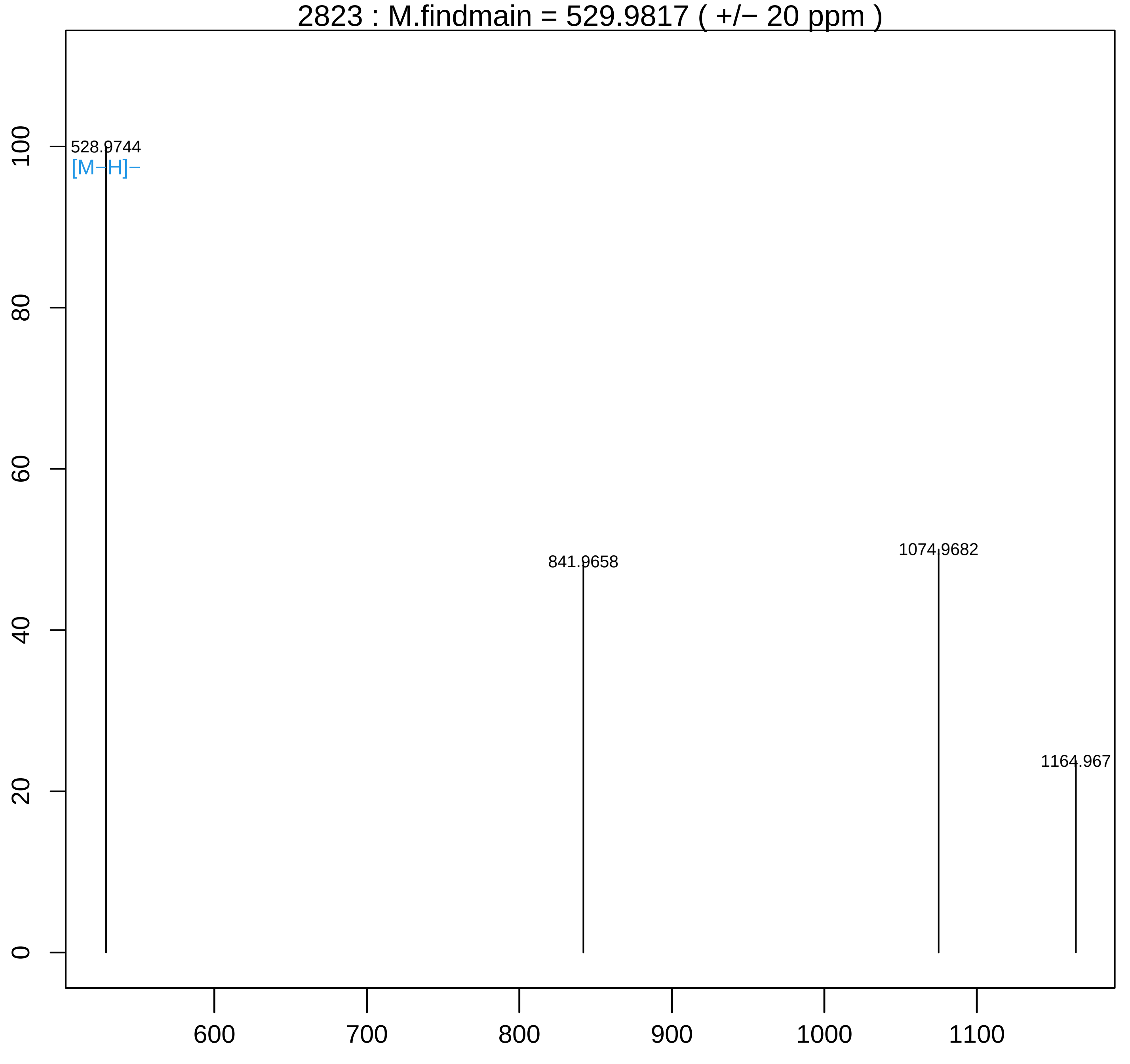

2823 : M.findmain = 529.9817 (+/− 20 ppm)
528.9744
[M−H]−
841.9658
1074.9682
1164.967
0
20
40
60
80
100
600
700
800
900
1000
1100

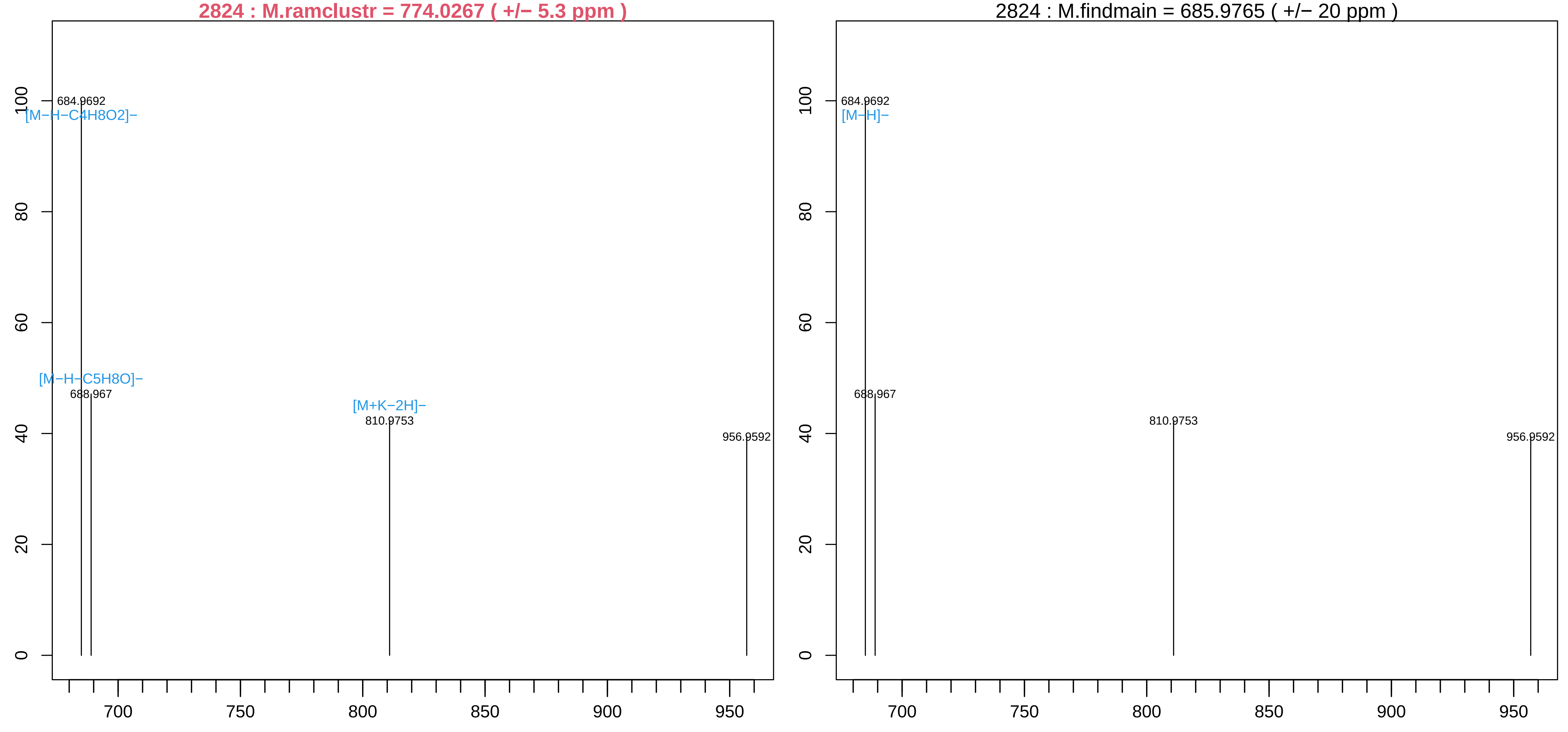

2824 : M.ramclustr = 774.0267 (+/− 5.3 ppm)
684.9692
[M−H−C4H8O2]−
[M−H−C5H8O]−
688.967
[M+K−2H]−
810.9753
956.9592
2824 : M.findmain = 685.9765 (+/− 20 ppm)
684.9692
[M−H]−
688.967
810.9753
956.9592

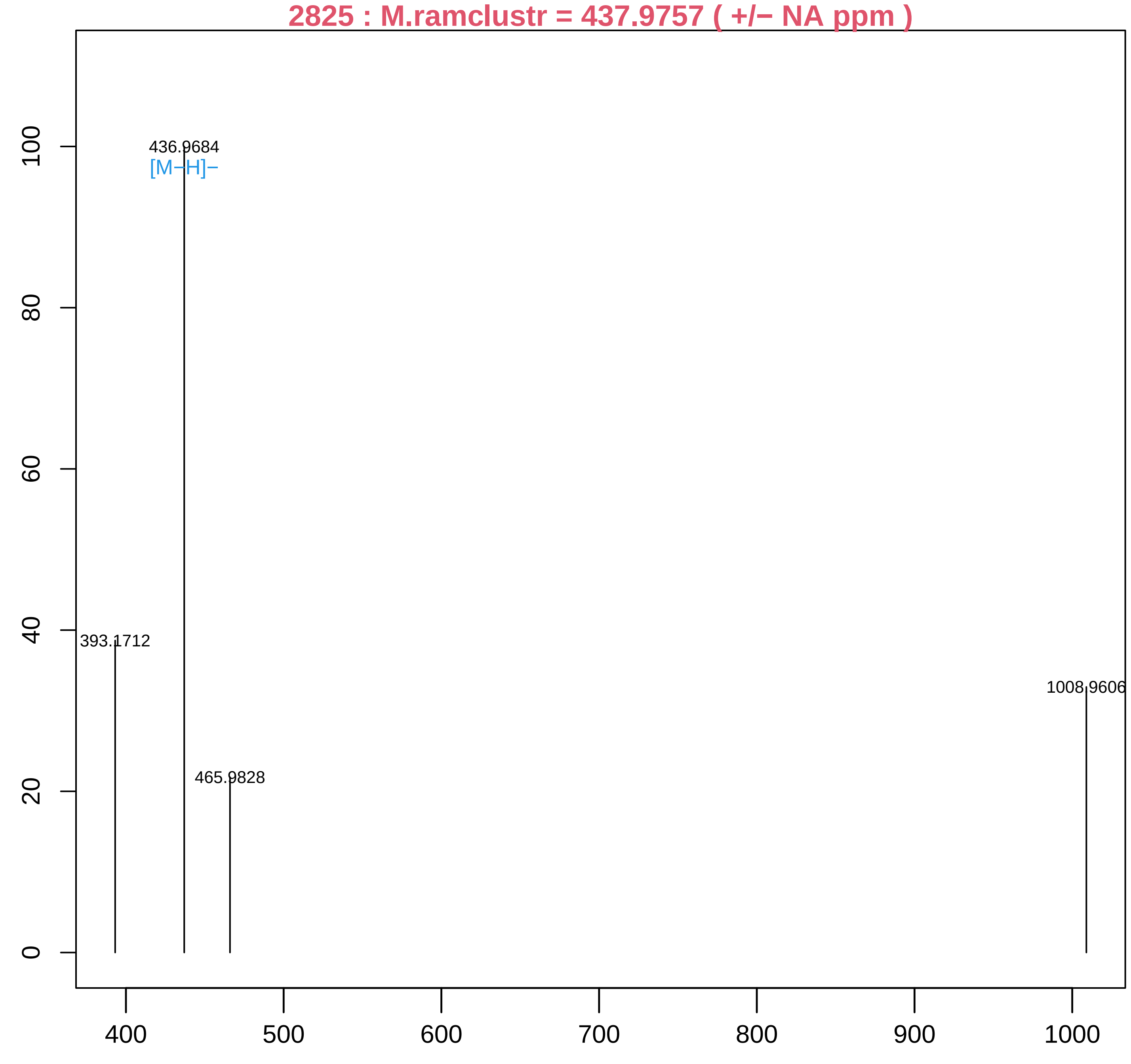
2825 : M.ramclustr = 437.9757 (+/− NA ppm)
436.9684
[M−H]−
393.1712
465.9828
1008.9606
0
20
40
60
80
100
400
500
600
700
800
900
1000

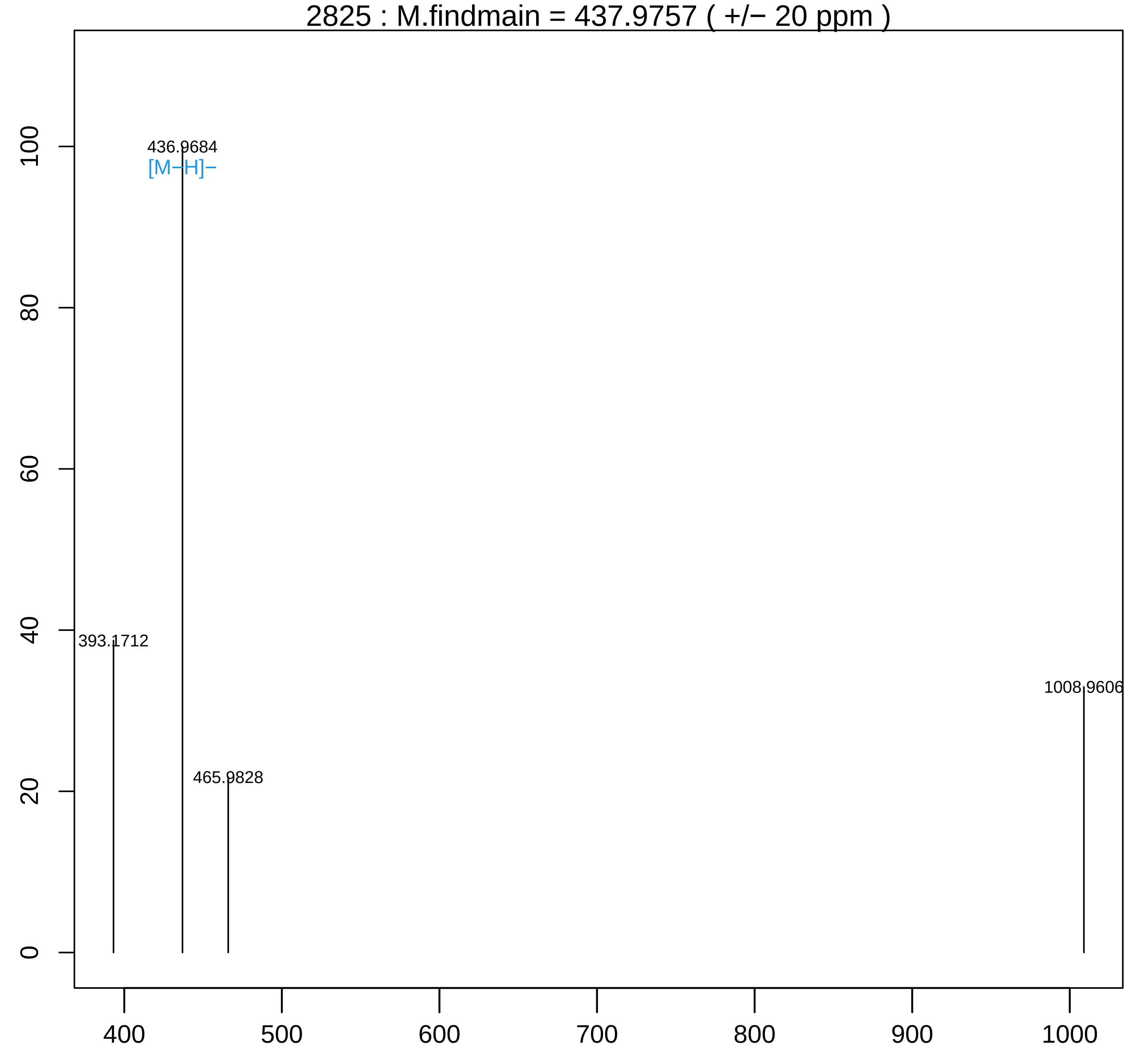
2825 : M.findmain = 437.9757 (+/− 20 ppm)
436.9684
[M−H]−
393.1712
465.9828
1008.9606
0
20
40
60
80
100
400
500
600
700
800
900
1000

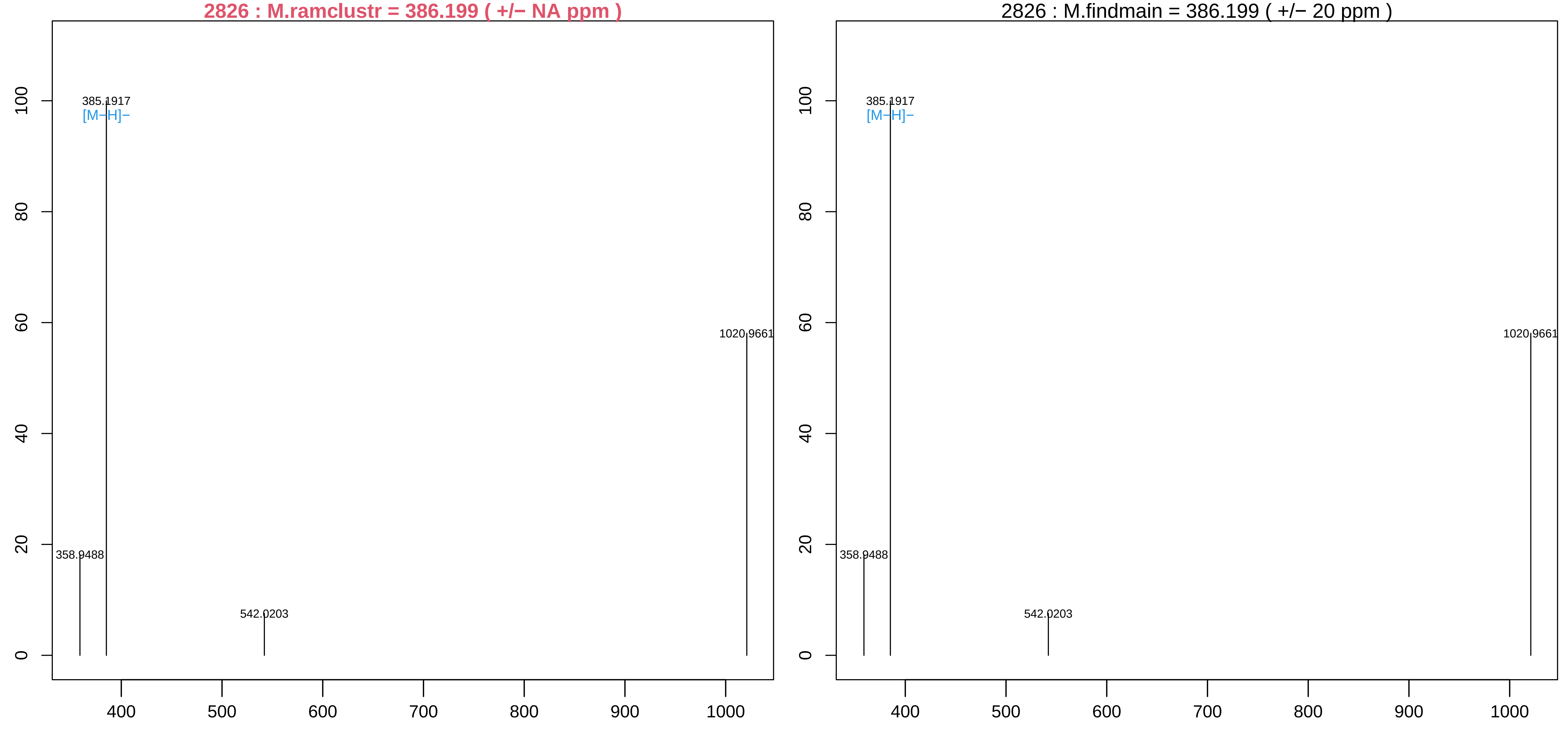
2826 : M.ramclustr = 386.199 (+/− NA ppm)
385.1917
[M−H]−
358.9488
542.0203
1020.9661
0
20
40
60
80
100
400
500
600
700
800
900
1000
2826 : M.findmain = 386.199 (+/− 20 ppm)
385.1917
[M−H]−
358.9488
542.0203
1020.9661
0
20
40
60
80
100
400
500
600
700
800
900
1000

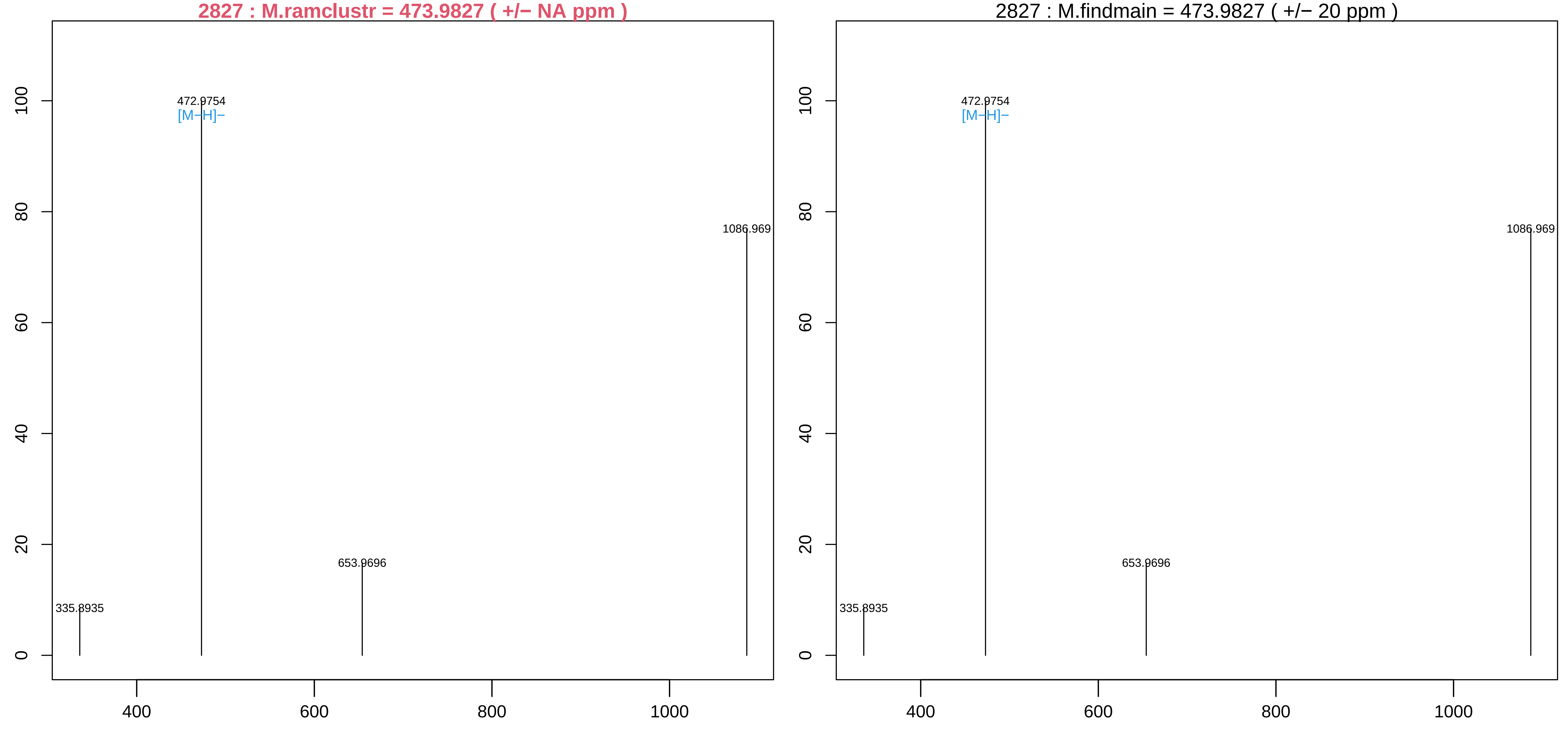
2827 : M.ramclustr = 473.9827 (+/− NA ppm)
2827 : M.findmain = 473.9827 (+/− 20 ppm)
472.9754
[M−H]−
1086.969
653.9696
335.8935
0
20
40
60
80
100
400
600
800
1000

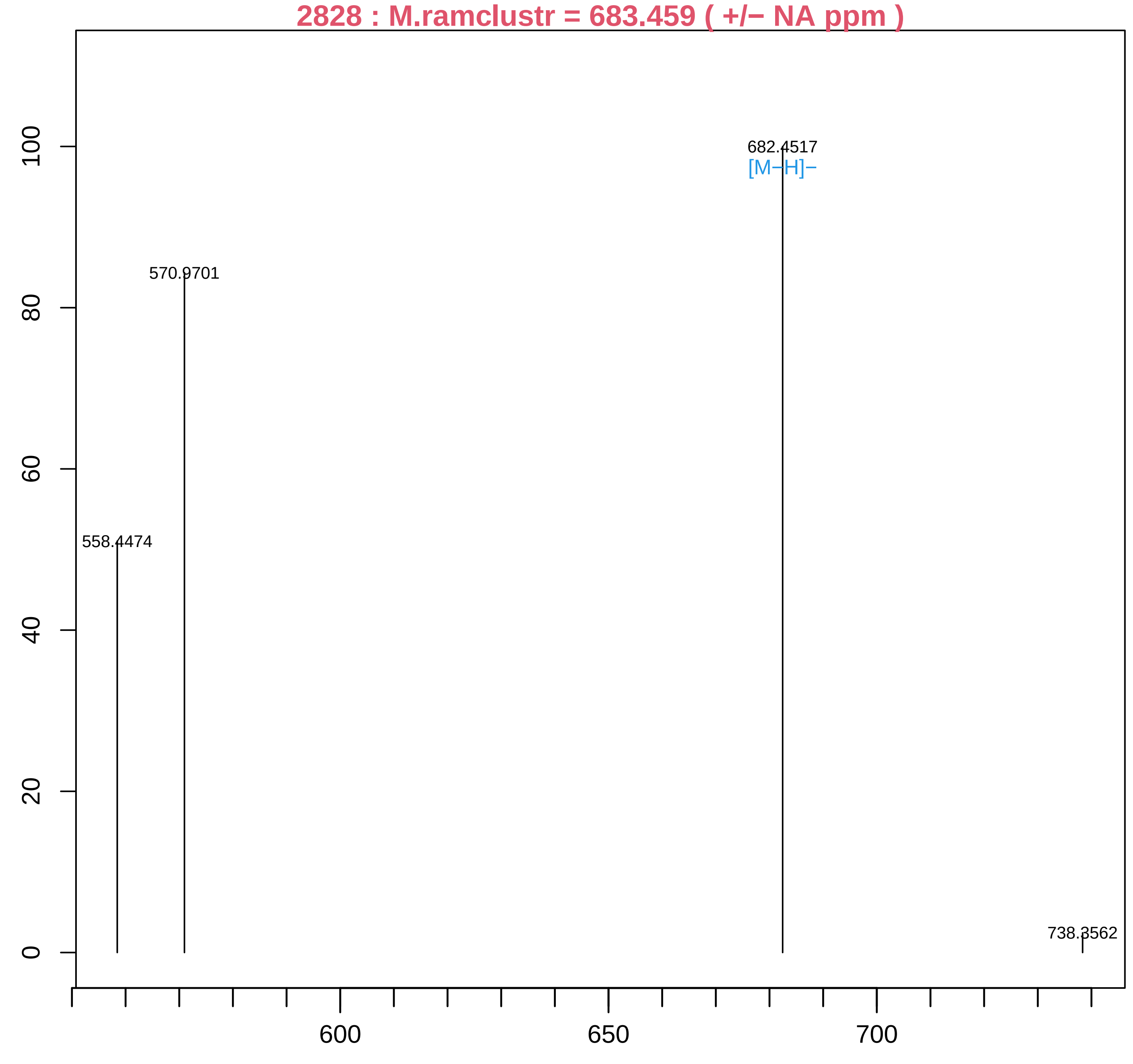
2828 : M.ramclustr = 683.459 (+/− NA ppm)
682.4517
[M−H]−
570.9701
558.4474
738.3562
0
20
40
60
80
100
600
650
700

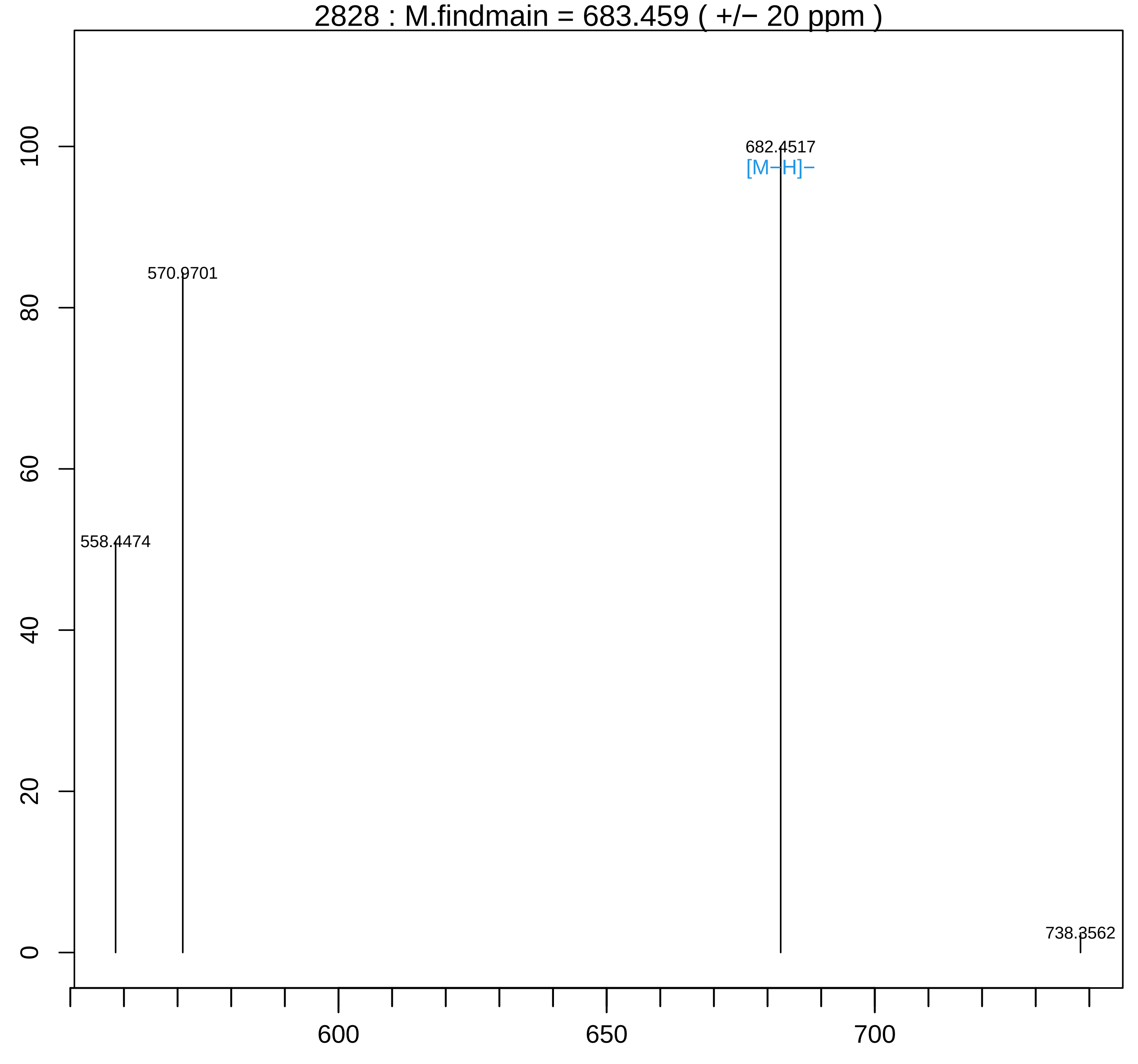
2828 : M.findmain = 683.459 (+/− 20 ppm)
682.4517
[M−H]−
570.9701
558.4474
738.3562
0
20
40
60
80
100
600
650
700

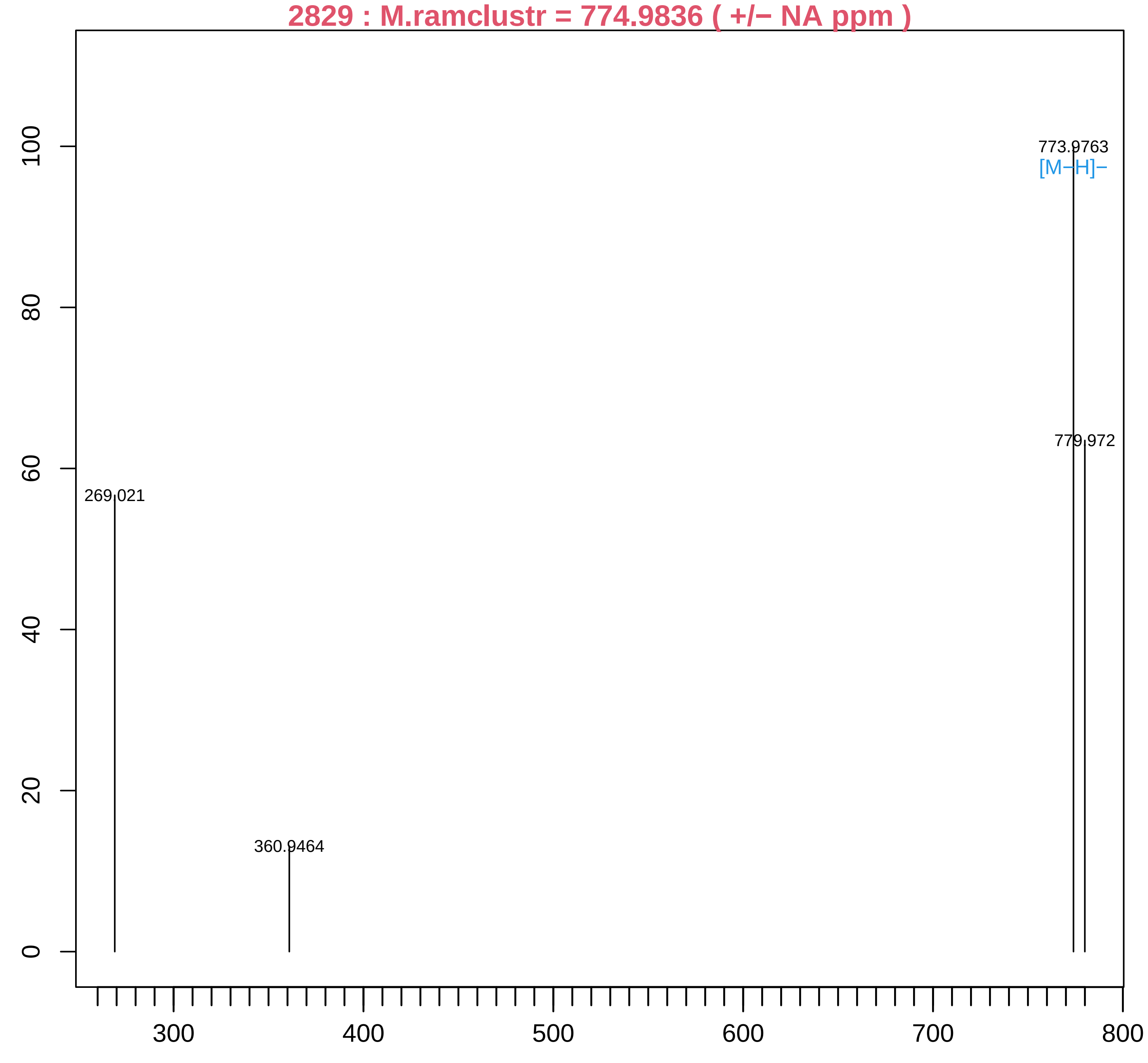
2829 : M.ramclustr = 774.9836 (+/− NA ppm)
773.9763
[M−H]−
779.972
269.021
360.9464
0
20
40
60
80
100
300
400
500
600
700
800

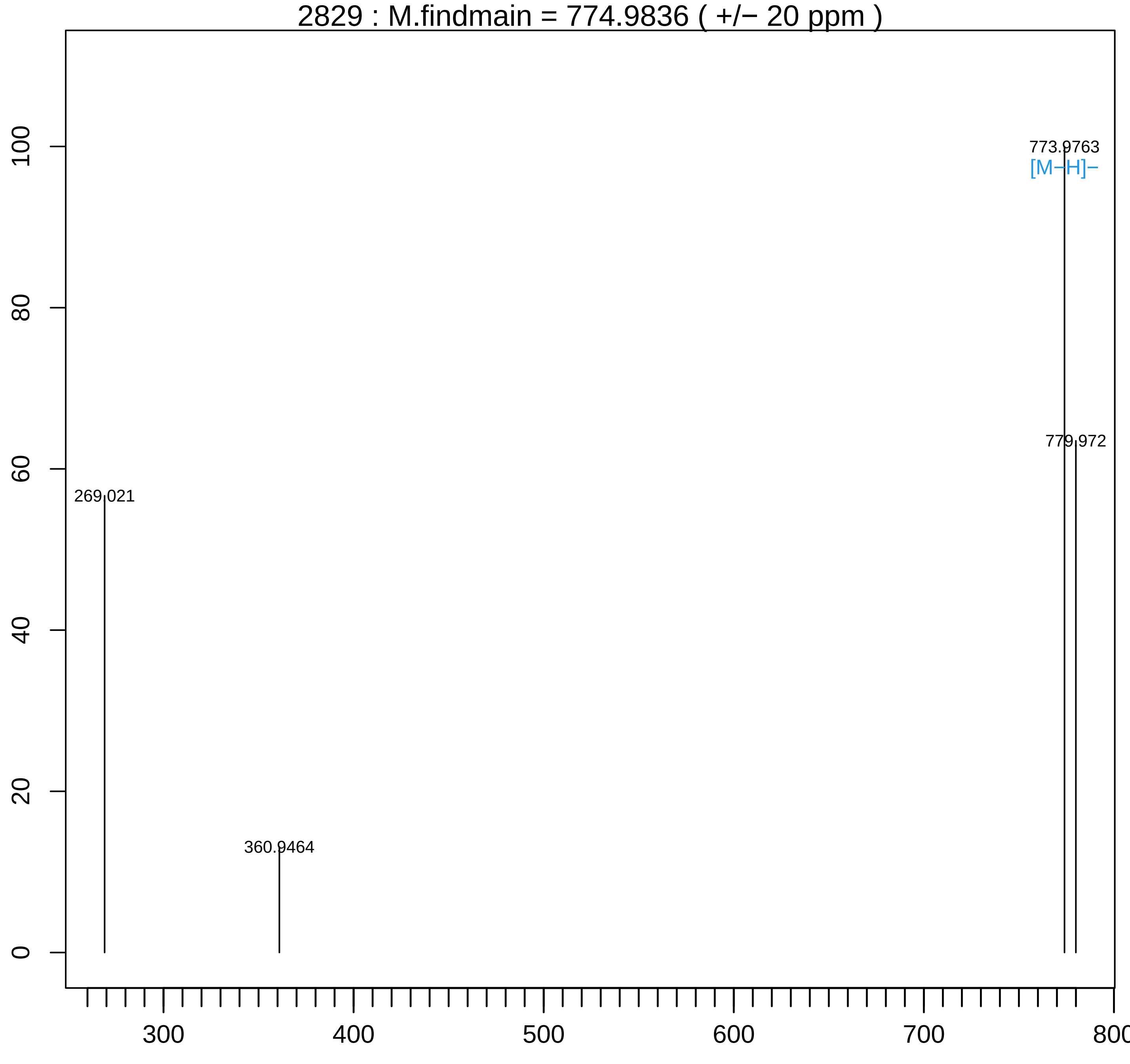
2829 : M.findmain = 774.9836 (+/− 20 ppm)
773.9763
[M−H]−
779.972
269.021
360.9464
0
20
40
60
80
100
300
400
500
600
700
800

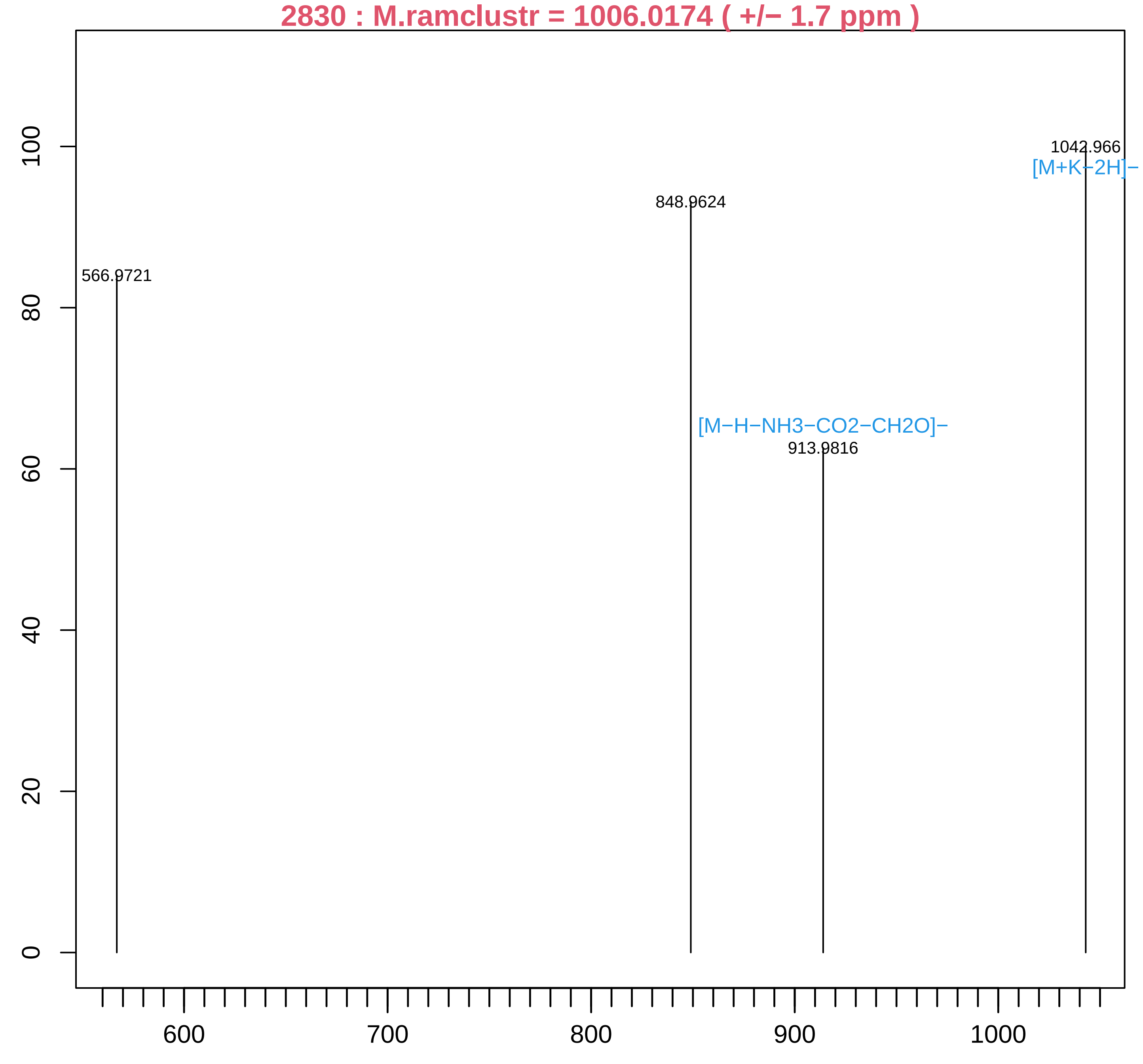

2830 : M.ramclustr = 1006.0174 (+/− 1.7 ppm)
1042.966
[M+K−2H]−
848.9624
566.9721
[M−H−NH3−CO2−CH2O]−
913.9816
0
20
40
60
80
100
600
700
800
900
1000

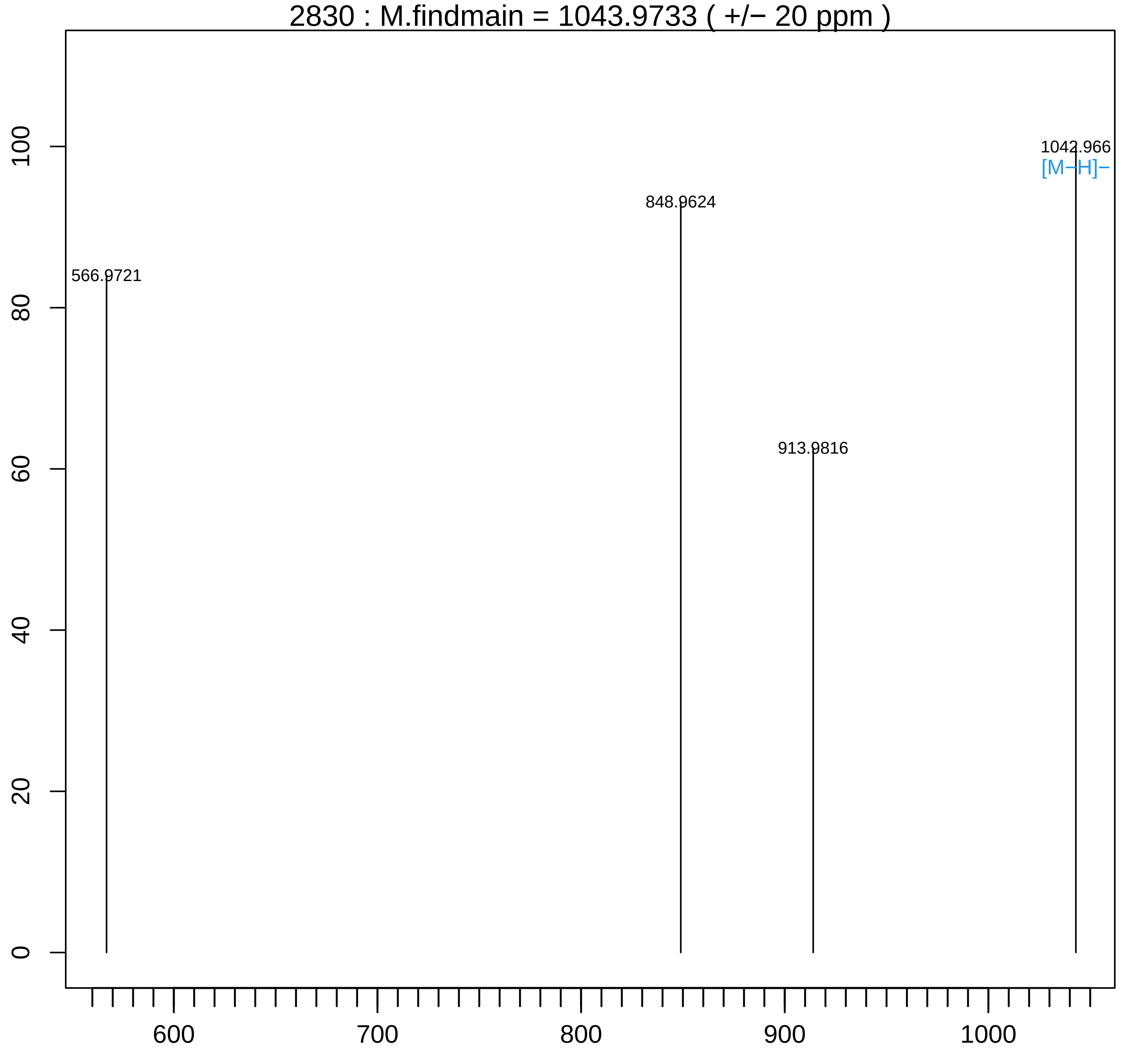

2830 : M.findmain = 1043.9733 (+/− 20 ppm)
1042.966
[M−H]−
848.9624
566.9721
913.9816
0
20
40
60
80
100
600
700
800
900
1000

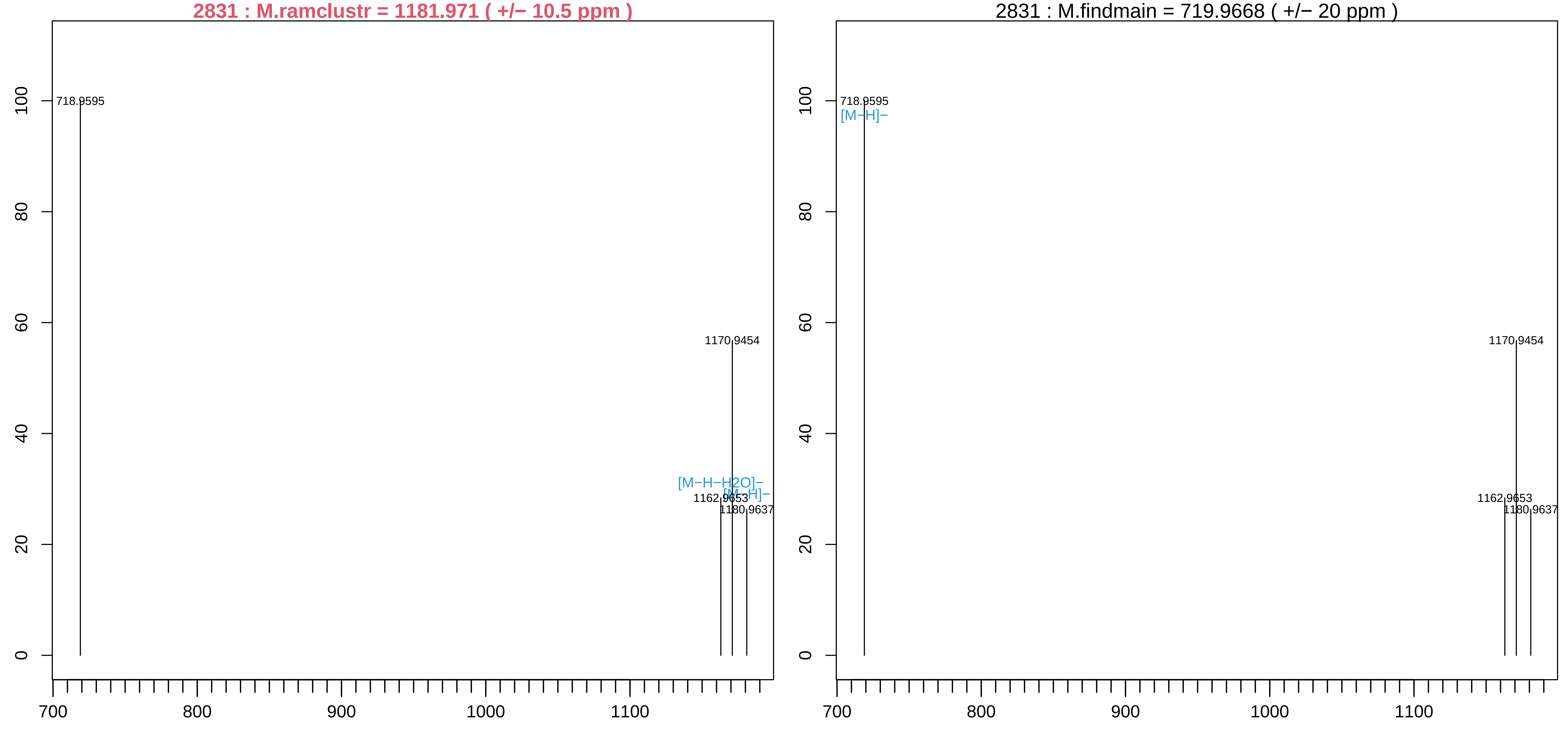
2831 : M.ramclustr = 1181.971 (+/− 10.5 ppm)
718.9595
1170.9454
[M−H−H2O]−
[M−H]−
1162.9653
1180.9637
2831 : M.findmain = 719.9668 (+/− 20 ppm)
718.9595
[M−H]−
1170.9454
1162.9653
1180.9637

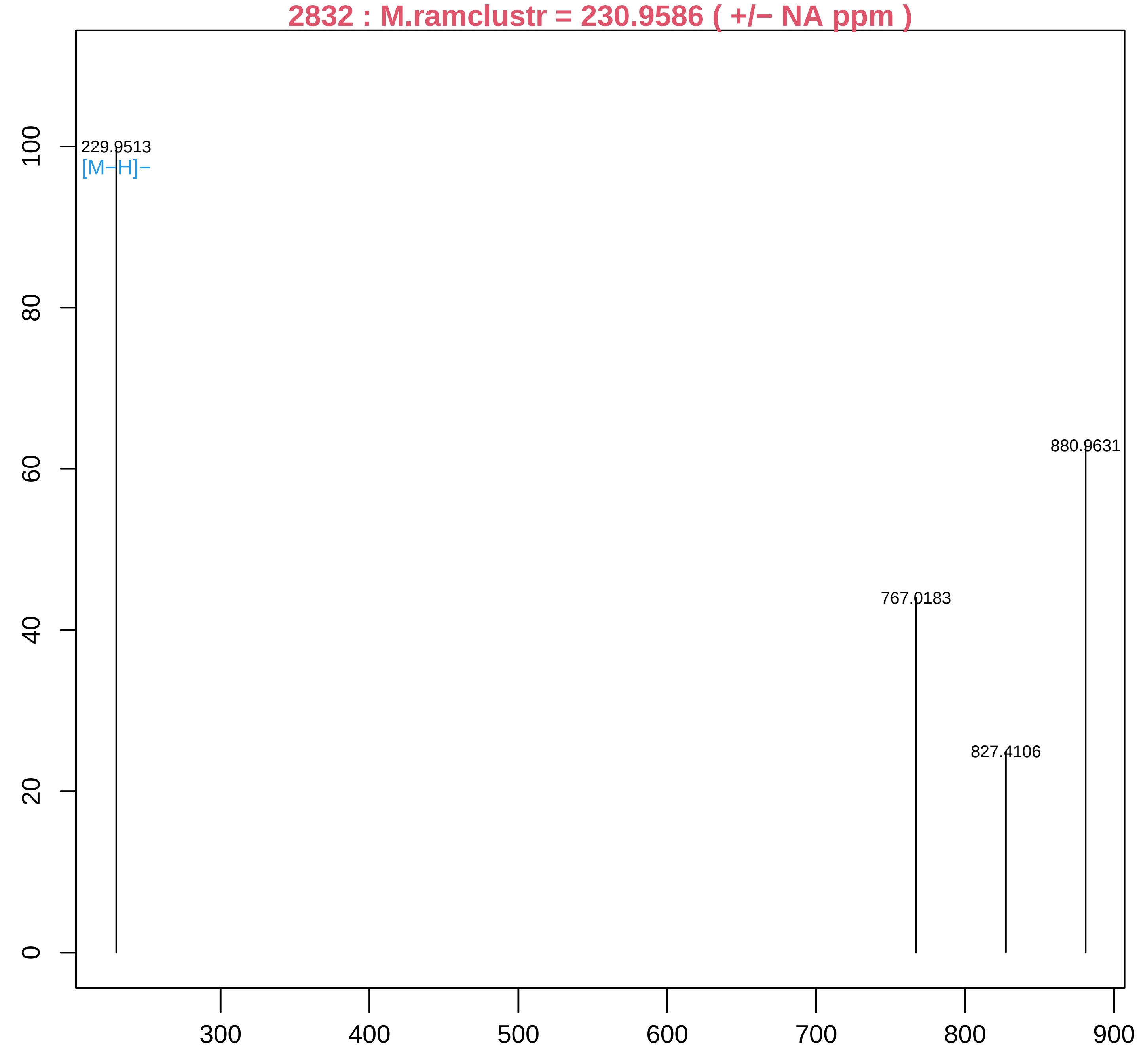
2832 : M.ramclustr = 230.9586 (+/− NA ppm)
229.9513
[M−H]−
767.0183
827.4106
880.9631
0
20
40
60
80
100
300
400
500
600
700
800
900

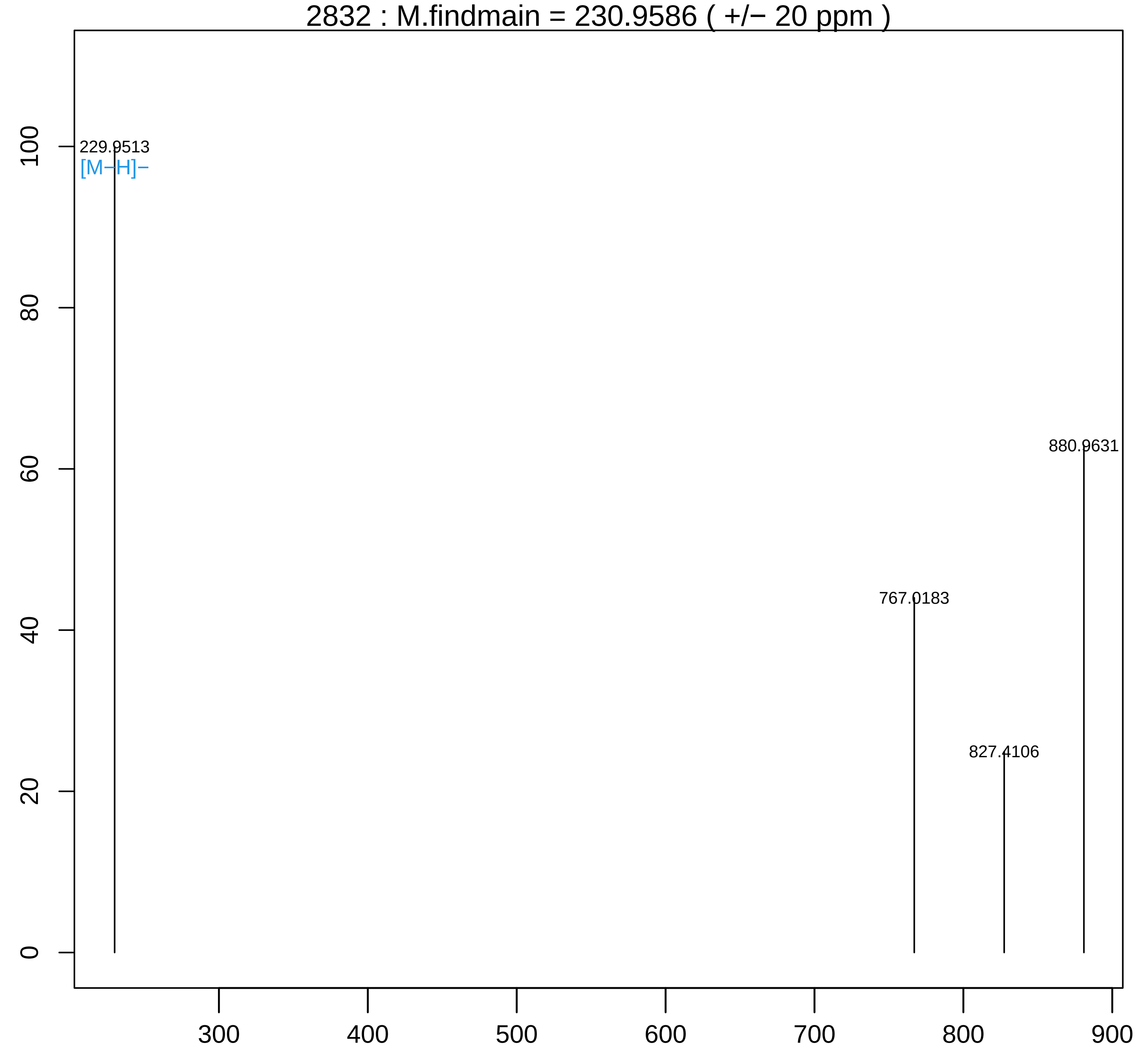
2832 : M.findmain = 230.9586 (+/− 20 ppm)
229.9513
[M−H]−
767.0183
827.4106
880.9631
0
20
40
60
80
100
300
400
500
600
700
800
900

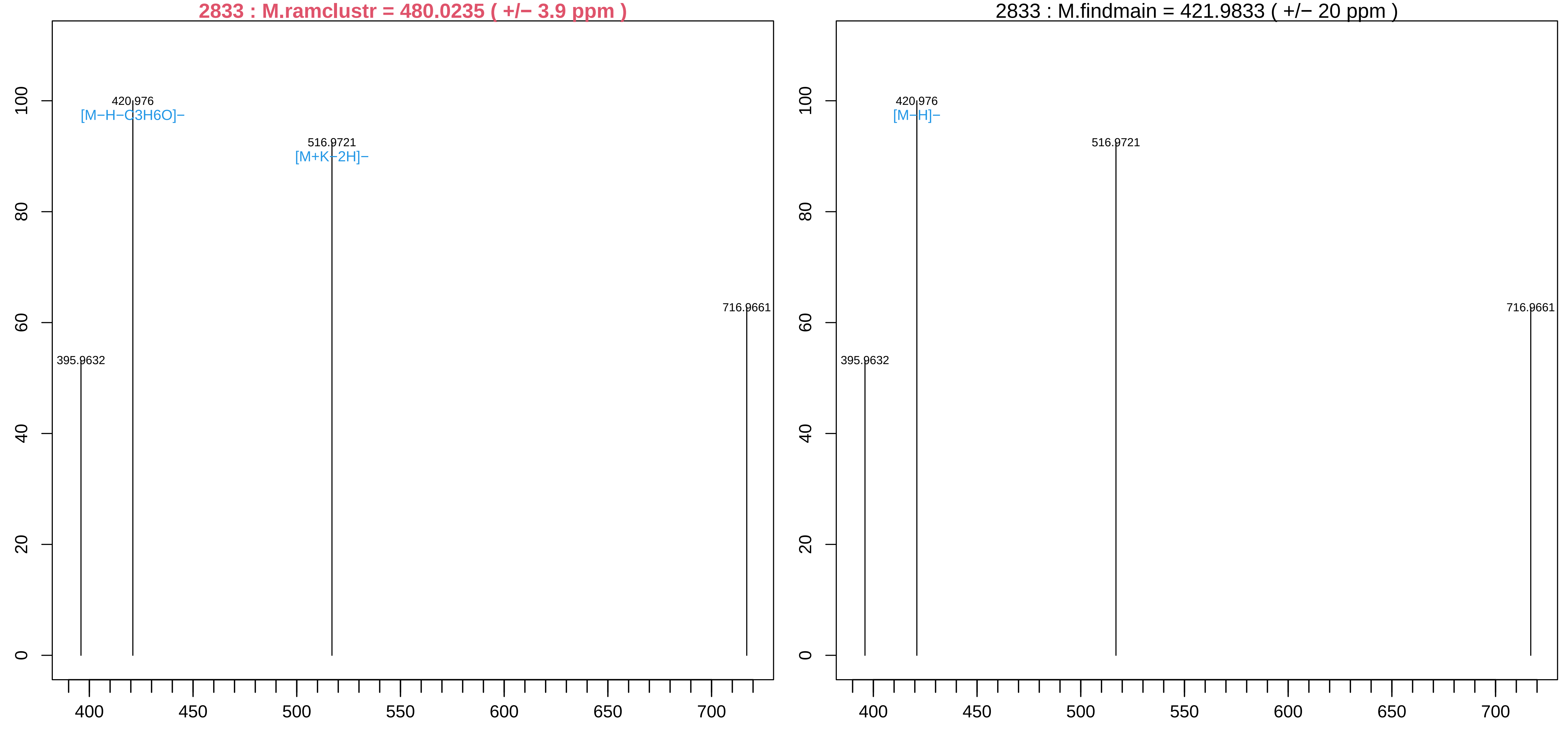

2833 : M.ramclustr = 480.0235 (+/− 3.9 ppm)
420.976
[M−H−C3H6O]−
516.9721
[M+K−2H]−
716.9661
395.9632
2833 : M.findmain = 421.9833 (+/− 20 ppm)
420.976
[M−H]−
516.9721
716.9661
395.9632

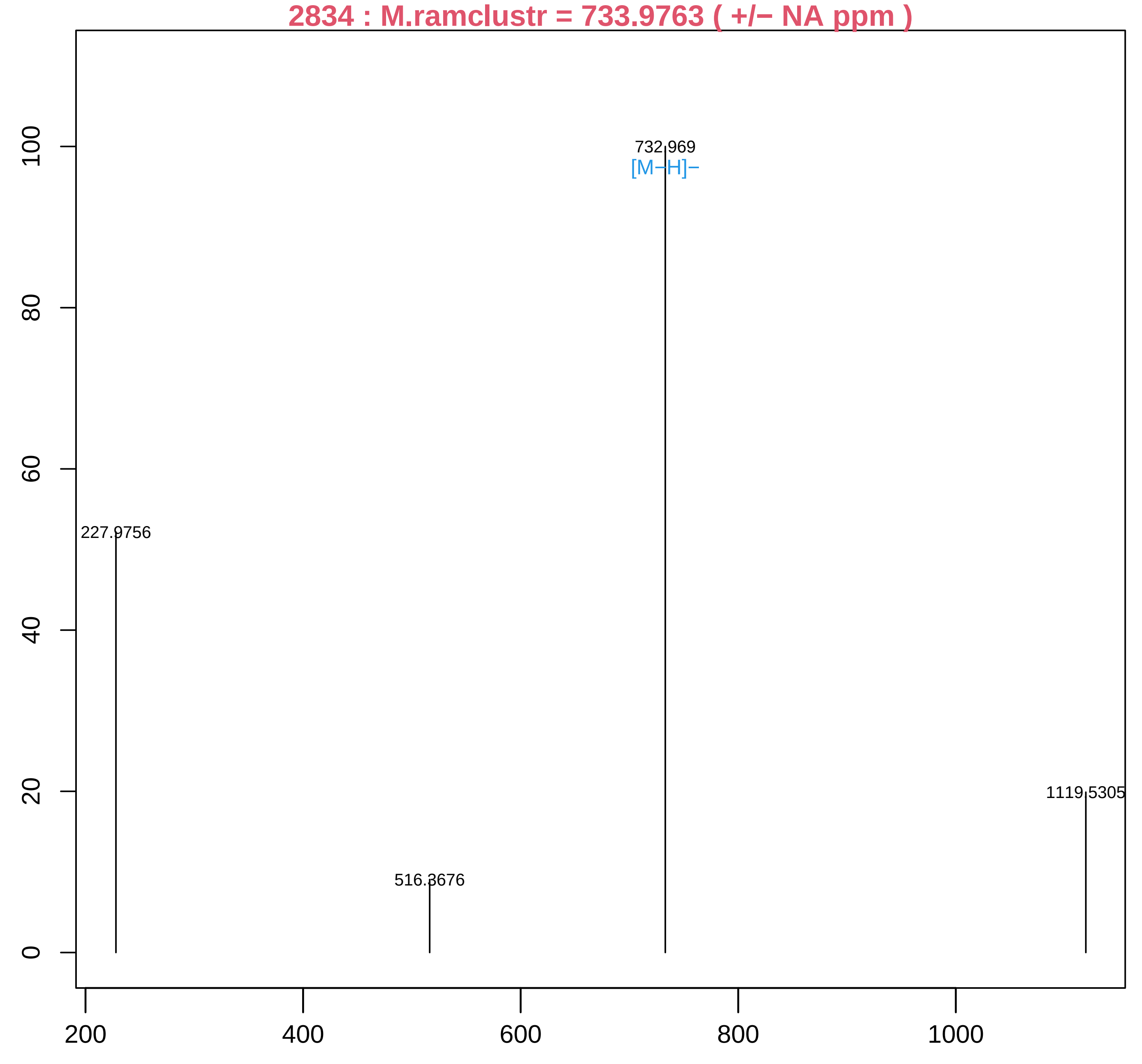
2834 : M.ramclustr = 733.9763 (+/− NA ppm)
732.969
[M−H]−
227.9756
516.3676
1119.5305
0
20
40
60
80
100
200
400
600
800
1000
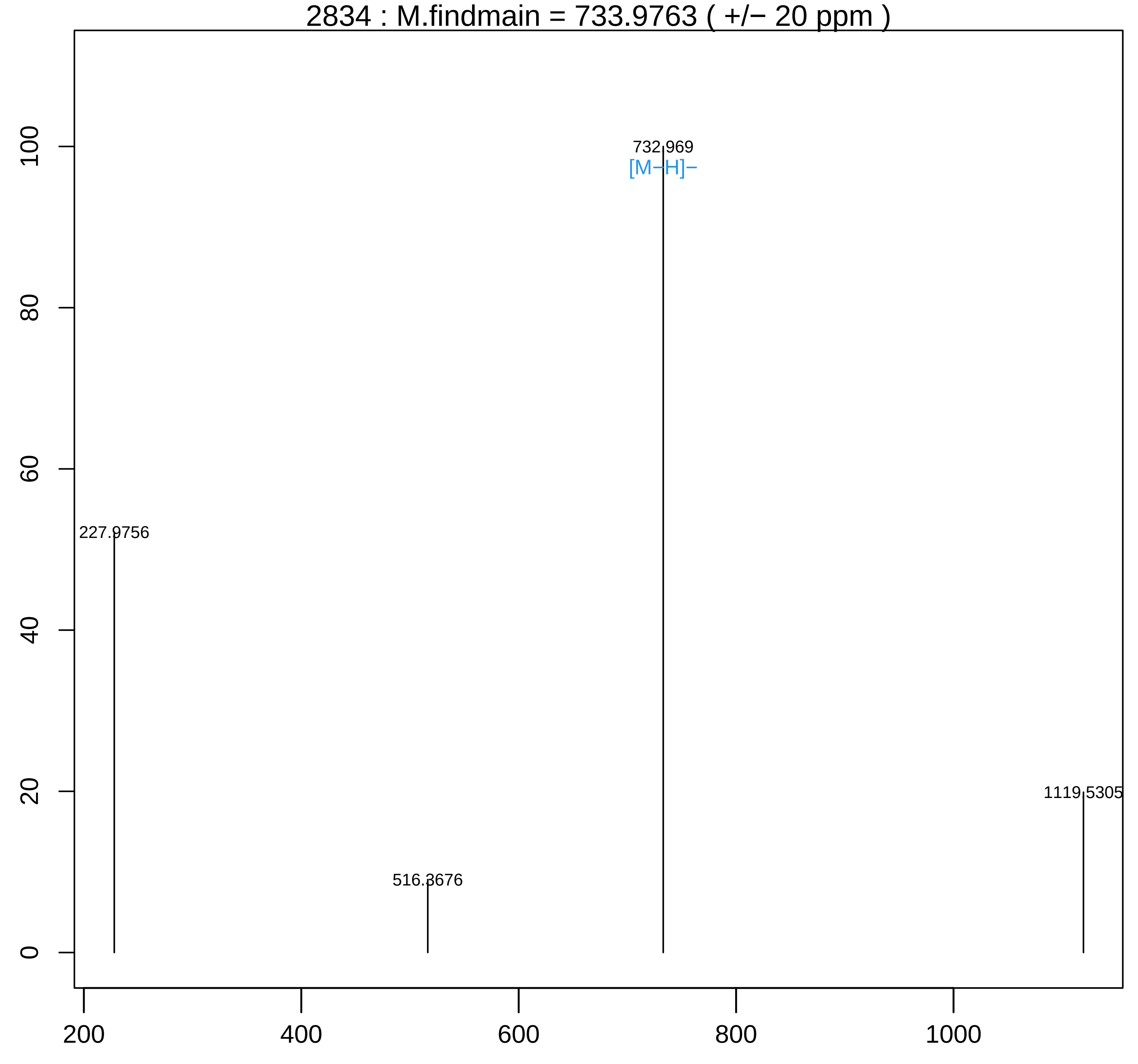
2834 : M.findmain = 733.9763 (+/− 20 ppm)
732.969
[M−H]−
227.9756
516.3676
1119.5305
0
20
40
60
80
100
200
400
600
800
1000

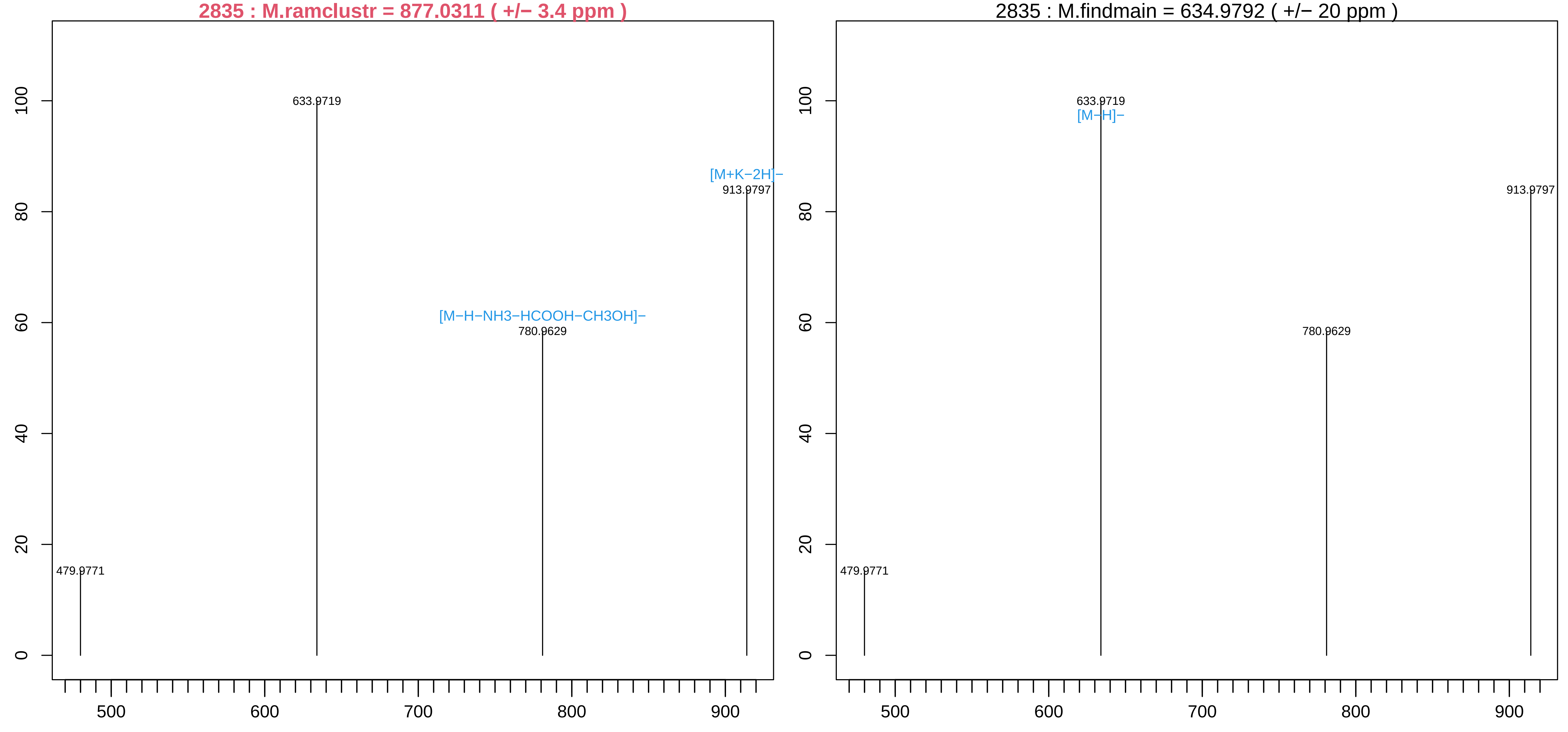
2835 : M.ramclustr = 877.0311 (+/− 3.4 ppm)
633.9719
[M+K−2H]−
913.9797
[M−H−NH3−HCOOH−CH3OH]−
780.9629
479.9771
2835 : M.findmain = 634.9792 (+/− 20 ppm)
633.9719
[M−H]−
913.9797
780.9629
479.9771
0
20
40
60
80
100
500
600
700
800
900

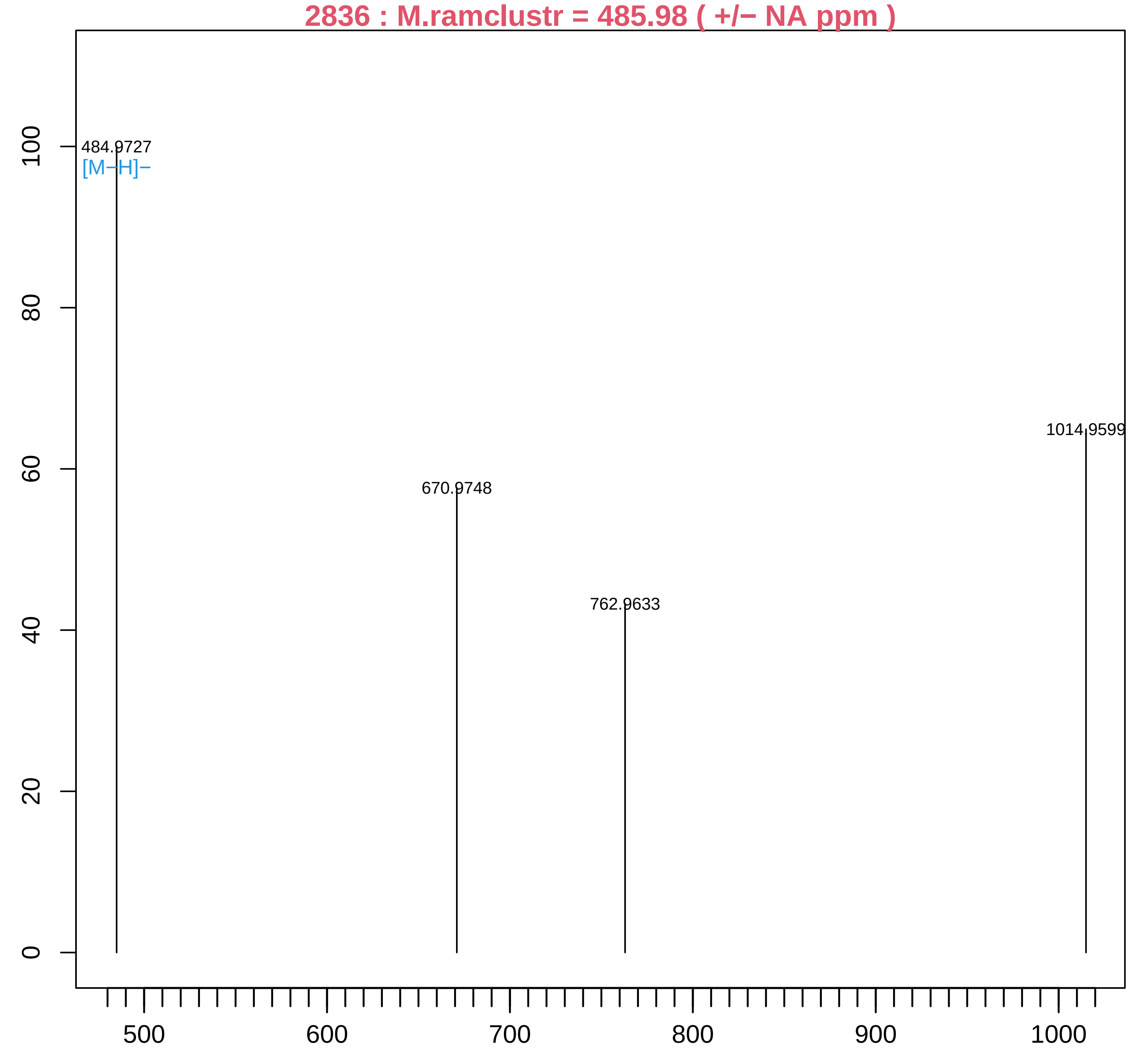
2836 : M.ramclustr = 485.98 (+/− NA ppm)
484.9727
[M−H]−
670.9748
762.9633
1014.9599
0
20
40
60
80
100
500
600
700
800
900
1000

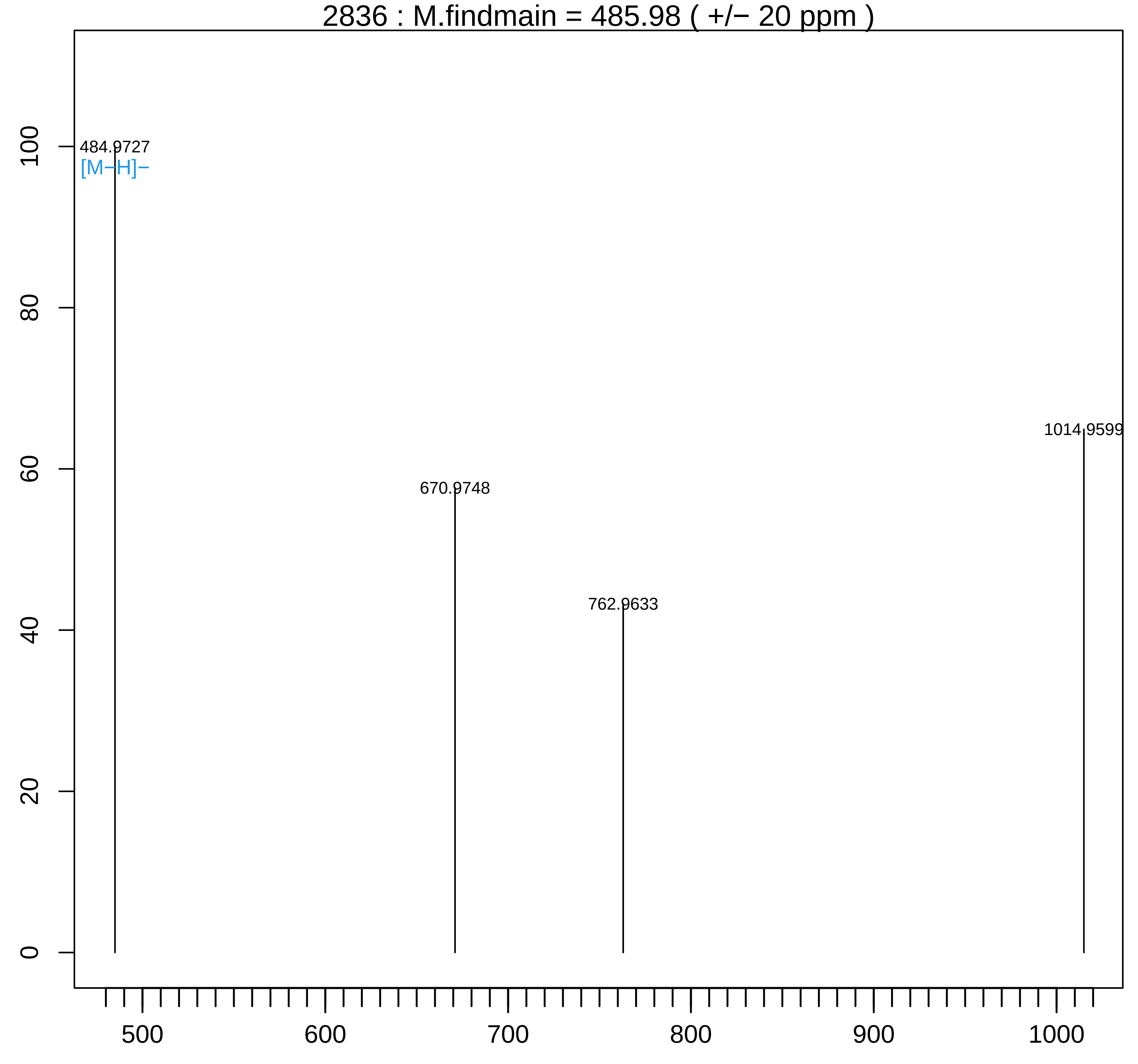
2836 : M.findmain = 485.98 (+/− 20 ppm)
484.9727
[M−H]−
670.9748
762.9633
1014.9599
0
20
40
60
80
100
500
600
700
800
900
1000

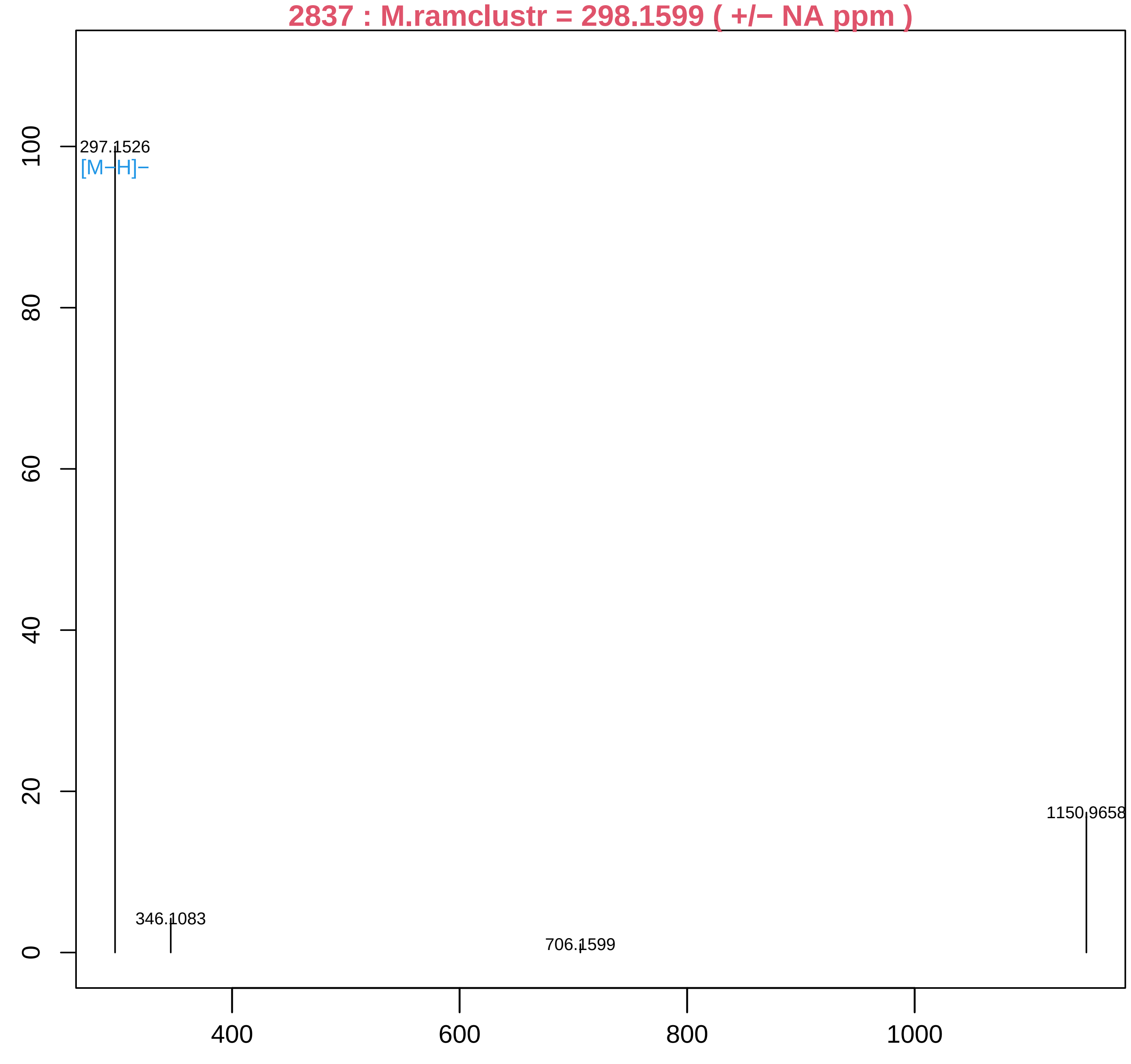
2837 : M.ramclustr = 298.1599 (+/− NA ppm)
297.1526
[M−H]−
346.1083
706.1599
1150.9658
0
20
40
60
80
100
400
600
800
1000

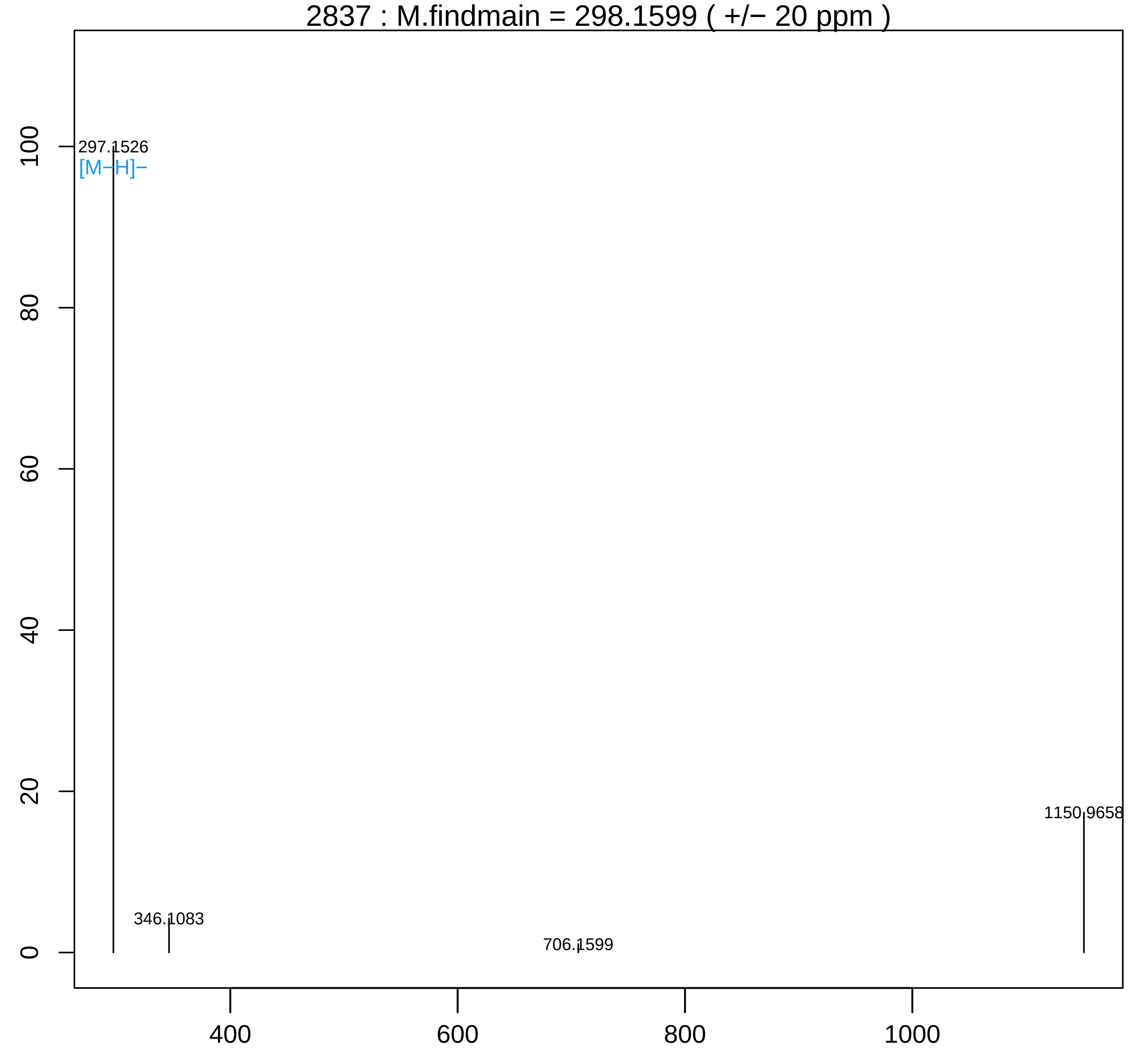
2837 : M.findmain = 298.1599 (+/− 20 ppm)
297.1526
[M−H]−
346.1083
706.1599
1150.9658
0
20
40
60
80
100
400
600
800
1000

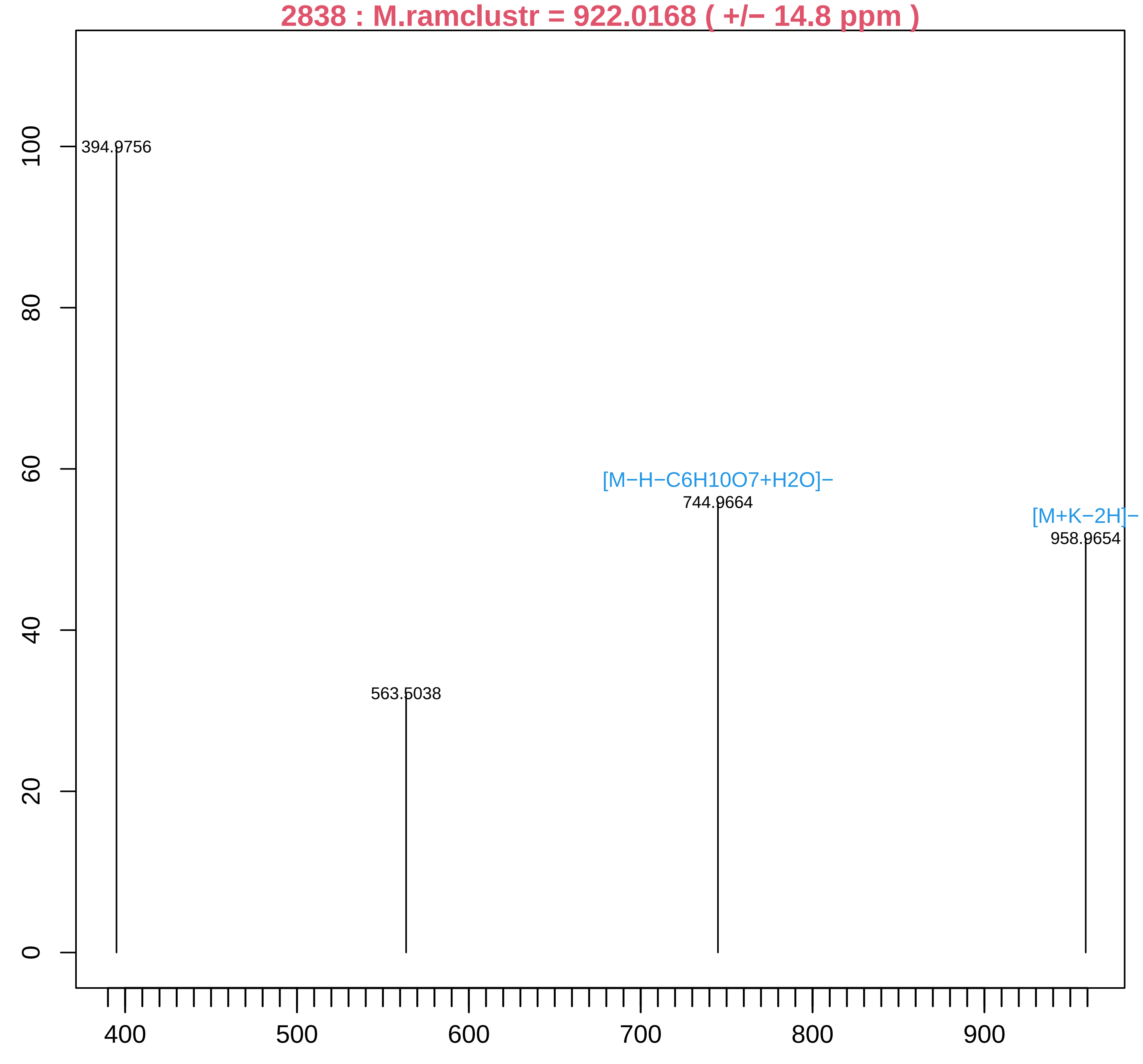

2838 : M.ramclustr = 922.0168 (+/− 14.8 ppm)
394.9756
563.5038
[M−H−C6H10O7+H2O]−
744.9664
[M+K−2H]−
958.9654
0
20
40
60
80
100
400
500
600
700
800
900

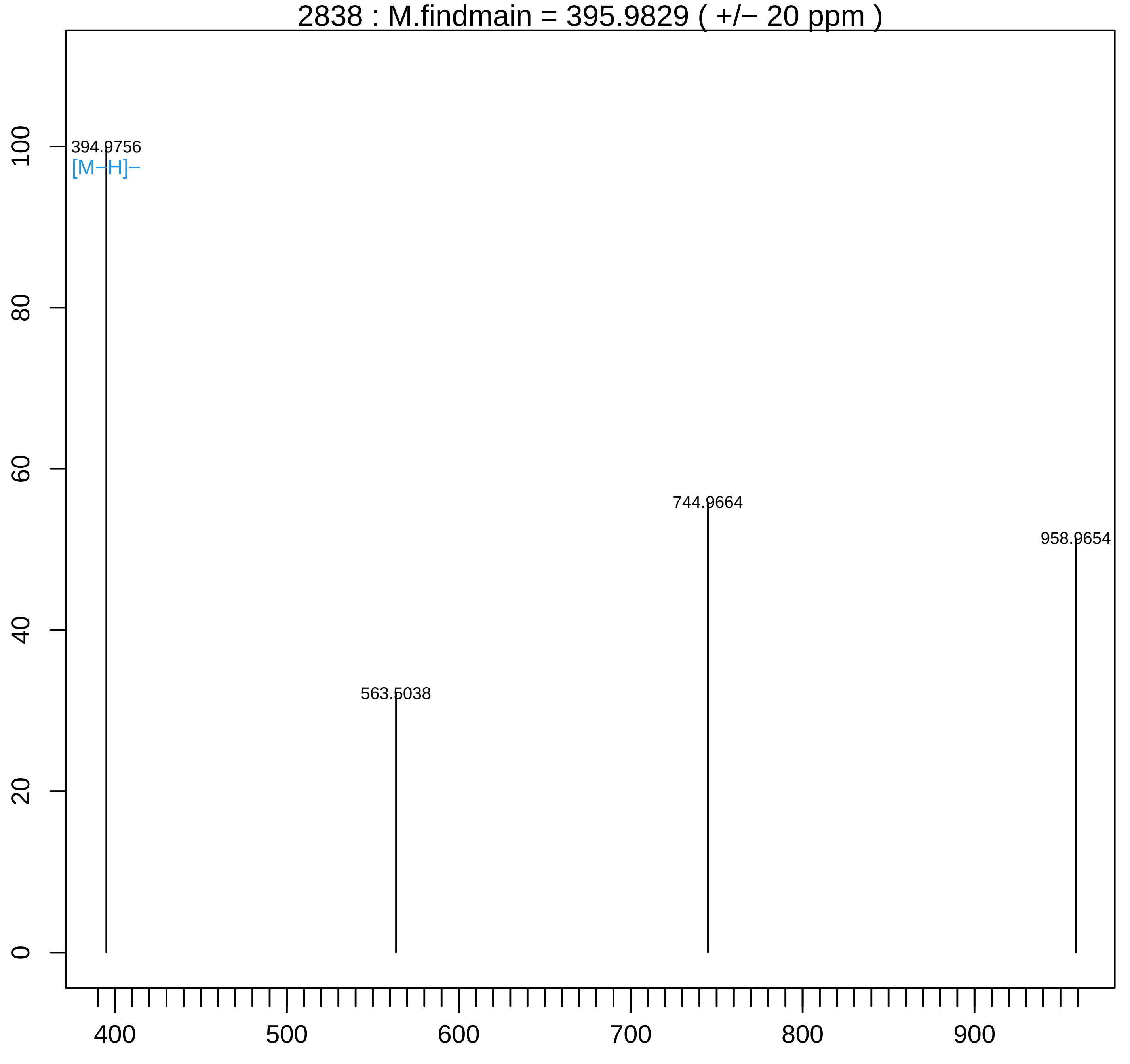

2838 : M.findmain = 395.9829 (+/− 20 ppm)
394.9756
[M−H]−
563.5038
744.9664
958.9654
0
20
40
60
80
100
400
500
600
700
800
900

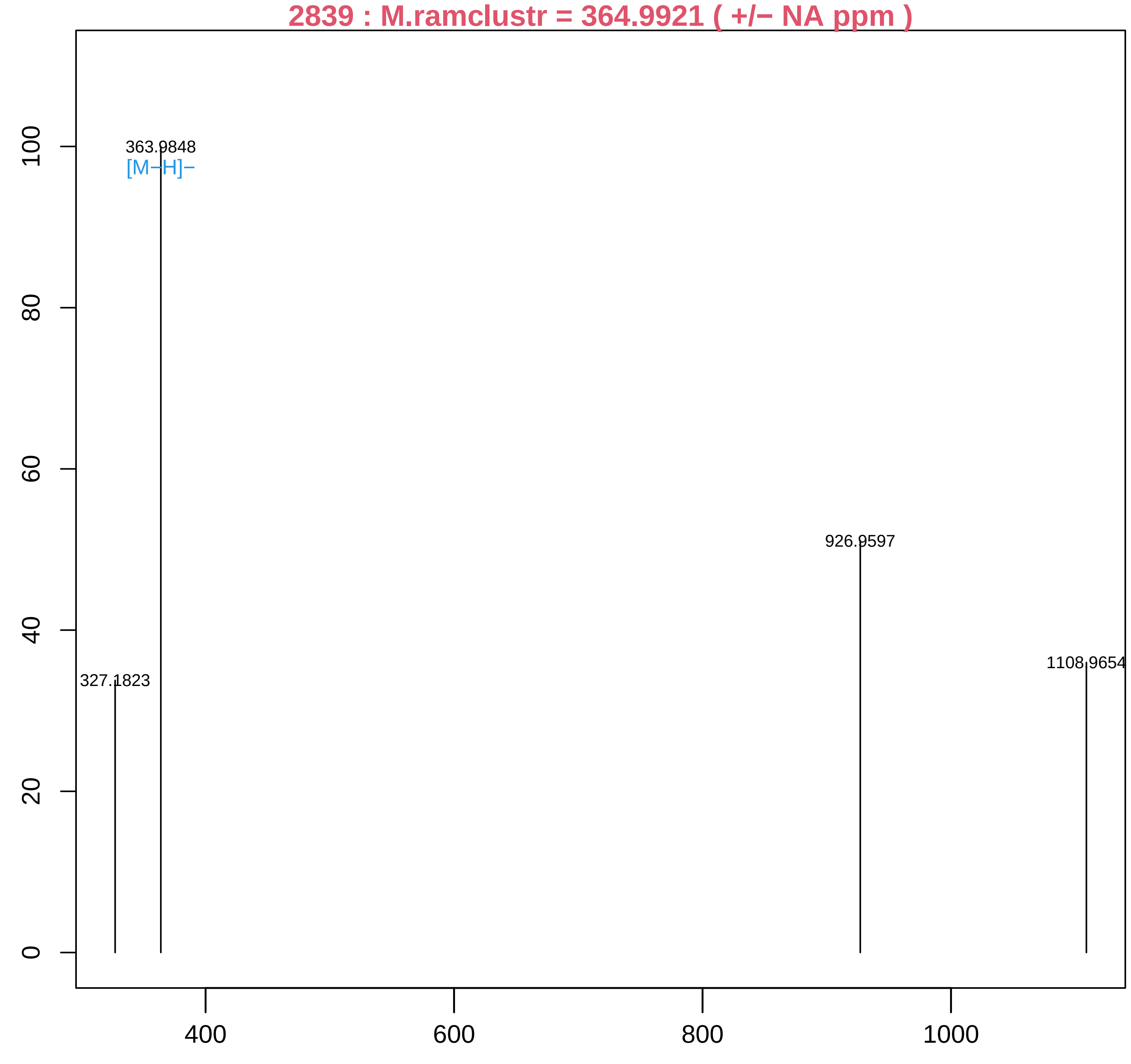

2839 : M.ramclustr = 364.9921 (+/− NA ppm)
363.9848
[M−H]−
926.9597
1108.9654
327.1823
0
20
40
60
80
100
400
600
800
1000

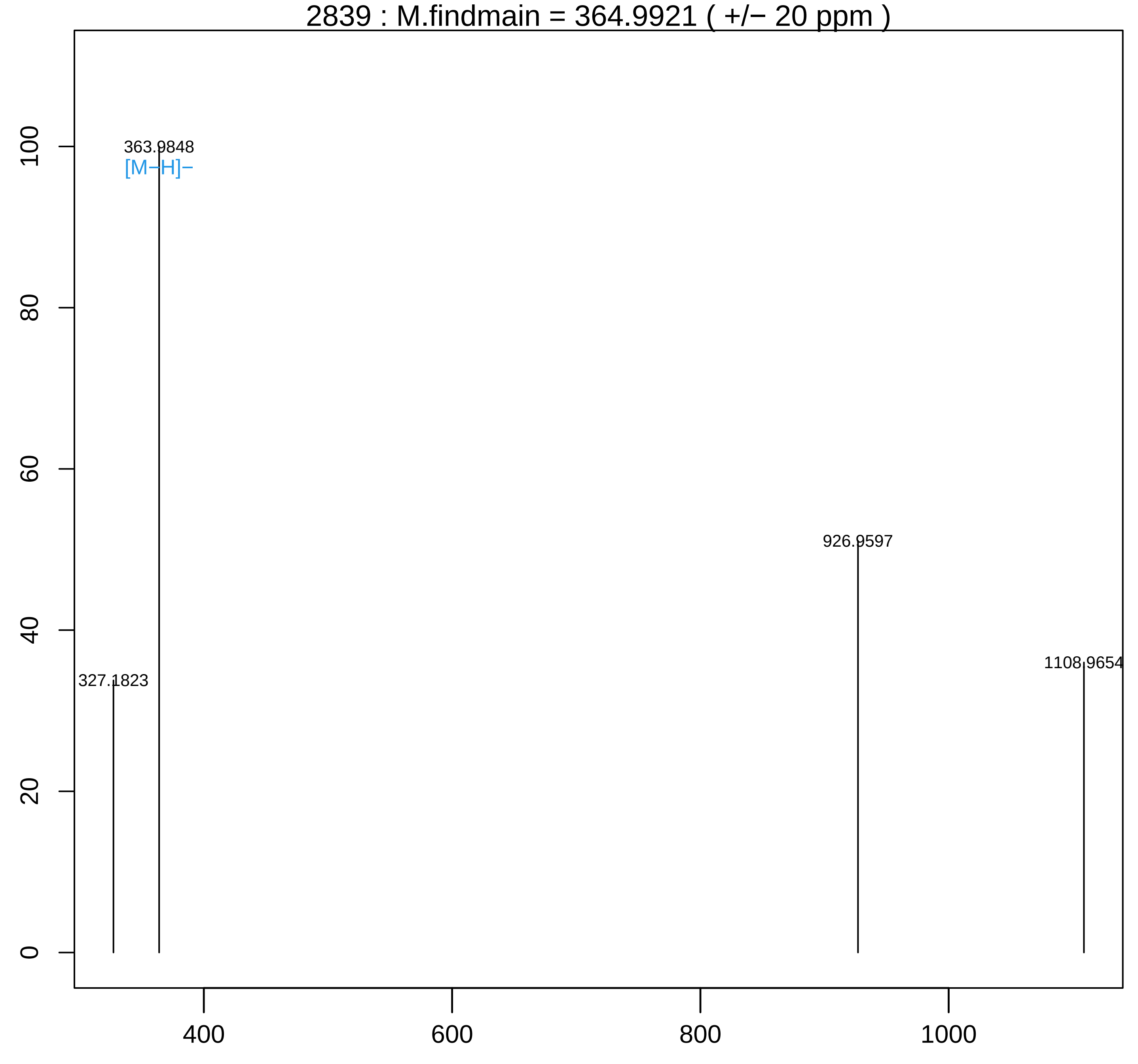

2839 : M.findmain = 364.9921 (+/− 20 ppm)
363.9848
[M−H]−
926.9597
1108.9654
327.1823
0
20
40
60
80
100
400
600
800
1000

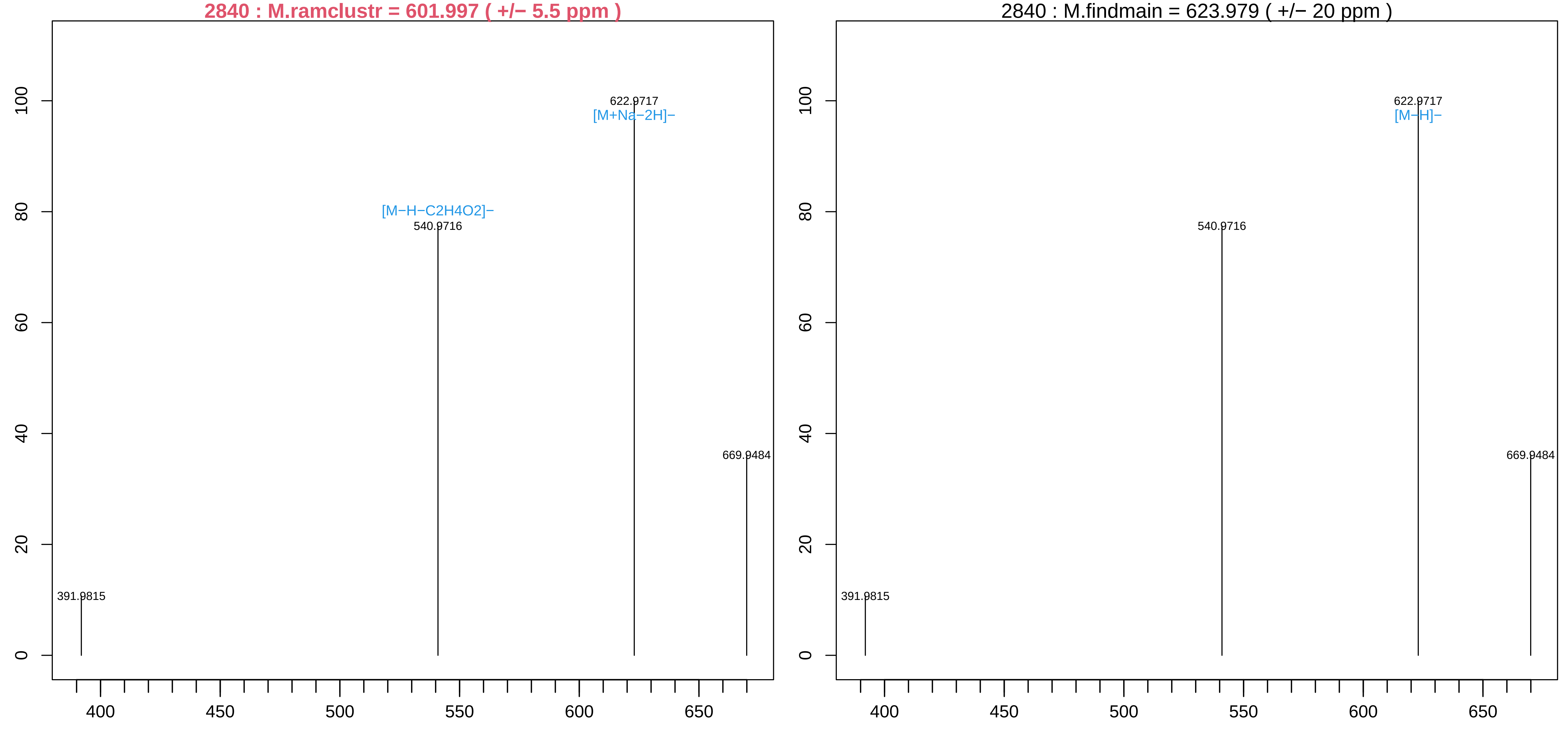
2840 : M.ramclustr = 601.997 (+/− 5.5 ppm)
622.9717
[M+Na−2H]−
[M−H−C2H4O2]−
540.9716
669.9484
391.9815
2840 : M.findmain = 623.979 (+/− 20 ppm)
622.9717
[M−H]−
540.9716
669.9484
391.9815
0
20
40
60
80
100
400
450
500
550
600
650

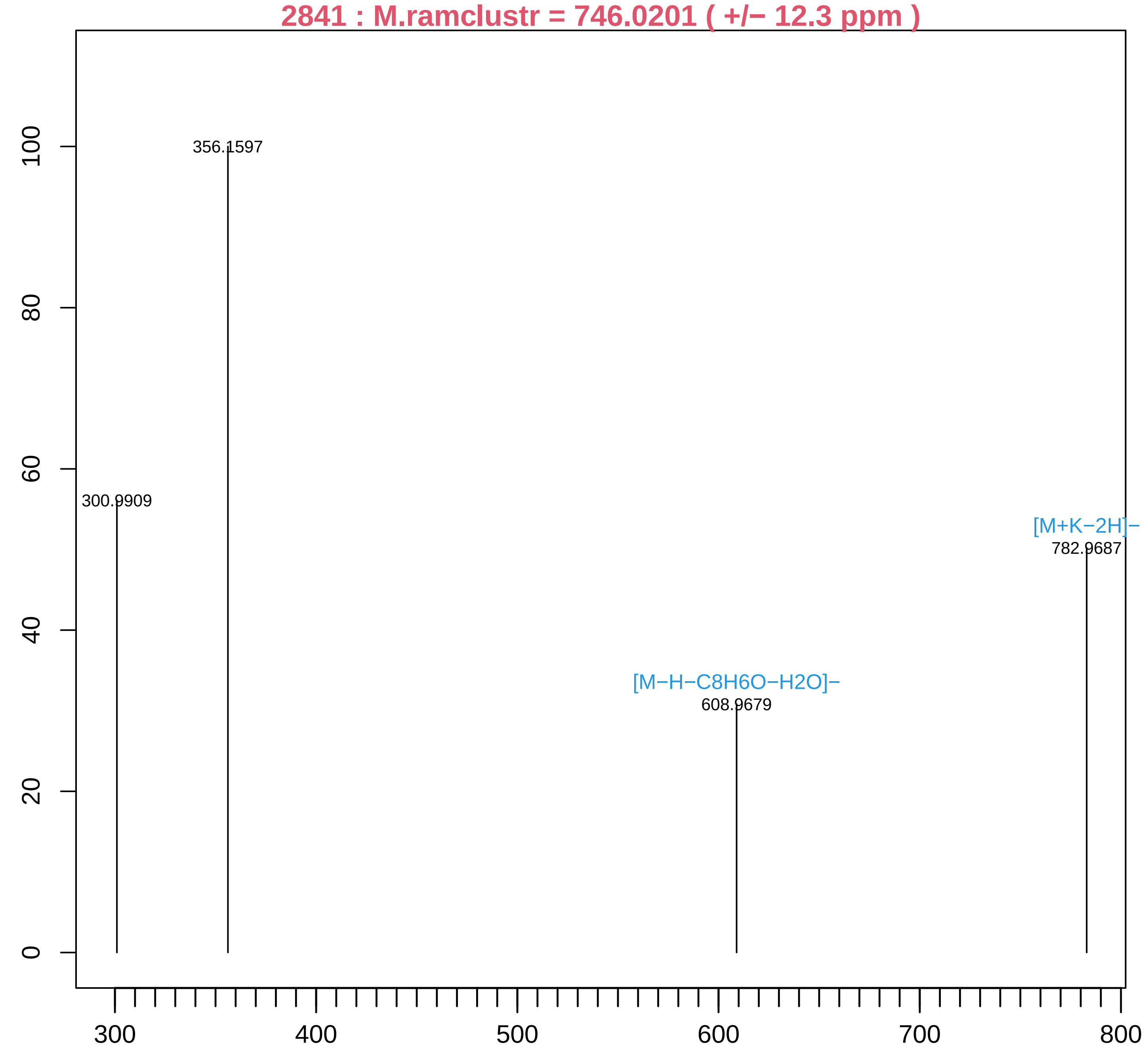
2841 : M.ramclustr = 746.0201 (+/− 12.3 ppm)
356.1597
300.9909
[M+K−2H]−
782.9687
[M−H−C8H6O−H2O]−
608.9679
0
20
40
60
80
100
300
400
500
600
700
800

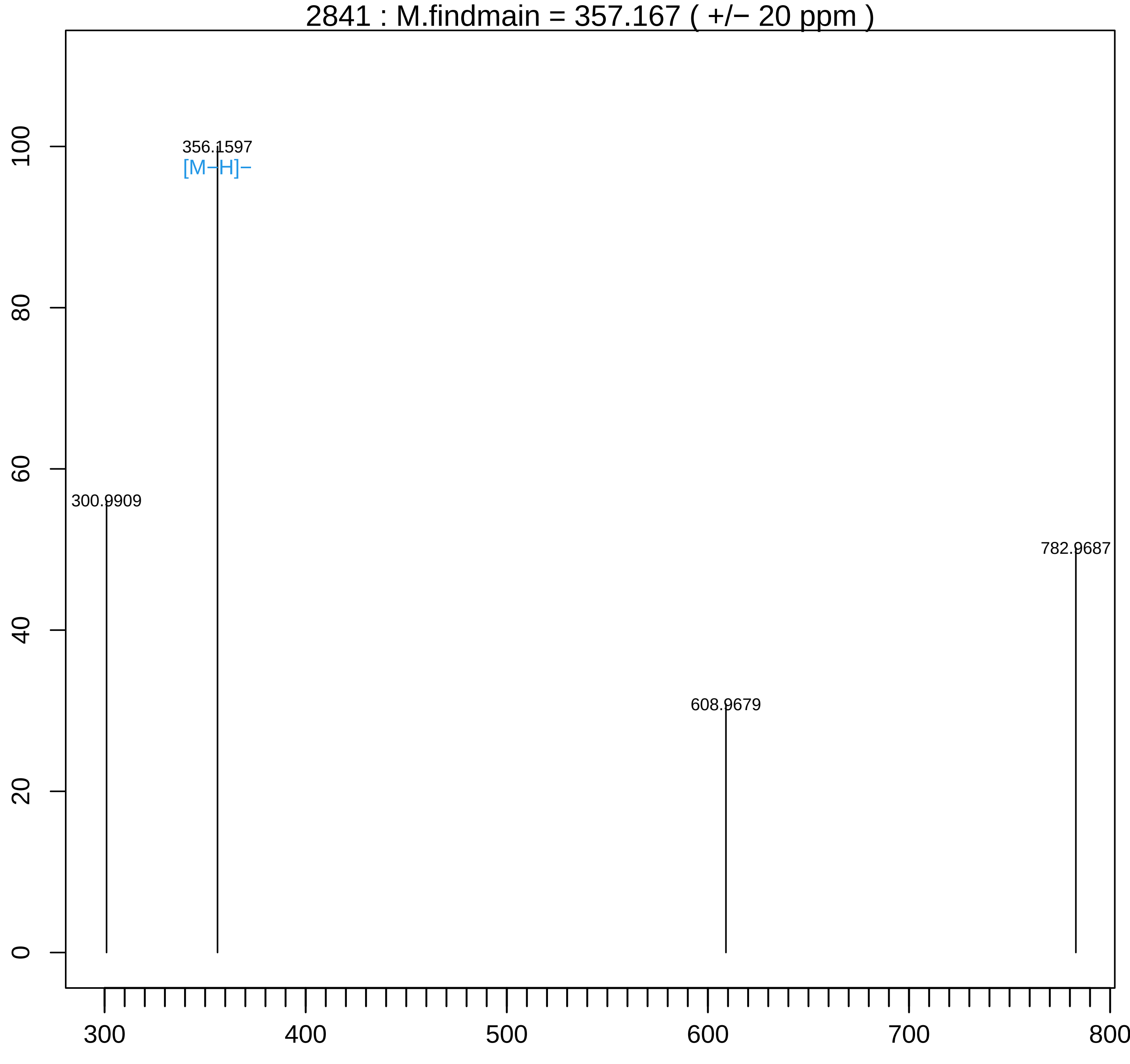
2841 : M.findmain = 357.167 (+/− 20 ppm)
356.1597
[M−H]−
300.9909
782.9687
608.9679
0
20
40
60
80
100
300
400
500
600
700
800

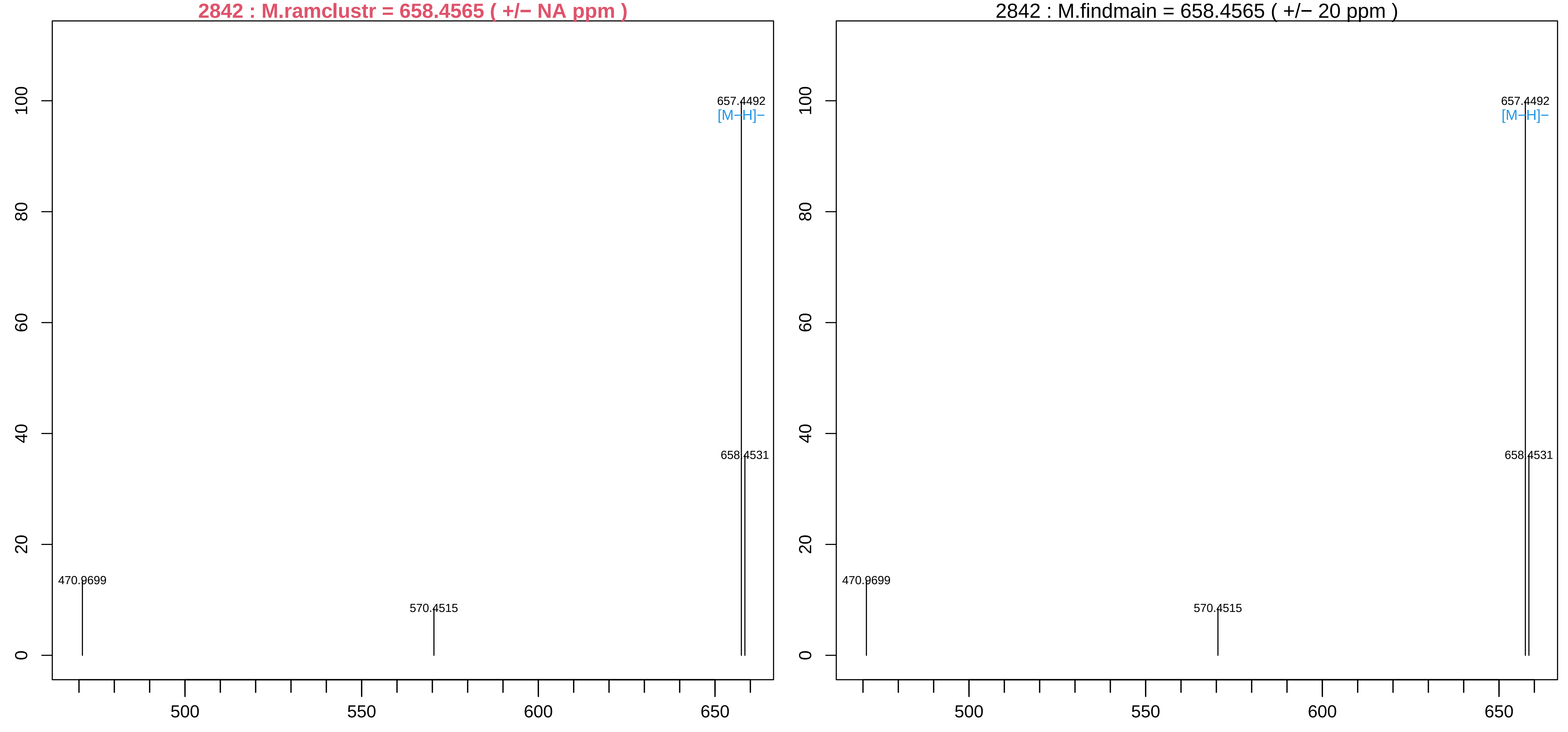

2842 : M.ramclustr = 658.4565 (+/− NA ppm)
657.4492
[M−H]−
658.4531
470.9699
570.4515
2842 : M.findmain = 658.4565 (+/− 20 ppm)
657.4492
[M−H]−
658.4531
470.9699
570.4515
0
20
40
60
80
100
500
550
600
650

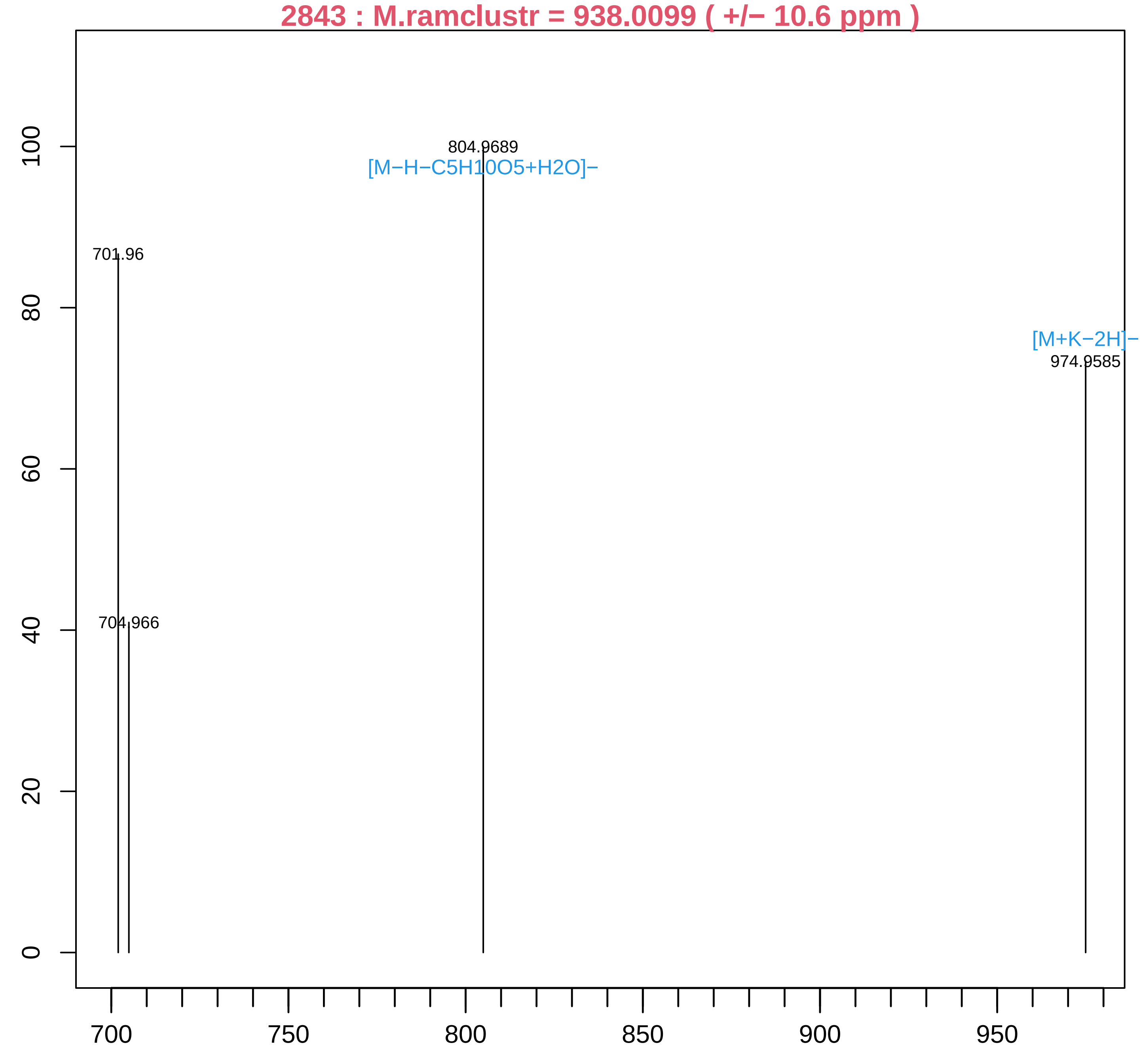
2843 : M.ramclustr = 938.0099 (+/− 10.6 ppm)
804.9689
[M−H−C5H10O5+H2O]−
701.96
704.966
[M+K−2H]−
974.9585
0
20
40
60
80
100
700
750
800
850
900
950

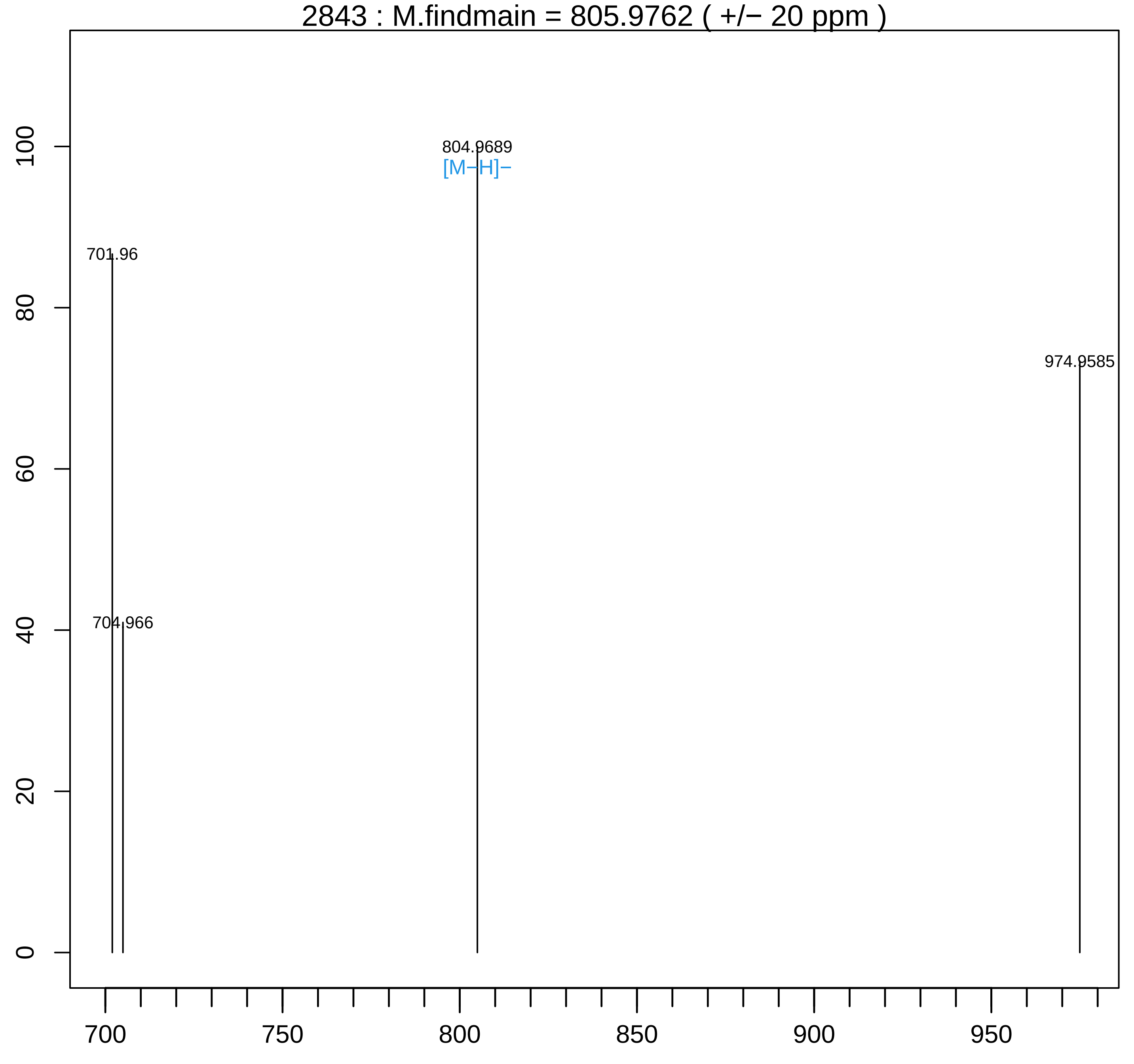
2843 : M.findmain = 805.9762 (+/− 20 ppm)
804.9689
[M−H]−
701.96
704.966
974.9585
0
20
40
60
80
100
700
750
800
850
900
950

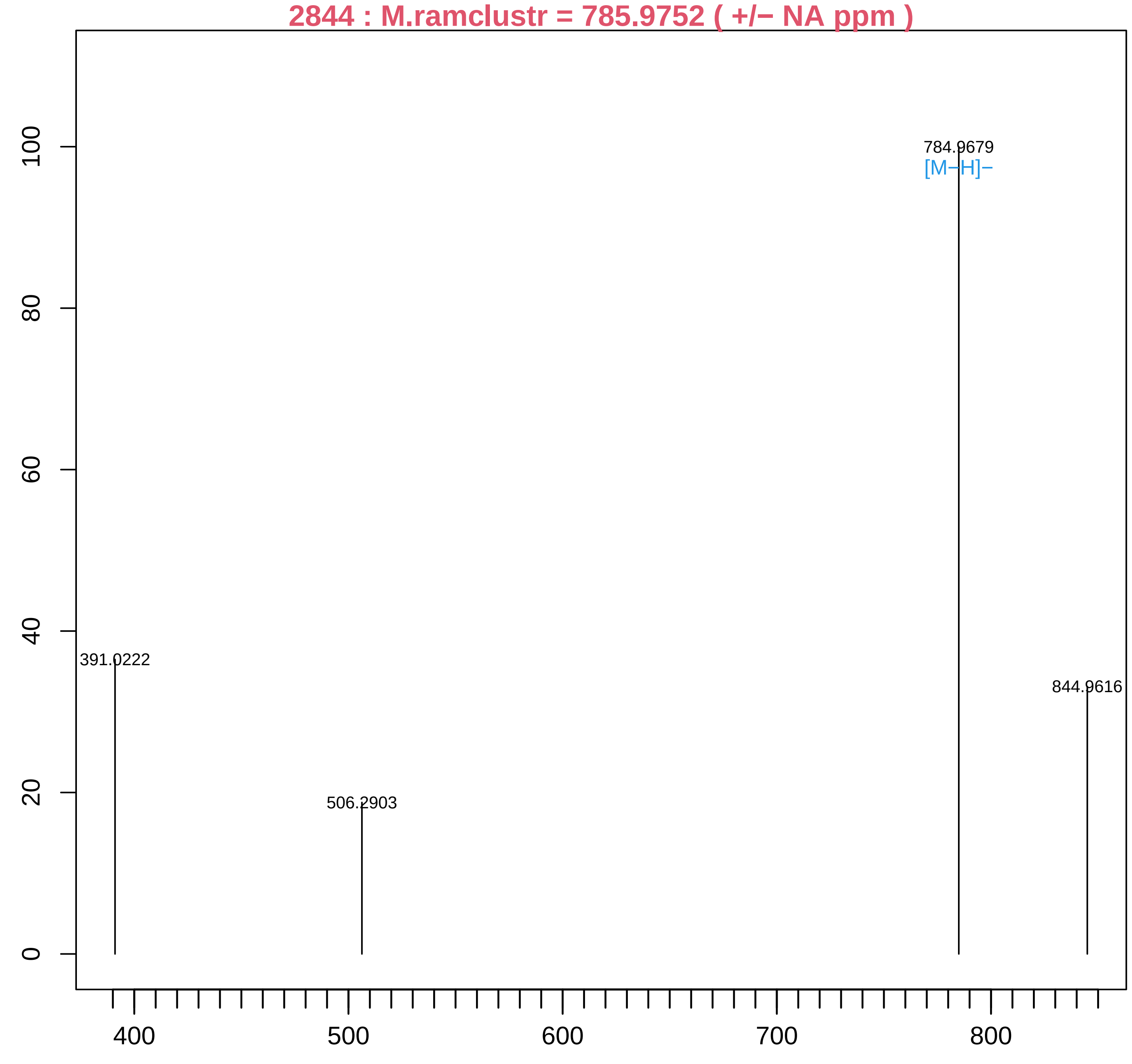
2844 : M.ramclustr = 785.9752 (+/− NA ppm)
784.9679
[M−H]−
391.0222
506.2903
844.9616
0
20
40
60
80
100
400
500
600
700
800
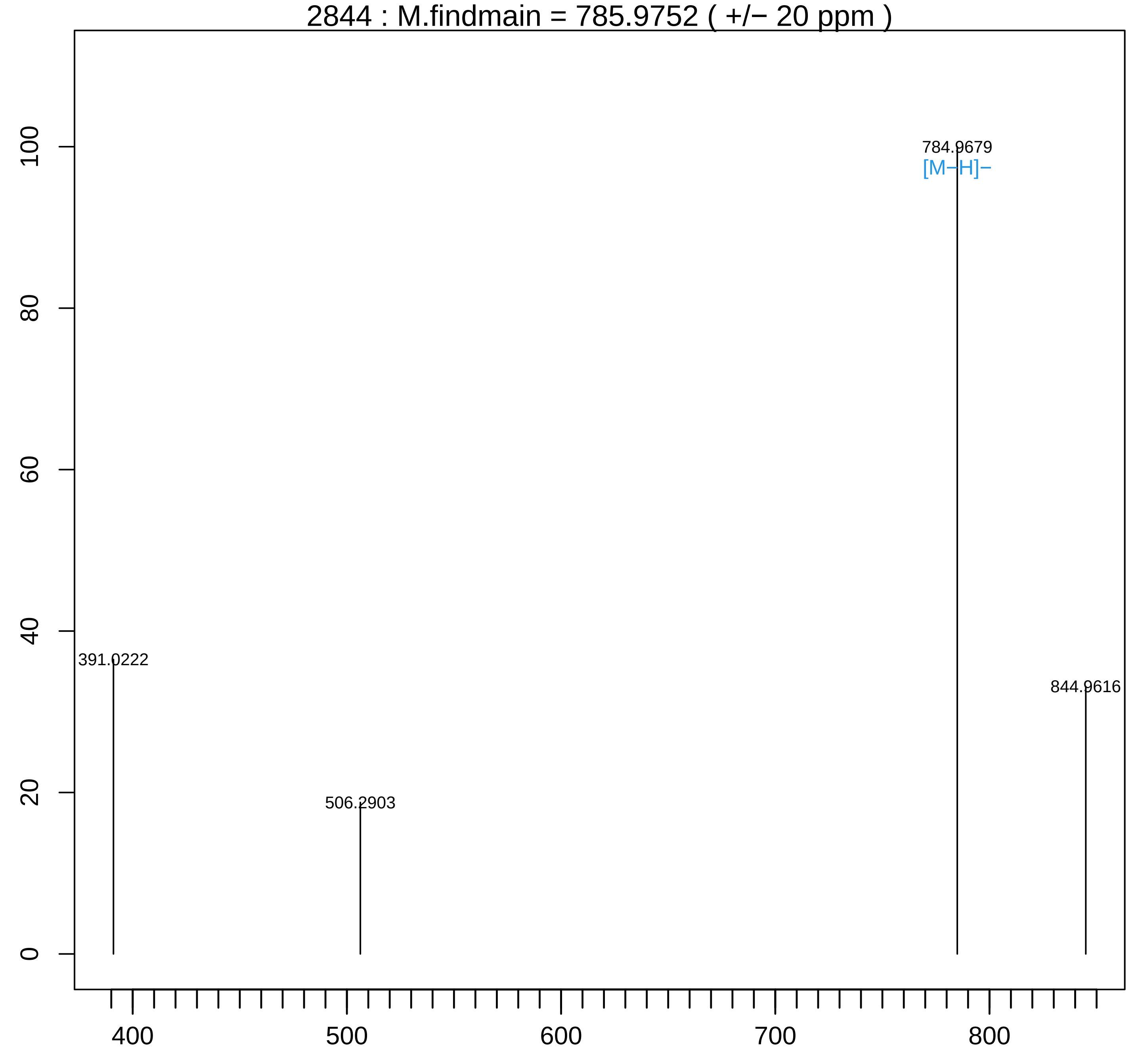
2844 : M.findmain = 785.9752 (+/− 20 ppm)
784.9679
[M−H]−
391.0222
506.2903
844.9616
0
20
40
60
80
100
400
500
600
700
800

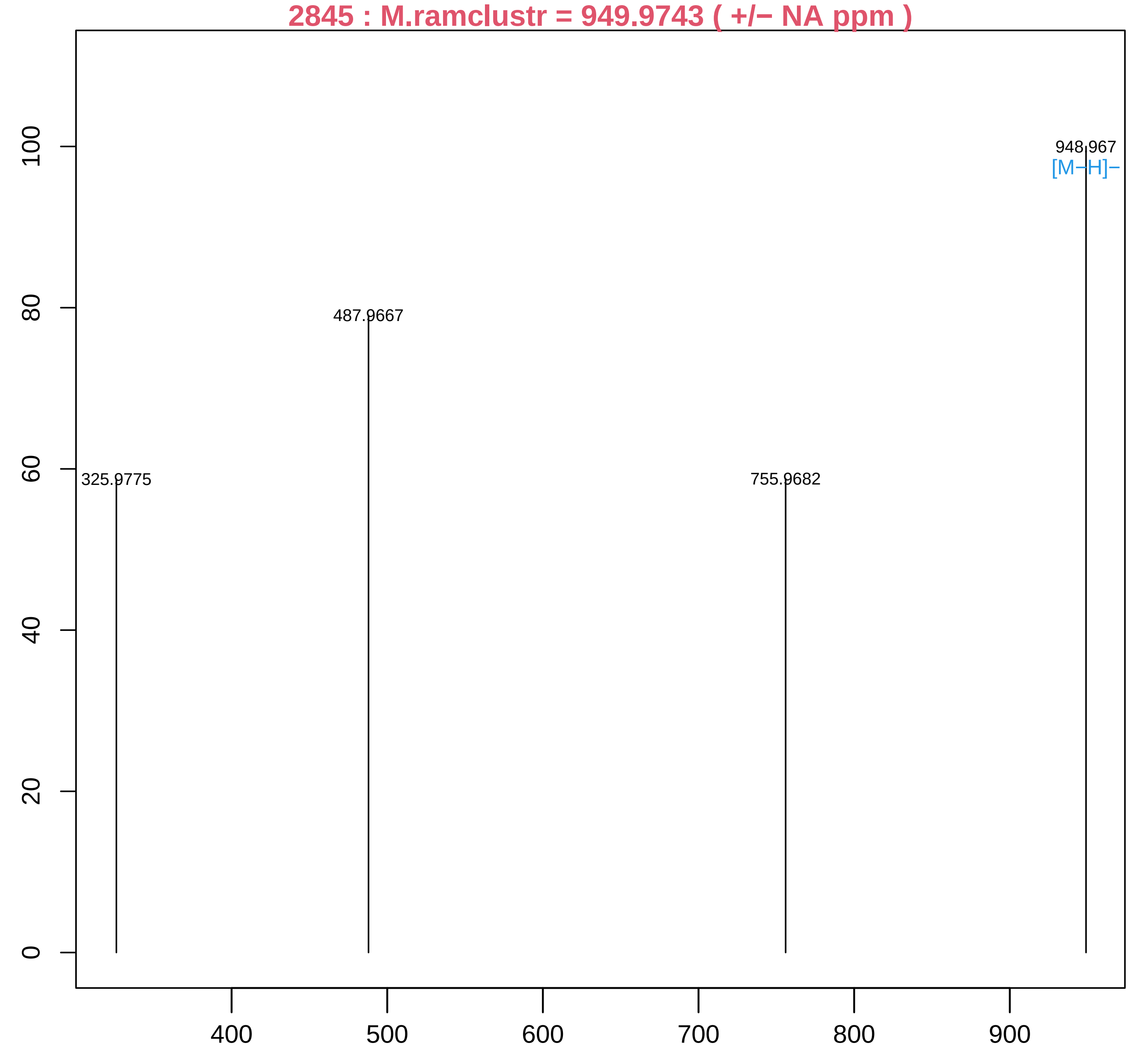
2845 : M.ramclustr = 949.9743 (+/− NA ppm)
948.967
[M−H]−
487.9667
325.9775
755.9682
0
20
40
60
80
100
400
500
600
700
800
900

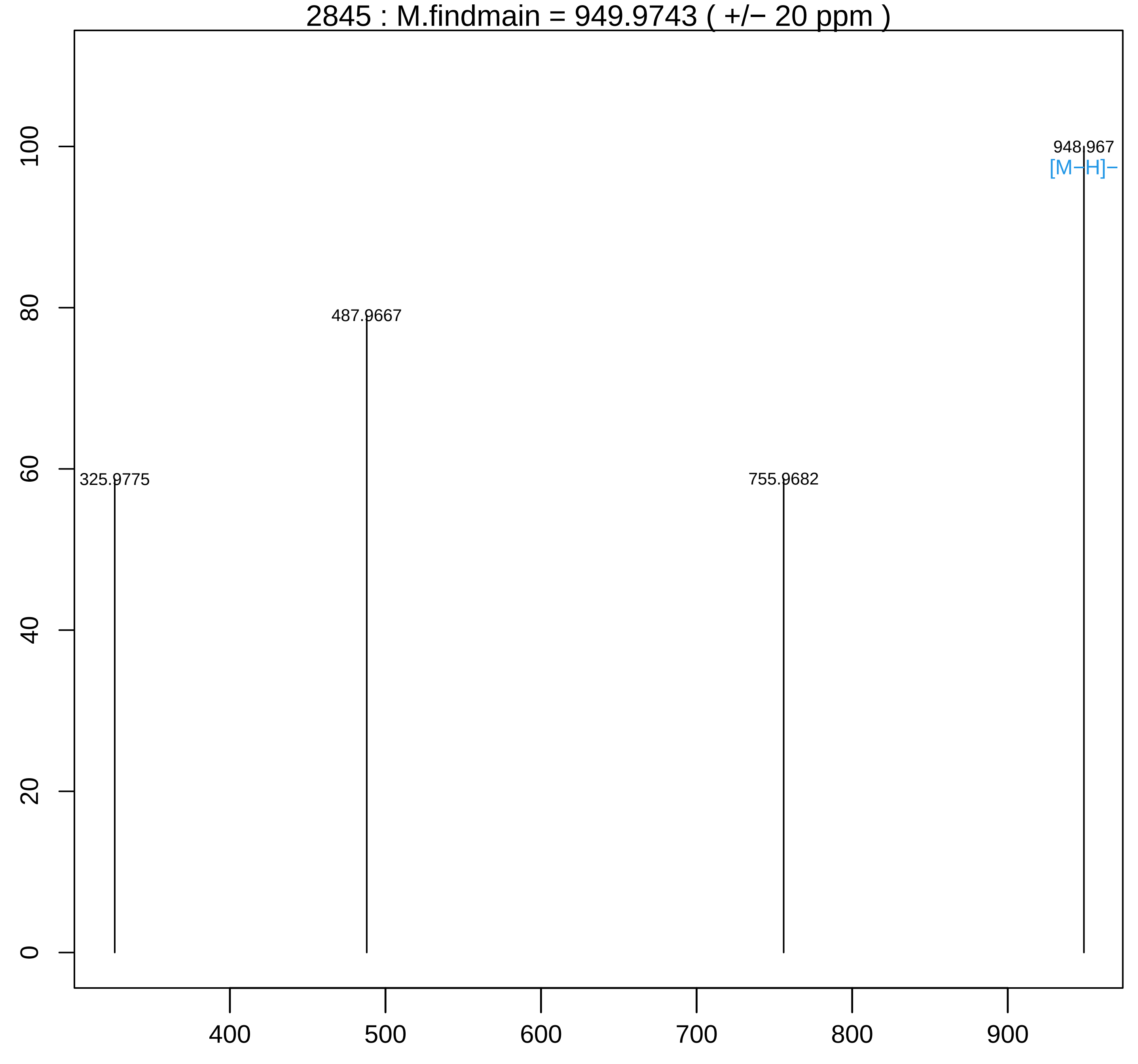
2845 : M.findmain = 949.9743 (+/− 20 ppm)
948.967
[M−H]−
487.9667
325.9775
755.9682
0
20
40
60
80
100
400
500
600
700
800
900

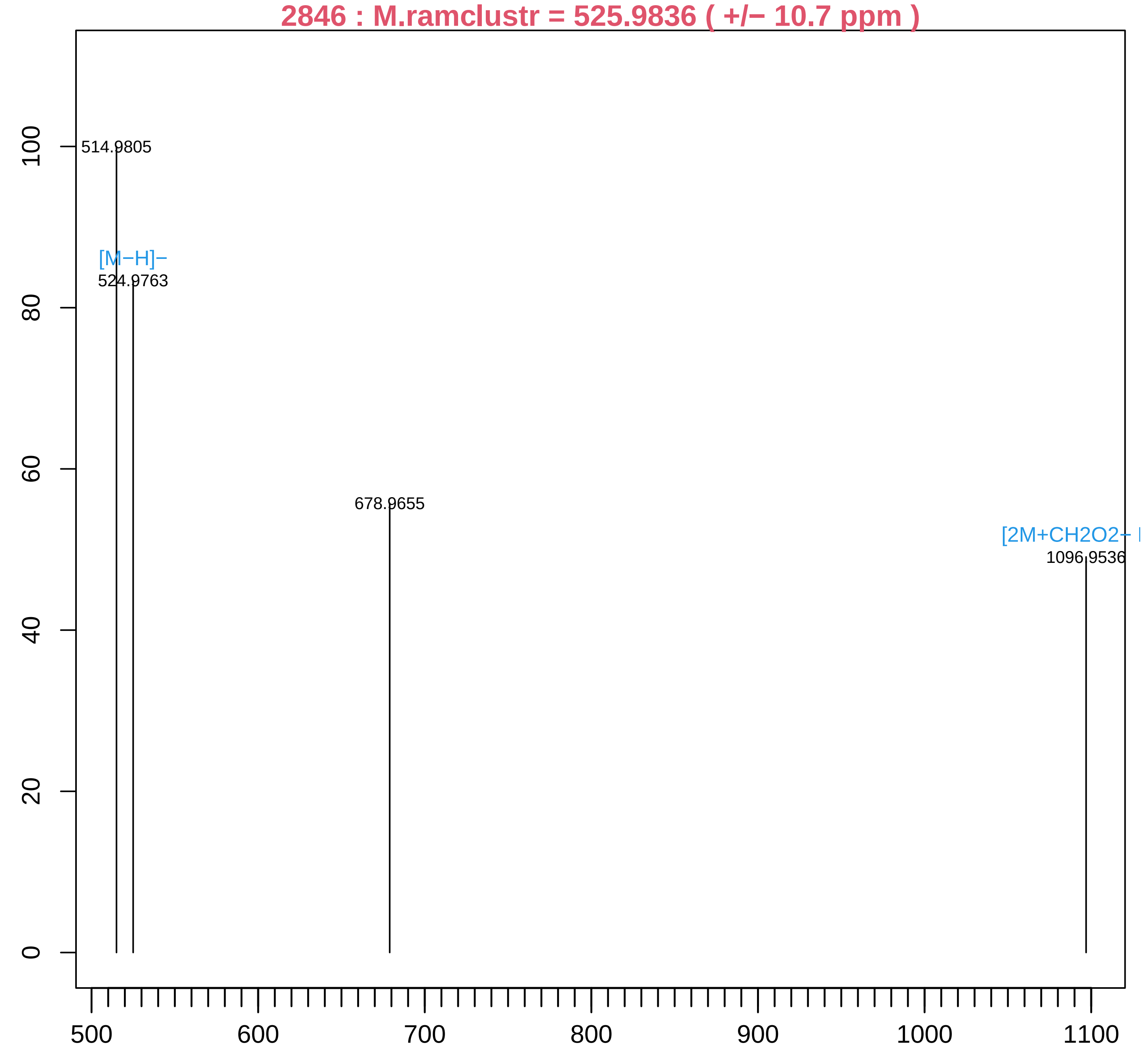

2846 : M.ramclustr = 525.9836 (+/− 10.7 ppm)
514.9805
[M−H]−
524.9763
678.9655
[2M+CH2O2−
1096.9536
0
20
40
60
80
100
500
600
700
800
900
1000
1100

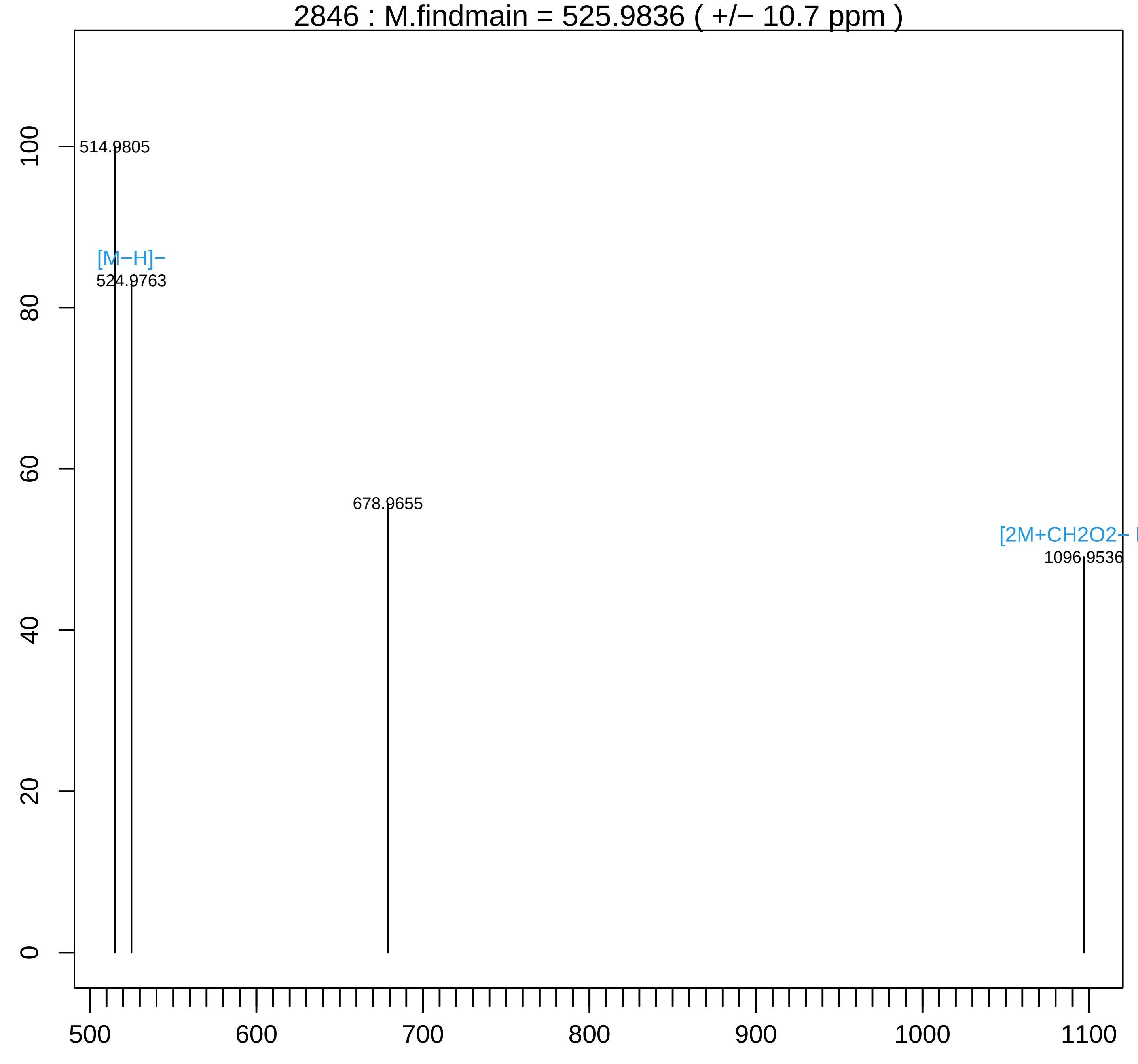

2846 : M.findmain = 525.9836 (+/− 10.7 ppm)
514.9805
[M−H]−
524.9763
678.9655
[2M+CH2O2−
1096.9536
0
20
40
60
80
100
500
600
700
800
900
1000
1100

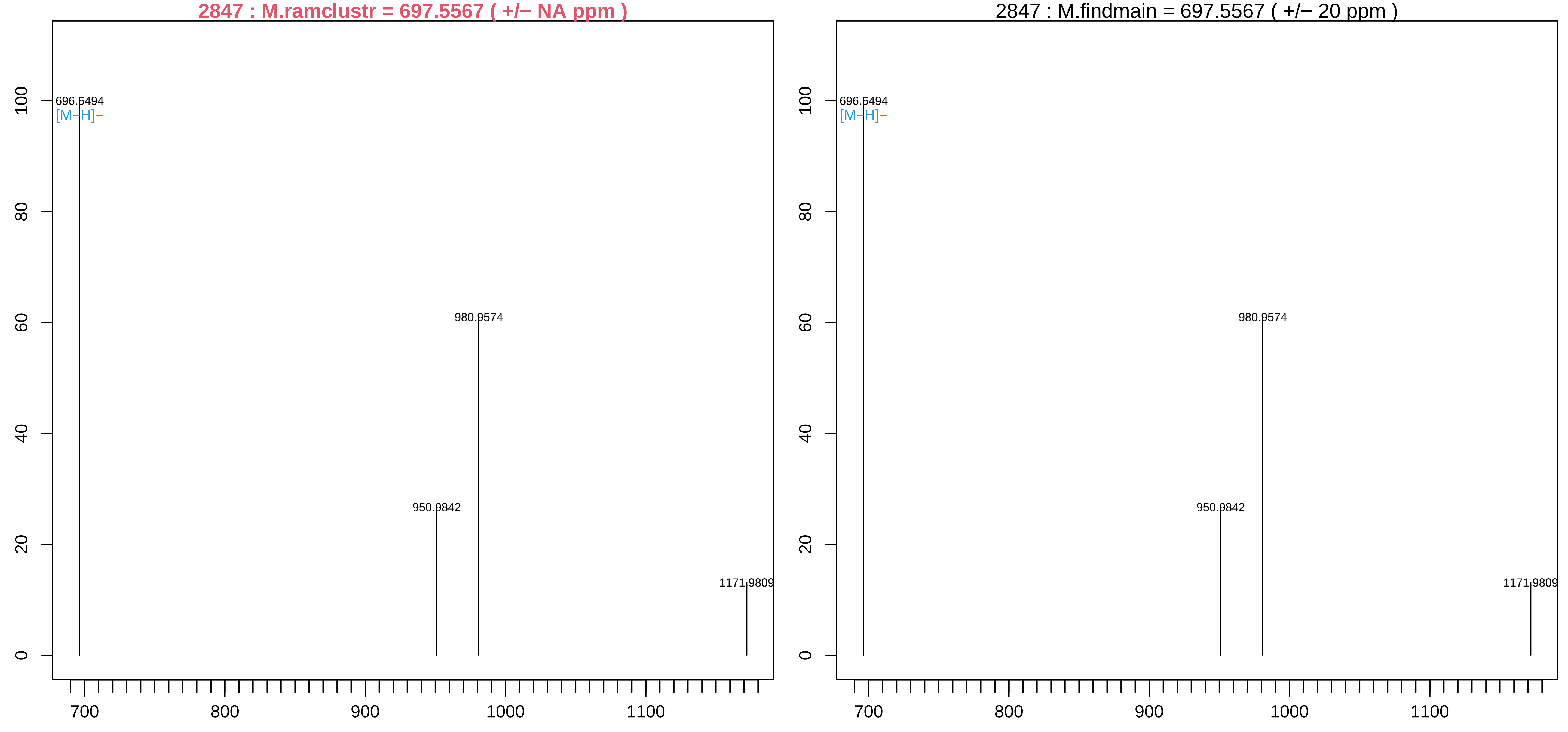

2847 : M.ramclustr = 697.5567 (+/− NA ppm)
696.5494
[M−H]−
980.9574
950.9842
1171.9809
0
20
40
60
80
100
700
800
900
1000
1100
2847 : M.findmain = 697.5567 (+/− 20 ppm)
696.5494
[M−H]−
980.9574
950.9842
1171.9809
0
20
40
60
80
100
700
800
900
1000
1100

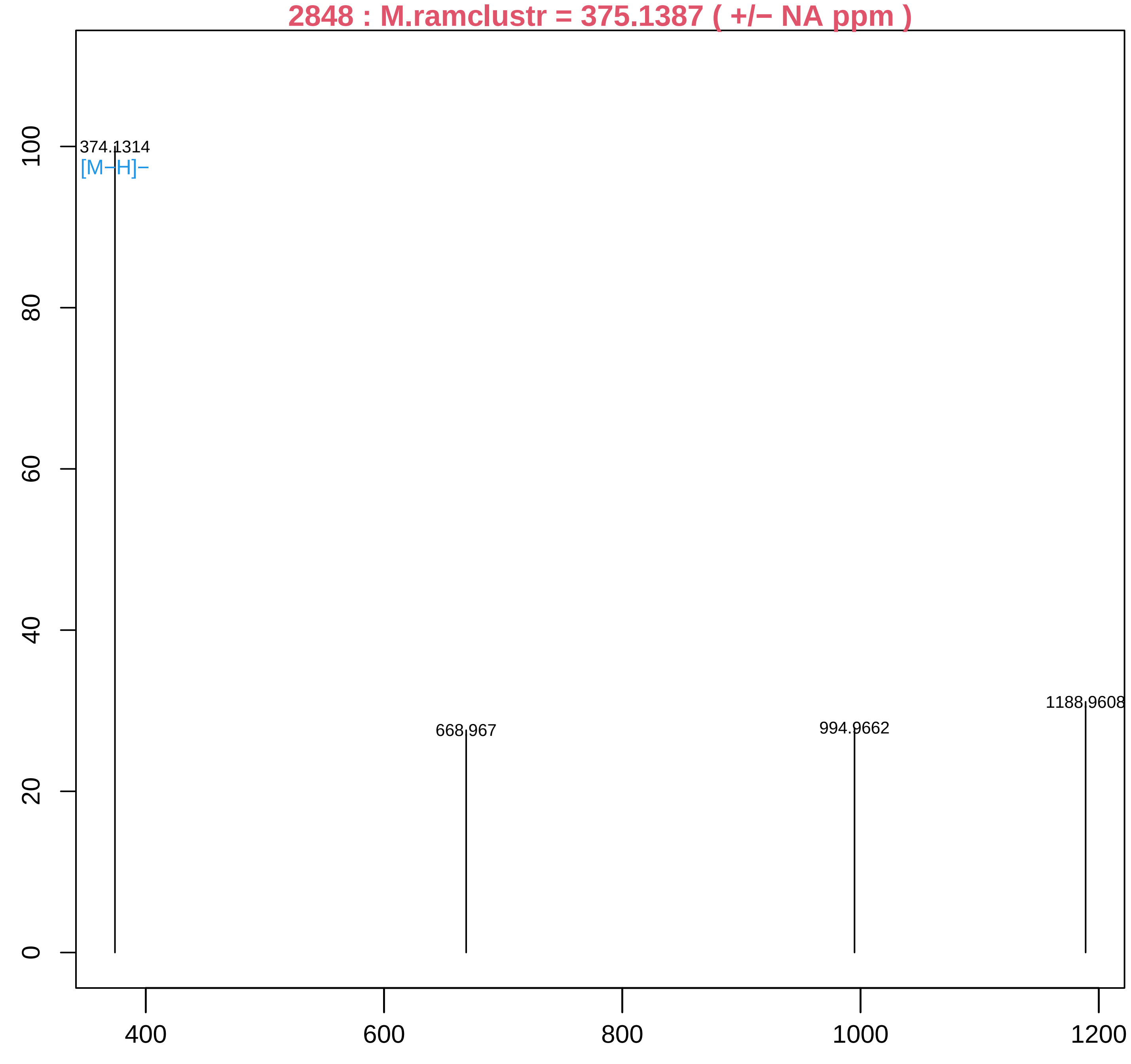
2848 : M.ramclustr = 375.1387 (+/− NA ppm)
374.1314
[M−H]−
668.967
994.9662
1188.9608
0
20
40
60
80
100
400
600
800
1000
1200

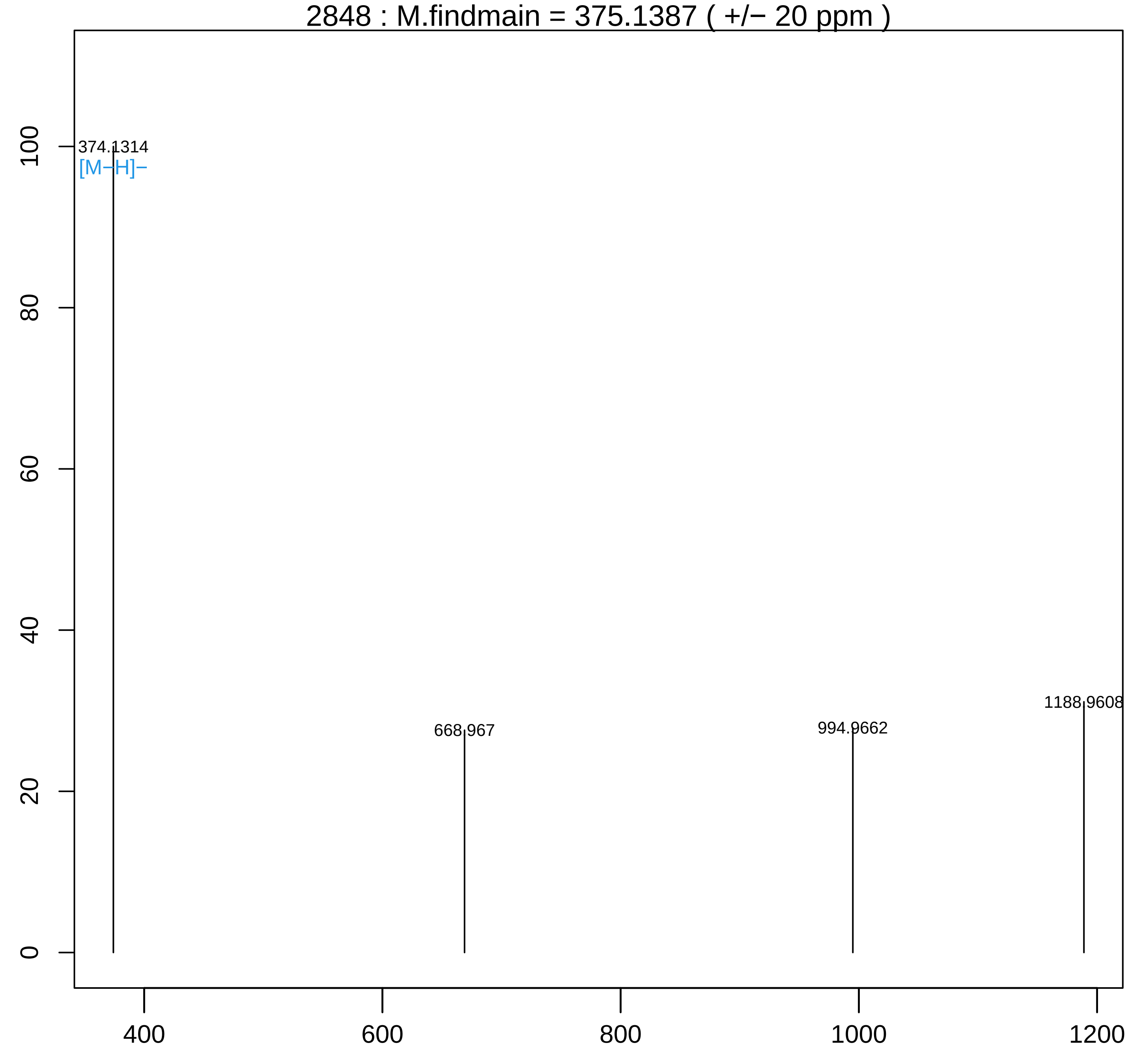
2848 : M.findmain = 375.1387 (+/− 20 ppm)
374.1314
[M−H]−
668.967
994.9662
1188.9608
0
20
40
60
80
100
400
600
800
1000
1200

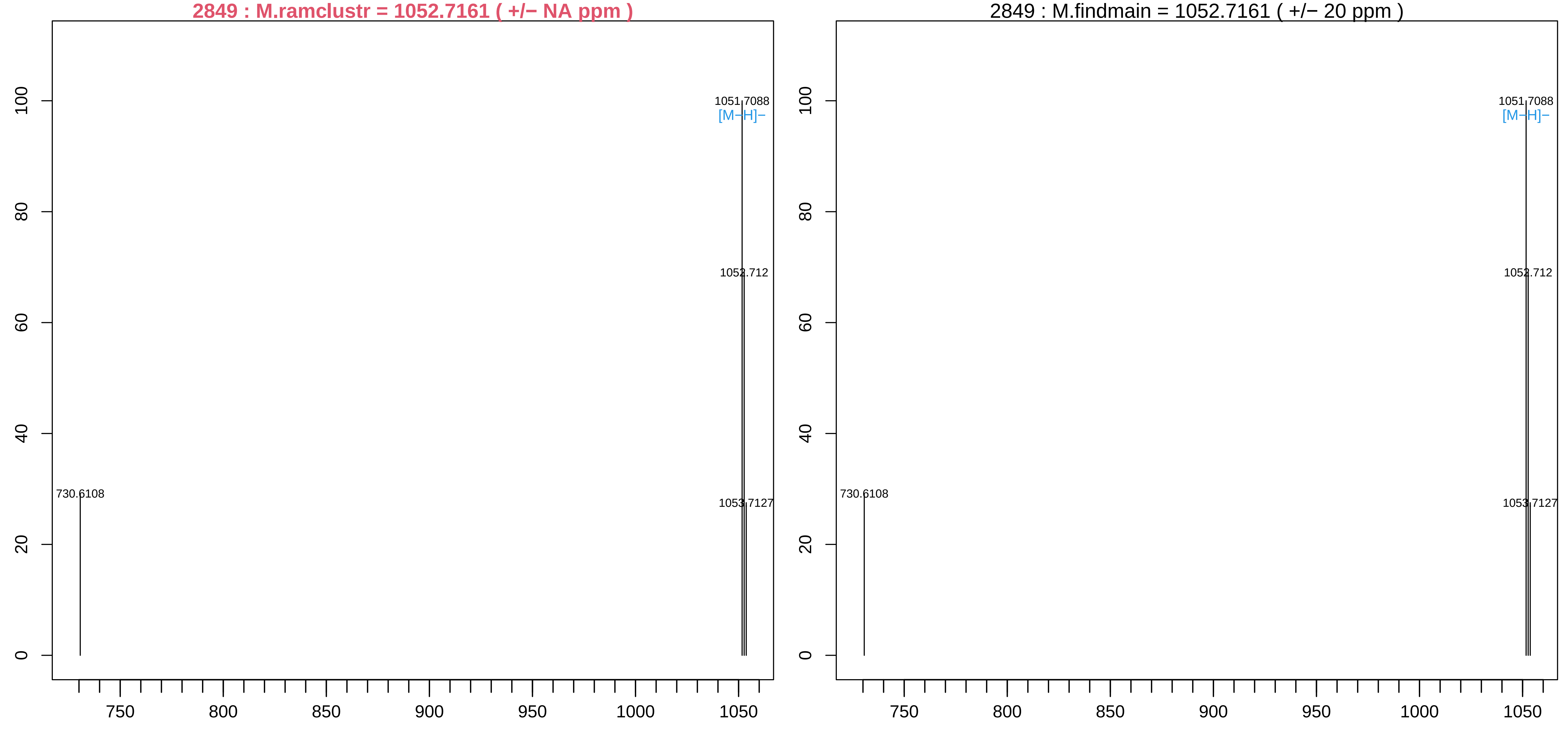
2849 : M.ramclustr = 1052.7161 (+/− NA ppm)
1051.7088
[M−H]−
1052.712
730.6108
1053.7127
0
20
40
60
80
100
750
800
850
900
950
1000
1050
2849 : M.findmain = 1052.7161 (+/− 20 ppm)
1051.7088
[M−H]−
1052.712
730.6108
1053.7127
0
20
40
60
80
100
750
800
850
900
950
1000
1050

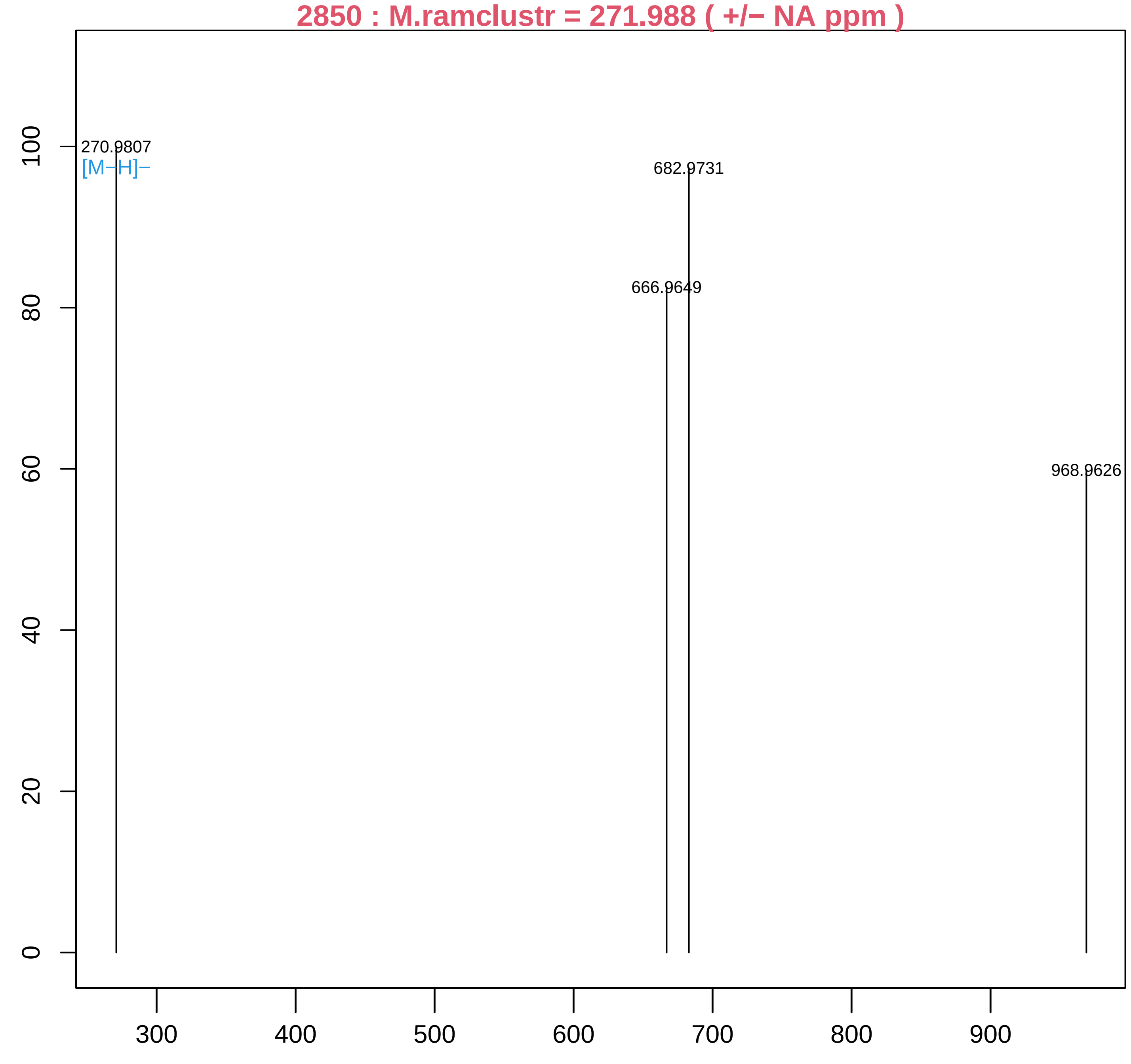
2850 : M.ramclustr = 271.988 (+/− NA ppm)
270.9807
[M−H]−
682.9731
666.9649
968.9626
0
20
40
60
80
100
300
400
500
600
700
800
900

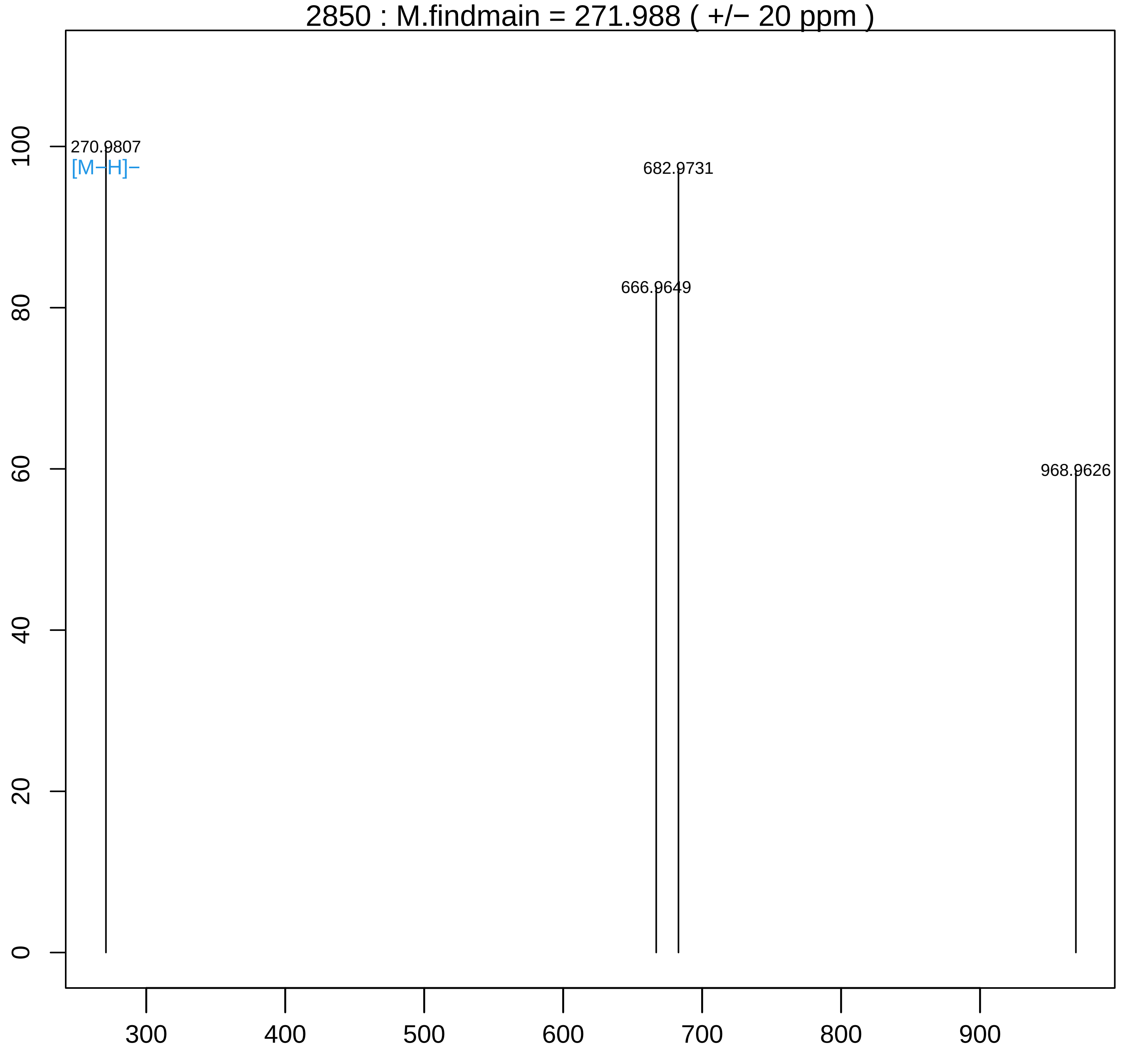
2850 : M.findmain = 271.988 (+/− 20 ppm)
270.9807
[M−H]−
682.9731
666.9649
968.9626
0
20
40
60
80
100
300
400
500
600
700
800
900

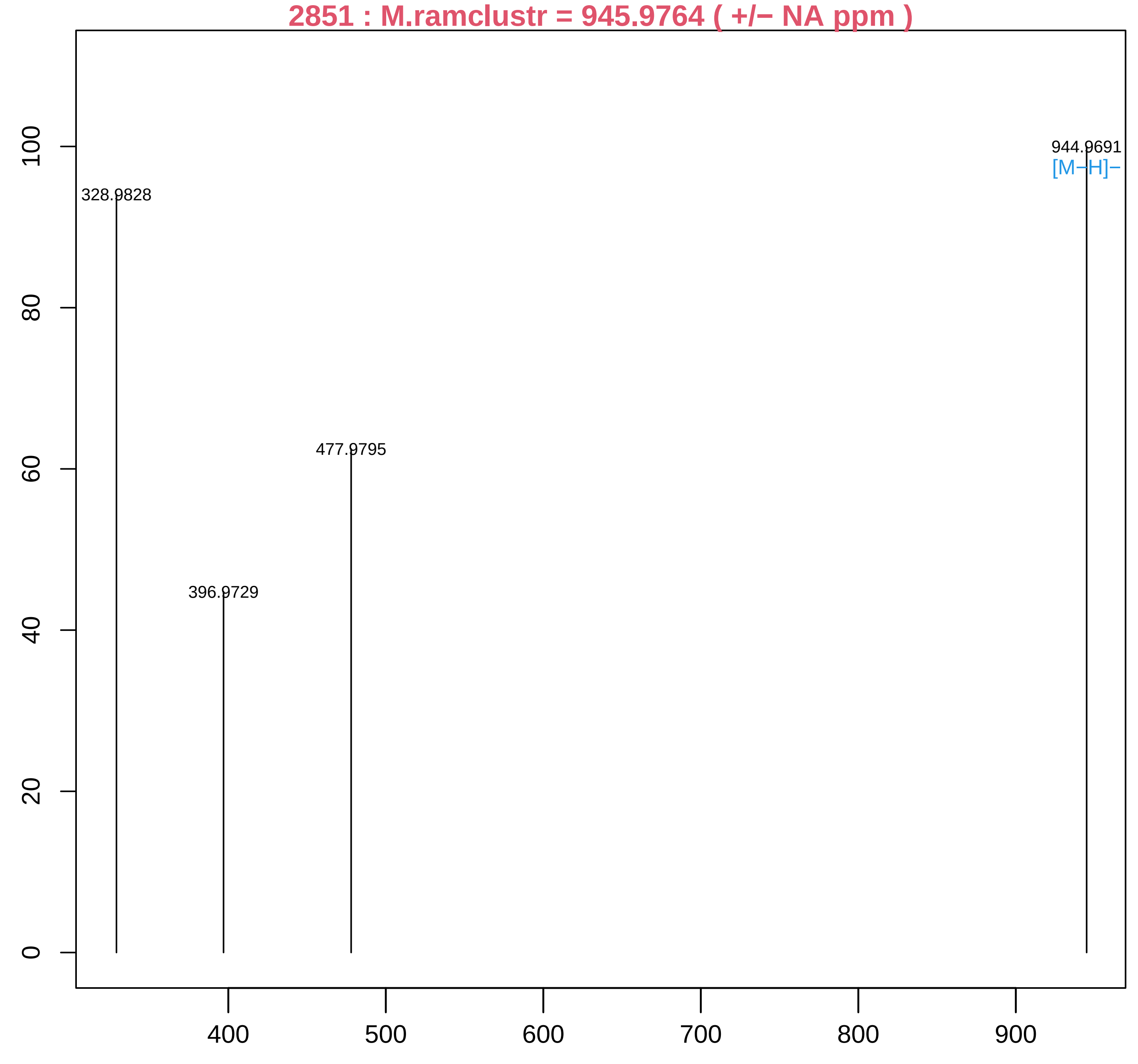
2851 : M.ramclustr = 945.9764 (+/− NA ppm)
328.9828
396.9729
477.9795
944.9691
[M−H]−
0
20
40
60
80
100
400
500
600
700
800
900

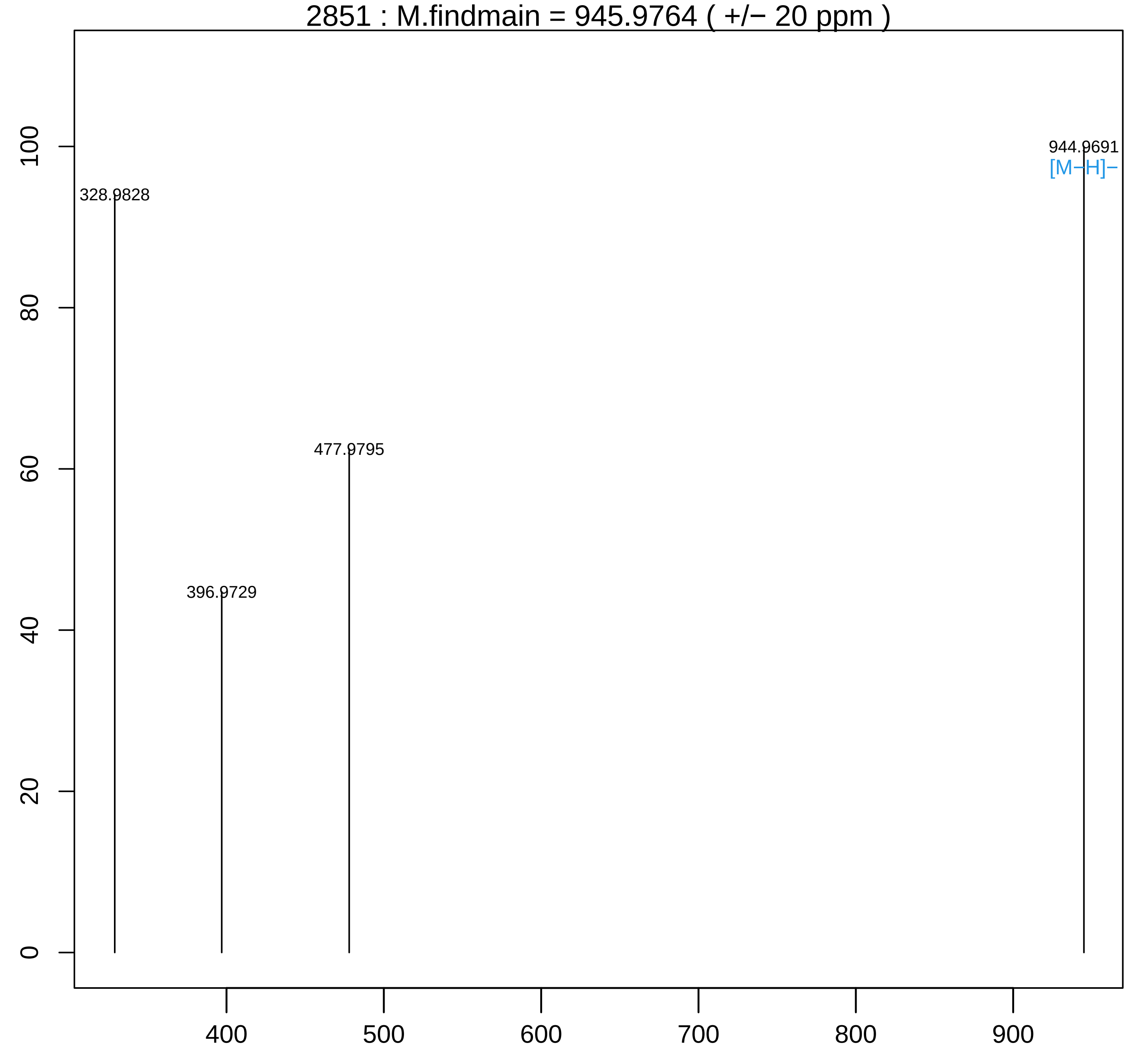
2851 : M.findmain = 945.9764 (+/− 20 ppm)
328.9828
396.9729
477.9795
944.9691
[M−H]−
0
20
40
60
80
100
400
500
600
700
800
900

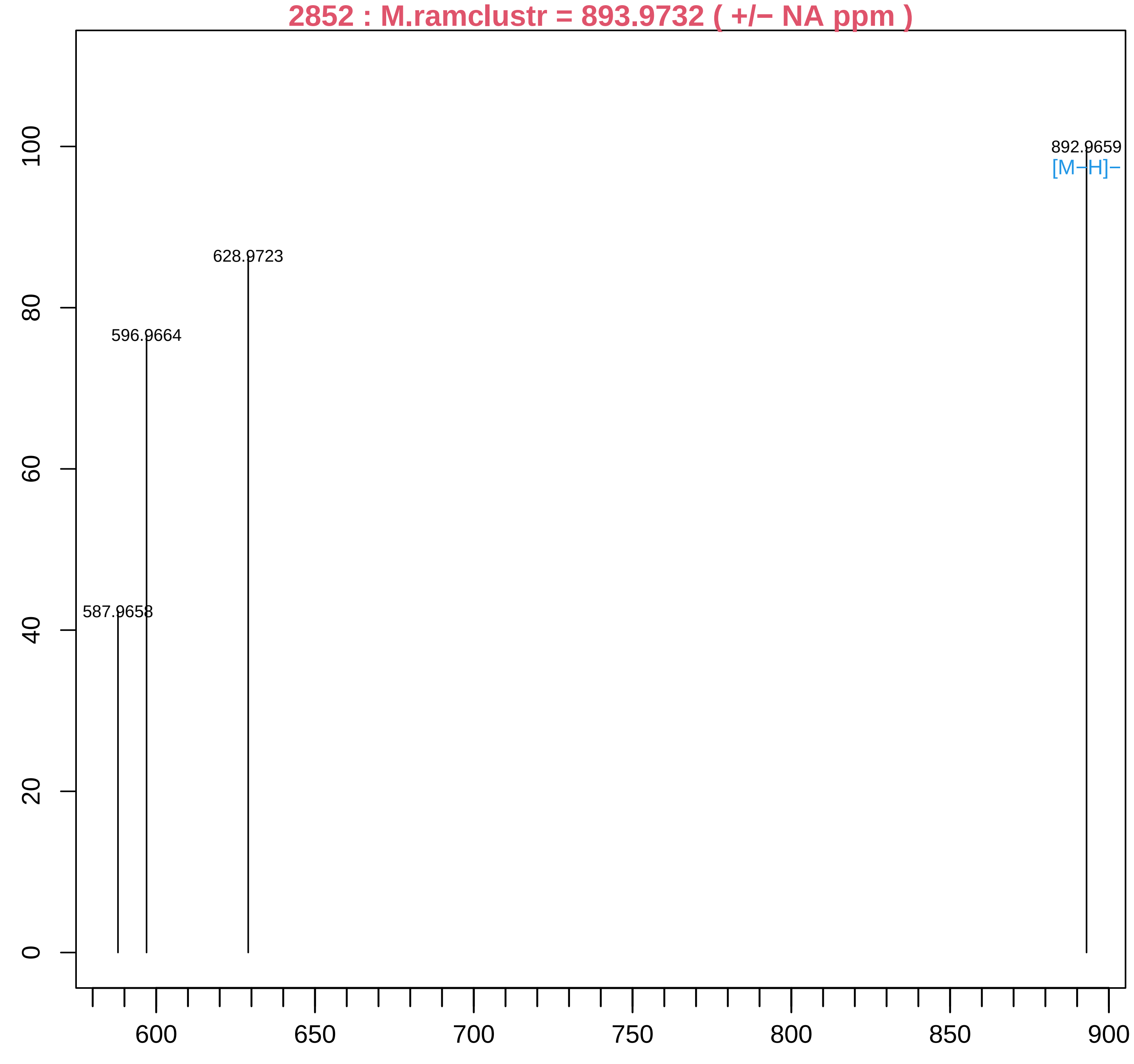
2852 : M.ramclustr = 893.9732 (+/− NA ppm)
892.9659
[M−H]−
628.9723
596.9664
587.9658
0
20
40
60
80
100
600
650
700
750
800
850
900

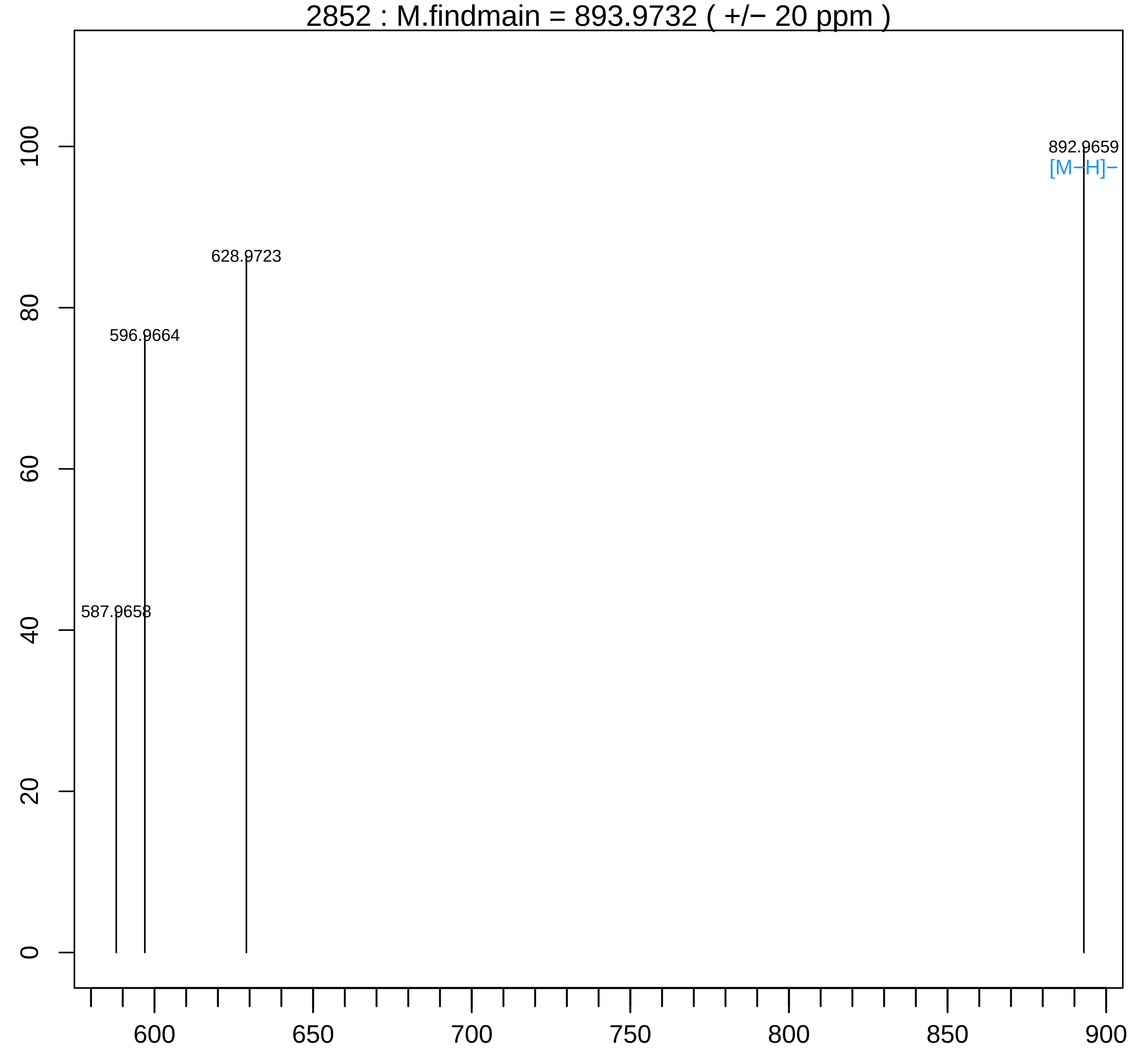
2852 : M.findmain = 893.9732 (+/− 20 ppm)
892.9659
[M−H]−
628.9723
596.9664
587.9658
0
20
40
60
80
100
600
650
700
750
800
850
900

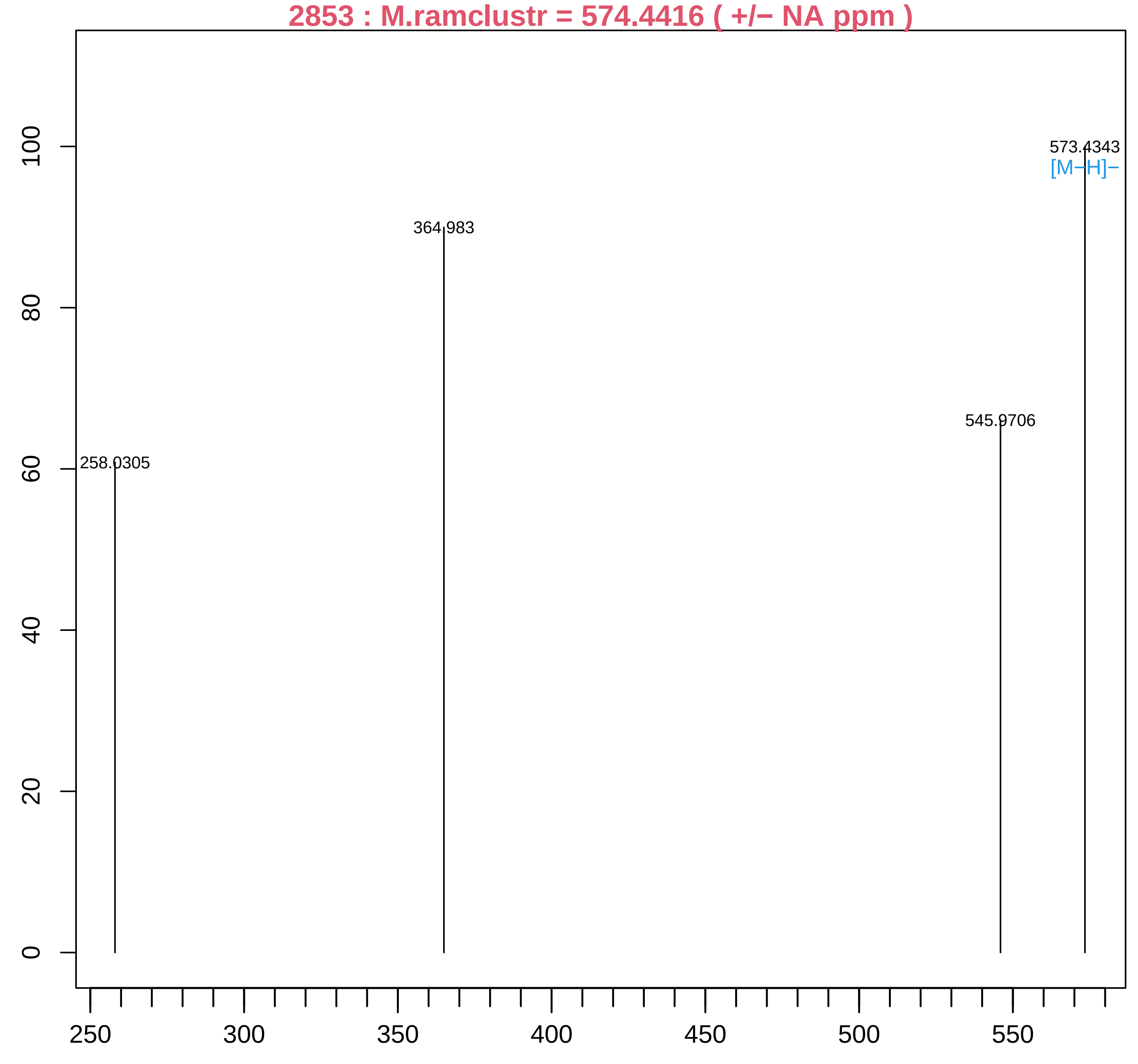
2853 : M.ramclustr = 574.4416 (+/− NA ppm)
258.0305
364.983
545.9706
573.4343
[M−H]−
0
20
40
60
80
100
250
300
350
400
450
500
550

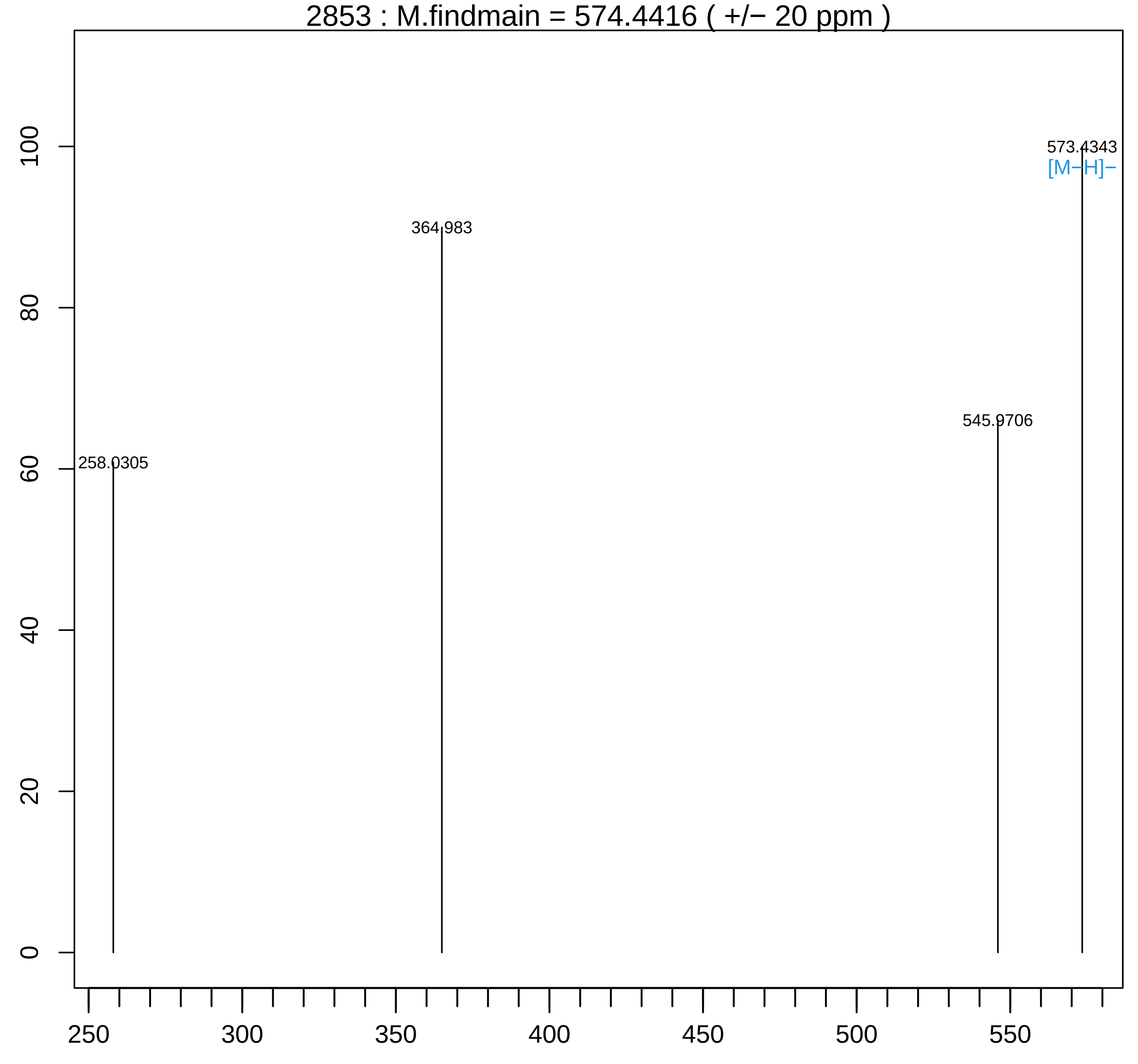
2853 : M.findmain = 574.4416 (+/− 20 ppm)
258.0305
364.983
545.9706
573.4343
[M−H]−
0
20
40
60
80
100
250
300
350
400
450
500
550

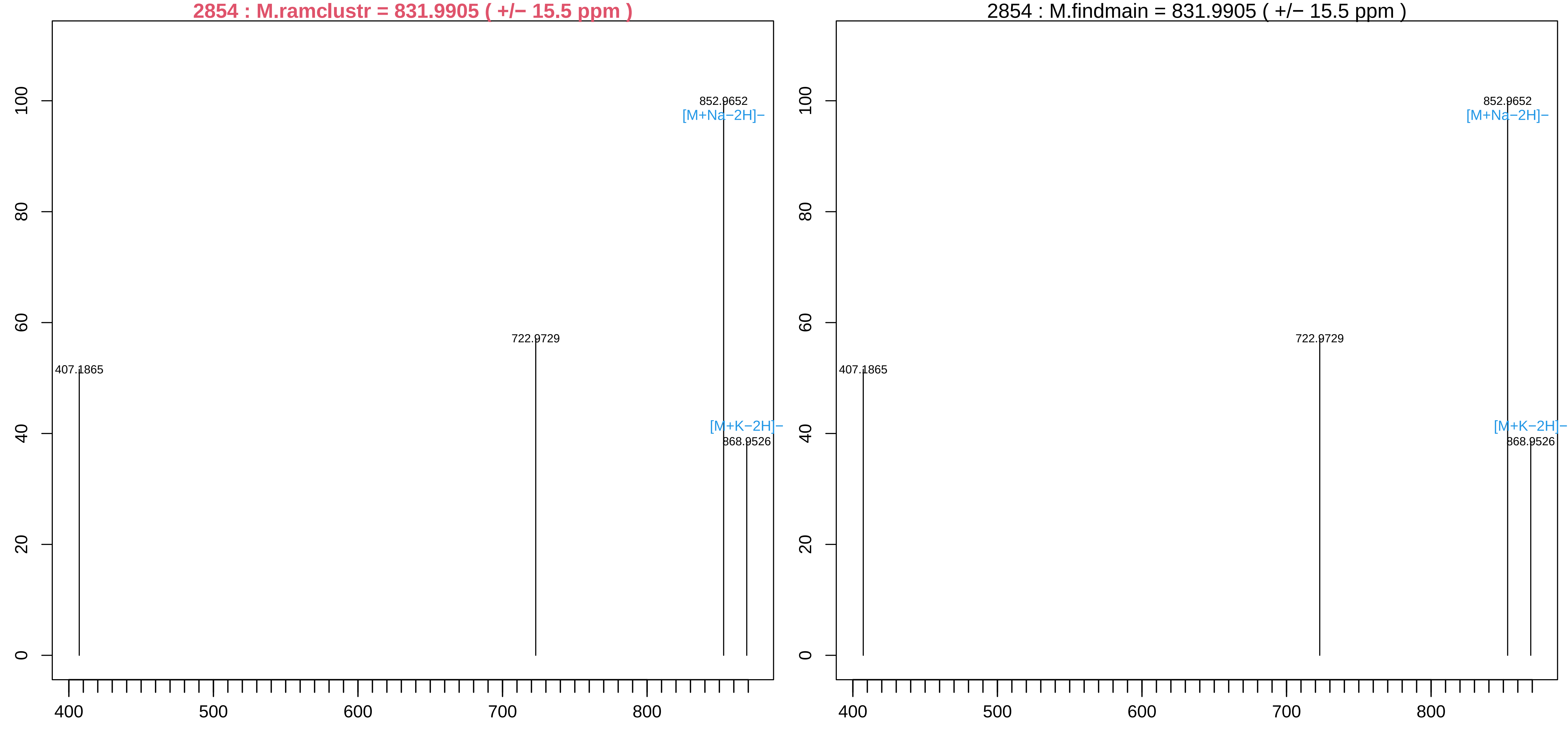
2854 : M.ramclustr = 831.9905 (+/− 15.5 ppm)
2854 : M.findmain = 831.9905 (+/− 15.5 ppm)
852.9652
[M+Na−2H]−
722.9729
407.1865
[M+K−2H]−
868.9526
0
20
40
60
80
100
400
500
600
700
800

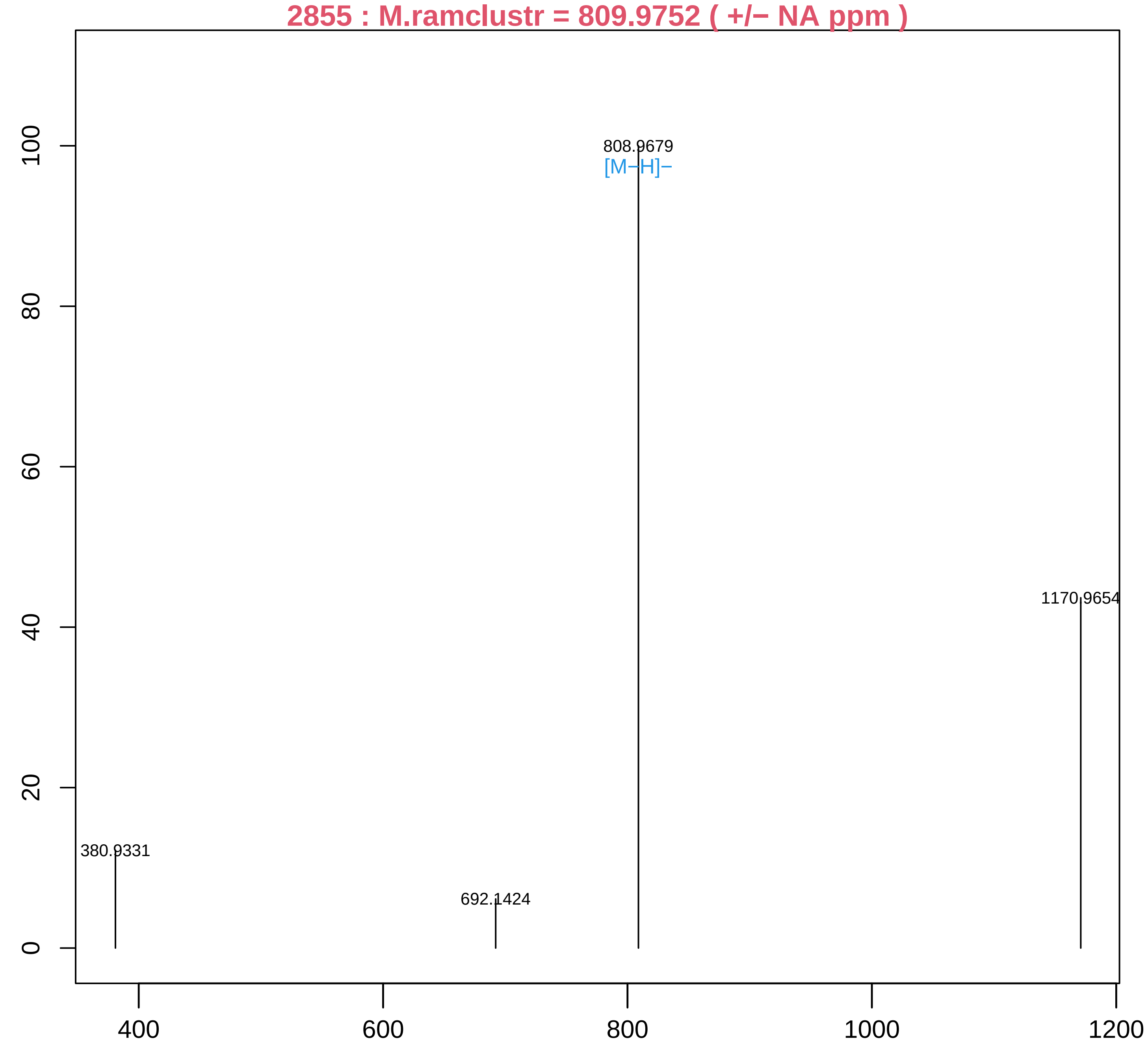

2855 : M.ramclustr = 809.9752 (+/− NA ppm)
808.9679
[M−H]−
1170.9654
380.9331
692.1424
0
20
40
60
80
100
400
600
800
1000
1200

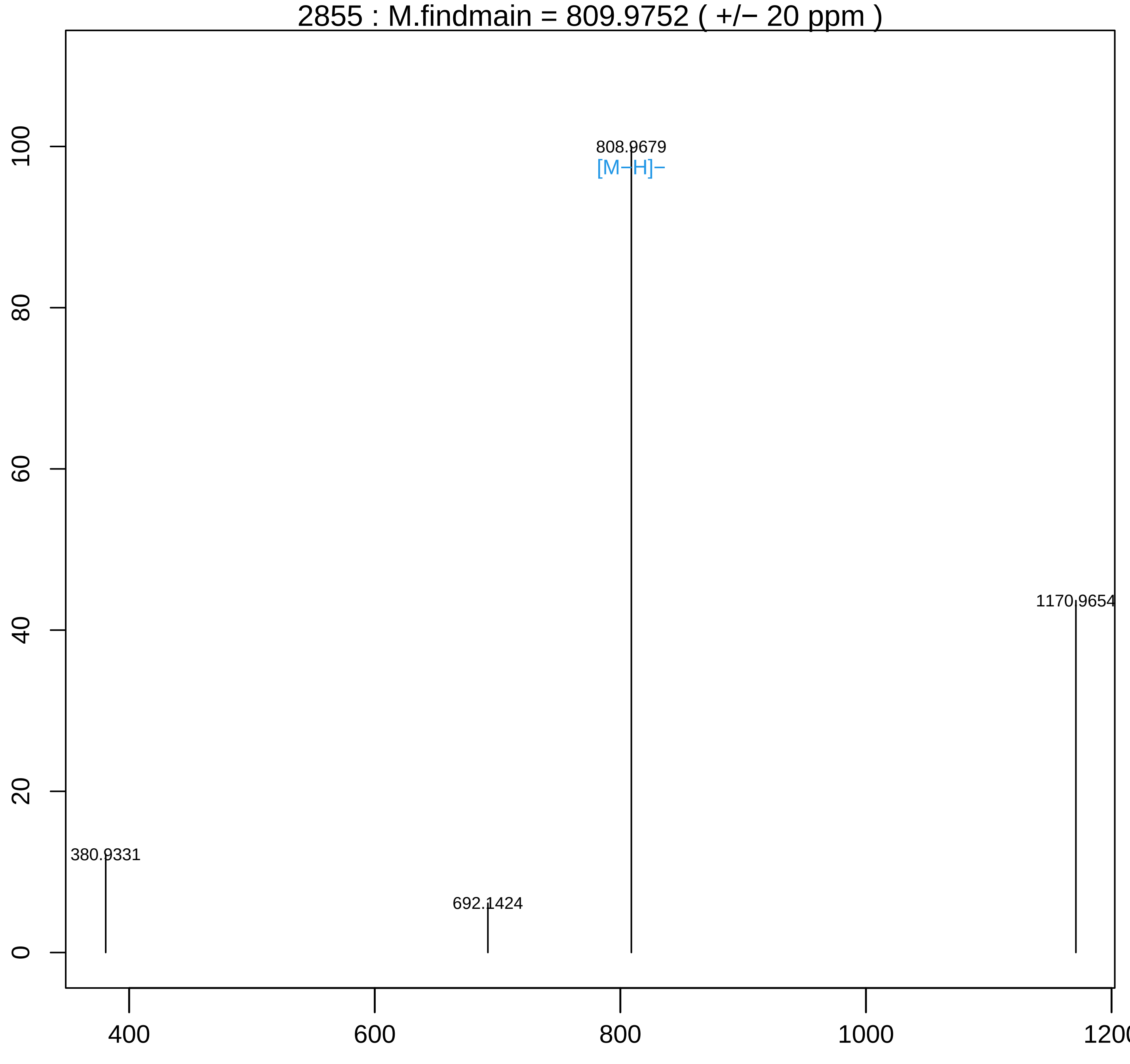

2855 : M.findmain = 809.9752 (+/− 20 ppm)
808.9679
[M−H]−
1170.9654
380.9331
692.1424
0
20
40
60
80
100
400
600
800
1000
1200

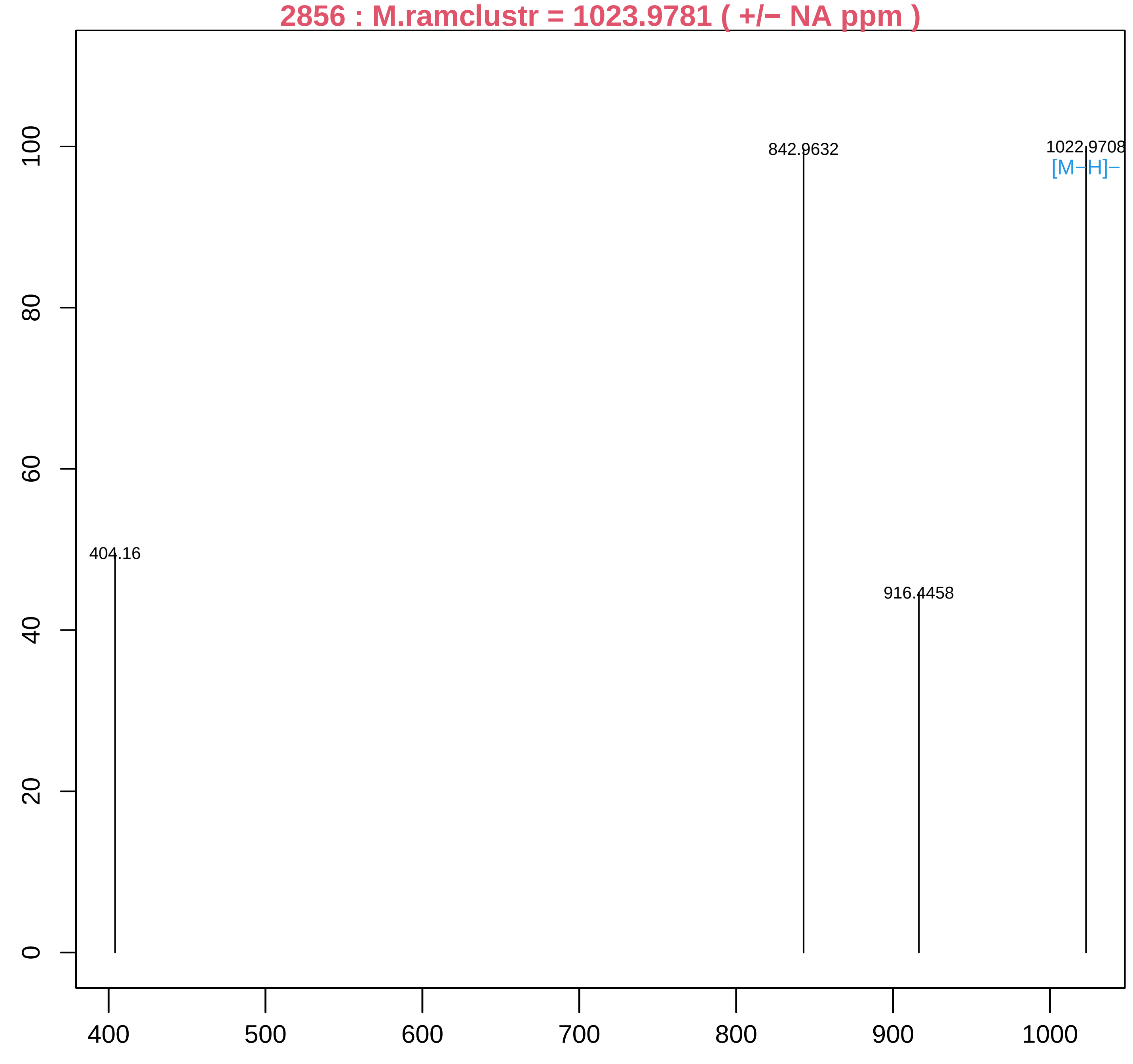

2856 : M.ramclustr = 1023.9781 (+/− NA ppm)
404.16
842.9632
916.4458
1022.9708
[M−H]−
0
20
40
60
80
100
400
500
600
700
800
900
1000

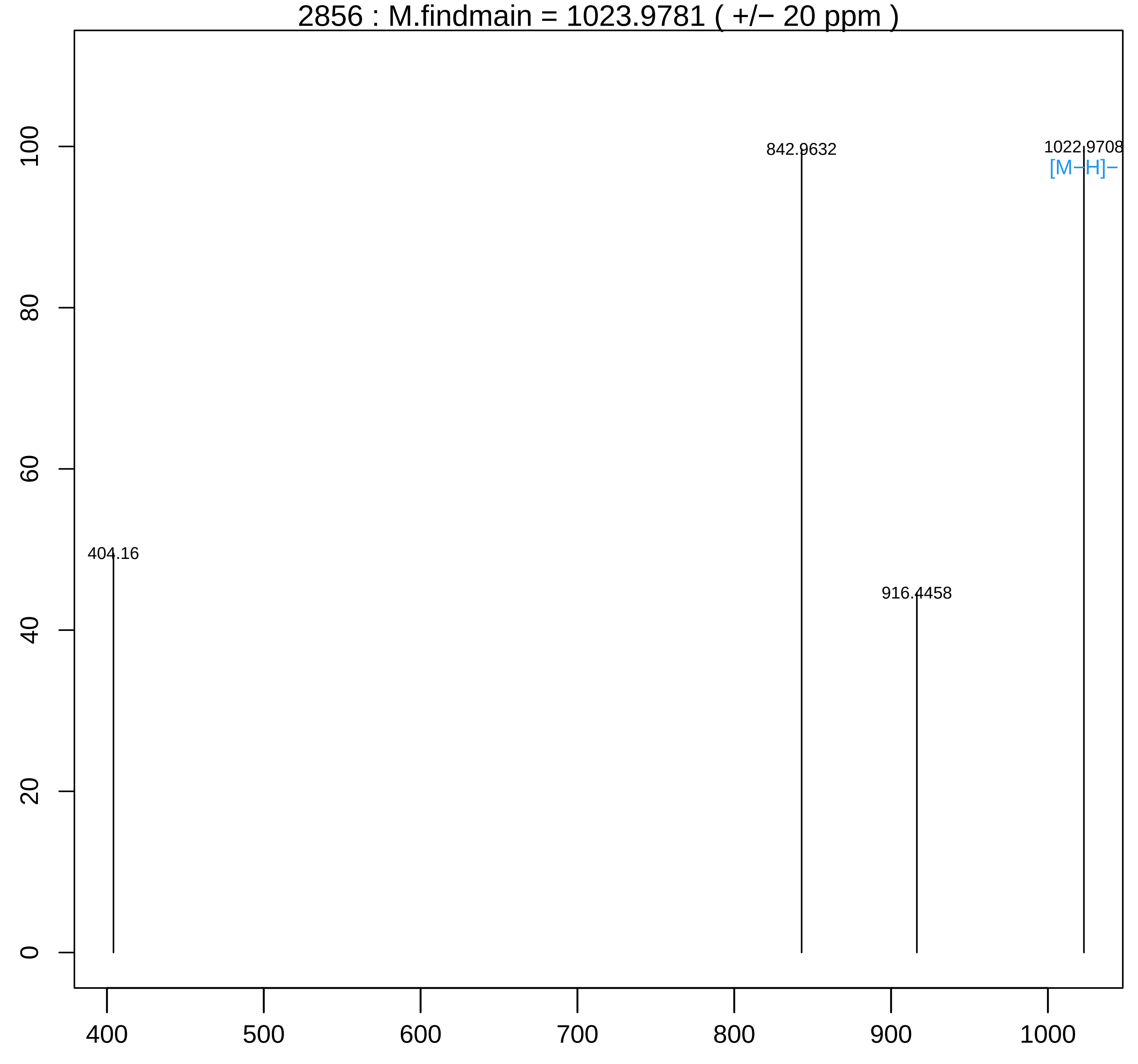

2856 : M.findmain = 1023.9781 (+/− 20 ppm)
404.16
842.9632
916.4458
1022.9708
[M−H]−
0
20
40
60
80
100
400
500
600
700
800
900
1000

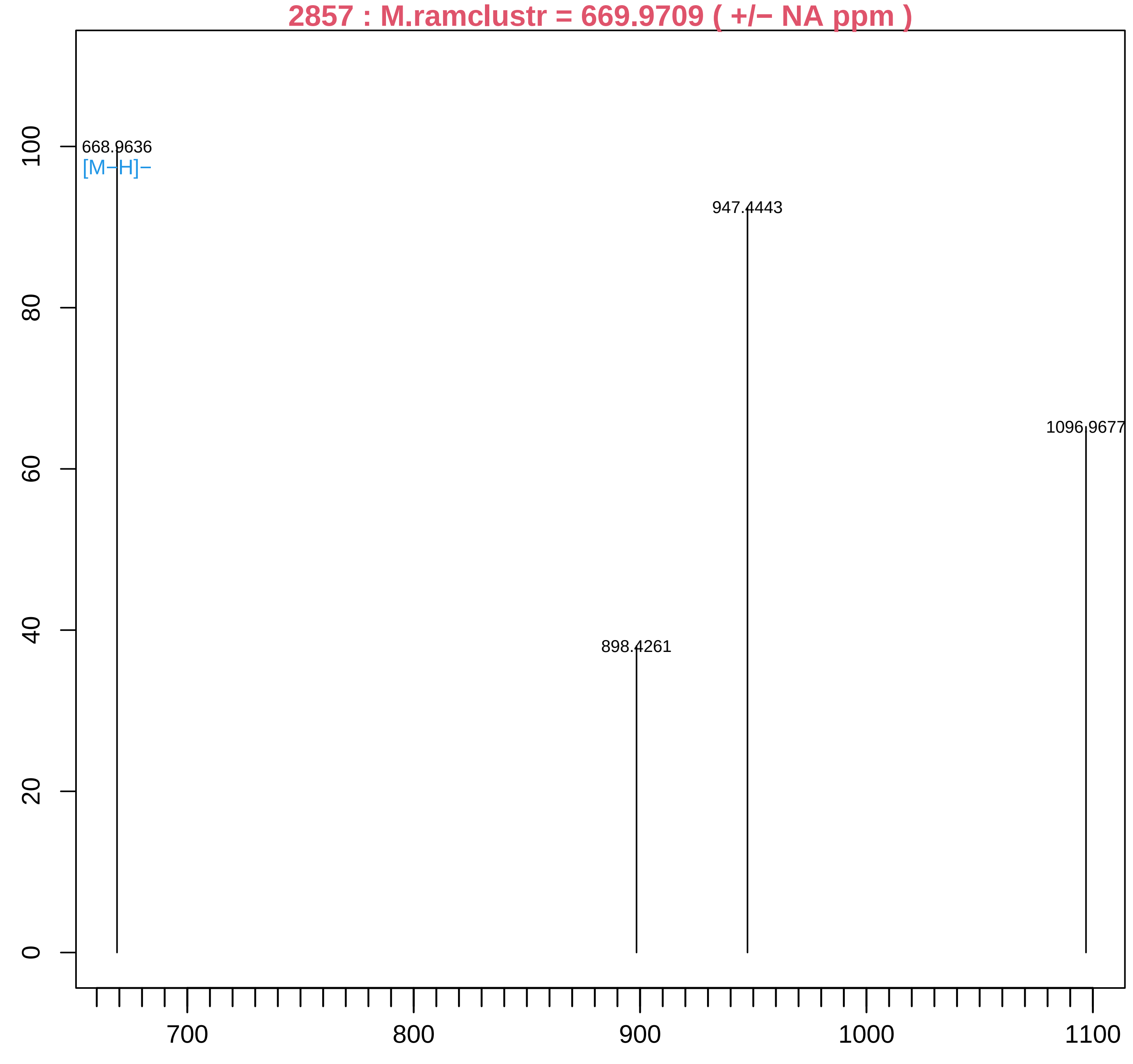

2857 : M.ramclustr = 669.9709 (+/− NA ppm)
668.9636
[M−H]−
947.4443
898.4261
1096.9677
0
20
40
60
80
100
700
800
900
1000
1100

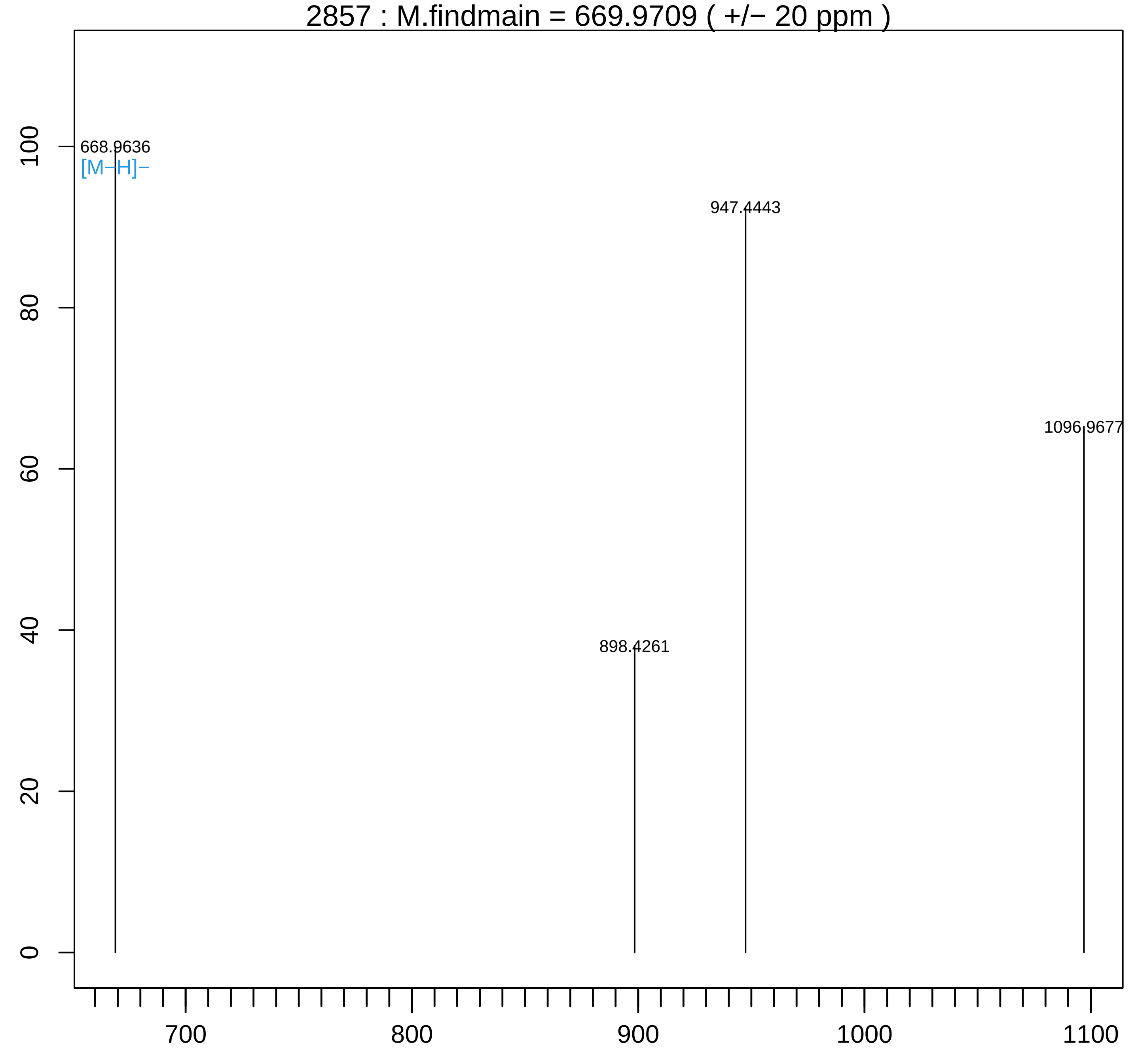

2857 : M.findmain = 669.9709 (+/− 20 ppm)
668.9636
[M−H]−
947.4443
898.4261
1096.9677
0
20
40
60
80
100
700
800
900
1000
1100

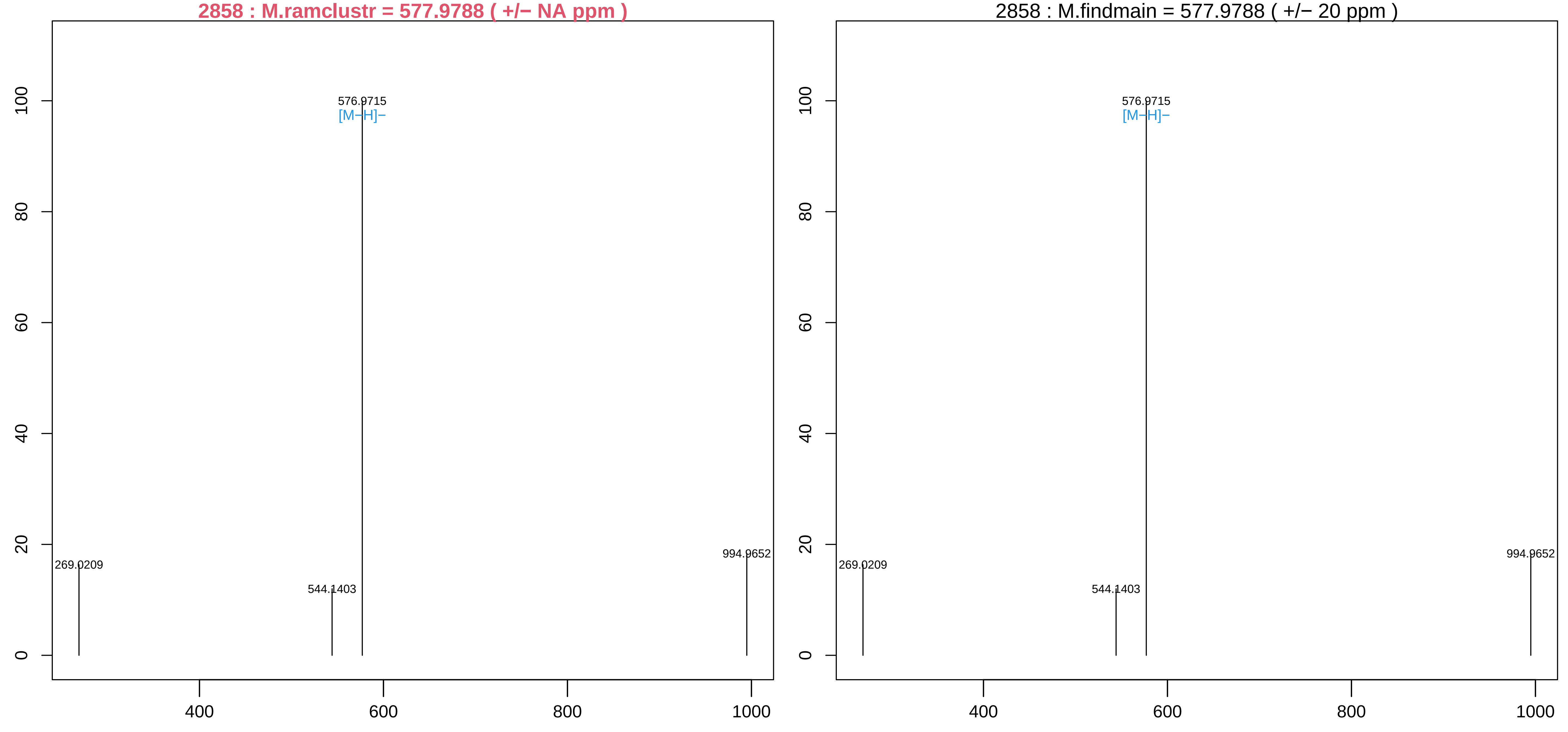
2858 : M.ramclustr = 577.9788 (+/− NA ppm)
576.9715
[M−H]−
269.0209
544.1403
994.9652
0
20
40
60
80
100
400
600
800
1000
2858 : M.findmain = 577.9788 (+/− 20 ppm)
576.9715
[M−H]−
269.0209
544.1403
994.9652
0
20
40
60
80
100
400
600
800
1000

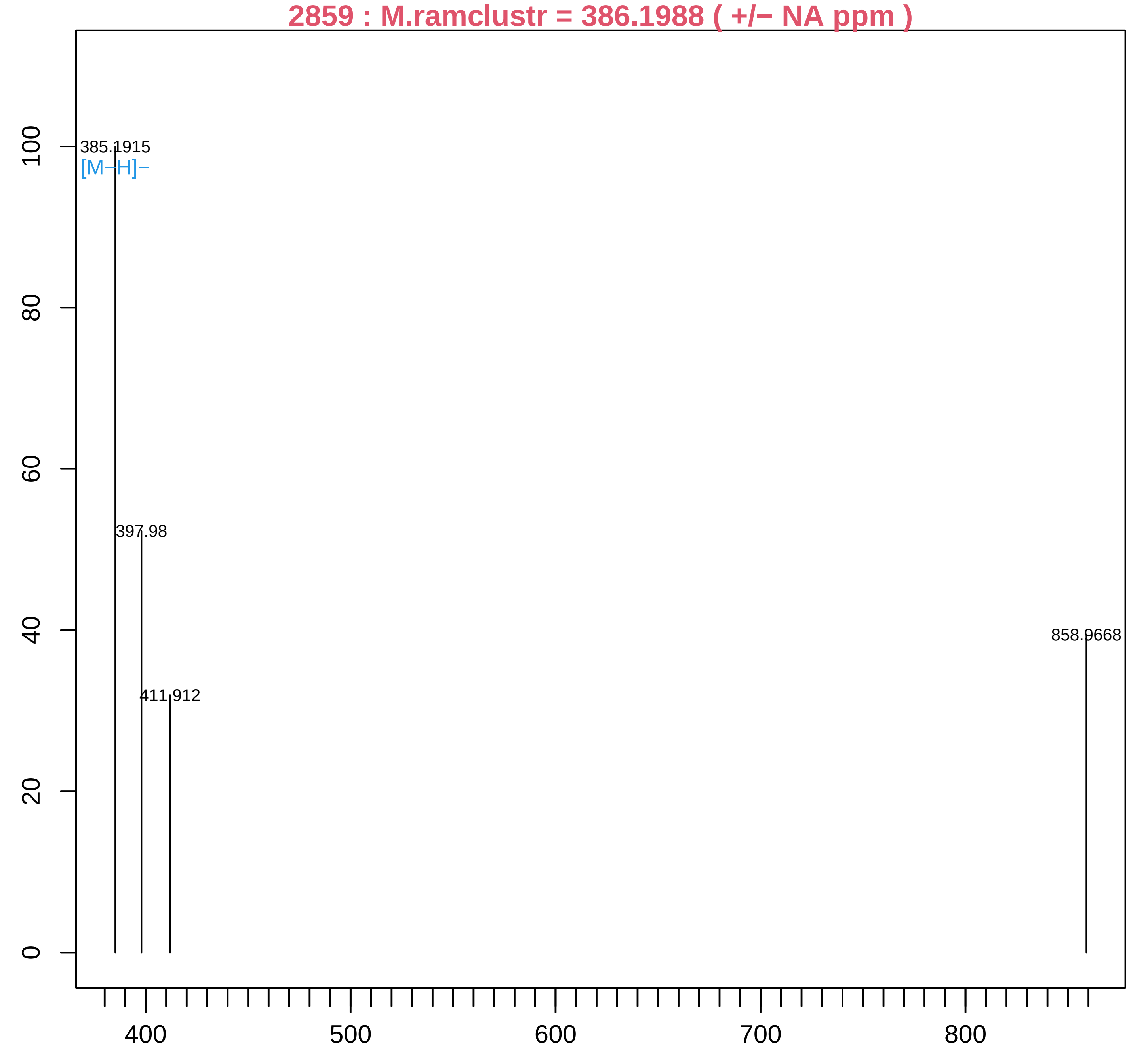

2859 : M.ramclustr = 386.1988 (+/− NA ppm)
385.1915
[M−H]−
397.98
411.912
858.9668
0
20
40
60
80
100
400
500
600
700
800

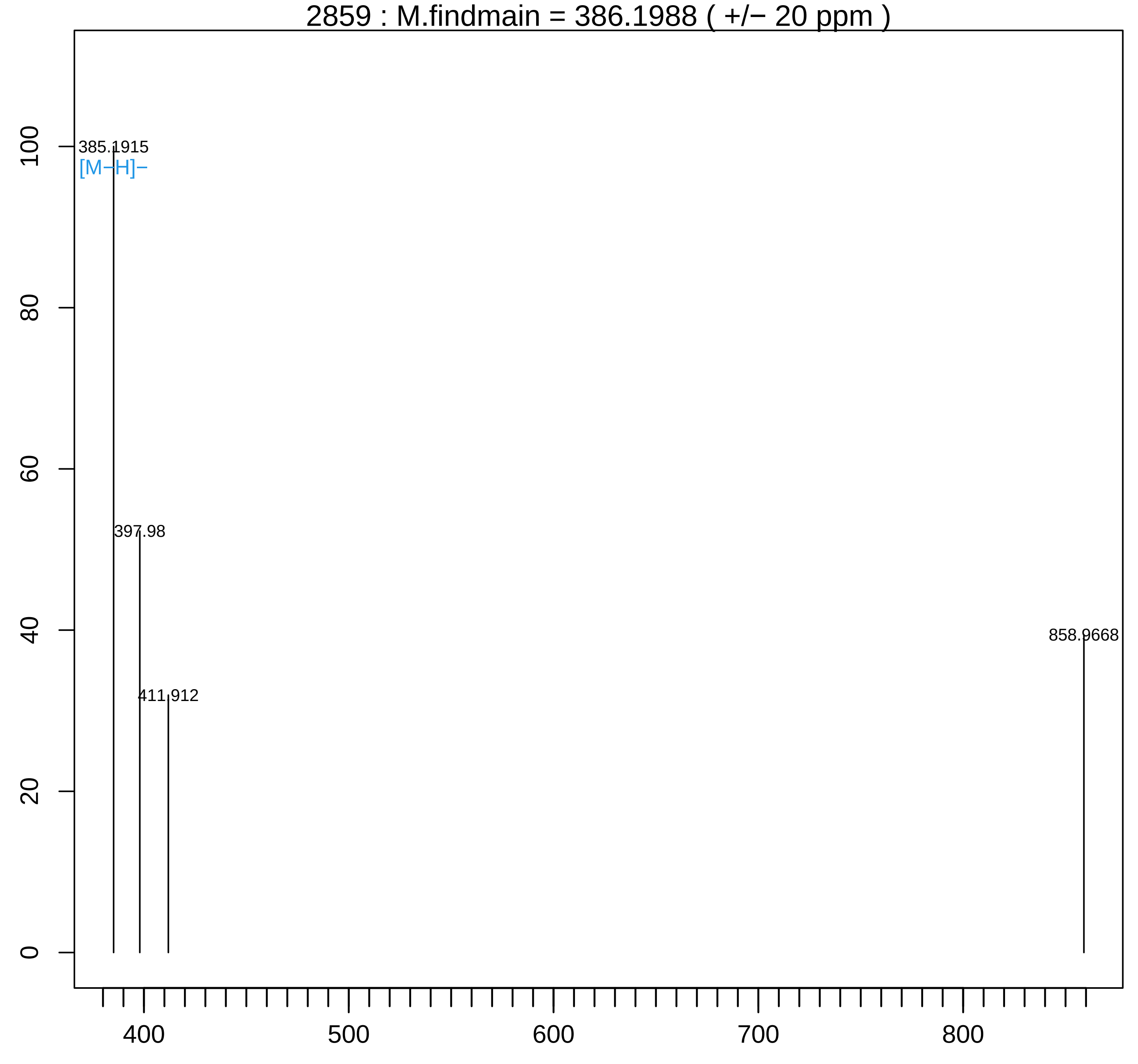

2859 : M.findmain = 386.1988 (+/− 20 ppm)
385.1915
[M−H]−
397.98
411.912
858.9668
0
20
40
60
80
100
400
500
600
700
800

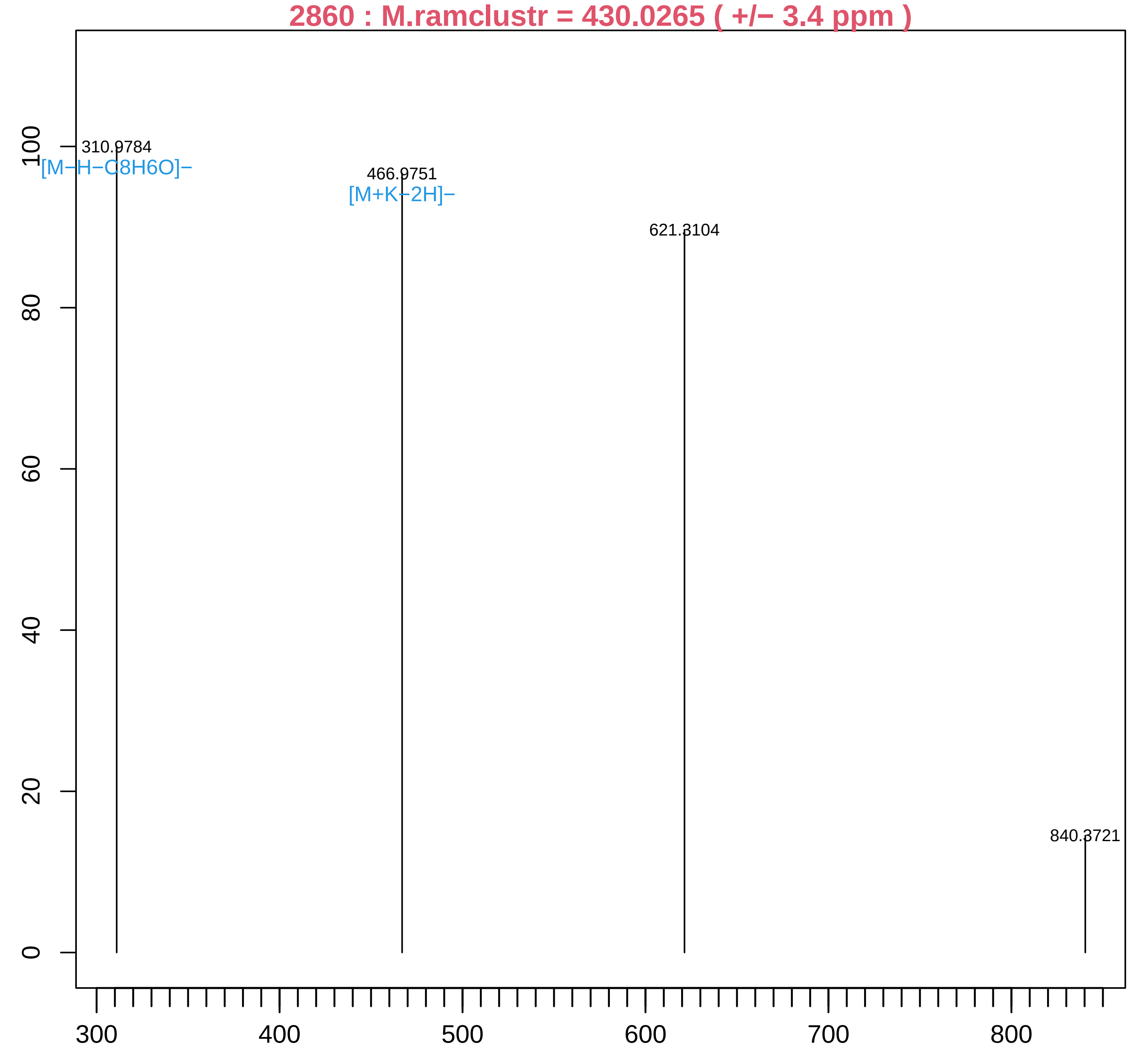

2860 : M.ramclustr = 430.0265 (+/− 3.4 ppm)
310.9784
[M−H−C8H6O]−
466.9751
[M+K−2H]−
621.3104
840.3721
0
20
40
60
80
100
300
400
500
600
700
800

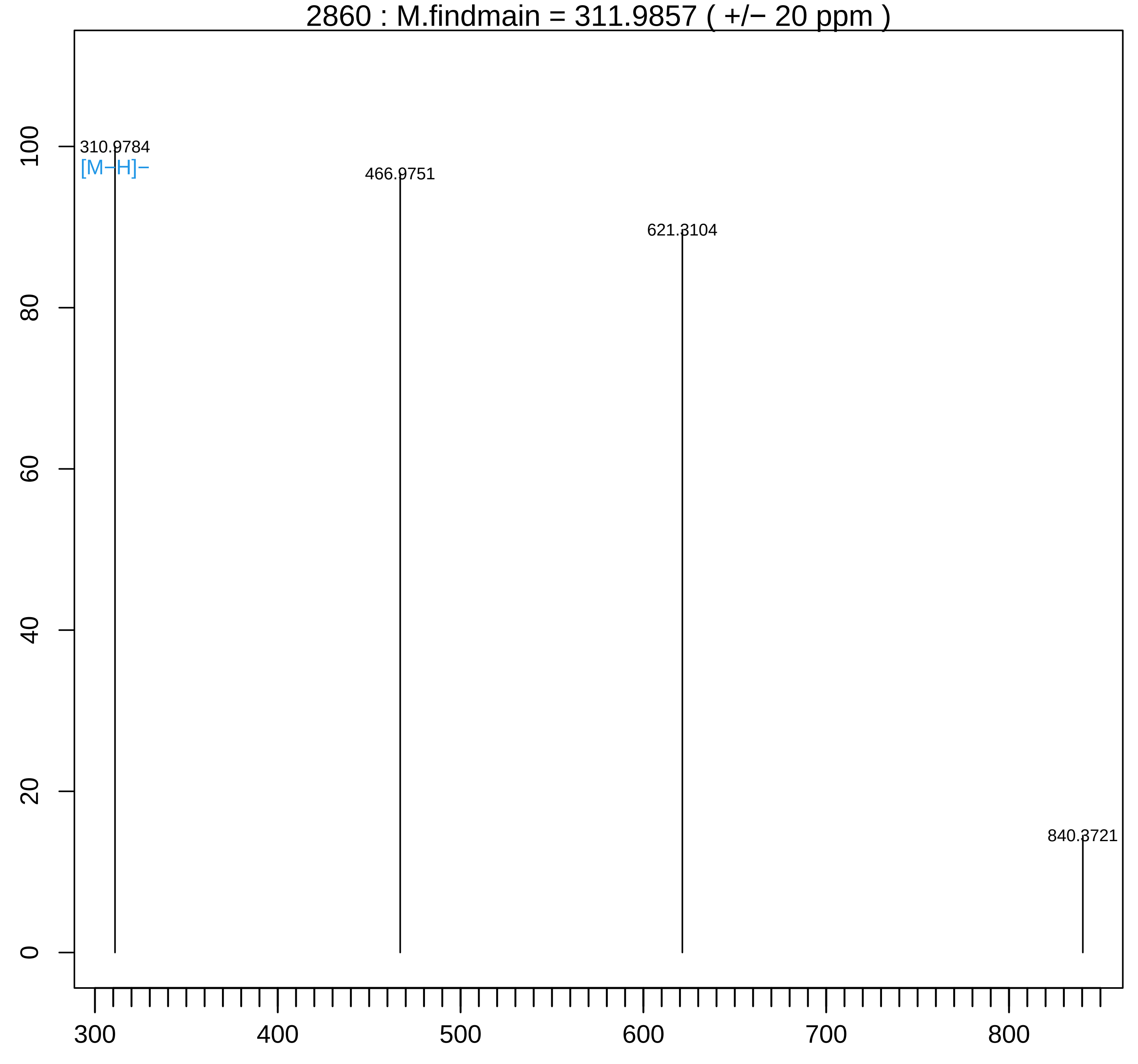

2860 : M.findmain = 311.9857 (+/− 20 ppm)
310.9784
[M−H]−
466.9751
621.3104
840.3721
0
20
40
60
80
100
300
400
500
600
700
800

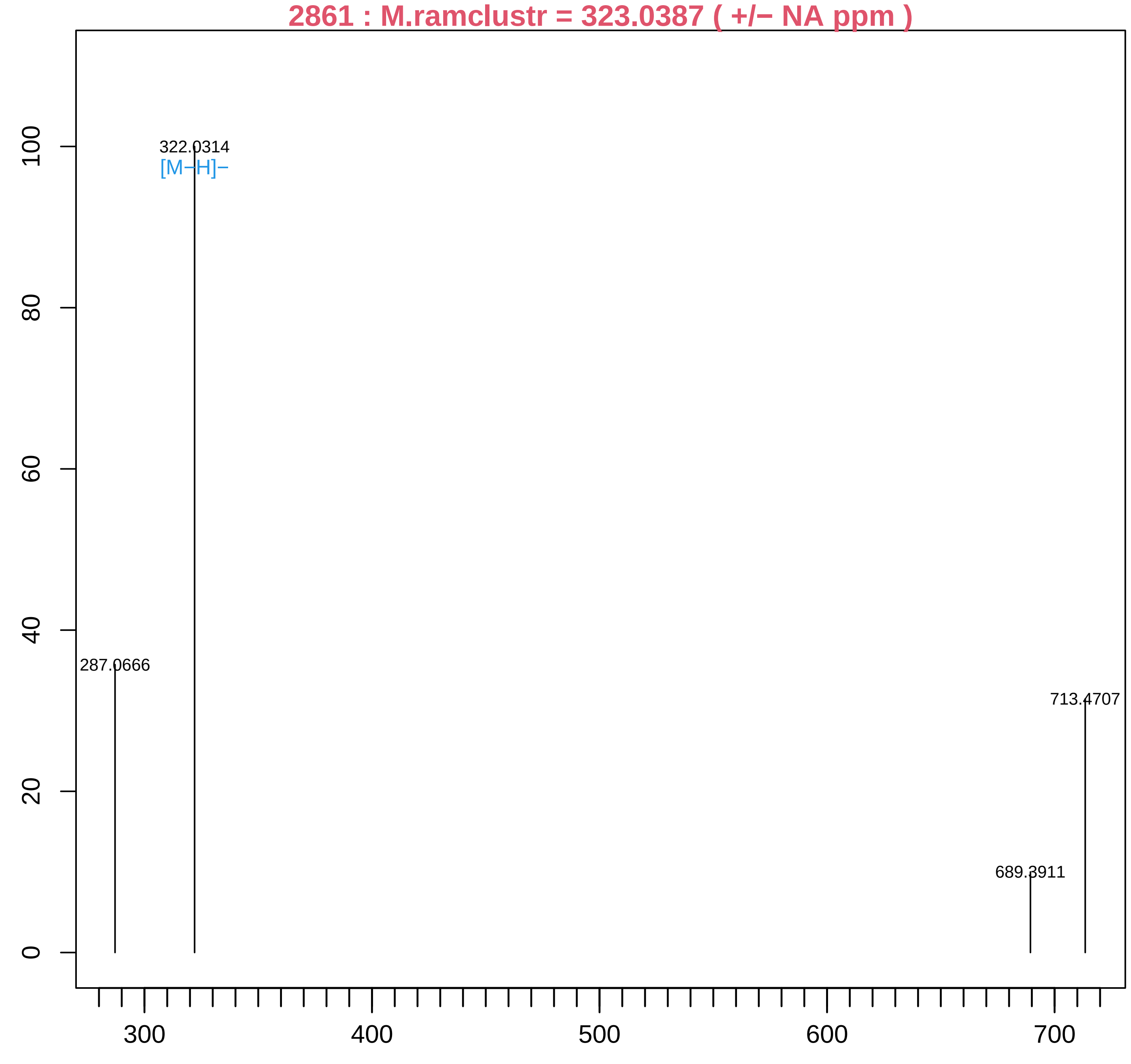

2861 : M.ramclustr = 323.0387 (+/− NA ppm)
322.0314
[M−H]−
287.0666
713.4707
689.3911
0
20
40
60
80
100
300
400
500
600
700

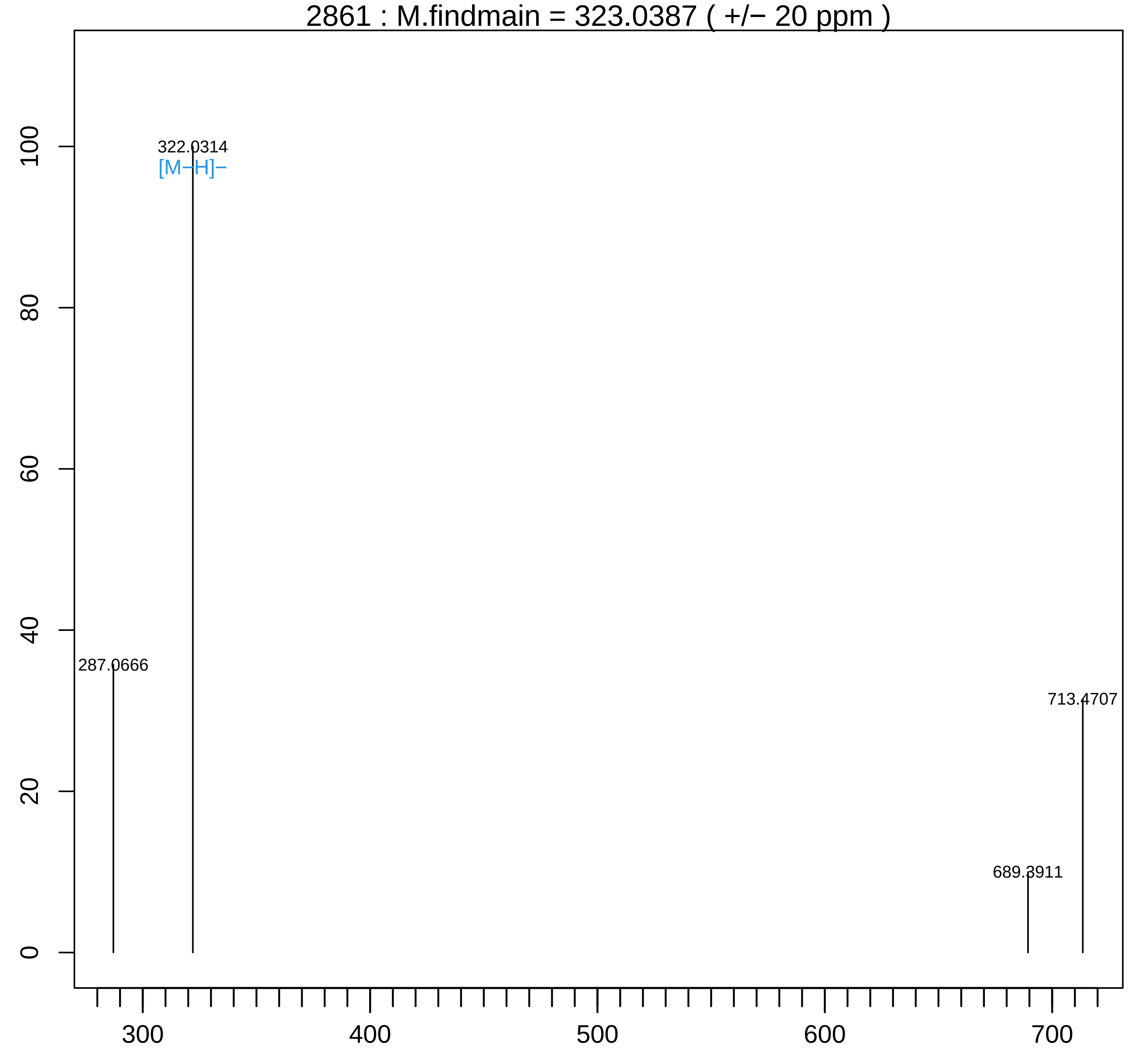

2861 : M.findmain = 323.0387 (+/− 20 ppm)
322.0314
[M−H]−
287.0666
713.4707
689.3911
0
20
40
60
80
100
300
400
500
600
700

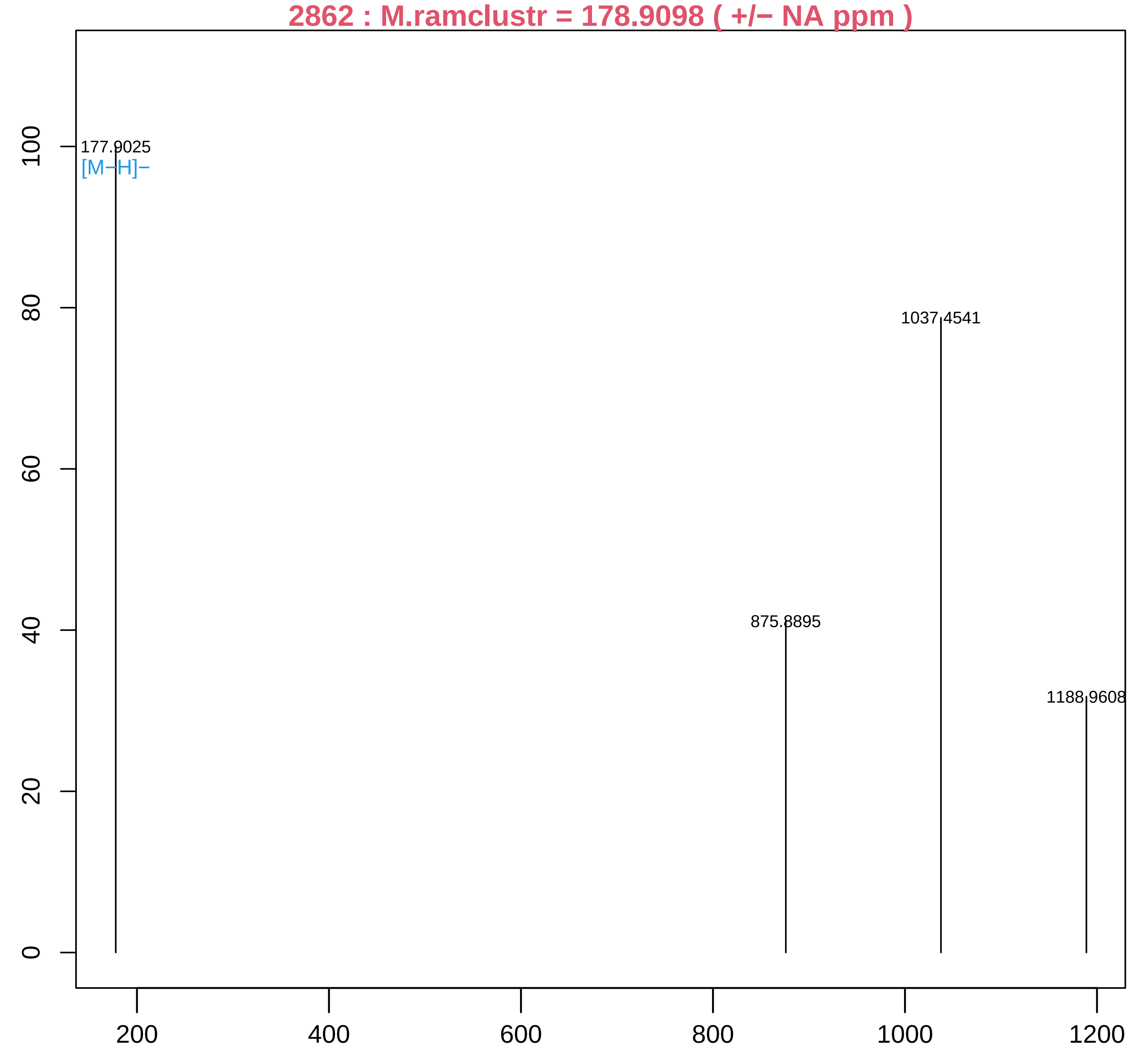
2862 : M.ramclustr = 178.9098 (+/− NA ppm)
177.9025
[M−H]−
1037.4541
875.8895
1188.9608
0
20
40
60
80
100
200
400
600
800
1000
1200

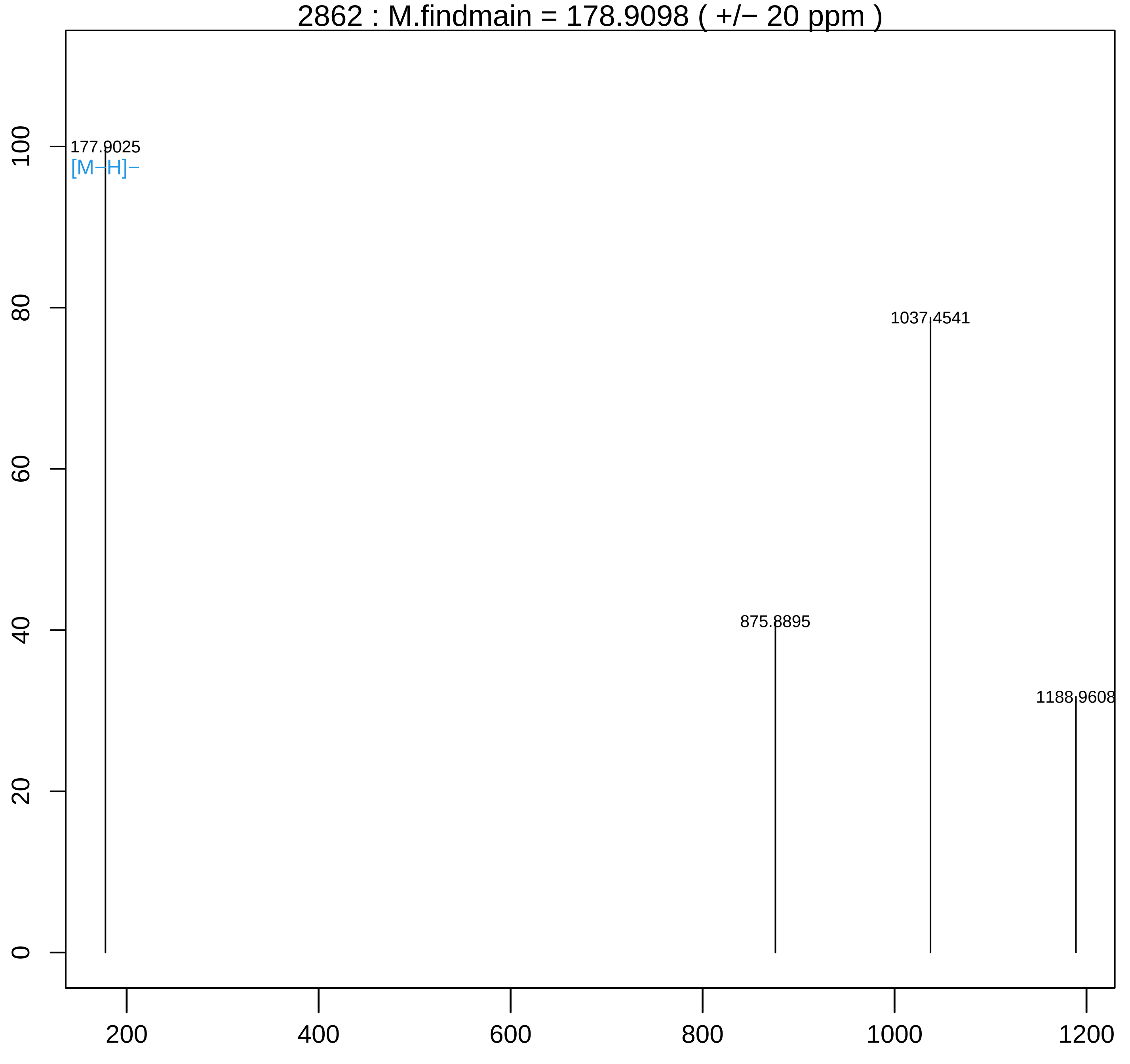
2862 : M.findmain = 178.9098 (+/− 20 ppm)
177.9025
[M−H]−
1037.4541
875.8895
1188.9608
0
20
40
60
80
100
200
400
600
800
1000
1200

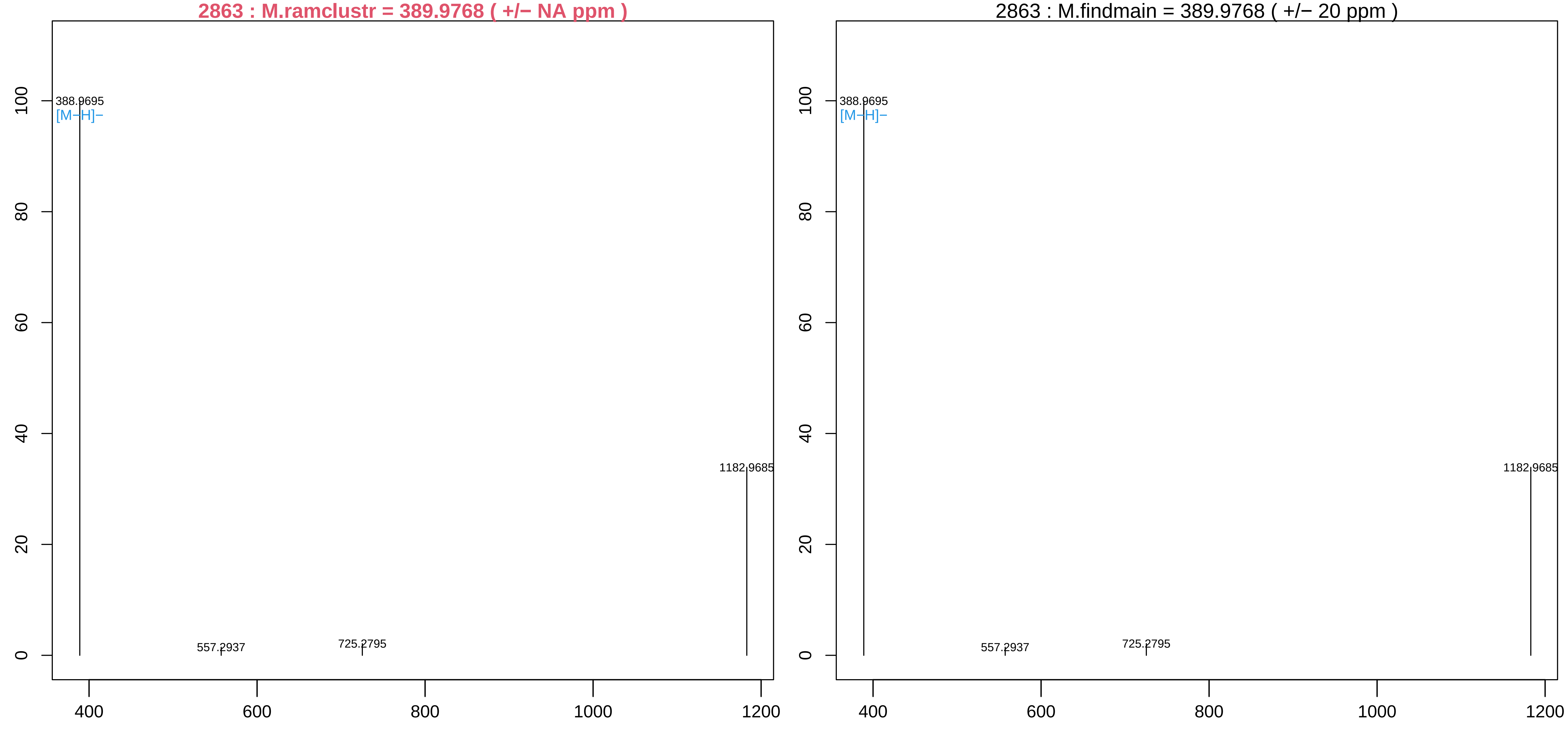
2863 : M.ramclustr = 389.9768 (+/− NA ppm)
2863 : M.findmain = 389.9768 (+/− 20 ppm)
388.9695
[M−H]−
557.2937
725.2795
1182.9685
0
20
40
60
80
100
400
600
800
1000
1200

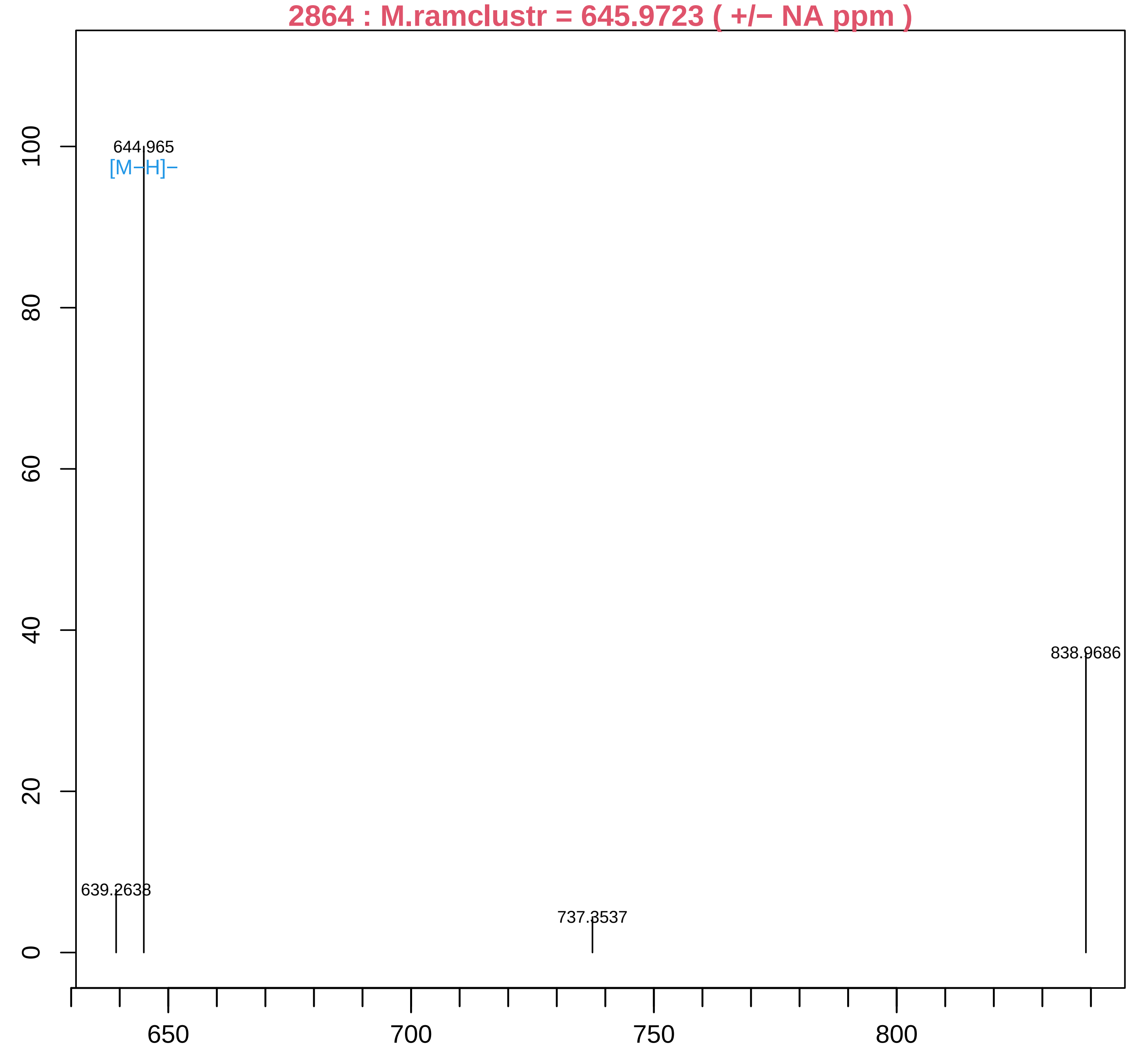
2864 : M.ramclustr = 645.9723 (+/− NA ppm)
644.965
[M−H]−
838.9686
639.2638
737.3537
0
20
40
60
80
100
650
700
750
800

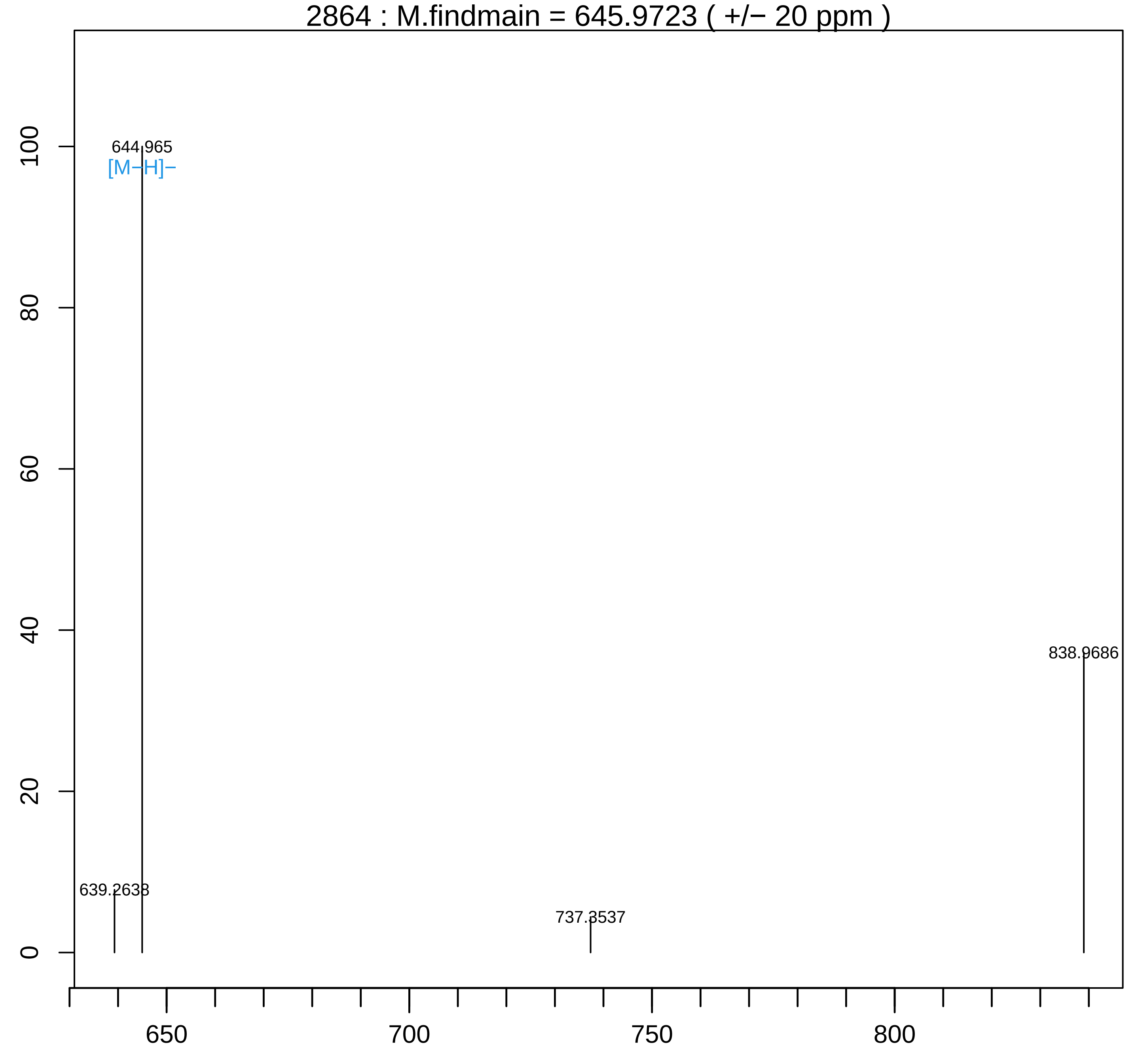
2864 : M.findmain = 645.9723 (+/− 20 ppm)
644.965
[M−H]−
838.9686
639.2638
737.3537
0
20
40
60
80
100
650
700
750
800

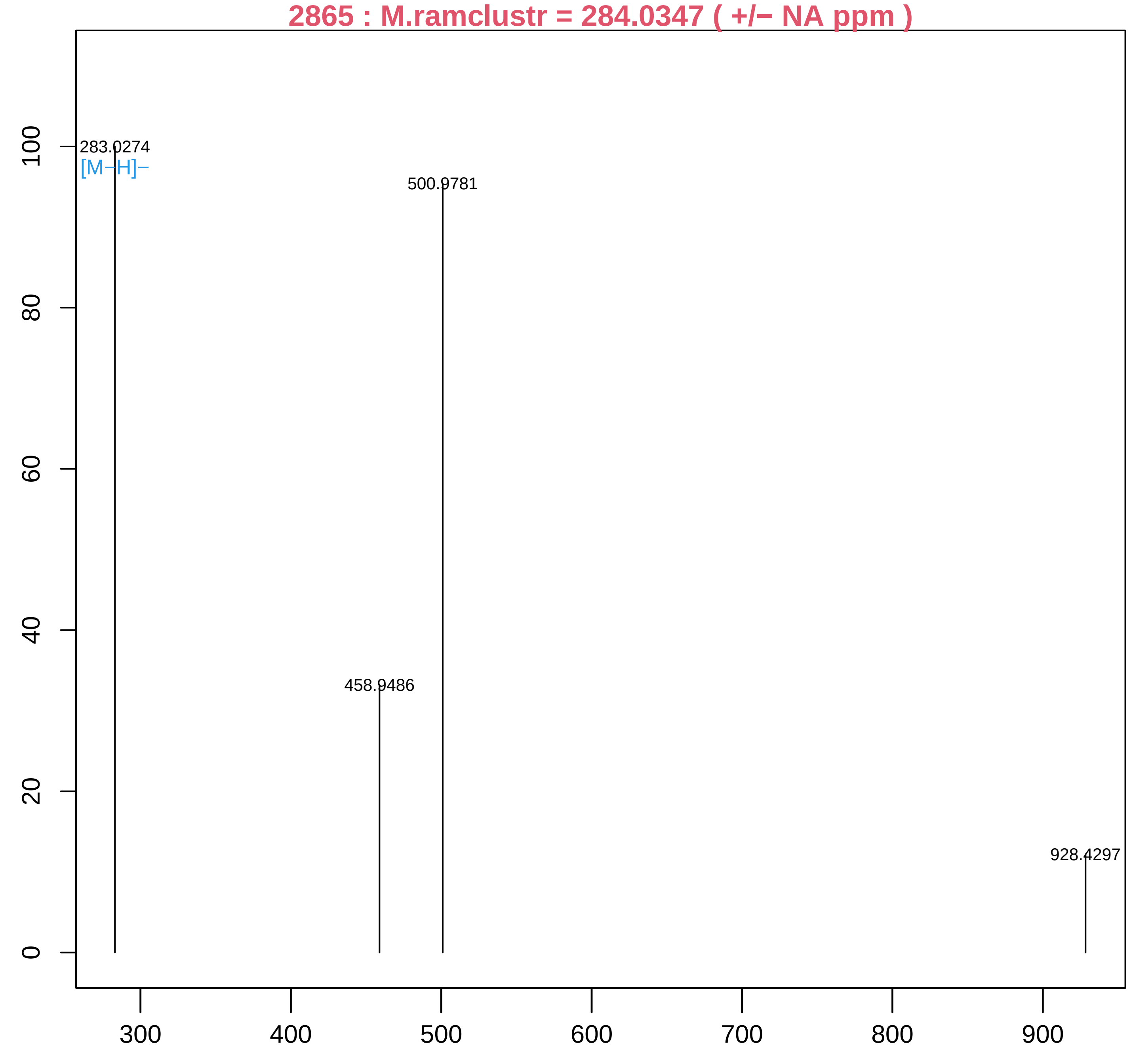
2865 : M.ramclustr = 284.0347 (+/− NA ppm)
283.0274
[M−H]−
500.9781
458.9486
928.4297
0
20
40
60
80
100
300
400
500
600
700
800
900

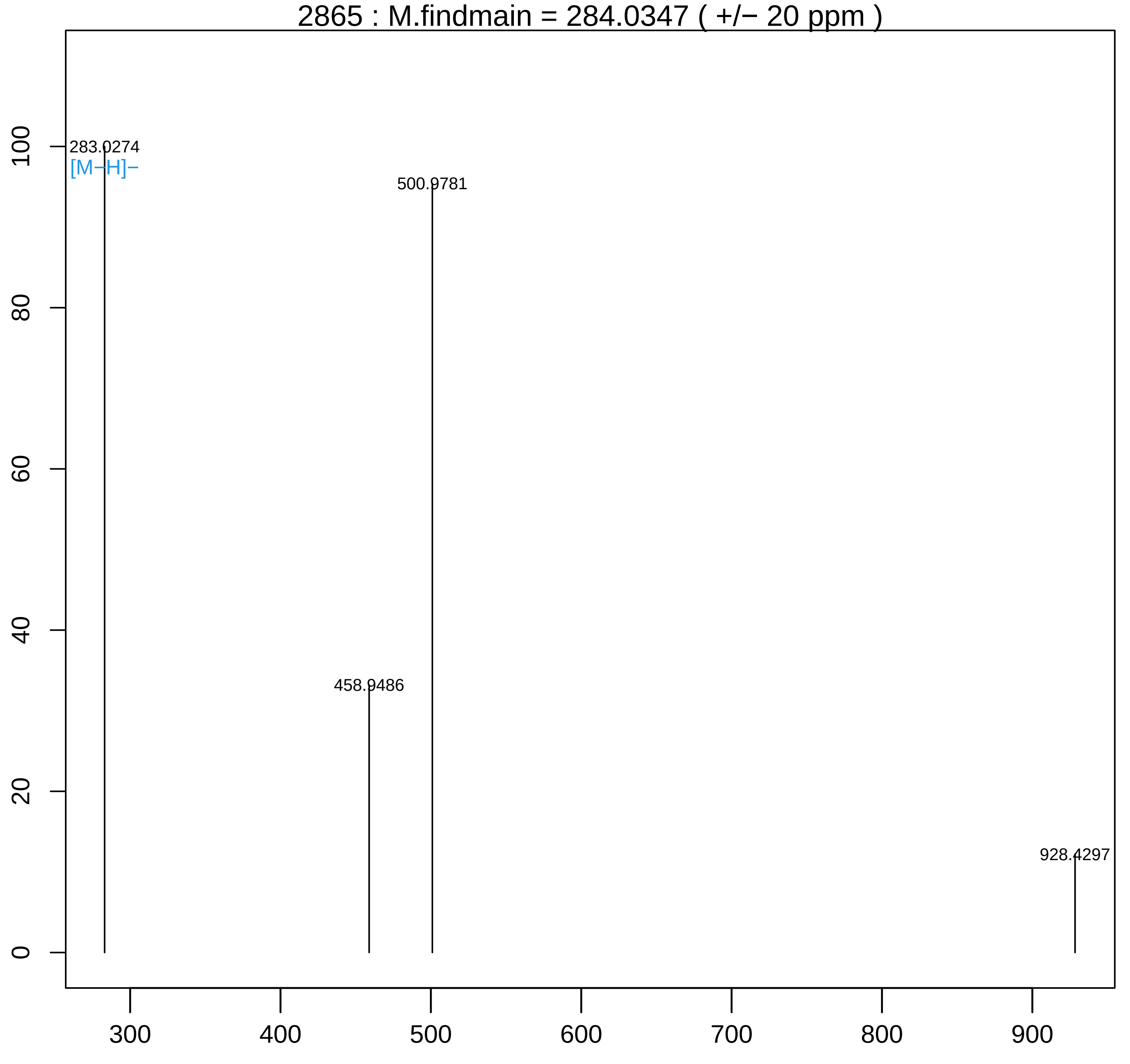
2865 : M.findmain = 284.0347 (+/− 20 ppm)
283.0274
[M−H]−
500.9781
458.9486
928.4297
0
20
40
60
80
100
300
400
500
600
700
800
900

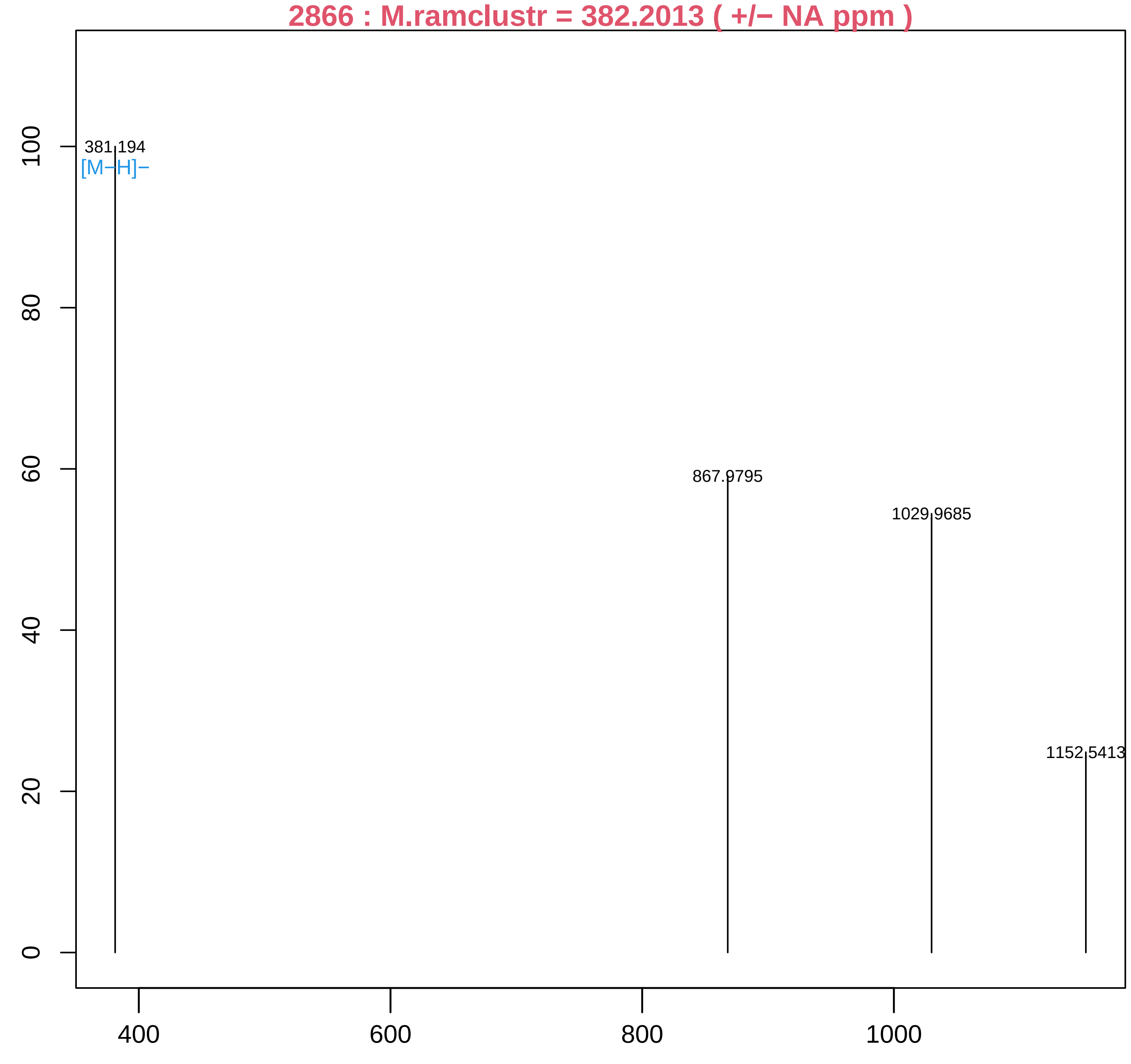

2866 : M.ramclustr = 382.2013 (+/− NA ppm)
381.194
[M−H]−
867.9795
1029.9685
1152.5413
0
20
40
60
80
100
400
600
800
1000

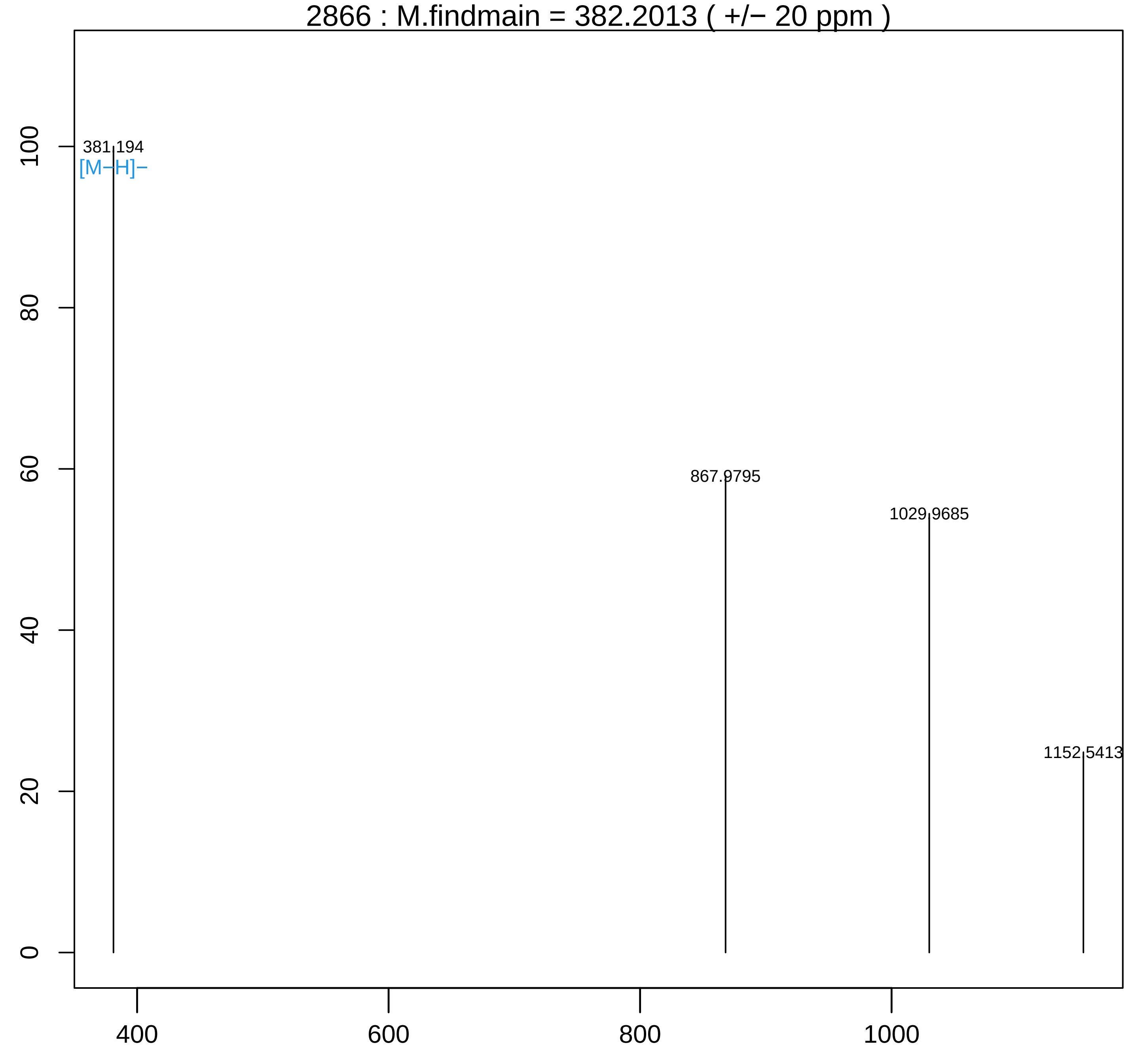

2866 : M.findmain = 382.2013 (+/− 20 ppm)
381.194
[M−H]−
867.9795
1029.9685
1152.5413
0
20
40
60
80
100
400
600
800
1000

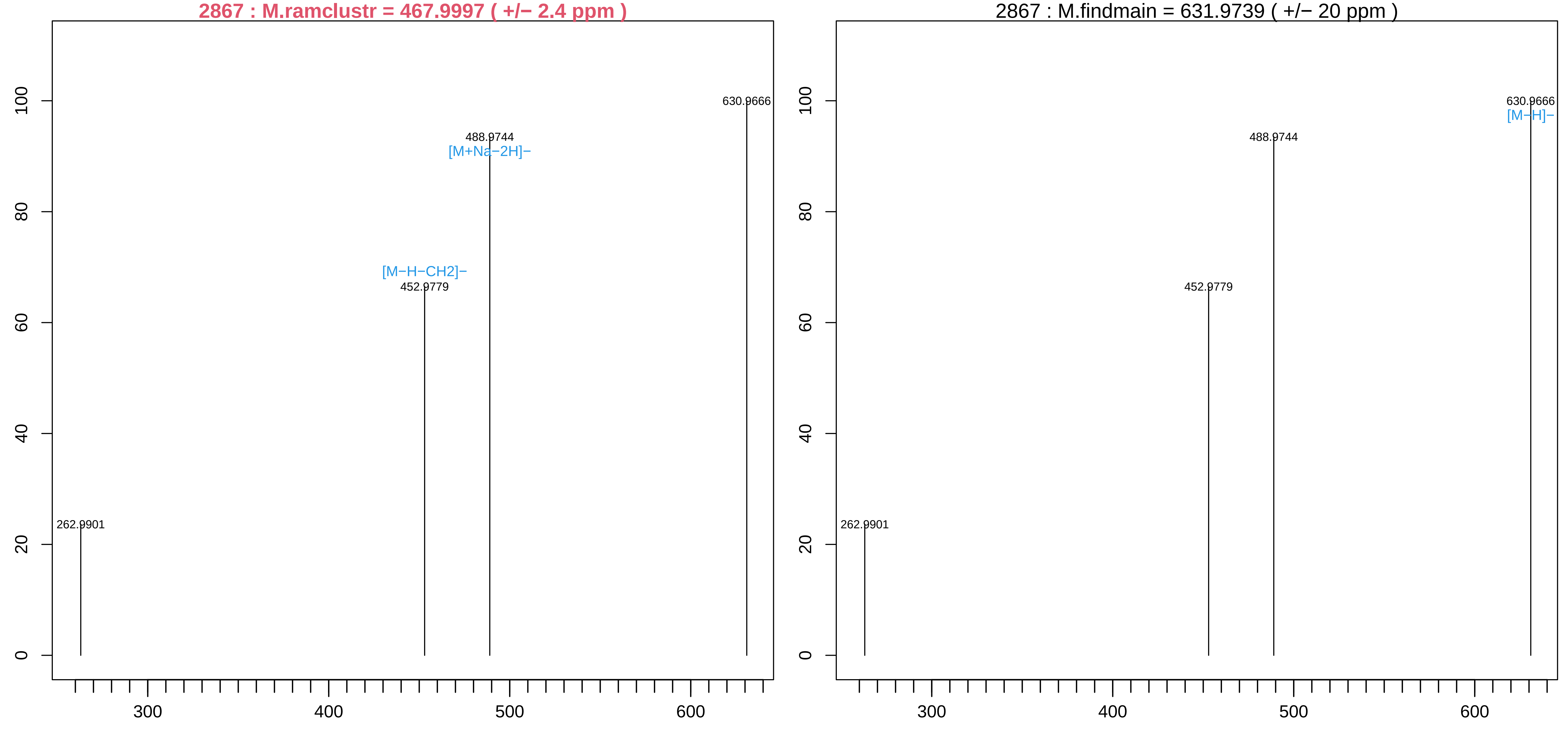
2867 : M.ramclustr = 467.9997 (+/− 2.4 ppm)
630.9666
488.9744
[M+Na−2H]−
[M−H−CH2]−
452.9779
262.9901
0
20
40
60
80
100
300
400
500
600
2867 : M.findmain = 631.9739 (+/− 20 ppm)
630.9666
[M−H]−
488.9744
452.9779
262.9901
0
20
40
60
80
100
300
400
500
600

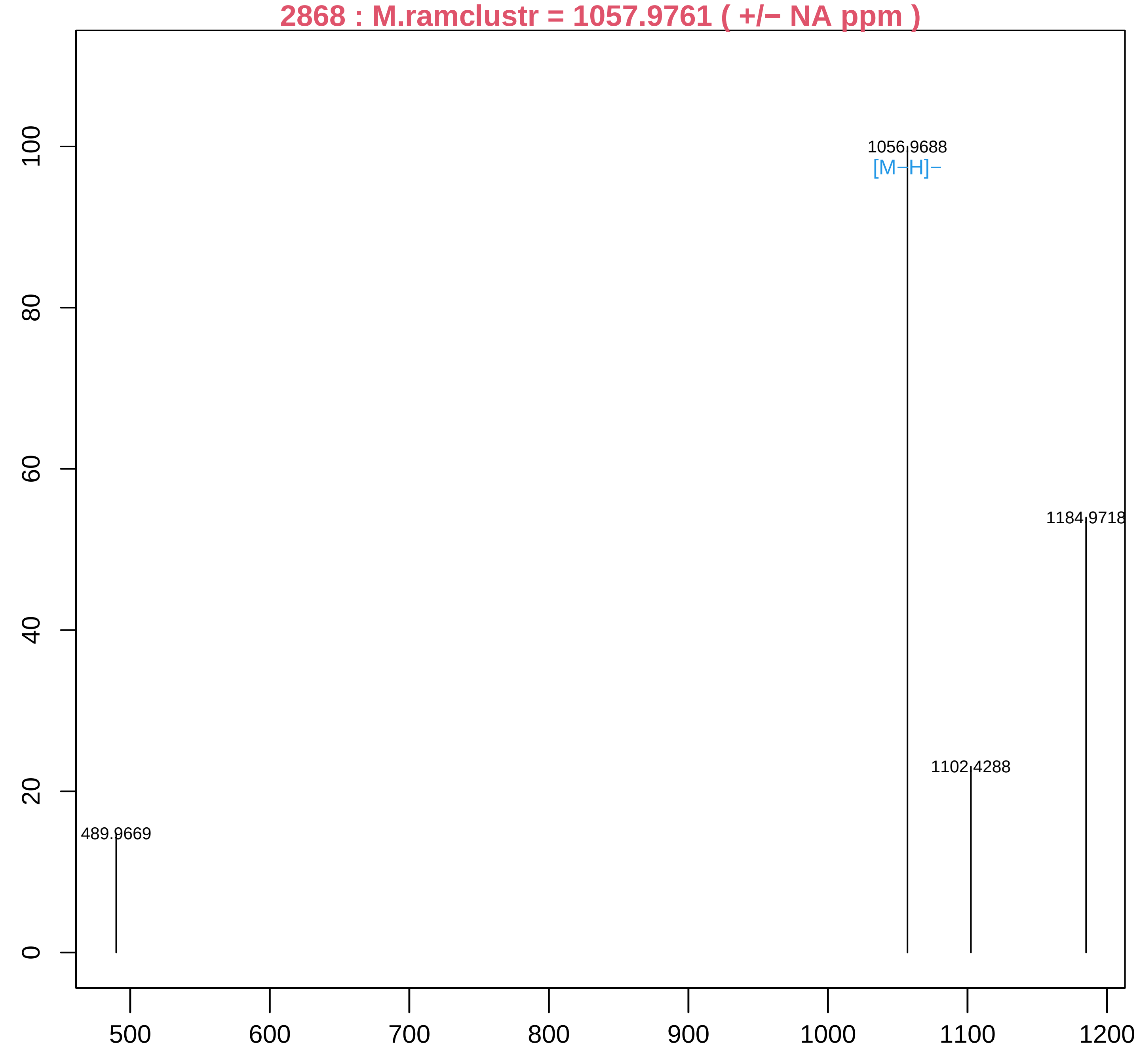
2868 : M.ramclustr = 1057.9761 (+/− NA ppm)
1056.9688
[M−H]−
1184.9718
1102.4288
489.9669
0
20
40
60
80
100
500
600
700
800
900
1000
1100
1200

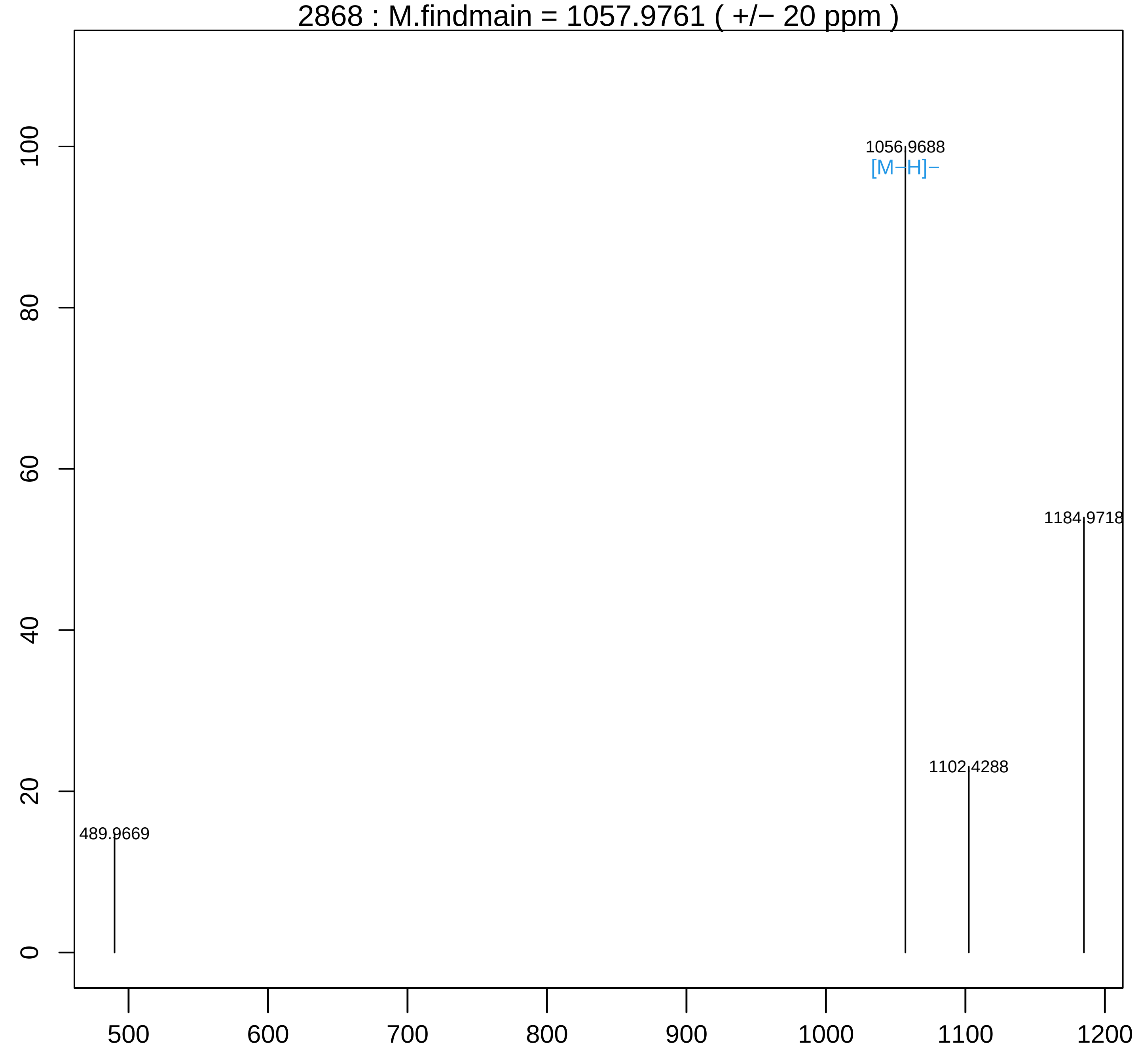
2868 : M.findmain = 1057.9761 (+/− 20 ppm)
1056.9688
[M−H]−
1184.9718
1102.4288
489.9669
0
20
40
60
80
100
500
600
700
800
900
1000
1100
1200

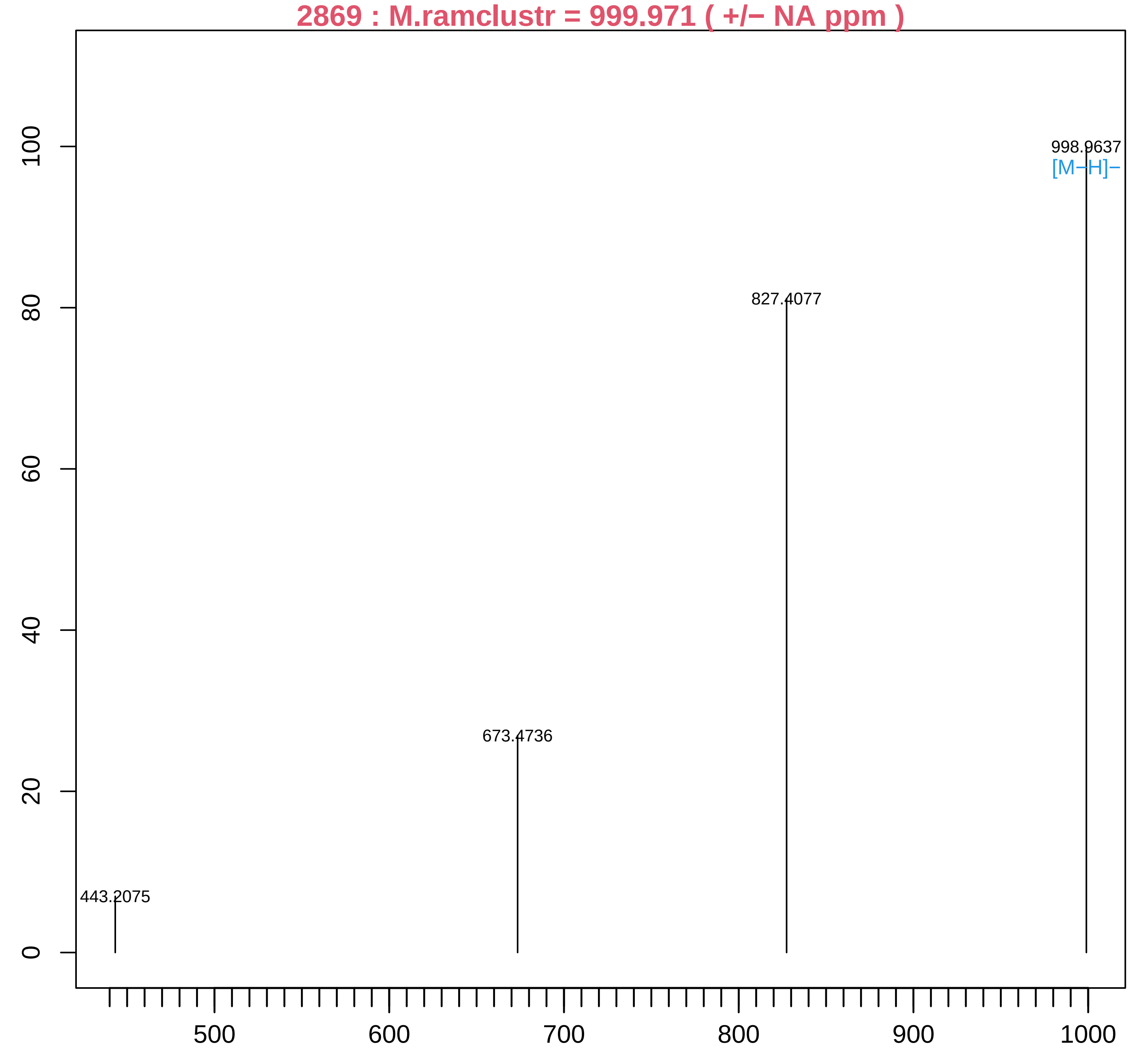
2869 : M.ramclustr = 999.971 (+/− NA ppm)
998.9637
[M−H]−
827.4077
673.4736
443.2075
0
20
40
60
80
100
500
600
700
800
900
1000

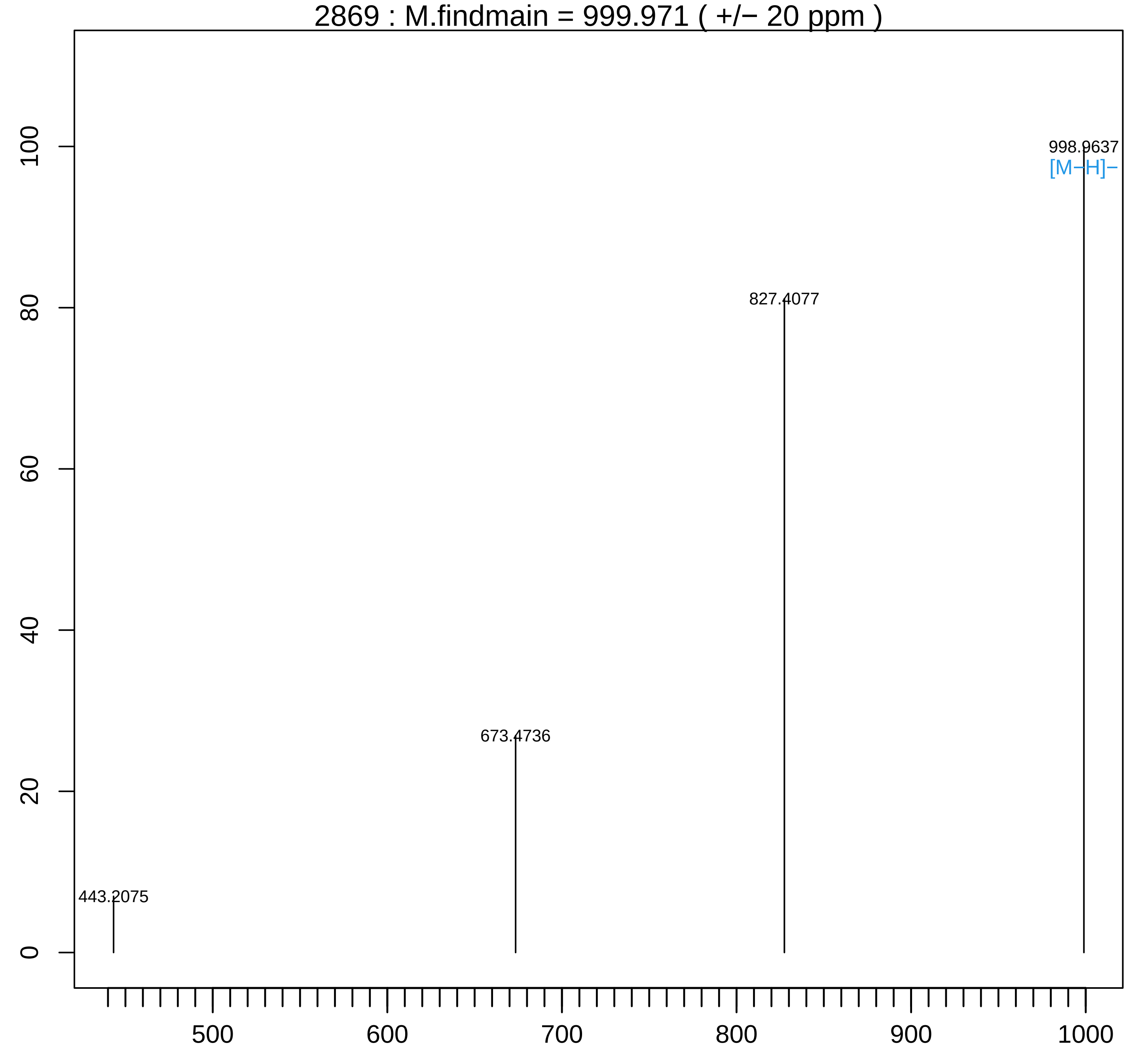
2869 : M.findmain = 999.971 (+/− 20 ppm)
998.9637
[M−H]−
827.4077
673.4736
443.2075
0
20
40
60
80
100
500
600
700
800
900
1000

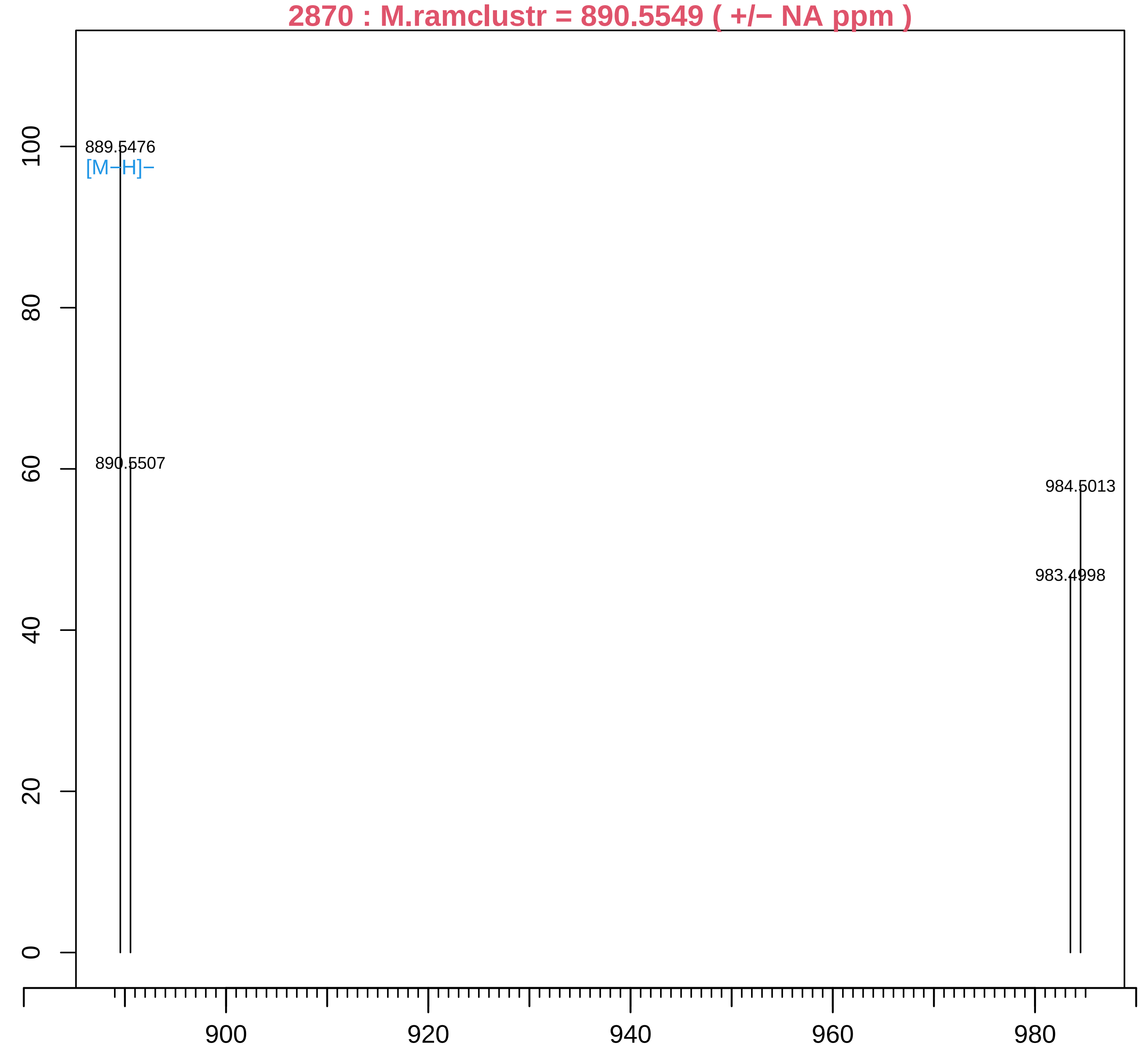
2870 : M.ramclustr = 890.5549 (+/− NA ppm)
889.5476
[M−H]−
890.5507
984.5013
983.4998
0
20
40
60
80
100
900
920
940
960
980

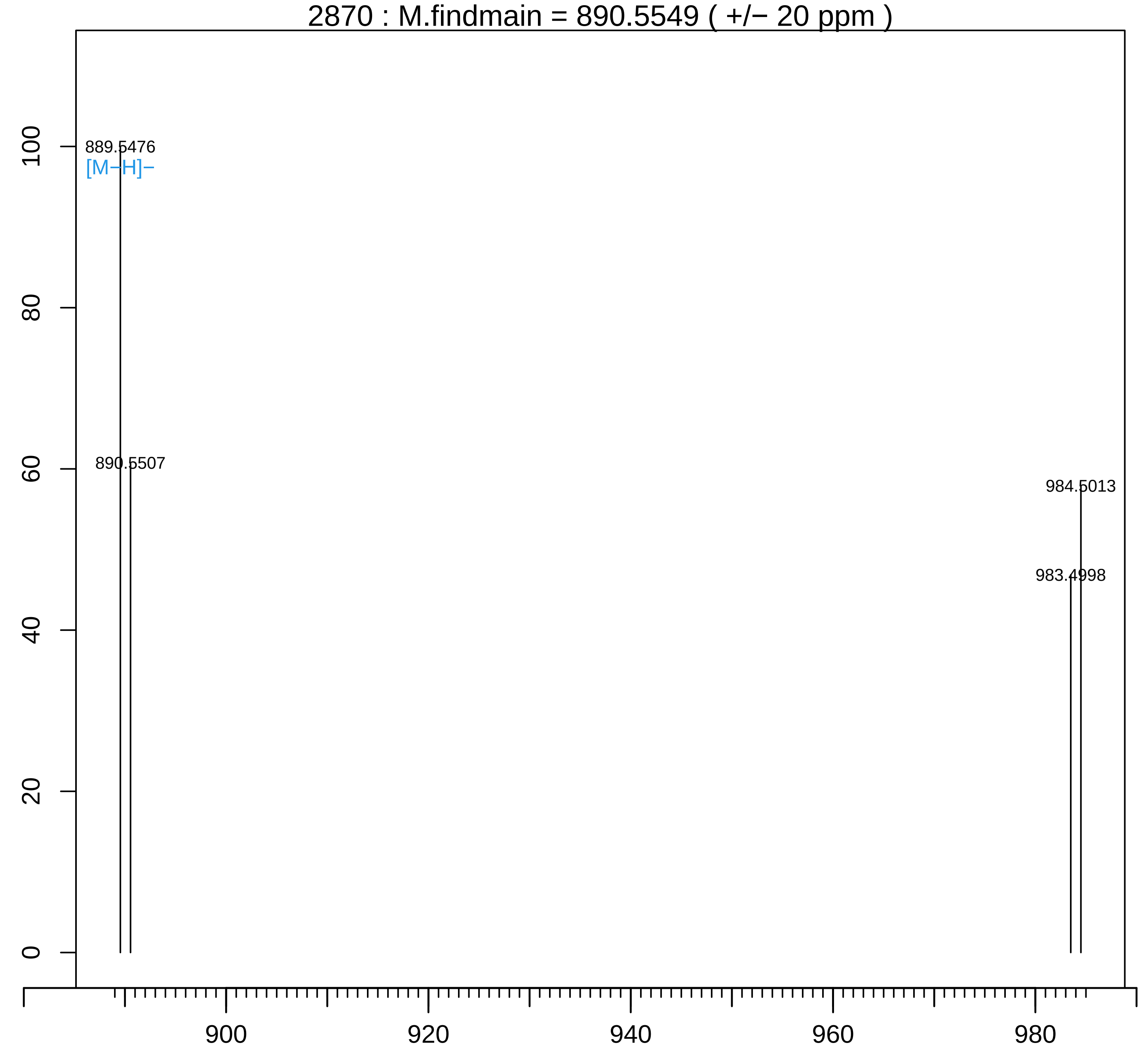
2870 : M.findmain = 890.5549 (+/− 20 ppm)
889.5476
[M−H]−
890.5507
984.5013
983.4998
0
20
40
60
80
100
900
920
940
960
980

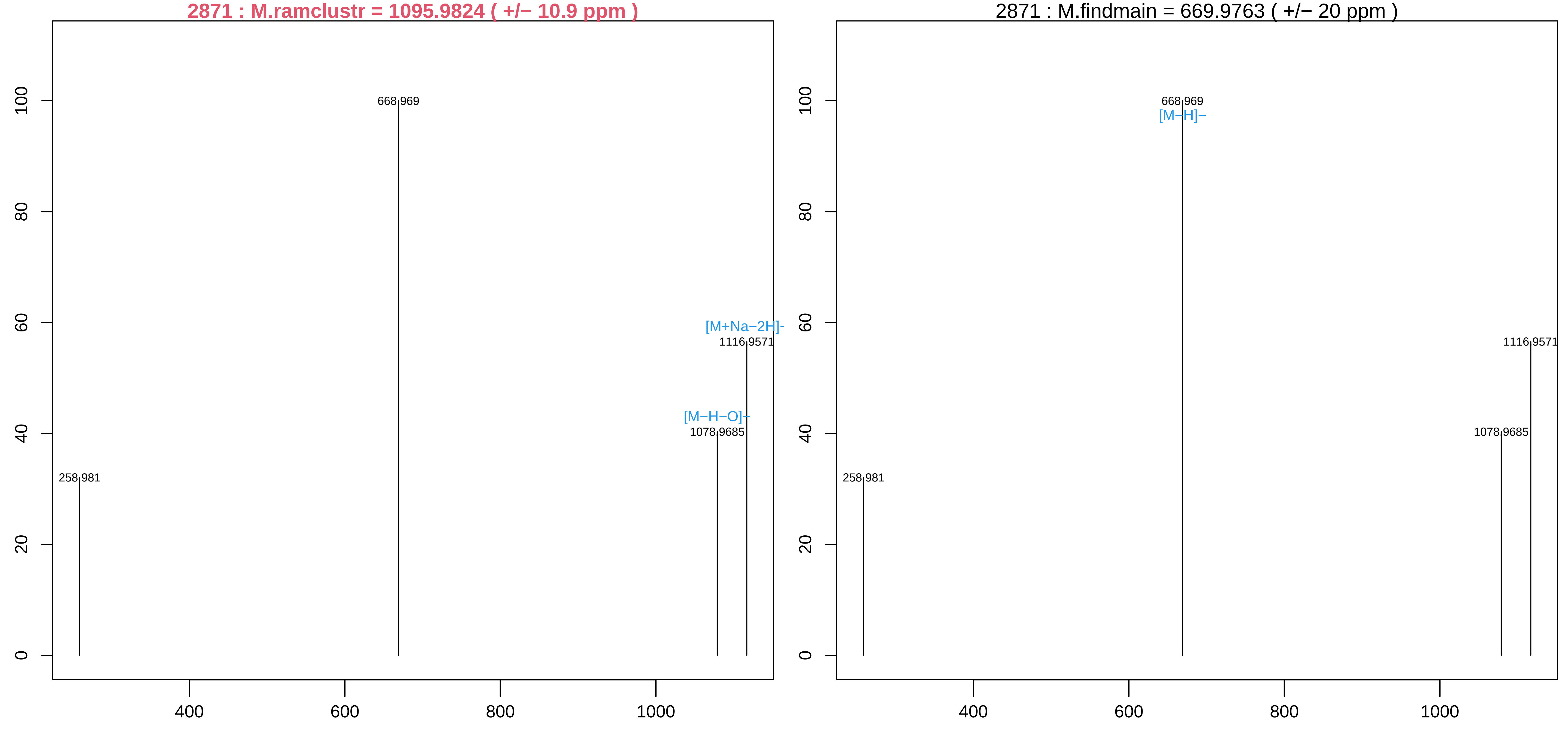
2871 : M.ramclustr = 1095.9824 (+/− 10.9 ppm)
668.969
[M+Na−2H]-
1116.9571
[M−H−O]-
1078.9685
258.981
2871 : M.findmain = 669.9763 (+/− 20 ppm)
668.969
[M−H]−
1116.9571
1078.9685
258.981

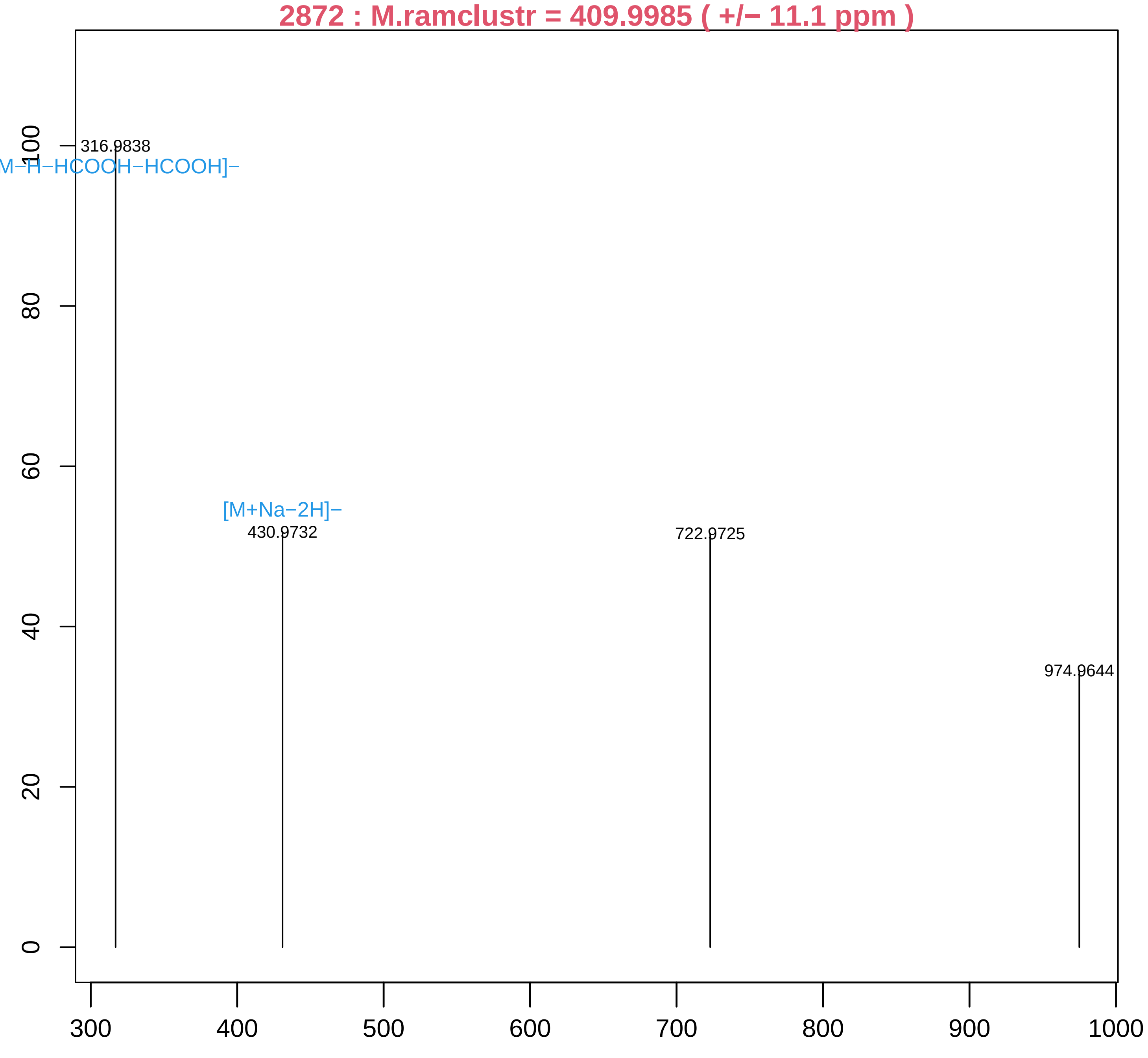
2872 : M.ramclustr = 409.9985 (+/− 11.1 ppm)
316.9838
[M−H−HCOOH−HCOOH]−
[M+Na−2H]−
430.9732
722.9725
974.9644

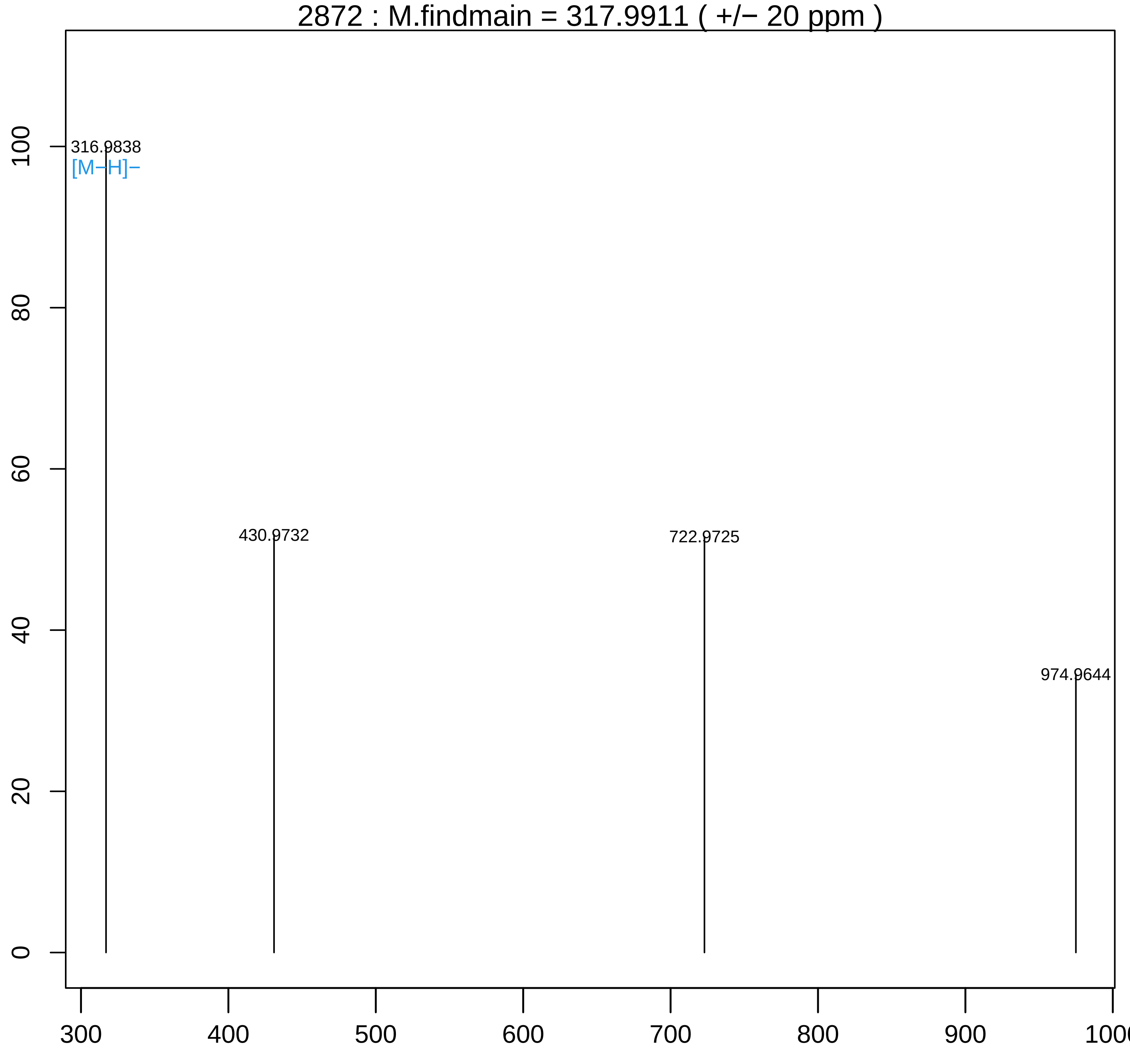
2872 : M.findmain = 317.9911 (+/− 20 ppm)
316.9838
[M−H]−
430.9732
722.9725
974.9644

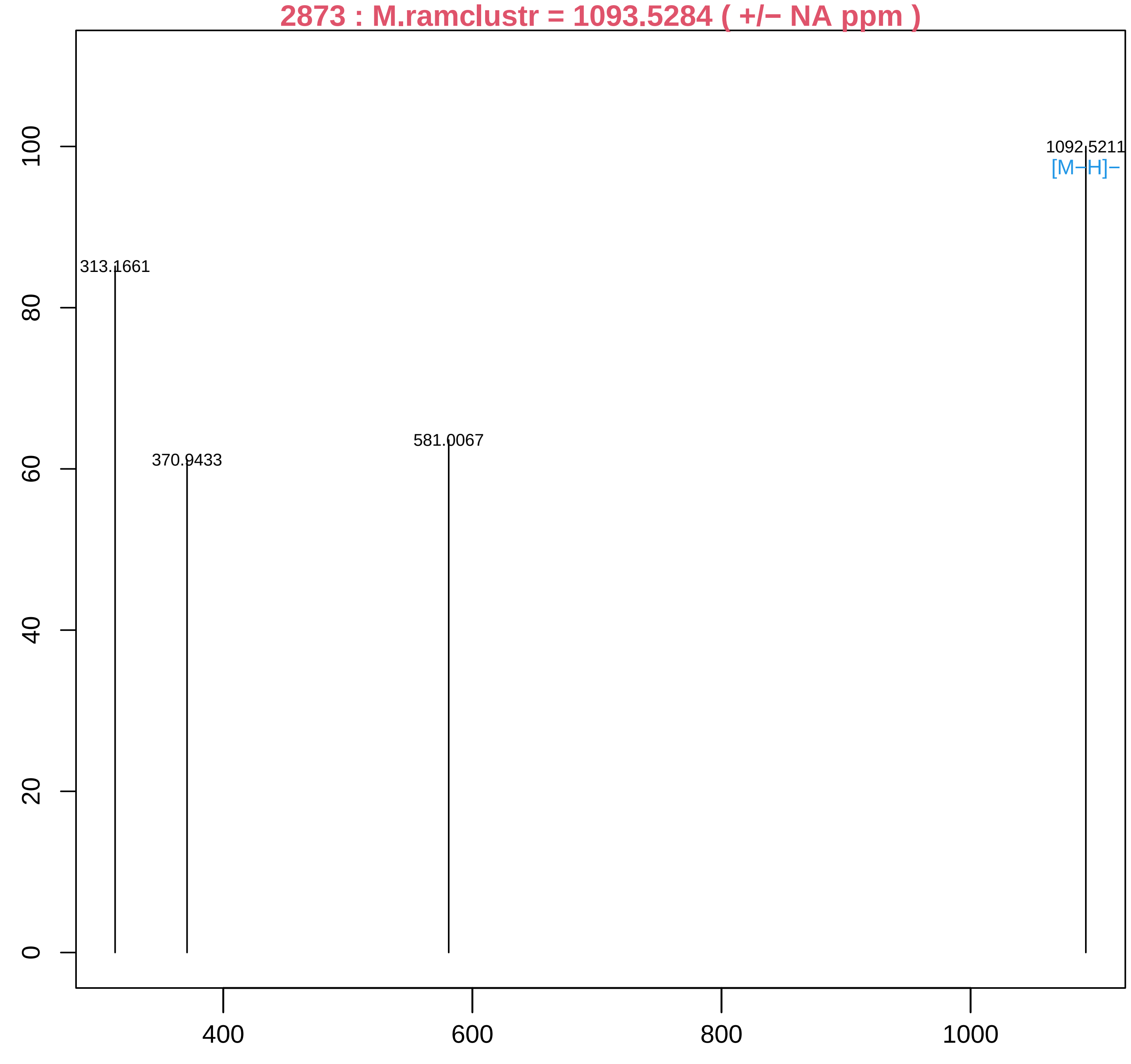
2873 : M.ramclustr = 1093.5284 (+/− NA ppm)
313.1661
370.9433
581.0067
1092.5211
[M−H]−
0
20
40
60
80
100
400
600
800
1000
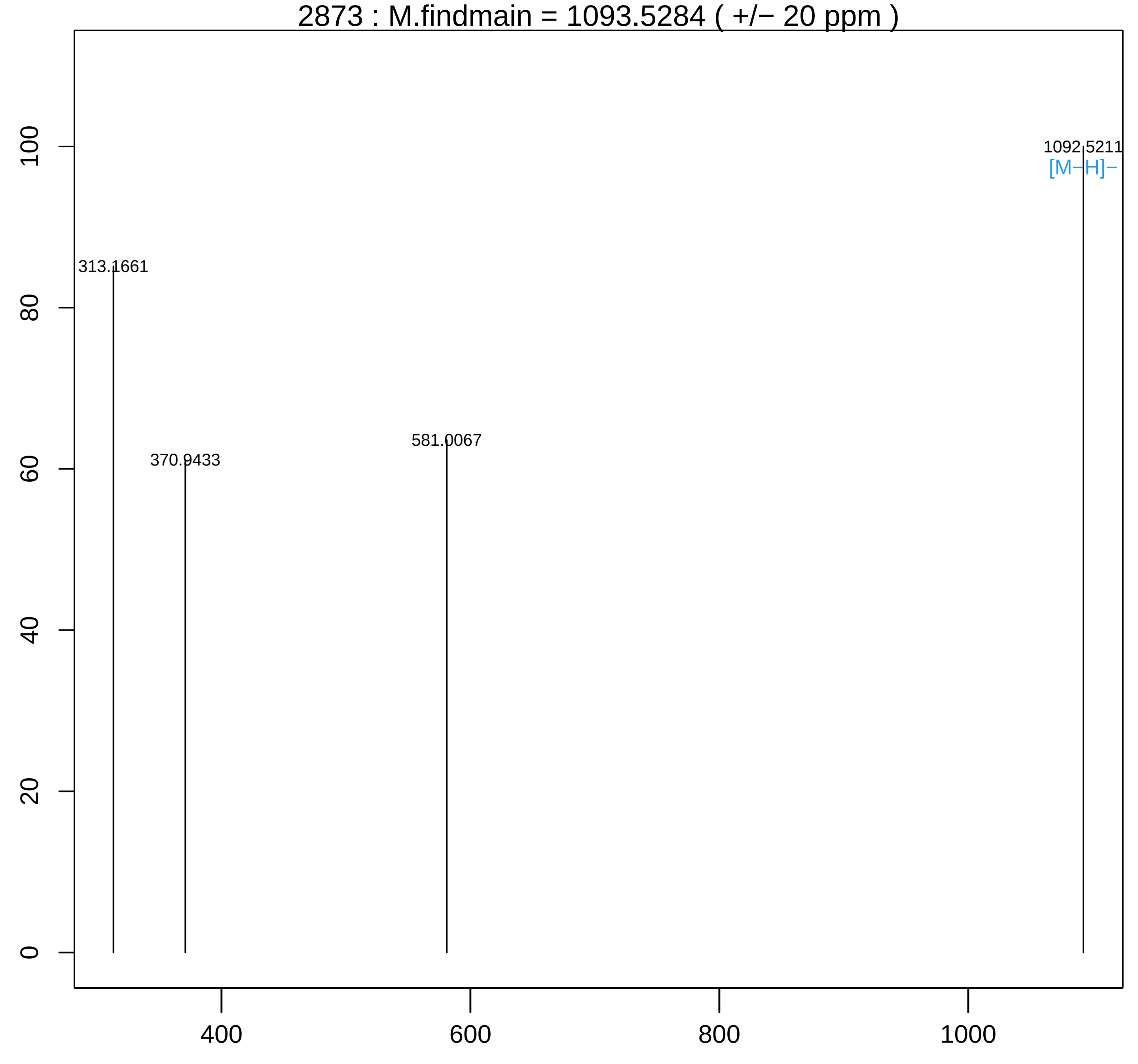
2873 : M.findmain = 1093.5284 (+/− 20 ppm)
313.1661
370.9433
581.0067
1092.5211
[M−H]−
0
20
40
60
80
100
400
600
800
1000

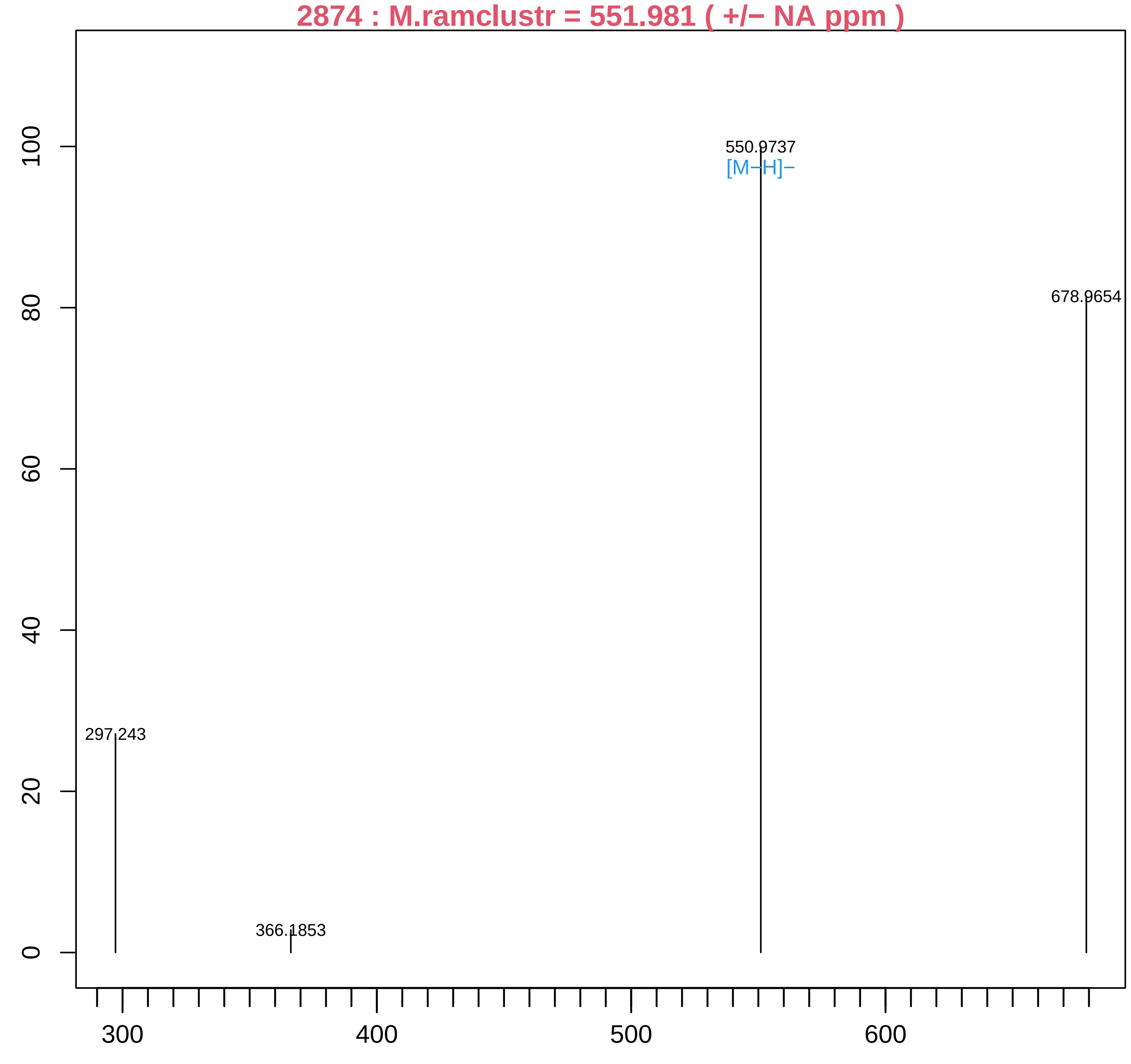
2874 : M.ramclustr = 551.981 (+/− NA ppm)
550.9737
[M−H]−
678.9654
297.243
366.1853
0
20
40
60
80
100
300
400
500
600
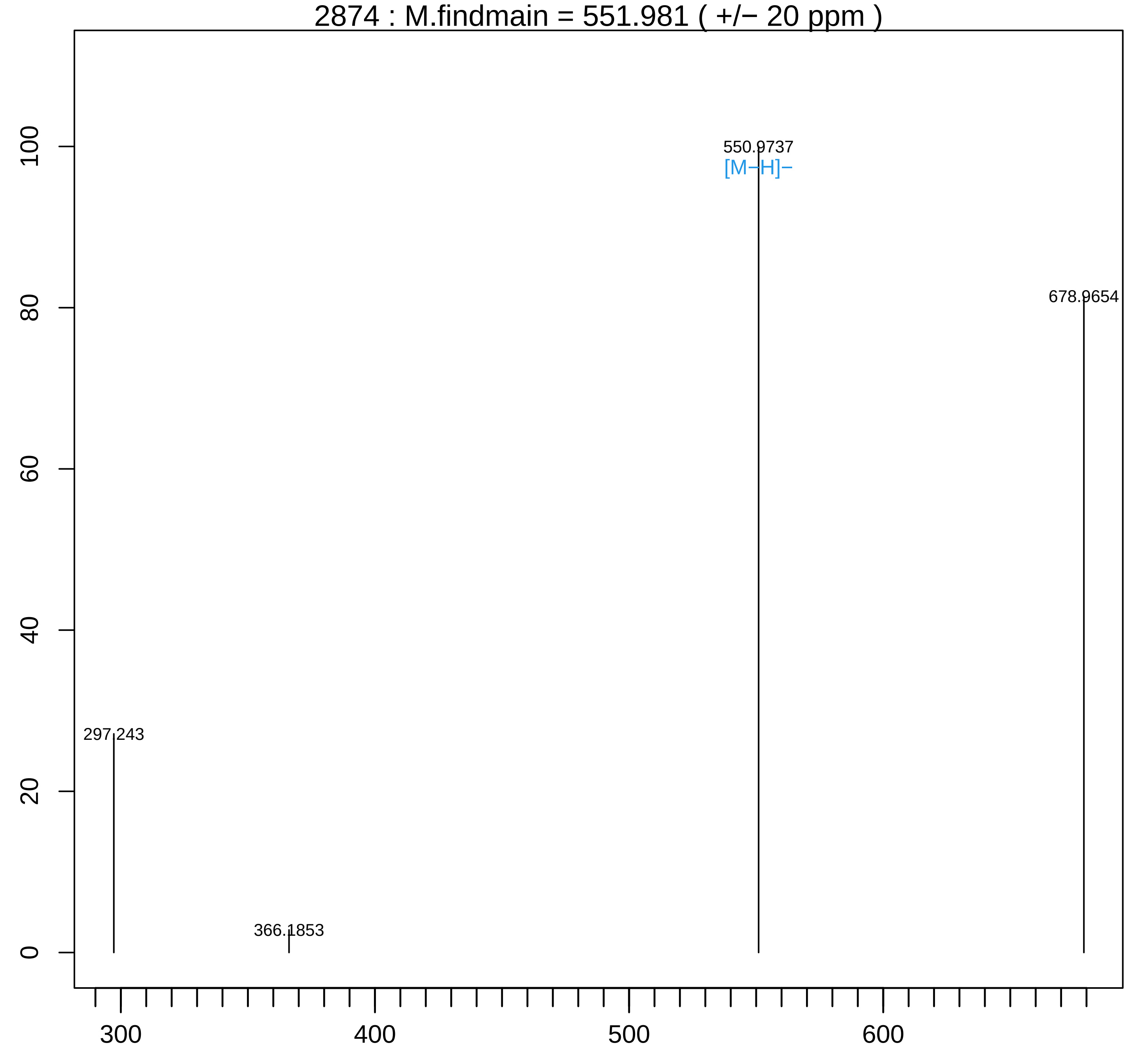
2874 : M.findmain = 551.981 (+/− 20 ppm)
550.9737
[M−H]−
678.9654
297.243
366.1853
0
20
40
60
80
100
300
400
500
600

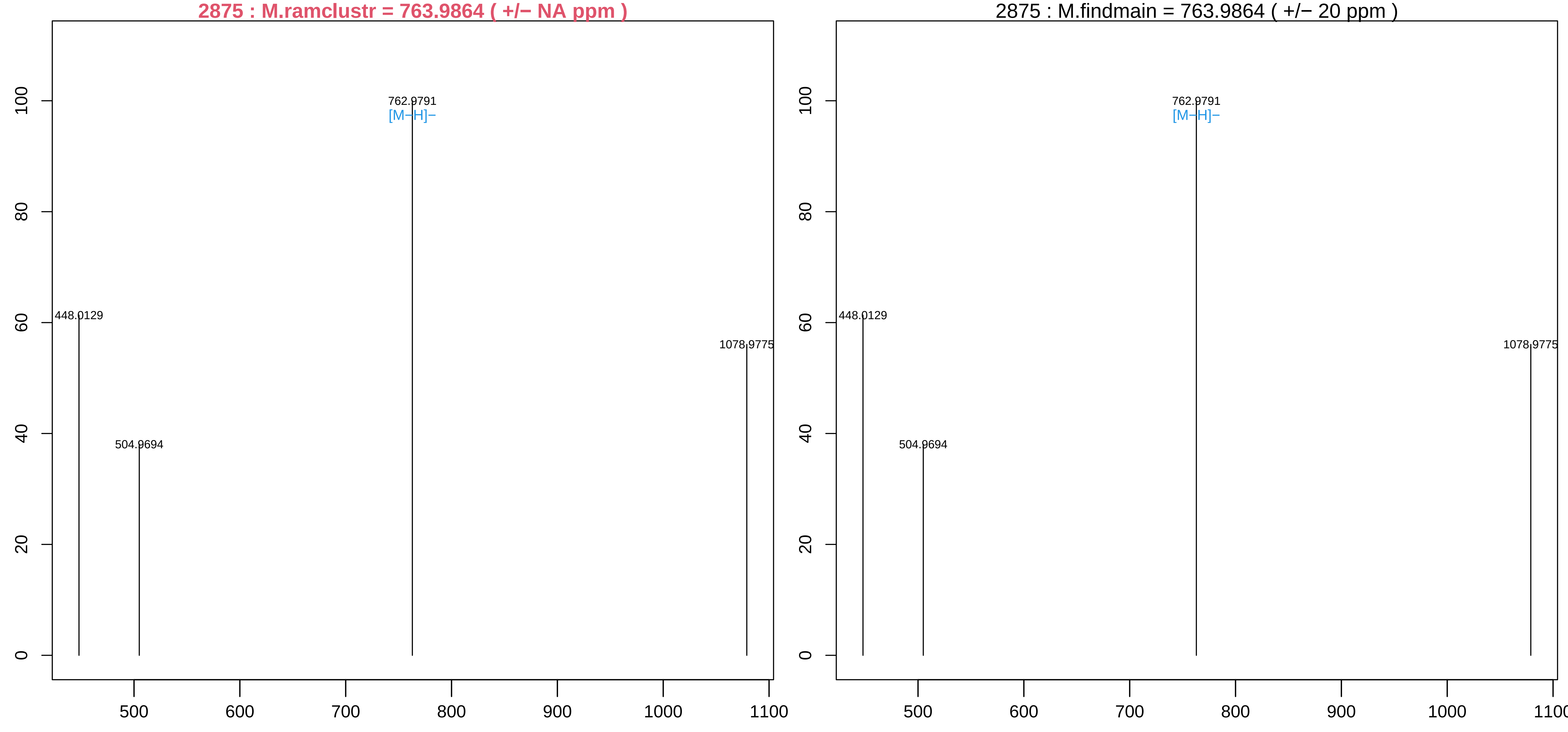
2875 : M.ramclustr = 763.9864 (+/− NA ppm)
2875 : M.findmain = 763.9864 (+/− 20 ppm)
762.9791
[M−H]−
448.0129
504.9694
1078.9775
0
20
40
60
80
100
500
600
700
800
900
1000
1100

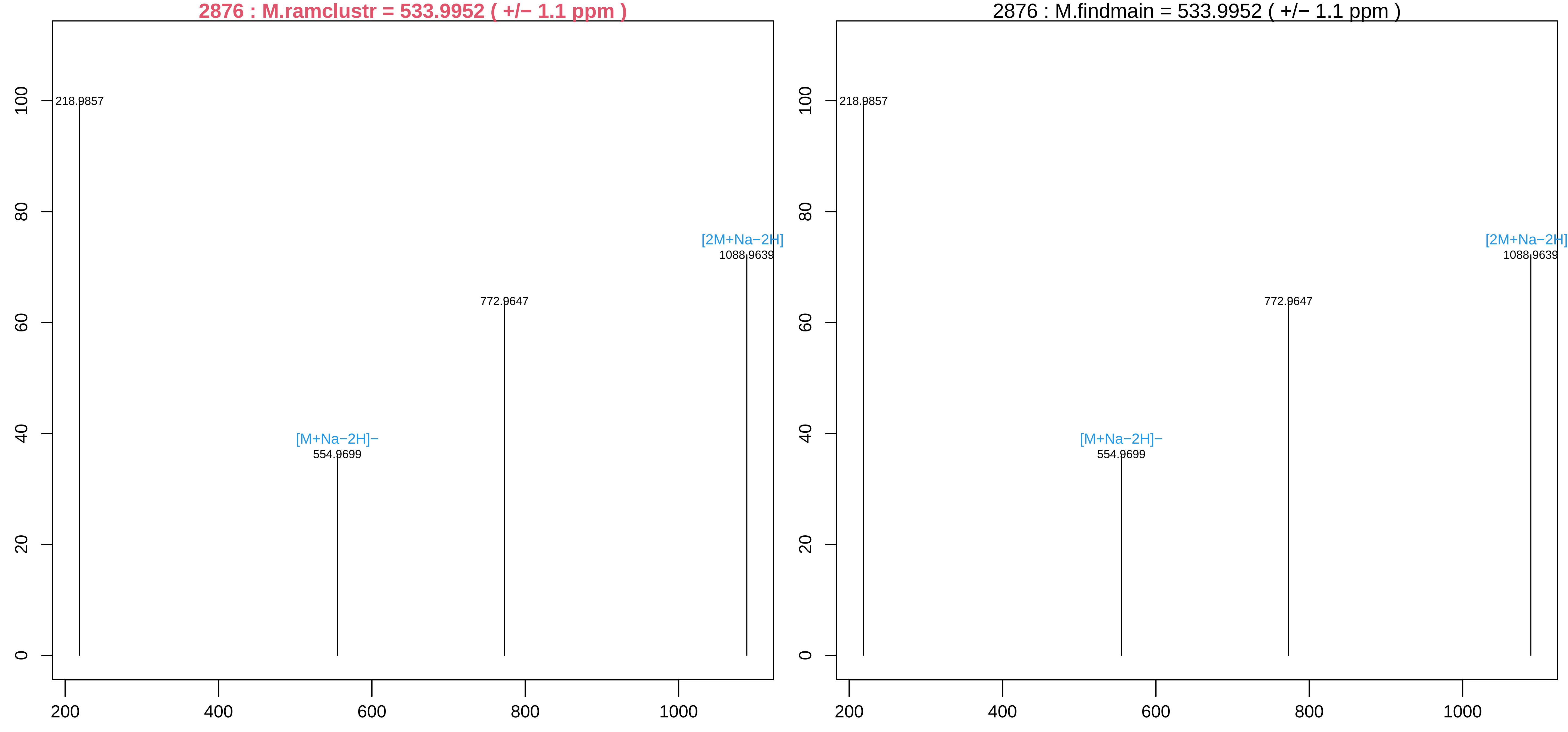

2876 : M.ramclustr = 533.9952 (+/− 1.1 ppm)
218.9857
[M+Na−2H]−
554.9699
772.9647
[2M+Na−2H]
1088.9639
0
20
40
60
80
100
200
400
600
800
1000
2876 : M.findmain = 533.9952 (+/− 1.1 ppm)
218.9857
[M+Na−2H]−
554.9699
772.9647
[2M+Na−2H]
1088.9639
0
20
40
60
80
100
200
400
600
800
1000

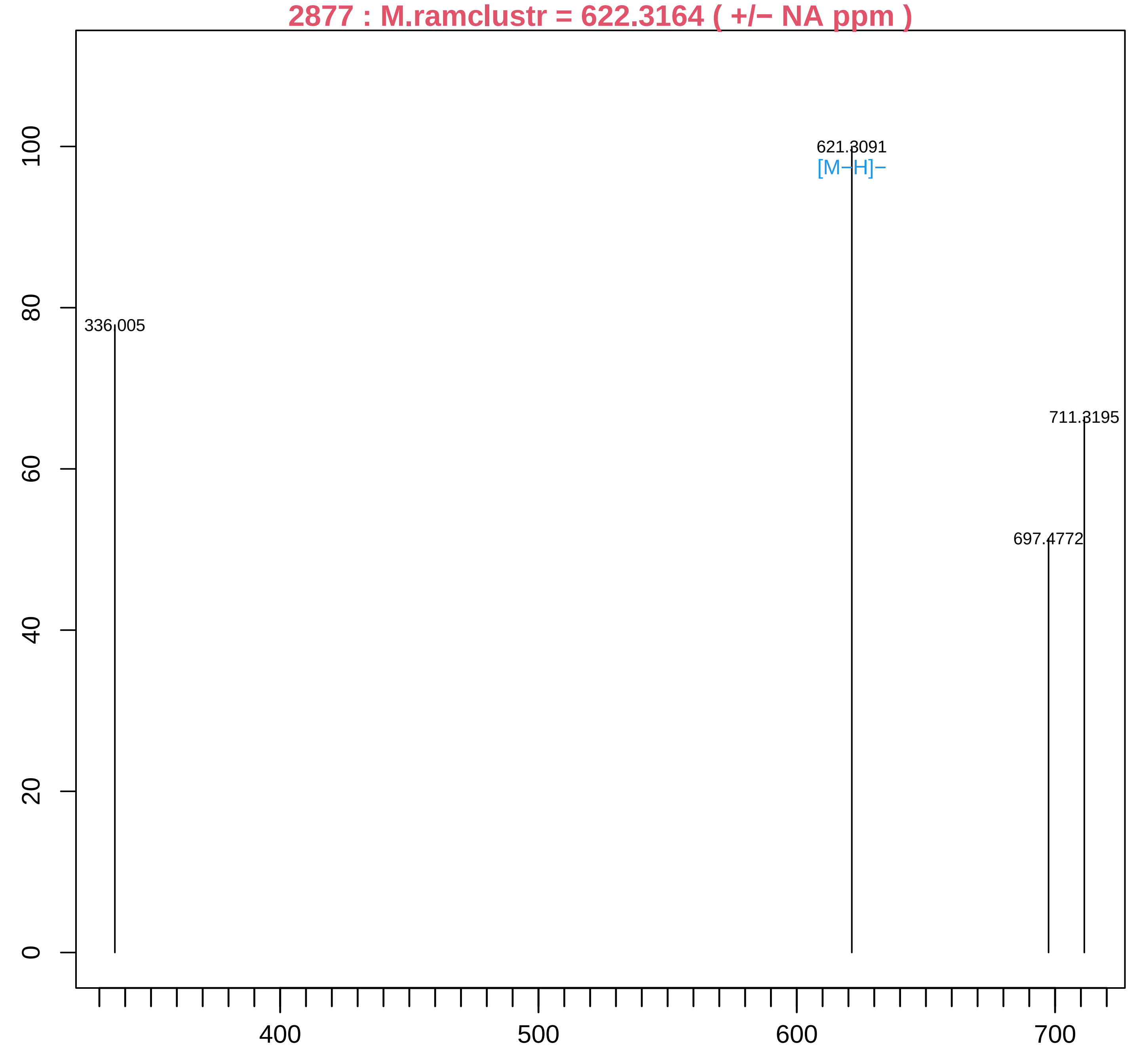
2877 : M.ramclustr = 622.3164 (+/− NA ppm)
336.005
621.3091
[M−H]−
697.4772
711.3195
0
20
40
60
80
100
400
500
600
700

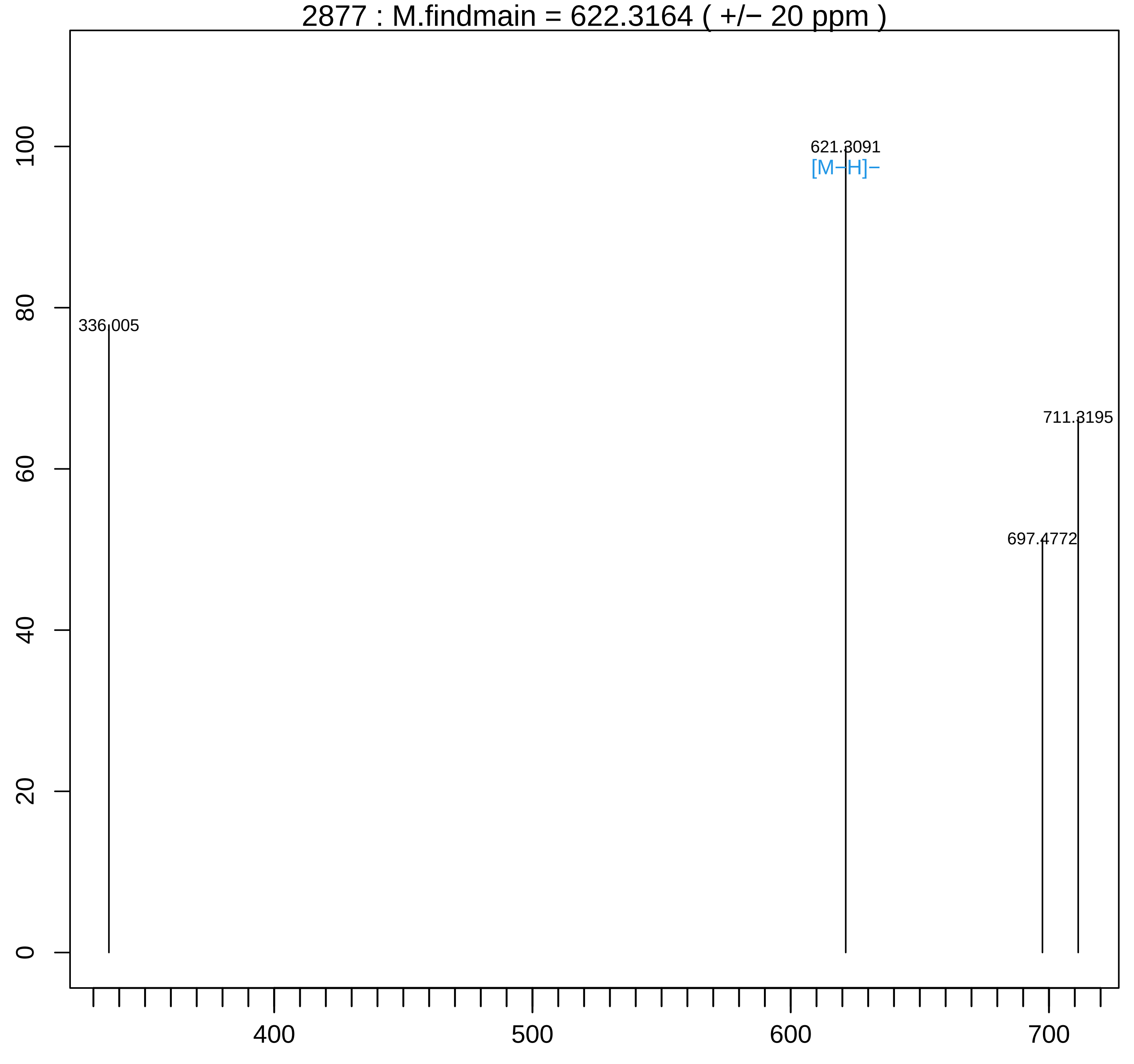
2877 : M.findmain = 622.3164 (+/− 20 ppm)
336.005
621.3091
[M−H]−
697.4772
711.3195
0
20
40
60
80
100
400
500
600
700

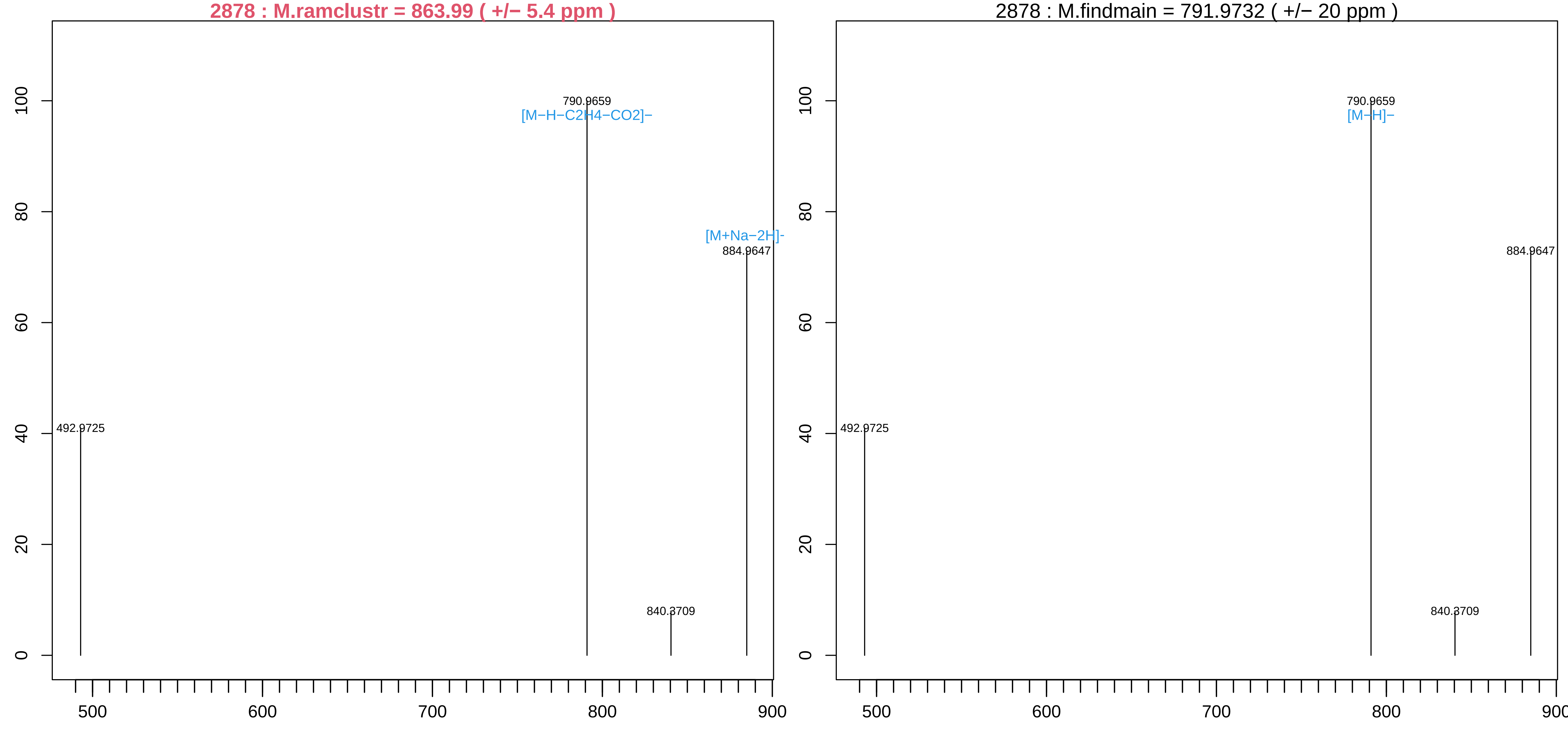
2878 : M.ramclustr = 863.99 (+/− 5.4 ppm)
790.9659
[M−H−C2H4−CO2]−
[M+Na−2H]-
884.9647
492.9725
840.3709
0
20
40
60
80
100
500
600
700
800
900
2878 : M.findmain = 791.9732 (+/− 20 ppm)
790.9659
[M−H]−
884.9647
492.9725
840.3709
0
20
40
60
80
100
500
600
700
800
900

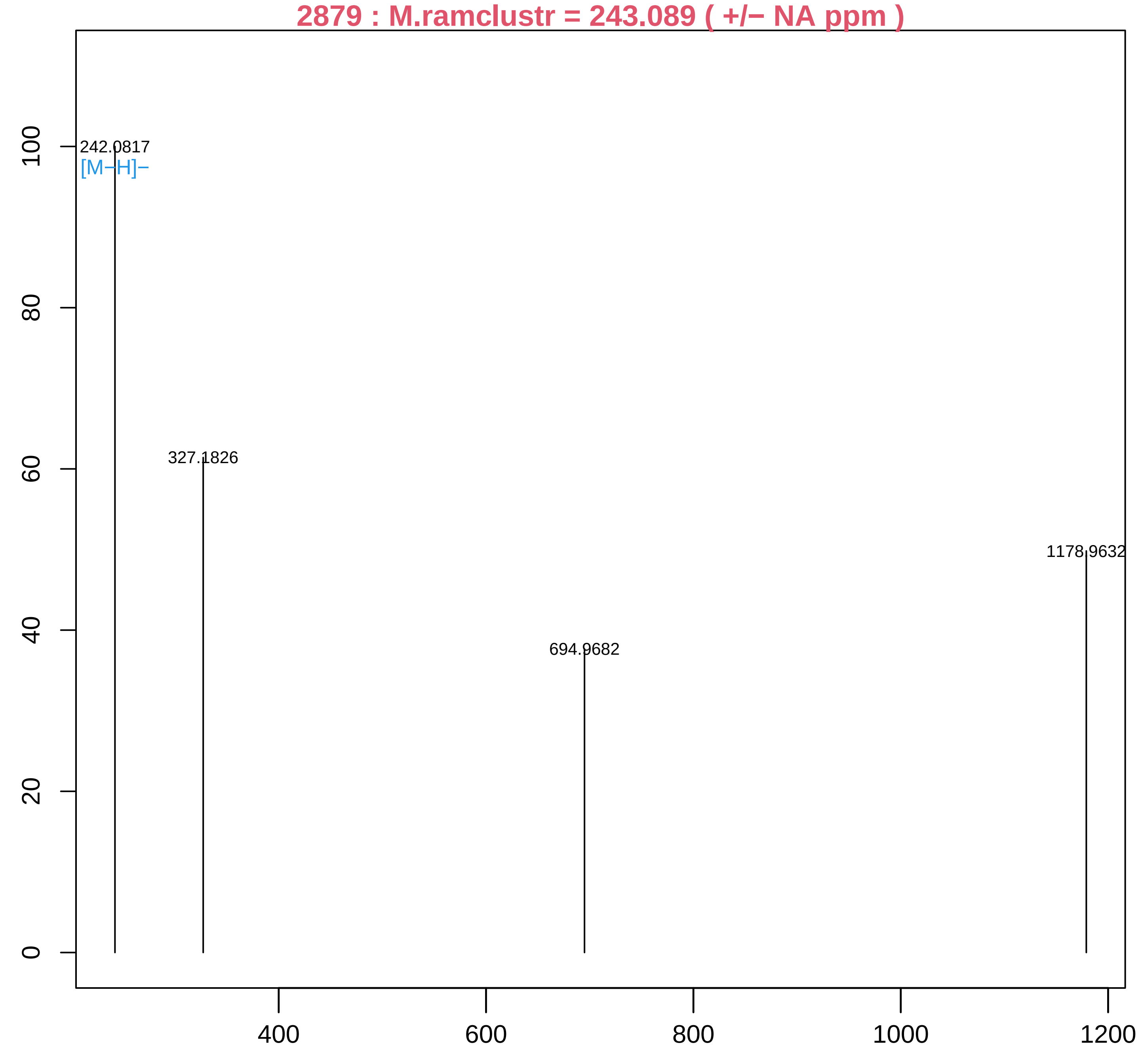

2879 : M.ramclustr = 243.089 (+/− NA ppm)
242.0817
[M−H]−
327.1826
694.9682
1178.9632
0
20
40
60
80
100
400
600
800
1000
1200

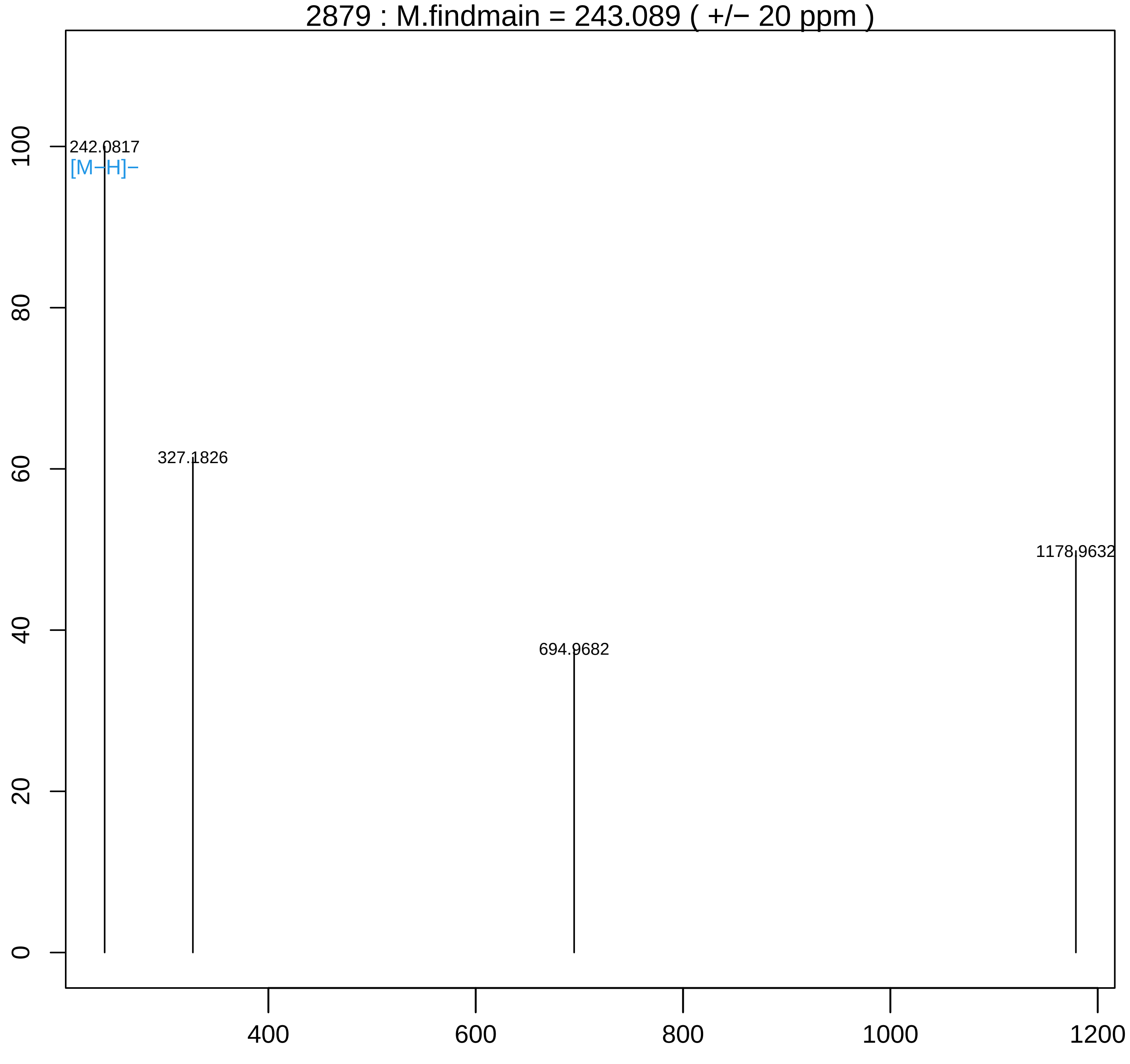

2879 : M.findmain = 243.089 (+/− 20 ppm)
242.0817
[M−H]−
327.1826
694.9682
1178.9632
0
20
40
60
80
100
400
600
800
1000
1200

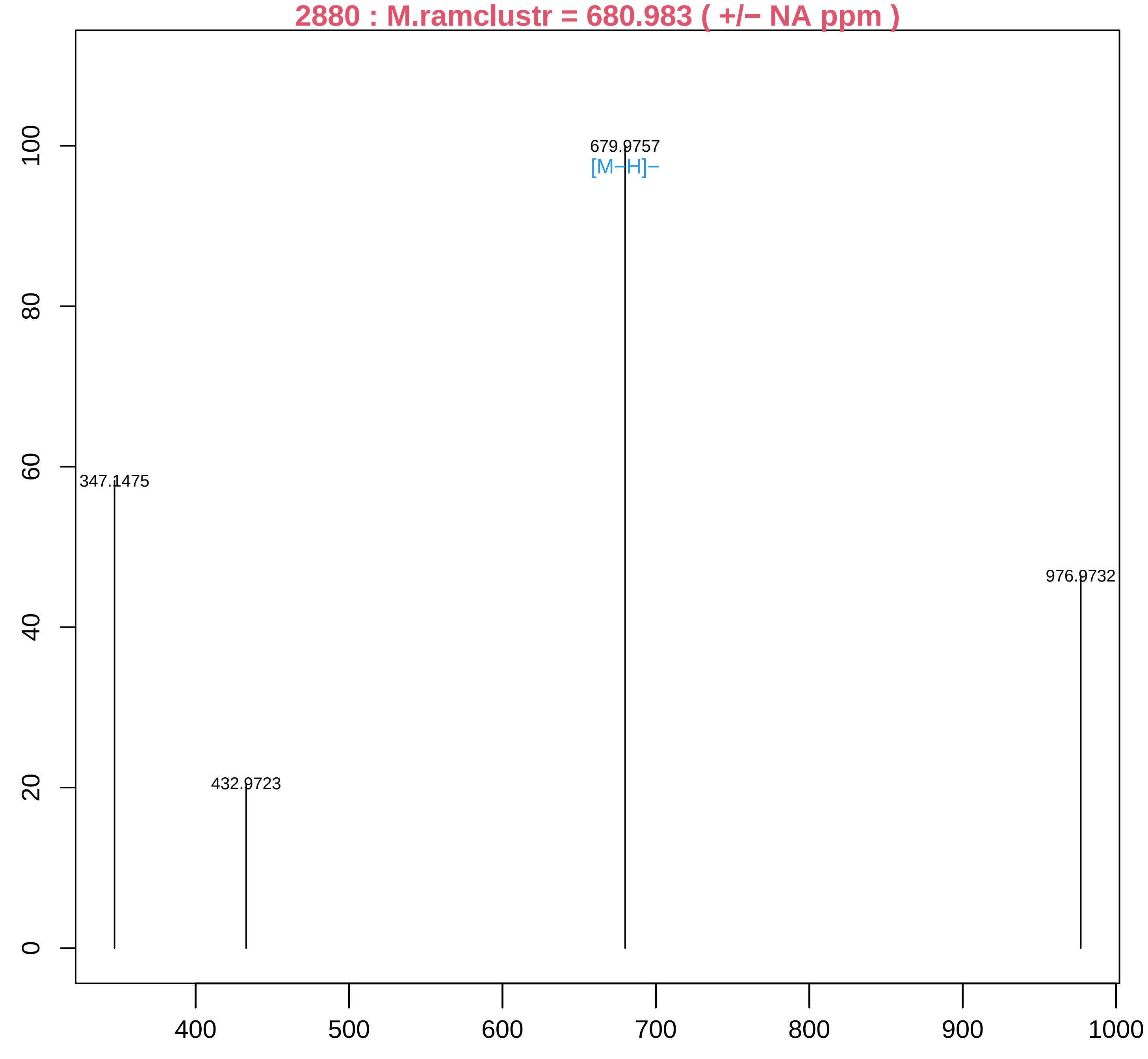
2880 : M.ramclustr = 680.983 (+/− NA ppm)
679.9757
[M−H]−
347.1475
432.9723
976.9732
0
20
40
60
80
100
400
500
600
700
800
900
1000

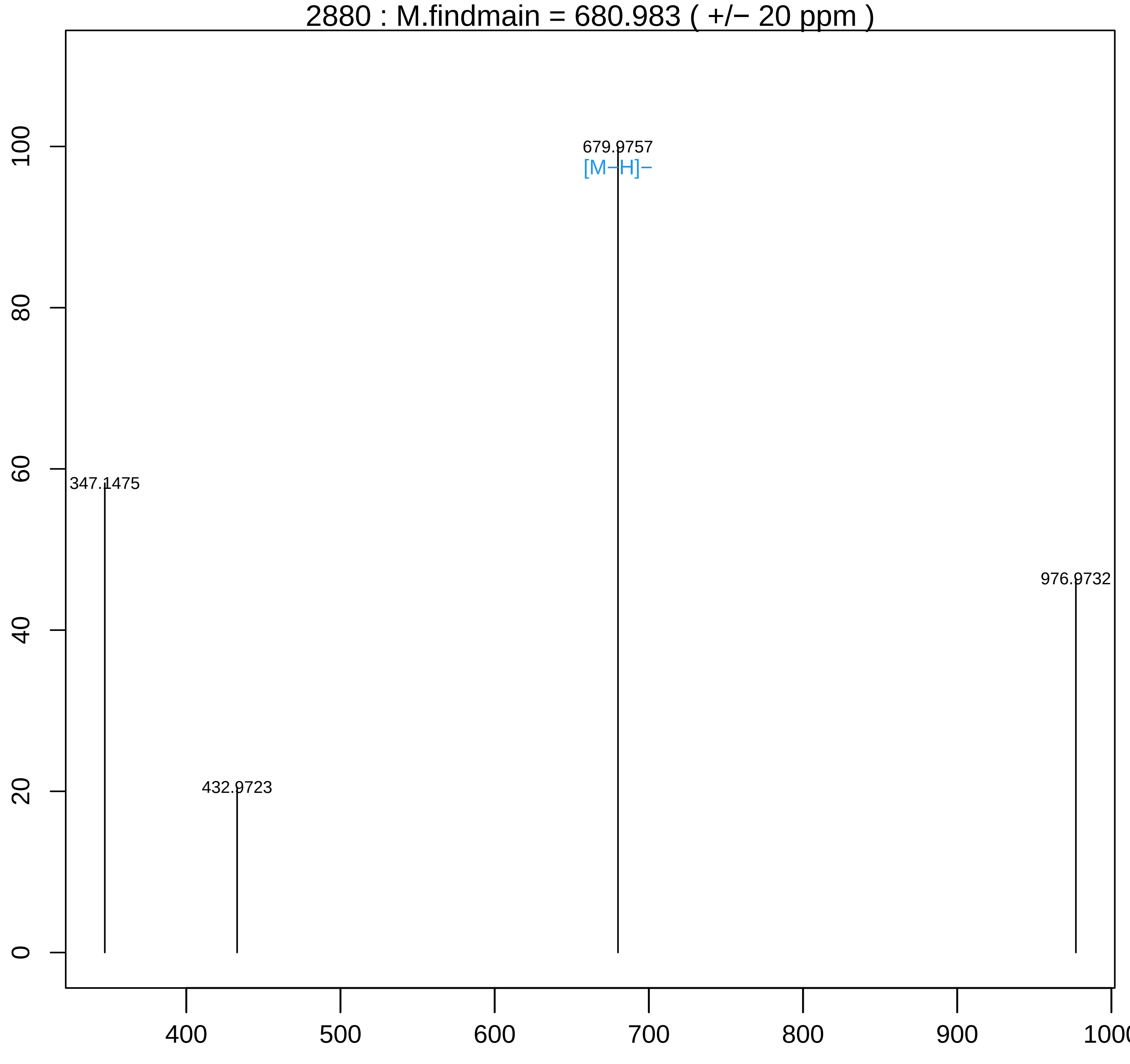
2880 : M.findmain = 680.983 (+/− 20 ppm)
679.9757
[M−H]−
347.1475
432.9723
976.9732
0
20
40
60
80
100
400
500
600
700
800
900
1000

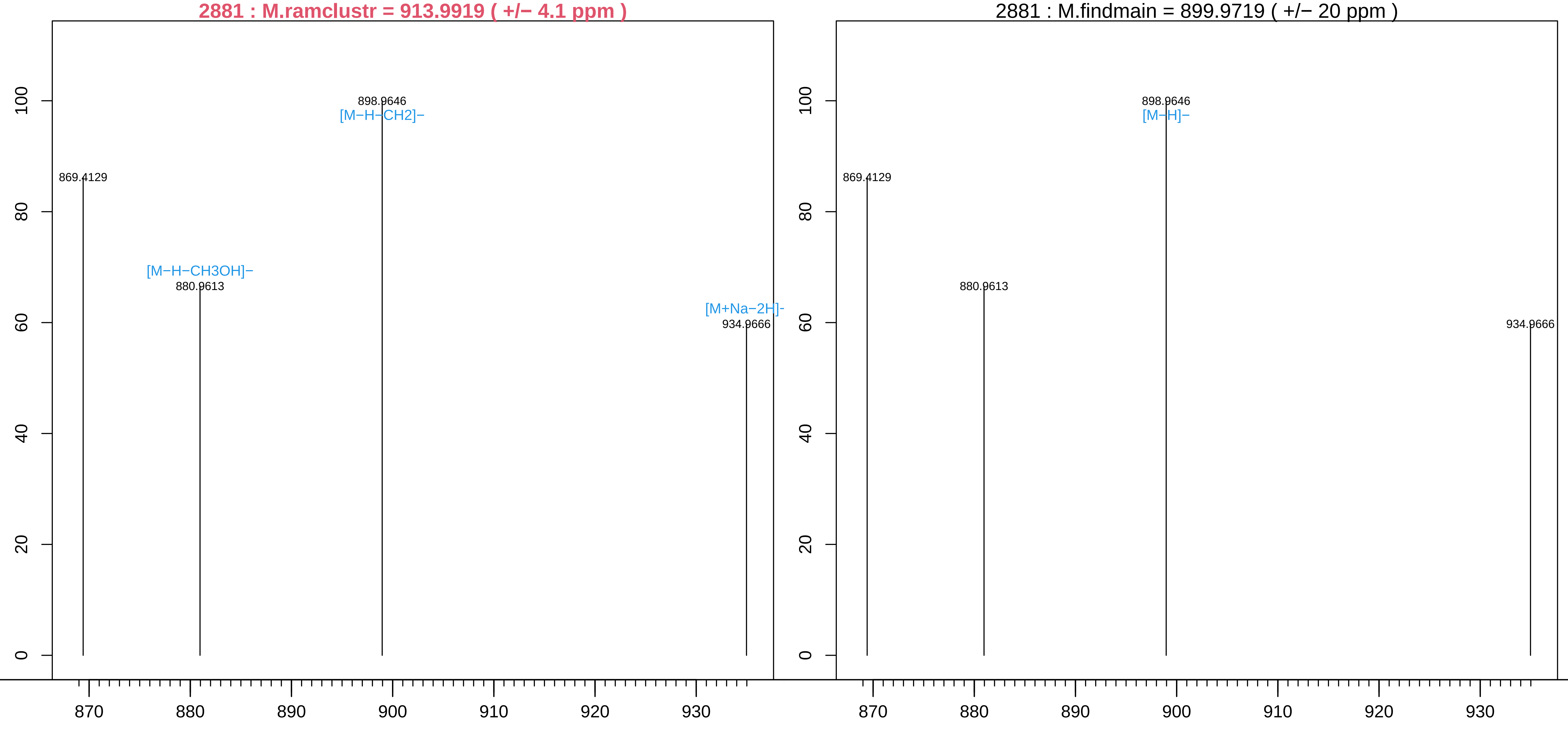
2881 : M.ramclustr = 913.9919 (+/− 4.1 ppm)
869.4129
[M−H−CH3OH]−
880.9613
898.9646
[M−H−CH2]−
[M+Na−2H]-
934.9666
2881 : M.findmain = 899.9719 (+/− 20 ppm)
869.4129
880.9613
898.9646
[M−H]−
934.9666
0
20
40
60
80
100
870
880
890
900
910
920
930

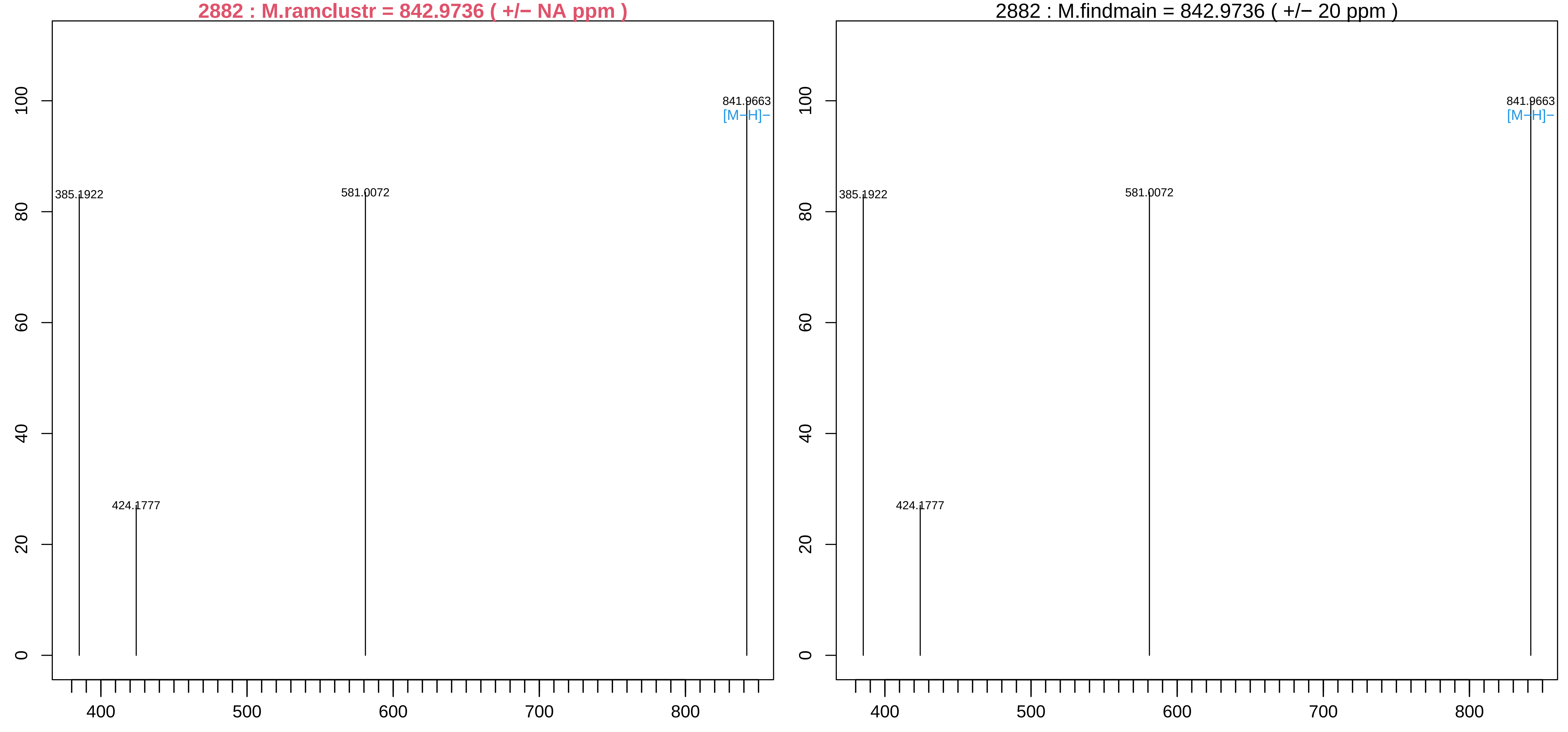

2882 : M.ramclustr = 842.9736 (+/− NA ppm)
2882 : M.findmain = 842.9736 (+/− 20 ppm)
385.1922
424.1777
581.0072
841.9663
[M−H]−
0
20
40
60
80
100
400
500
600
700
800

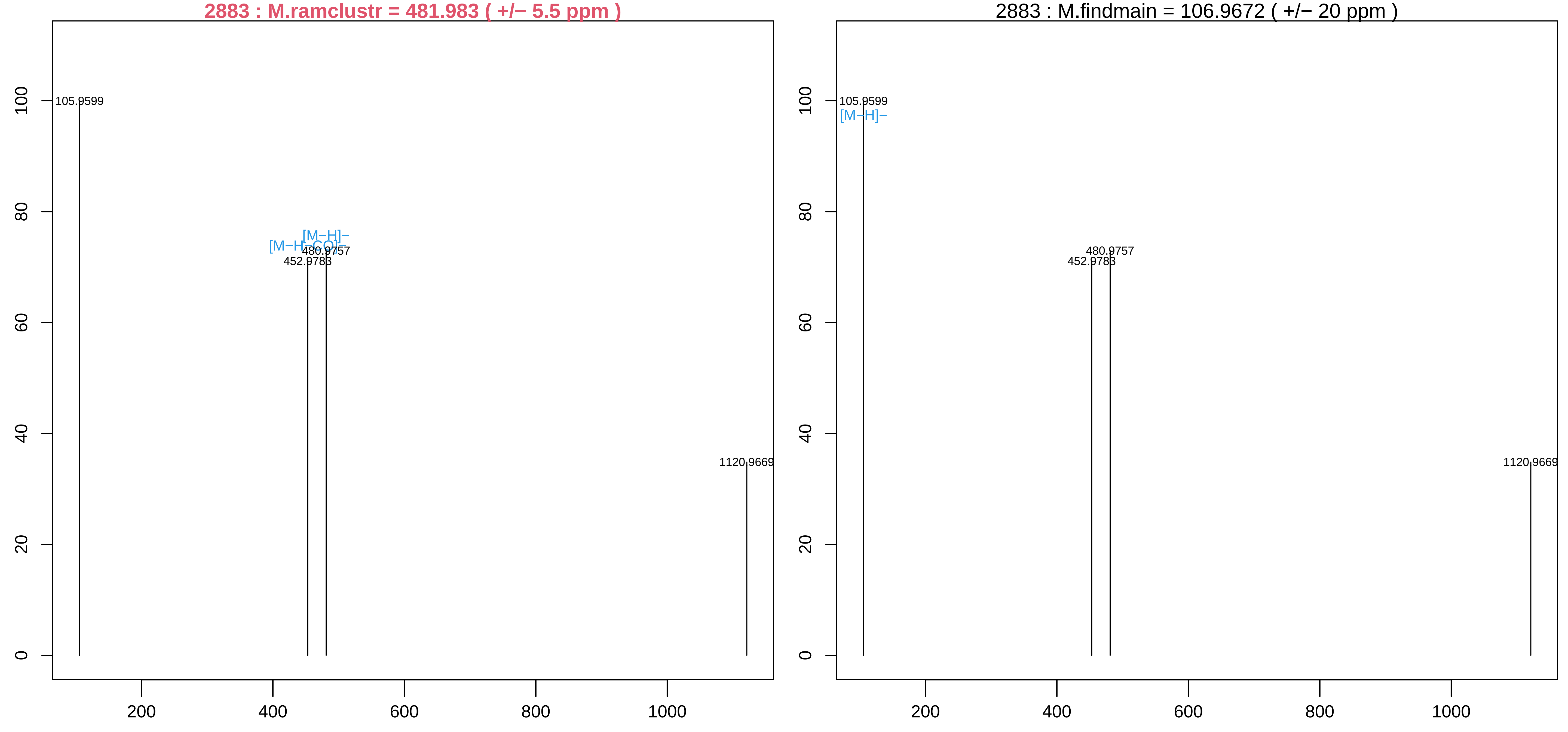
2883 : M.ramclustr = 481.983 (+/− 5.5 ppm)
105.9599
[M−H]−
[M−H−CO]−
480.9757
452.9783
1120.9669
0
20
40
60
80
100
200
400
600
800
1000
2883 : M.findmain = 106.9672 (+/− 20 ppm)
105.9599
[M−H]−
480.9757
452.9783
1120.9669
0
20
40
60
80
100
200
400
600
800
1000

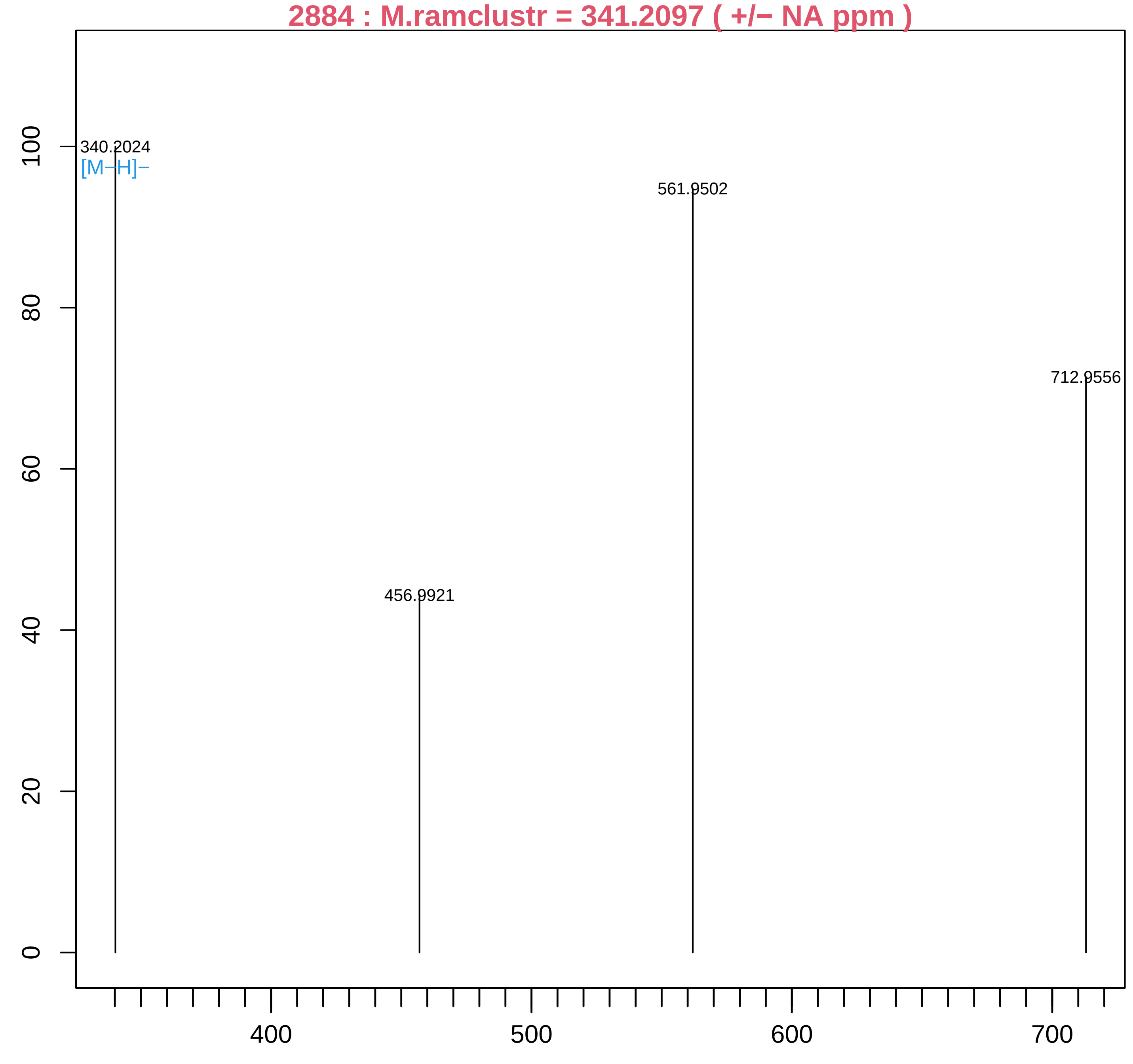
2884 : M.ramclustr = 341.2097 (+/− NA ppm)
340.2024
[M−H]−
456.9921
561.9502
712.9556
0
20
40
60
80
100
400
500
600
700

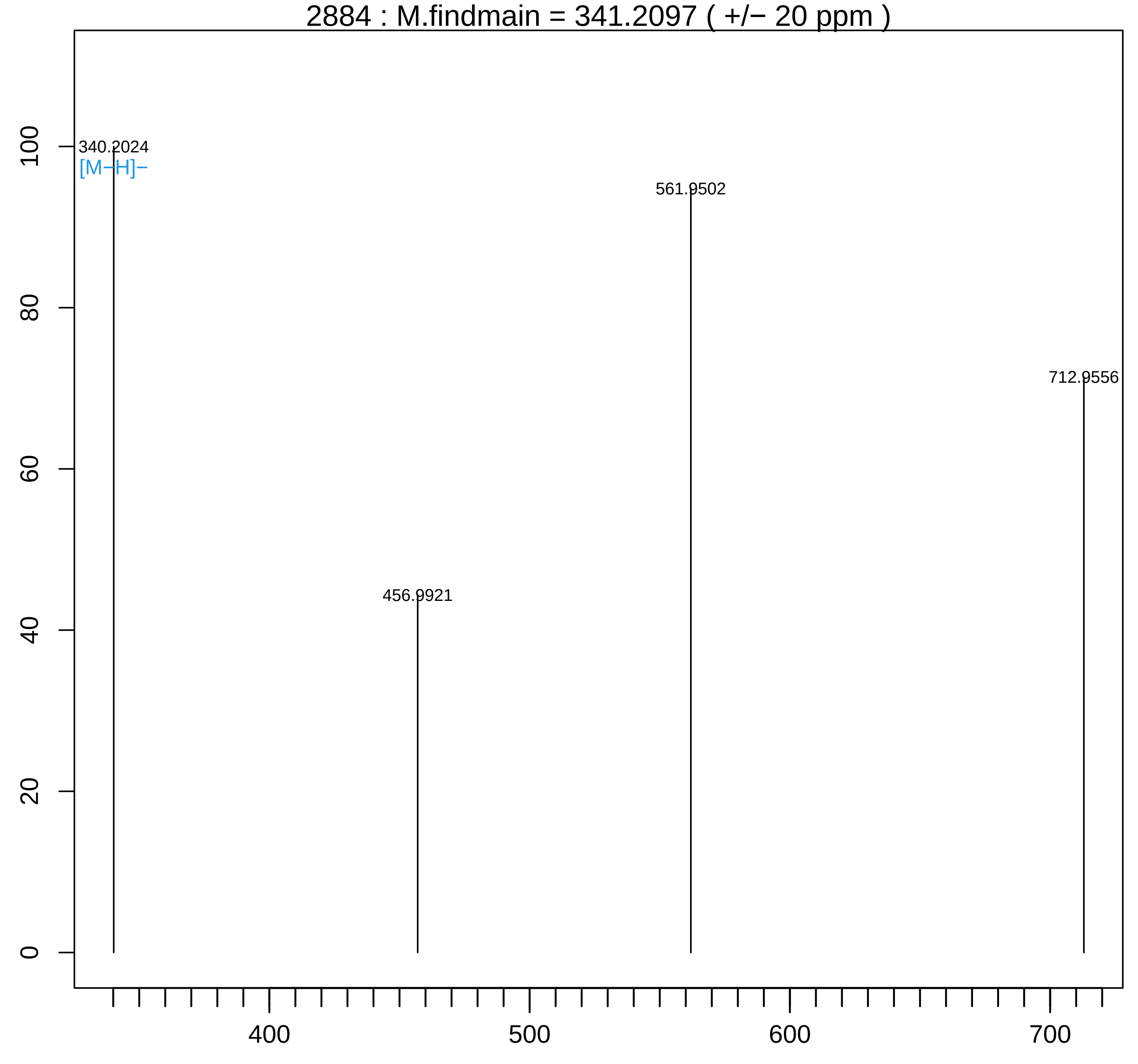
2884 : M.findmain = 341.2097 (+/− 20 ppm)
340.2024
[M−H]−
456.9921
561.9502
712.9556
0
20
40
60
80
100
400
500
600
700

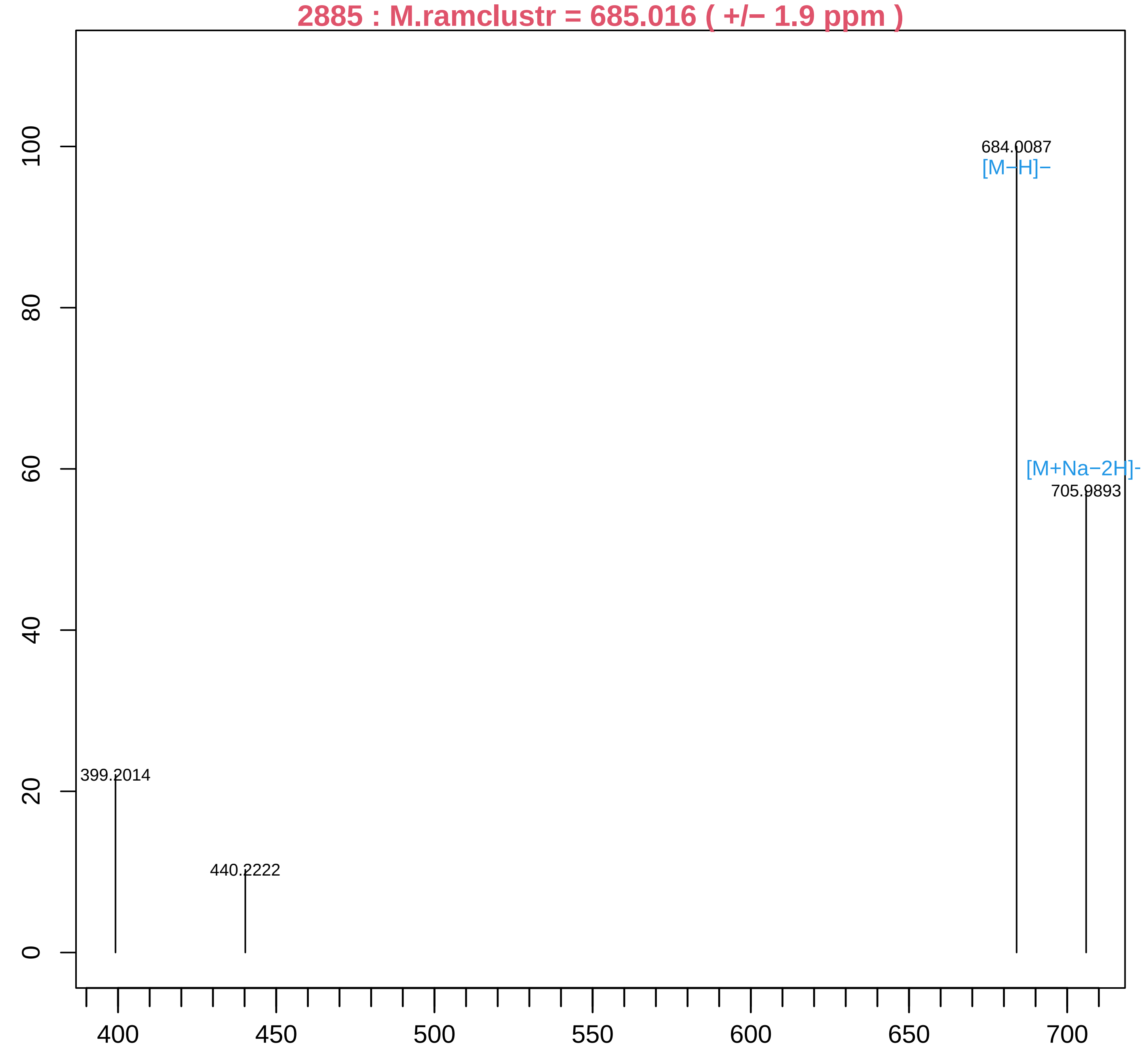

2885 : M.ramclustr = 685.016 (+/− 1.9 ppm)
684.0087
[M−H]−
[M+Na−2H]-
705.9893
399.2014
440.2222
0
20
40
60
80
100
400
450
500
550
600
650
700

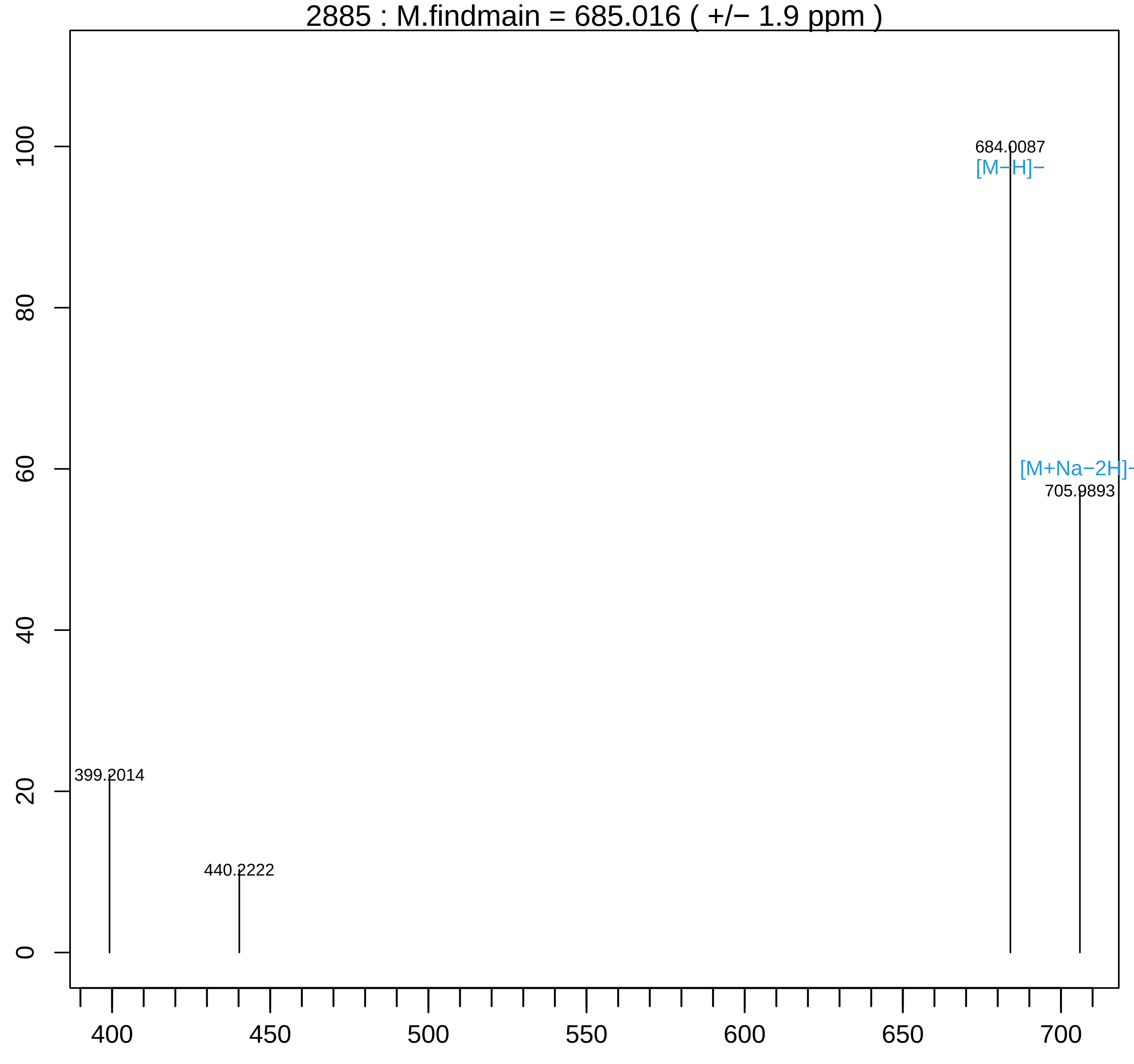

2885 : M.findmain = 685.016 (+/− 1.9 ppm)
684.0087
[M−H]−
[M+Na−2H]-
705.9893
399.2014
440.2222
0
20
40
60
80
100
400
450
500
550
600
650
700

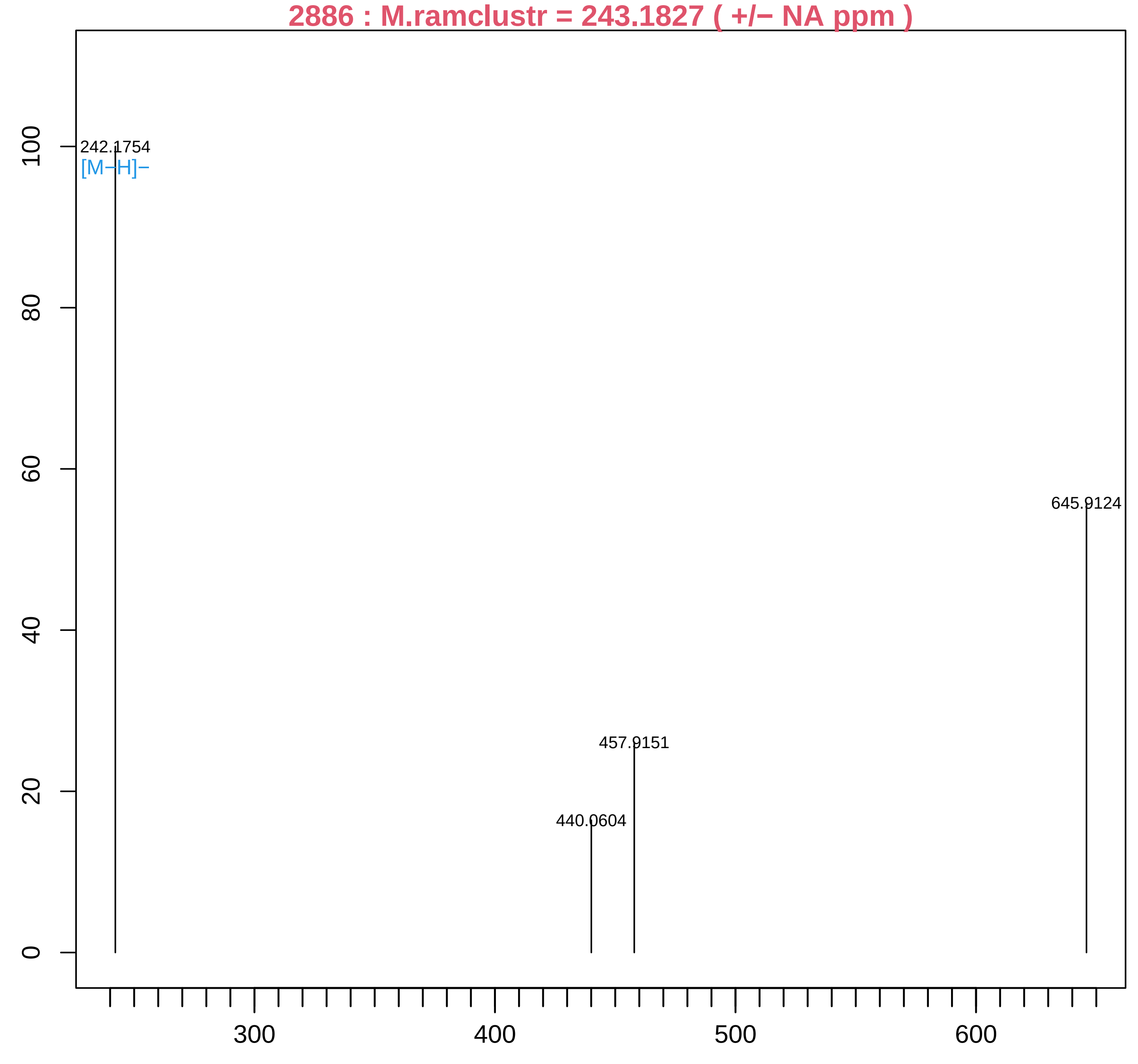
2886 : M.ramclustr = 243.1827 (+/− NA ppm)
242.1754
[M−H]−
440.0604
457.9151
645.9124
0
20
40
60
80
100
300
400
500
600

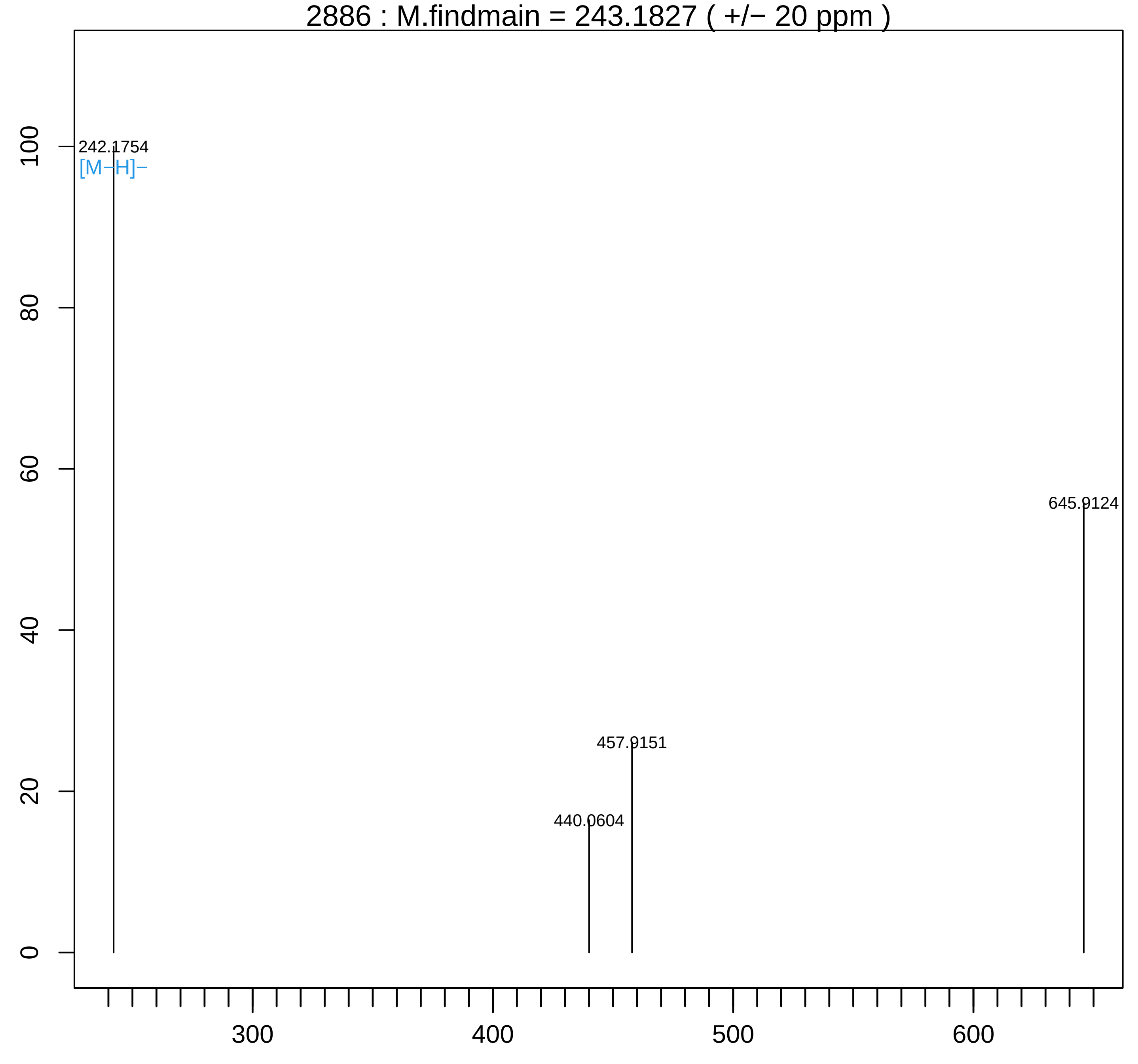
2886 : M.findmain = 243.1827 (+/− 20 ppm)
242.1754
[M−H]−
440.0604
457.9151
645.9124
0
20
40
60
80
100
300
400
500
600

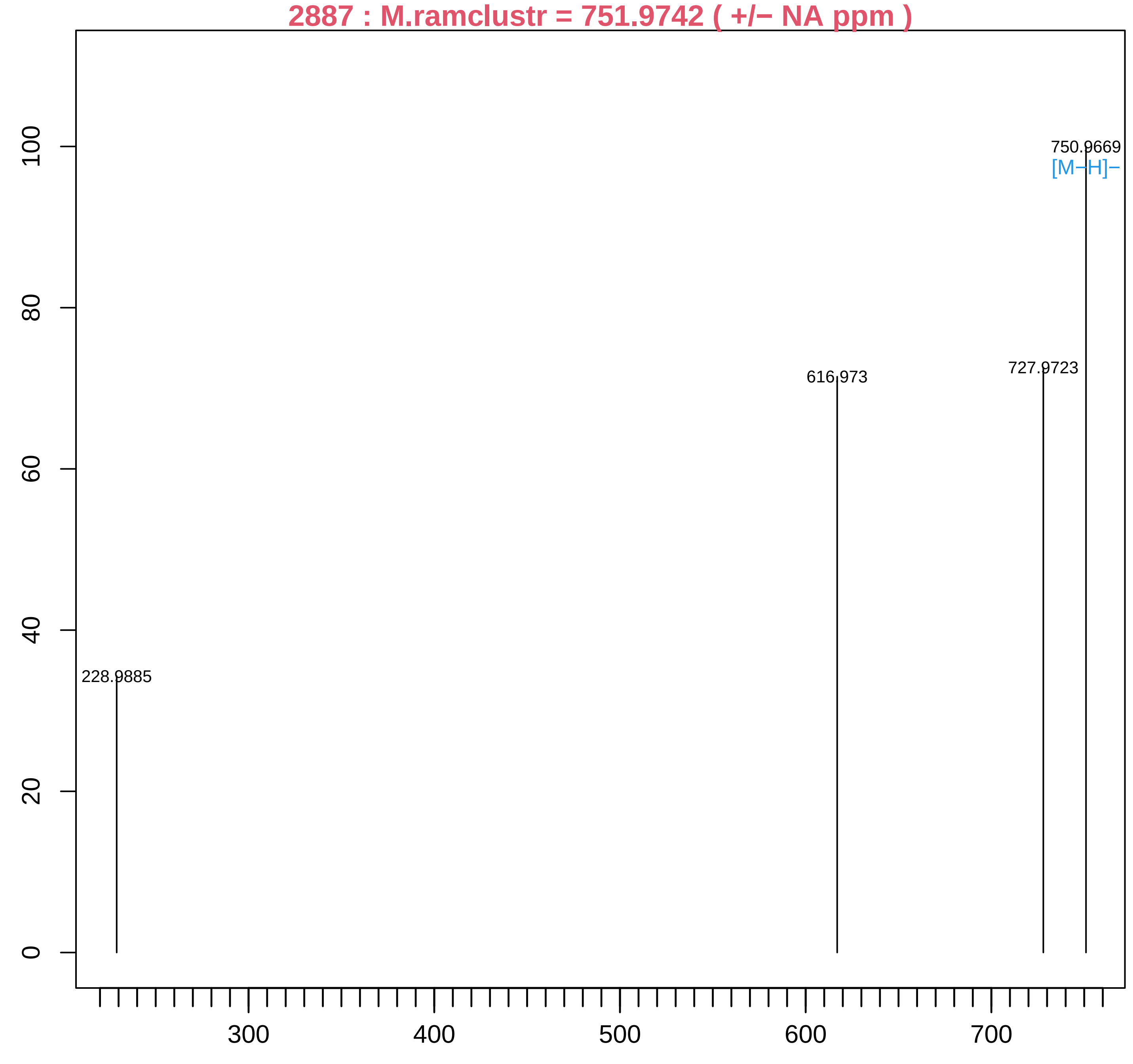
2887 : M.ramclustr = 751.9742 (+/− NA ppm)
750.9669
[M−H]−
727.9723
616.973
228.9885
0
20
40
60
80
100
300
400
500
600
700

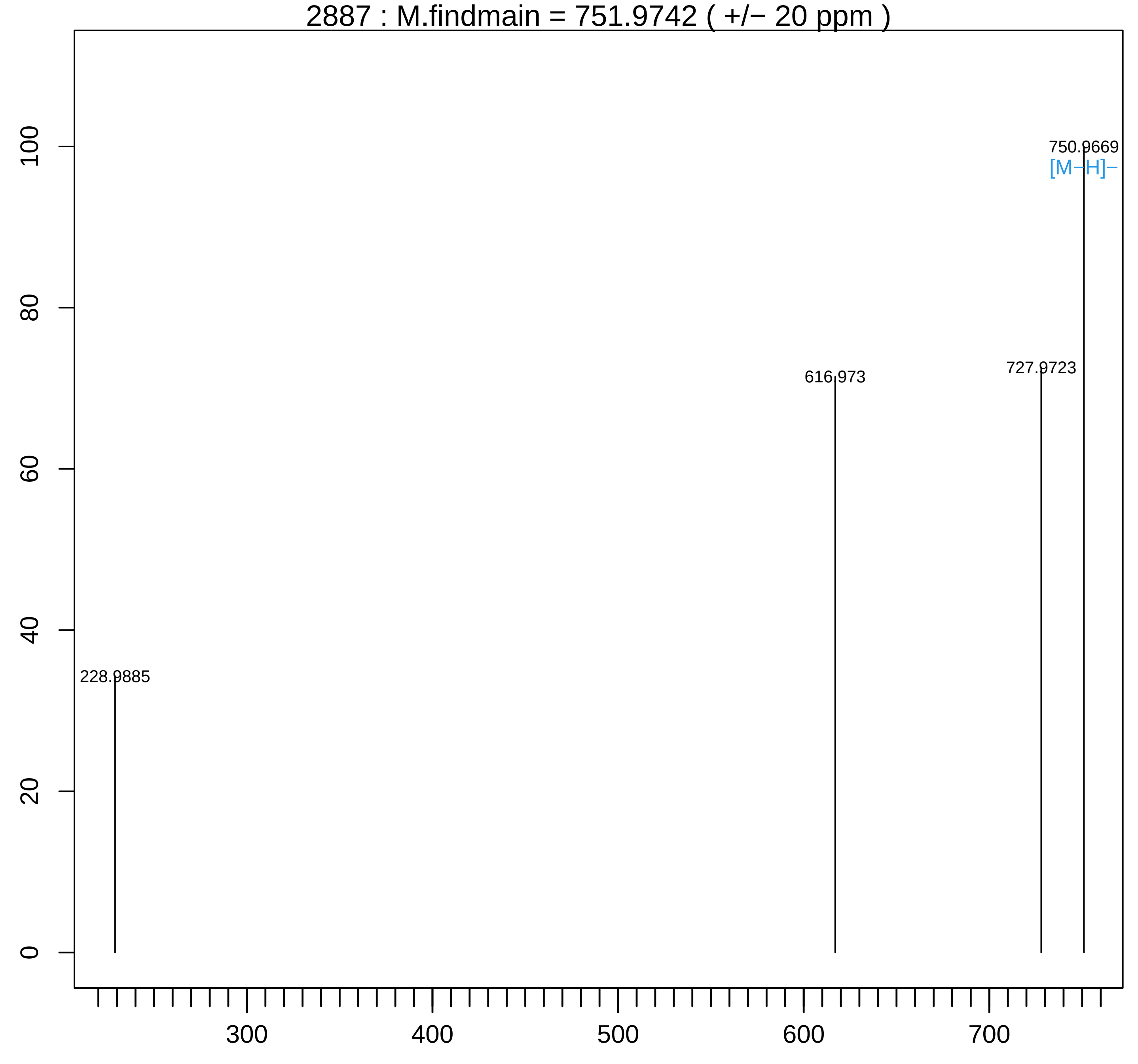
2887 : M.findmain = 751.9742 (+/− 20 ppm)
750.9669
[M−H]−
727.9723
616.973
228.9885
0
20
40
60
80
100
300
400
500
600
700

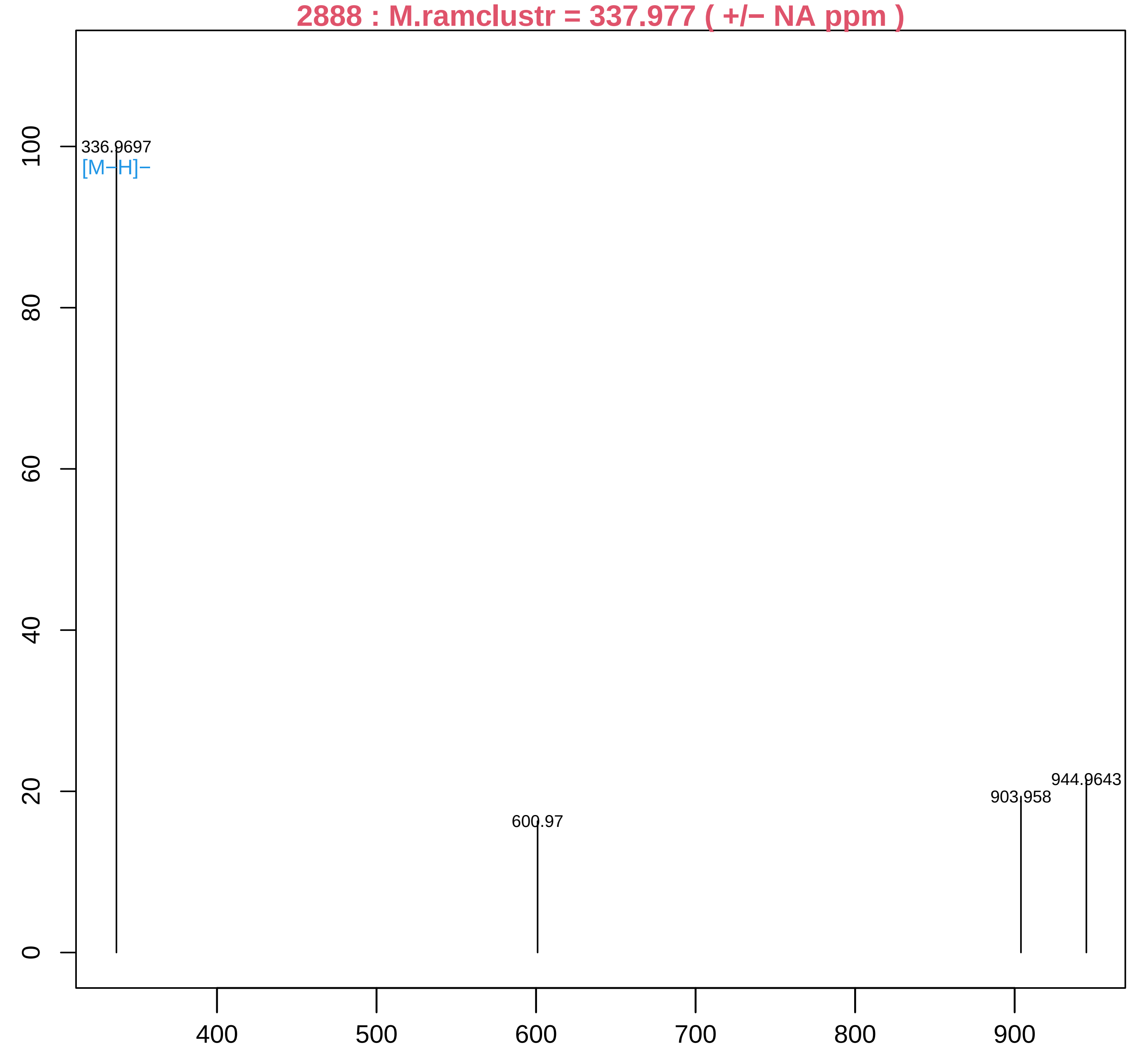

2888 : M.ramclustr = 337.977 (+/− NA ppm)
336.9697
[M−H]−
600.97
903.958
944.9643
0
20
40
60
80
100
400
500
600
700
800
900

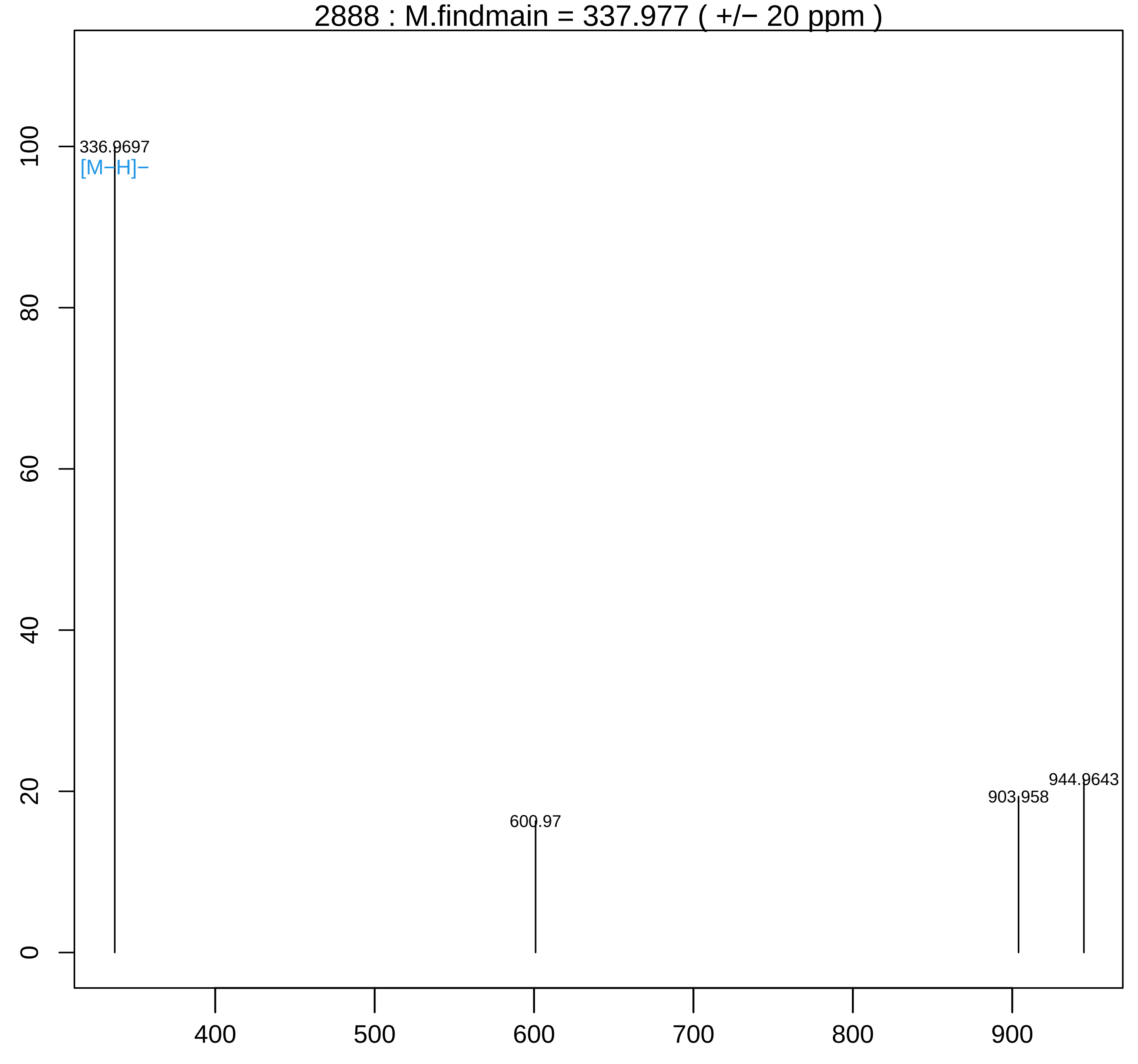

2888 : M.findmain = 337.977 (+/− 20 ppm)
336.9697
[M−H]−
600.97
903.958
944.9643
0
20
40
60
80
100
400
500
600
700
800
900

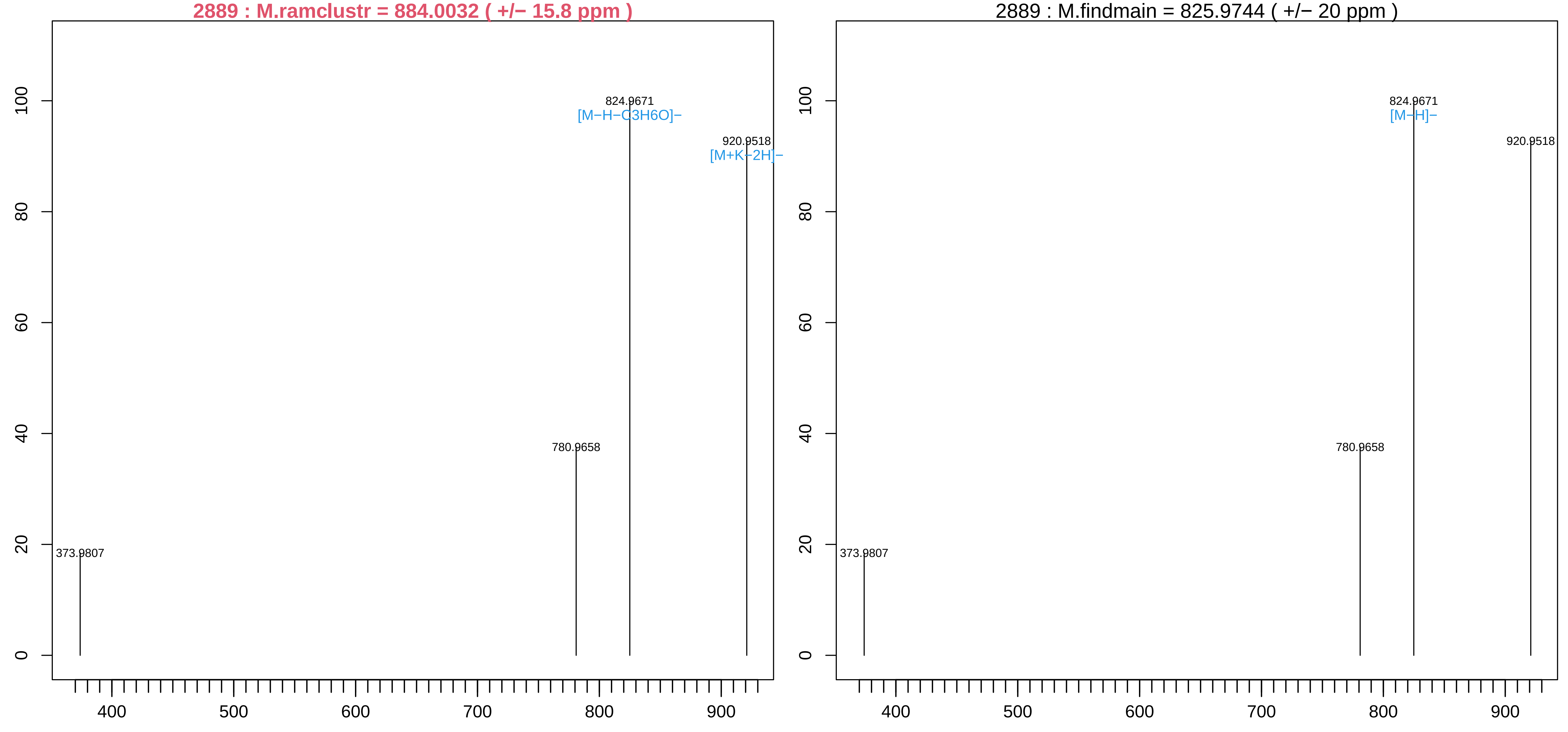
2889 : M.ramclustr = 884.0032 (+/− 15.8 ppm)
824.9671
[M−H−C3H6O]−
920.9518
[M+K−2H]−
780.9658
373.9807
2889 : M.findmain = 825.9744 (+/− 20 ppm)
824.9671
[M−H]−
920.9518
780.9658
373.9807

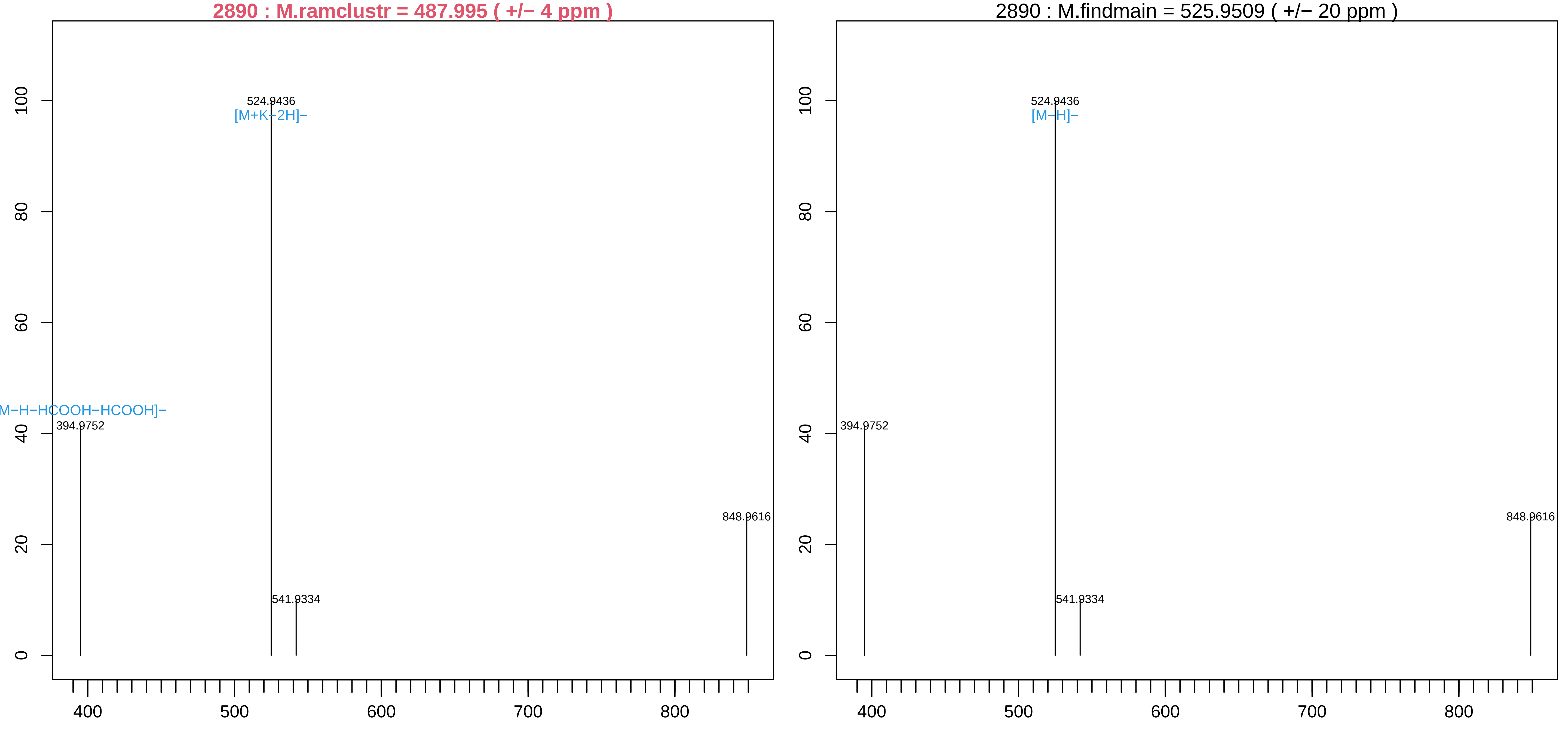
2890 : M.ramclustr = 487.995 (+/− 4 ppm)
524.9436
[M+K−2H]−
[M−H−HCOOH−HCOOH]−
394.9752
848.9616
541.9334
0
20
40
60
80
100
400
500
600
700
800
2890 : M.findmain = 525.9509 (+/− 20 ppm)
524.9436
[M−H]−
394.9752
848.9616
541.9334

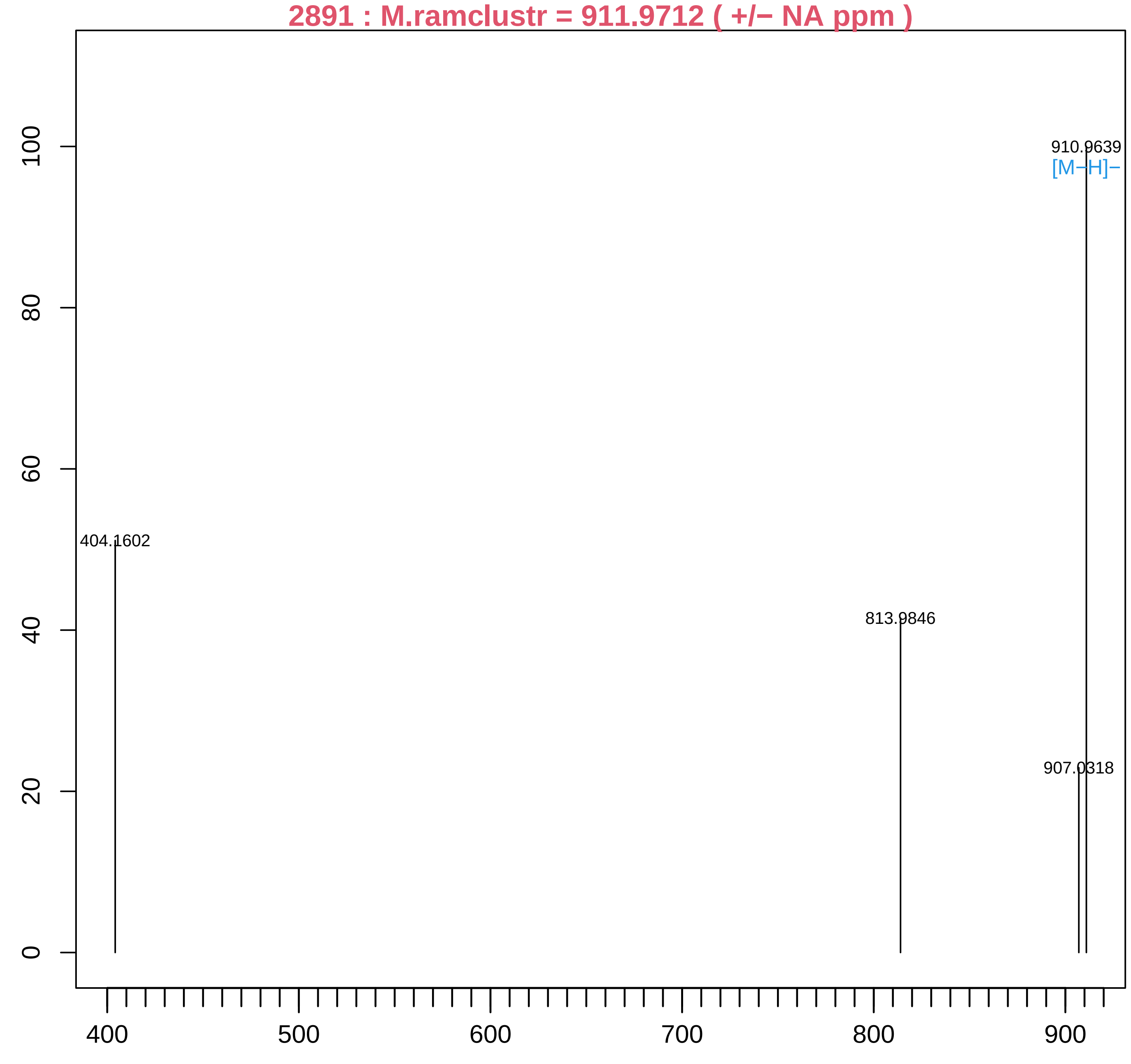

2891 : M.ramclustr = 911.9712 (+/− NA ppm)
910.9639
[M−H]−
404.1602
813.9846
907.0318
0
20
40
60
80
100
400
500
600
700
800
900

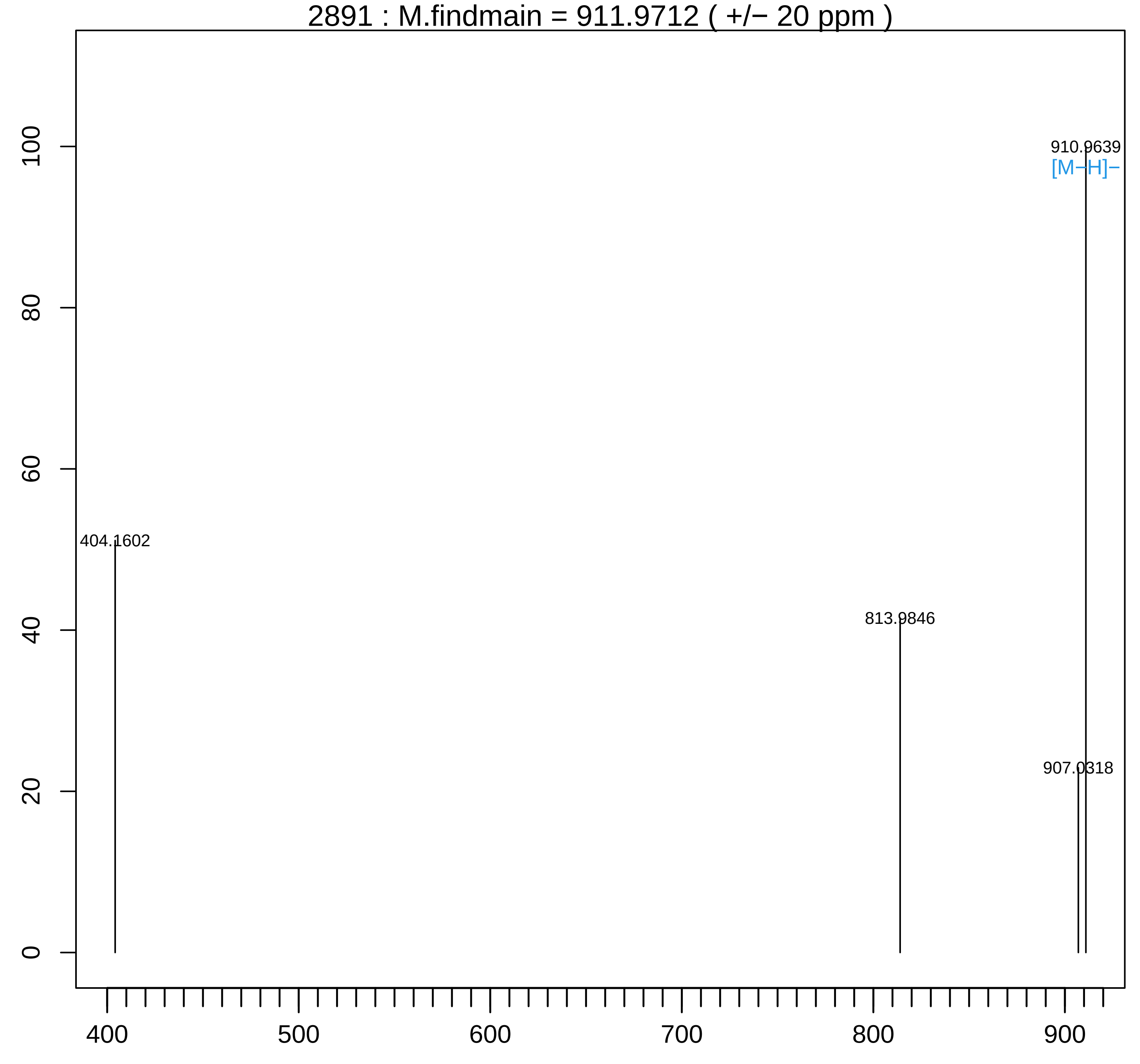

2891 : M.findmain = 911.9712 (+/− 20 ppm)
910.9639
[M−H]−
404.1602
813.9846
907.0318
0
20
40
60
80
100
400
500
600
700
800
900

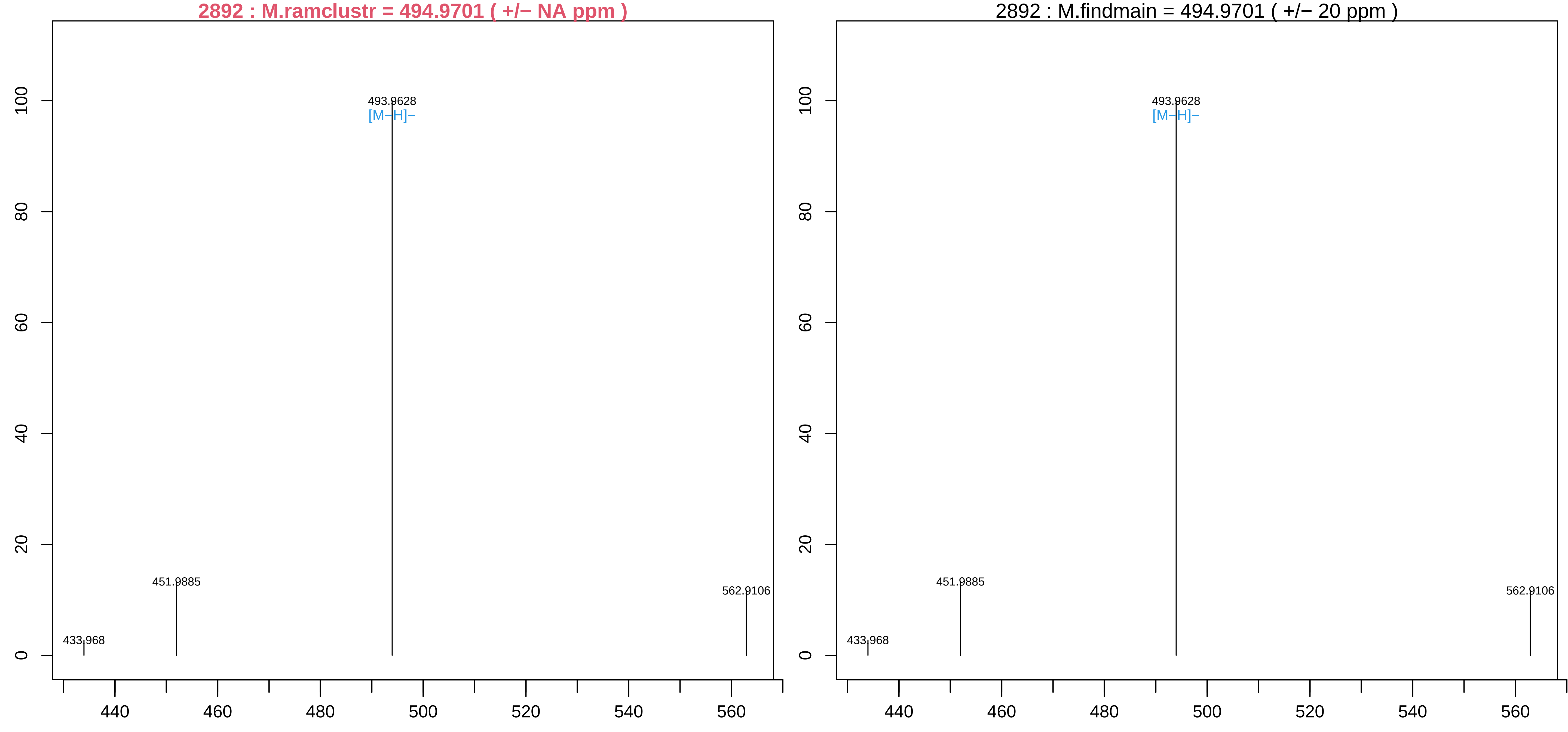
2892 : M.ramclustr = 494.9701 (+/− NA ppm)
493.9628
[M−H]−
451.9885
562.9106
433.968
2892 : M.findmain = 494.9701 (+/− 20 ppm)
493.9628
[M−H]−
451.9885
562.9106
433.968

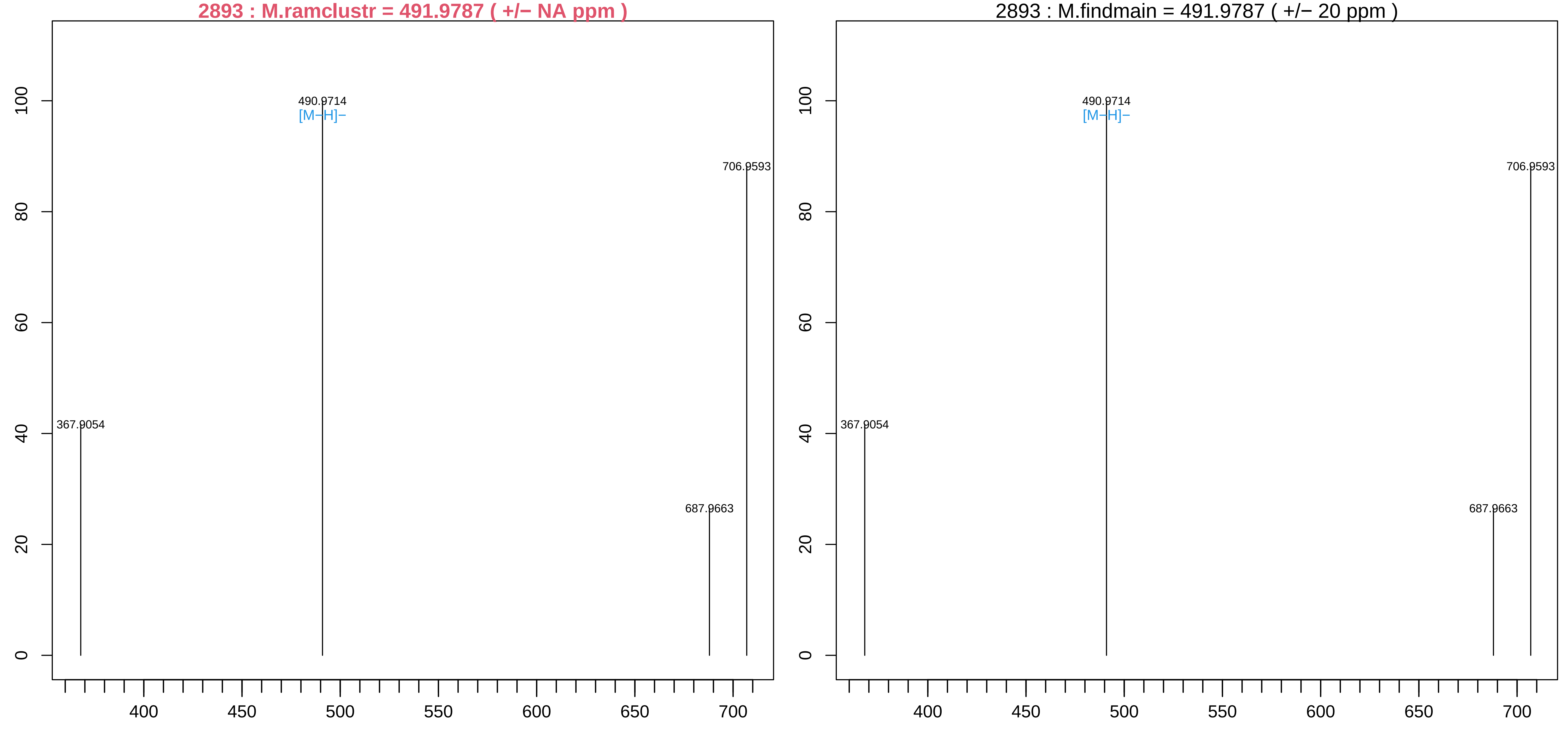

2893 : M.ramclustr = 491.9787 (+/− NA ppm)
2893 : M.findmain = 491.9787 (+/− 20 ppm)
367.9054
490.9714
[M−H]−
687.9663
706.9593
0
20
40
60
80
100
400
450
500
550
600
650
700

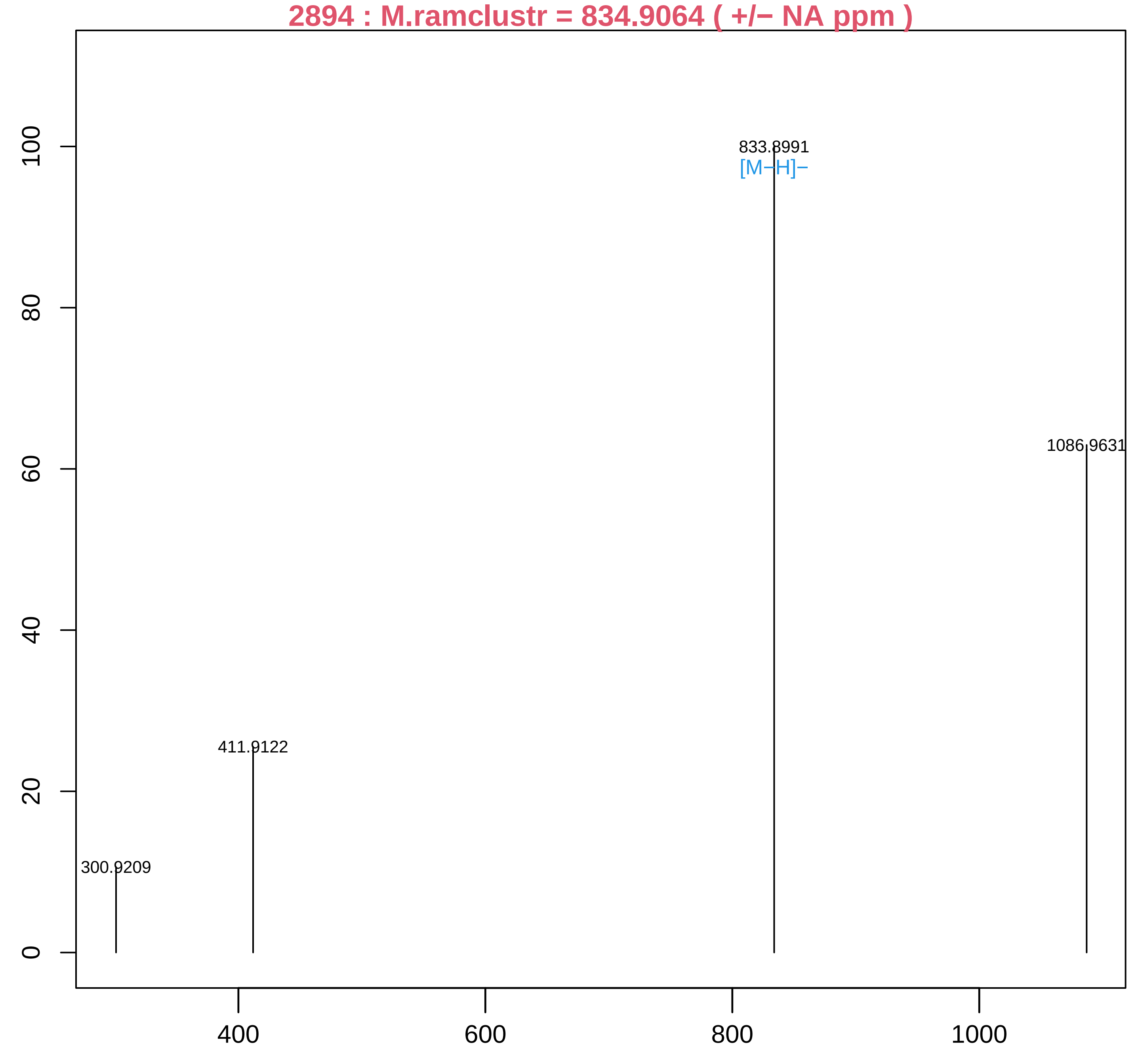

2894 : M.ramclustr = 834.9064 (+/− NA ppm)
833.8991
[M−H]−
1086.9631
411.9122
300.9209
0
20
40
60
80
100
400
600
800
1000

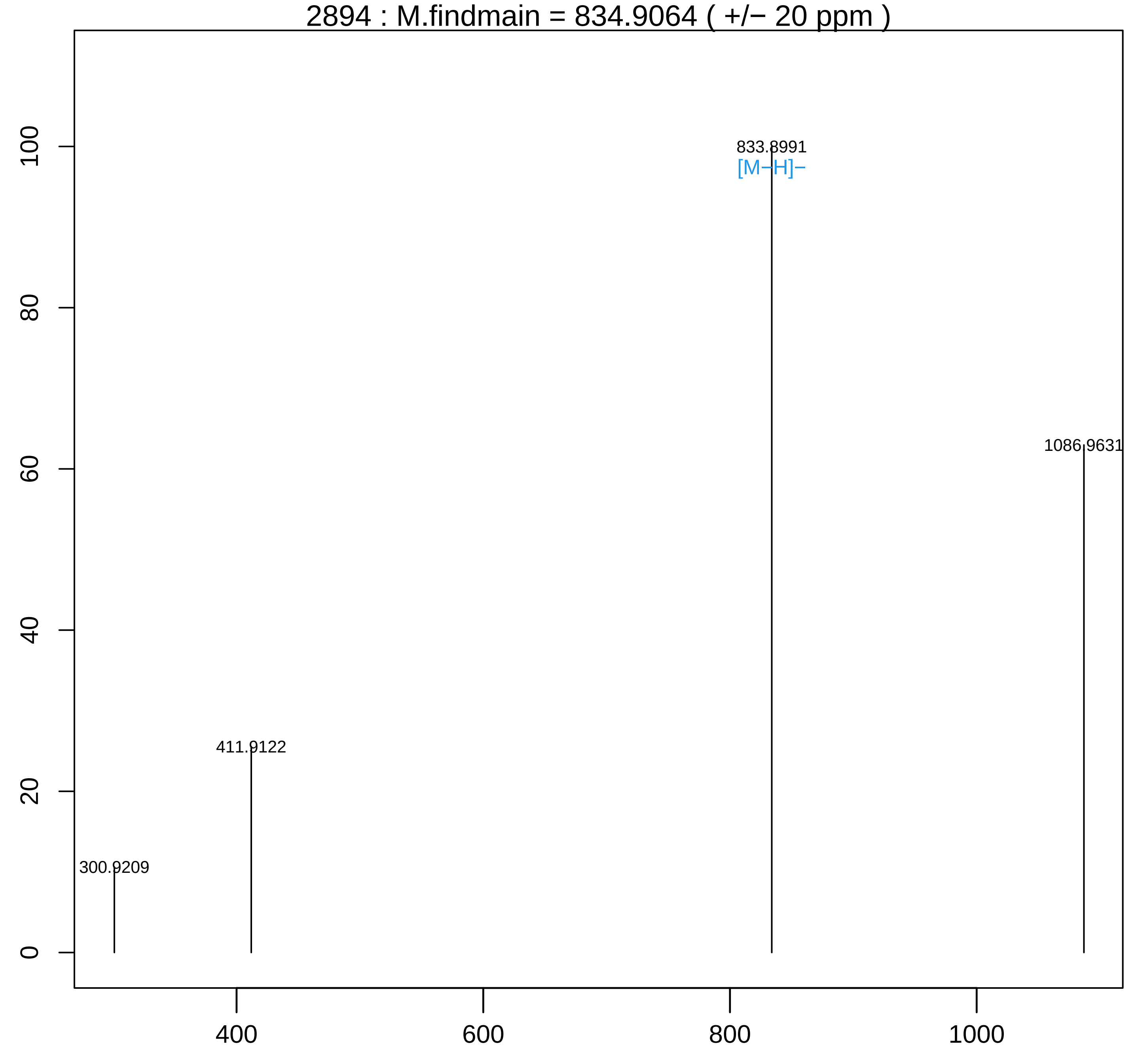

2894 : M.findmain = 834.9064 (+/− 20 ppm)
833.8991
[M−H]−
1086.9631
411.9122
300.9209
0
20
40
60
80
100
400
600
800
1000

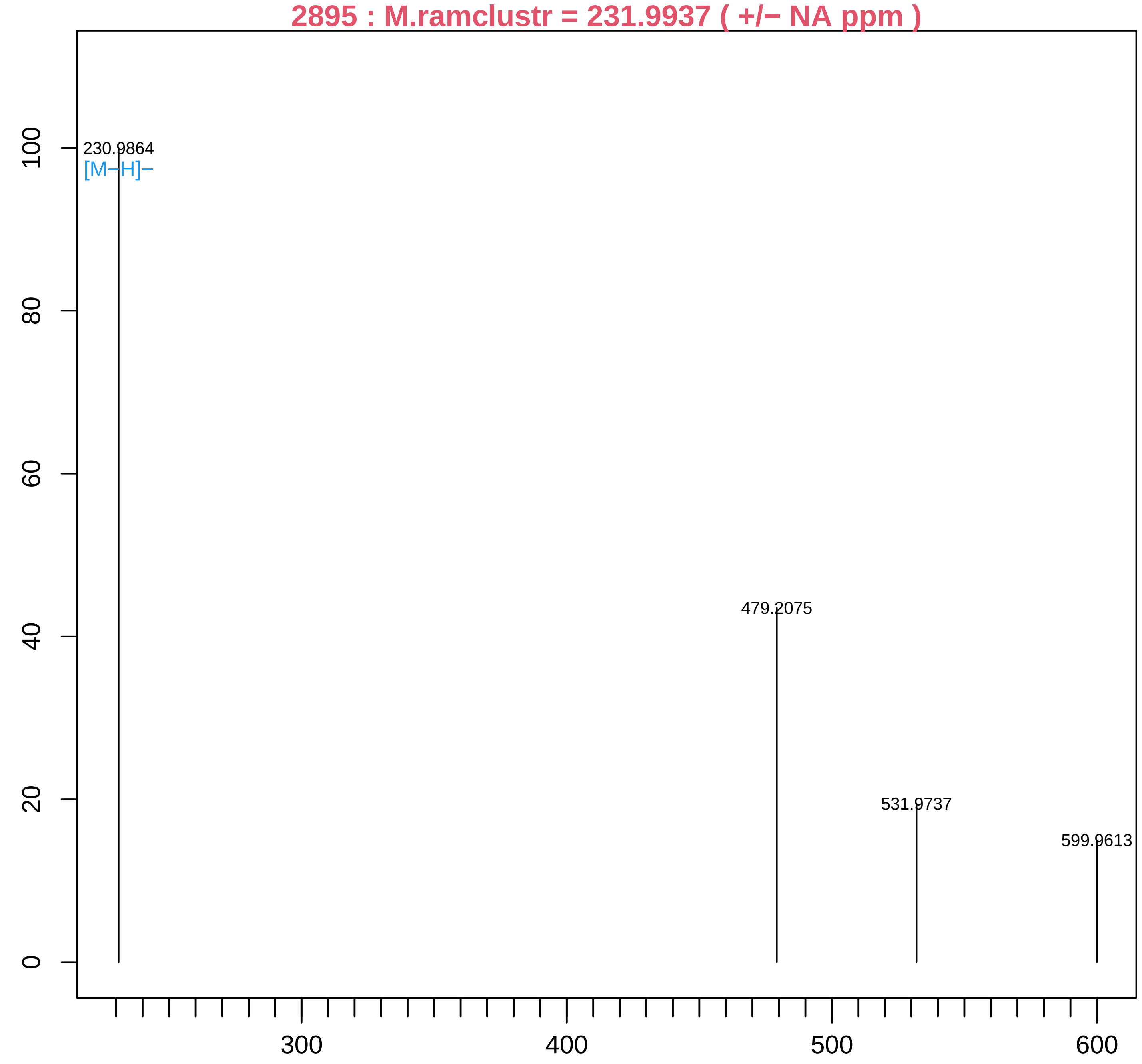
2895 : M.ramclustr = 231.9937 (+/− NA ppm)
230.9864
[M−H]−
479.2075
531.9737
599.9613
0
20
40
60
80
100
300
400
500
600

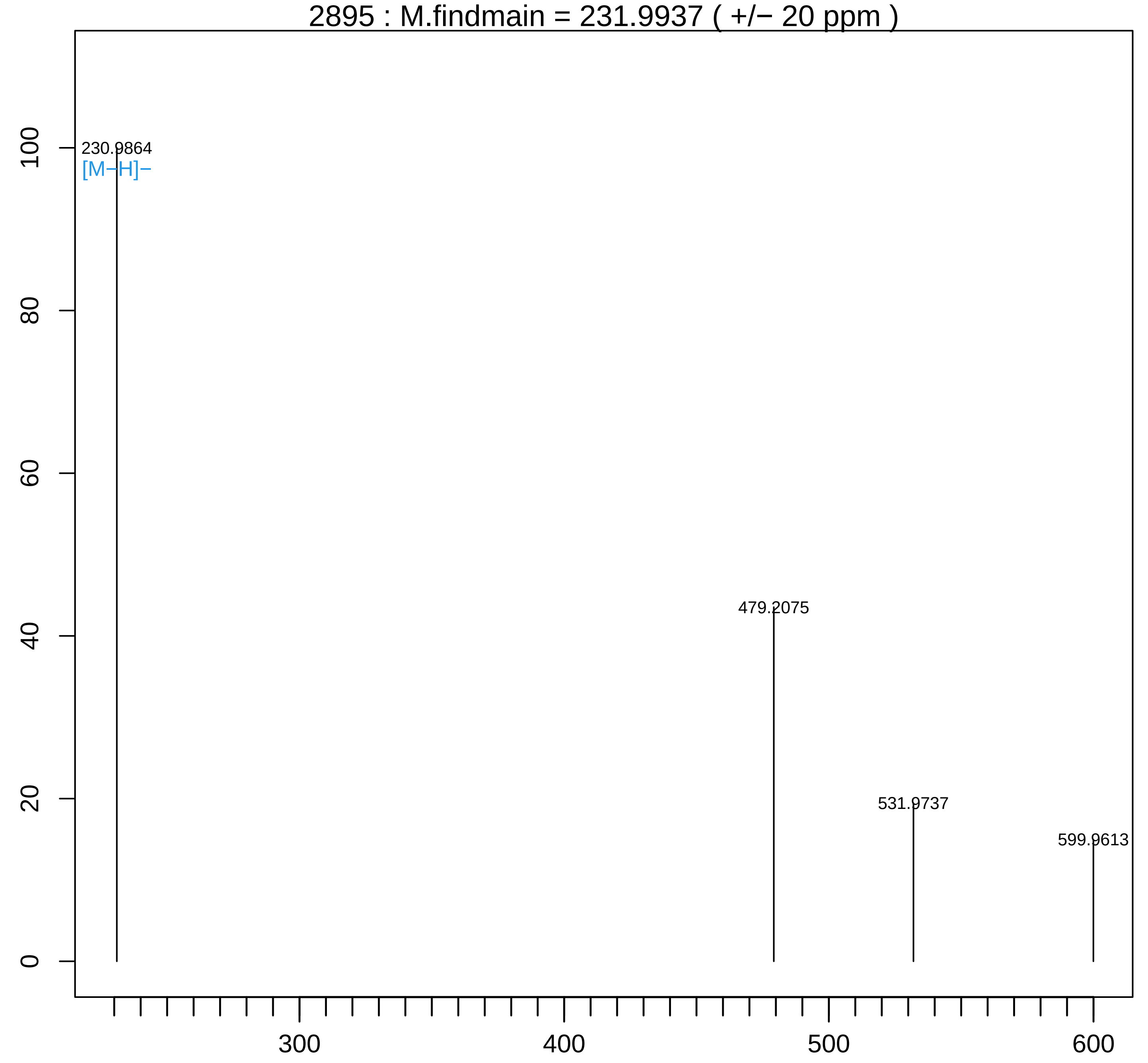
2895 : M.findmain = 231.9937 (+/− 20 ppm)
230.9864
[M−H]−
479.2075
531.9737
599.9613
0
20
40
60
80
100
300
400
500
600

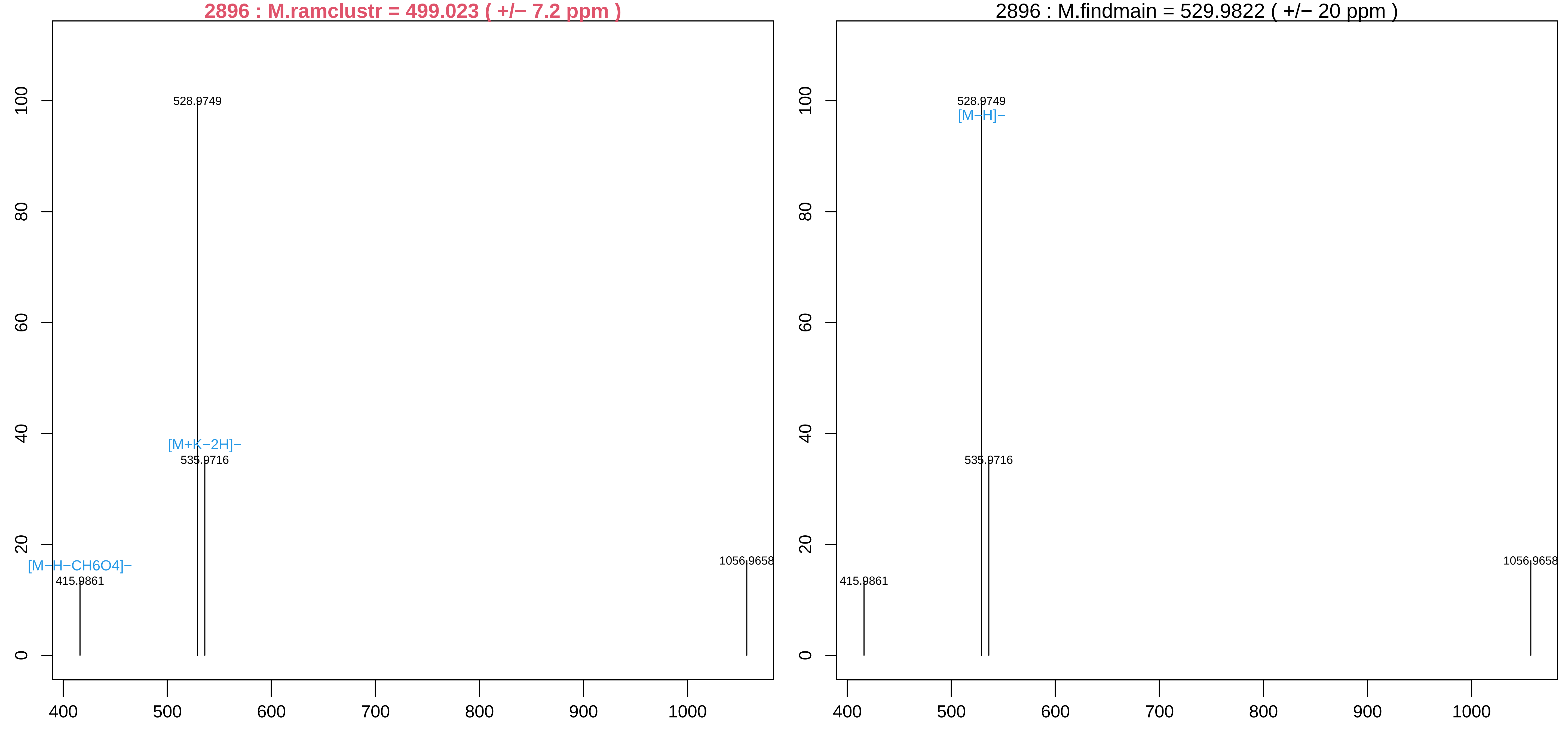
2896 : M.ramclustr = 499.023 (+/− 7.2 ppm)
528.9749
[M+K−2H]−
535.9716
[M−H−CH6O4]−
415.9861
1056.9658
2896 : M.findmain = 529.9822 (+/− 20 ppm)
528.9749
[M−H]−
535.9716
415.9861
1056.9658
0
20
40
60
80
100
400
500
600
700
800
900
1000

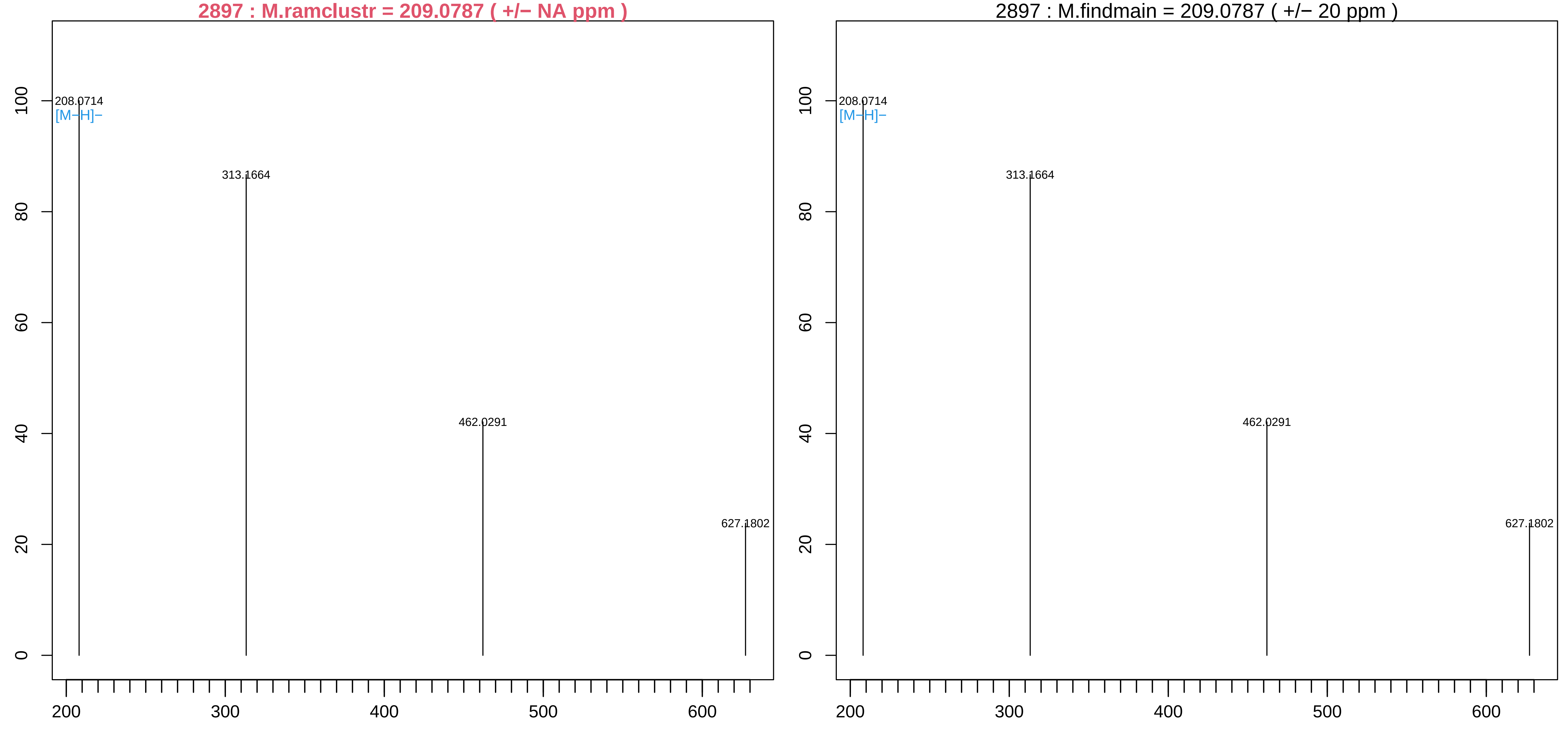

2897 : M.ramclustr = 209.0787 (+/− NA ppm)
208.0714
[M−H]−
313.1664
462.0291
627.1802
2897 : M.findmain = 209.0787 (+/− 20 ppm)
208.0714
[M−H]−
313.1664
462.0291
627.1802

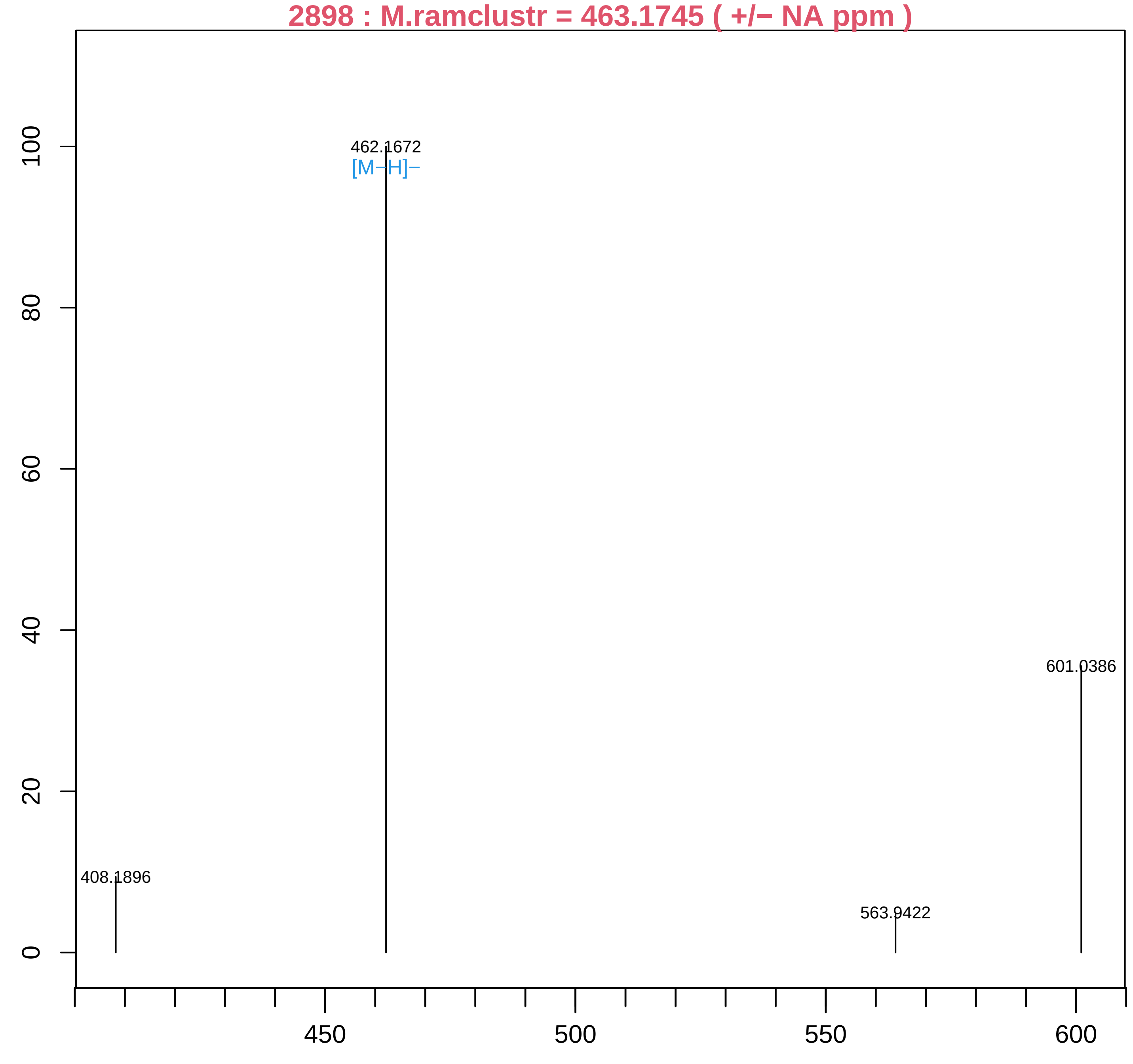
2898 : M.ramclustr = 463.1745 (+/− NA ppm)
462.1672
[M−H]−
601.0386
408.1896
563.9422
0
20
40
60
80
100
450
500
550
600

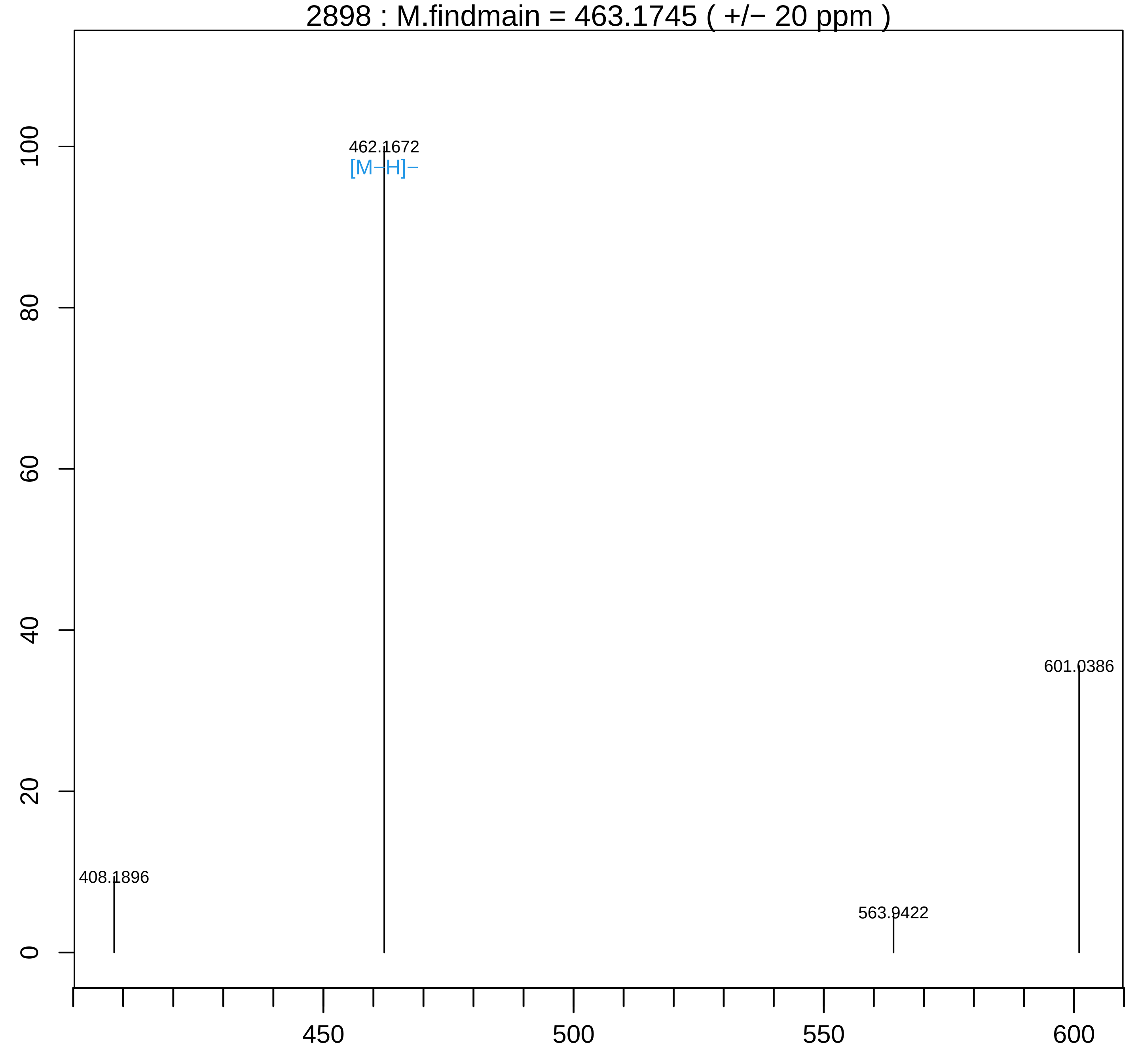
2898 : M.findmain = 463.1745 (+/− 20 ppm)
462.1672
[M−H]−
601.0386
408.1896
563.9422
0
20
40
60
80
100
450
500
550
600

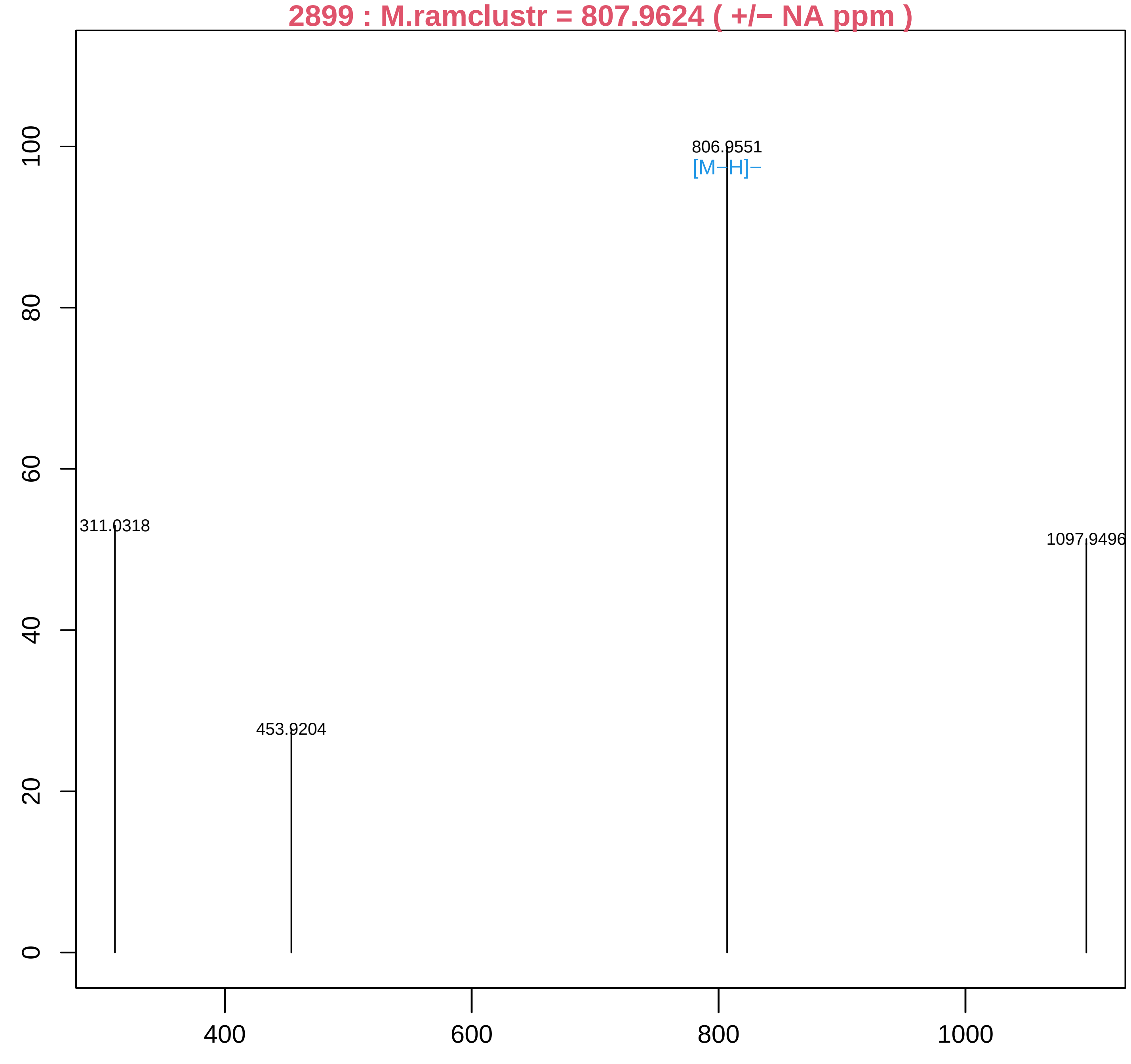
2899 : M.ramclustr = 807.9624 (+/− NA ppm)
806.9551
[M−H]−
311.0318
453.9204
1097.9496
0
20
40
60
80
100
400
600
800
1000

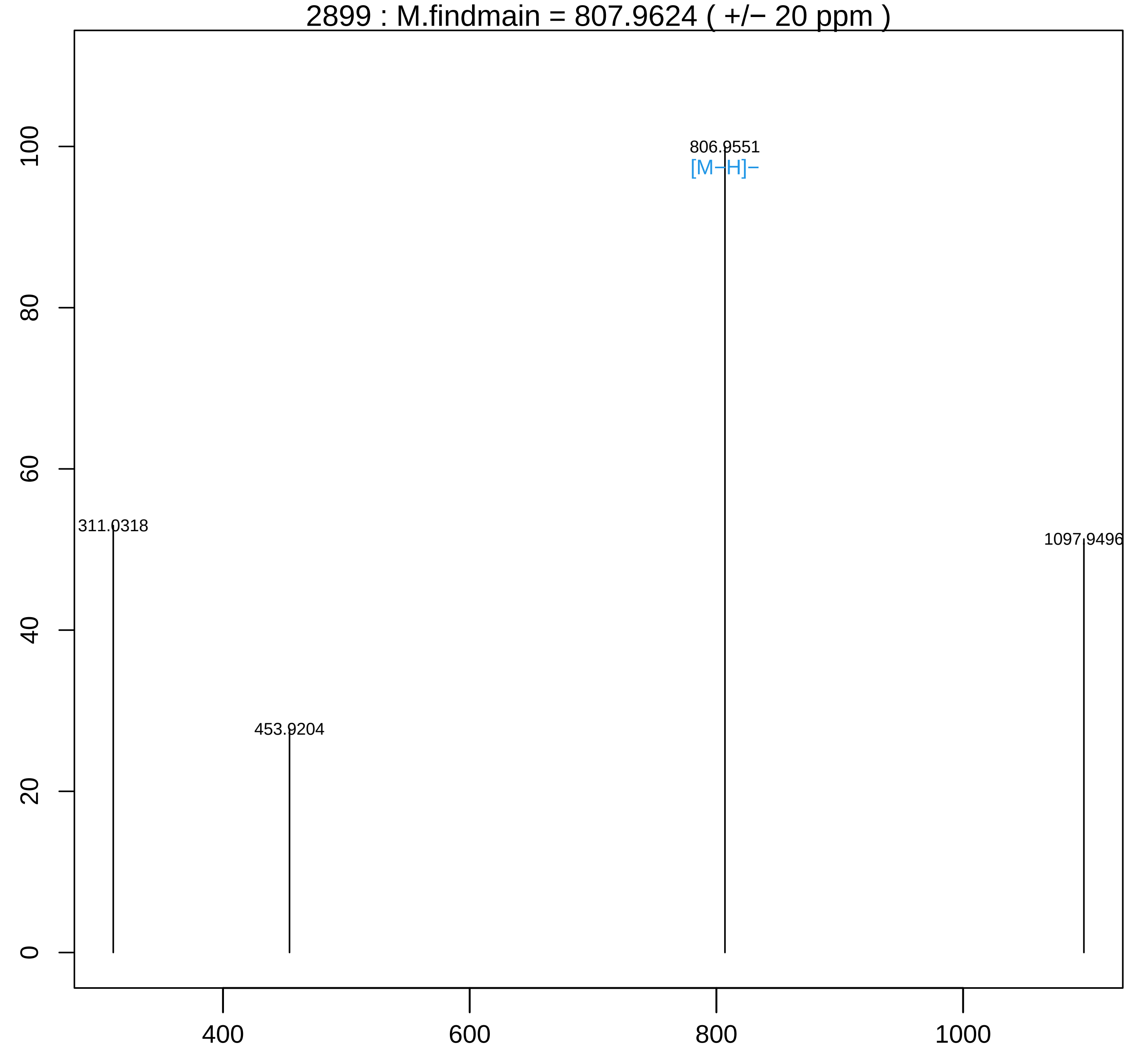
2899 : M.findmain = 807.9624 (+/− 20 ppm)
806.9551
[M−H]−
311.0318
453.9204
1097.9496
0
20
40
60
80
100
400
600
800
1000

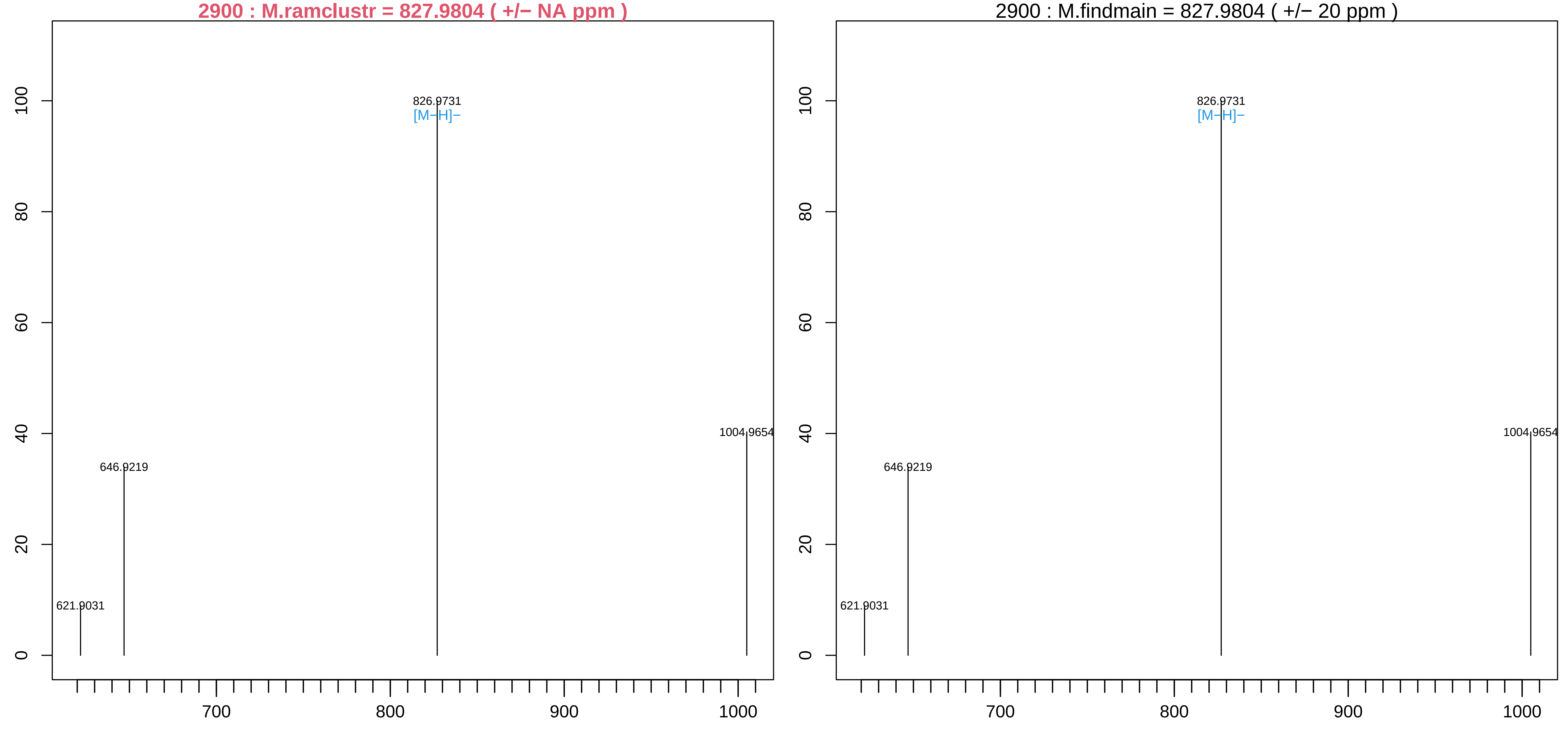

2900 : M.ramclustr = 827.9804 (+/− NA ppm)
2900 : M.findmain = 827.9804 (+/− 20 ppm)
826.9731
[M−H]−
646.9219
621.9031
1004.9654
0
20
40
60
80
100
700
800
900
1000

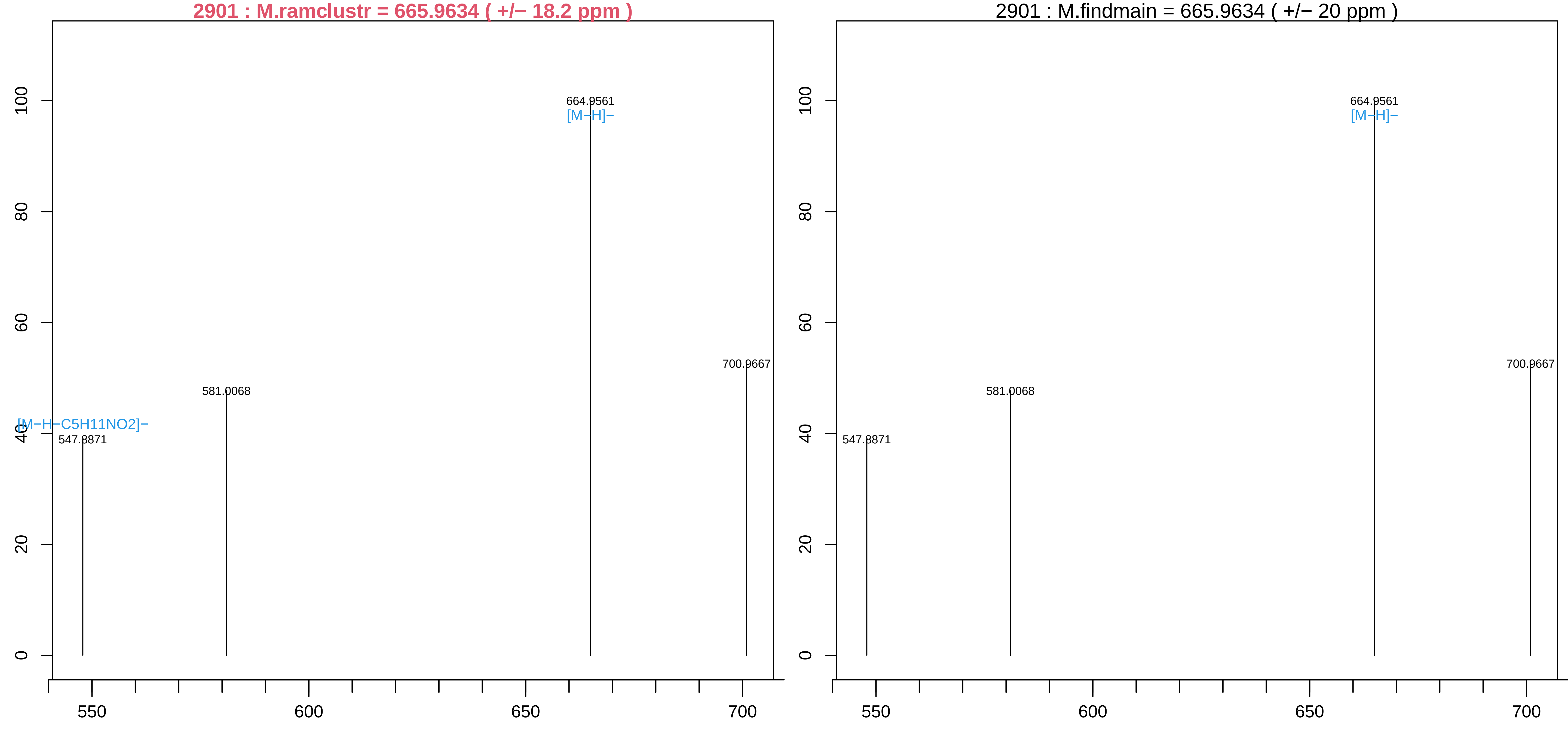

2901 : M.ramclustr = 665.9634 (+/− 18.2 ppm)
664.9561
[M−H]−
581.0068
700.9667
[M−H−C5H11NO2]−
547.8871
0
20
40
60
80
100
550
600
650
700
2901 : M.findmain = 665.9634 (+/− 20 ppm)
664.9561
[M−H]−
581.0068
700.9667
547.8871
0
20
40
60
80
100
550
600
650
700

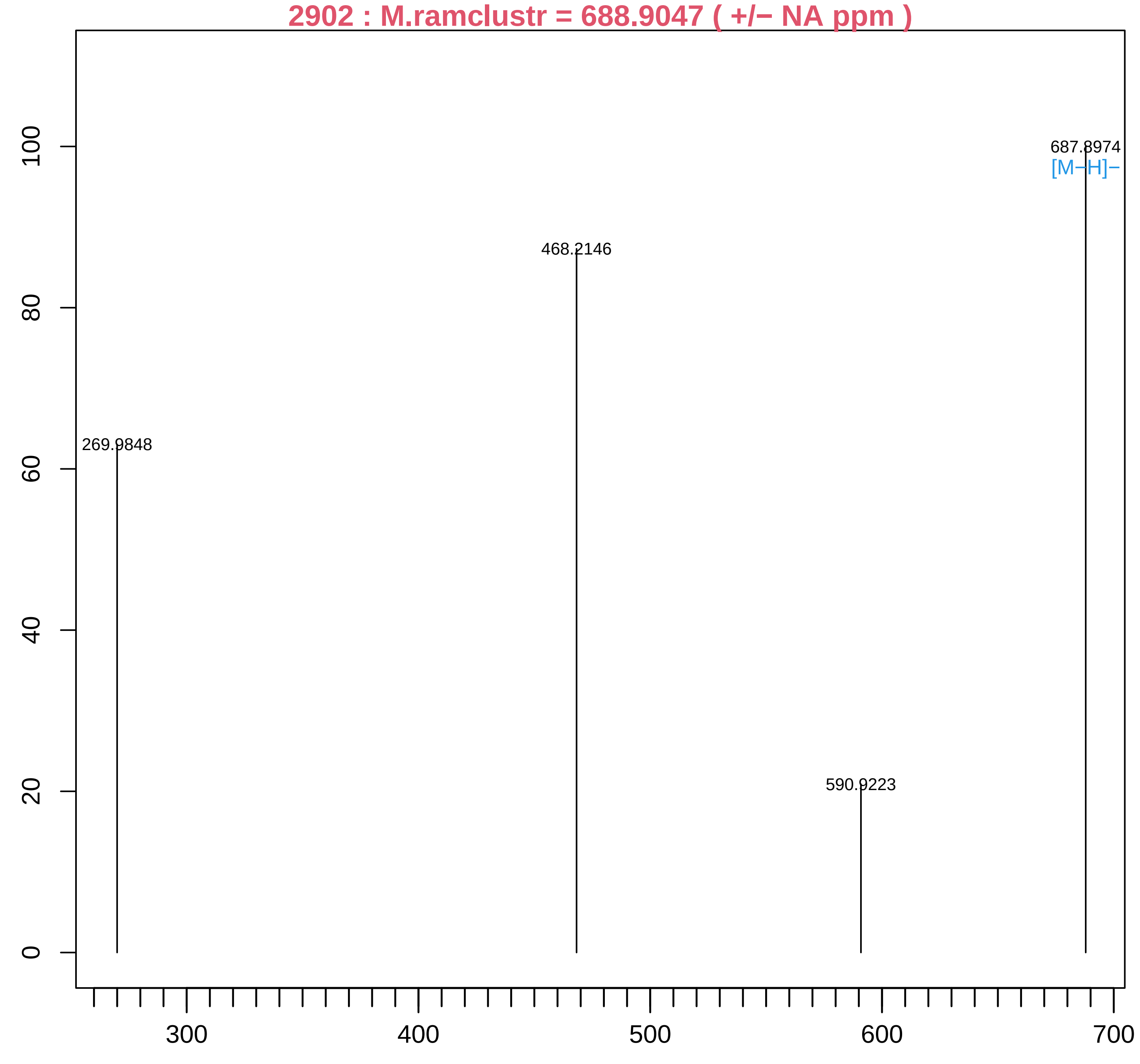
2902 : M.ramclustr = 688.9047 (+/− NA ppm)
687.8974
[M−H]−
468.2146
269.9848
590.9223
0
20
40
60
80
100
300
400
500
600
700

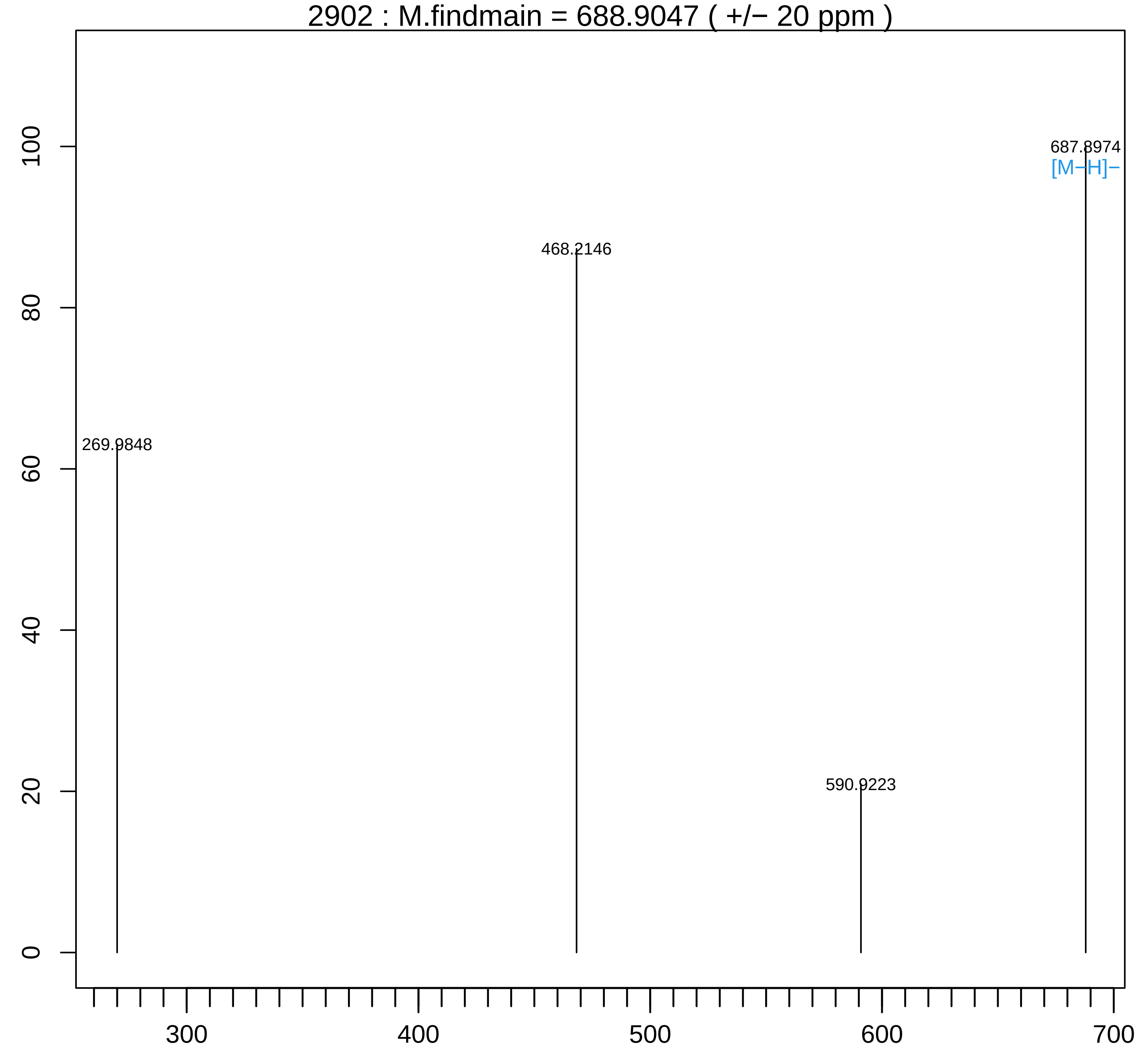
2902 : M.findmain = 688.9047 (+/− 20 ppm)
687.8974
[M−H]−
468.2146
269.9848
590.9223
0
20
40
60
80
100
300
400
500
600
700

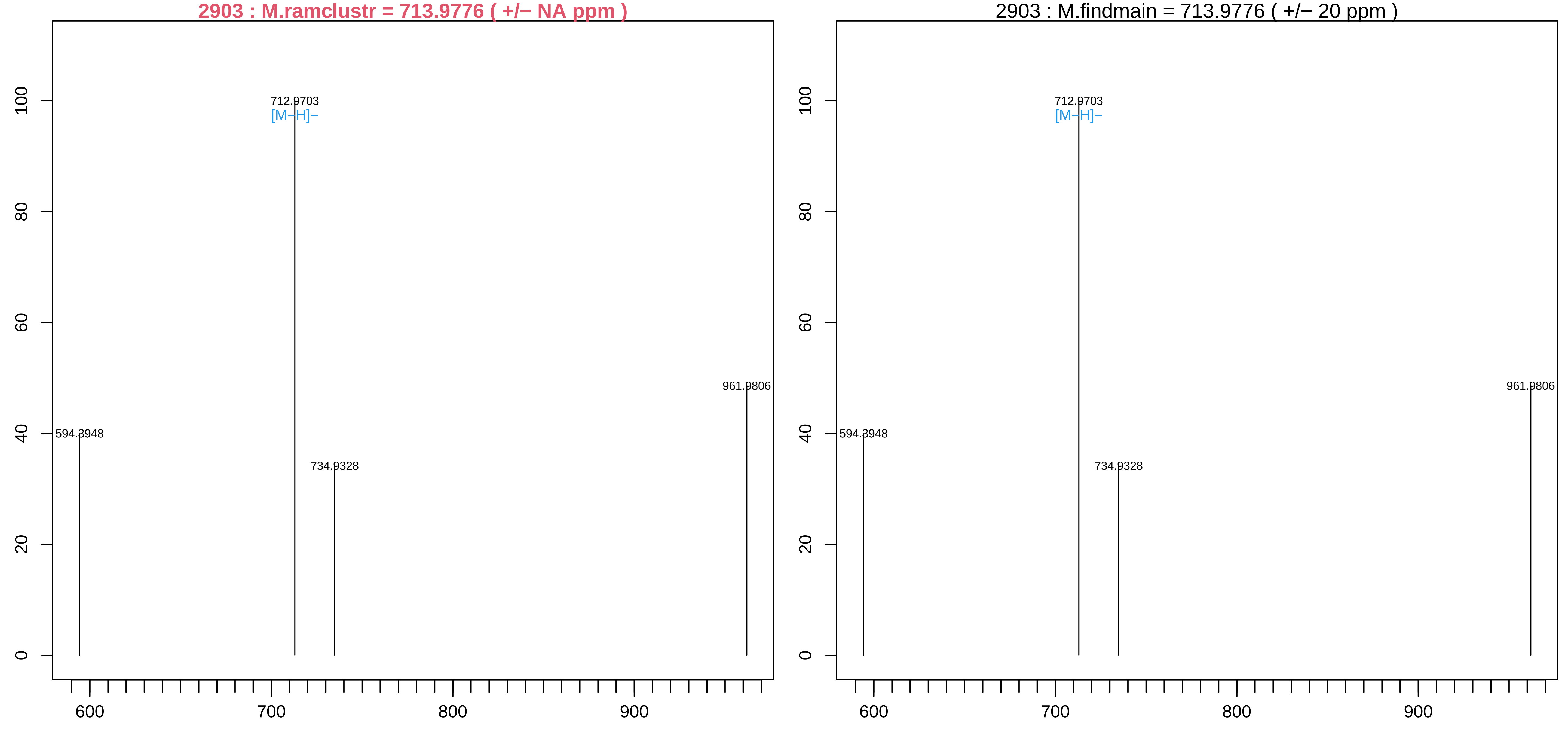
2903 : M.ramclustr = 713.9776 (+/− NA ppm)
712.9703
[M−H]−
594.3948
734.9328
961.9806
2903 : M.findmain = 713.9776 (+/− 20 ppm)
712.9703
[M−H]−
594.3948
734.9328
961.9806
0
20
40
60
80
100
600
700
800
900

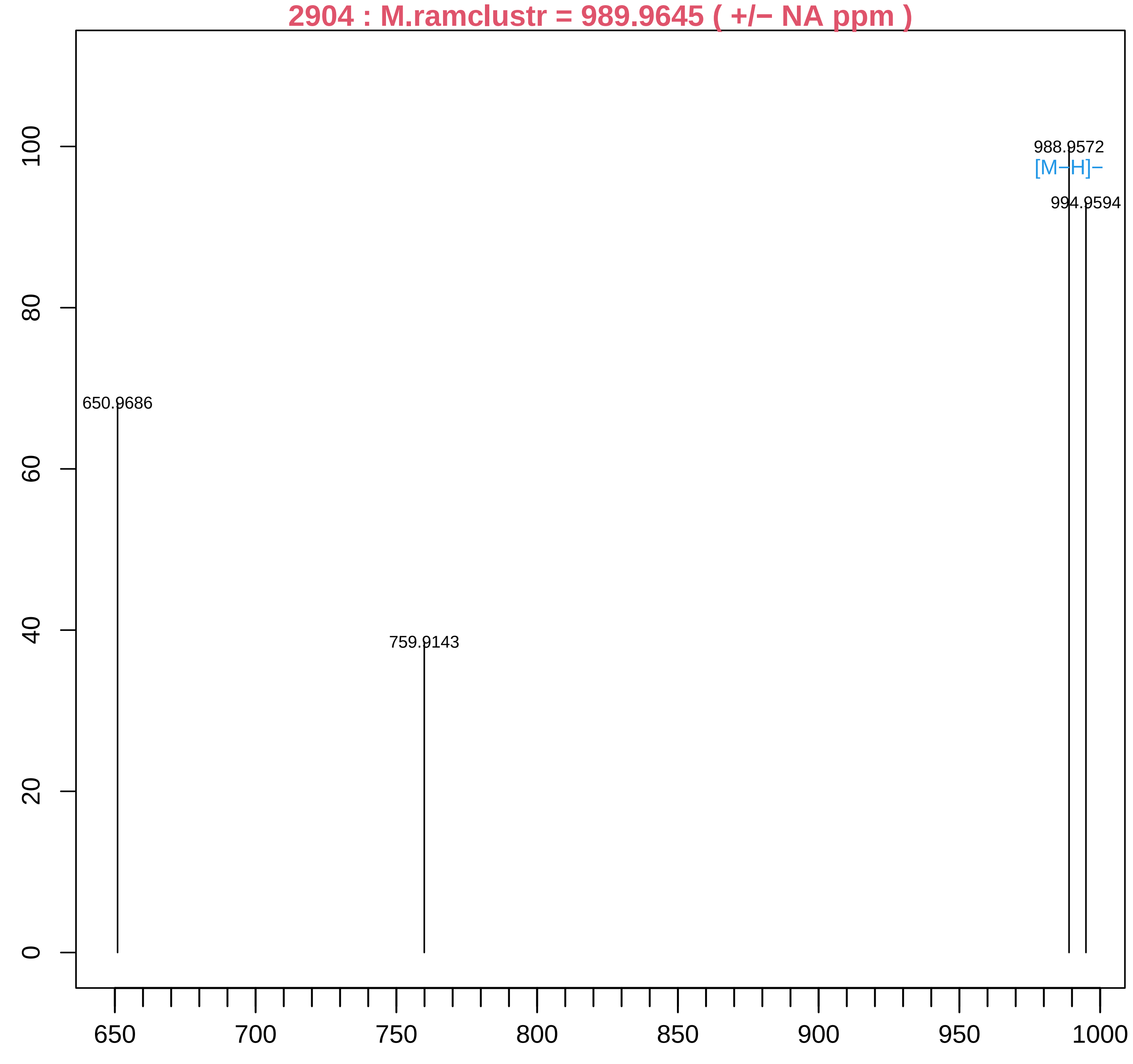

2904 : M.ramclustr = 989.9645 (+/− NA ppm)
988.9572
[M−H]−
994.9594
650.9686
759.9143
0
20
40
60
80
100
650
700
750
800
850
900
950
1000

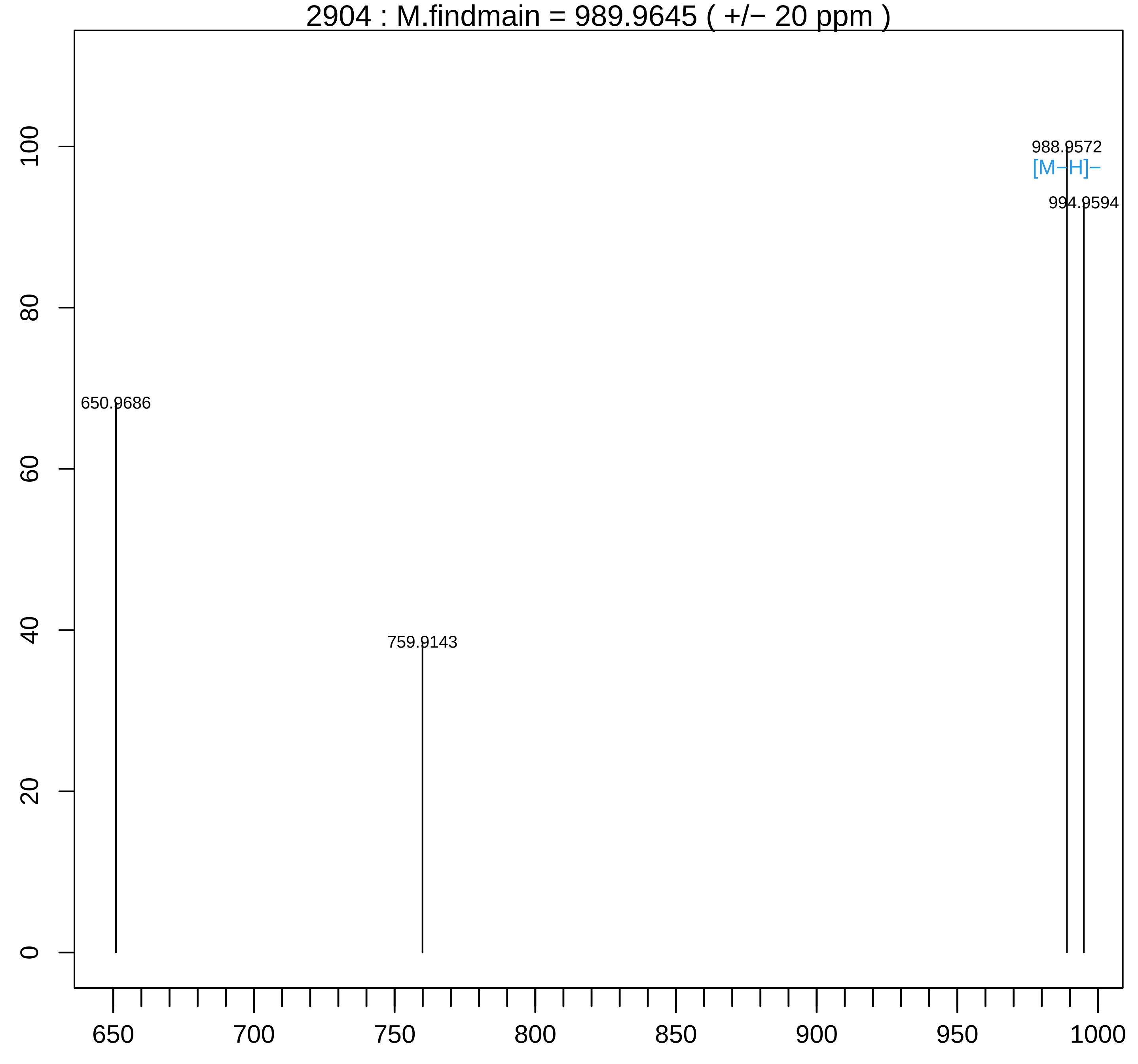

2904 : M.findmain = 989.9645 (+/− 20 ppm)
988.9572
[M−H]−
994.9594
650.9686
759.9143
0
20
40
60
80
100
650
700
750
800
850
900
950
1000

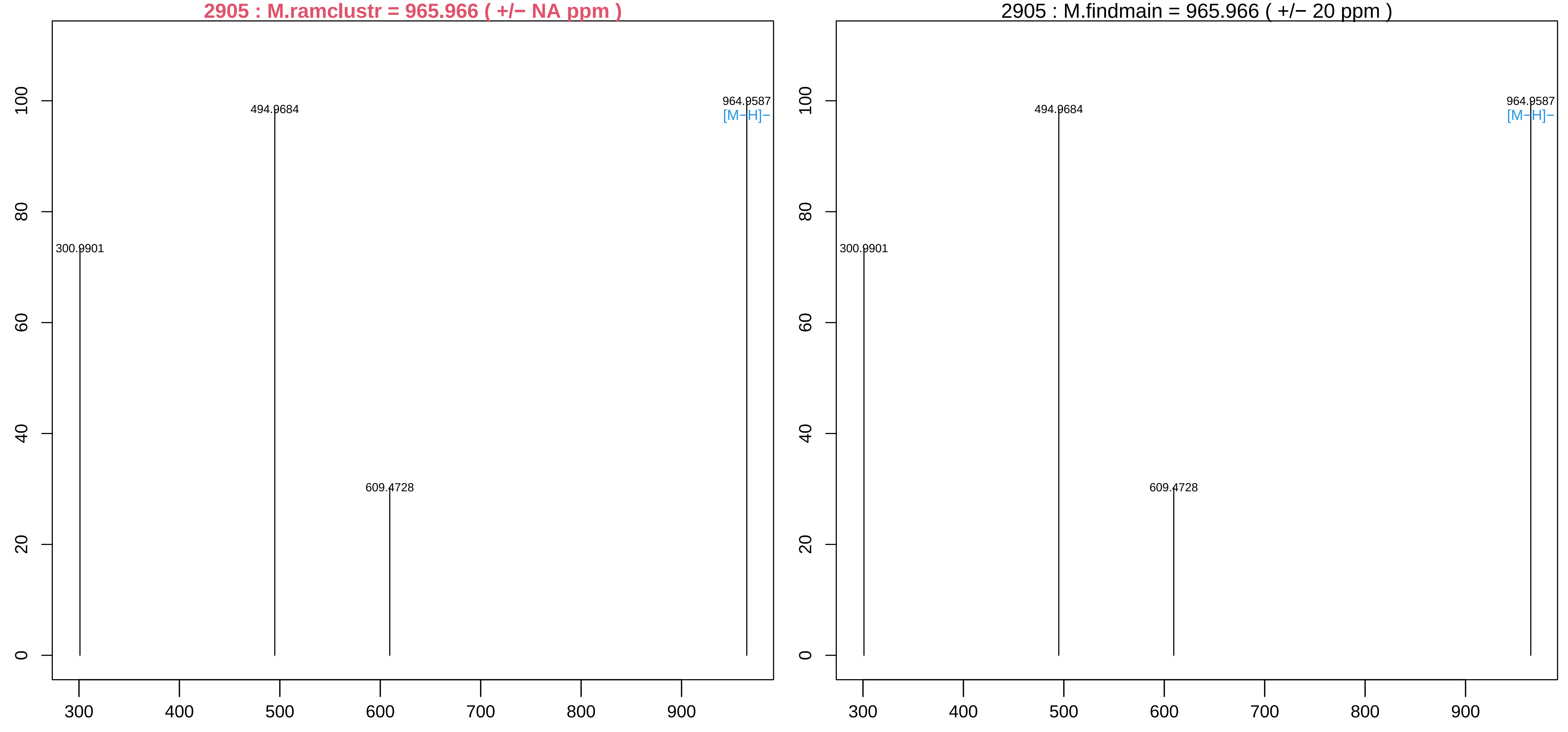
2905 : M.ramclustr = 965.966 (+/− NA ppm)
300.9901
494.9684
609.4728
964.9587
[M−H]−
2905 : M.findmain = 965.966 (+/− 20 ppm)
300.9901
494.9684
609.4728
964.9587
[M−H]−
0
20
40
60
80
100
300
400
500
600
700
800
900

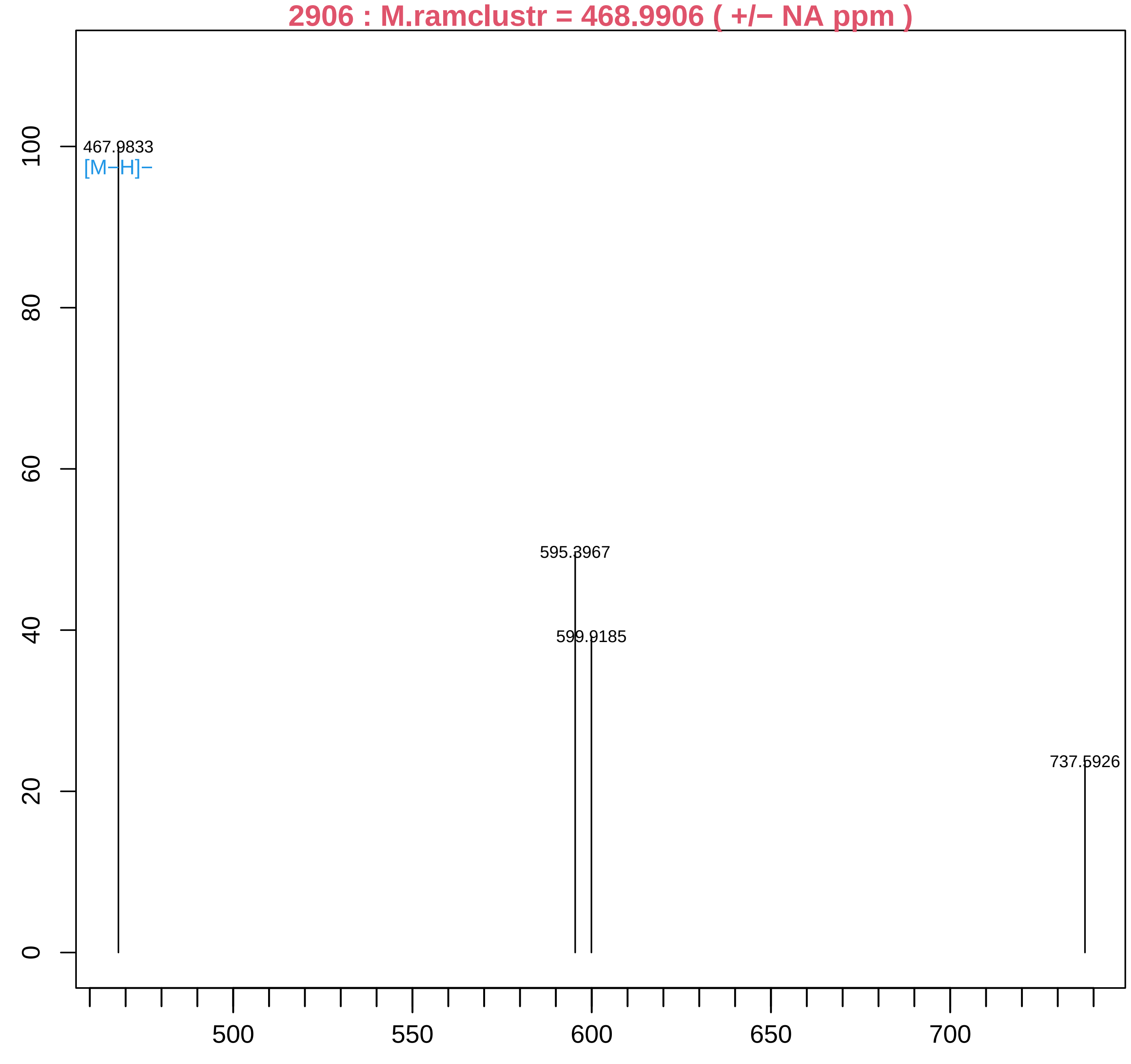

2906 : M.ramclustr = 468.9906 (+/− NA ppm)
467.9833
[M−H]−
595.3967
599.9185
737.5926
0
20
40
60
80
100
500
550
600
650
700

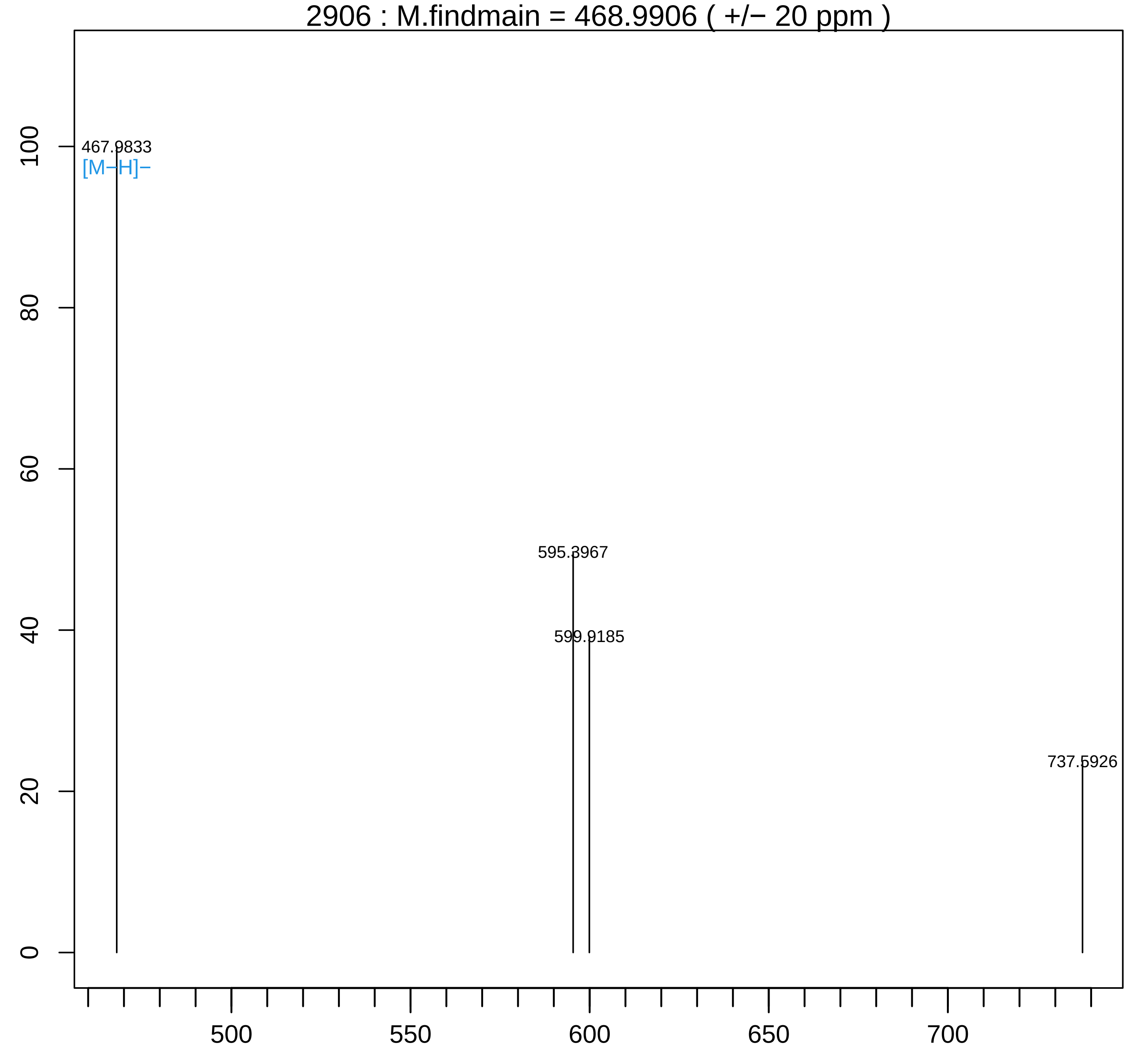

2906 : M.findmain = 468.9906 (+/− 20 ppm)
467.9833
[M−H]−
595.3967
599.9185
737.5926
0
20
40
60
80
100
500
550
600
650
700

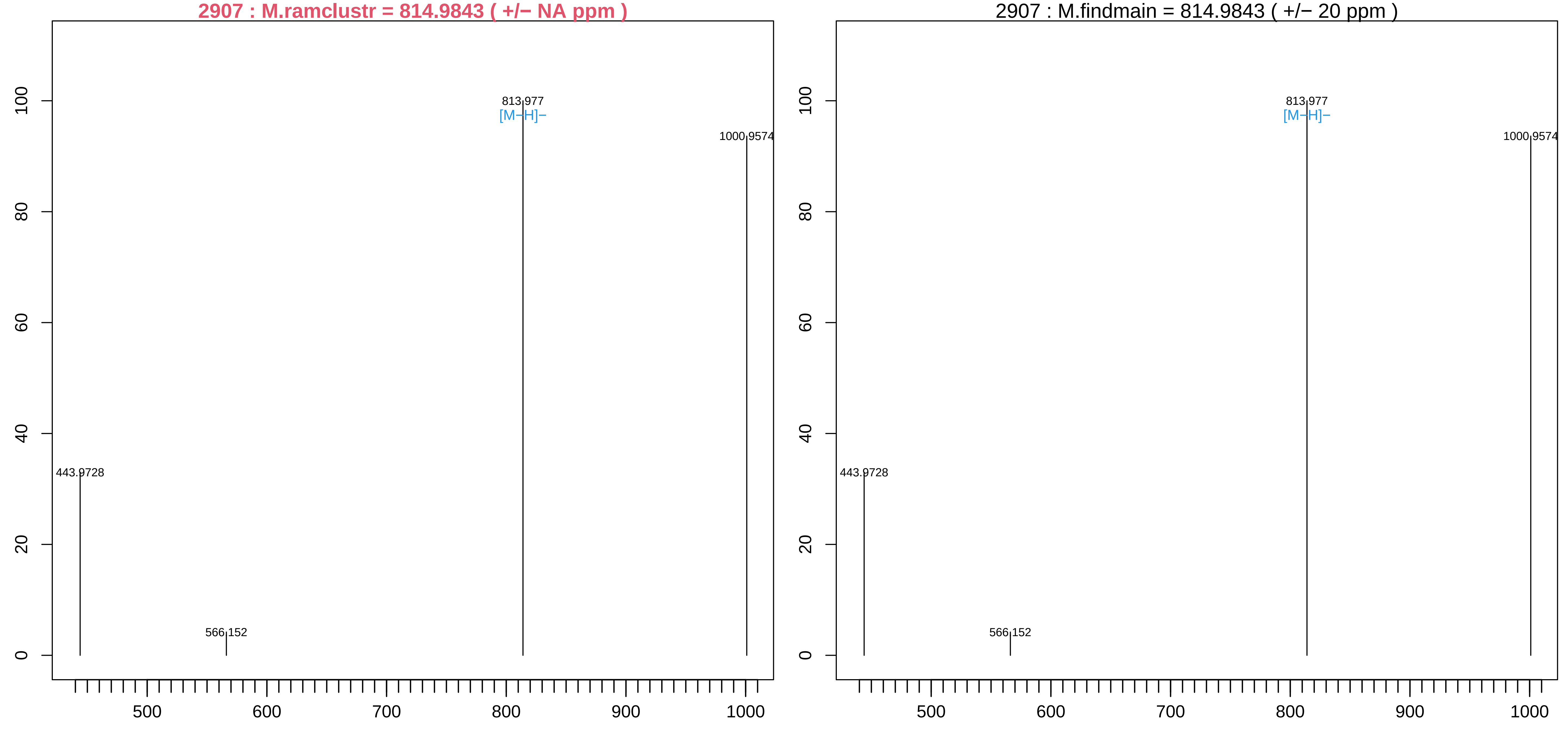
2907 : M.ramclustr = 814.9843 (+/− NA ppm)
813.977
[M−H]−
1000.9574
443.9728
566.152
0
20
40
60
80
100
500
600
700
800
900
1000
2907 : M.findmain = 814.9843 (+/− 20 ppm)
813.977
[M−H]−
1000.9574
443.9728
566.152
0
20
40
60
80
100
500
600
700
800
900
1000

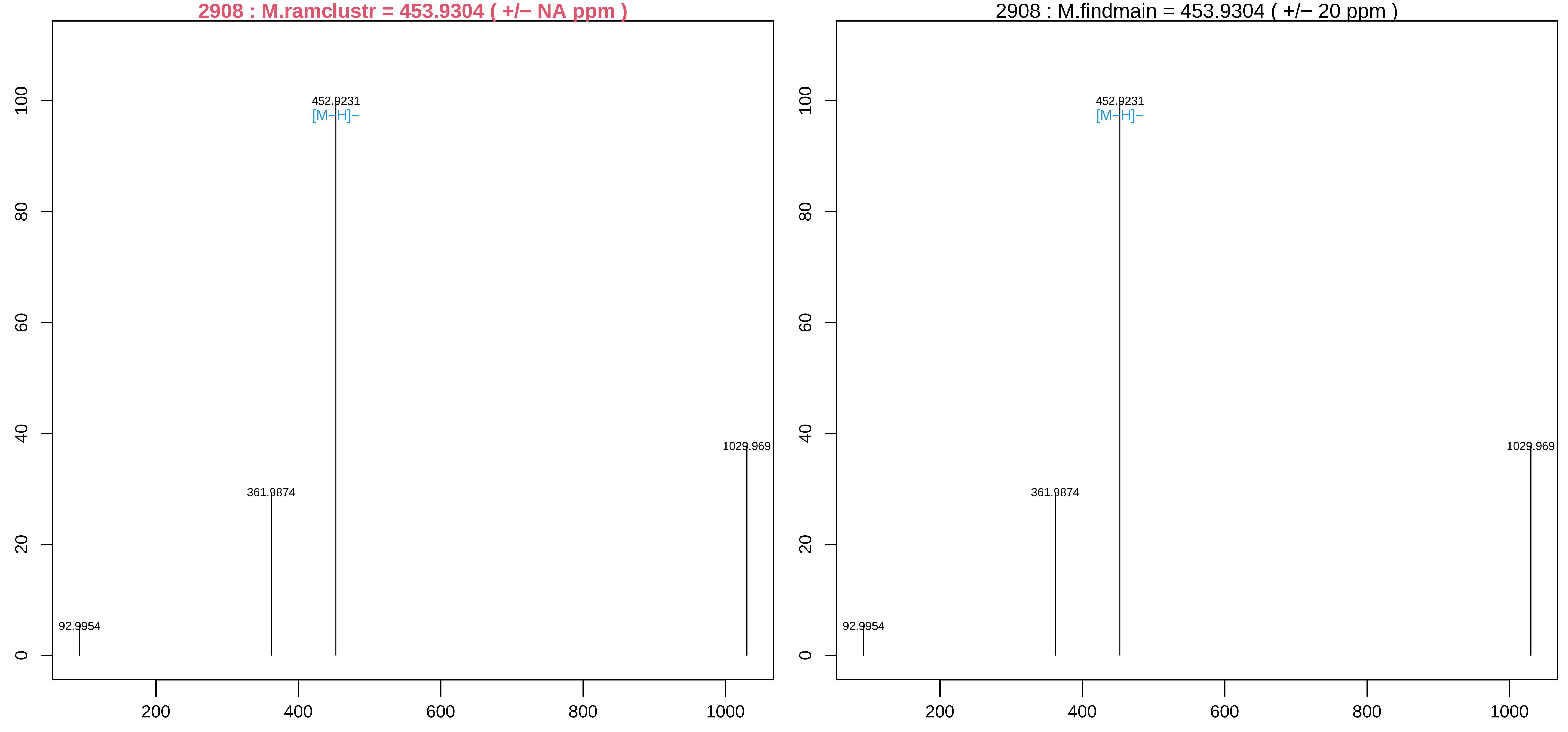
2908 : M.ramclustr = 453.9304 (+/− NA ppm)
452.9231
[M−H]−
361.9874
1029.969
92.9954
0
20
40
60
80
100
200
400
600
800
1000
2908 : M.findmain = 453.9304 (+/− 20 ppm)
452.9231
[M−H]−
361.9874
1029.969
92.9954
0
20
40
60
80
100
200
400
600
800
1000

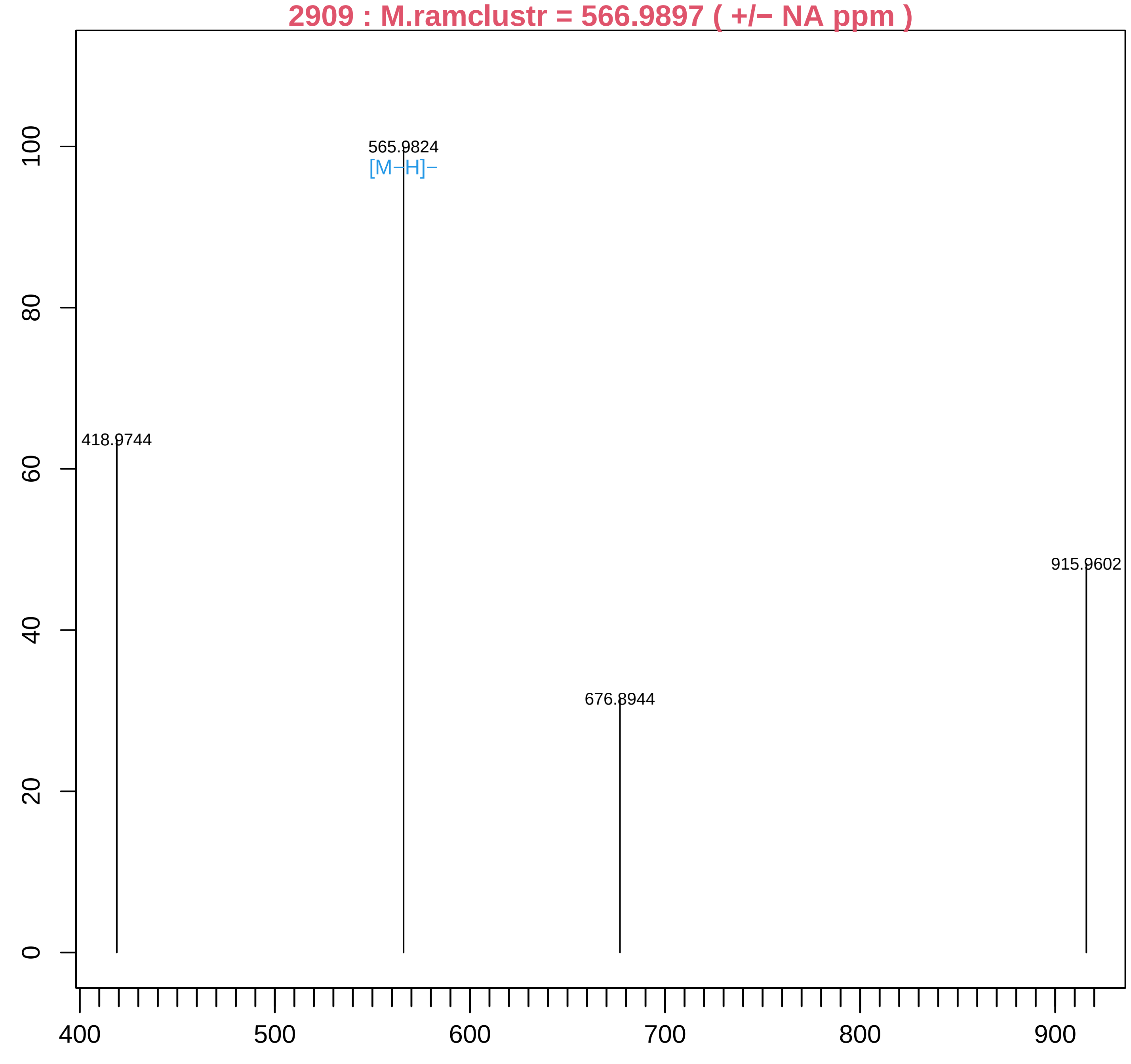

2909 : M.ramclustr = 566.9897 (+/− NA ppm)
565.9824
[M−H]−
418.9744
915.9602
676.8944
0
20
40
60
80
100
400
500
600
700
800
900

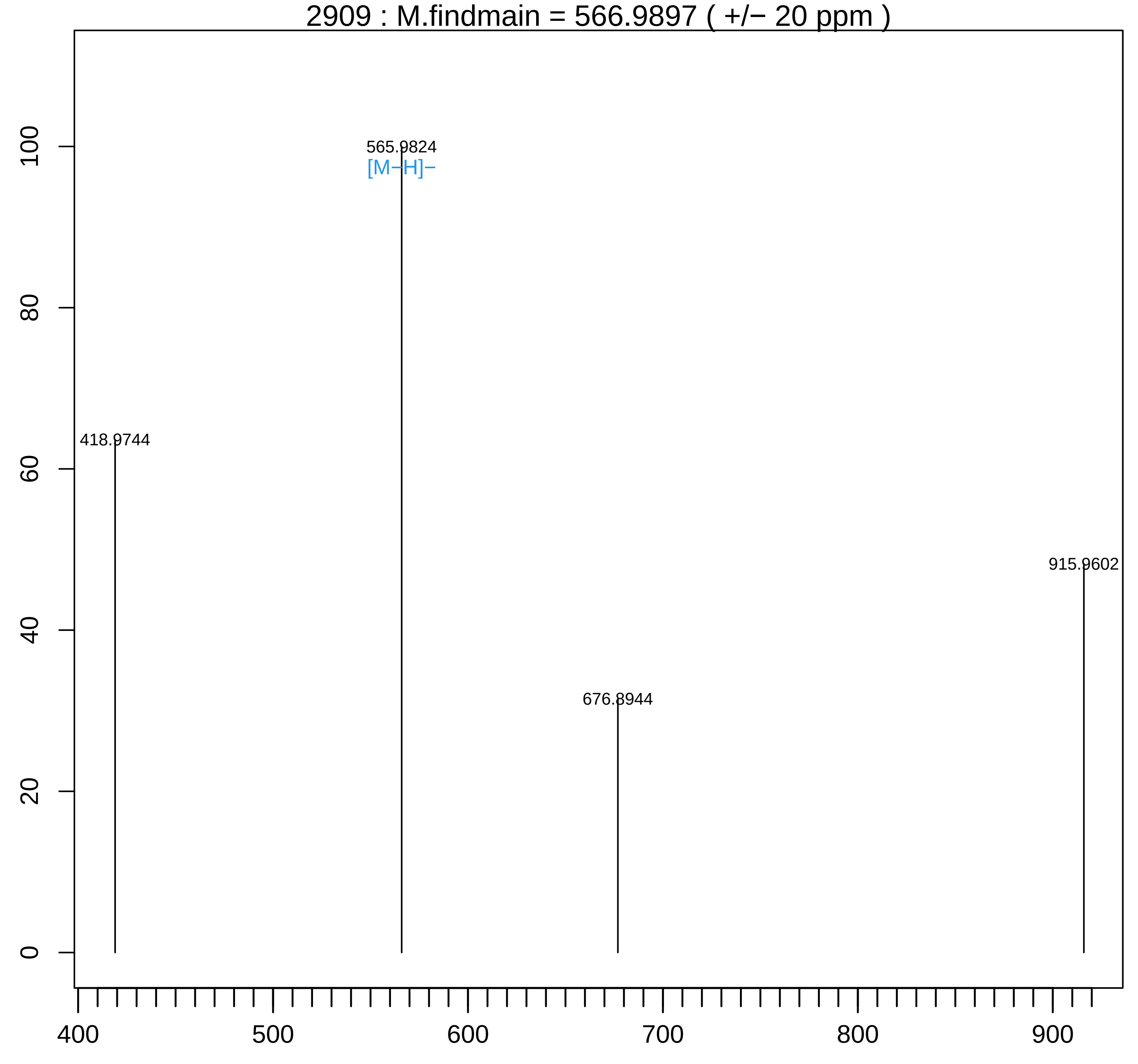

2909 : M.findmain = 566.9897 (+/− 20 ppm)
565.9824
[M−H]−
418.9744
915.9602
676.8944
0
20
40
60
80
100
400
500
600
700
800
900

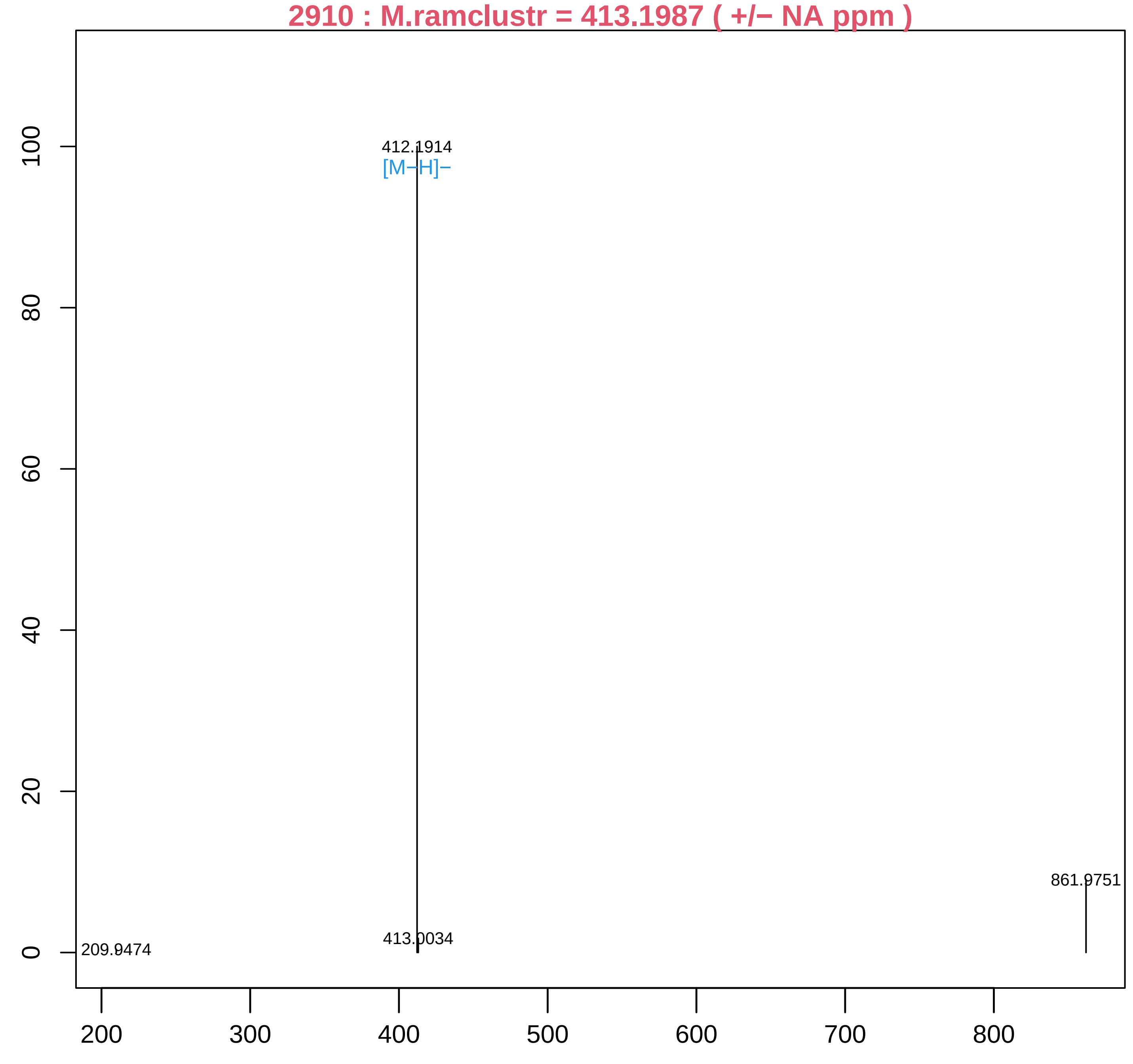
2910 : M.ramclustr = 413.1987 (+/− NA ppm)
412.1914
[M−H]−
413.0034
209.9474
861.9751
0
20
40
60
80
100
200
300
400
500
600
700
800

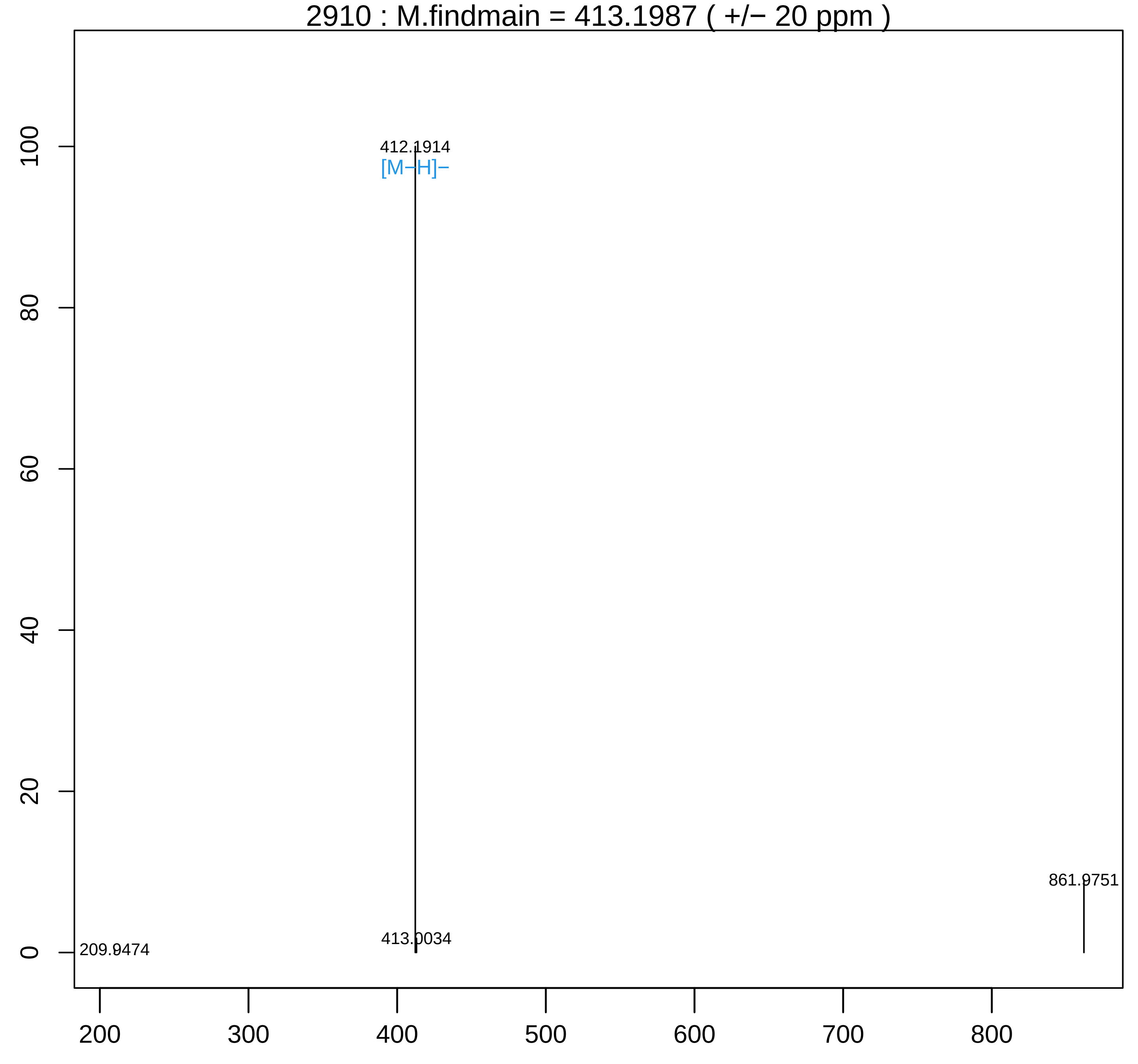
2910 : M.findmain = 413.1987 (+/− 20 ppm)
412.1914
[M−H]−
413.0034
209.9474
861.9751
0
20
40
60
80
100
200
300
400
500
600
700
800

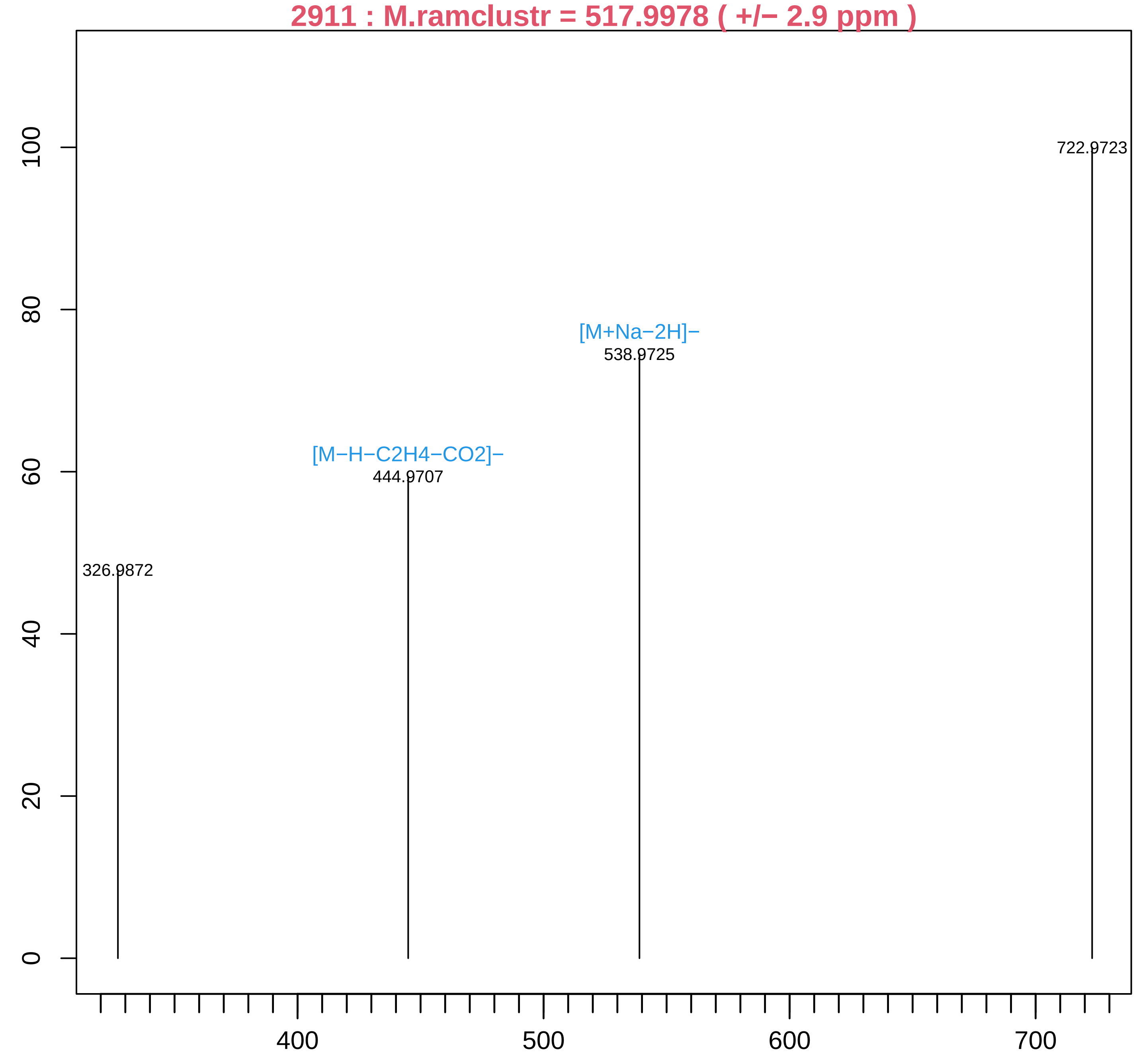
2911 : M.ramclustr = 517.9978 (+/− 2.9 ppm)
722.9723
[M+Na−2H]−
538.9725
[M−H−C2H4−CO2]−
444.9707
326.9872
0
20
40
60
80
100
400
500
600
700

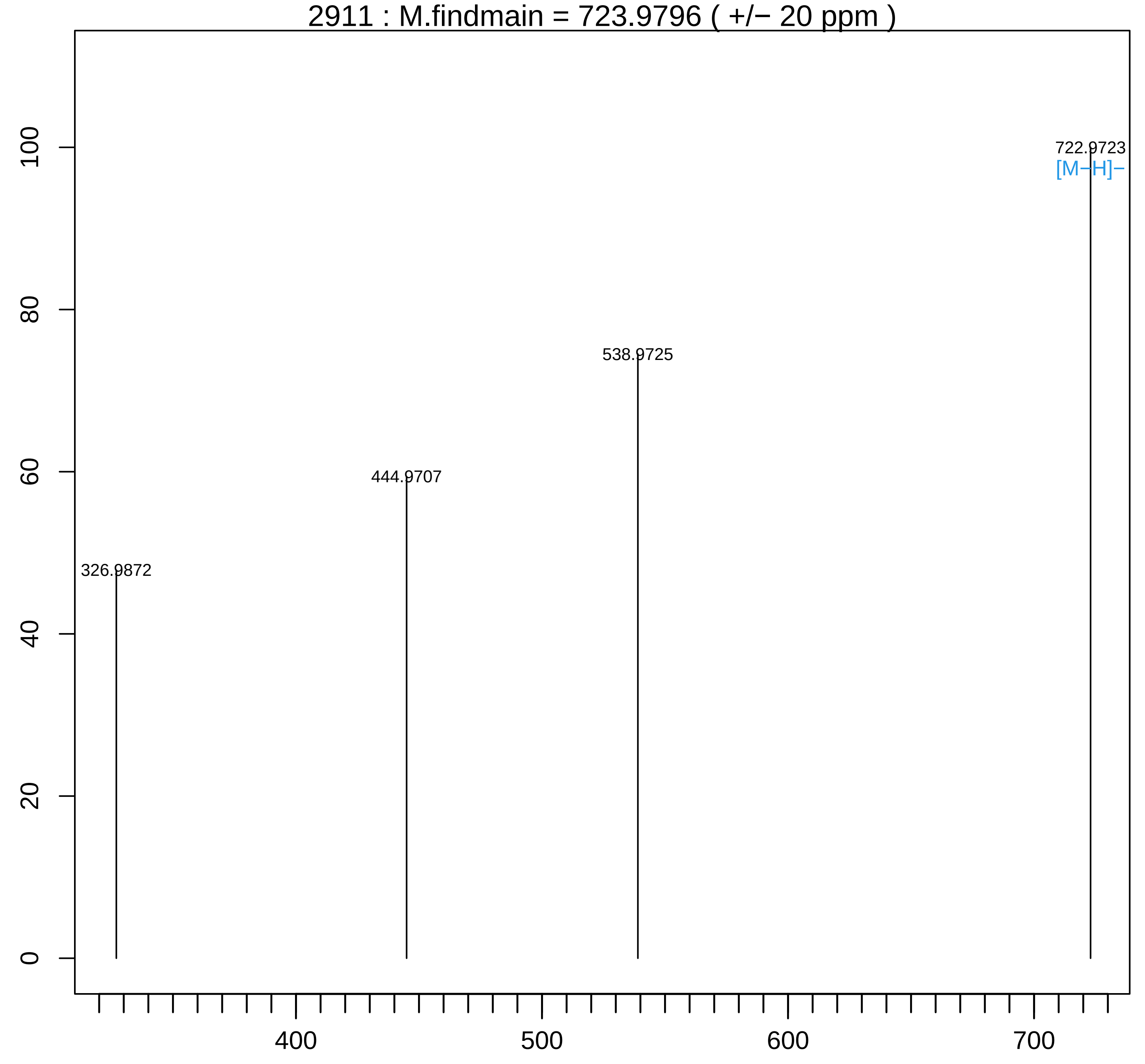
2911 : M.findmain = 723.9796 (+/− 20 ppm)
722.9723
[M−H]−
538.9725
444.9707
326.9872
0
20
40
60
80
100
400
500
600
700

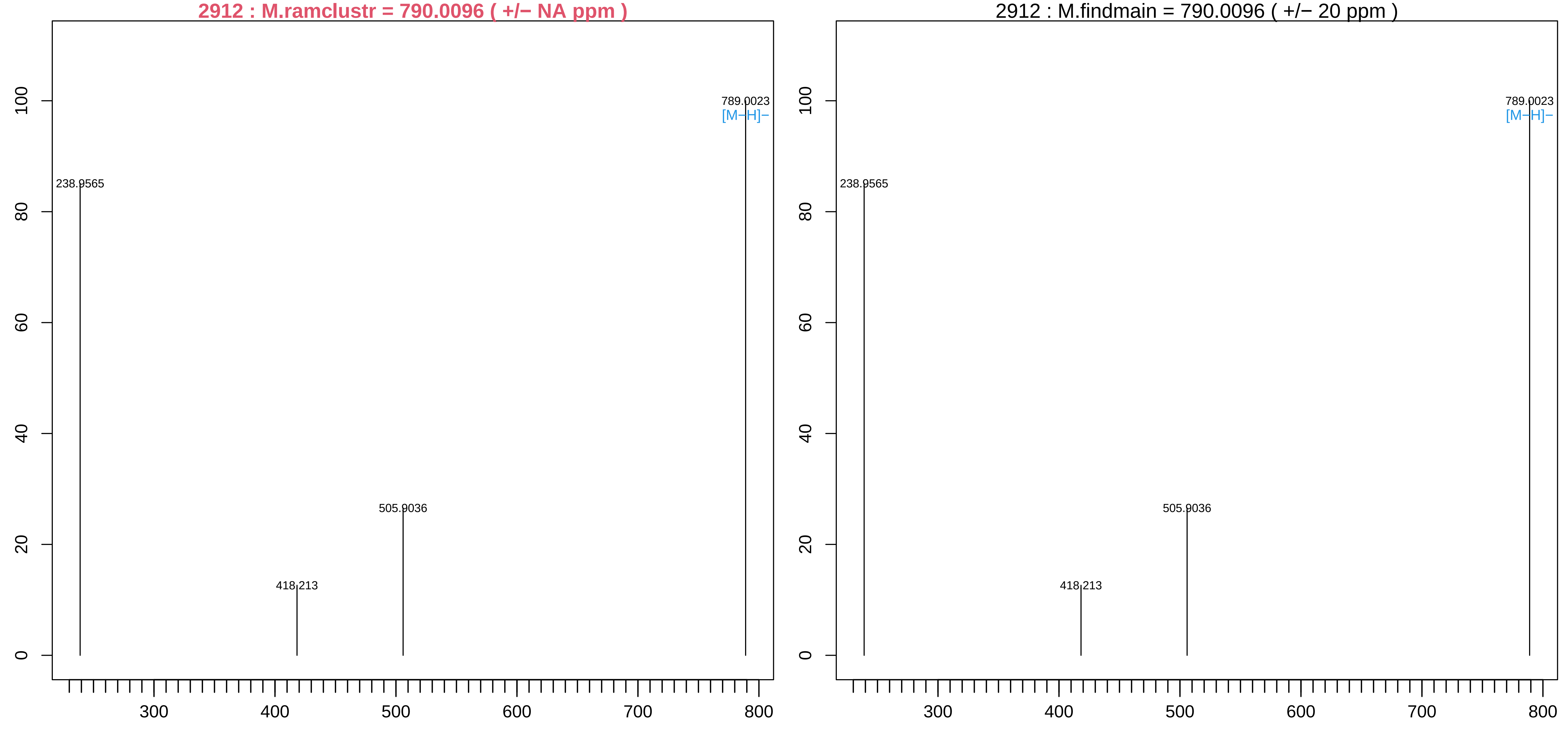
2912 : M.ramclustr = 790.0096 (+/− NA ppm)
789.0023
[M−H]−
238.9565
505.9036
418.213
2912 : M.findmain = 790.0096 (+/− 20 ppm)
789.0023
[M−H]−
238.9565
505.9036
418.213

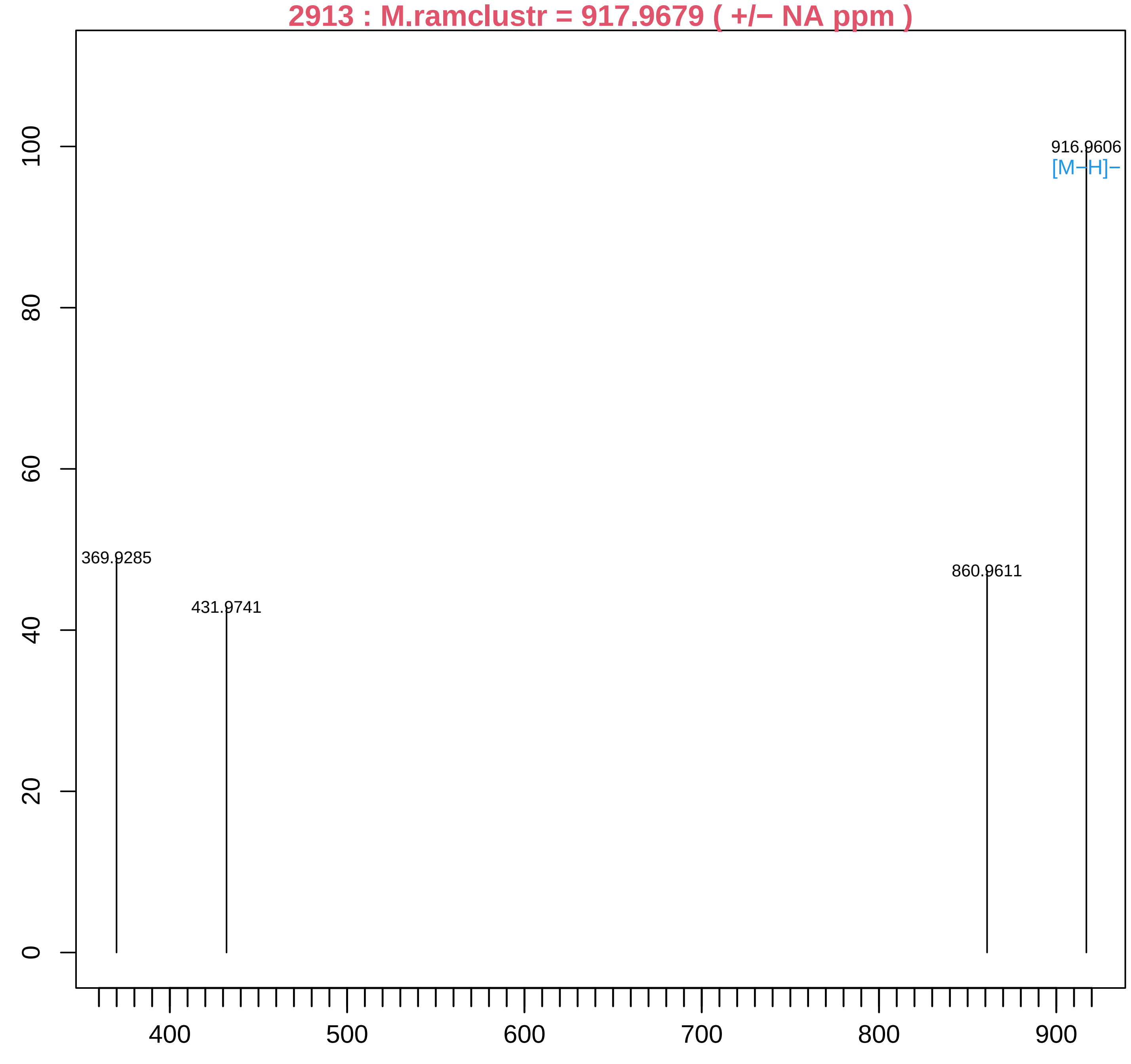
2913 : M.ramclustr = 917.9679 (+/− NA ppm)
916.9606
[M−H]−
369.9285
431.9741
860.9611
0
20
40
60
80
100
400
500
600
700
800
900

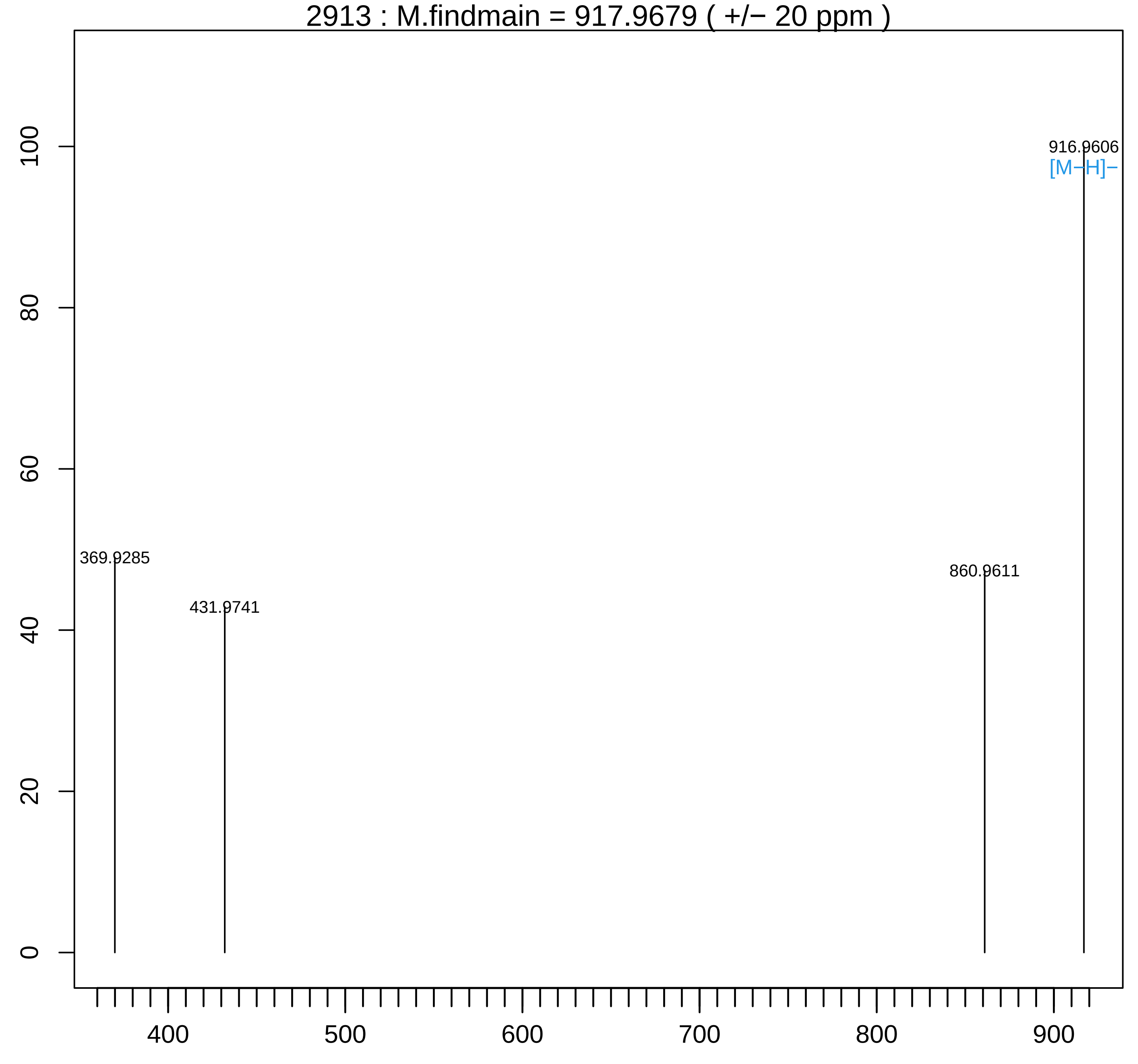
2913 : M.findmain = 917.9679 (+/− 20 ppm)
916.9606
[M−H]−
369.9285
431.9741
860.9611
0
20
40
60
80
100
400
500
600
700
800
900

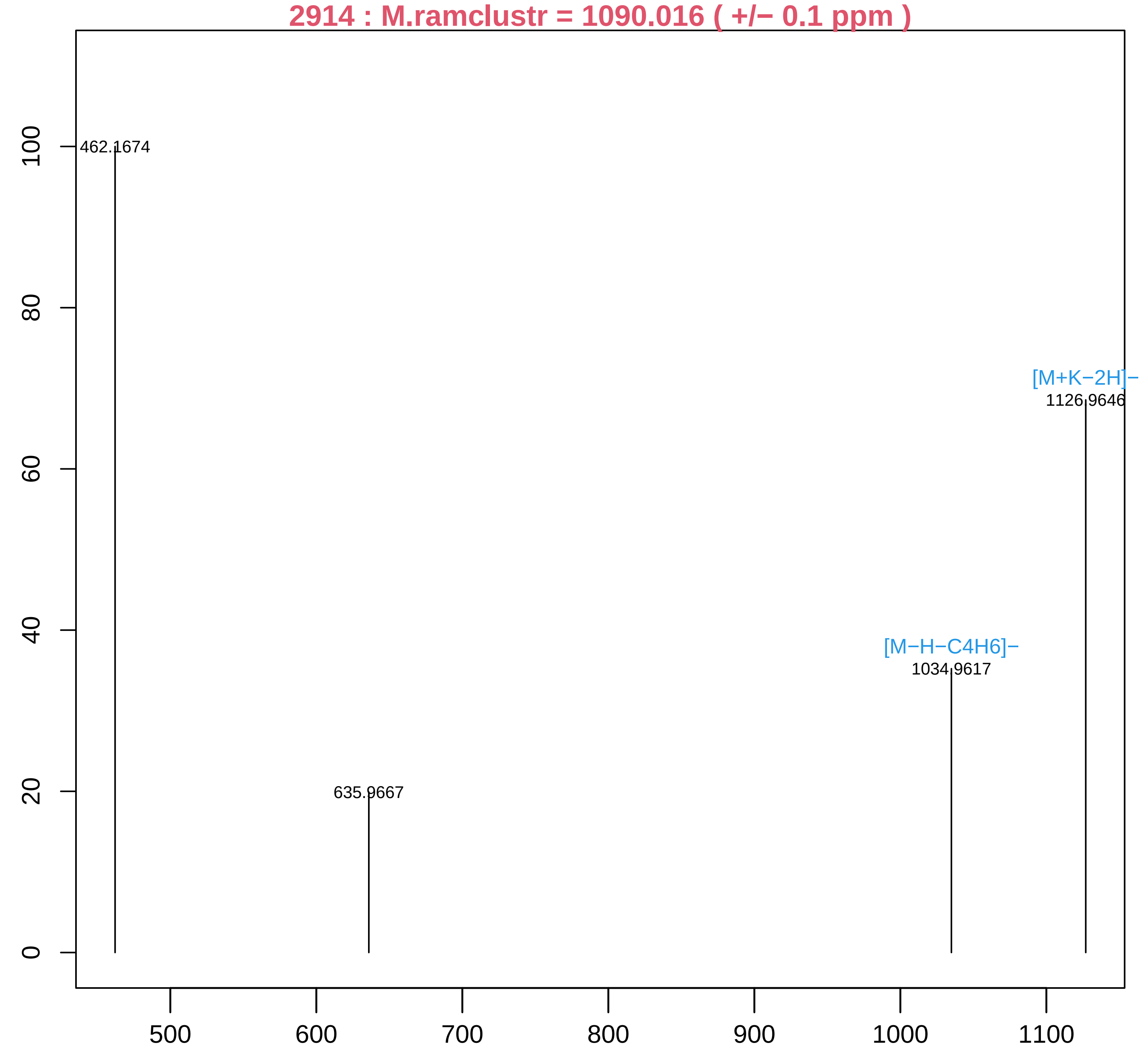
2914 : M.ramclustr = 1090.016 (+/− 0.1 ppm)
462.1674
635.9667
[M−H−C4H6]−
1034.9617
[M+K−2H]−
1126.9646
0
20
40
60
80
100
500
600
700
800
900
1000
1100

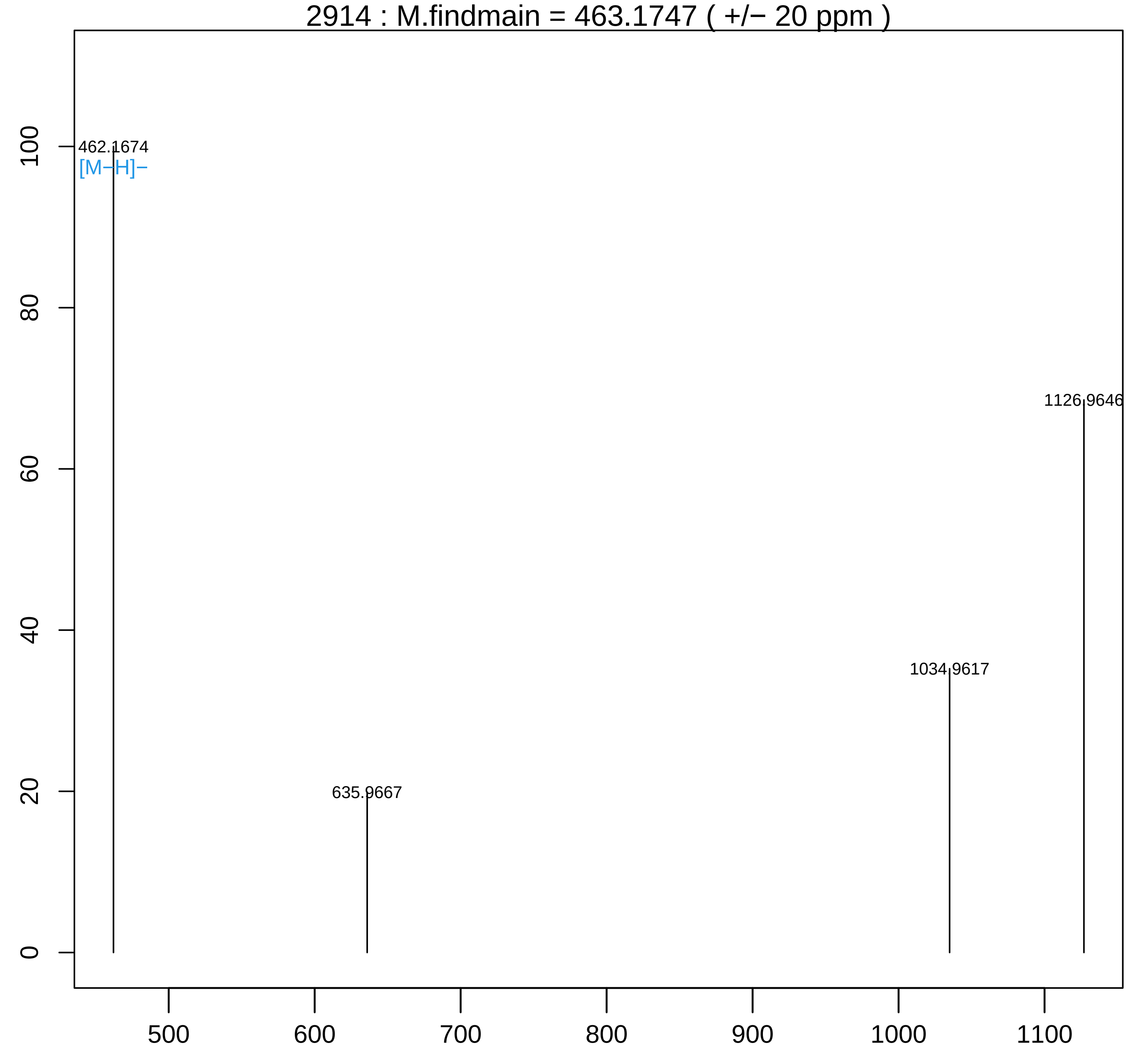
2914 : M.findmain = 463.1747 (+/− 20 ppm)
462.1674
[M−H]−
635.9667
1034.9617
1126.9646
0
20
40
60
80
100
500
600
700
800
900
1000
1100

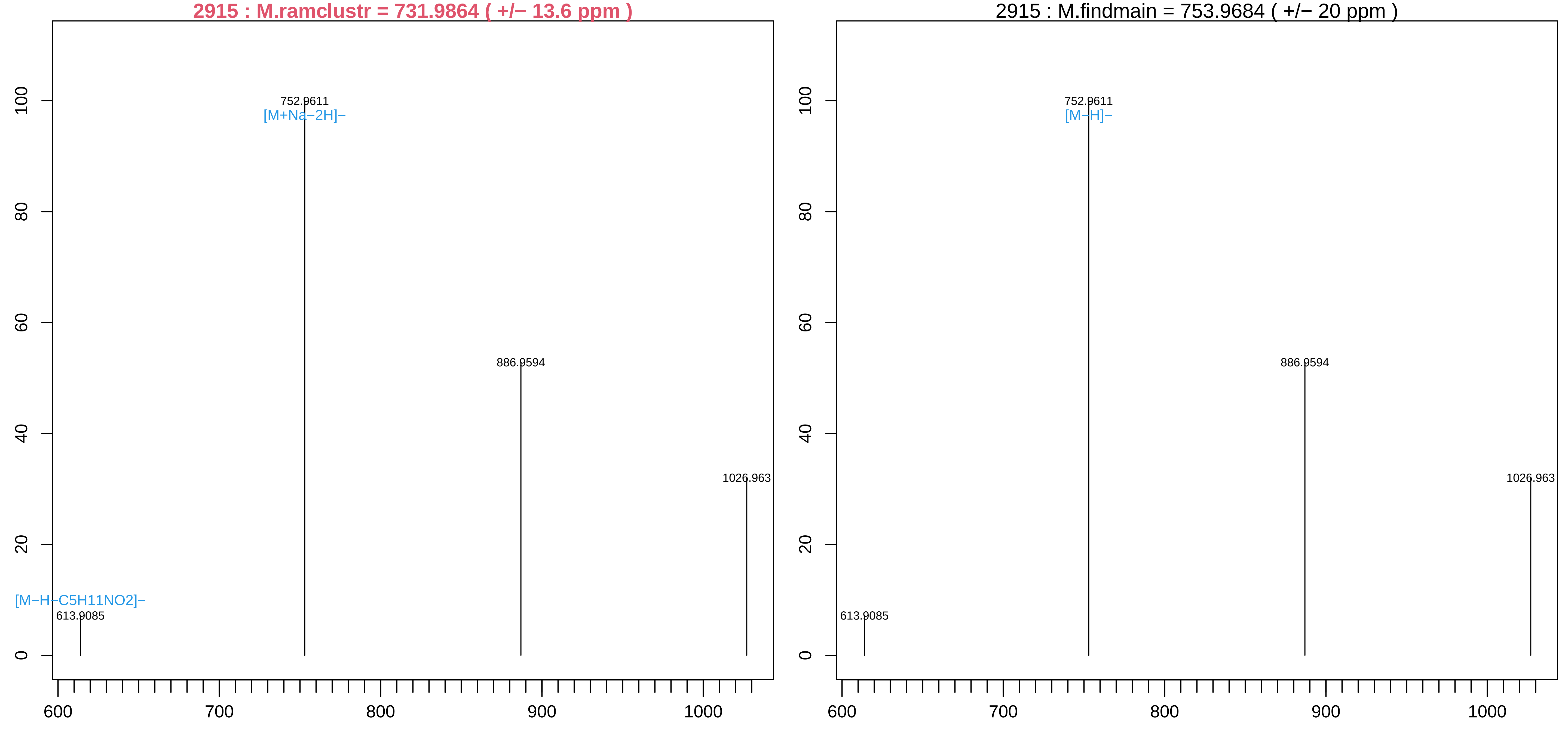
2915 : M.ramclustr = 731.9864 (+/− 13.6 ppm)
752.9611
[M+Na−2H]−
886.9594
1026.963
[M−H−C5H11NO2]−
613.9085
2915 : M.findmain = 753.9684 (+/− 20 ppm)
752.9611
[M−H]−
886.9594
1026.963
613.9085

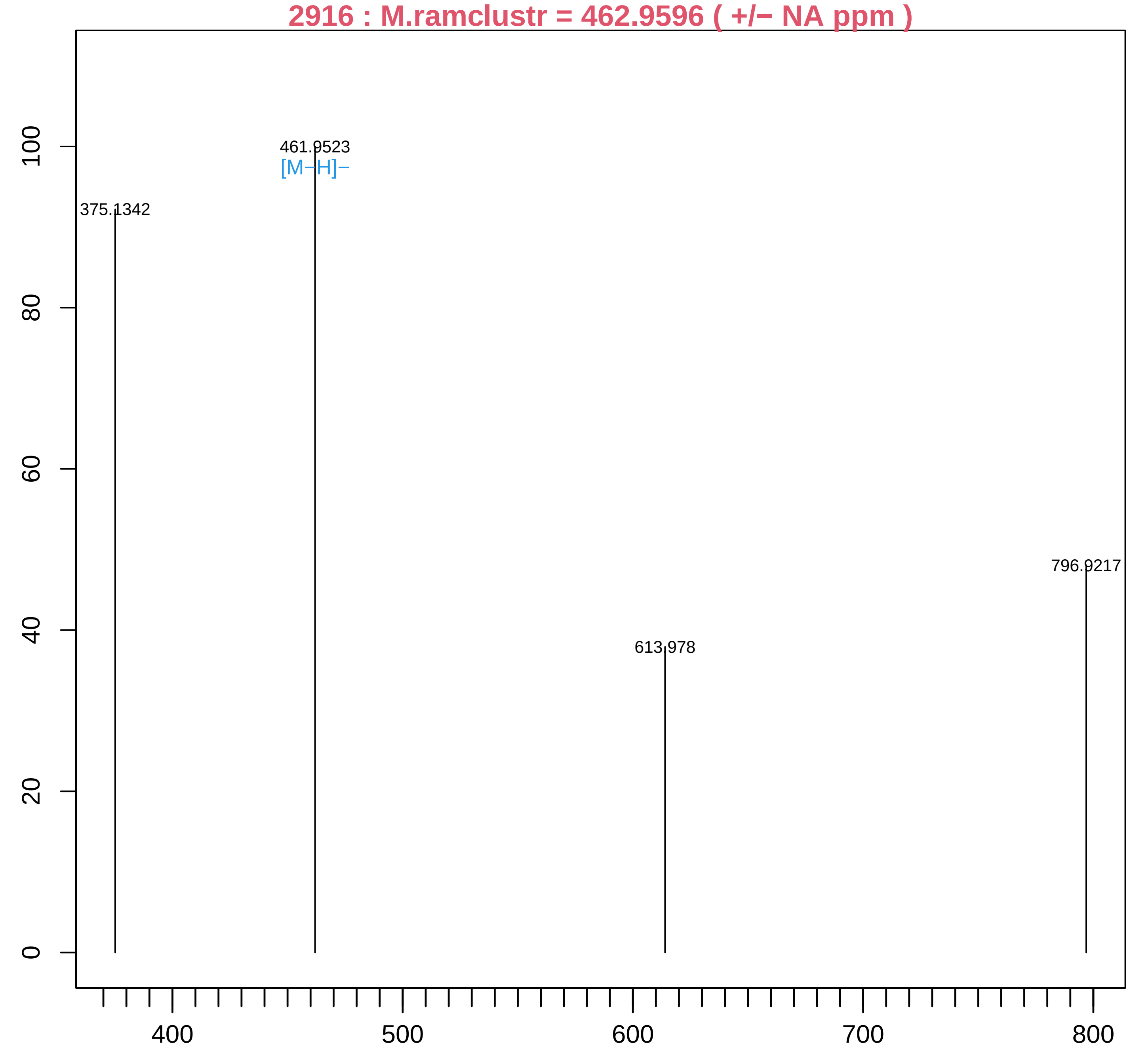
2916 : M.ramclustr = 462.9596 (+/− NA ppm)
375.1342
461.9523
[M−H]−
613.978
796.9217
0
20
40
60
80
100
400
500
600
700
800

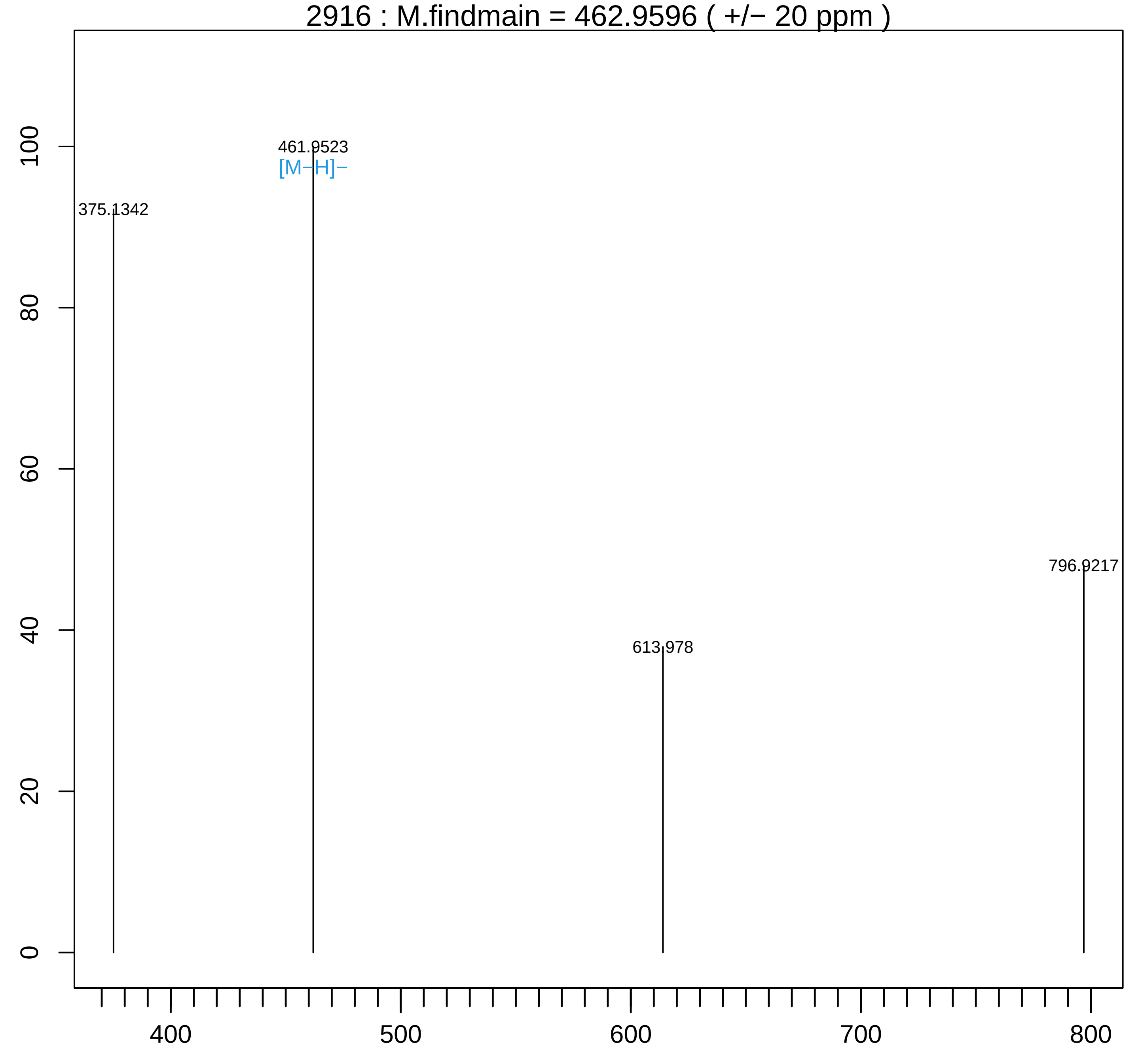
2916 : M.findmain = 462.9596 (+/− 20 ppm)
375.1342
461.9523
[M−H]−
613.978
796.9217
0
20
40
60
80
100
400
500
600
700
800

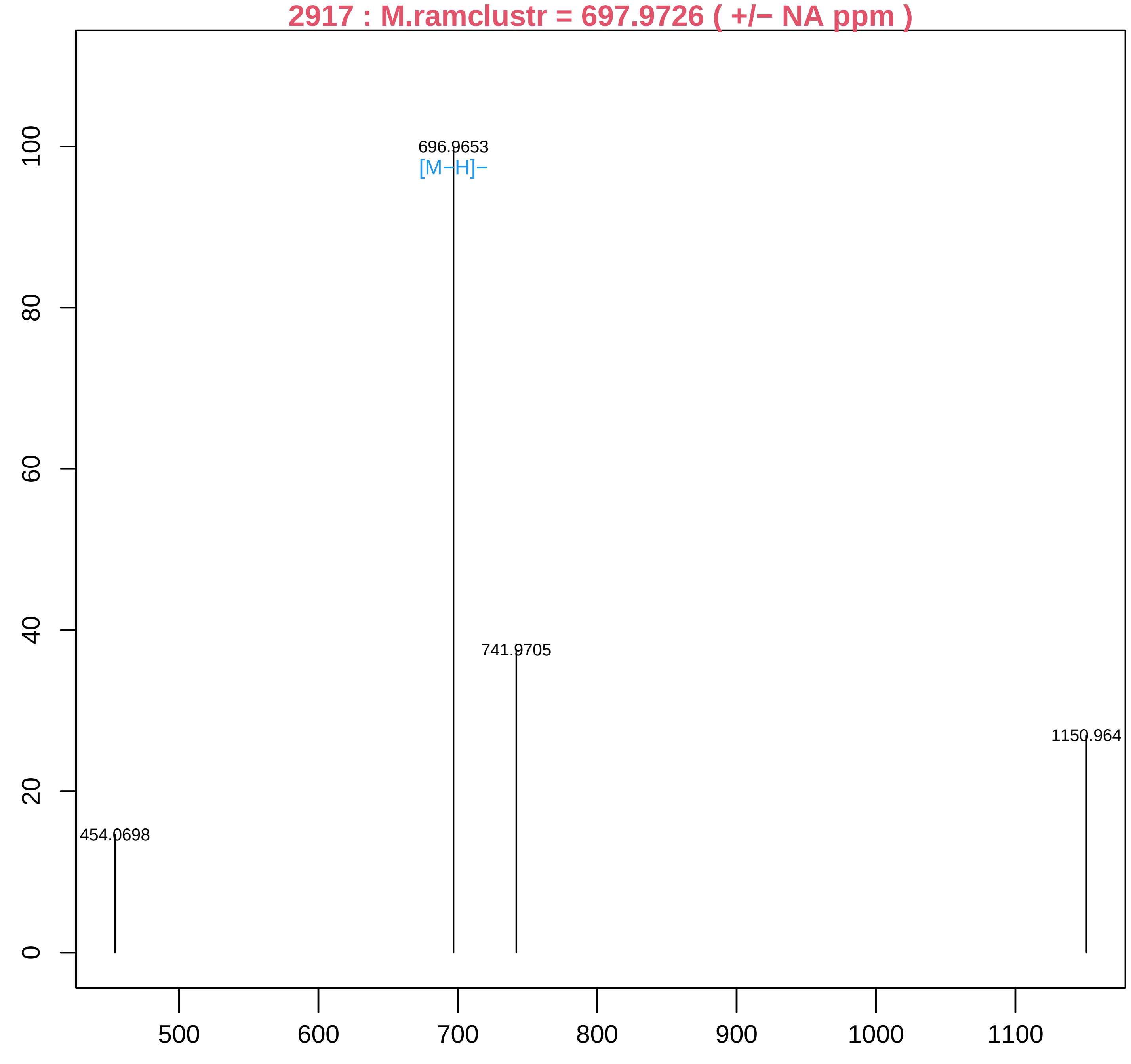
2917 : M.ramclustr = 697.9726 (+/− NA ppm)
696.9653
[M−H]−
741.9705
1150.964
454.0698
0
20
40
60
80
100
500
600
700
800
900
1000
1100

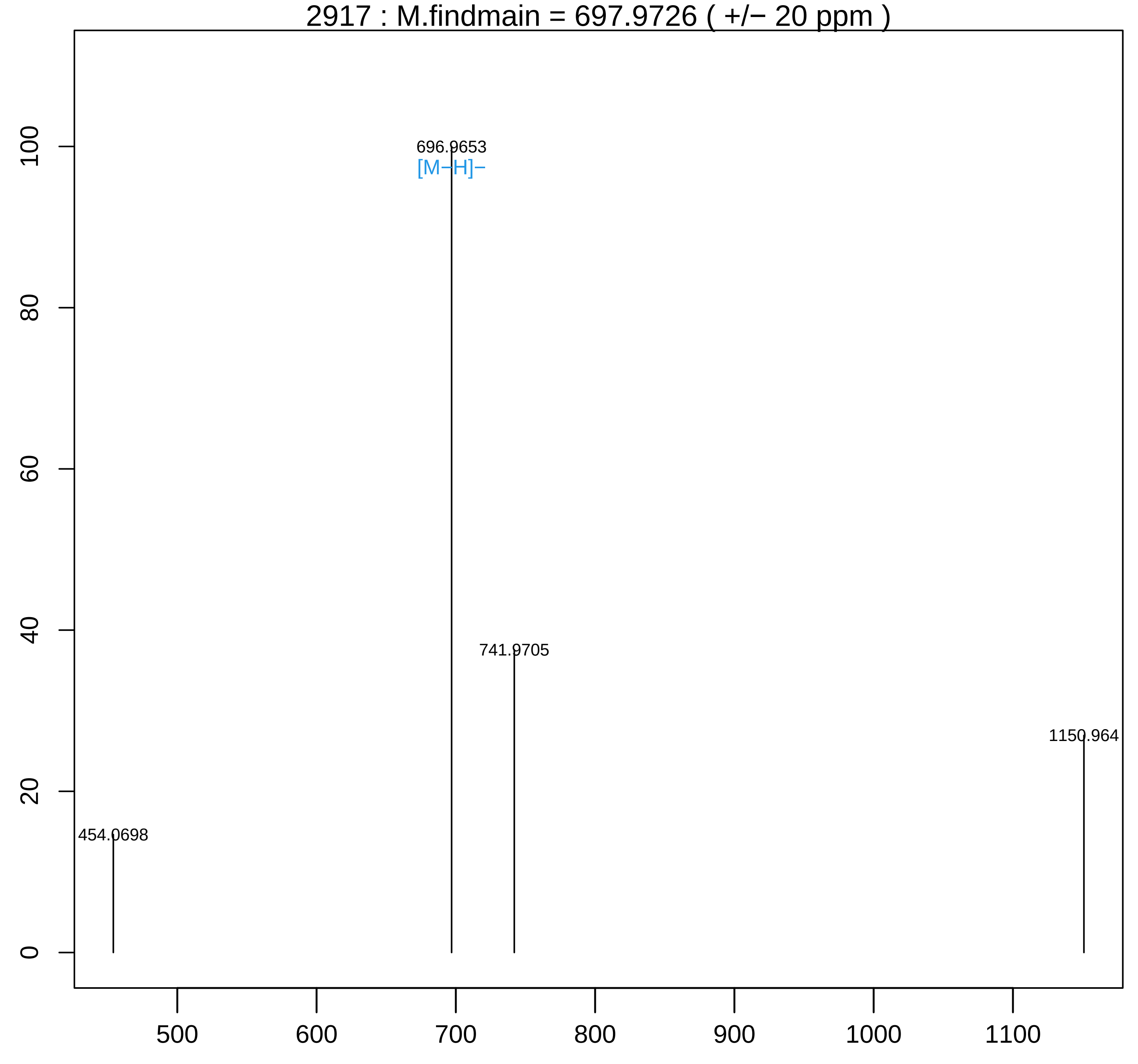
2917 : M.findmain = 697.9726 (+/− 20 ppm)
696.9653
[M−H]−
741.9705
1150.964
454.0698
0
20
40
60
80
100
500
600
700
800
900
1000
1100

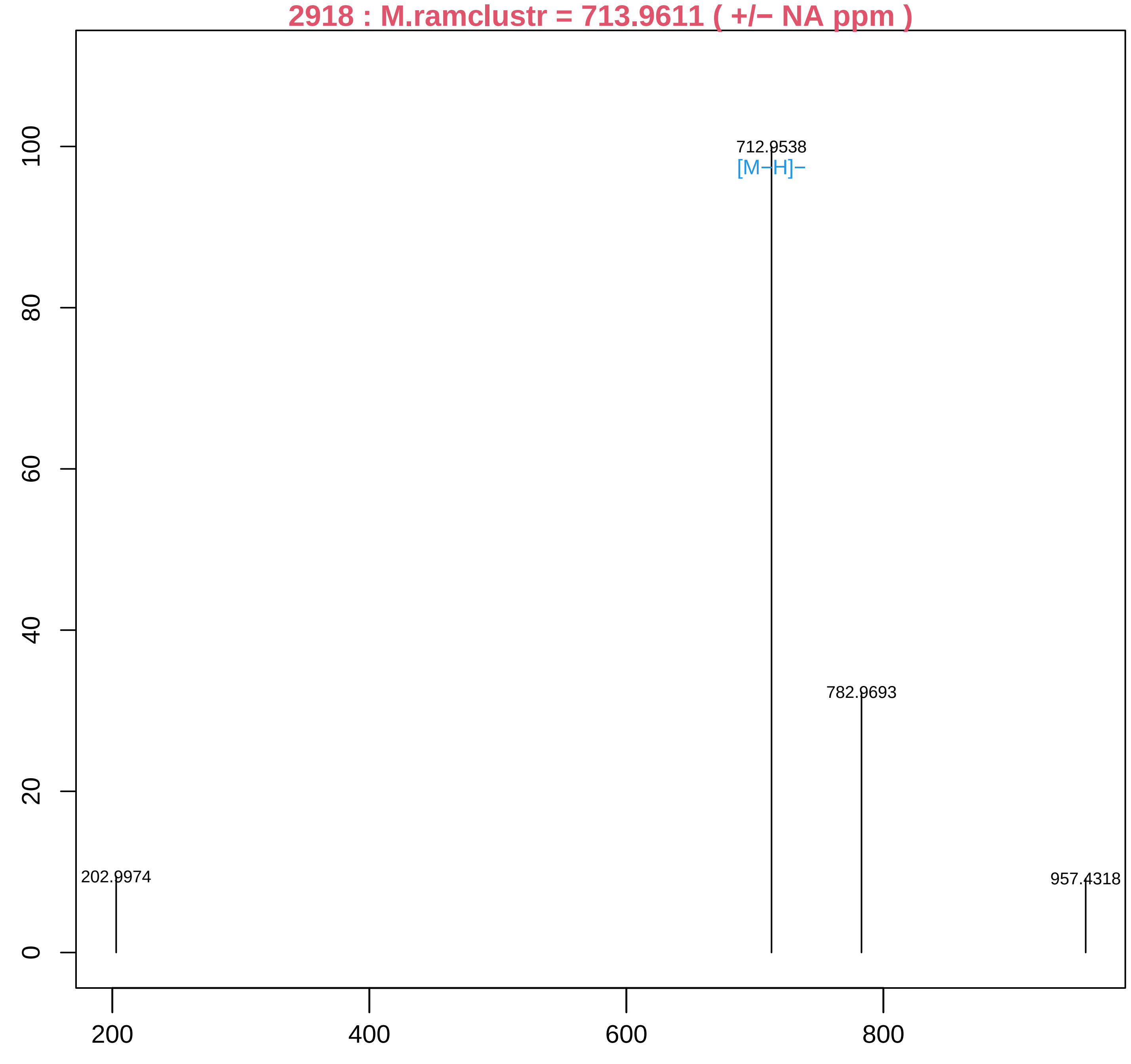

2918 : M.ramclustr = 713.9611 (+/− NA ppm)
712.9538
[M−H]−
782.9693
202.9974
957.4318
0
20
40
60
80
100
200
400
600
800

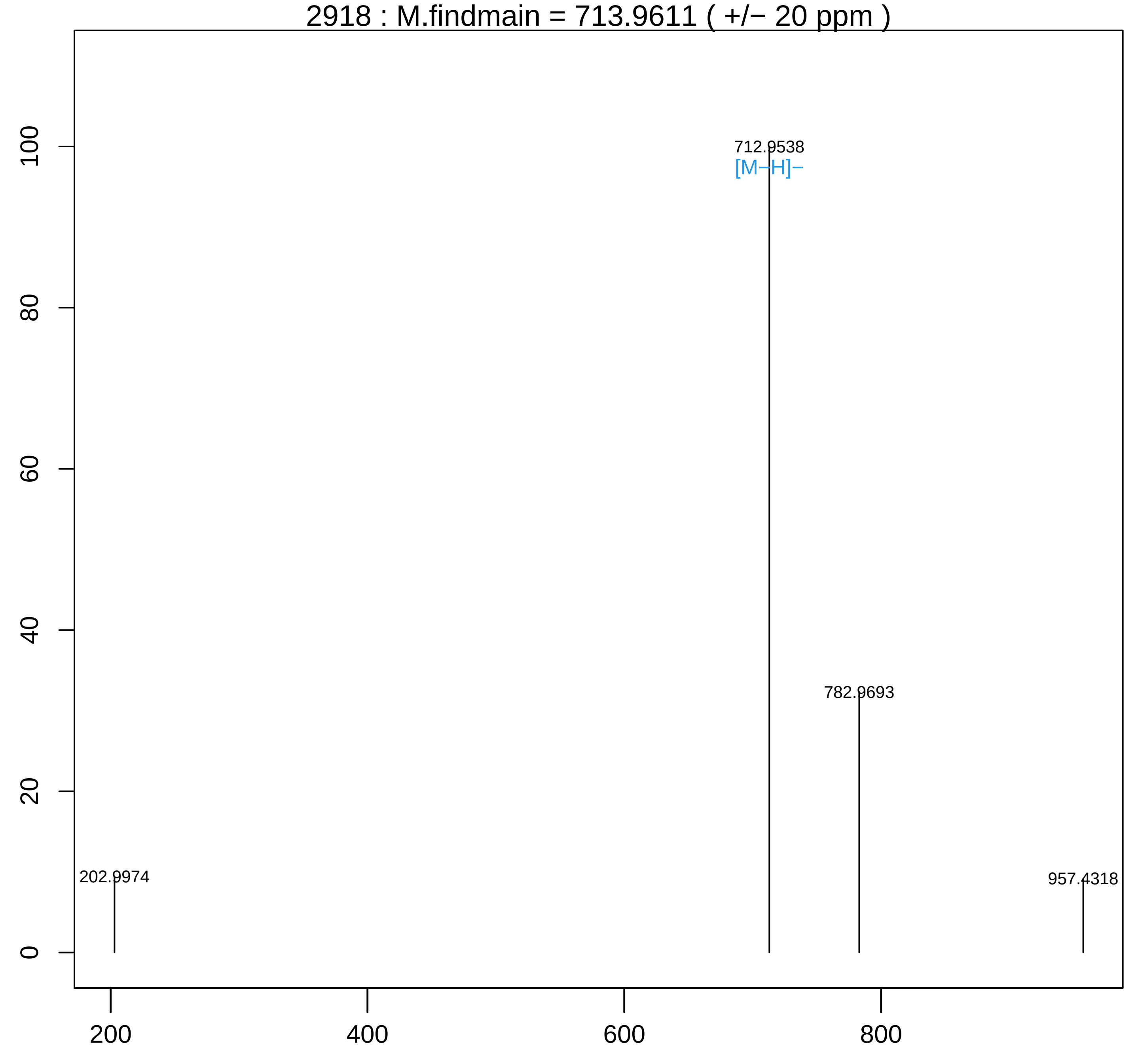

2918 : M.findmain = 713.9611 (+/− 20 ppm)
712.9538
[M−H]−
782.9693
202.9974
957.4318
0
20
40
60
80
100
200
400
600
800

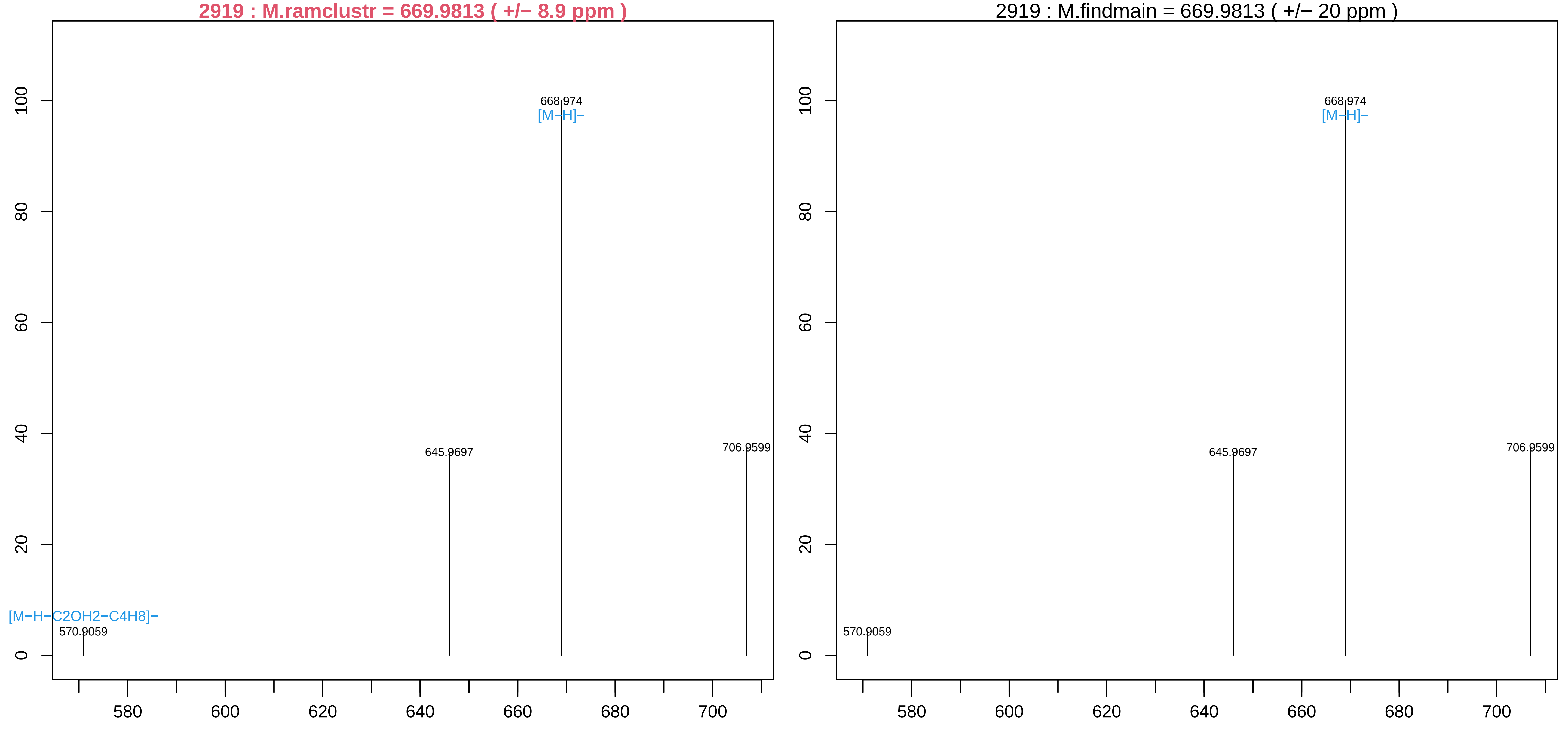

2919 : M.ramclustr = 669.9813 (+/− 8.9 ppm)
668.974
[M−H]−
645.9697
706.9599
[M−H−C2OH2−C4H8]−
570.9059
0
20
40
60
80
100
580
600
620
640
660
680
700
2919 : M.findmain = 669.9813 (+/− 20 ppm)
668.974
[M−H]−
645.9697
706.9599
570.9059
0
20
40
60
80
100
580
600
620
640
660
680
700

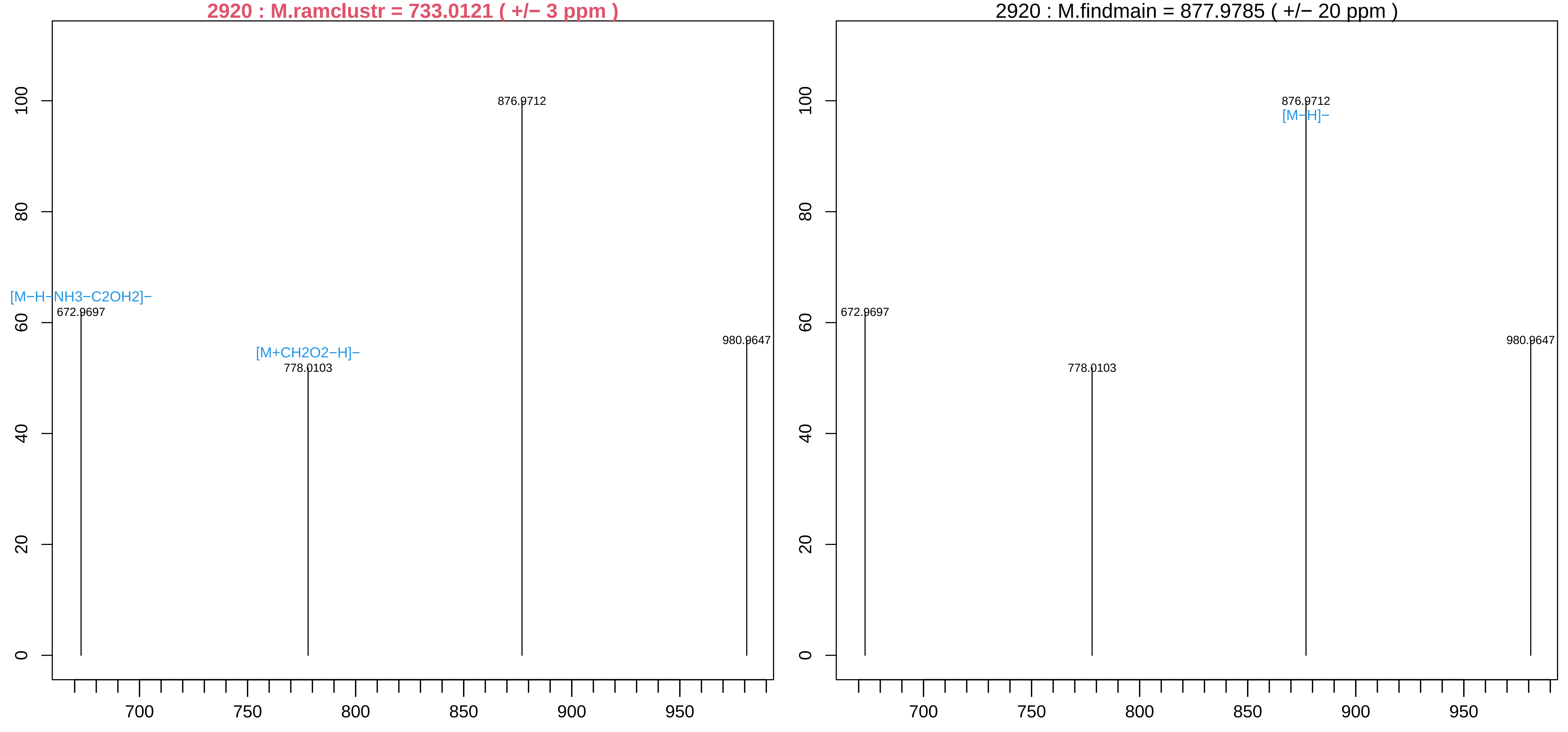
2920 : M.ramclustr = 733.0121 (+/− 3 ppm)
[M−H−NH3−C2OH2]−
672.9697
[M+CH2O2−H]−
778.0103
876.9712
980.9647
2920 : M.findmain = 877.9785 (+/− 20 ppm)
672.9697
778.0103
876.9712
[M−H]−
980.9647
0
20
40
60
80
100
700
750
800
850
900
950

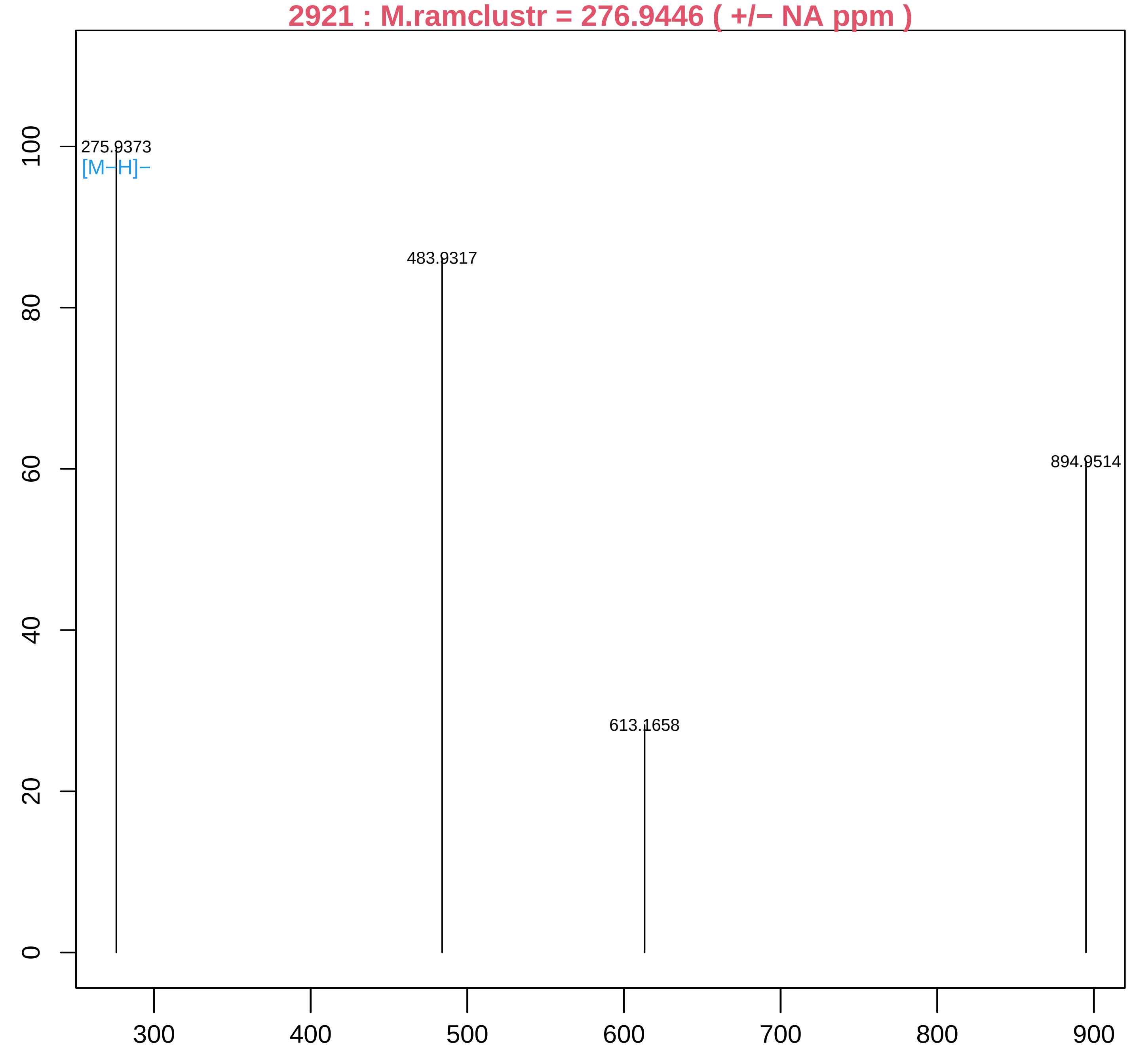

2921 : M.ramclustr = 276.9446 (+/− NA ppm)
275.9373
[M−H]−
483.9317
613.1658
894.9514
0
20
40
60
80
100
300
400
500
600
700
800
900

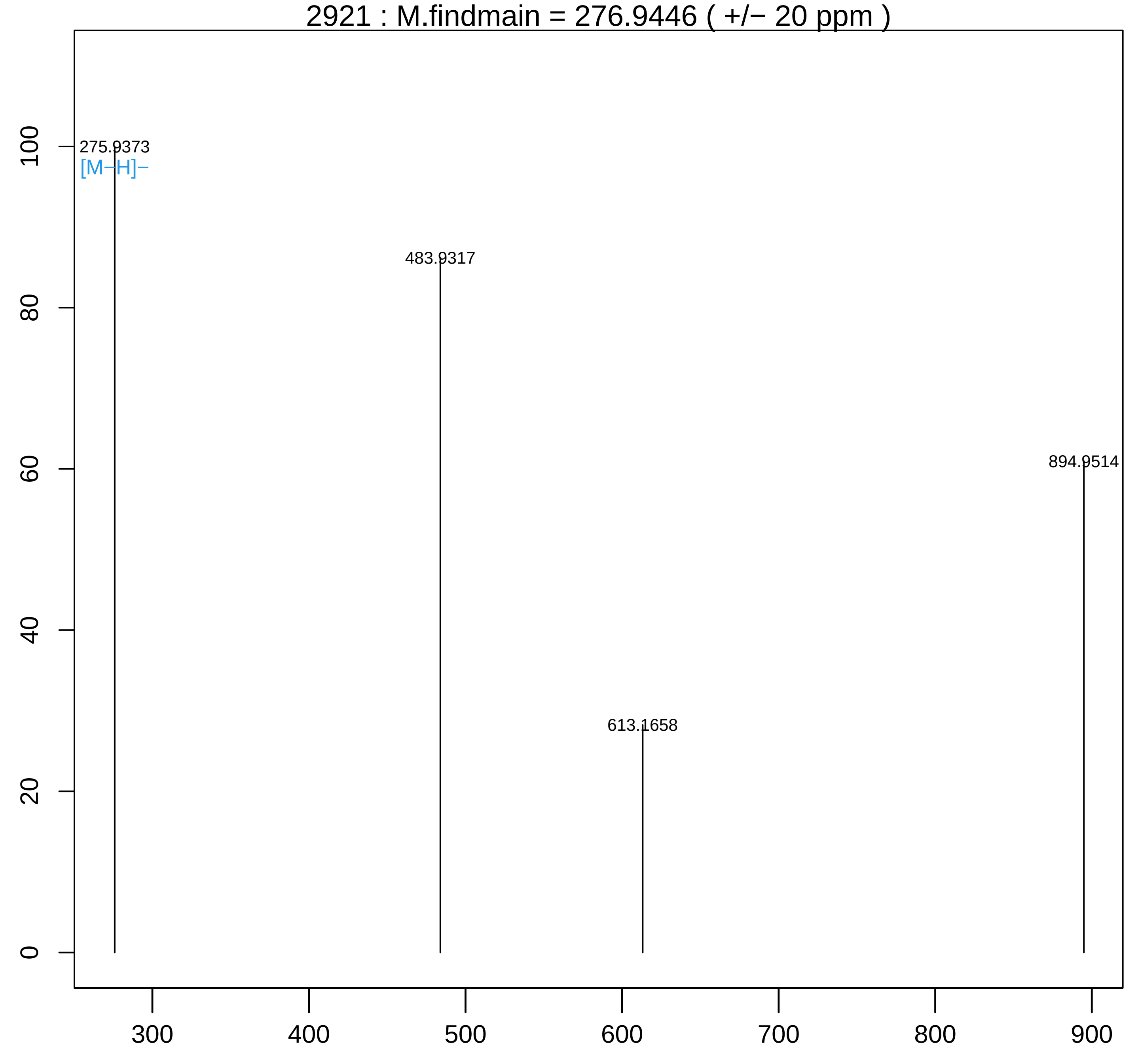

2921 : M.findmain = 276.9446 (+/− 20 ppm)
275.9373
[M−H]−
483.9317
613.1658
894.9514
0
20
40
60
80
100
300
400
500
600
700
800
900

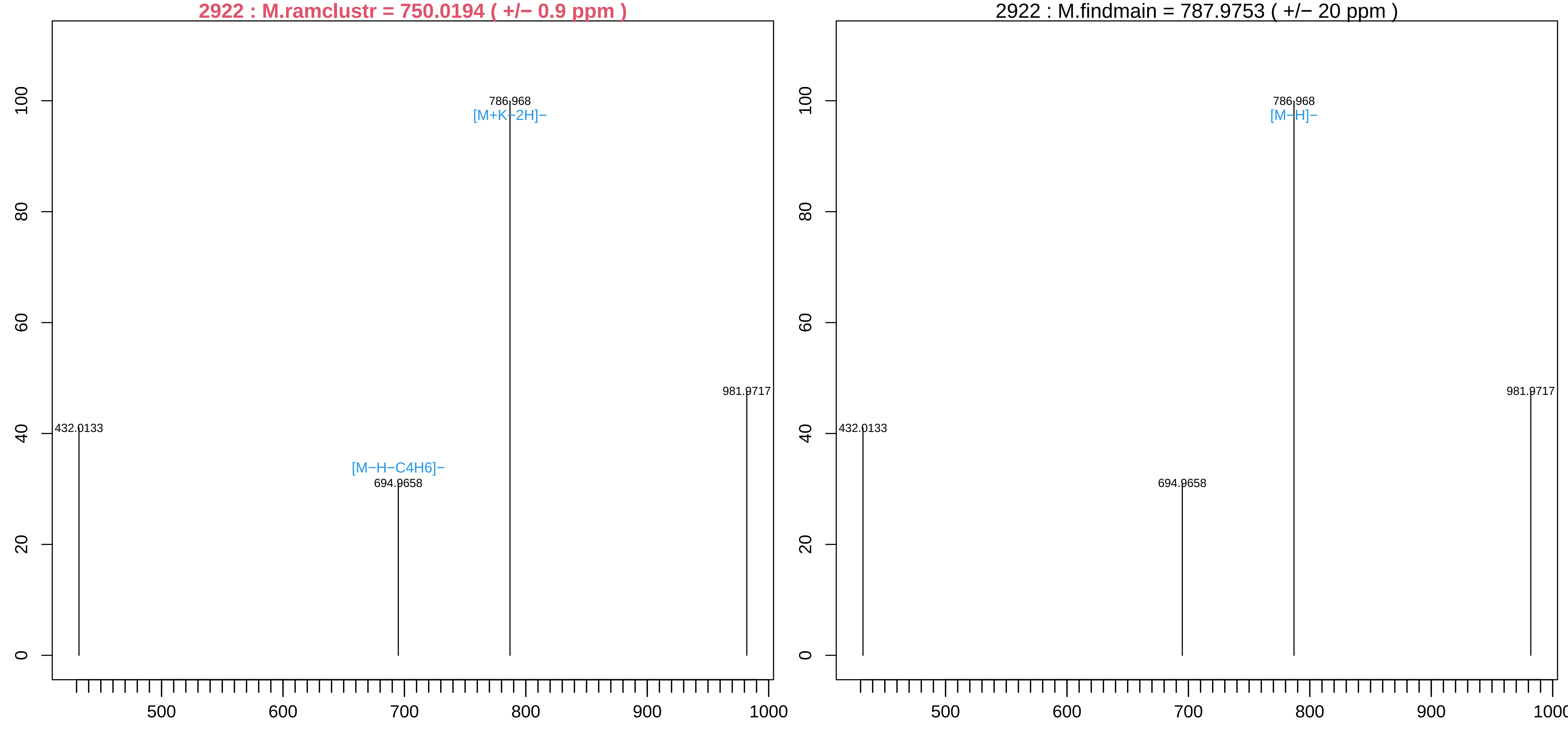
2922 : M.ramclustr = 750.0194 (+/− 0.9 ppm)
786.968
[M+K−2H]−
981.9717
432.0133
[M−H−C4H6]−
694.9658
2922 : M.findmain = 787.9753 (+/− 20 ppm)
786.968
[M−H]−
981.9717
432.0133
694.9658

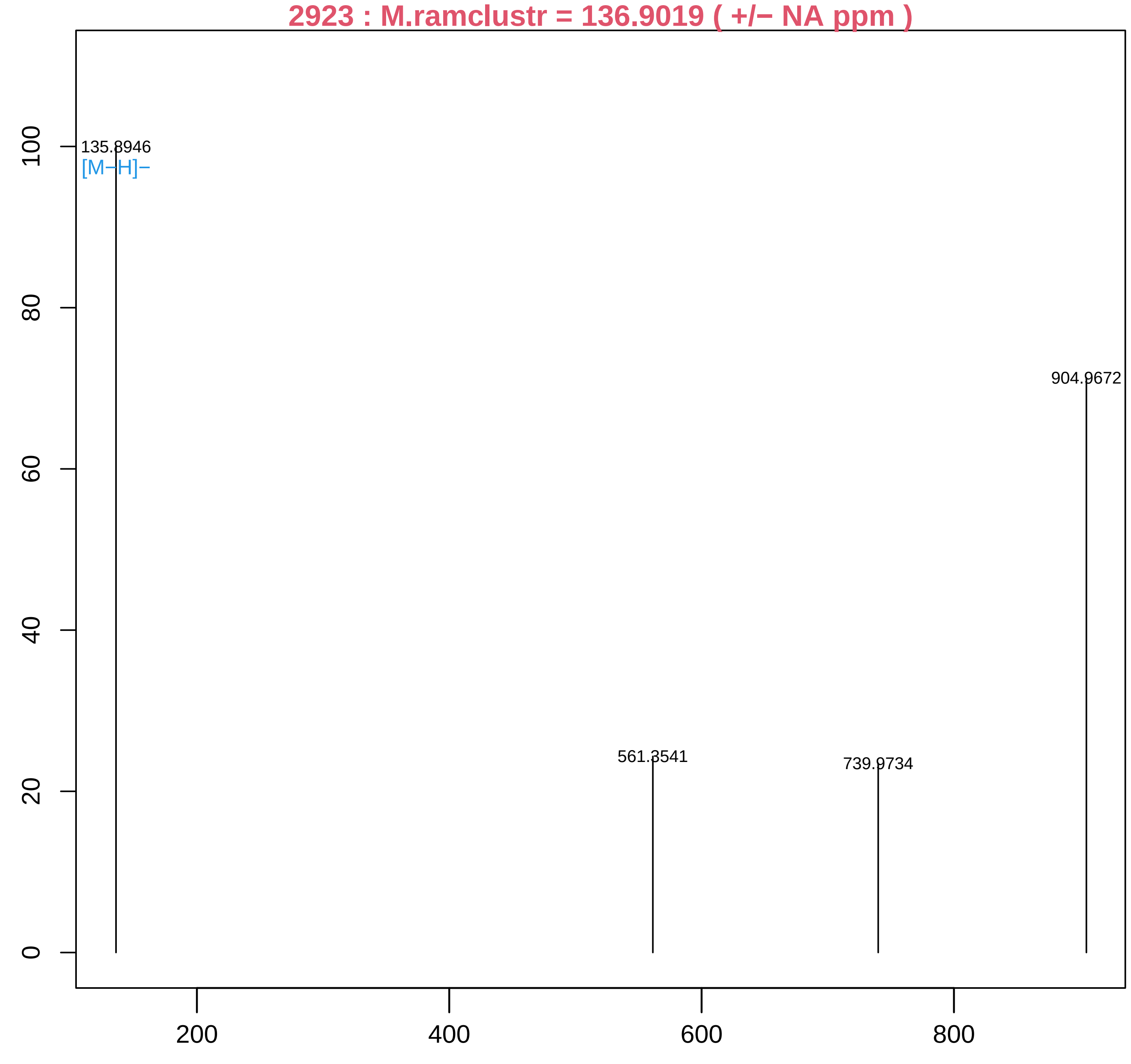

2923 : M.ramclustr = 136.9019 (+/− NA ppm)
135.8946
[M−H]−
904.9672
561.3541
739.9734
0
20
40
60
80
100
200
400
600
800

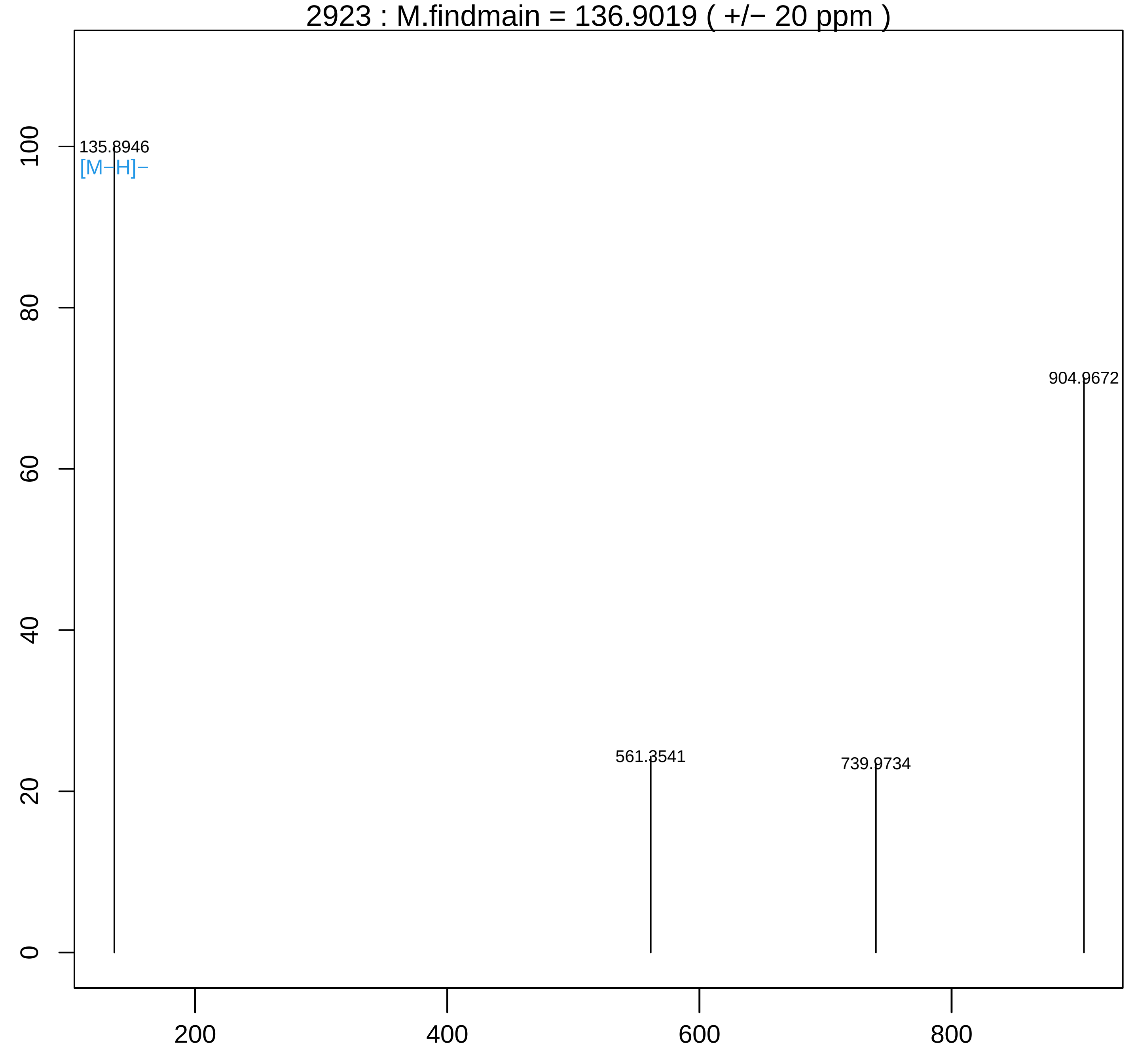

2923 : M.findmain = 136.9019 (+/− 20 ppm)
135.8946
[M−H]−
904.9672
561.3541
739.9734
0
20
40
60
80
100
200
400
600
800

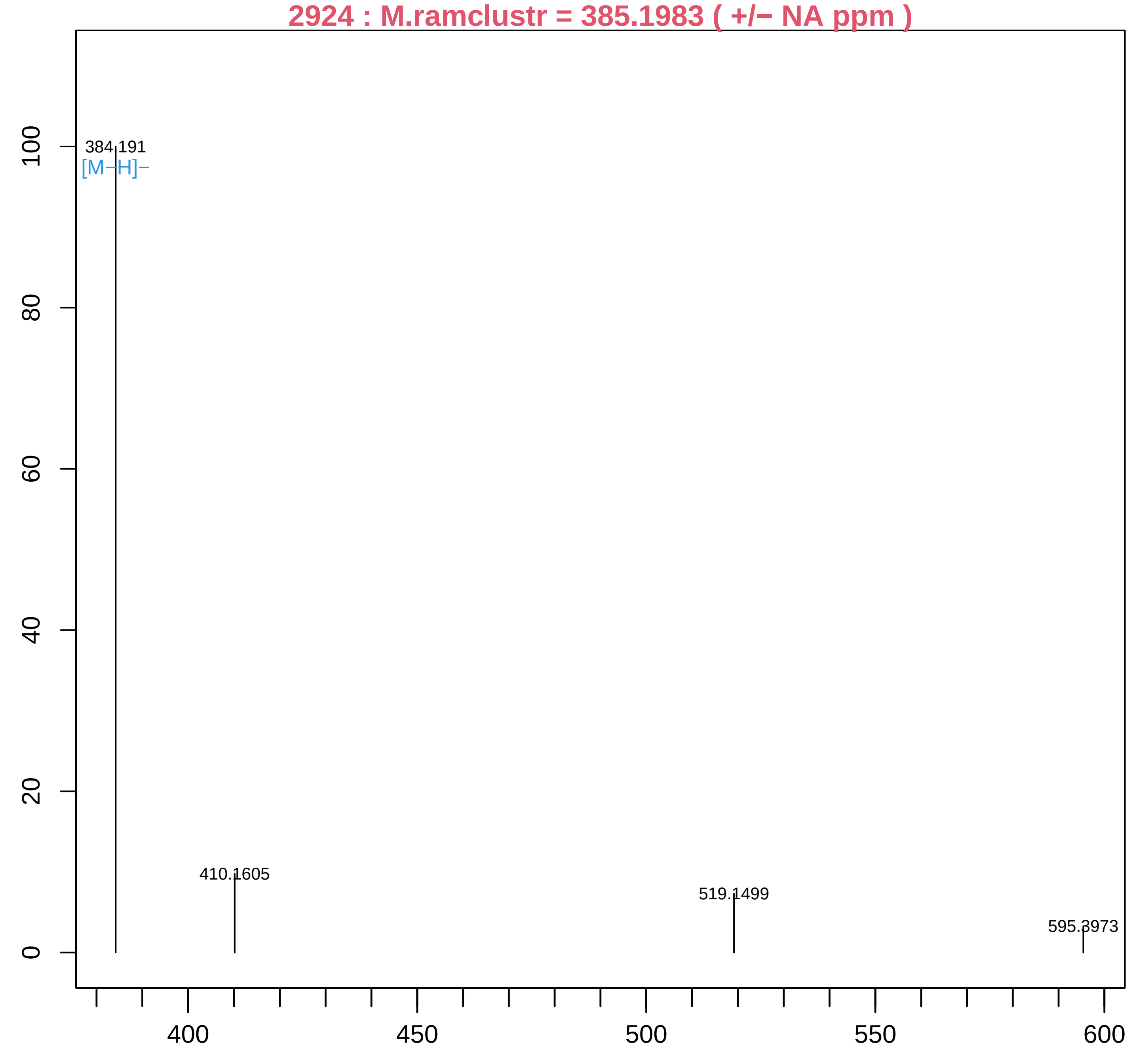
2924 : M.ramclustr = 385.1983 (+/− NA ppm)
384.191
[M−H]−
410.1605
519.1499
595.3973
0
20
40
60
80
100
400
450
500
550
600

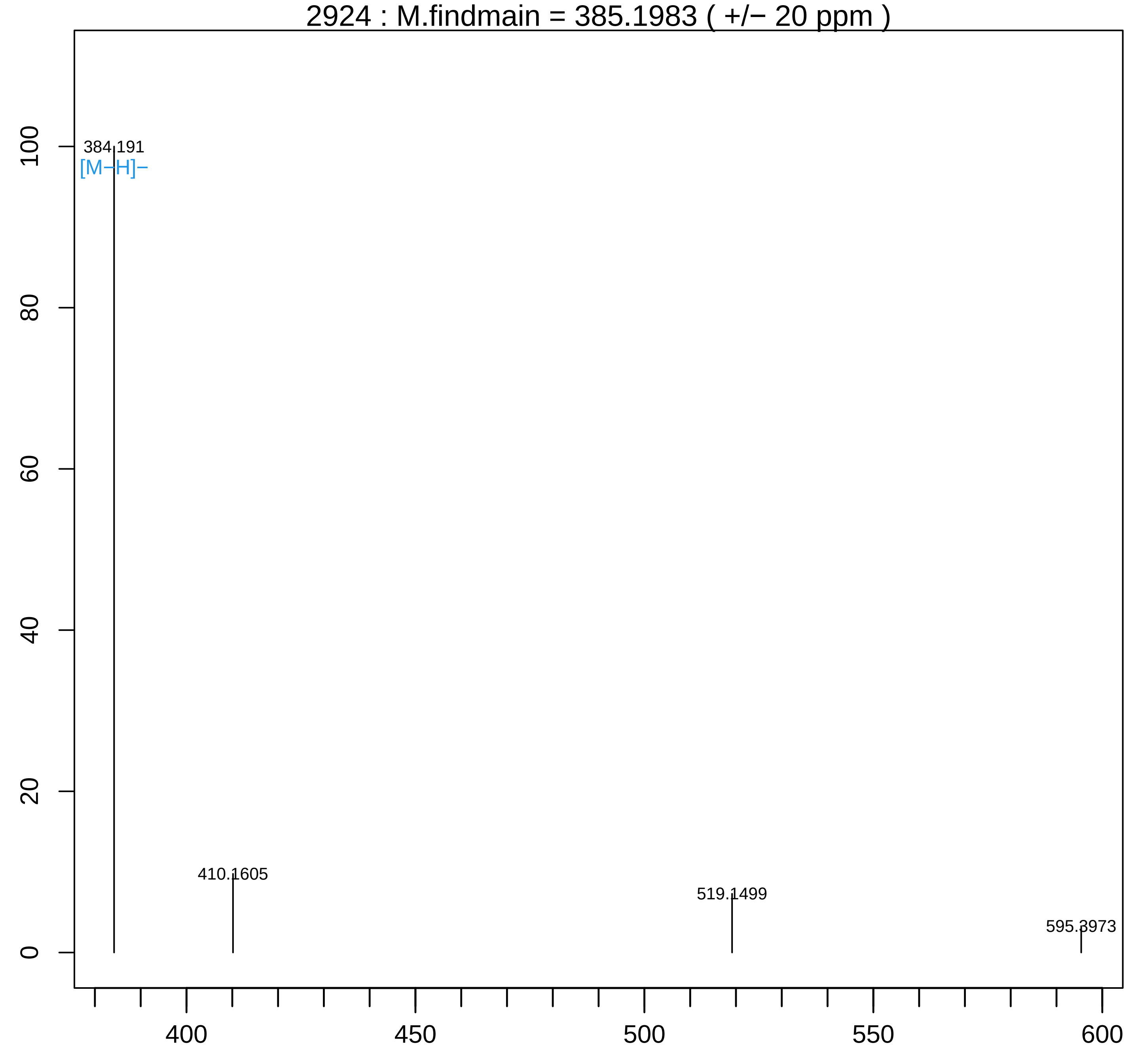
2924 : M.findmain = 385.1983 (+/− 20 ppm)
384.191
[M−H]−
410.1605
519.1499
595.3973
0
20
40
60
80
100
400
450
500
550
600

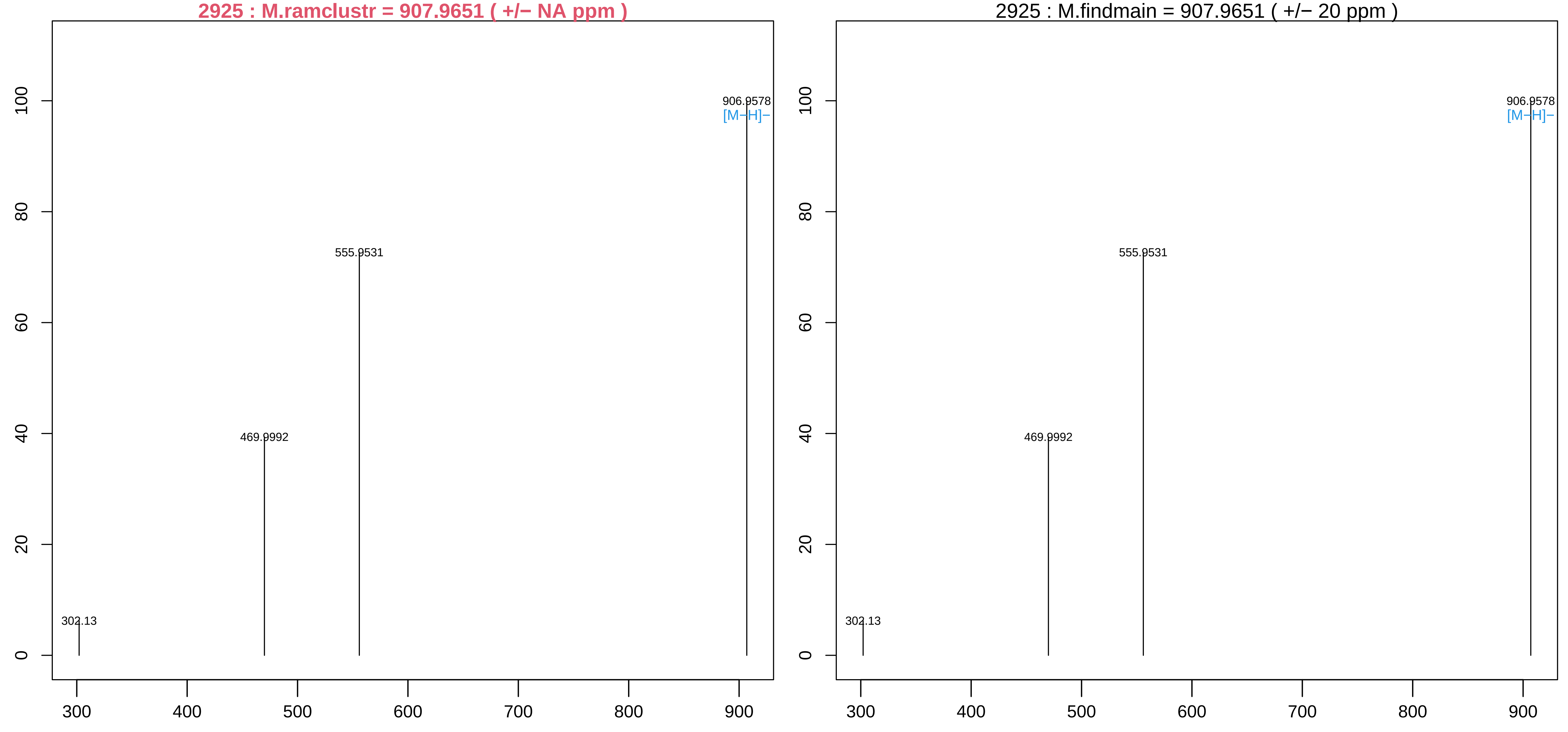
2925 : M.ramclustr = 907.9651 (+/− NA ppm)
906.9578
[M−H]−
555.9531
469.9992
302.13
2925 : M.findmain = 907.9651 (+/− 20 ppm)
906.9578
[M−H]−
555.9531
469.9992
302.13

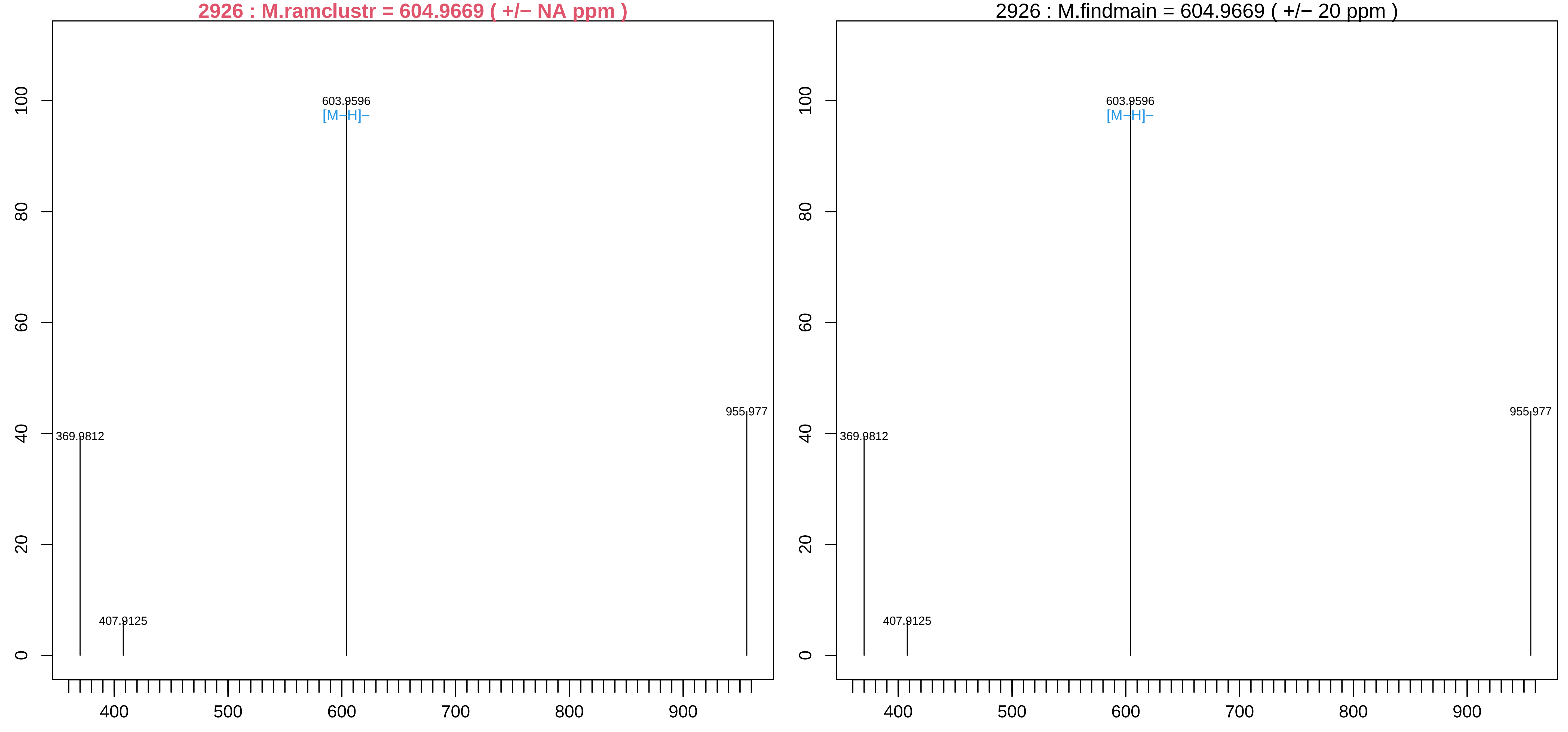
2926 : M.ramclustr = 604.9669 (+/− NA ppm)
2926 : M.findmain = 604.9669 (+/− 20 ppm)
603.9596
[M−H]−
369.9812
407.9125
955.977
0
20
40
60
80
100
400
500
600
700
800
900

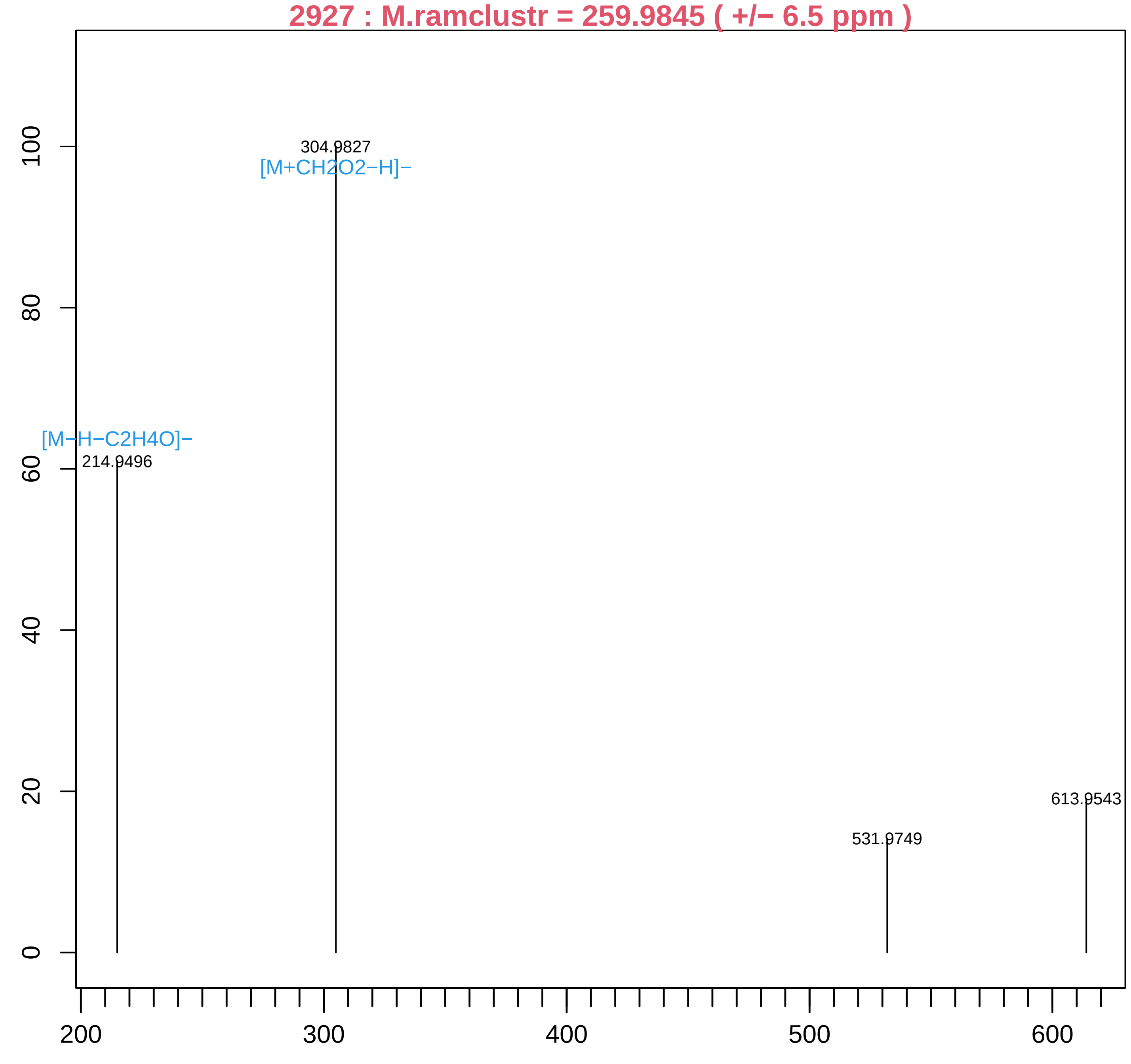
2927 : M.ramclustr = 259.9845 (+/− 6.5 ppm)
304.9827
[M+CH2O2−H]−
[M−H−C2H4O]−
214.9496
531.9749
613.9543
0
20
40
60
80
100
200
300
400
500
600

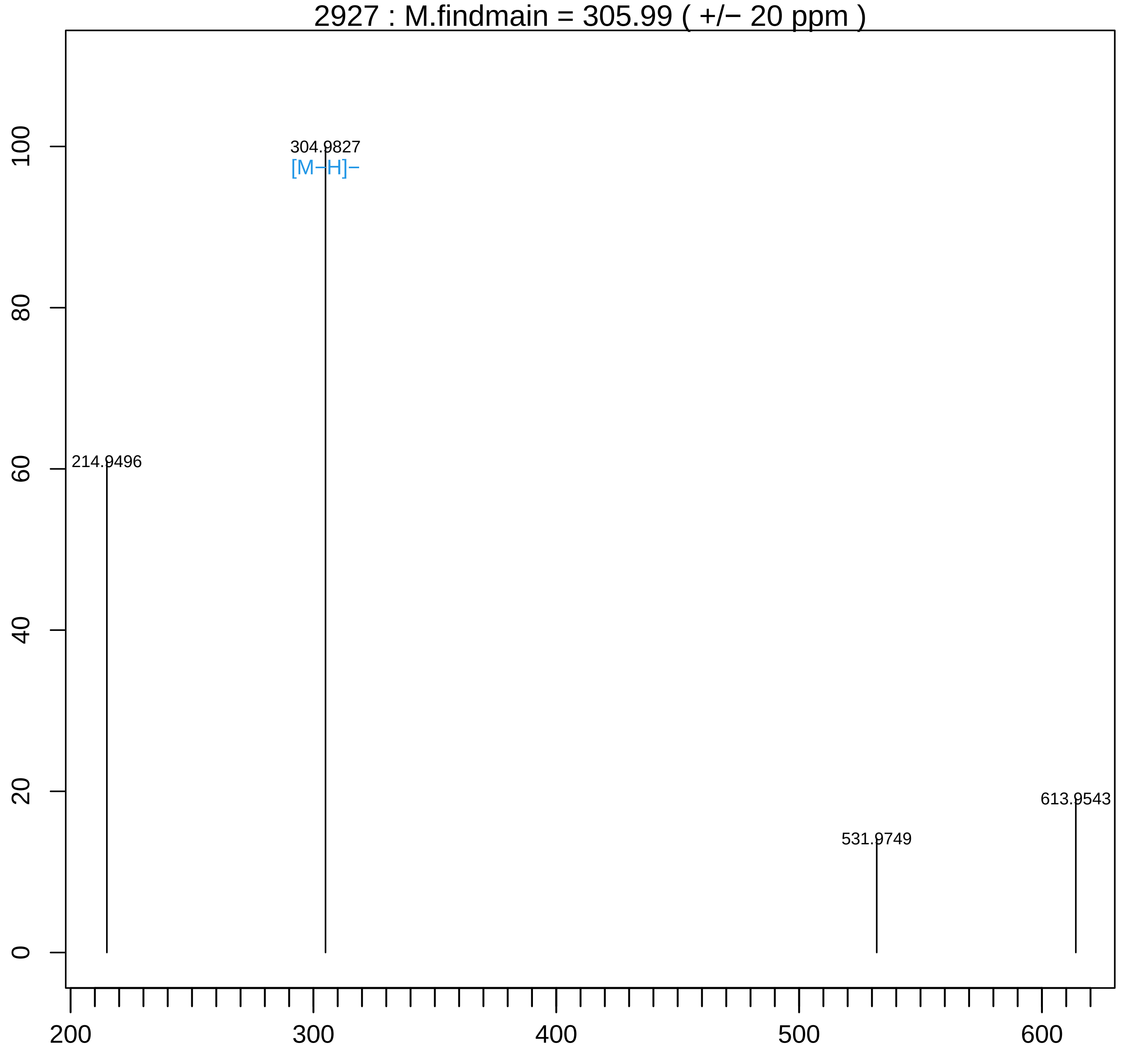
2927 : M.findmain = 305.99 (+/− 20 ppm)
304.9827
[M−H]−
214.9496
531.9749
613.9543
0
20
40
60
80
100
200
300
400
500
600

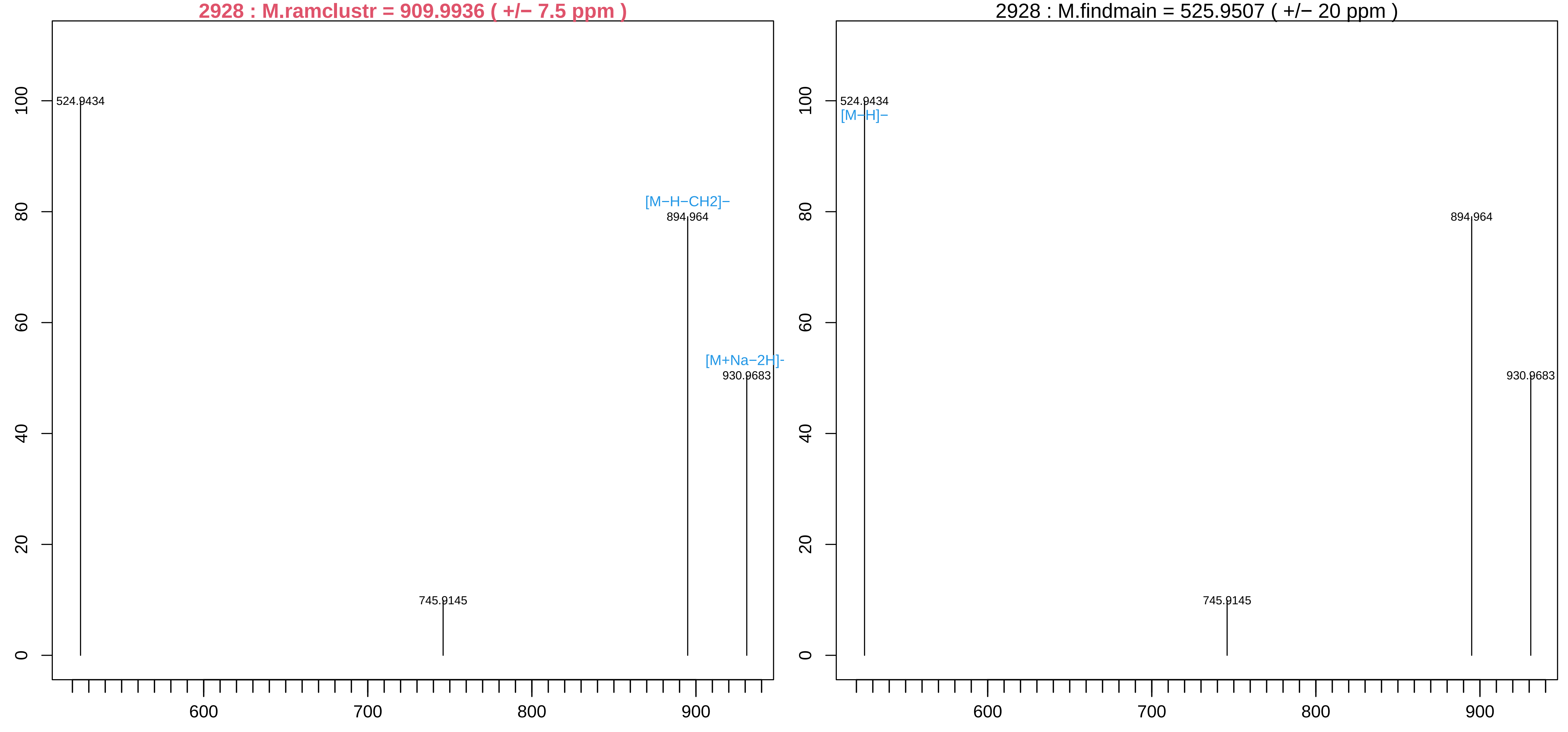
2928 : M.ramclustr = 909.9936 (+/− 7.5 ppm)
2928 : M.findmain = 525.9507 (+/− 20 ppm)
524.9434
[M−H]−
745.9145
[M−H−CH2]−
894.964
[M+Na−2H]-
930.9683
0
20
40
60
80
100
600
700
800
900

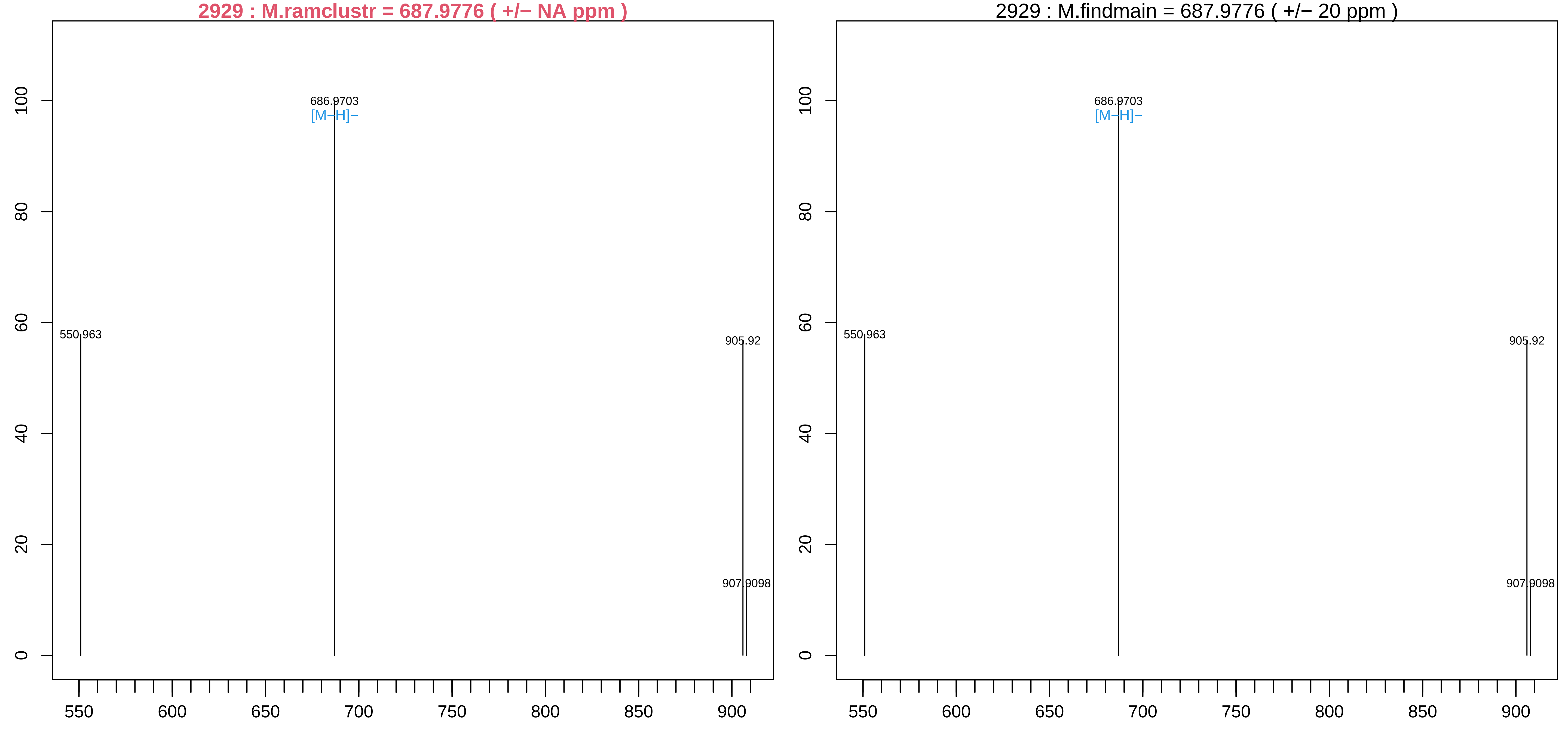

2929 : M.ramclustr = 687.9776 (+/− NA ppm)
686.9703
[M−H]−
550.963
905.92
907.9098
2929 : M.findmain = 687.9776 (+/− 20 ppm)
686.9703
[M−H]−
550.963
905.92
907.9098
0
20
40
60
80
100
550
600
650
700
750
800
850
900

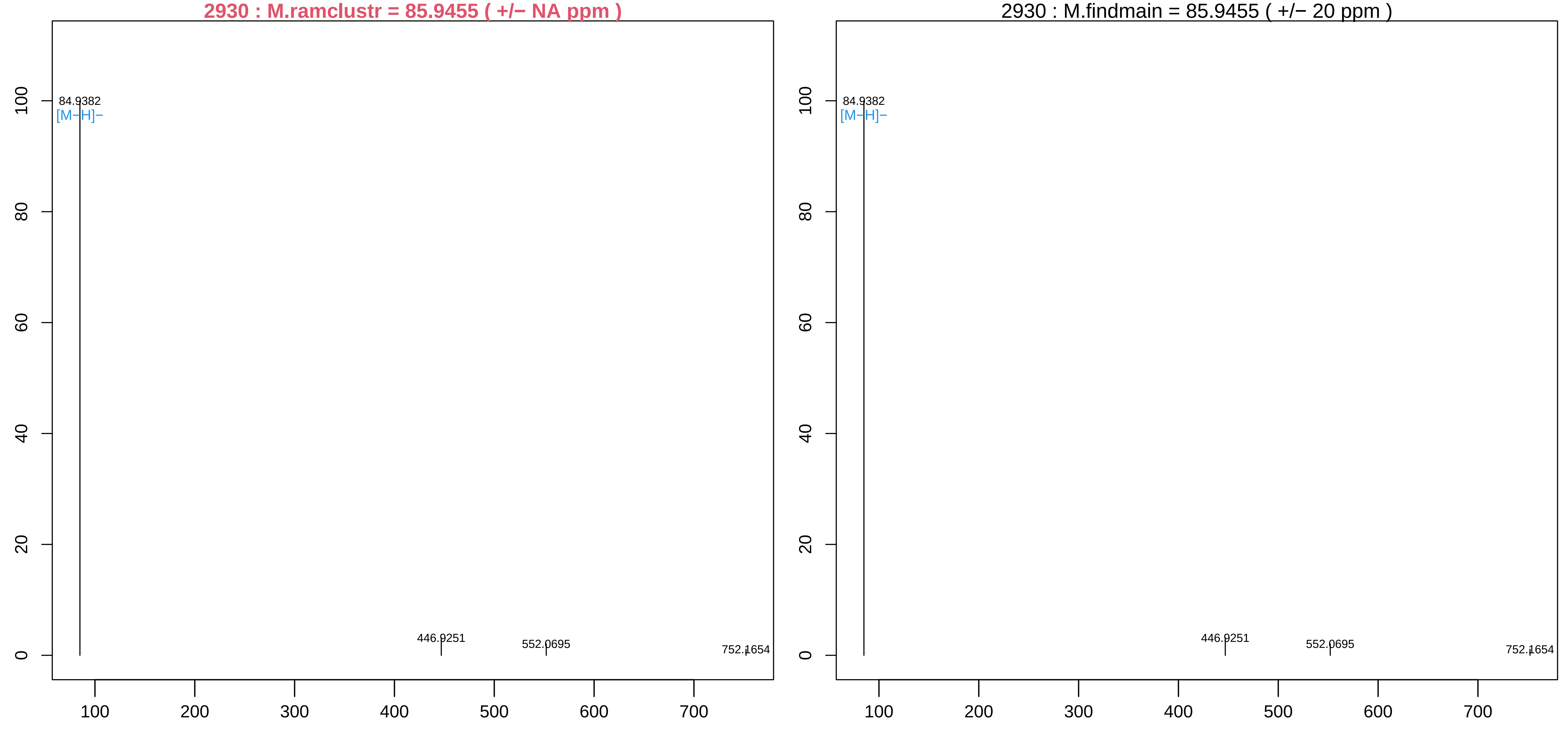
2930 : M.ramclustr = 85.9455 (+/− NA ppm)
84.9382
[M−H]−
446.9251
552.0695
752.1654
2930 : M.findmain = 85.9455 (+/− 20 ppm)
84.9382
[M−H]−
446.9251
552.0695
752.1654

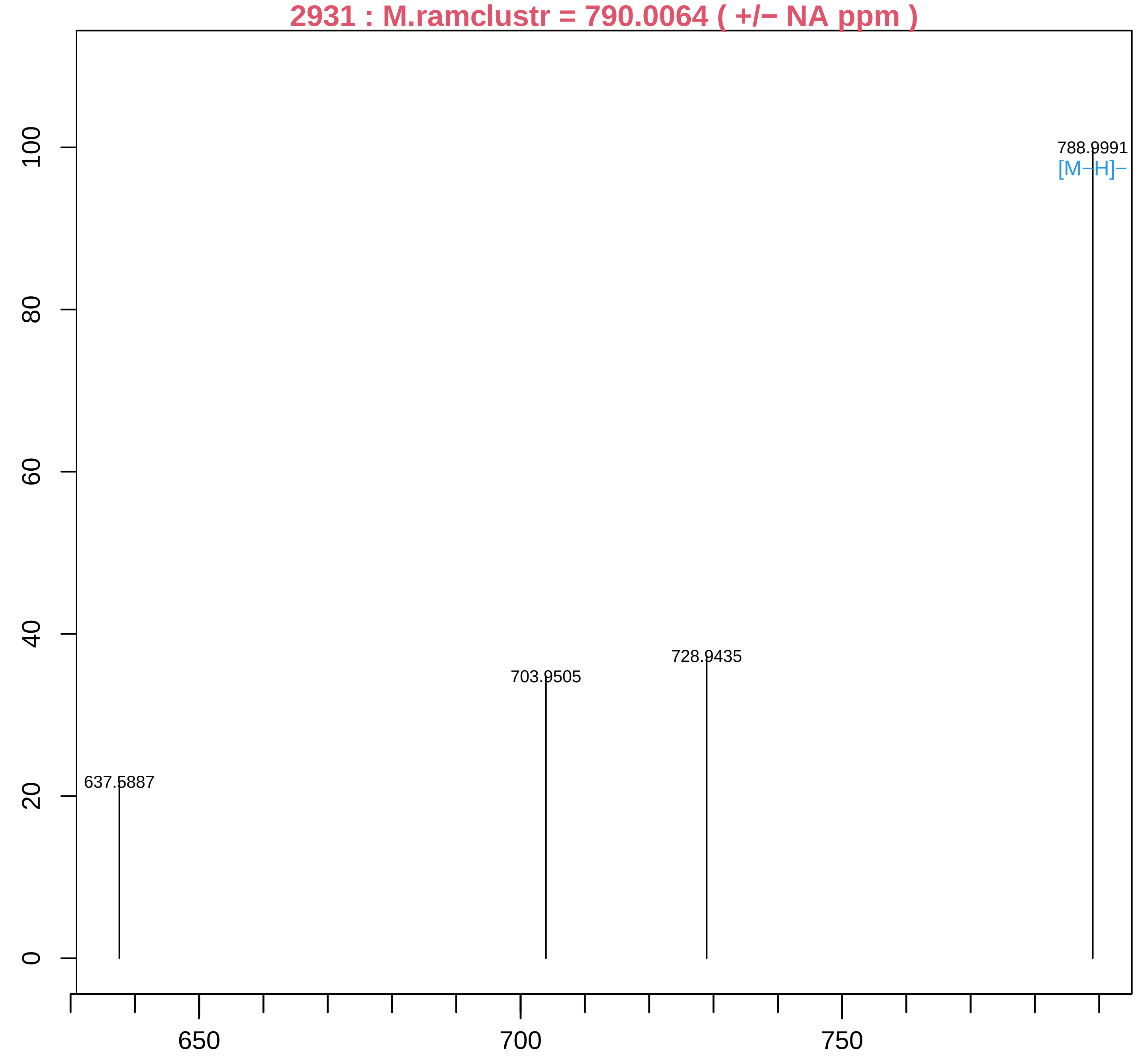
2931 : M.ramclustr = 790.0064 (+/− NA ppm)
788.9991
[M−H]−
728.9435
703.9505
637.5887
0
20
40
60
80
100
650
700
750

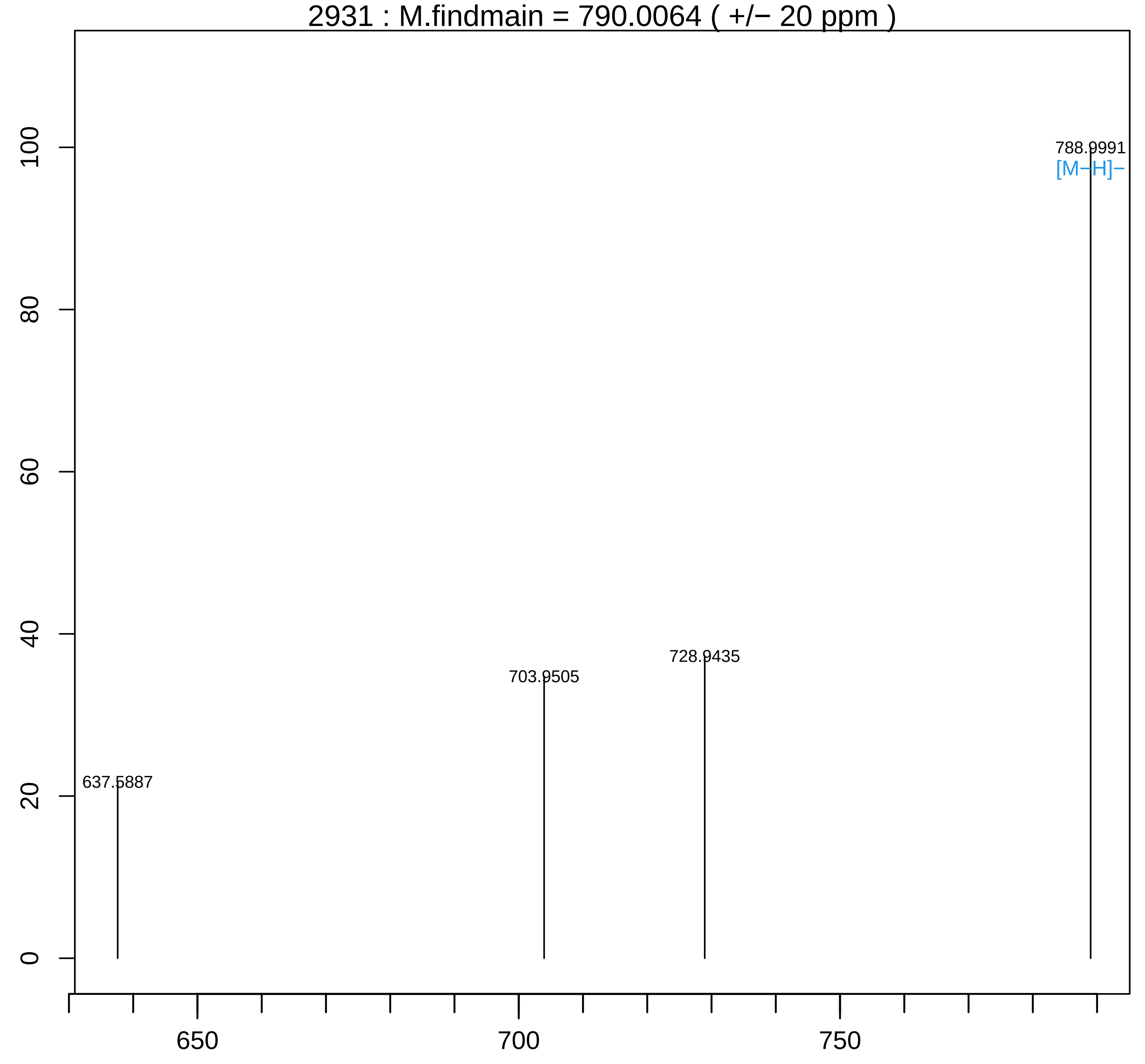
2931 : M.findmain = 790.0064 (+/− 20 ppm)
788.9991
[M−H]−
728.9435
703.9505
637.5887
0
20
40
60
80
100
650
700
750

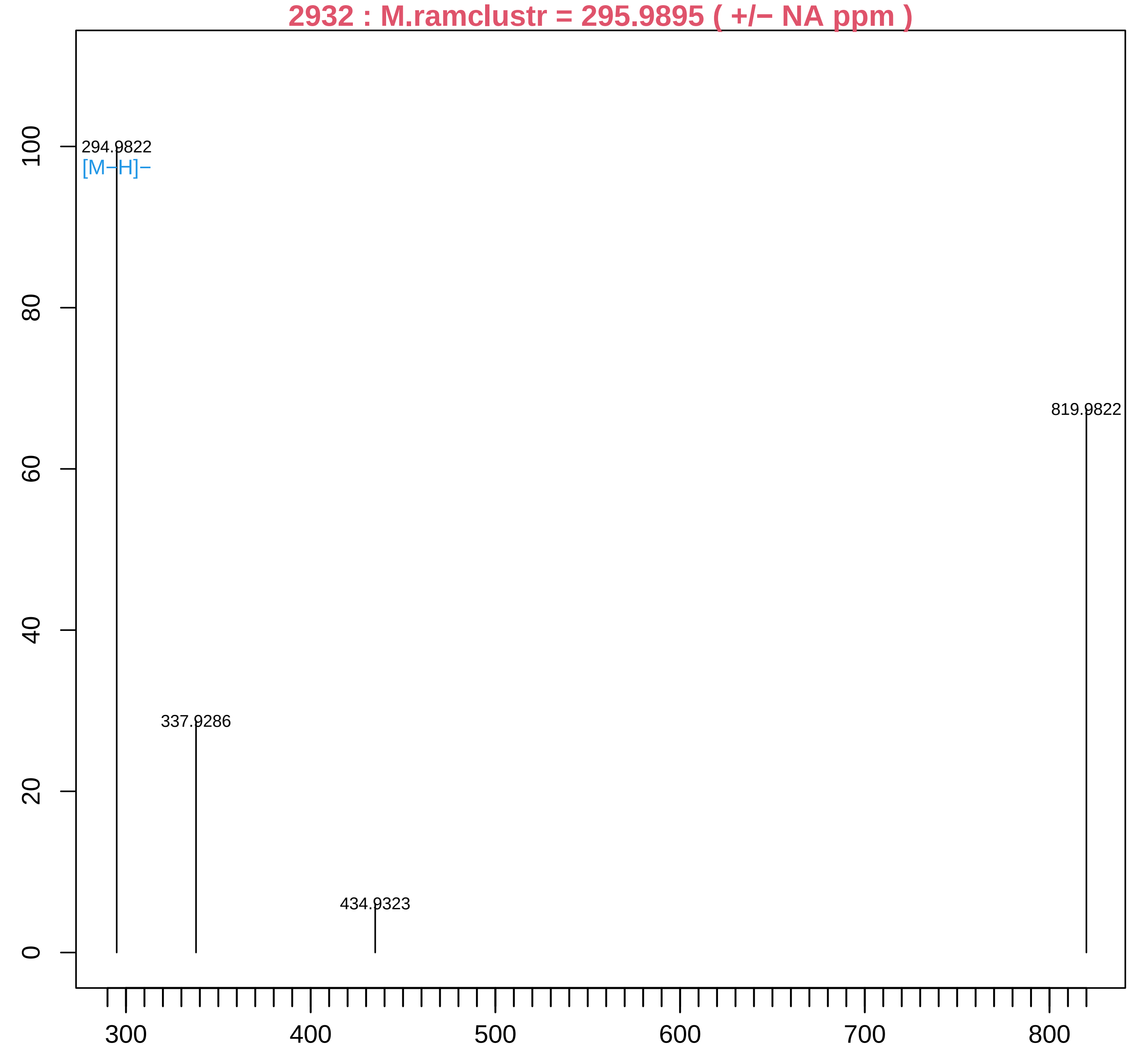

2932 : M.ramclustr = 295.9895 (+/− NA ppm)
294.9822
[M−H]−
337.9286
434.9323
819.9822
0
20
40
60
80
100
300
400
500
600
700
800

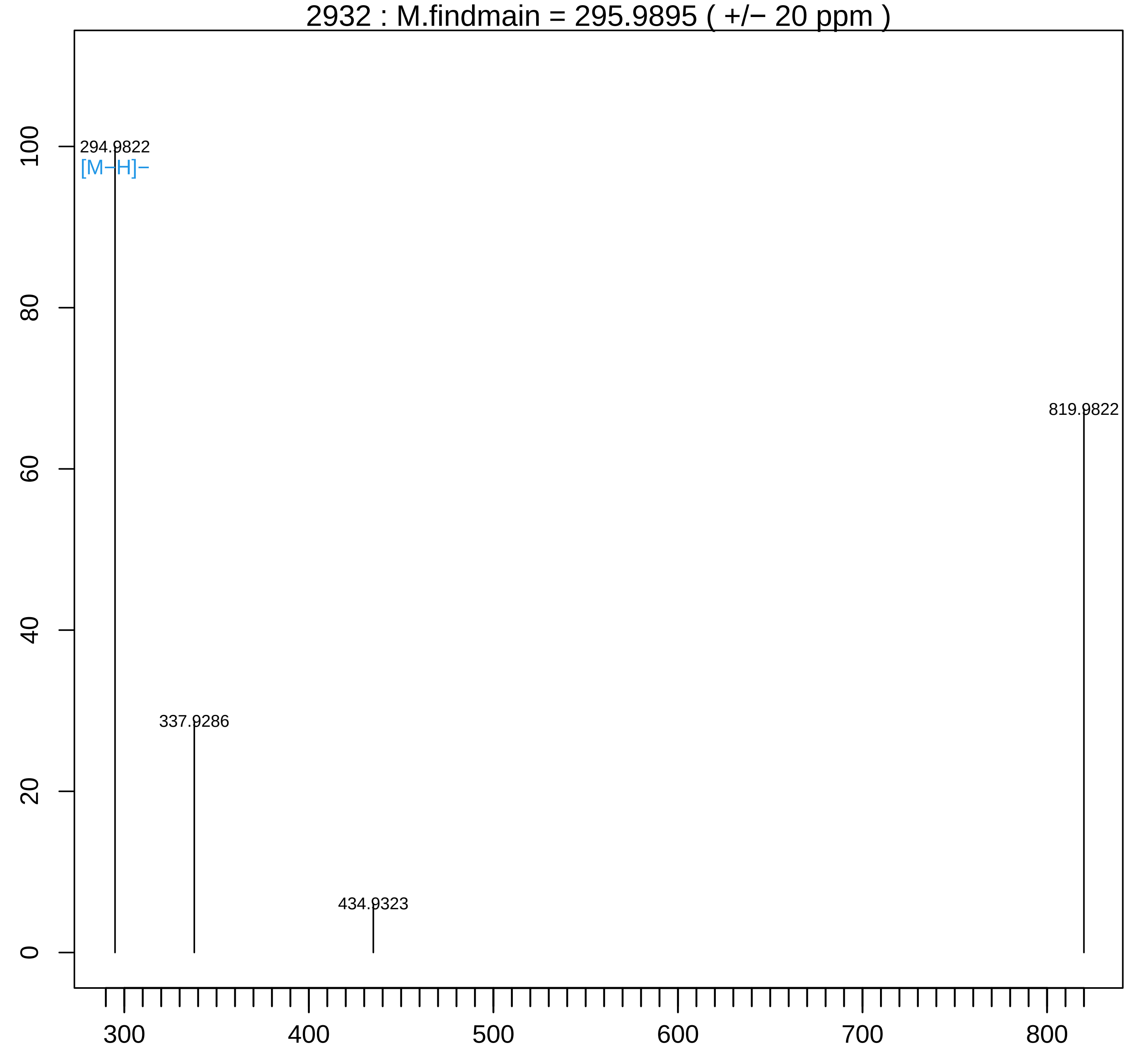

2932 : M.findmain = 295.9895 (+/− 20 ppm)
294.9822
[M−H]−
337.9286
434.9323
819.9822
0
20
40
60
80
100
300
400
500
600
700
800

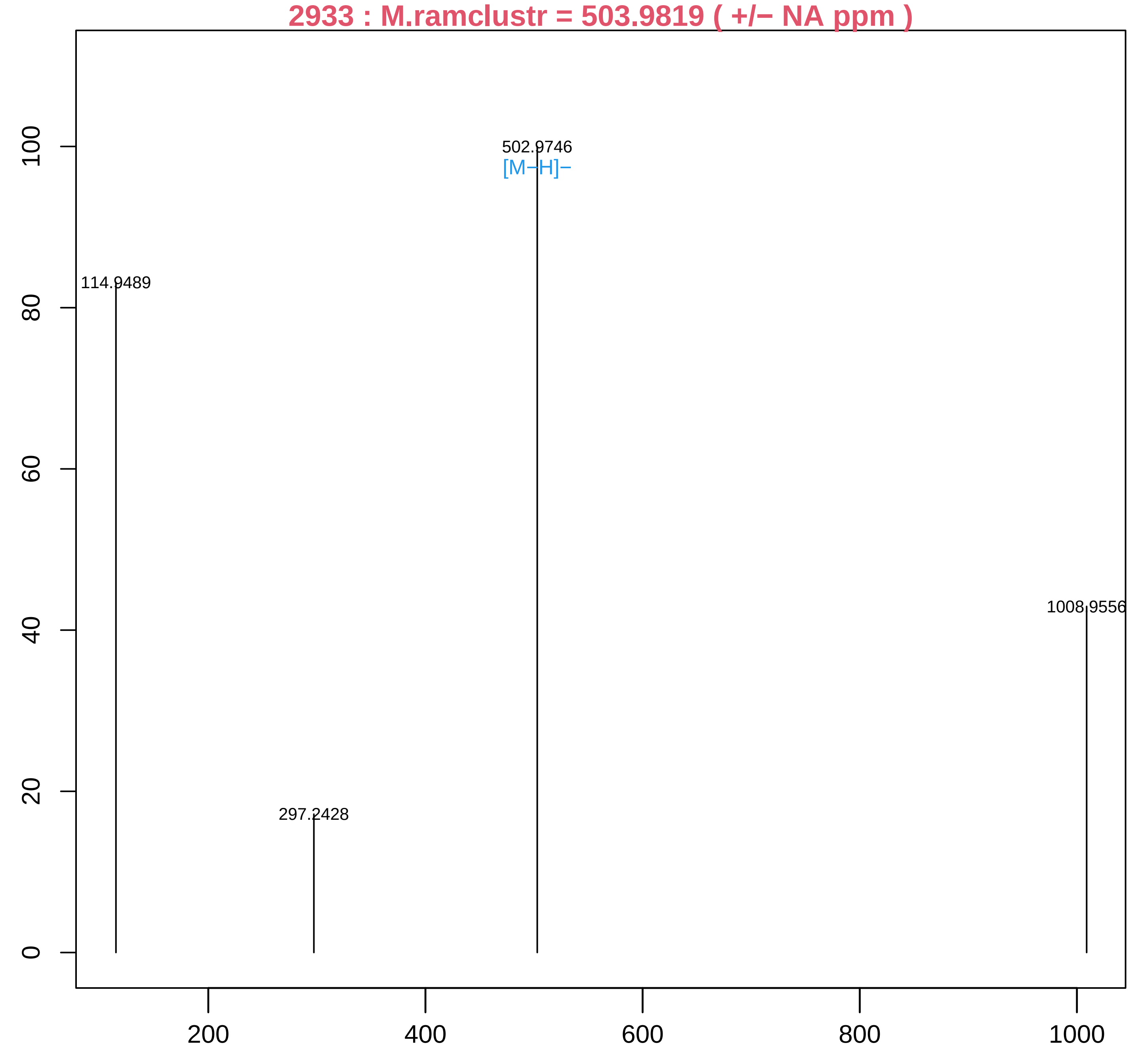
2933 : M.ramclustr = 503.9819 (+/− NA ppm)
502.9746
[M−H]−
114.9489
297.2428
1008.9556
0
20
40
60
80
100
200
400
600
800
1000

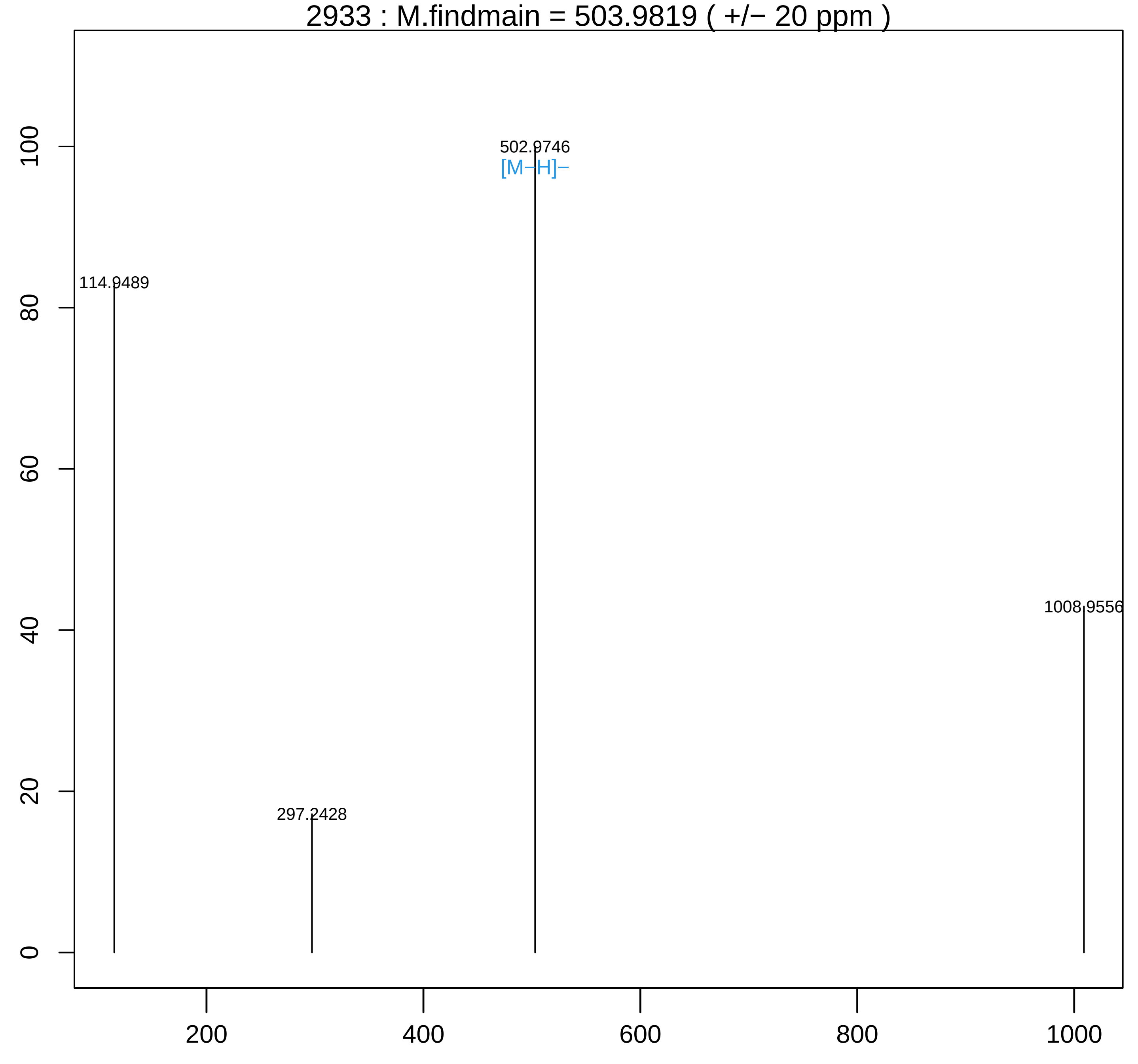
2933 : M.findmain = 503.9819 (+/− 20 ppm)
502.9746
[M−H]−
114.9489
297.2428
1008.9556
0
20
40
60
80
100
200
400
600
800
1000

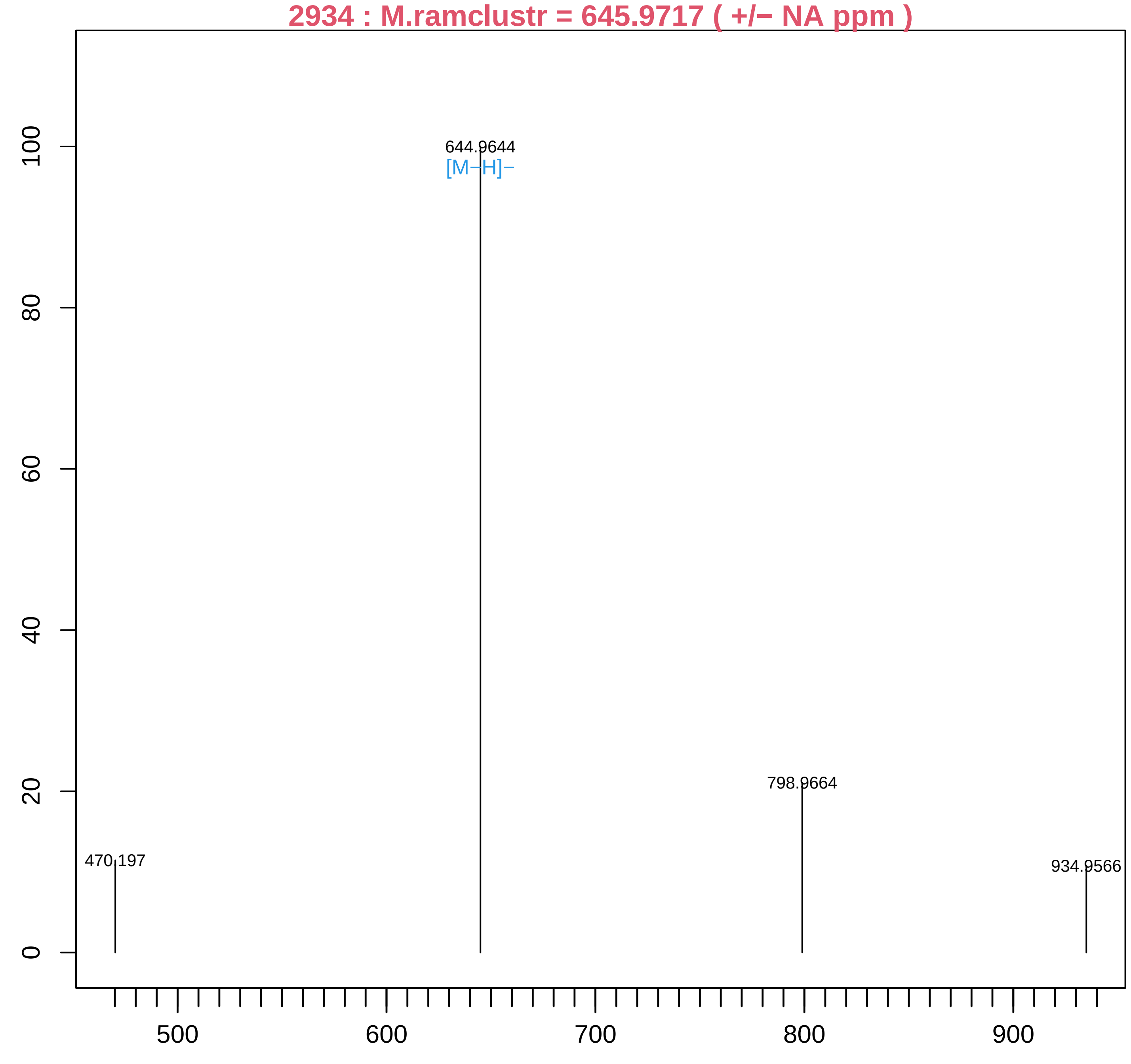
2934 : M.ramclustr = 645.9717 (+/− NA ppm)
644.9644
[M−H]−
798.9664
470.197
934.9566
0
20
40
60
80
100
500
600
700
800
900

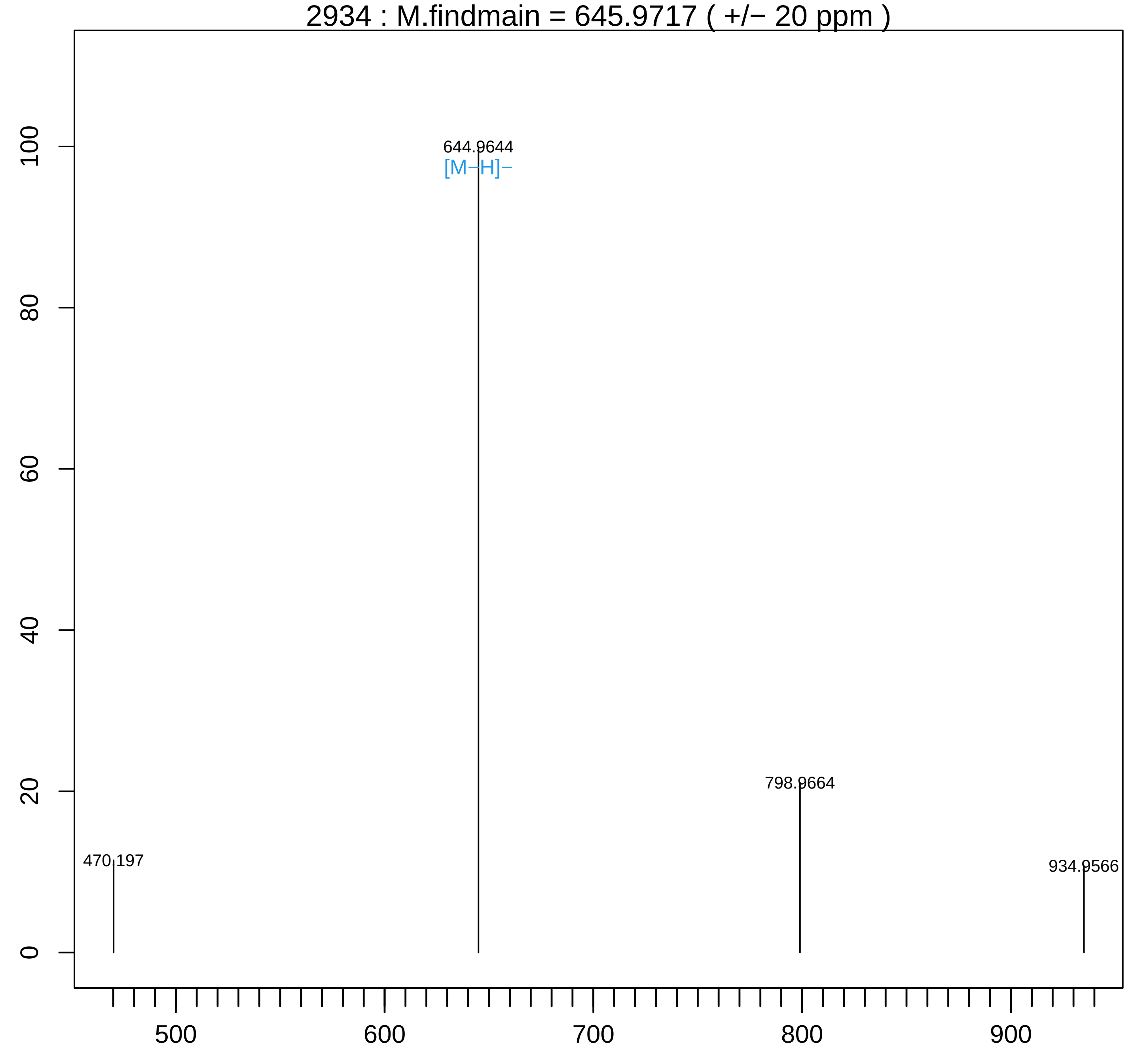
2934 : M.findmain = 645.9717 (+/− 20 ppm)
644.9644
[M−H]−
798.9664
470.197
934.9566
0
20
40
60
80
100
500
600
700
800
900

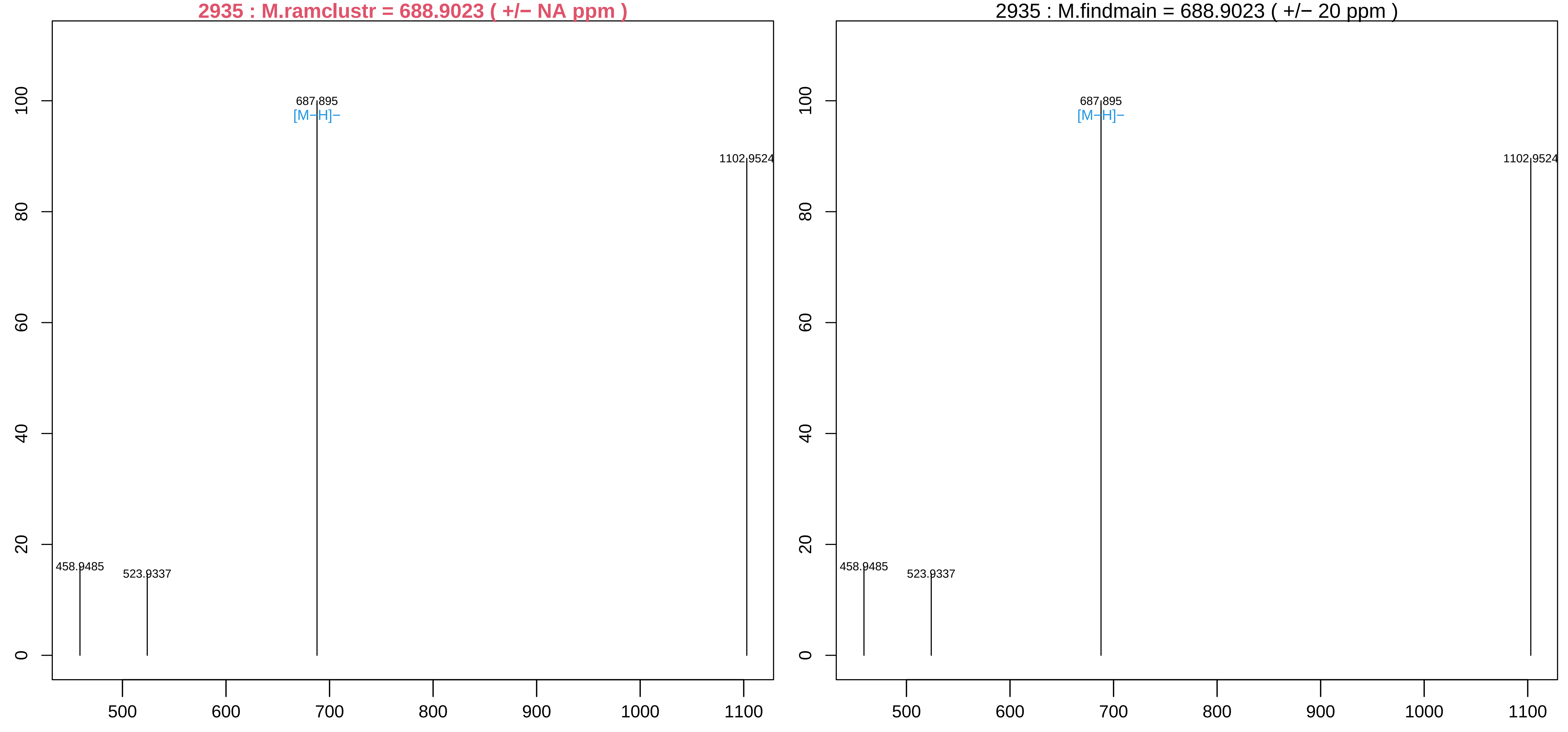
2935 : M.ramclustr = 688.9023 (+/− NA ppm)
687.895
[M−H]−
1102.9524
458.9485
523.9337
2935 : M.findmain = 688.9023 (+/− 20 ppm)
687.895
[M−H]−
1102.9524
458.9485
523.9337
0
20
40
60
80
100
500
600
700
800
900
1000
1100

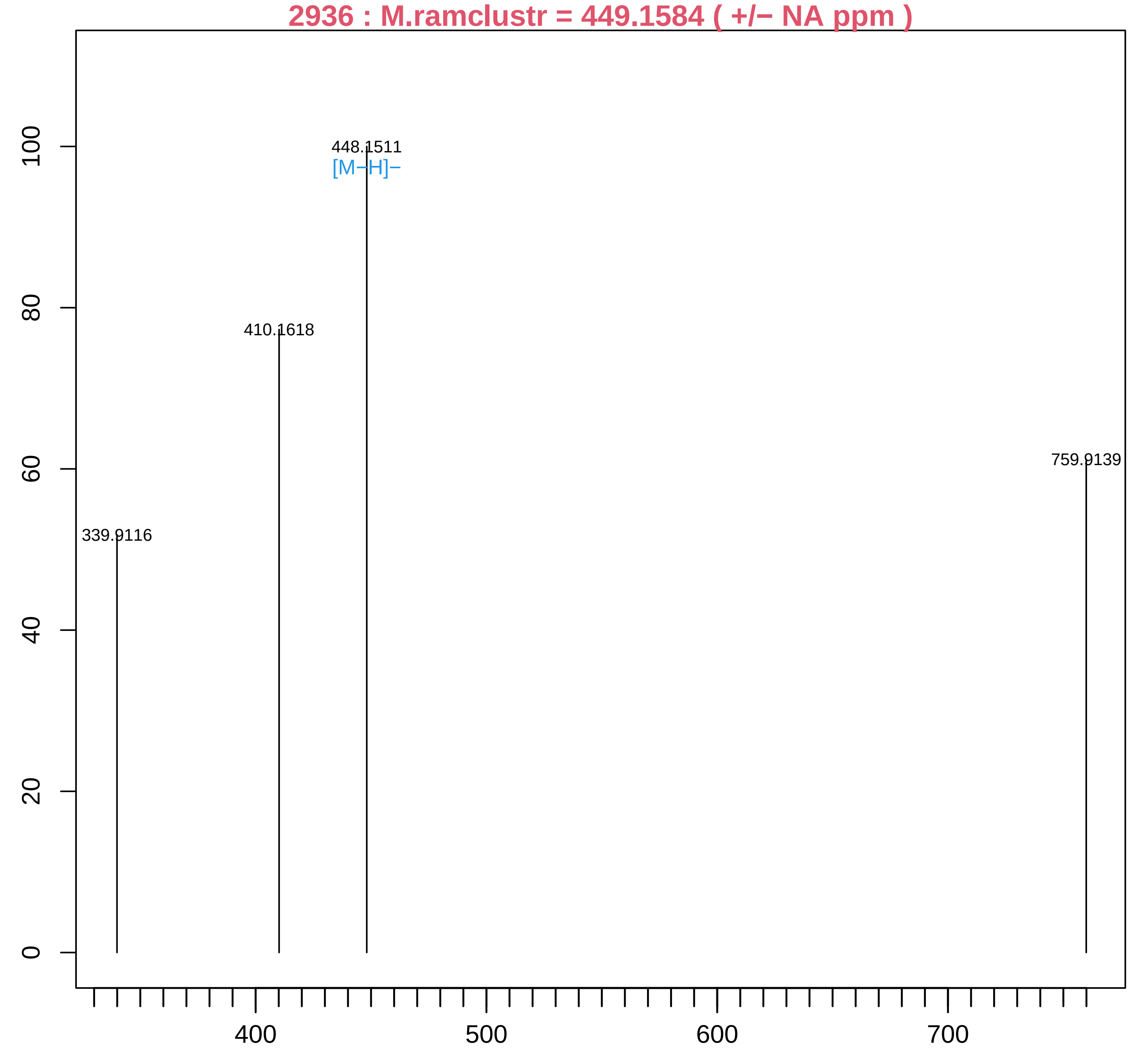

2936 : M.ramclustr = 449.1584 (+/− NA ppm)
448.1511
[M−H]−
410.1618
759.9139
339.9116
0
20
40
60
80
100
400
500
600
700

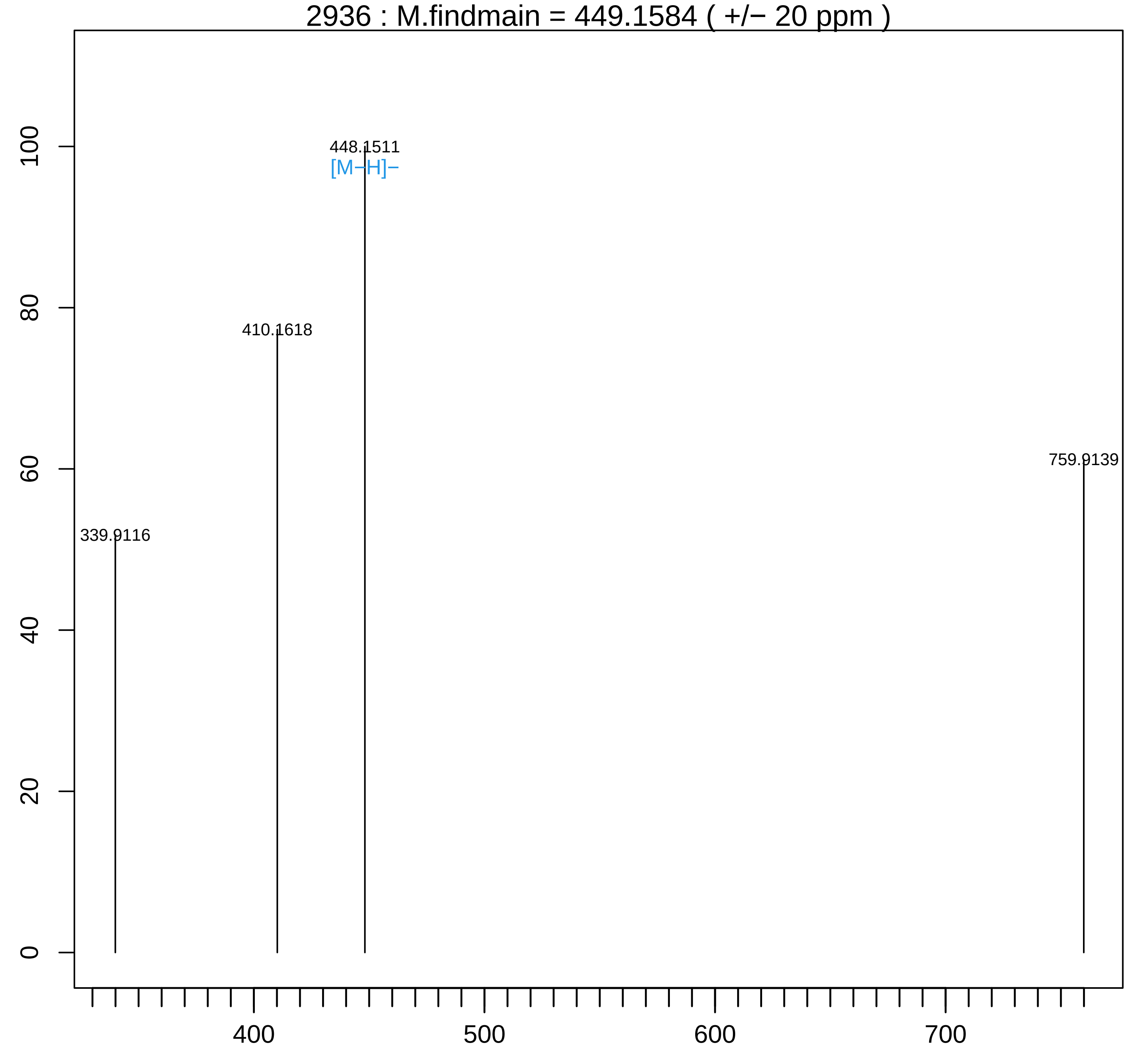

2936 : M.findmain = 449.1584 (+/− 20 ppm)
448.1511
[M−H]−
410.1618
759.9139
339.9116
0
20
40
60
80
100
400
500
600
700

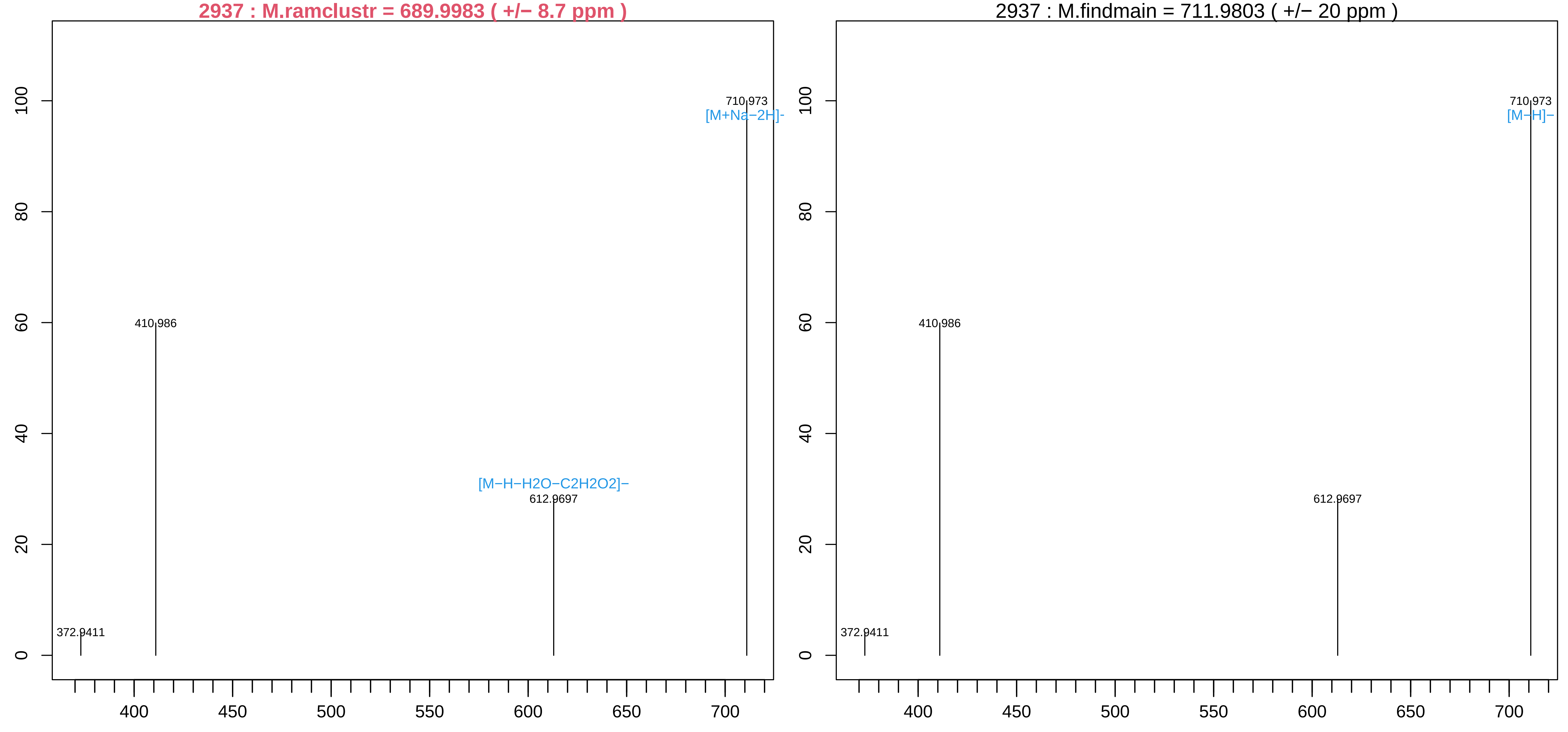

2937 : M.ramclustr = 689.9983 (+/− 8.7 ppm)
710.973
[M+Na−2H]-
410.986
[M−H−H2O−C2H2O2]−
612.9697
372.9411
2937 : M.findmain = 711.9803 (+/− 20 ppm)
710.973
[M−H]−
410.986
612.9697
372.9411
0
20
40
60
80
100
400
450
500
550
600
650
700

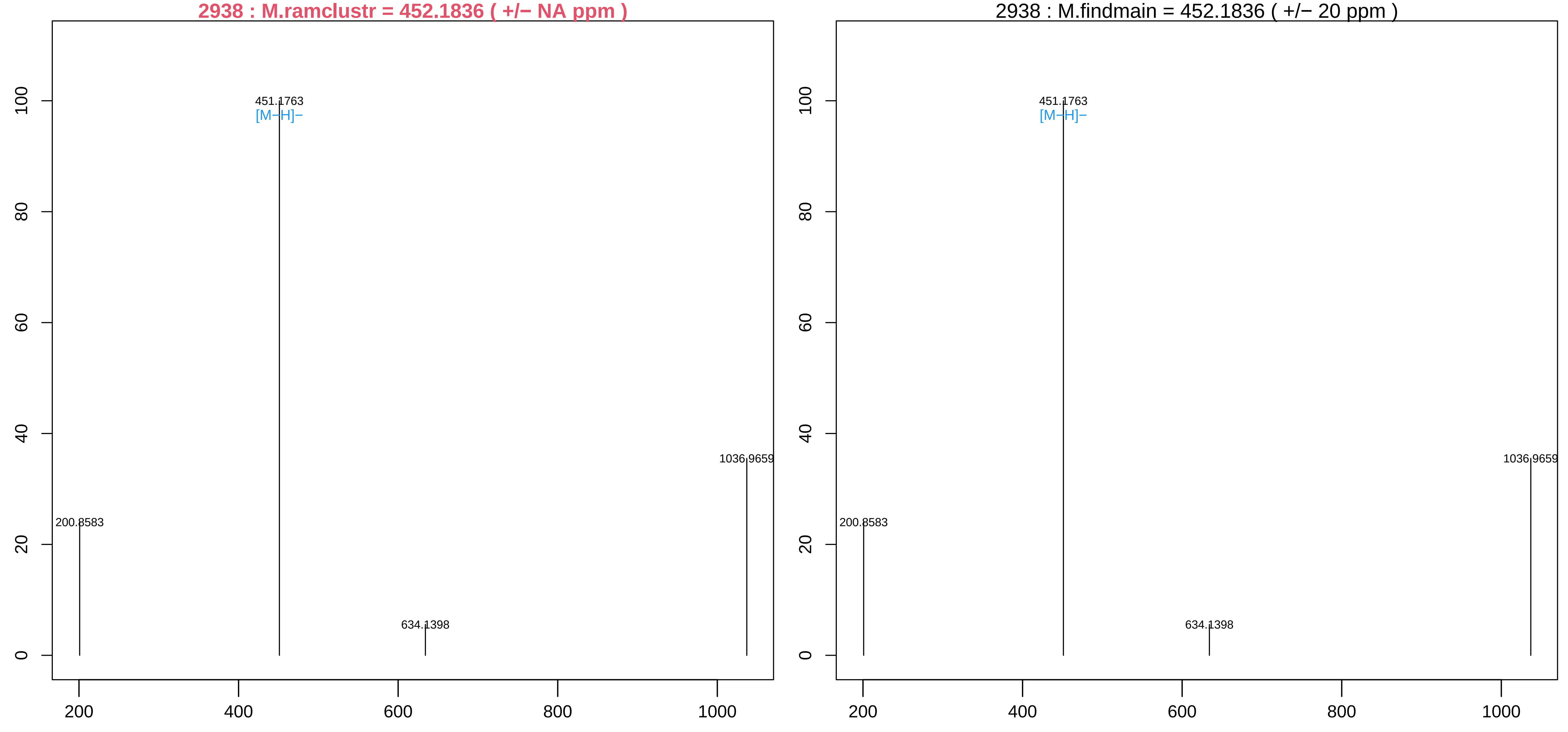
2938 : M.ramclustr = 452.1836 (+/− NA ppm)
451.1763
[M−H]−
200.8583
634.1398
1036.9659
2938 : M.findmain = 452.1836 (+/− 20 ppm)
451.1763
[M−H]−
200.8583
634.1398
1036.9659
0
20
40
60
80
100
200
400
600
800
1000

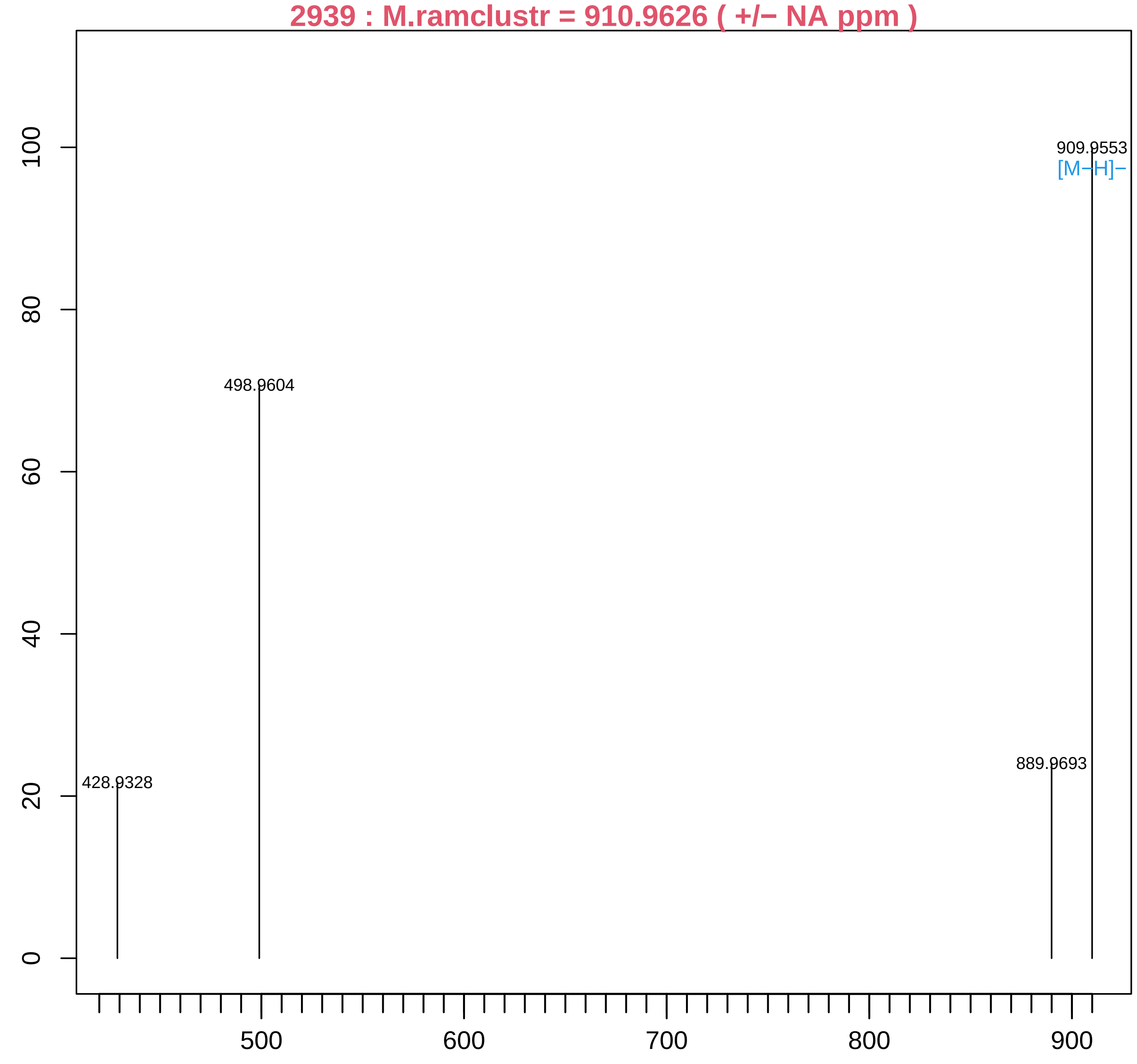
2939 : M.ramclustr = 910.9626 (+/− NA ppm)
909.9553
[M−H]−
498.9604
889.9693
428.9328
0
20
40
60
80
100
500
600
700
800
900

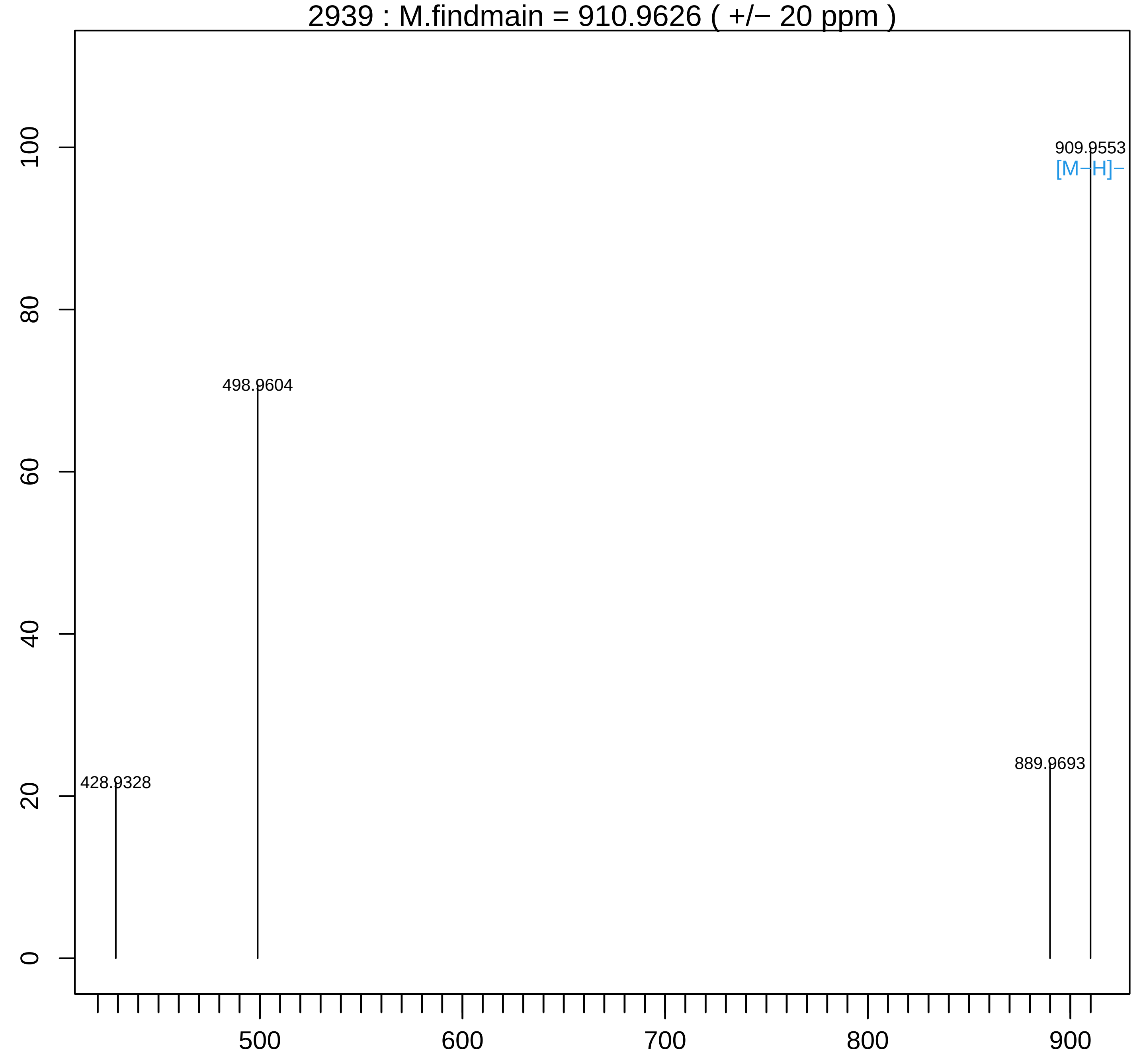
2939 : M.findmain = 910.9626 (+/− 20 ppm)
909.9553
[M−H]−
498.9604
889.9693
428.9328
0
20
40
60
80
100
500
600
700
800
900

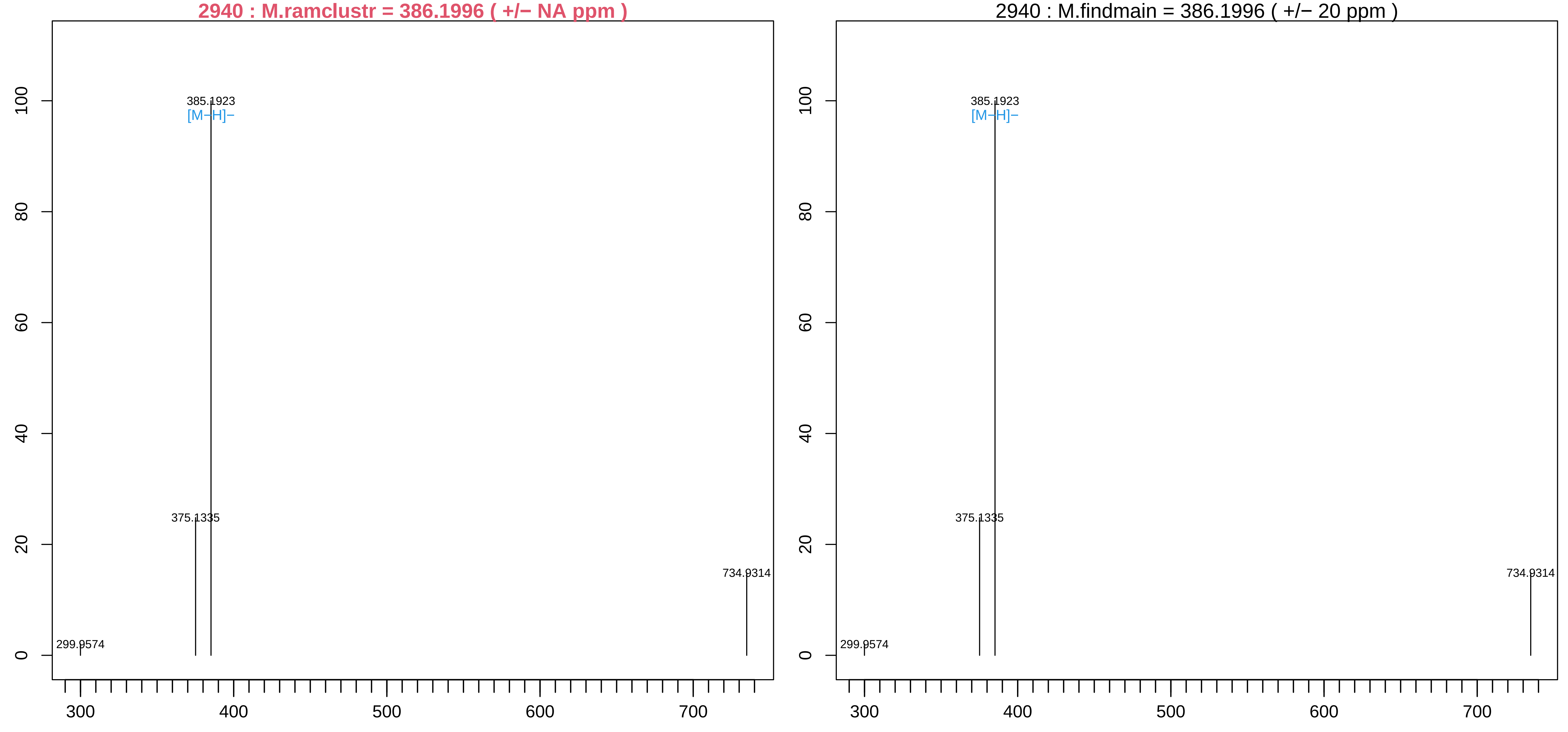

2940 : M.ramclustr = 386.1996 (+/− NA ppm)
385.1923
[M−H]−
375.1335
734.9314
299.9574
2940 : M.findmain = 386.1996 (+/− 20 ppm)
385.1923
[M−H]−
375.1335
734.9314
299.9574

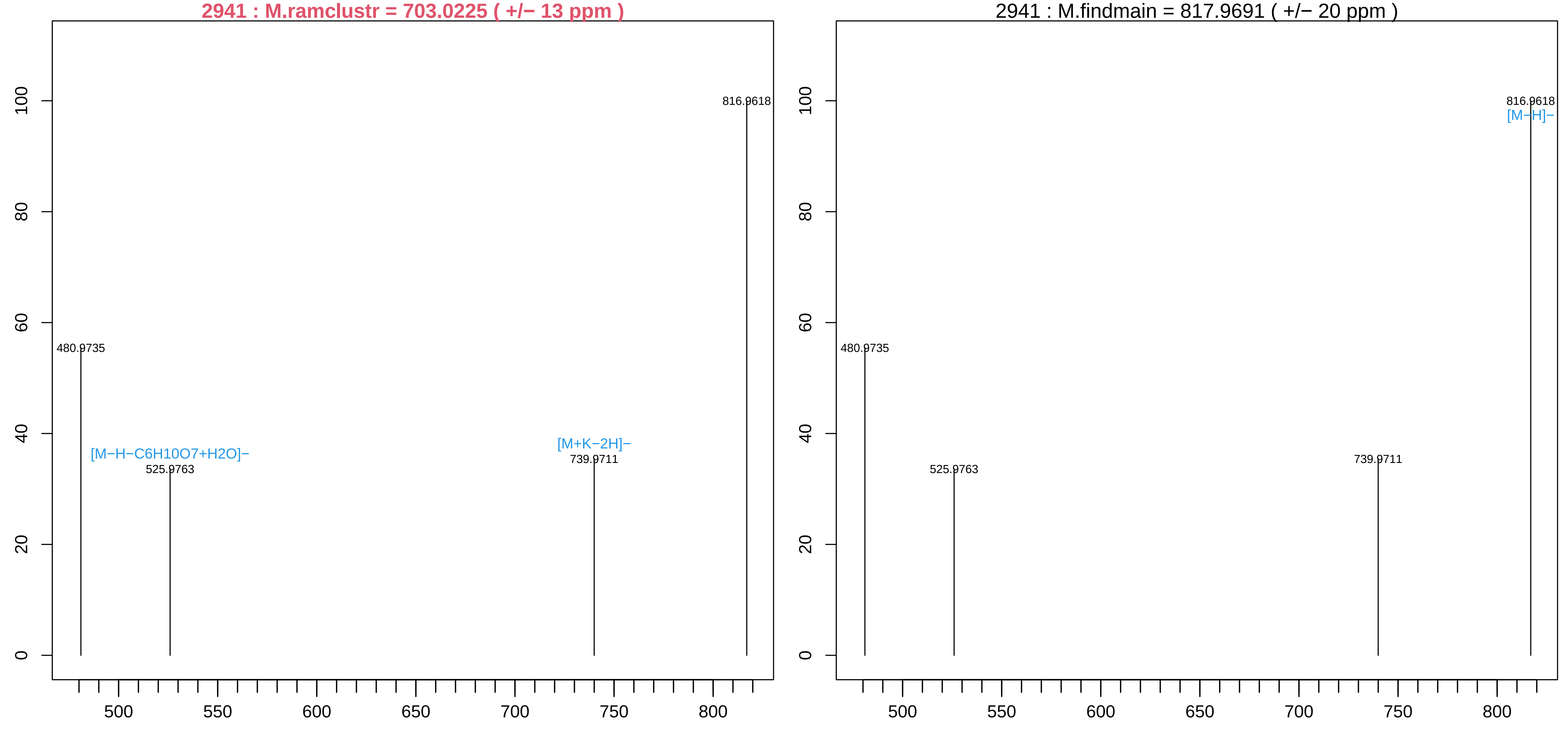

2941 : M.ramclustr = 703.0225 (+/− 13 ppm)
816.9618
480.9735
[M−H−C6H10O7+H2O]−
525.9763
[M+K−2H]−
739.9711
2941 : M.findmain = 817.9691 (+/− 20 ppm)
816.9618
[M−H]−
480.9735
525.9763
739.9711
0
20
40
60
80
100
500
550
600
650
700
750
800

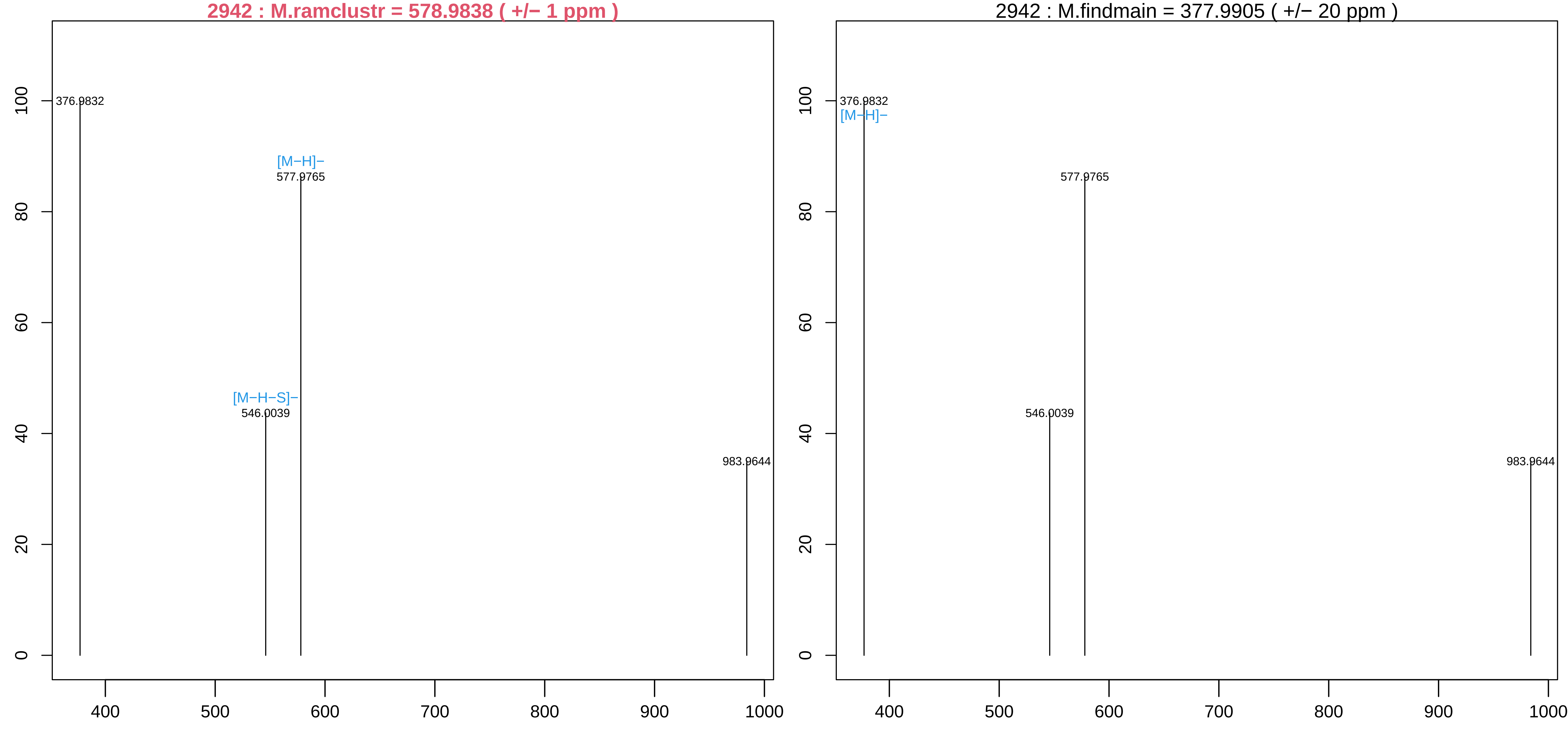

2942 : M.ramclustr = 578.9838 (+/− 1 ppm)
376.9832
[M−H]−
577.9765
[M−H−S]−
546.0039
983.9644
2942 : M.findmain = 377.9905 (+/− 20 ppm)
376.9832
[M−H]−
577.9765
546.0039
983.9644

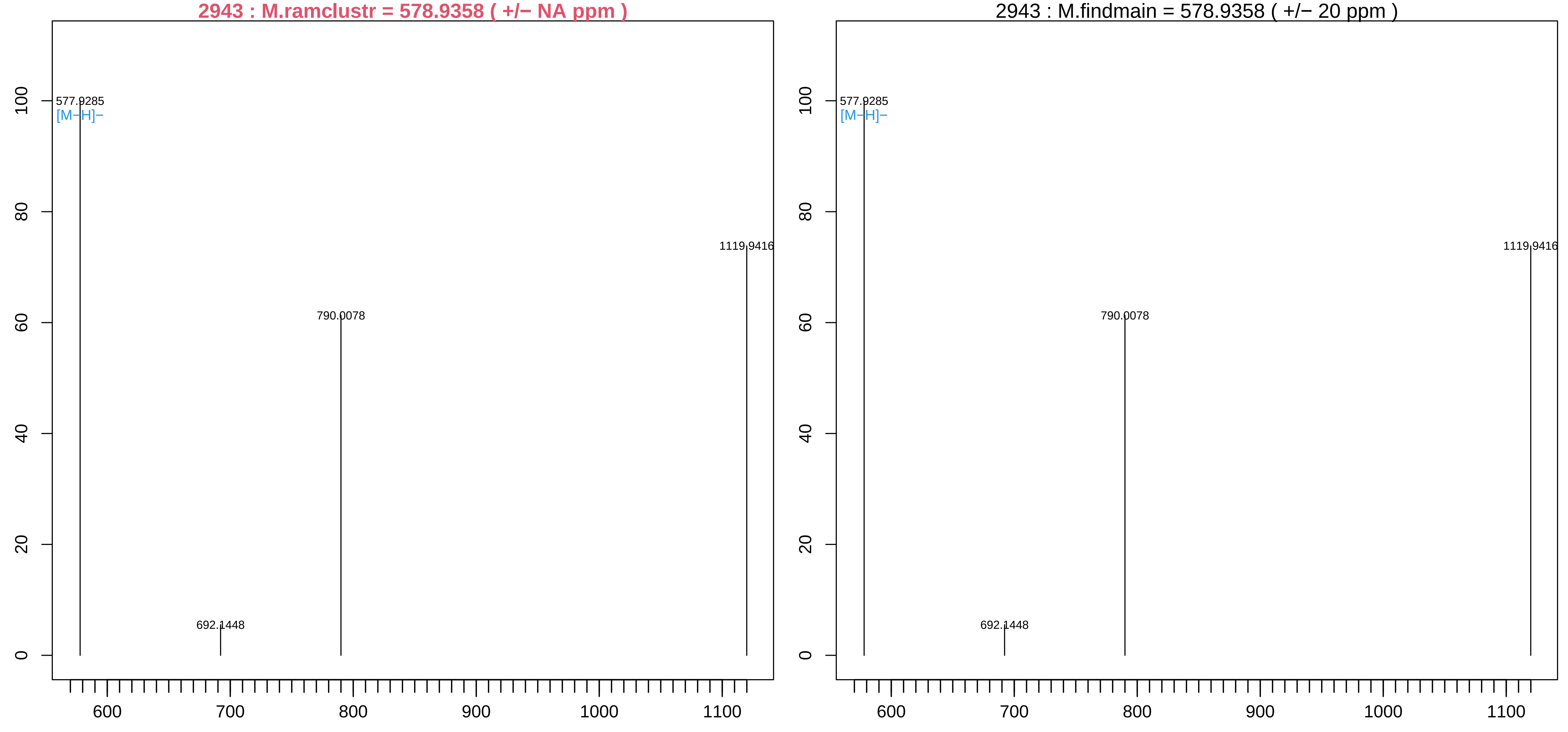
2943 : M.ramclustr = 578.9358 (+/− NA ppm)
2943 : M.findmain = 578.9358 (+/− 20 ppm)
577.9285
[M−H]−
692.1448
790.0078
1119.9416
0
20
40
60
80
100
600
700
800
900
1000
1100

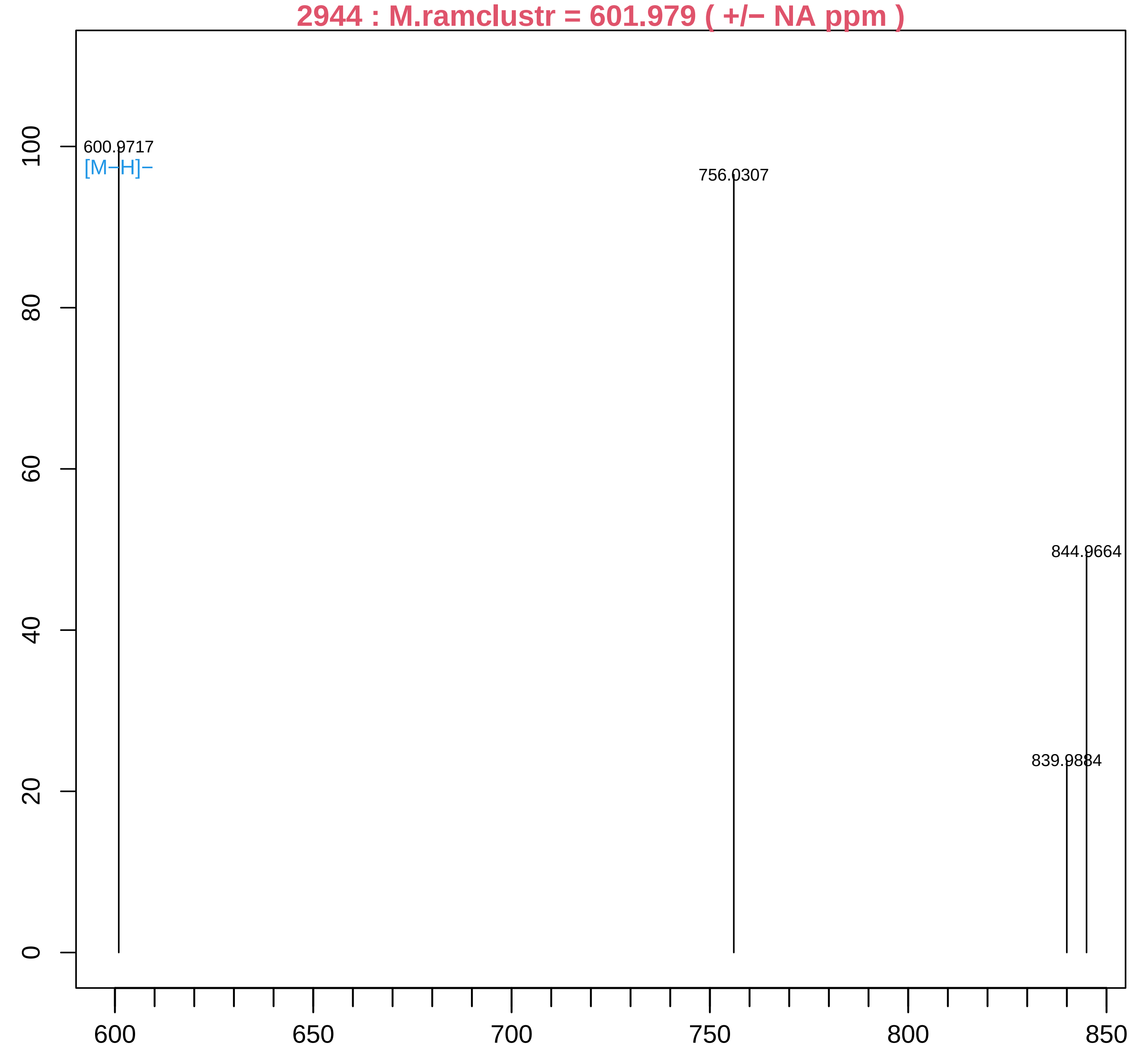

2944 : M.ramclustr = 601.979 (+/− NA ppm)
600.9717
[M−H]−
756.0307
844.9664
839.9884
0
20
40
60
80
100
600
650
700
750
800
850

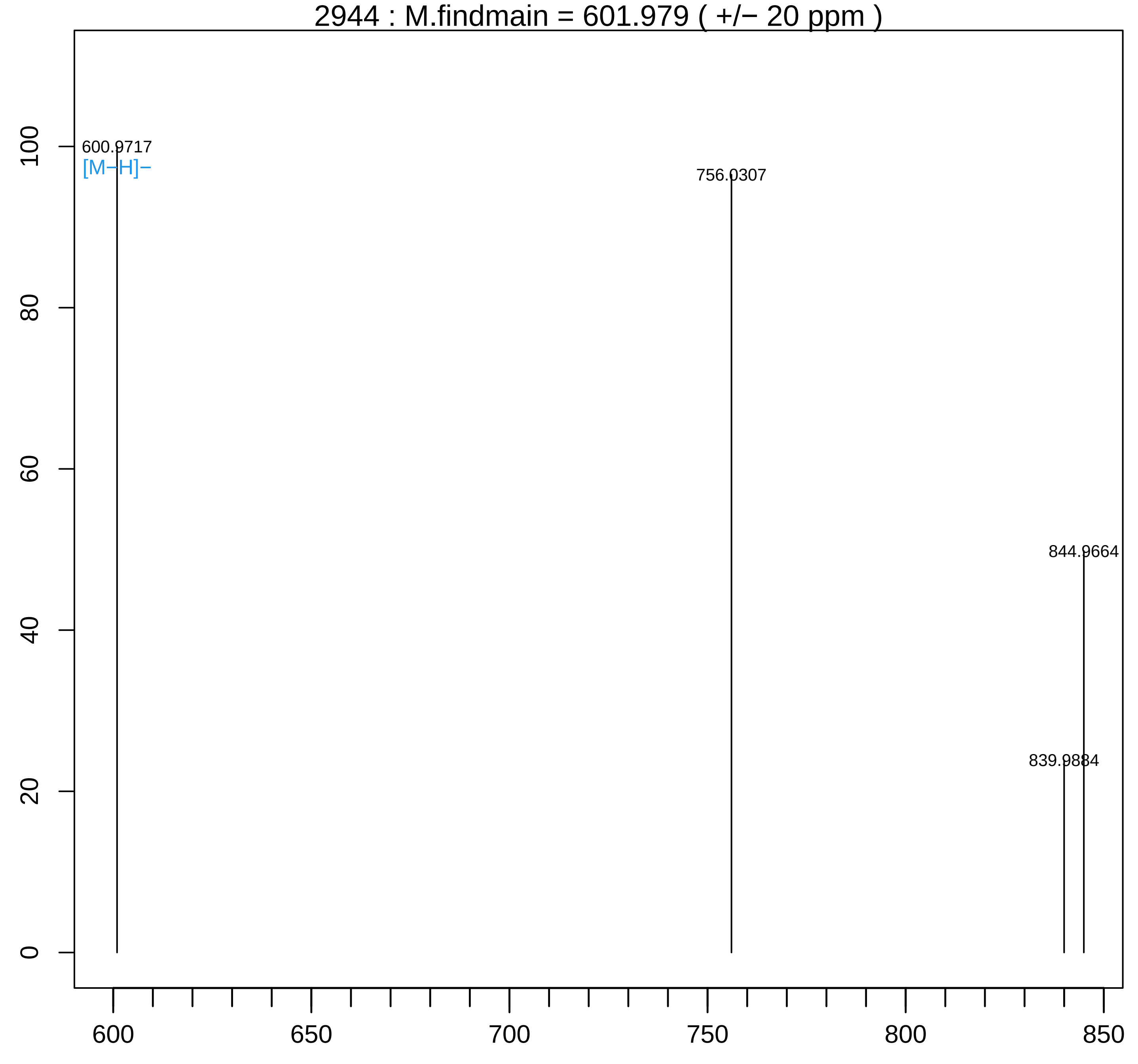

2944 : M.findmain = 601.979 (+/− 20 ppm)
600.9717
[M−H]−
756.0307
844.9664
839.9884
0
20
40
60
80
100
600
650
700
750
800
850

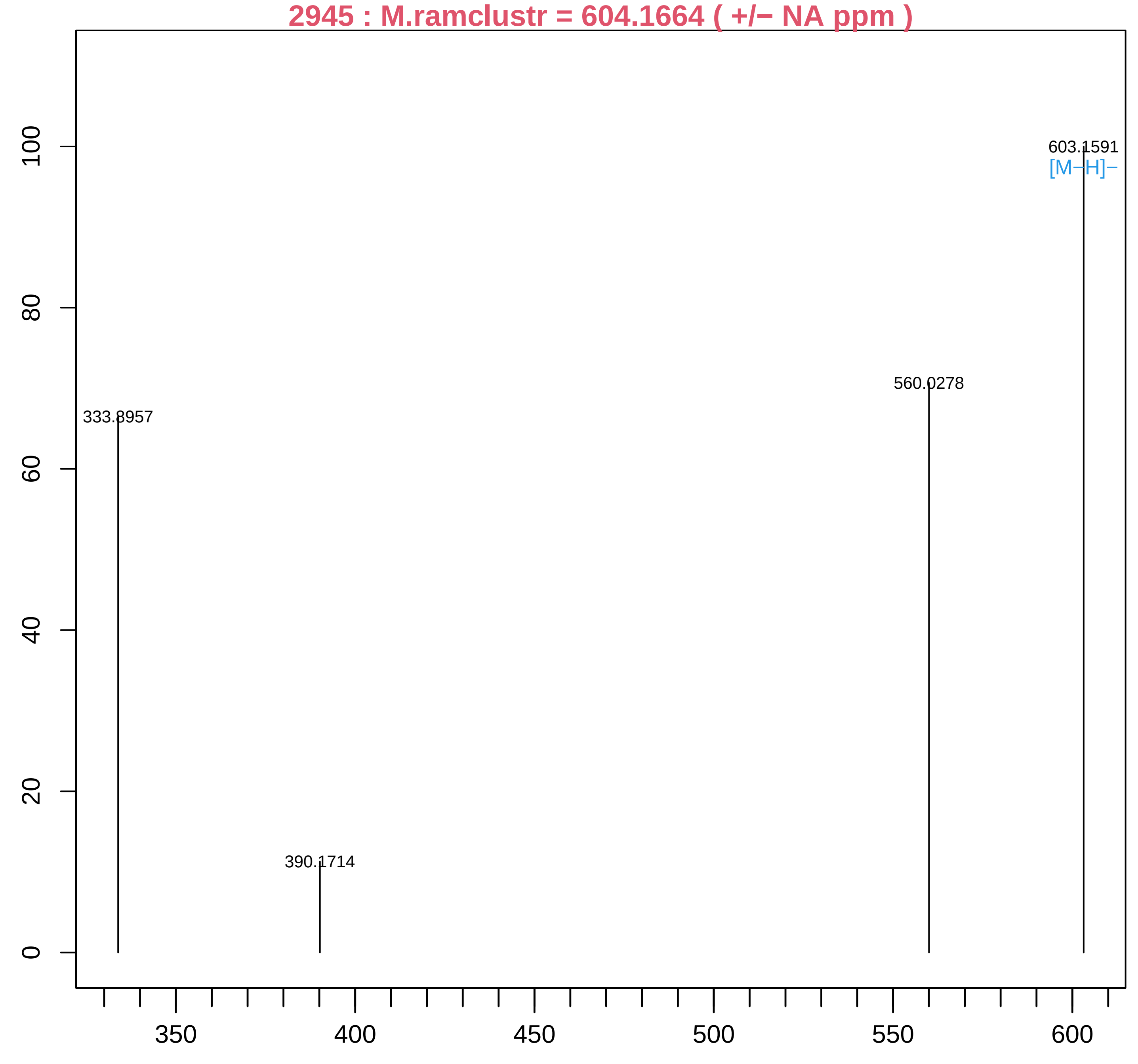
2945 : M.ramclustr = 604.1664 (+/− NA ppm)
603.1591
[M−H]−
560.0278
333.8957
390.1714
0
20
40
60
80
100
350
400
450
500
550
600

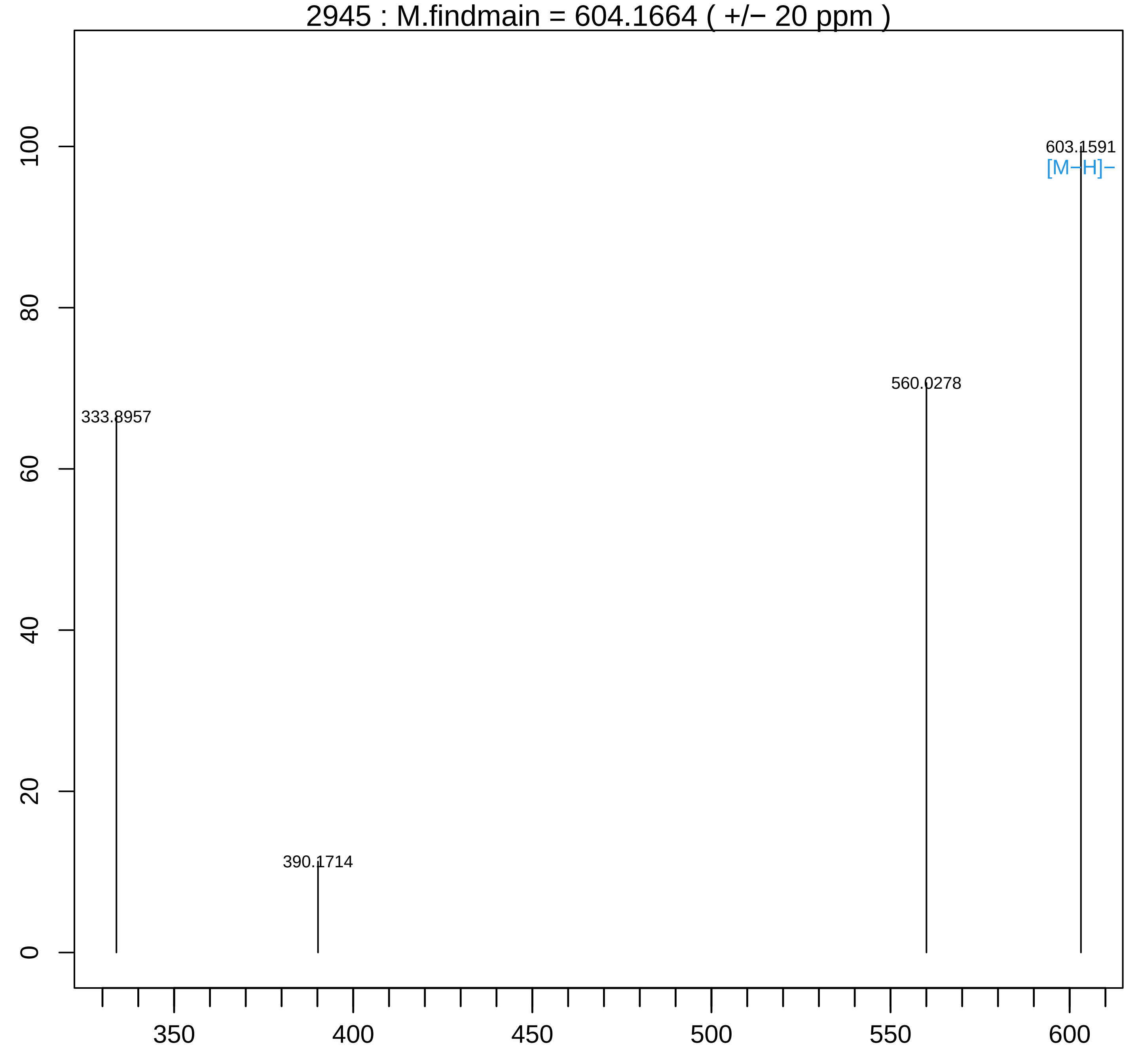
2945 : M.findmain = 604.1664 (+/− 20 ppm)
603.1591
[M−H]−
560.0278
333.8957
390.1714
0
20
40
60
80
100
350
400
450
500
550
600

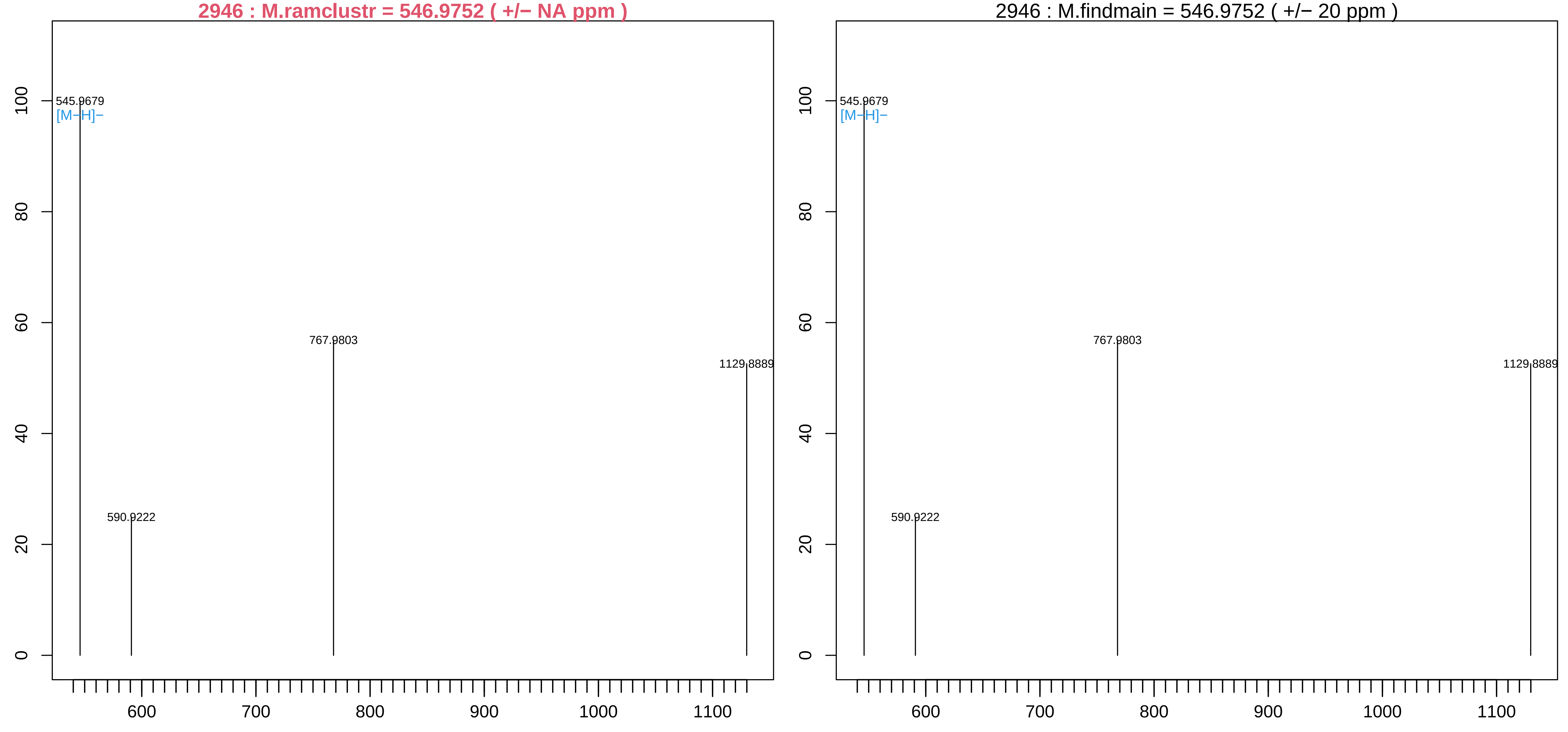

2946 : M.ramclustr = 546.9752 (+/− NA ppm)
545.9679
[M−H]−
590.9222
767.9803
1129.8889
2946 : M.findmain = 546.9752 (+/− 20 ppm)
545.9679
[M−H]−
590.9222
767.9803
1129.8889

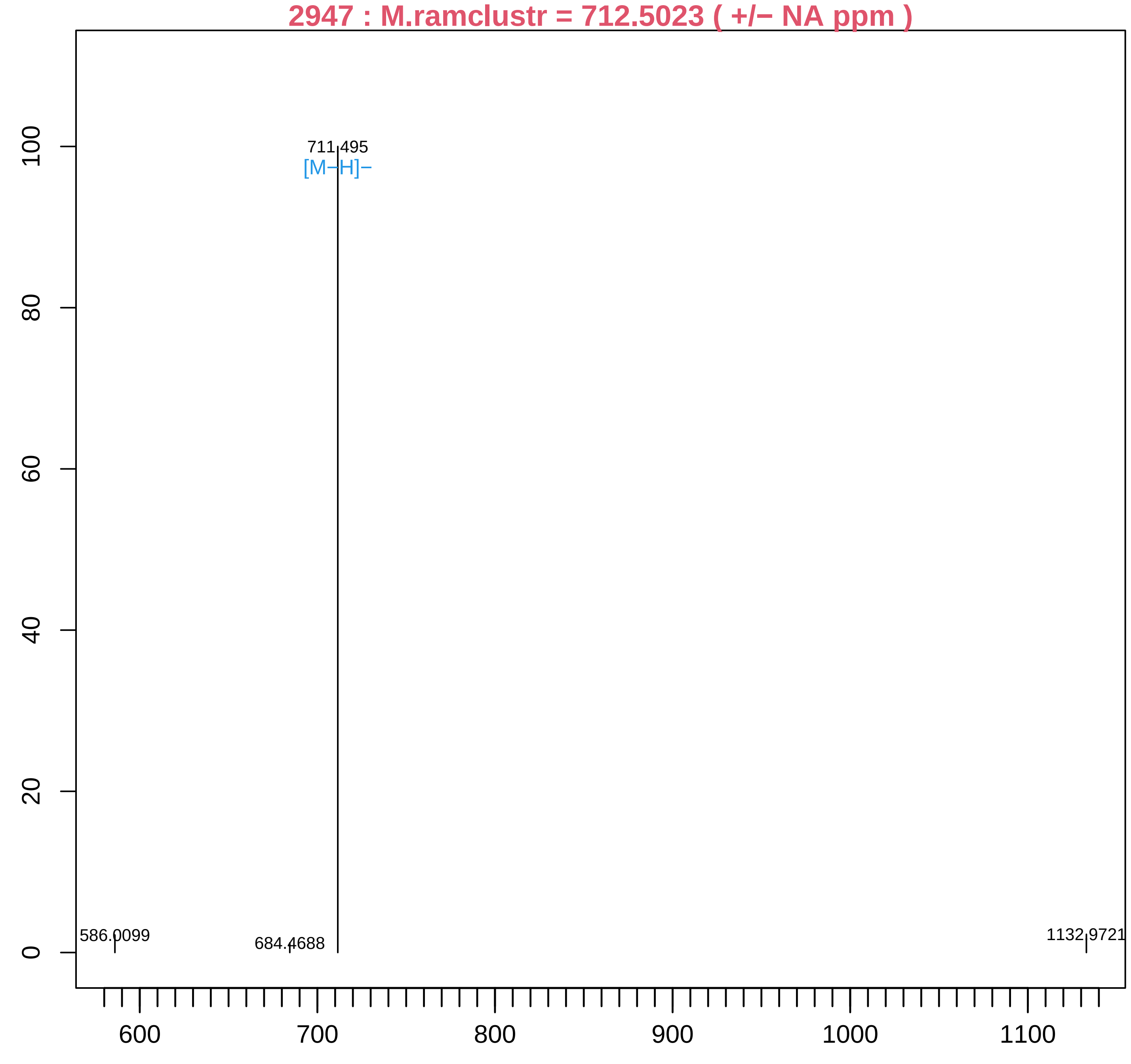
2947 : M.ramclustr = 712.5023 (+/− NA ppm)
711.495
[M−H]−
586.0099
684.4688
1132.9721
0
20
40
60
80
100
600
700
800
900
1000
1100

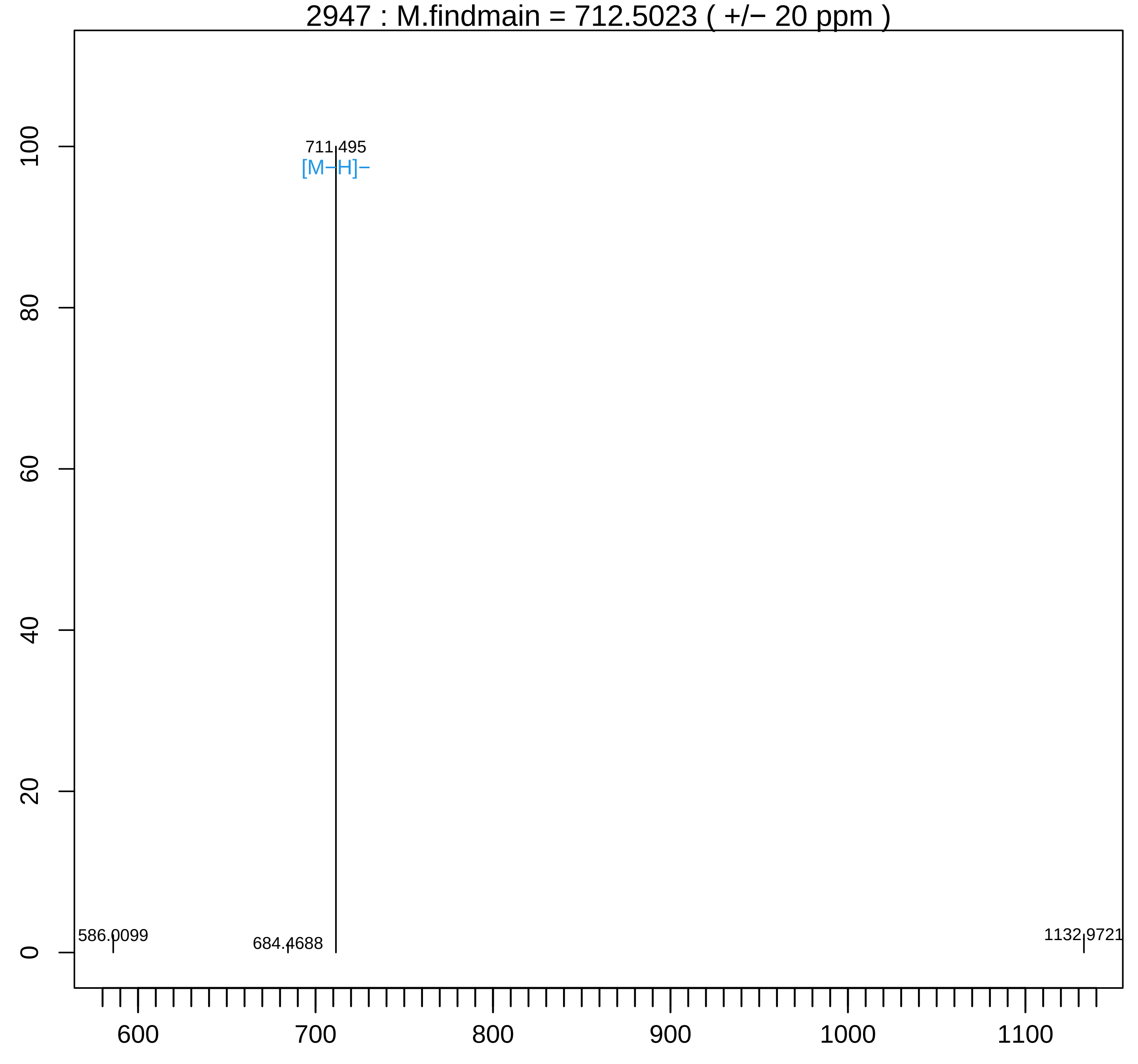
2947 : M.findmain = 712.5023 (+/− 20 ppm)
711.495
[M−H]−
586.0099
684.4688
1132.9721
0
20
40
60
80
100
600
700
800
900
1000
1100

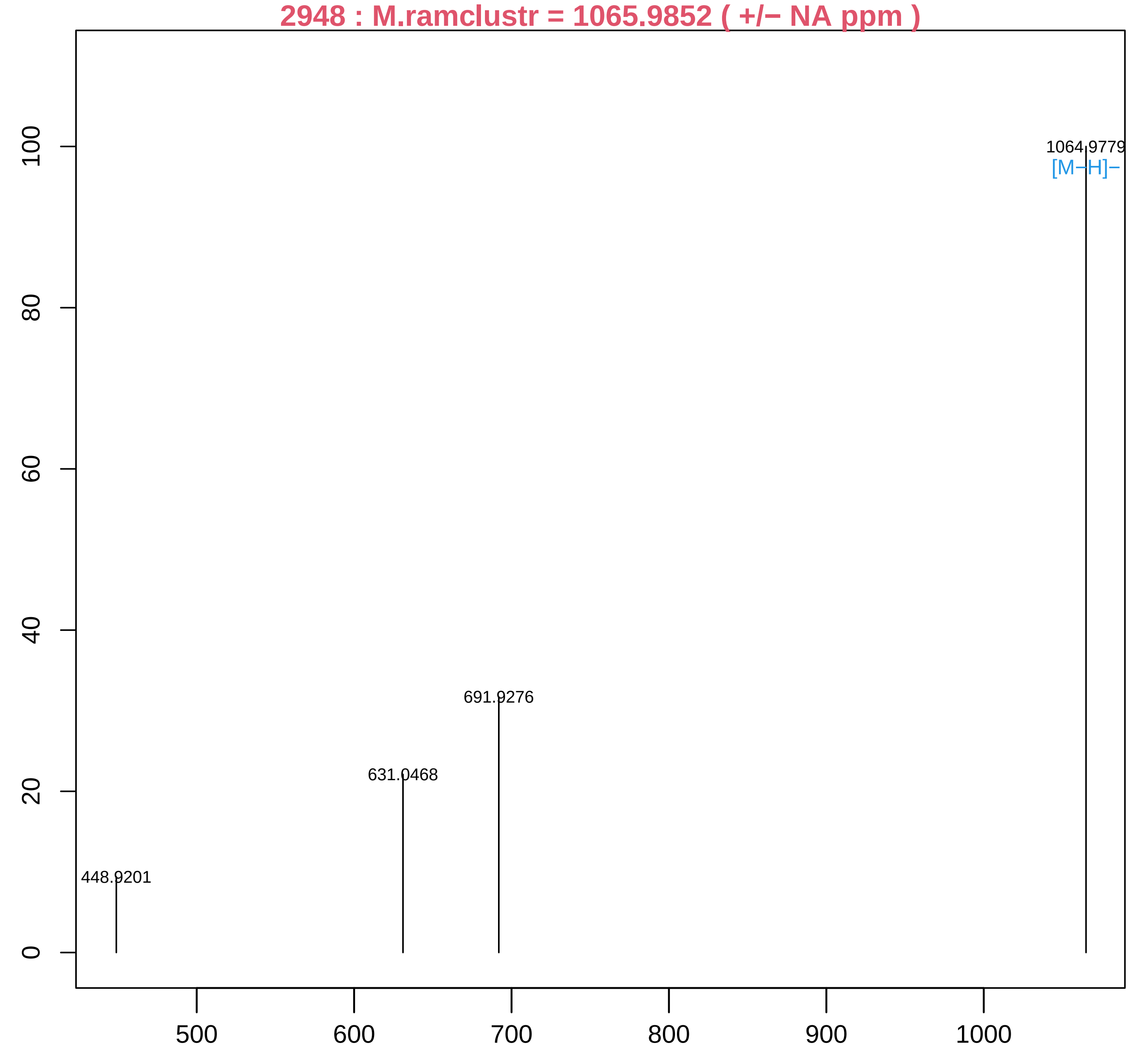

2948 : M.ramclustr = 1065.9852 (+/− NA ppm)
1064.9779
[M−H]−
691.9276
631.0468
448.9201
0
20
40
60
80
100
500
600
700
800
900
1000

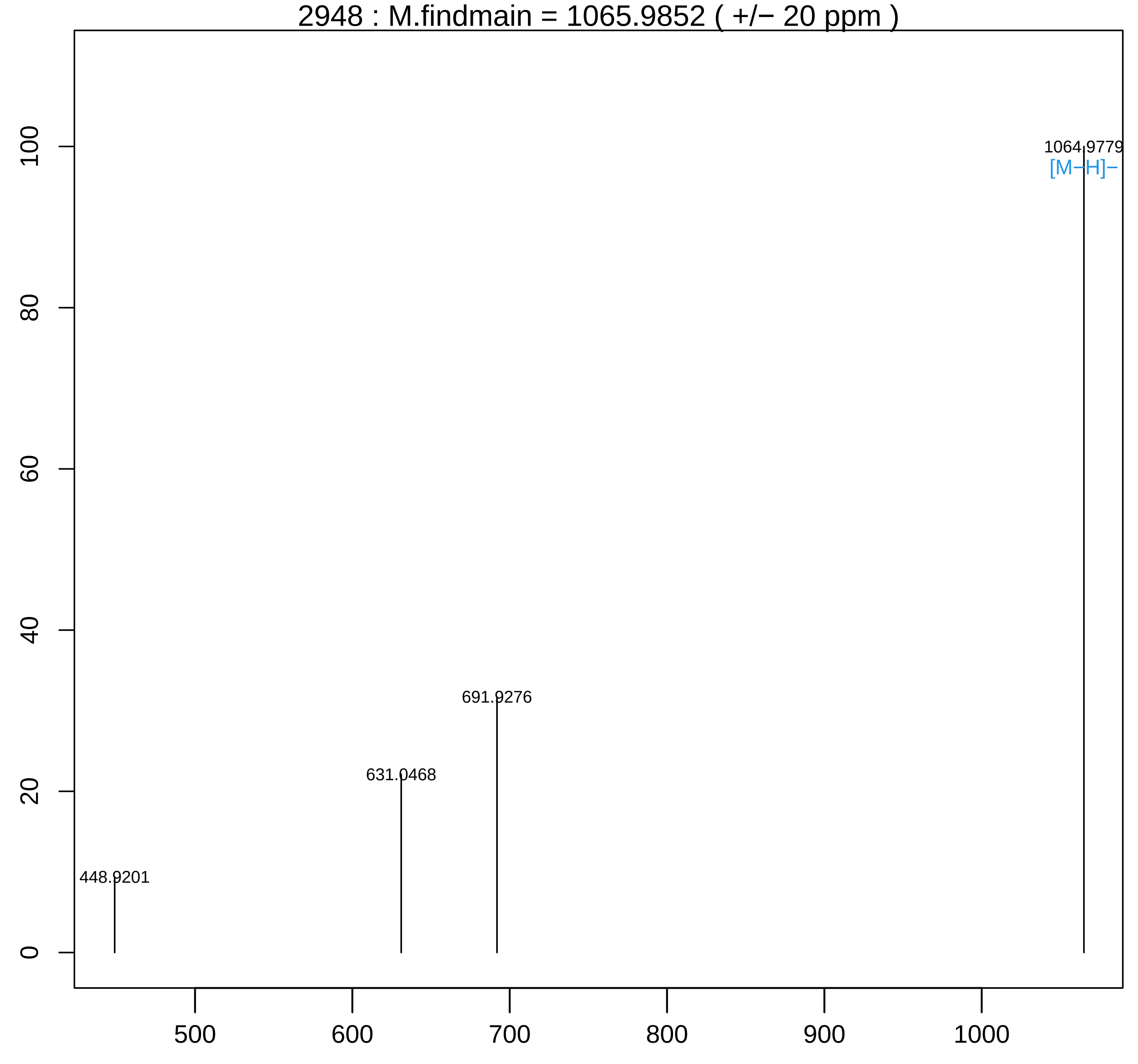

2948 : M.findmain = 1065.9852 (+/− 20 ppm)
1064.9779
[M−H]−
691.9276
631.0468
448.9201
0
20
40
60
80
100
500
600
700
800
900
1000

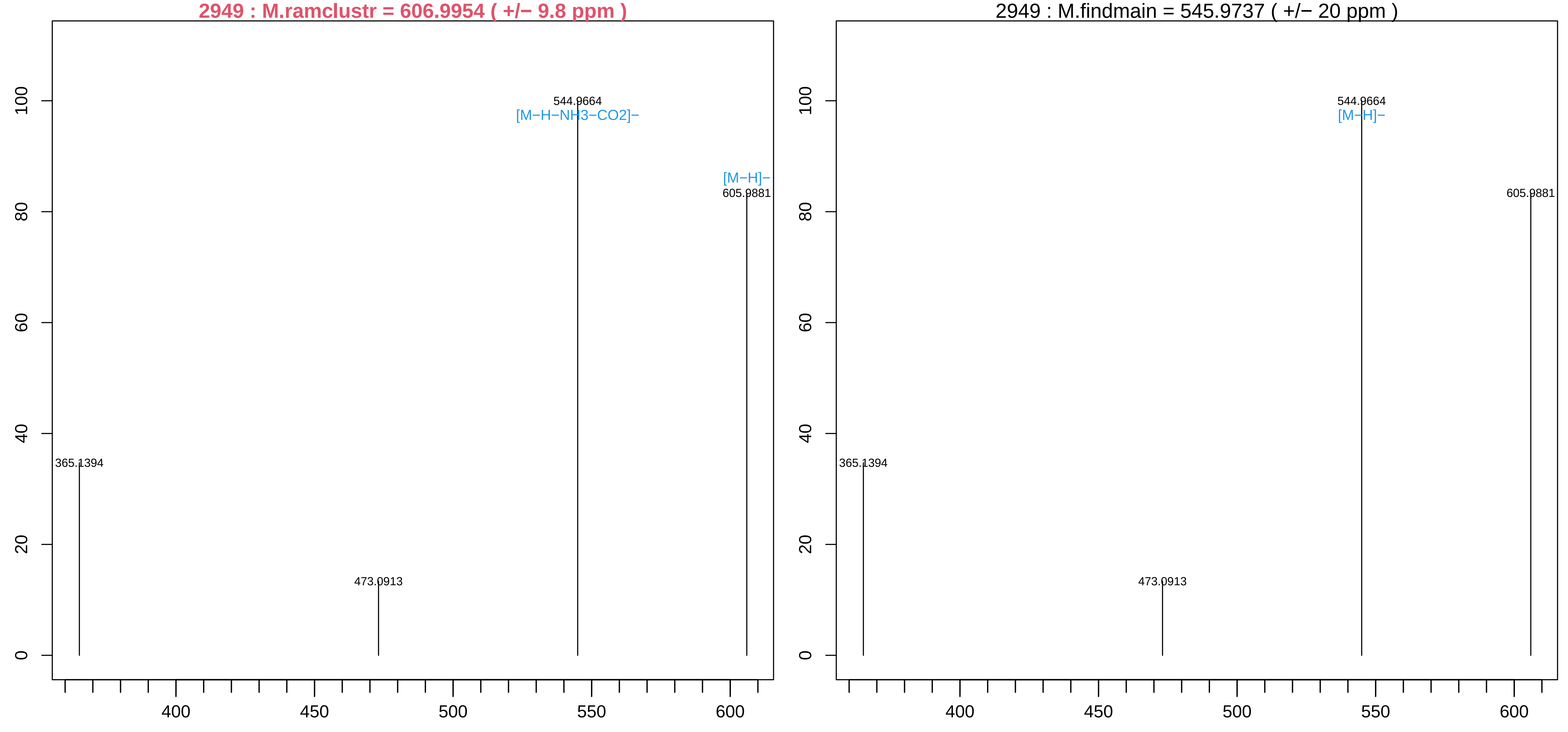

2949 : M.ramclustr = 606.9954 (+/− 9.8 ppm)
544.9664
[M−H−NH3−CO2]−
[M−H]−
605.9881
365.1394
473.0913
0
20
40
60
80
100
400
450
500
550
600
2949 : M.findmain = 545.9737 (+/− 20 ppm)
544.9664
[M−H]−
605.9881
365.1394
473.0913
0
20
40
60
80
100
400
450
500
550
600

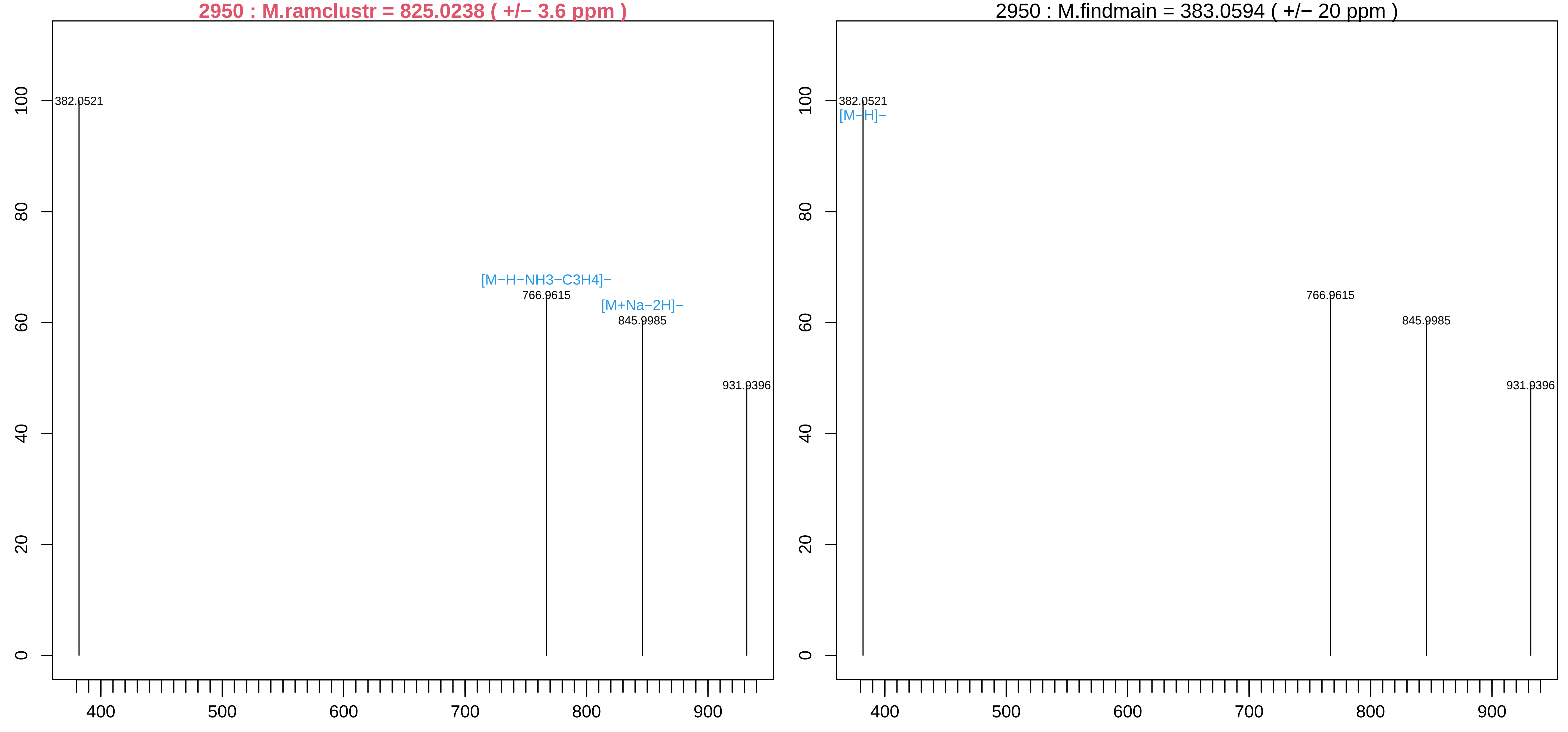
2950 : M.ramclustr = 825.0238 (+/− 3.6 ppm)
382.0521
[M−H−NH3−C3H4]−
766.9615
[M+Na−2H]−
845.9985
931.9396
0
20
40
60
80
100
400
500
600
700
800
900
2950 : M.findmain = 383.0594 (+/− 20 ppm)
382.0521
[M−H]−
766.9615
845.9985
931.9396
0
20
40
60
80
100
400
500
600
700
800
900

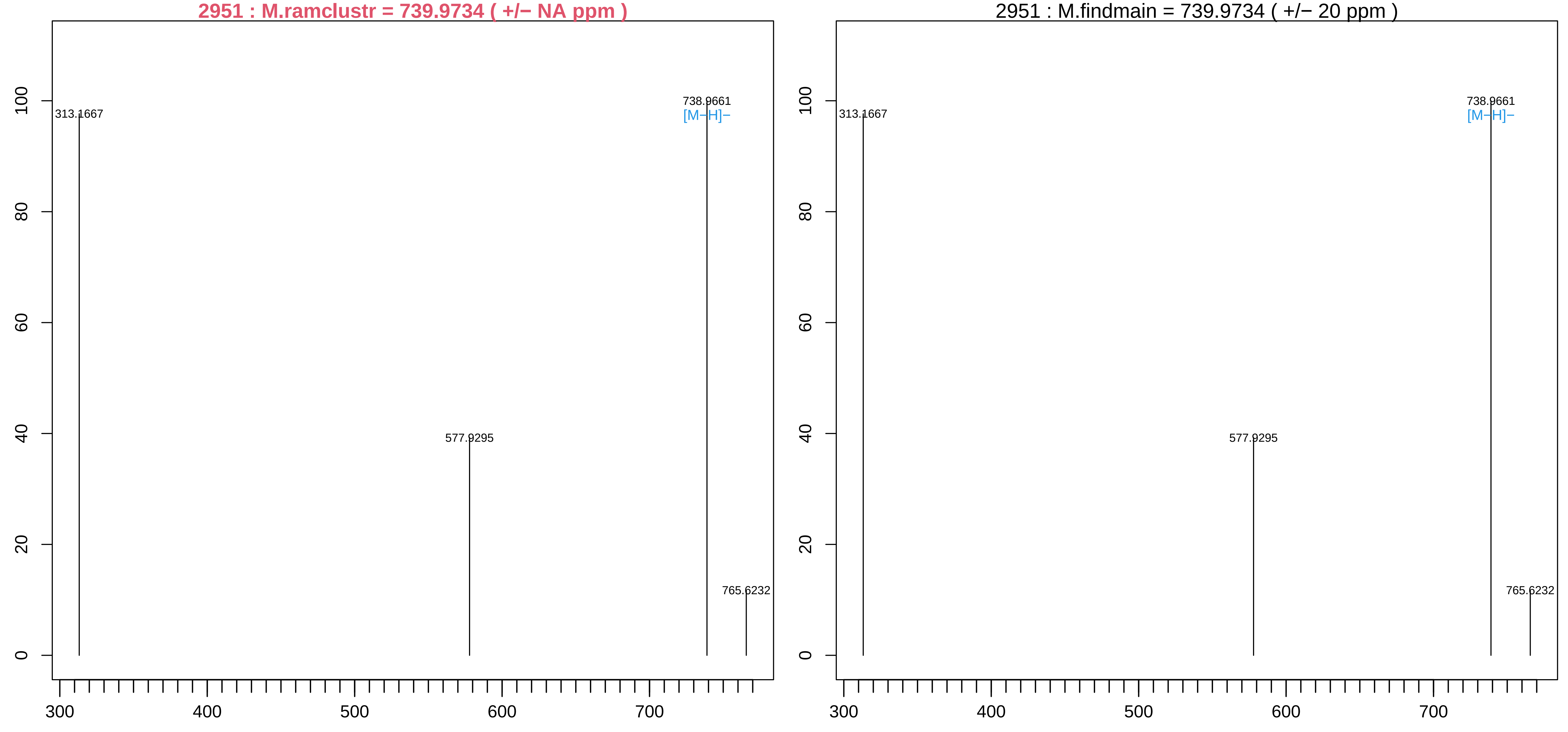
2951 : M.ramclustr = 739.9734 (+/− NA ppm)
313.1667
577.9295
738.9661
[M−H]−
765.6232
0
20
40
60
80
100
300
400
500
600
700
2951 : M.findmain = 739.9734 (+/− 20 ppm)
313.1667
577.9295
738.9661
[M−H]−
765.6232
0
20
40
60
80
100
300
400
500
600
700

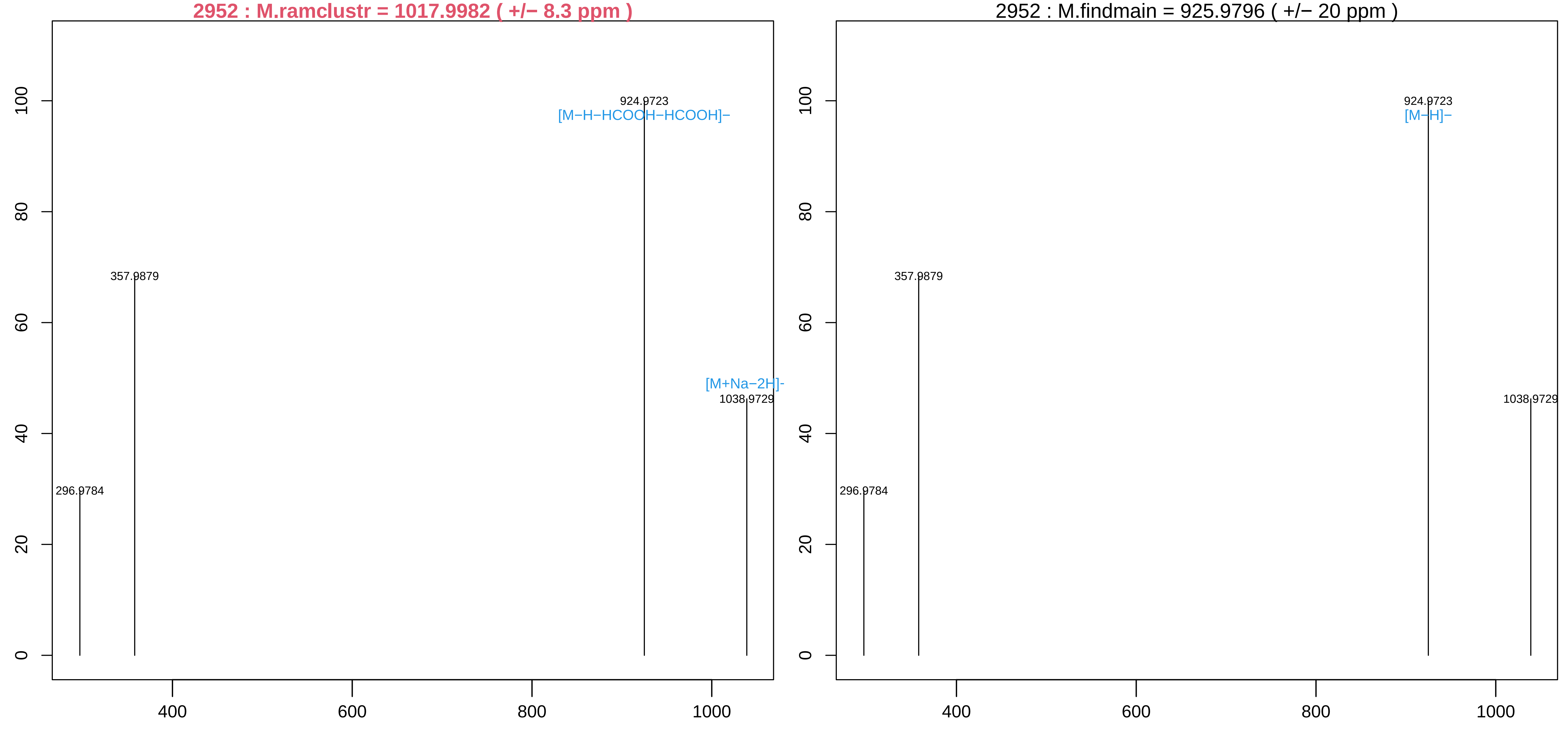
2952 : M.ramclustr = 1017.9982 (+/− 8.3 ppm)
924.9723
[M−H−HCOOH−HCOOH]−
357.9879
296.9784
[M+Na−2H]-
1038.9729
2952 : M.findmain = 925.9796 (+/− 20 ppm)
924.9723
[M−H]−
357.9879
296.9784
1038.9729
0
20
40
60
80
100
400
600
800
1000

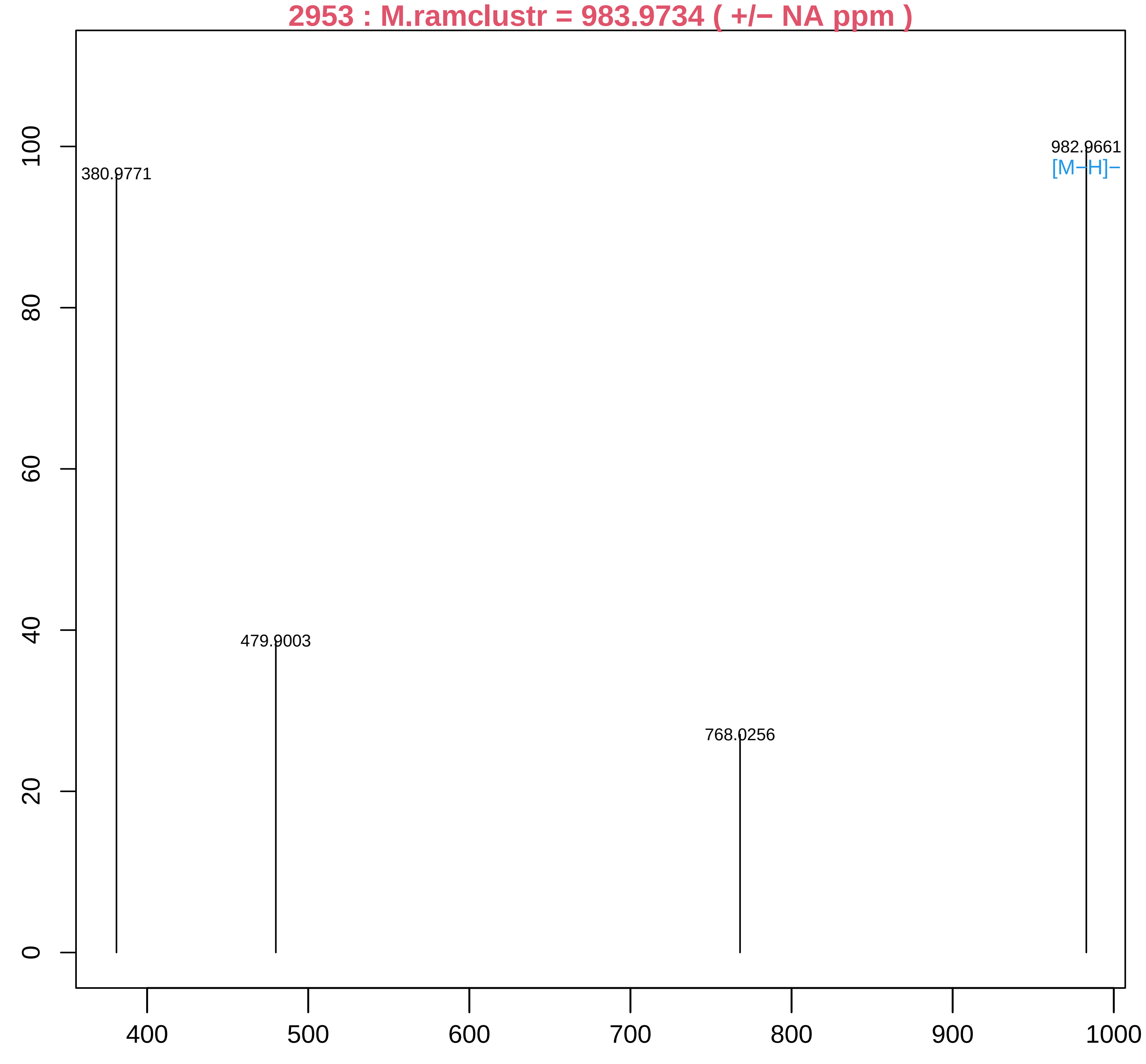

2953 : M.ramclustr = 983.9734 (+/− NA ppm)
380.9771
479.9003
768.0256
982.9661
[M−H]−
0
20
40
60
80
100
400
500
600
700
800
900
1000

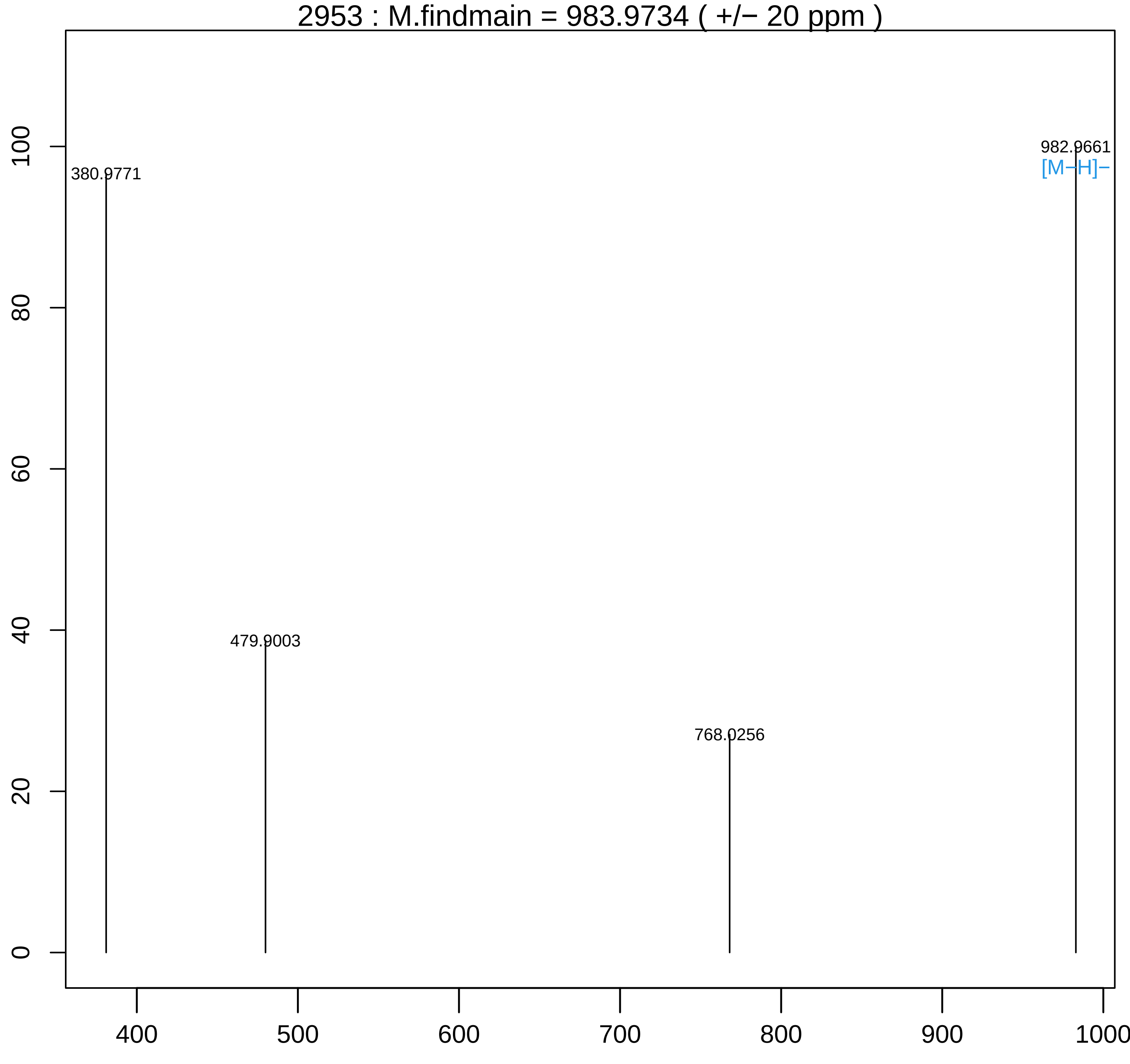

2953 : M.findmain = 983.9734 (+/− 20 ppm)
380.9771
479.9003
768.0256
982.9661
[M−H]−
0
20
40
60
80
100
400
500
600
700
800
900
1000

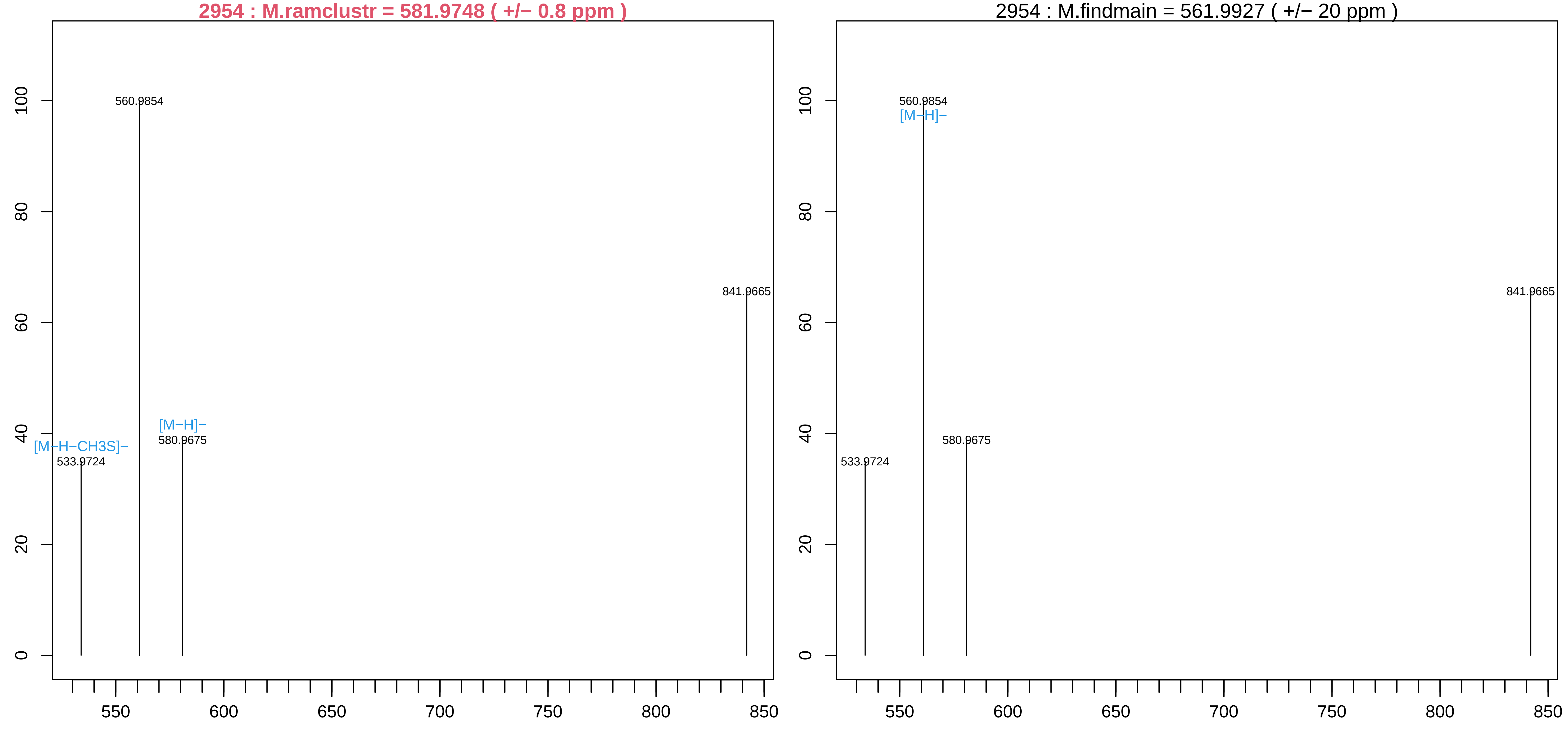
2954 : M.ramclustr = 581.9748 (+/− 0.8 ppm)
560.9854
841.9665
[M−H]−
580.9675
[M−H−CH3S]−
533.9724
2954 : M.findmain = 561.9927 (+/− 20 ppm)
560.9854
[M−H]−
841.9665
580.9675
533.9724
0
20
40
60
80
100
550
600
650
700
750
800
850

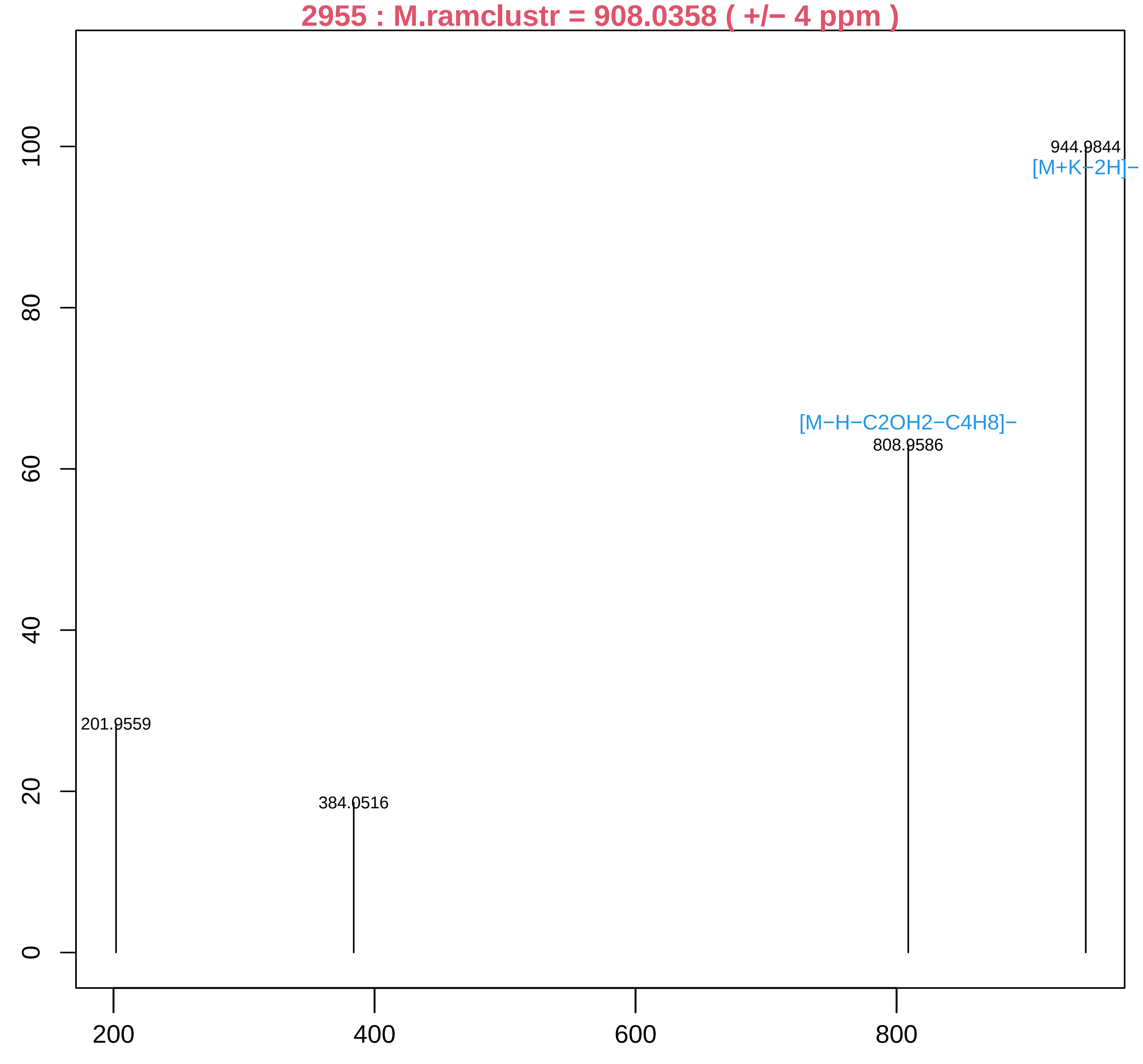
2955 : M.ramclustr = 908.0358 (+/− 4 ppm)
944.9844
[M+K−2H]−
[M−H−C2OH2−C4H8]−
808.9586
201.9559
384.0516
0
20
40
60
80
100
200
400
600
800

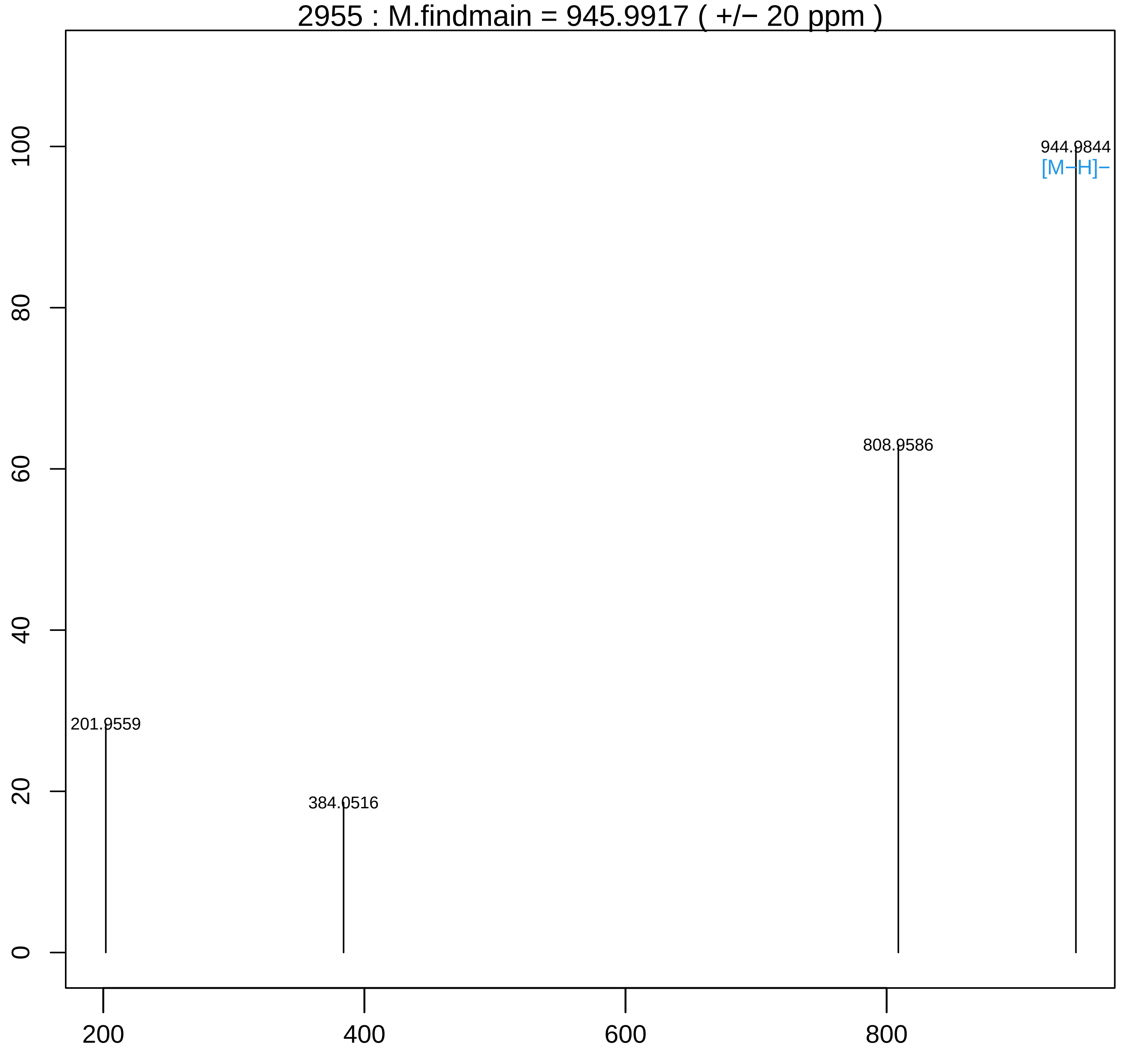
2955 : M.findmain = 945.9917 (+/− 20 ppm)
944.9844
[M−H]−
808.9586
201.9559
384.0516
0
20
40
60
80
100
200
400
600
800

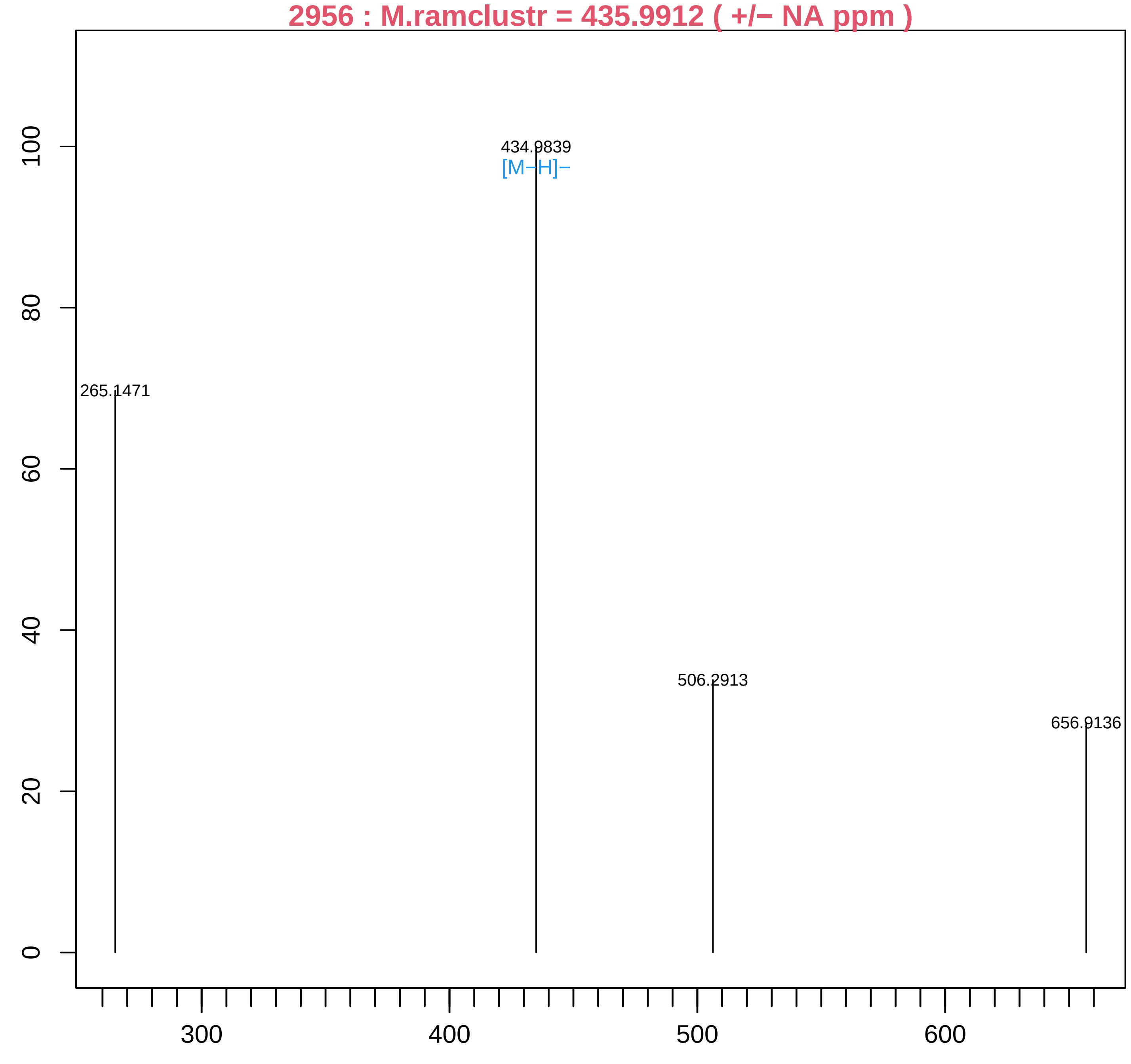

2956 : M.ramclustr = 435.9912 (+/− NA ppm)
434.9839
[M−H]−
265.1471
506.2913
656.9136
0
20
40
60
80
100
300
400
500
600

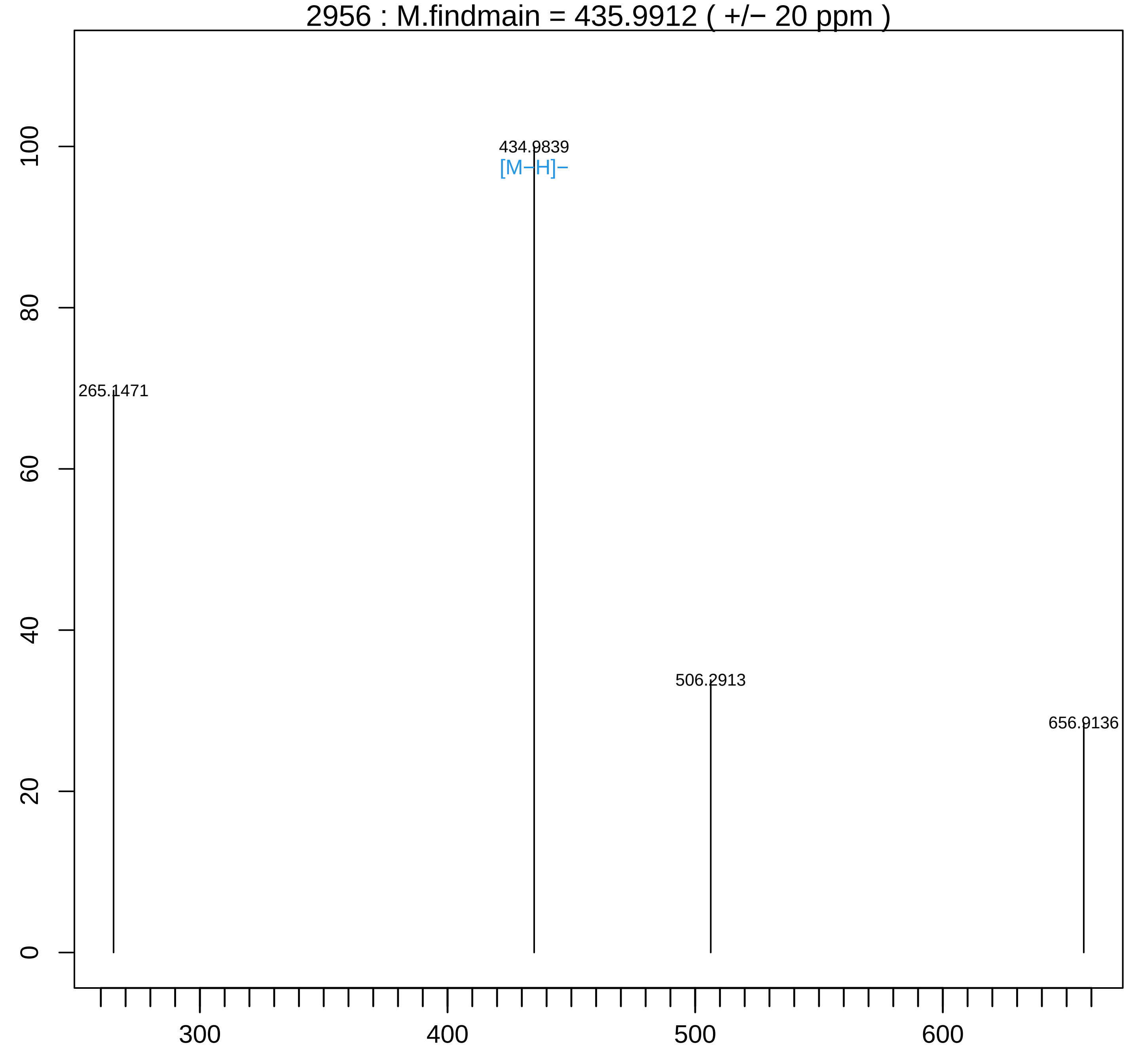

2956 : M.findmain = 435.9912 (+/− 20 ppm)
434.9839
[M−H]−
265.1471
506.2913
656.9136
0
20
40
60
80
100
300
400
500
600

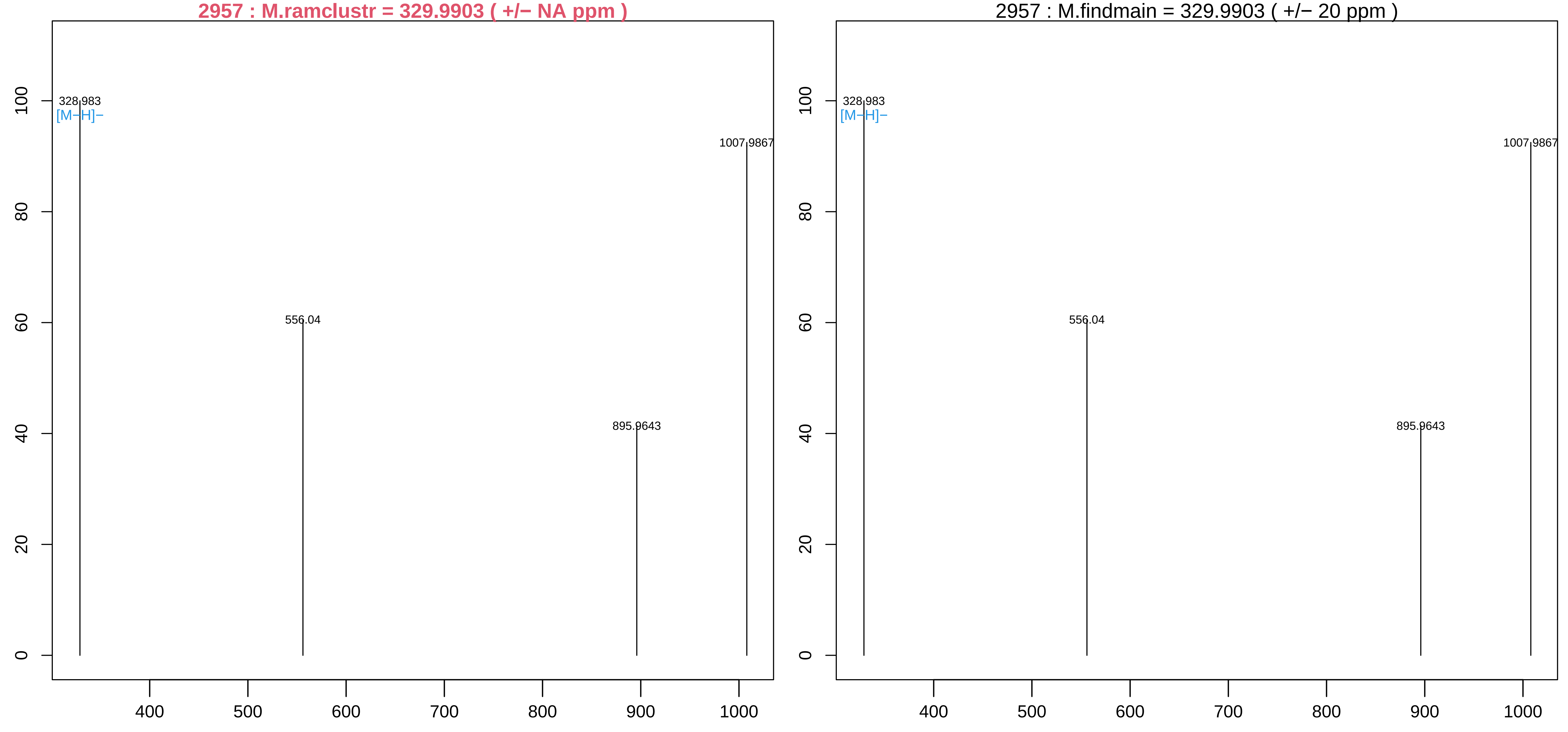
2957 : M.ramclustr = 329.9903 (+/− NA ppm)
328.983
[M−H]−
556.04
895.9643
1007.9867
2957 : M.findmain = 329.9903 (+/− 20 ppm)
328.983
[M−H]−
556.04
895.9643
1007.9867
0
20
40
60
80
100
400
500
600
700
800
900
1000

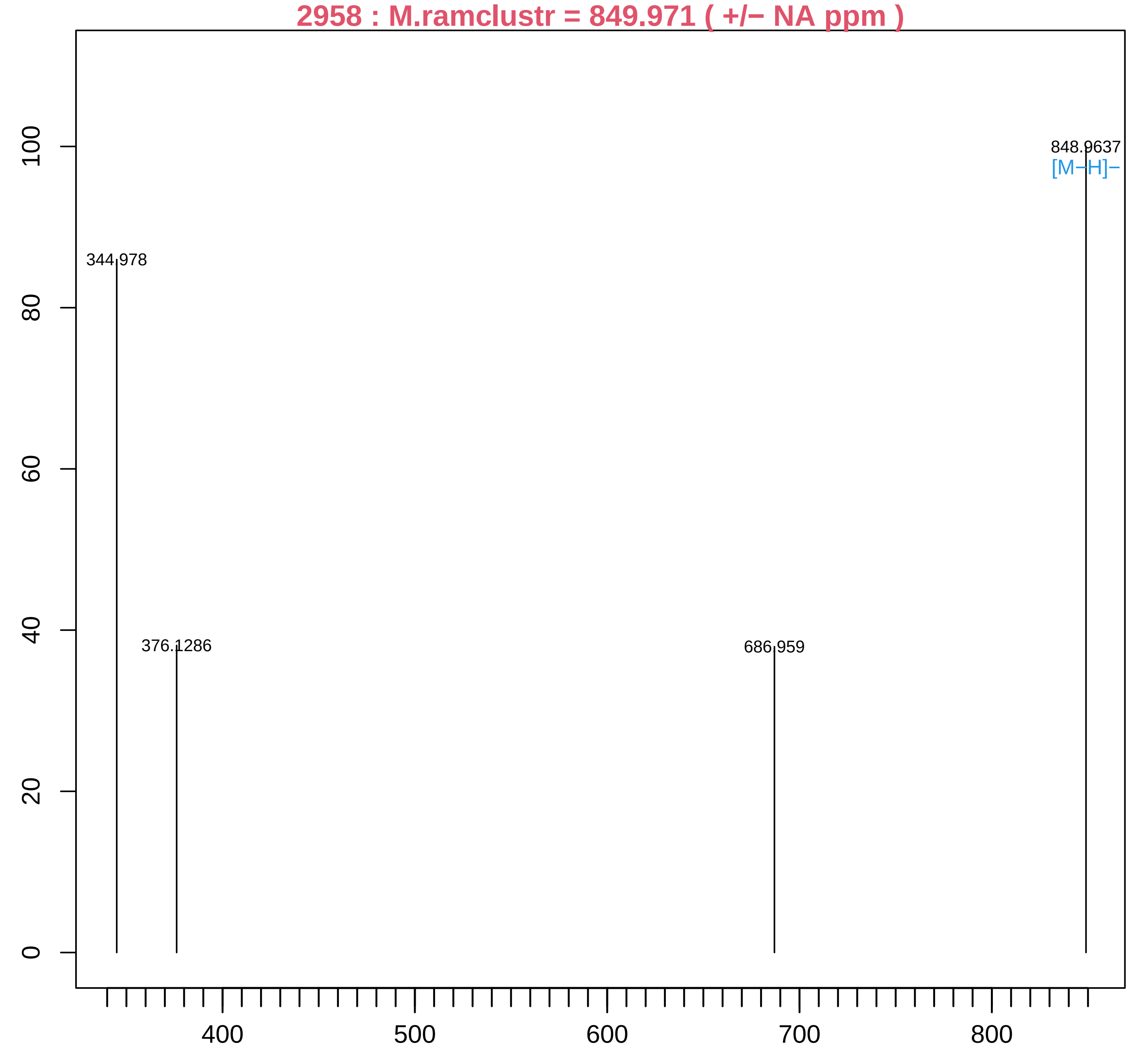
2958 : M.ramclustr = 849.971 (+/− NA ppm)
848.9637
[M−H]−
344.978
376.1286
686.959
0
20
40
60
80
100
400
500
600
700
800

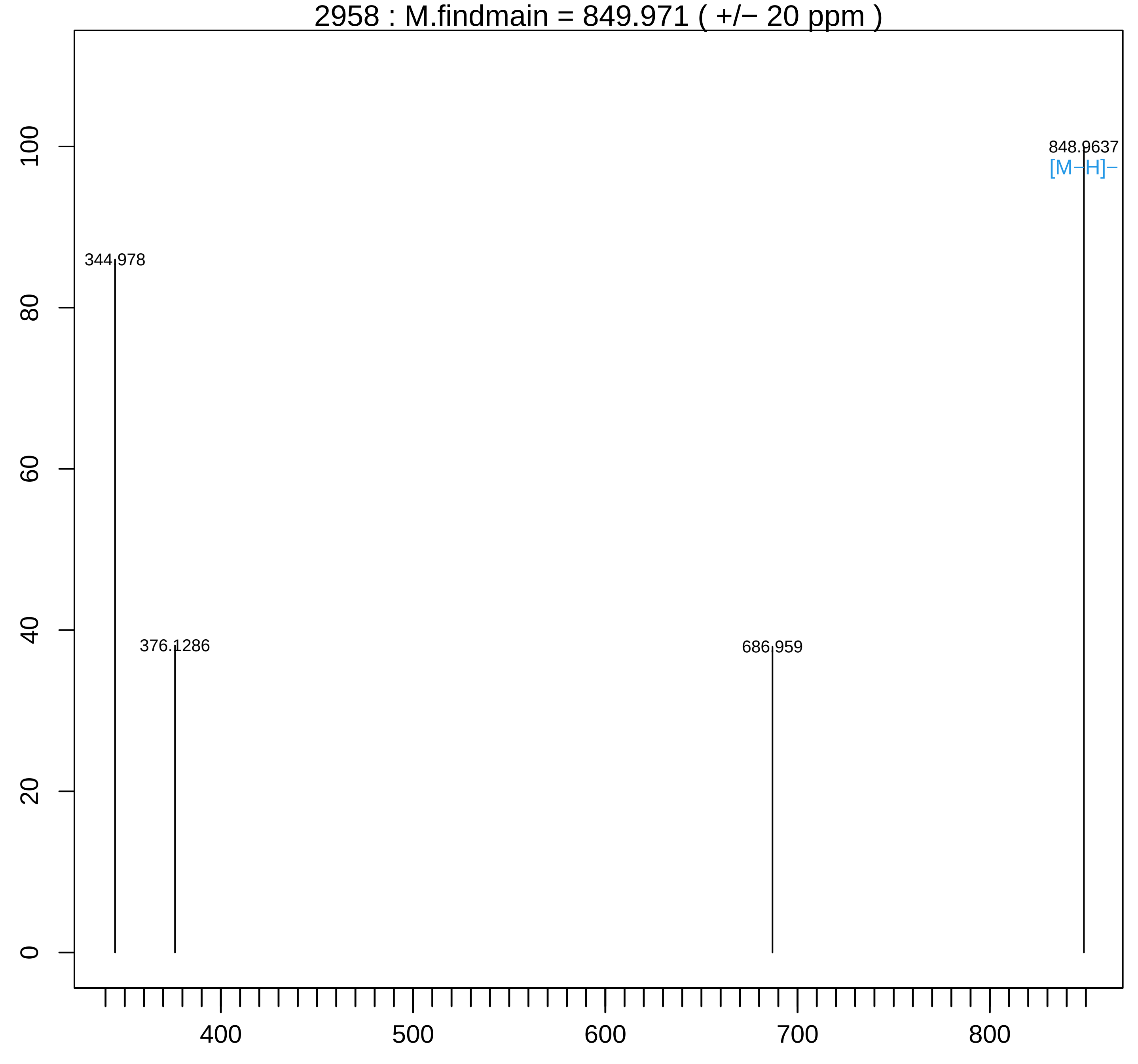
2958 : M.findmain = 849.971 (+/− 20 ppm)
848.9637
[M−H]−
344.978
376.1286
686.959
0
20
40
60
80
100
400
500
600
700
800

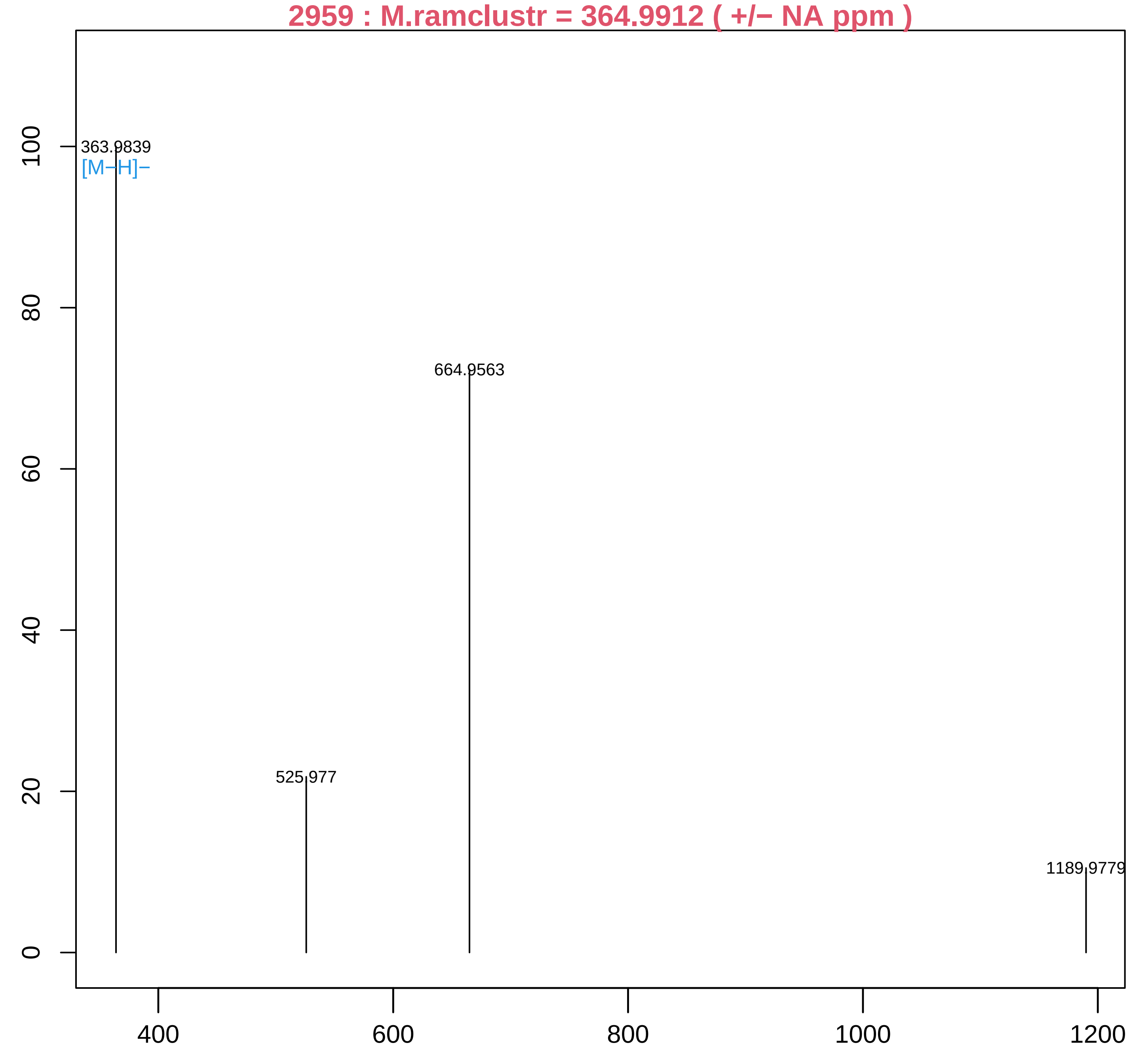
2959 : M.ramclustr = 364.9912 (+/− NA ppm)
363.9839
[M−H]−
664.9563
525.977
1189.9779
0
20
40
60
80
100
400
600
800
1000
1200
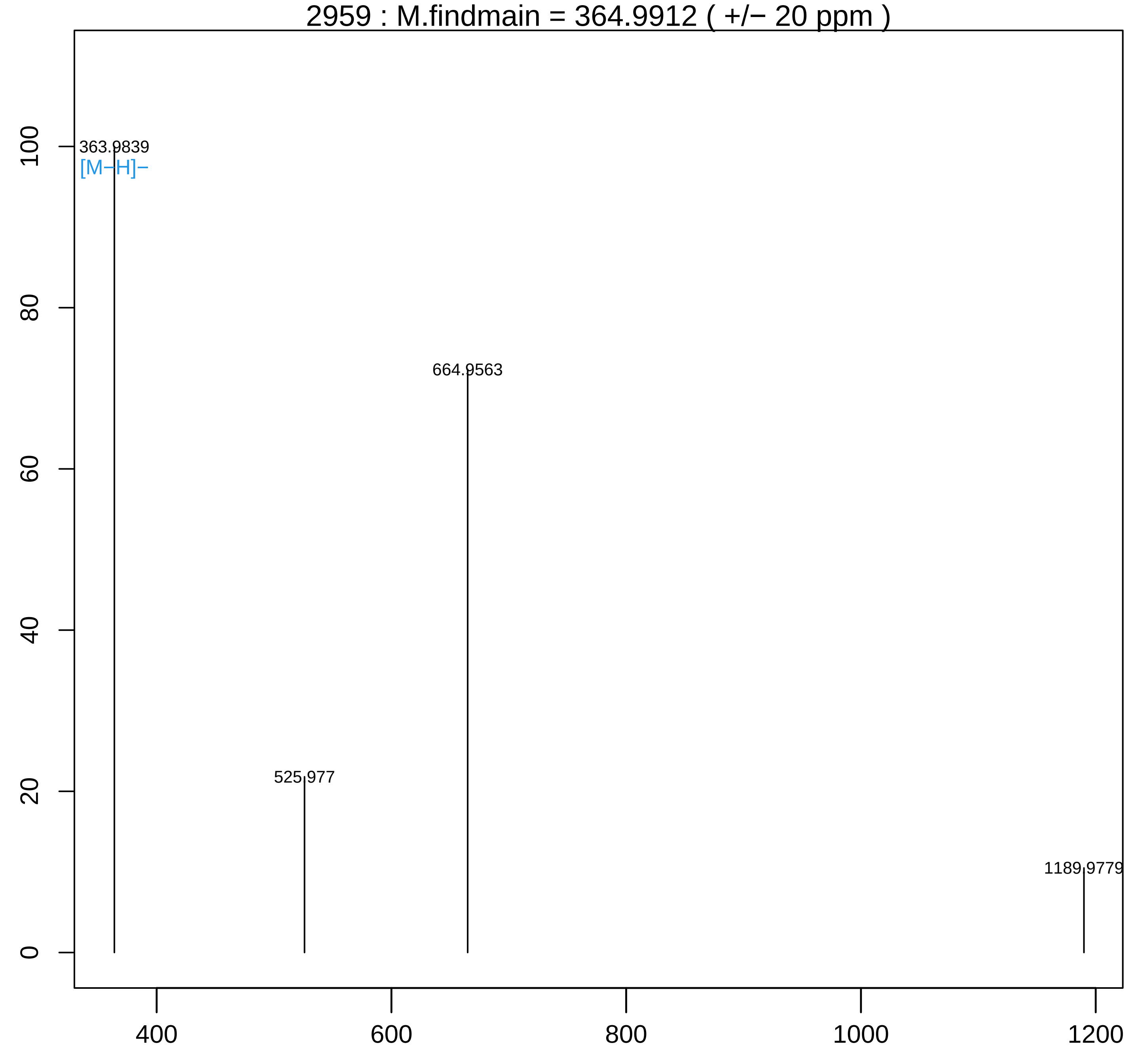
2959 : M.findmain = 364.9912 (+/− 20 ppm)
363.9839
[M−H]−
664.9563
525.977
1189.9779
0
20
40
60
80
100
400
600
800
1000
1200

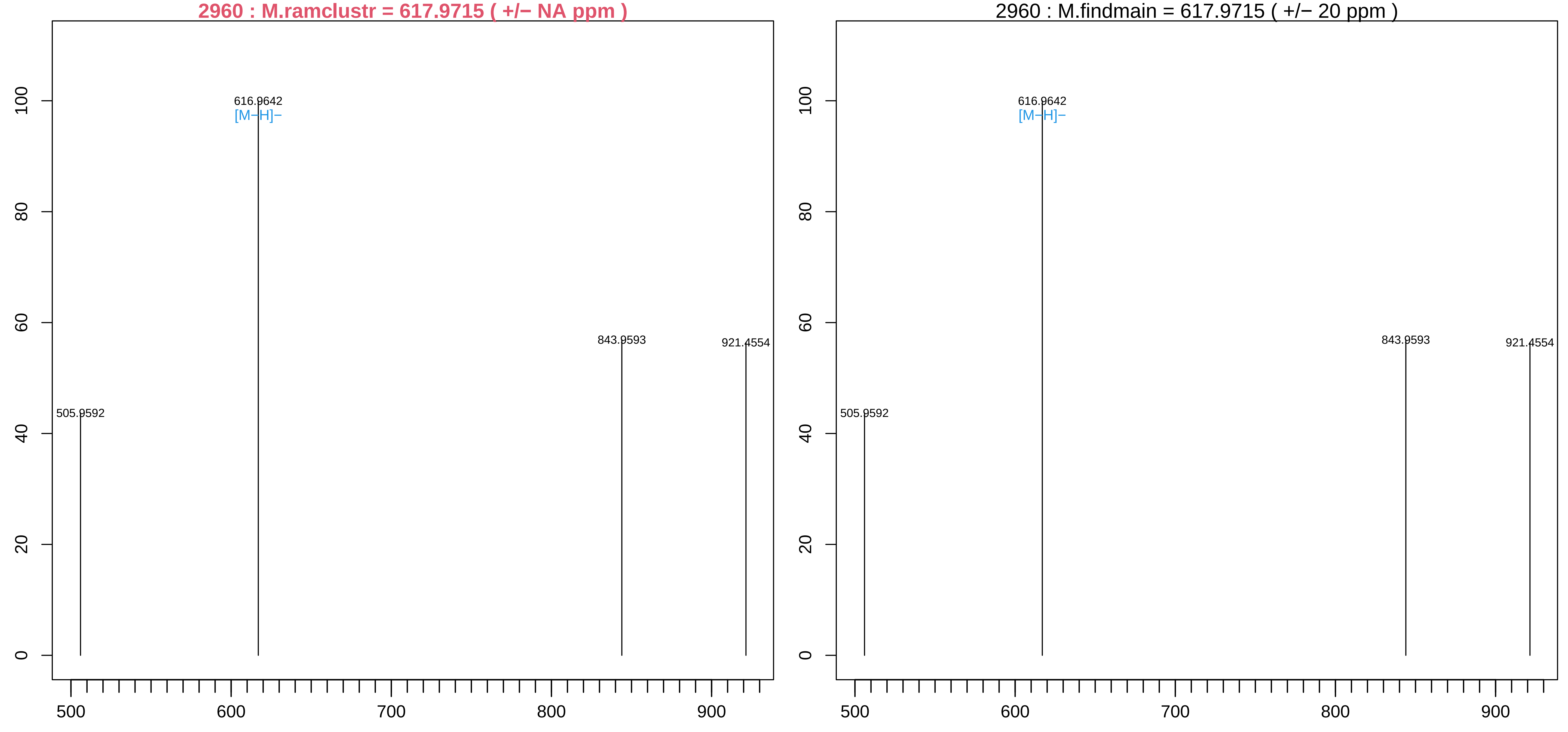
2960 : M.ramclustr = 617.9715 (+/− NA ppm)
505.9592
616.9642
[M−H]−
843.9593
921.4554
2960 : M.findmain = 617.9715 (+/− 20 ppm)
505.9592
616.9642
[M−H]−
843.9593
921.4554

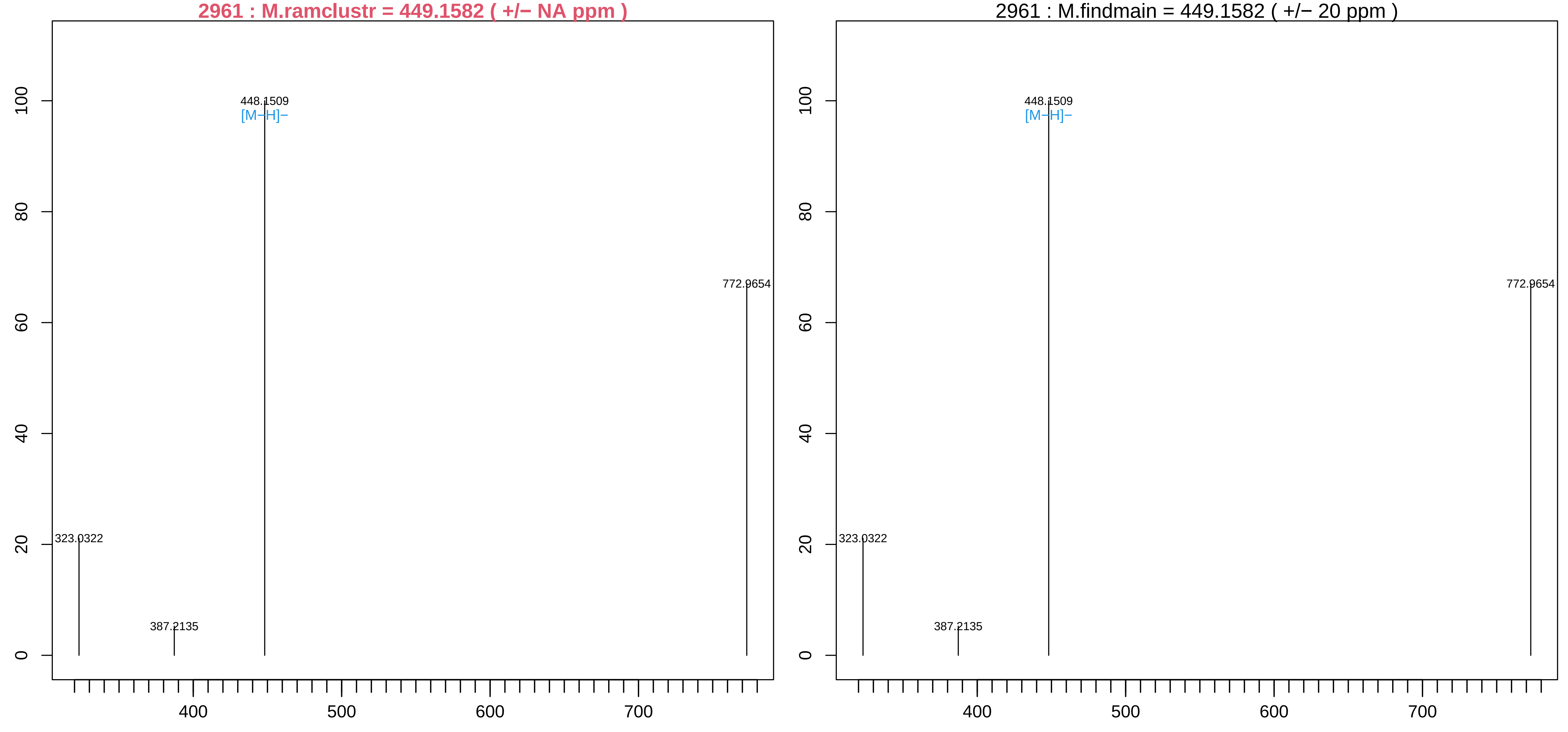

2961 : M.ramclustr = 449.1582 (+/− NA ppm)
448.1509
[M−H]−
772.9654
323.0322
387.2135
2961 : M.findmain = 449.1582 (+/− 20 ppm)
448.1509
[M−H]−
772.9654
323.0322
387.2135
0
20
40
60
80
100
400
500
600
700

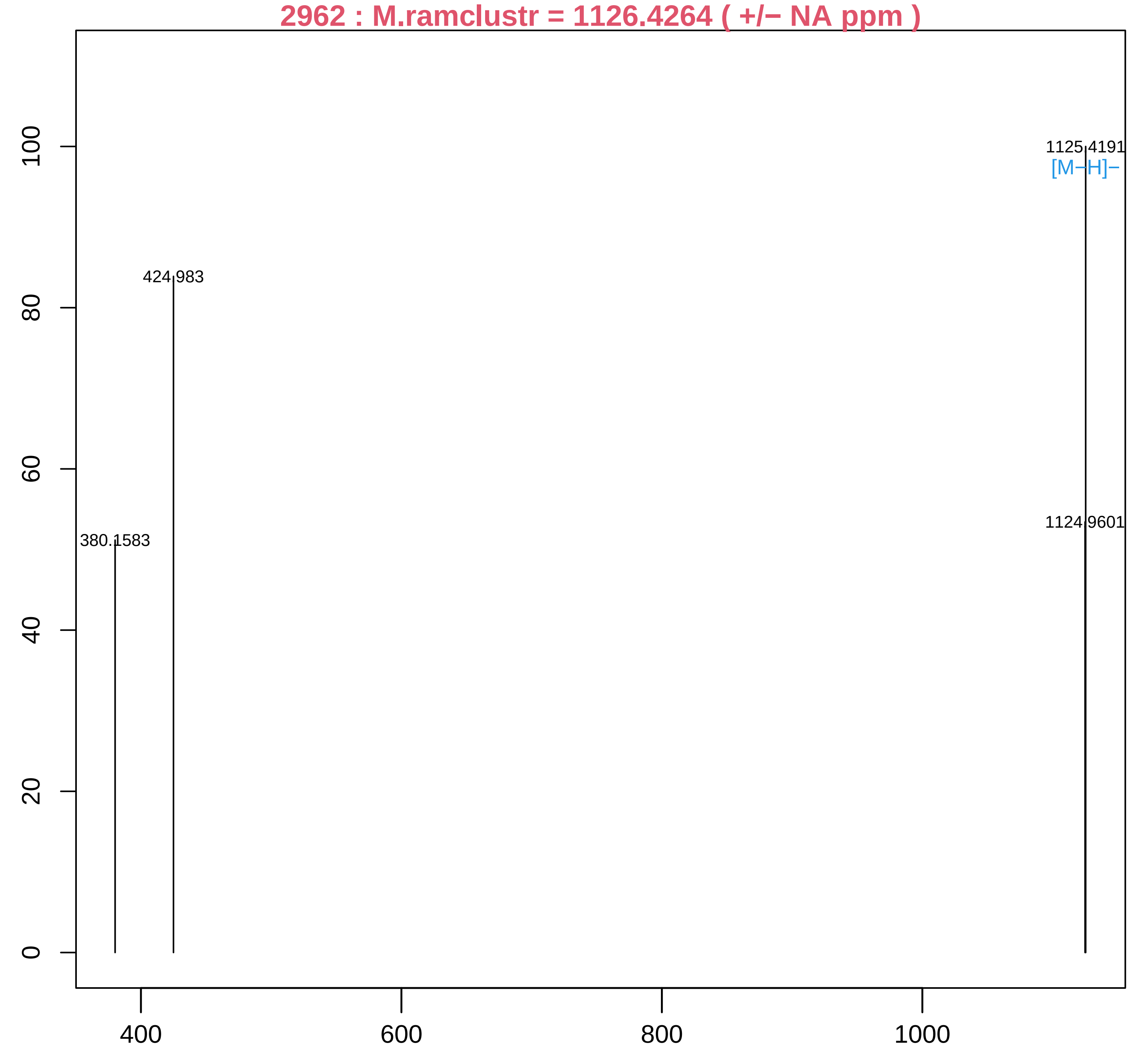

2962 : M.ramclustr = 1126.4264 (+/− NA ppm)
1125.4191
[M−H]−
424.983
380.1583
1124.9601
0
20
40
60
80
100
400
600
800
1000

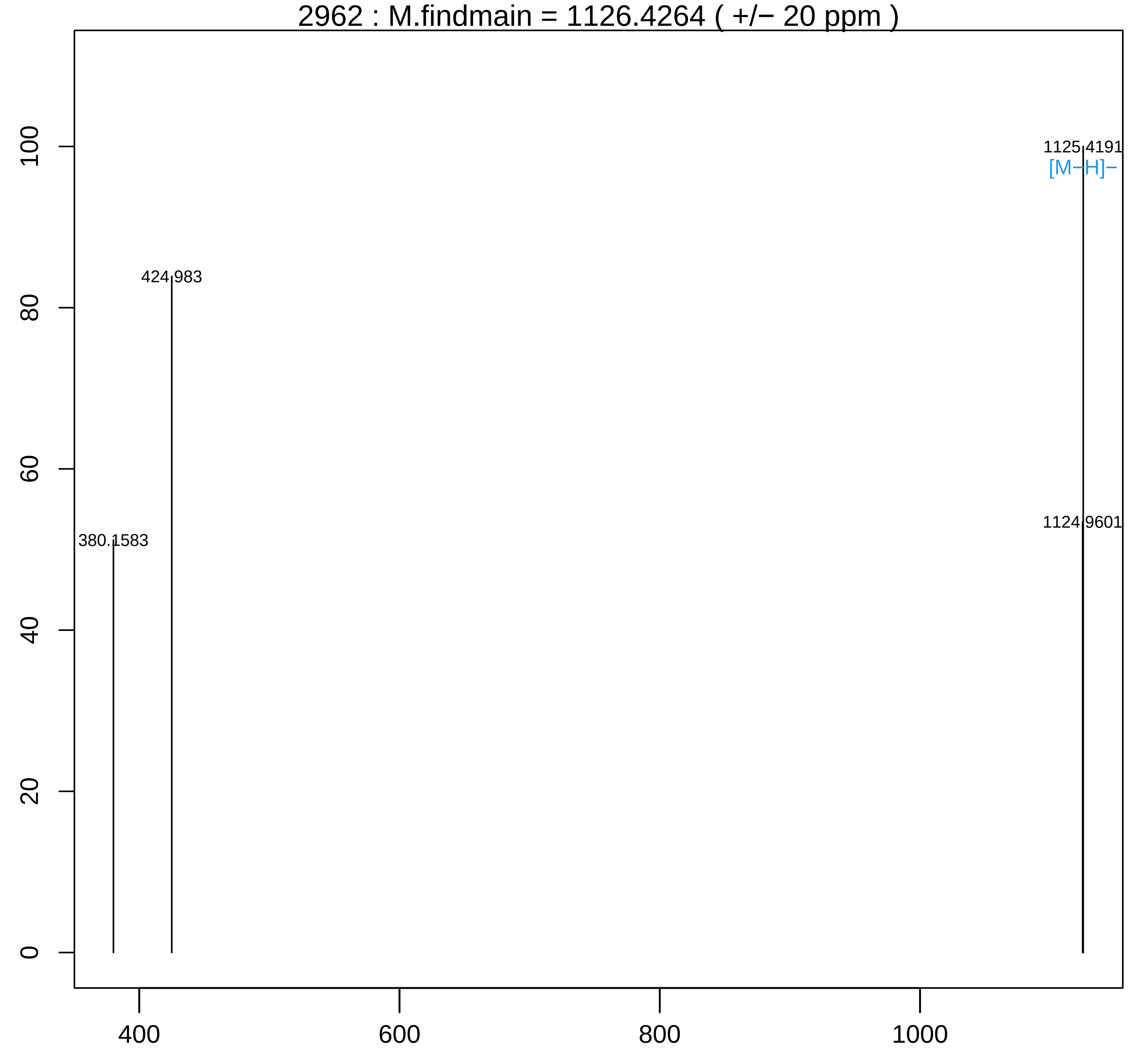

2962 : M.findmain = 1126.4264 (+/− 20 ppm)
1125.4191
[M−H]−
424.983
380.1583
1124.9601
0
20
40
60
80
100
400
600
800
1000

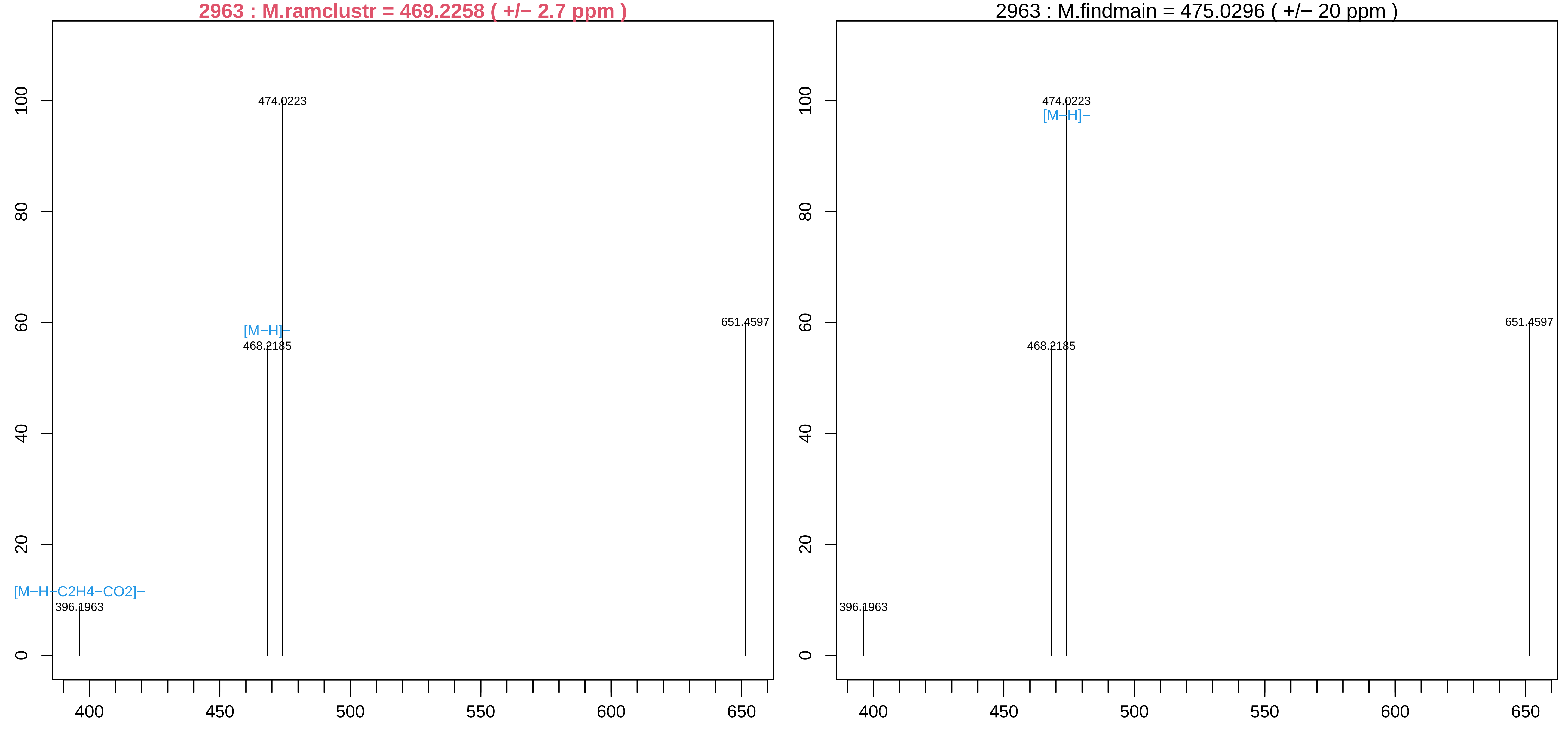
2963 : M.ramclustr = 469.2258 (+/− 2.7 ppm)
474.0223
[M−H]−
468.2185
651.4597
[M−H−C2H4−CO2]−
396.1963
2963 : M.findmain = 475.0296 (+/− 20 ppm)
474.0223
[M−H]−
468.2185
651.4597
396.1963
0
20
40
60
80
100
400
450
500
550
600
650

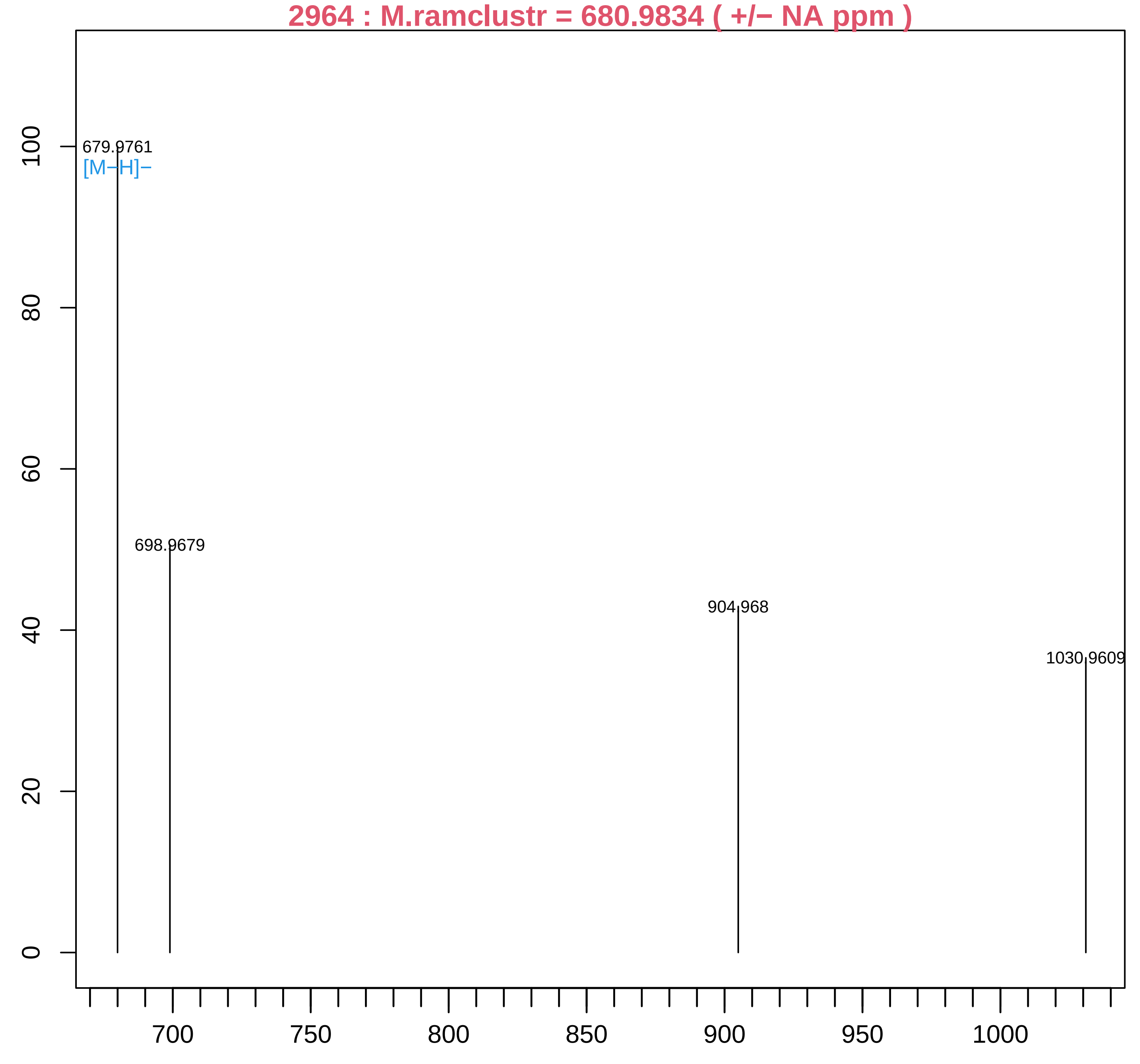
2964 : M.ramclustr = 680.9834 (+/− NA ppm)
679.9761
[M−H]−
698.9679
904.968
1030.9609
0
20
40
60
80
100
700
750
800
850
900
950
1000

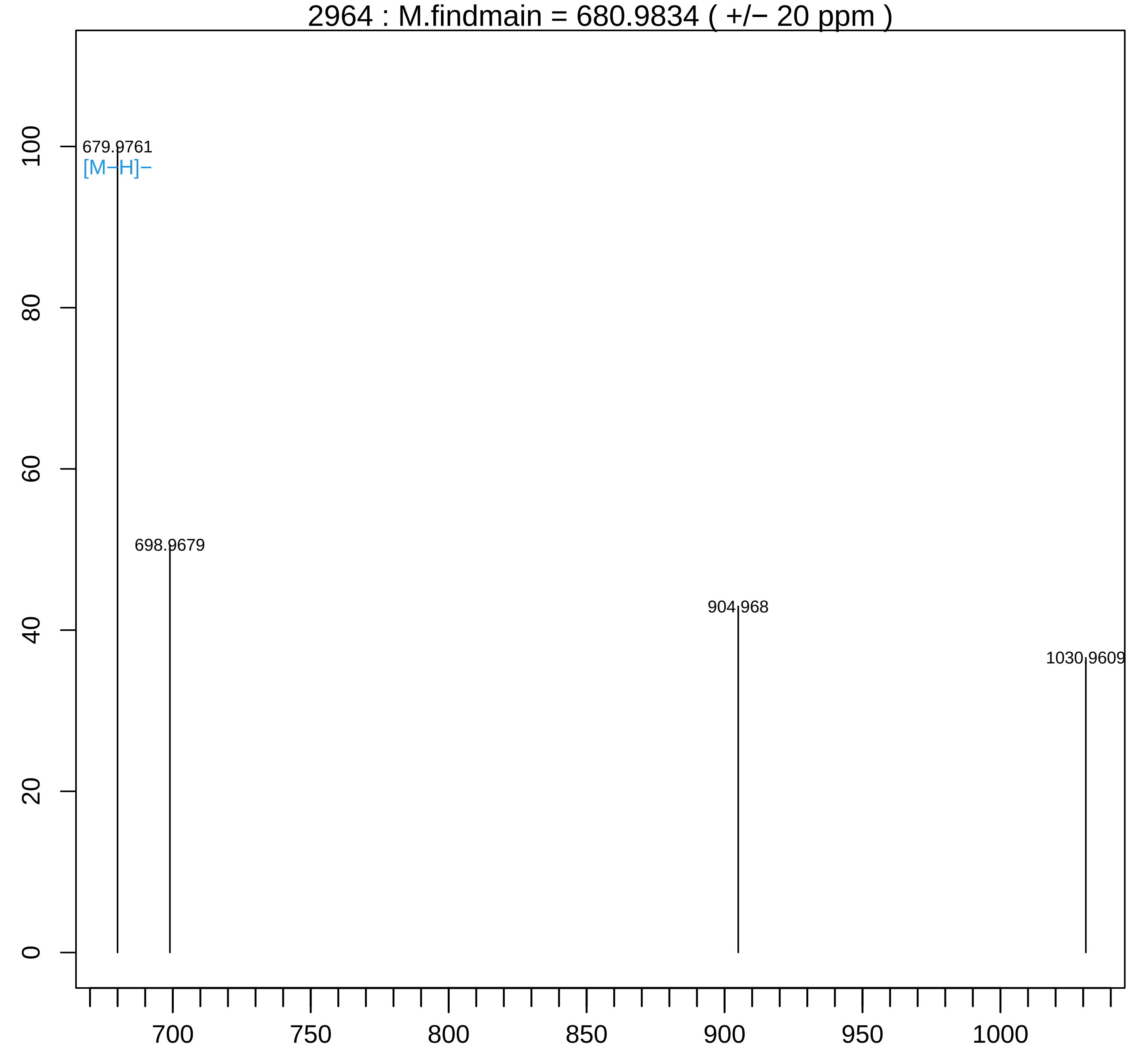
2964 : M.findmain = 680.9834 (+/− 20 ppm)
679.9761
[M−H]−
698.9679
904.968
1030.9609
0
20
40
60
80
100
700
750
800
850
900
950
1000

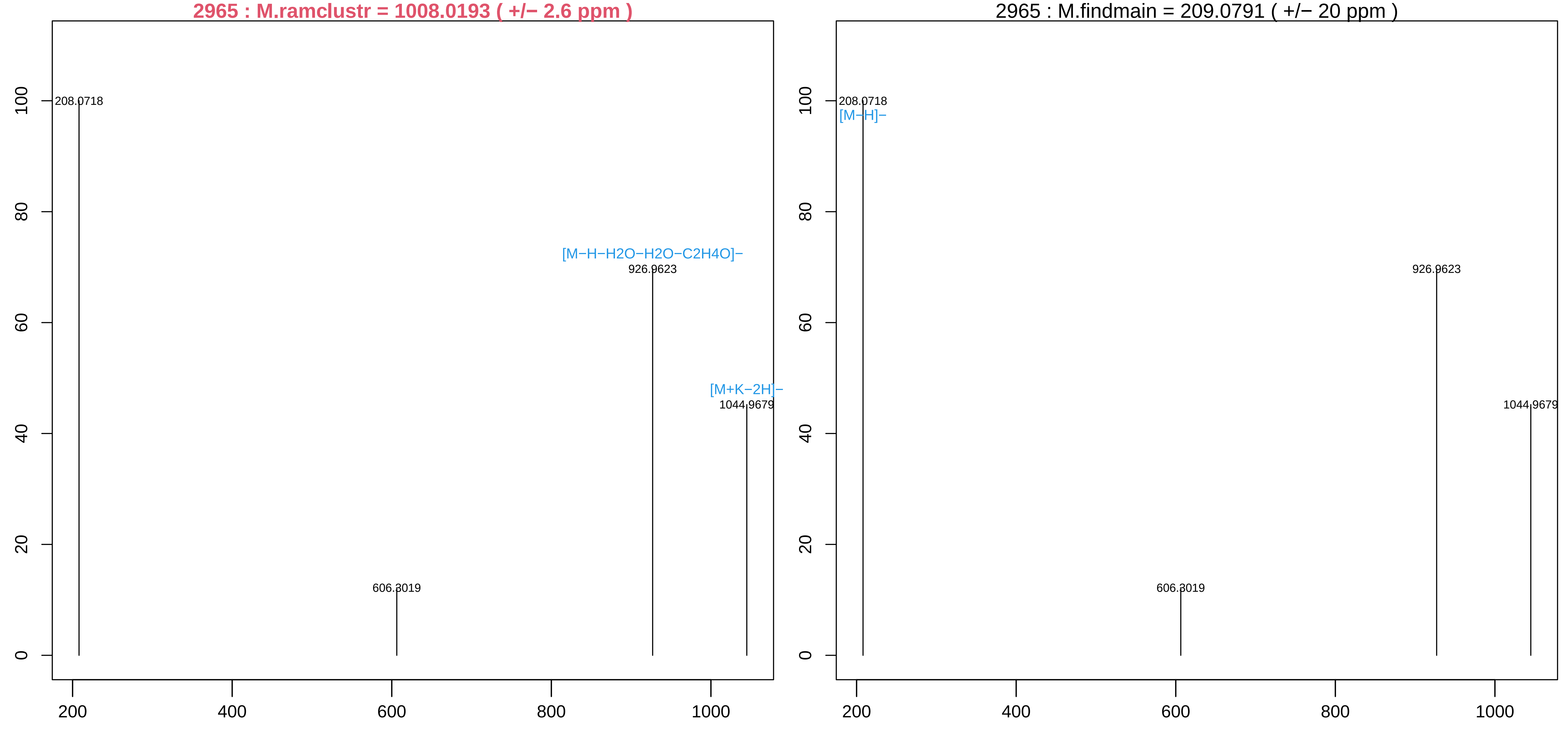

2965 : M.ramclustr = 1008.0193 (+/− 2.6 ppm)
208.0718
[M−H−H2O−H2O−C2H4O]−
926.9623
[M+K−2H]−
1044.9679
606.3019
2965 : M.findmain = 209.0791 (+/− 20 ppm)
208.0718
[M−H]−
926.9623
1044.9679
606.3019
0
20
40
60
80
100
200
400
600
800
1000

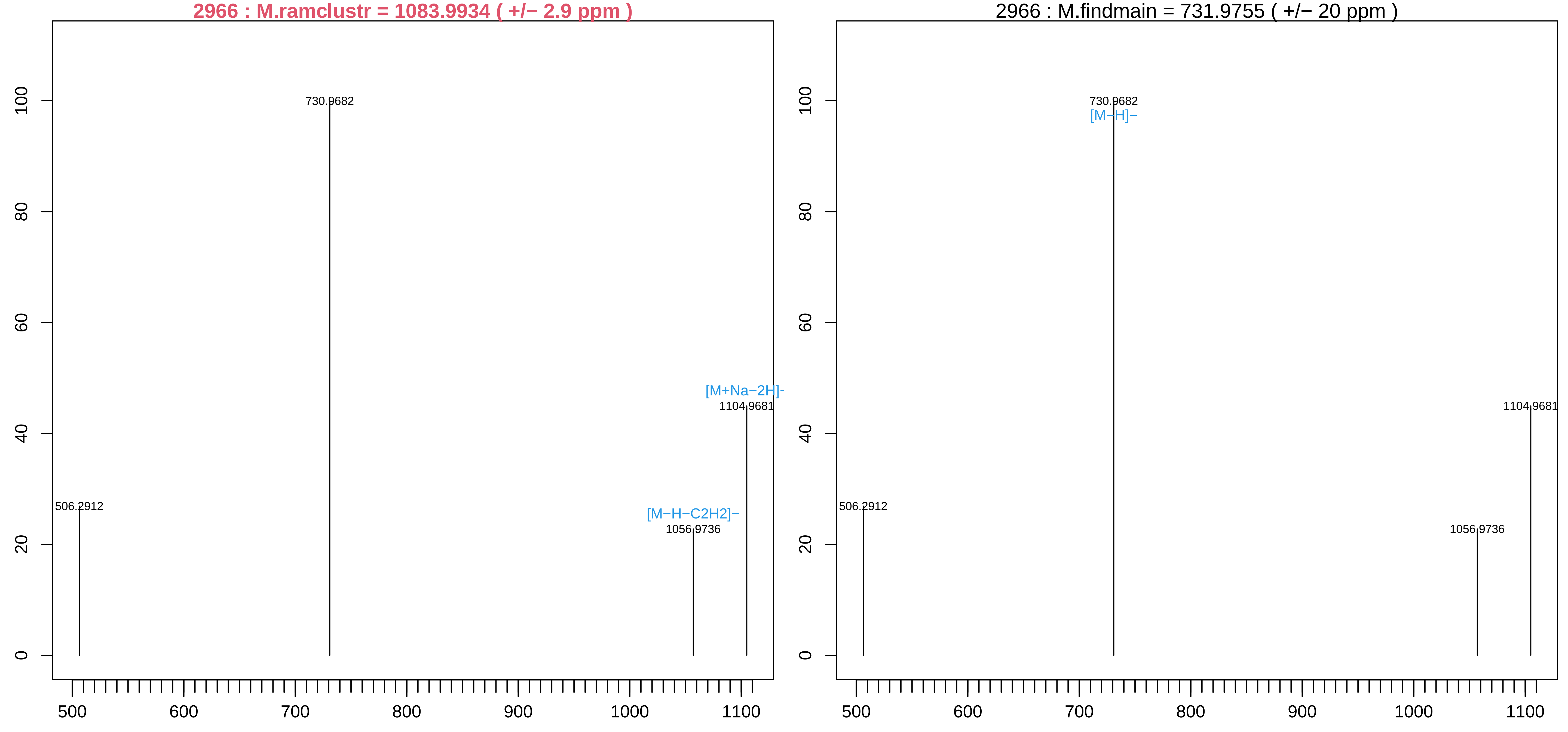
2966 : M.ramclustr = 1083.9934 (+/− 2.9 ppm)
730.9682
[M+Na−2H]-
1104.9681
[M−H−C2H2]−
1056.9736
506.2912
2966 : M.findmain = 731.9755 (+/− 20 ppm)
730.9682
[M−H]−
1104.9681
506.2912
1056.9736
0
20
40
60
80
100
500
600
700
800
900
1000
1100

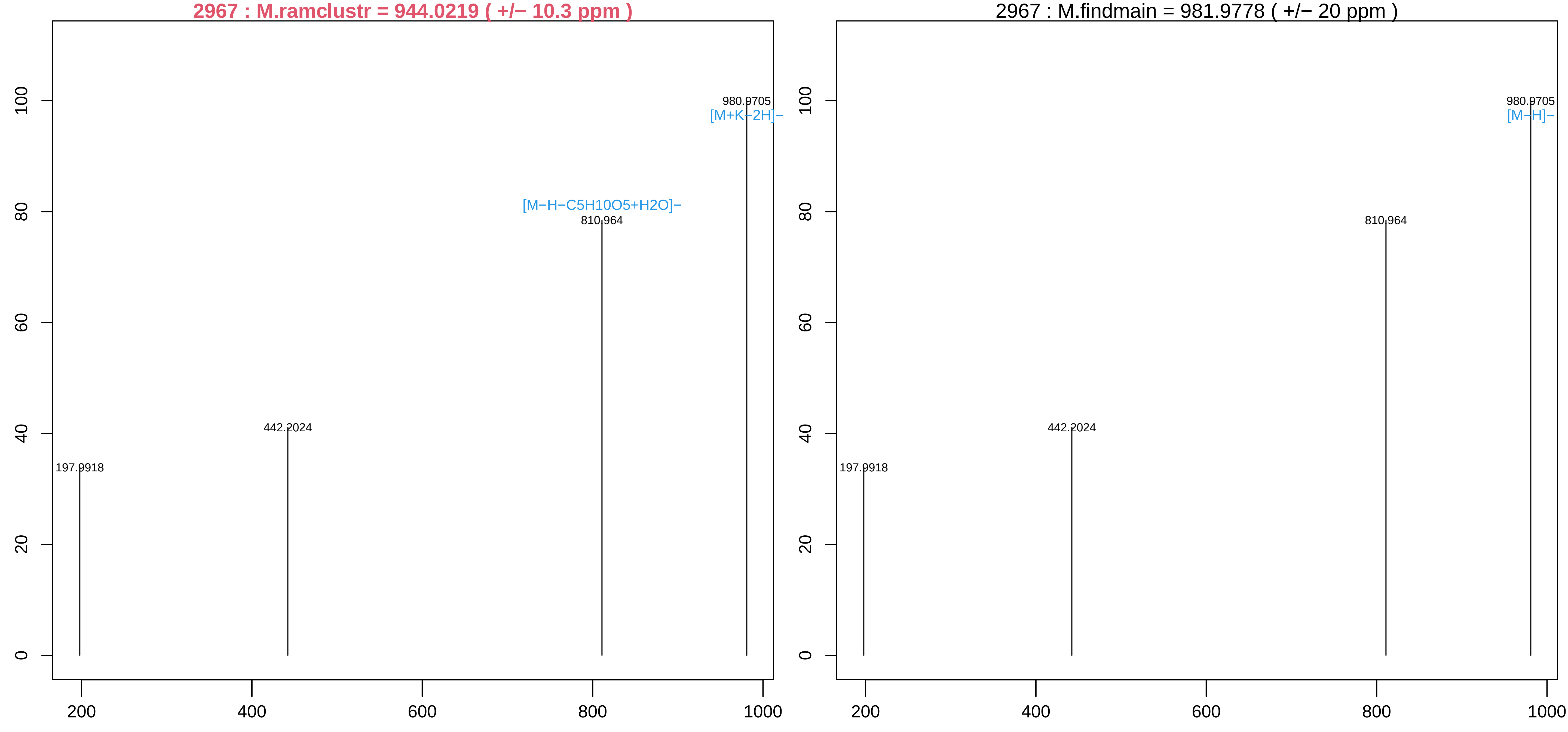
2967 : M.ramclustr = 944.0219 (+/− 10.3 ppm)
980.9705
[M+K−2H]−
[M−H−C5H10O5+H2O]−
810.964
442.2024
197.9918
2967 : M.findmain = 981.9778 (+/− 20 ppm)
980.9705
[M−H]−
810.964
442.2024
197.9918
0
20
40
60
80
100
200
400
600
800
1000

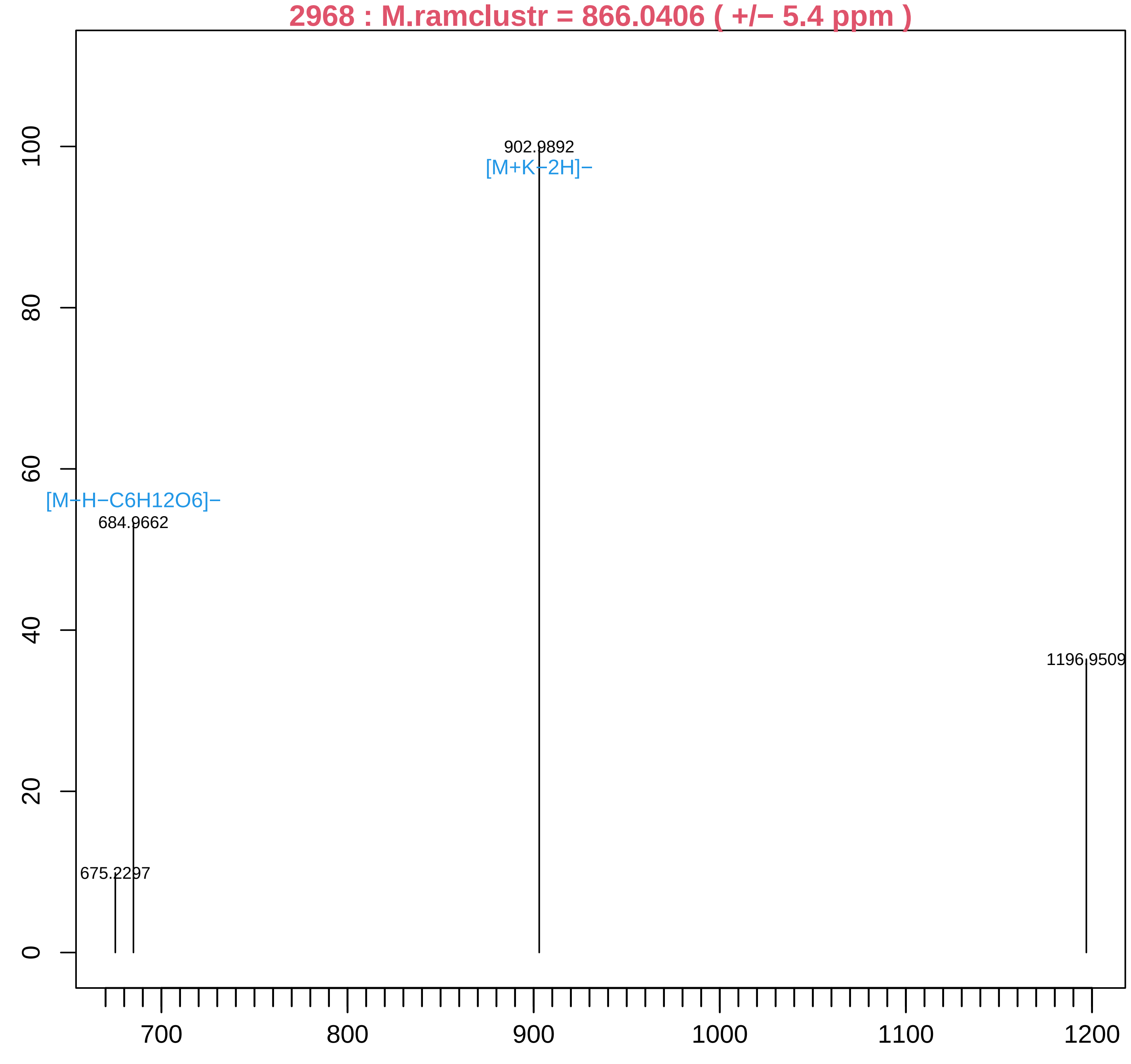
2968 : M.ramclustr = 866.0406 (+/− 5.4 ppm)
902.9892
[M+K−2H]−
[M−H−C6H12O6]−
684.9662
675.2297
1196.9509
0
20
40
60
80
100
700
800
900
1000
1100
1200

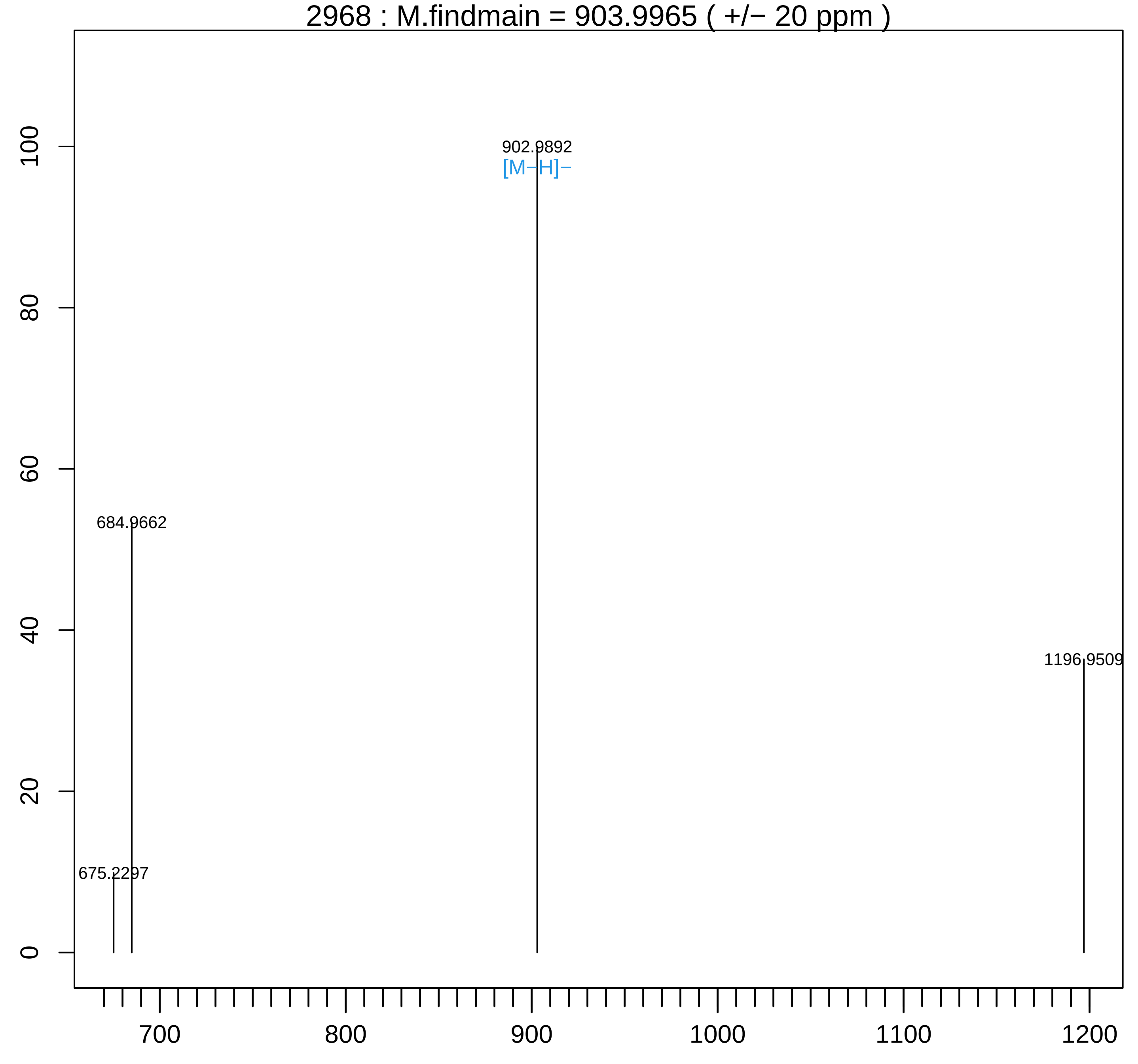
2968 : M.findmain = 903.9965 (+/− 20 ppm)
902.9892
[M−H]−
684.9662
675.2297
1196.9509
0
20
40
60
80
100
700
800
900
1000
1100
1200

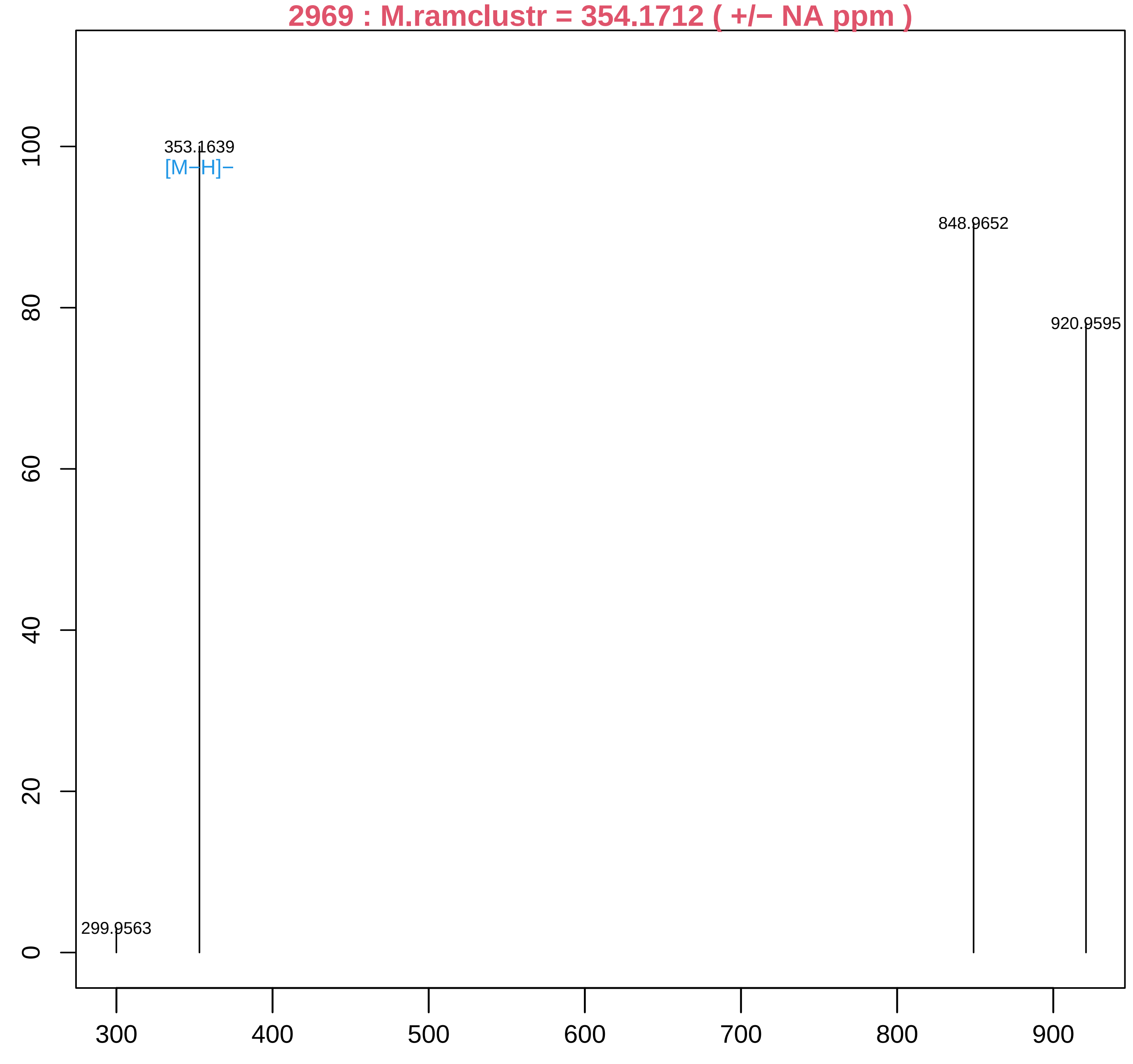
2969 : M.ramclustr = 354.1712 (+/− NA ppm)
353.1639
[M−H]−
848.9652
920.9595
299.9563
0
20
40
60
80
100
300
400
500
600
700
800
900

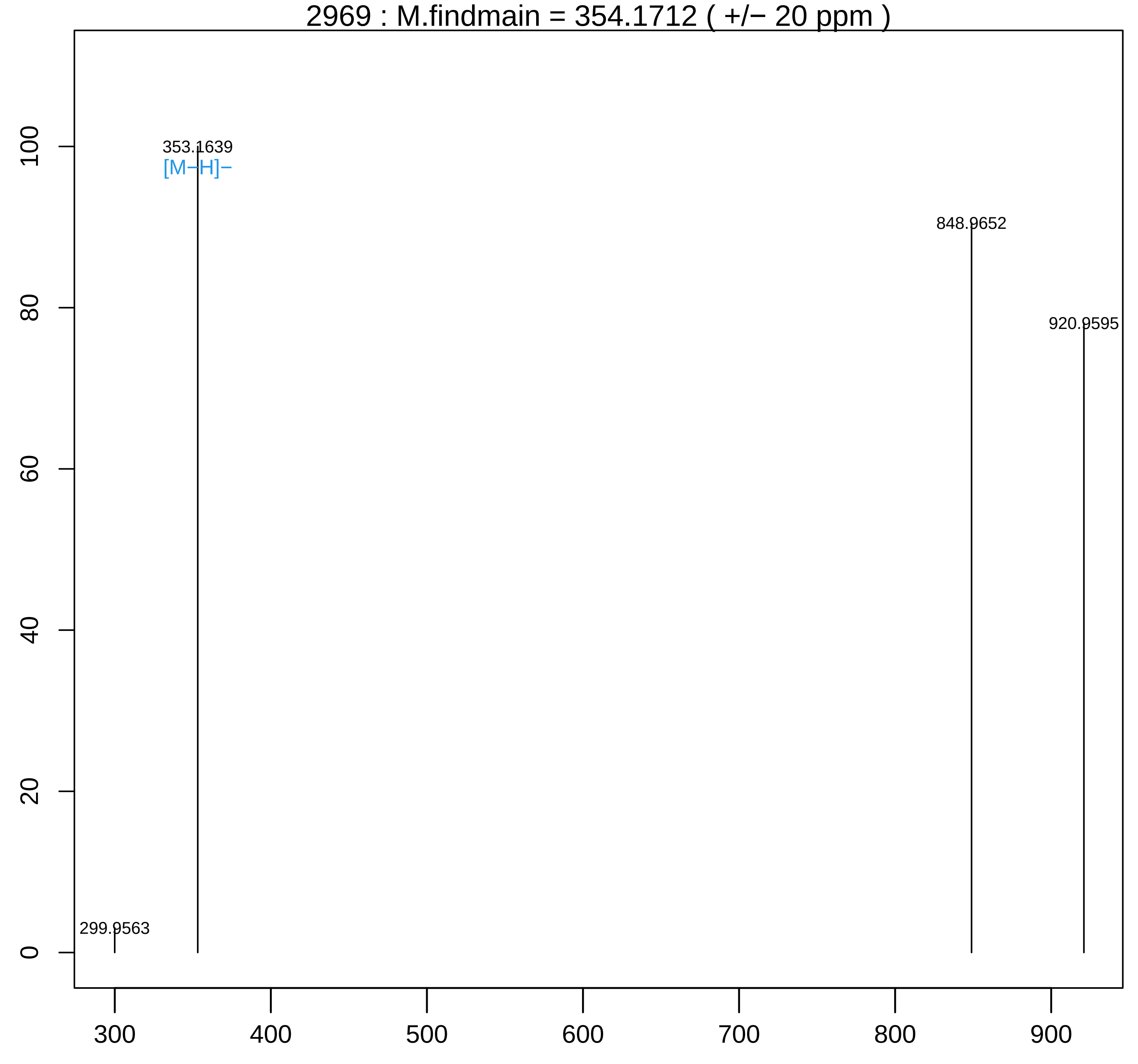
2969 : M.findmain = 354.1712 (+/− 20 ppm)
353.1639
[M−H]−
848.9652
920.9595
299.9563
0
20
40
60
80
100
300
400
500
600
700
800
900

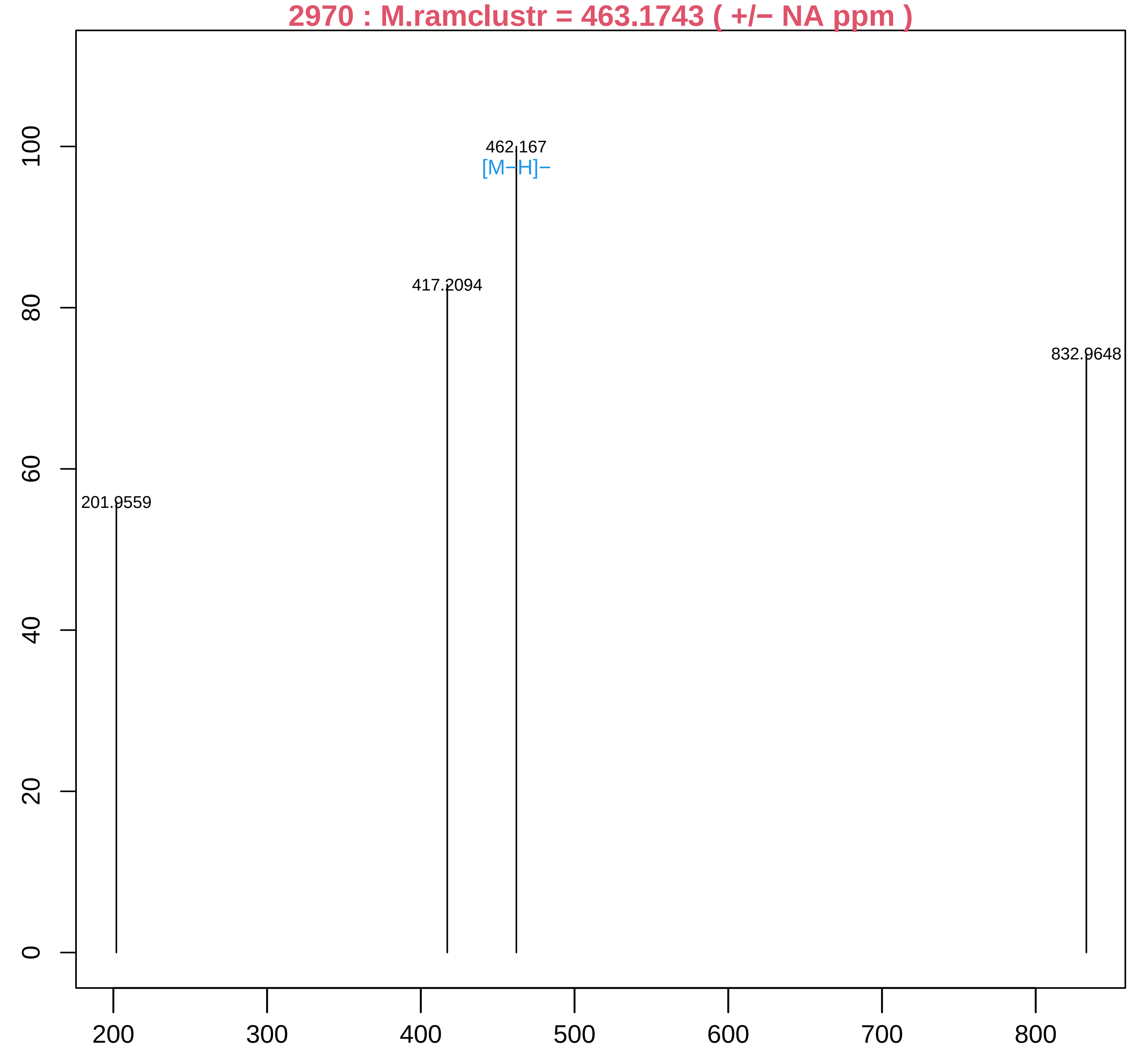
2970 : M.ramclustr = 463.1743 (+/− NA ppm)
462.167
[M−H]−
417.2094
832.9648
201.9559
0
20
40
60
80
100
200
300
400
500
600
700
800

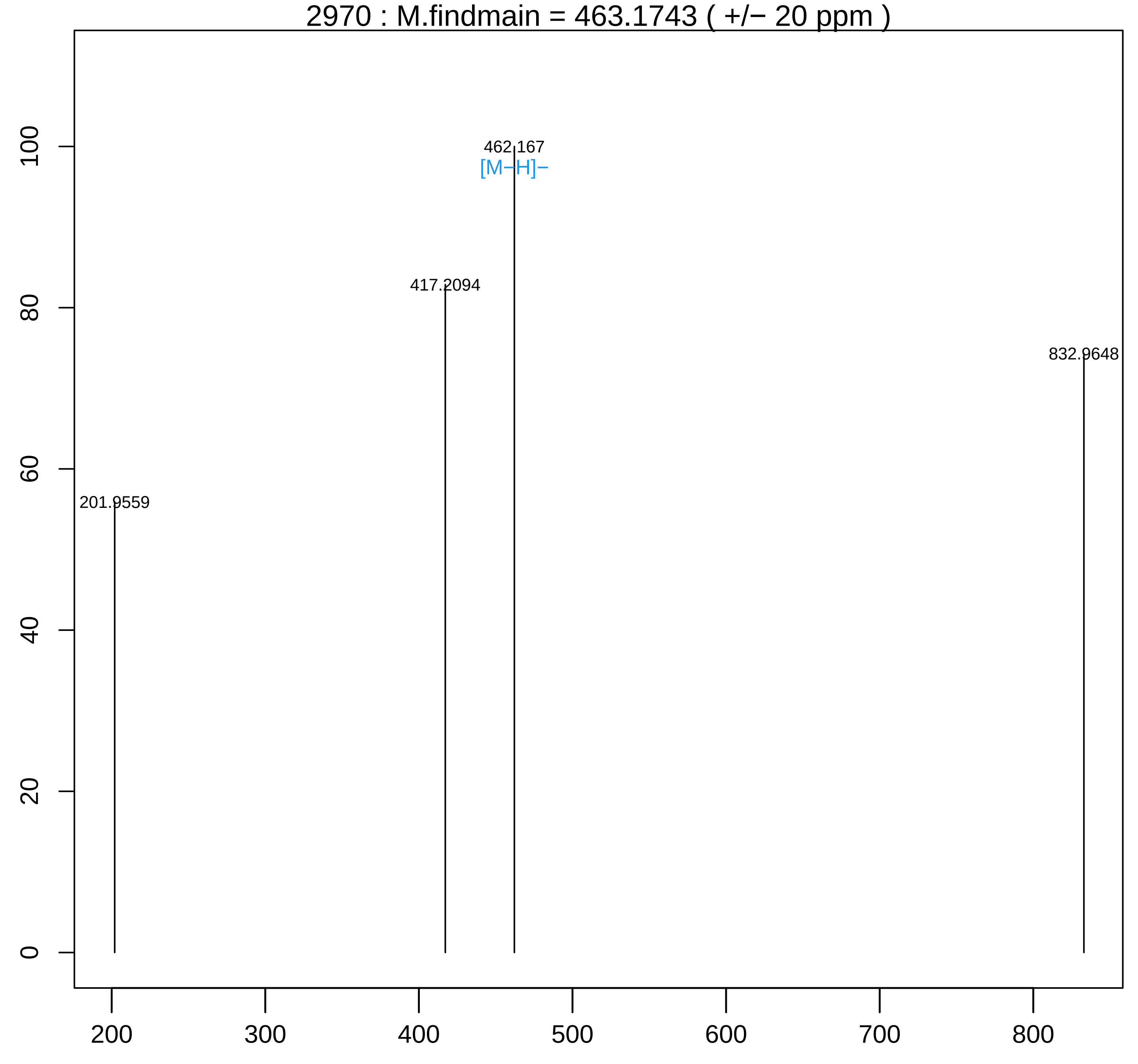
2970 : M.findmain = 463.1743 (+/− 20 ppm)
462.167
[M−H]−
417.2094
832.9648
201.9559
0
20
40
60
80
100
200
300
400
500
600
700
800

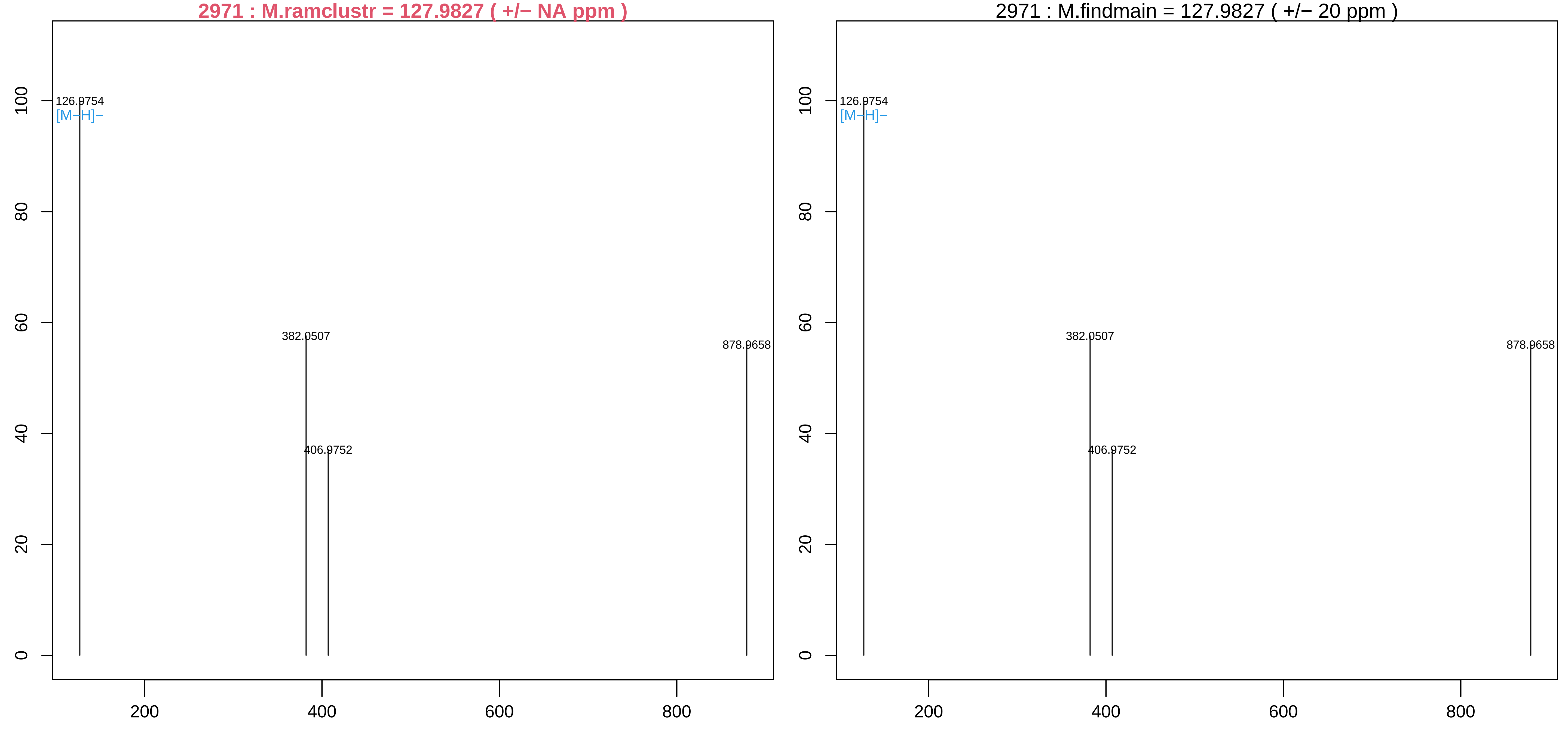
2971 : M.ramclustr = 127.9827 (+/− NA ppm)
2971 : M.findmain = 127.9827 (+/− 20 ppm)
126.9754
[M−H]−
382.0507
406.9752
878.9658
0
20
40
60
80
100
200
400
600
800

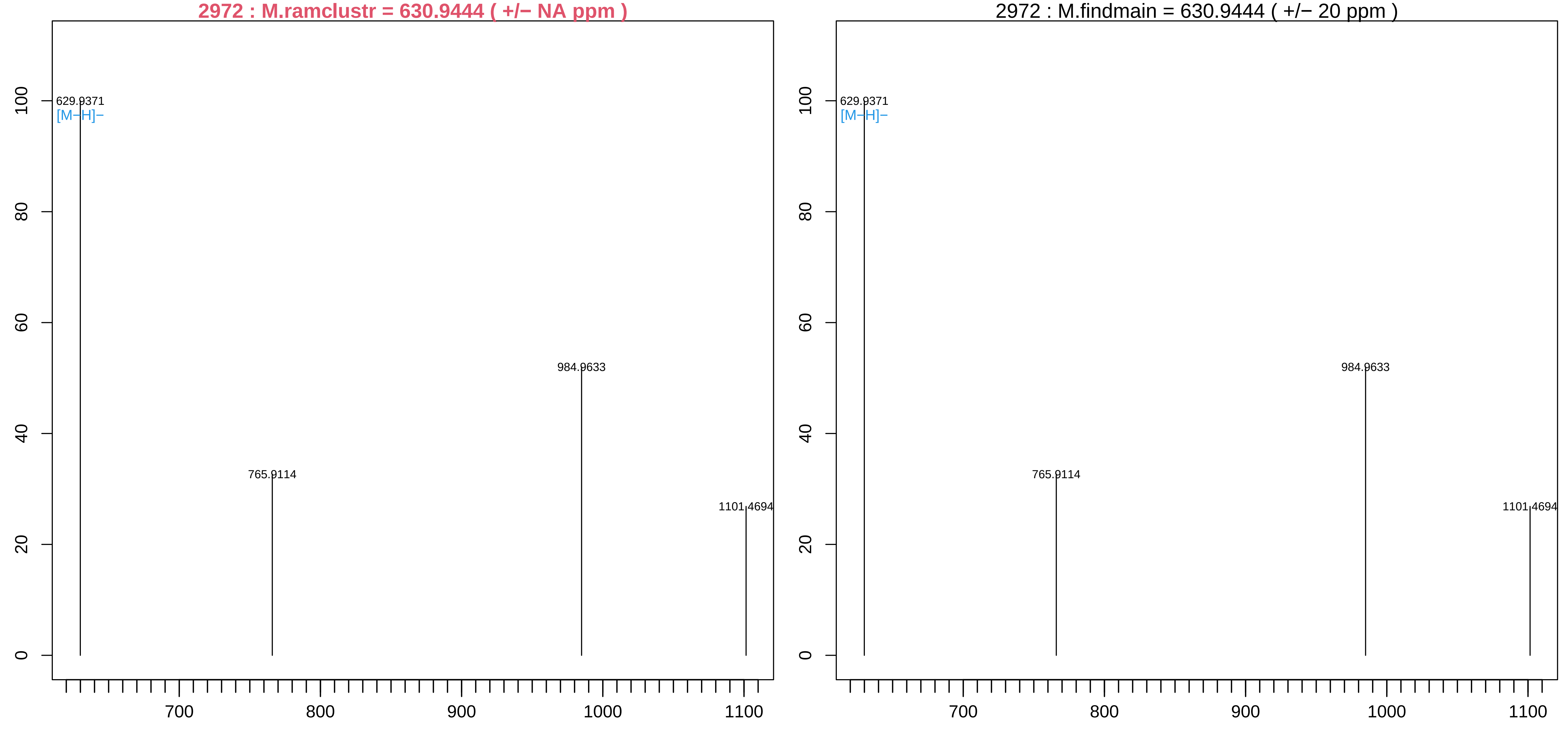

2972 : M.ramclustr = 630.9444 (+/− NA ppm)
629.9371
[M−H]−
765.9114
984.9633
1101.4694
0
20
40
60
80
100
700
800
900
1000
1100
2972 : M.findmain = 630.9444 (+/− 20 ppm)
629.9371
[M−H]−
765.9114
984.9633
1101.4694
0
20
40
60
80
100
700
800
900
1000
1100

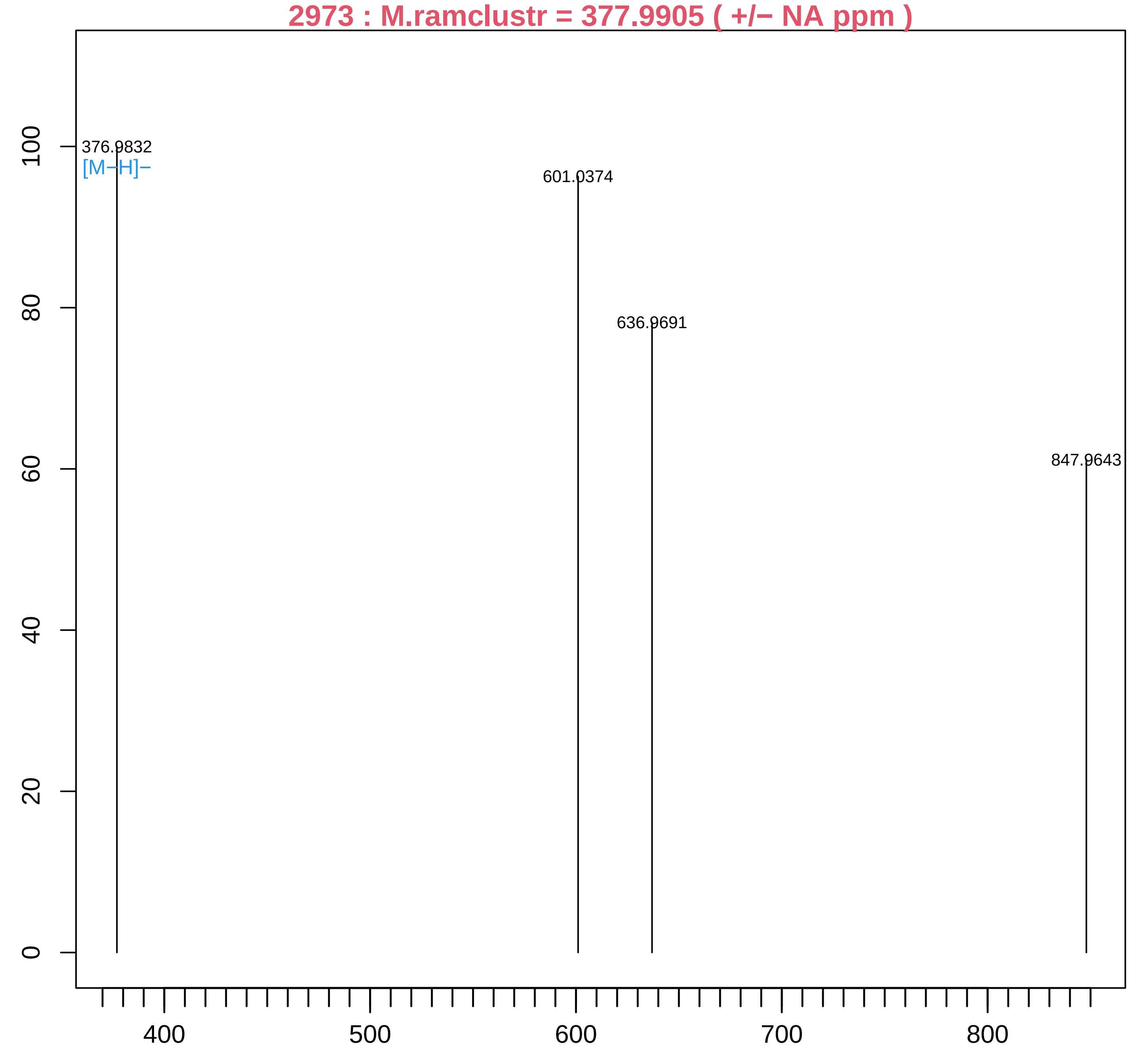
2973 : M.ramclustr = 377.9905 (+/− NA ppm)
376.9832
[M−H]−
601.0374
636.9691
847.9643
0
20
40
60
80
100
400
500
600
700
800

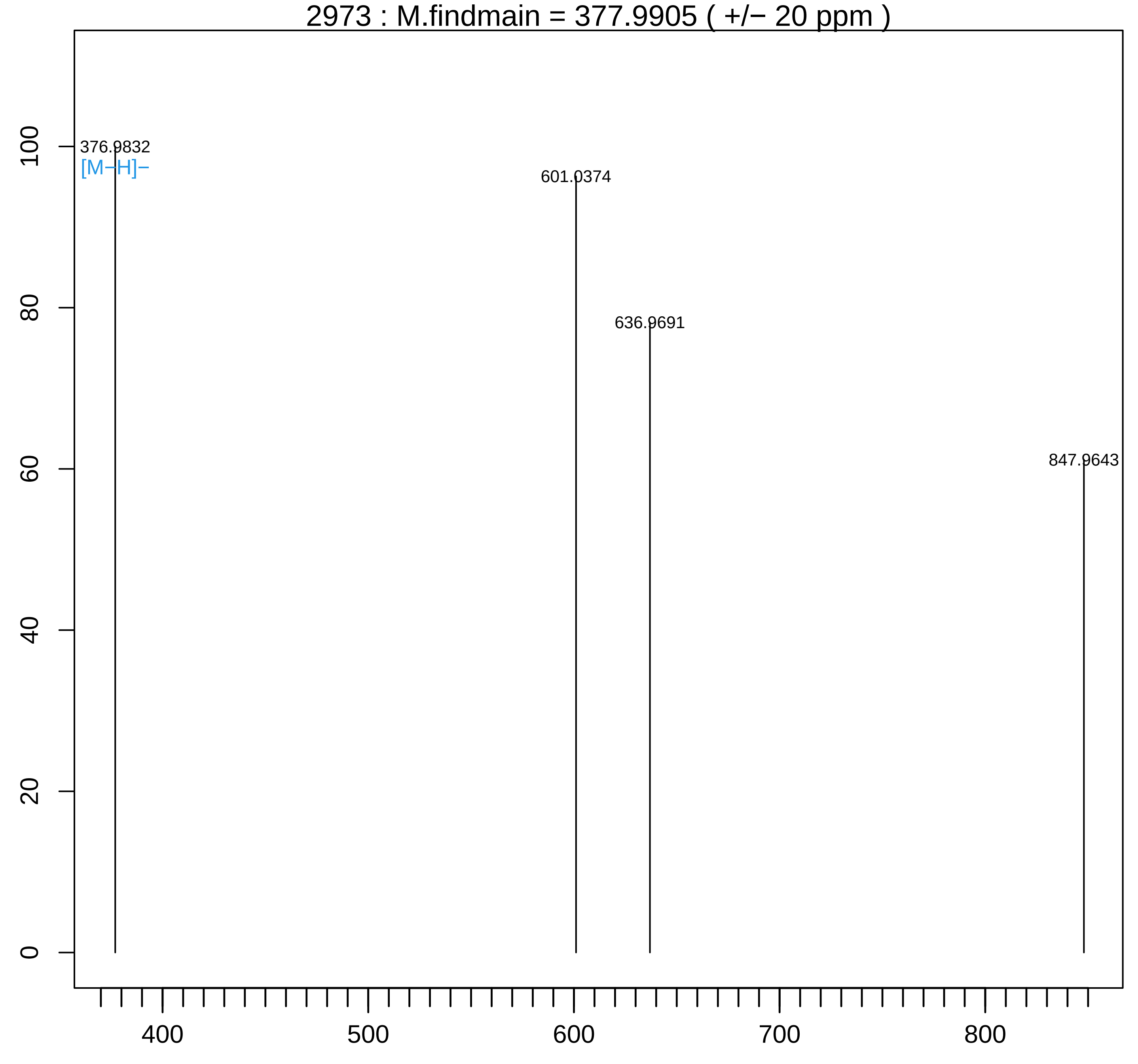
2973 : M.findmain = 377.9905 (+/− 20 ppm)
376.9832
[M−H]−
601.0374
636.9691
847.9643
0
20
40
60
80
100
400
500
600
700
800

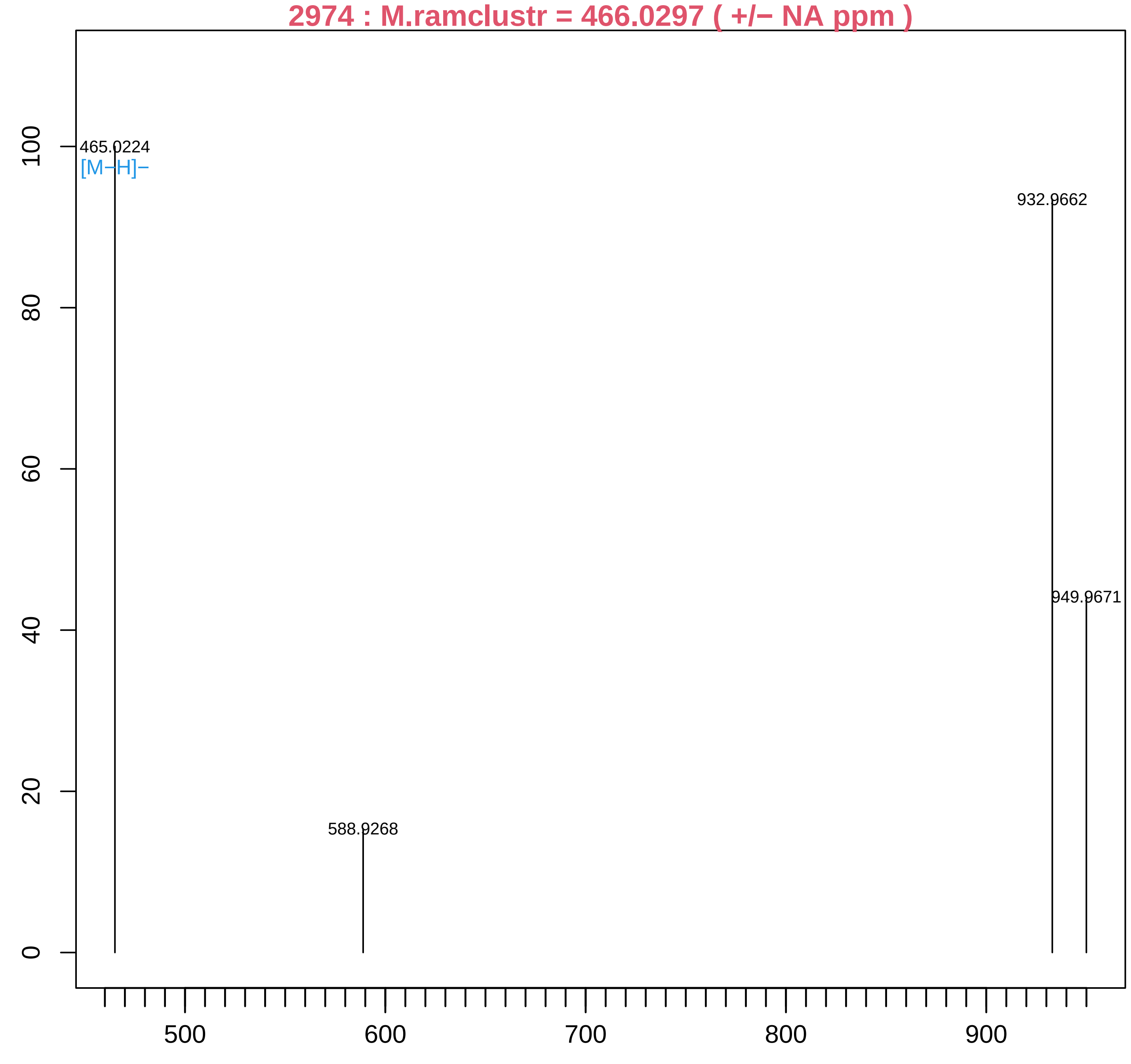

2974 : M.ramclustr = 466.0297 (+/− NA ppm)
465.0224
[M−H]−
932.9662
949.9671
588.9268
0
20
40
60
80
100
500
600
700
800
900

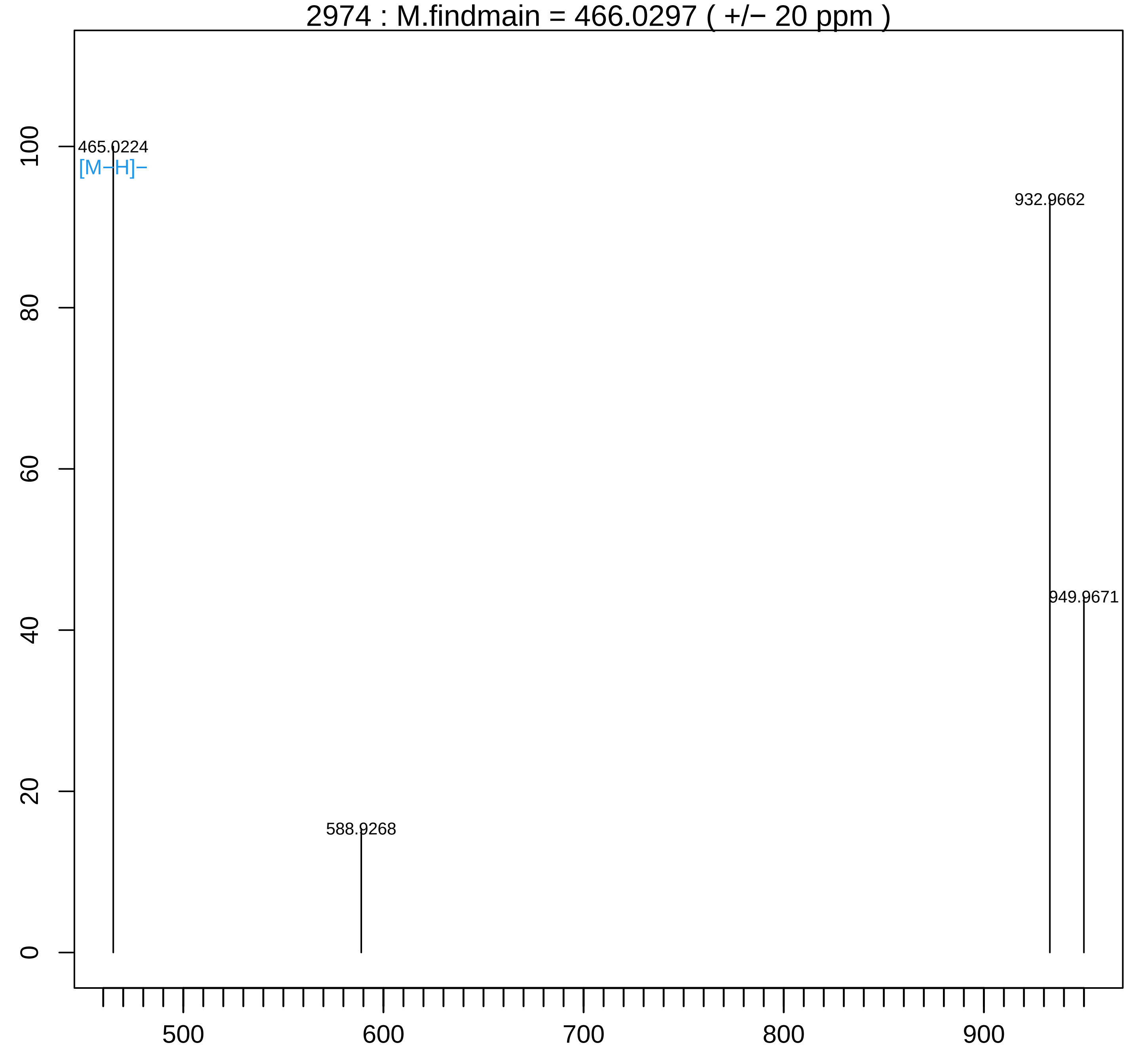

2974 : M.findmain = 466.0297 (+/− 20 ppm)
465.0224
[M−H]−
932.9662
949.9671
588.9268
0
20
40
60
80
100
500
600
700
800
900

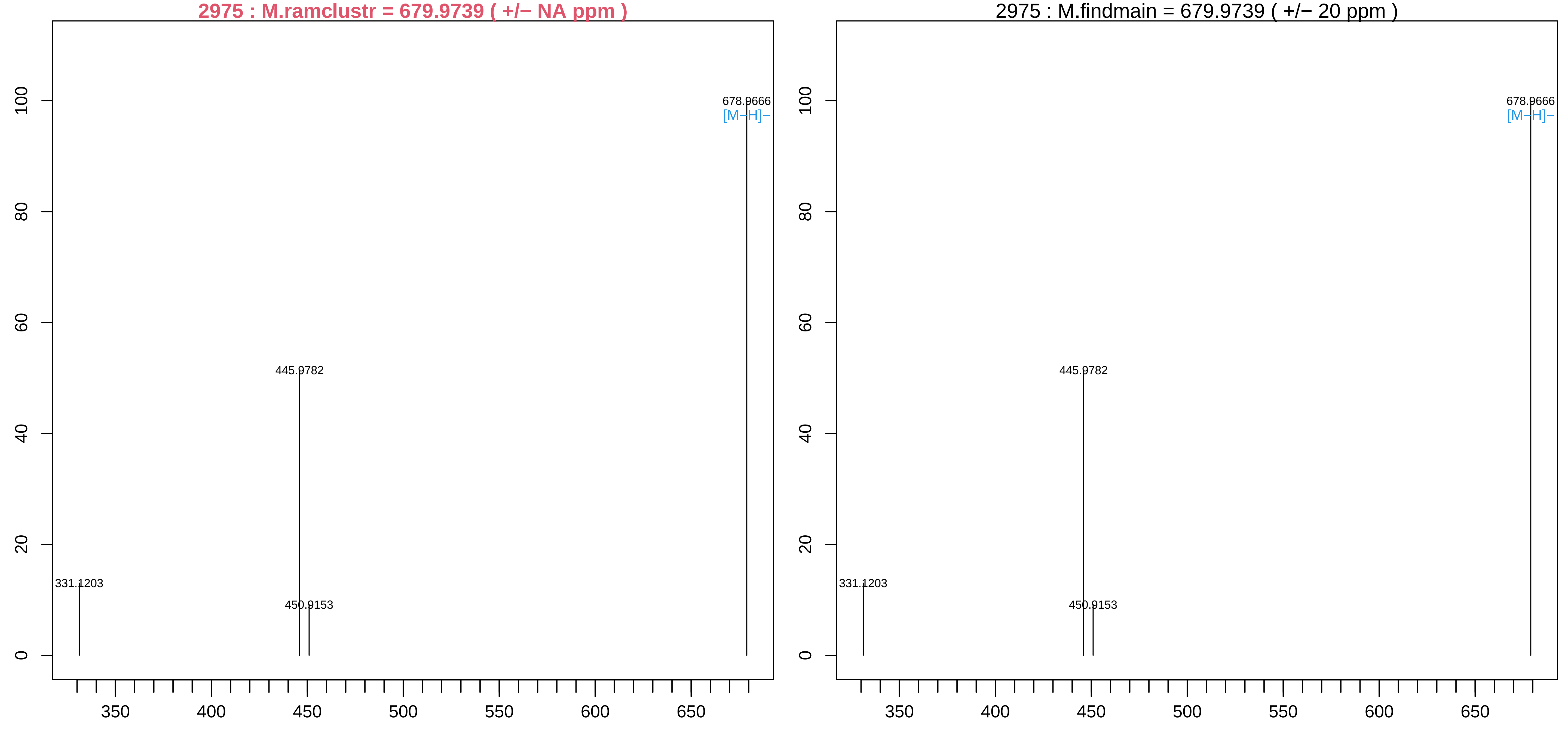
2975 : M.ramclustr = 679.9739 (+/− NA ppm)
678.9666
[M−H]−
445.9782
331.1203
450.9153
2975 : M.findmain = 679.9739 (+/− 20 ppm)
678.9666
[M−H]−
445.9782
331.1203
450.9153
0
20
40
60
80
100
350
400
450
500
550
600
650

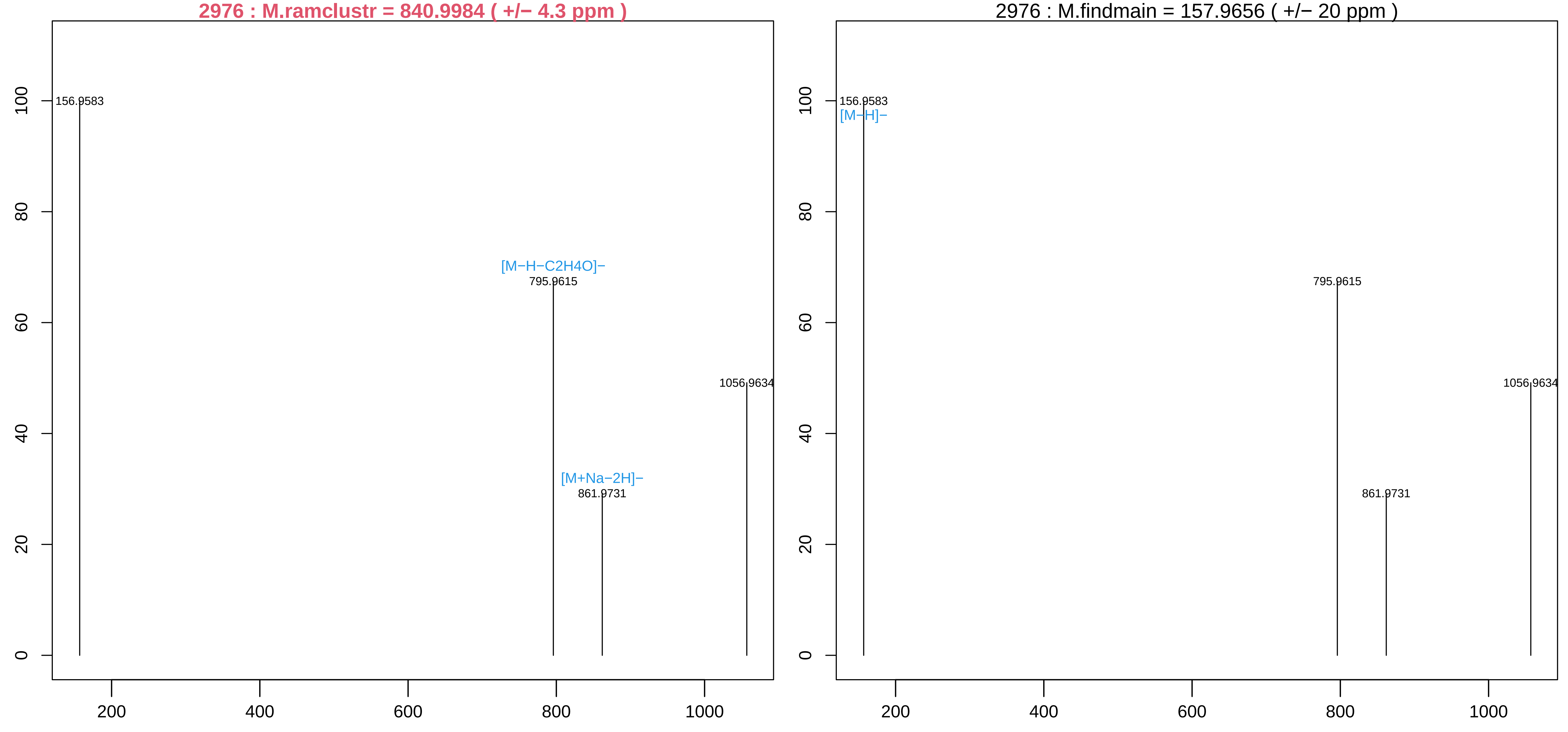

2976 : M.ramclustr = 840.9984 (+/− 4.3 ppm)
156.9583
[M−H−C2H4O]−
795.9615
[M+Na−2H]−
861.9731
1056.9634
2976 : M.findmain = 157.9656 (+/− 20 ppm)
156.9583
[M−H]−
795.9615
861.9731
1056.9634
0
20
40
60
80
100
200
400
600
800
1000

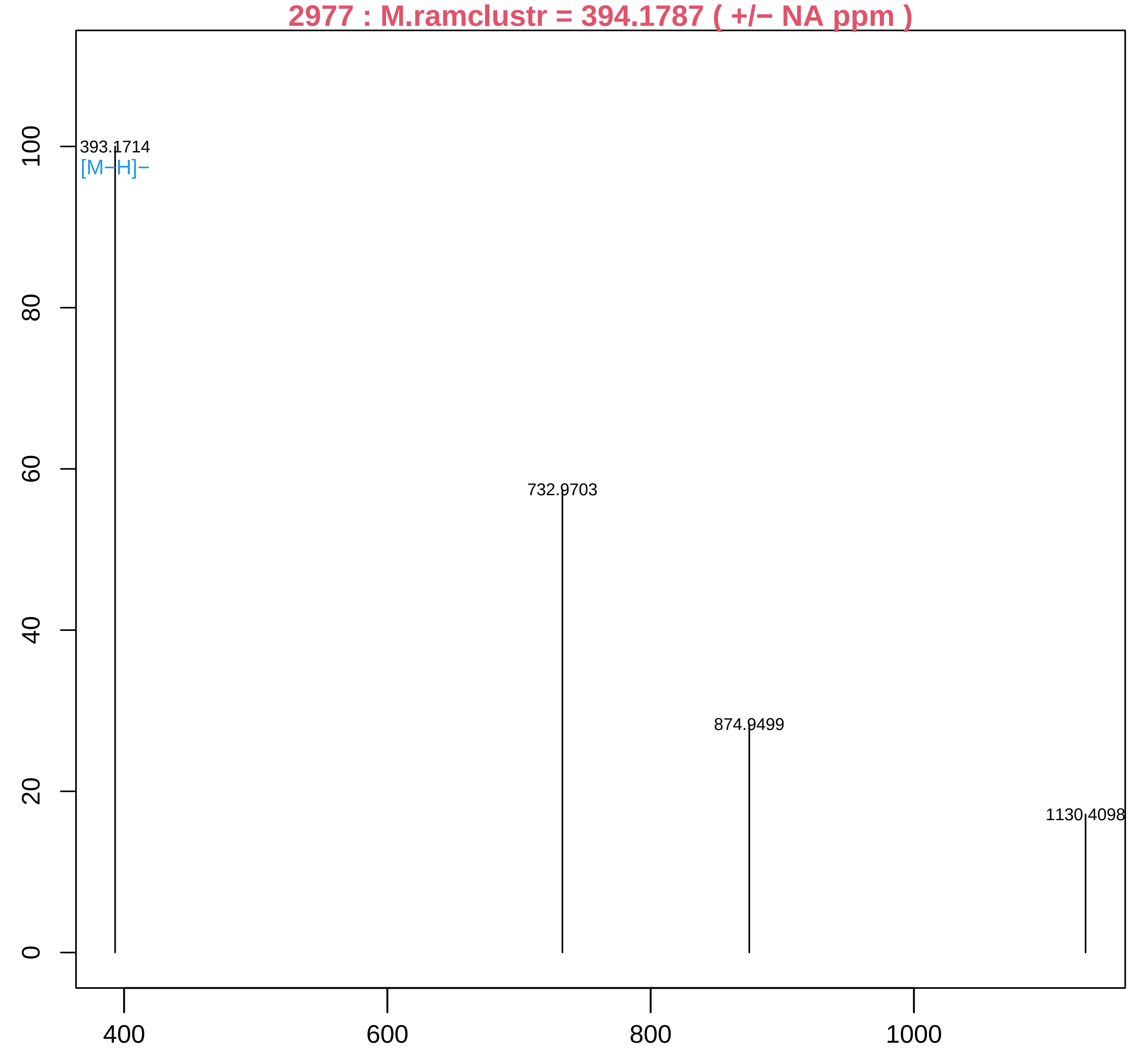
2977 : M.ramclustr = 394.1787 (+/− NA ppm)
393.1714
[M−H]−
732.9703
874.9499
1130.4098
0
20
40
60
80
100
400
600
800
1000

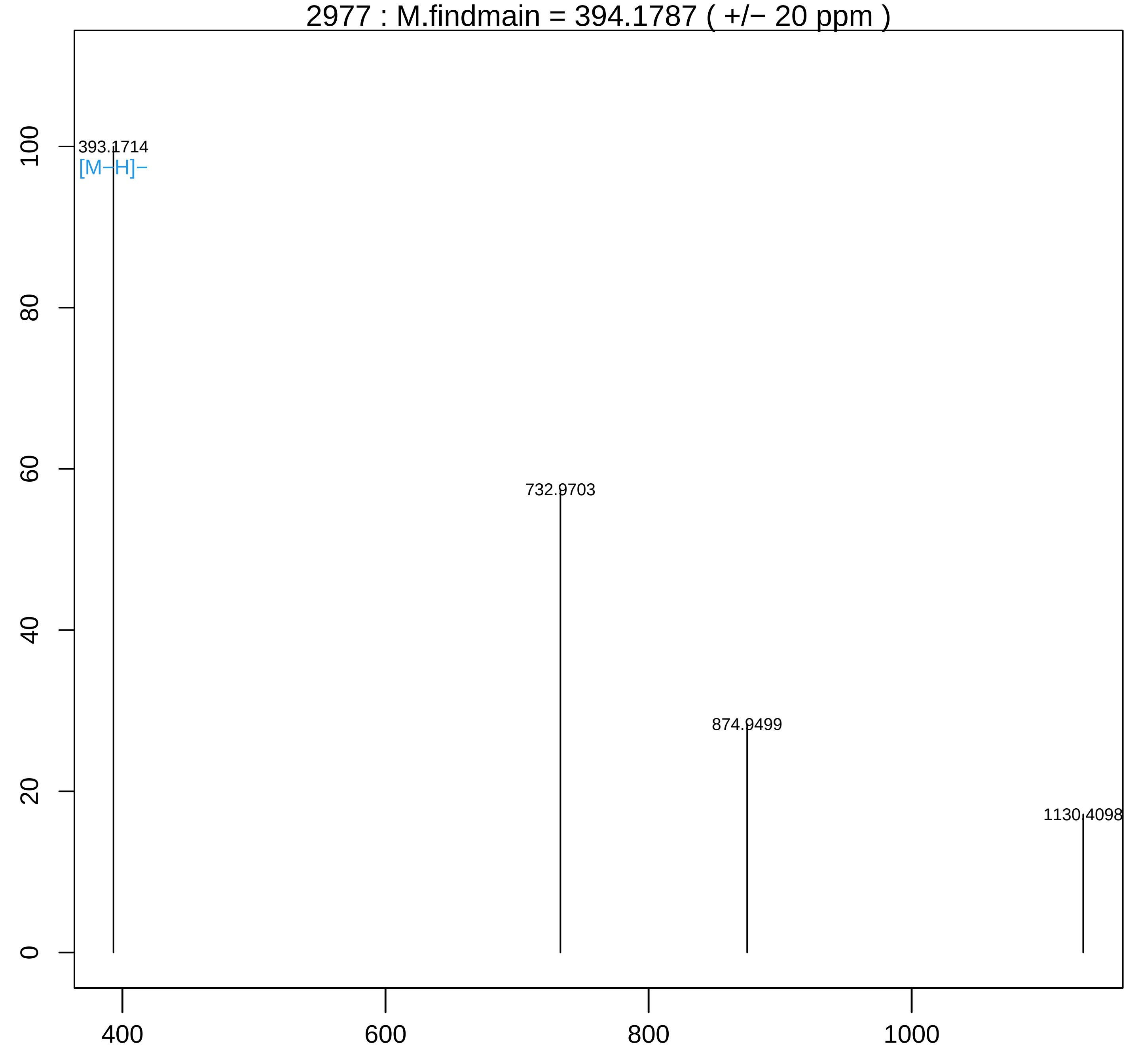
2977 : M.findmain = 394.1787 (+/− 20 ppm)
393.1714
[M−H]−
732.9703
874.9499
1130.4098
0
20
40
60
80
100
400
600
800
1000

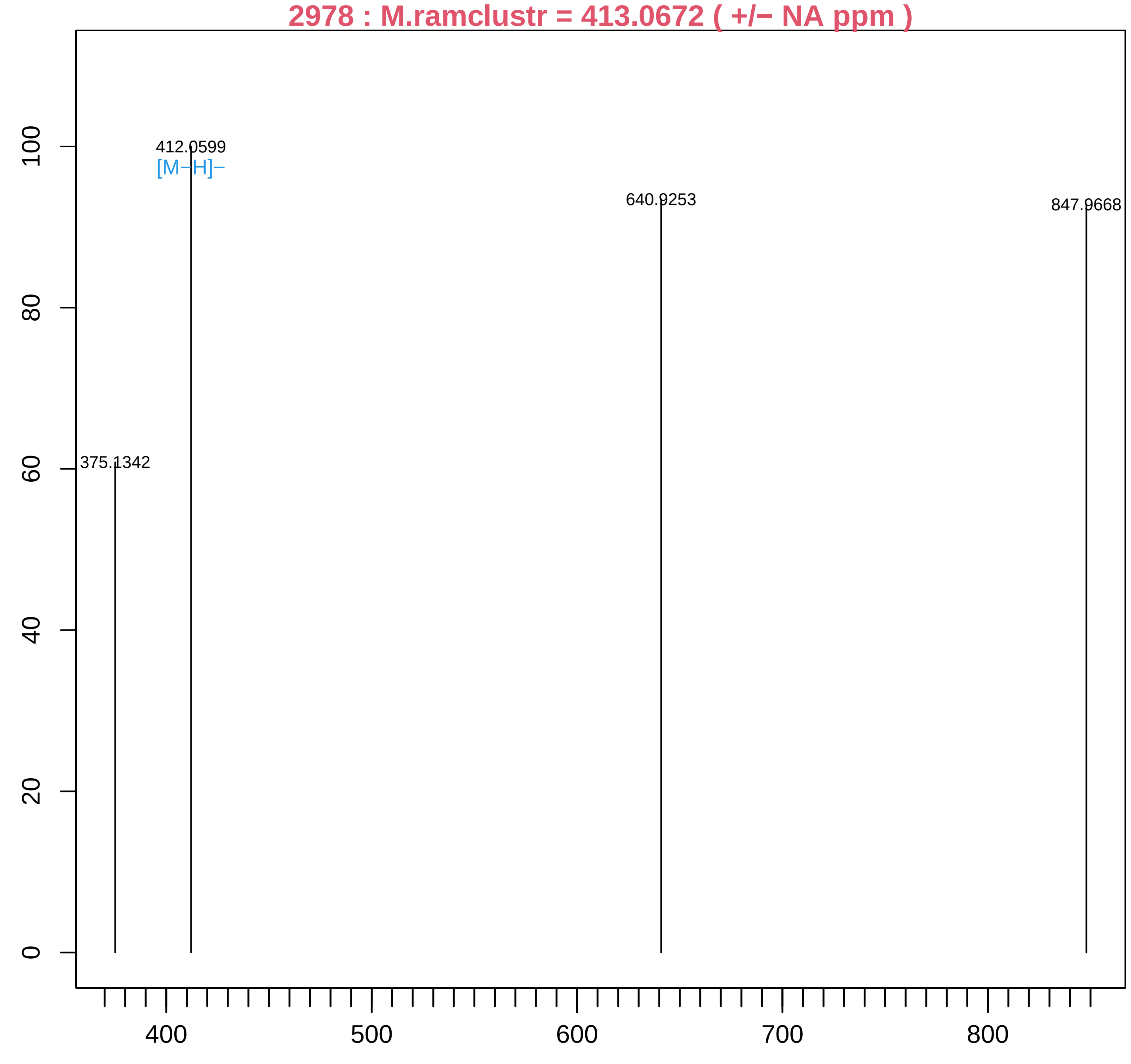
2978 : M.ramclustr = 413.0672 (+/− NA ppm)
412.0599
[M−H]−
640.9253
847.9668
375.1342
0
20
40
60
80
100
400
500
600
700
800

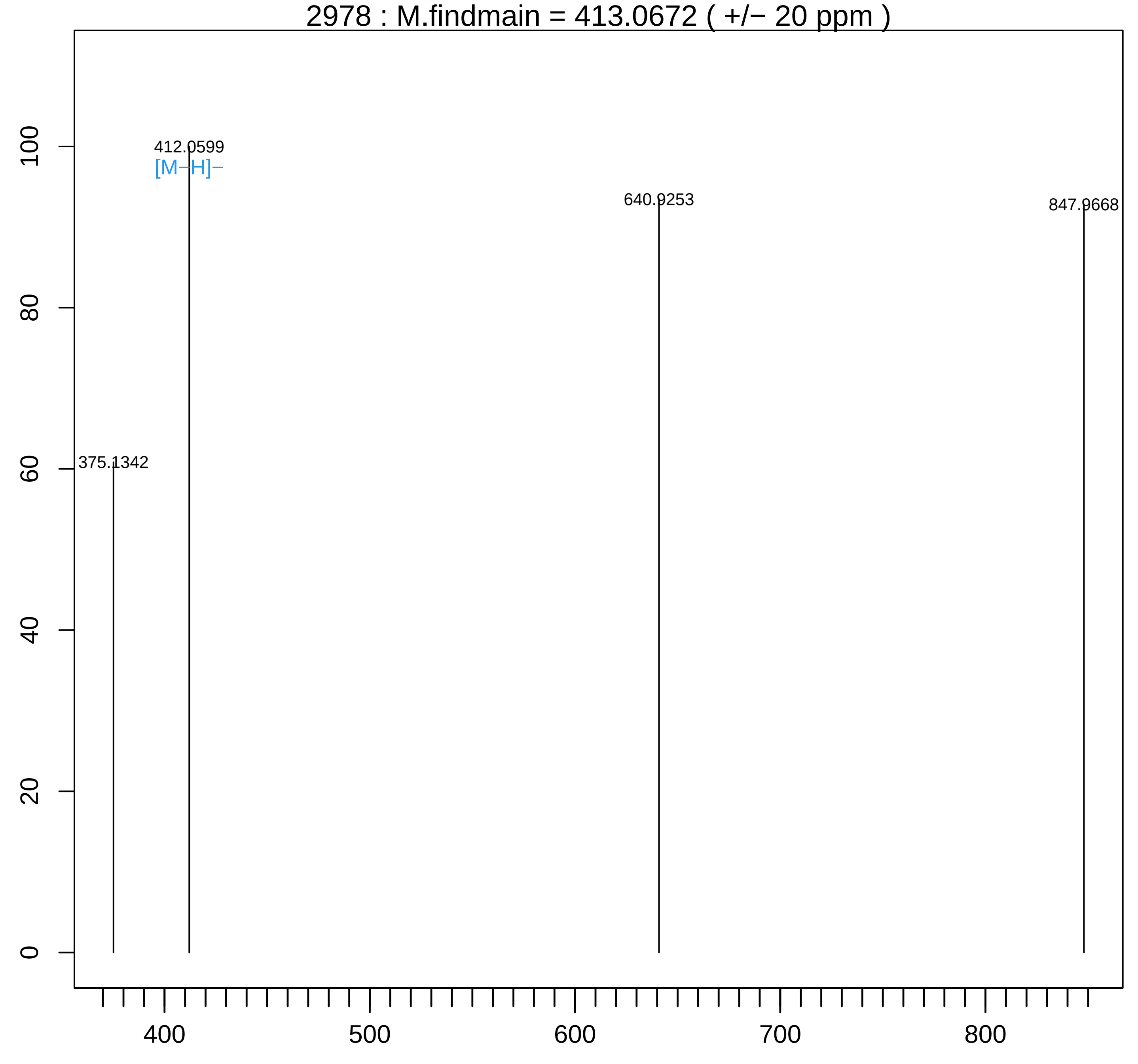
2978 : M.findmain = 413.0672 (+/− 20 ppm)
412.0599
[M−H]−
640.9253
847.9668
375.1342
0
20
40
60
80
100
400
500
600
700
800

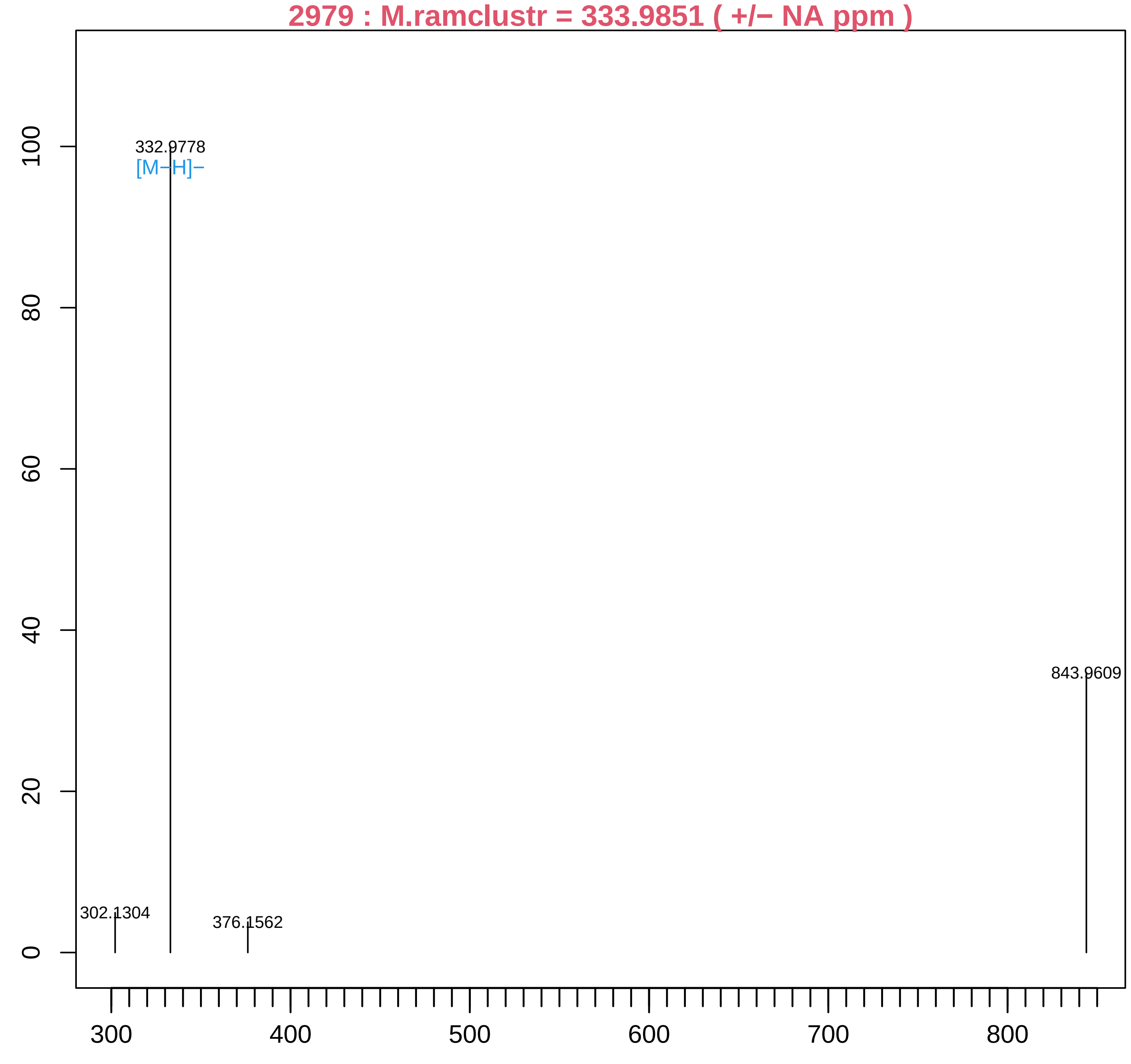
2979 : M.ramclustr = 333.9851 (+/− NA ppm)
332.9778
[M−H]−
843.9609
302.1304
376.1562
0
20
40
60
80
100
300
400
500
600
700
800

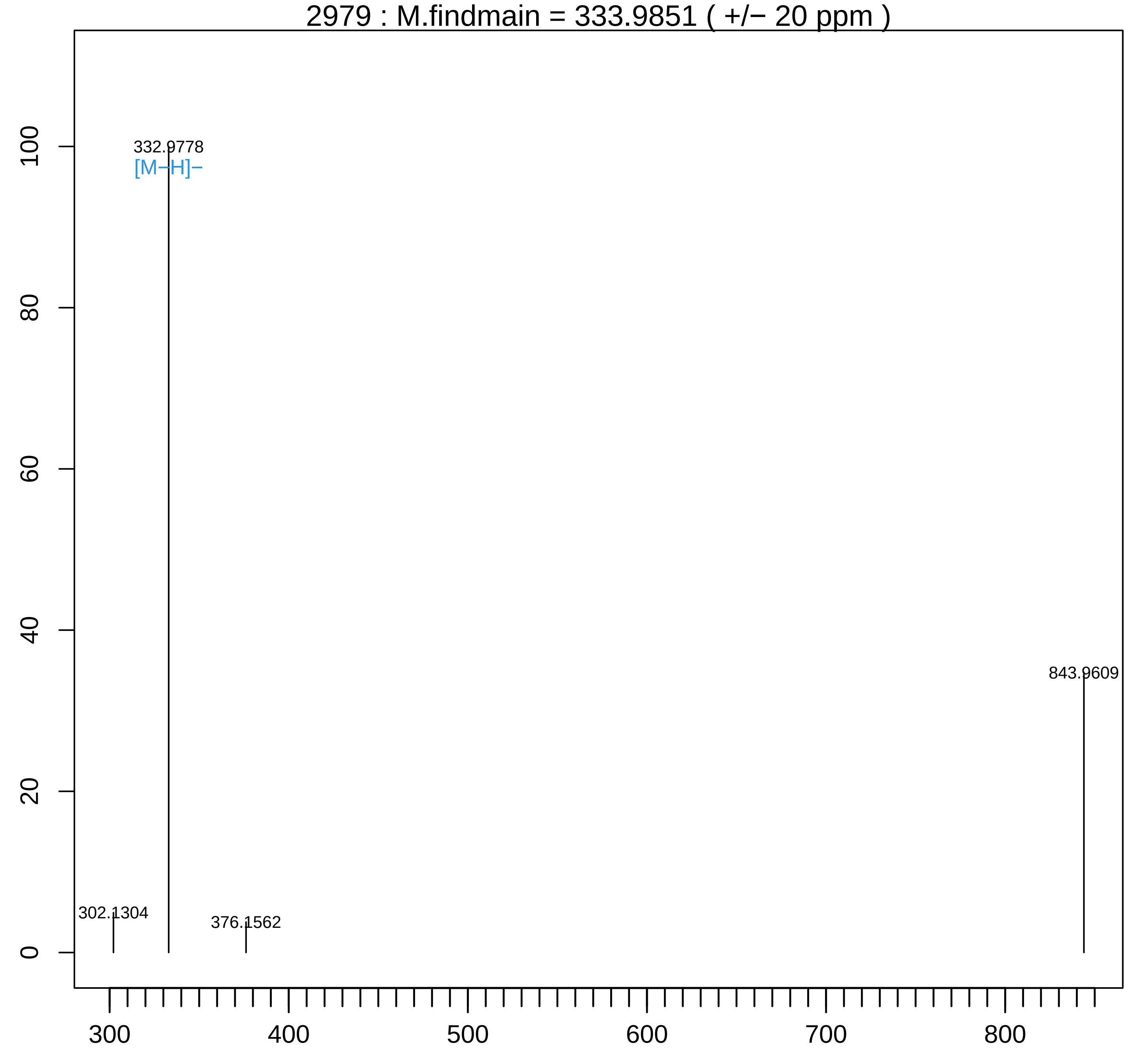
2979 : M.findmain = 333.9851 (+/− 20 ppm)
332.9778
[M−H]−
843.9609
302.1304
376.1562
0
20
40
60
80
100
300
400
500
600
700
800

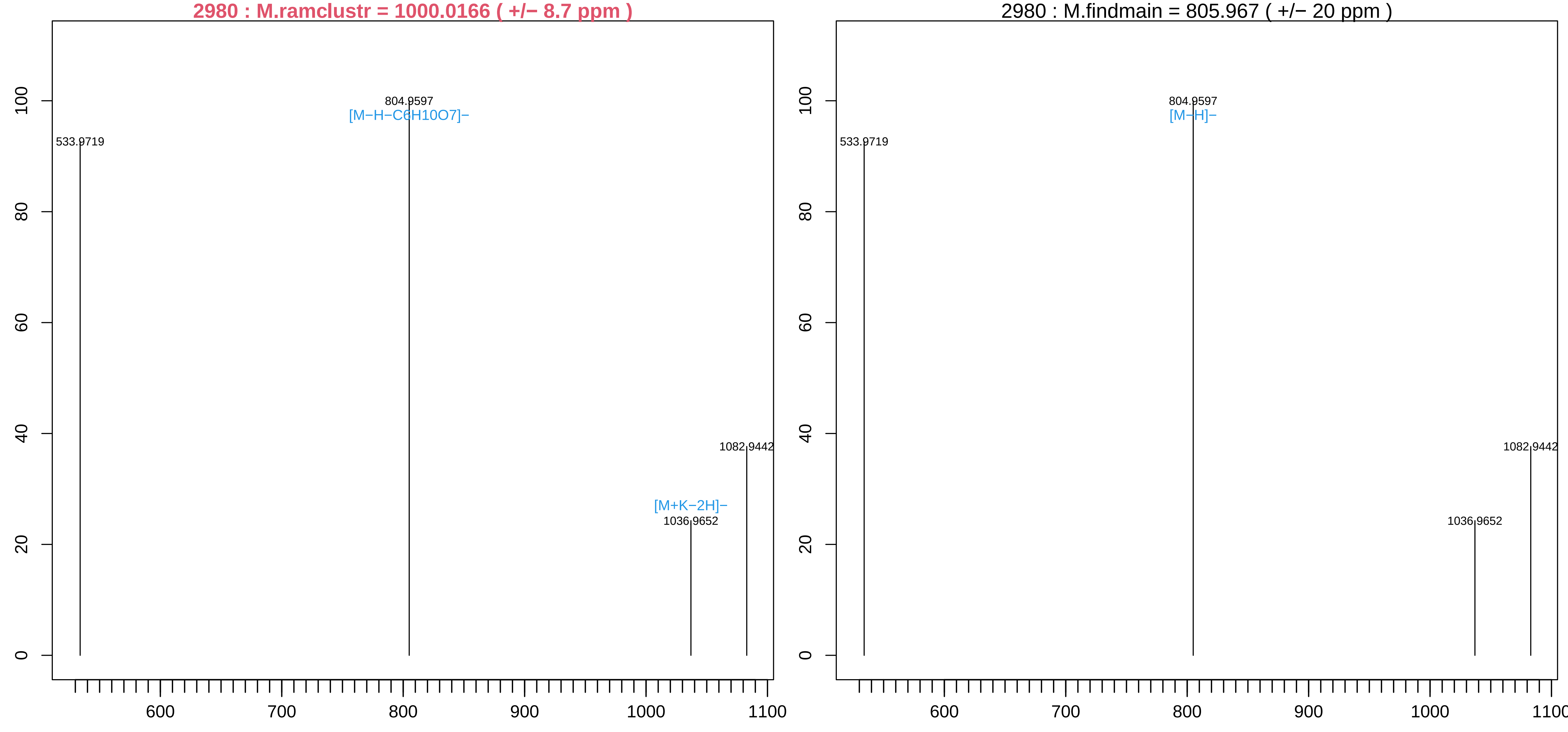
2980 : M.ramclustr = 1000.0166 (+/− 8.7 ppm)
804.9597
[M−H−C6H10O7]−
533.9719
1082.9442
[M+K−2H]−
1036.9652
2980 : M.findmain = 805.967 (+/− 20 ppm)
804.9597
[M−H]−
533.9719
1082.9442
1036.9652
0
20
40
60
80
100
600
700
800
900
1000
1100

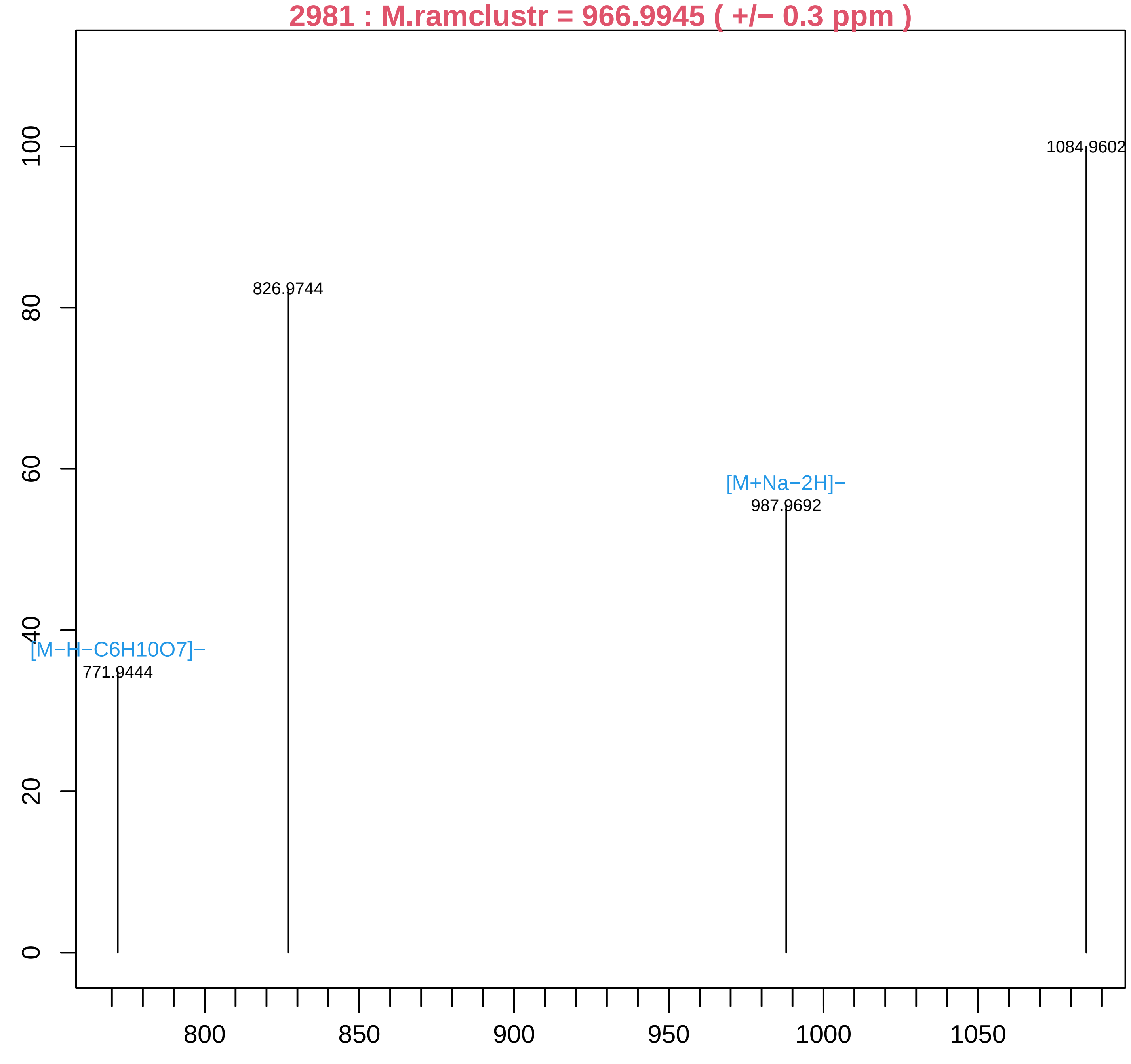
2981 : M.ramclustr = 966.9945 (+/− 0.3 ppm)
[M−H−C6H10O7]−
771.9444
826.9744
[M+Na−2H]−
987.9692
1084.9602
0
20
40
60
80
100
800
850
900
950
1000
1050

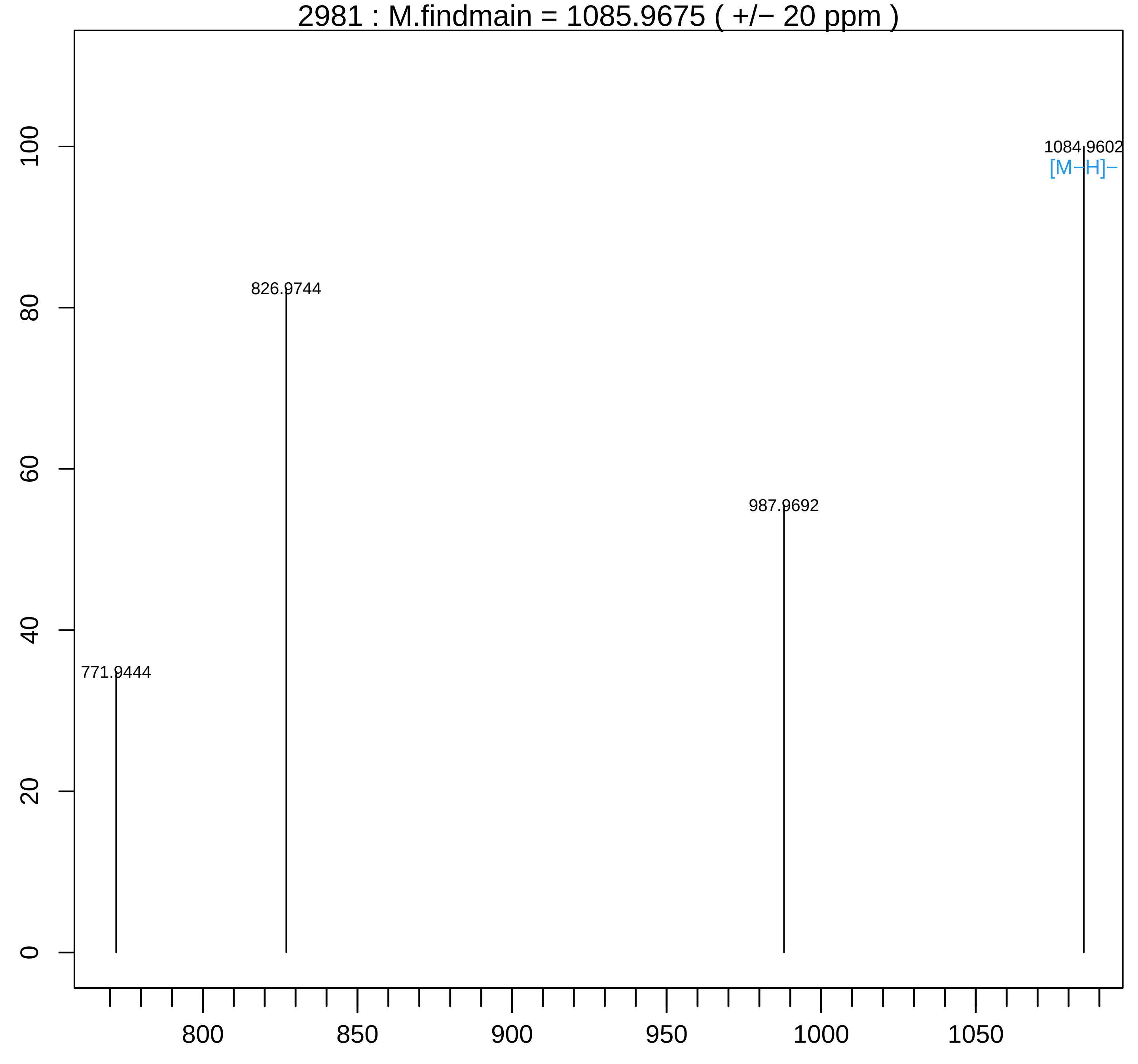
2981 : M.findmain = 1085.9675 (+/− 20 ppm)
771.9444
826.9744
987.9692
1084.9602
[M−H]−
0
20
40
60
80
100
800
850
900
950
1000
1050

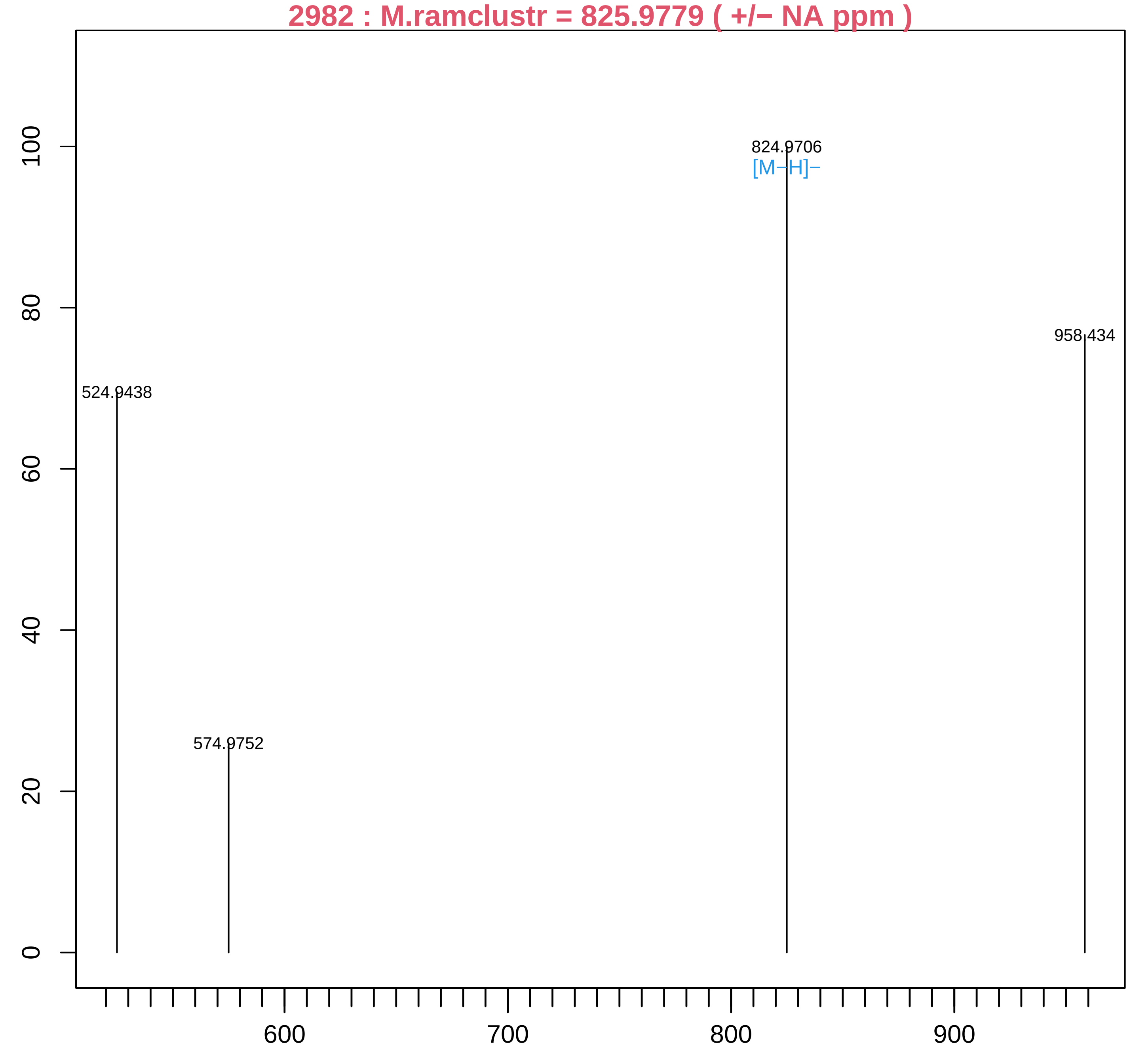
2982 : M.ramclustr = 825.9779 (+/− NA ppm)
824.9706
[M−H]−
958.434
524.9438
574.9752
0
20
40
60
80
100
600
700
800
900

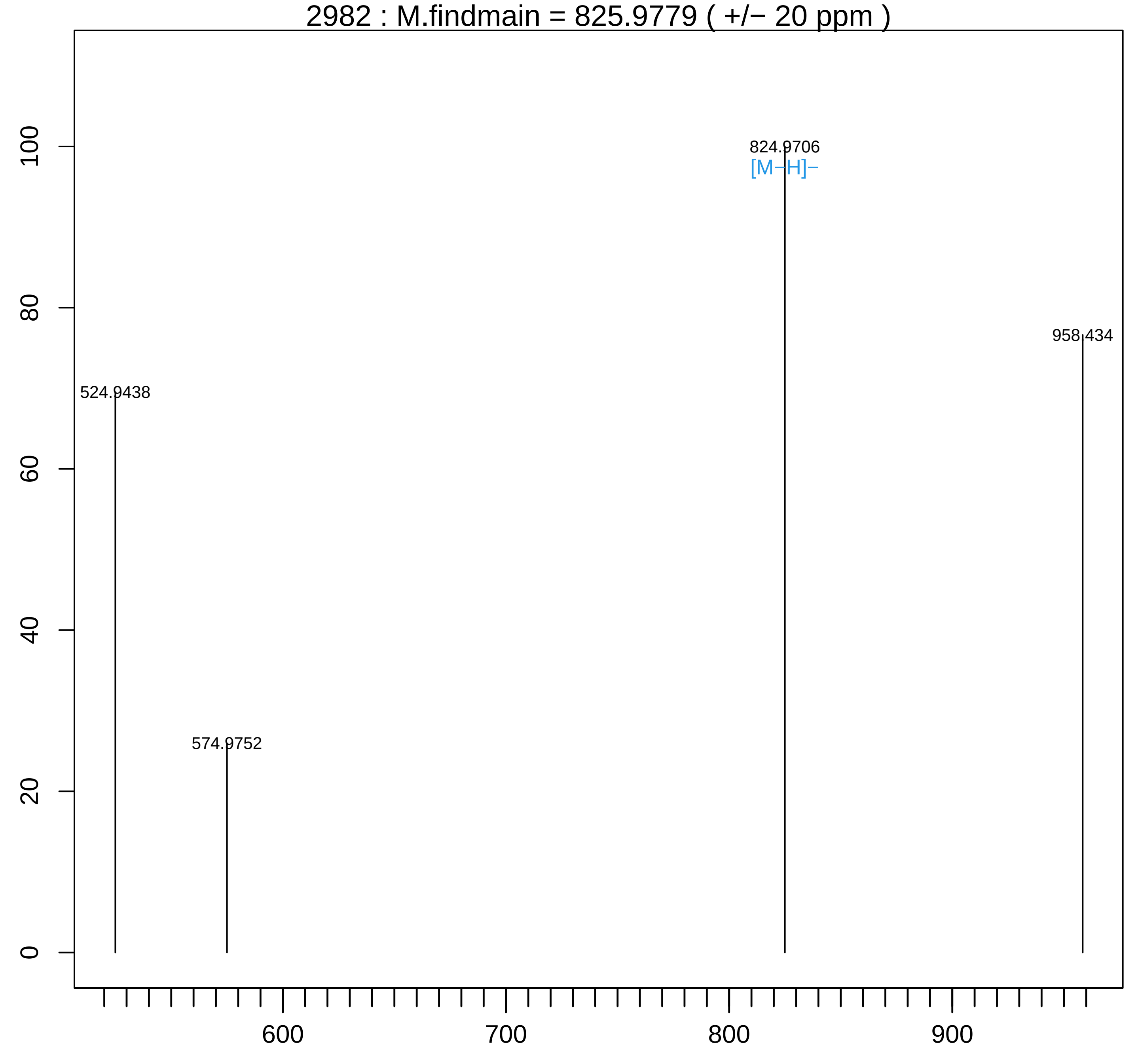
2982 : M.findmain = 825.9779 (+/− 20 ppm)
824.9706
[M−H]−
958.434
524.9438
574.9752
0
20
40
60
80
100
600
700
800
900

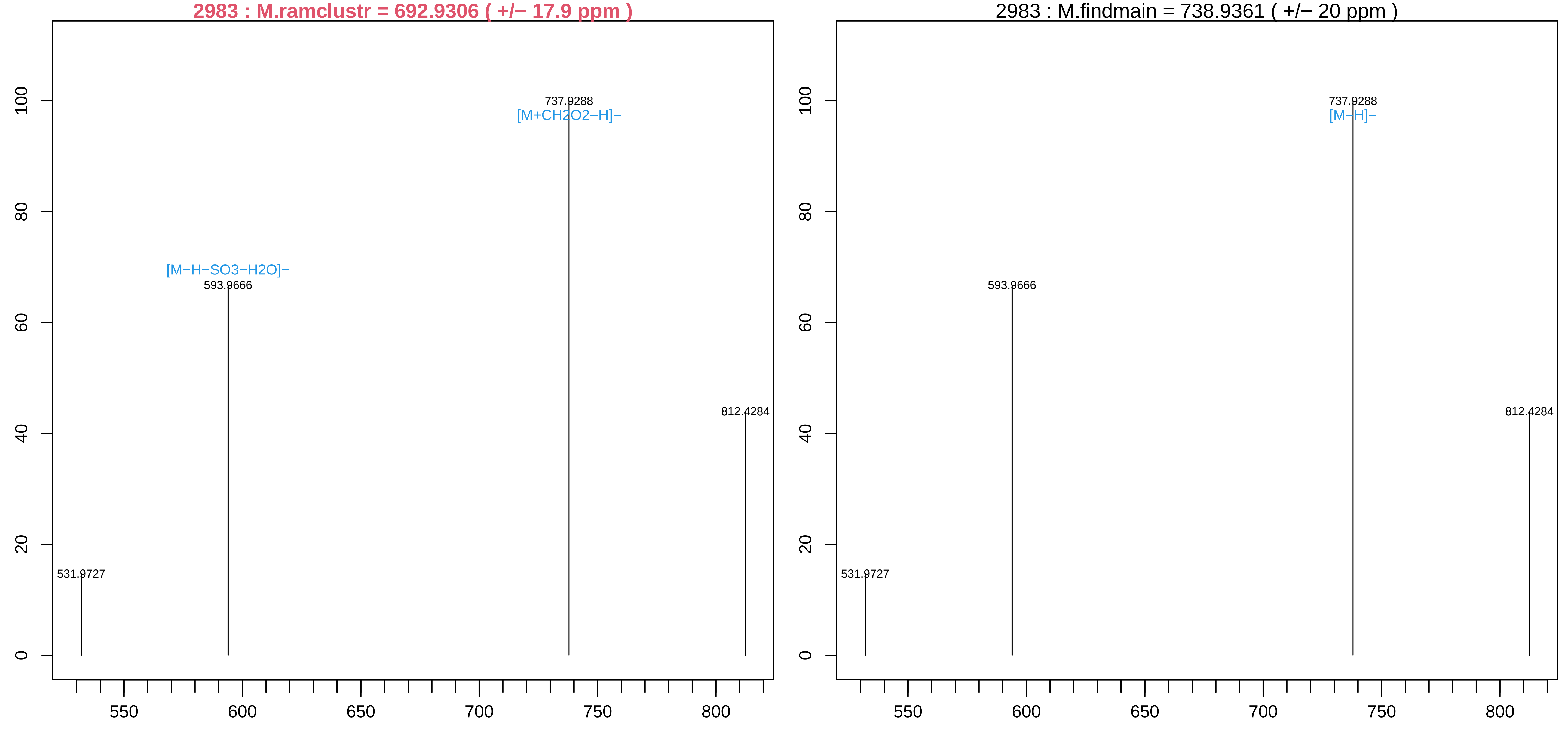
2983 : M.ramclustr = 692.9306 (+/− 17.9 ppm)
737.9288
[M+CH2O2−H]−
[M−H−SO3−H2O]−
593.9666
812.4284
531.9727
0
20
40
60
80
100
550
600
650
700
750
800
2983 : M.findmain = 738.9361 (+/− 20 ppm)
737.9288
[M−H]−
593.9666
812.4284
531.9727
0
20
40
60
80
100
550
600
650
700
750
800

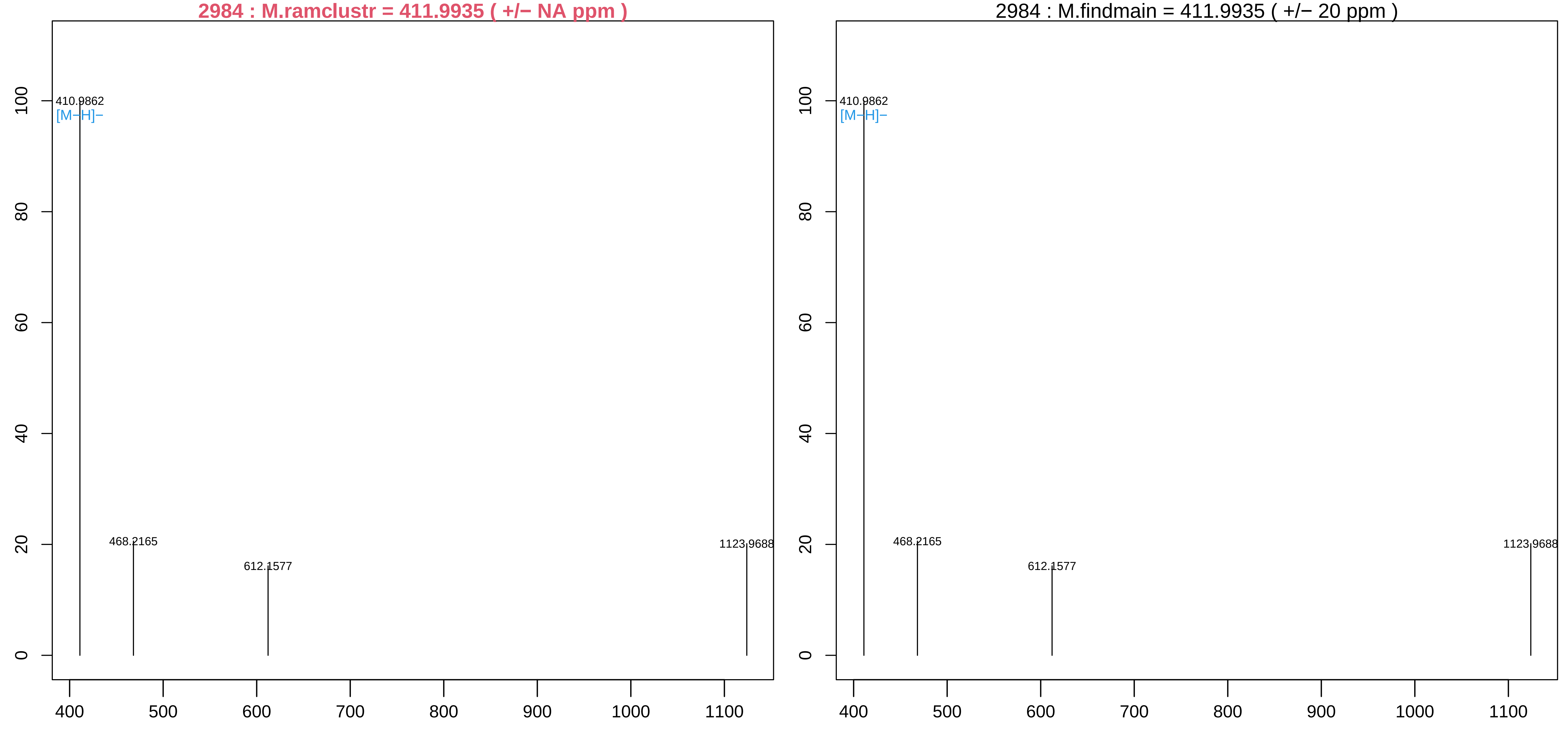

2984 : M.ramclustr = 411.9935 (+/− NA ppm)
410.9862
[M−H]−
468.2165
612.1577
1123.9688
0
20
40
60
80
100
400
500
600
700
800
900
1000
1100
2984 : M.findmain = 411.9935 (+/− 20 ppm)
410.9862
[M−H]−
468.2165
612.1577
1123.9688
0
20
40
60
80
100
400
500
600
700
800
900
1000
1100

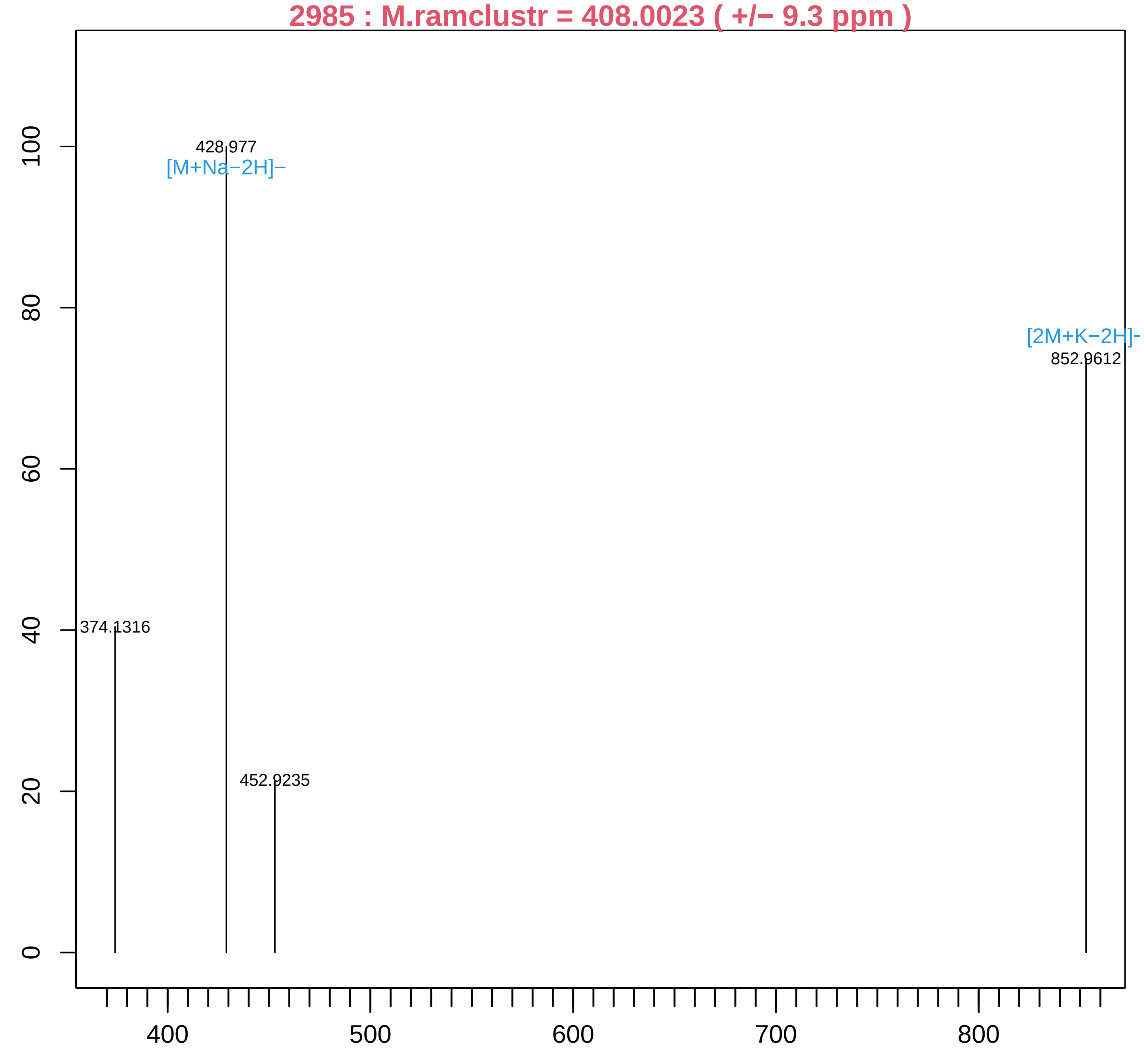
2985 : M.ramclustr = 408.0023 (+/− 9.3 ppm)
428.977
[M+Na−2H]−
[2M+K−2H]-
852.9612
374.1316
452.9235
0
20
40
60
80
100
400
500
600
700
800

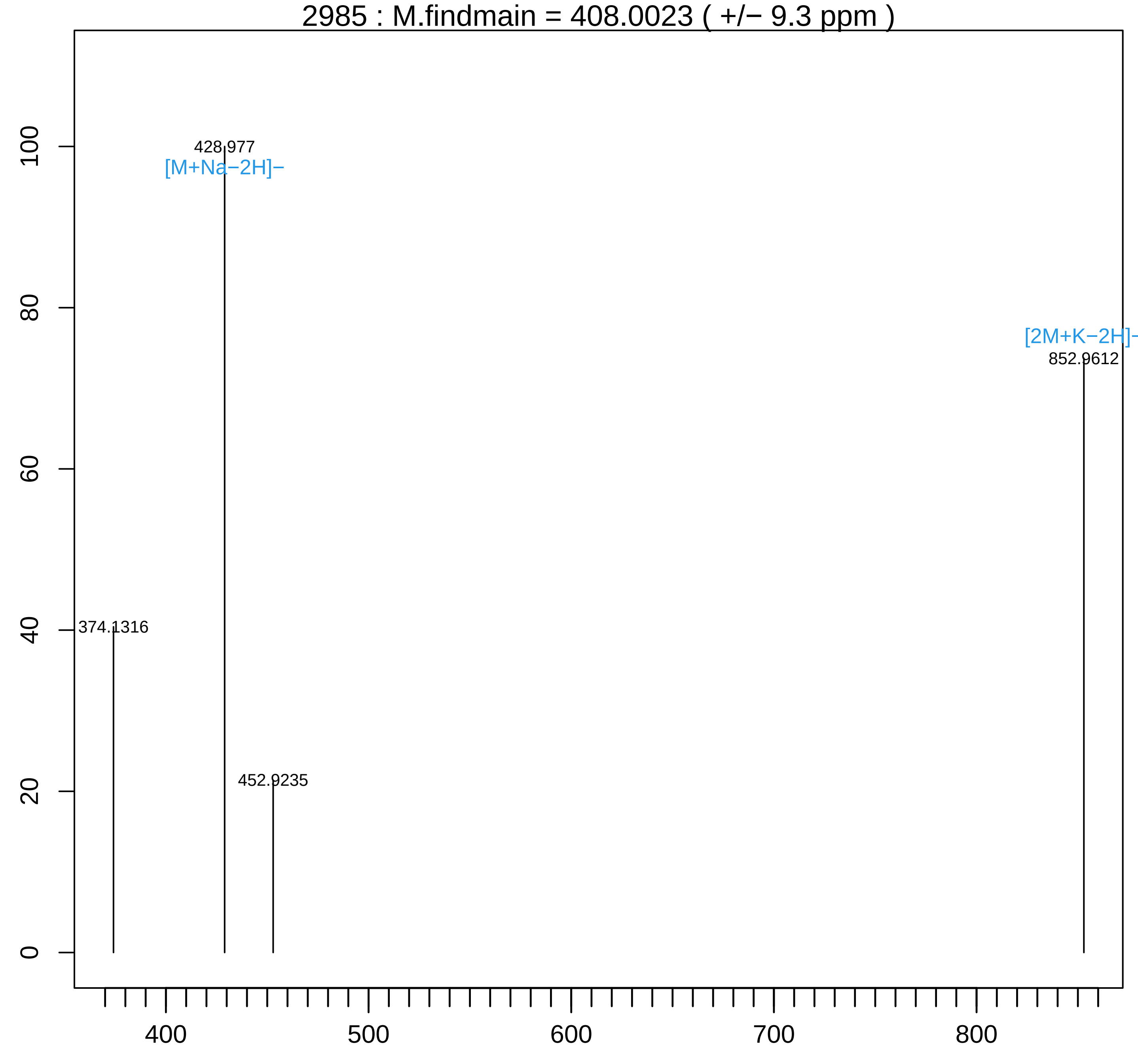
2985 : M.findmain = 408.0023 (+/− 9.3 ppm)
428.977
[M+Na−2H]−
[2M+K−2H]-
852.9612
374.1316
452.9235
0
20
40
60
80
100
400
500
600
700
800

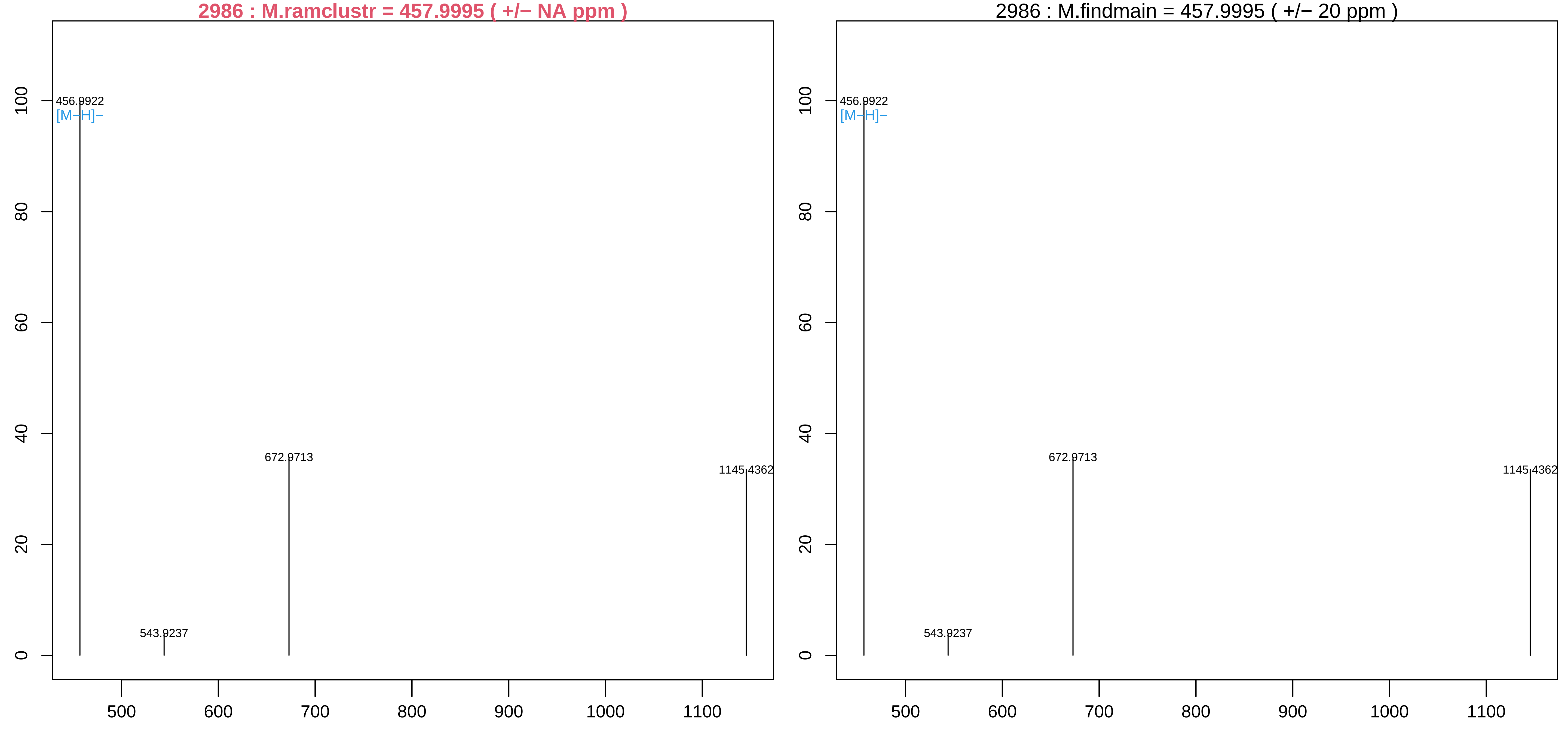
2986 : M.ramclustr = 457.9995 (+/− NA ppm)
2986 : M.findmain = 457.9995 (+/− 20 ppm)
456.9922
[M−H]−
543.9237
672.9713
1145.4362
0
20
40
60
80
100
500
600
700
800
900
1000
1100

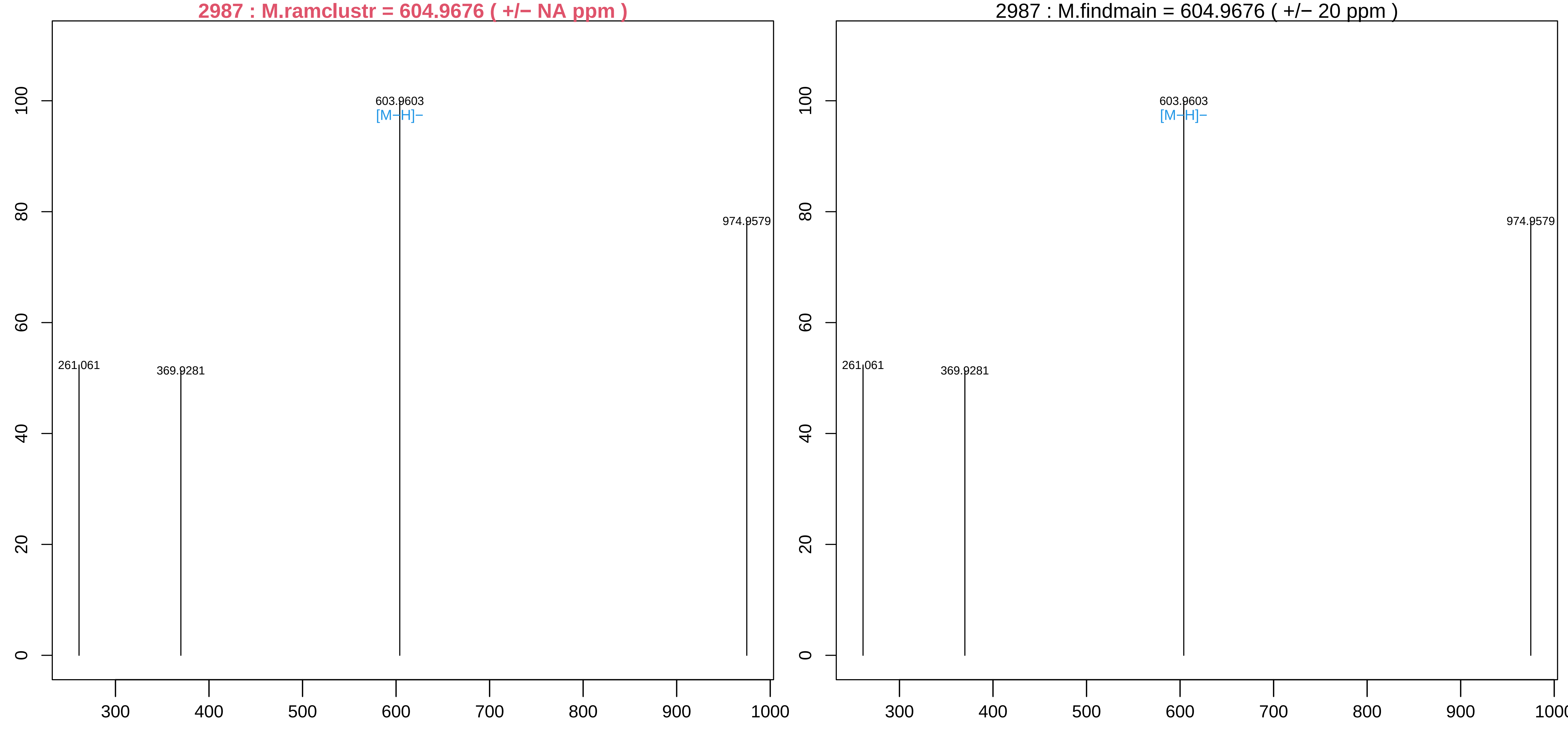
2987 : M.ramclustr = 604.9676 (+/− NA ppm)
2987 : M.findmain = 604.9676 (+/− 20 ppm)
603.9603
[M−H]−
974.9579
261.061
369.9281
0
20
40
60
80
100
300
400
500
600
700
800
900
1000

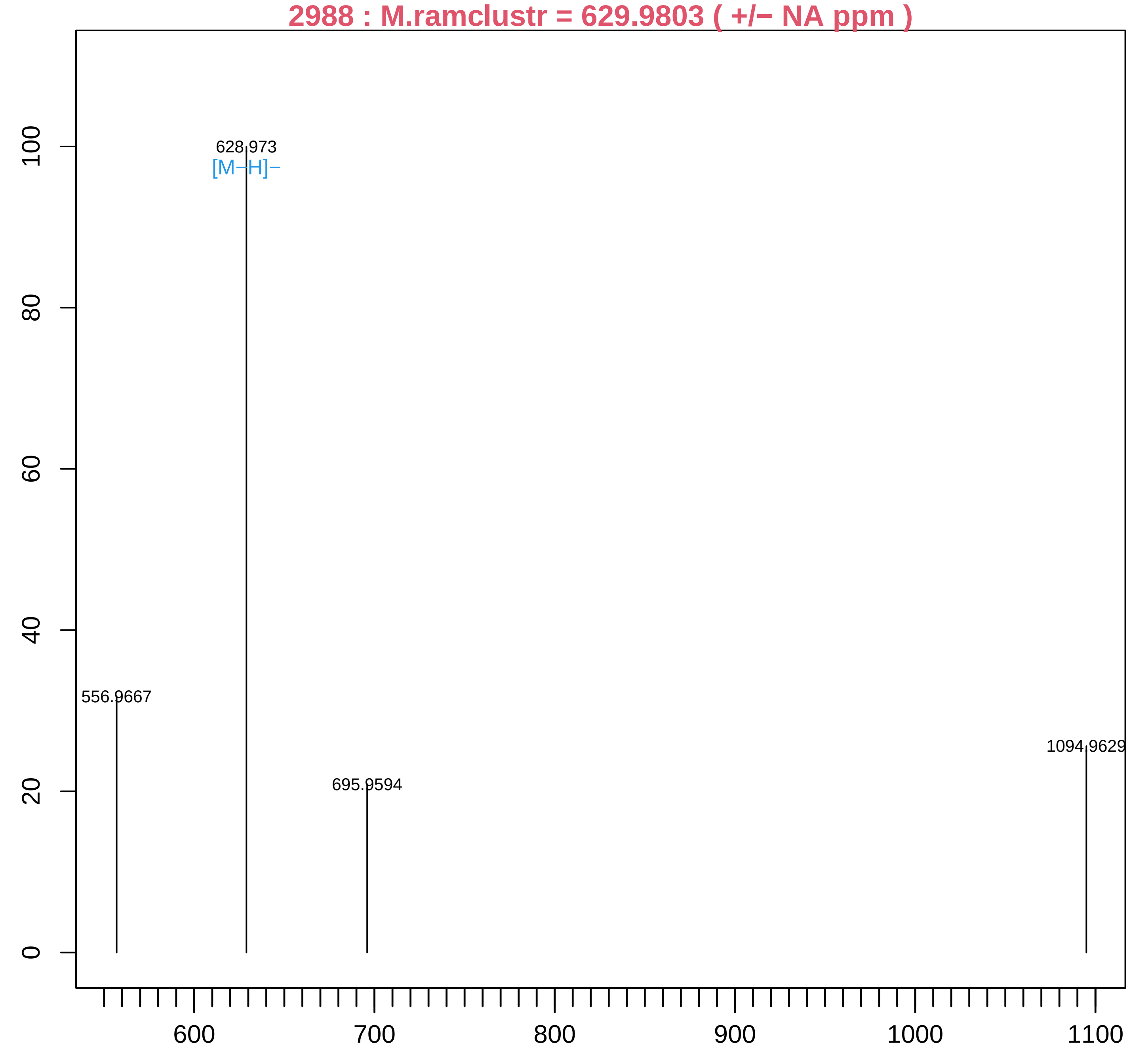
2988 : M.ramclustr = 629.9803 (+/− NA ppm)
628.973
[M−H]−
556.9667
695.9594
1094.9629
0
20
40
60
80
100
600
700
800
900
1000
1100

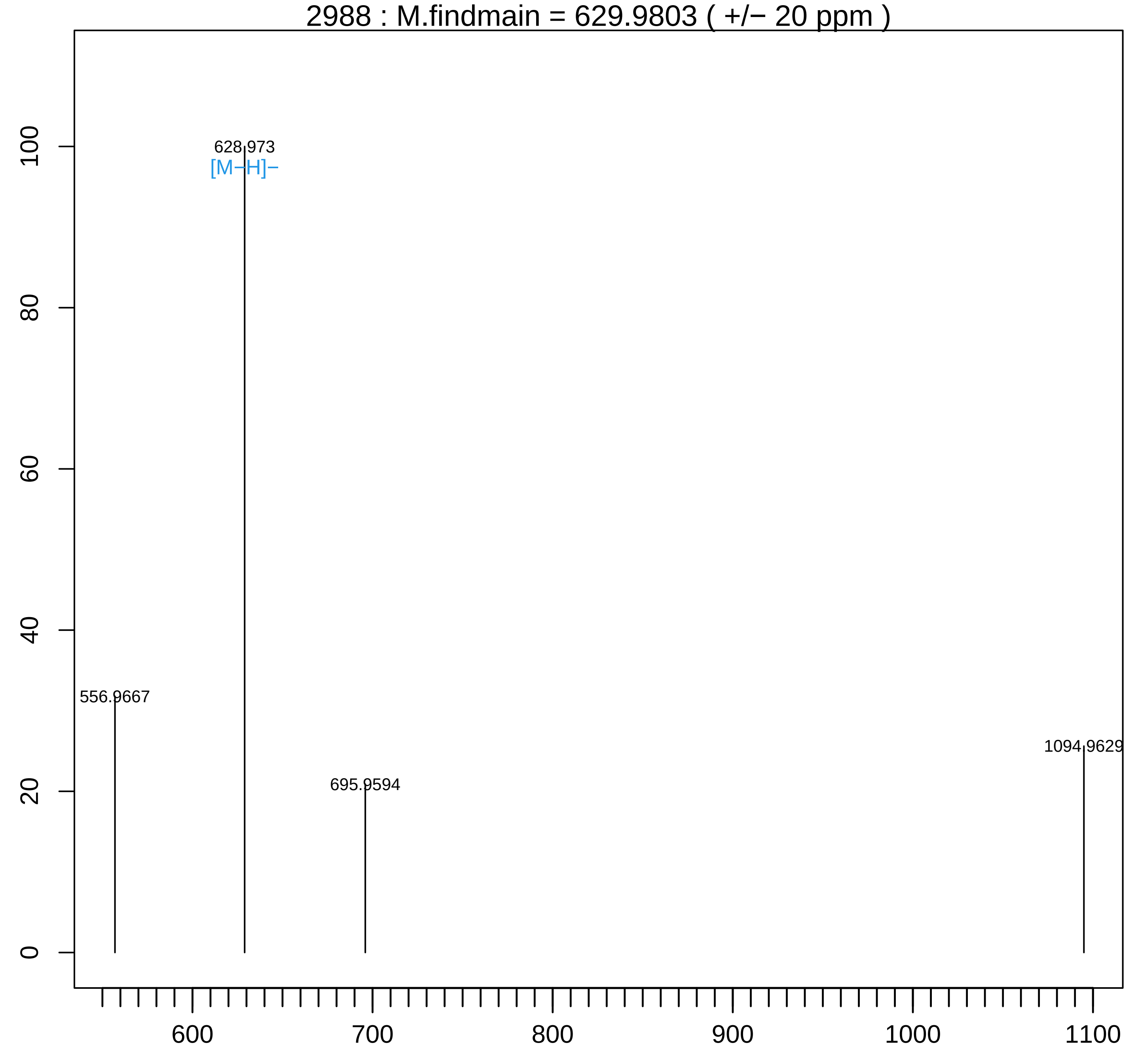
2988 : M.findmain = 629.9803 (+/− 20 ppm)
628.973
[M−H]−
556.9667
695.9594
1094.9629
0
20
40
60
80
100
600
700
800
900
1000
1100

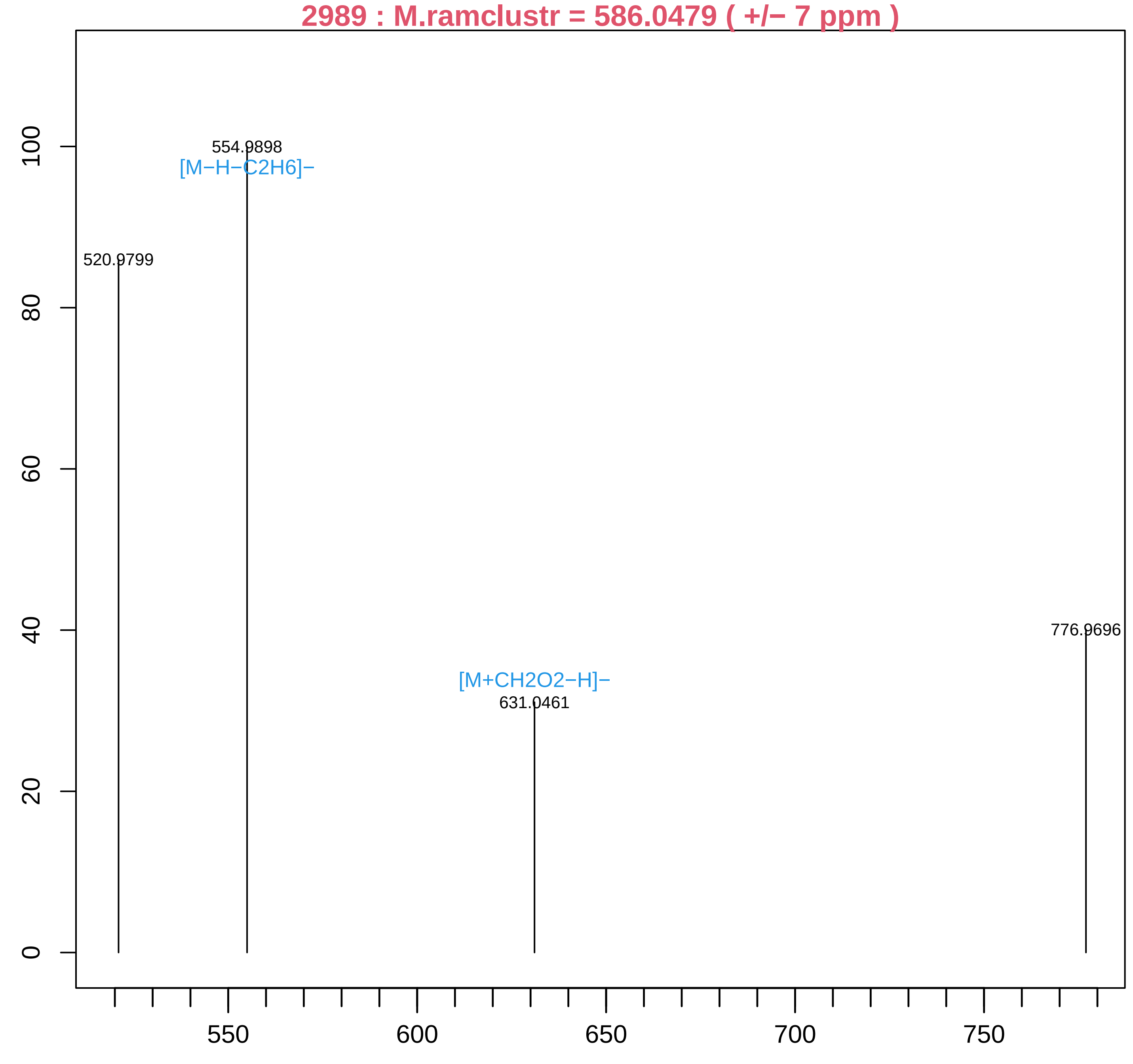
2989 : M.ramclustr = 586.0479 (+/− 7 ppm)
554.9898
[M−H−C2H6]−
520.9799
776.9696
[M+CH2O2−H]−
631.0461
0
20
40
60
80
100
550
600
650
700
750

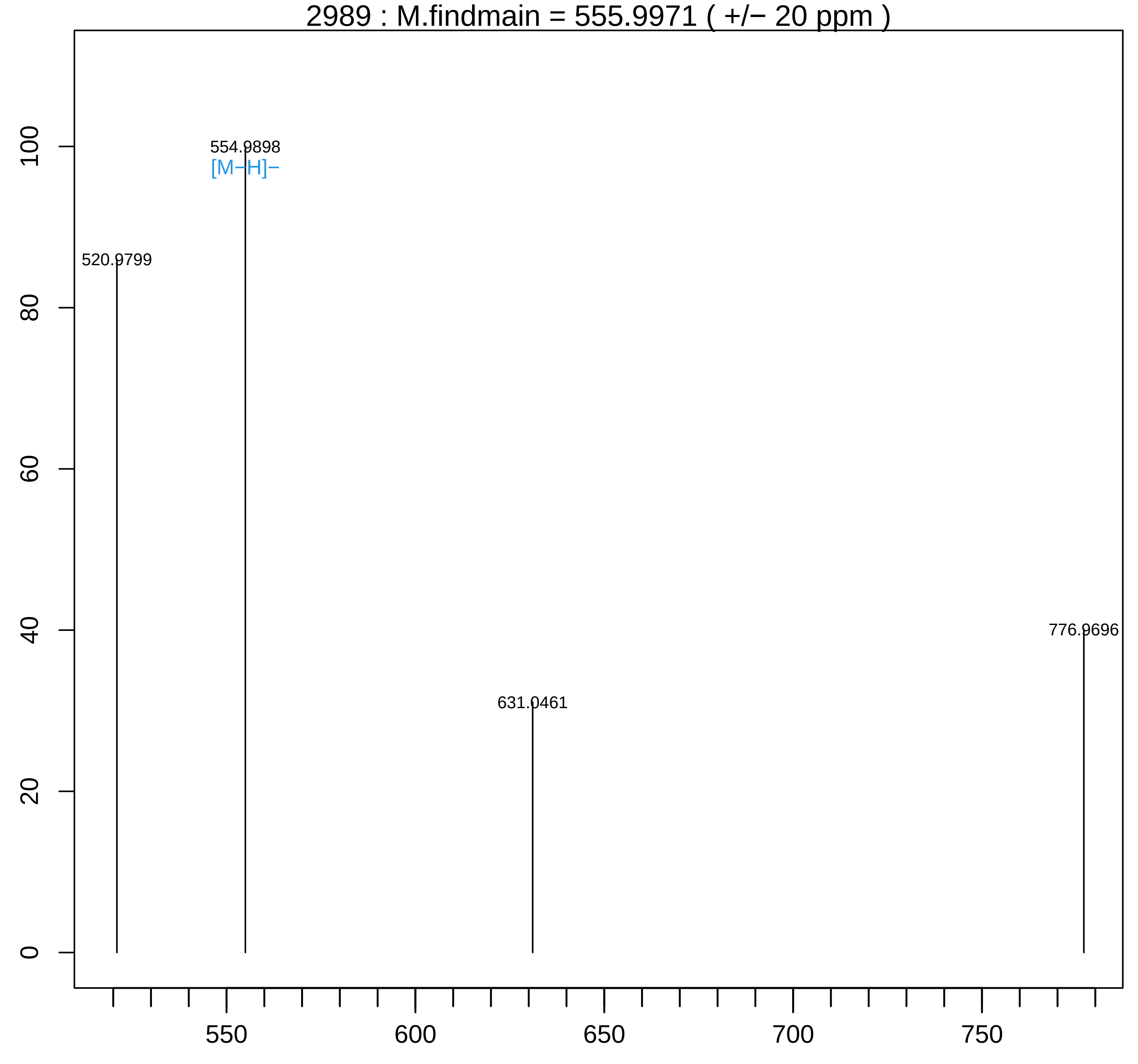
2989 : M.findmain = 555.9971 (+/− 20 ppm)
554.9898
[M−H]−
520.9799
776.9696
631.0461
0
20
40
60
80
100
550
600
650
700
750

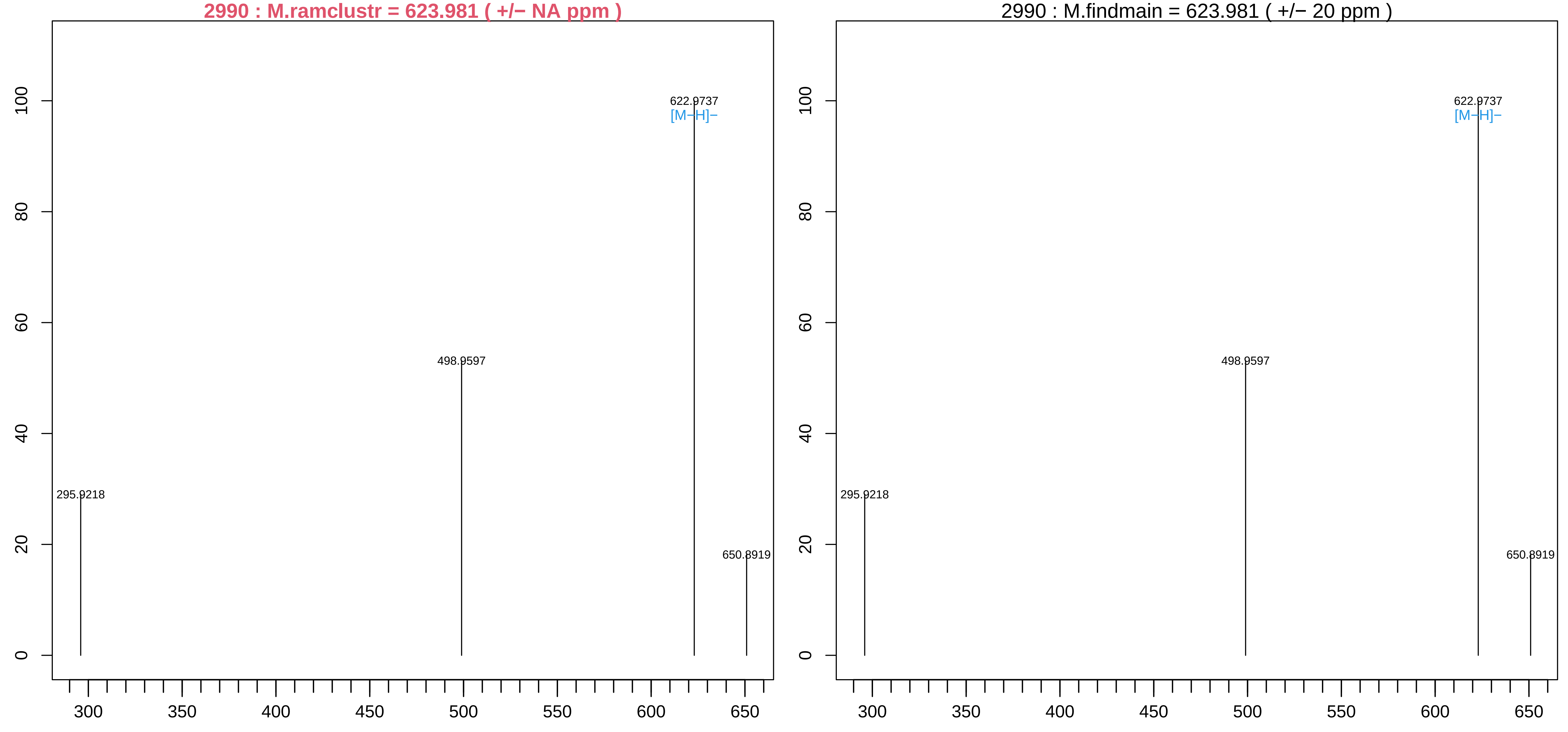

2990 : M.ramclustr = 623.981 (+/− NA ppm)
2990 : M.findmain = 623.981 (+/− 20 ppm)
622.9737
[M−H]−
498.9597
295.9218
650.8919
0
20
40
60
80
100
300
350
400
450
500
550
600
650

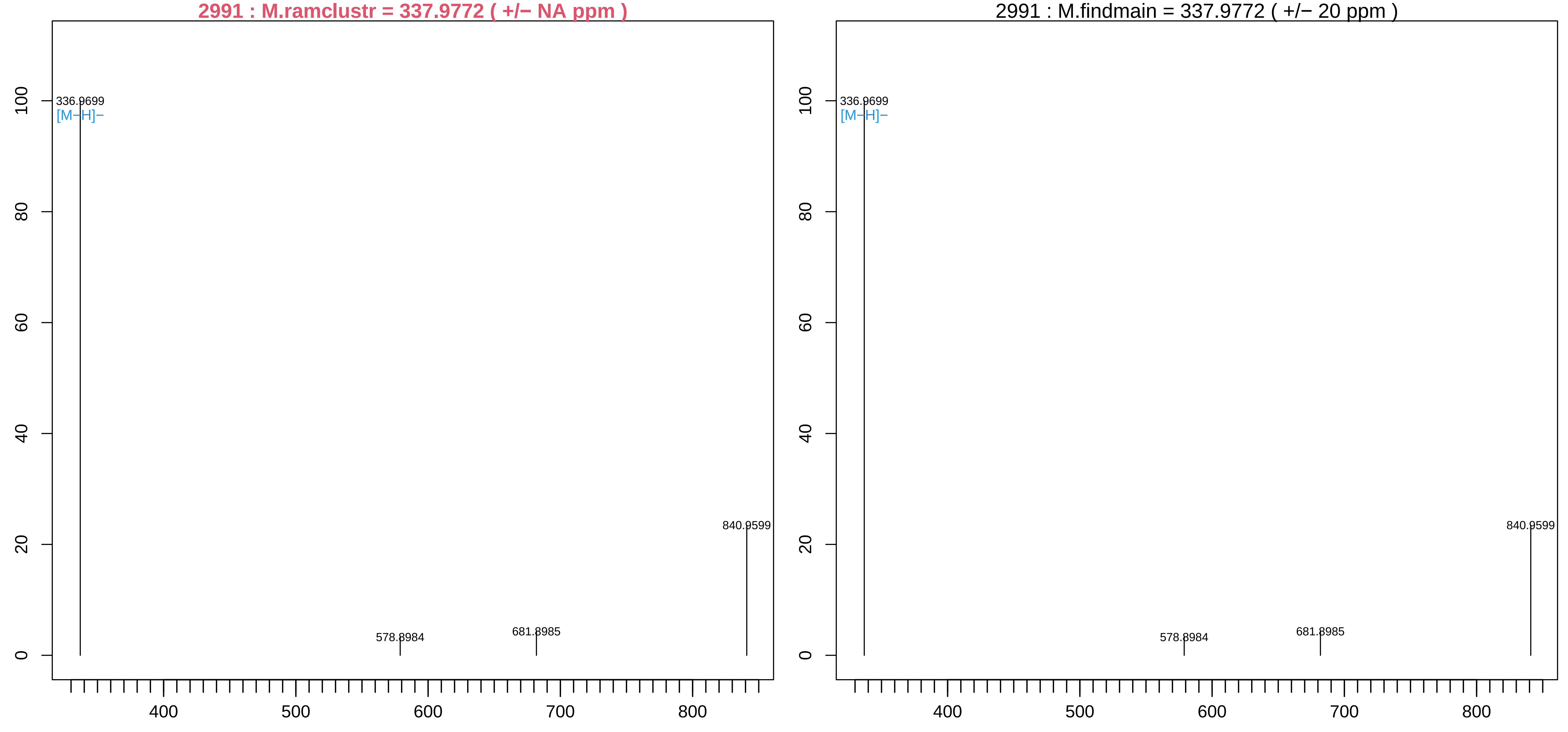
2991 : M.ramclustr = 337.9772 (+/− NA ppm)
336.9699
[M−H]−
578.8984
681.8985
840.9599
2991 : M.findmain = 337.9772 (+/− 20 ppm)
336.9699
[M−H]−
578.8984
681.8985
840.9599
0
20
40
60
80
100
400
500
600
700
800

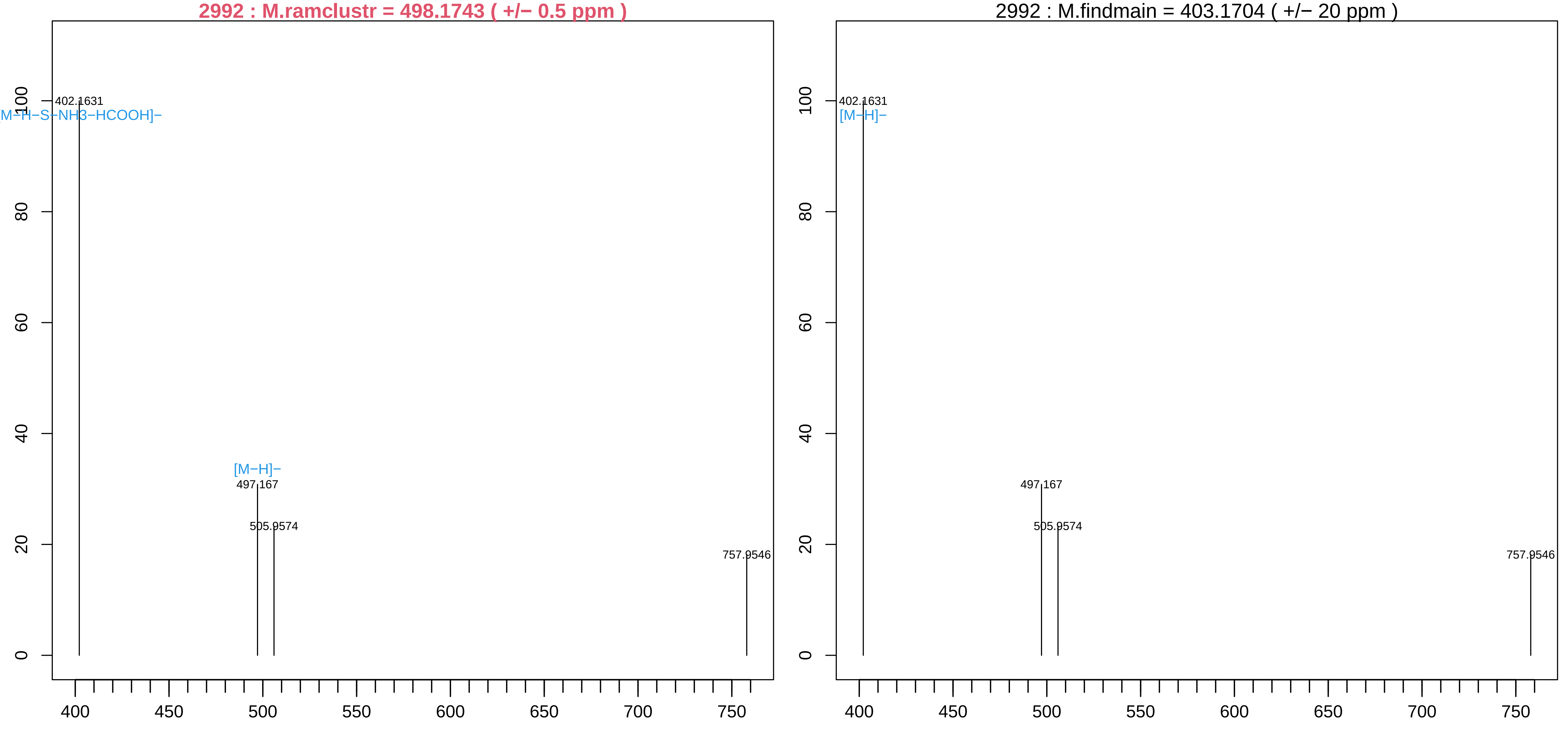

2992 : M.ramclustr = 498.1743 (+/− 0.5 ppm)
402.1631
[M−H−S−NH3−HCOOH]−
[M−H]−
497.167
505.9574
757.9546
2992 : M.findmain = 403.1704 (+/− 20 ppm)
402.1631
[M−H]−
497.167
505.9574
757.9546
0
20
40
60
80
100
400
450
500
550
600
650
700
750

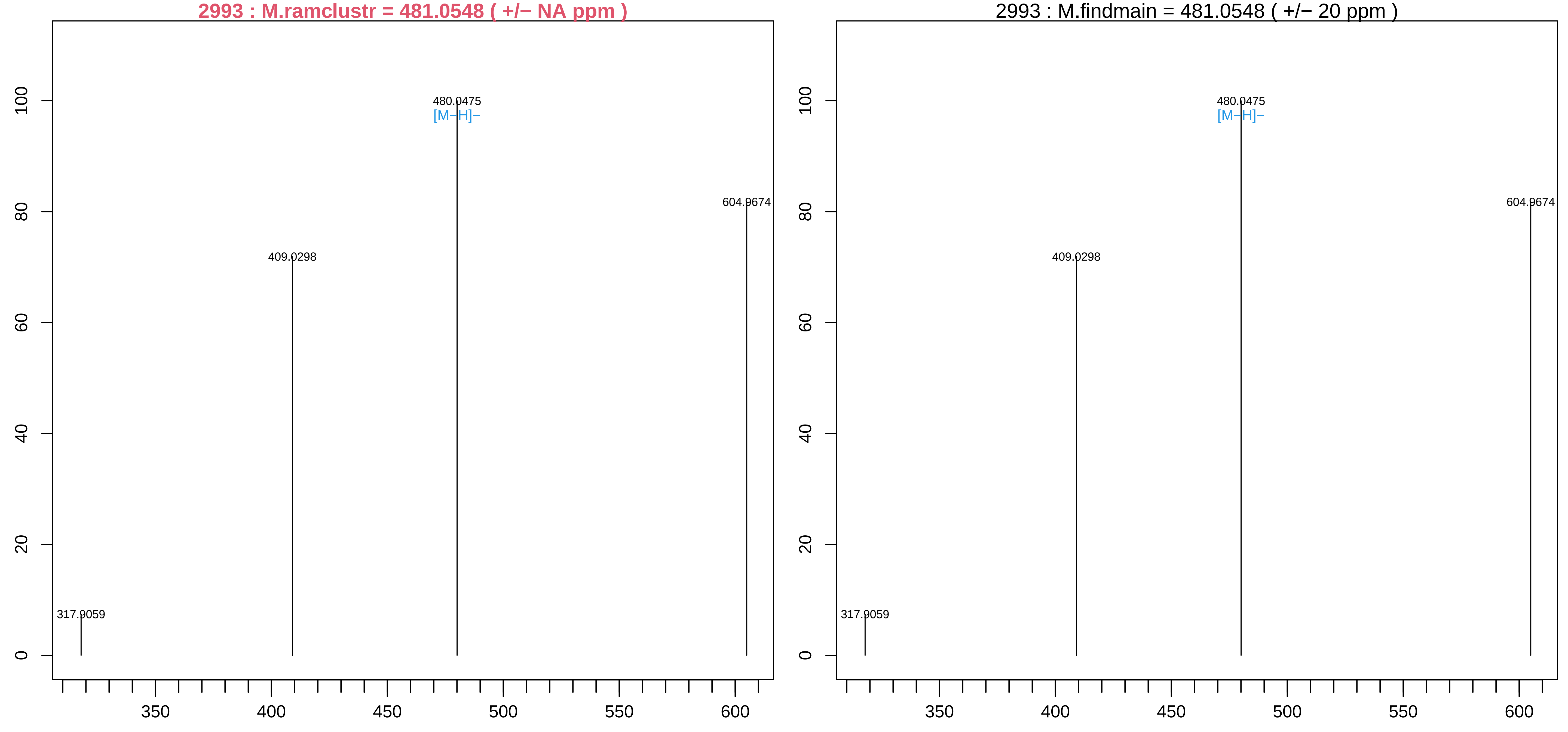
2993 : M.ramclustr = 481.0548 (+/− NA ppm)
2993 : M.findmain = 481.0548 (+/− 20 ppm)
480.0475
[M−H]−
604.9674
409.0298
317.9059
0
20
40
60
80
100
350
400
450
500
550
600

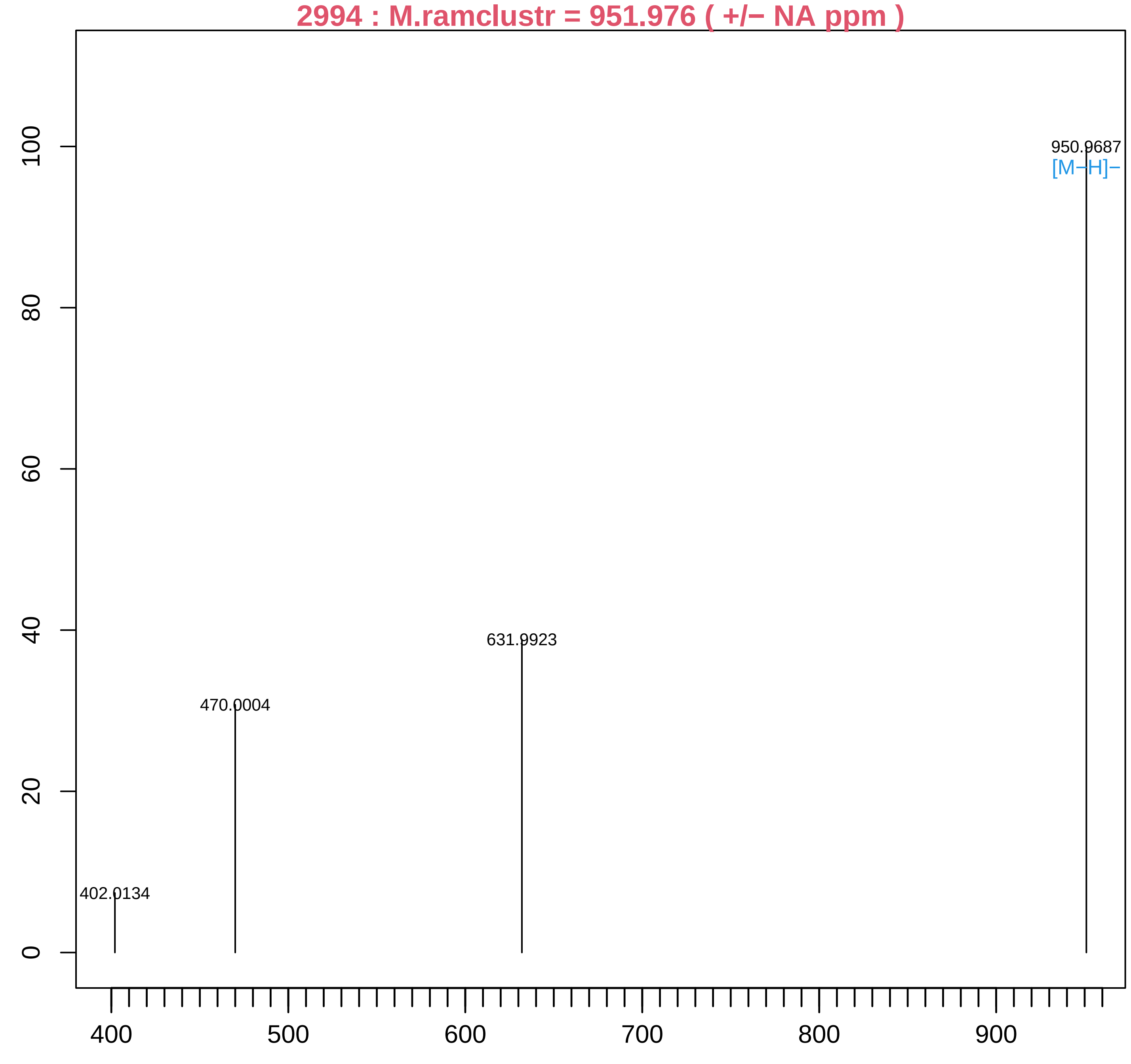
2994 : M.ramclustr = 951.976 (+/− NA ppm)
950.9687
[M−H]−
631.9923
470.0004
402.0134
0
20
40
60
80
100
400
500
600
700
800
900

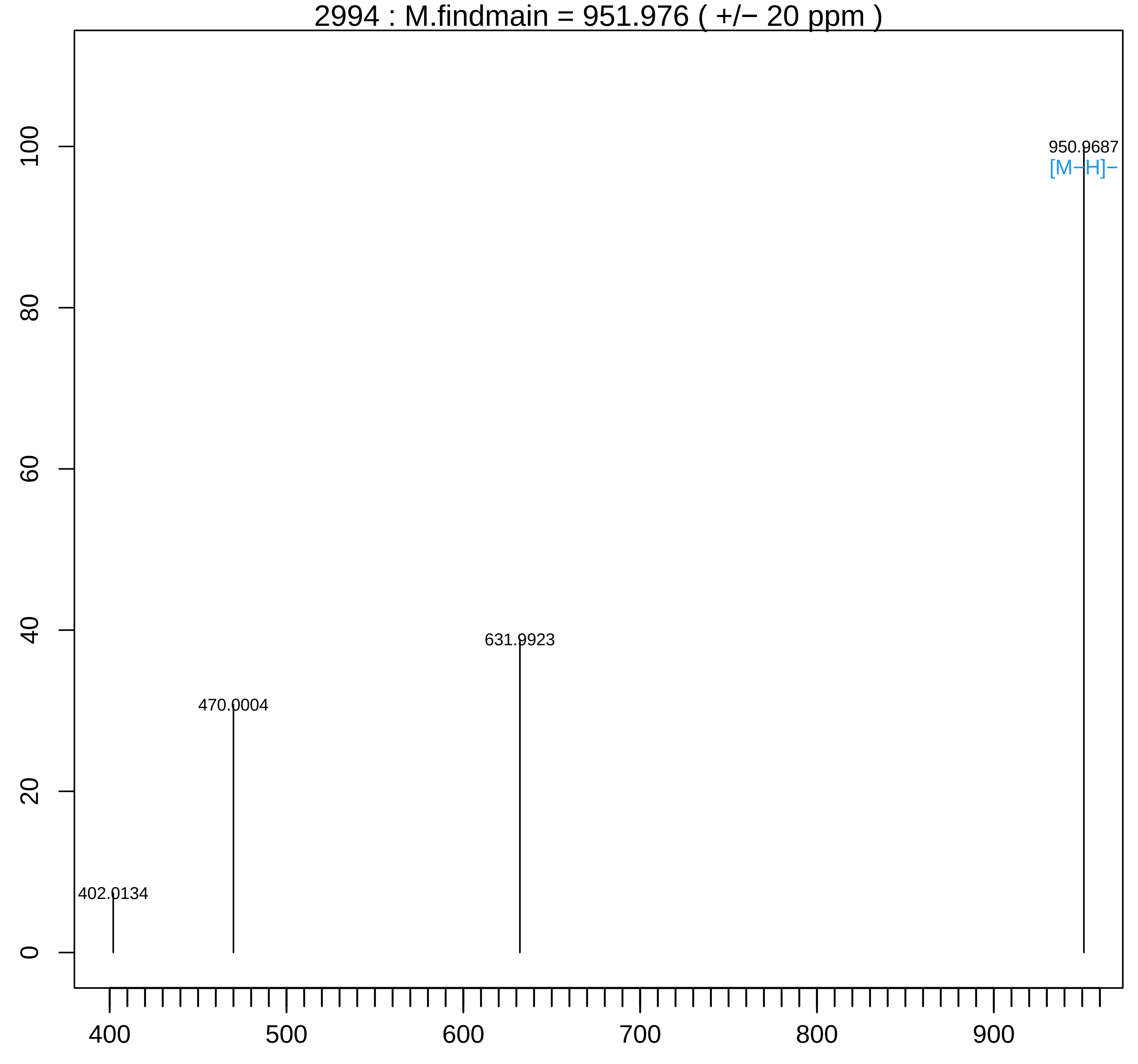
2994 : M.findmain = 951.976 (+/− 20 ppm)
950.9687
[M−H]−
631.9923
470.0004
402.0134
0
20
40
60
80
100
400
500
600
700
800
900

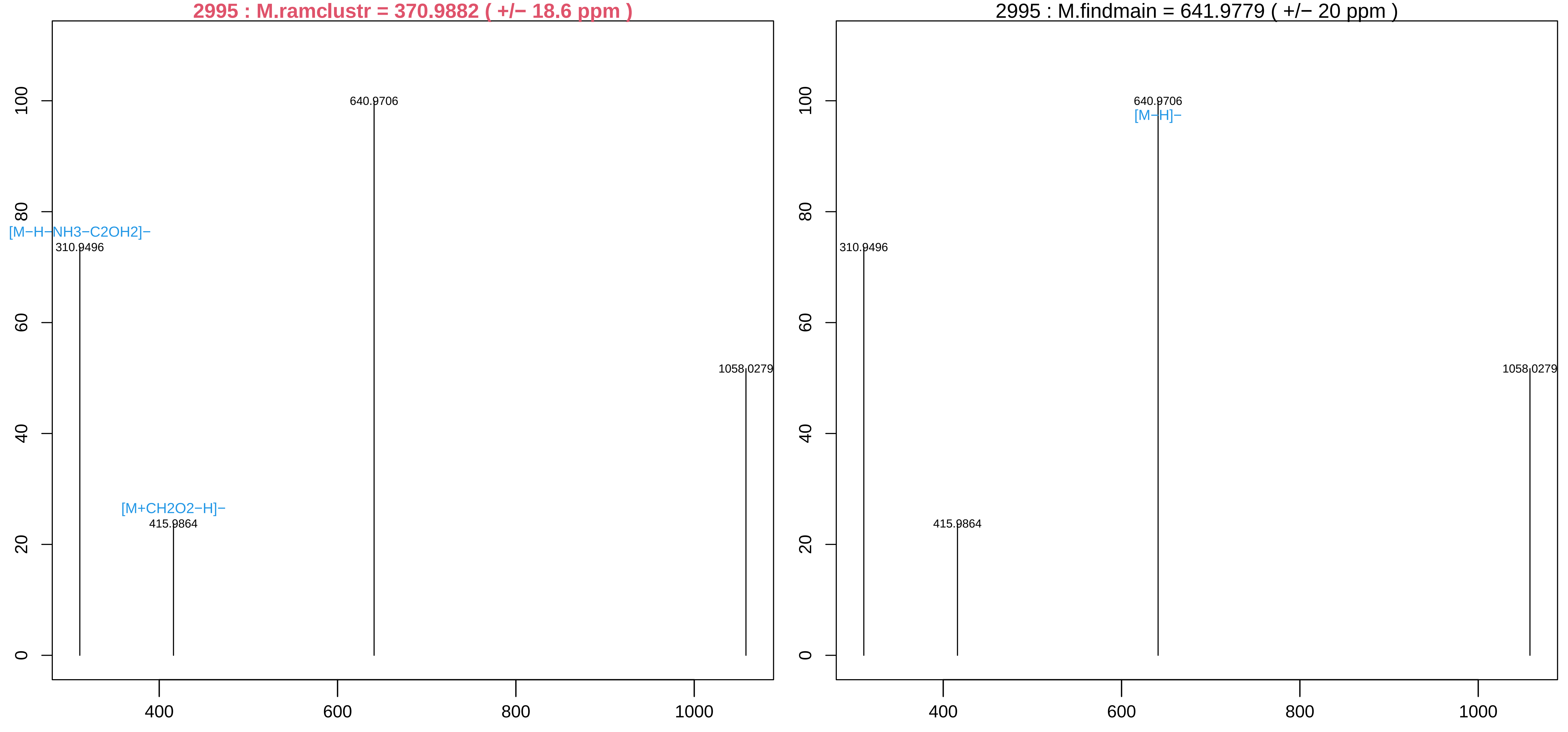
2995 : M.ramclustr = 370.9882 (+/− 18.6 ppm)
[M−H−NH3−C2OH2]−
310.9496
[M+CH2O2−H]−
415.9864
640.9706
1058.0279
0
20
40
60
80
100
400
600
800
1000
2995 : M.findmain = 641.9779 (+/− 20 ppm)
310.9496
415.9864
640.9706
[M−H]−
1058.0279

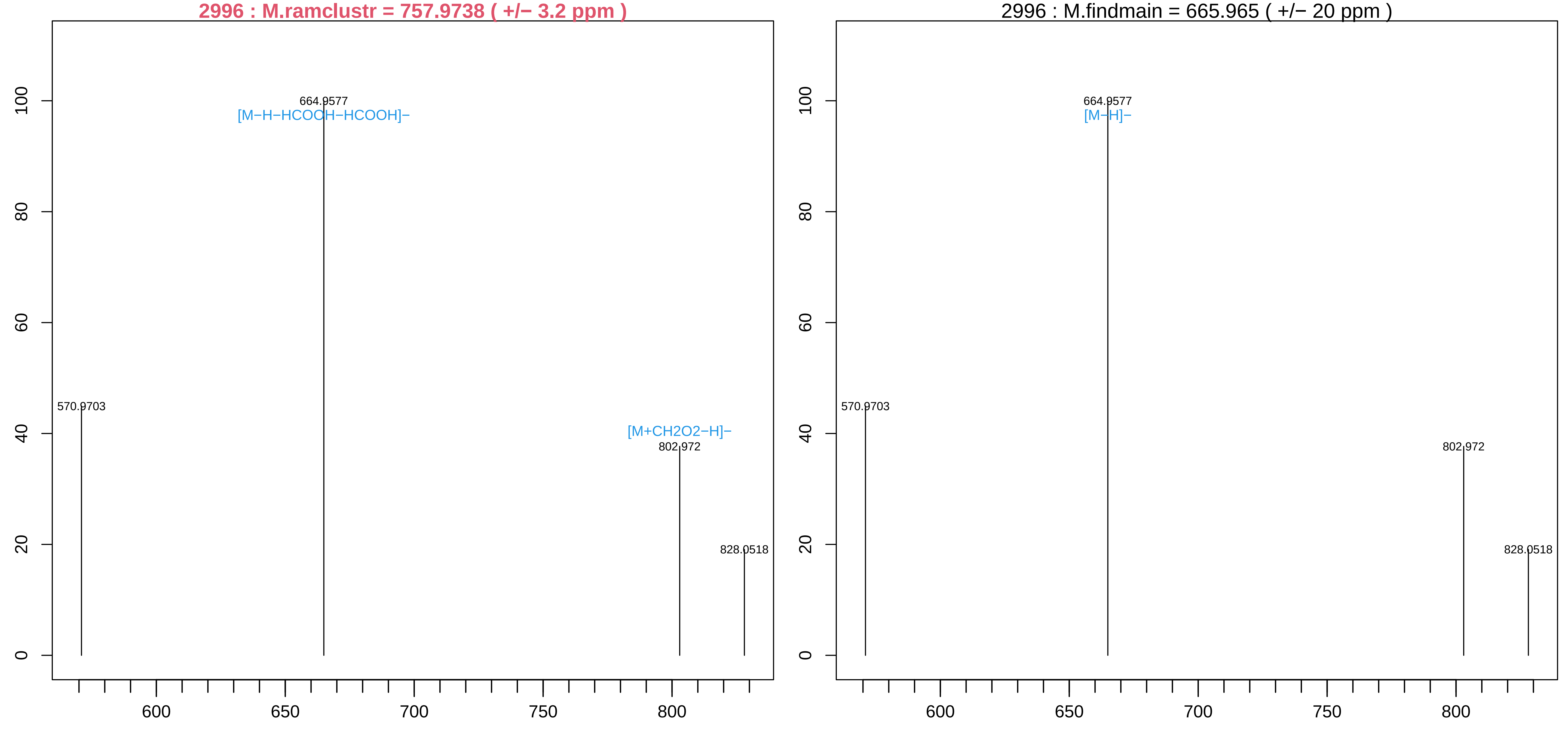

2996 : M.ramclustr = 757.9738 (+/− 3.2 ppm)
664.9577
[M−H−HCOOH−HCOOH]−
570.9703
[M+CH2O2−H]−
802.972
828.0518
0
20
40
60
80
100
600
650
700
750
800
2996 : M.findmain = 665.965 (+/− 20 ppm)
664.9577
[M−H]−
570.9703
802.972
828.0518

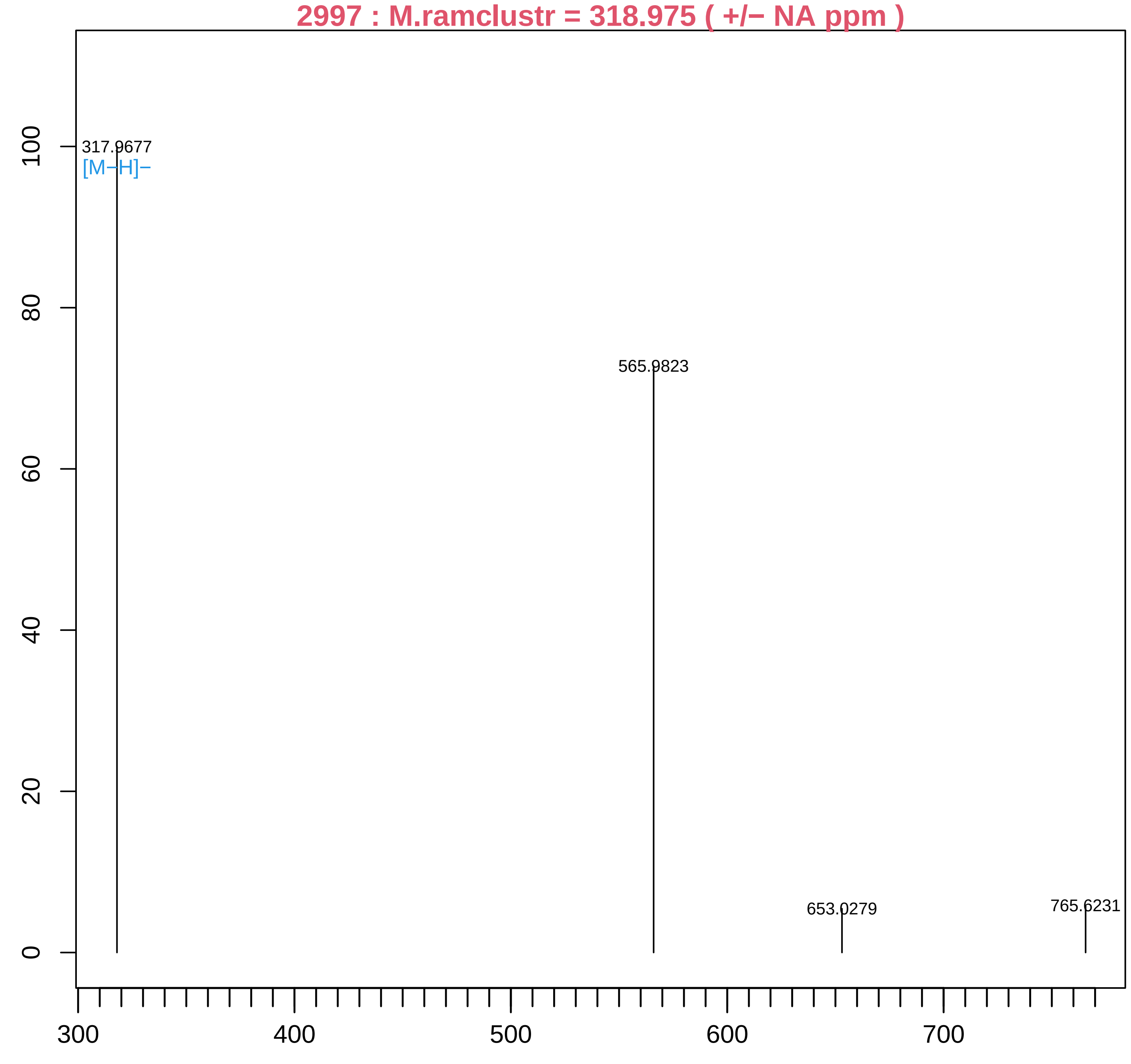
2997 : M.ramclustr = 318.975 (+/− NA ppm)
317.9677
[M−H]−
565.9823
653.0279
765.6231
0
20
40
60
80
100
300
400
500
600
700

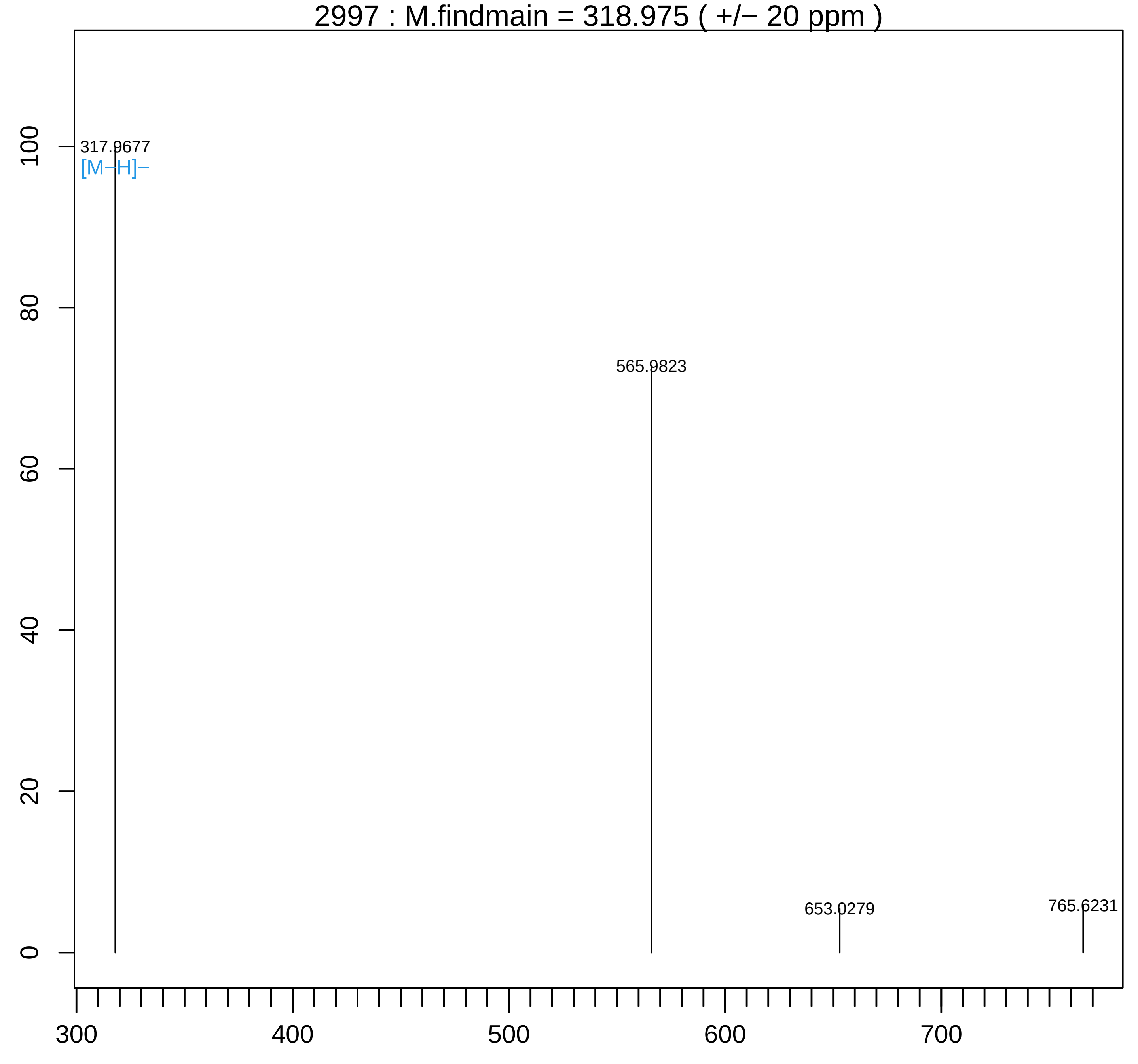
2997 : M.findmain = 318.975 (+/− 20 ppm)
317.9677
[M−H]−
565.9823
653.0279
765.6231
0
20
40
60
80
100
300
400
500
600
700

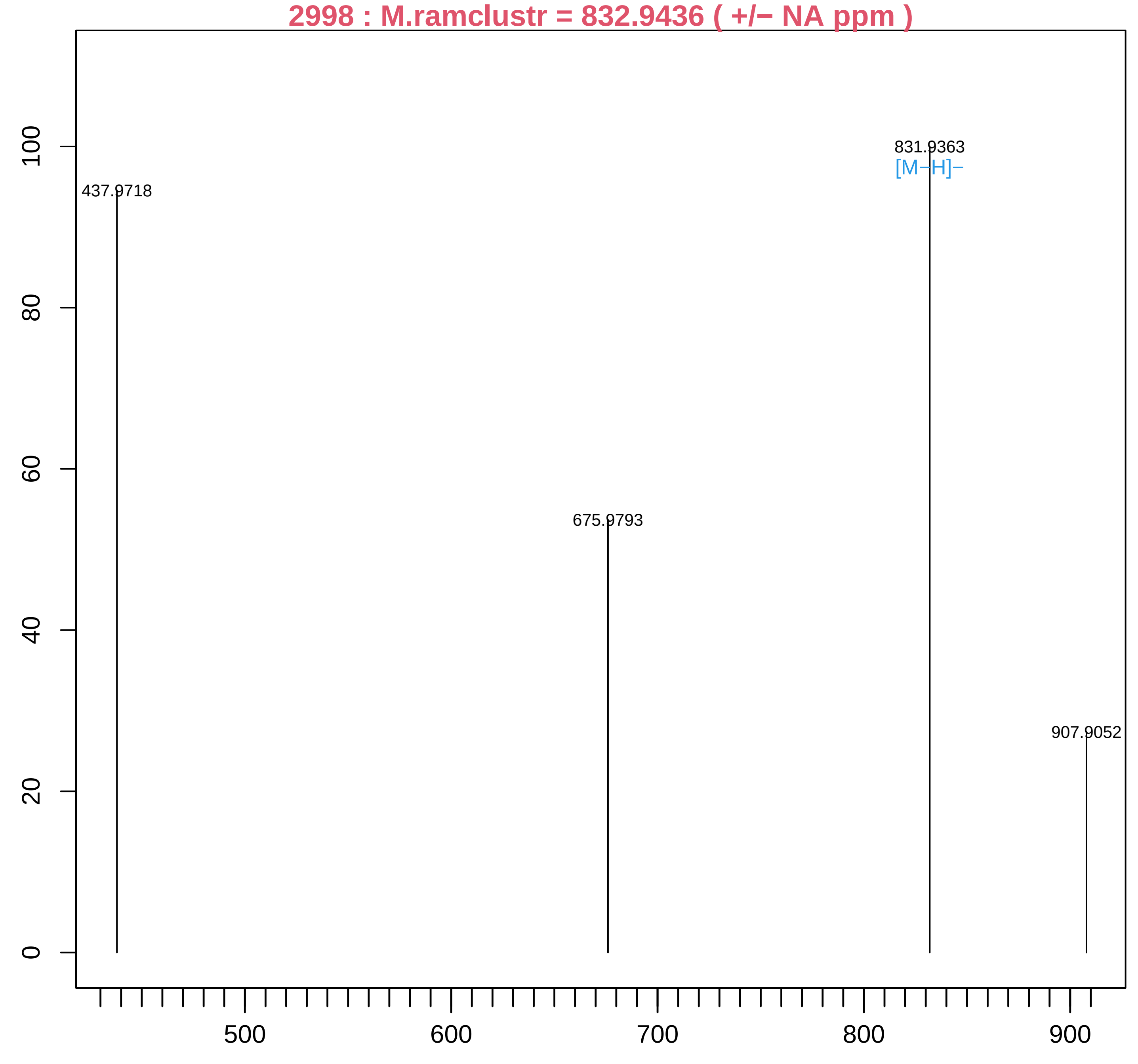
2998 : M.ramclustr = 832.9436 (+/− NA ppm)
437.9718
675.9793
831.9363
[M−H]−
907.9052
0
20
40
60
80
100
500
600
700
800
900

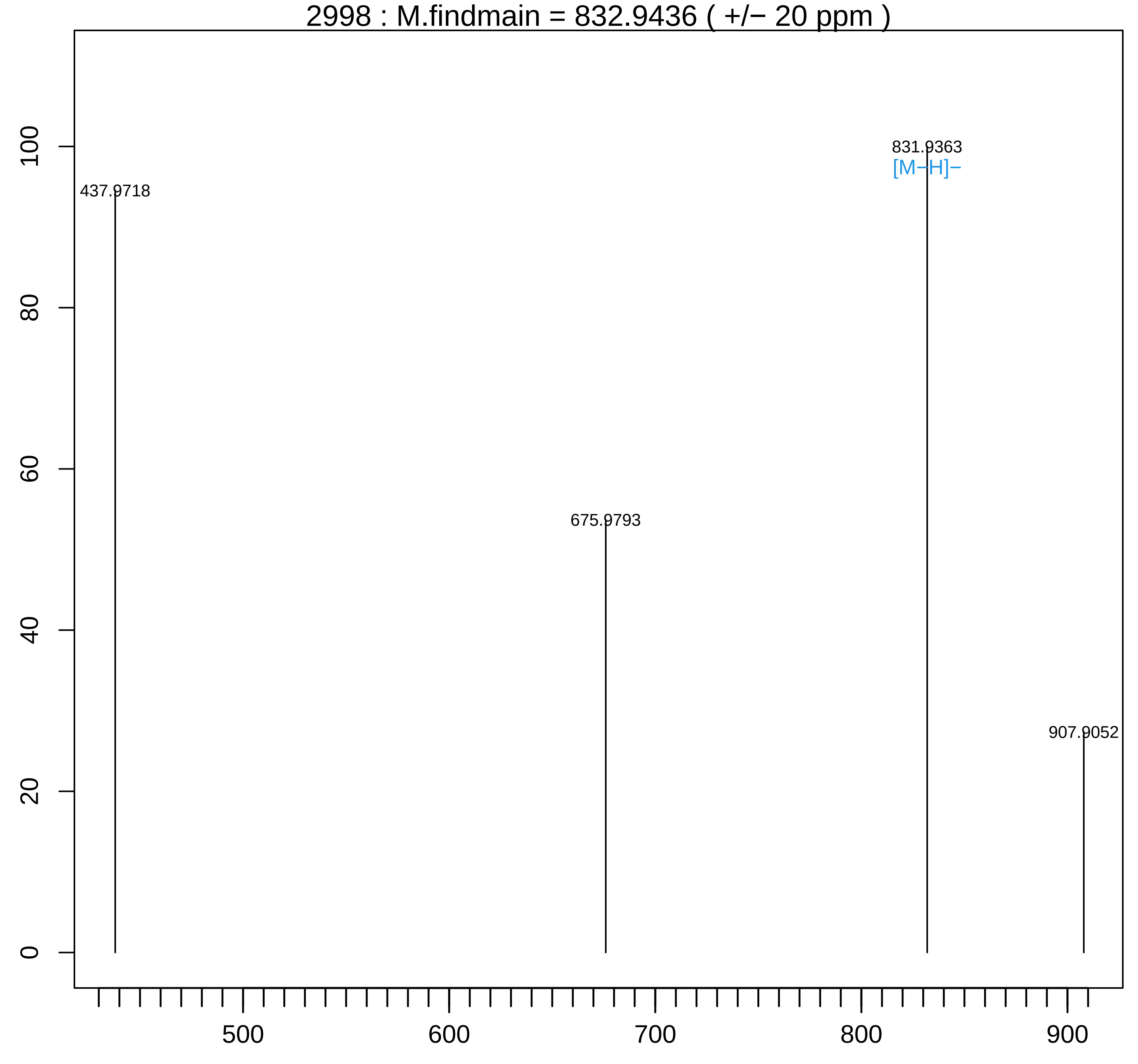
2998 : M.findmain = 832.9436 (+/− 20 ppm)
437.9718
675.9793
831.9363
[M−H]−
907.9052
0
20
40
60
80
100
500
600
700
800
900

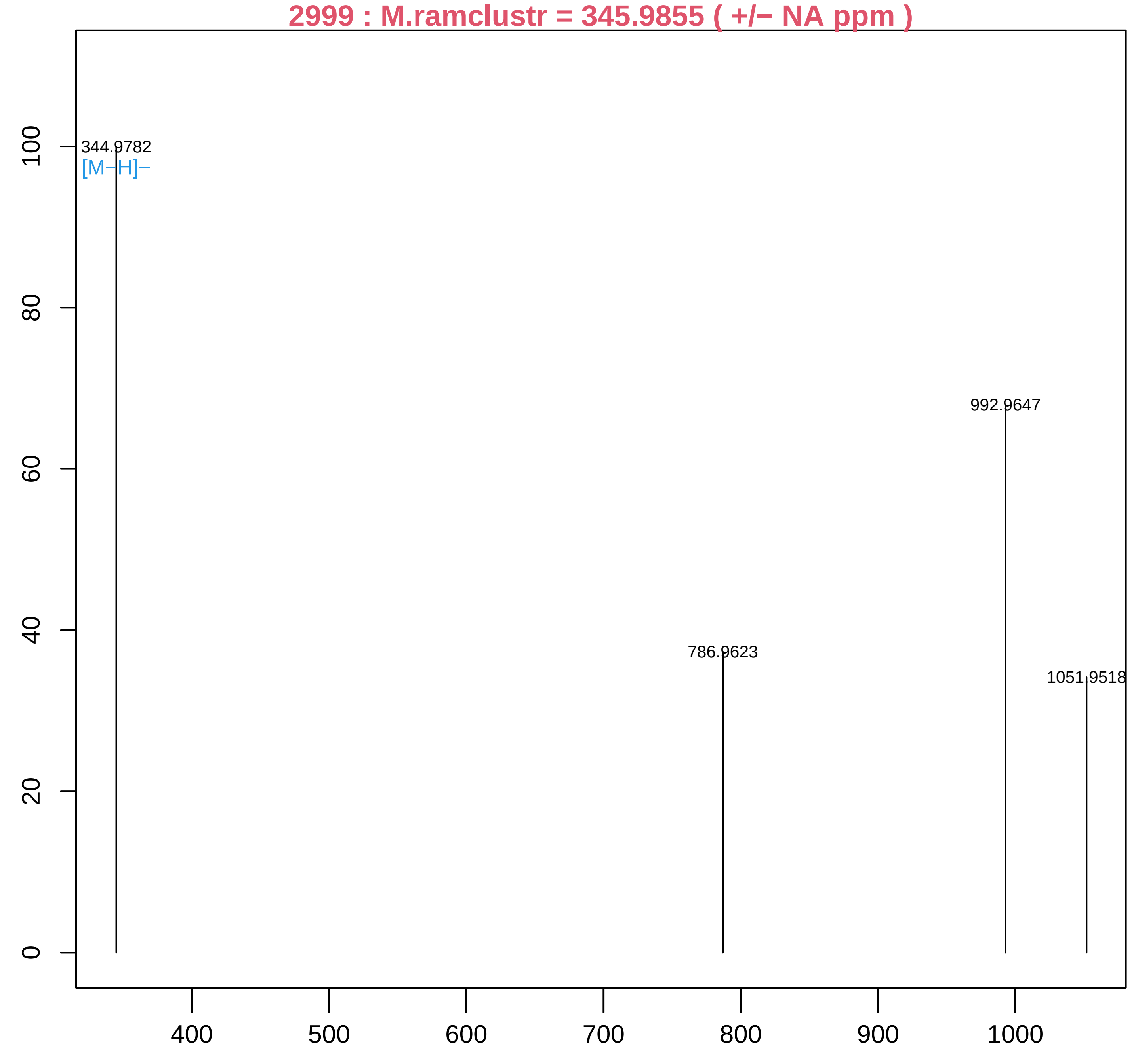
2999 : M.ramclustr = 345.9855 (+/− NA ppm)
344.9782
[M−H]−
992.9647
786.9623
1051.9518
0
20
40
60
80
100
400
500
600
700
800
900
1000

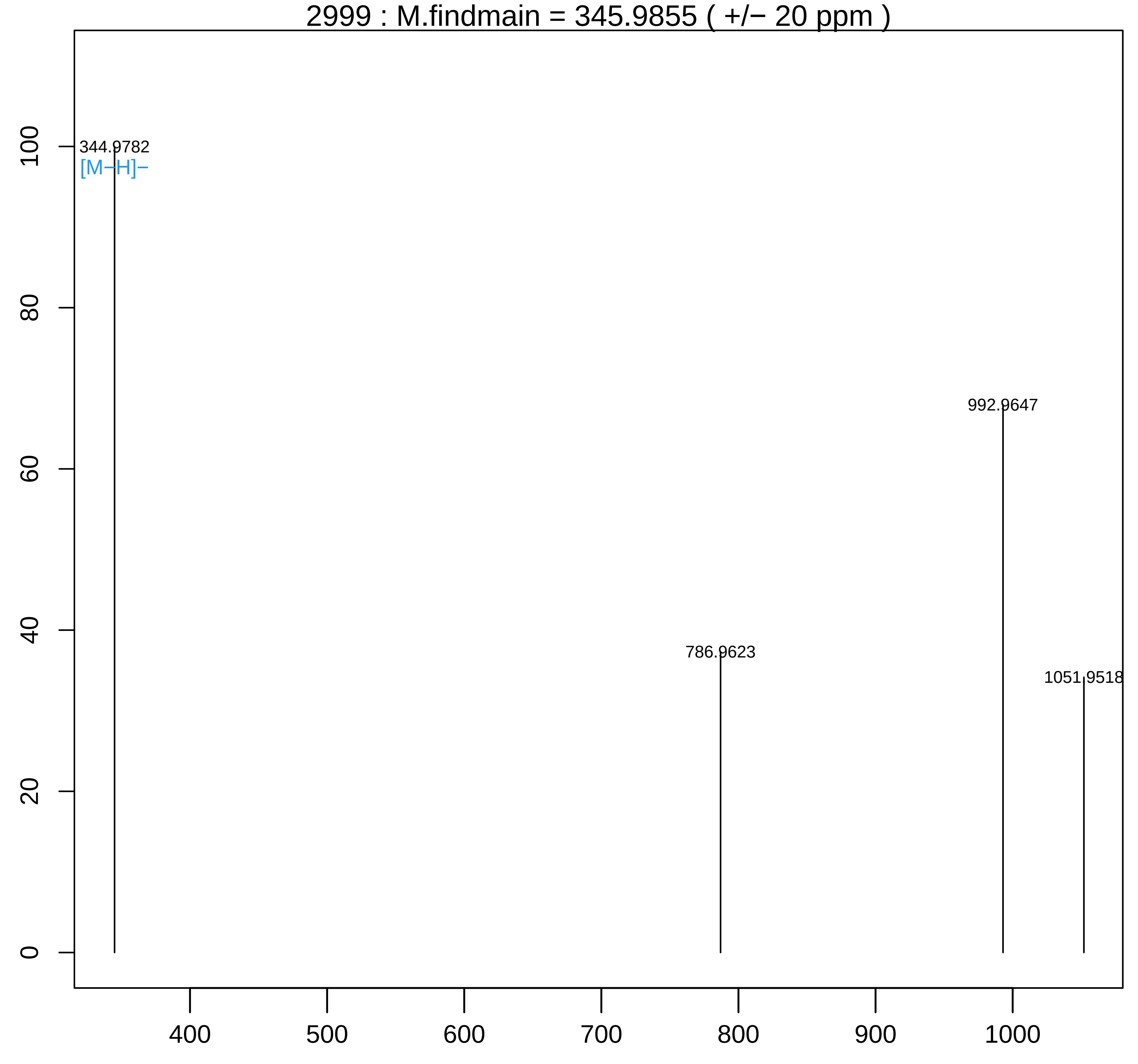
2999 : M.findmain = 345.9855 (+/− 20 ppm)
344.9782
[M−H]−
992.9647
786.9623
1051.9518
0
20
40
60
80
100
400
500
600
700
800
900
1000

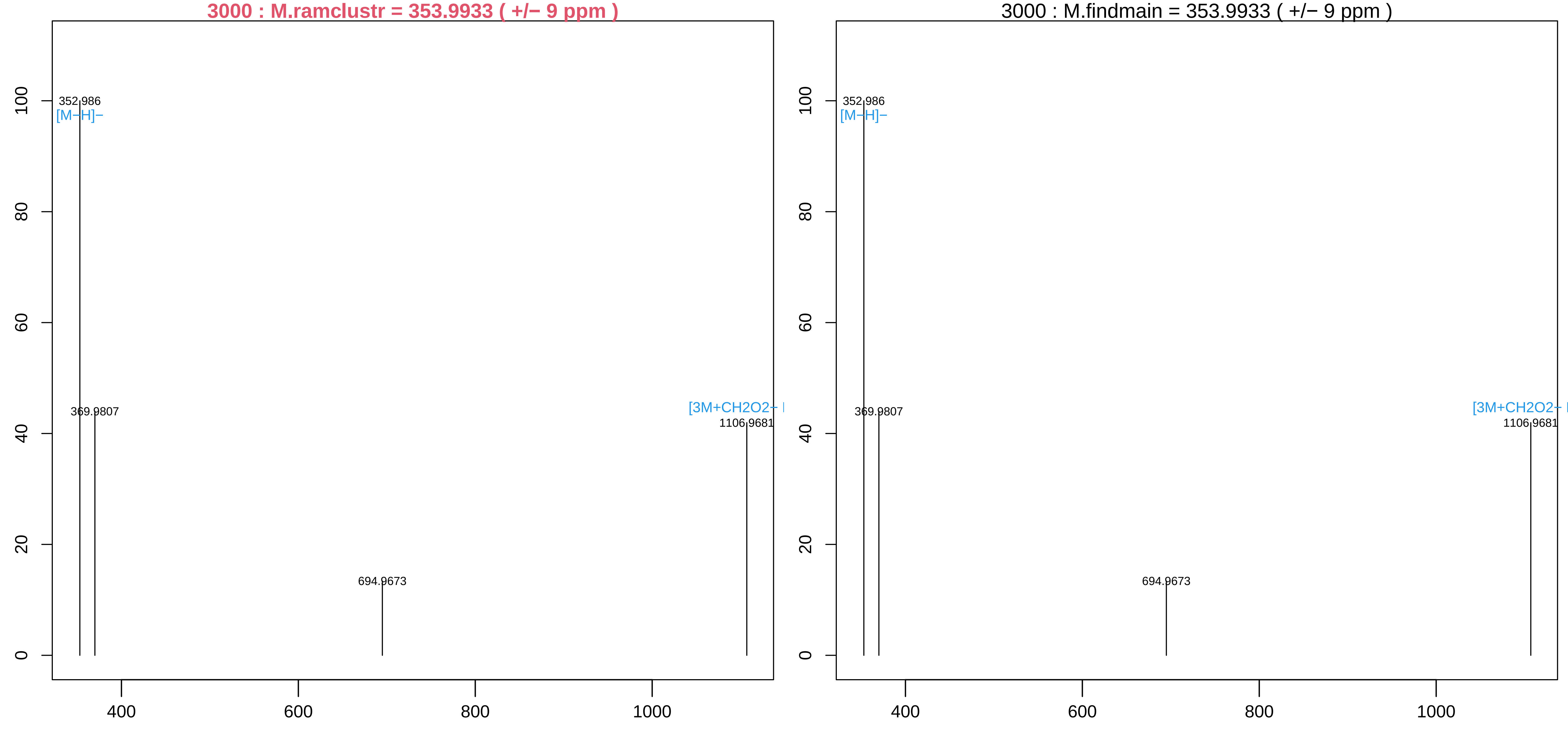
3000 : M.ramclustr = 353.9933 (+/− 9 ppm)
352.986
[M−H]−
369.9807
694.9673
[3M+CH2O2−
1106.9681
0
20
40
60
80
100
400
600
800
1000
3000 : M.findmain = 353.9933 (+/− 9 ppm)
352.986
[M−H]−
369.9807
694.9673
[3M+CH2O2−
1106.9681
0
20
40
60
80
100
400
600
800
1000

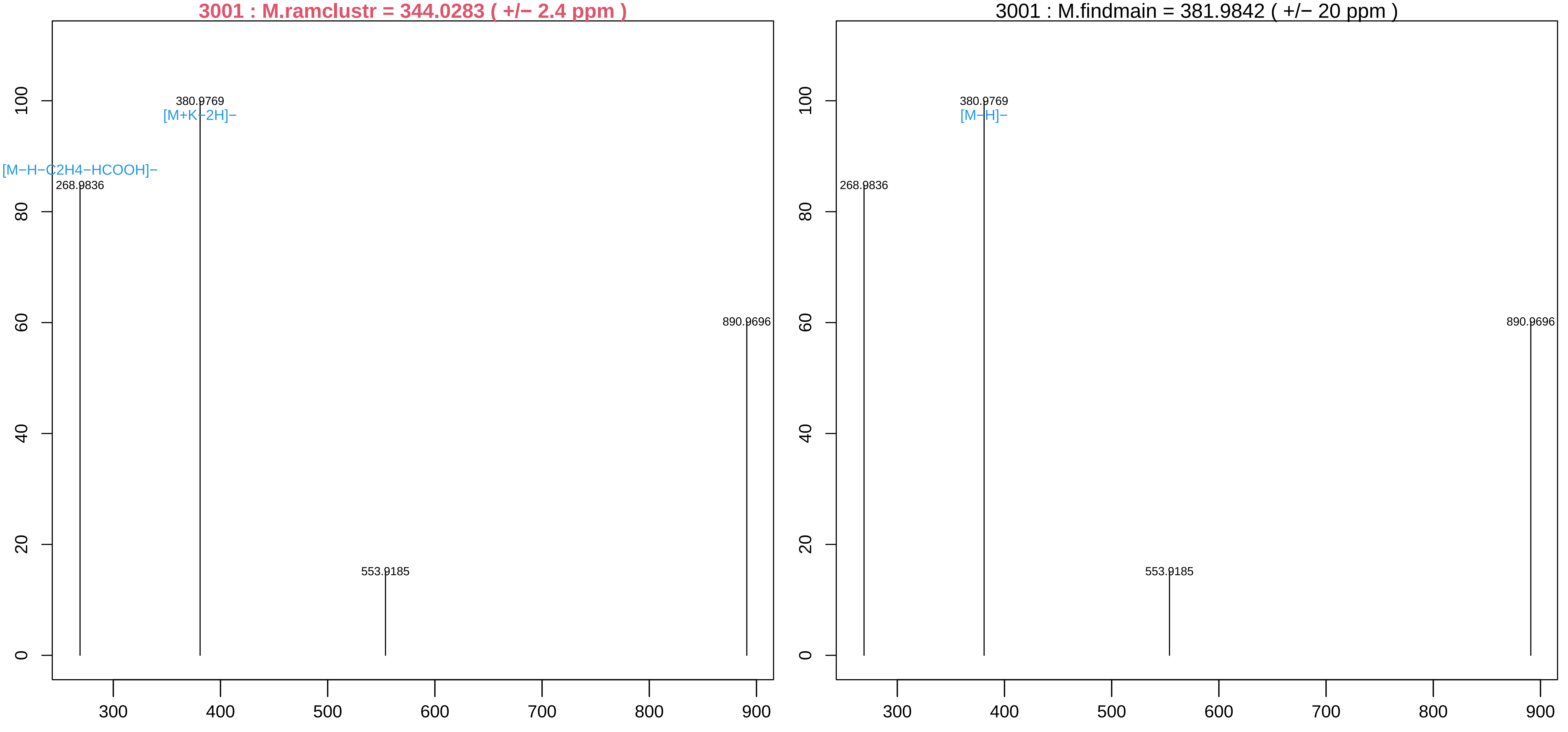
3001 : M.ramclustr = 344.0283 (+/− 2.4 ppm)
380.9769
[M+K−2H]−
[M−H−C2H4−HCOOH]−
268.9836
890.9696
553.9185
0
20
40
60
80
100
300
400
500
600
700
800
900
3001 : M.findmain = 381.9842 (+/− 20 ppm)
380.9769
[M−H]−
268.9836
890.9696
553.9185
0
20
40
60
80
100
300
400
500
600
700
800
900

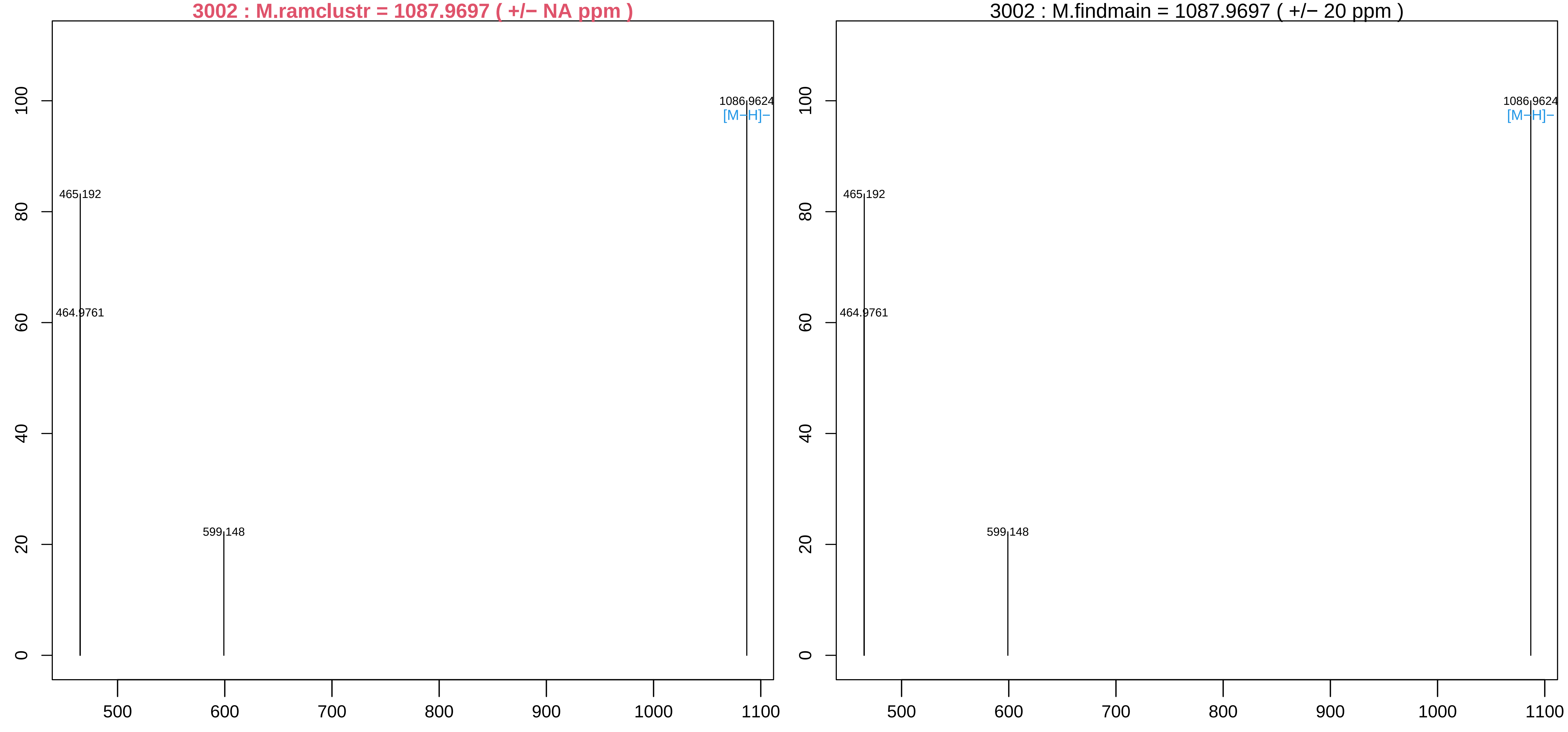

3002 : M.ramclustr = 1087.9697 (+/− NA ppm)
3002 : M.findmain = 1087.9697 (+/− 20 ppm)
1086.9624
[M−H]−
465.192
464.9761
599.148
0
20
40
60
80
100
500
600
700
800
900
1000
1100

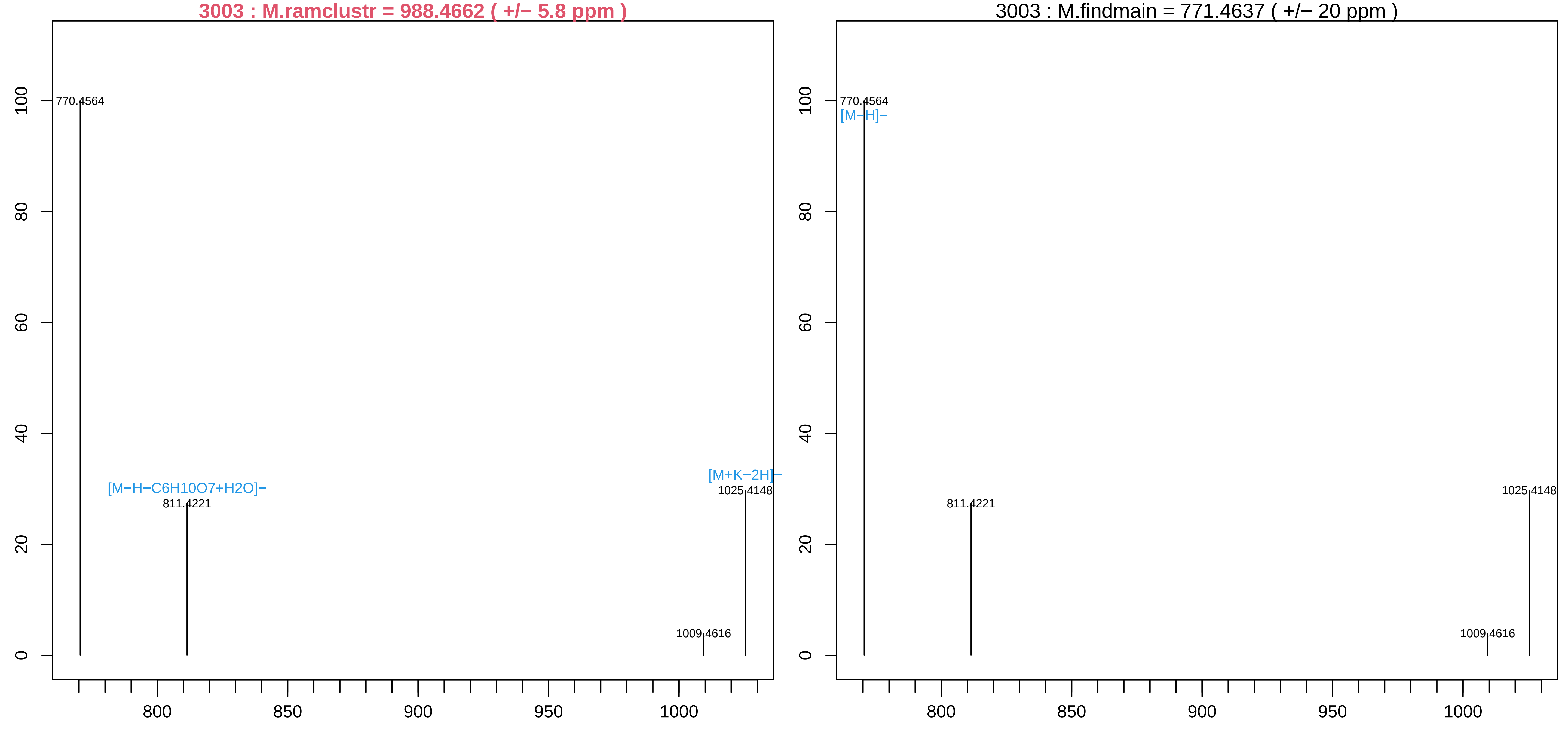

3003 : M.ramclustr = 988.4662 (+/− 5.8 ppm)
770.4564
[M−H−C6H10O7+H2O]−
811.4221
[M+K−2H]−
1025.4148
1009.4616
3003 : M.findmain = 771.4637 (+/− 20 ppm)
770.4564
[M−H]−
811.4221
1025.4148
1009.4616
0
20
40
60
80
100
800
850
900
950
1000

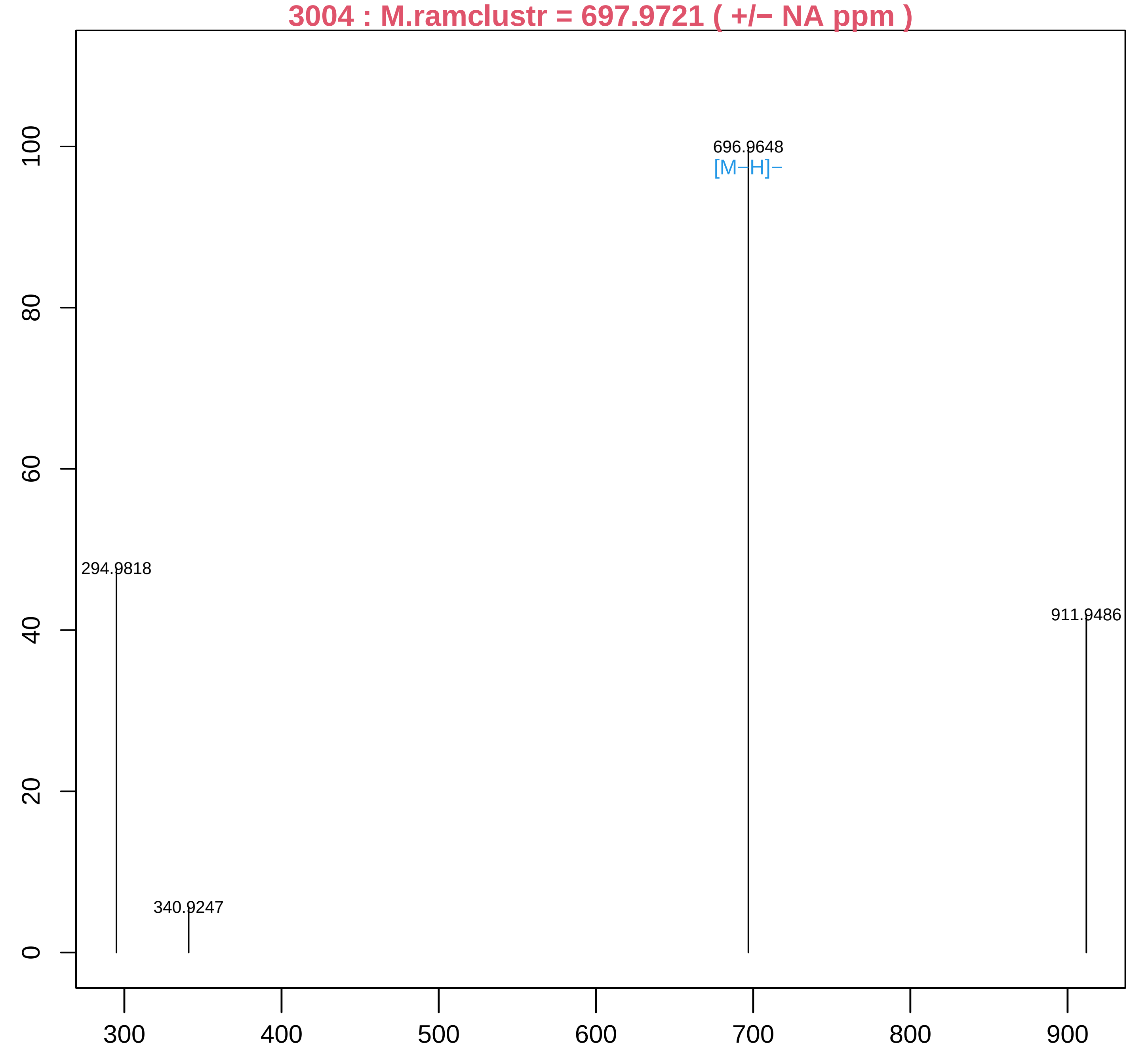
3004 : M.ramclustr = 697.9721 (+/− NA ppm)
696.9648
[M−H]−
294.9818
340.9247
911.9486
0
20
40
60
80
100
300
400
500
600
700
800
900

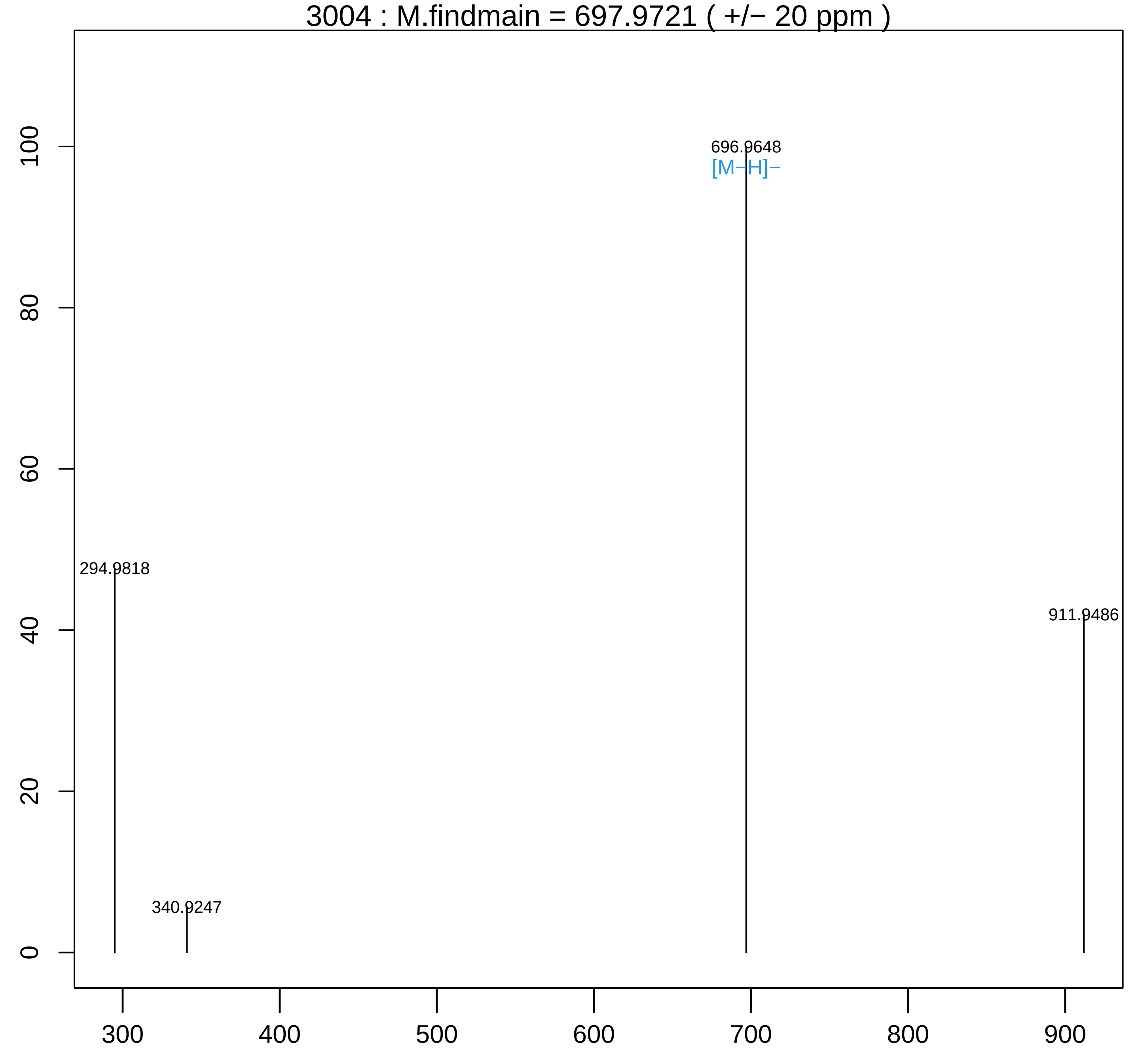
3004 : M.findmain = 697.9721 (+/− 20 ppm)
696.9648
[M−H]−
294.9818
340.9247
911.9486
0
20
40
60
80
100
300
400
500
600
700
800
900

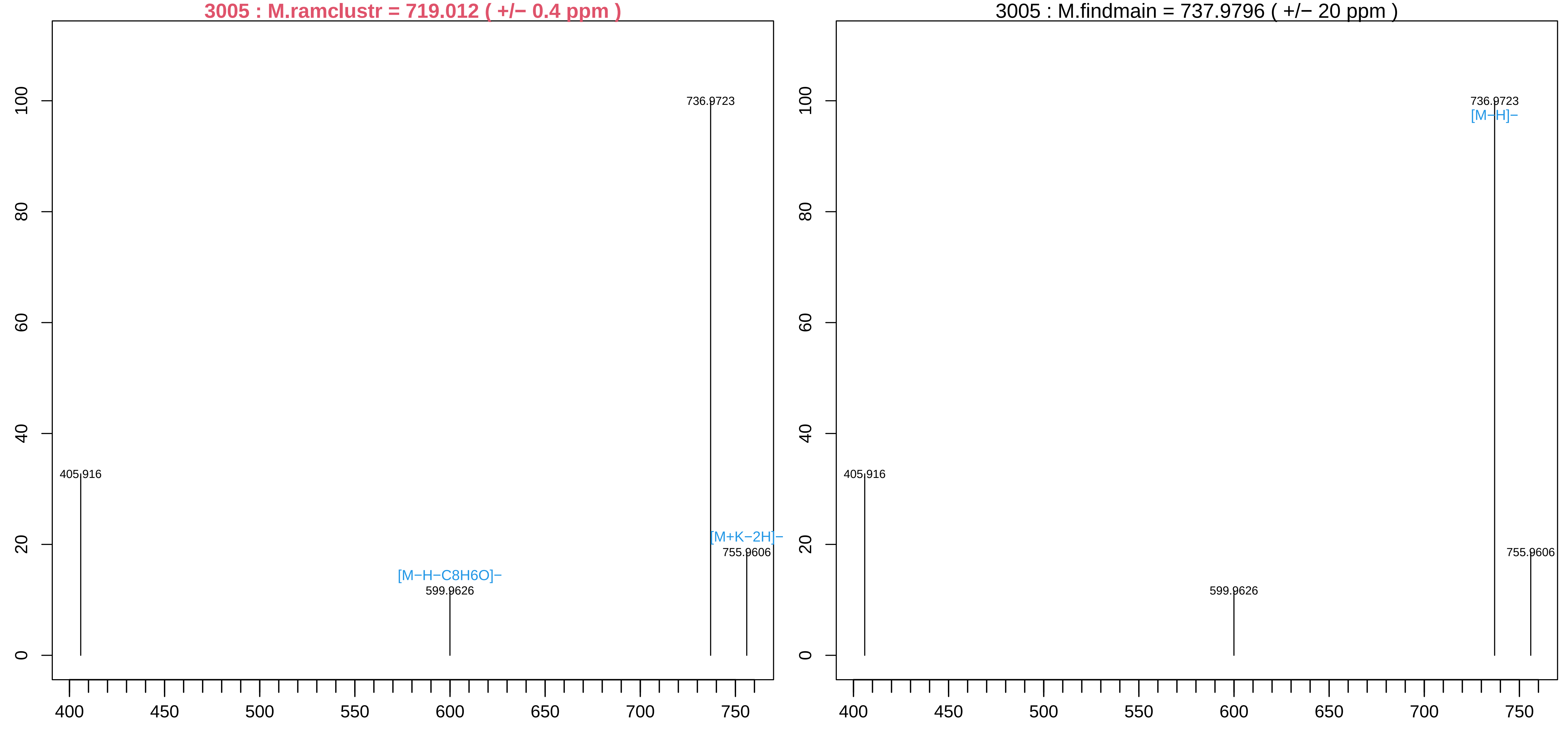
3005 : M.ramclustr = 719.012 (+/− 0.4 ppm)
3005 : M.findmain = 737.9796 (+/− 20 ppm)
405.916
599.9626
[M−H−C8H6O]−
736.9723
755.9606
[M+K−2H]−
405.916
599.9626
736.9723
[M−H]−
755.9606
0
20
40
60
80
100
400
450
500
550
600
650
700
750

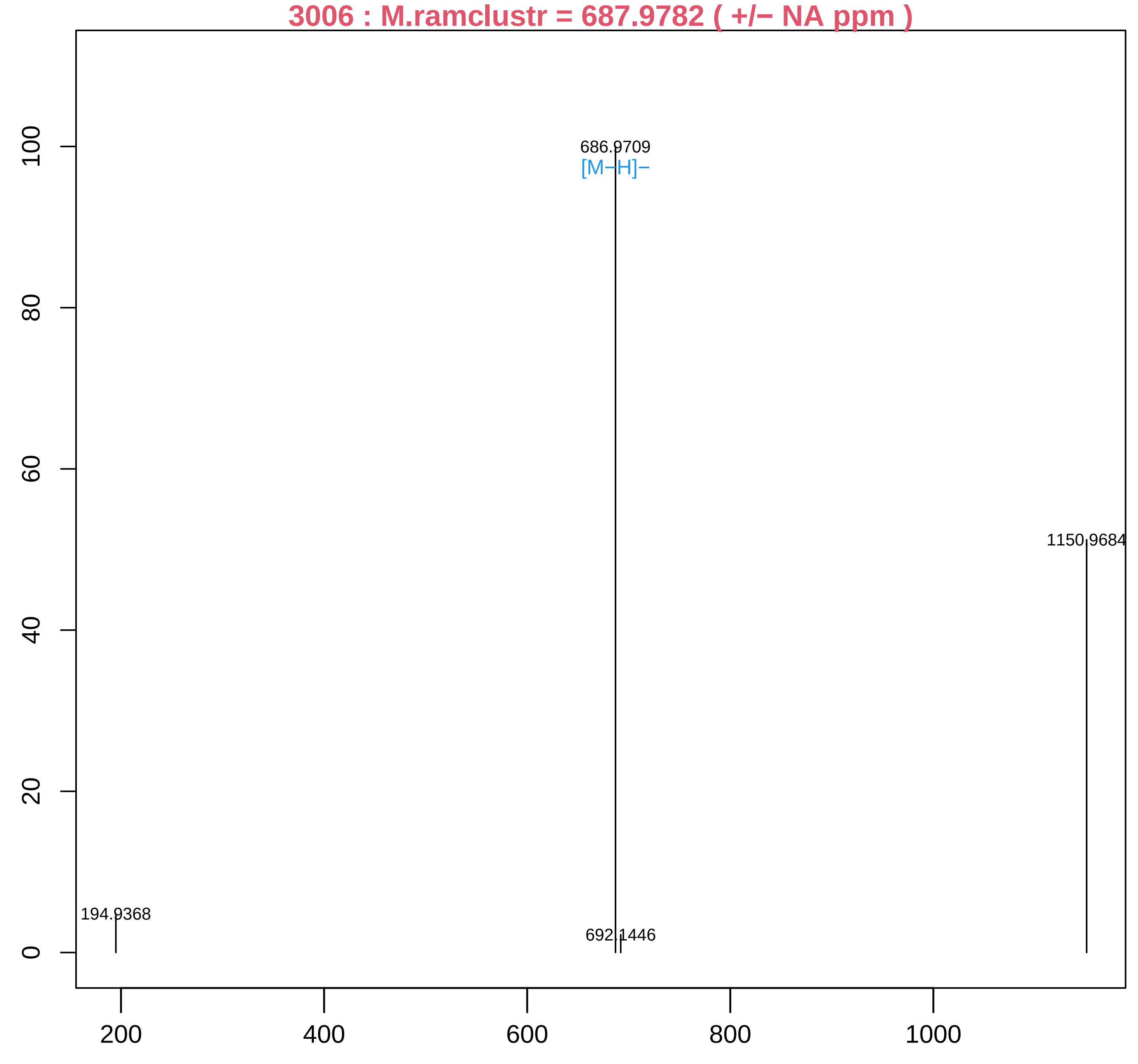
3006 : M.ramclustr = 687.9782 (+/− NA ppm)
686.9709
[M−H]−
1150.9684
194.9368
692.1446
0
20
40
60
80
100
200
400
600
800
1000

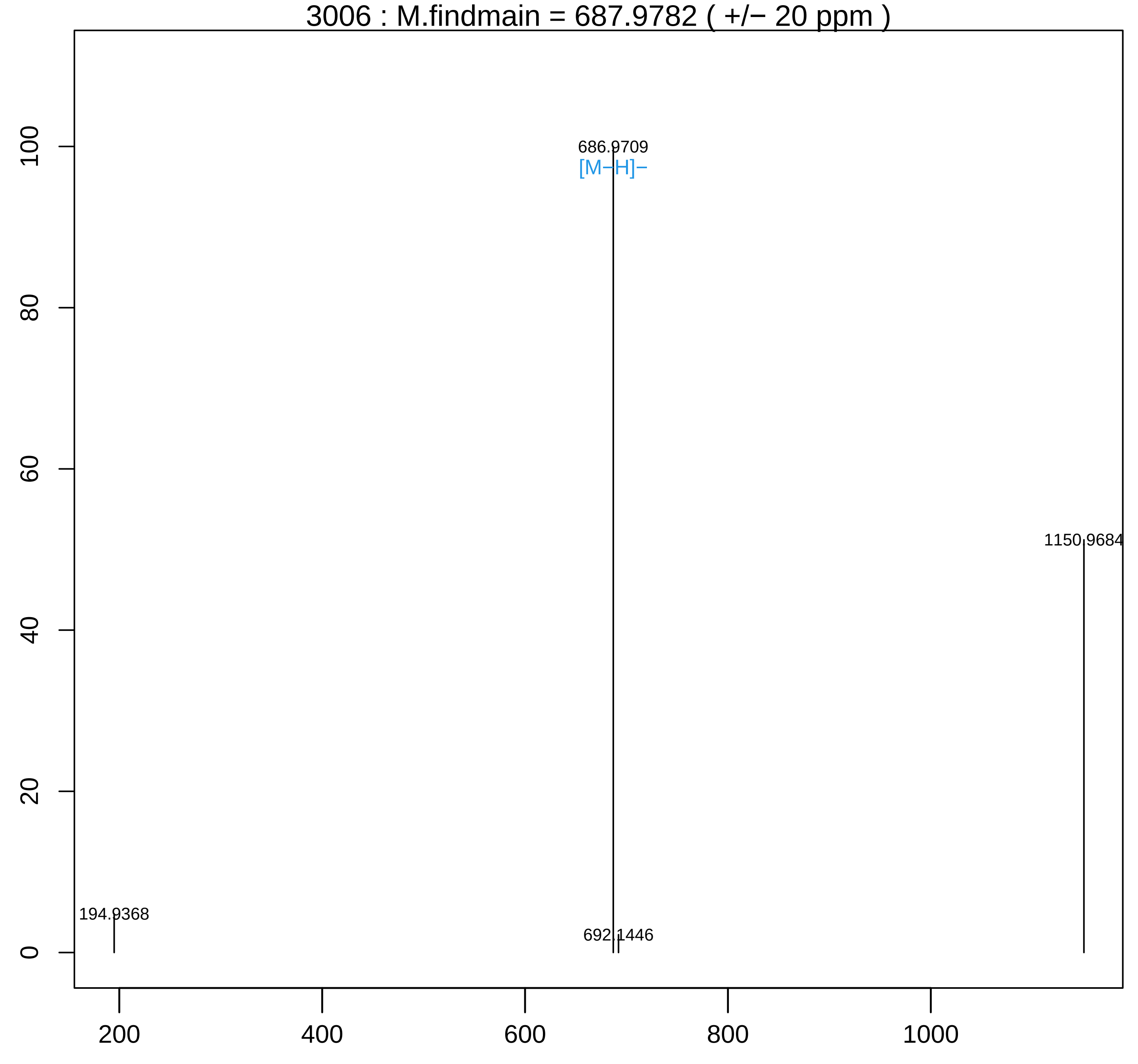
3006 : M.findmain = 687.9782 (+/− 20 ppm)
686.9709
[M−H]−
1150.9684
194.9368
692.1446
0
20
40
60
80
100
200
400
600
800
1000

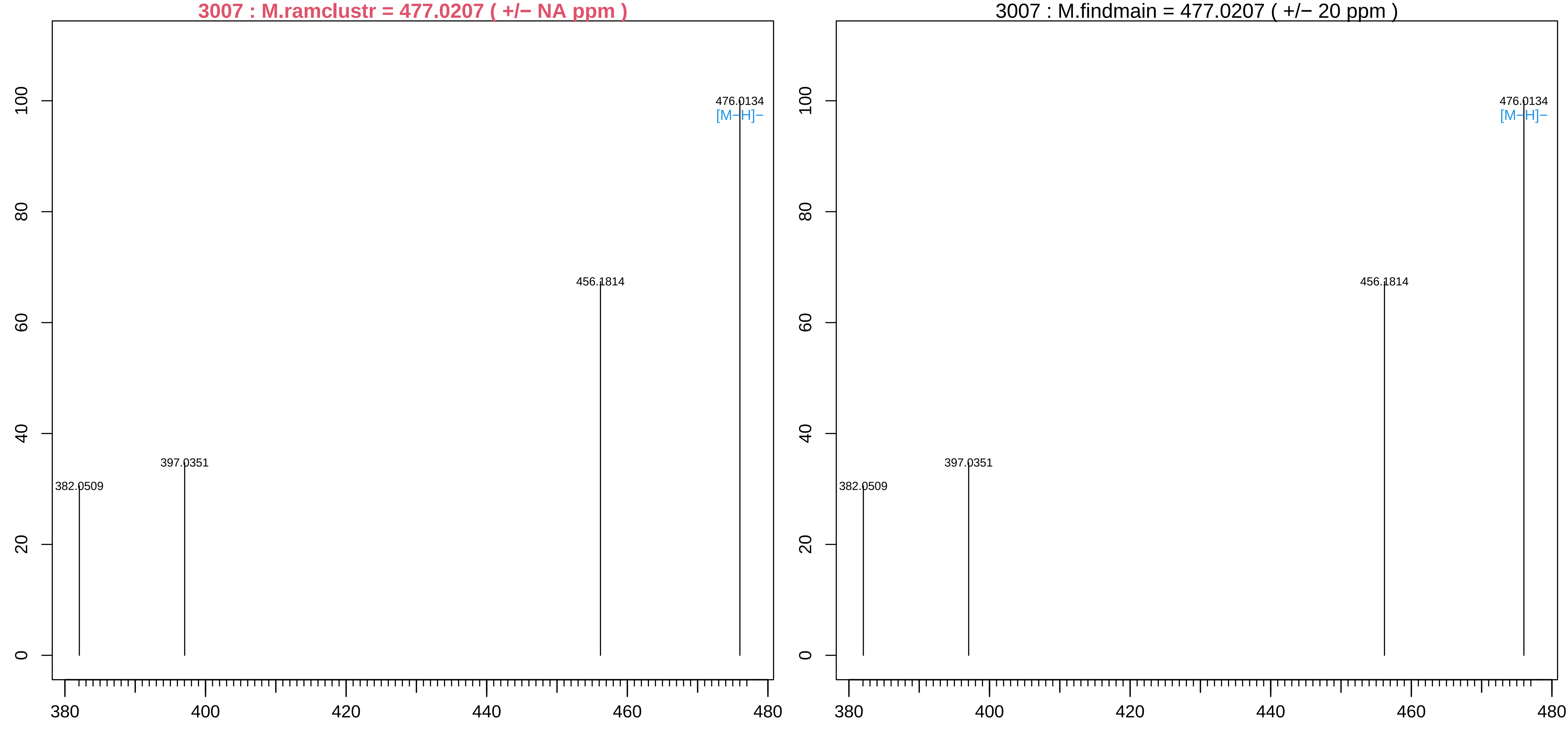

3007 : M.ramclustr = 477.0207 (+/− NA ppm)
476.0134
[M−H]−
456.1814
397.0351
382.0509
0
20
40
60
80
100
380
400
420
440
460
480
3007 : M.findmain = 477.0207 (+/− 20 ppm)
476.0134
[M−H]−
456.1814
397.0351
382.0509
0
20
40
60
80
100
380
400
420
440
460
480

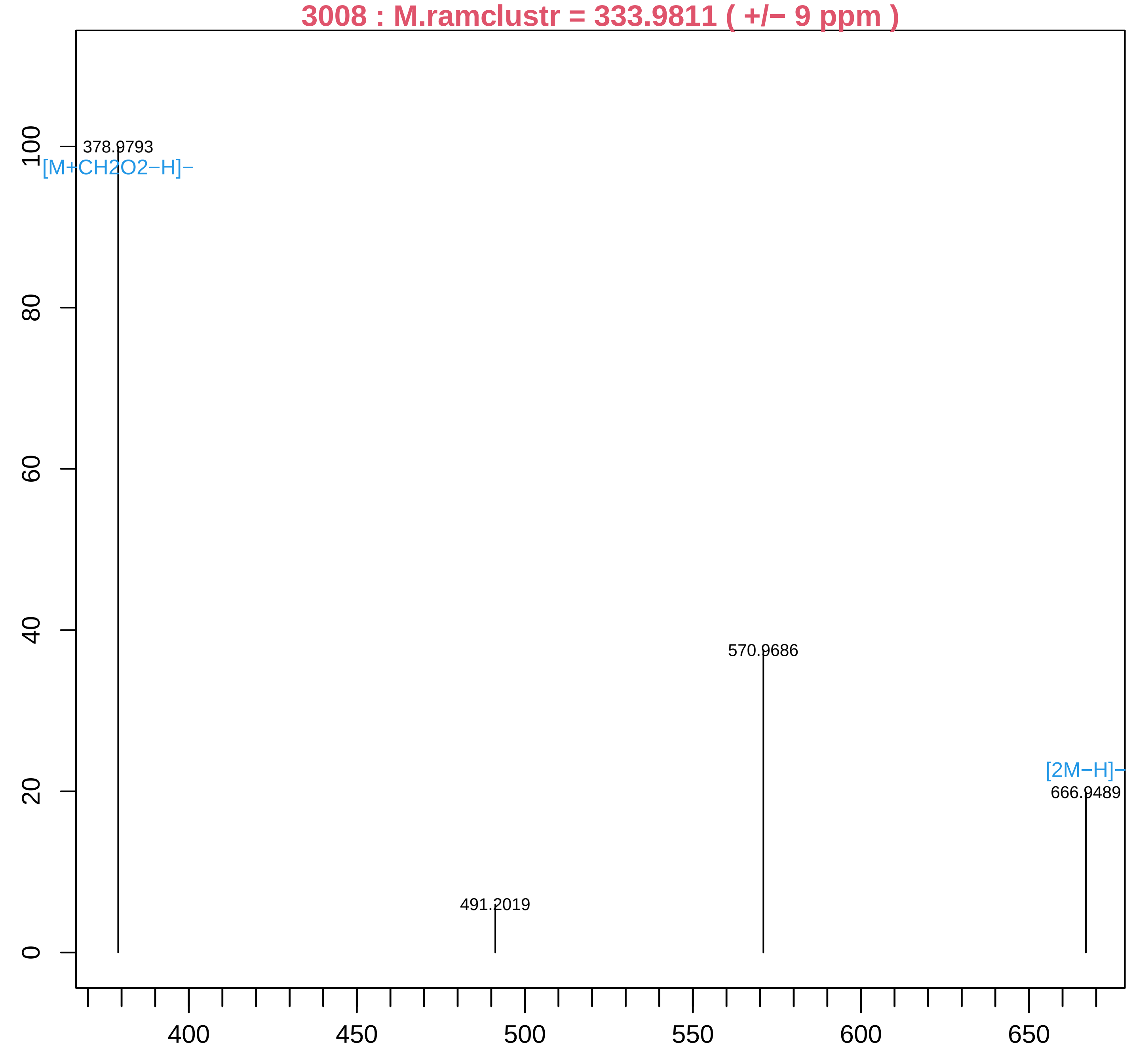
3008 : M.ramclustr = 333.9811 (+/− 9 ppm)
378.9793
[M+CH2O2−H]−
570.9686
[2M−H]−
666.9489
491.2019
0
20
40
60
80
100
400
450
500
550
600
650

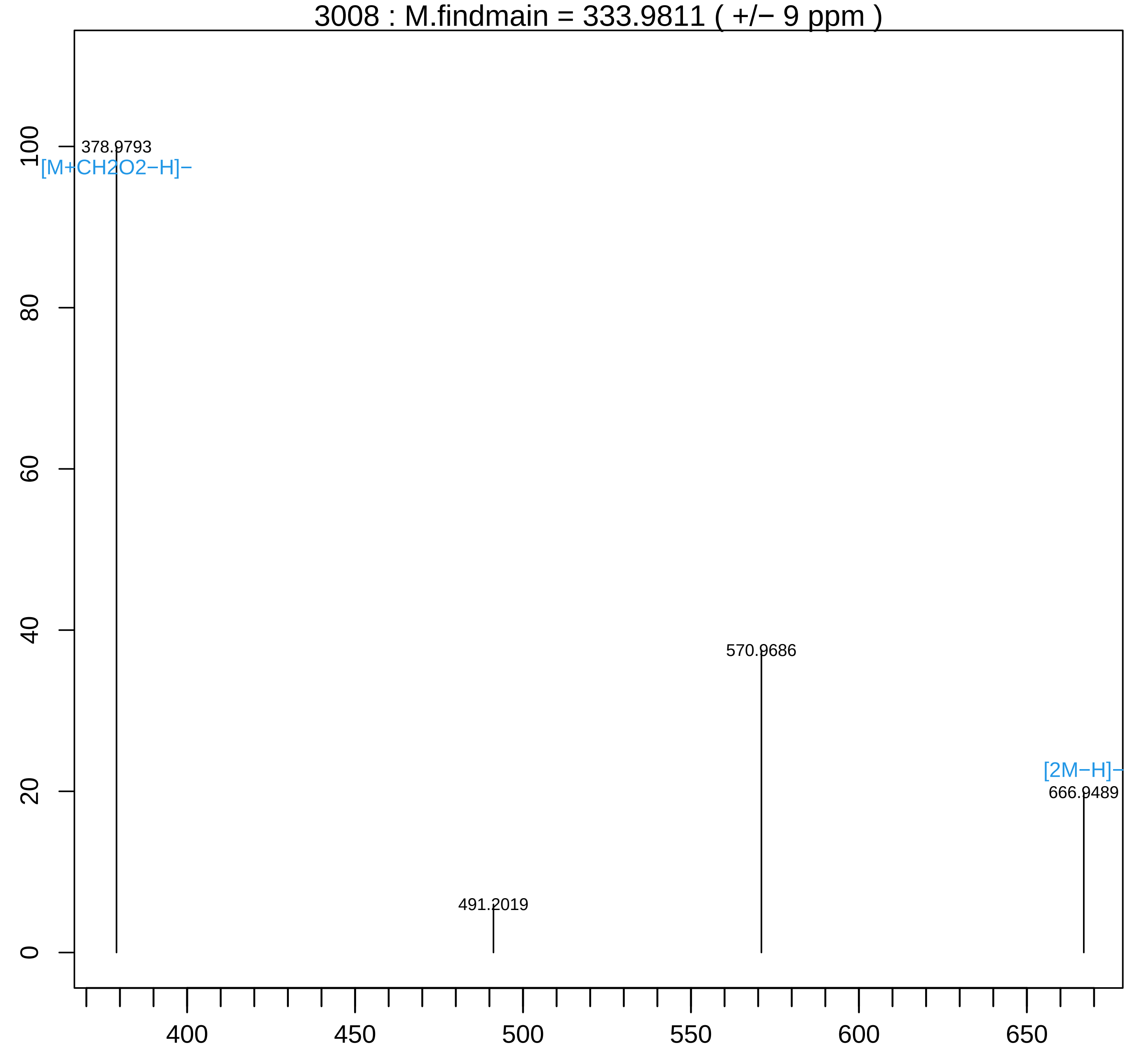
3008 : M.findmain = 333.9811 (+/− 9 ppm)
378.9793
[M+CH2O2−H]−
570.9686
[2M−H]−
666.9489
491.2019
0
20
40
60
80
100
400
450
500
550
600
650

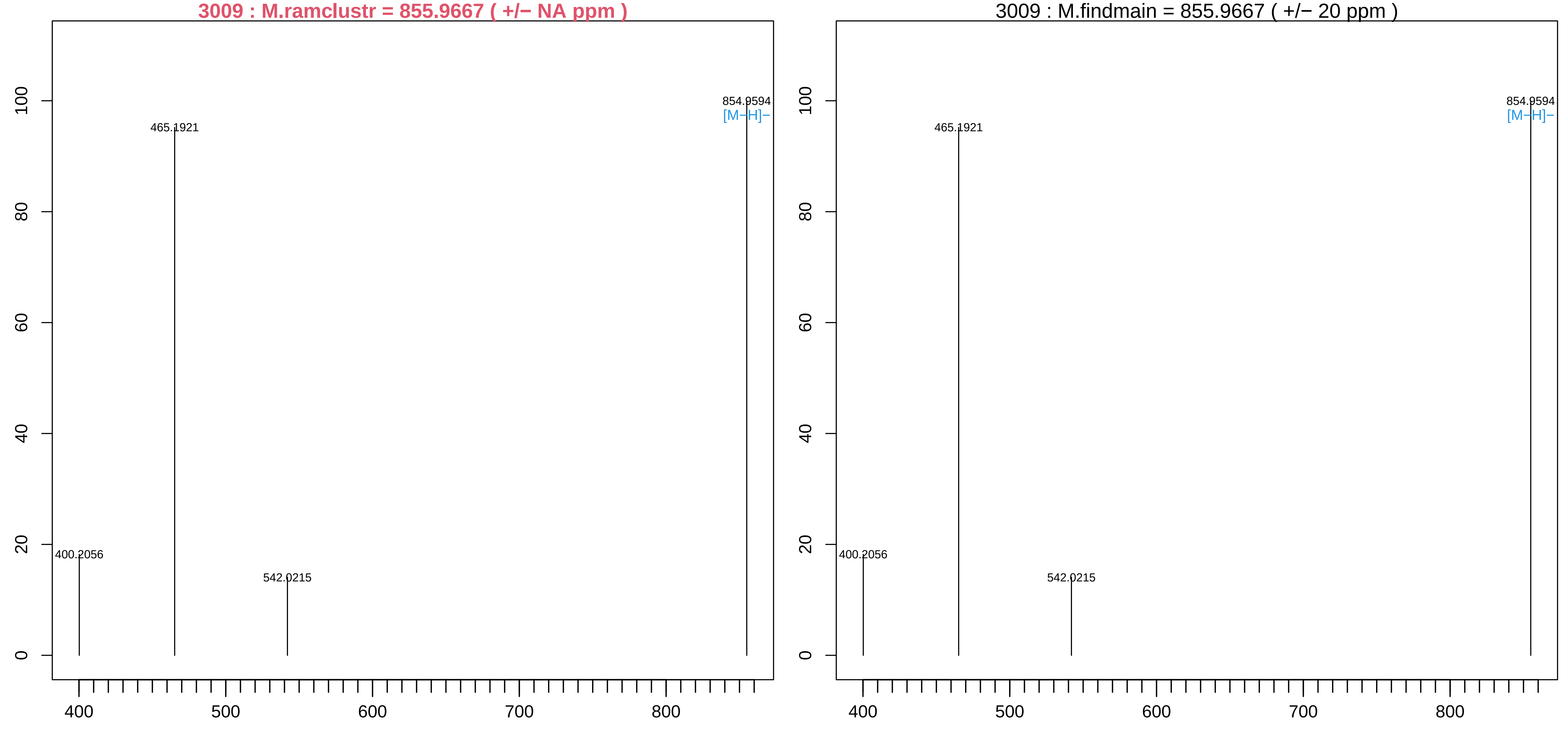
3009 : M.ramclustr = 855.9667 (+/− NA ppm)
3009 : M.findmain = 855.9667 (+/− 20 ppm)
854.9594
[M−H]−
465.1921
542.0215
400.2056
0
20
40
60
80
100
400
500
600
700
800

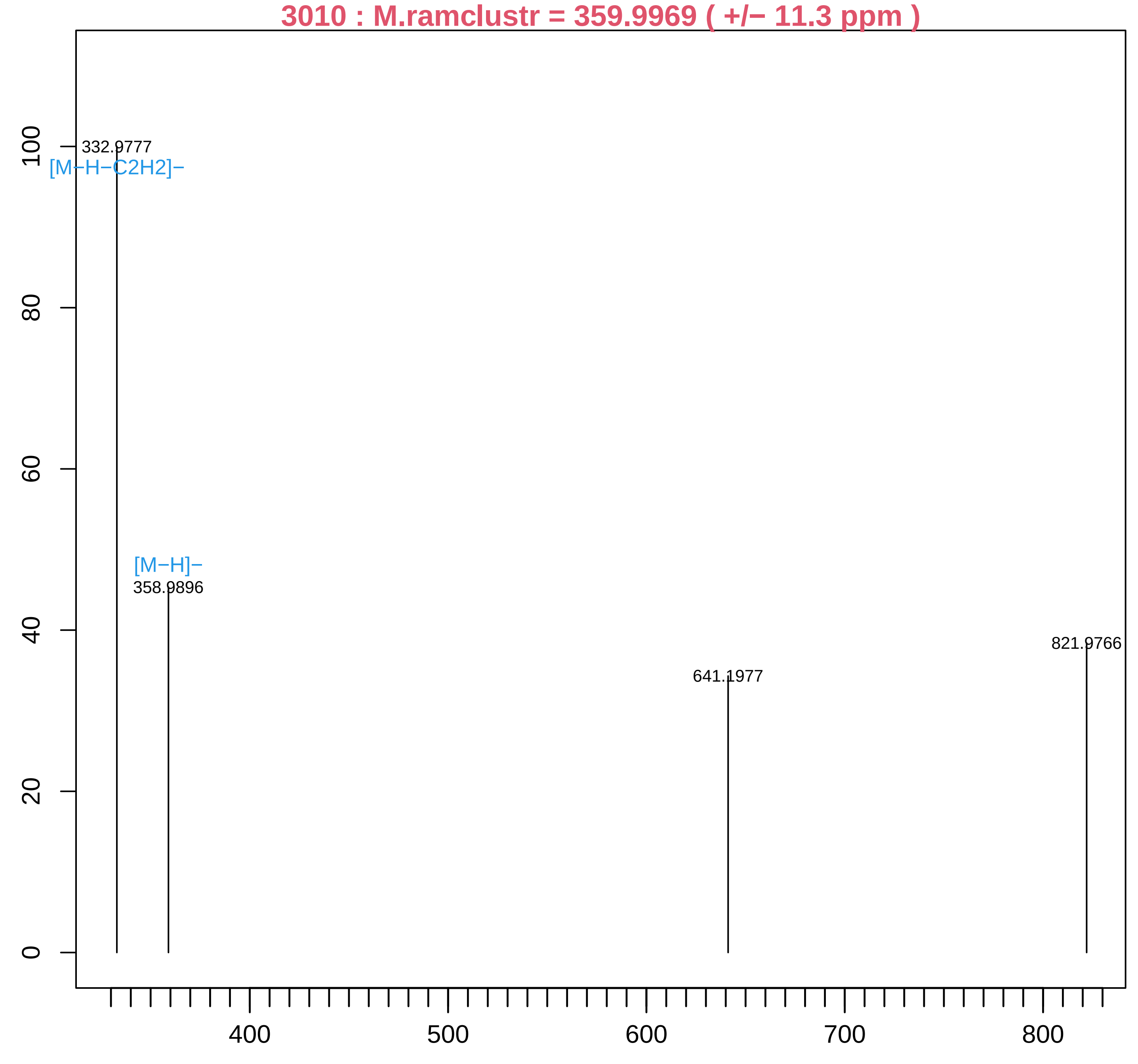

3010 : M.ramclustr = 359.9969 (+/− 11.3 ppm)
332.9777
[M−H−C2H2]−
[M−H]−
358.9896
641.1977
821.9766

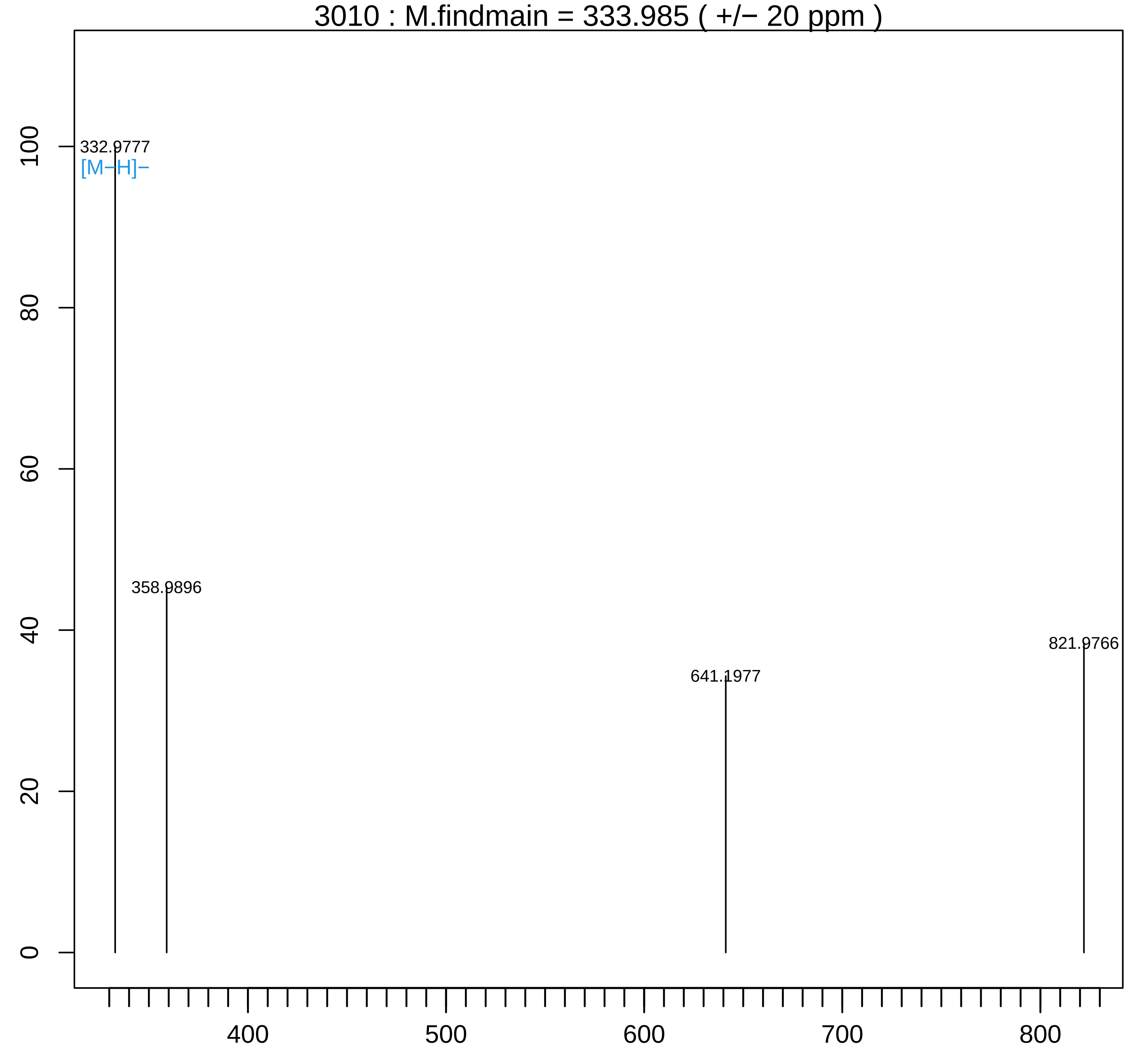

3010 : M.findmain = 333.985 (+/− 20 ppm)
332.9777
[M−H]−
358.9896
641.1977
821.9766

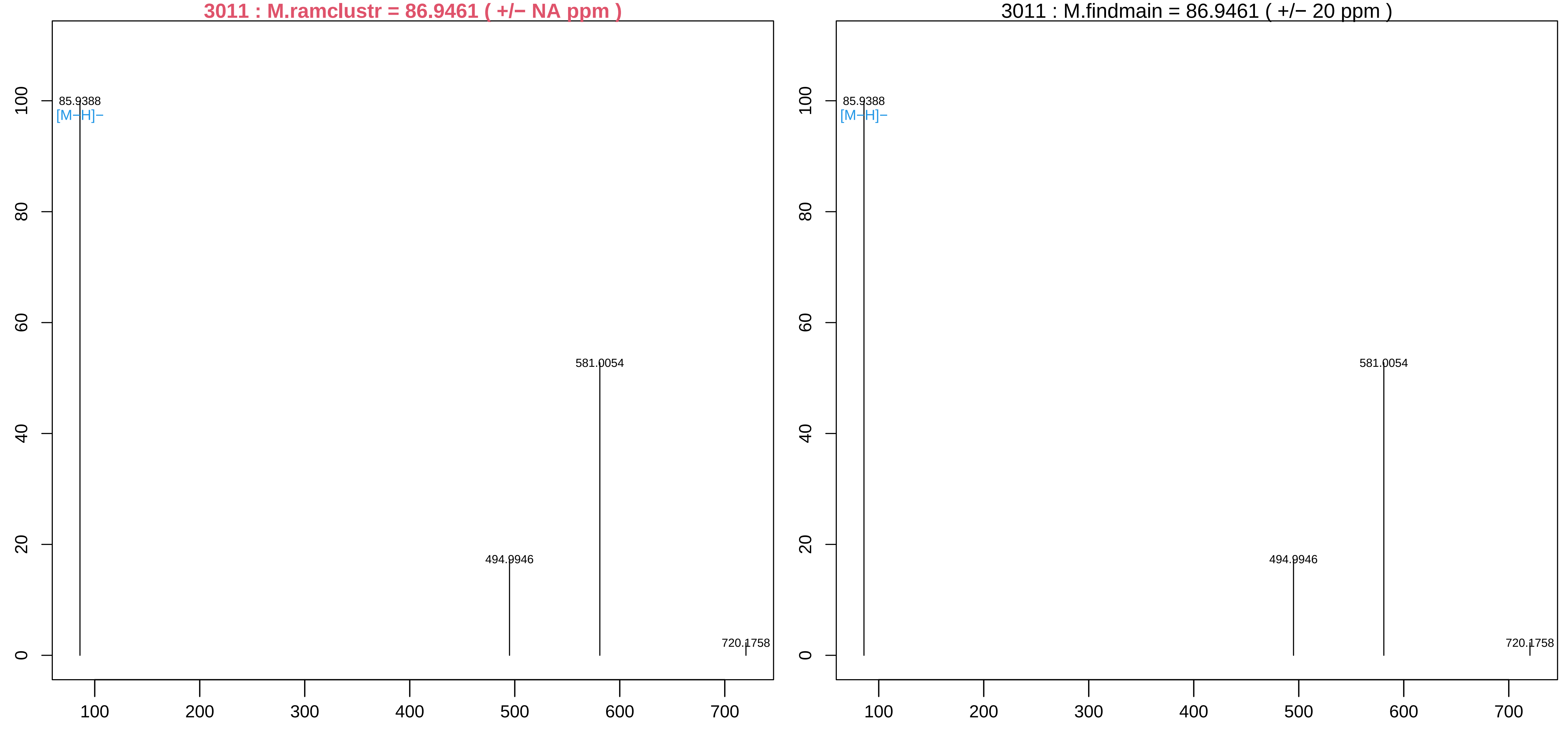

3011 : M.ramclustr = 86.9461 (+/− NA ppm)
85.9388
[M−H]−
581.0054
494.9946
720.1758
0
20
40
60
80
100
100
200
300
400
500
600
700
3011 : M.findmain = 86.9461 (+/− 20 ppm)
85.9388
[M−H]−
581.0054
494.9946
720.1758
0
20
40
60
80
100
100
200
300
400
500
600
700

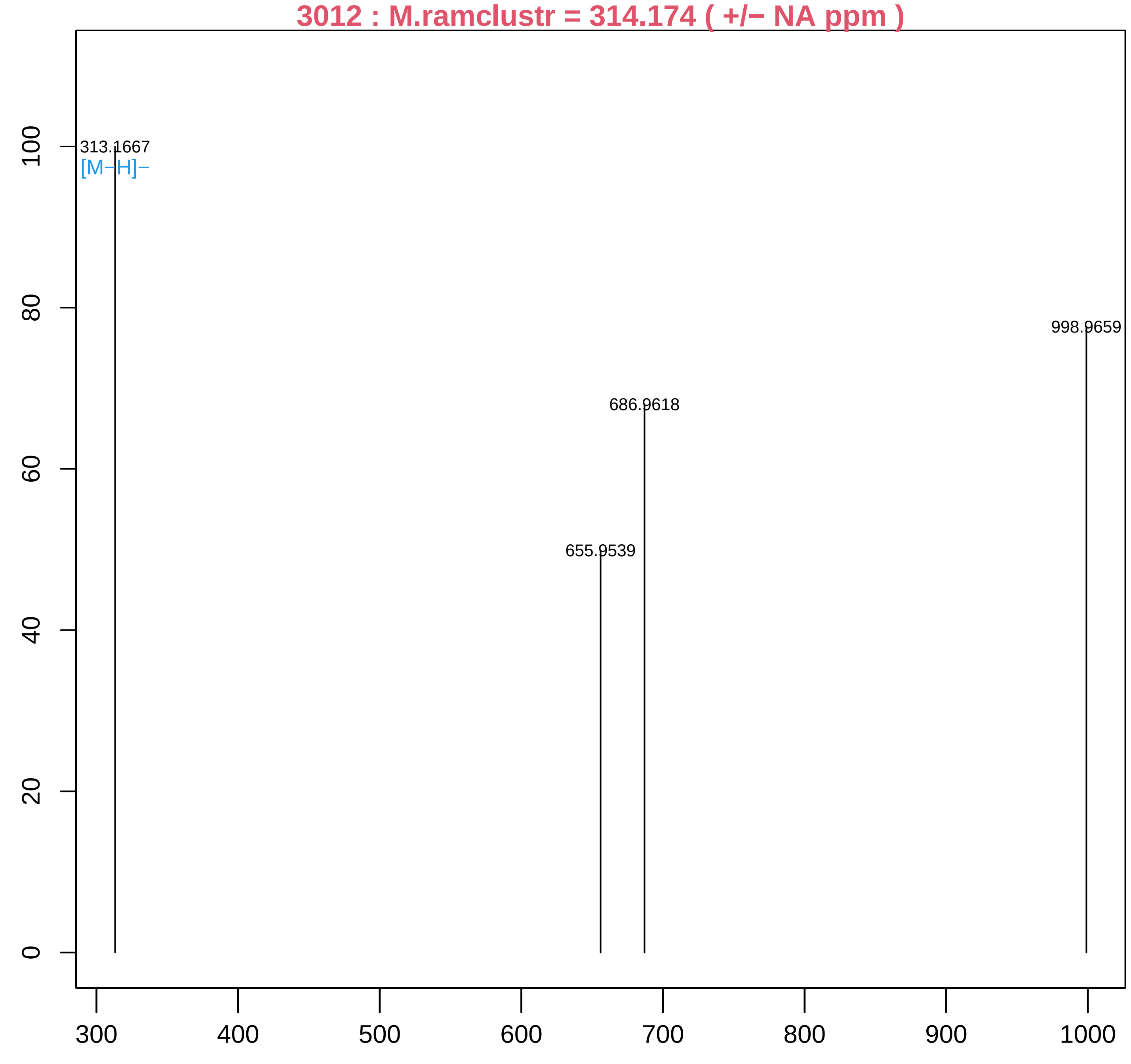
3012 : M.ramclustr = 314.174 (+/− NA ppm)
313.1667
[M−H]−
655.9539
686.9618
998.9659
0
20
40
60
80
100
300
400
500
600
700
800
900
1000

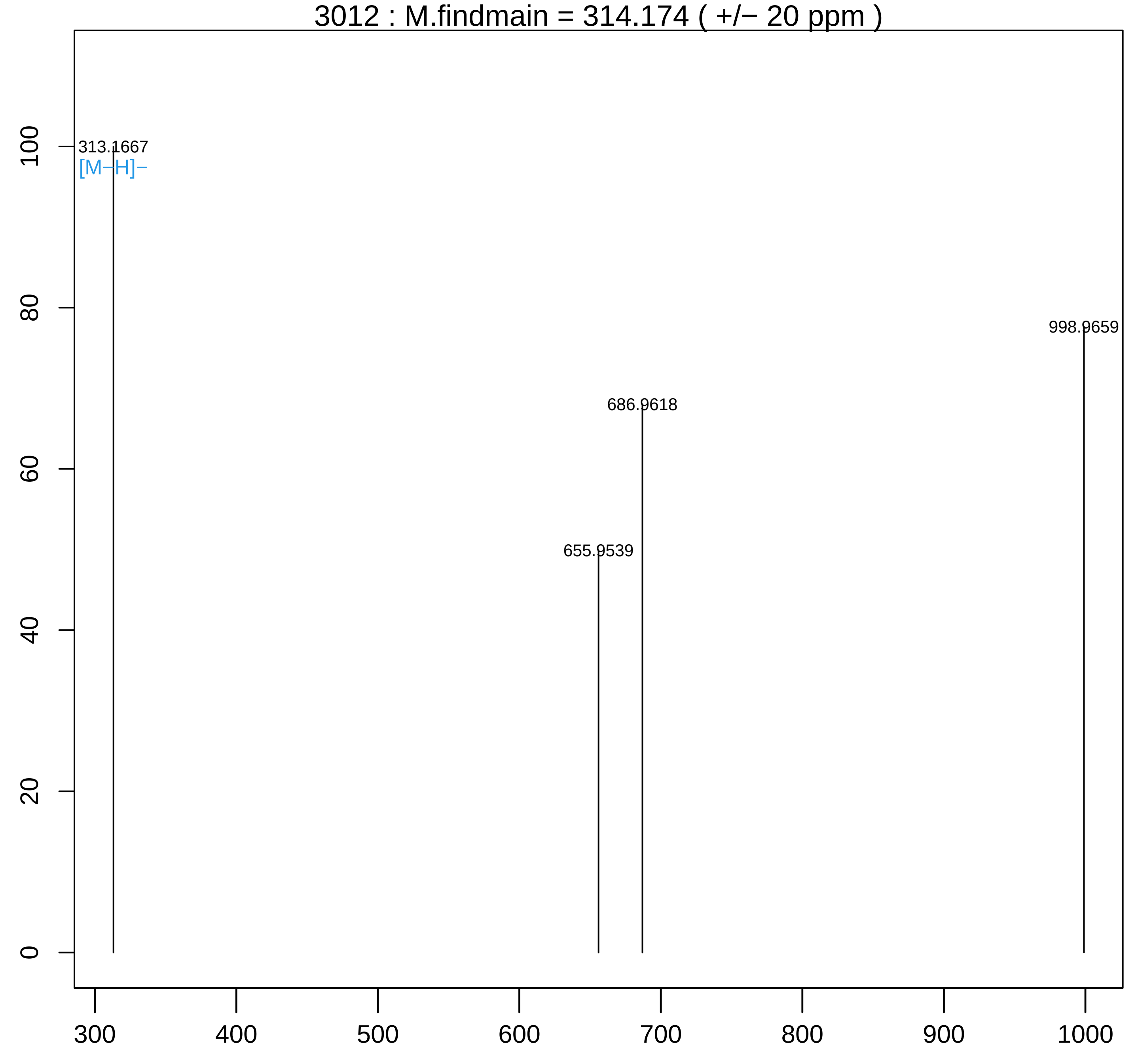
3012 : M.findmain = 314.174 (+/− 20 ppm)
313.1667
[M−H]−
655.9539
686.9618
998.9659
0
20
40
60
80
100
300
400
500
600
700
800
900
1000

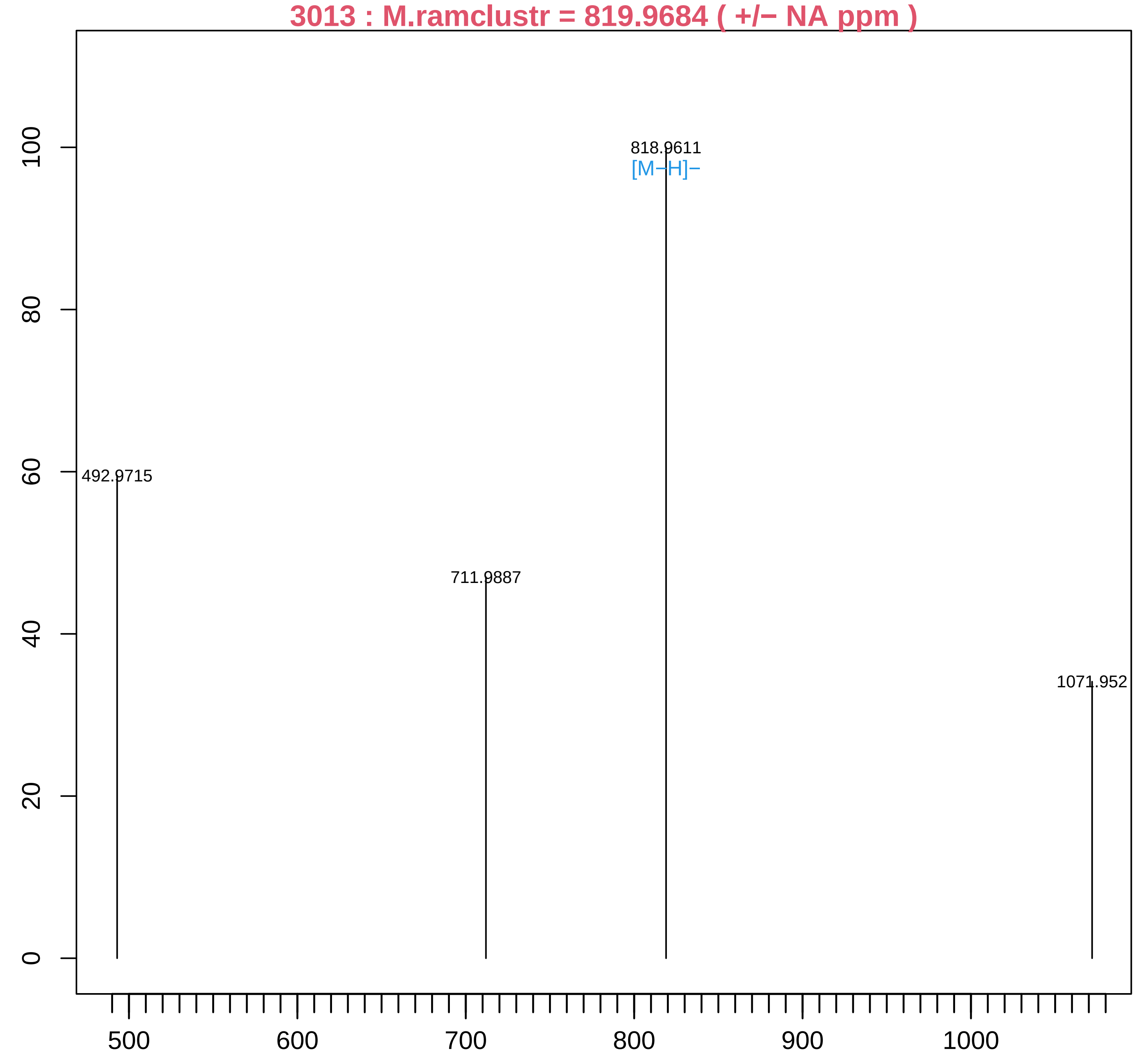
3013 : M.ramclustr = 819.9684 (+/− NA ppm)
492.9715
711.9887
818.9611
[M−H]−
1071.952
0
20
40
60
80
100
500
600
700
800
900
1000

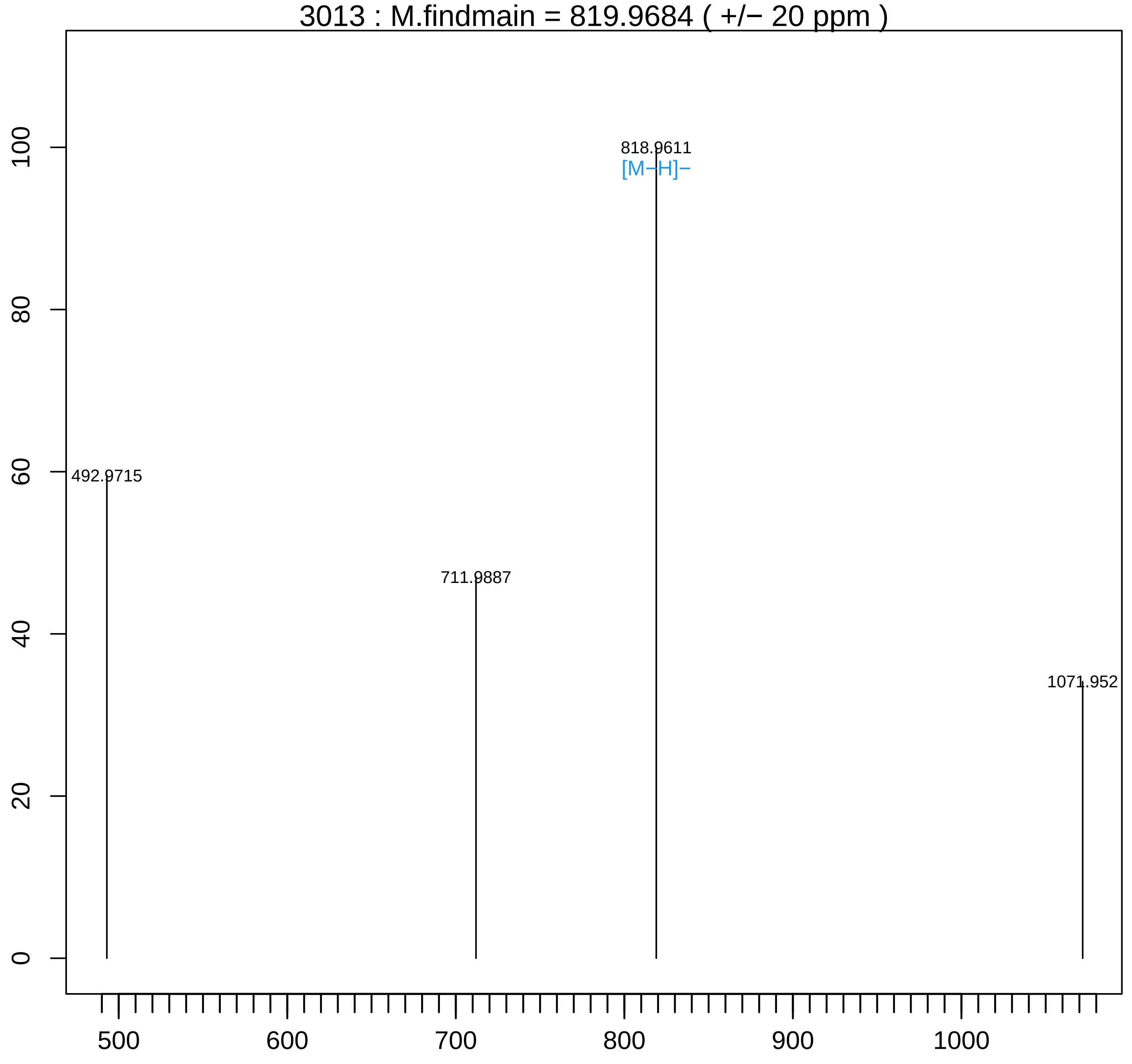
3013 : M.findmain = 819.9684 (+/− 20 ppm)
492.9715
711.9887
818.9611
[M−H]−
1071.952
0
20
40
60
80
100
500
600
700
800
900
1000

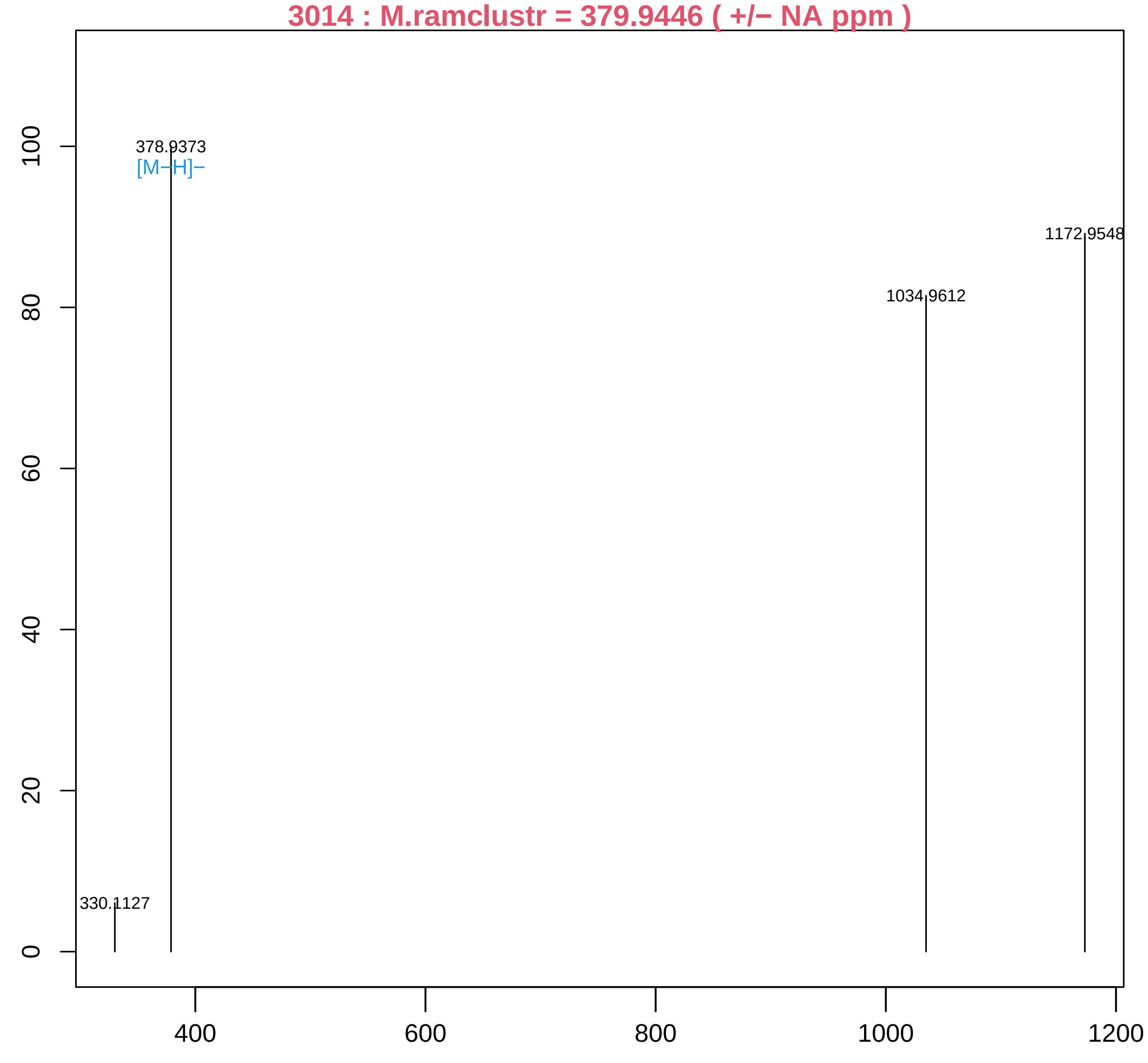
3014 : M.ramclustr = 379.9446 (+/− NA ppm)
378.9373
[M−H]−
1172.9548
1034.9612
330.1127
0
20
40
60
80
100
400
600
800
1000
1200

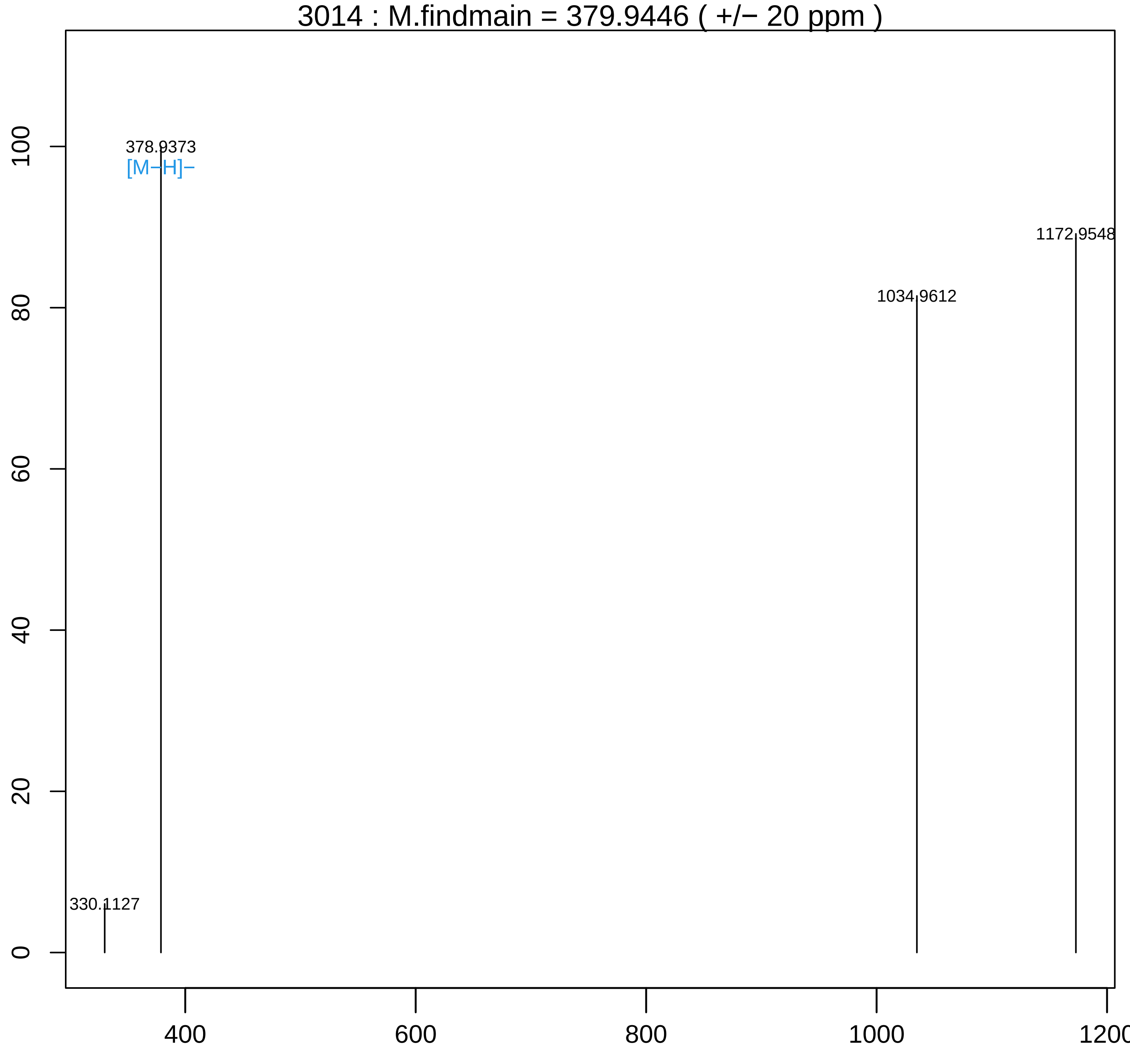
3014 : M.findmain = 379.9446 (+/− 20 ppm)
378.9373
[M−H]−
1172.9548
1034.9612
330.1127
0
20
40
60
80
100
400
600
800
1000
1200

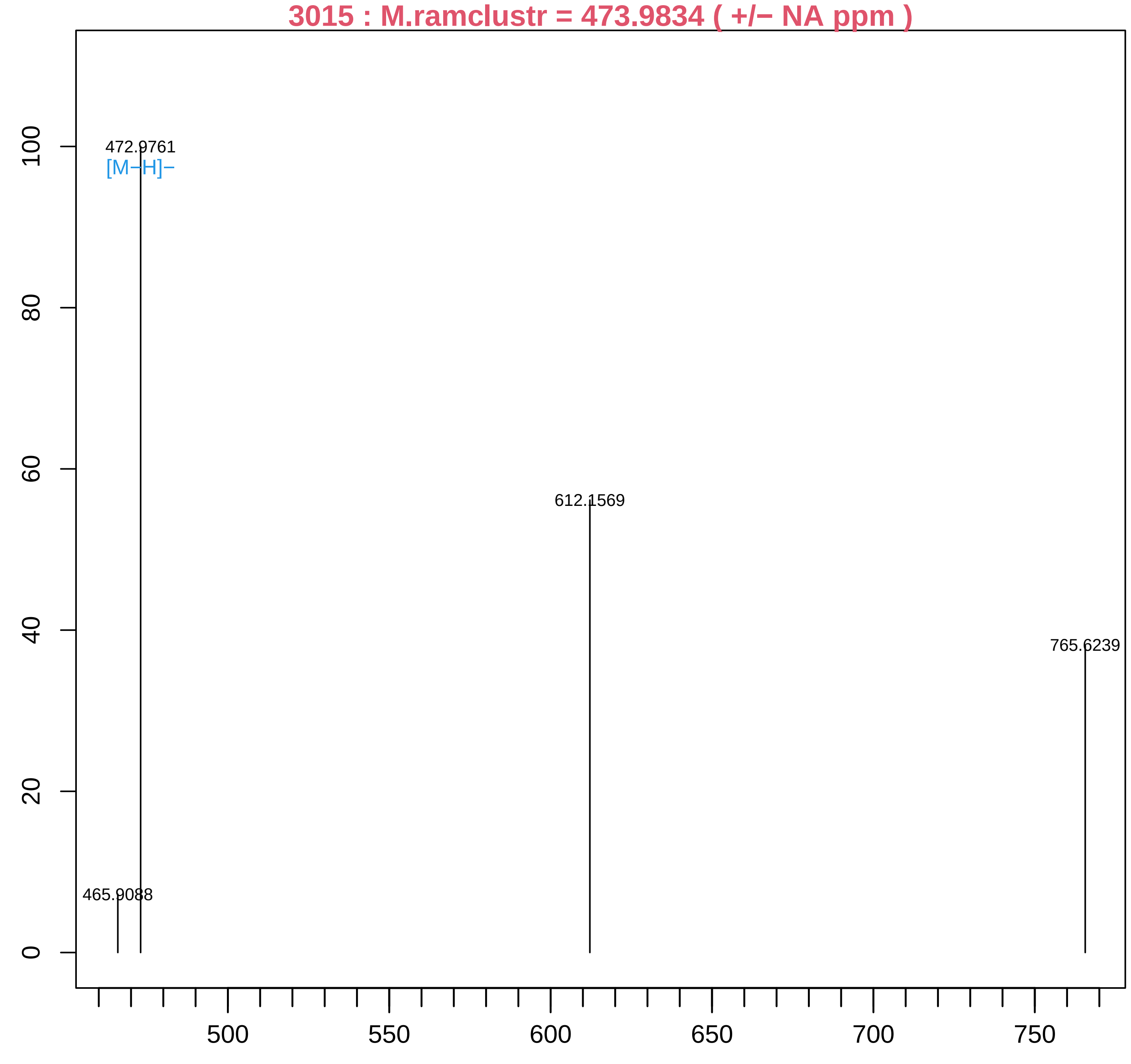
3015 : M.ramclustr = 473.9834 (+/− NA ppm)
472.9761
[M−H]−
612.1569
765.6239
465.9088
0
20
40
60
80
100
500
550
600
650
700
750

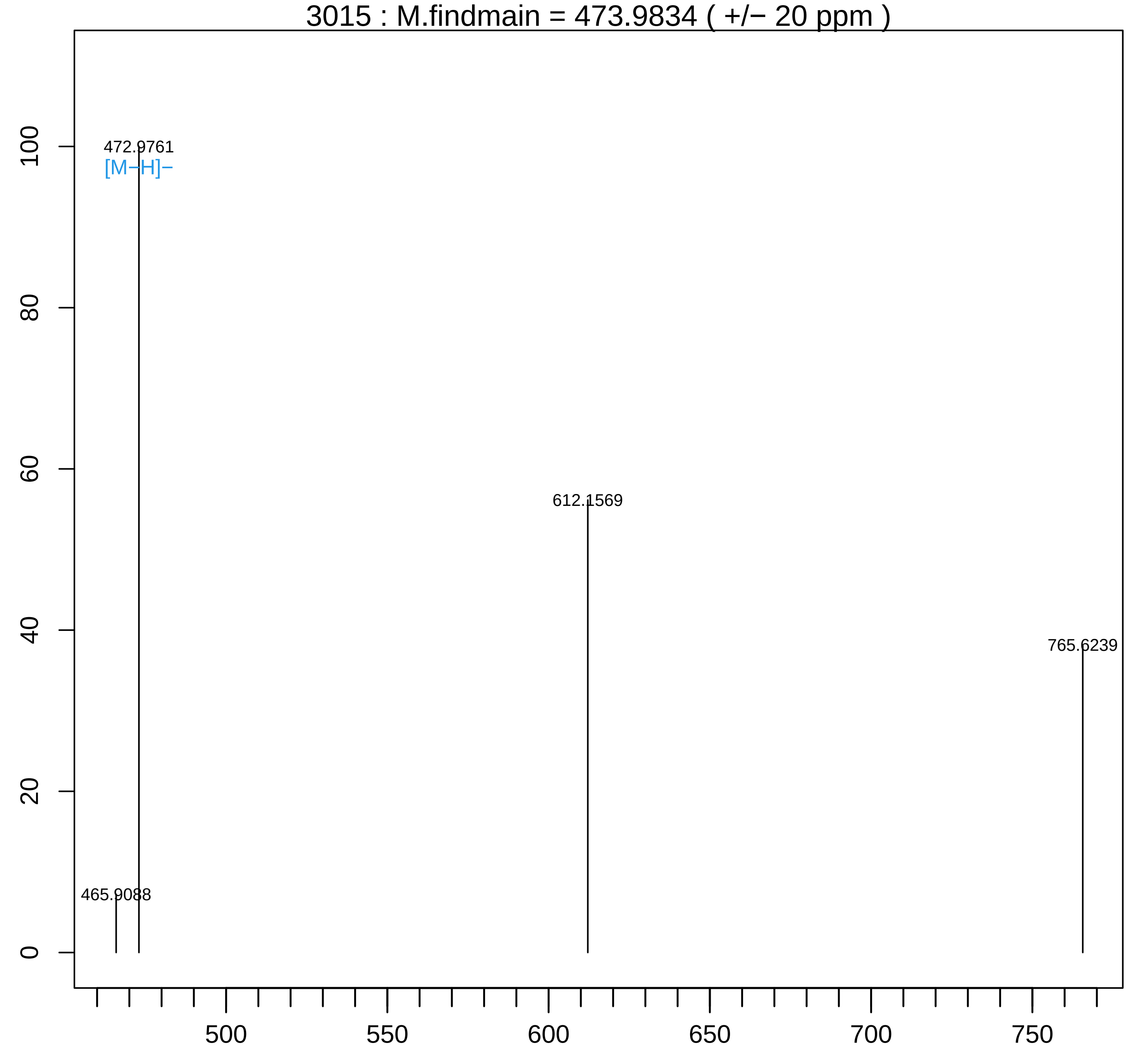
3015 : M.findmain = 473.9834 (+/− 20 ppm)
472.9761
[M−H]−
612.1569
765.6239
465.9088
0
20
40
60
80
100
500
550
600
650
700
750

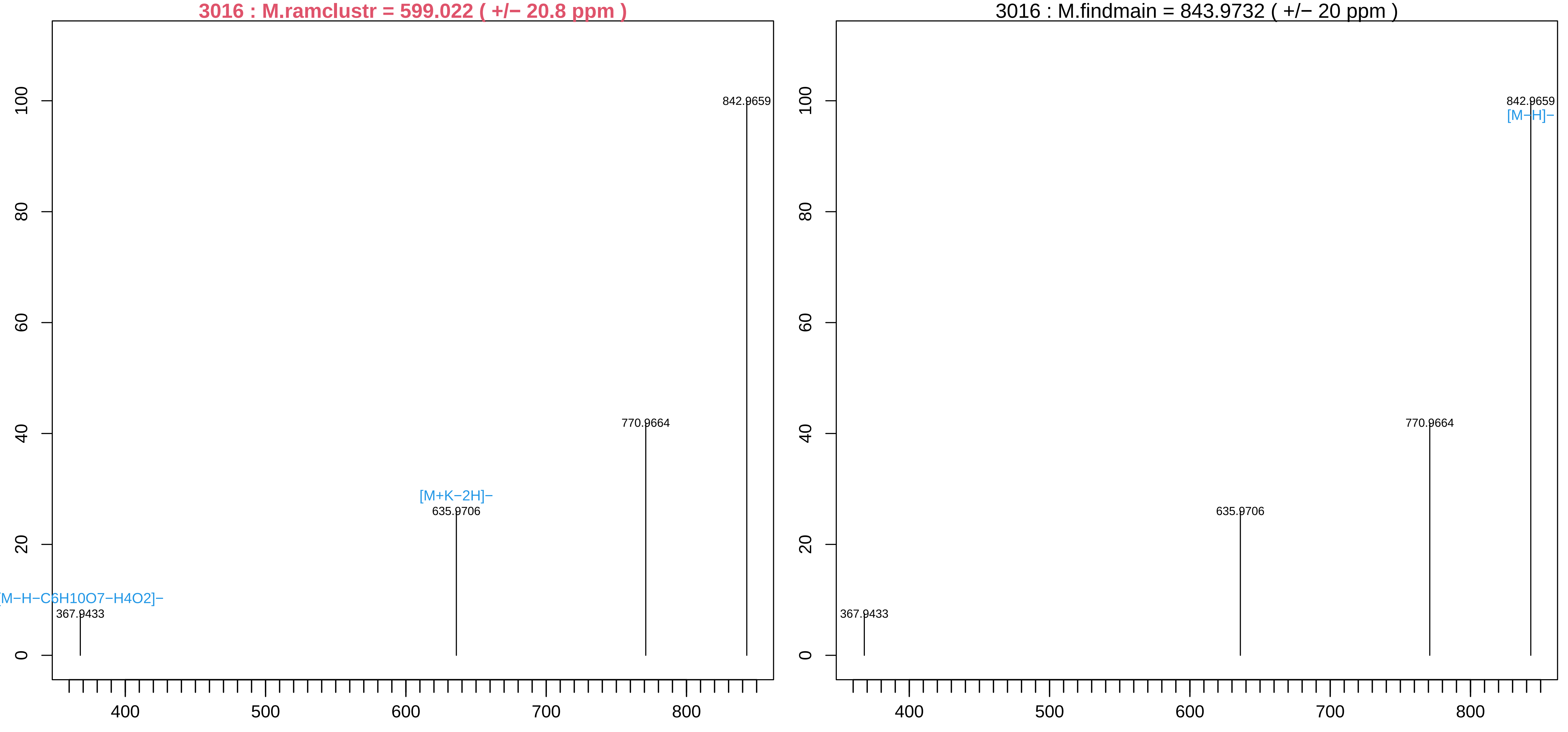

3016 : M.ramclustr = 599.022 (+/− 20.8 ppm)
842.9659
770.9664
[M+K−2H]−
635.9706
[M−H−C6H10O7−H4O2]−
367.9433
3016 : M.findmain = 843.9732 (+/− 20 ppm)
842.9659
[M−H]−
770.9664
635.9706
367.9433
0
20
40
60
80
100
400
500
600
700
800

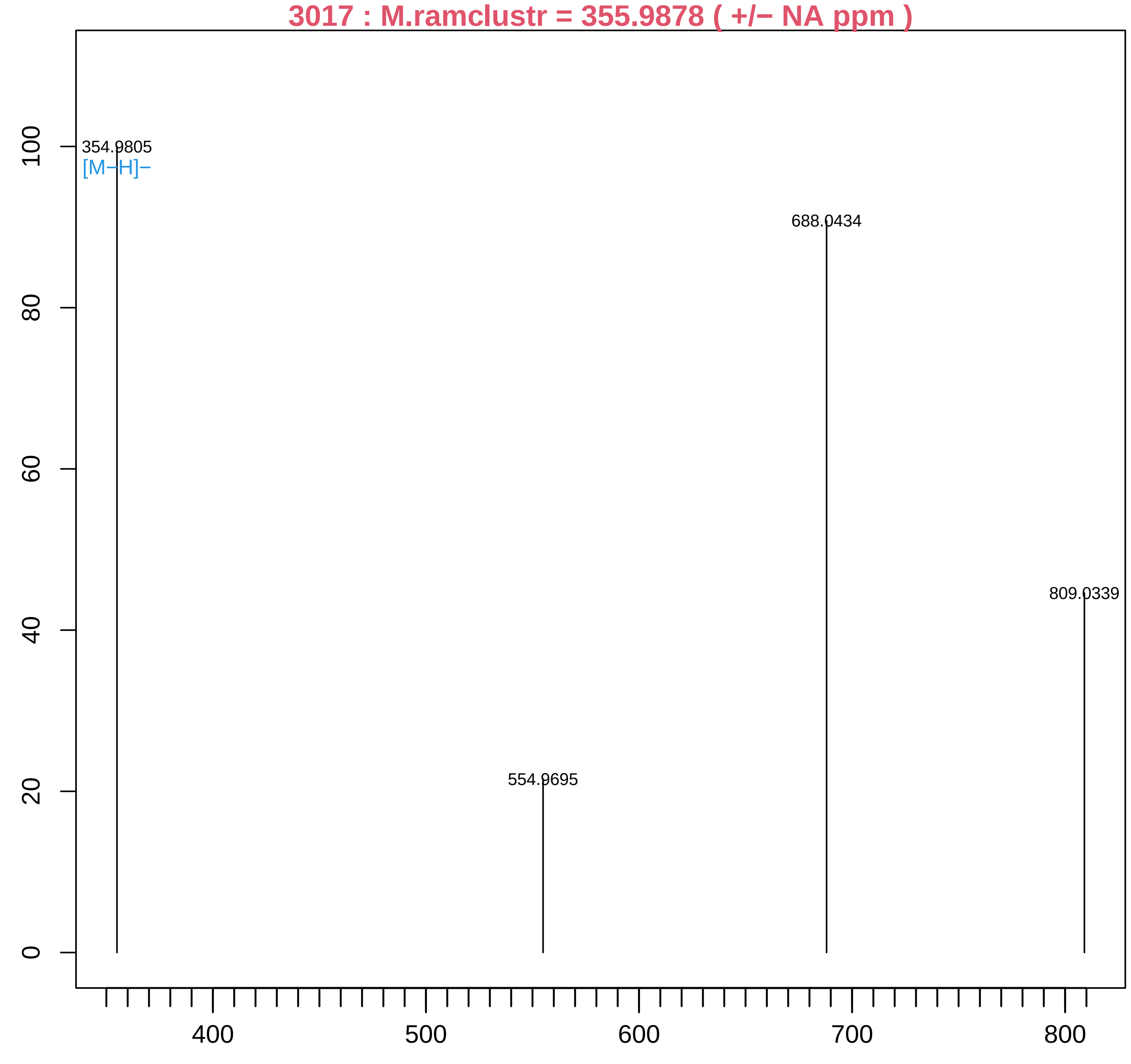
3017 : M.ramclustr = 355.9878 (+/− NA ppm)
354.9805
[M−H]−
688.0434
809.0339
554.9695
0
20
40
60
80
100
400
500
600
700
800

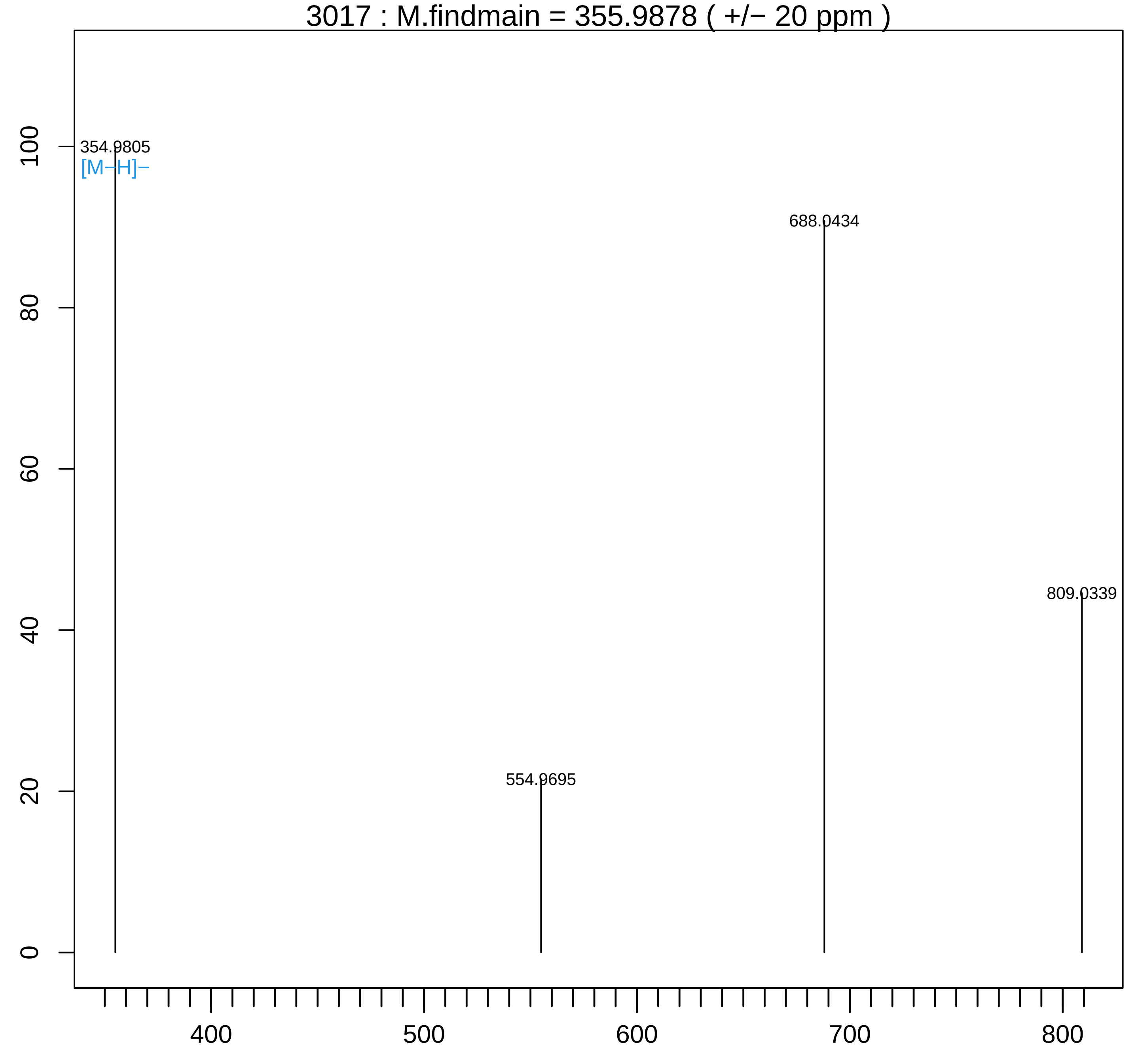
3017 : M.findmain = 355.9878 (+/− 20 ppm)
354.9805
[M−H]−
688.0434
809.0339
554.9695
0
20
40
60
80
100
400
500
600
700
800

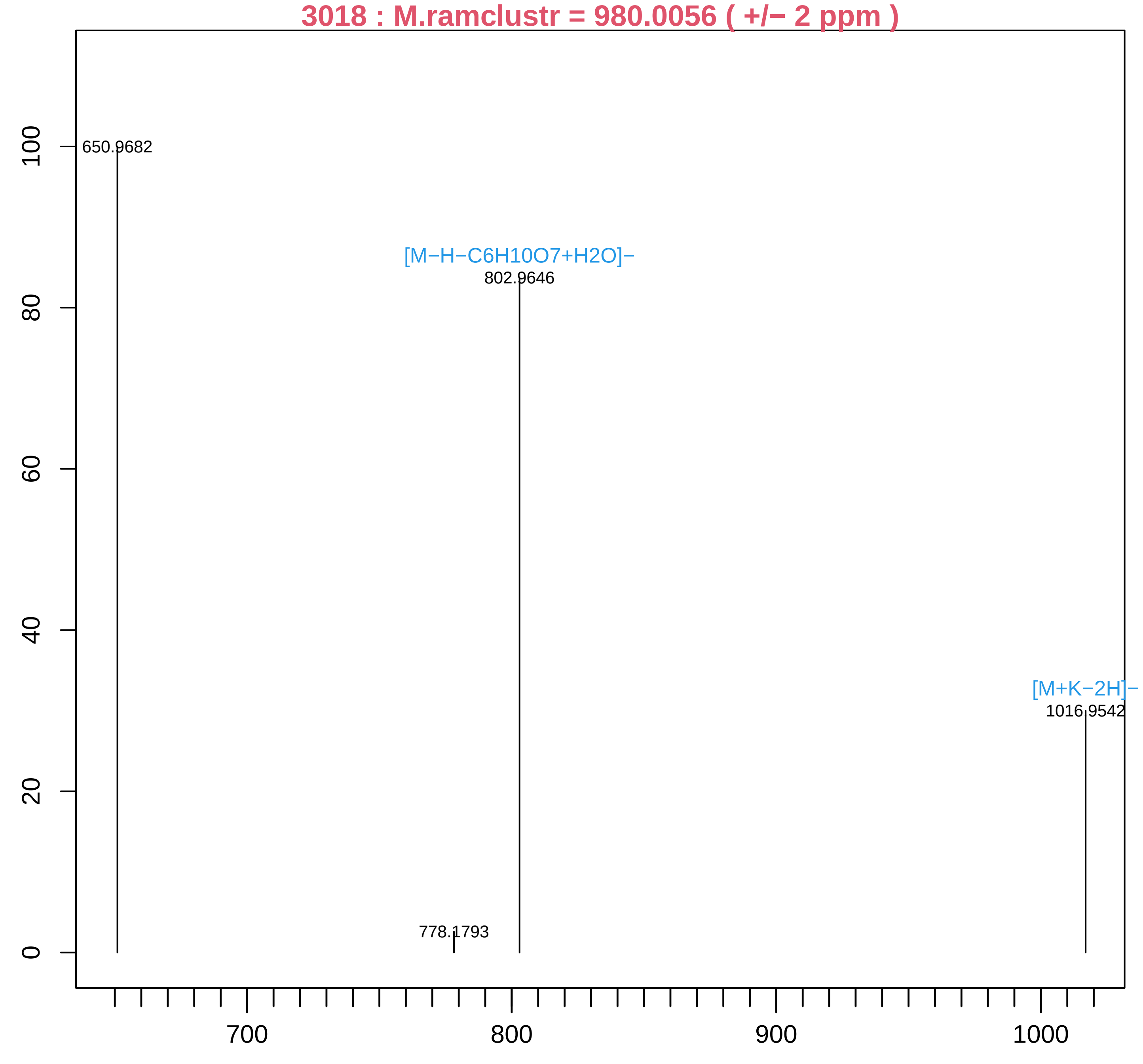
3018 : M.ramclustr = 980.0056 (+/− 2 ppm)
650.9682
[M−H−C6H10O7+H2O]−
802.9646
778.1793
[M+K−2H]−
1016.9542
0
20
40
60
80
100
700
800
900
1000

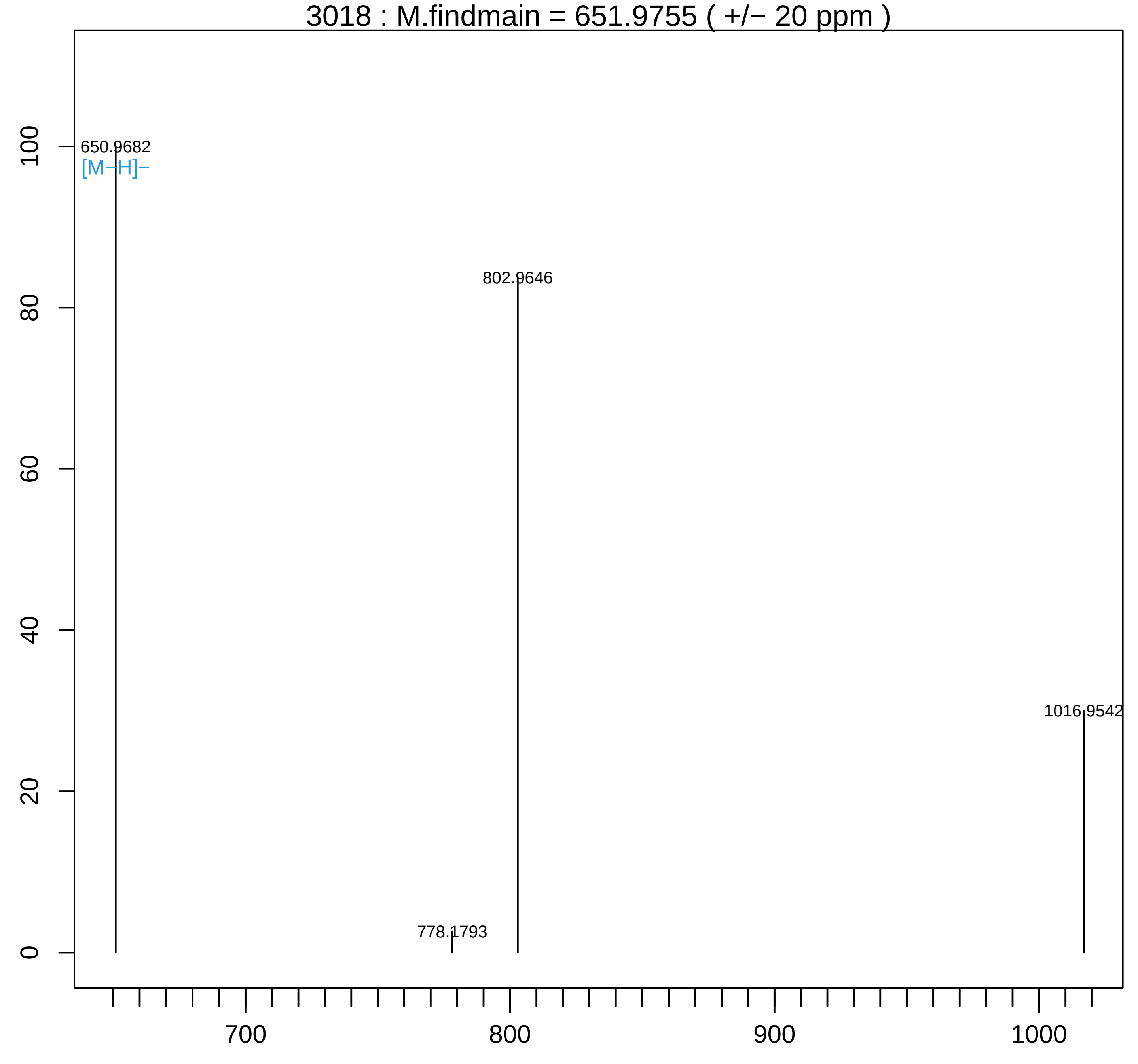
3018 : M.findmain = 651.9755 (+/− 20 ppm)
650.9682
[M−H]−
802.9646
778.1793
1016.9542
0
20
40
60
80
100
700
800
900
1000

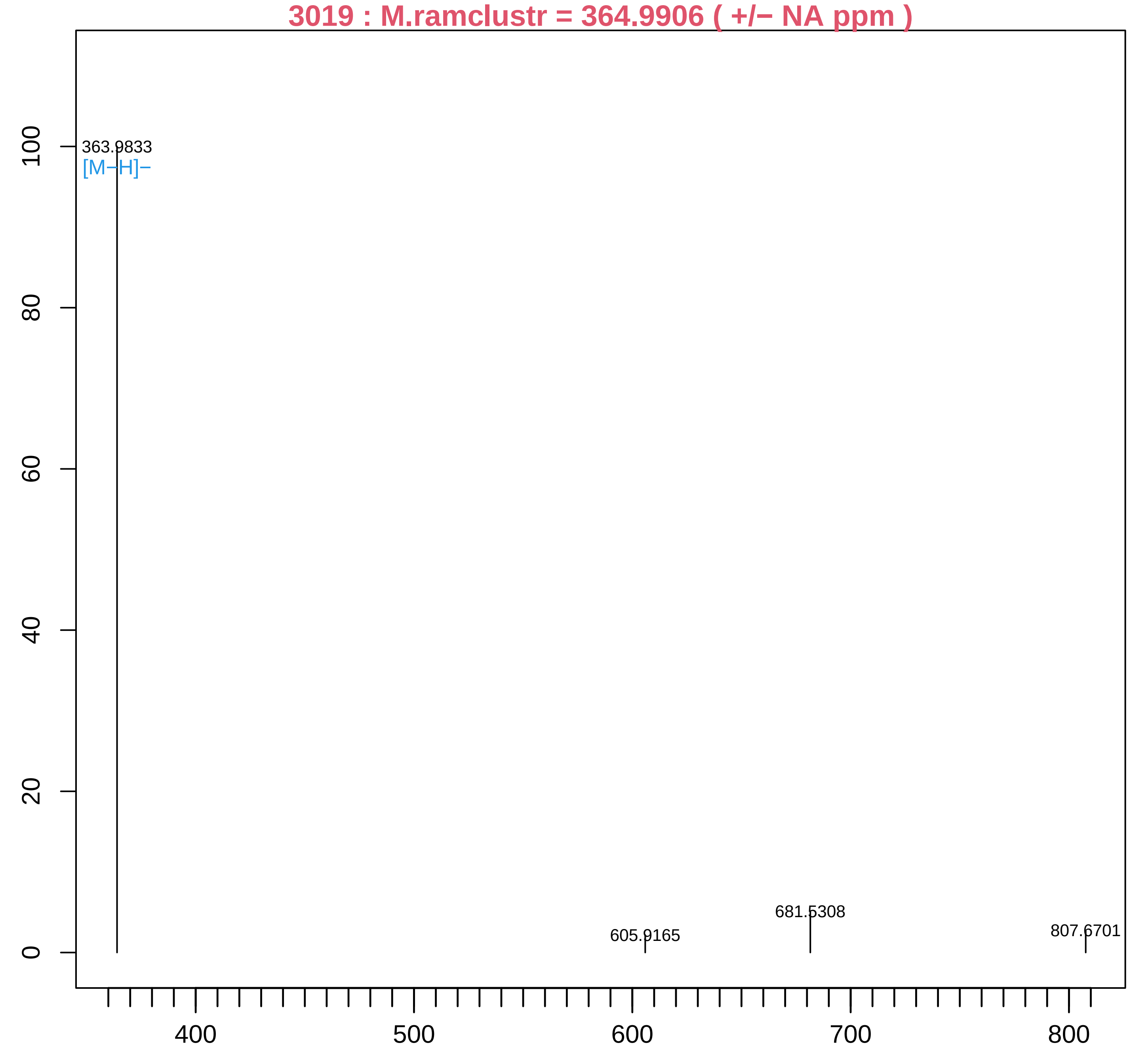
3019 : M.ramclustr = 364.9906 (+/− NA ppm)
363.9833
[M−H]−
605.9165
681.5308
807.6701
0
20
40
60
80
100
400
500
600
700
800

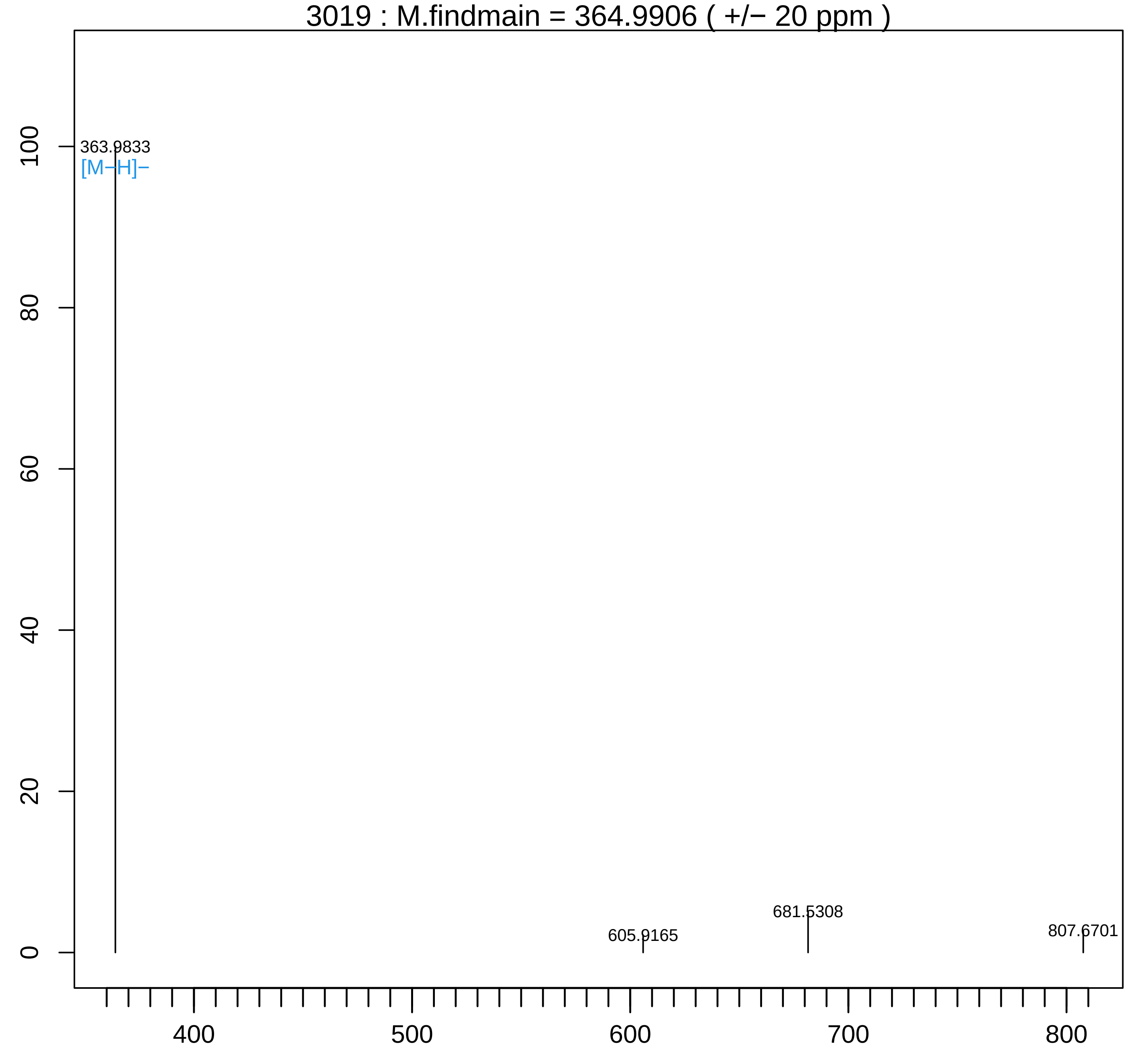
3019 : M.findmain = 364.9906 (+/− 20 ppm)
363.9833
[M−H]−
605.9165
681.5308
807.6701
0
20
40
60
80
100
400
500
600
700
800

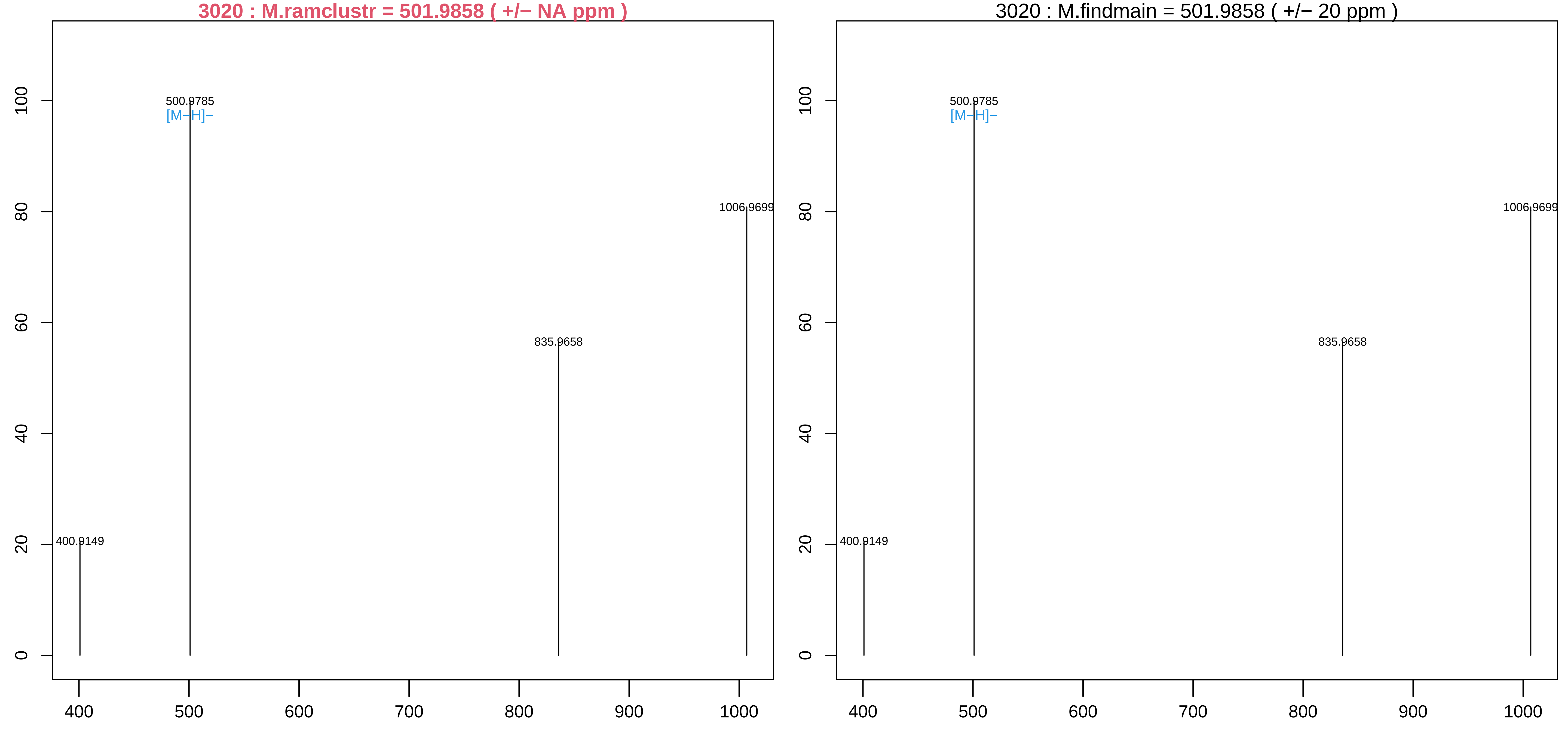
3020 : M.ramclustr = 501.9858 (+/− NA ppm)
500.9785
[M−H]−
1006.9699
835.9658
400.9149
3020 : M.findmain = 501.9858 (+/− 20 ppm)
500.9785
[M−H]−
1006.9699
835.9658
400.9149

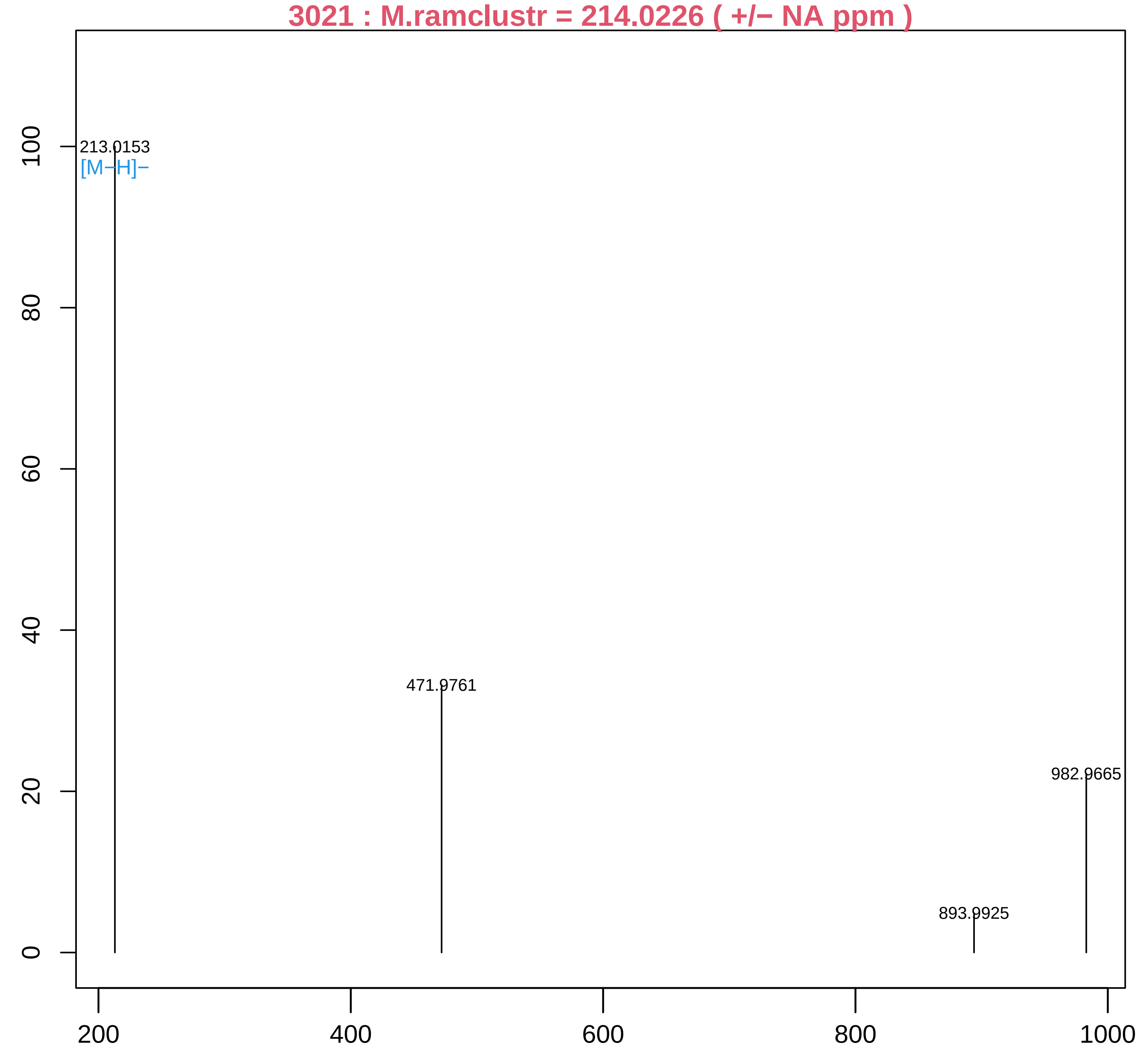

3021 : M.ramclustr = 214.0226 (+/− NA ppm)
213.0153
[M−H]−
471.9761
893.9925
982.9665
0
20
40
60
80
100
200
400
600
800
1000

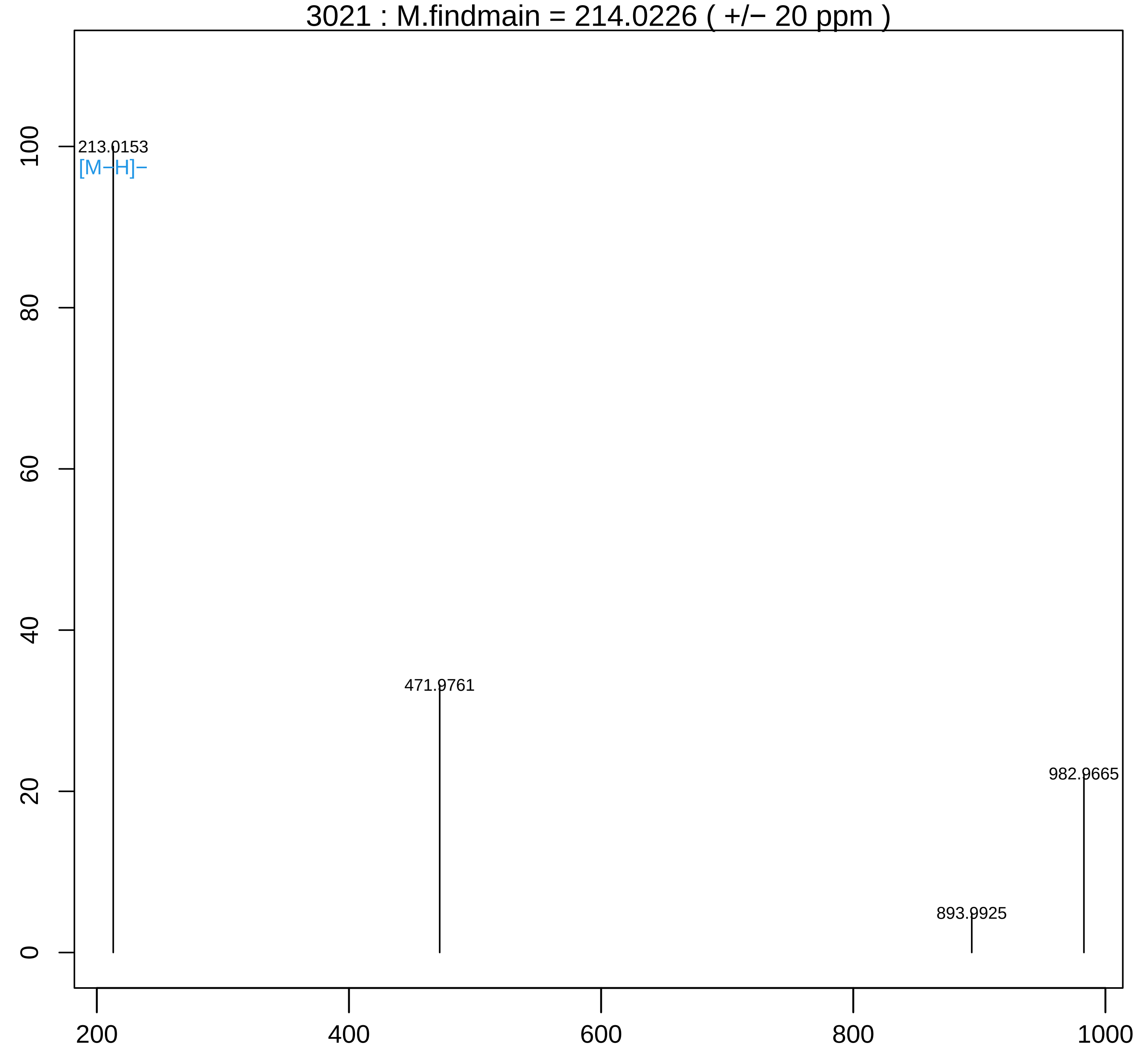

3021 : M.findmain = 214.0226 (+/− 20 ppm)
213.0153
[M−H]−
471.9761
893.9925
982.9665
0
20
40
60
80
100
200
400
600
800
1000

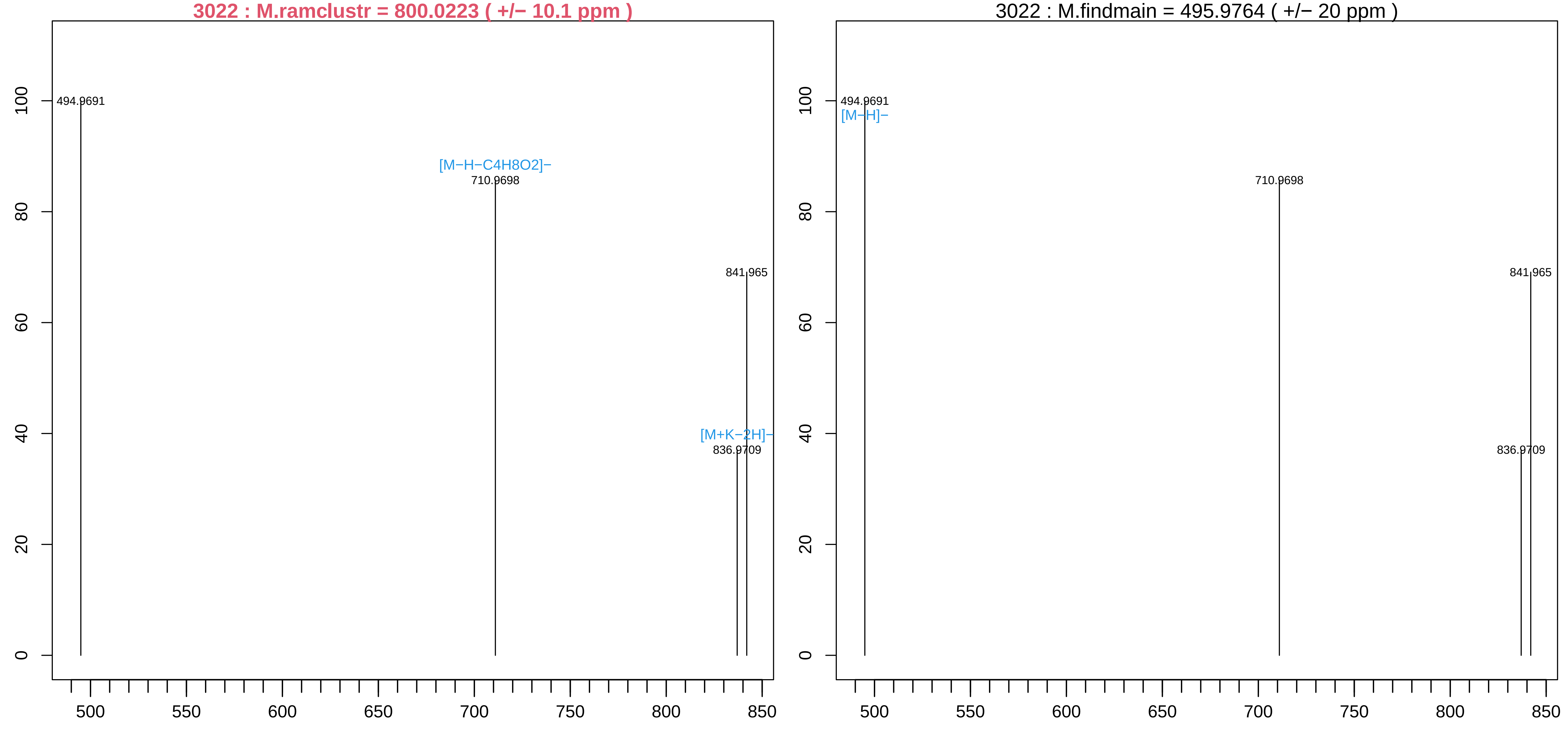

3022 : M.ramclustr = 800.0223 (+/− 10.1 ppm)
494.9691
[M−H−C4H8O2]−
710.9698
841.965
[M+K−2H]−
836.9709
3022 : M.findmain = 495.9764 (+/− 20 ppm)
494.9691
[M−H]−
710.9698
841.965
836.9709
0
20
40
60
80
100
500
550
600
650
700
750
800
850

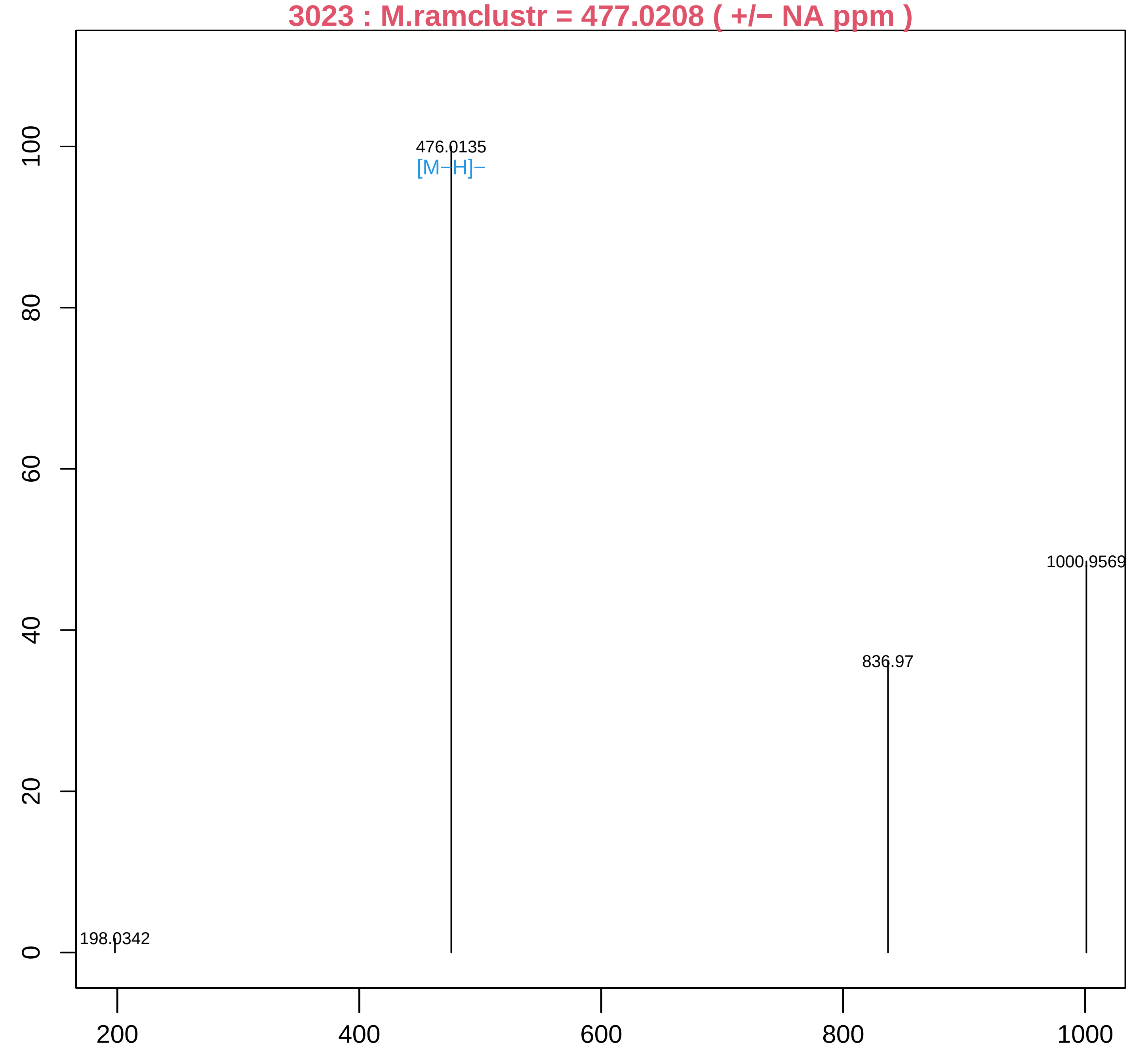
3023 : M.ramclustr = 477.0208 (+/− NA ppm)
476.0135
[M−H]−
1000.9569
836.97
198.0342
0
20
40
60
80
100
200
400
600
800
1000

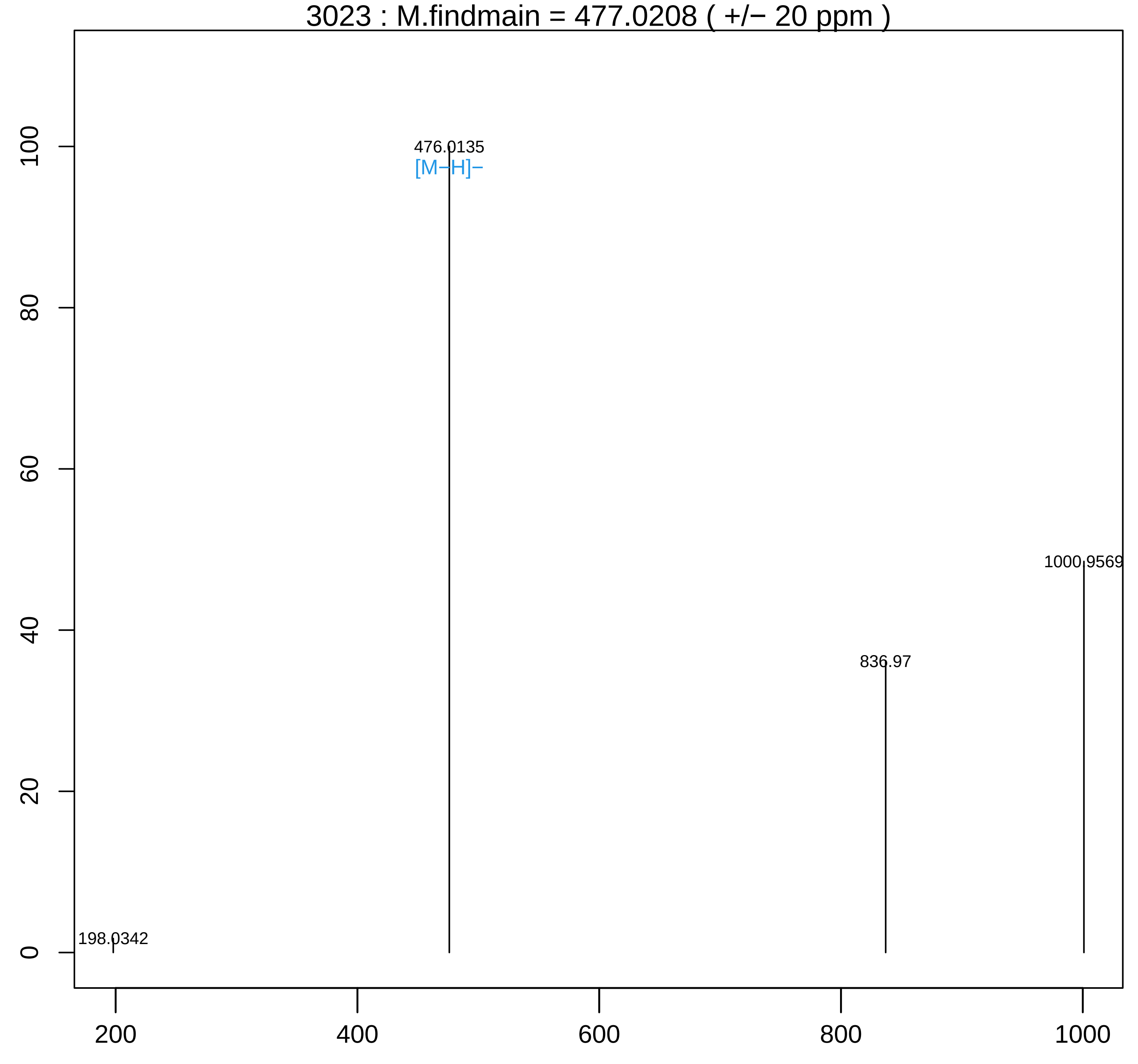
3023 : M.findmain = 477.0208 (+/− 20 ppm)
476.0135
[M−H]−
1000.9569
836.97
198.0342
0
20
40
60
80
100
200
400
600
800
1000

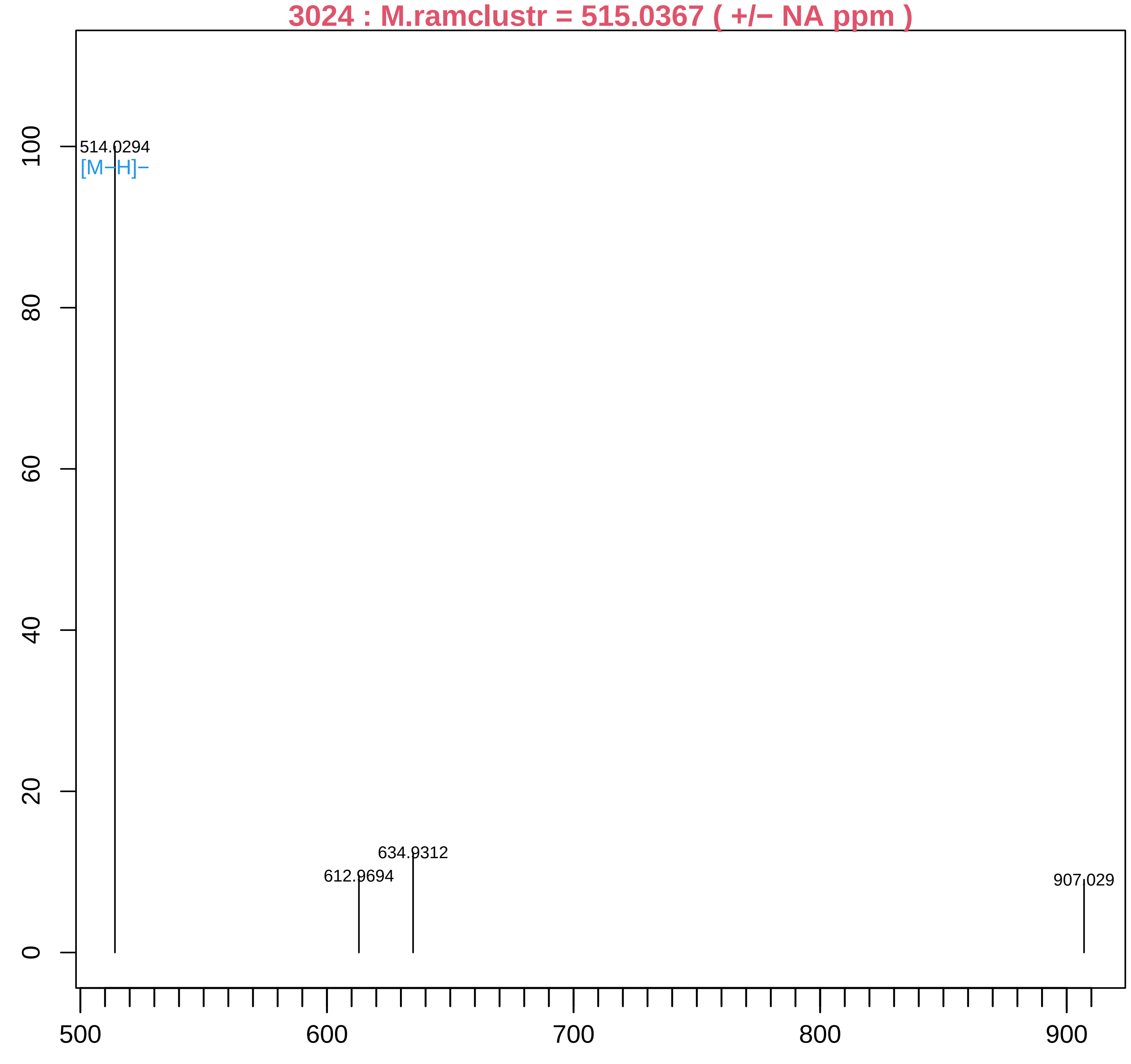

3024 : M.ramclustr = 515.0367 (+/− NA ppm)
514.0294
[M−H]−
634.9312
612.9694
907.029
0
20
40
60
80
100
500
600
700
800
900

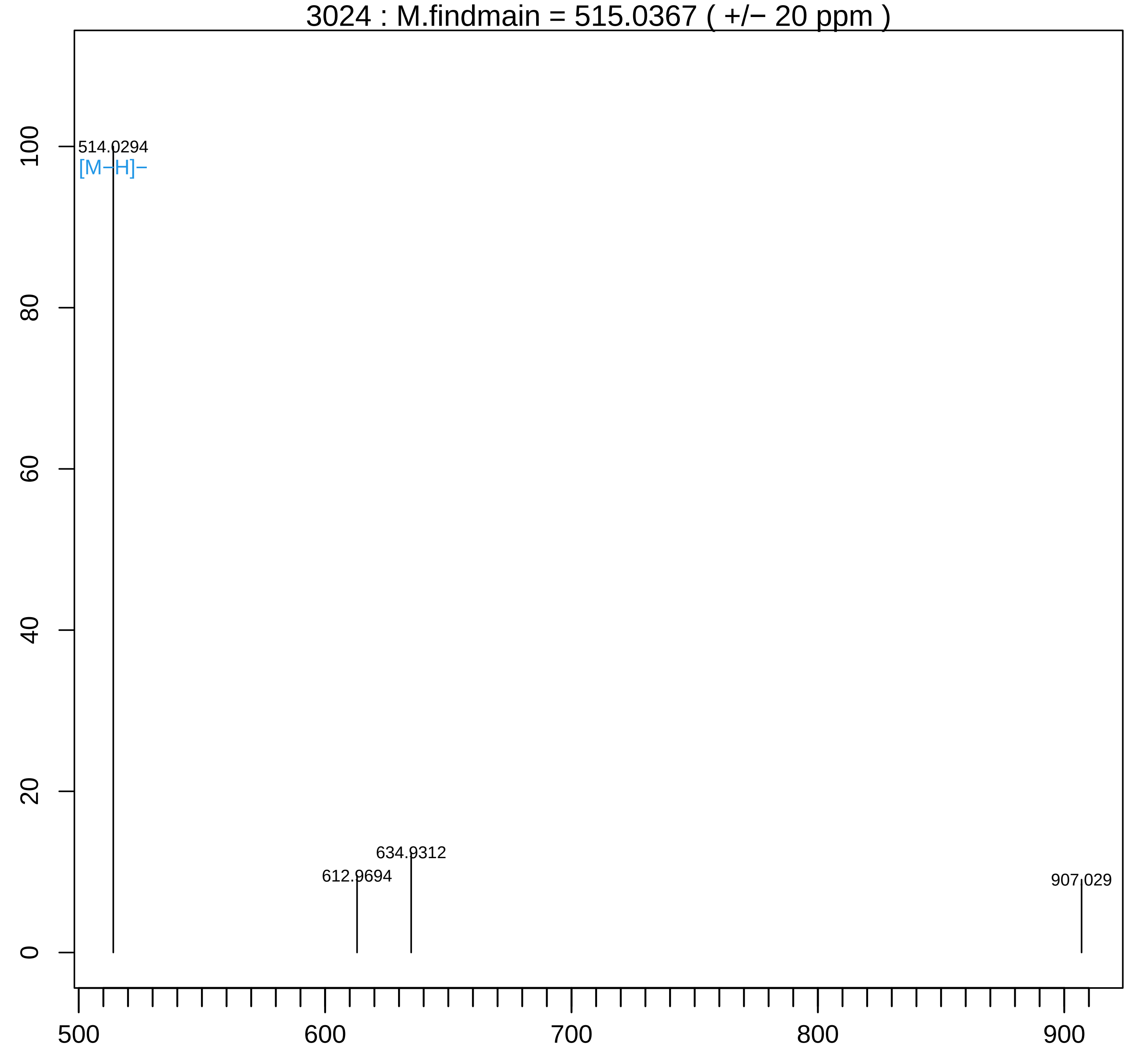

3024 : M.findmain = 515.0367 (+/− 20 ppm)
514.0294
[M−H]−
634.9312
612.9694
907.029
0
20
40
60
80
100
500
600
700
800
900

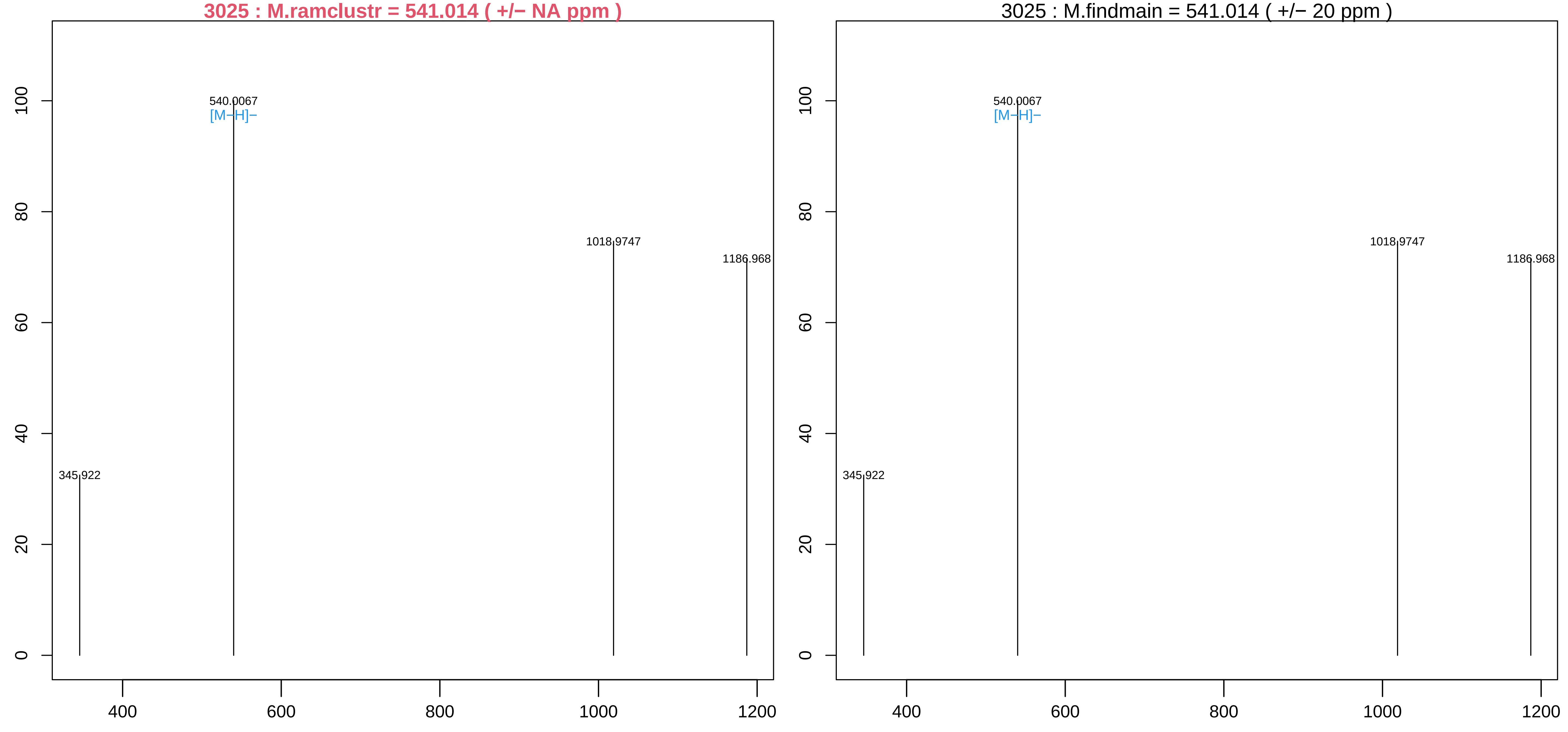
3025 : M.ramclustr = 541.014 (+/− NA ppm)
3025 : M.findmain = 541.014 (+/− 20 ppm)
540.0067
[M−H]−
1018.9747
1186.968
345.922
0
20
40
60
80
100
400
600
800
1000
1200

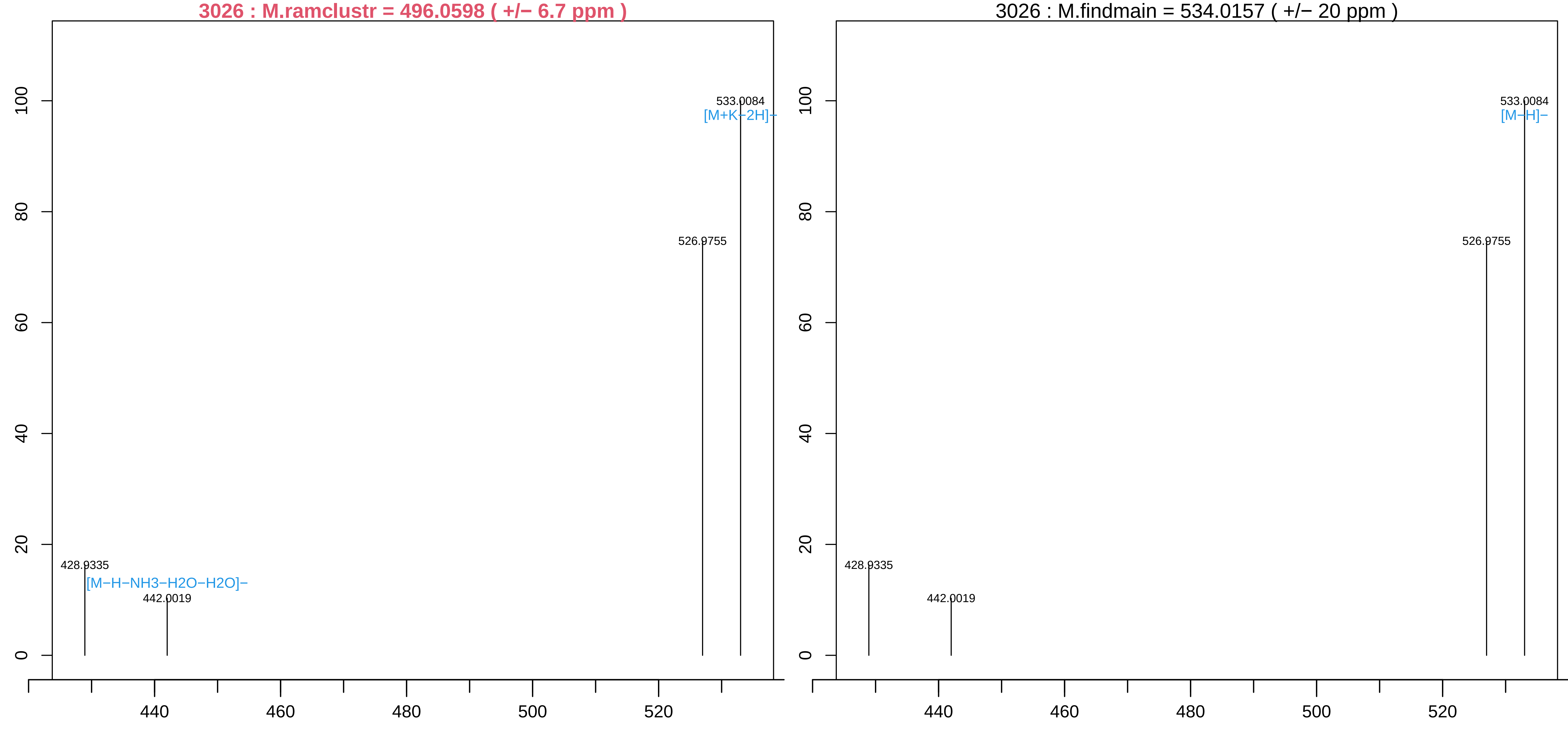

3026 : M.ramclustr = 496.0598 (+/− 6.7 ppm)
533.0084
[M+K−2H]−
526.9755
428.9335
[M−H−NH3−H2O−H2O]−
442.0019
3026 : M.findmain = 534.0157 (+/− 20 ppm)
533.0084
[M−H]−
526.9755
428.9335
442.0019

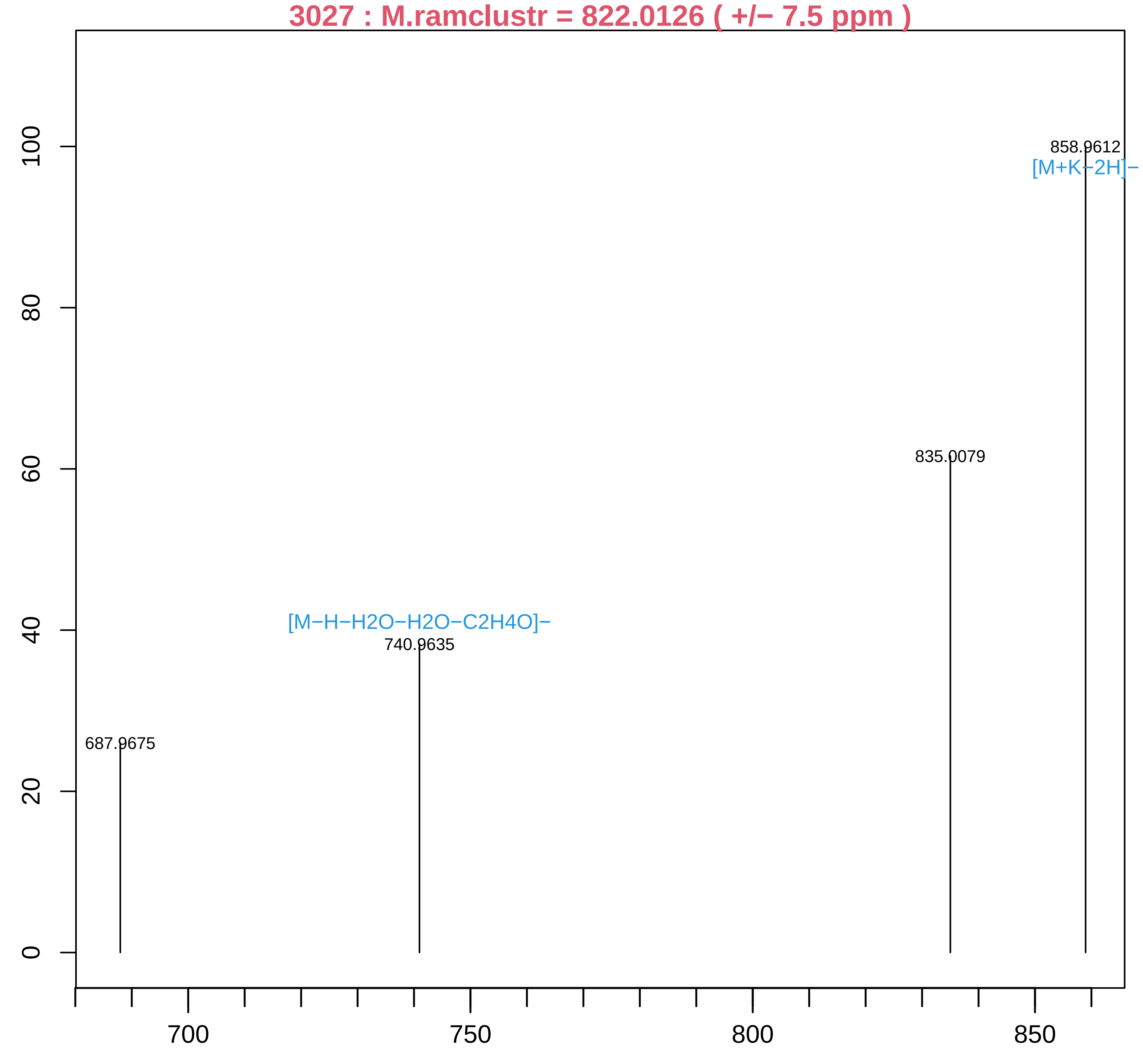

3027 : M.ramclustr = 822.0126 (+/− 7.5 ppm)
858.9612
[M+K−2H]−
835.0079
[M−H−H2O−H2O−C2H4O]−
740.9635
687.9675
0
20
40
60
80
100
700
750
800
850

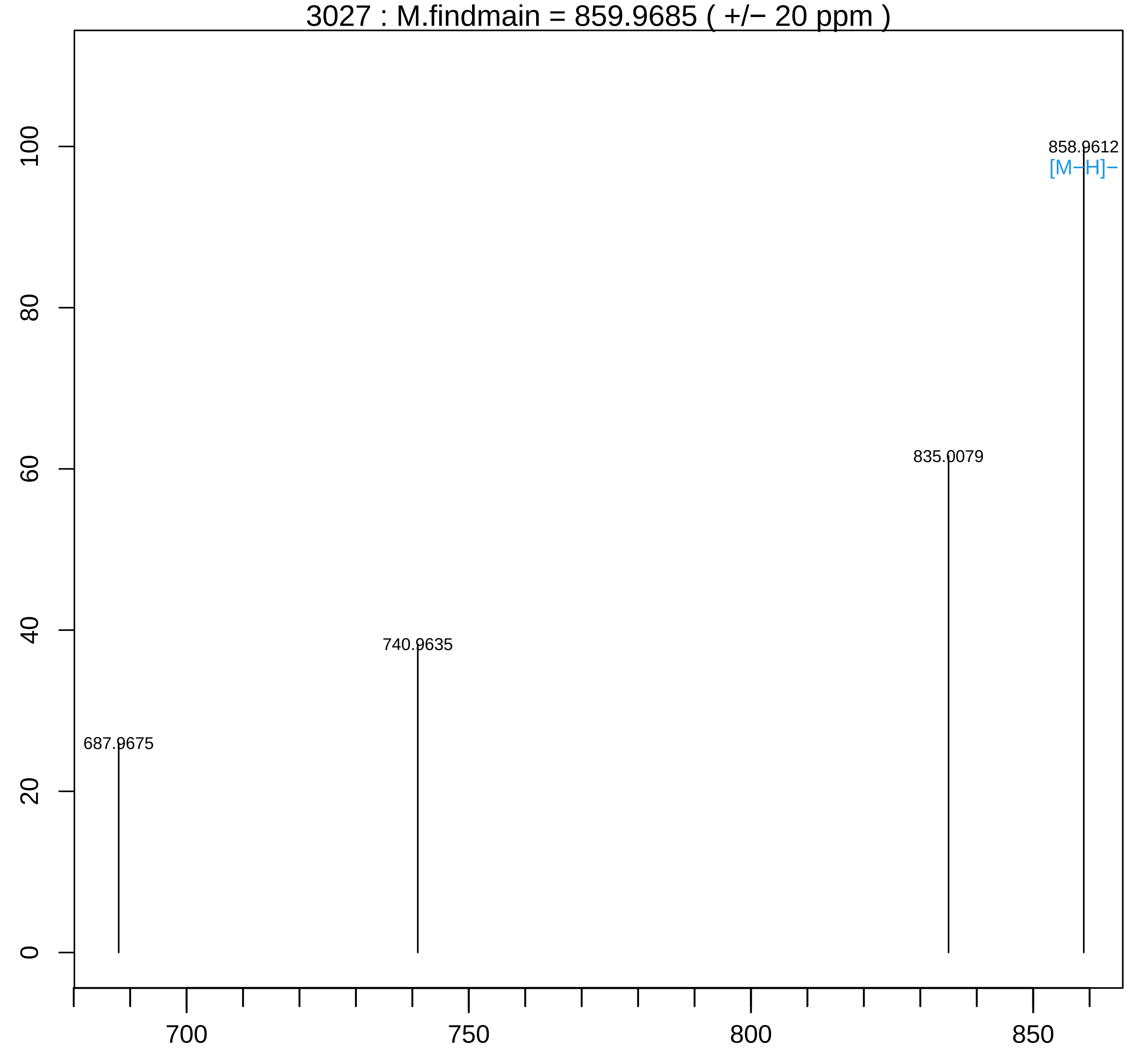

3027 : M.findmain = 859.9685 (+/− 20 ppm)
858.9612
[M−H]−
835.0079
740.9635
687.9675
0
20
40
60
80
100
700
750
800
850

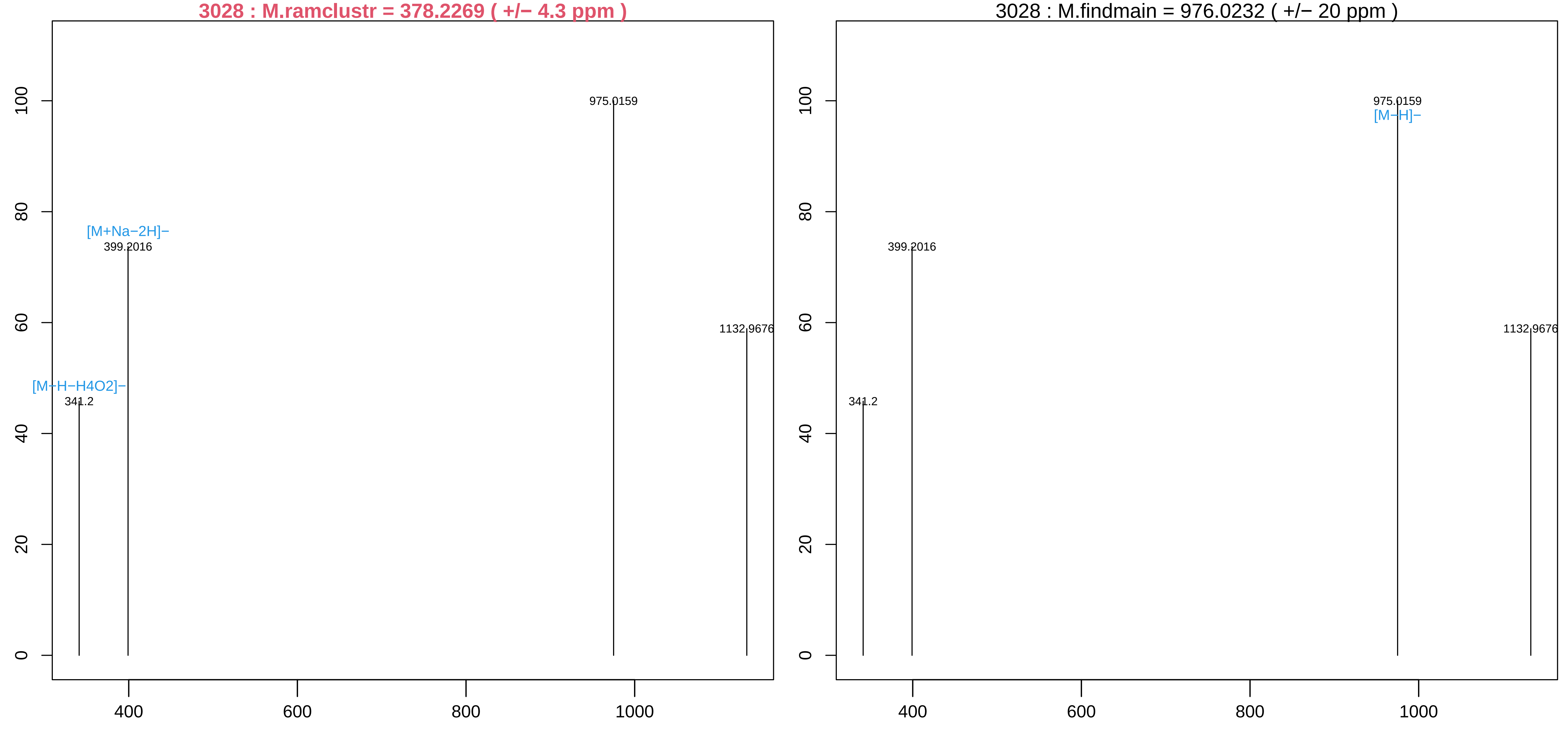
3028 : M.ramclustr = 378.2269 (+/− 4.3 ppm)
975.0159
[M+Na−2H]−
399.2016
1132.9676
[M+H−H4O2]−
341.2
3028 : M.findmain = 976.0232 (+/− 20 ppm)
975.0159
[M−H]−
399.2016
1132.9676
341.2
0
20
40
60
80
100
400
600
800
1000

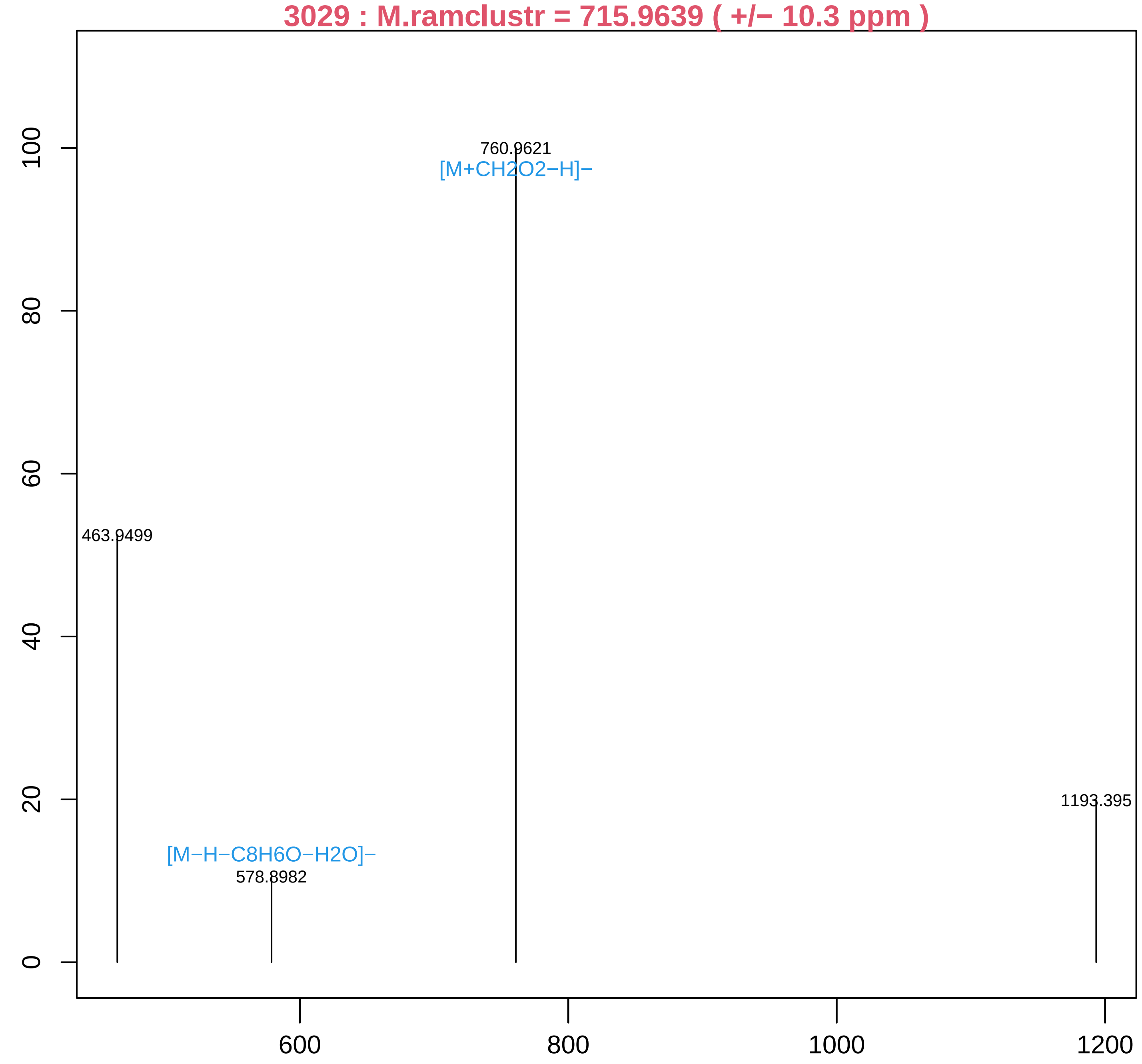
3029 : M.ramclustr = 715.9639 (+/− 10.3 ppm)
760.9621
[M+CH2O2−H]−
463.9499
[M−H−C8H6O−H2O]−
578.8982
1193.395
0
20
40
60
80
100
600
800
1000
1200

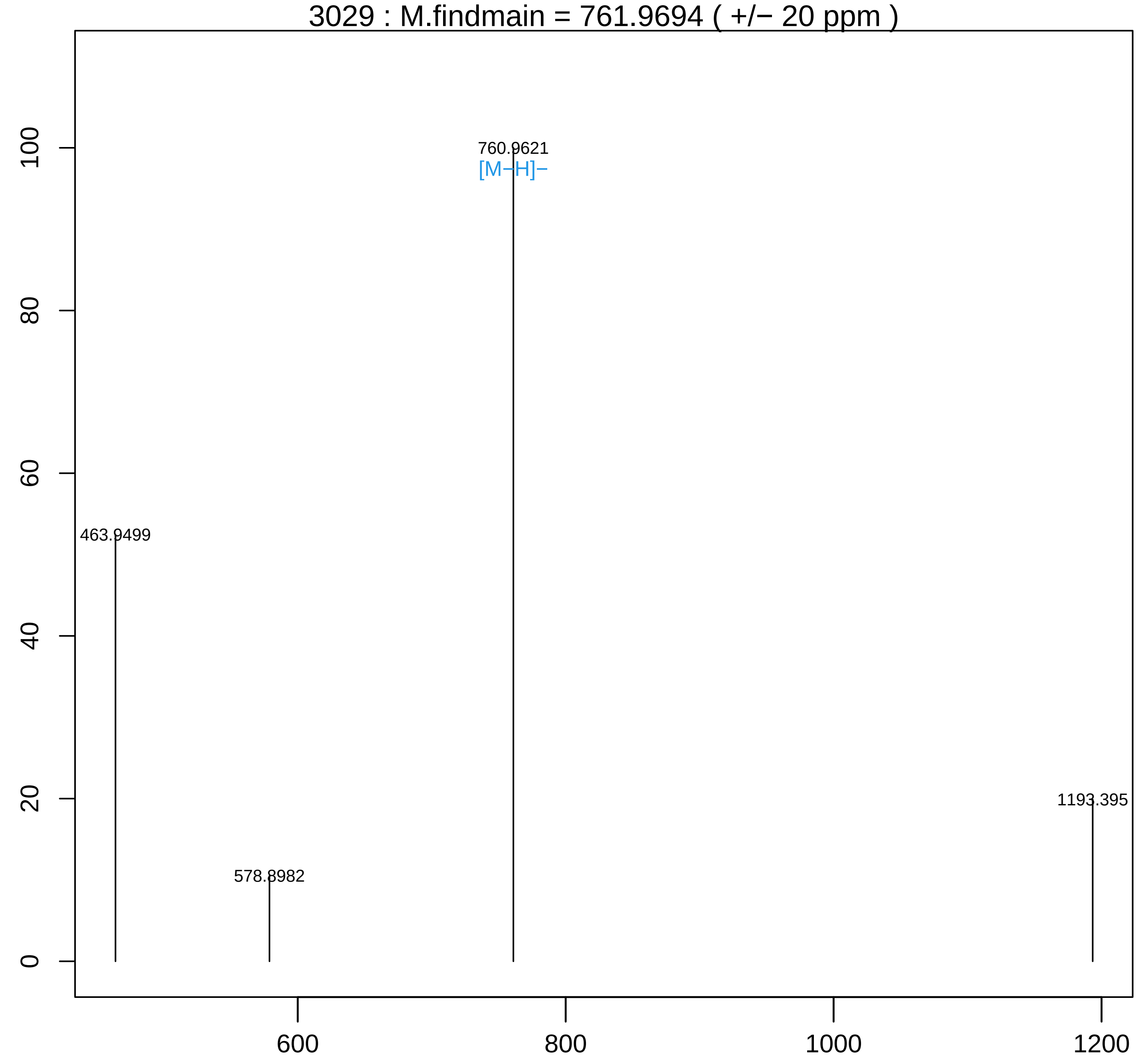
3029 : M.findmain = 761.9694 (+/− 20 ppm)
760.9621
[M−H]−
463.9499
578.8982
1193.395
0
20
40
60
80
100
600
800
1000
1200

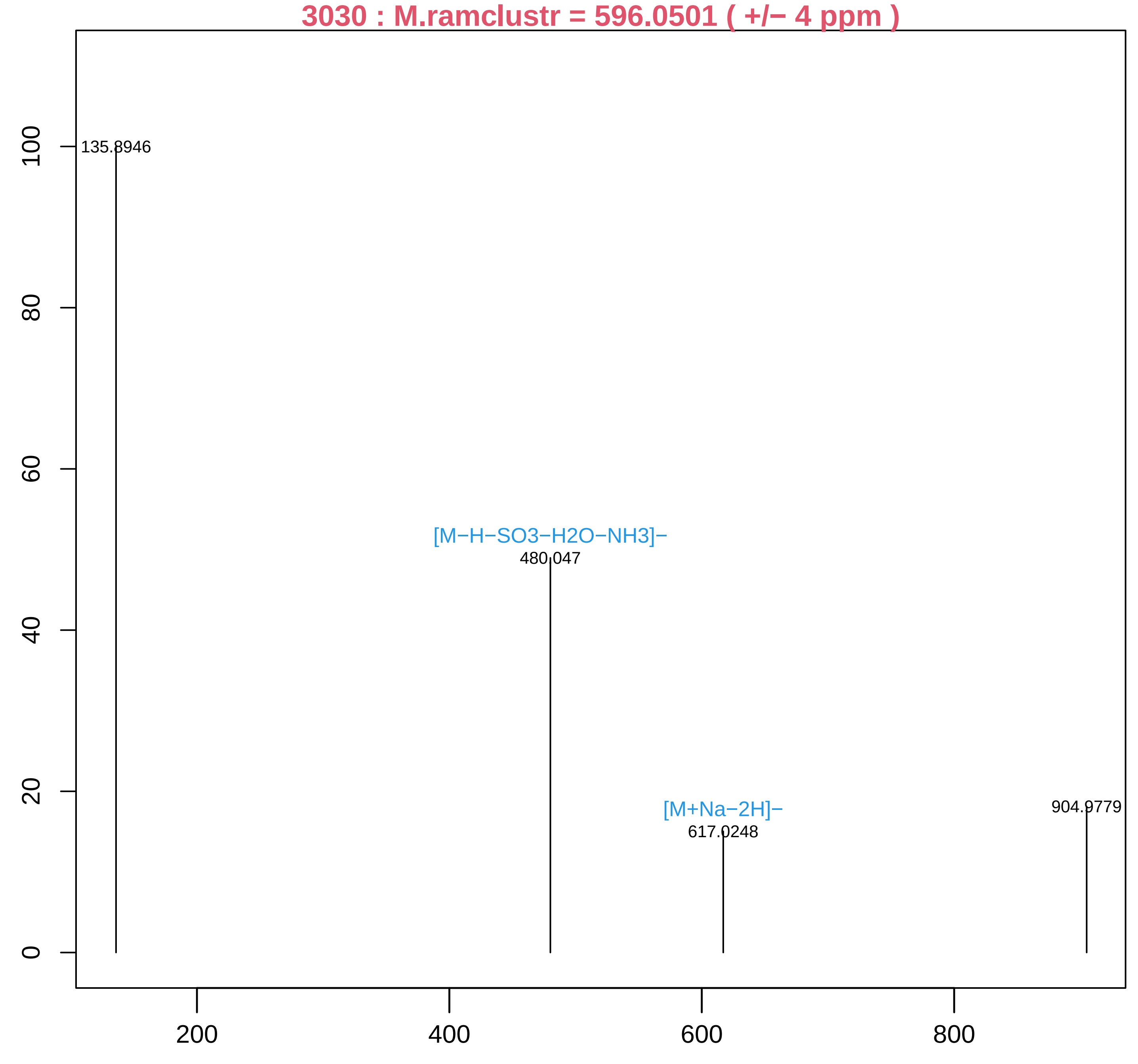
3030 : M.ramclustr = 596.0501 (+/− 4 ppm)
135.8946
[M−H−SO3−H2O−NH3]−
480.047
[M+Na−2H]−
617.0248
904.9779
0
20
40
60
80
100
200
400
600
800

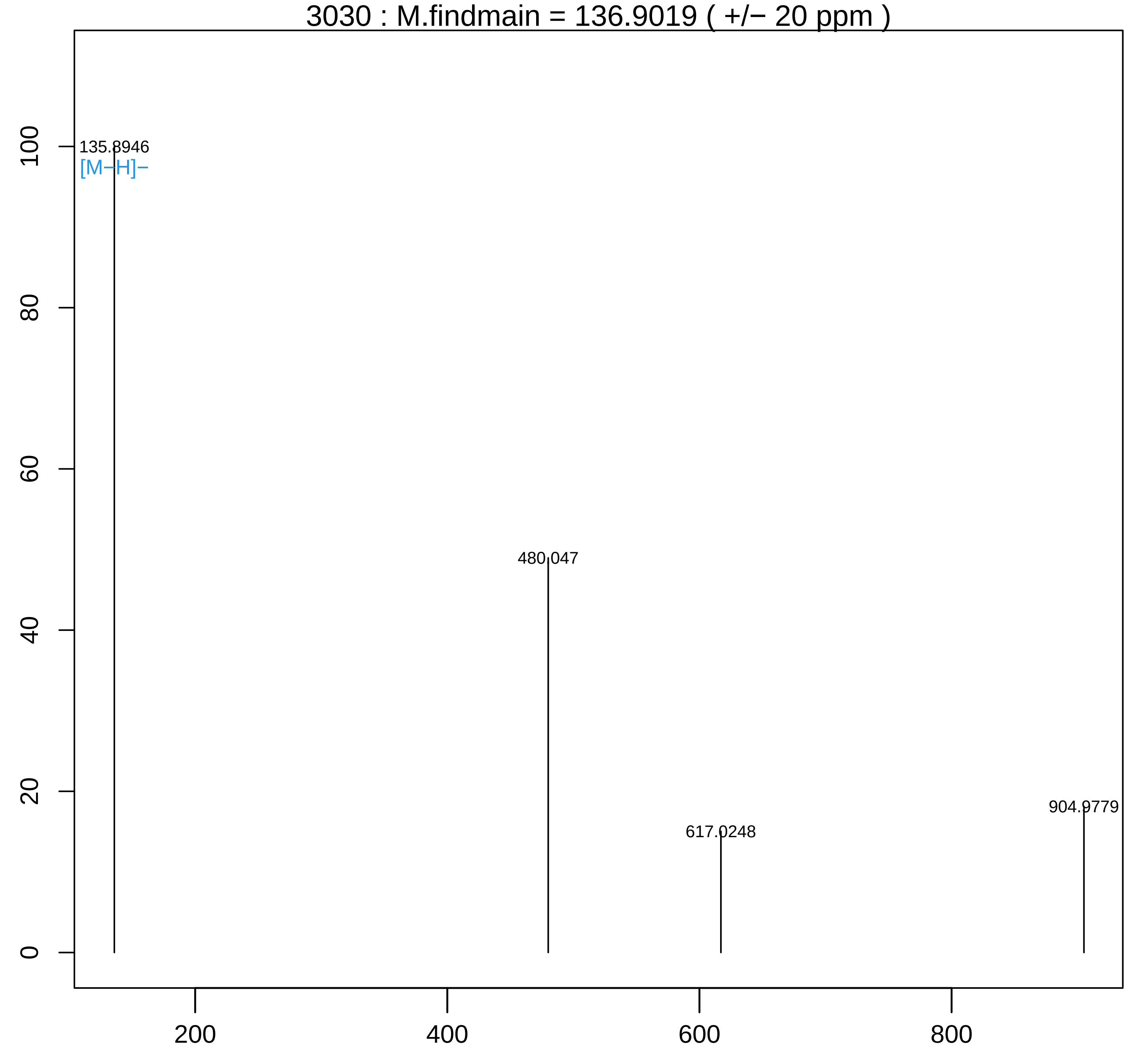
3030 : M.findmain = 136.9019 (+/− 20 ppm)
135.8946
[M−H]−
480.047
617.0248
904.9779
0
20
40
60
80
100
200
400
600
800

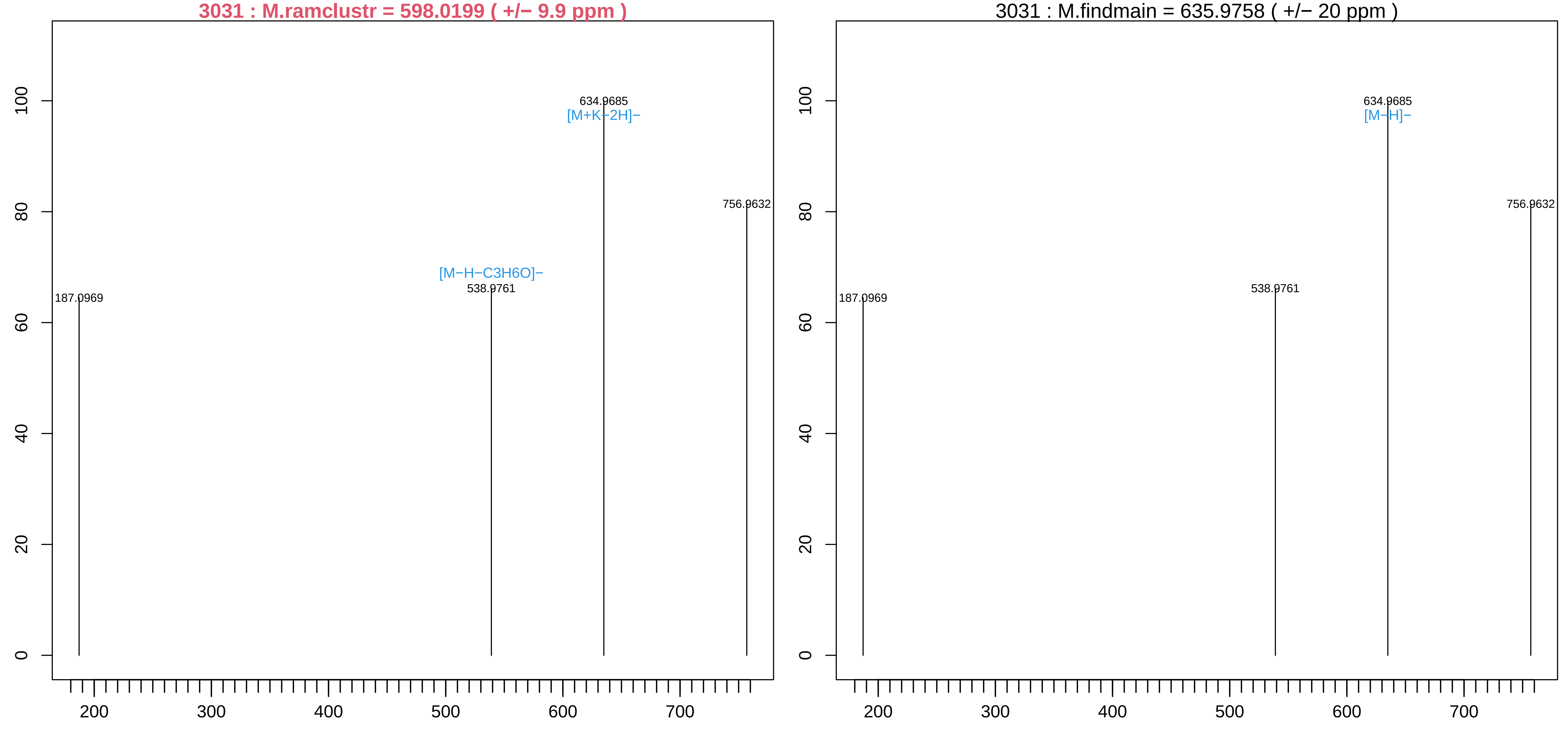

3031 : M.ramclustr = 598.0199 (+/− 9.9 ppm)
634.9685
[M+K−2H]−
756.9632
[M−H−C3H6O]−
538.9761
187.0969
3031 : M.findmain = 635.9758 (+/− 20 ppm)
634.9685
[M−H]−
756.9632
538.9761
187.0969
0
20
40
60
80
100
200
300
400
500
600
700

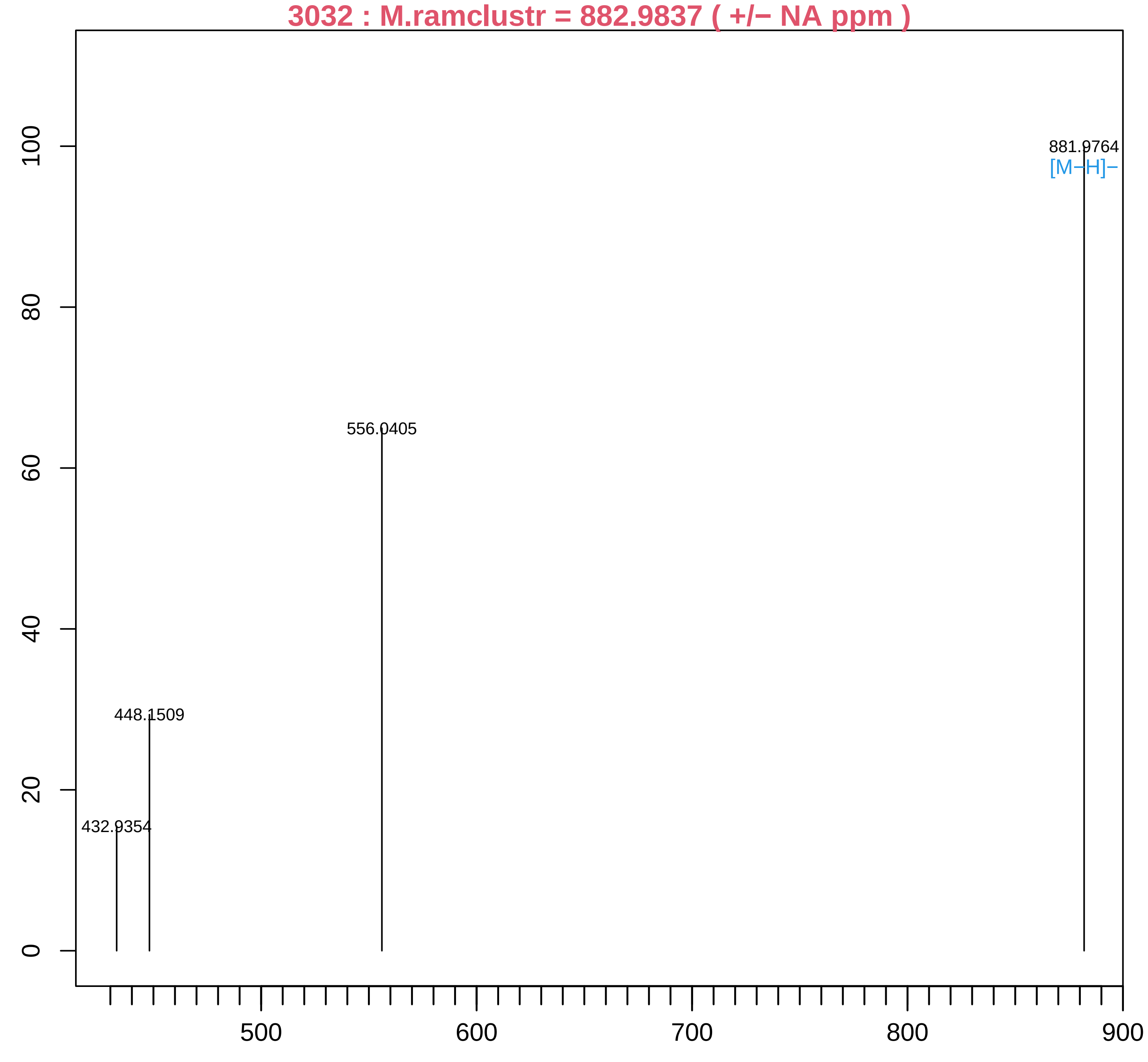

3032 : M.ramclustr = 882.9837 (+/− NA ppm)
881.9764
[M−H]−
556.0405
448.1509
432.9354
0
20
40
60
80
100
500
600
700
800
900

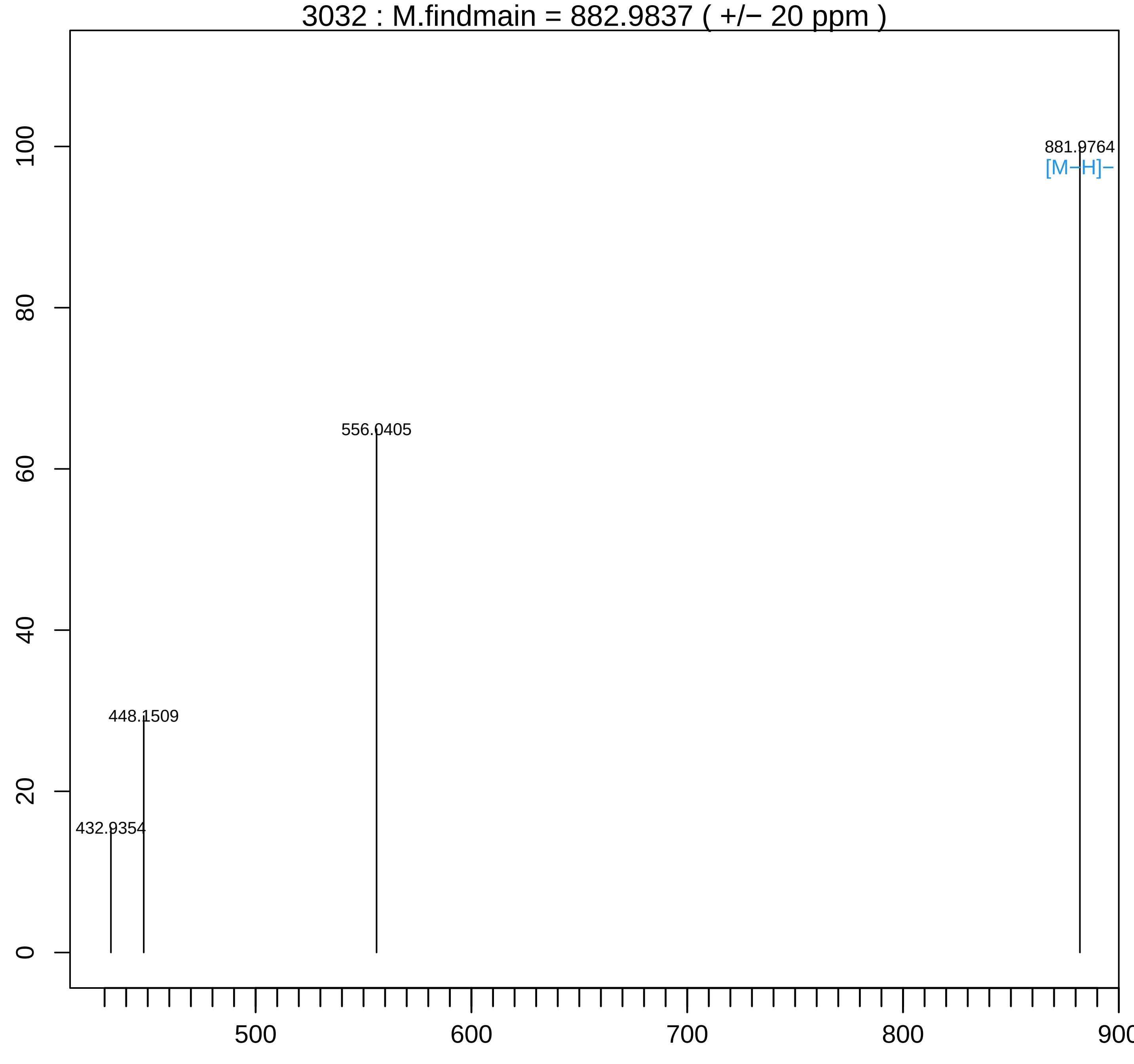

3032 : M.findmain = 882.9837 (+/− 20 ppm)
881.9764
[M−H]−
556.0405
448.1509
432.9354
0
20
40
60
80
100
500
600
700
800
900

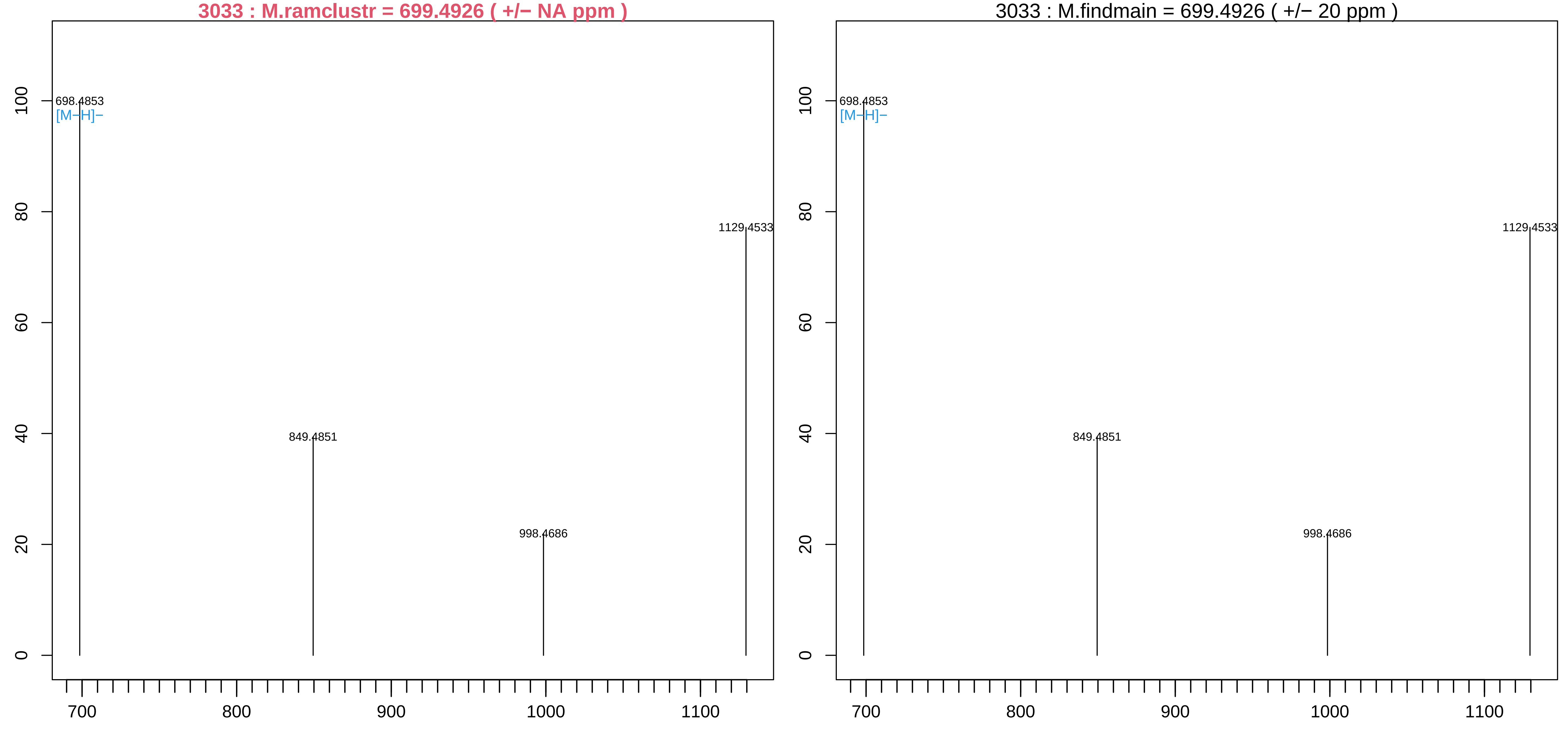

3033 : M.ramclustr = 699.4926 (+/− NA ppm)
698.4853
[M−H]−
849.4851
998.4686
1129.4533
0
20
40
60
80
100
700
800
900
1000
1100
3033 : M.findmain = 699.4926 (+/− 20 ppm)
698.4853
[M−H]−
849.4851
998.4686
1129.4533
0
20
40
60
80
100
700
800
900
1000
1100

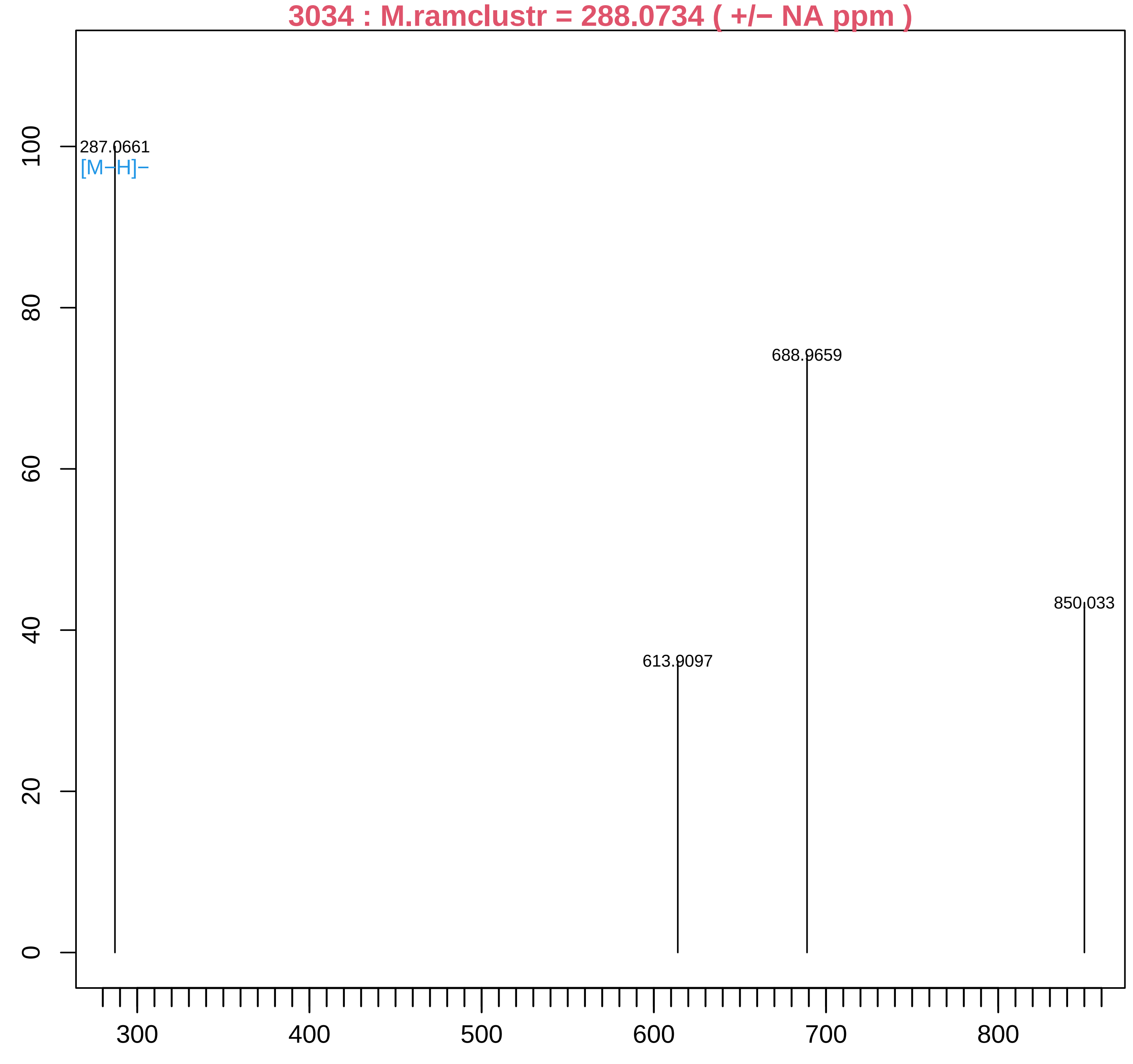
3034 : M.ramclustr = 288.0734 (+/− NA ppm)
287.0661
[M−H]−
688.9659
613.9097
850.033
0
20
40
60
80
100
300
400
500
600
700
800
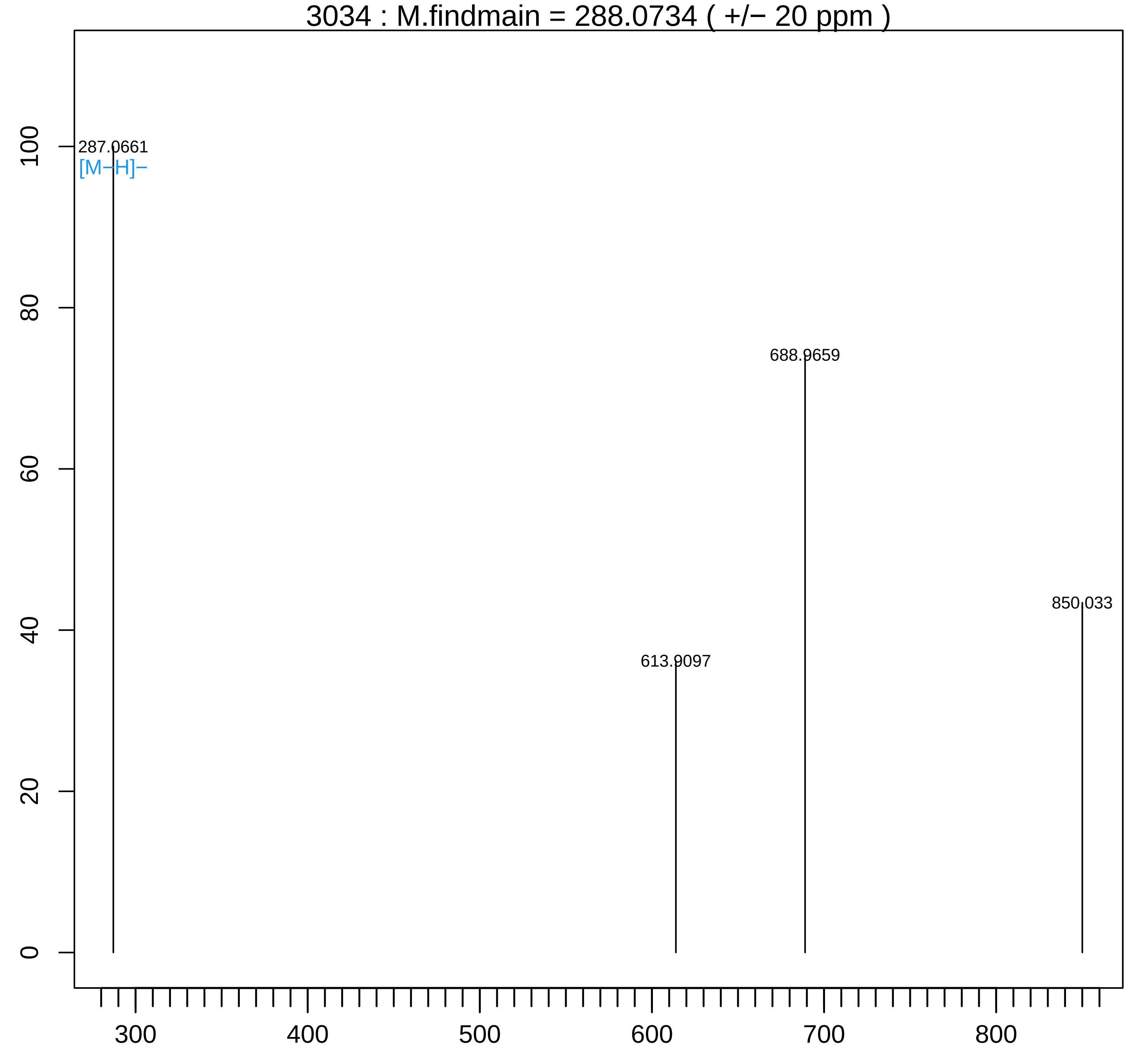
3034 : M.findmain = 288.0734 (+/− 20 ppm)
287.0661
[M−H]−
688.9659
613.9097
850.033
0
20
40
60
80
100
300
400
500
600
700
800

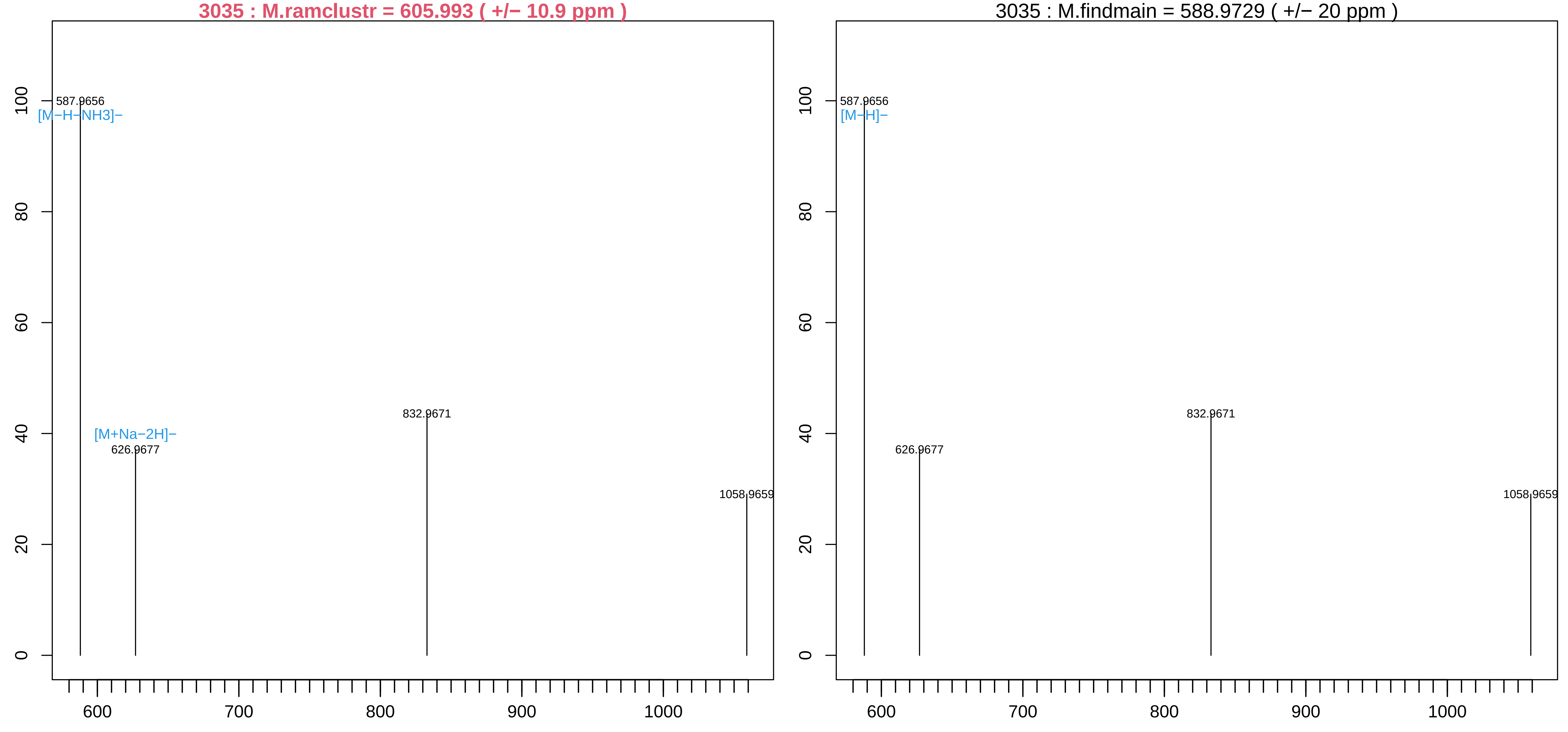

3035 : M.ramclustr = 605.993 (+/− 10.9 ppm)
587.9656
[M−H−NH3]−
[M+Na−2H]−
626.9677
832.9671
1058.9659
3035 : M.findmain = 588.9729 (+/− 20 ppm)
587.9656
[M−H]−
626.9677
832.9671
1058.9659

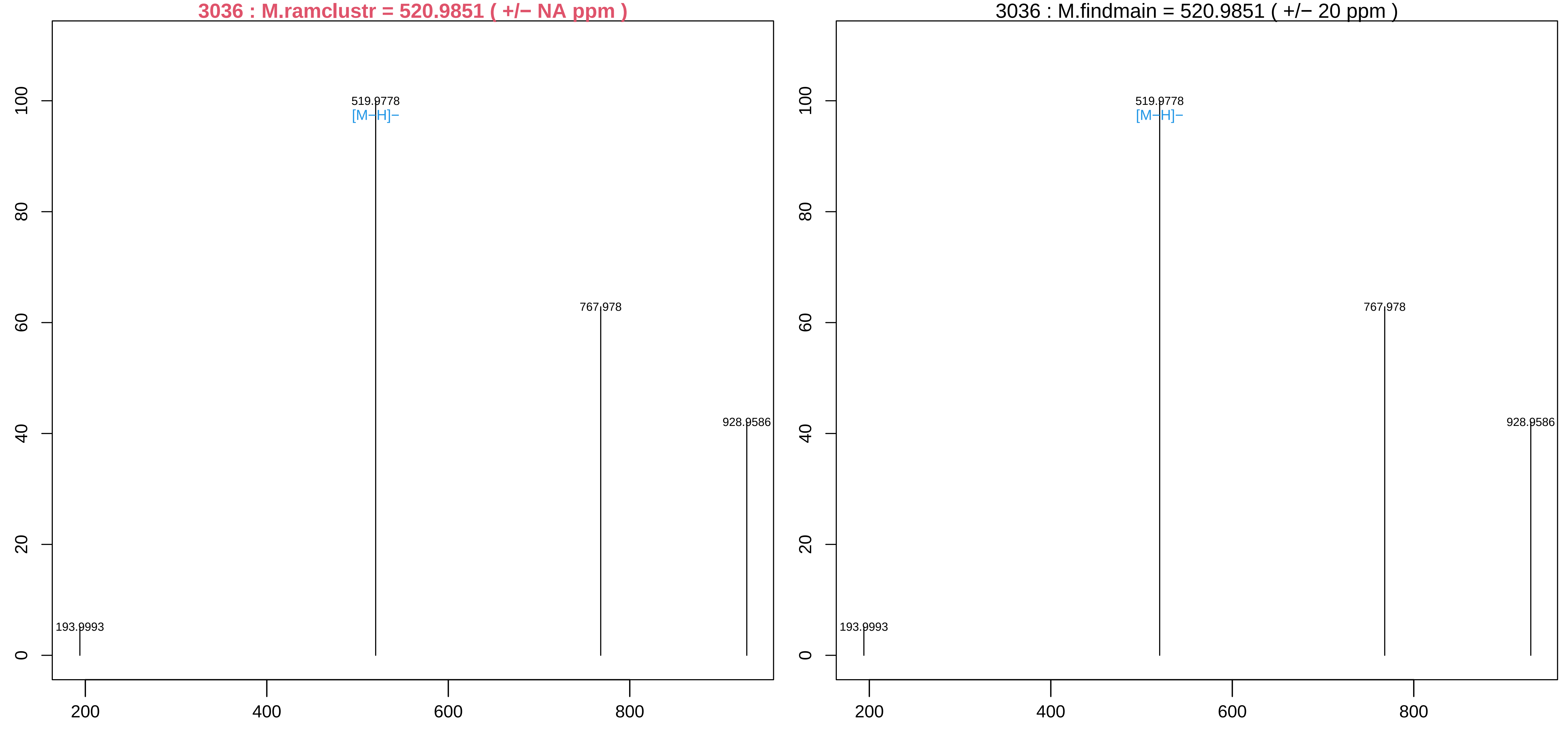

3036 : M.ramclustr = 520.9851 (+/− NA ppm)
519.9778
[M−H]−
767.978
928.9586
193.9993
0
20
40
60
80
100
200
400
600
800
3036 : M.findmain = 520.9851 (+/− 20 ppm)
519.9778
[M−H]−
767.978
928.9586
193.9993
0
20
40
60
80
100
200
400
600
800

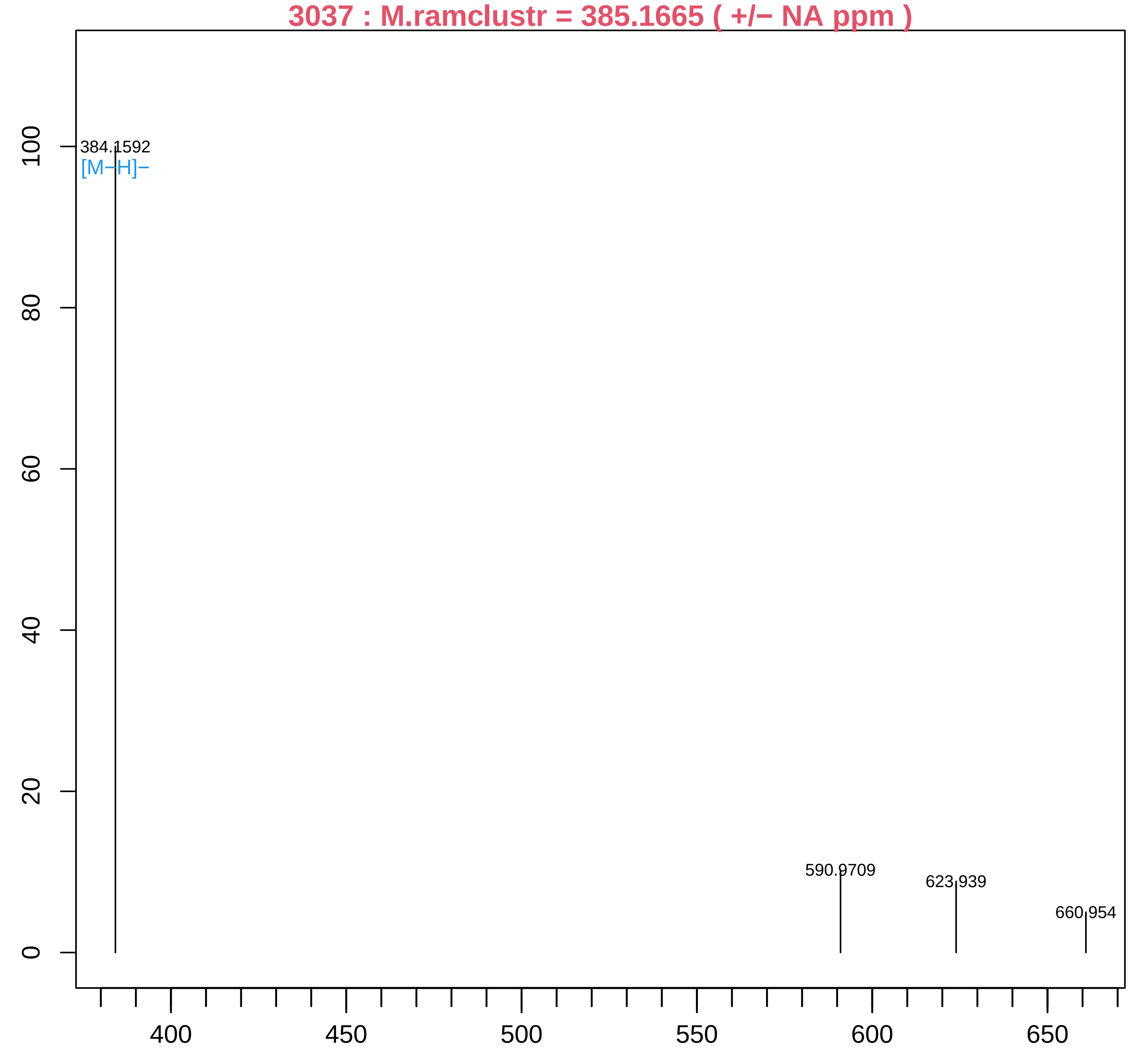
3037 : M.ramclustr = 385.1665 (+/− NA ppm)
100
80
60
40
20
0
384.1592
[M−H]−
590.9709
623.939
660.954
400
450
500
550
600
650
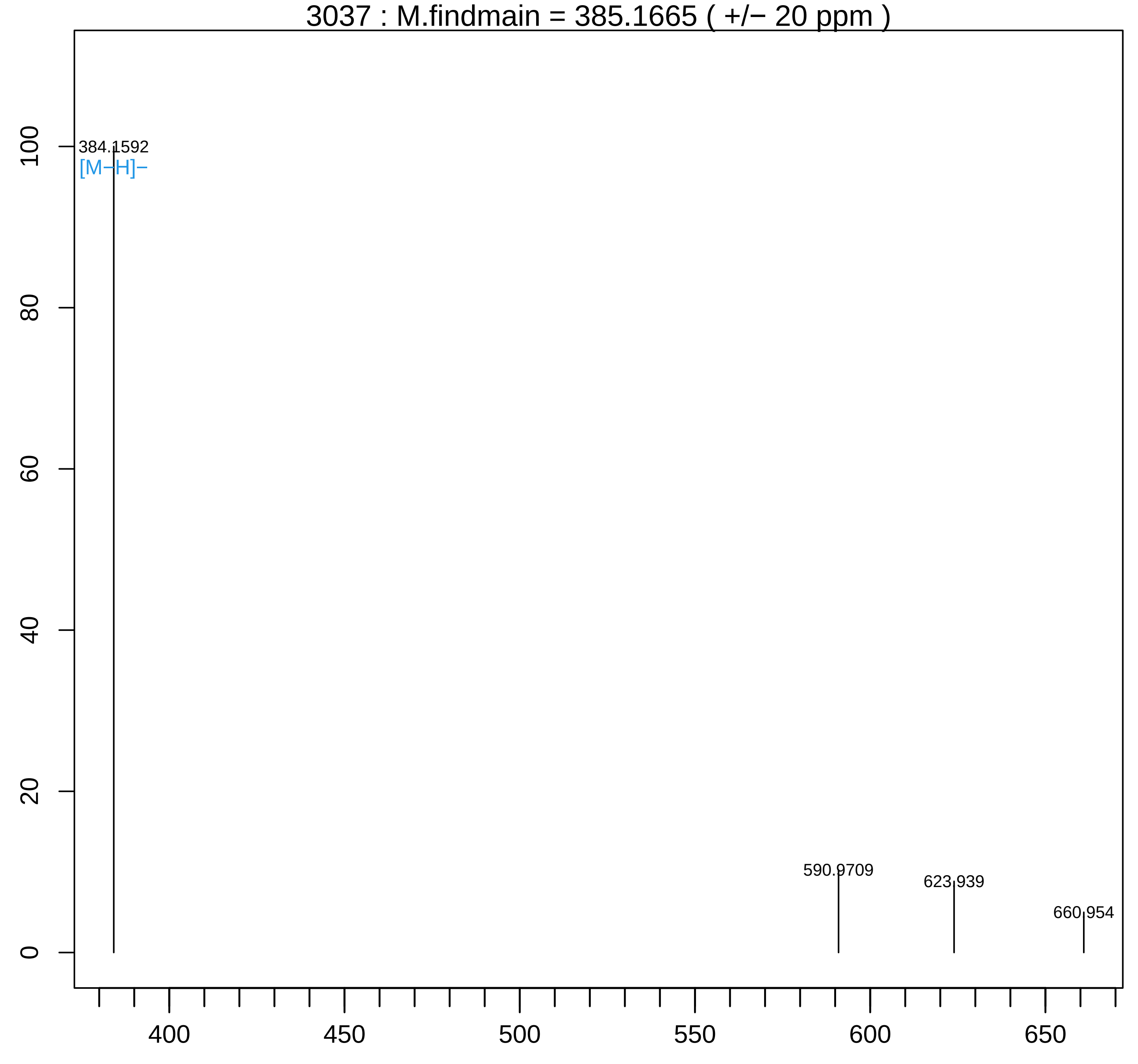
3037 : M.findmain = 385.1665 (+/− 20 ppm)
100
80
60
40
20
0
384.1592
[M−H]−
590.9709
623.939
660.954
400
450
500
550
600
650

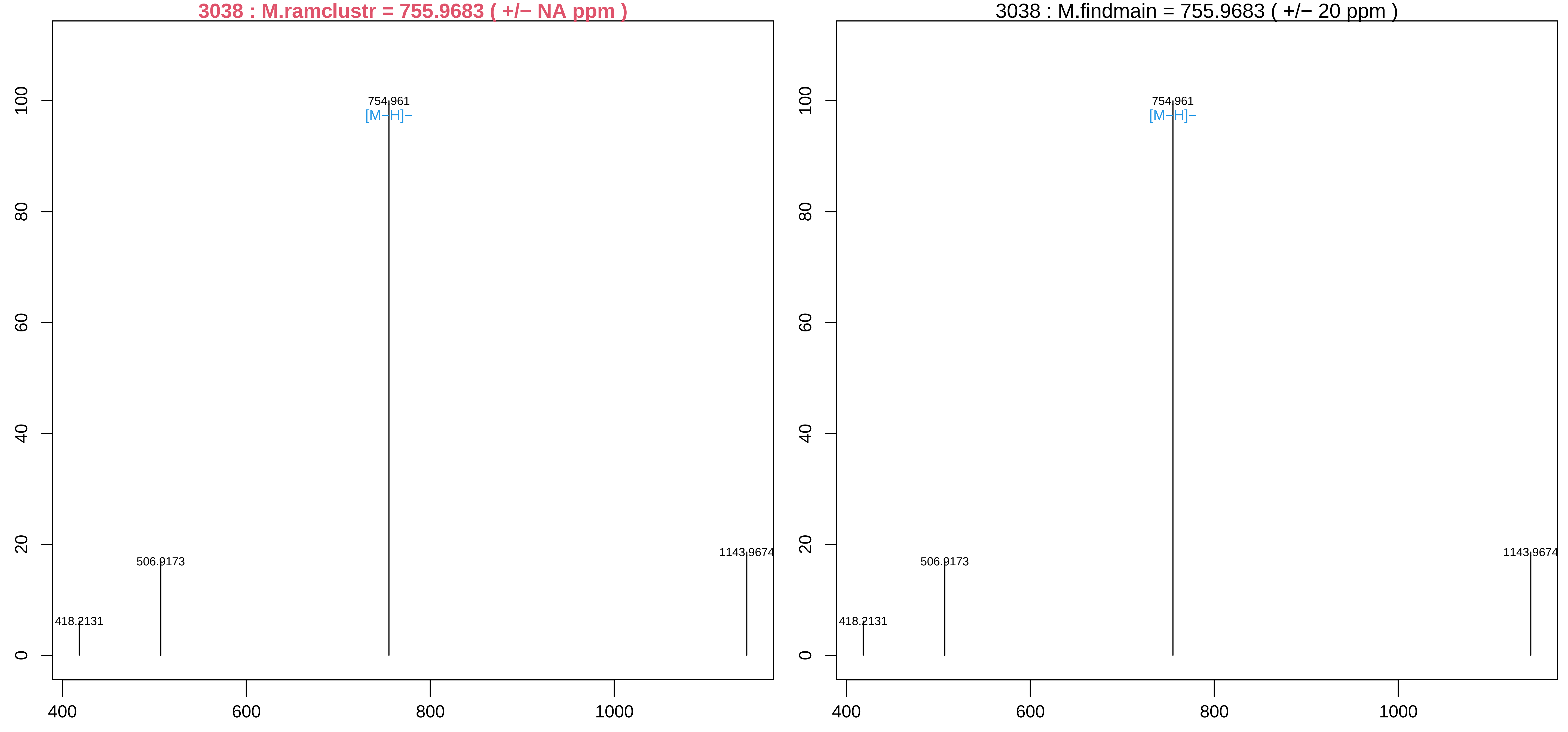
3038 : M.ramclustr = 755.9683 (+/− NA ppm)
3038 : M.findmain = 755.9683 (+/− 20 ppm)
754.961
[M−H]−
506.9173
418.2131
1143.9674
0
20
40
60
80
100
400
600
800
1000

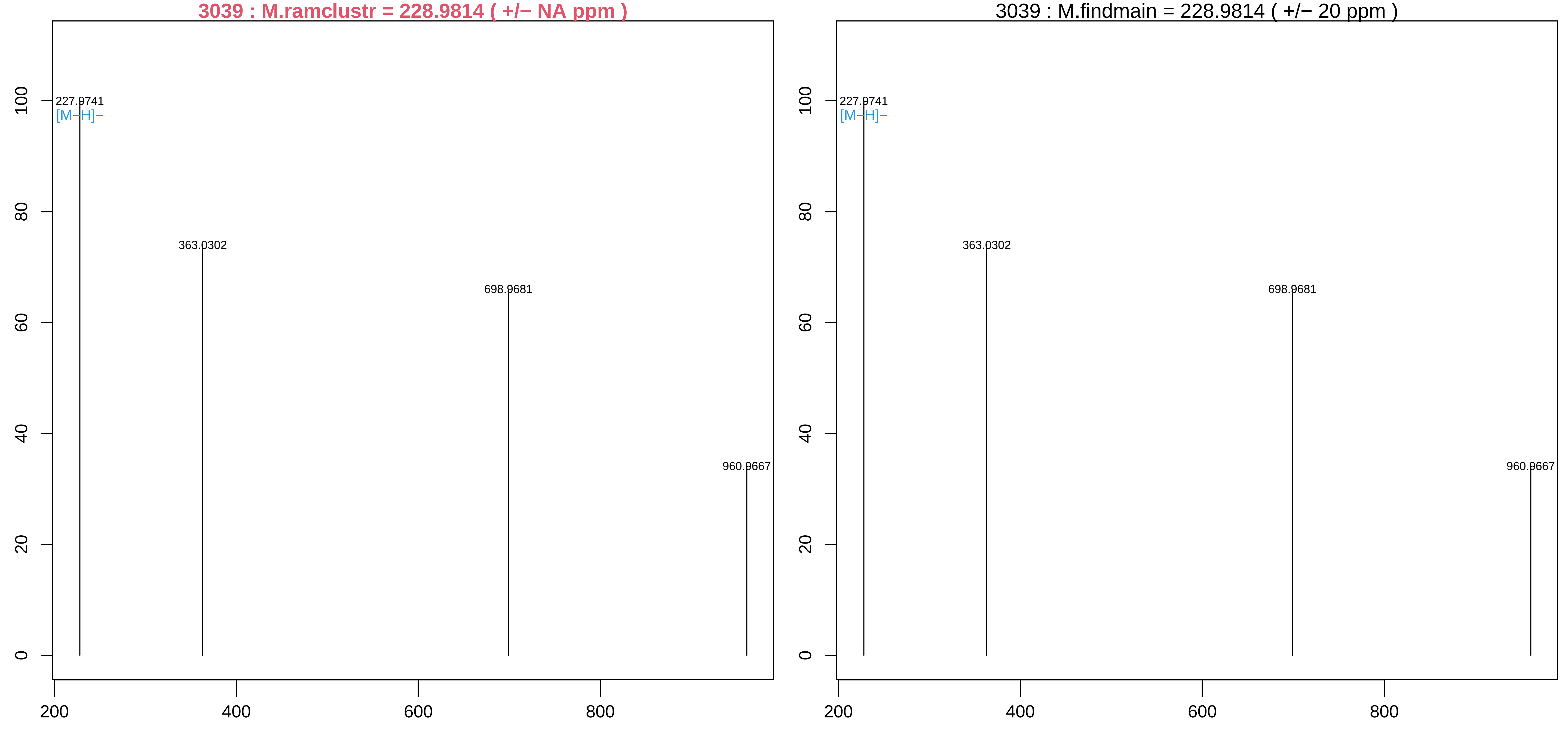
3039 : M.ramclustr = 228.9814 (+/− NA ppm)
227.9741
[M−H]−
363.0302
698.9681
960.9667
3039 : M.findmain = 228.9814 (+/− 20 ppm)
227.9741
[M−H]−
363.0302
698.9681
960.9667
0
20
40
60
80
100
200
400
600
800

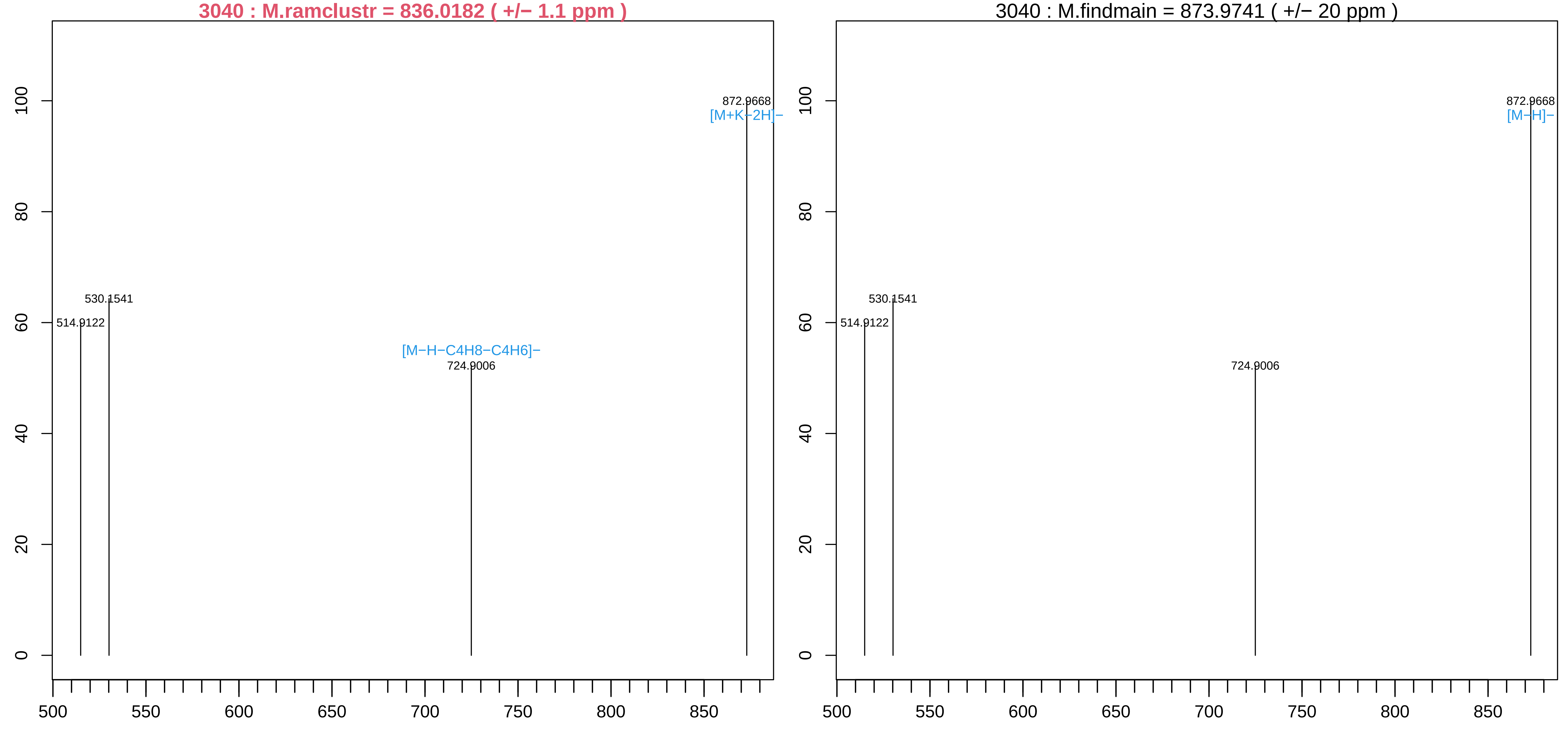

3040 : M.ramclustr = 836.0182 (+/− 1.1 ppm)
872.9668
[M+K−2H]−
530.1541
514.9122
[M−H−C4H8−C4H6]−
724.9006
3040 : M.findmain = 873.9741 (+/− 20 ppm)
872.9668
[M−H]−
530.1541
514.9122
724.9006
0
20
40
60
80
100
500
550
600
650
700
750
800
850

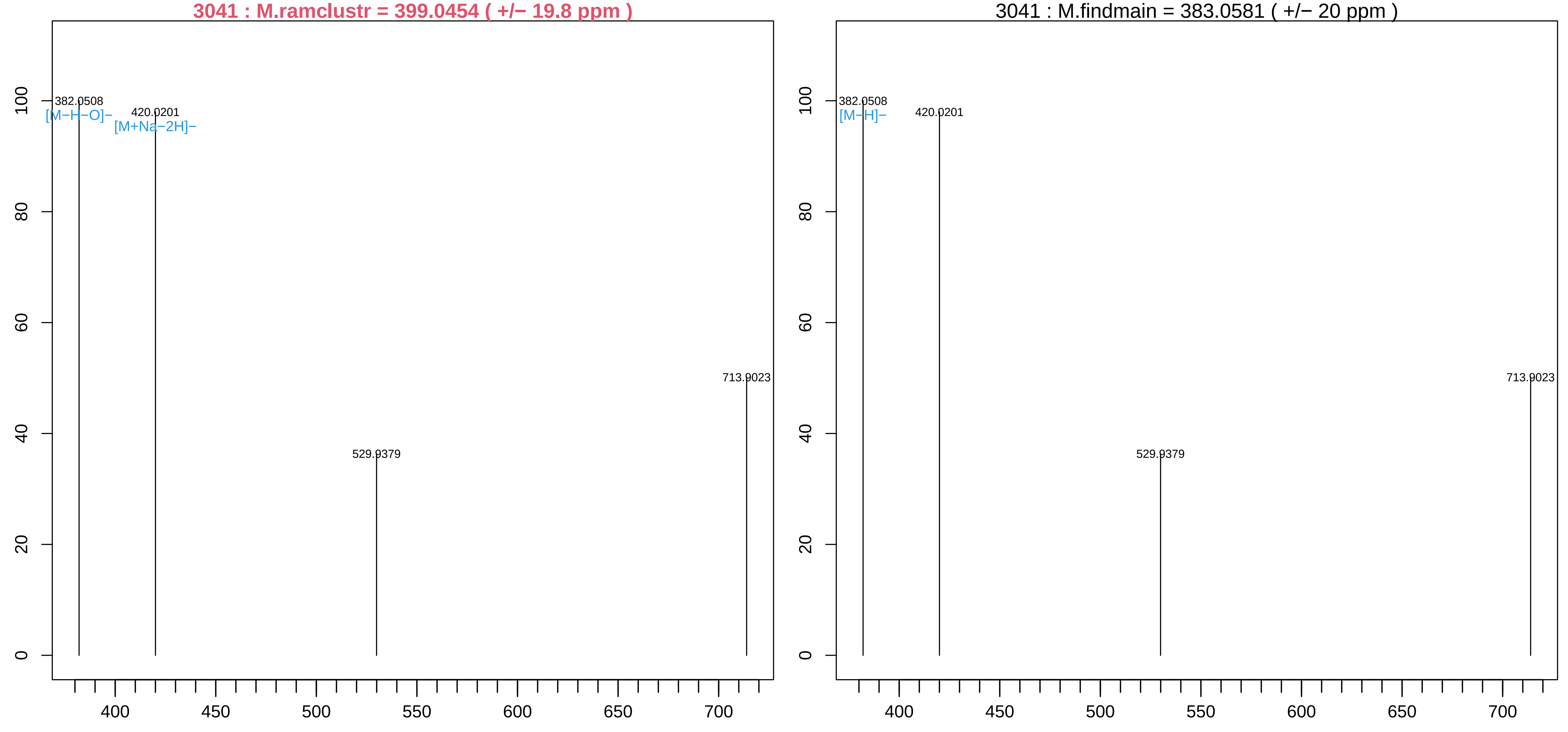

3041 : M.ramclustr = 399.0454 (+/− 19.8 ppm)
382.0508
[M−H−O]−
420.0201
[M+Na−2H]−
529.9379
713.9023
3041 : M.findmain = 383.0581 (+/− 20 ppm)
382.0508
[M−H]−
420.0201
529.9379
713.9023
0
20
40
60
80
100
400
450
500
550
600
650
700

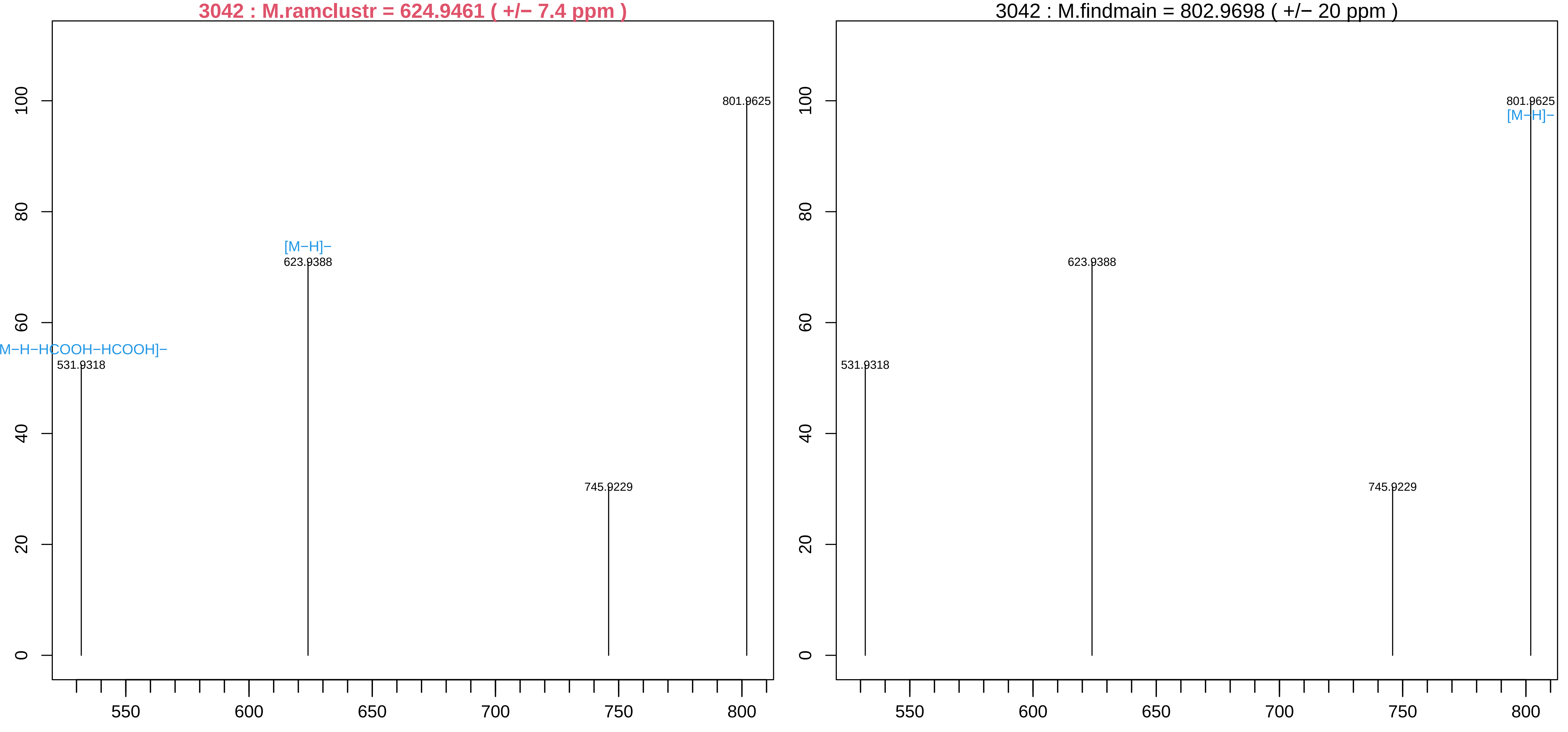
3042 : M.ramclustr = 624.9461 (+/− 7.4 ppm)
M−H−HCOOH−HCOOH]−
531.9318
[M−H]−
623.9388
745.9229
801.9625
0
20
40
60
80
100
550
600
650
700
750
800
3042 : M.findmain = 802.9698 (+/− 20 ppm)
531.9318
623.9388
745.9229
801.9625
[M−H]−
0
20
40
60
80
100
550
600
650
700
750
800

3043 : M.ramclustr = 371.951 (+/− NA ppm)

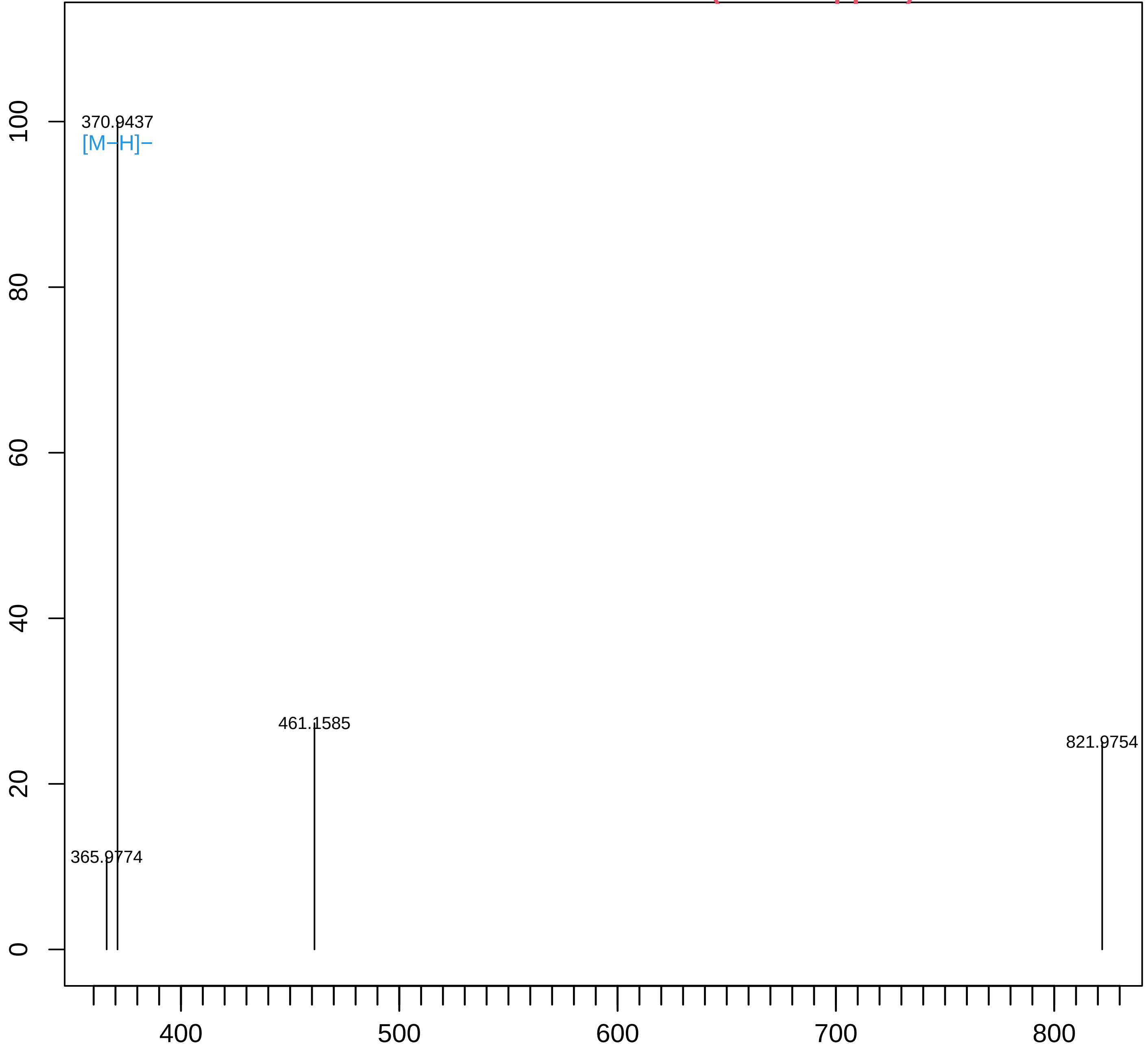

3043 : M.findmain = 371.951 (+/− 20 ppm)

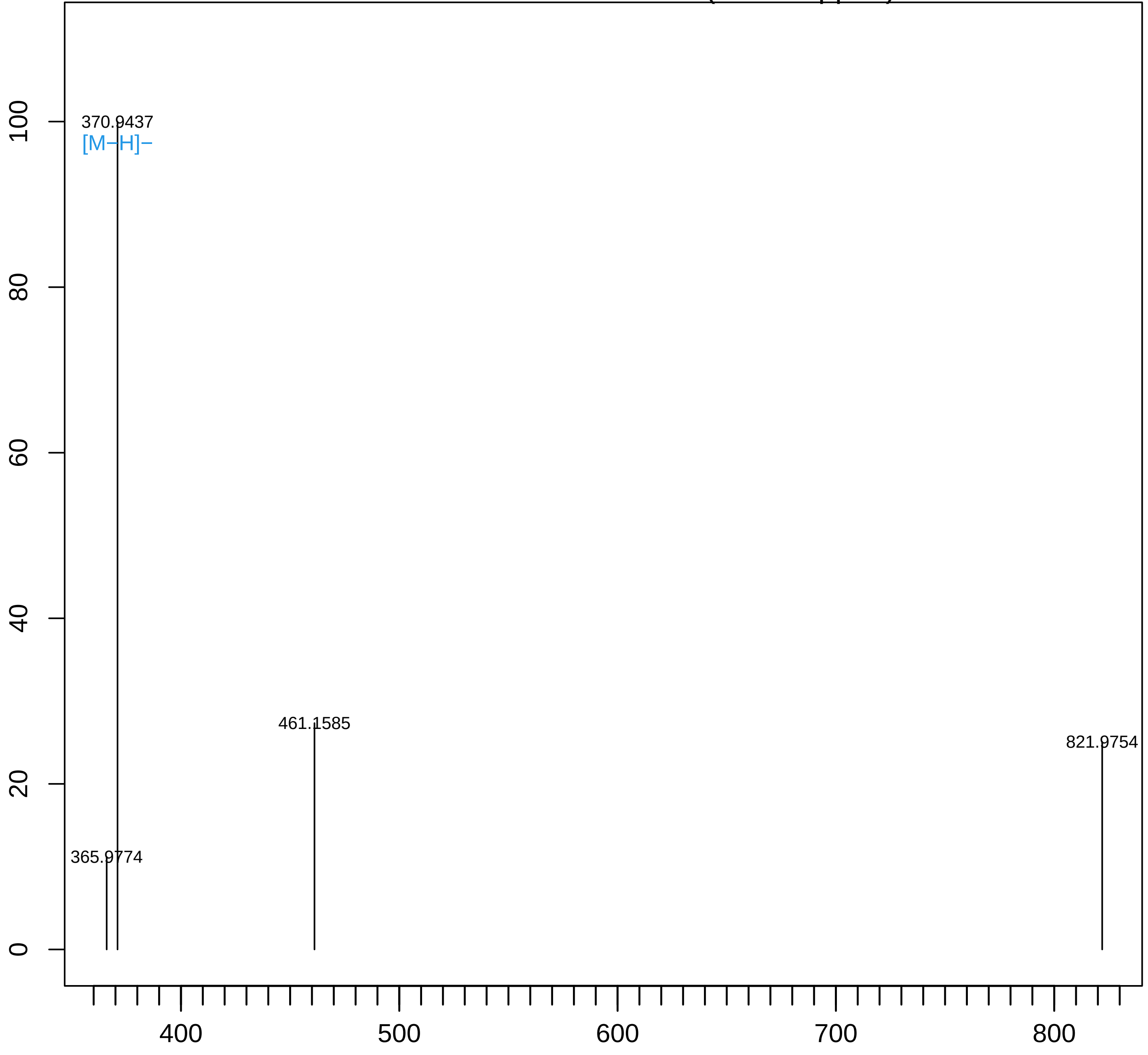

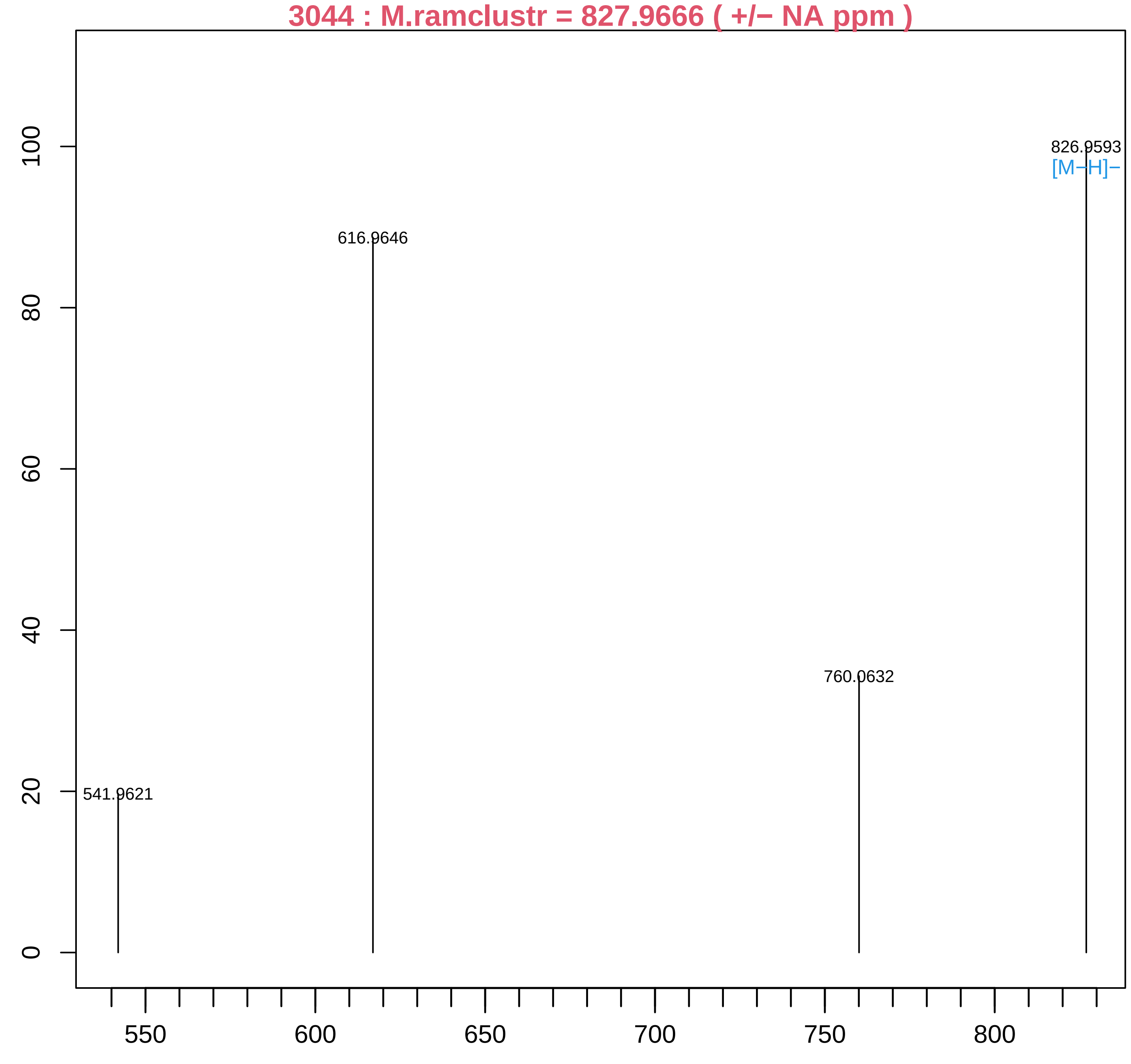
3044 : M.ramclustr = 827.9666 (+/− NA ppm)
826.9593
[M−H]−
616.9646
760.0632
541.9621
0
20
40
60
80
100
550
600
650
700
750
800

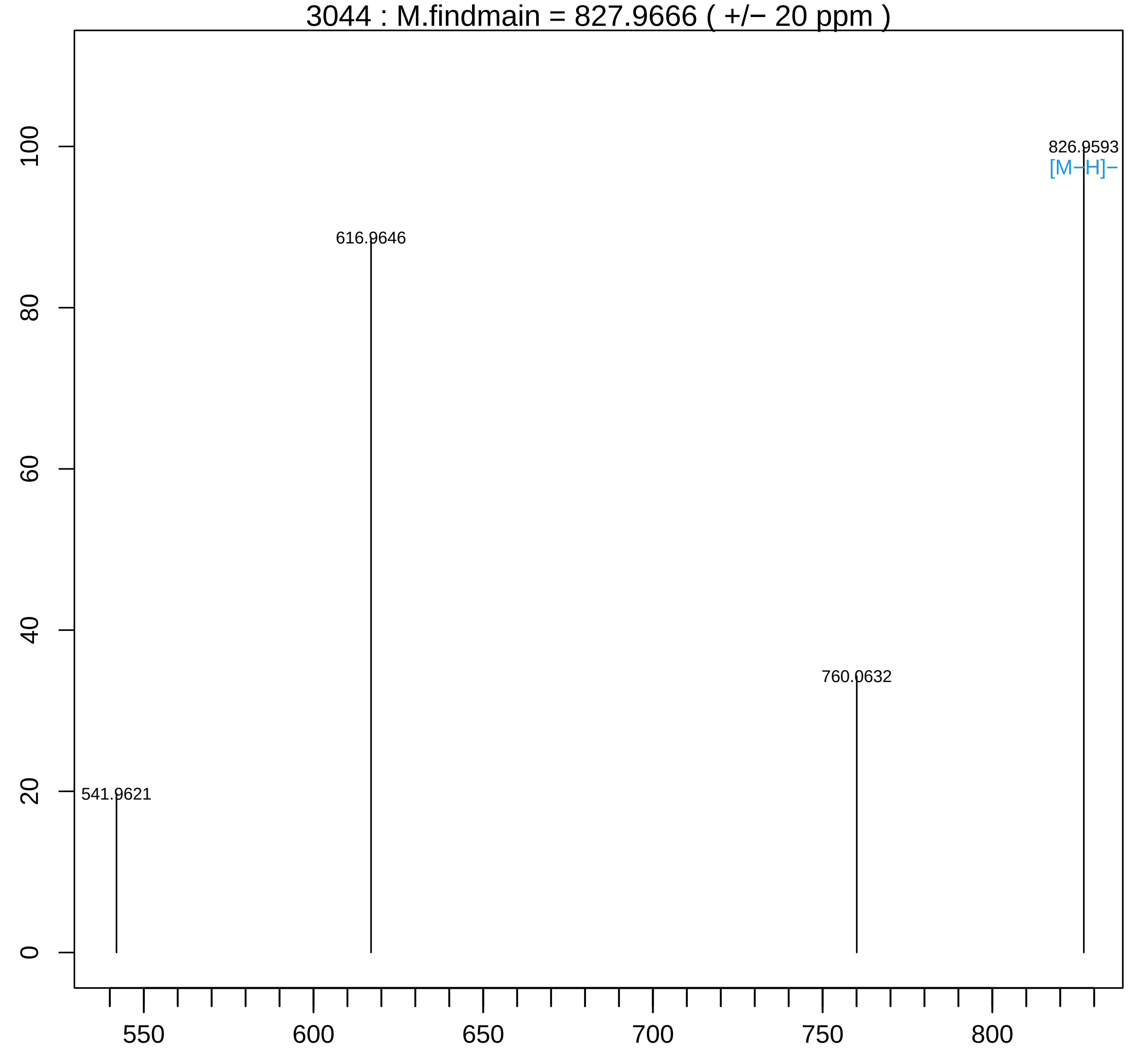
3044 : M.findmain = 827.9666 (+/− 20 ppm)
826.9593
[M−H]−
616.9646
760.0632
541.9621
0
20
40
60
80
100
550
600
650
700
750
800

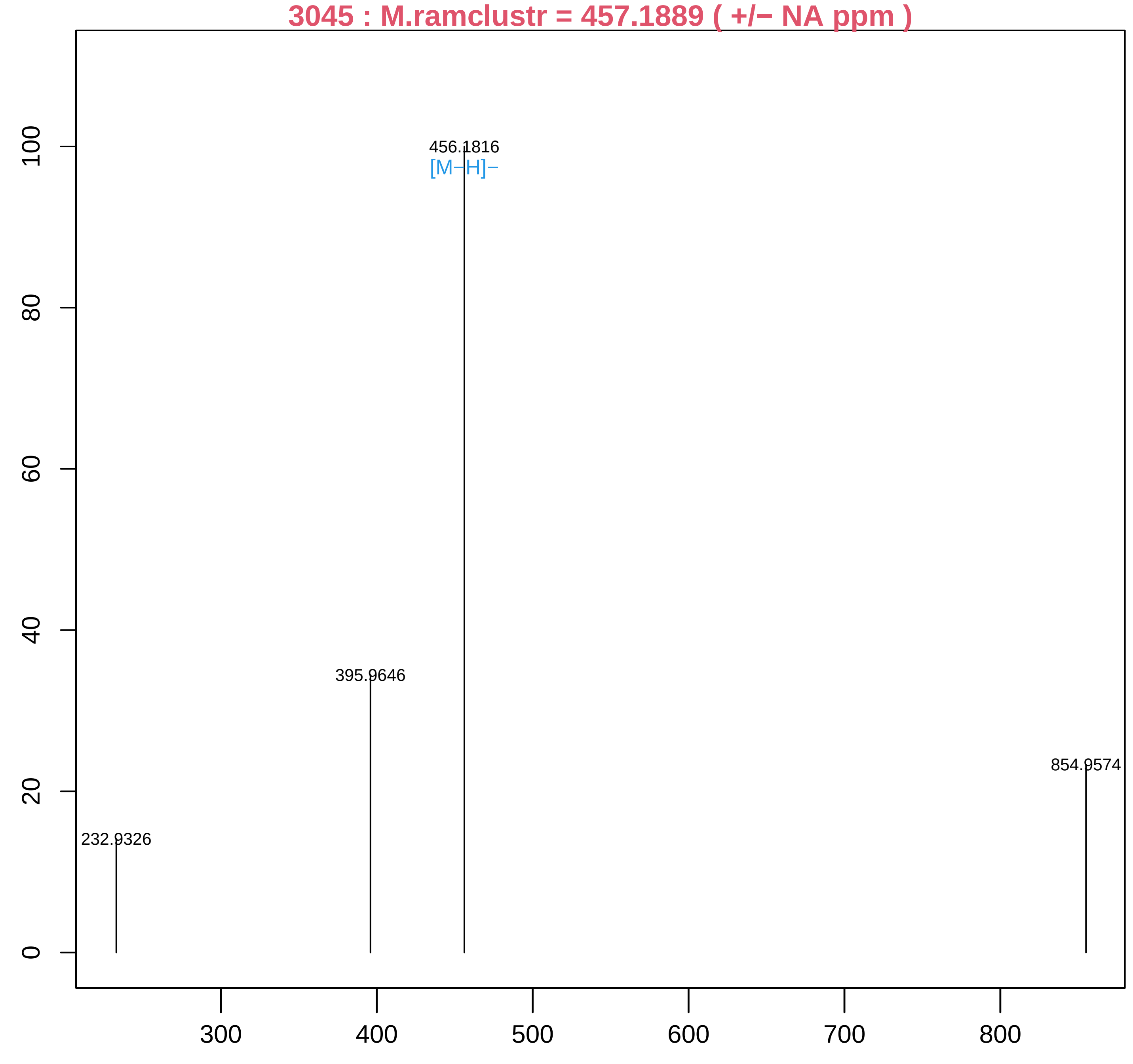
3045 : M.ramclustr = 457.1889 (+/− NA ppm)
456.1816
[M−H]−
395.9646
854.9574
232.9326
0
20
40
60
80
100
300
400
500
600
700
800

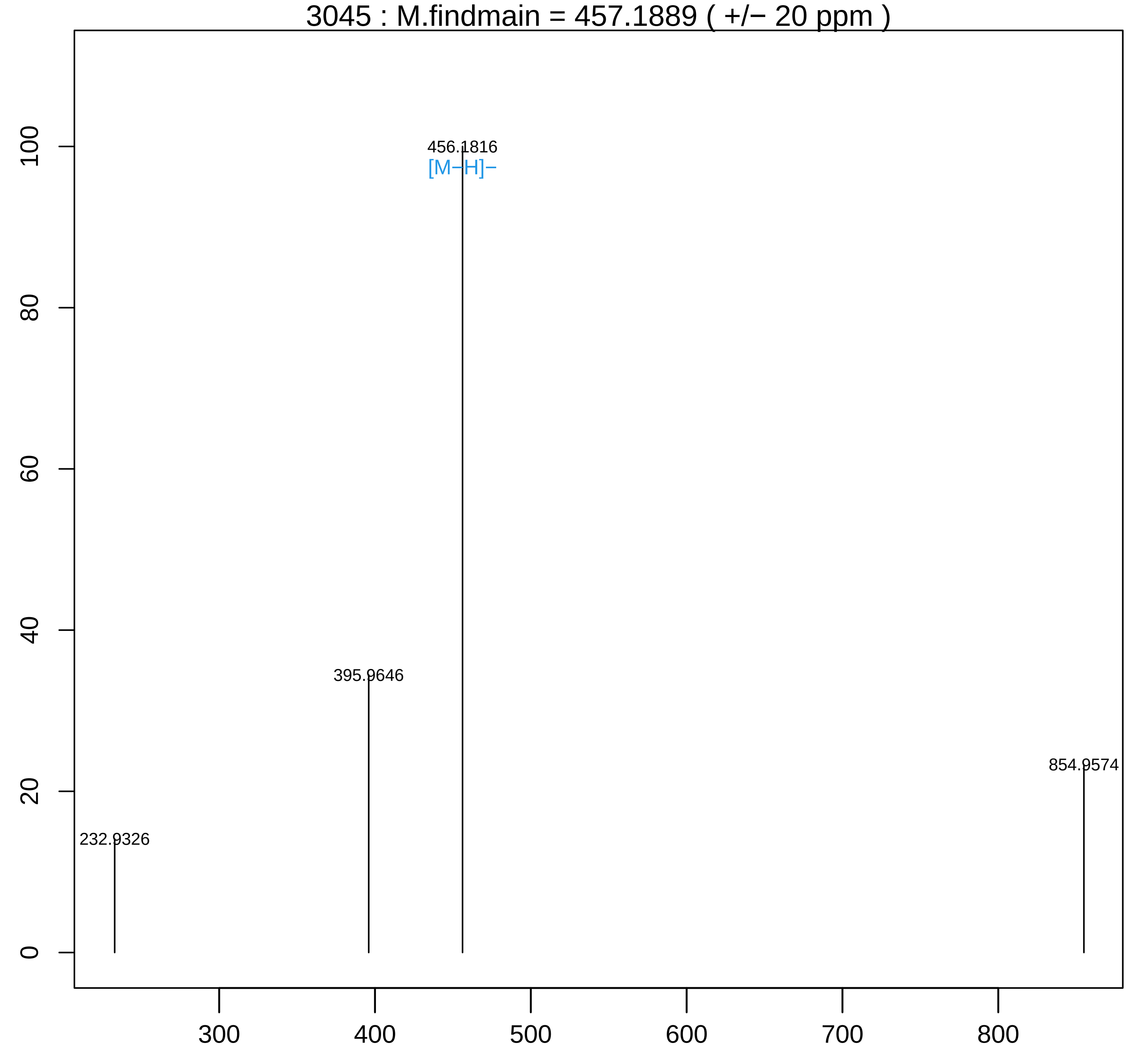
3045 : M.findmain = 457.1889 (+/− 20 ppm)
456.1816
[M−H]−
395.9646
854.9574
232.9326
0
20
40
60
80
100
300
400
500
600
700
800

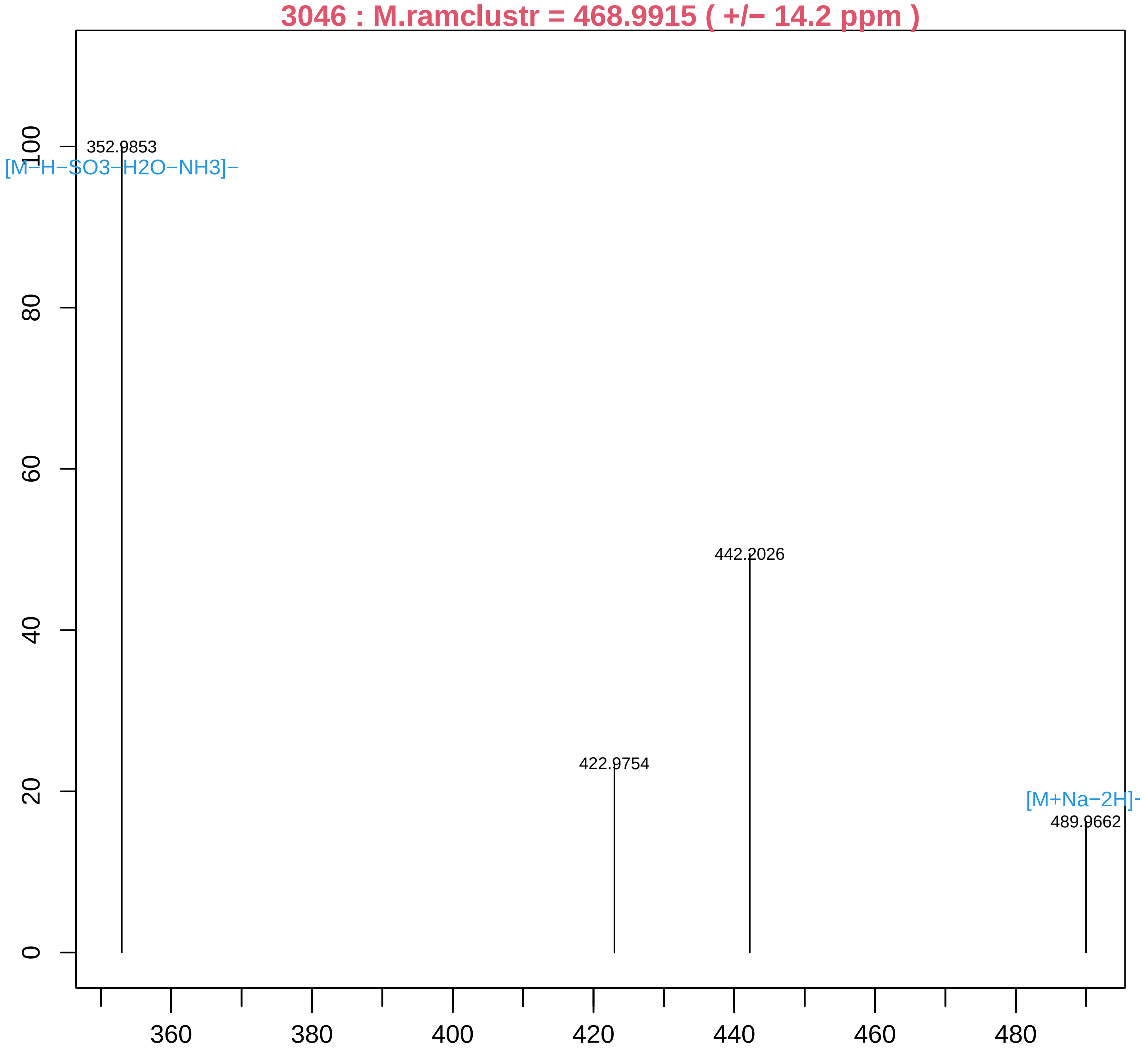
3046 : M.ramclustr = 468.9915 (+/− 14.2 ppm)
352.9853
[M−H−SO3−H2O−NH3]−
422.9754
442.2026
[M+Na−2H]-
489.9662
0
20
40
60
80
100
360
380
400
420
440
460
480

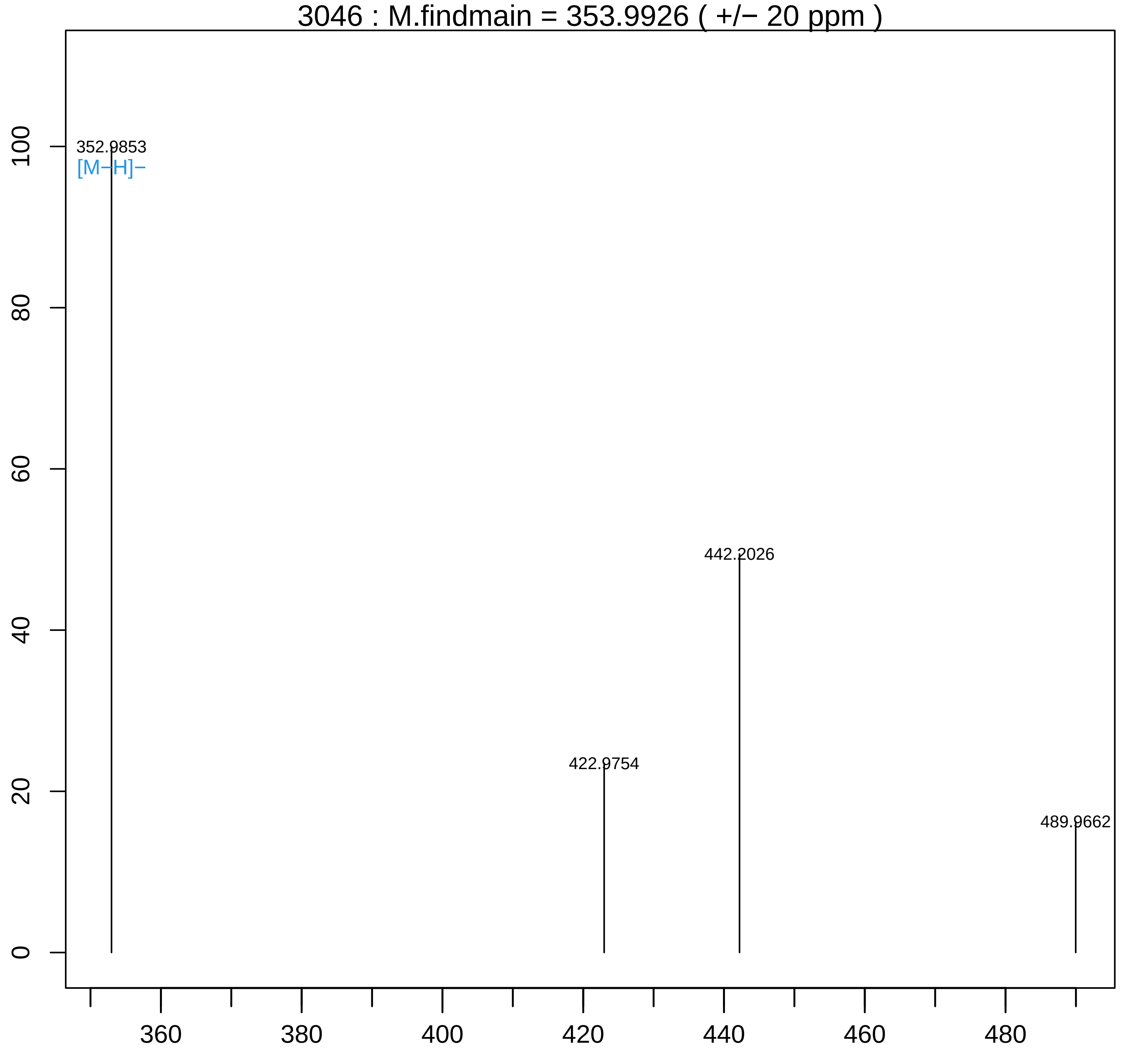
3046 : M.findmain = 353.9926 (+/− 20 ppm)
352.9853
[M−H]−
422.9754
442.2026
489.9662
0
20
40
60
80
100
360
380
400
420
440
460
480

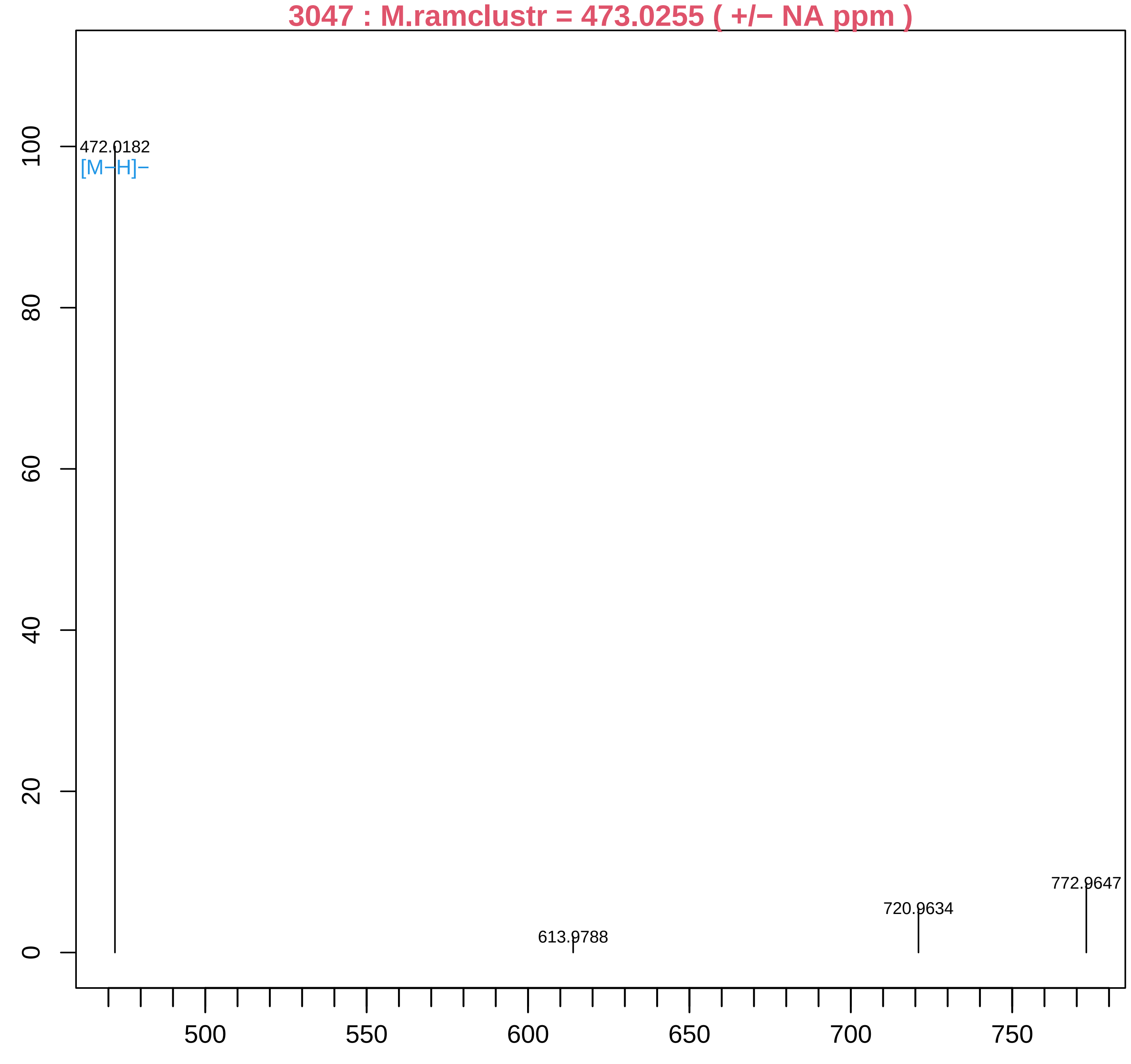
3047 : M.ramclustr = 473.0255 (+/− NA ppm)
472.0182
[M−H]−
613.9788
720.9634
772.9647
0
20
40
60
80
100
500
550
600
650
700
750

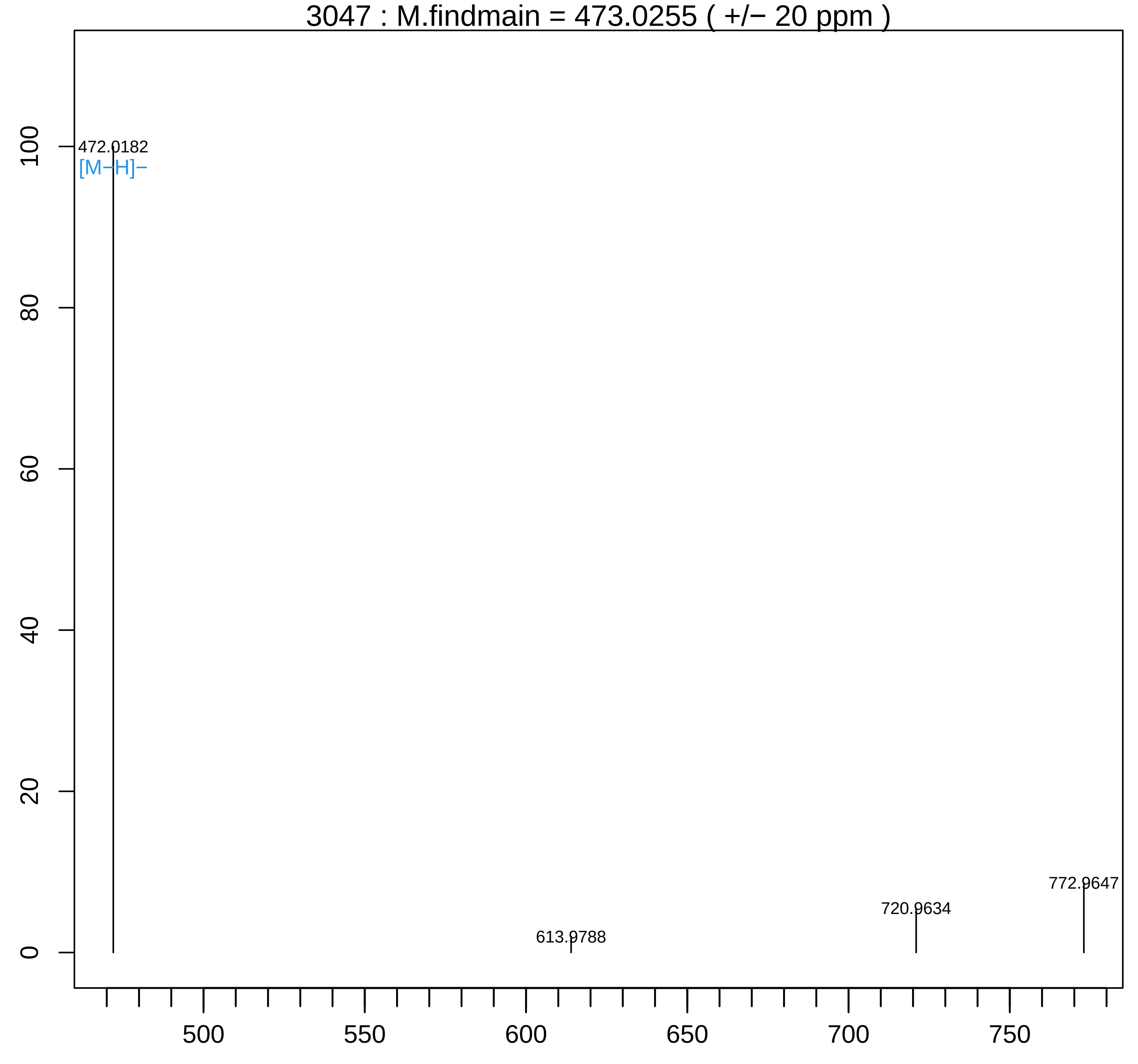
3047 : M.findmain = 473.0255 (+/− 20 ppm)
472.0182
[M−H]−
613.9788
720.9634
772.9647
0
20
40
60
80
100
500
550
600
650
700
750

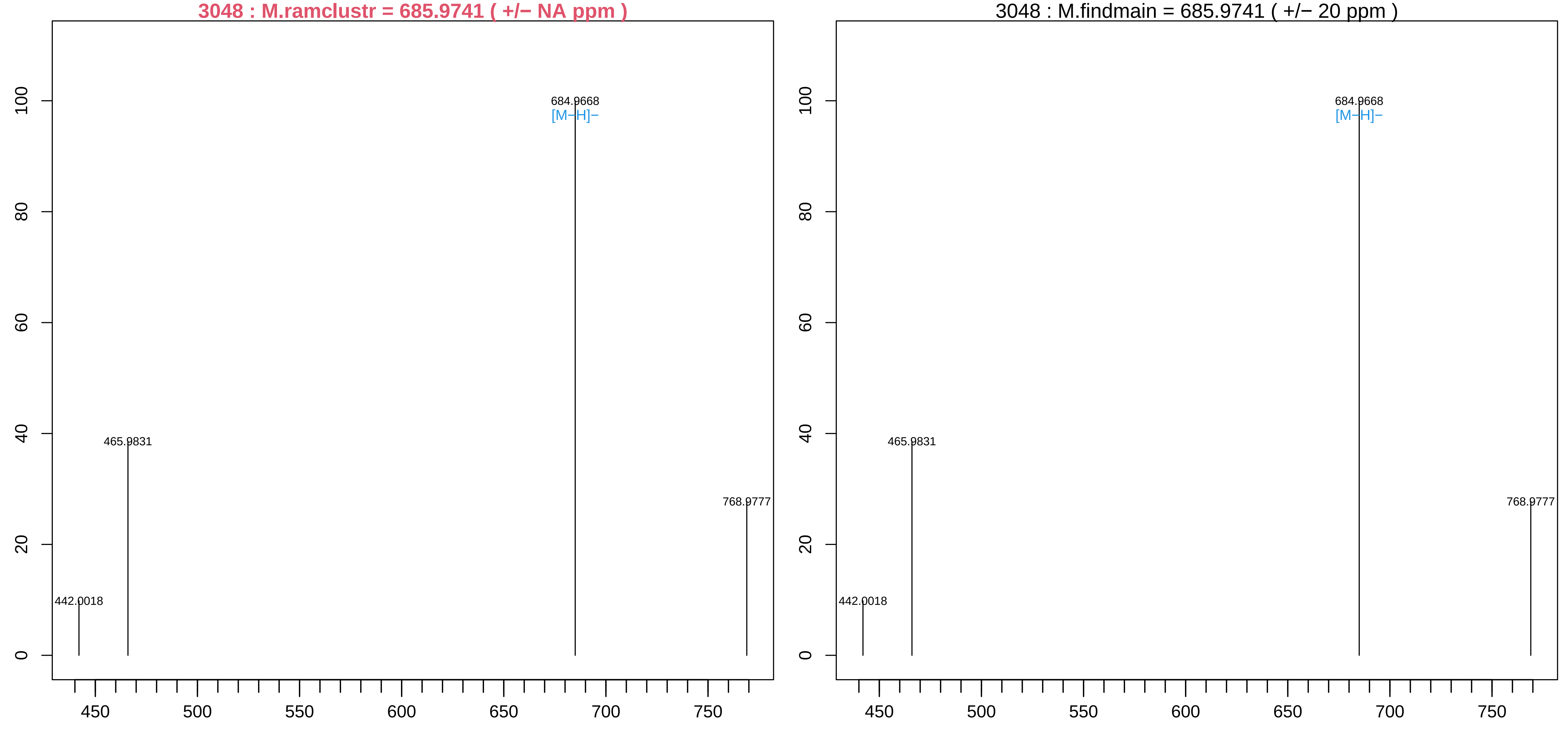
3048 : M.ramclustr = 685.9741 (+/− NA ppm)
3048 : M.findmain = 685.9741 (+/− 20 ppm)
684.9668
[M−H]−
465.9831
442.0018
768.9777
0
20
40
60
80
100
450
500
550
600
650
700
750

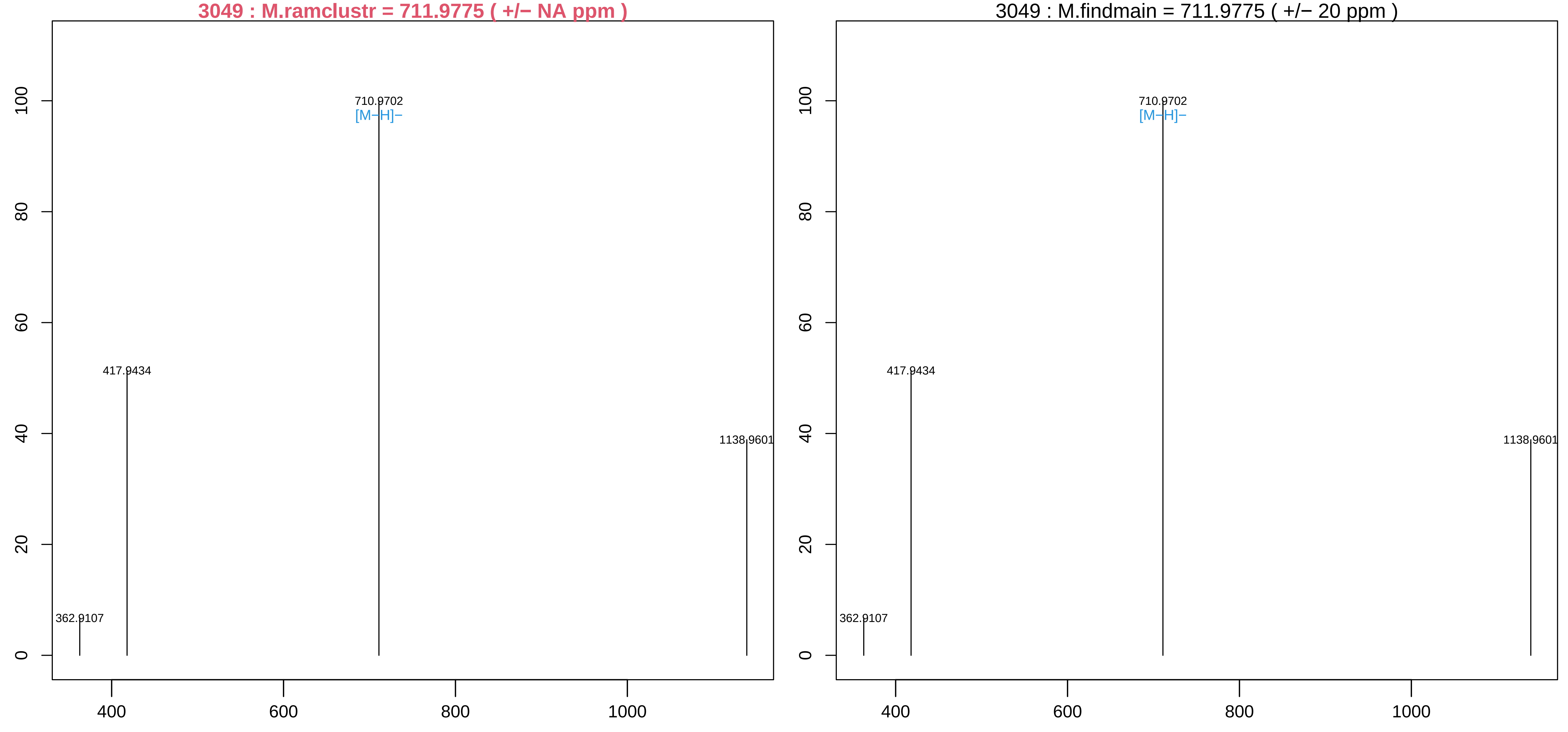
3049 : M.ramclustr = 711.9775 (+/− NA ppm)
3049 : M.findmain = 711.9775 (+/− 20 ppm)
710.9702
[M−H]−
417.9434
1138.9601
362.9107
0
20
40
60
80
100
400
600
800
1000

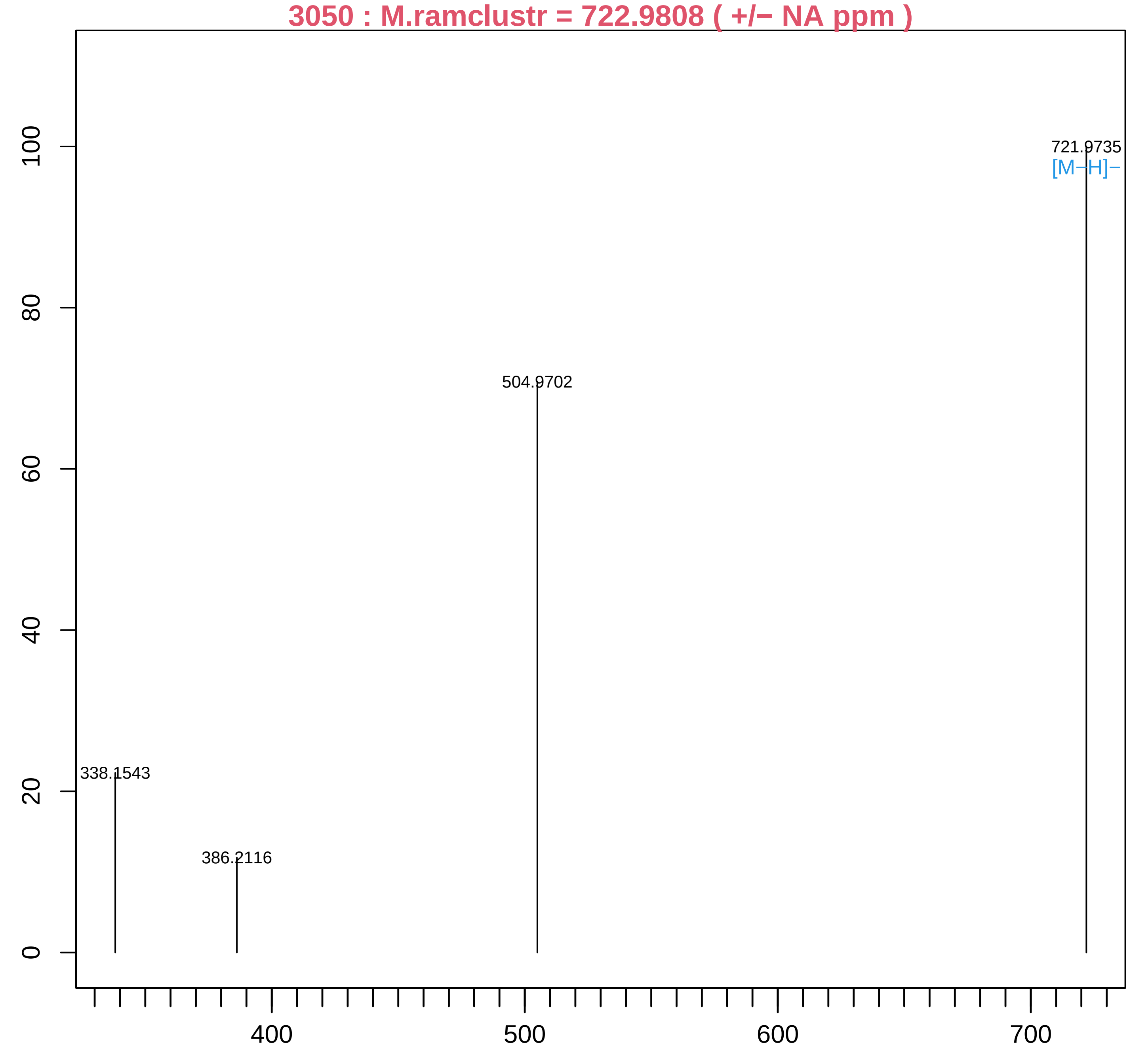
3050 : M.ramclustr = 722.9808 (+/− NA ppm)
721.9735
[M−H]−
504.9702
338.1543
386.2116
0
20
40
60
80
100
400
500
600
700

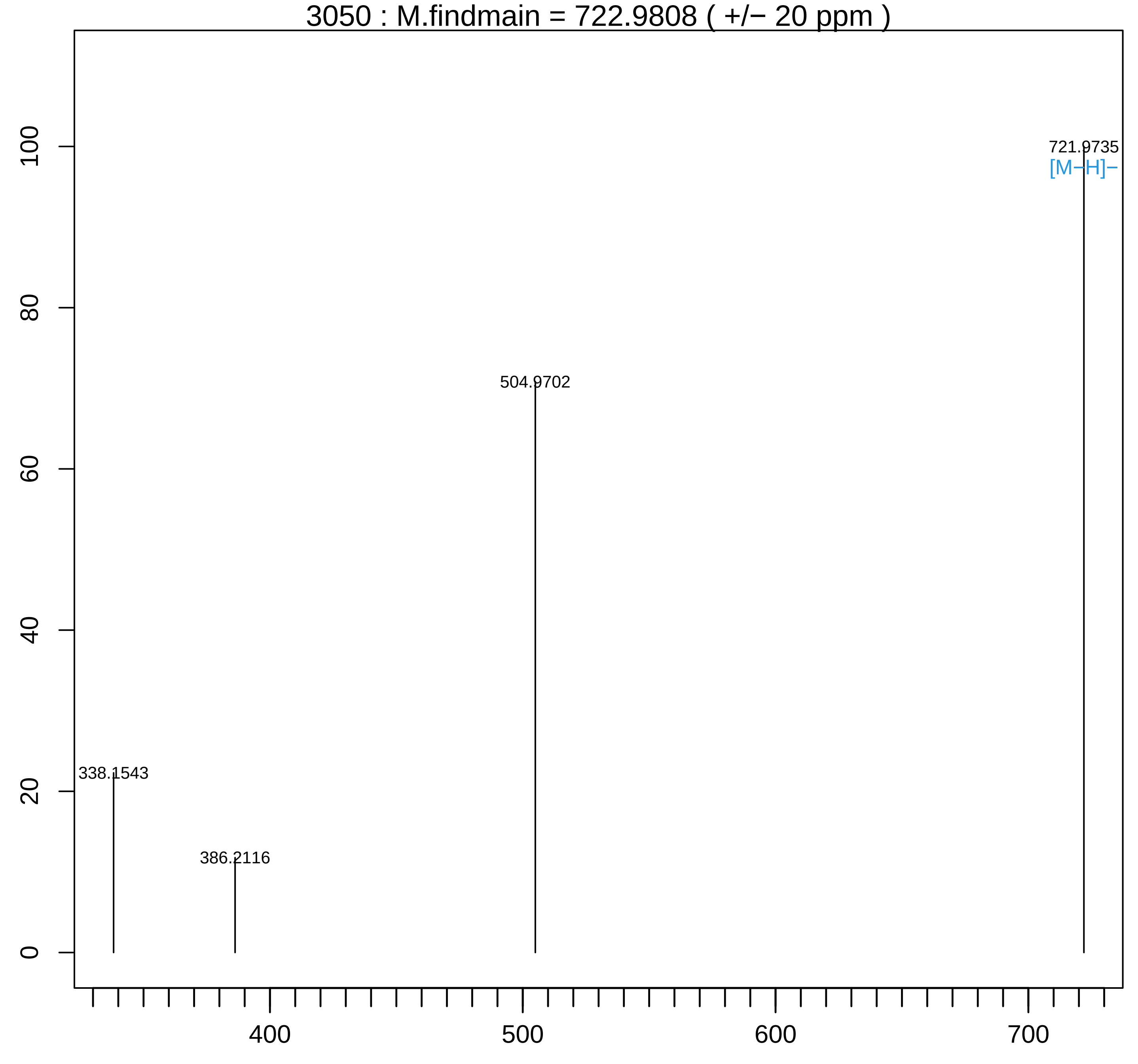
3050 : M.findmain = 722.9808 (+/− 20 ppm)
721.9735
[M−H]−
504.9702
338.1543
386.2116
0
20
40
60
80
100
400
500
600
700

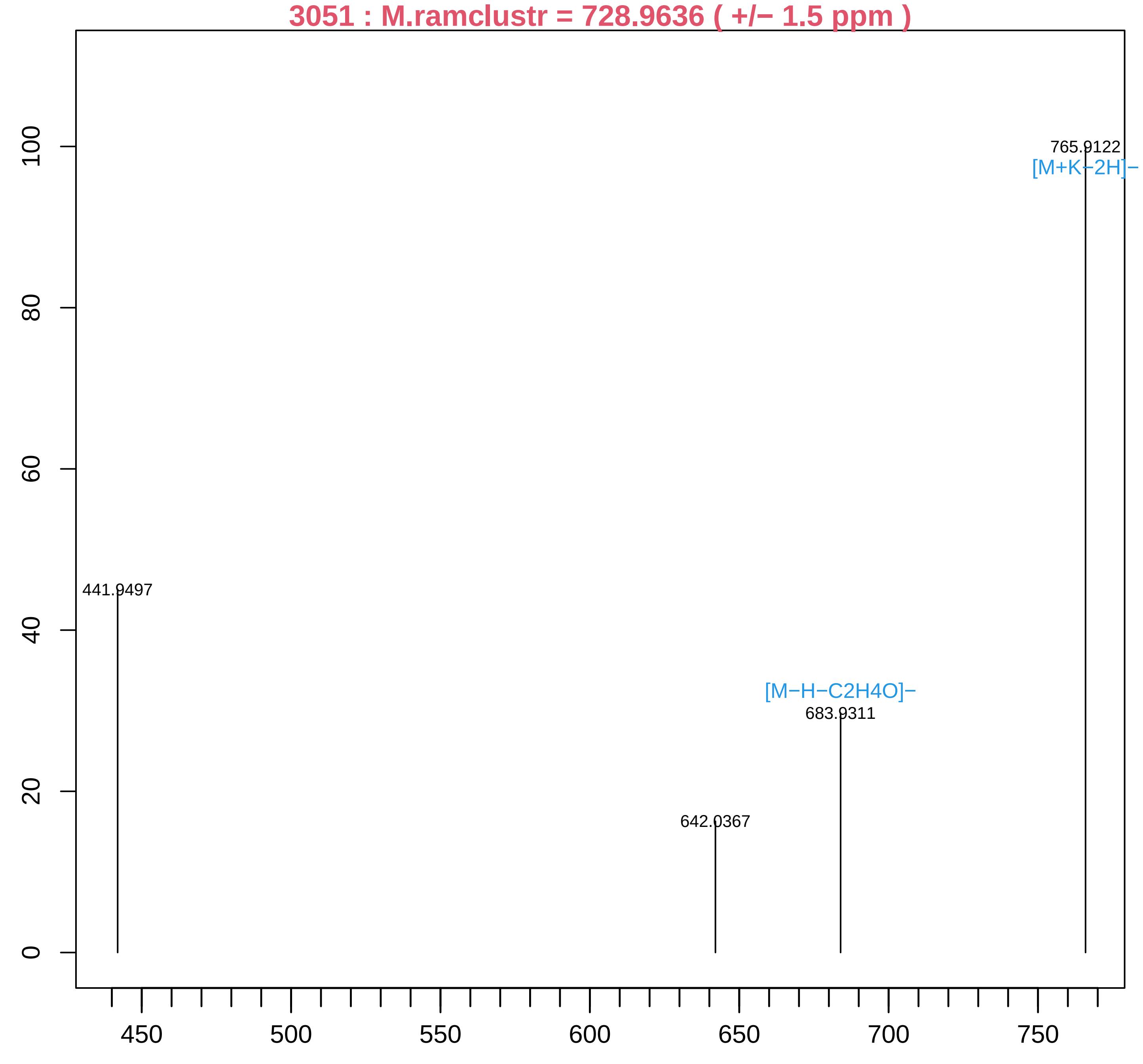
3051 : M.ramclustr = 728.9636 (+/− 1.5 ppm)
765.9122
[M+K−2H]−
441.9497
[M−H−C2H4O]−
683.9311
642.0367
0
20
40
60
80
100
450
500
550
600
650
700
750

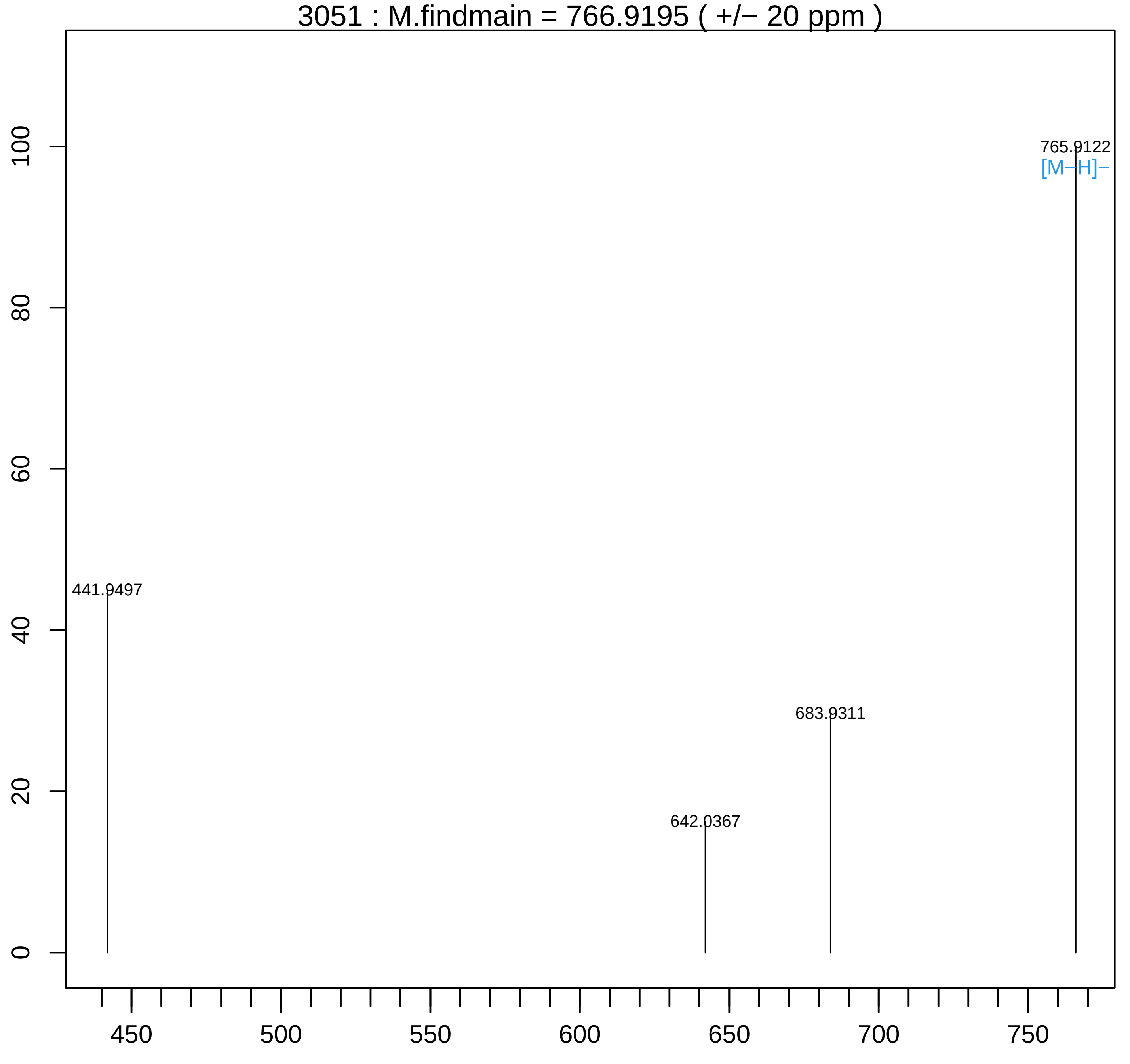
3051 : M.findmain = 766.9195 (+/− 20 ppm)
765.9122
[M−H]−
441.9497
683.9311
642.0367
0
20
40
60
80
100
450
500
550
600
650
700
750

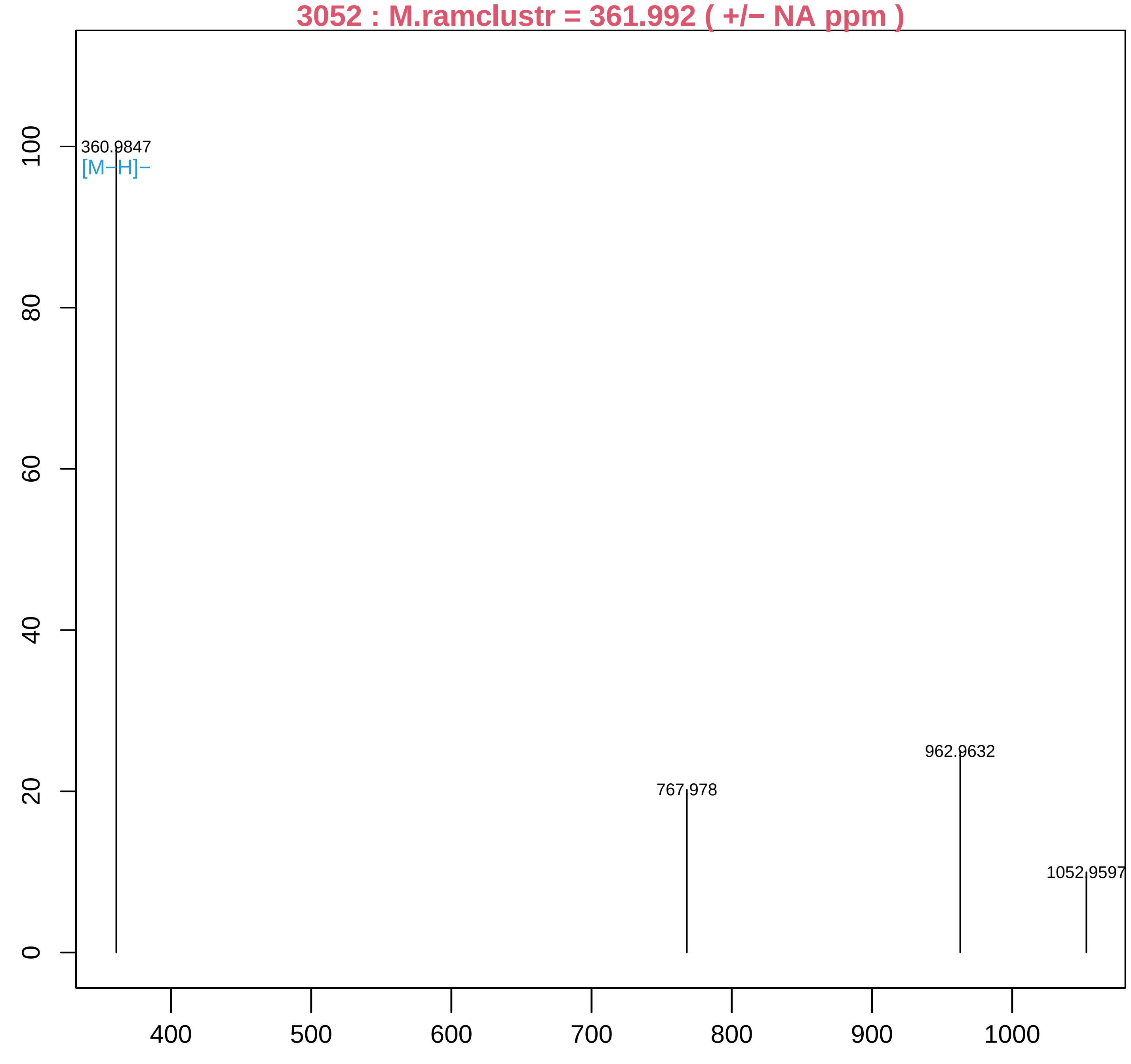
3052 : M.ramclustr = 361.992 (+/− NA ppm)
360.9847
[M−H]−
767.978
962.9632
1052.9597
0
20
40
60
80
100
400
500
600
700
800
900
1000

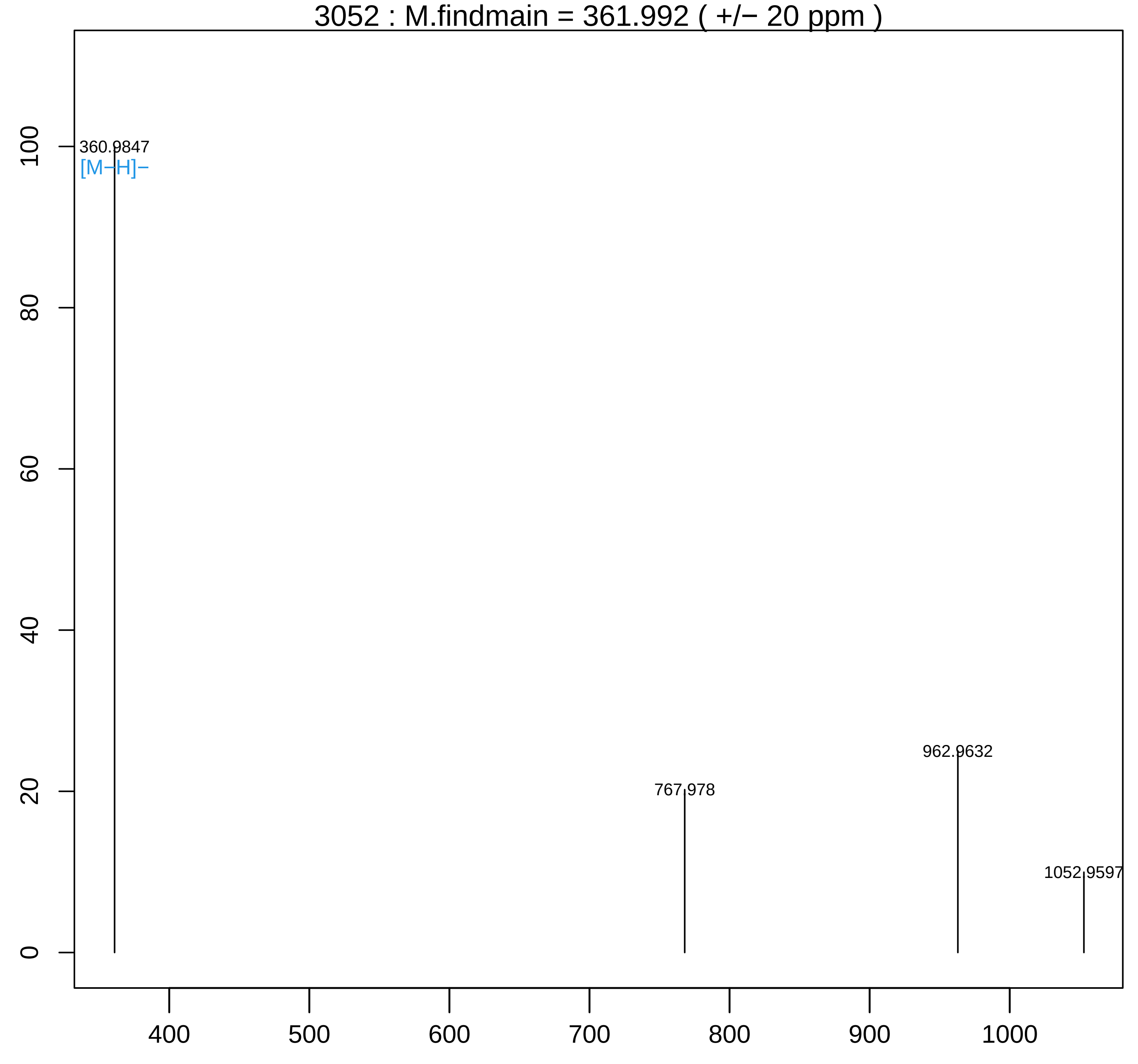
3052 : M.findmain = 361.992 (+/− 20 ppm)
360.9847
[M−H]−
767.978
962.9632
1052.9597
0
20
40
60
80
100
400
500
600
700
800
900
1000

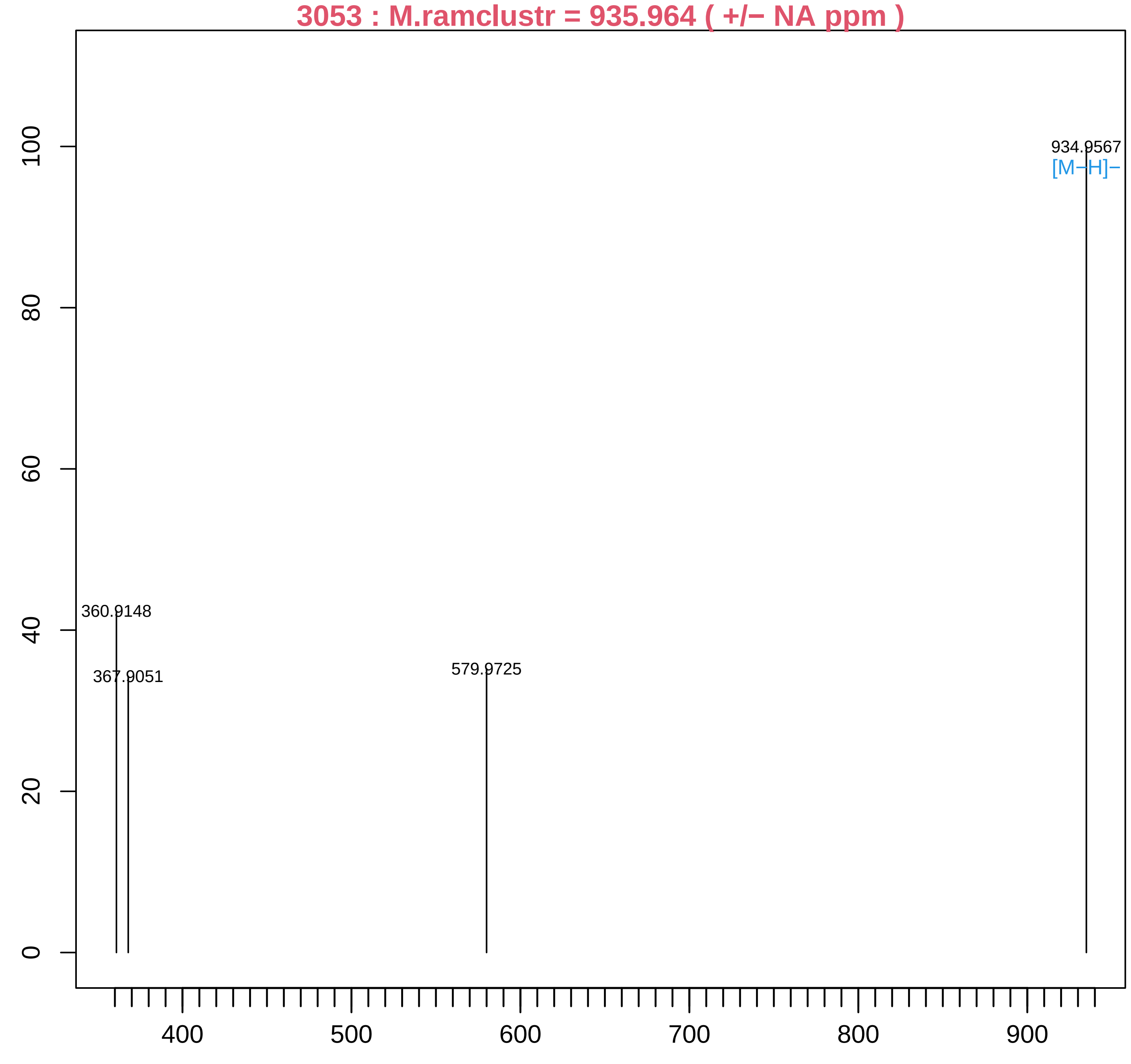
3053 : M.ramclustr = 935.964 (+/− NA ppm)
934.9567
[M−H]−
360.9148
367.9051
579.9725
0
20
40
60
80
100
400
500
600
700
800
900

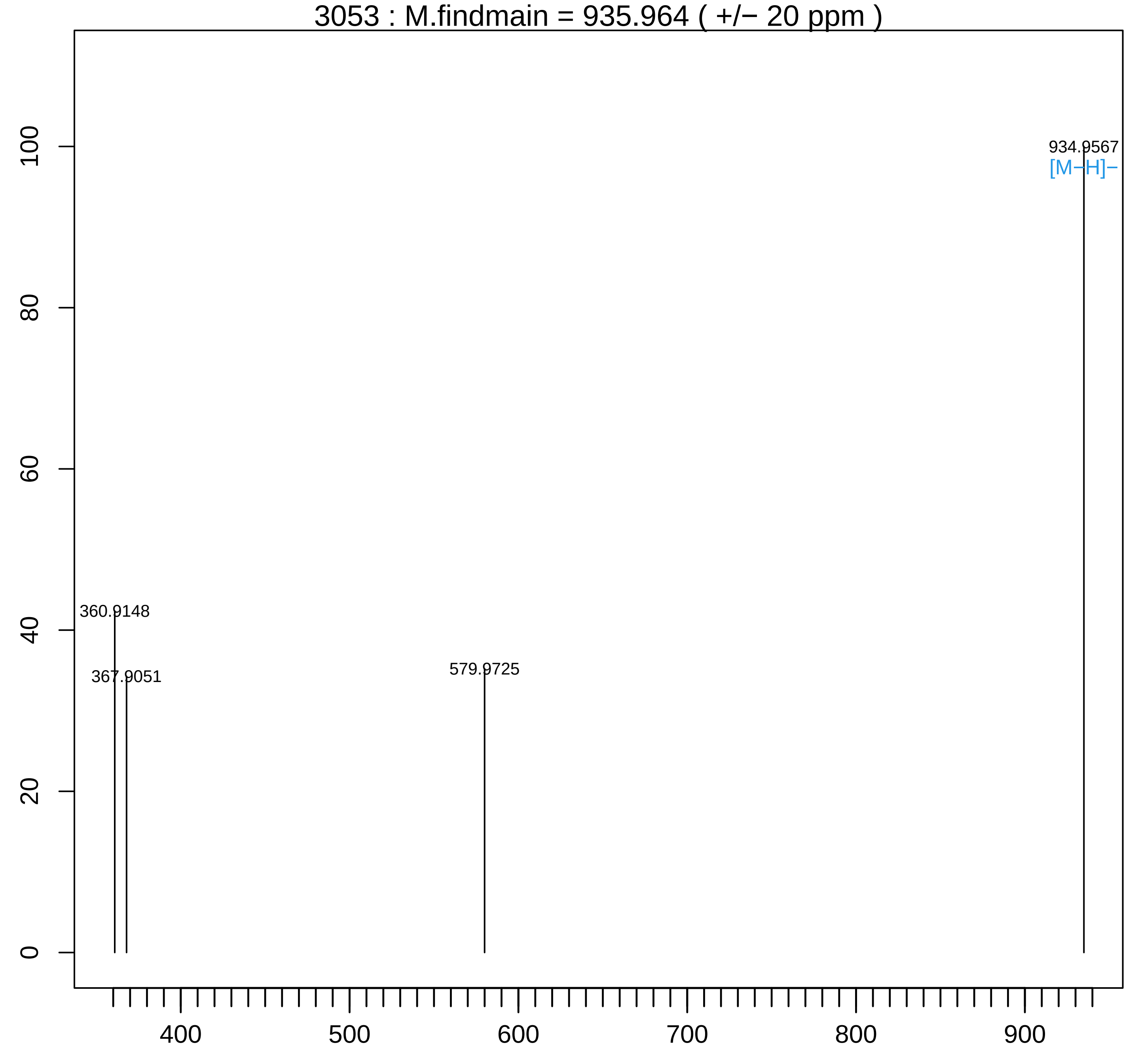
3053 : M.findmain = 935.964 (+/− 20 ppm)
934.9567
[M−H]−
360.9148
367.9051
579.9725
0
20
40
60
80
100
400
500
600
700
800
900

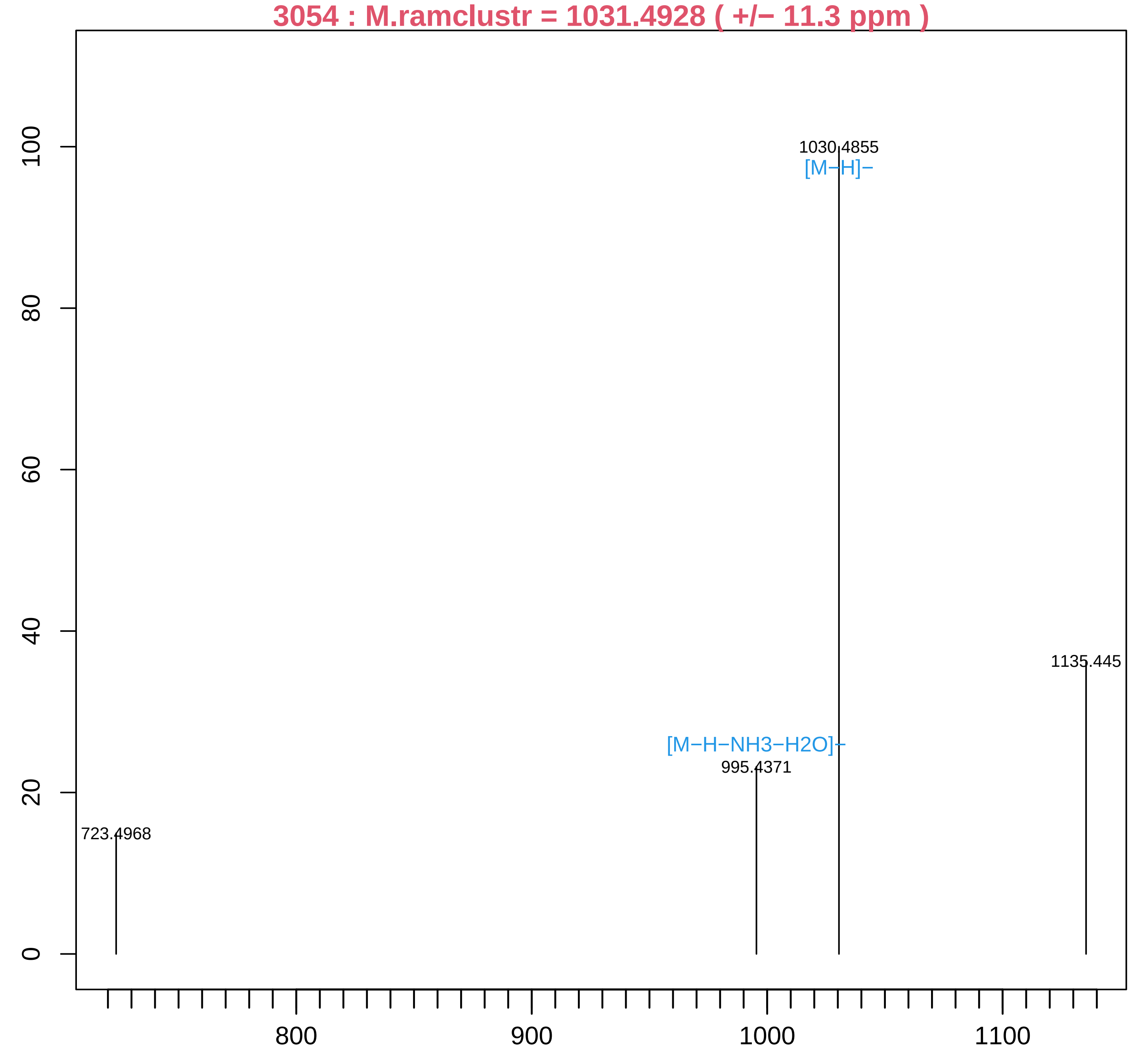
3054 : M.ramclustr = 1031.4928 (+/− 11.3 ppm)
1030.4855
[M−H]−
1135.445
[M−H−NH3−H2O]−
995.4371
723.4968
0
20
40
60
80
100
800
900
1000
1100

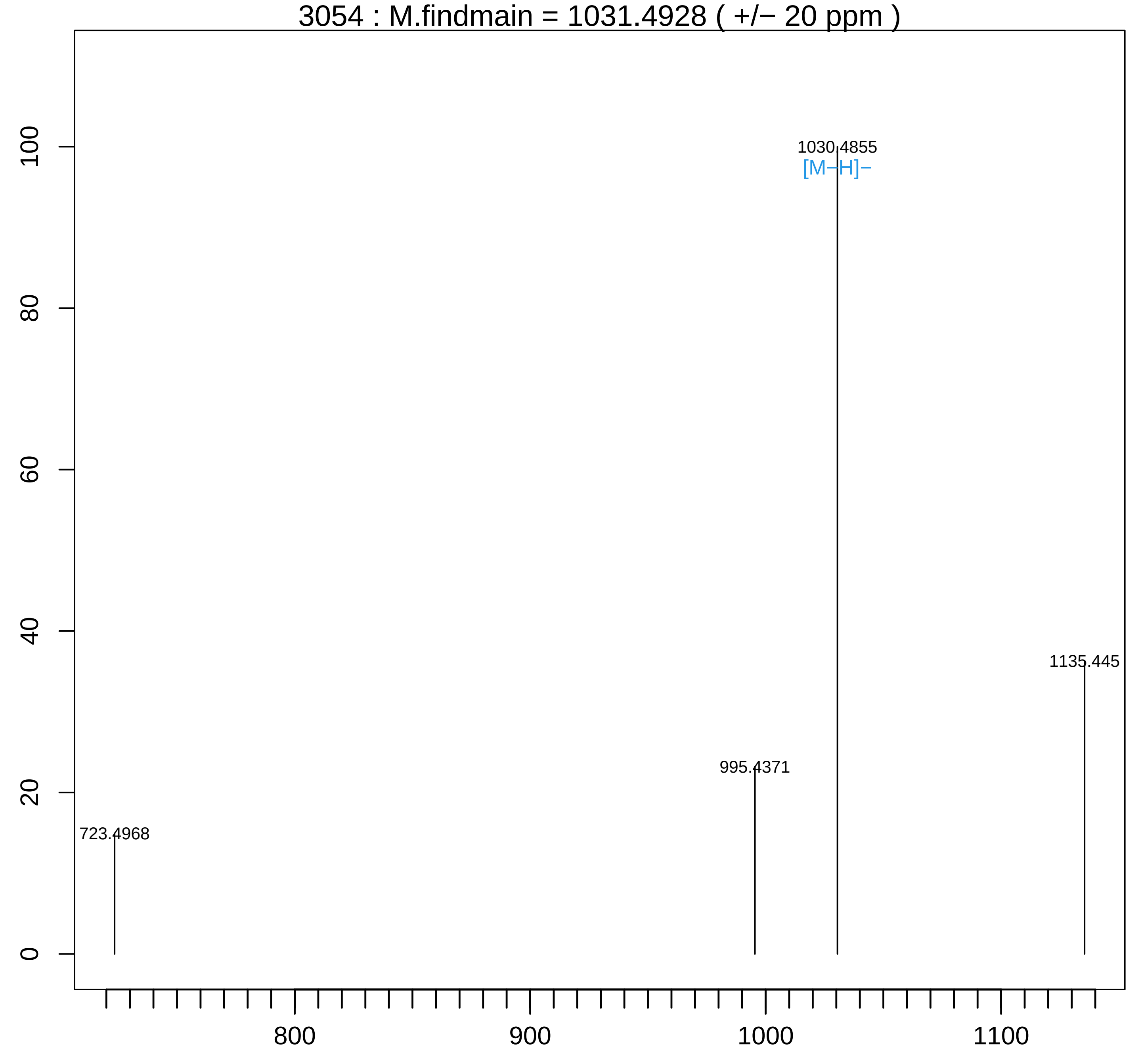
3054 : M.findmain = 1031.4928 (+/− 20 ppm)
1030.4855
[M−H]−
1135.445
995.4371
723.4968
0
20
40
60
80
100
800
900
1000
1100

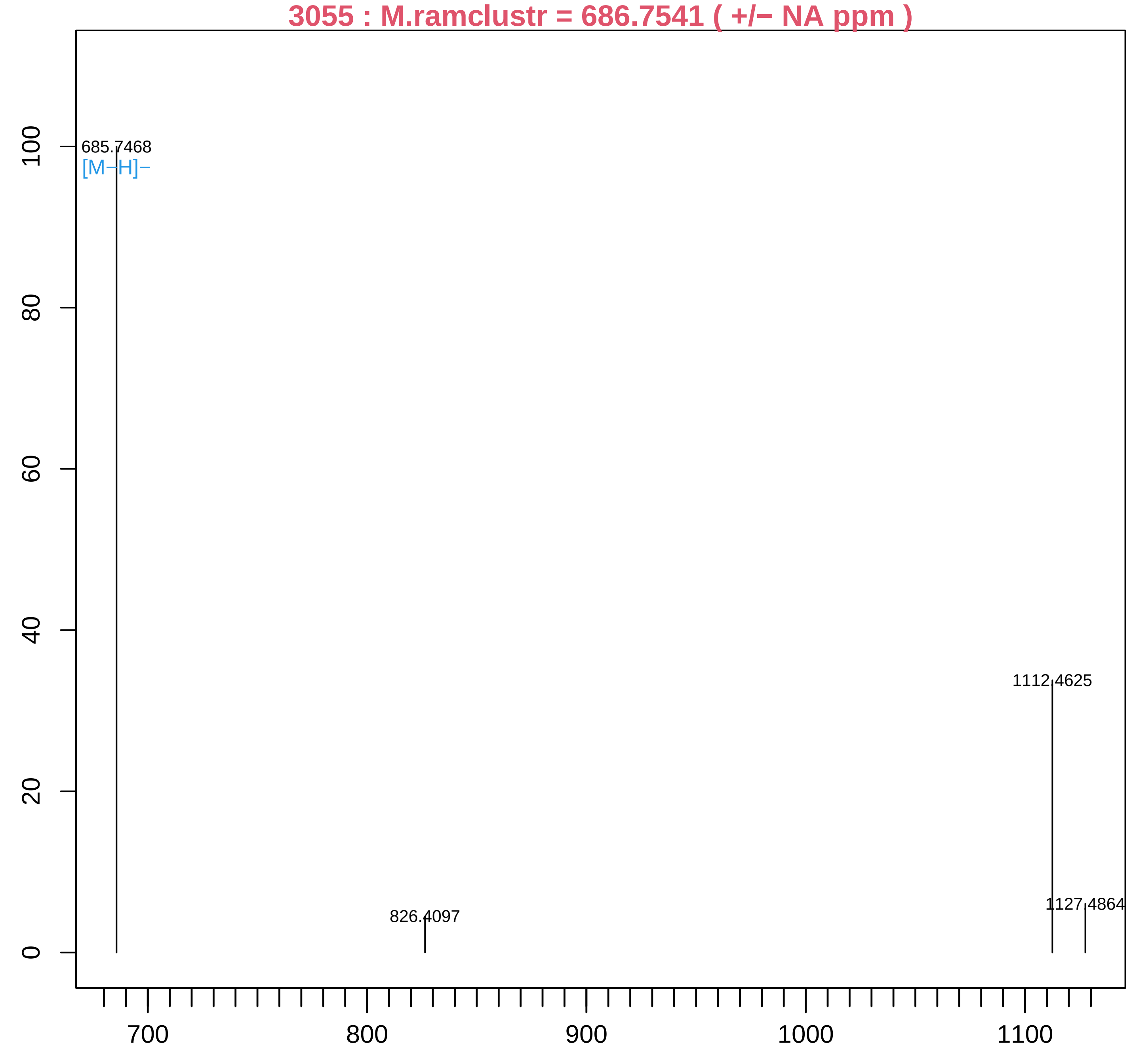
3055 : M.ramclustr = 686.7541 (+/− NA ppm)
685.7468
[M−H]−
826.4097
1112.4625
1127.4864
0
20
40
60
80
100
700
800
900
1000
1100

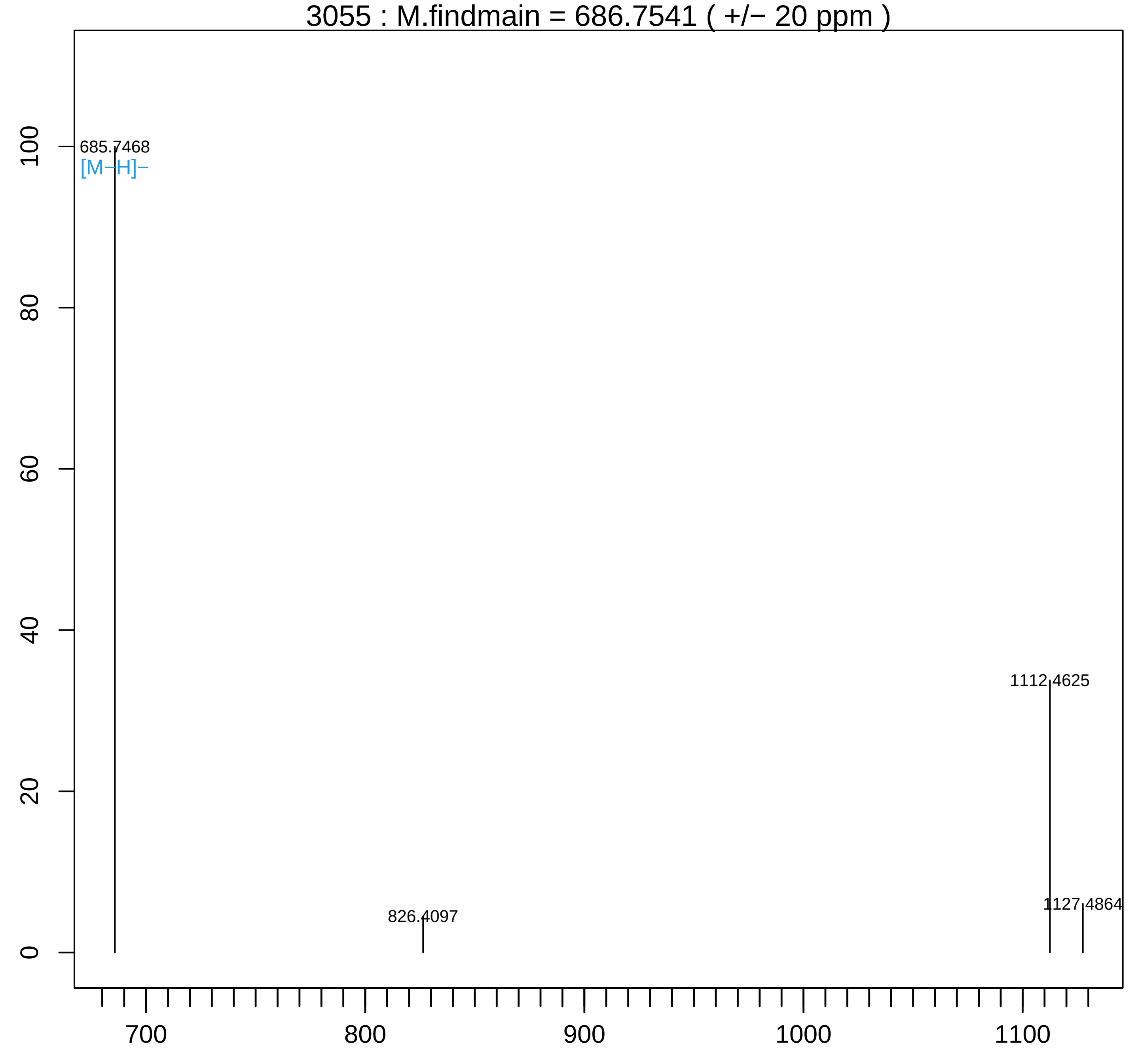
3055 : M.findmain = 686.7541 (+/− 20 ppm)
685.7468
[M−H]−
826.4097
1112.4625
1127.4864
0
20
40
60
80
100
700
800
900
1000
1100

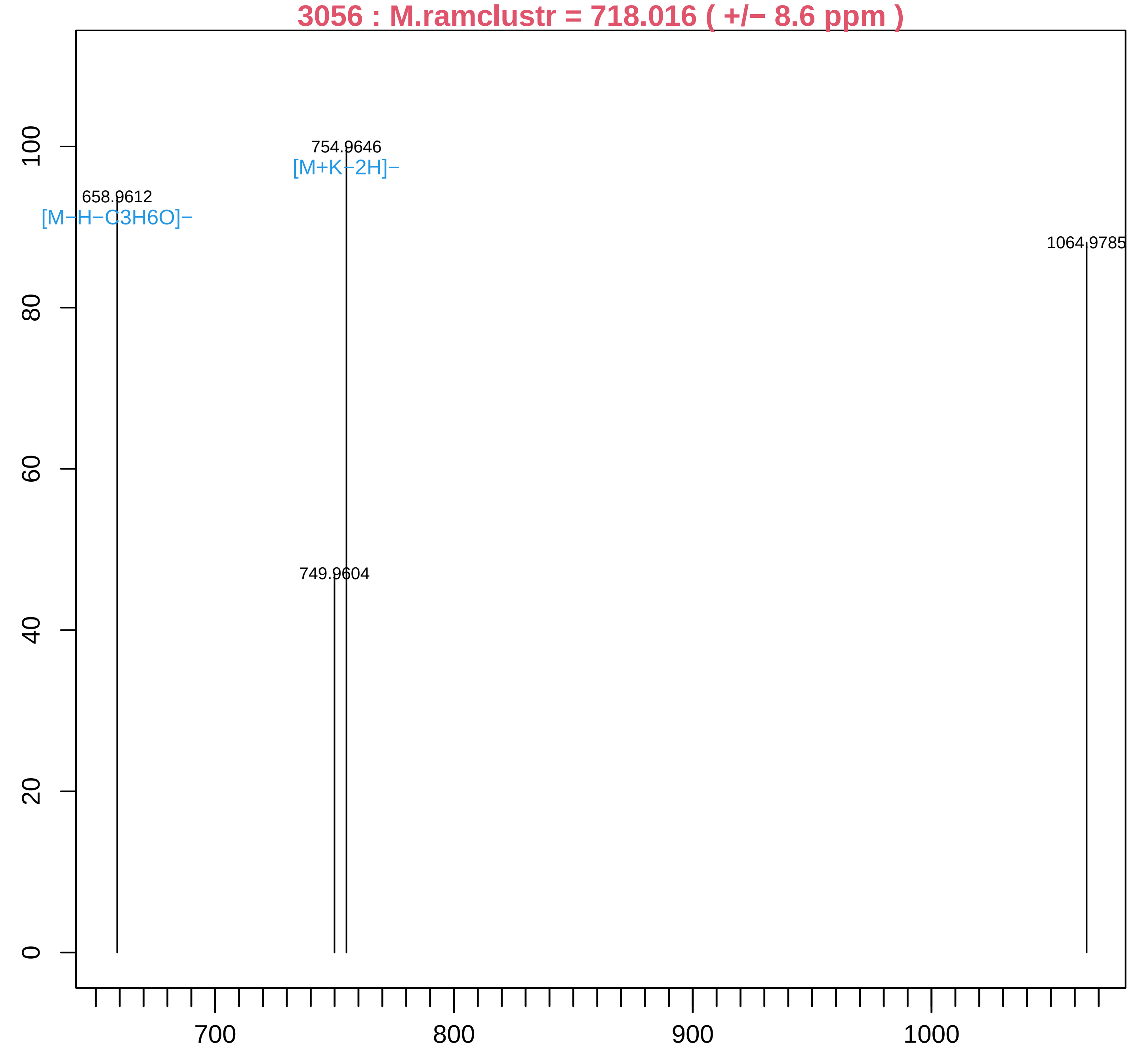
3056 : M.ramclustr = 718.016 (+/− 8.6 ppm)
754.9646
[M+K−2H]−
658.9612
[M−H−C3H6O]−
1064.9785
749.9604
0
20
40
60
80
100
700
800
900
1000

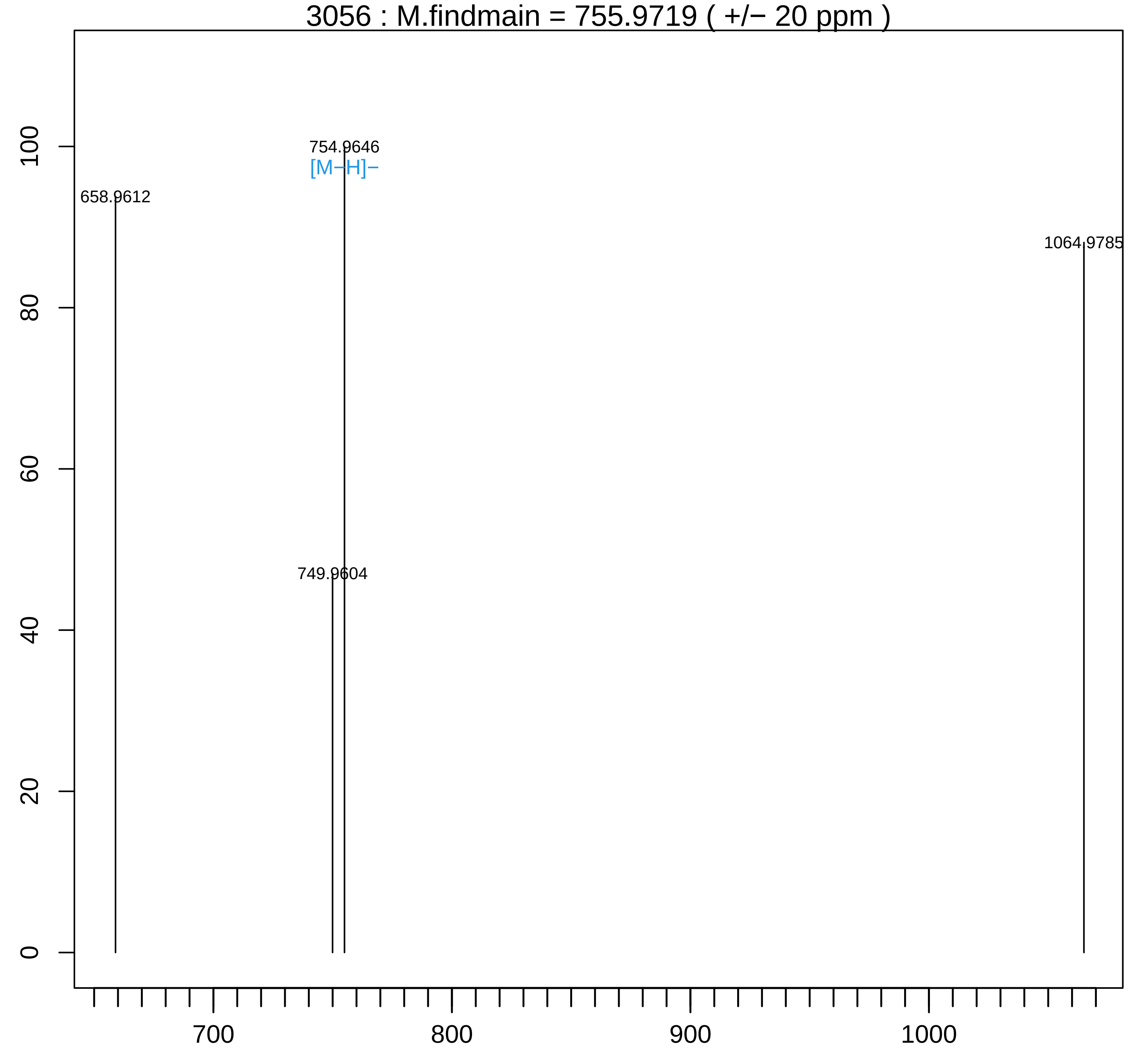
3056 : M.findmain = 755.9719 (+/− 20 ppm)
754.9646
[M−H]−
658.9612
1064.9785
749.9604
0
20
40
60
80
100
700
800
900
1000

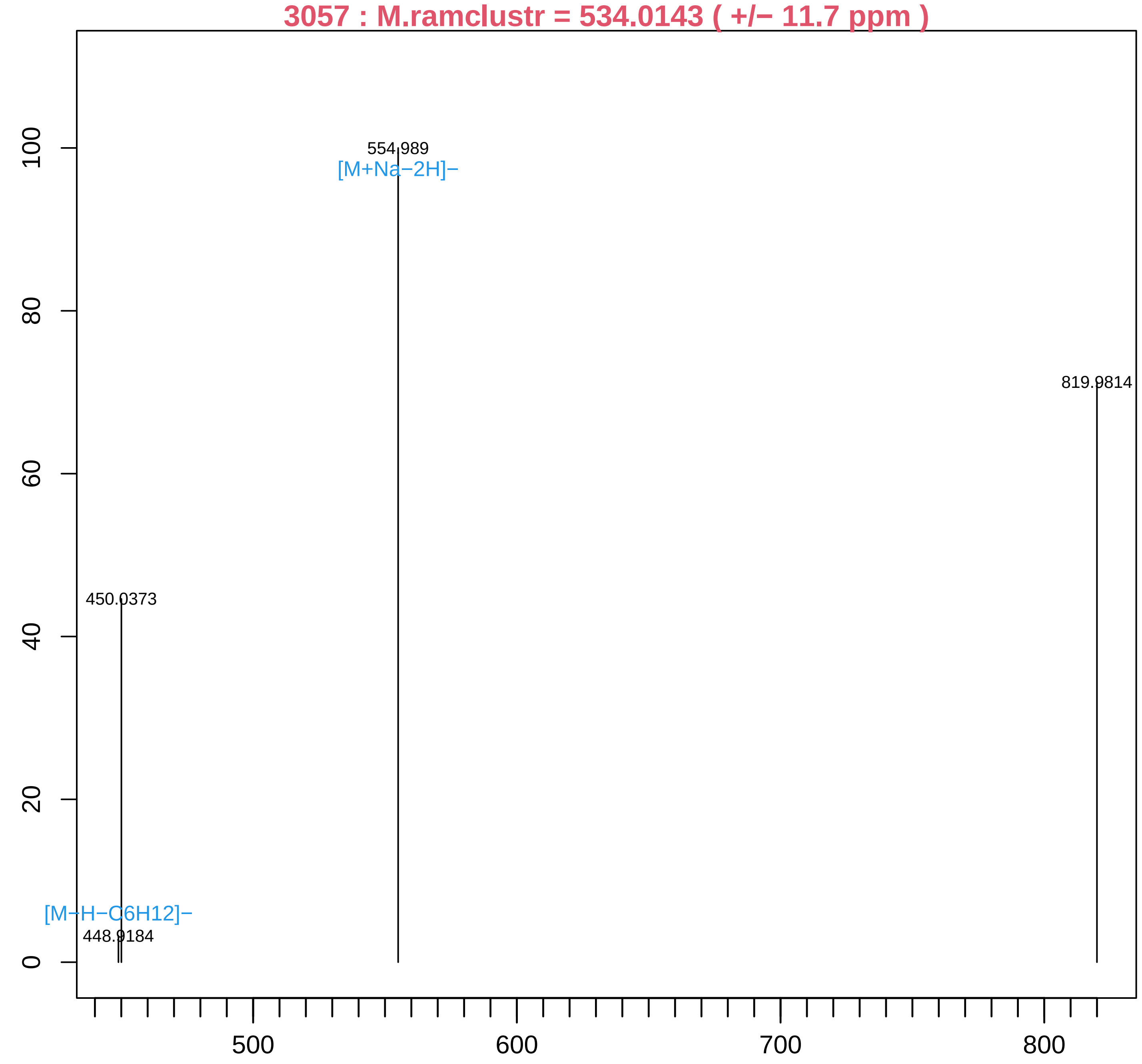

3057 : M.ramclustr = 534.0143 (+/− 11.7 ppm)
554.989
[M+Na−2H]−
819.9814
450.0373
[M−H−C6H12]−
448.9184
0
20
40
60
80
100
500
600
700
800

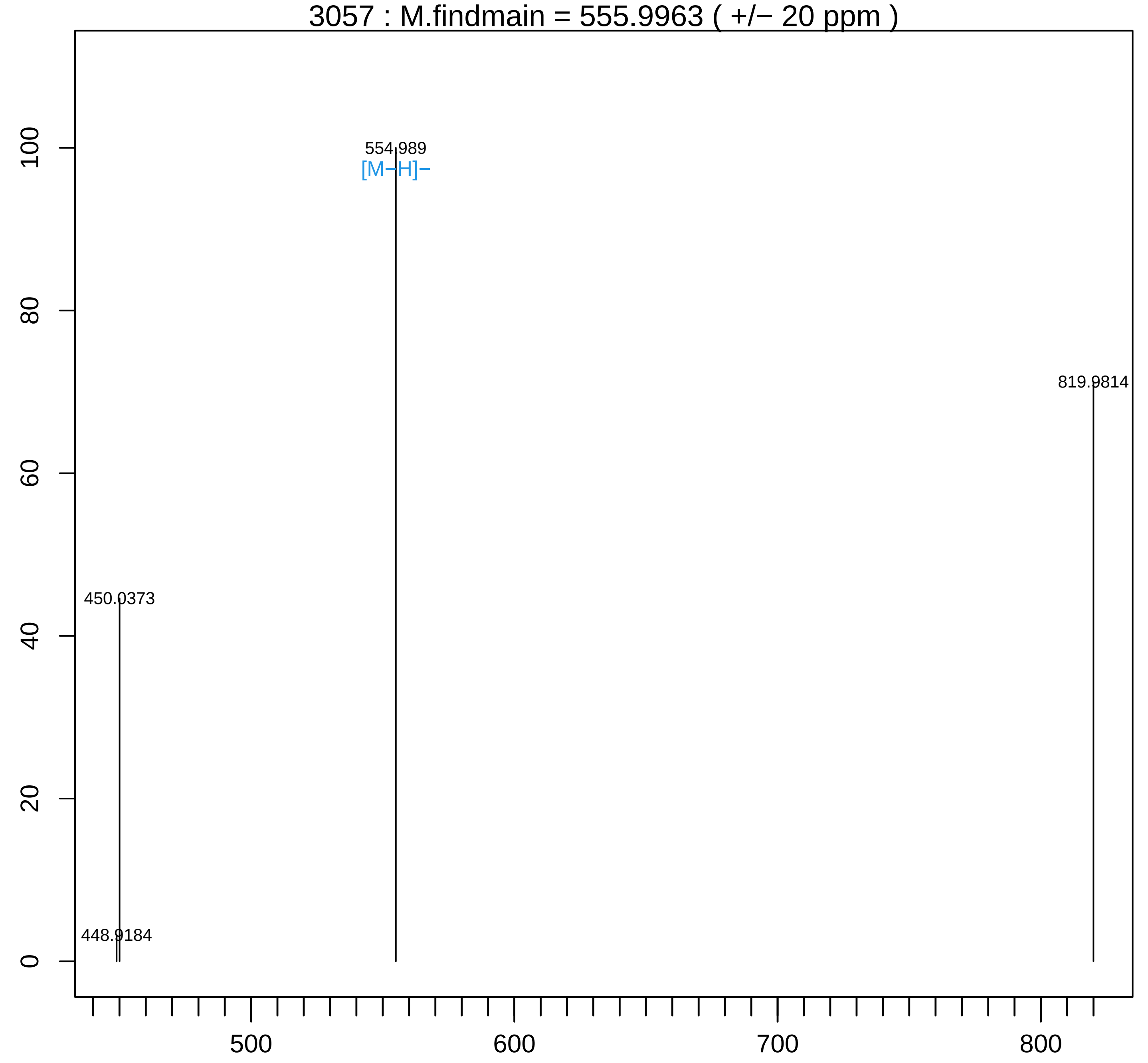

3057 : M.findmain = 555.9963 (+/− 20 ppm)
554.989
[M−H]−
819.9814
450.0373
448.9184
0
20
40
60
80
100
500
600
700
800

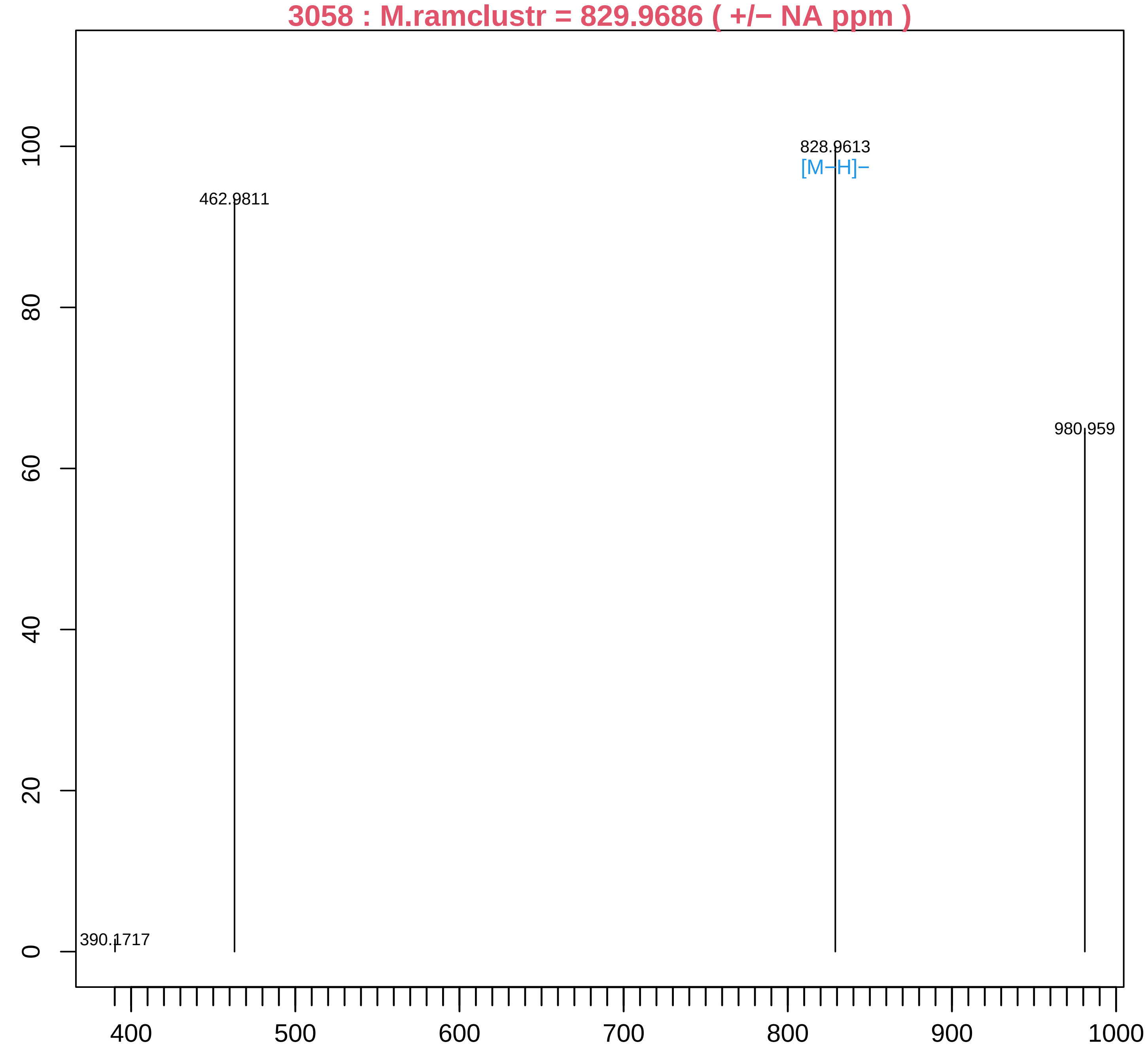
3058 : M.ramclustr = 829.9686 (+/− NA ppm)
828.9613
[M−H]−
462.9811
980.959
390.1717
0
20
40
60
80
100
400
500
600
700
800
900
1000

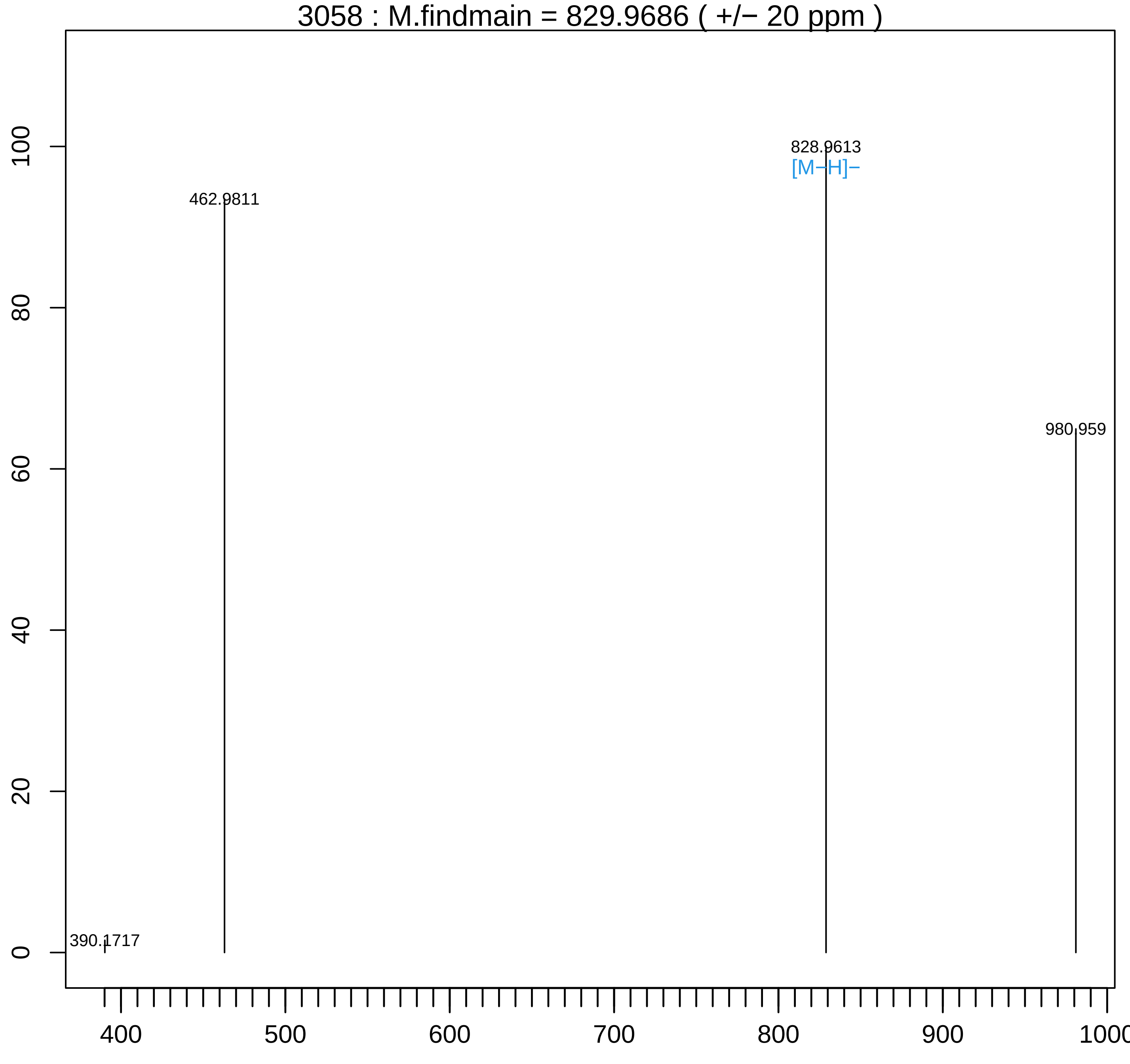
3058 : M.findmain = 829.9686 (+/− 20 ppm)
828.9613
[M−H]−
462.9811
980.959
390.1717
0
20
40
60
80
100
400
500
600
700
800
900
1000

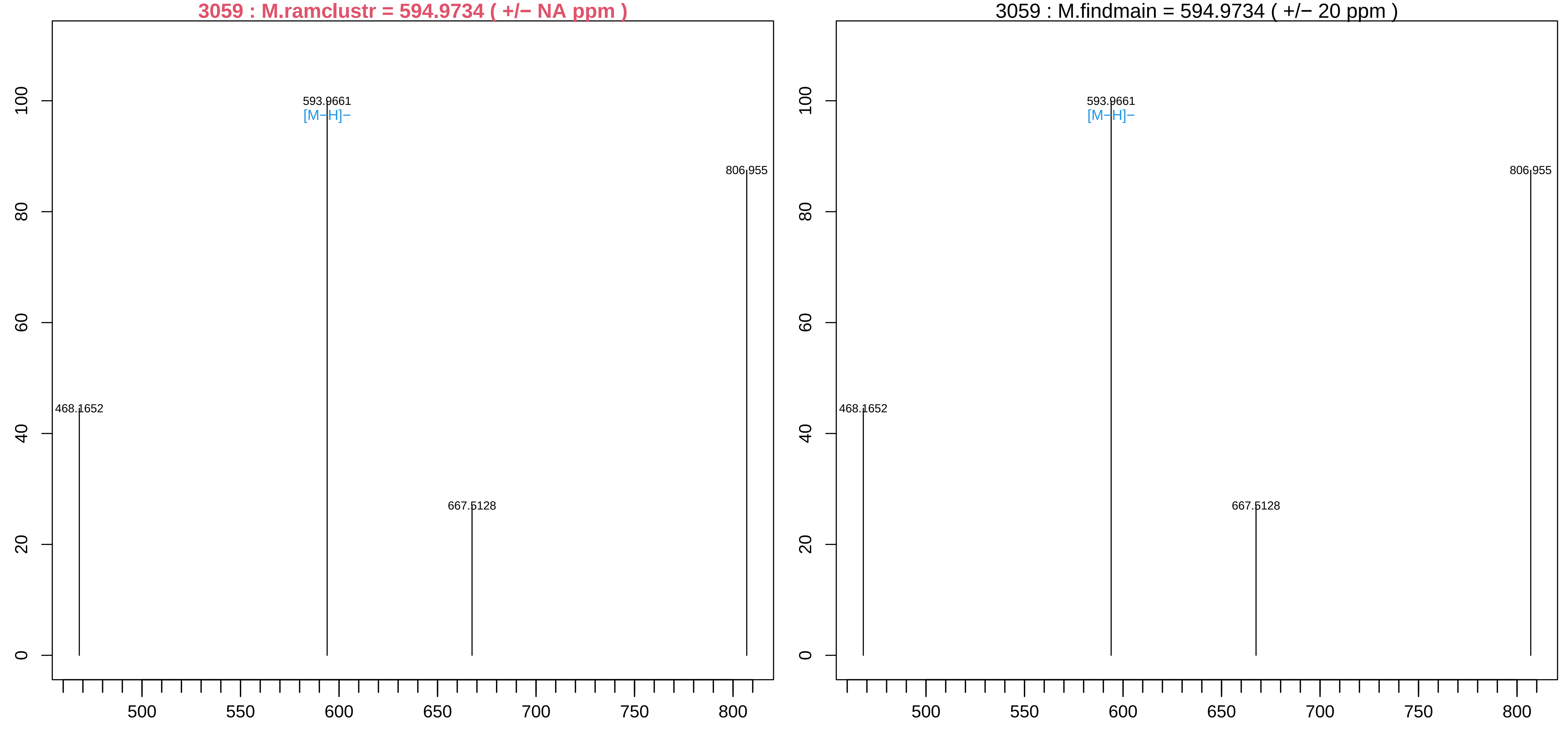
3059 : M.ramclustr = 594.9734 (+/− NA ppm)
3059 : M.findmain = 594.9734 (+/− 20 ppm)
593.9661
[M−H]−
806.955
468.1652
667.5128
593.9661
[M−H]−
806.955
468.1652
667.5128
0
20
40
60
80
100
500
550
600
650
700
750
800

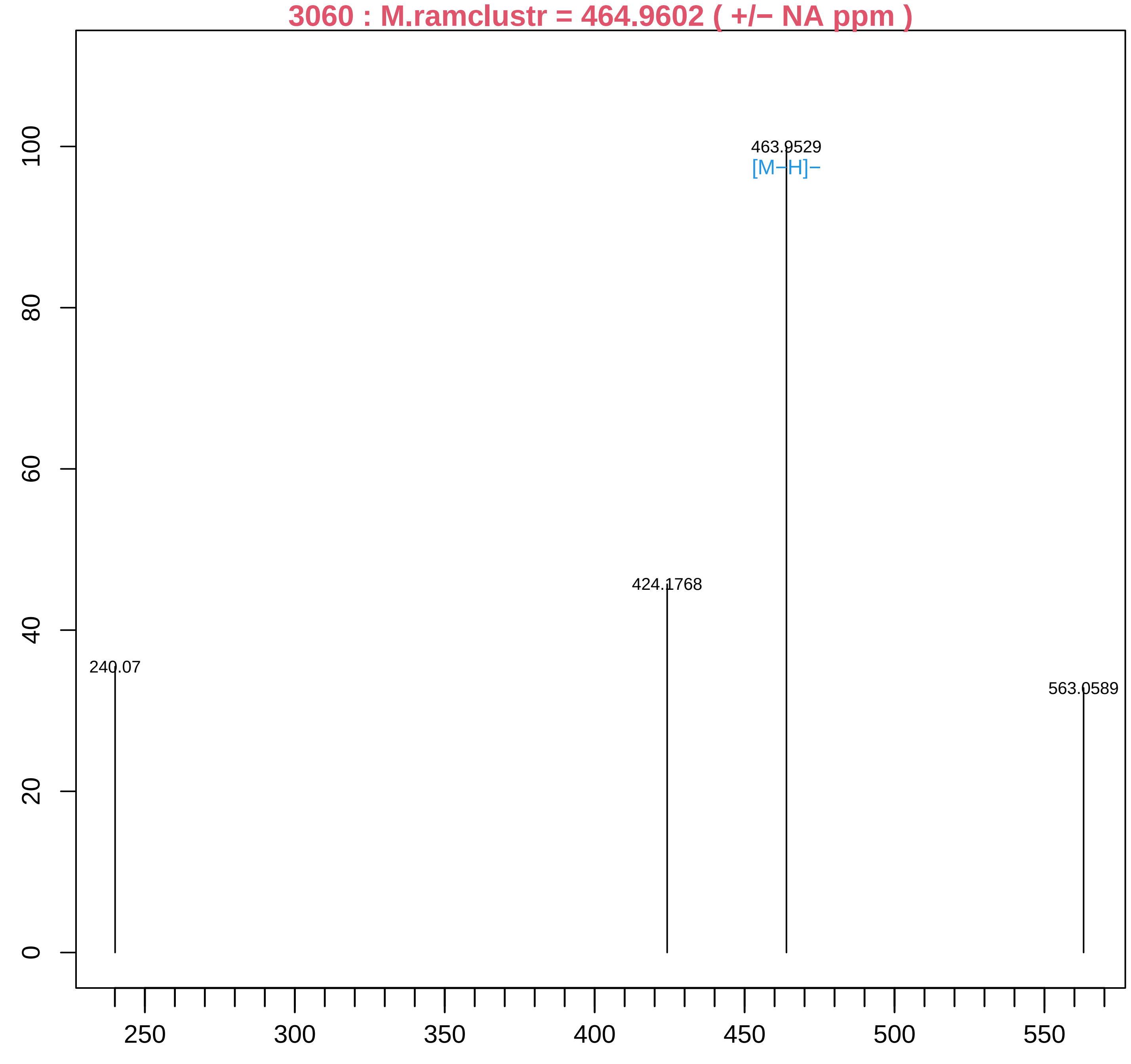
3060 : M.ramclustr = 464.9602 (+/− NA ppm)
463.9529
[M−H]−
424.1768
240.07
563.0589
0
20
40
60
80
100
250
300
350
400
450
500
550

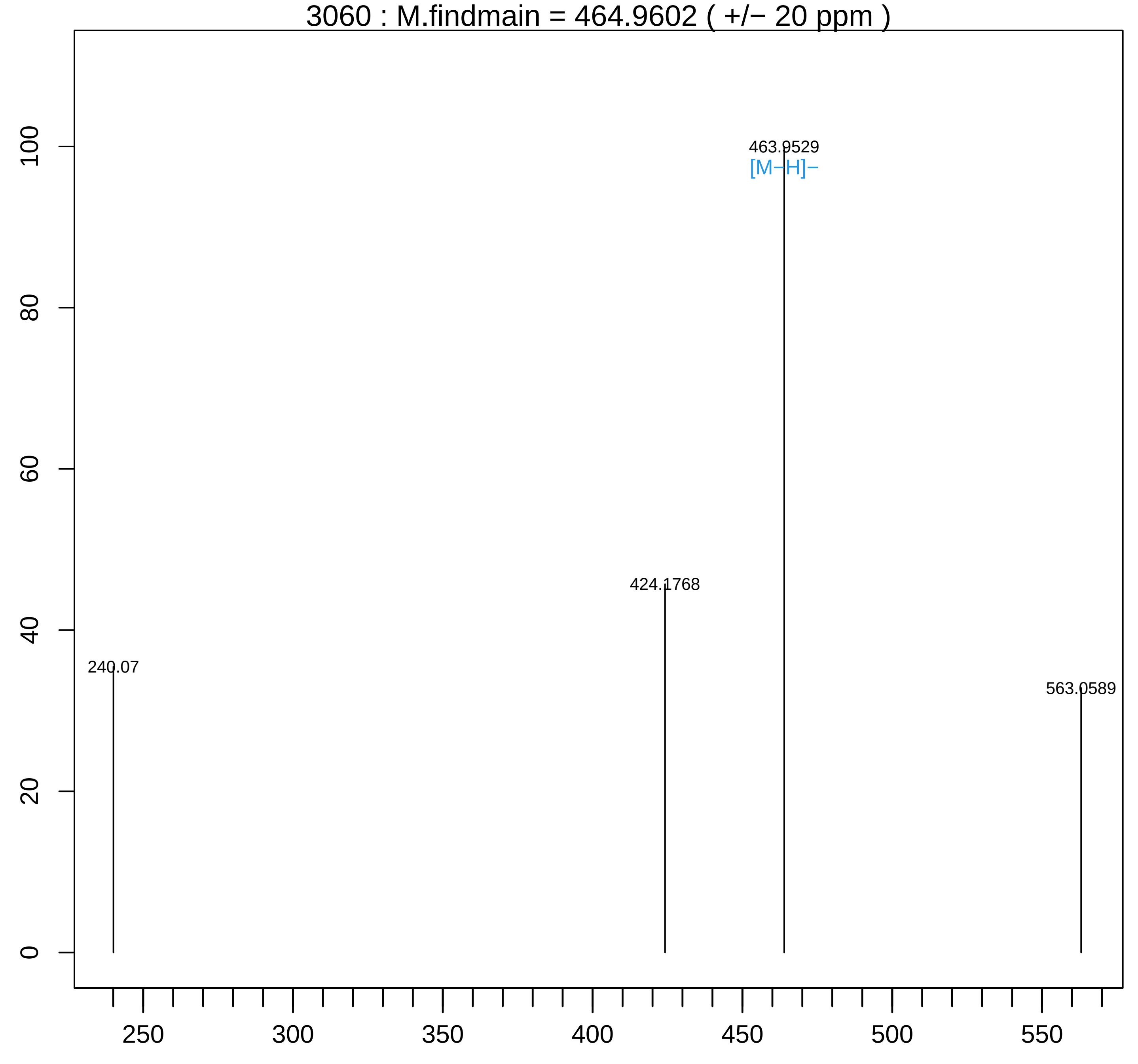
3060 : M.findmain = 464.9602 (+/− 20 ppm)
463.9529
[M−H]−
424.1768
240.07
563.0589
0
20
40
60
80
100
250
300
350
400
450
500
550

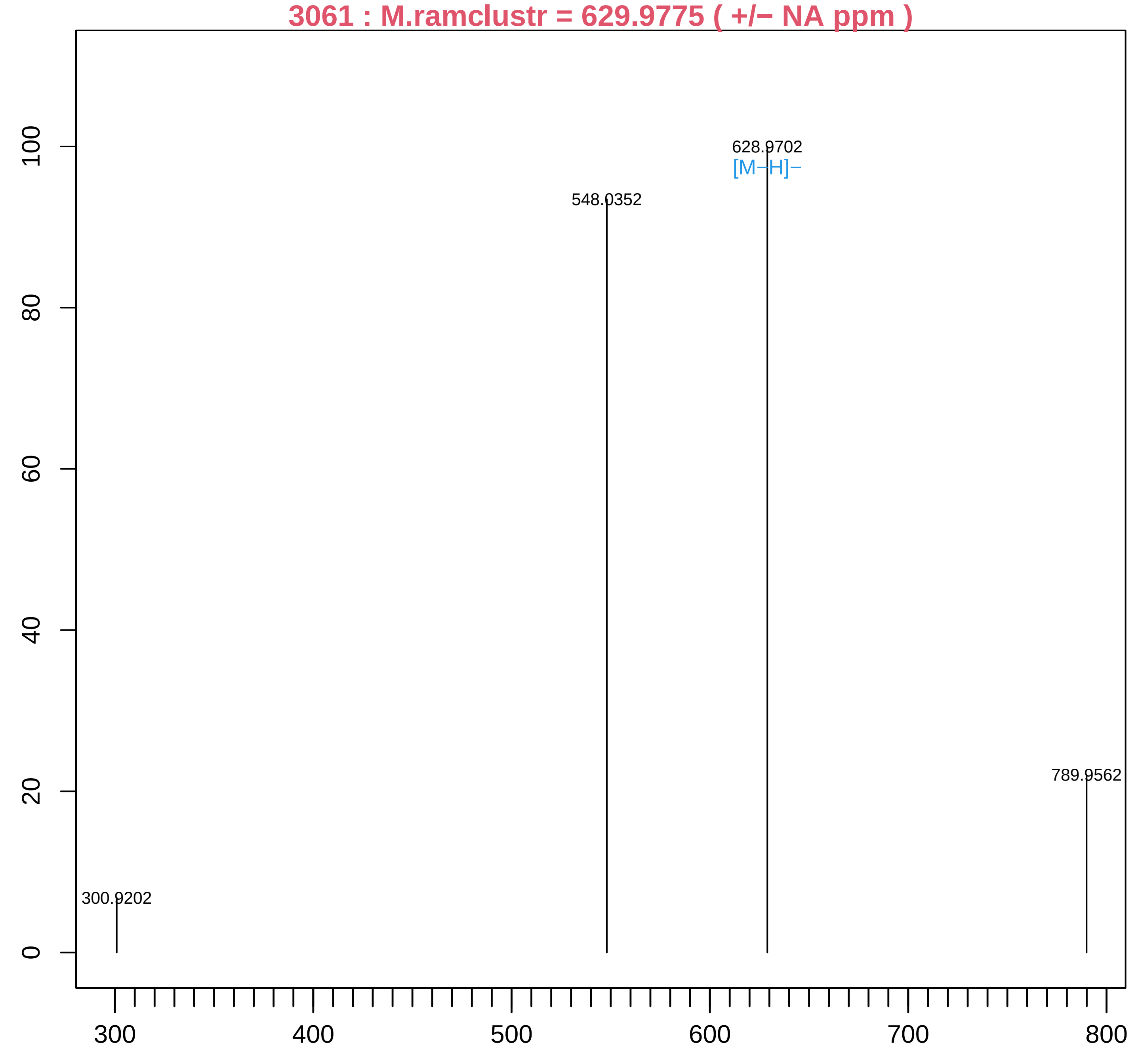
3061 : M.ramclustr = 629.9775 (+/− NA ppm)
628.9702
[M−H]−
548.0352
789.9562
300.9202
0
20
40
60
80
100
300
400
500
600
700
800

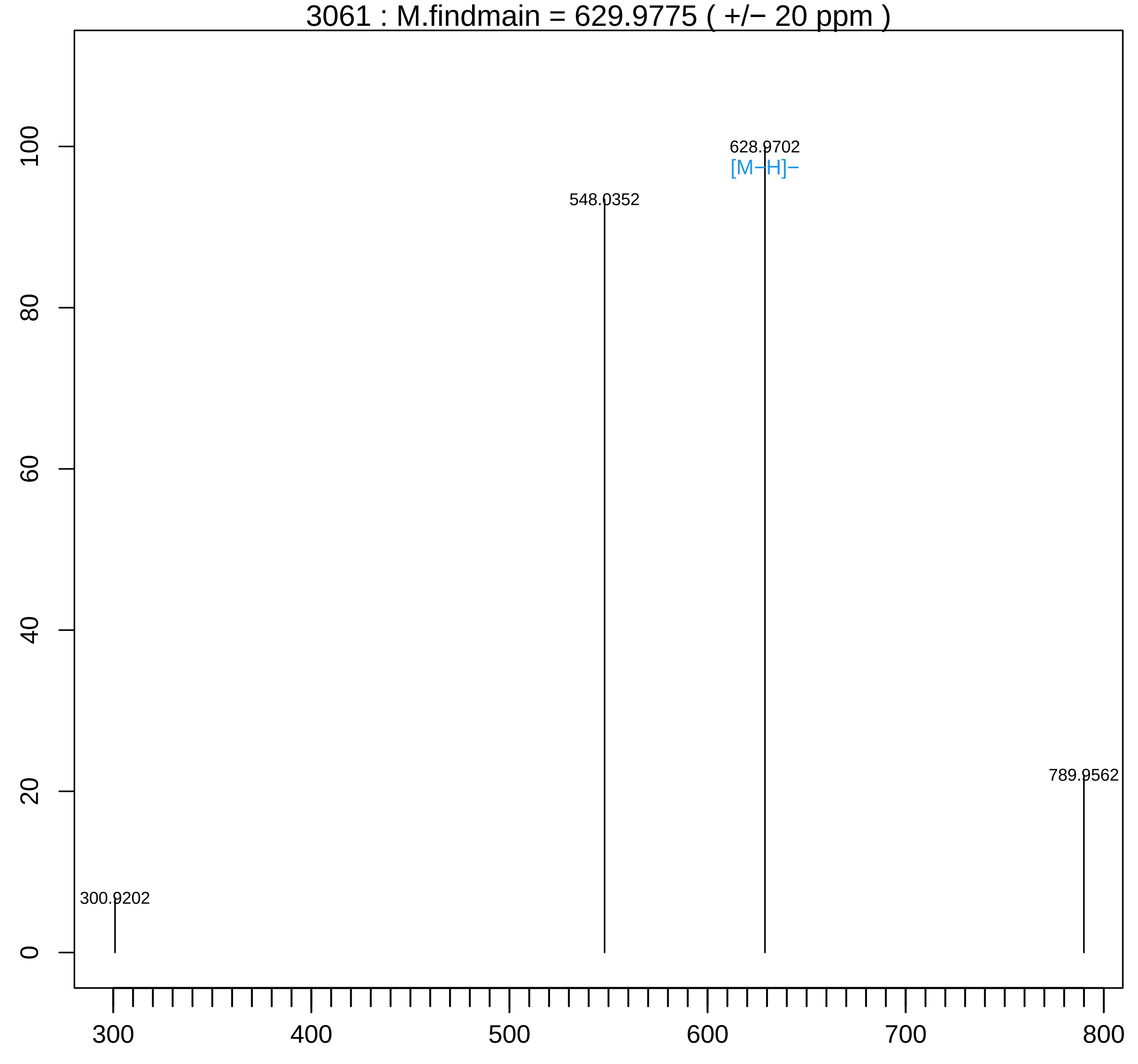
3061 : M.findmain = 629.9775 (+/− 20 ppm)
628.9702
[M−H]−
548.0352
789.9562
300.9202
0
20
40
60
80
100
300
400
500
600
700
800

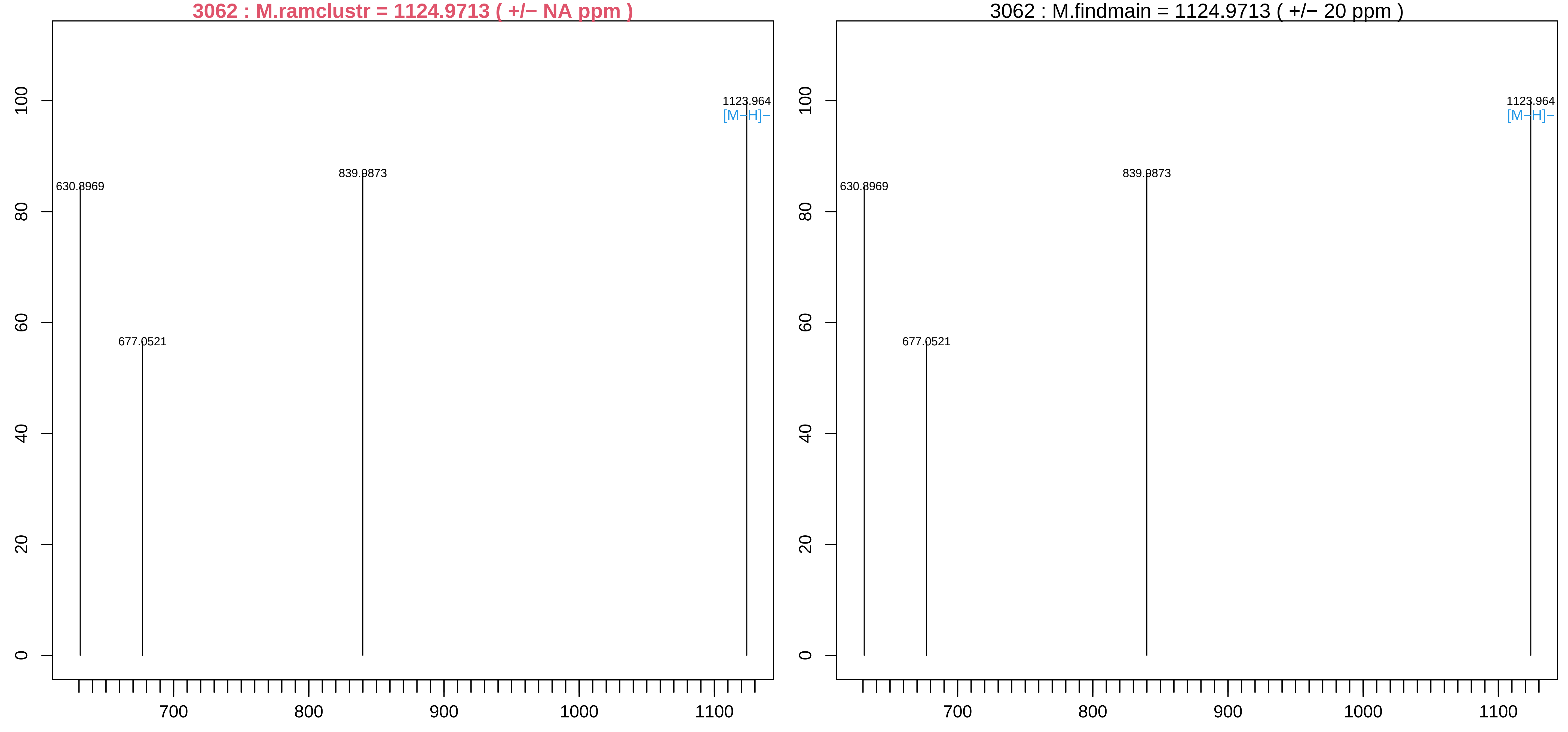
3062 : M.ramclustr = 1124.9713 (+/− NA ppm)
3062 : M.findmain = 1124.9713 (+/− 20 ppm)
1123.964
[M−H]−
839.9873
630.8969
677.0521
0
20
40
60
80
100
700
800
900
1000
1100

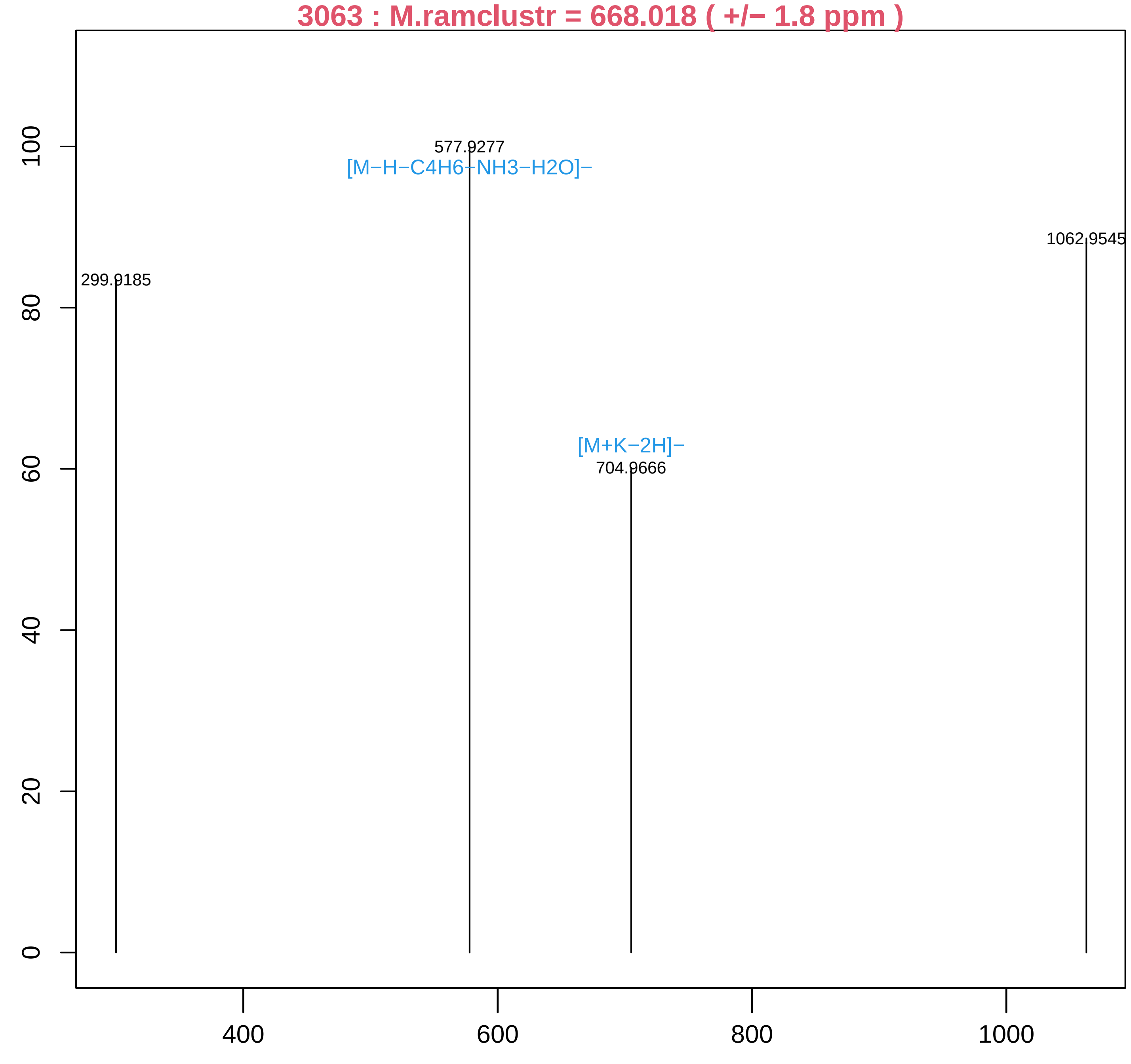
3063 : M.ramclustr = 668.018 (+/− 1.8 ppm)
577.9277
[M−H−C4H6−NH3−H2O]−
1062.9545
299.9185
[M+K−2H]−
704.9666
0
20
40
60
80
100
400
600
800
1000

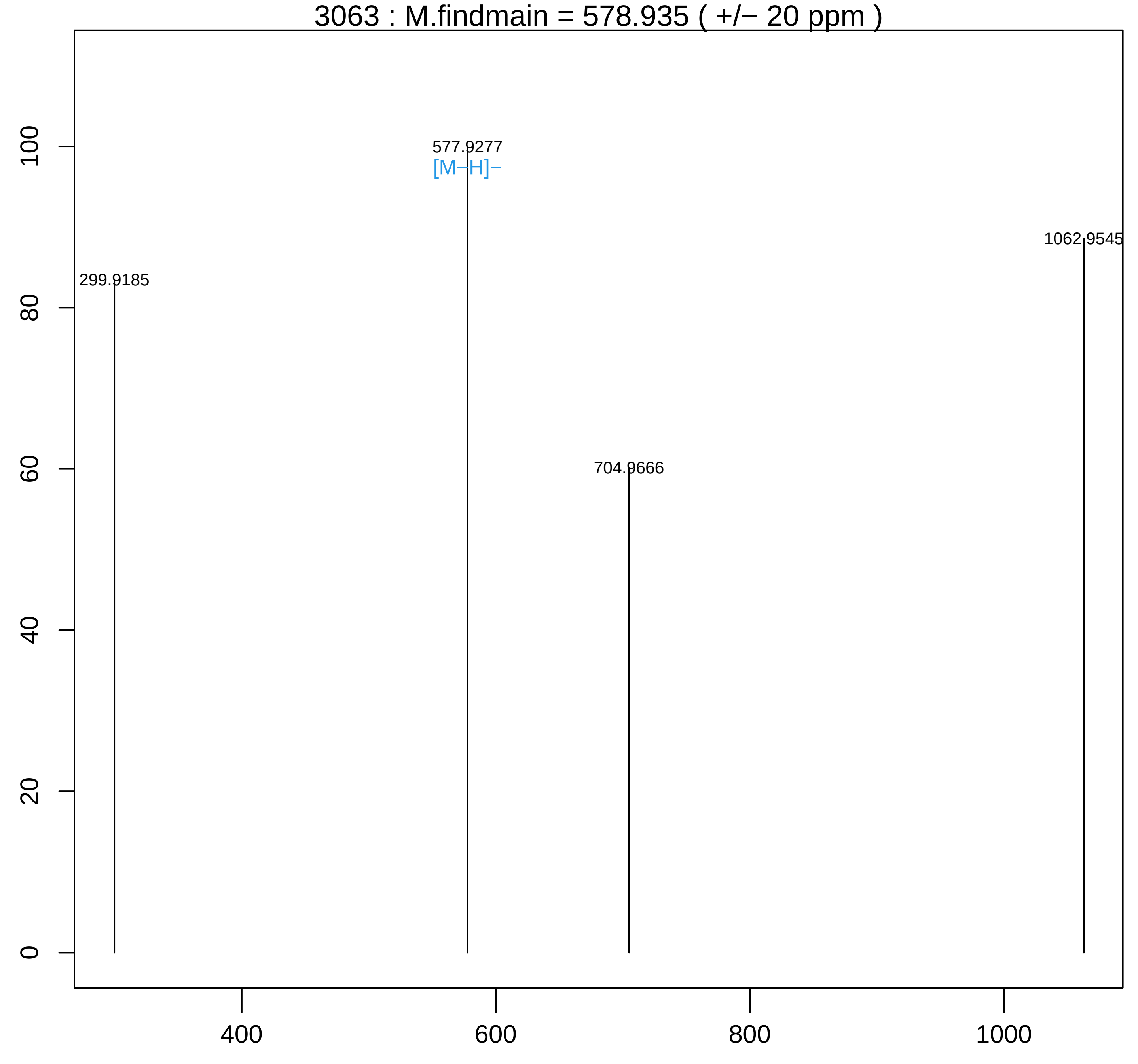
3063 : M.findmain = 578.935 (+/− 20 ppm)
577.9277
[M−H]−
1062.9545
299.9185
704.9666
0
20
40
60
80
100
400
600
800
1000

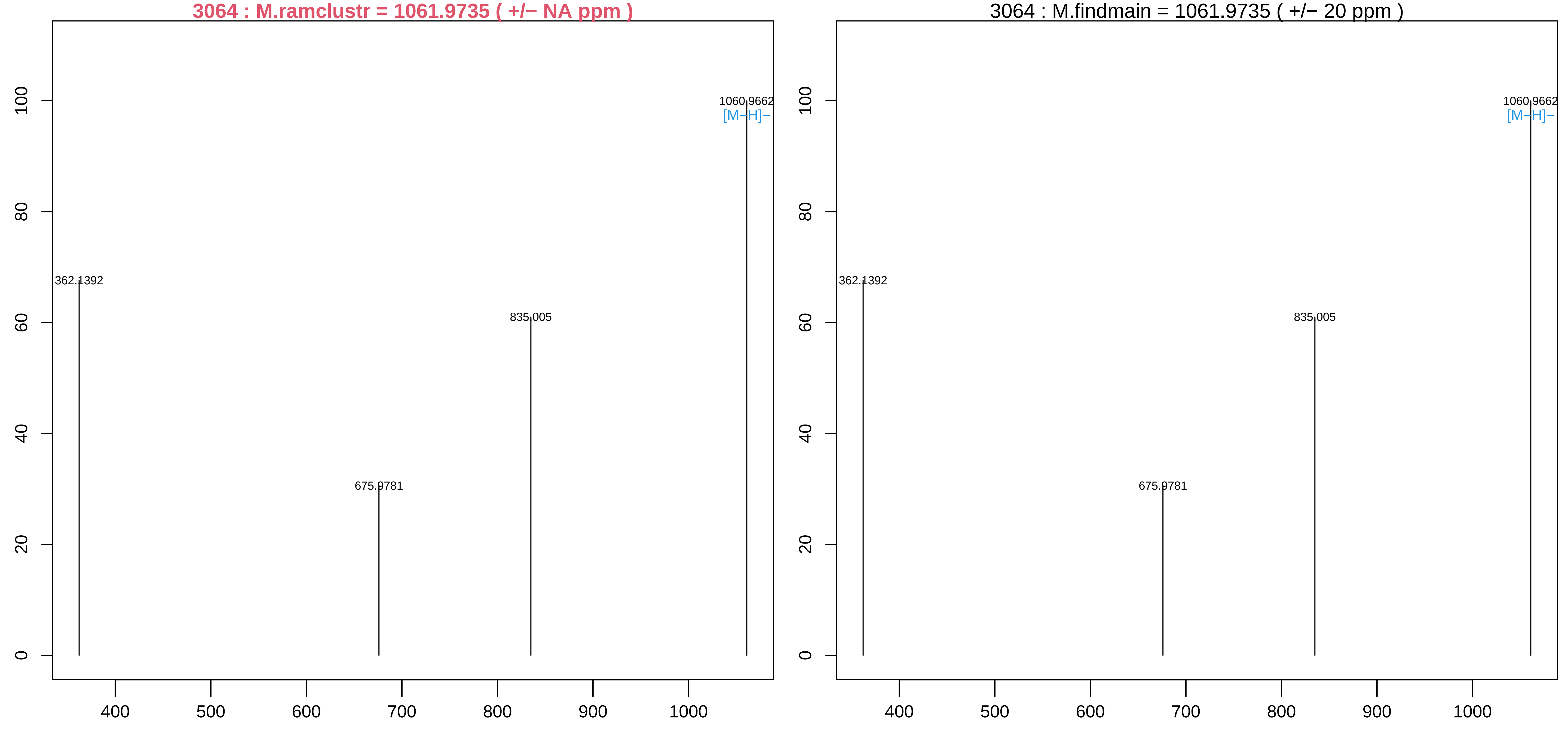
3064 : M.ramclustr = 1061.9735 (+/− NA ppm)
3064 : M.findmain = 1061.9735 (+/− 20 ppm)
1060.9662
[M−H]−
362.1392
835.005
675.9781
0
20
40
60
80
100
400
500
600
700
800
900
1000

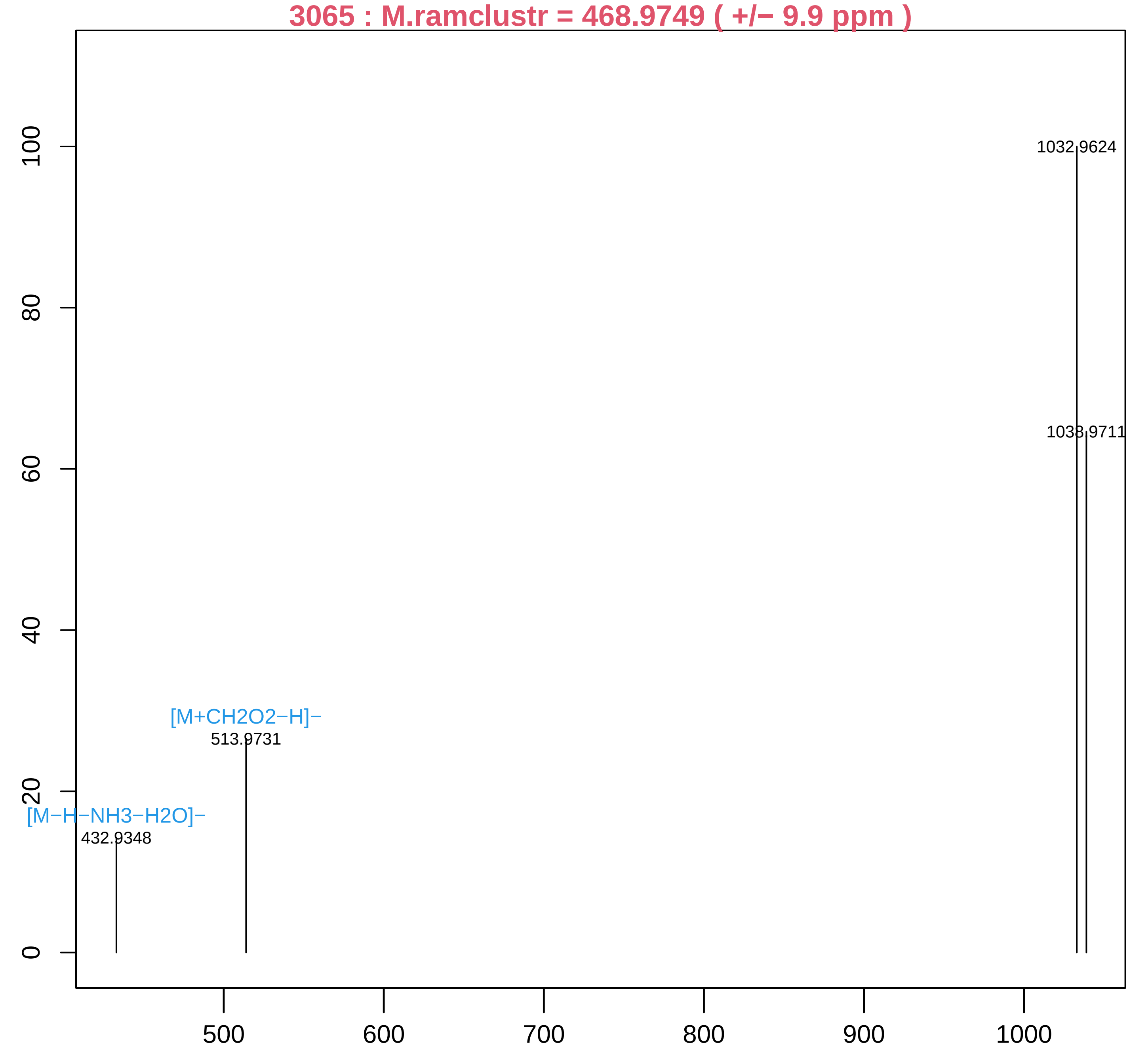
3065 : M.ramclustr = 468.9749 (+/− 9.9 ppm)
1032.9624
1033.9711
[M+CH2O2−H]−
513.9731
[M−H−NH3−H2O]−
432.9348
0
20
40
60
80
100
500
600
700
800
900
1000

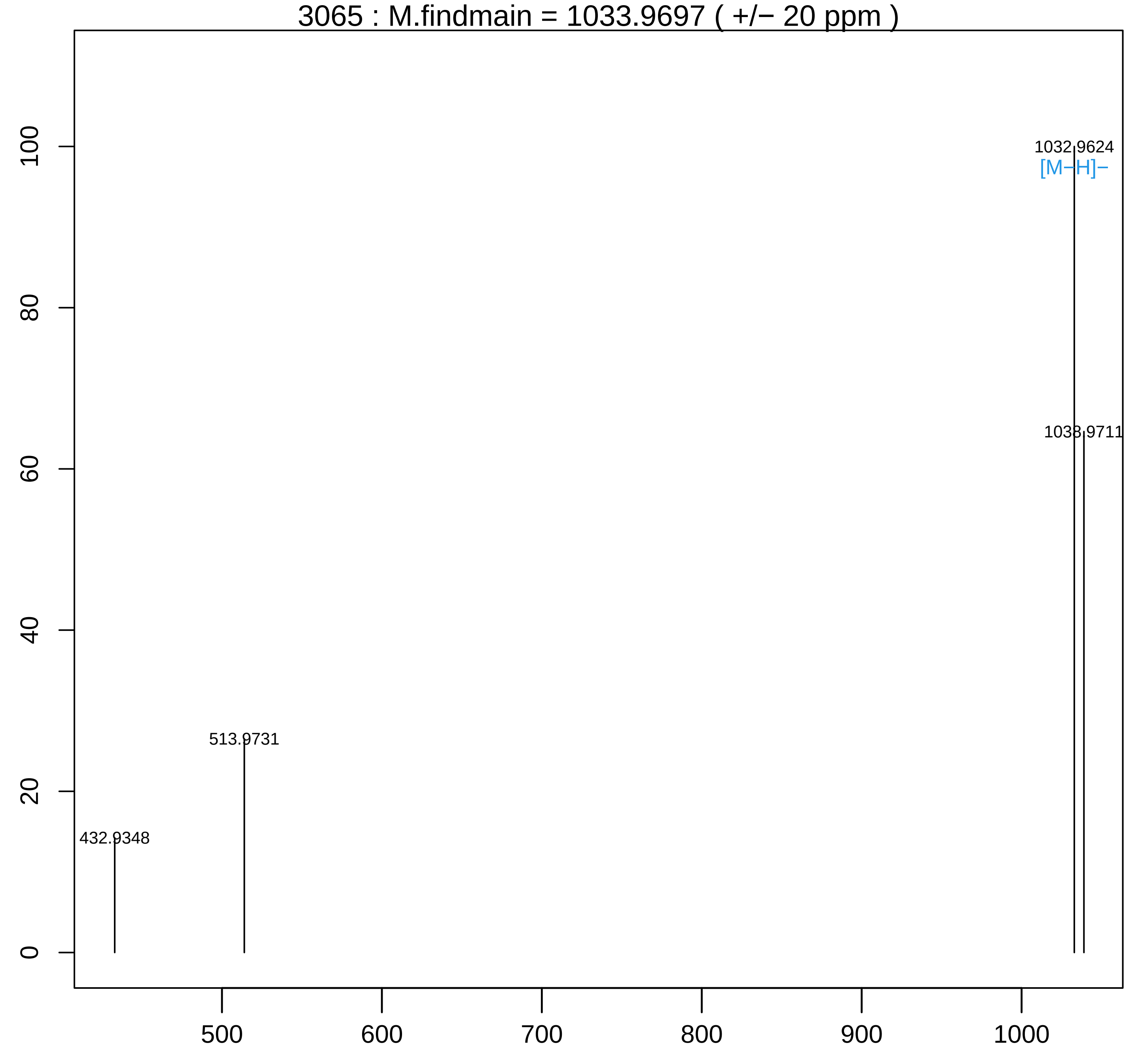
3065 : M.findmain = 1033.9697 (+/− 20 ppm)
1032.9624
[M−H]−
1033.9711
513.9731
432.9348
0
20
40
60
80
100
500
600
700
800
900
1000

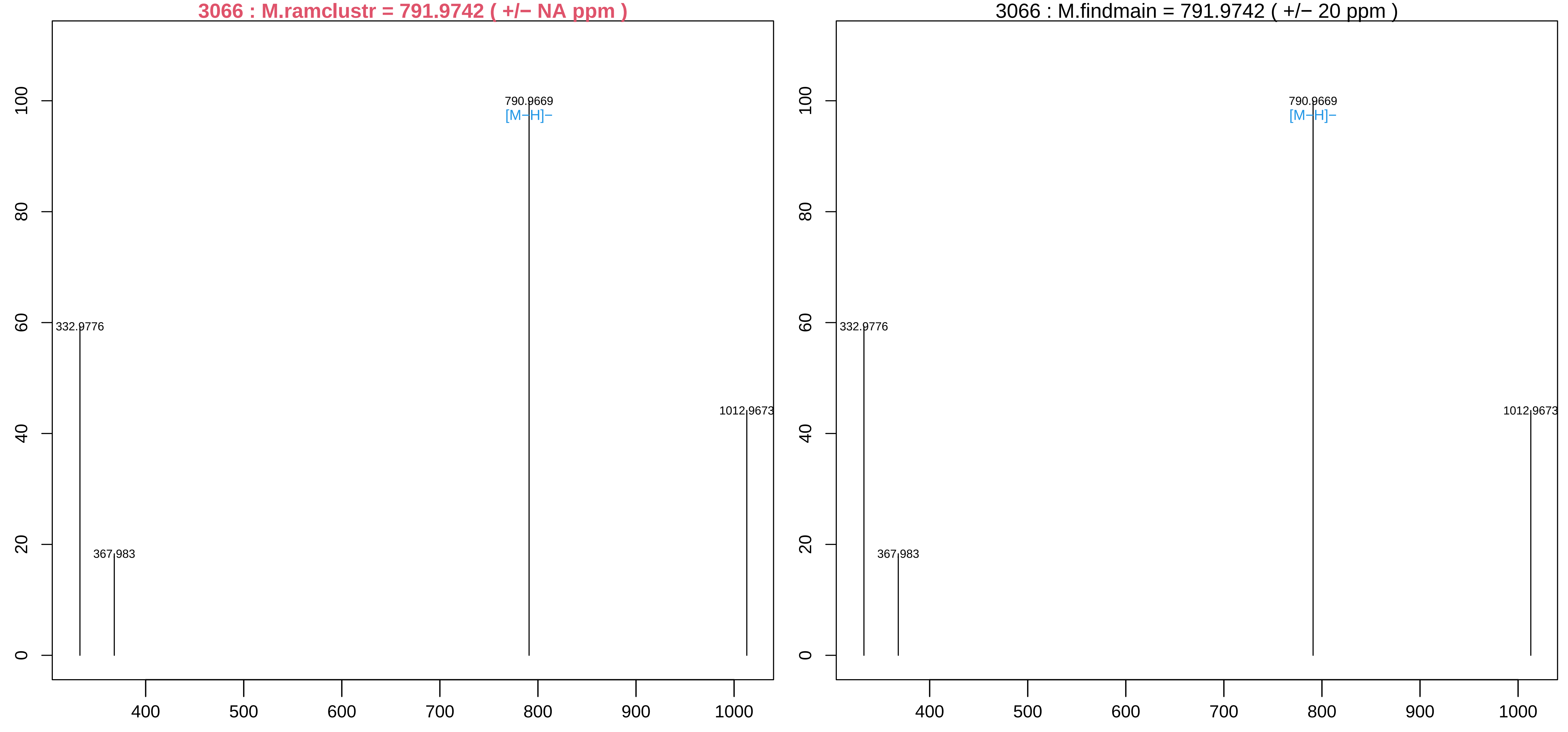

3066 : M.ramclustr = 791.9742 (+/− NA ppm)
790.9669
[M−H]−
332.9776
367.983
1012.9673
0
20
40
60
80
100
400
500
600
700
800
900
1000
3066 : M.findmain = 791.9742 (+/− 20 ppm)
790.9669
[M−H]−
332.9776
367.983
1012.9673
0
20
40
60
80
100
400
500
600
700
800
900
1000

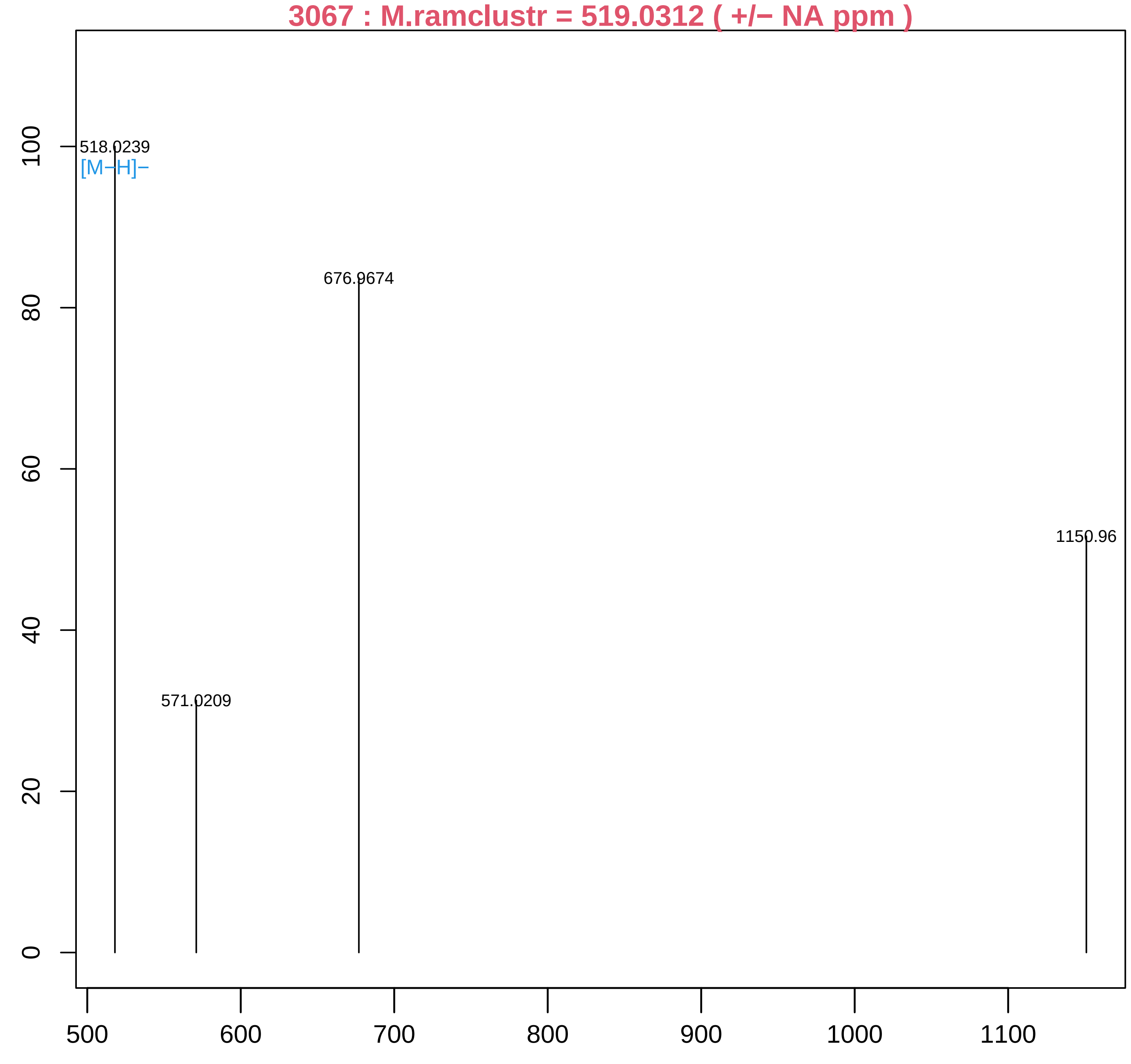
3067 : M.ramclustr = 519.0312 (+/− NA ppm)
518.0239
[M−H]−
676.9674
571.0209
1150.96
0
20
40
60
80
100
500
600
700
800
900
1000
1100

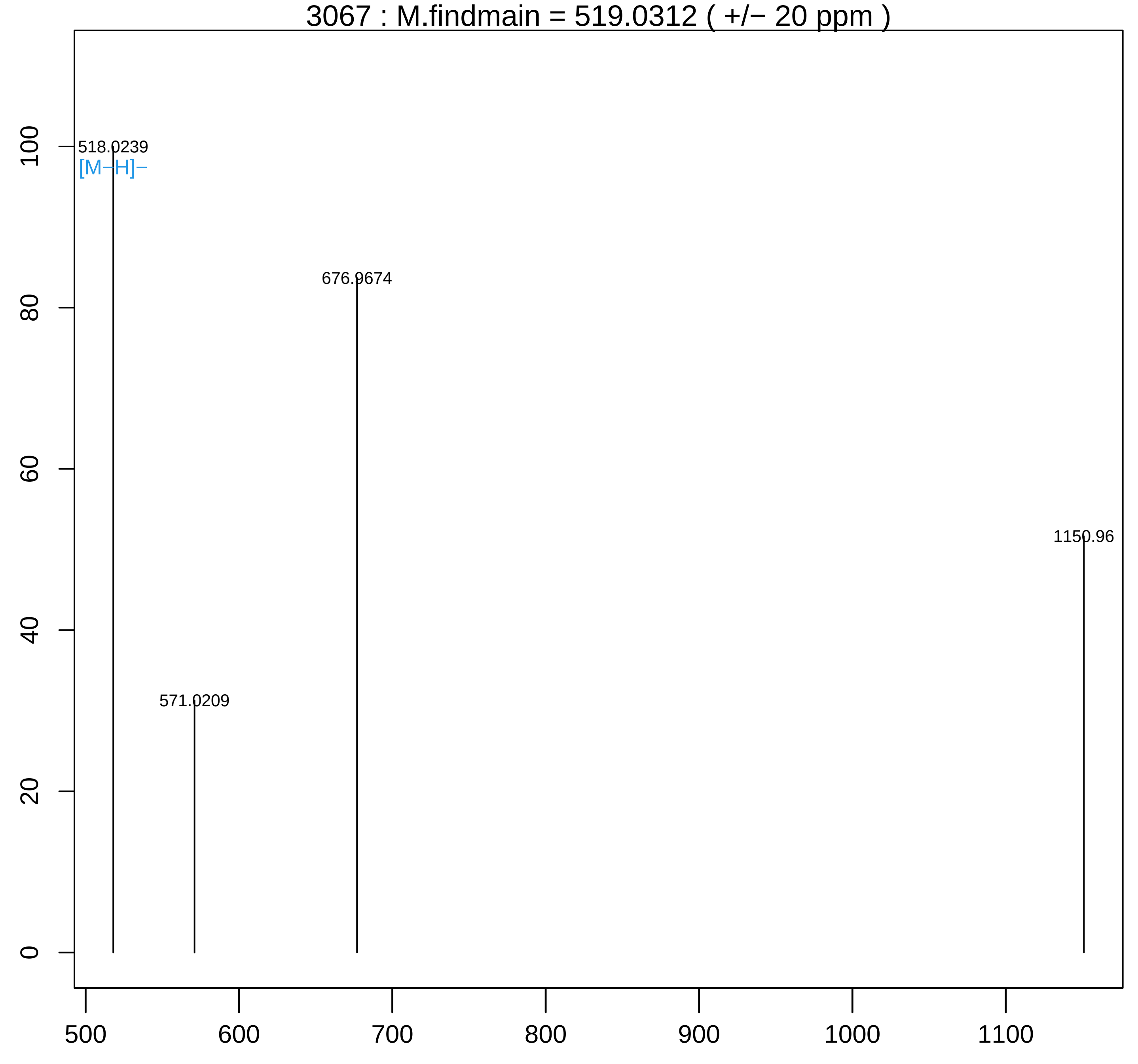
3067 : M.findmain = 519.0312 (+/− 20 ppm)
518.0239
[M−H]−
676.9674
571.0209
1150.96
0
20
40
60
80
100
500
600
700
800
900
1000
1100

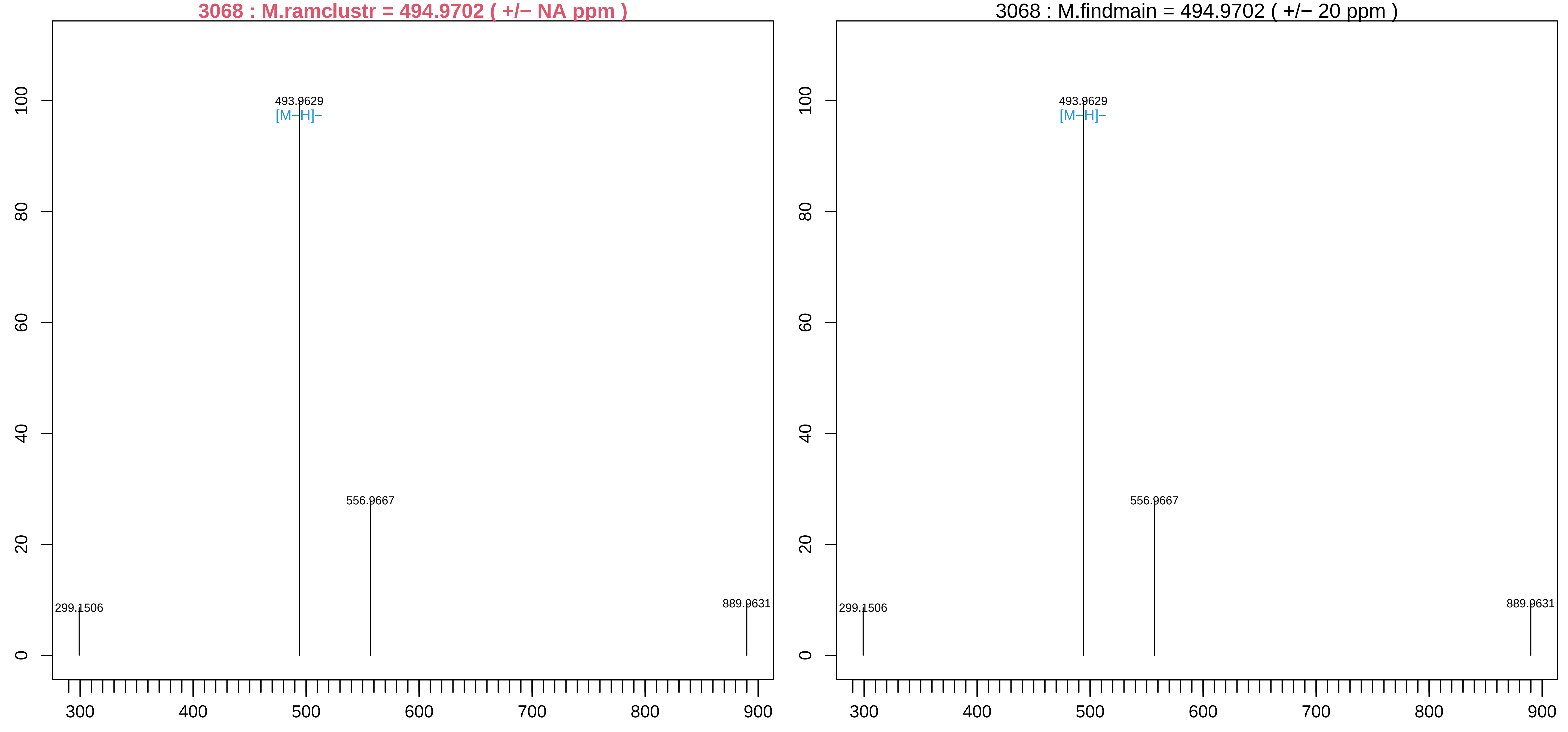
3068 : M.ramclustr = 494.9702 (+/− NA ppm)
493.9629
[M−H]−
556.9667
299.1506
889.9631
3068 : M.findmain = 494.9702 (+/− 20 ppm)
493.9629
[M−H]−
556.9667
299.1506
889.9631

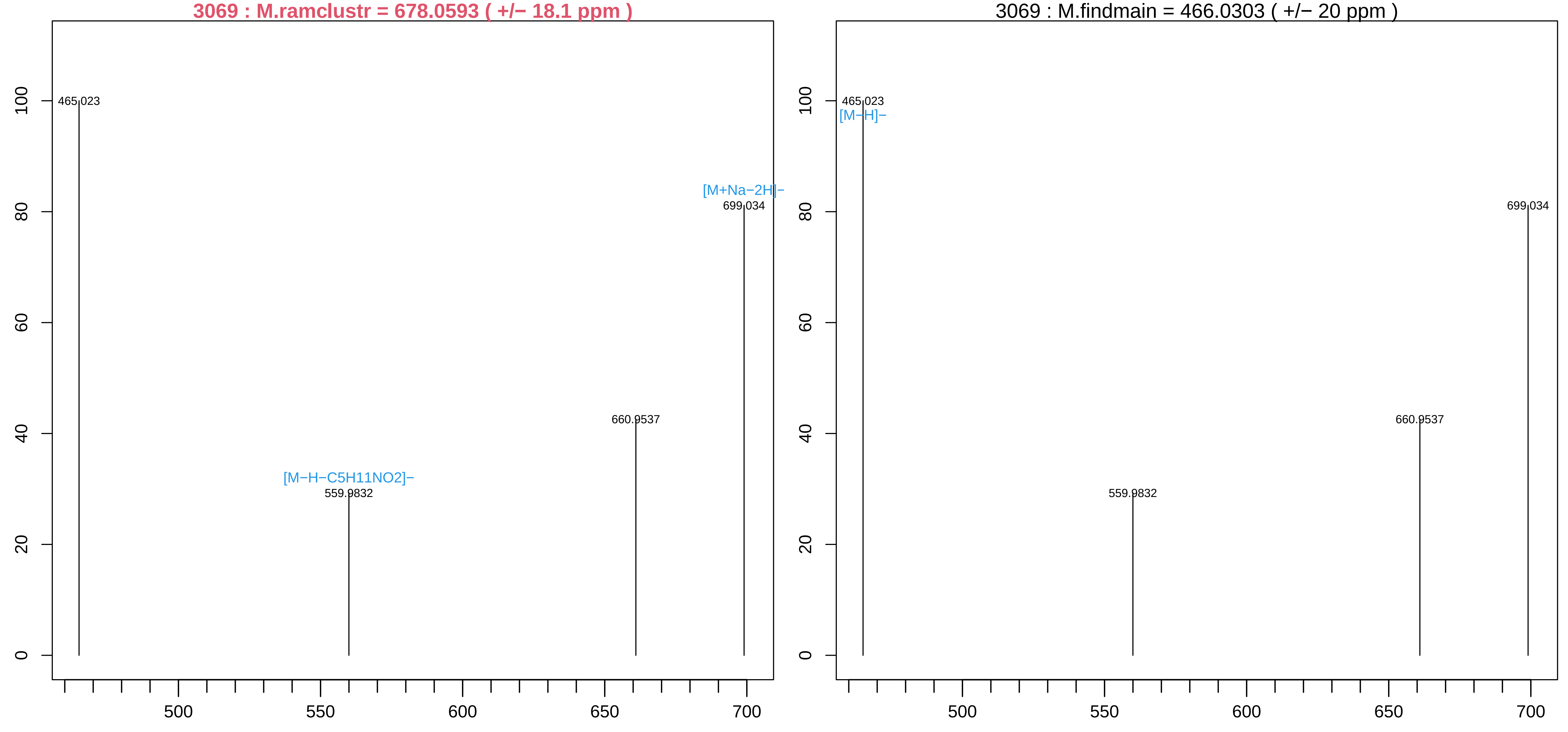
3069 : M.ramclustr = 678.0593 (+/− 18.1 ppm)
465.023
[M+Na−2H]−
699.034
660.9537
[M−H−C5H11NO2]−
559.9832
0
20
40
60
80
100
500
550
600
650
700
3069 : M.findmain = 466.0303 (+/− 20 ppm)
465.023
[M−H]−
699.034
660.9537
559.9832
0
20
40
60
80
100
500
550
600
650
700

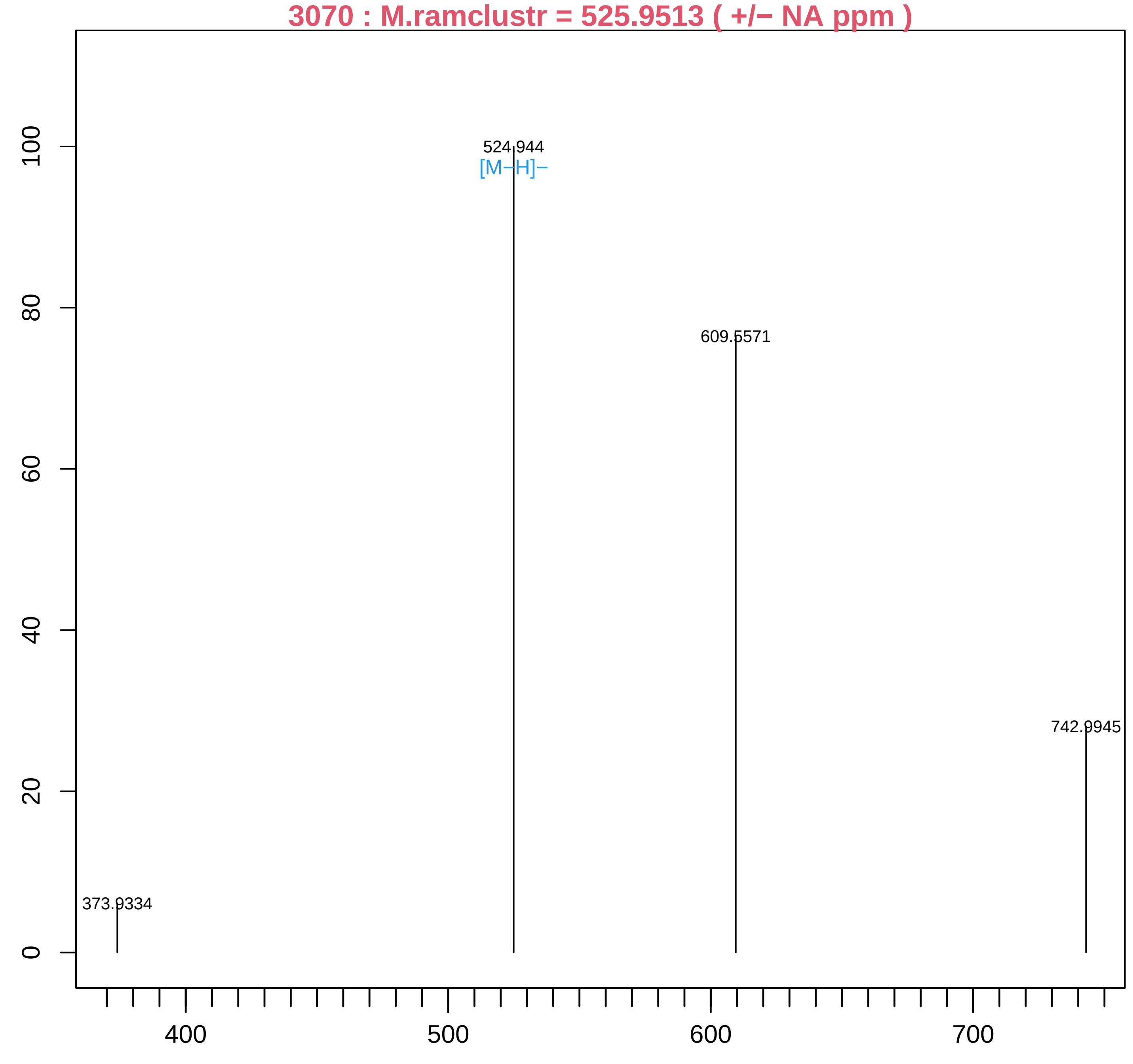
3070 : M.ramclustr = 525.9513 (+/− NA ppm)
524.944
[M−H]−
609.5571
742.9945
373.9334
0
20
40
60
80
100
400
500
600
700

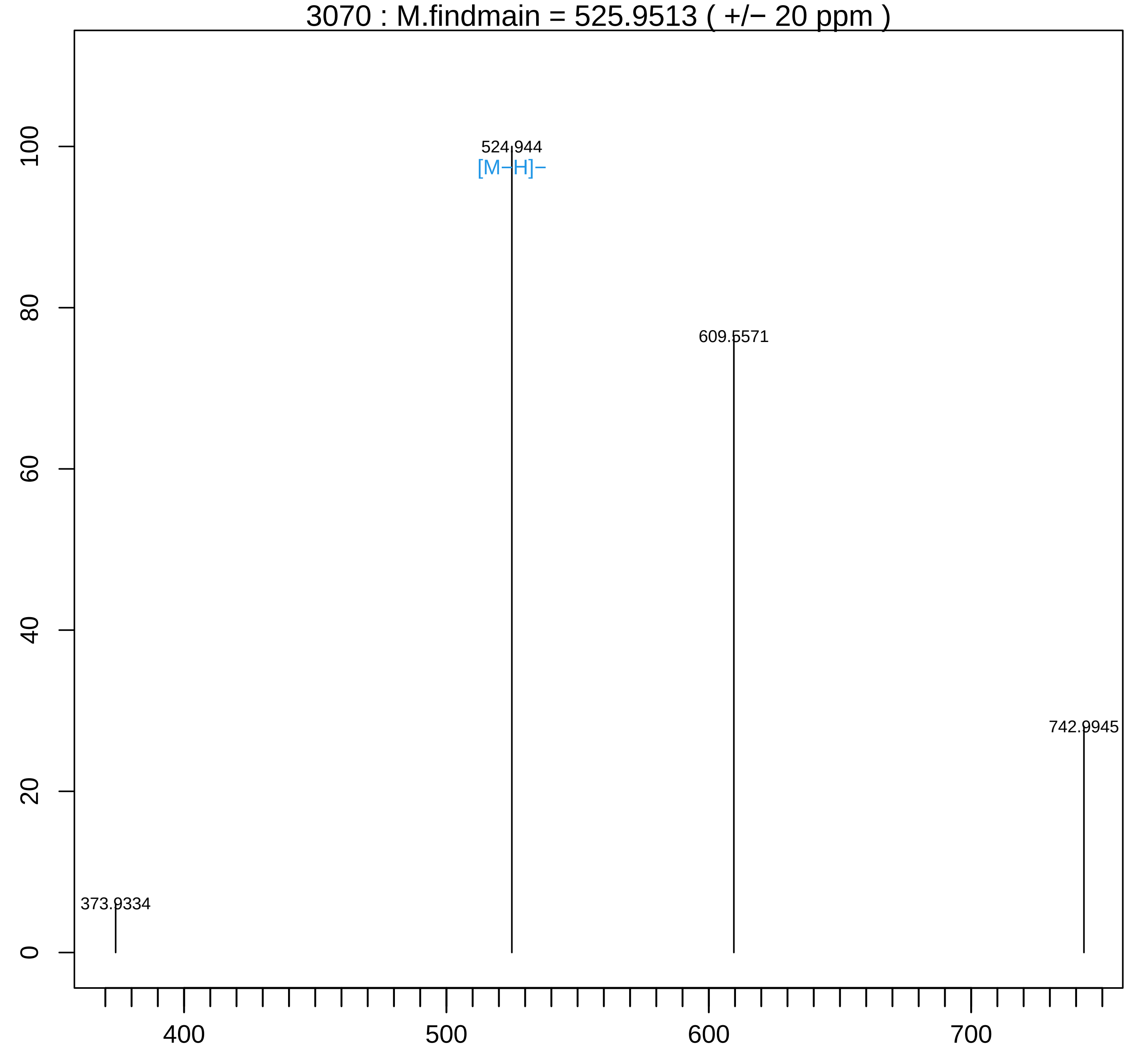
3070 : M.findmain = 525.9513 (+/− 20 ppm)
524.944
[M−H]−
609.5571
742.9945
373.9334
0
20
40
60
80
100
400
500
600
700

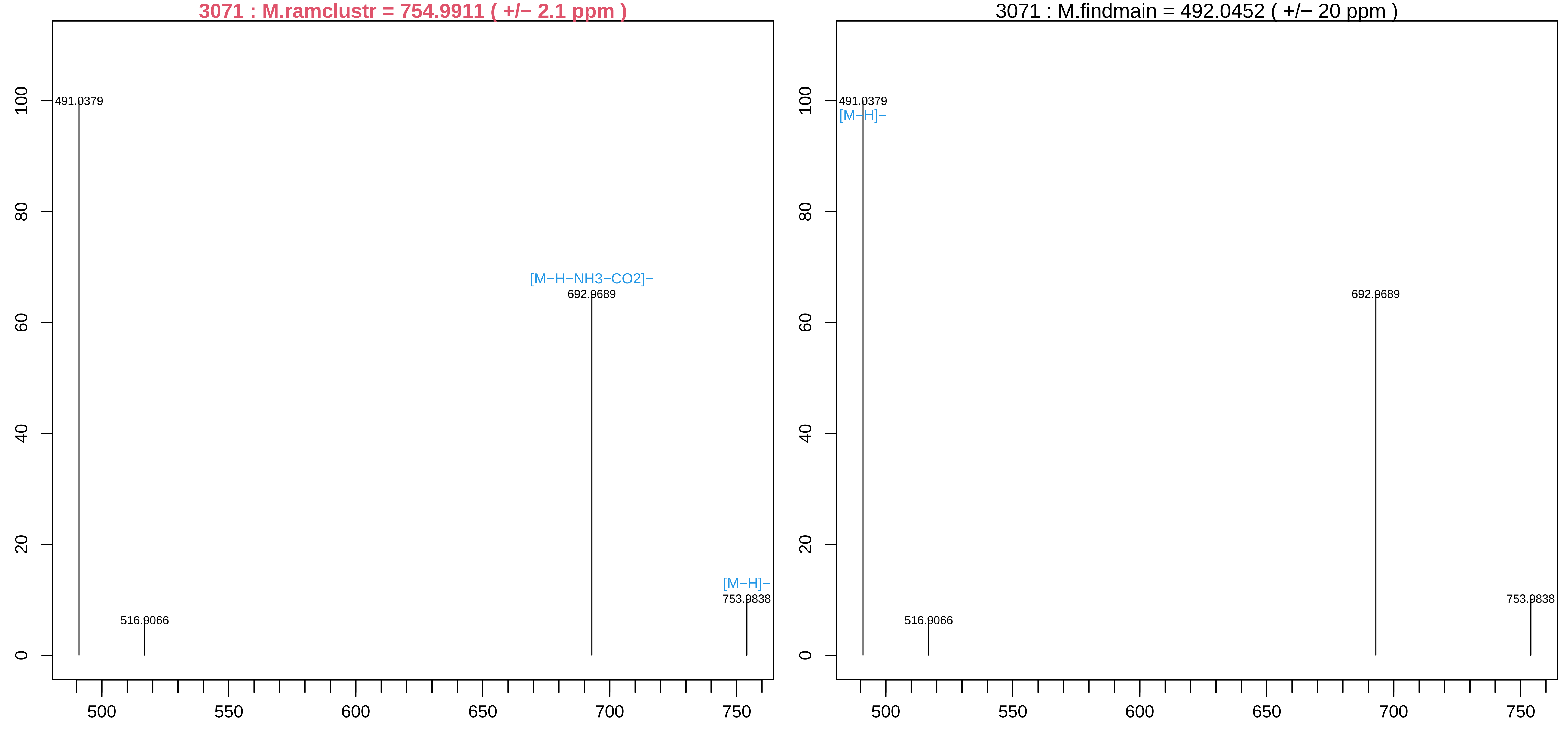
3071 : M.ramclustr = 754.9911 (+/− 2.1 ppm)
491.0379
516.9066
[M−H−NH3−CO2]−
692.9689
[M−H]−
753.9838
0
20
40
60
80
100
500
550
600
650
700
750
3071 : M.findmain = 492.0452 (+/− 20 ppm)
491.0379
[M−H]−
516.9066
692.9689
753.9838
0
20
40
60
80
100
500
550
600
650
700
750

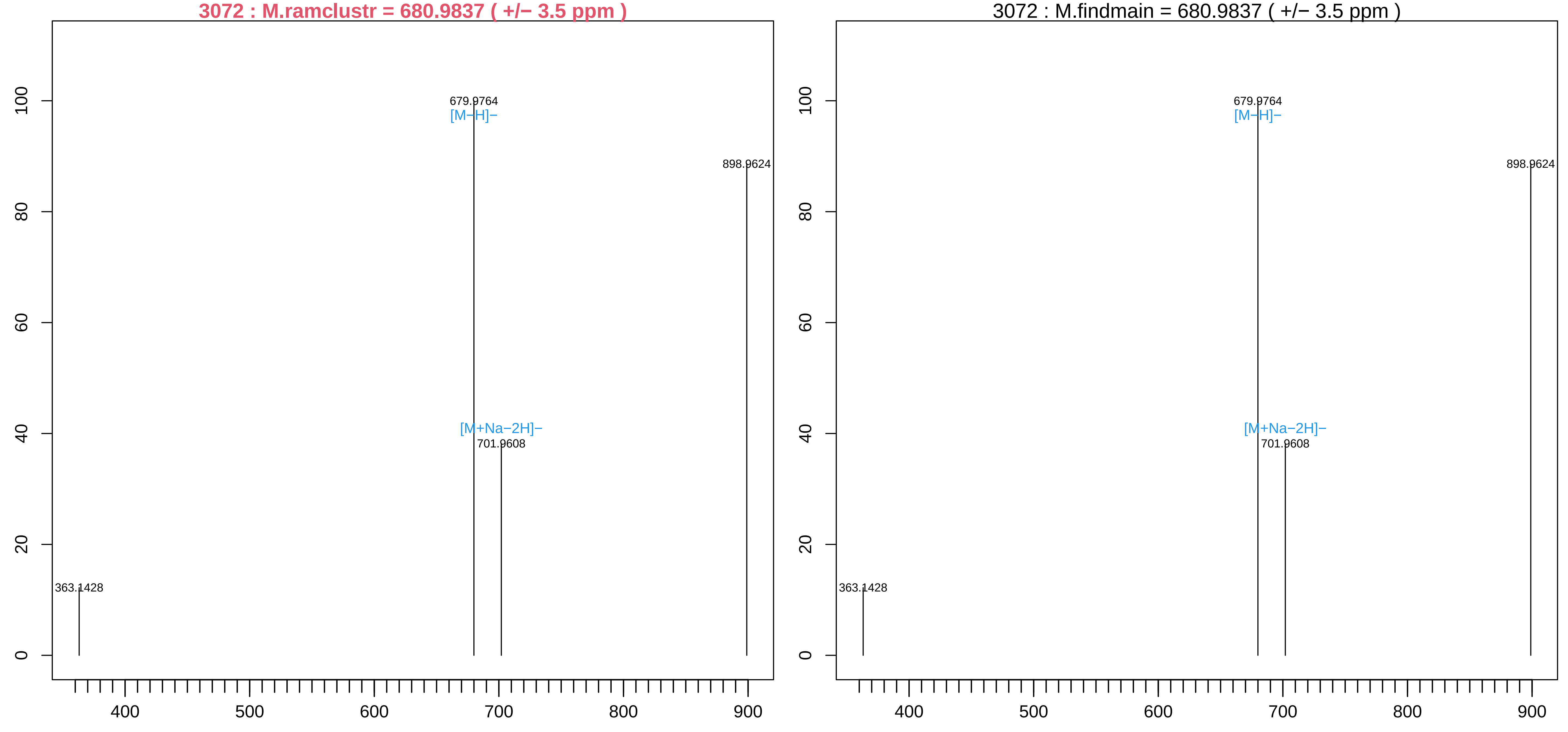
3072 : M.ramclustr = 680.9837 (+/− 3.5 ppm)
679.9764
[M−H]−
898.9624
[M+Na−2H]−
701.9608
363.1428
0
20
40
60
80
100
400
500
600
700
800
900
3072 : M.findmain = 680.9837 (+/− 3.5 ppm)
679.9764
[M−H]−
898.9624
[M+Na−2H]−
701.9608
363.1428
0
20
40
60
80
100
400
500
600
700
800
900

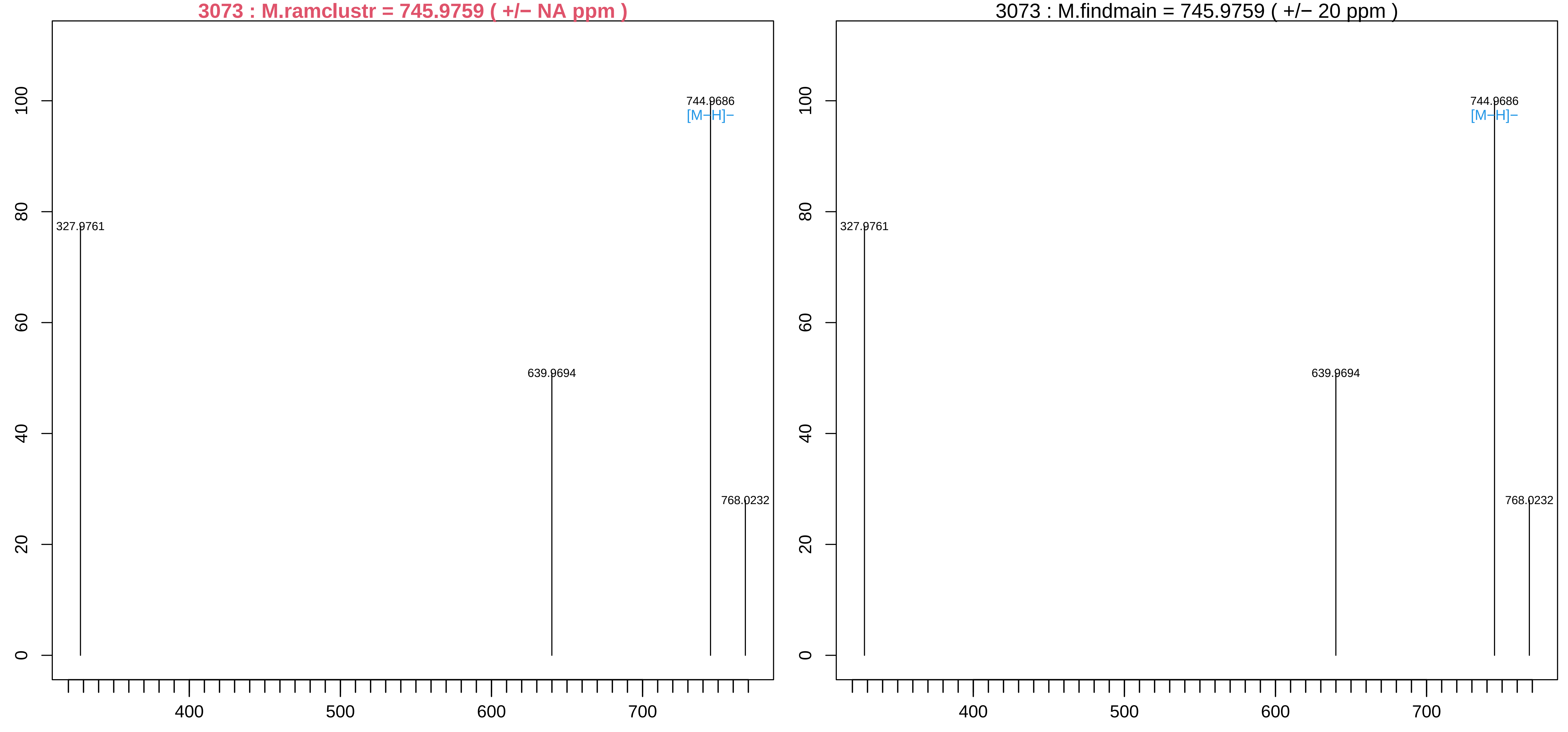
3073 : M.ramclustr = 745.9759 (+/− NA ppm)
3073 : M.findmain = 745.9759 (+/− 20 ppm)
0
20
40
60
80
100
400
500
600
700
327.9761
639.9694
744.9686
[M−H]−
768.0232

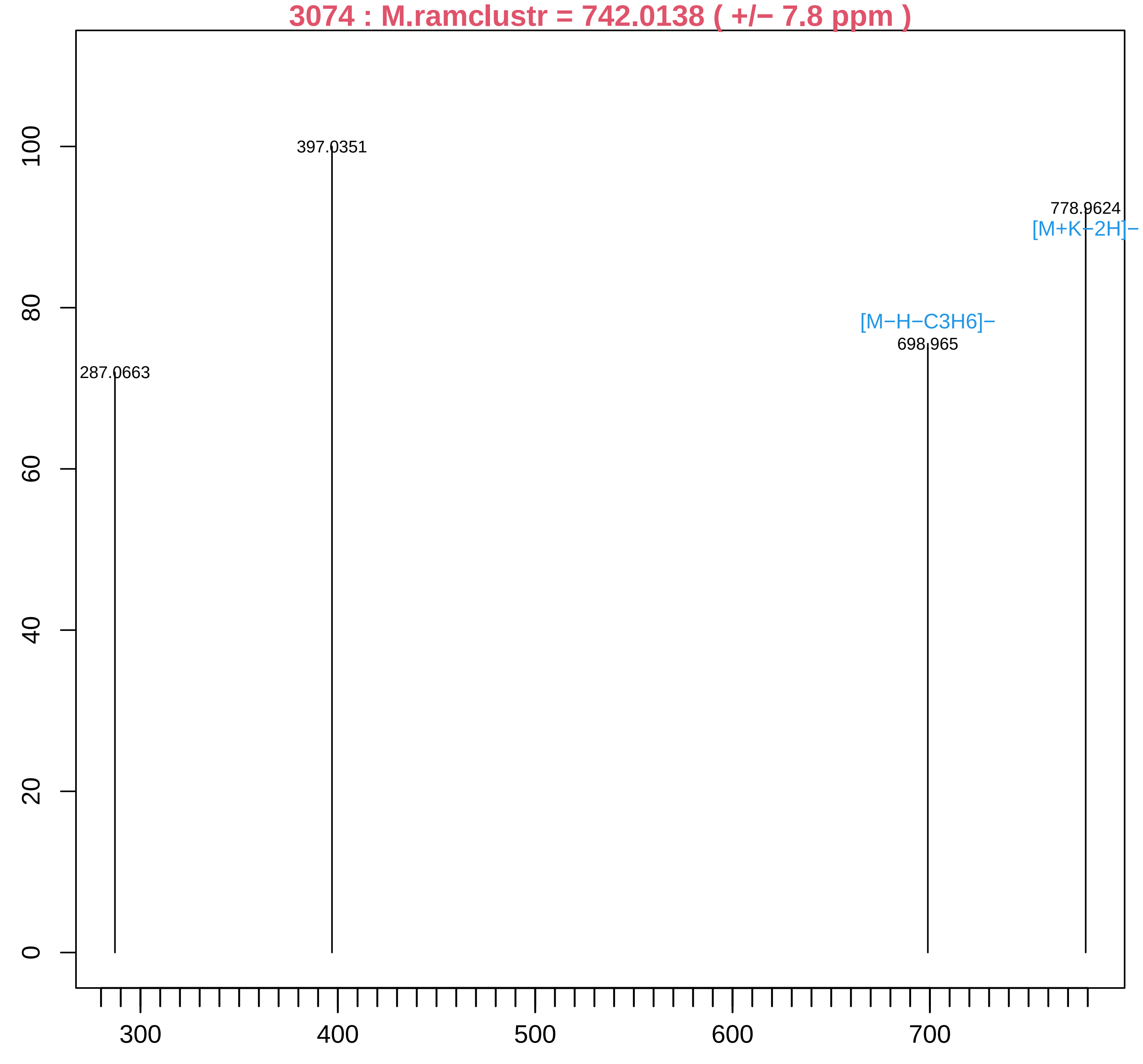
3074 : M.ramclustr = 742.0138 (+/− 7.8 ppm)
397.0351
287.0663
698.965
[M−H−C3H6]−
778.9624
[M+K−2H]−
0
20
40
60
80
100
300
400
500
600
700

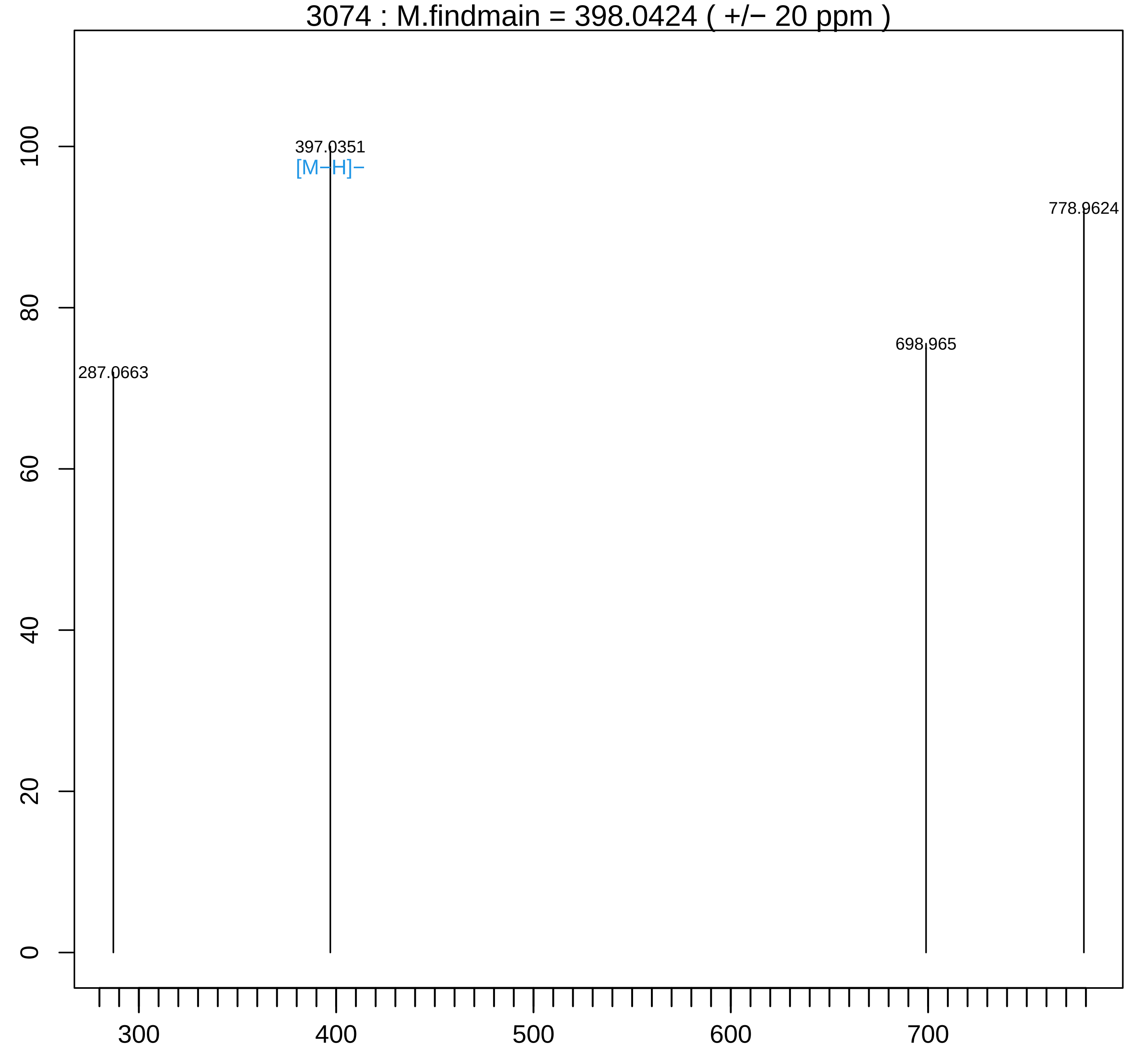
3074 : M.findmain = 398.0424 (+/− 20 ppm)
397.0351
[M−H]−
287.0663
698.965
778.9624
0
20
40
60
80
100
300
400
500
600
700

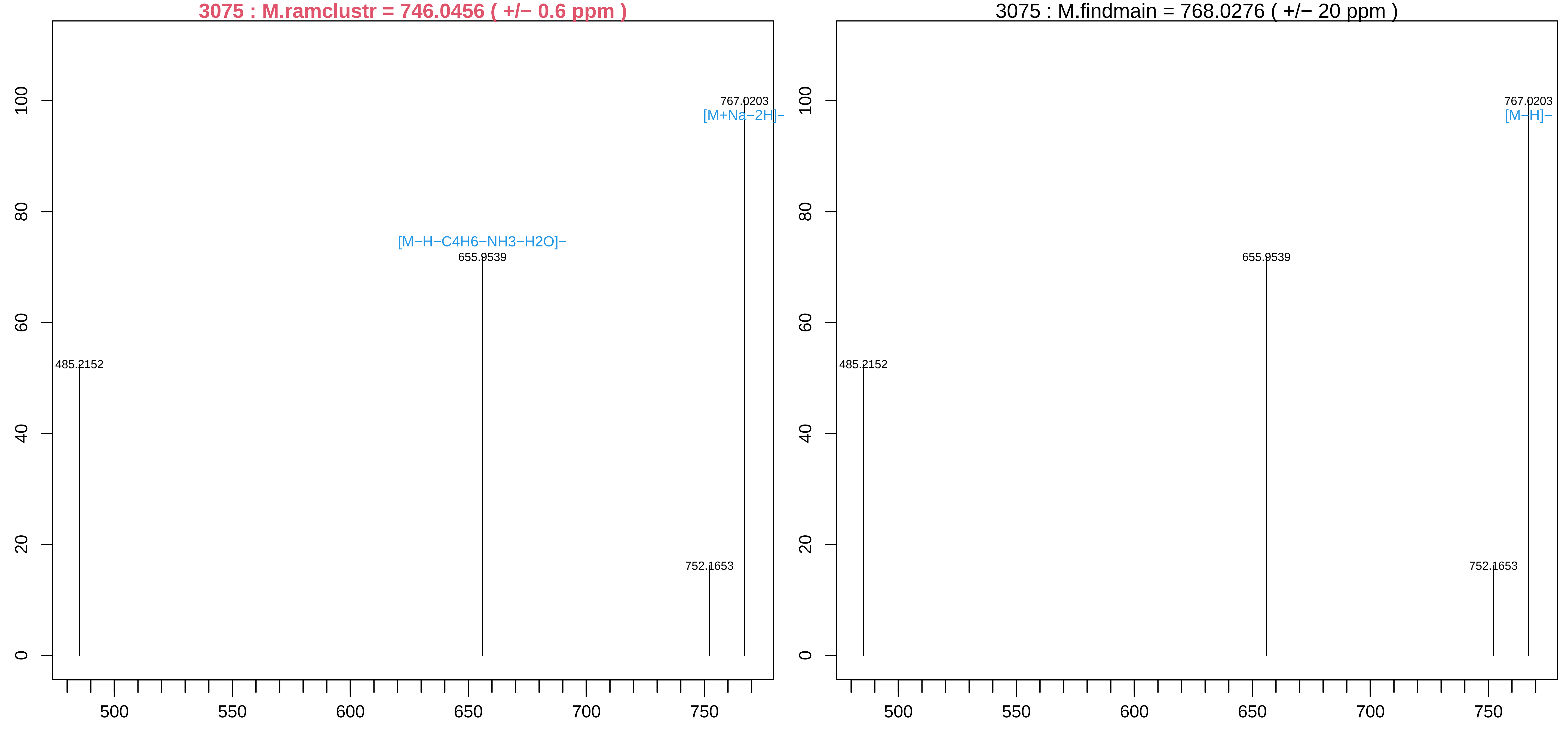

3075 : M.ramclustr = 746.0456 (+/− 0.6 ppm)
767.0203
[M+Na−2H]−
[M−H−C4H6−NH3−H2O]−
655.9539
485.2152
752.1653
3075 : M.findmain = 768.0276 (+/− 20 ppm)
767.0203
[M−H]−
655.9539
485.2152
752.1653

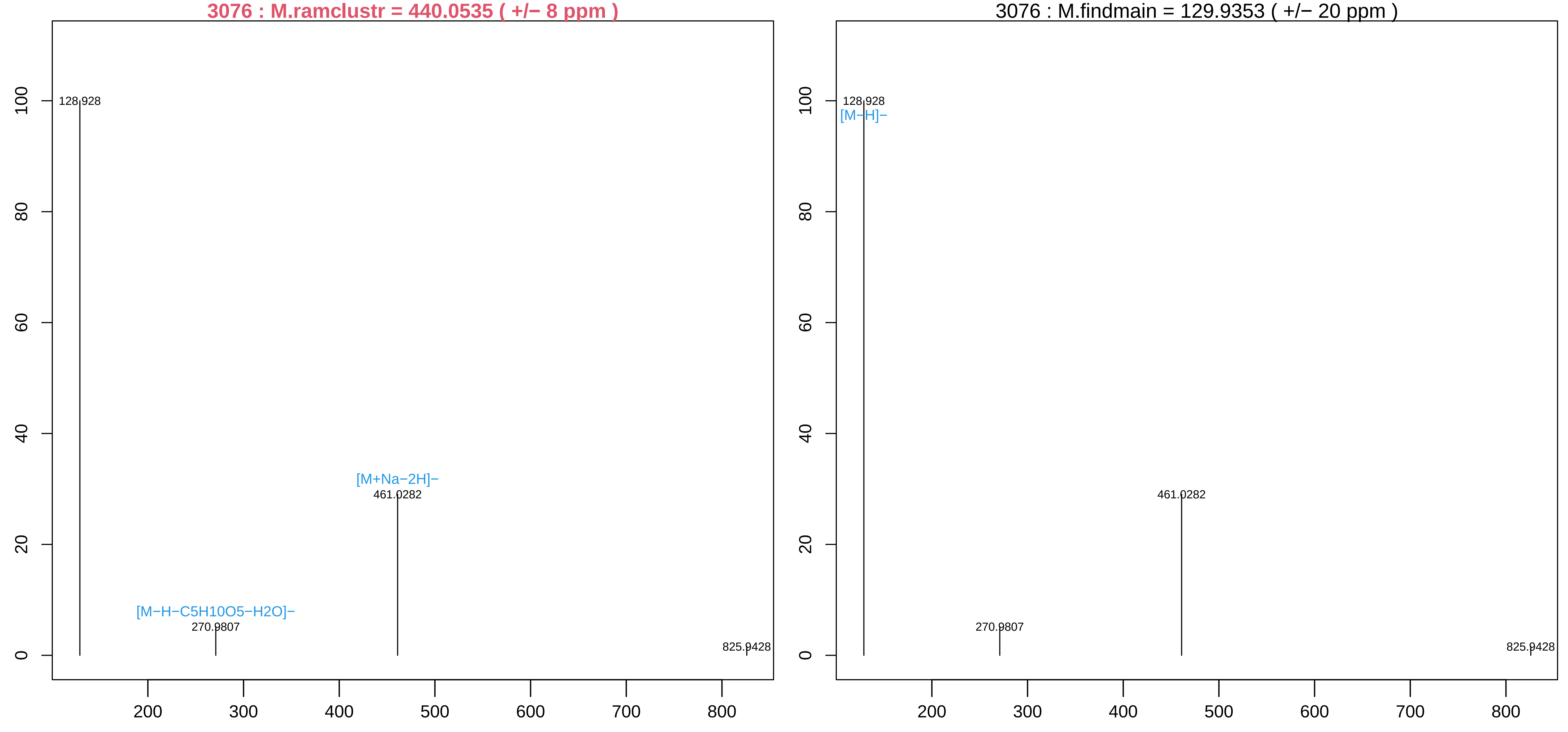

3076 : M.ramclustr = 440.0535 (+/− 8 ppm)
128.928
[M−H−C5H10O5−H2O]−
270.9807
[M+Na−2H]−
461.0282
825.9428
3076 : M.findmain = 129.9353 (+/− 20 ppm)
128.928
[M−H]−
270.9807
461.0282
825.9428
0
20
40
60
80
100
200
300
400
500
600
700
800

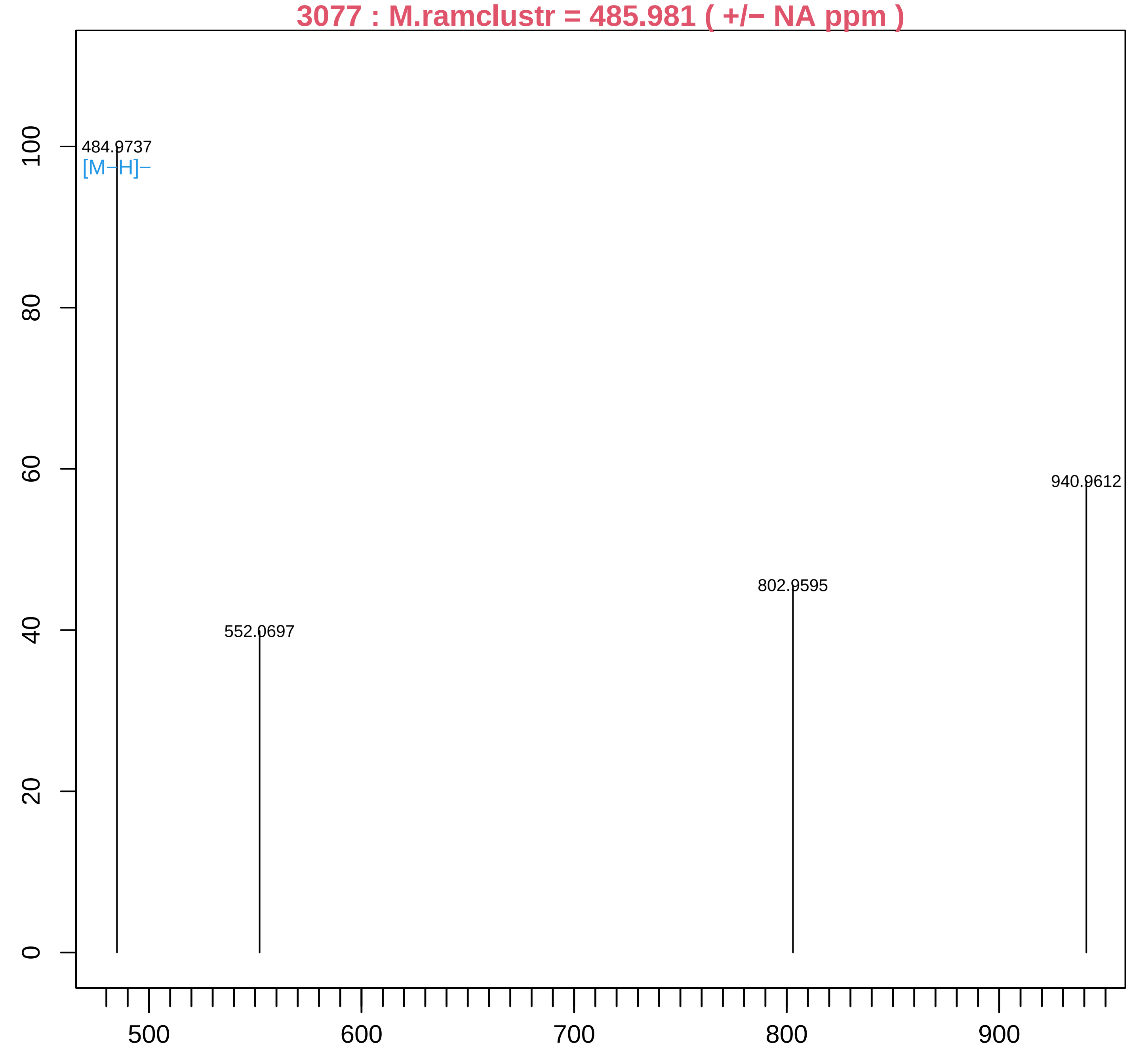
3077 : M.ramclustr = 485.981 (+/− NA ppm)
484.9737
[M−H]−
552.0697
802.9595
940.9612
0
20
40
60
80
100
500
600
700
800
900

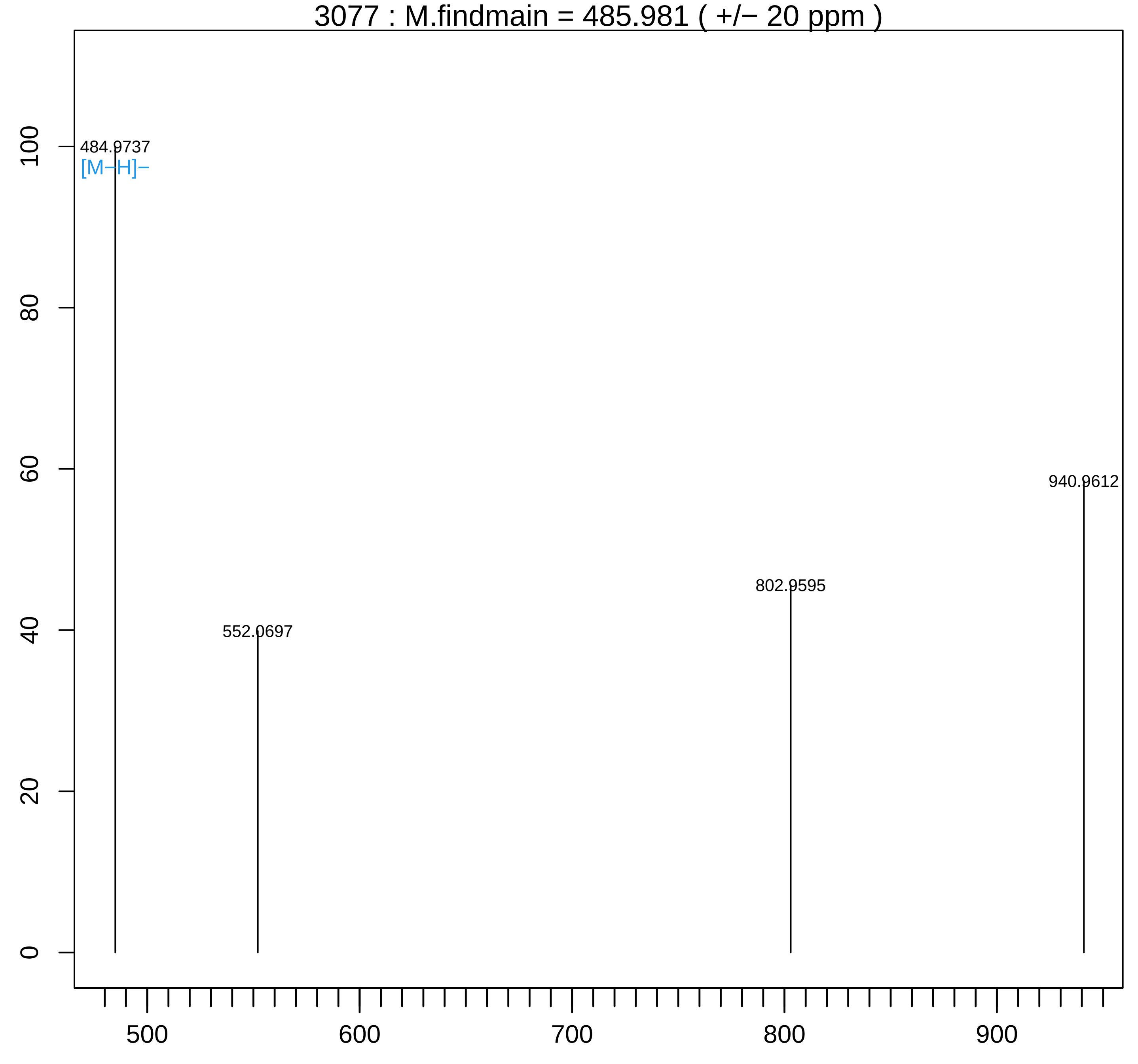
3077 : M.findmain = 485.981 (+/− 20 ppm)
484.9737
[M−H]−
552.0697
802.9595
940.9612
0
20
40
60
80
100
500
600
700
800
900

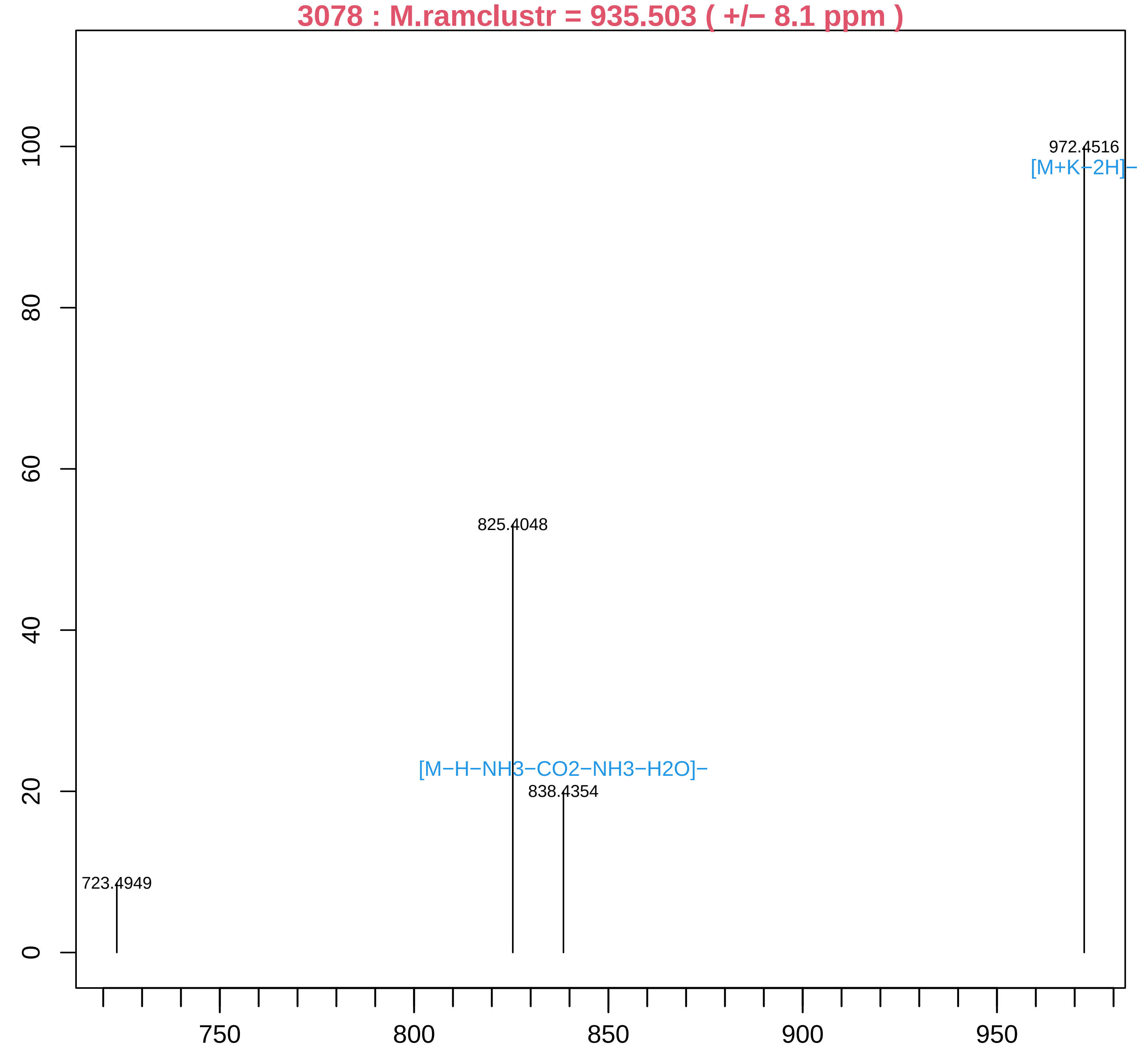
3078 : M.ramclustr = 935.503 (+/− 8.1 ppm)
972.4516
[M+K−2H]−
825.4048
[M−H−NH3−CO2−NH3−H2O]−
838.4354
723.4949
0
20
40
60
80
100
750
800
850
900
950

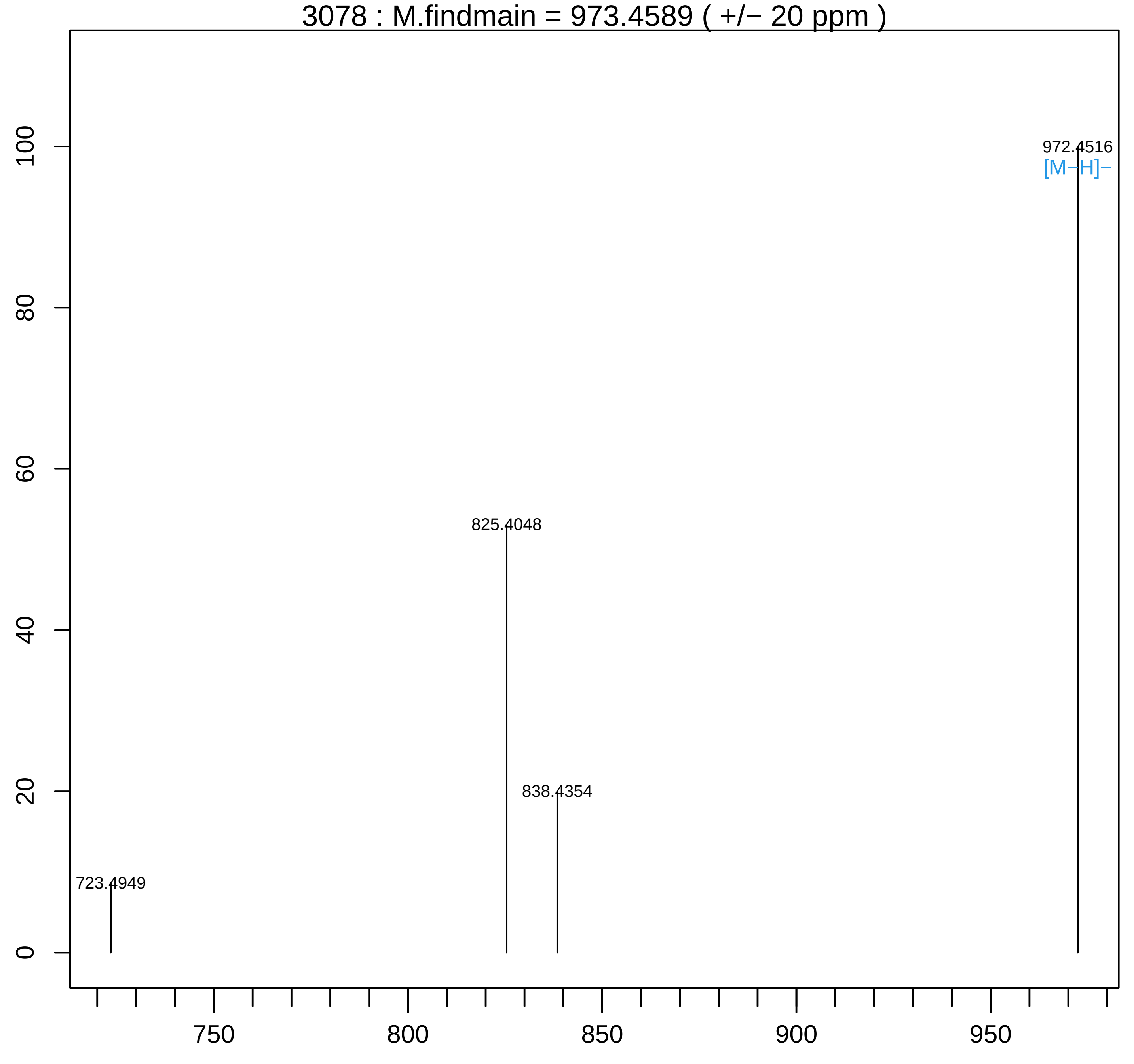
3078 : M.findmain = 973.4589 (+/− 20 ppm)
972.4516
[M−H]−
825.4048
838.4354
723.4949
0
20
40
60
80
100
750
800
850
900
950

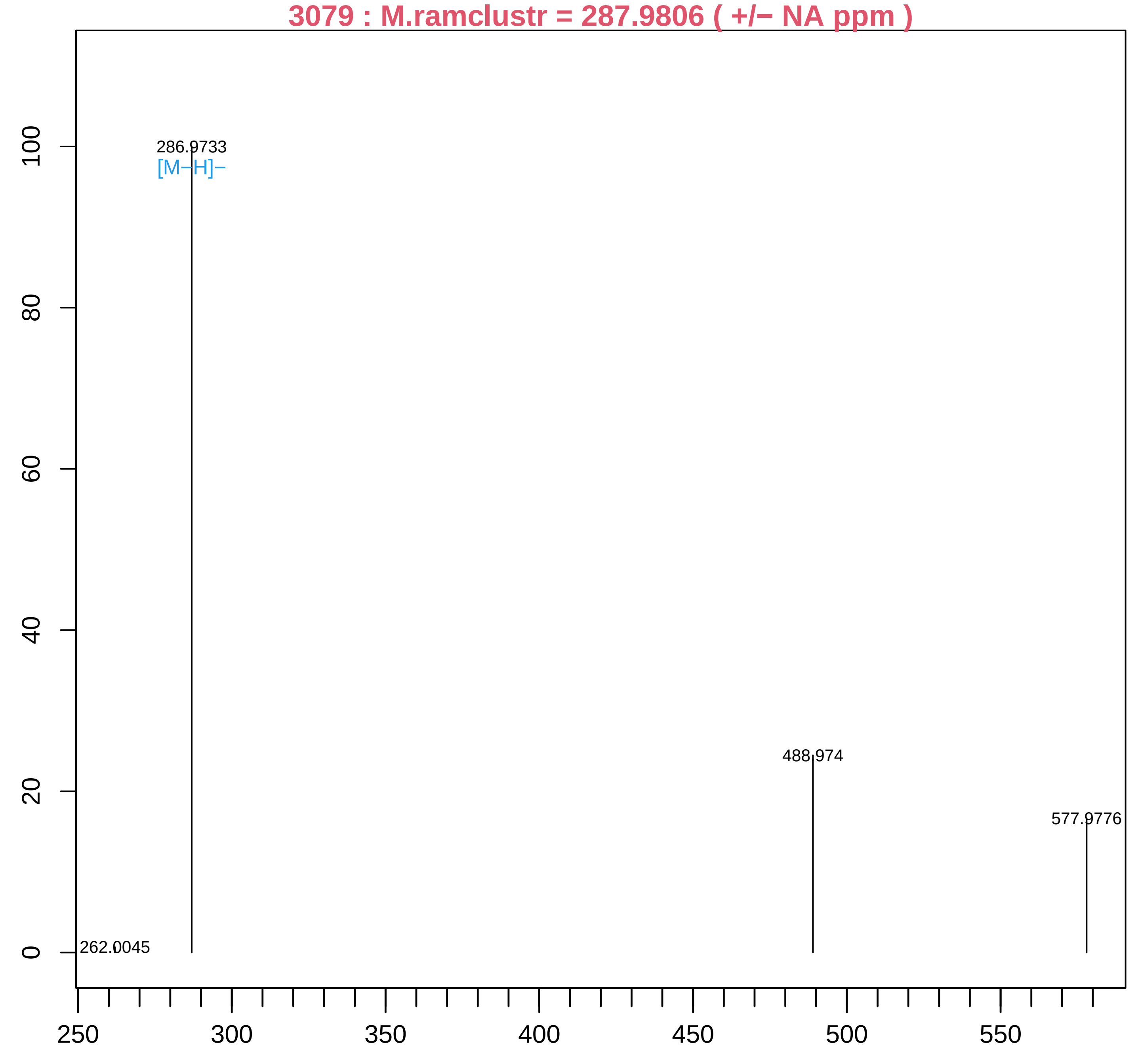
3079 : M.ramclustr = 287.9806 (+/− NA ppm)
286.9733
[M−H]−
488.974
577.9776
262.0045
0
20
40
60
80
100
250
300
350
400
450
500
550

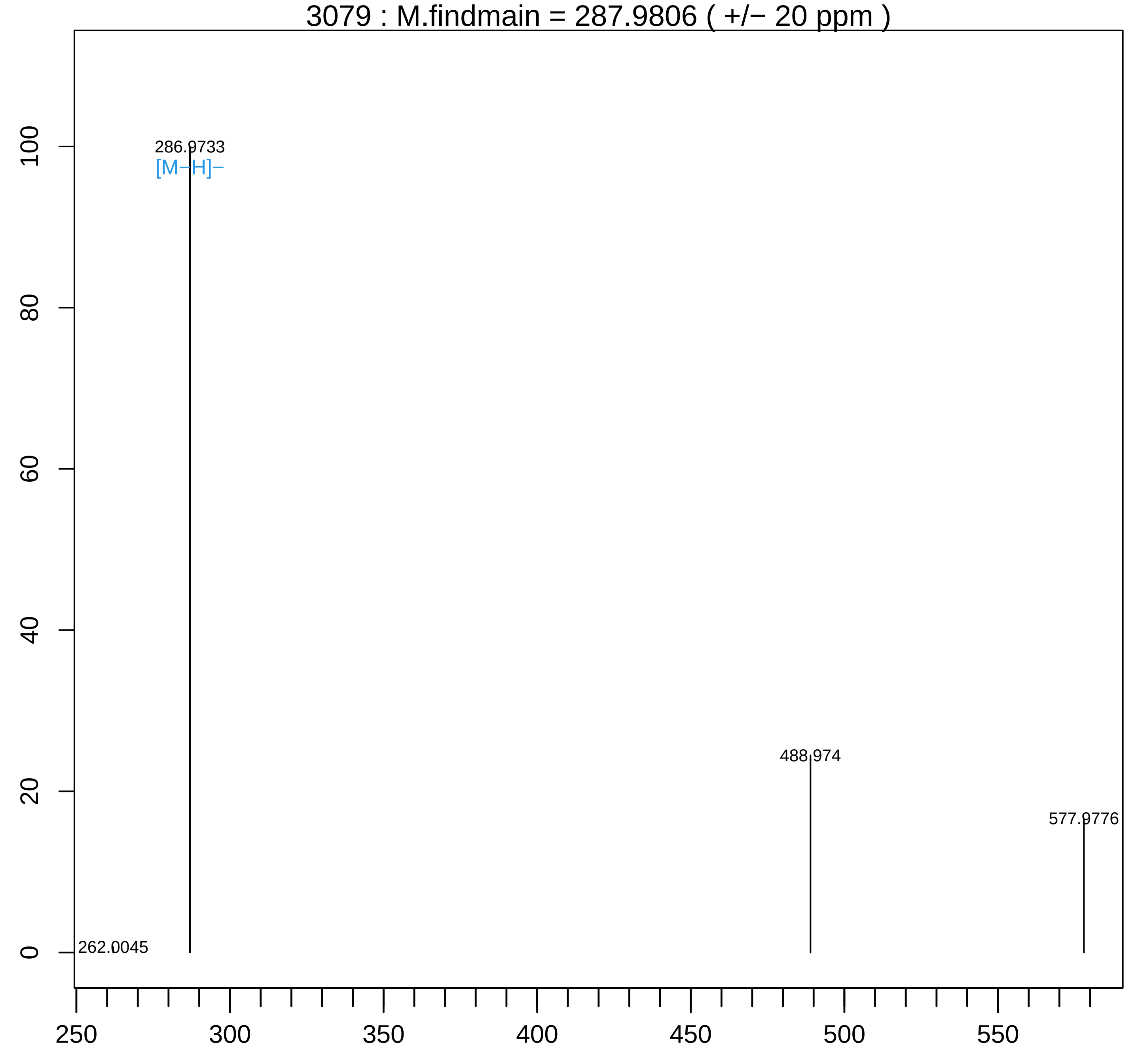
3079 : M.findmain = 287.9806 (+/− 20 ppm)
286.9733
[M−H]−
488.974
577.9776
262.0045
0
20
40
60
80
100
250
300
350
400
450
500
550

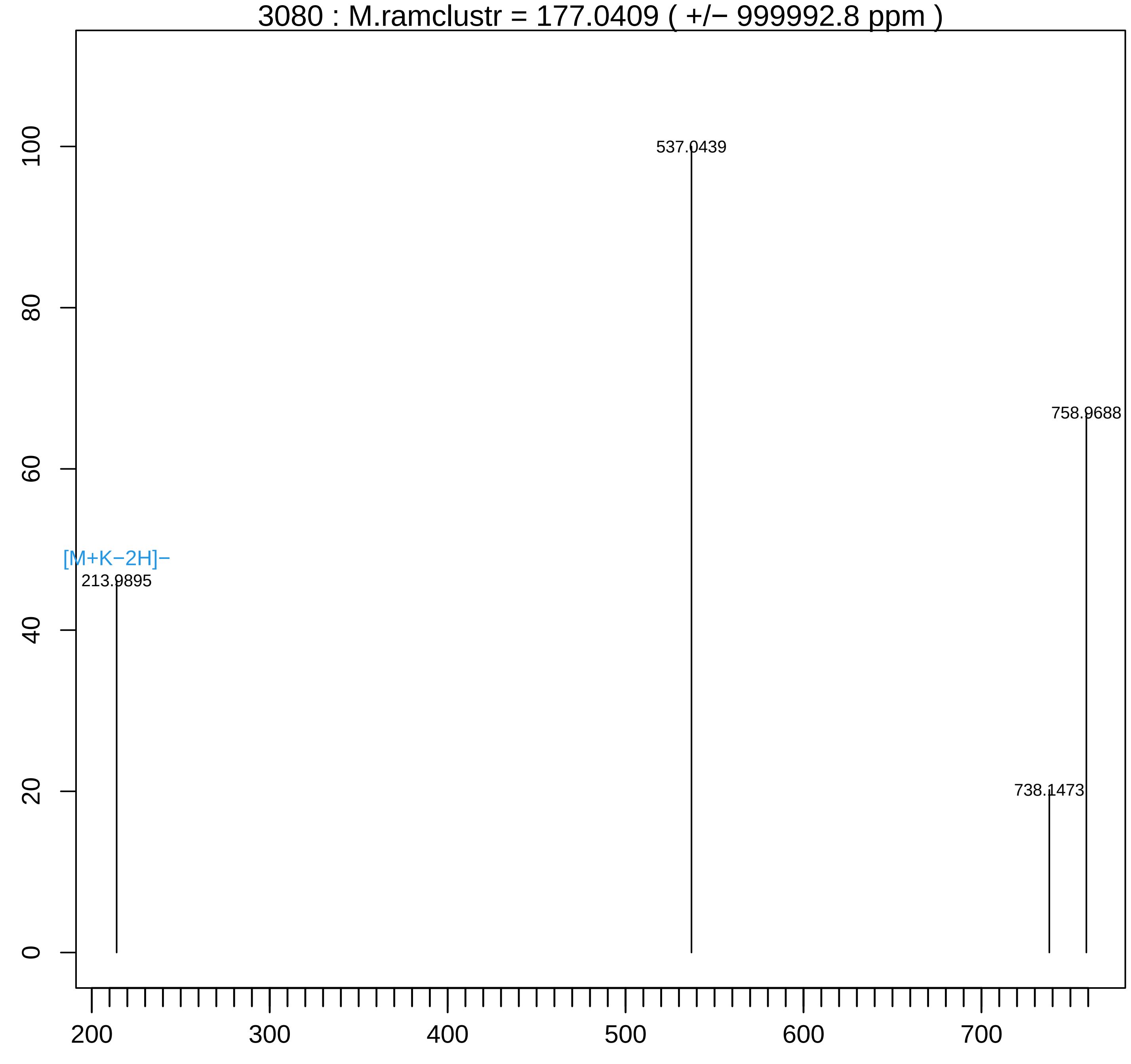

3080 : M.ramclustr = 177.0409 (+/− 999992.8 ppm)
537.0439
758.9688
[M+K−2H]−
213.9895
738.1473
0
20
40
60
80
100
200
300
400
500
600
700

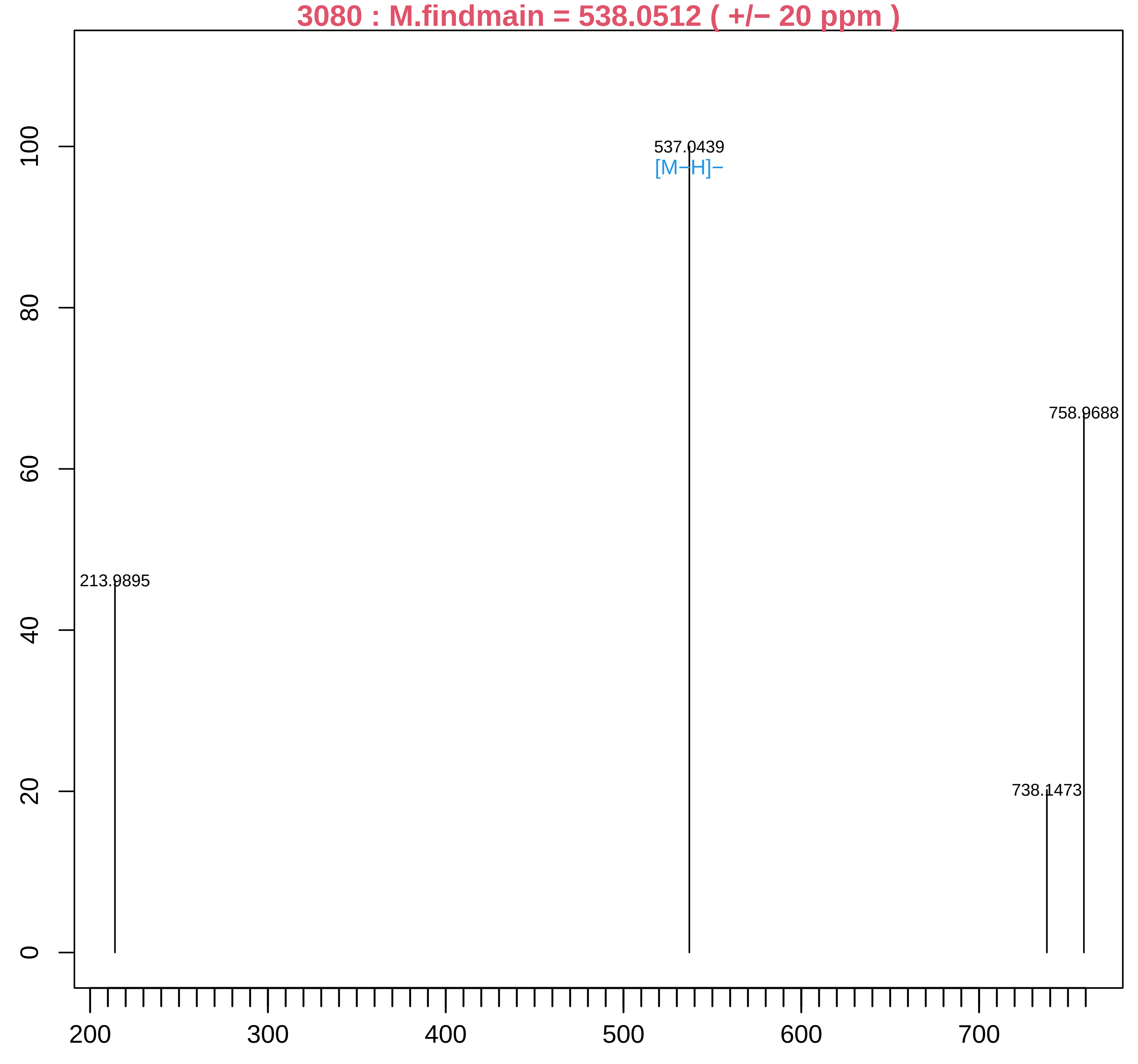

3080 : M.findmain = 538.0512 (+/− 20 ppm)
537.0439
[M−H]−
758.9688
213.9895
738.1473
0
20
40
60
80
100
200
300
400
500
600
700

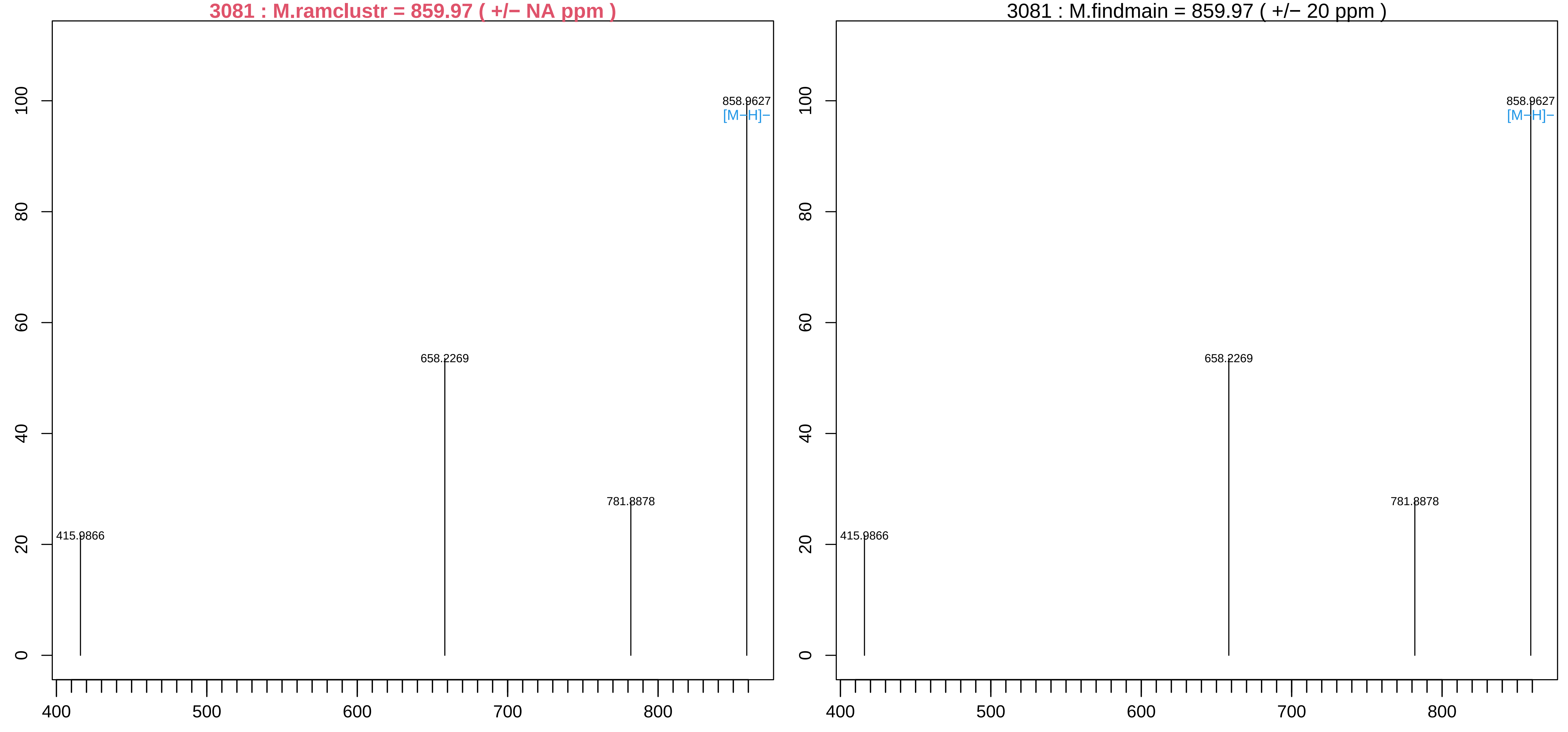
3081 : M.ramclustr = 859.97 (+/− NA ppm)
858.9627
[M−H]−
658.2269
781.8878
415.9866
3081 : M.findmain = 859.97 (+/− 20 ppm)
858.9627
[M−H]−
658.2269
781.8878
415.9866

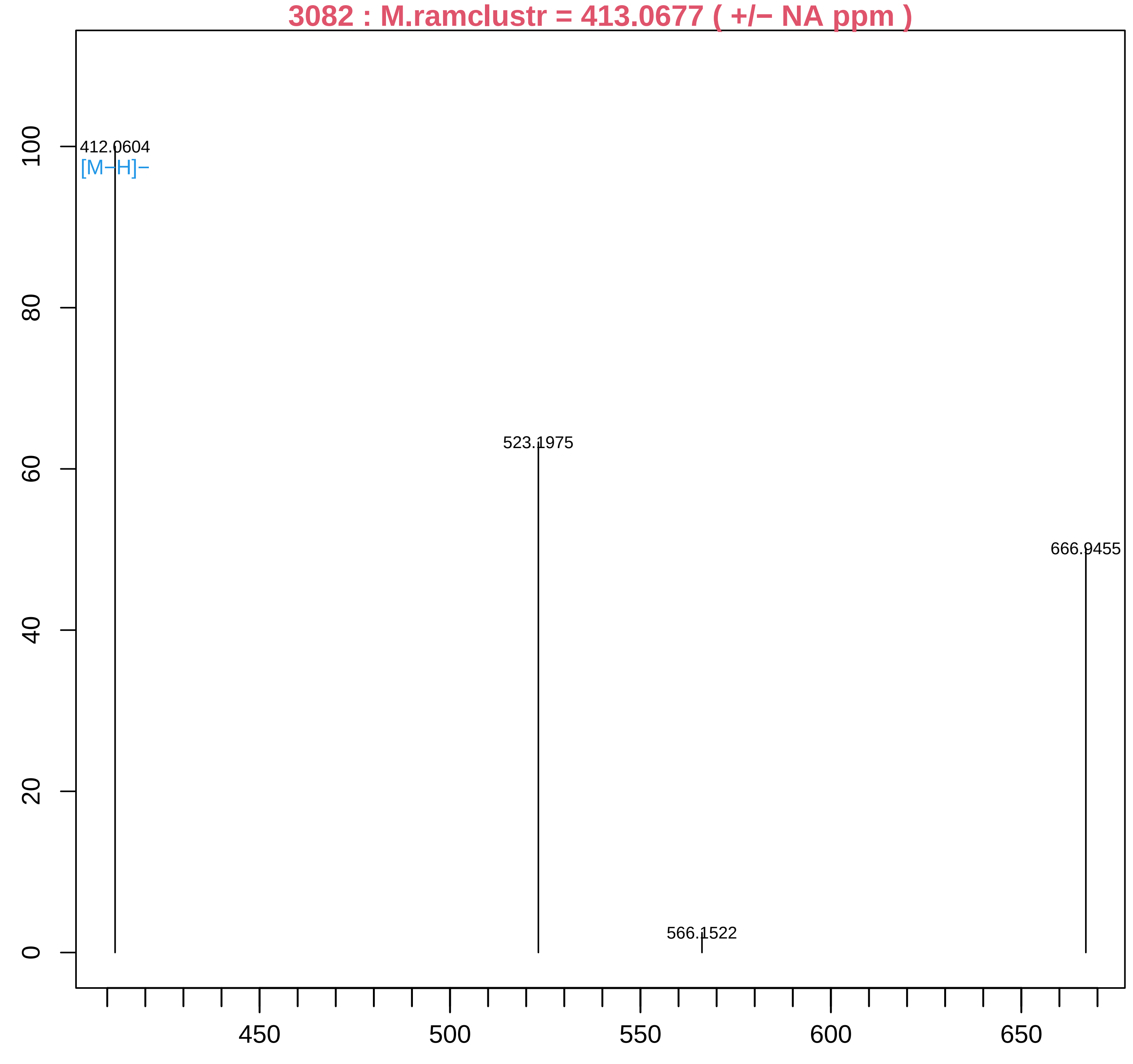
3082 : M.ramclustr = 413.0677 (+/− NA ppm)
412.0604
[M−H]−
523.1975
566.1522
666.9455
0
20
40
60
80
100
450
500
550
600
650

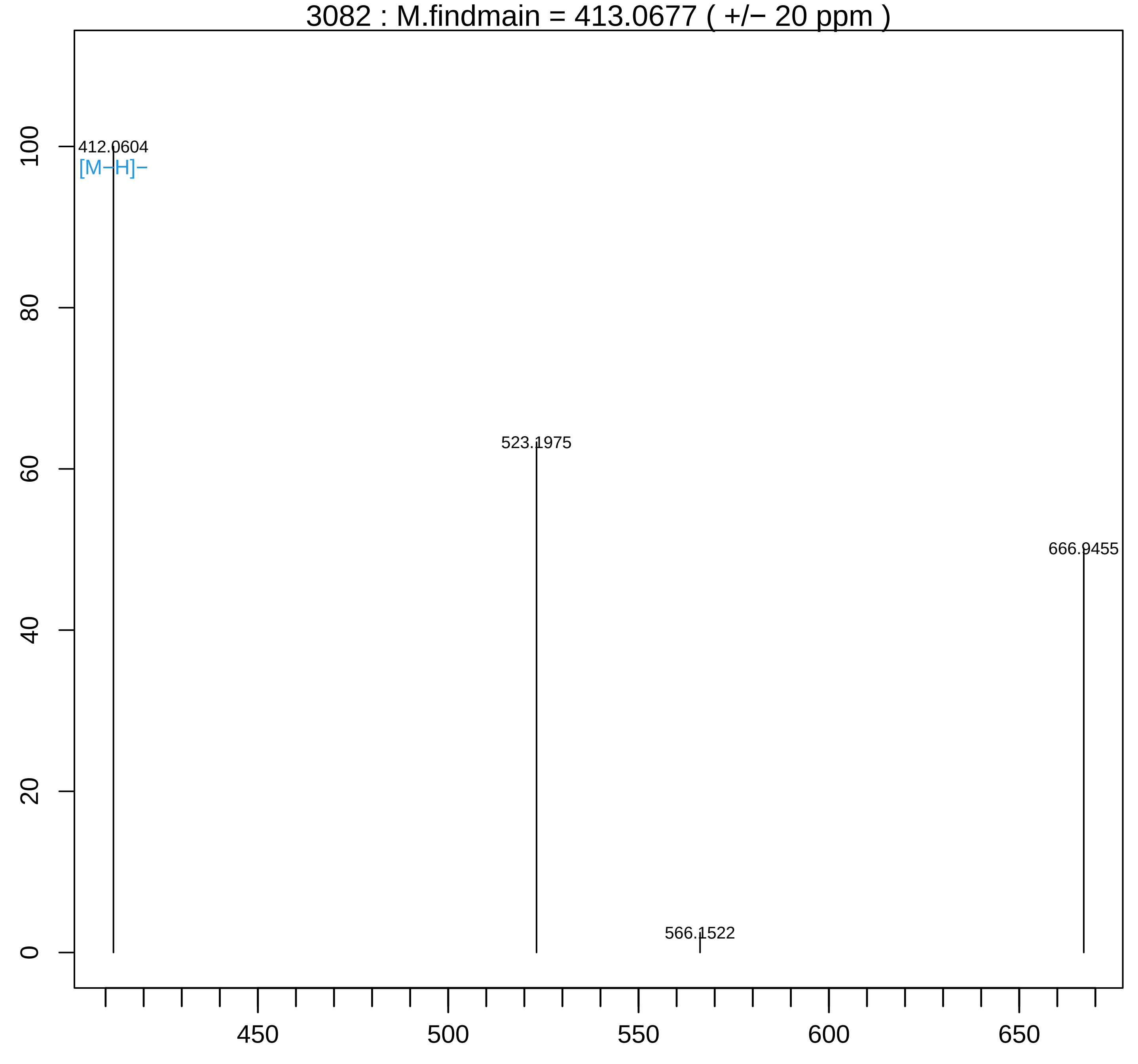
3082 : M.findmain = 413.0677 (+/− 20 ppm)
412.0604
[M−H]−
523.1975
566.1522
666.9455
0
20
40
60
80
100
450
500
550
600
650

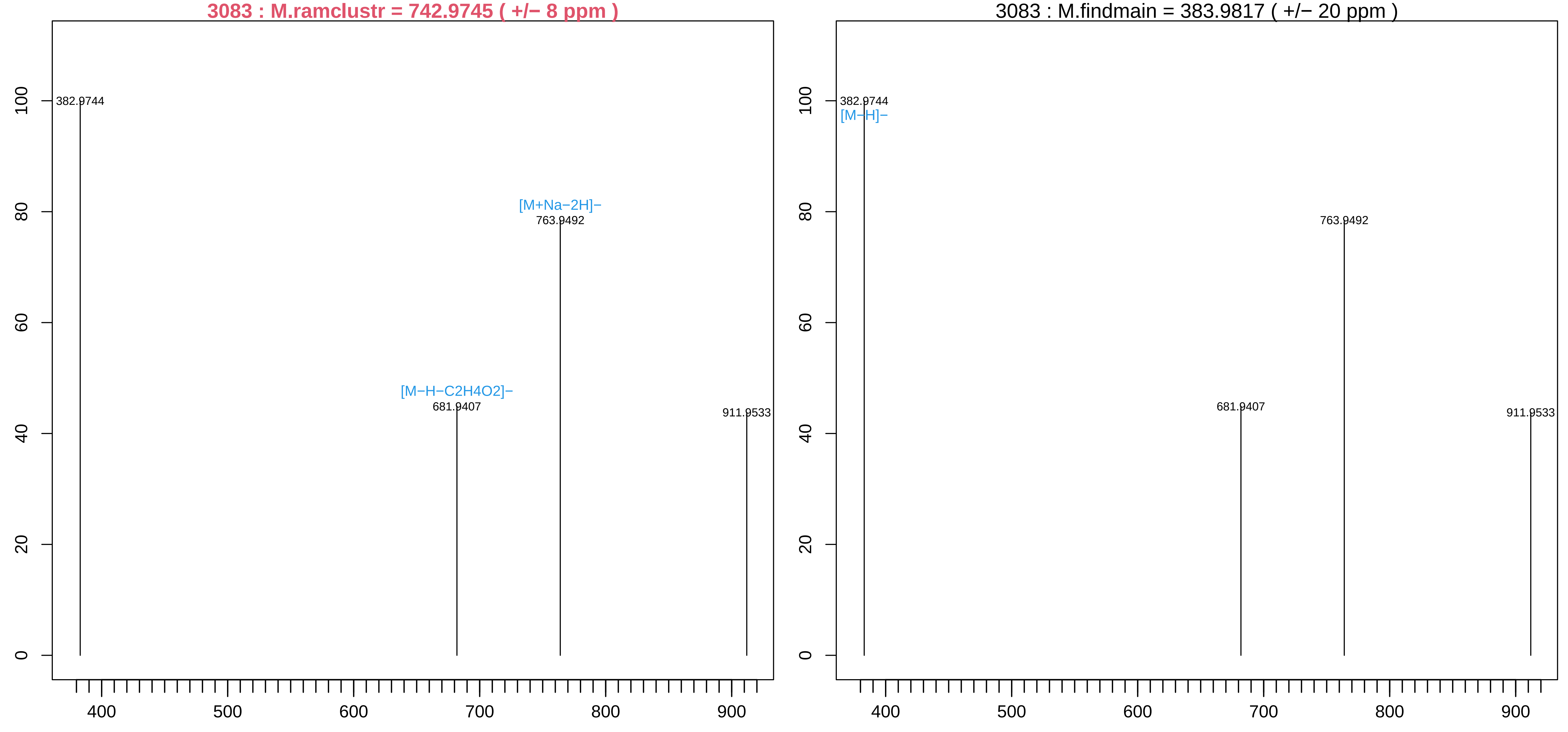
3083 : M.ramclustr = 742.9745 (+/− 8 ppm)
382.9744
[M+Na−2H]−
763.9492
[M−H−C2H4O2]−
681.9407
911.9533
3083 : M.findmain = 383.9817 (+/− 20 ppm)
382.9744
[M−H]−
763.9492
681.9407
911.9533
0
20
40
60
80
100
400
500
600
700
800
900

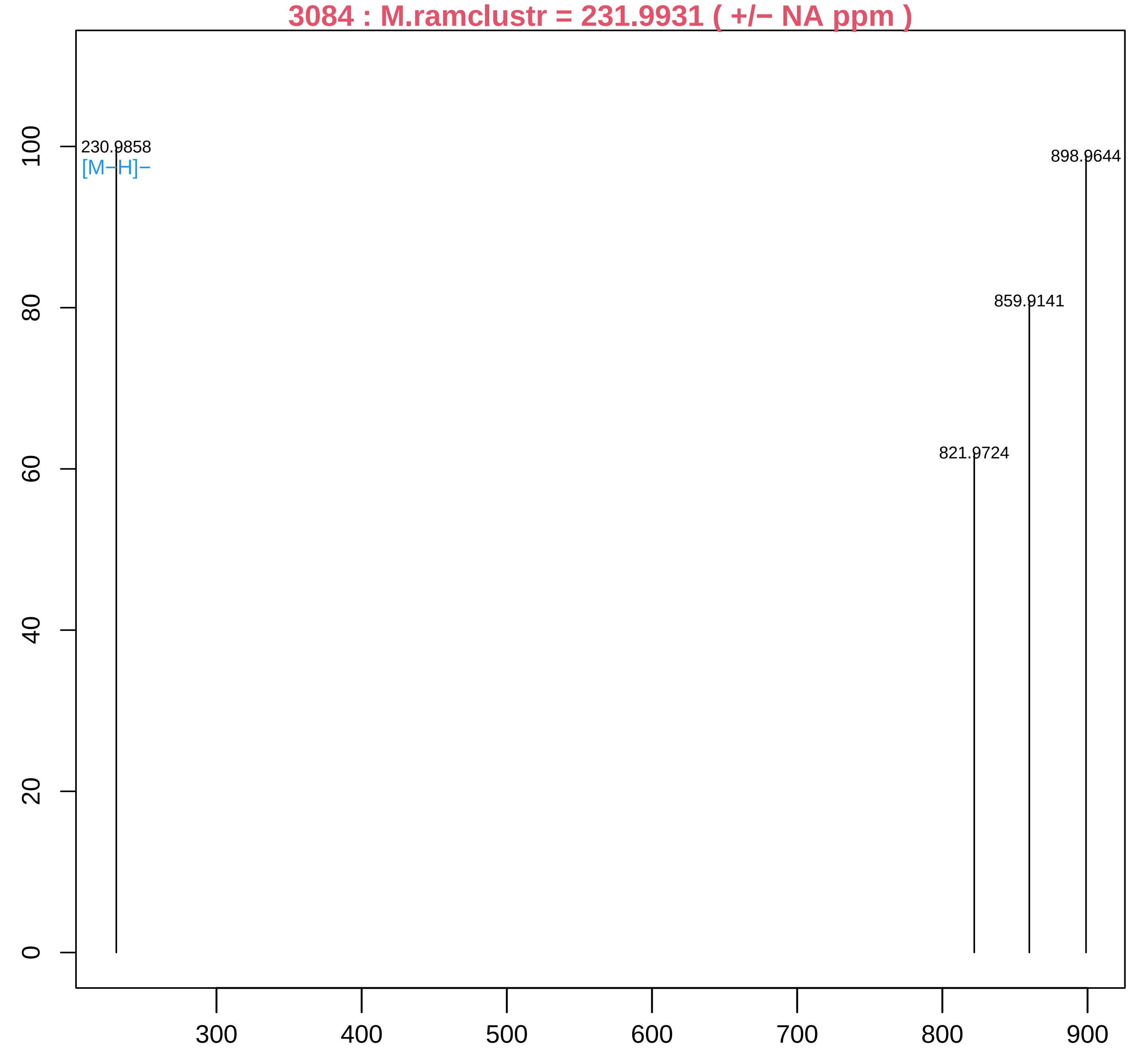
3084 : M.ramclustr = 231.9931 (+/− NA ppm)
230.9858
[M−H]−
898.9644
859.9141
821.9724
0
20
40
60
80
100
300
400
500
600
700
800
900

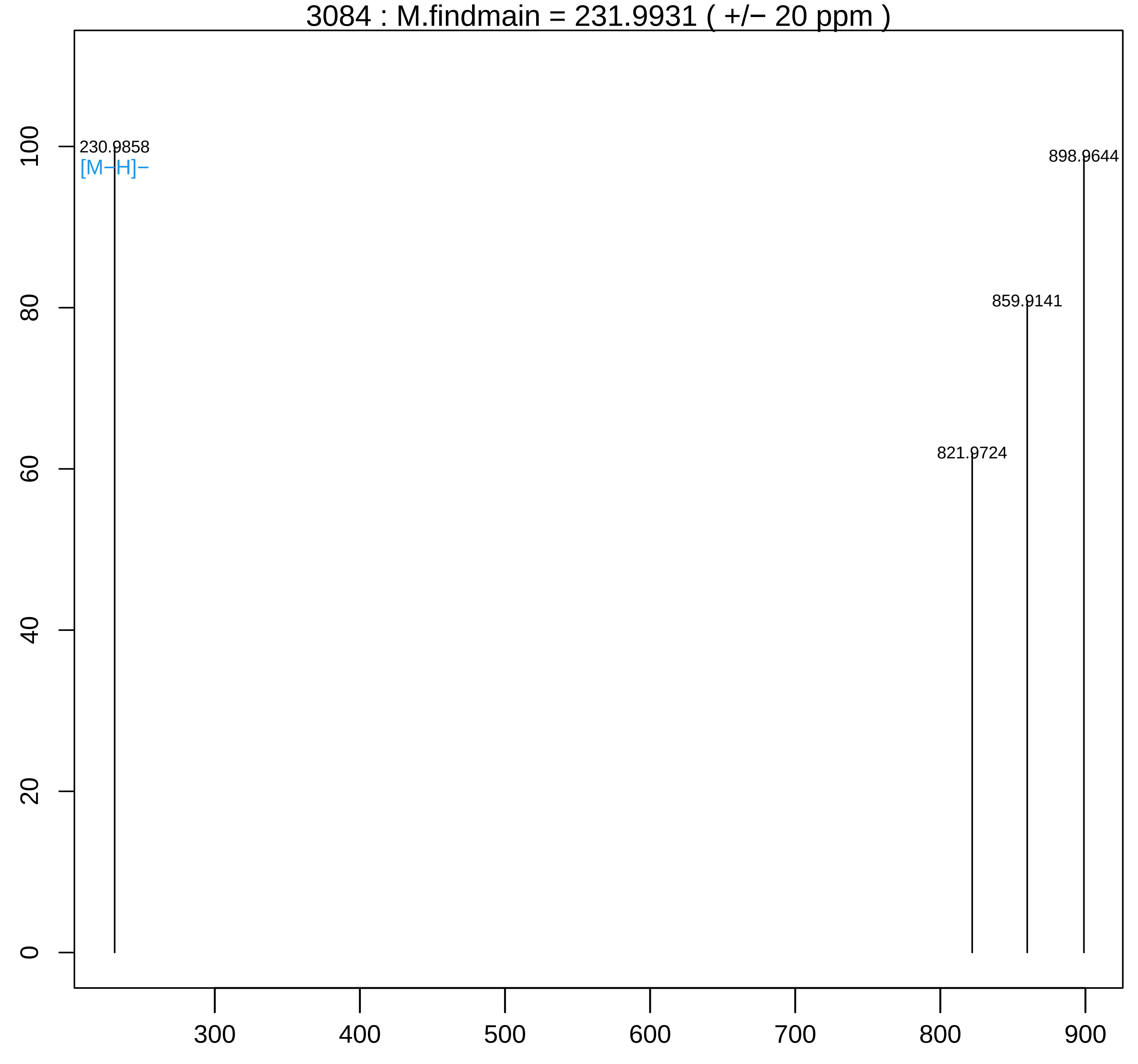
3084 : M.findmain = 231.9931 (+/− 20 ppm)
230.9858
[M−H]−
898.9644
859.9141
821.9724
0
20
40
60
80
100
300
400
500
600
700
800
900

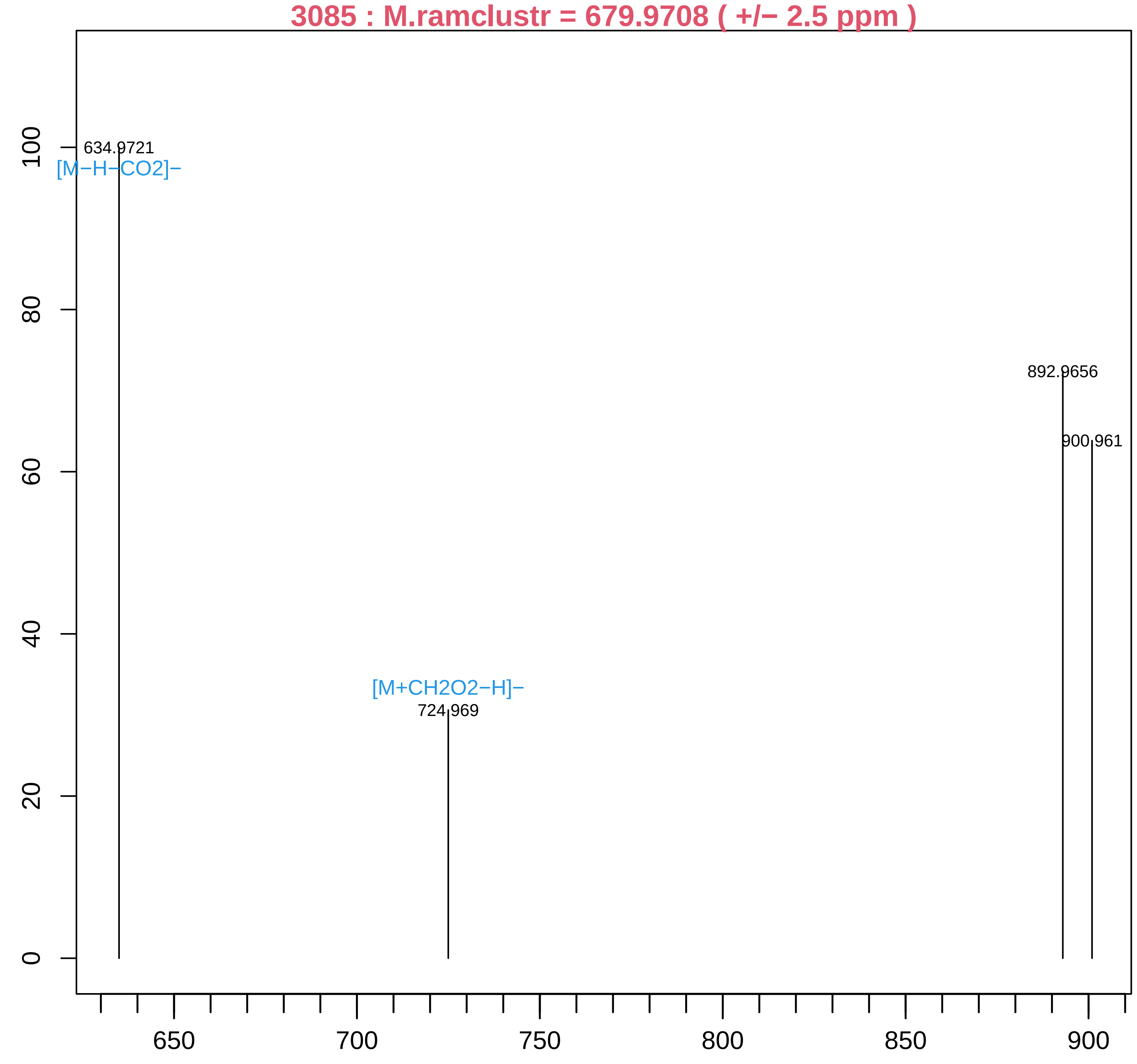

3085 : M.ramclustr = 679.9708 (+/− 2.5 ppm)
634.9721
[M−H−CO2]−
[M+CH2O2−H]−
724.969
892.9656
900.961
0
20
40
60
80
100
650
700
750
800
850
900

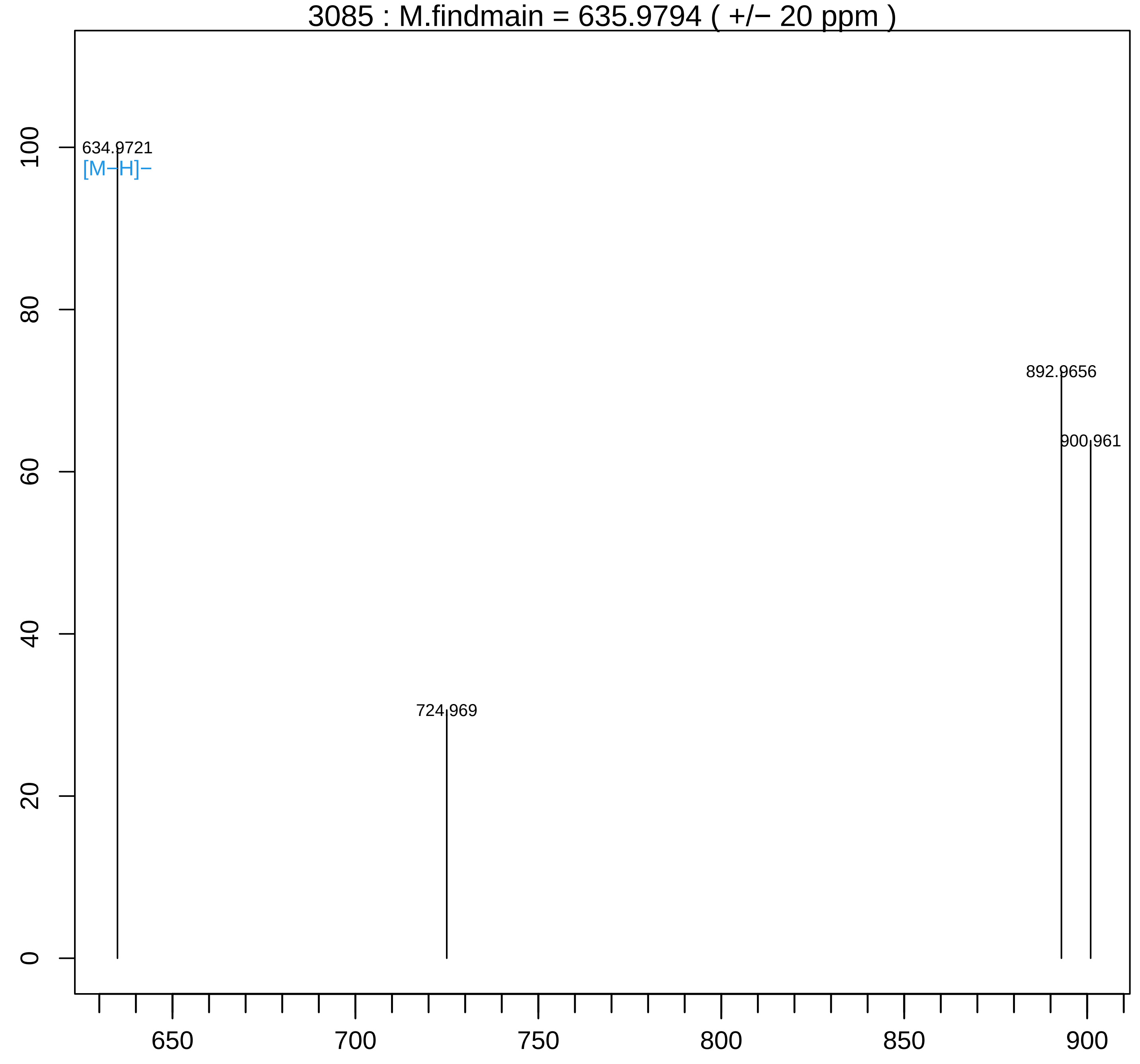

3085 : M.findmain = 635.9794 (+/− 20 ppm)
634.9721
[M−H]−
724.969
892.9656
900.961
0
20
40
60
80
100
650
700
750
800
850
900

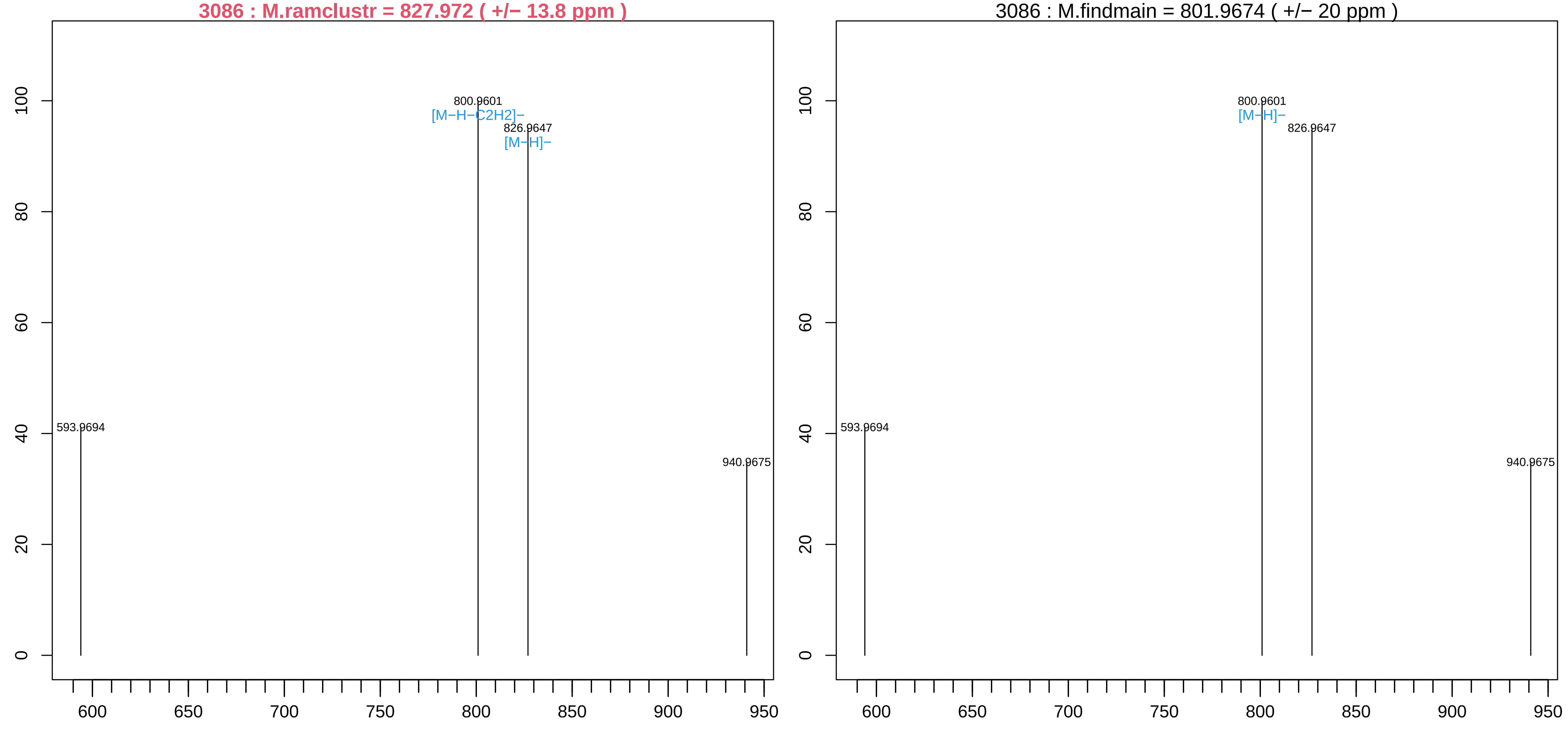

3086 : M.ramclustr = 827.972 (+/− 13.8 ppm)
800.9601
[M−H−C2H2]−
826.9647
[M−H]−
593.9694
940.9675
3086 : M.findmain = 801.9674 (+/− 20 ppm)
800.9601
[M−H]−
826.9647
593.9694
940.9675
0
20
40
60
80
100
600
650
700
750
800
850
900
950

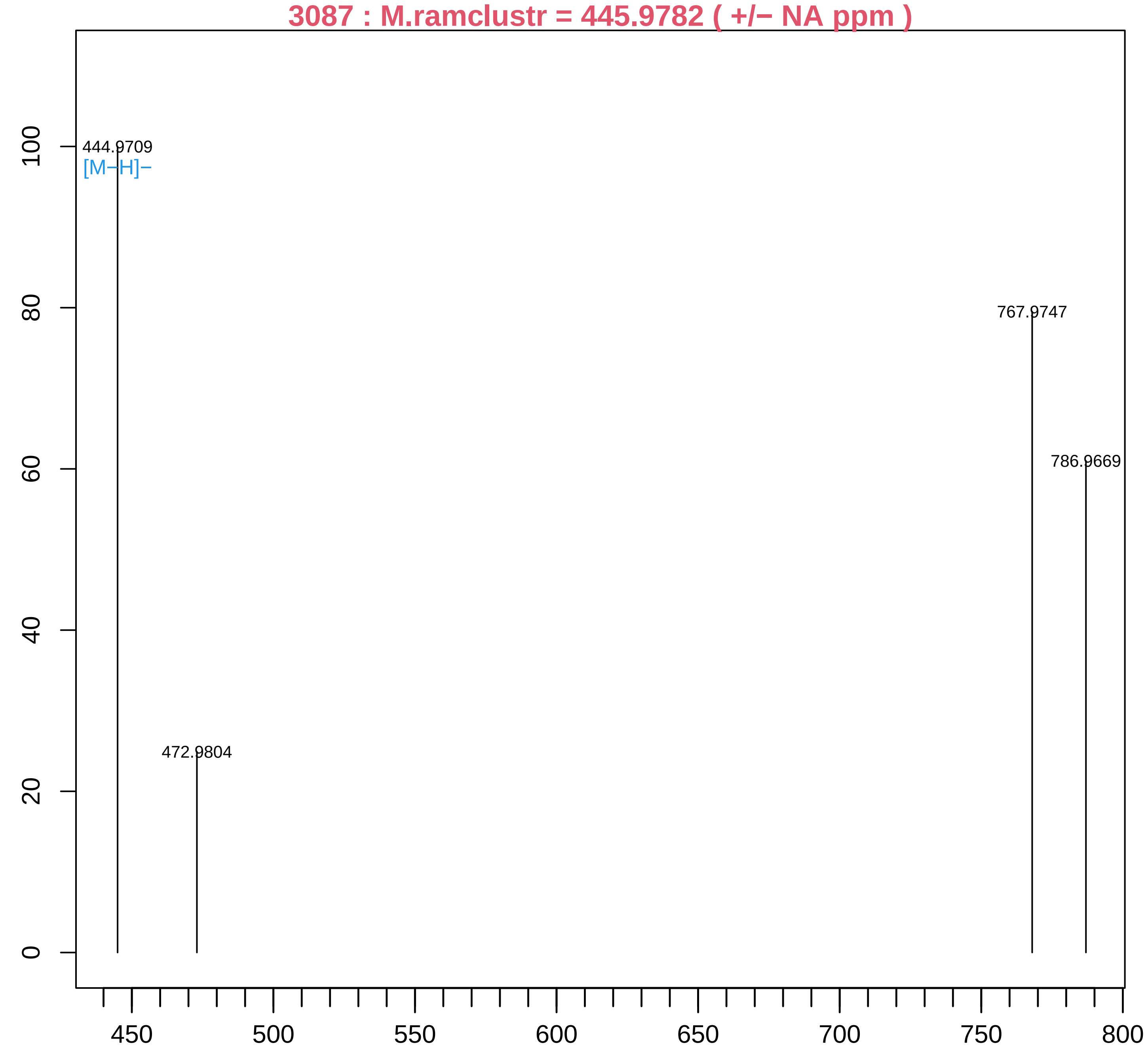
3087 : M.ramclustr = 445.9782 (+/− NA ppm)
444.9709
[M−H]−
472.9804
767.9747
786.9669
0
20
40
60
80
100
450
500
550
600
650
700
750
800

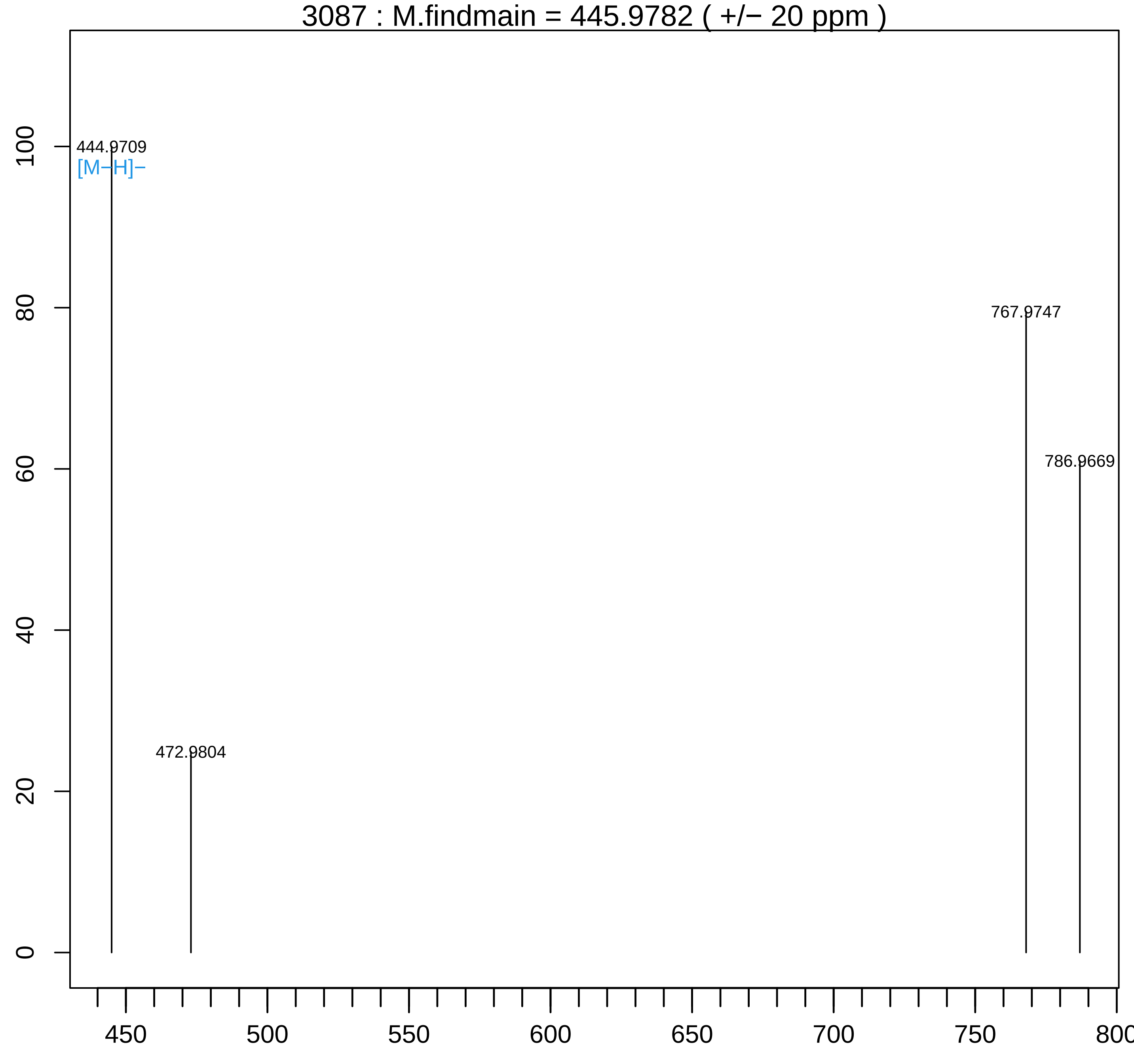
3087 : M.findmain = 445.9782 (+/− 20 ppm)
444.9709
[M−H]−
472.9804
767.9747
786.9669
0
20
40
60
80
100
450
500
550
600
650
700
750
800

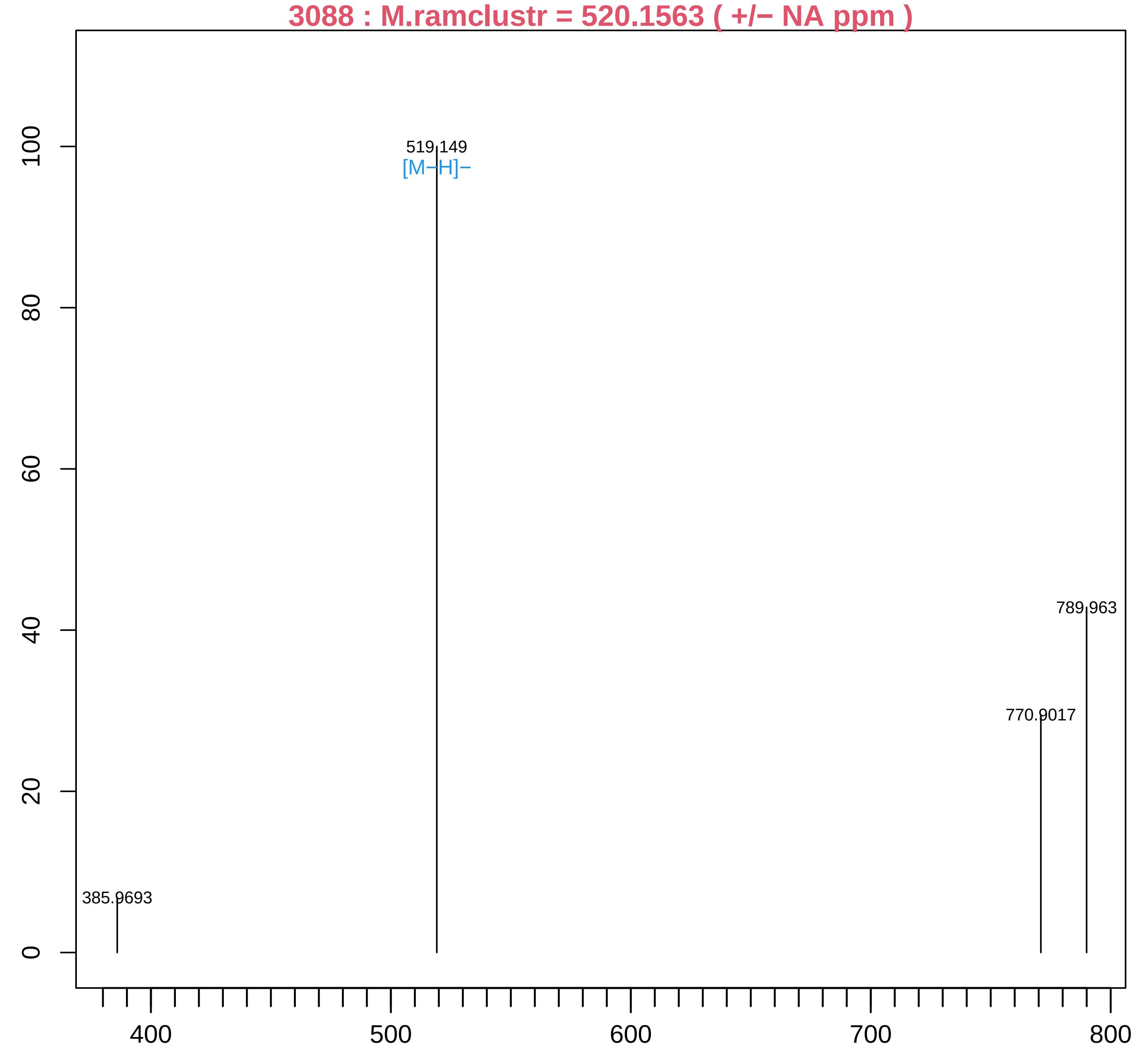
3088 : M.ramclustr = 520.1563 (+/− NA ppm)
519.149
[M−H]−
789.963
770.9017
385.9693
0
20
40
60
80
100
400
500
600
700
800

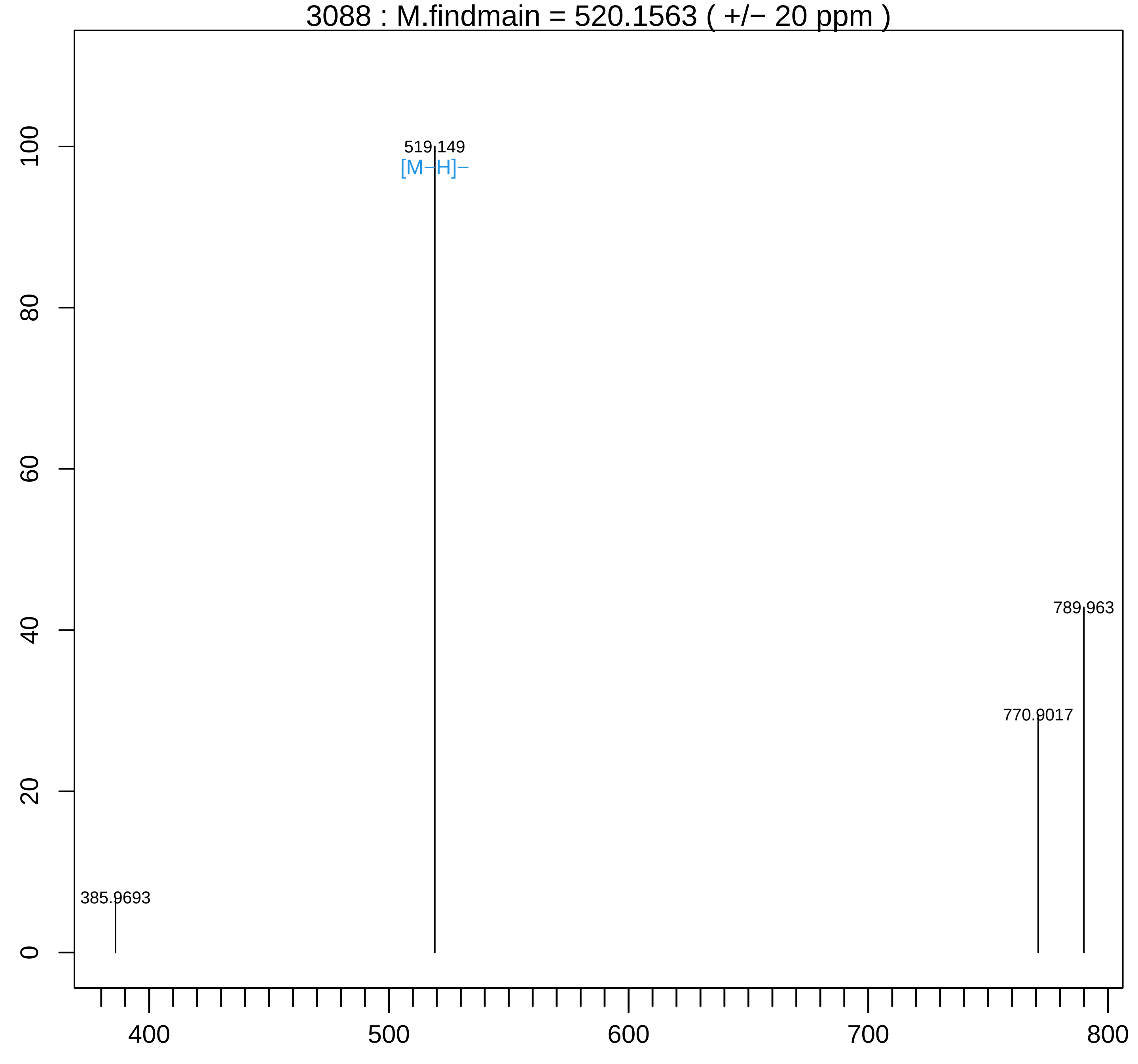
3088 : M.findmain = 520.1563 (+/− 20 ppm)
519.149
[M−H]−
789.963
770.9017
385.9693
0
20
40
60
80
100
400
500
600
700
800

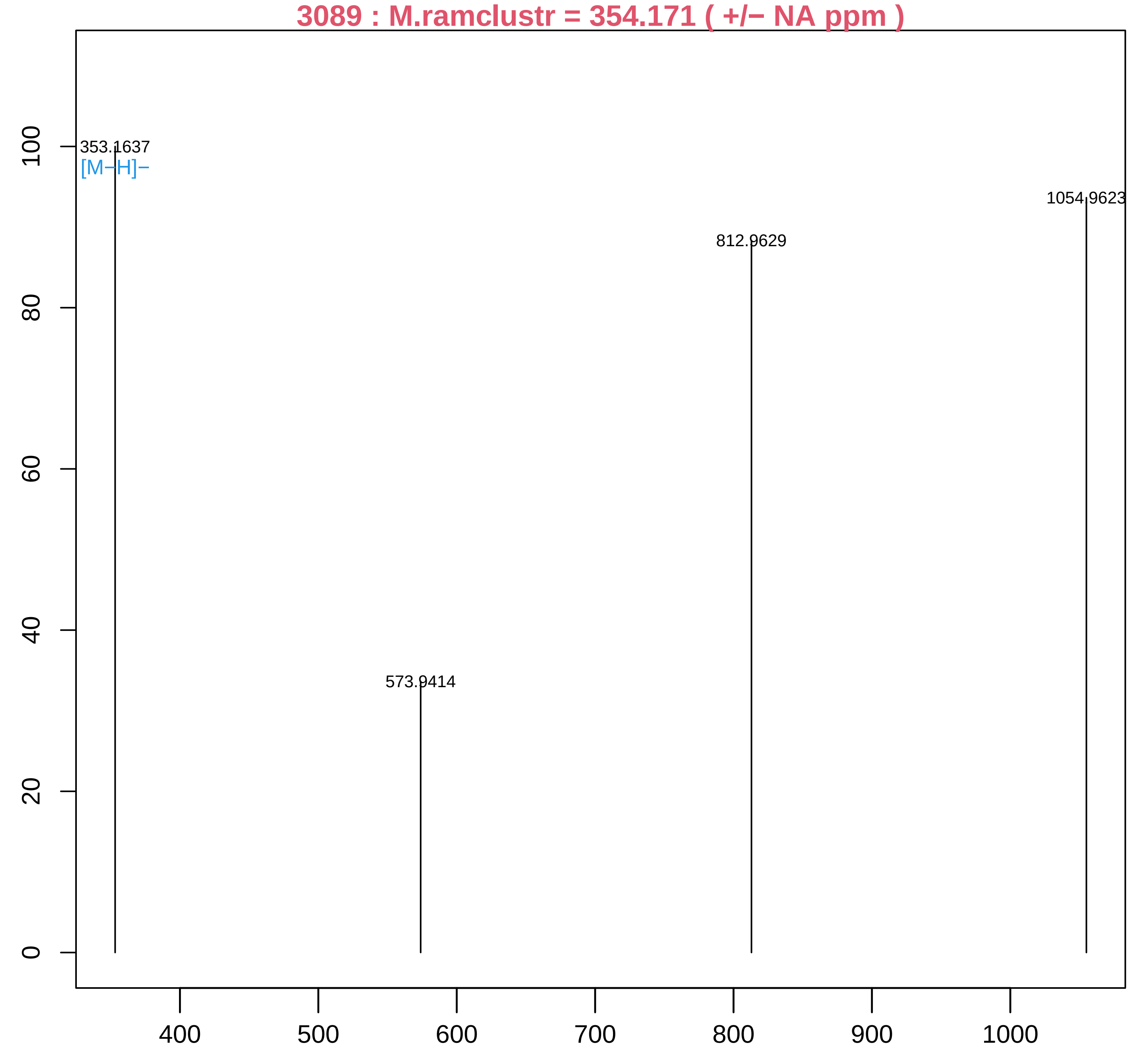
3089 : M.ramclustr = 354.171 (+/− NA ppm)
353.1637
[M−H]−
573.9414
812.9629
1054.9623
0
20
40
60
80
100
400
500
600
700
800
900
1000

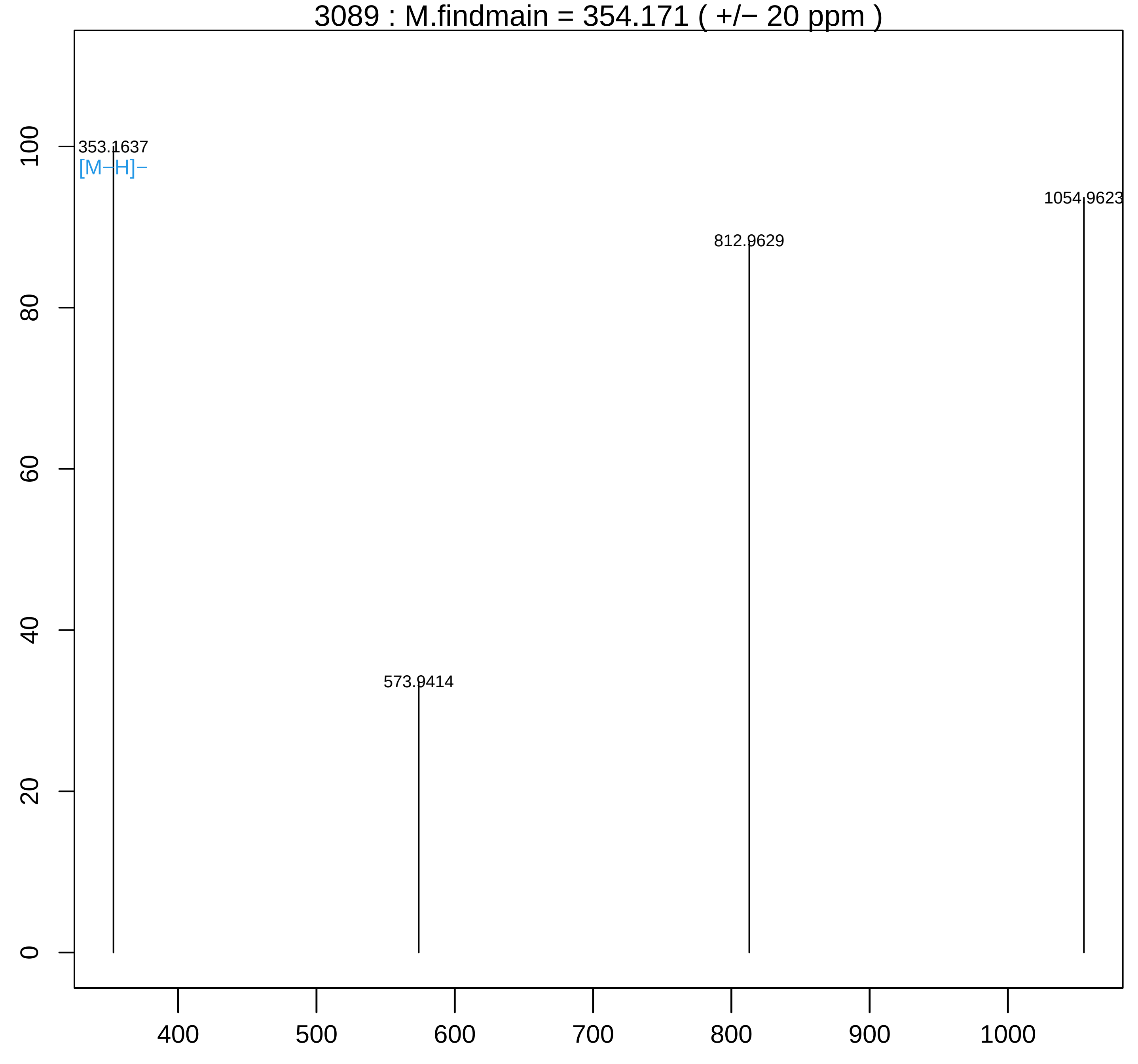
3089 : M.findmain = 354.171 (+/− 20 ppm)
353.1637
[M−H]−
573.9414
812.9629
1054.9623
0
20
40
60
80
100
400
500
600
700
800
900
1000

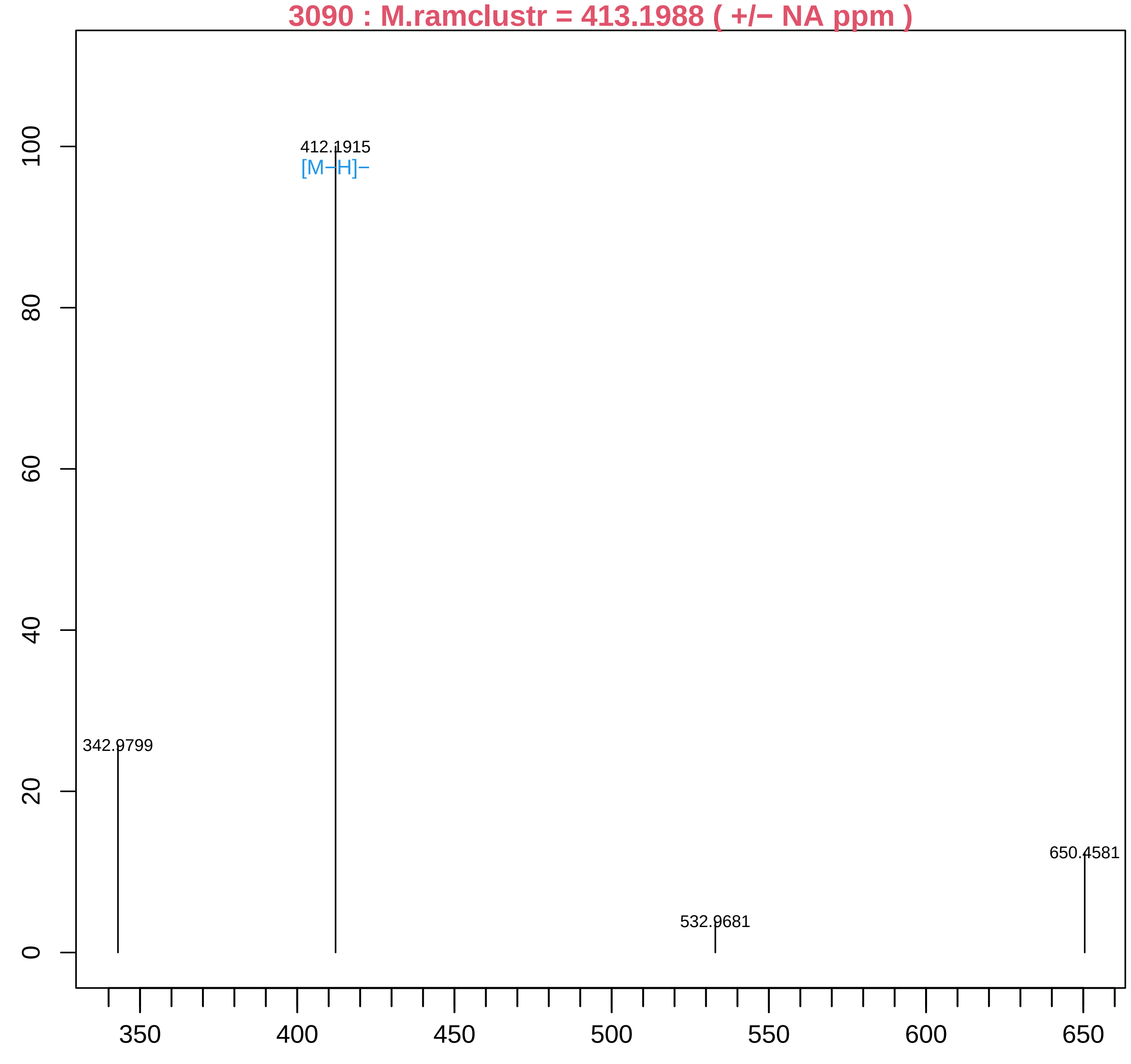

3090 : M.ramclustr = 413.1988 (+/− NA ppm)
412.1915
[M−H]−
342.9799
532.9681
650.4581
0
20
40
60
80
100
350
400
450
500
550
600
650

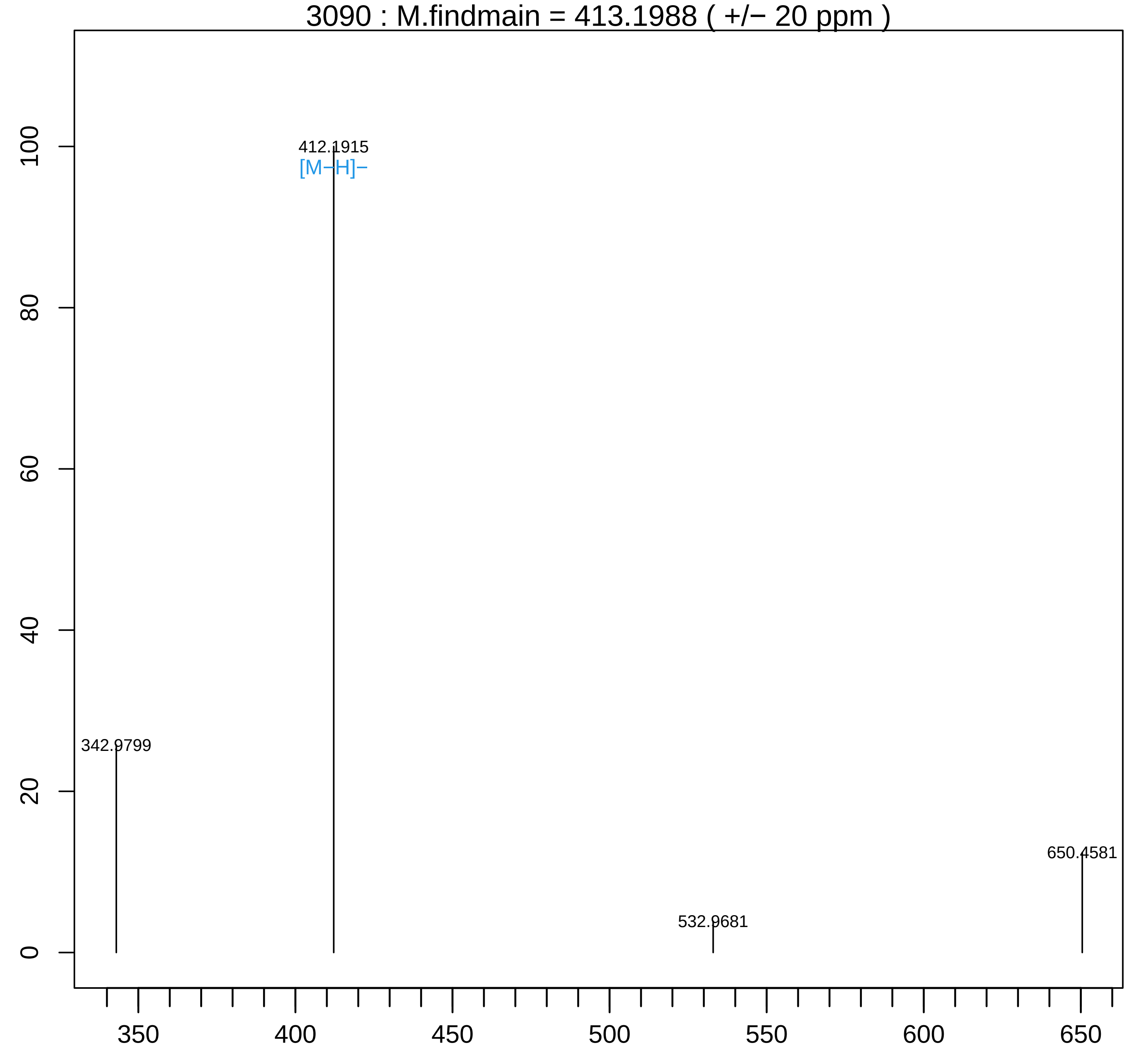

3090 : M.findmain = 413.1988 (+/− 20 ppm)
412.1915
[M−H]−
342.9799
532.9681
650.4581
0
20
40
60
80
100
350
400
450
500
550
600
650

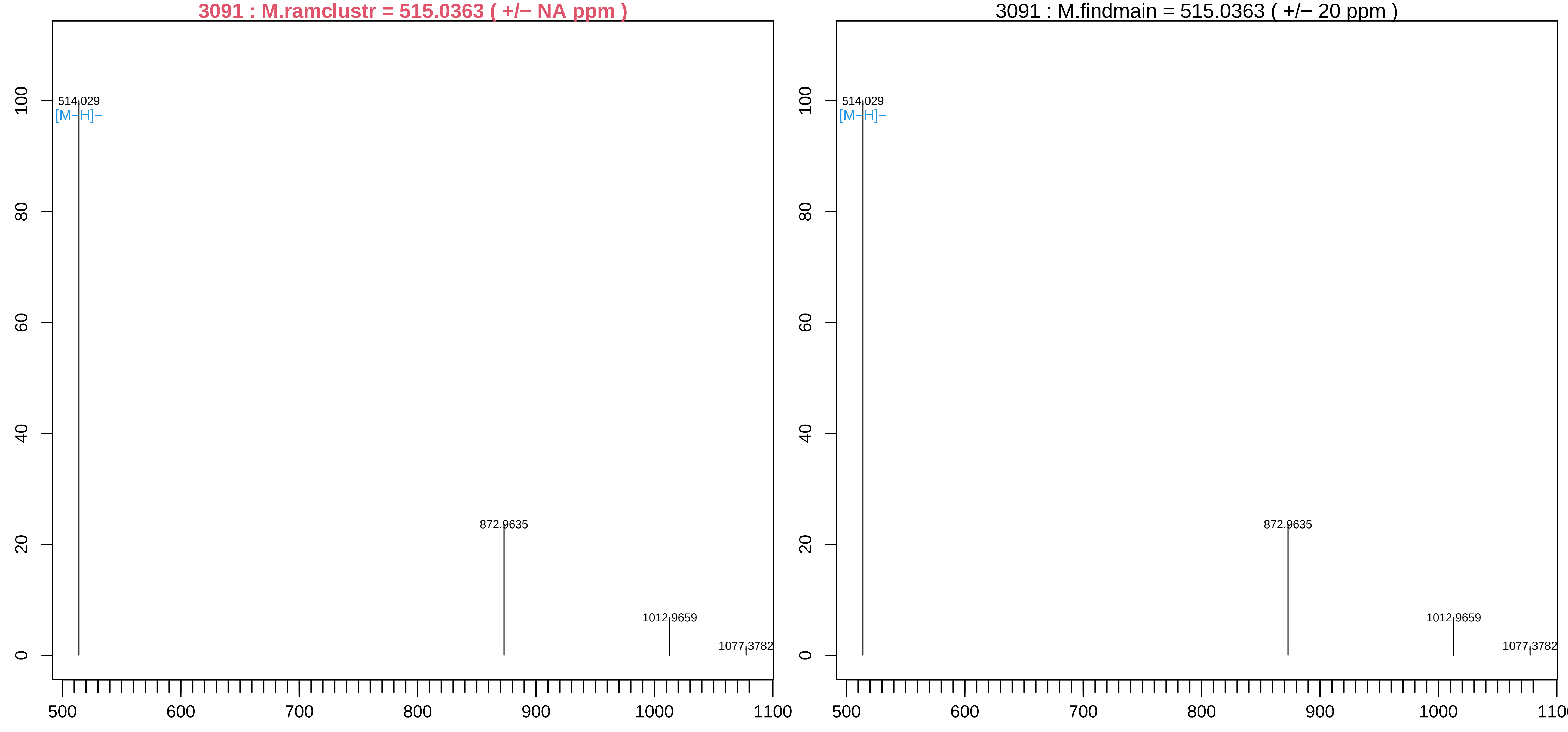

3091 : M.ramclustr = 515.0363 (+/− NA ppm)
3091 : M.findmain = 515.0363 (+/− 20 ppm)
514.029
[M−H]−
872.9635
1012.9659
1077.3782
0
20
40
60
80
100
500
600
700
800
900
1000
1100

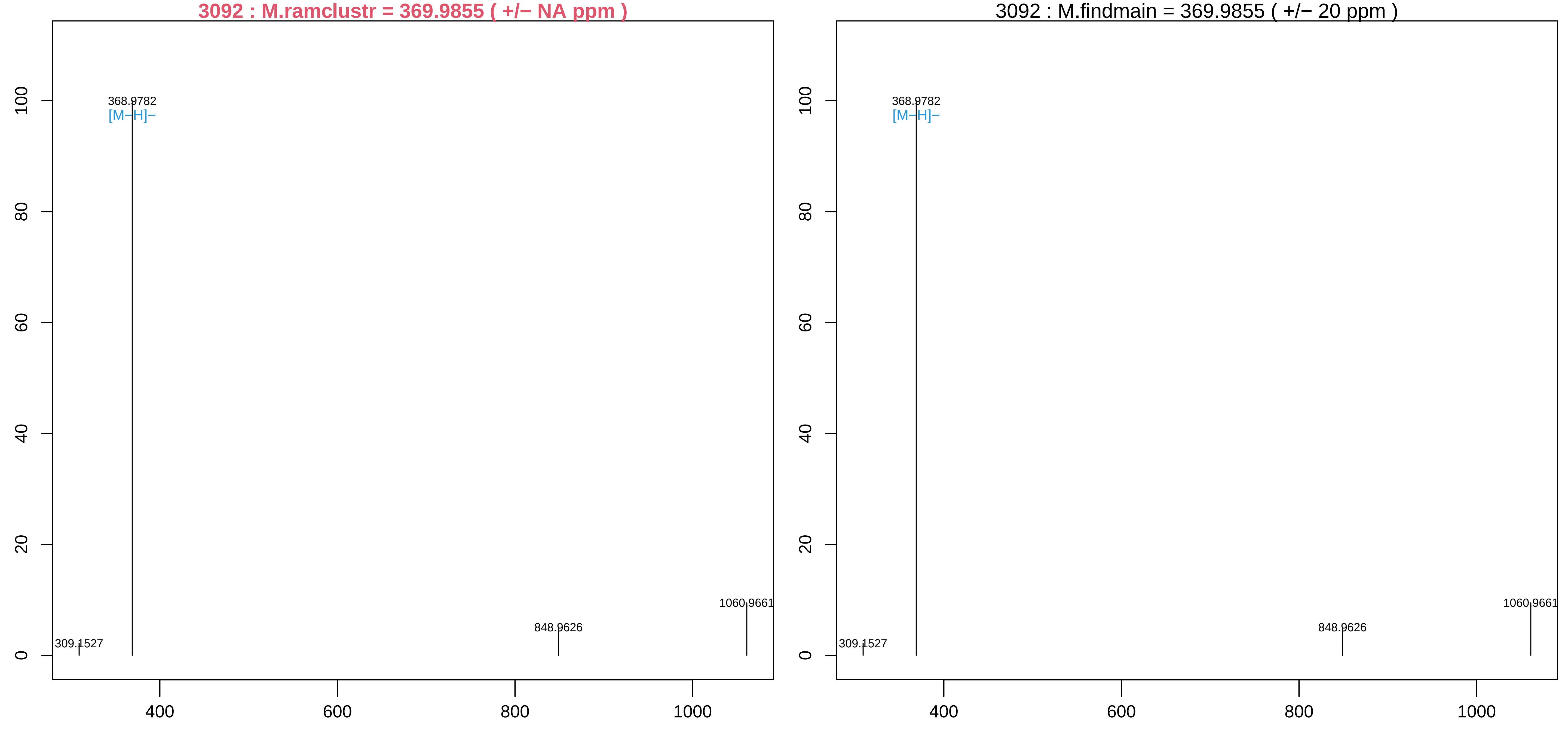
3092 : M.ramclustr = 369.9855 (+/− NA ppm)
368.9782
[M−H]−
309.1527
848.9626
1060.9661
3092 : M.findmain = 369.9855 (+/− 20 ppm)
368.9782
[M−H]−
309.1527
848.9626
1060.9661
0
20
40
60
80
100
400
600
800
1000

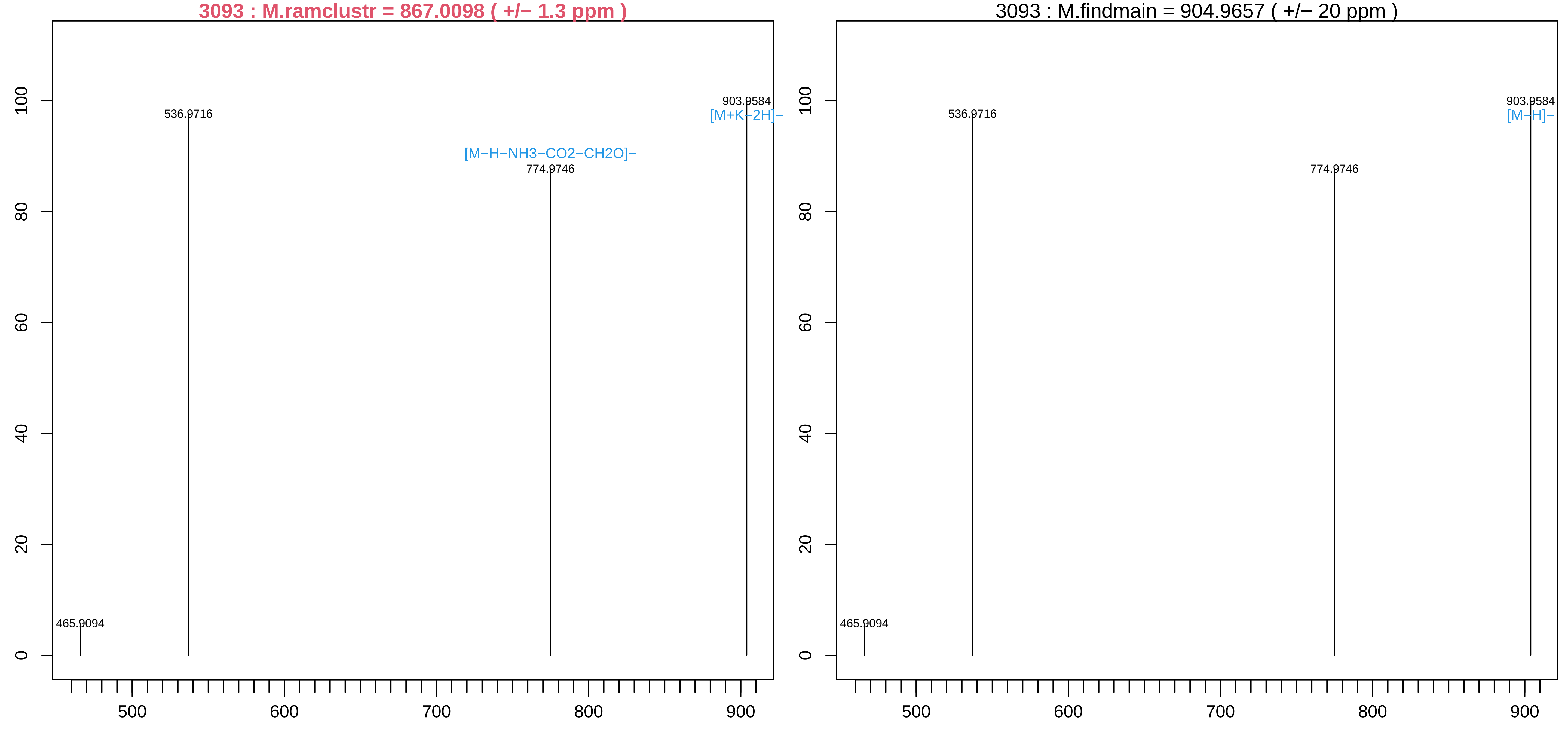

3093 : M.ramclustr = 867.0098 (+/− 1.3 ppm)
903.9584
[M+K−2H]−
536.9716
[M−H−NH3−CO2−CH2O]−
774.9746
465.9094
3093 : M.findmain = 904.9657 (+/− 20 ppm)
903.9584
[M−H]−
536.9716
774.9746
465.9094
0
20
40
60
80
100
500
600
700
800
900

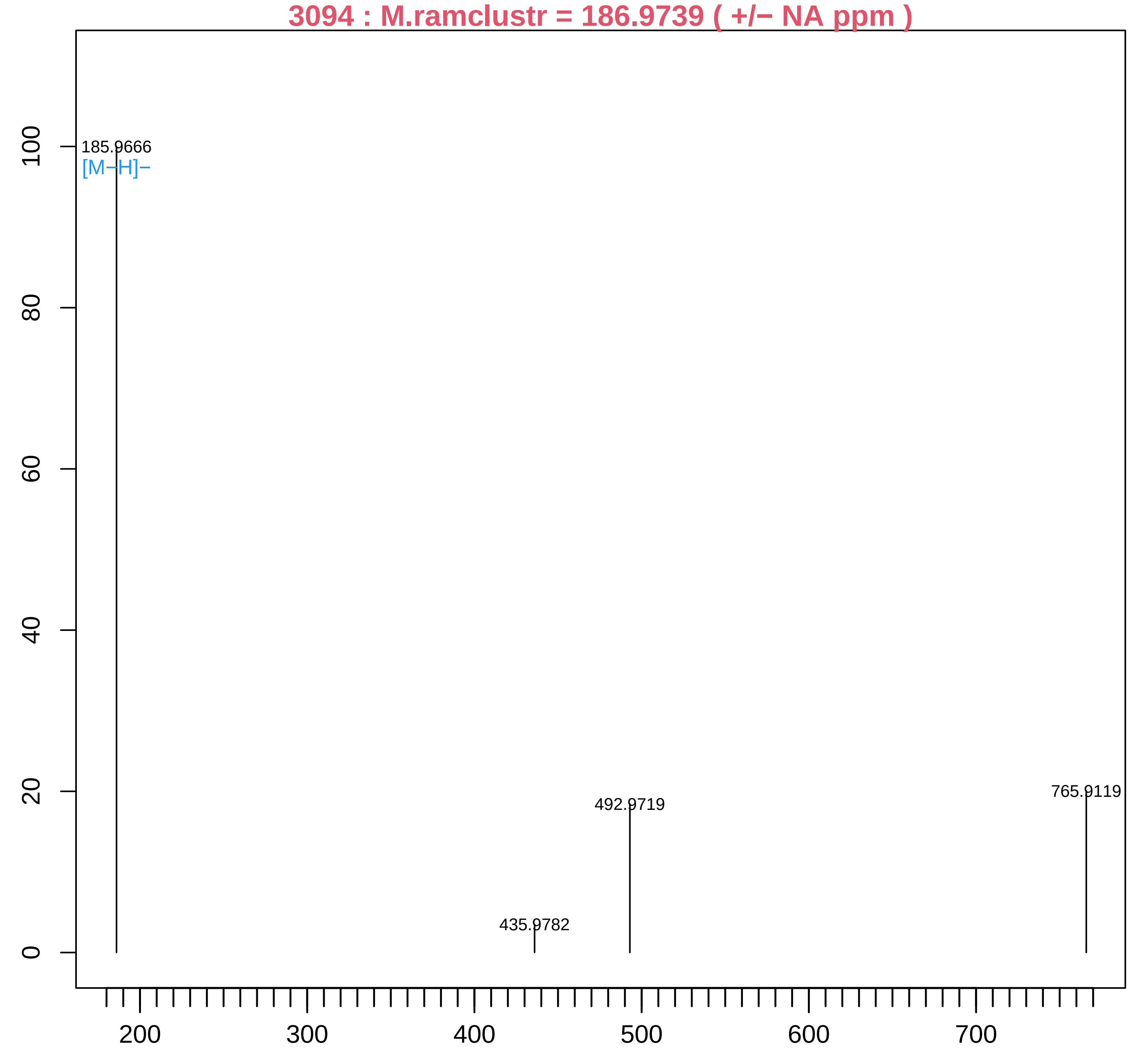
3094 : M.ramclustr = 186.9739 (+/− NA ppm)
185.9666
[M−H]−
435.9782
492.9719
765.9119
0
20
40
60
80
100
200
300
400
500
600
700

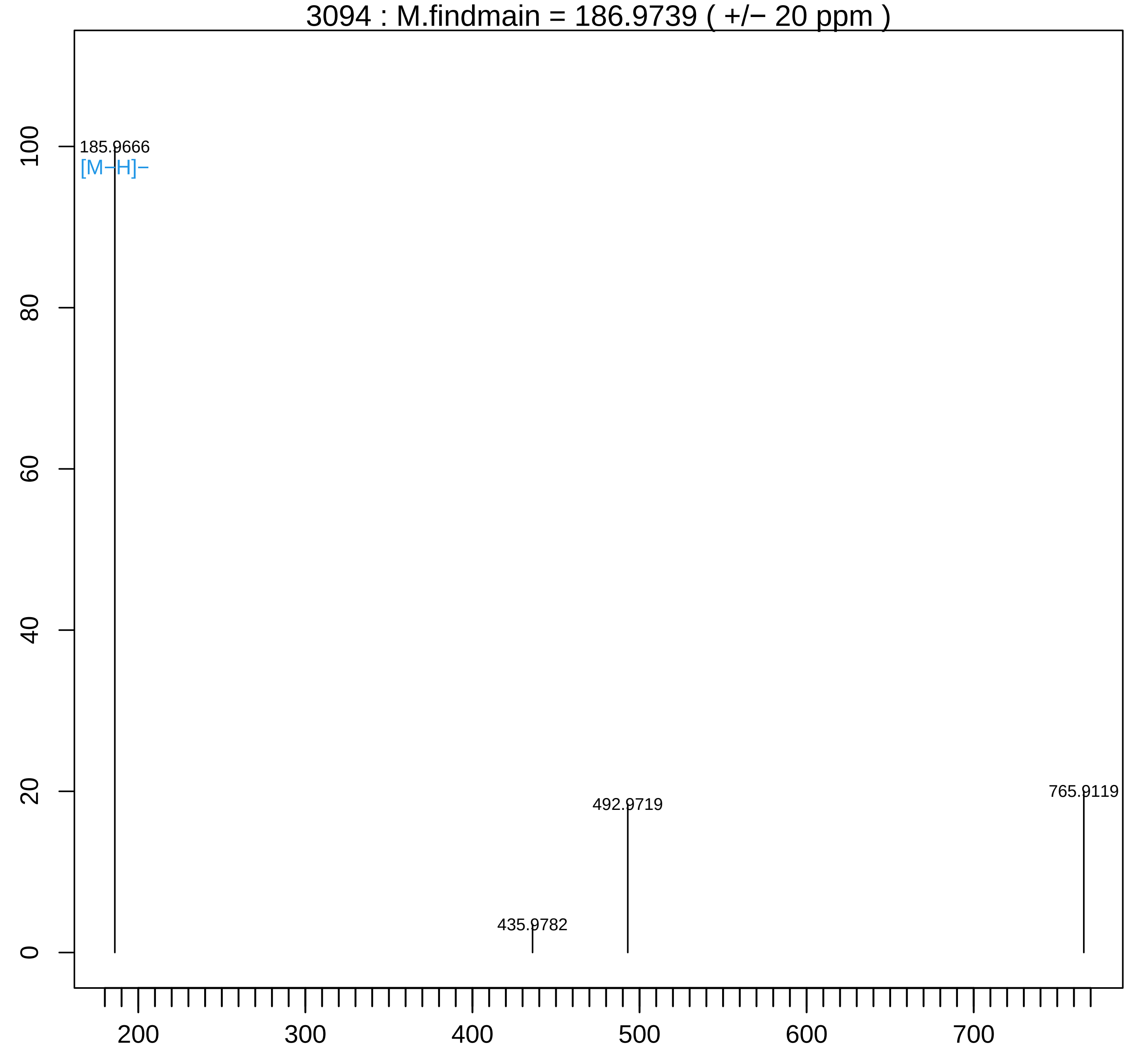
3094 : M.findmain = 186.9739 (+/− 20 ppm)
185.9666
[M−H]−
435.9782
492.9719
765.9119
0
20
40
60
80
100
200
300
400
500
600
700

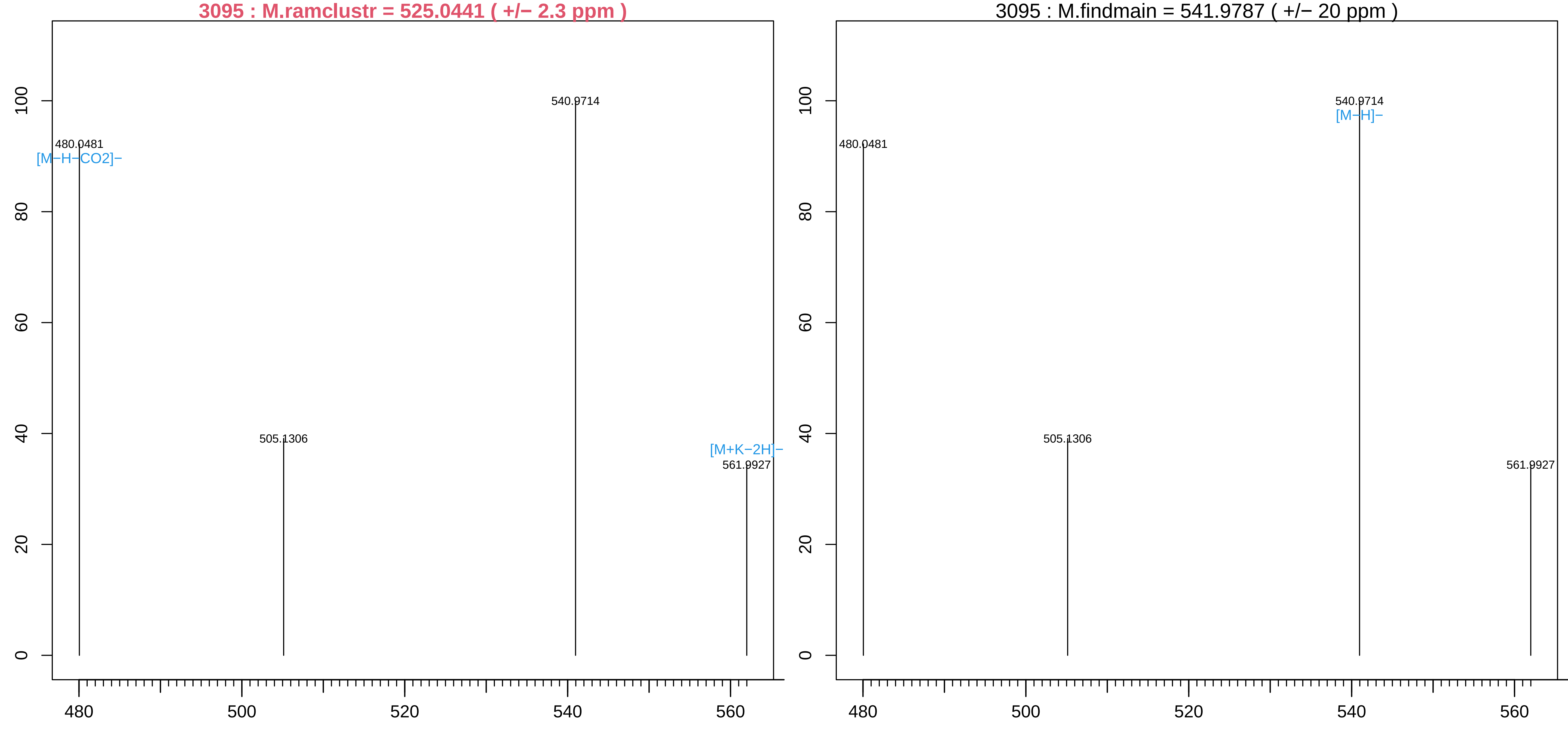
3095 : M.ramclustr = 525.0441 (+/− 2.3 ppm)
480.0481
[M−H−CO2]−
505.1306
540.9714
[M+K−2H]−
561.9927
3095 : M.findmain = 541.9787 (+/− 20 ppm)
480.0481
505.1306
540.9714
[M−H]−
561.9927
0
20
40
60
80
100
480
500
520
540
560

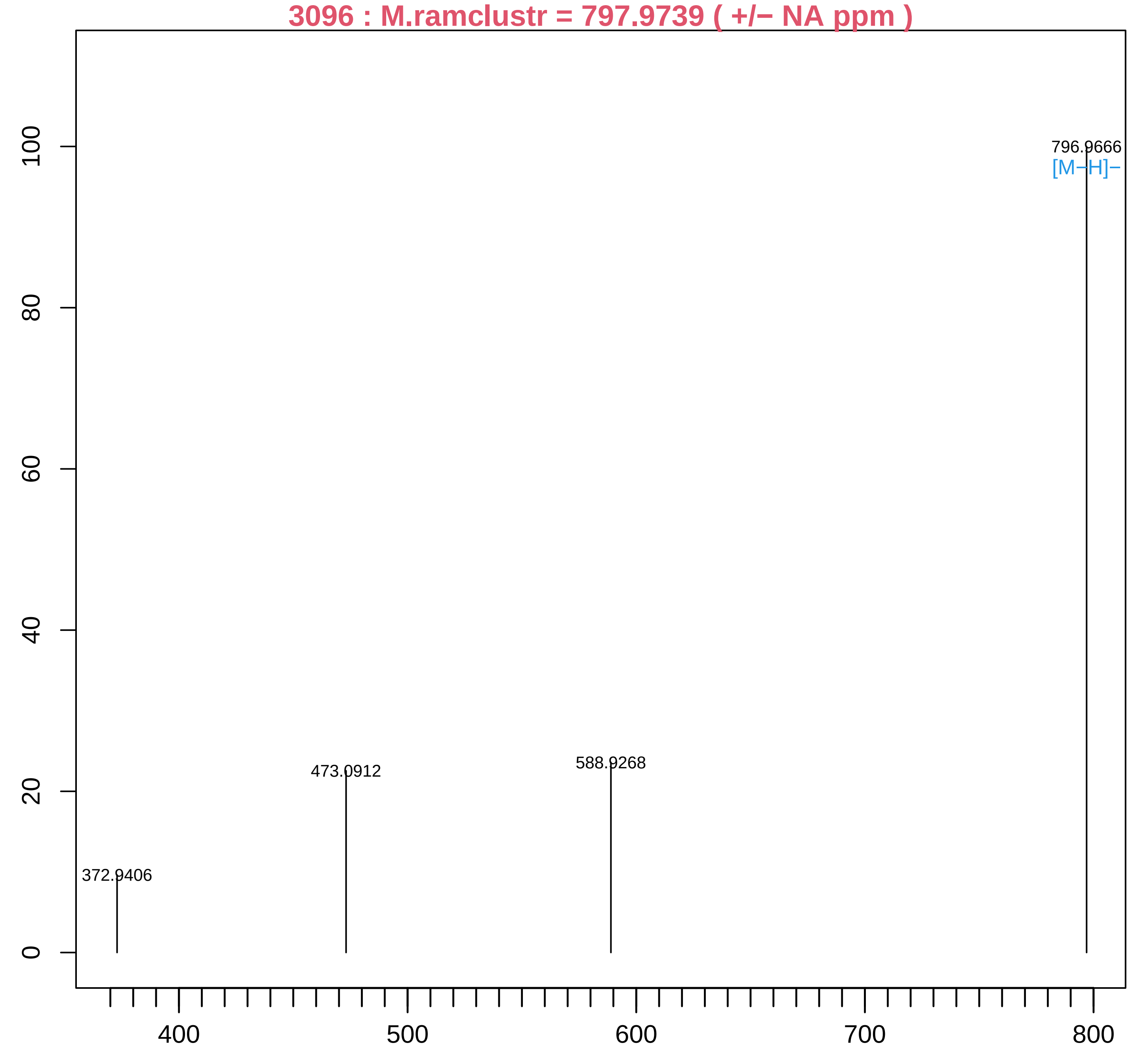

3096 : M.ramclustr = 797.9739 (+/− NA ppm)
796.9666
[M−H]−
473.0912
588.9268
372.9406

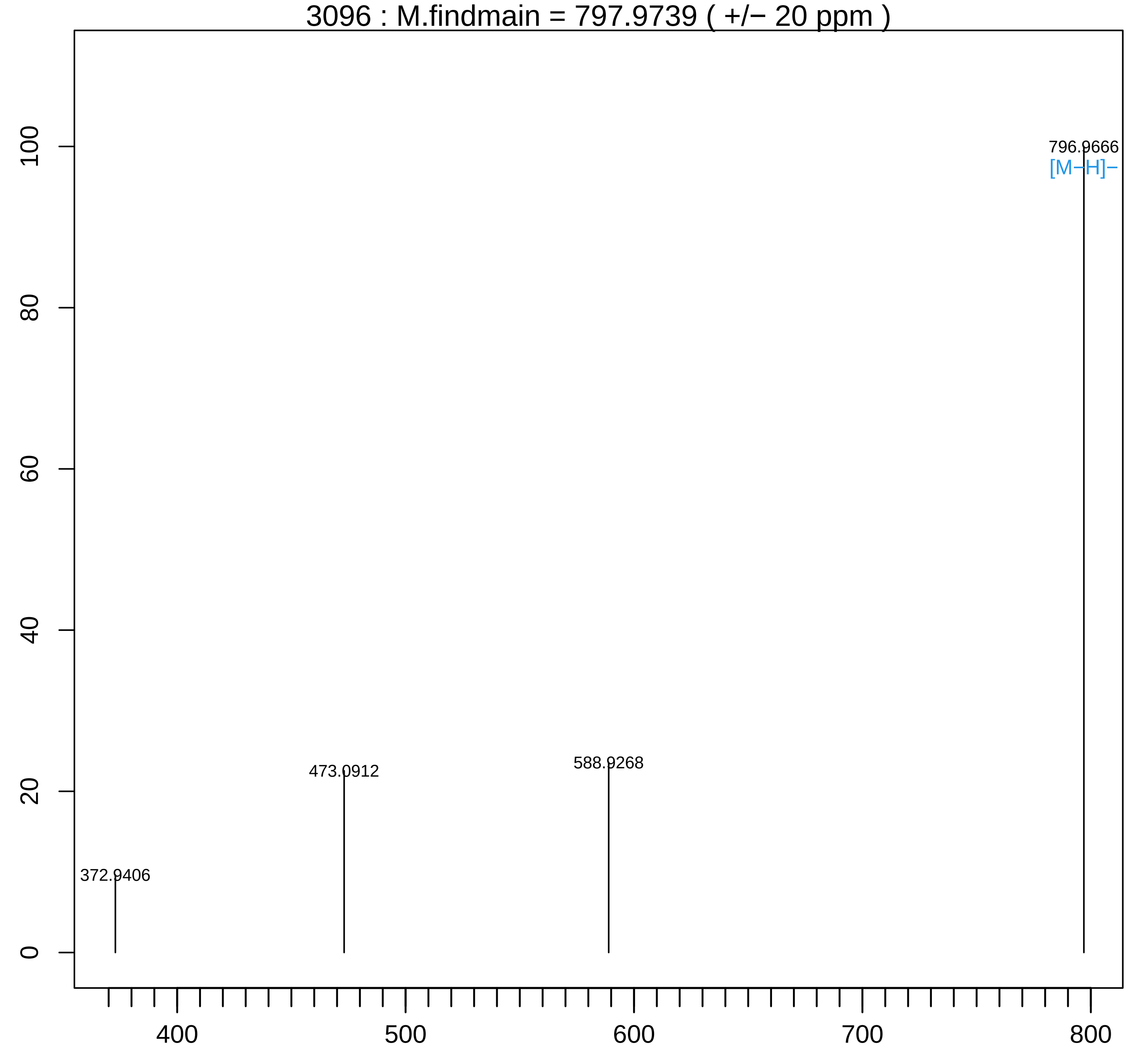

3096 : M.findmain = 797.9739 (+/− 20 ppm)
796.9666
[M−H]−
473.0912
588.9268
372.9406

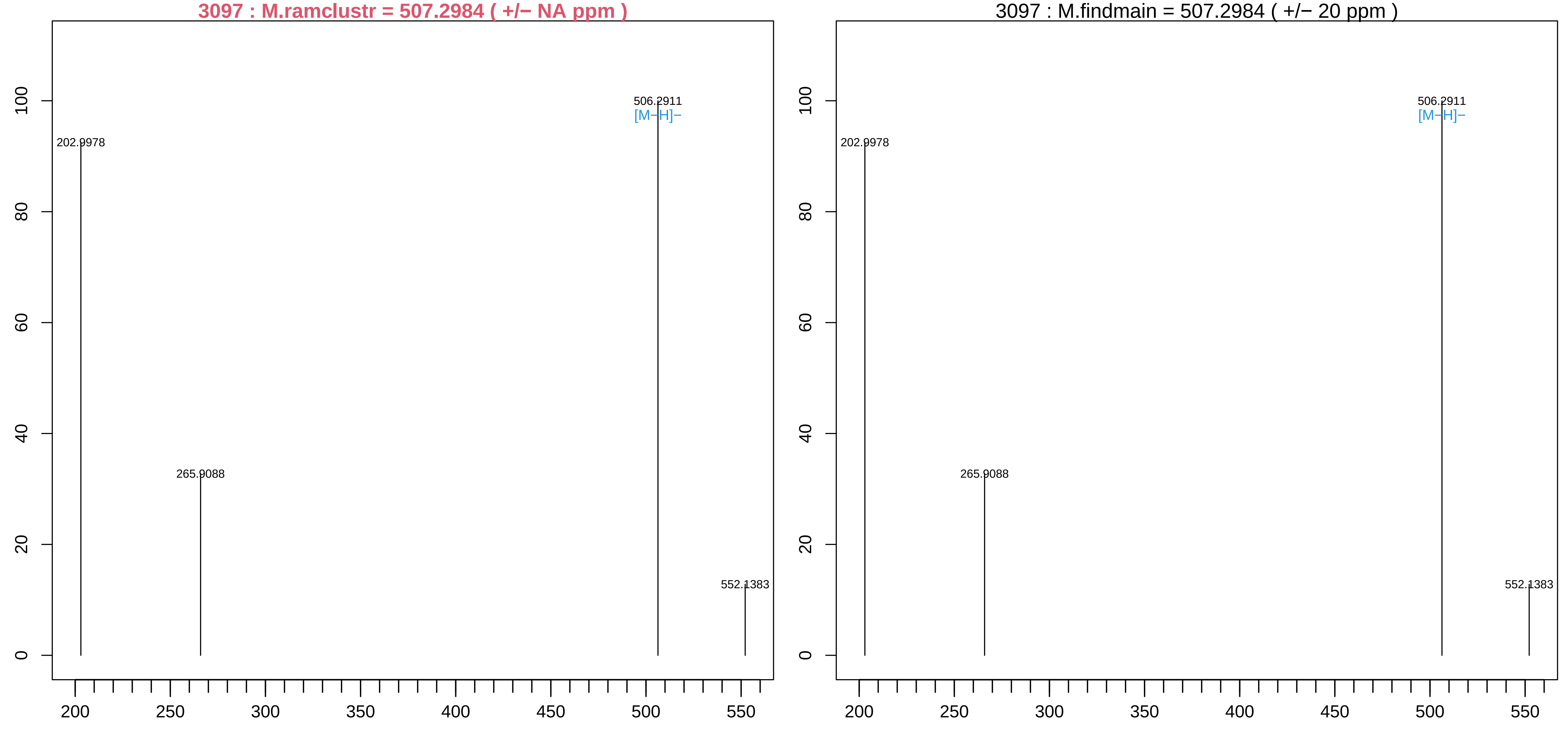
3097 : M.ramclustr = 507.2984 (+/− NA ppm)
202.9978
265.9088
506.2911
[M−H]−
552.1383
0
20
40
60
80
100
200
250
300
350
400
450
500
550
3097 : M.findmain = 507.2984 (+/− 20 ppm)
202.9978
265.9088
506.2911
[M−H]−
552.1383
0
20
40
60
80
100
200
250
300
350
400
450
500
550

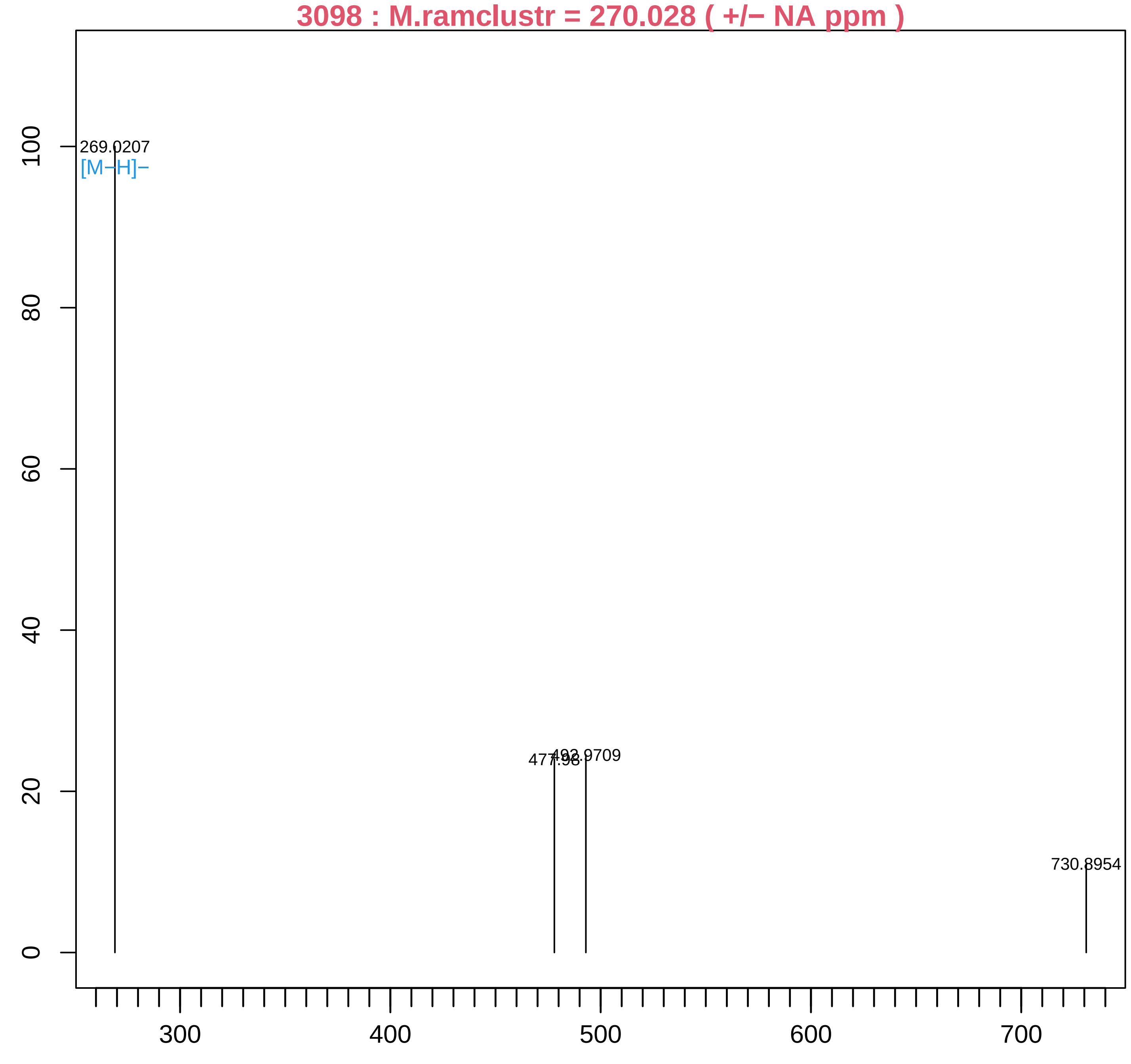

3098 : M.ramclustr = 270.028 (+/− NA ppm)
269.0207
[M−H]−
477.98
492.9709
730.8954
0
20
40
60
80
100
300
400
500
600
700

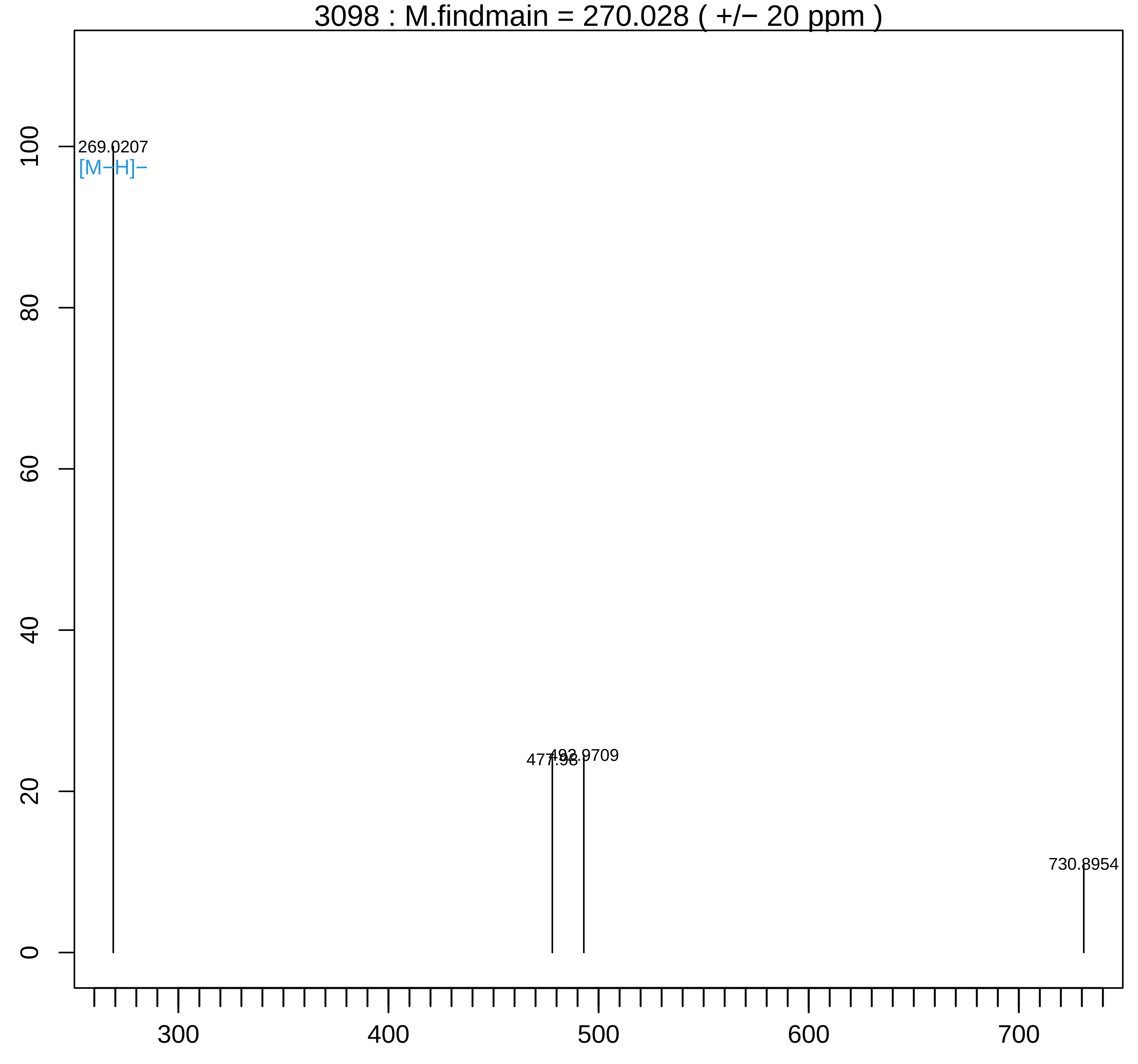

3098 : M.findmain = 270.028 (+/− 20 ppm)
269.0207
[M−H]−
477.98
492.9709
730.8954
0
20
40
60
80
100
300
400
500
600
700

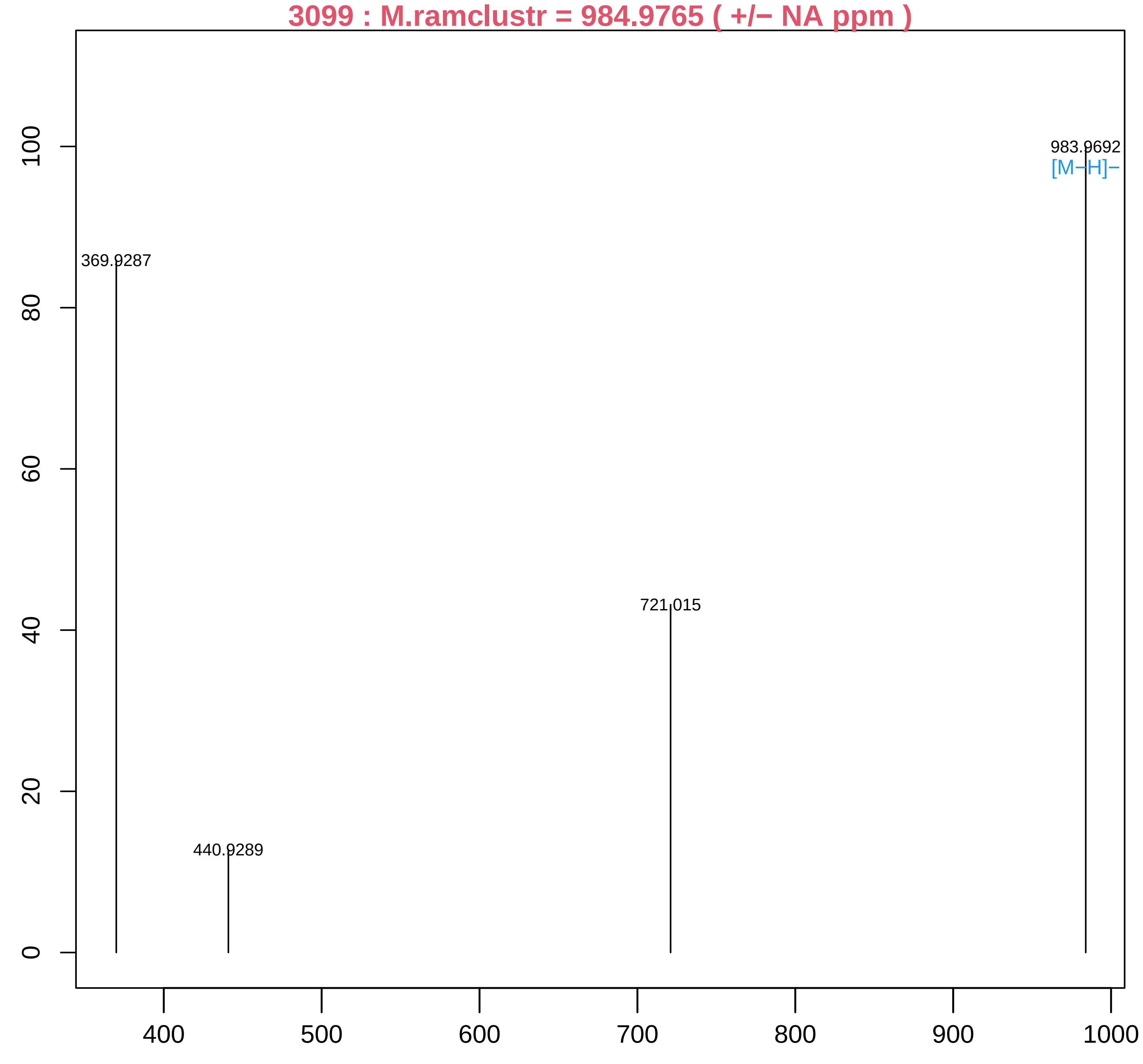
3099 : M.ramclustr = 984.9765 (+/− NA ppm)
983.9692
[M−H]−
369.9287
721.015
440.9289
0
20
40
60
80
100
400
500
600
700
800
900
1000

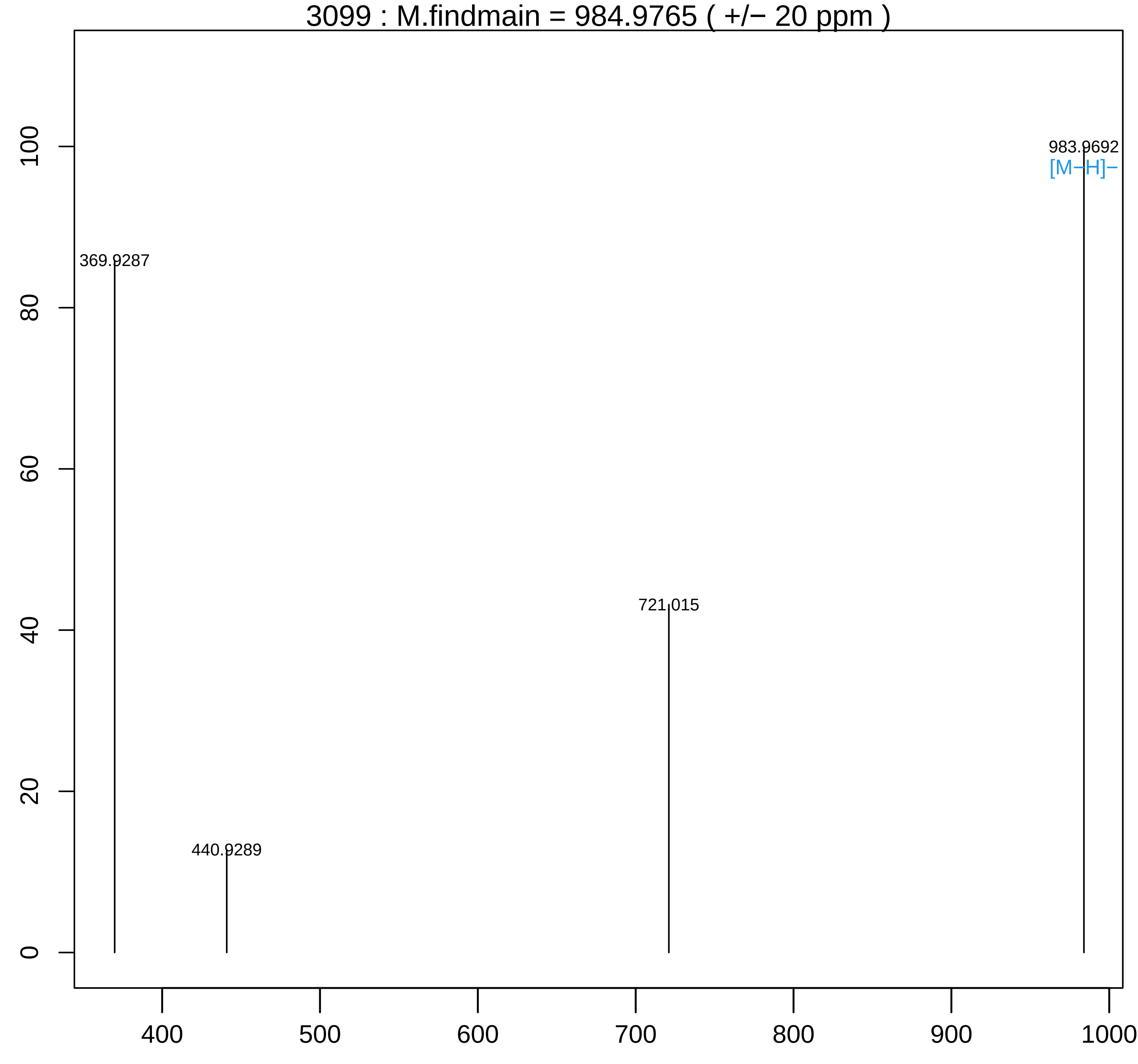
3099 : M.findmain = 984.9765 (+/− 20 ppm)
983.9692
[M−H]−
369.9287
721.015
440.9289
0
20
40
60
80
100
400
500
600
700
800
900
1000

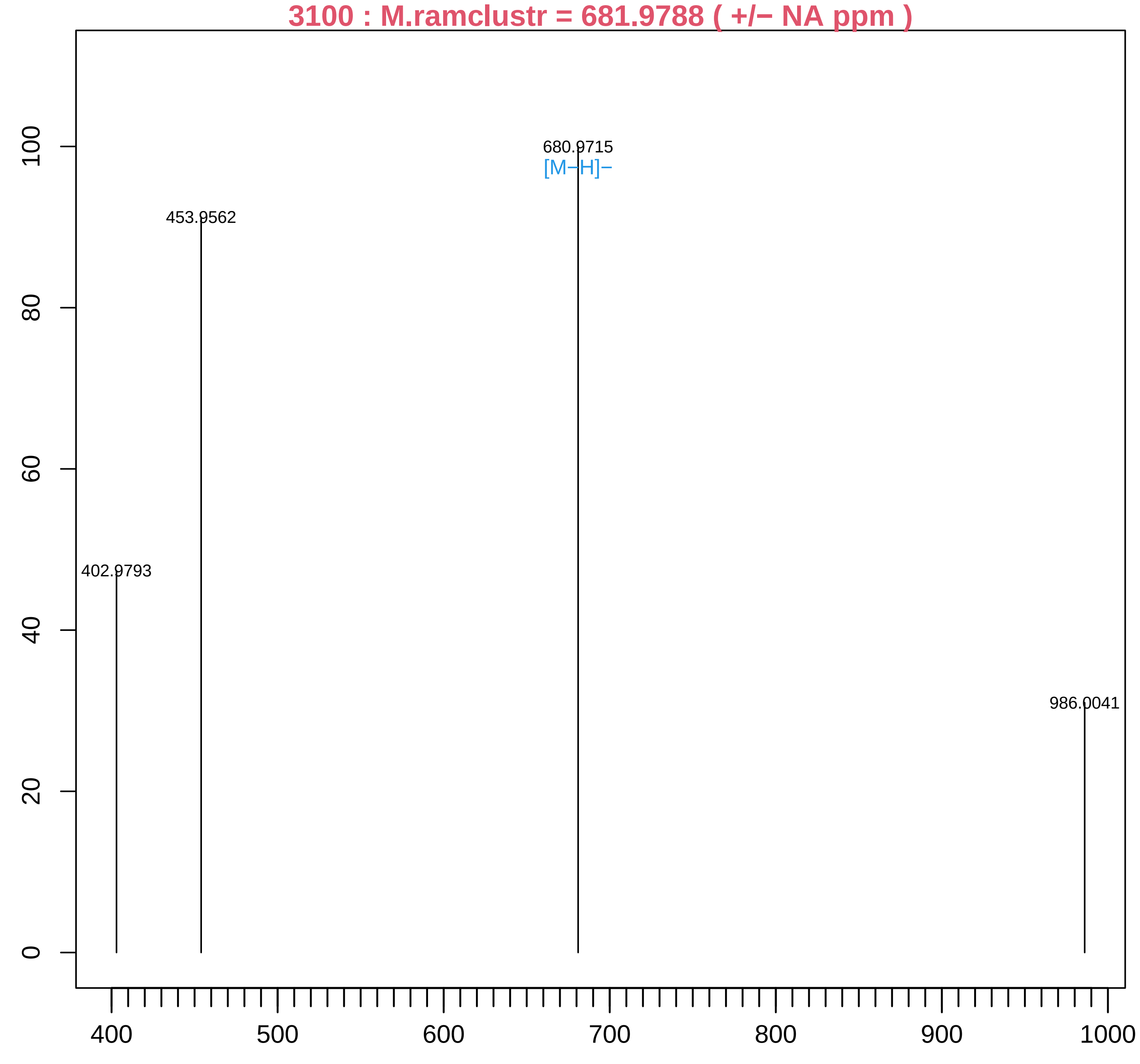

3100 : M.ramclustr = 681.9788 (+/− NA ppm)
680.9715
[M−H]−
453.9562
402.9793
986.0041

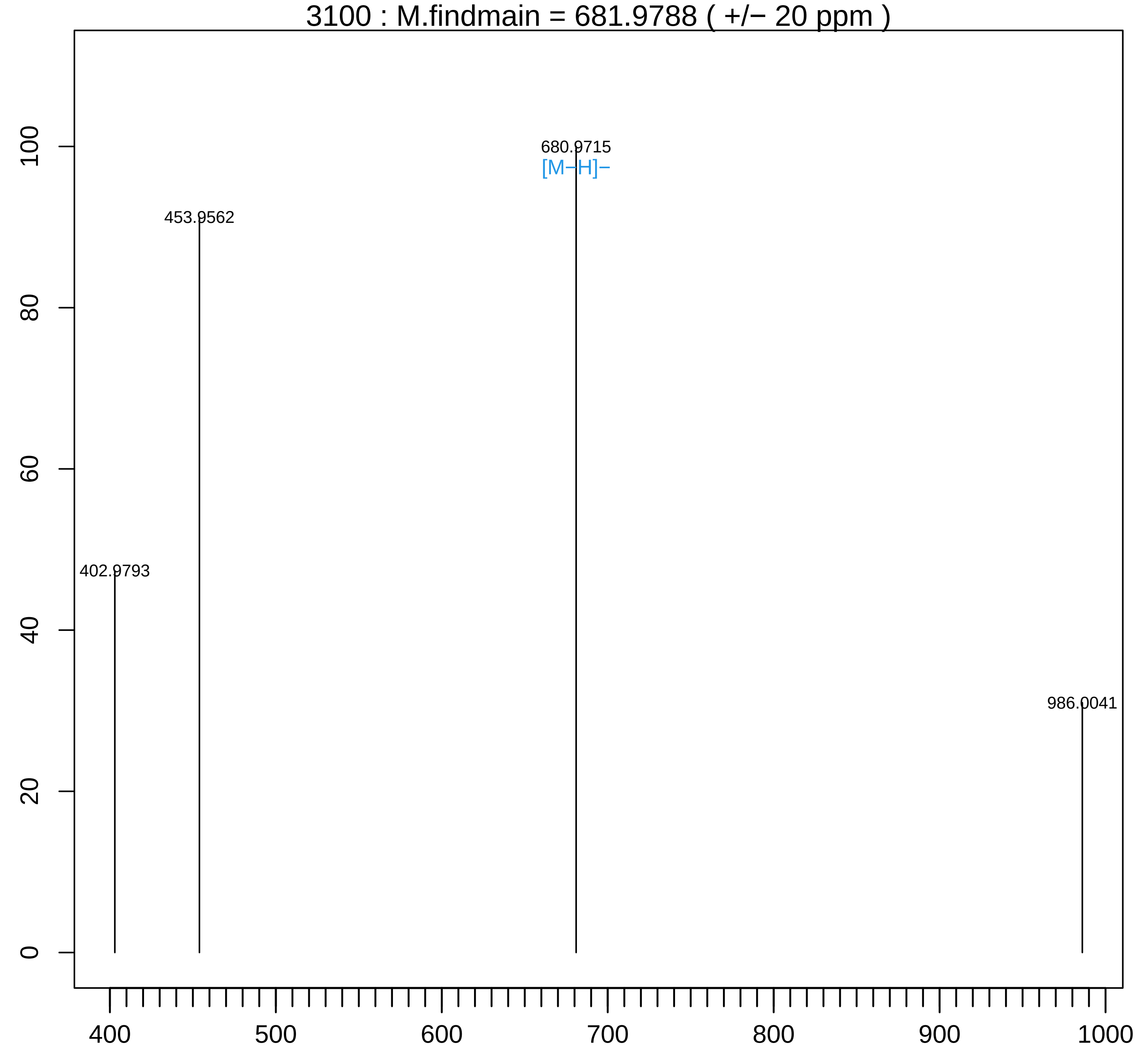

3100 : M.findmain = 681.9788 (+/− 20 ppm)
680.9715
[M−H]−
453.9562
402.9793
986.0041

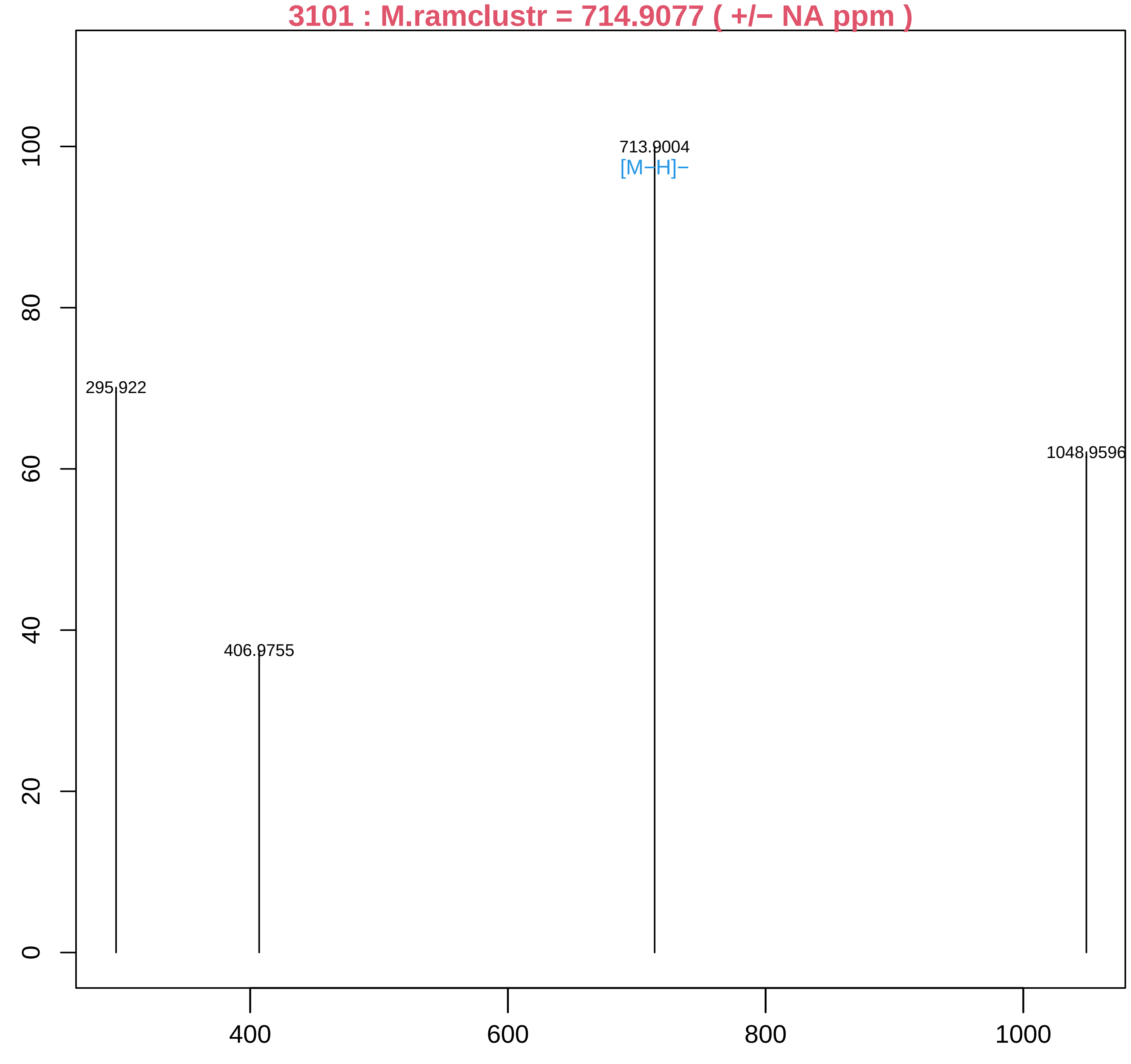
3101 : M.ramclustr = 714.9077 (+/− NA ppm)
713.9004
[M−H]−
295.922
1048.9596
406.9755
0
20
40
60
80
100
400
600
800
1000

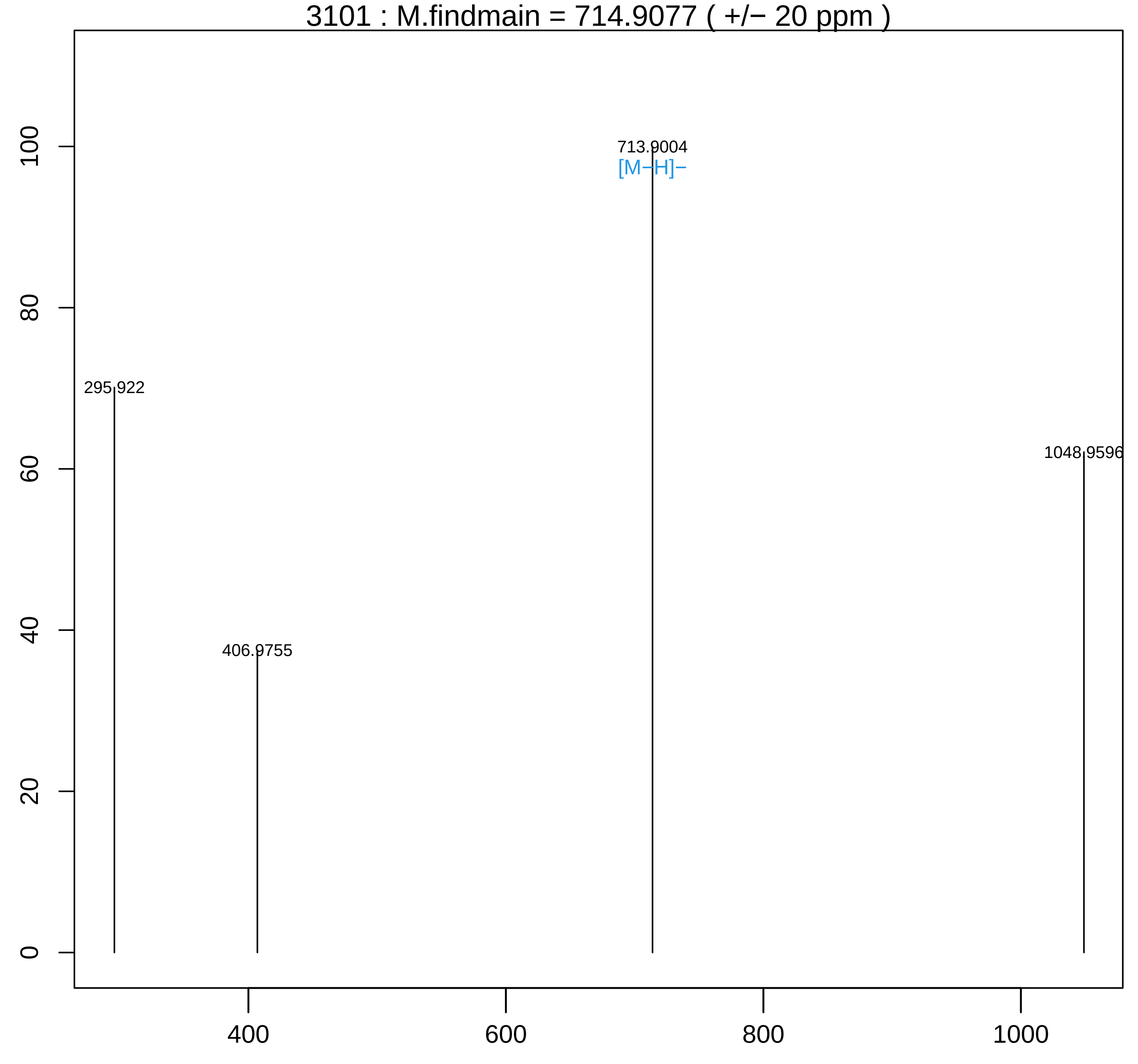
3101 : M.findmain = 714.9077 (+/− 20 ppm)
713.9004
[M−H]−
295.922
1048.9596
406.9755
0
20
40
60
80
100
400
600
800
1000

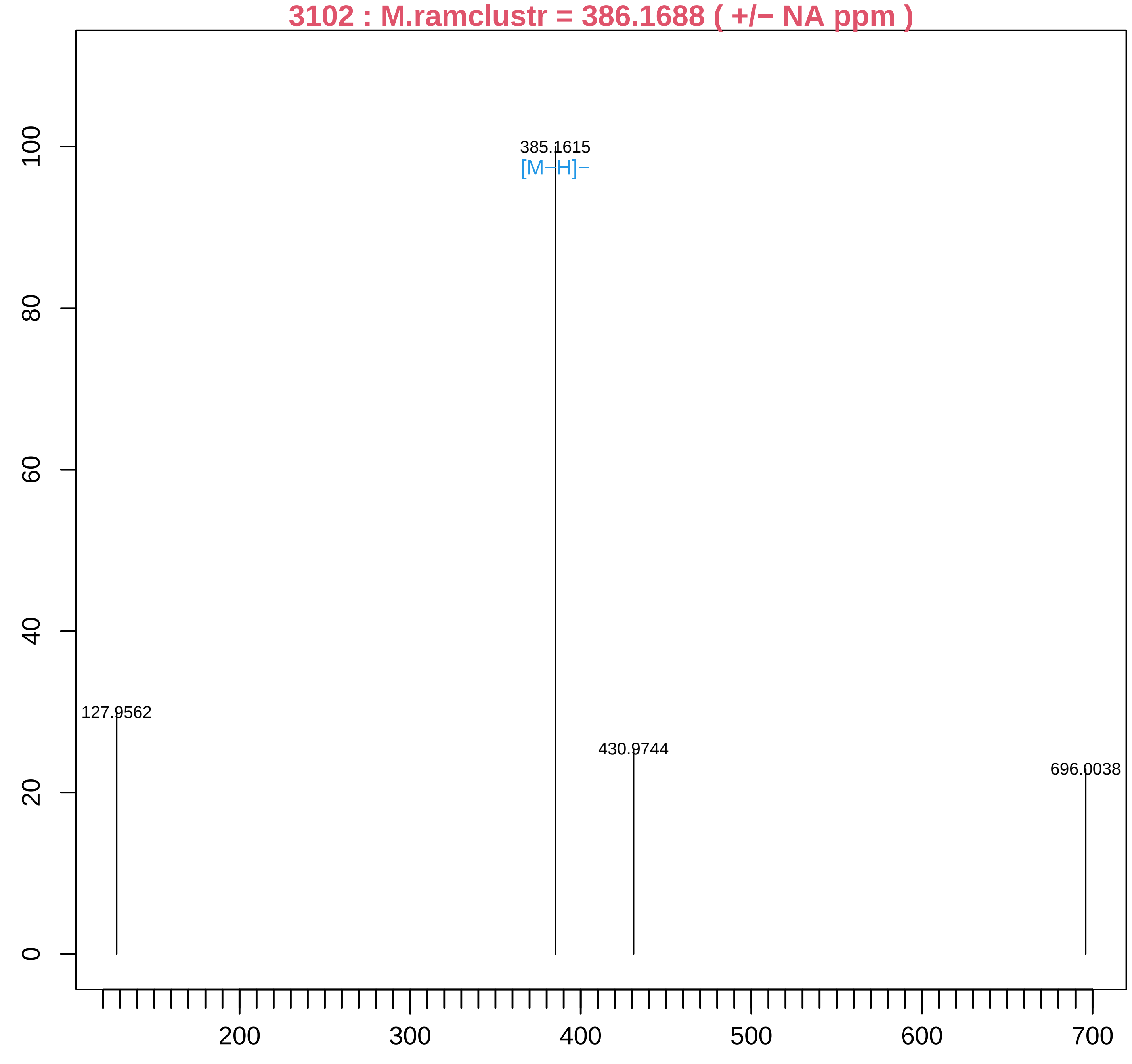
3102 : M.ramclustr = 386.1688 (+/− NA ppm)
385.1615
[M−H]−
127.9562
430.9744
696.0038
0
20
40
60
80
100
200
300
400
500
600
700

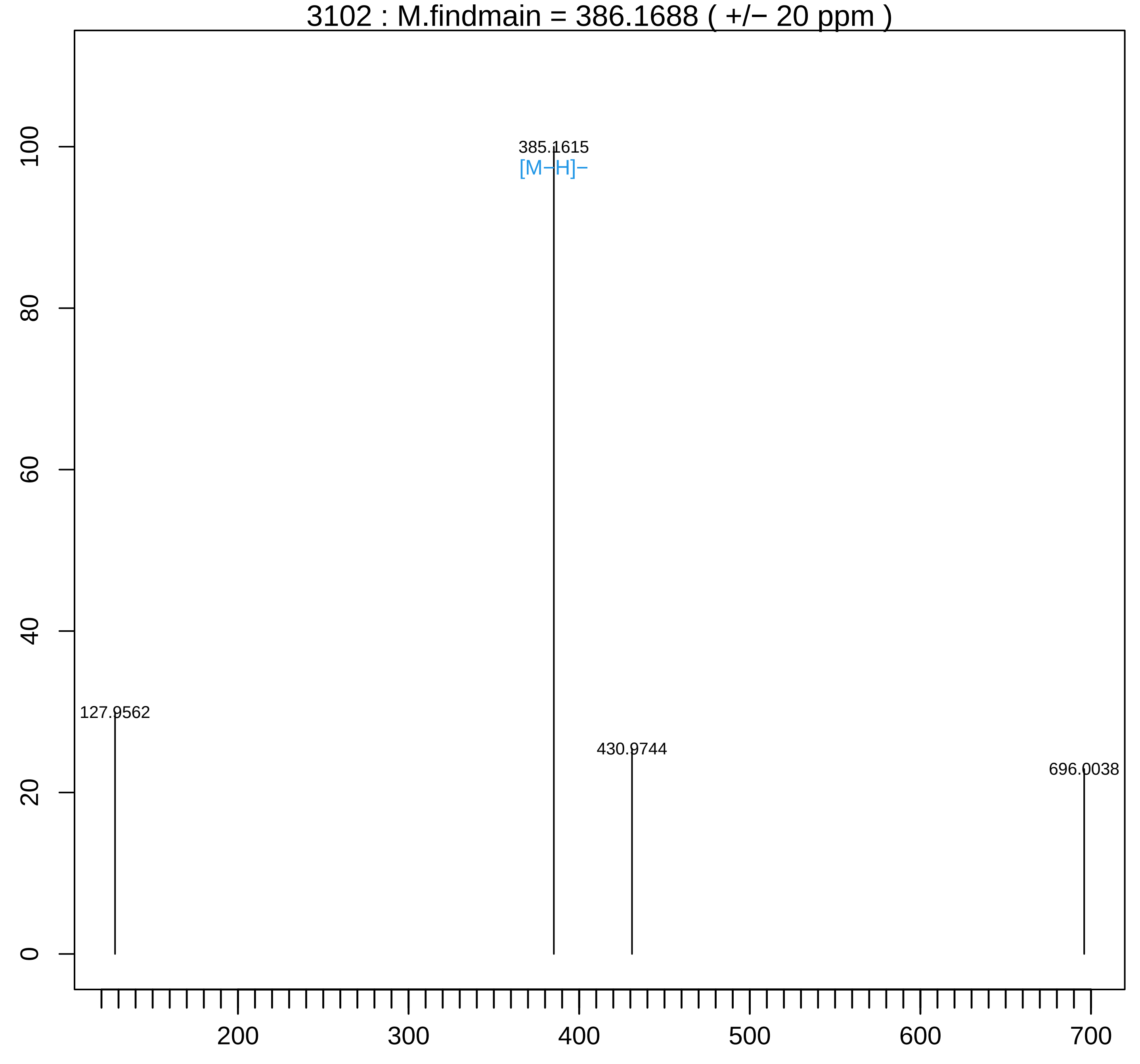
3102 : M.findmain = 386.1688 (+/− 20 ppm)
385.1615
[M−H]−
127.9562
430.9744
696.0038
0
20
40
60
80
100
200
300
400
500
600
700

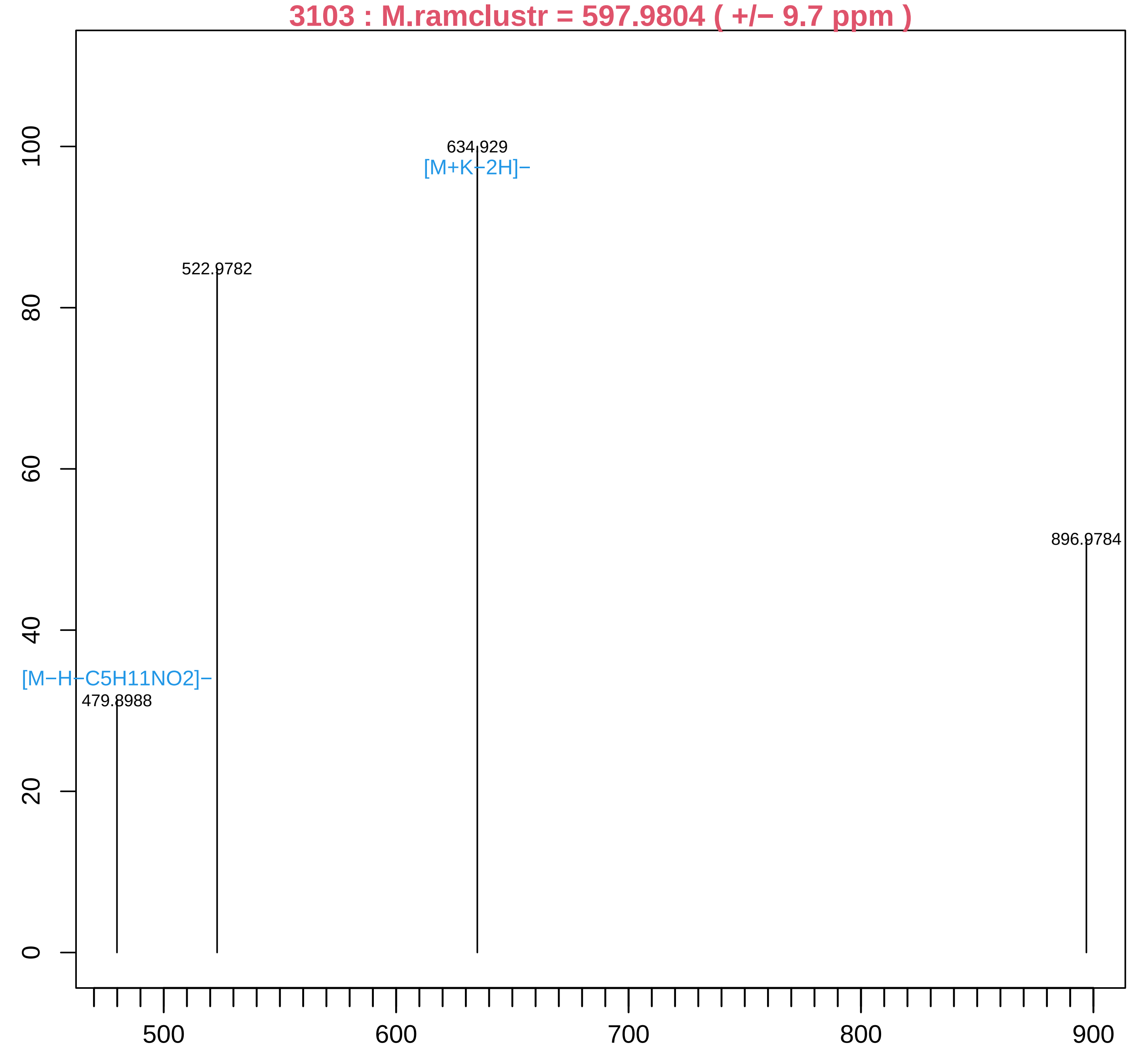

3103 : M.ramclustr = 597.9804 (+/− 9.7 ppm)
634.929
[M+K−2H]−
522.9782
896.9784
[M−H−C5H11NO2]−
479.8988
0
20
40
60
80
100
500
600
700
800
900

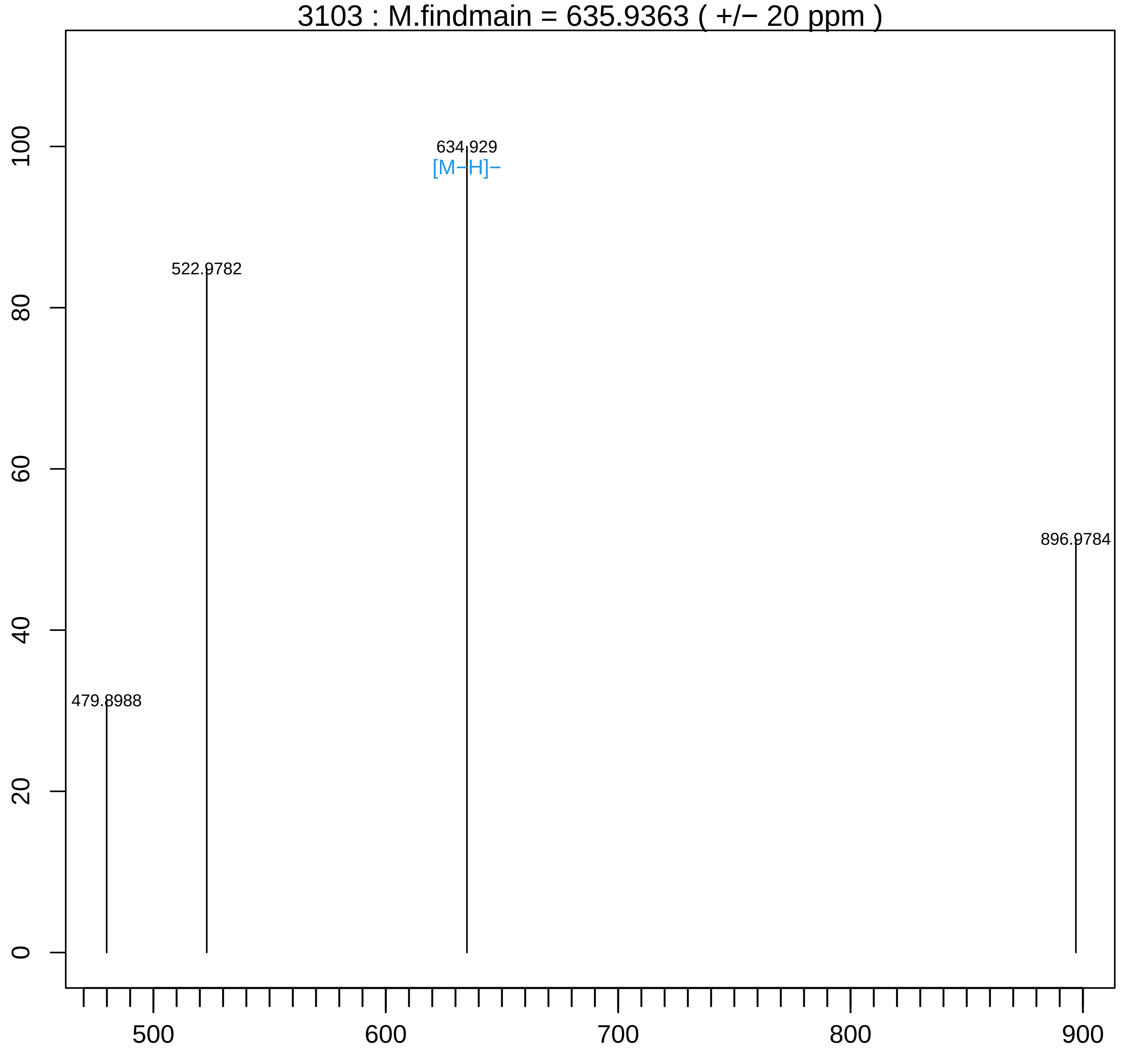

3103 : M.findmain = 635.9363 (+/− 20 ppm)
634.929
[M−H]−
522.9782
896.9784
479.8988
0
20
40
60
80
100
500
600
700
800
900

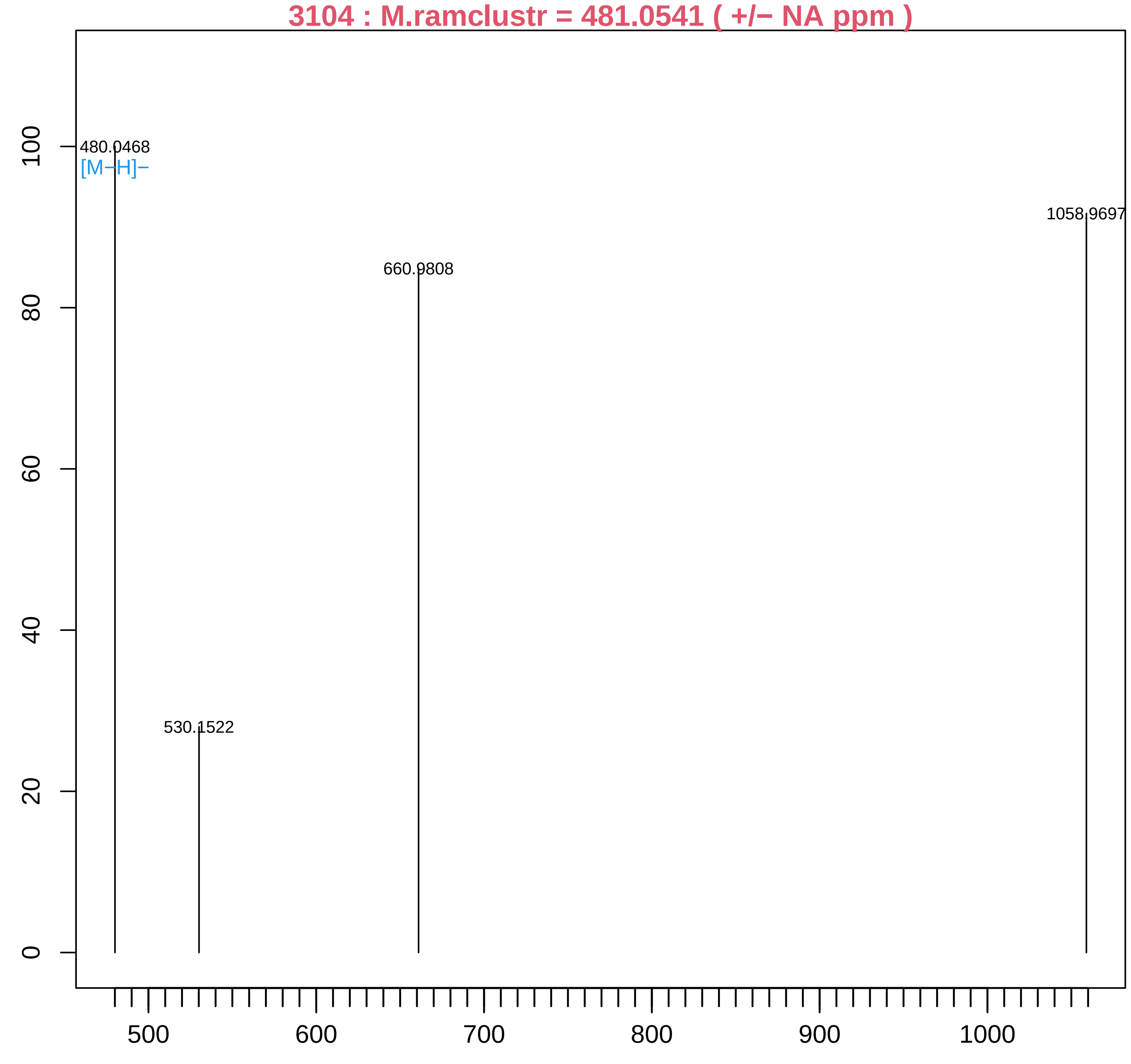
3104 : M.ramclustr = 481.0541 (+/− NA ppm)
480.0468
[M−H]−
530.1522
660.9808
1058.9697
0
20
40
60
80
100
500
600
700
800
900
1000
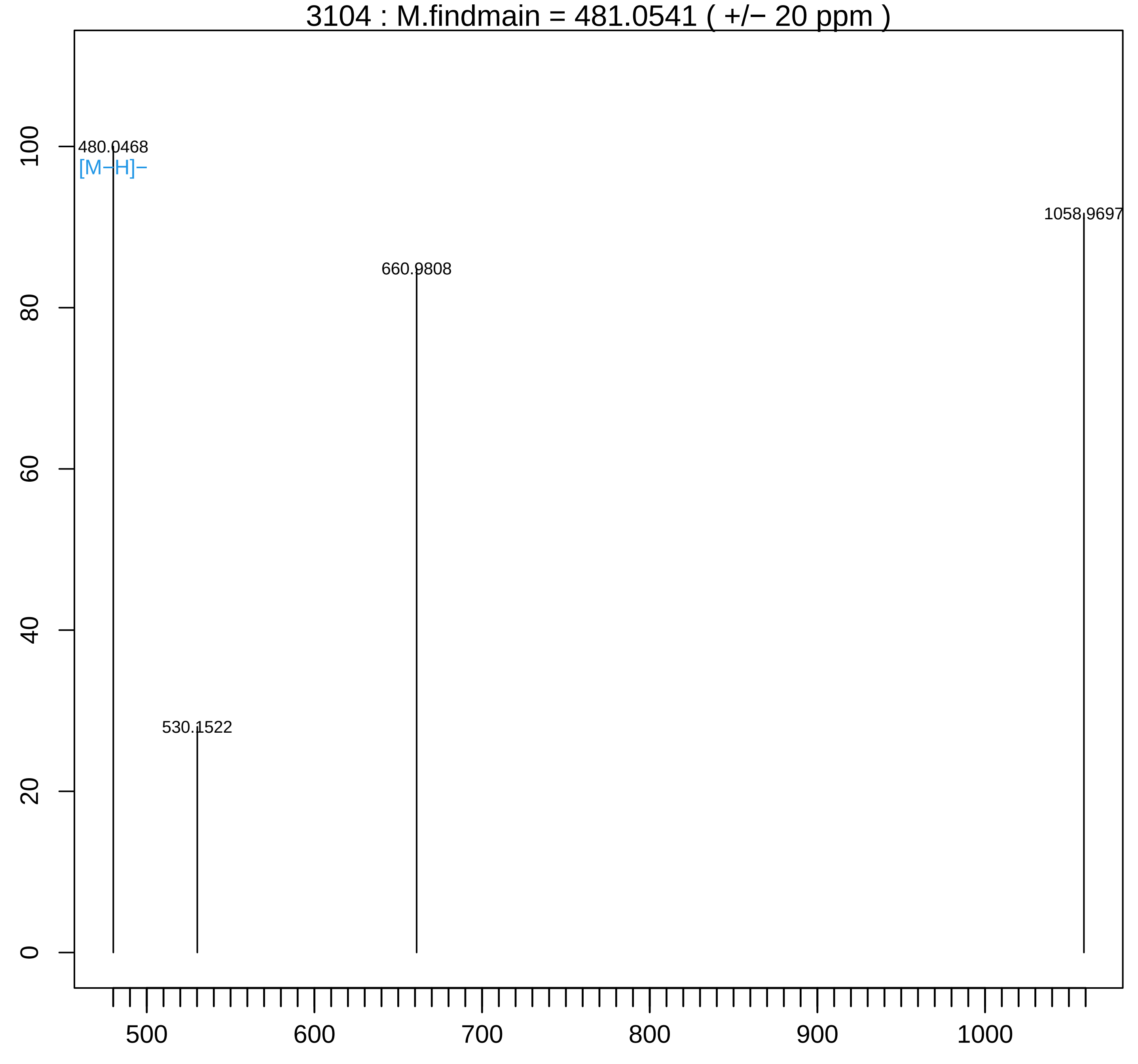
3104 : M.findmain = 481.0541 (+/− 20 ppm)
480.0468
[M−H]−
530.1522
660.9808
1058.9697
0
20
40
60
80
100
500
600
700
800
900
1000

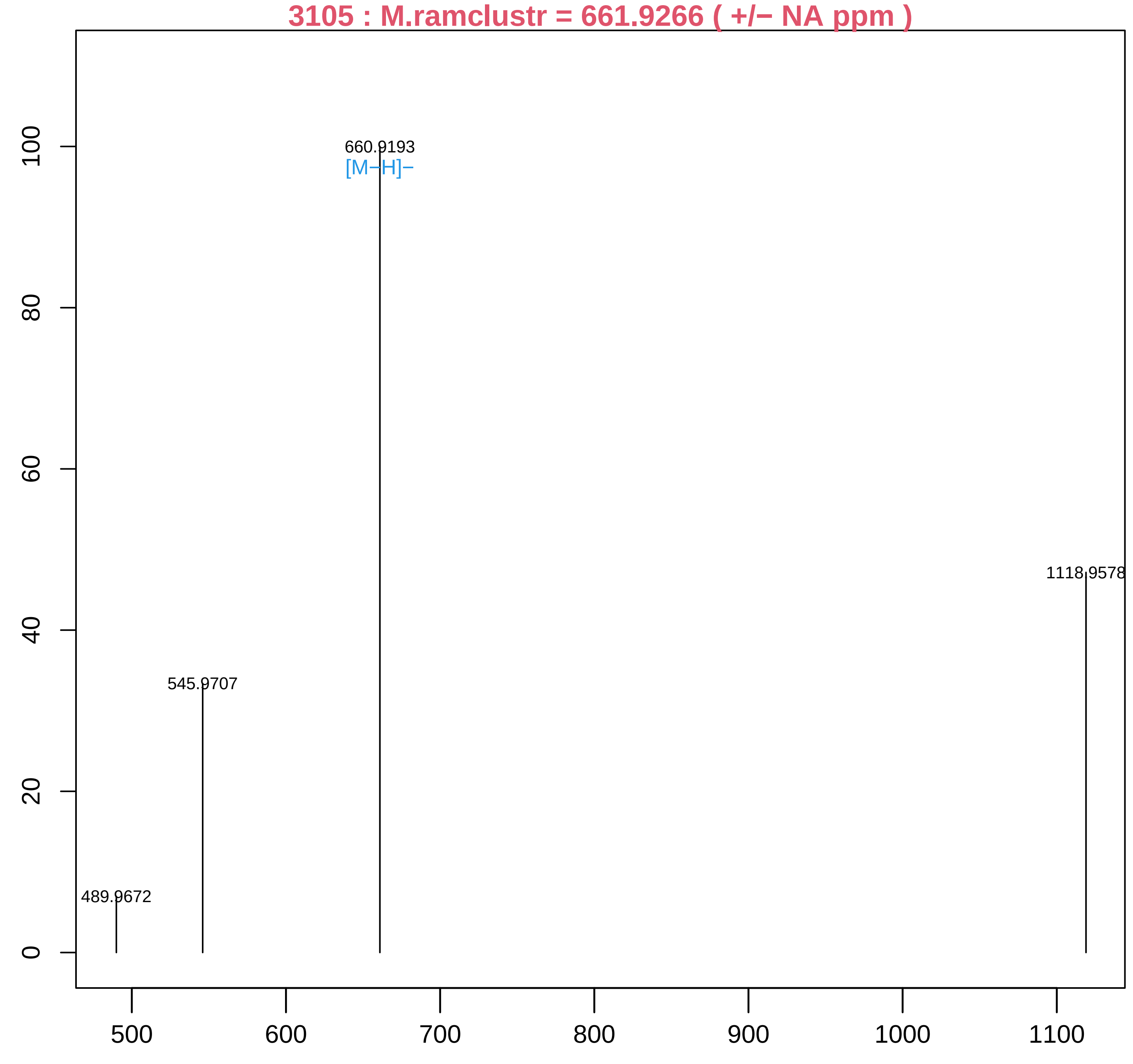
3105 : M.ramclustr = 661.9266 (+/− NA ppm)
660.9193
[M−H]−
545.9707
489.9672
1118.9578
0
20
40
60
80
100
500
600
700
800
900
1000
1100

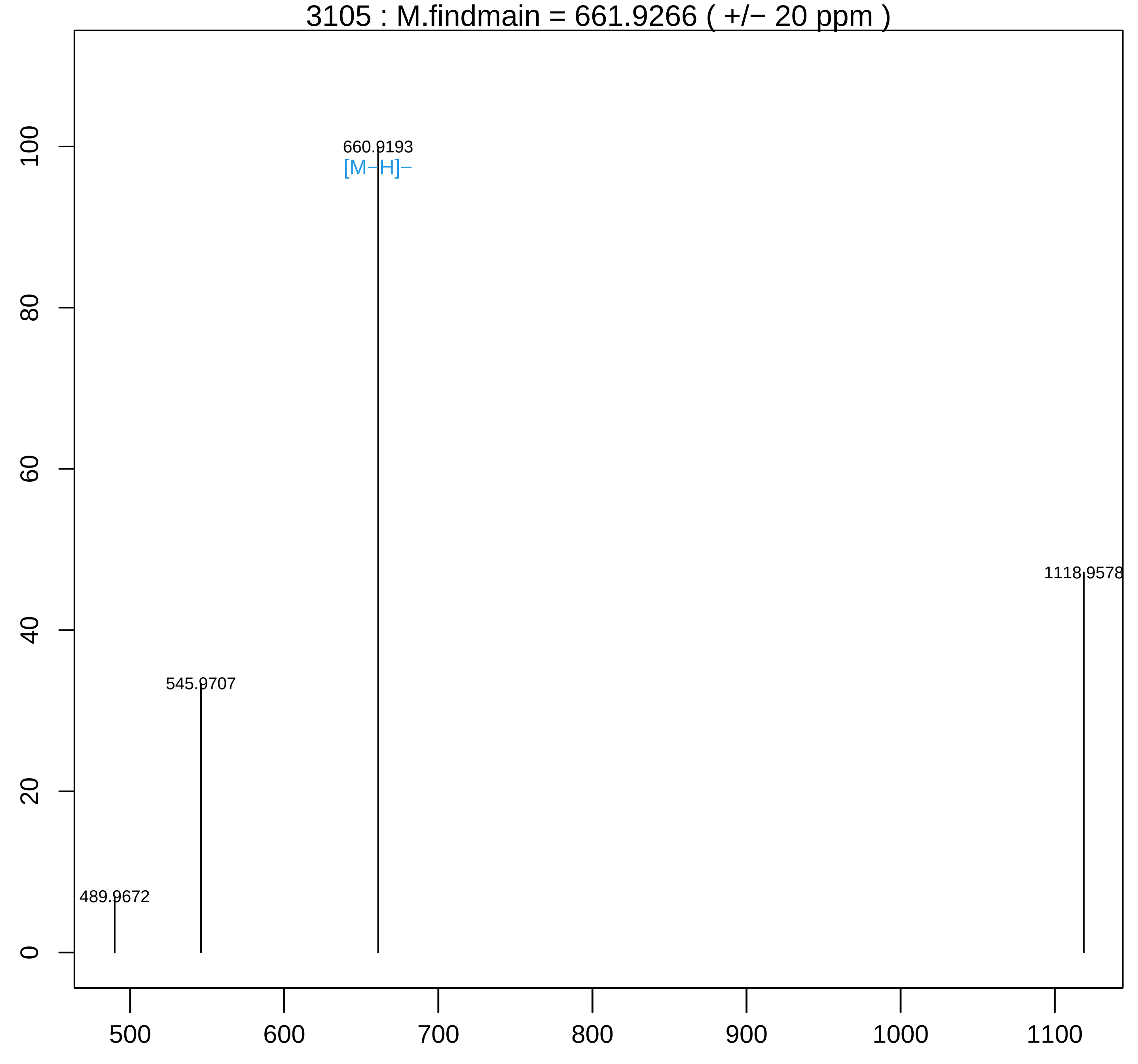
3105 : M.findmain = 661.9266 (+/− 20 ppm)
660.9193
[M−H]−
545.9707
489.9672
1118.9578
0
20
40
60
80
100
500
600
700
800
900
1000
1100

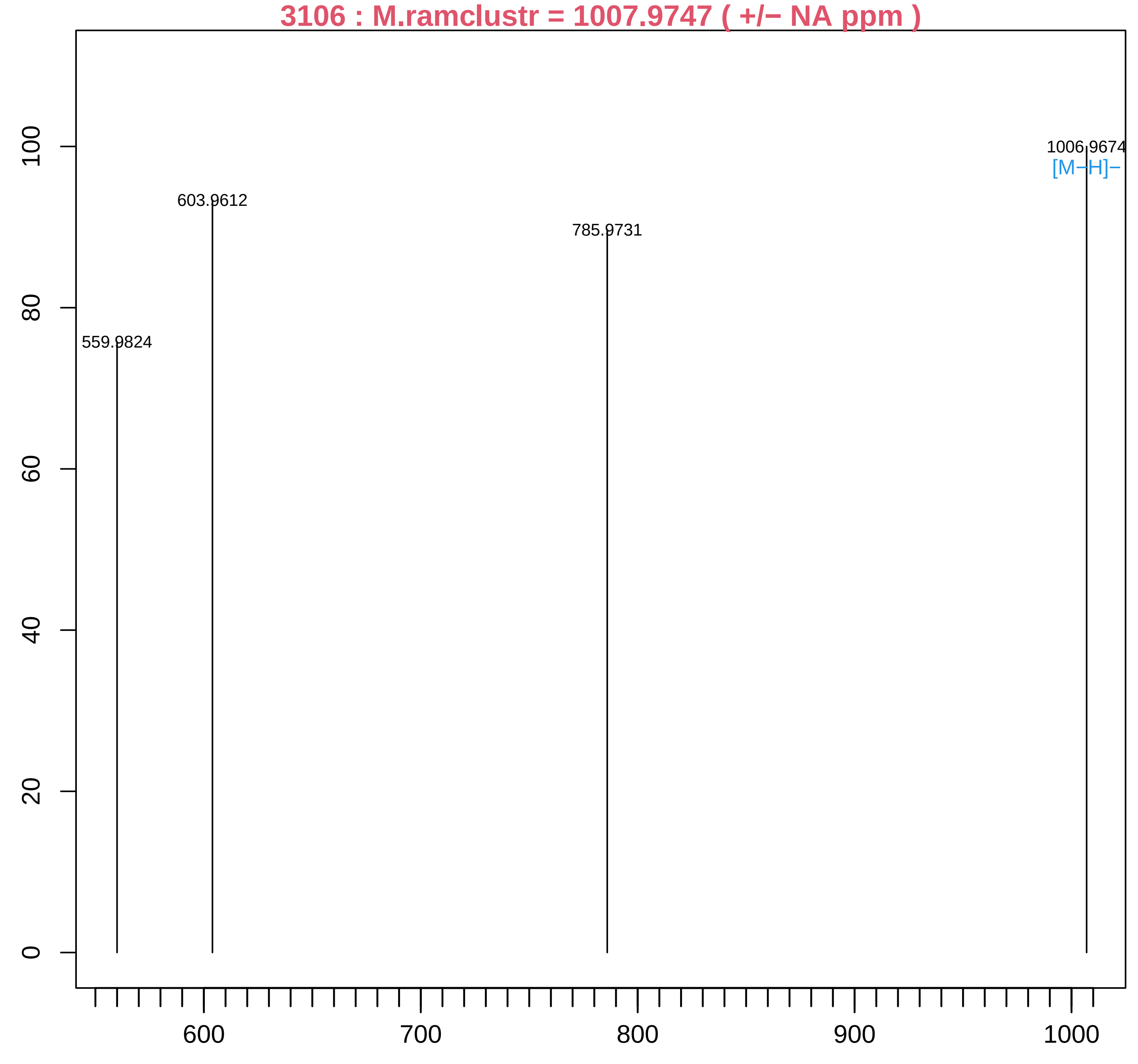
3106 : M.ramclustr = 1007.9747 (+/− NA ppm)
1006.9674
[M−H]−
603.9612
785.9731
559.9824
0
20
40
60
80
100
600
700
800
900
1000

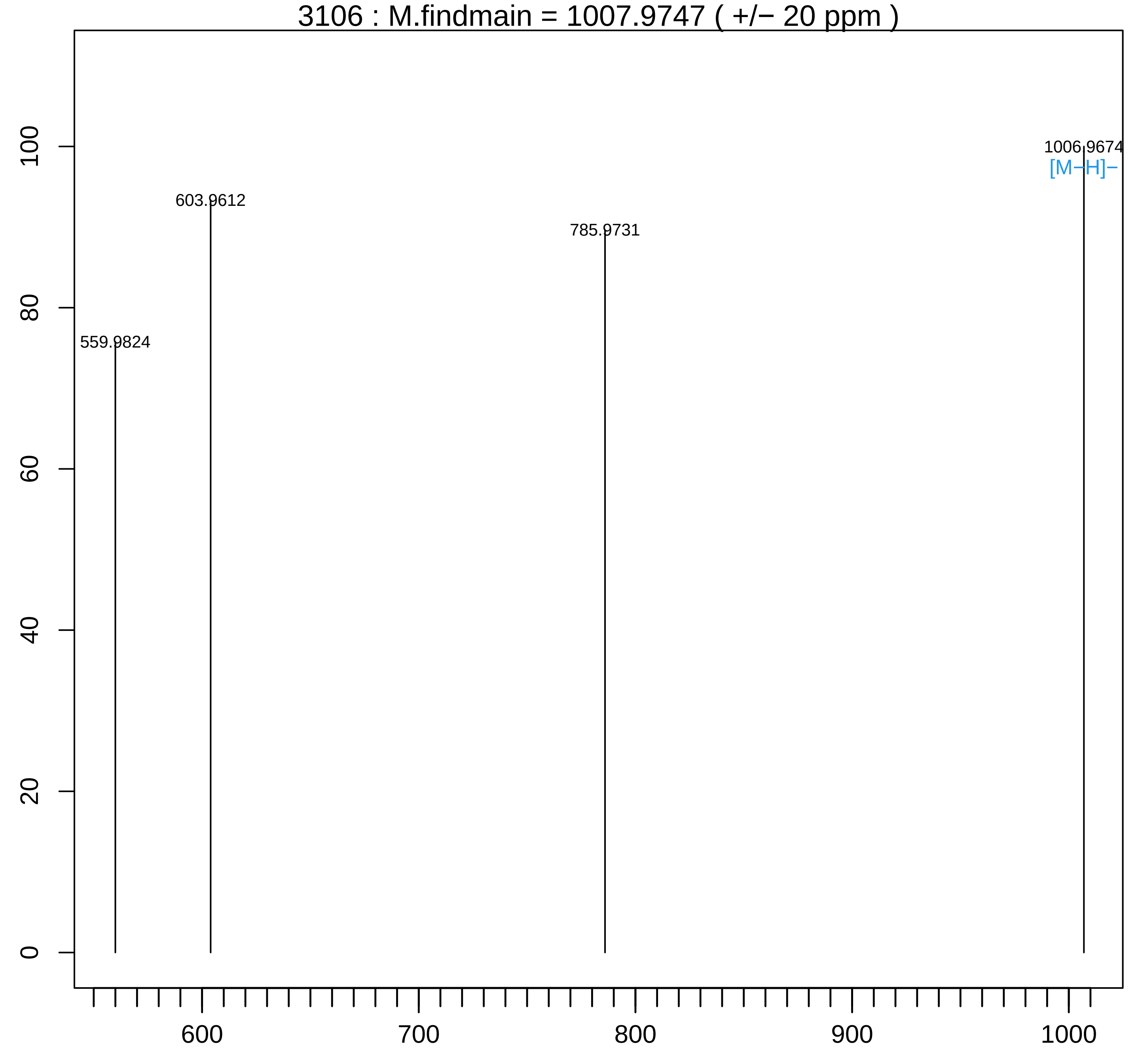
3106 : M.findmain = 1007.9747 (+/− 20 ppm)
1006.9674
[M−H]−
603.9612
785.9731
559.9824
0
20
40
60
80
100
600
700
800
900
1000

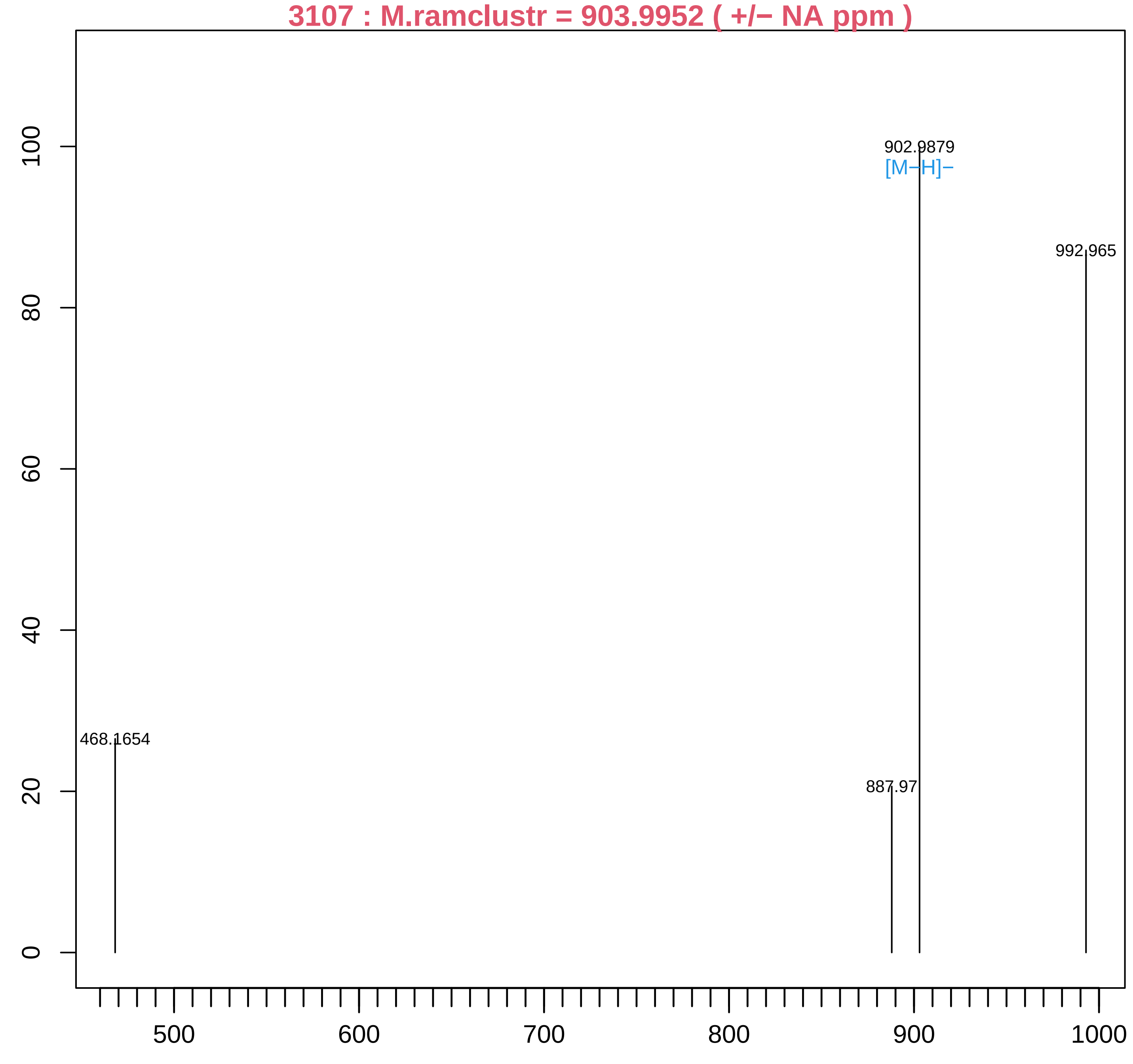
3107 : M.ramclustr = 903.9952 (+/− NA ppm)
902.9879
[M−H]−
992.965
468.1654
887.97
0
20
40
60
80
100
500
600
700
800
900
1000

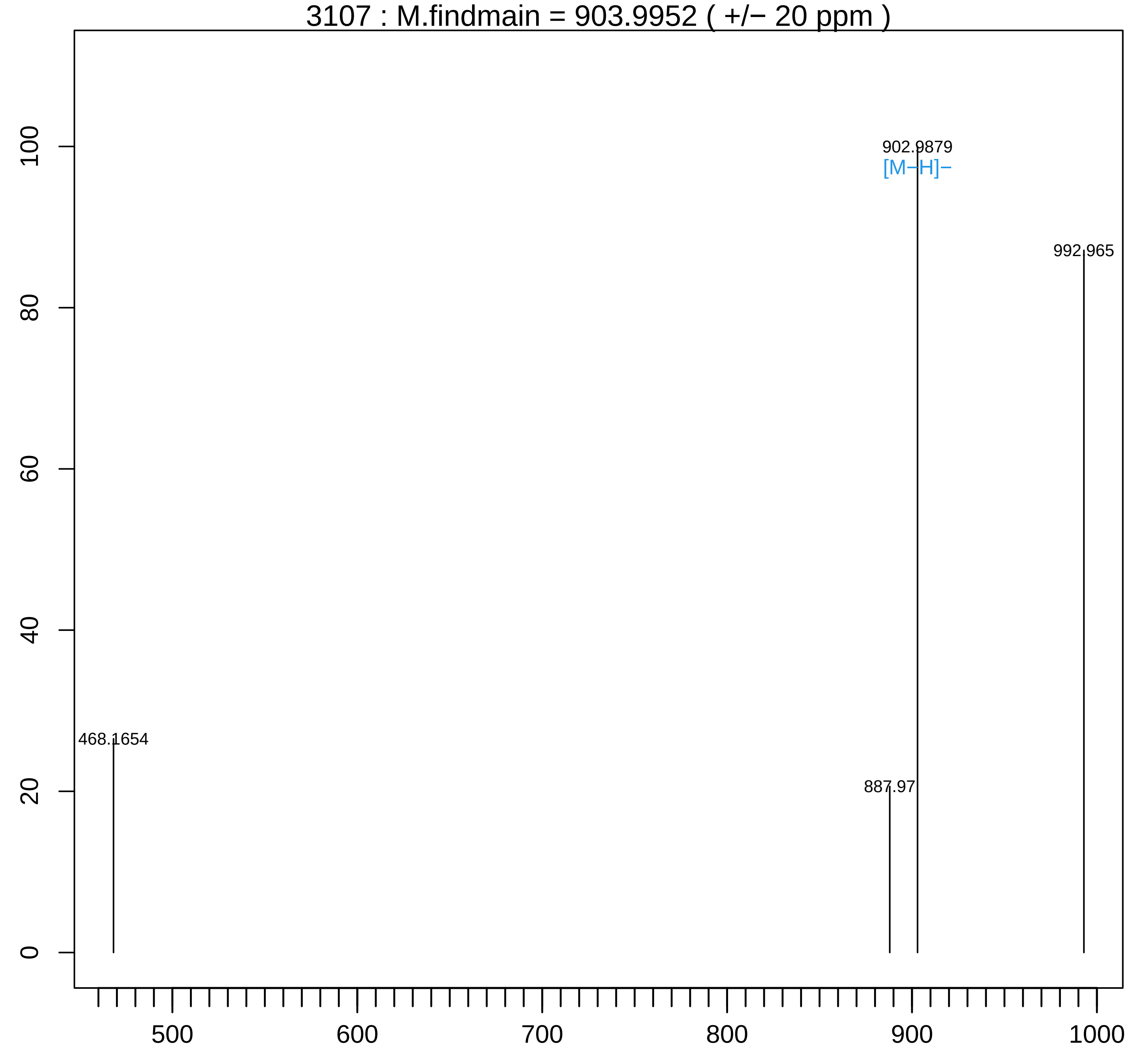
3107 : M.findmain = 903.9952 (+/− 20 ppm)
902.9879
[M−H]−
992.965
468.1654
887.97
0
20
40
60
80
100
500
600
700
800
900
1000

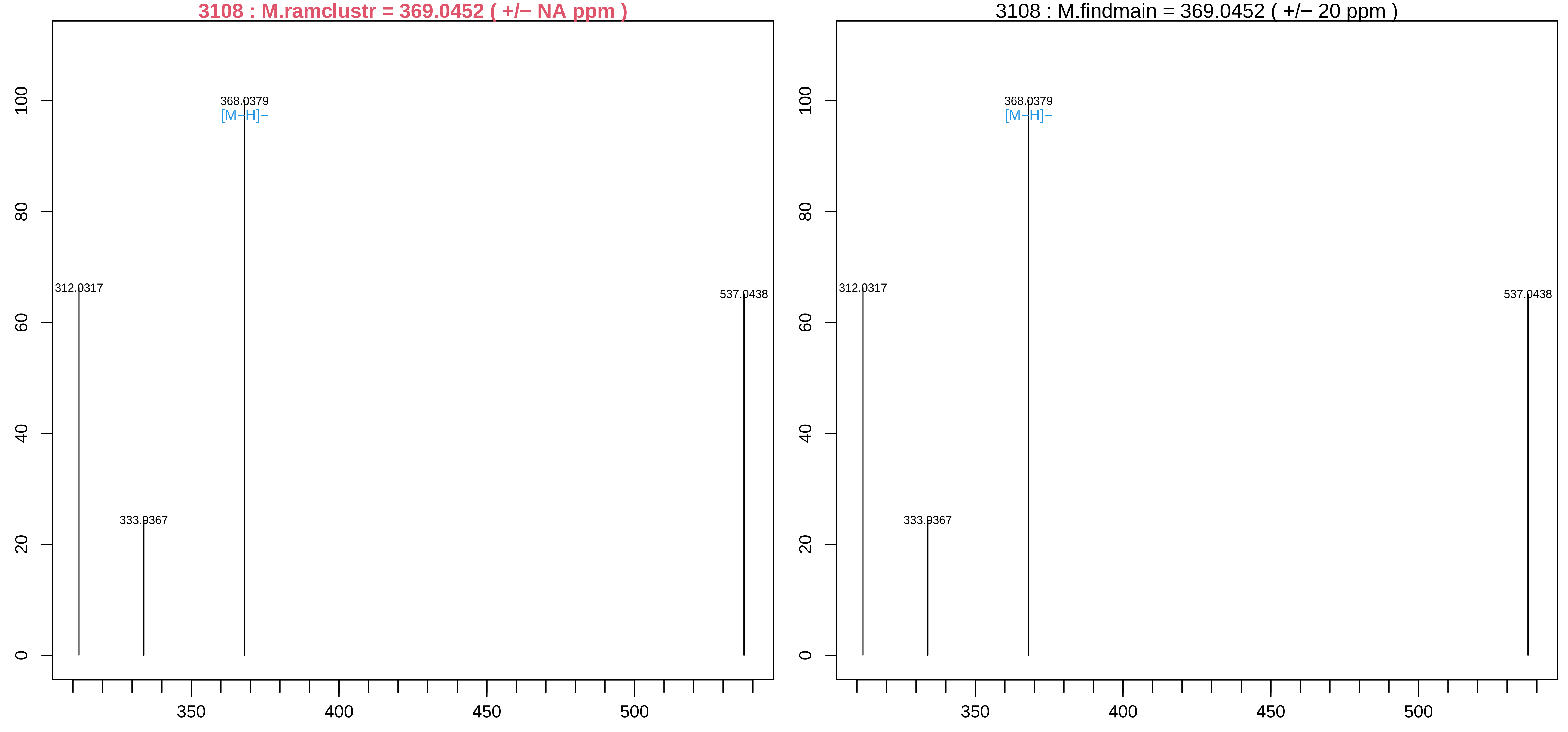

3108 : M.ramclustr = 369.0452 (+/− NA ppm)
312.0317
333.9367
368.0379
[M−H]−
537.0438
0
20
40
60
80
100
350
400
450
500
3108 : M.findmain = 369.0452 (+/− 20 ppm)
312.0317
333.9367
368.0379
[M−H]−
537.0438
0
20
40
60
80
100
350
400
450
500

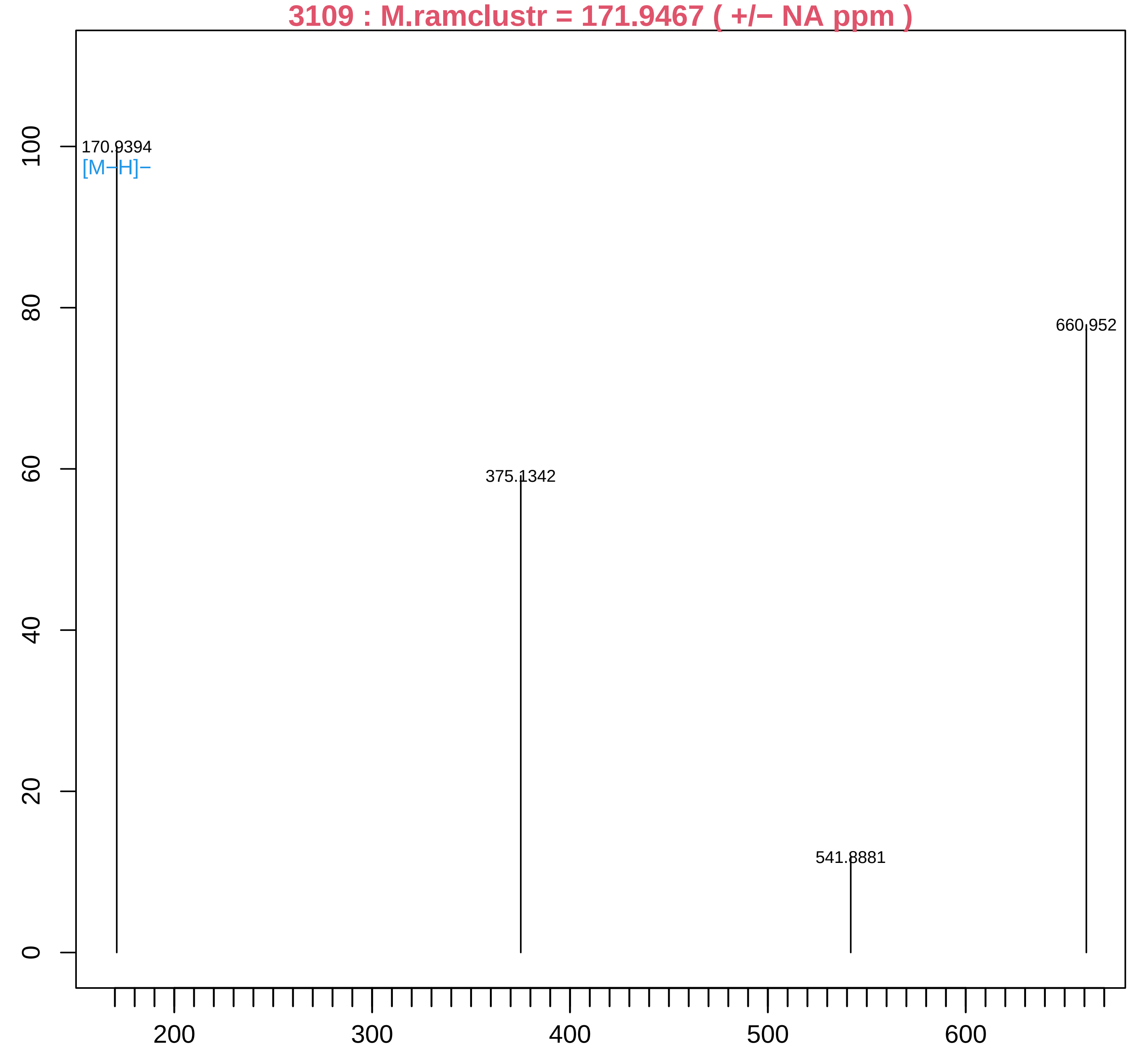
3109 : M.ramclustr = 171.9467 (+/− NA ppm)
170.9394
[M−H]−
375.1342
541.8881
660.952
0
20
40
60
80
100
200
300
400
500
600

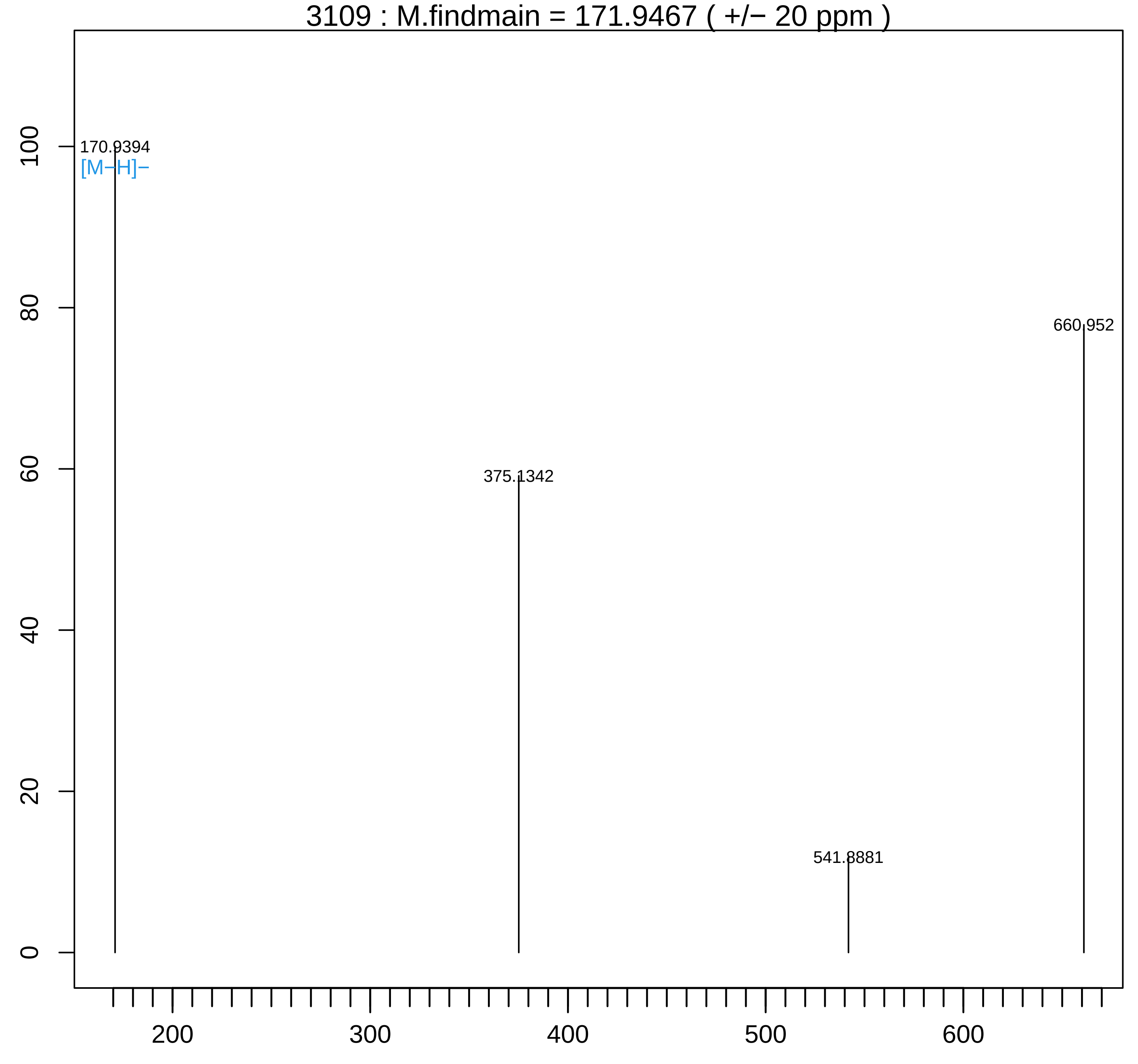
3109 : M.findmain = 171.9467 (+/− 20 ppm)
170.9394
[M−H]−
375.1342
541.8881
660.952
0
20
40
60
80
100
200
300
400
500
600

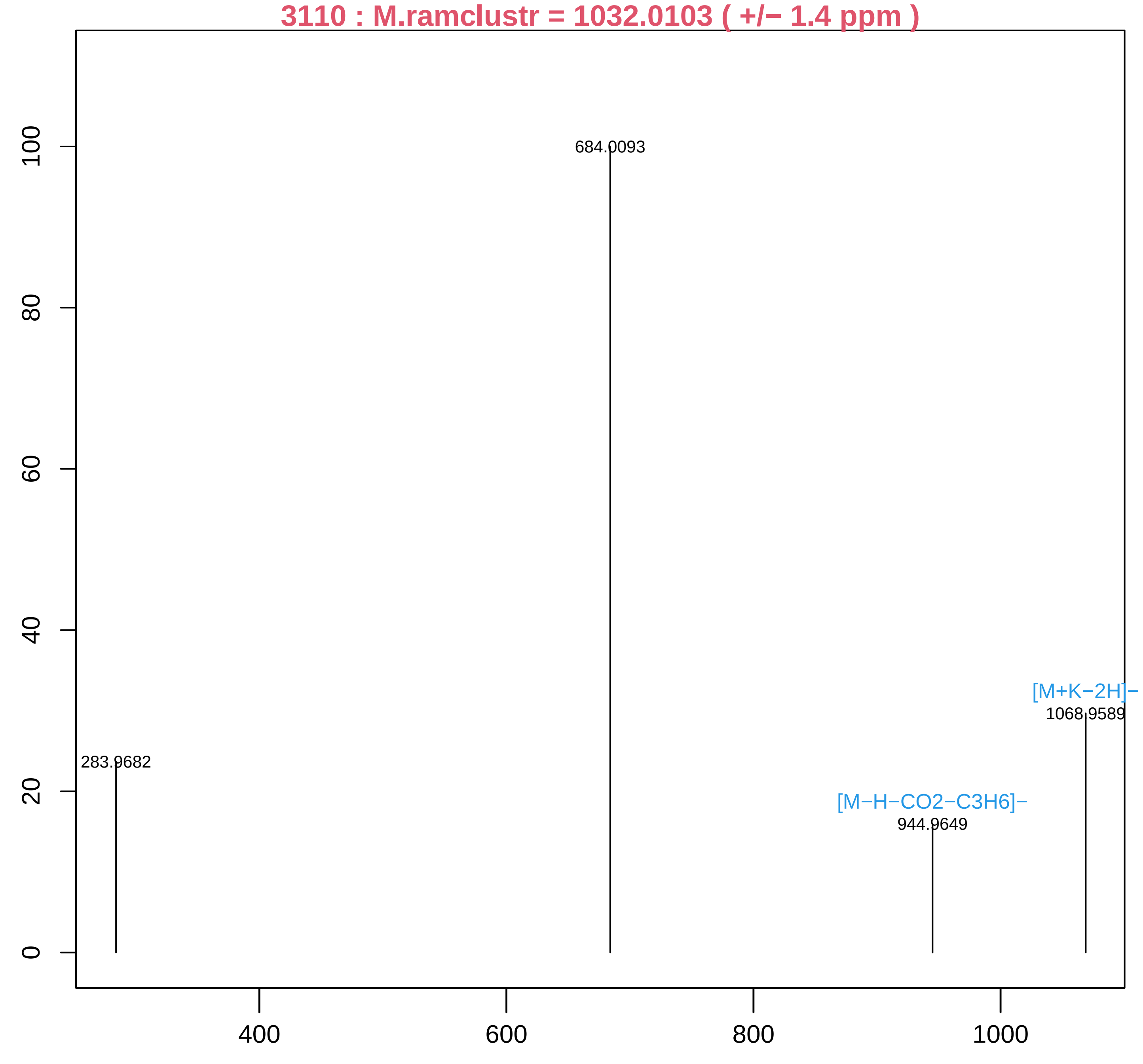

3110 : M.ramclustr = 1032.0103 (+/− 1.4 ppm)
684.0093
283.9682
[M−H−CO2−C3H6]−
944.9649
[M+K−2H]−
1068.9589
0
20
40
60
80
100
400
600
800
1000

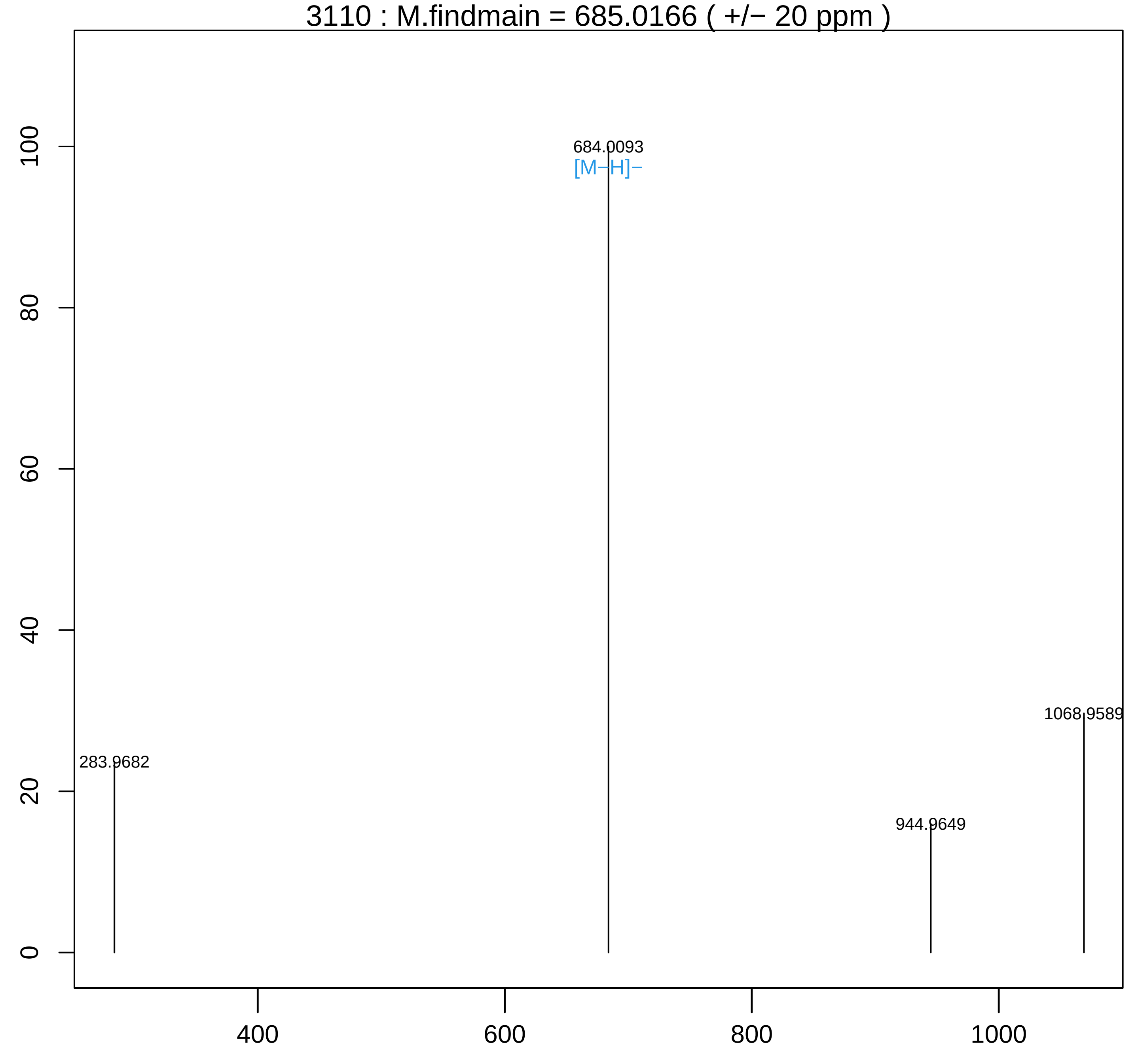

3110 : M.findmain = 685.0166 (+/− 20 ppm)
684.0093
[M−H]−
283.9682
944.9649
1068.9589
0
20
40
60
80
100
400
600
800
1000

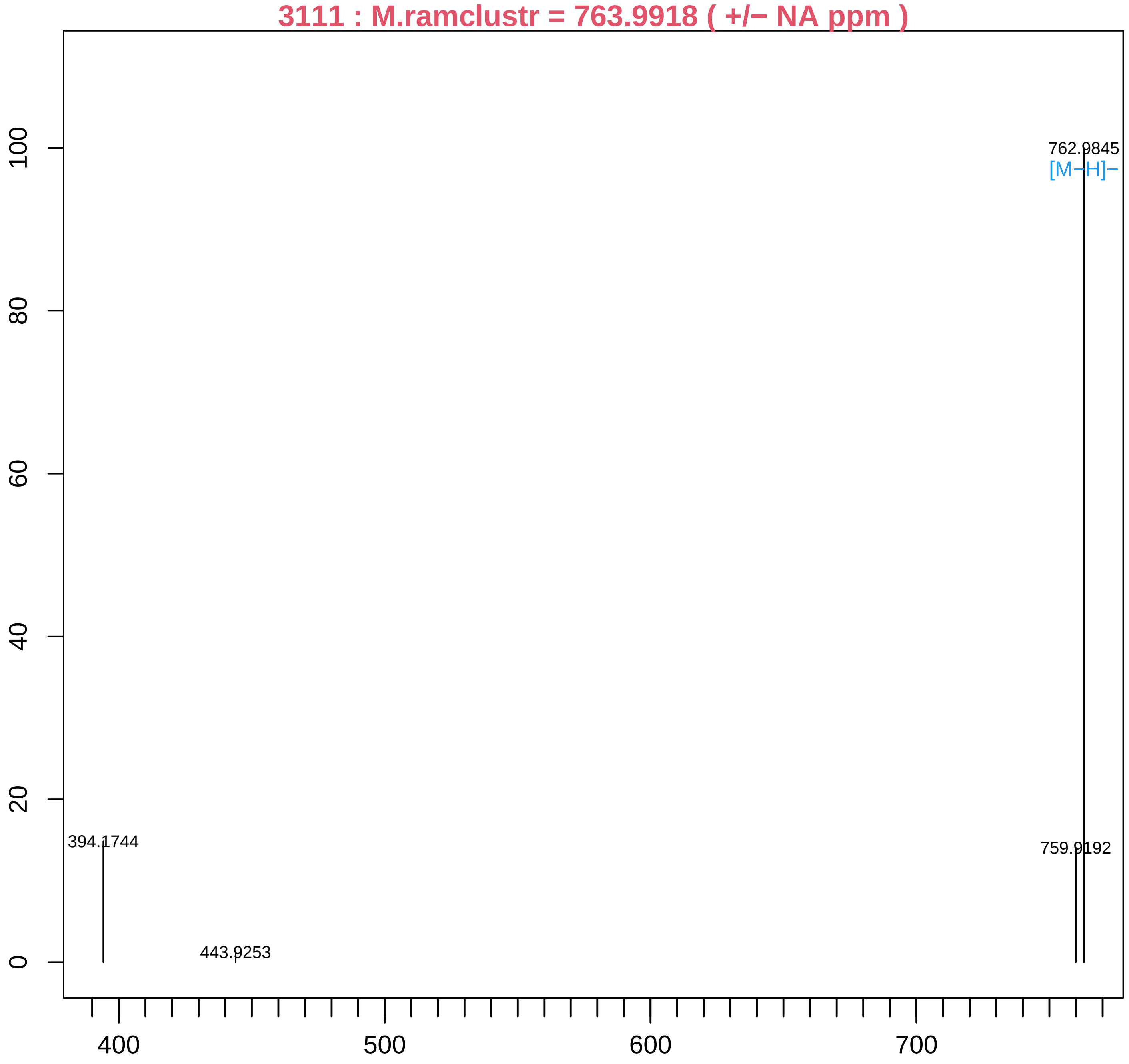
3111 : M.ramclustr = 763.9918 (+/− NA ppm)
100
80
60
40
20
0
400
500
600
700
762.9845
[M−H]−
394.1744
443.9253
759.9192
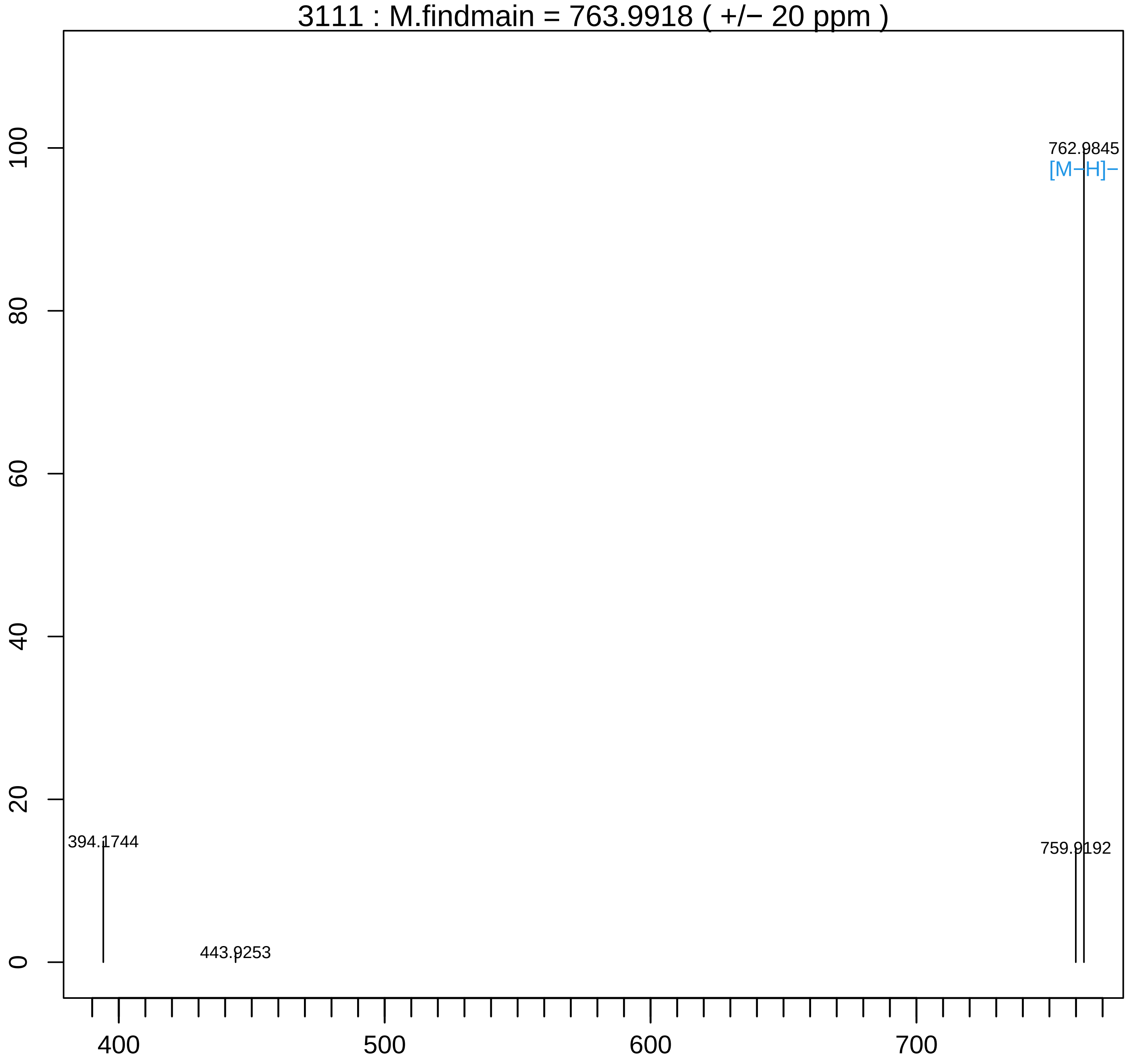
3111 : M.findmain = 763.9918 (+/− 20 ppm)
100
80
60
40
20
0
400
500
600
700
762.9845
[M−H]−
394.1744
443.9253
759.9192

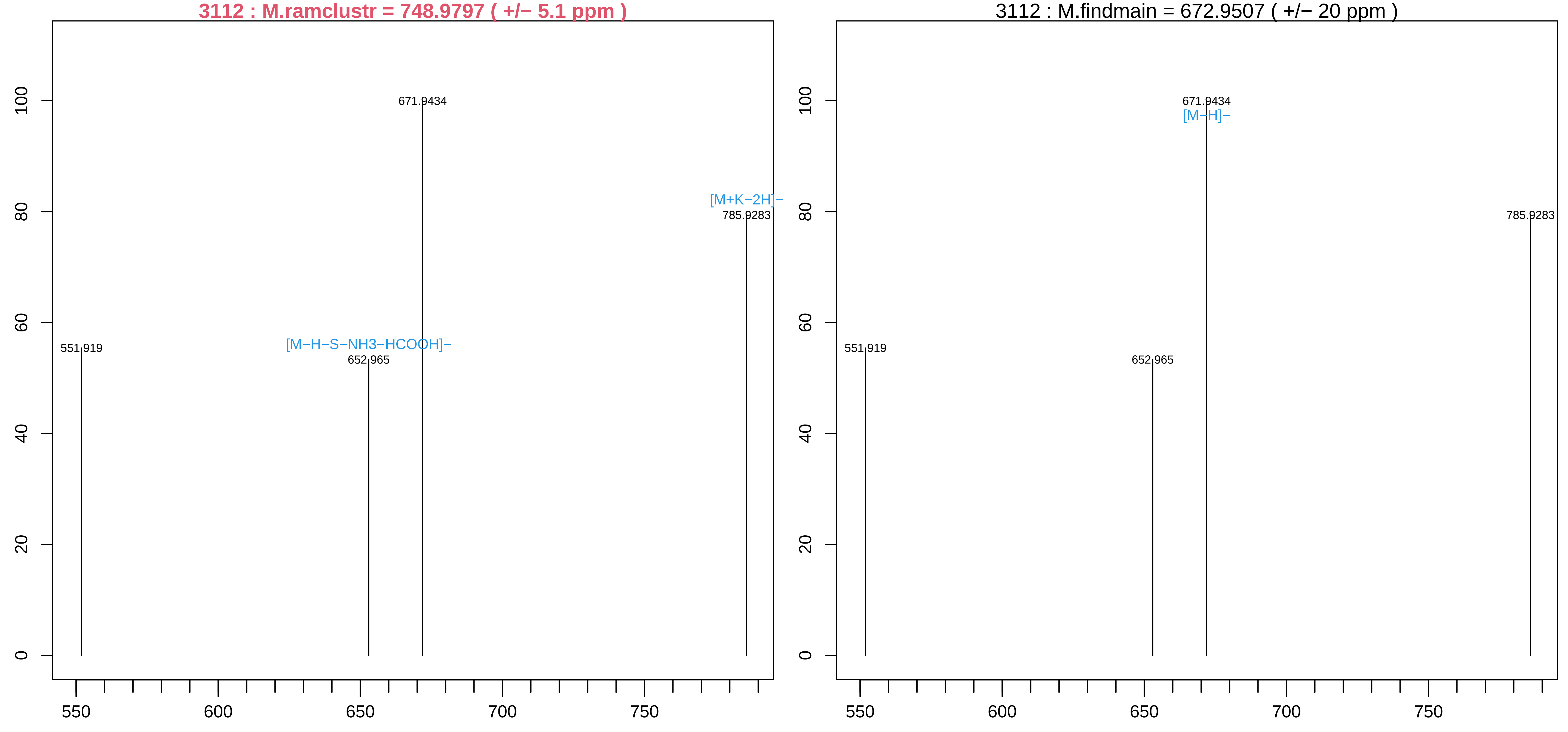
3112 : M.ramclustr = 748.9797 (+/− 5.1 ppm)
671.9434
[M+K−2H]−
785.9283
551.919
[M−H−S−NH3−HCOOH]−
652.965
3112 : M.findmain = 672.9507 (+/− 20 ppm)
671.9434
[M−H]−
785.9283
551.919
652.965
0
20
40
60
80
100
550
600
650
700
750

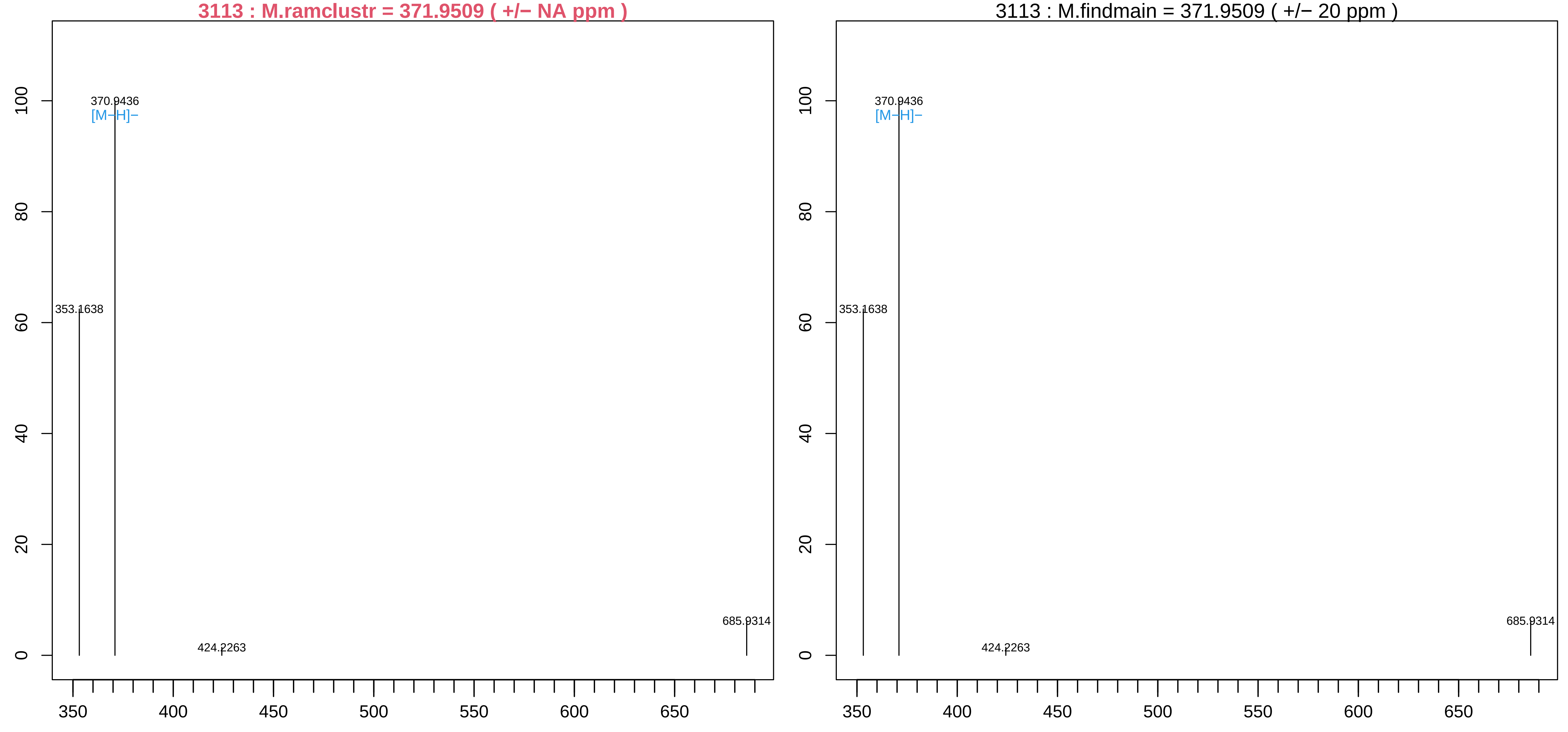
3113 : M.ramclustr = 371.9509 (+/− NA ppm)
370.9436
[M−H]−
353.1638
424.2263
685.9314
3113 : M.findmain = 371.9509 (+/− 20 ppm)
370.9436
[M−H]−
353.1638
424.2263
685.9314
0
20
40
60
80
100
350
400
450
500
550
600
650

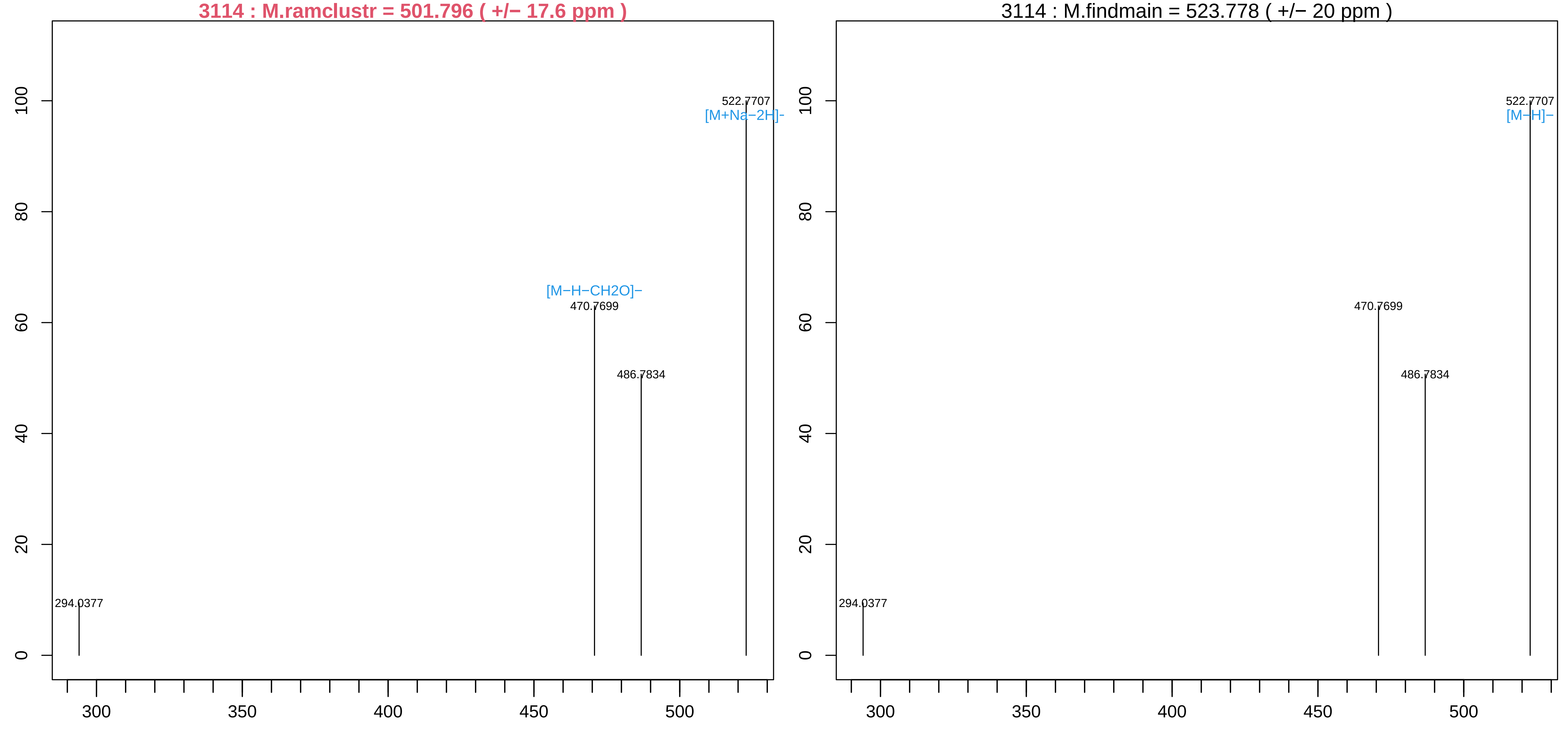

3114 : M.ramclustr = 501.796 (+/− 17.6 ppm)
522.7707
[M+Na−2H]-
[M−H−CH2O]−
470.7699
486.7834
294.0377
3114 : M.findmain = 523.778 (+/− 20 ppm)
522.7707
[M−H]−
470.7699
486.7834
294.0377
0
20
40
60
80
100
300
350
400
450
500

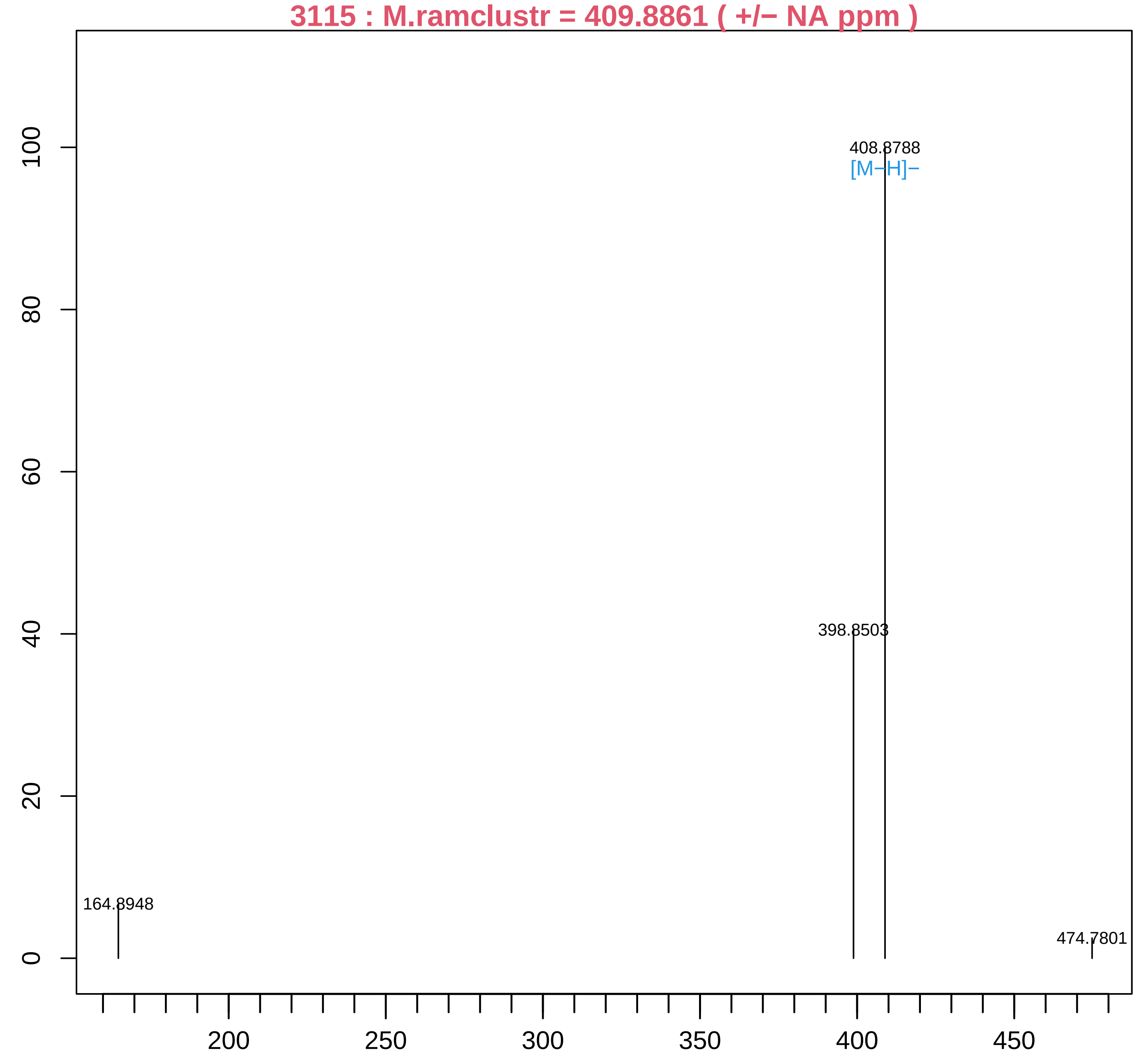
3115 : M.ramclustr = 409.8861 (+/− NA ppm)
408.8788
[M−H]−
398.8503
164.8948
474.7801
0
20
40
60
80
100
200
250
300
350
400
450

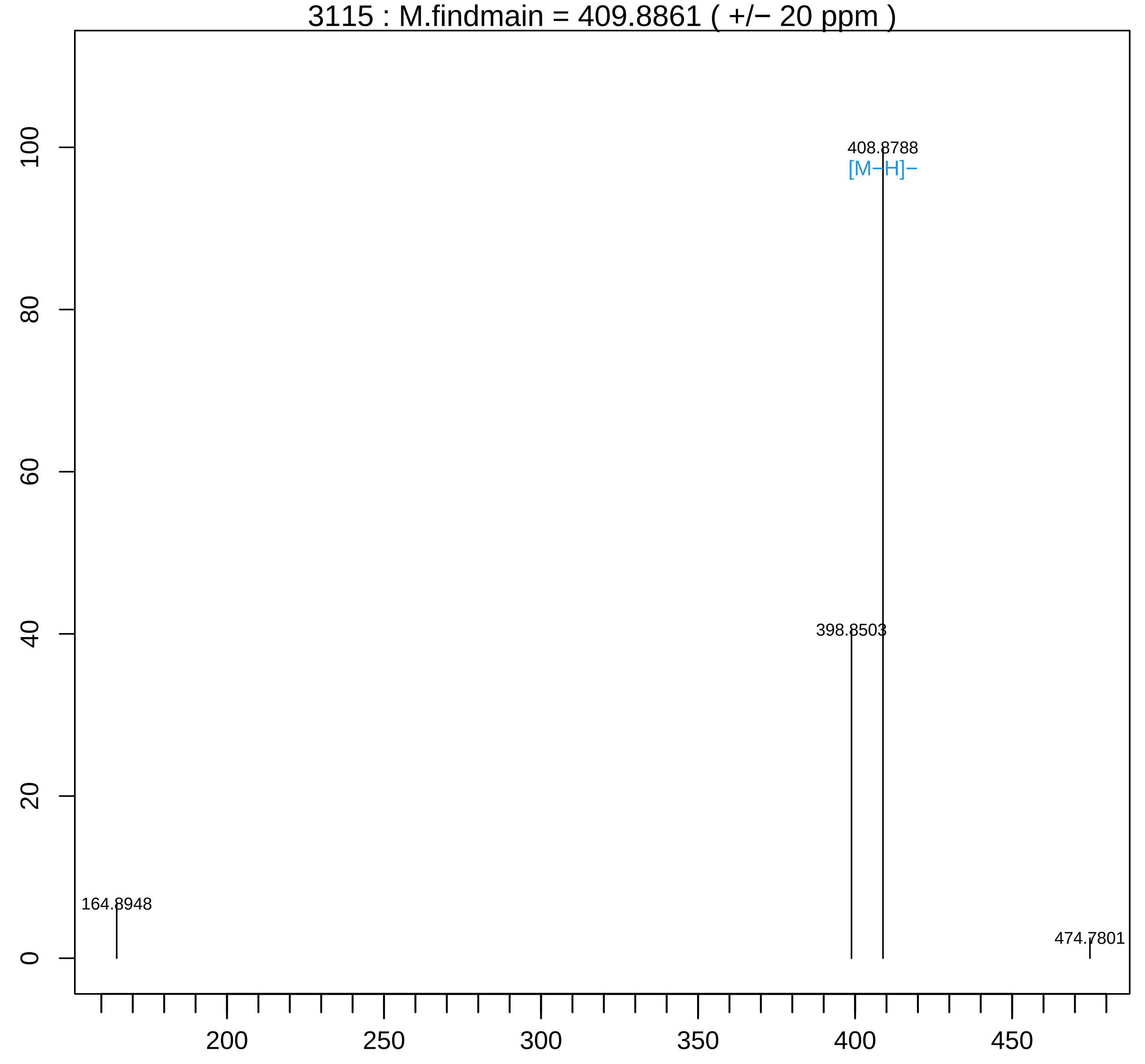
3115 : M.findmain = 409.8861 (+/− 20 ppm)
408.8788
[M−H]−
398.8503
164.8948
474.7801
0
20
40
60
80
100
200
250
300
350
400
450

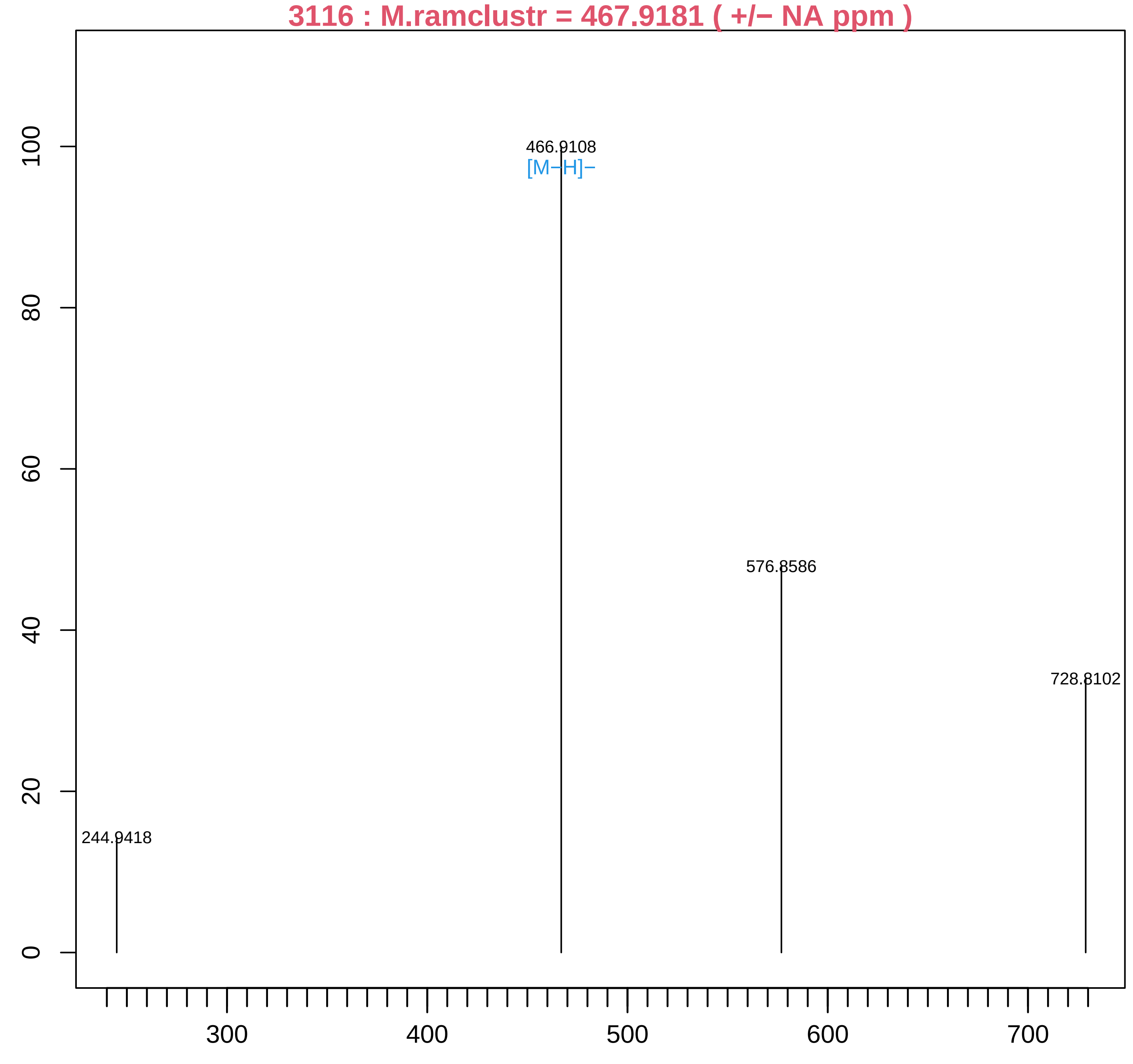
3116 : M.ramclustr = 467.9181 (+/− NA ppm)
466.9108
[M−H]−
576.8586
728.8102
244.9418
0
20
40
60
80
100
300
400
500
600
700

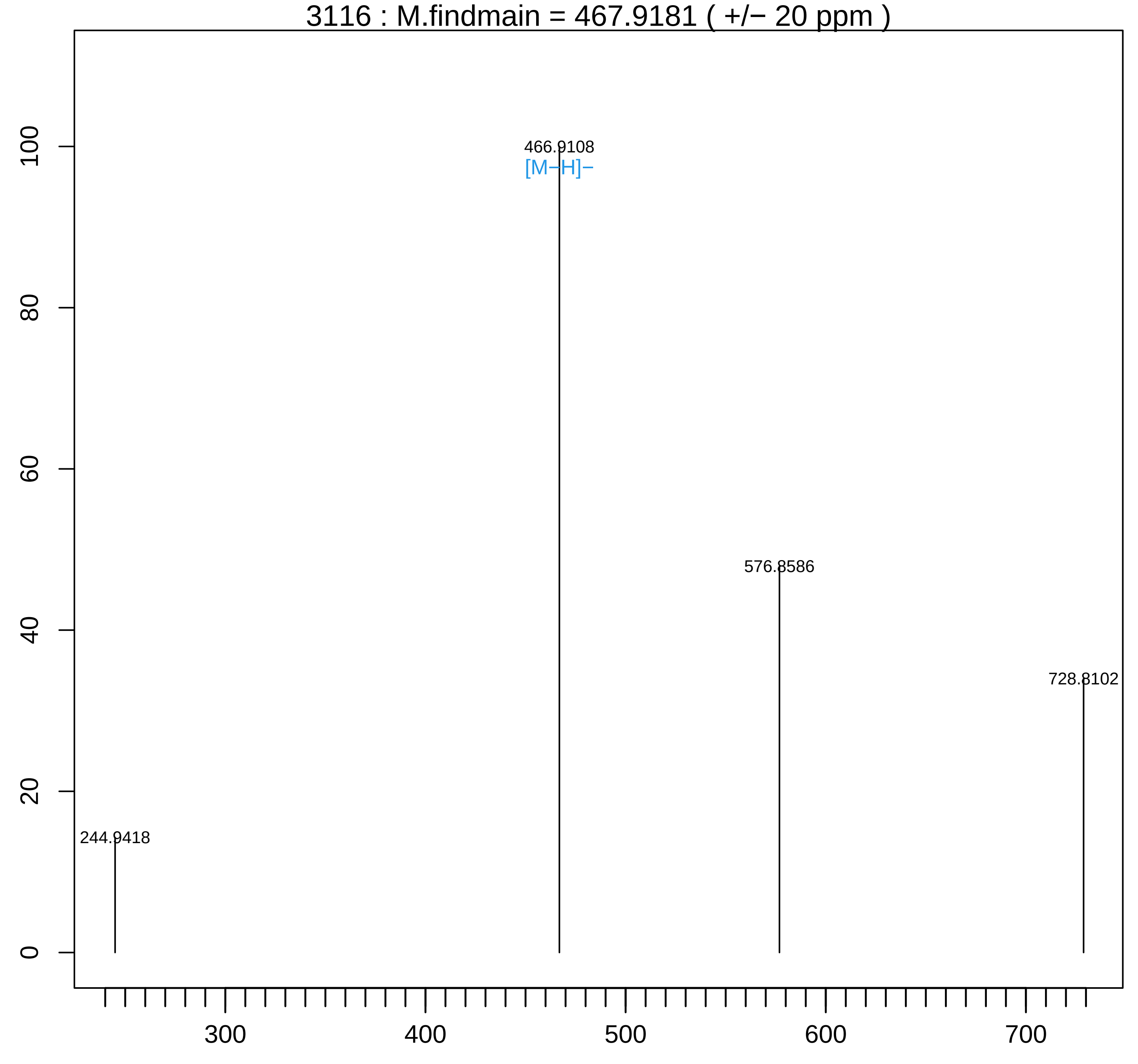
3116 : M.findmain = 467.9181 (+/− 20 ppm)
466.9108
[M−H]−
576.8586
728.8102
244.9418
0
20
40
60
80
100
300
400
500
600
700

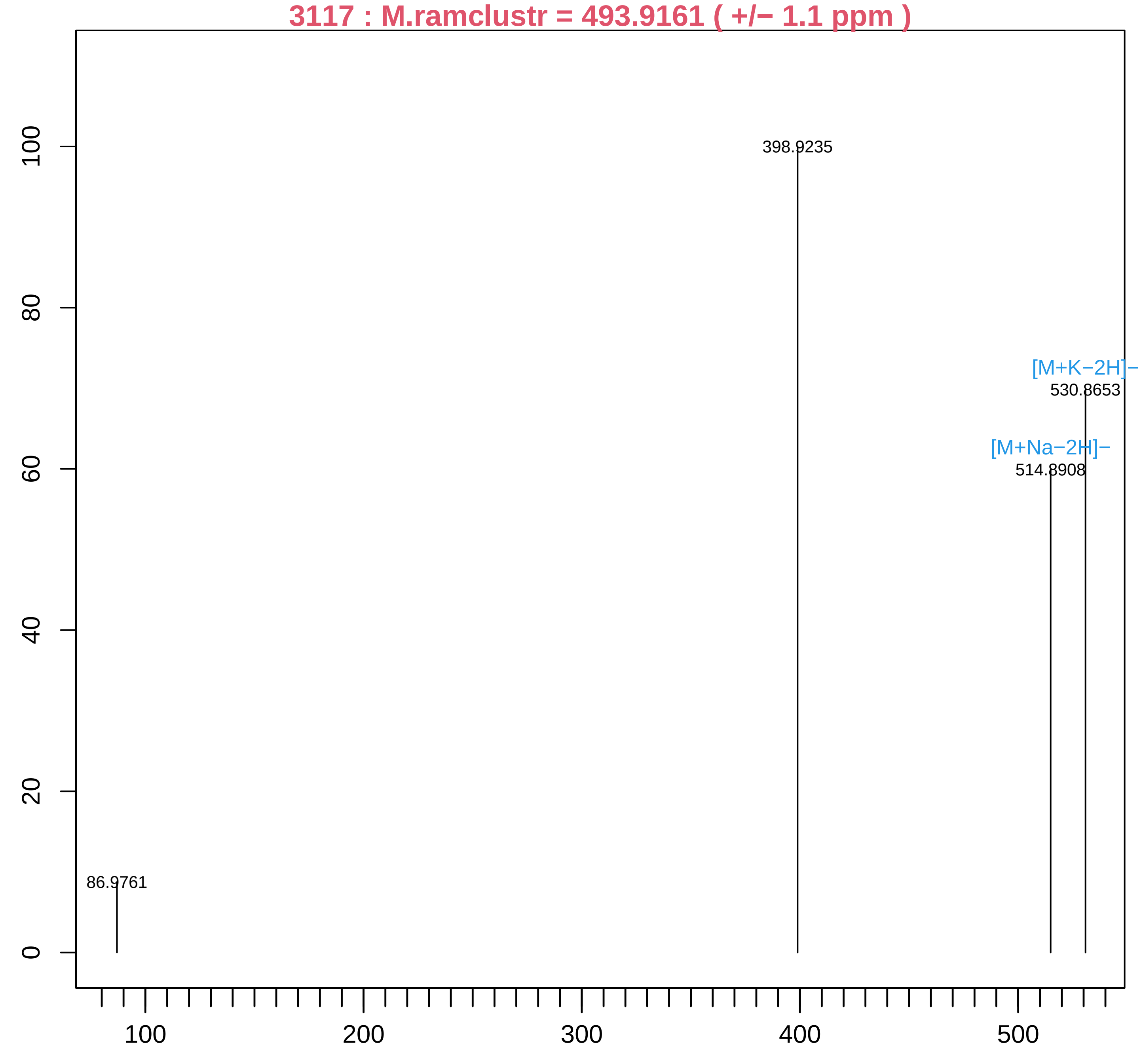

3117 : M.ramclustr = 493.9161 (+/− 1.1 ppm)
398.9235
[M+K−2H]−
530.8653
[M+Na−2H]−
514.8908
86.9761
0
20
40
60
80
100
100
200
300
400
500

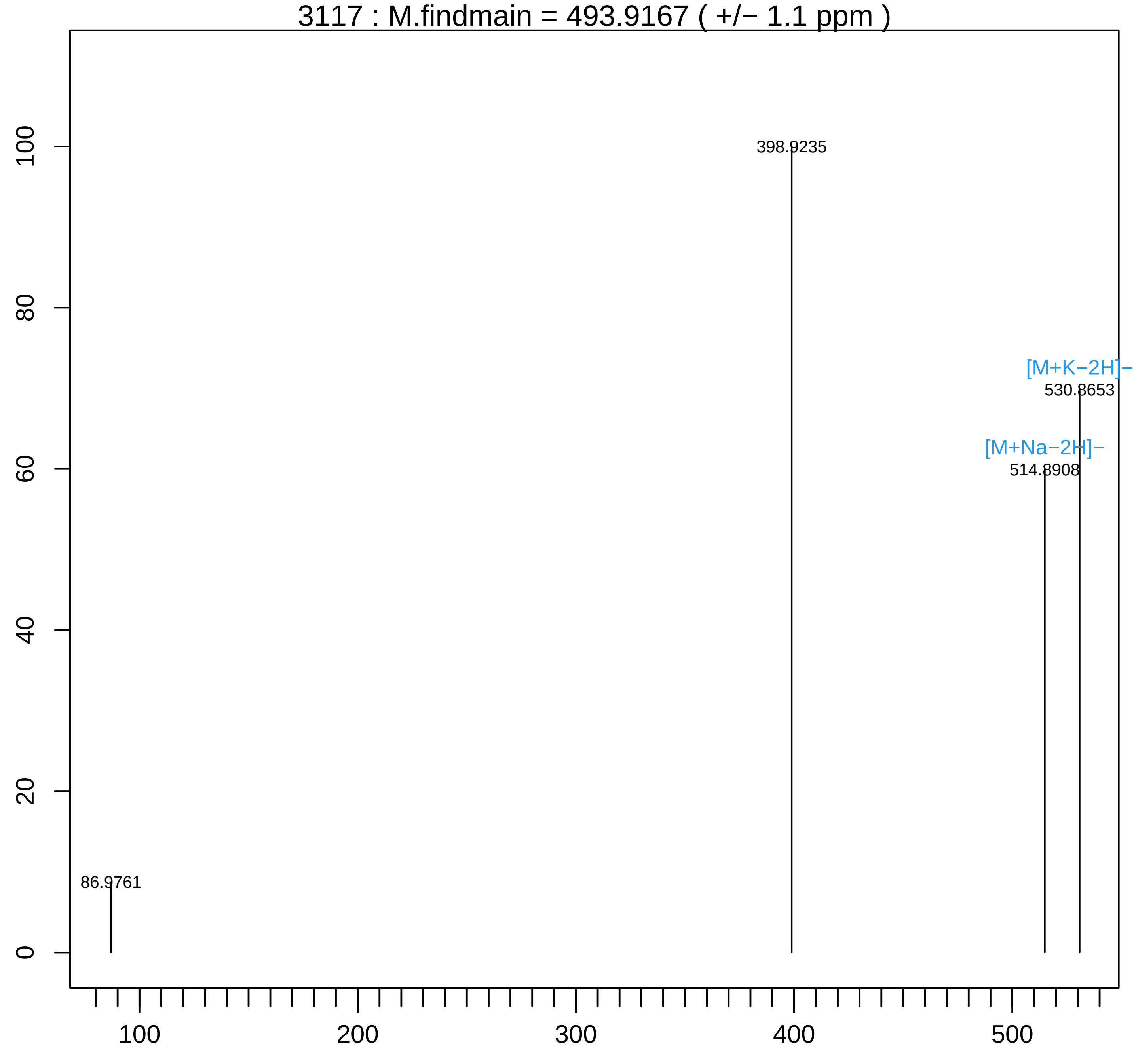

3117 : M.findmain = 493.9167 (+/− 1.1 ppm)
398.9235
[M+K−2H]−
530.8653
[M+Na−2H]−
514.8908
86.9761
0
20
40
60
80
100
100
200
300
400
500

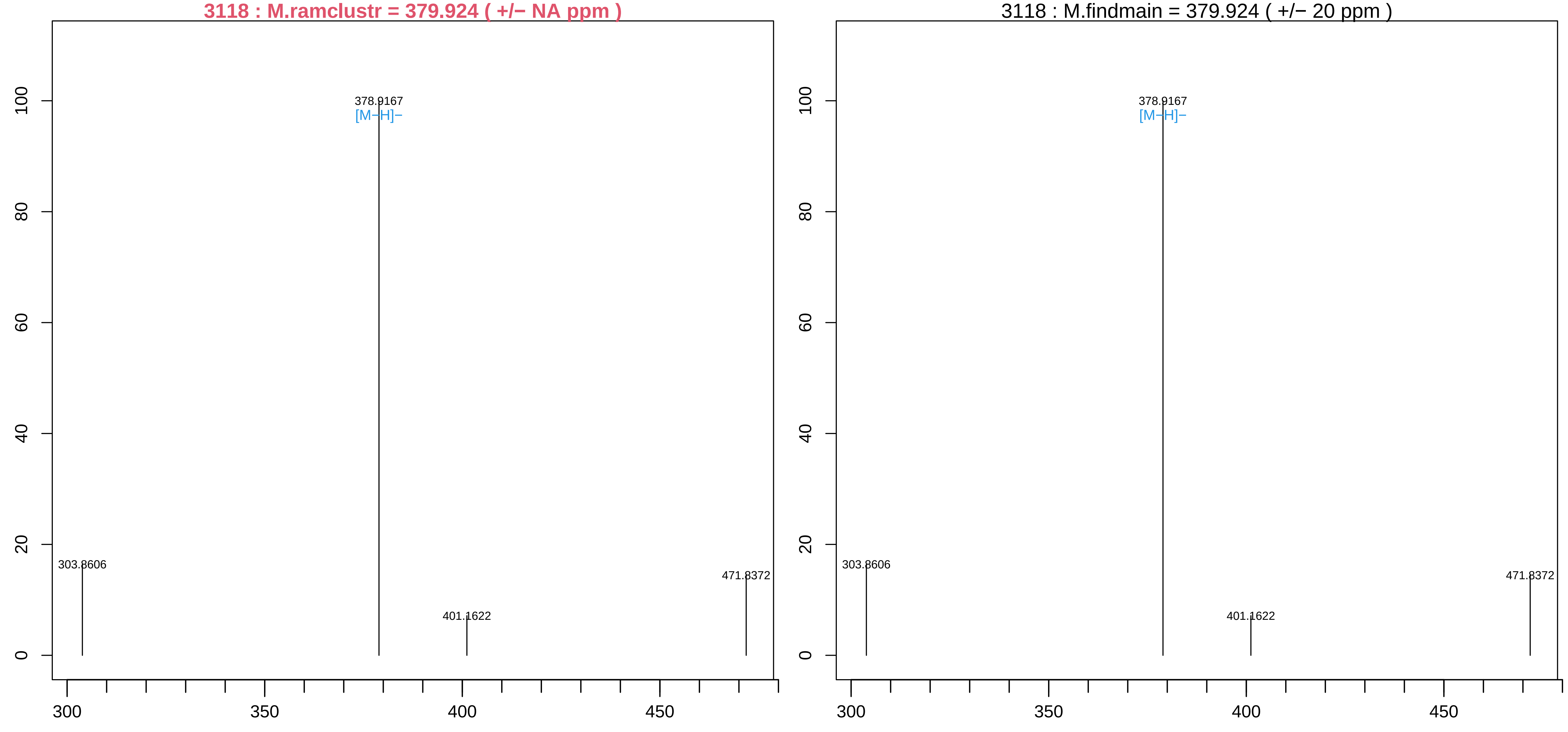

3118 : M.ramclustr = 379.924 (+/− NA ppm)
3118 : M.findmain = 379.924 (+/− 20 ppm)
378.9167
[M−H]−
303.8606
401.1622
471.8372
0
20
40
60
80
100
300
350
400
450

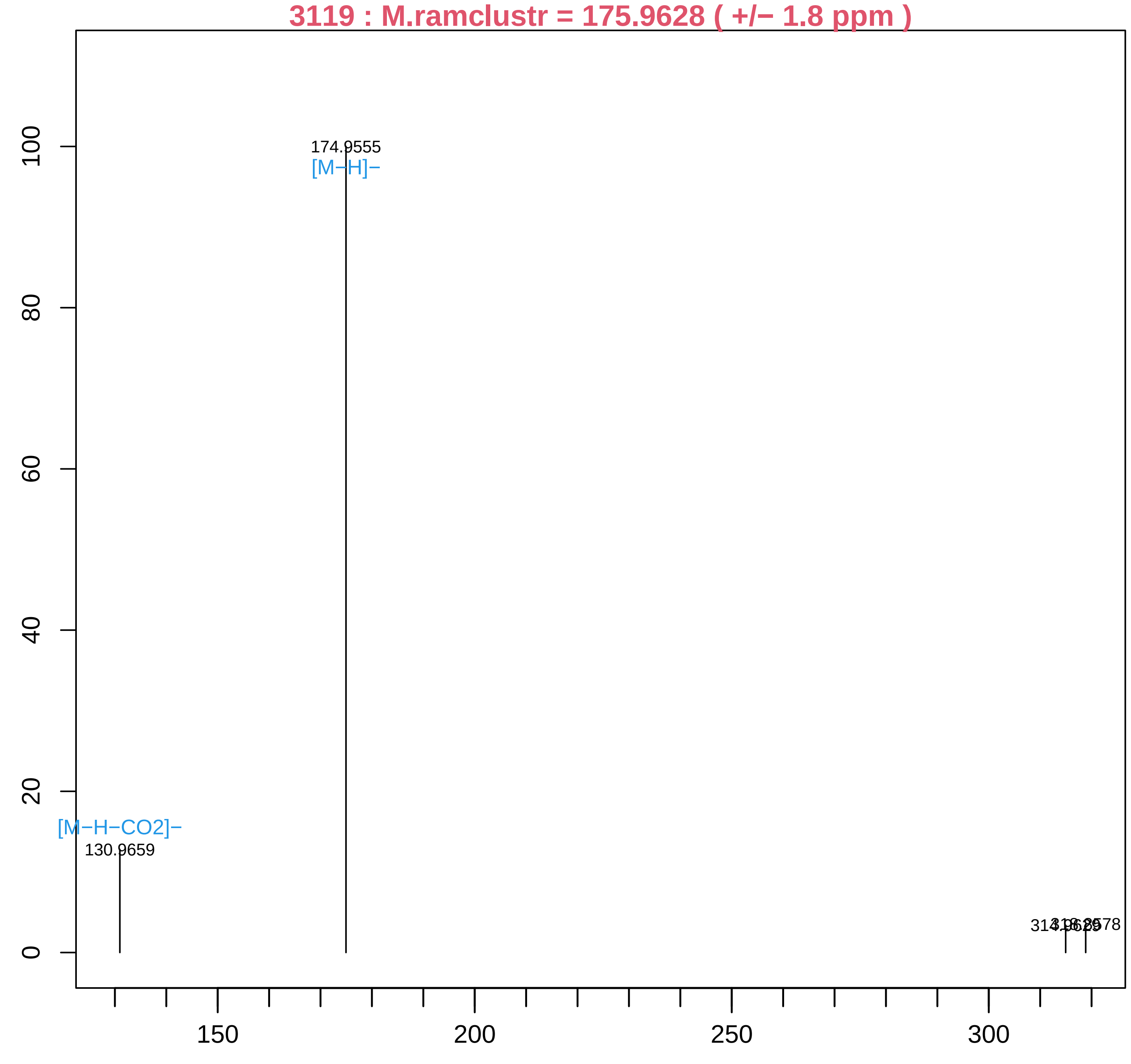
3119 : M.ramclustr = 175.9628 (+/− 1.8 ppm)
174.9555
[M−H]−
[M−H−CO2]−
130.9659
0
20
40
60
80
100
150
200
250
300

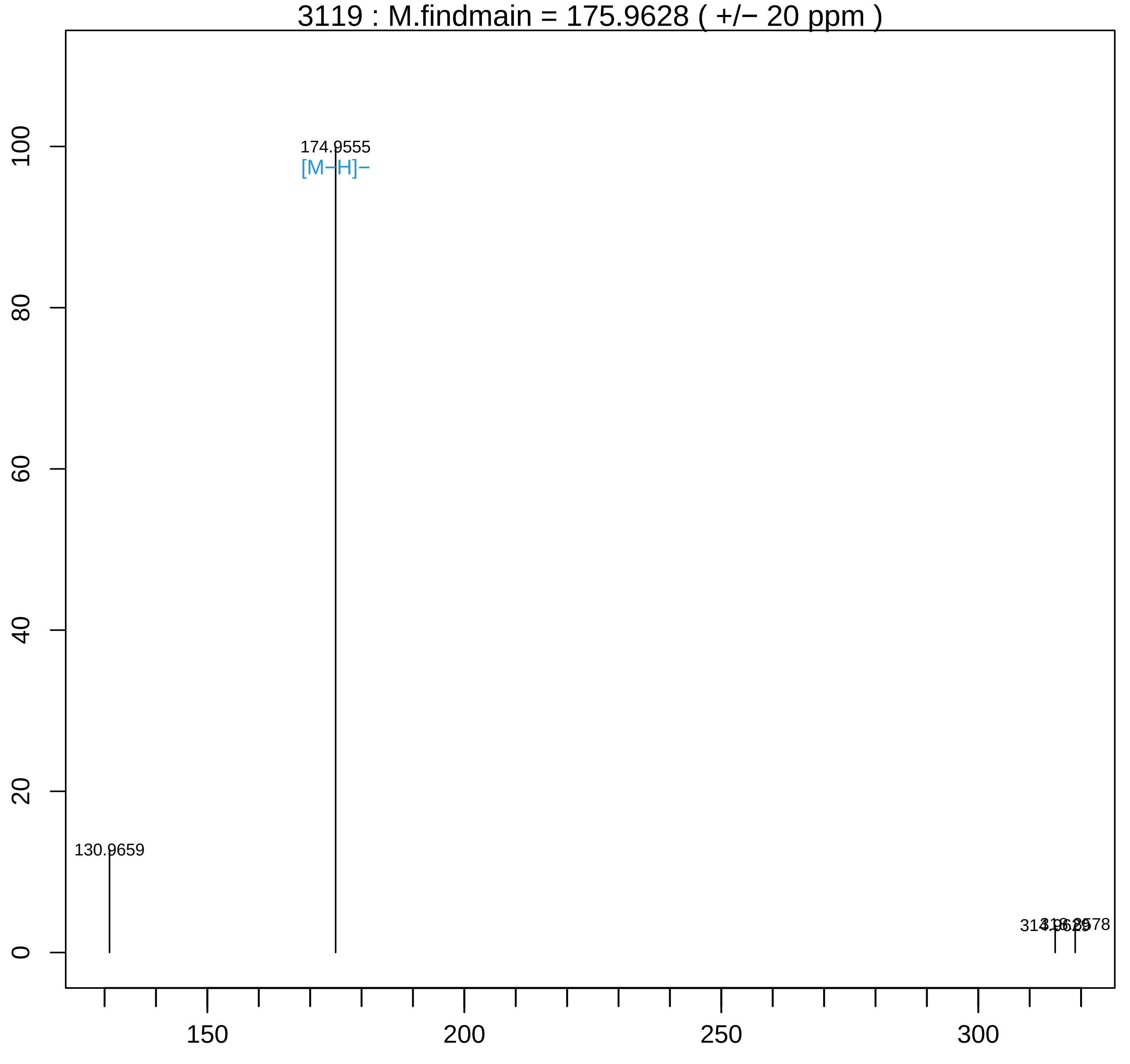
3119 : M.findmain = 175.9628 (+/− 20 ppm)
174.9555
[M−H]−
130.9659
0
20
40
60
80
100
150
200
250
300

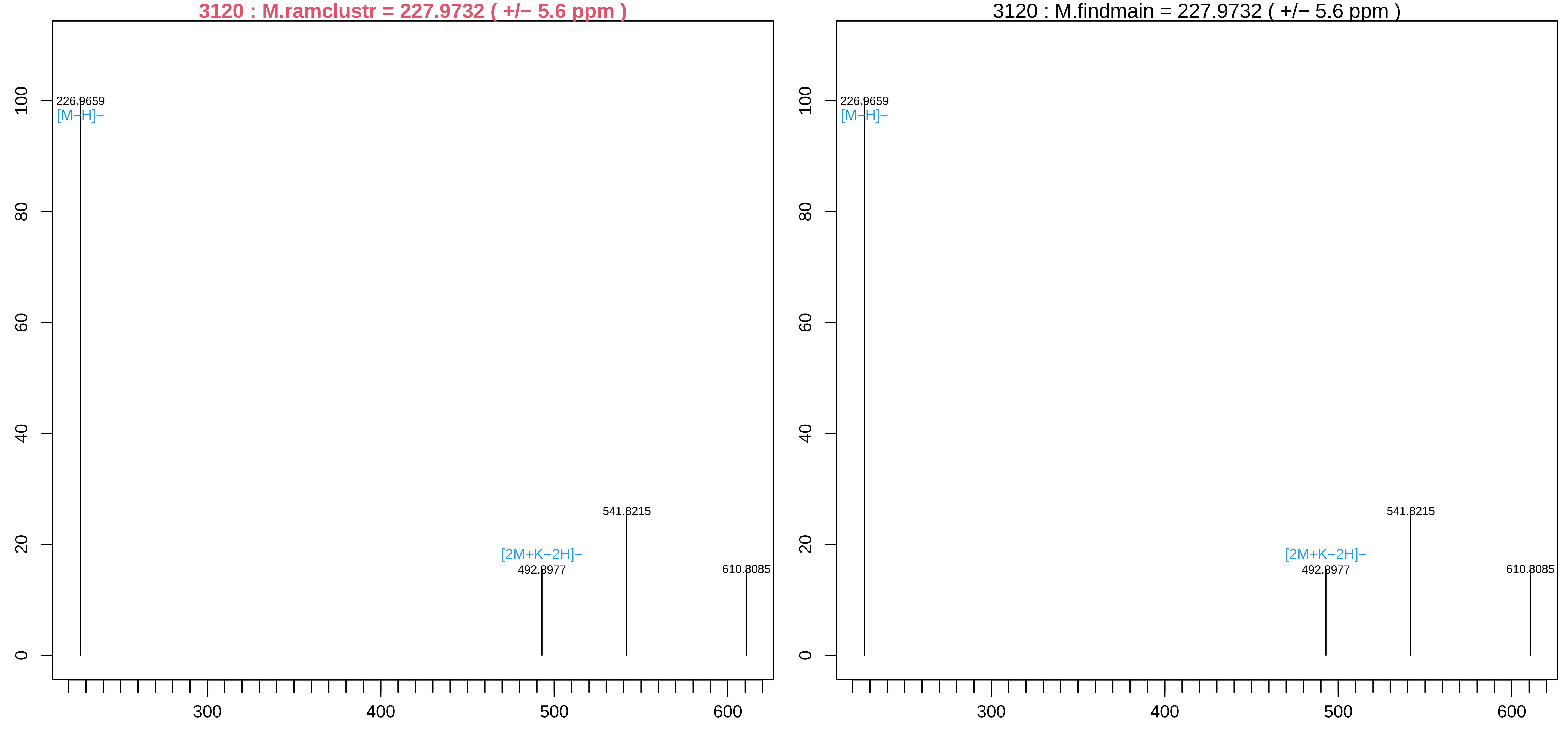

3120 : M.ramclustr = 227.9732 (+/− 5.6 ppm)
226.9659
[M−H]−
[2M+K−2H]−
492.8977
541.8215
610.8085
3120 : M.findmain = 227.9732 (+/− 5.6 ppm)
226.9659
[M−H]−
[2M+K−2H]−
492.8977
541.8215
610.8085

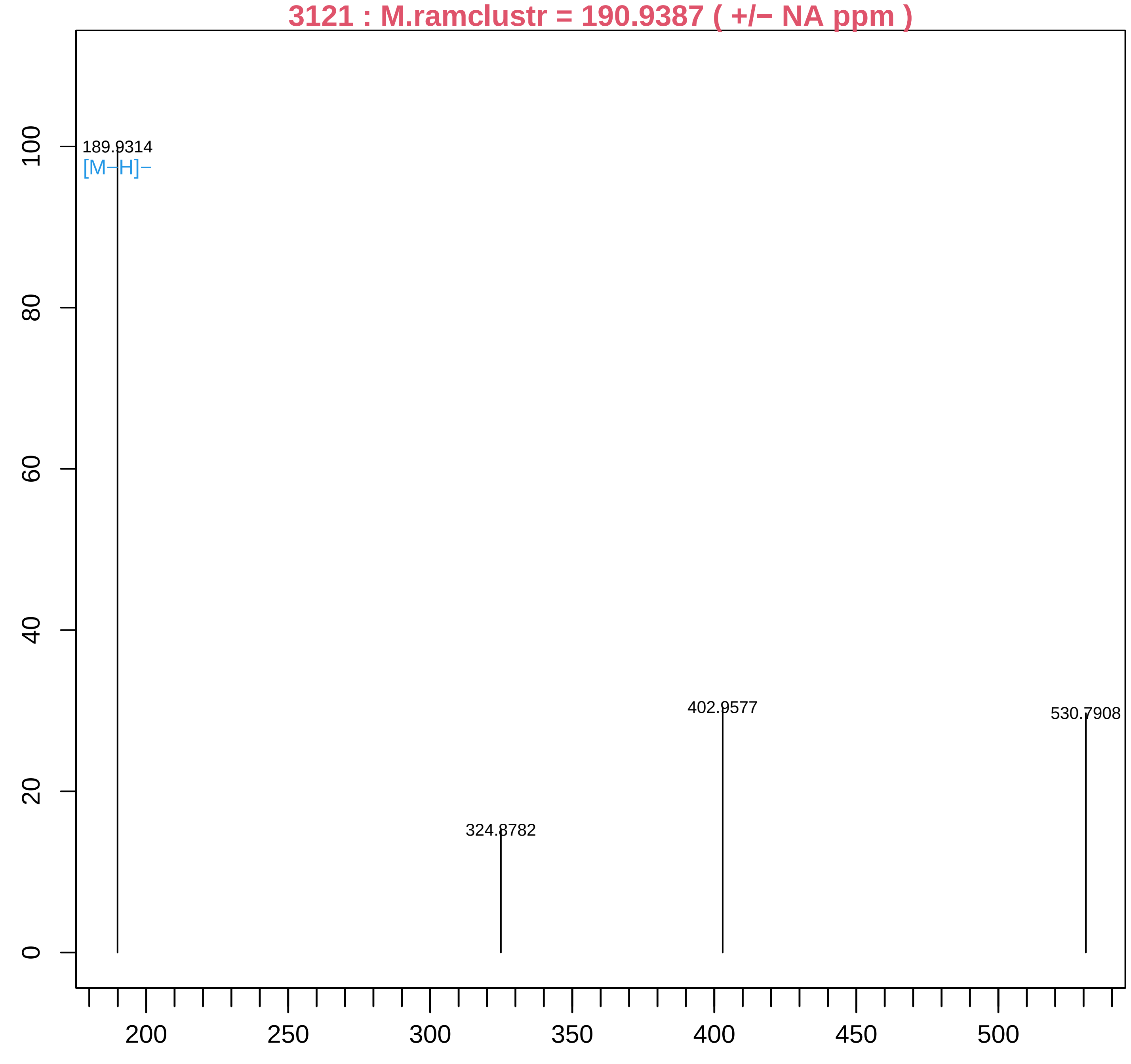
3121 : M.ramclustr = 190.9387 (+/− NA ppm)
189.9314
[M−H]−
324.8782
402.9577
530.7908
0
20
40
60
80
100
200
250
300
350
400
450
500

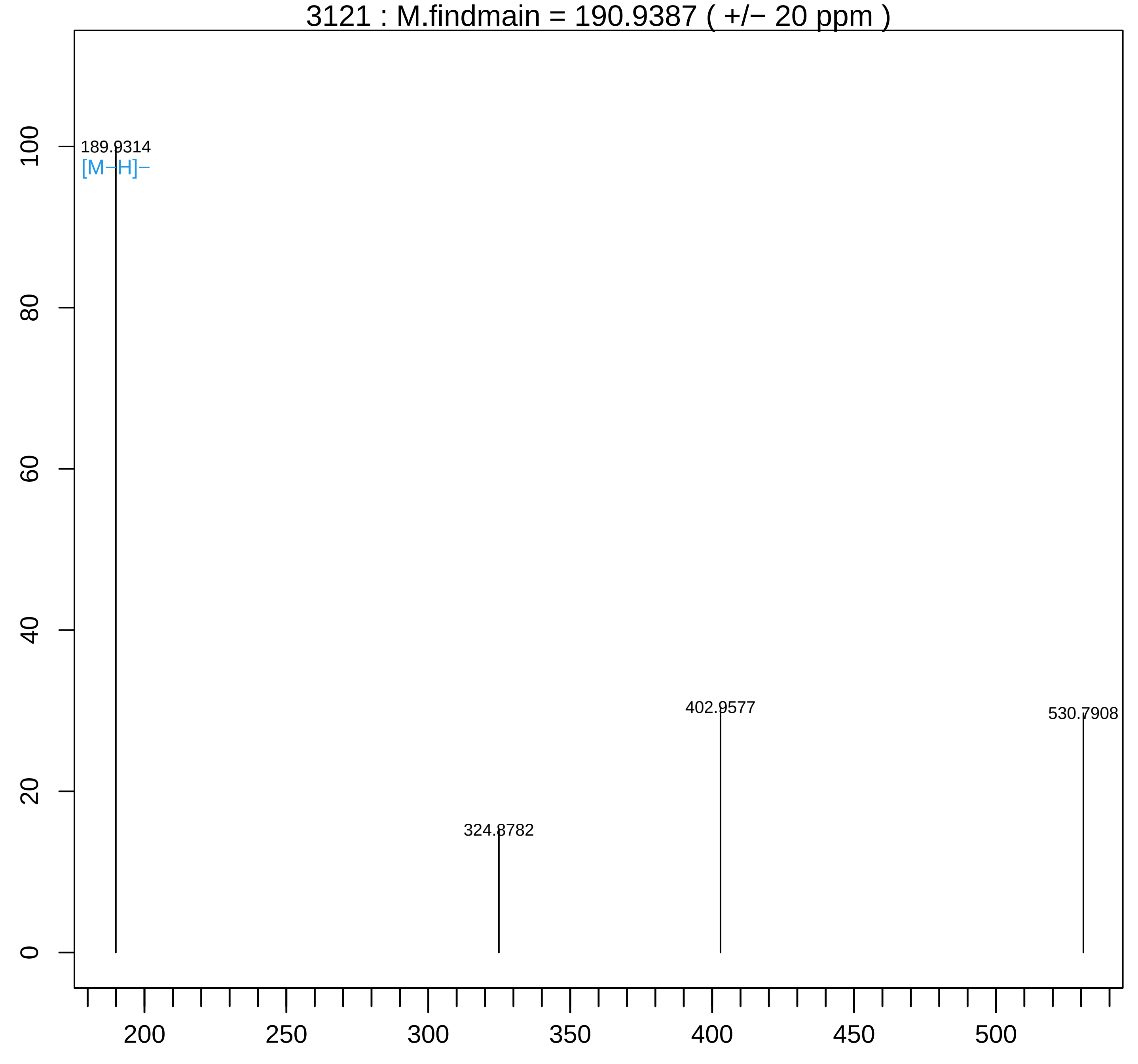
3121 : M.findmain = 190.9387 (+/− 20 ppm)
189.9314
[M−H]−
324.8782
402.9577
530.7908
0
20
40
60
80
100
200
250
300
350
400
450
500

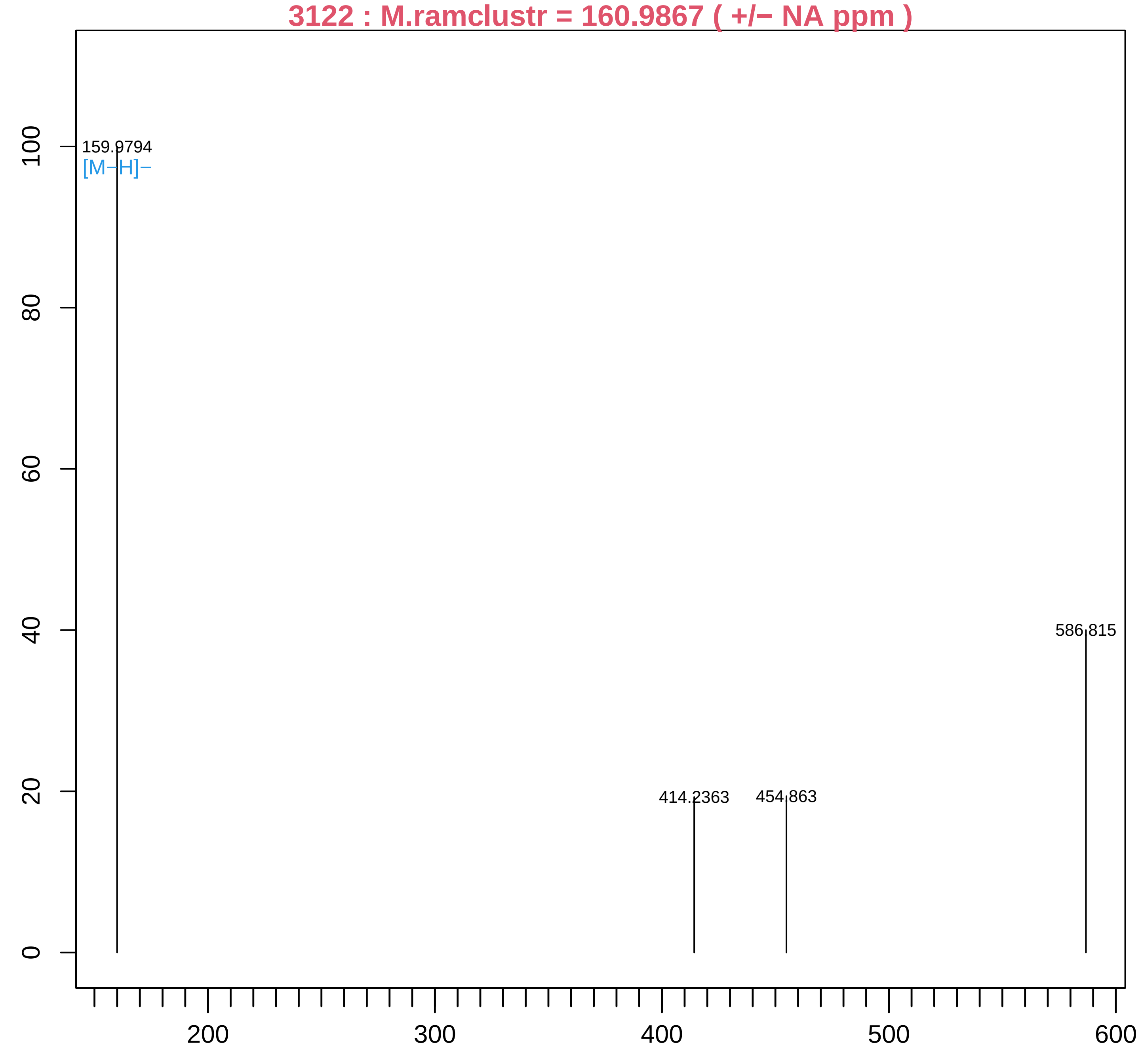
3122 : M.ramclustr = 160.9867 (+/− NA ppm)
159.9794
[M−H]−
414.2363
454.863
586.815
0
20
40
60
80
100
200
300
400
500
600

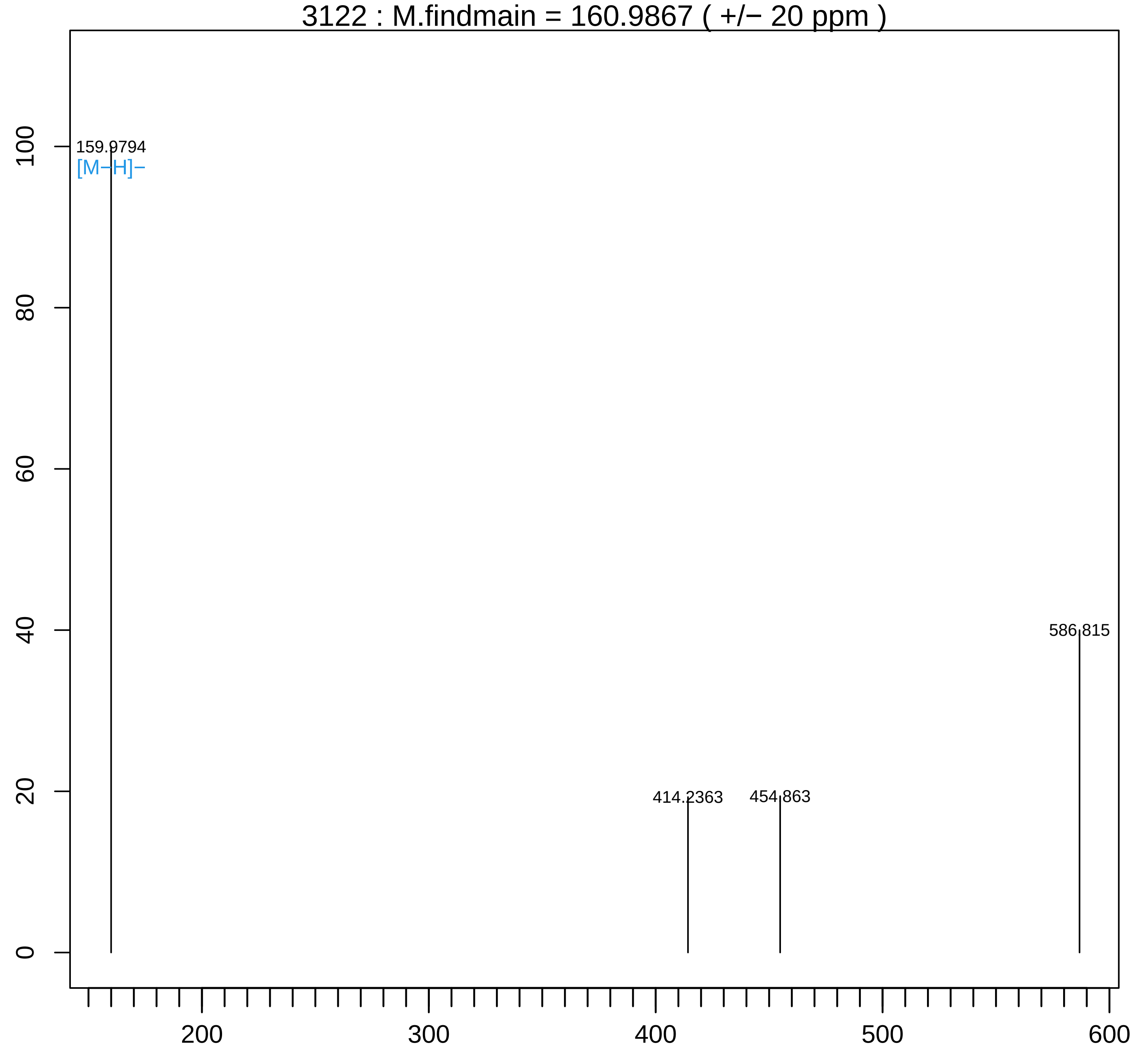
3122 : M.findmain = 160.9867 (+/− 20 ppm)
159.9794
[M−H]−
414.2363
454.863
586.815
0
20
40
60
80
100
200
300
400
500
600

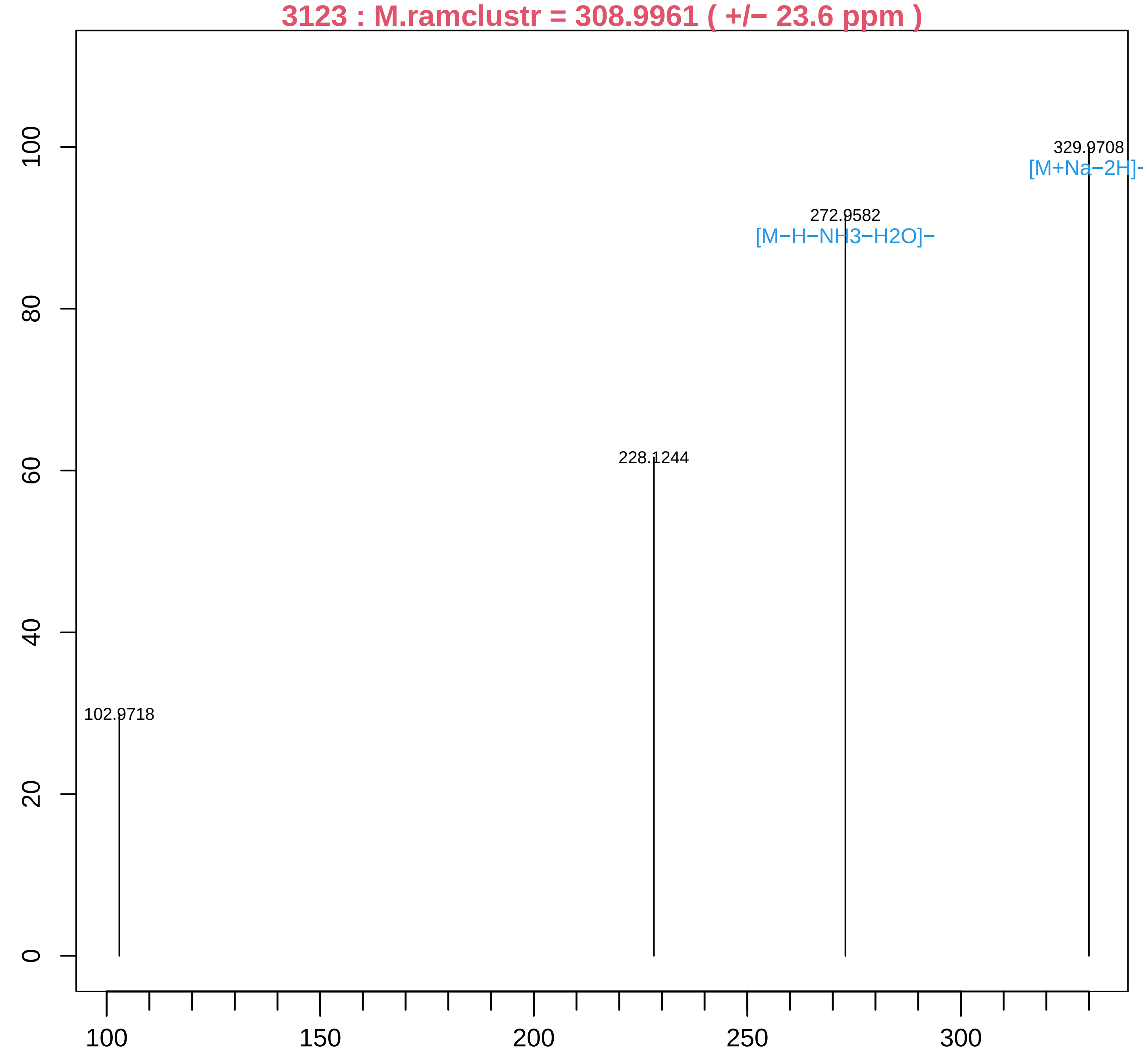

3123 : M.ramclustr = 308.9961 (+/− 23.6 ppm)
329.9708
[M+Na−2H]-
272.9582
[M−H−NH3−H2O]−
228.1244
102.9718
0
20
40
60
80
100
100
150
200
250
300

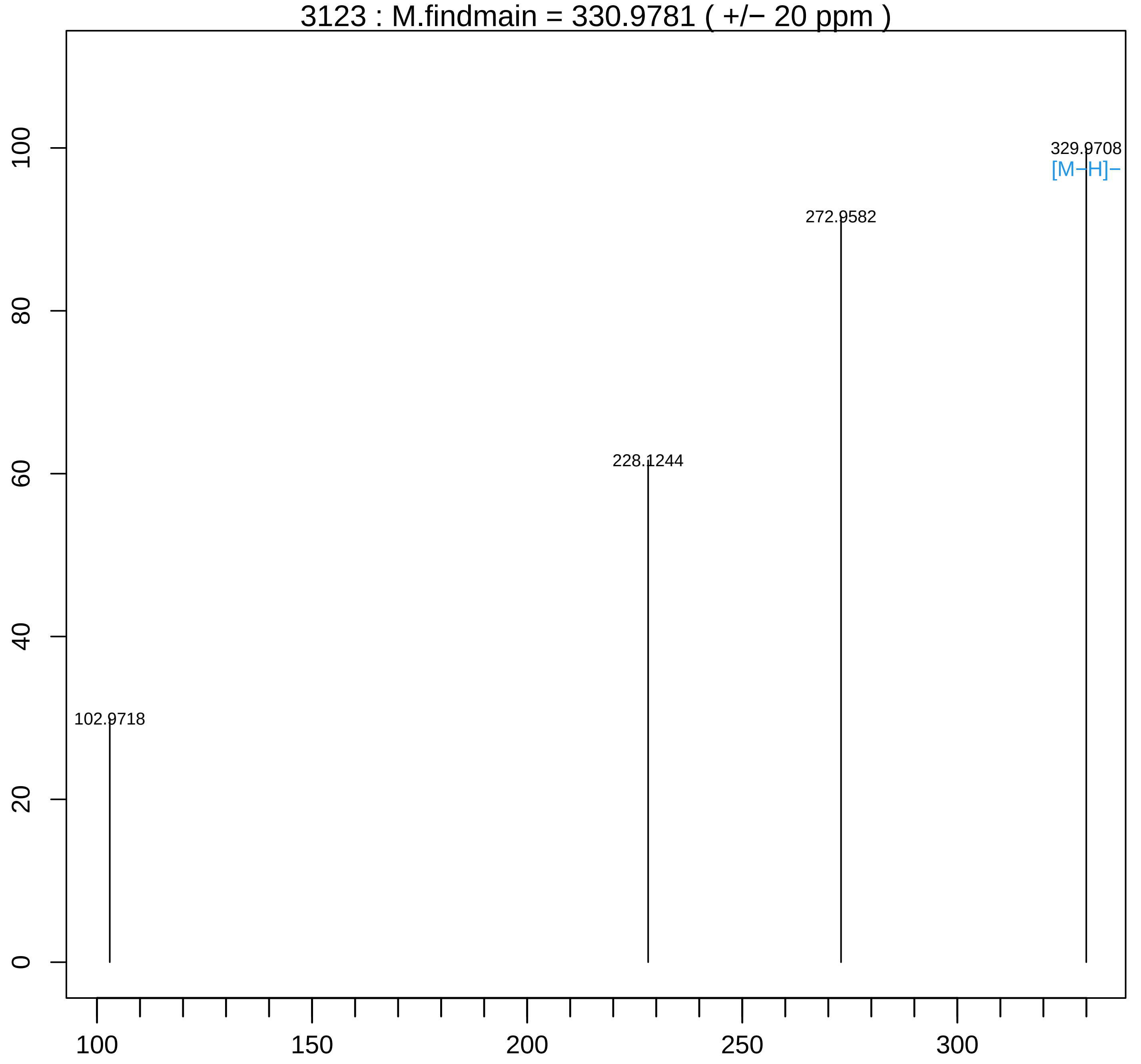

3123 : M.findmain = 330.9781 (+/− 20 ppm)
329.9708
[M−H]−
272.9582
228.1244
102.9718
0
20
40
60
80
100
100
150
200
250
300

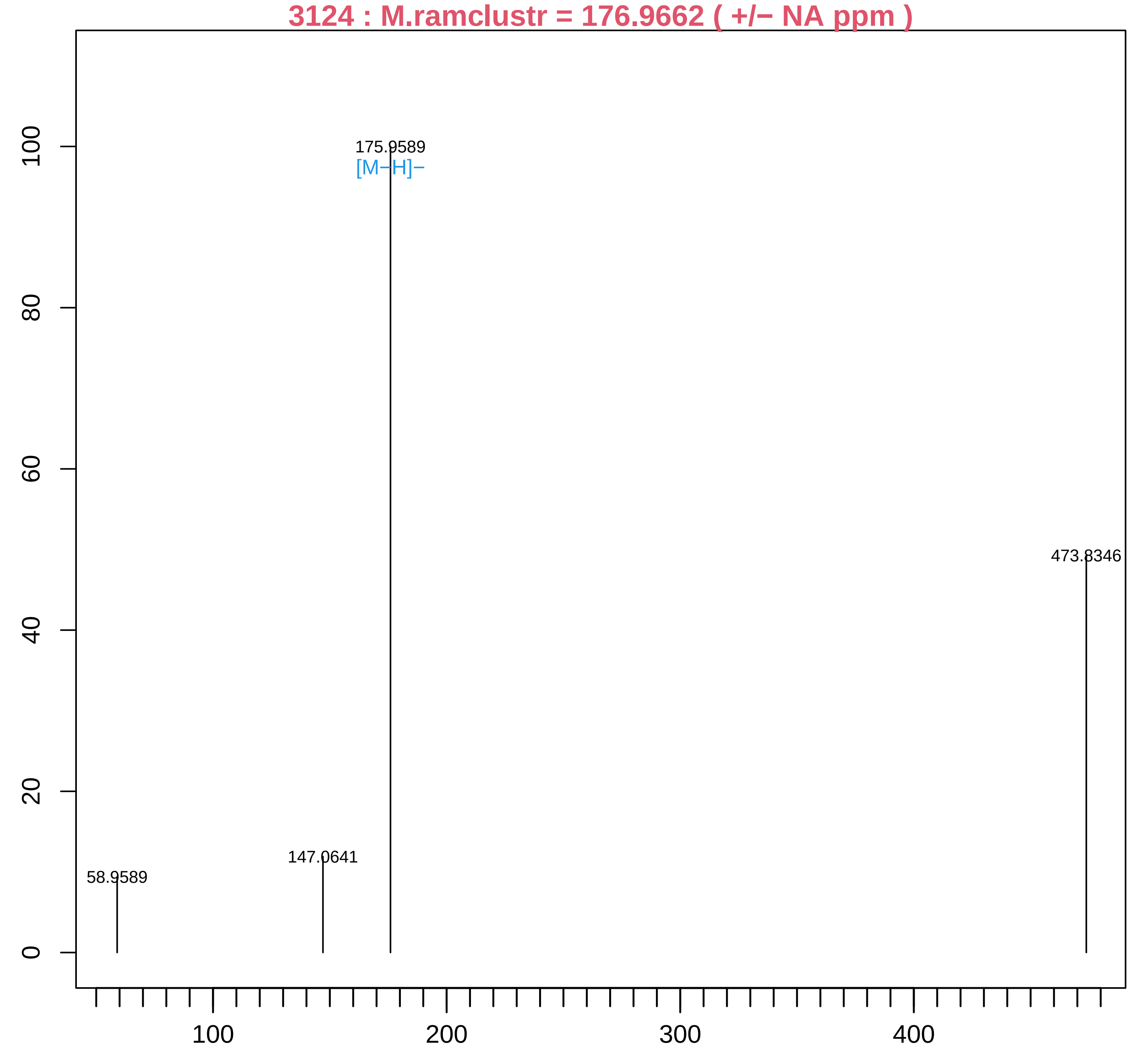
3124 : M.ramclustr = 176.9662 (+/− NA ppm)
175.9589
[M−H]−
473.8346
147.0641
58.9589
0
20
40
60
80
100
100
200
300
400

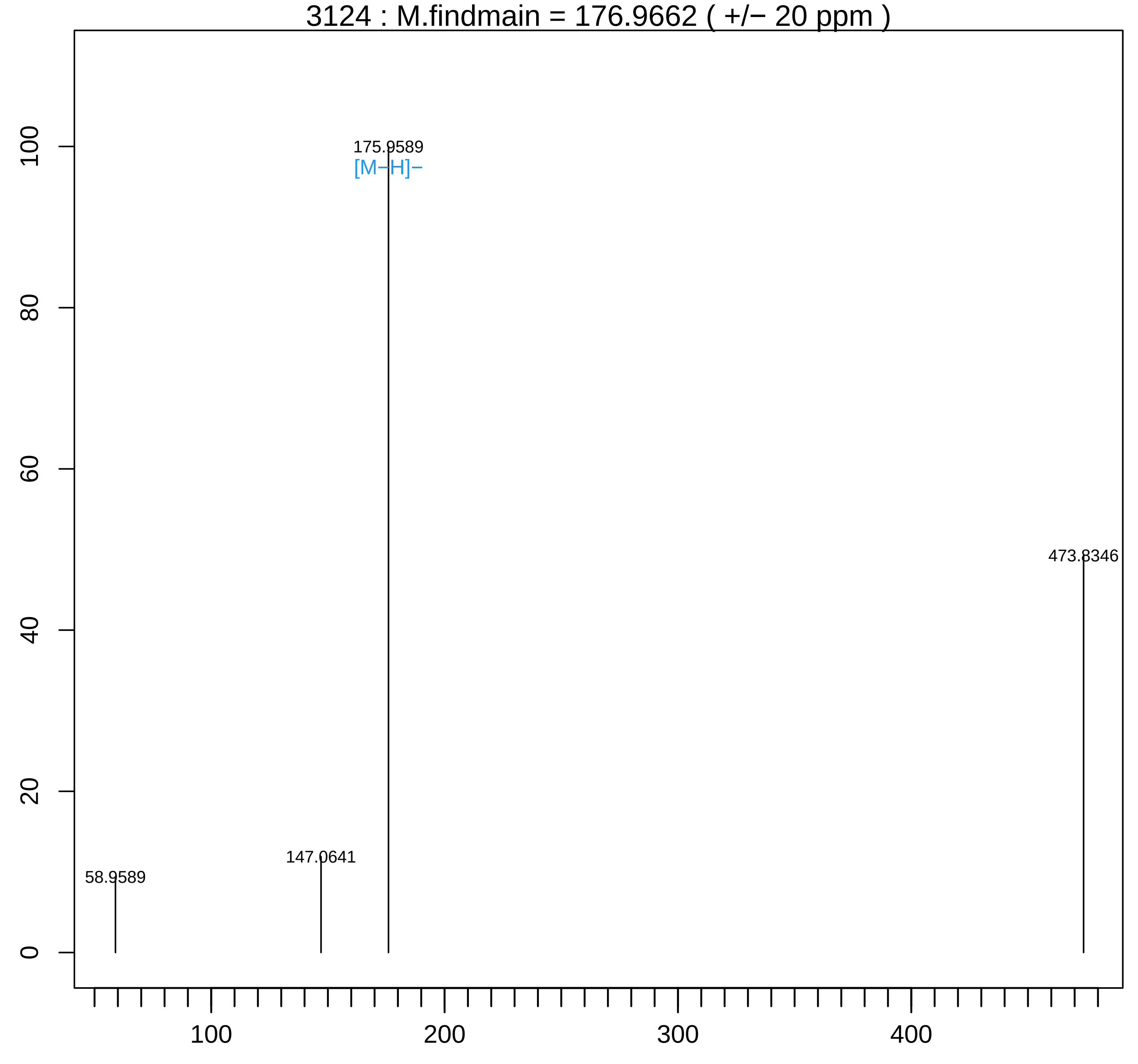
3124 : M.findmain = 176.9662 (+/− 20 ppm)
175.9589
[M−H]−
473.8346
147.0641
58.9589
0
20
40
60
80
100
100
200
300
400

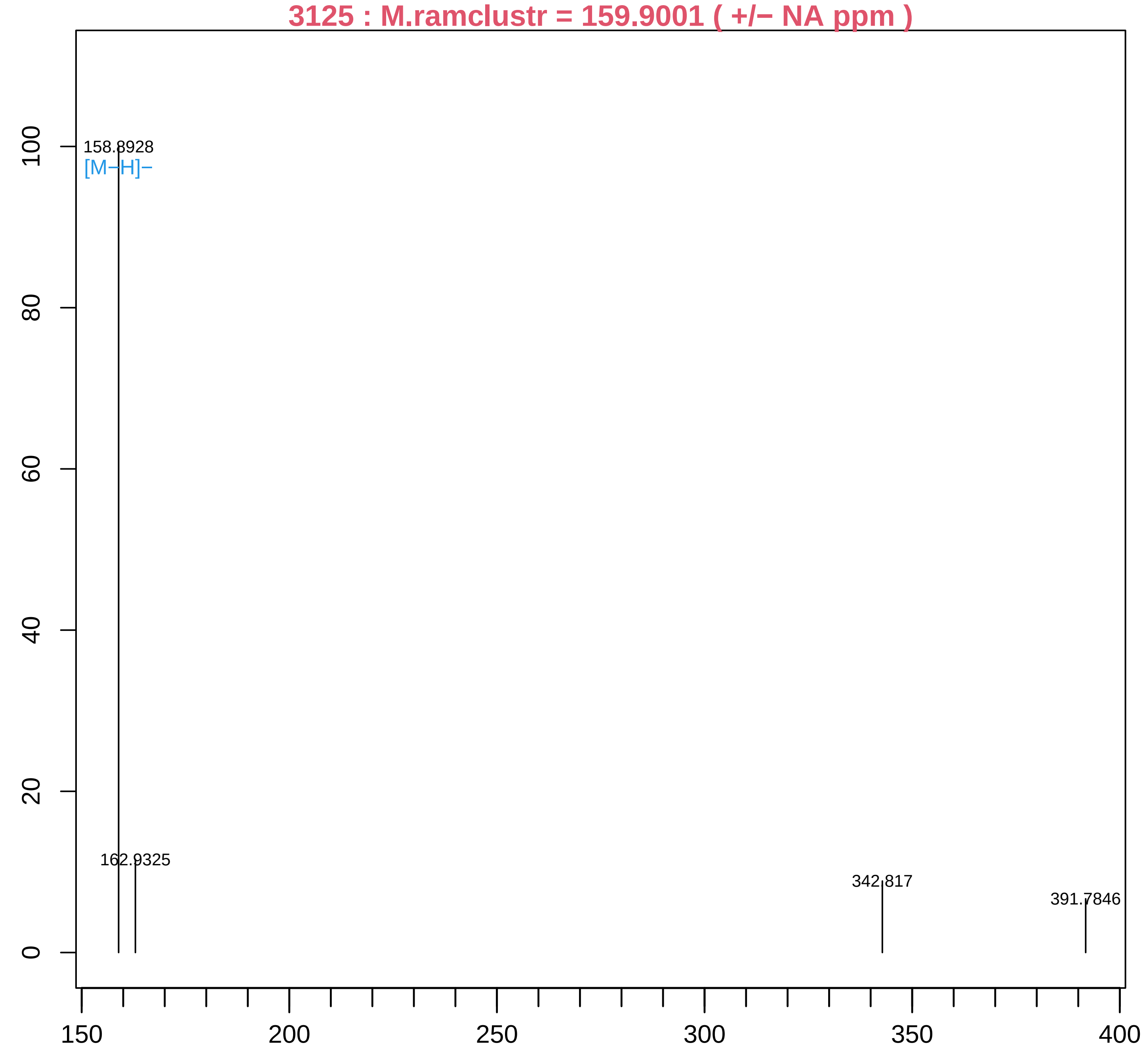
3125 : M.ramclustr = 159.9001 (+/− NA ppm)
158.8928
[M−H]−
162.9325
342.817
391.7846

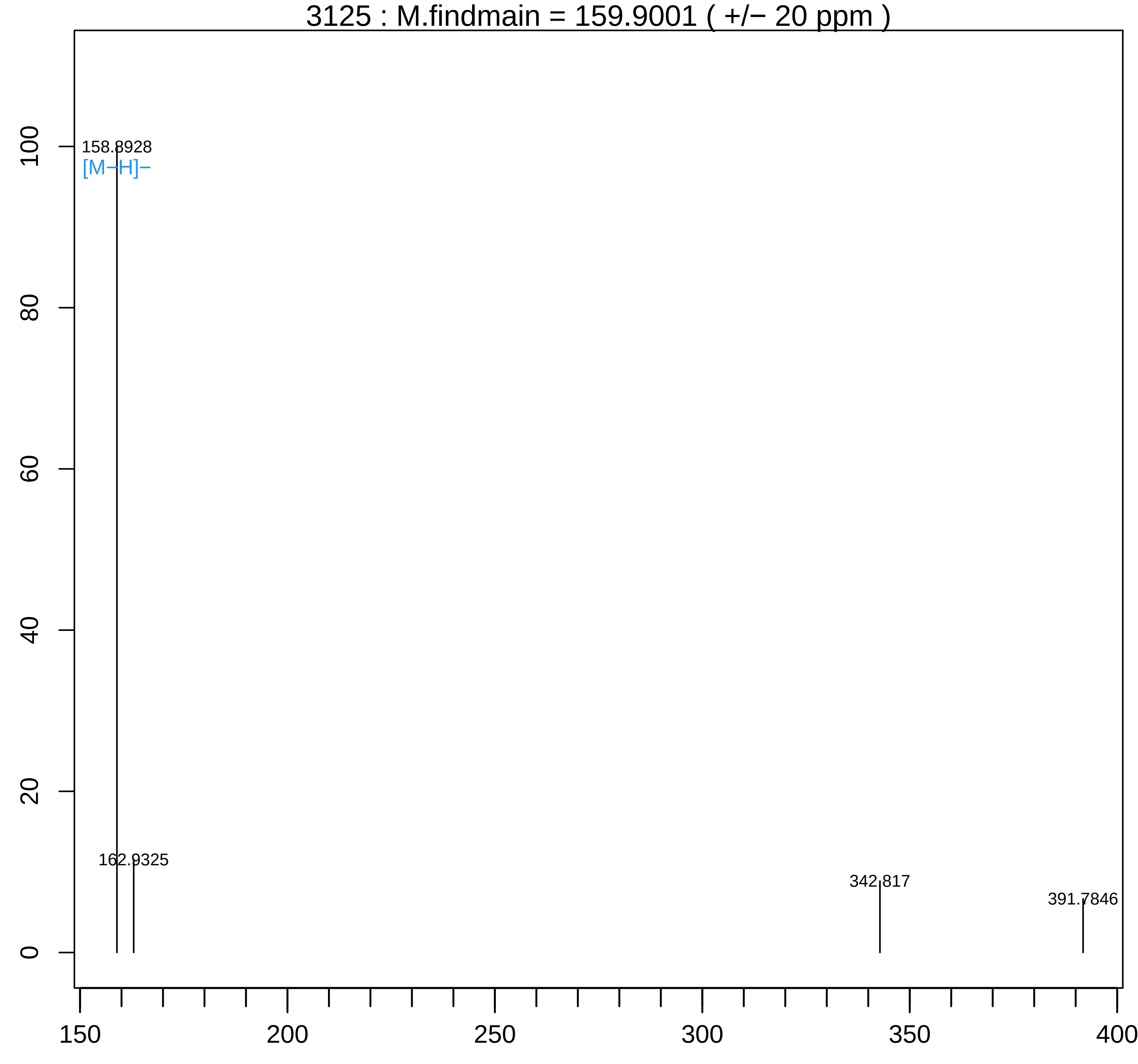
3125 : M.findmain = 159.9001 (+/− 20 ppm)
158.8928
[M−H]−
162.9325
342.817
391.7846

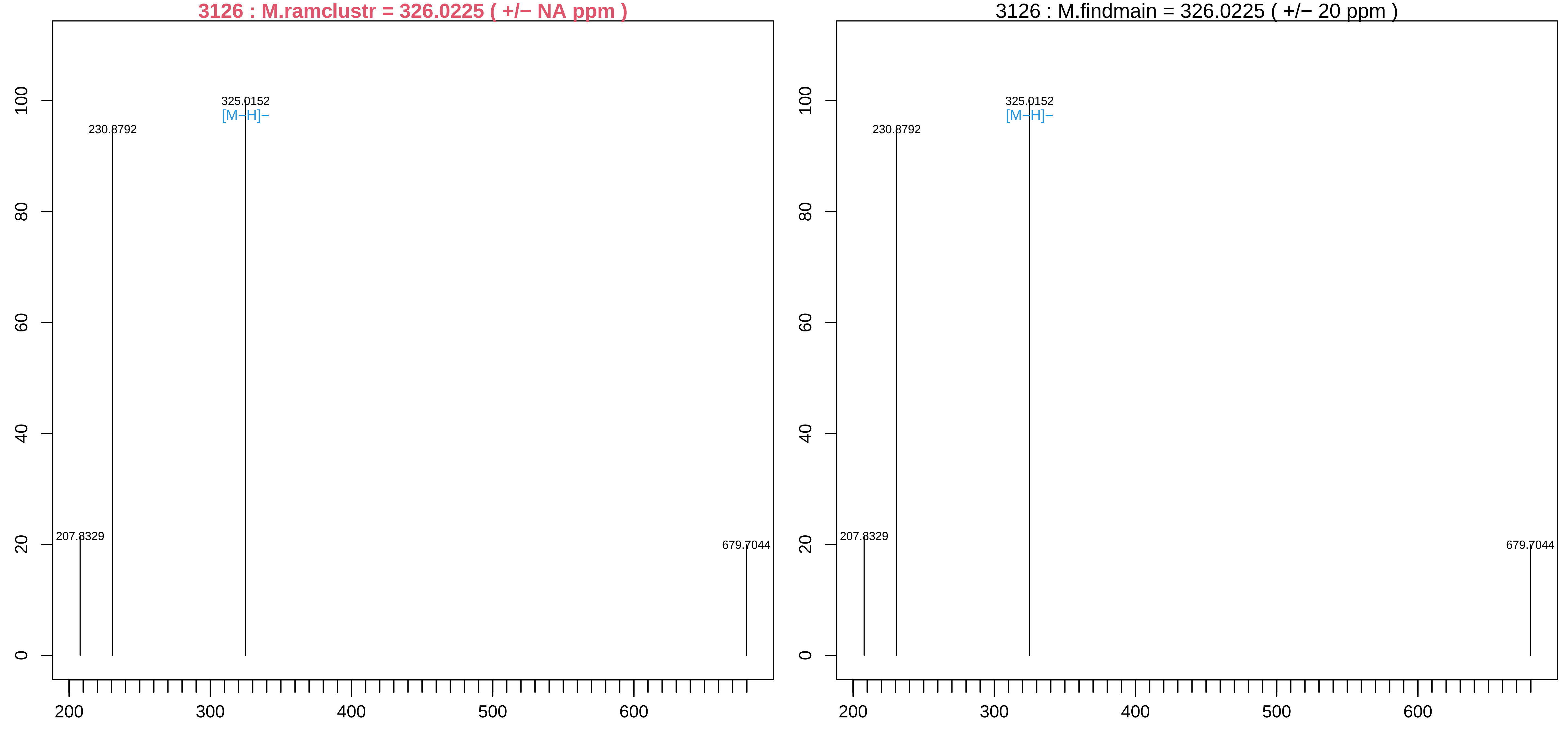
3126 : M.ramclustr = 326.0225 (+/− NA ppm)
325.0152
[M−H]−
230.8792
207.8329
679.7044
3126 : M.findmain = 326.0225 (+/− 20 ppm)
325.0152
[M−H]−
230.8792
207.8329
679.7044
0
20
40
60
80
100
200
300
400
500
600

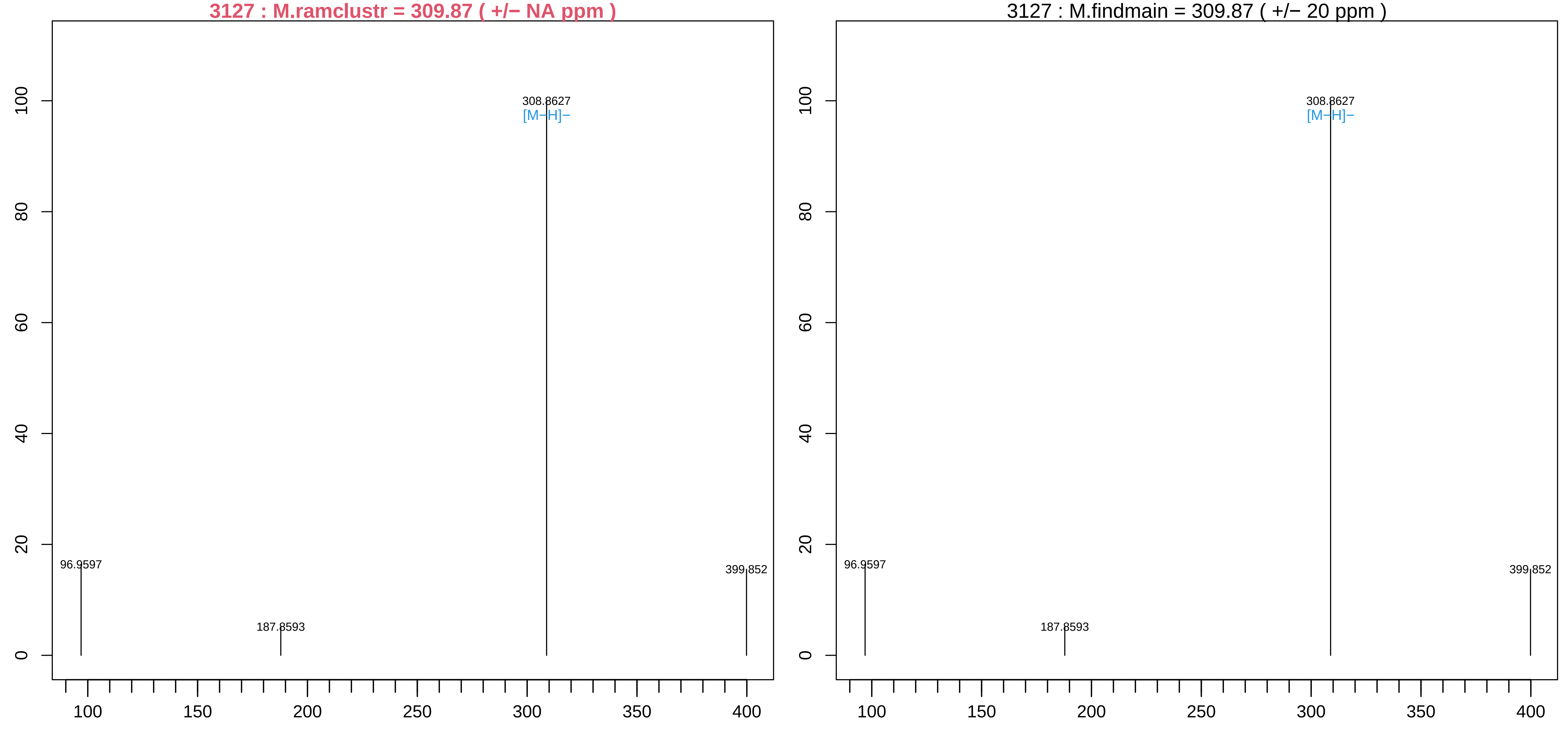
3127 : M.ramclustr = 309.87 (+/− NA ppm)
3127 : M.findmain = 309.87 (+/− 20 ppm)
308.8627
[M−H]−
96.9597
187.8593
399.852
0
20
40
60
80
100
100
150
200
250
300
350
400

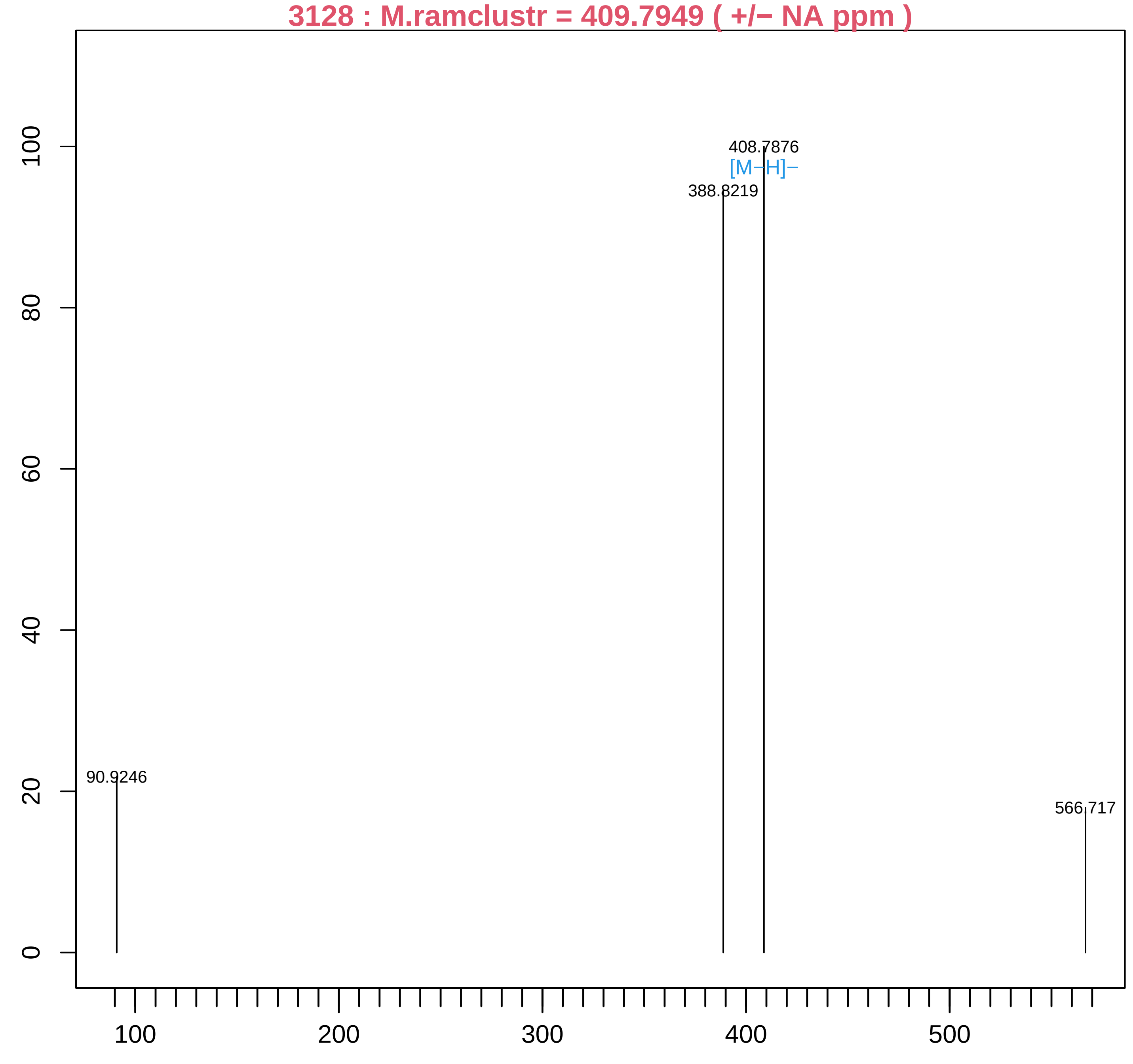
3128 : M.ramclustr = 409.7949 (+/− NA ppm)
408.7876
[M−H]−
388.8219
90.9246
566.717
0
20
40
60
80
100
100
200
300
400
500

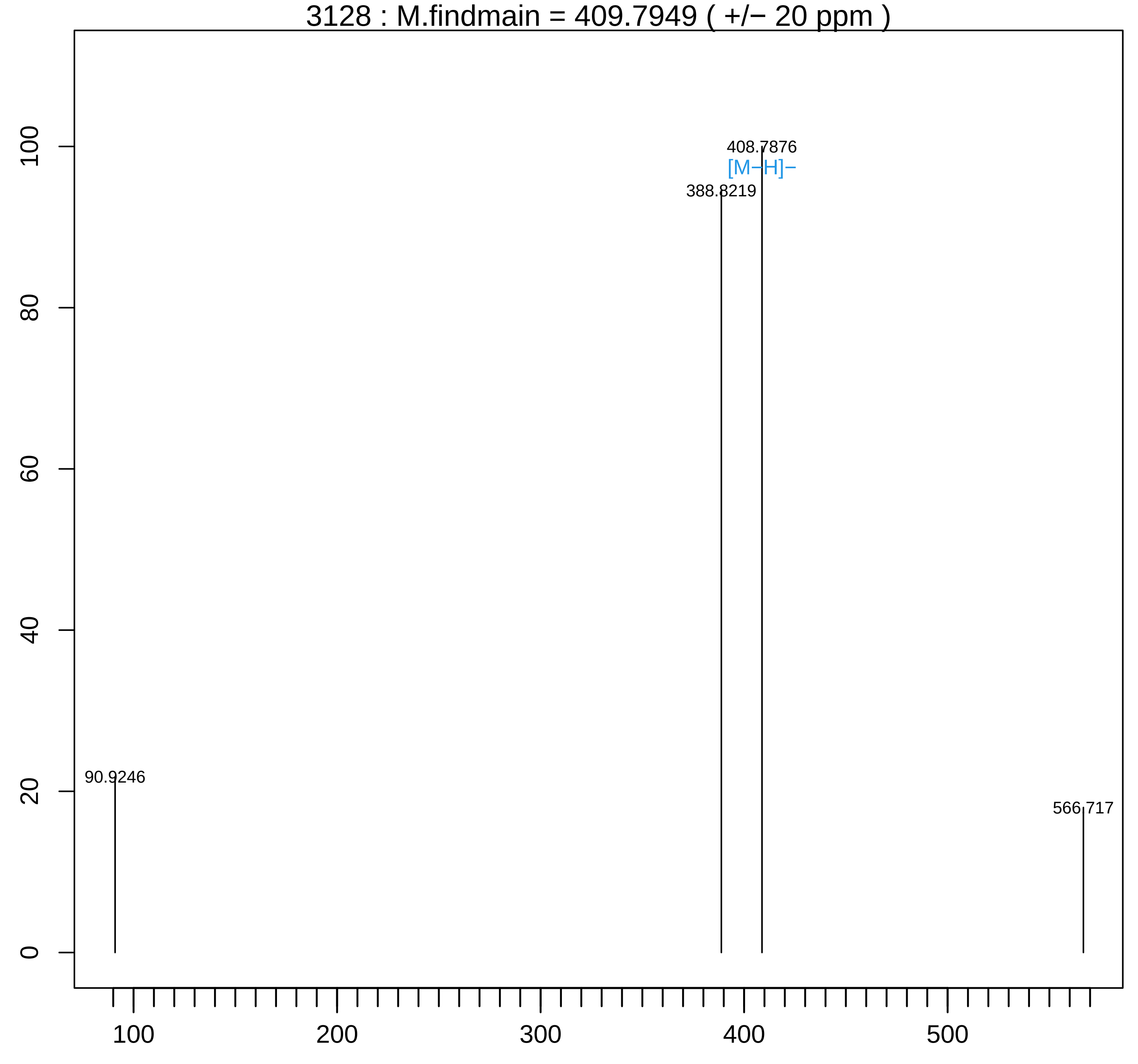
3128 : M.findmain = 409.7949 (+/− 20 ppm)
408.7876
[M−H]−
388.8219
90.9246
566.717
0
20
40
60
80
100
100
200
300
400
500

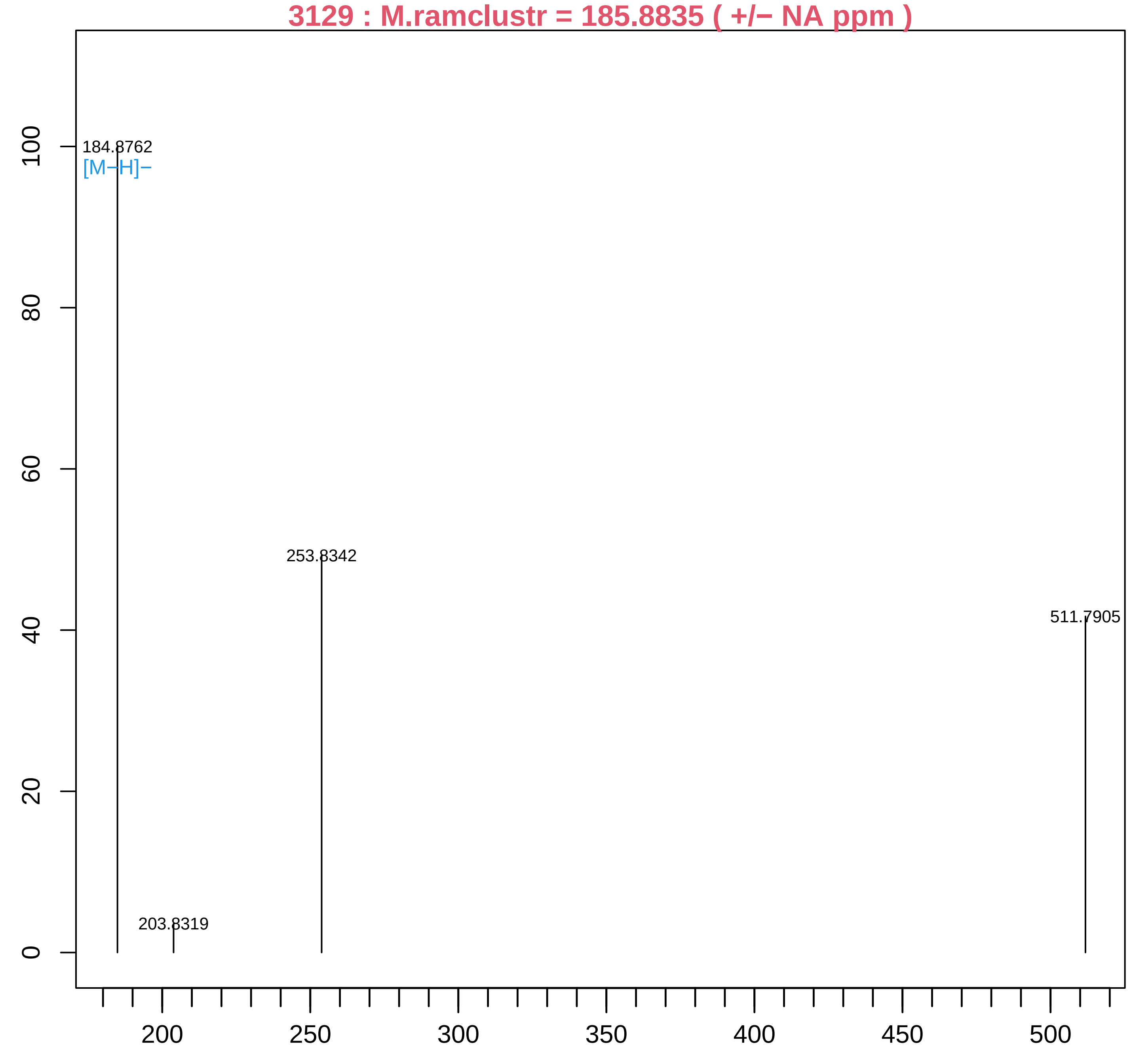
3129 : M.ramclustr = 185.8835 (+/− NA ppm)
184.8762
[M−H]−
203.8319
253.8342
511.7905
0
20
40
60
80
100
200
250
300
350
400
450
500

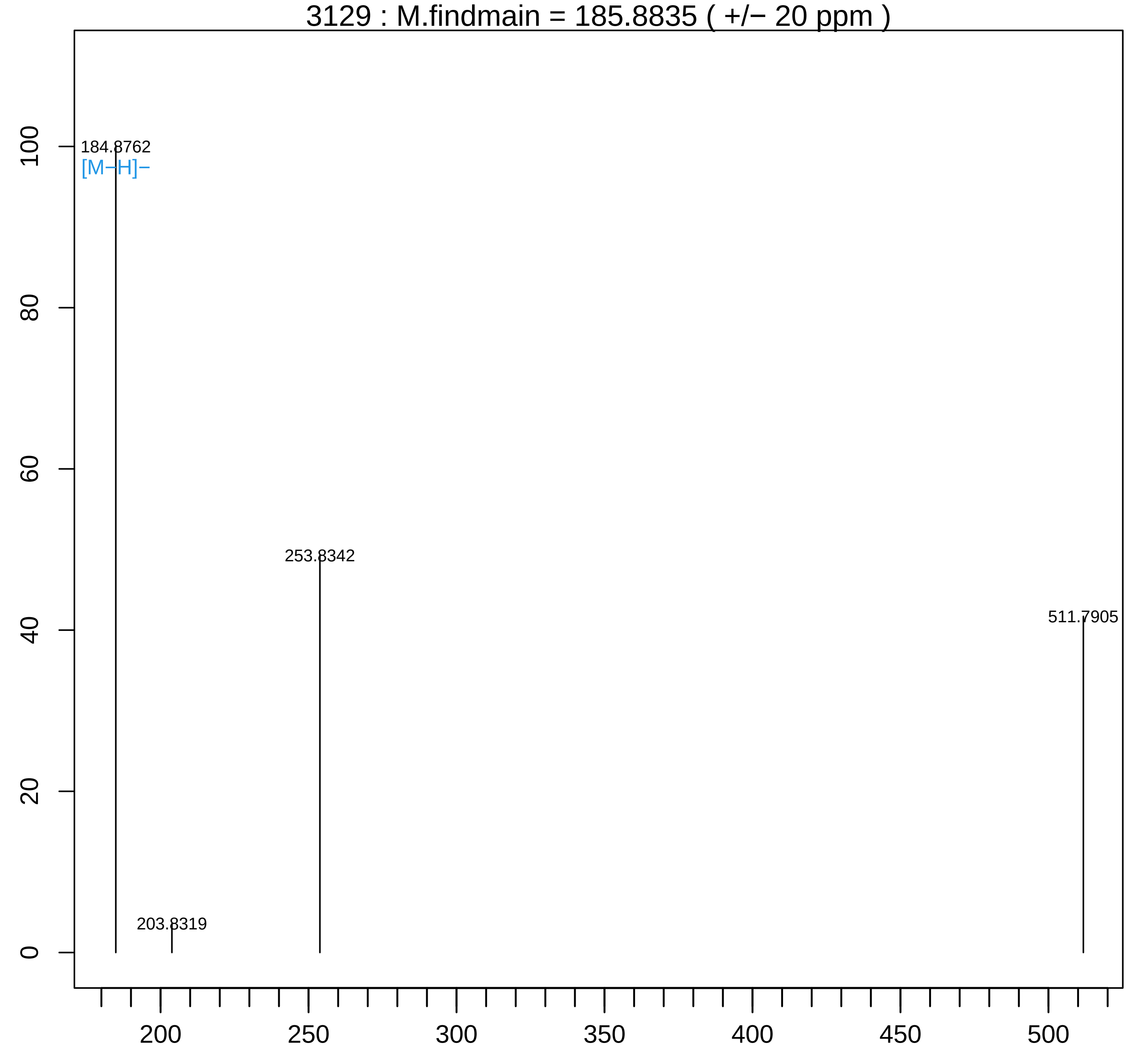
3129 : M.findmain = 185.8835 (+/− 20 ppm)
184.8762
[M−H]−
203.8319
253.8342
511.7905
0
20
40
60
80
100
200
250
300
350
400
450
500

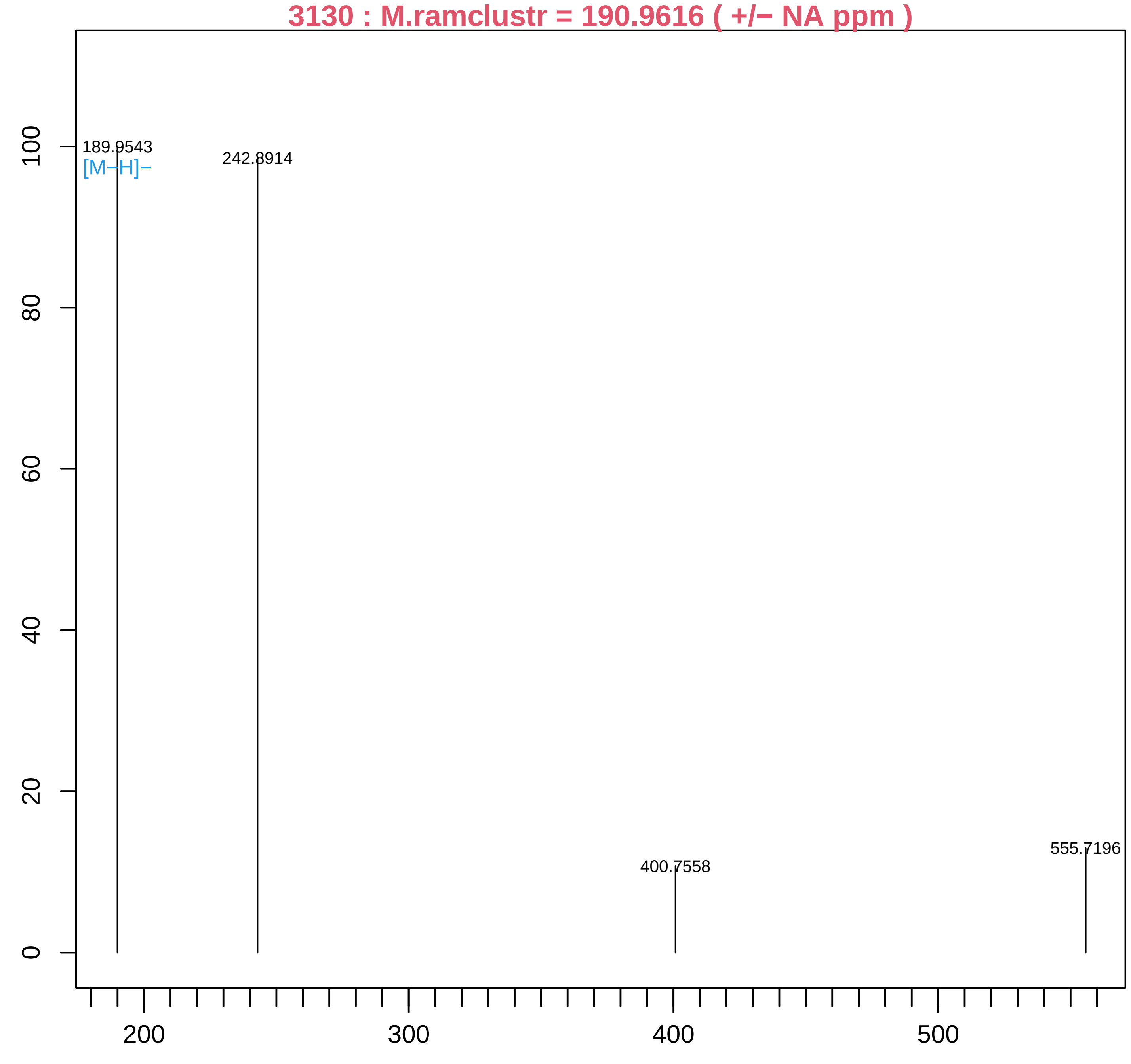
3130 : M.ramclustr = 190.9616 (+/− NA ppm)
189.9543
[M−H]−
242.8914
400.7558
555.7196
0
20
40
60
80
100
200
300
400
500

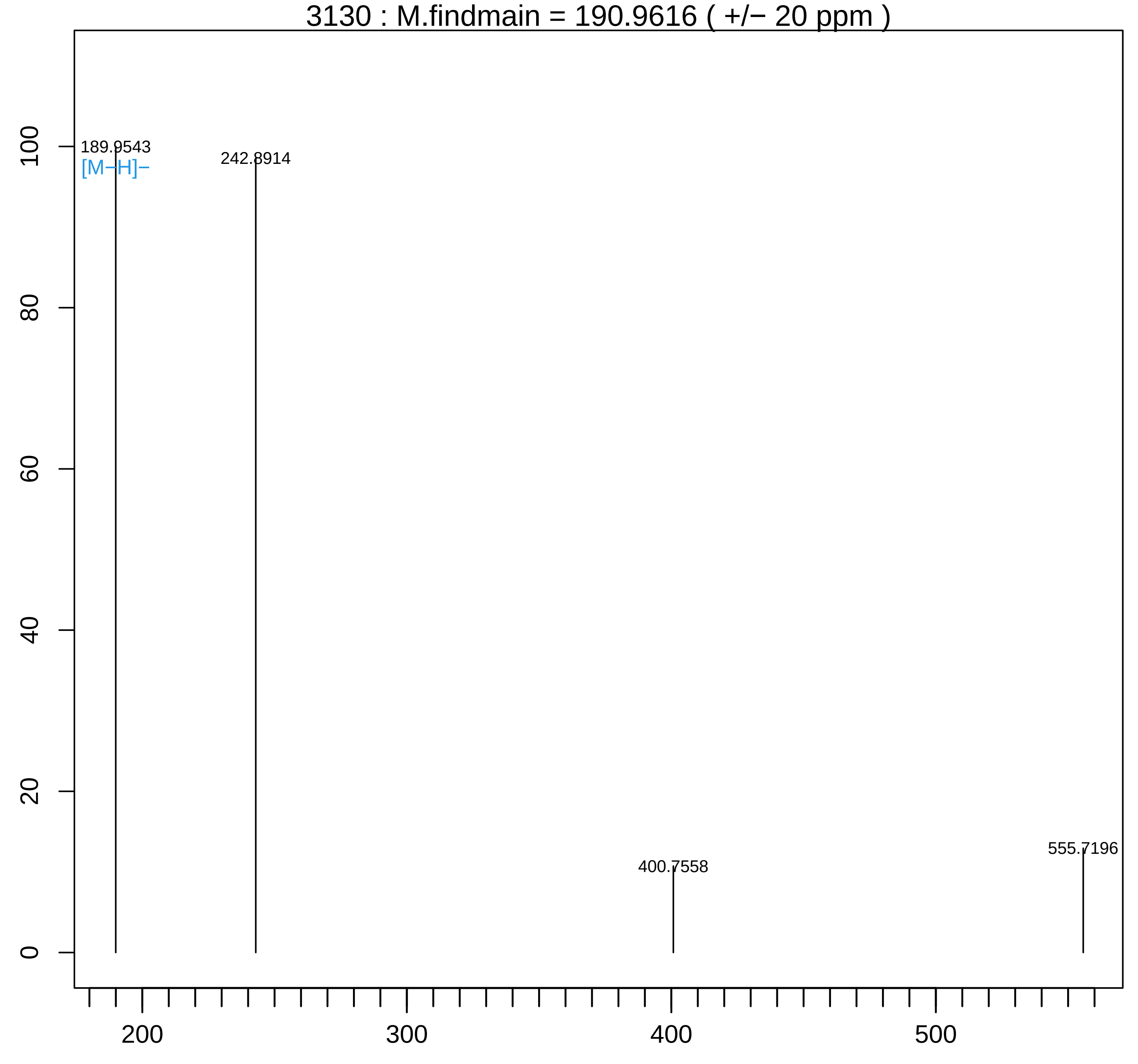
3130 : M.findmain = 190.9616 (+/− 20 ppm)
189.9543
[M−H]−
242.8914
400.7558
555.7196
0
20
40
60
80
100
200
300
400
500

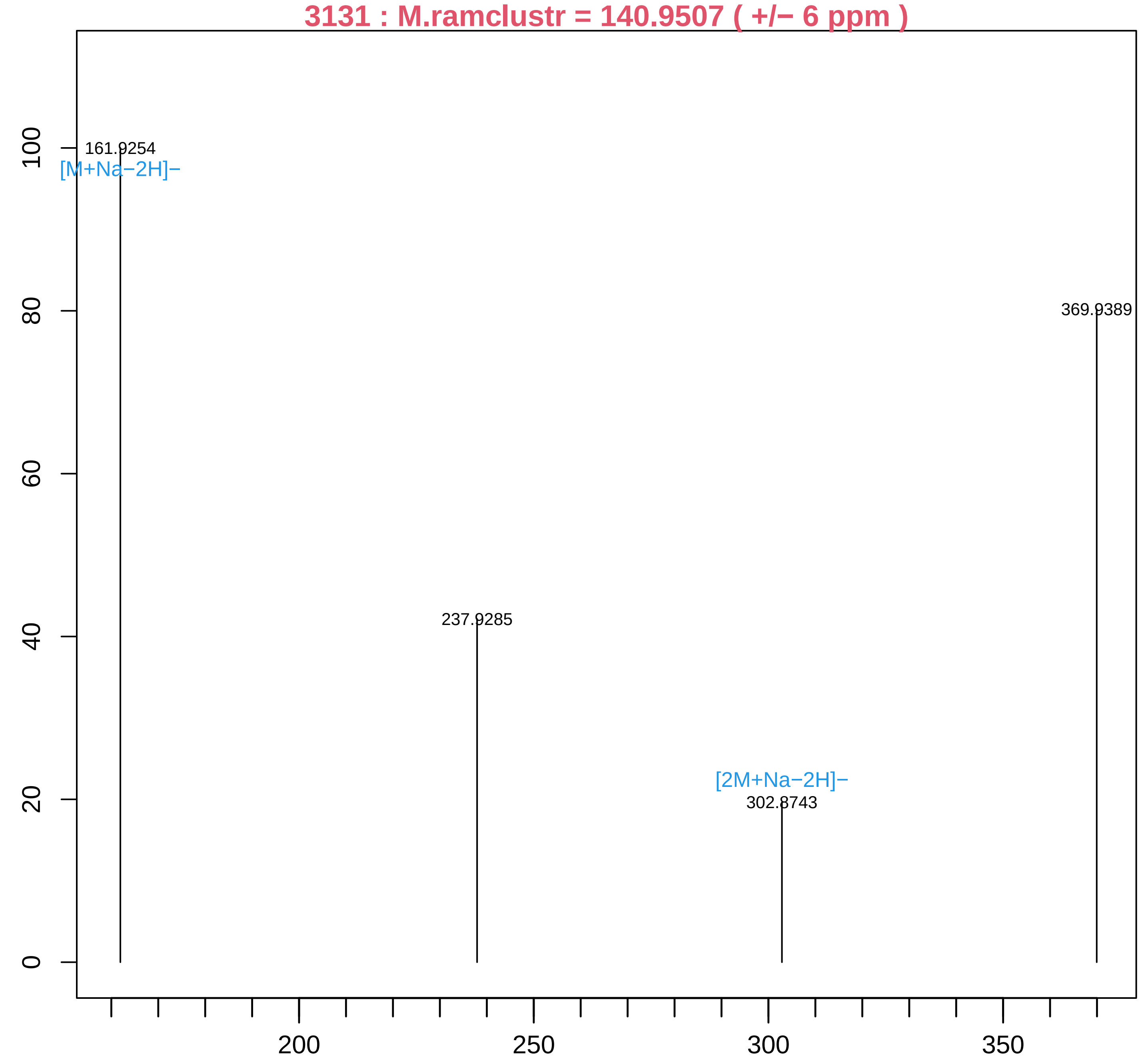
3131 : M.ramclustr = 140.9507 (+/− 6 ppm)
161.9254
[M+Na−2H]−
369.9389
237.9285
[2M+Na−2H]−
302.8743
0
20
40
60
80
100
200
250
300
350

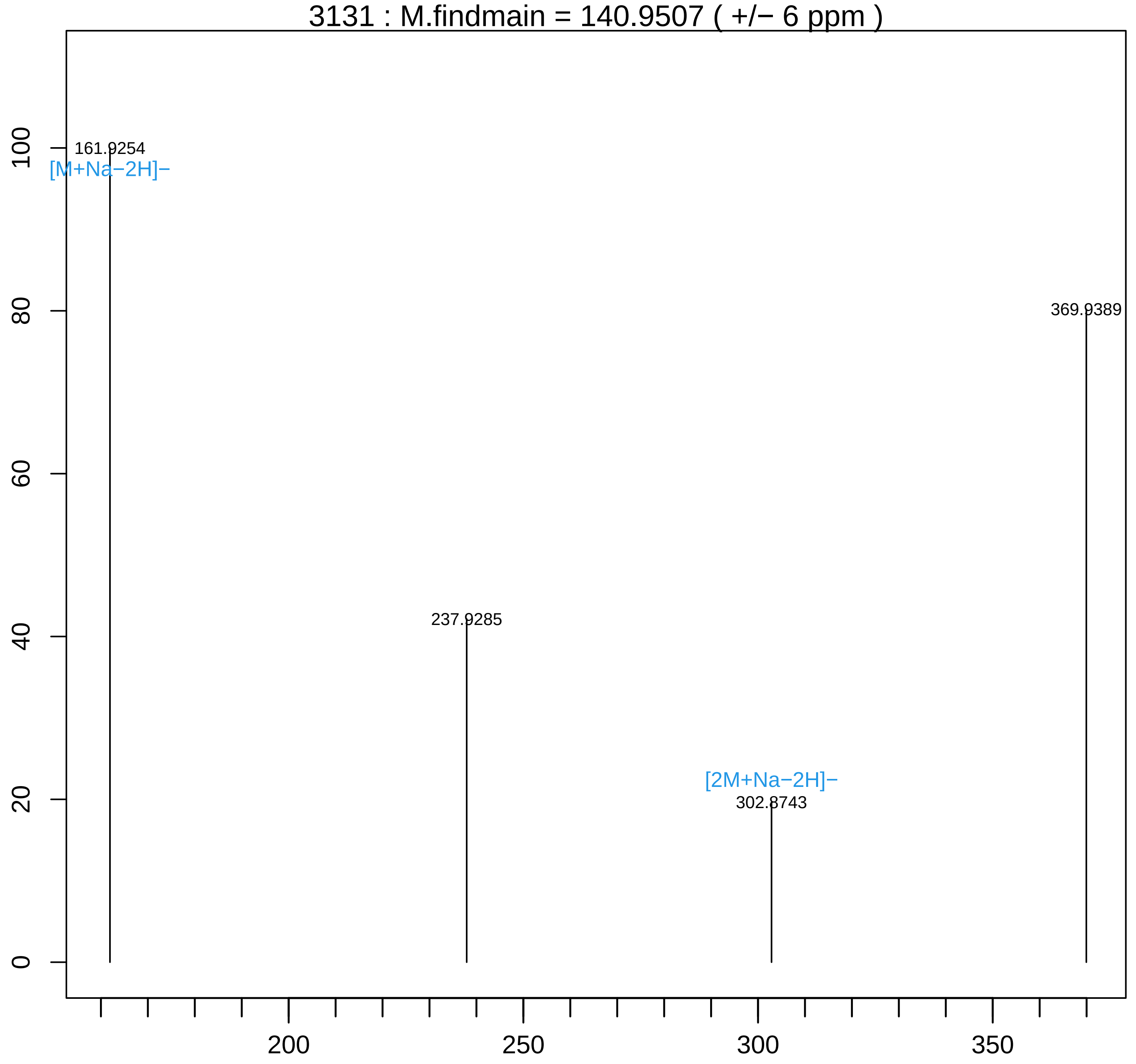
3131 : M.findmain = 140.9507 (+/− 6 ppm)
161.9254
[M+Na−2H]−
369.9389
237.9285
[2M+Na−2H]−
302.8743
0
20
40
60
80
100
200
250
300
350

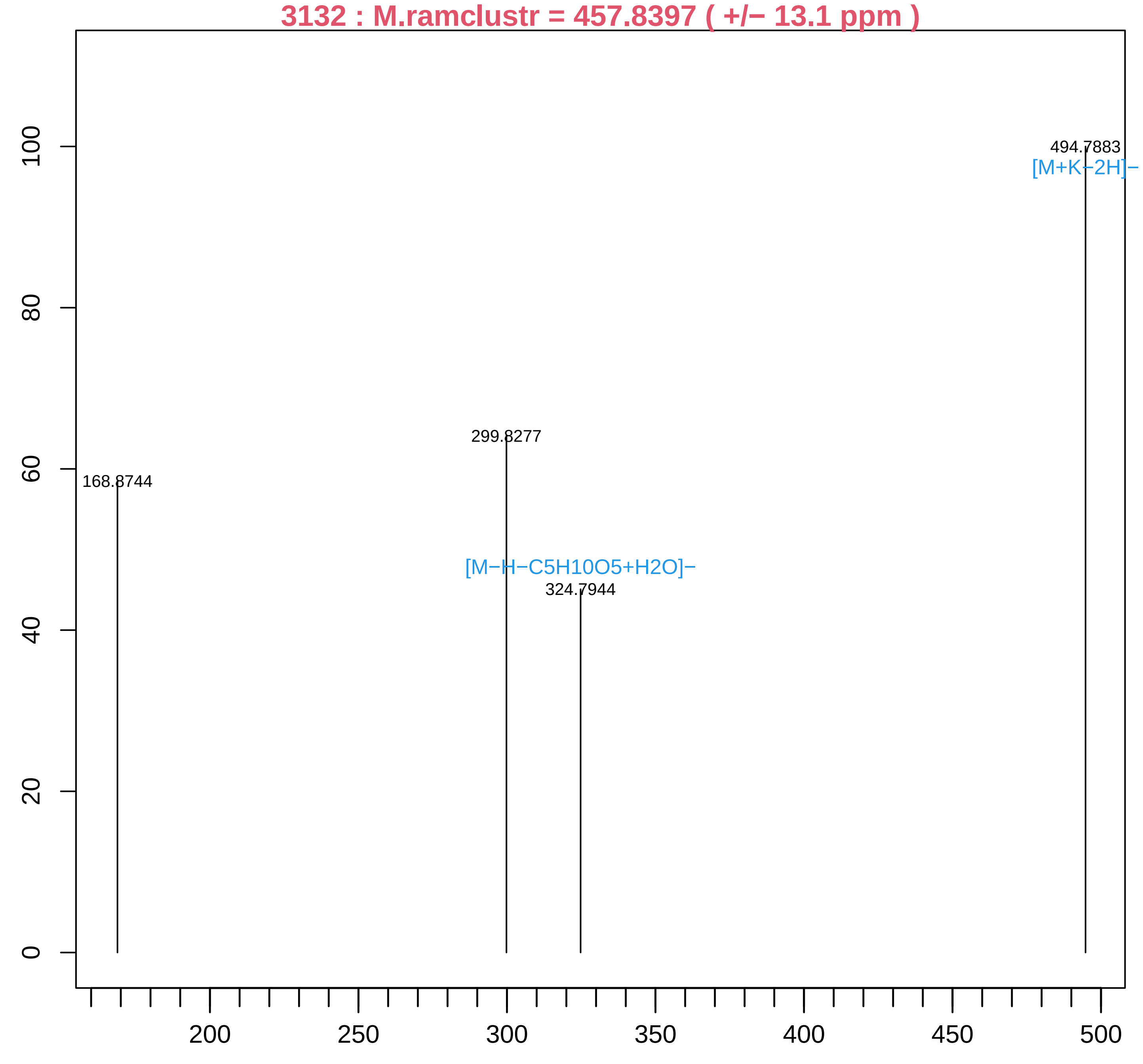
3132 : M.ramclustr = 457.8397 (+/− 13.1 ppm)
494.7883
[M+K−2H]−
299.8277
168.8744
[M−H−C5H10O5+H2O]−
324.7944
0
20
40
60
80
100
200
250
300
350
400
450
500

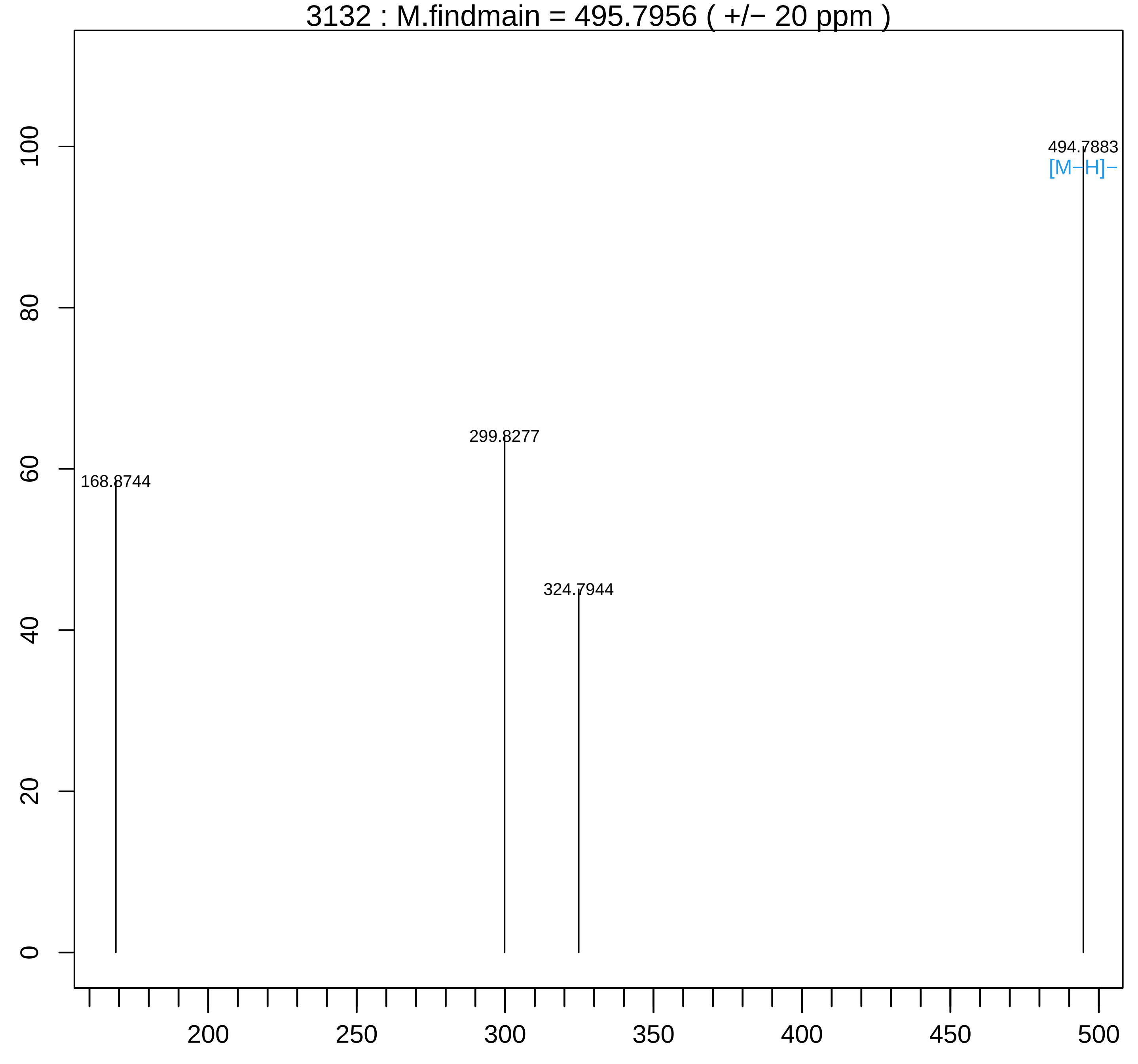
3132 : M.findmain = 495.7956 (+/− 20 ppm)
494.7883
[M−H]−
299.8277
168.8744
324.7944
0
20
40
60
80
100
200
250
300
350
400
450
500

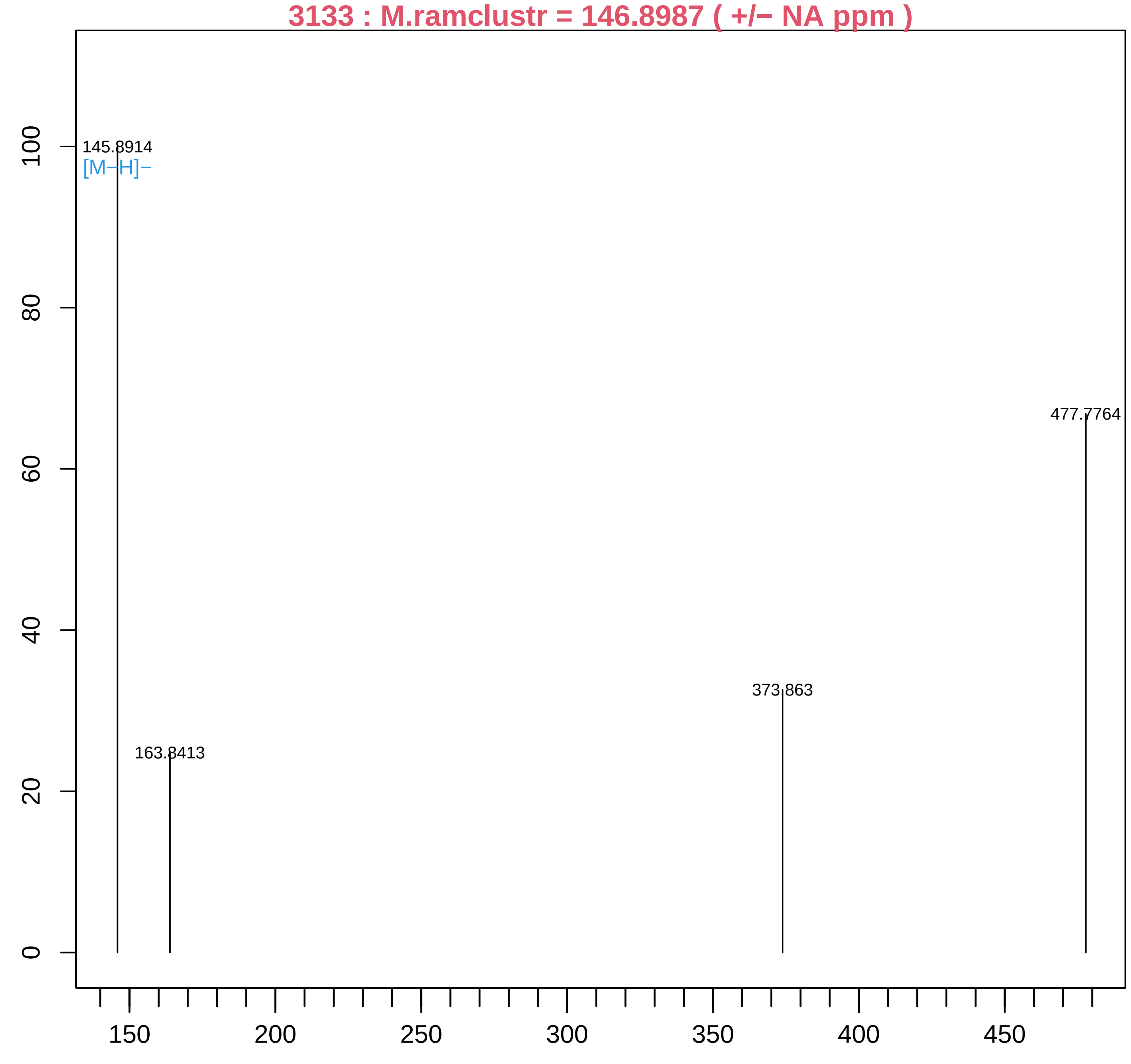
3133 : M.ramclustr = 146.8987 (+/− NA ppm)
145.8914
[M−H]−
163.8413
373.863
477.7764
0
20
40
60
80
100
150
200
250
300
350
400
450

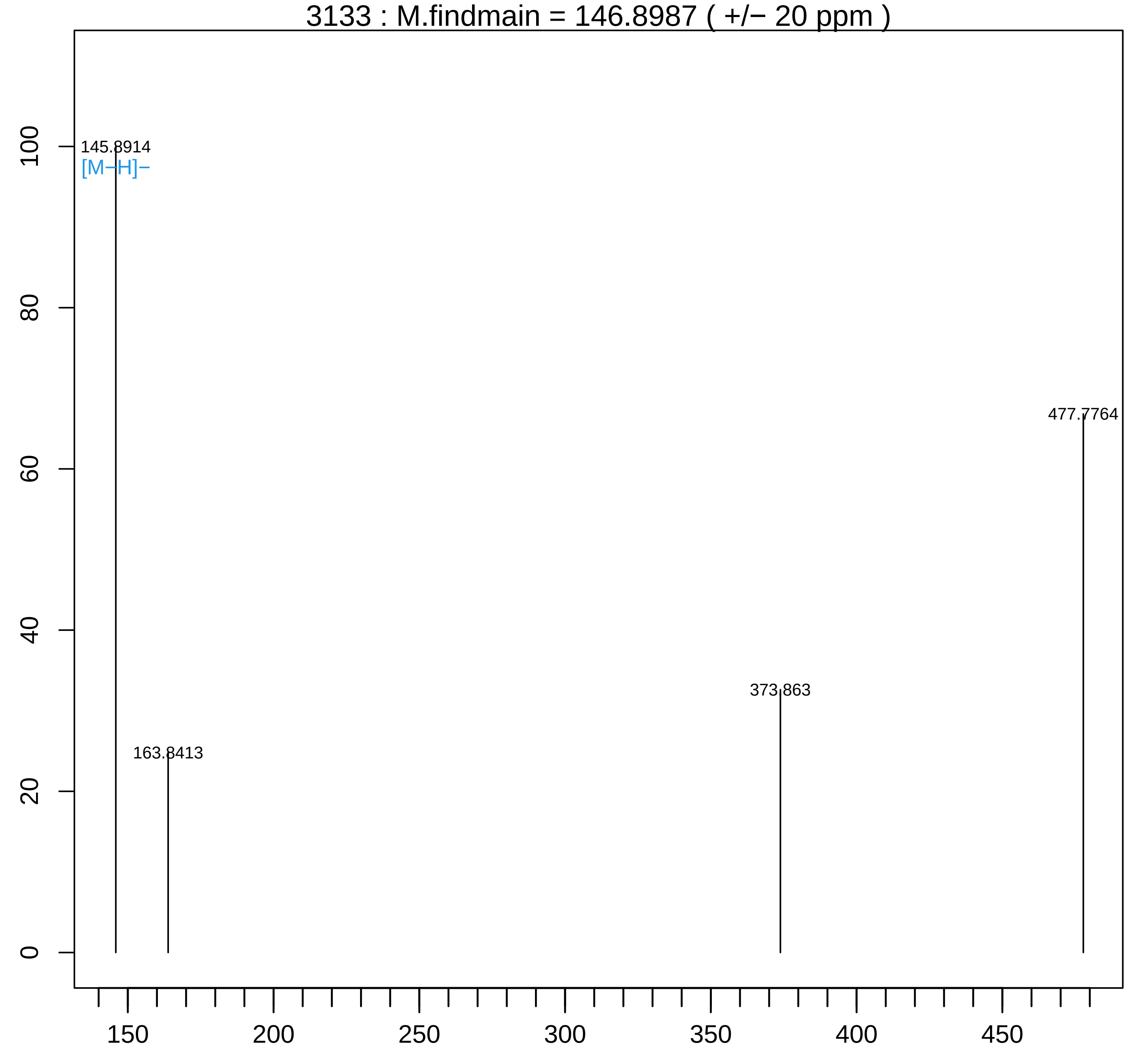
3133 : M.findmain = 146.8987 (+/− 20 ppm)
145.8914
[M−H]−
163.8413
373.863
477.7764
0
20
40
60
80
100
150
200
250
300
350
400
450

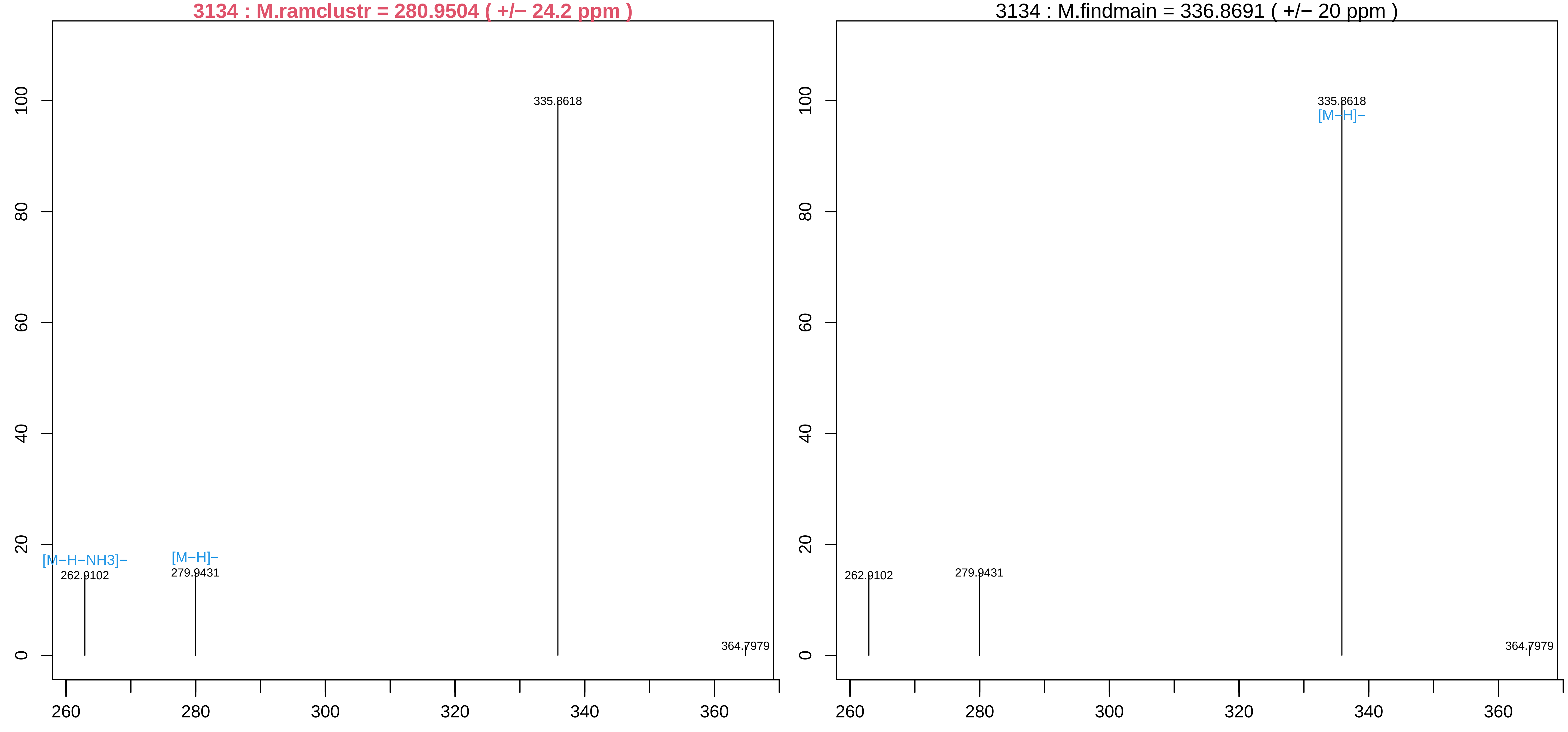
3134 : M.ramclustr = 280.9504 (+/− 24.2 ppm)
[M−H−NH3]−
262.9102
[M−H]−
279.9431
335.8618
364.7979
3134 : M.findmain = 336.8691 (+/− 20 ppm)
262.9102
279.9431
335.8618
[M−H]−
364.7979
0
20
40
60
80
100
260
280
300
320
340
360

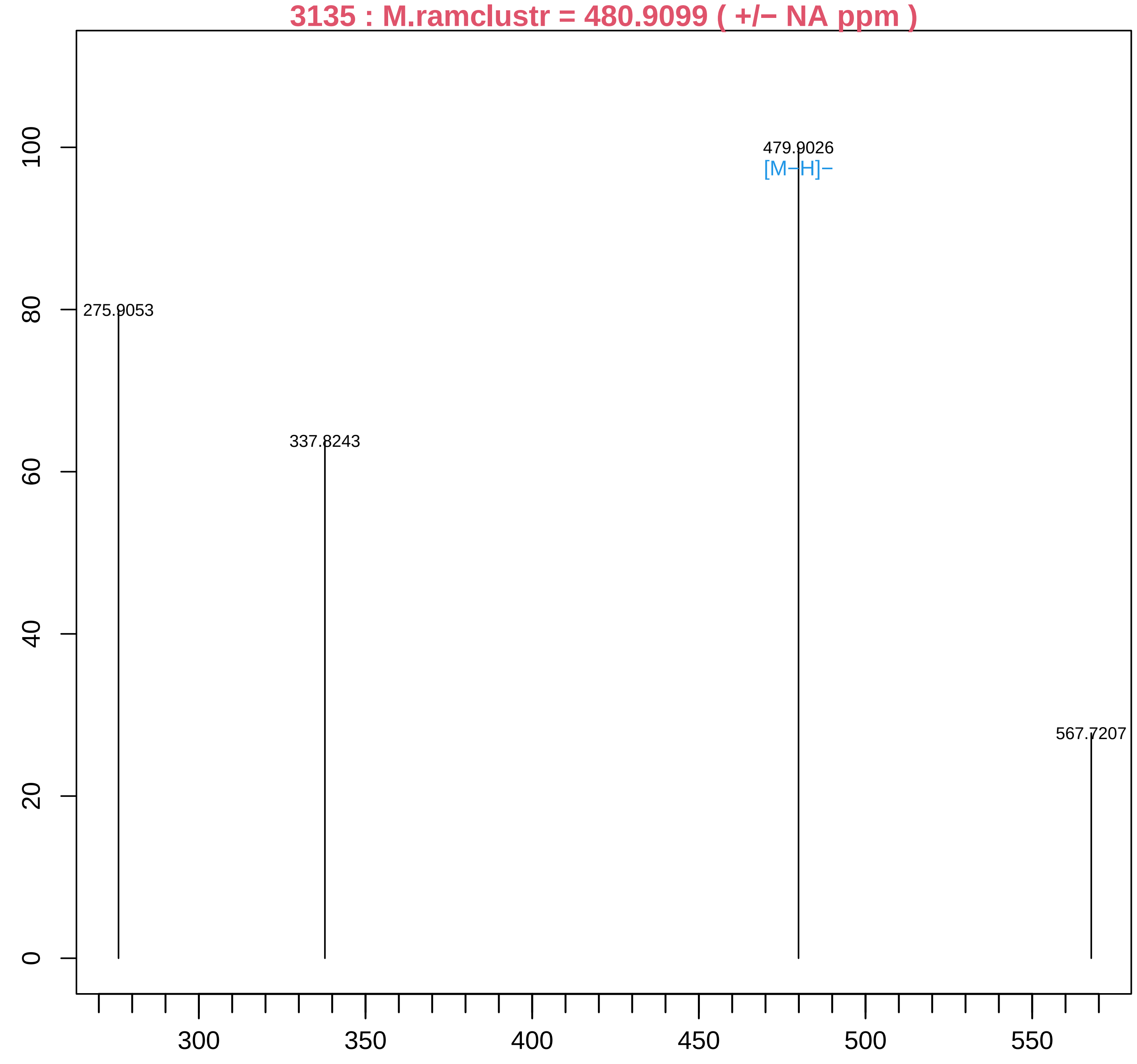
3135 : M.ramclustr = 480.9099 (+/− NA ppm)
479.9026
[M−H]−
275.9053
337.8243
567.7207
0
20
40
60
80
100
300
350
400
450
500
550

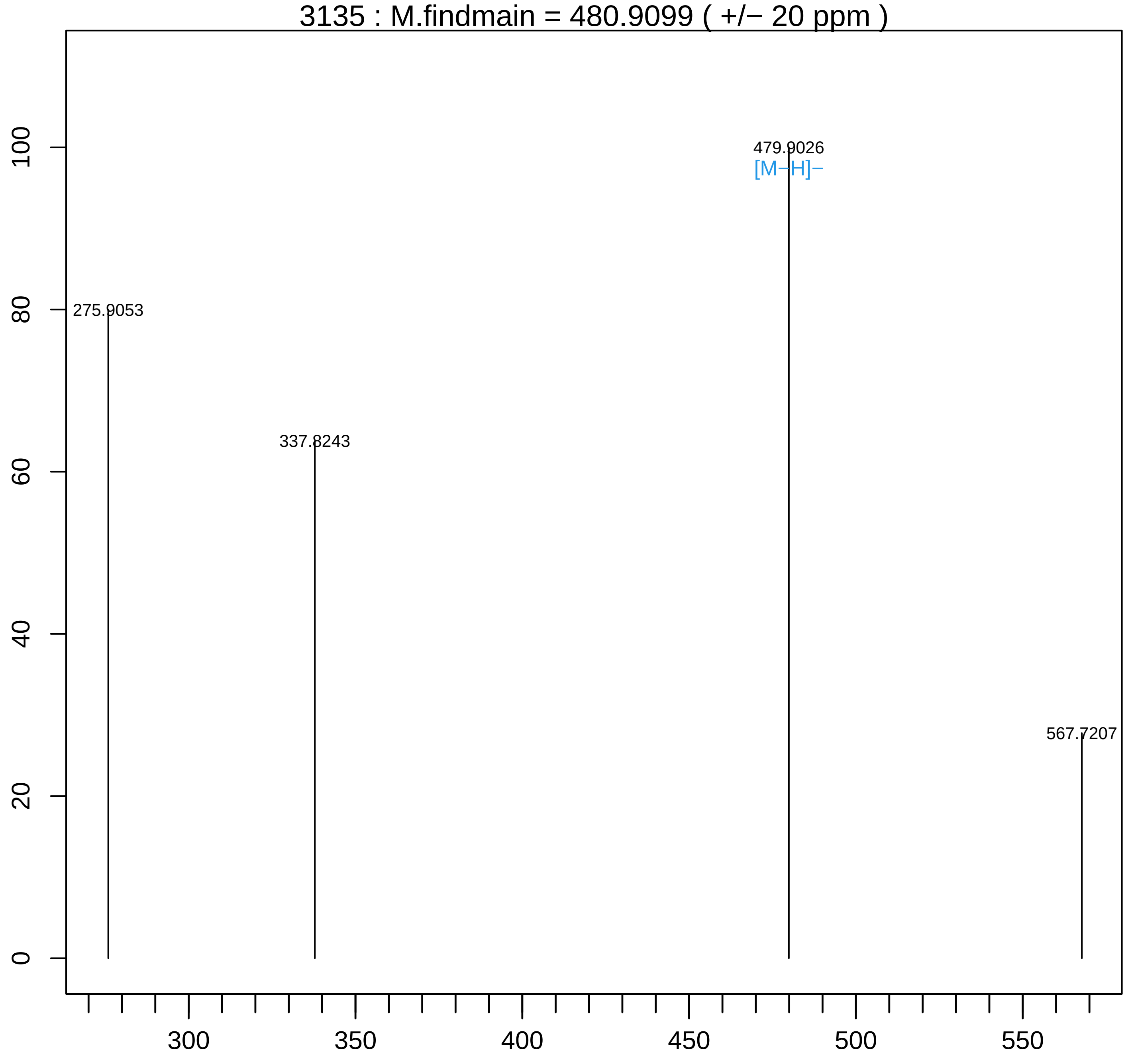
3135 : M.findmain = 480.9099 (+/− 20 ppm)
479.9026
[M−H]−
275.9053
337.8243
567.7207
0
20
40
60
80
100
300
350
400
450
500
550

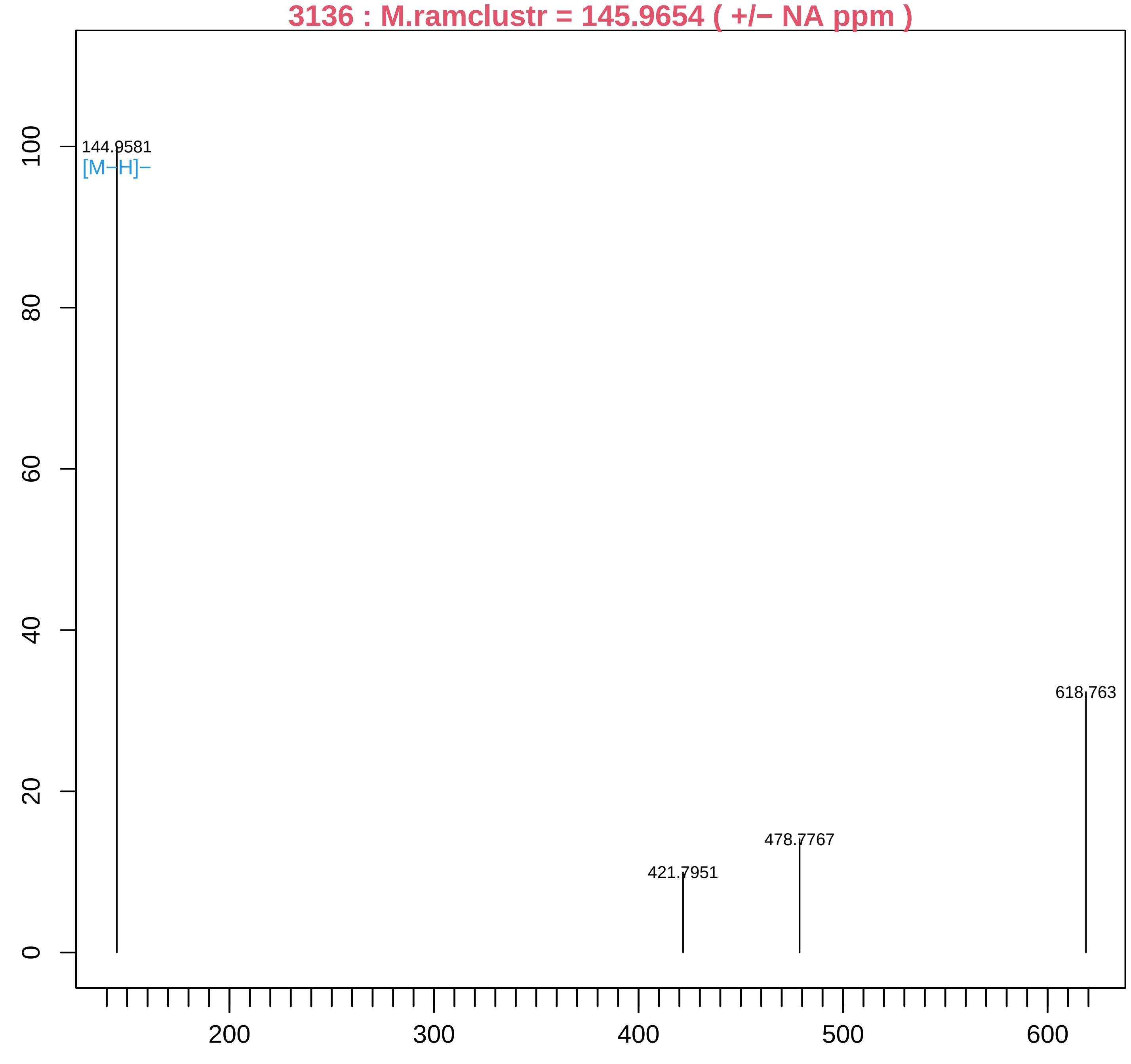
3136 : M.ramclustr = 145.9654 (+/− NA ppm)
144.9581
[M−H]−
421.7951
478.7767
618.763
0
20
40
60
80
100
200
300
400
500
600
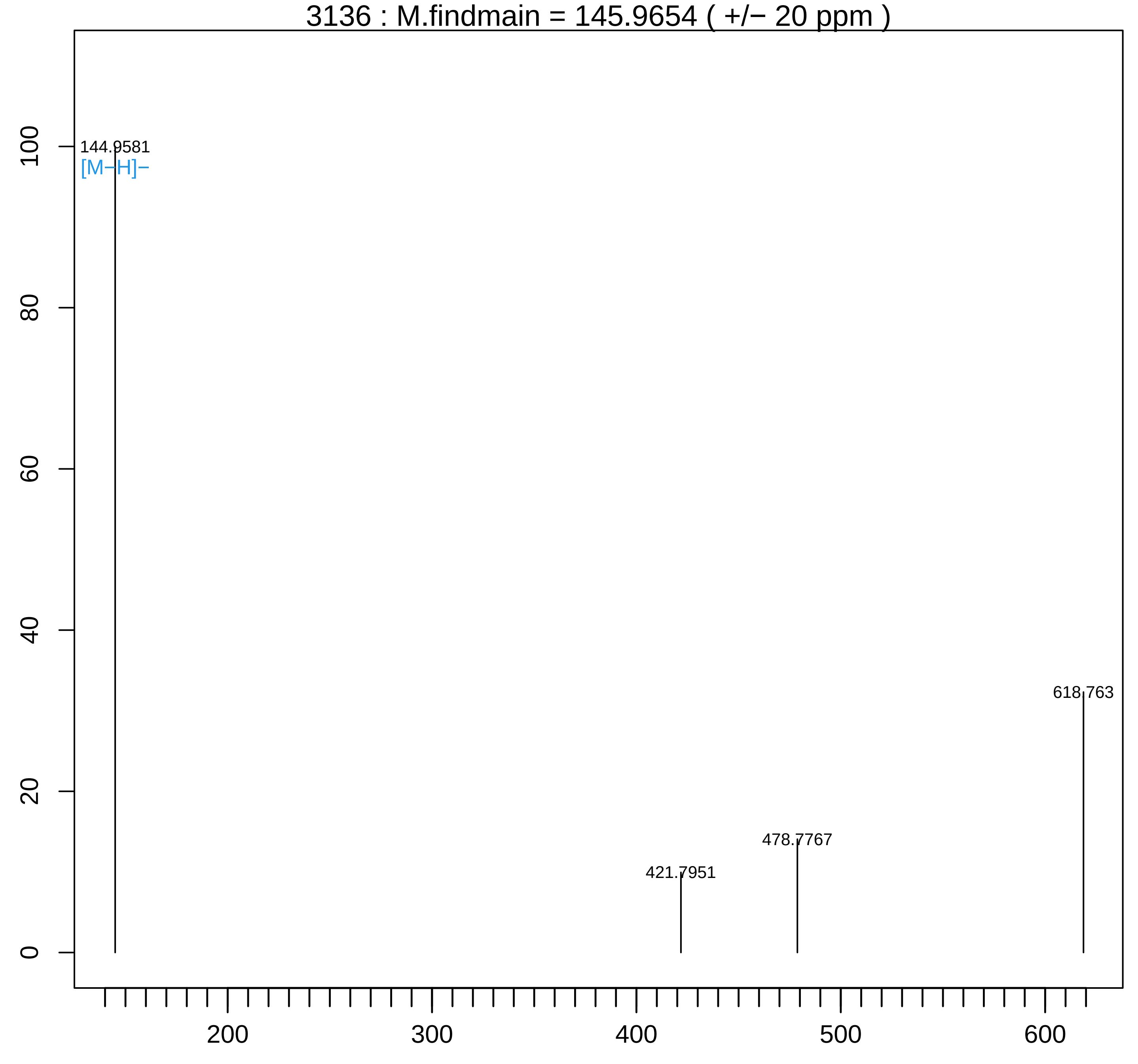
3136 : M.findmain = 145.9654 (+/− 20 ppm)
144.9581
[M−H]−
421.7951
478.7767
618.763
0
20
40
60
80
100
200
300
400
500
600

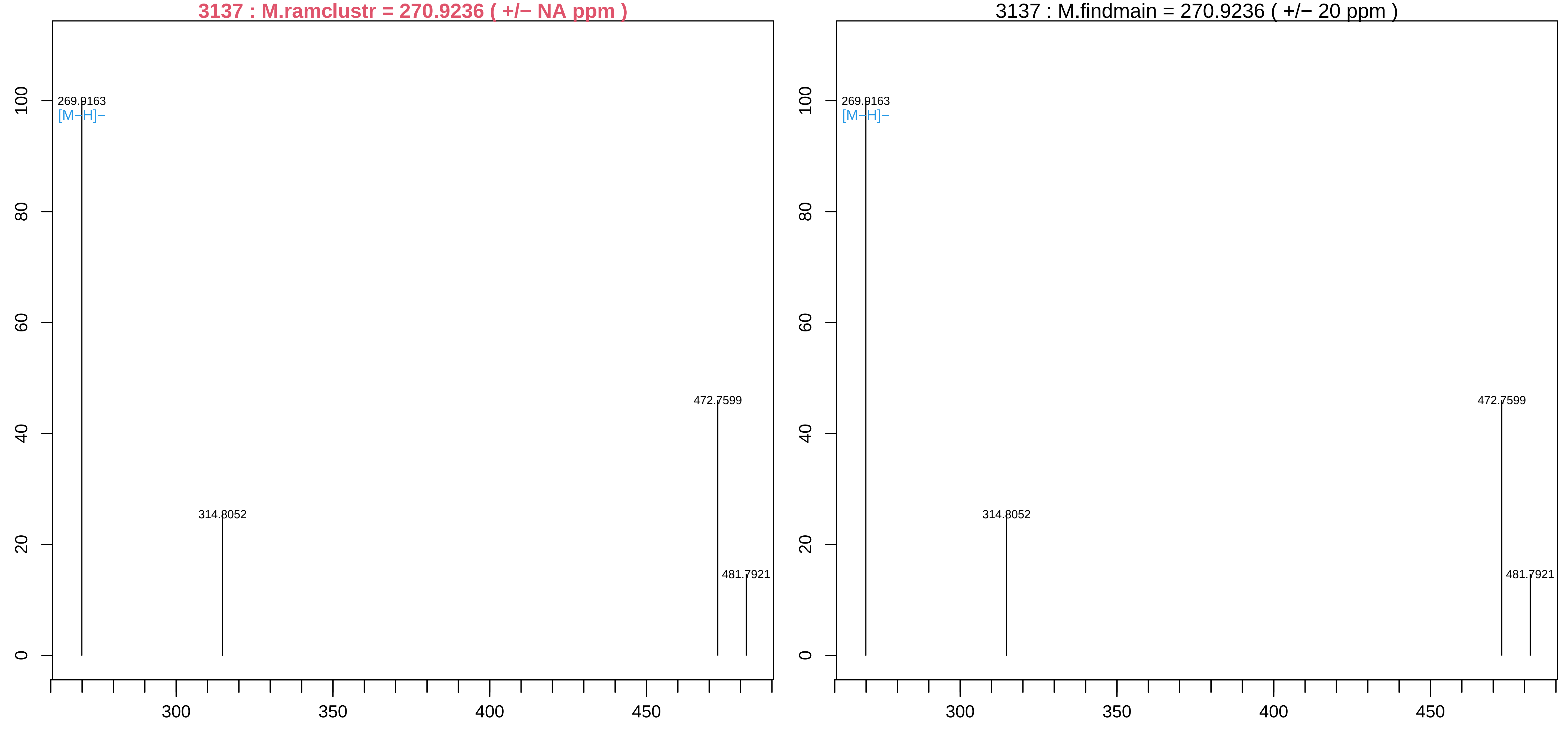
3137 : M.ramclustr = 270.9236 (+/− NA ppm)
269.9163
[M−H]−
314.8052
472.7599
481.7921
0
20
40
60
80
100
300
350
400
450
3137 : M.findmain = 270.9236 (+/− 20 ppm)
269.9163
[M−H]−
314.8052
472.7599
481.7921
0
20
40
60
80
100
300
350
400
450

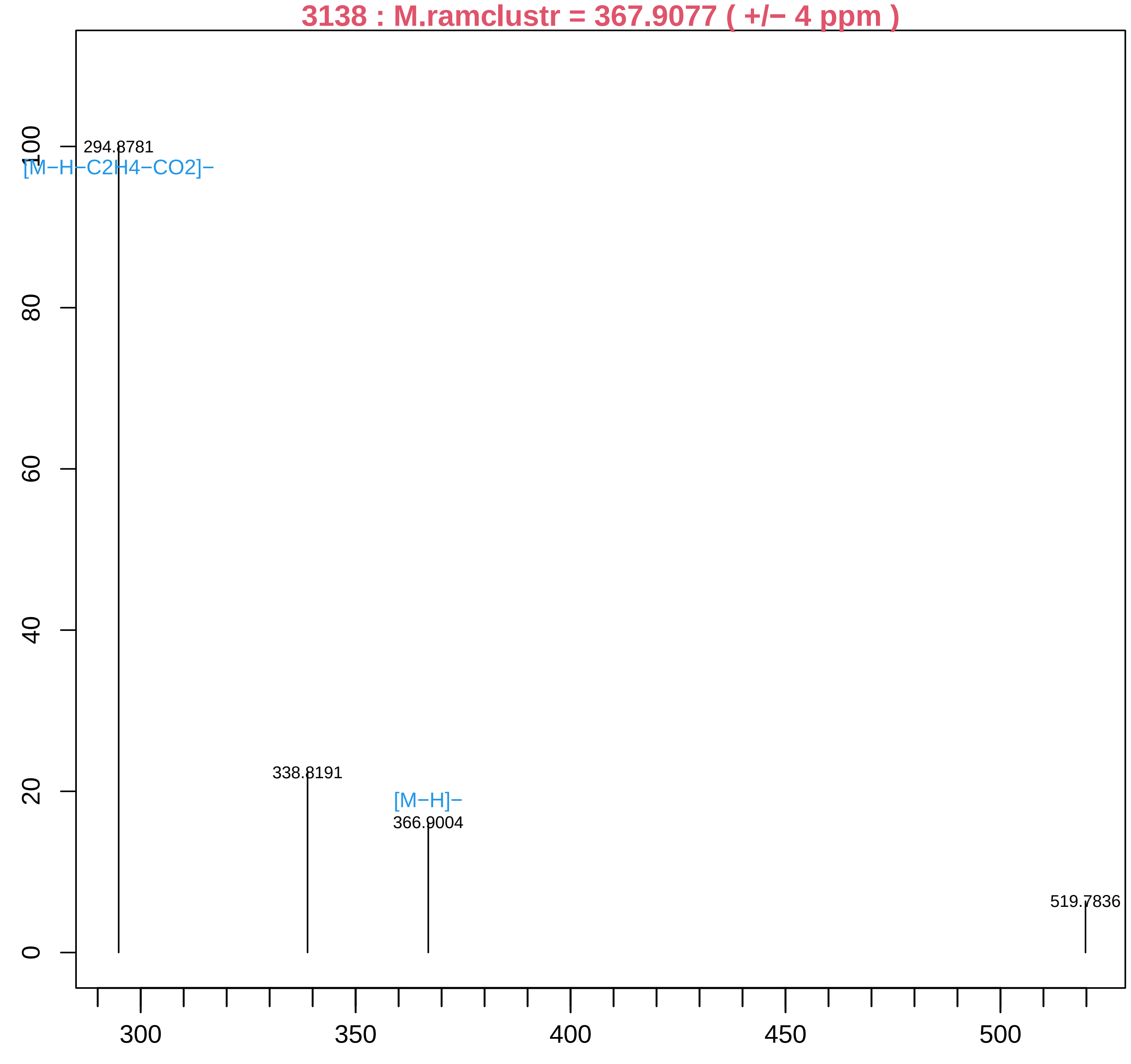
3138 : M.ramclustr = 367.9077 (+/− 4 ppm)
294.8781
[M−H−C2H4−CO2]−
338.8191
[M−H]−
366.9004
519.7836
0
20
40
60
80
100
300
350
400
450
500

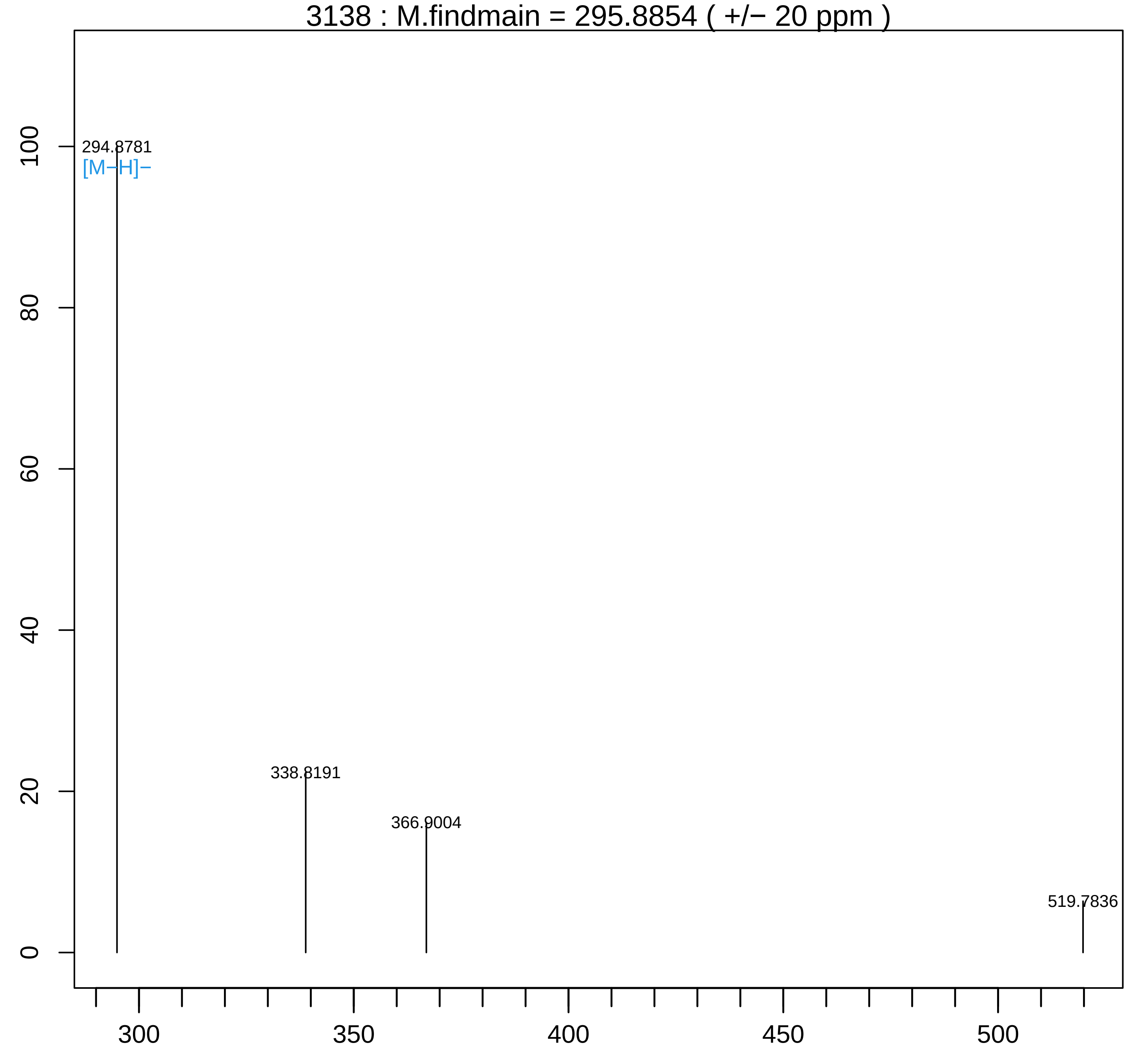
3138 : M.findmain = 295.8854 (+/− 20 ppm)
294.8781
[M−H]−
338.8191
366.9004
519.7836
0
20
40
60
80
100
300
350
400
450
500

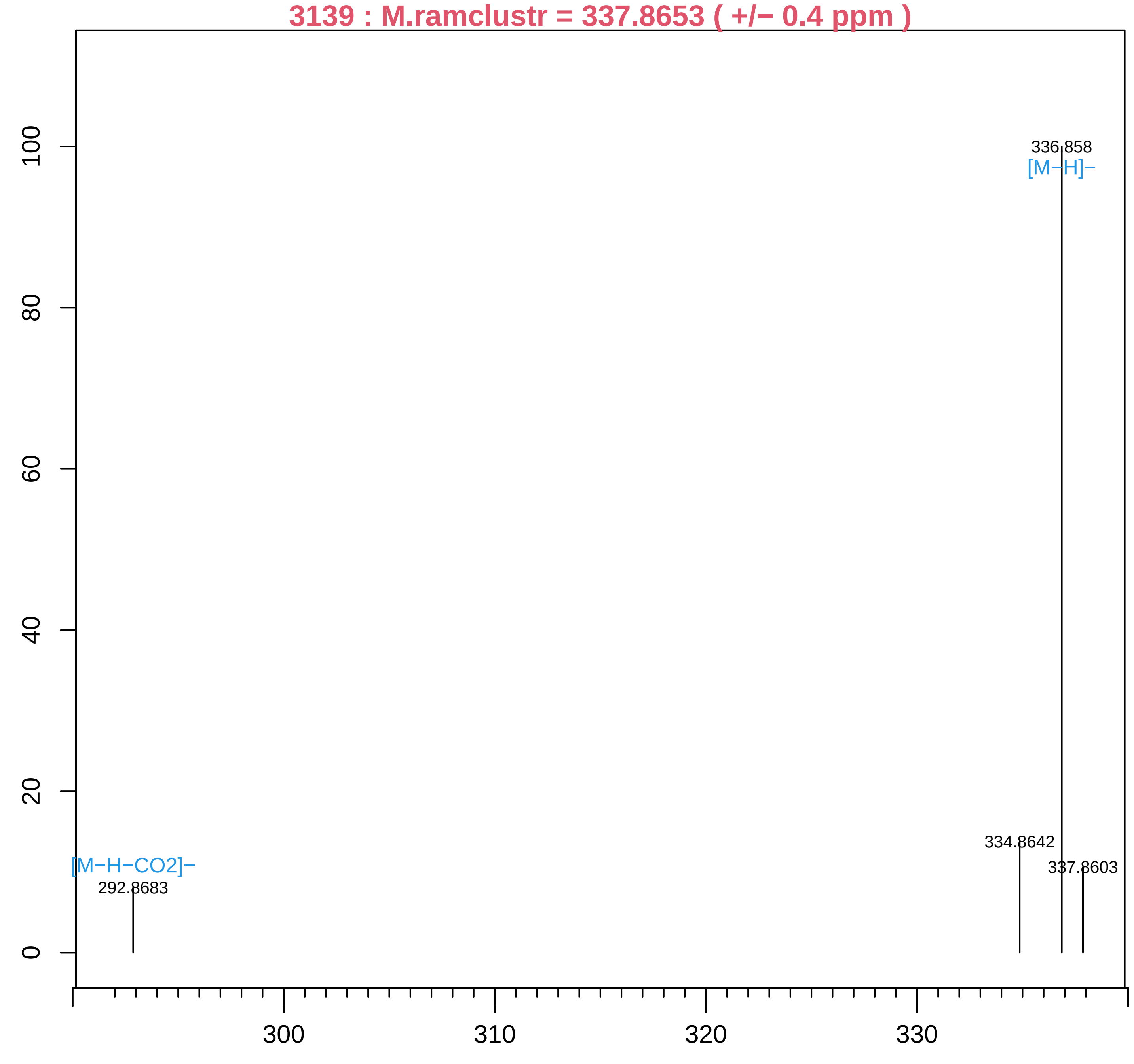

3139 : M.ramclustr = 337.8653 (+/− 0.4 ppm)
336.858
[M−H]−
334.8642
337.8603
[M−H−CO2]−
292.8683
0
20
40
60
80
100
300
310
320
330

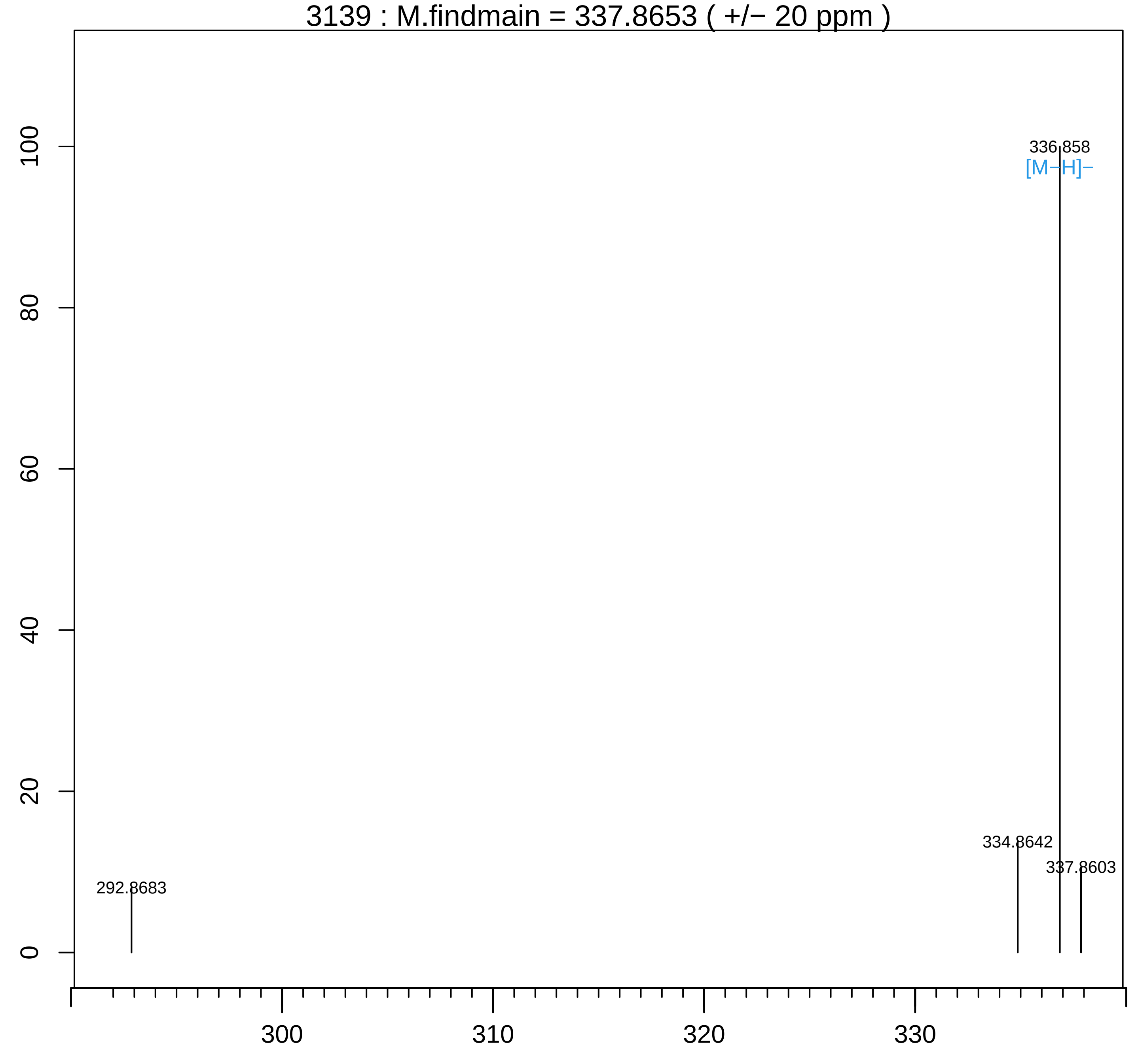

3139 : M.findmain = 337.8653 (+/− 20 ppm)
336.858
[M−H]−
334.8642
337.8603
292.8683
0
20
40
60
80
100
300
310
320
330

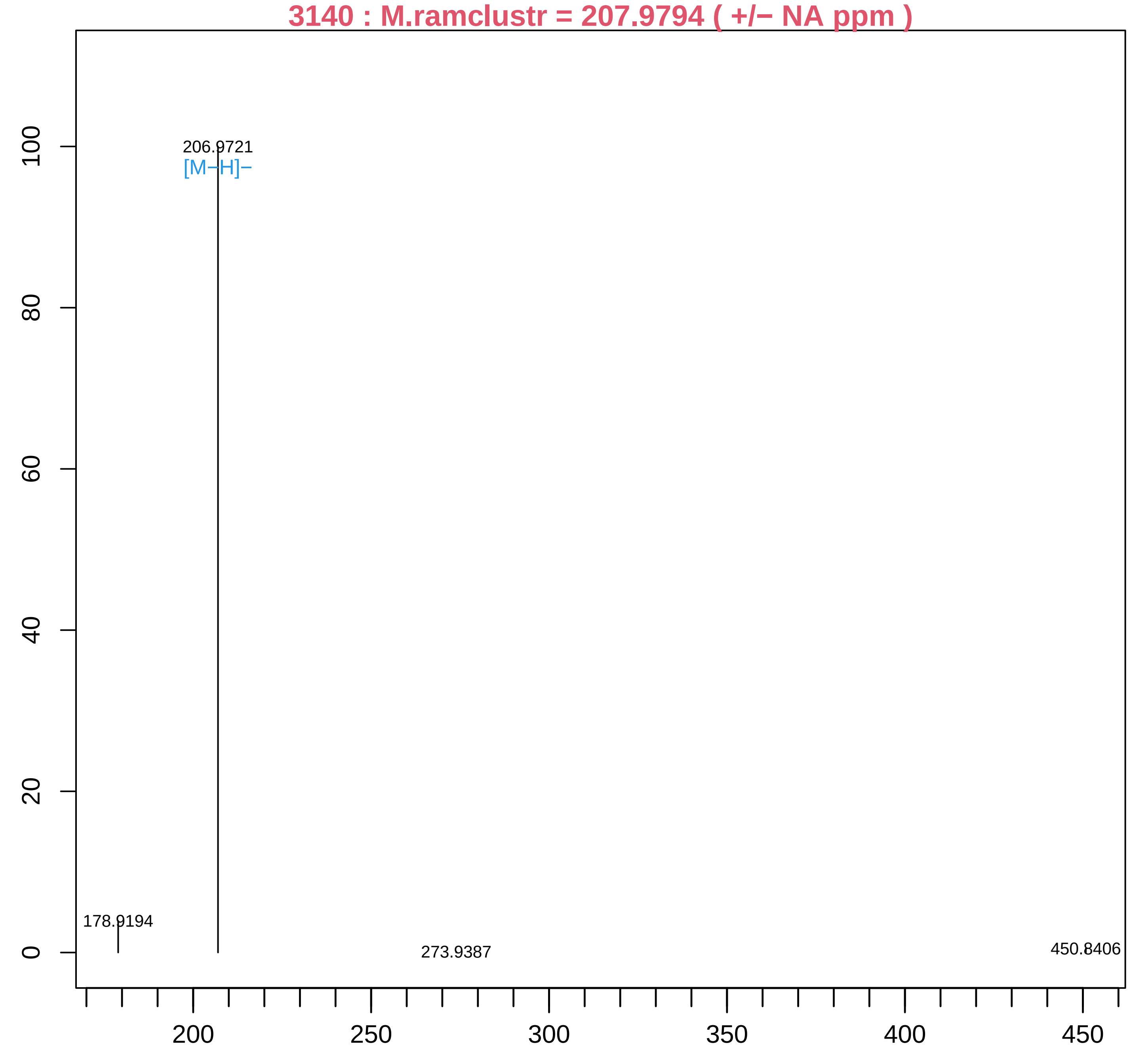
3140 : M.ramclustr = 207.9794 (+/− NA ppm)
206.9721
[M−H]−
178.9194
273.9387
450.8406
0
20
40
60
80
100
200
250
300
350
400
450
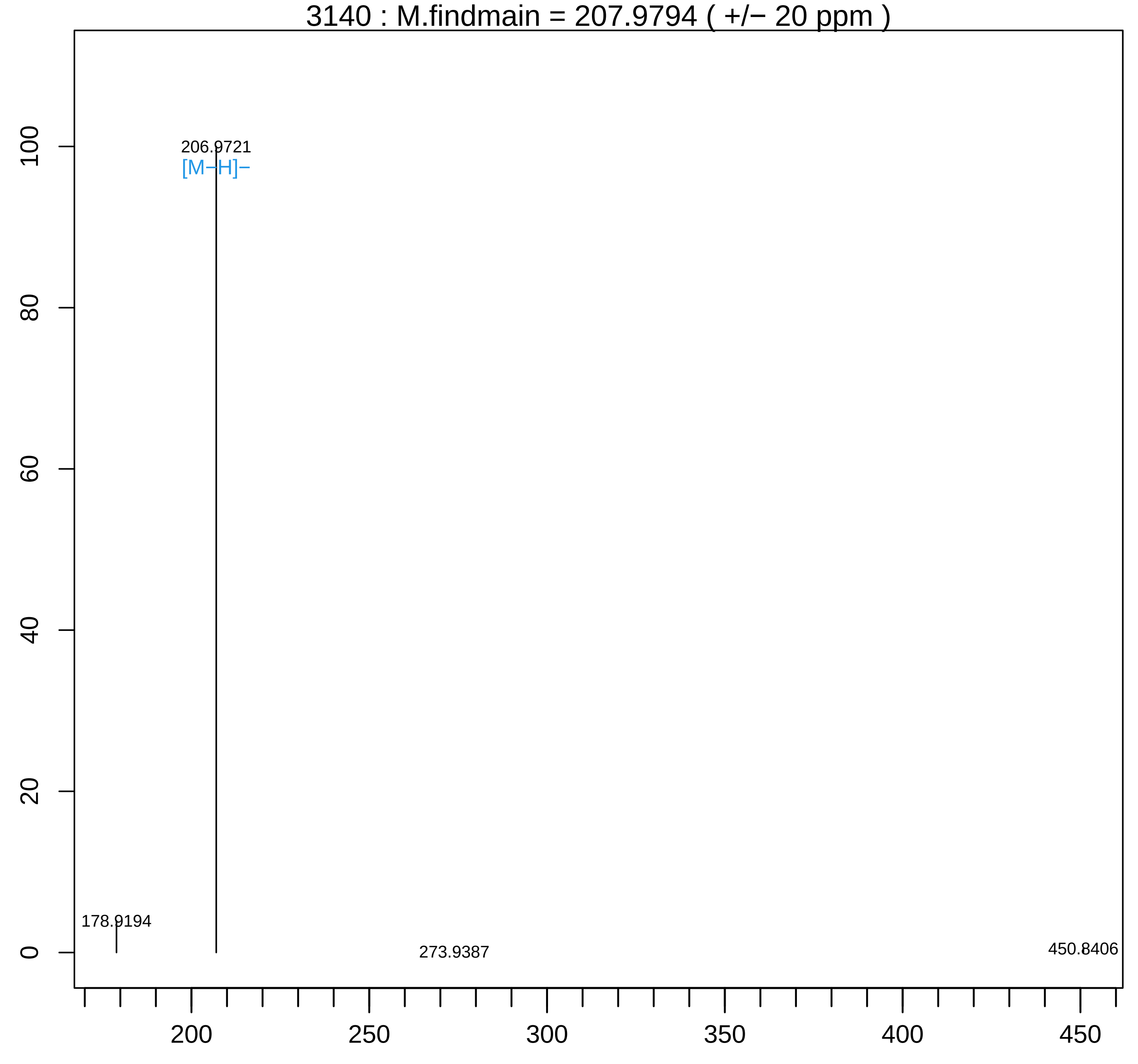
3140 : M.findmain = 207.9794 (+/− 20 ppm)
206.9721
[M−H]−
178.9194
273.9387
450.8406
0
20
40
60
80
100
200
250
300
350
400
450

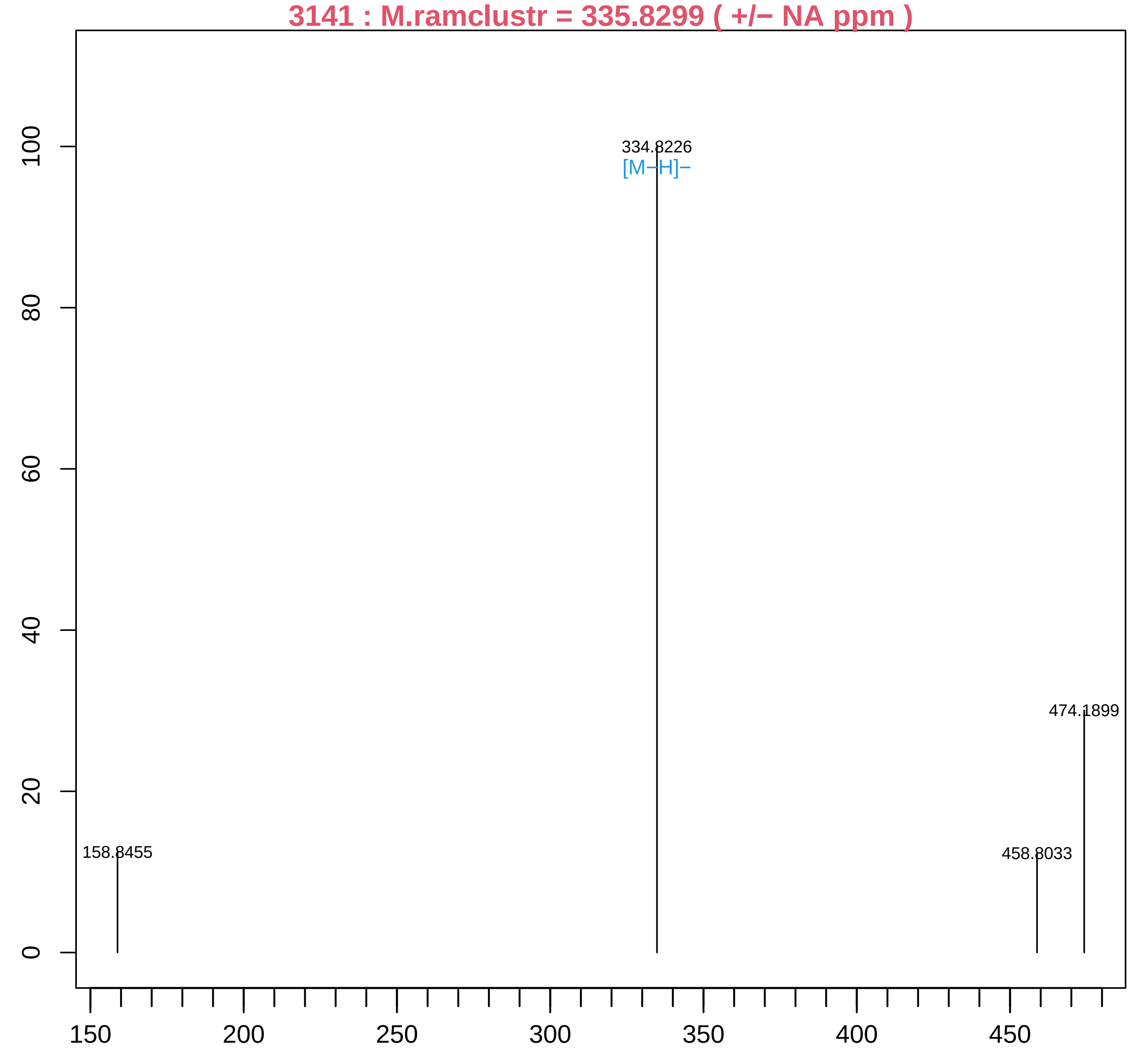
3141 : M.ramclustr = 335.8299 (+/− NA ppm)
334.8226
[M−H]−
158.8455
458.8033
474.1899
0
20
40
60
80
100
150
200
250
300
350
400
450

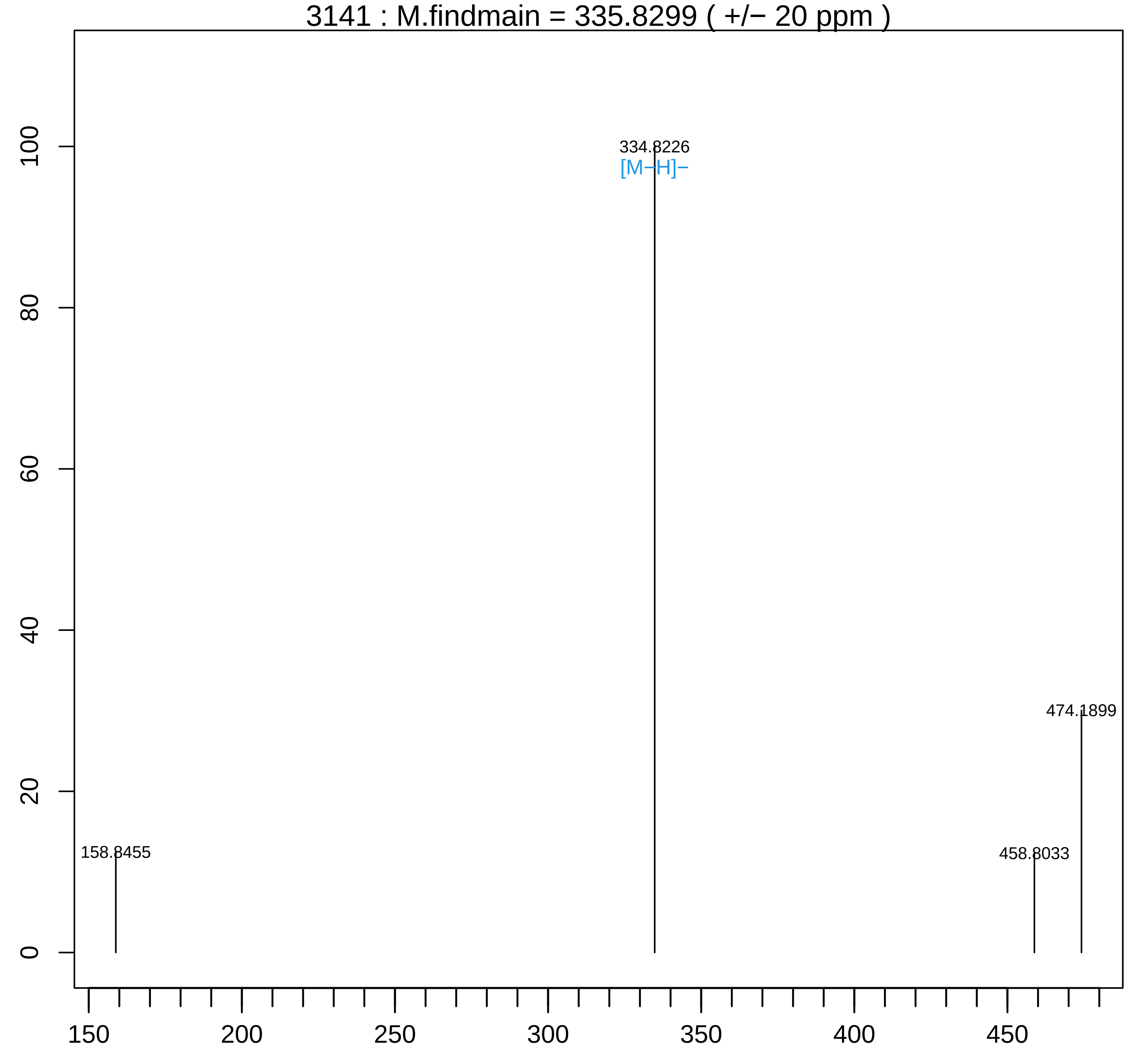
3141 : M.findmain = 335.8299 (+/− 20 ppm)
334.8226
[M−H]−
158.8455
458.8033
474.1899
0
20
40
60
80
100
150
200
250
300
350
400
450

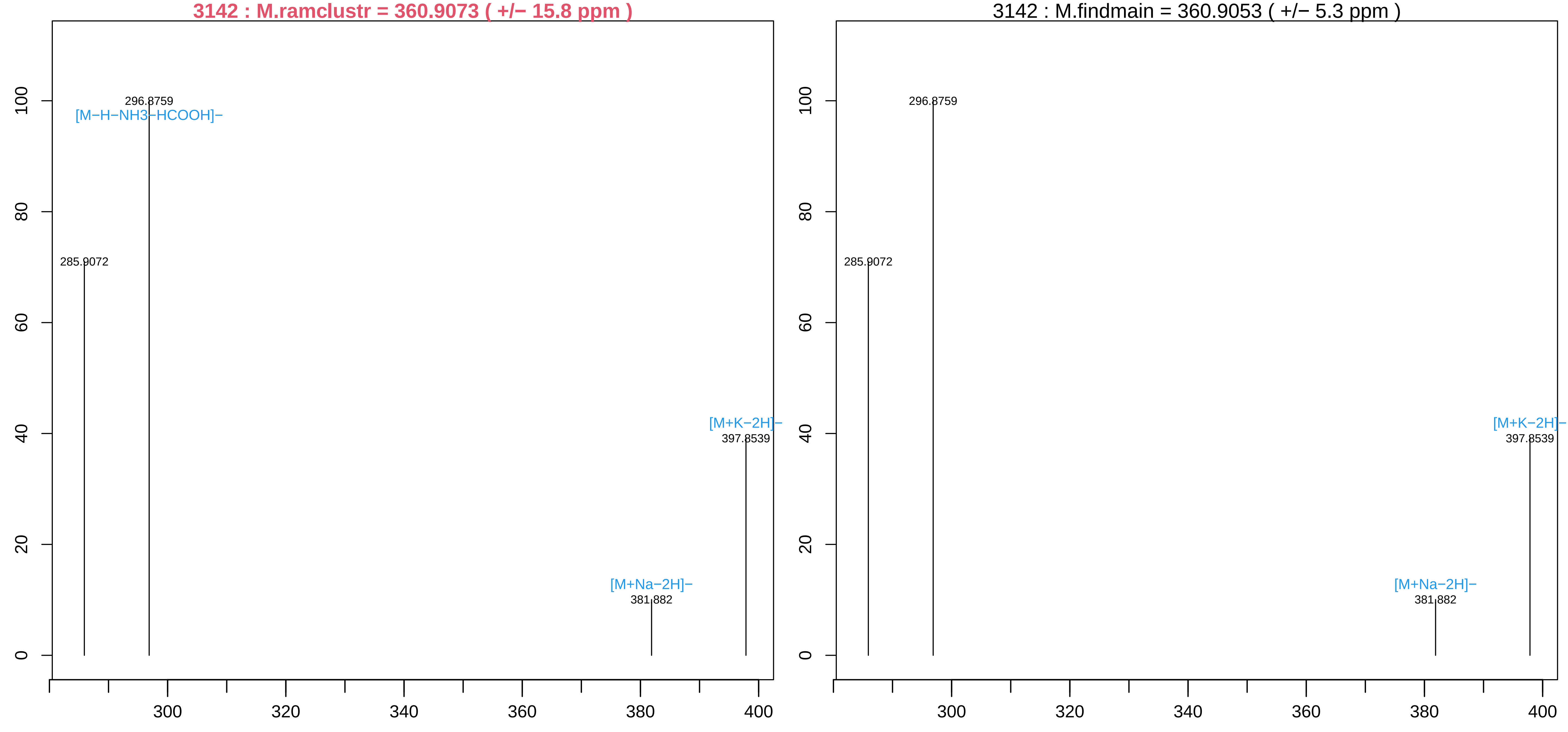
3142 : M.ramclustr = 360.9073 (+/− 15.8 ppm)
296.8759
[M−H−NH3−HCOOH]−
285.9072
[M+K−2H]−
397.8539
[M+Na−2H]−
381.882
3142 : M.findmain = 360.9053 (+/− 5.3 ppm)
296.8759
285.9072
[M+K−2H]−
397.8539
[M+Na−2H]−
381.882

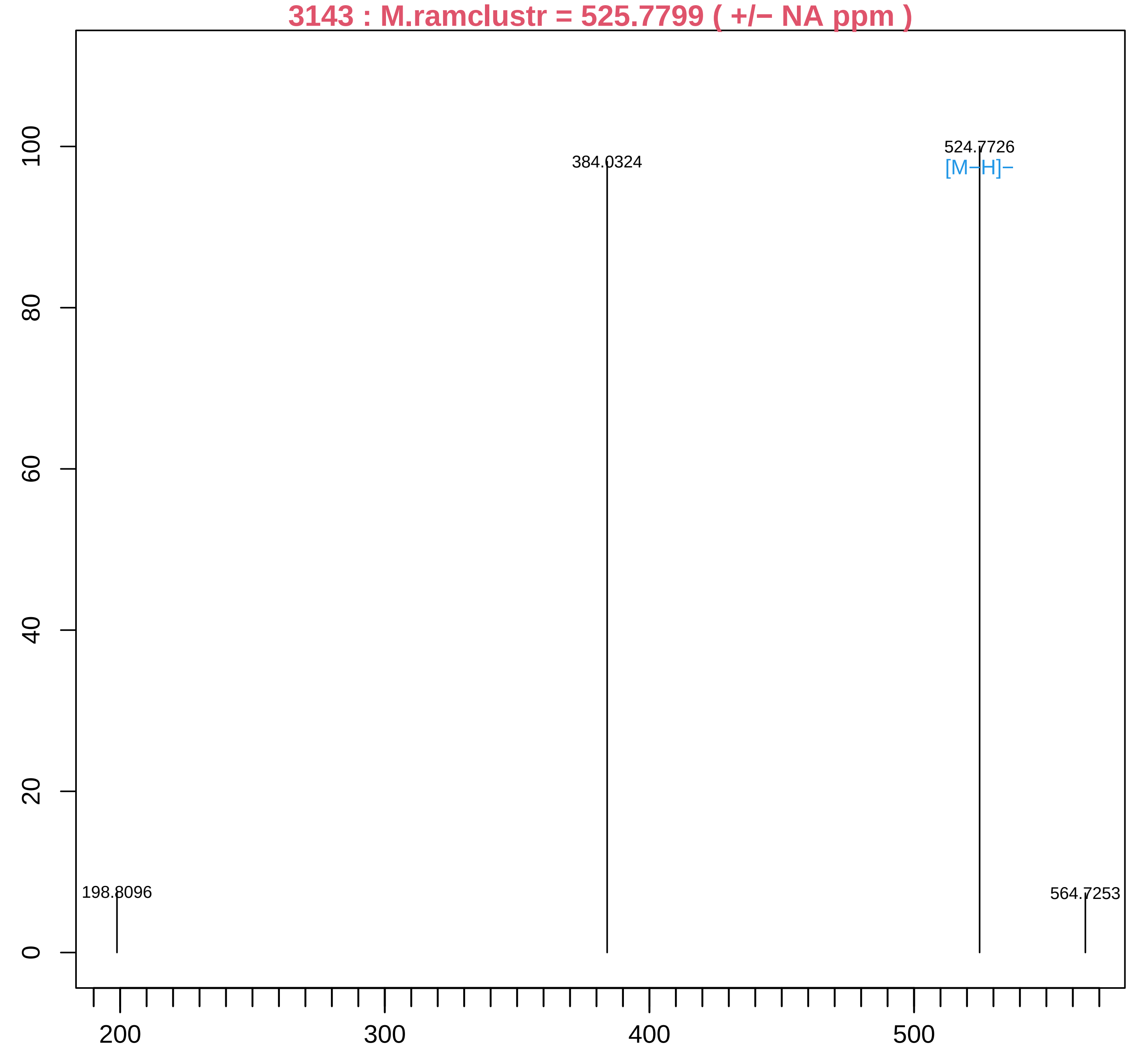
3143 : M.ramclustr = 525.7799 (+/− NA ppm)
384.0324
524.7726
[M−H]−
198.8096
564.7253

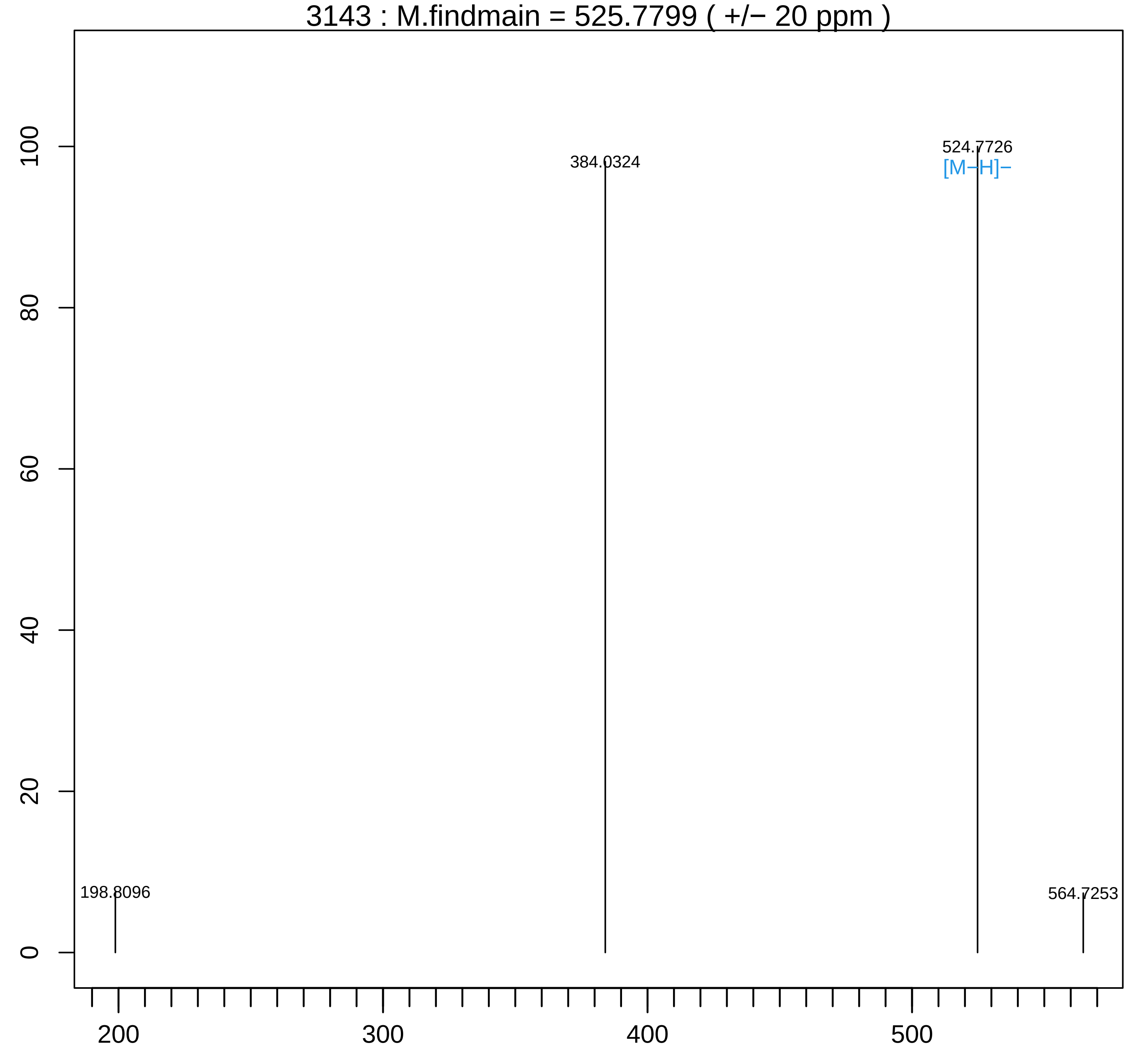
3143 : M.findmain = 525.7799 (+/− 20 ppm)
384.0324
524.7726
[M−H]−
198.8096
564.7253

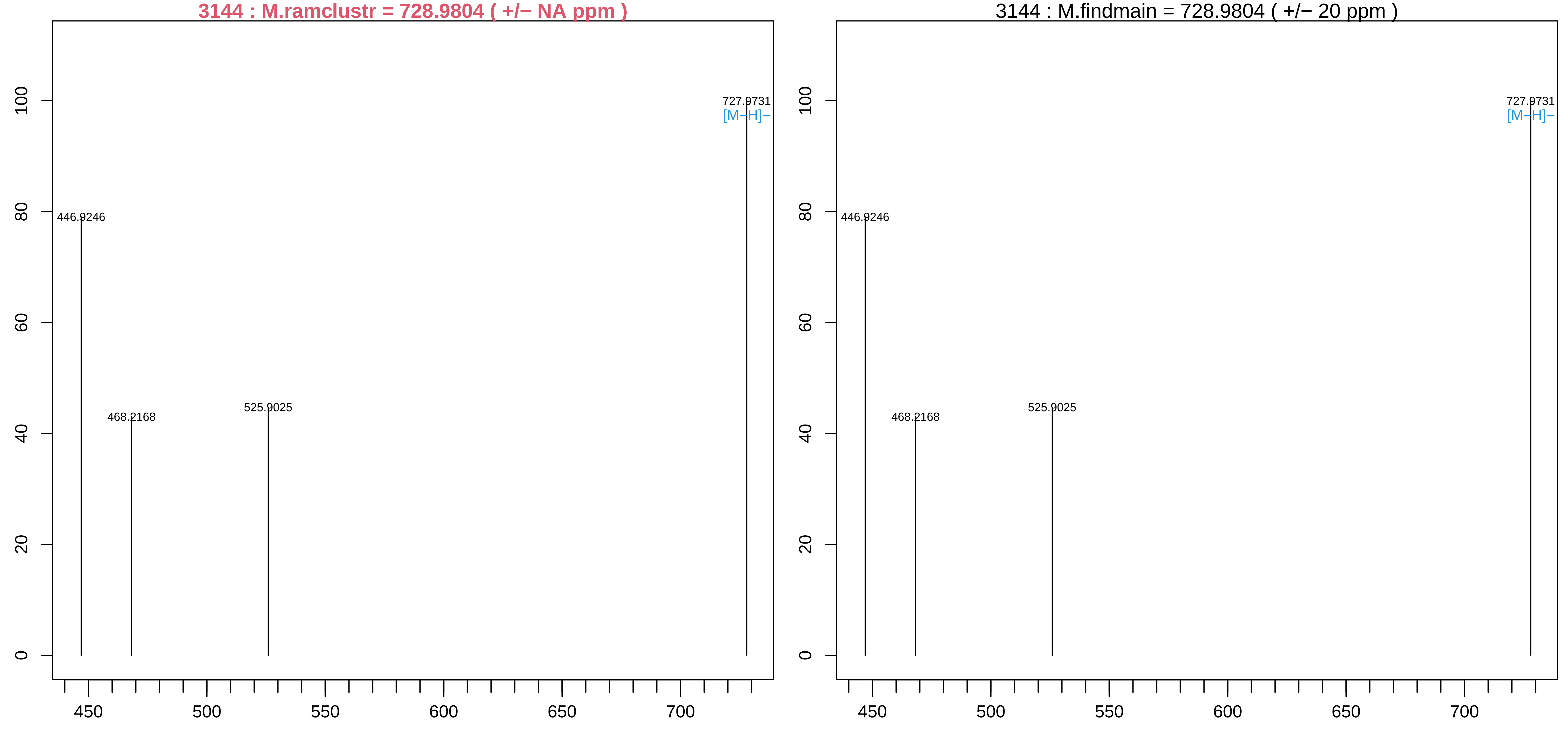
3144 : M.ramclustr = 728.9804 (+/− NA ppm)
3144 : M.findmain = 728.9804 (+/− 20 ppm)
727.9731
[M−H]−
446.9246
468.2168
525.9025
0
20
40
60
80
100
450
500
550
600
650
700

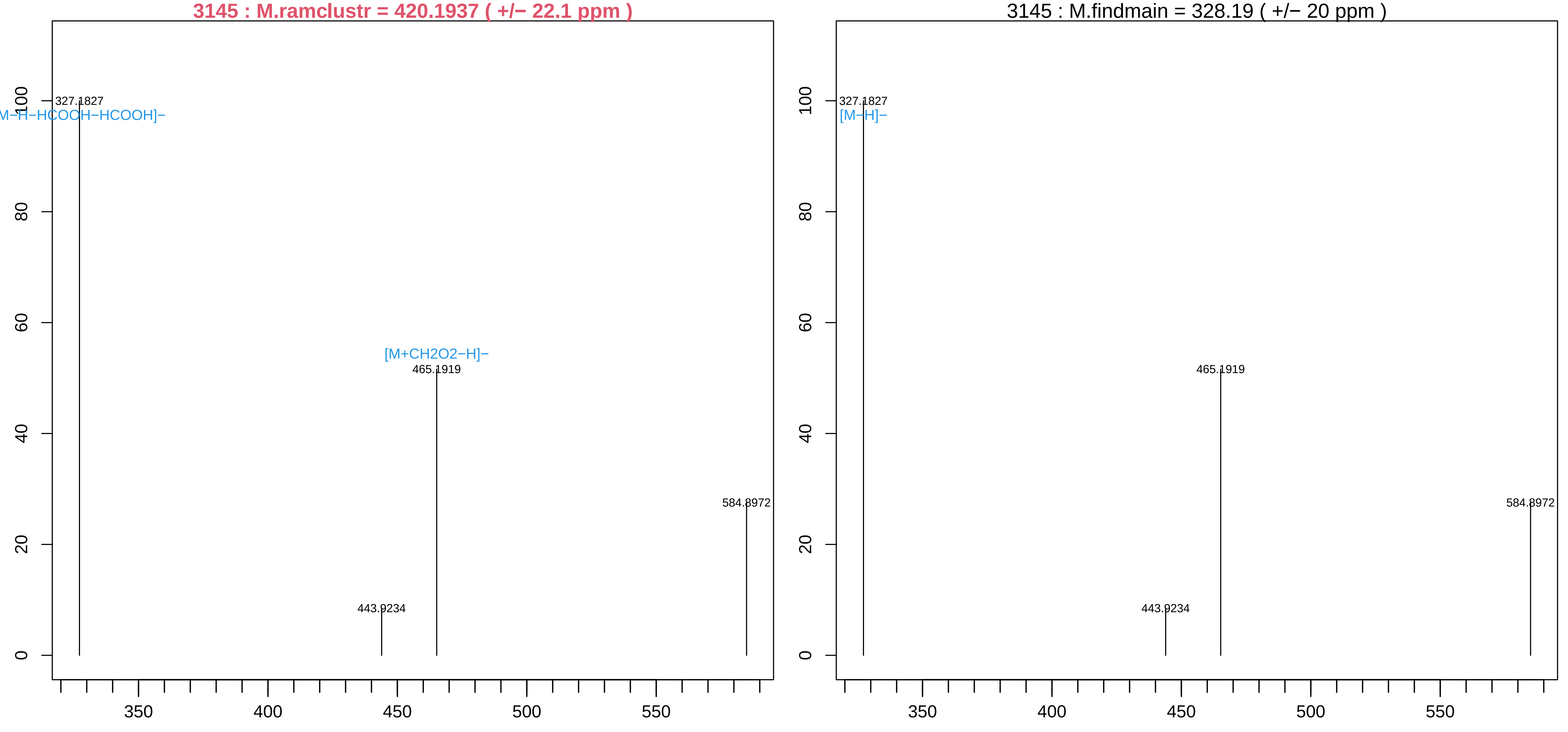
3145 : M.ramclustr = 420.1937 (+/− 22.1 ppm)
327.1827
[M−H−HCOOH−HCOOH]−
[M+CH2O2−H]−
465.1919
443.9234
584.8972
3145 : M.findmain = 328.19 (+/− 20 ppm)
327.1827
[M−H]−
465.1919
443.9234
584.8972

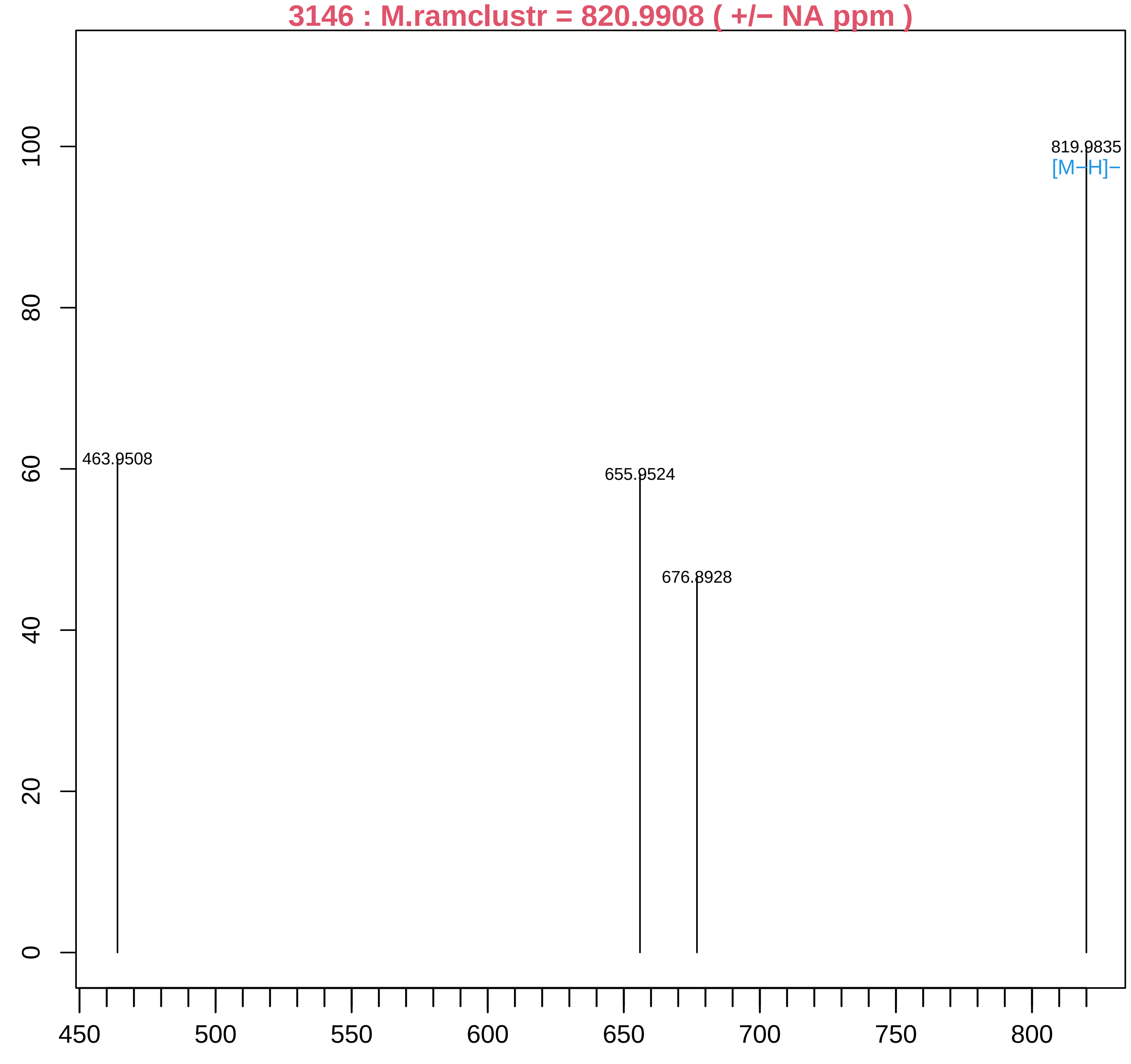
3146 : M.ramclustr = 820.9908 (+/− NA ppm)
819.9835
[M−H]−
463.9508
655.9524
676.8928
0
20
40
60
80
100
450
500
550
600
650
700
750
800

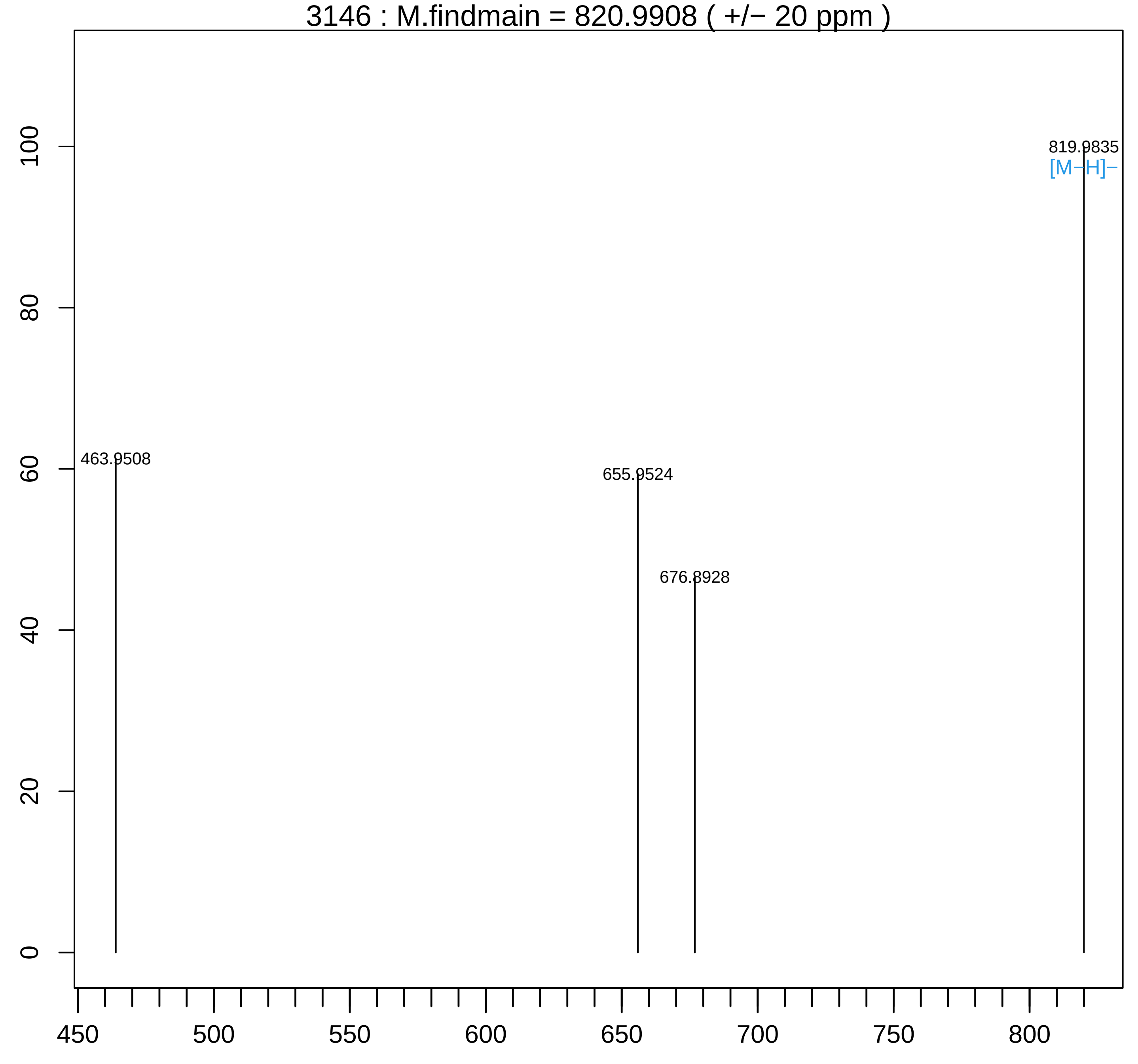
3146 : M.findmain = 820.9908 (+/− 20 ppm)
819.9835
[M−H]−
463.9508
655.9524
676.8928
0
20
40
60
80
100
450
500
550
600
650
700
750
800

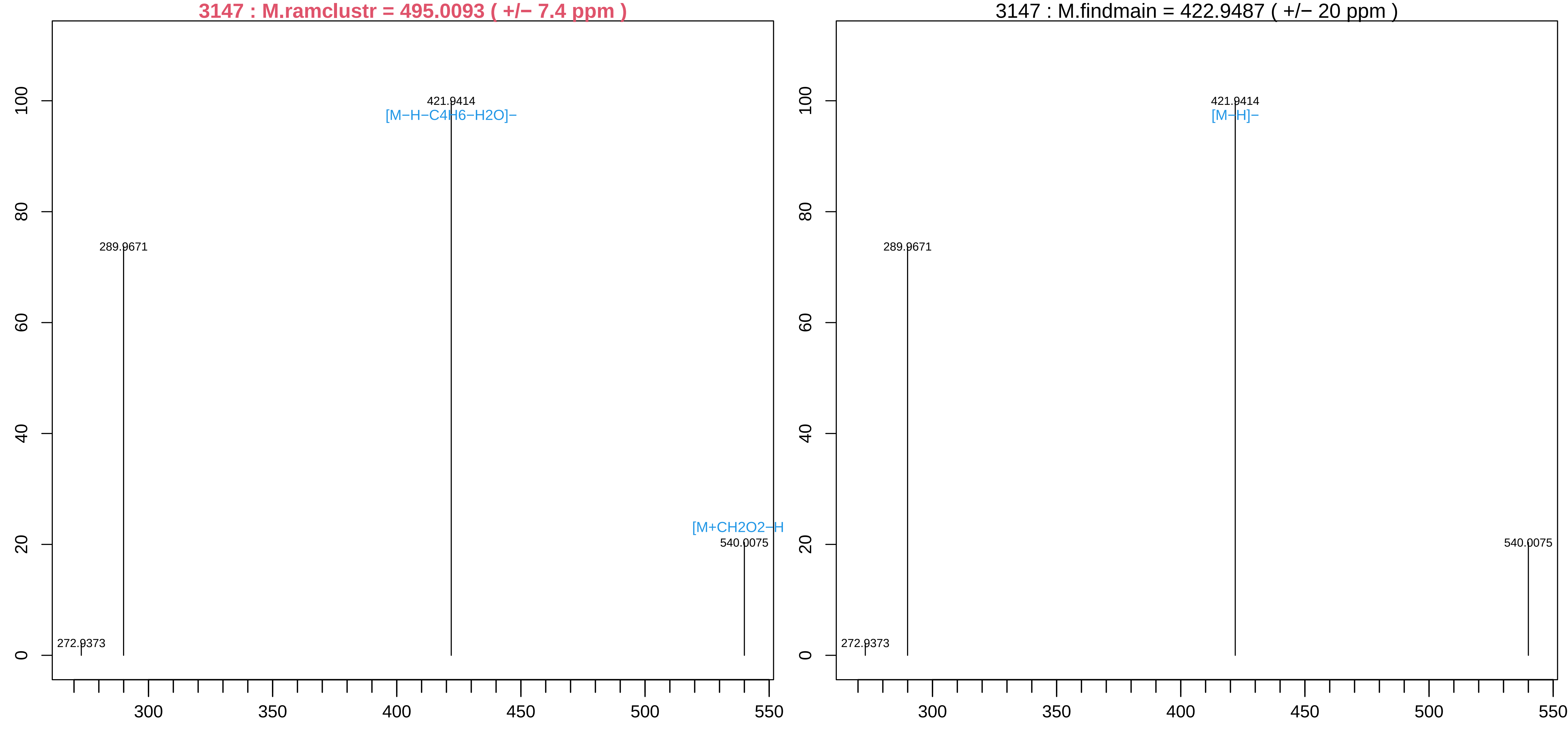
3147 : M.ramclustr = 495.0093 (+/− 7.4 ppm)
421.9414
[M−H−C4H6−H2O]−
289.9671
[M+CH2O2−H
540.0075
272.9373
0
20
40
60
80
100
300
350
400
450
500
550
3147 : M.findmain = 422.9487 (+/− 20 ppm)
421.9414
[M−H]−
289.9671
540.0075
272.9373

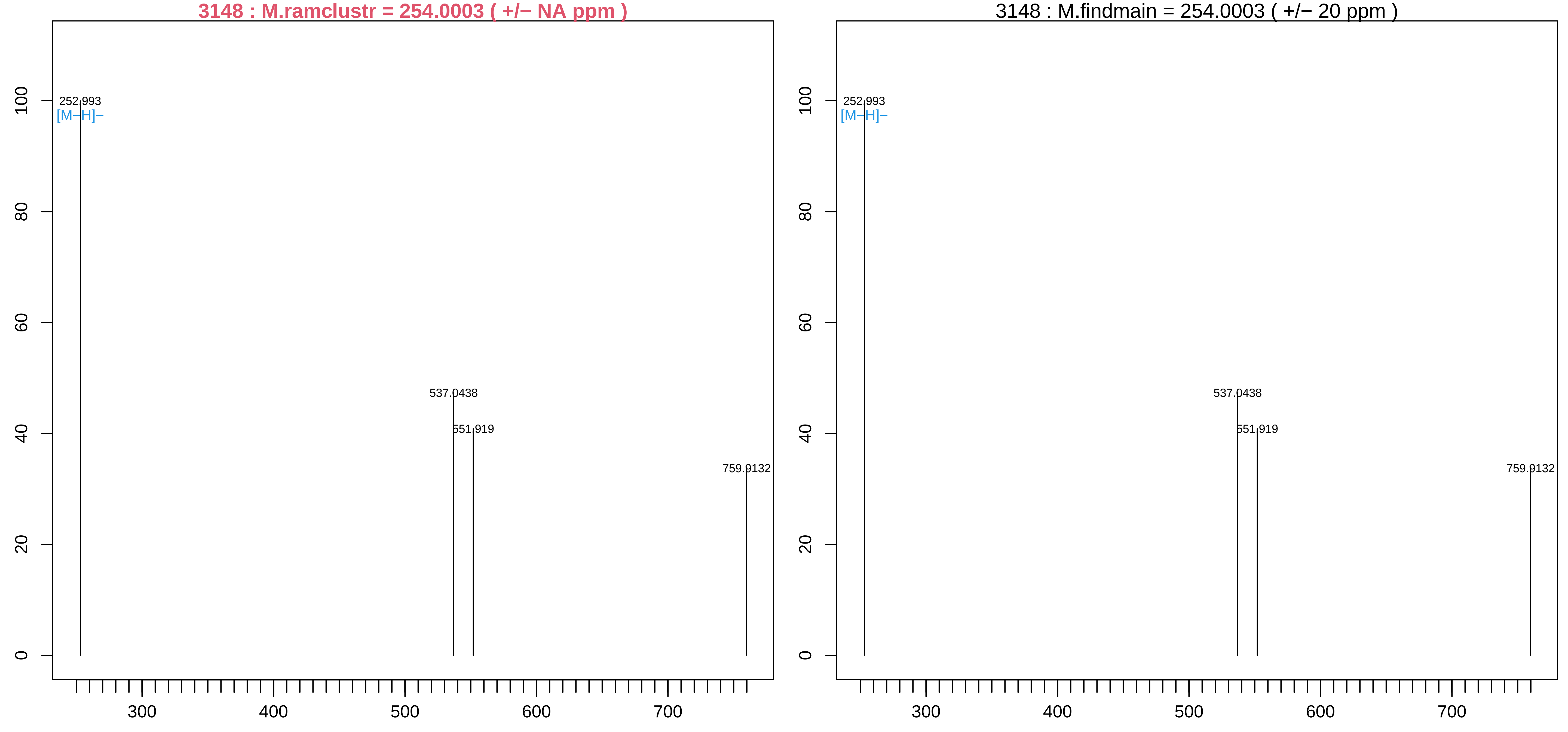
3148 : M.ramclustr = 254.0003 (+/− NA ppm)
3148 : M.findmain = 254.0003 (+/− 20 ppm)
252.993
[M−H]−
537.0438
551.919
759.9132
0
20
40
60
80
100
300
400
500
600
700

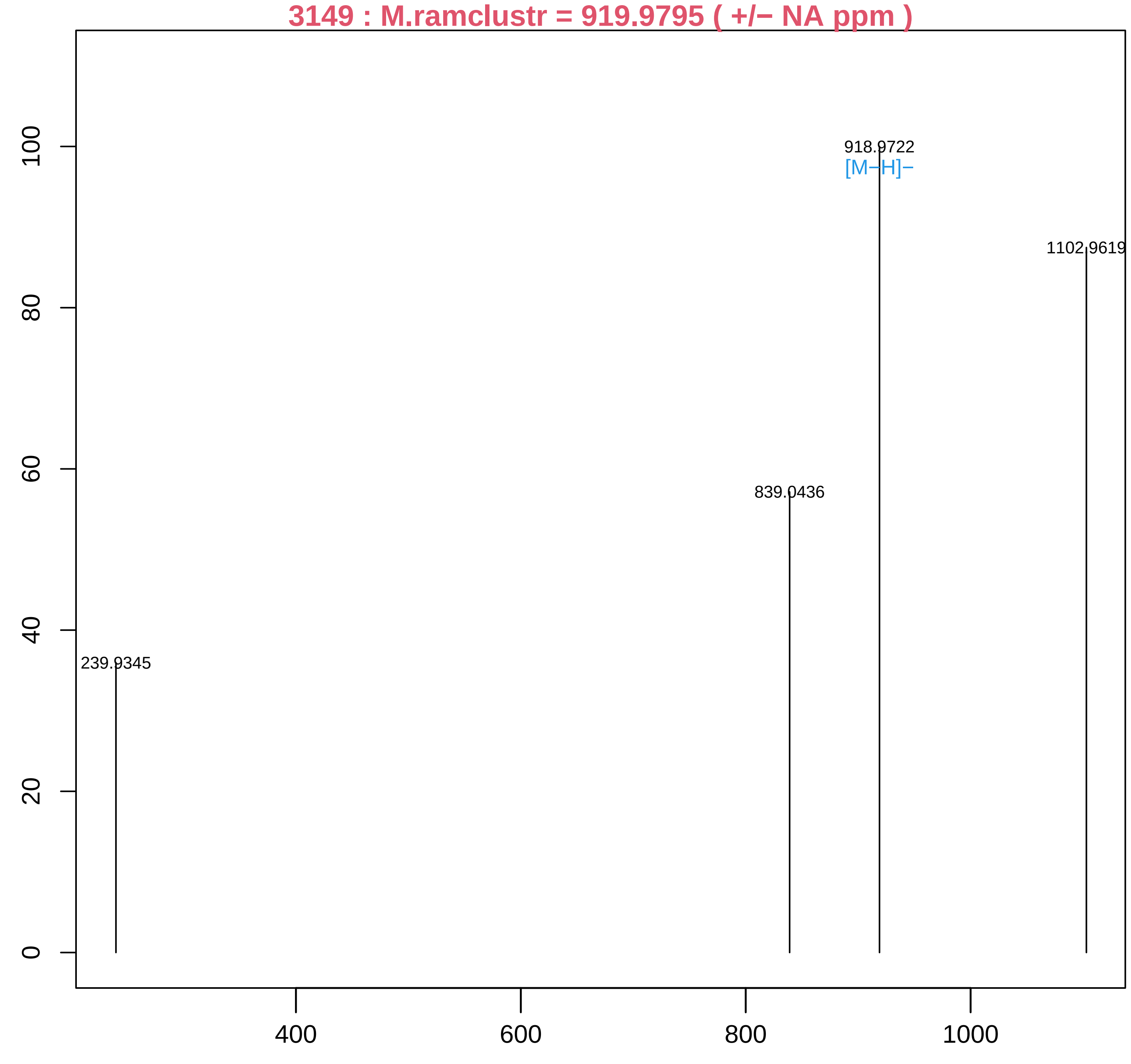

3149 : M.ramclustr = 919.9795 (+/− NA ppm)
918.9722
[M−H]−
1102.9619
839.0436
239.9345
0
20
40
60
80
100
400
600
800
1000

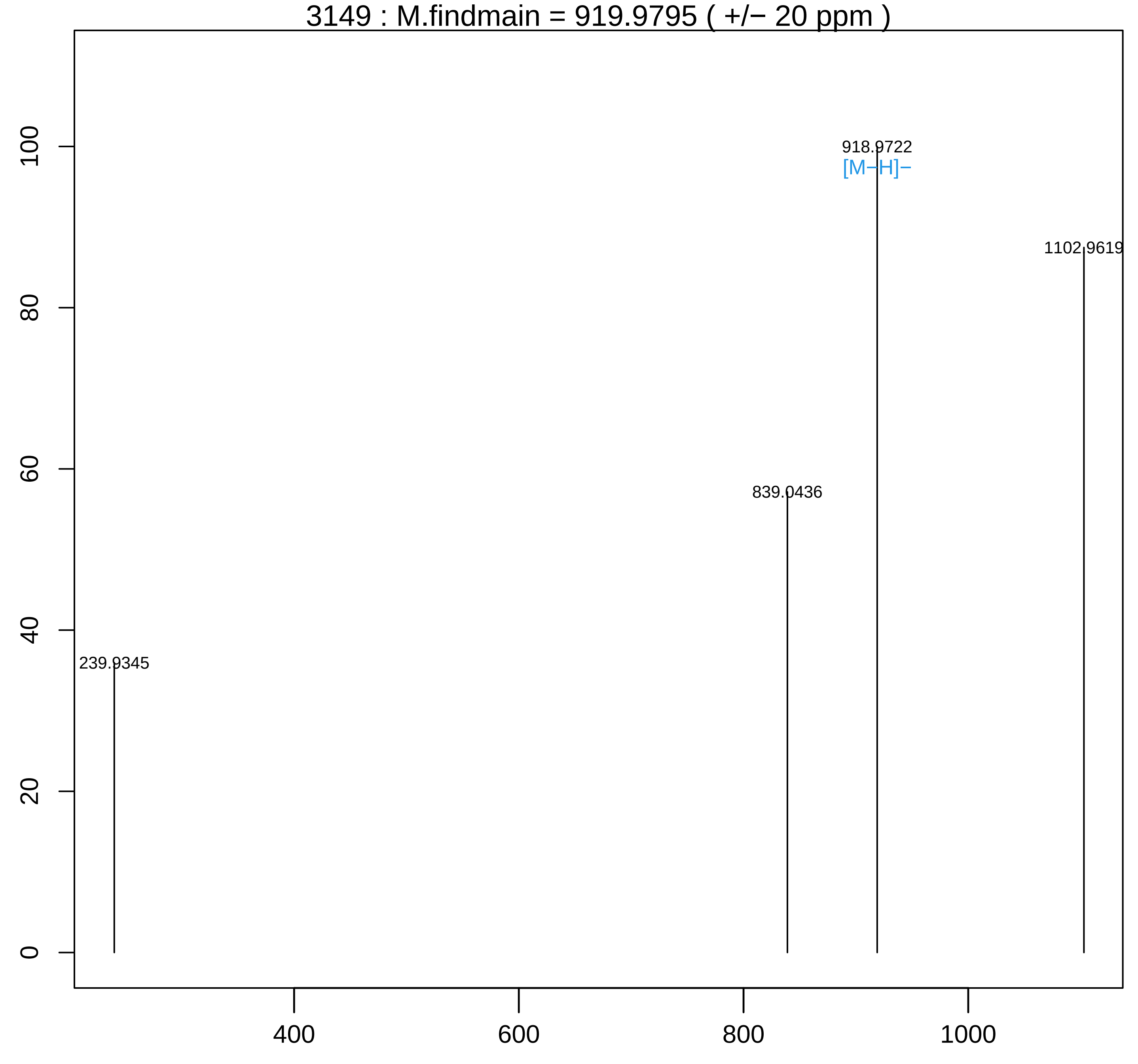

3149 : M.findmain = 919.9795 (+/− 20 ppm)
918.9722
[M−H]−
1102.9619
839.0436
239.9345
0
20
40
60
80
100
400
600
800
1000

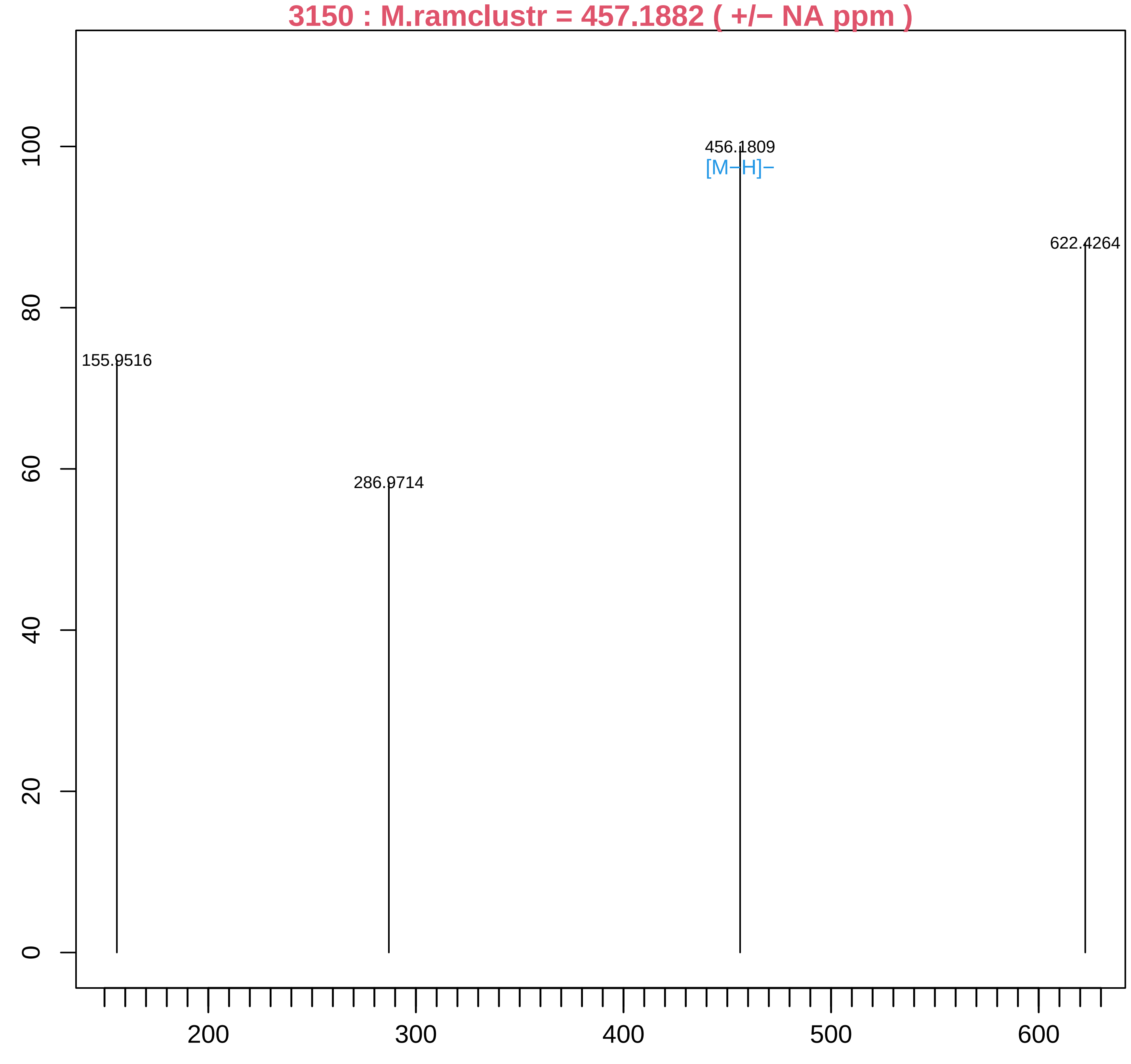
3150 : M.ramclustr = 457.1882 (+/− NA ppm)
155.9516
286.9714
456.1809
[M−H]−
622.4264
0
20
40
60
80
100
200
300
400
500
600
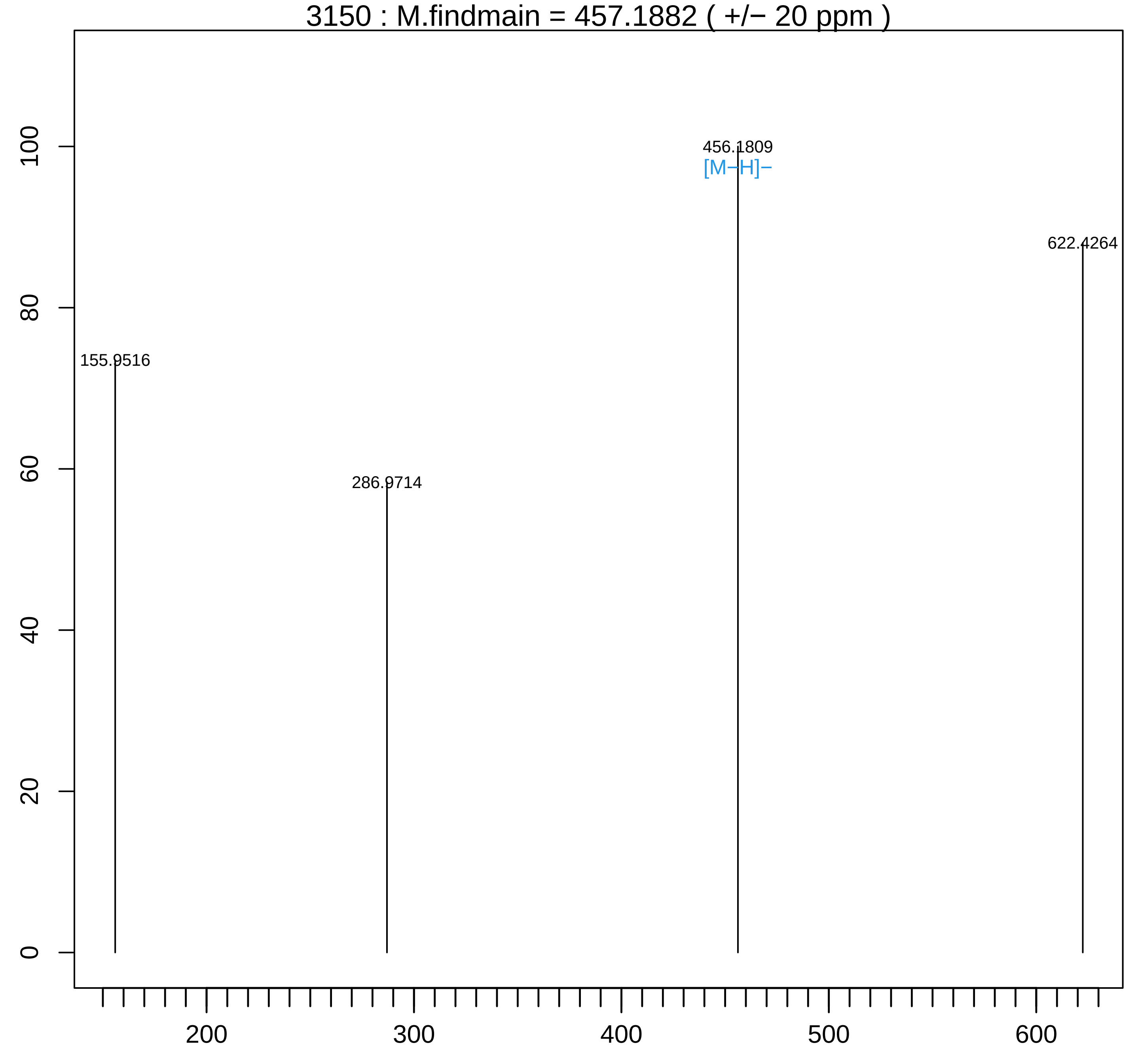
3150 : M.findmain = 457.1882 (+/− 20 ppm)
155.9516
286.9714
456.1809
[M−H]−
622.4264
0
20
40
60
80
100
200
300
400
500
600

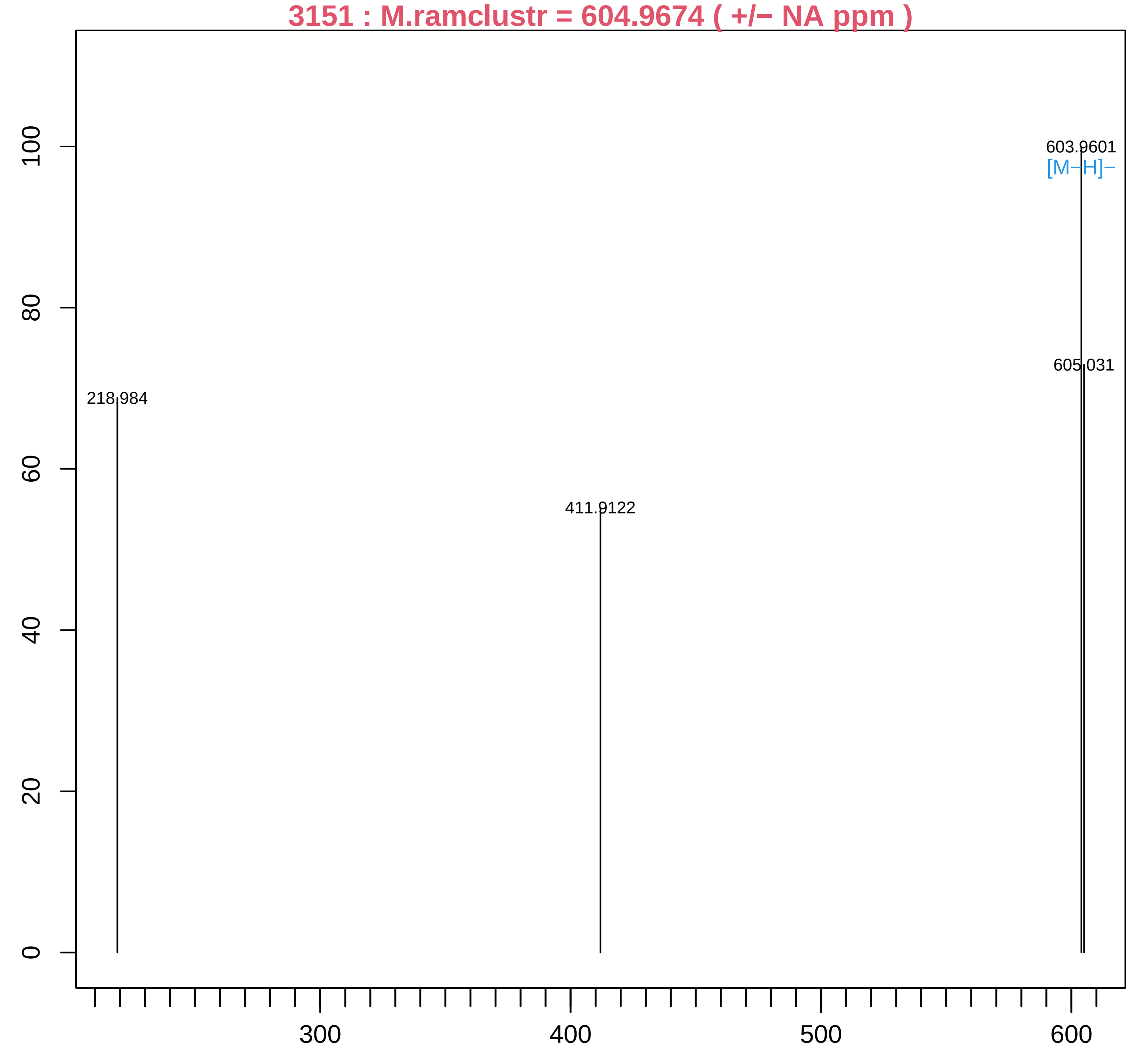
3151 : M.ramclustr = 604.9674 (+/− NA ppm)
603.9601
[M−H]−
605.031
218.984
411.9122
0
20
40
60
80
100
300
400
500
600
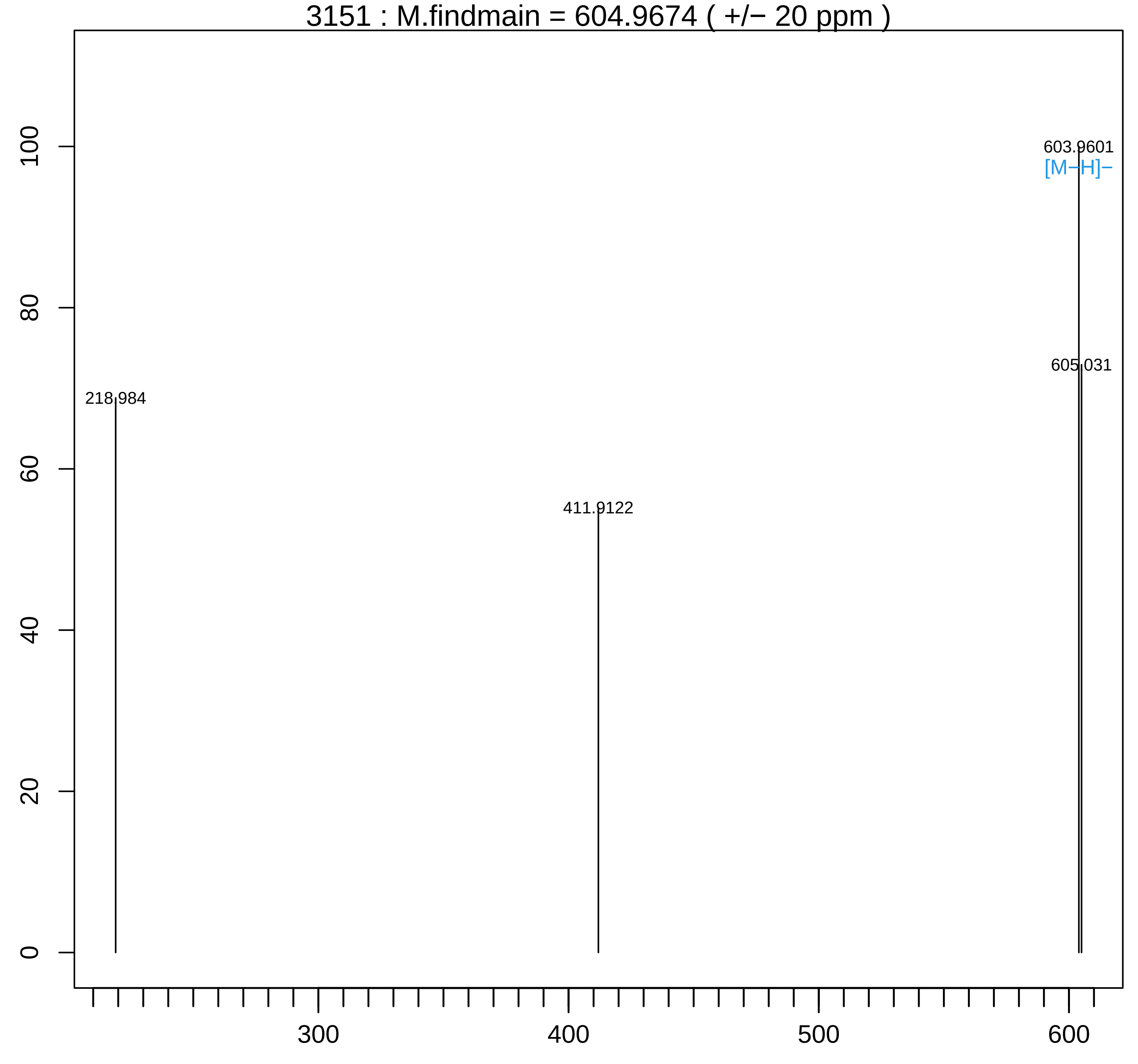
3151 : M.findmain = 604.9674 (+/− 20 ppm)
603.9601
[M−H]−
605.031
218.984
411.9122
0
20
40
60
80
100
300
400
500
600

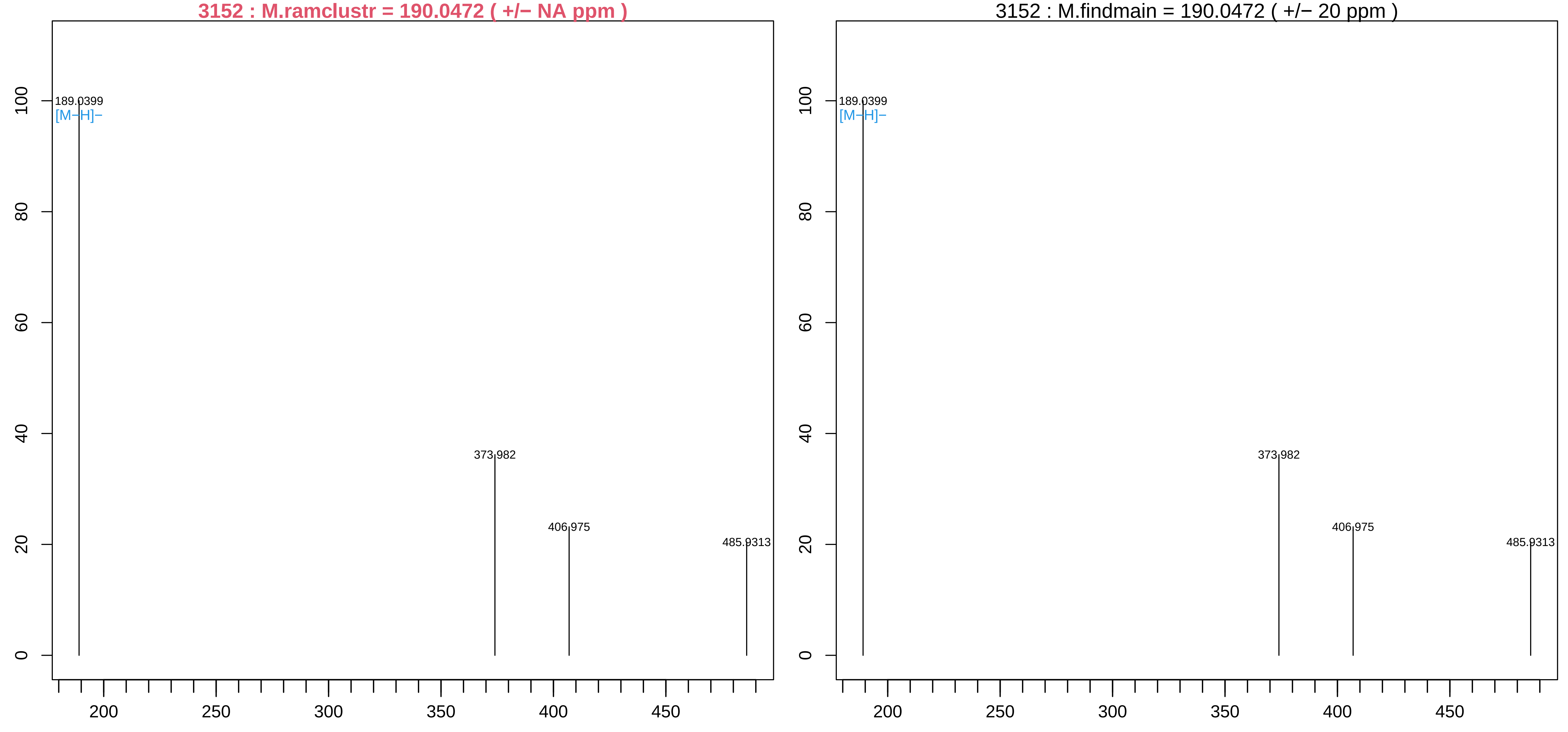

3152 : M.ramclustr = 190.0472 (+/− NA ppm)
189.0399
[M−H]−
373.982
406.975
485.9313
3152 : M.findmain = 190.0472 (+/− 20 ppm)
189.0399
[M−H]−
373.982
406.975
485.9313

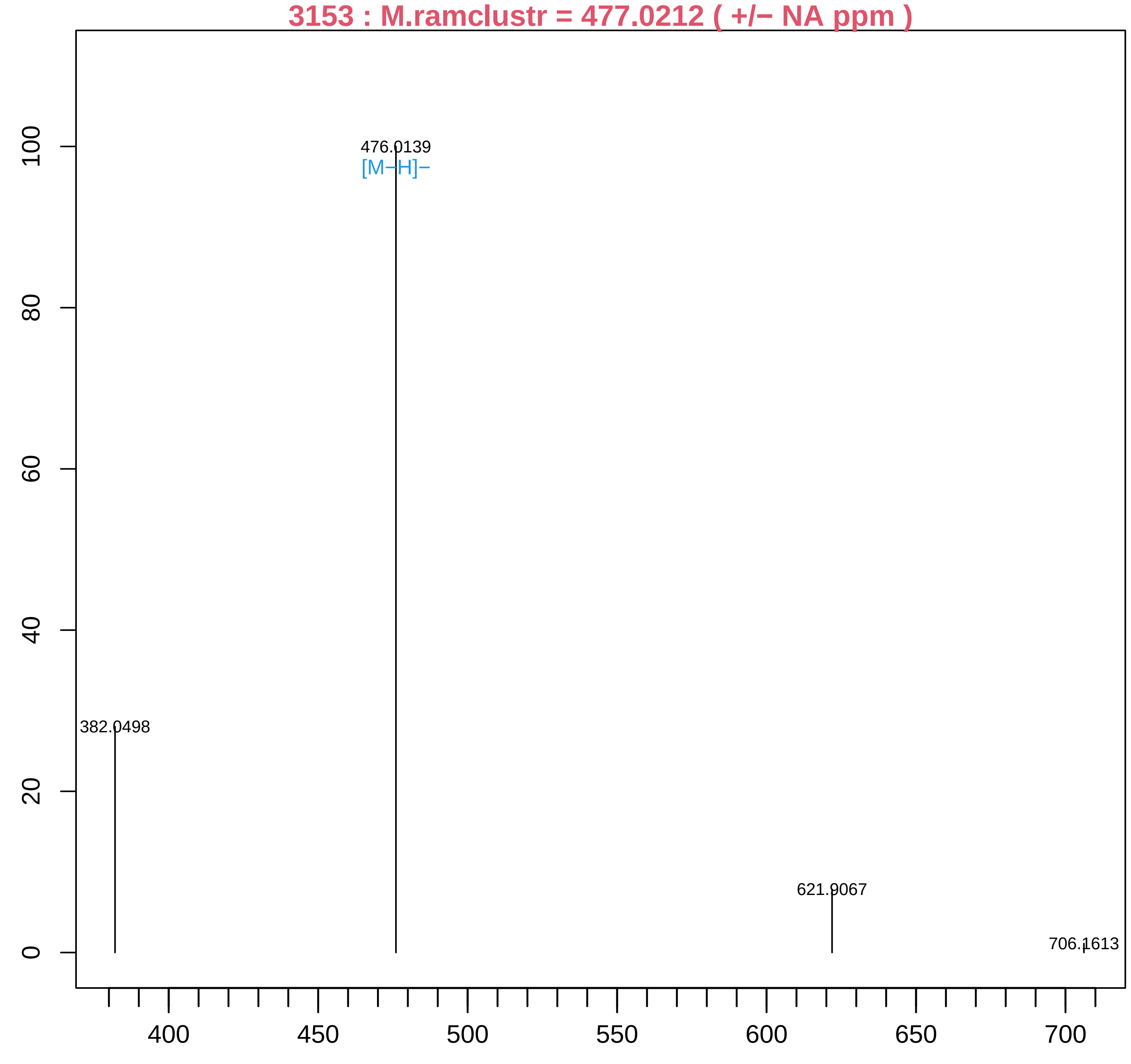
3153 : M.ramclustr = 477.0212 (+/− NA ppm)
476.0139
[M−H]−
382.0498
621.9067
706.1613
0
20
40
60
80
100
400
450
500
550
600
650
700

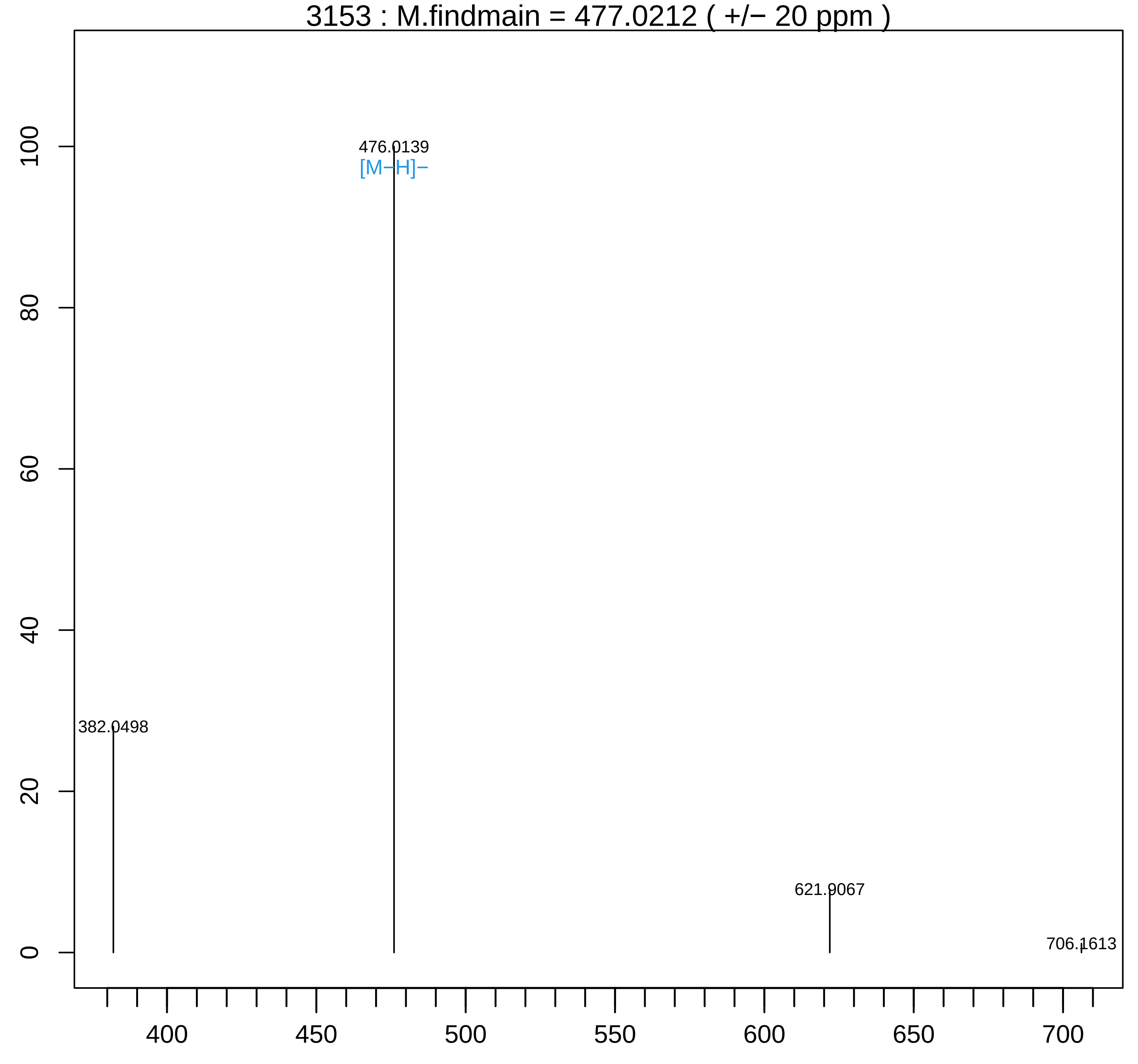
3153 : M.findmain = 477.0212 (+/− 20 ppm)
476.0139
[M−H]−
382.0498
621.9067
706.1613
0
20
40
60
80
100
400
450
500
550
600
650
700

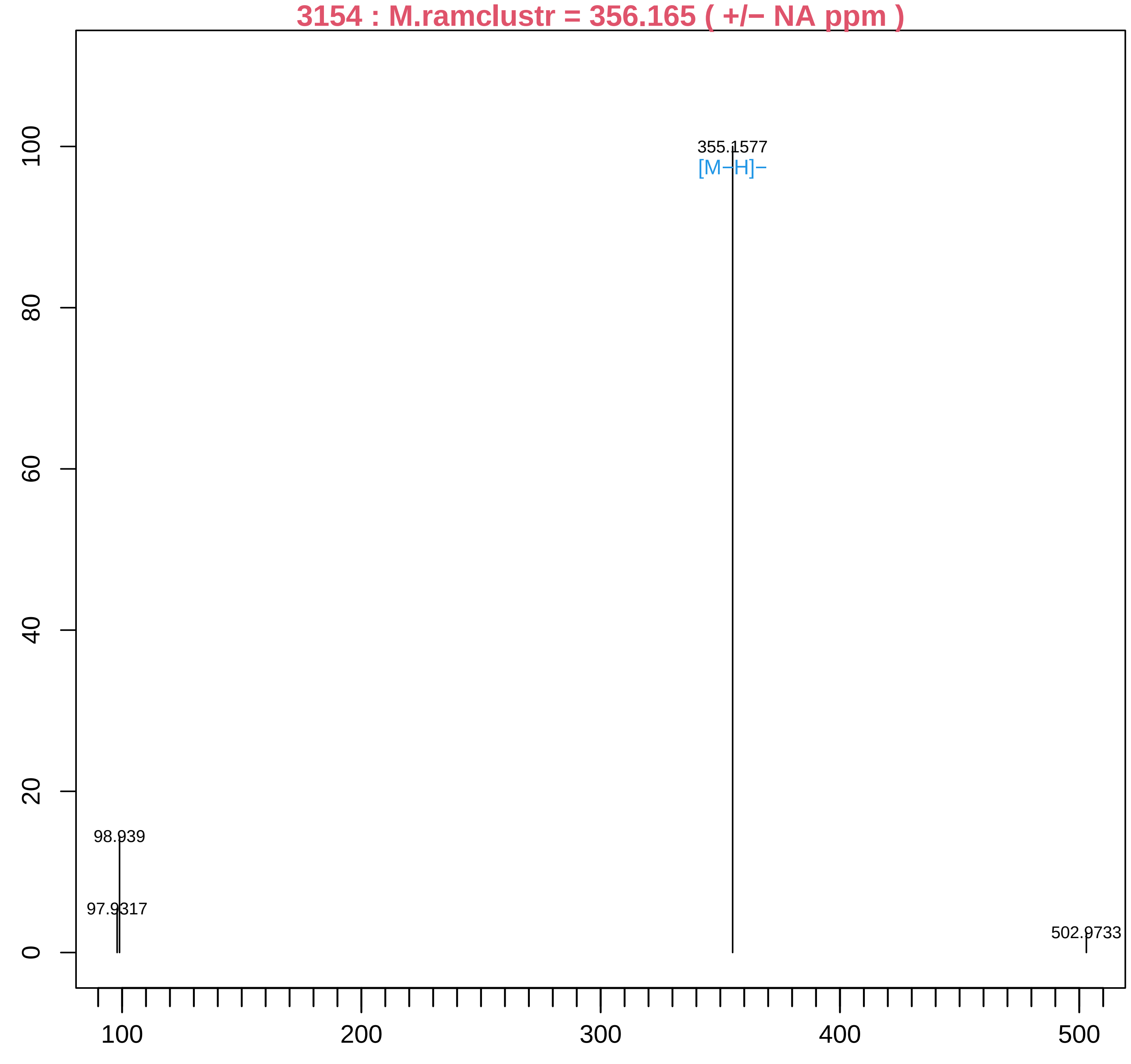
3154 : M.ramclustr = 356.165 (+/− NA ppm)
355.1577
[M−H]−
98.939
97.9317
502.9733
0
20
40
60
80
100
100
200
300
400
500

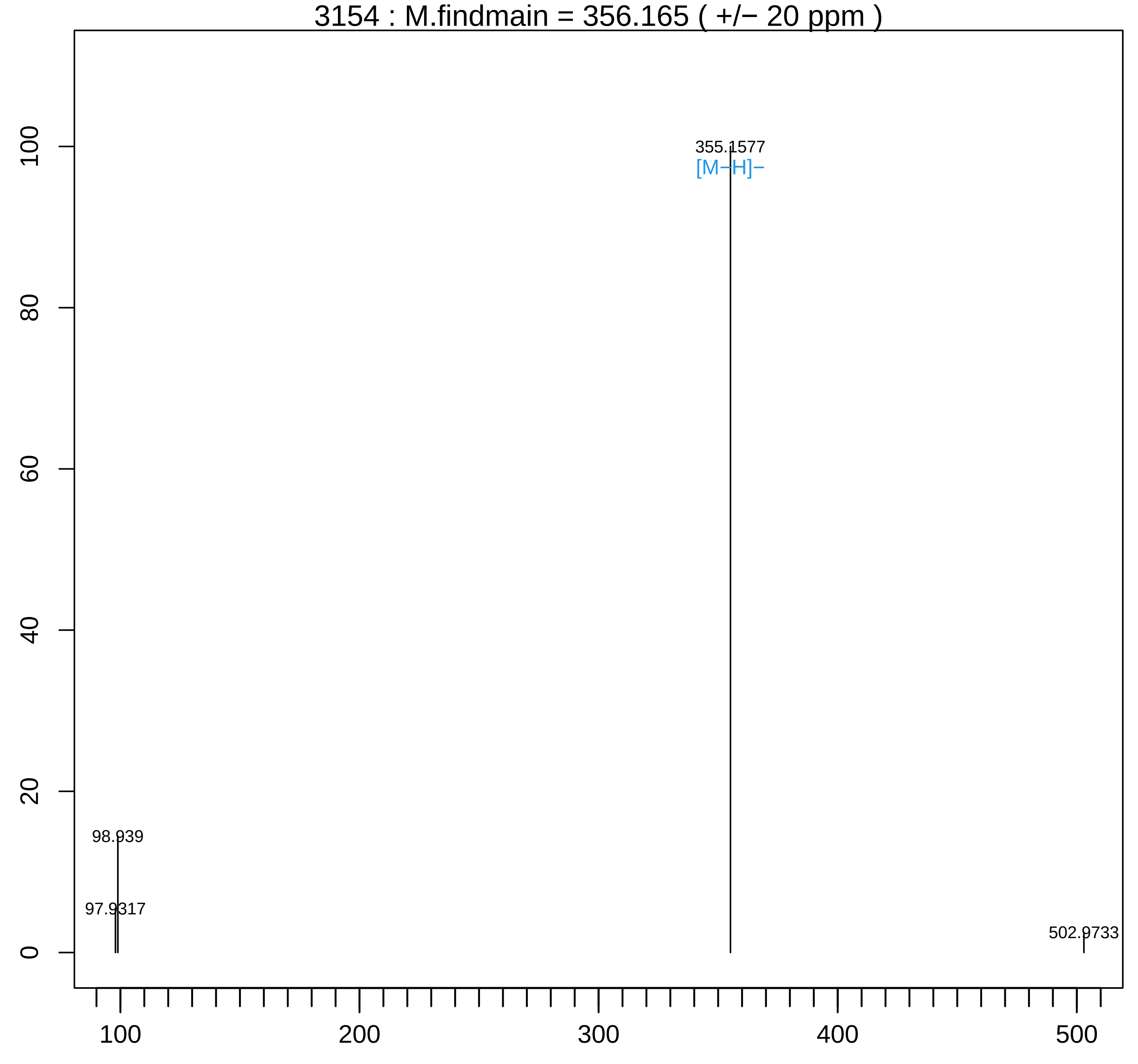
3154 : M.findmain = 356.165 (+/− 20 ppm)
355.1577
[M−H]−
98.939
97.9317
502.9733
0
20
40
60
80
100
100
200
300
400
500

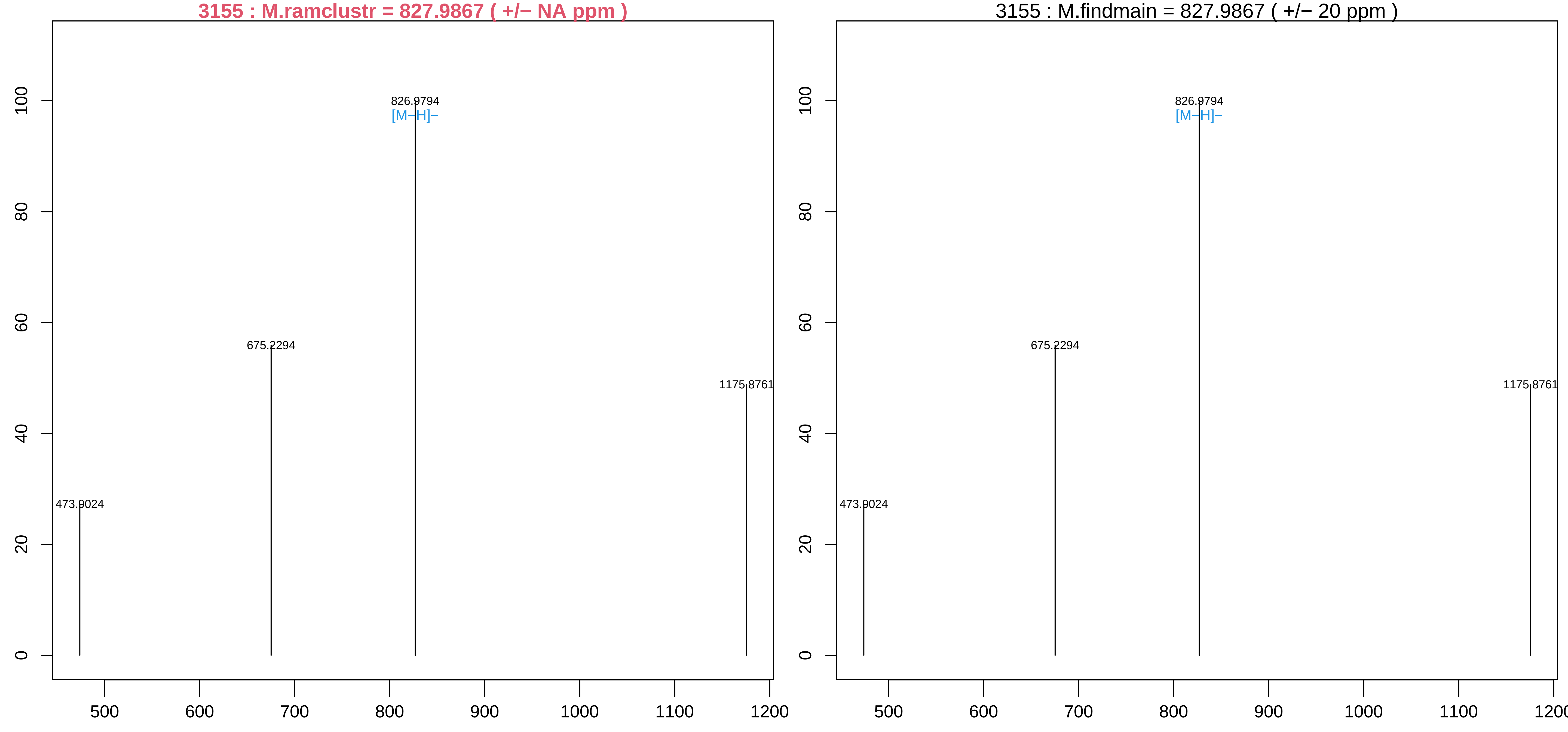
3155 : M.ramclustr = 827.9867 (+/− NA ppm)
3155 : M.findmain = 827.9867 (+/− 20 ppm)
826.9794
[M−H]−
675.2294
1175.8761
473.9024
0
20
40
60
80
100
500
600
700
800
900
1000
1100
1200

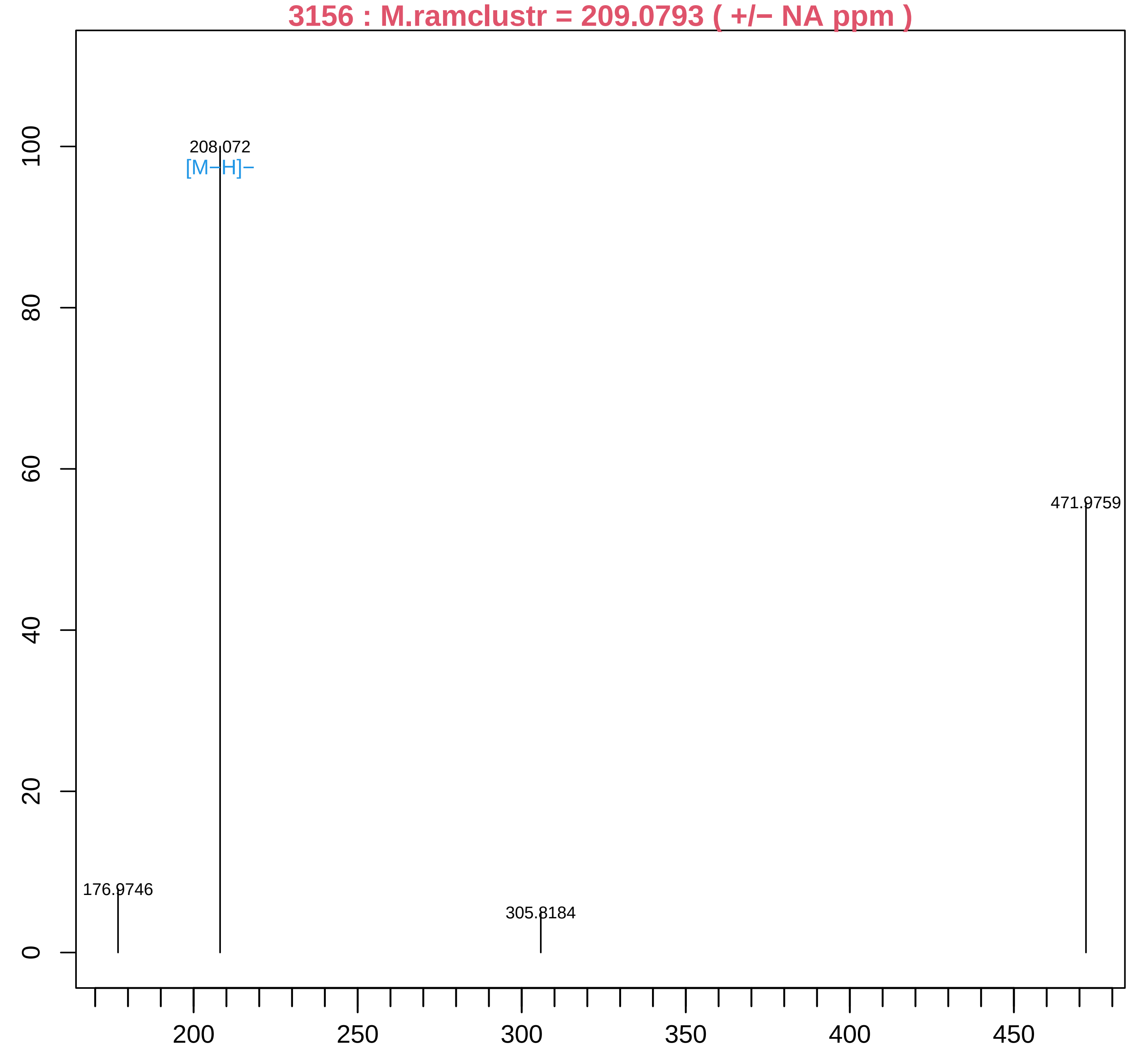
3156 : M.ramclustr = 209.0793 (+/− NA ppm)
208.072
[M−H]−
471.9759
176.9746
305.8184
0
20
40
60
80
100
200
250
300
350
400
450

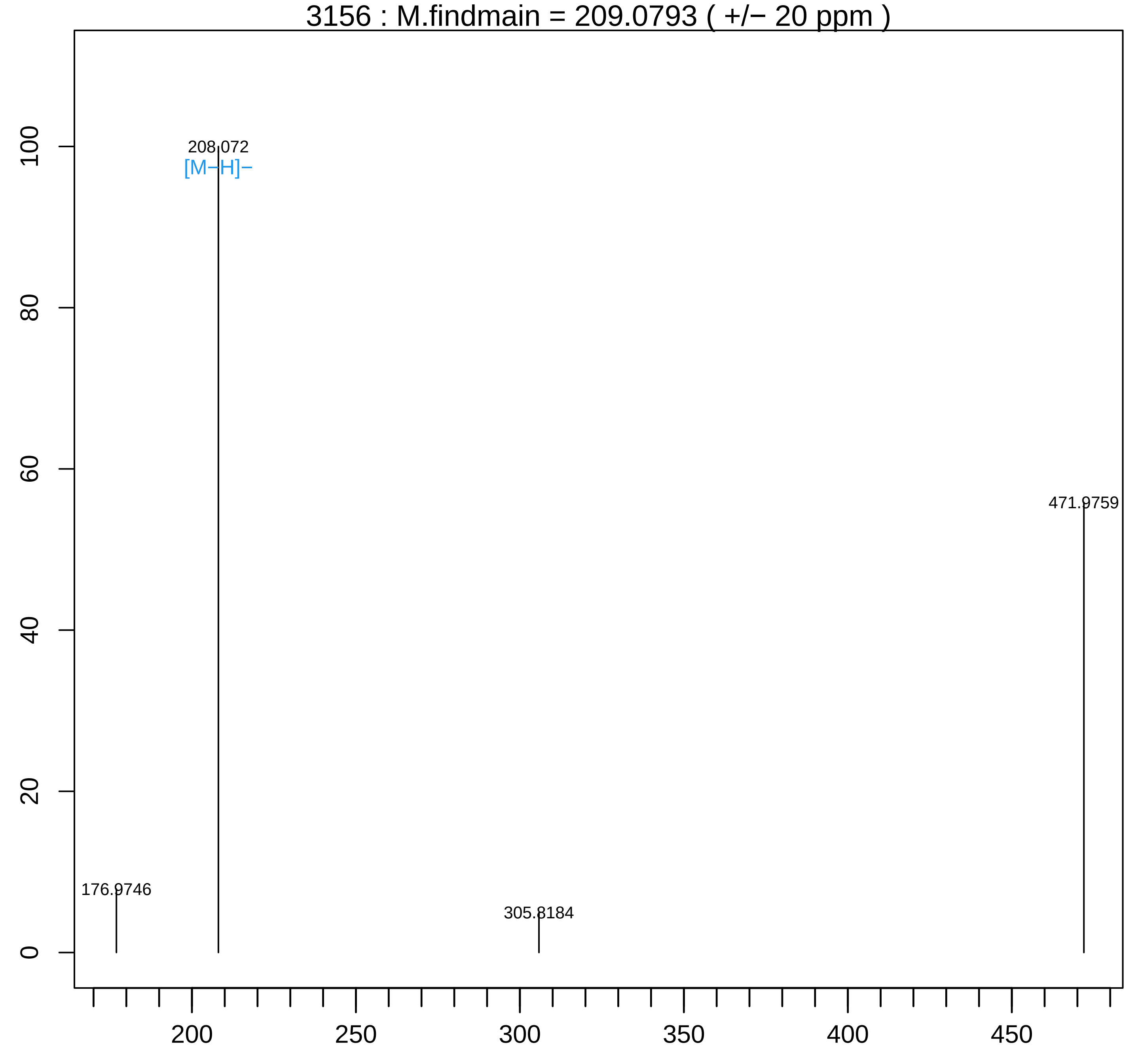
3156 : M.findmain = 209.0793 (+/− 20 ppm)
208.072
[M−H]−
471.9759
176.9746
305.8184
0
20
40
60
80
100
200
250
300
350
400
450

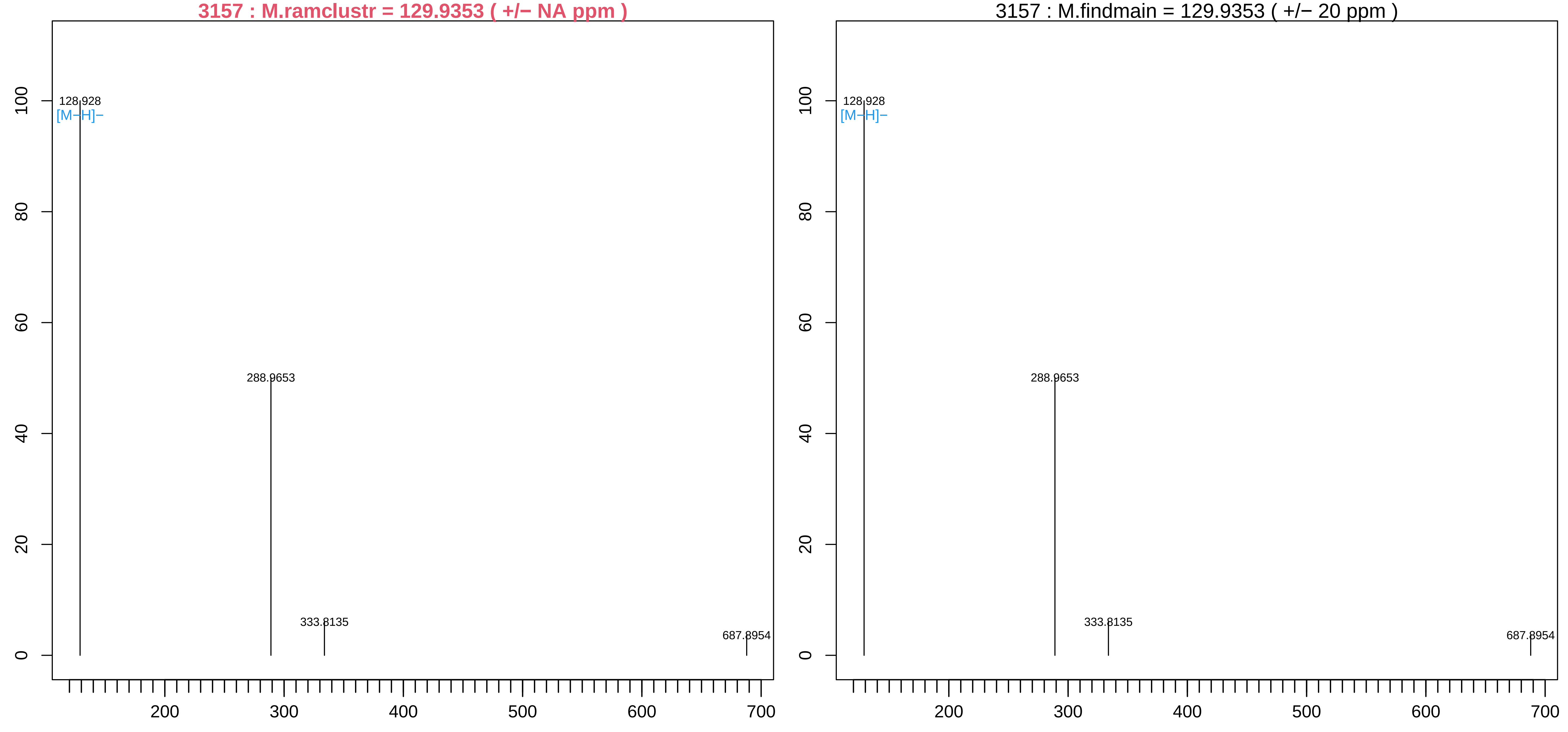
3157 : M.ramclustr = 129.9353 (+/− NA ppm)
128.928
[M−H]−
288.9653
333.8135
687.8954
0
20
40
60
80
100
200
300
400
500
600
700
3157 : M.findmain = 129.9353 (+/− 20 ppm)
128.928
[M−H]−
288.9653
333.8135
687.8954
0
20
40
60
80
100
200
300
400
500
600
700

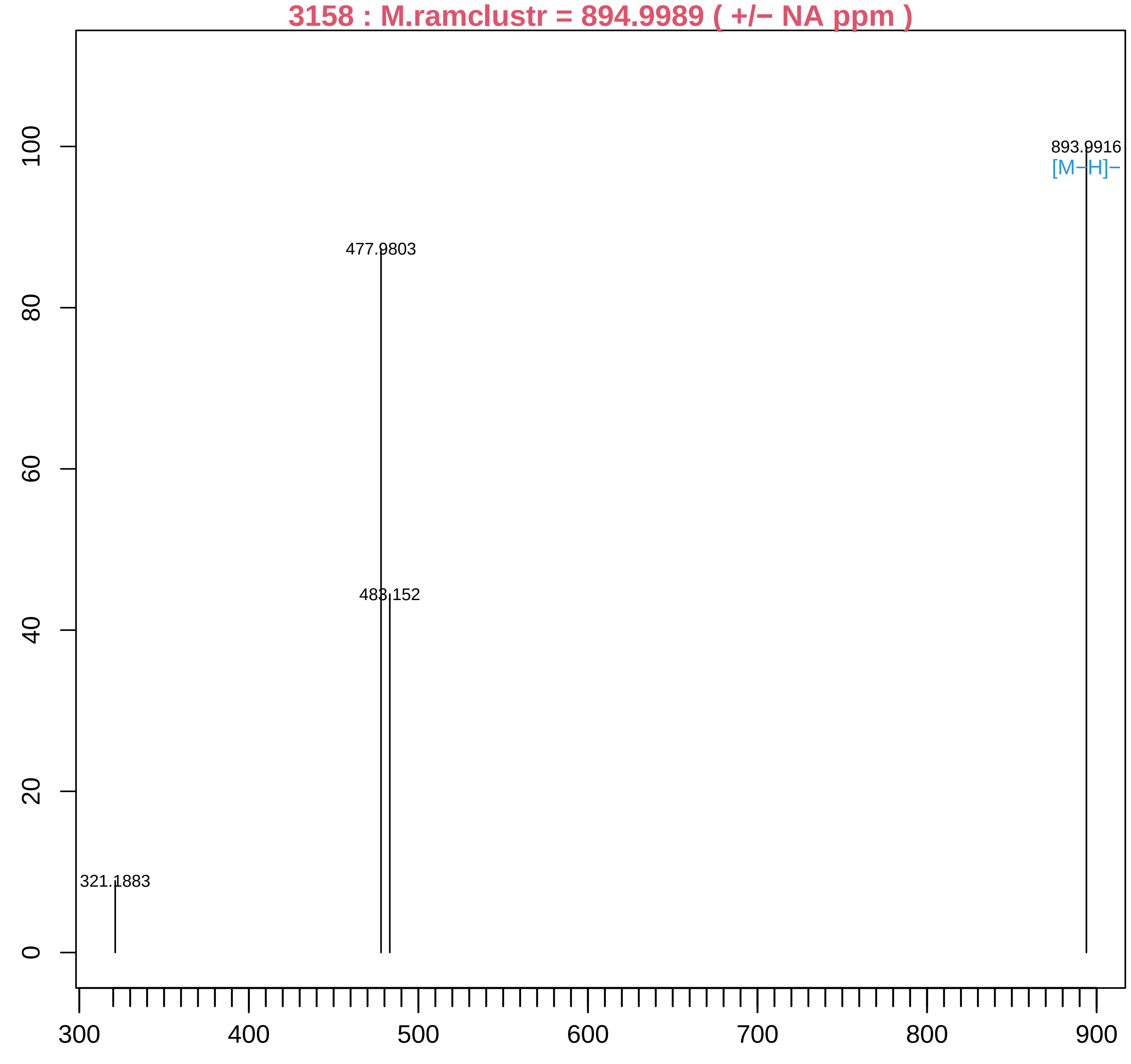
3158 : M.ramclustr = 894.9989 (+/− NA ppm)
893.9916
[M−H]−
477.9803
483.152
321.1883
0
20
40
60
80
100
300
400
500
600
700
800
900

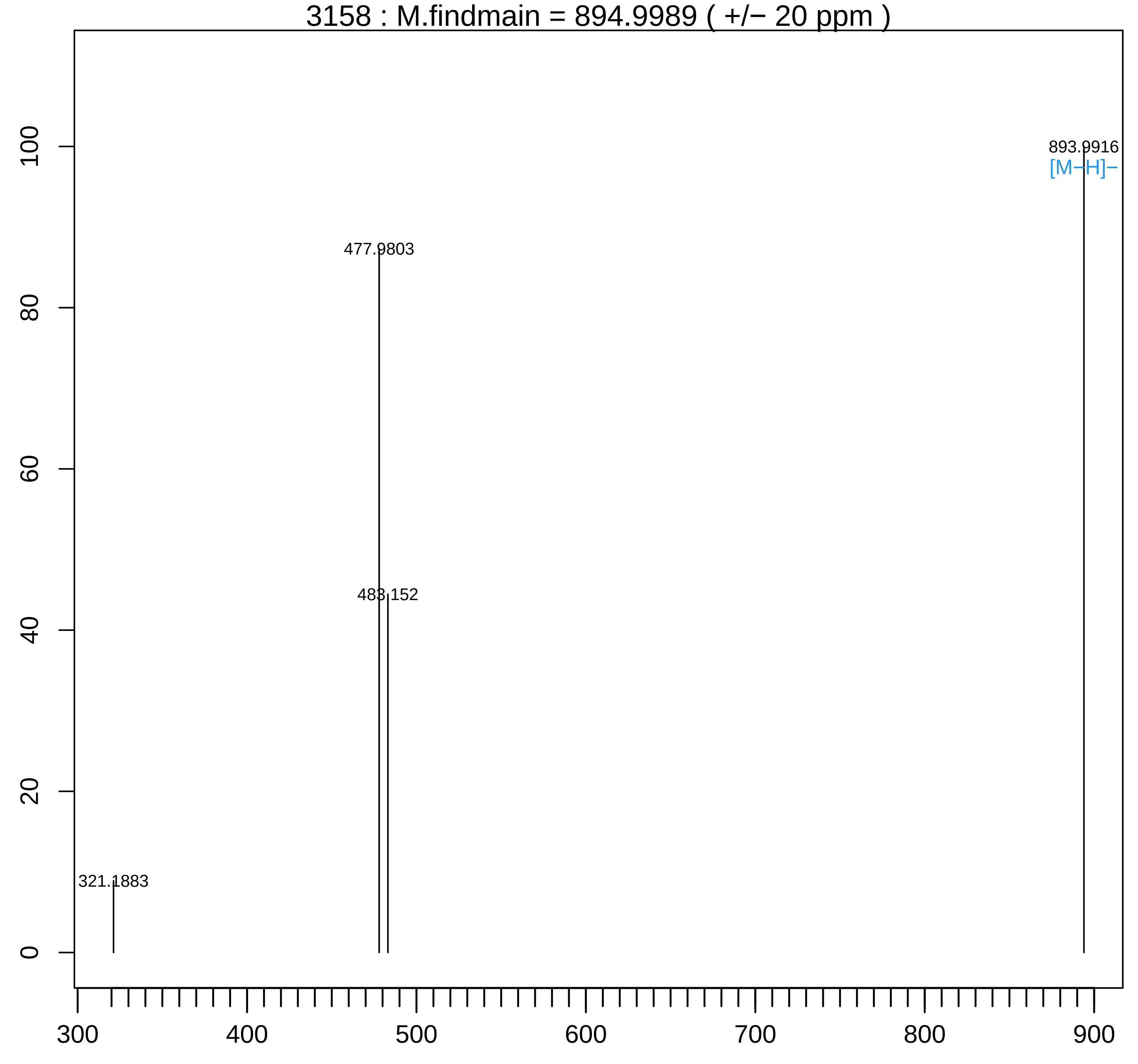
3158 : M.findmain = 894.9989 (+/− 20 ppm)
893.9916
[M−H]−
477.9803
483.152
321.1883
0
20
40
60
80
100
300
400
500
600
700
800
900

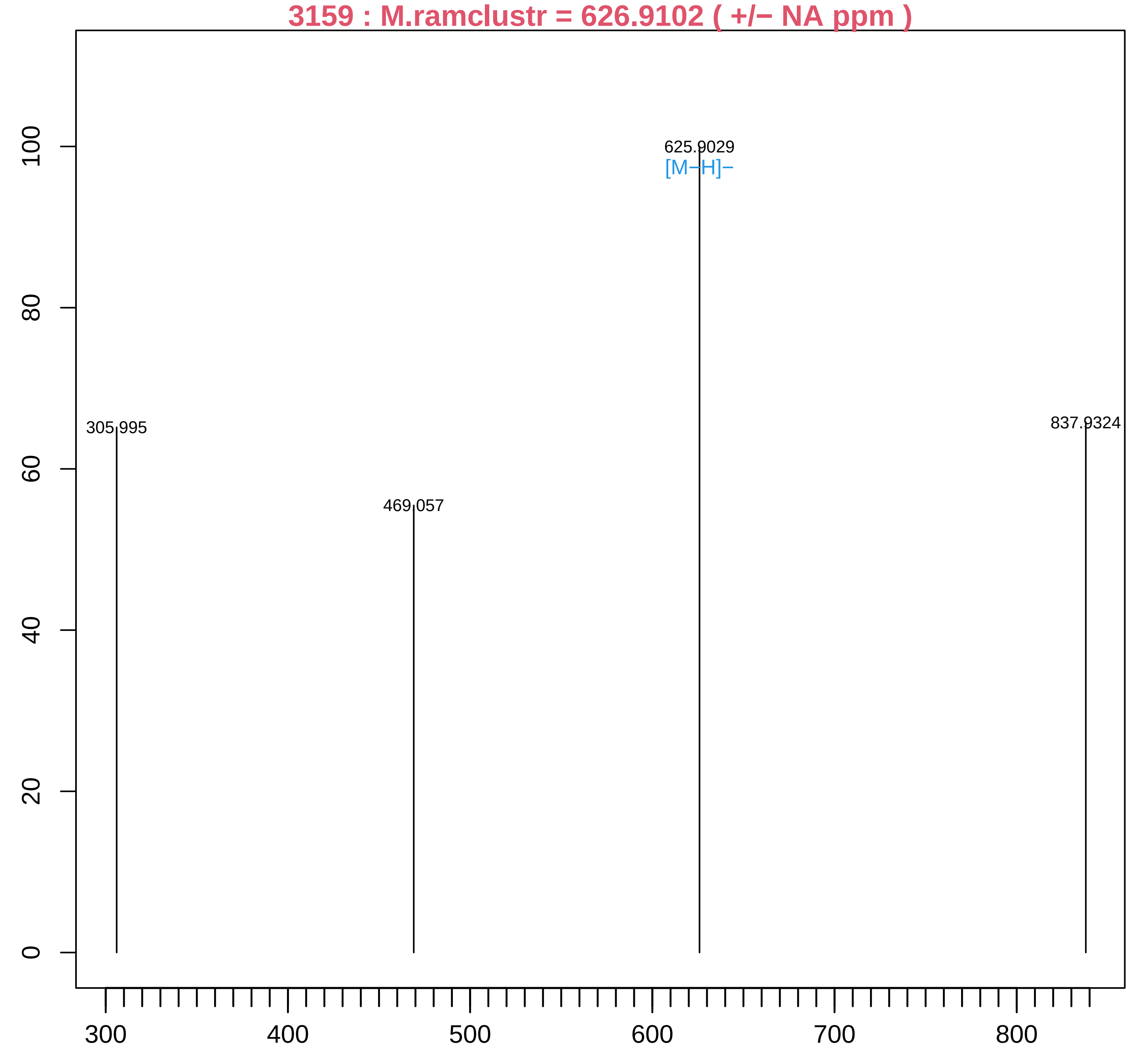
3159 : M.ramclustr = 626.9102 (+/− NA ppm)
625.9029
[M−H]−
305.995
469.057
837.9324
0
20
40
60
80
100
300
400
500
600
700
800

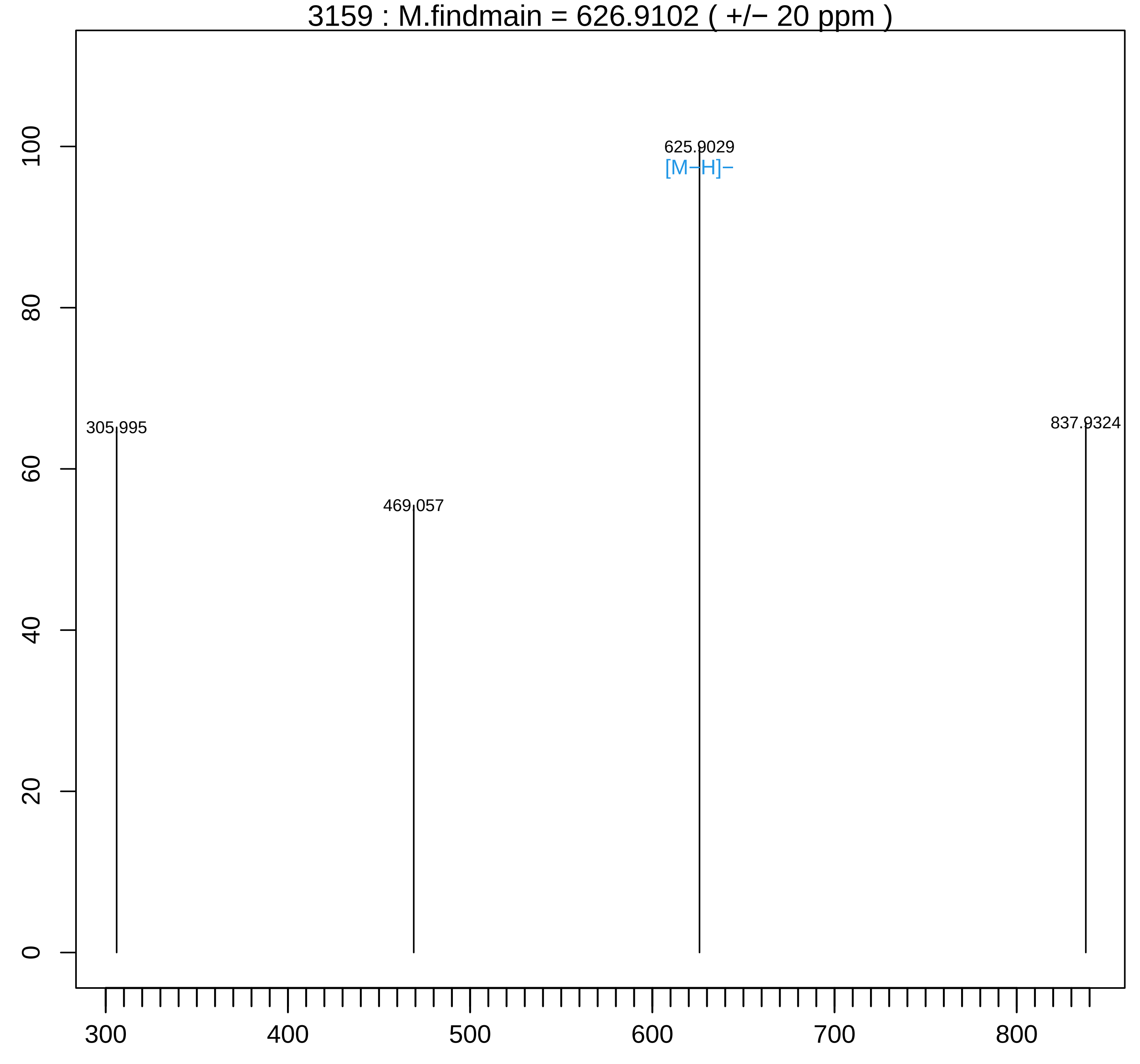
3159 : M.findmain = 626.9102 (+/− 20 ppm)
625.9029
[M−H]−
305.995
469.057
837.9324
0
20
40
60
80
100
300
400
500
600
700
800

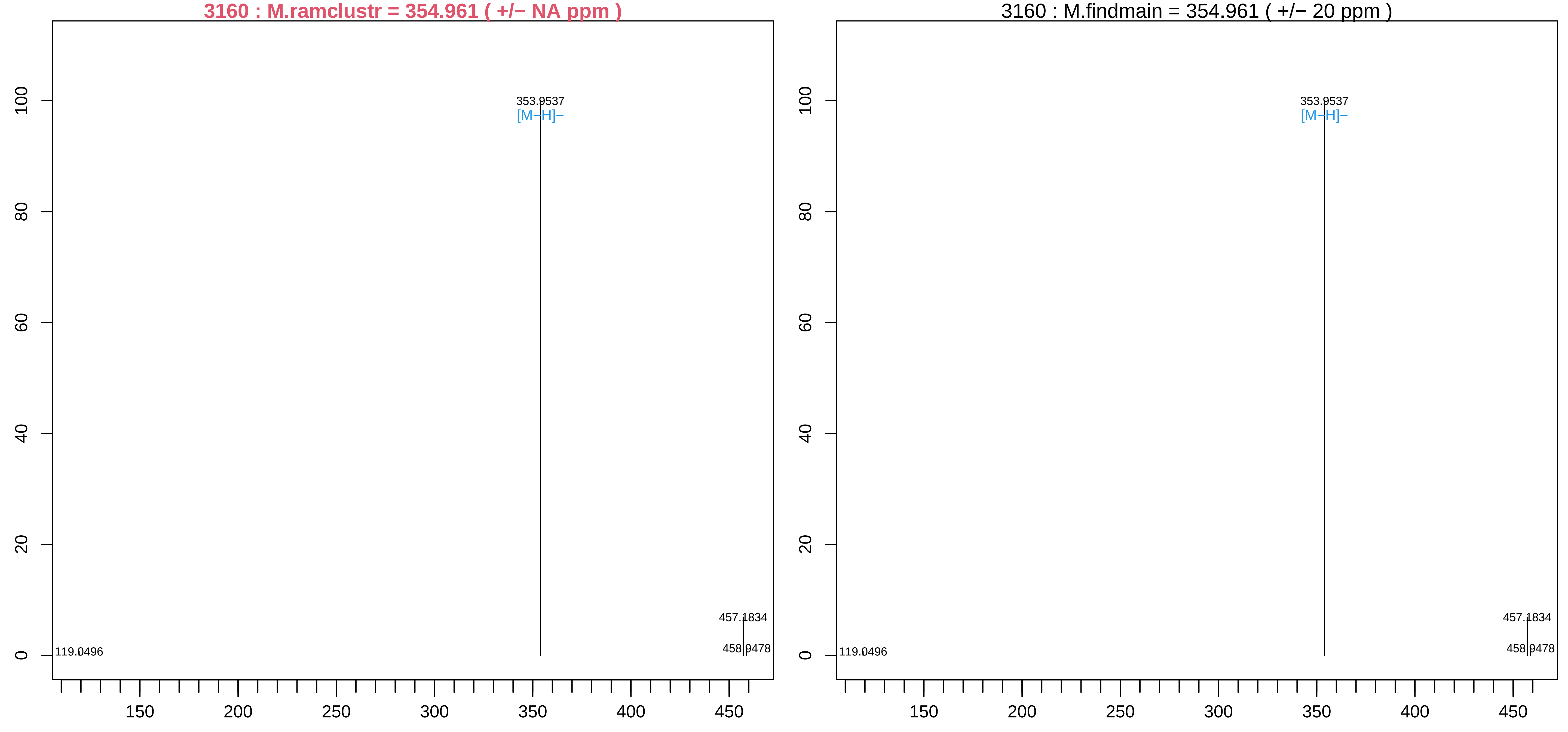

3160 : M.ramclustr = 354.961 (+/− NA ppm)
353.9537
[M−H]−
457.1834
458.9478
119.0496
3160 : M.findmain = 354.961 (+/− 20 ppm)
353.9537
[M−H]−
457.1834
458.9478
119.0496

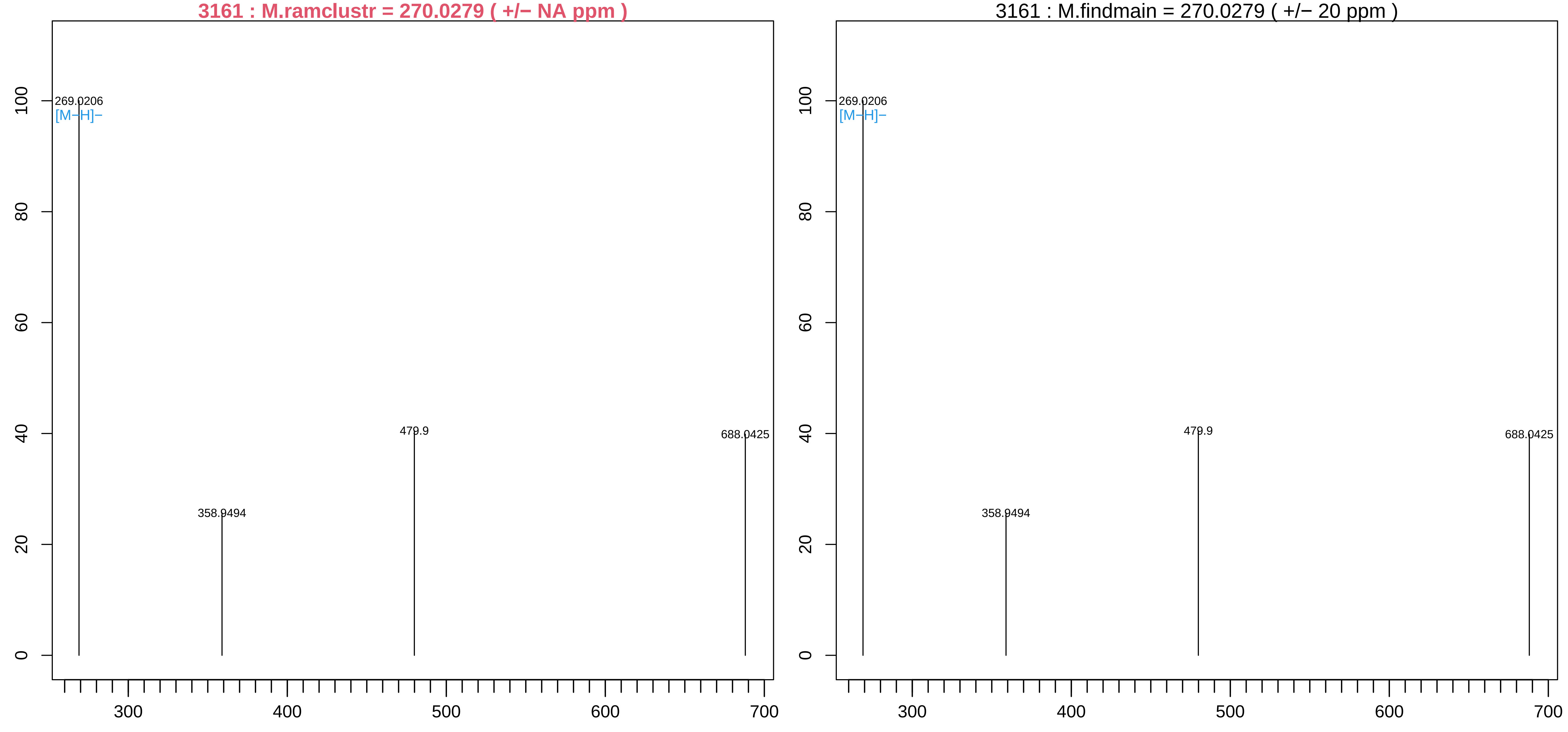
3161 : M.ramclustr = 270.0279 (+/− NA ppm)
269.0206
[M−H]−
358.9494
479.9
688.0425
3161 : M.findmain = 270.0279 (+/− 20 ppm)
269.0206
[M−H]−
358.9494
479.9
688.0425
0
20
40
60
80
100
300
400
500
600
700

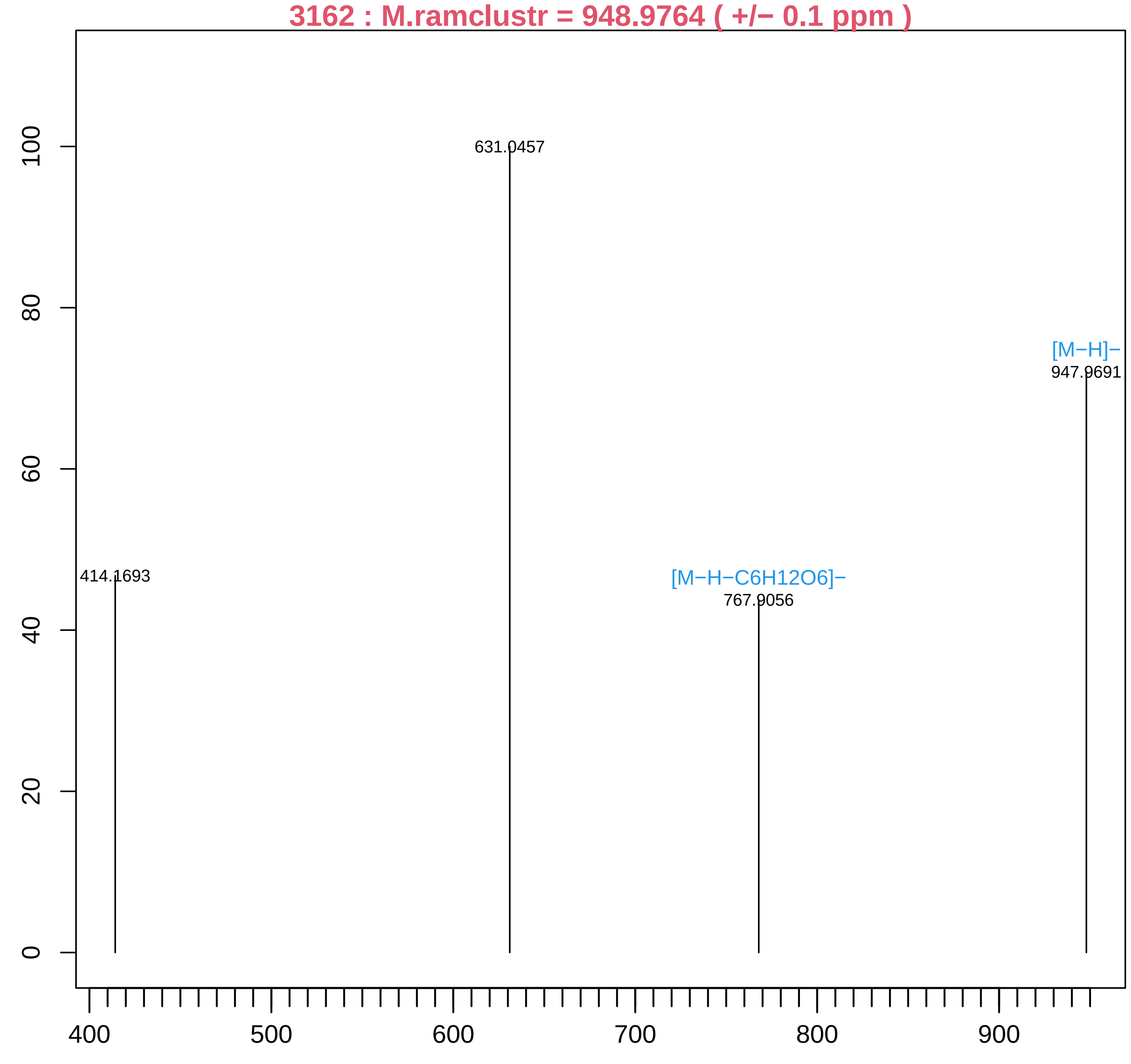
3162 : M.ramclustr = 948.9764 (+/− 0.1 ppm)
631.0457
[M−H]−
947.9691
414.1693
[M−H−C6H12O6]−
767.9056
0
20
40
60
80
100
400
500
600
700
800
900

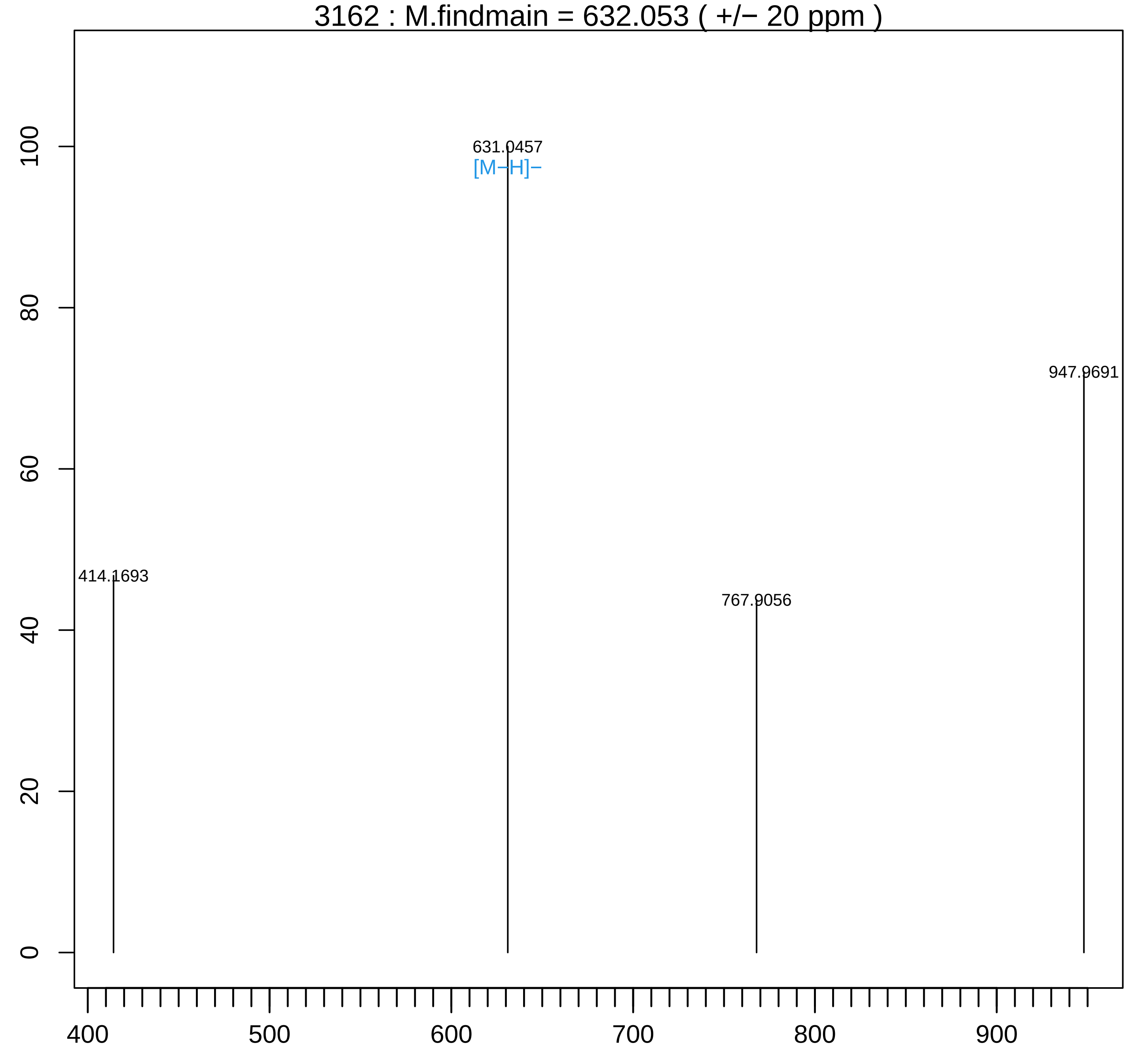
3162 : M.findmain = 632.053 (+/− 20 ppm)
631.0457
[M−H]−
947.9691
414.1693
767.9056
0
20
40
60
80
100
400
500
600
700
800
900

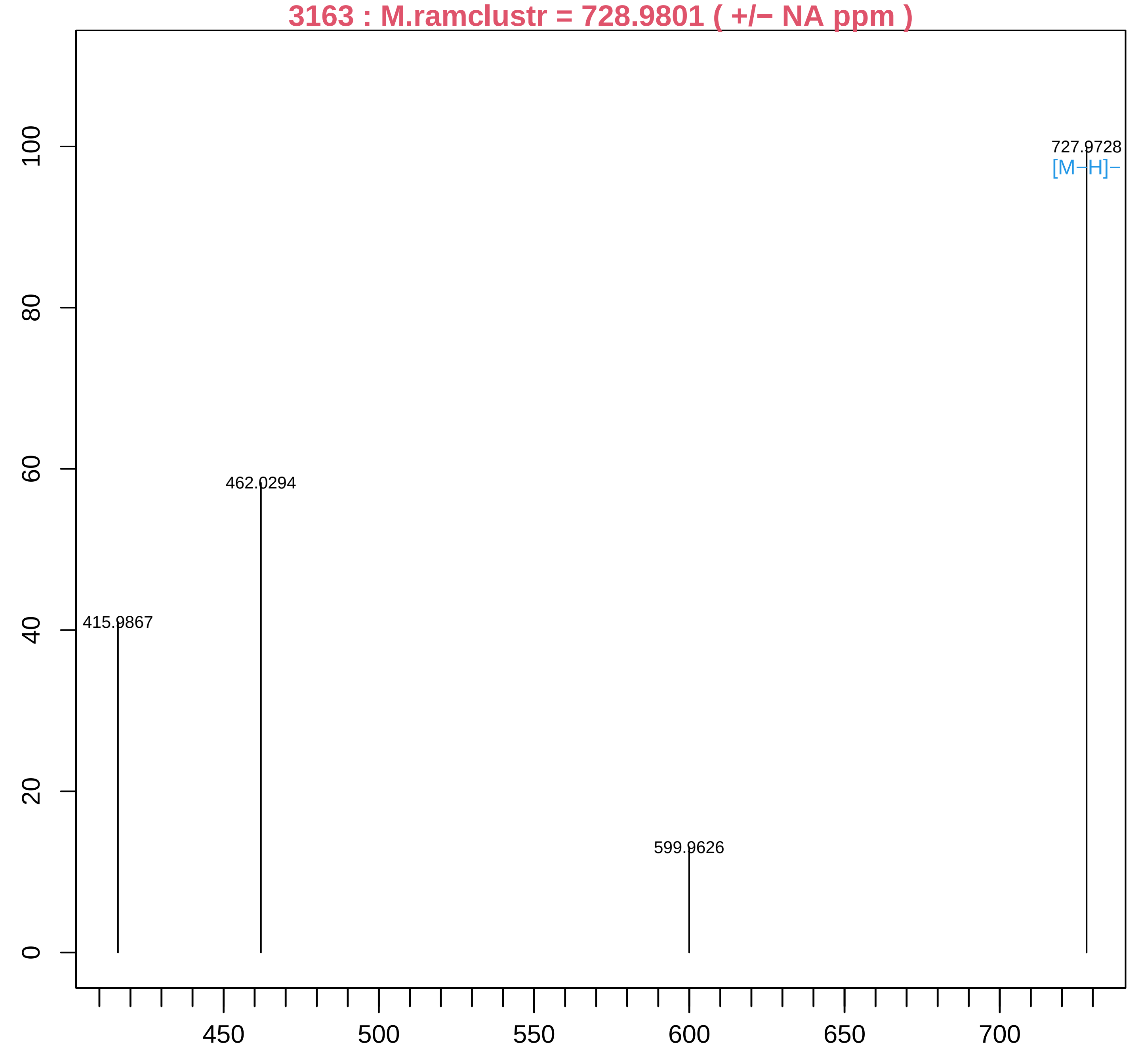
3163 : M.ramclustr = 728.9801 (+/− NA ppm)
727.9728
[M−H]−
462.0294
415.9867
599.9626
0
20
40
60
80
100
450
500
550
600
650
700

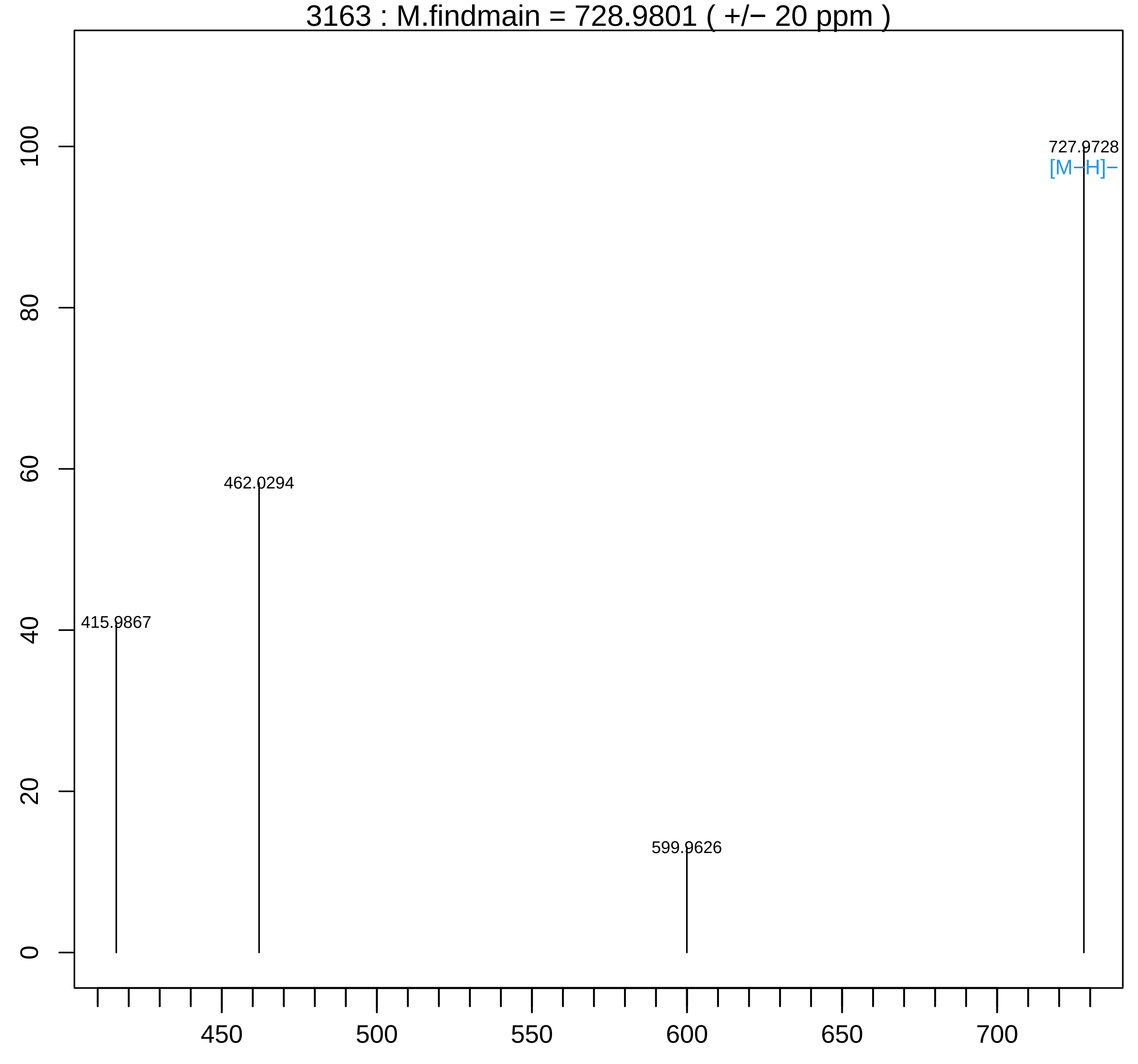
3163 : M.findmain = 728.9801 (+/− 20 ppm)
727.9728
[M−H]−
462.0294
415.9867
599.9626
0
20
40
60
80
100
450
500
550
600
650
700

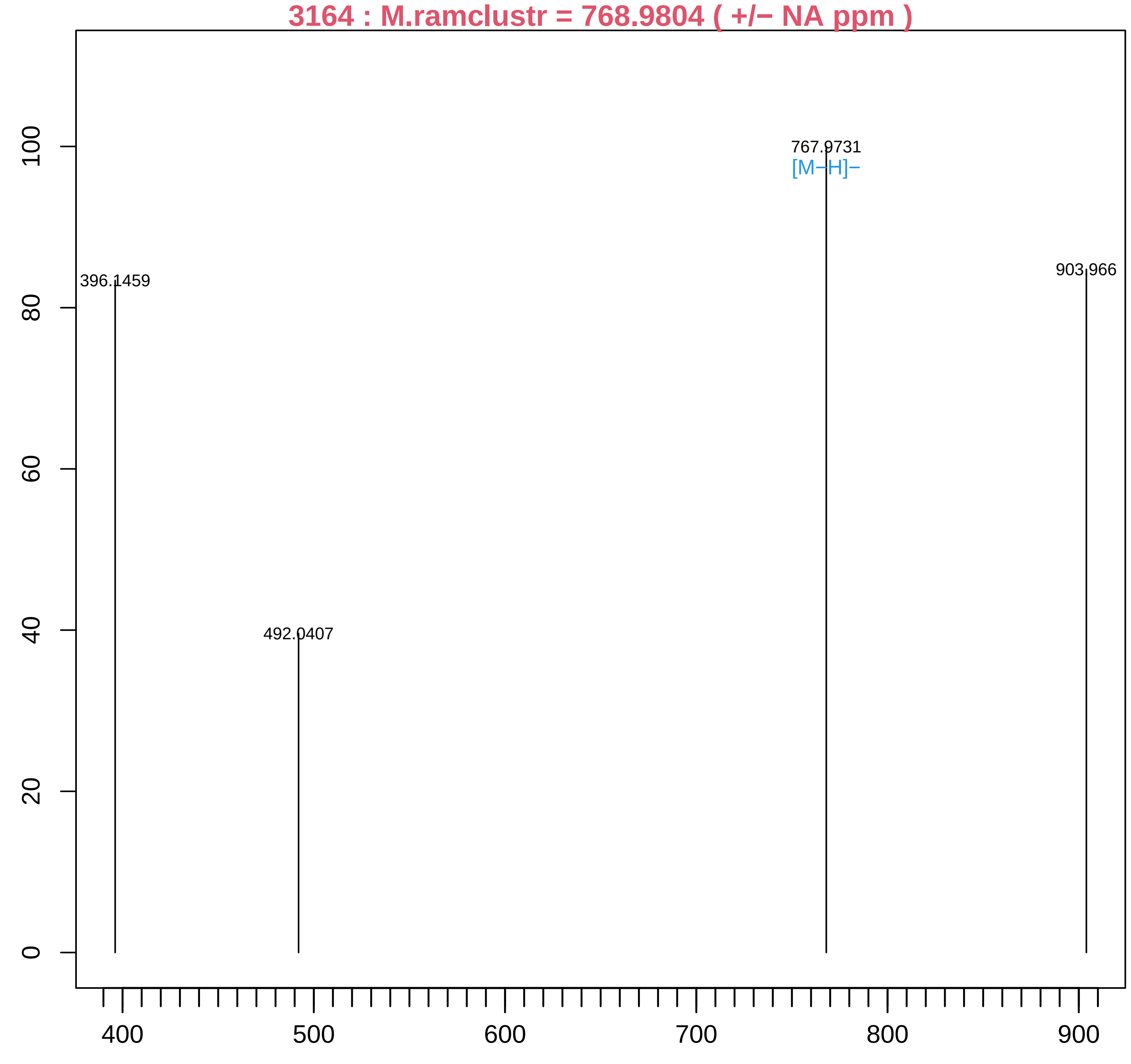

3164 : M.ramclustr = 768.9804 (+/− NA ppm)
396.1459
492.0407
767.9731
[M−H]−
903.966
0
20
40
60
80
100
400
500
600
700
800
900

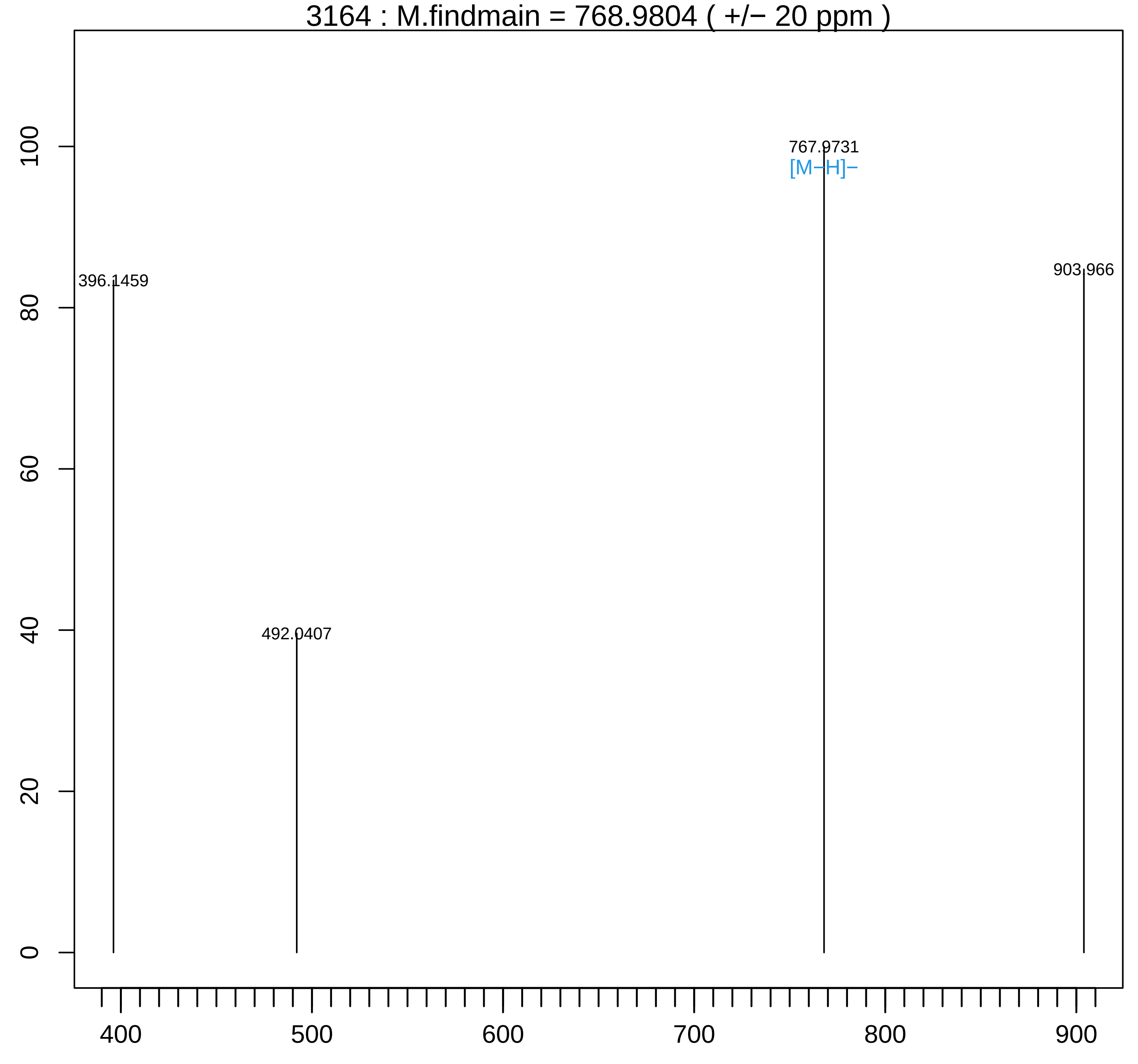

3164 : M.findmain = 768.9804 (+/− 20 ppm)
396.1459
492.0407
767.9731
[M−H]−
903.966
0
20
40
60
80
100
400
500
600
700
800
900

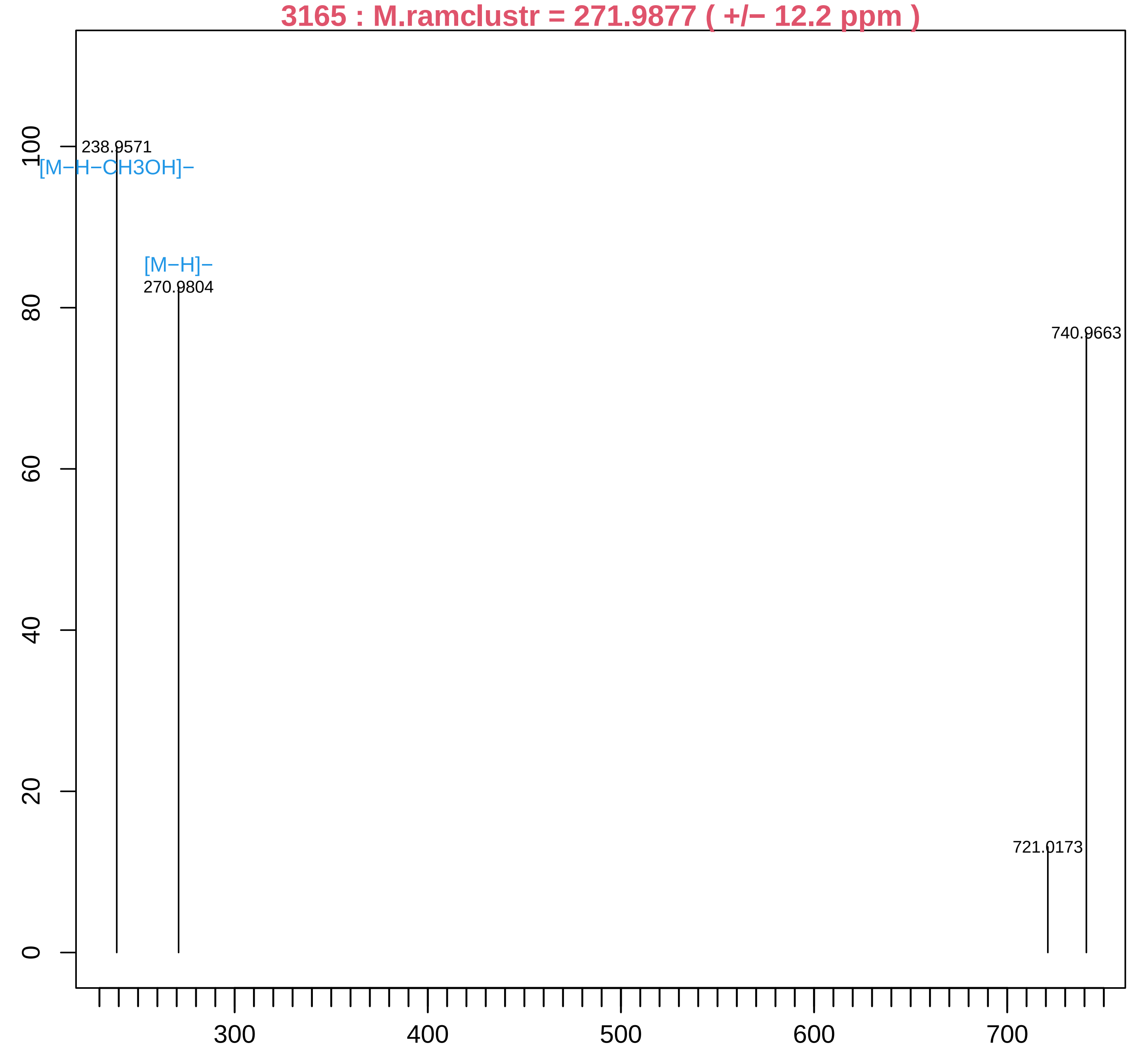
3165 : M.ramclustr = 271.9877 (+/− 12.2 ppm)
238.9571
[M−H−CH3OH]−
[M−H]−
270.9804
740.9663
721.0173
0
20
40
60
80
100
300
400
500
600
700

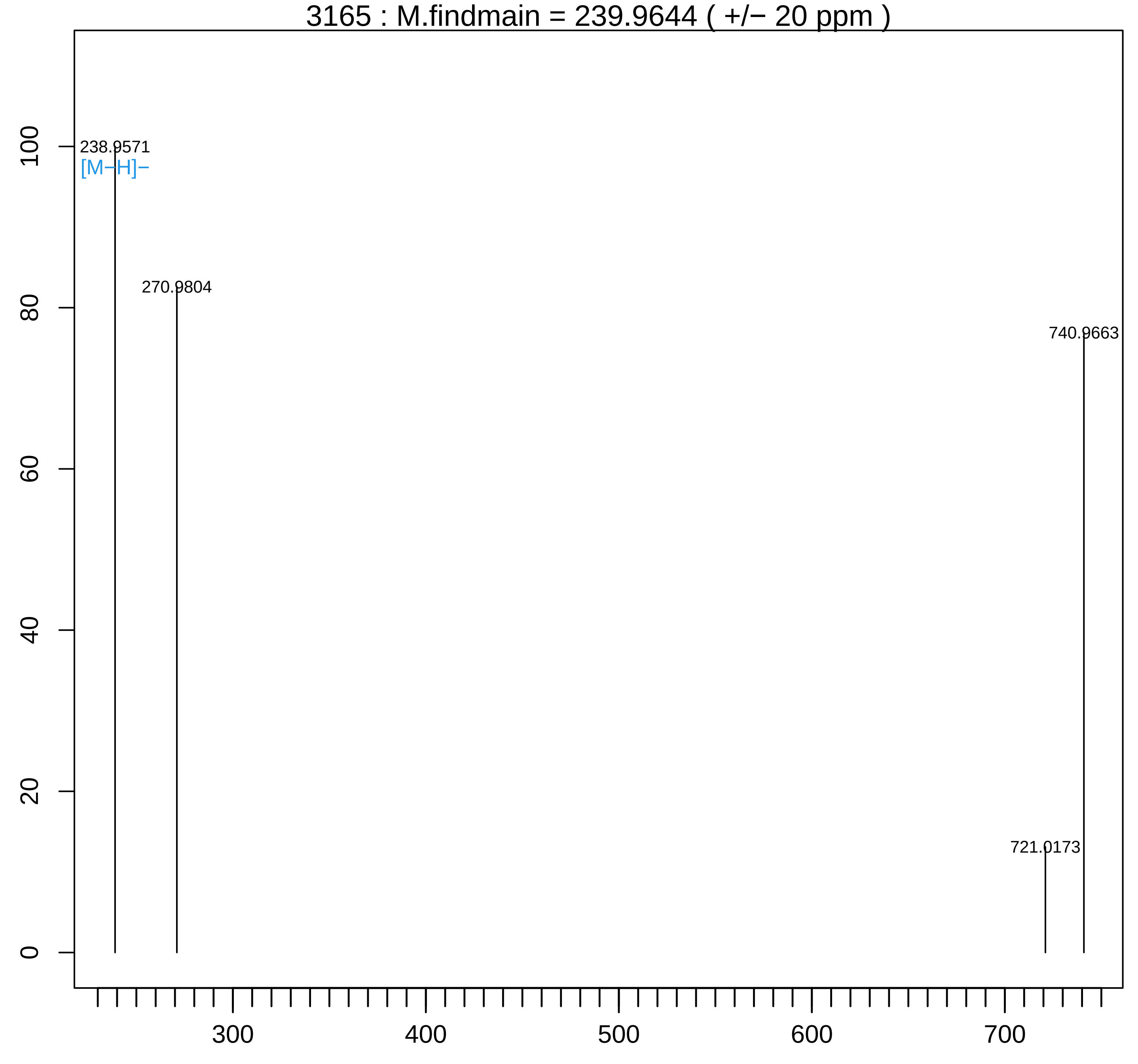
3165 : M.findmain = 239.9644 (+/− 20 ppm)
238.9571
[M−H]−
270.9804
740.9663
721.0173
0
20
40
60
80
100
300
400
500
600
700

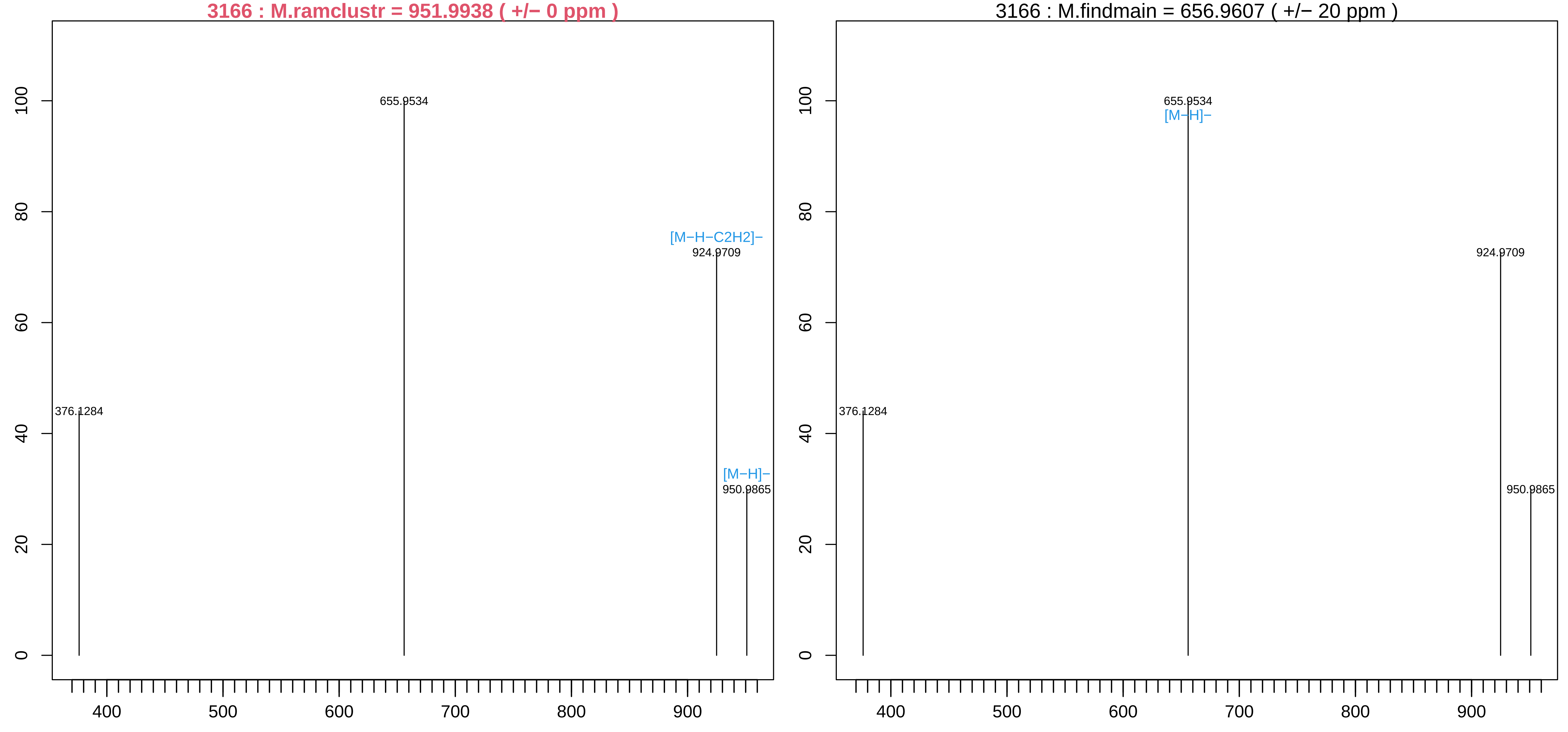

3166 : M.ramclustr = 951.9938 (+/− 0 ppm)
655.9534
[M−H−C2H2]−
924.9709
376.1284
[M−H]−
950.9865
3166 : M.findmain = 656.9607 (+/− 20 ppm)
655.9534
[M−H]−
924.9709
376.1284
950.9865
0
20
40
60
80
100
400
500
600
700
800
900

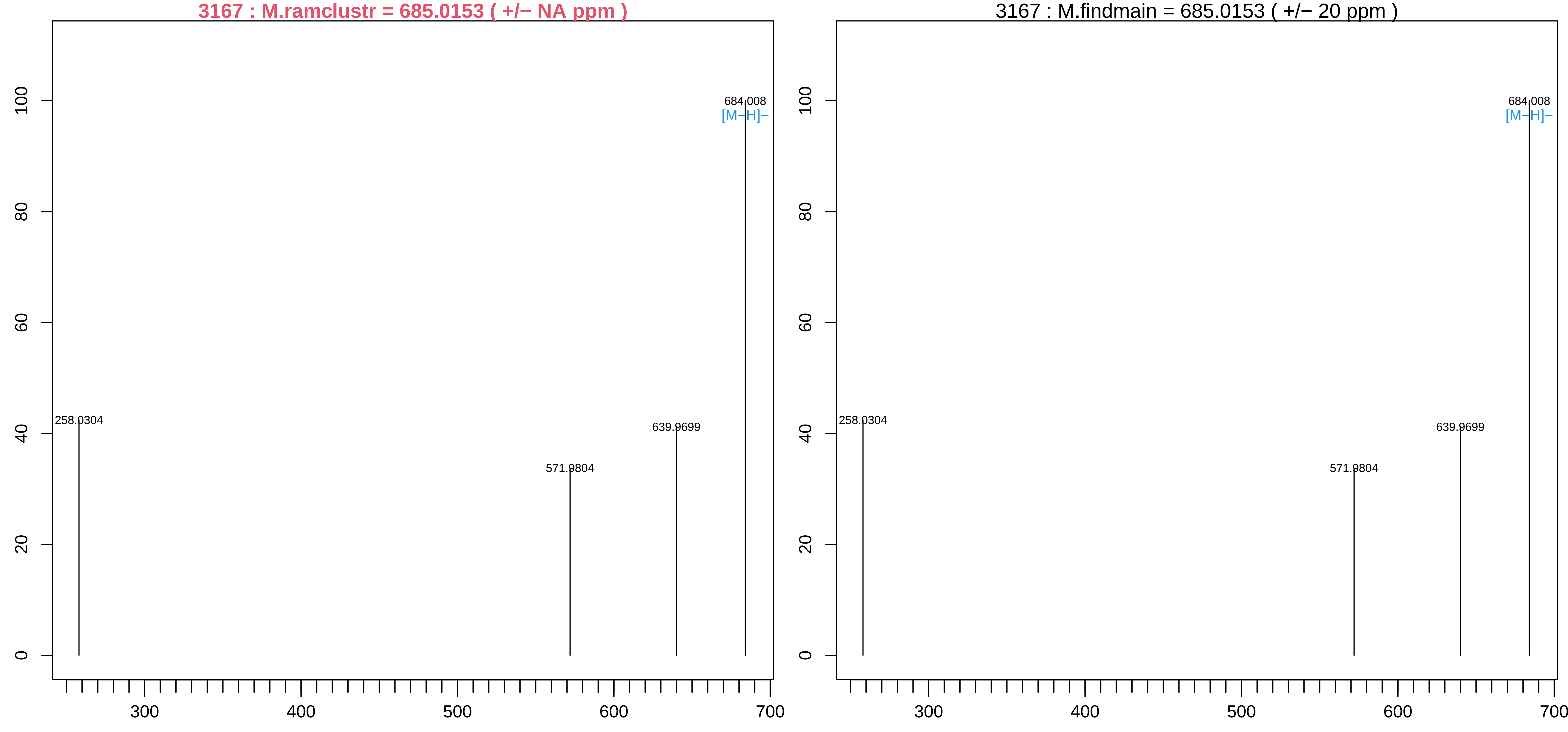
3167 : M.ramclustr = 685.0153 (+/− NA ppm)
3167 : M.findmain = 685.0153 (+/− 20 ppm)
684.008
[M−H]−
258.0304
571.9804
639.9699
0
20
40
60
80
100
300
400
500
600
700

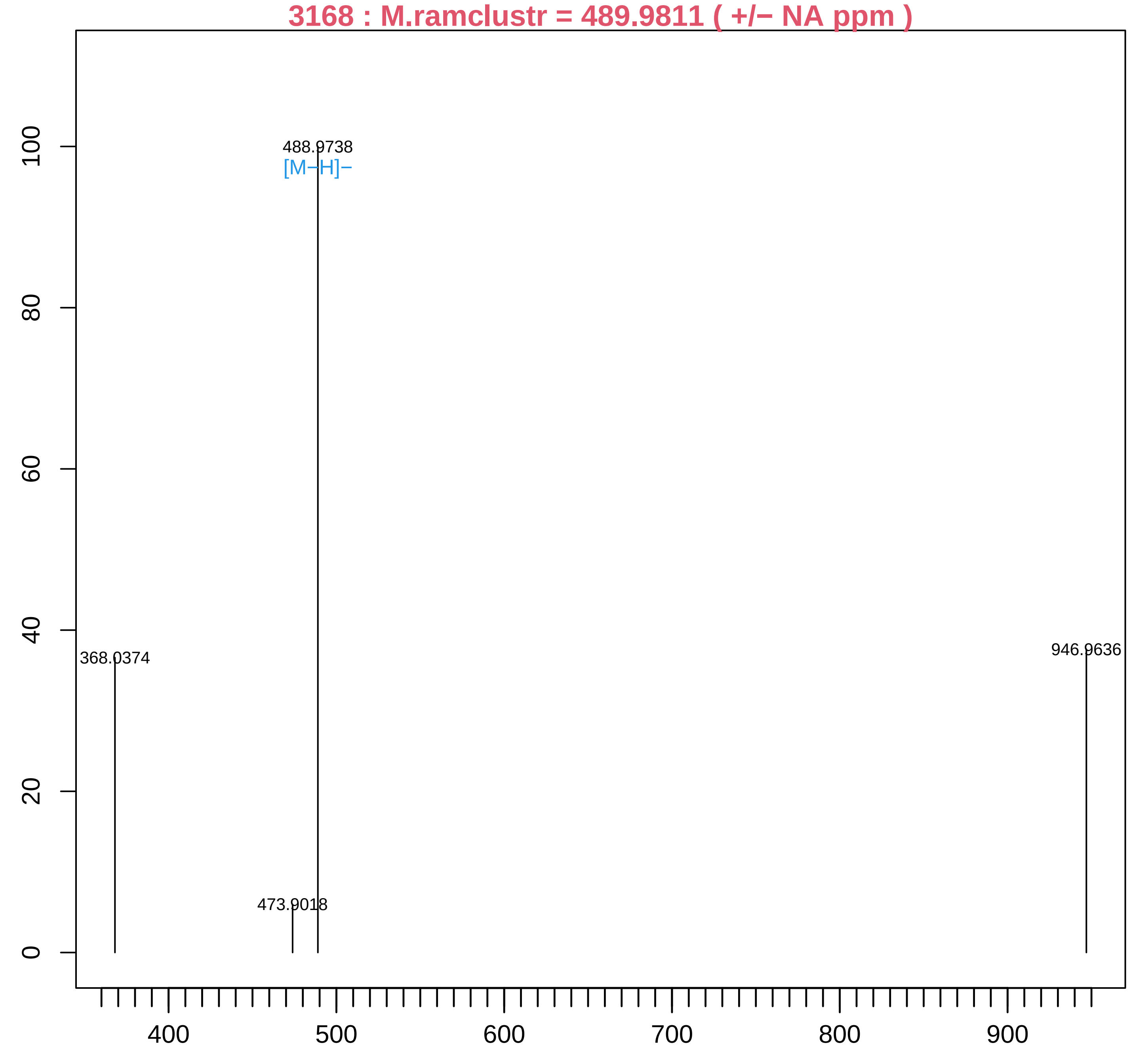

3168 : M.ramclustr = 489.9811 (+/− NA ppm)
488.9738
[M−H]−
368.0374
473.9018
946.9636
0
20
40
60
80
100
400
500
600
700
800
900

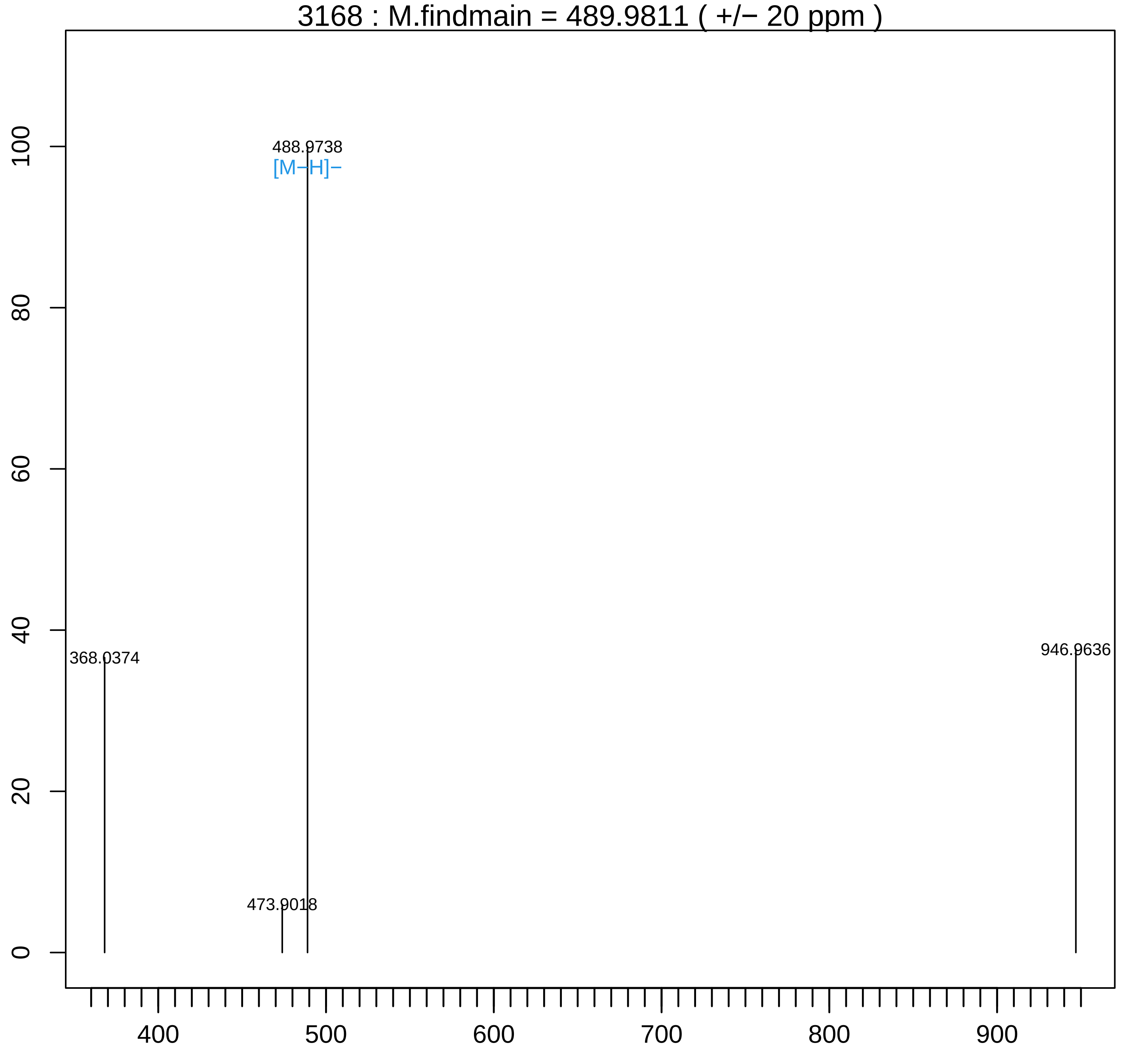

3168 : M.findmain = 489.9811 (+/− 20 ppm)
488.9738
[M−H]−
368.0374
473.9018
946.9636
0
20
40
60
80
100
400
500
600
700
800
900

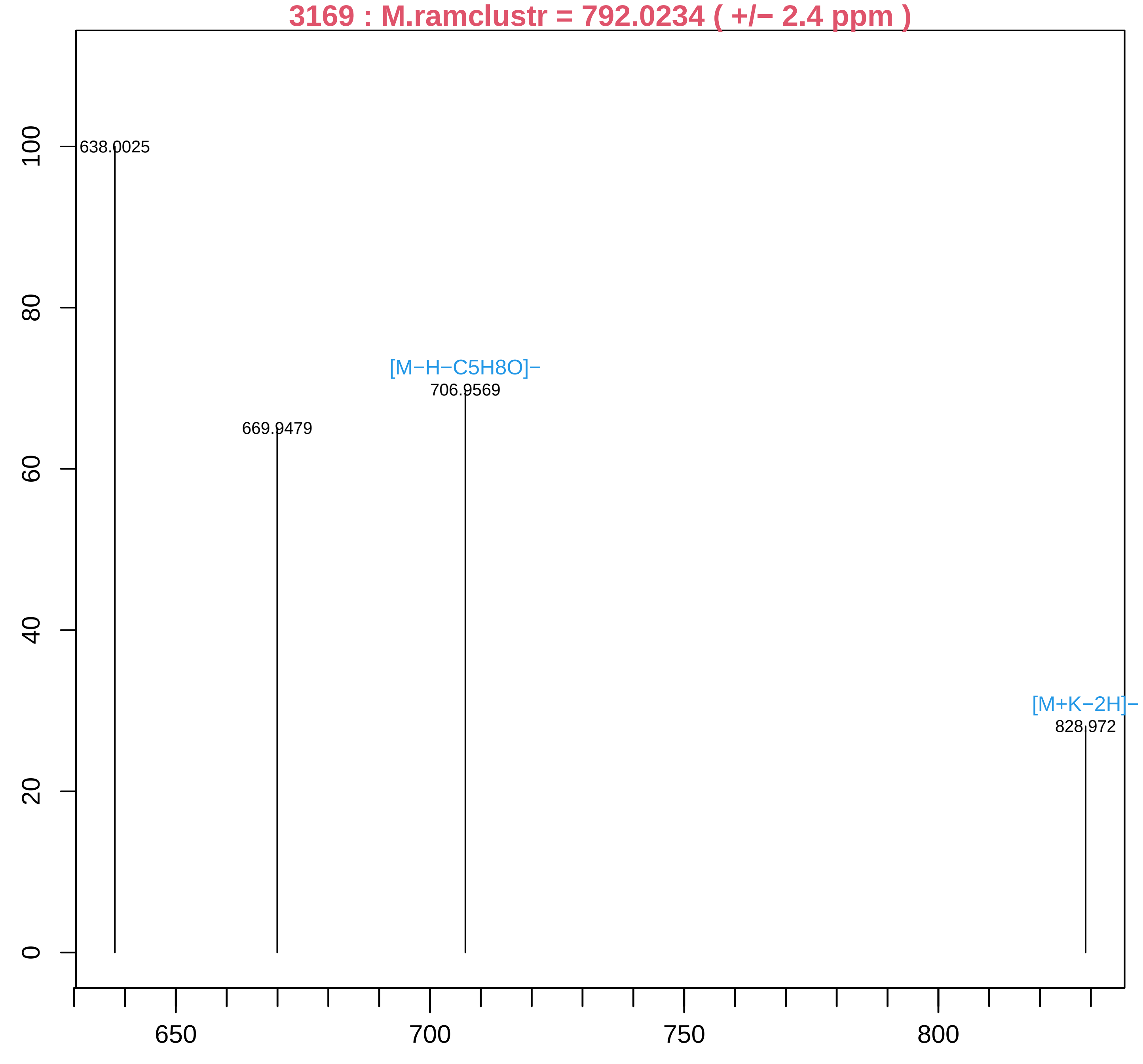
3169 : M.ramclustr = 792.0234 (+/− 2.4 ppm)
638.0025
669.9479
[M−H−C5H8O]−
706.9569
[M+K−2H]−
828.972
0
20
40
60
80
100
650
700
750
800

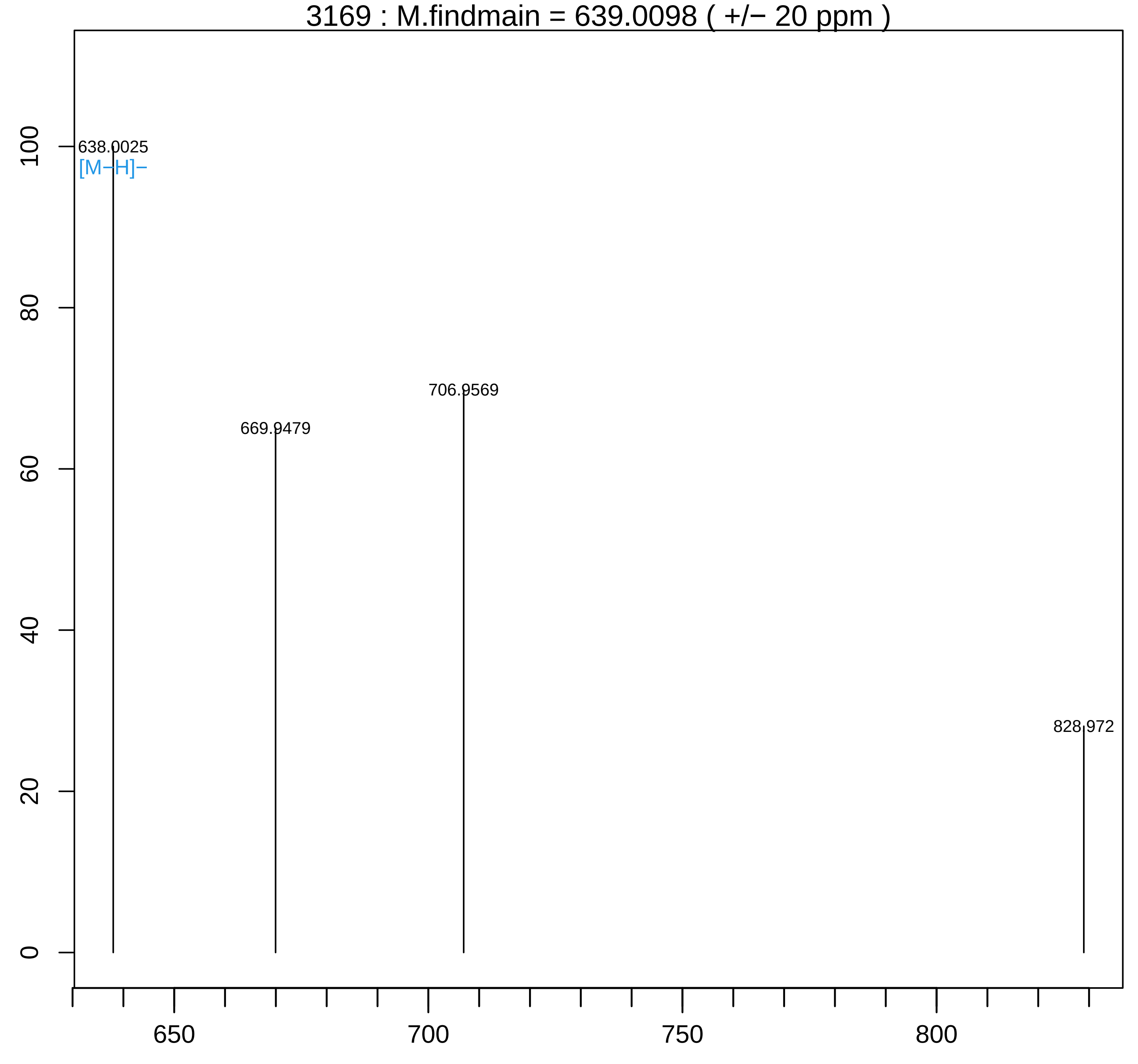
3169 : M.findmain = 639.0098 (+/− 20 ppm)
638.0025
[M−H]−
669.9479
706.9569
828.972
0
20
40
60
80
100
650
700
750
800

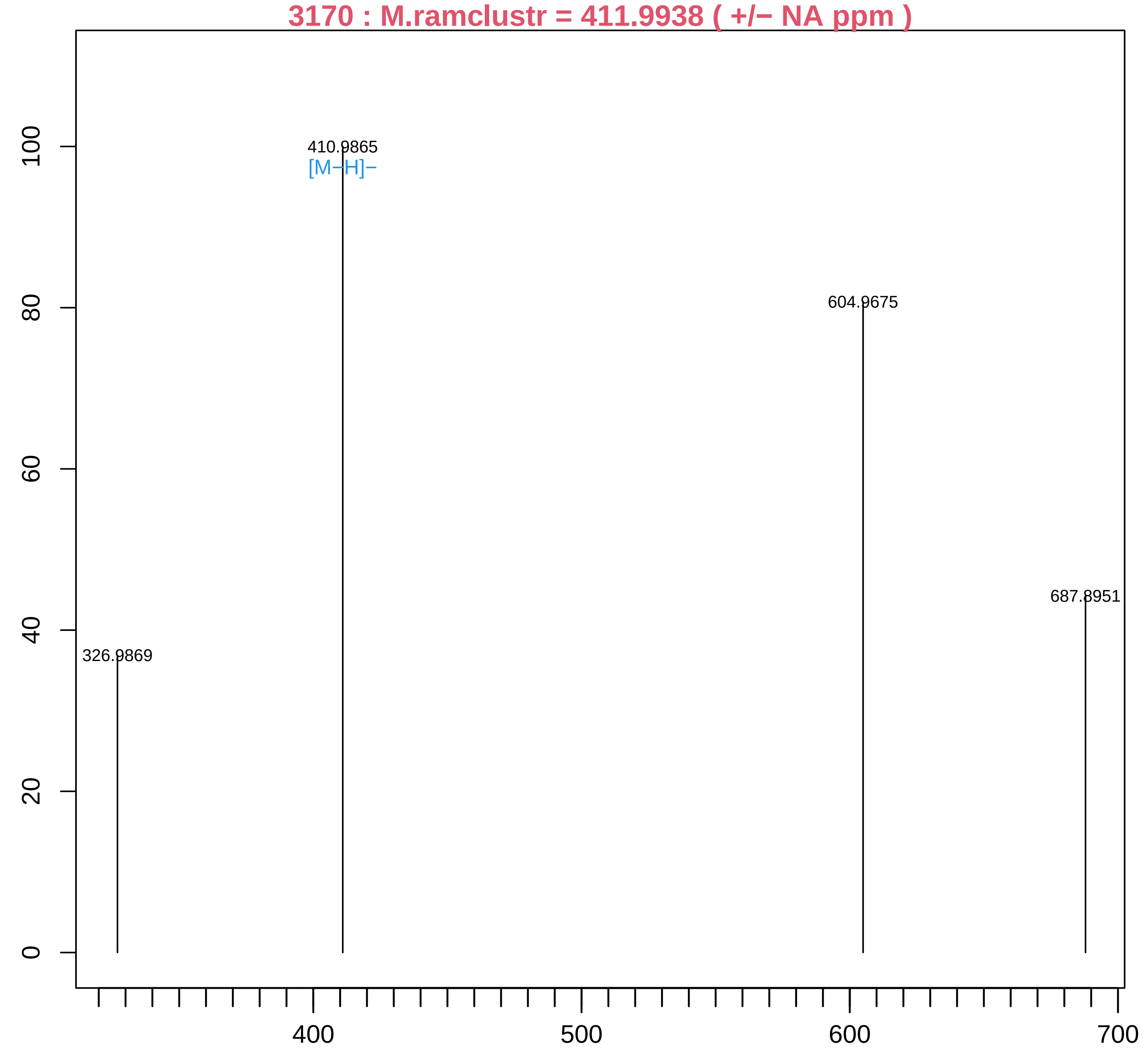
3170 : M.ramclustr = 411.9938 (+/− NA ppm)
410.9865
[M−H]−
604.9675
687.8951
326.9869
0
20
40
60
80
100
400
500
600
700

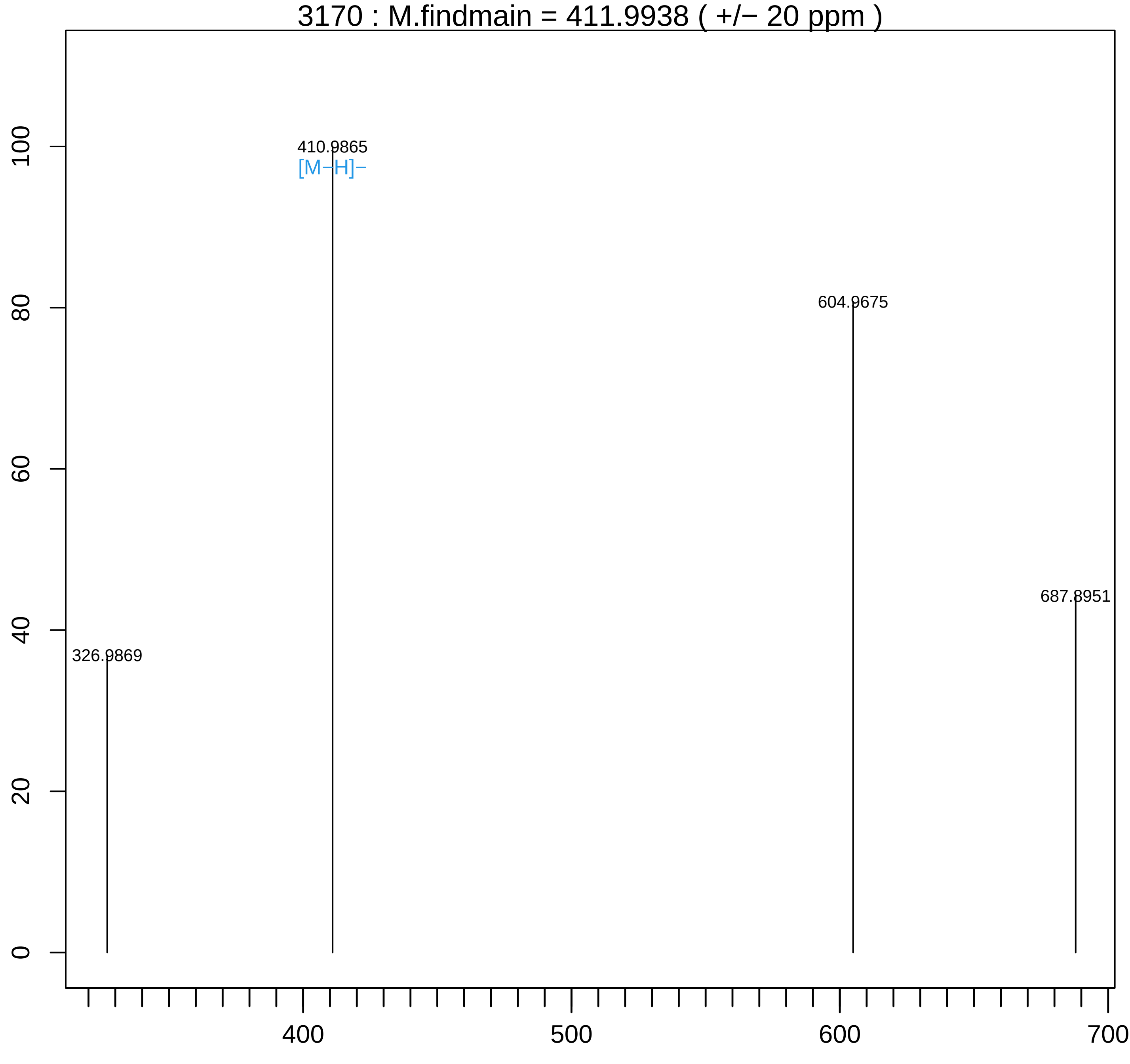
3170 : M.findmain = 411.9938 (+/− 20 ppm)
410.9865
[M−H]−
604.9675
687.8951
326.9869
0
20
40
60
80
100
400
500
600
700

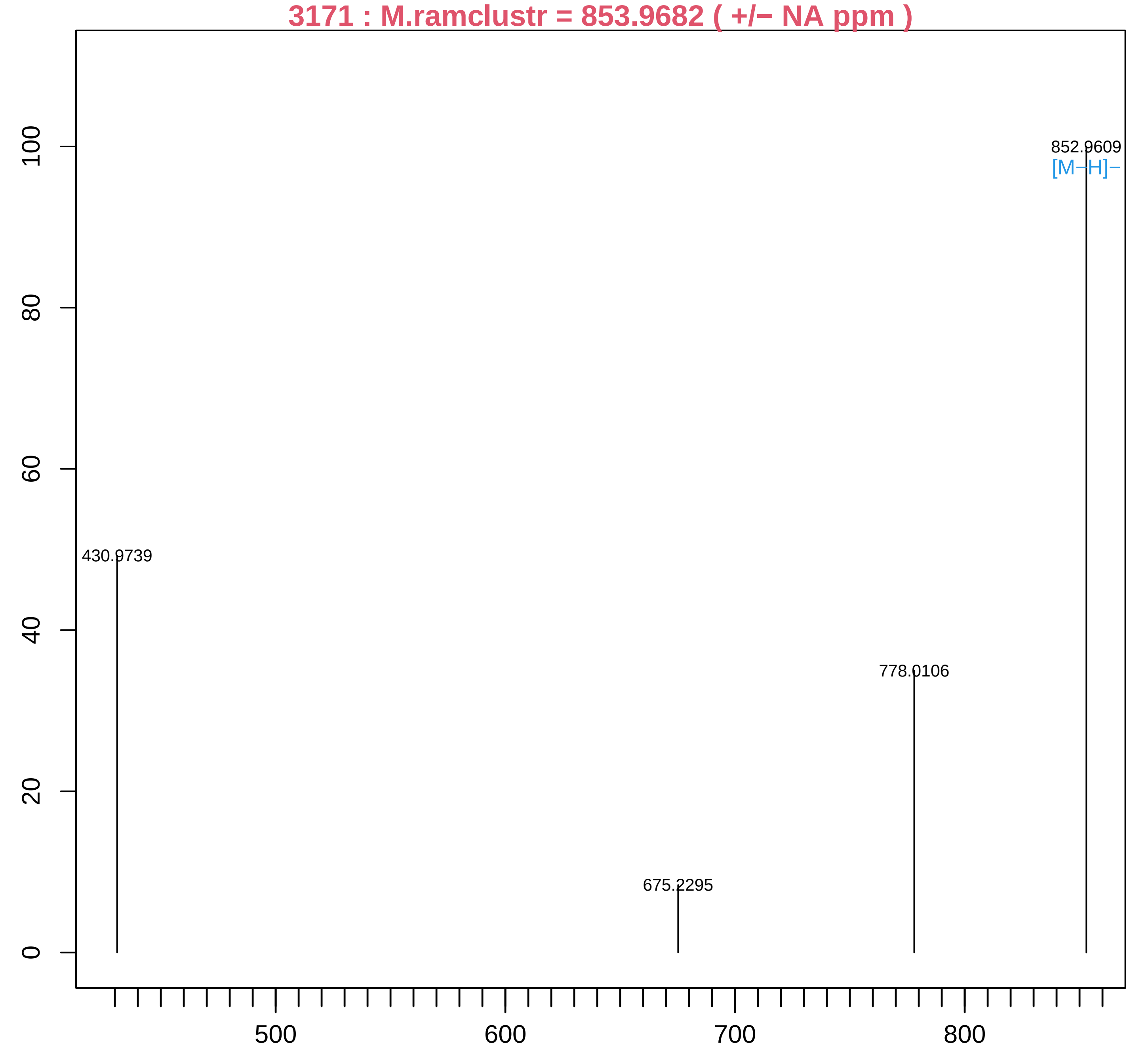
3171 : M.ramclustr = 853.9682 (+/− NA ppm)
0
20
40
60
80
100
500
600
700
800
430.9739
675.2295
778.0106
852.9609
[M−H]−

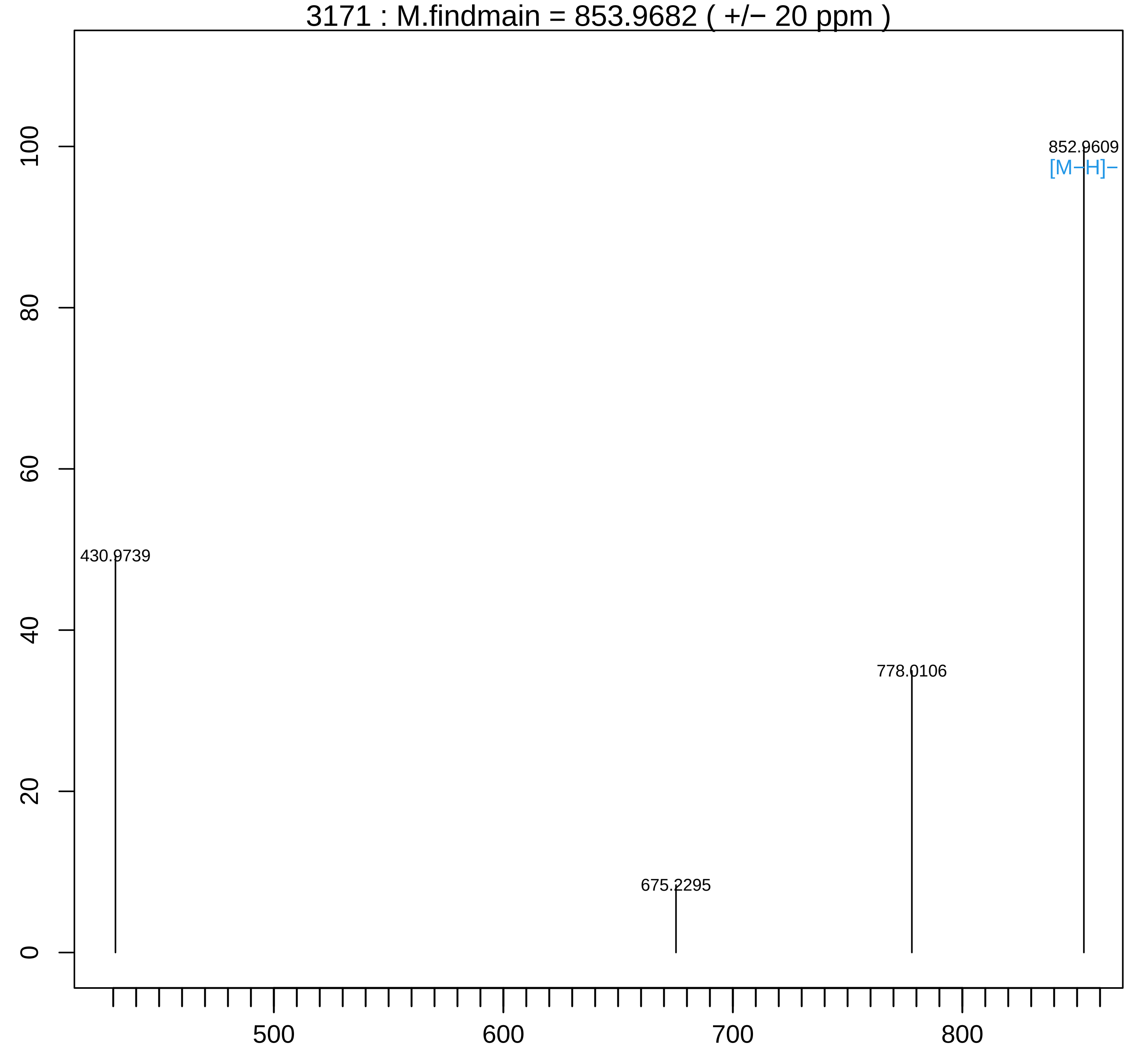
3171 : M.findmain = 853.9682 (+/− 20 ppm)
0
20
40
60
80
100
500
600
700
800
430.9739
675.2295
778.0106
852.9609
[M−H]−

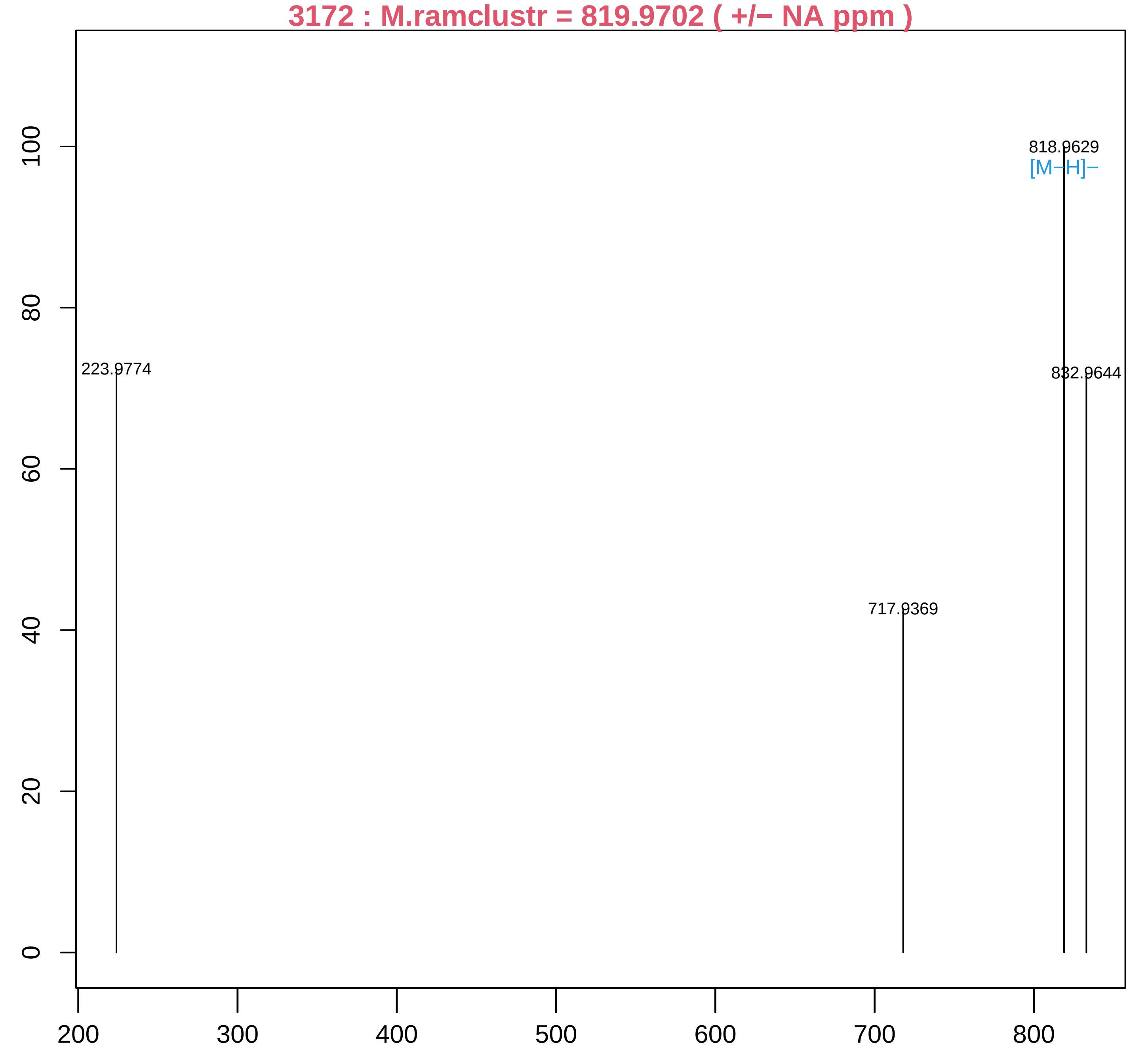
3172 : M.ramclustr = 819.9702 (+/− NA ppm)
223.9774
717.9369
818.9629
[M−H]−
832.9644
0
20
40
60
80
100
200
300
400
500
600
700
800

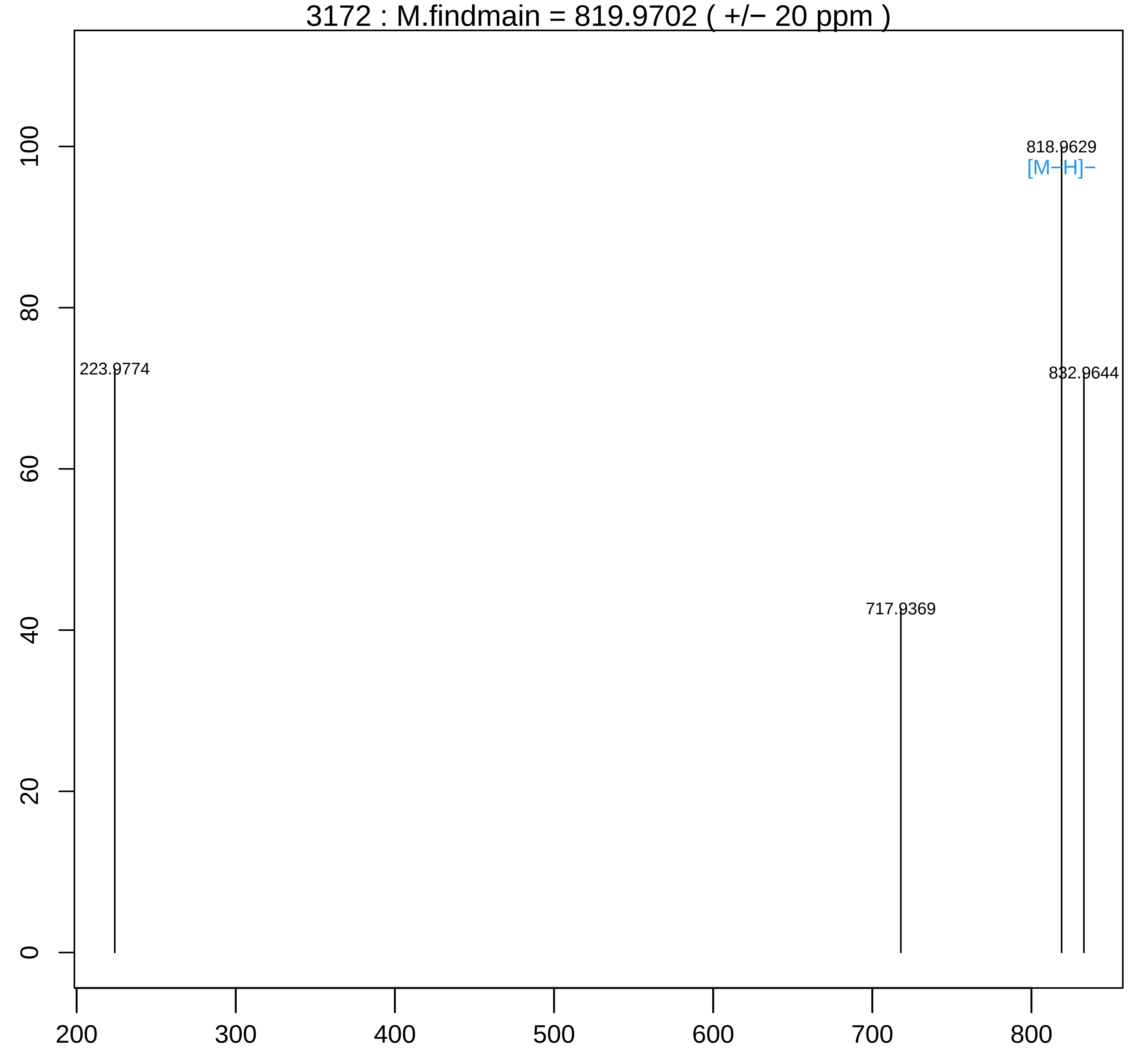
3172 : M.findmain = 819.9702 (+/− 20 ppm)
223.9774
717.9369
818.9629
[M−H]−
832.9644
0
20
40
60
80
100
200
300
400
500
600
700
800

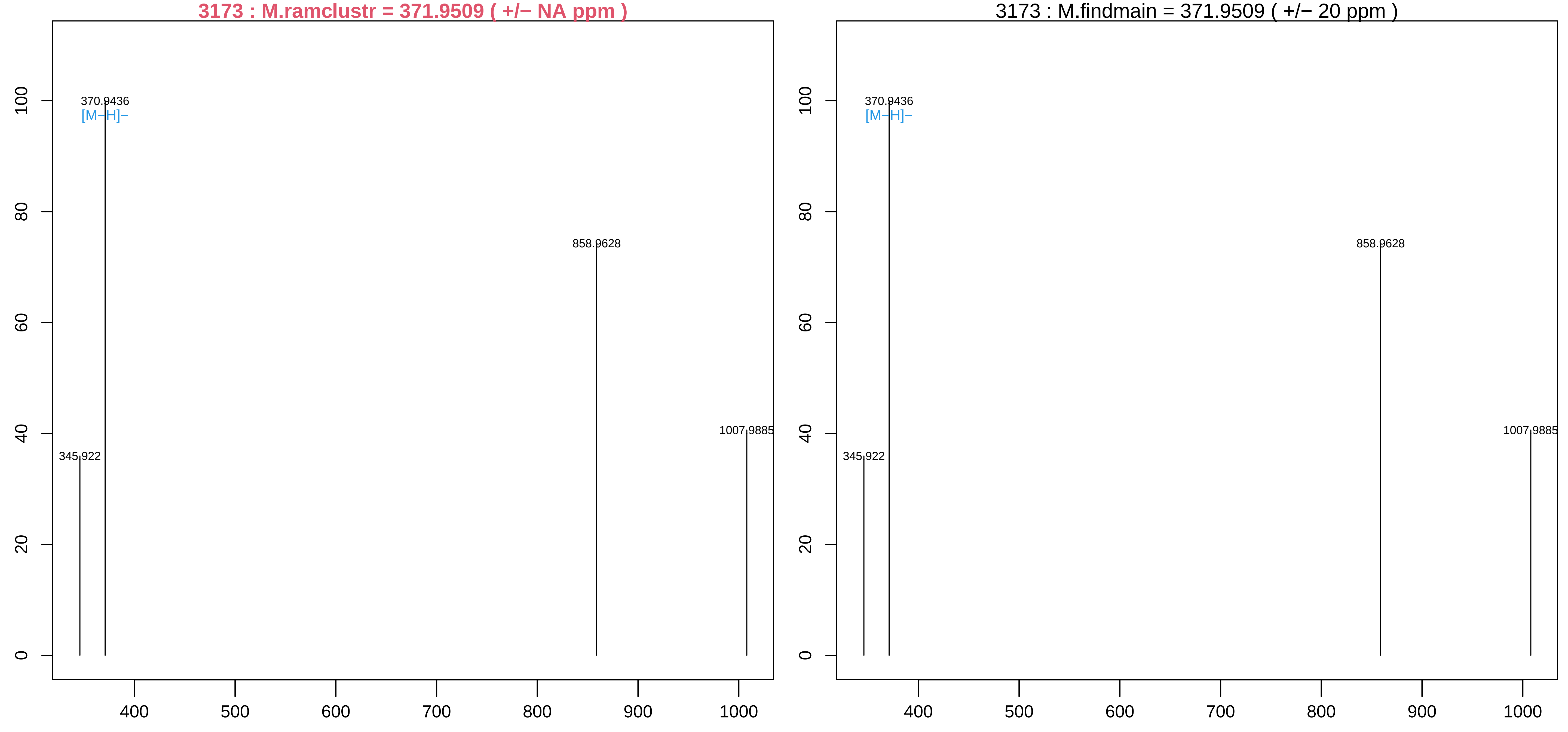

3173 : M.ramclustr = 371.9509 (+/− NA ppm)
370.9436
[M−H]−
858.9628
1007.9885
345.922
0
20
40
60
80
100
400
500
600
700
800
900
1000
3173 : M.findmain = 371.9509 (+/− 20 ppm)
370.9436
[M−H]−
858.9628
1007.9885
345.922
0
20
40
60
80
100
400
500
600
700
800
900
1000

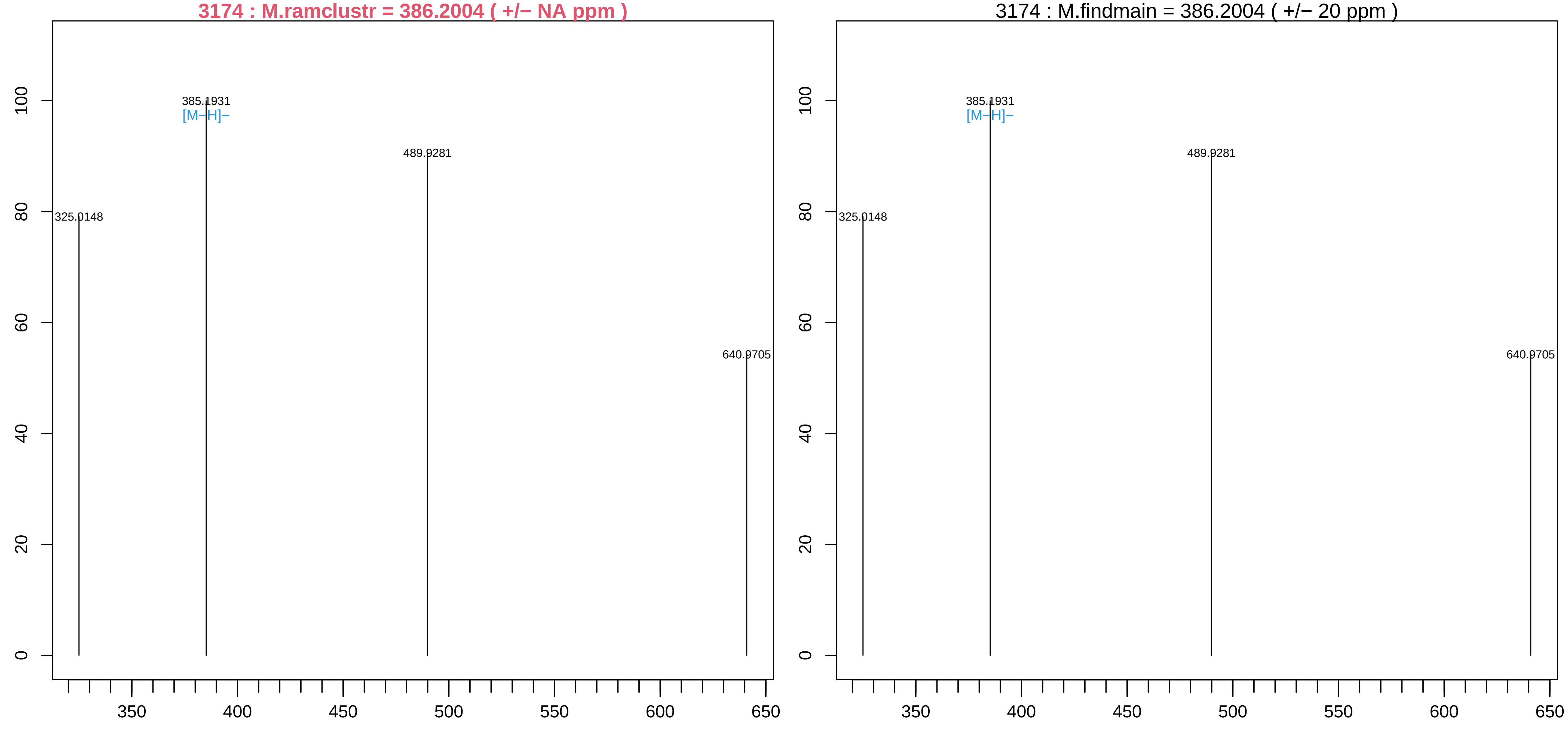

3174 : M.ramclustr = 386.2004 (+/− NA ppm)
325.0148
385.1931
[M−H]−
489.9281
640.9705
3174 : M.findmain = 386.2004 (+/− 20 ppm)
325.0148
385.1931
[M−H]−
489.9281
640.9705

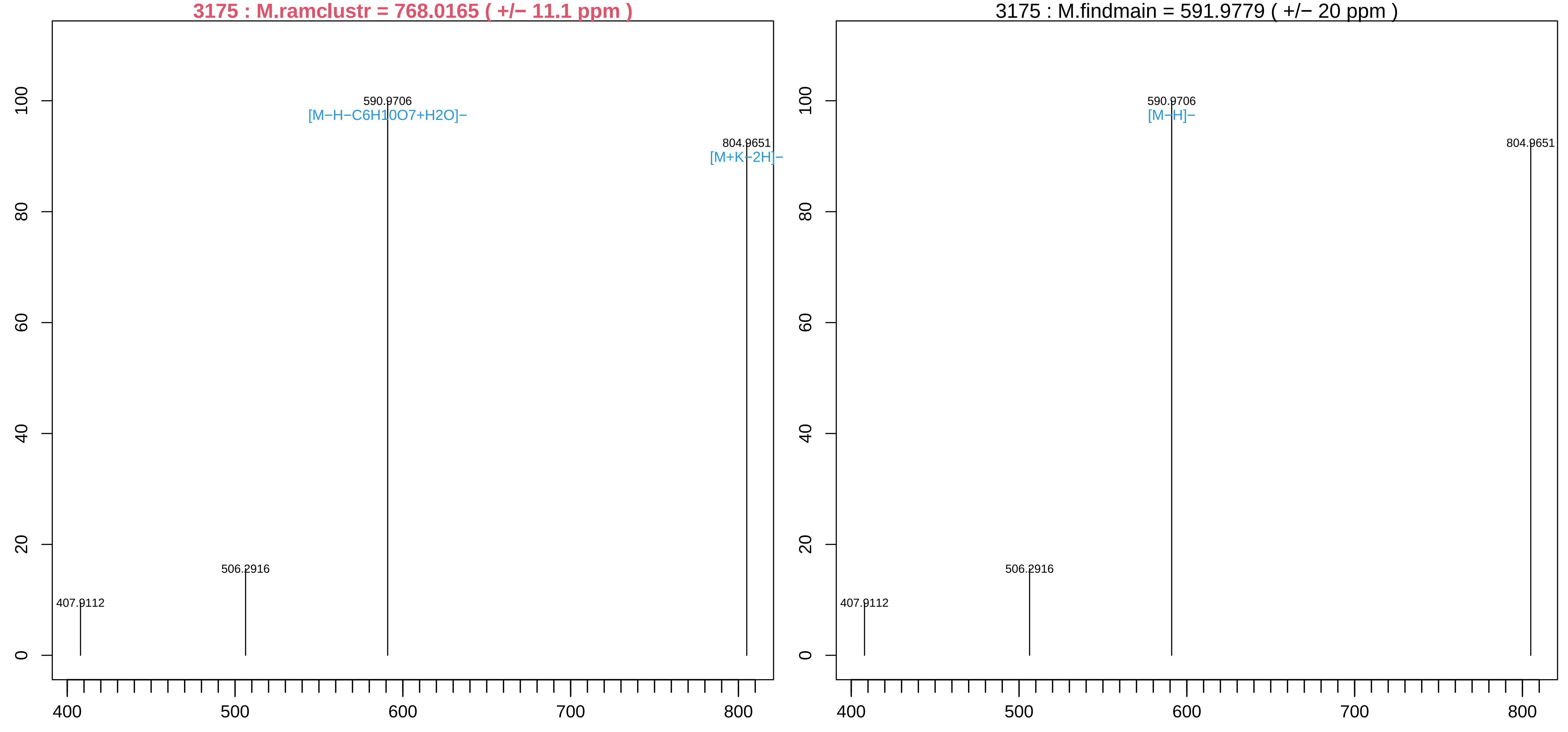
3175 : M.ramclustr = 768.0165 (+/− 11.1 ppm)
590.9706
[M−H−C6H10O7+H2O]−
804.9651
[M+K−2H]−
506.2916
407.9112
0
20
40
60
80
100
400
500
600
700
800
3175 : M.findmain = 591.9779 (+/− 20 ppm)
590.9706
[M−H]−
804.9651
506.2916
407.9112
0
20
40
60
80
100
400
500
600
700
800

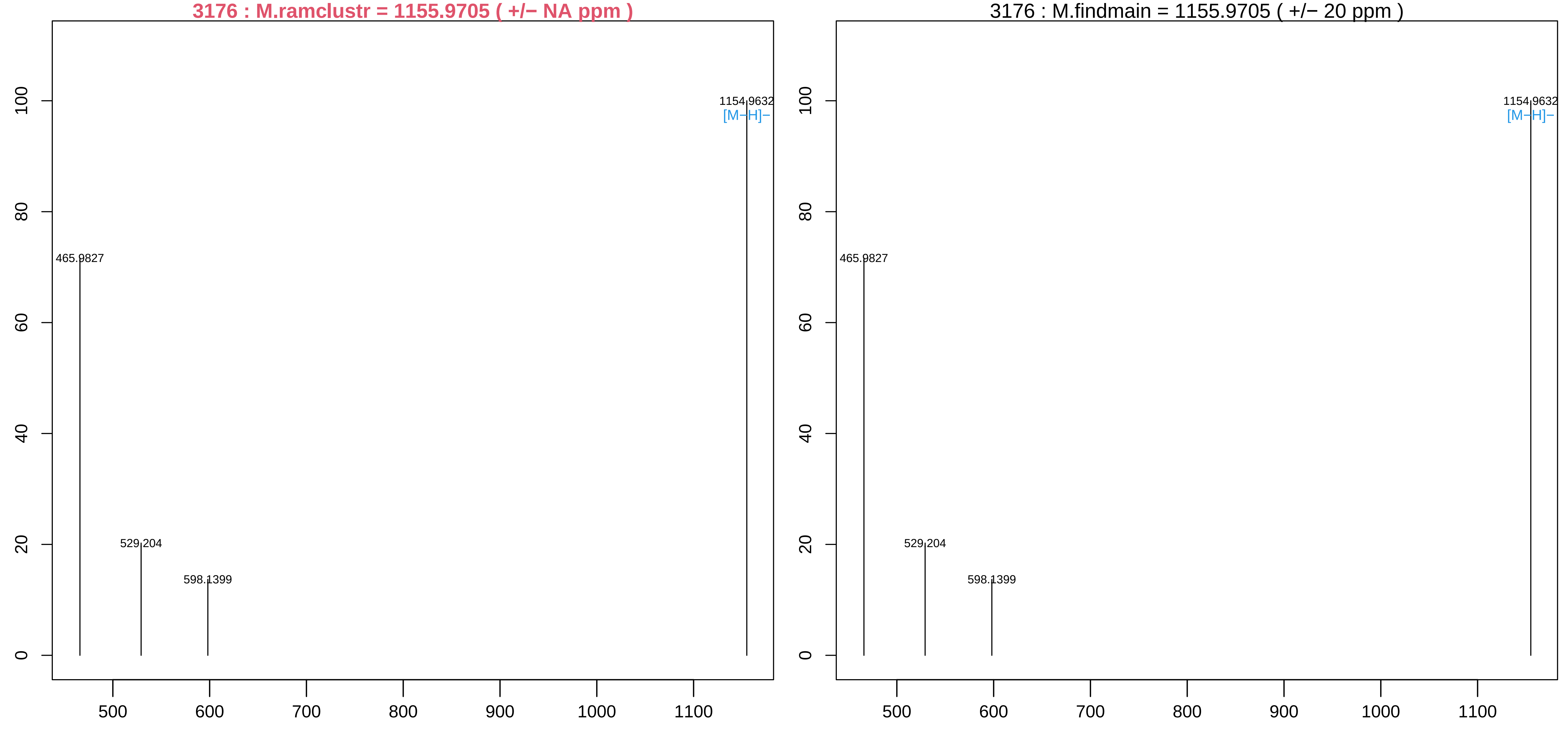
3176 : M.ramclustr = 1155.9705 (+/− NA ppm)
1154.9632
[M−H]−
465.9827
529.204
598.1399
3176 : M.findmain = 1155.9705 (+/− 20 ppm)
1154.9632
[M−H]−
465.9827
529.204
598.1399

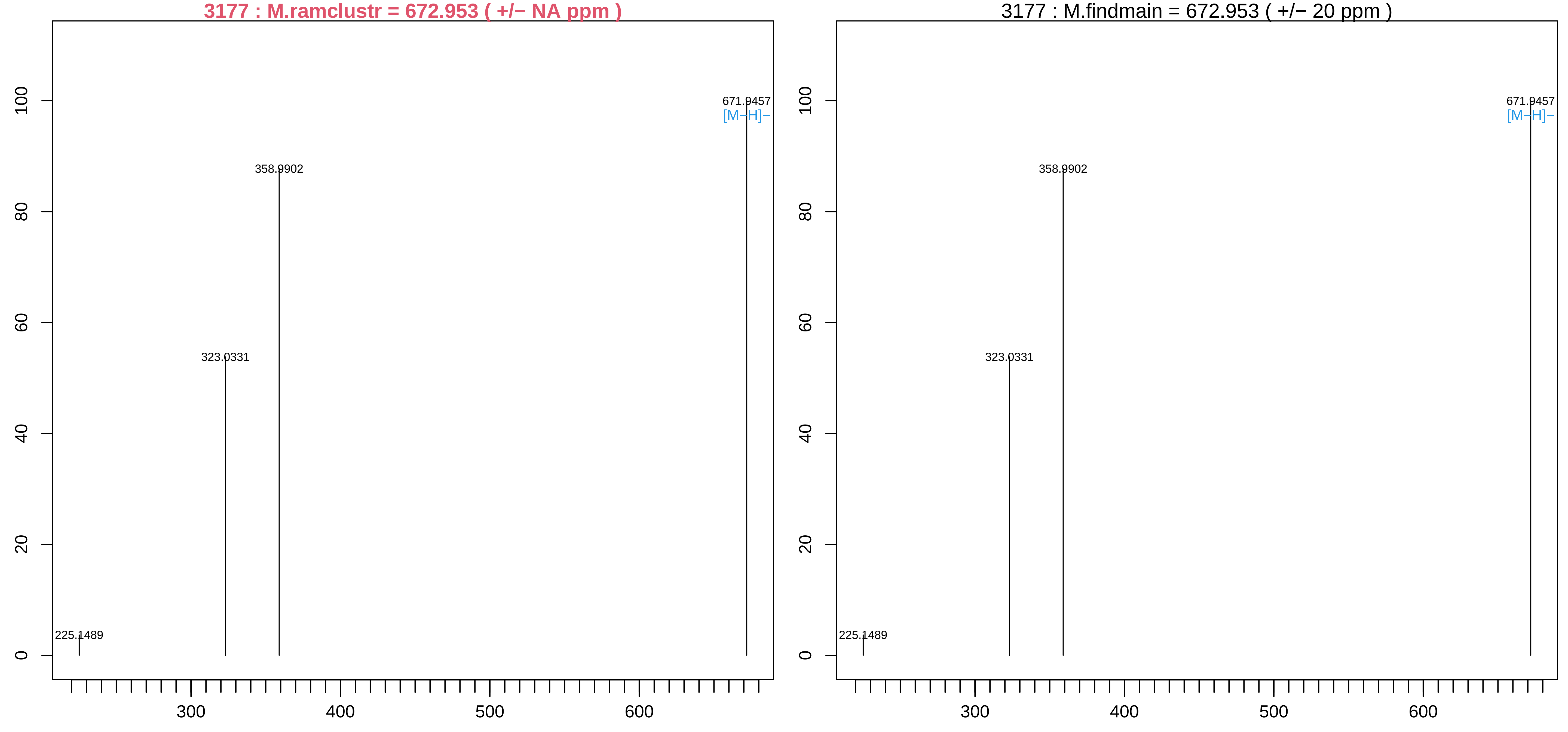

3177 : M.ramclustr = 672.953 (+/− NA ppm)
3177 : M.findmain = 672.953 (+/− 20 ppm)
671.9457
[M−H]−
358.9902
323.0331
225.1489
0
20
40
60
80
100
300
400
500
600

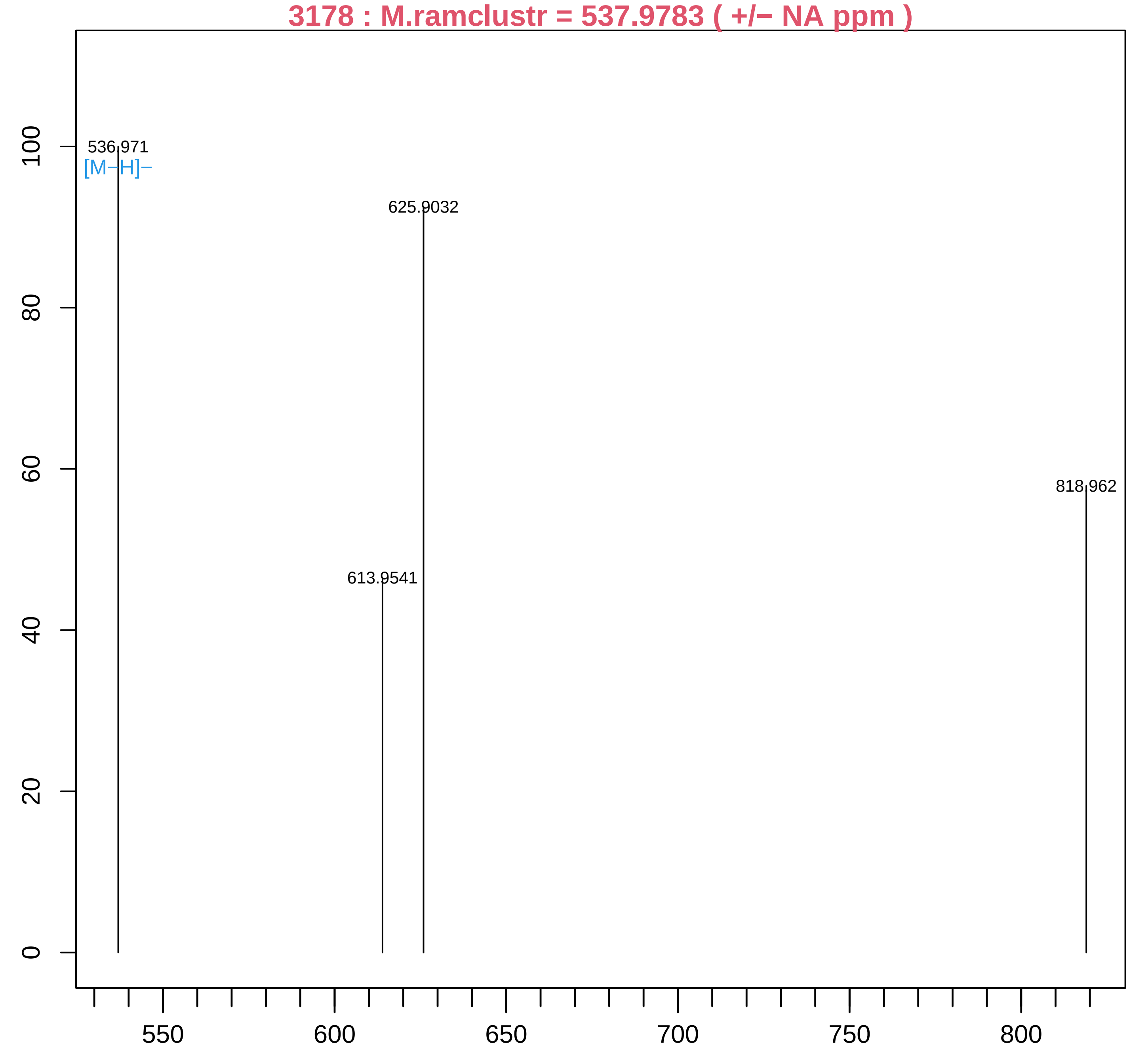
3178 : M.ramclustr = 537.9783 (+/− NA ppm)
536.971
[M−H]−
625.9032
613.9541
818.962
0
20
40
60
80
100
550
600
650
700
750
800

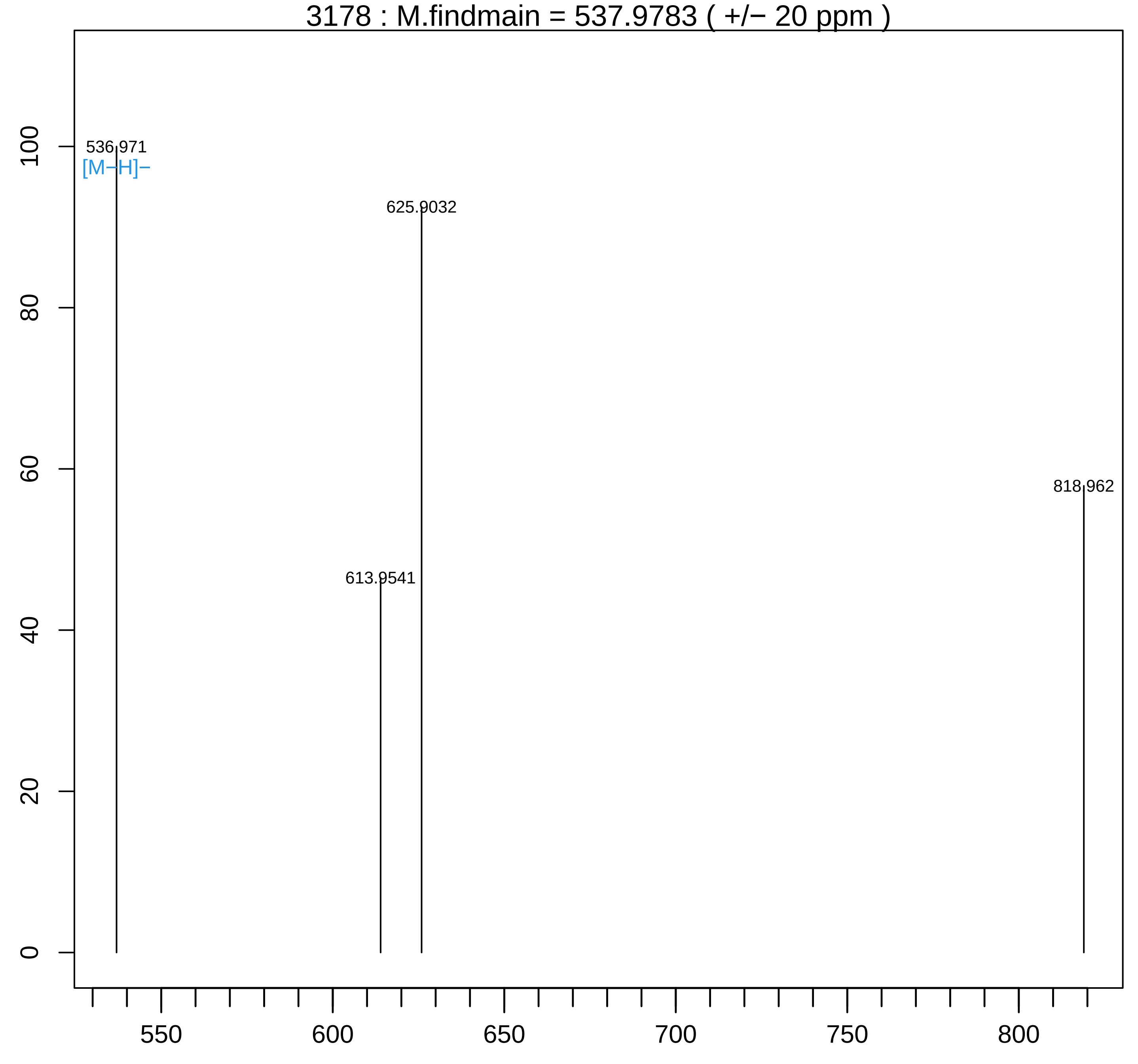
3178 : M.findmain = 537.9783 (+/− 20 ppm)
536.971
[M−H]−
625.9032
613.9541
818.962
0
20
40
60
80
100
550
600
650
700
750
800

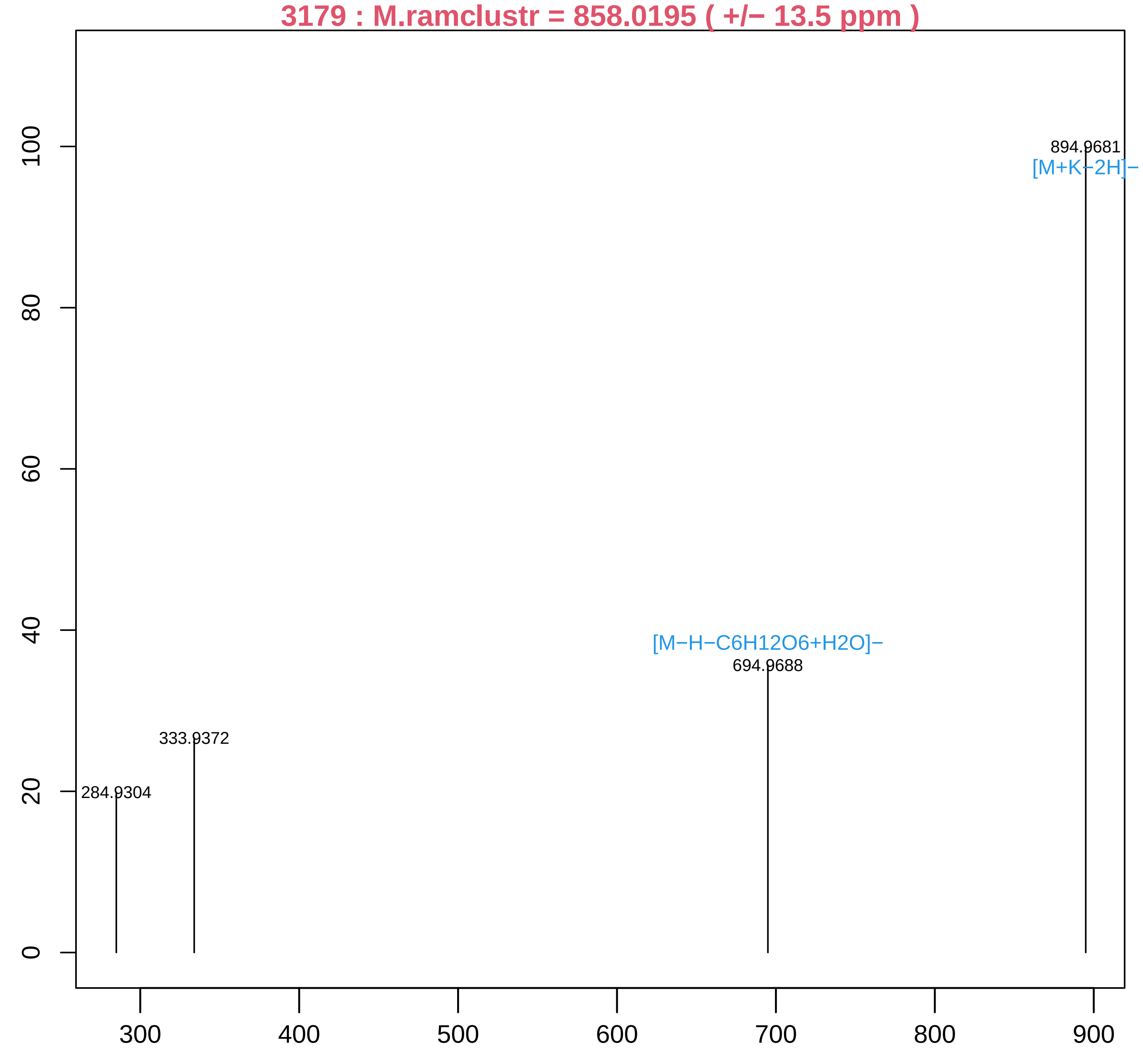
3179 : M.ramclustr = 858.0195 (+/− 13.5 ppm)
894.9681
[M+K−2H]−
[M−H−C6H12O6+H2O]−
694.9688
333.9372
284.9304
0
20
40
60
80
100
300
400
500
600
700
800
900

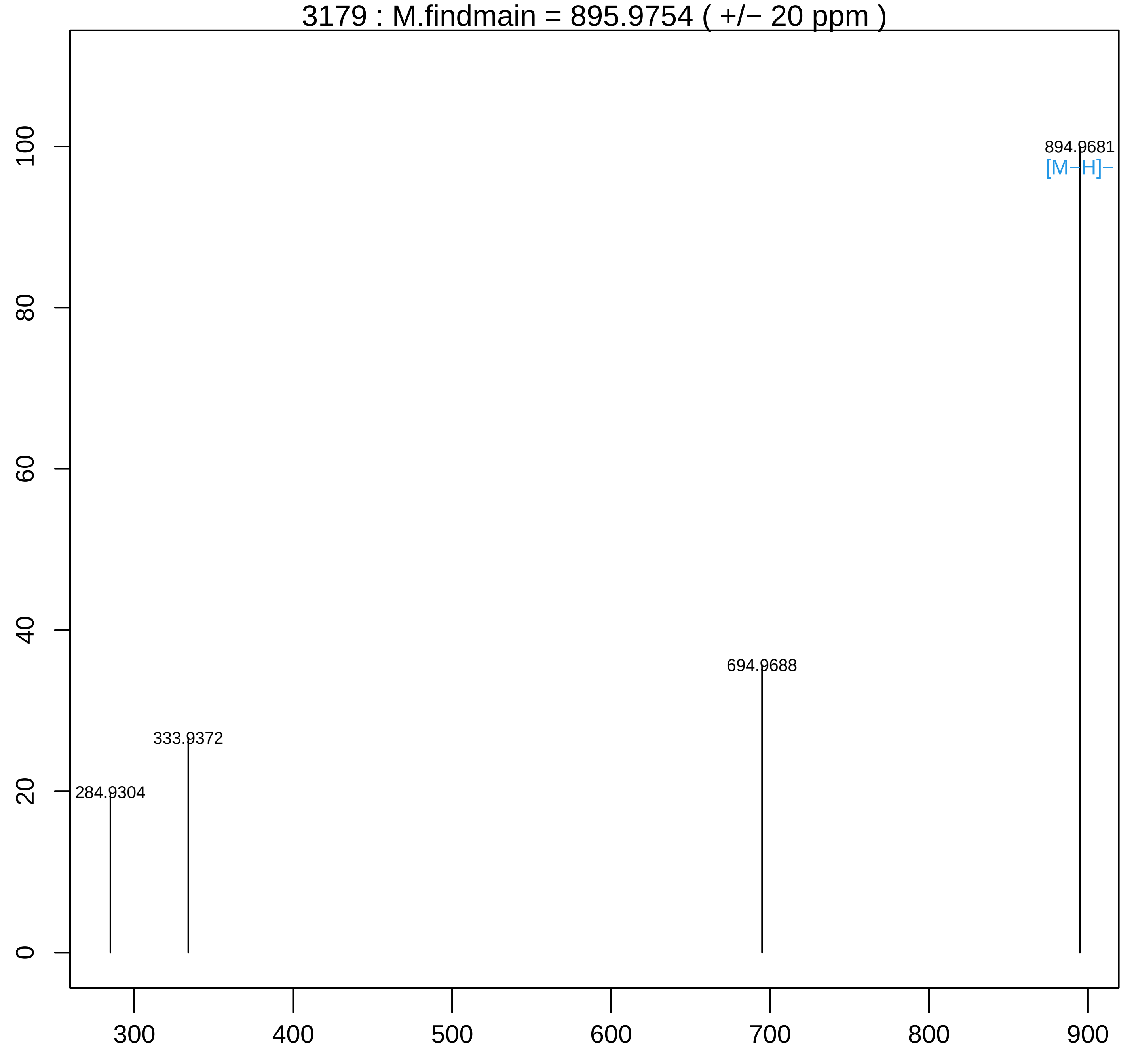
3179 : M.findmain = 895.9754 (+/− 20 ppm)
894.9681
[M−H]−
694.9688
333.9372
284.9304
0
20
40
60
80
100
300
400
500
600
700
800
900

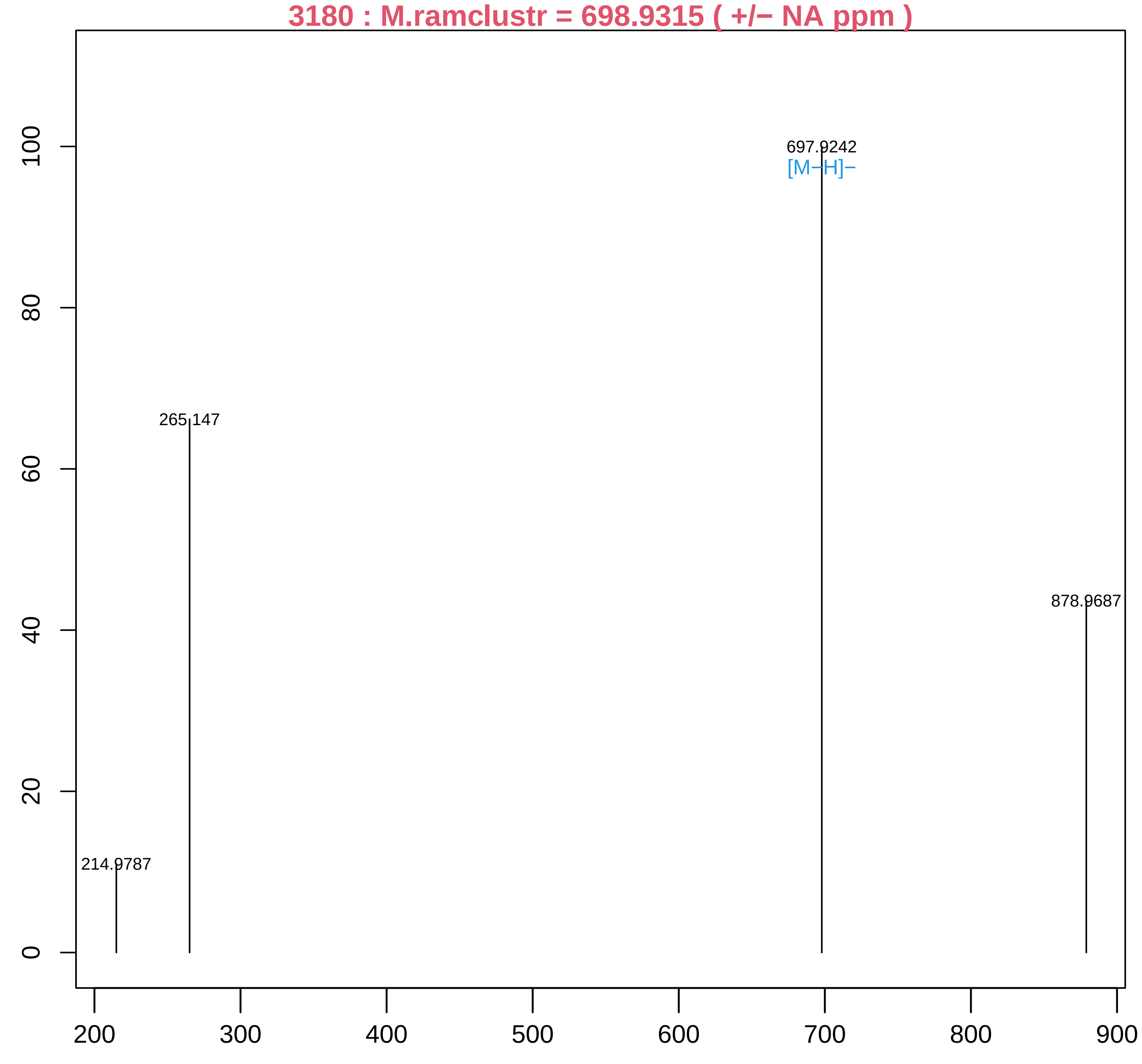
3180 : M.ramclustr = 698.9315 (+/− NA ppm)
697.9242
[M−H]−
265.147
878.9687
214.9787
0
20
40
60
80
100
200
300
400
500
600
700
800
900

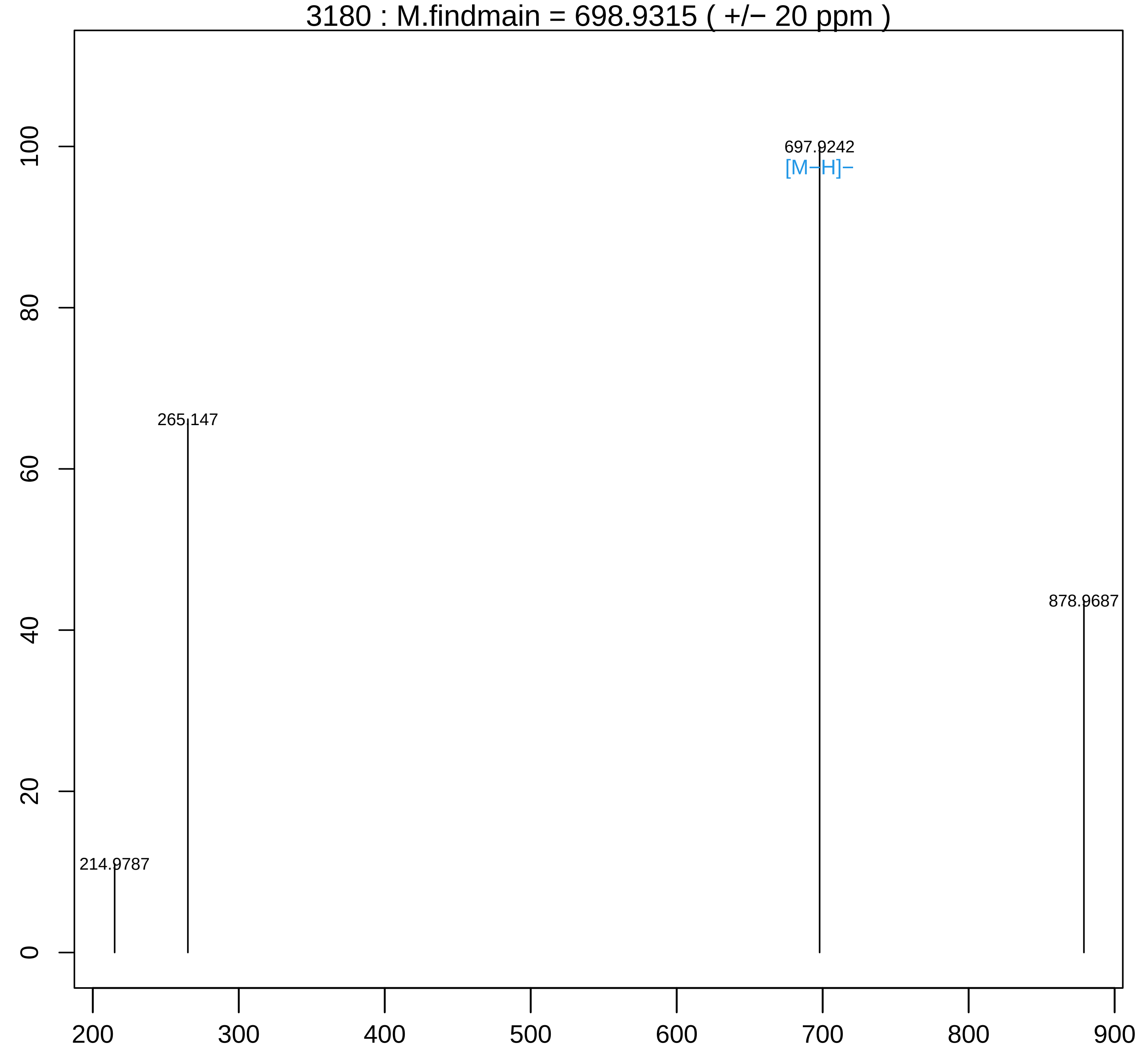
3180 : M.findmain = 698.9315 (+/− 20 ppm)
697.9242
[M−H]−
265.147
878.9687
214.9787
0
20
40
60
80
100
200
300
400
500
600
700
800
900

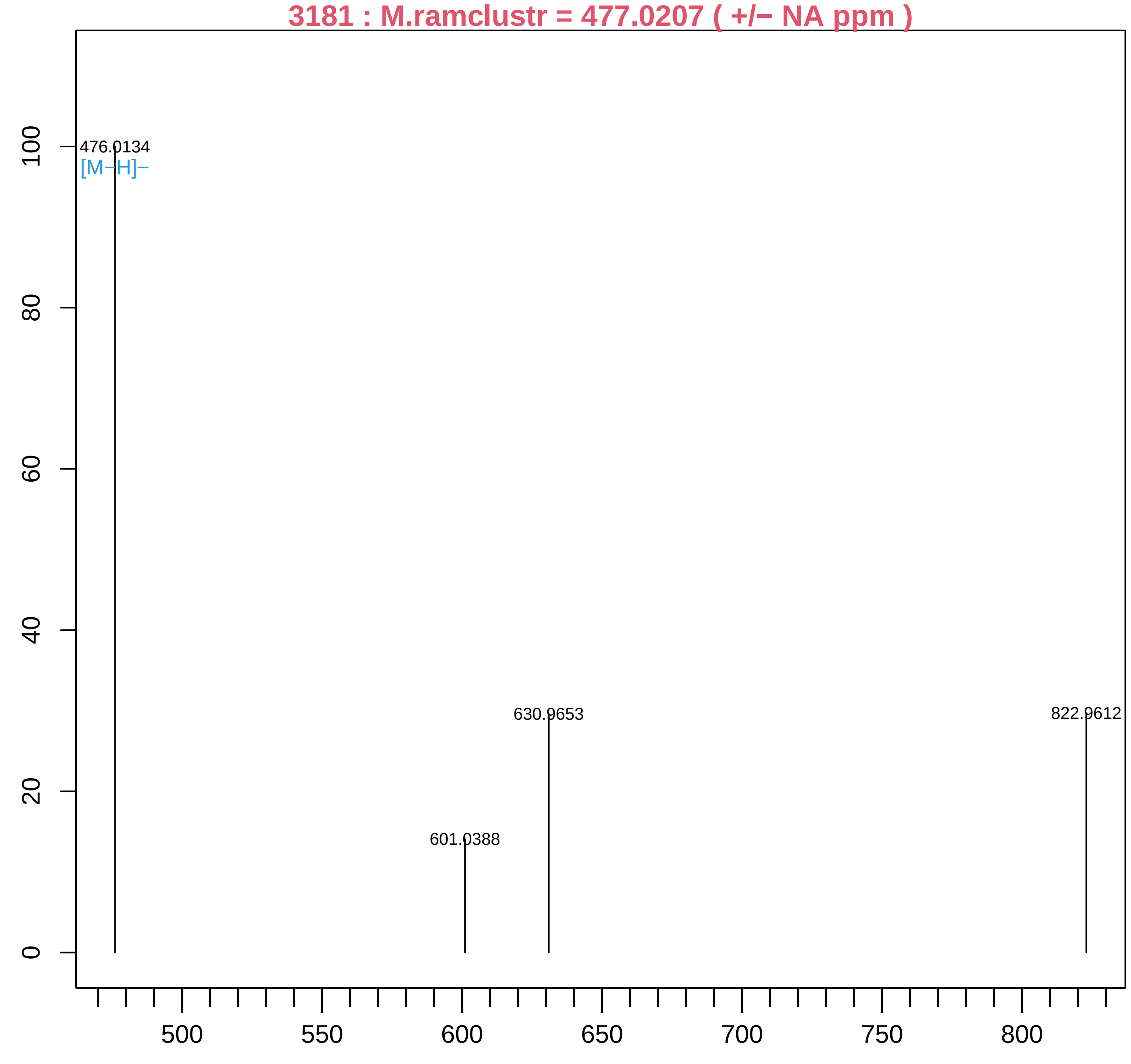
3181 : M.ramclustr = 477.0207 (+/− NA ppm)
476.0134
[M−H]−
601.0388
630.9653
822.9612
0
20
40
60
80
100
500
550
600
650
700
750
800

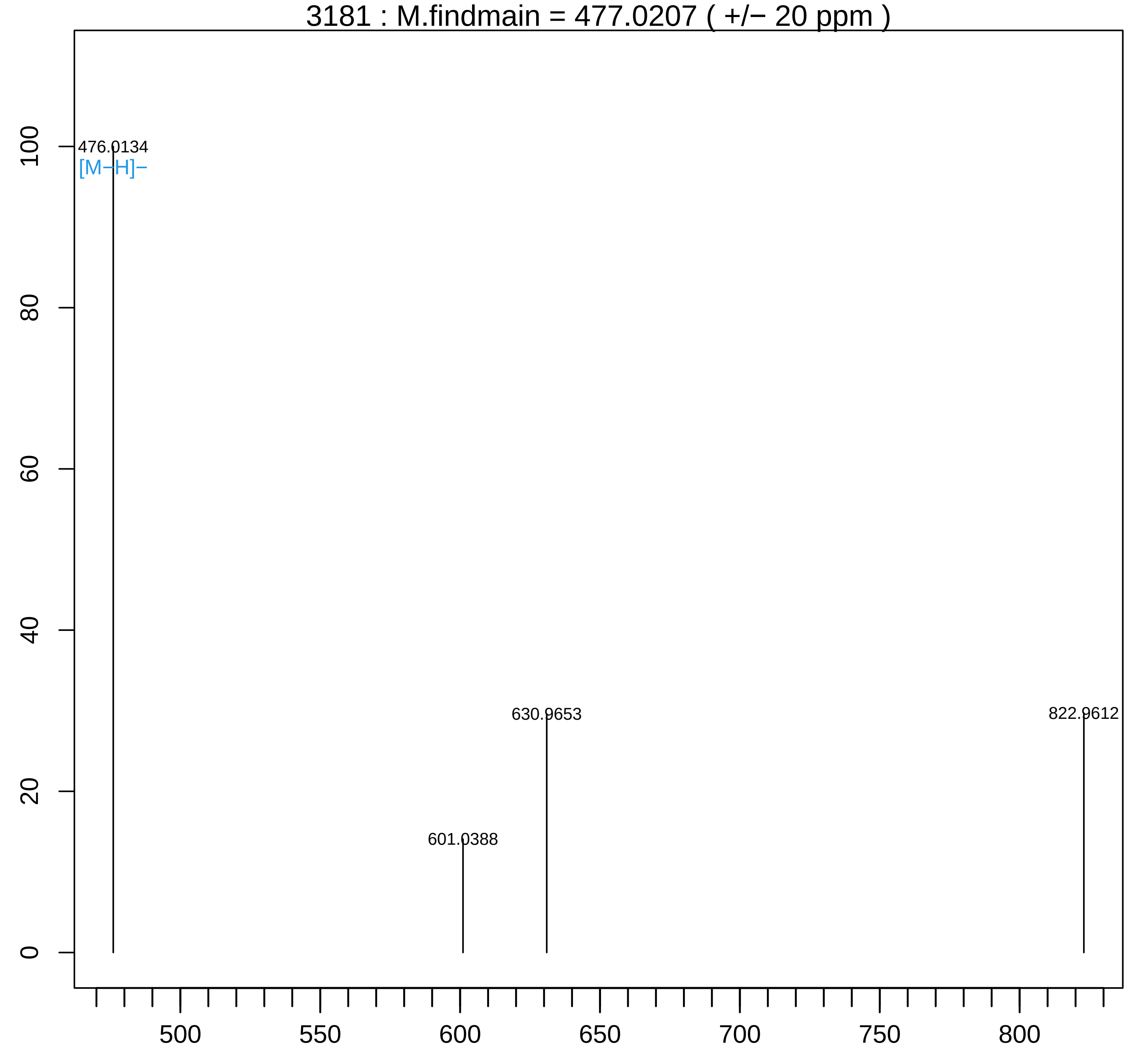
3181 : M.findmain = 477.0207 (+/− 20 ppm)
476.0134
[M−H]−
601.0388
630.9653
822.9612
0
20
40
60
80
100
500
550
600
650
700
750
800

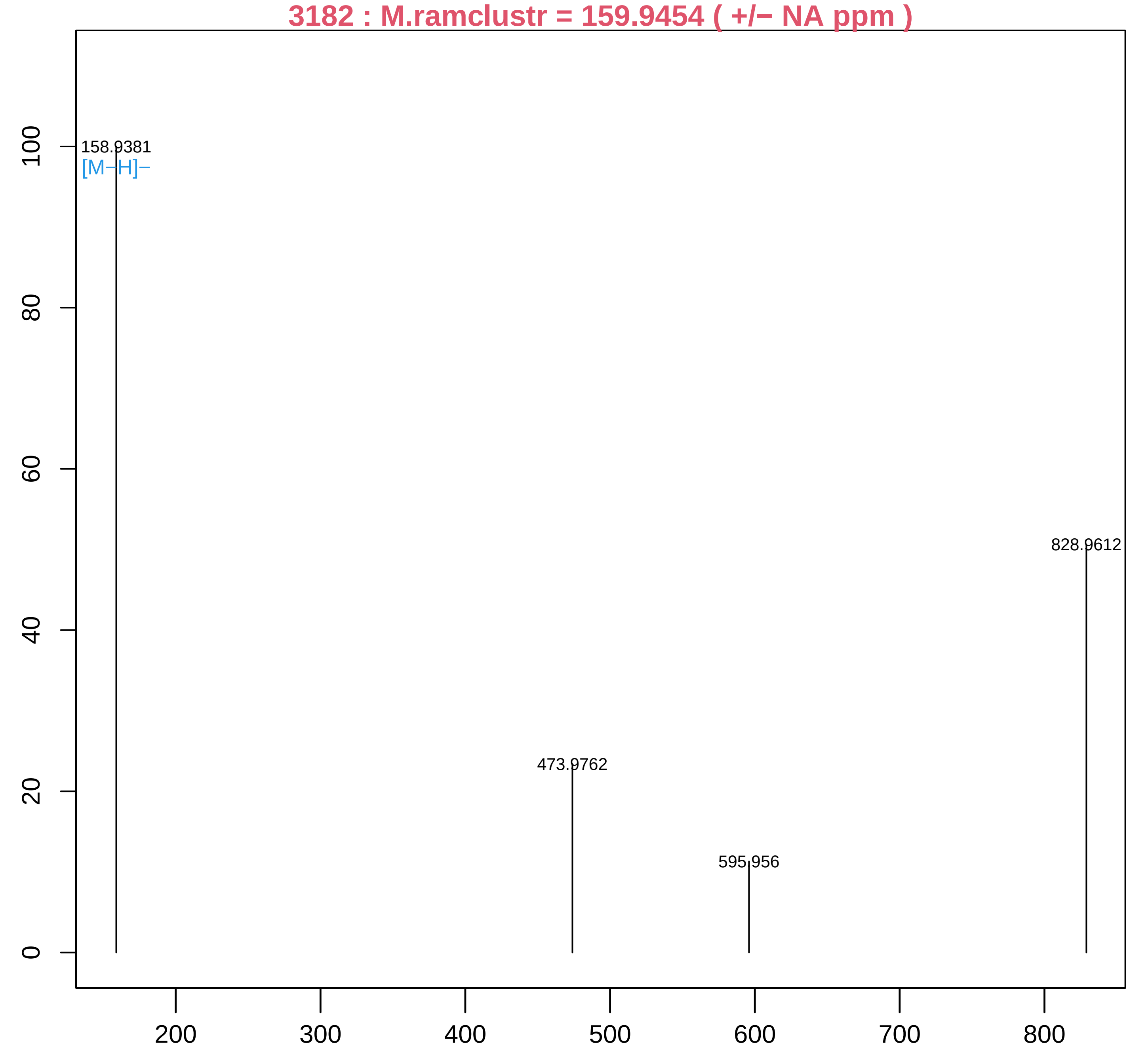
3182 : M.ramclustr = 159.9454 (+/− NA ppm)
158.9381
[M−H]−
473.9762
595.956
828.9612
0
20
40
60
80
100
200
300
400
500
600
700
800

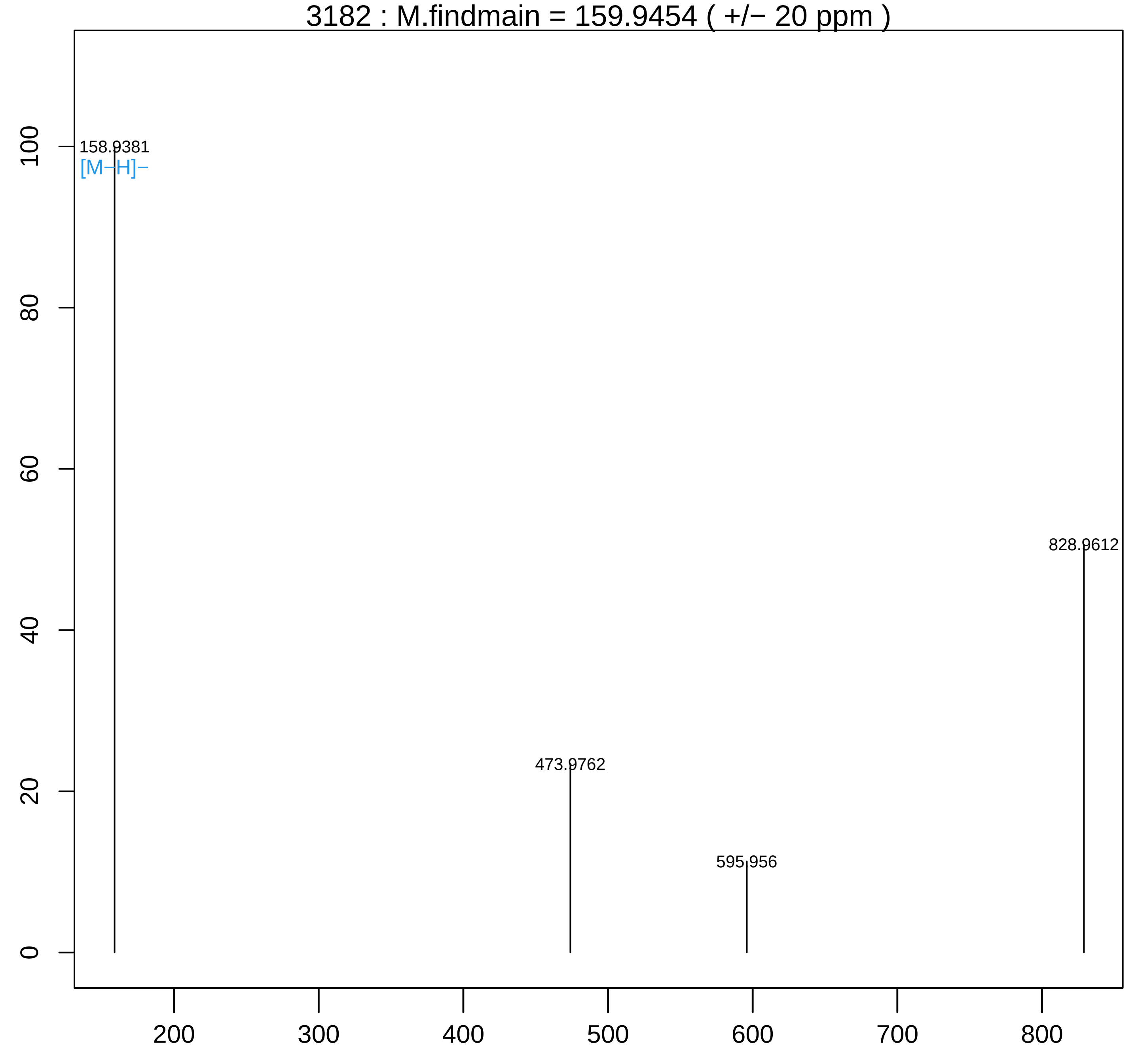
3182 : M.findmain = 159.9454 (+/− 20 ppm)
158.9381
[M−H]−
473.9762
595.956
828.9612
0
20
40
60
80
100
200
300
400
500
600
700
800

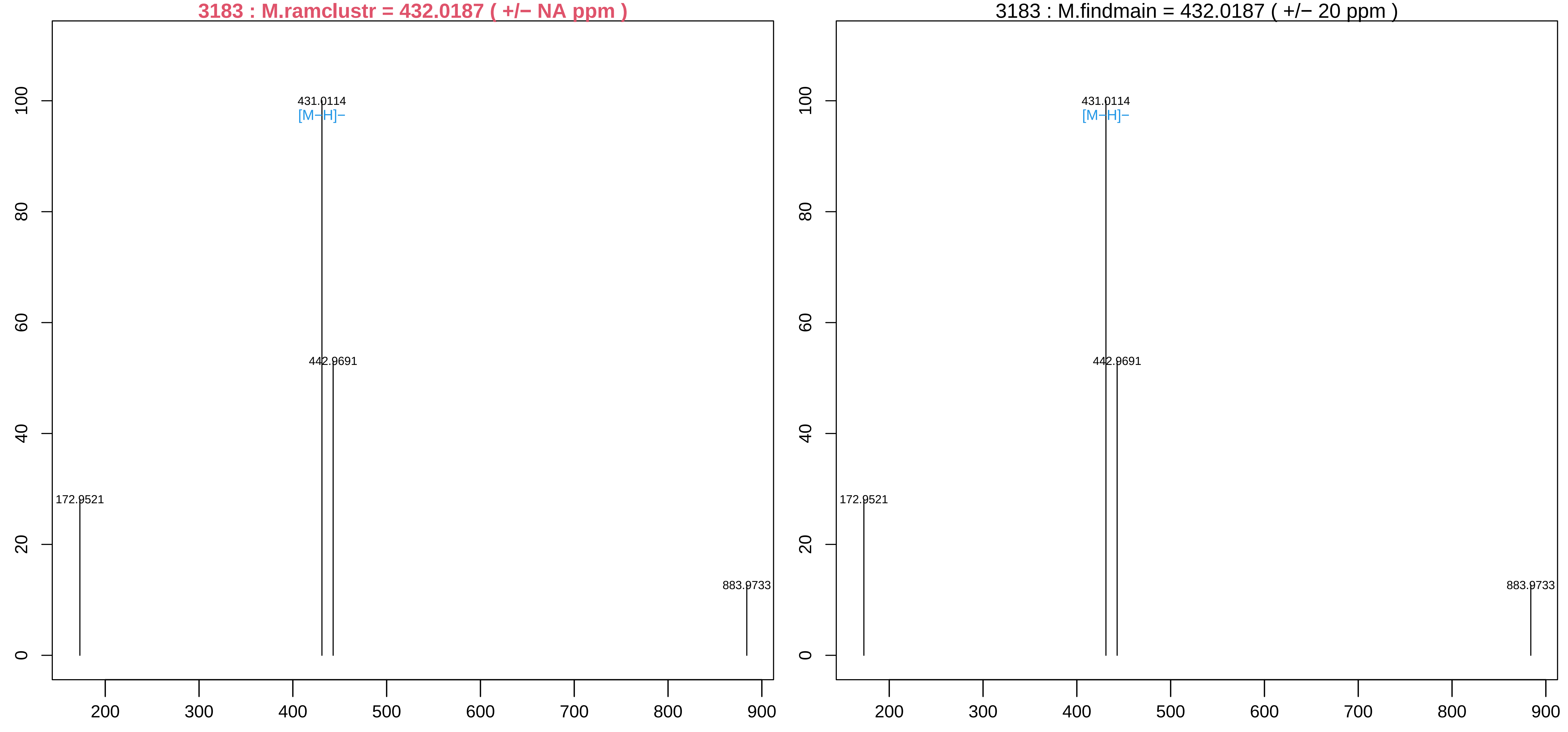
3183 : M.ramclustr = 432.0187 (+/− NA ppm)
431.0114
[M−H]−
442.9691
172.9521
883.9733
3183 : M.findmain = 432.0187 (+/− 20 ppm)
431.0114
[M−H]−
442.9691
172.9521
883.9733
0
20
40
60
80
100
200
300
400
500
600
700
800
900

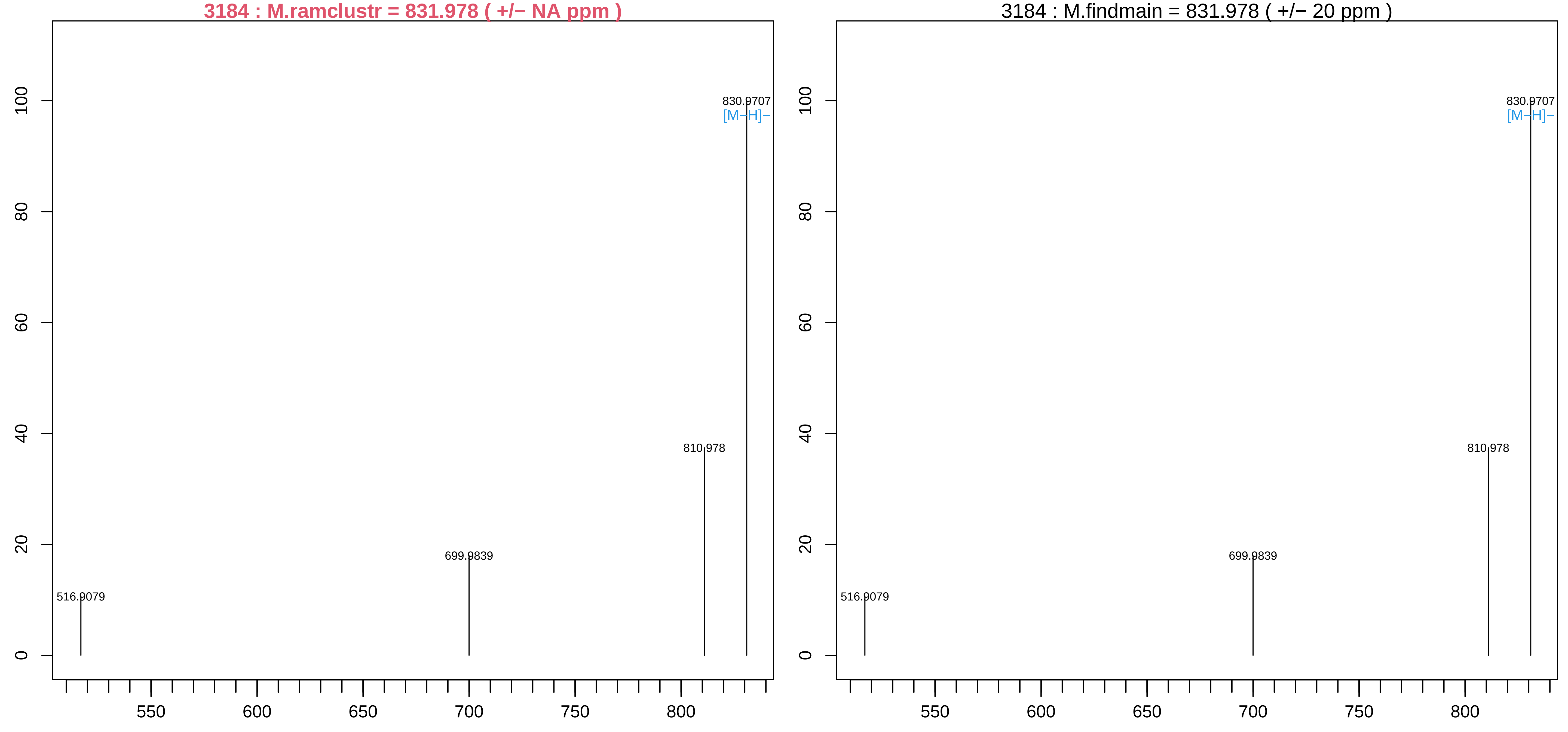
3184 : M.ramclustr = 831.978 (+/− NA ppm)
830.9707
[M−H]−
810.978
699.9839
516.9079
3184 : M.findmain = 831.978 (+/− 20 ppm)
830.9707
[M−H]−
810.978
699.9839
516.9079

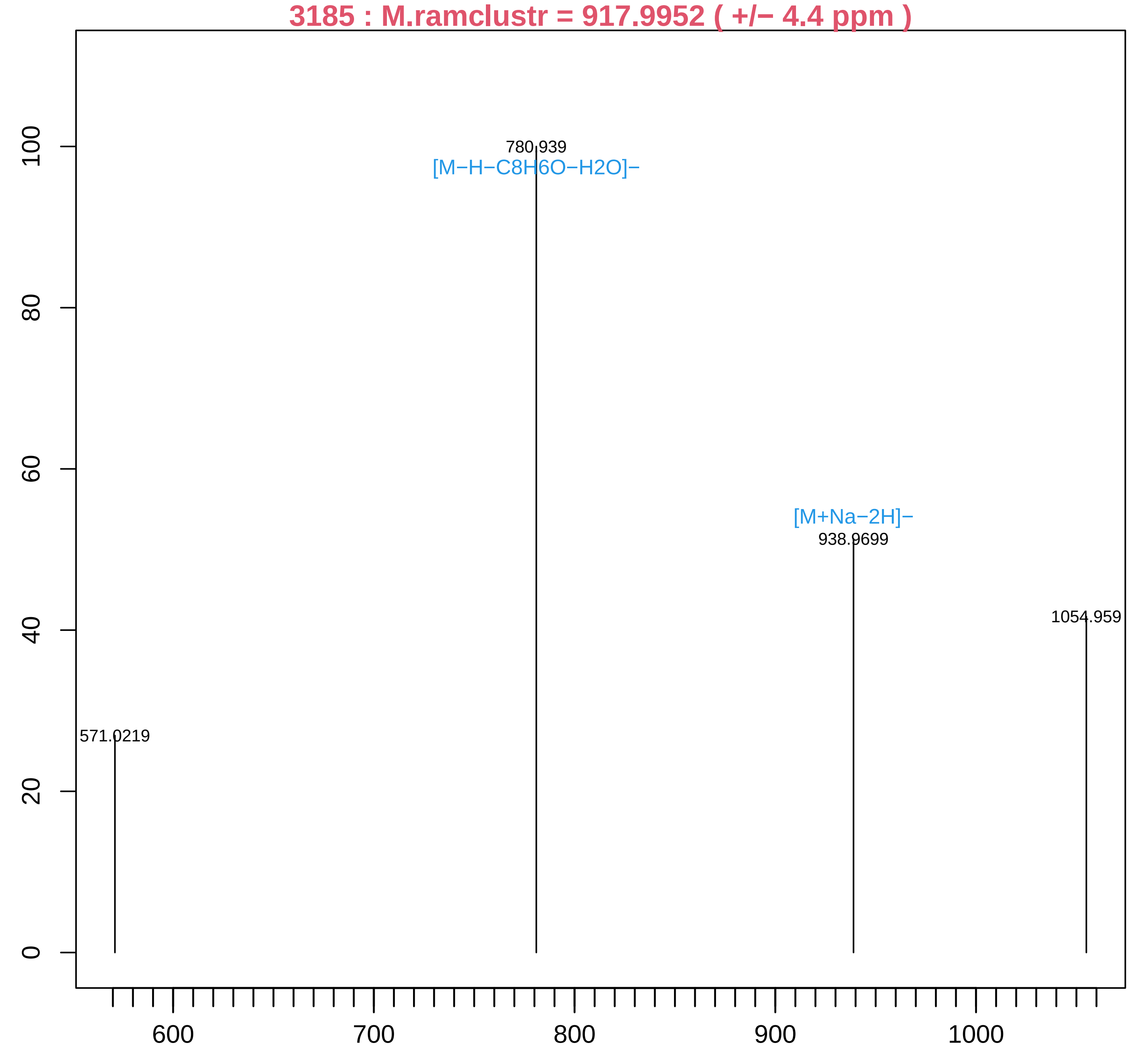

3185 : M.ramclustr = 917.9952 (+/− 4.4 ppm)
780.939
[M−H−C8H6O−H2O]−
[M+Na−2H]−
938.9699
1054.959
571.0219
0
20
40
60
80
100
600
700
800
900
1000

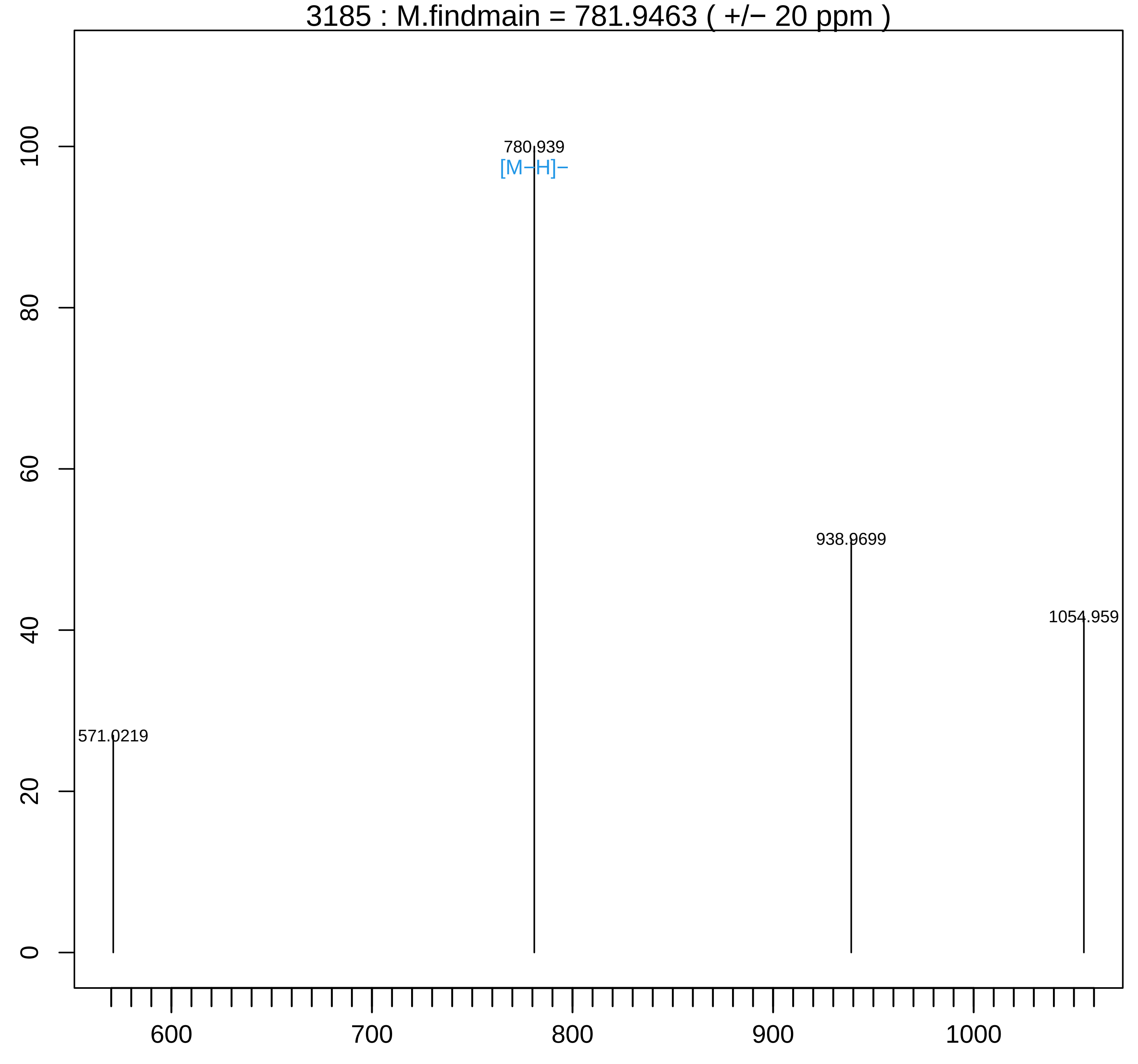

3185 : M.findmain = 781.9463 (+/− 20 ppm)
780.939
[M−H]−
938.9699
1054.959
571.0219
0
20
40
60
80
100
600
700
800
900
1000

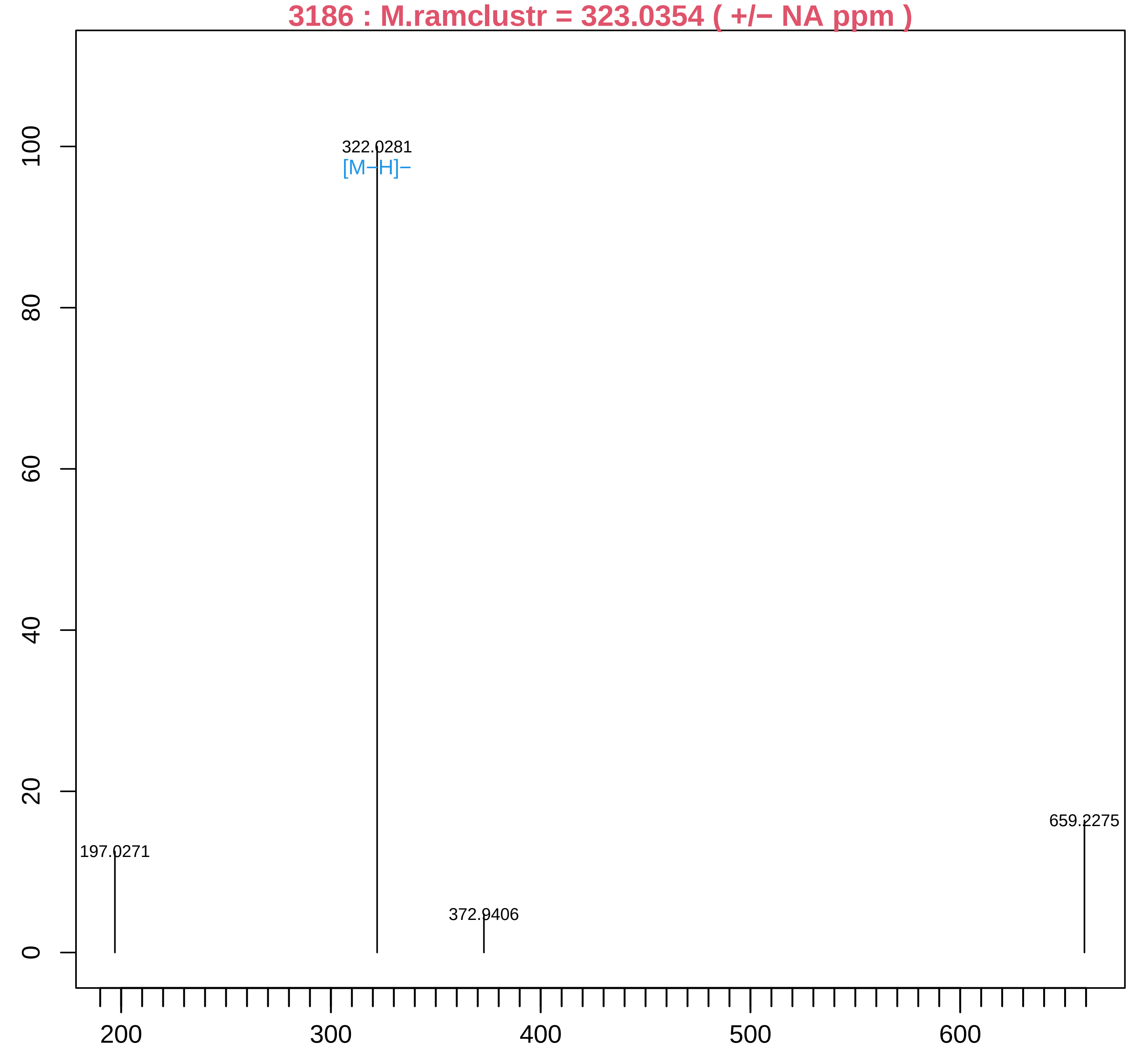
3186 : M.ramclustr = 323.0354 (+/− NA ppm)
322.0281
[M−H]−
197.0271
372.9406
659.2275
0
20
40
60
80
100
200
300
400
500
600

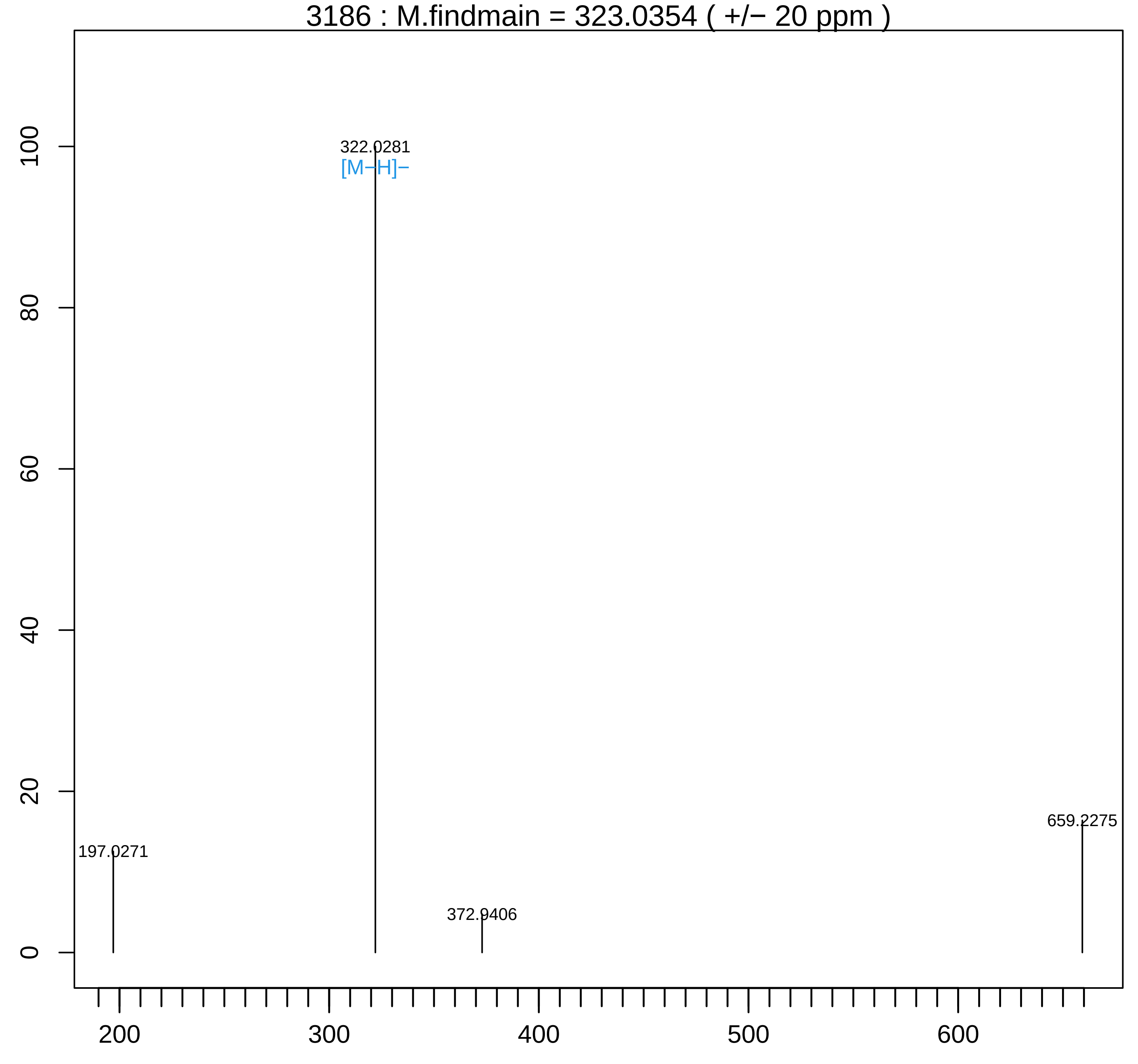
3186 : M.findmain = 323.0354 (+/− 20 ppm)
322.0281
[M−H]−
197.0271
372.9406
659.2275
0
20
40
60
80
100
200
300
400
500
600

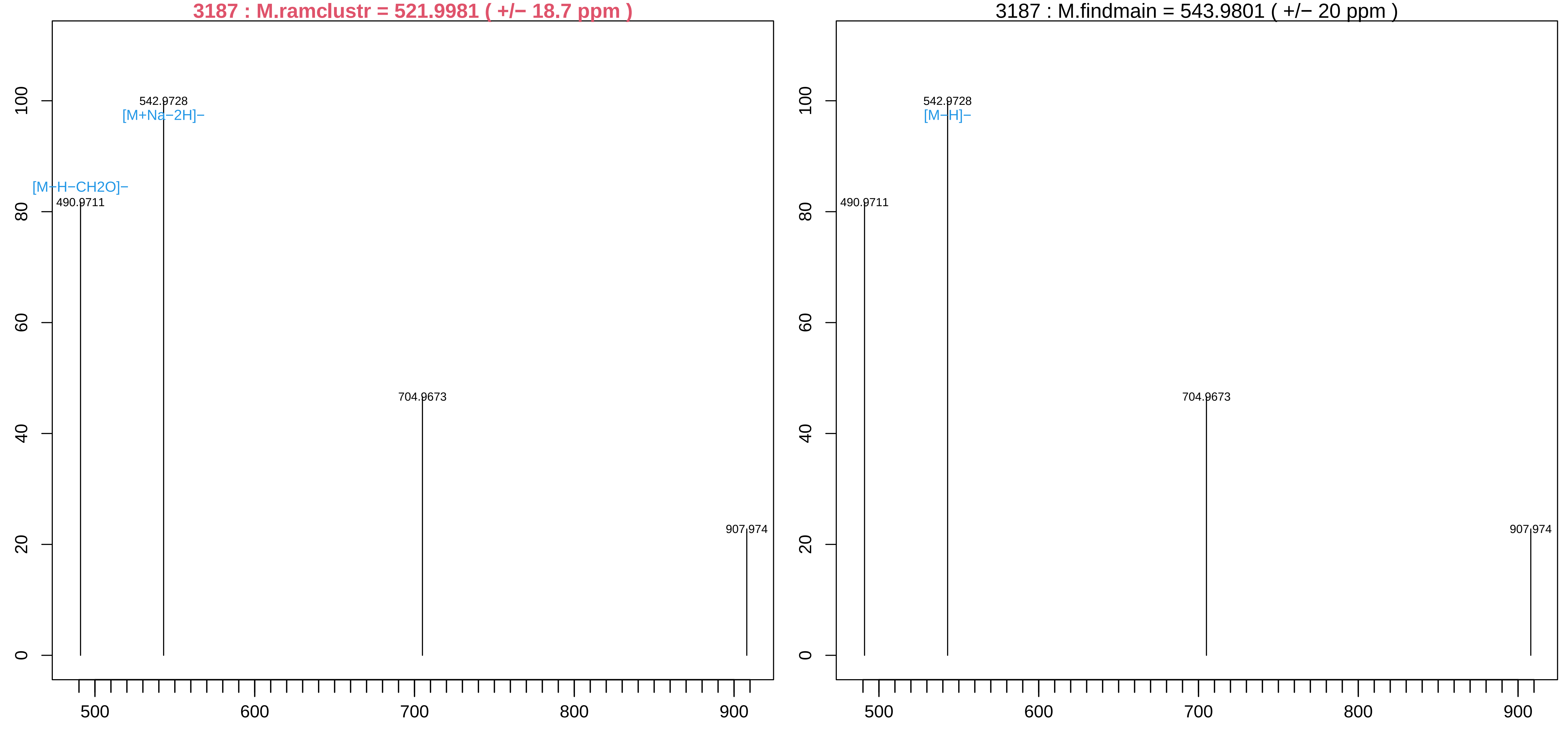
3187 : M.ramclustr = 521.9981 (+/− 18.7 ppm)
542.9728
[M+Na−2H]−
[M−H−CH2O]−
490.9711
704.9673
907.974
3187 : M.findmain = 543.9801 (+/− 20 ppm)
542.9728
[M−H]−
490.9711
704.9673
907.974

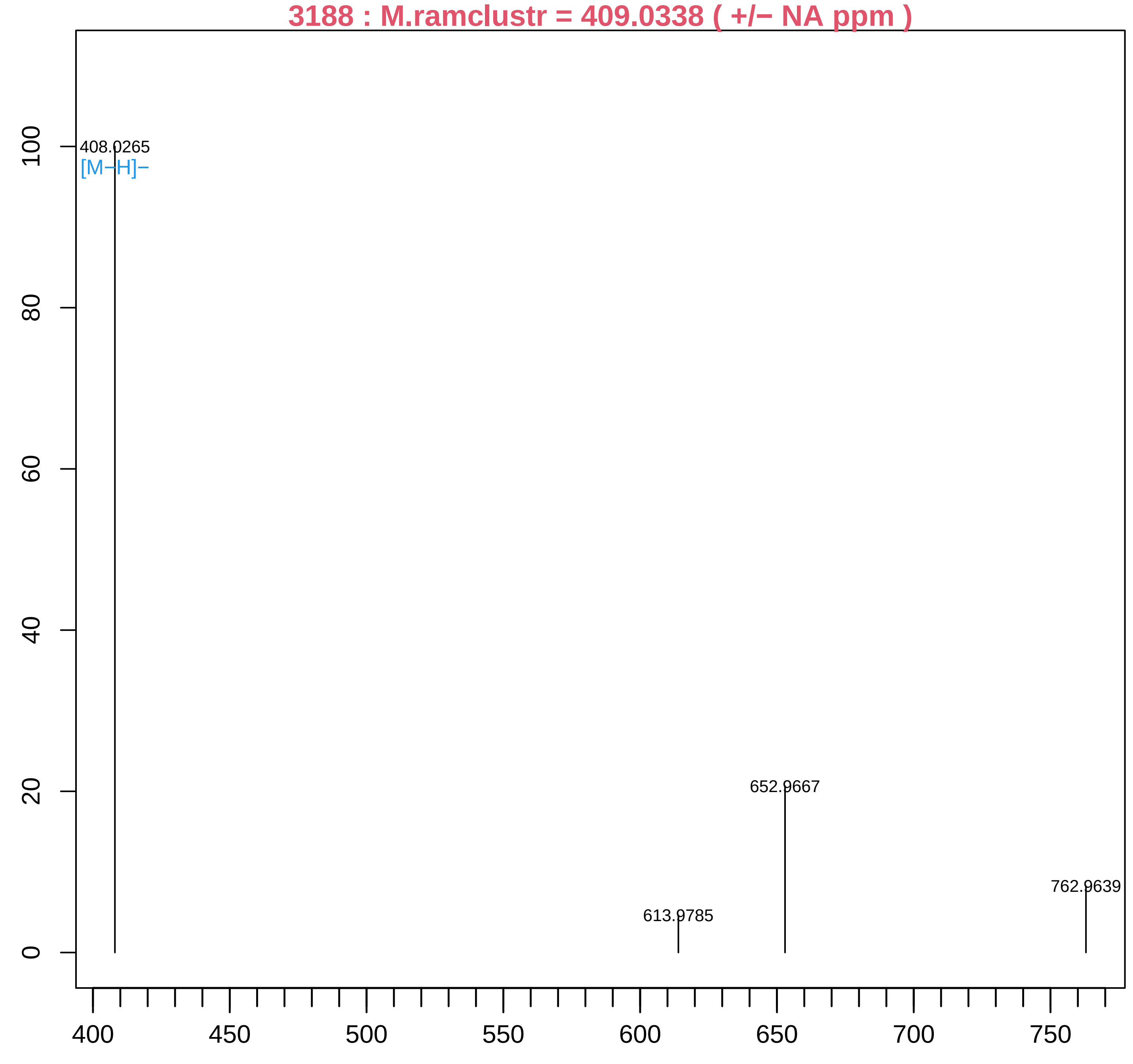

3188 : M.ramclustr = 409.0338 (+/− NA ppm)
408.0265
[M−H]−
613.9785
652.9667
762.9639
0
20
40
60
80
100
400
450
500
550
600
650
700
750

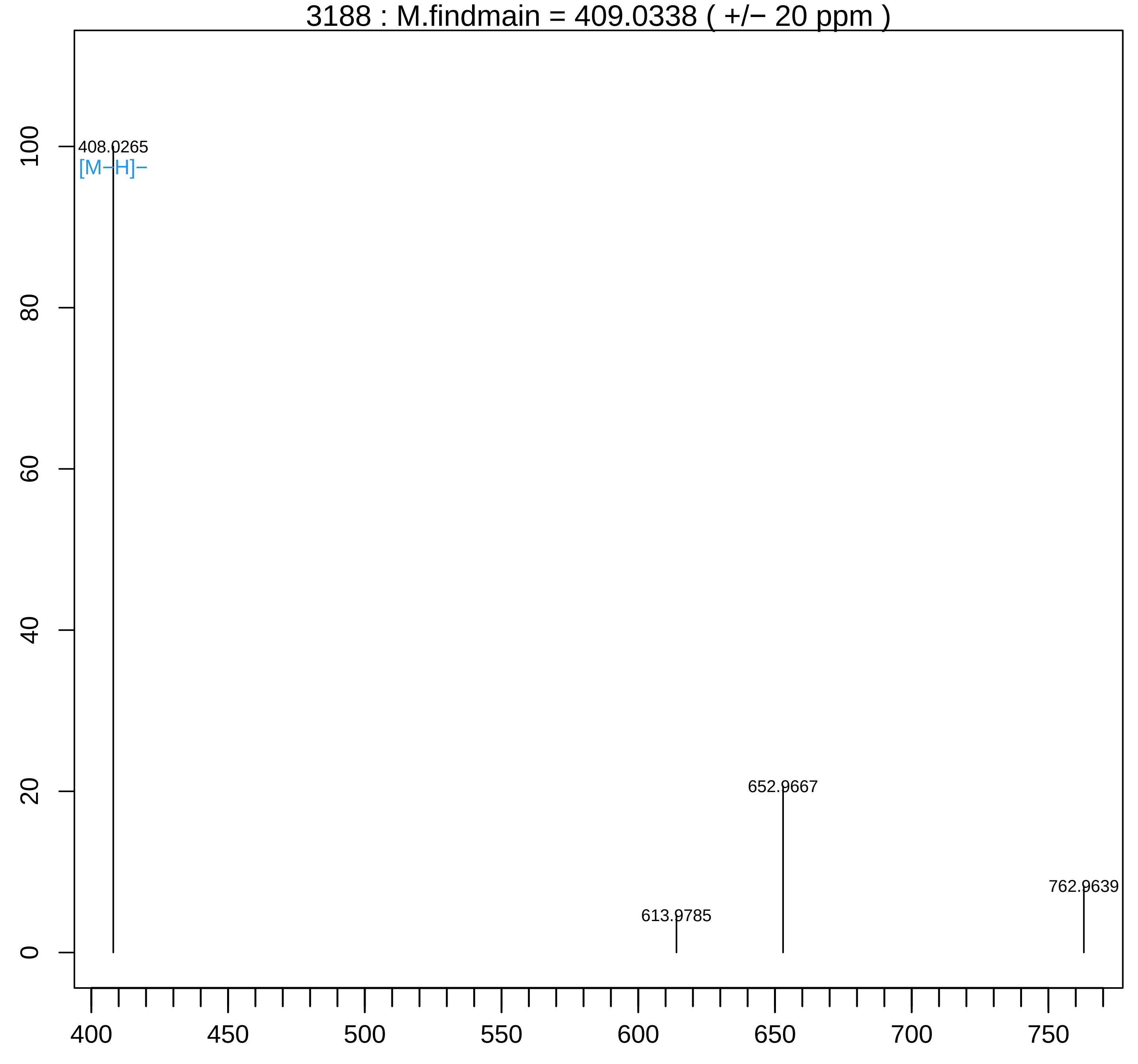

3188 : M.findmain = 409.0338 (+/− 20 ppm)
408.0265
[M−H]−
613.9785
652.9667
762.9639
0
20
40
60
80
100
400
450
500
550
600
650
700
750

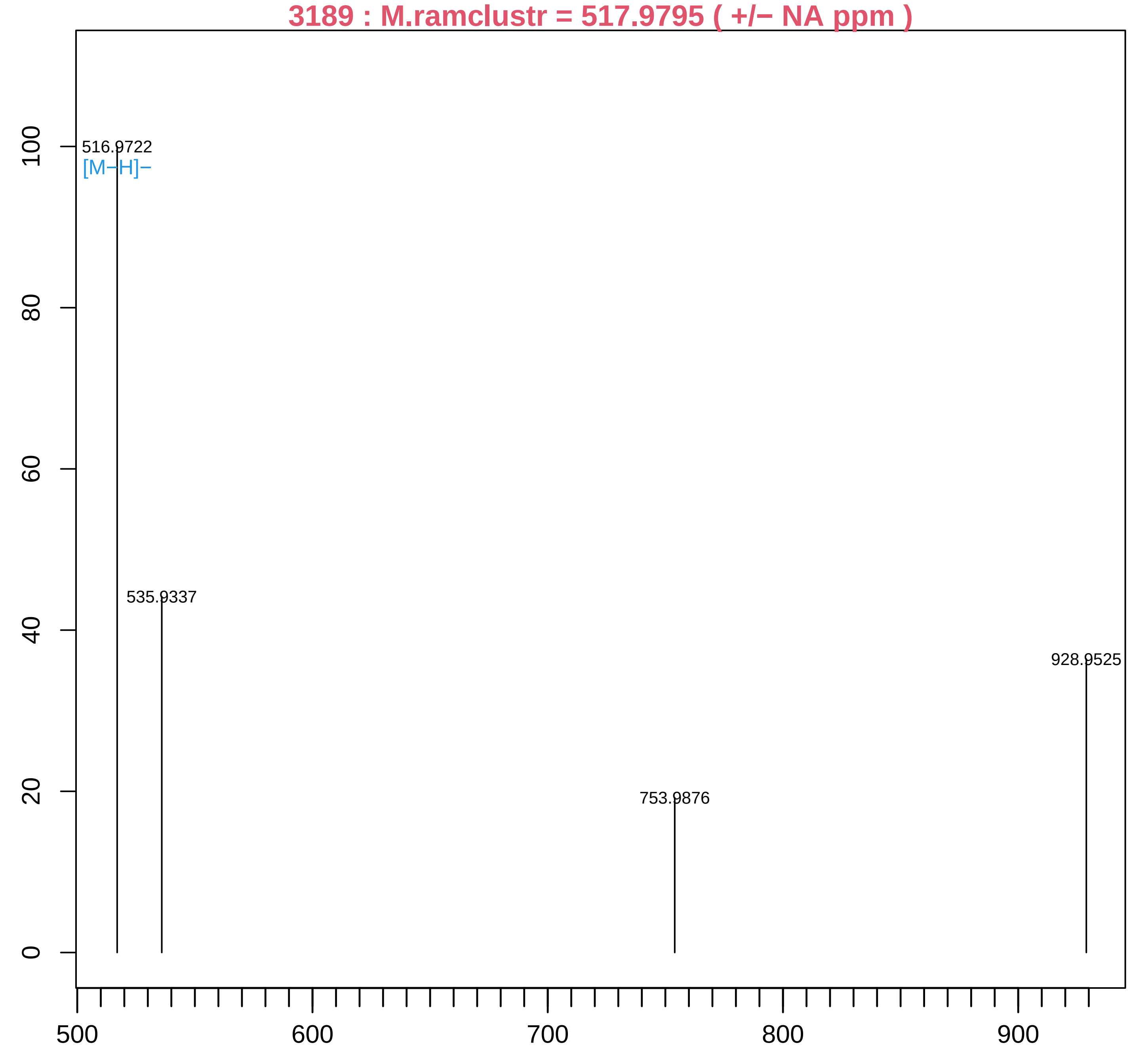
3189 : M.ramclustr = 517.9795 (+/− NA ppm)
516.9722
[M−H]−
535.9337
753.9876
928.9525
0
20
40
60
80
100
500
600
700
800
900

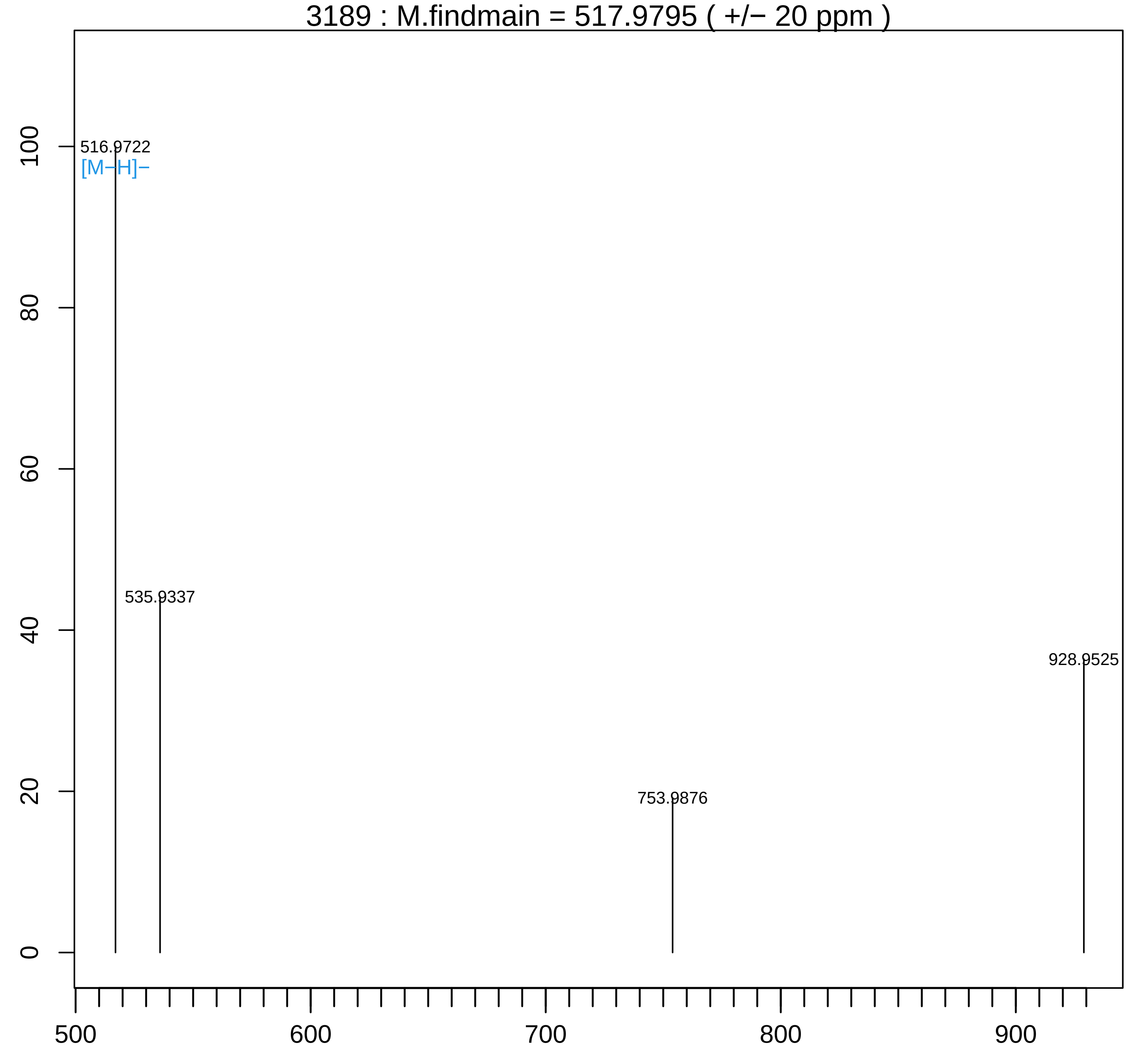
3189 : M.findmain = 517.9795 (+/− 20 ppm)
516.9722
[M−H]−
535.9337
753.9876
928.9525
0
20
40
60
80
100
500
600
700
800
900

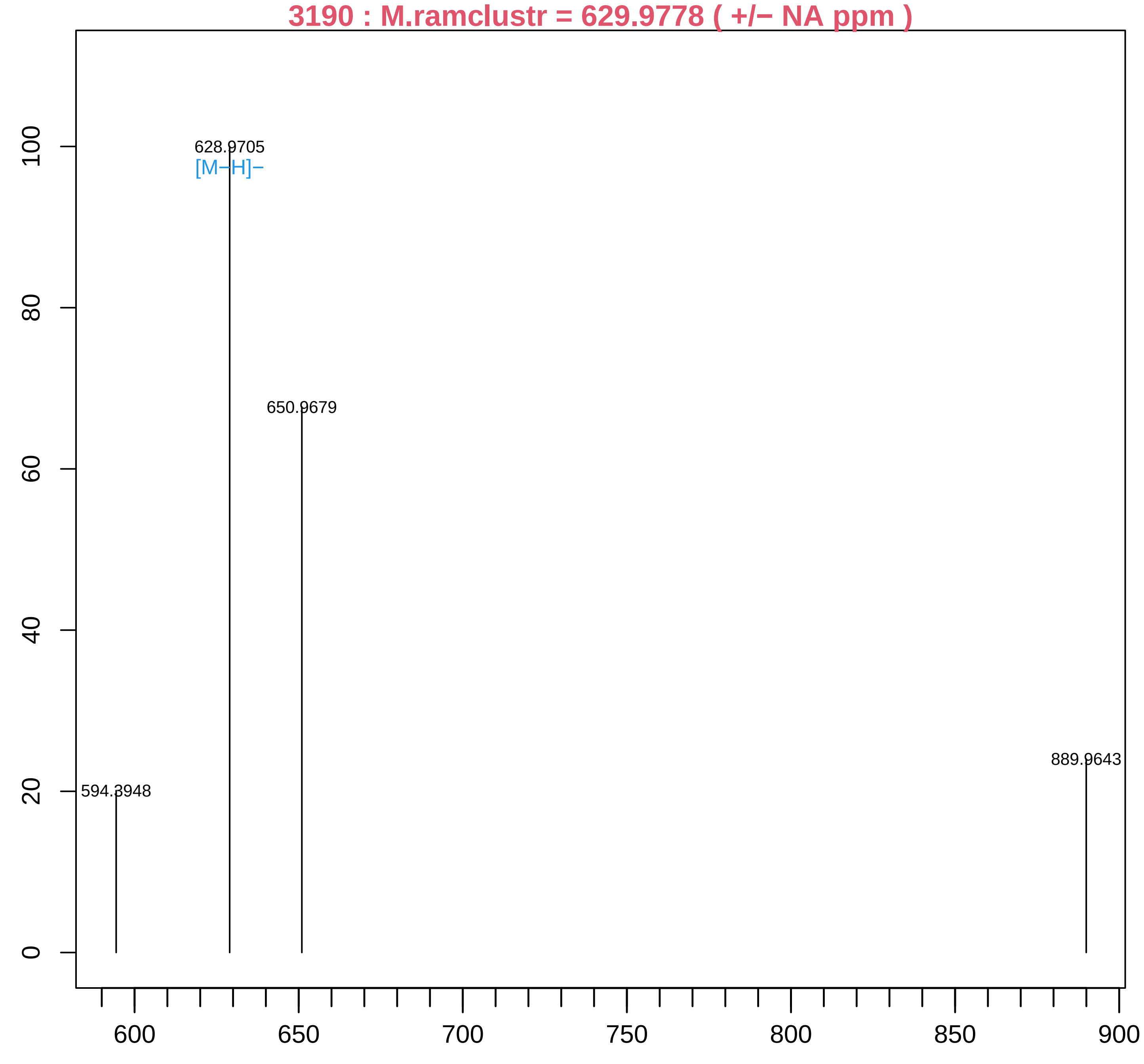
3190 : M.ramclustr = 629.9778 (+/− NA ppm)
628.9705
[M−H]−
650.9679
594.3948
889.9643
0
20
40
60
80
100
600
650
700
750
800
850
900

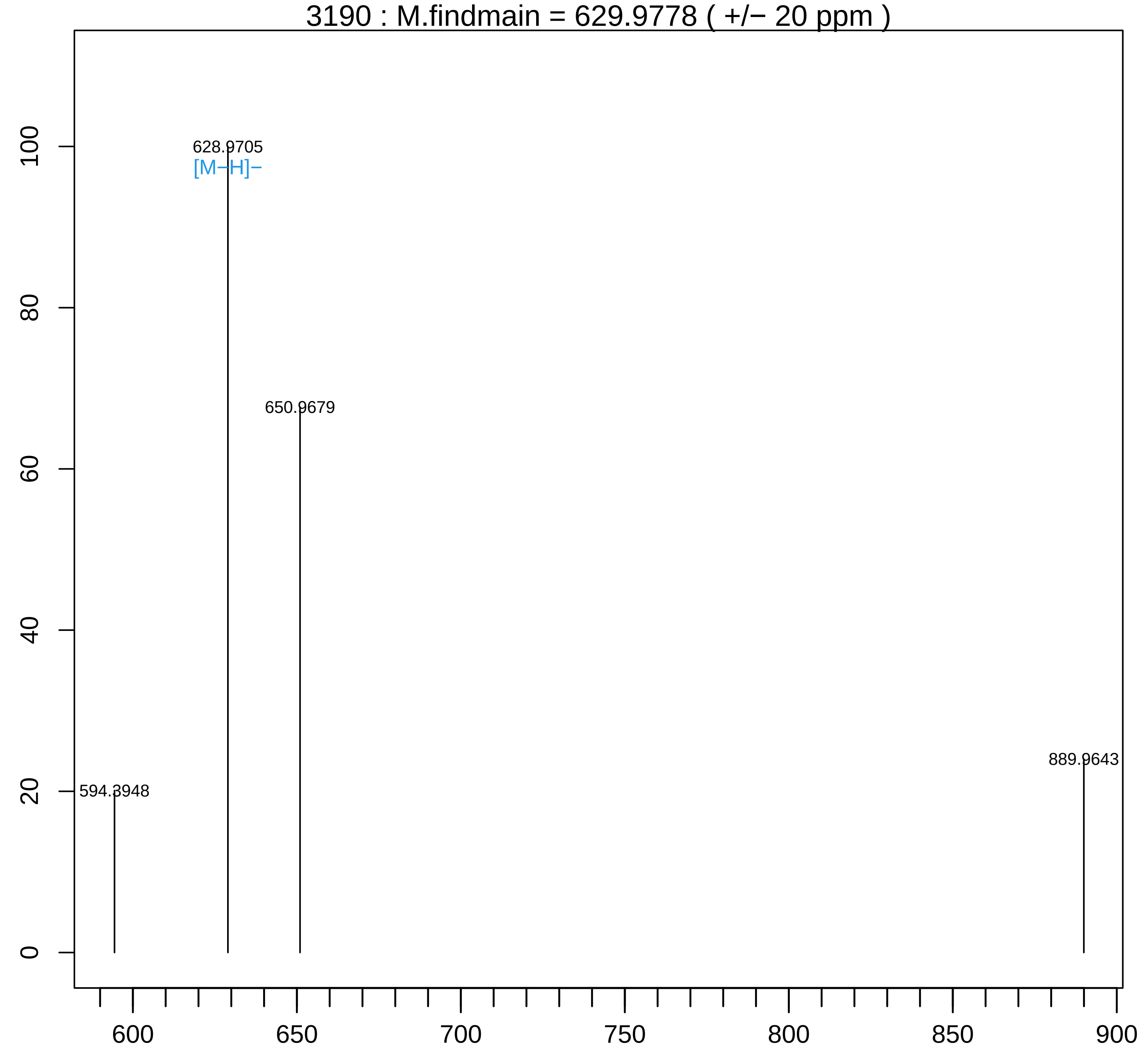
3190 : M.findmain = 629.9778 (+/− 20 ppm)
628.9705
[M−H]−
650.9679
594.3948
889.9643
0
20
40
60
80
100
600
650
700
750
800
850
900

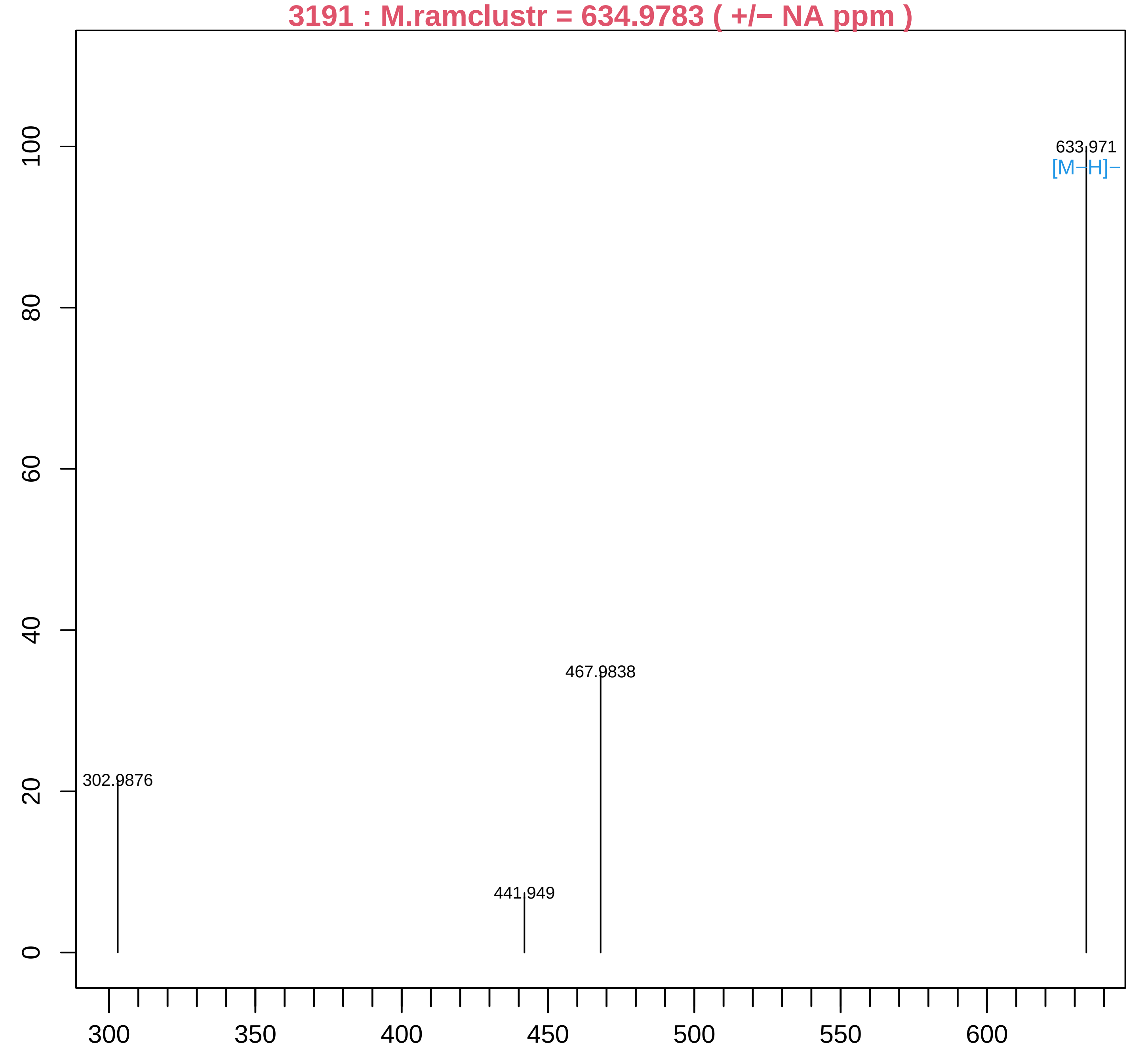
3191 : M.ramclustr = 634.9783 (+/− NA ppm)
633.971
[M−H]−
467.9838
441.949
302.9876
0
20
40
60
80
100
300
350
400
450
500
550
600

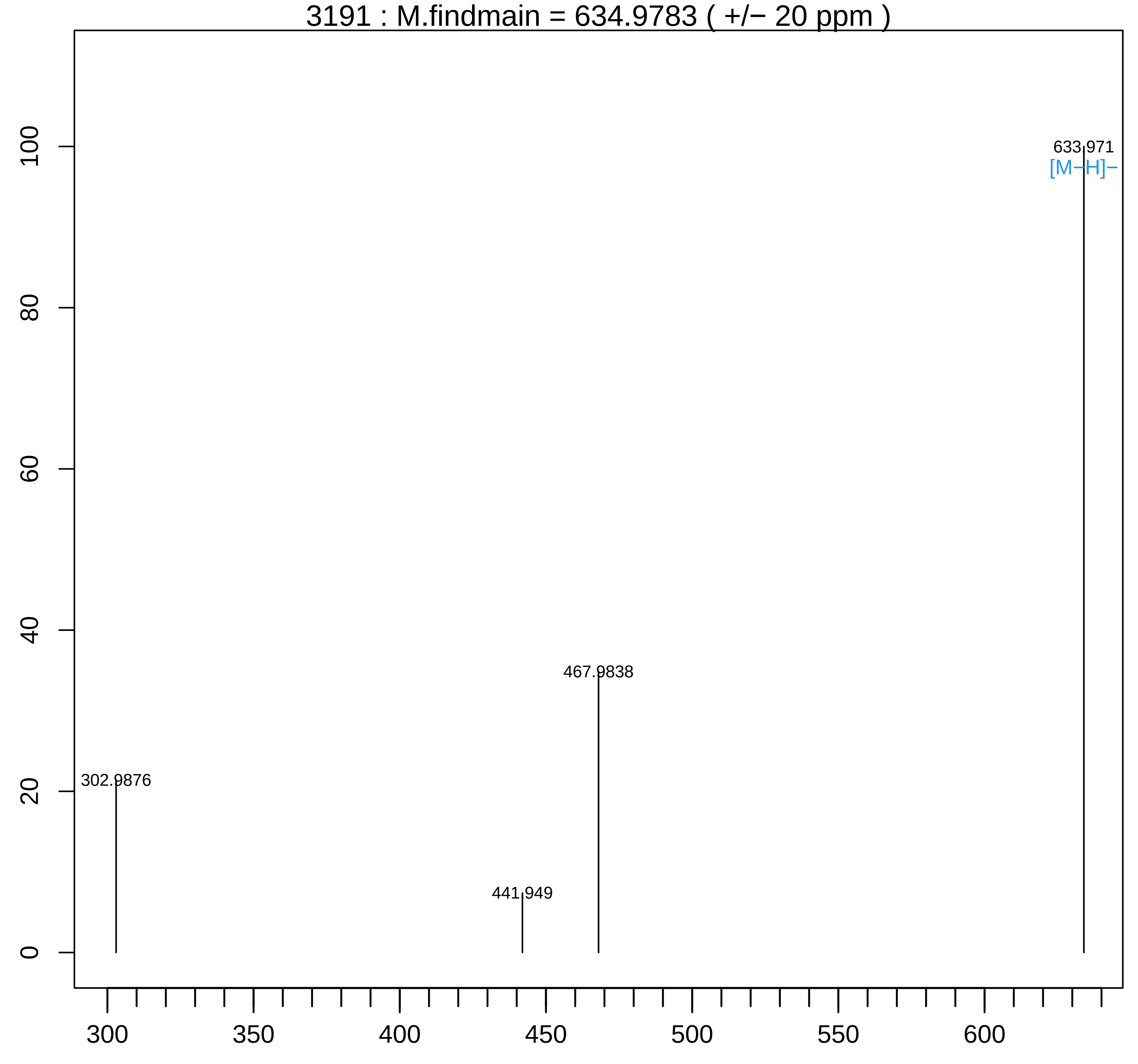
3191 : M.findmain = 634.9783 (+/− 20 ppm)
633.971
[M−H]−
467.9838
441.949
302.9876
0
20
40
60
80
100
300
350
400
450
500
550
600

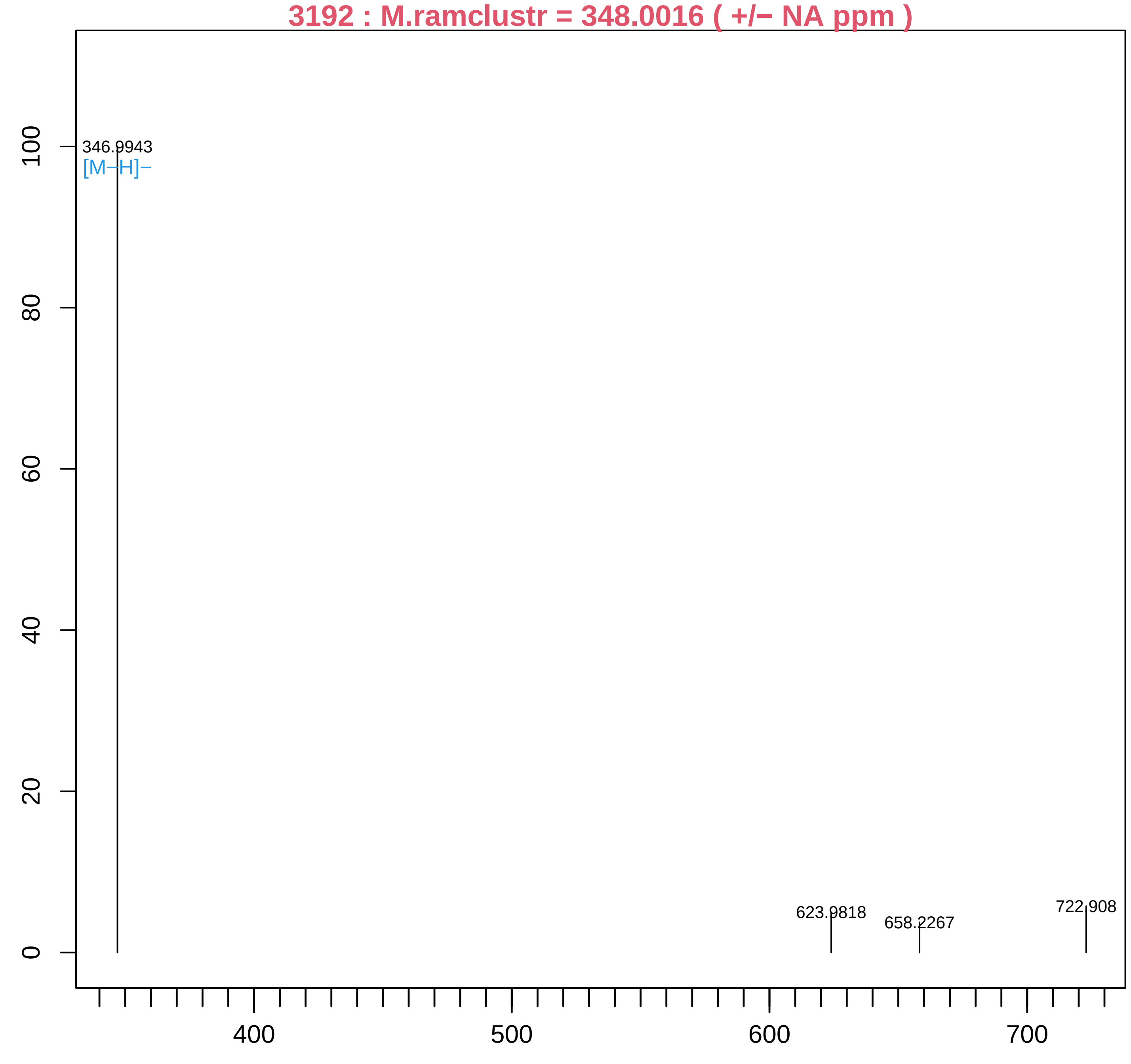

3192 : M.ramclustr = 348.0016 (+/− NA ppm)
346.9943
[M−H]−
623.9818
658.2267
722.908
0
20
40
60
80
100
400
500
600
700

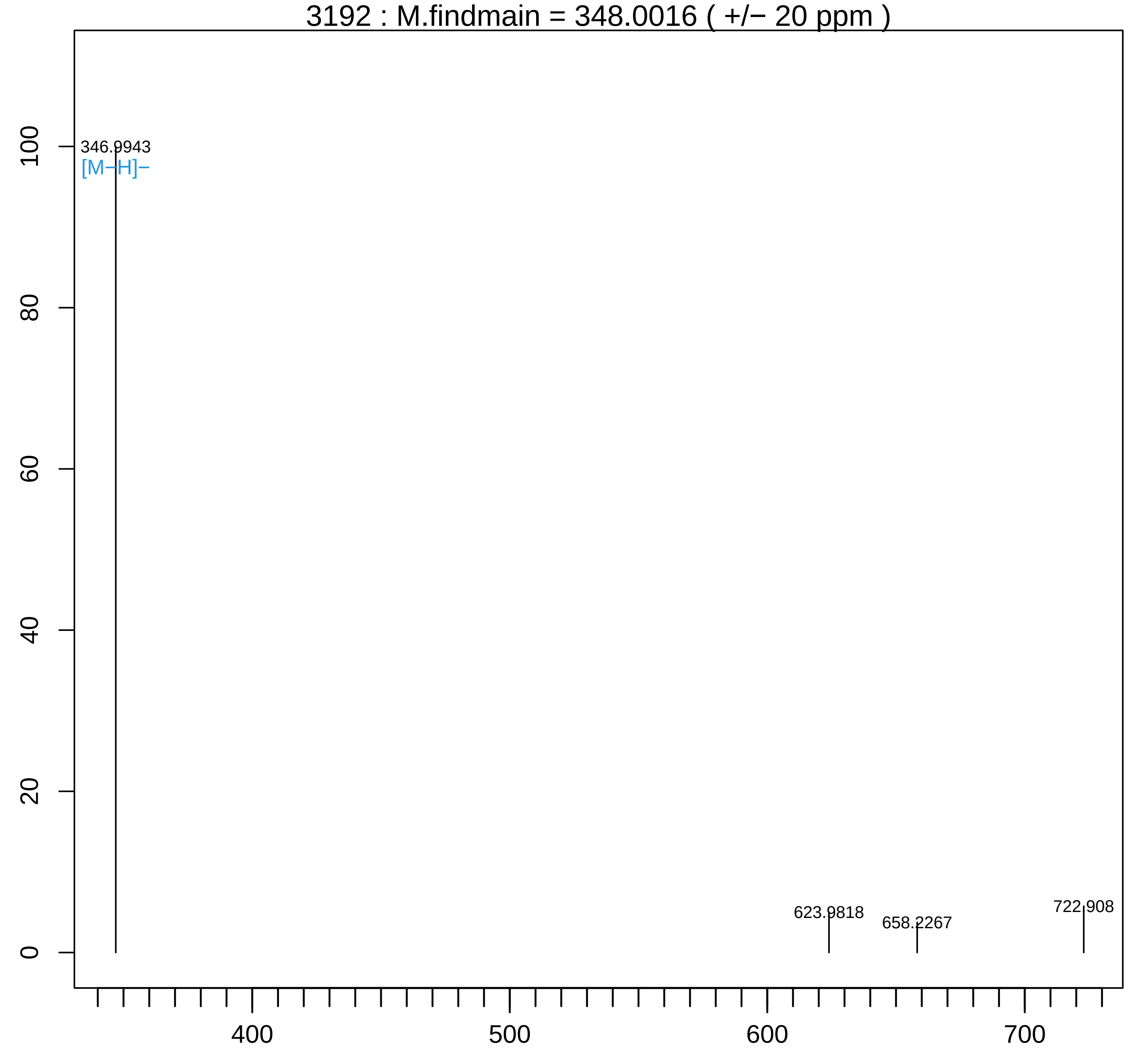

3192 : M.findmain = 348.0016 (+/− 20 ppm)
346.9943
[M−H]−
623.9818
658.2267
722.908
0
20
40
60
80
100
400
500
600
700

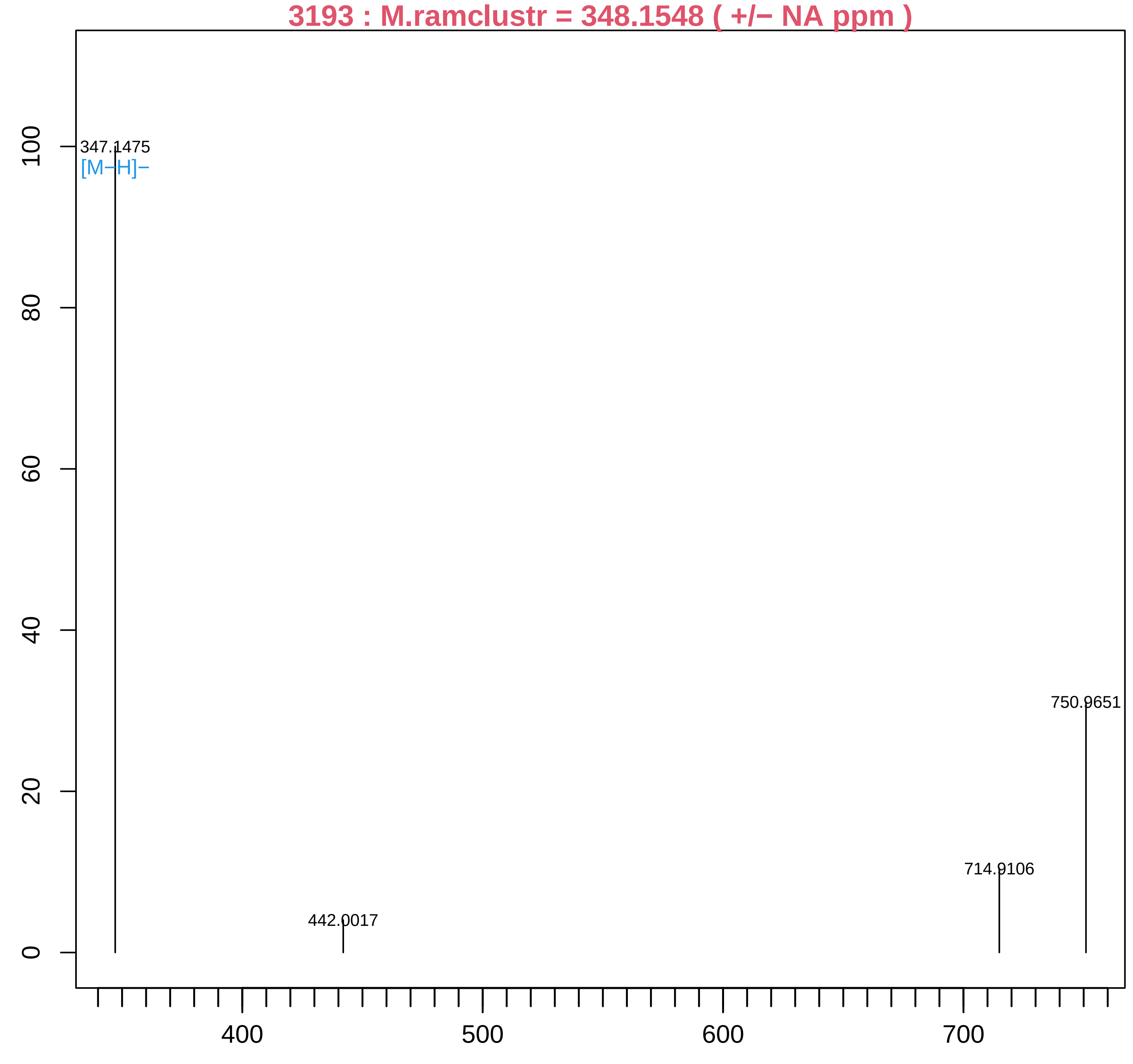
3193 : M.ramclustr = 348.1548 (+/− NA ppm)
347.1475
[M−H]−
442.0017
714.9106
750.9651
0
20
40
60
80
100
400
500
600
700

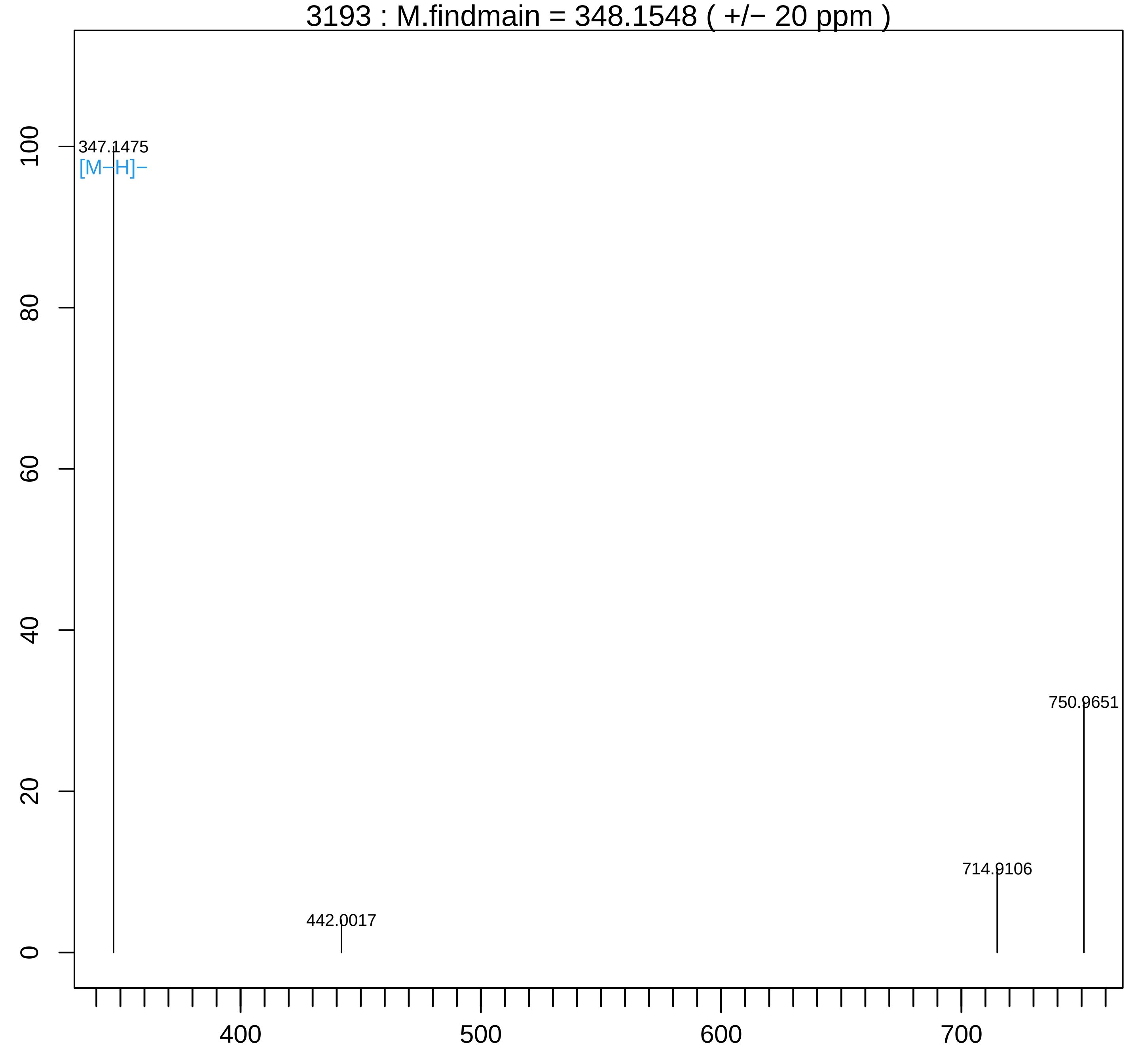
3193 : M.findmain = 348.1548 (+/− 20 ppm)
347.1475
[M−H]−
442.0017
714.9106
750.9651
0
20
40
60
80
100
400
500
600
700

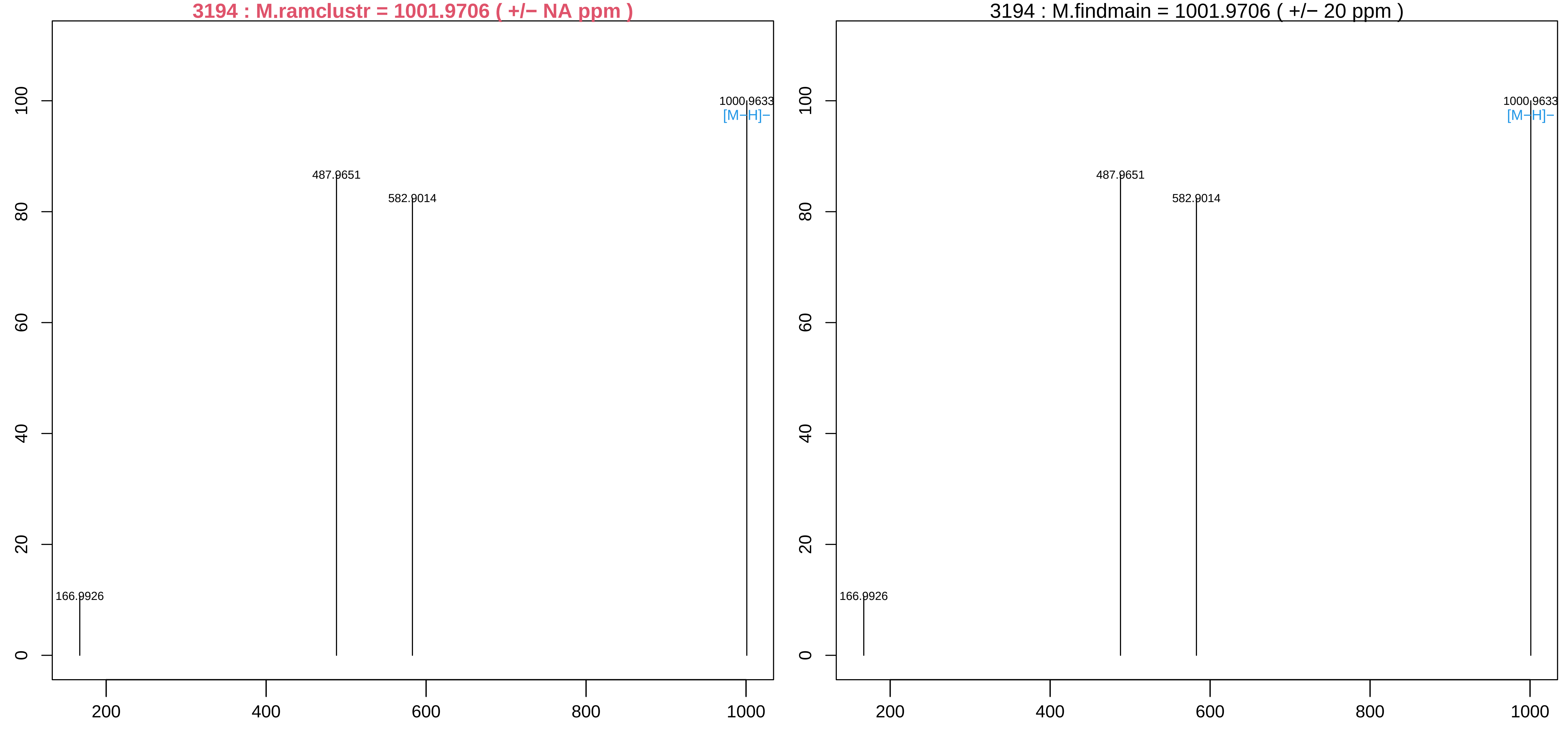
3194 : M.ramclustr = 1001.9706 (+/− NA ppm)
3194 : M.findmain = 1001.9706 (+/− 20 ppm)
166.9926
487.9651
582.9014
1000.9633
[M−H]−
0
20
40
60
80
100
200
400
600
800
1000

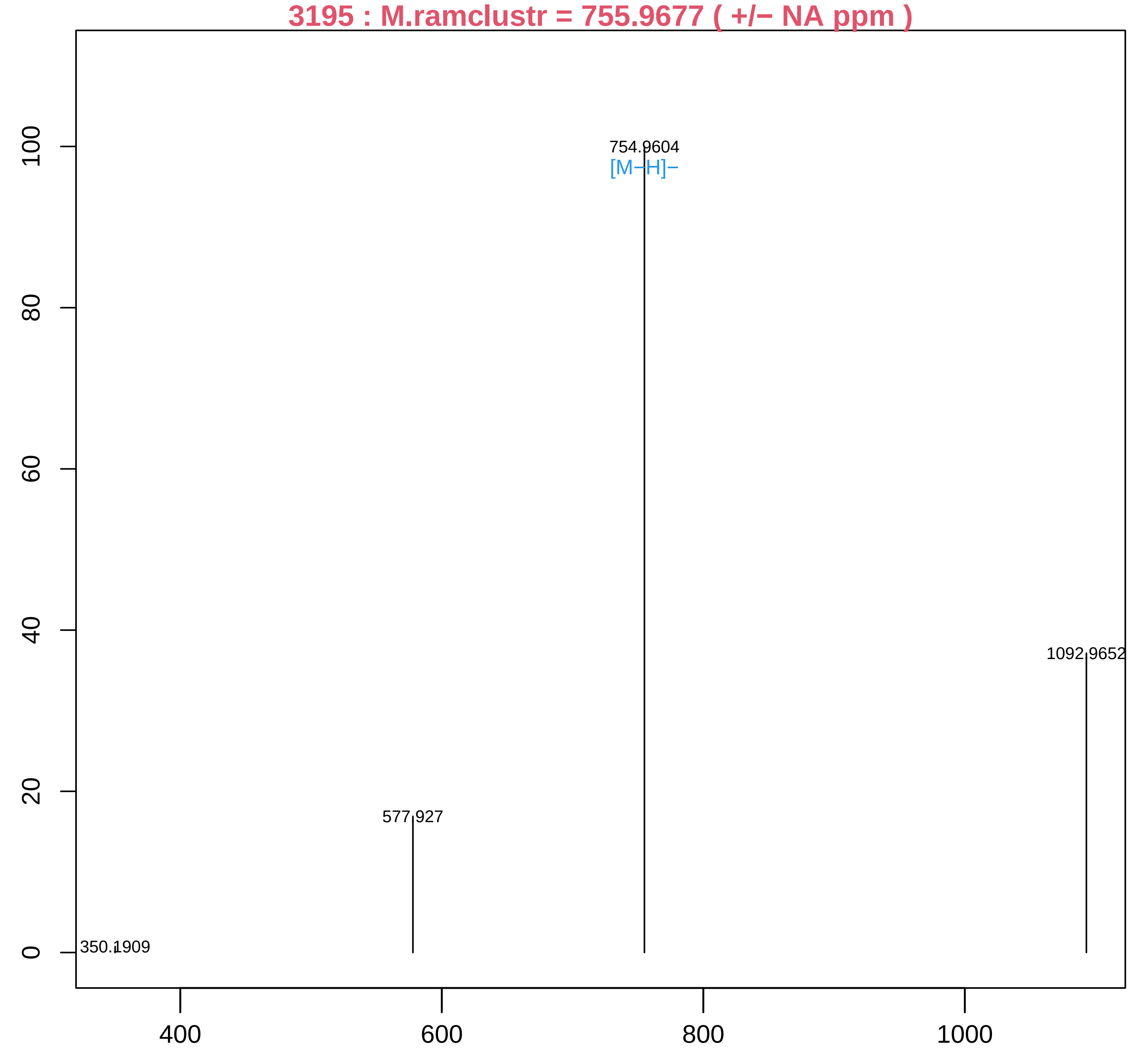

3195 : M.ramclustr = 755.9677 (+/− NA ppm)
754.9604
[M−H]−
1092.9652
577.927
350.1909
0
20
40
60
80
100
400
600
800
1000

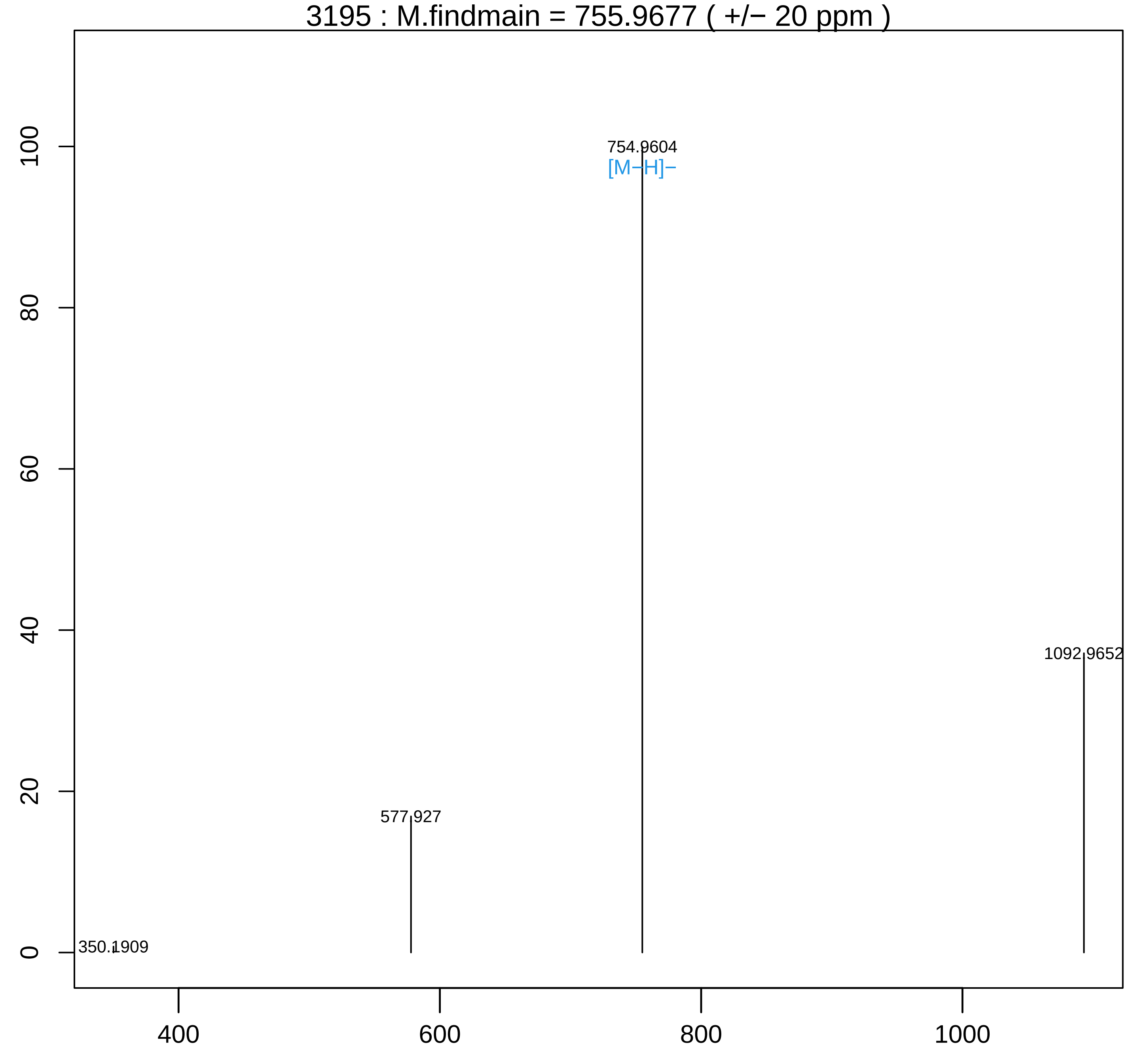

3195 : M.findmain = 755.9677 (+/− 20 ppm)
754.9604
[M−H]−
1092.9652
577.927
350.1909
0
20
40
60
80
100
400
600
800
1000

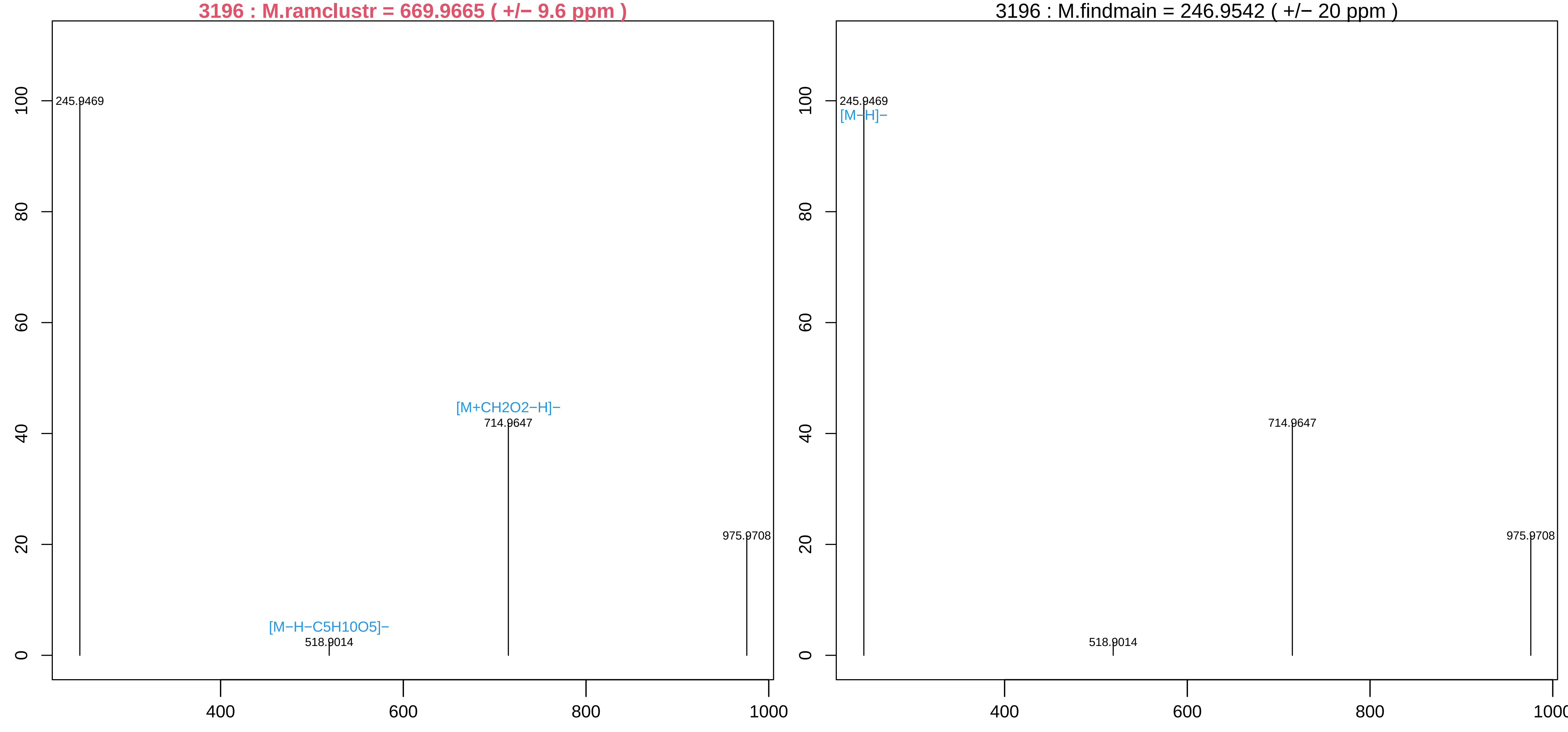
3196 : M.ramclustr = 669.9665 (+/− 9.6 ppm)
245.9469
[M+CH2O2−H]−
714.9647
975.9708
[M−H−C5H10O5]−
518.9014
0
20
40
60
80
100
400
600
800
1000
3196 : M.findmain = 246.9542 (+/− 20 ppm)
245.9469
[M−H]−
714.9647
975.9708
518.9014
0
20
40
60
80
100
400
600
800
1000

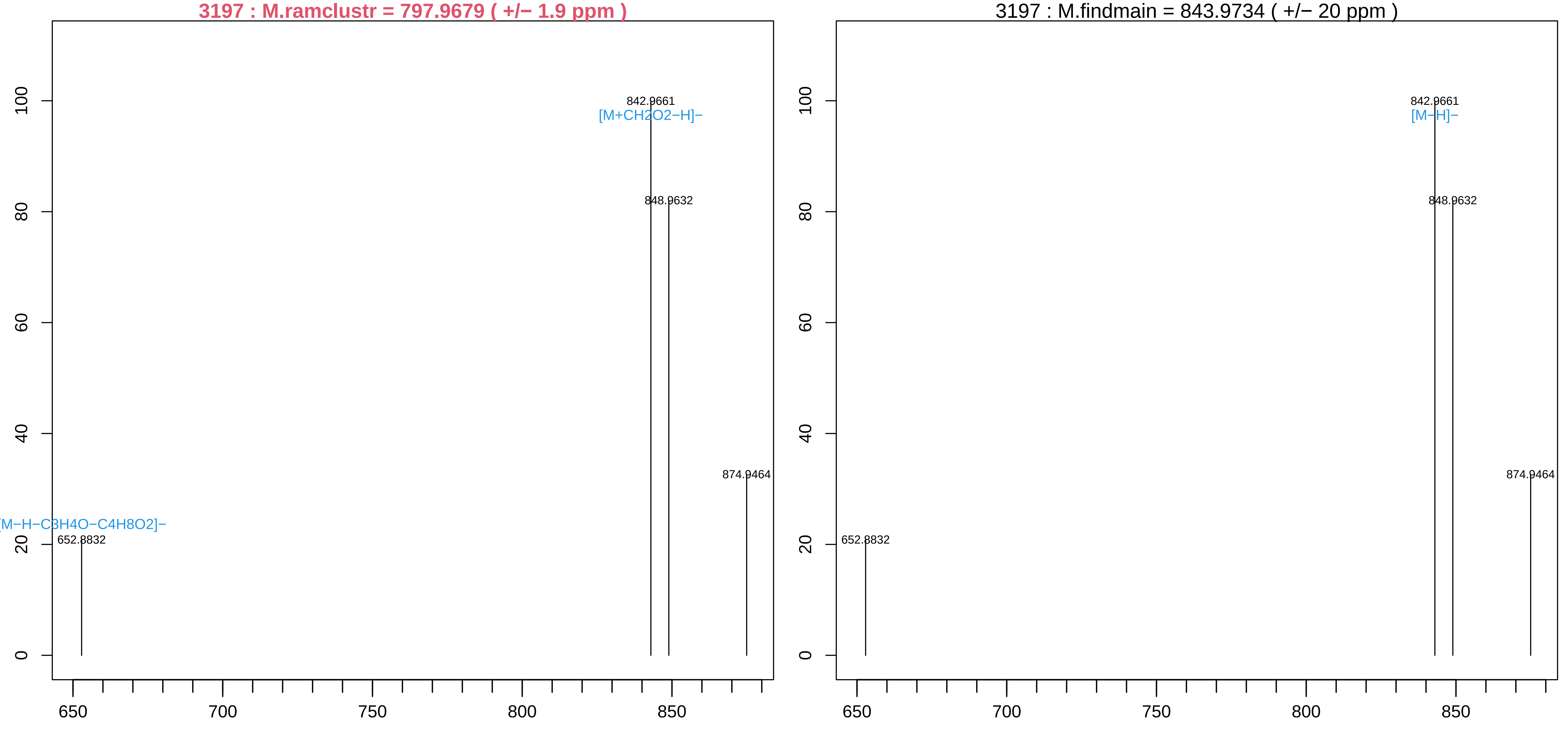

3197 : M.ramclustr = 797.9679 (+/− 1.9 ppm)
842.9661
[M+CH2O2−H]−
848.9632
874.9464
[M−H−C3H4O−C4H8O2]−
652.8832
3197 : M.findmain = 843.9734 (+/− 20 ppm)
842.9661
[M−H]−
848.9632
874.9464
652.8832
0
20
40
60
80
100
650
700
750
800
850

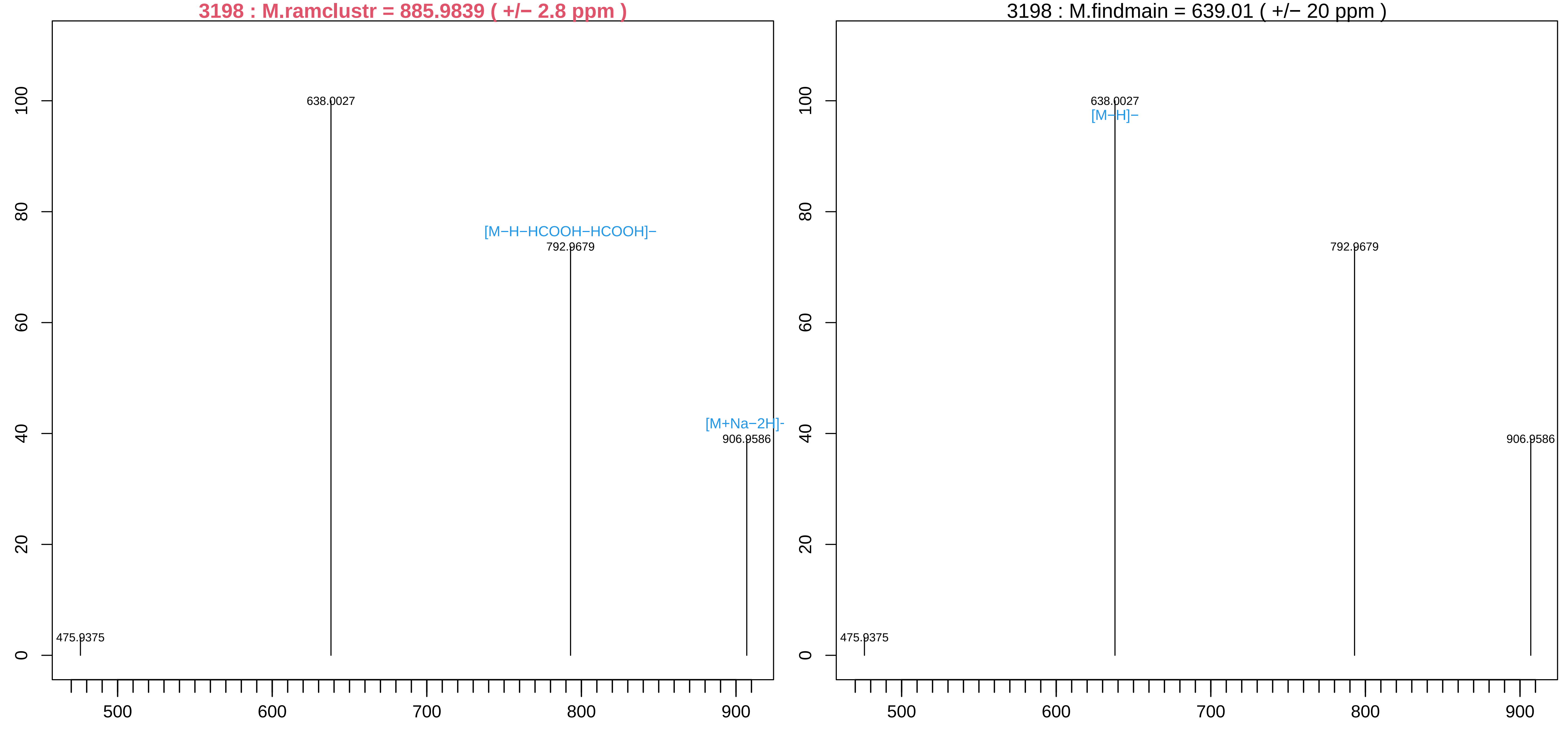
3198 : M.ramclustr = 885.9839 (+/− 2.8 ppm)
638.0027
[M−H−HCOOH−HCOOH]−
792.9679
[M+Na−2H]-
906.9586
475.9375
3198 : M.findmain = 639.01 (+/− 20 ppm)
638.0027
[M−H]−
792.9679
906.9586
475.9375
0
20
40
60
80
100
500
600
700
800
900

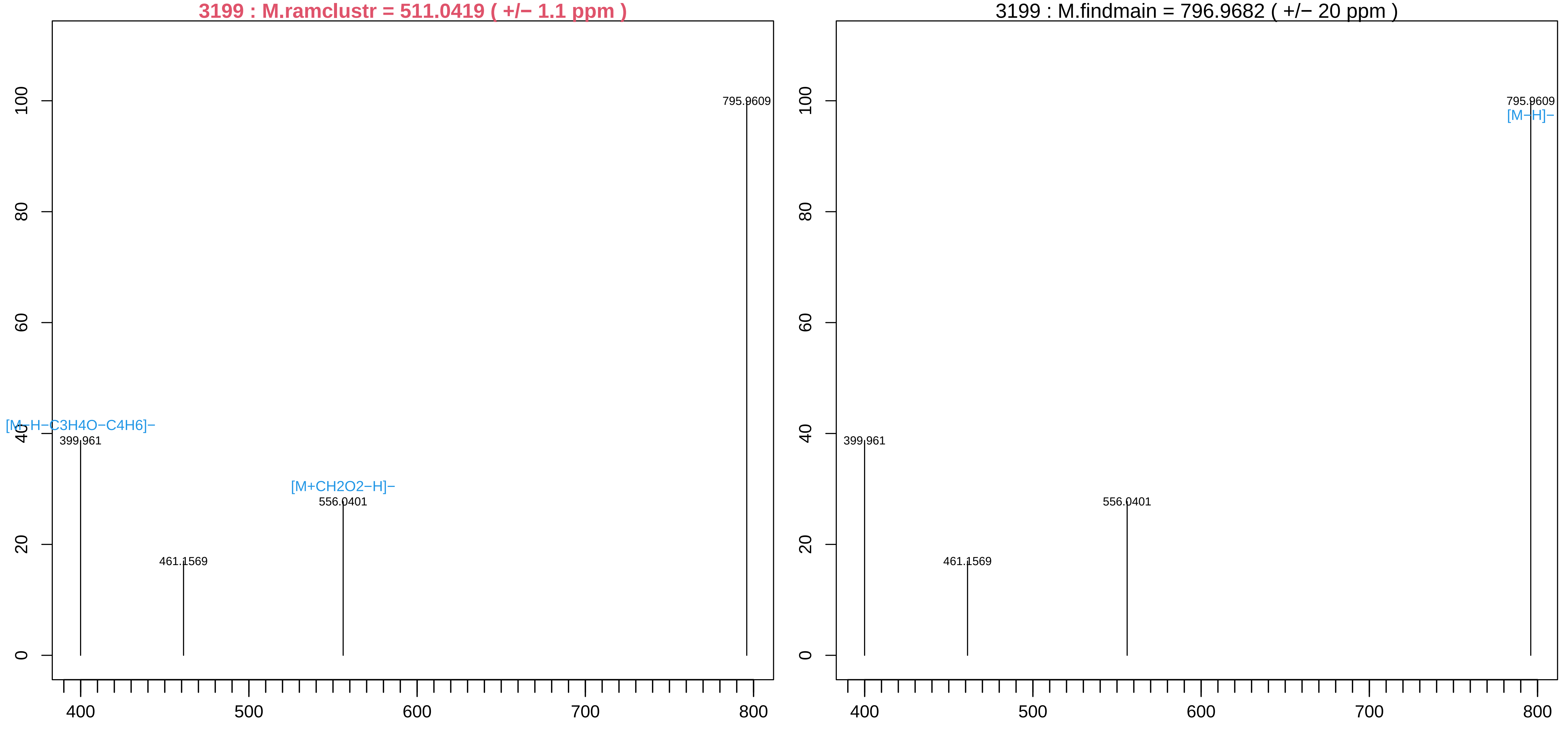
3199 : M.ramclustr = 511.0419 (+/− 1.1 ppm)
[M−H−C3H4O−C4H6]−
399.961
461.1569
[M+CH2O2−H]−
556.0401
795.9609
3199 : M.findmain = 796.9682 (+/− 20 ppm)
399.961
461.1569
556.0401
795.9609
[M−H]−

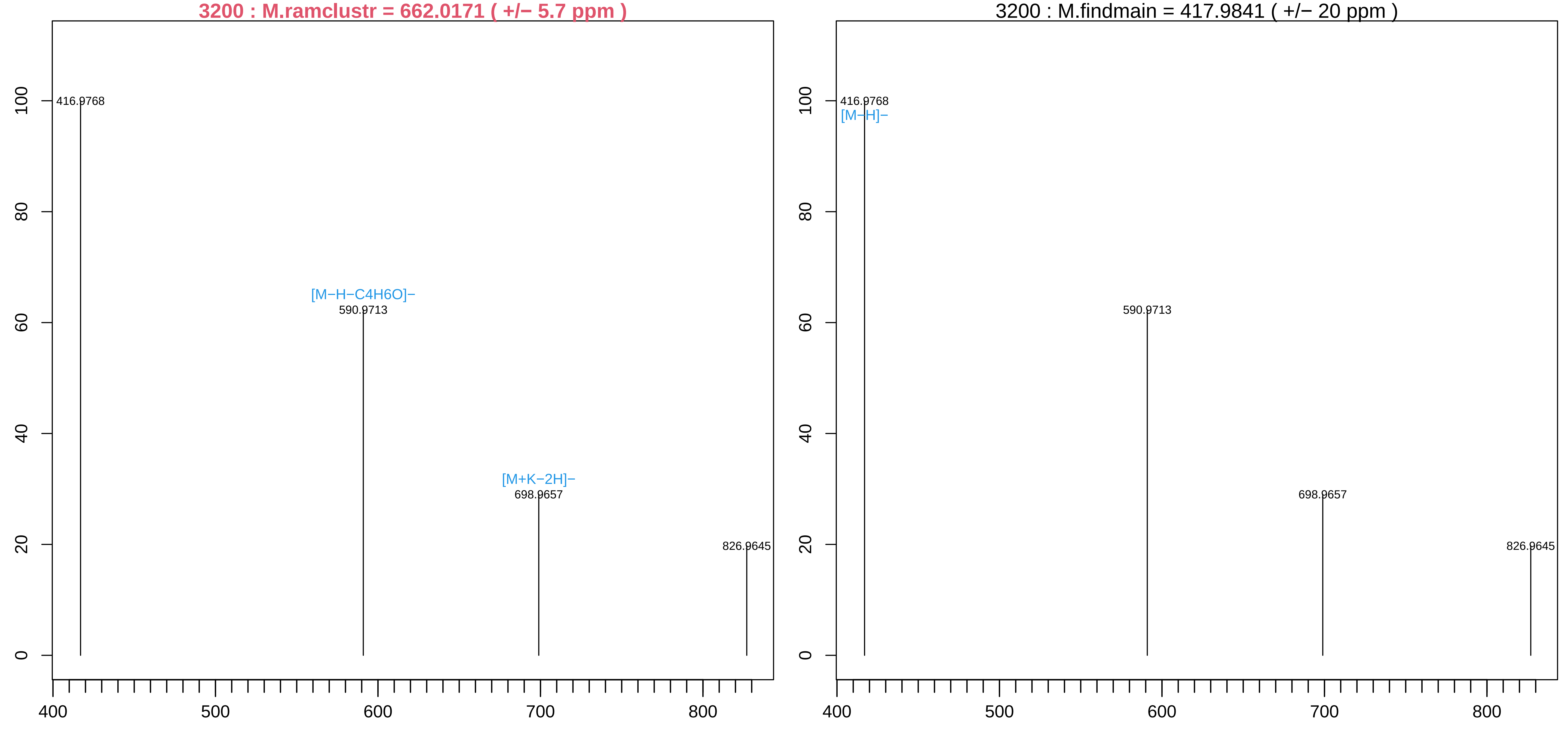
3200 : M.ramclustr = 662.0171 (+/− 5.7 ppm)
416.9768
[M−H−C4H6O]−
590.9713
[M+K−2H]−
698.9657
826.9645
3200 : M.findmain = 417.9841 (+/− 20 ppm)
416.9768
[M−H]−
590.9713
698.9657
826.9645
0
20
40
60
80
100
400
500
600
700
800

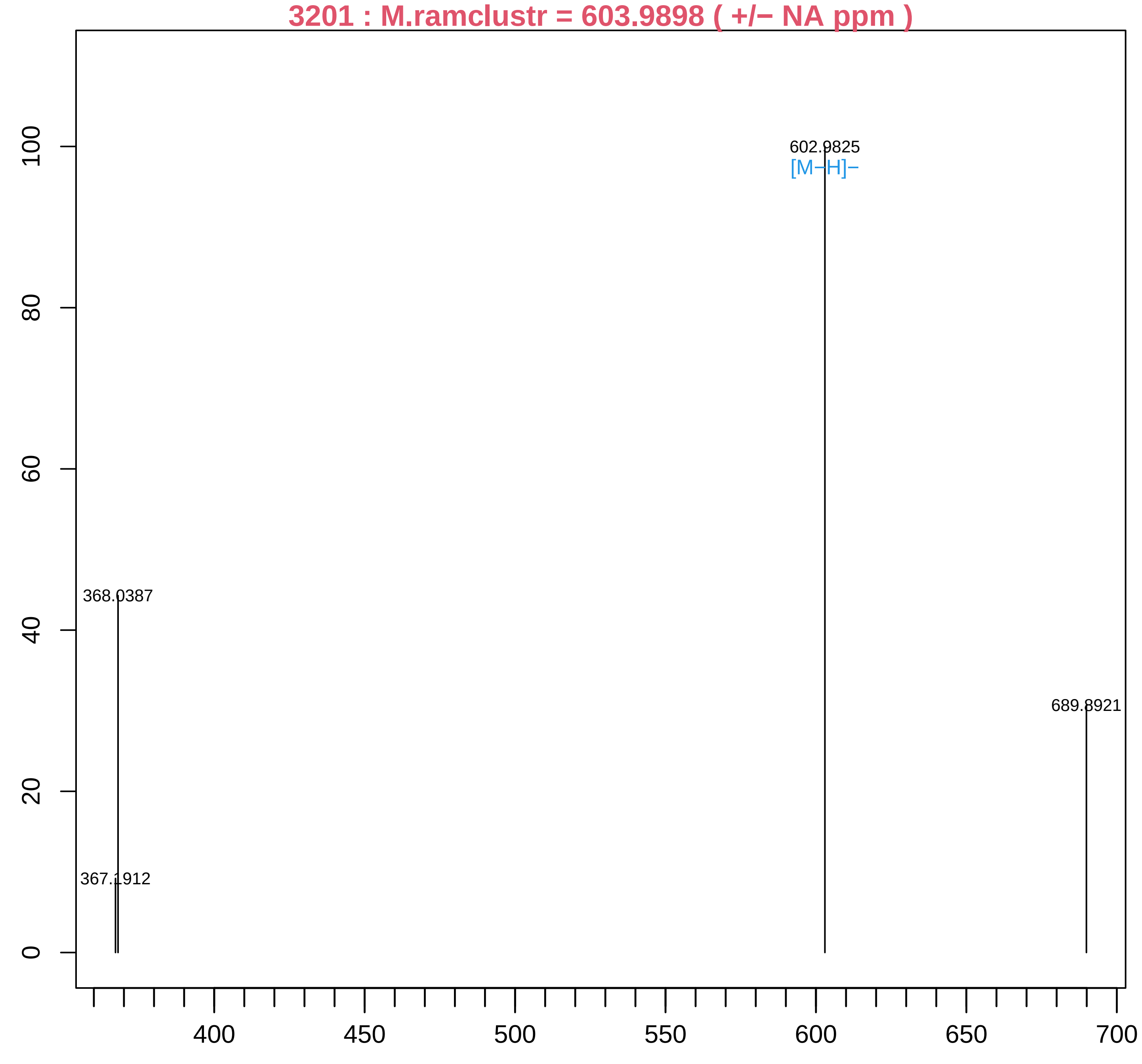
3201 : M.ramclustr = 603.9898 (+/− NA ppm)
602.9825
[M−H]−
368.0387
689.8921
367.1912
0
20
40
60
80
100
400
450
500
550
600
650
700

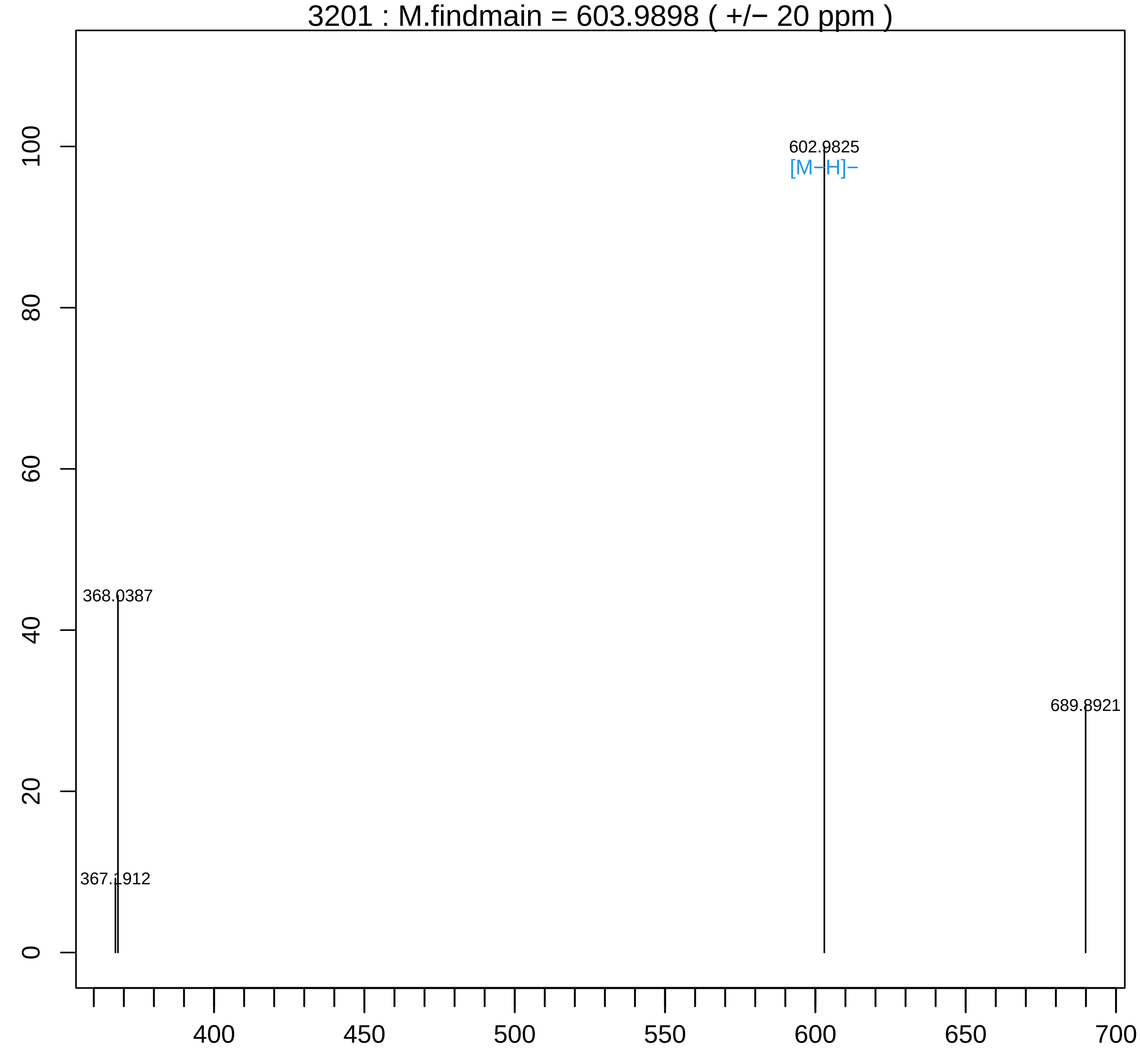
3201 : M.findmain = 603.9898 (+/− 20 ppm)
602.9825
[M−H]−
368.0387
689.8921
367.1912
0
20
40
60
80
100
400
450
500
550
600
650
700

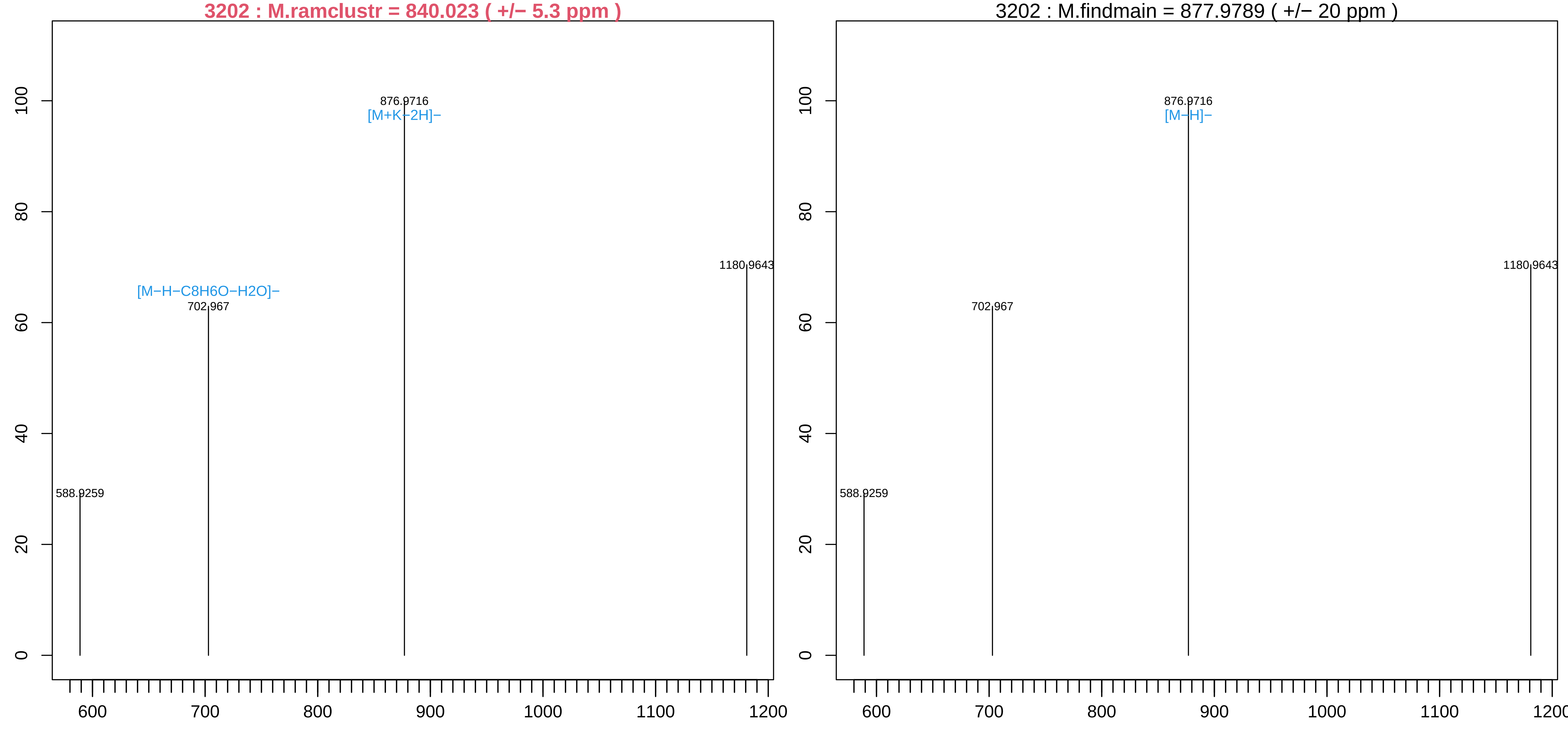
3202 : M.ramclustr = 840.023 (+/− 5.3 ppm)
876.9716
[M+K−2H]−
[M−H−C8H6O−H2O]−
702.967
1180.9643
588.9259
3202 : M.findmain = 877.9789 (+/− 20 ppm)
876.9716
[M−H]−
702.967
1180.9643
588.9259
0
20
40
60
80
100
600
700
800
900
1000
1100
1200

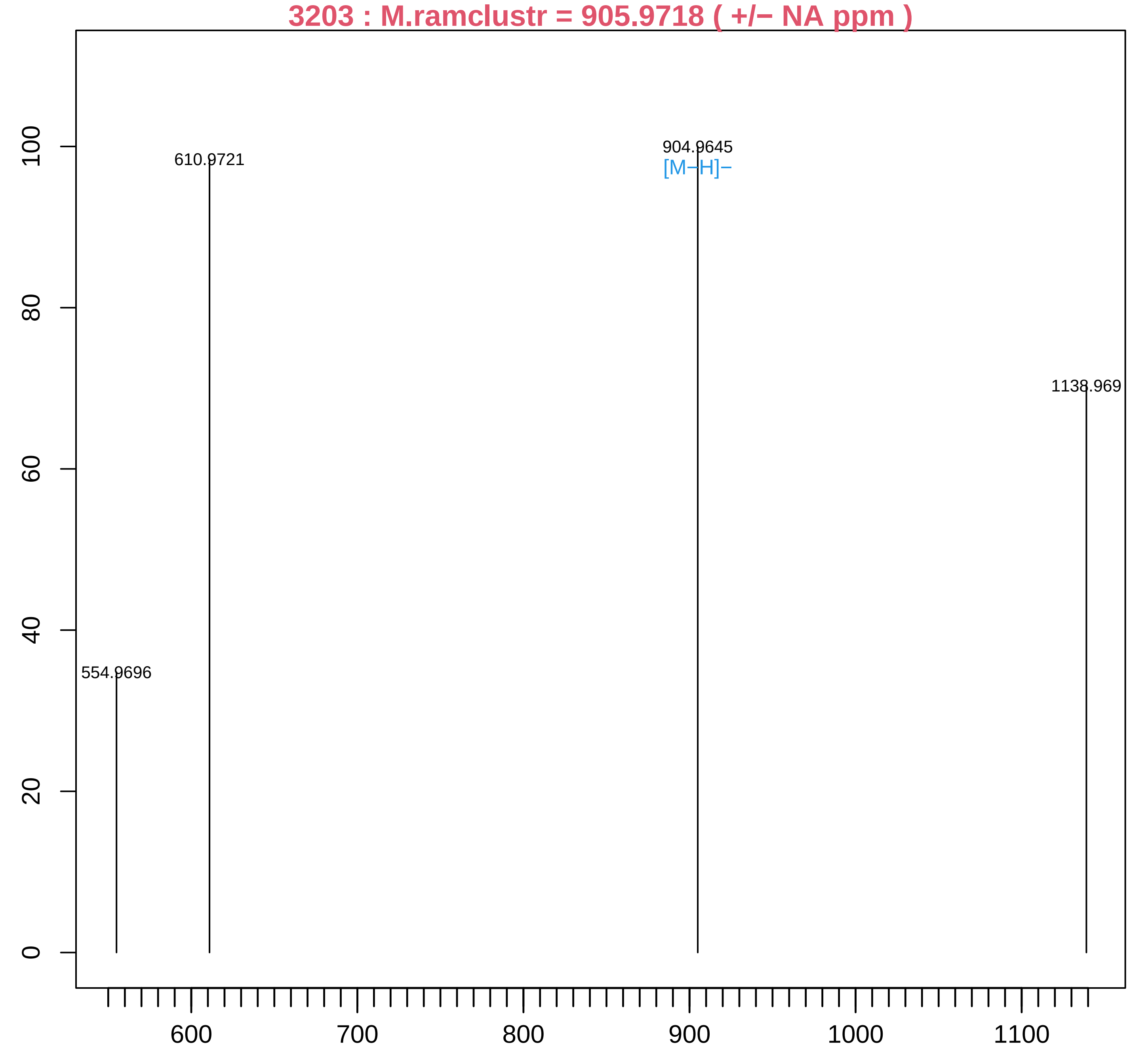
3203 : M.ramclustr = 905.9718 (+/− NA ppm)
554.9696
610.9721
904.9645
[M−H]−
1138.969
0
20
40
60
80
100
600
700
800
900
1000
1100

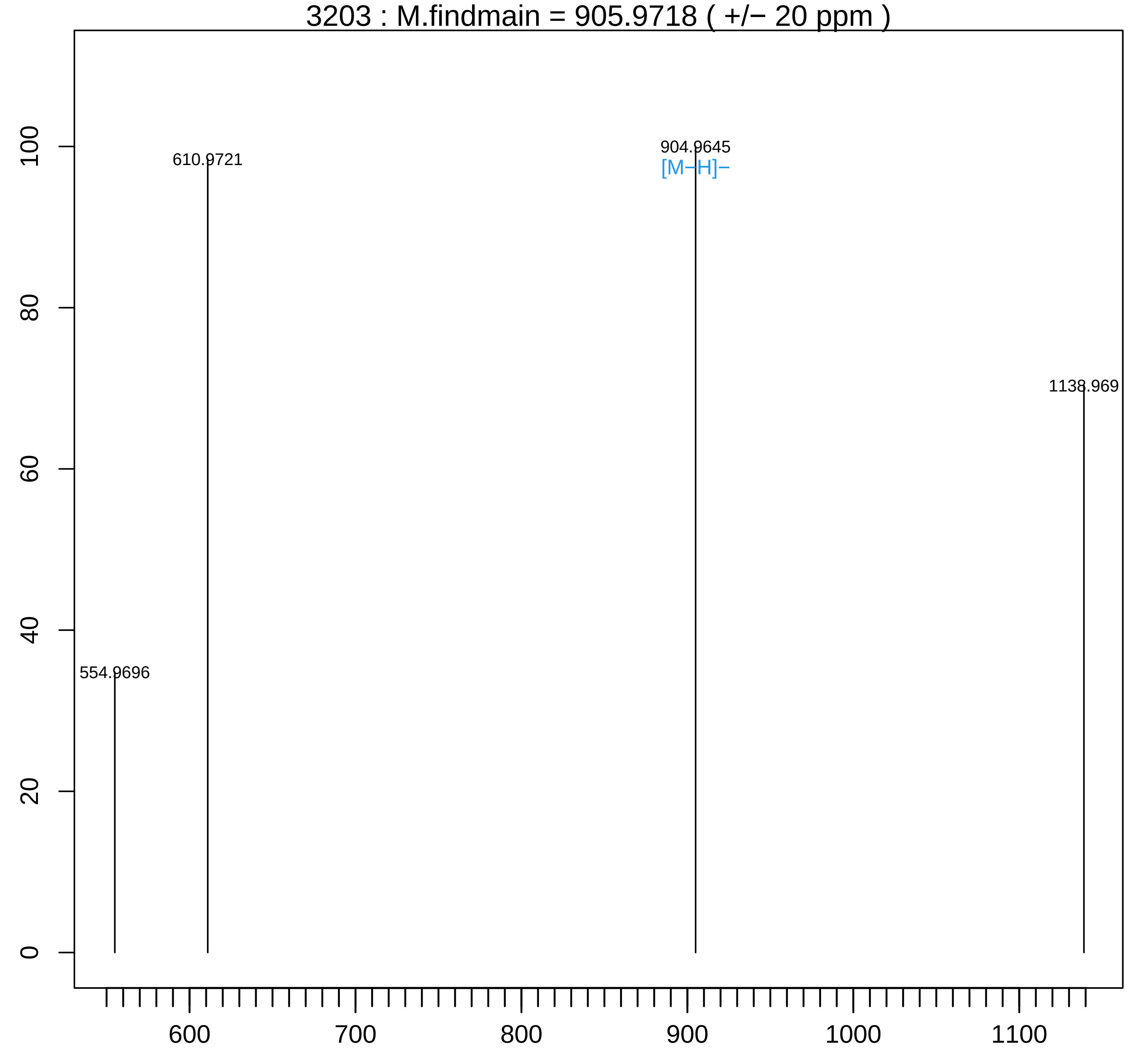
3203 : M.findmain = 905.9718 (+/− 20 ppm)
554.9696
610.9721
904.9645
[M−H]−
1138.969
0
20
40
60
80
100
600
700
800
900
1000
1100

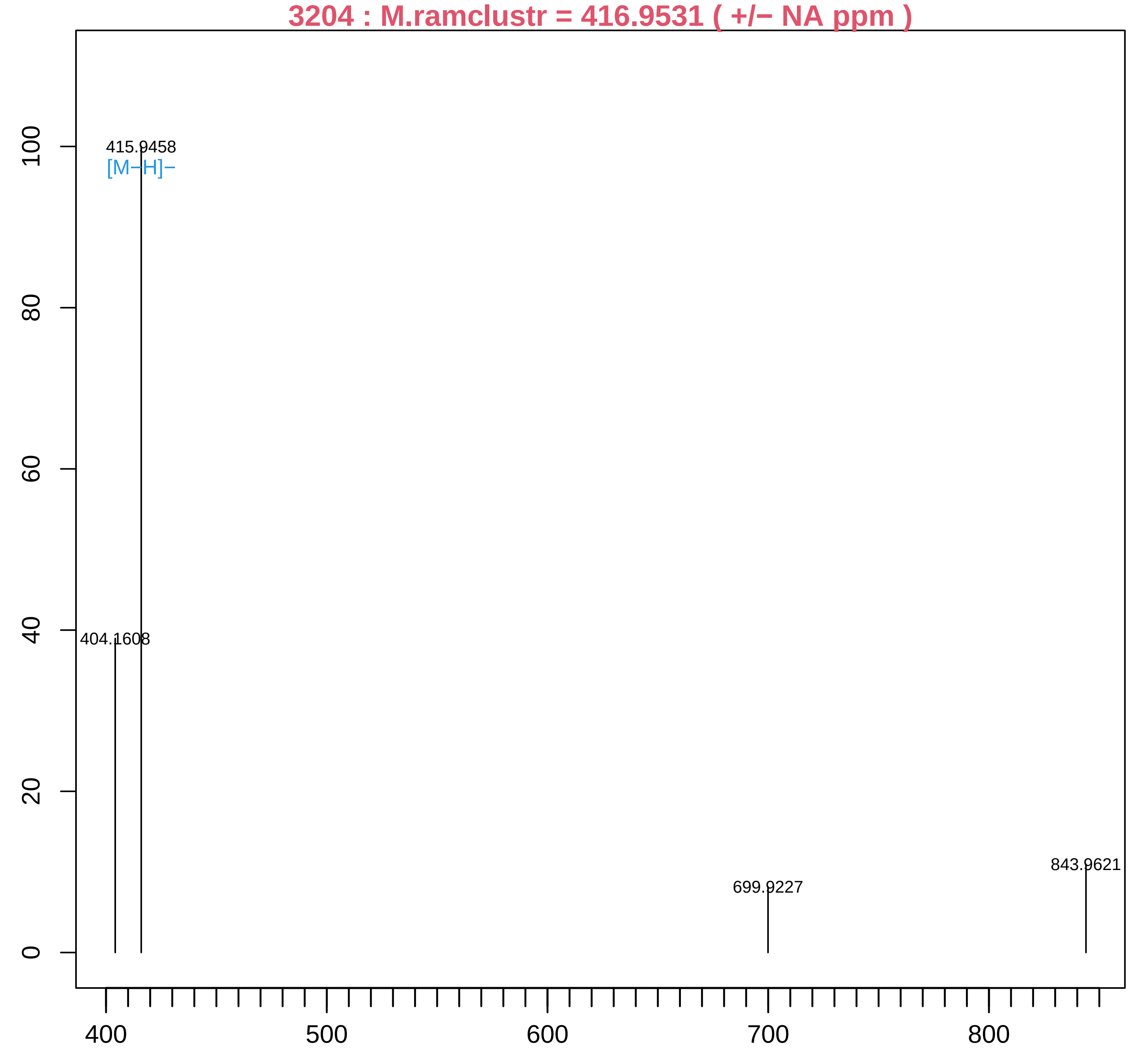
3204 : M.ramclustr = 416.9531 (+/− NA ppm)
415.9458
[M−H]−
404.1608
699.9227
843.9621
0
20
40
60
80
100
400
500
600
700
800

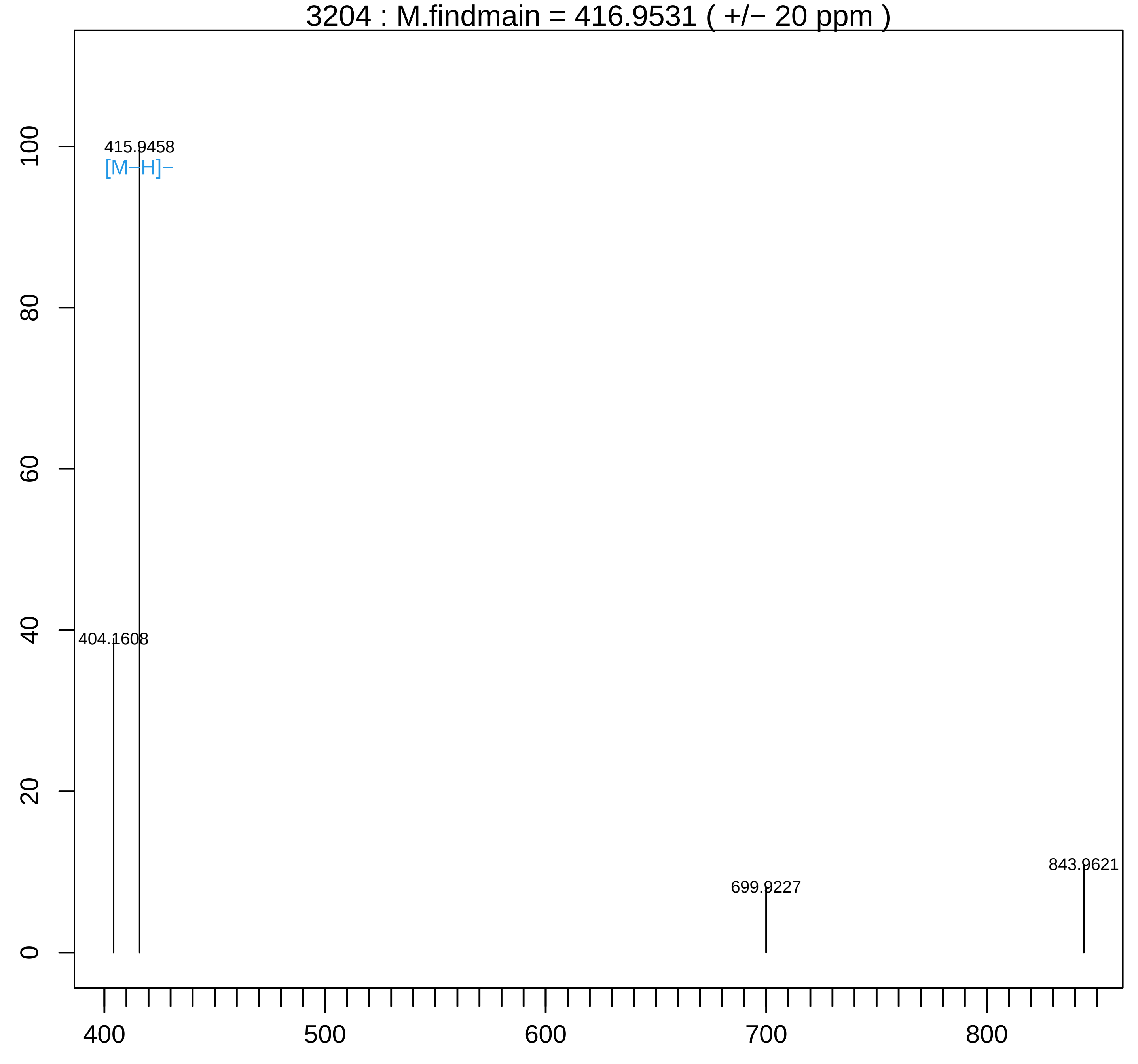
3204 : M.findmain = 416.9531 (+/− 20 ppm)
415.9458
[M−H]−
404.1608
699.9227
843.9621
0
20
40
60
80
100
400
500
600
700
800

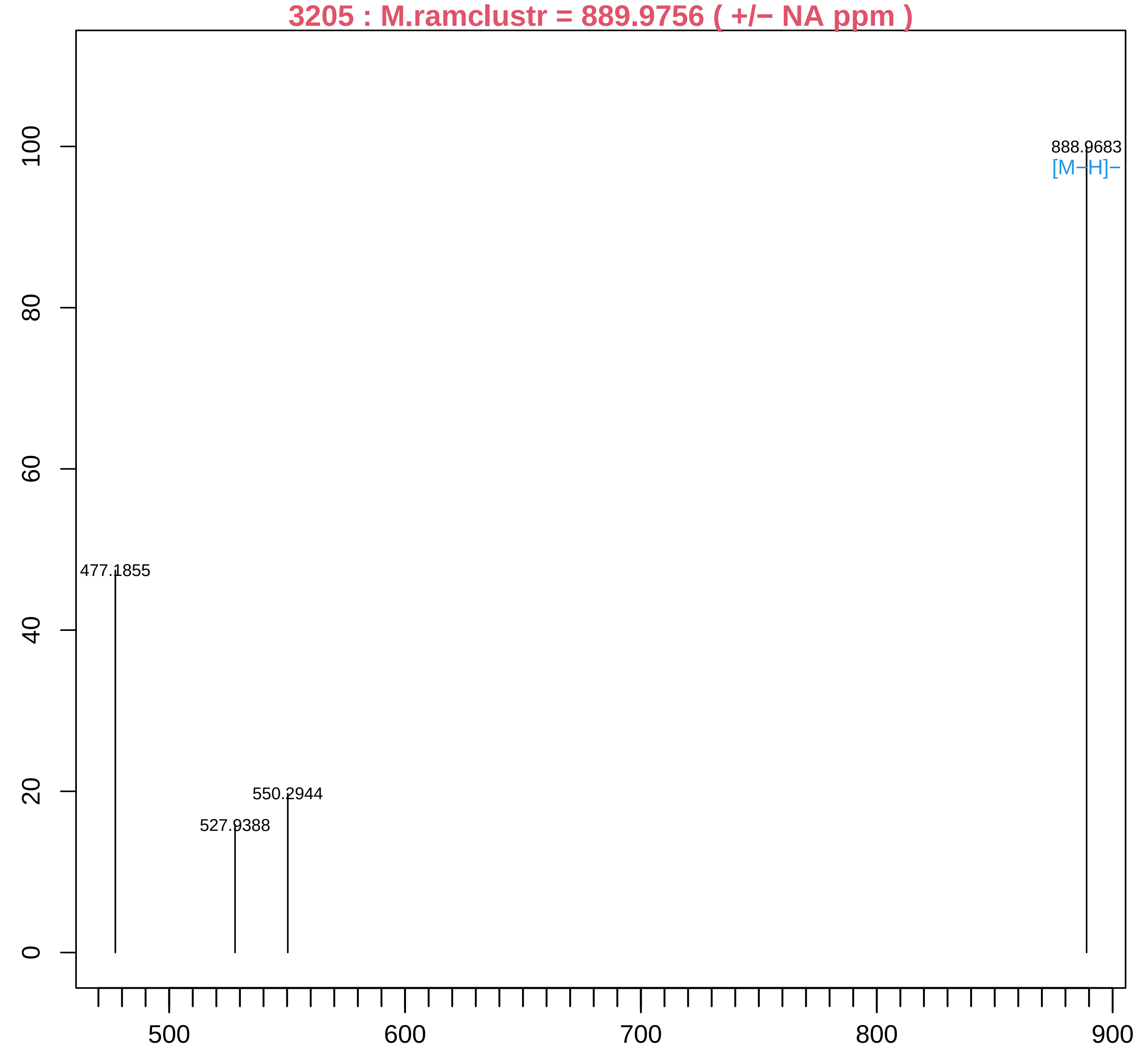
3205 : M.ramclustr = 889.9756 (+/− NA ppm)
888.9683
[M−H]−
477.1855
550.2944
527.9388
0
20
40
60
80
100
500
600
700
800
900

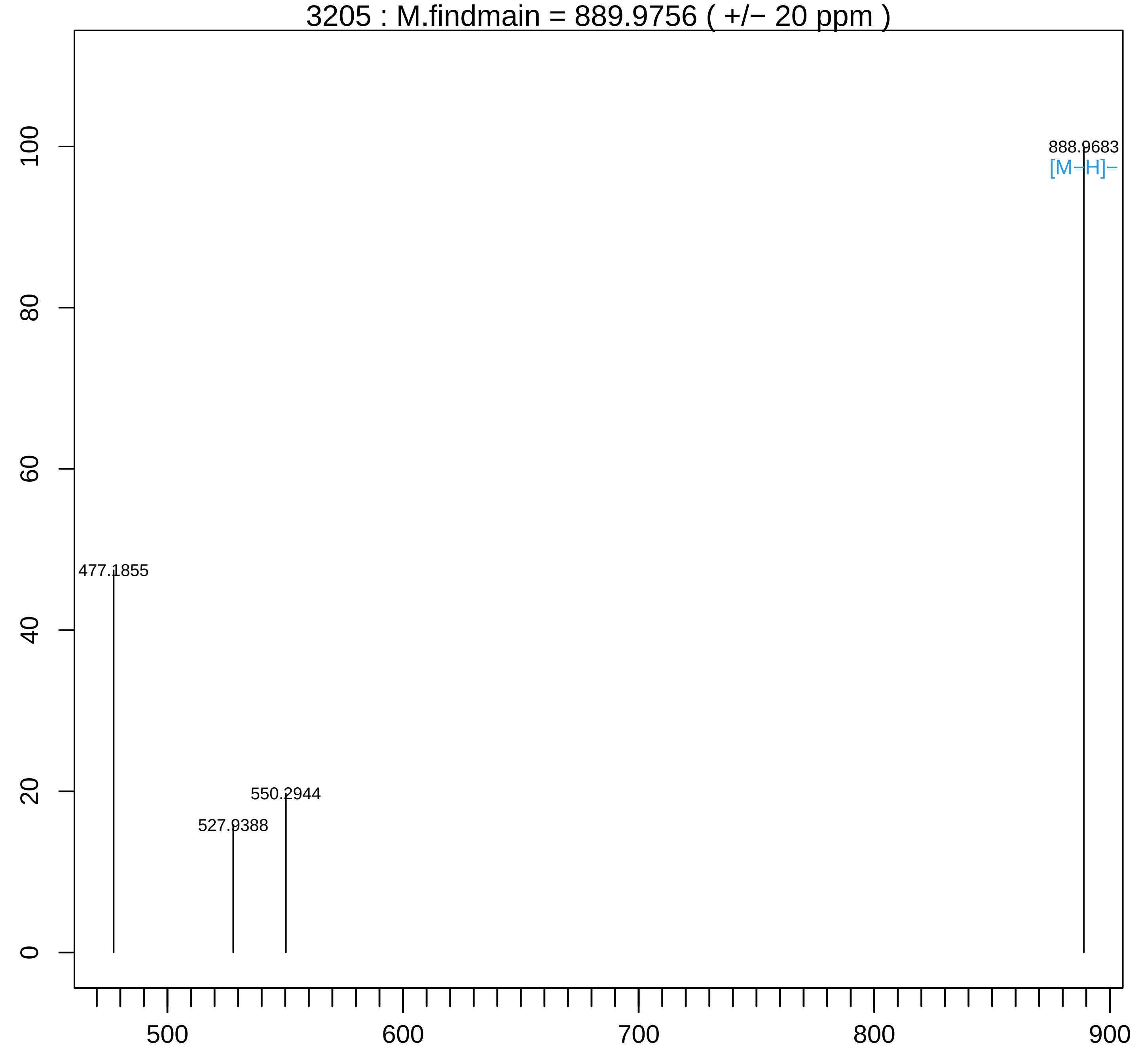
3205 : M.findmain = 889.9756 (+/− 20 ppm)
888.9683
[M−H]−
477.1855
550.2944
527.9388
0
20
40
60
80
100
500
600
700
800
900

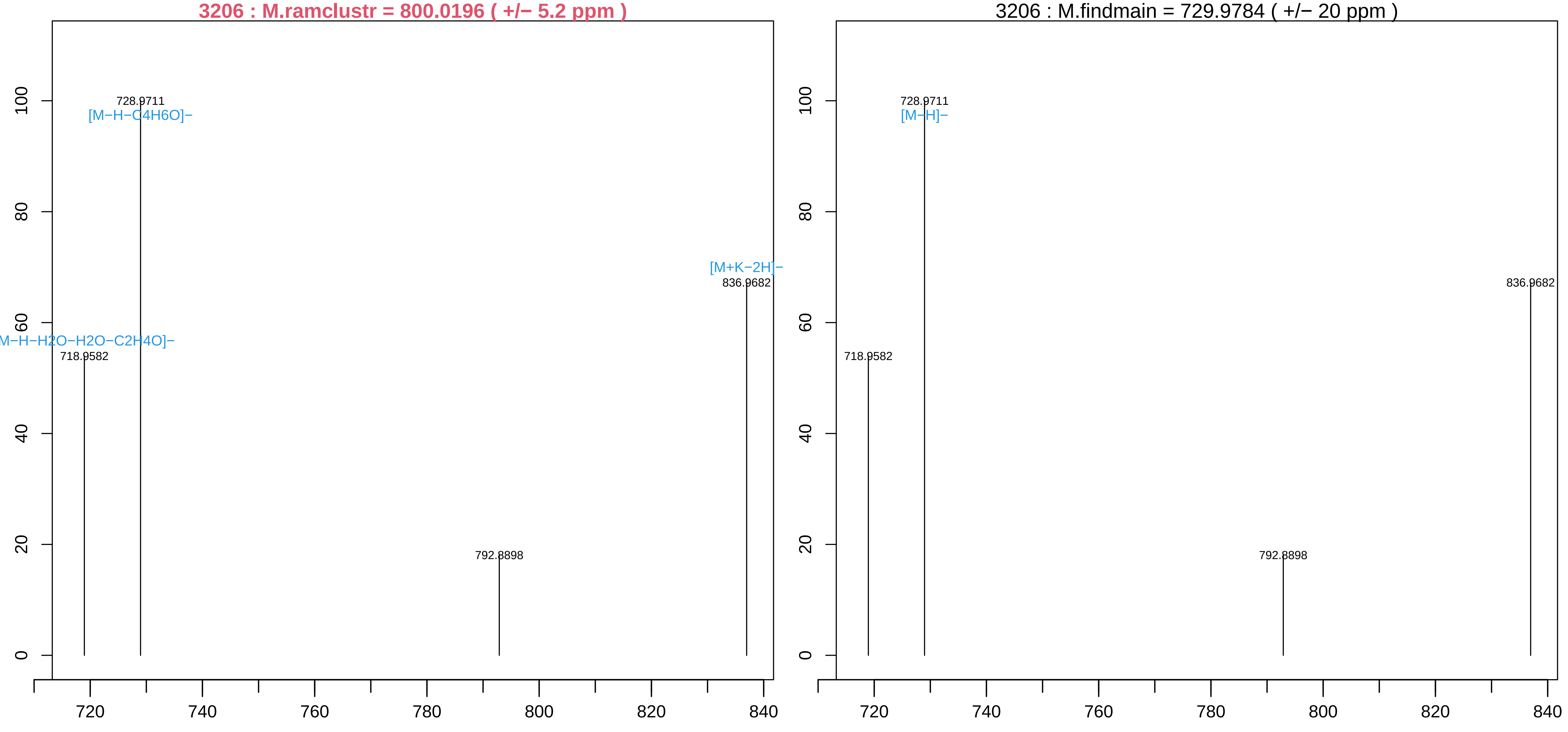
3206 : M.ramclustr = 800.0196 (+/− 5.2 ppm)
728.9711
[M−H−C4H6O]−
[M+K−2H]−
836.9682
M−H−H2O−H2O−C2H4O]−
718.9582
792.8898
0
20
40
60
80
100
720
740
760
780
800
820
840
3206 : M.findmain = 729.9784 (+/− 20 ppm)
728.9711
[M−H]−
836.9682
718.9582
792.8898

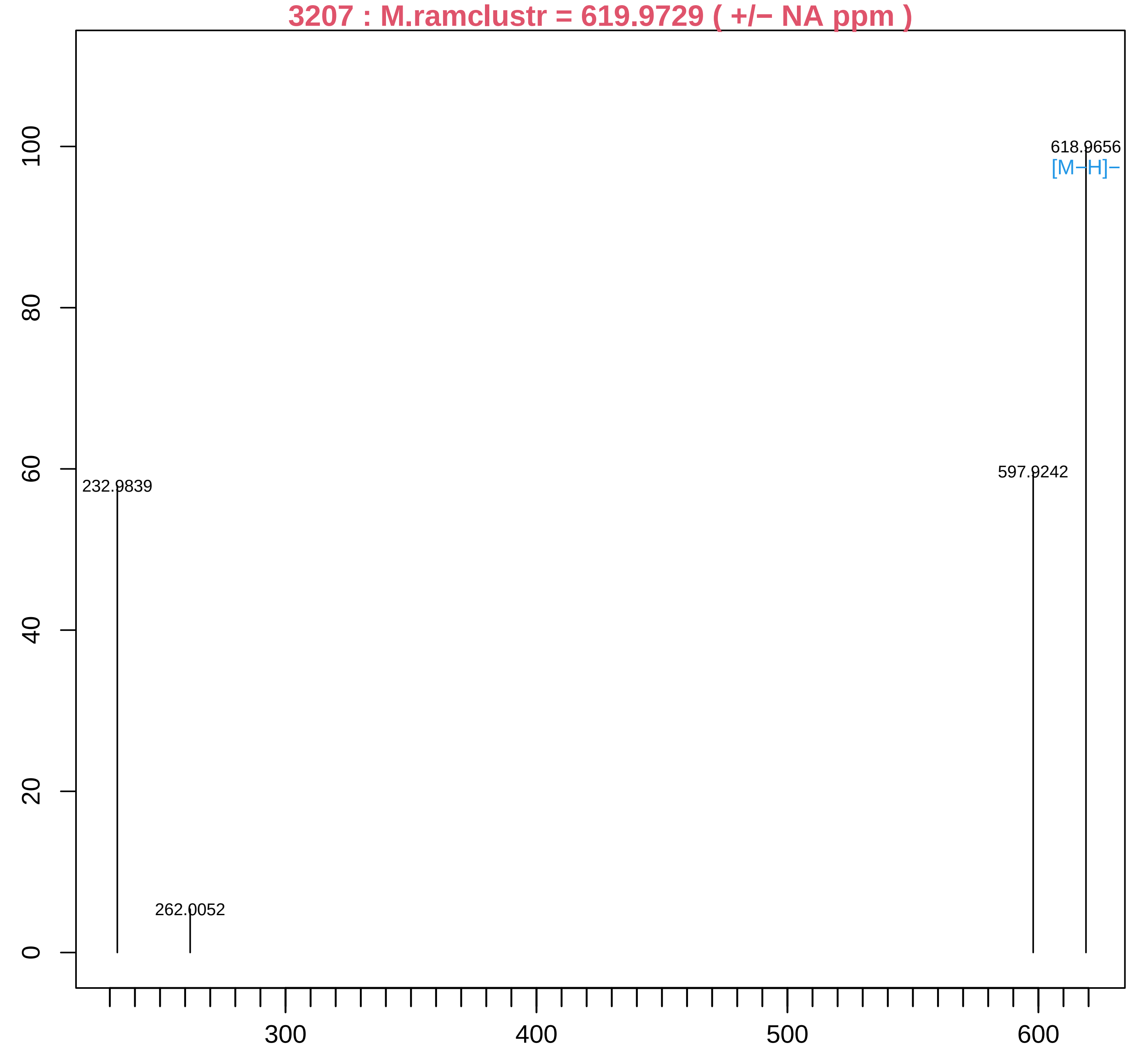
3207 : M.ramclustr = 619.9729 (+/− NA ppm)
618.9656
[M−H]−
597.9242
232.9839
262.0052
0
20
40
60
80
100
300
400
500
600

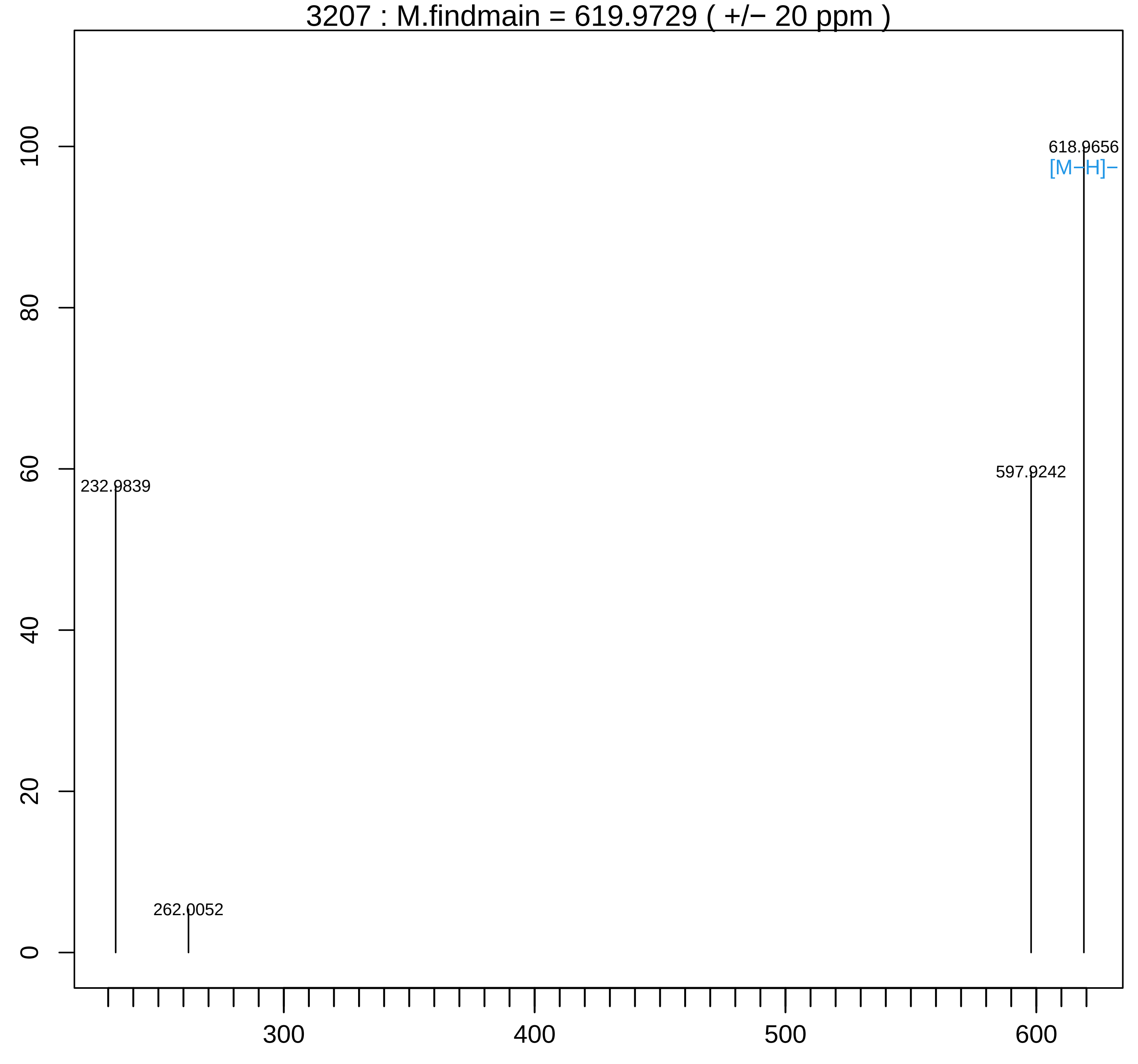
3207 : M.findmain = 619.9729 (+/− 20 ppm)
618.9656
[M−H]−
597.9242
232.9839
262.0052
0
20
40
60
80
100
300
400
500
600

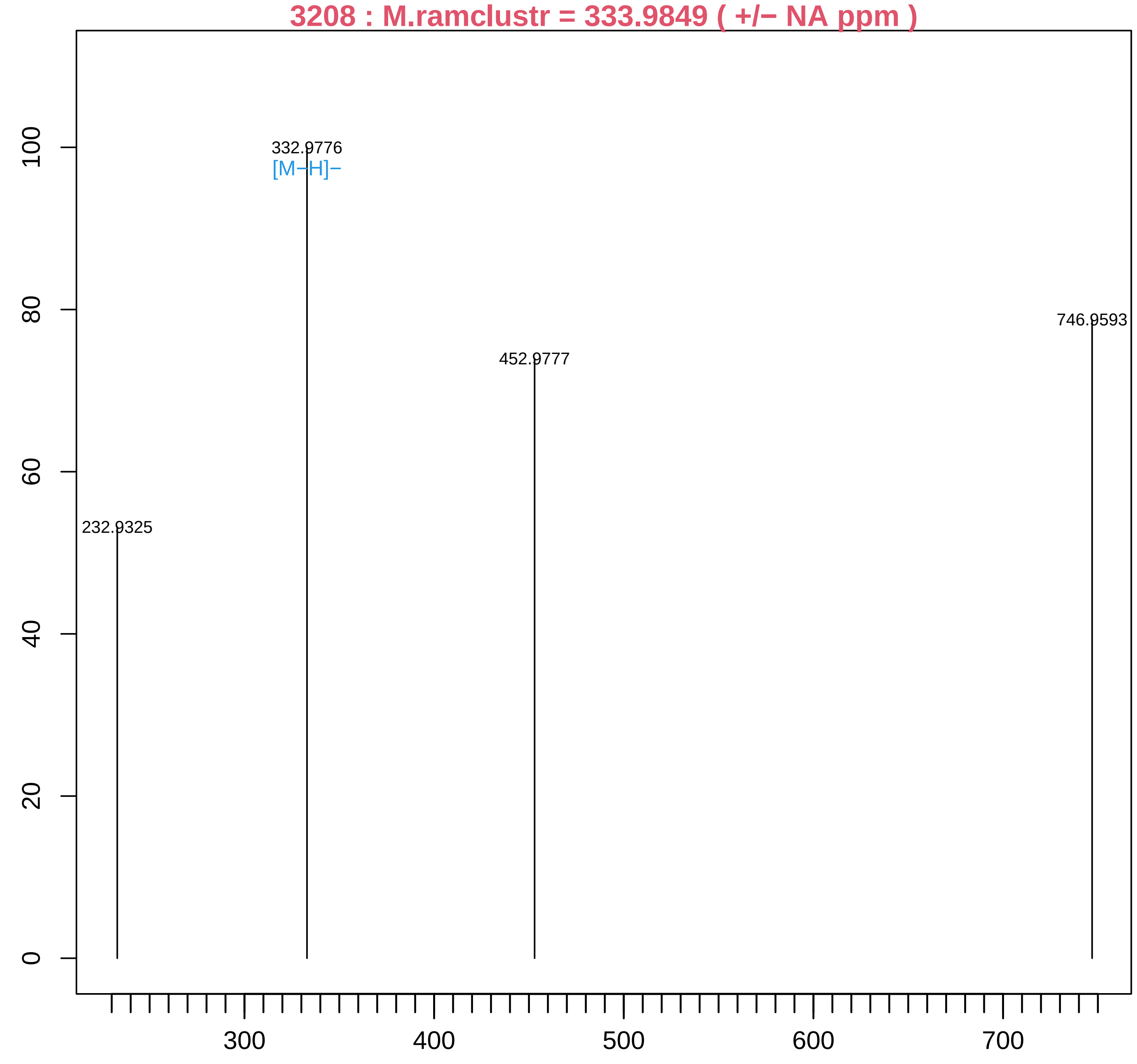
3208 : M.ramclustr = 333.9849 (+/− NA ppm)
332.9776
[M−H]−
452.9777
746.9593
232.9325
0
20
40
60
80
100
300
400
500
600
700

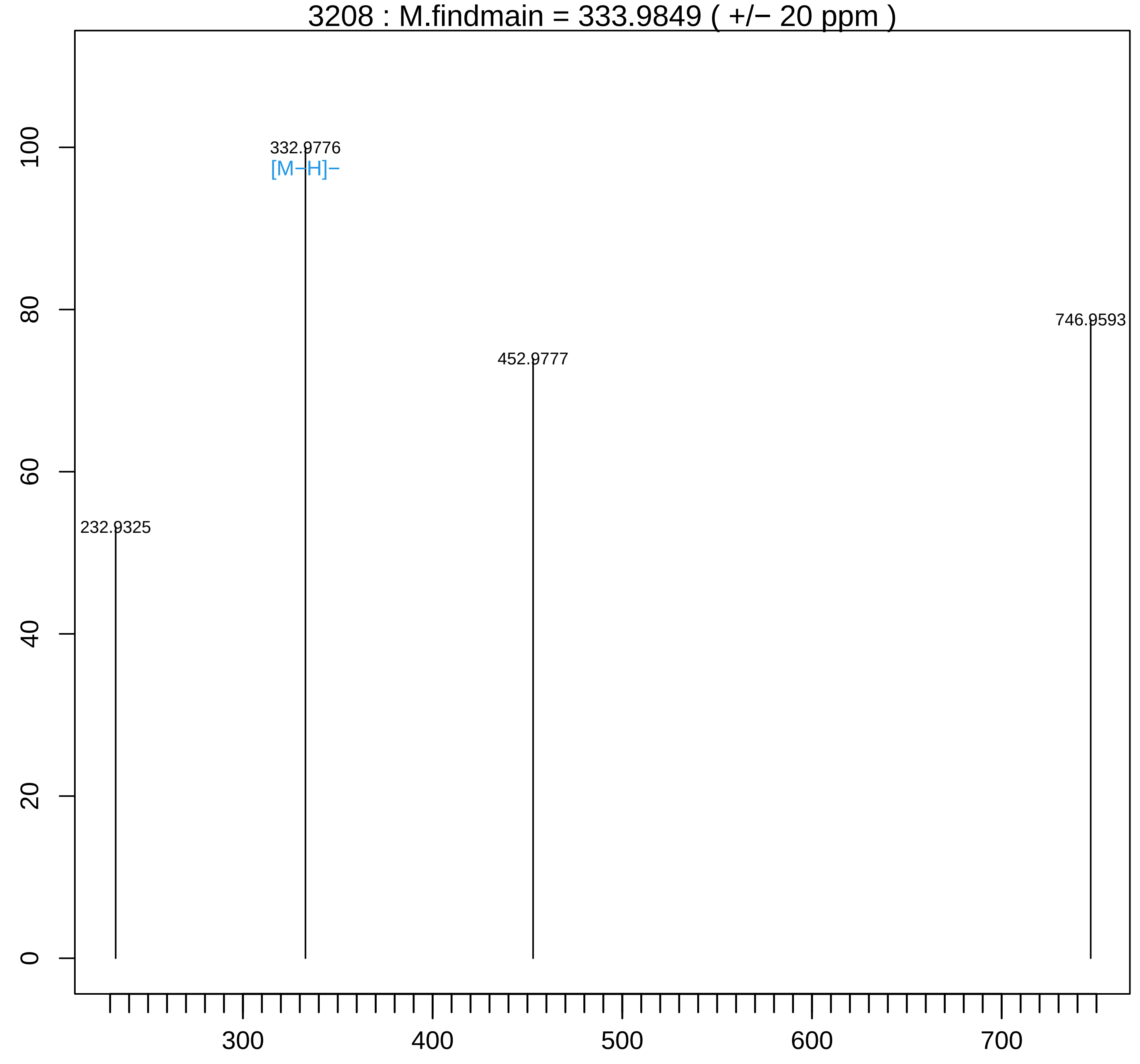
3208 : M.findmain = 333.9849 (+/− 20 ppm)
332.9776
[M−H]−
452.9777
746.9593
232.9325
0
20
40
60
80
100
300
400
500
600
700

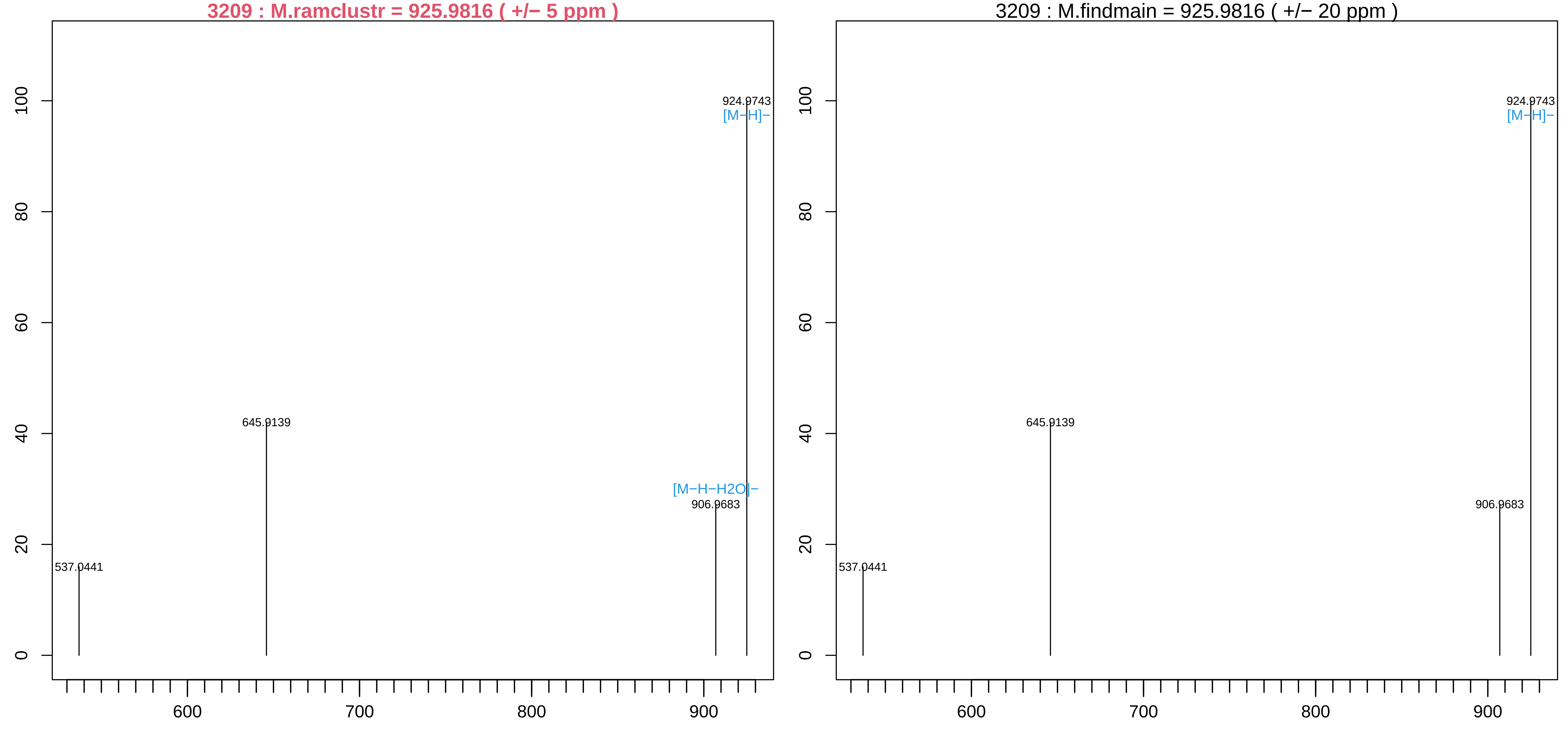
3209 : M.ramclustr = 925.9816 (+/− 5 ppm)
924.9743
[M−H]−
645.9139
[M−H−H2O]−
906.9683
537.0441
3209 : M.findmain = 925.9816 (+/− 20 ppm)
924.9743
[M−H]−
645.9139
906.9683
537.0441
0
20
40
60
80
100
600
700
800
900

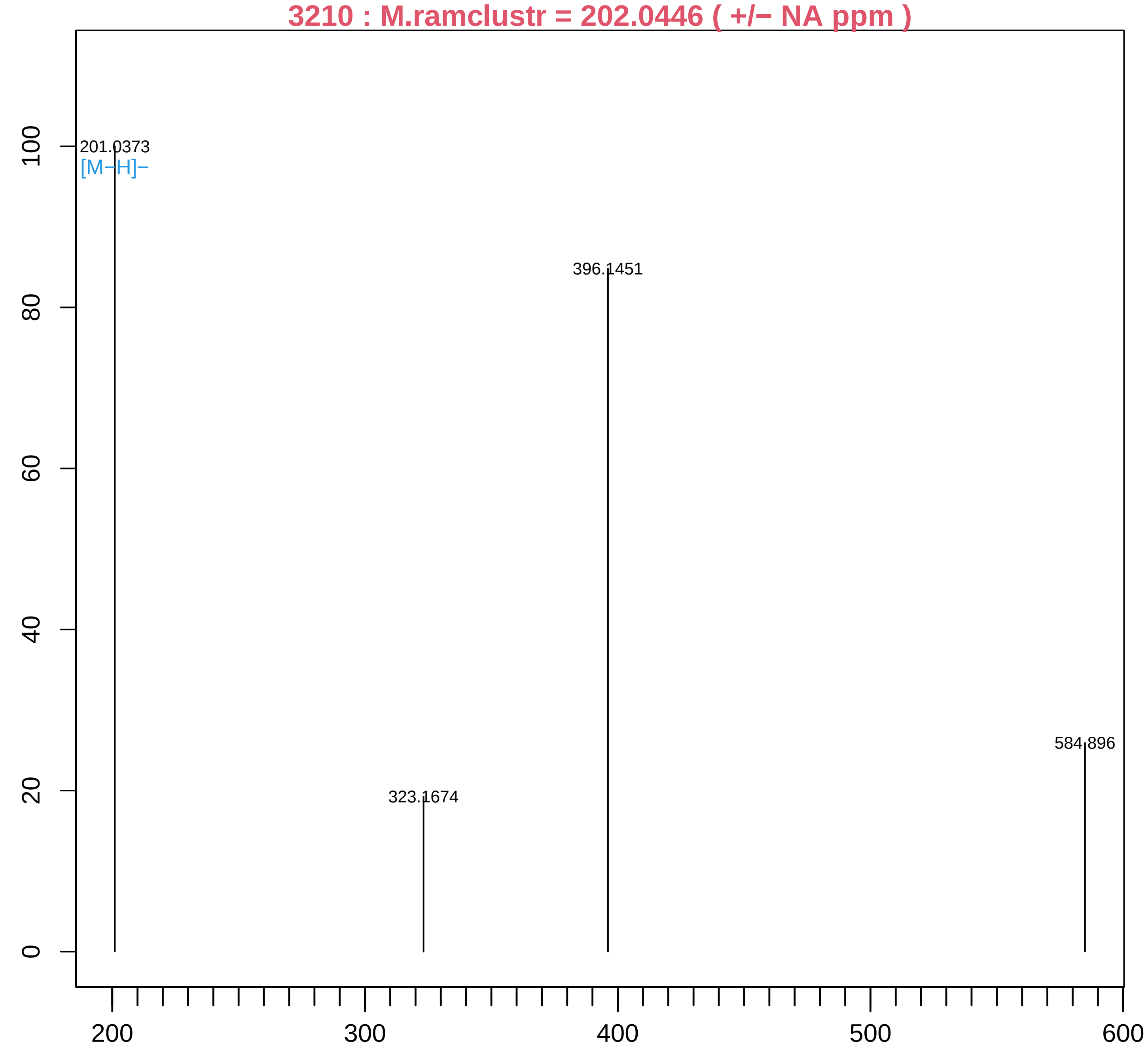
3210 : M.ramclustr = 202.0446 (+/− NA ppm)
201.0373
[M−H]−
396.1451
323.1674
584.896
0
20
40
60
80
100
200
300
400
500
600

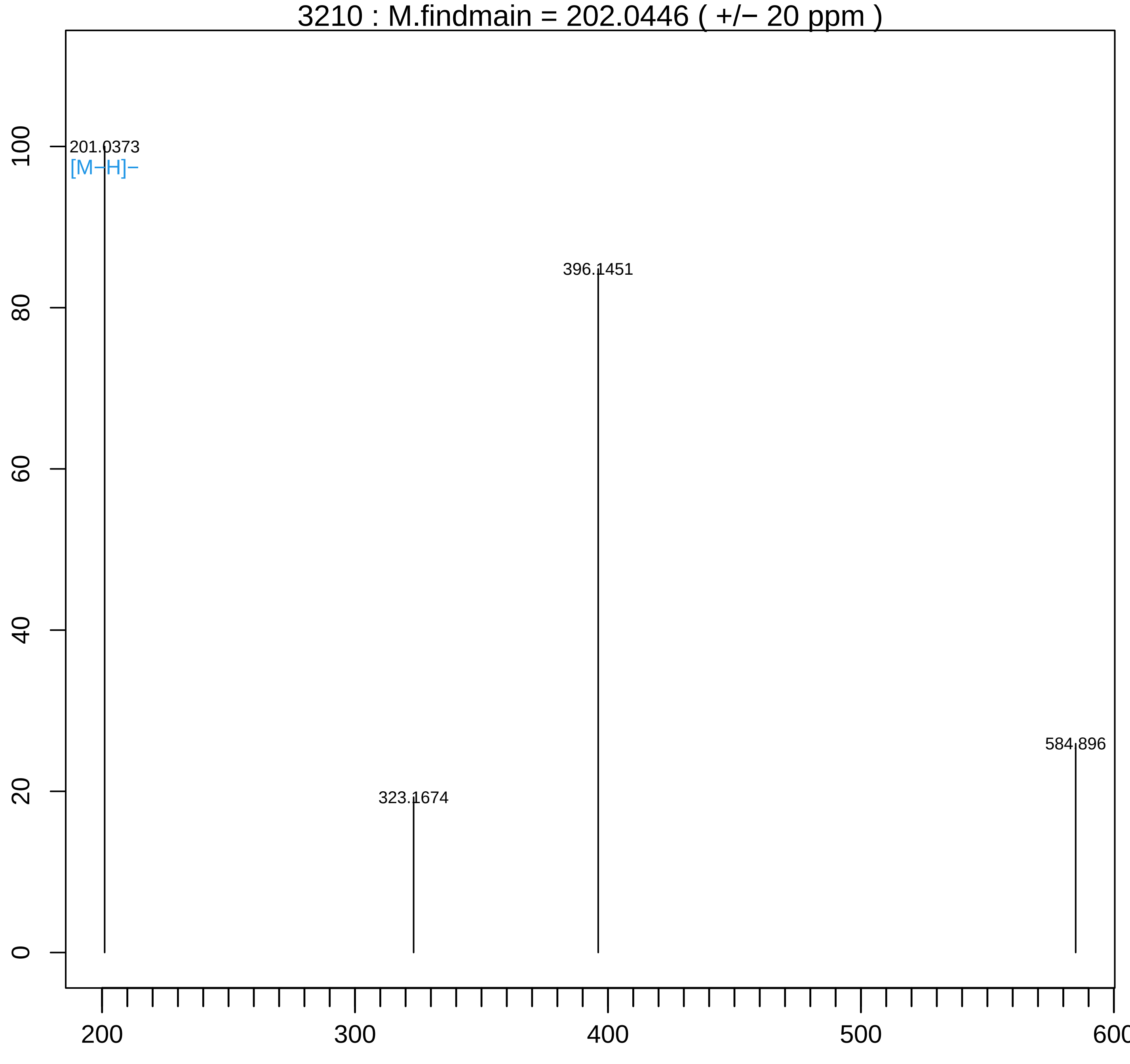
3210 : M.findmain = 202.0446 (+/− 20 ppm)
201.0373
[M−H]−
396.1451
323.1674
584.896
0
20
40
60
80
100
200
300
400
500
600

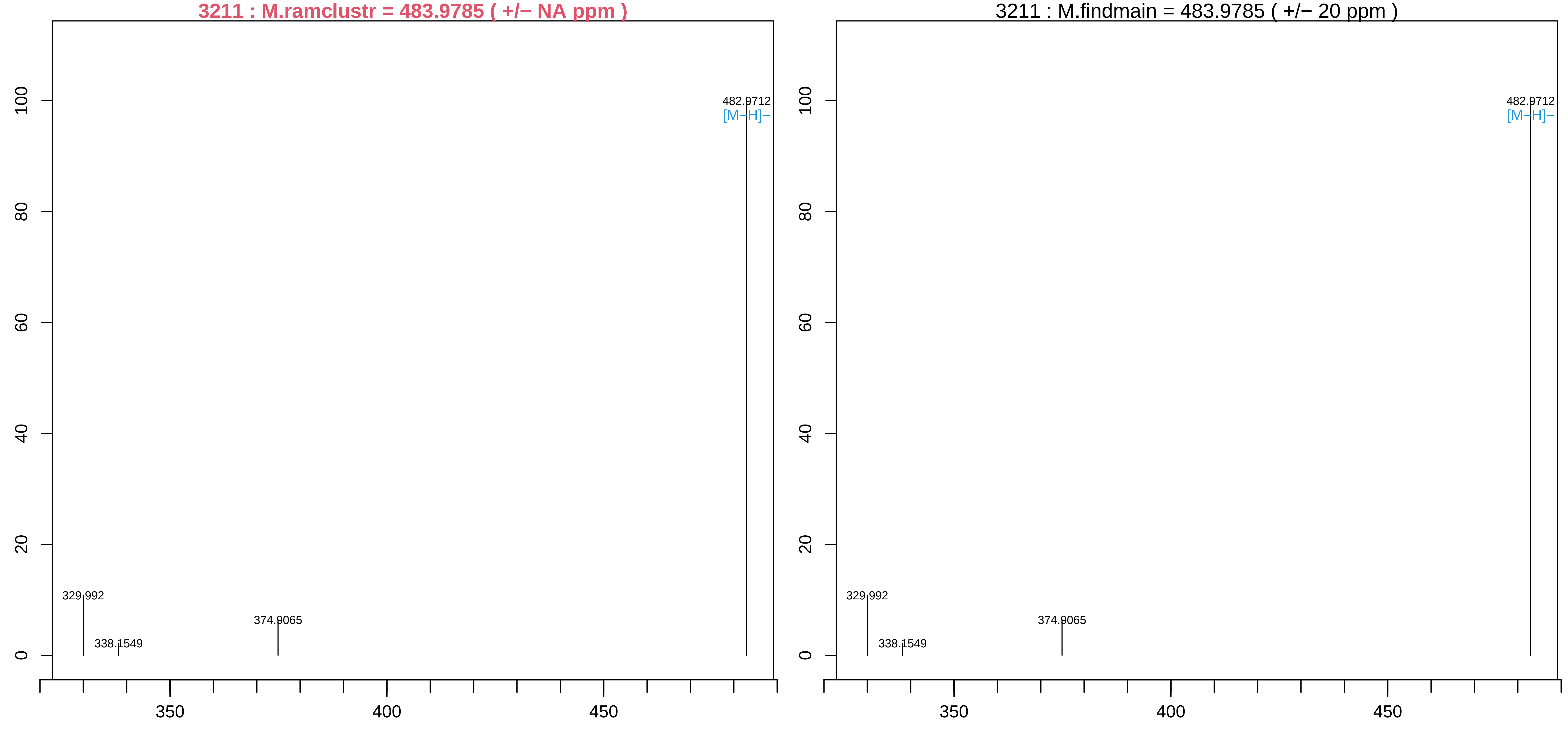
3211 : M.ramclustr = 483.9785 (+/− NA ppm)
482.9712
[M−H]−
329.992
338.1549
374.9065
3211 : M.findmain = 483.9785 (+/− 20 ppm)
482.9712
[M−H]−
329.992
338.1549
374.9065
0
20
40
60
80
100
350
400
450

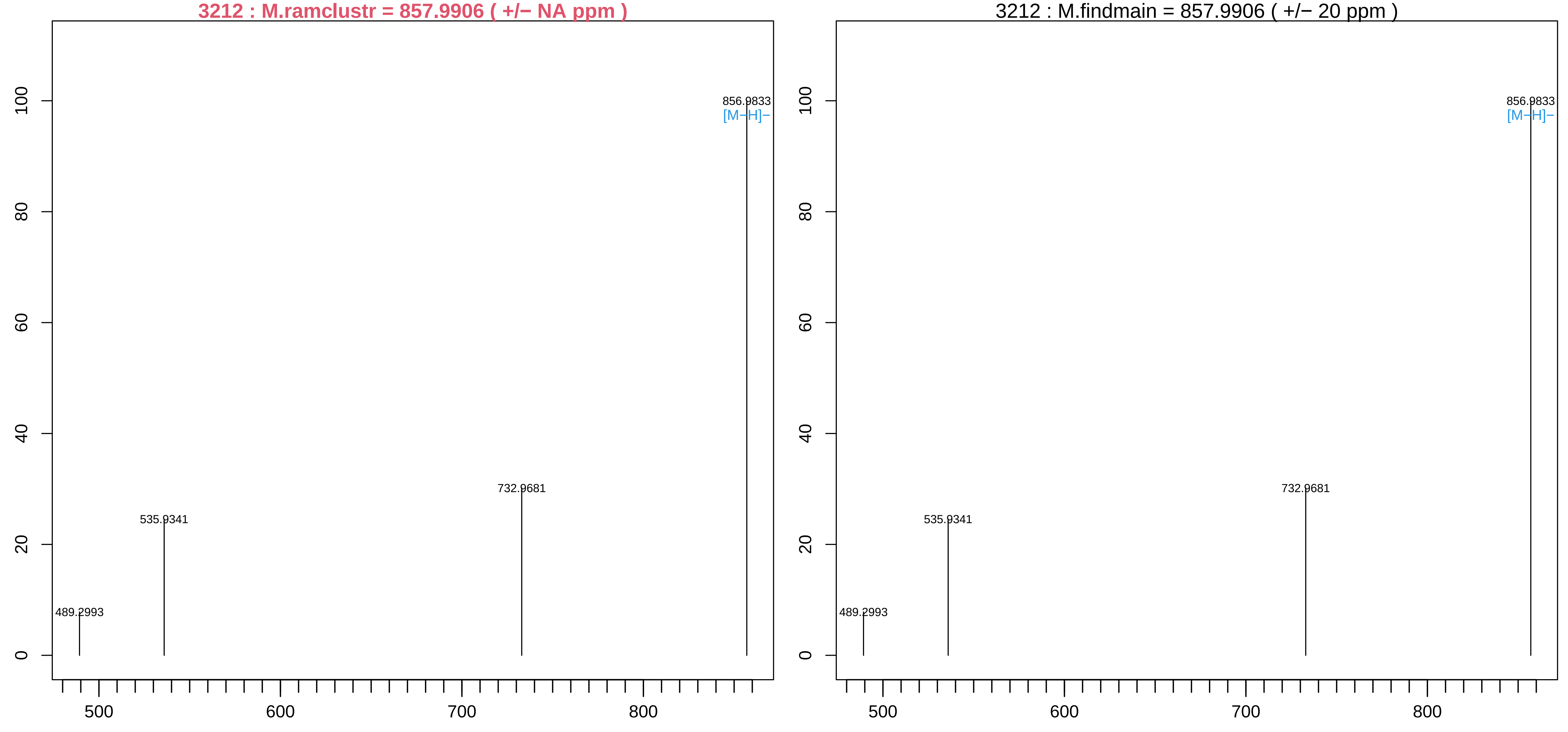
3212 : M.ramclustr = 857.9906 (+/− NA ppm)
3212 : M.findmain = 857.9906 (+/− 20 ppm)
856.9833
[M−H]−
732.9681
535.9341
489.2993
0
20
40
60
80
100
500
600
700
800

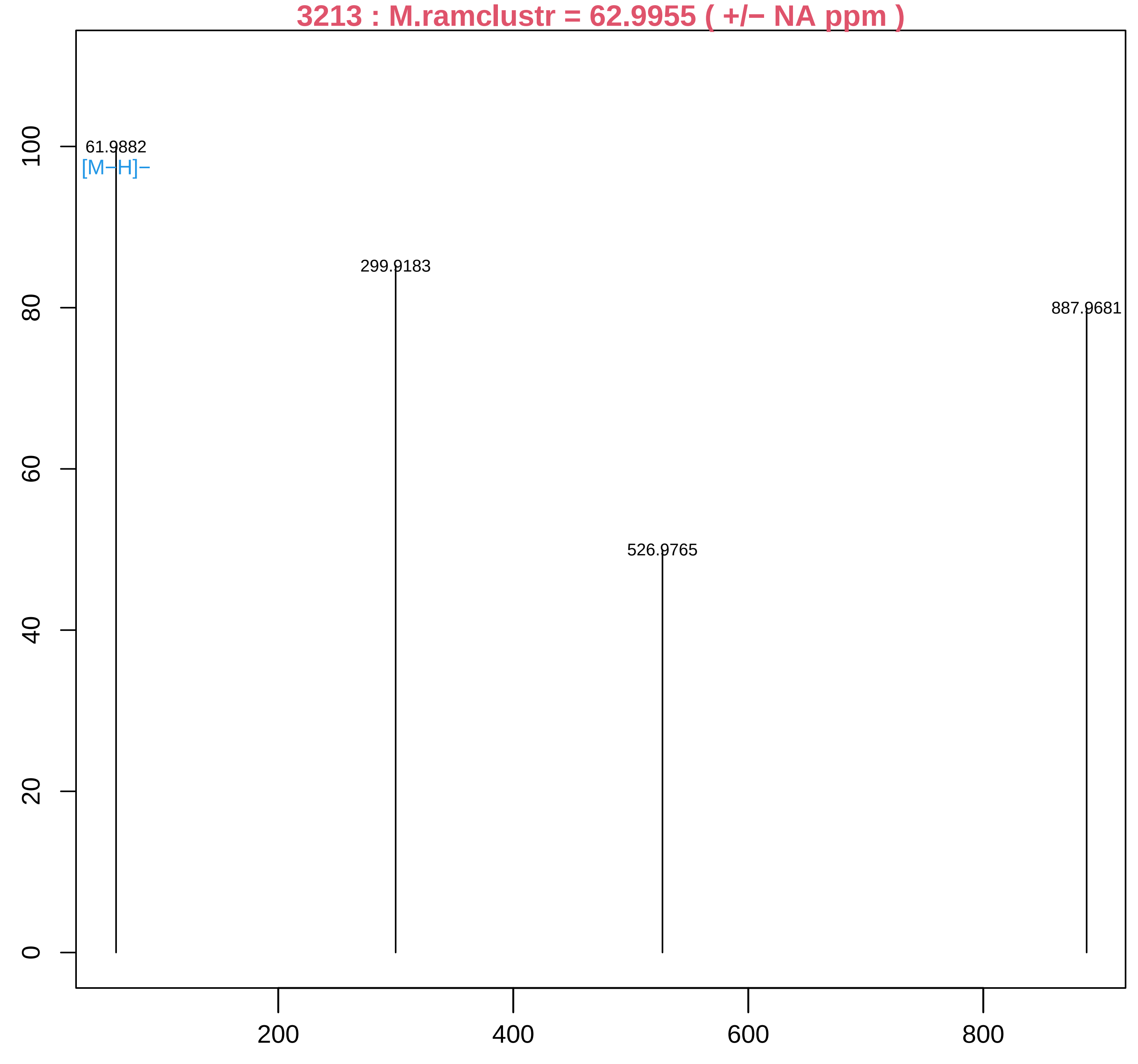
3213 : M.ramclustr = 62.9955 (+/− NA ppm)
61.9882
[M−H]−
299.9183
526.9765
887.9681
0
20
40
60
80
100
200
400
600
800

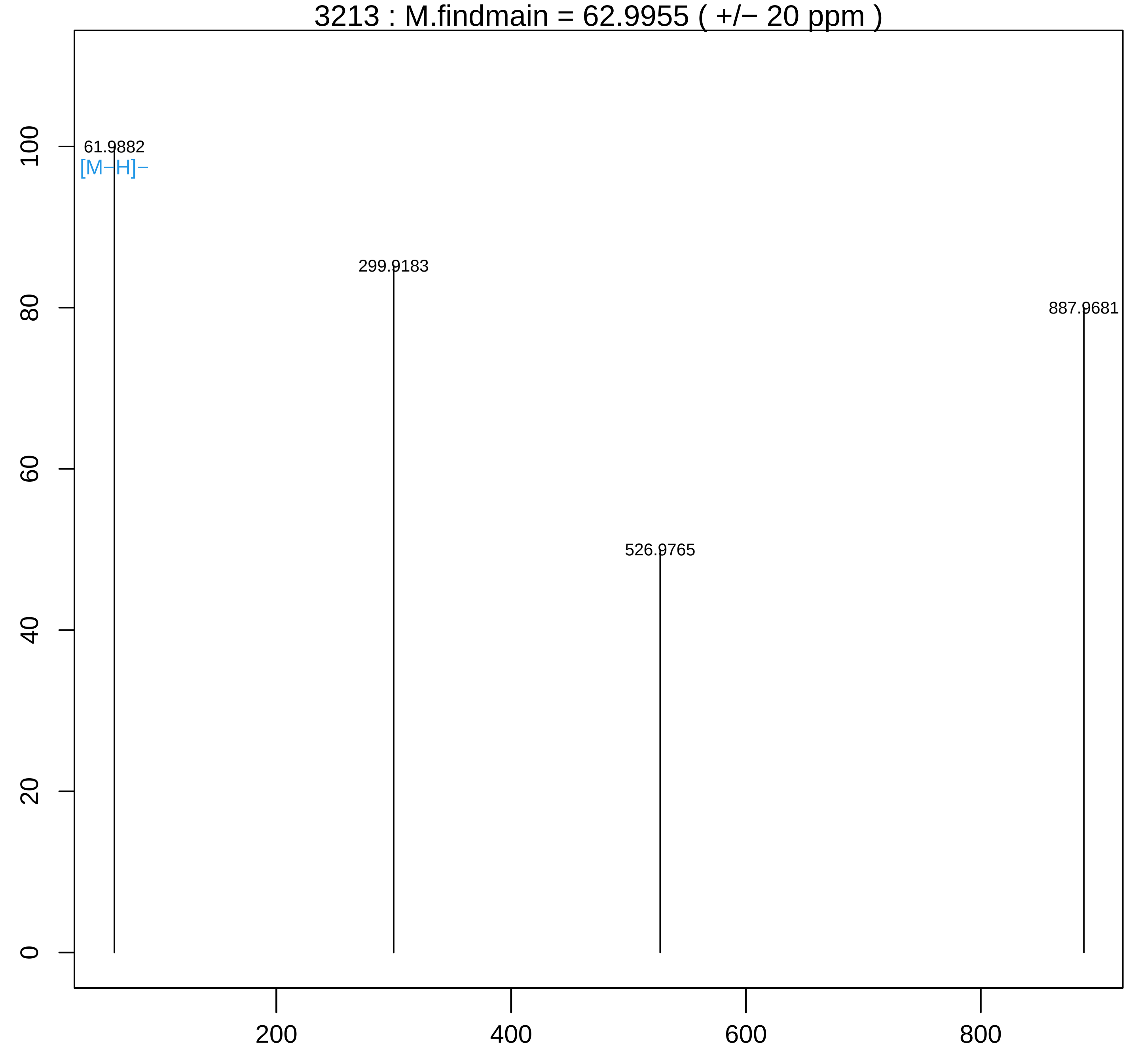
3213 : M.findmain = 62.9955 (+/− 20 ppm)
61.9882
[M−H]−
299.9183
526.9765
887.9681
0
20
40
60
80
100
200
400
600
800

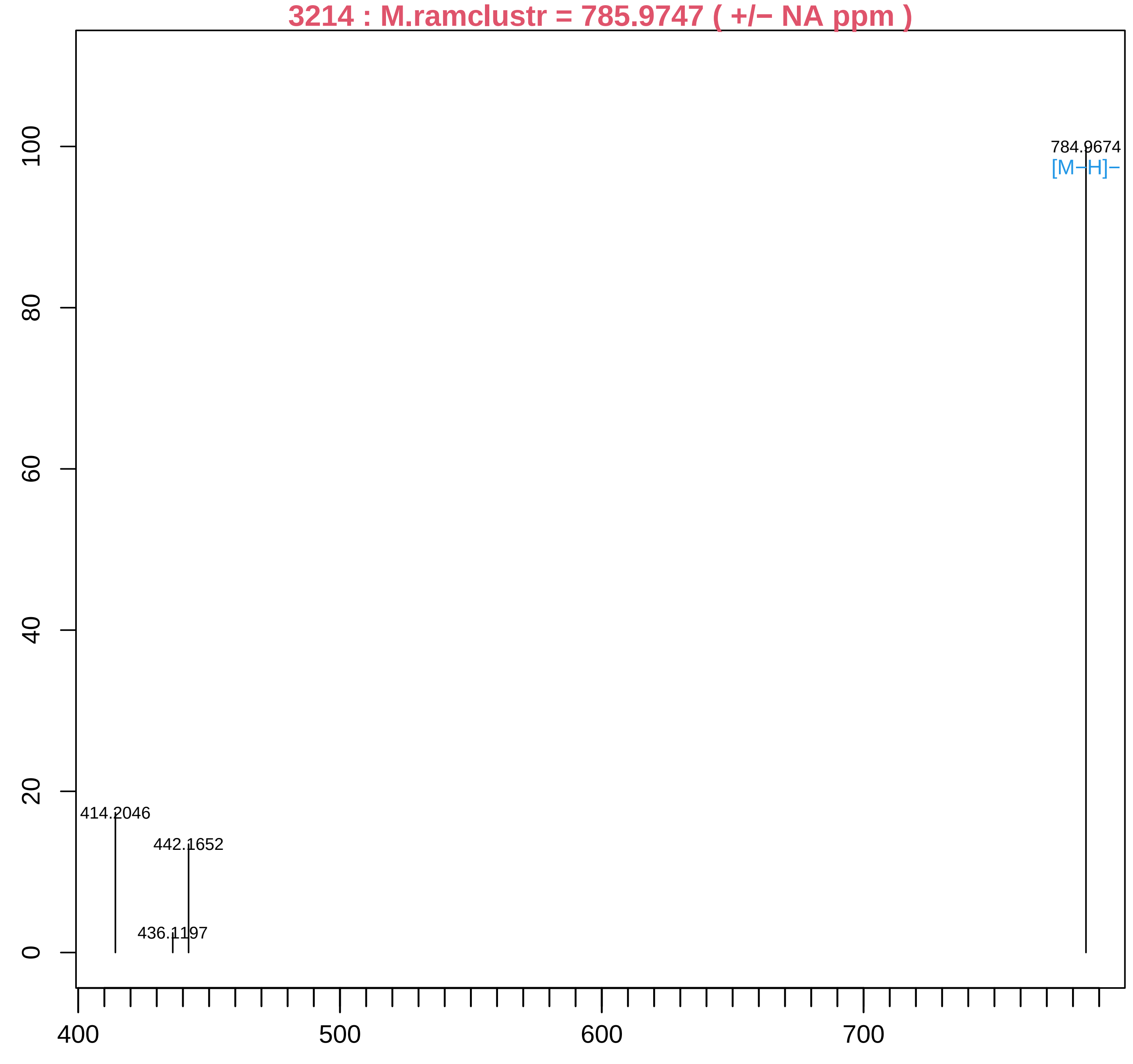
3214 : M.ramclustr = 785.9747 (+/− NA ppm)
784.9674
[M−H]−
414.2046
442.1652
436.1197
0
20
40
60
80
100
400
500
600
700

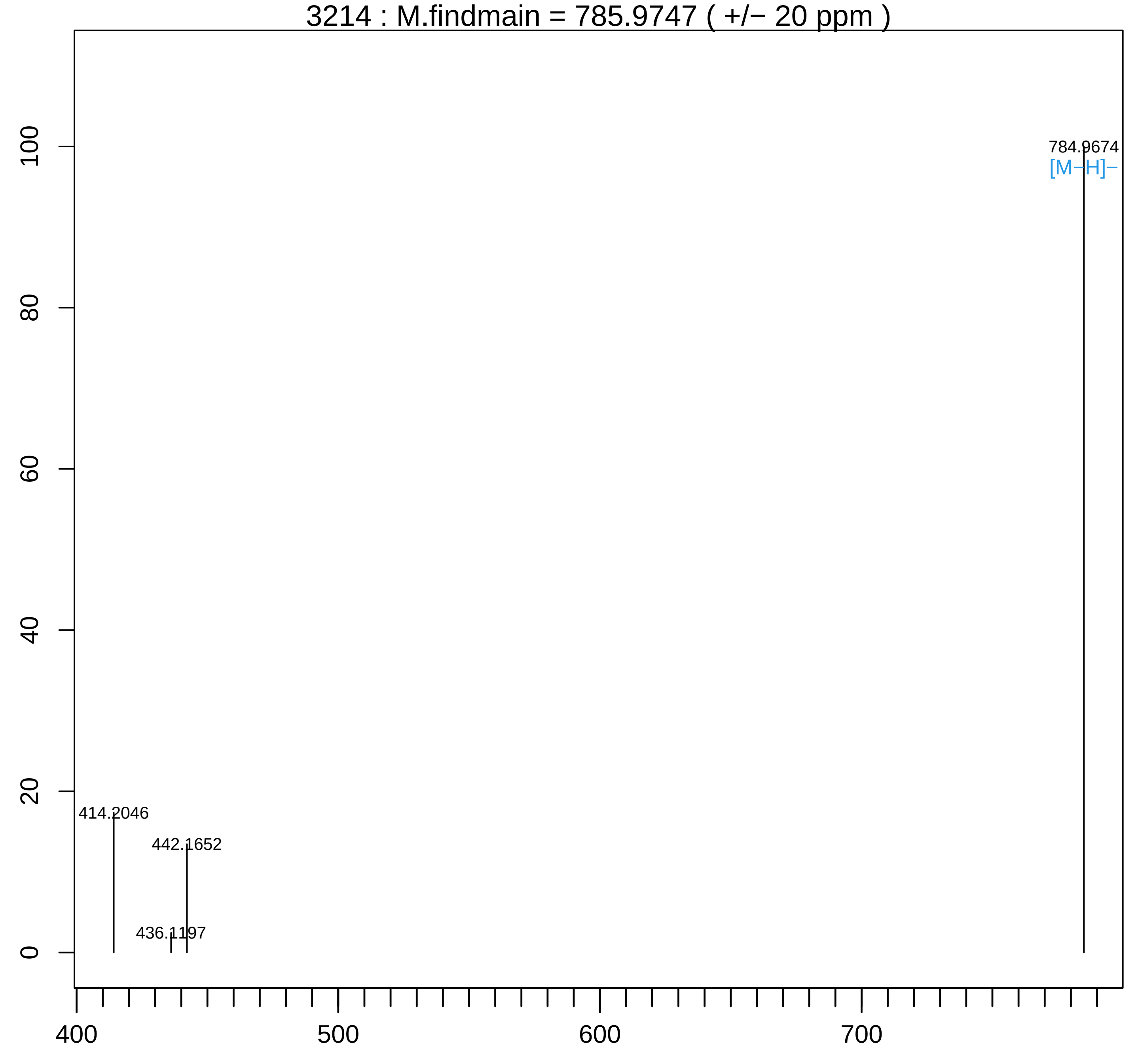
3214 : M.findmain = 785.9747 (+/− 20 ppm)
784.9674
[M−H]−
414.2046
442.1652
436.1197
0
20
40
60
80
100
400
500
600
700

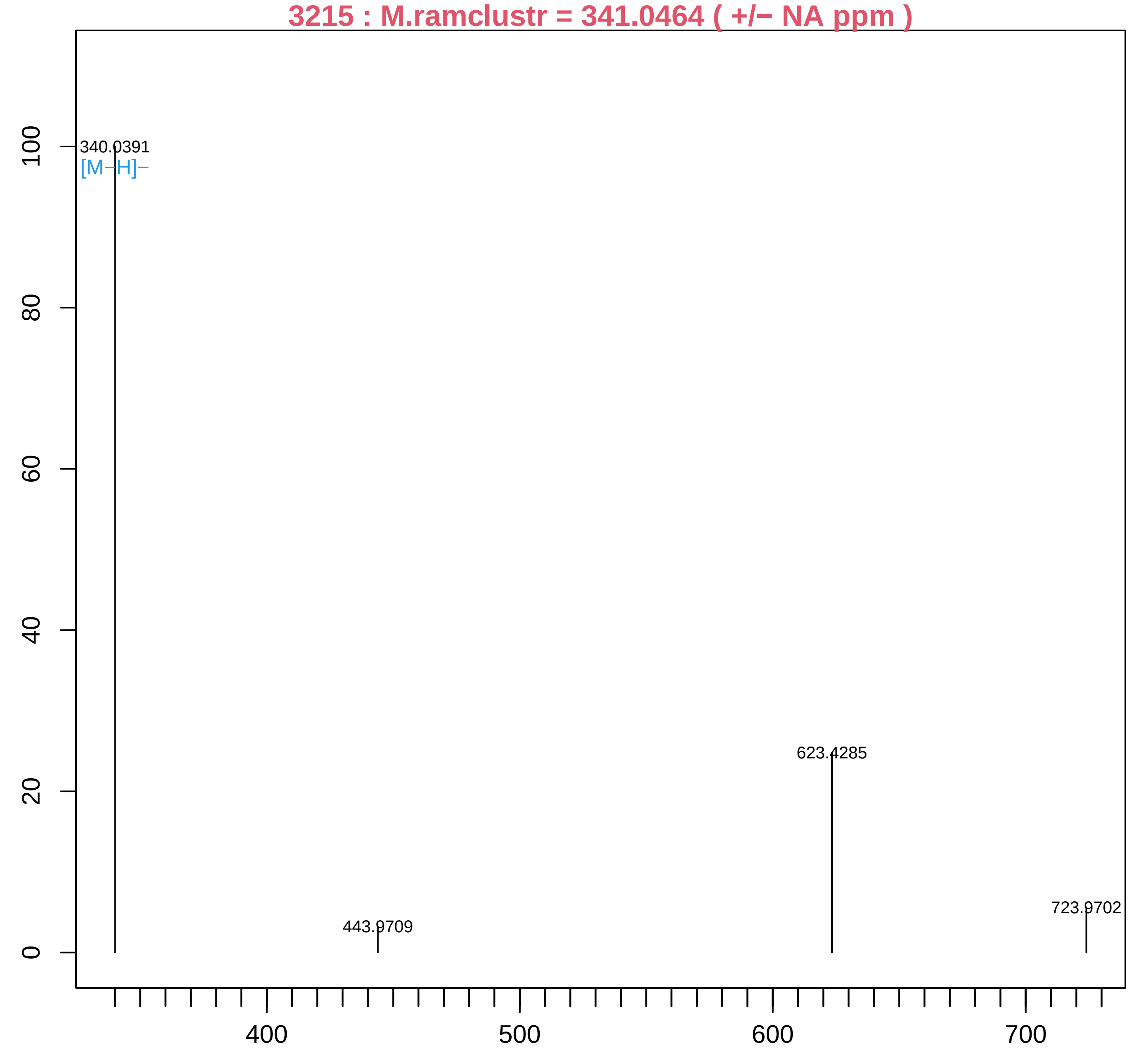
3215 : M.ramclustr = 341.0464 (+/− NA ppm)
340.0391
[M−H]−
443.9709
623.4285
723.9702
0
20
40
60
80
100
400
500
600
700

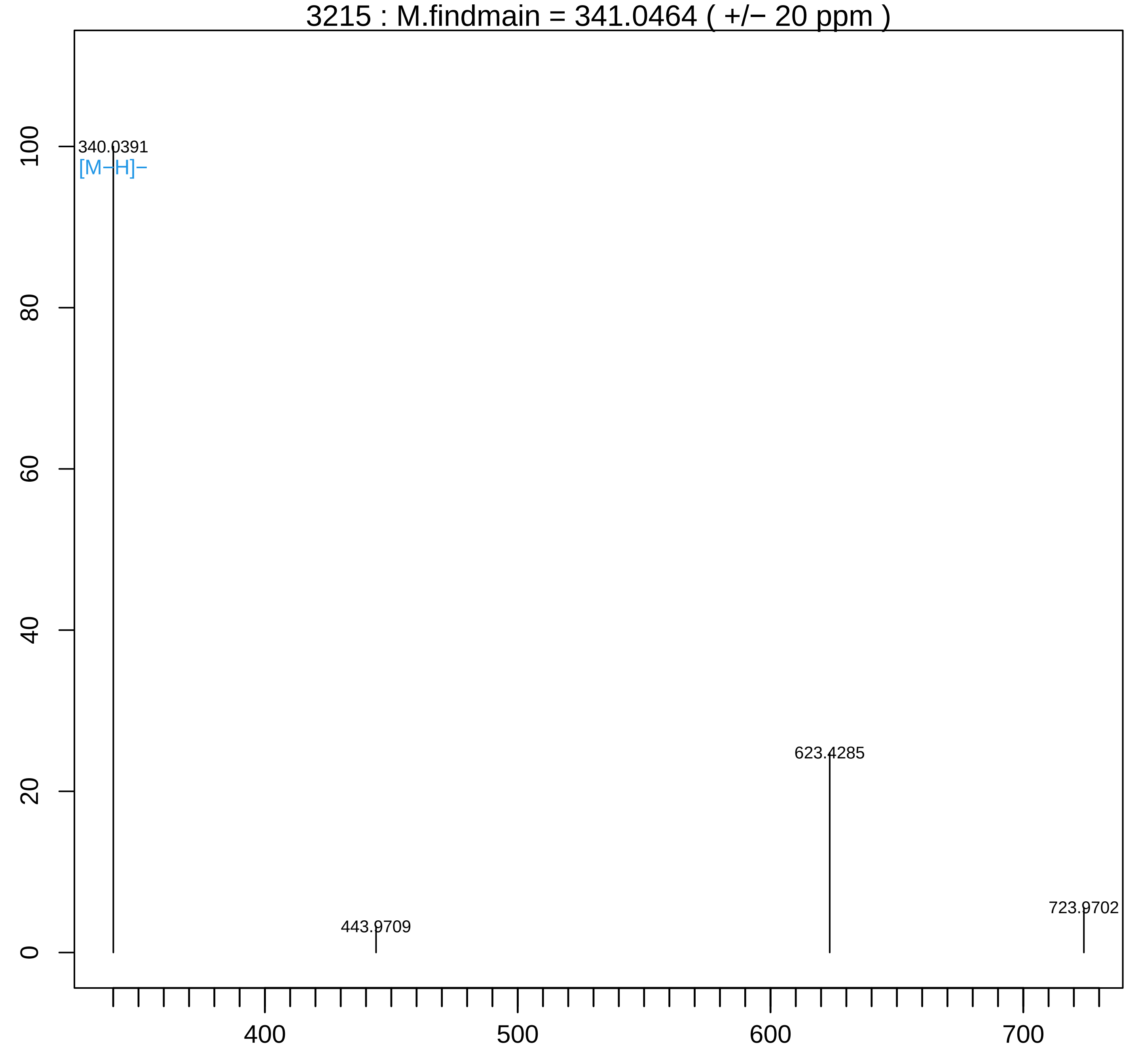
3215 : M.findmain = 341.0464 (+/− 20 ppm)
340.0391
[M−H]−
443.9709
623.4285
723.9702
0
20
40
60
80
100
400
500
600
700

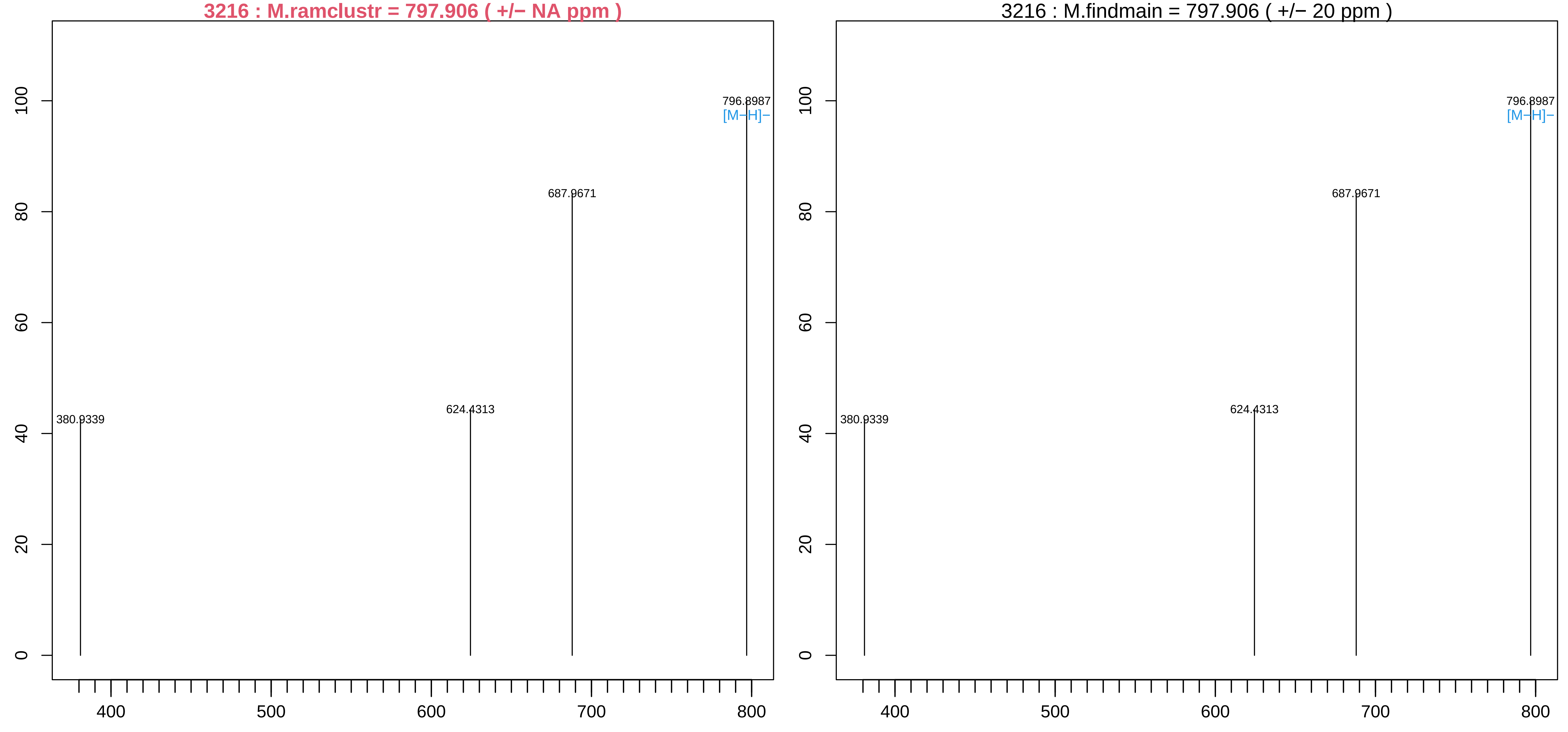
3216 : M.ramclustr = 797.906 (+/− NA ppm)
3216 : M.findmain = 797.906 (+/− 20 ppm)
796.8987
[M−H]−
687.9671
624.4313
380.9339
0
20
40
60
80
100
400
500
600
700
800

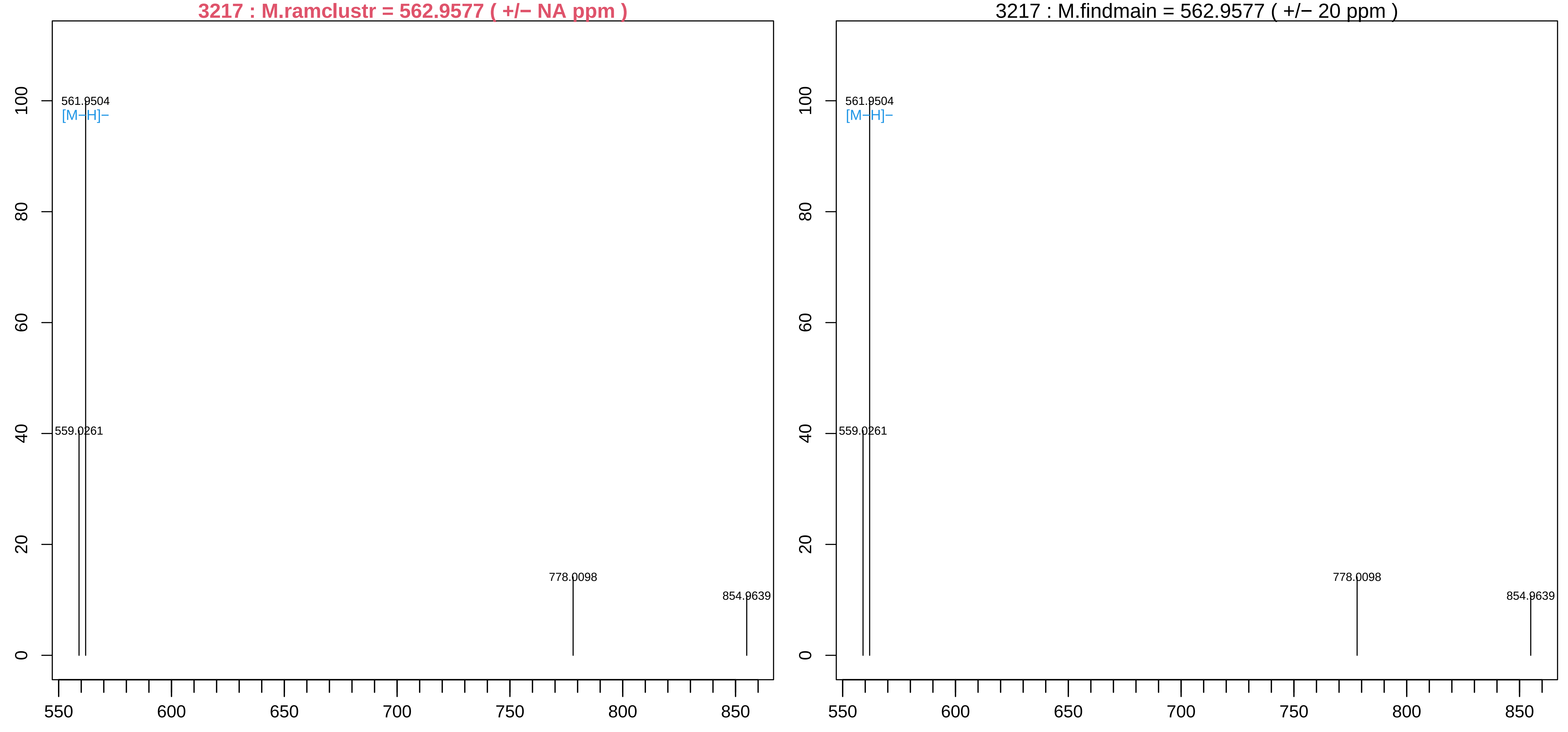

3217 : M.ramclustr = 562.9577 (+/− NA ppm)
561.9504
[M−H]−
559.0261
778.0098
854.9639
3217 : M.findmain = 562.9577 (+/− 20 ppm)
561.9504
[M−H]−
559.0261
778.0098
854.9639

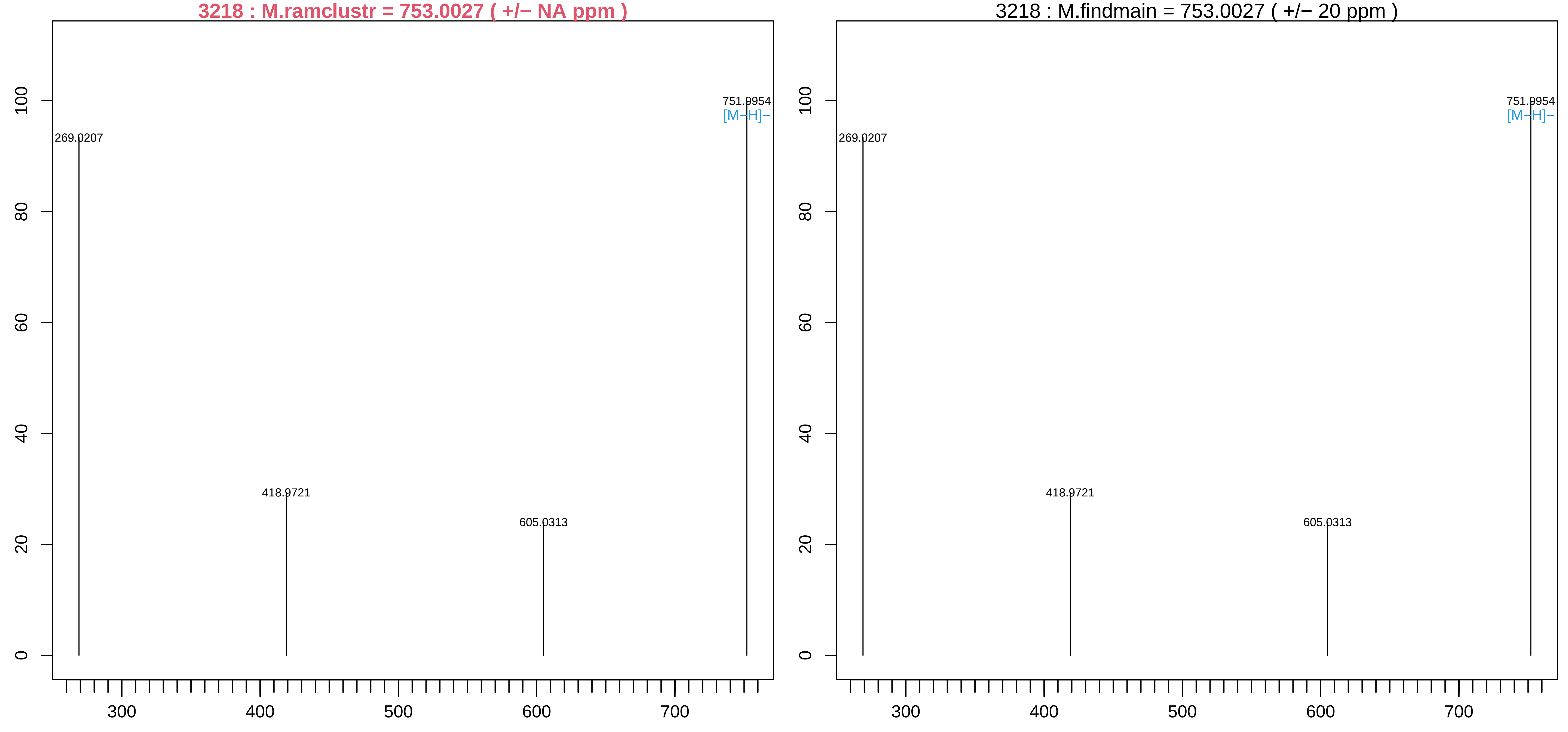
3218 : M.ramclustr = 753.0027 (+/− NA ppm)
269.0207
418.9721
605.0313
751.9954
[M−H]−
3218 : M.findmain = 753.0027 (+/− 20 ppm)
269.0207
418.9721
605.0313
751.9954
[M−H]−
0
20
40
60
80
100
300
400
500
600
700

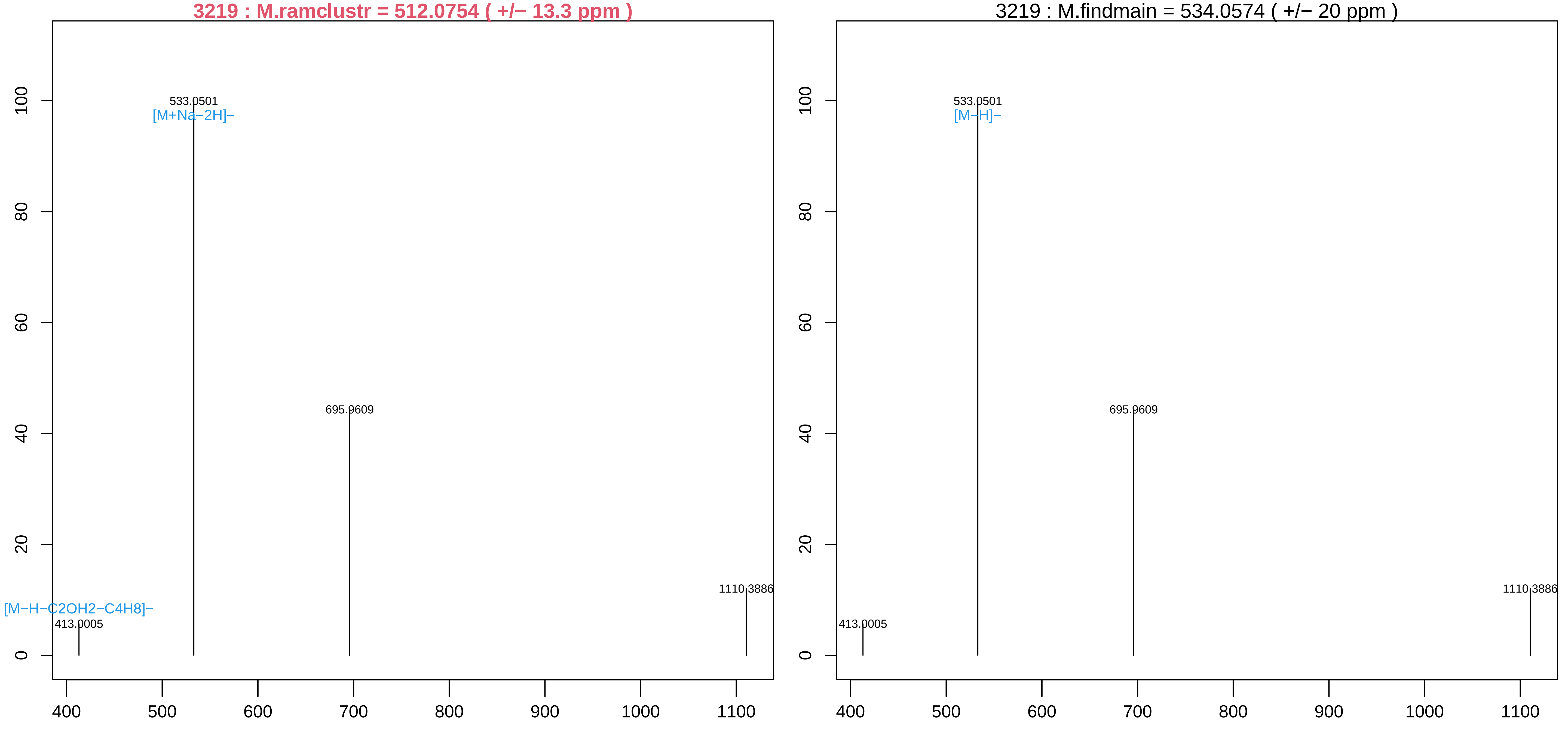
3219 : M.ramclustr = 512.0754 (+/− 13.3 ppm)
533.0501
[M+Na−2H]−
695.9609
1110.3886
[M−H−C2OH2−C4H8]−
413.0005
0
20
40
60
80
100
400
500
600
700
800
900
1000
1100
3219 : M.findmain = 534.0574 (+/− 20 ppm)
533.0501
[M−H]−
695.9609
1110.3886
413.0005

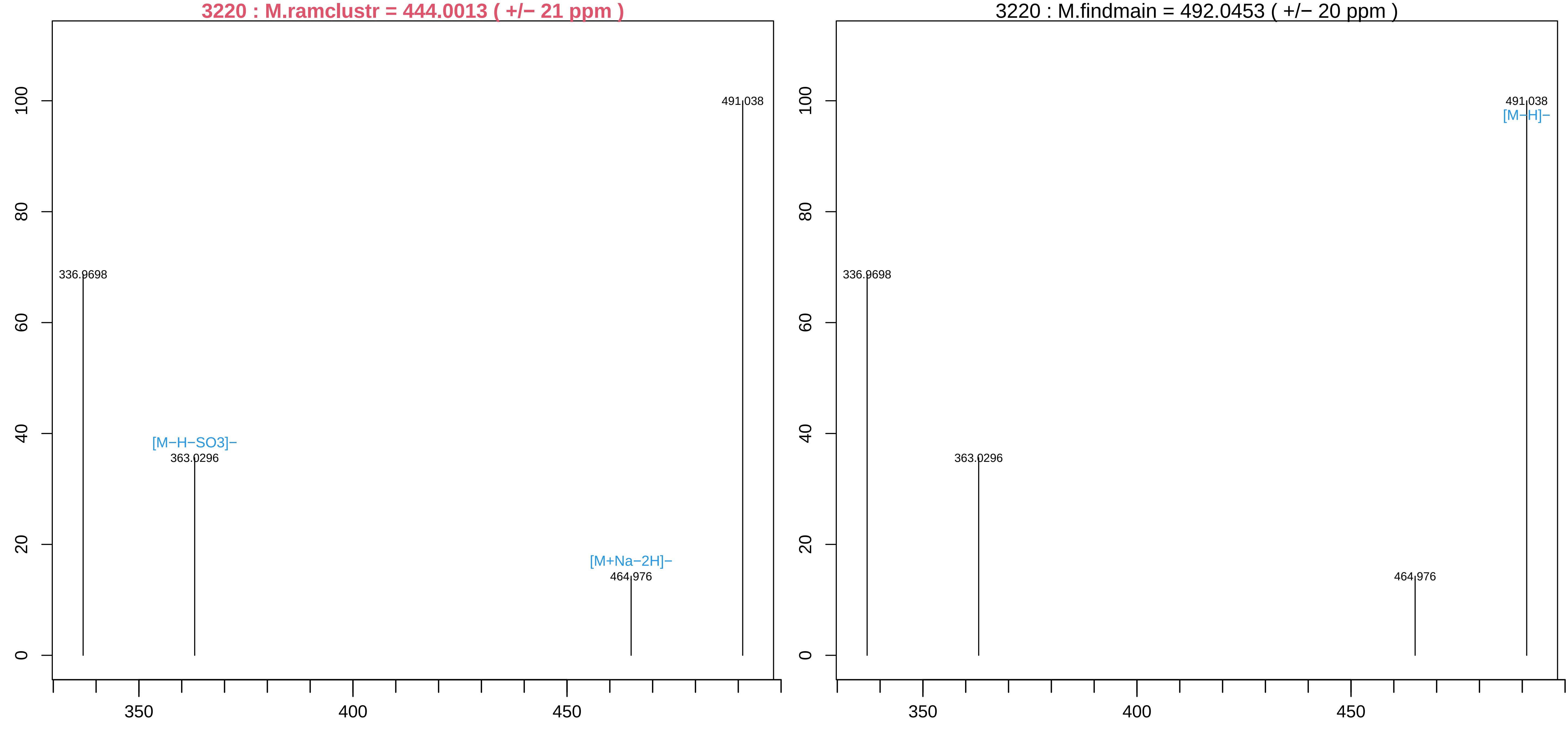
3220 : M.ramclustr = 444.0013 (+/− 21 ppm)
491.038
336.9698
[M−H−SO3]−
363.0296
[M+Na−2H]−
464.976
0
20
40
60
80
100
350
400
450
3220 : M.findmain = 492.0453 (+/− 20 ppm)
491.038
[M−H]−
336.9698
363.0296
464.976
0
20
40
60
80
100
350
400
450

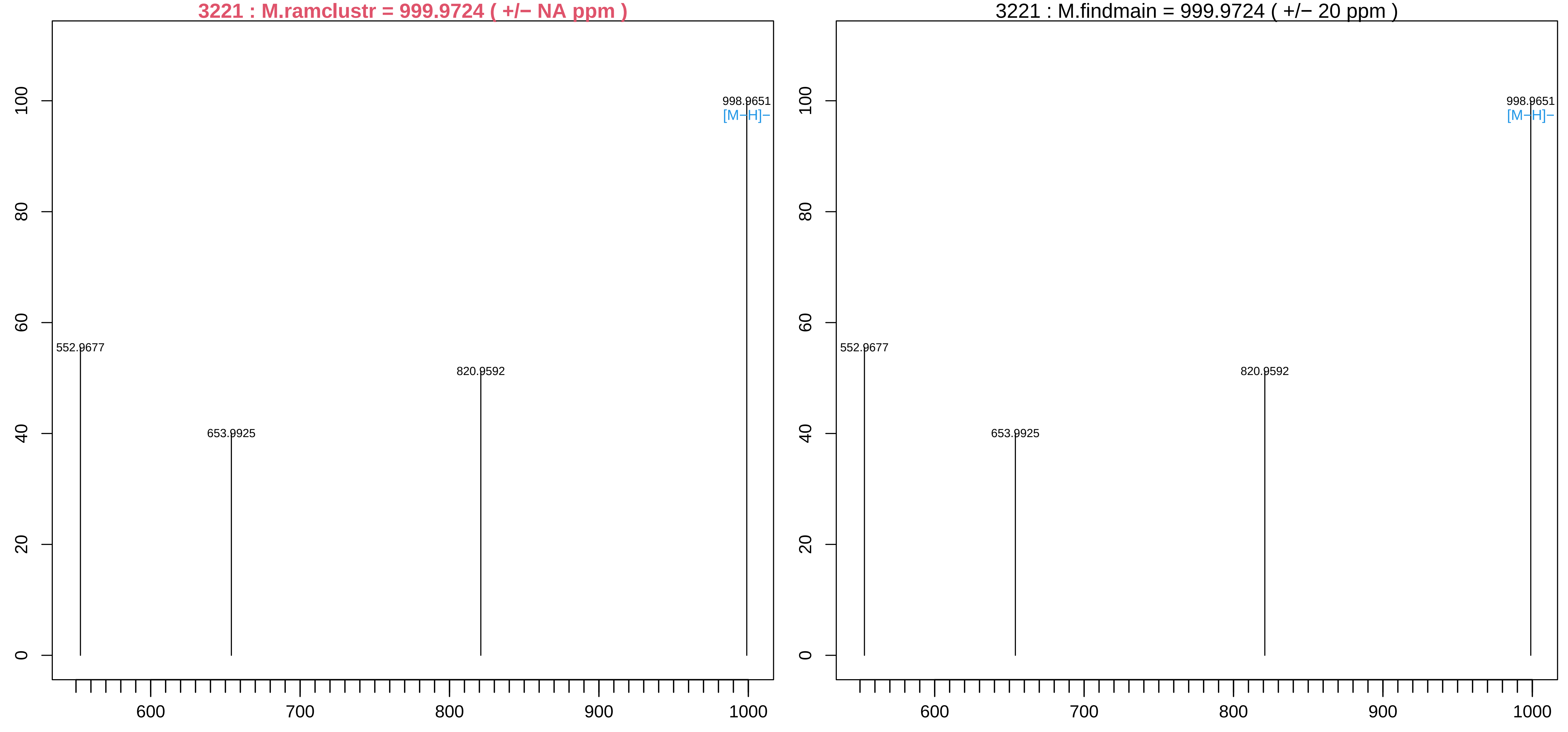
3221 : M.ramclustr = 999.9724 (+/− NA ppm)
552.9677
653.9925
820.9592
998.9651
[M−H]−
0
20
40
60
80
100
600
700
800
900
1000
3221 : M.findmain = 999.9724 (+/− 20 ppm)
552.9677
653.9925
820.9592
998.9651
[M−H]−
0
20
40
60
80
100
600
700
800
900
1000

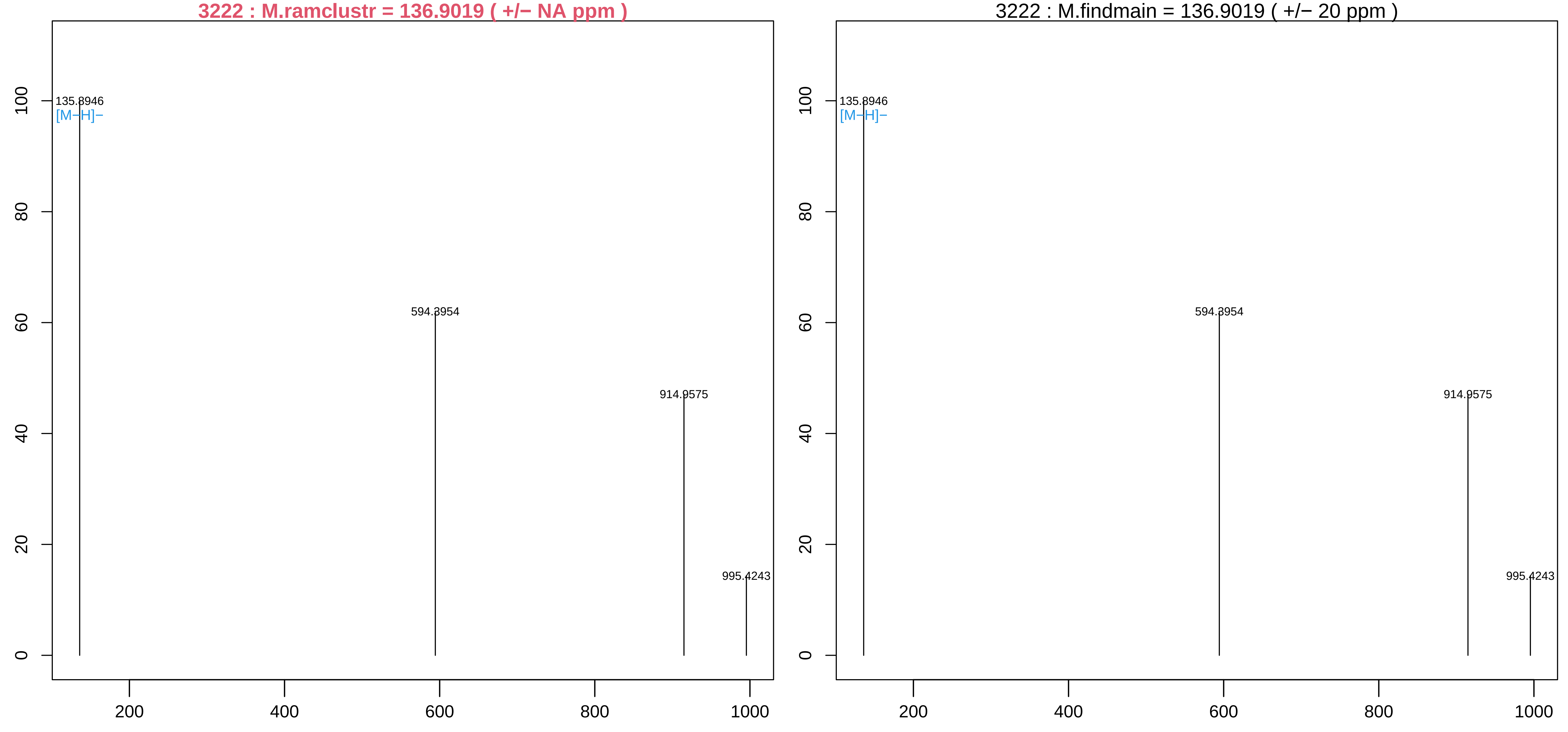
3222 : M.ramclustr = 136.9019 (+/− NA ppm)
135.8946
[M−H]−
594.3954
914.9575
995.4243
3222 : M.findmain = 136.9019 (+/− 20 ppm)
135.8946
[M−H]−
594.3954
914.9575
995.4243
0
20
40
60
80
100
200
400
600
800
1000

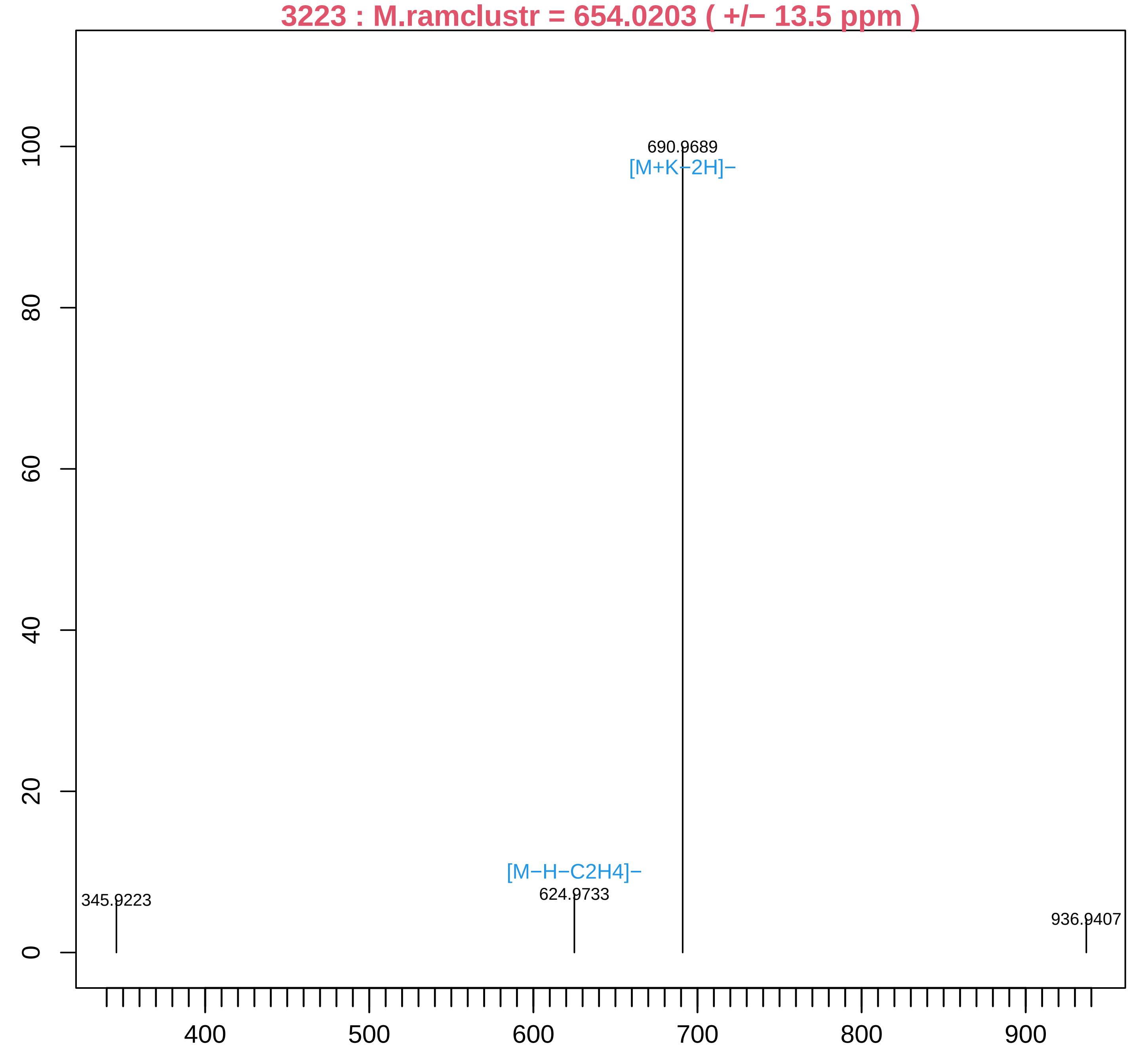
3223 : M.ramclustr = 654.0203 (+/− 13.5 ppm)
690.9689
[M+K−2H]−
[M−H−C2H4]−
345.9223
624.9733
936.9407
0
20
40
60
80
100
400
500
600
700
800
900

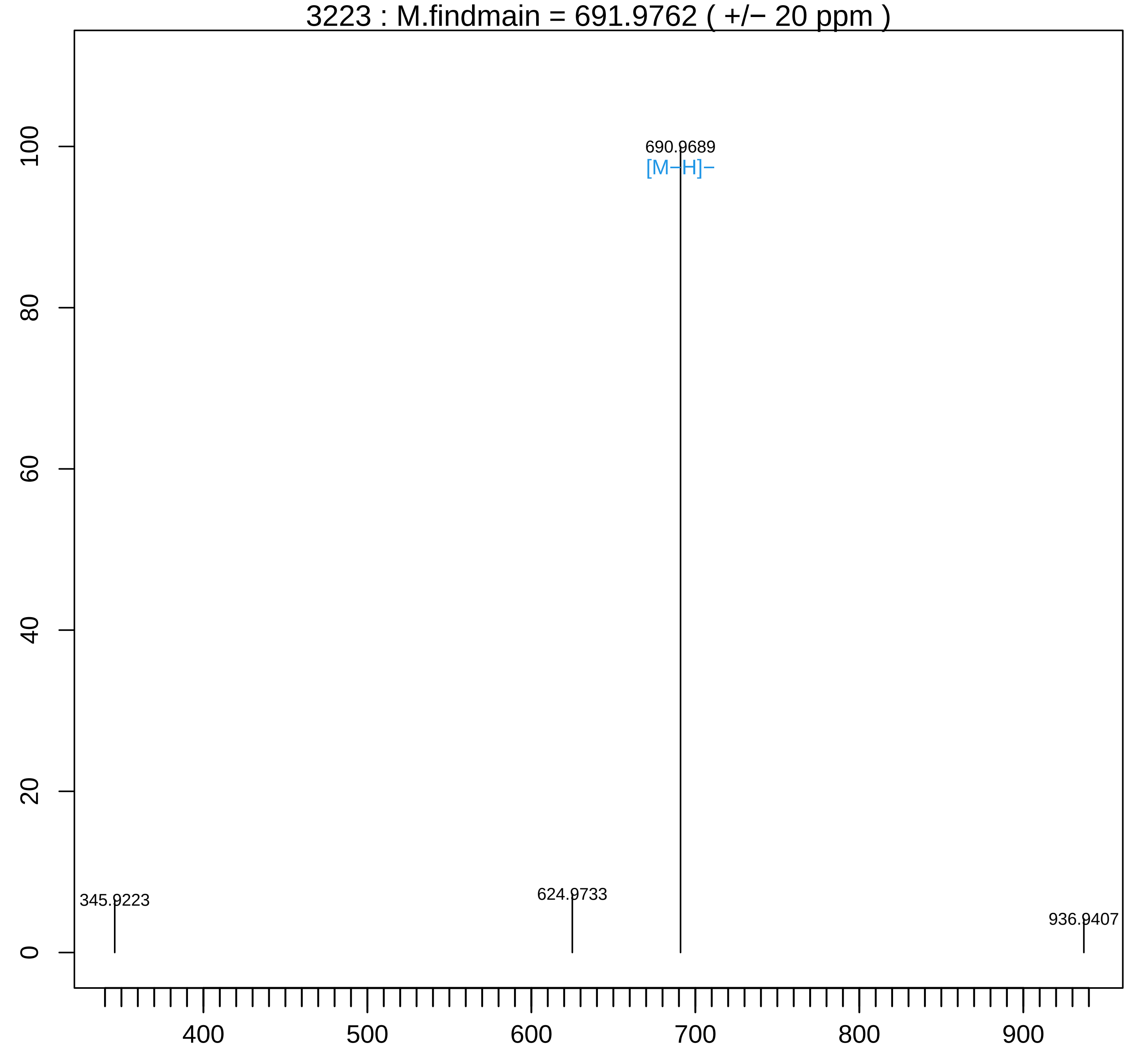
3223 : M.findmain = 691.9762 (+/− 20 ppm)
690.9689
[M−H]−
345.9223
624.9733
936.9407
0
20
40
60
80
100
400
500
600
700
800
900

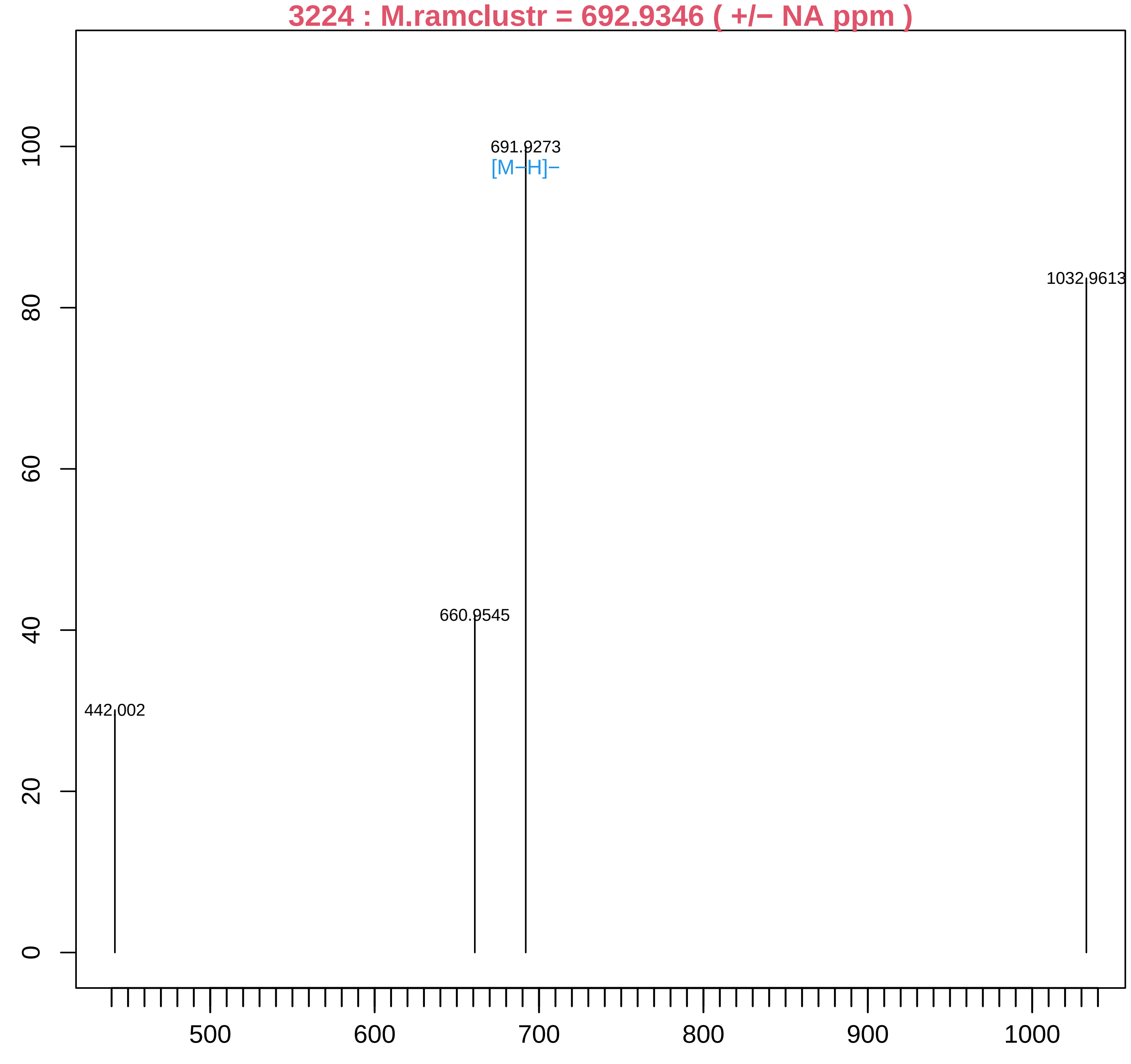

3224 : M.ramclustr = 692.9346 (+/− NA ppm)
691.9273
[M−H]−
1032.9613
660.9545
442.002
0
20
40
60
80
100
500
600
700
800
900
1000

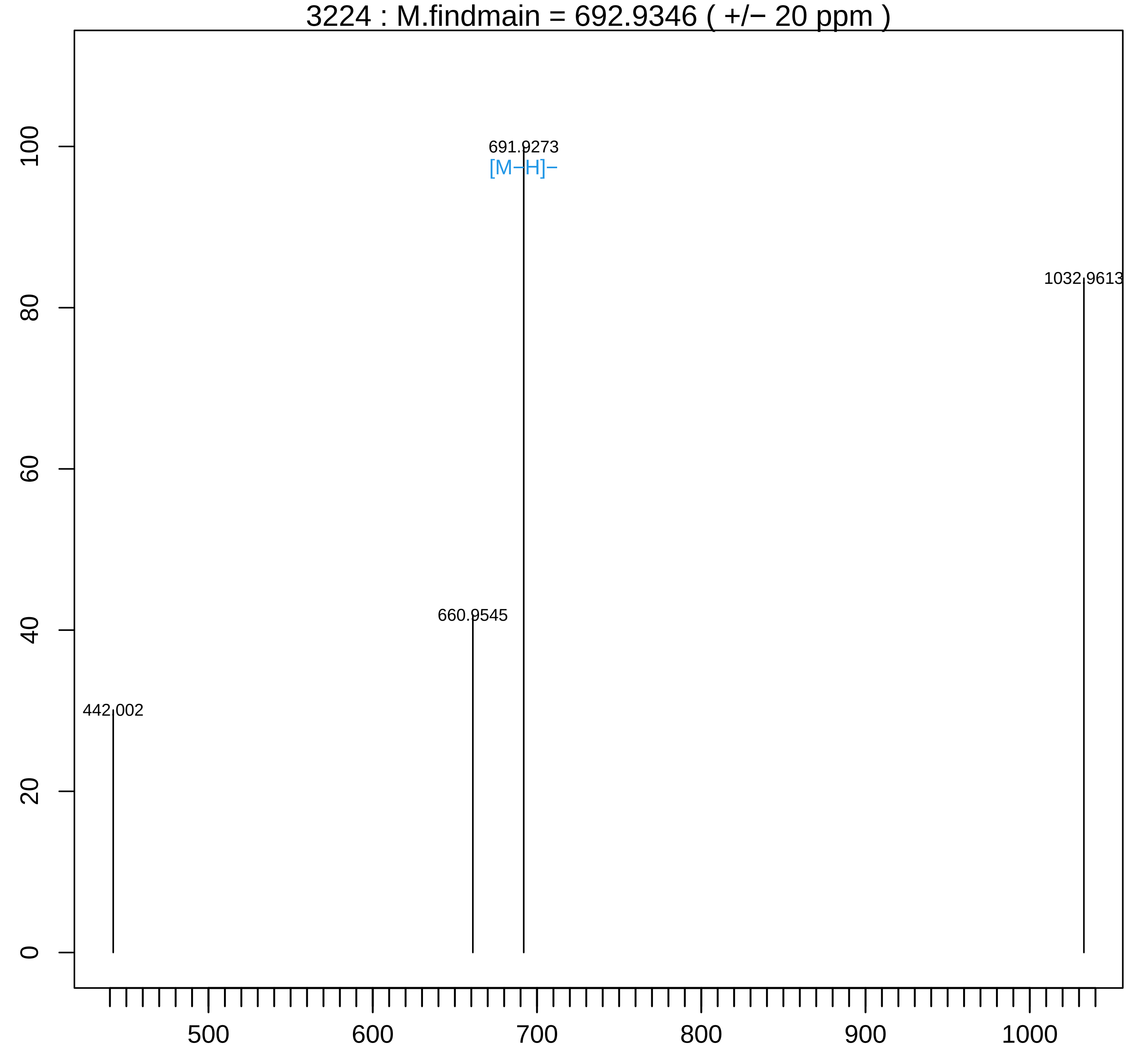

3224 : M.findmain = 692.9346 (+/− 20 ppm)
691.9273
[M−H]−
1032.9613
660.9545
442.002
0
20
40
60
80
100
500
600
700
800
900
1000

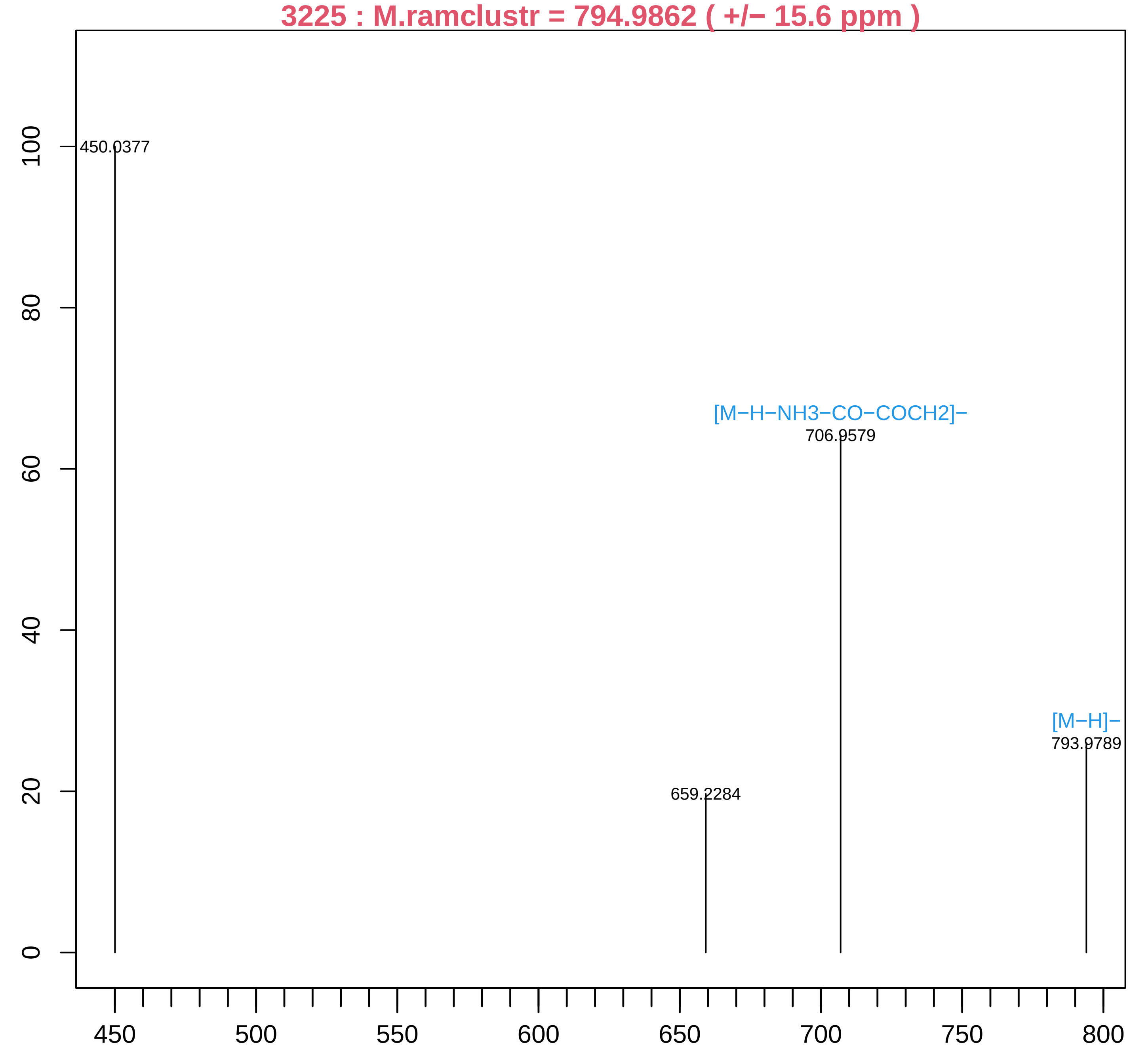

3225 : M.ramclustr = 794.9862 (+/− 15.6 ppm)
450.0377
[M−H−NH3−CO−COCH2]−
706.9579
[M−H]−
793.9789
659.2284
0
20
40
60
80
100
450
500
550
600
650
700
750
800

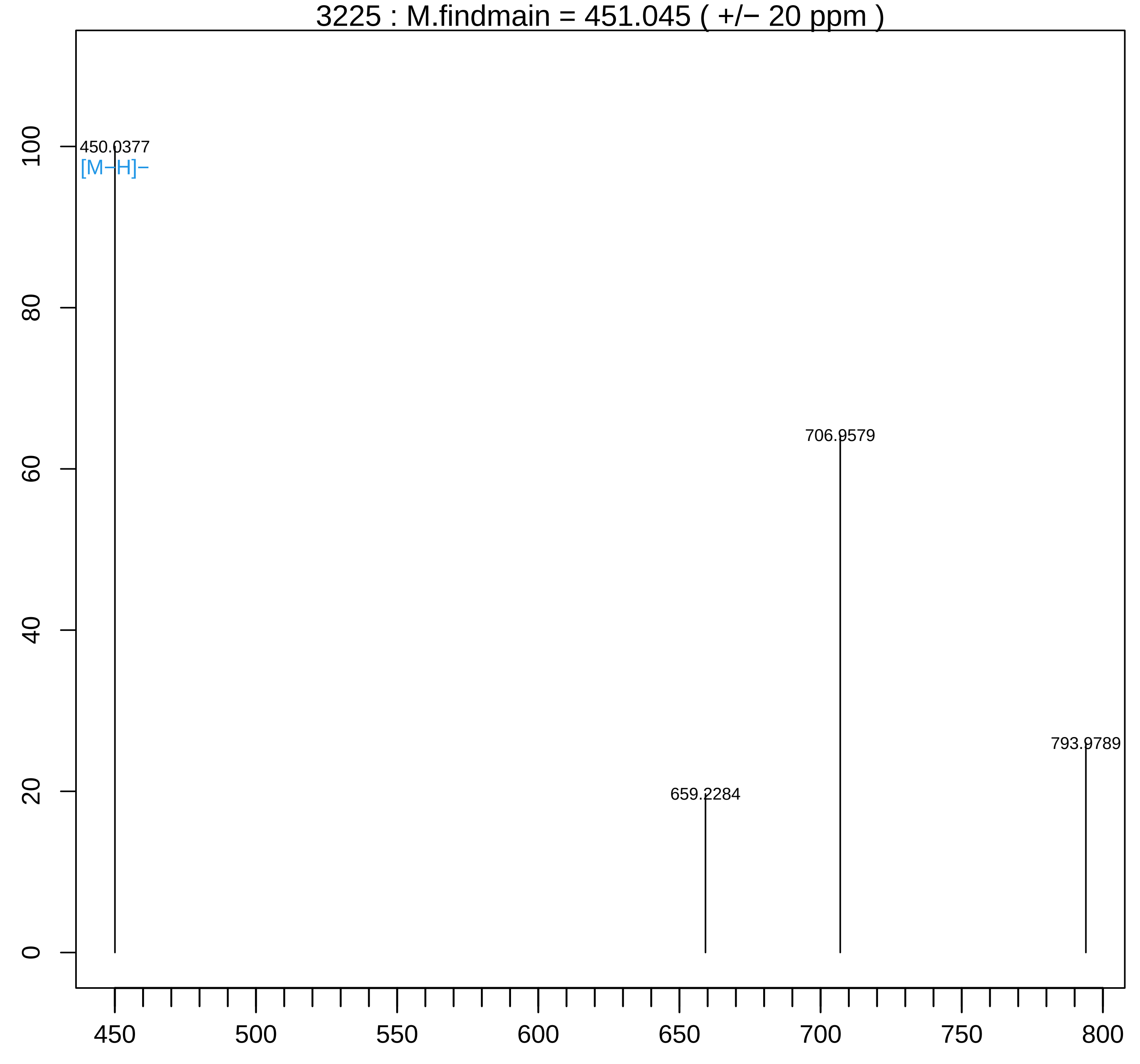

3225 : M.findmain = 451.045 (+/− 20 ppm)
450.0377
[M−H]−
706.9579
659.2284
793.9789
0
20
40
60
80
100
450
500
550
600
650
700
750
800

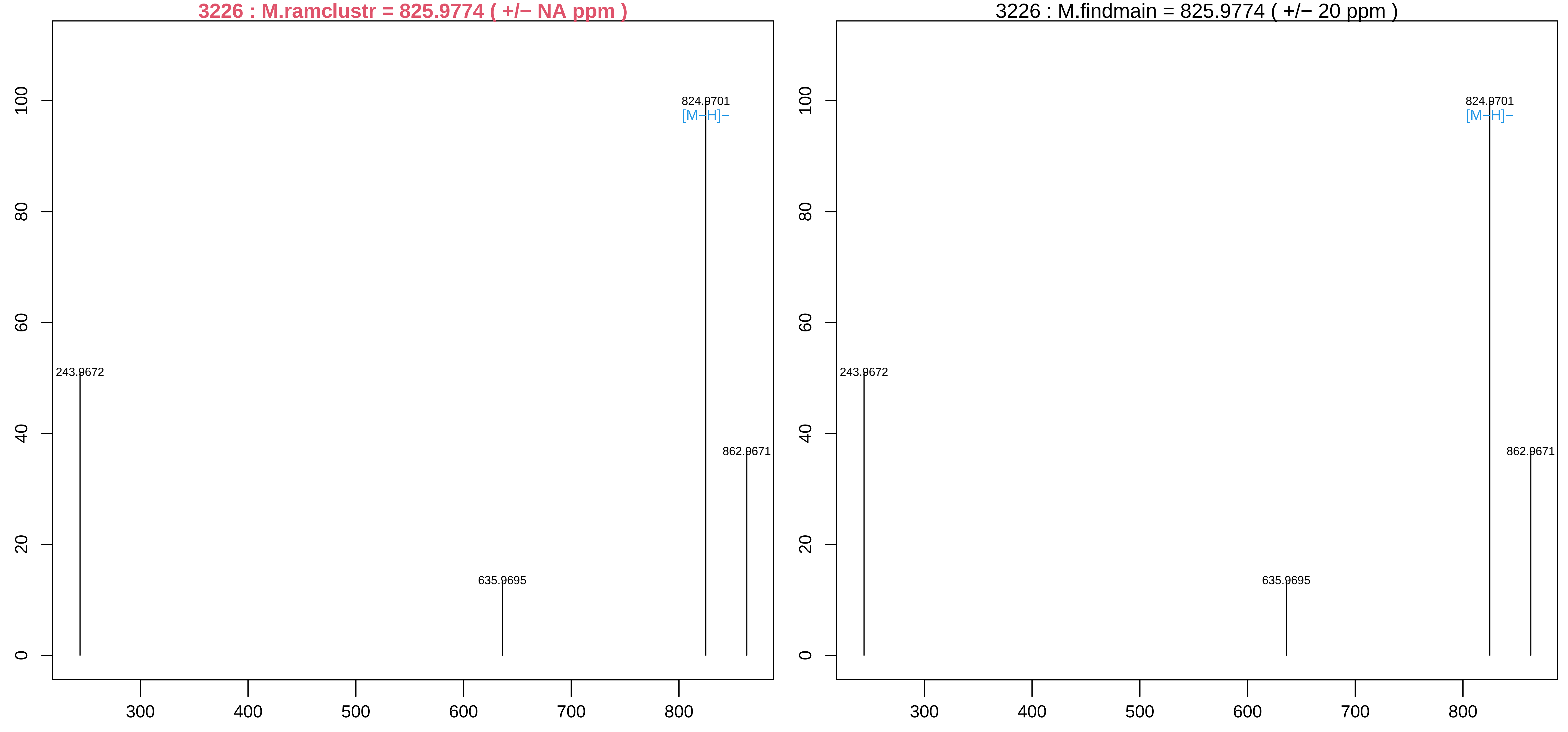
3226 : M.ramclustr = 825.9774 (+/− NA ppm)
3226 : M.findmain = 825.9774 (+/− 20 ppm)
243.9672
635.9695
824.9701
[M−H]−
862.9671
0
20
40
60
80
100
300
400
500
600
700
800

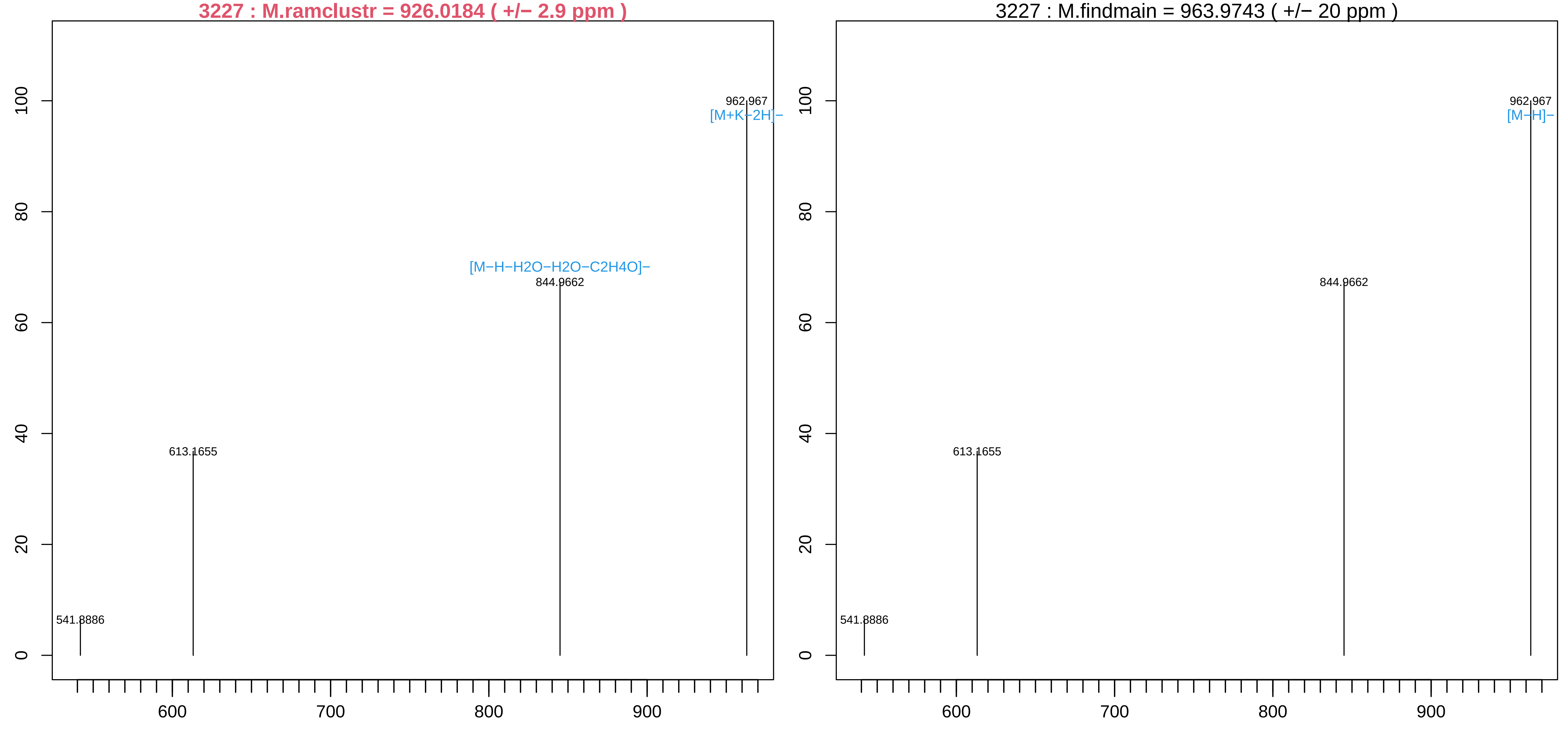
3227 : M.ramclustr = 926.0184 (+/− 2.9 ppm)
962.967
[M+K−2H]−
[M−H−H2O−H2O−C2H4O]−
844.9662
613.1655
541.8886
3227 : M.findmain = 963.9743 (+/− 20 ppm)
962.967
[M−H]−
844.9662
613.1655
541.8886

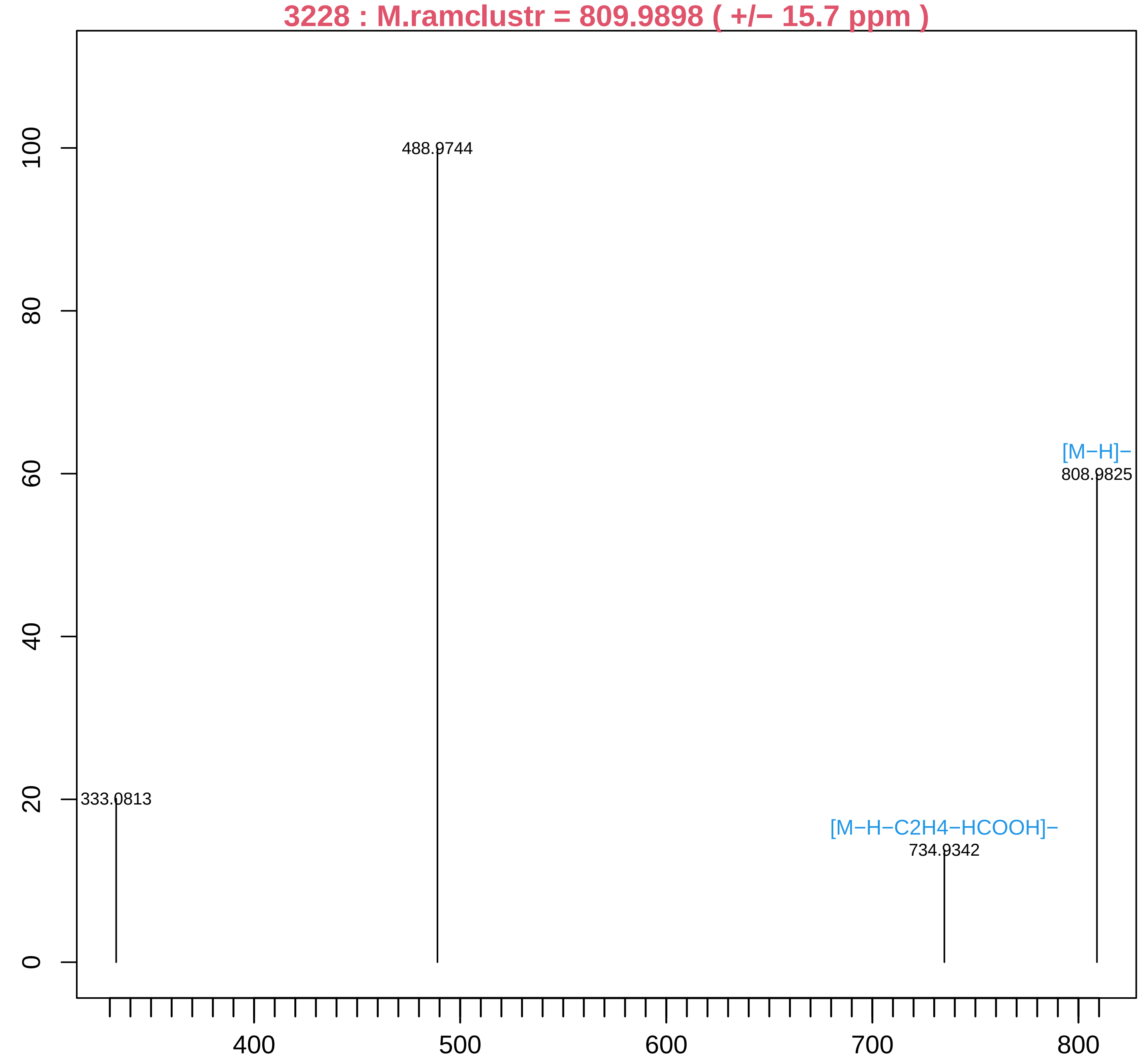
3228 : M.ramclustr = 809.9898 (+/− 15.7 ppm)
488.9744
[M−H]−
808.9825
333.0813
[M−H−C2H4−HCOOH]−
734.9342
0
20
40
60
80
100
400
500
600
700
800

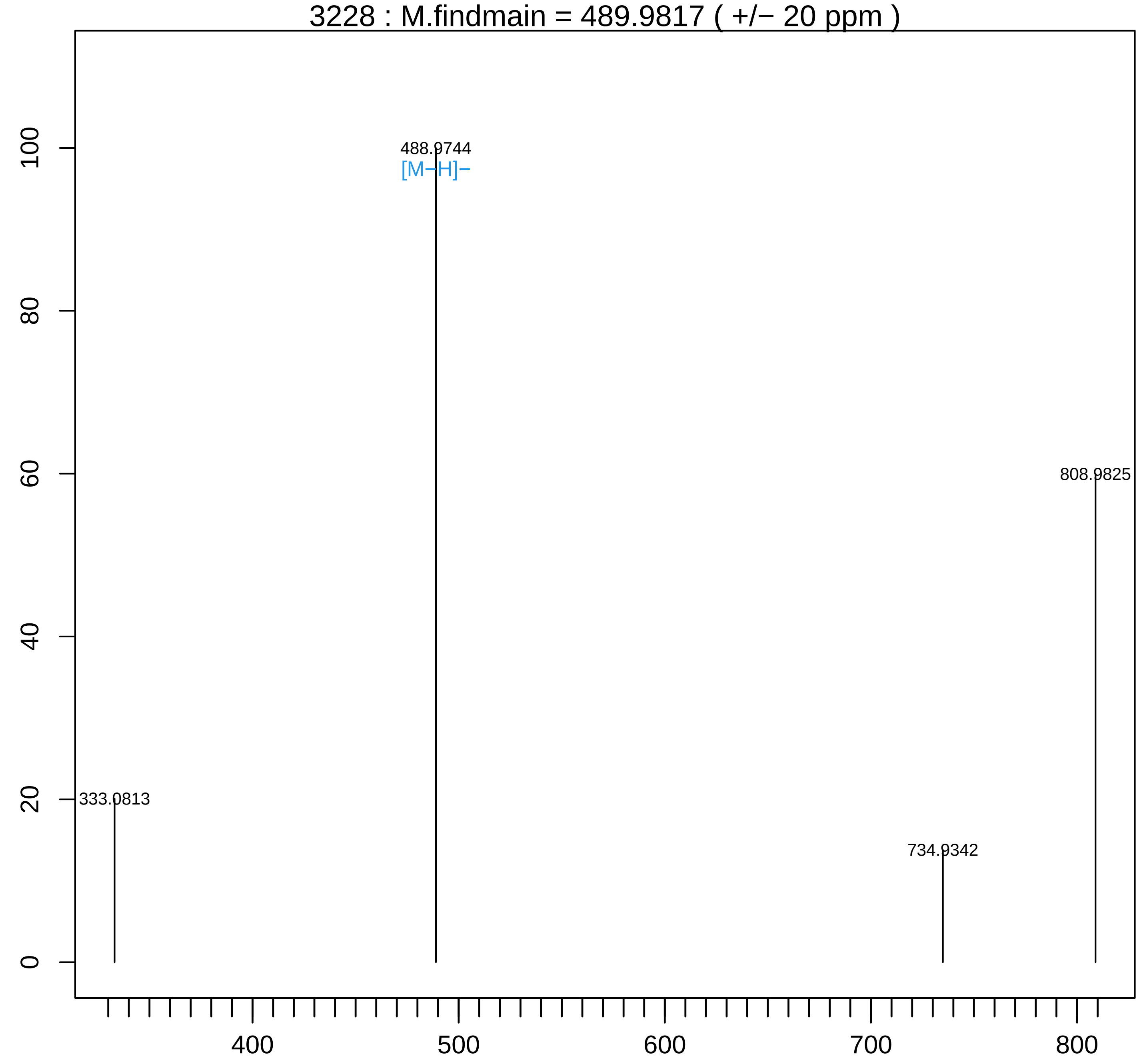
3228 : M.findmain = 489.9817 (+/− 20 ppm)
488.9744
[M−H]−
808.9825
333.0813
734.9342
0
20
40
60
80
100
400
500
600
700
800

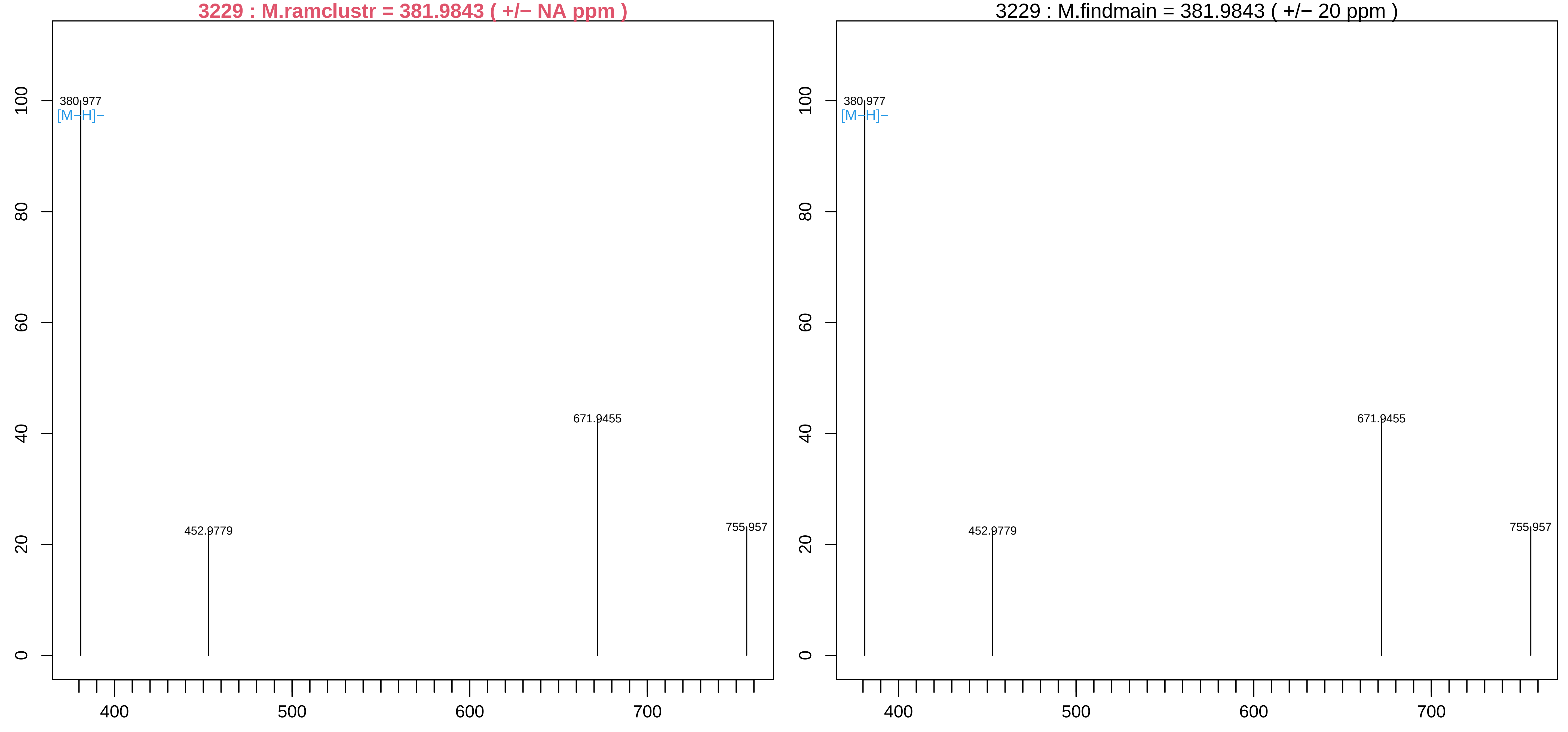

3229 : M.ramclustr = 381.9843 (+/− NA ppm)
380.977
[M−H]−
452.9779
671.9455
755.957
0
20
40
60
80
100
400
500
600
700
3229 : M.findmain = 381.9843 (+/− 20 ppm)
380.977
[M−H]−
452.9779
671.9455
755.957
0
20
40
60
80
100
400
500
600
700

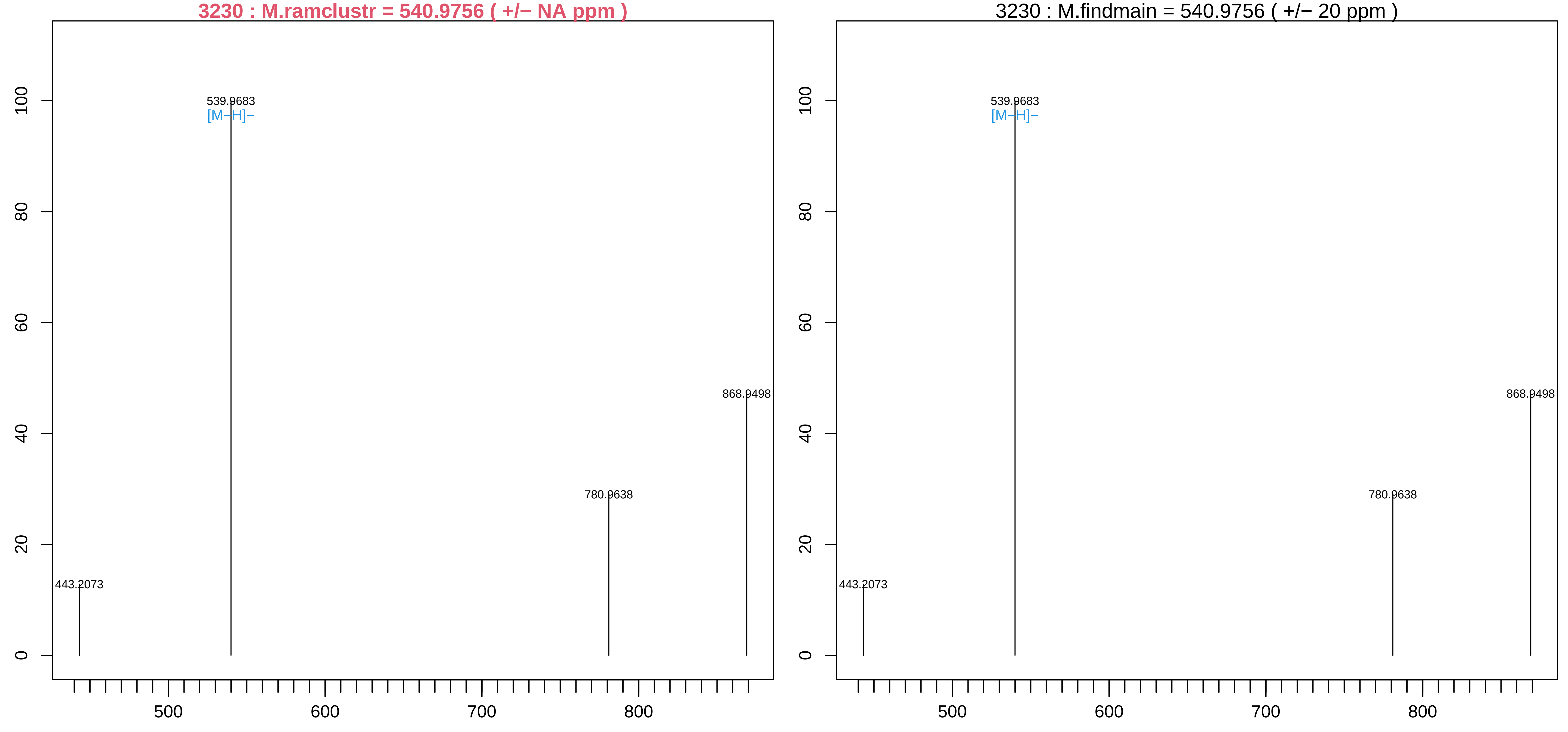
3230 : M.ramclustr = 540.9756 (+/− NA ppm)
539.9683
[M−H]−
868.9498
780.9638
443.2073
3230 : M.findmain = 540.9756 (+/− 20 ppm)
539.9683
[M−H]−
868.9498
780.9638
443.2073
0
20
40
60
80
100
500
600
700
800

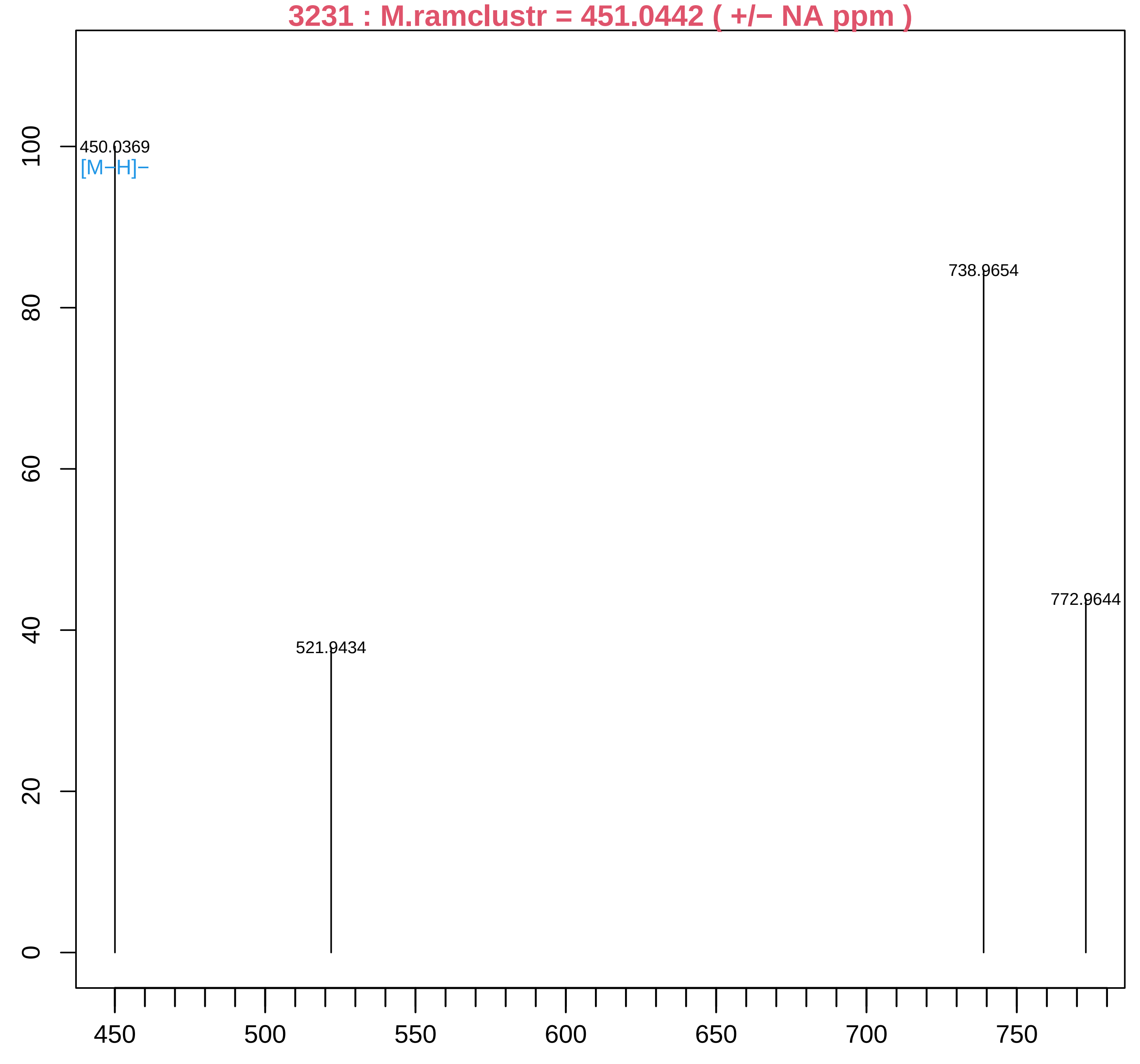
3231 : M.ramclustr = 451.0442 (+/− NA ppm)
450.0369
[M−H]−
521.9434
738.9654
772.9644
0
20
40
60
80
100
450
500
550
600
650
700
750

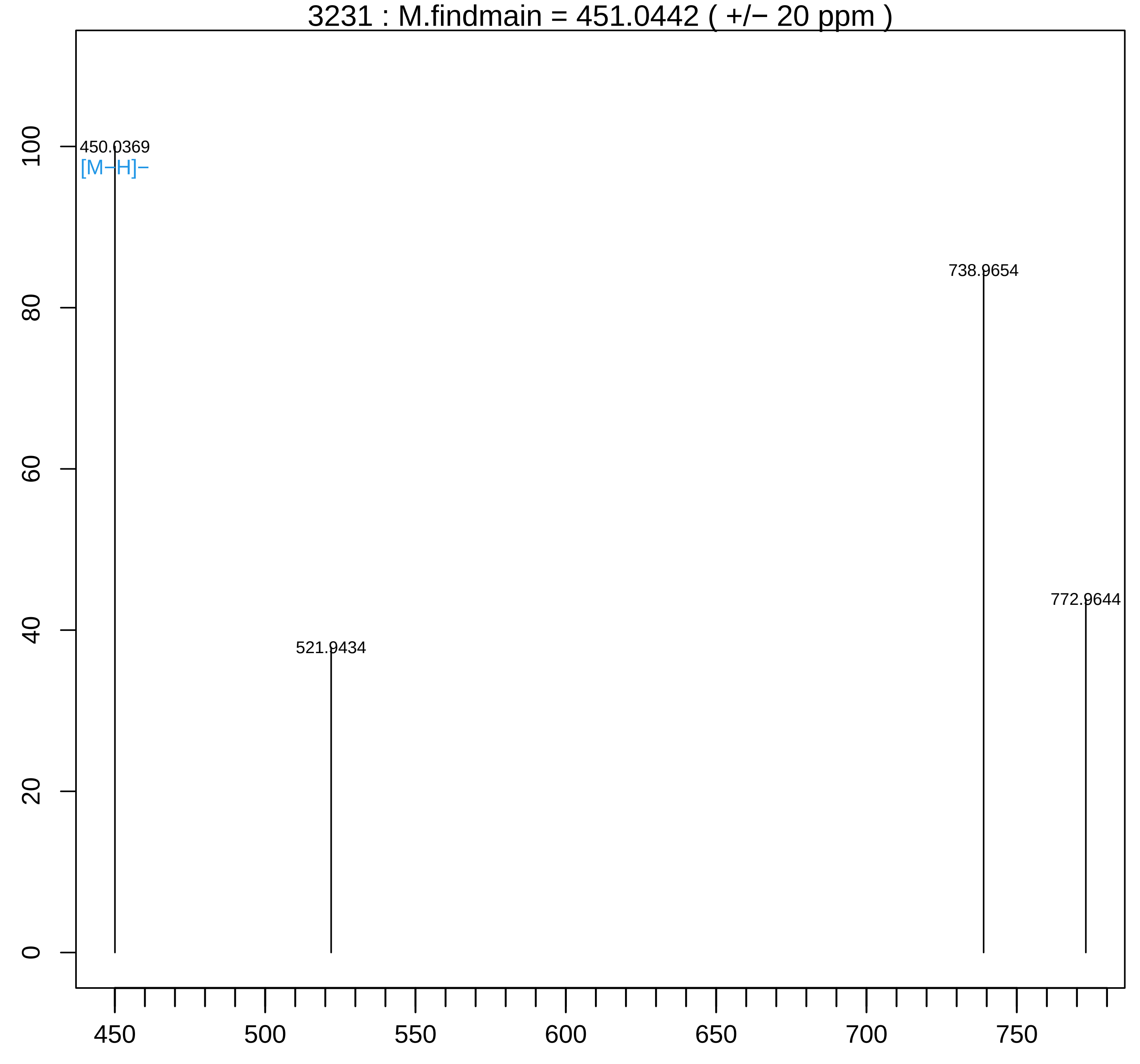
3231 : M.findmain = 451.0442 (+/− 20 ppm)
450.0369
[M−H]−
521.9434
738.9654
772.9644
0
20
40
60
80
100
450
500
550
600
650
700
750

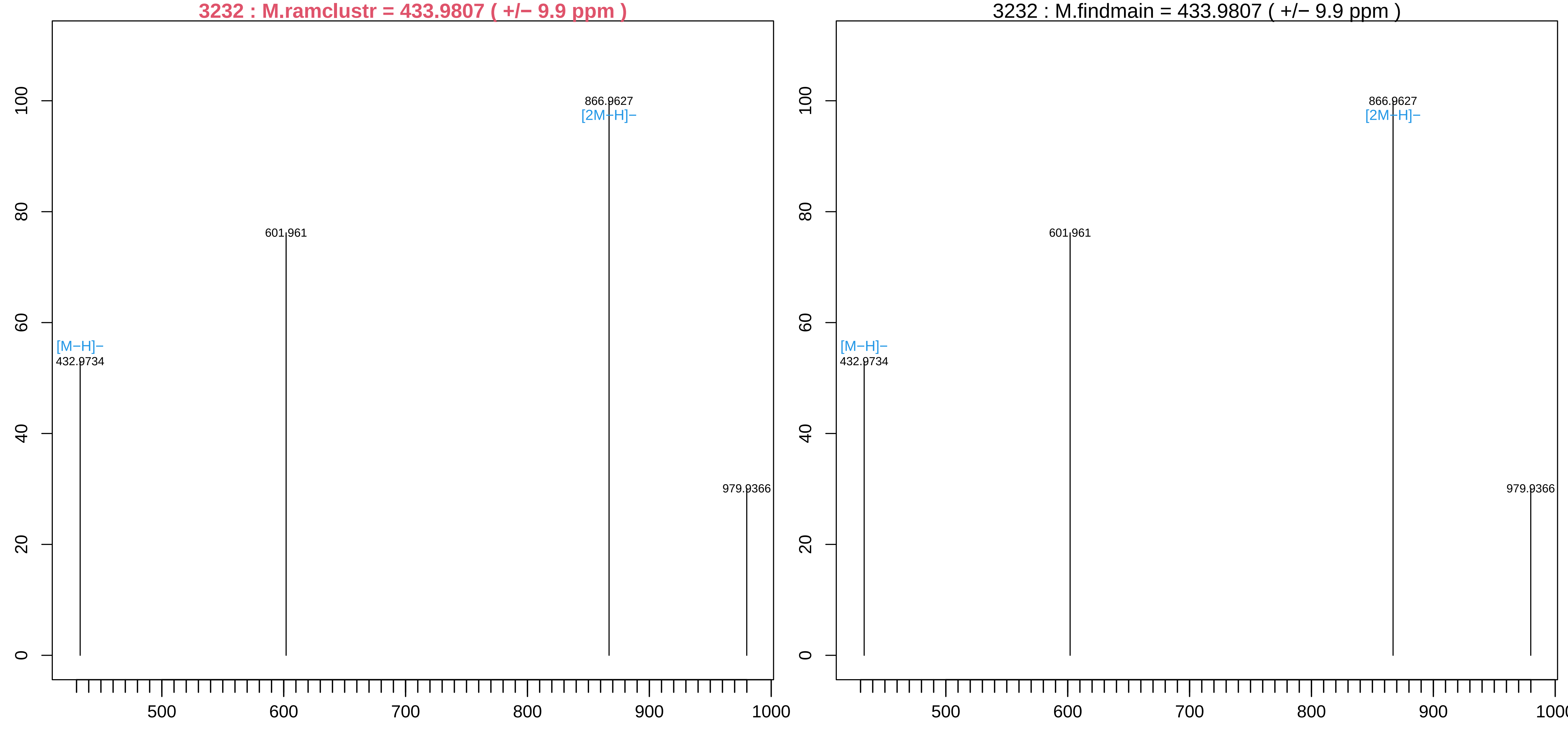
3232 : M.ramclustr = 433.9807 (+/− 9.9 ppm)
[M−H]−
432.9734
601.961
866.9627
[2M−H]−
979.9366
3232 : M.findmain = 433.9807 (+/− 9.9 ppm)
[M−H]−
432.9734
601.961
866.9627
[2M−H]−
979.9366

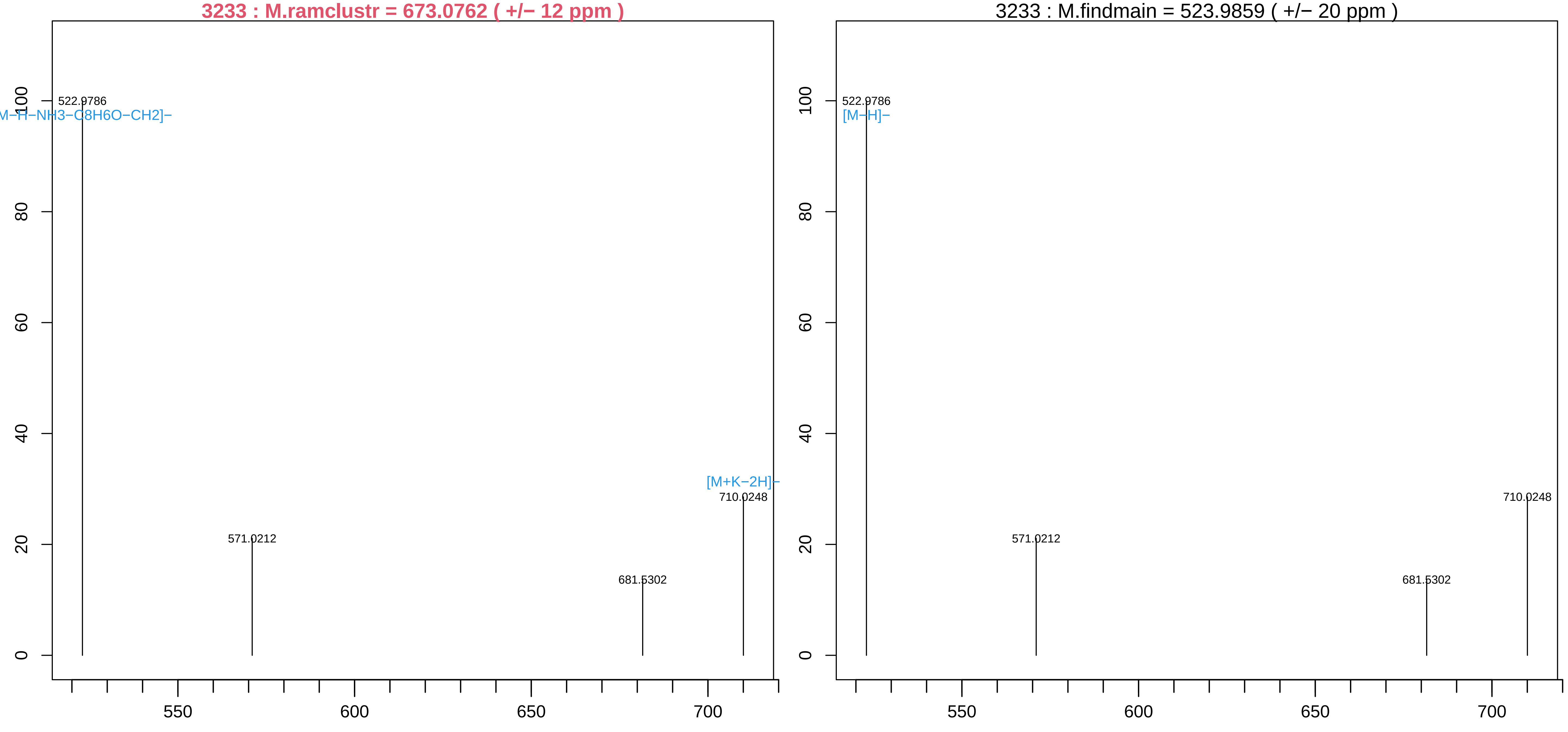
3233 : M.ramclustr = 673.0762 (+/− 12 ppm)
522.9786
[M−H−NH3−C8H6O−CH2]−
571.0212
681.5302
710.0248
[M+K−2H]−
3233 : M.findmain = 523.9859 (+/− 20 ppm)
522.9786
[M−H]−
571.0212
681.5302
710.0248
0
20
40
60
80
100
550
600
650
700

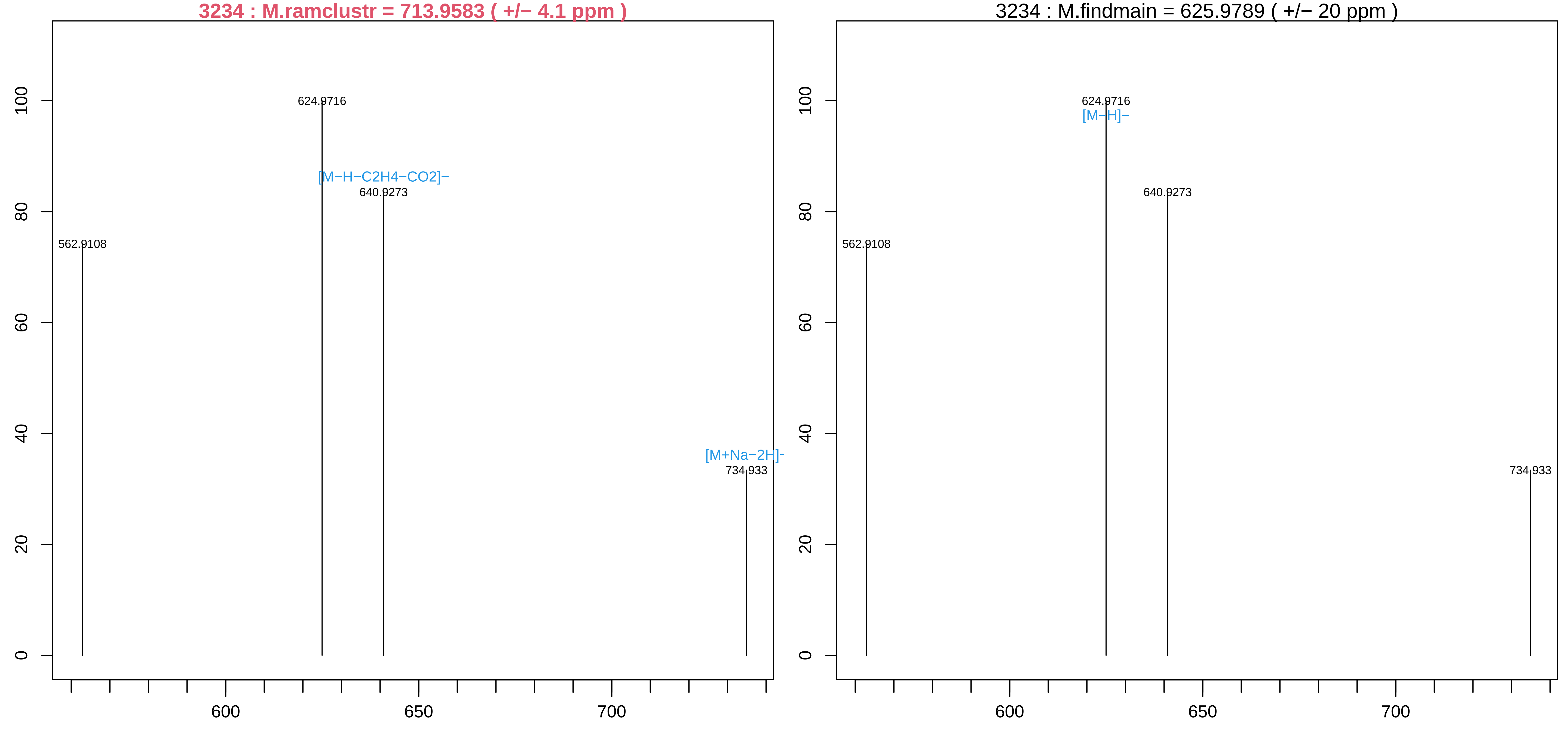
3234 : M.ramclustr = 713.9583 (+/− 4.1 ppm)
624.9716
[M−H−C2H4−CO2]−
640.9273
562.9108
[M+Na−2H]-
734.933
0
20
40
60
80
100
600
650
700
3234 : M.findmain = 625.9789 (+/− 20 ppm)
624.9716
[M−H]−
640.9273
562.9108
734.933
0
20
40
60
80
100
600
650
700

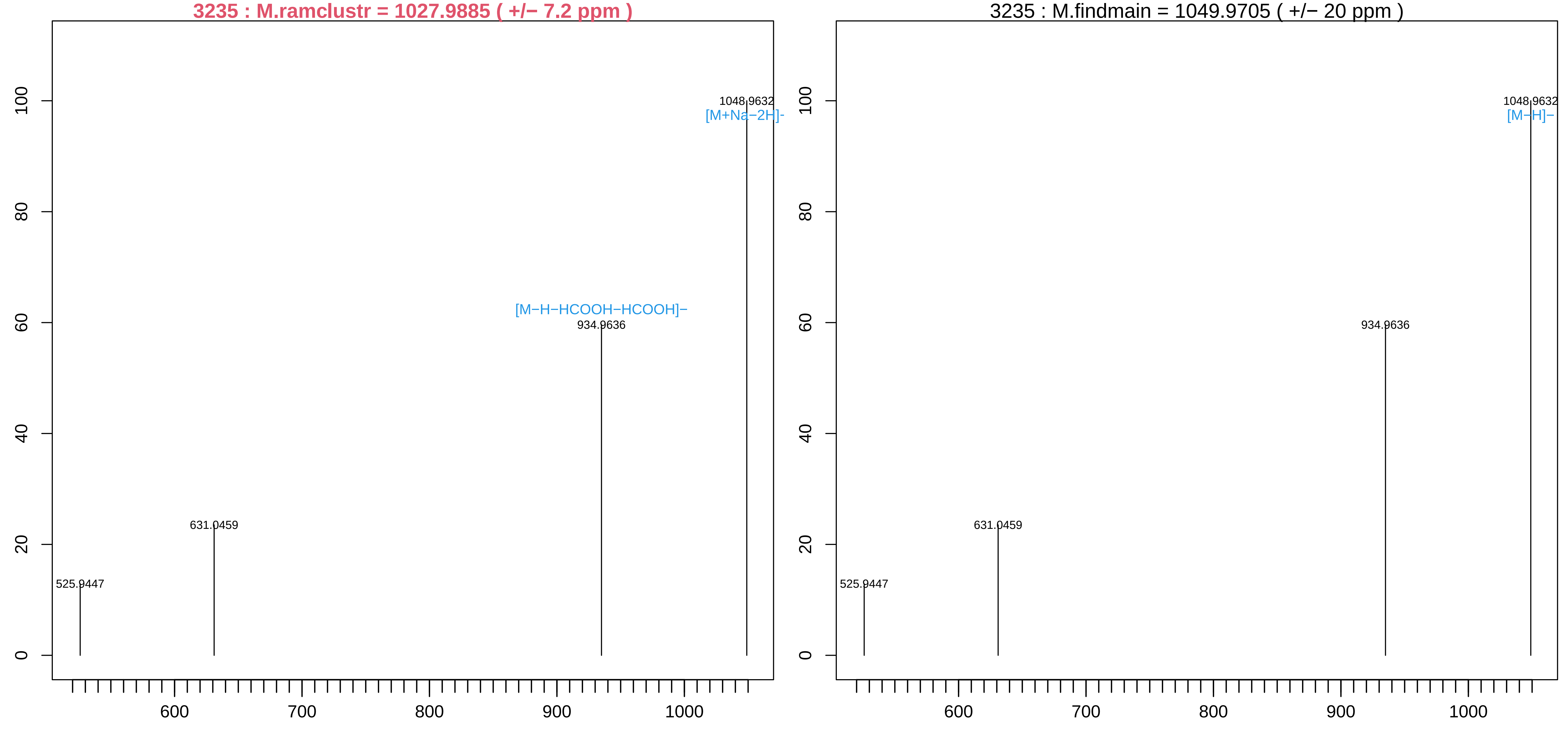
3235 : M.ramclustr = 1027.9885 (+/− 7.2 ppm)
1048.9632
[M+Na−2H]-
[M−H−HCOOH−HCOOH]−
934.9636
631.0459
525.9447
3235 : M.findmain = 1049.9705 (+/− 20 ppm)
1048.9632
[M−H]−
934.9636
631.0459
525.9447

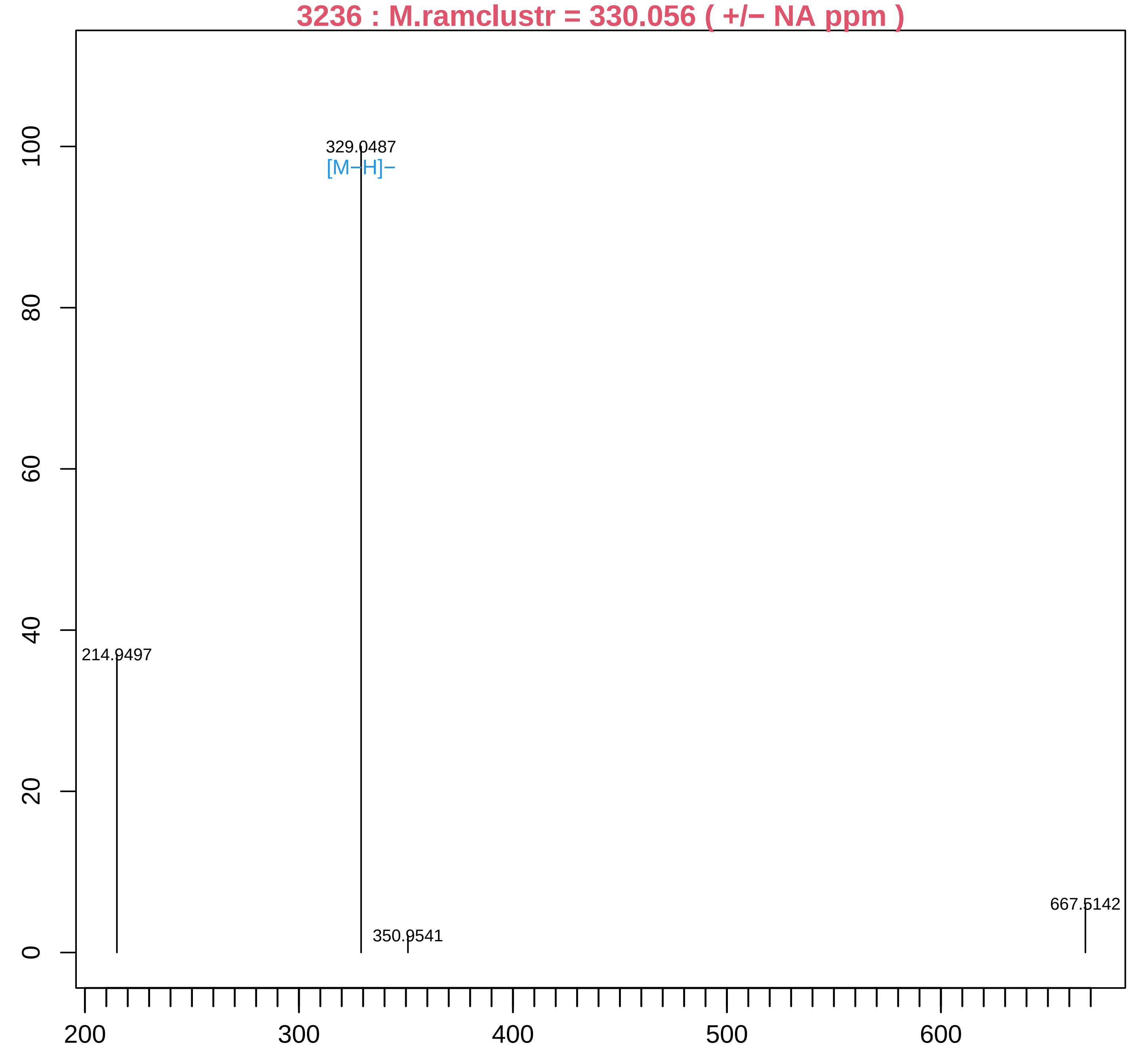
3236 : M.ramclustr = 330.056 (+/− NA ppm)
329.0487
[M−H]−
214.9497
350.9541
667.5142
0
20
40
60
80
100
200
300
400
500
600

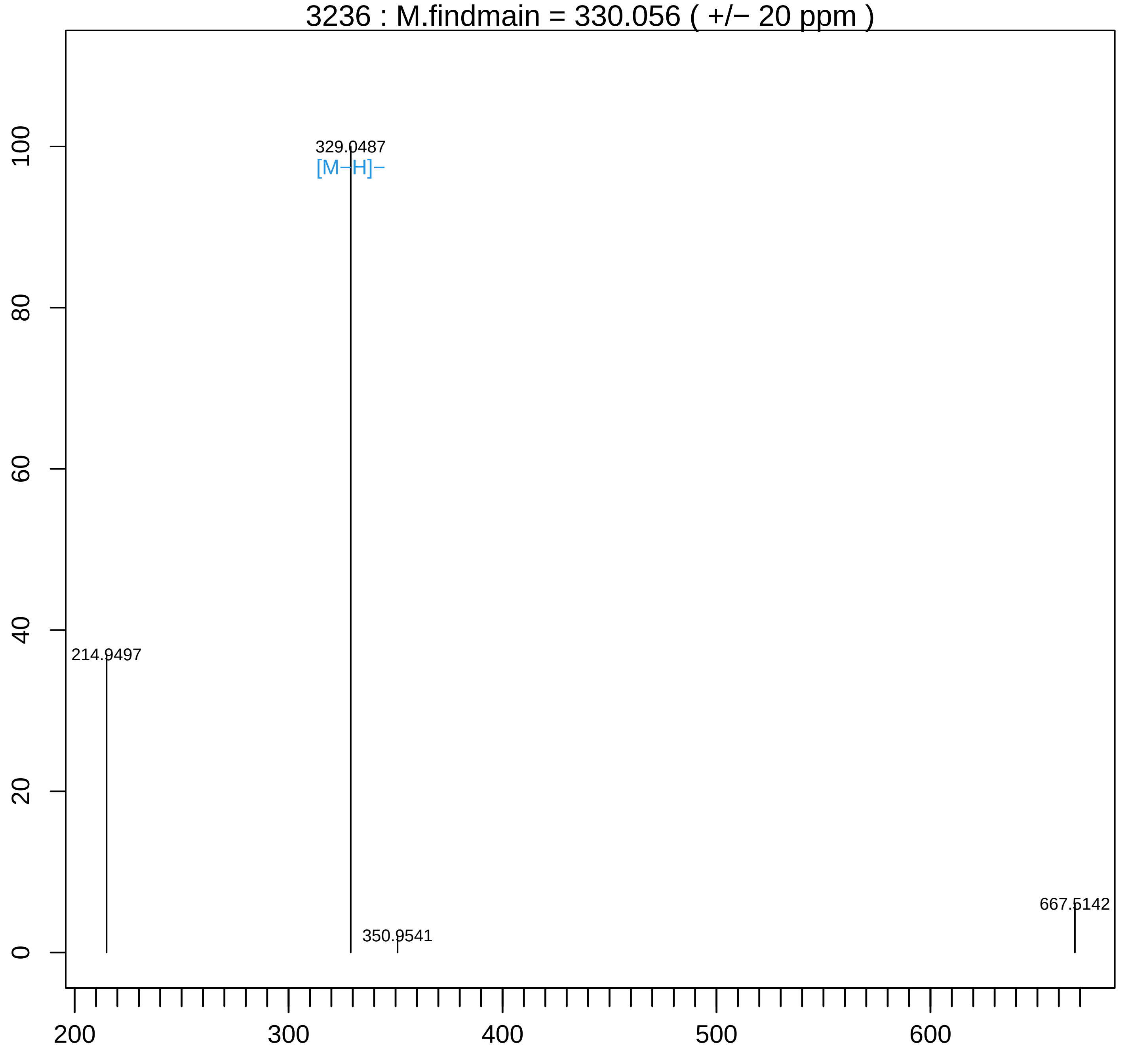
3236 : M.findmain = 330.056 (+/− 20 ppm)
329.0487
[M−H]−
214.9497
350.9541
667.5142
0
20
40
60
80
100
200
300
400
500
600

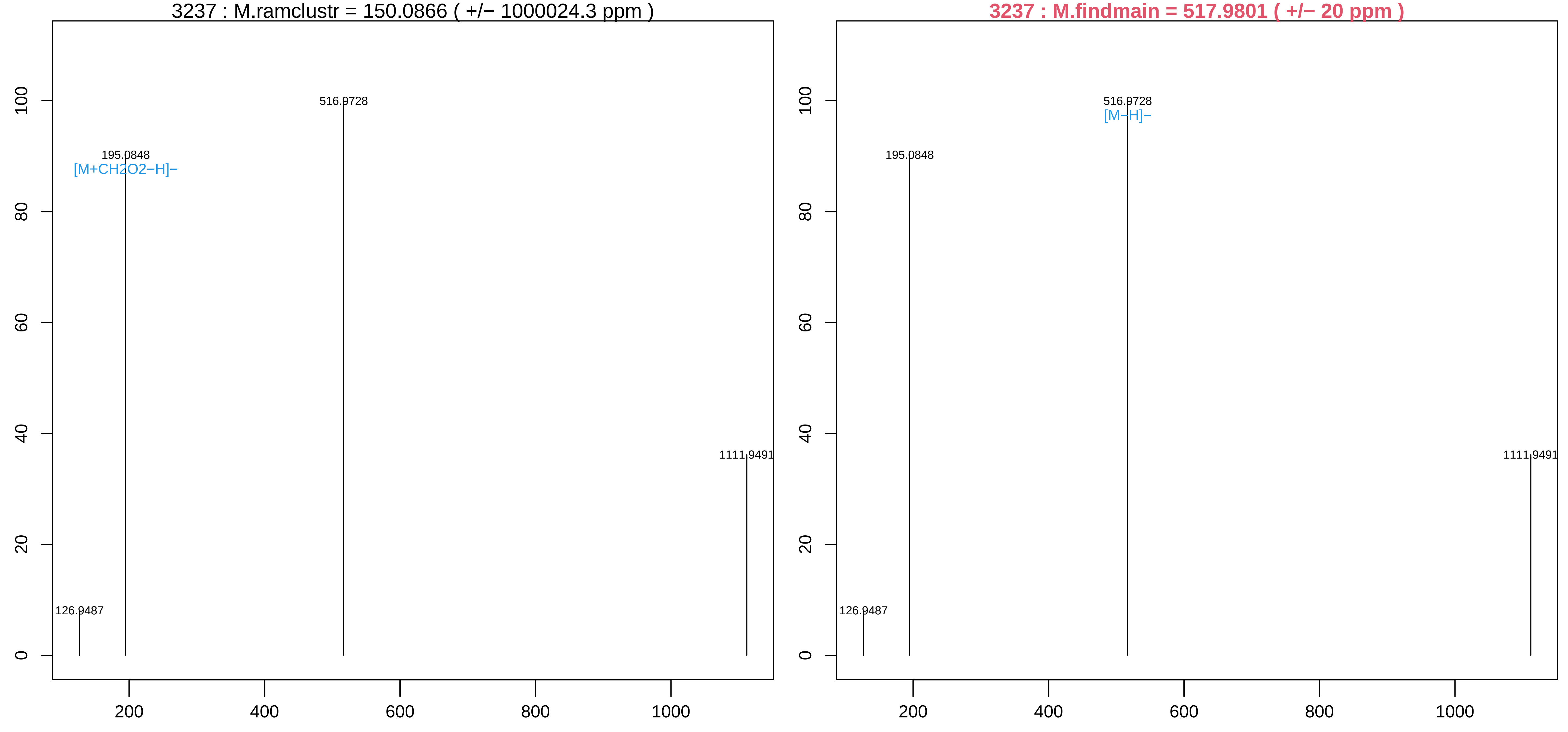

3237 : M.ramclustr = 150.0866 (+/− 1000024.3 ppm)
516.9728
195.0848
[M+CH2O2−H]−
1111.9491
126.9487
3237 : M.findmain = 517.9801 (+/− 20 ppm)
516.9728
[M−H]−
195.0848
1111.9491
126.9487
0
20
40
60
80
100
200
400
600
800
1000

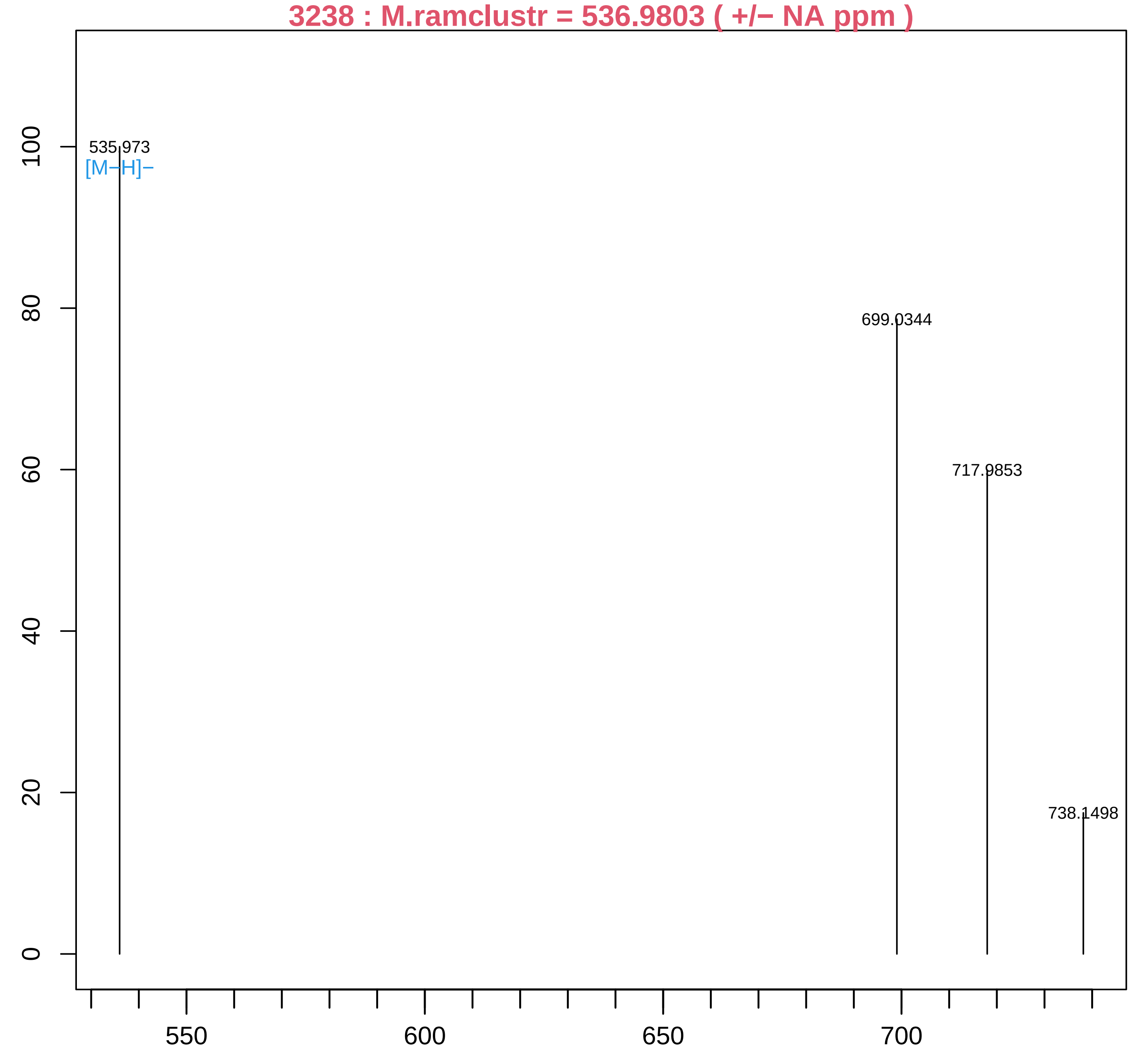
3238 : M.ramclustr = 536.9803 (+/− NA ppm)
535.973
[M−H]−
699.0344
717.9853
738.1498
0
20
40
60
80
100
550
600
650
700
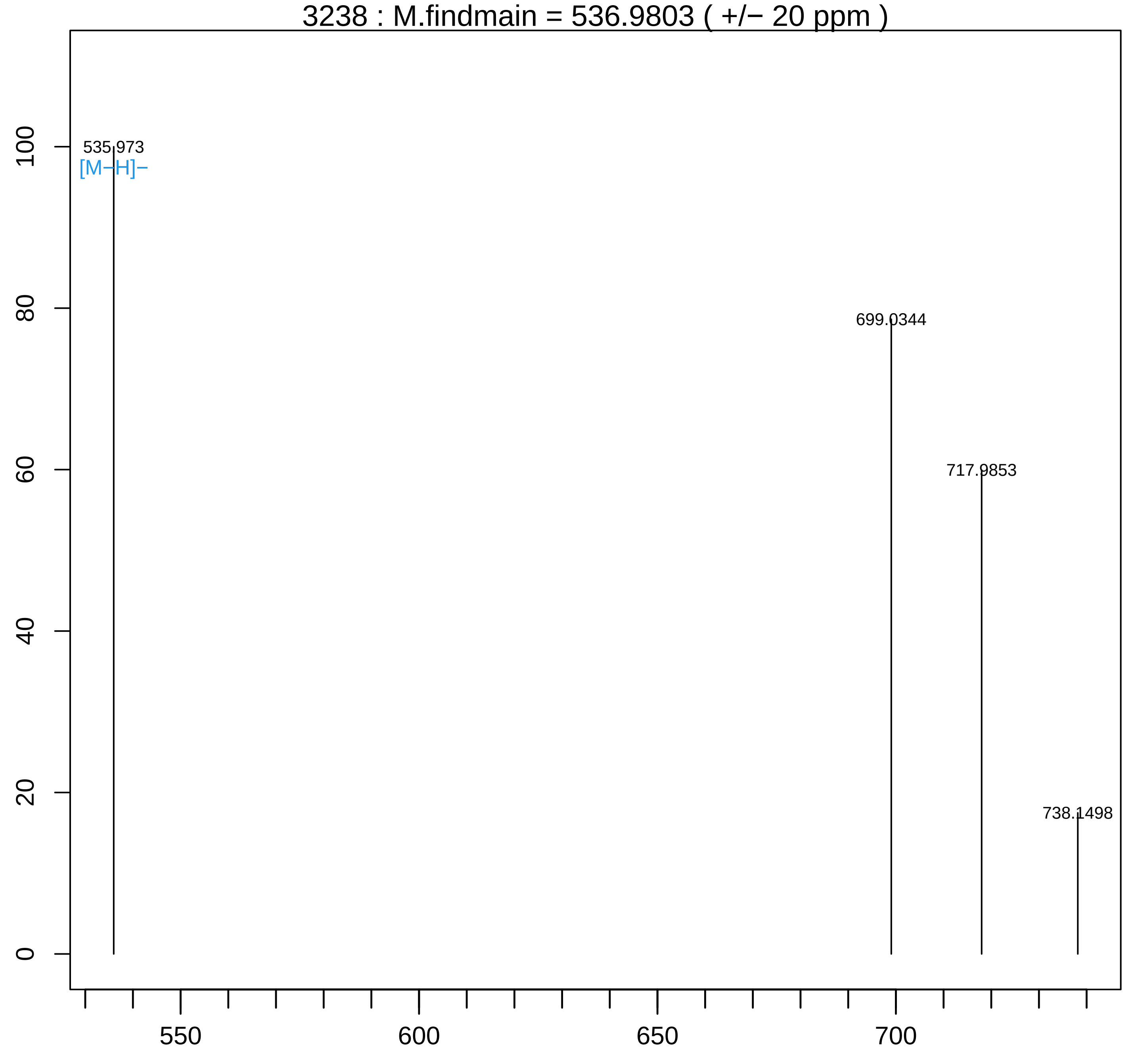
3238 : M.findmain = 536.9803 (+/− 20 ppm)
535.973
[M−H]−
699.0344
717.9853
738.1498
0
20
40
60
80
100
550
600
650
700

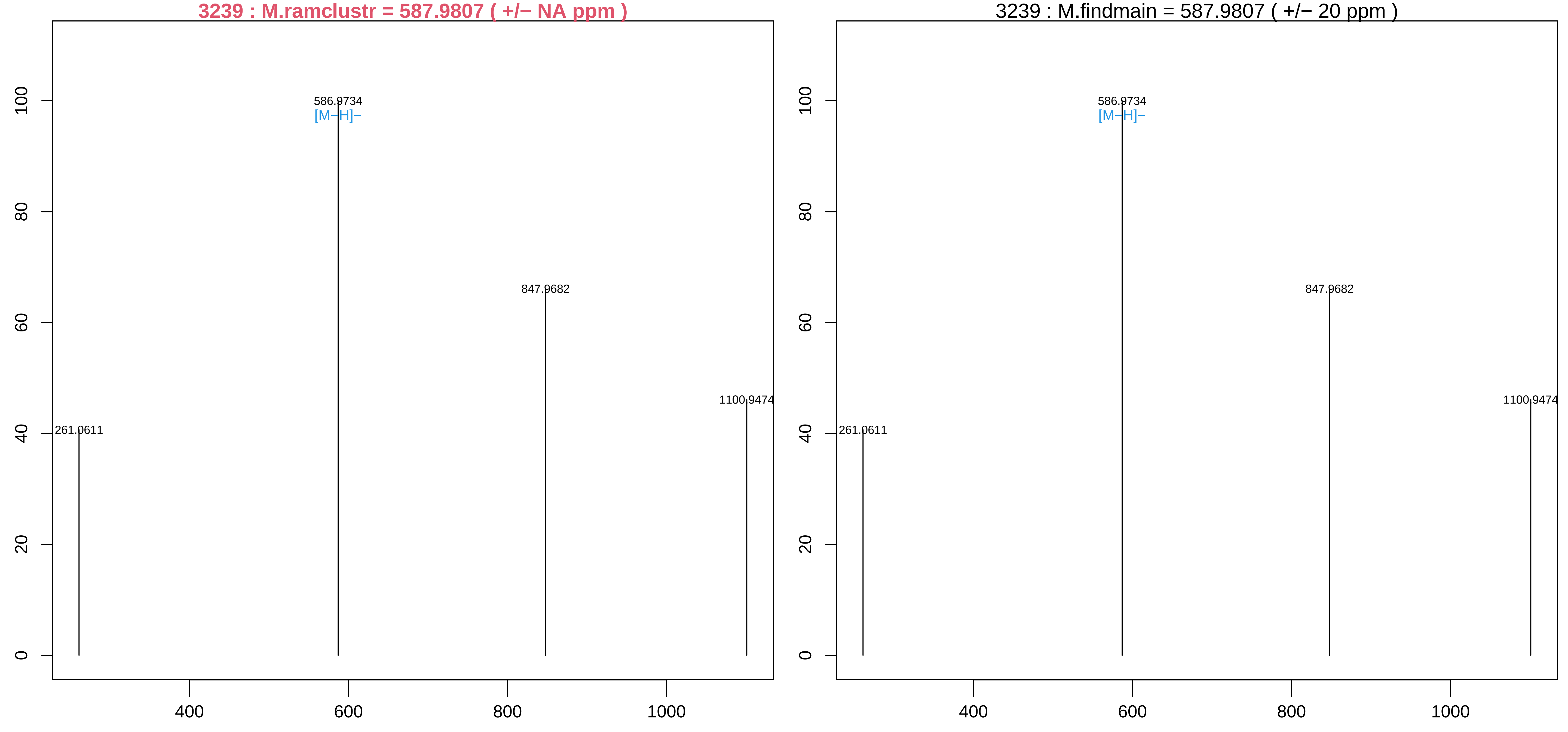

3239 : M.ramclustr = 587.9807 (+/− NA ppm)
586.9734
[M−H]−
847.9682
1100.9474
261.0611
0
20
40
60
80
100
400
600
800
1000
3239 : M.findmain = 587.9807 (+/− 20 ppm)
586.9734
[M−H]−
847.9682
1100.9474
261.0611
0
20
40
60
80
100
400
600
800
1000

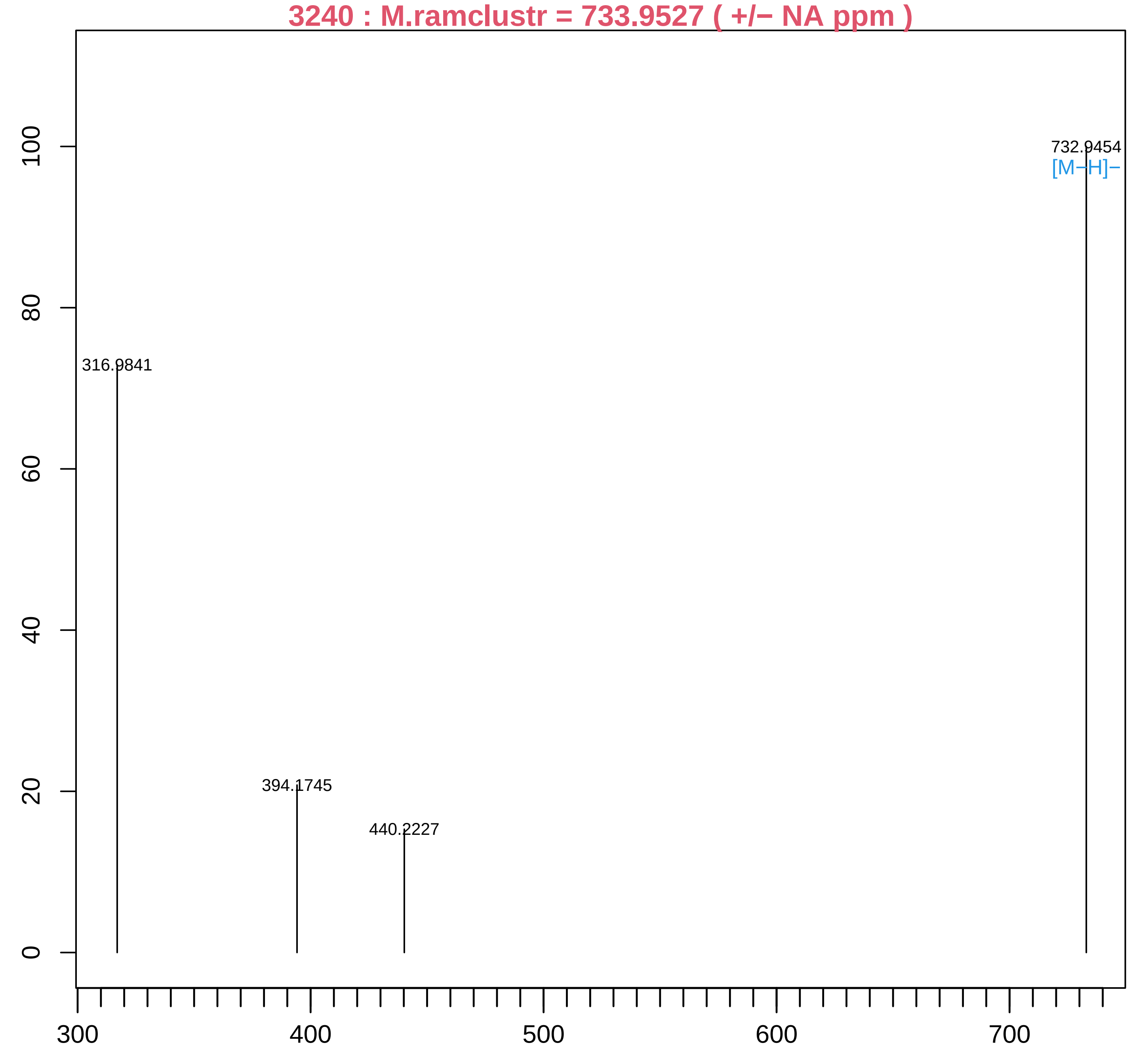
3240 : M.ramclustr = 733.9527 (+/− NA ppm)
732.9454
[M−H]−
316.9841
394.1745
440.2227
0
20
40
60
80
100
300
400
500
600
700

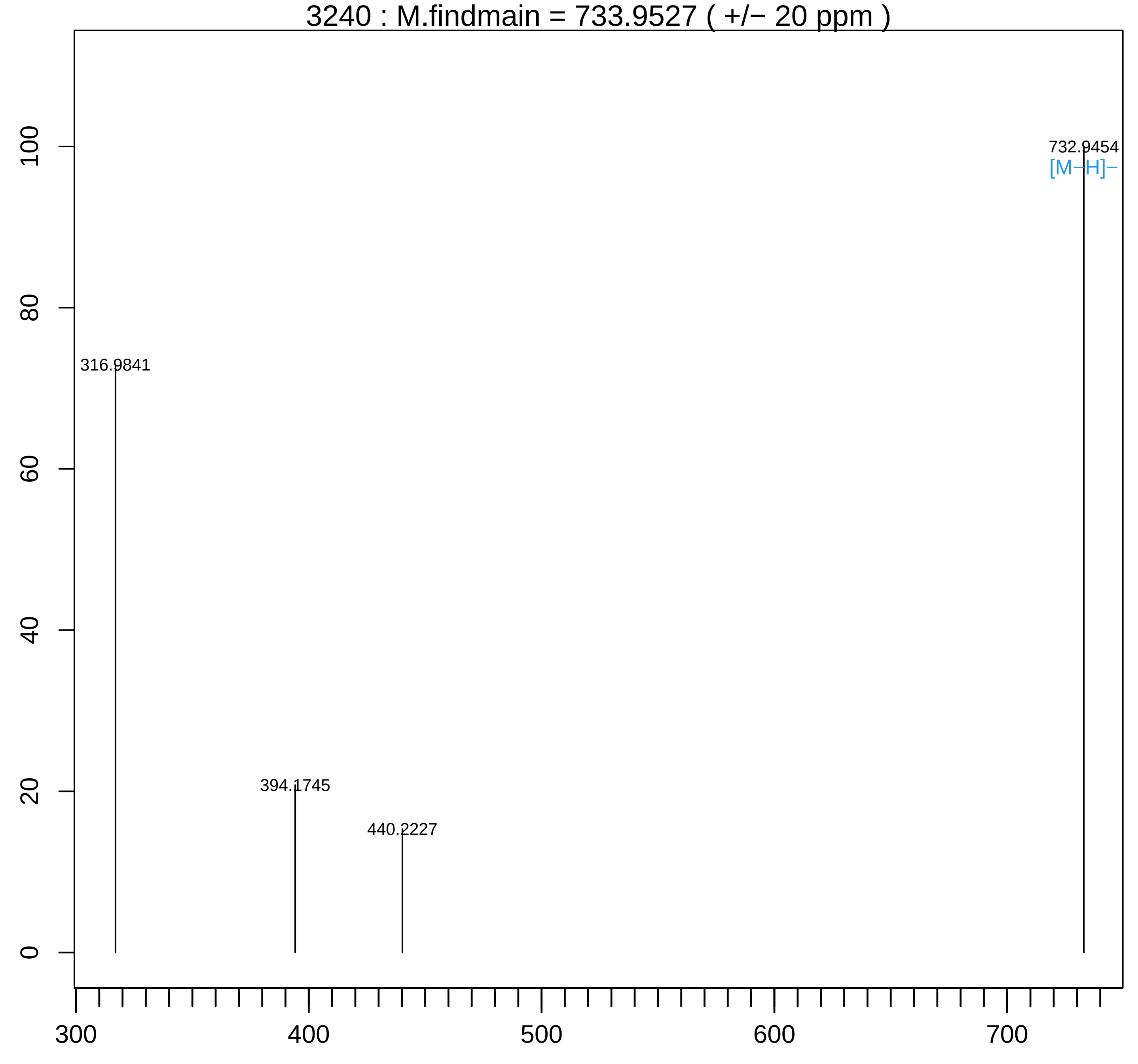
3240 : M.findmain = 733.9527 (+/− 20 ppm)
732.9454
[M−H]−
316.9841
394.1745
440.2227
0
20
40
60
80
100
300
400
500
600
700

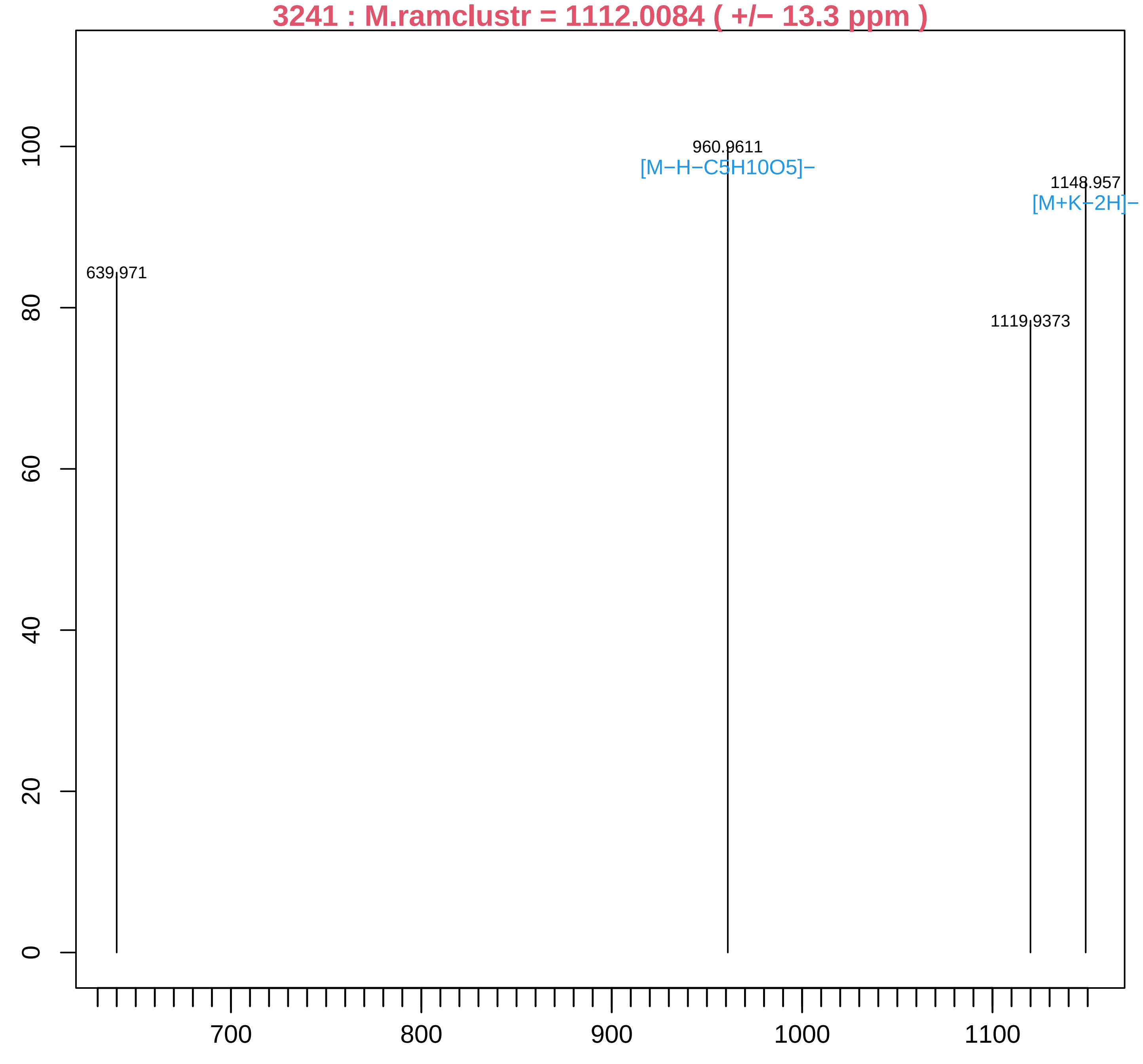
3241 : M.ramclustr = 1112.0084 (+/− 13.3 ppm)
960.9611
[M−H−C5H10O5]−
1148.957
[M+K−2H]−
639.971
1119.9373
0
20
40
60
80
100
700
800
900
1000
1100

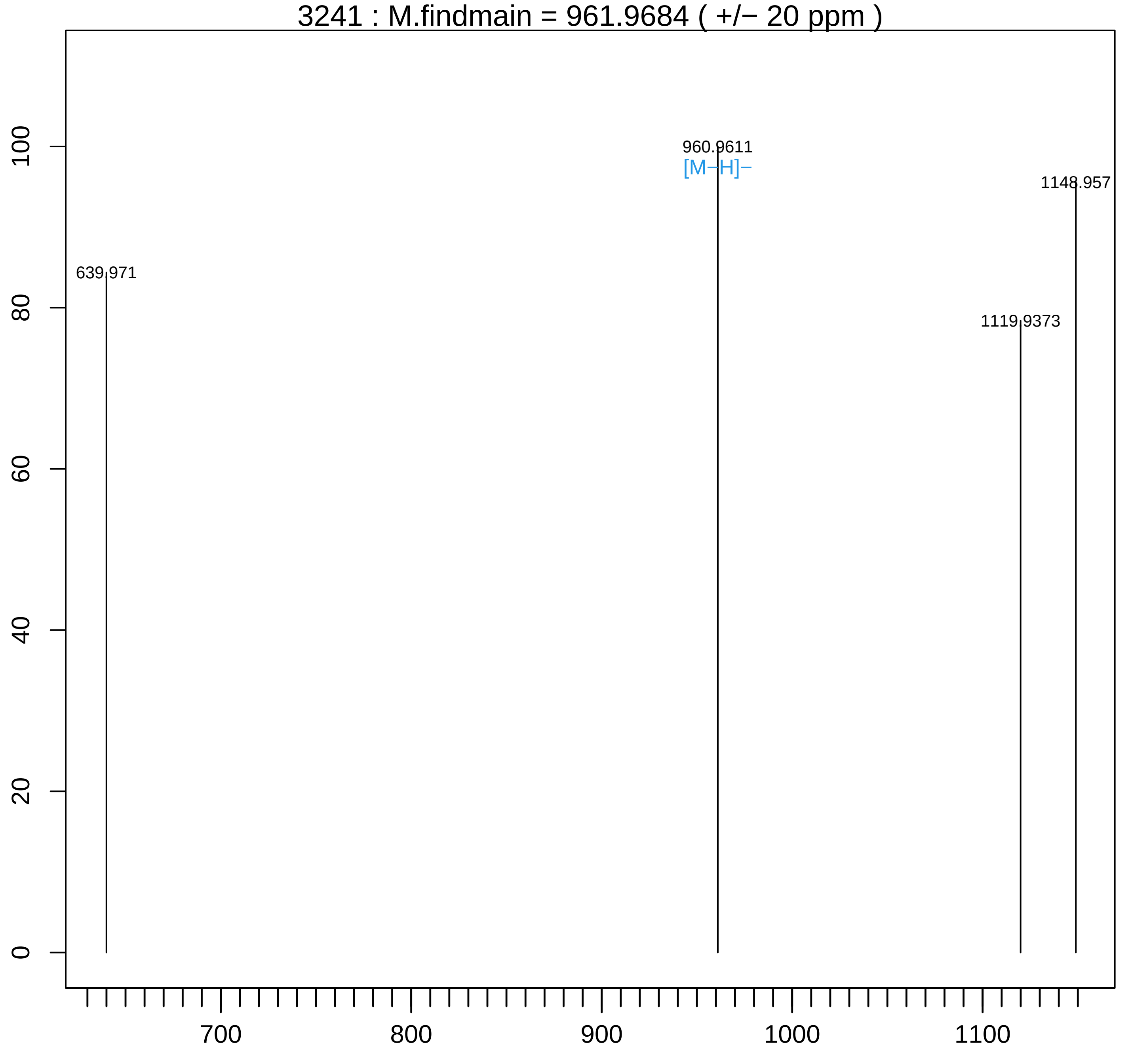
3241 : M.findmain = 961.9684 (+/− 20 ppm)
960.9611
[M−H]−
1148.957
639.971
1119.9373
0
20
40
60
80
100
700
800
900
1000
1100

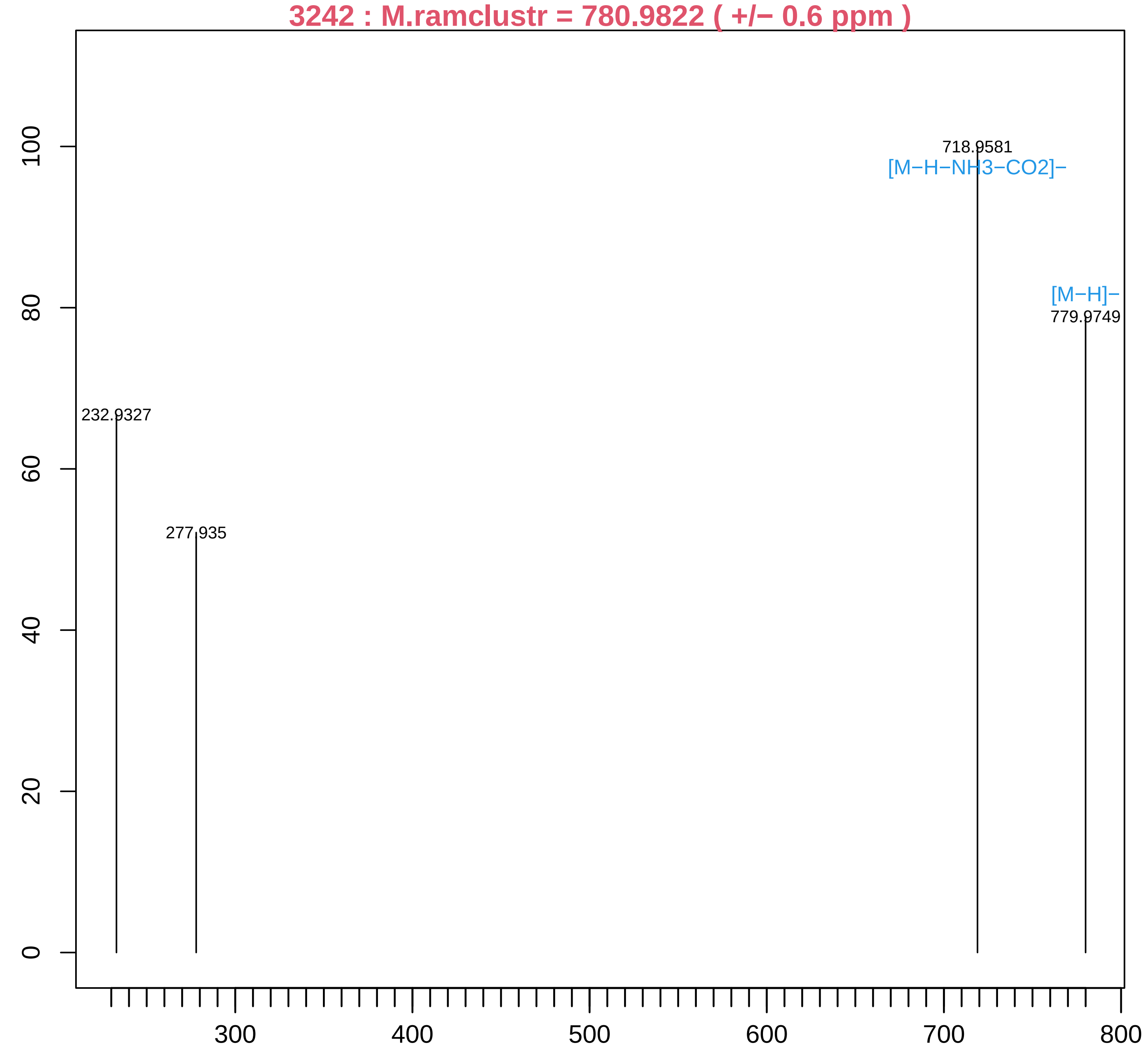
3242 : M.ramclustr = 780.9822 (+/− 0.6 ppm)
718.9581
[M−H−NH3−CO2]−
[M−H]−
779.9749
232.9327
277.935
0
20
40
60
80
100
300
400
500
600
700
800

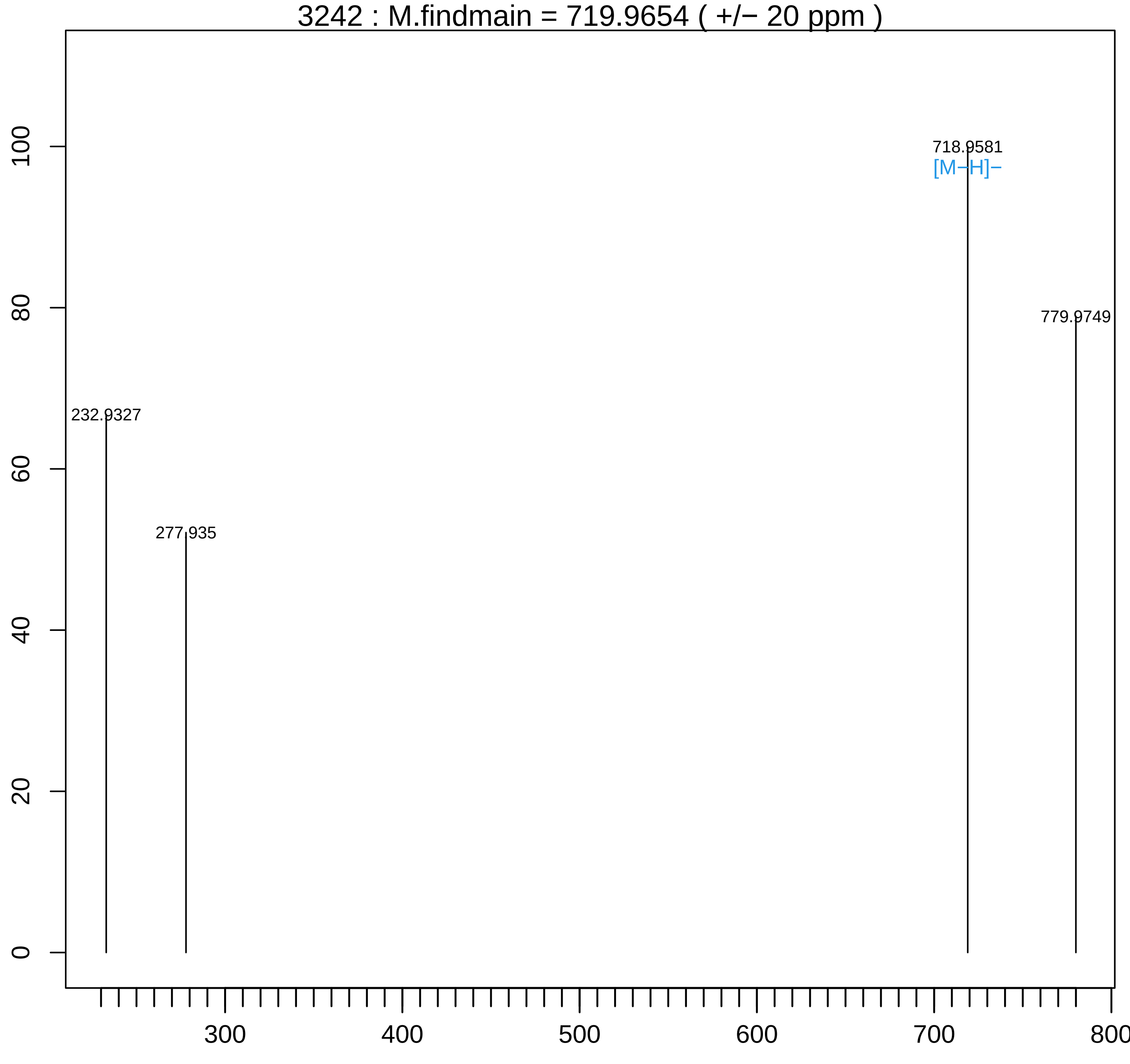
3242 : M.findmain = 719.9654 (+/− 20 ppm)
718.9581
[M−H]−
779.9749
232.9327
277.935
0
20
40
60
80
100
300
400
500
600
700
800

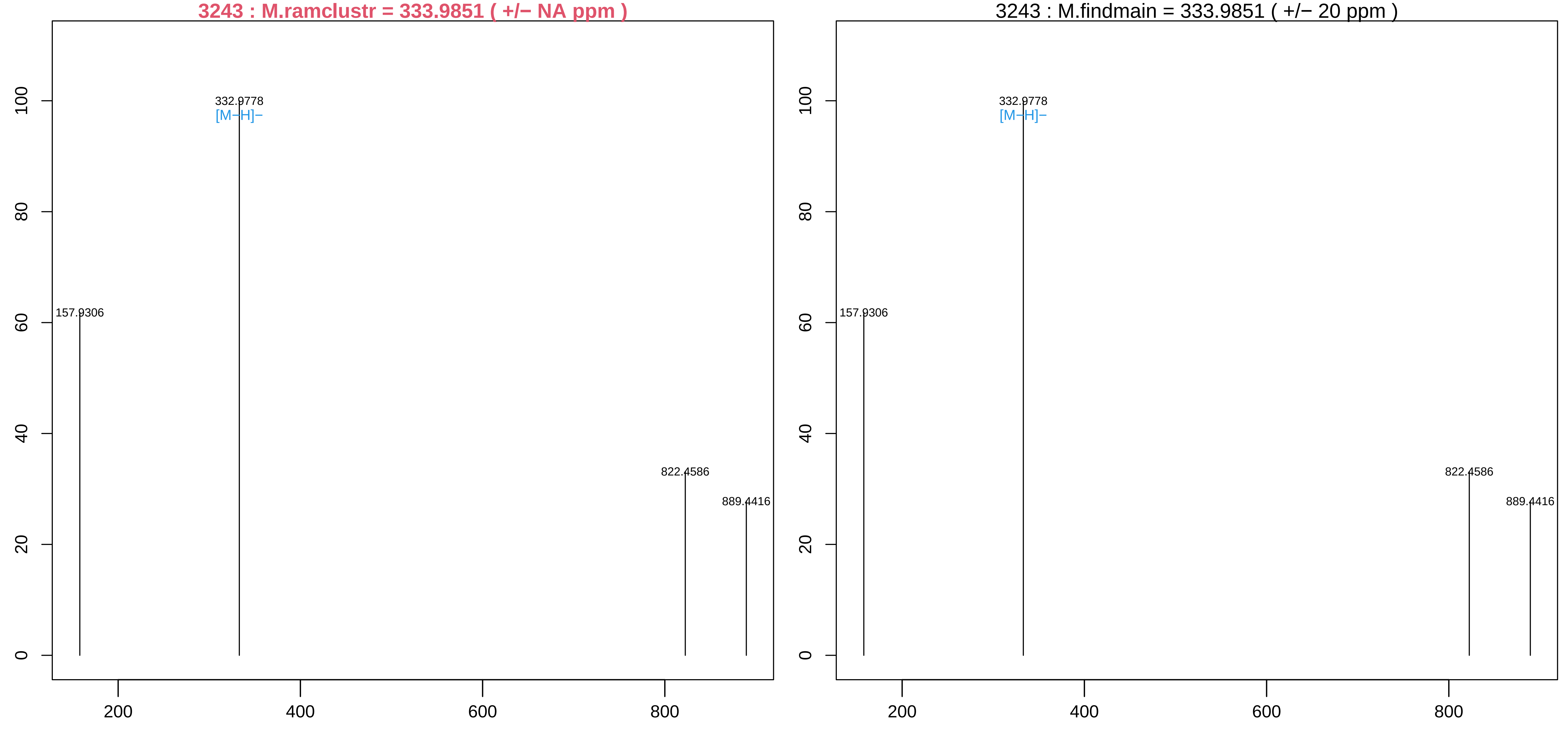
3243 : M.ramclustr = 333.9851 (+/− NA ppm)
332.9778
[M−H]−
157.9306
822.4586
889.4416
3243 : M.findmain = 333.9851 (+/− 20 ppm)
332.9778
[M−H]−
157.9306
822.4586
889.4416
0
20
40
60
80
100
200
400
600
800

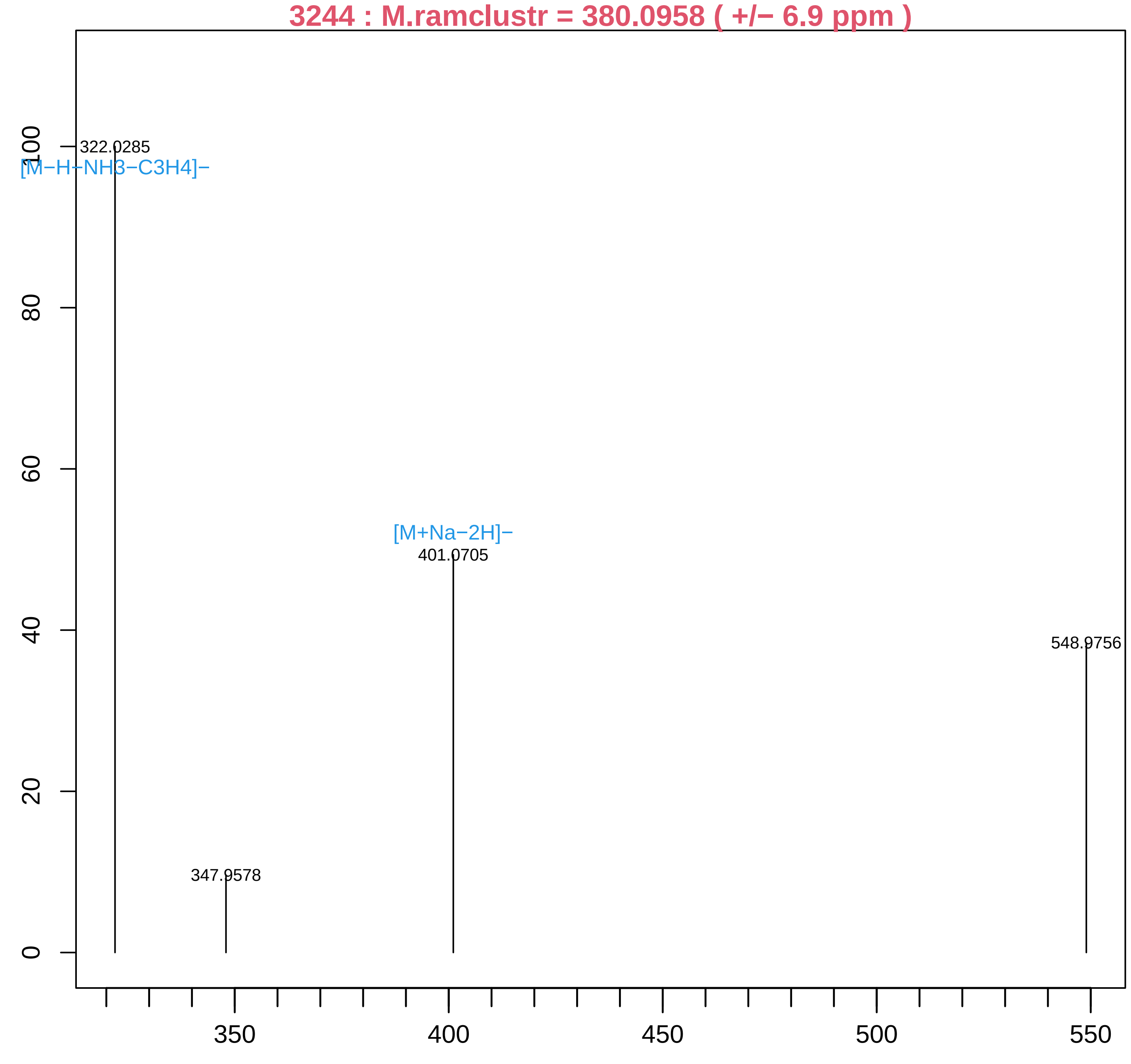
3244 : M.ramclustr = 380.0958 (+/− 6.9 ppm)
322.0285
[M−H−NH3−C3H4]−
347.9578
[M+Na−2H]−
401.0705
548.9756
0
20
40
60
80
100
350
400
450
500
550

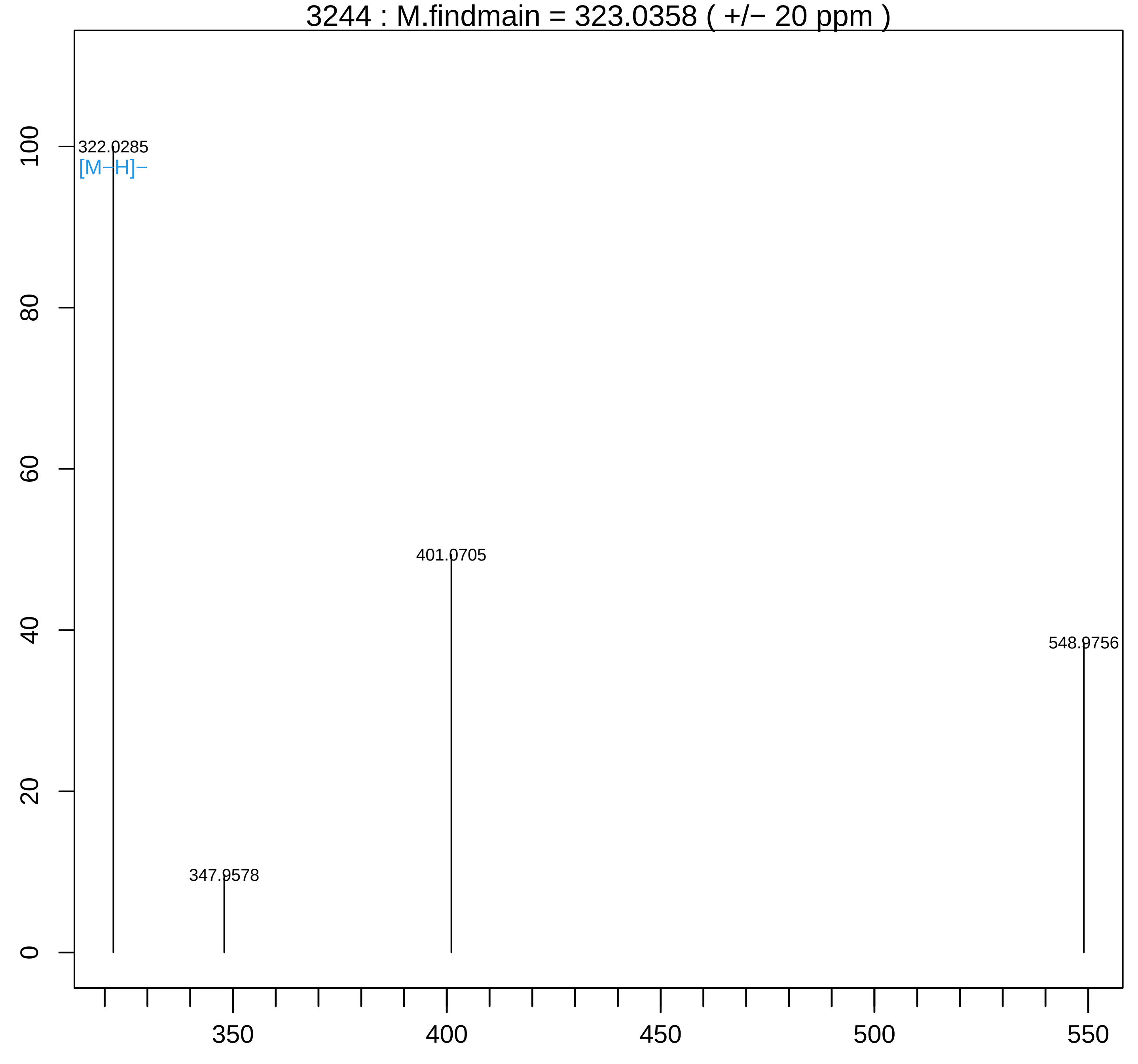
3244 : M.findmain = 323.0358 (+/− 20 ppm)
322.0285
[M−H]−
347.9578
401.0705
548.9756
0
20
40
60
80
100
350
400
450
500
550

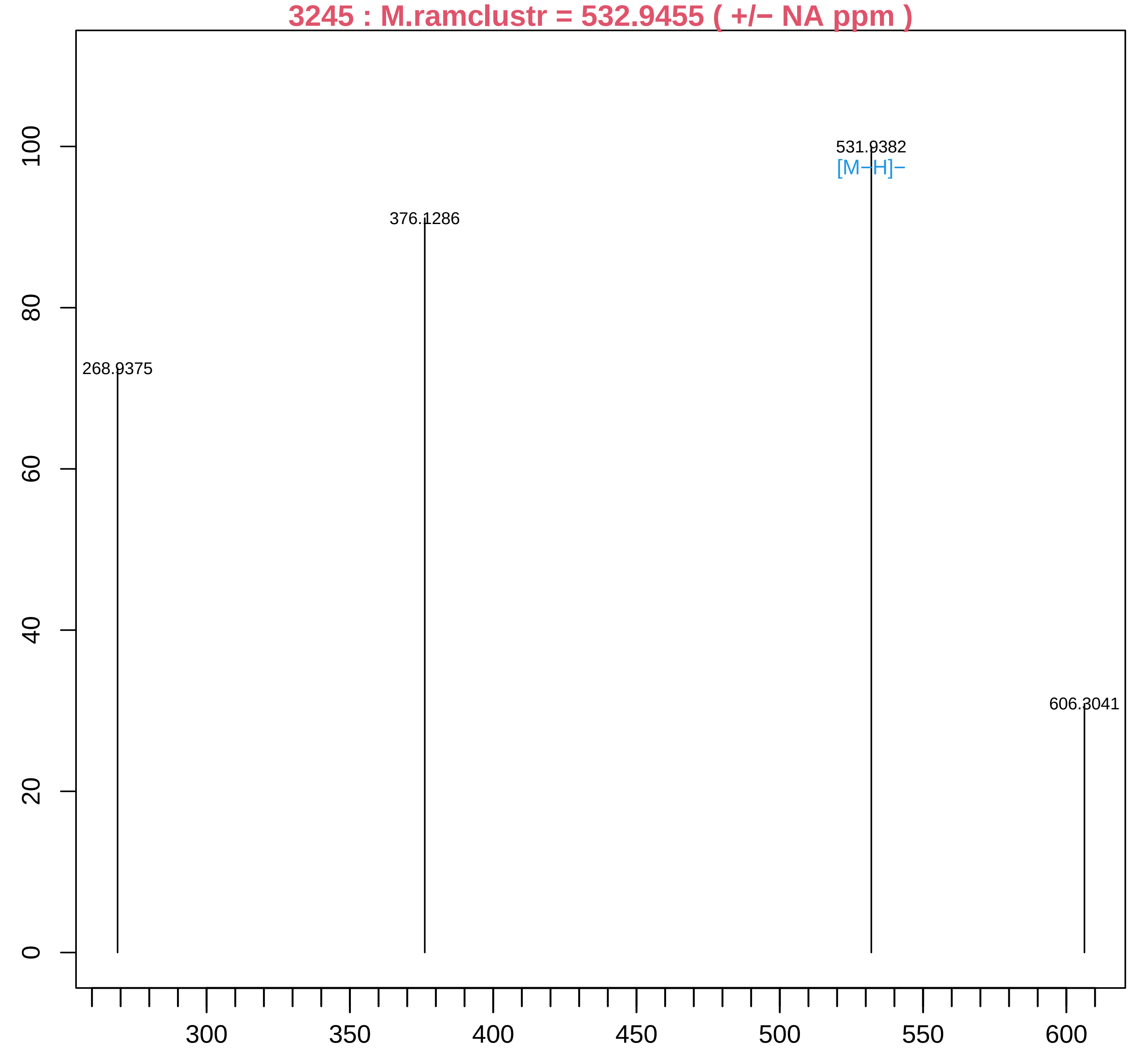

3245 : M.ramclustr = 532.9455 (+/− NA ppm)
531.9382
[M−H]−
376.1286
268.9375
606.3041
0
20
40
60
80
100
300
350
400
450
500
550
600

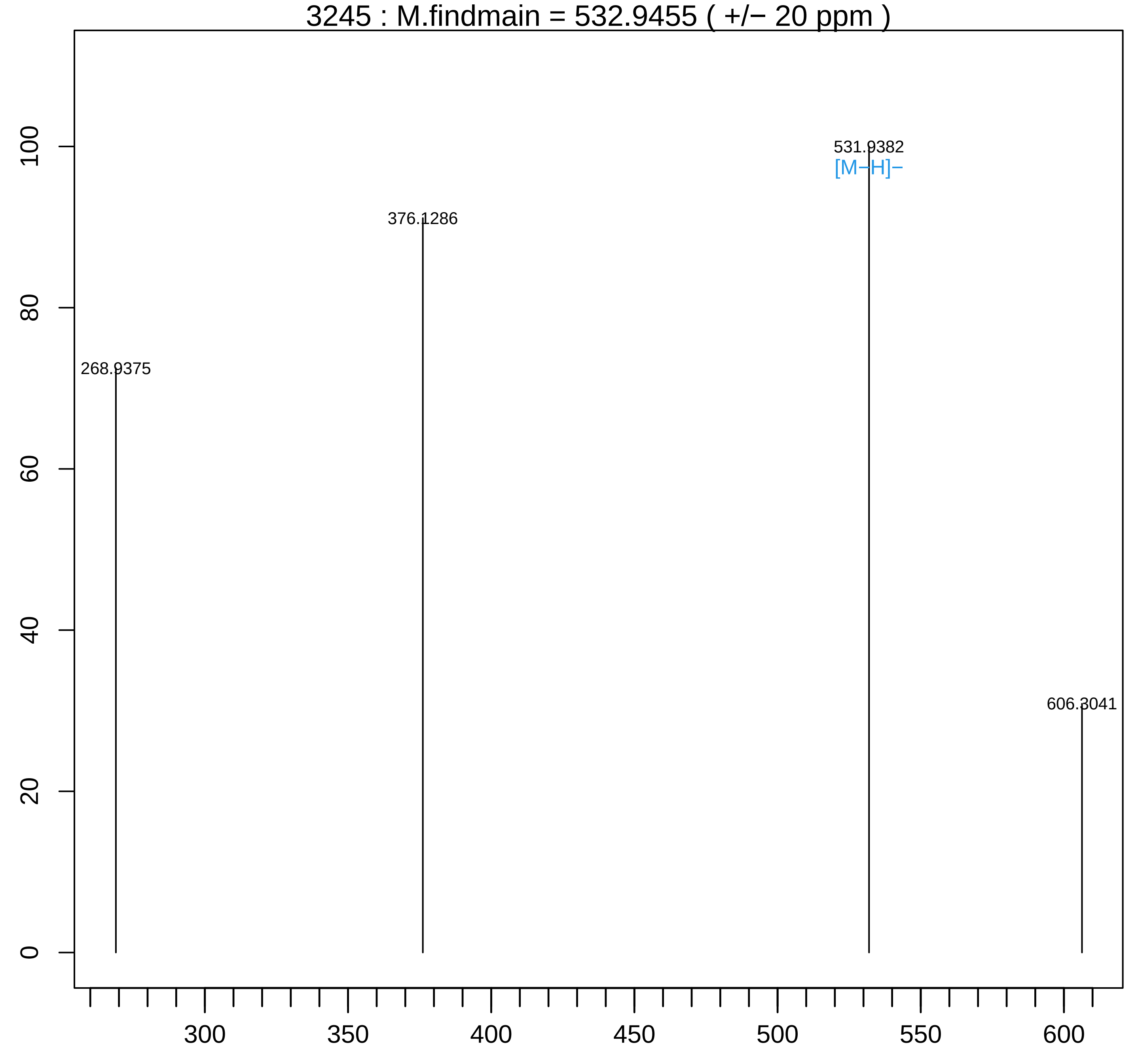

3245 : M.findmain = 532.9455 (+/− 20 ppm)
531.9382
[M−H]−
376.1286
268.9375
606.3041
0
20
40
60
80
100
300
350
400
450
500
550
600

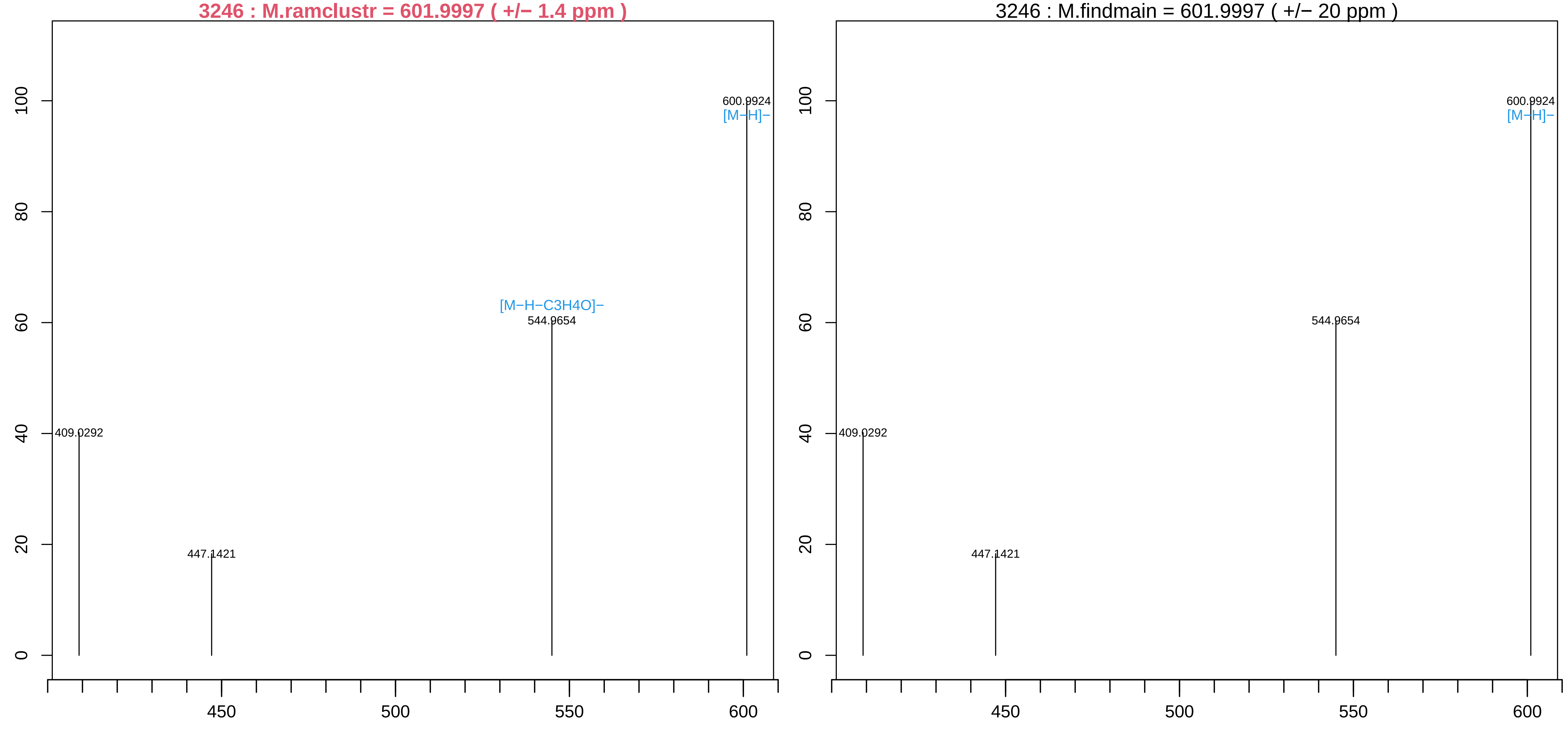
3246 : M.ramclustr = 601.9997 (+/− 1.4 ppm)
600.9924
[M−H]−
[M−H−C3H4O]−
544.9654
409.0292
447.1421
3246 : M.findmain = 601.9997 (+/− 20 ppm)
600.9924
[M−H]−
544.9654
409.0292
447.1421

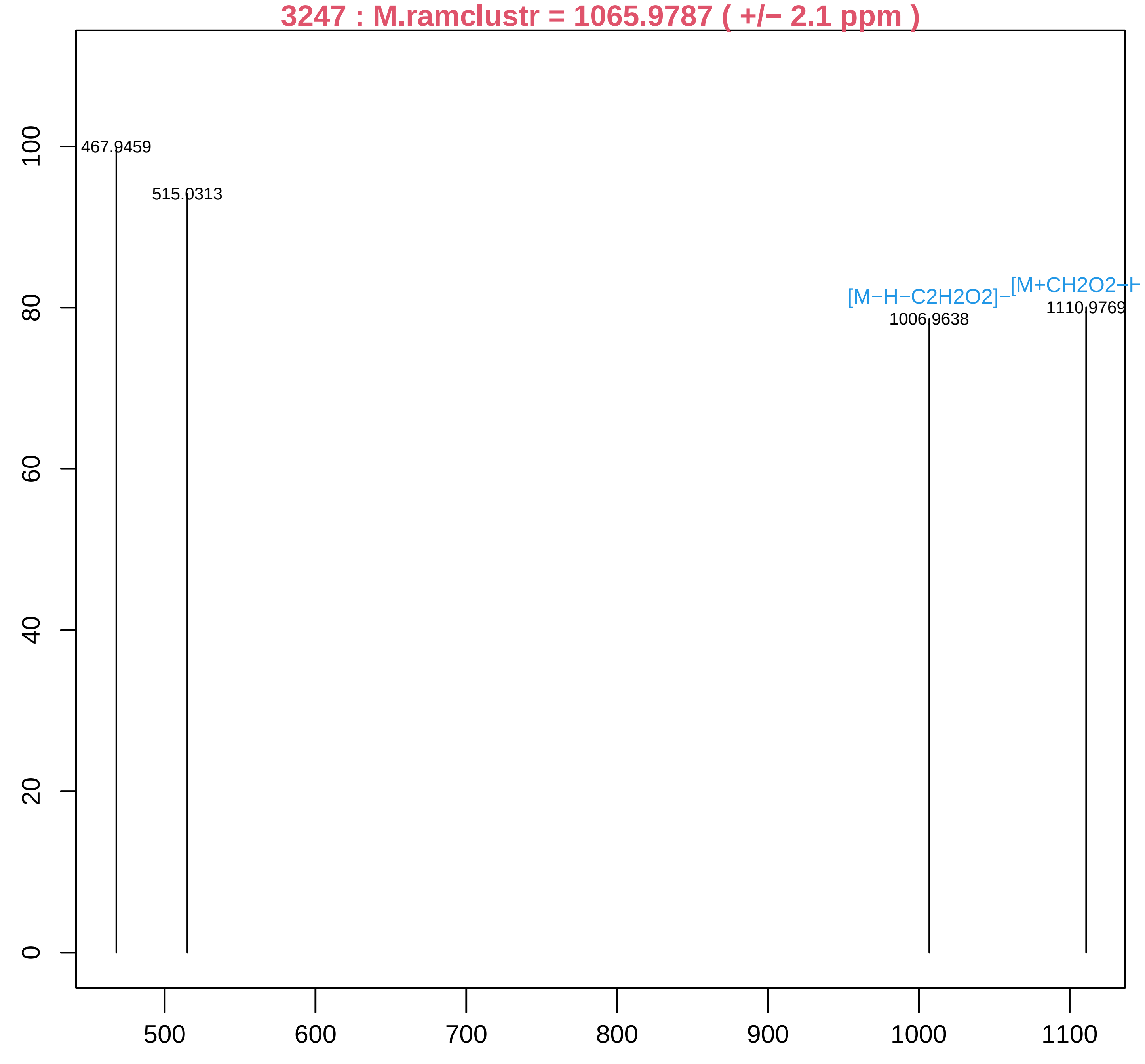
3247 : M.ramclustr = 1065.9787 (+/− 2.1 ppm)
467.9459
515.0313
[M−H−C2H2O2]−
1006.9638
[M+CH2O2−H
1110.9769
0
20
40
60
80
100
500
600
700
800
900
1000
1100

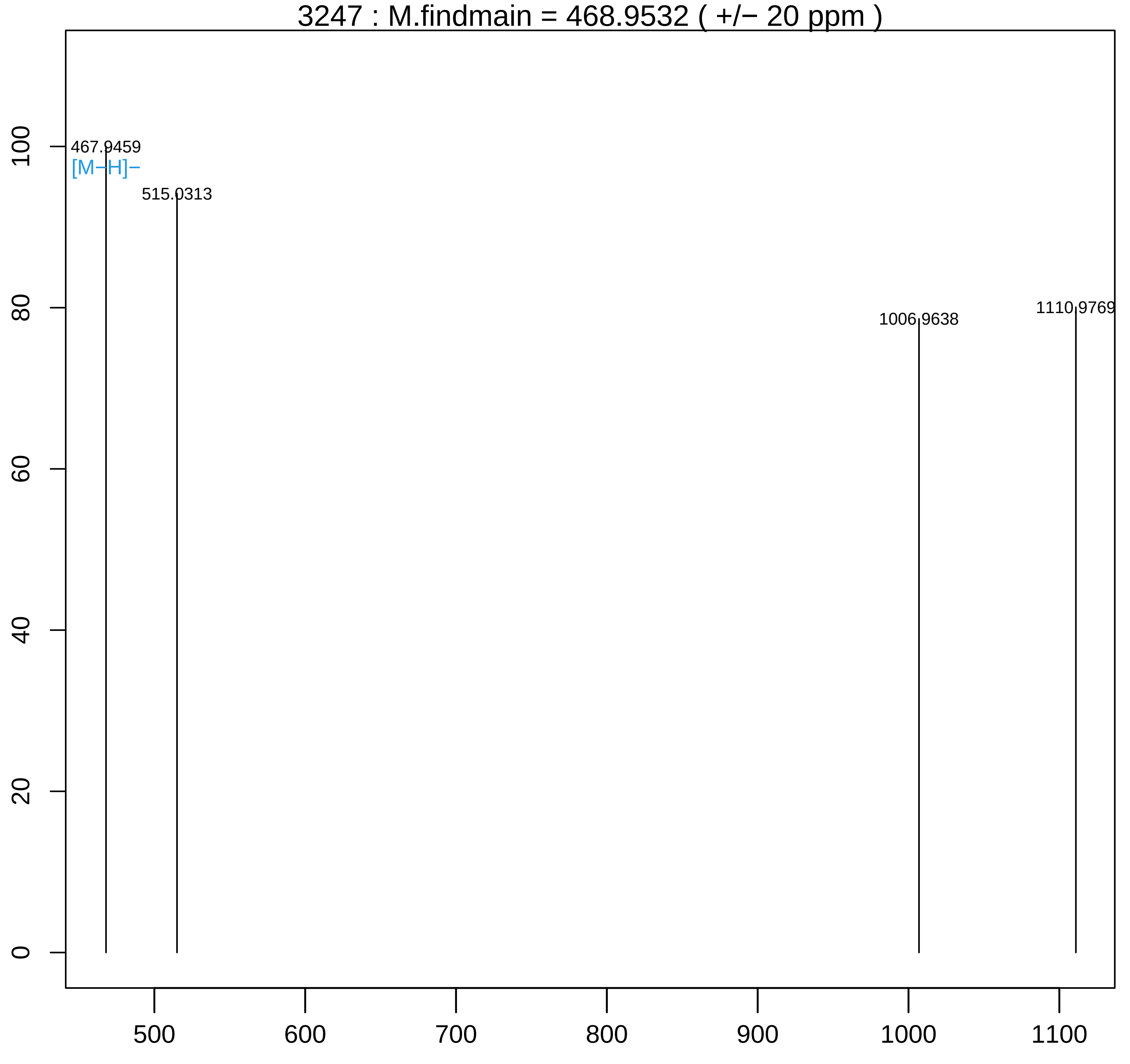
3247 : M.findmain = 468.9532 (+/− 20 ppm)
467.9459
[M−H]−
515.0313
1006.9638
1110.9769
0
20
40
60
80
100
500
600
700
800
900
1000
1100

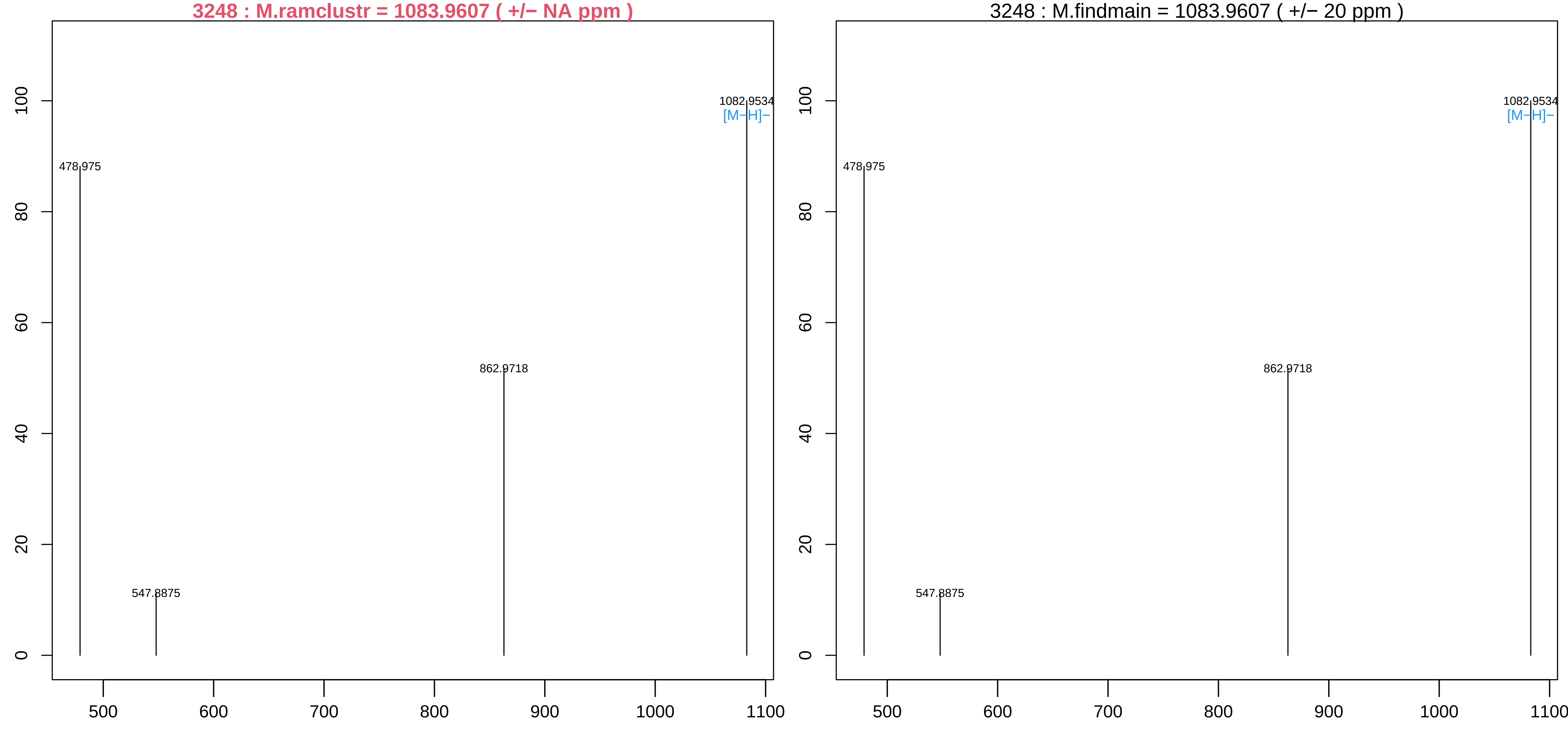
3248 : M.ramclustr = 1083.9607 (+/− NA ppm)
3248 : M.findmain = 1083.9607 (+/− 20 ppm)
1082.9534
[M−H]−
478.975
547.8875
862.9718
0
20
40
60
80
100
500
600
700
800
900
1000
1100

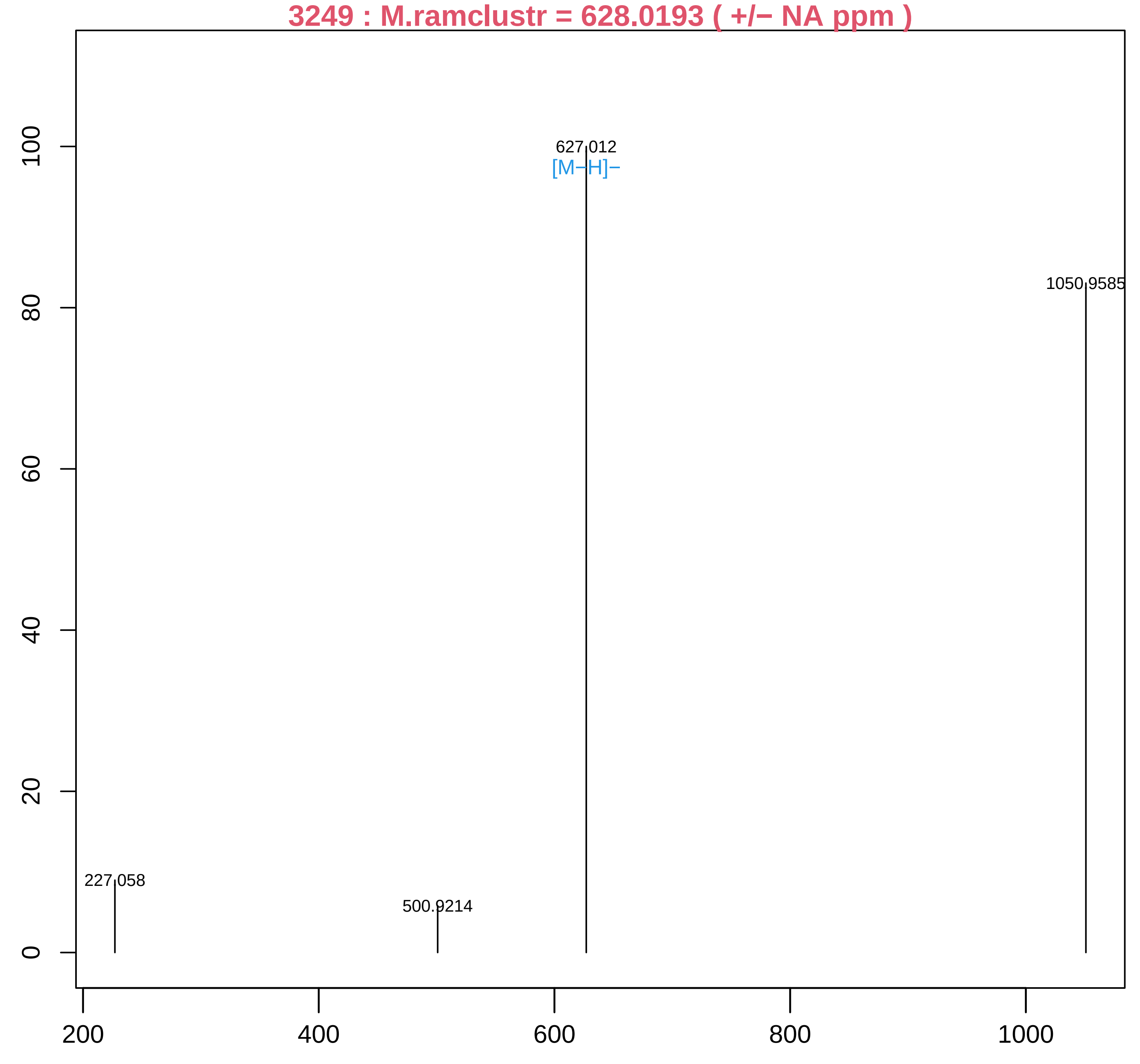
3249 : M.ramclustr = 628.0193 (+/− NA ppm)
627.012
[M−H]−
1050.9585
227.058
500.9214
0
20
40
60
80
100
200
400
600
800
1000

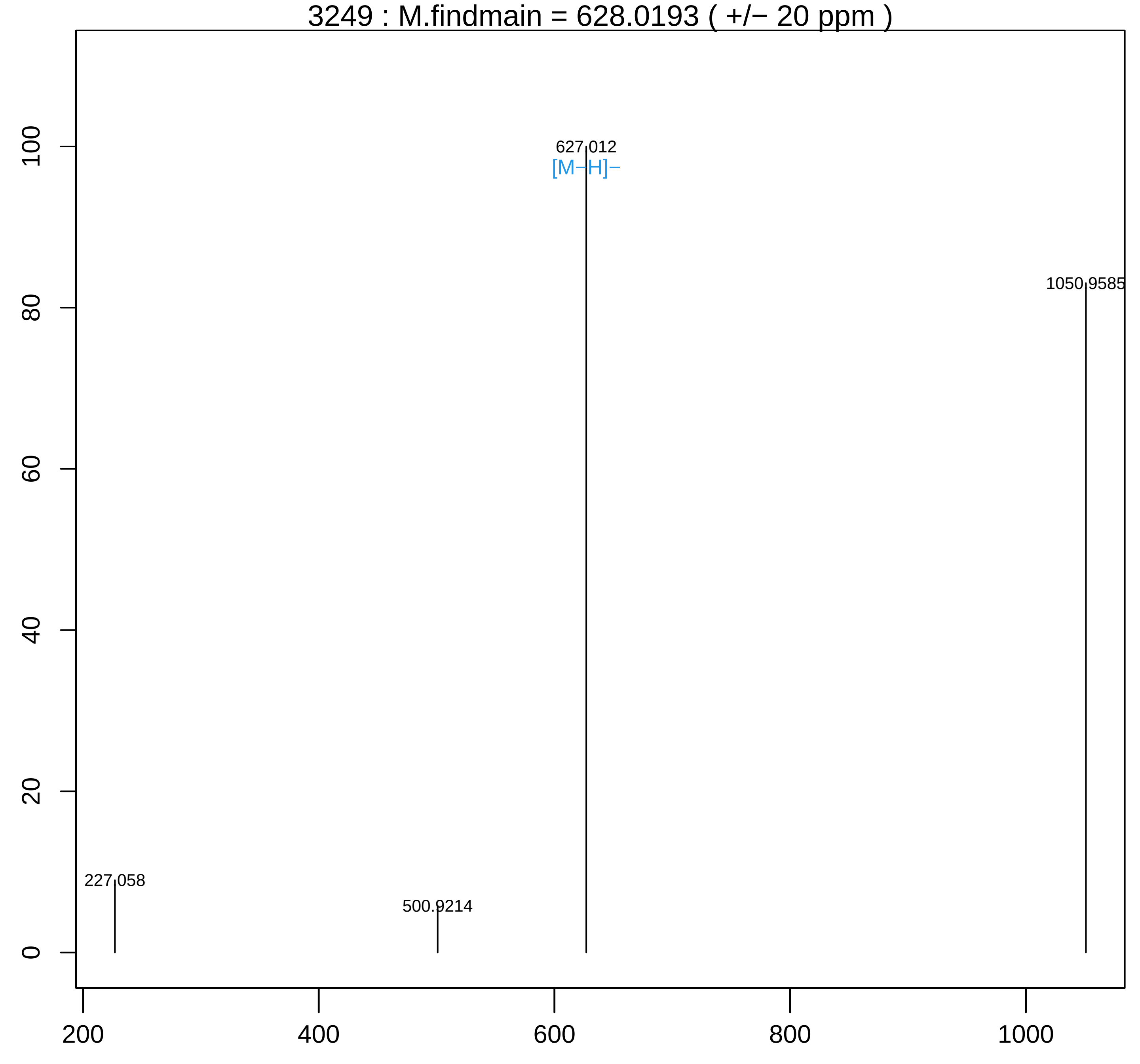
3249 : M.findmain = 628.0193 (+/− 20 ppm)
627.012
[M−H]−
1050.9585
227.058
500.9214
0
20
40
60
80
100
200
400
600
800
1000

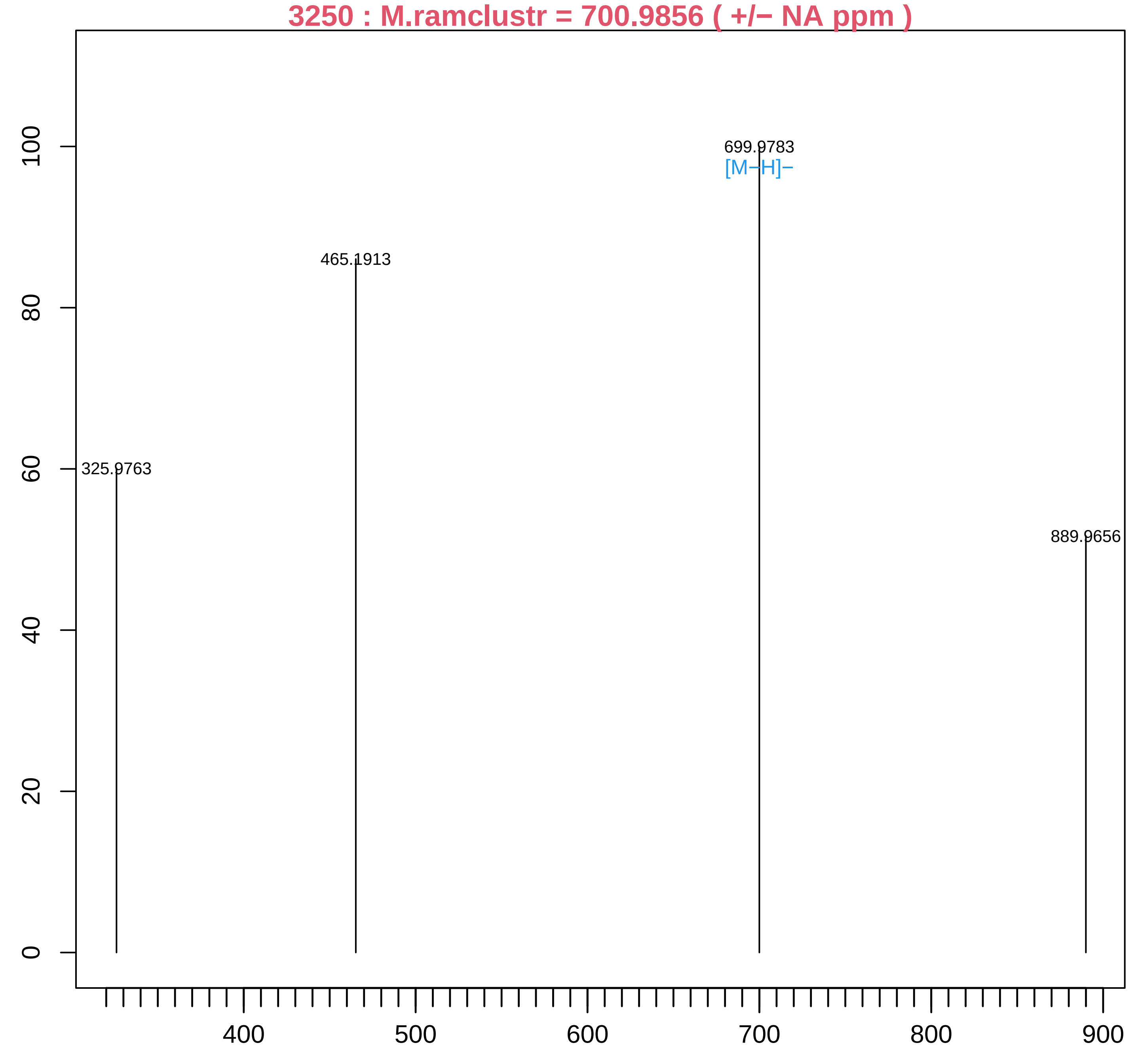
3250 : M.ramclustr = 700.9856 (+/− NA ppm)
699.9783
[M−H]−
465.1913
325.9763
889.9656
0
20
40
60
80
100
400
500
600
700
800
900

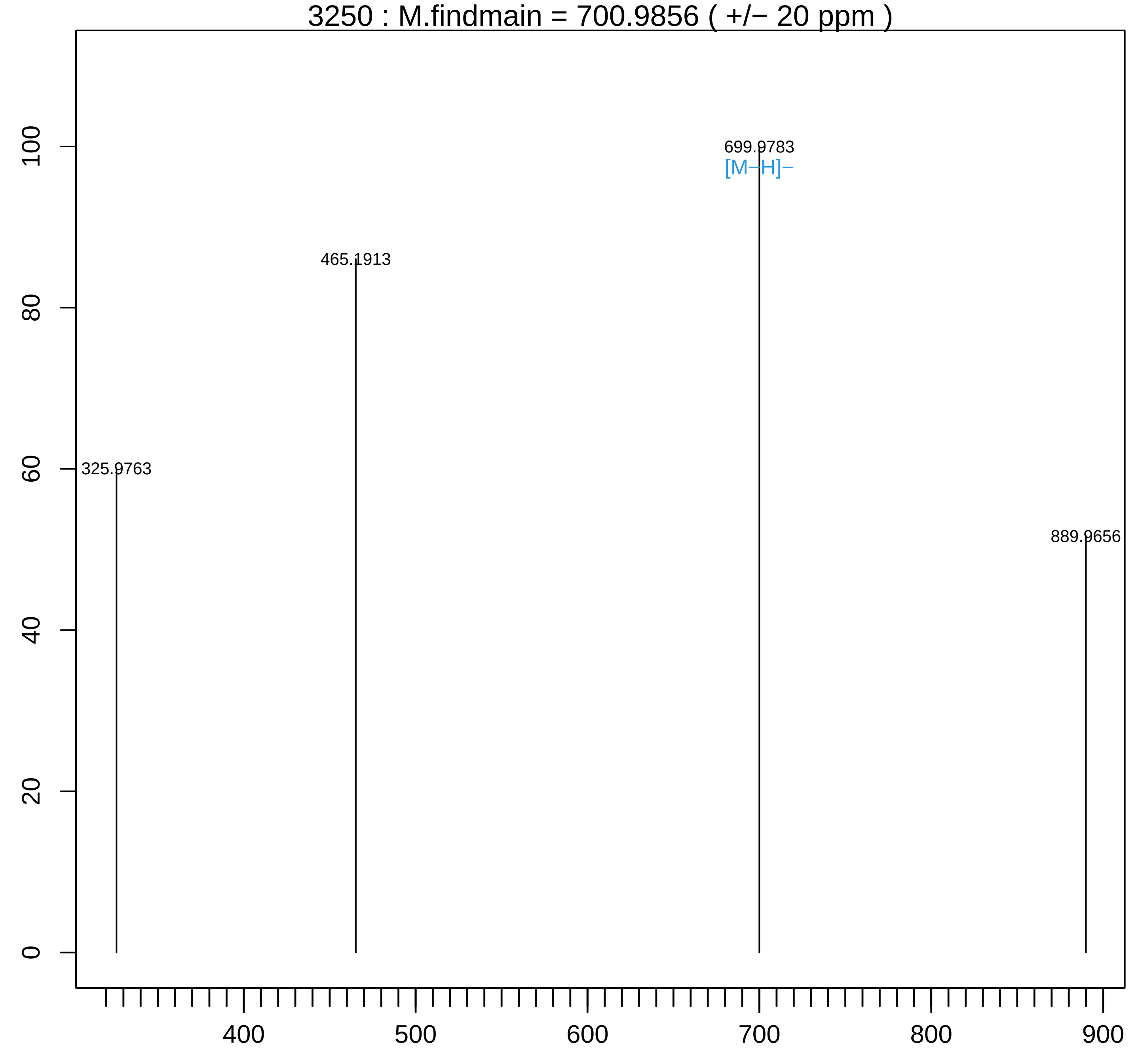
3250 : M.findmain = 700.9856 (+/− 20 ppm)
699.9783
[M−H]−
465.1913
325.9763
889.9656
0
20
40
60
80
100
400
500
600
700
800
900

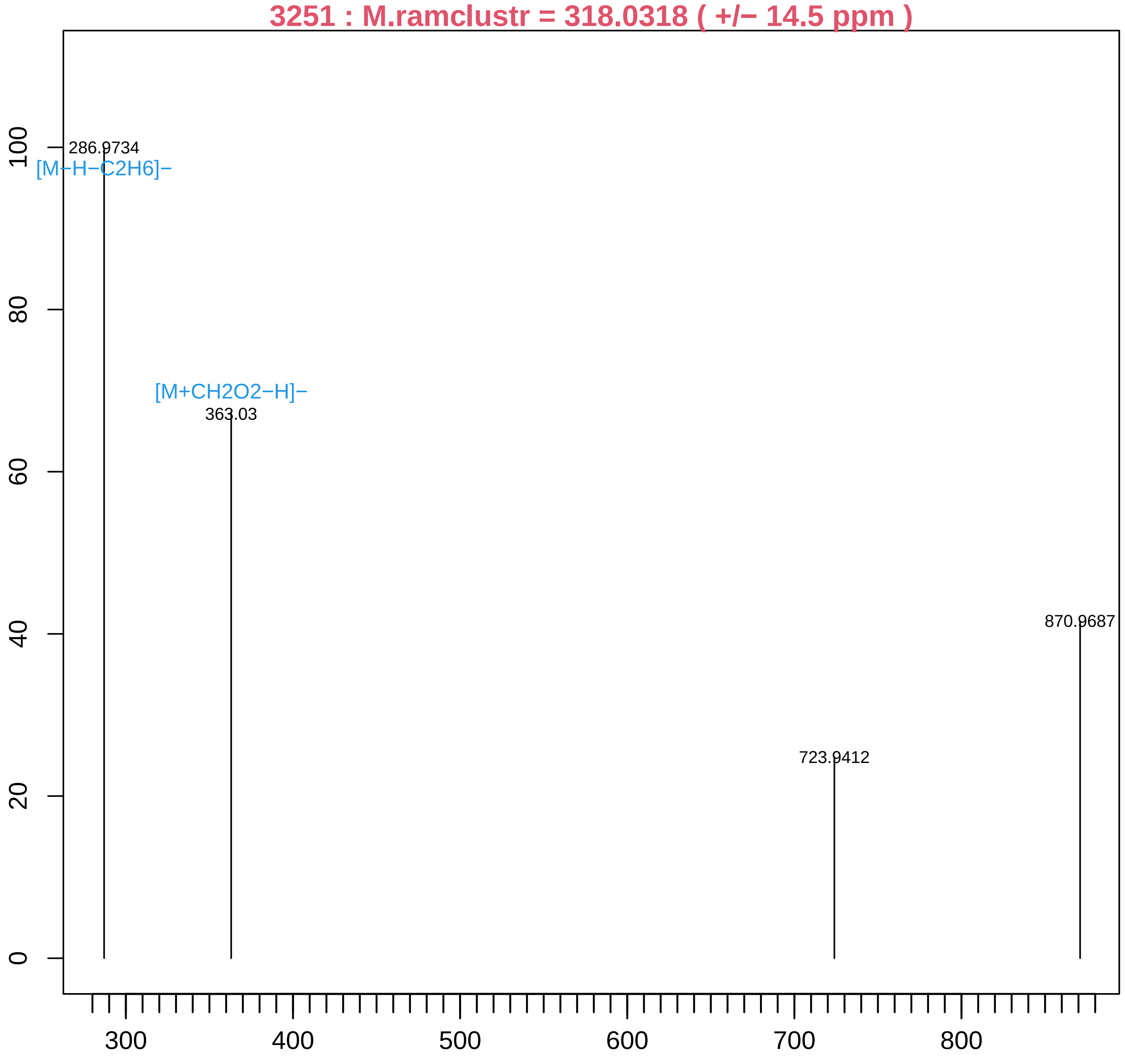
3251 : M.ramclustr = 318.0318 (+/− 14.5 ppm)
286.9734
[M−H−C2H6]−
[M+CH2O2−H]−
363.03
723.9412
870.9687
0
20
40
60
80
100
300
400
500
600
700
800

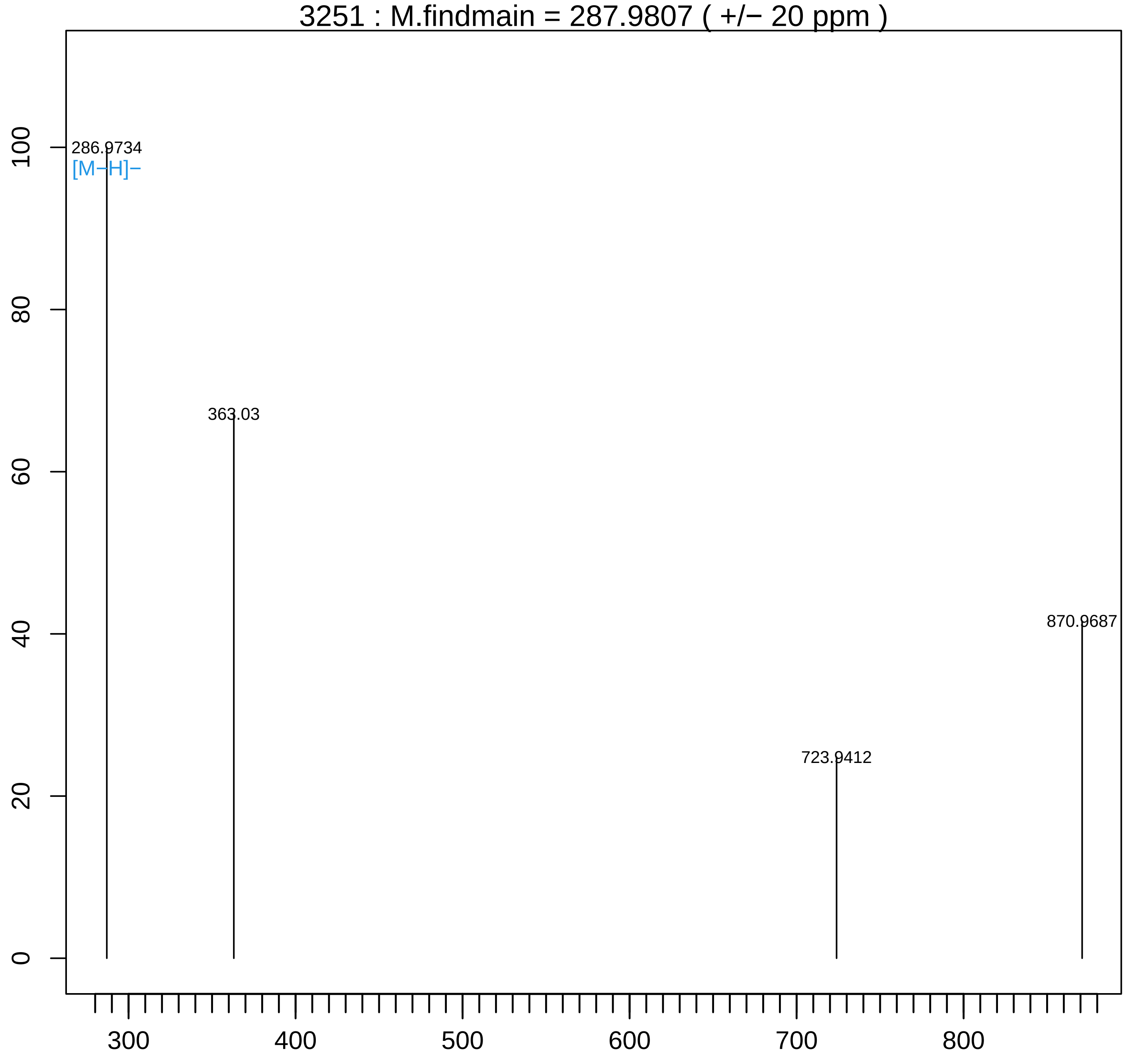
3251 : M.findmain = 287.9807 (+/− 20 ppm)
286.9734
[M−H]−
363.03
723.9412
870.9687
0
20
40
60
80
100
300
400
500
600
700
800

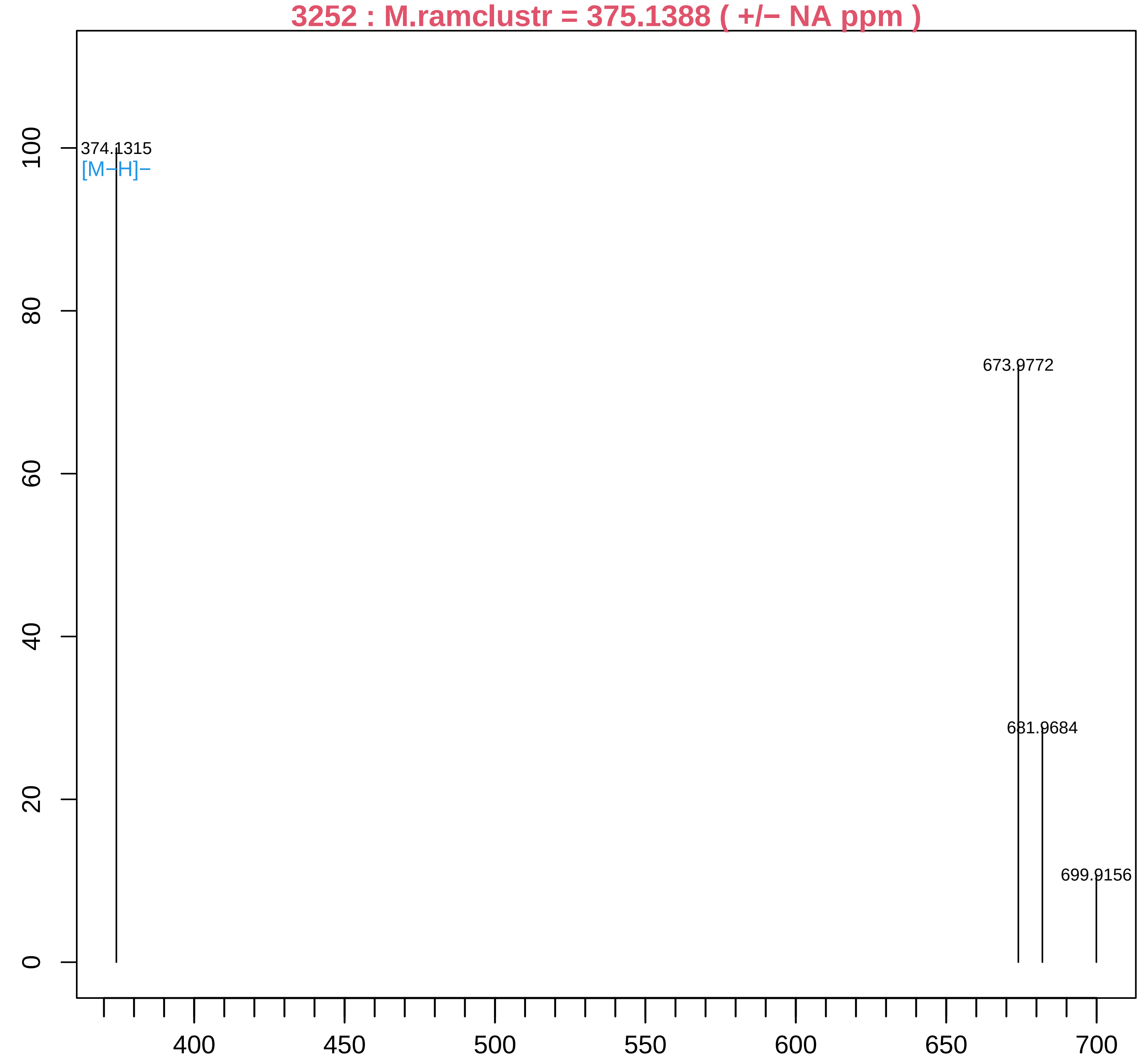
3252 : M.ramclustr = 375.1388 (+/− NA ppm)
374.1315
[M−H]−
673.9772
681.9684
699.9156
0
20
40
60
80
100
400
450
500
550
600
650
700

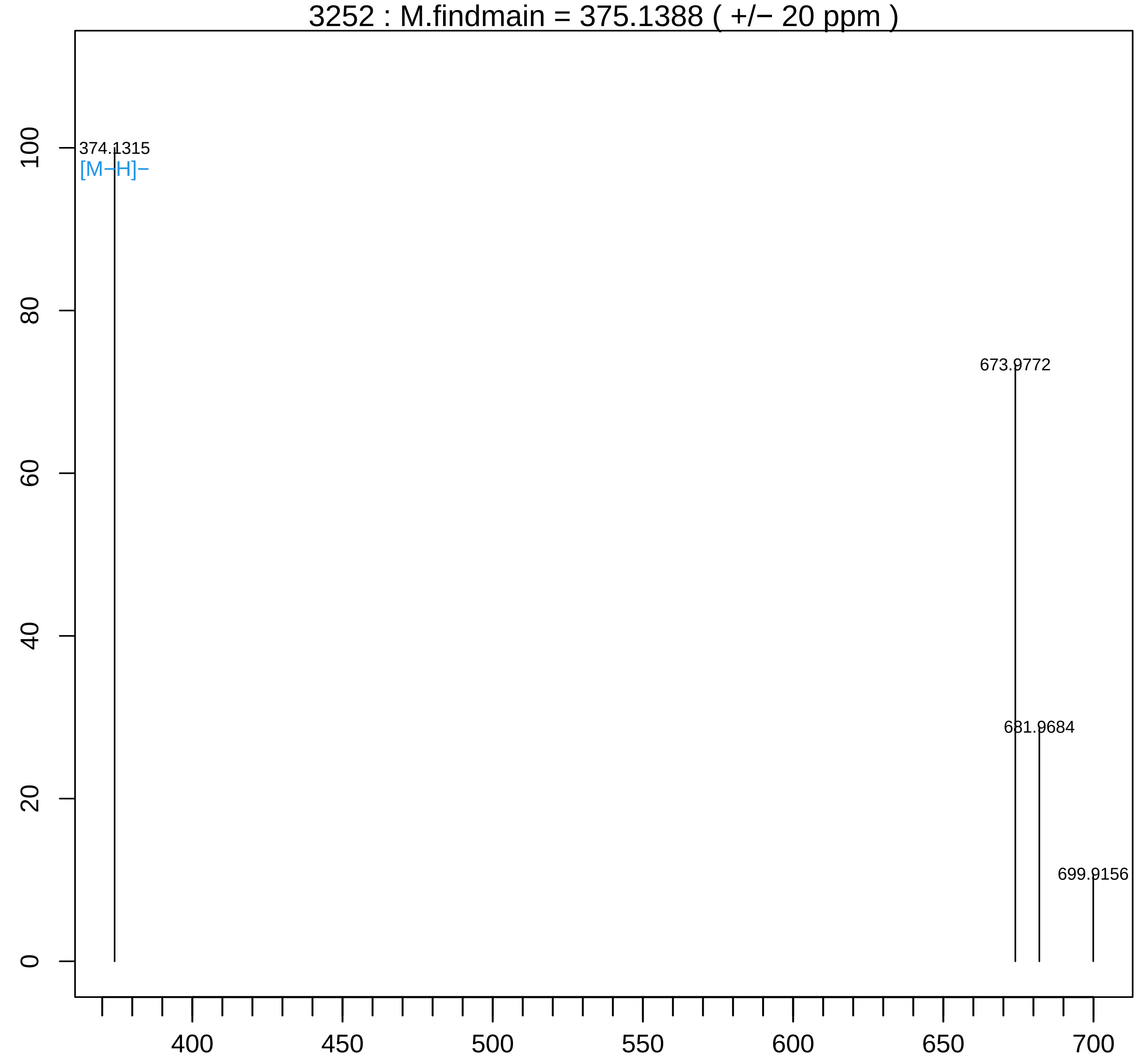
3252 : M.findmain = 375.1388 (+/− 20 ppm)
374.1315
[M−H]−
673.9772
681.9684
699.9156
0
20
40
60
80
100
400
450
500
550
600
650
700

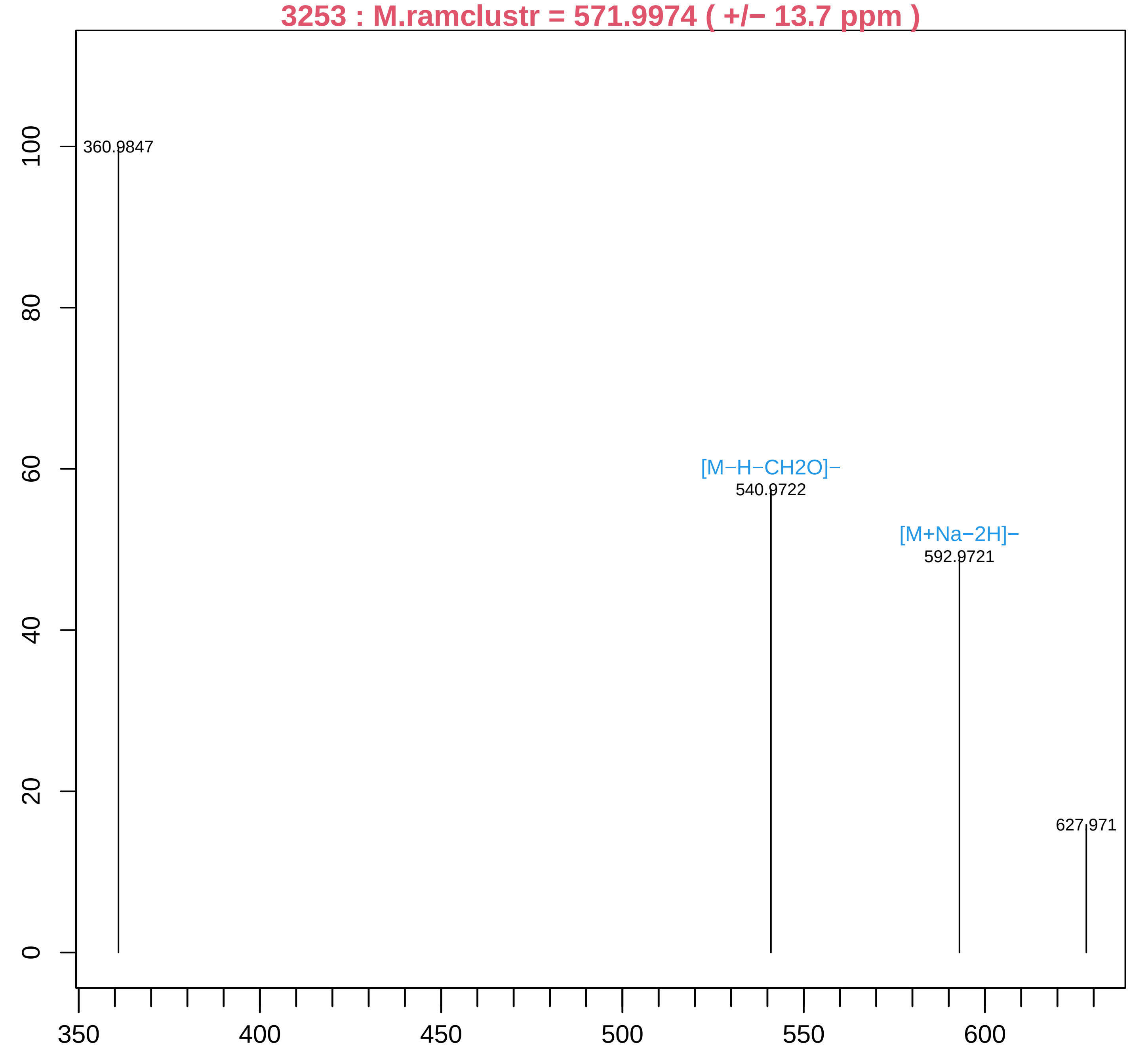
3253 : M.ramclustr = 571.9974 (+/− 13.7 ppm)
360.9847
[M−H−CH2O]−
540.9722
[M+Na−2H]−
592.9721
627.971
0
20
40
60
80
100
350
400
450
500
550
600

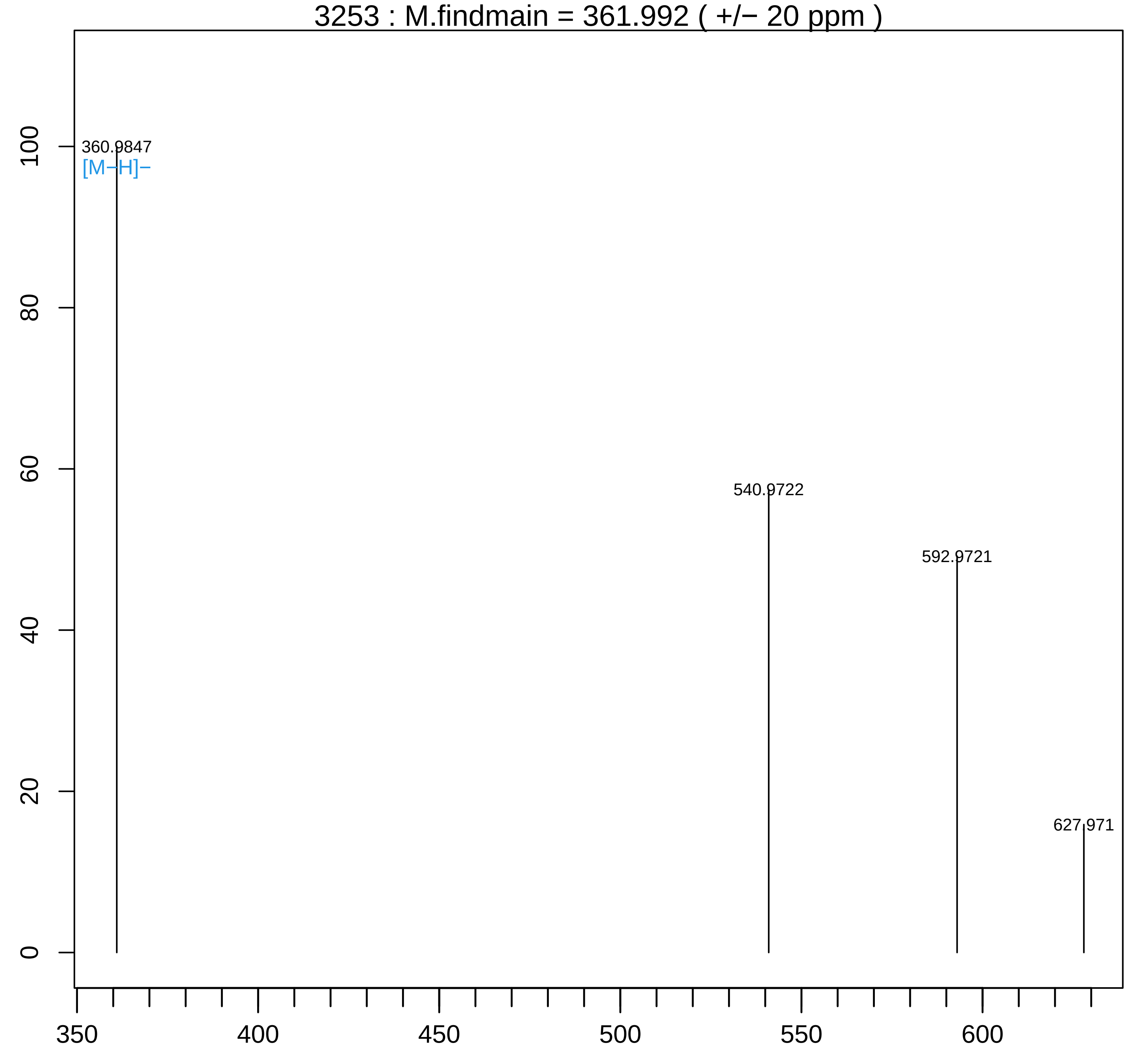
3253 : M.findmain = 361.992 (+/− 20 ppm)
360.9847
[M−H]−
540.9722
592.9721
627.971
0
20
40
60
80
100
350
400
450
500
550
600

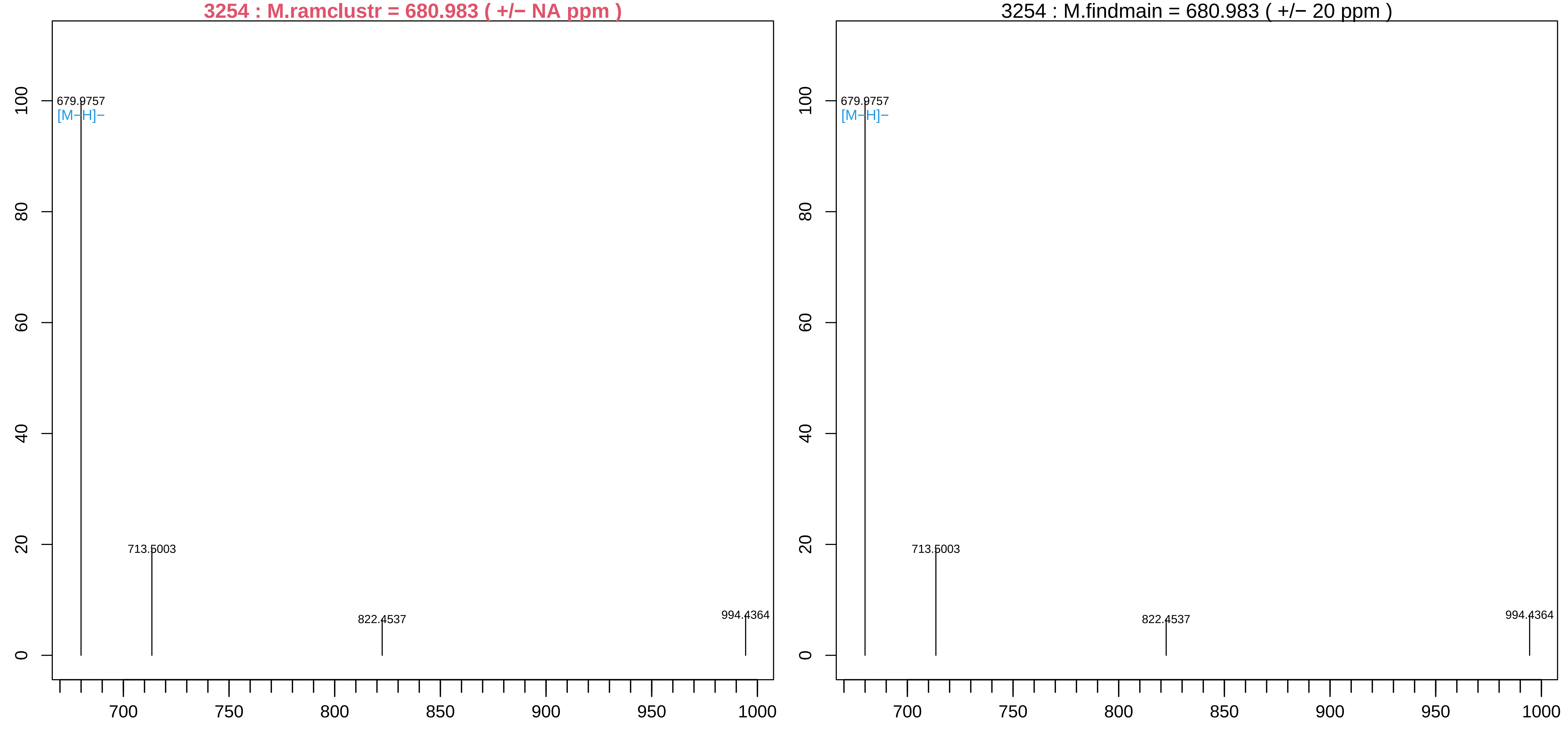
3254 : M.ramclustr = 680.983 (+/− NA ppm)
3254 : M.findmain = 680.983 (+/− 20 ppm)
679.9757
[M−H]−
713.5003
822.4537
994.4364
0
20
40
60
80
100
700
750
800
850
900
950
1000

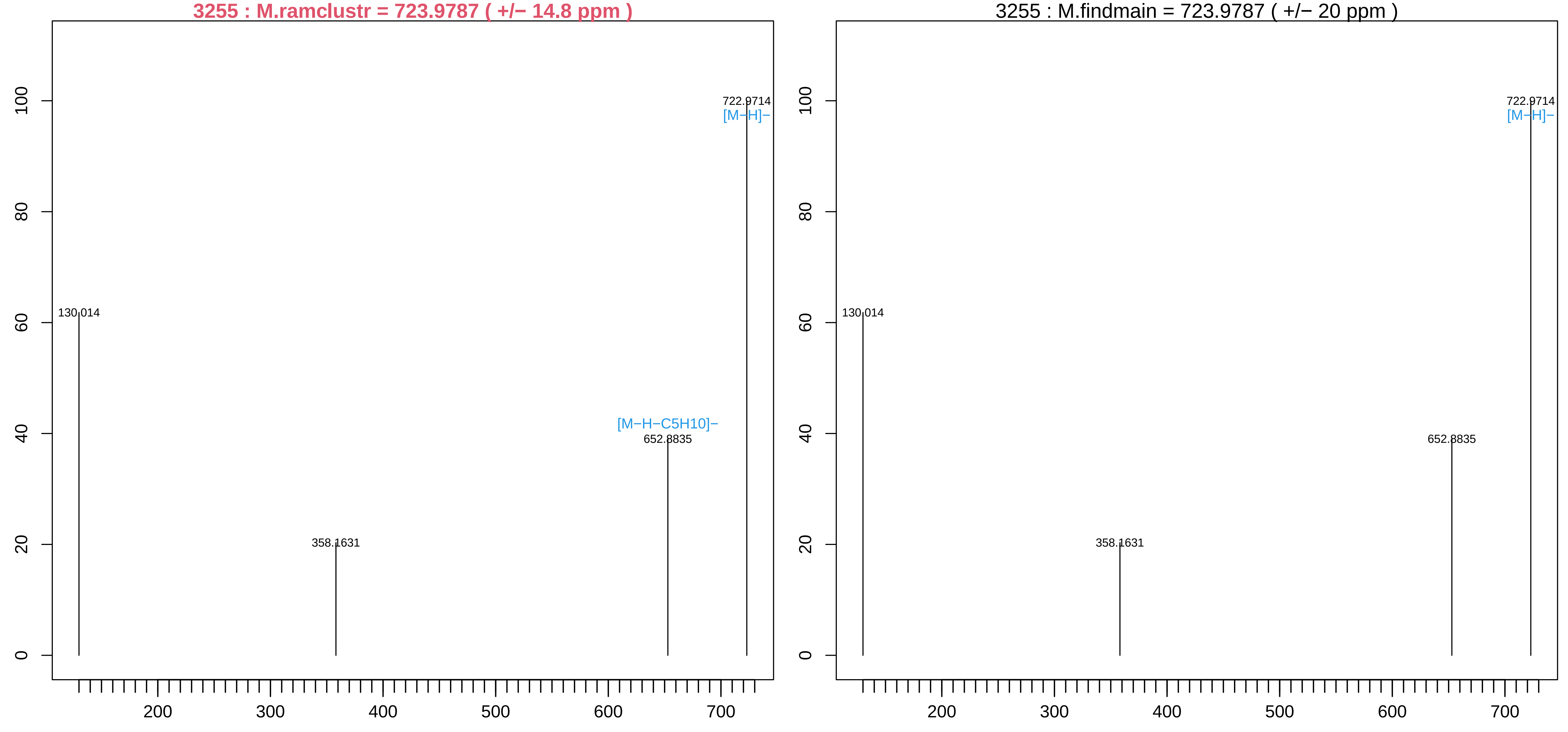
3255 : M.ramclustr = 723.9787 (+/− 14.8 ppm)
722.9714
[M−H]−
130.014
[M−H−C5H10]−
652.8835
358.1631
3255 : M.findmain = 723.9787 (+/− 20 ppm)
722.9714
[M−H]−
130.014
652.8835
358.1631
0
20
40
60
80
100
200
300
400
500
600
700

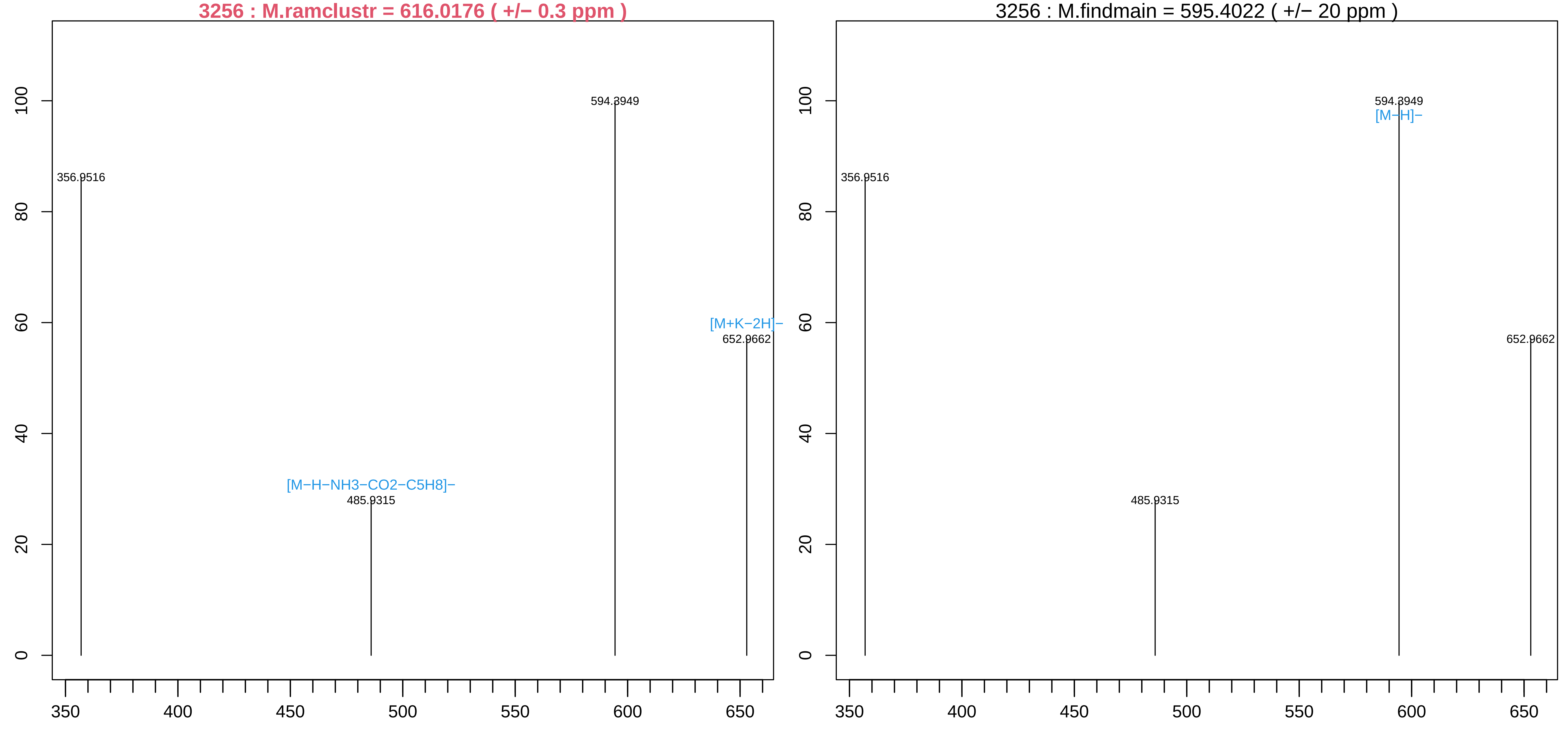

3256 : M.ramclustr = 616.0176 (+/− 0.3 ppm)
356.9516
[M−H−NH3−CO2−C5H8]−
485.9315
594.3949
[M+K−2H]−
652.9662
0
20
40
60
80
100
350
400
450
500
550
600
650
3256 : M.findmain = 595.4022 (+/− 20 ppm)
356.9516
485.9315
594.3949
[M−H]−
652.9662

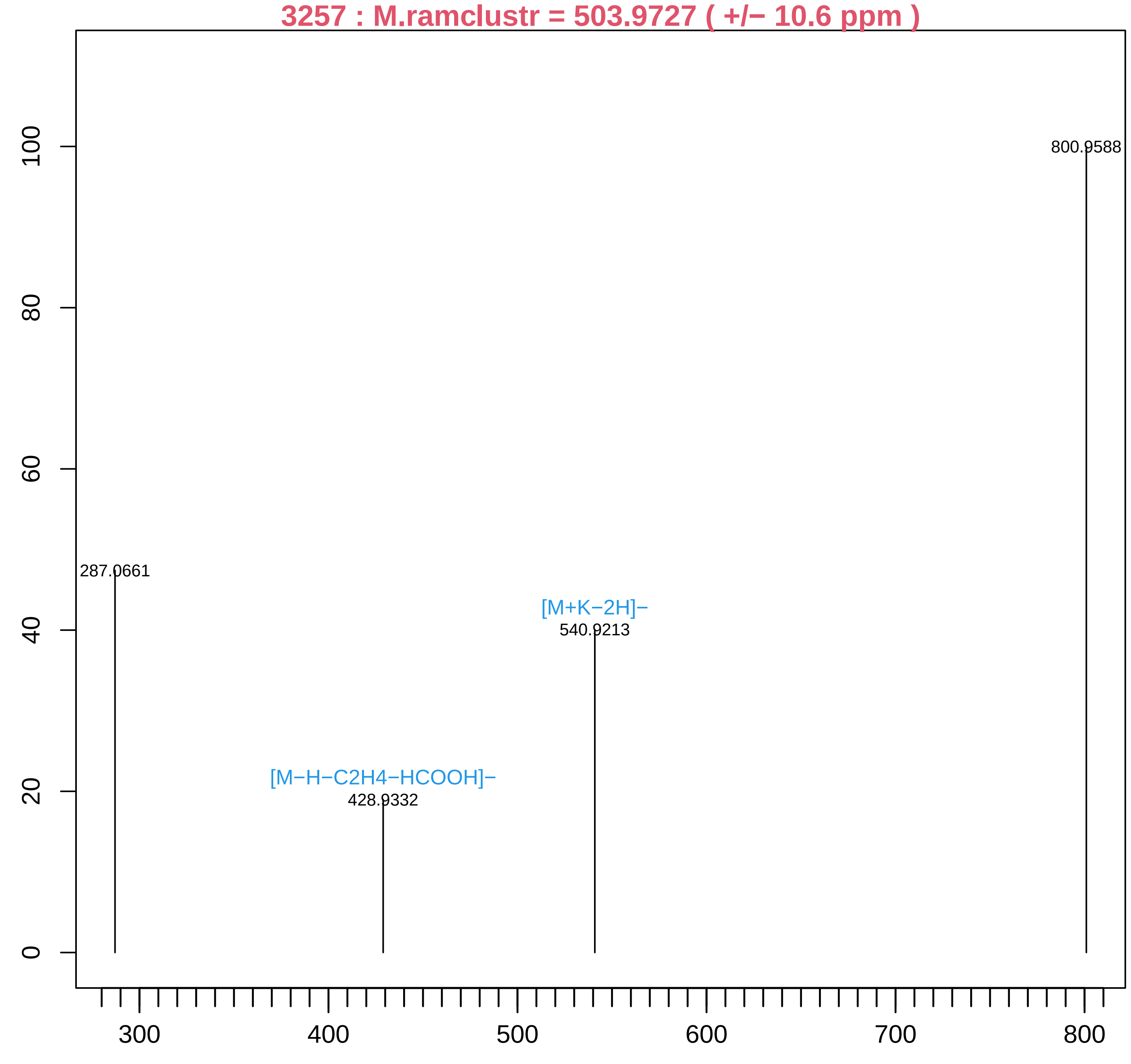
3257 : M.ramclustr = 503.9727 (+/− 10.6 ppm)
800.9588
287.0661
[M+K−2H]−
540.9213
[M−H−C2H4−HCOOH]−
428.9332
0
20
40
60
80
100
300
400
500
600
700
800

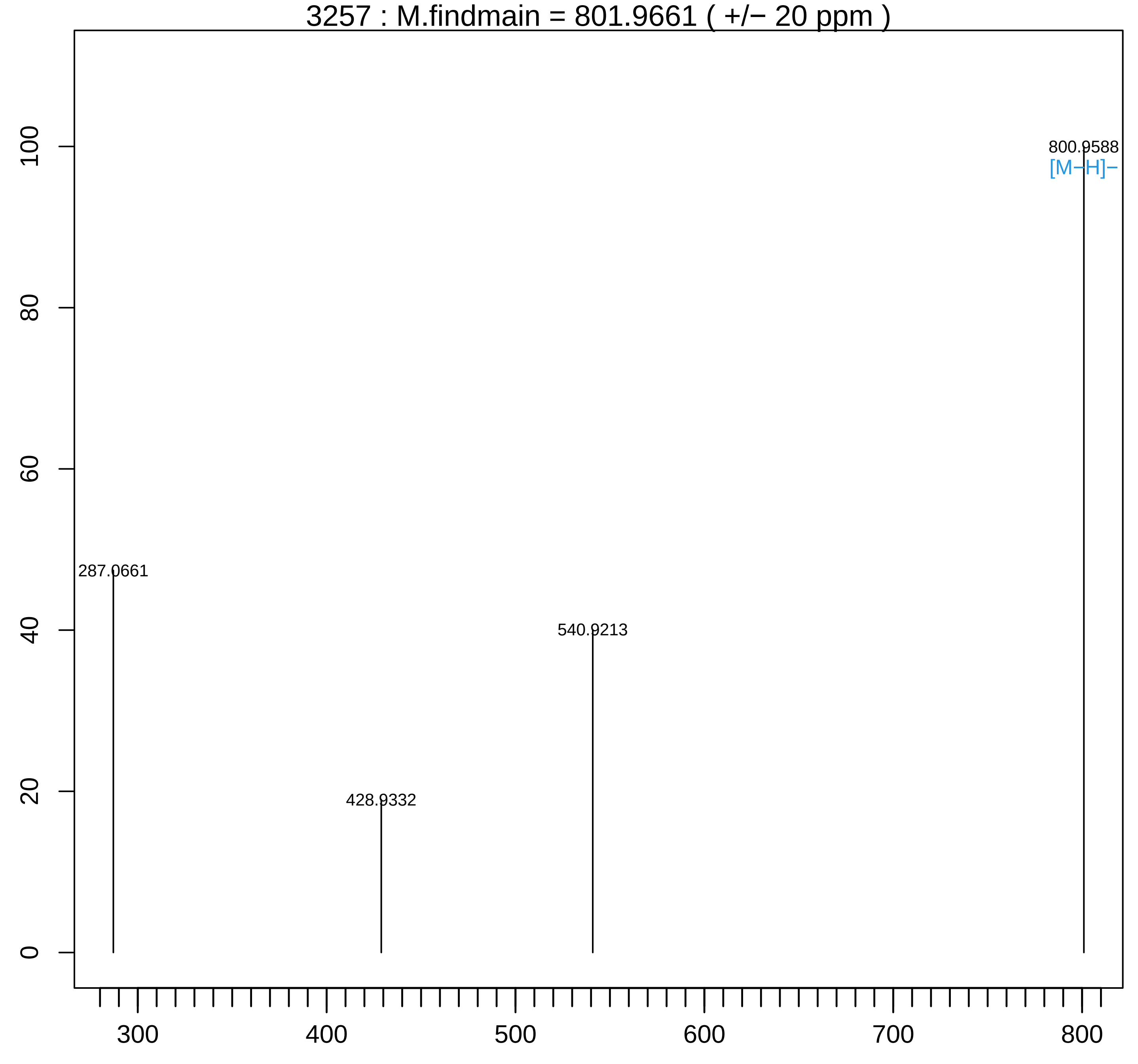
3257 : M.findmain = 801.9661 (+/− 20 ppm)
800.9588
[M−H]−
287.0661
540.9213
428.9332
0
20
40
60
80
100
300
400
500
600
700
800

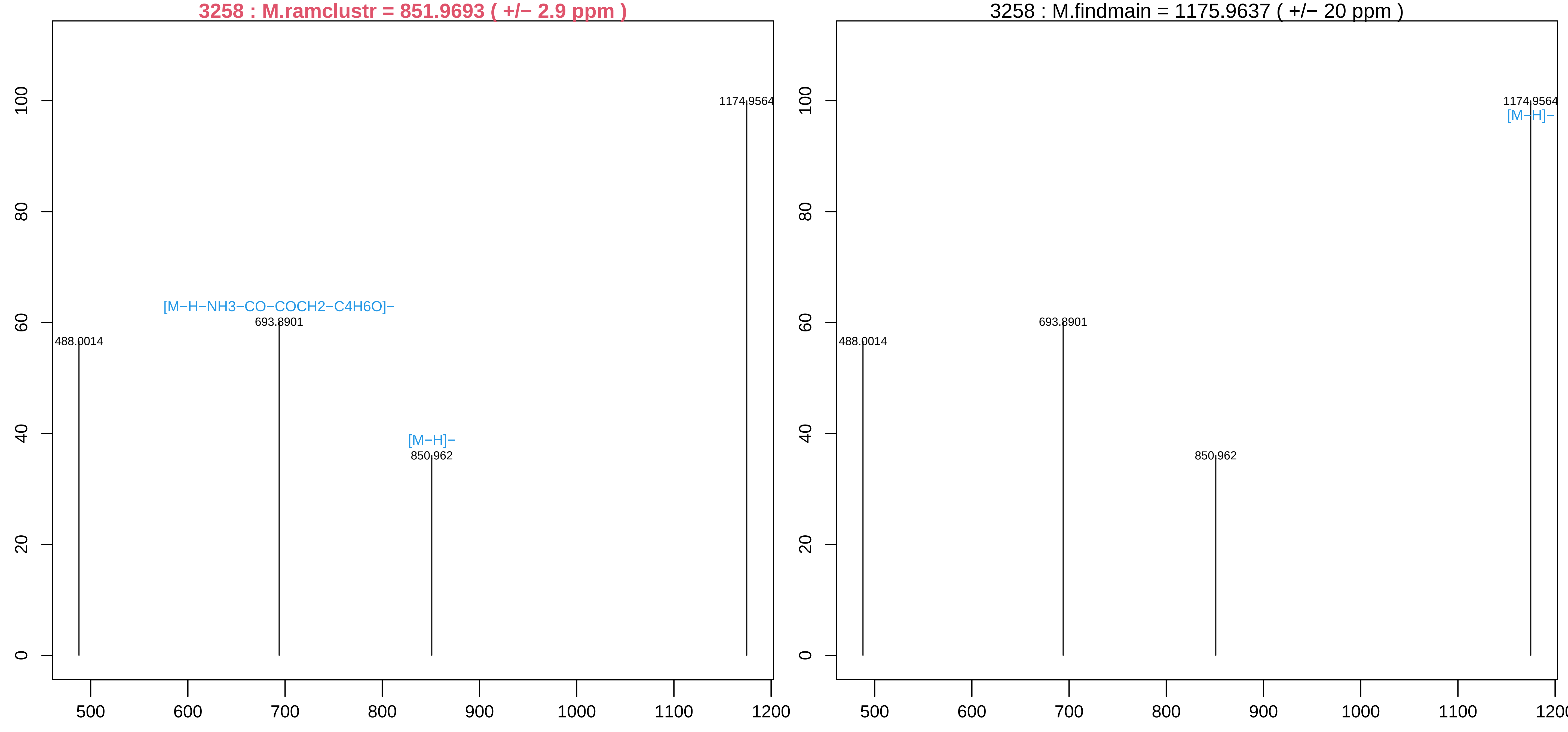

3258 : M.ramclustr = 851.9693 (+/− 2.9 ppm)
1174.9564
[M−H−NH3−CO−COCH2−C4H6O]−
693.8901
488.0014
[M−H]−
850.962
3258 : M.findmain = 1175.9637 (+/− 20 ppm)
1174.9564
[M−H]−
693.8901
488.0014
850.962

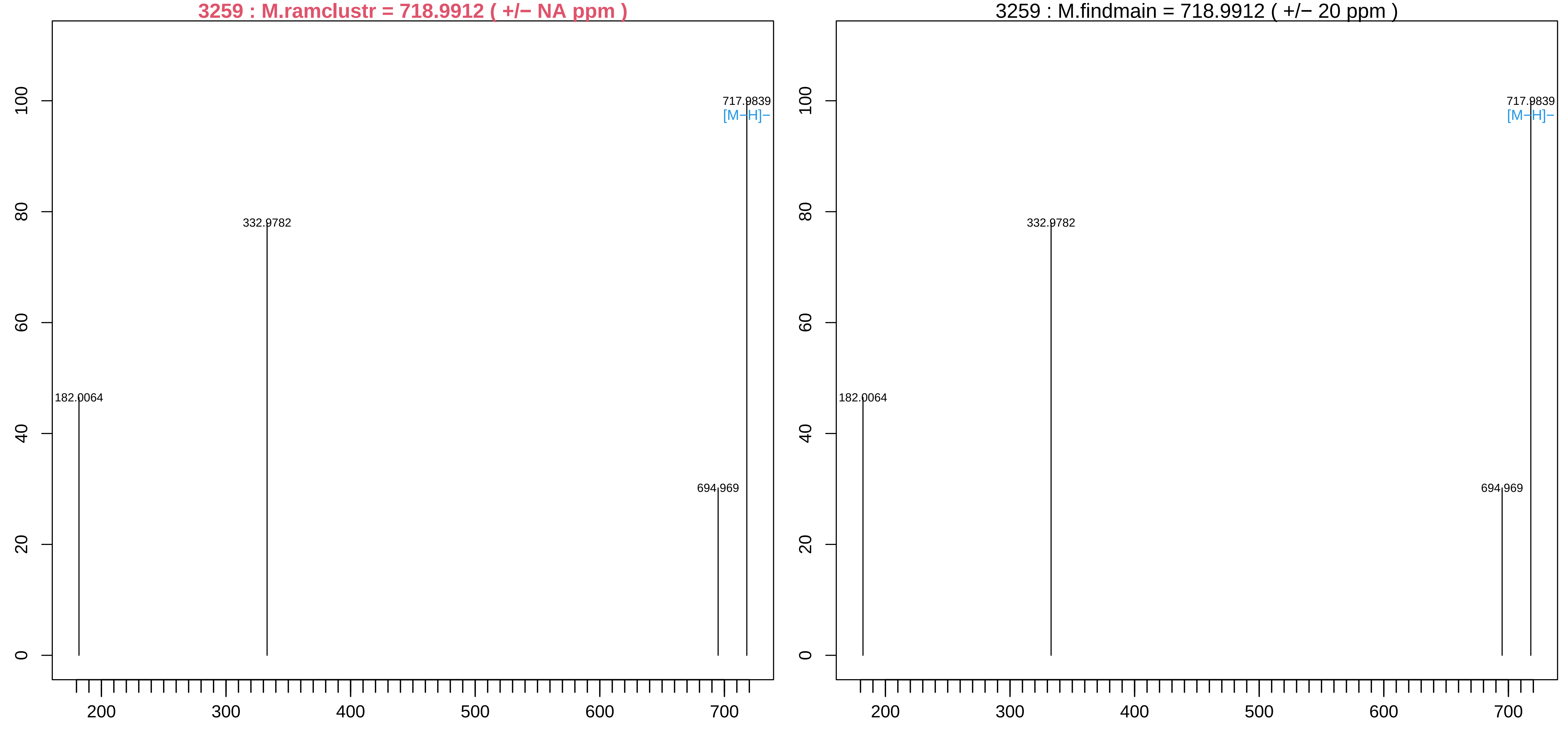
3259 : M.ramclustr = 718.9912 (+/− NA ppm)
3259 : M.findmain = 718.9912 (+/− 20 ppm)
182.0064
332.9782
694.969
717.9839
[M−H]−
0
20
40
60
80
100
200
300
400
500
600
700

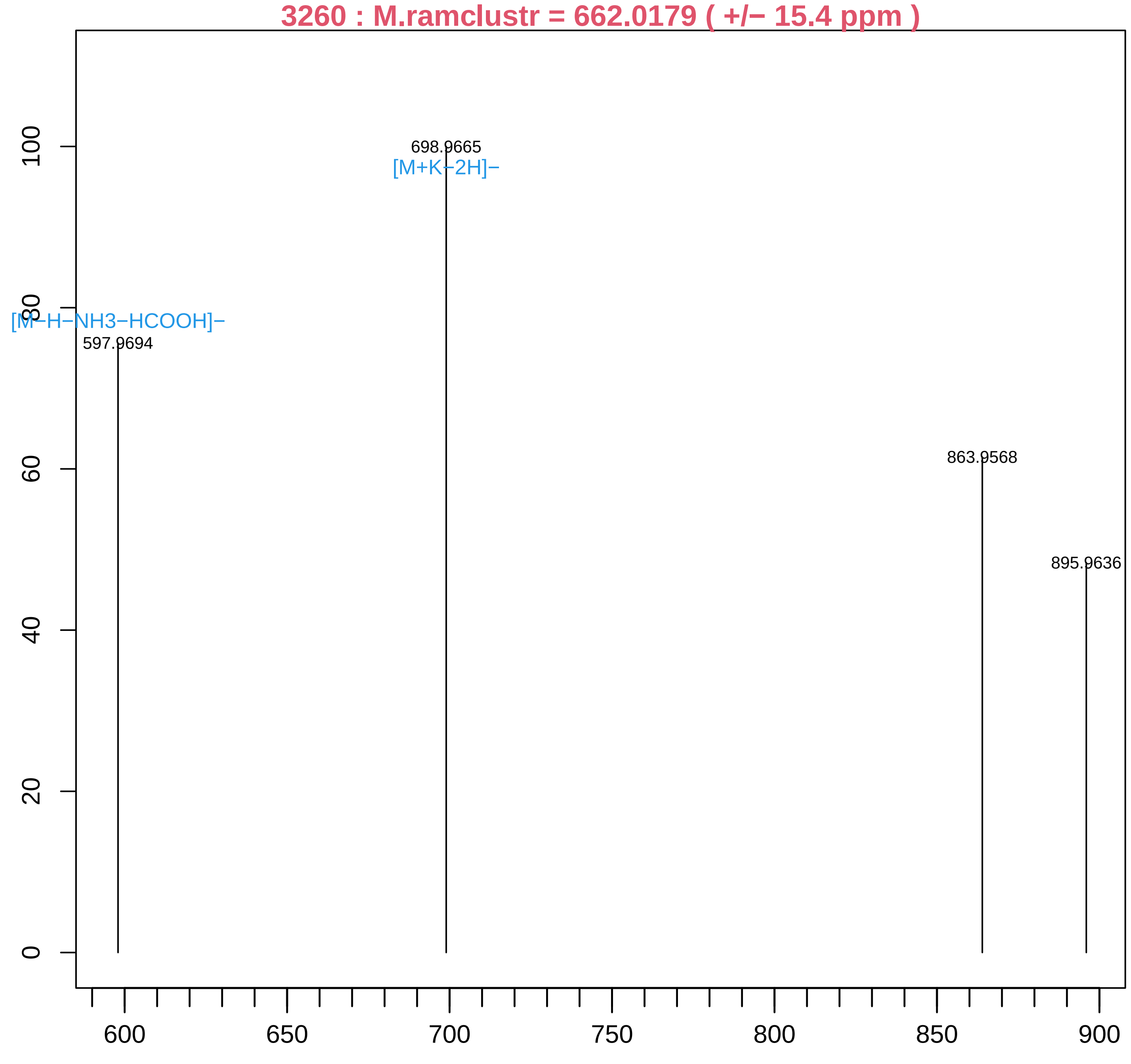
3260 : M.ramclustr = 662.0179 (+/− 15.4 ppm)
[M−H−NH3−HCOOH]−
597.9694
698.9665
[M+K−2H]−
863.9568
895.9636
0
20
40
60
80
100
600
650
700
750
800
850
900

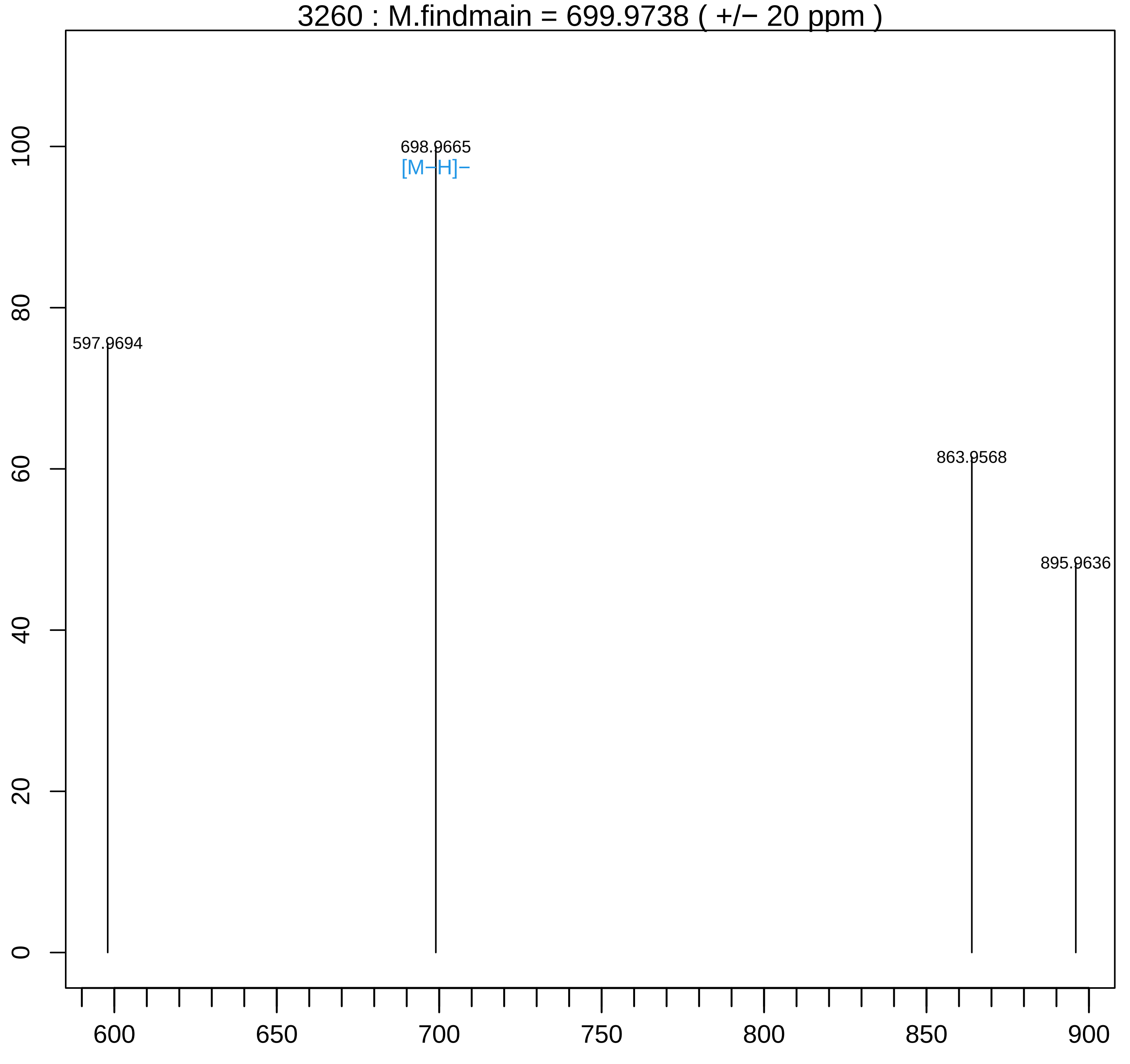
3260 : M.findmain = 699.9738 (+/− 20 ppm)
597.9694
698.9665
[M−H]−
863.9568
895.9636
0
20
40
60
80
100
600
650
700
750
800
850
900

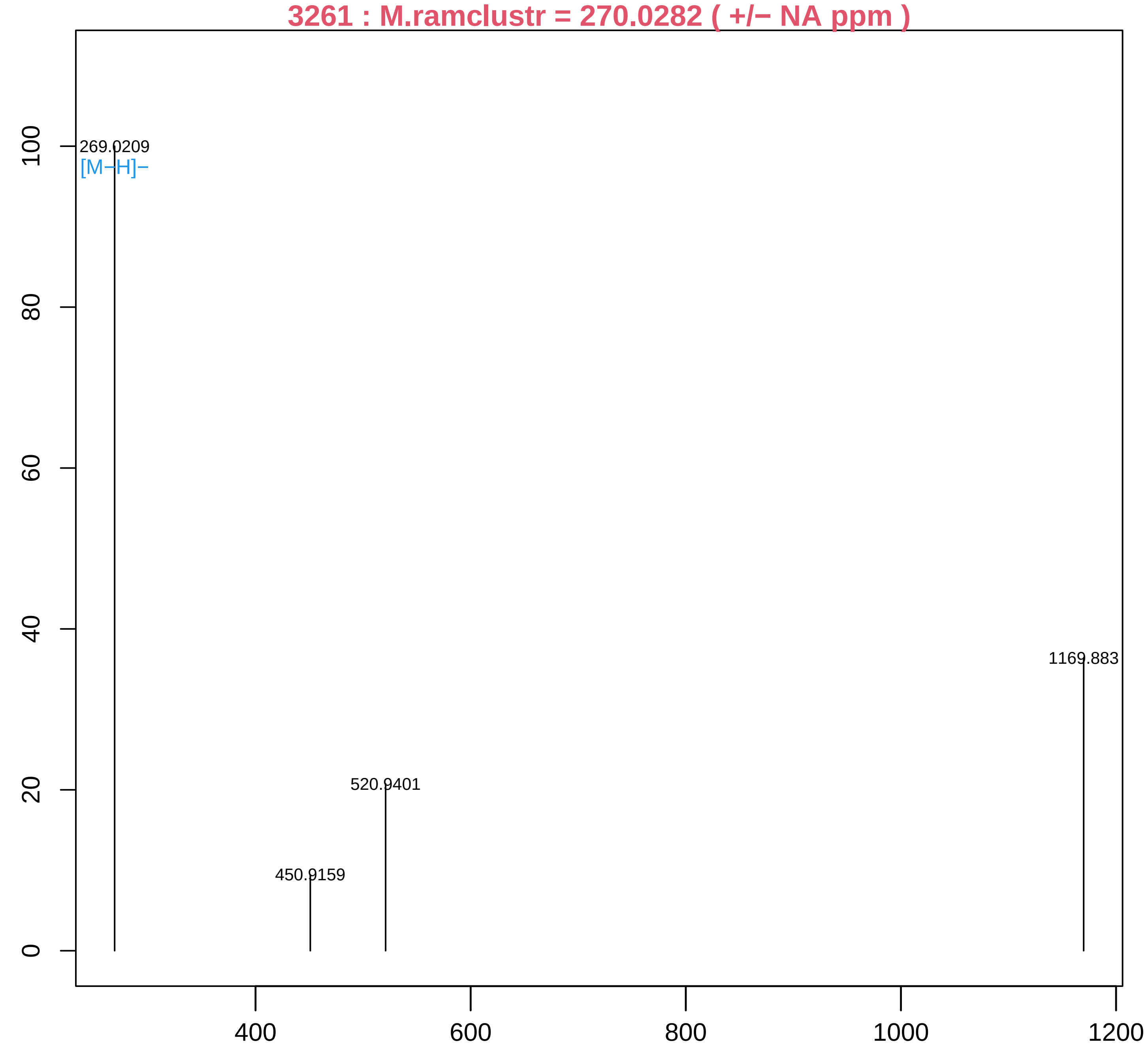
3261 : M.ramclustr = 270.0282 (+/− NA ppm)
269.0209
[M−H]−
450.9159
520.9401
1169.883
0
20
40
60
80
100
400
600
800
1000
1200

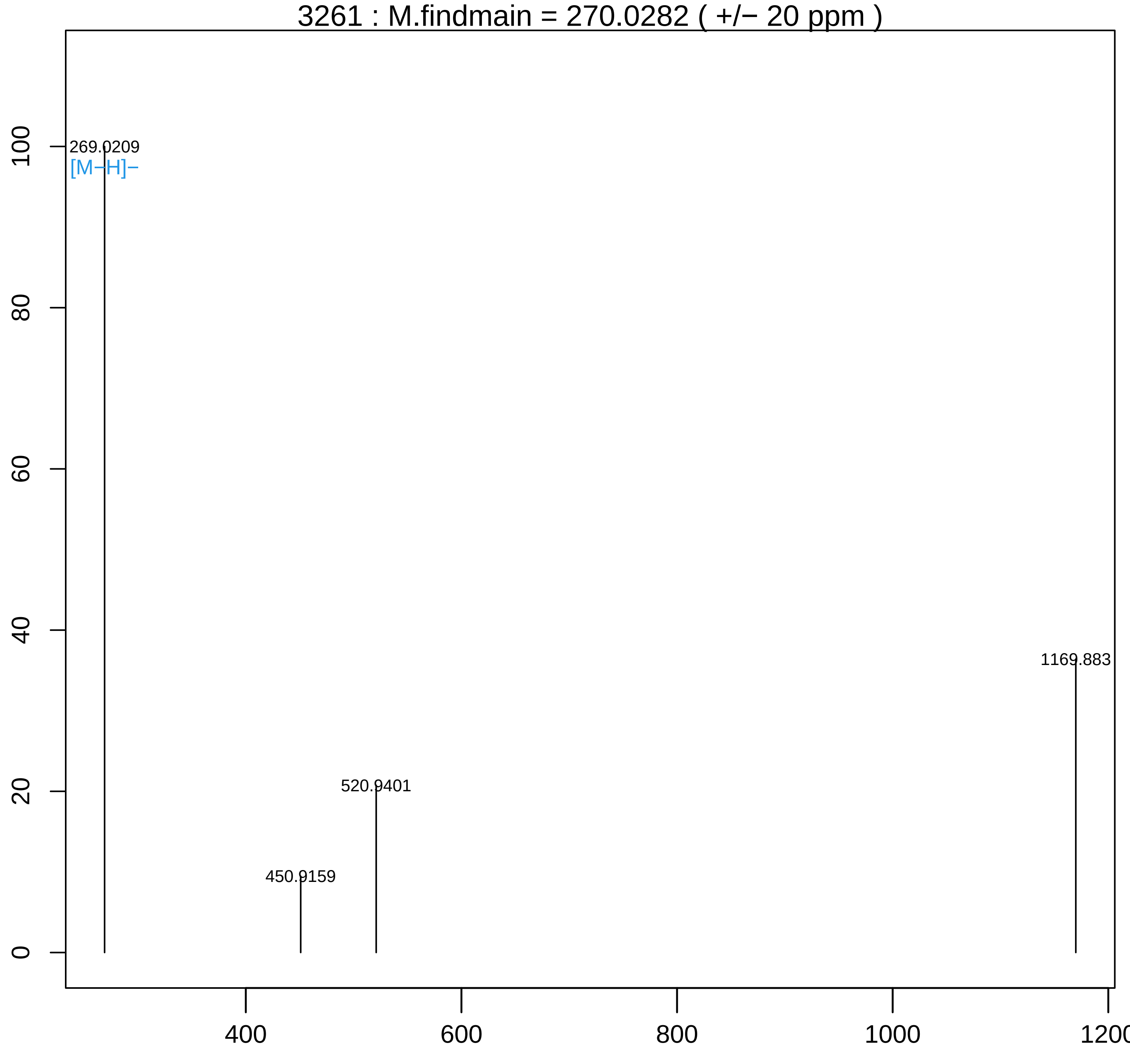
3261 : M.findmain = 270.0282 (+/− 20 ppm)
269.0209
[M−H]−
450.9159
520.9401
1169.883
0
20
40
60
80
100
400
600
800
1000
1200

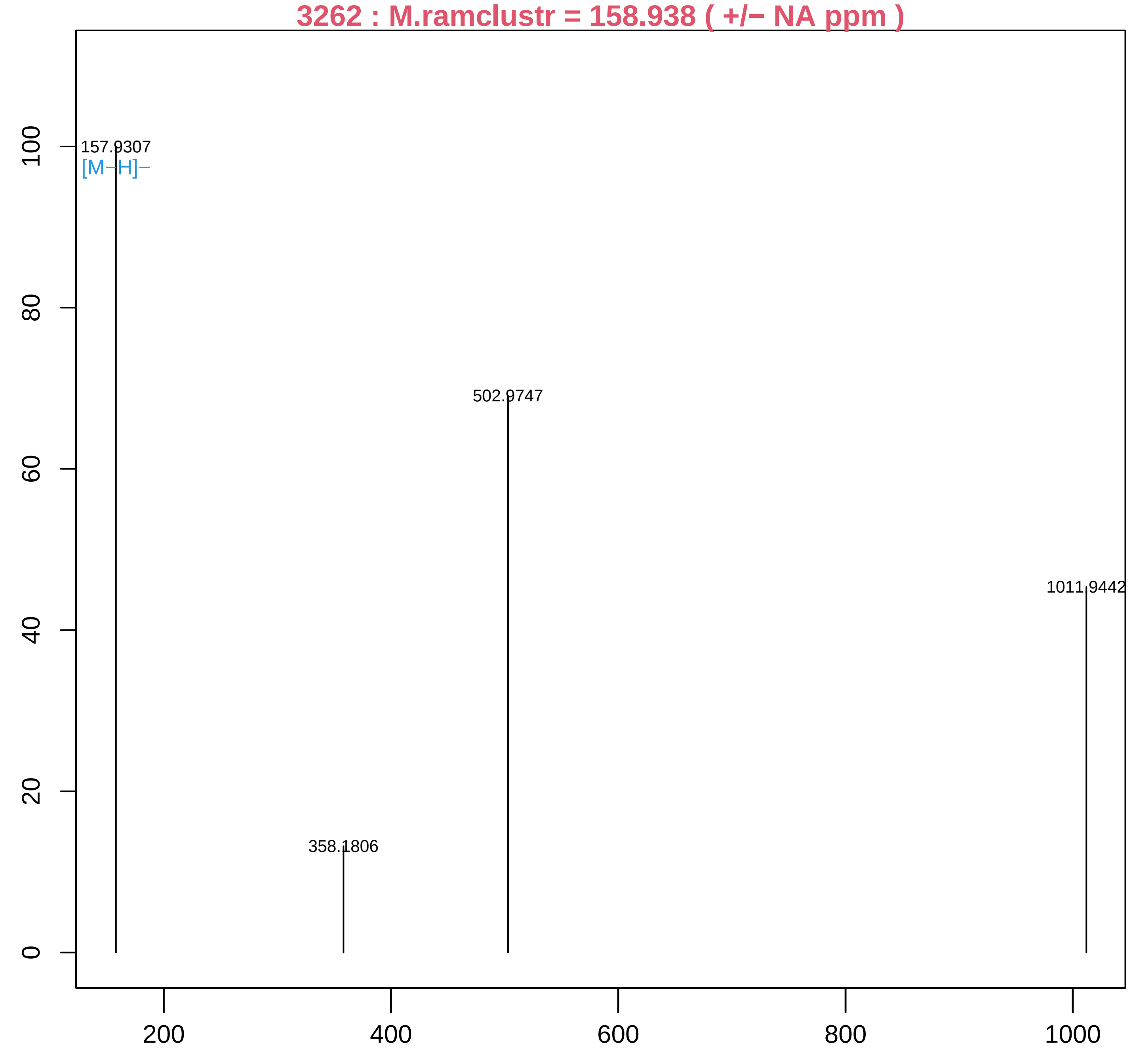
3262 : M.ramclustr = 158.938 (+/− NA ppm)
157.9307
[M−H]−
502.9747
1011.9442
358.1806
0
20
40
60
80
100
200
400
600
800
1000

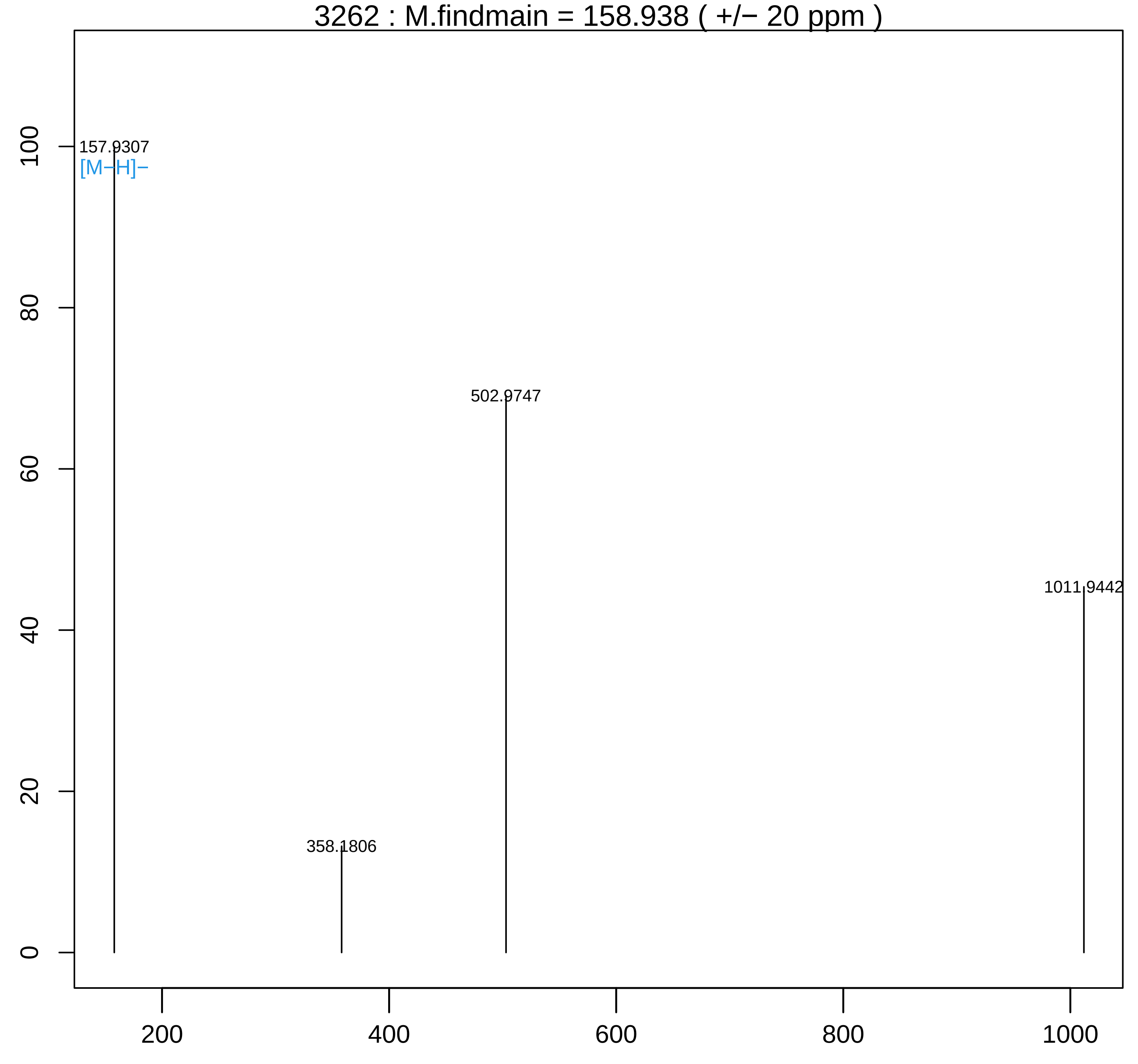
3262 : M.findmain = 158.938 (+/− 20 ppm)
157.9307
[M−H]−
502.9747
1011.9442
358.1806
0
20
40
60
80
100
200
400
600
800
1000

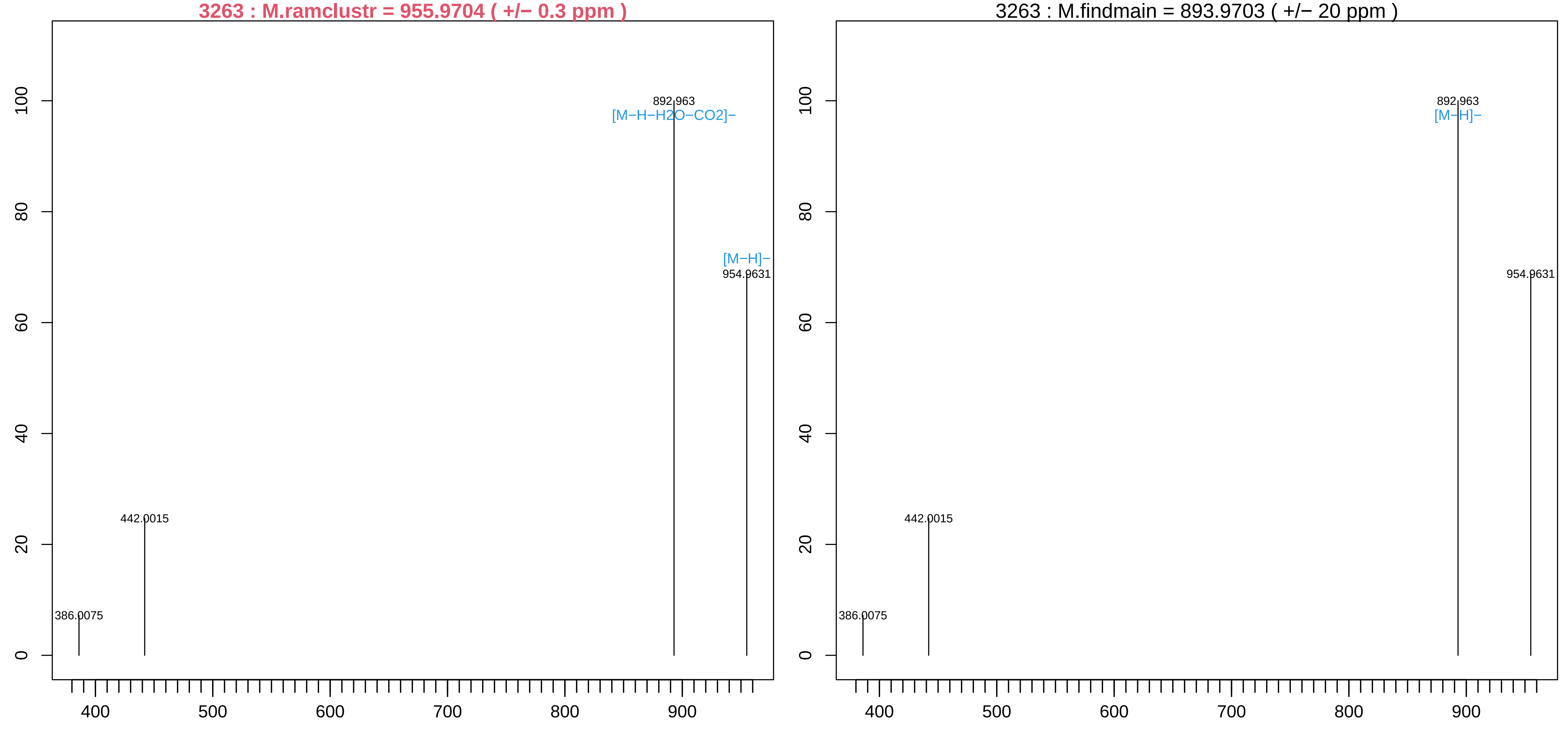
3263 : M.ramclustr = 955.9704 (+/− 0.3 ppm)
892.963
[M−H−H2O−CO2]−
[M−H]−
954.9631
442.0015
386.0075
3263 : M.findmain = 893.9703 (+/− 20 ppm)
892.963
[M−H]−
954.9631
442.0015
386.0075

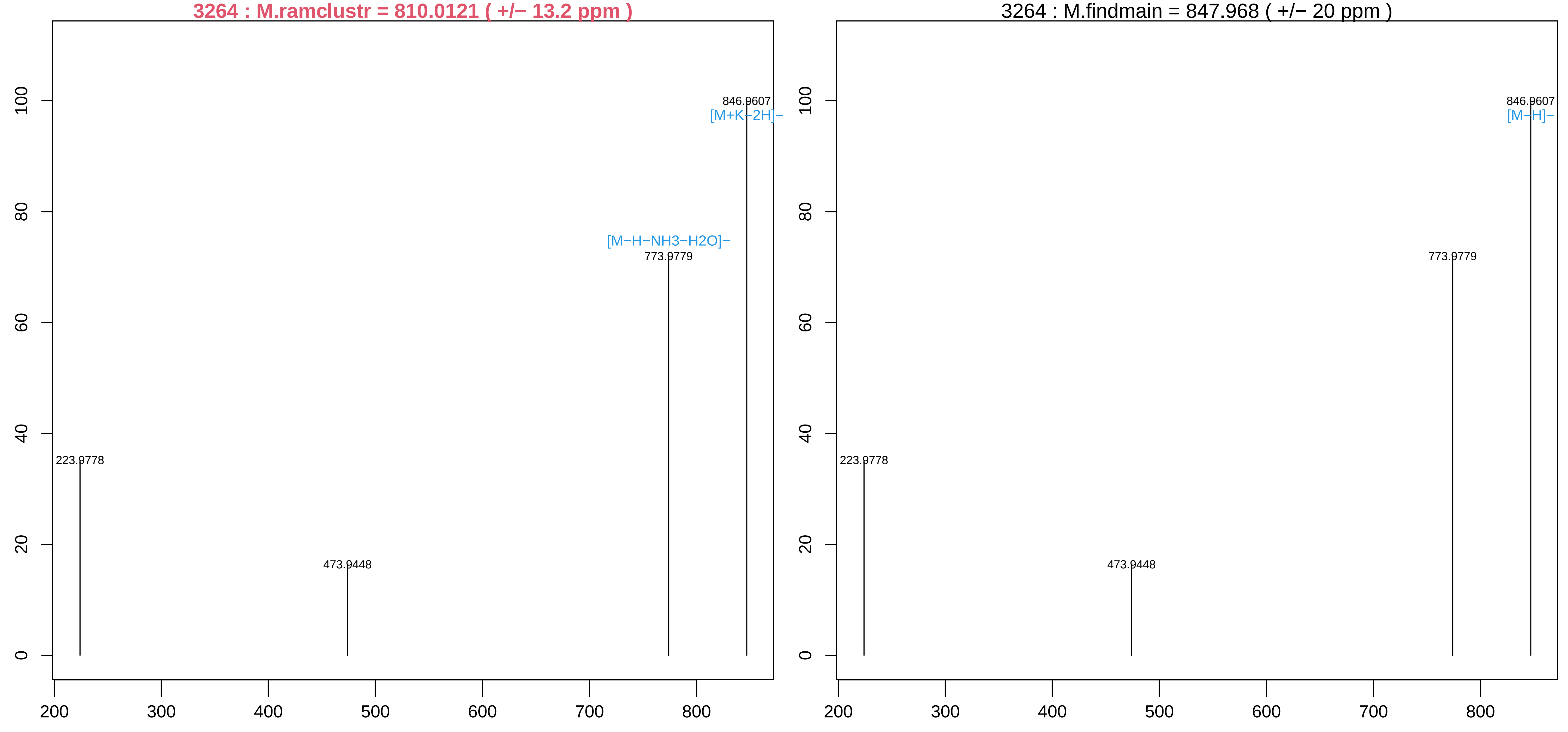

3264 : M.ramclustr = 810.0121 (+/− 13.2 ppm)
846.9607
[M+K−2H]−
[M−H−NH3−H2O]−
773.9779
223.9778
473.9448
0
20
40
60
80
100
200
300
400
500
600
700
800
3264 : M.findmain = 847.968 (+/− 20 ppm)
846.9607
[M−H]−
773.9779
223.9778
473.9448
0
20
40
60
80
100
200
300
400
500
600
700
800

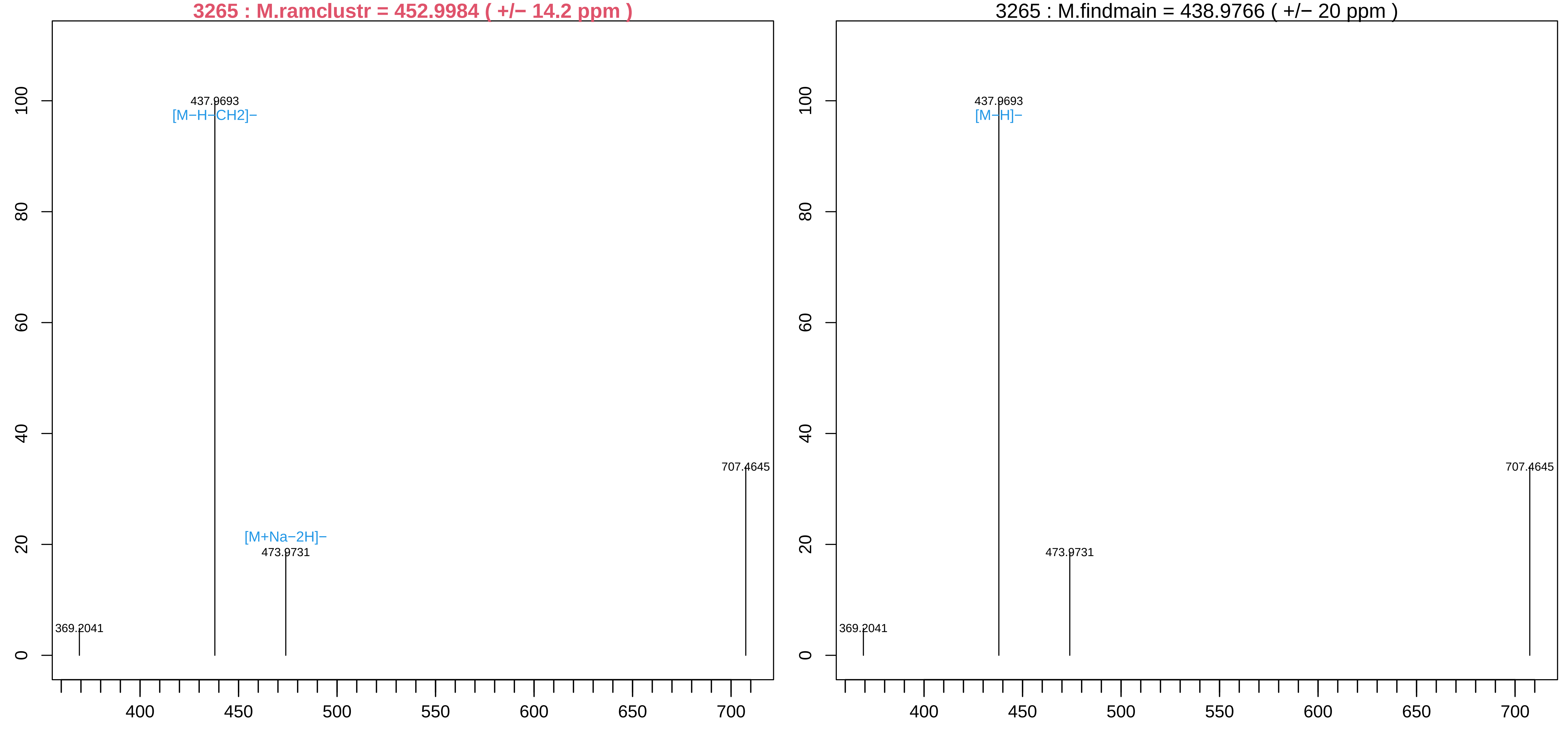
3265 : M.ramclustr = 452.9984 (+/− 14.2 ppm)
437.9693
[M−H−CH2]−
[M+Na−2H]−
473.9731
707.4645
369.2041
3265 : M.findmain = 438.9766 (+/− 20 ppm)
437.9693
[M−H]−
473.9731
707.4645
369.2041
0
20
40
60
80
100
400
450
500
550
600
650
700

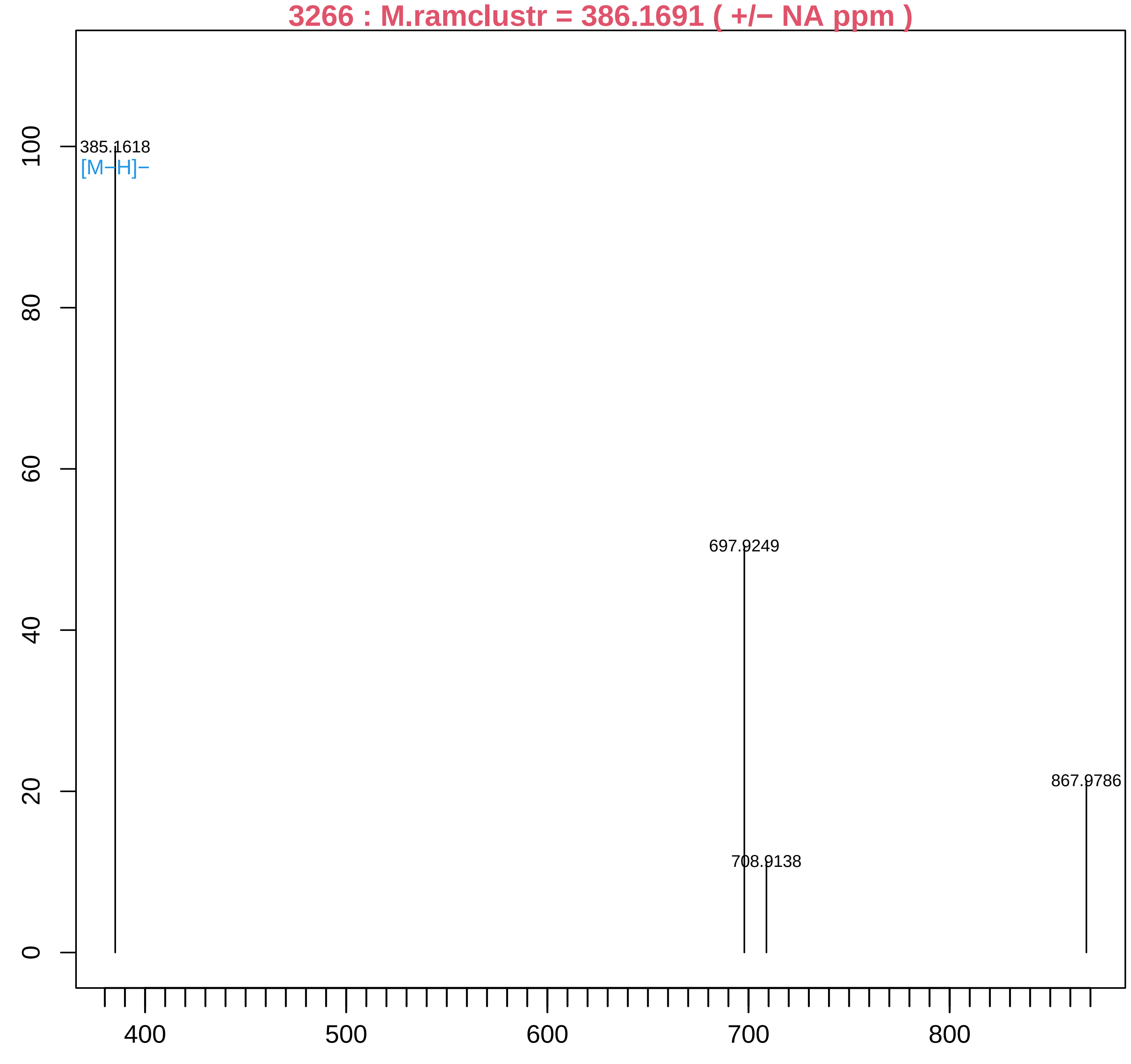
3266 : M.ramclustr = 386.1691 (+/− NA ppm)
385.1618
[M−H]−
697.9249
708.9138
867.9786
0
20
40
60
80
100
400
500
600
700
800

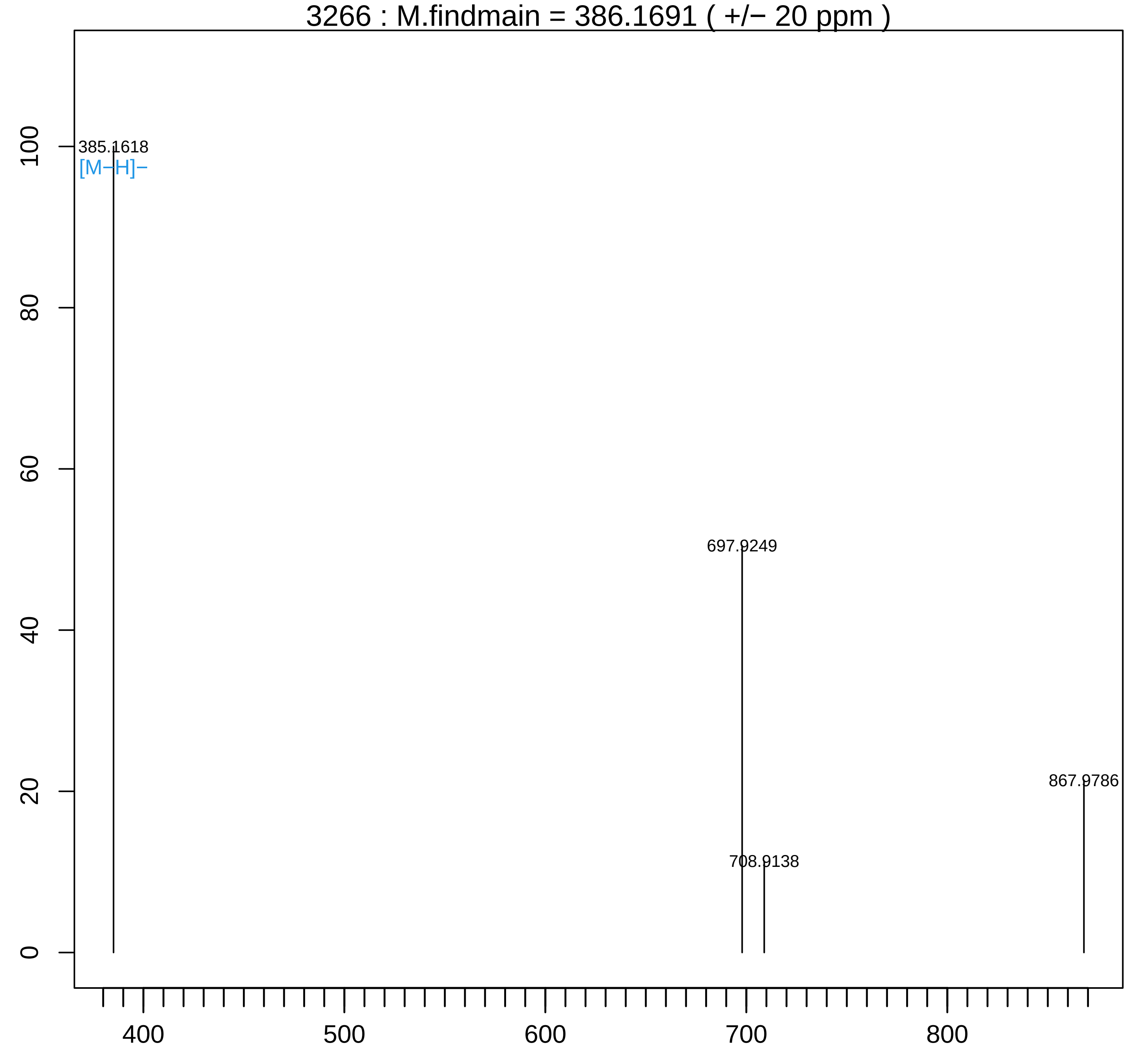
3266 : M.findmain = 386.1691 (+/− 20 ppm)
385.1618
[M−H]−
697.9249
708.9138
867.9786
0
20
40
60
80
100
400
500
600
700
800

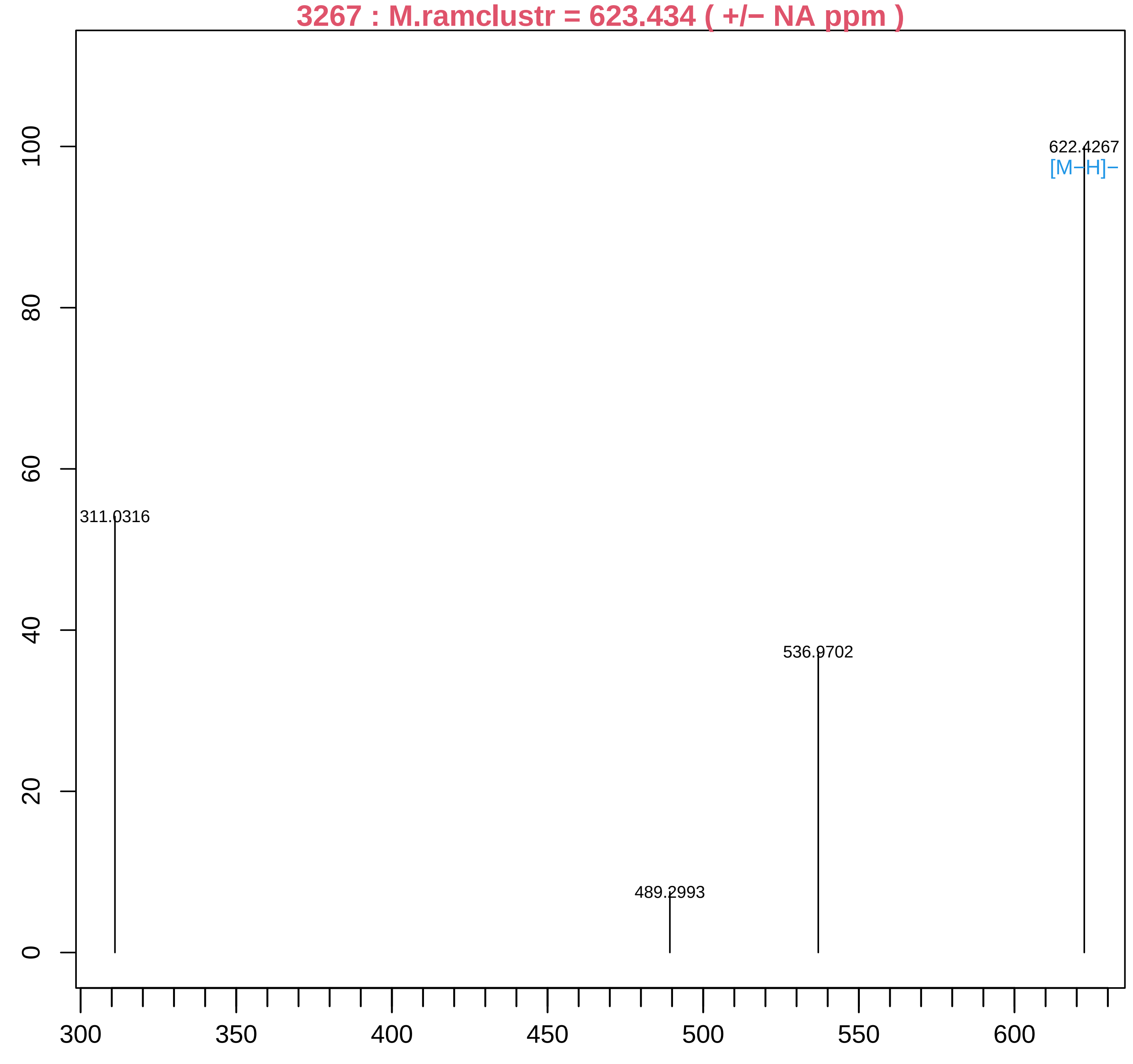
3267 : M.ramclustr = 623.434 (+/− NA ppm)
622.4267
[M−H]−
311.0316
536.9702
489.2993
0
20
40
60
80
100
300
350
400
450
500
550
600

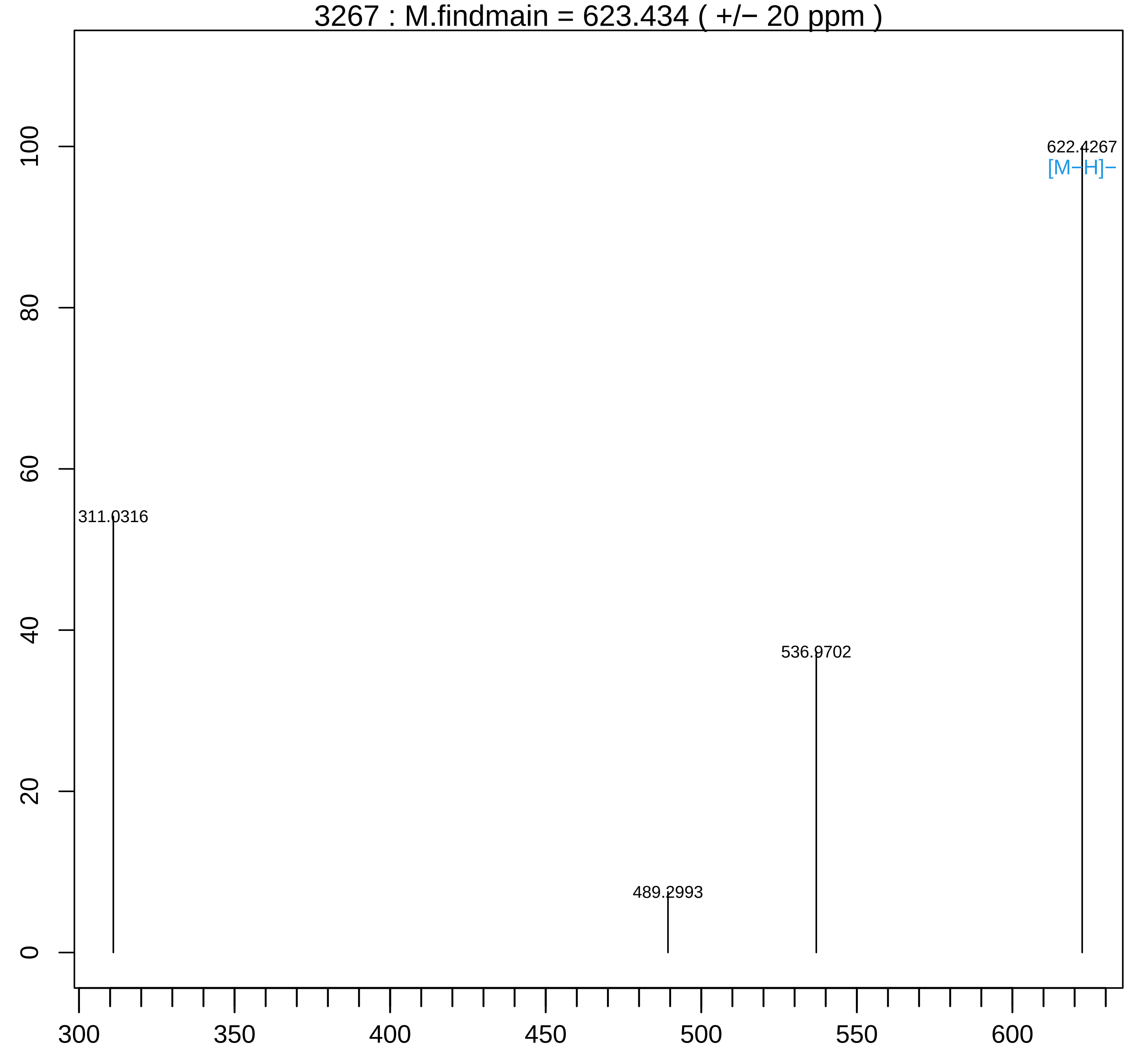
3267 : M.findmain = 623.434 (+/− 20 ppm)
622.4267
[M−H]−
311.0316
536.9702
489.2993
0
20
40
60
80
100
300
350
400
450
500
550
600

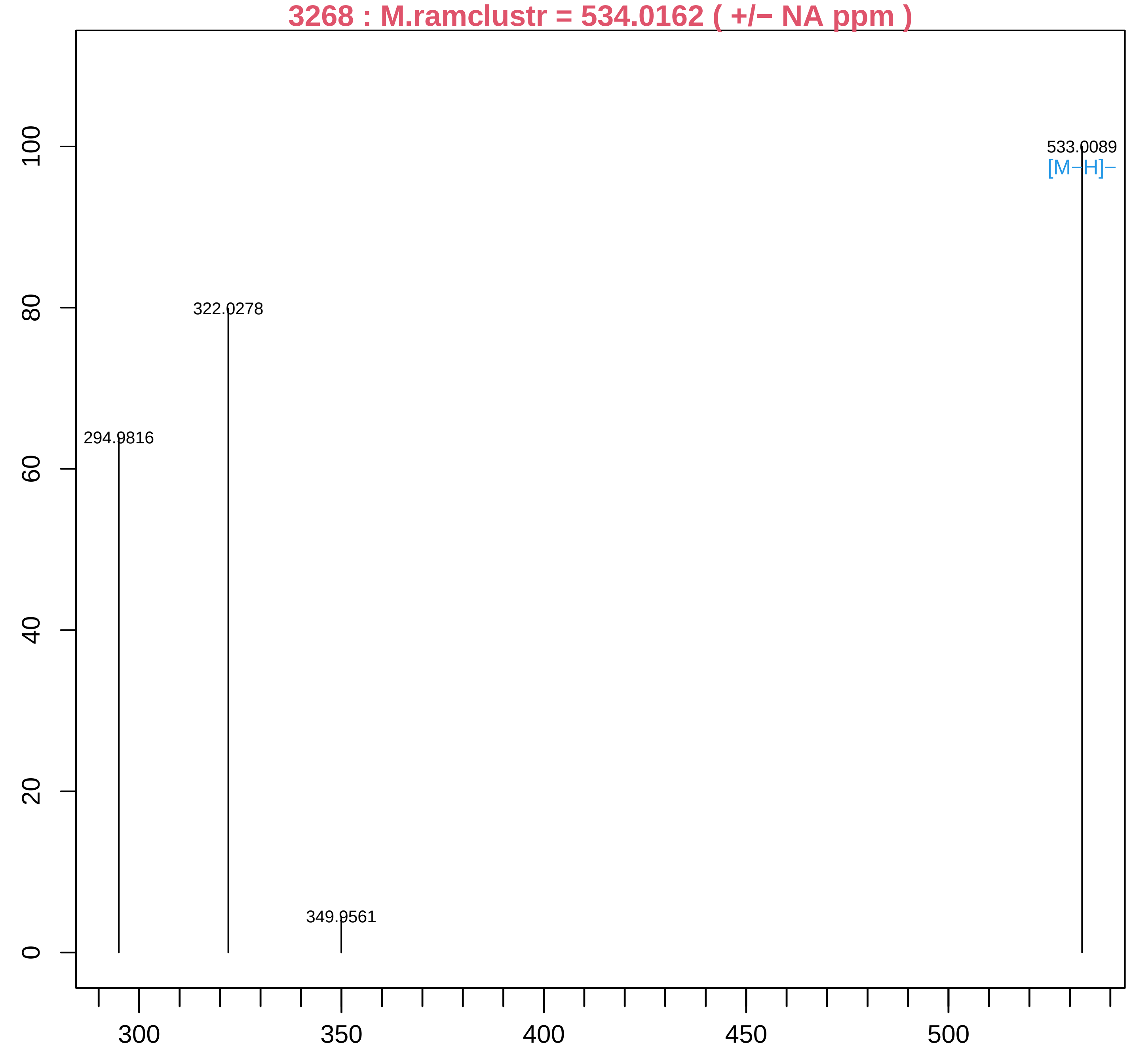
3268 : M.ramclustr = 534.0162 (+/− NA ppm)
533.0089
[M−H]−
322.0278
294.9816
349.9561
0
20
40
60
80
100
300
350
400
450
500

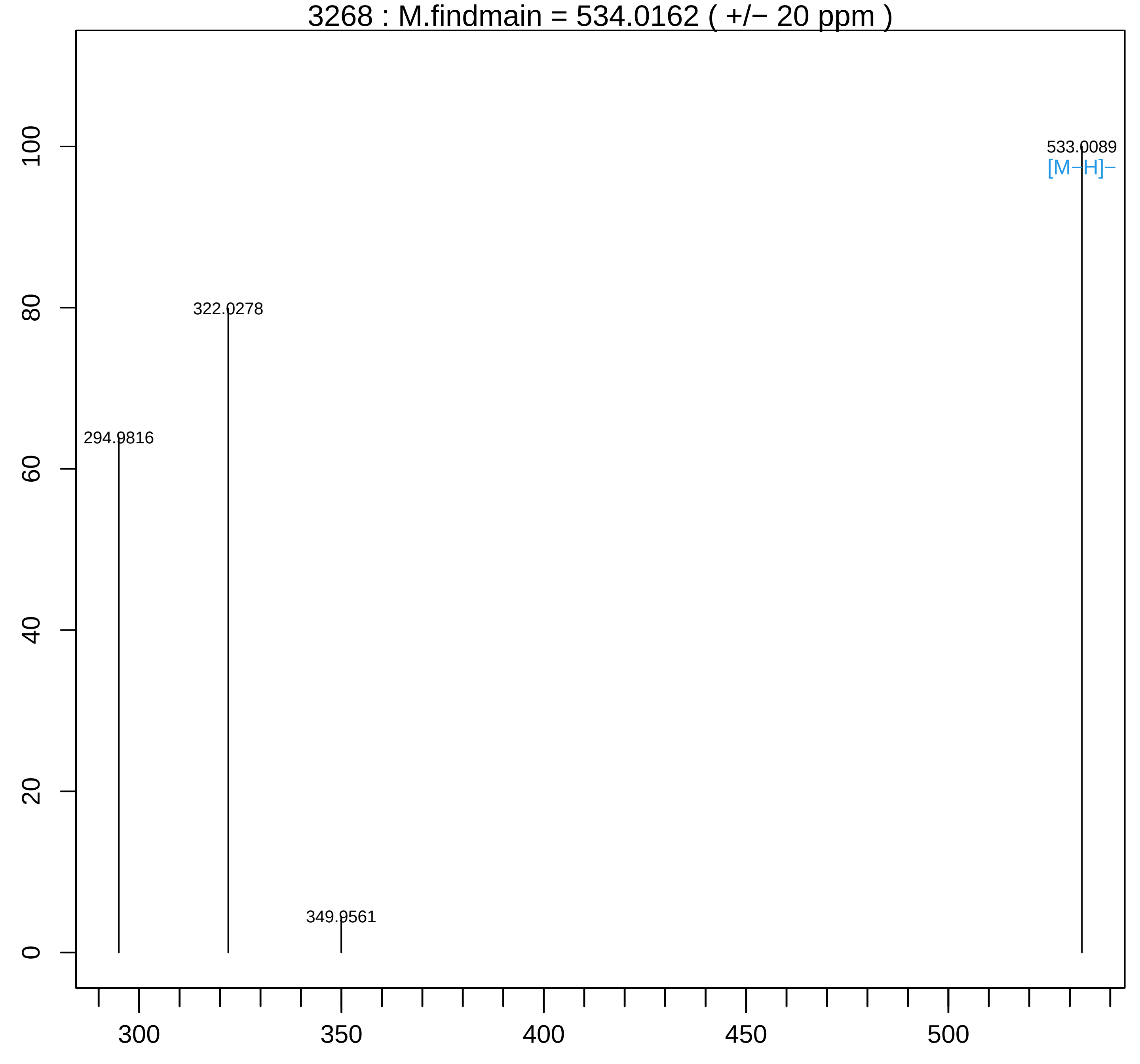
3268 : M.findmain = 534.0162 (+/− 20 ppm)
533.0089
[M−H]−
322.0278
294.9816
349.9561
0
20
40
60
80
100
300
350
400
450
500

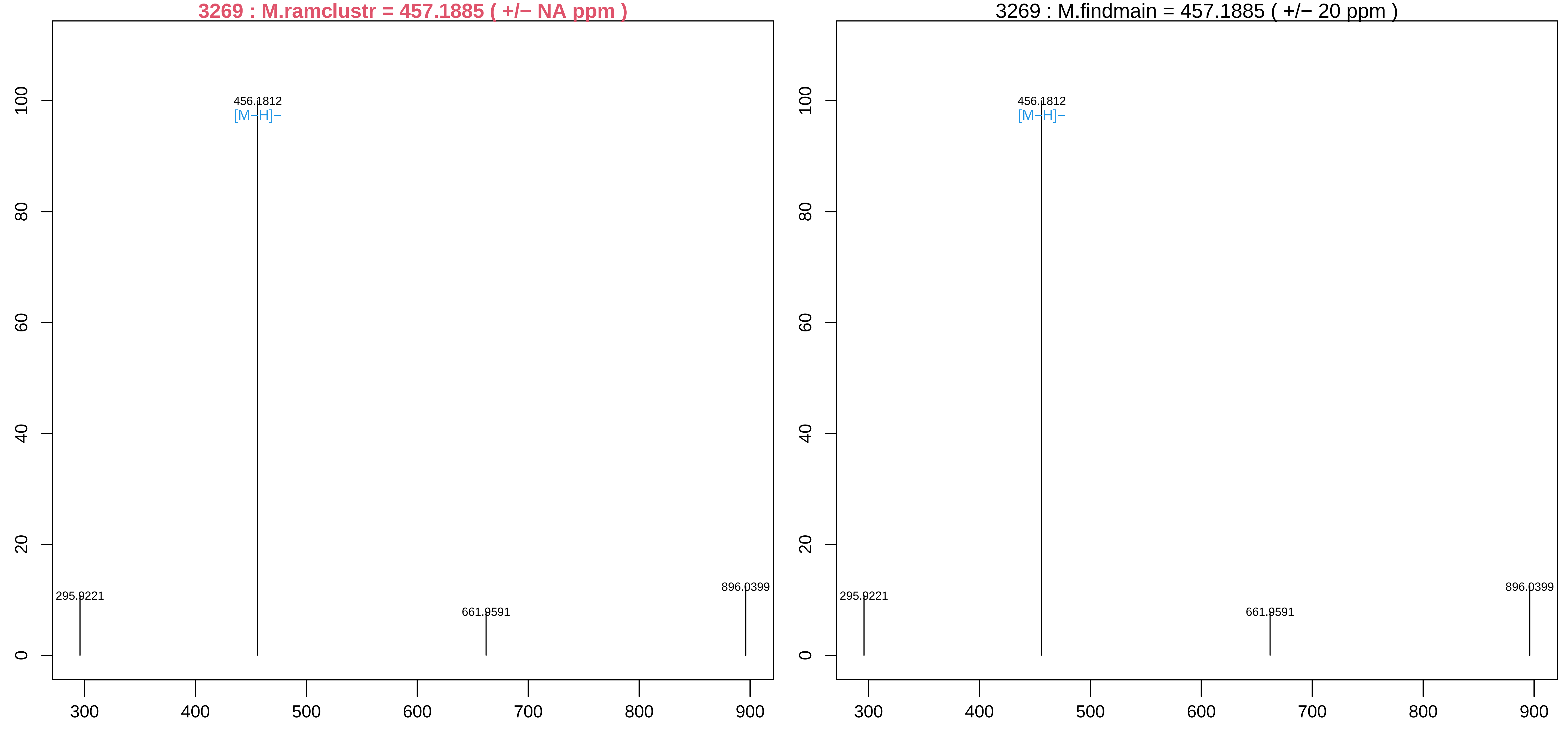
3269 : M.ramclustr = 457.1885 (+/− NA ppm)
456.1812
[M−H]−
295.9221
661.9591
896.0399
3269 : M.findmain = 457.1885 (+/− 20 ppm)
456.1812
[M−H]−
295.9221
661.9591
896.0399
0
20
40
60
80
100
300
400
500
600
700
800
900

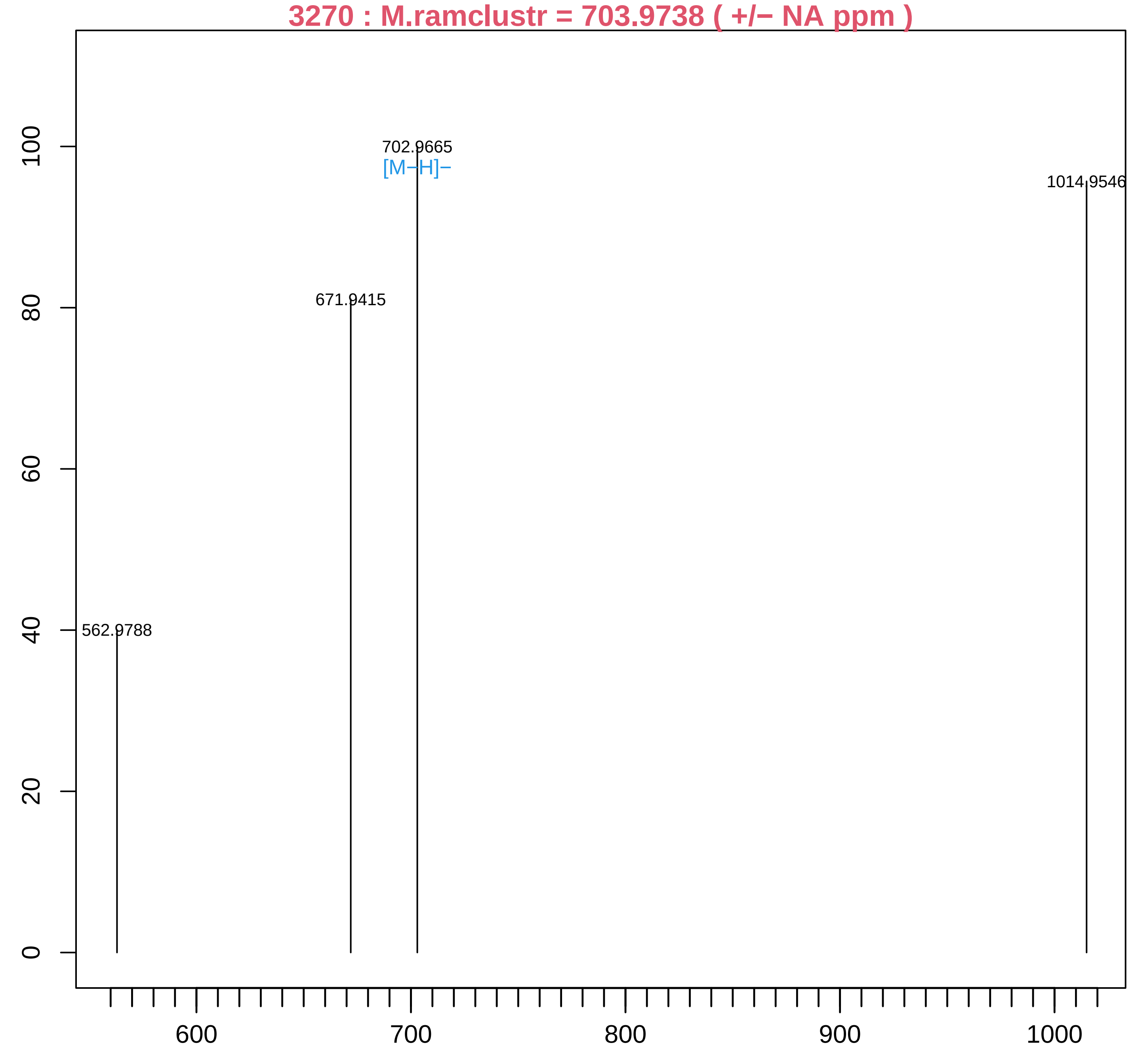
3270 : M.ramclustr = 703.9738 (+/− NA ppm)
702.9665
[M−H]−
1014.9546
671.9415
562.9788
0
20
40
60
80
100
600
700
800
900
1000

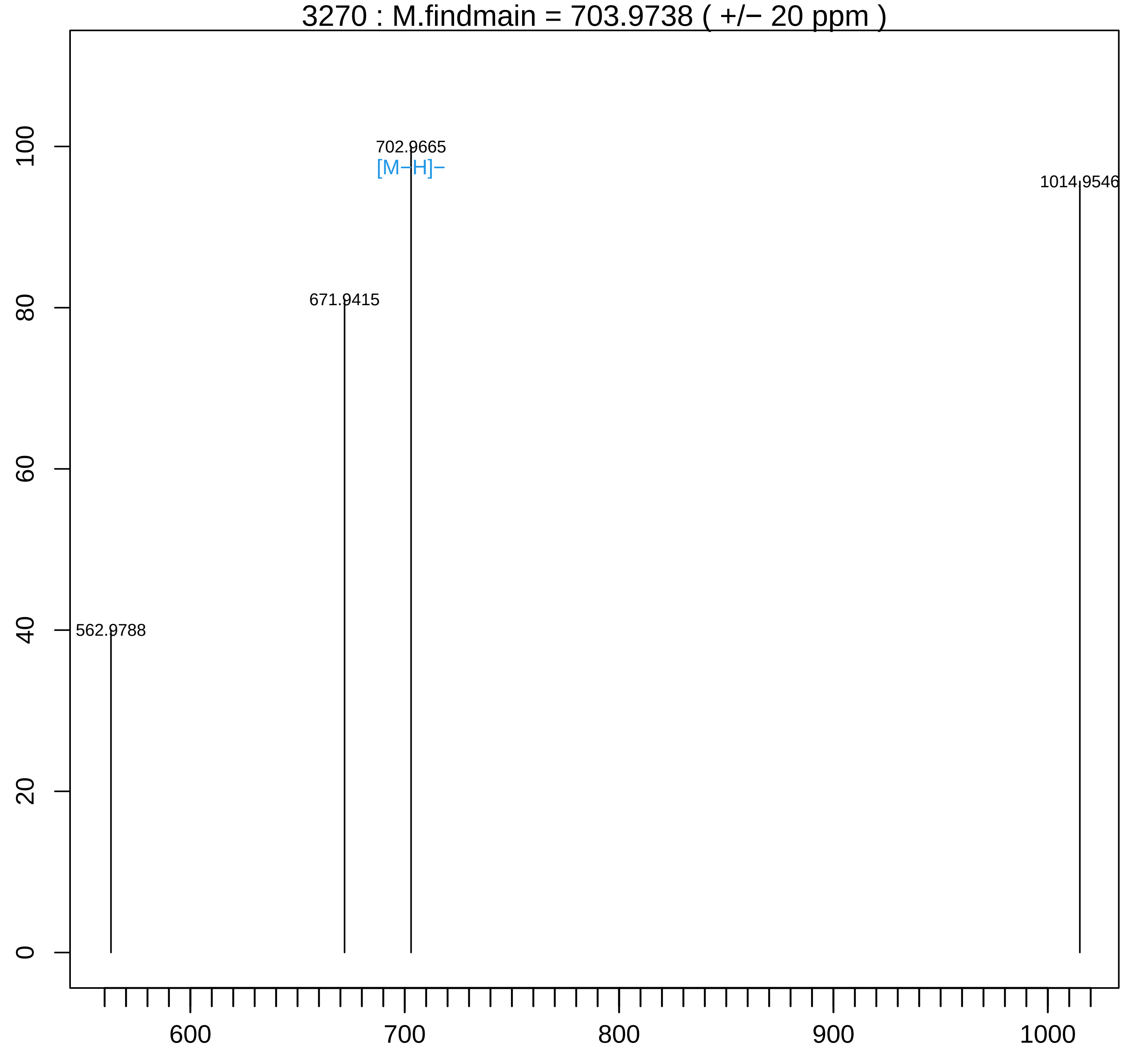
3270 : M.findmain = 703.9738 (+/− 20 ppm)
702.9665
[M−H]−
1014.9546
671.9415
562.9788
0
20
40
60
80
100
600
700
800
900
1000

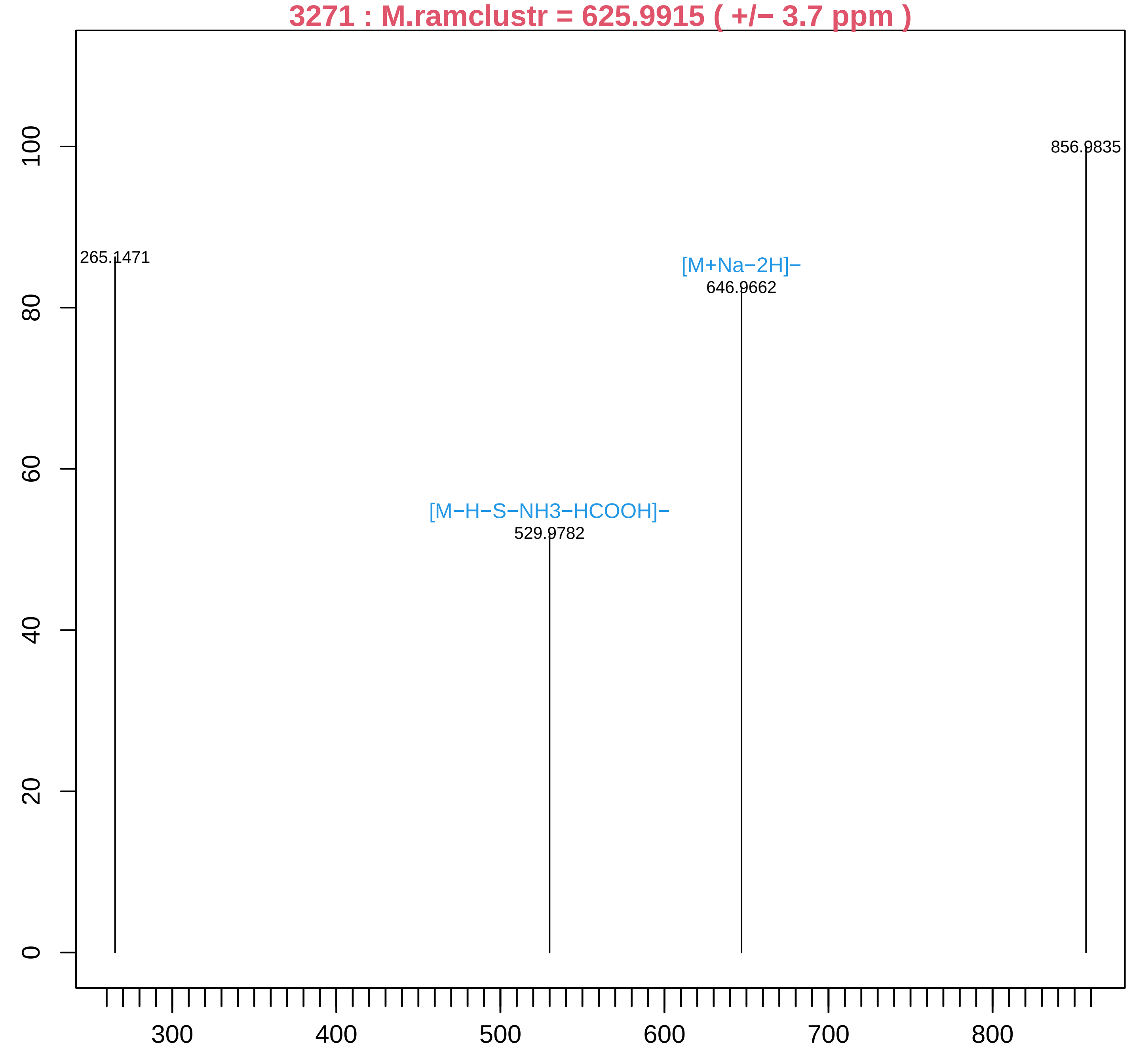
3271 : M.ramclustr = 625.9915 (+/− 3.7 ppm)
265.1471
[M−H−S−NH3−HCOOH]−
529.9782
[M+Na−2H]−
646.9662
856.9835
0
20
40
60
80
100
300
400
500
600
700
800

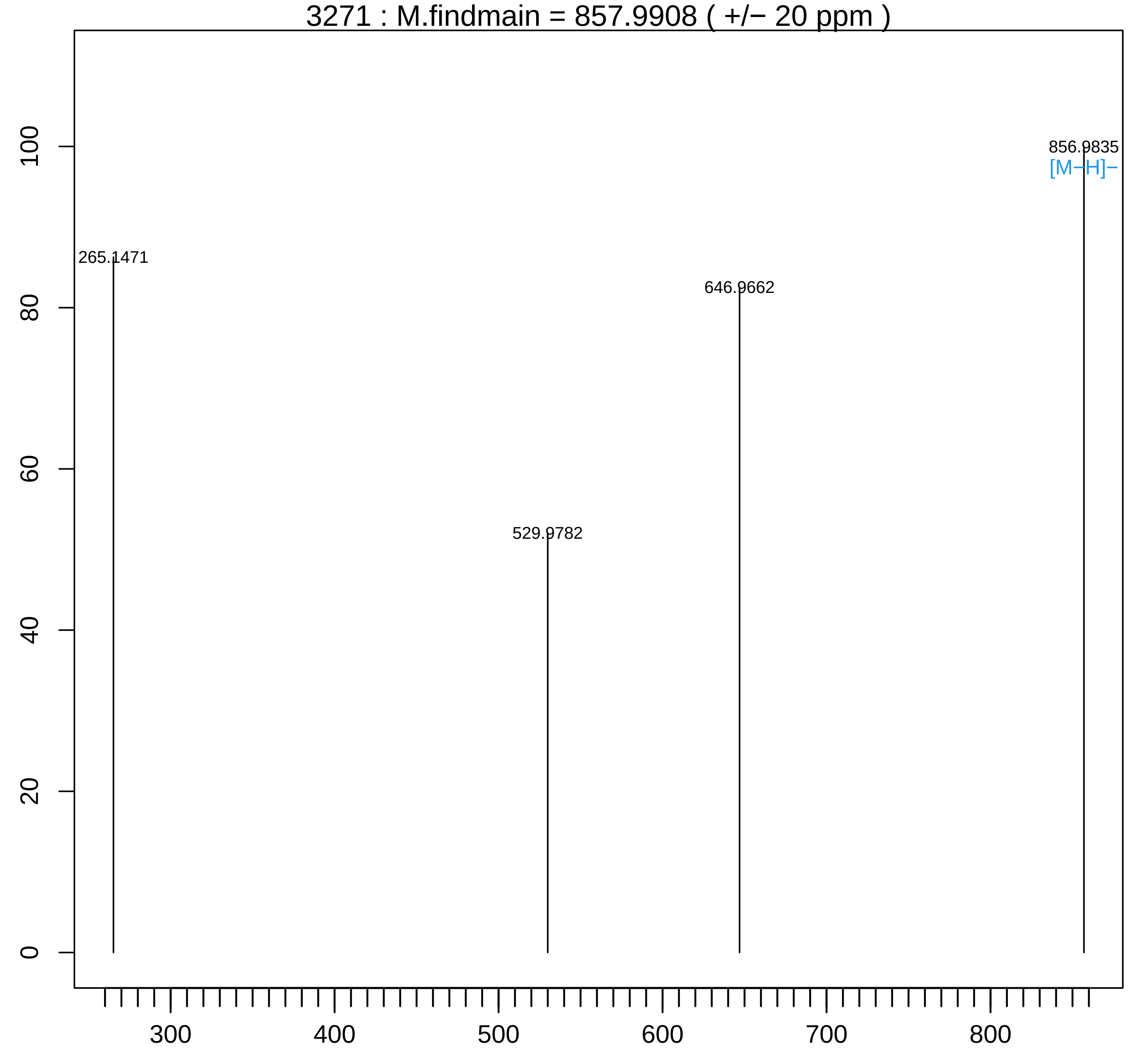
3271 : M.findmain = 857.9908 (+/− 20 ppm)
265.1471
529.9782
646.9662
856.9835
[M−H]−
0
20
40
60
80
100
300
400
500
600
700
800

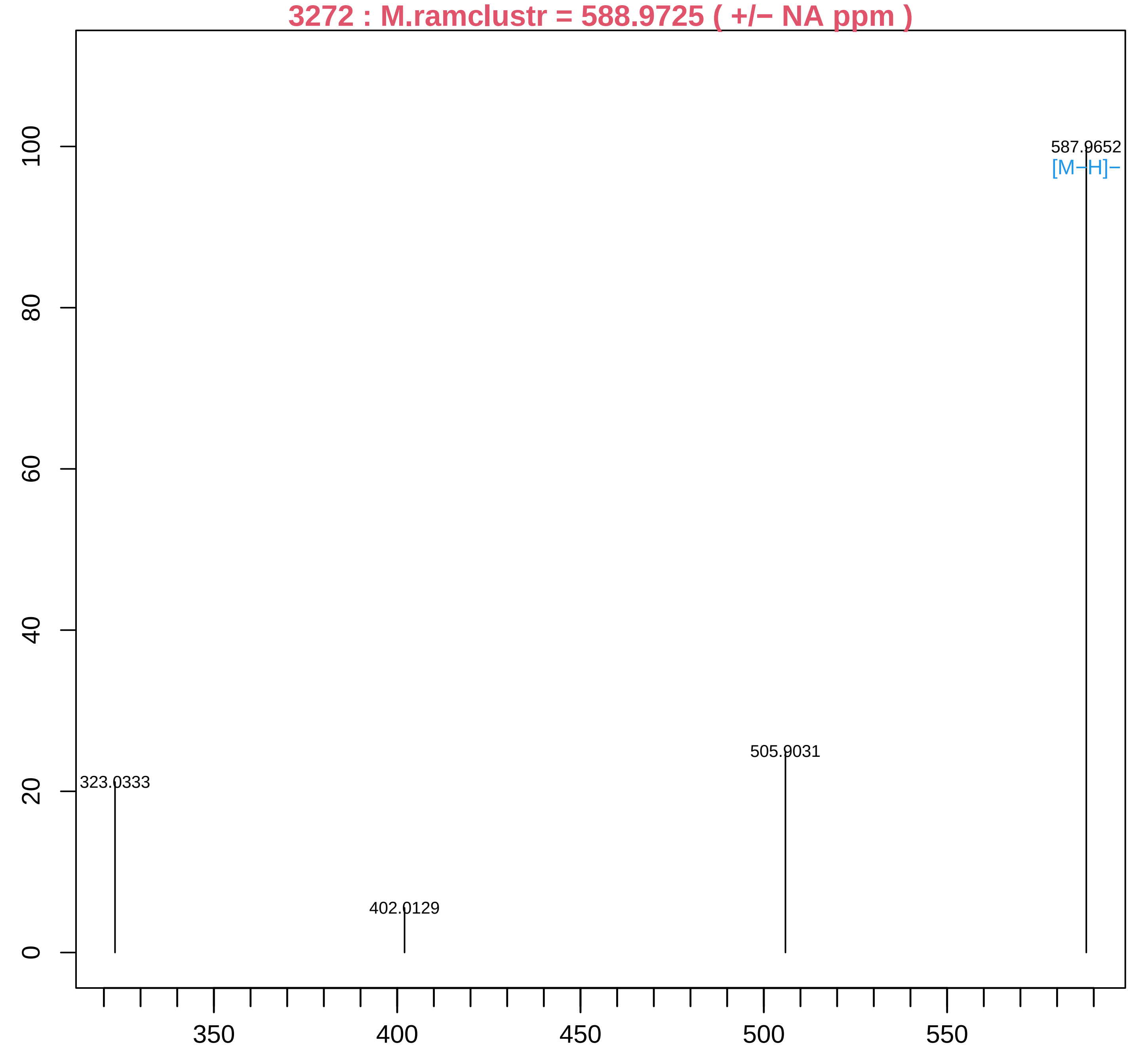
3272 : M.ramclustr = 588.9725 (+/− NA ppm)
587.9652
[M−H]−
505.9031
323.0333
402.0129
0
20
40
60
80
100
350
400
450
500
550

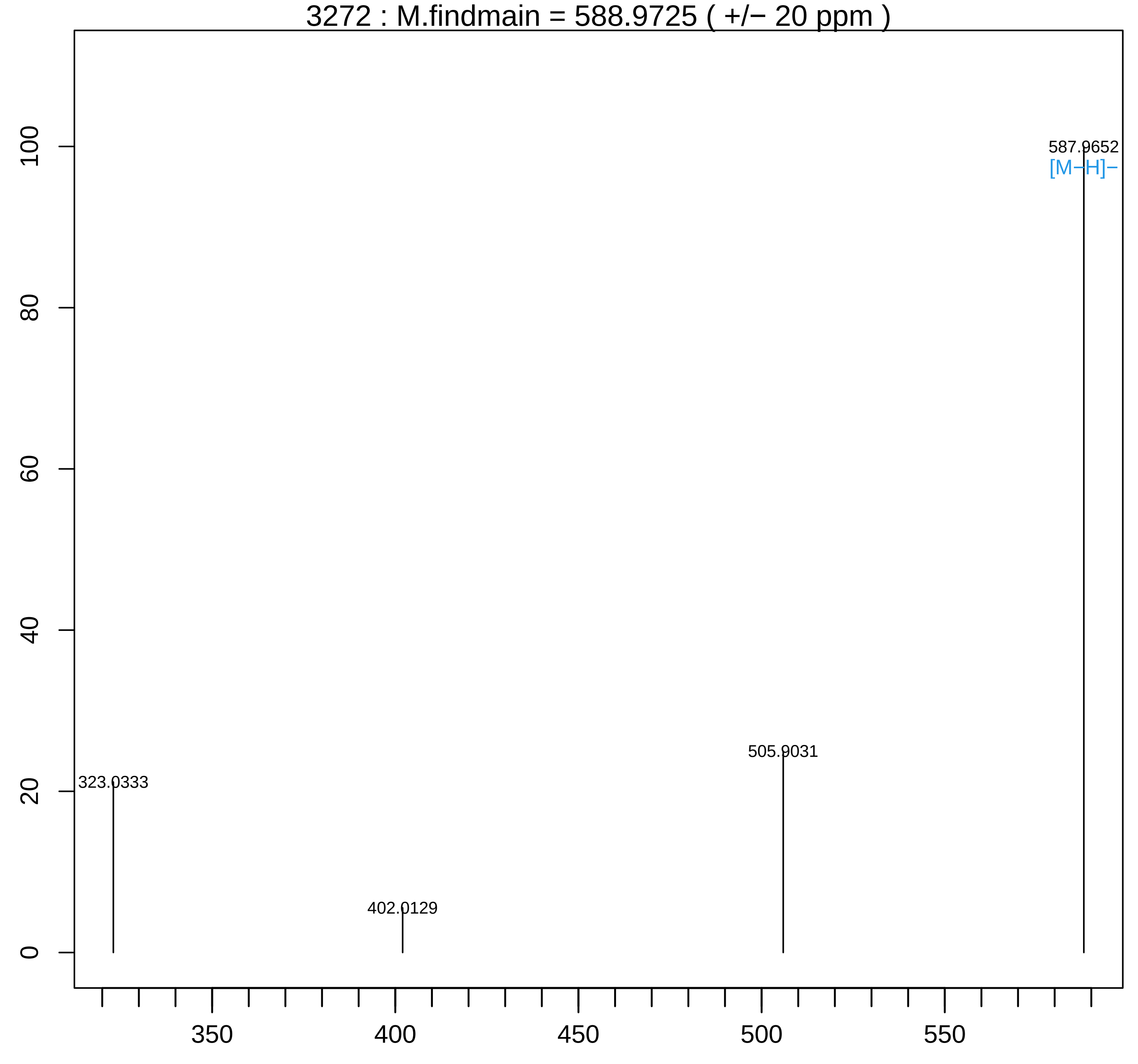
3272 : M.findmain = 588.9725 (+/− 20 ppm)
587.9652
[M−H]−
505.9031
323.0333
402.0129
0
20
40
60
80
100
350
400
450
500
550

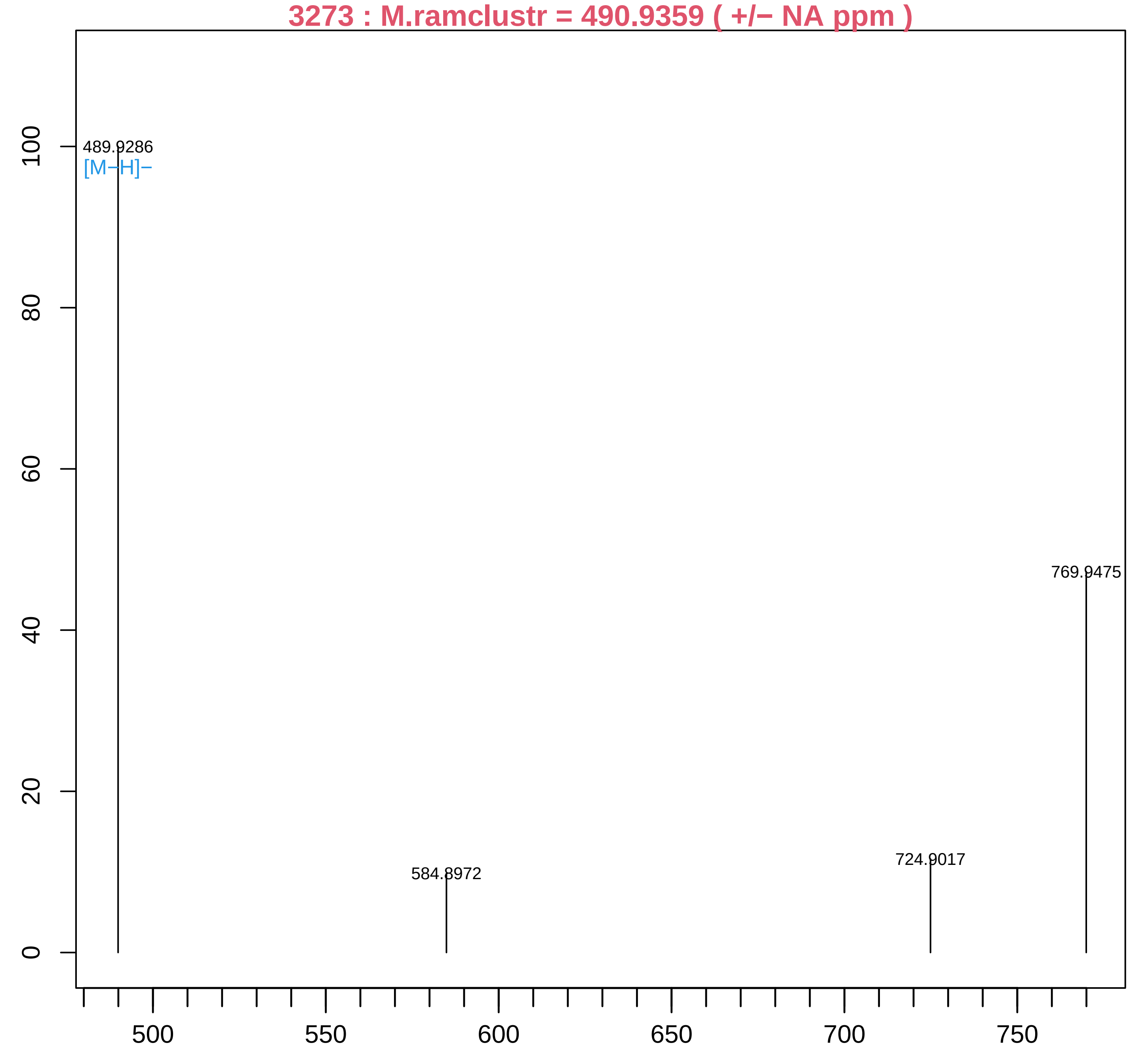
3273 : M.ramclustr = 490.9359 (+/− NA ppm)
489.9286
[M−H]−
584.8972
724.9017
769.9475
0
20
40
60
80
100
500
550
600
650
700
750

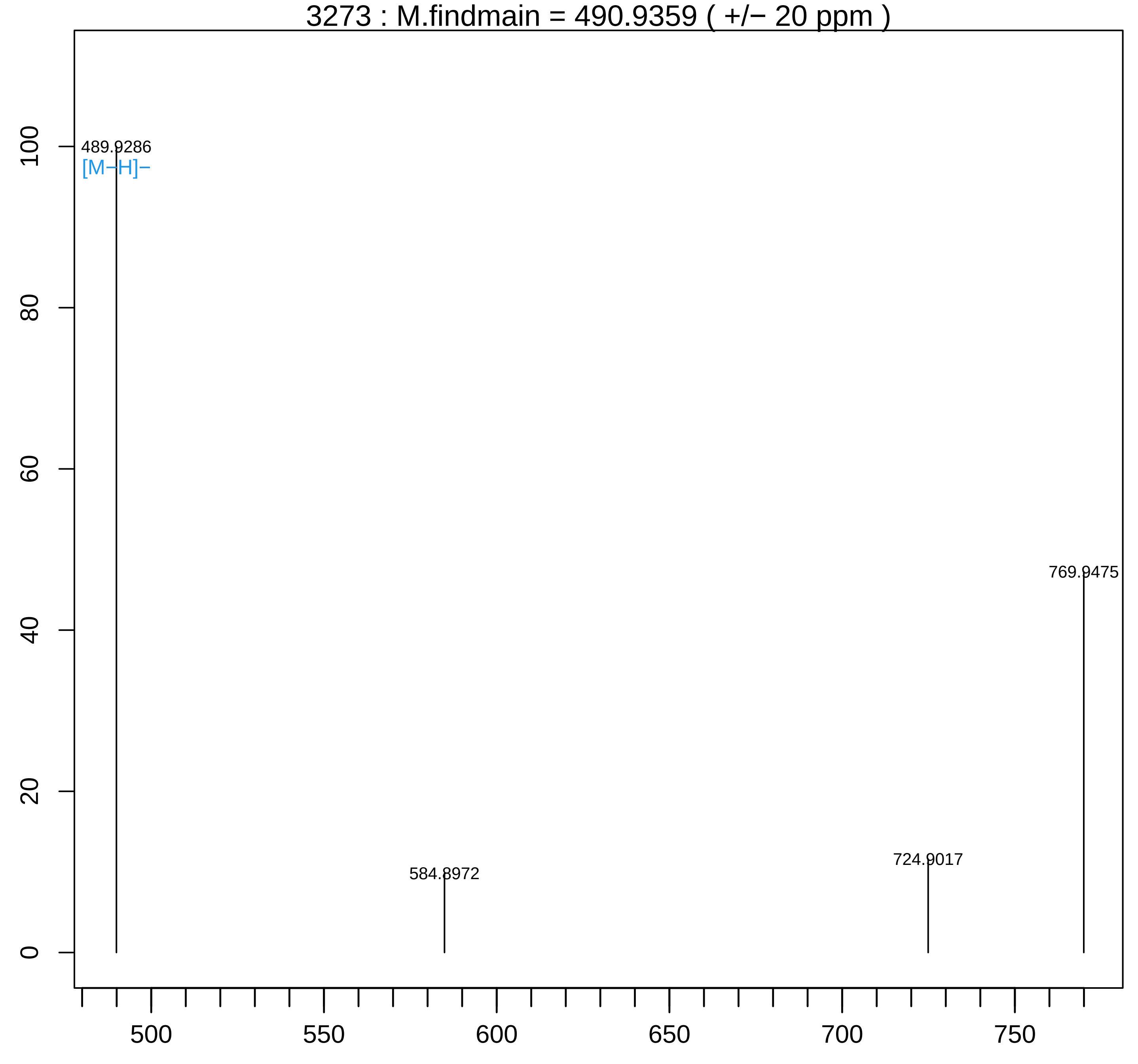
3273 : M.findmain = 490.9359 (+/− 20 ppm)
489.9286
[M−H]−
584.8972
724.9017
769.9475
0
20
40
60
80
100
500
550
600
650
700
750

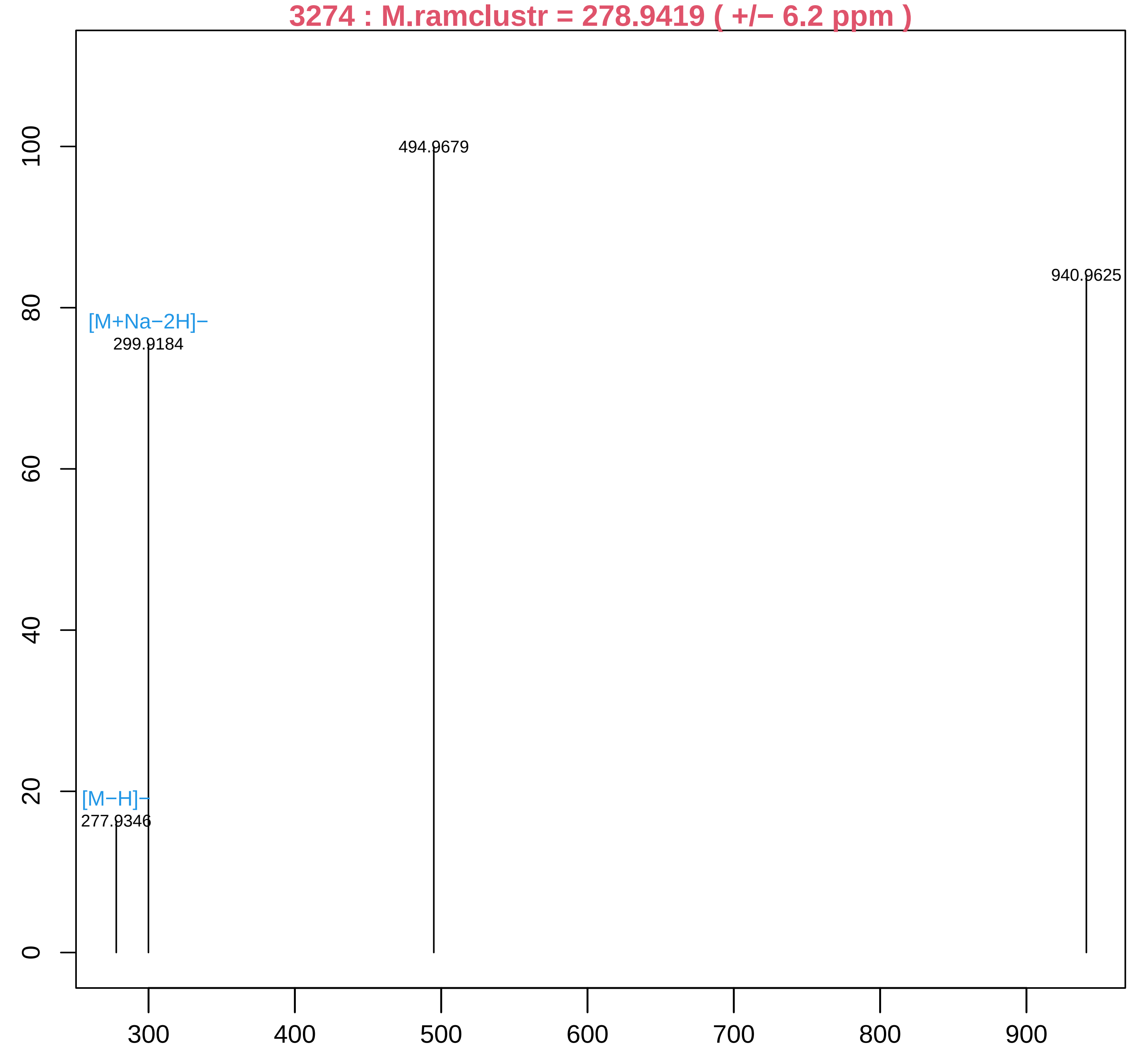
3274 : M.ramclustr = 278.9419 (+/− 6.2 ppm)
494.9679
940.9625
[M+Na−2H]−
299.9184
[M−H]−
277.9346
0
20
40
60
80
100
300
400
500
600
700
800
900

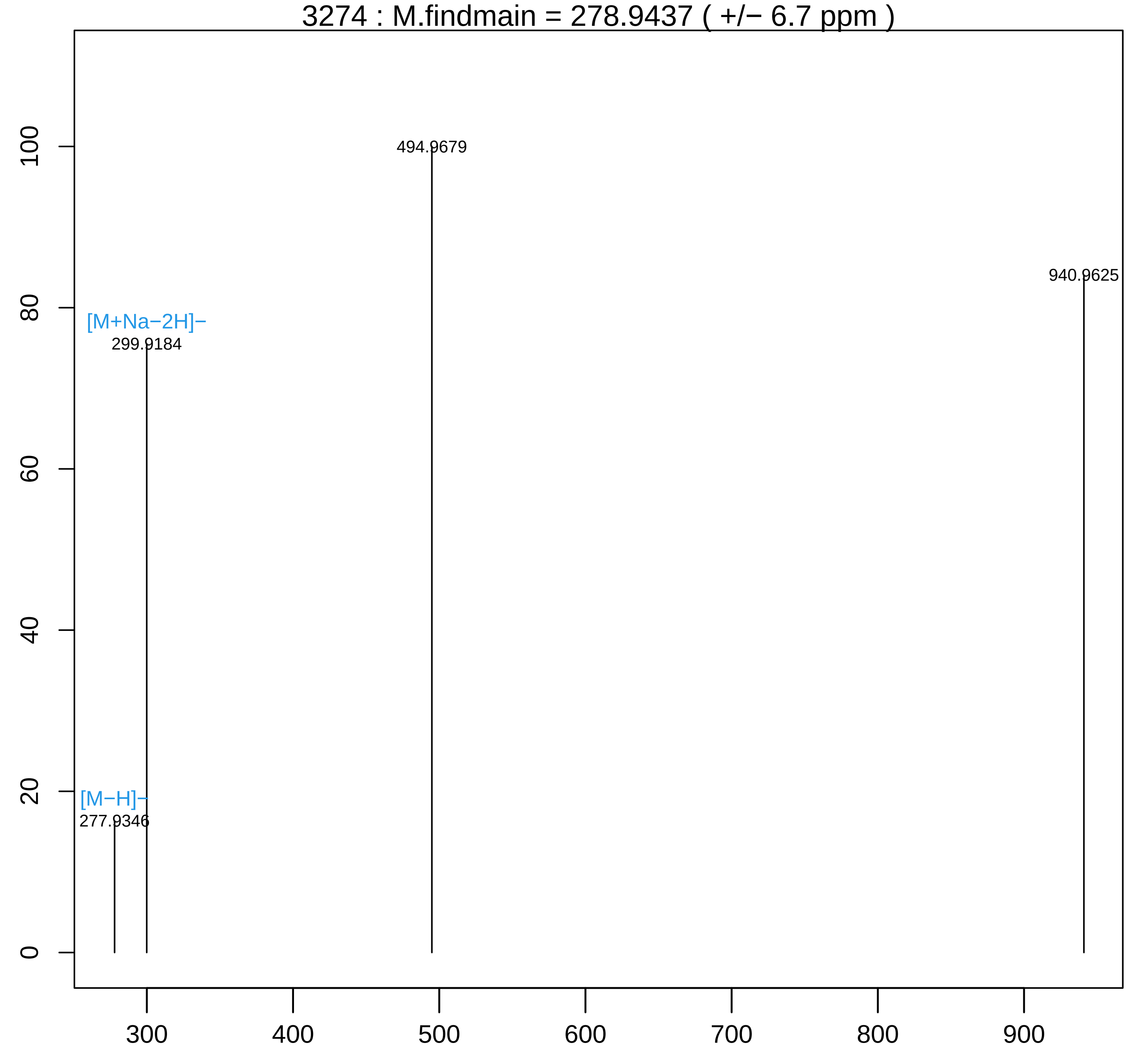
3274 : M.findmain = 278.9437 (+/− 6.7 ppm)
494.9679
940.9625
[M+Na−2H]−
299.9184
[M−H]−
277.9346
0
20
40
60
80
100
300
400
500
600
700
800
900

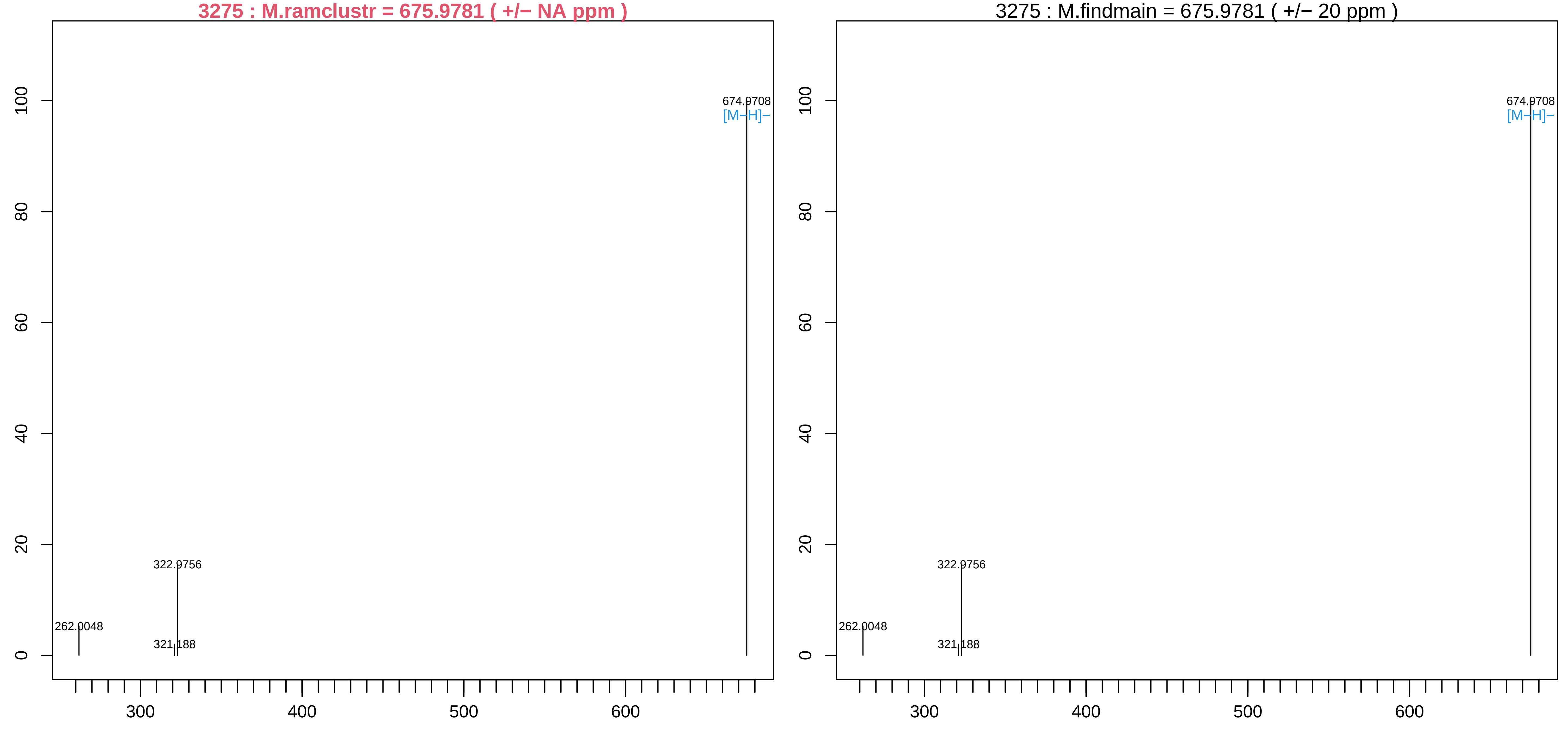
3275 : M.ramclustr = 675.9781 (+/− NA ppm)
3275 : M.findmain = 675.9781 (+/− 20 ppm)
674.9708
[M−H]−
322.9756
262.0048
321.188
0
20
40
60
80
100
300
400
500
600

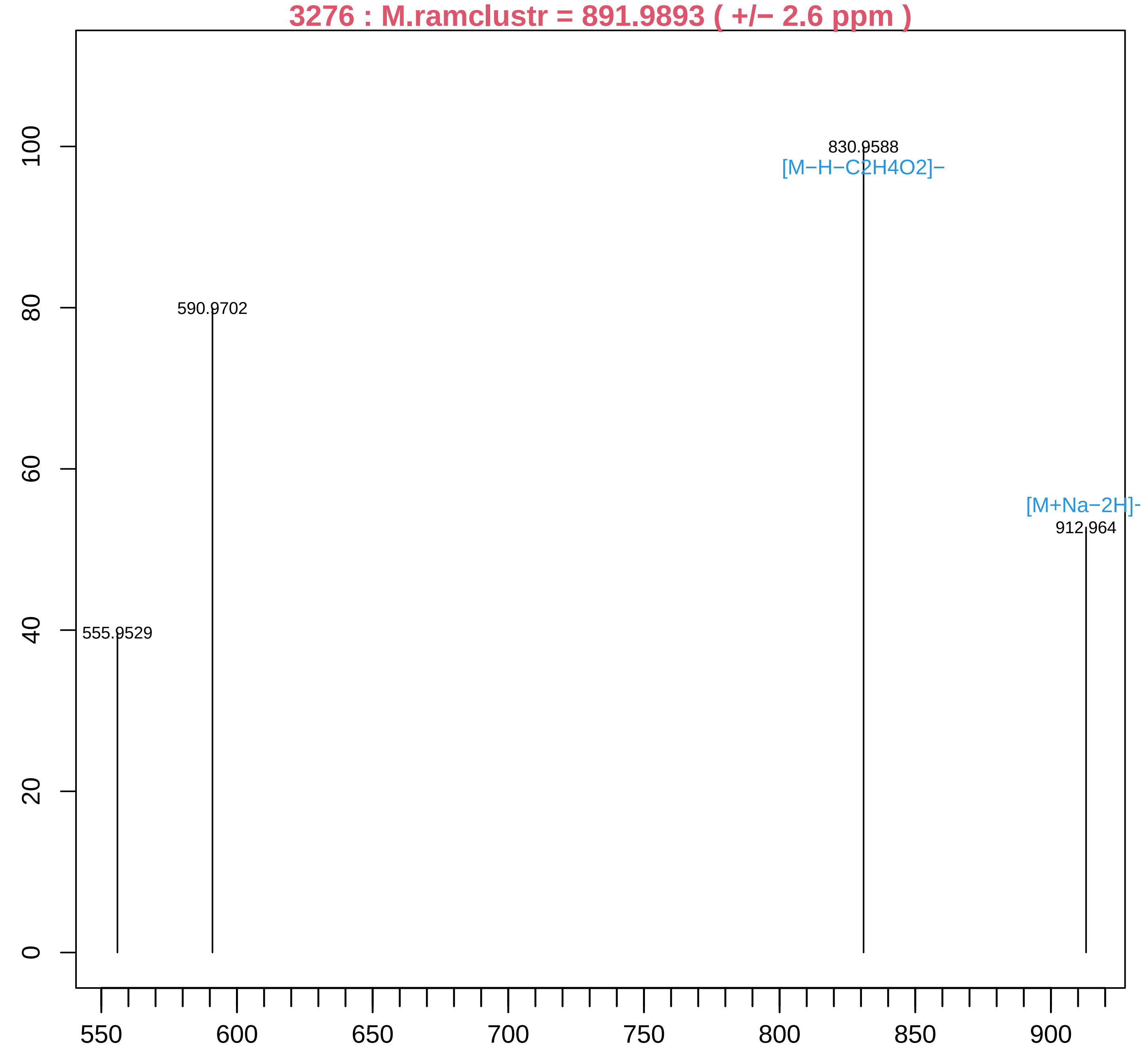
3276 : M.ramclustr = 891.9893 (+/− 2.6 ppm)
830.9588
[M−H−C2H4O2]−
590.9702
555.9529
[M+Na−2H]-
912.964
0
20
40
60
80
100
550
600
650
700
750
800
850
900

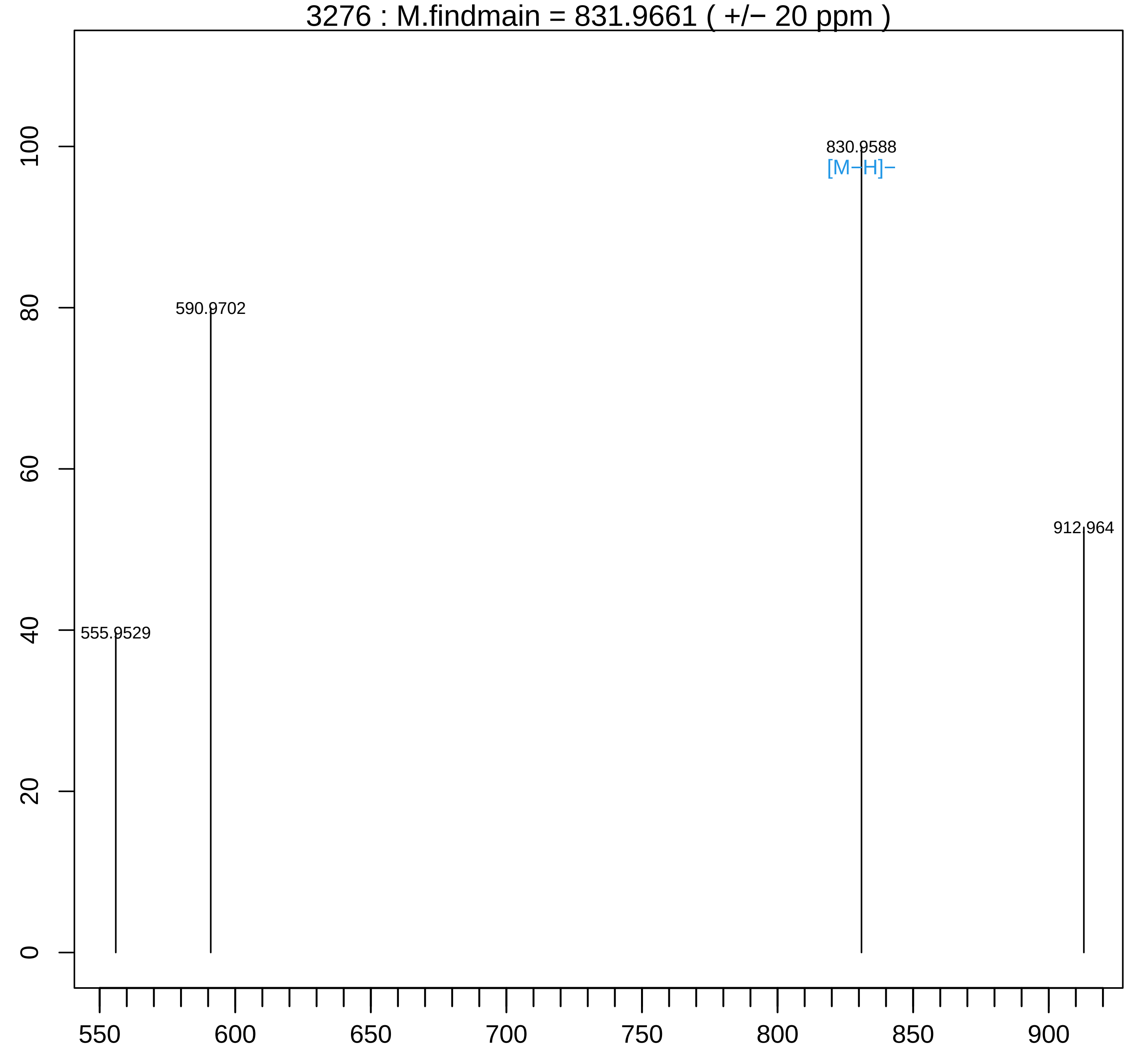
3276 : M.findmain = 831.9661 (+/− 20 ppm)
830.9588
[M−H]−
590.9702
555.9529
912.964
0
20
40
60
80
100
550
600
650
700
750
800
850
900

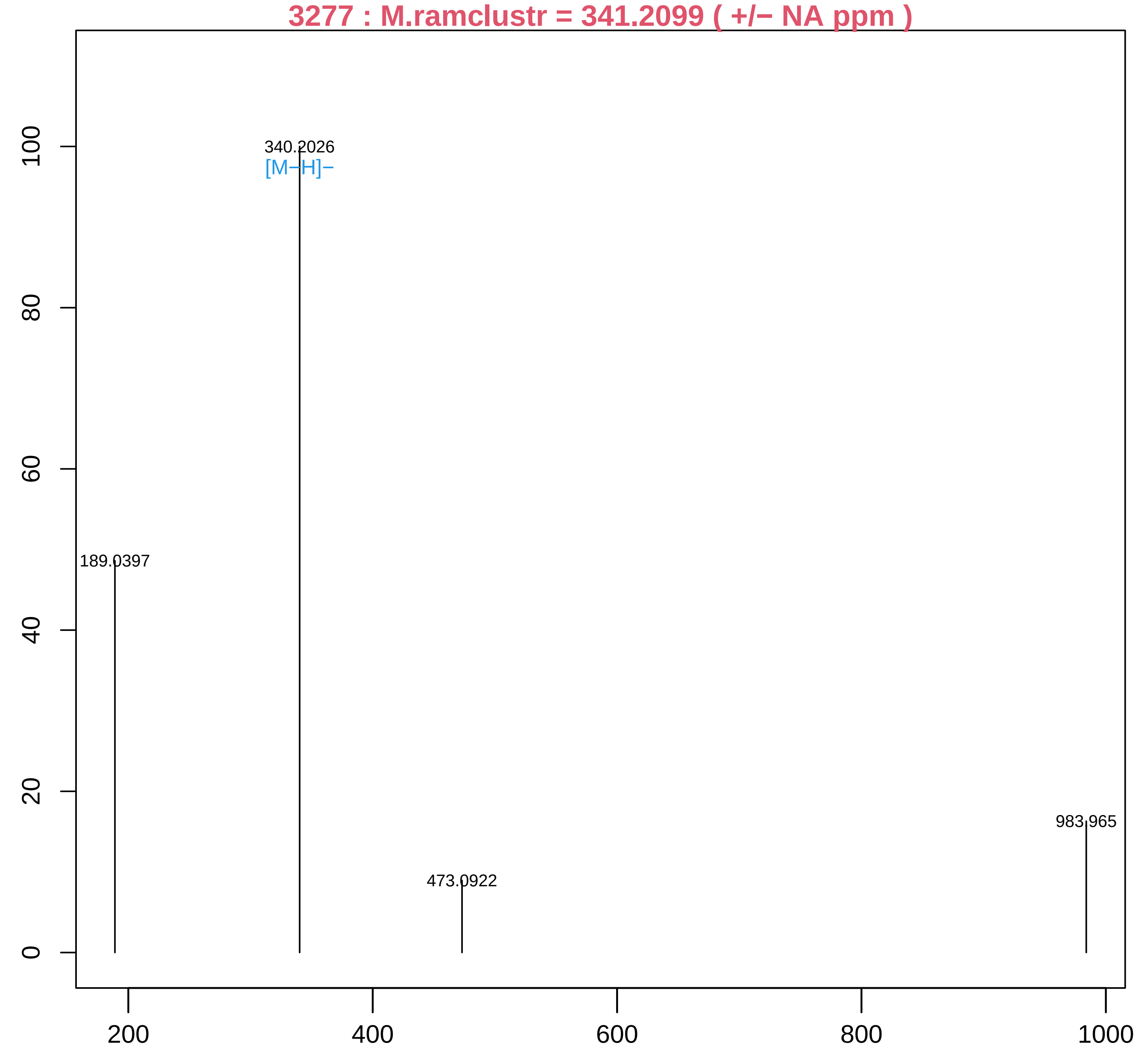

3277 : M.ramclustr = 341.2099 (+/− NA ppm)
340.2026
[M−H]−
189.0397
473.0922
983.965
0
20
40
60
80
100
200
400
600
800
1000

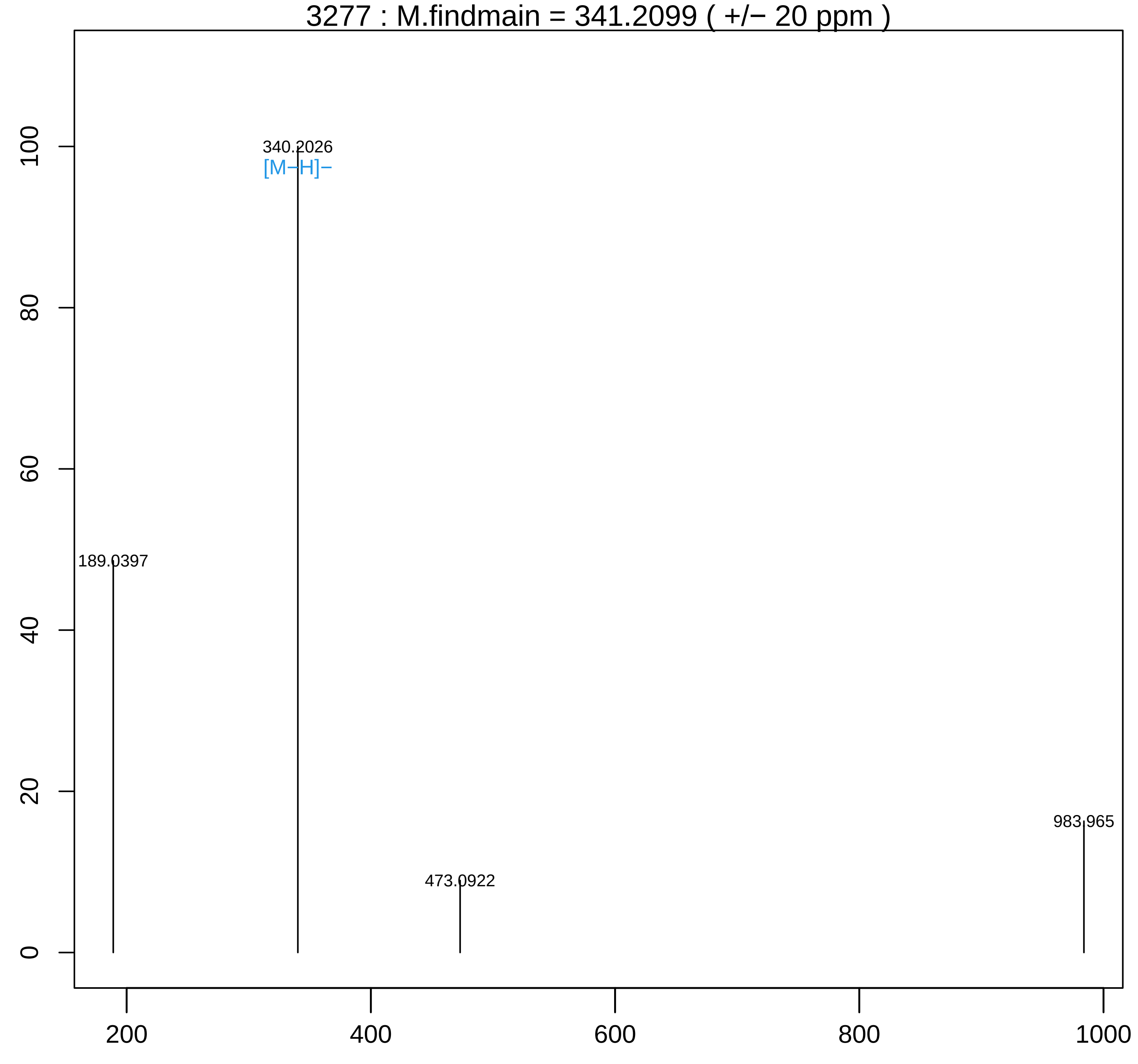

3277 : M.findmain = 341.2099 (+/− 20 ppm)
340.2026
[M−H]−
189.0397
473.0922
983.965
0
20
40
60
80
100
200
400
600
800
1000

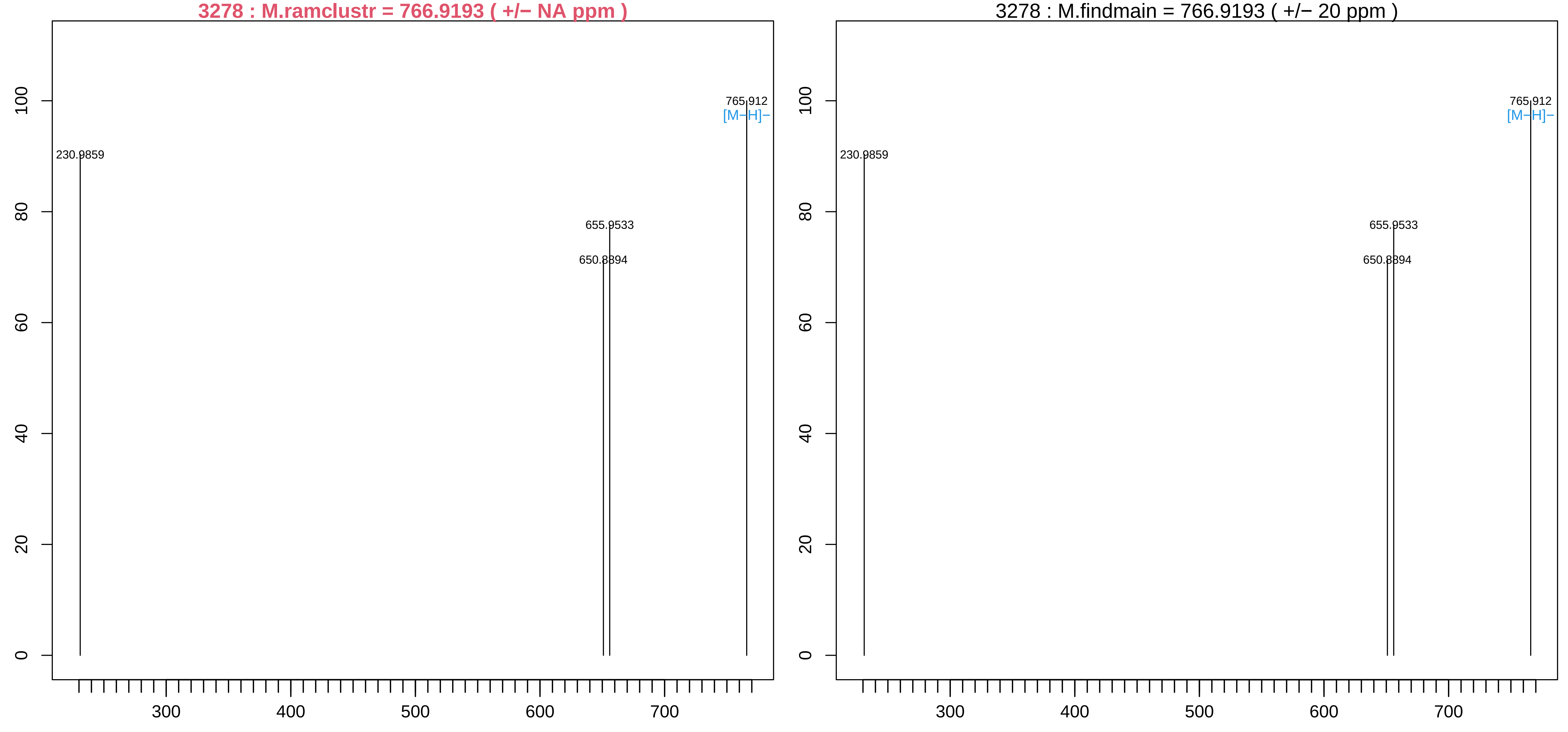

3278 : M.ramclustr = 766.9193 (+/− NA ppm)
765.912
[M−H]−
230.9859
655.9533
650.8394
0
20
40
60
80
100
300
400
500
600
700
3278 : M.findmain = 766.9193 (+/− 20 ppm)
765.912
[M−H]−
230.9859
655.9533
650.8394
0
20
40
60
80
100
300
400
500
600
700

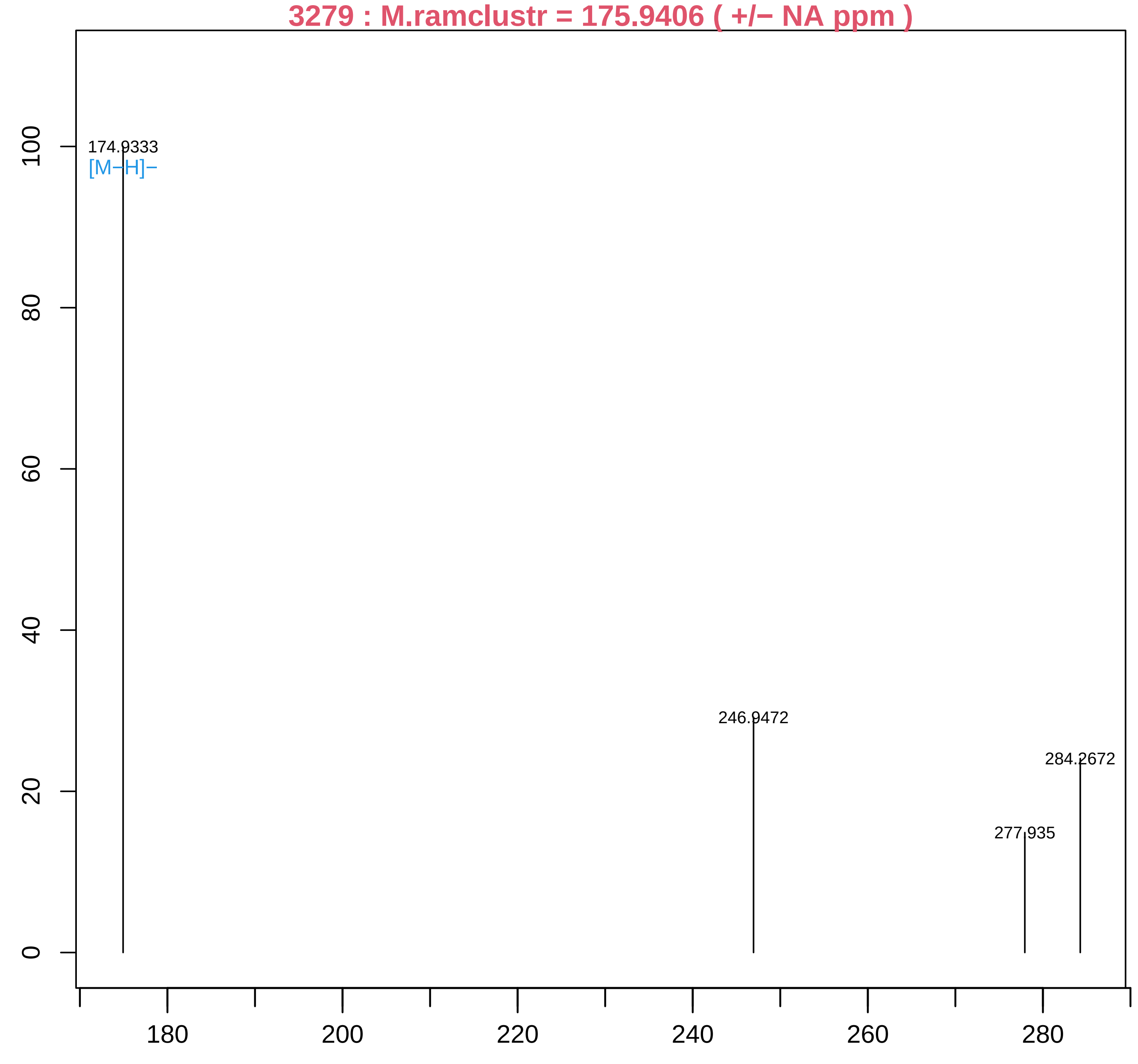
3279 : M.ramclustr = 175.9406 (+/− NA ppm)
174.9333
[M−H]−
246.9472
277.935
284.2672
0
20
40
60
80
100
180
200
220
240
260
280

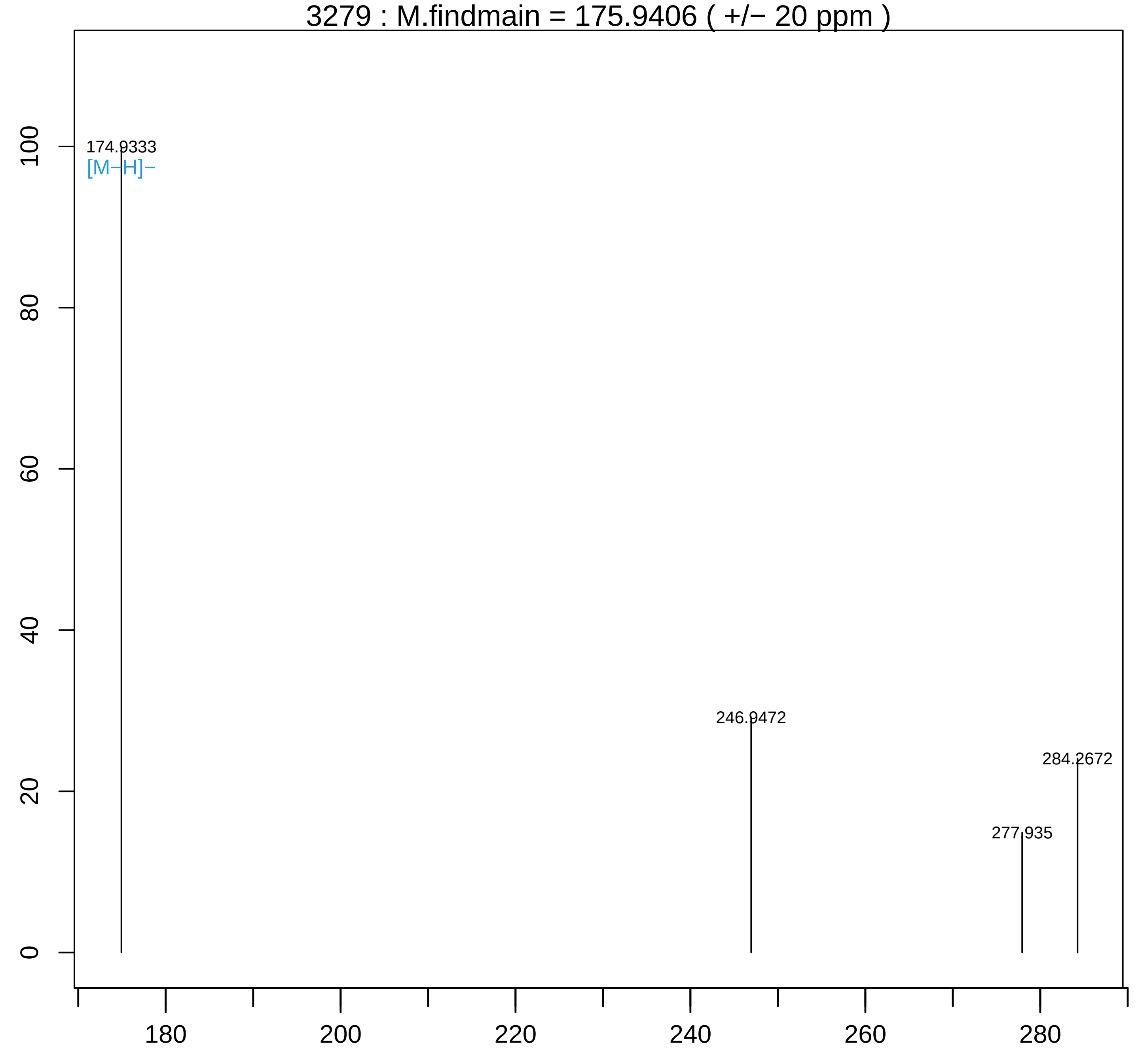
3279 : M.findmain = 175.9406 (+/− 20 ppm)
174.9333
[M−H]−
246.9472
277.935
284.2672
0
20
40
60
80
100
180
200
220
240
260
280

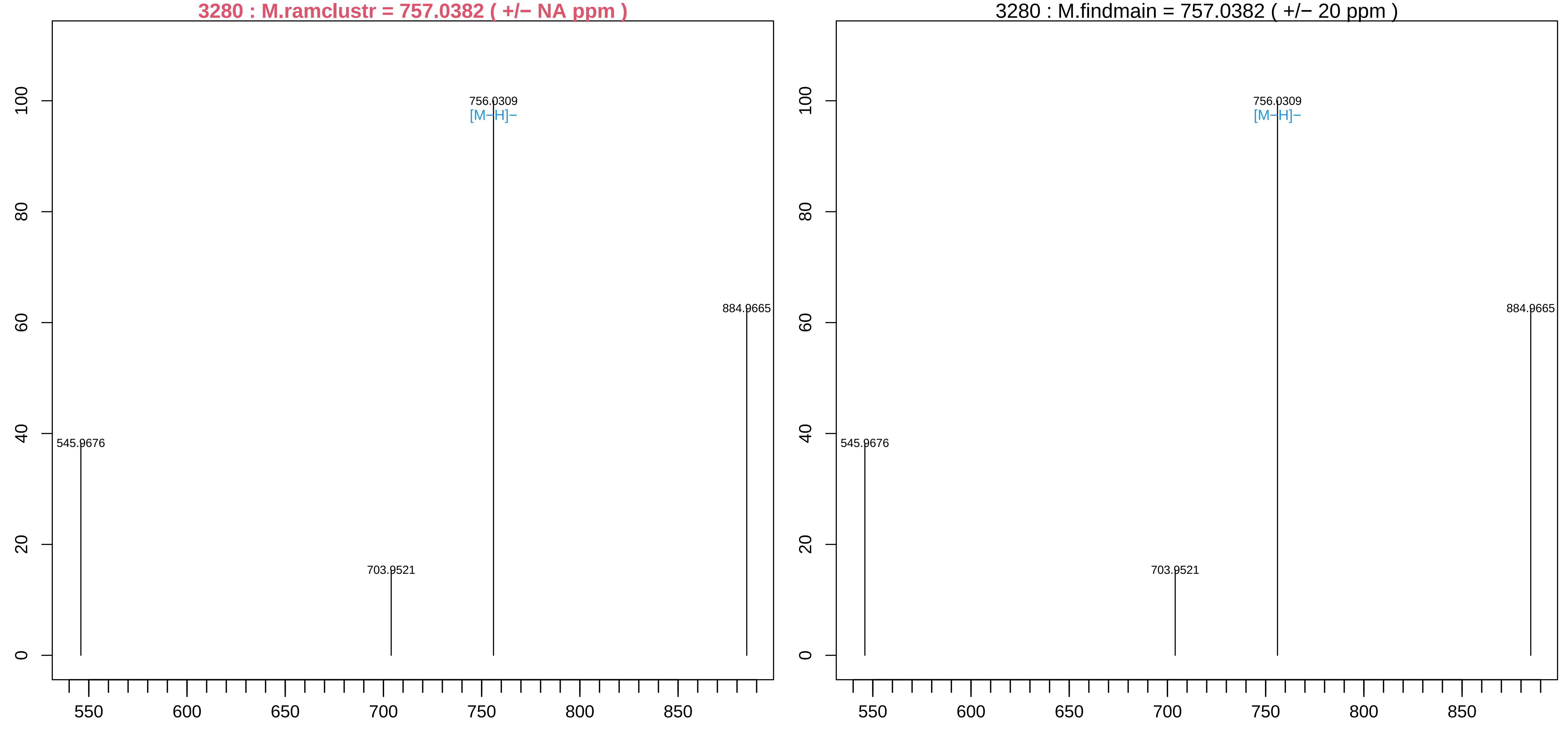

3280 : M.ramclustr = 757.0382 (+/− NA ppm)
3280 : M.findmain = 757.0382 (+/− 20 ppm)
545.9676
703.9521
756.0309
[M−H]−
884.9665
0
20
40
60
80
100
550
600
650
700
750
800
850

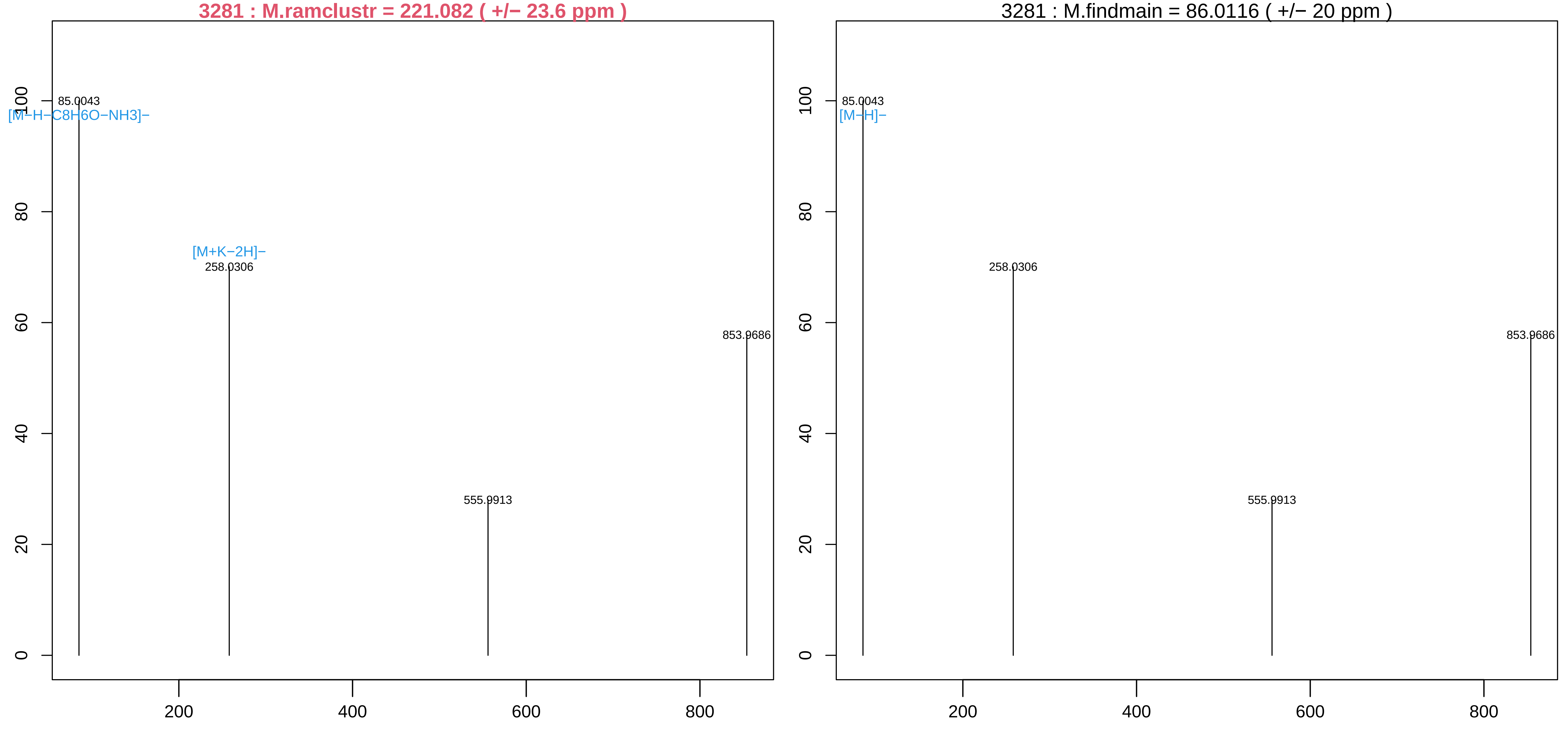
3281 : M.ramclustr = 221.082 (+/− 23.6 ppm)
85.0043
[M−H−C8H6O−NH3]−
[M+K−2H]−
258.0306
853.9686
555.9913
3281 : M.findmain = 86.0116 (+/− 20 ppm)
85.0043
[M−H]−
258.0306
853.9686
555.9913
0
20
40
60
80
100
200
400
600
800

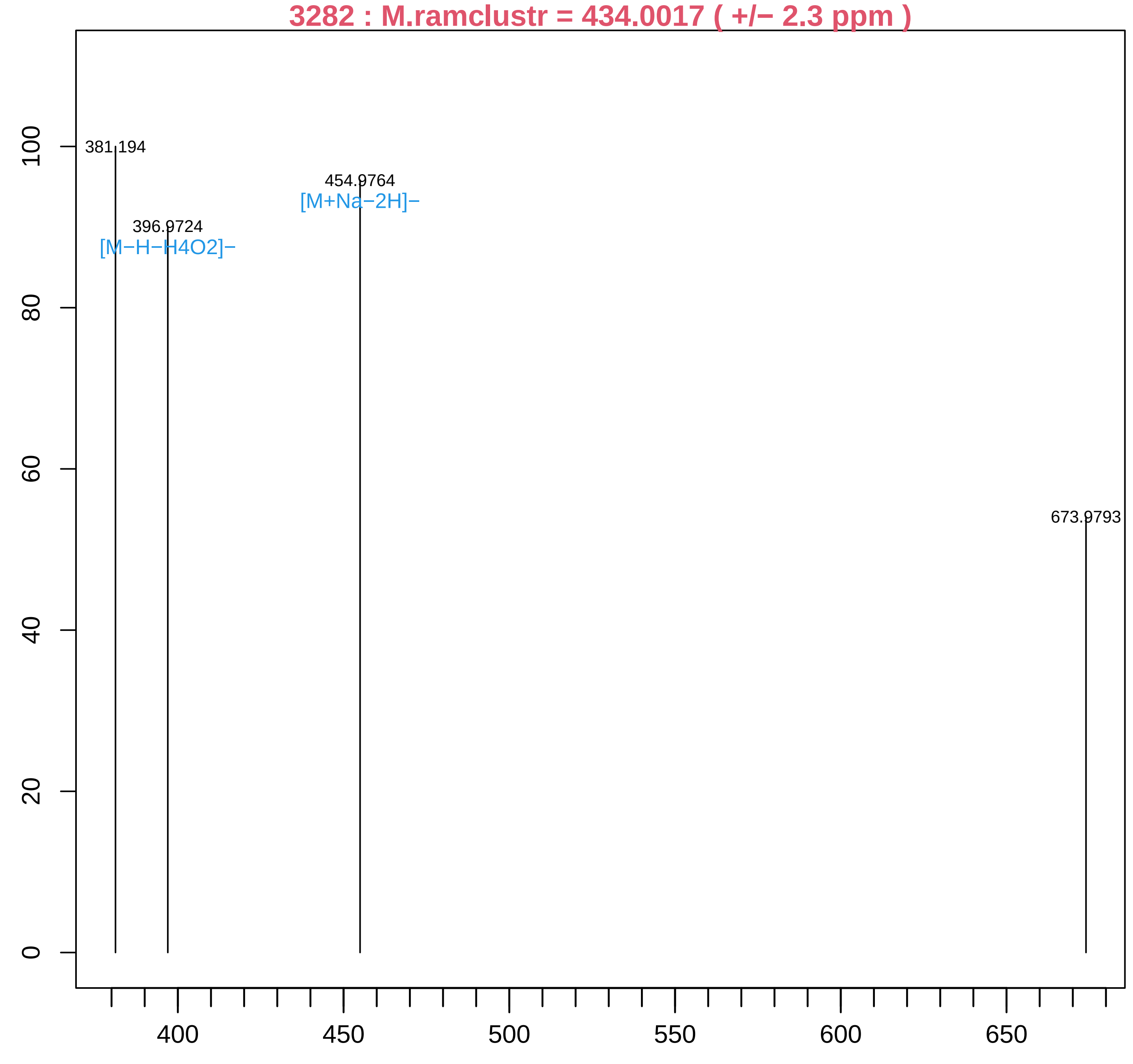
3282 : M.ramclustr = 434.0017 (+/− 2.3 ppm)
381.194
396.9724
[M−H−H4O2]−
454.9764
[M+Na−2H]−
673.9793
0
20
40
60
80
100
400
450
500
550
600
650

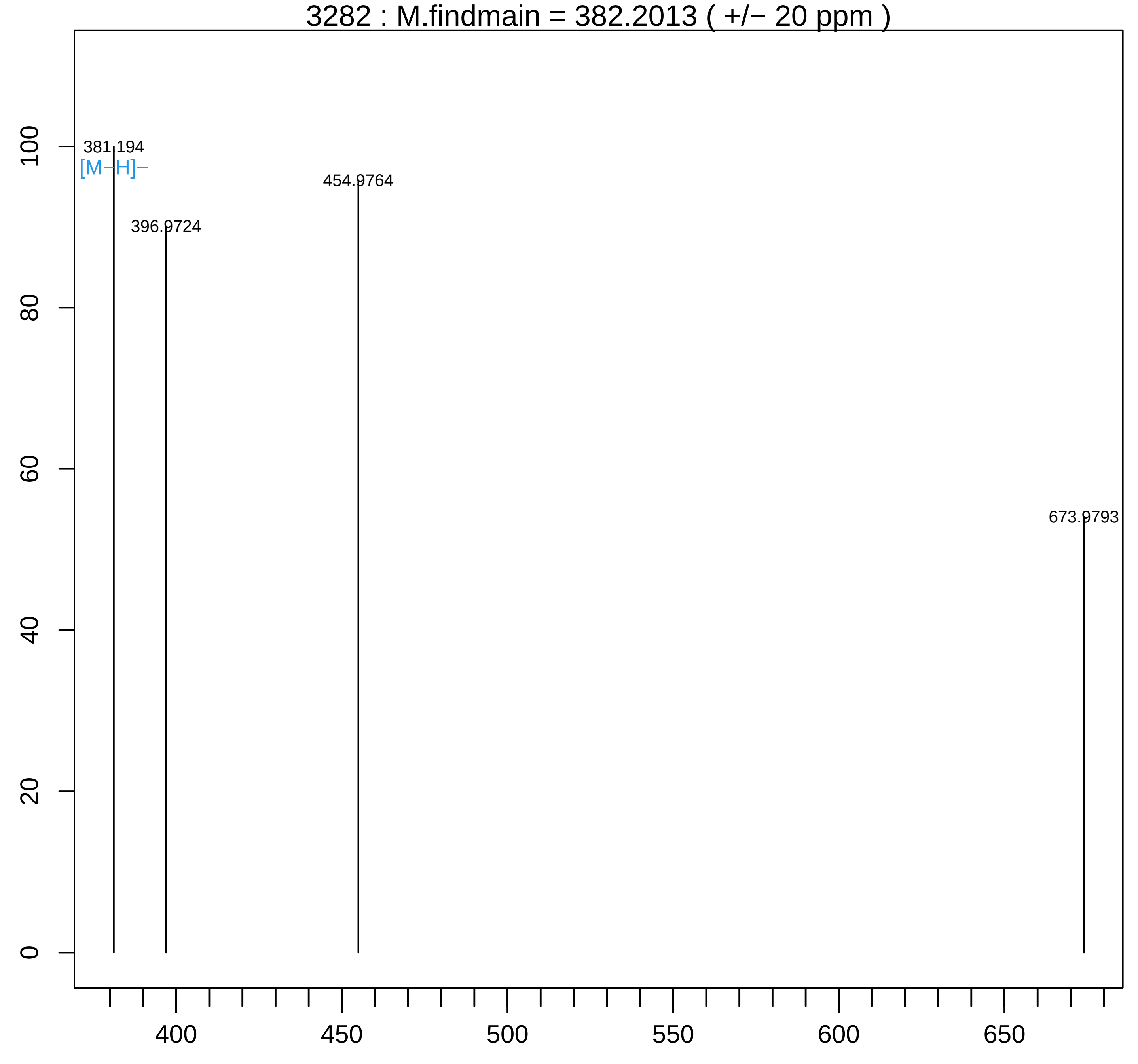
3282 : M.findmain = 382.2013 (+/− 20 ppm)
381.194
[M−H]−
396.9724
454.9764
673.9793
0
20
40
60
80
100
400
450
500
550
600
650

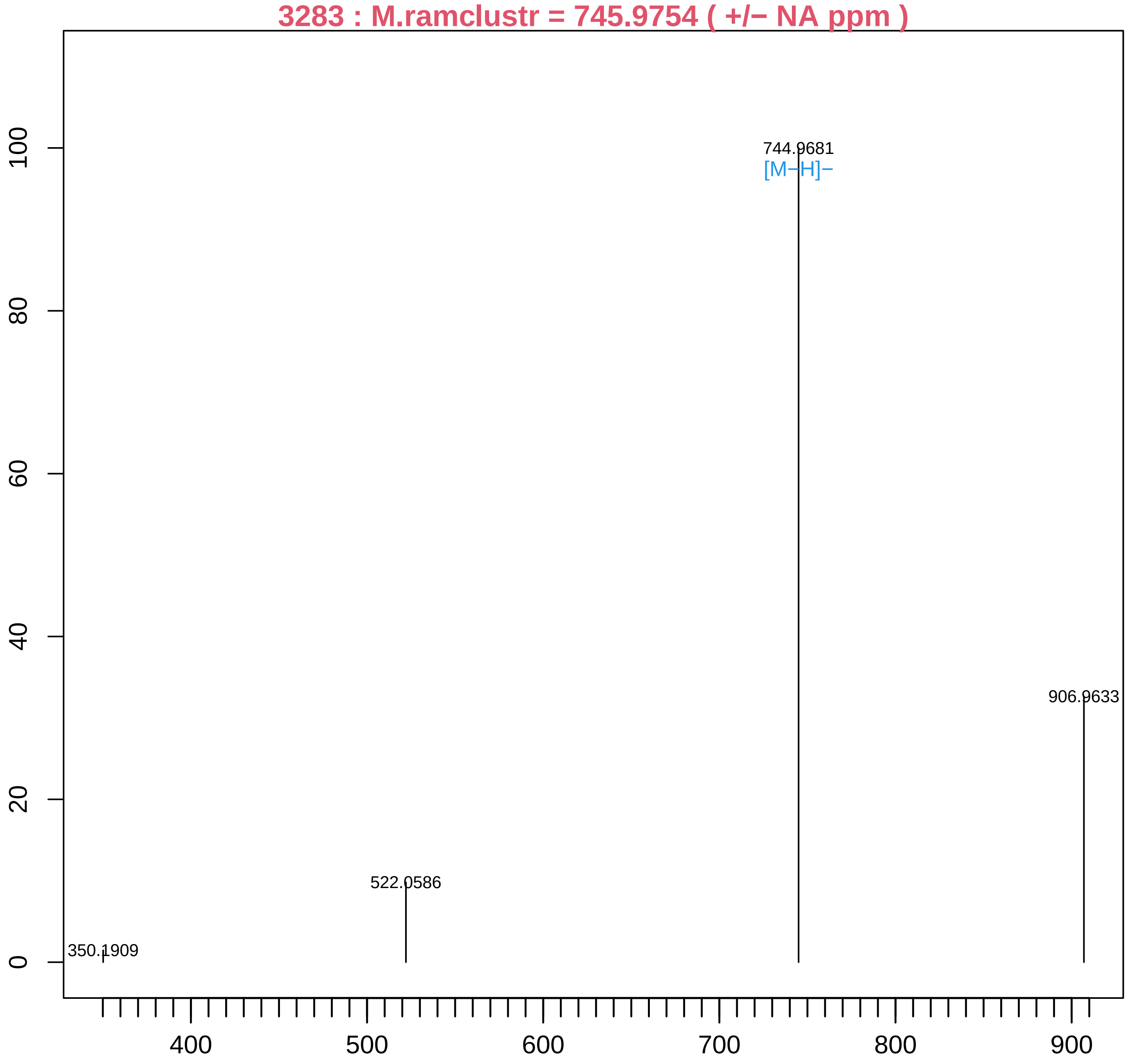
3283 : M.ramclustr = 745.9754 (+/− NA ppm)
744.9681
[M−H]−
906.9633
522.0586
350.1909
0
20
40
60
80
100
400
500
600
700
800
900

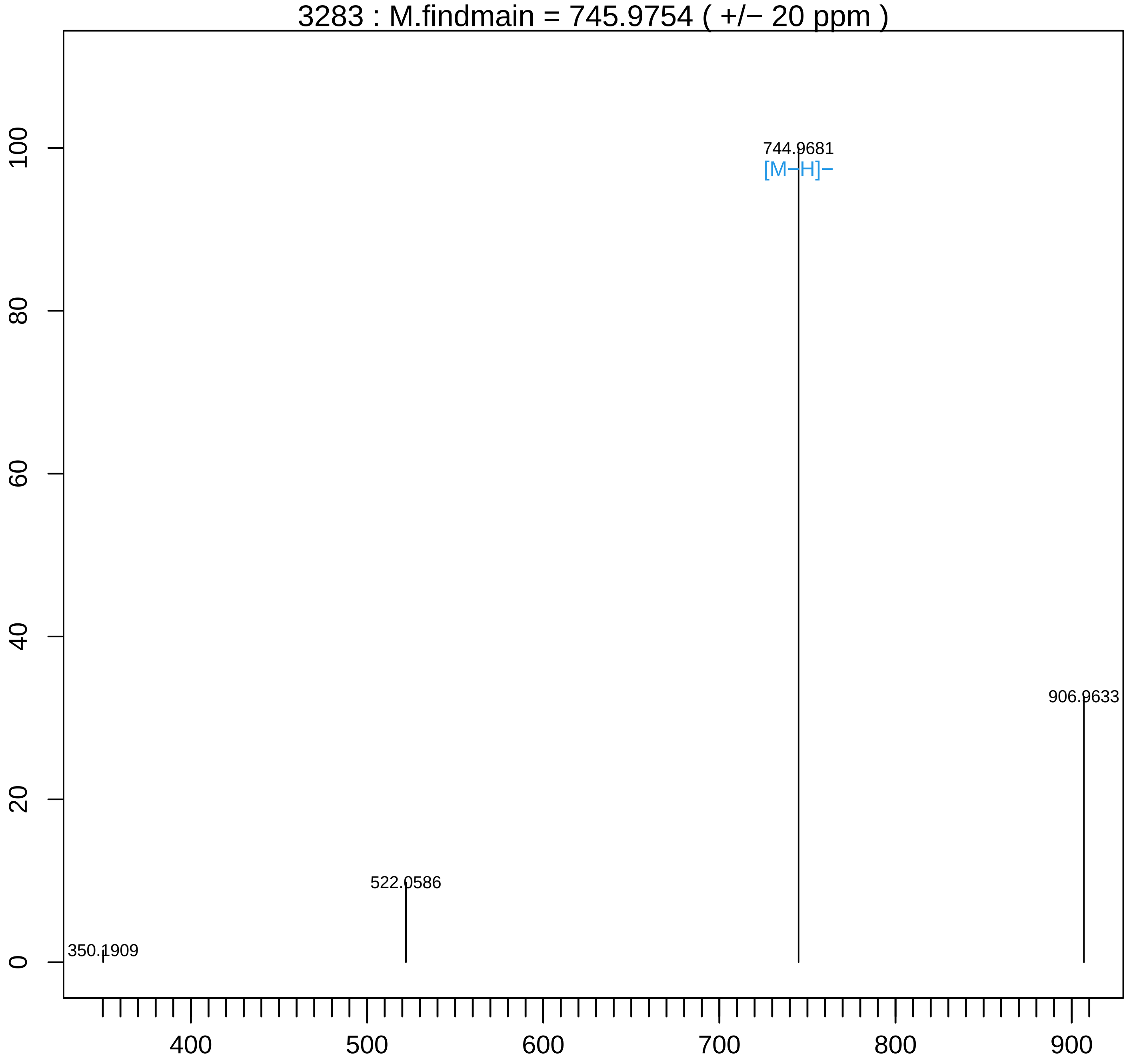
3283 : M.findmain = 745.9754 (+/− 20 ppm)
744.9681
[M−H]−
906.9633
522.0586
350.1909
0
20
40
60
80
100
400
500
600
700
800
900

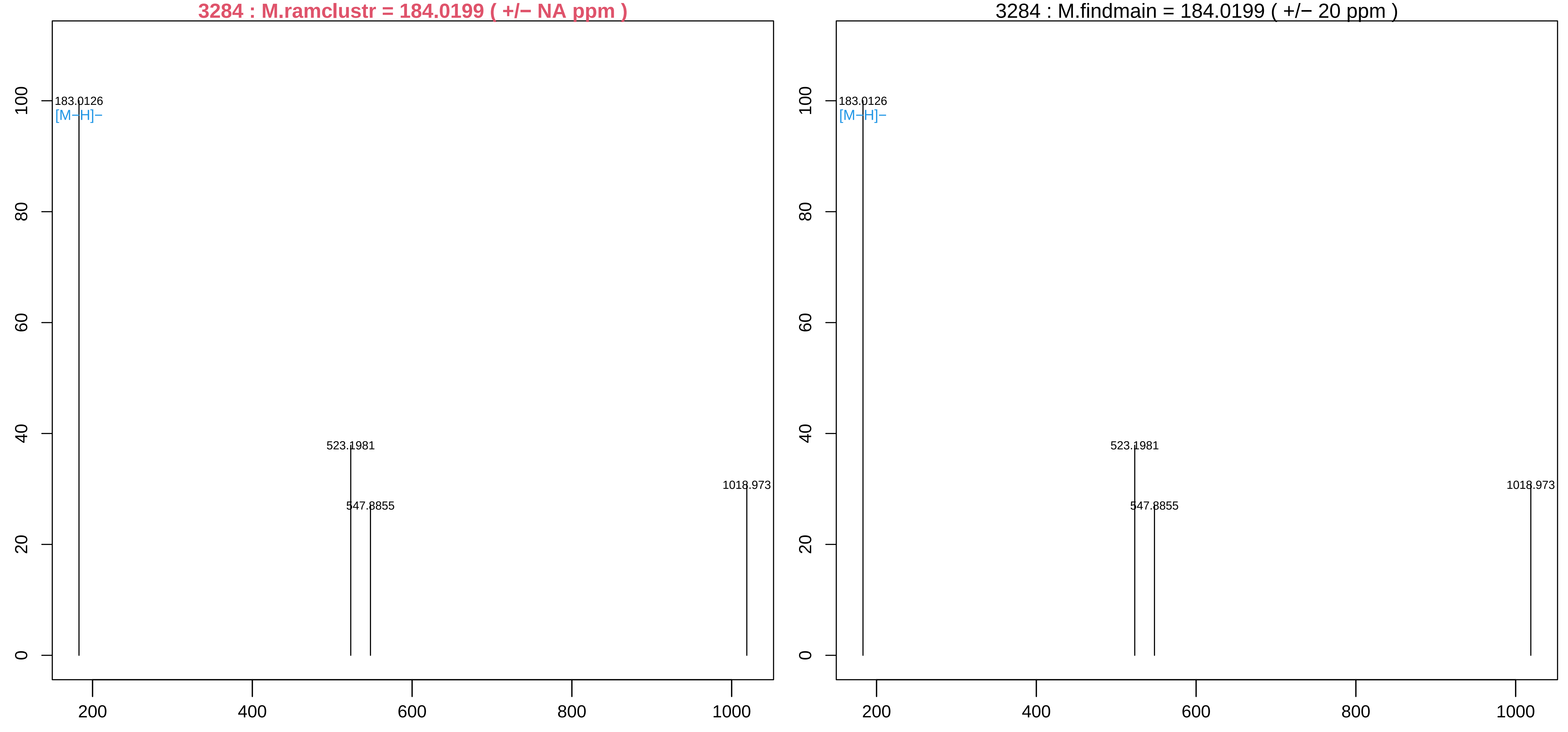
3284 : M.ramclustr = 184.0199 (+/− NA ppm)
3284 : M.findmain = 184.0199 (+/− 20 ppm)
183.0126
[M−H]−
523.1981
547.8855
1018.973
0
20
40
60
80
100
200
400
600
800
1000

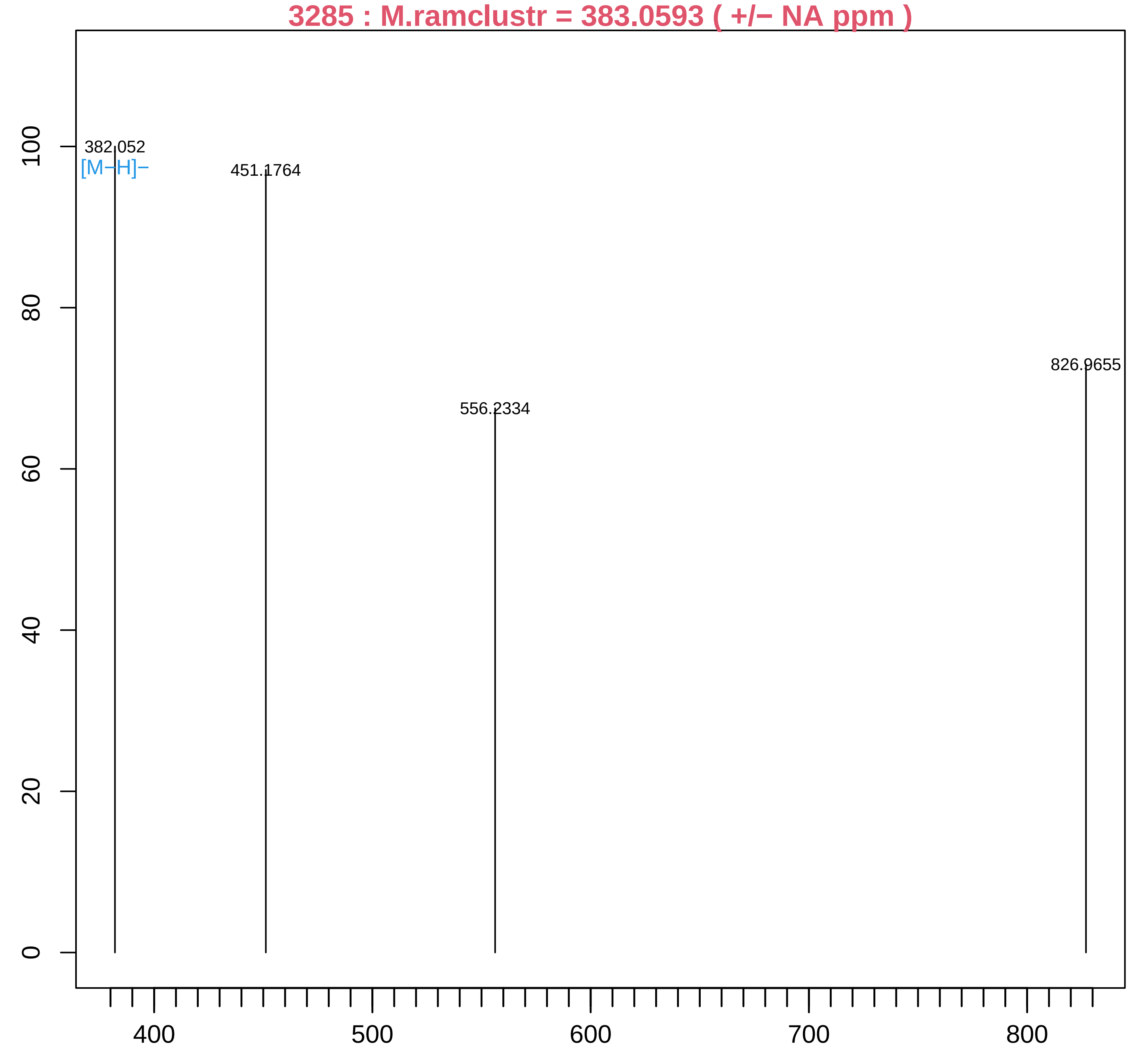
3285 : M.ramclustr = 383.0593 (+/− NA ppm)
382.052
[M−H]−
451.1764
556.2334
826.9655
0
20
40
60
80
100
400
500
600
700
800

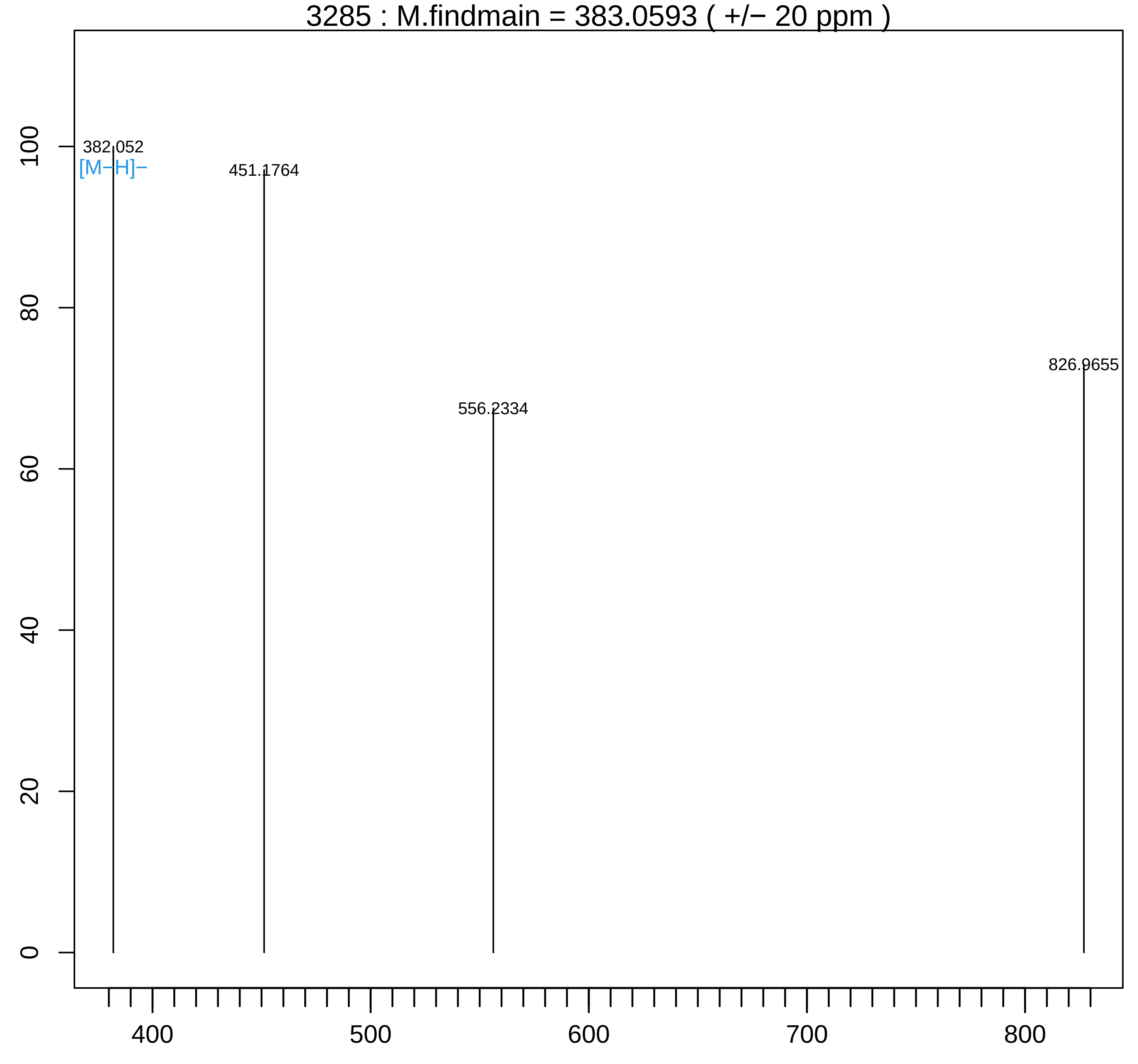
3285 : M.findmain = 383.0593 (+/− 20 ppm)
382.052
[M−H]−
451.1764
556.2334
826.9655
0
20
40
60
80
100
400
500
600
700
800

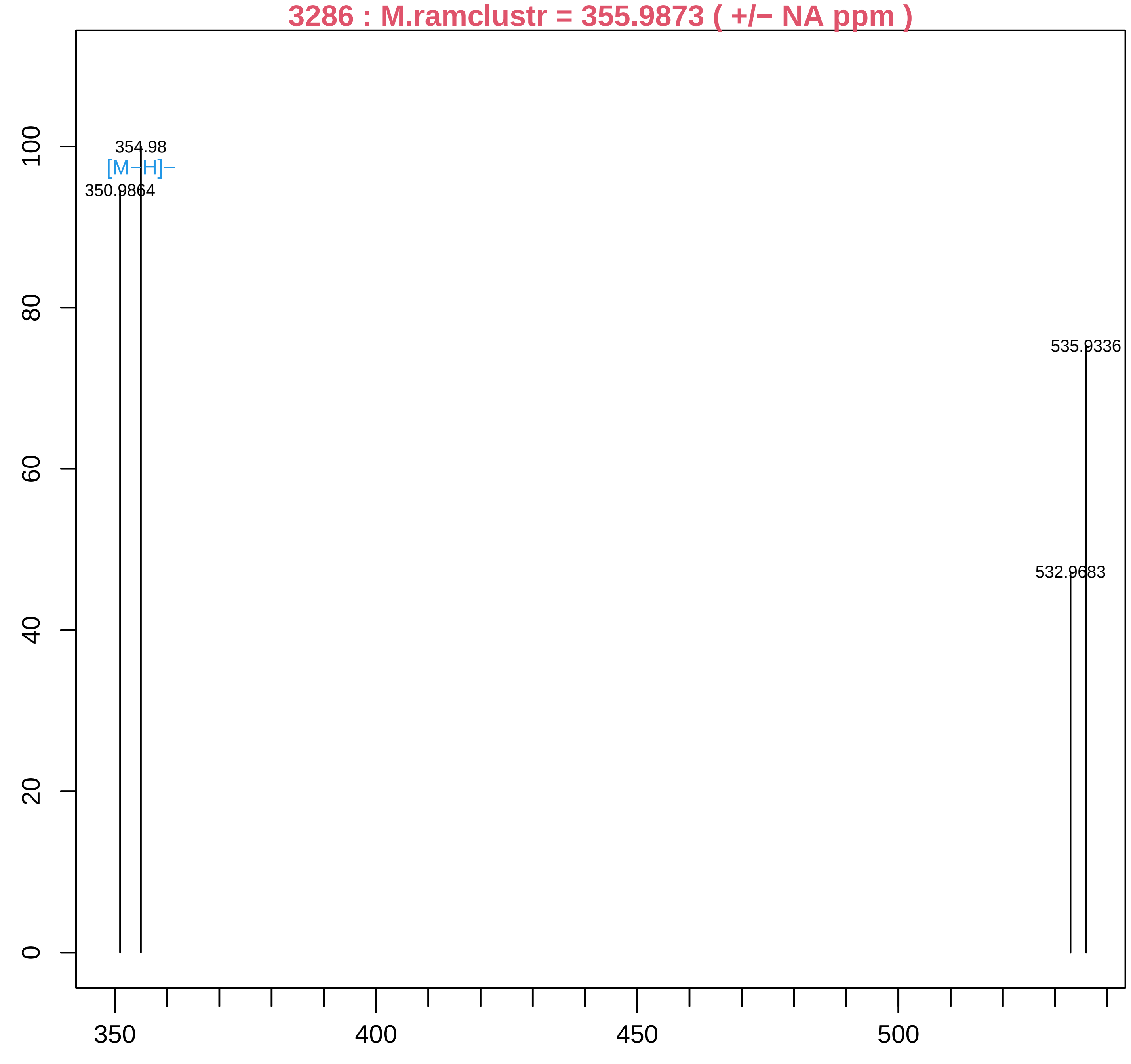
3286 : M.ramclustr = 355.9873 (+/− NA ppm)
354.98
[M−H]−
350.9864
535.9336
532.9683
0
20
40
60
80
100
350
400
450
500

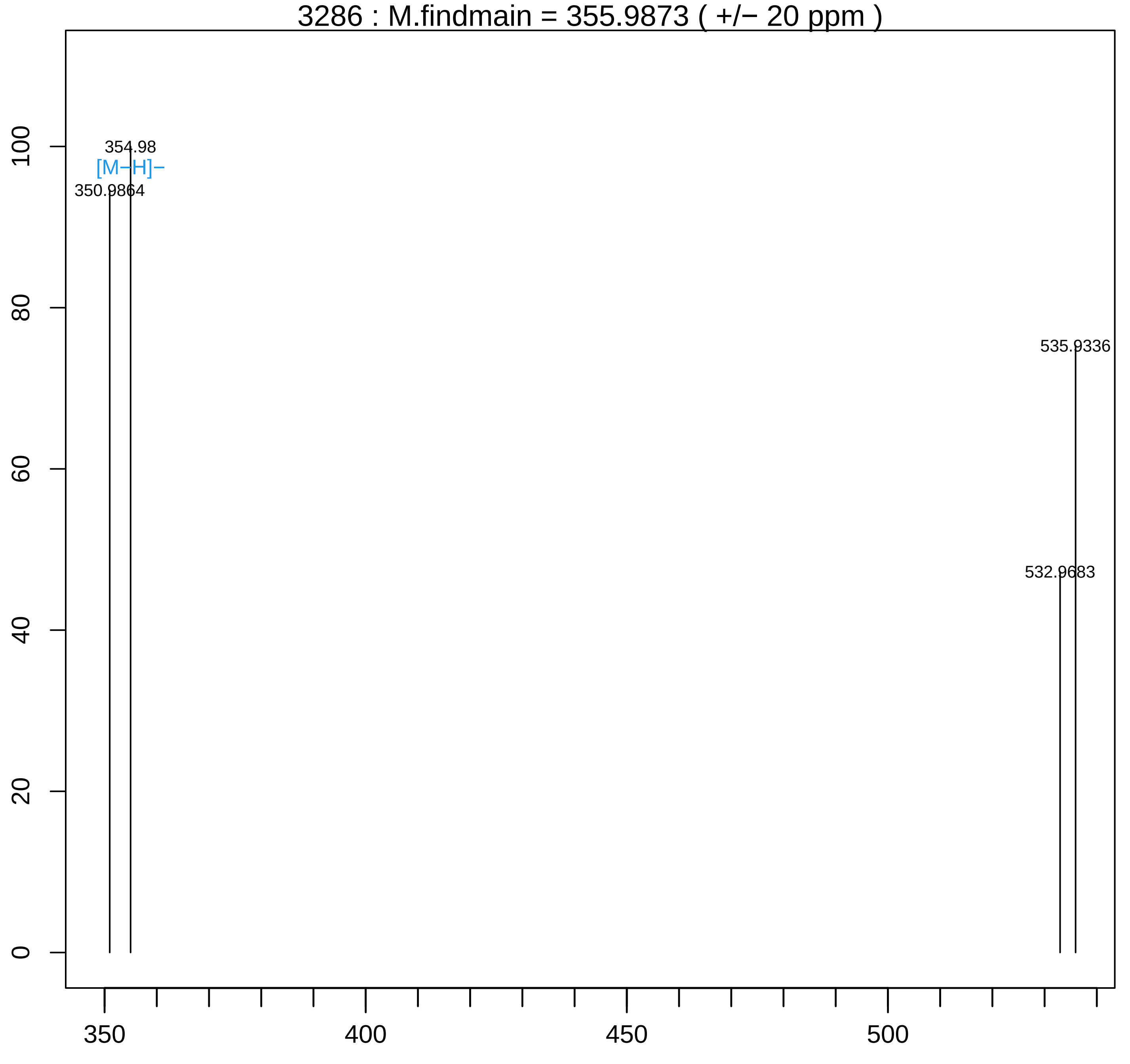
3286 : M.findmain = 355.9873 (+/− 20 ppm)
354.98
[M−H]−
350.9864
535.9336
532.9683
0
20
40
60
80
100
350
400
450
500

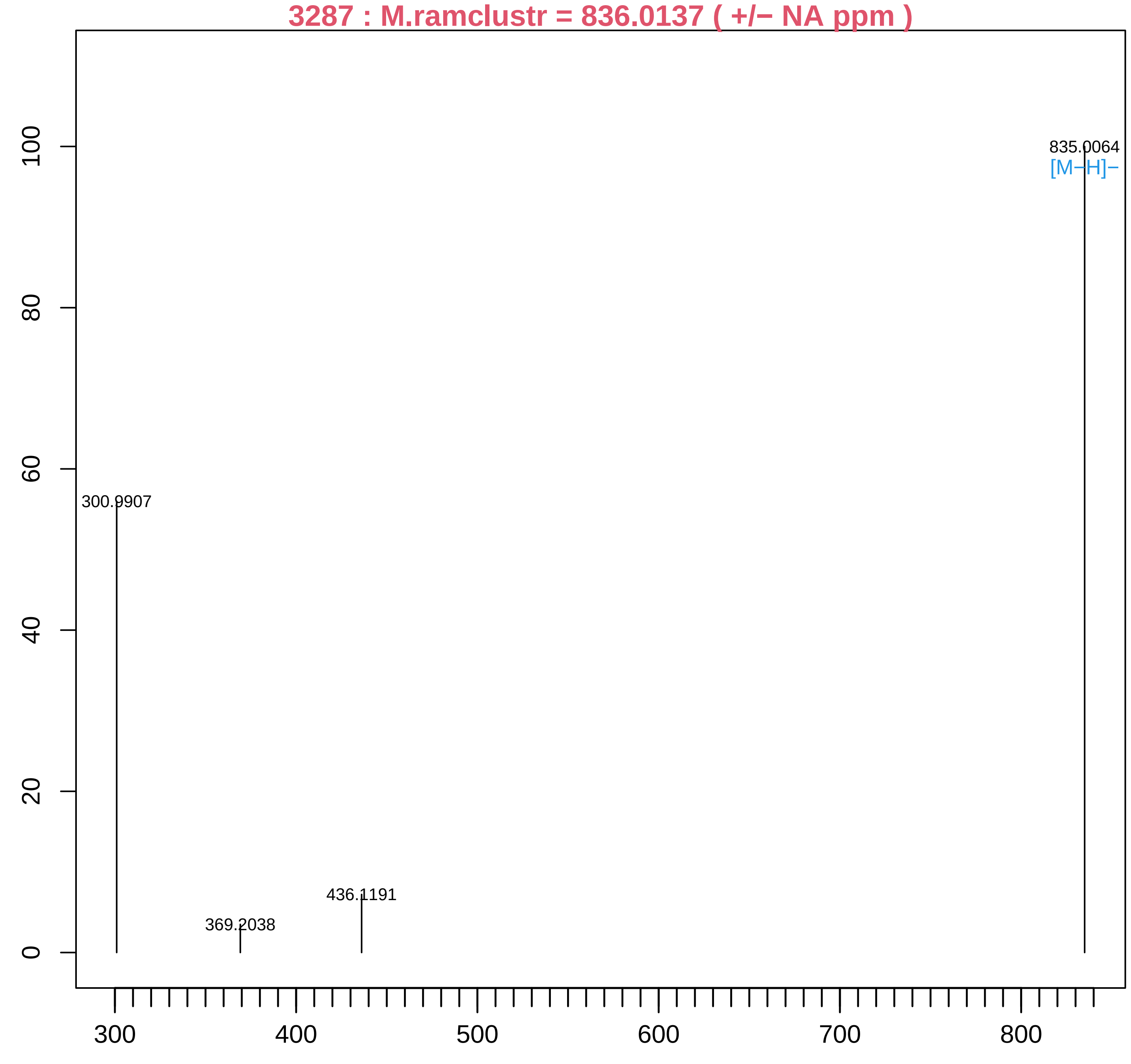
3287 : M.ramclustr = 836.0137 (+/− NA ppm)
835.0064
[M−H]−
300.9907
369.2038
436.1191
0
20
40
60
80
100
300
400
500
600
700
800

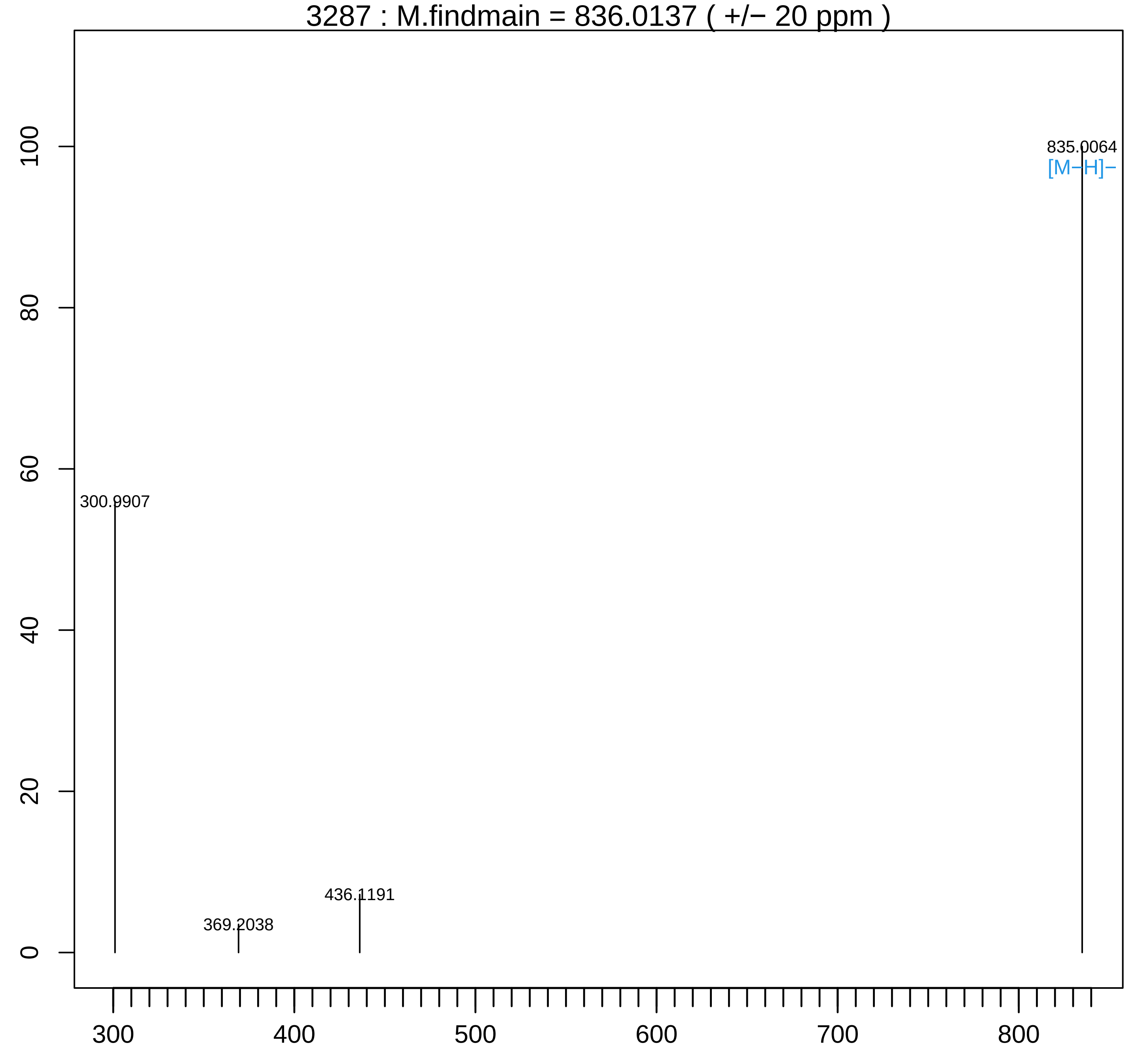
3287 : M.findmain = 836.0137 (+/− 20 ppm)
835.0064
[M−H]−
300.9907
369.2038
436.1191
0
20
40
60
80
100
300
400
500
600
700
800

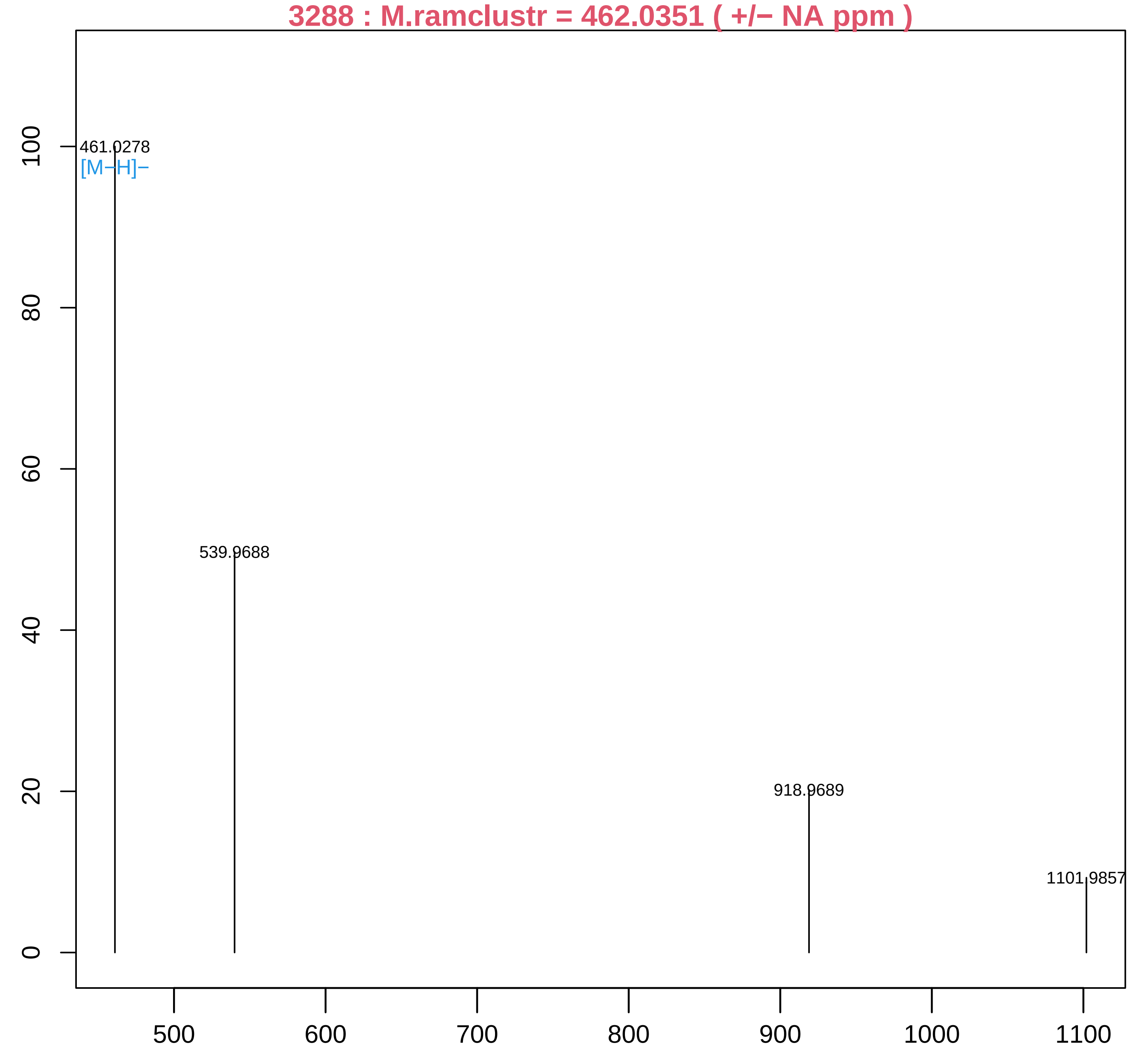
3288 : M.ramclustr = 462.0351 (+/− NA ppm)
461.0278
[M−H]−
539.9688
918.9689
1101.9857
0
20
40
60
80
100
500
600
700
800
900
1000
1100

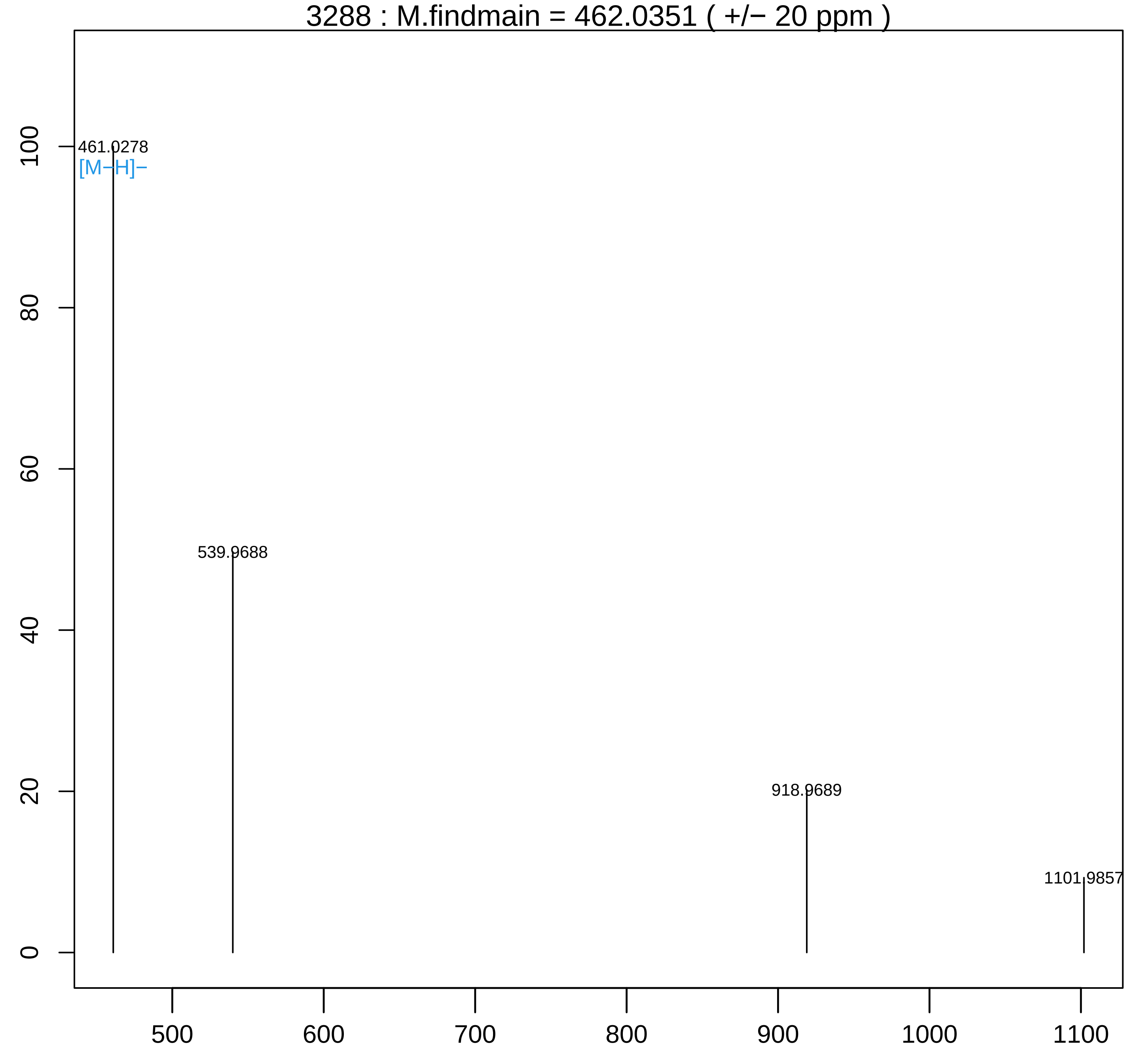
3288 : M.findmain = 462.0351 (+/− 20 ppm)
461.0278
[M−H]−
539.9688
918.9689
1101.9857
0
20
40
60
80
100
500
600
700
800
900
1000
1100

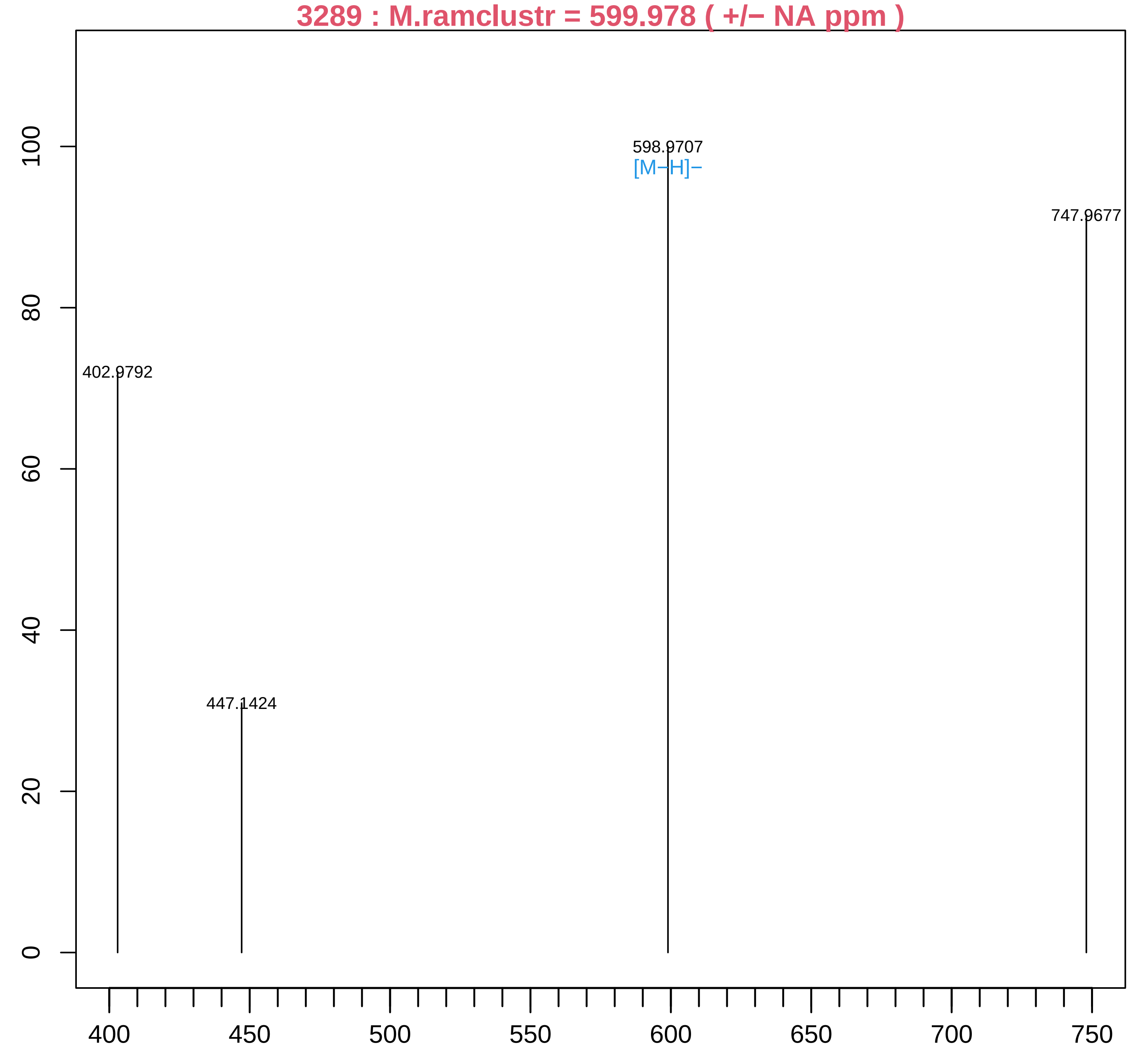
3289 : M.ramclustr = 599.978 (+/− NA ppm)
402.9792
447.1424
598.9707
[M−H]−
747.9677
0
20
40
60
80
100
400
450
500
550
600
650
700
750

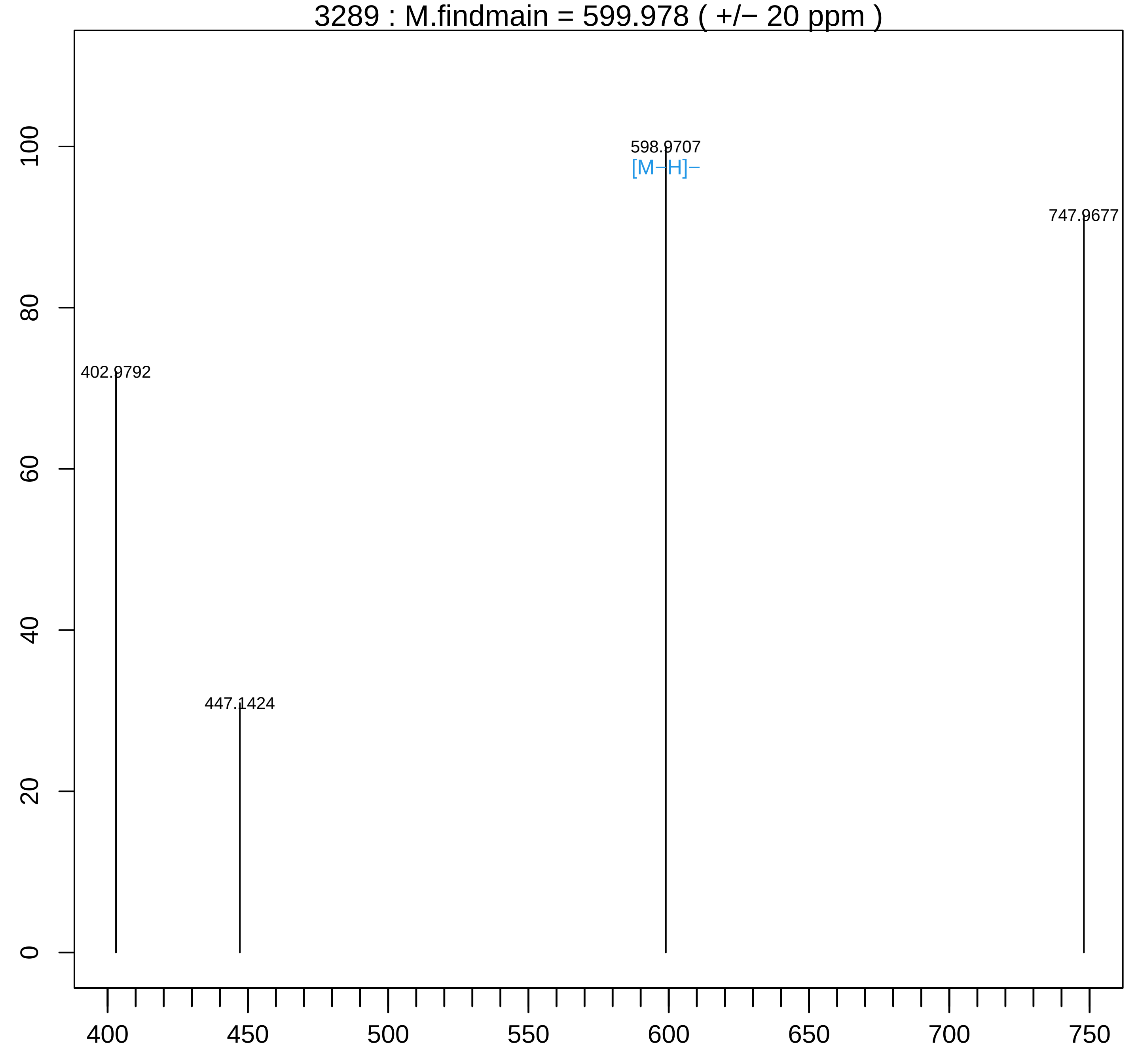
3289 : M.findmain = 599.978 (+/− 20 ppm)
402.9792
447.1424
598.9707
[M−H]−
747.9677
0
20
40
60
80
100
400
450
500
550
600
650
700
750

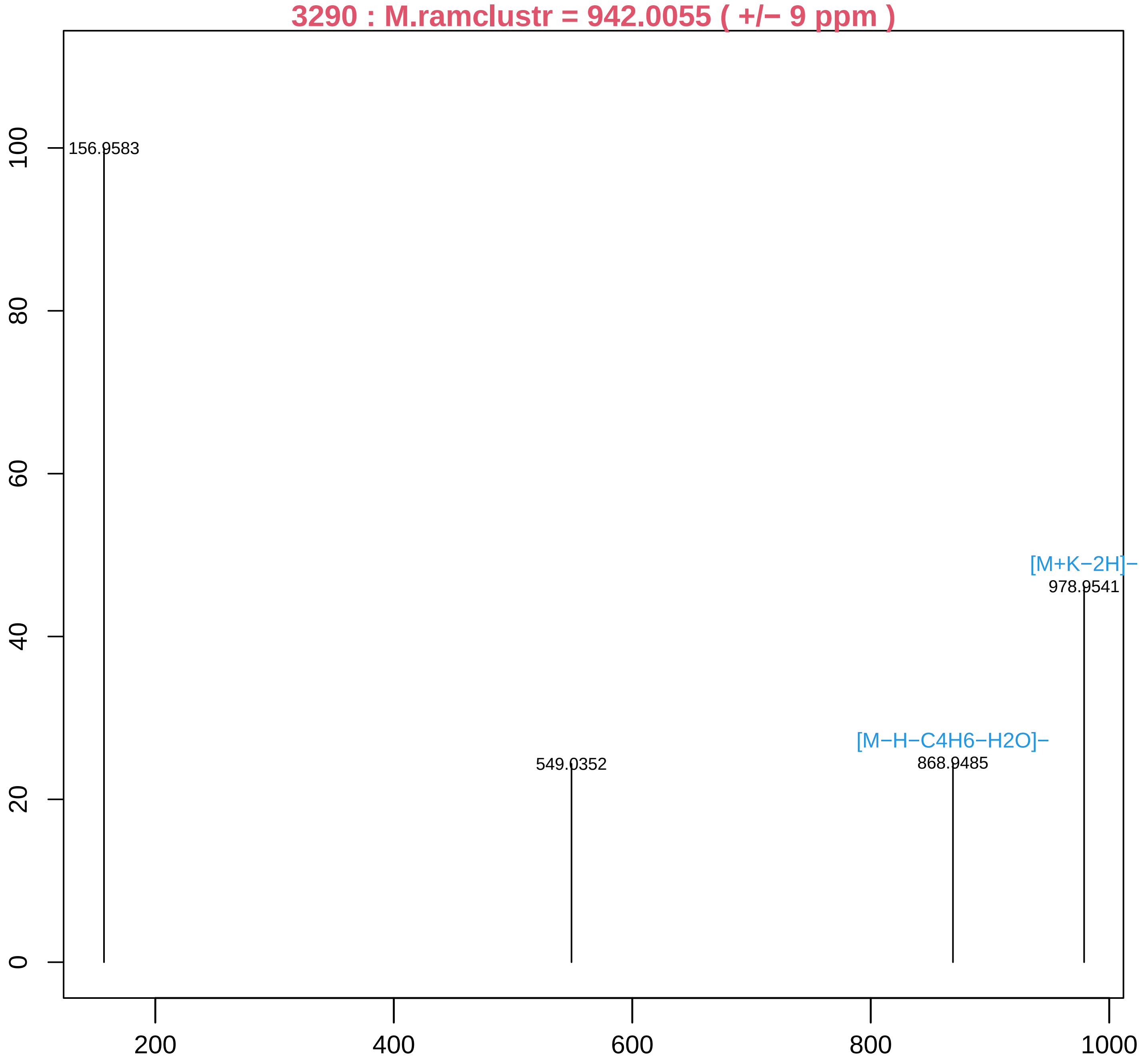
3290 : M.ramclustr = 942.0055 (+/− 9 ppm)
156.9583
549.0352
[M−H−C4H6−H2O]−
868.9485
[M+K−2H]−
978.9541
0
20
40
60
80
100
200
400
600
800
1000

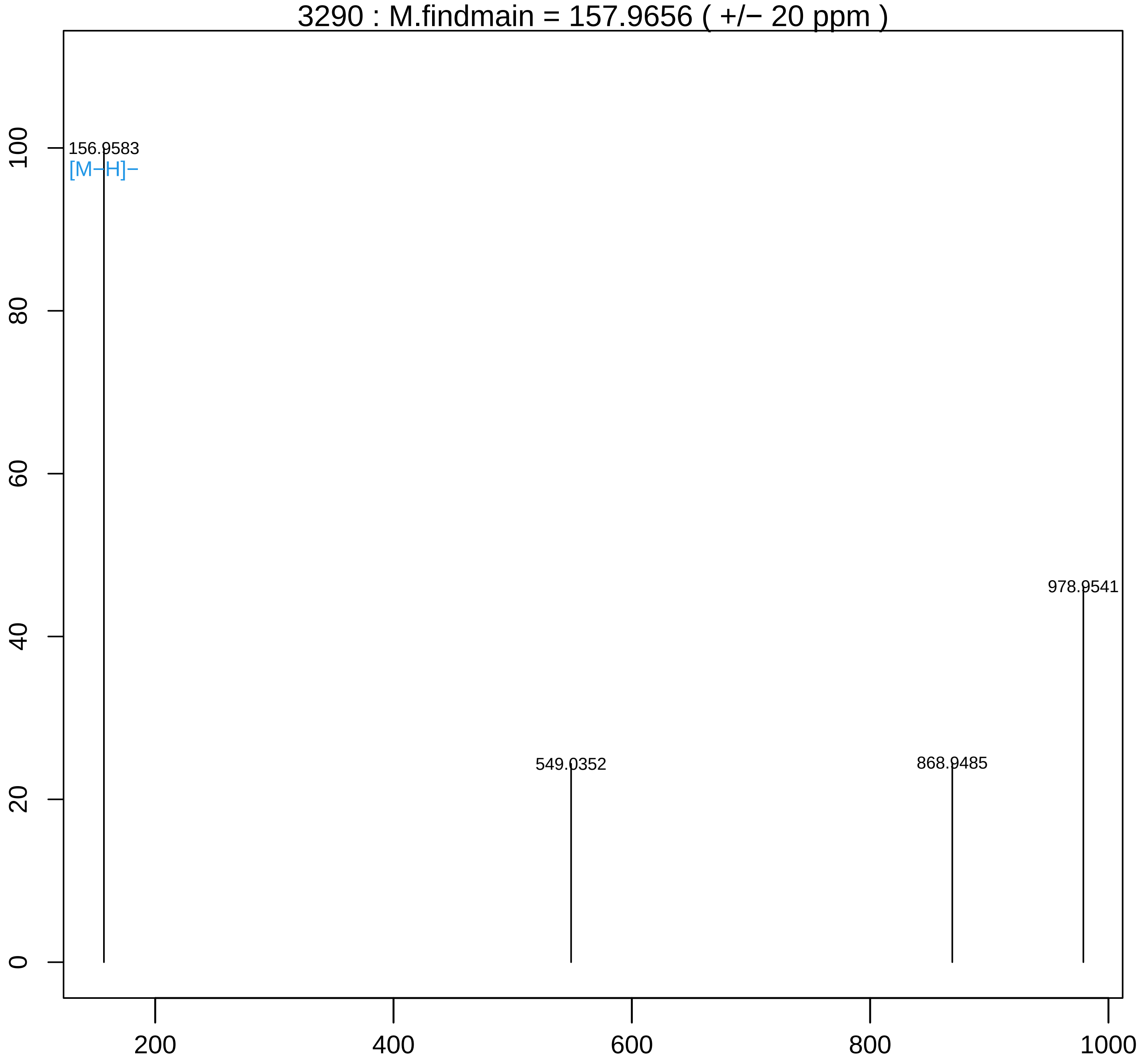
3290 : M.findmain = 157.9656 (+/− 20 ppm)
156.9583
[M−H]−
549.0352
868.9485
978.9541
0
20
40
60
80
100
200
400
600
800
1000

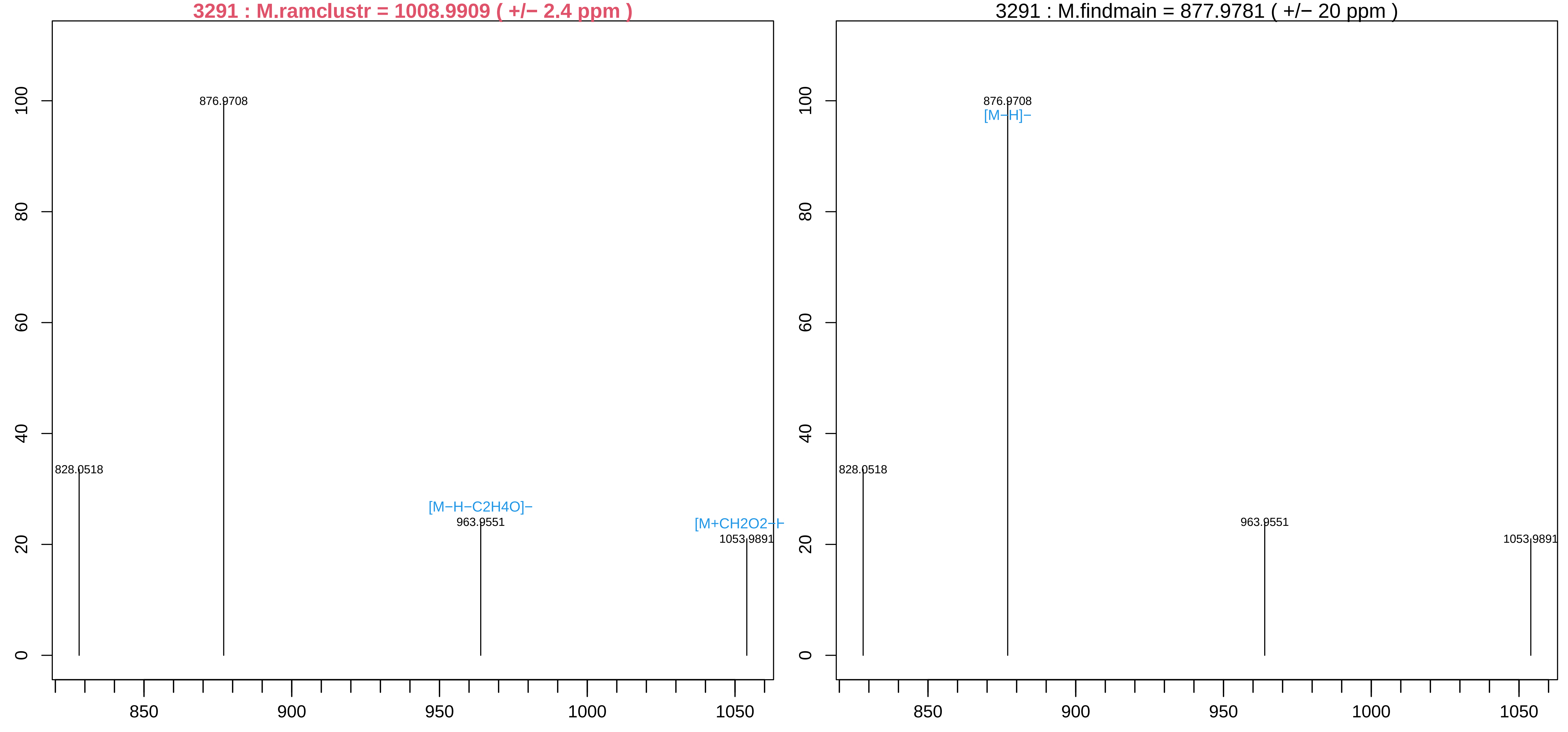
3291 : M.ramclustr = 1008.9909 (+/− 2.4 ppm)
876.9708
828.0518
[M−H−C2H4O]−
963.9551
[M+CH2O2−H
1053.9891
3291 : M.findmain = 877.9781 (+/− 20 ppm)
876.9708
[M−H]−
828.0518
963.9551
1053.9891
0
20
40
60
80
100
850
900
950
1000
1050

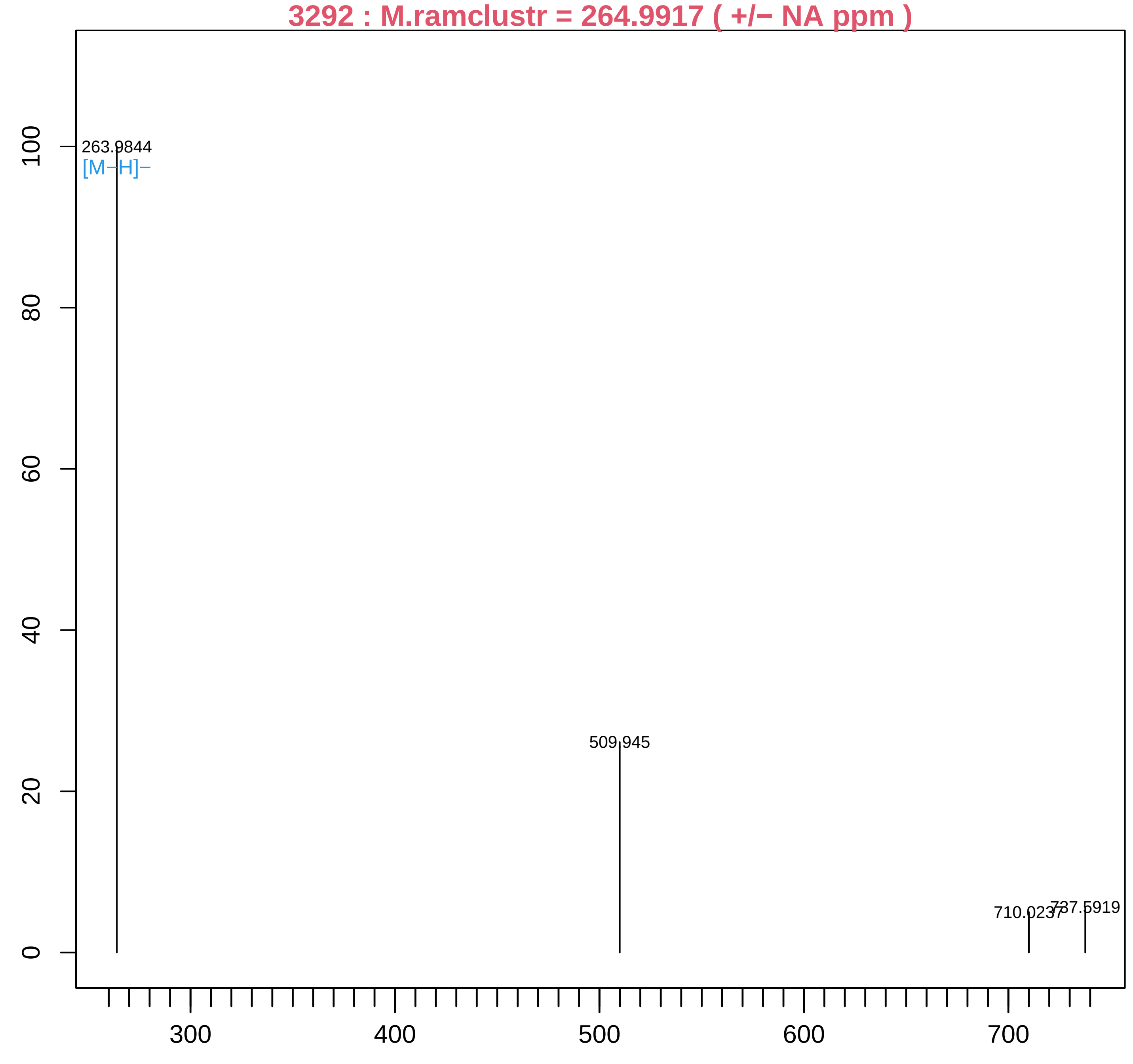
3292 : M.ramclustr = 264.9917 (+/− NA ppm)
263.9844
[M−H]−
509.945
710.0237
737.5919
0
20
40
60
80
100
300
400
500
600
700

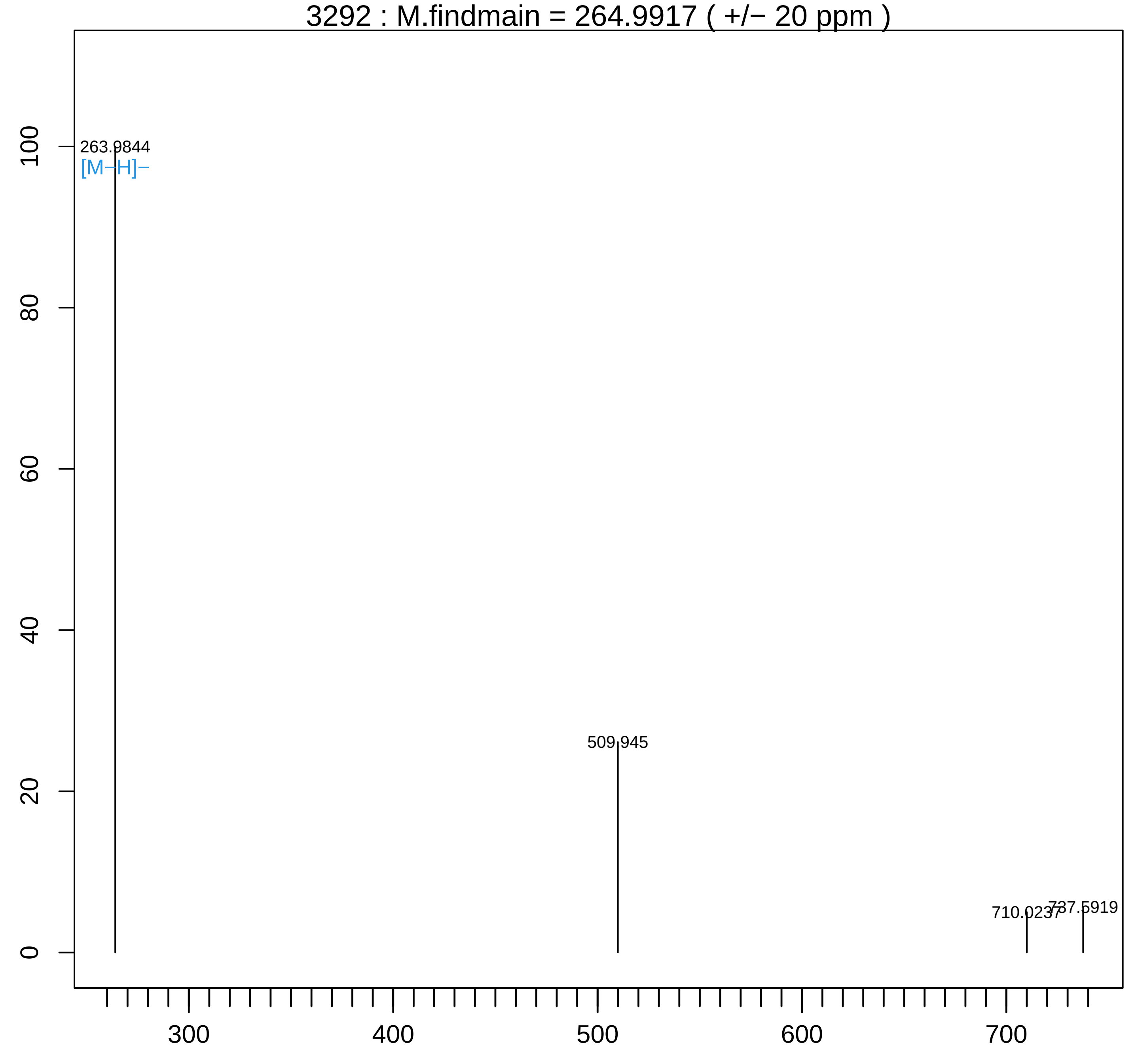
3292 : M.findmain = 264.9917 (+/− 20 ppm)
263.9844
[M−H]−
509.945
710.0237
737.5919
0
20
40
60
80
100
300
400
500
600
700

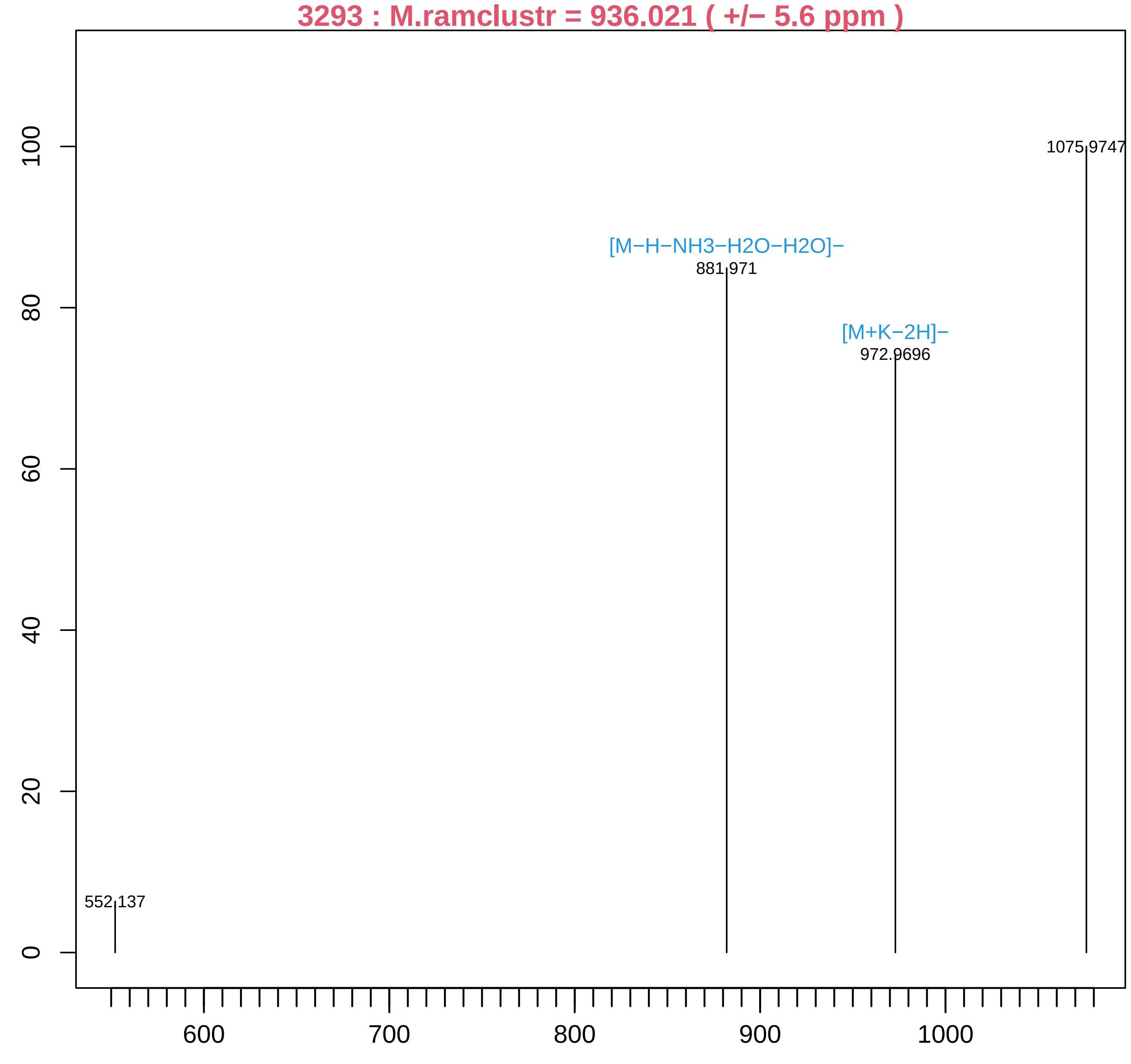
3293 : M.ramclustr = 936.021 (+/− 5.6 ppm)
[M−H−NH3−H2O−H2O]−
881.971
[M+K−2H]−
972.9696
1075.9747
552.137
0
20
40
60
80
100
600
700
800
900
1000

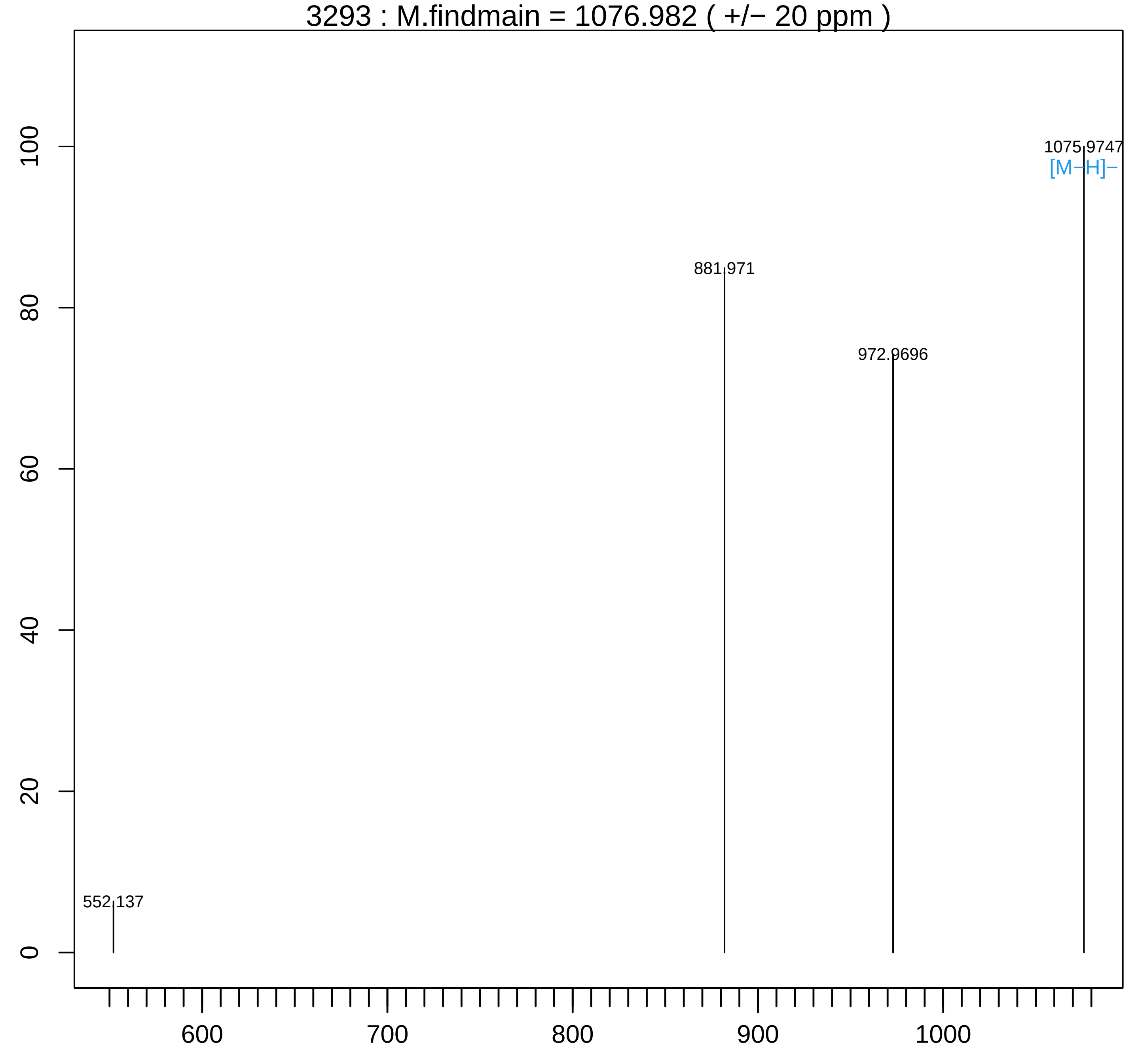
3293 : M.findmain = 1076.982 (+/− 20 ppm)
1075.9747
[M−H]−
881.971
972.9696
552.137
0
20
40
60
80
100
600
700
800
900
1000

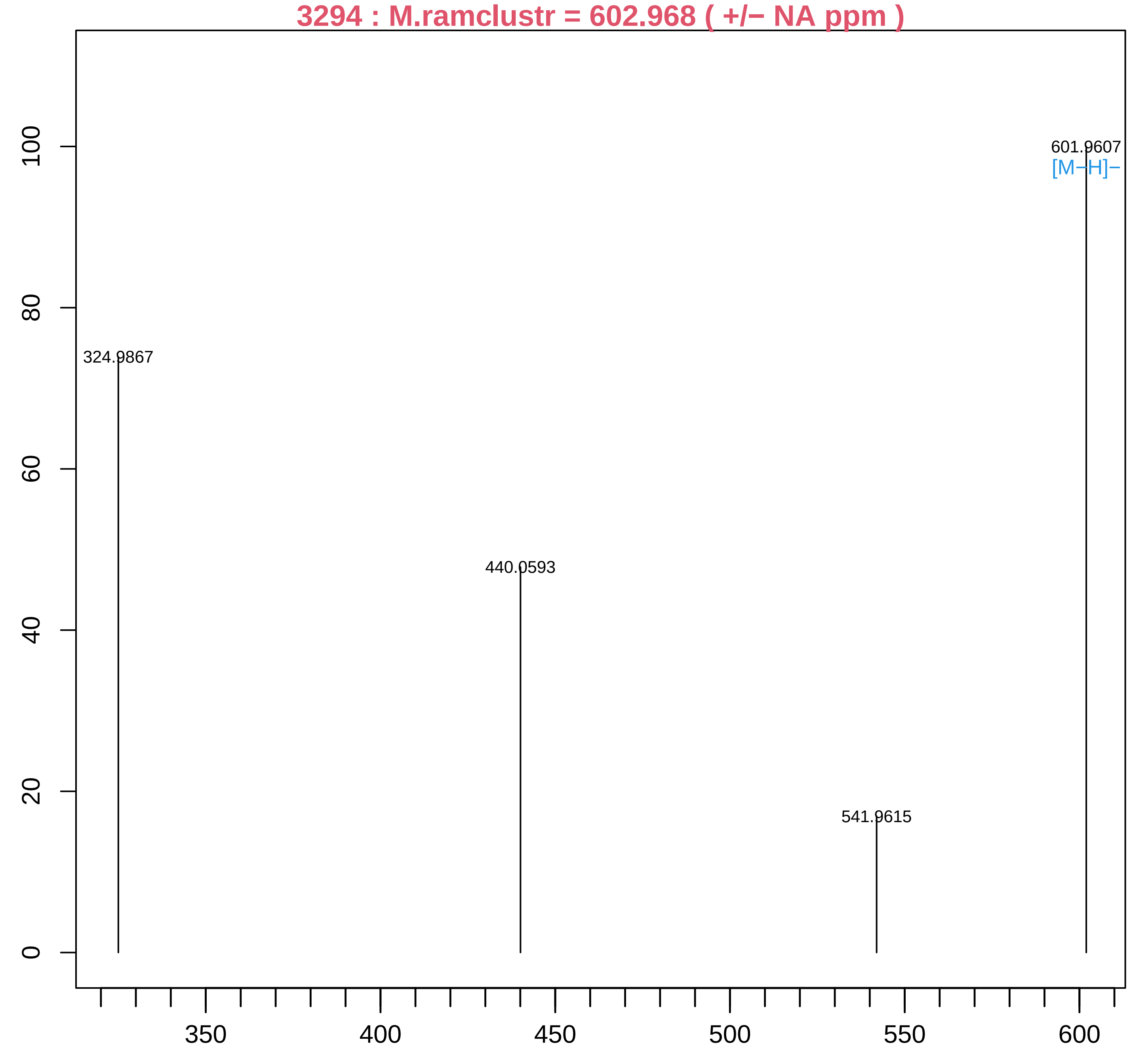

3294 : M.ramclustr = 602.968 (+/− NA ppm)
601.9607
[M−H]−
324.9867
440.0593
541.9615
0
20
40
60
80
100
350
400
450
500
550
600

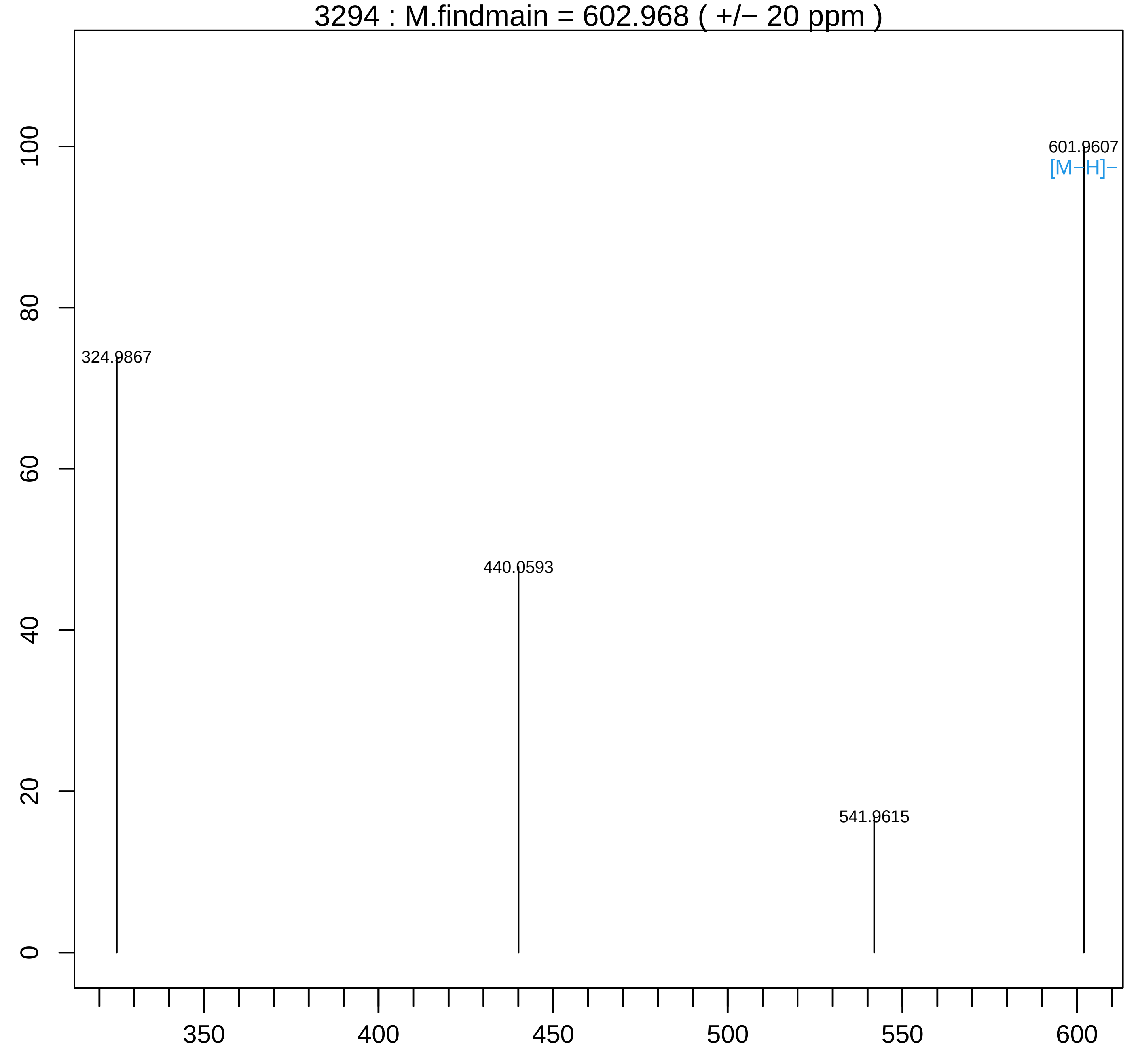

3294 : M.findmain = 602.968 (+/− 20 ppm)
601.9607
[M−H]−
324.9867
440.0593
541.9615
0
20
40
60
80
100
350
400
450
500
550
600

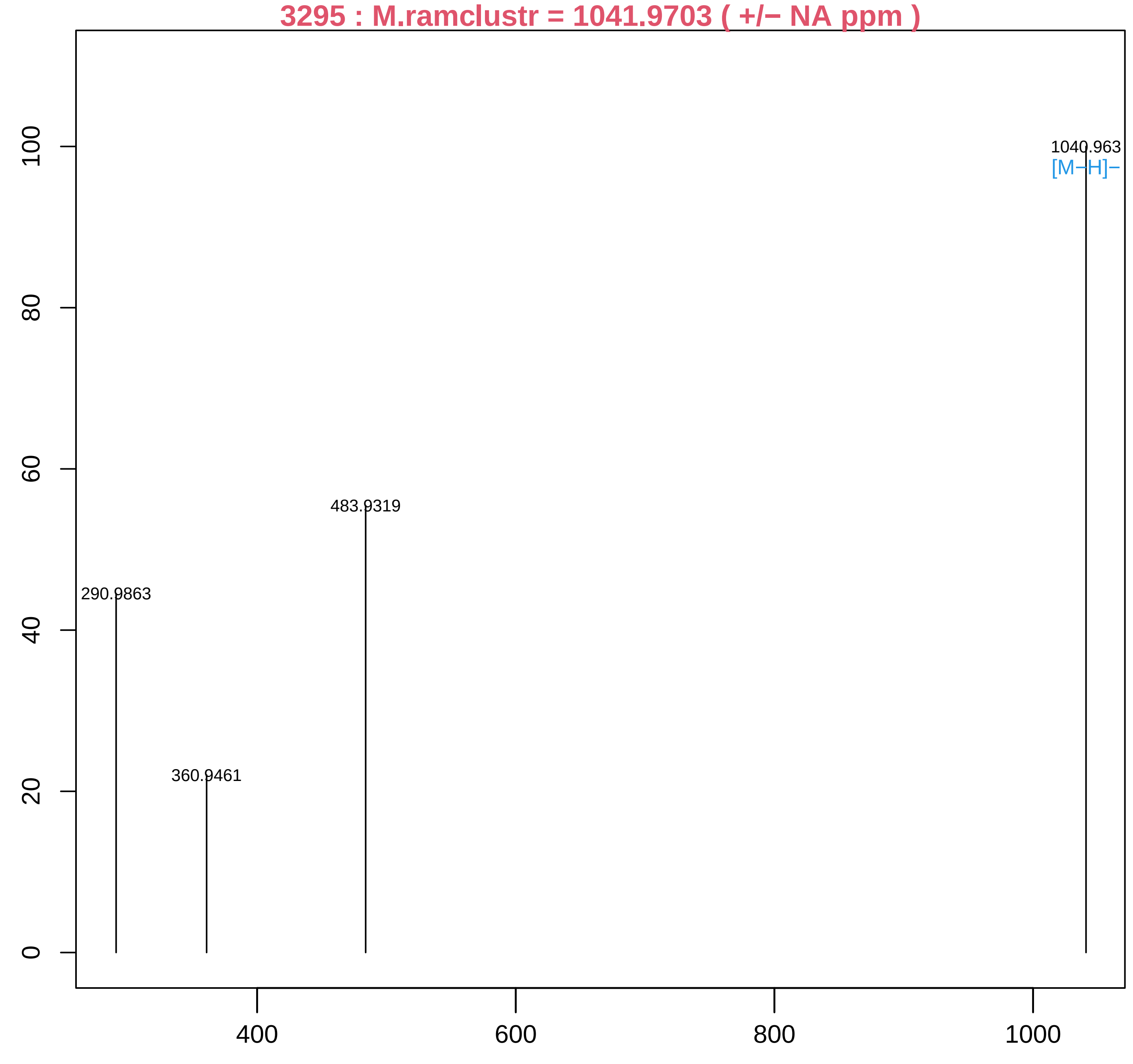

3295 : M.ramclustr = 1041.9703 (+/− NA ppm)
1040.963
[M−H]−
483.9319
290.9863
360.9461
0
20
40
60
80
100
400
600
800
1000

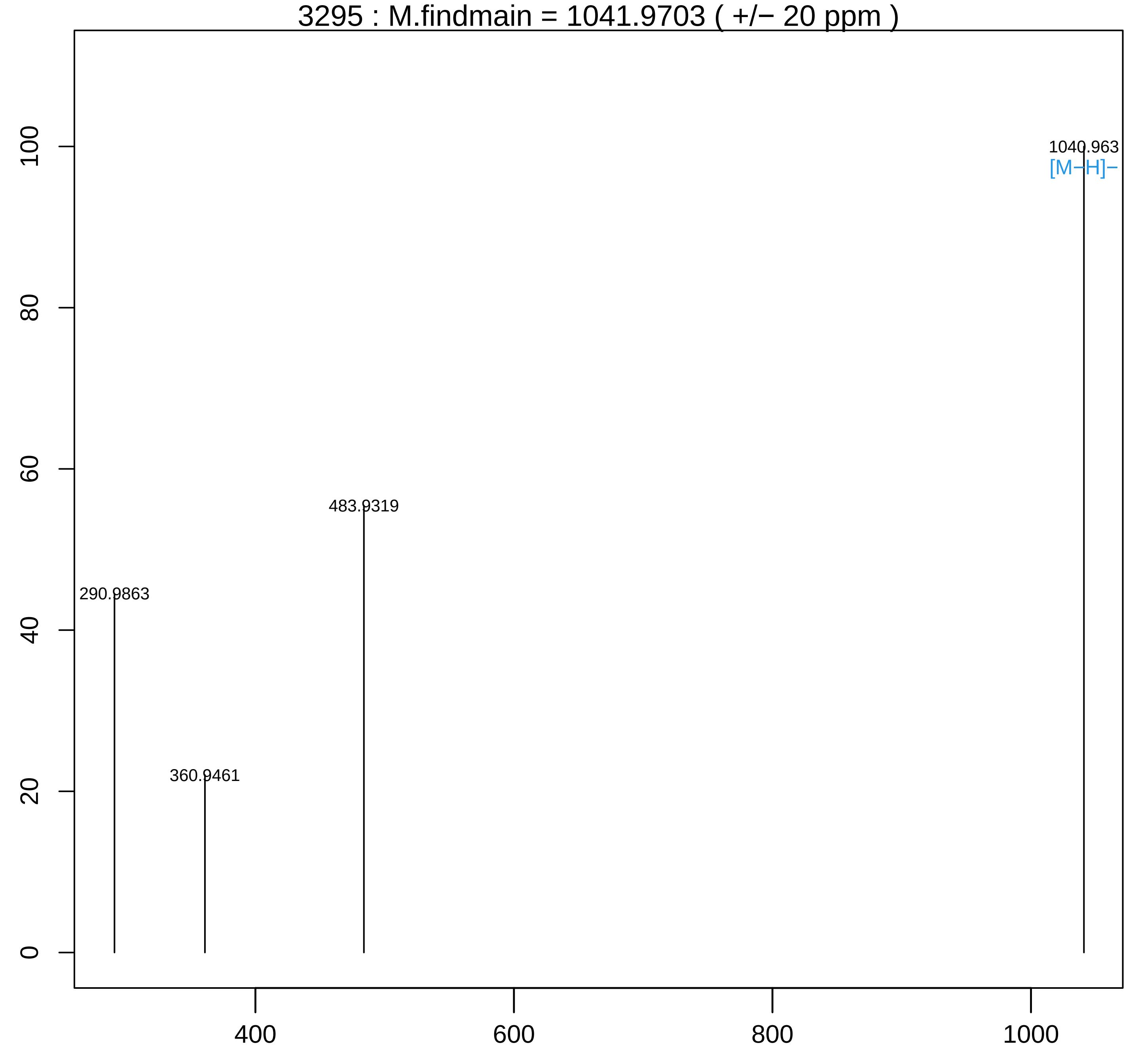

3295 : M.findmain = 1041.9703 (+/− 20 ppm)
1040.963
[M−H]−
483.9319
290.9863
360.9461
0
20
40
60
80
100
400
600
800
1000

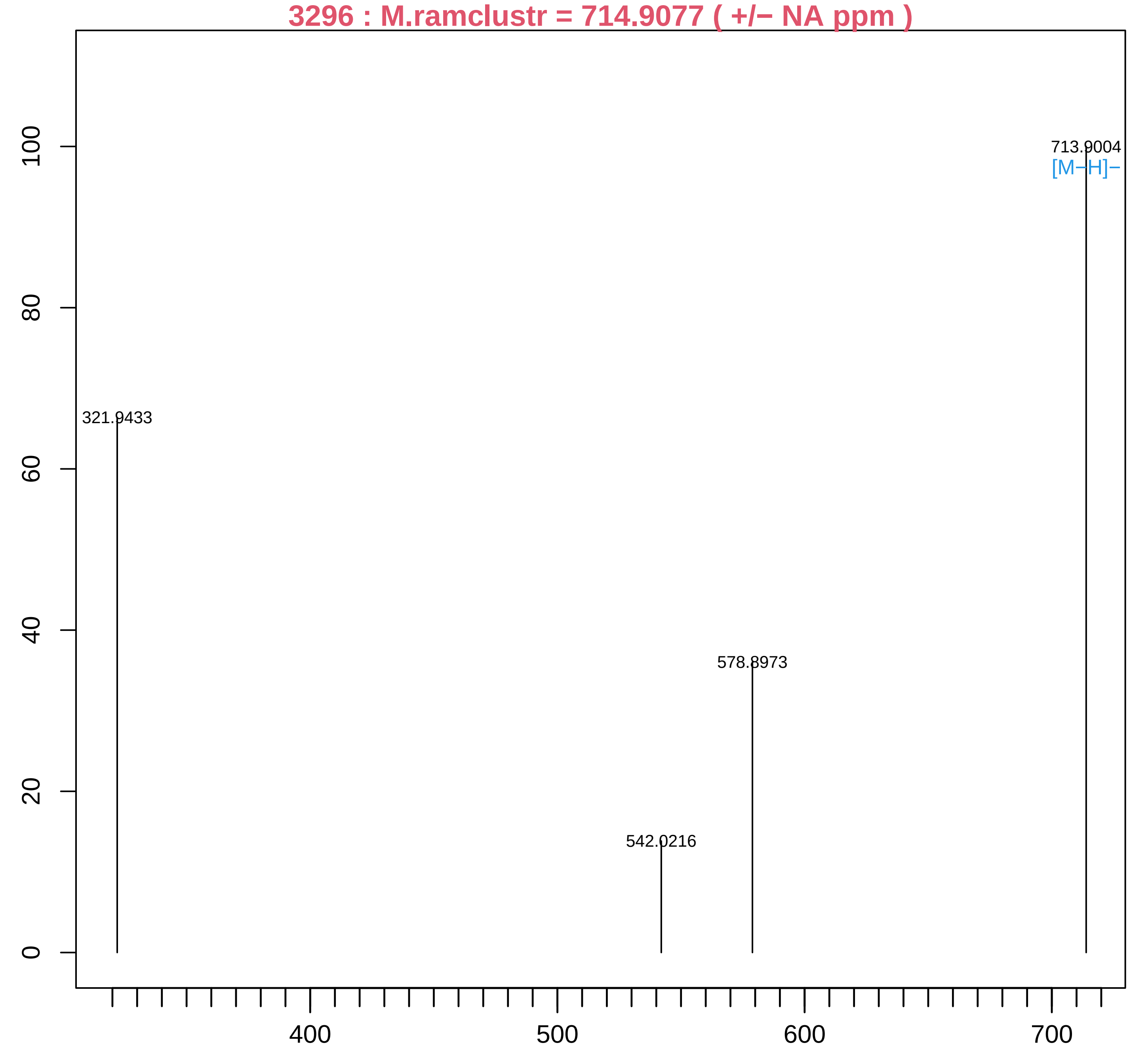
3296 : M.ramclustr = 714.9077 (+/− NA ppm)
713.9004
[M−H]−
321.9433
578.8973
542.0216
0
20
40
60
80
100
400
500
600
700

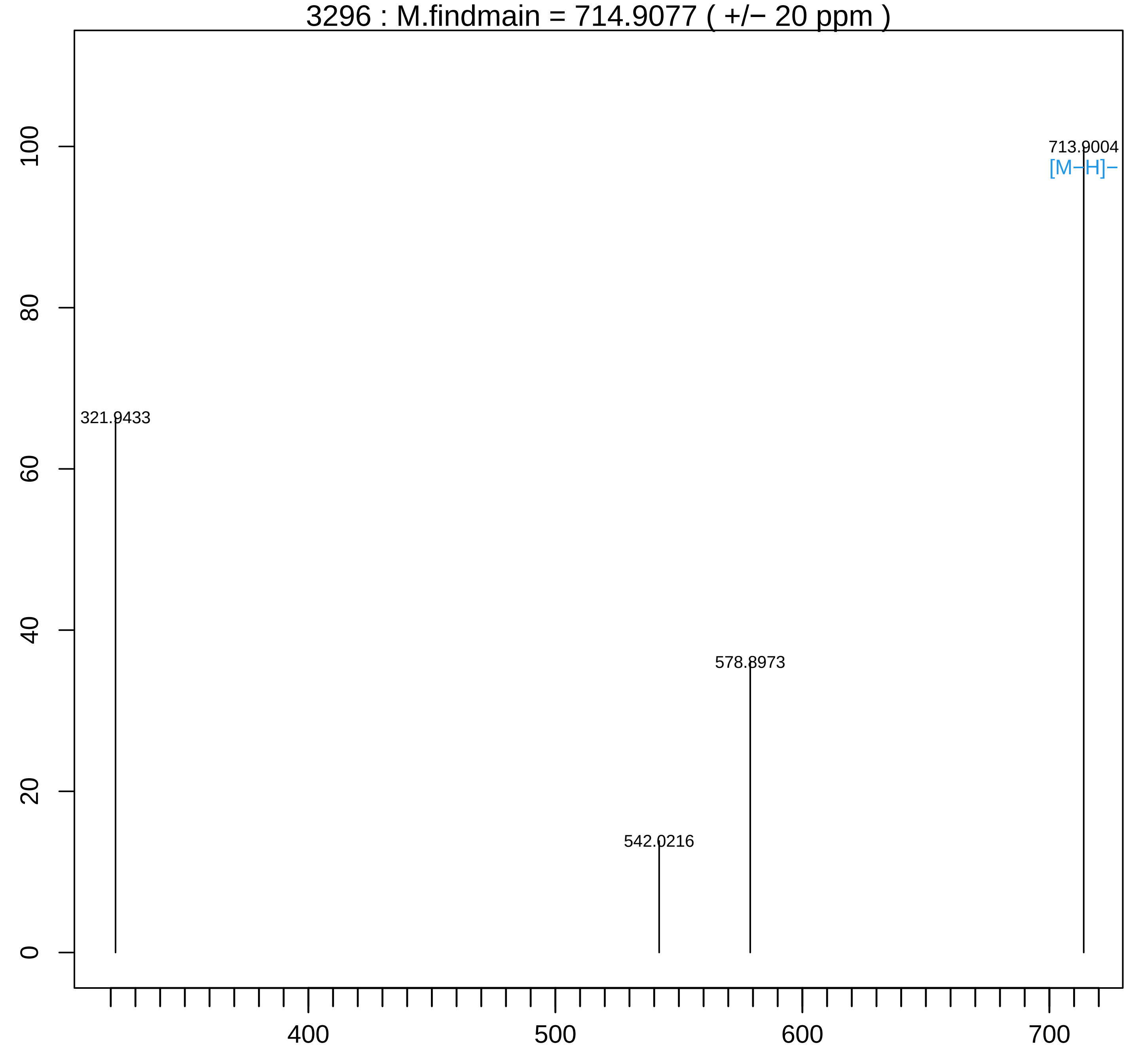
3296 : M.findmain = 714.9077 (+/− 20 ppm)
713.9004
[M−H]−
321.9433
578.8973
542.0216
0
20
40
60
80
100
400
500
600
700

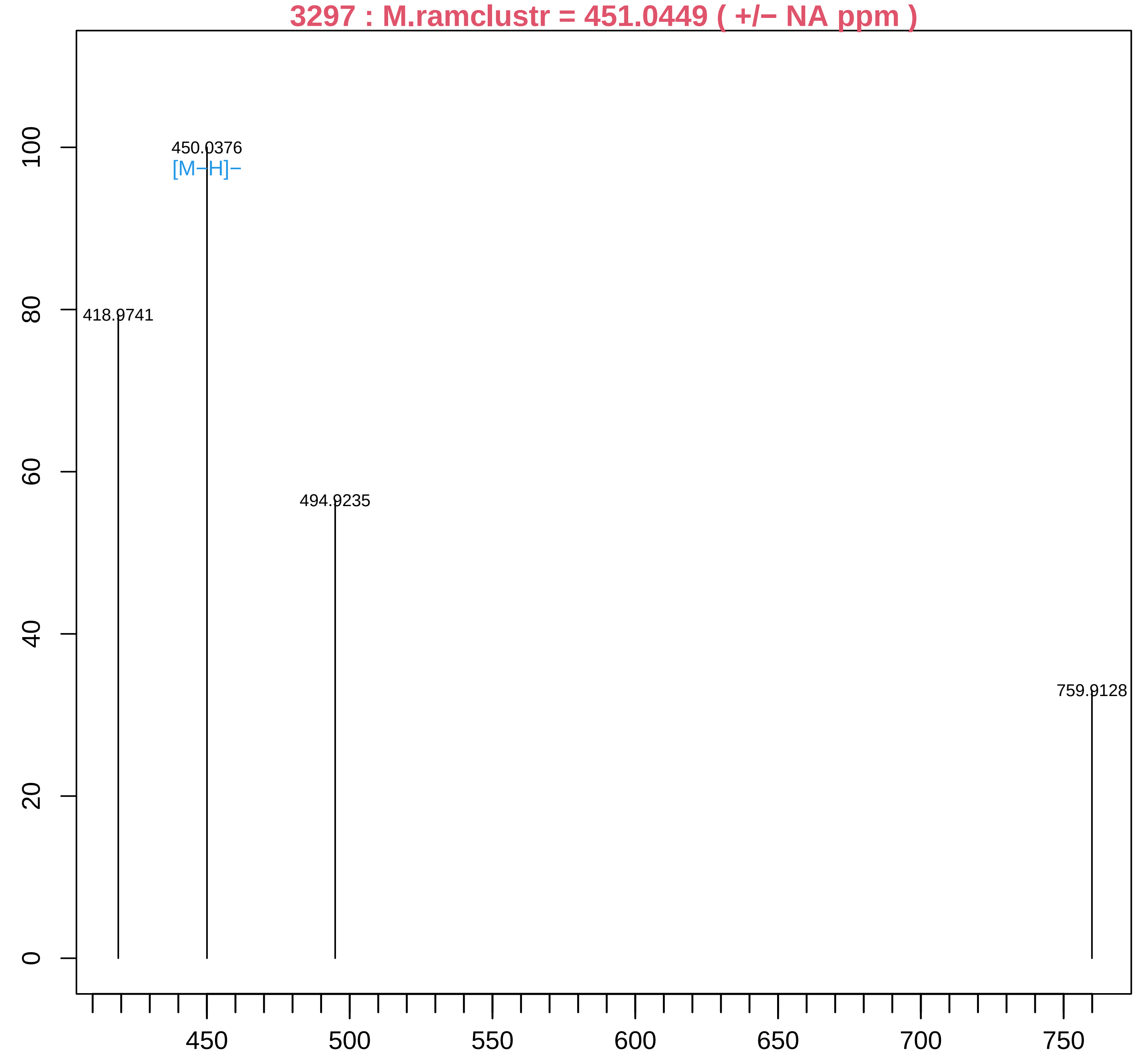

3297 : M.ramclustr = 451.0449 (+/− NA ppm)
450.0376
[M−H]−
418.9741
494.9235
759.9128
0
20
40
60
80
100
450
500
550
600
650
700
750

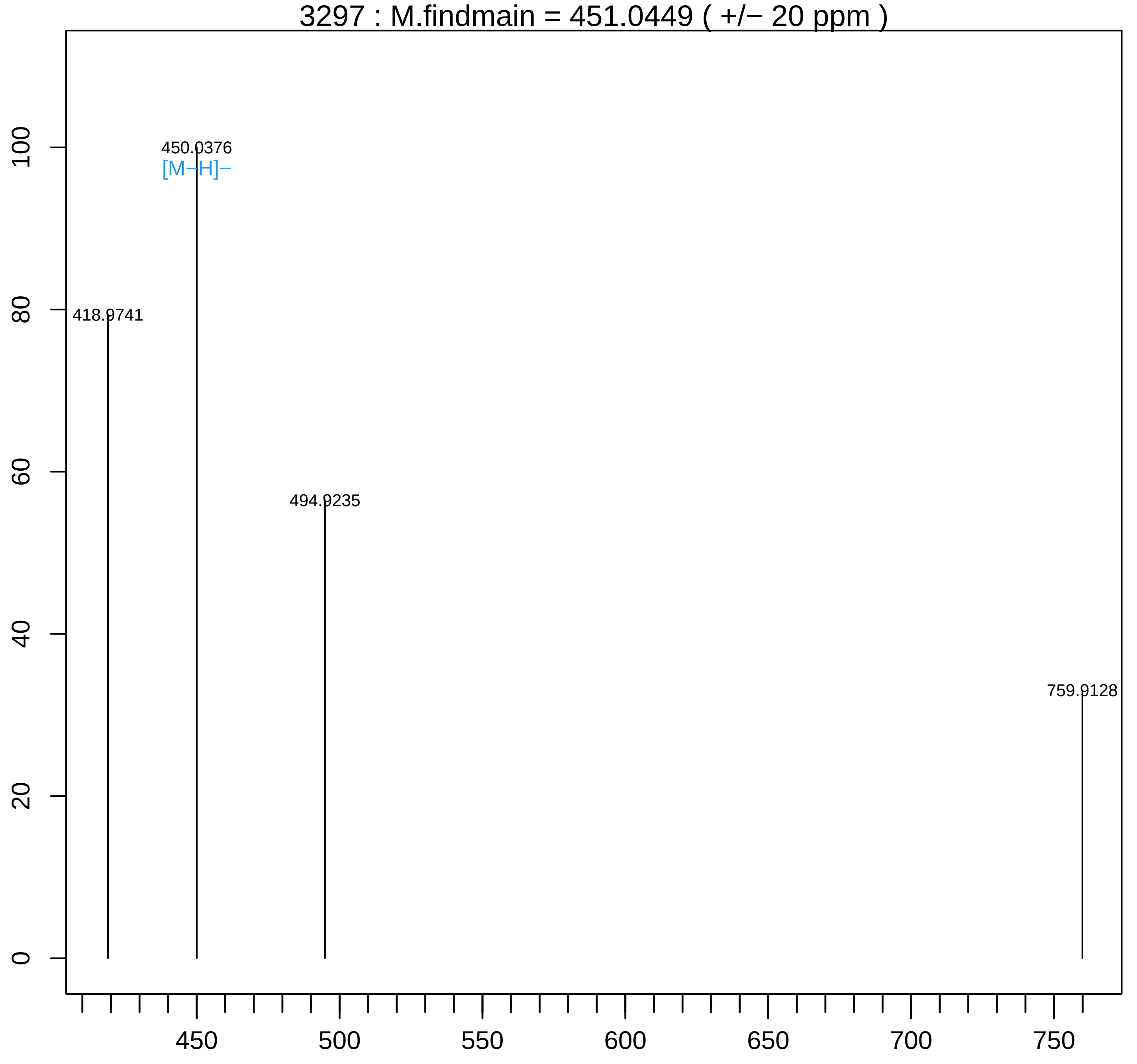

3297 : M.findmain = 451.0449 (+/− 20 ppm)
450.0376
[M−H]−
418.9741
494.9235
759.9128
0
20
40
60
80
100
450
500
550
600
650
700
750

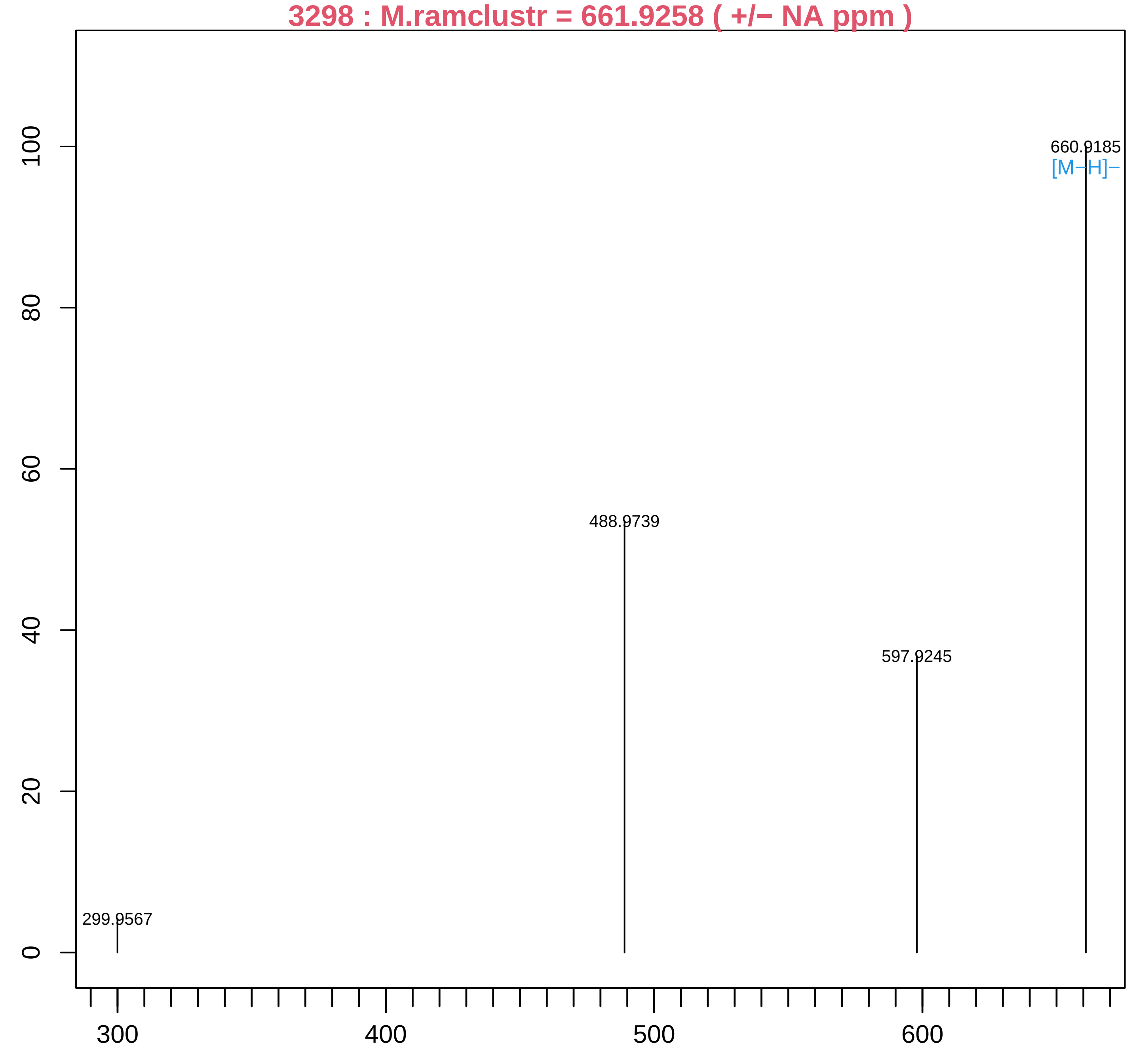

3298 : M.ramclustr = 661.9258 (+/− NA ppm)
660.9185
[M−H]−
488.9739
597.9245
299.9567
0
20
40
60
80
100
300
400
500
600

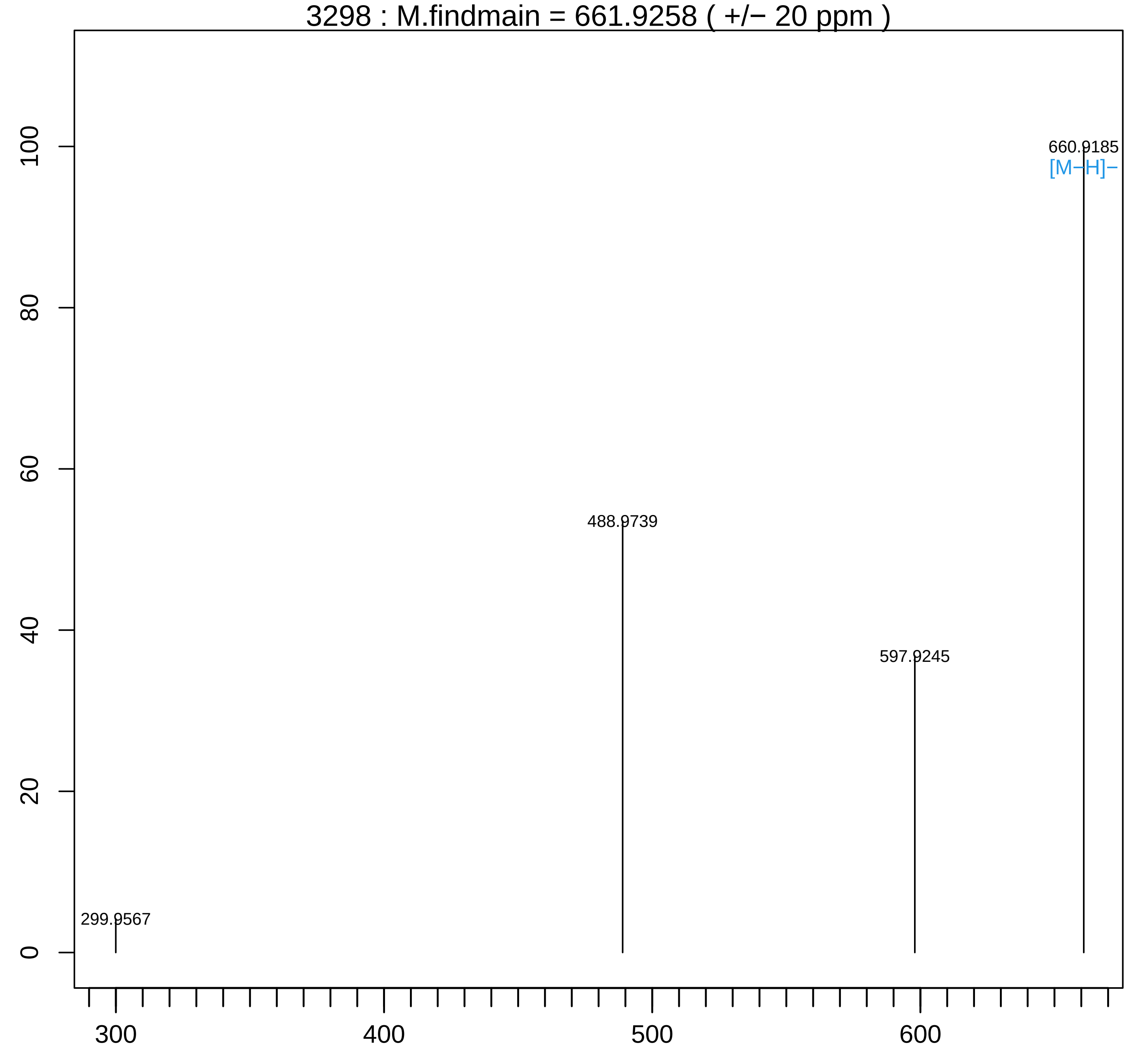

3298 : M.findmain = 661.9258 (+/− 20 ppm)
660.9185
[M−H]−
488.9739
597.9245
299.9567
0
20
40
60
80
100
300
400
500
600

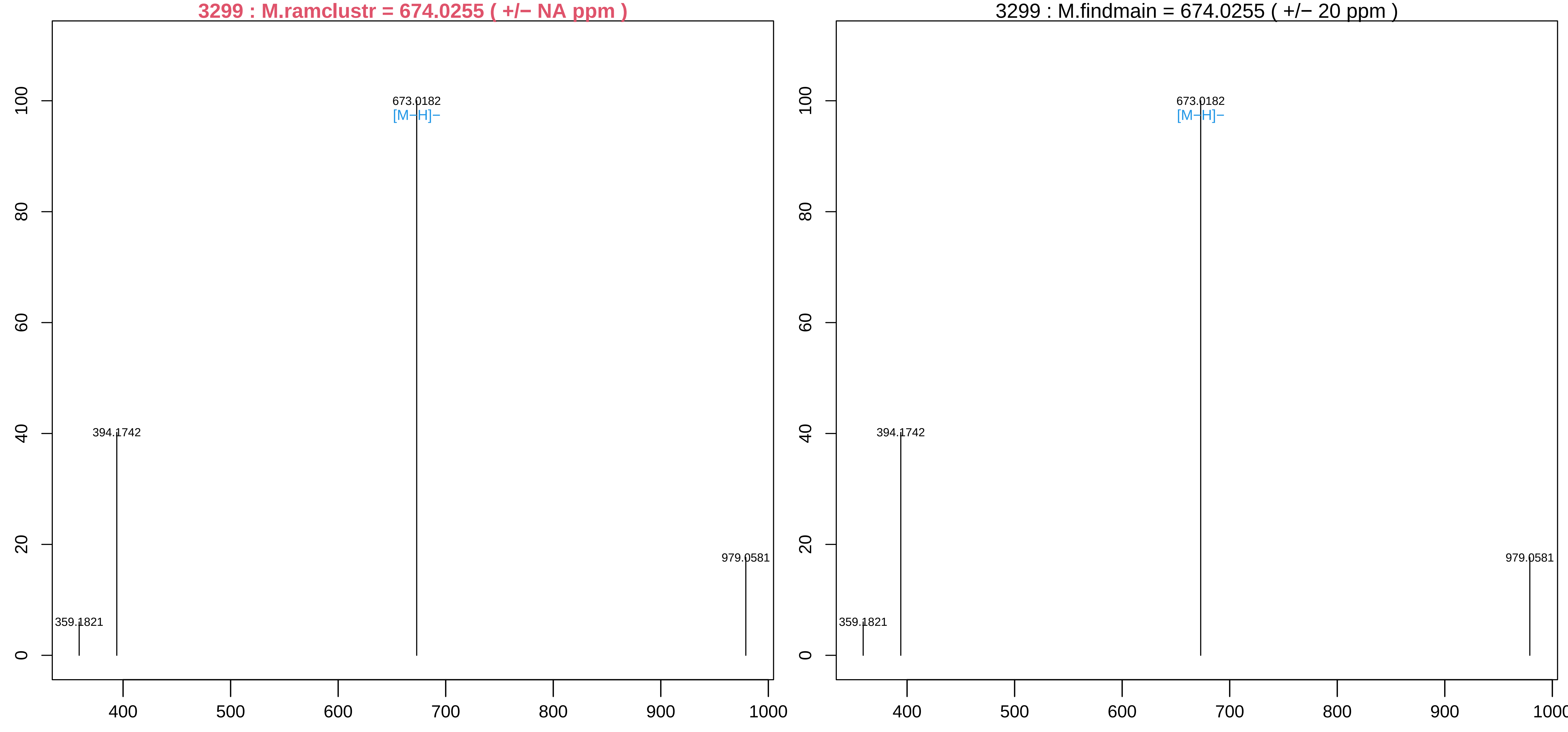
3299 : M.ramclustr = 674.0255 (+/− NA ppm)
673.0182
[M−H]−
394.1742
979.0581
359.1821
3299 : M.findmain = 674.0255 (+/− 20 ppm)
673.0182
[M−H]−
394.1742
979.0581
359.1821
0
20
40
60
80
100
400
500
600
700
800
900
1000

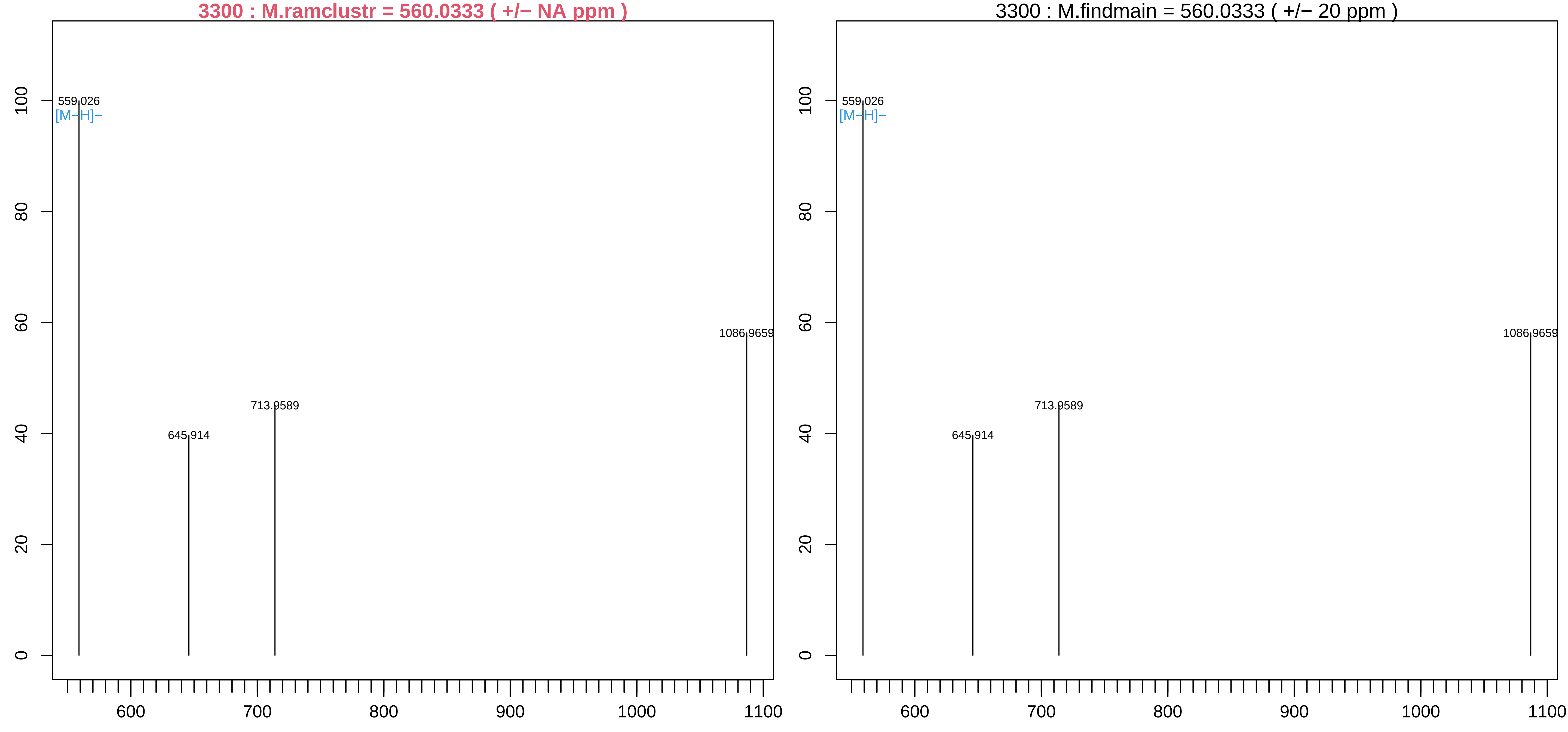
3300 : M.ramclustr = 560.0333 (+/− NA ppm)
559.026
[M−H]−
645.914
713.9589
1086.9659
3300 : M.findmain = 560.0333 (+/− 20 ppm)
559.026
[M−H]−
645.914
713.9589
1086.9659

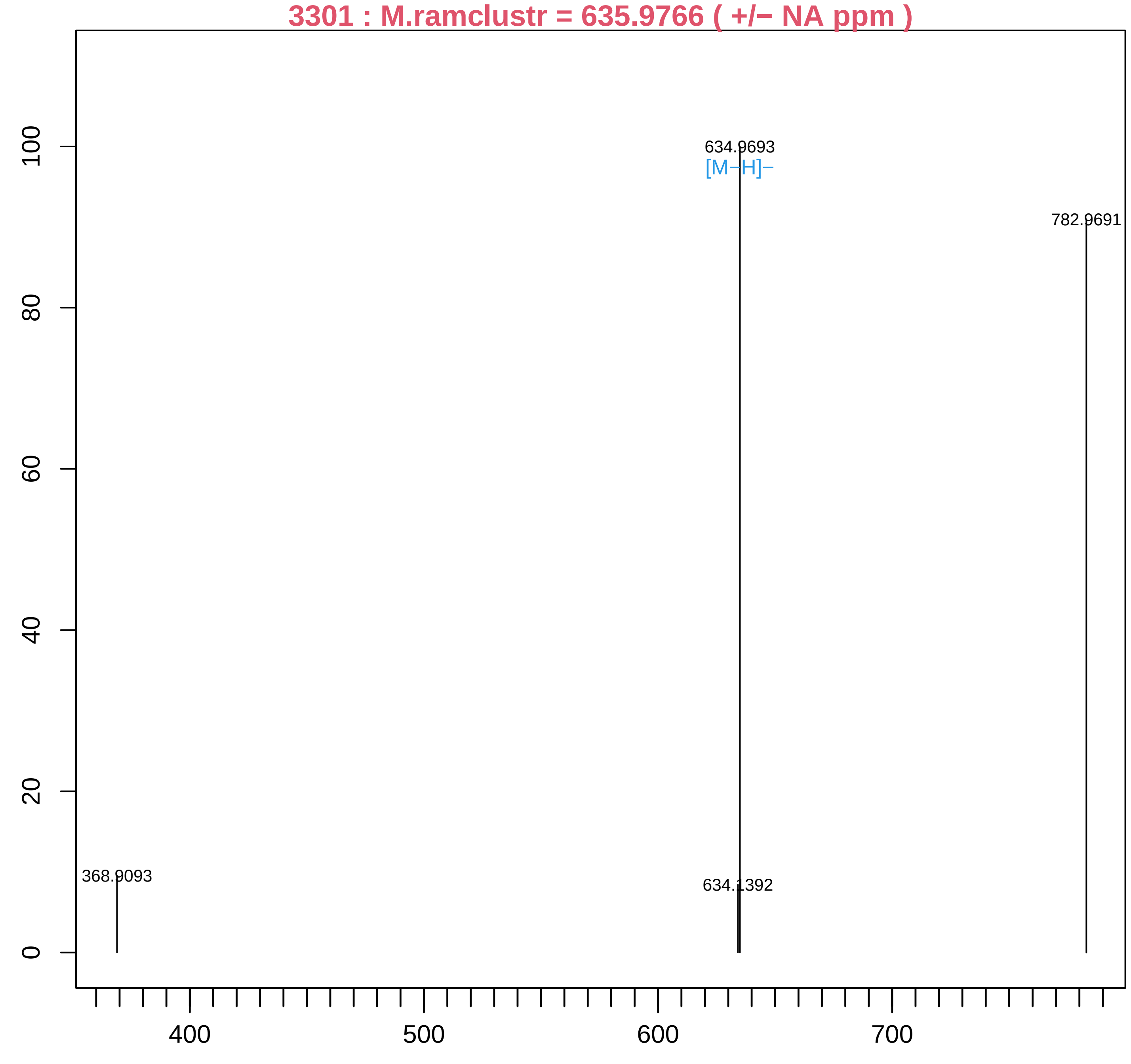
3301 : M.ramclustr = 635.9766 (+/− NA ppm)
634.9693
[M−H]−
782.9691
368.9093
634.1392
0
20
40
60
80
100
400
500
600
700

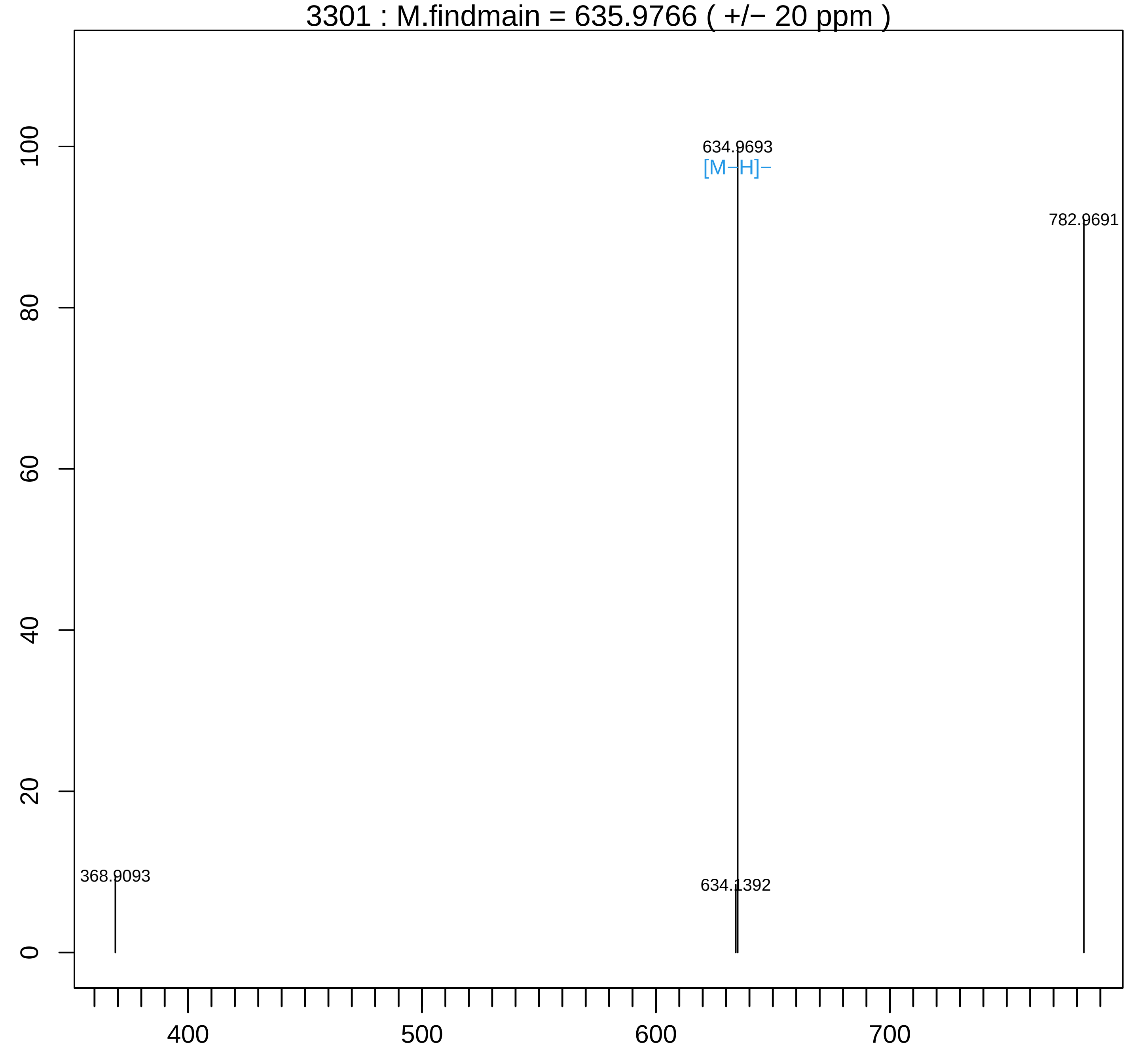
3301 : M.findmain = 635.9766 (+/− 20 ppm)
634.9693
[M−H]−
782.9691
368.9093
634.1392
0
20
40
60
80
100
400
500
600
700

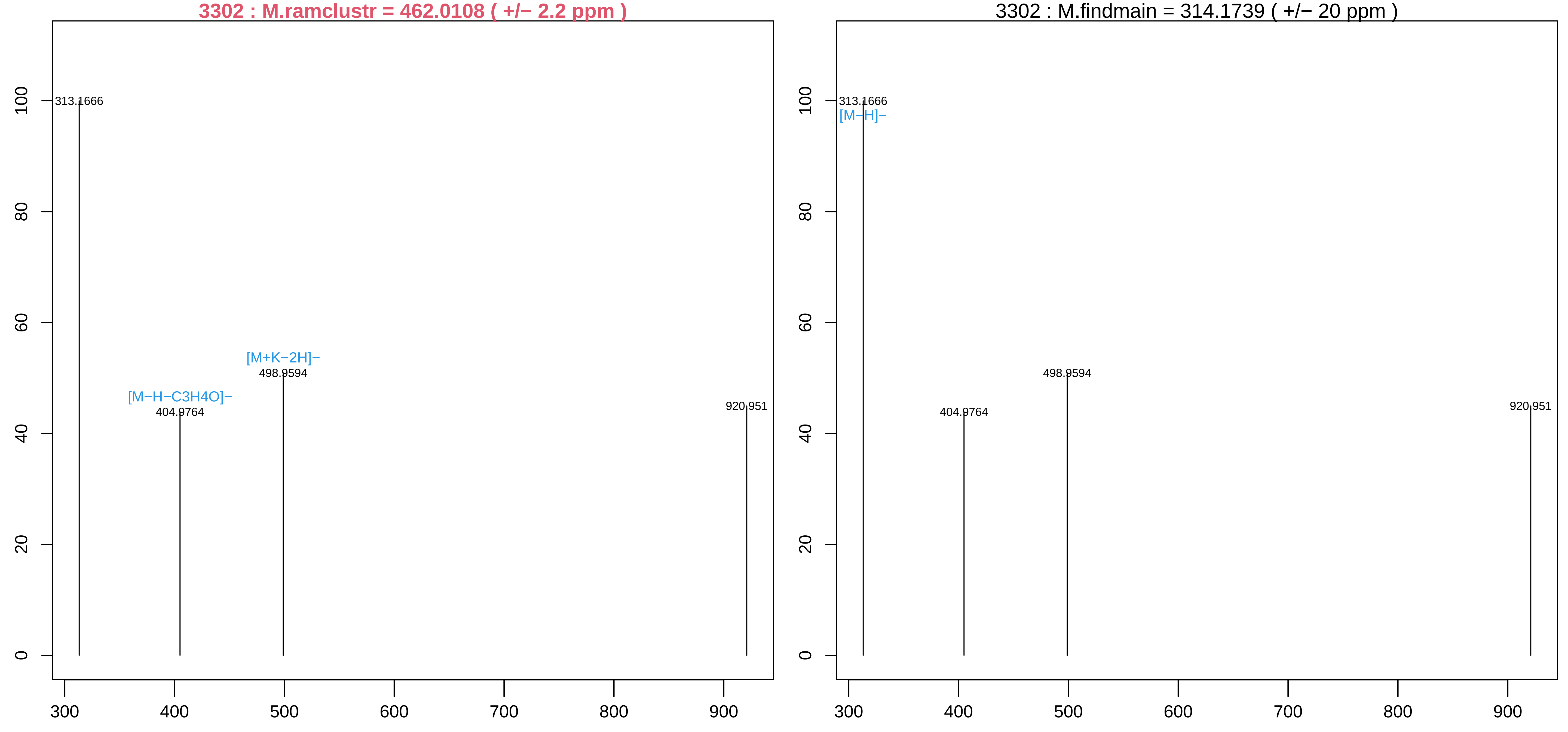
3302 : M.ramclustr = 462.0108 (+/− 2.2 ppm)
313.1666
[M−H−C3H4O]−
404.9764
[M+K−2H]−
498.9594
920.951
3302 : M.findmain = 314.1739 (+/− 20 ppm)
313.1666
[M−H]−
404.9764
498.9594
920.951
0
20
40
60
80
100
300
400
500
600
700
800
900

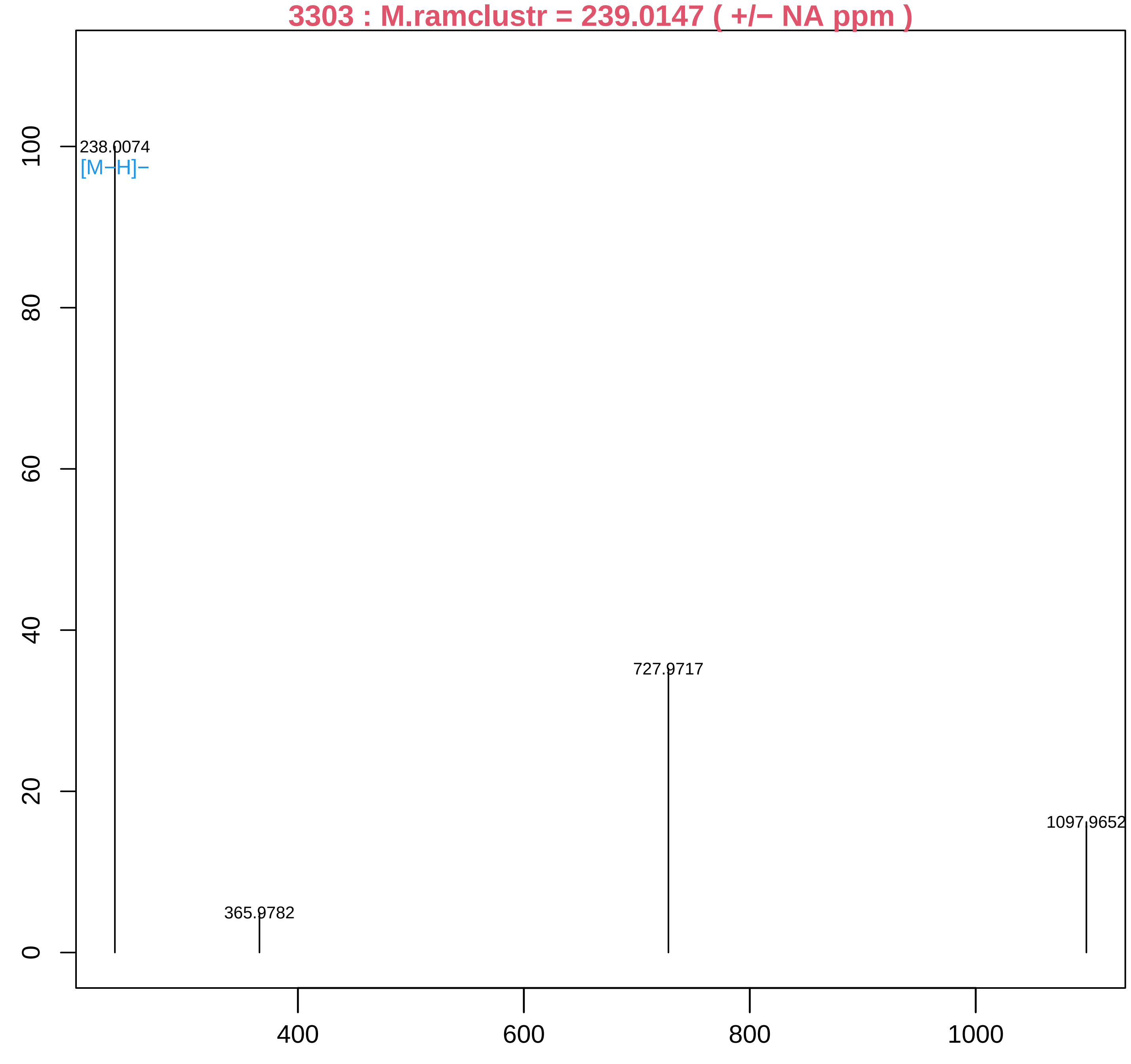
3303 : M.ramclustr = 239.0147 (+/− NA ppm)
238.0074
[M−H]−
365.9782
727.9717
1097.9652
0
20
40
60
80
100
400
600
800
1000

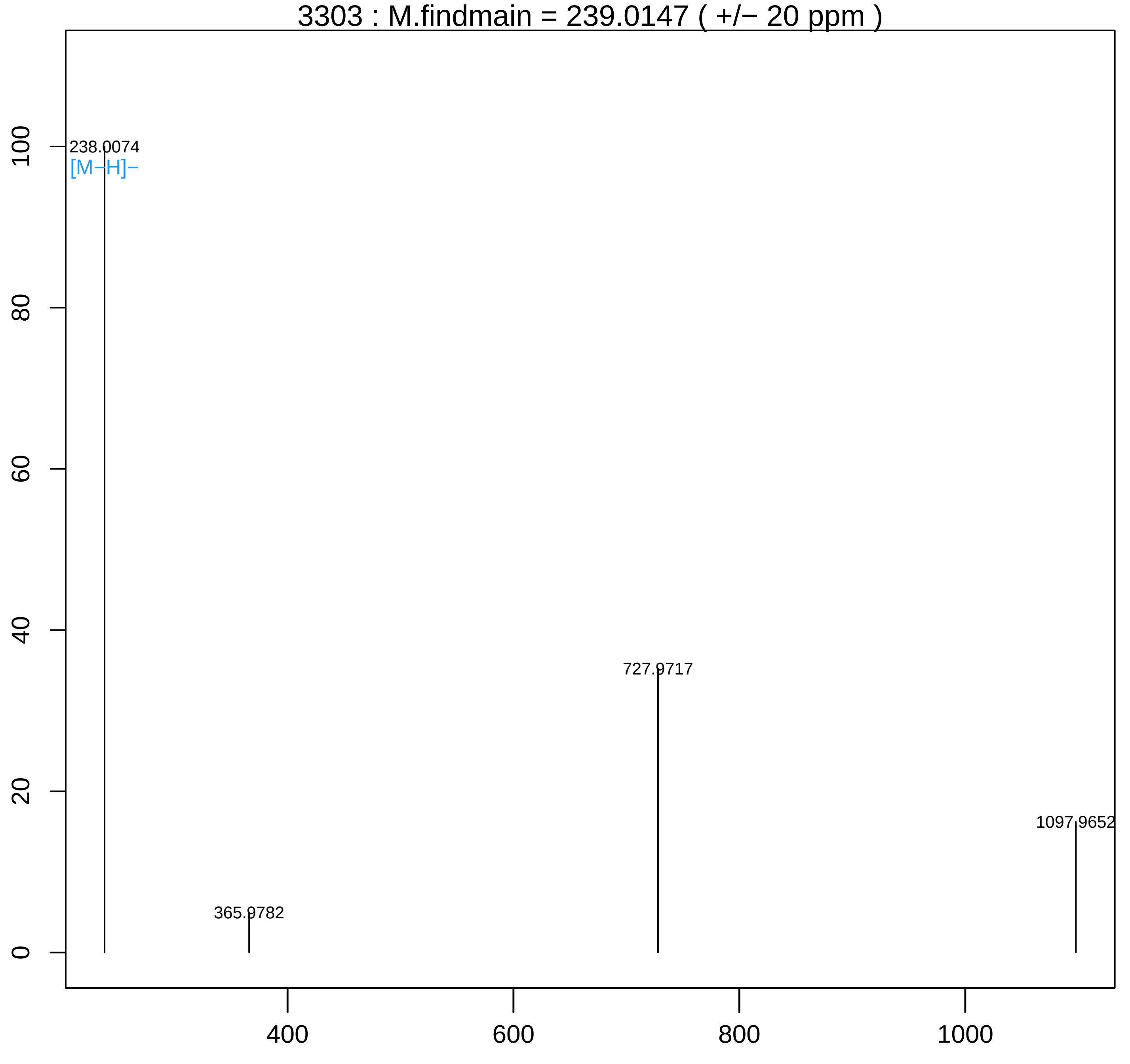
3303 : M.findmain = 239.0147 (+/− 20 ppm)
238.0074
[M−H]−
365.9782
727.9717
1097.9652
0
20
40
60
80
100
400
600
800
1000

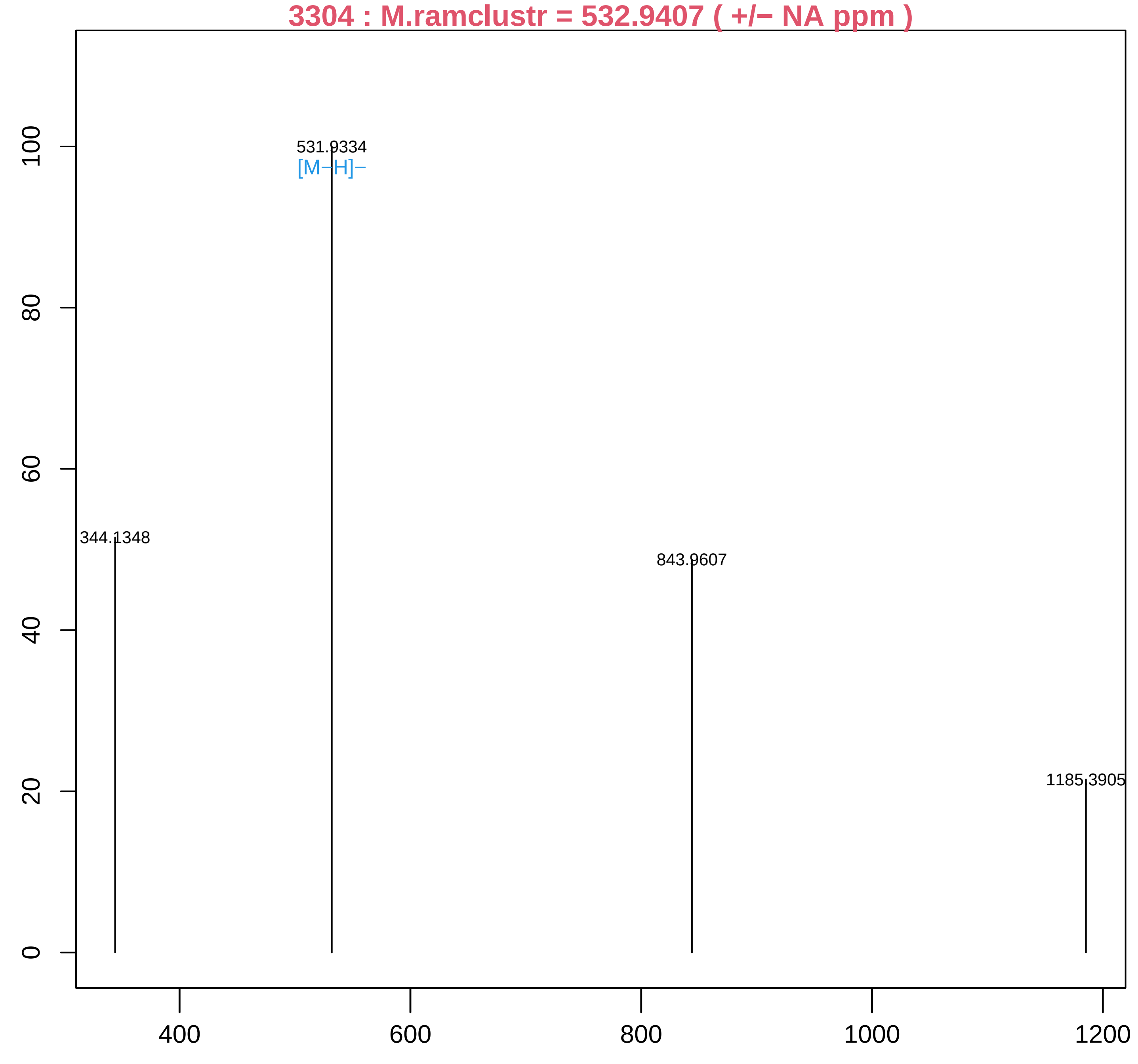
3304 : M.ramclustr = 532.9407 (+/− NA ppm)
531.9334
[M−H]−
344.1348
843.9607
1185.3905
0
20
40
60
80
100
400
600
800
1000
1200

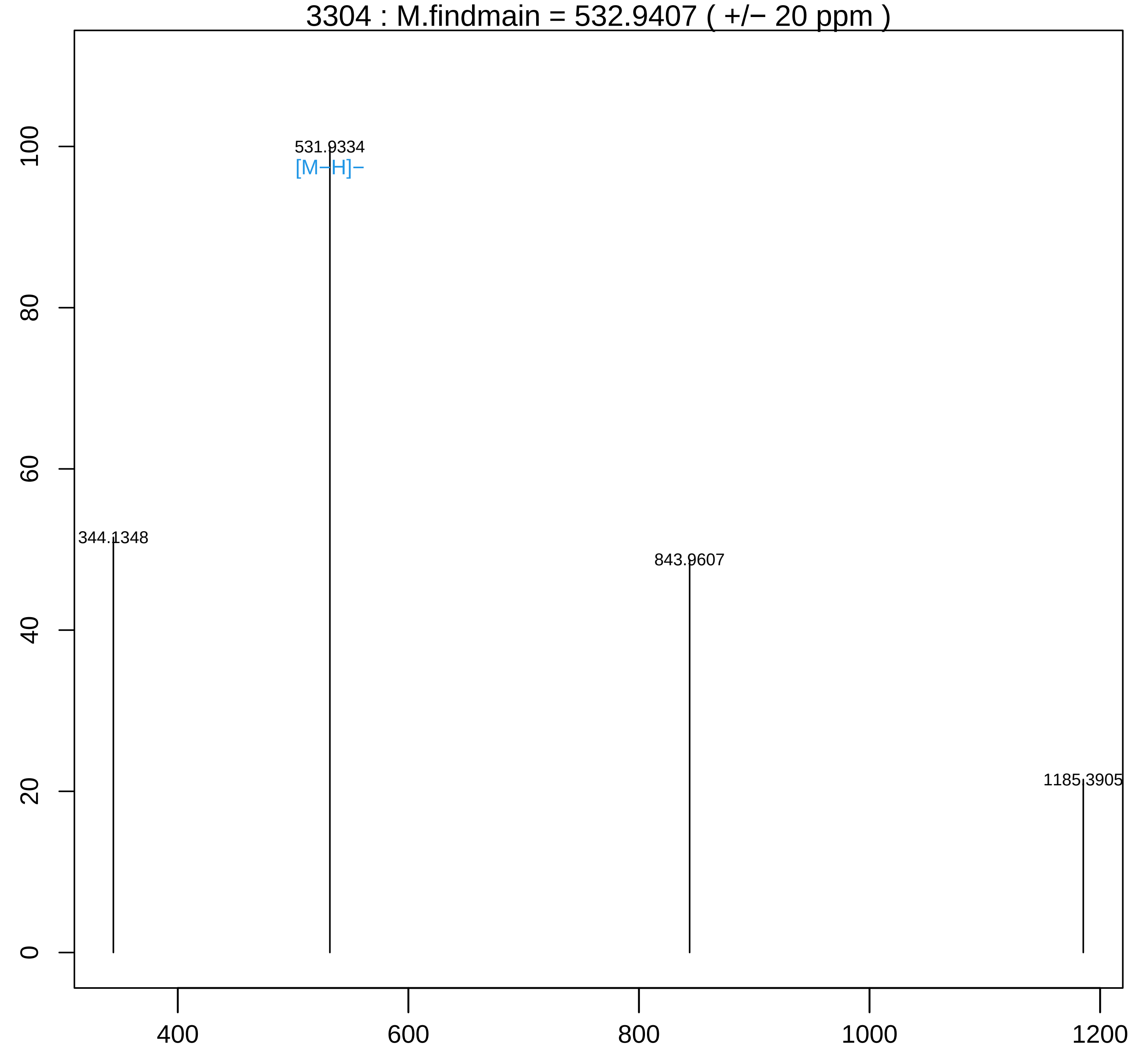
3304 : M.findmain = 532.9407 (+/− 20 ppm)
531.9334
[M−H]−
344.1348
843.9607
1185.3905
0
20
40
60
80
100
400
600
800
1000
1200

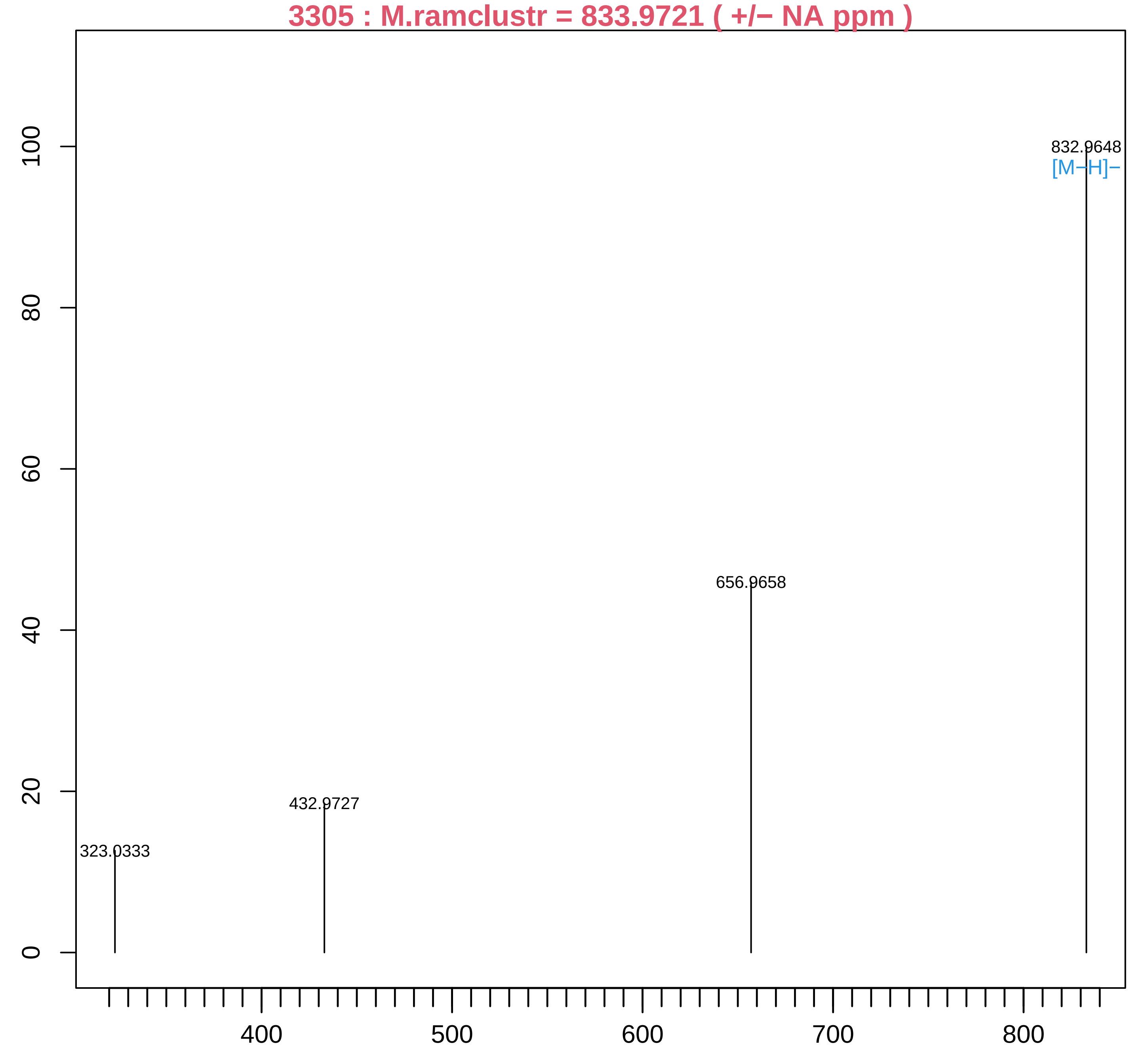

3305 : M.ramclustr = 833.9721 (+/− NA ppm)
832.9648
[M−H]−
656.9658
432.9727
323.0333

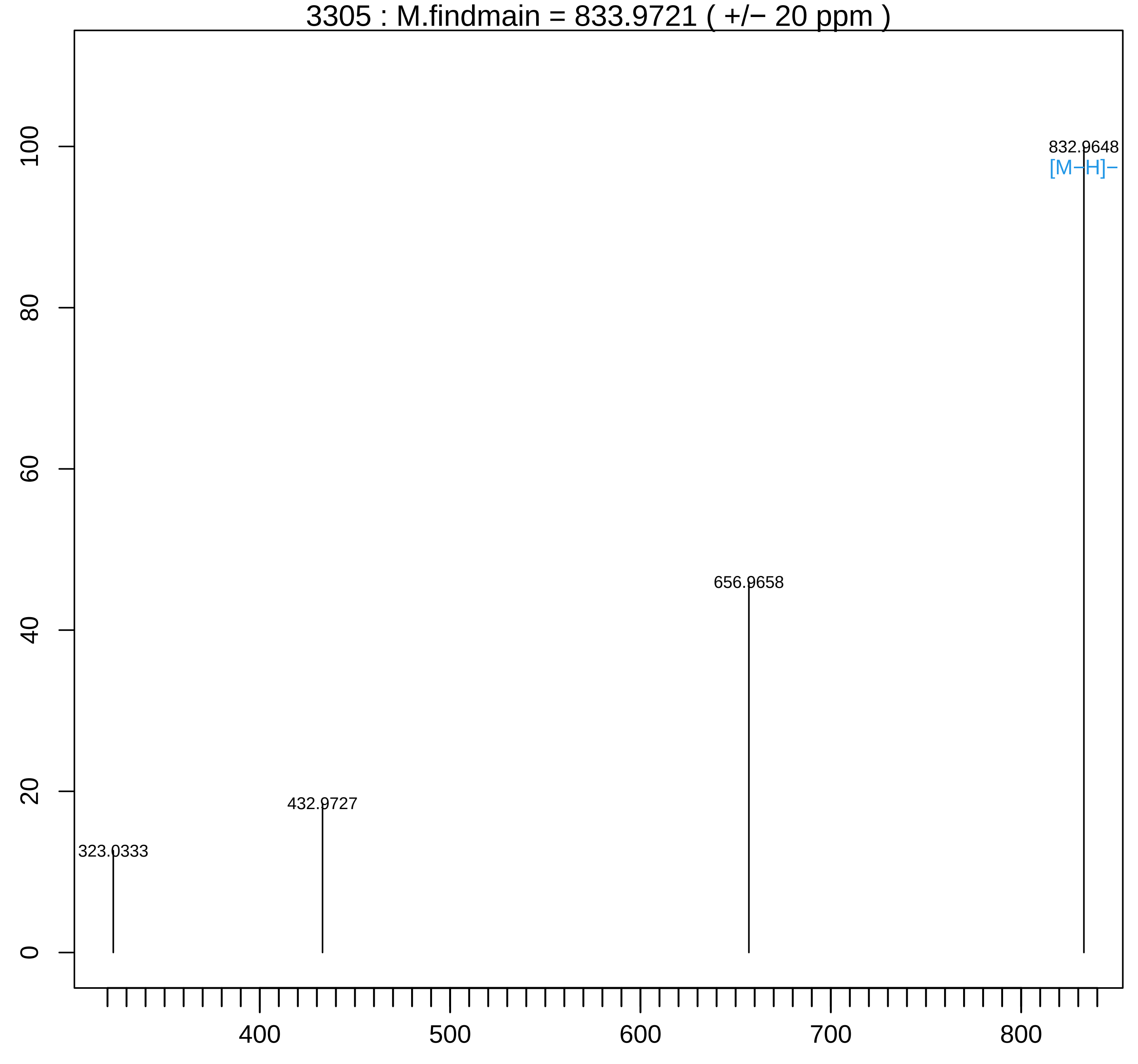

3305 : M.findmain = 833.9721 (+/− 20 ppm)
832.9648
[M−H]−
656.9658
432.9727
323.0333

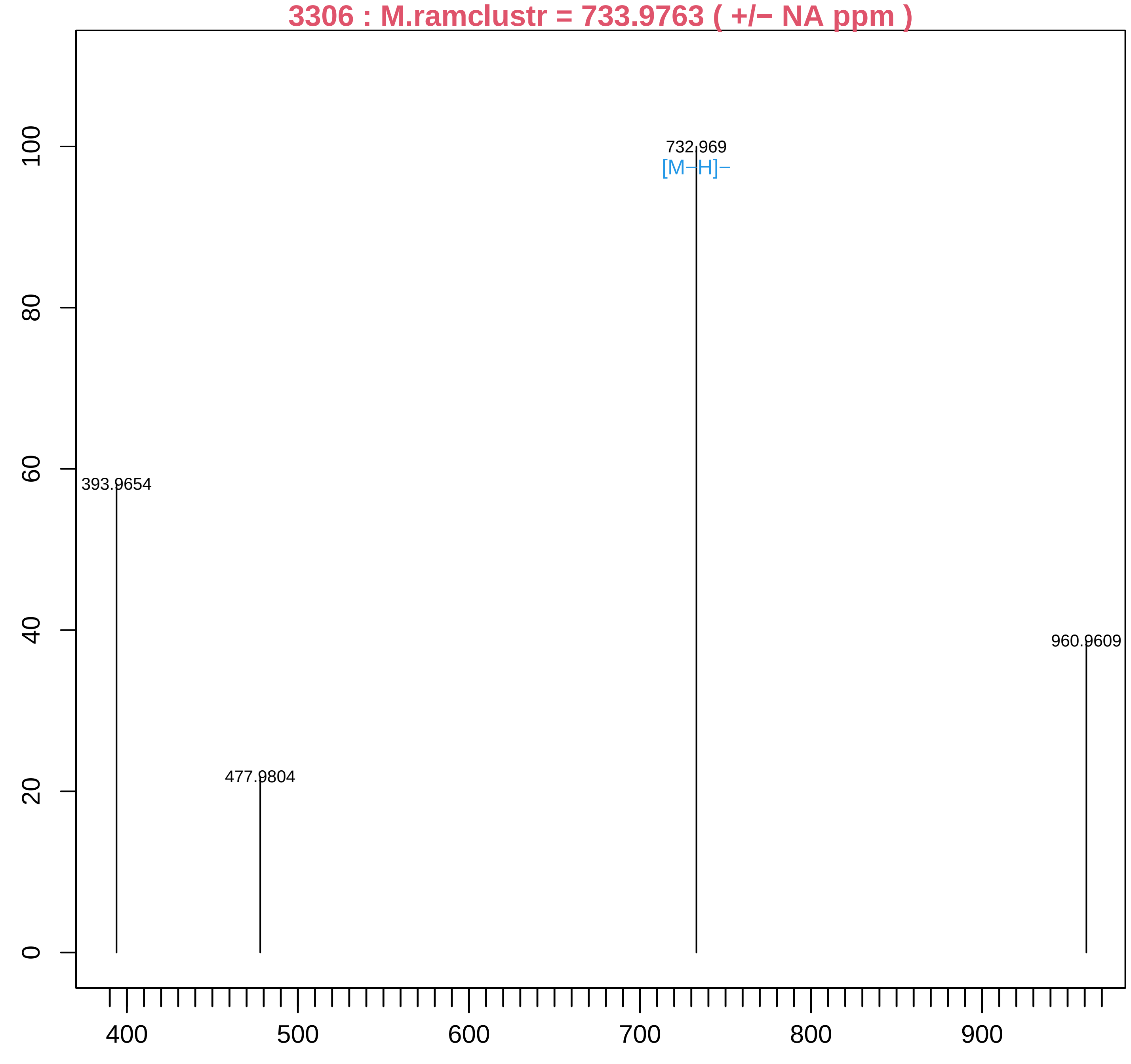

3306 : M.ramclustr = 733.9763 (+/− NA ppm)
732.969
[M−H]−
393.9654
477.9804
960.9609
0
20
40
60
80
100
400
500
600
700
800
900

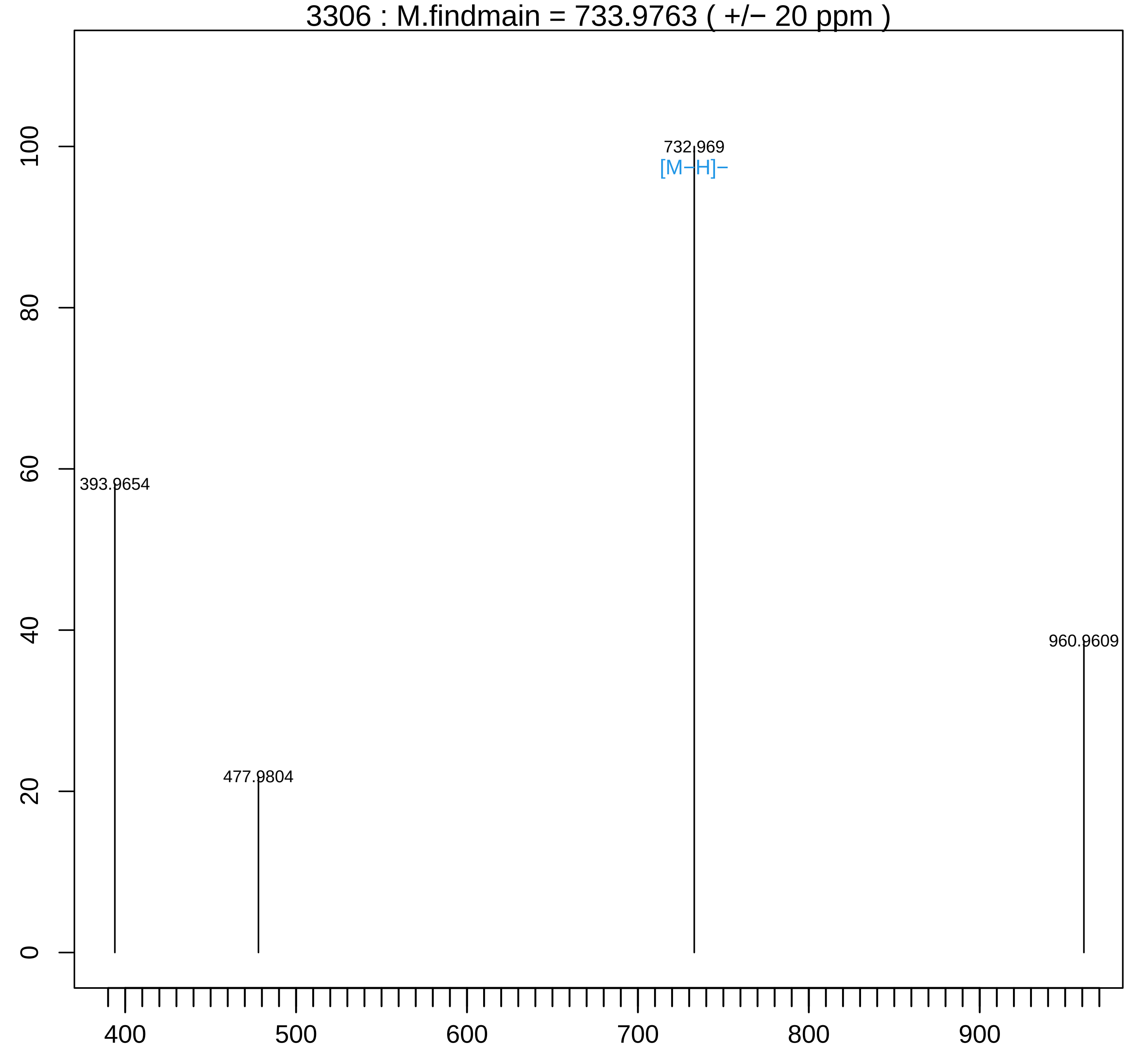

3306 : M.findmain = 733.9763 (+/− 20 ppm)
732.969
[M−H]−
393.9654
477.9804
960.9609
0
20
40
60
80
100
400
500
600
700
800
900

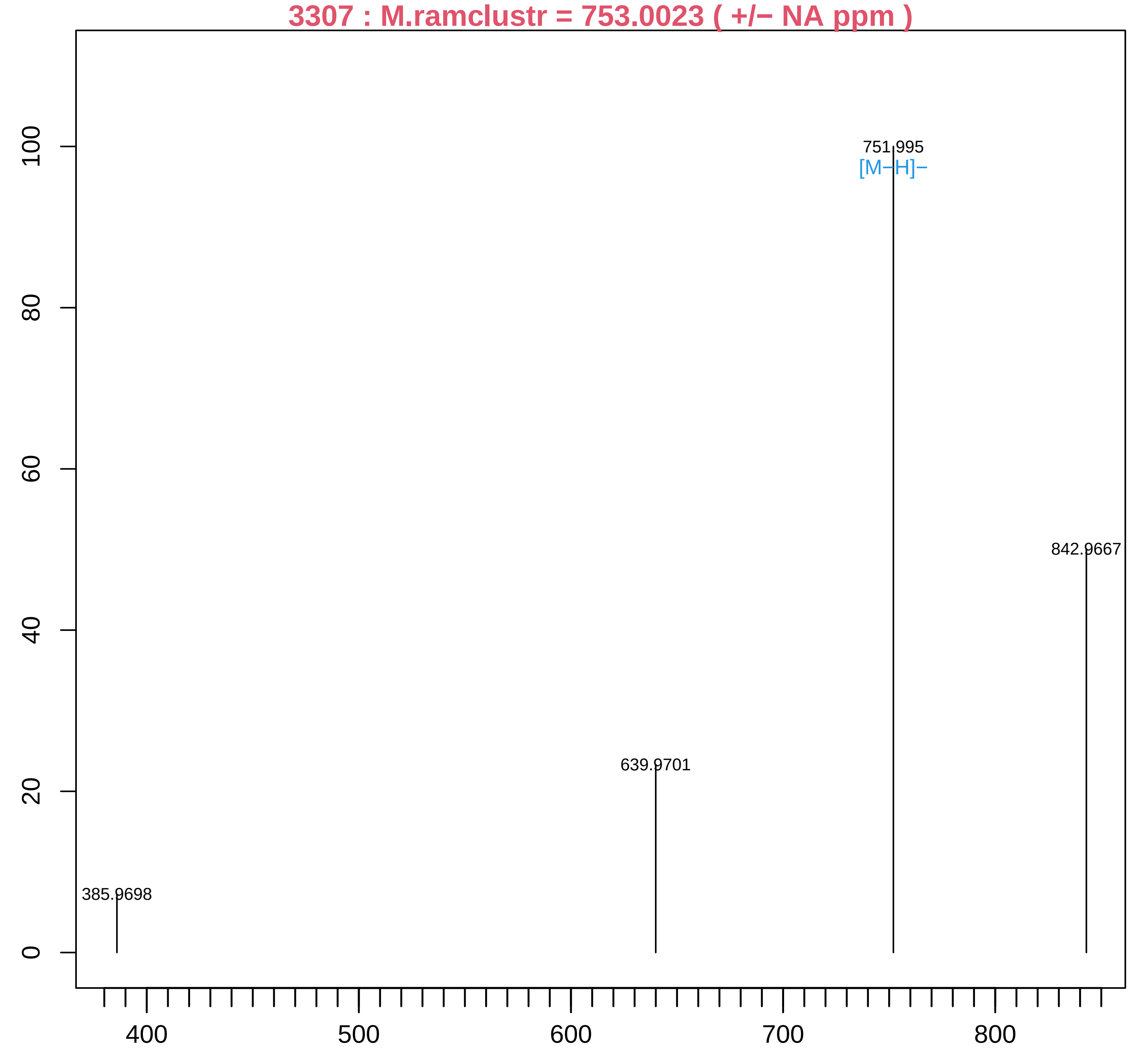
3307 : M.ramclustr = 753.0023 (+/− NA ppm)
751.995
[M−H]−
842.9667
639.9701
385.9698
0
20
40
60
80
100
400
500
600
700
800

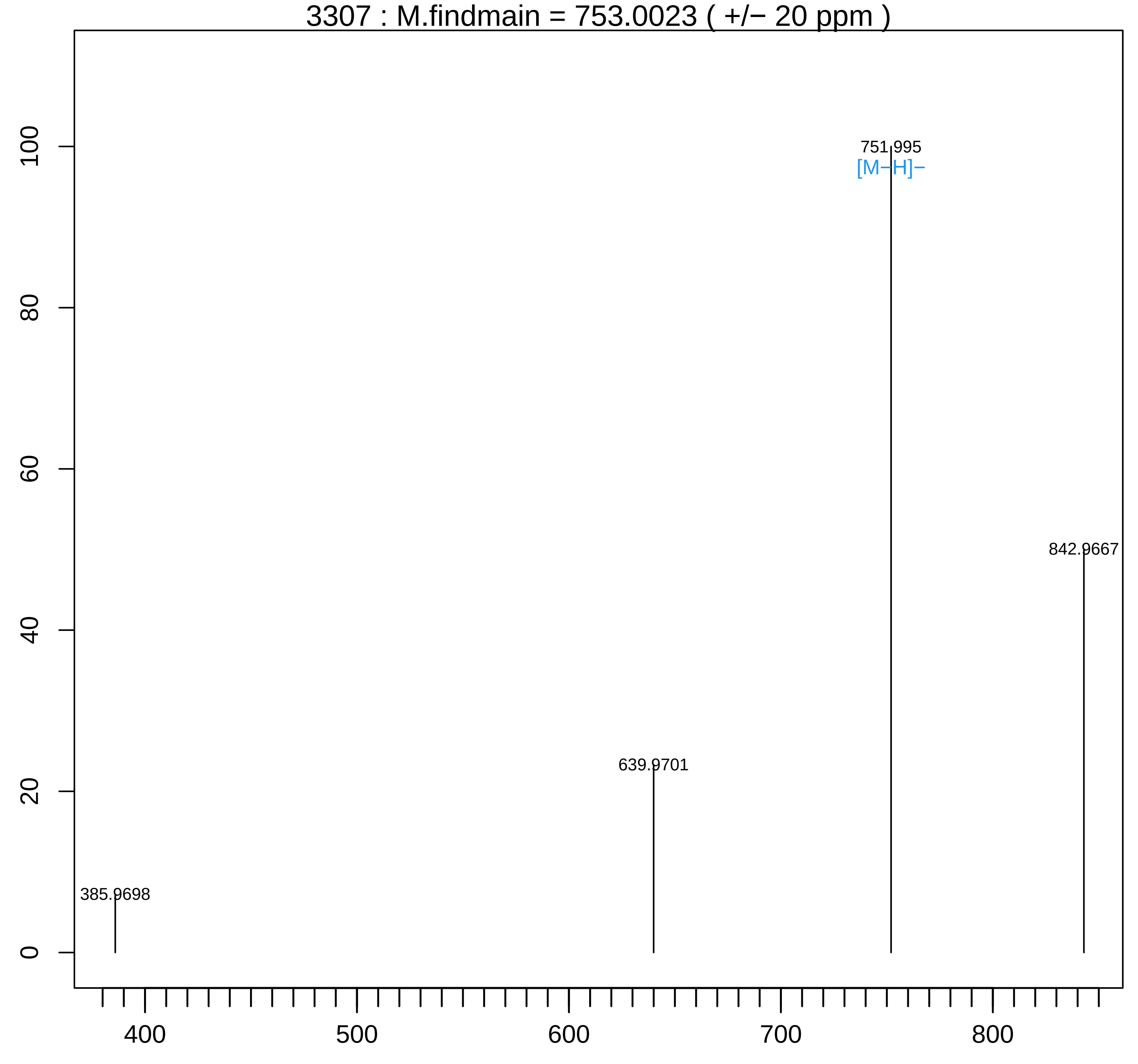
3307 : M.findmain = 753.0023 (+/− 20 ppm)
751.995
[M−H]−
842.9667
639.9701
385.9698
0
20
40
60
80
100
400
500
600
700
800

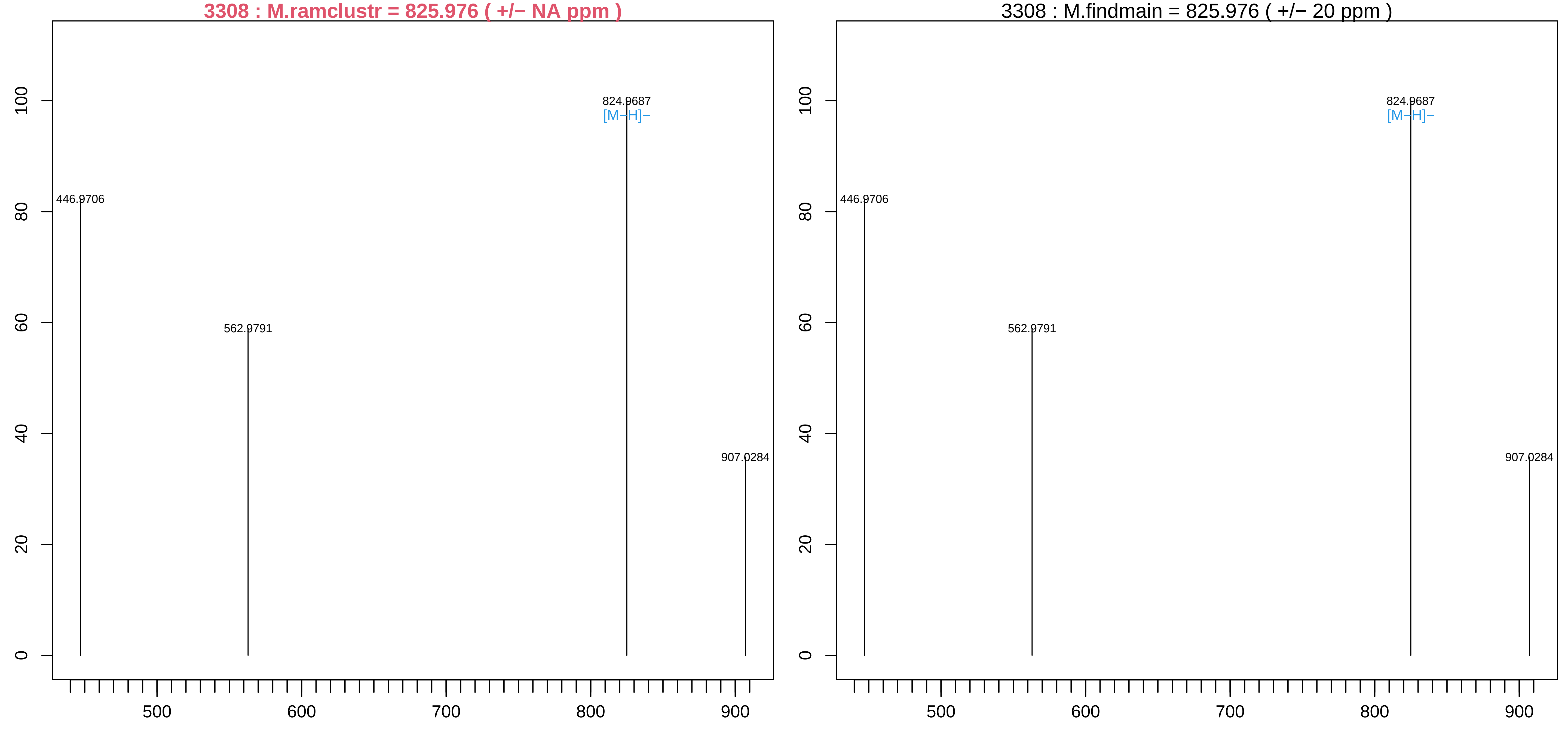
3308 : M.ramclustr = 825.976 (+/− NA ppm)
3308 : M.findmain = 825.976 (+/− 20 ppm)
446.9706
562.9791
824.9687
[M−H]−
907.0284
0
20
40
60
80
100
500
600
700
800
900

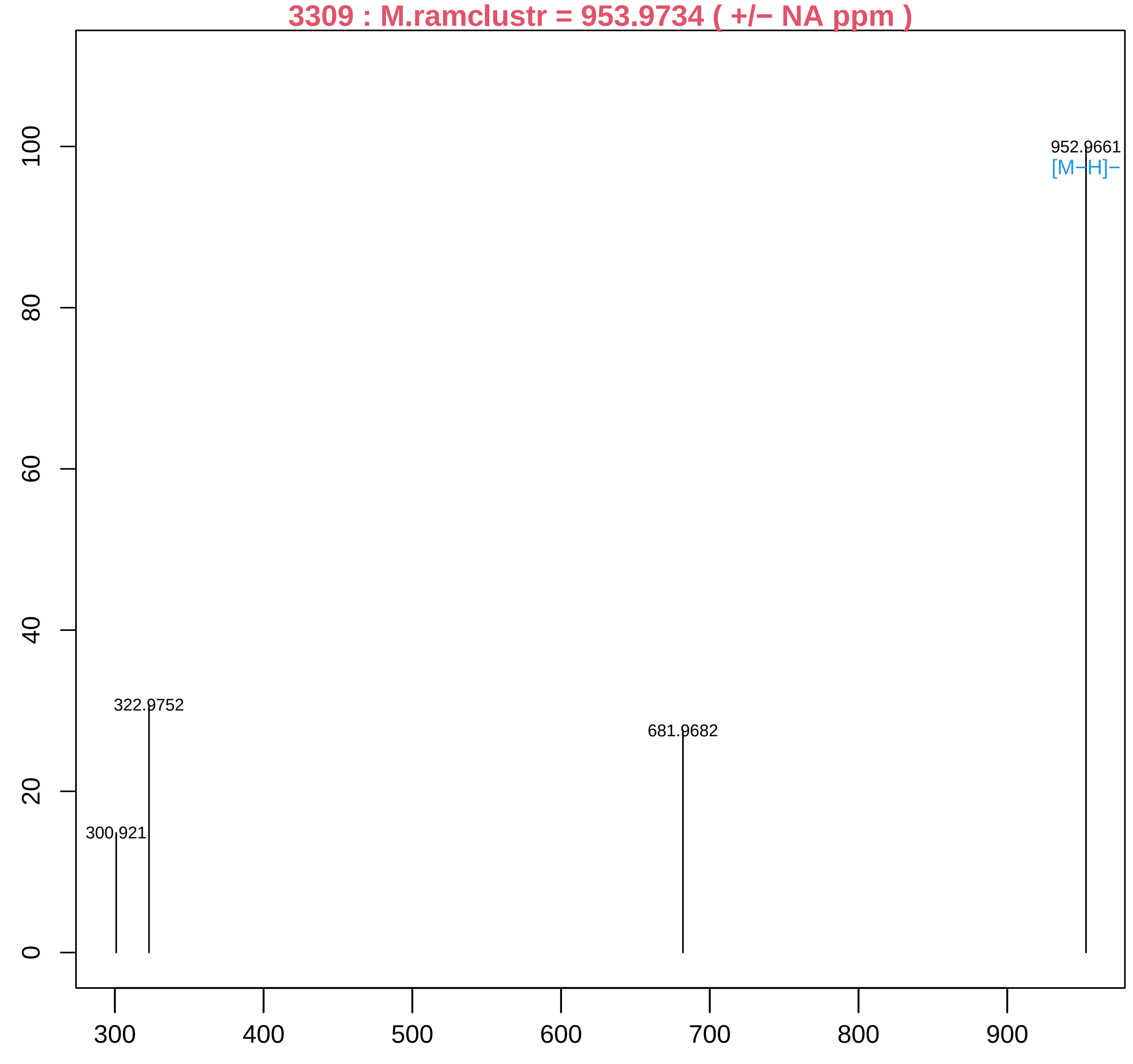

3309 : M.ramclustr = 953.9734 (+/− NA ppm)
952.9661
[M−H]−
322.9752
681.9682
300.921

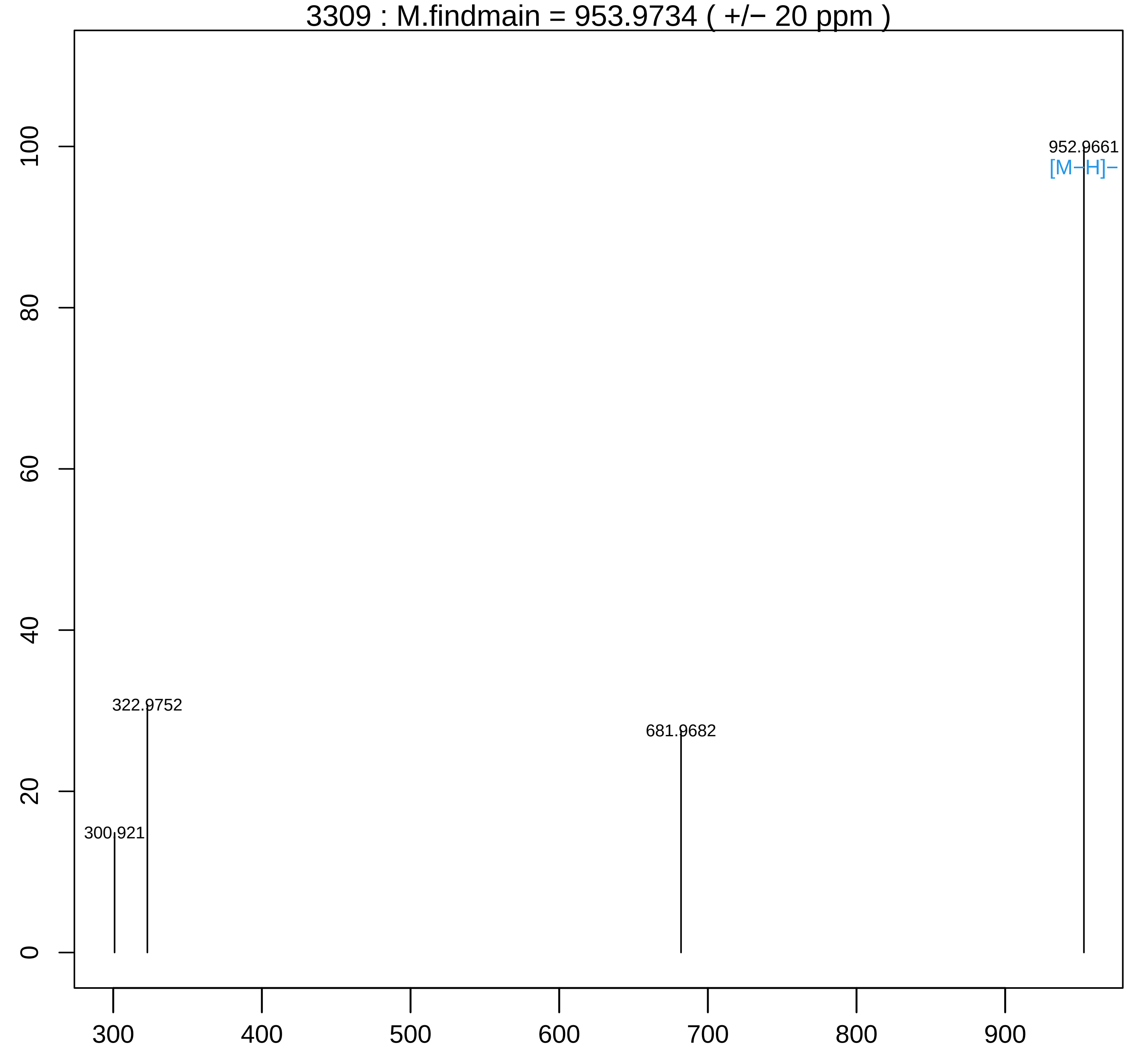

3309 : M.findmain = 953.9734 (+/− 20 ppm)
952.9661
[M−H]−
322.9752
681.9682
300.921

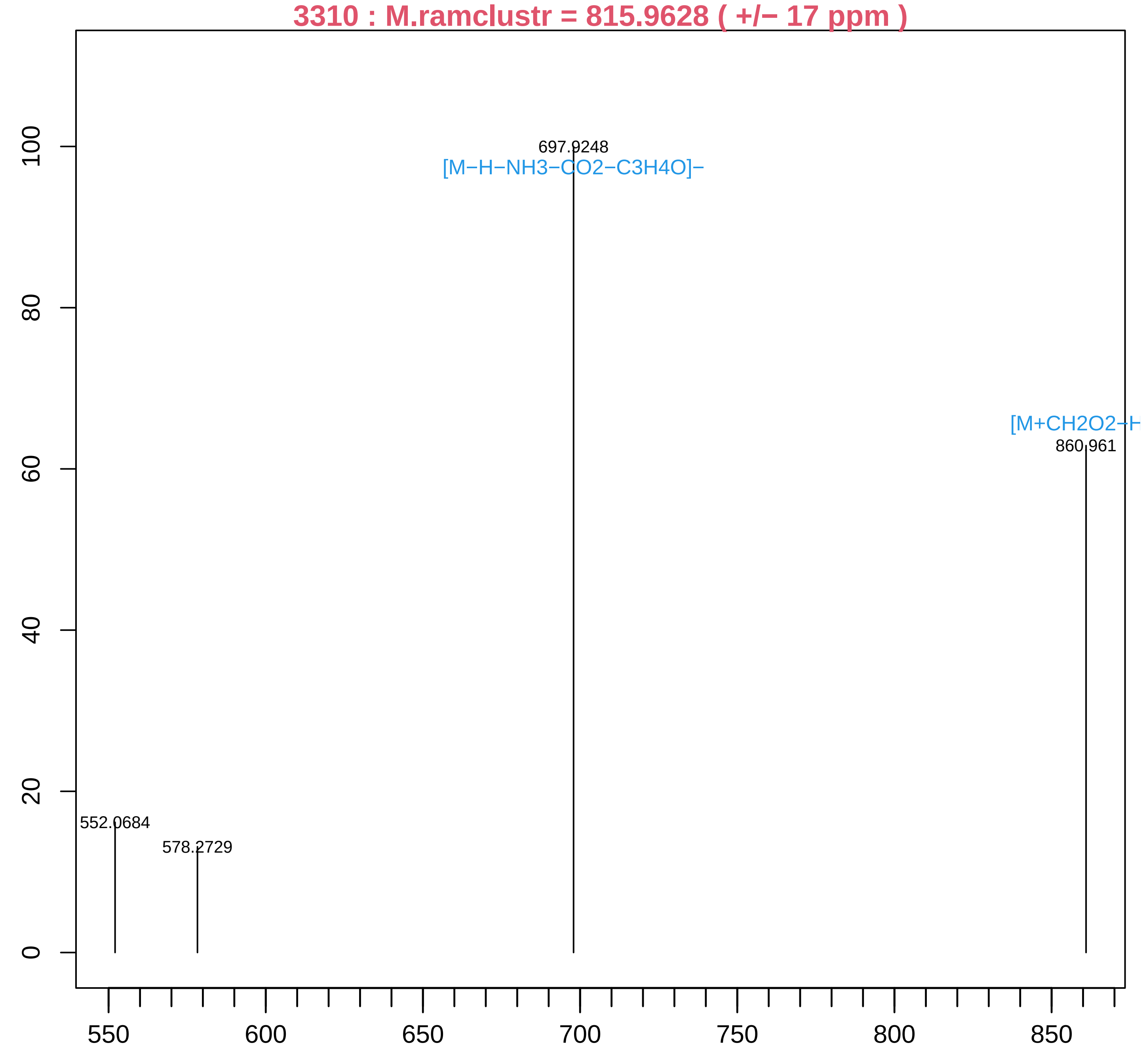
3310 : M.ramclustr = 815.9628 (+/− 17 ppm)
697.9248
[M−H−NH3−CO2−C3H4O]−
[M+CH2O2−H
860.961
552.0684
578.2729
0
20
40
60
80
100
550
600
650
700
750
800
850

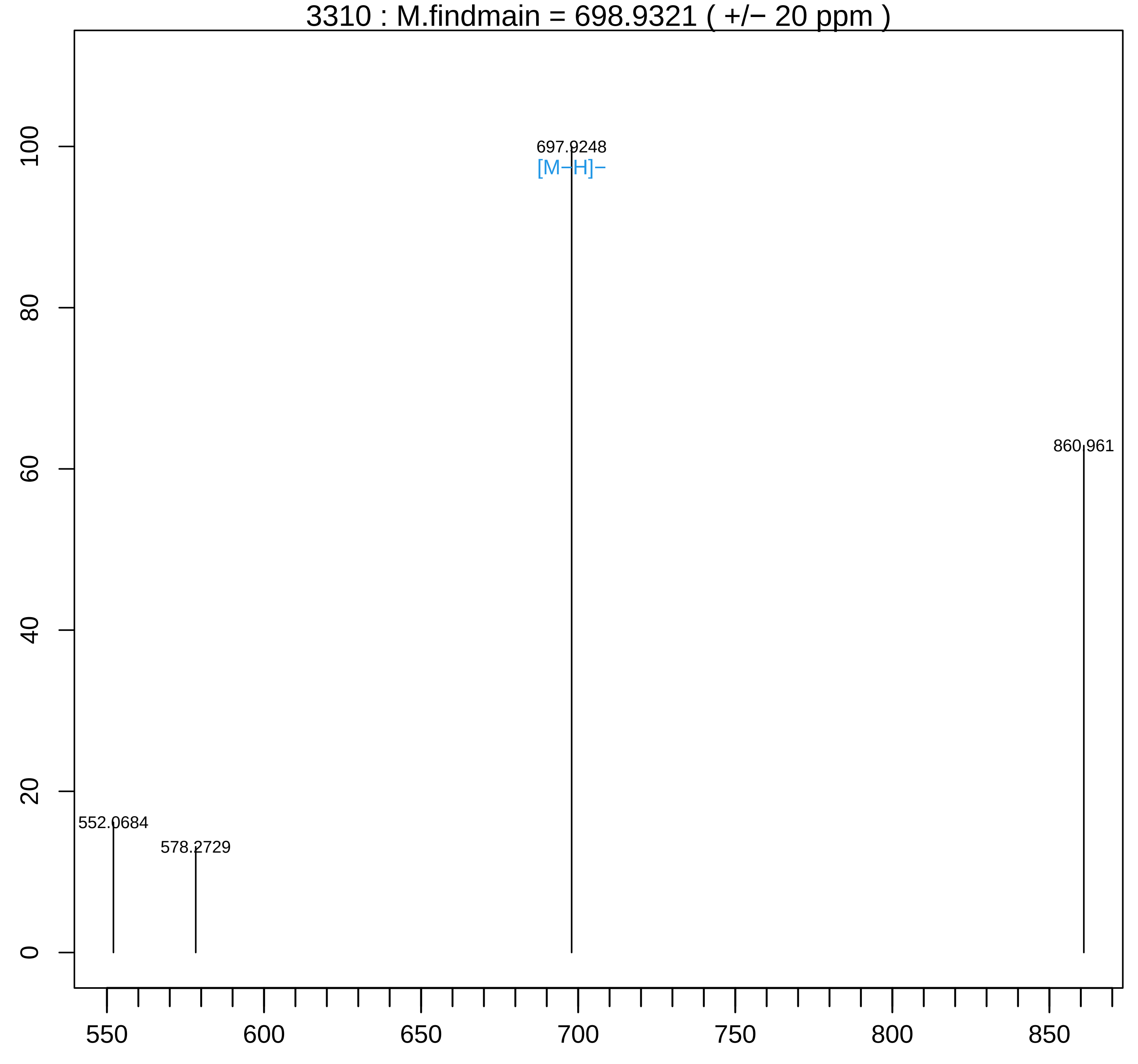
3310 : M.findmain = 698.9321 (+/− 20 ppm)
697.9248
[M−H]−
860.961
552.0684
578.2729
0
20
40
60
80
100
550
600
650
700
750
800
850

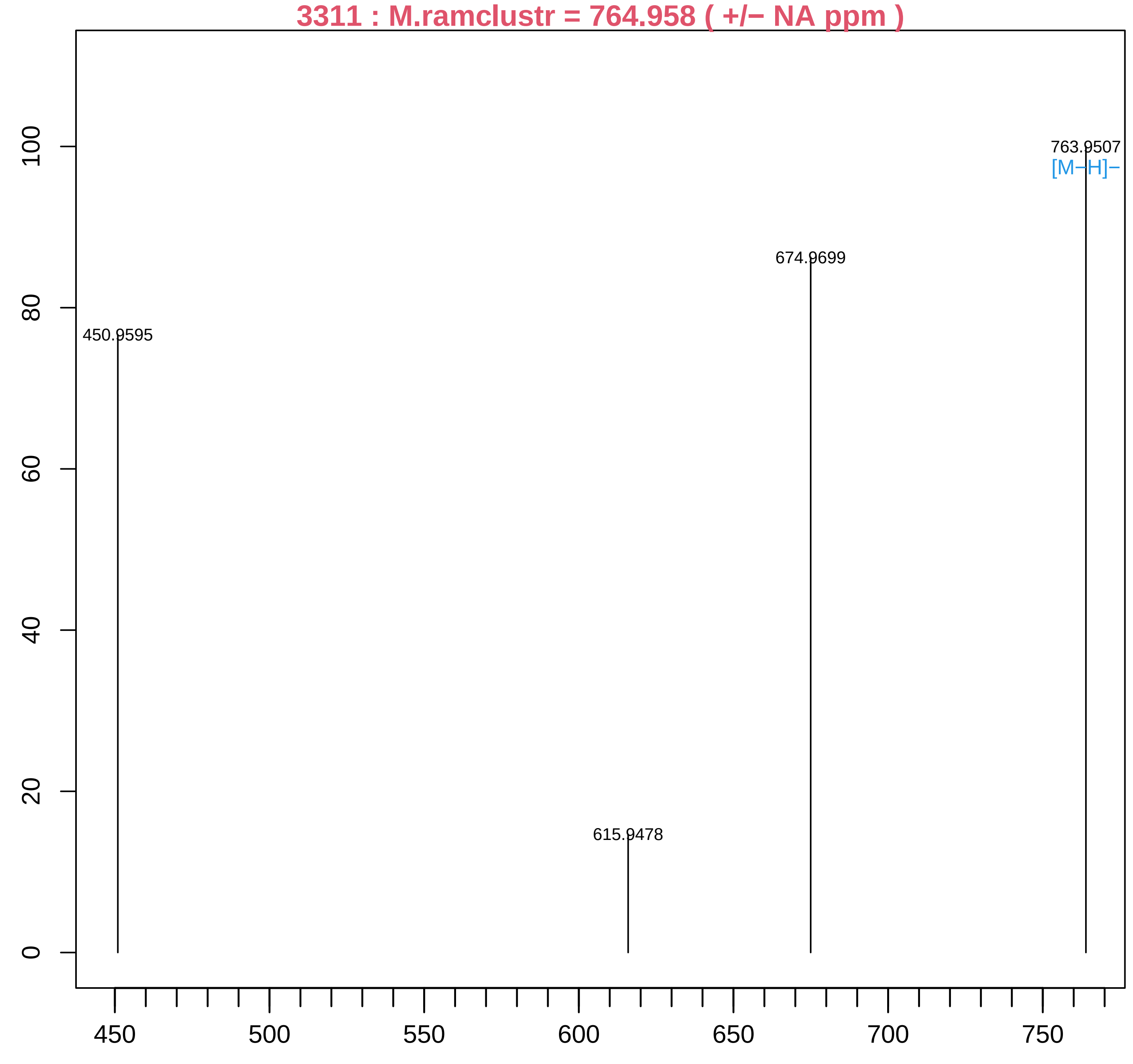
3311 : M.ramclustr = 764.958 (+/− NA ppm)
763.9507
[M−H]−
674.9699
450.9595
615.9478

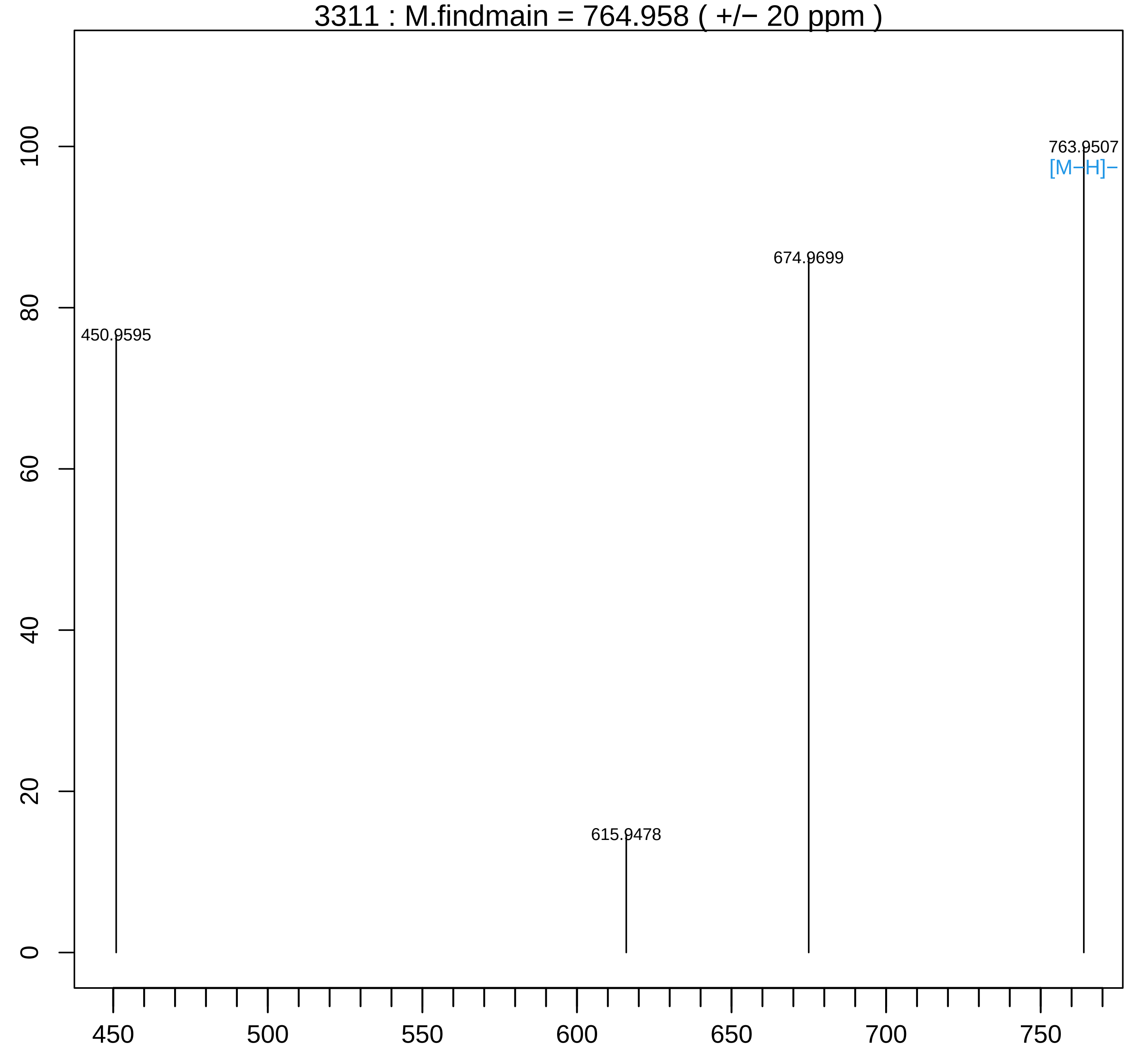
3311 : M.findmain = 764.958 (+/− 20 ppm)
763.9507
[M−H]−
674.9699
450.9595
615.9478

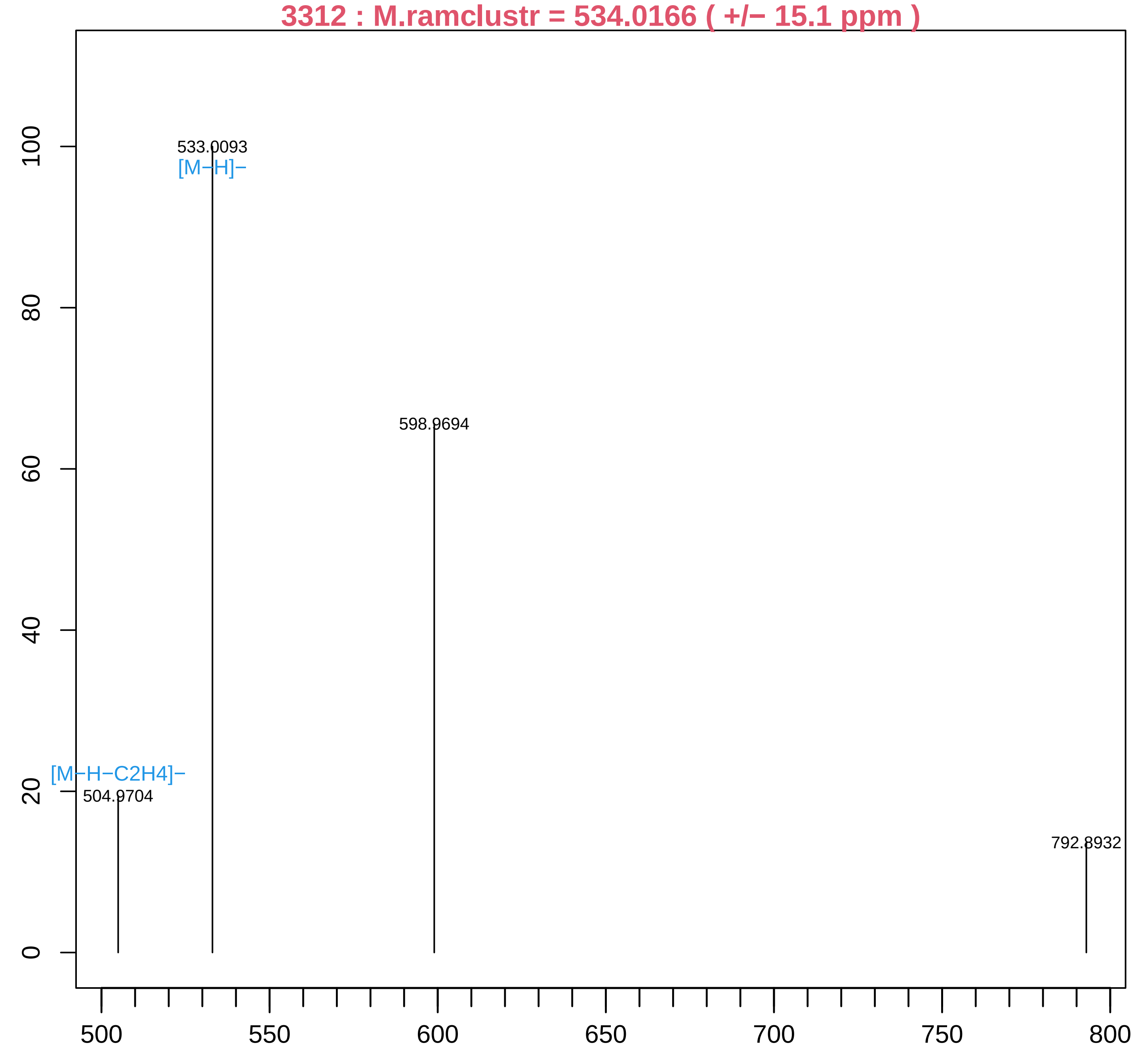
3312 : M.ramclustr = 534.0166 (+/− 15.1 ppm)
533.0093
[M−H]−
598.9694
[M−H−C2H4]−
504.9704
792.8932
0
20
40
60
80
100
500
550
600
650
700
750
800

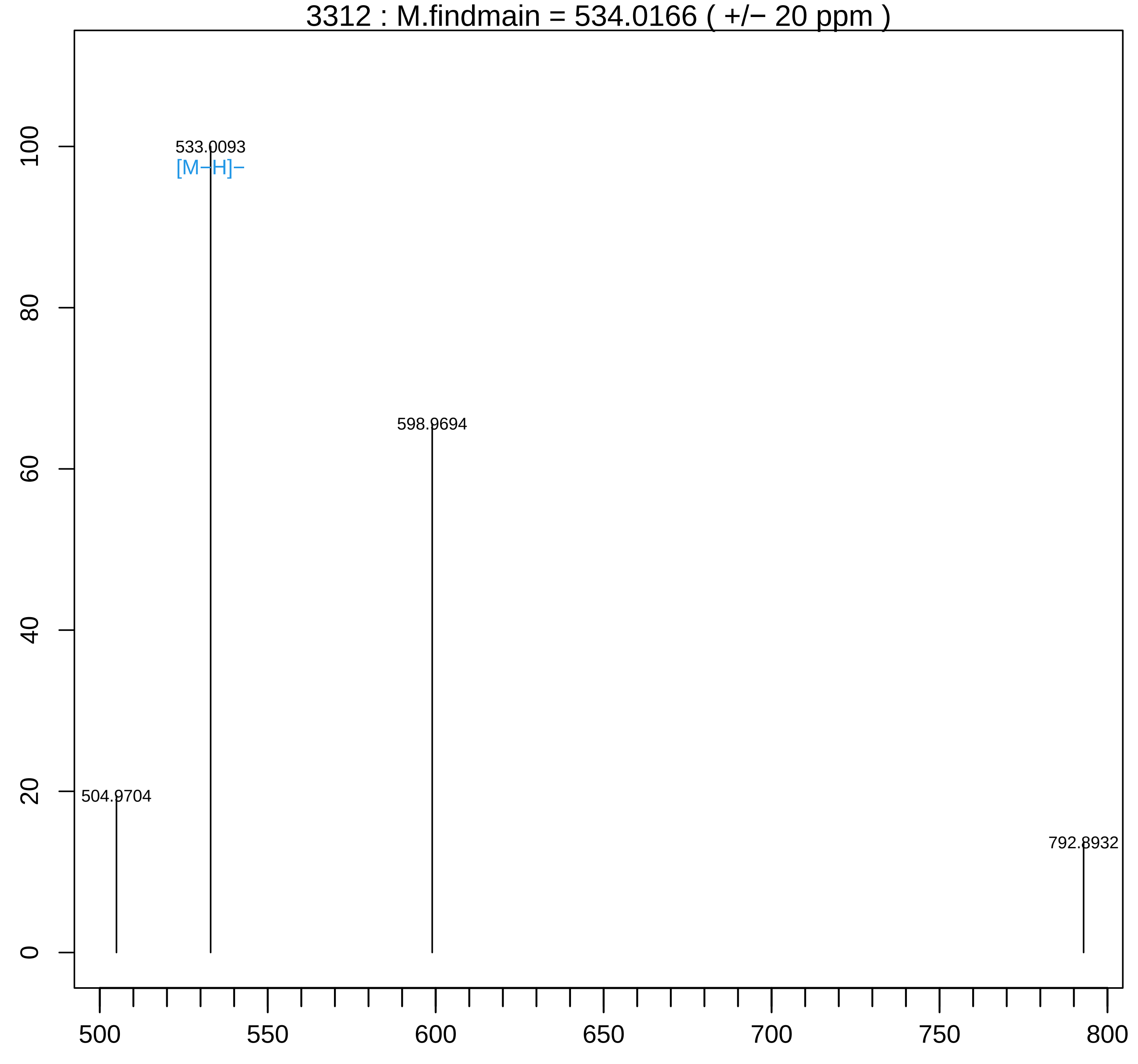
3312 : M.findmain = 534.0166 (+/− 20 ppm)
533.0093
[M−H]−
598.9694
504.9704
792.8932
0
20
40
60
80
100
500
550
600
650
700
750
800

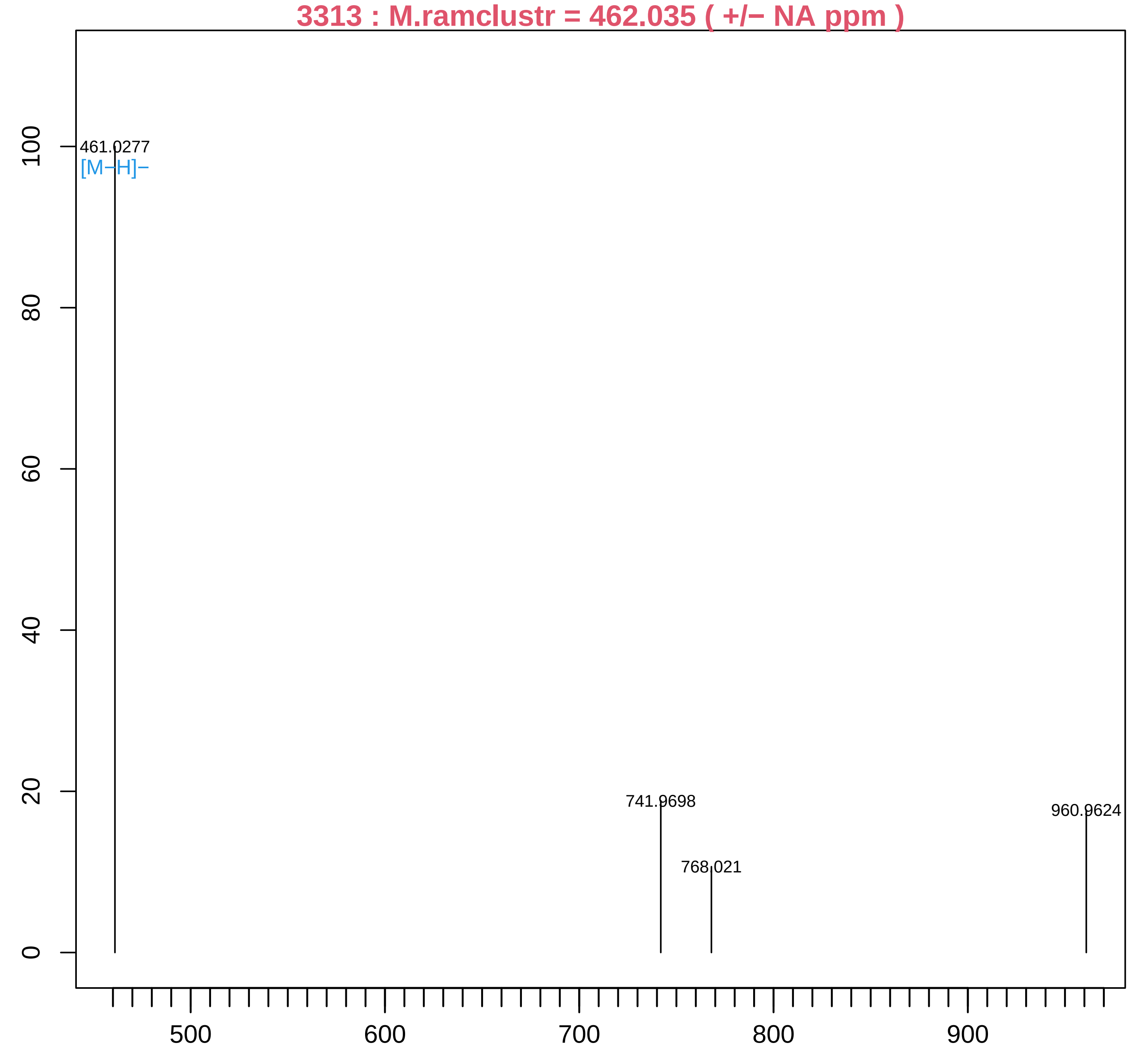
3313 : M.ramclustr = 462.035 (+/− NA ppm)
461.0277
[M−H]−
741.9698
768.021
960.9624
0
20
40
60
80
100
500
600
700
800
900

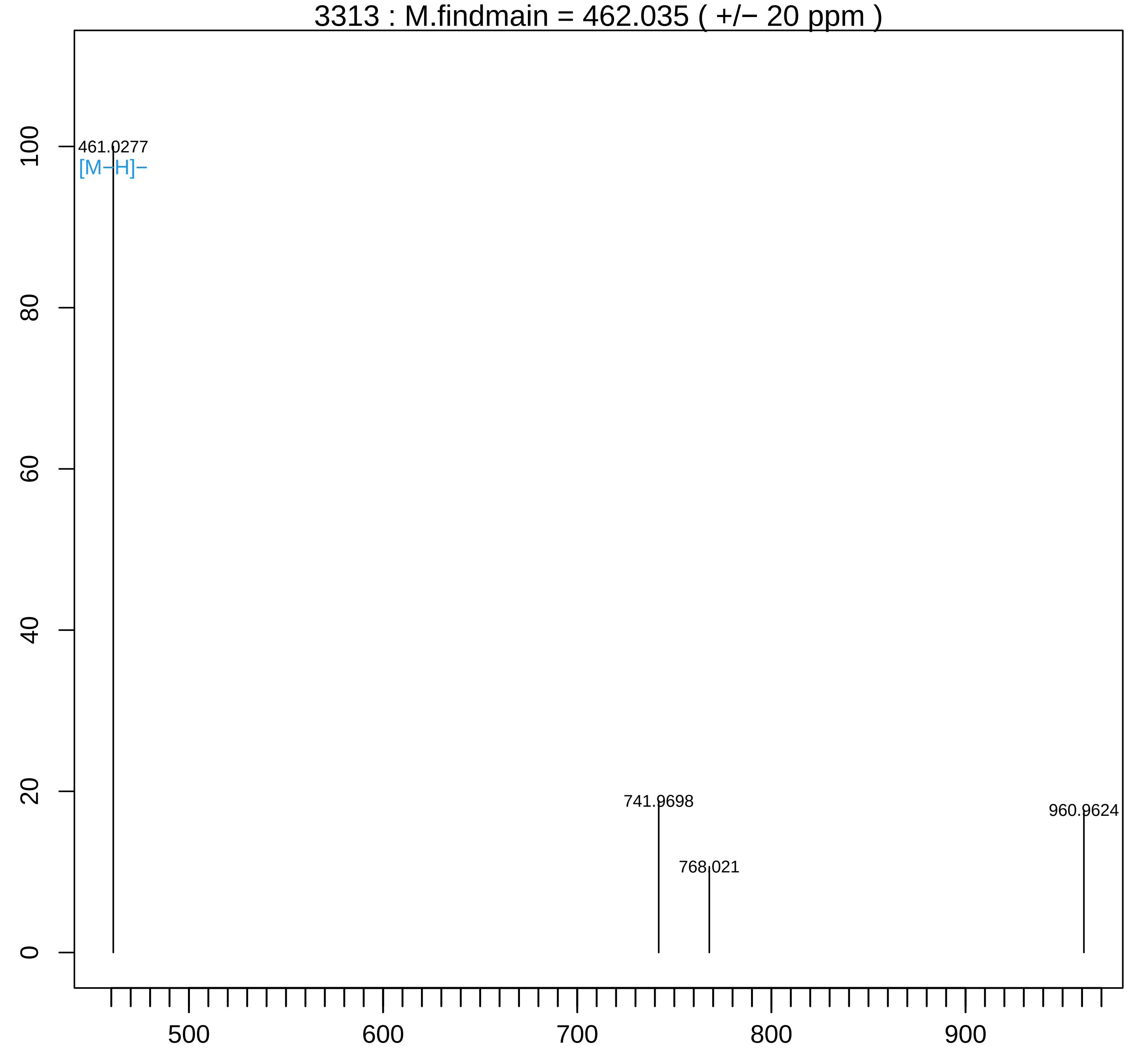
3313 : M.findmain = 462.035 (+/− 20 ppm)
461.0277
[M−H]−
741.9698
768.021
960.9624
0
20
40
60
80
100
500
600
700
800
900

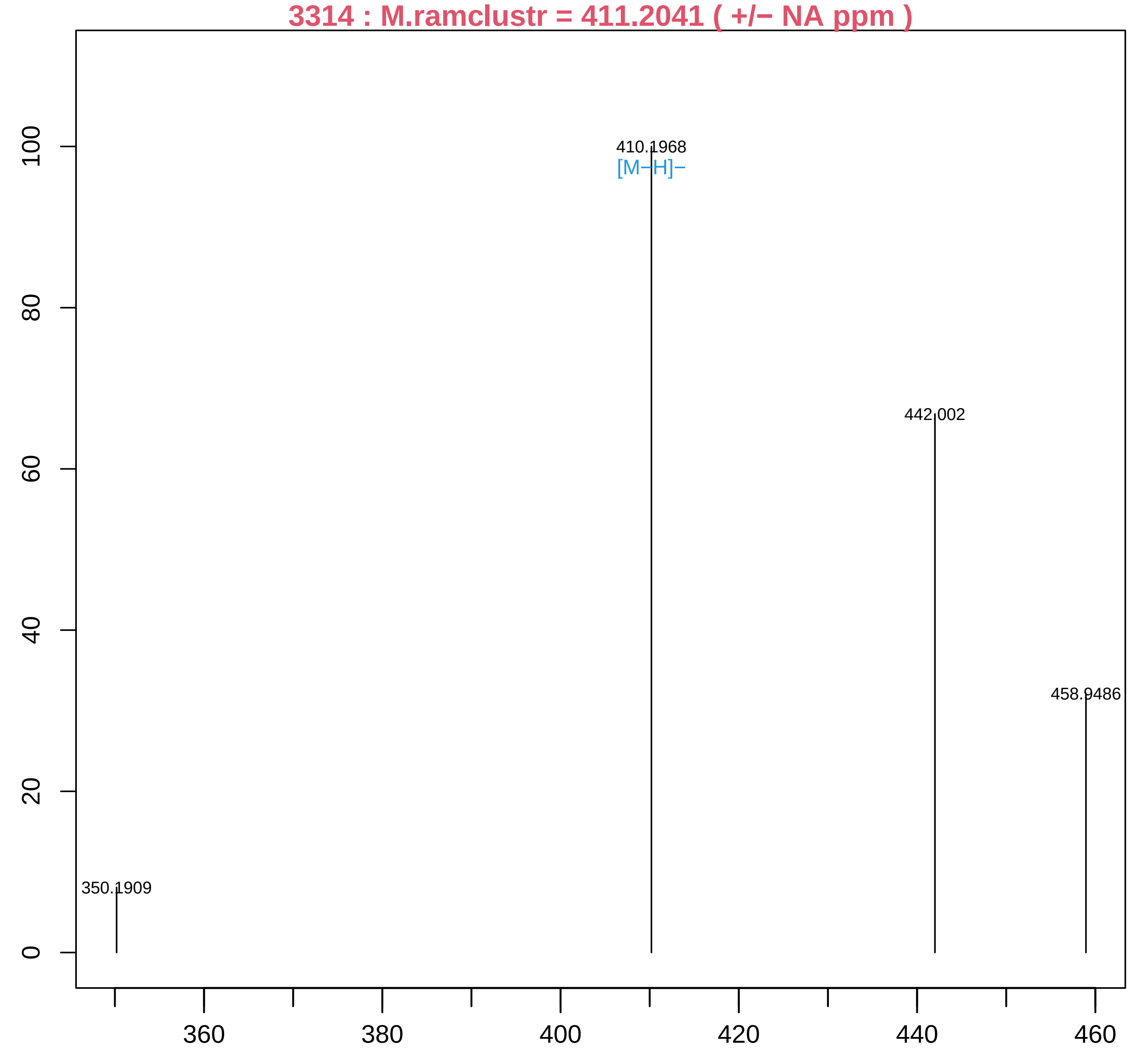
3314 : M.ramclustr = 411.2041 (+/− NA ppm)
410.1968
[M−H]−
442.002
458.9486
350.1909
0
20
40
60
80
100
360
380
400
420
440
460

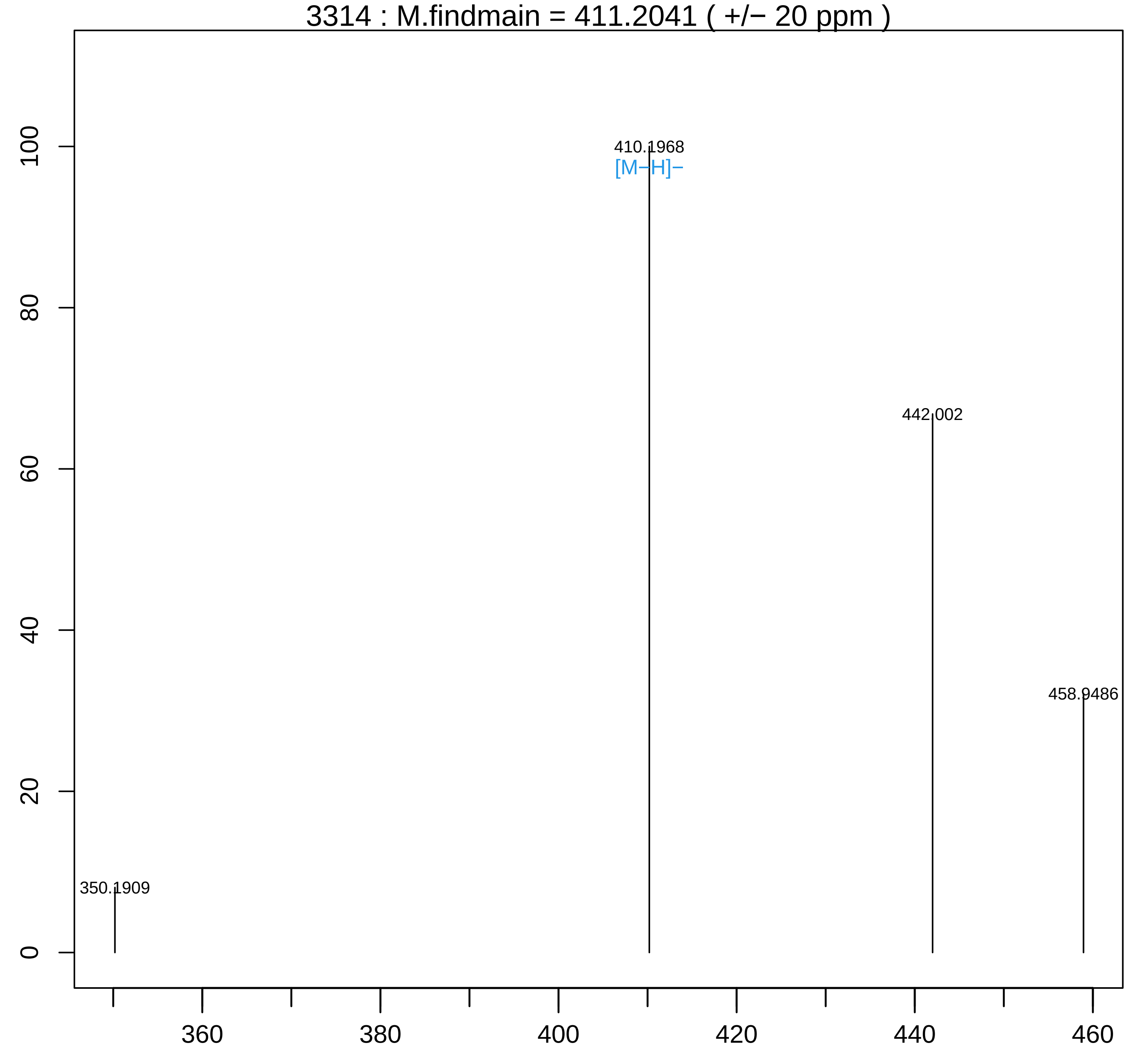
3314 : M.findmain = 411.2041 (+/− 20 ppm)
410.1968
[M−H]−
442.002
458.9486
350.1909
0
20
40
60
80
100
360
380
400
420
440
460

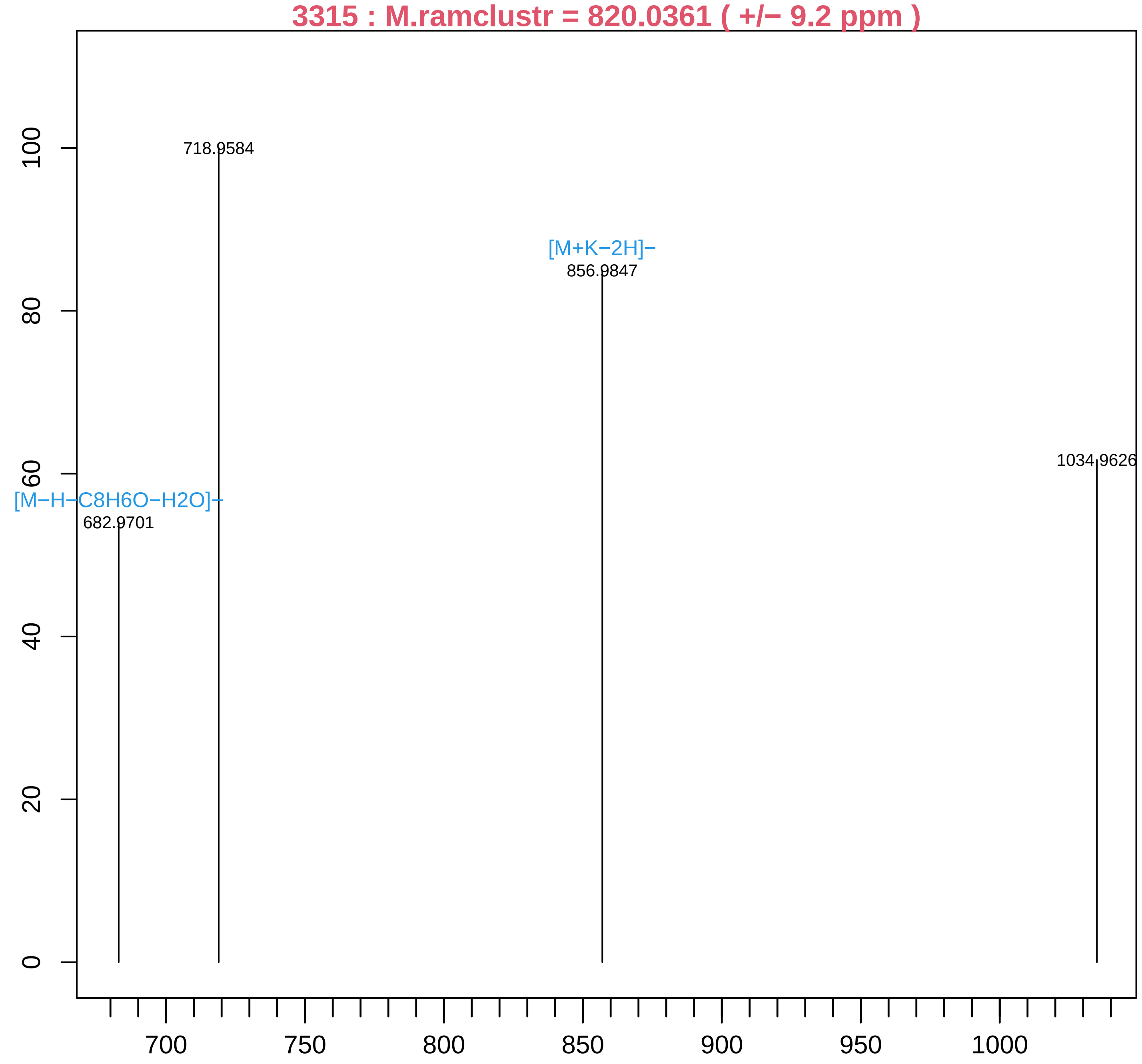
3315 : M.ramclustr = 820.0361 (+/− 9.2 ppm)
718.9584
[M+K−2H]−
856.9847
1034.9626
[M−H−C8H6O−H2O]−
682.9701

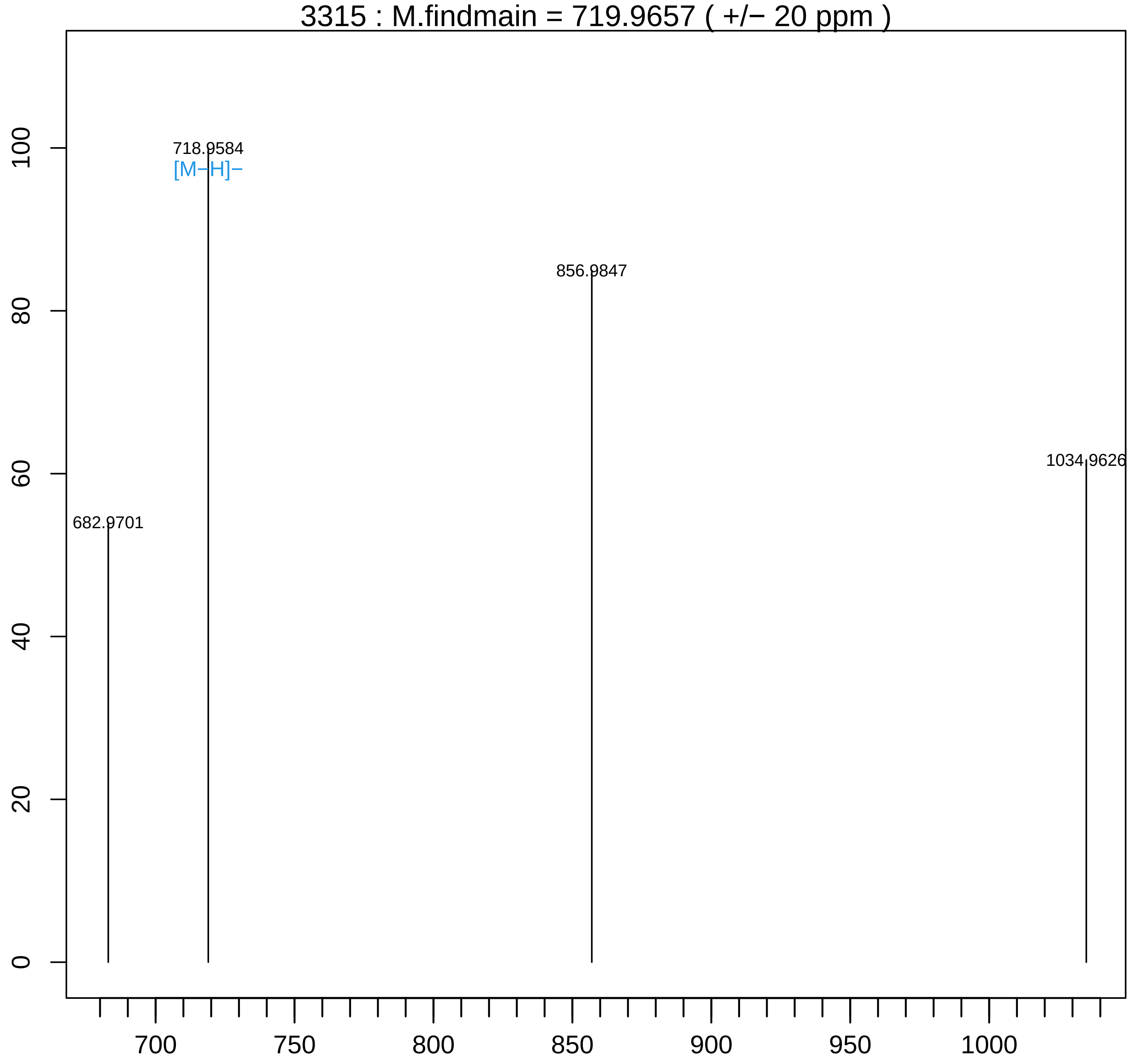
3315 : M.findmain = 719.9657 (+/− 20 ppm)
718.9584
[M−H]−
856.9847
1034.9626
682.9701

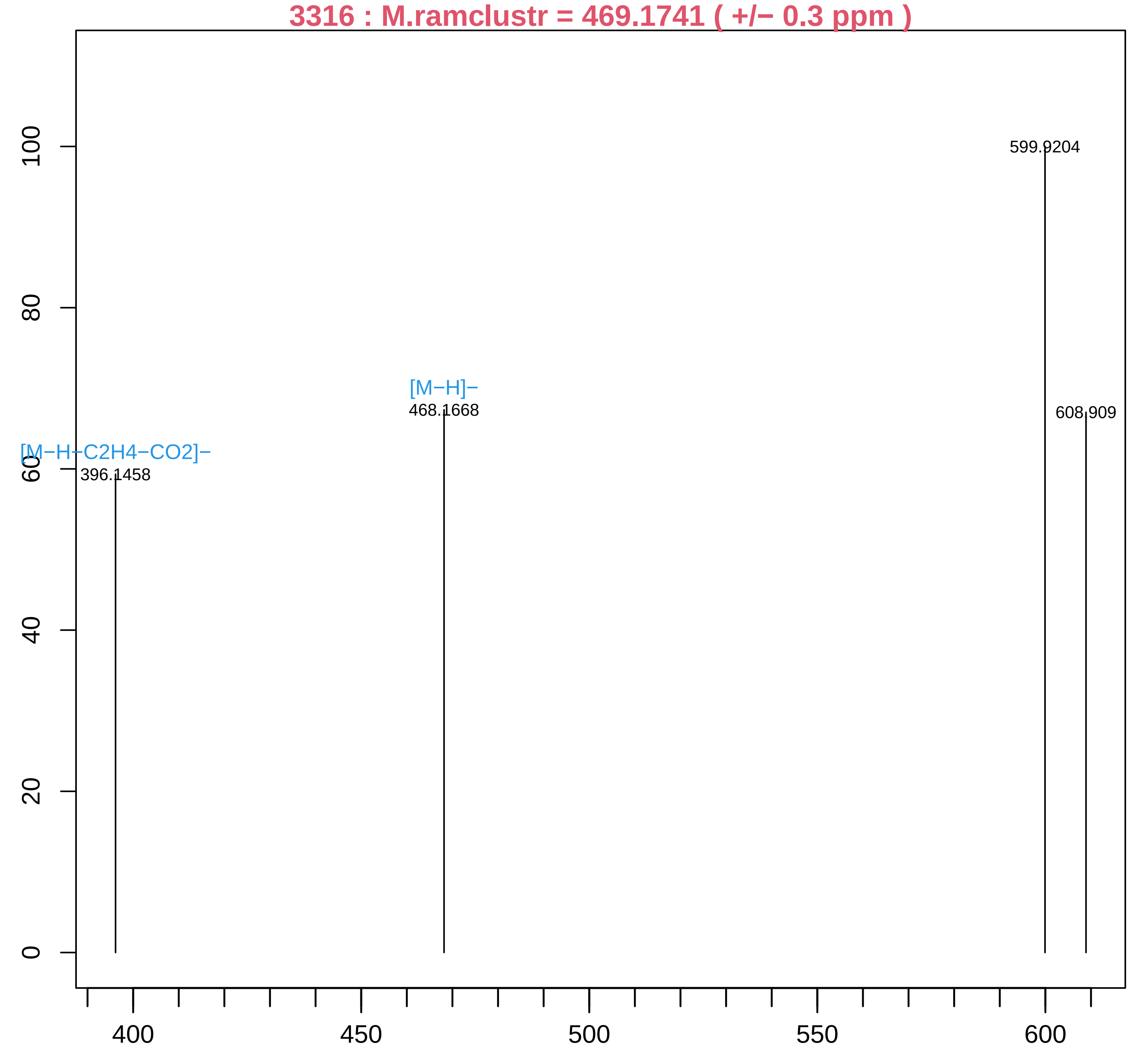
3316 : M.ramclustr = 469.1741 (+/− 0.3 ppm)
[M−H]−
468.1668
[M−H−C2H4−CO2]−
396.1458
599.9204
608.909
0
20
40
60
80
100
400
450
500
550
600

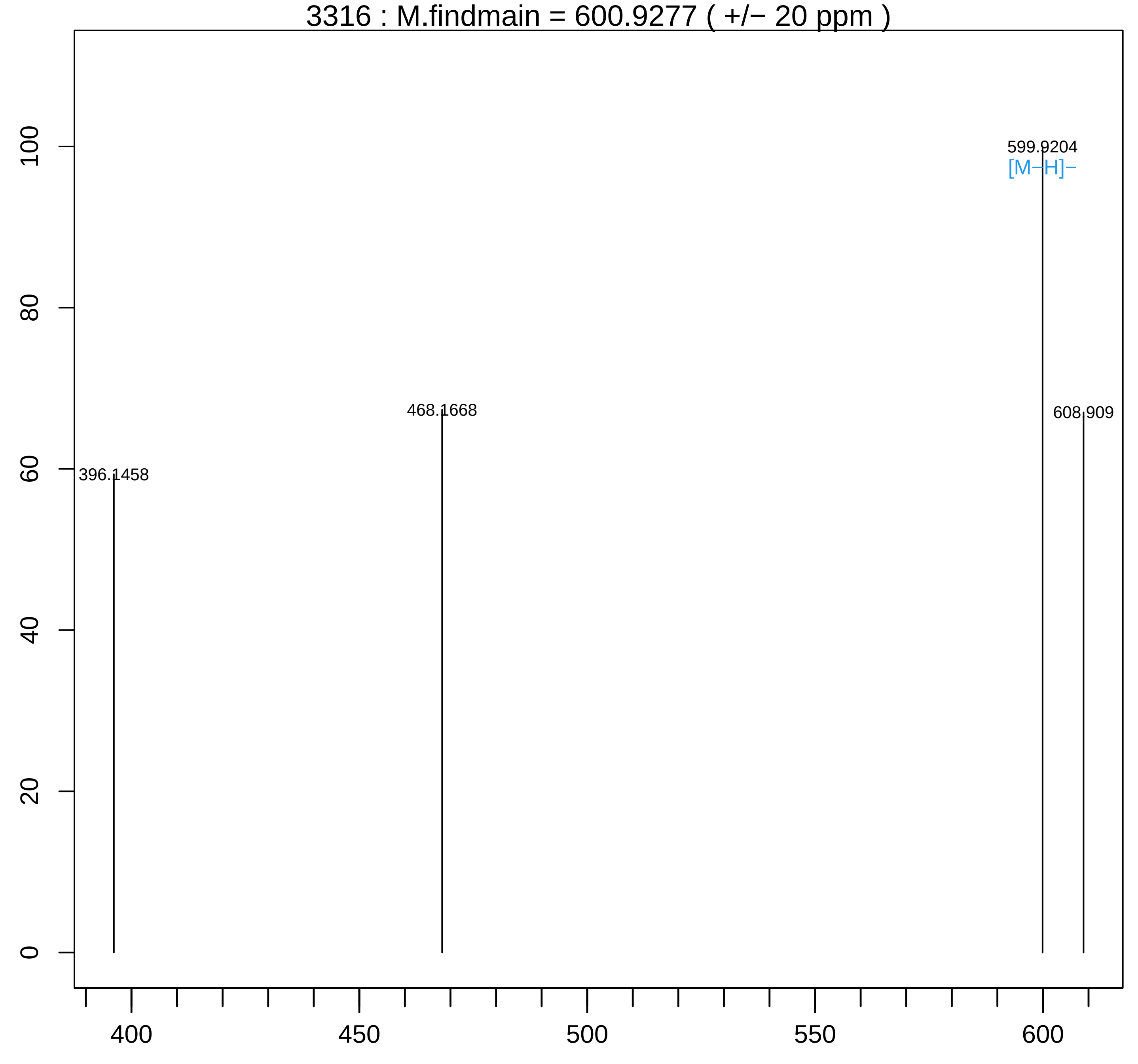
3316 : M.findmain = 600.9277 (+/− 20 ppm)
599.9204
[M−H]−
468.1668
396.1458
608.909
0
20
40
60
80
100
400
450
500
550
600

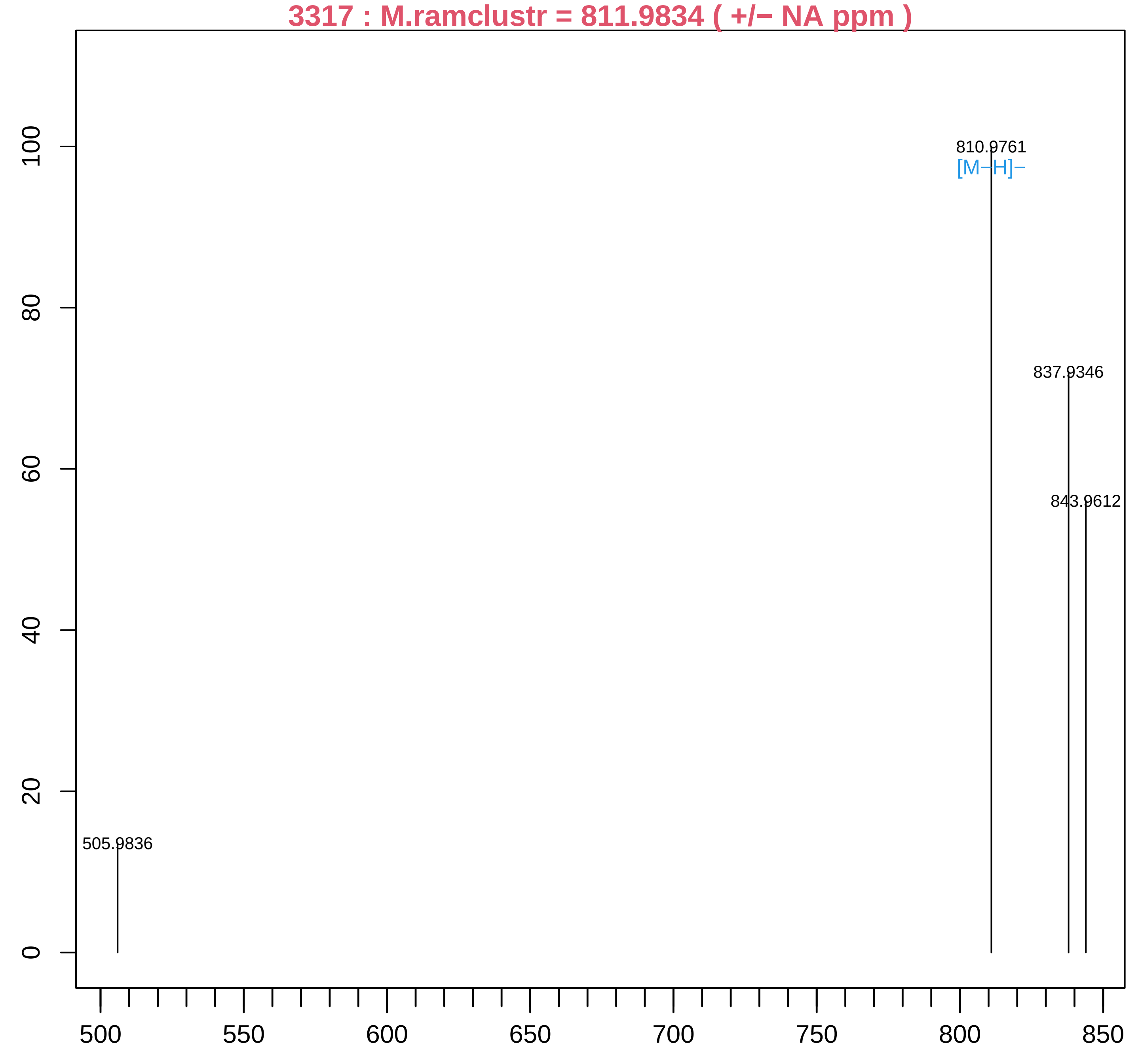
3317 : M.ramclustr = 811.9834 (+/− NA ppm)
810.9761
[M−H]−
837.9346
843.9612
505.9836
0
20
40
60
80
100
500
550
600
650
700
750
800
850

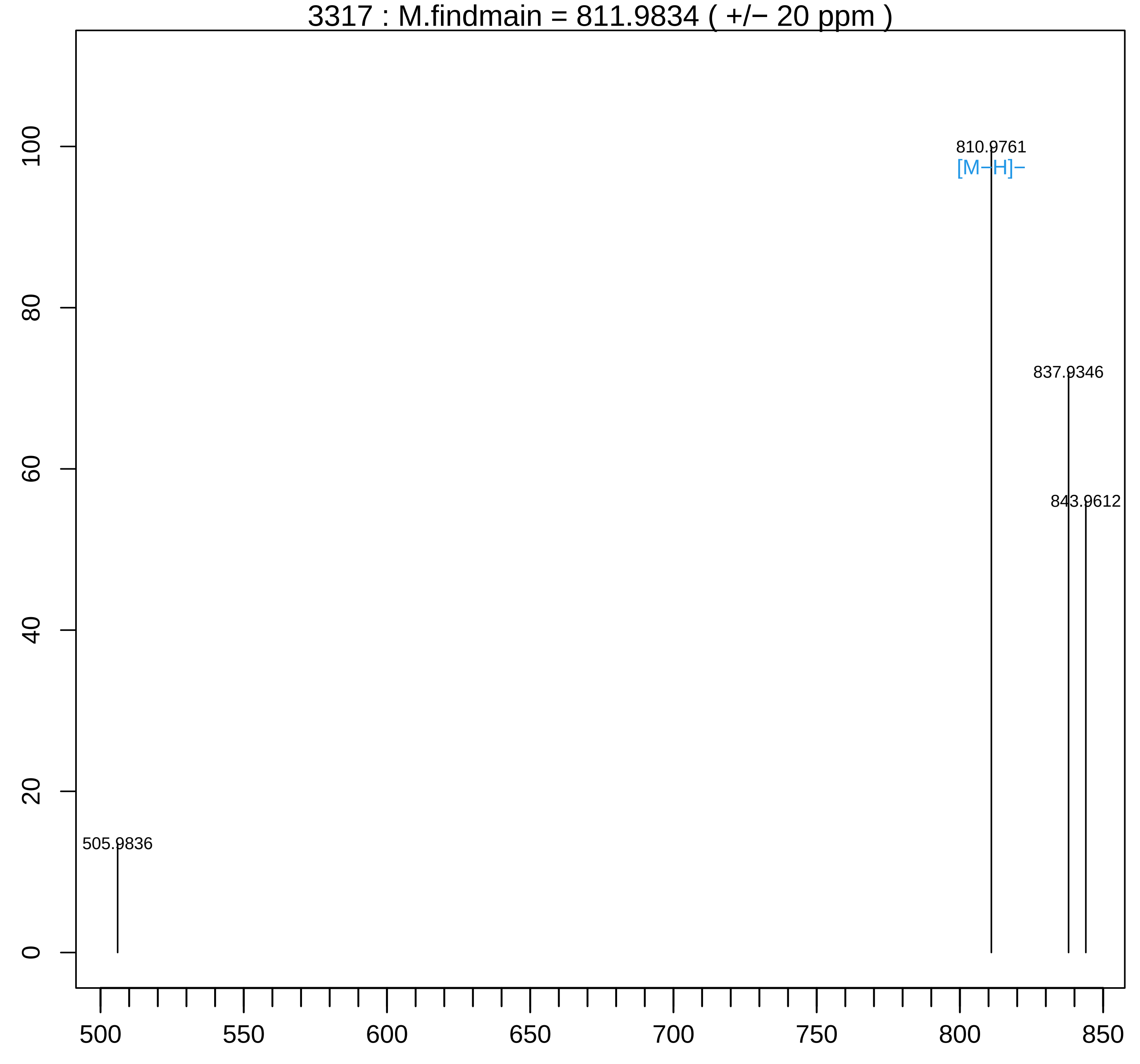
3317 : M.findmain = 811.9834 (+/− 20 ppm)
810.9761
[M−H]−
837.9346
843.9612
505.9836
0
20
40
60
80
100
500
550
600
650
700
750
800
850

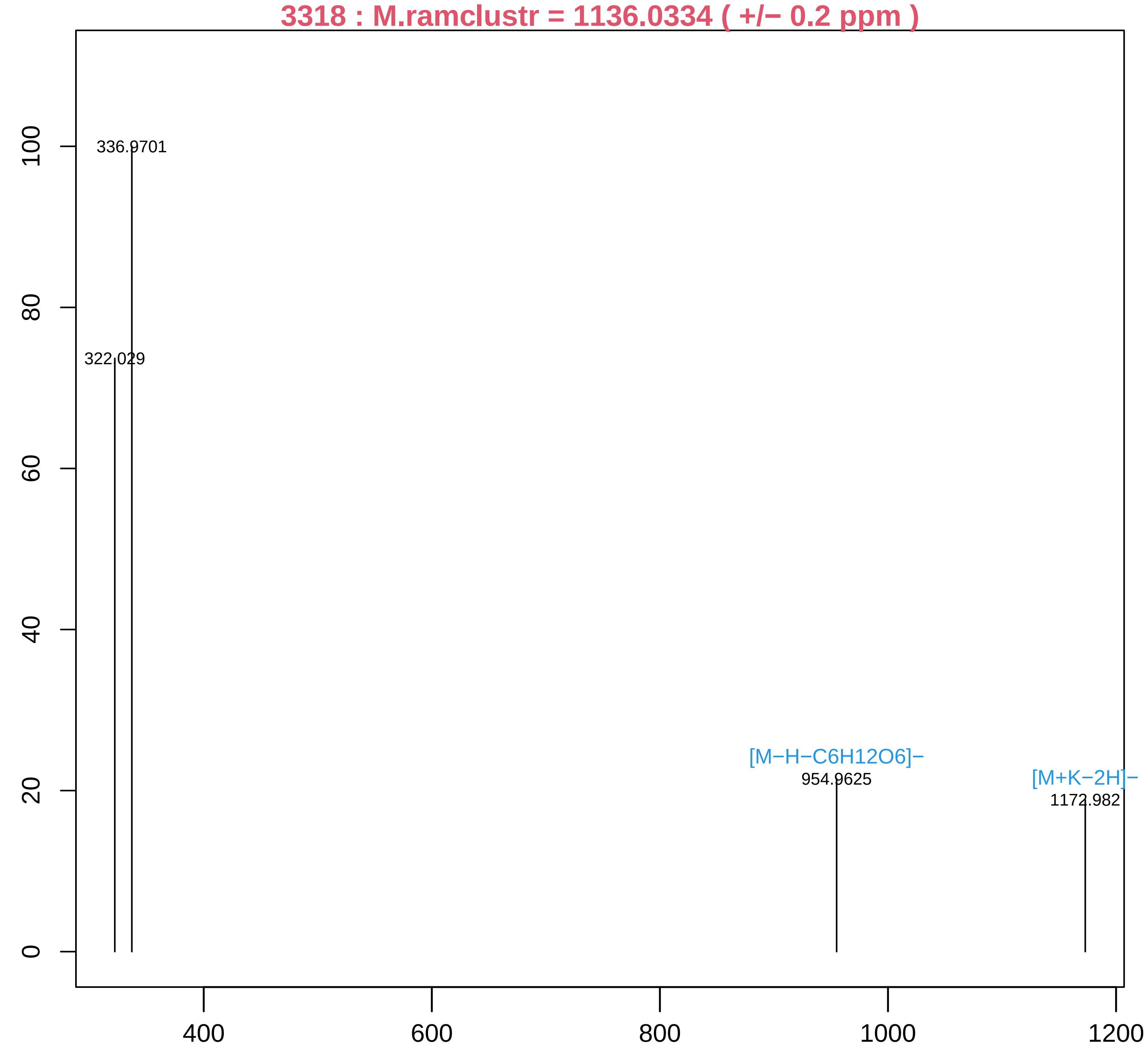

3318 : M.ramclustr = 1136.0334 (+/− 0.2 ppm)
336.9701
322.029
[M−H−C6H12O6]−
954.9625
[M+K−2H]−
1172.982
0
20
40
60
80
100
400
600
800
1000
1200

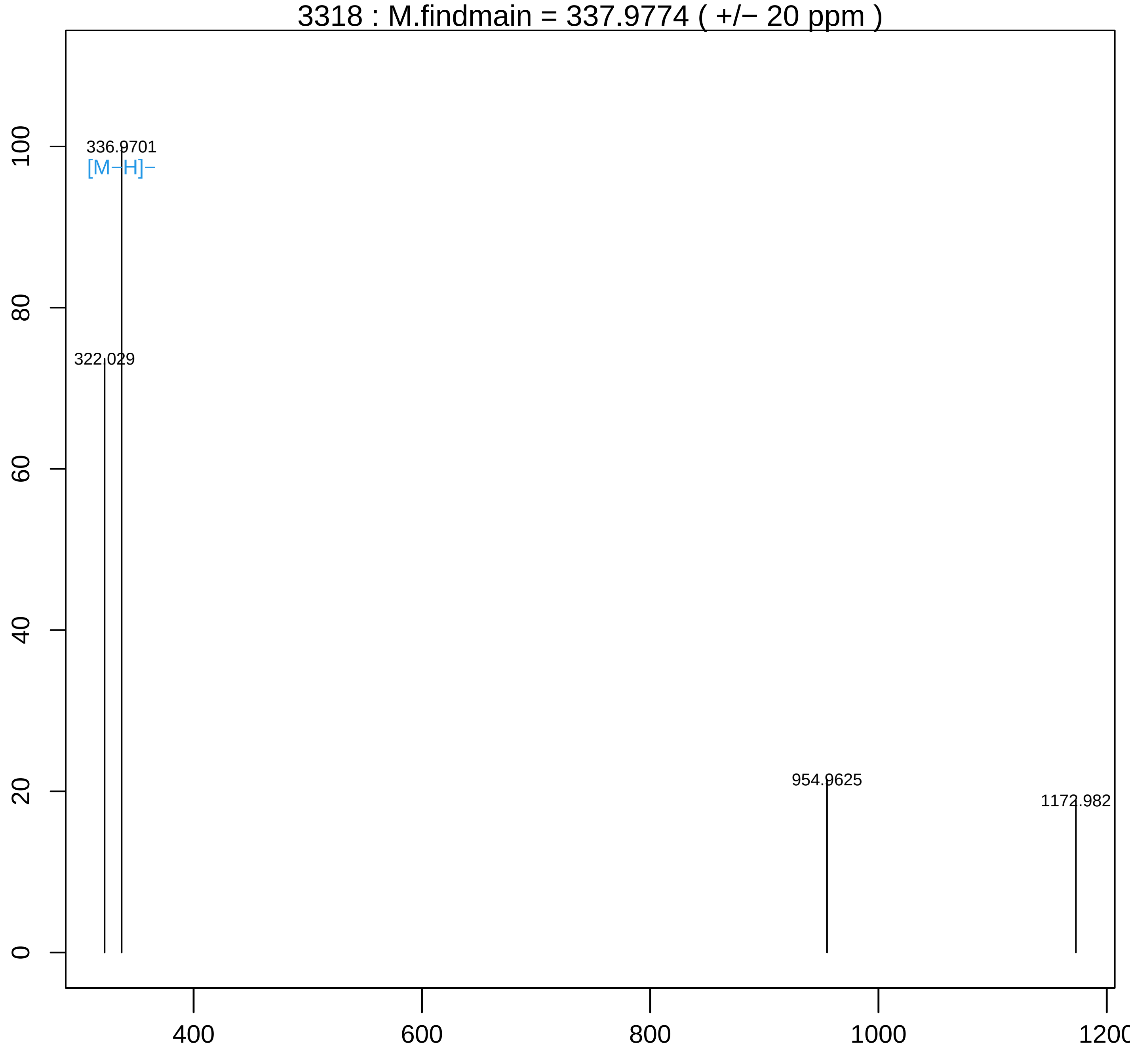

3318 : M.findmain = 337.9774 (+/− 20 ppm)
336.9701
[M−H]−
322.029
954.9625
1172.982
0
20
40
60
80
100
400
600
800
1000
1200

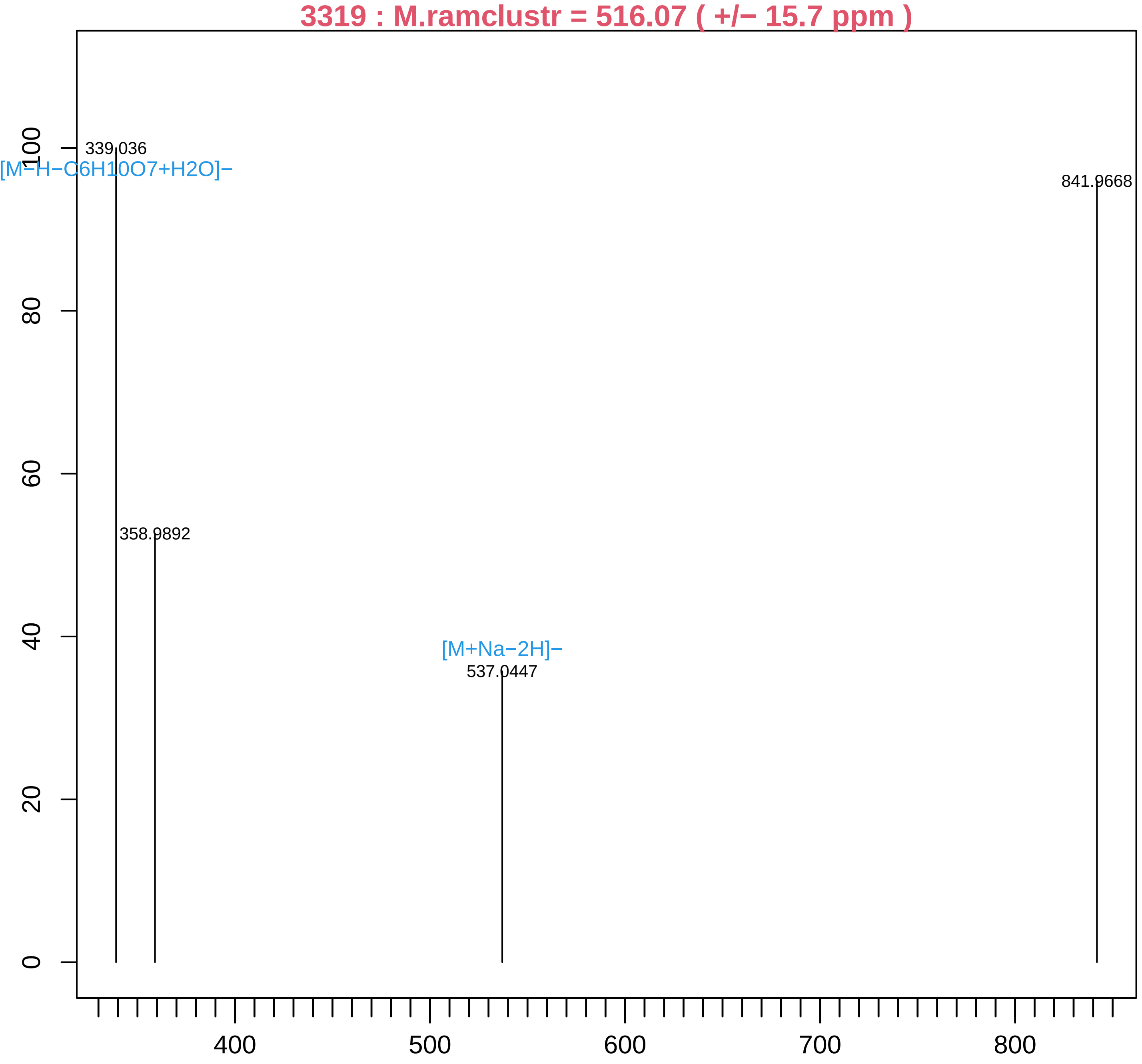
3319 : M.ramclustr = 516.07 (+/− 15.7 ppm)
339.036
[M−H−C6H10O7+H2O]−
358.9892
[M+Na−2H]−
537.0447
841.9668
0
20
40
60
80
100
400
500
600
700
800

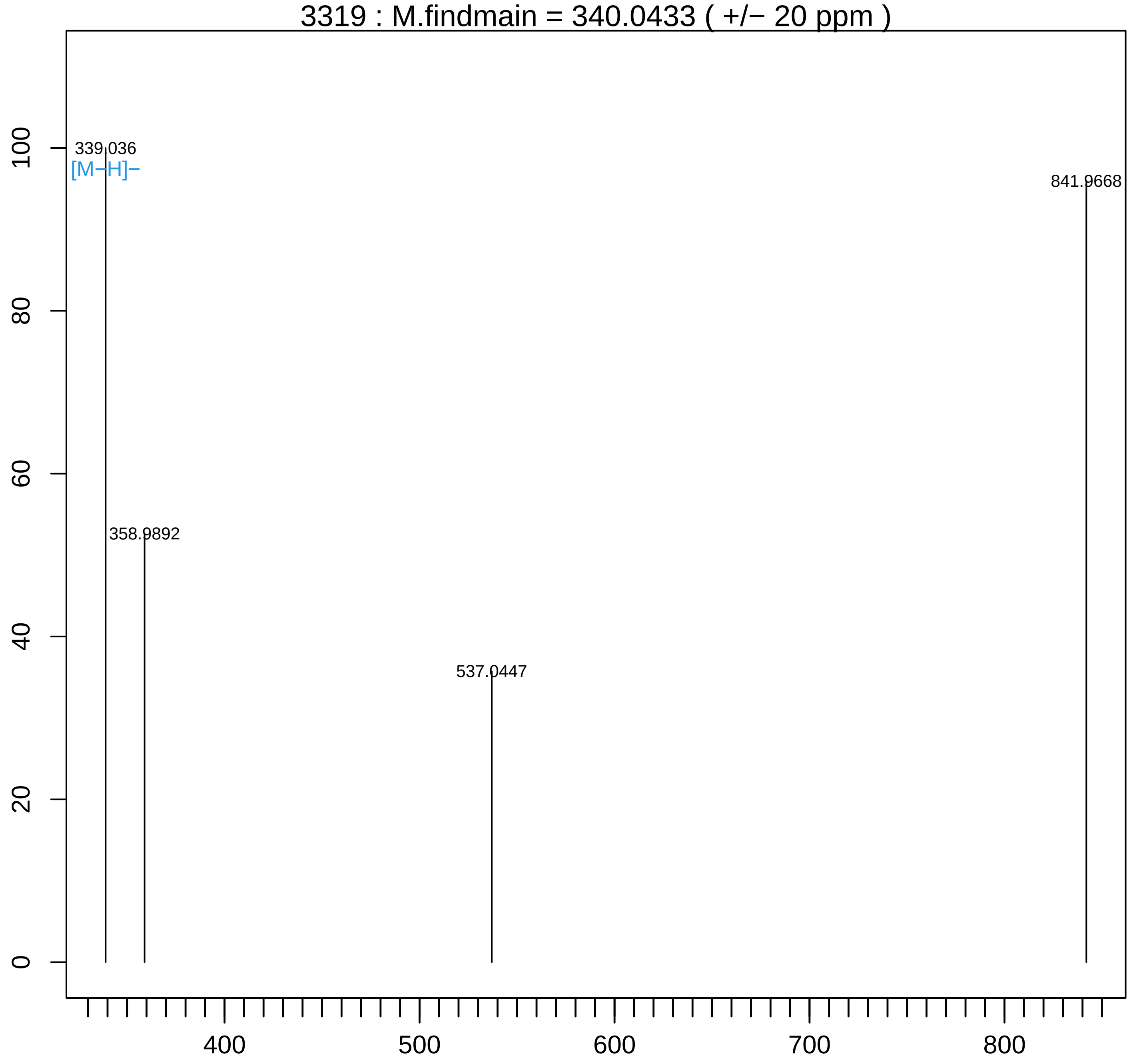
3319 : M.findmain = 340.0433 (+/− 20 ppm)
339.036
[M−H]−
358.9892
537.0447
841.9668
0
20
40
60
80
100
400
500
600
700
800

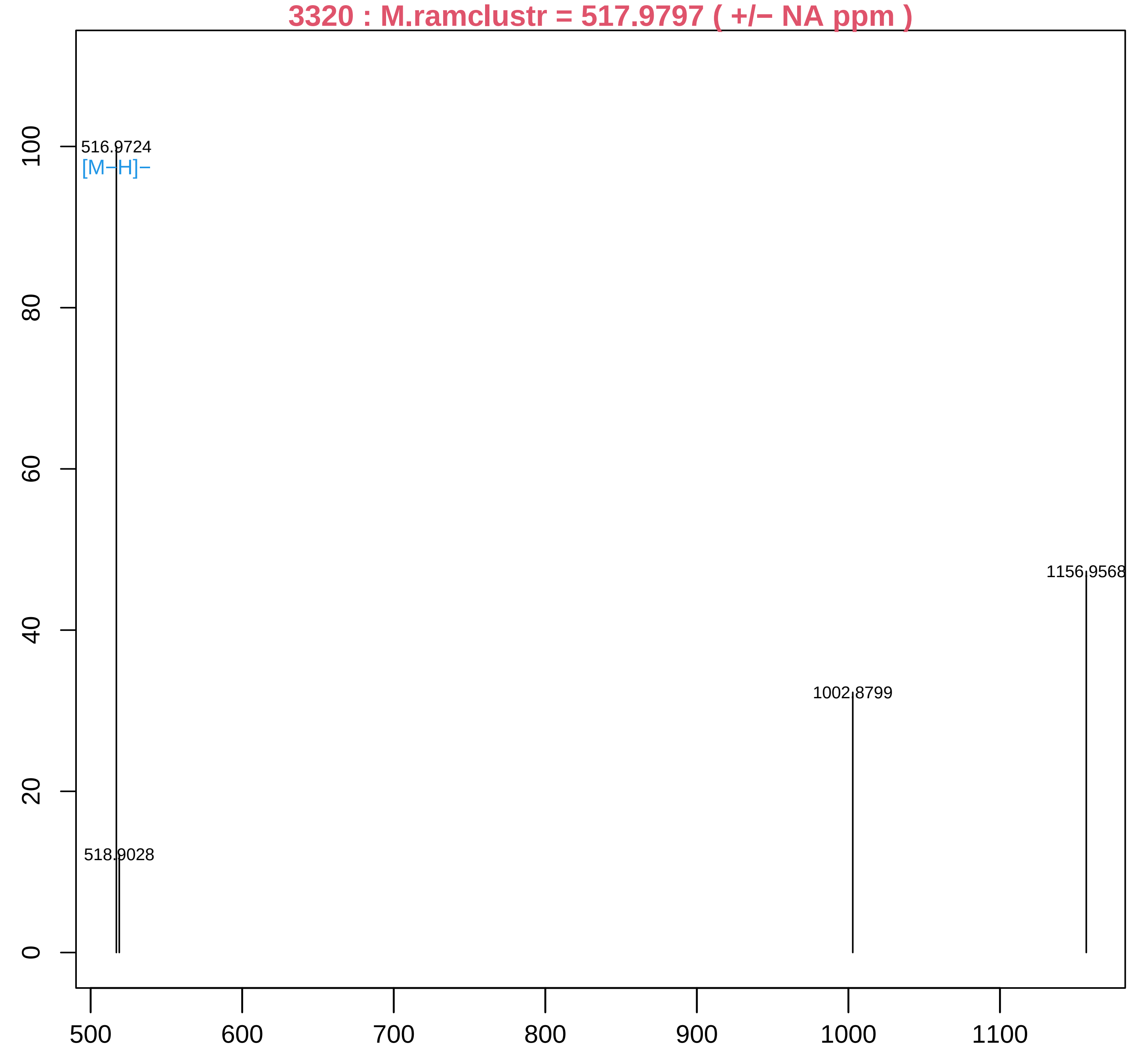

3320 : M.ramclustr = 517.9797 (+/− NA ppm)
516.9724
[M−H]−
518.9028
1002.8799
1156.9568
0
20
40
60
80
100
500
600
700
800
900
1000
1100

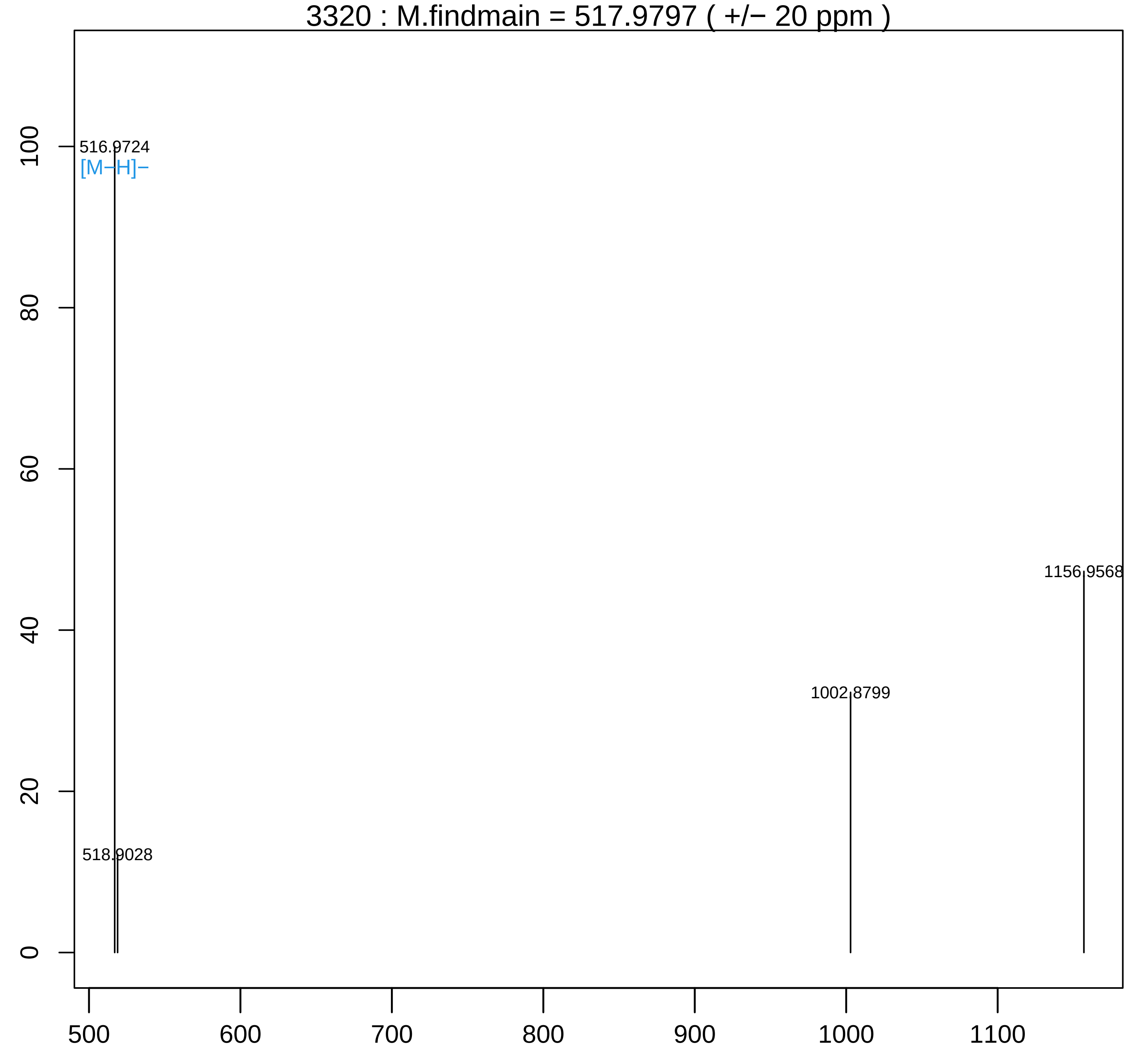

3320 : M.findmain = 517.9797 (+/− 20 ppm)
516.9724
[M−H]−
518.9028
1002.8799
1156.9568
0
20
40
60
80
100
500
600
700
800
900
1000
1100

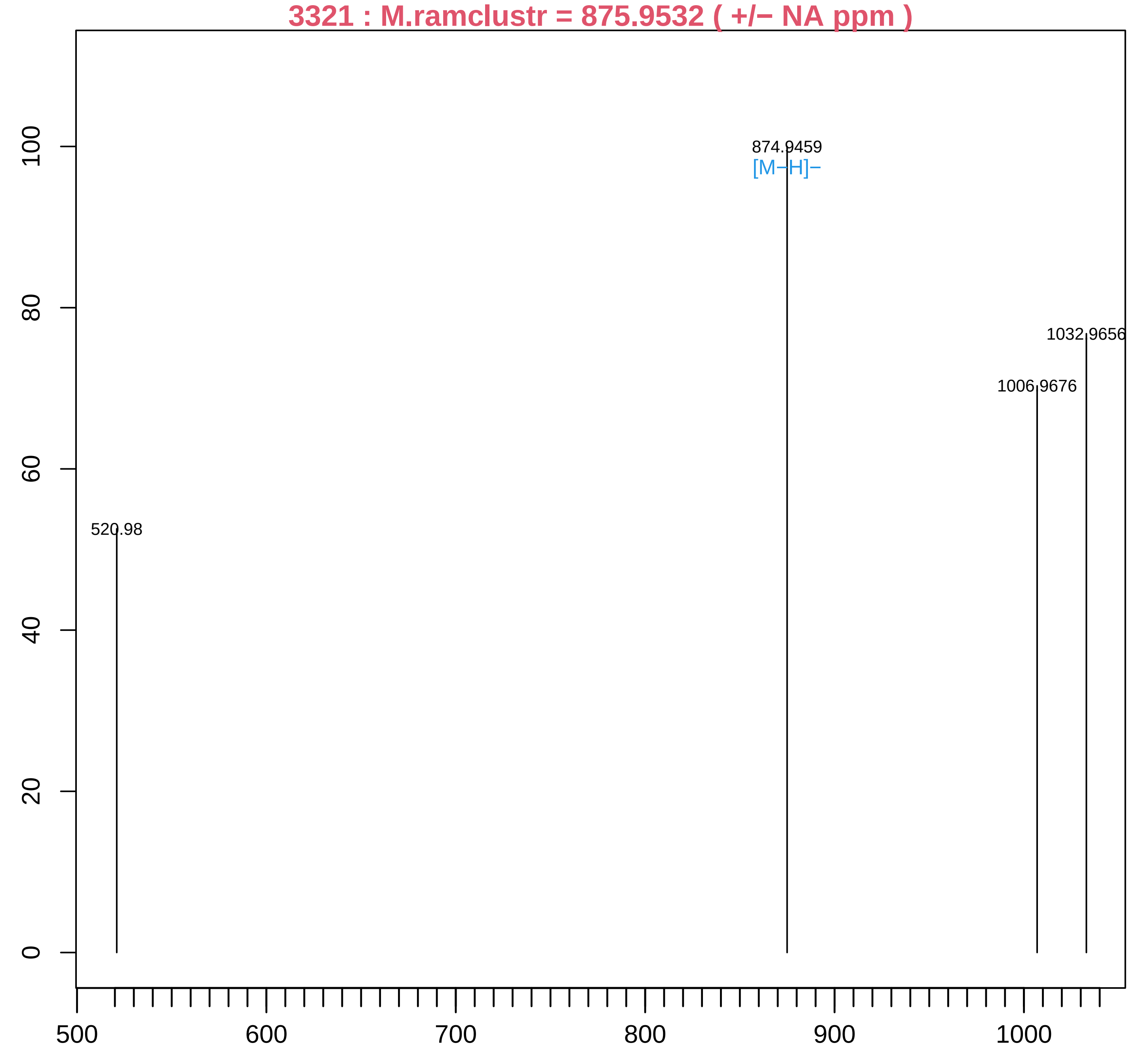
3321 : M.ramclustr = 875.9532 (+/− NA ppm)
874.9459
[M−H]−
1032.9656
1006.9676
520.98
0
20
40
60
80
100
500
600
700
800
900
1000

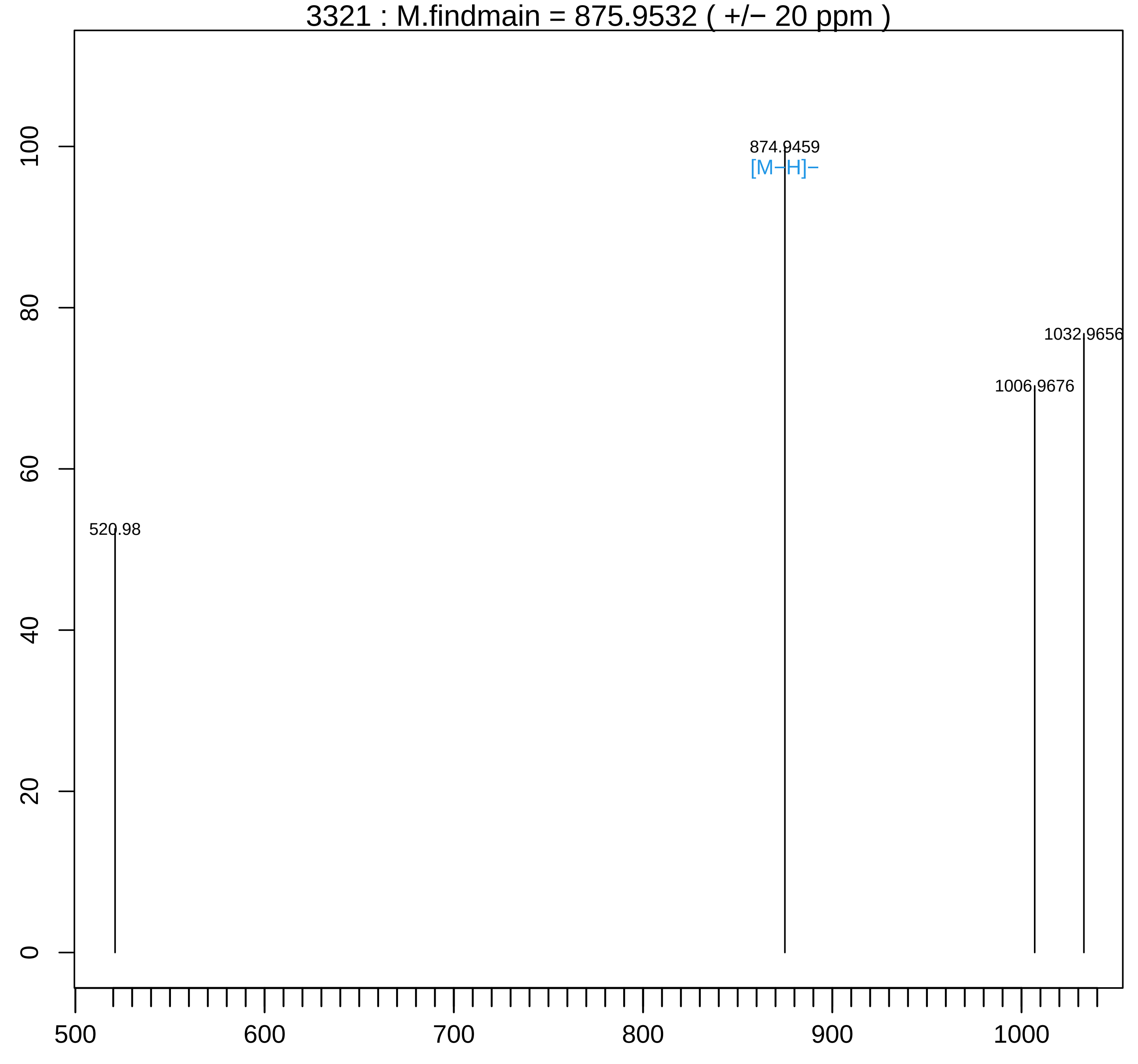
3321 : M.findmain = 875.9532 (+/− 20 ppm)
874.9459
[M−H]−
1032.9656
1006.9676
520.98
0
20
40
60
80
100
500
600
700
800
900
1000

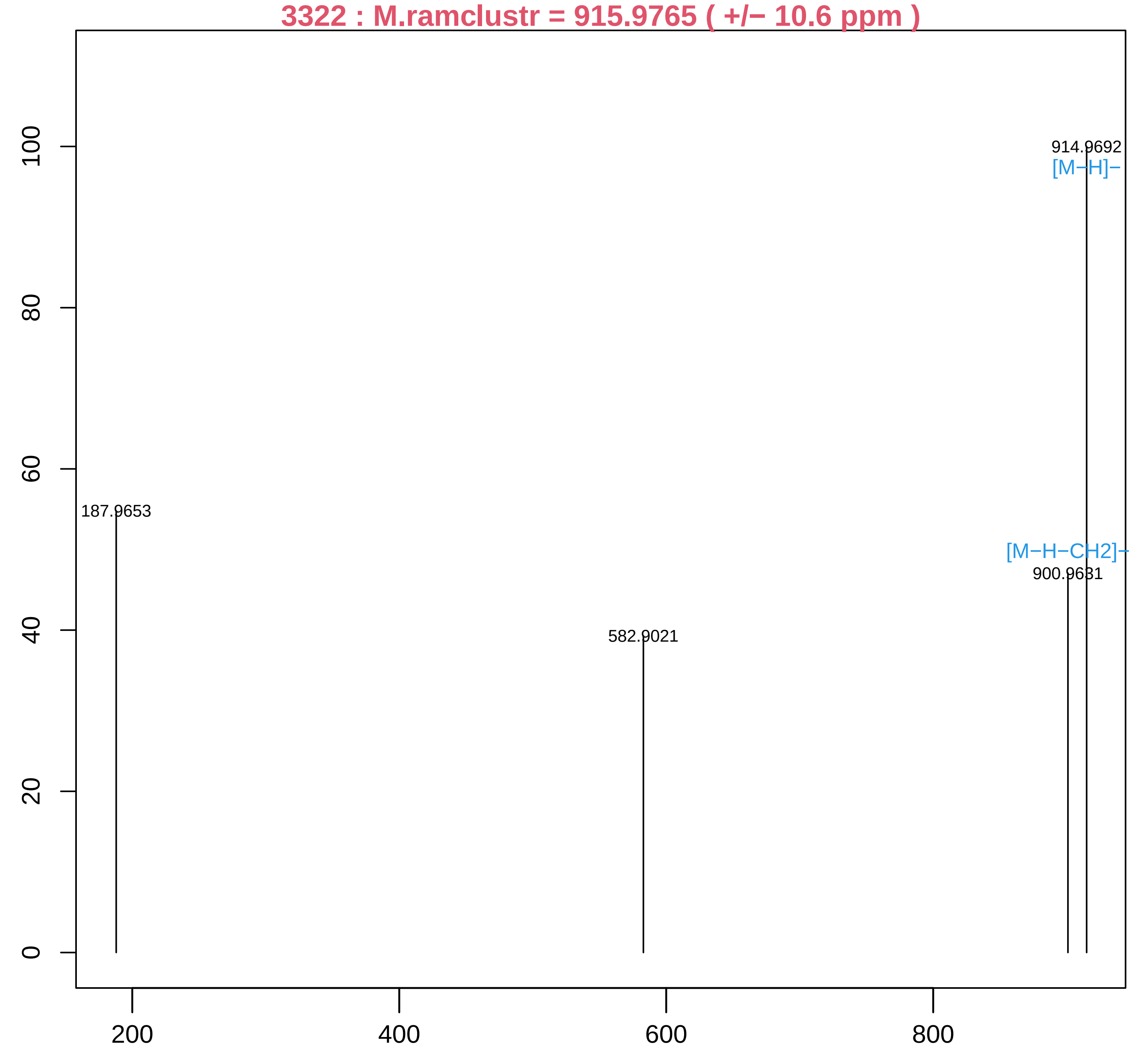
3322 : M.ramclustr = 915.9765 (+/− 10.6 ppm)
914.9692
[M−H]−
187.9653
[M−H−CH2]−
900.9631
582.9021
0
20
40
60
80
100
200
400
600
800

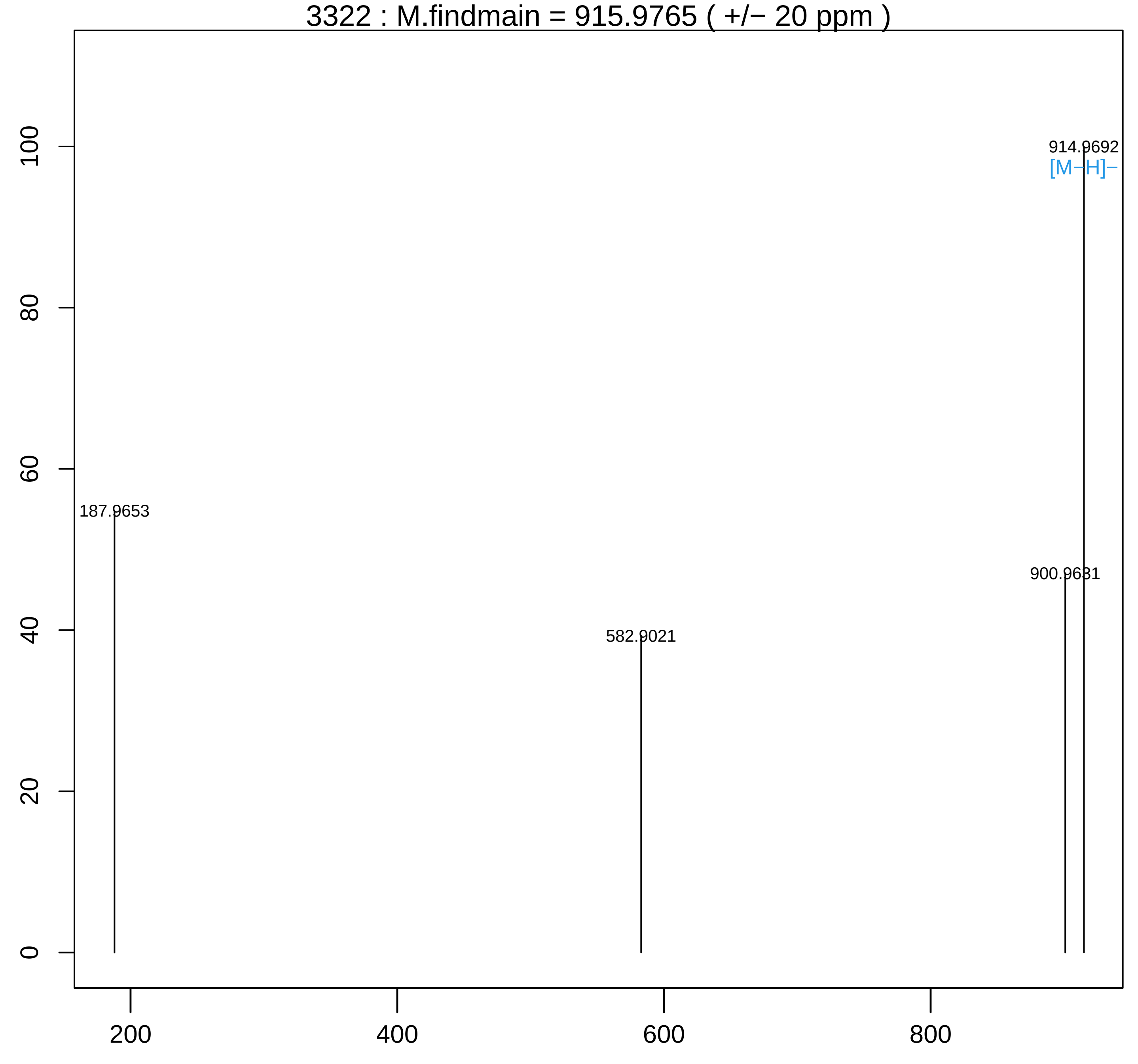
3322 : M.findmain = 915.9765 (+/− 20 ppm)
914.9692
[M−H]−
187.9653
900.9631
582.9021
0
20
40
60
80
100
200
400
600
800

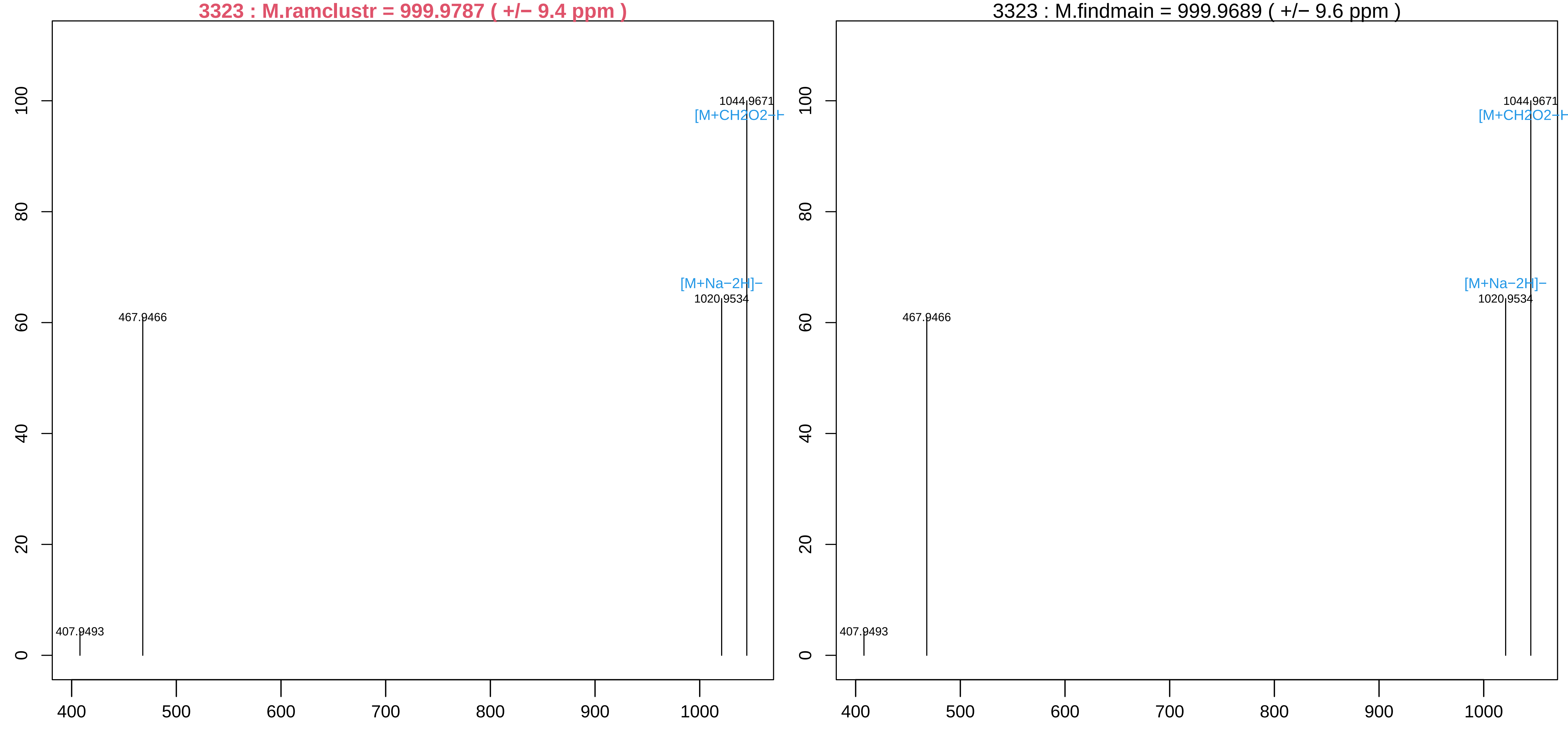

3323 : M.ramclustr = 999.9787 (+/− 9.4 ppm)
1044.9671
[M+CH2O2−H
[M+Na−2H]−
1020.9534
467.9466
407.9493
0
20
40
60
80
100
400
500
600
700
800
900
1000
3323 : M.findmain = 999.9689 (+/− 9.6 ppm)
1044.9671
[M+CH2O2−H
[M+Na−2H]−
1020.9534
467.9466
407.9493

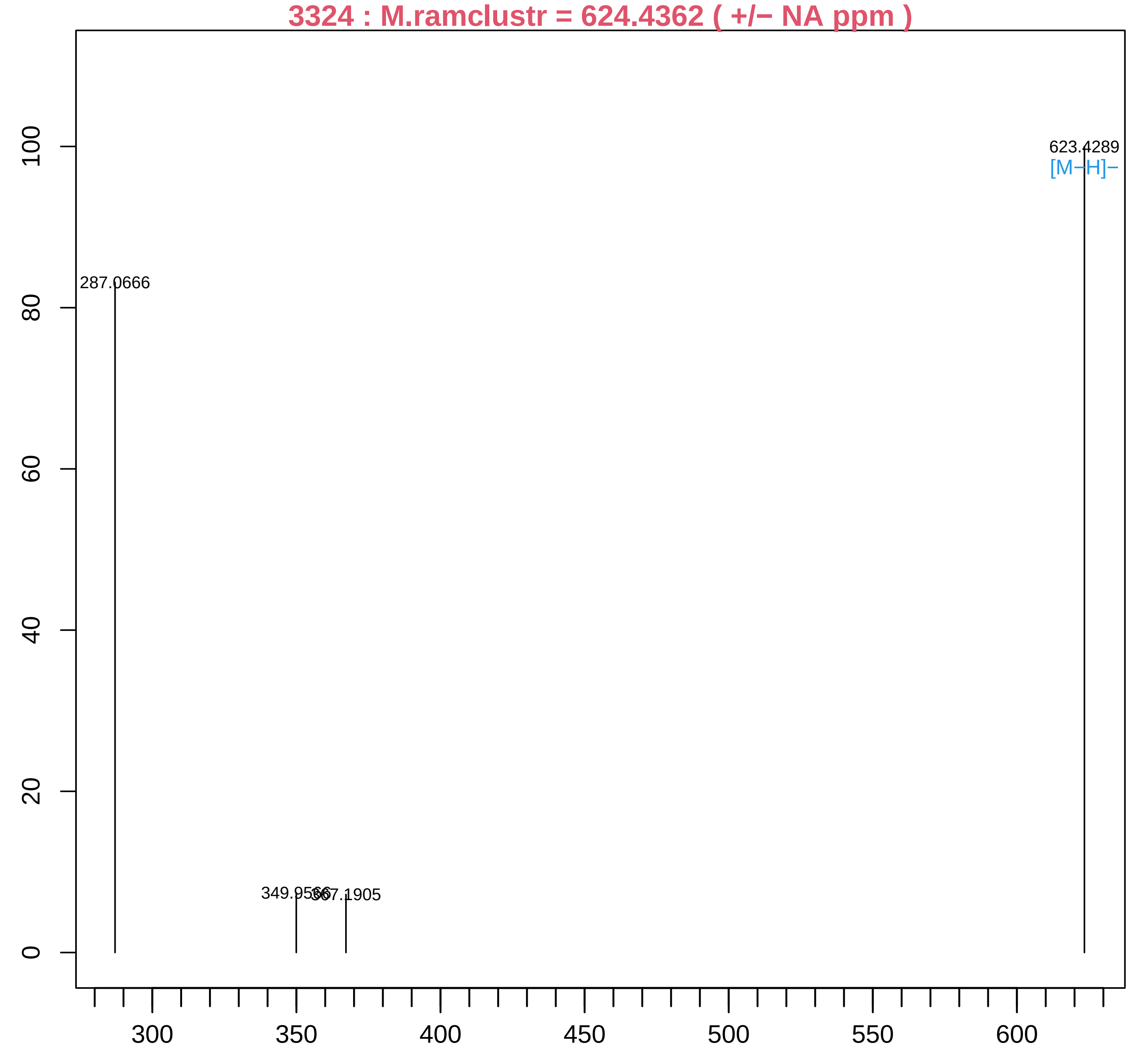
3324 : M.ramclustr = 624.4362 (+/− NA ppm)
623.4289
[M−H]−
287.0666
349.9566
367.1905
0
20
40
60
80
100
300
350
400
450
500
550
600
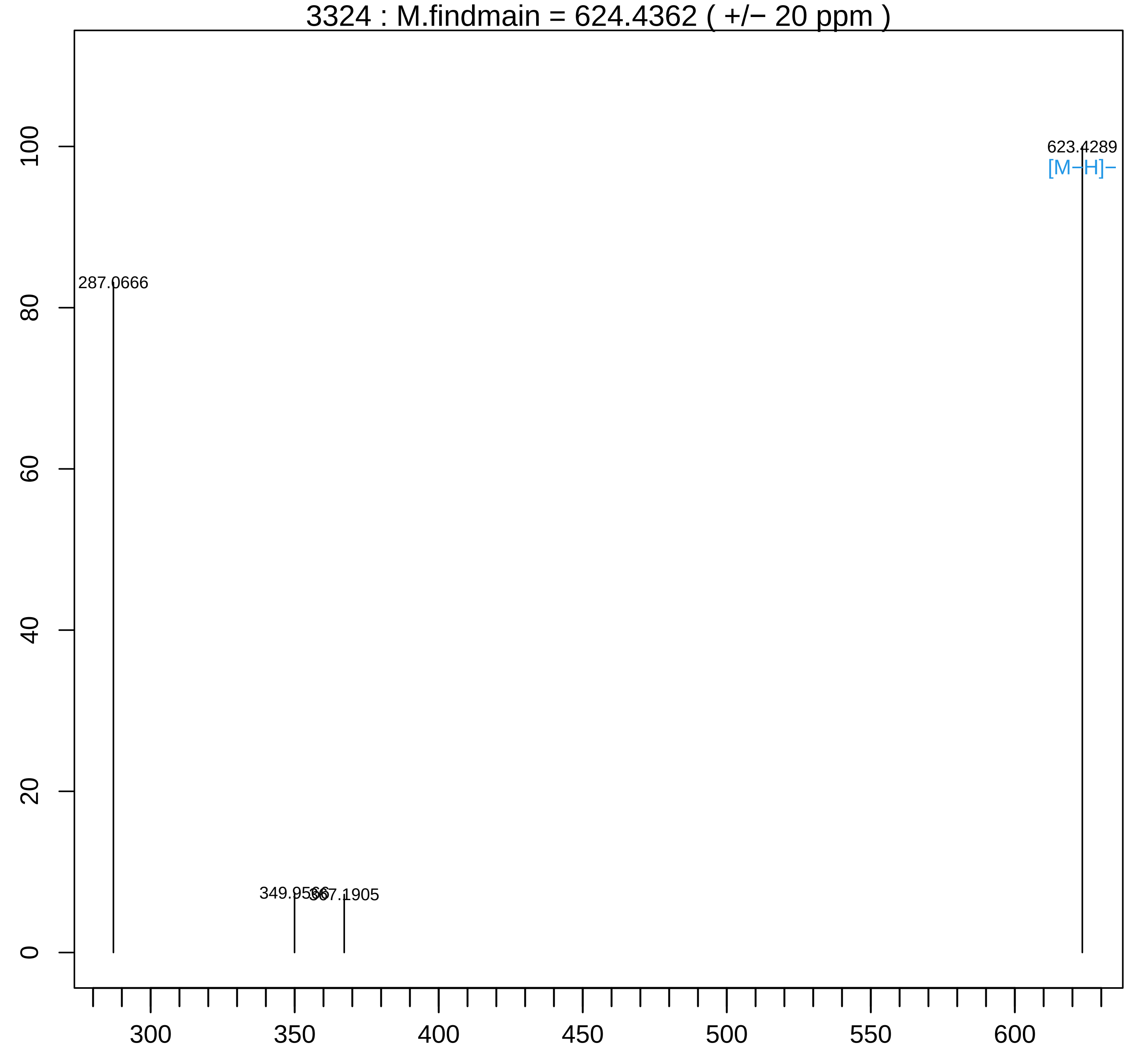
3324 : M.findmain = 624.4362 (+/− 20 ppm)
623.4289
[M−H]−
287.0666
349.9566
367.1905
0
20
40
60
80
100
300
350
400
450
500
550
600

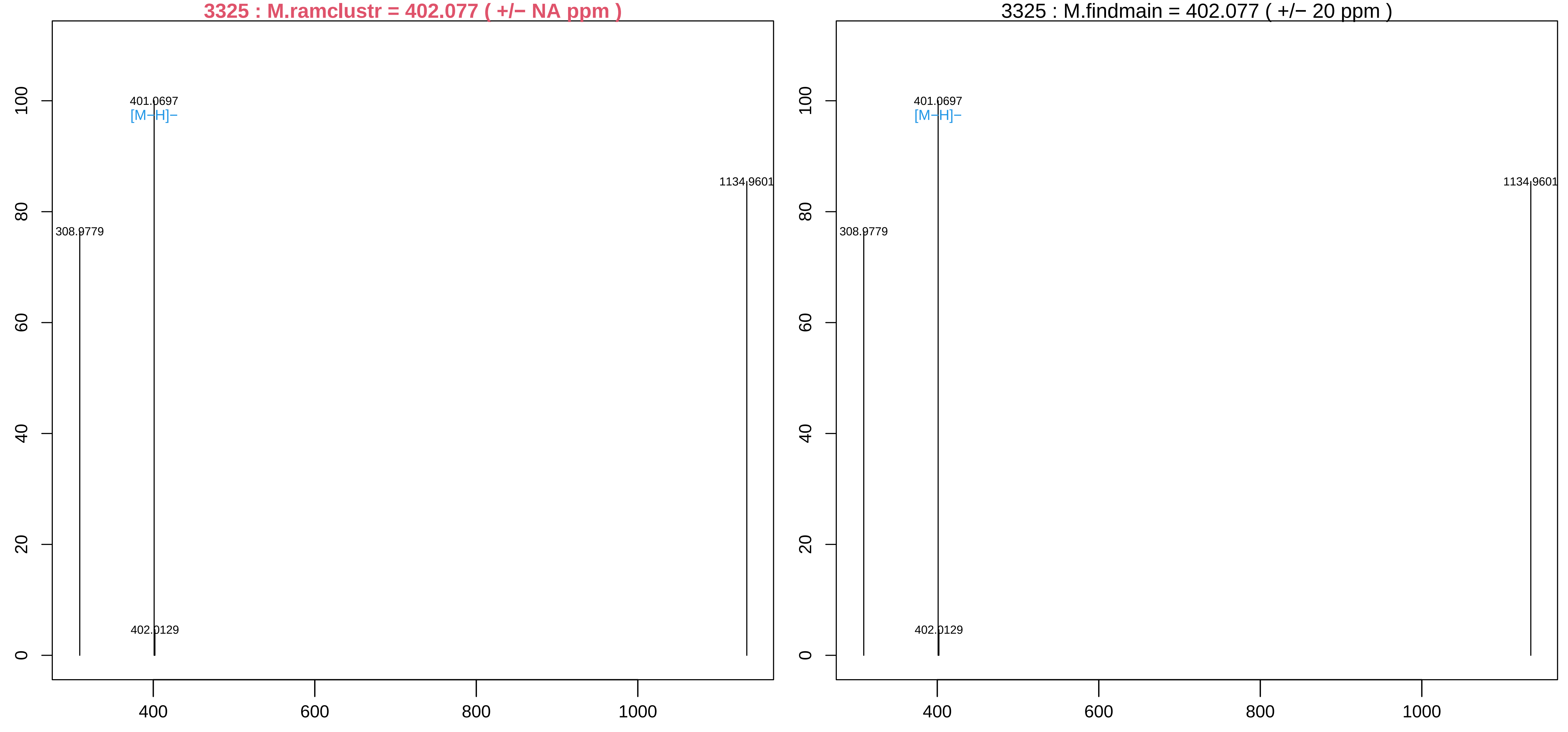
3325 : M.ramclustr = 402.077 (+/− NA ppm)
401.0697
[M−H]−
1134.9601
308.9779
402.0129
3325 : M.findmain = 402.077 (+/− 20 ppm)
401.0697
[M−H]−
1134.9601
308.9779
402.0129

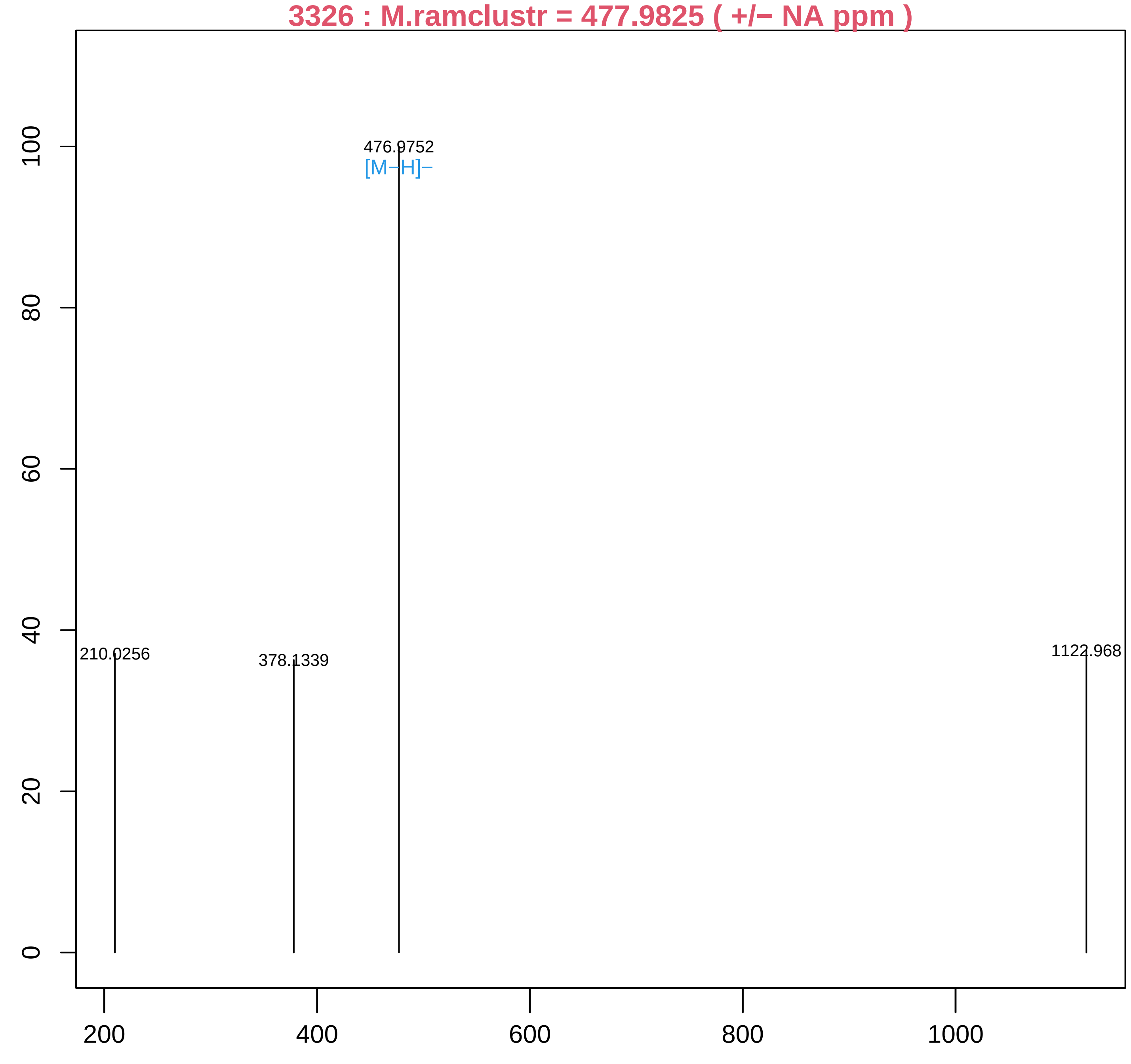

3326 : M.ramclustr = 477.9825 (+/− NA ppm)
476.9752
[M−H]−
210.0256
378.1339
1122.968
0
20
40
60
80
100
200
400
600
800
1000

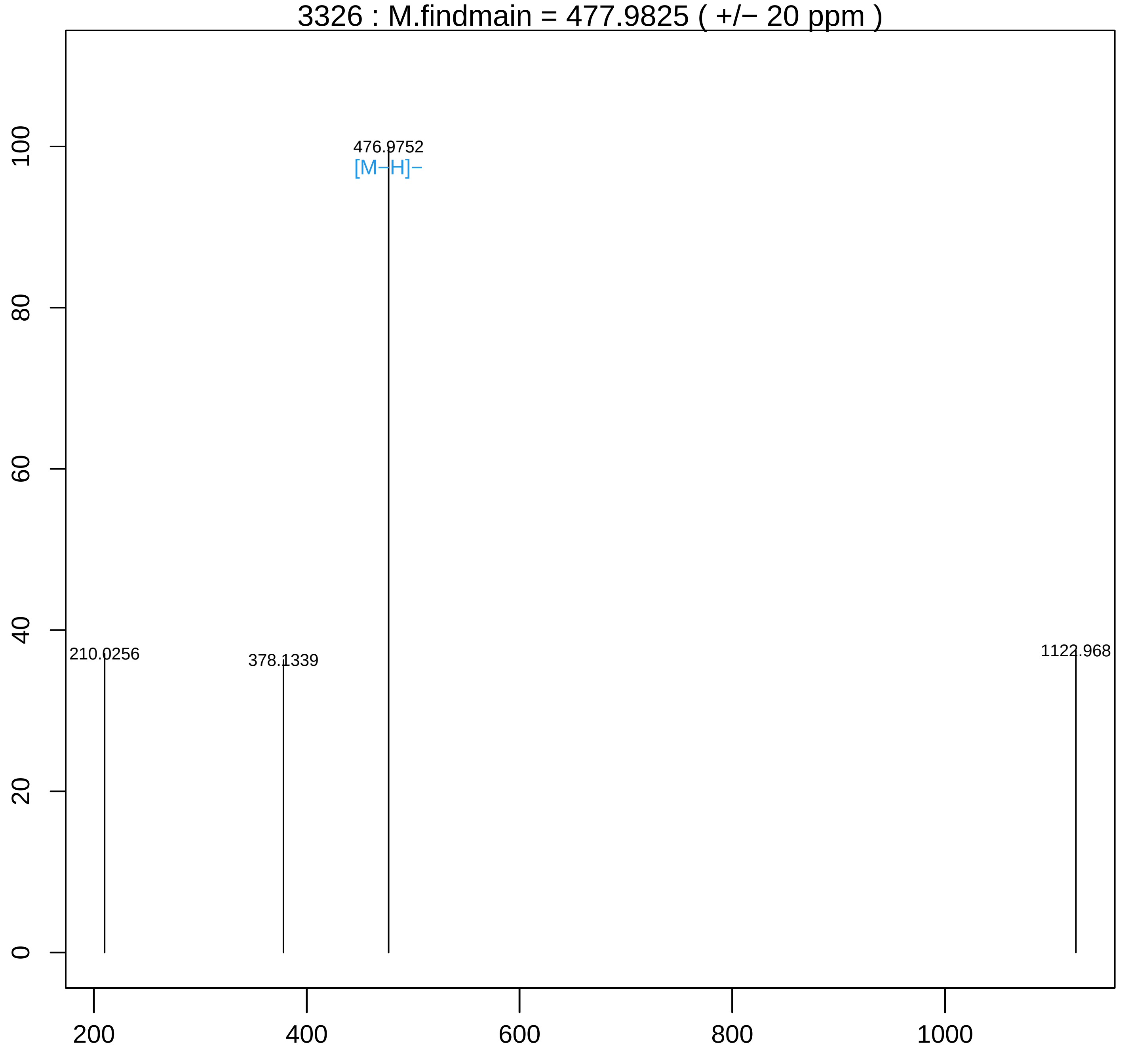

3326 : M.findmain = 477.9825 (+/− 20 ppm)
476.9752
[M−H]−
210.0256
378.1339
1122.968
0
20
40
60
80
100
200
400
600
800
1000

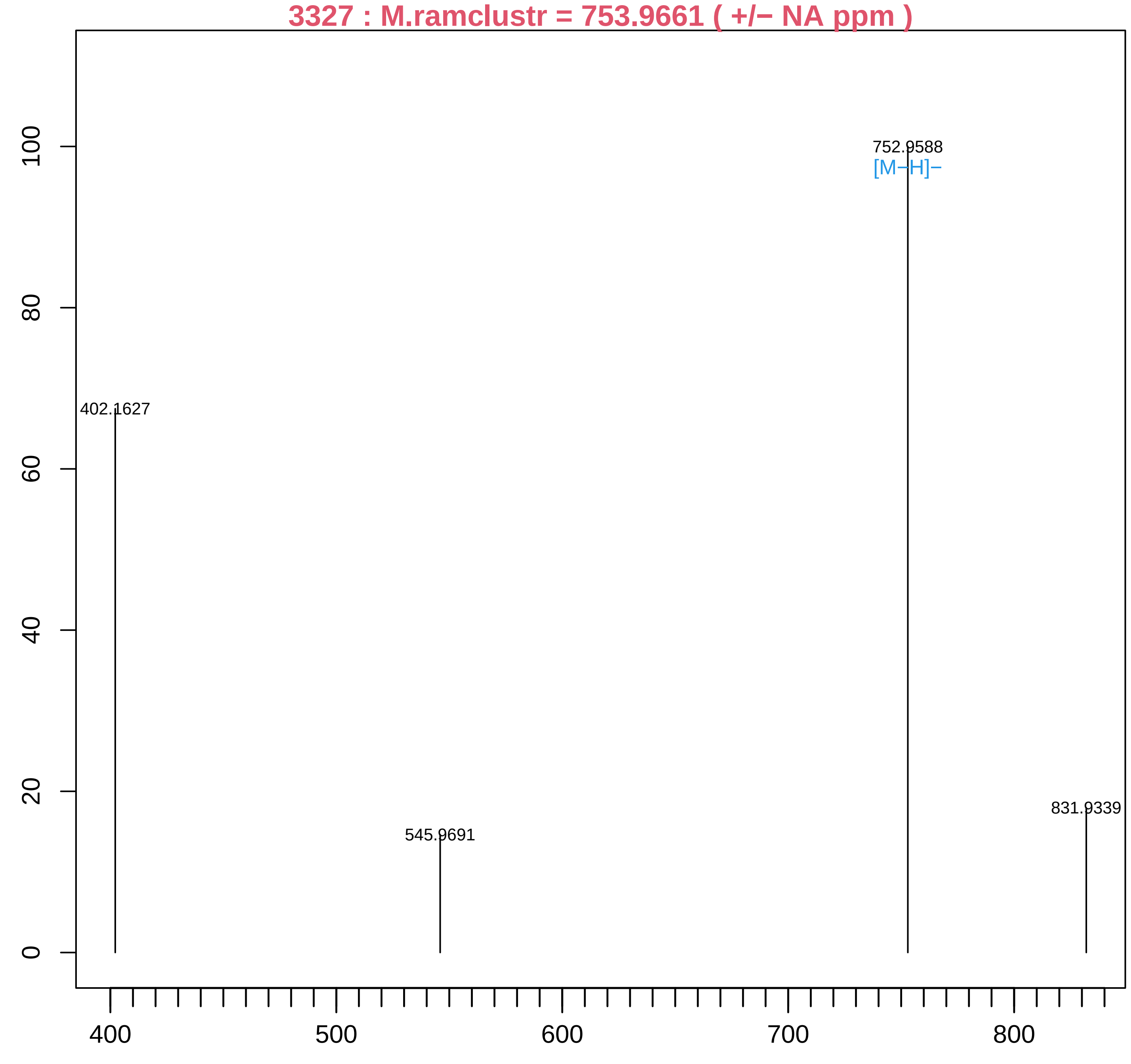
3327 : M.ramclustr = 753.9661 (+/− NA ppm)
402.1627
545.9691
752.9588
[M−H]−
831.9339
0
20
40
60
80
100
400
500
600
700
800

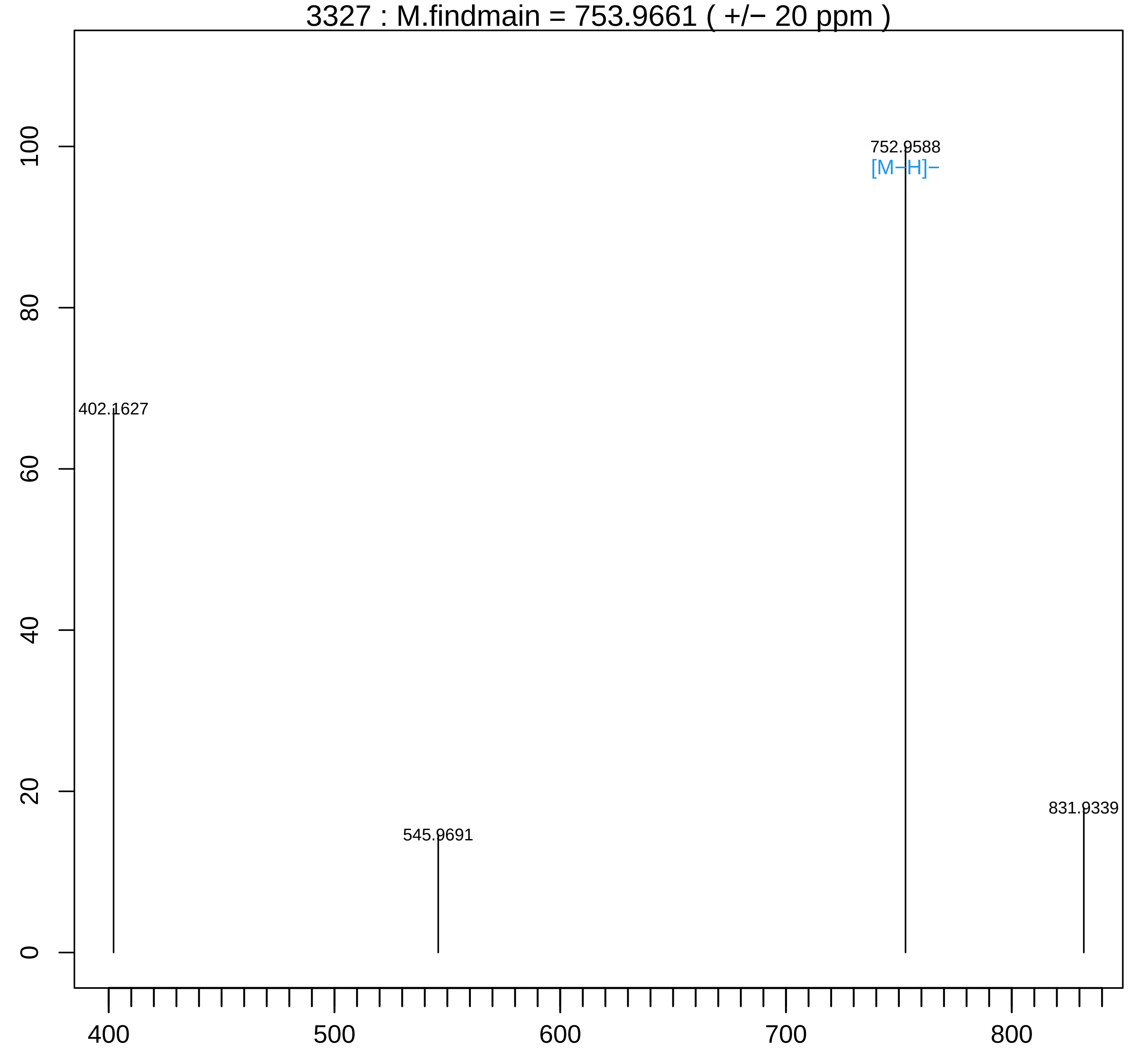
3327 : M.findmain = 753.9661 (+/− 20 ppm)
402.1627
545.9691
752.9588
[M−H]−
831.9339
0
20
40
60
80
100
400
500
600
700
800

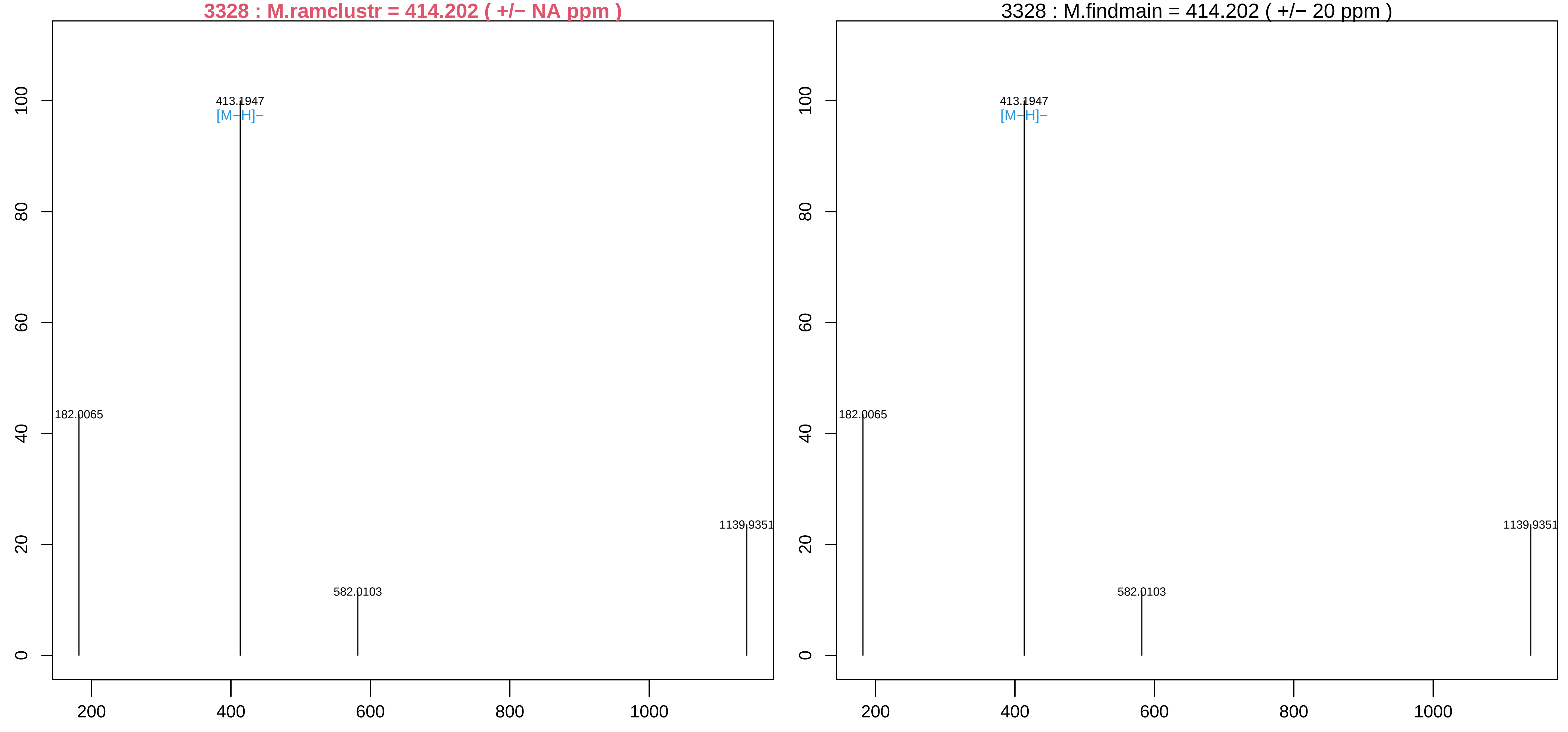
3328 : M.ramclustr = 414.202 (+/− NA ppm)
413.1947
[M−H]−
182.0065
582.0103
1139.9351
3328 : M.findmain = 414.202 (+/− 20 ppm)
413.1947
[M−H]−
182.0065
582.0103
1139.9351
0
20
40
60
80
100
200
400
600
800
1000

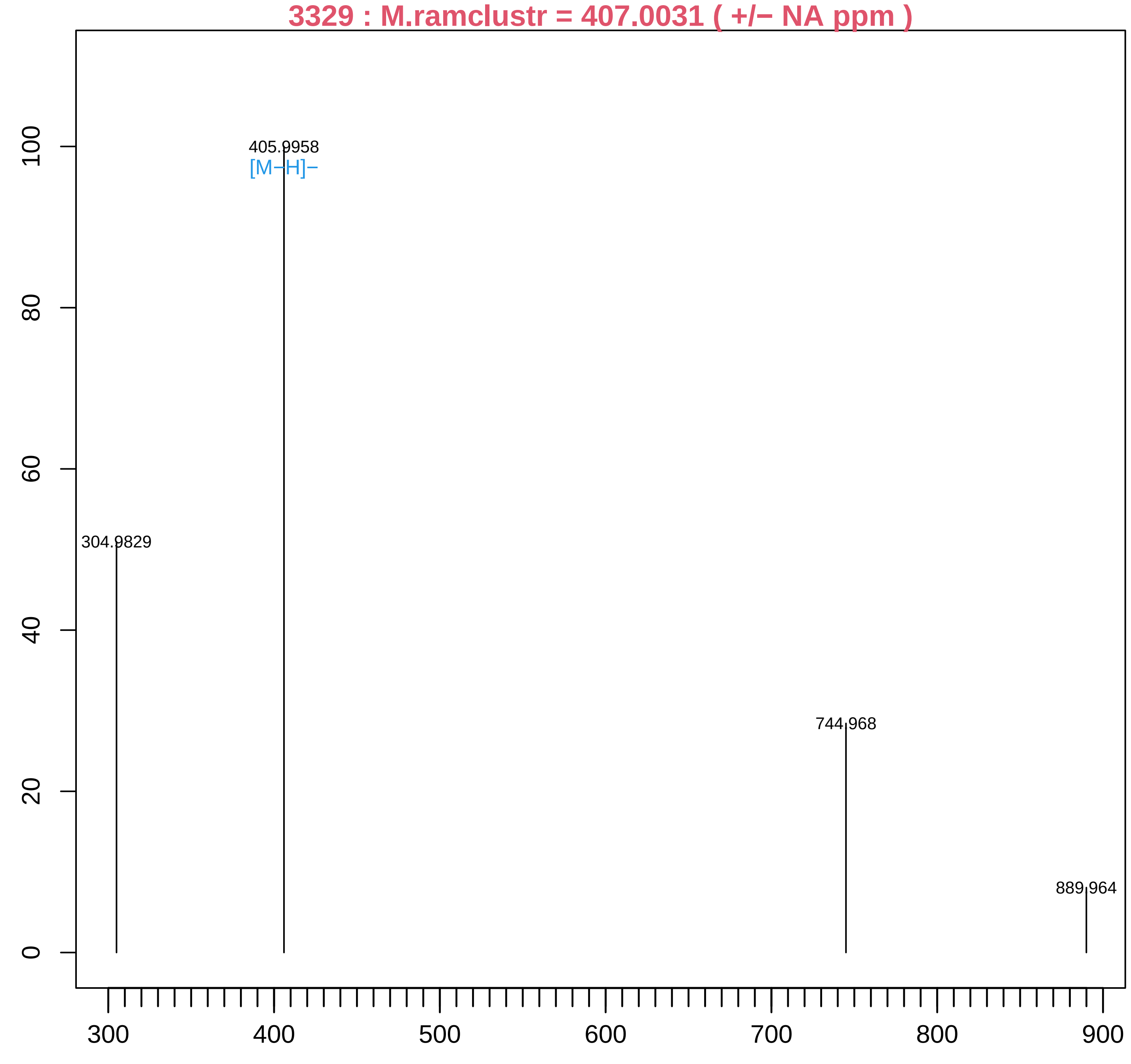
3329 : M.ramclustr = 407.0031 (+/− NA ppm)
405.9958
[M−H]−
304.9829
744.968
889.964
0
20
40
60
80
100
300
400
500
600
700
800
900

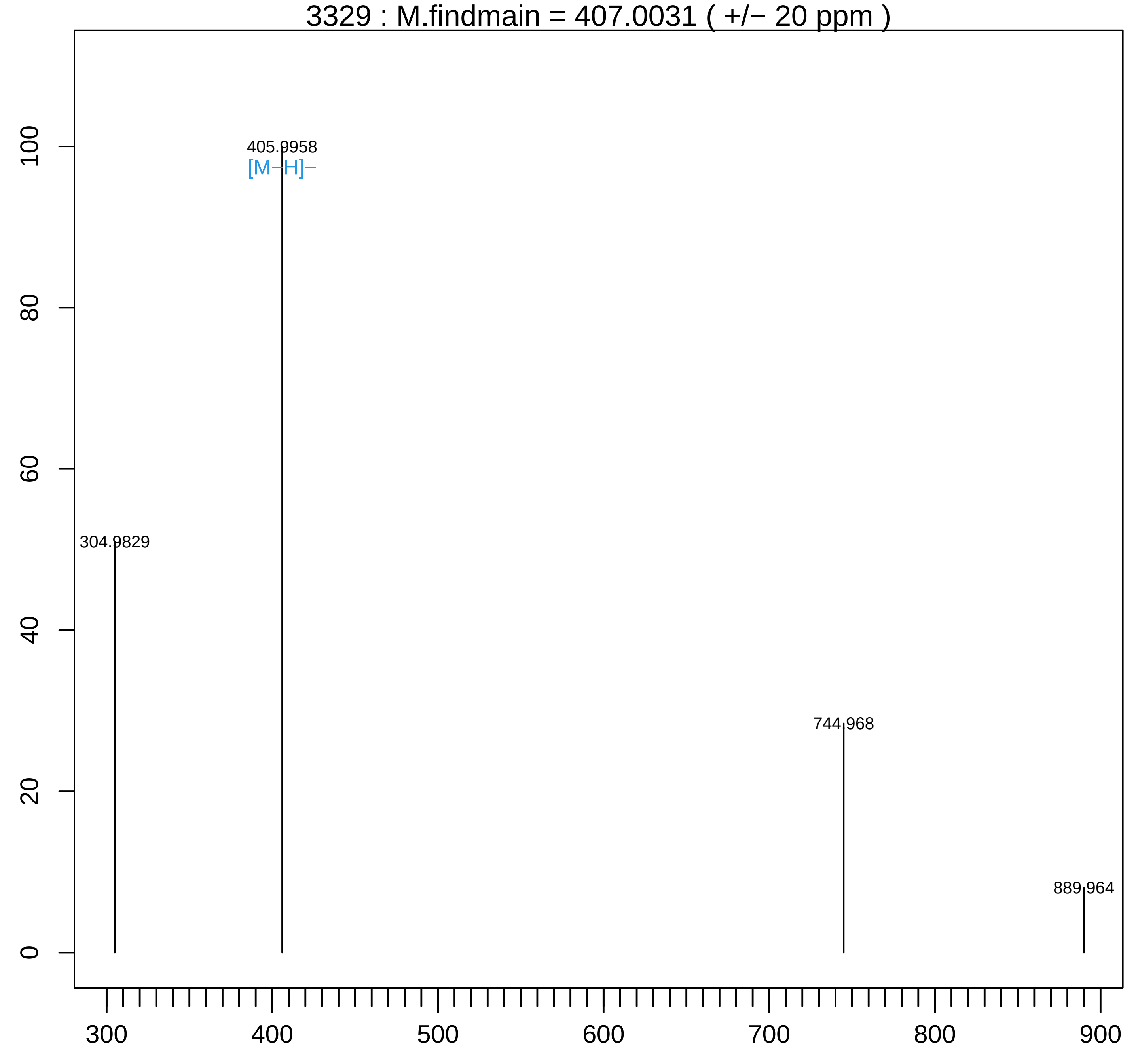
3329 : M.findmain = 407.0031 (+/− 20 ppm)
405.9958
[M−H]−
304.9829
744.968
889.964
0
20
40
60
80
100
300
400
500
600
700
800
900

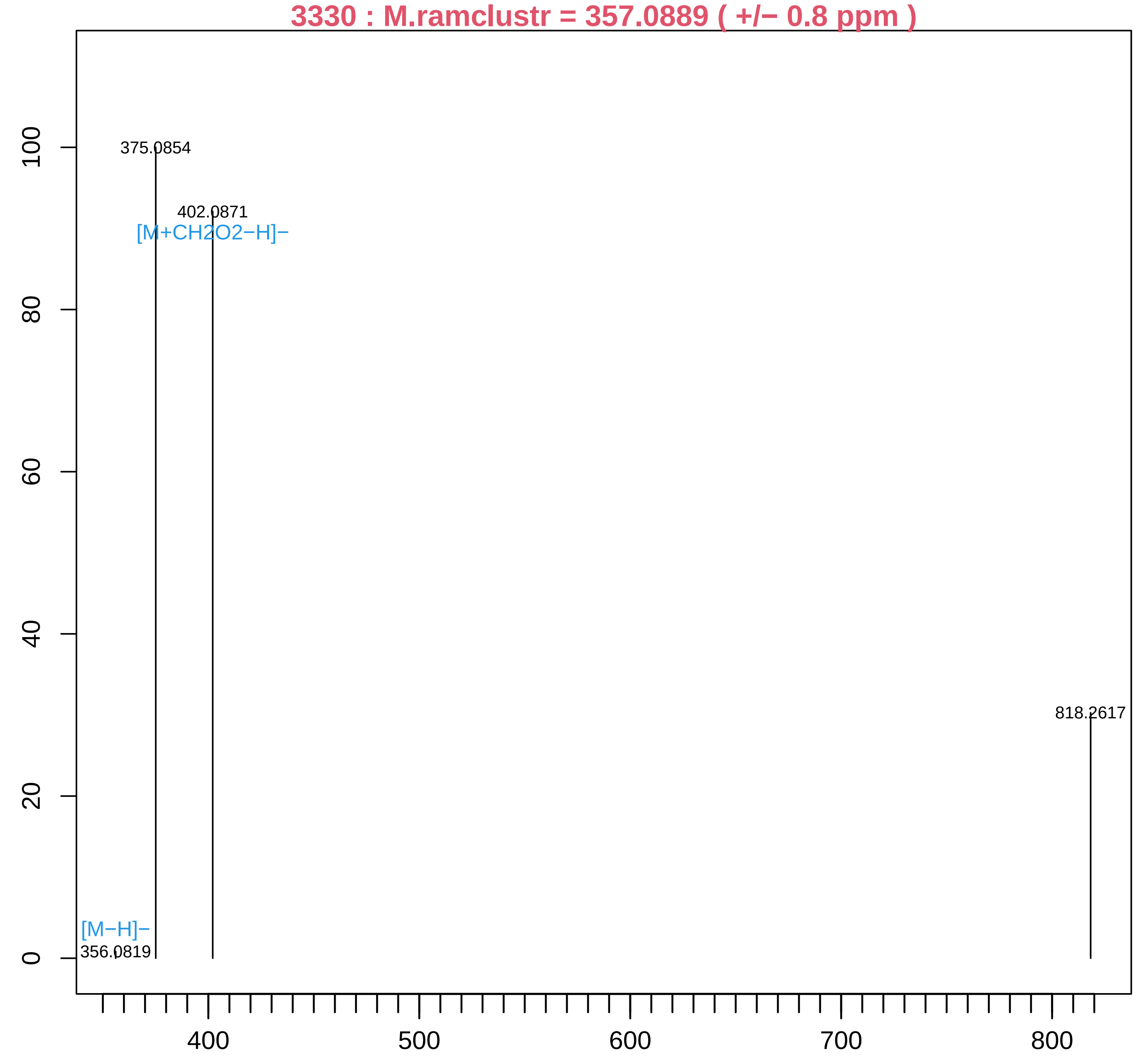
3330 : M.ramclustr = 357.0889 (+/− 0.8 ppm)
375.0854
402.0871
[M+CH2O2−H]−
818.2617
[M−H]−
356.0819
0
20
40
60
80
100
400
500
600
700
800

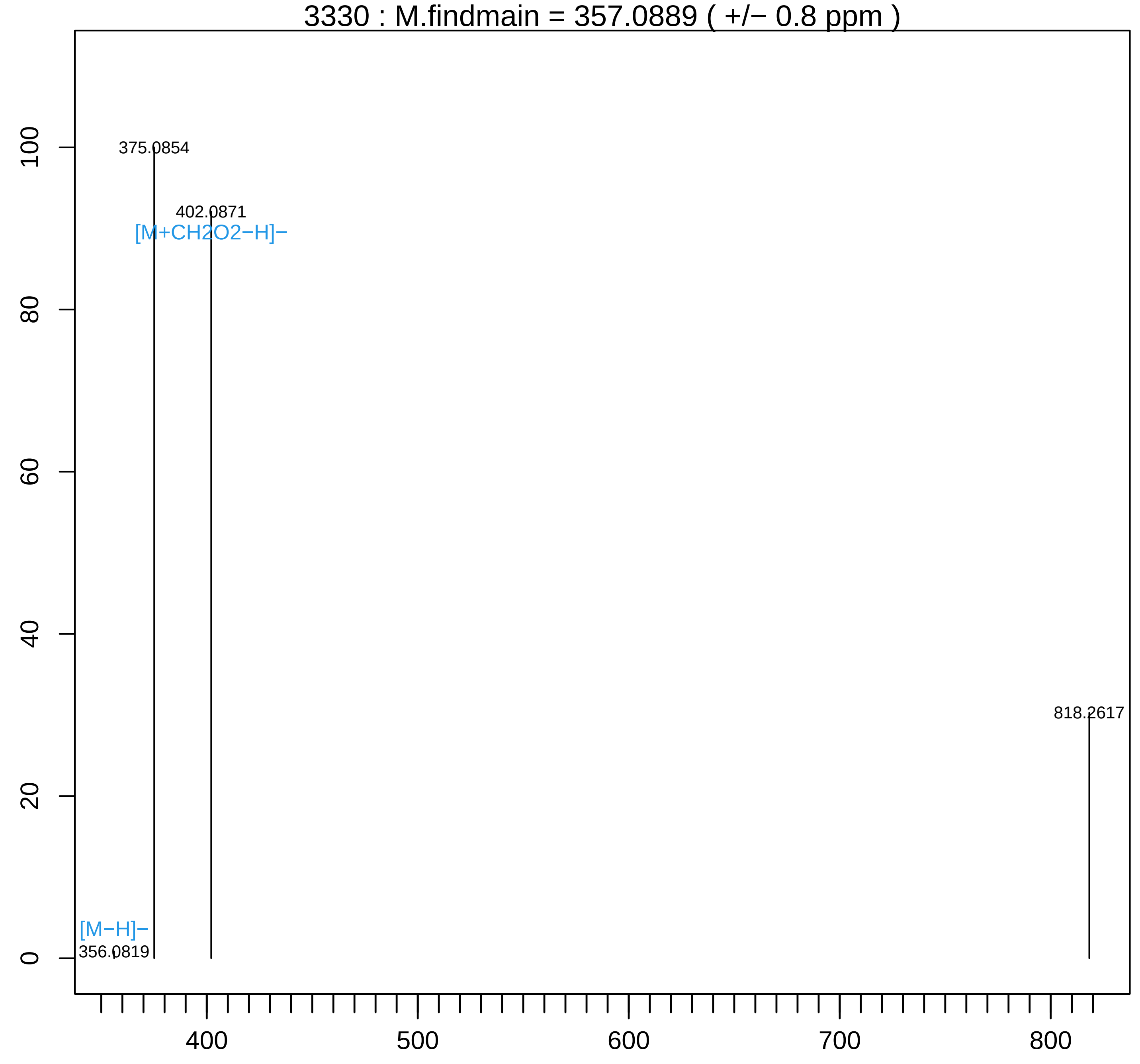
3330 : M.findmain = 357.0889 (+/− 0.8 ppm)
375.0854
402.0871
[M+CH2O2−H]−
818.2617
[M−H]−
356.0819
0
20
40
60
80
100
400
500
600
700
800

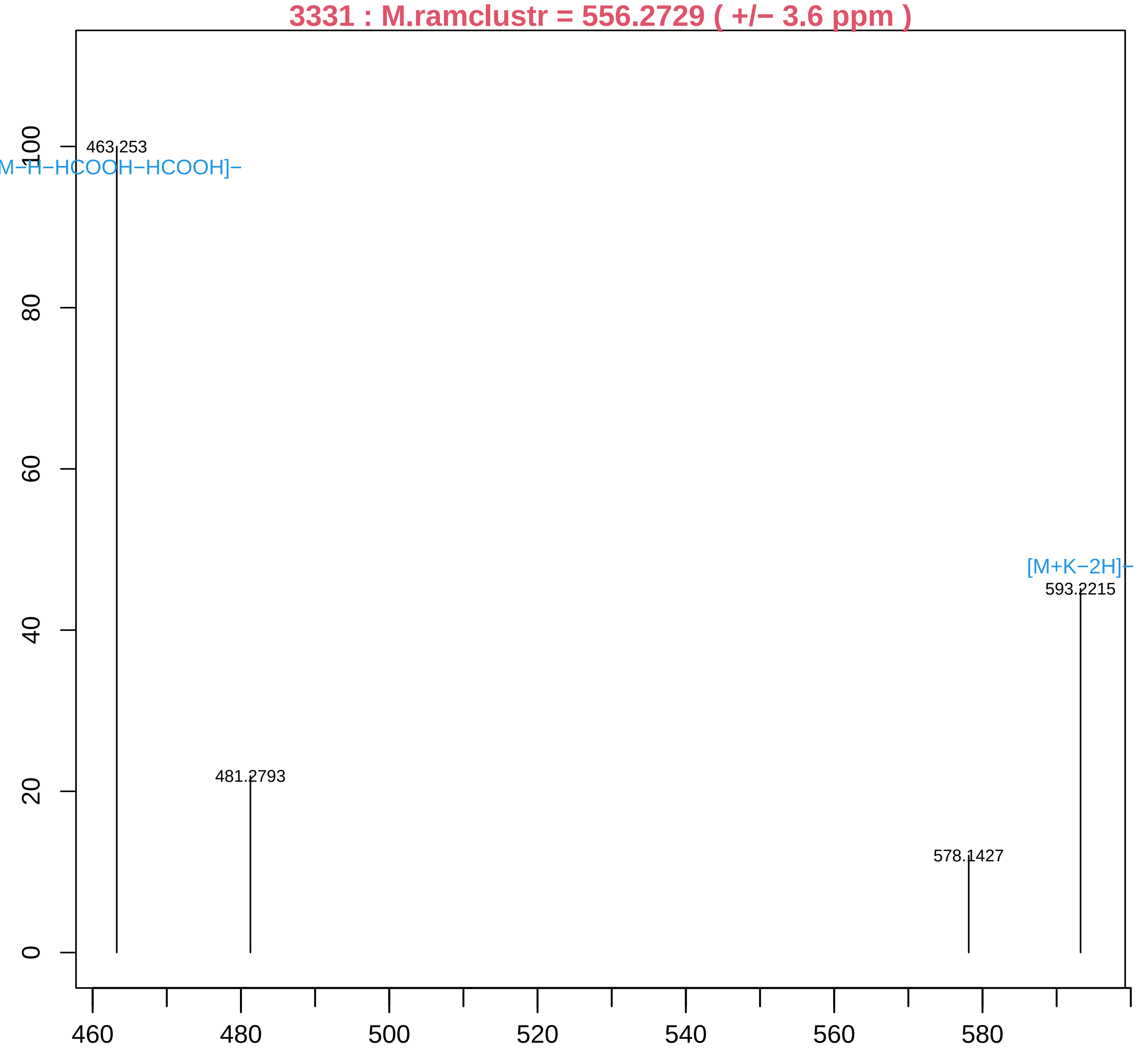
3331 : M.ramclustr = 556.2729 (+/− 3.6 ppm)
463.253
[M−H−HCOOH−HCOOH]−
481.2793
578.1427
[M+K−2H]−
593.2215
0
20
40
60
80
100
460
480
500
520
540
560
580

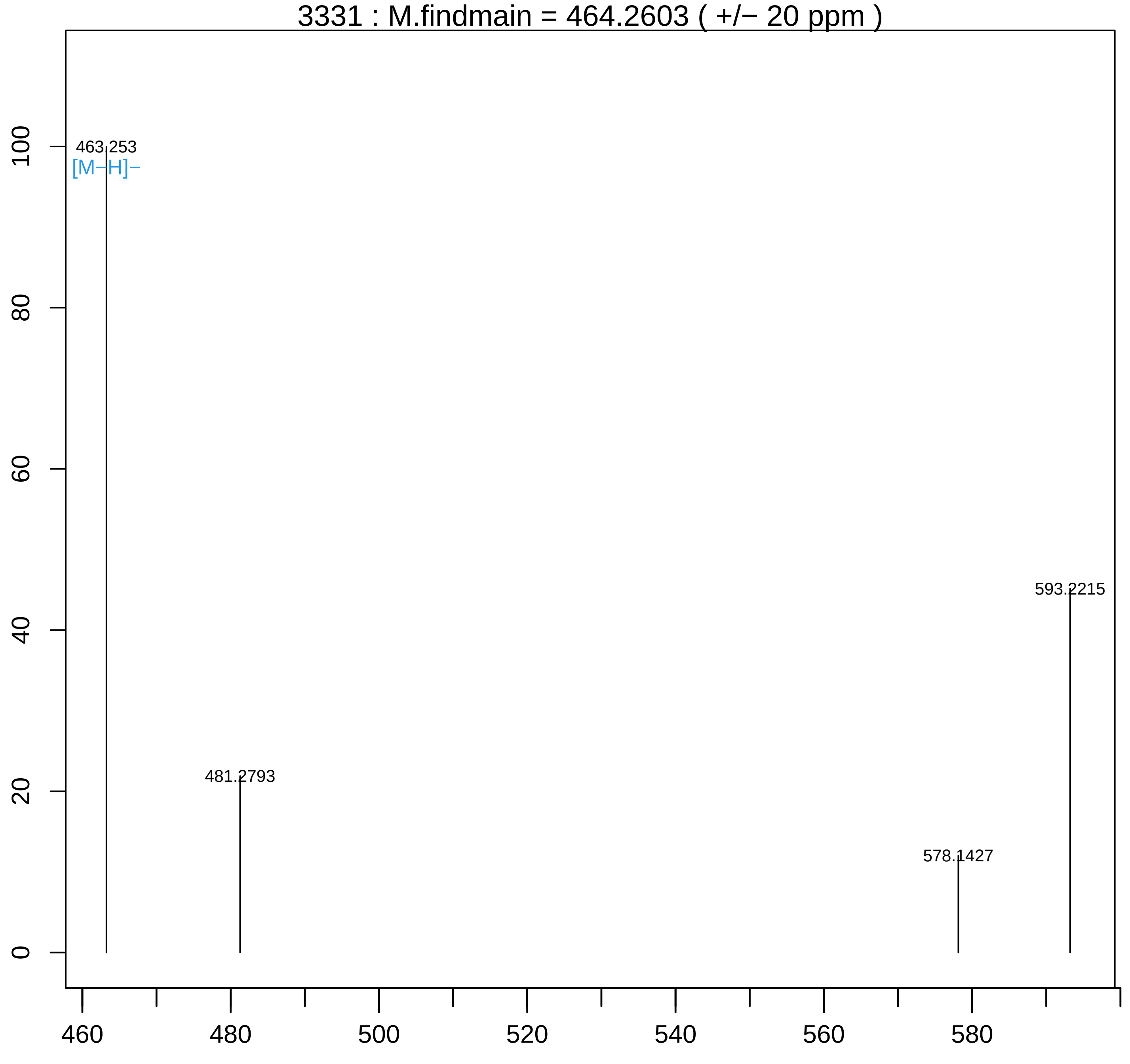
3331 : M.findmain = 464.2603 (+/− 20 ppm)
463.253
[M−H]−
481.2793
578.1427
593.2215
0
20
40
60
80
100
460
480
500
520
540
560
580

3332 : M.ramclustr = 969.5299 (+/− 1.9 ppm)

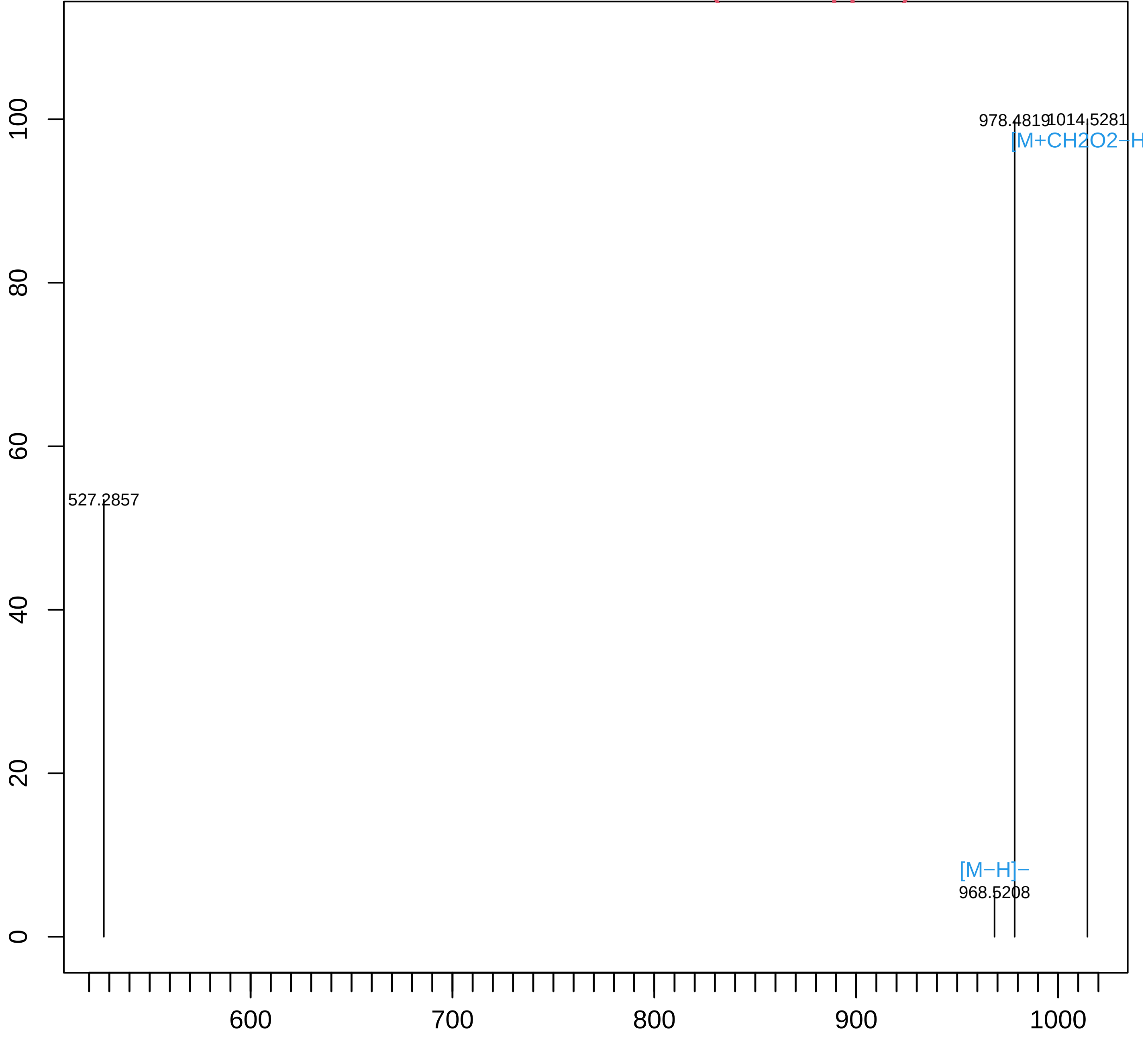

3332 : M.findmain = 969.5299 (+/− 1.9 ppm)

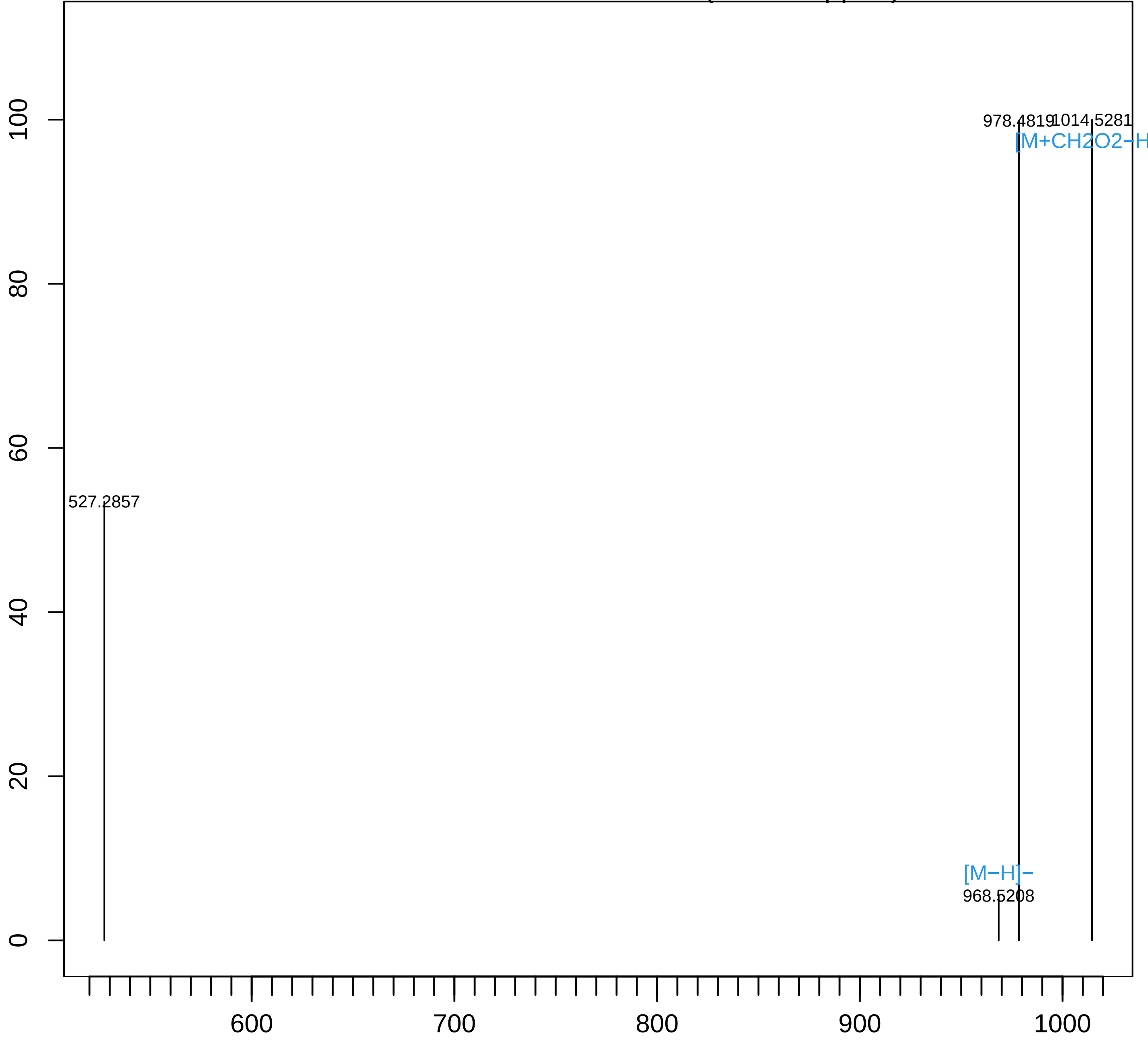

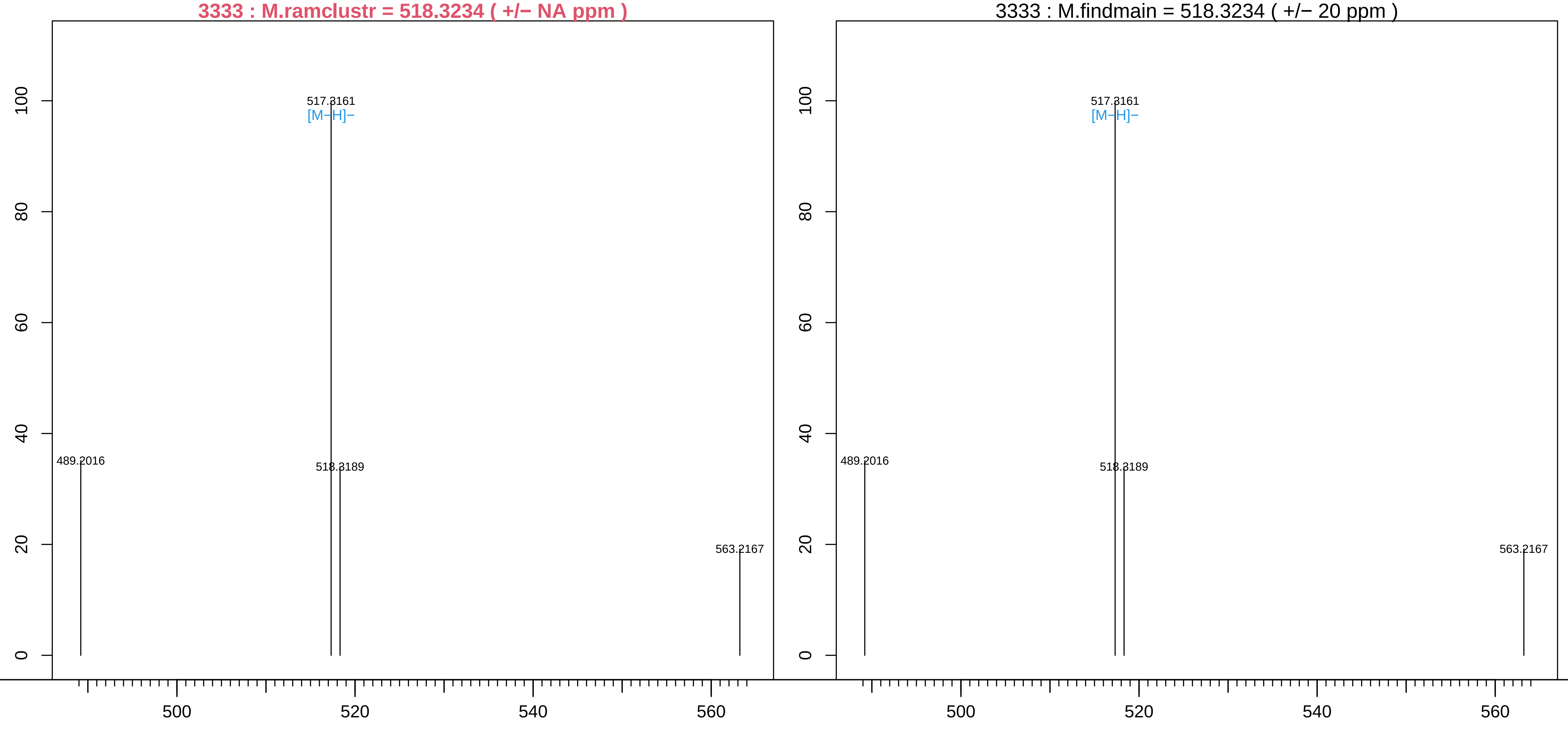
3333 : M.ramclustr = 518.3234 (+/− NA ppm)
517.3161
[M−H]−
489.2016
518.3189
563.2167
3333 : M.findmain = 518.3234 (+/− 20 ppm)
517.3161
[M−H]−
489.2016
518.3189
563.2167
0
20
40
60
80
100
500
520
540
560

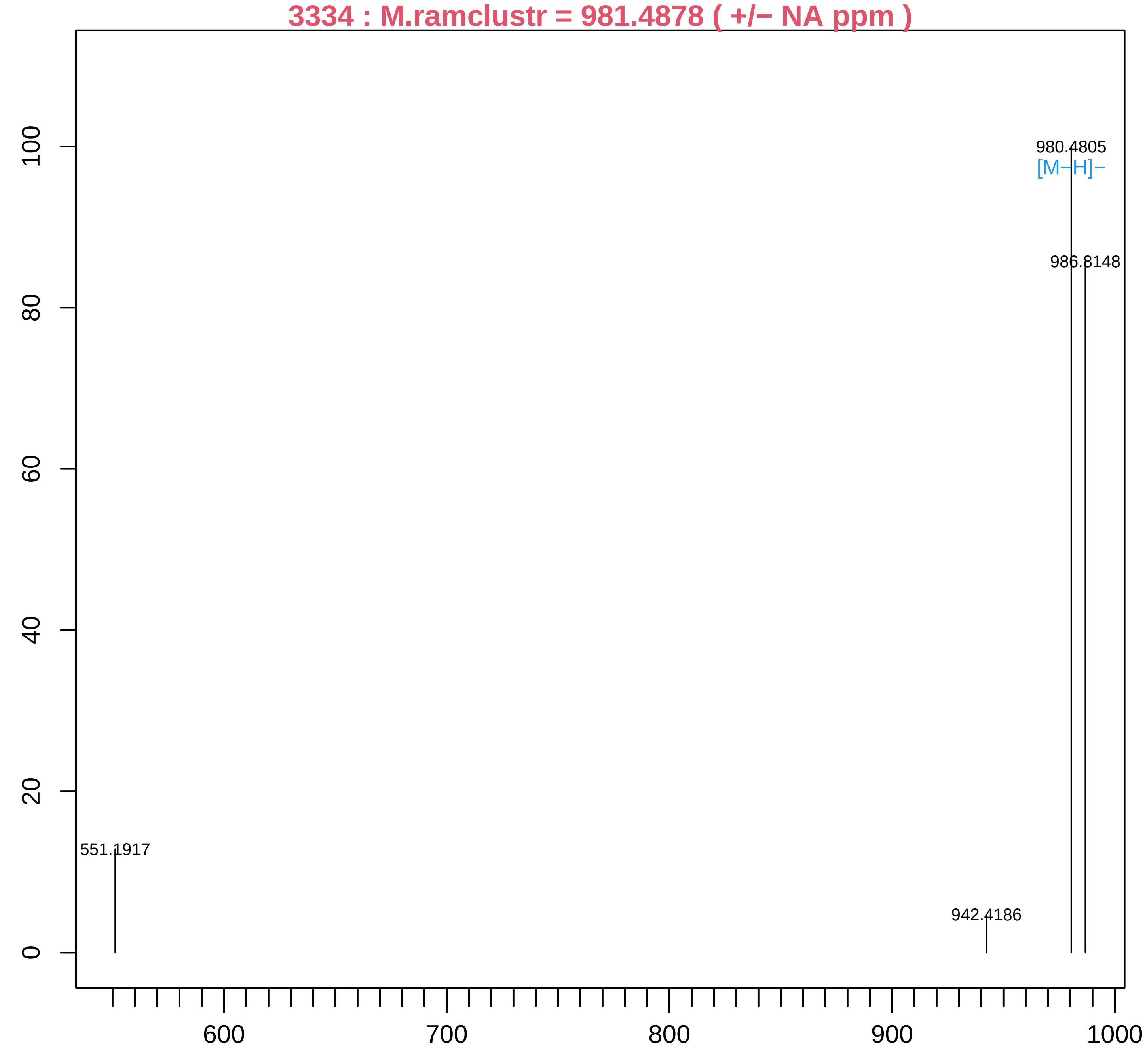
3334 : M.ramclustr = 981.4878 (+/− NA ppm)
980.4805
[M−H]−
986.8148
551.1917
942.4186
0
20
40
60
80
100
600
700
800
900
1000

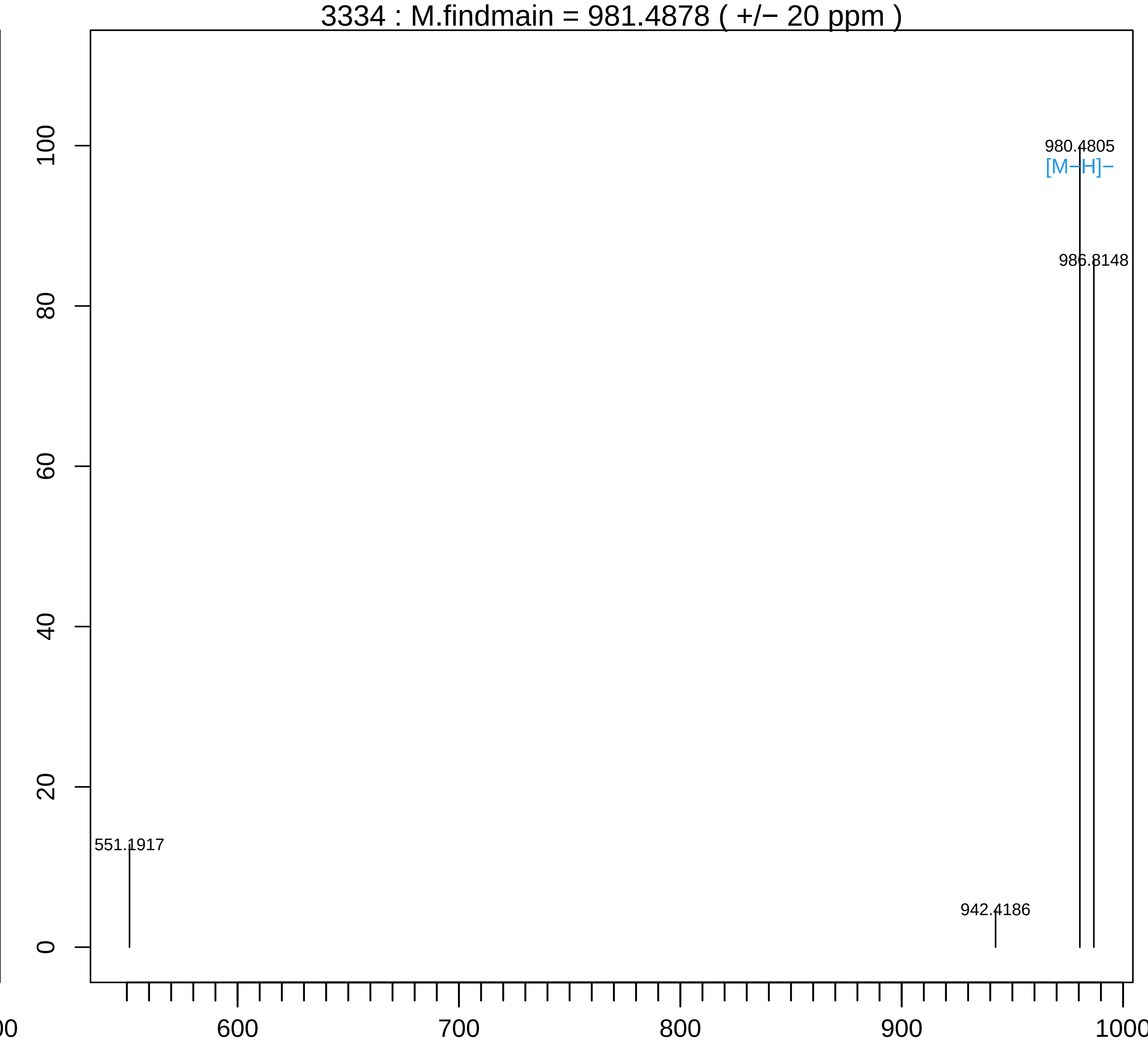
3334 : M.findmain = 981.4878 (+/− 20 ppm)
980.4805
[M−H]−
986.8148
551.1917
942.4186
0
20
40
60
80
100
600
700
800
900
1000

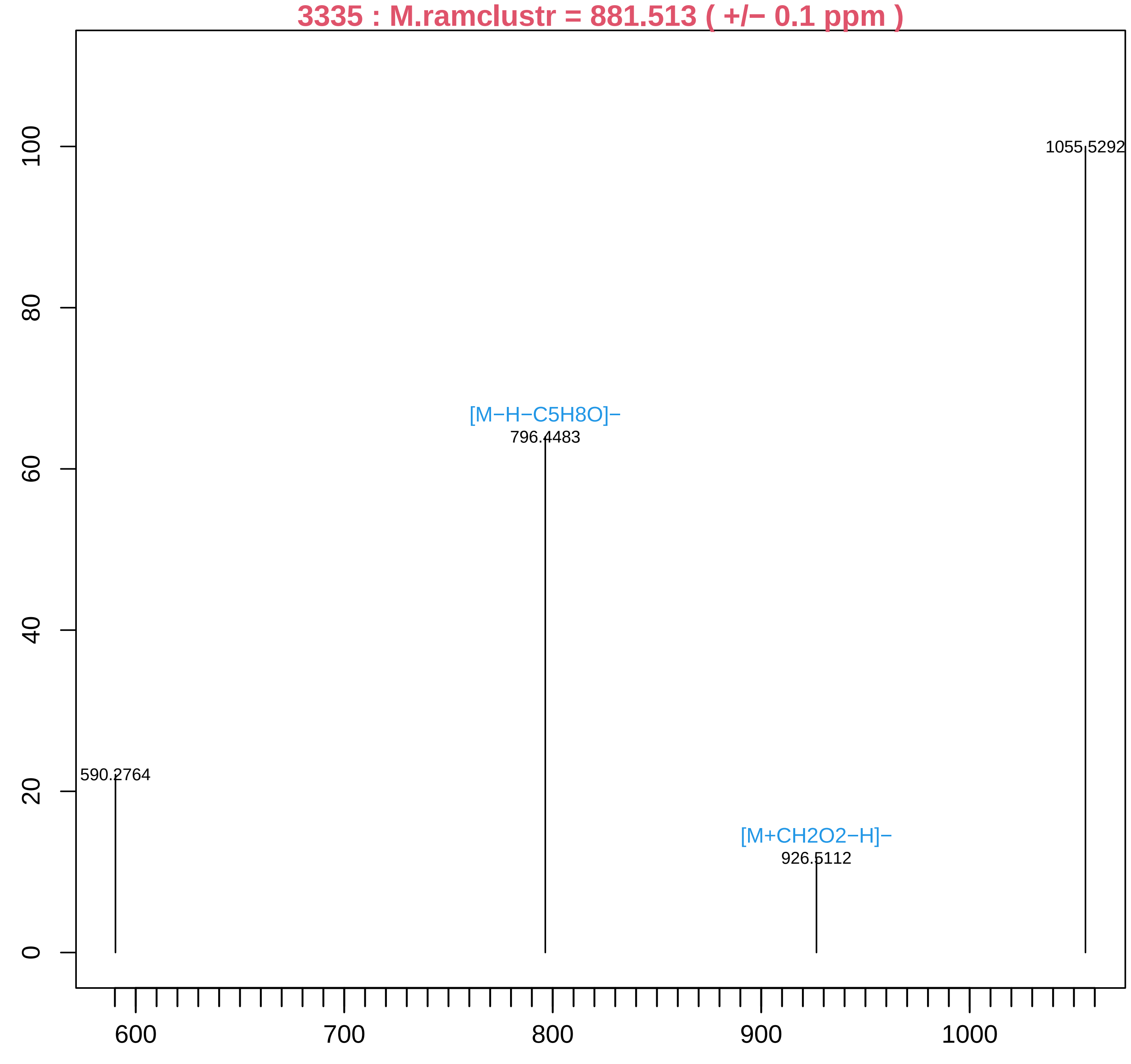
3335 : M.ramclustr = 881.513 (+/− 0.1 ppm)
1055.5292
[M−H−C5H8O]−
796.4483
590.2764
[M+CH2O2−H]−
926.5112
0
20
40
60
80
100
600
700
800
900
1000

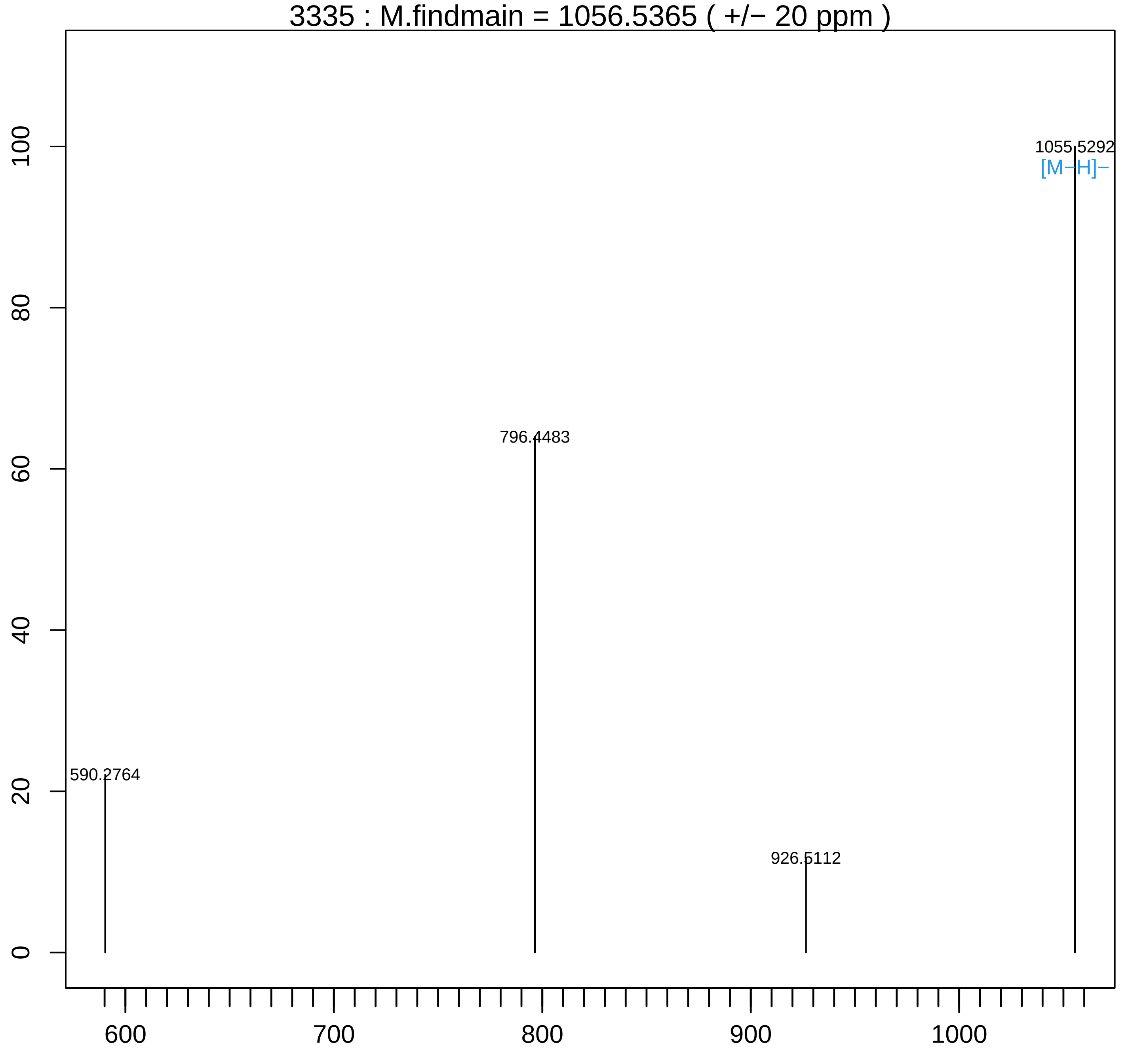
3335 : M.findmain = 1056.5365 (+/− 20 ppm)
1055.5292
[M−H]−
796.4483
590.2764
926.5112
0
20
40
60
80
100
600
700
800
900
1000

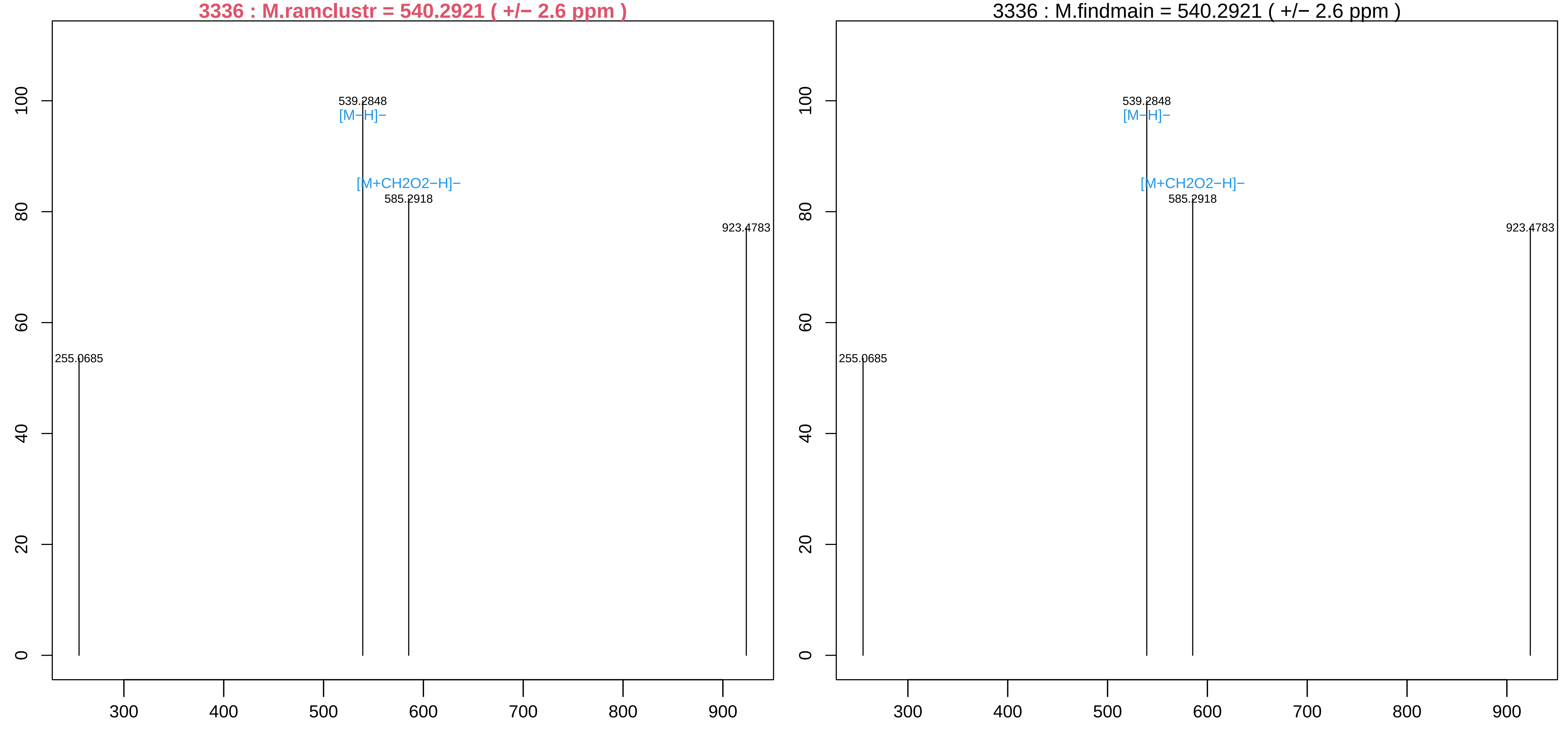
3336 : M.ramclustr = 540.2921 (+/− 2.6 ppm)
3336 : M.findmain = 540.2921 (+/− 2.6 ppm)
539.2848
[M−H]−
[M+CH2O2−H]−
585.2918
923.4783
255.0685
0
20
40
60
80
100
300
400
500
600
700
800
900

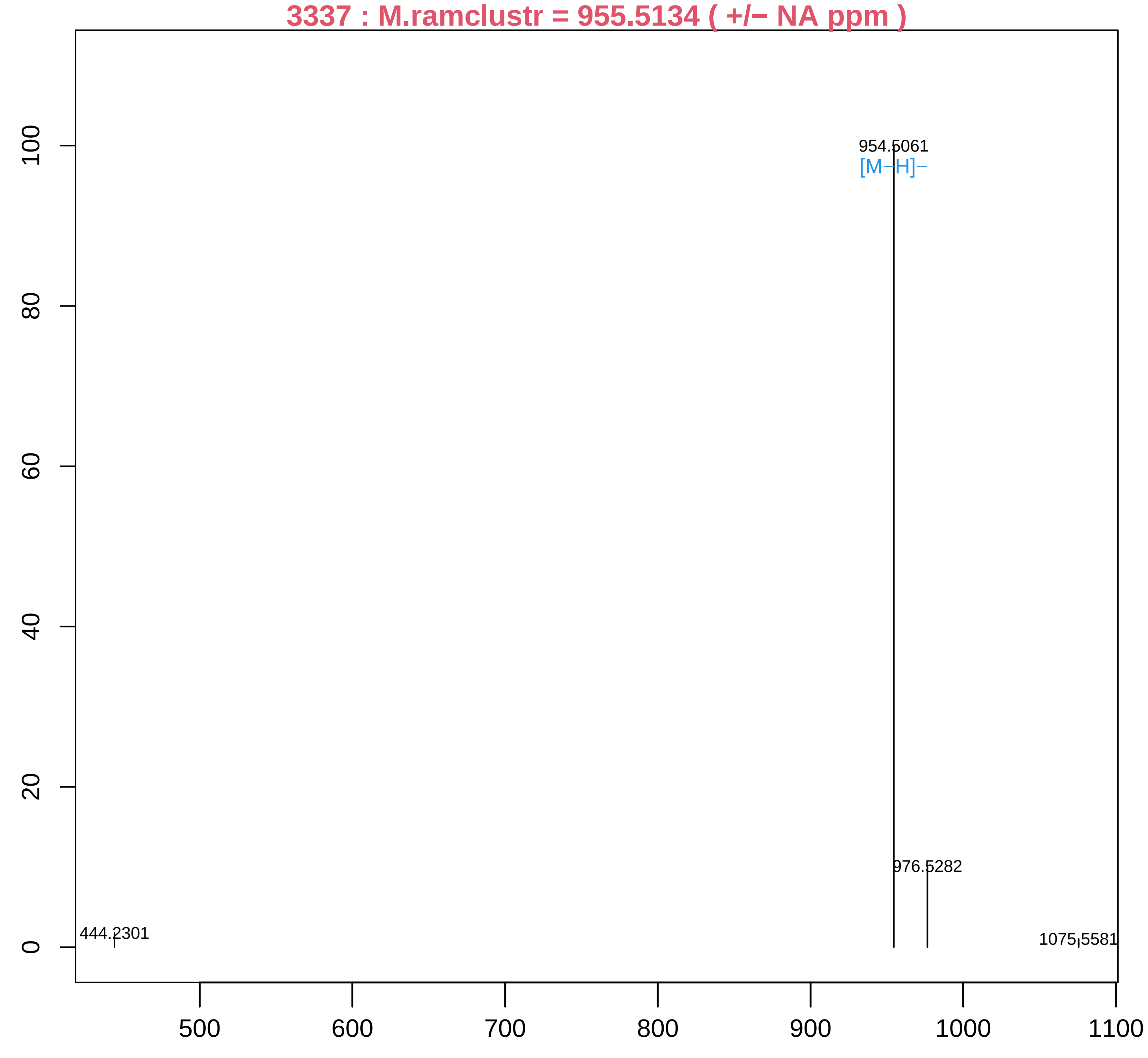
3337 : M.ramclustr = 955.5134 (+/− NA ppm)
954.5061
[M−H]−
976.5282
444.2301
1075.5581
0
20
40
60
80
100
500
600
700
800
900
1000
1100

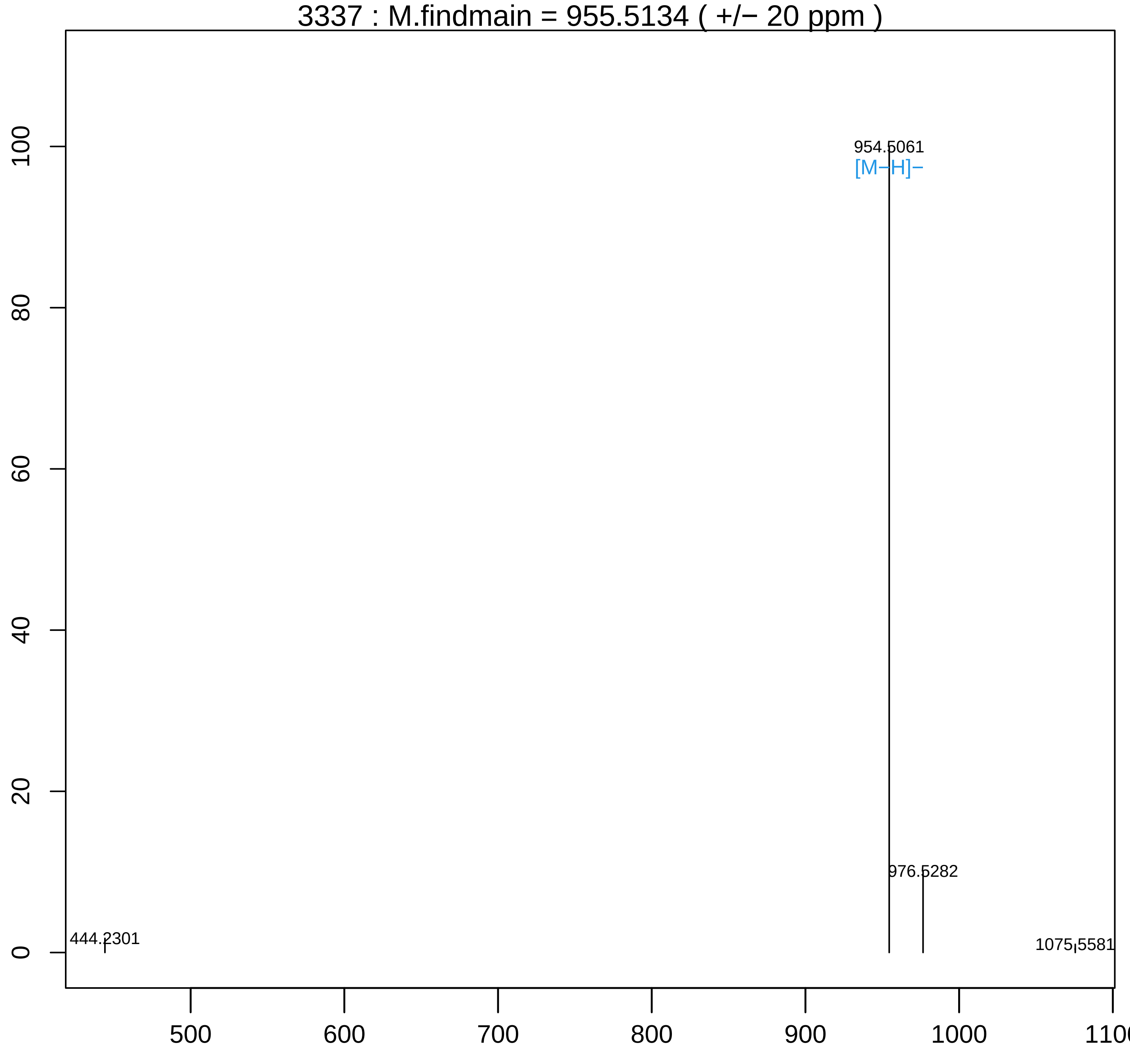
3337 : M.findmain = 955.5134 (+/− 20 ppm)
954.5061
[M−H]−
976.5282
444.2301
1075.5581
0
20
40
60
80
100
500
600
700
800
900
1000
1100

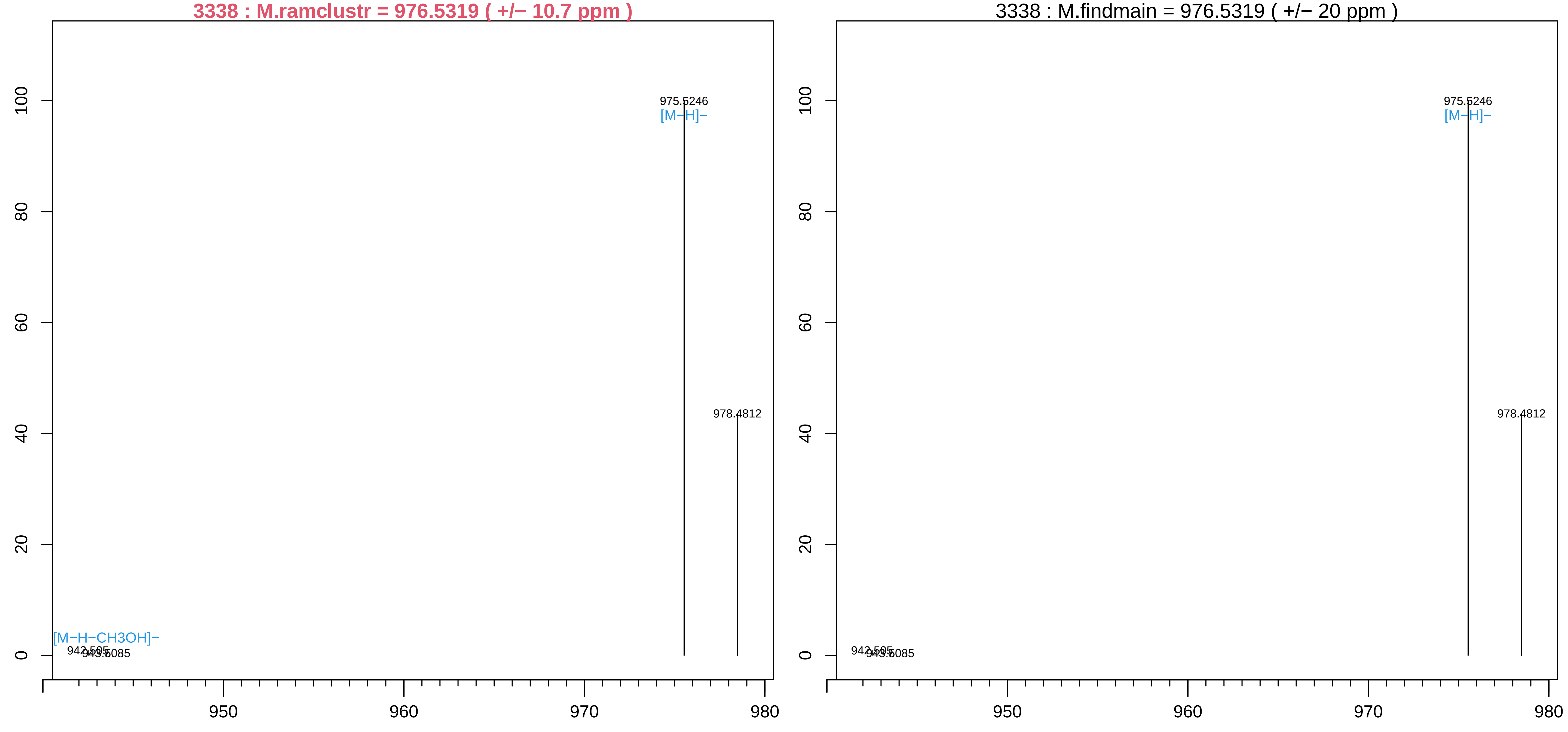
3338 : M.ramclustr = 976.5319 (+/− 10.7 ppm)
975.5246
[M−H]−
978.4812
[M−H−CH3OH]−
942.505
943.5085
0
20
40
60
80
100
950
960
970
980
3338 : M.findmain = 976.5319 (+/− 20 ppm)
975.5246
[M−H]−
978.4812
942.505
943.5085
0
20
40
60
80
100
950
960
970
980

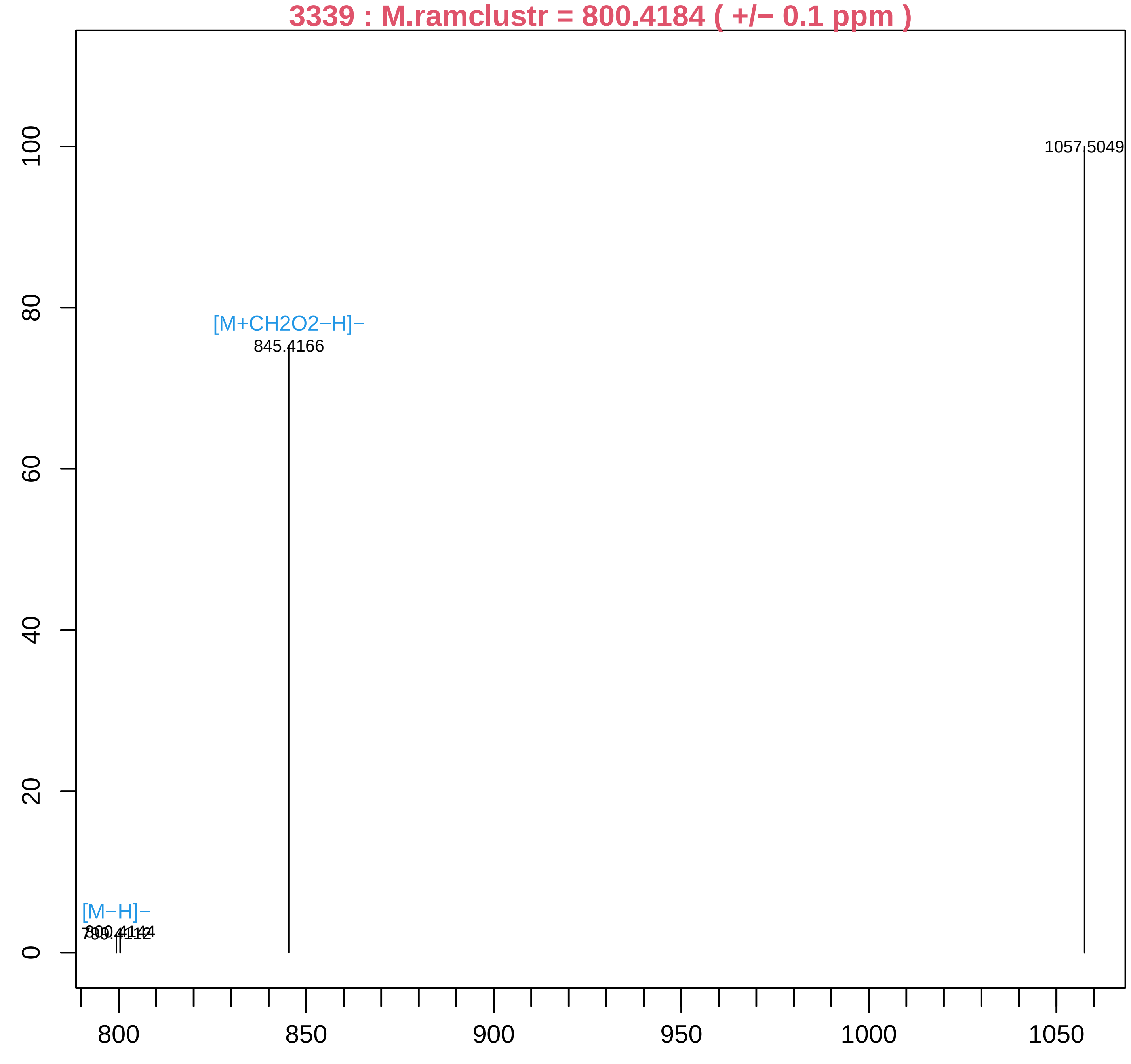
3339 : M.ramclustr = 800.4184 (+/− 0.1 ppm)
[M+CH2O2−H]−
845.4166
1057.5049
[M−H]−
800.4144
799.4112
0
20
40
60
80
100
800
850
900
950
1000
1050

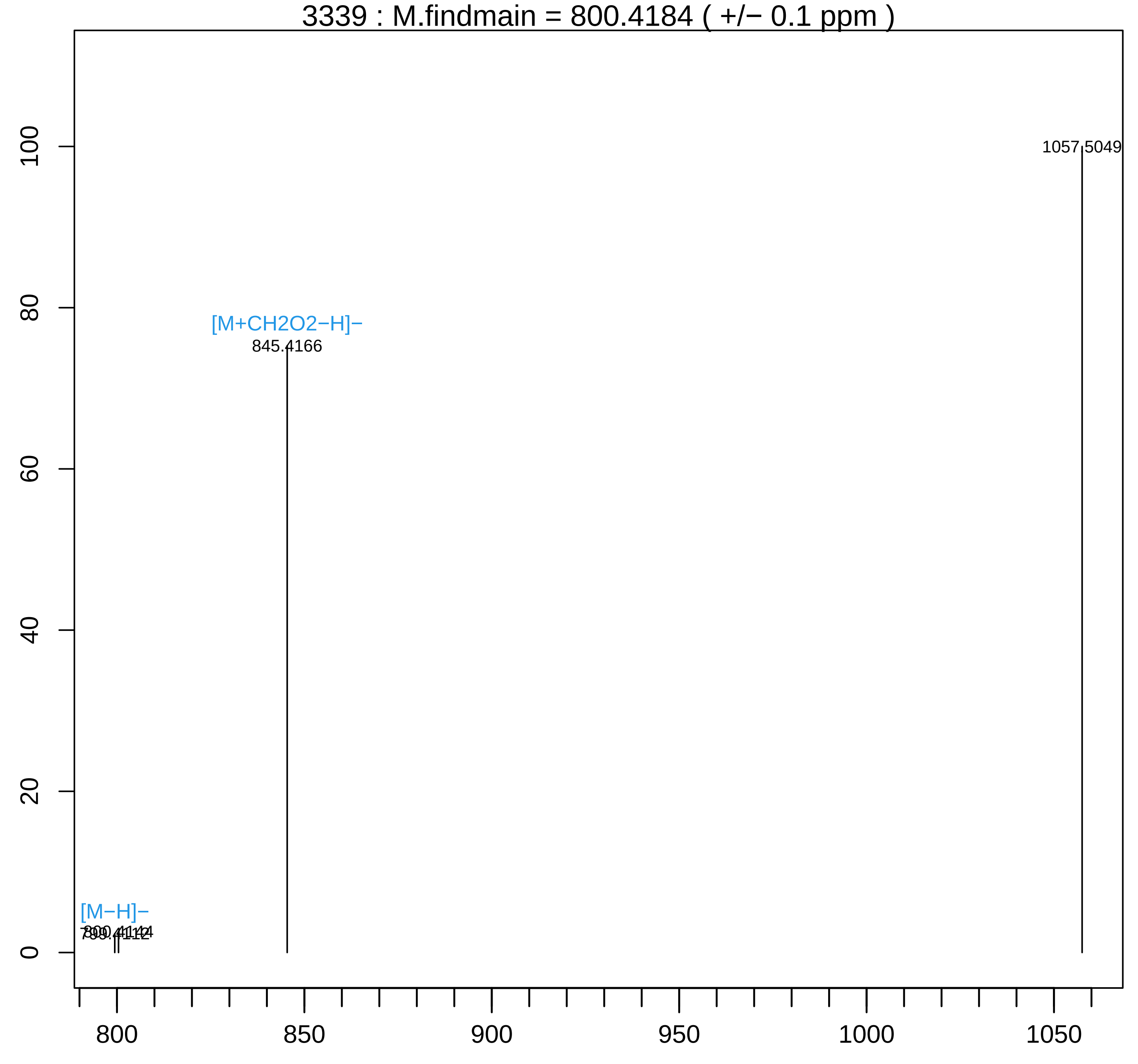
3339 : M.findmain = 800.4184 (+/− 0.1 ppm)
[M+CH2O2−H]−
845.4166
1057.5049
[M−H]−
800.4144
799.4112
0
20
40
60
80
100
800
850
900
950
1000
1050

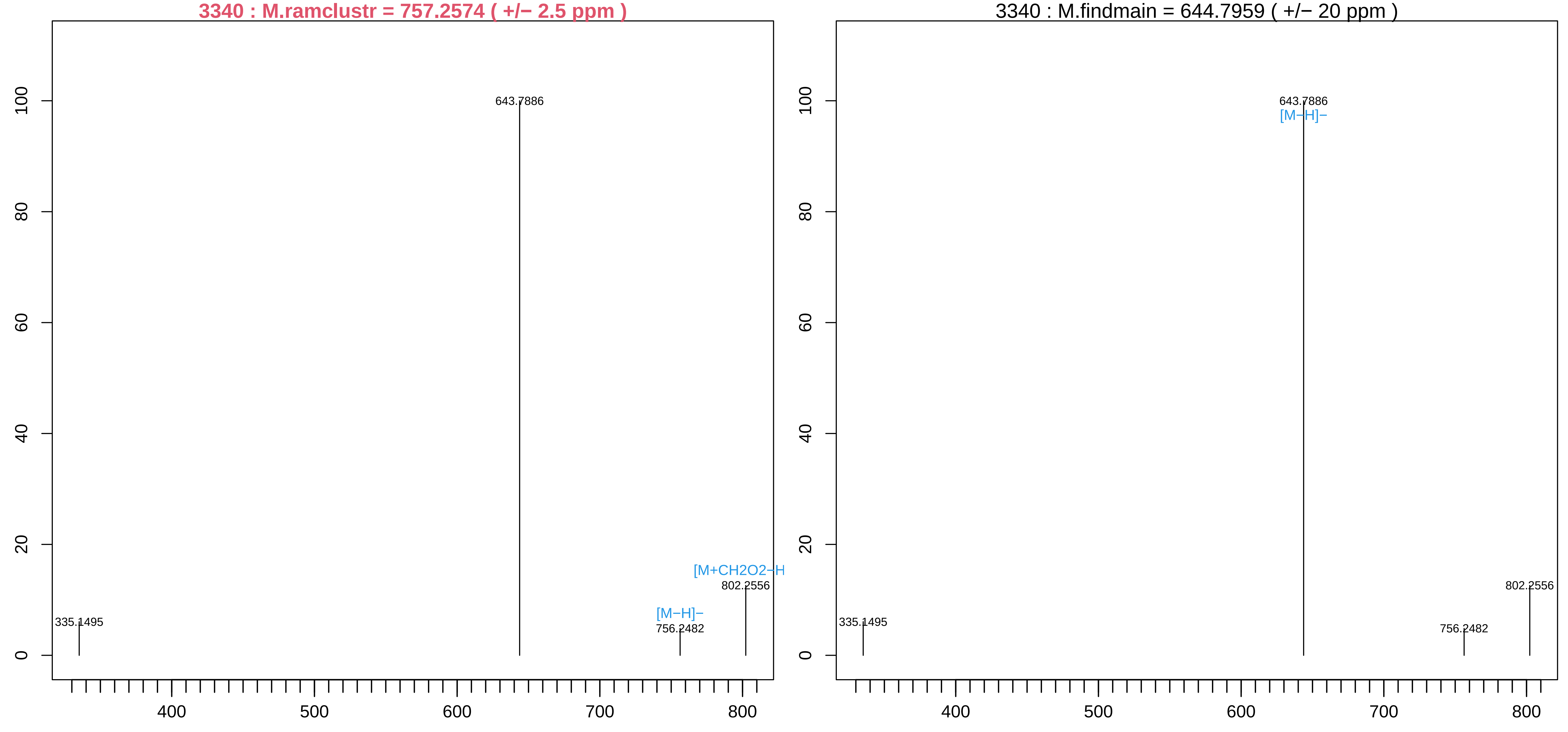
3340 : M.ramclustr = 757.2574 (+/− 2.5 ppm)
643.7886
[M+CH2O2−H
802.2556
[M−H]−
756.2482
335.1495
3340 : M.findmain = 644.7959 (+/− 20 ppm)
643.7886
[M−H]−
335.1495
756.2482
802.2556
0
20
40
60
80
100
400
500
600
700
800

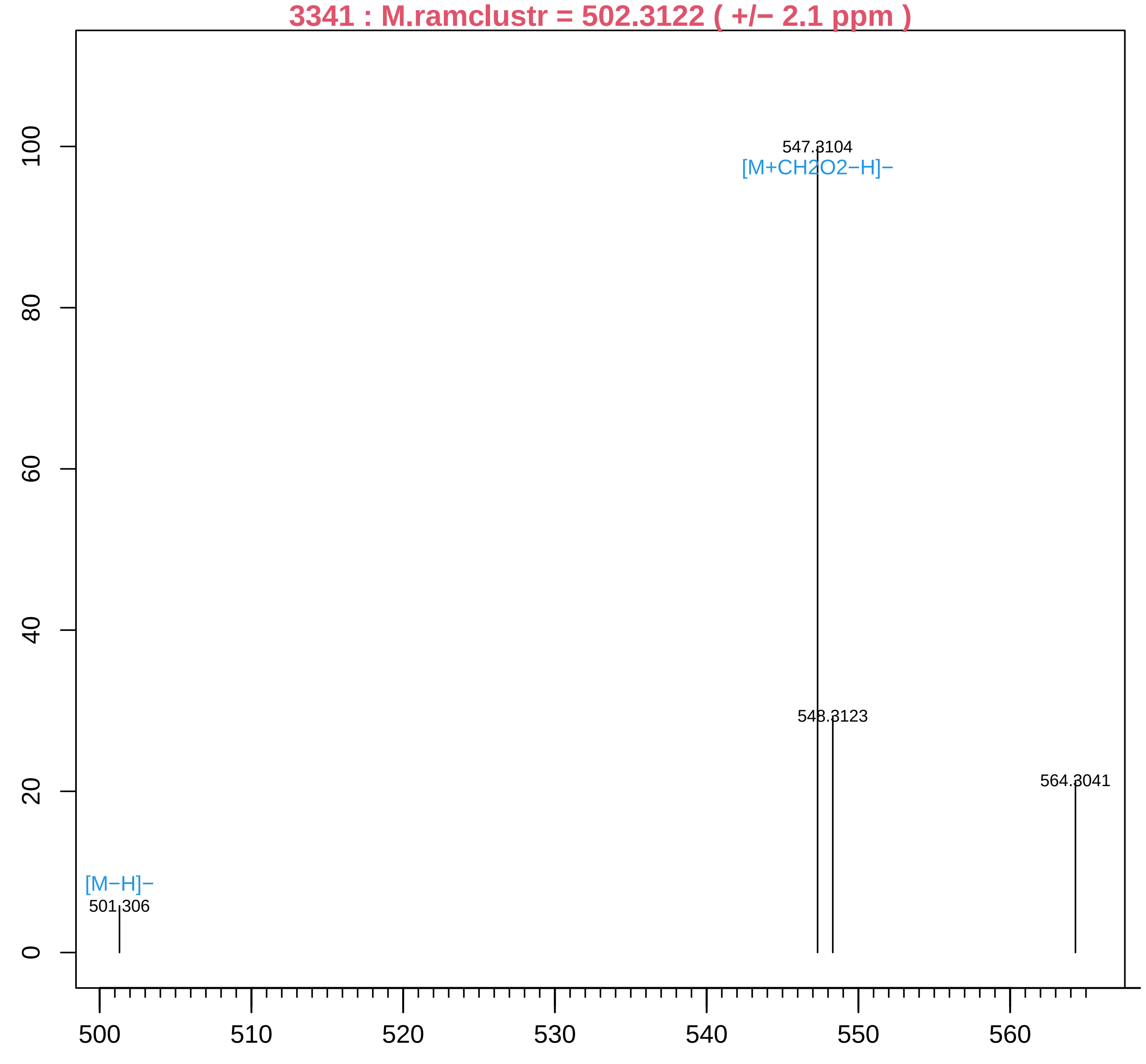
3341 : M.ramclustr = 502.3122 (+/− 2.1 ppm)
547.3104
[M+CH2O2−H]−
548.3123
564.3041
[M−H]−
501.306
0
20
40
60
80
100
500
510
520
530
540
550
560

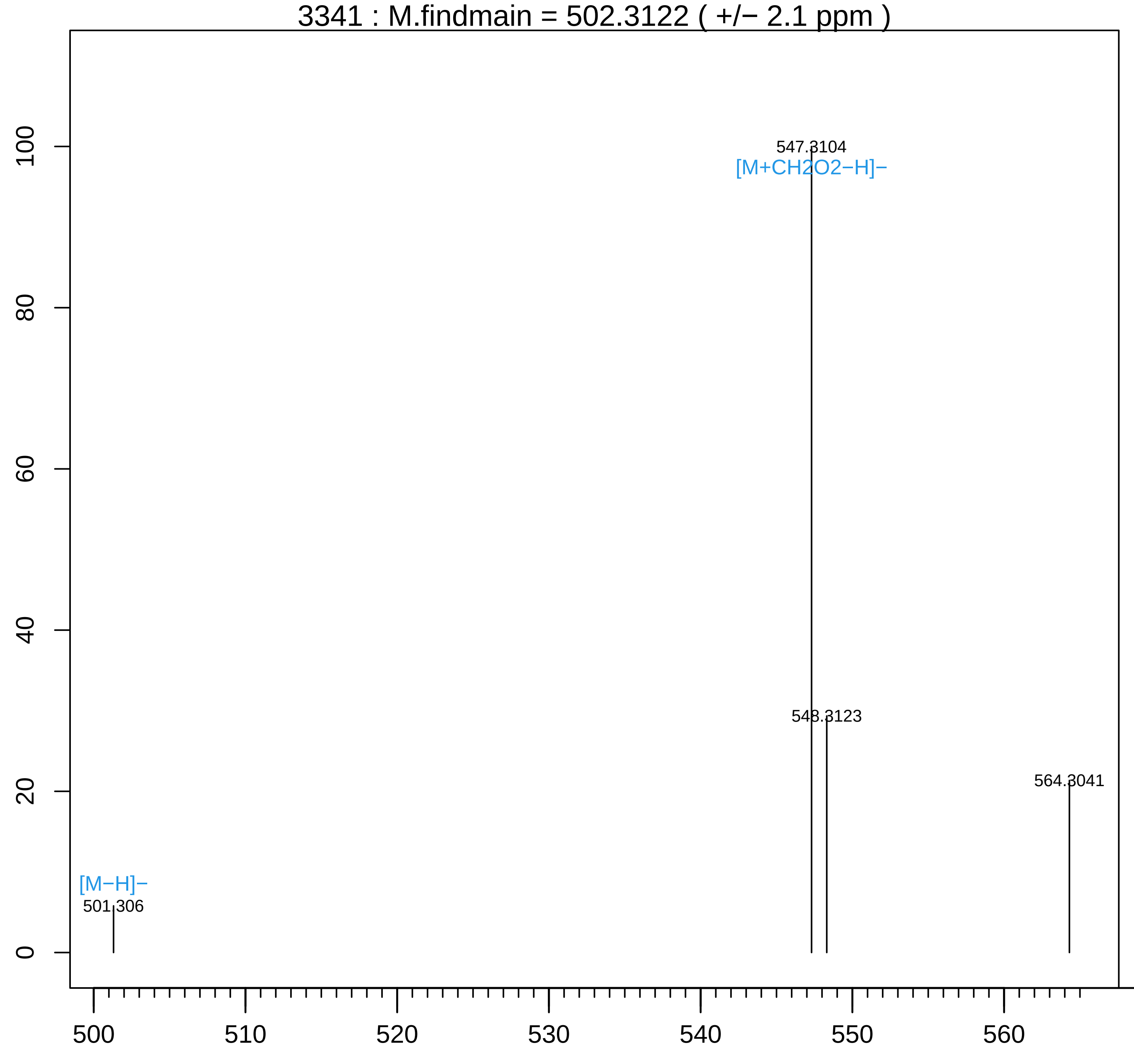
3341 : M.findmain = 502.3122 (+/− 2.1 ppm)
547.3104
[M+CH2O2−H]−
548.3123
564.3041
[M−H]−
501.306
0
20
40
60
80
100
500
510
520
530
540
550
560

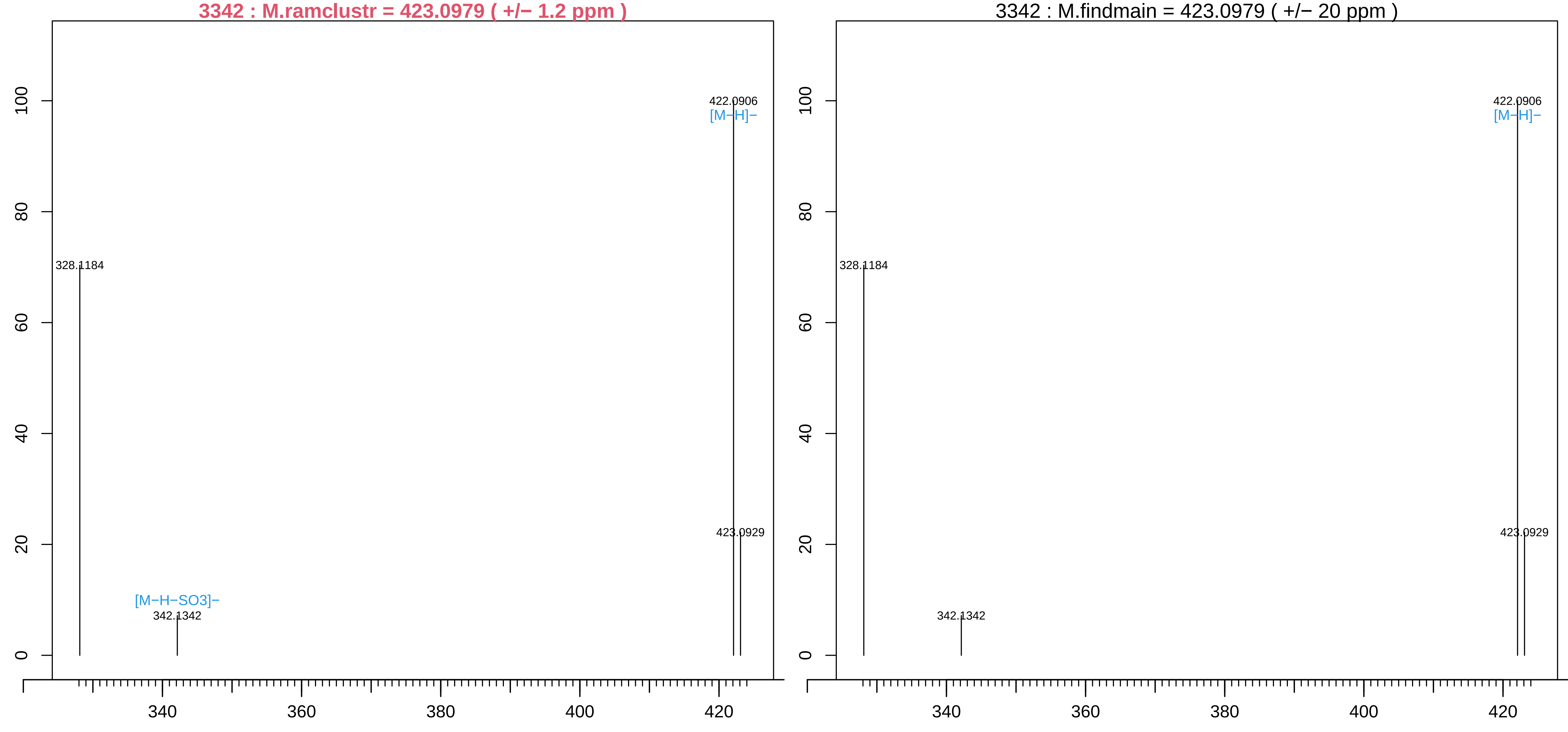
3342 : M.ramclustr = 423.0979 (+/− 1.2 ppm)
328.1184
[M−H−SO3]−
342.1342
422.0906
[M−H]−
423.0929
0
20
40
60
80
100
340
360
380
400
420
3342 : M.findmain = 423.0979 (+/− 20 ppm)
328.1184
342.1342
422.0906
[M−H]−
423.0929
0
20
40
60
80
100
340
360
380
400
420

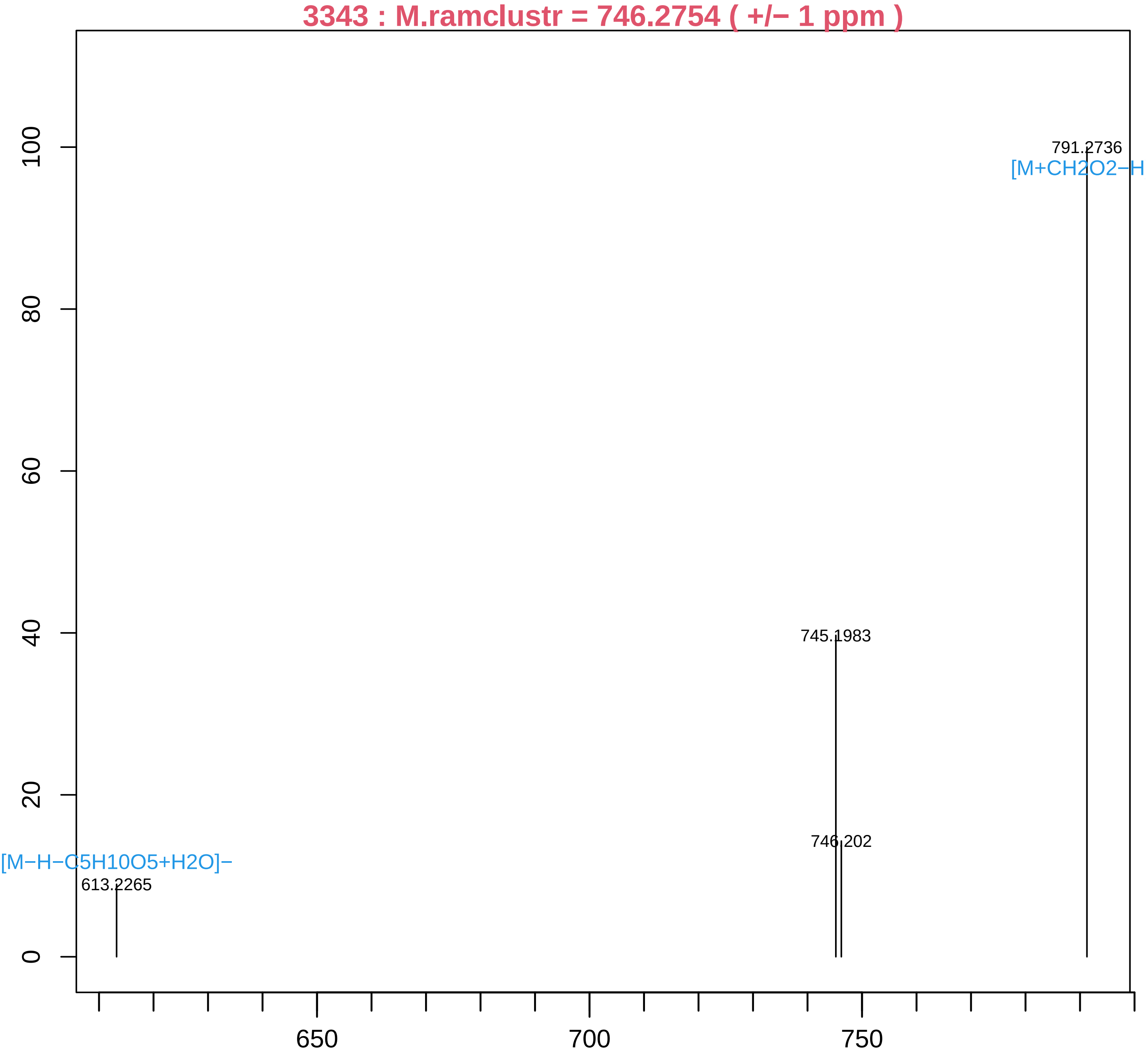
3343 : M.ramclustr = 746.2754 (+/− 1 ppm)
791.2736
[M+CH2O2−H
745.1983
746.202
[M−H−C5H10O5+H2O]−
613.2265
0
20
40
60
80
100
650
700
750

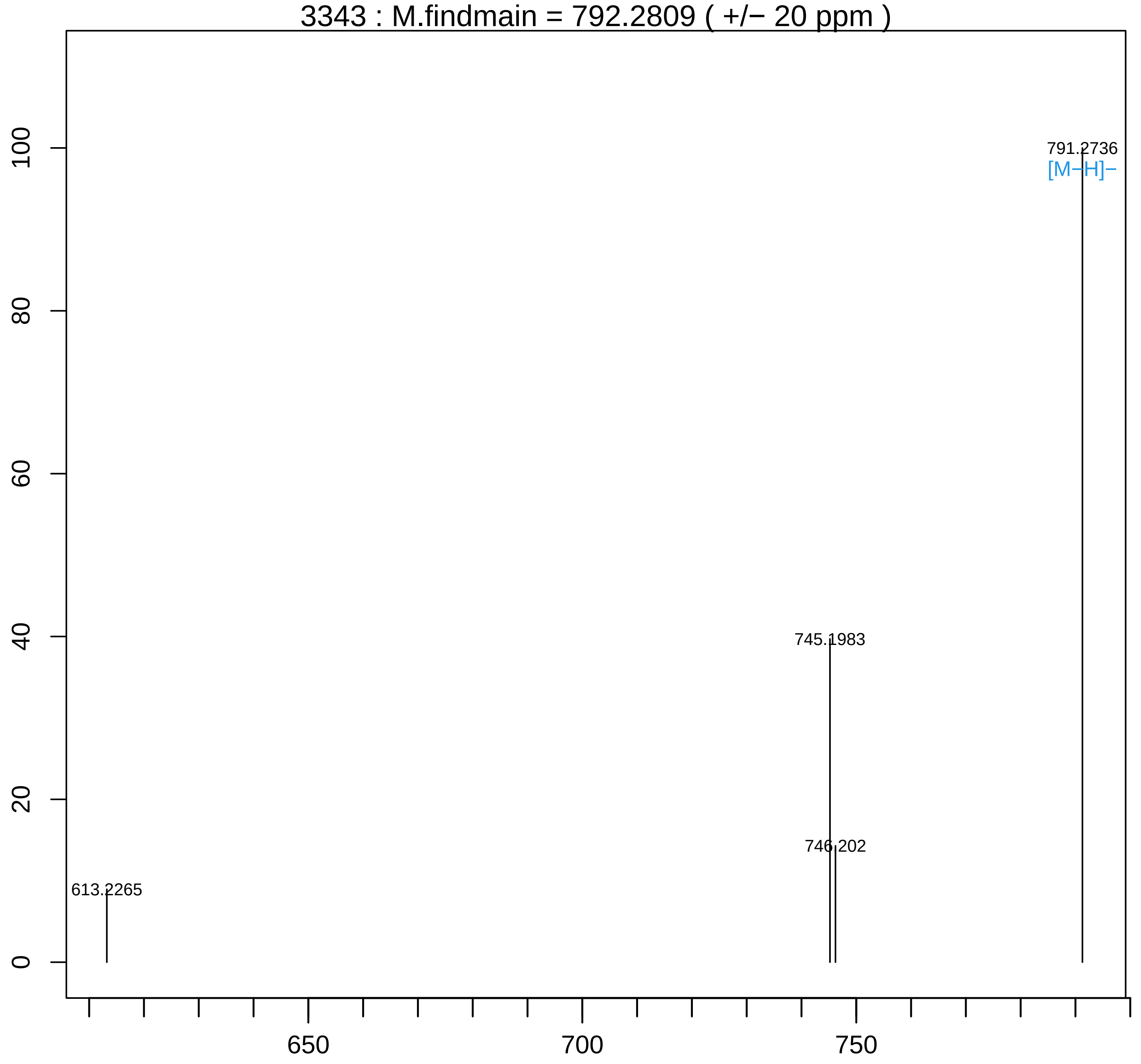
3343 : M.findmain = 792.2809 (+/− 20 ppm)
791.2736
[M−H]−
745.1983
746.202
613.2265
0
20
40
60
80
100
650
700
750

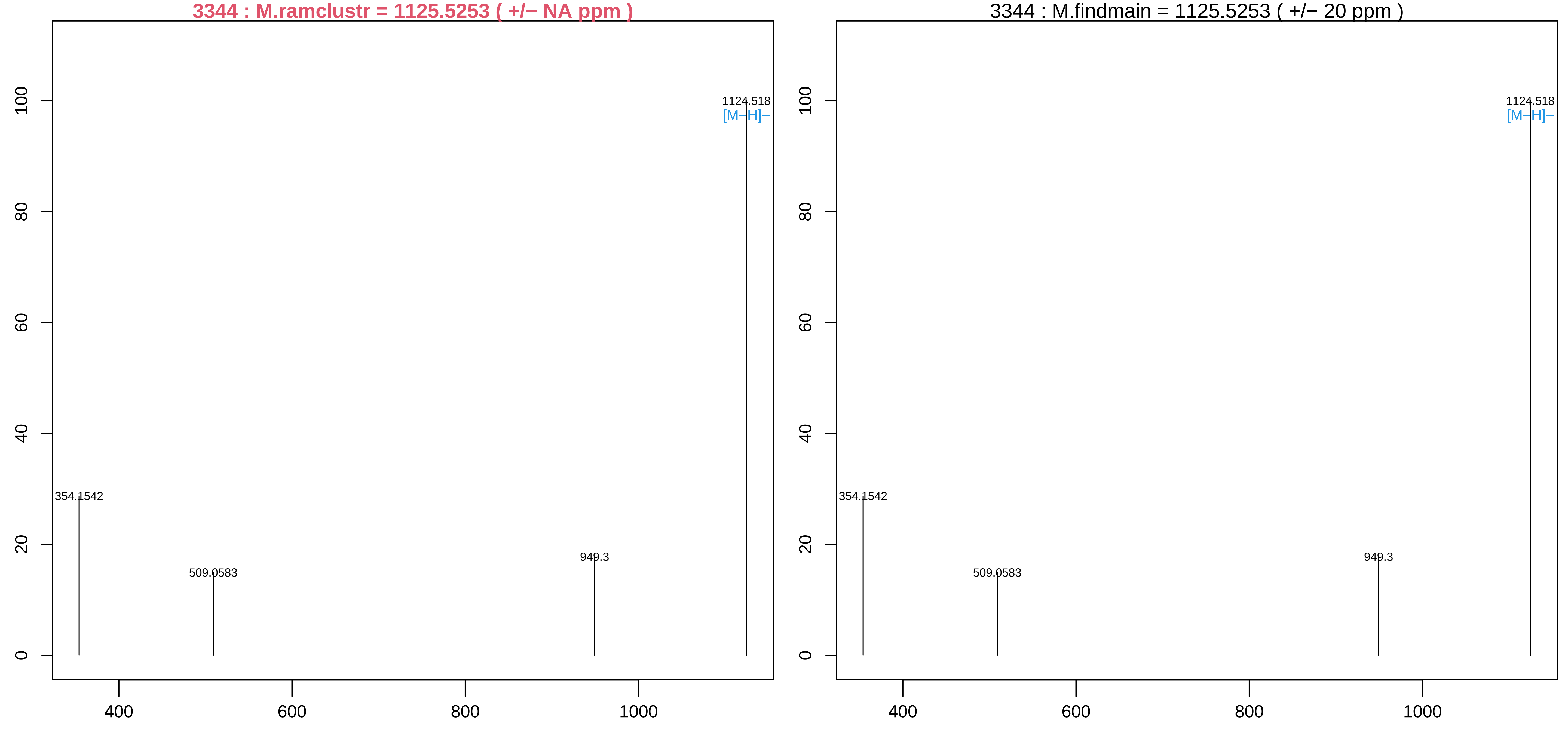

3344 : M.ramclustr = 1125.5253 (+/− NA ppm)
3344 : M.findmain = 1125.5253 (+/− 20 ppm)
1124.518
[M−H]−
354.1542
509.0583
949.3
0
20
40
60
80
100
400
600
800
1000

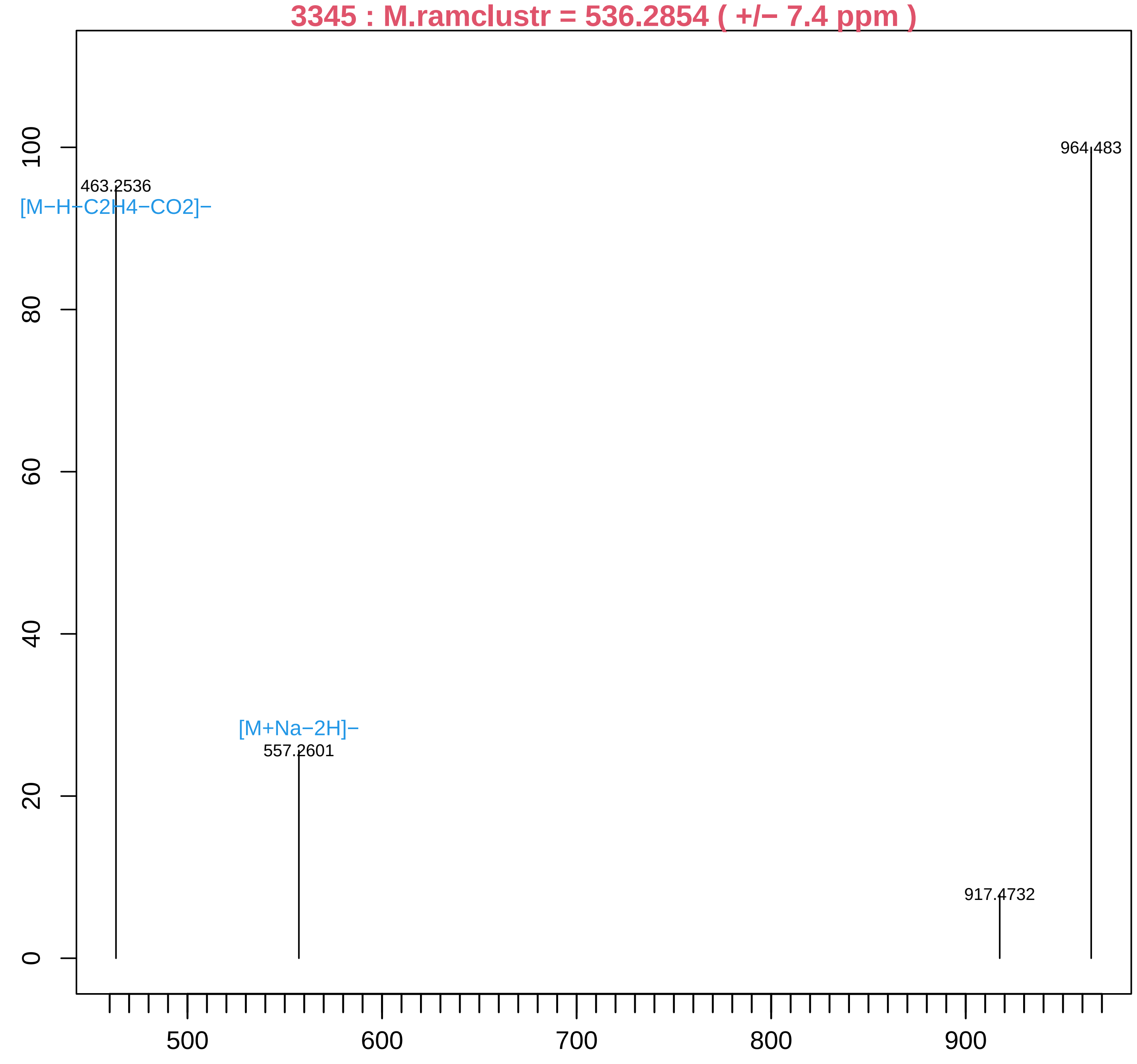
3345 : M.ramclustr = 536.2854 (+/− 7.4 ppm)
463.2536
[M−H−C2H4−CO2]−
[M+Na−2H]−
557.2601
917.4732
964.483
0
20
40
60
80
100
500
600
700
800
900

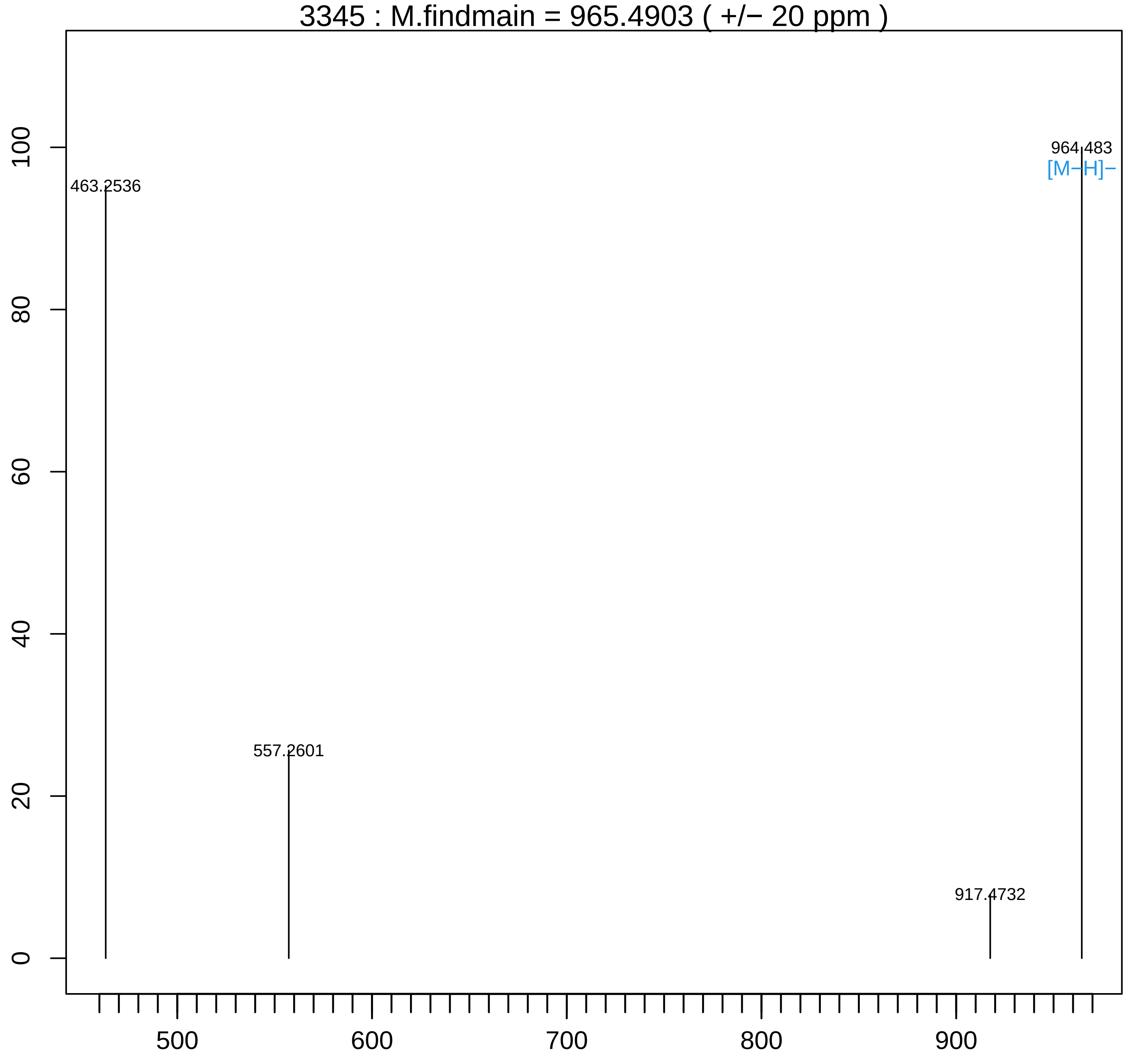
3345 : M.findmain = 965.4903 (+/− 20 ppm)
463.2536
557.2601
917.4732
964.483
[M−H]−
0
20
40
60
80
100
500
600
700
800
900

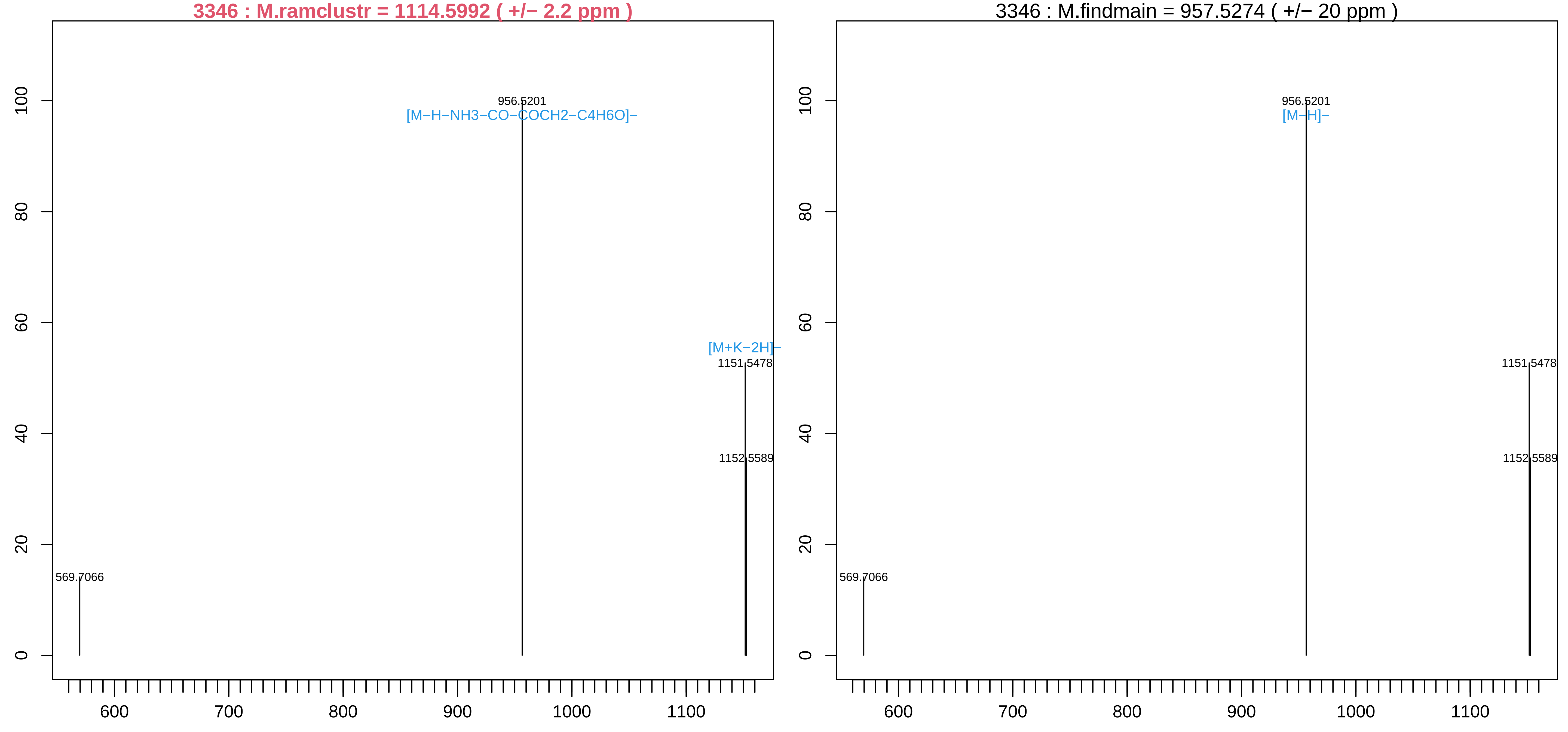

3346 : M.ramclustr = 1114.5992 (+/− 2.2 ppm)
956.5201
[M−H−NH3−CO−COCH2−C4H6O]−
[M+K−2H]−
1151.5478
1152.5589
569.7066
3346 : M.findmain = 957.5274 (+/− 20 ppm)
956.5201
[M−H]−
1151.5478
1152.5589
569.7066

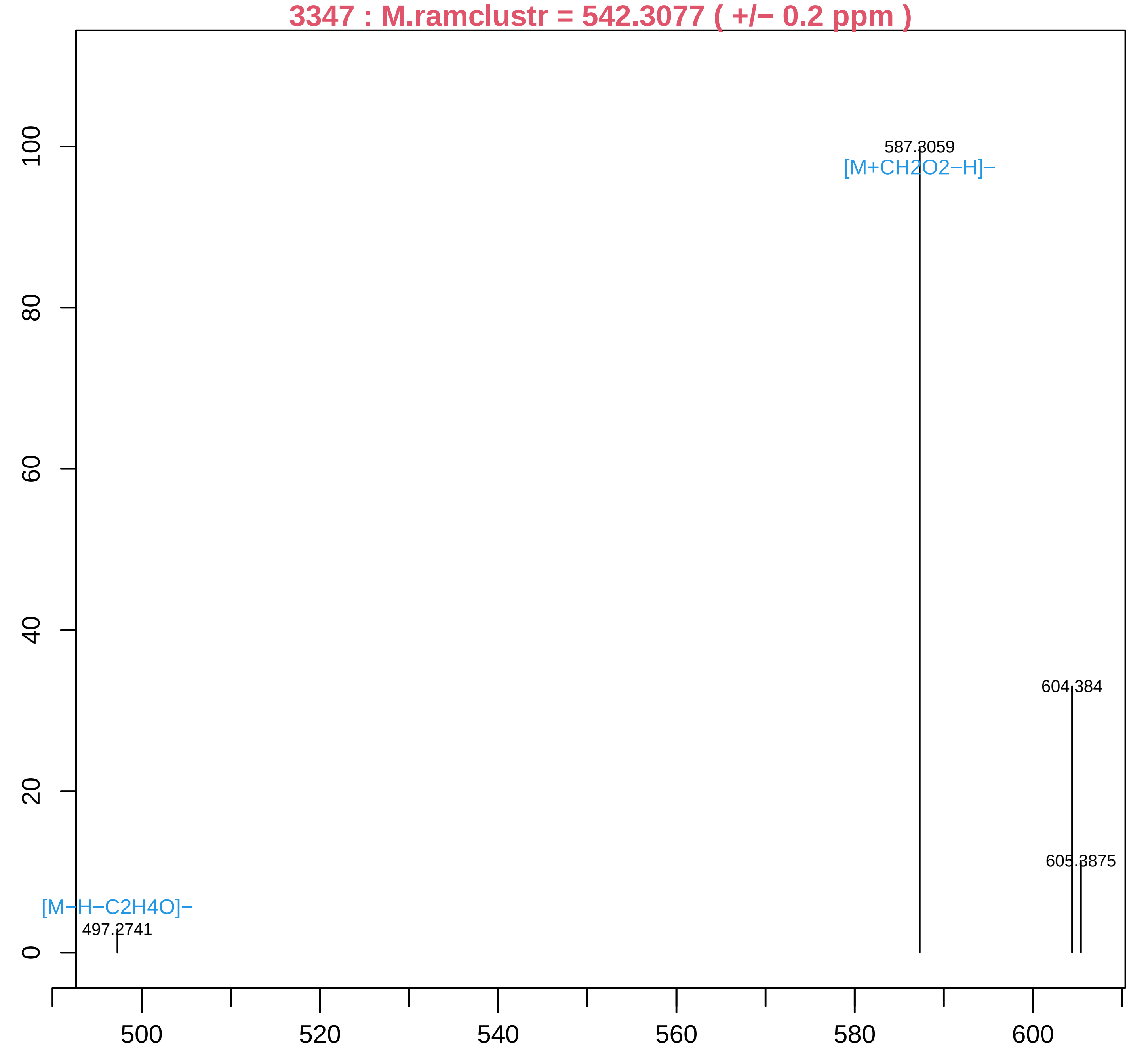
3347 : M.ramclustr = 542.3077 (+/− 0.2 ppm)
587.3059
[M+CH2O2−H]−
604.384
605.3875
[M−H−C2H4O]−
497.2741
0
20
40
60
80
100
500
520
540
560
580
600

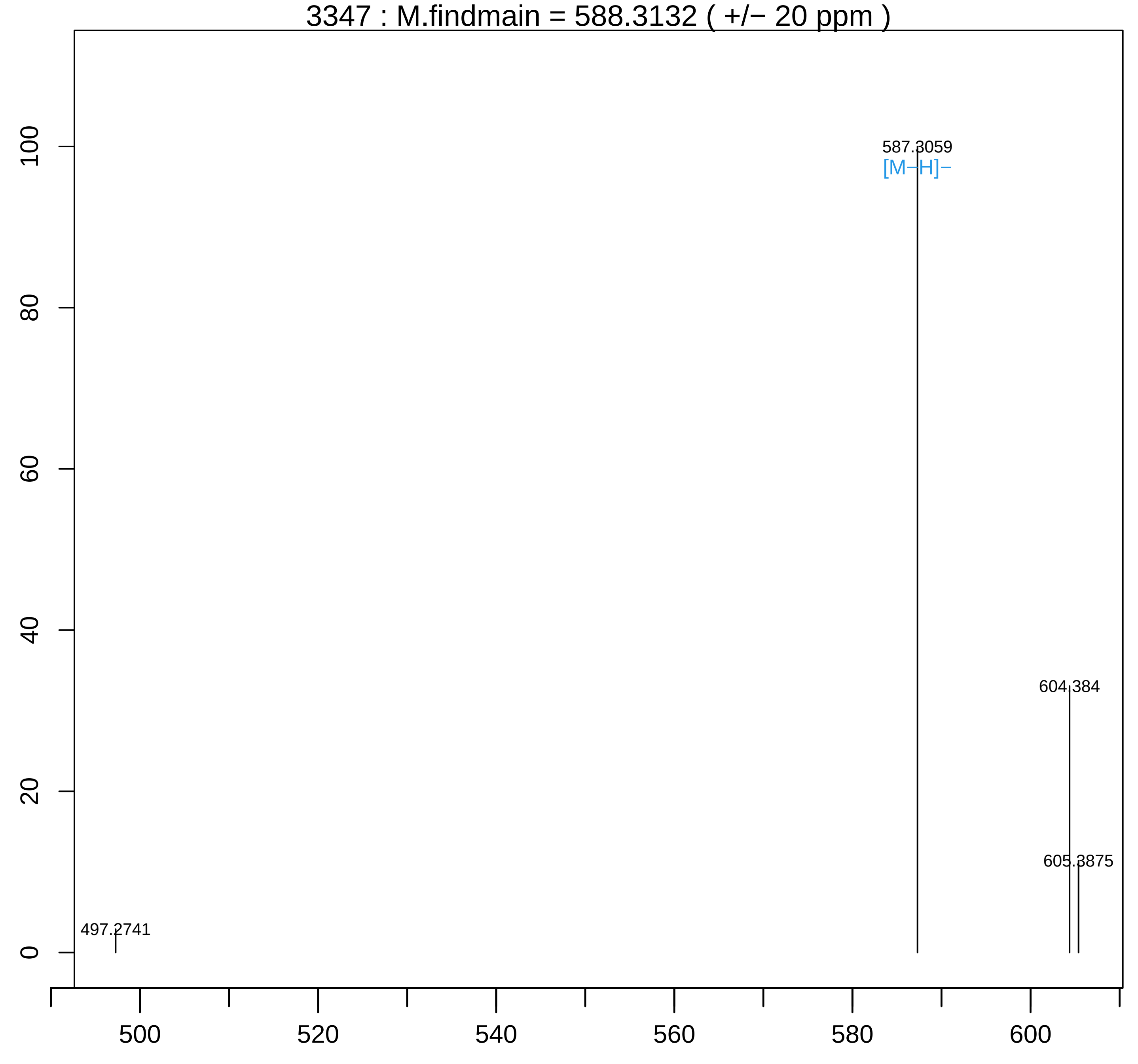
3347 : M.findmain = 588.3132 (+/− 20 ppm)
587.3059
[M−H]−
604.384
605.3875
497.2741
0
20
40
60
80
100
500
520
540
560
580
600

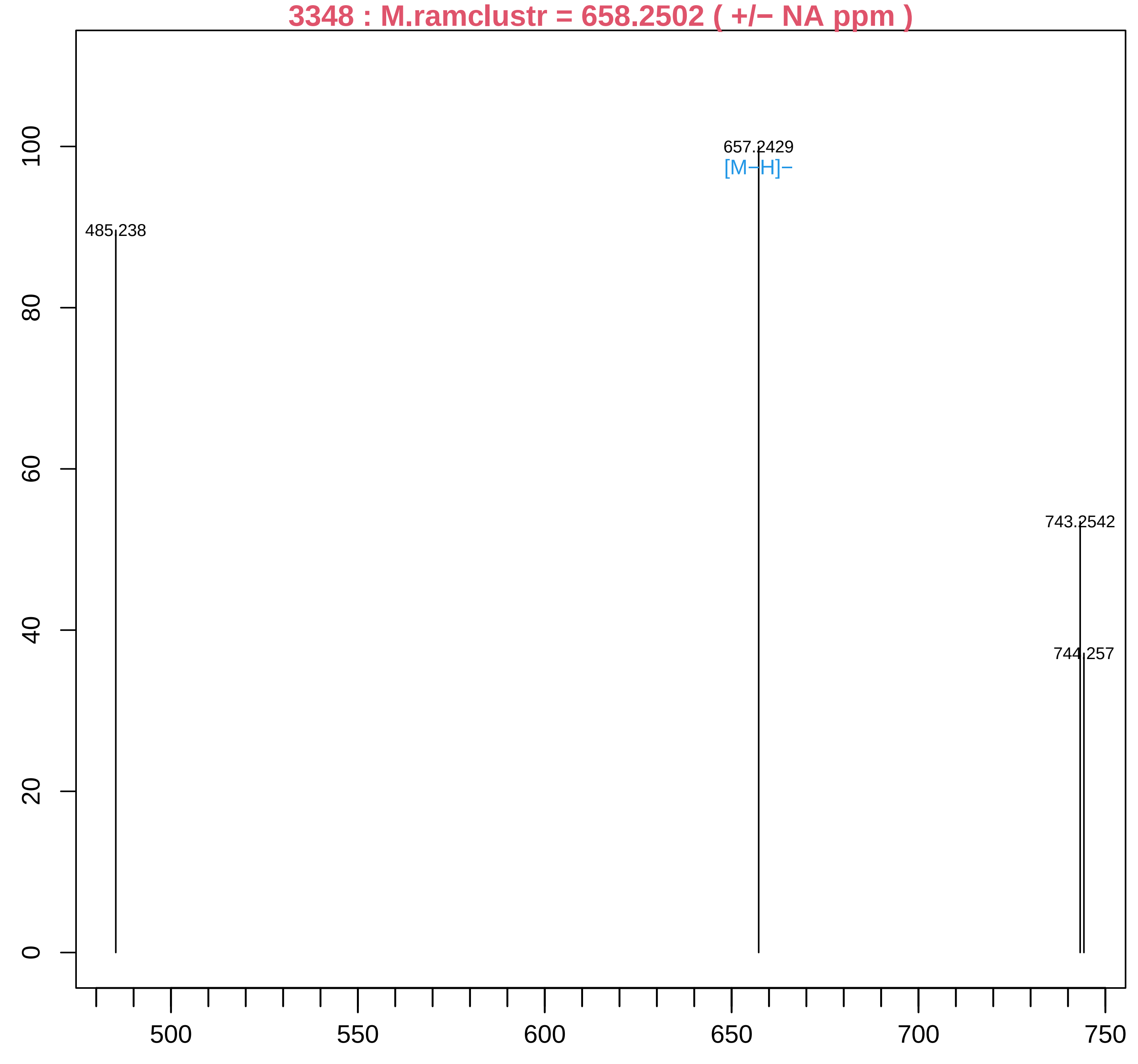
3348 : M.ramclustr = 658.2502 (+/− NA ppm)
657.2429
[M−H]−
485.238
743.2542
744.257
0
20
40
60
80
100
500
550
600
650
700
750

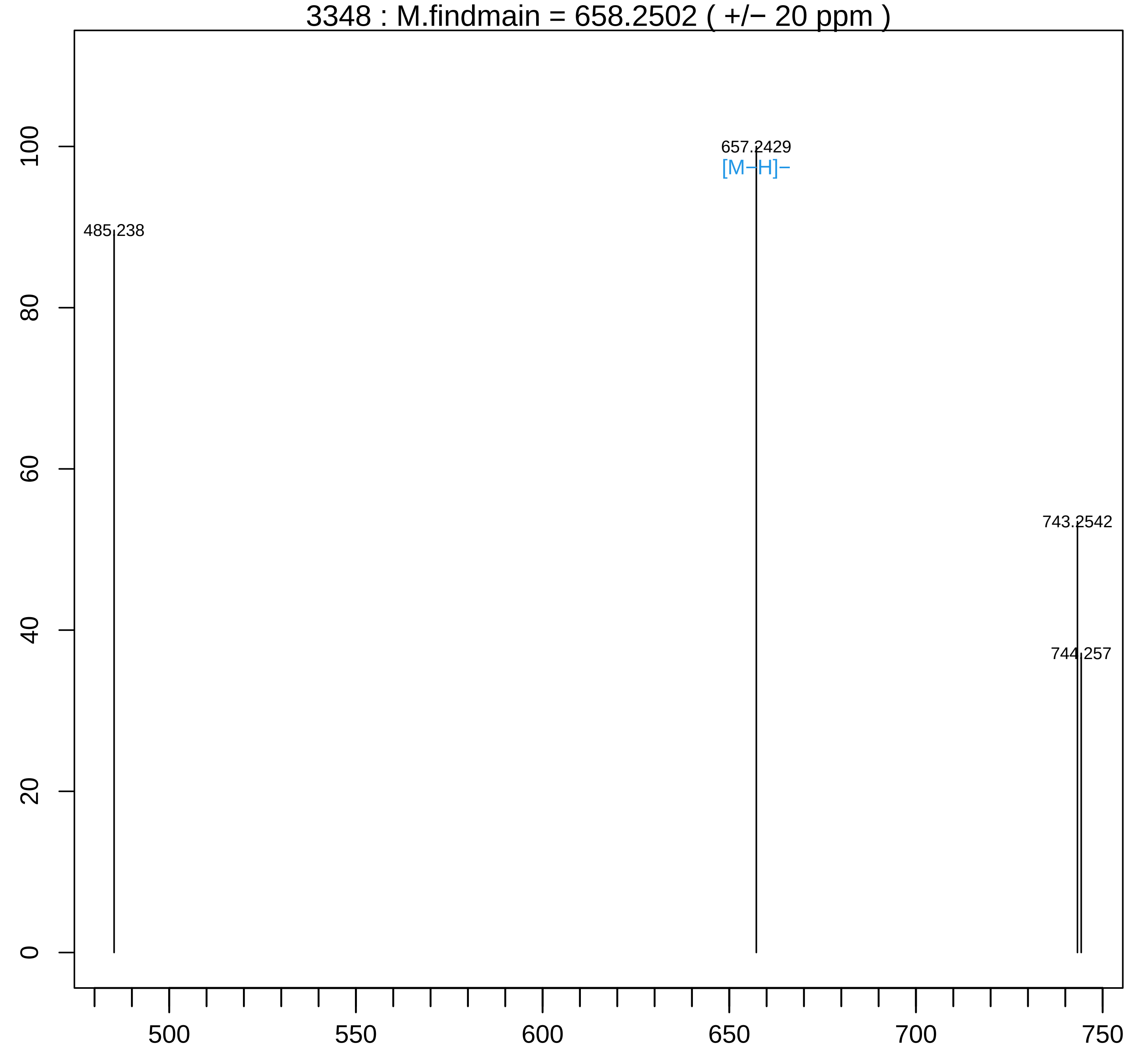
3348 : M.findmain = 658.2502 (+/− 20 ppm)
657.2429
[M−H]−
485.238
743.2542
744.257
0
20
40
60
80
100
500
550
600
650
700
750

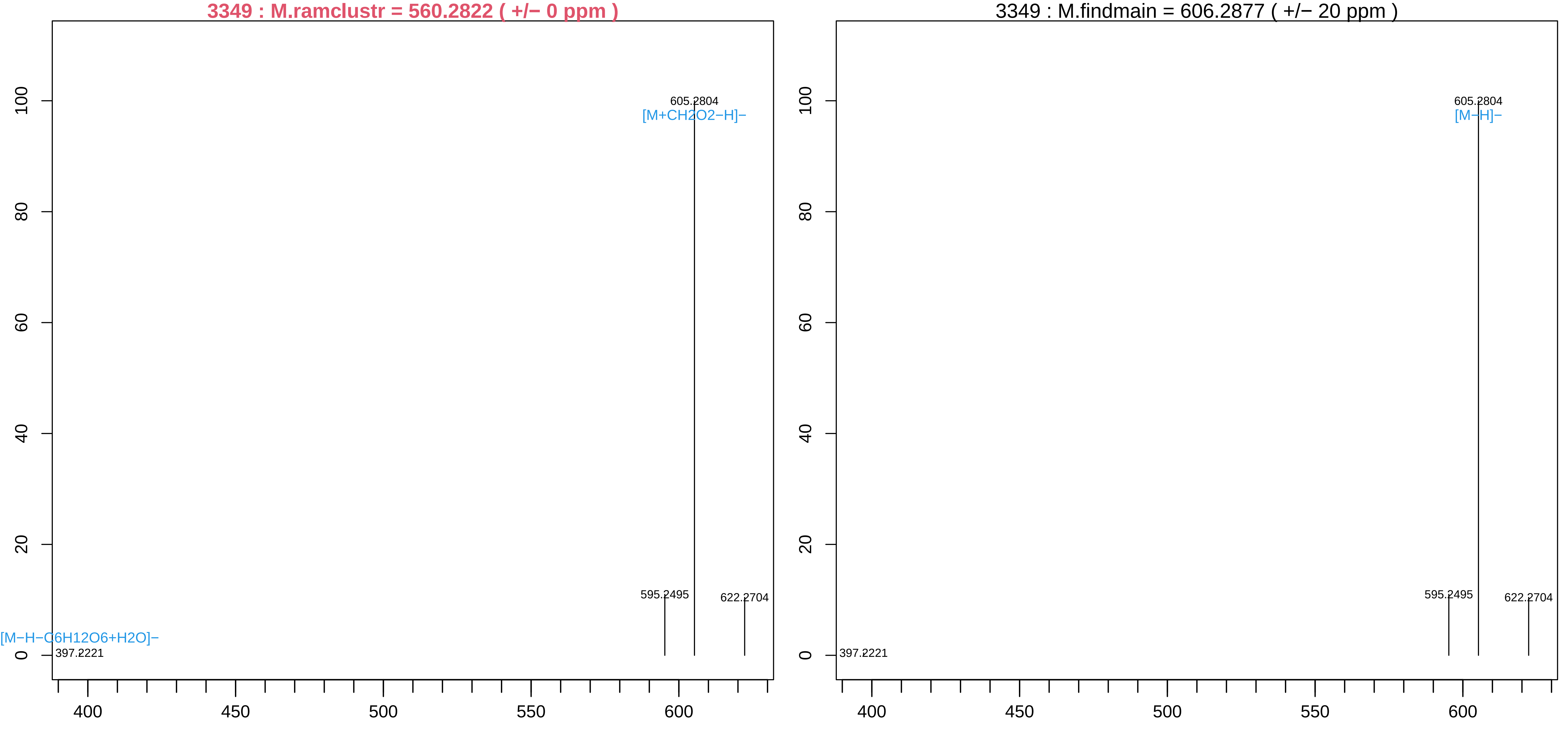
3349 : M.ramclustr = 560.2822 (+/− 0 ppm)
605.2804
[M+CH2O2−H]−
595.2495
622.2704
[M−H−C6H12O6+H2O]−
397.2221
3349 : M.findmain = 606.2877 (+/− 20 ppm)
605.2804
[M−H]−
595.2495
622.2704
397.2221

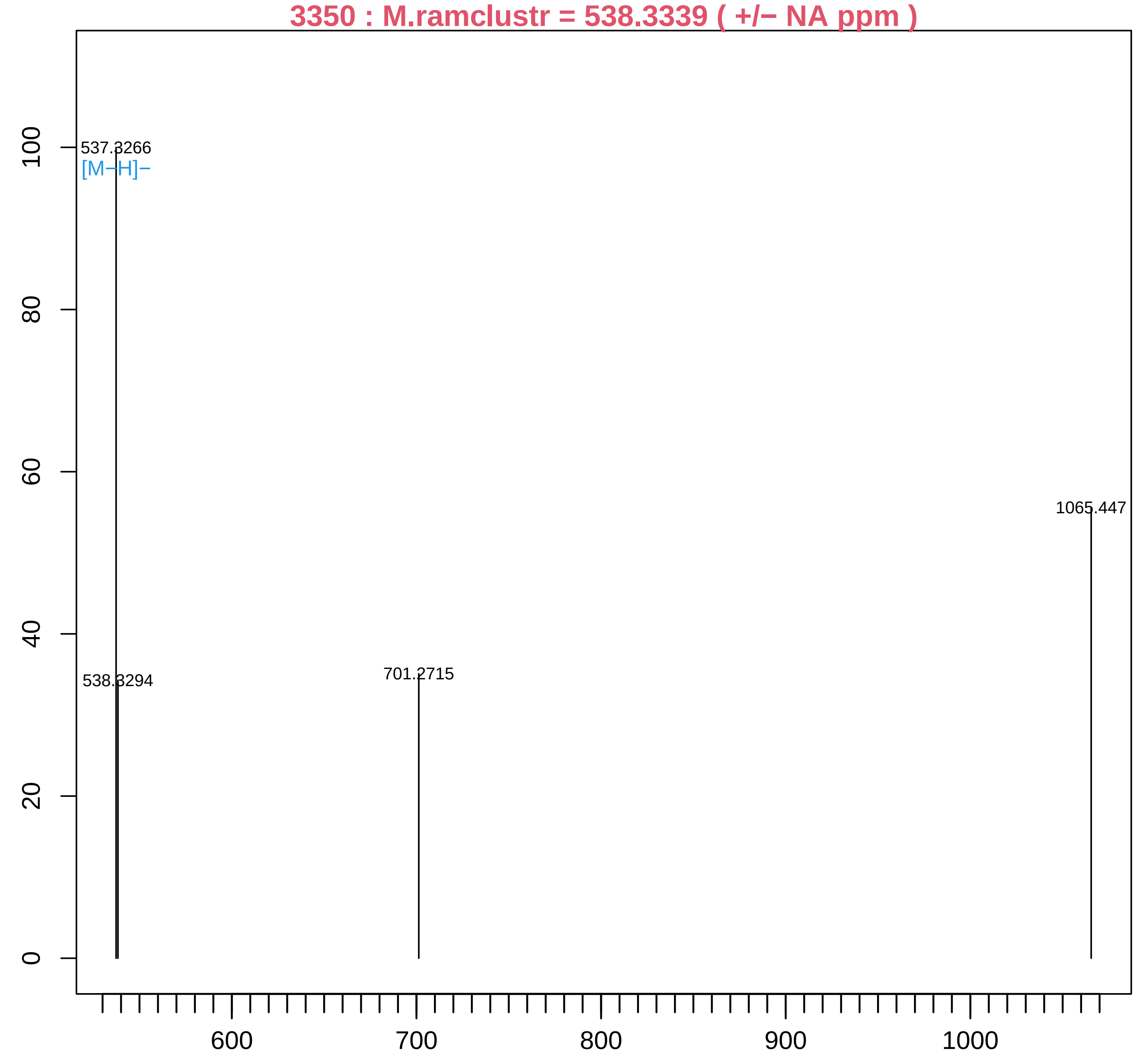
3350 : M.ramclustr = 538.3339 (+/− NA ppm)
537.3266
[M−H]−
538.3294
701.2715
1065.447
0
20
40
60
80
100
600
700
800
900
1000

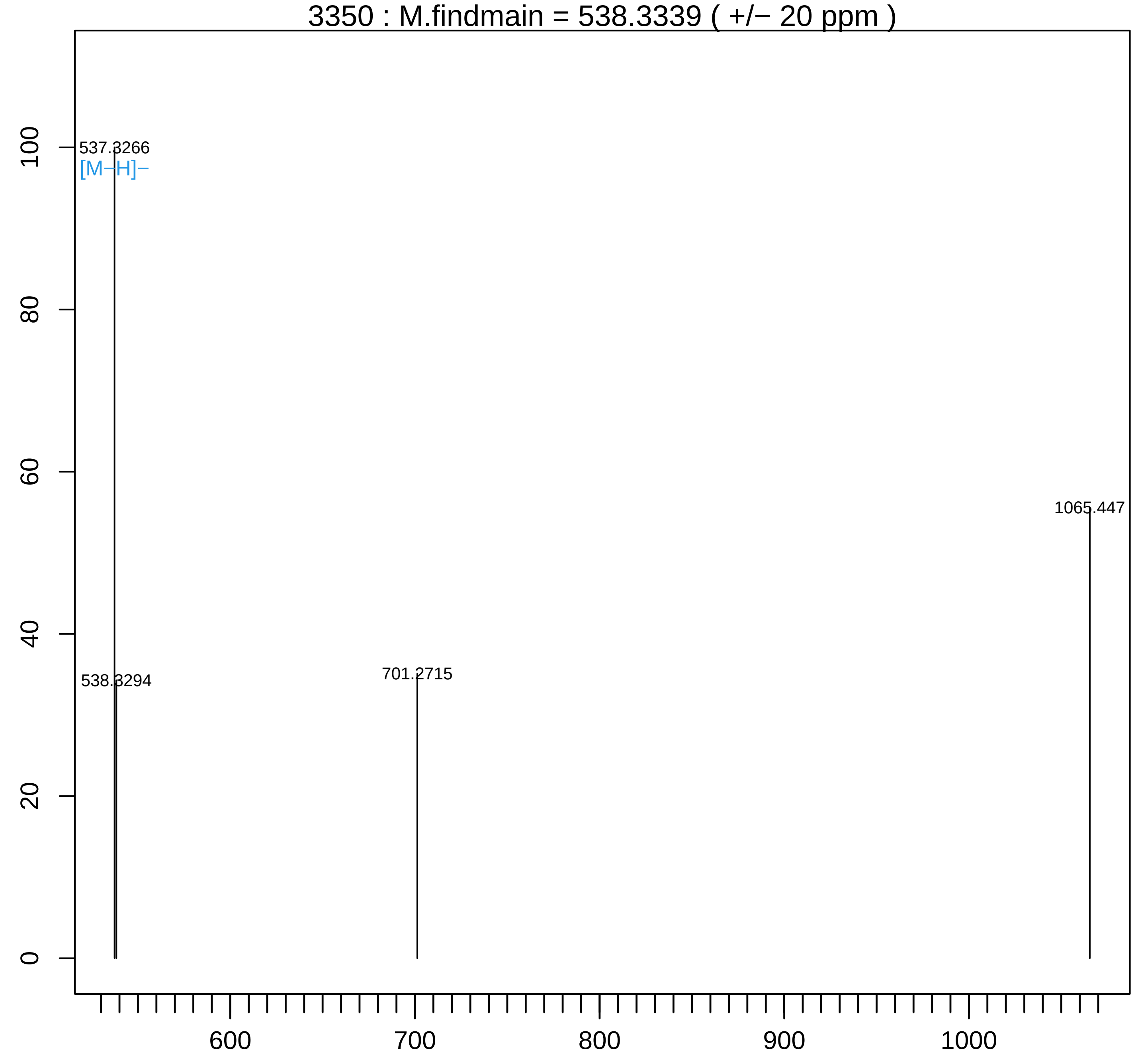
3350 : M.findmain = 538.3339 (+/− 20 ppm)
537.3266
[M−H]−
538.3294
701.2715
1065.447
0
20
40
60
80
100
600
700
800
900
1000

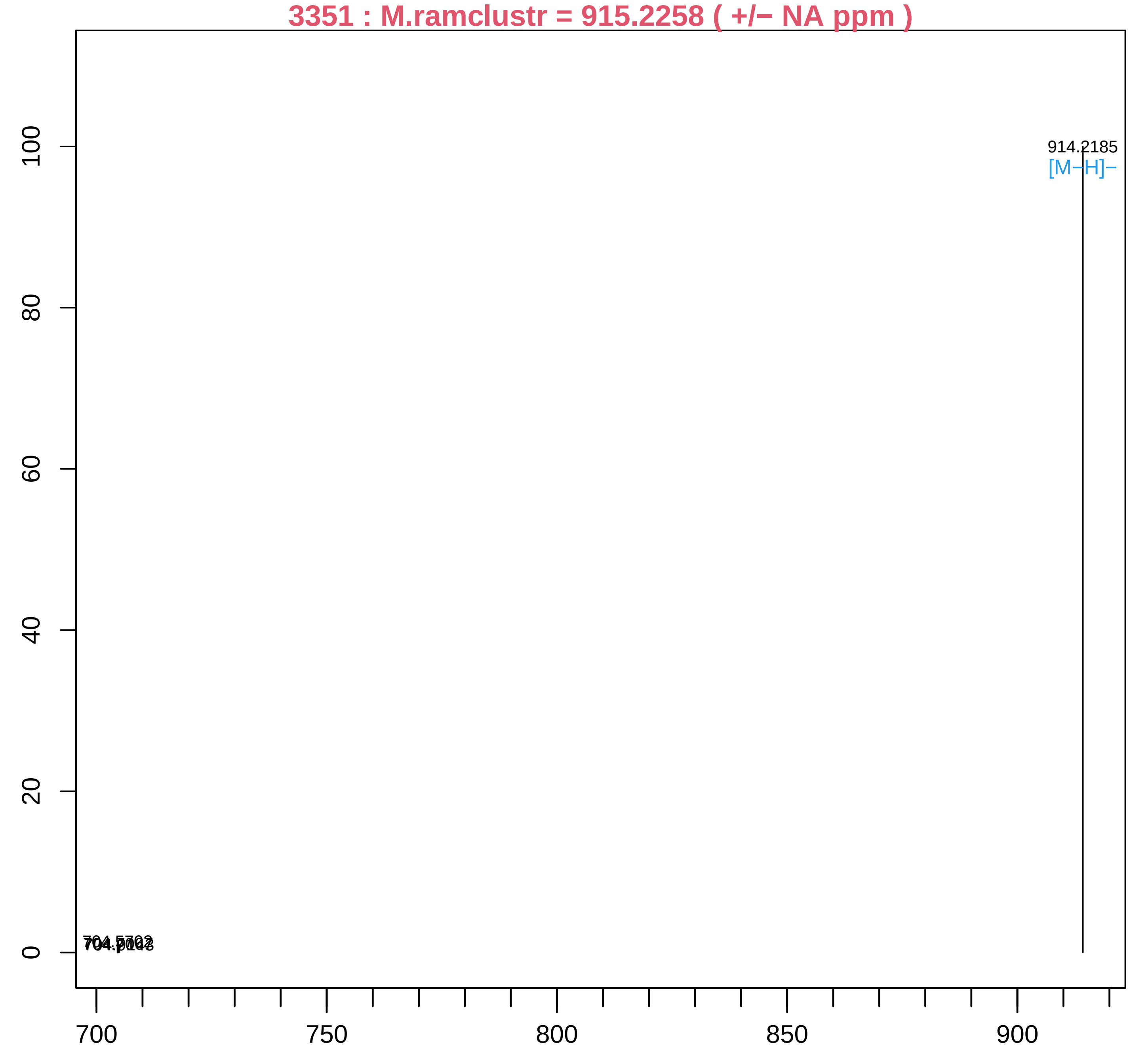
3351 : M.ramclustr = 915.2258 (+/− NA ppm)
914.2185
[M−H]−
0
20
40
60
80
100
700
750
800
850
900

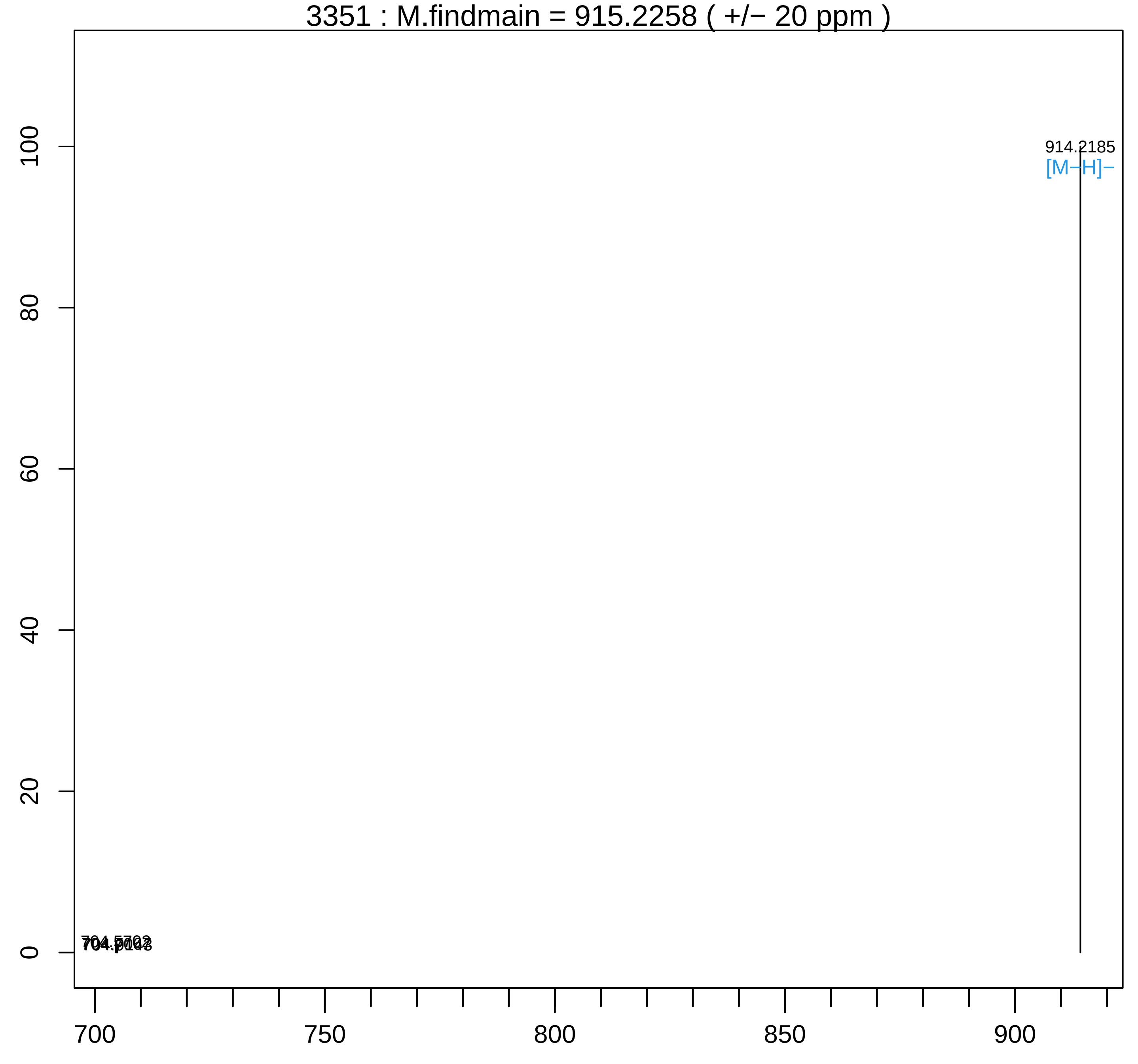
3351 : M.findmain = 915.2258 (+/− 20 ppm)
914.2185
[M−H]−
0
20
40
60
80
100
700
750
800
850
900

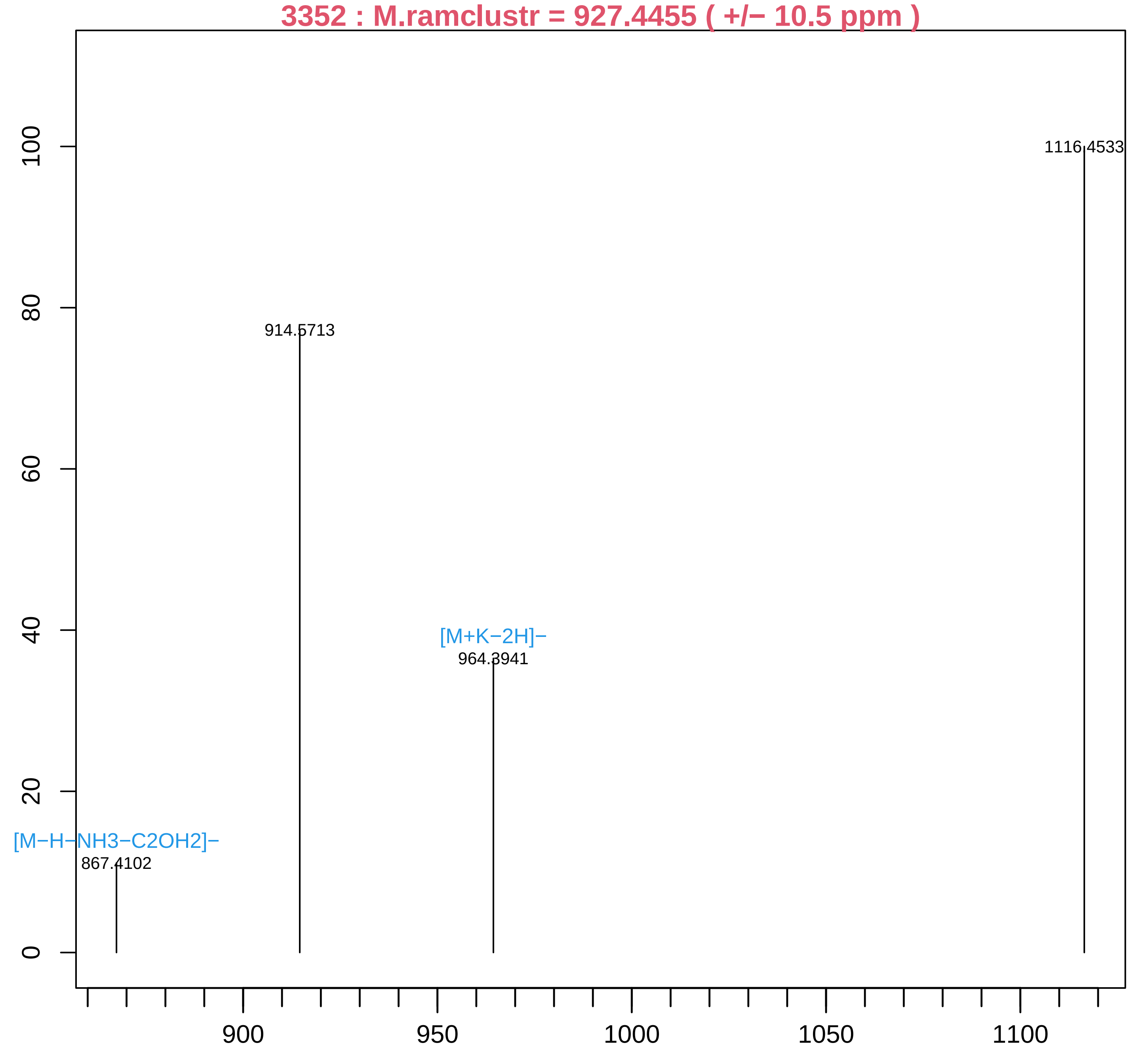
3352 : M.ramclustr = 927.4455 (+/− 10.5 ppm)
1116.4533
914.5713
[M+K−2H]−
964.3941
[M−H−NH3−C2OH2]−
867.4102
0
20
40
60
80
100
900
950
1000
1050
1100

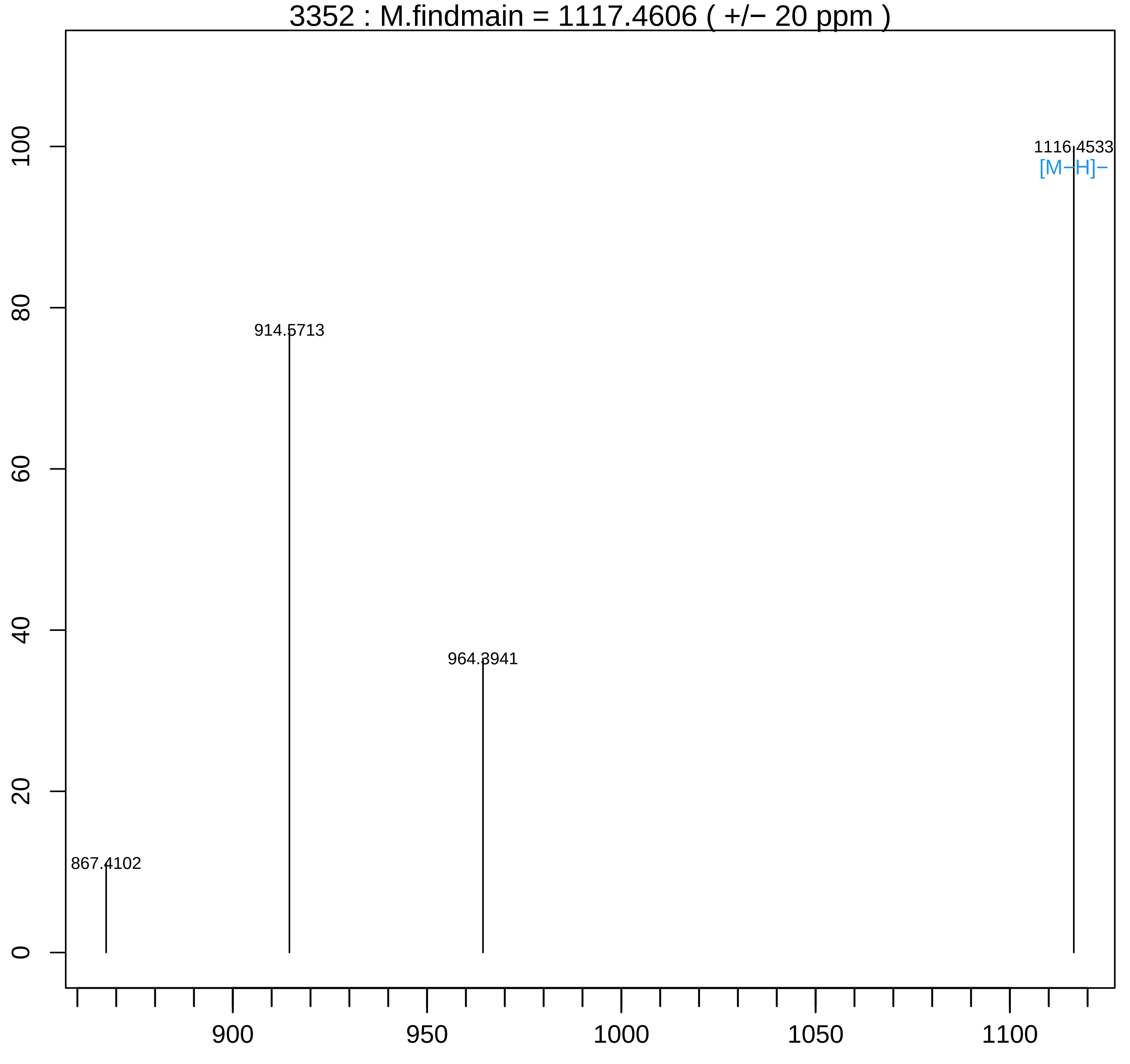
3352 : M.findmain = 1117.4606 (+/− 20 ppm)
1116.4533
[M−H]−
914.5713
964.3941
867.4102
0
20
40
60
80
100
900
950
1000
1050
1100

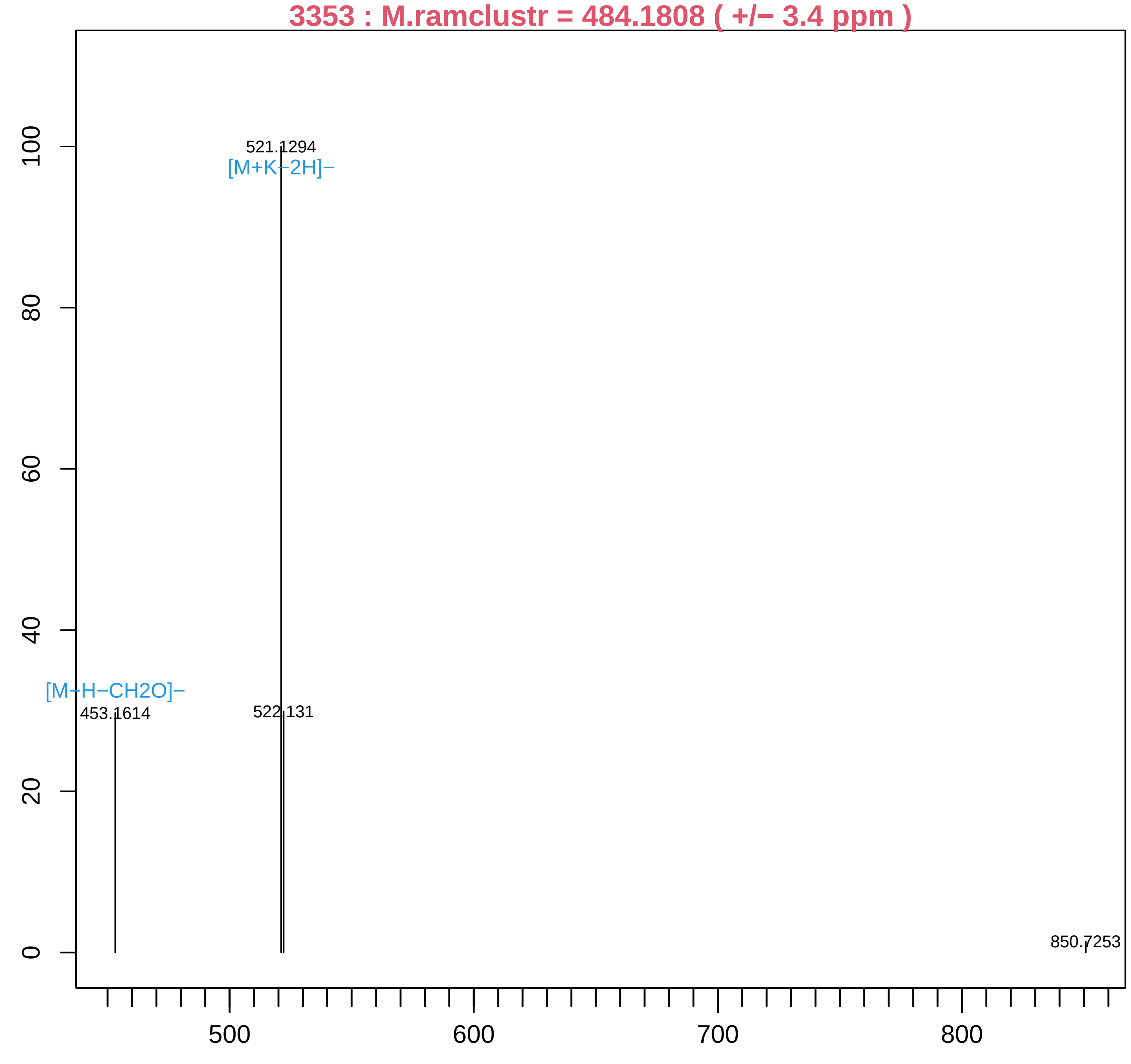

3353 : M.ramclustr = 484.1808 (+/− 3.4 ppm)
521.1294
[M+K−2H]−
[M−H−CH2O]−
453.1614
522.131
850.7253
0
20
40
60
80
100
500
600
700
800

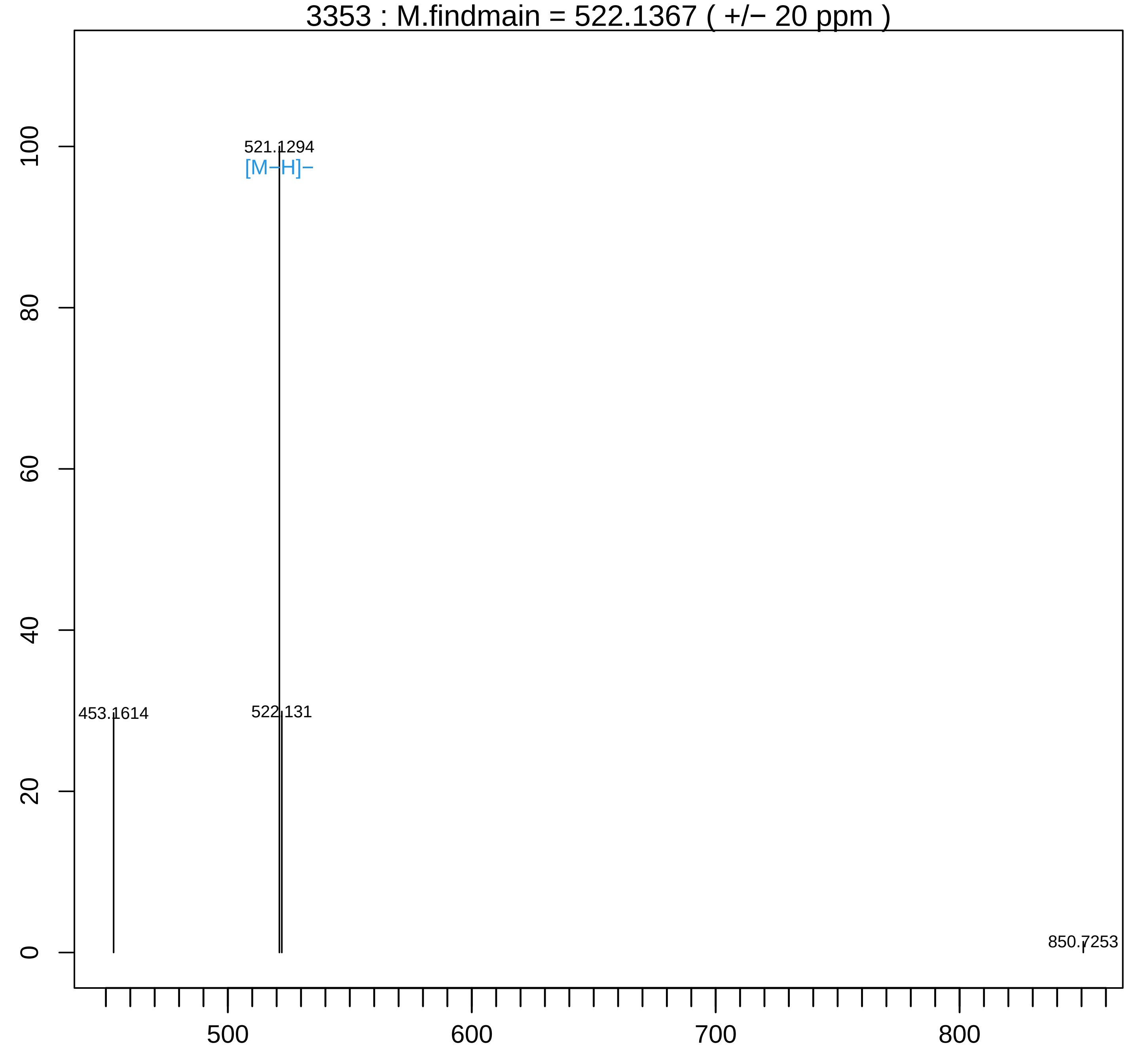

3353 : M.findmain = 522.1367 (+/− 20 ppm)
521.1294
[M−H]−
453.1614
522.131
850.7253
0
20
40
60
80
100
500
600
700
800

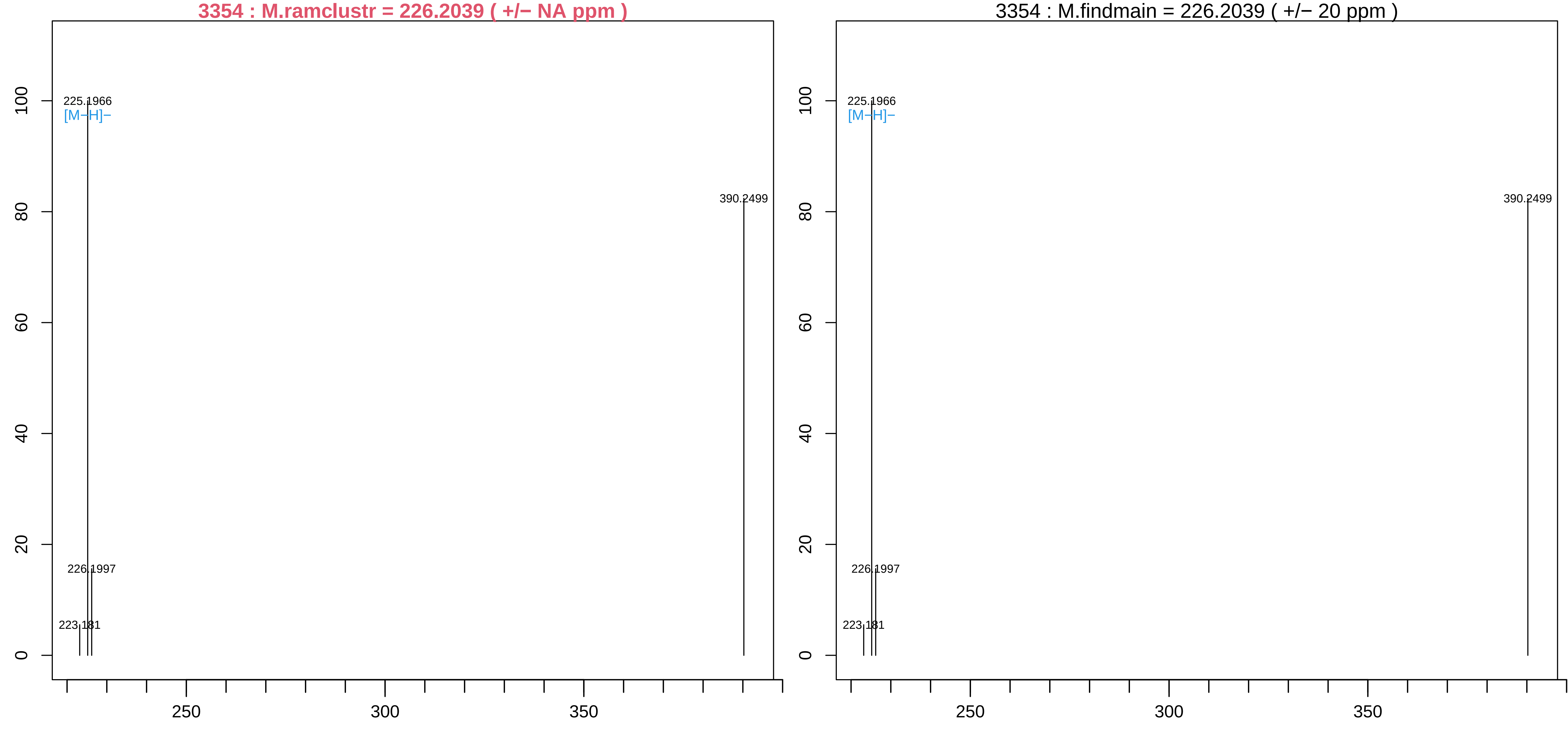
3354 : M.ramclustr = 226.2039 (+/− NA ppm)
3354 : M.findmain = 226.2039 (+/− 20 ppm)
225.1966
[M−H]−
390.2499
226.1997
223.181
0
20
40
60
80
100
250
300
350

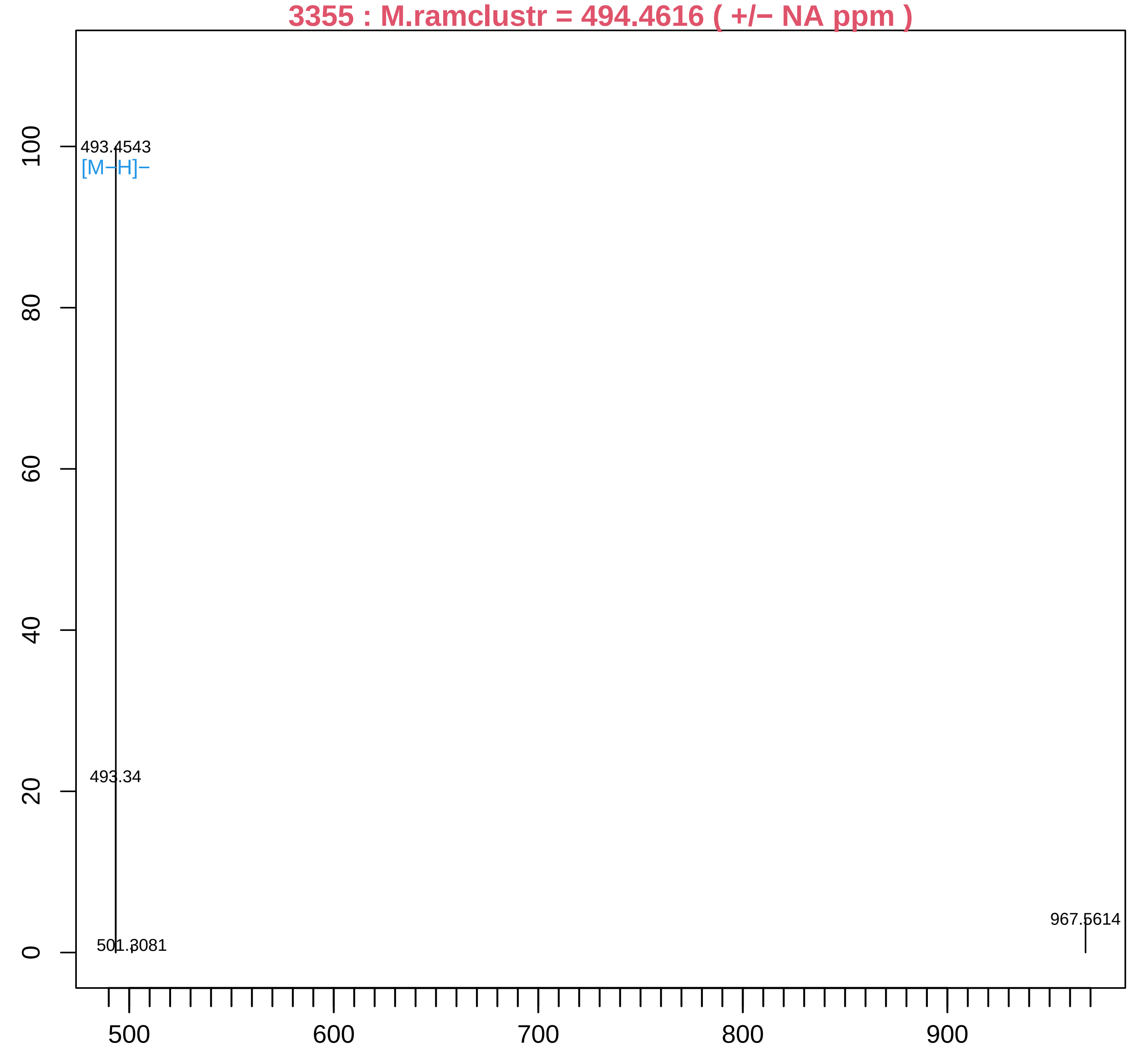

3355 : M.ramclustr = 494.4616 (+/− NA ppm)
493.4543
[M−H]−
493.34
501.3081
967.5614
0
20
40
60
80
100
500
600
700
800
900

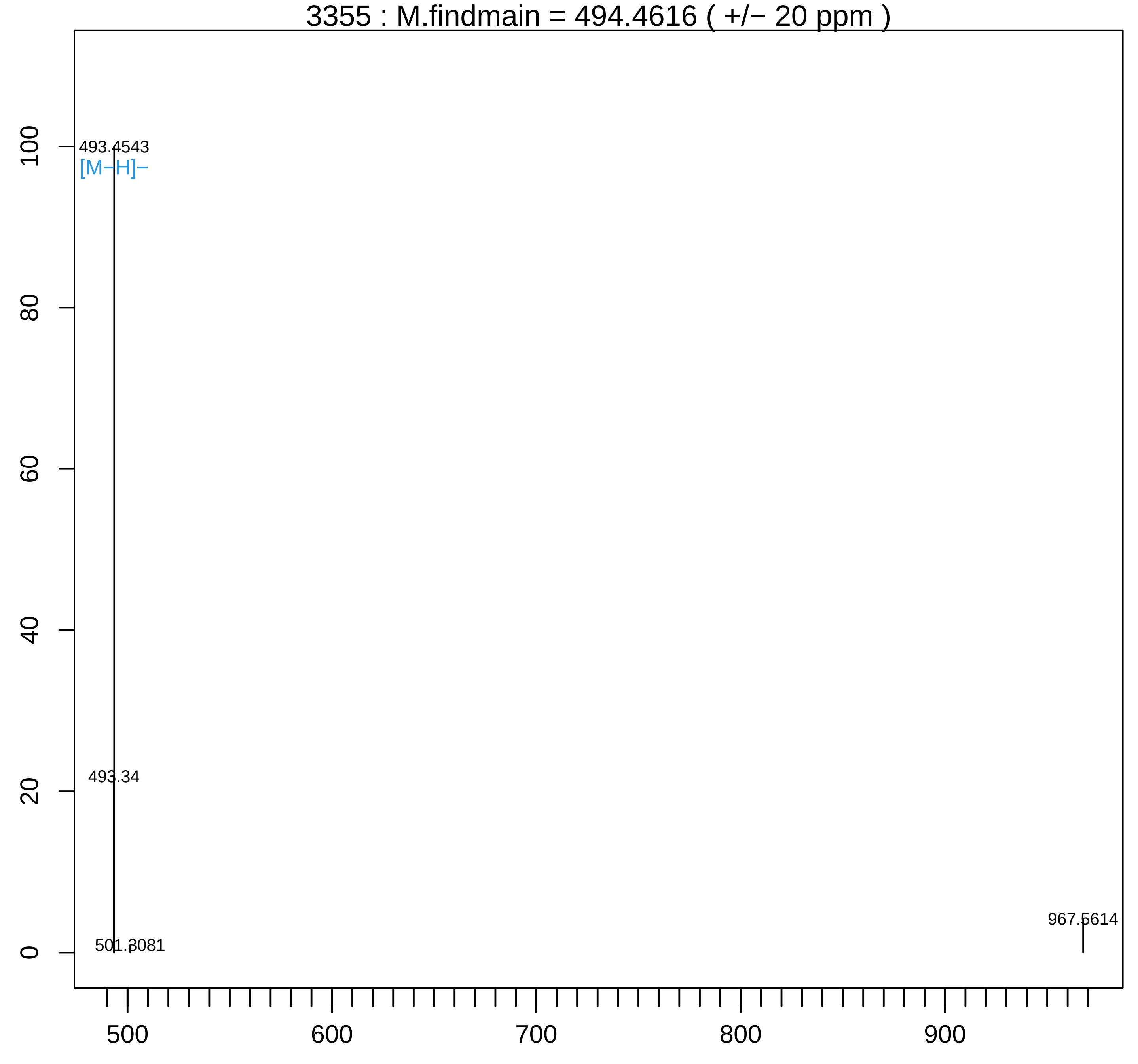

3355 : M.findmain = 494.4616 (+/− 20 ppm)
493.4543
[M−H]−
493.34
501.3081
967.5614
0
20
40
60
80
100
500
600
700
800
900

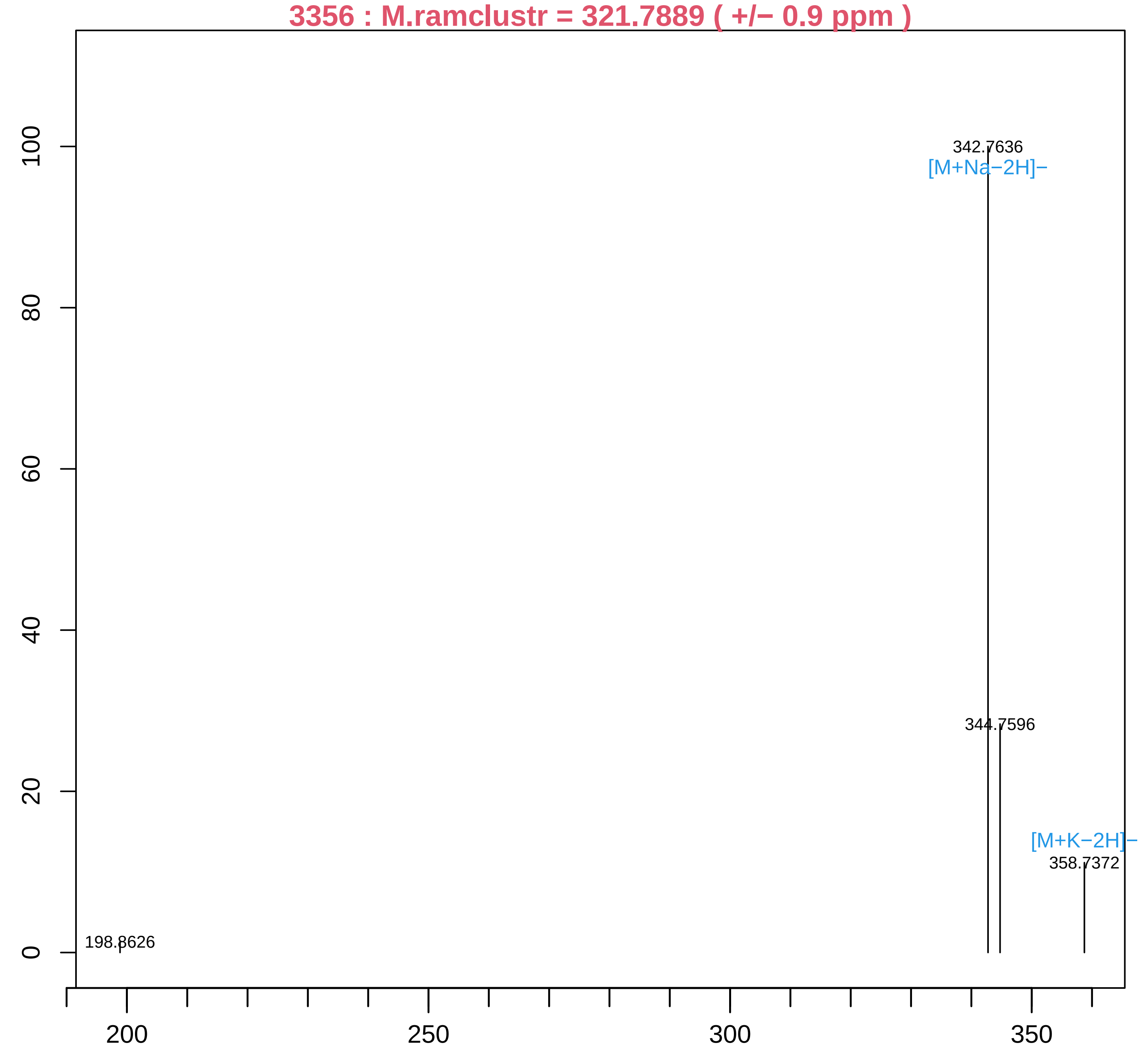
3356 : M.ramclustr = 321.7889 (+/− 0.9 ppm)
342.7636
[M+Na−2H]−
344.7596
[M+K−2H]−
358.7372
198.8626
0
20
40
60
80
100
200
250
300
350

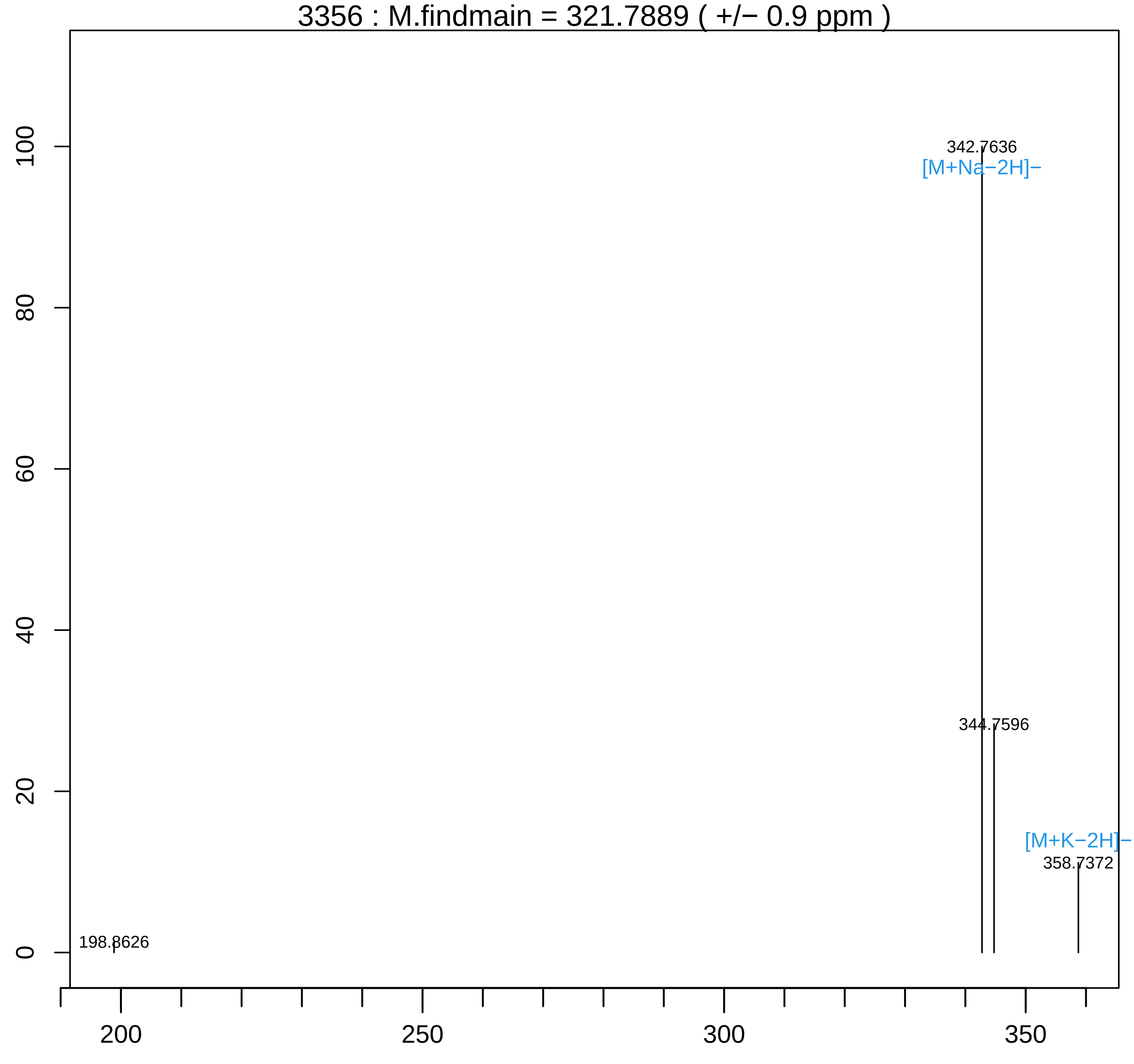
3356 : M.findmain = 321.7889 (+/− 0.9 ppm)
342.7636
[M+Na−2H]−
344.7596
[M+K−2H]−
358.7372
198.8626
0
20
40
60
80
100
200
250
300
350

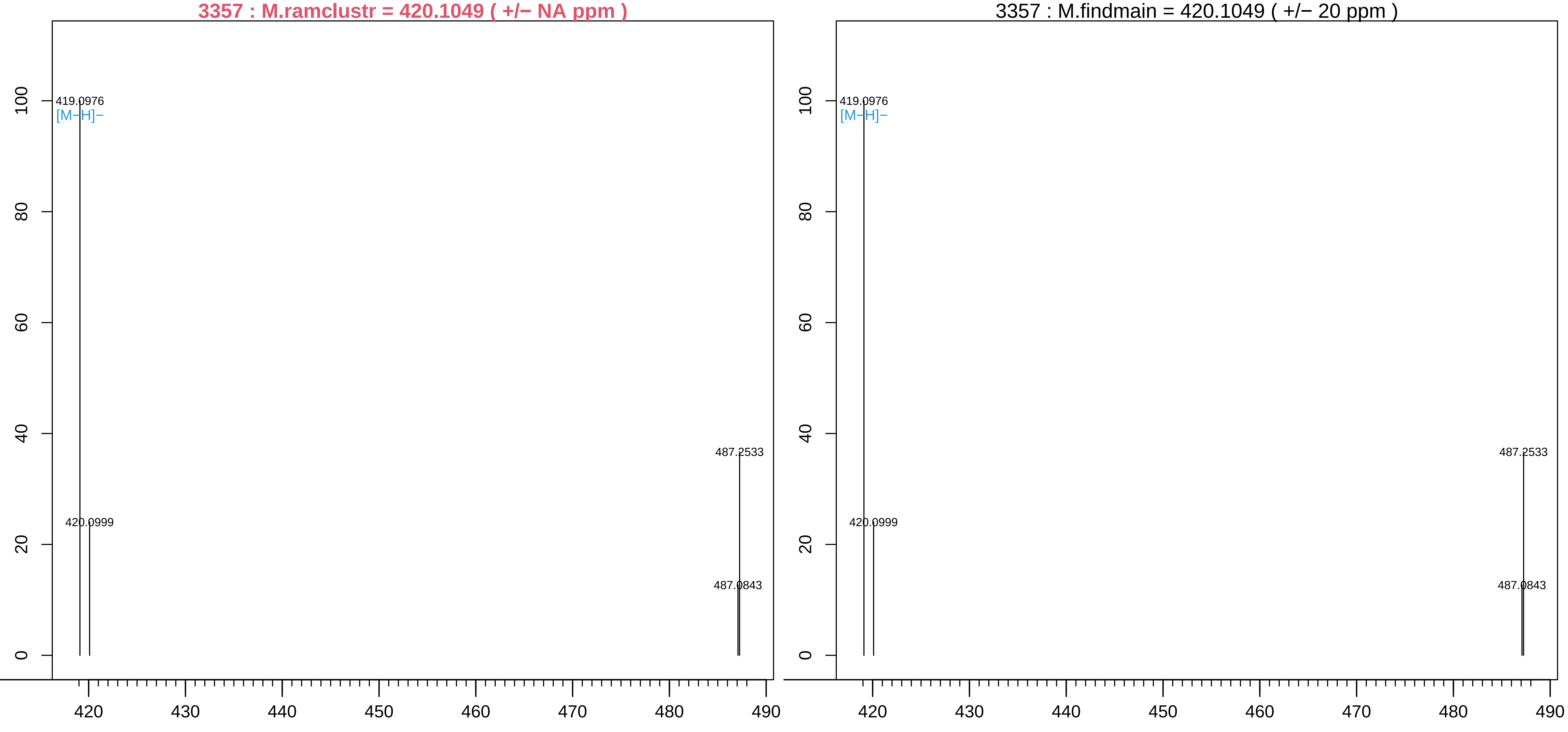

3357 : M.ramclustr = 420.1049 (+/− NA ppm)
419.0976
[M−H]−
420.0999
487.2533
487.0843
3357 : M.findmain = 420.1049 (+/− 20 ppm)
419.0976
[M−H]−
420.0999
487.2533
487.0843
0
20
40
60
80
100
420
430
440
450
460
470
480
490

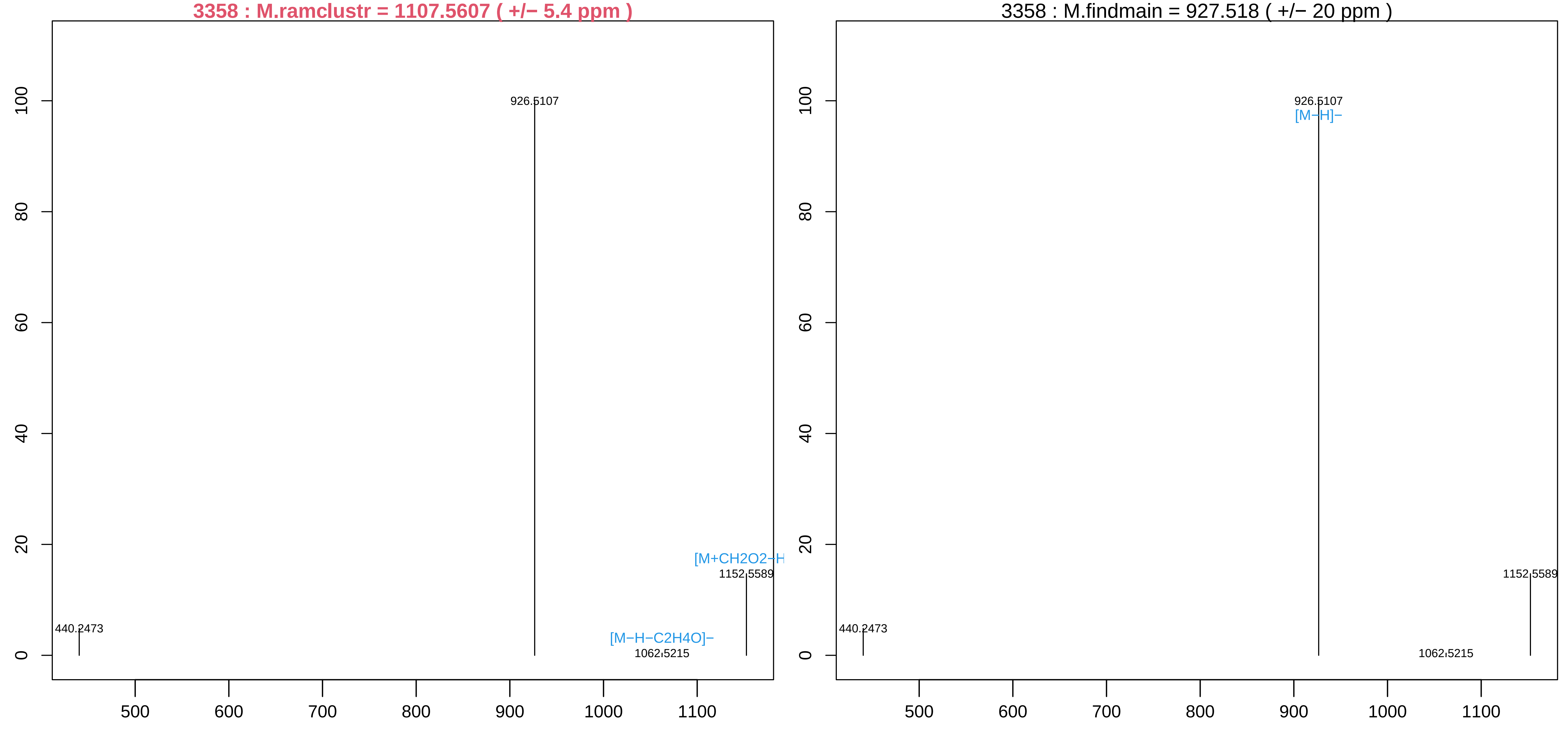

3358 : M.ramclustr = 1107.5607 (+/− 5.4 ppm)
926.5107
440.2473
[M−H−C2H4O]−
1062.5215
[M+CH2O2−H
1152.5589
3358 : M.findmain = 927.518 (+/− 20 ppm)
926.5107
[M−H]−
440.2473
1062.5215
1152.5589
0
20
40
60
80
100
500
600
700
800
900
1000
1100

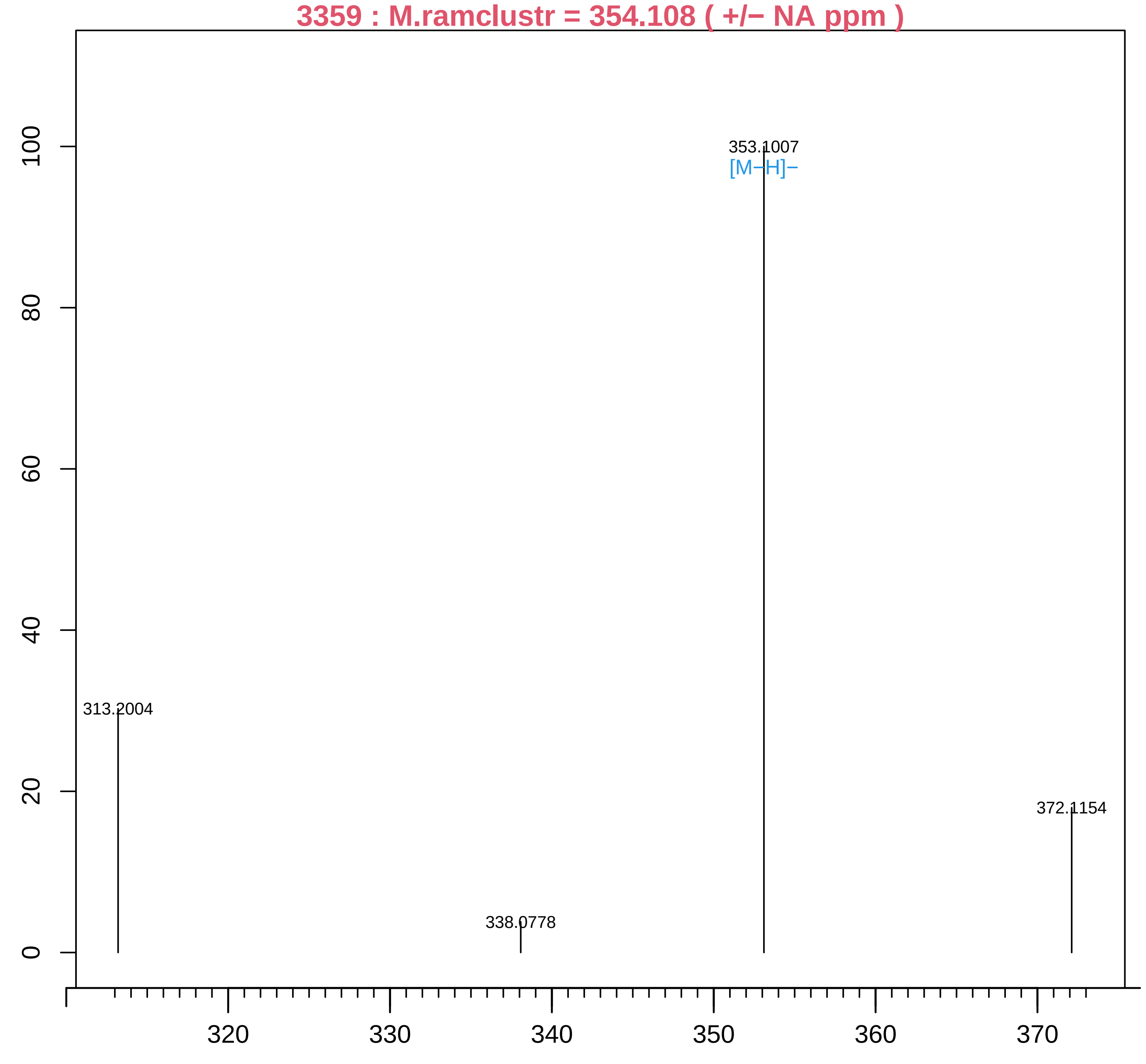
3359 : M.ramclustr = 354.108 (+/− NA ppm)
353.1007
[M−H]−
313.2004
338.0778
372.1154
0
20
40
60
80
100
320
330
340
350
360
370
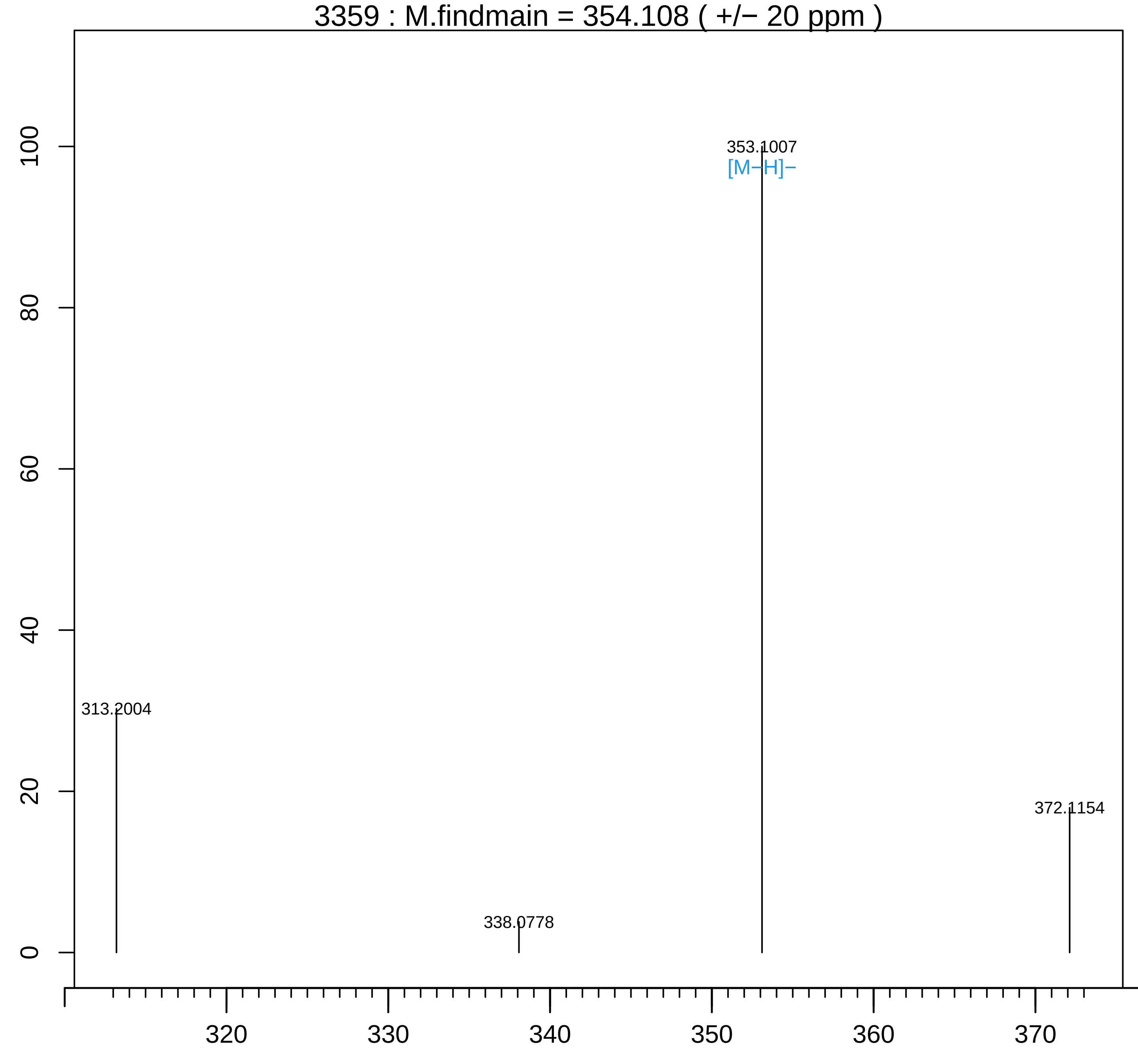
3359 : M.findmain = 354.108 (+/− 20 ppm)
353.1007
[M−H]−
313.2004
338.0778
372.1154
0
20
40
60
80
100
320
330
340
350
360
370

3360 : M.ramclustr = 941.4971 (+/− 0.4 ppm)

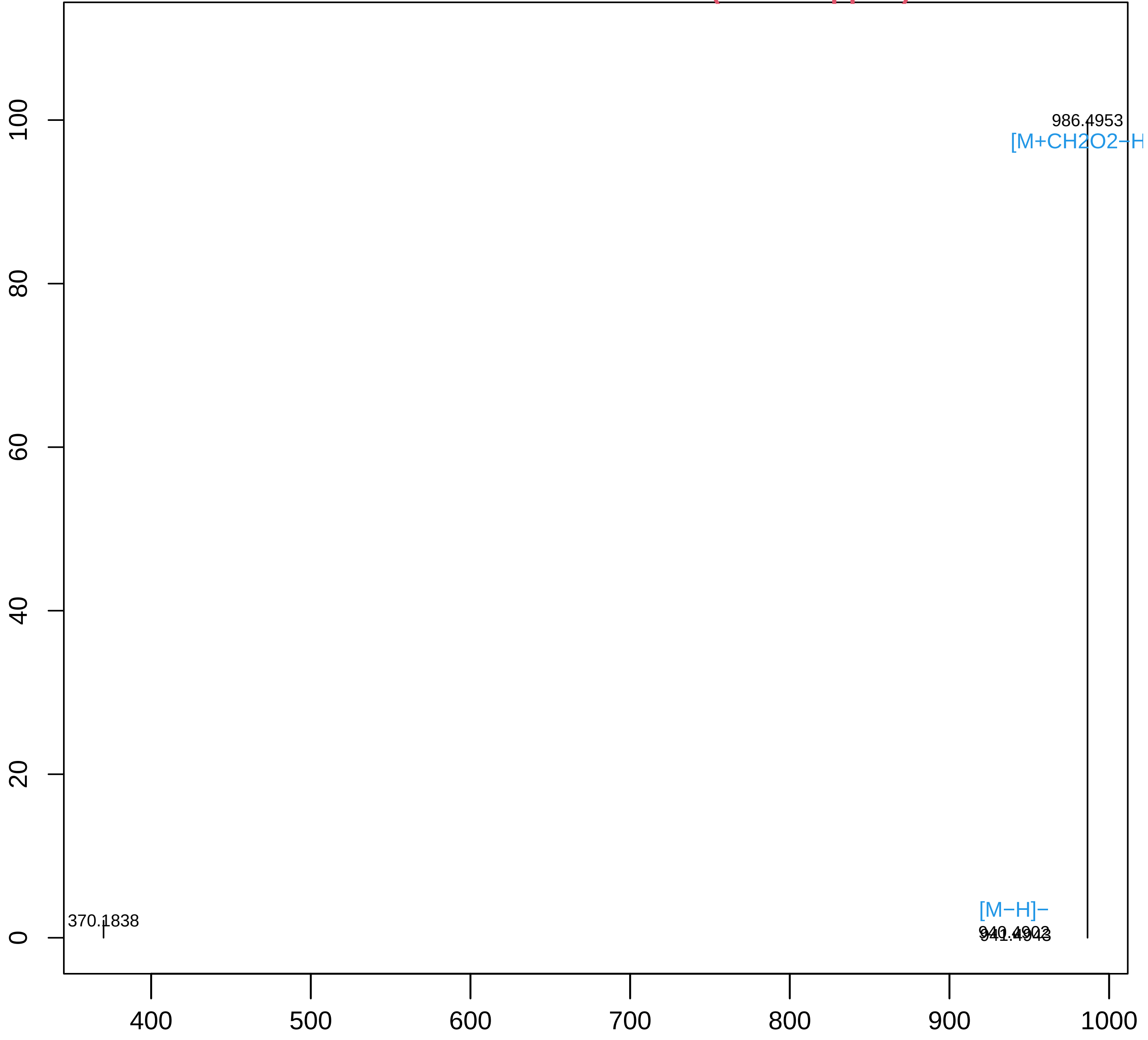

3360 : M.findmain = 941.4971 (+/− 0.4 ppm)

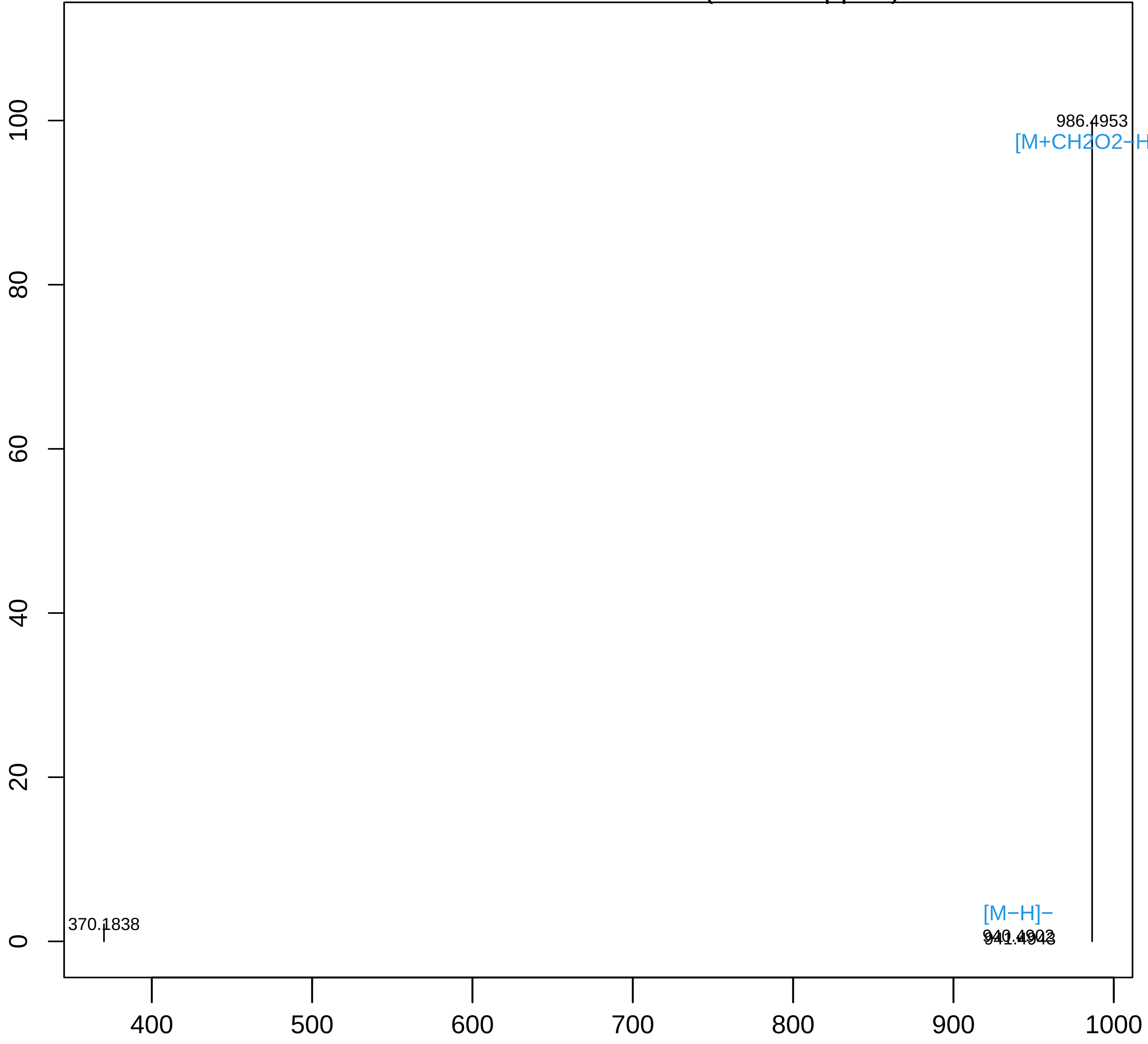

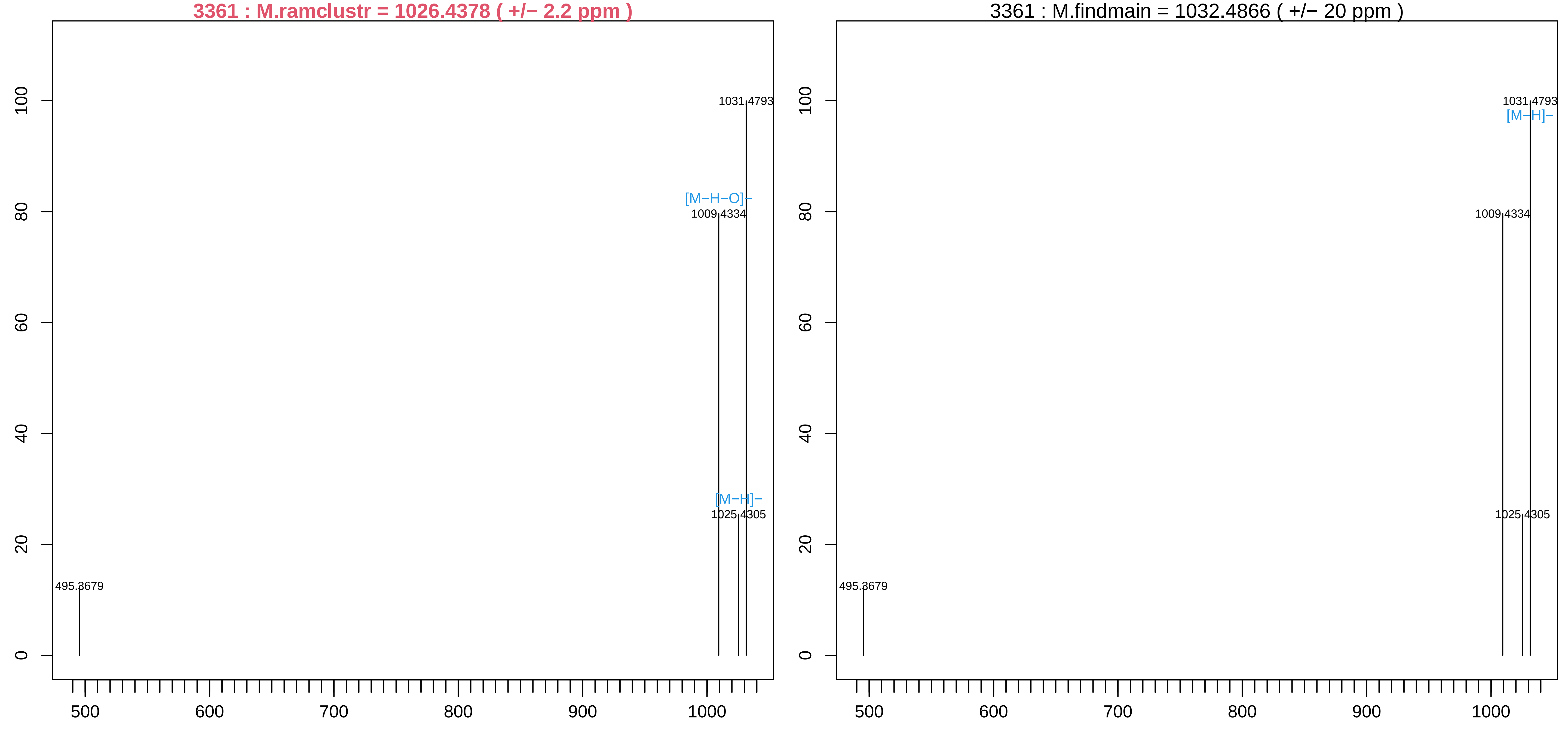
3361 : M.ramclustr = 1026.4378 (+/− 2.2 ppm)
1031.4793
[M−H−O]−
1009.4334
[M−H]−
1025.4305
495.3679
3361 : M.findmain = 1032.4866 (+/− 20 ppm)
1031.4793
[M−H]−
1009.4334
1025.4305
495.3679

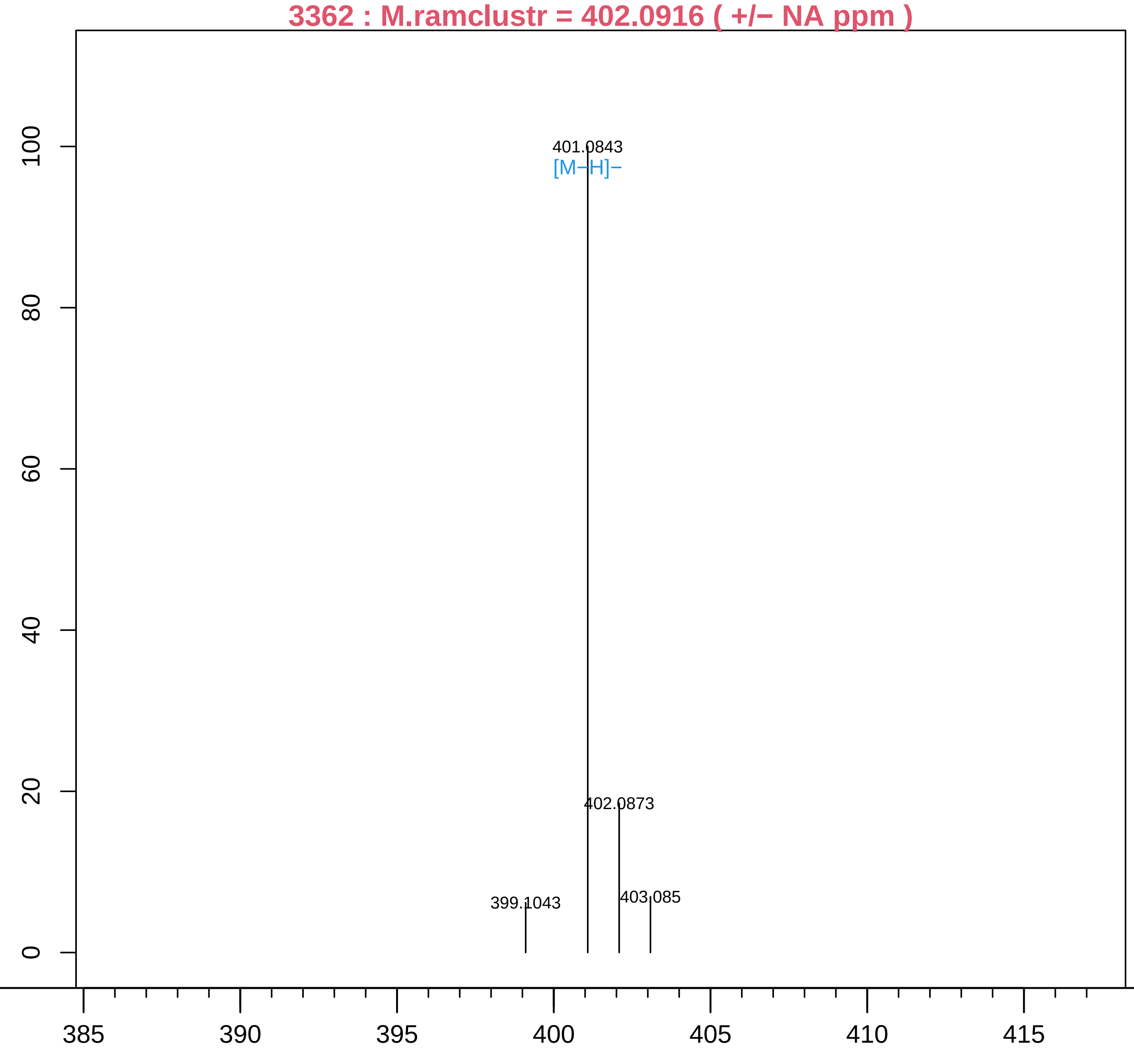
3362 : M.ramclustr = 402.0916 (+/− NA ppm)
401.0843
[M−H]−
402.0873
399.1043
403.085
0
20
40
60
80
100
385
390
395
400
405
410
415

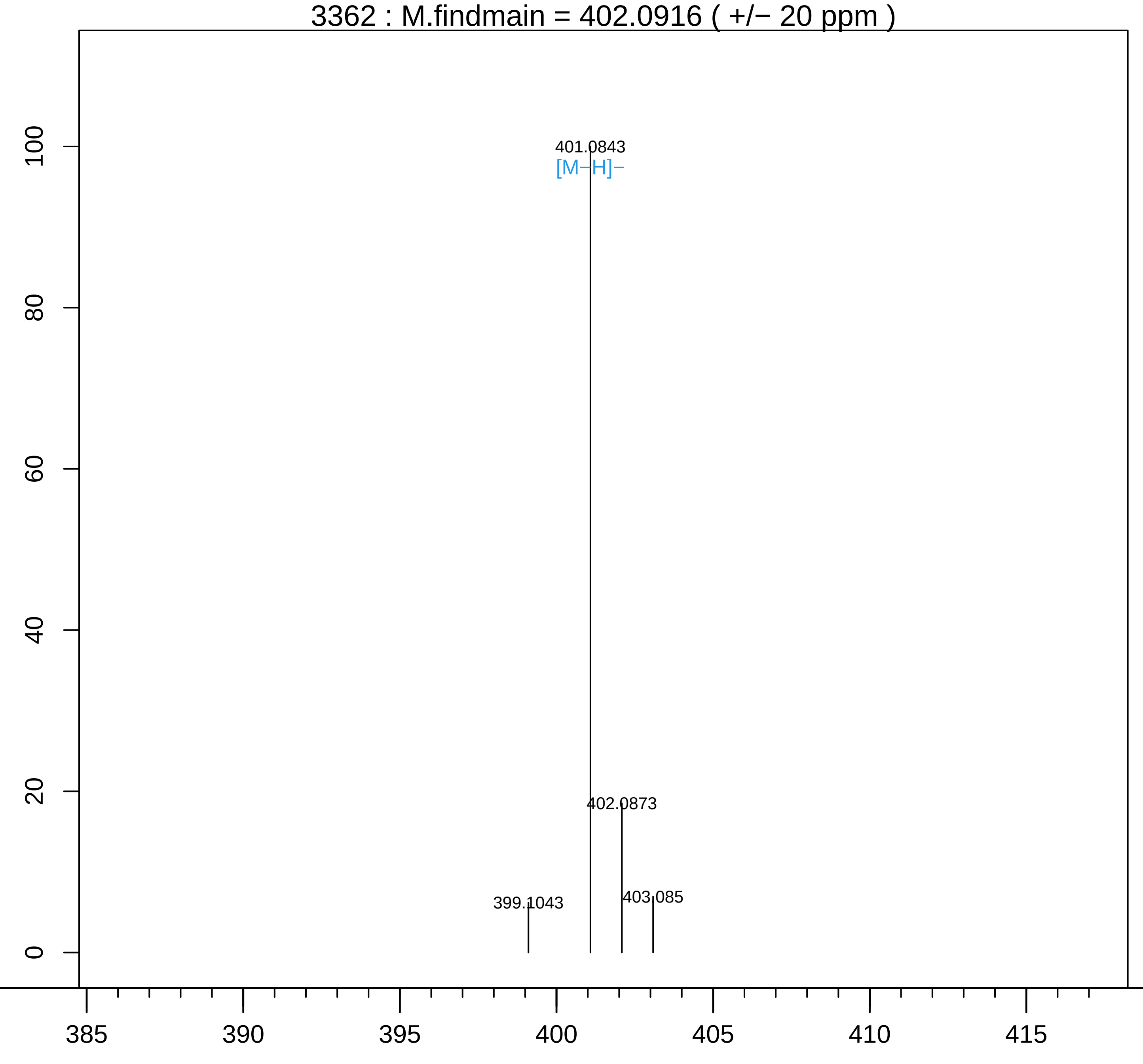
3362 : M.findmain = 402.0916 (+/− 20 ppm)
401.0843
[M−H]−
402.0873
399.1043
403.085
0
20
40
60
80
100
385
390
395
400
405
410
415

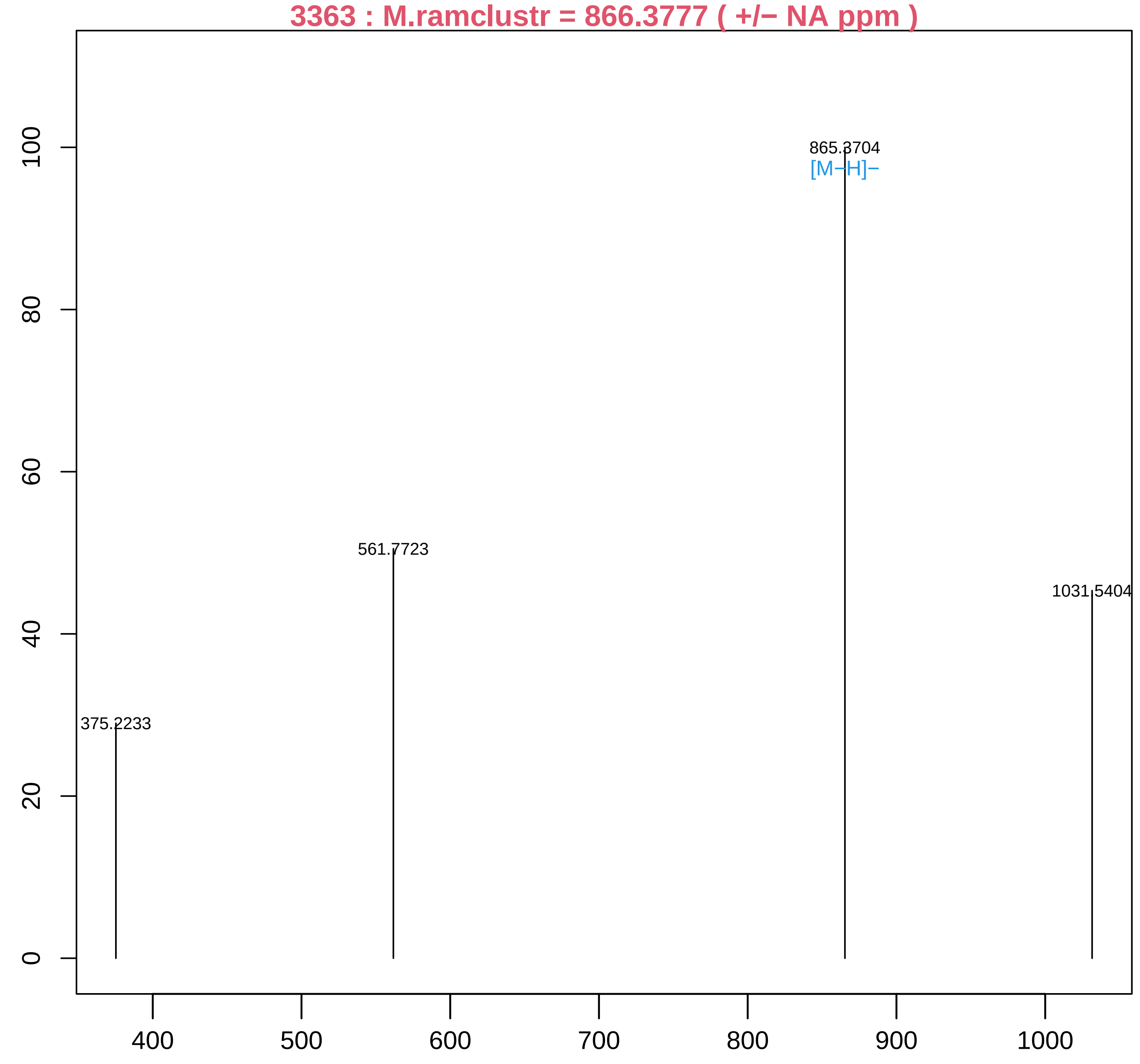
3363 : M.ramclustr = 866.3777 (+/− NA ppm)
865.3704
[M−H]−
561.7723
1031.5404
375.2233
0
20
40
60
80
100
400
500
600
700
800
900
1000

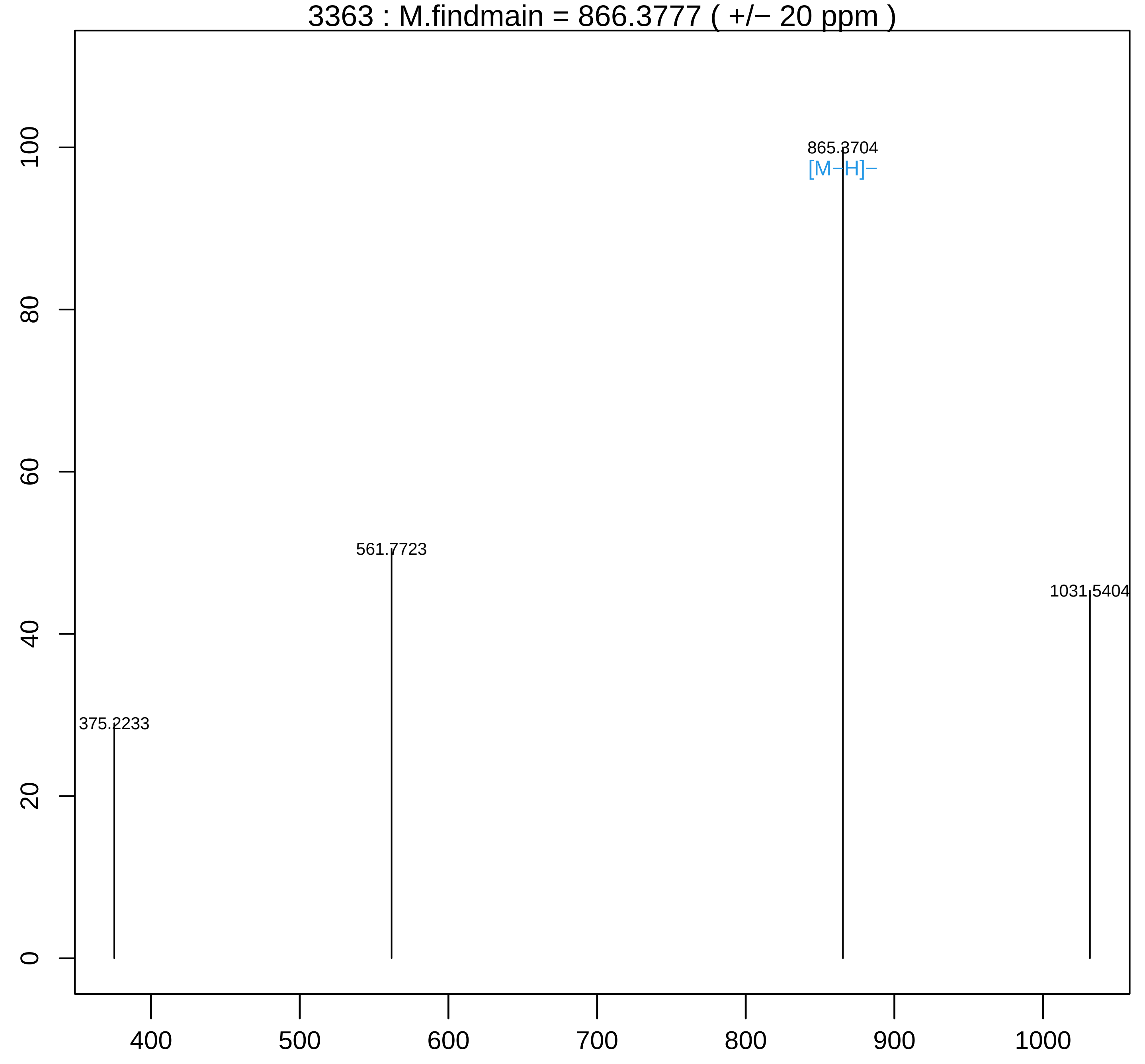
3363 : M.findmain = 866.3777 (+/− 20 ppm)
865.3704
[M−H]−
561.7723
1031.5404
375.2233
0
20
40
60
80
100
400
500
600
700
800
900
1000

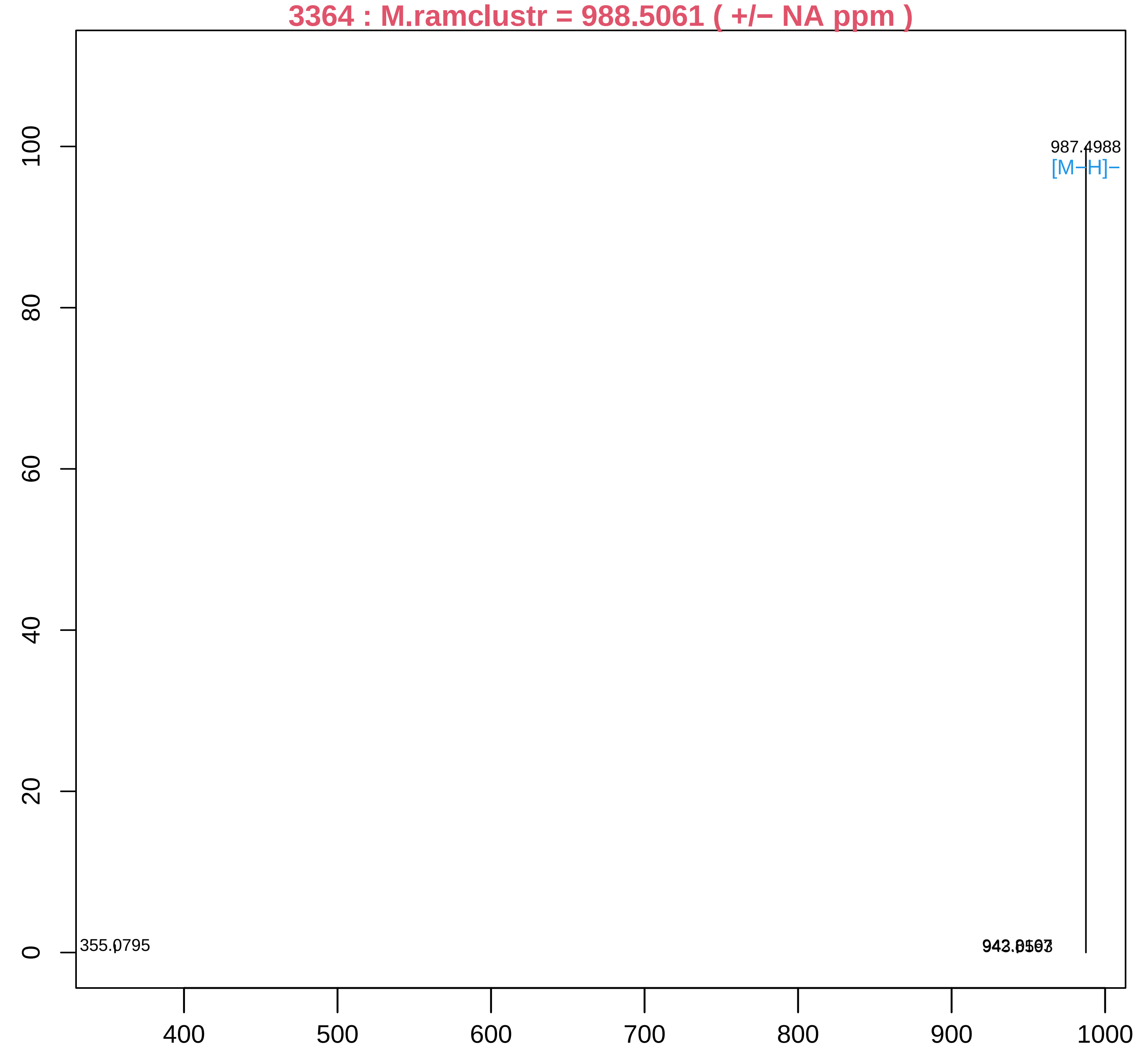
3364 : M.ramclustr = 988.5061 (+/− NA ppm)
987.4988
[M−H]−
355.0795
943.0507
942.9163
0
20
40
60
80
100
400
500
600
700
800
900
1000

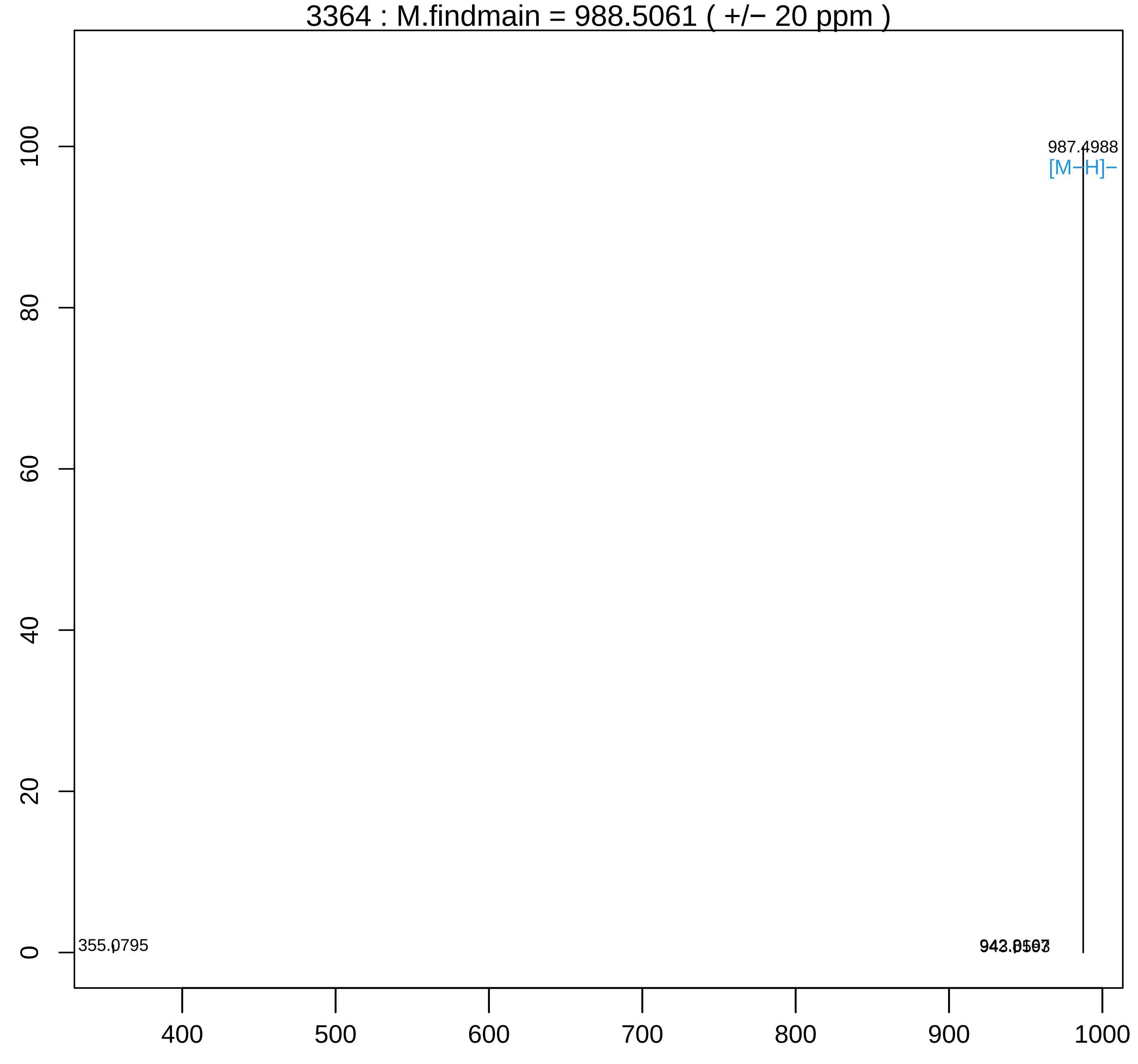
3364 : M.findmain = 988.5061 (+/− 20 ppm)
987.4988
[M−H]−
355.0795
943.0507
942.9163
0
20
40
60
80
100
400
500
600
700
800
900
1000

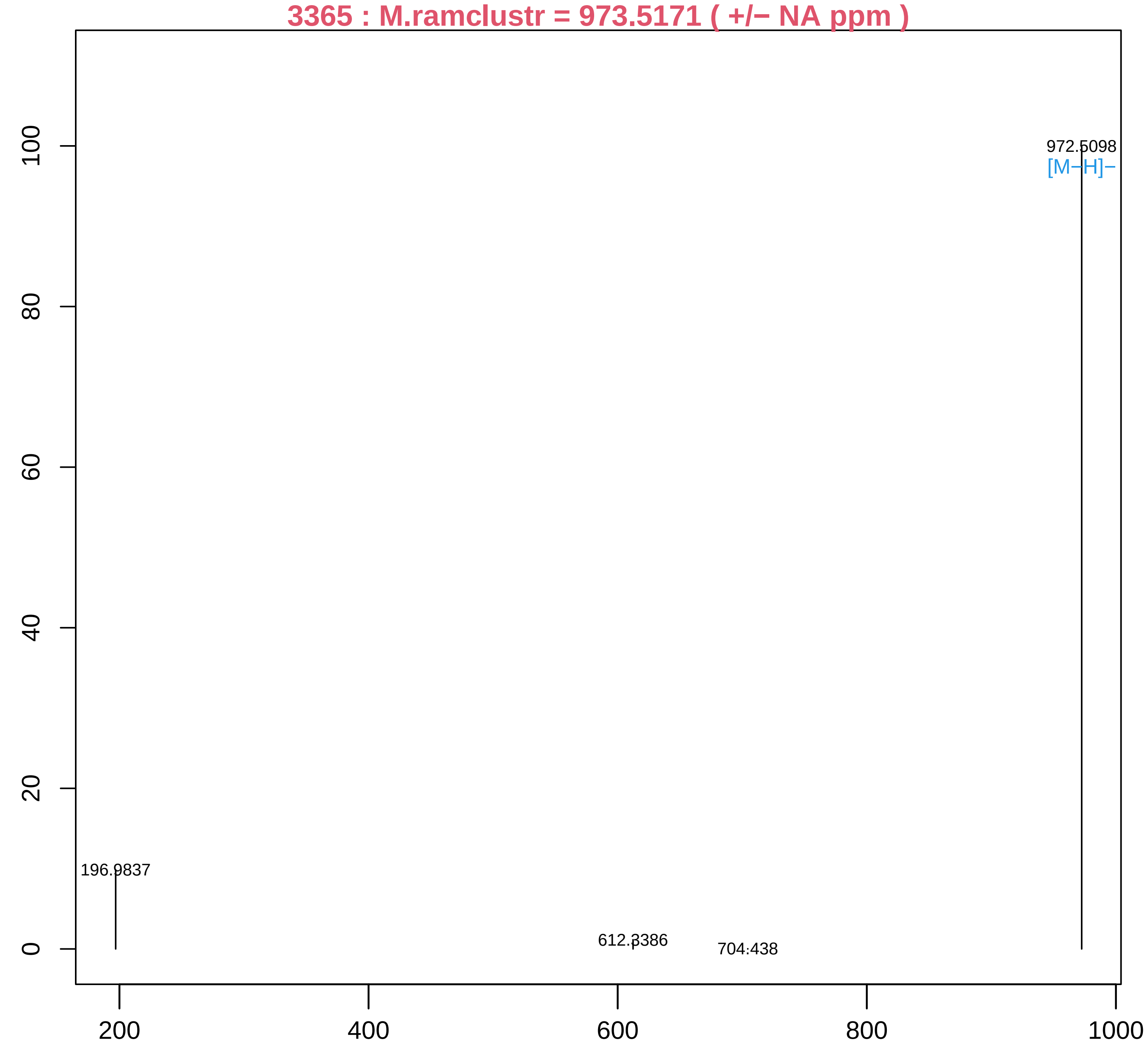
3365 : M.ramclustr = 973.5171 (+/− NA ppm)
972.5098
[M−H]−
196.9837
612.3386
704.438
0
20
40
60
80
100
200
400
600
800
1000

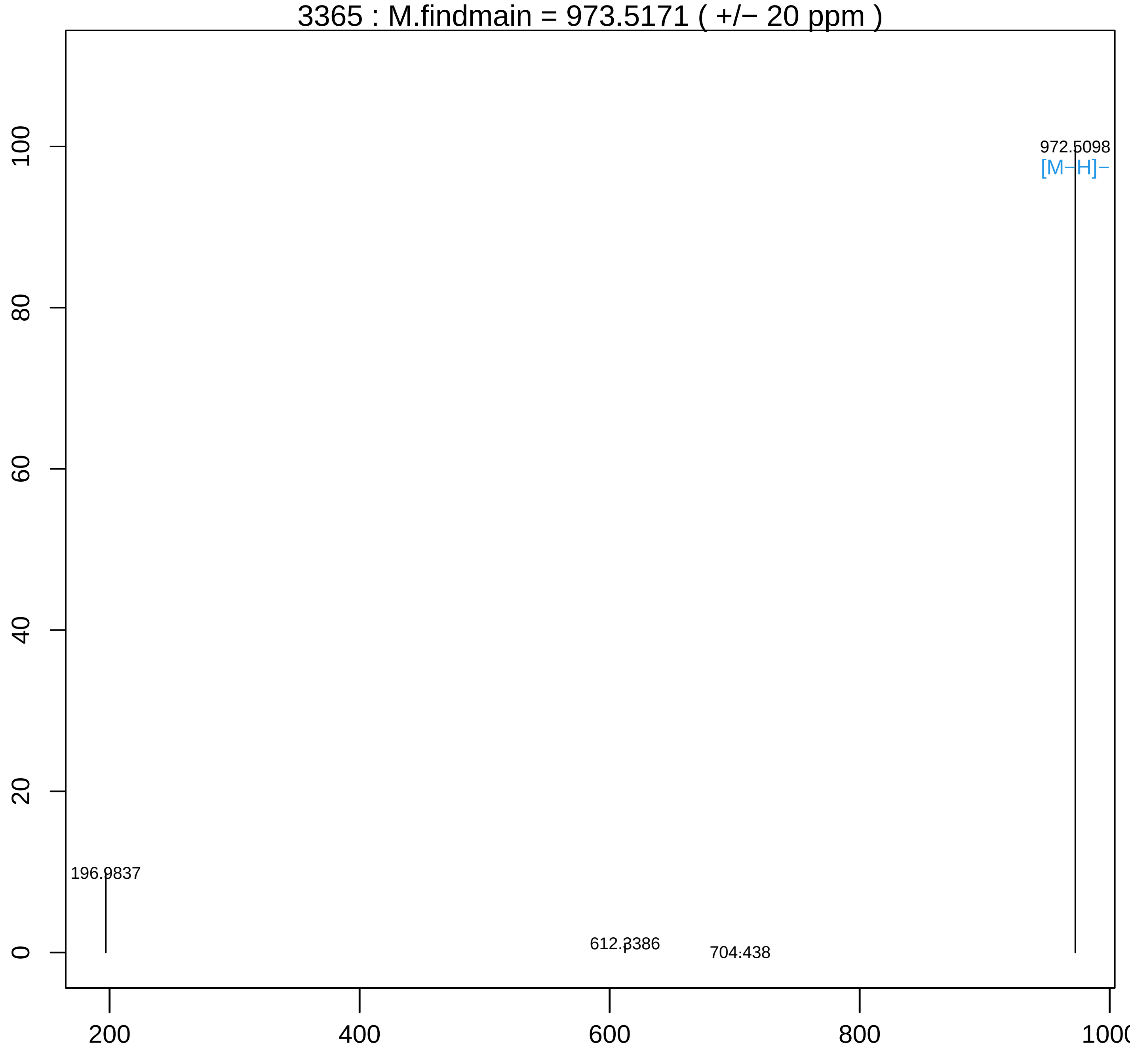
3365 : M.findmain = 973.5171 (+/− 20 ppm)
972.5098
[M−H]−
196.9837
612.3386
704.438
0
20
40
60
80
100
200
400
600
800
1000

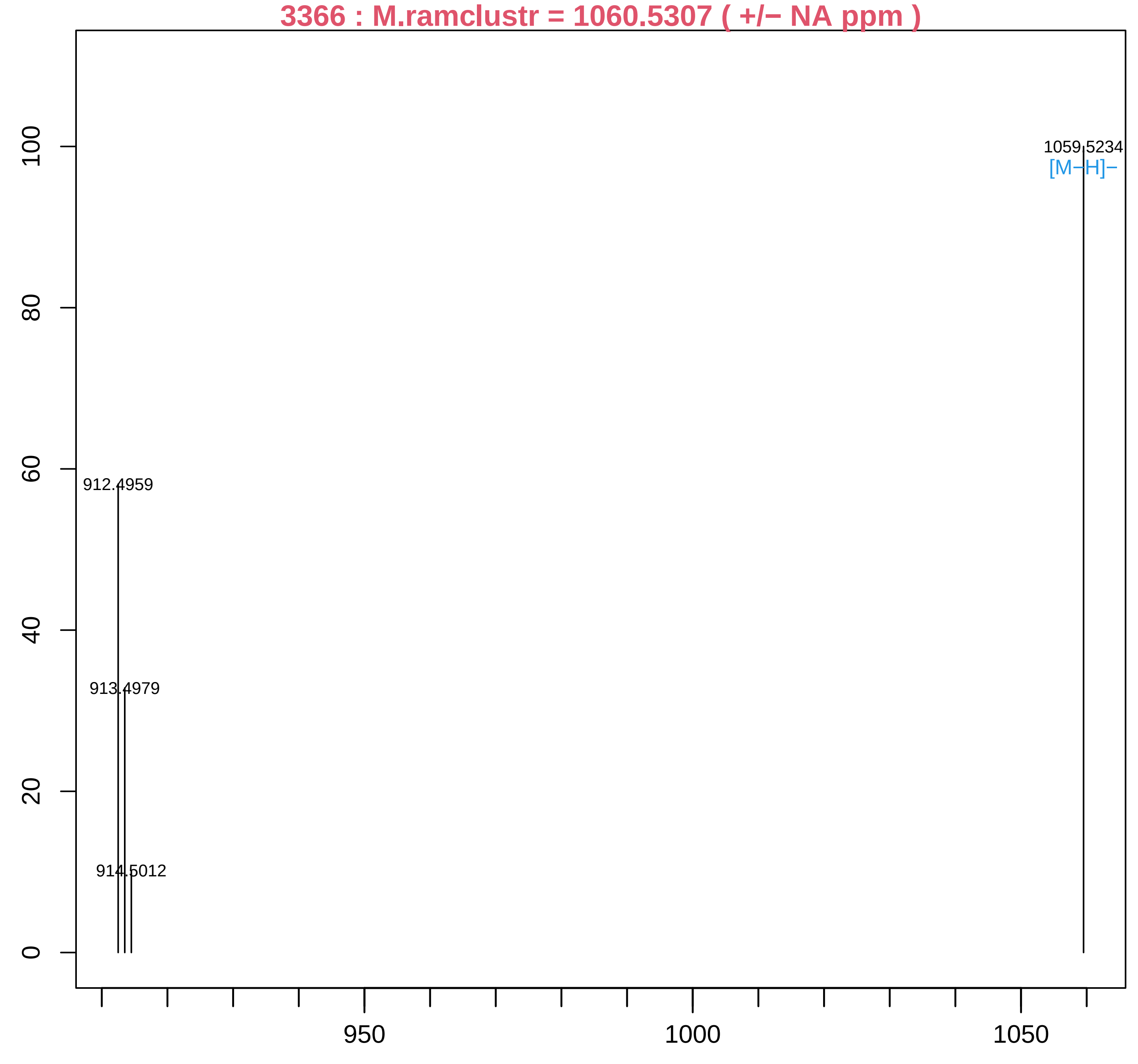
3366 : M.ramclustr = 1060.5307 (+/− NA ppm)
1059.5234
[M−H]−
912.4959
913.4979
914.5012
0
20
40
60
80
100
950
1000
1050
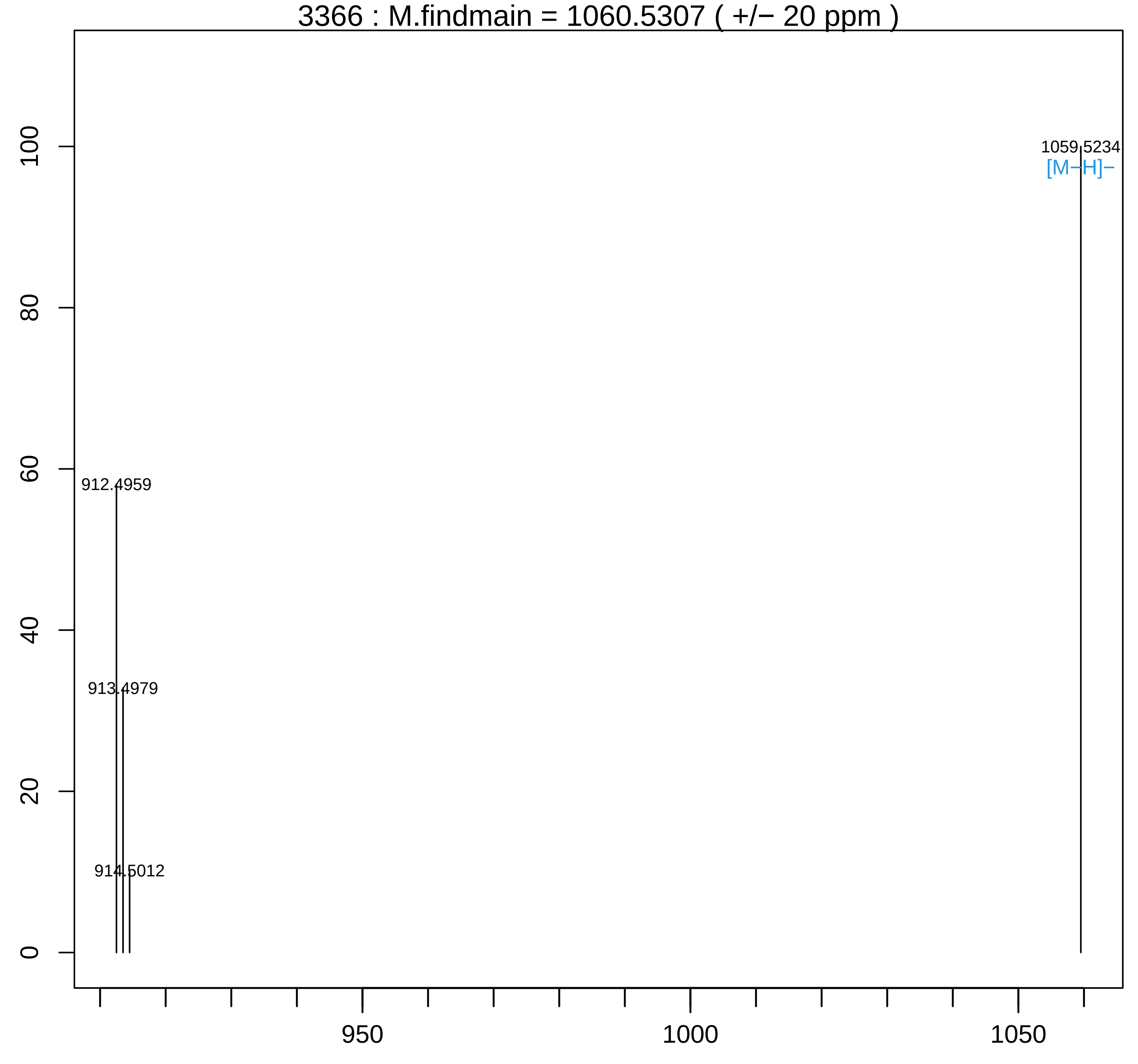
3366 : M.findmain = 1060.5307 (+/− 20 ppm)
1059.5234
[M−H]−
912.4959
913.4979
914.5012
0
20
40
60
80
100
950
1000
1050

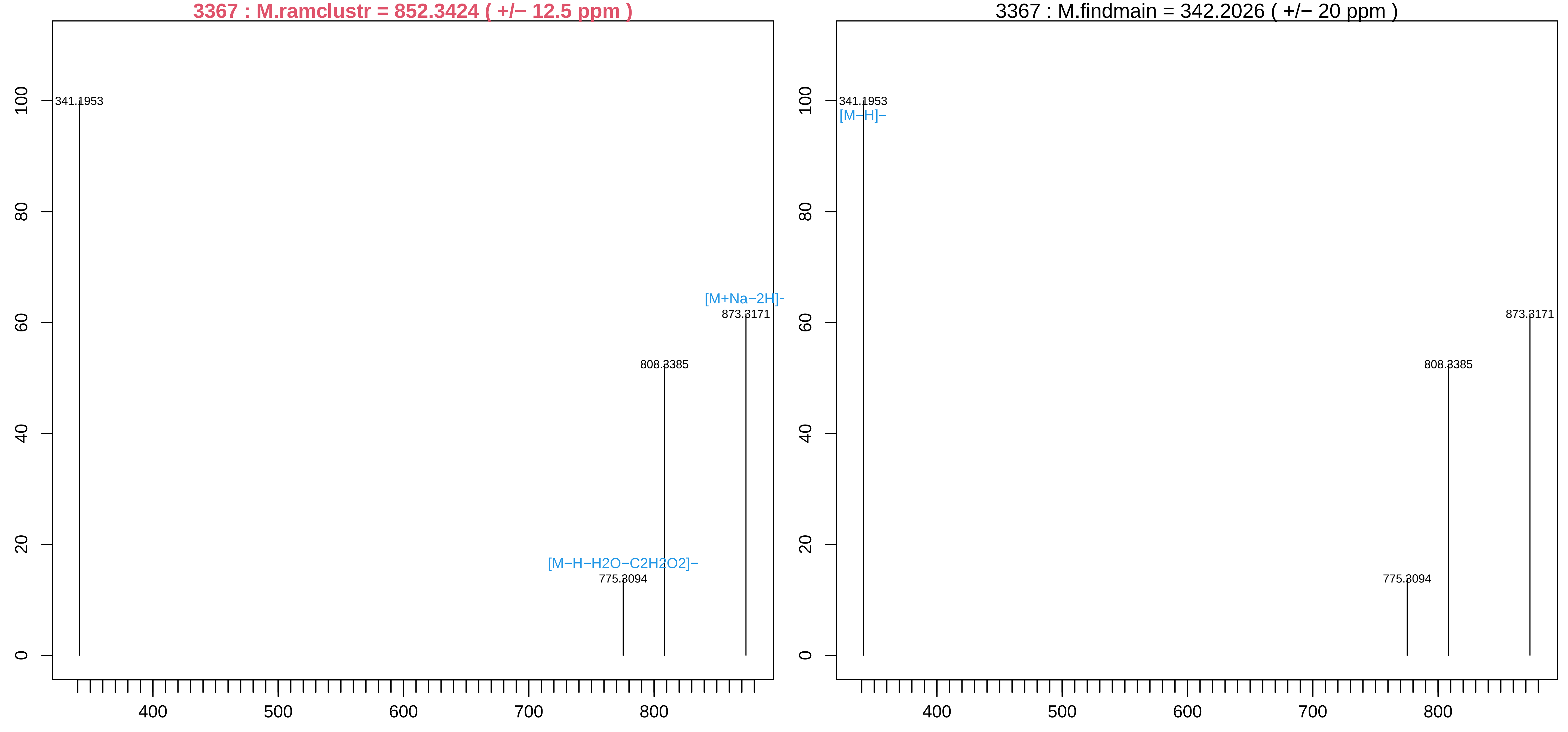

3367 : M.ramclustr = 852.3424 (+/− 12.5 ppm)
341.1953
[M+Na−2H]-
873.3171
808.3385
[M−H−H2O−C2H2O2]−
775.3094
0
20
40
60
80
100
400
500
600
700
800
3367 : M.findmain = 342.2026 (+/− 20 ppm)
341.1953
[M−H]−
873.3171
808.3385
775.3094
0
20
40
60
80
100
400
500
600
700
800

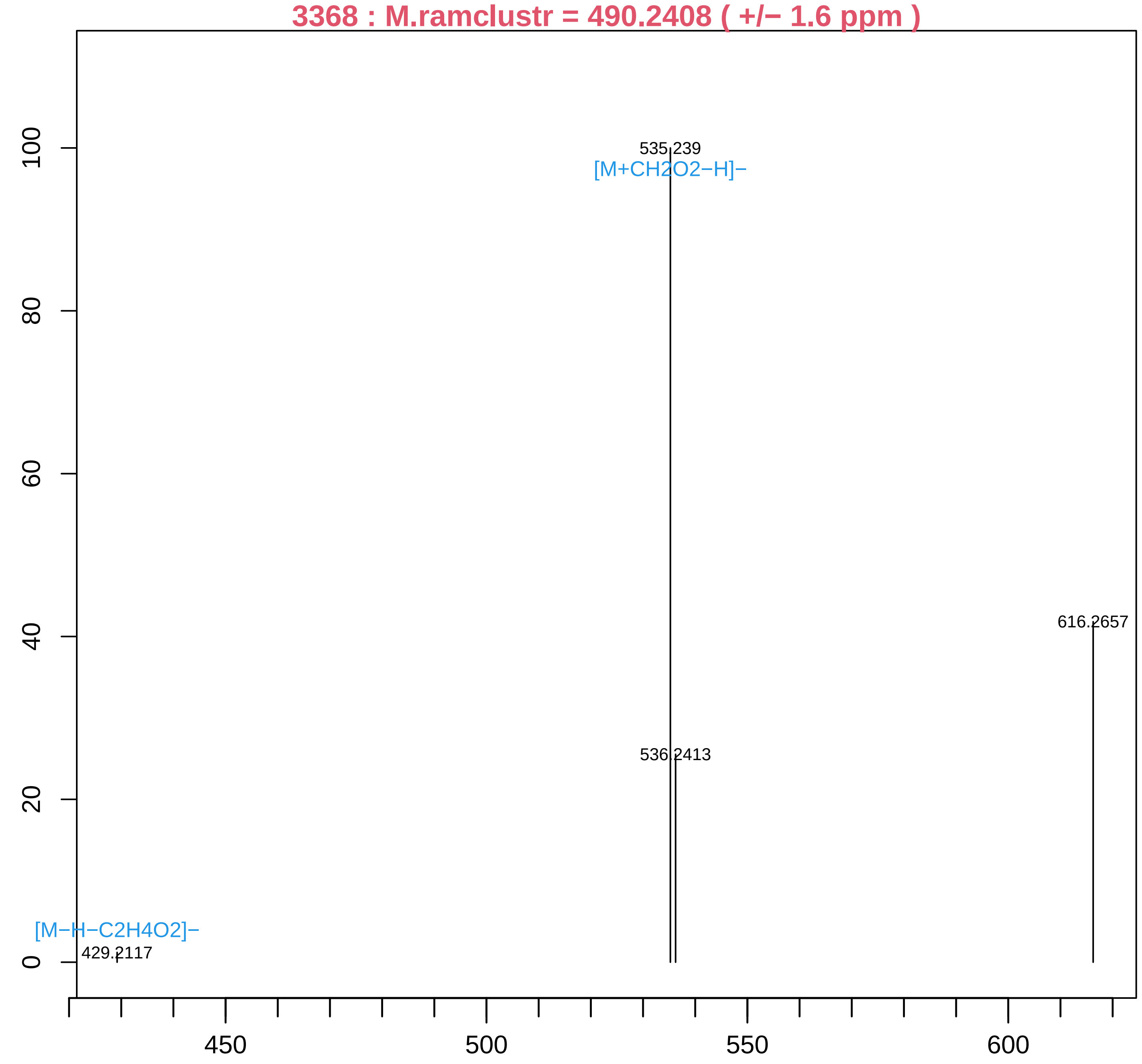

3368 : M.ramclustr = 490.2408 (+/− 1.6 ppm)
535.239
[M+CH2O2−H]−
616.2657
536.2413
[M−H−C2H4O2]−
429.2117
0
20
40
60
80
100
450
500
550
600

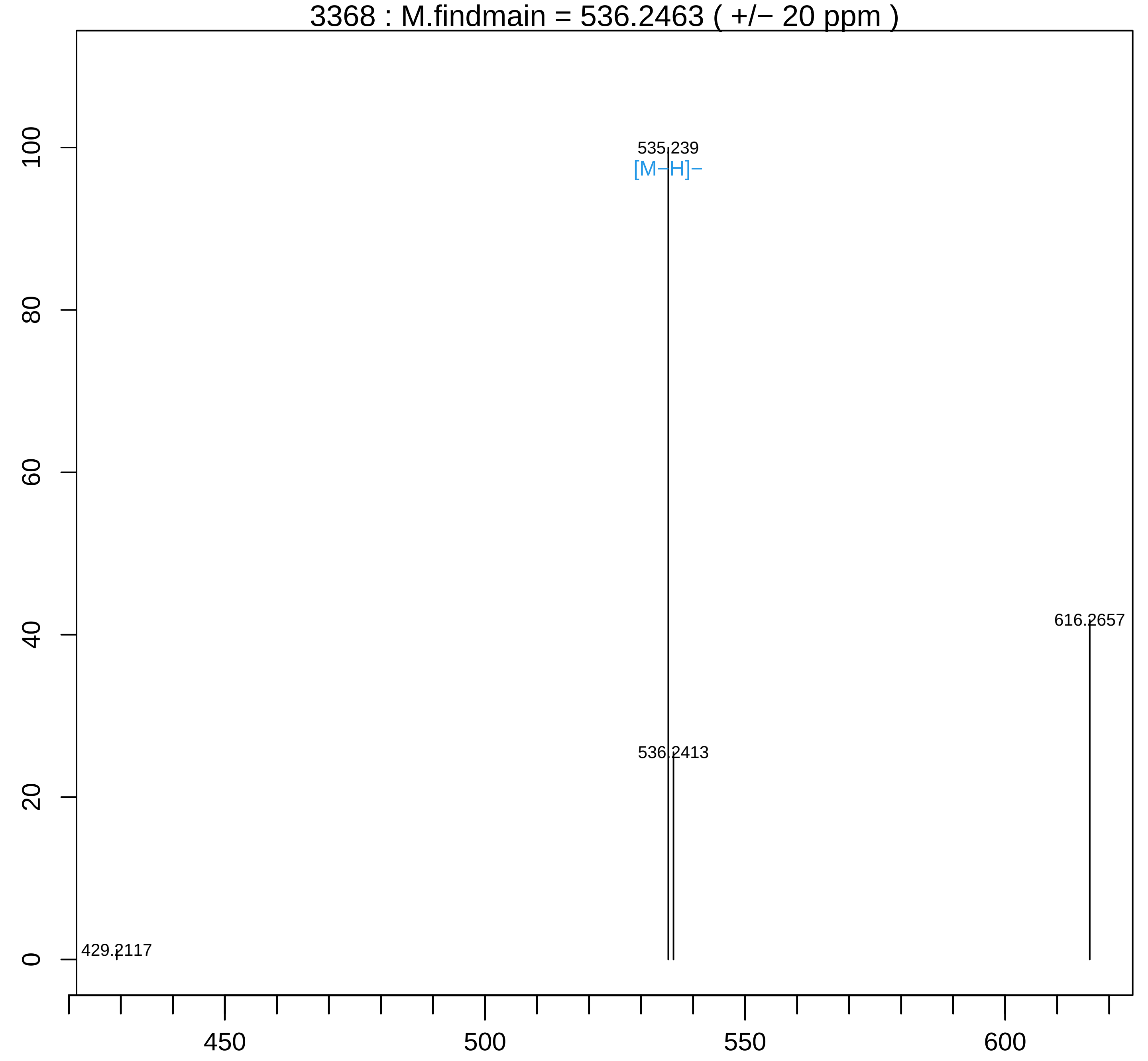

3368 : M.findmain = 536.2463 (+/− 20 ppm)
535.239
[M−H]−
616.2657
536.2413
429.2117
0
20
40
60
80
100
450
500
550
600

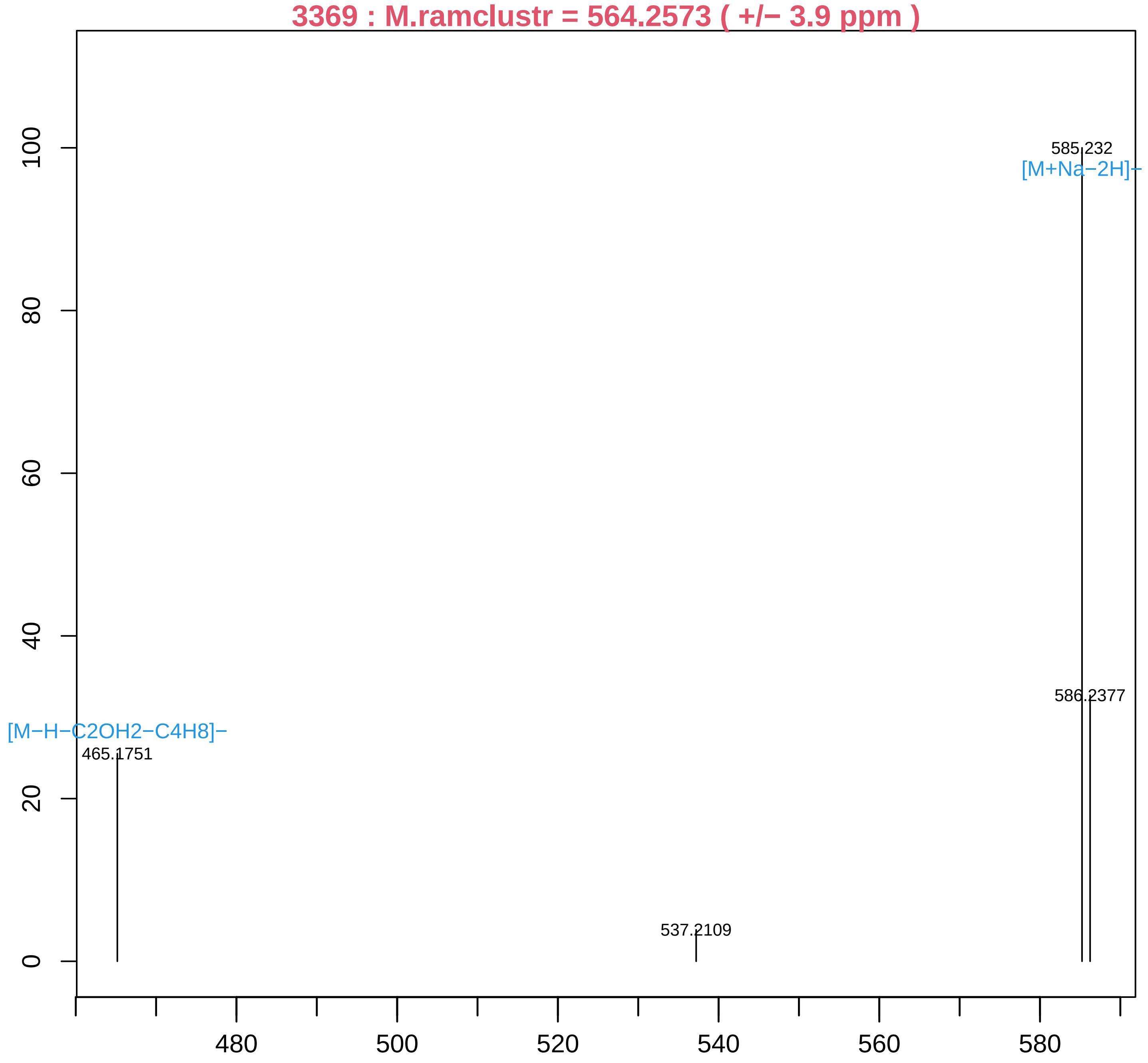
3369 : M.ramclustr = 564.2573 (+/− 3.9 ppm)
585.232
[M+Na−2H]−
586.2377
[M−H−C2OH2−C4H8]−
465.1751
537.2109
0
20
40
60
80
100
480
500
520
540
560
580

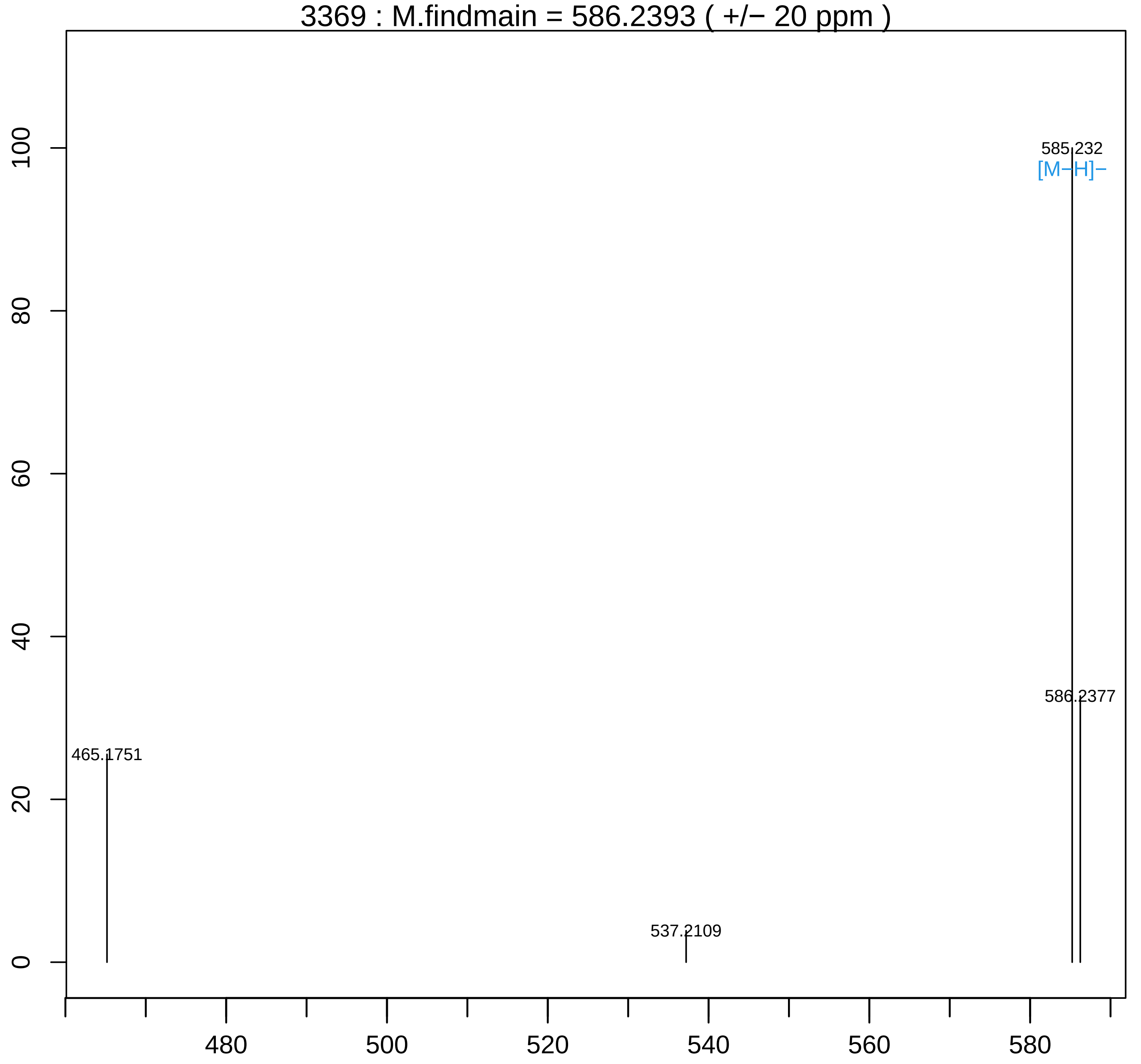
3369 : M.findmain = 586.2393 (+/− 20 ppm)
585.232
[M−H]−
586.2377
465.1751
537.2109
0
20
40
60
80
100
480
500
520
540
560
580

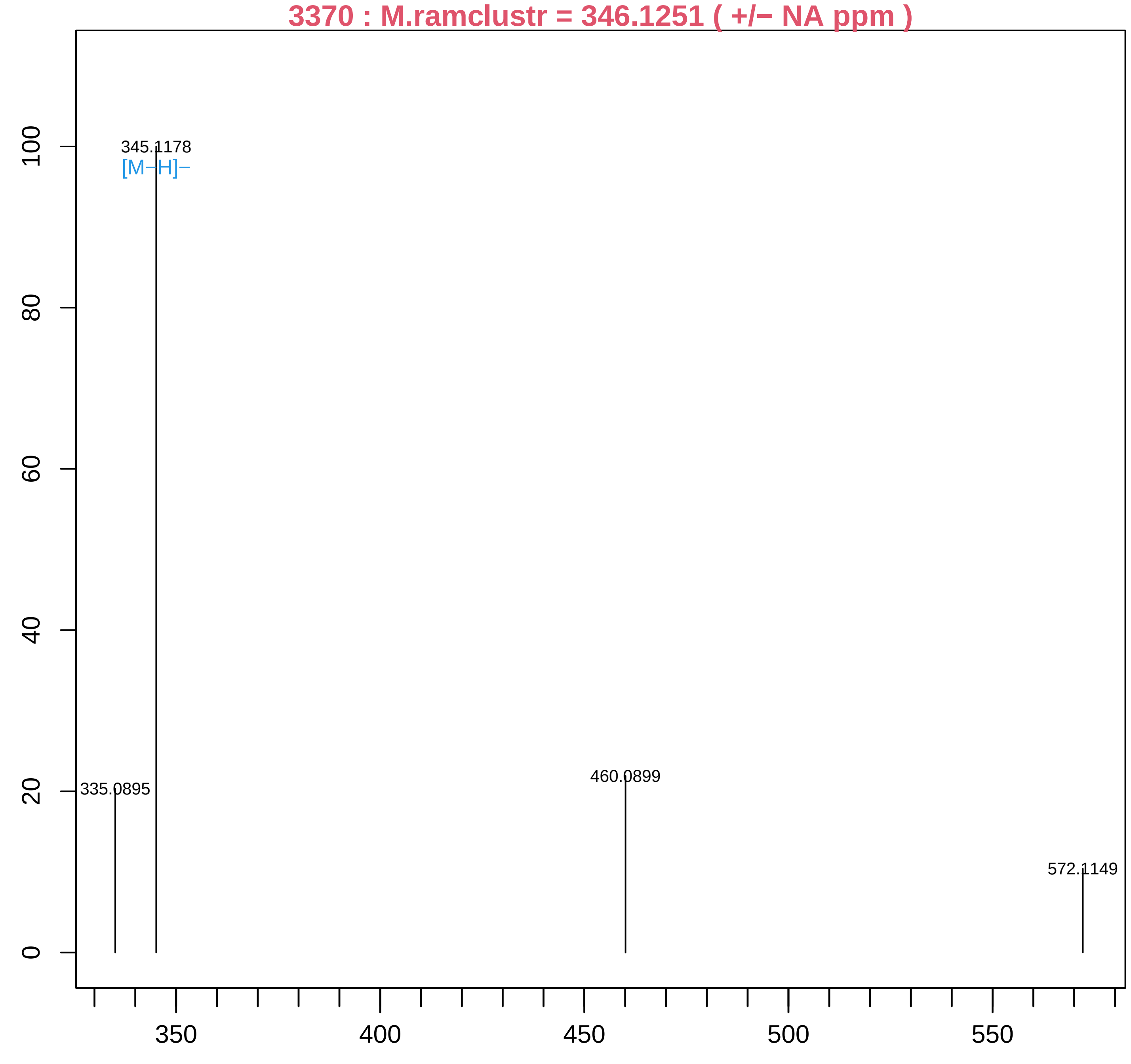

3370 : M.ramclustr = 346.1251 (+/− NA ppm)
345.1178
[M−H]−
335.0895
460.0899
572.1149
0
20
40
60
80
100
350
400
450
500
550

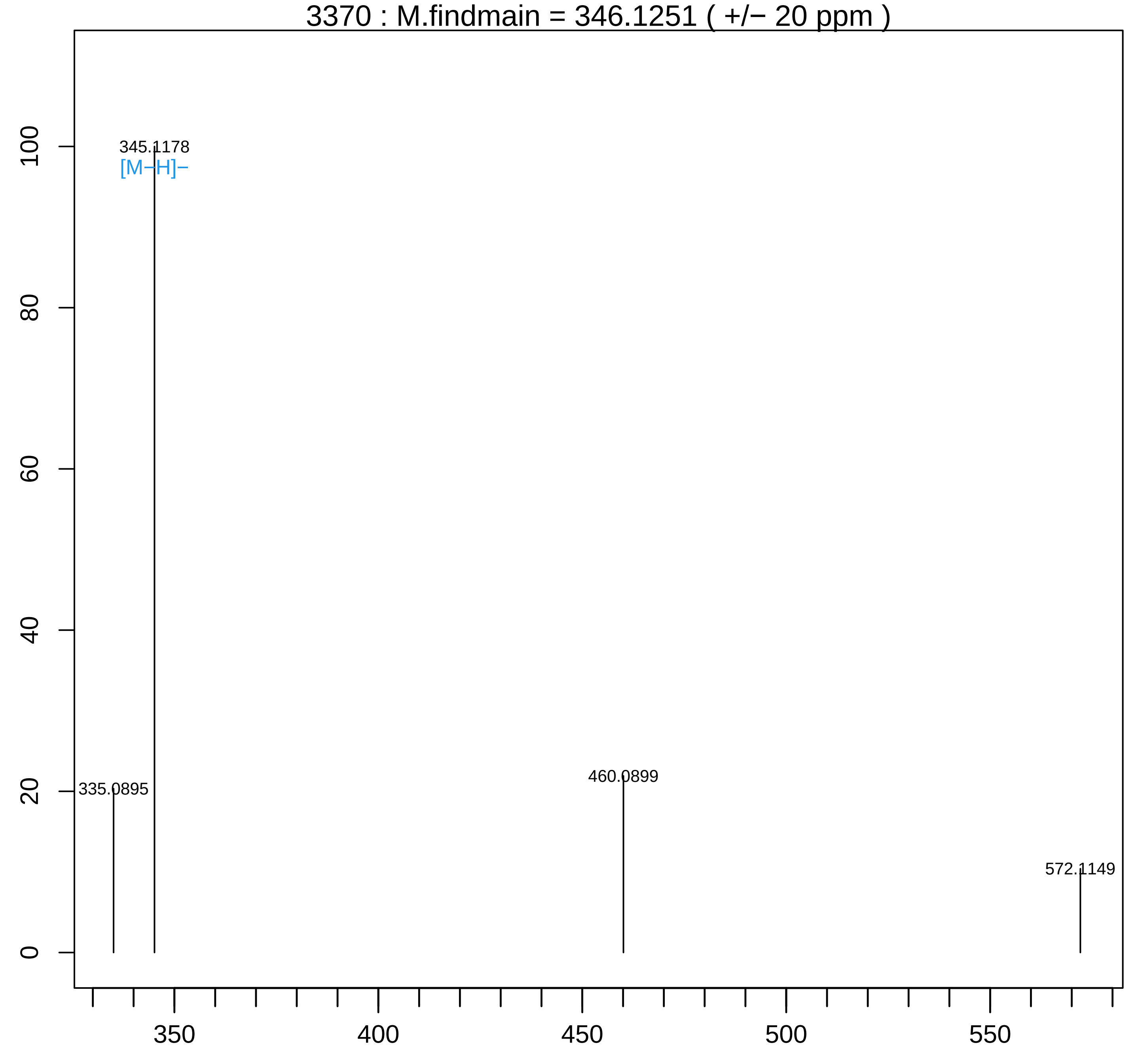

3370 : M.findmain = 346.1251 (+/− 20 ppm)
345.1178
[M−H]−
335.0895
460.0899
572.1149
0
20
40
60
80
100
350
400
450
500
550

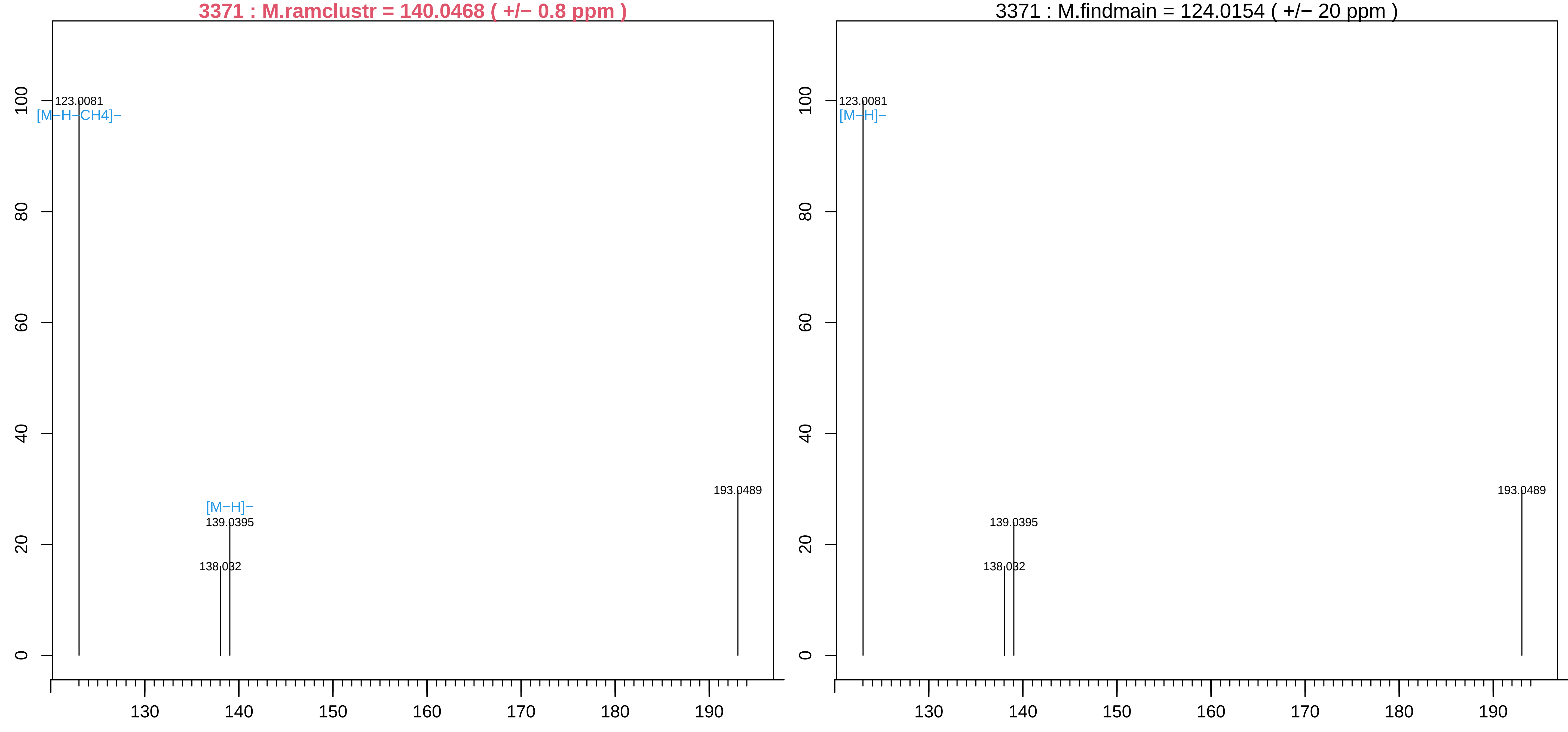
3371 : M.ramclustr = 140.0468 (+/− 0.8 ppm)
123.0081
[M−H−CH4]−
[M−H]−
139.0395
138.032
193.0489
3371 : M.findmain = 124.0154 (+/− 20 ppm)
123.0081
[M−H]−
139.0395
138.032
193.0489
0
20
40
60
80
100
130
140
150
160
170
180
190

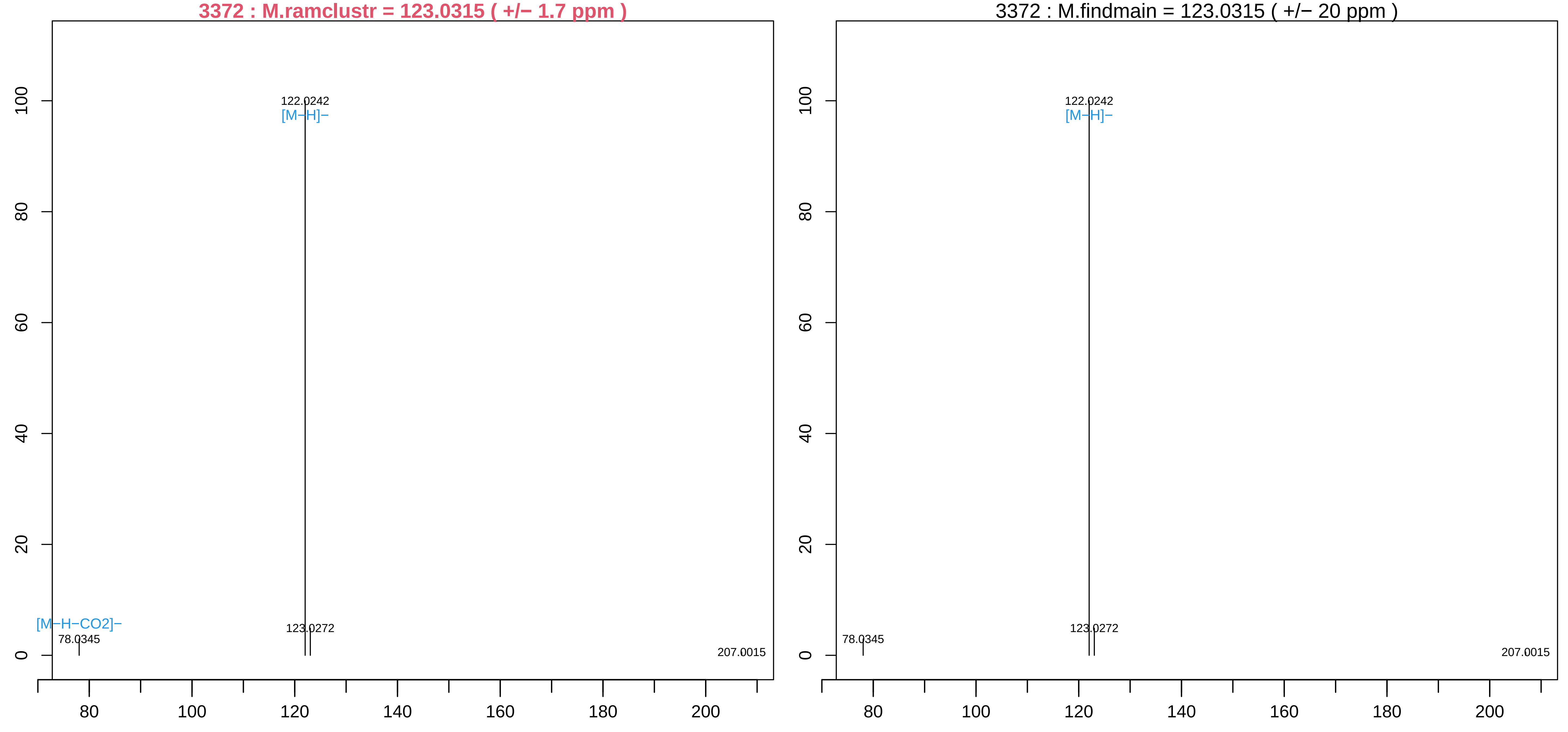
3372 : M.ramclustr = 123.0315 (+/− 1.7 ppm)
122.0242
[M−H]−
[M−H−CO2]−
78.0345
123.0272
207.0015
3372 : M.findmain = 123.0315 (+/− 20 ppm)
122.0242
[M−H]−
78.0345
123.0272
207.0015
0
20
40
60
80
100
80
100
120
140
160
180
200

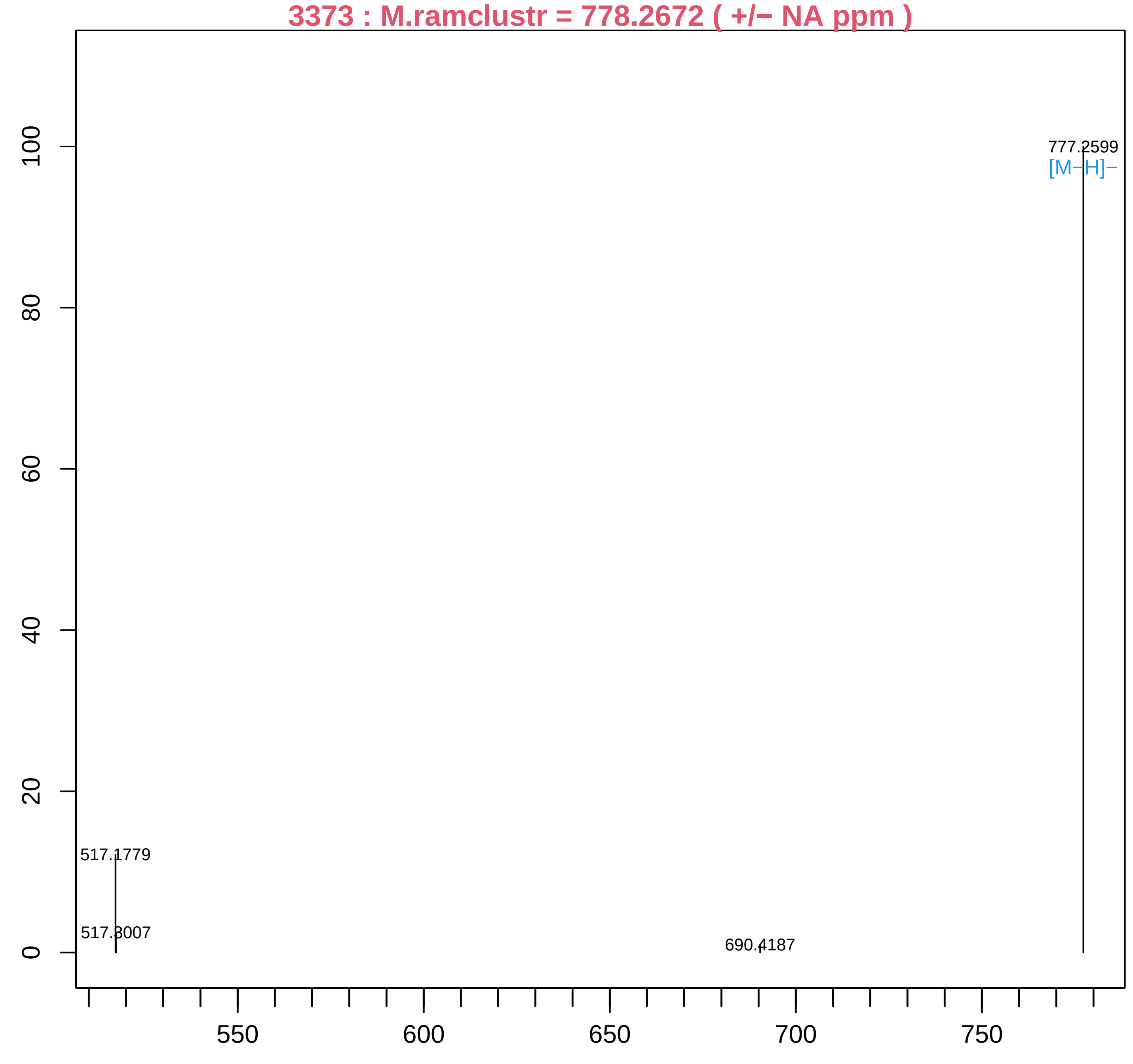
3373 : M.ramclustr = 778.2672 (+/− NA ppm)
777.2599
[M−H]−
517.1779
517.3007
690.4187
0
20
40
60
80
100
550
600
650
700
750

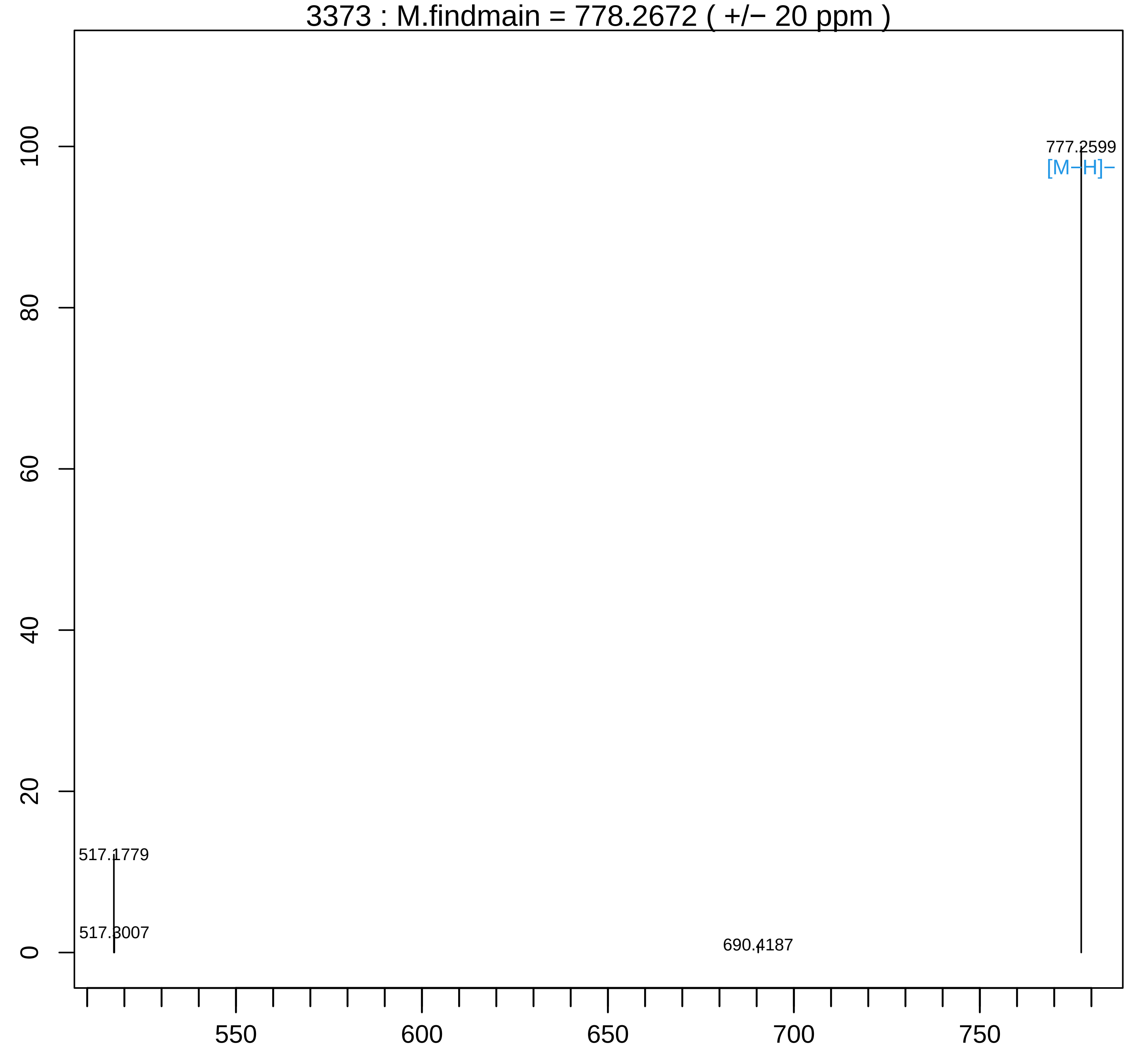
3373 : M.findmain = 778.2672 (+/− 20 ppm)
777.2599
[M−H]−
517.1779
517.3007
690.4187
0
20
40
60
80
100
550
600
650
700
750

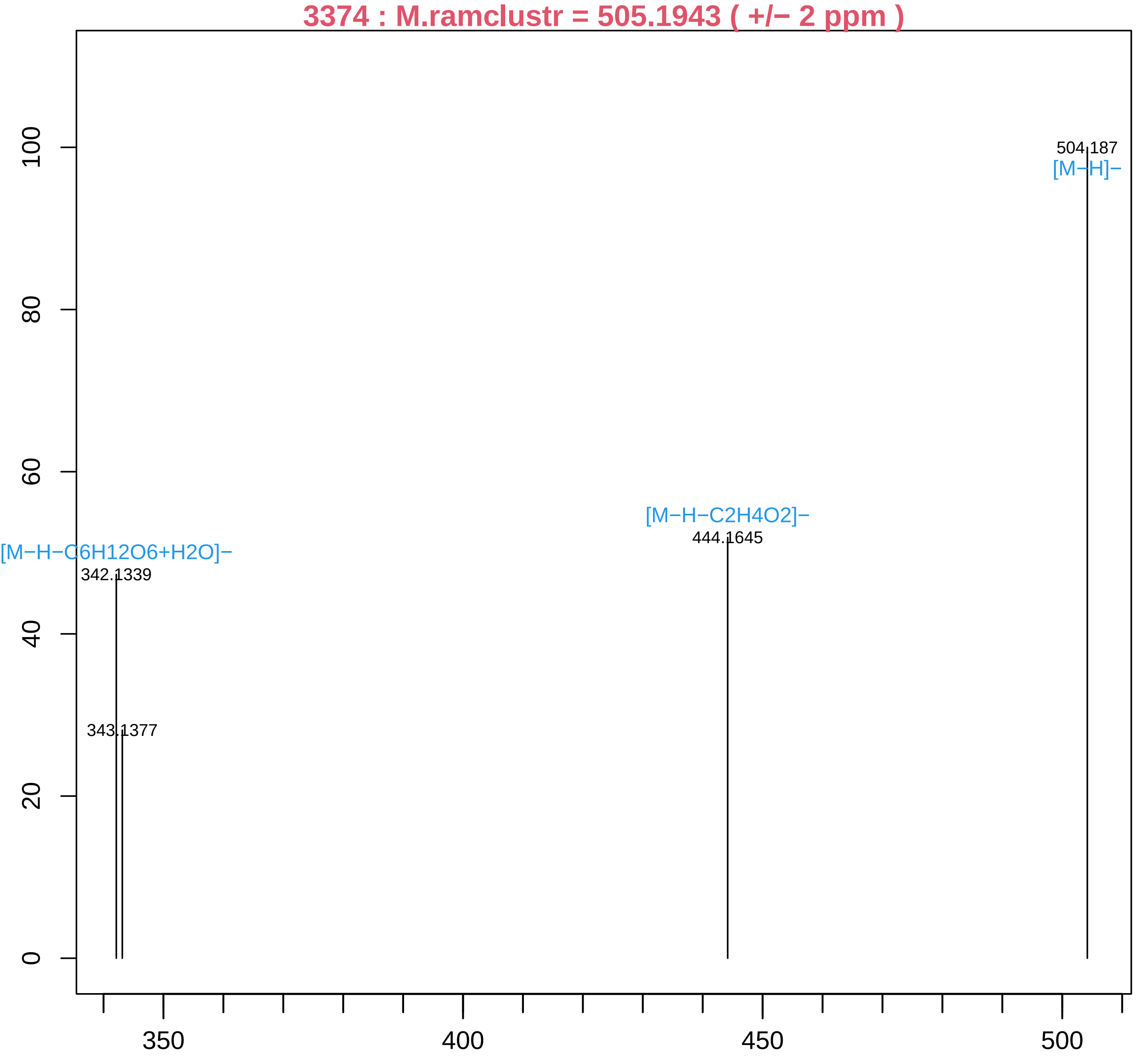
3374 : M.ramclustr = 505.1943 (+/− 2 ppm)
504.187
[M−H]−
[M−H−C2H4O2]−
444.1645
[M−H−C6H12O6+H2O]−
342.1339
343.1377
0
20
40
60
80
100
350
400
450
500

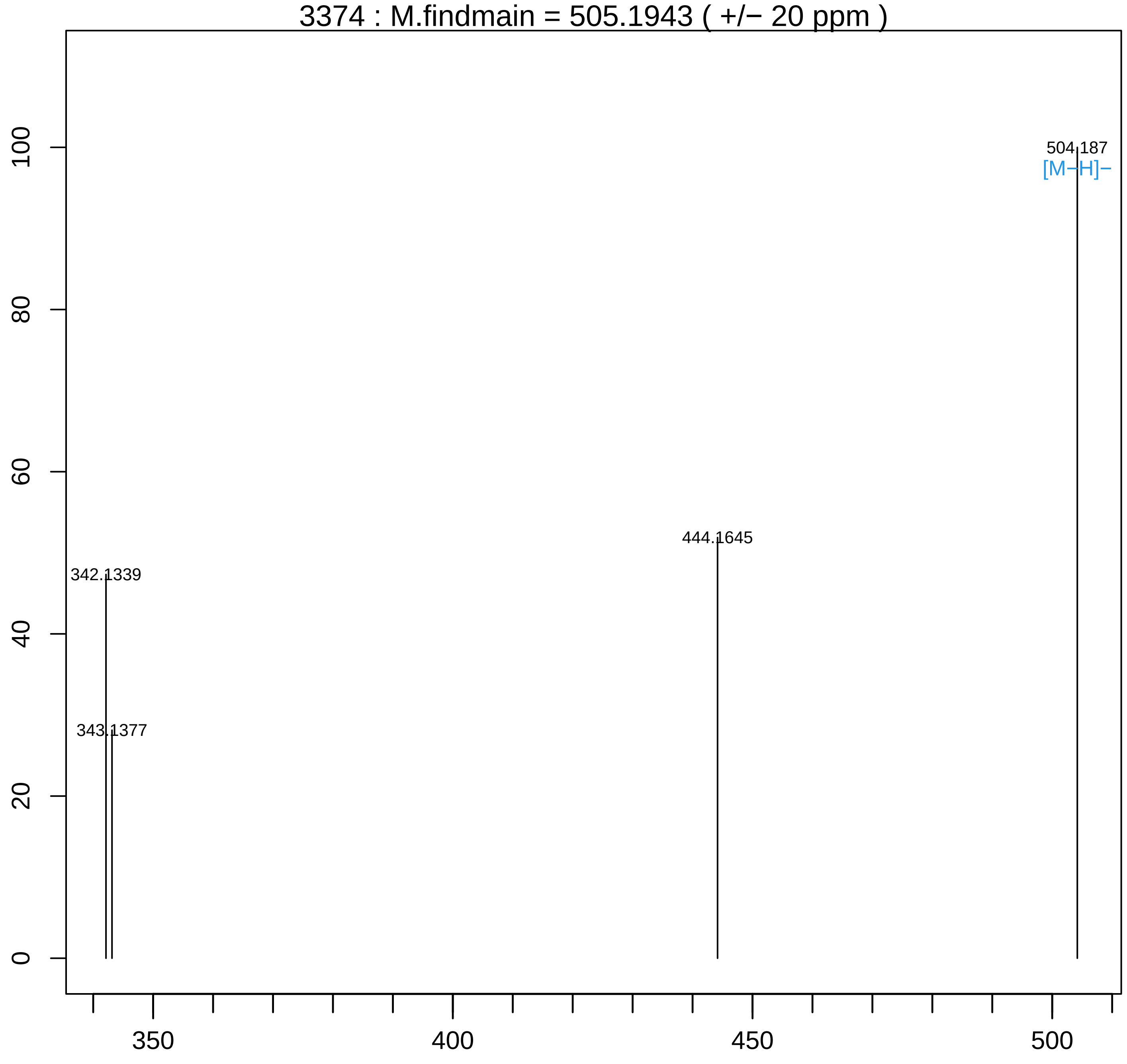
3374 : M.findmain = 505.1943 (+/− 20 ppm)
504.187
[M−H]−
444.1645
342.1339
343.1377
0
20
40
60
80
100
350
400
450
500

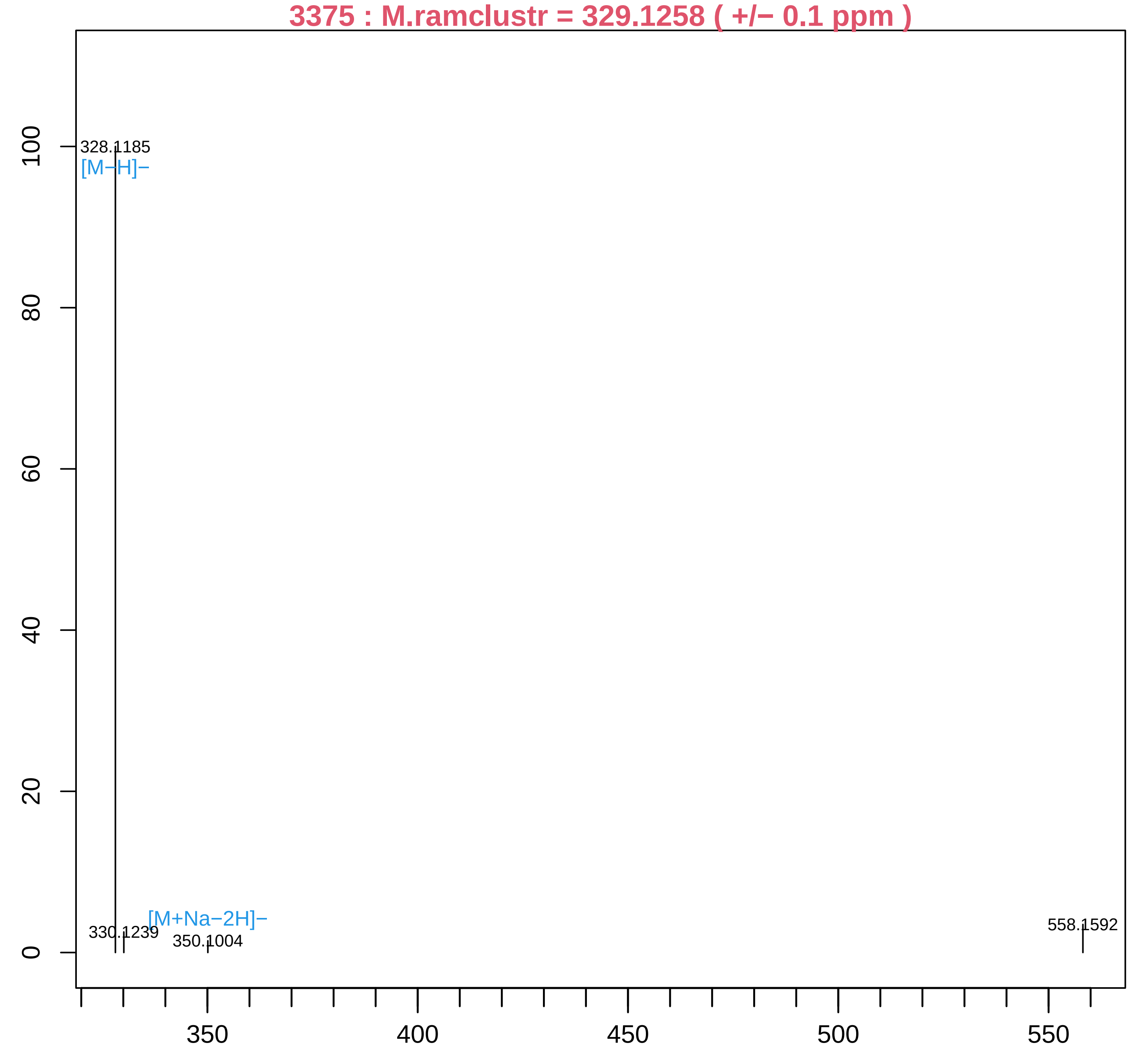
3375 : M.ramclustr = 329.1258 (+/− 0.1 ppm)
328.1185
[M−H]−
330.1239
[M+Na−2H]−
350.1004
558.1592
0
20
40
60
80
100
350
400
450
500
550

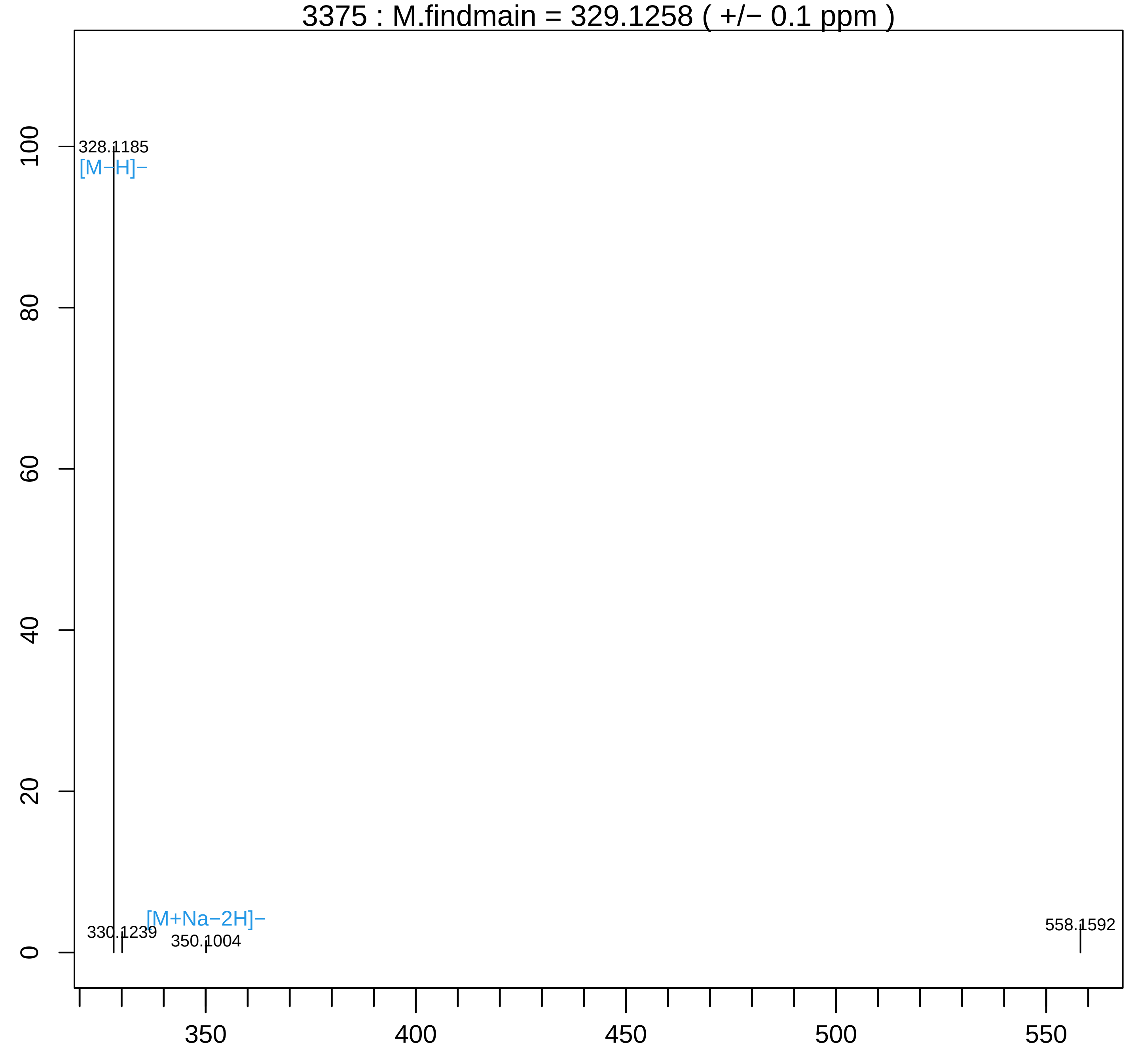
3375 : M.findmain = 329.1258 (+/− 0.1 ppm)
328.1185
[M−H]−
330.1239
[M+Na−2H]−
350.1004
558.1592
0
20
40
60
80
100
350
400
450
500
550

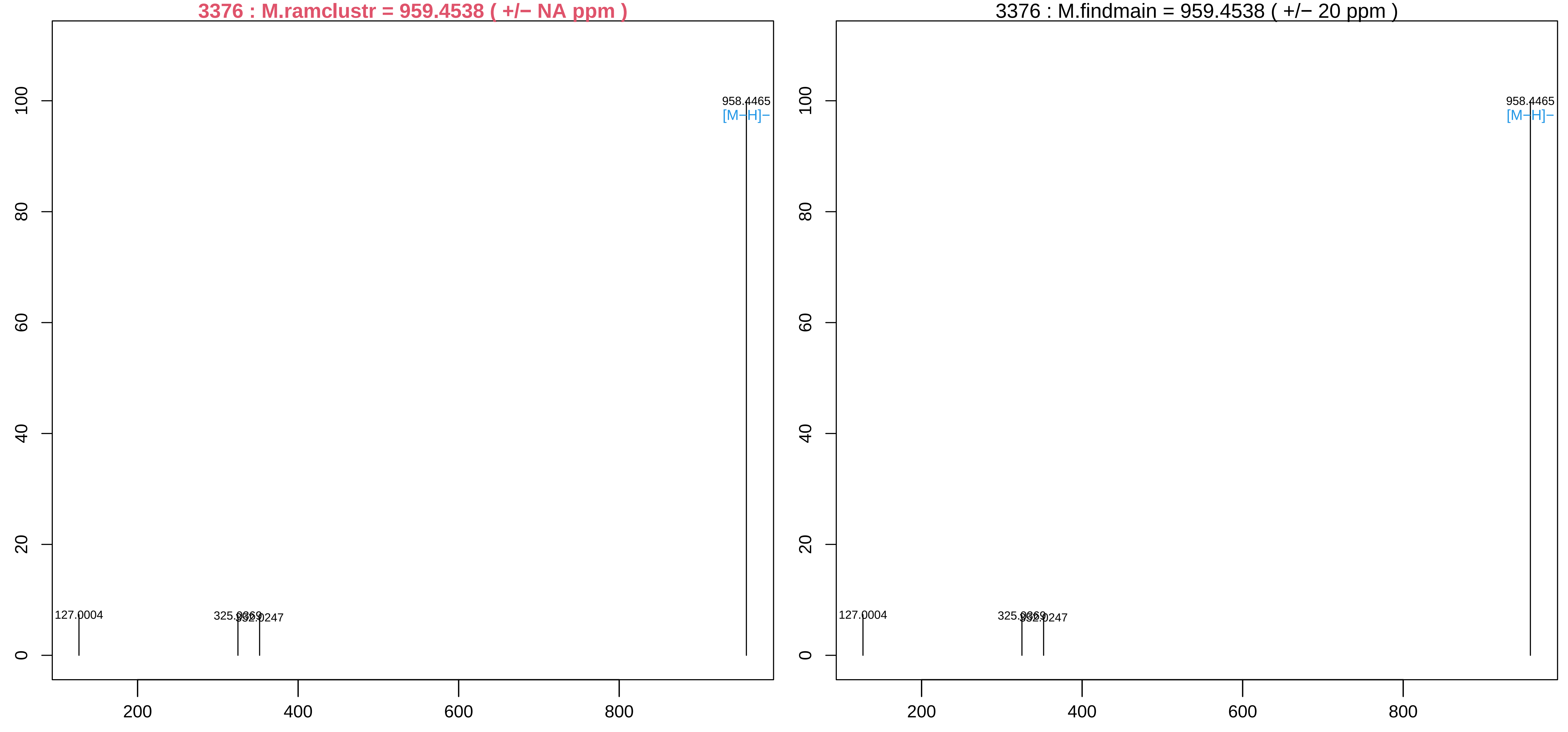
3376 : M.ramclustr = 959.4538 (+/− NA ppm)
958.4465
[M−H]−
127.0004
325.9869
352.0247
3376 : M.findmain = 959.4538 (+/− 20 ppm)
958.4465
[M−H]−
127.0004
325.9869
352.0247
0
20
40
60
80
100
200
400
600
800

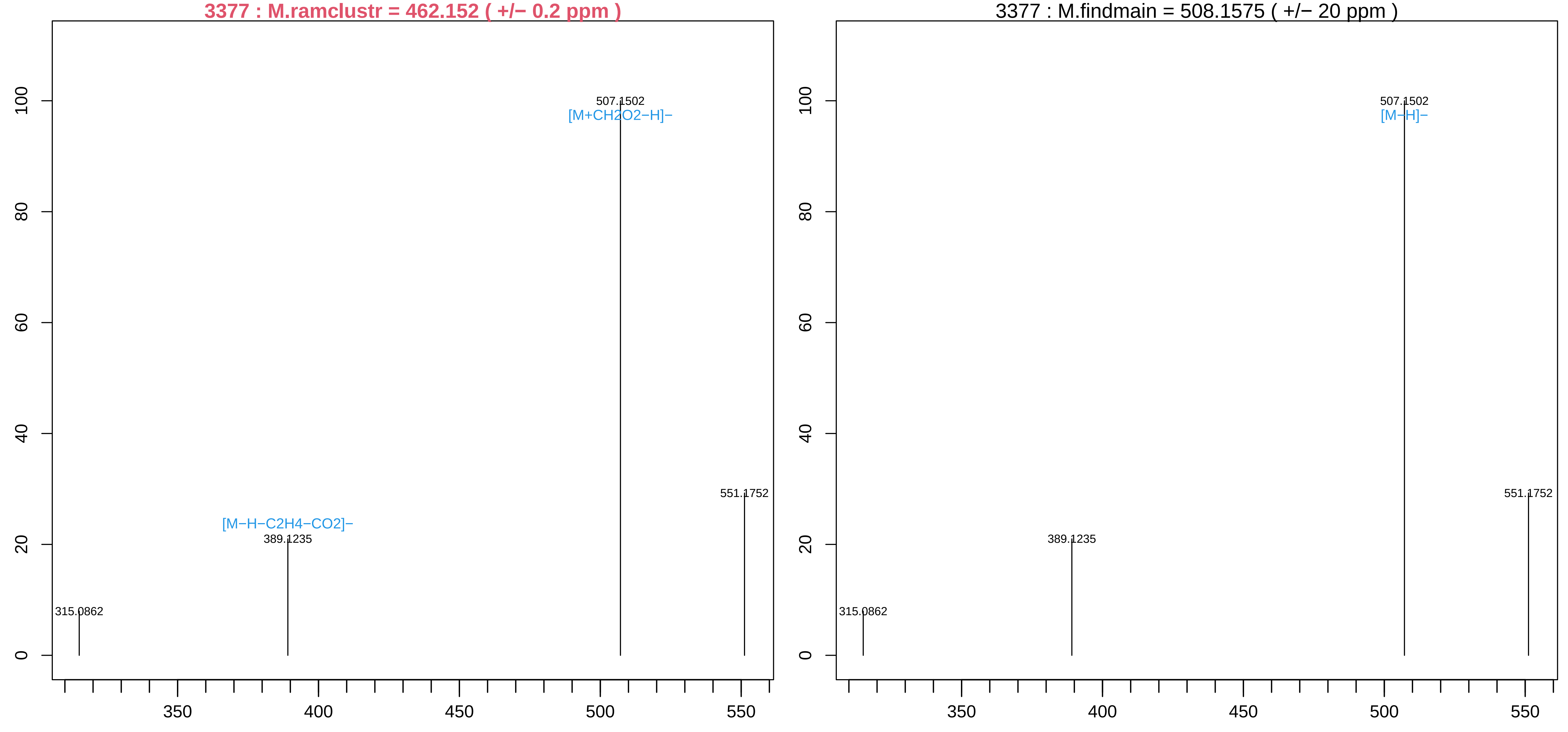

3377 : M.ramclustr = 462.152 (+/− 0.2 ppm)
507.1502
[M+CH2O2−H]−
551.1752
[M−H−C2H4−CO2]−
389.1235
315.0862
3377 : M.findmain = 508.1575 (+/− 20 ppm)
507.1502
[M−H]−
551.1752
389.1235
315.0862
0
20
40
60
80
100
350
400
450
500
550

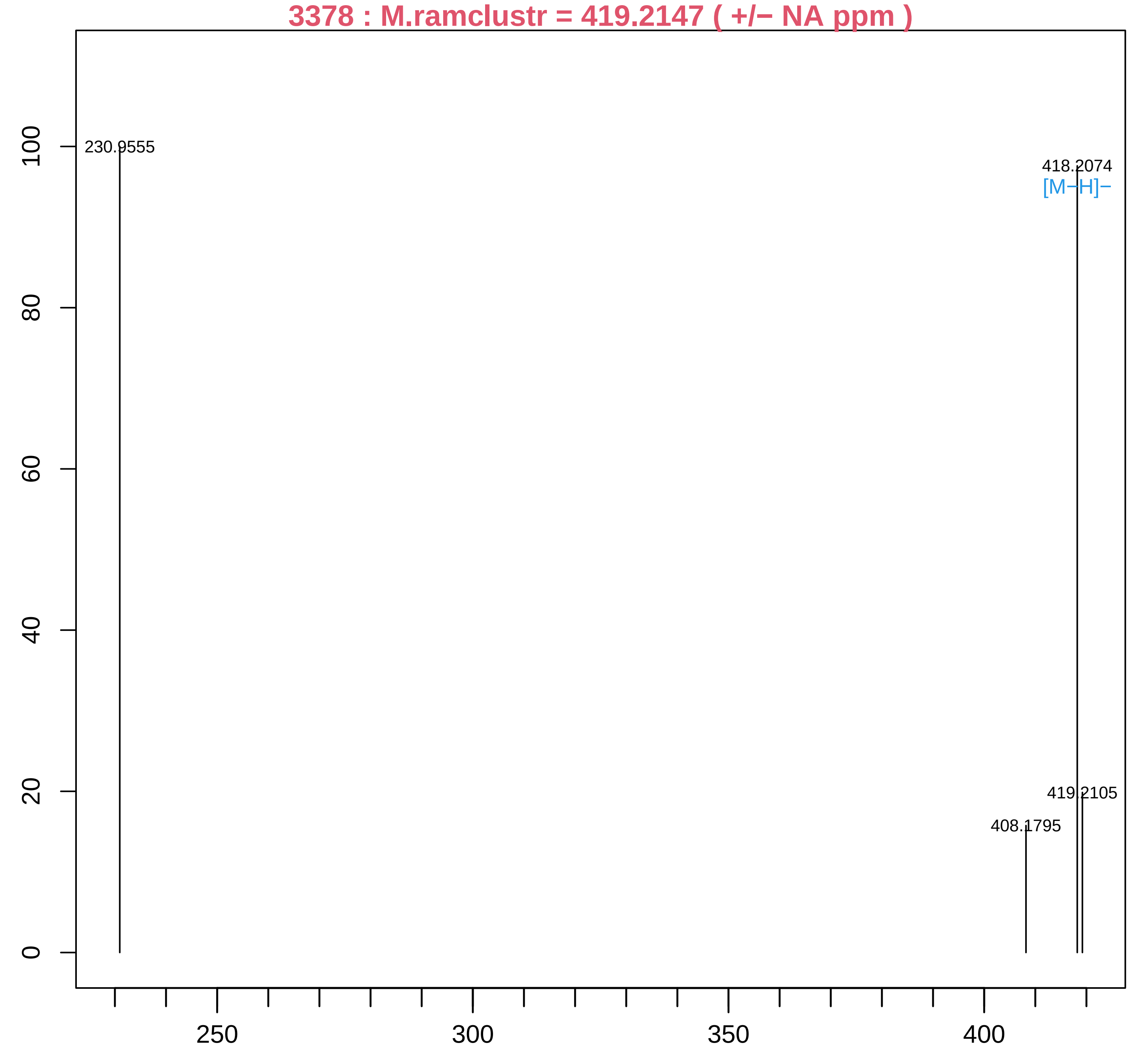
3378 : M.ramclustr = 419.2147 (+/− NA ppm)
230.9555
418.2074
[M−H]−
419.2105
408.1795
0
20
40
60
80
100
250
300
350
400

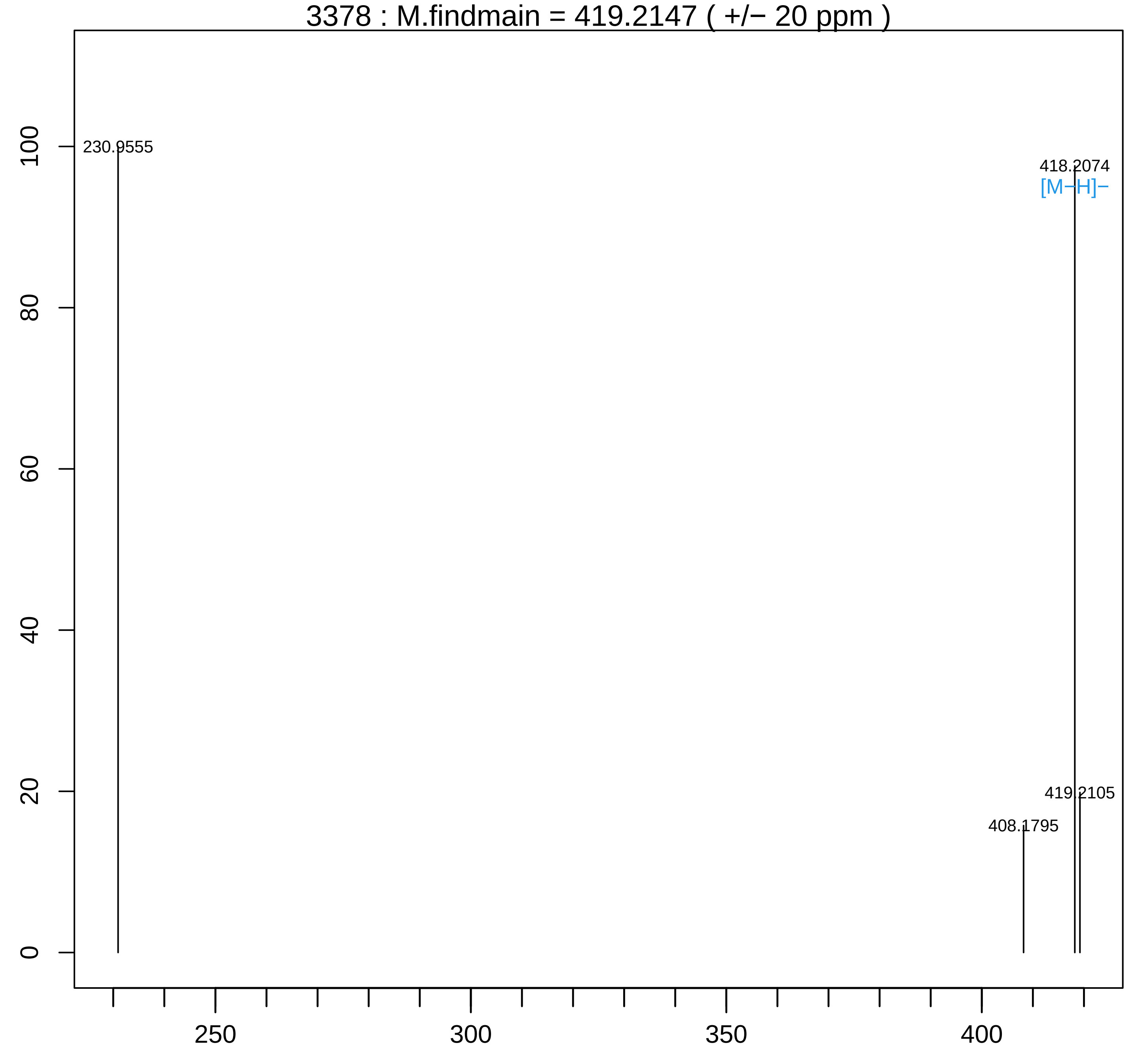
3378 : M.findmain = 419.2147 (+/− 20 ppm)
230.9555
418.2074
[M−H]−
419.2105
408.1795
0
20
40
60
80
100
250
300
350
400

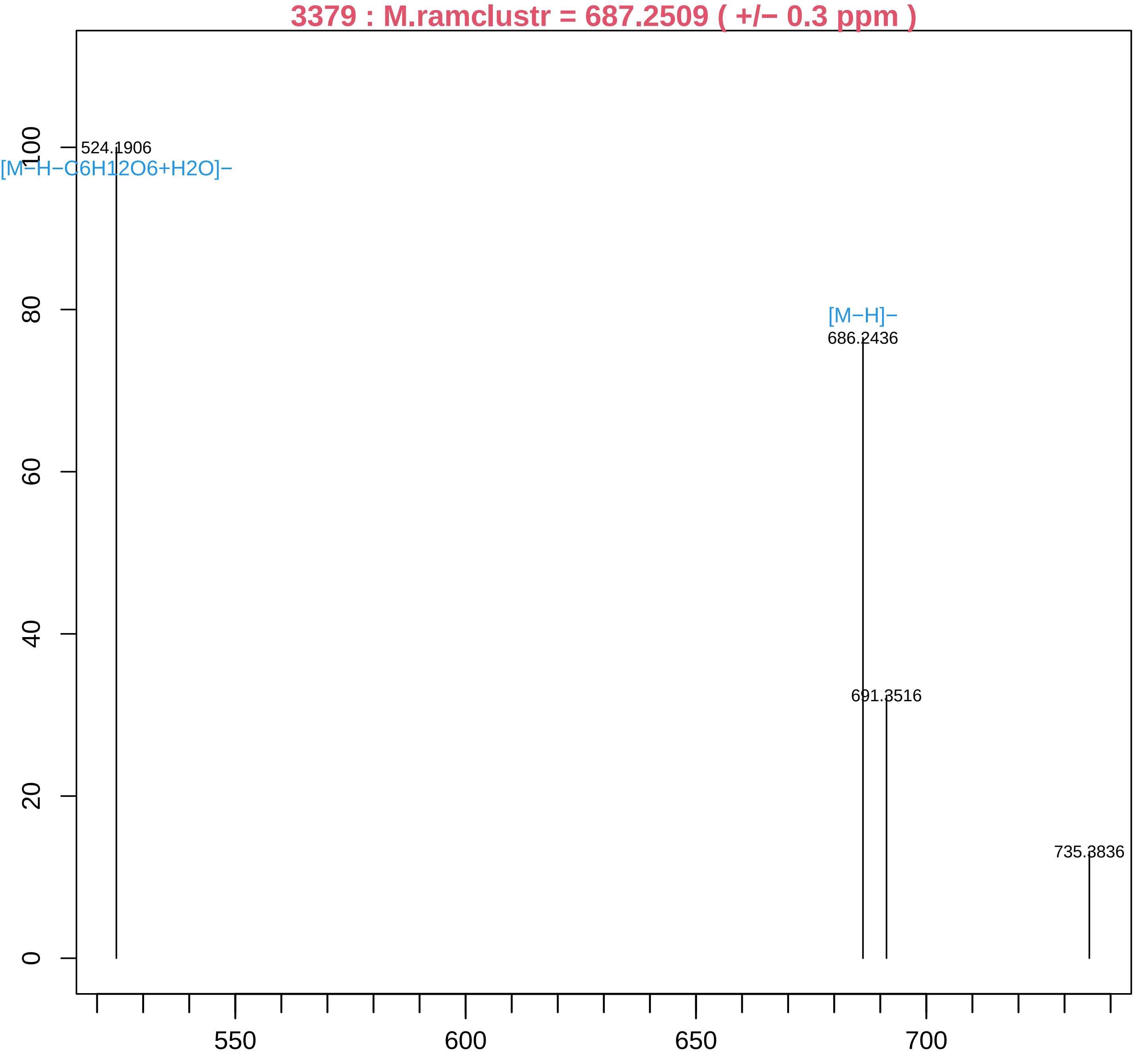
3379 : M.ramclustr = 687.2509 (+/− 0.3 ppm)
524.1906
[M−H−C6H12O6+H2O]−
[M−H]−
686.2436
691.3516
735.3836
0
20
40
60
80
100
550
600
650
700

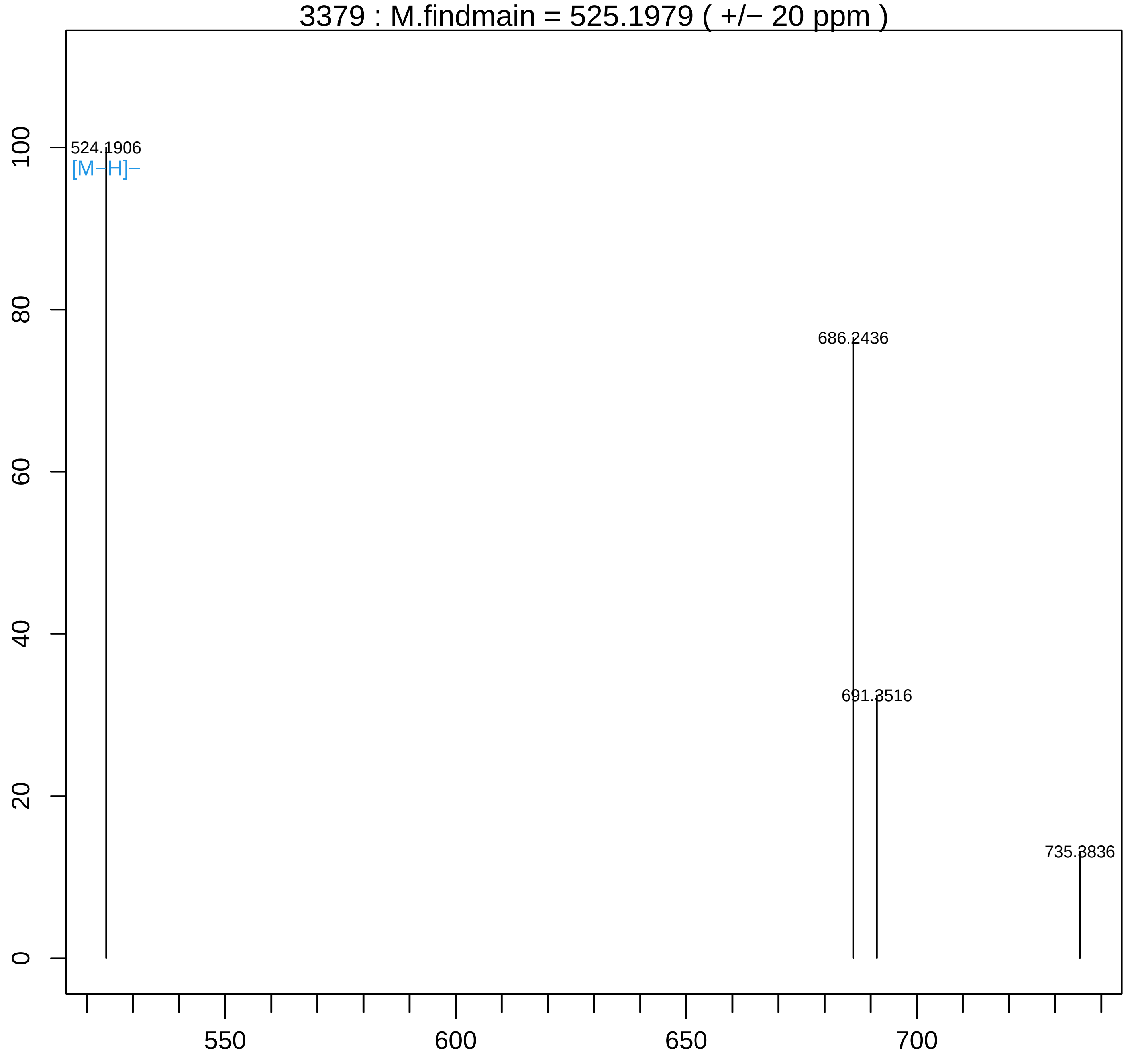
3379 : M.findmain = 525.1979 (+/− 20 ppm)
524.1906
[M−H]−
686.2436
691.3516
735.3836
0
20
40
60
80
100
550
600
650
700

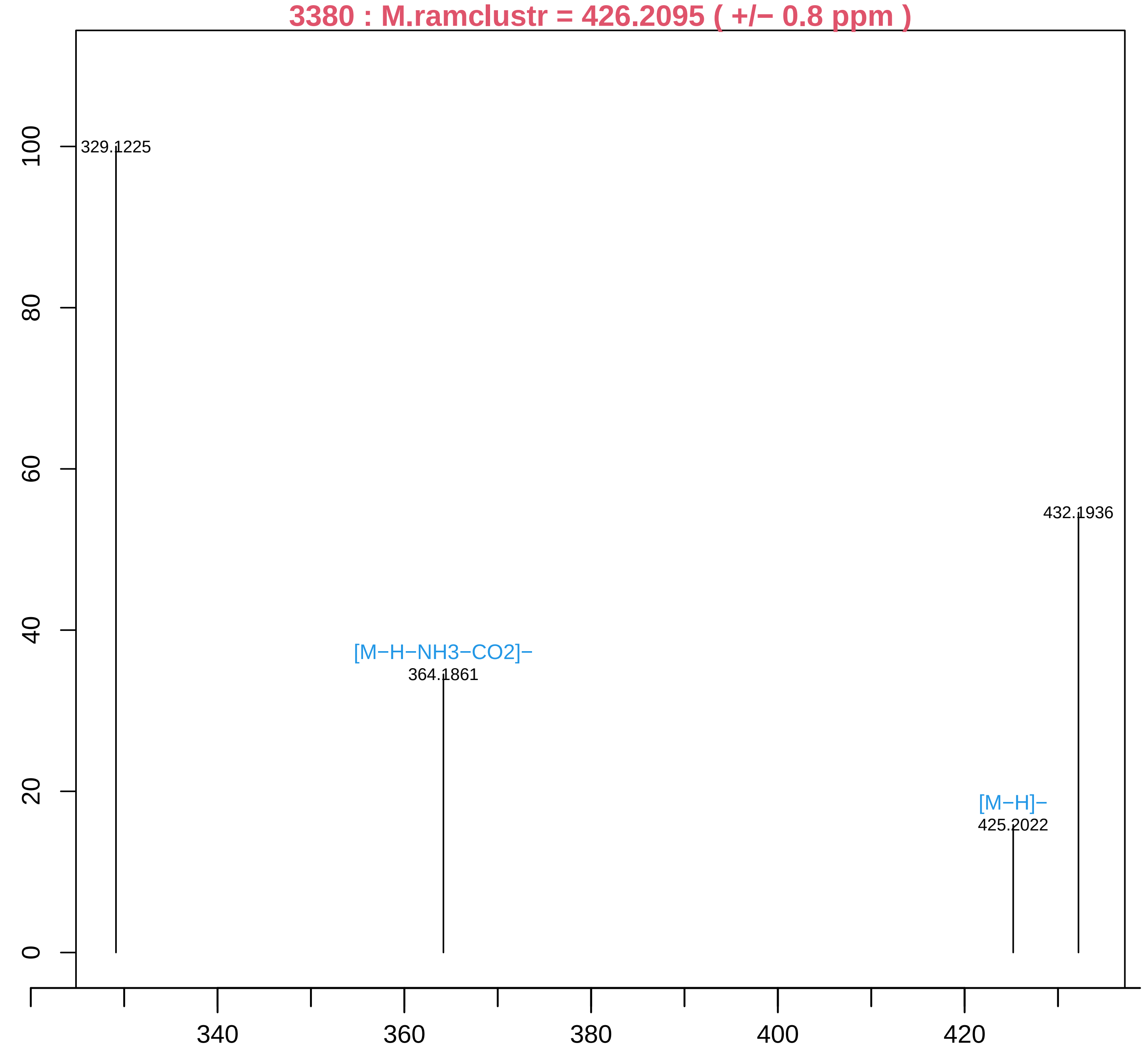
3380 : M.ramclustr = 426.2095 (+/− 0.8 ppm)
329.1225
[M−H−NH3−CO2]−
364.1861
[M−H]−
425.2022
432.1936
0
20
40
60
80
100
340
360
380
400
420

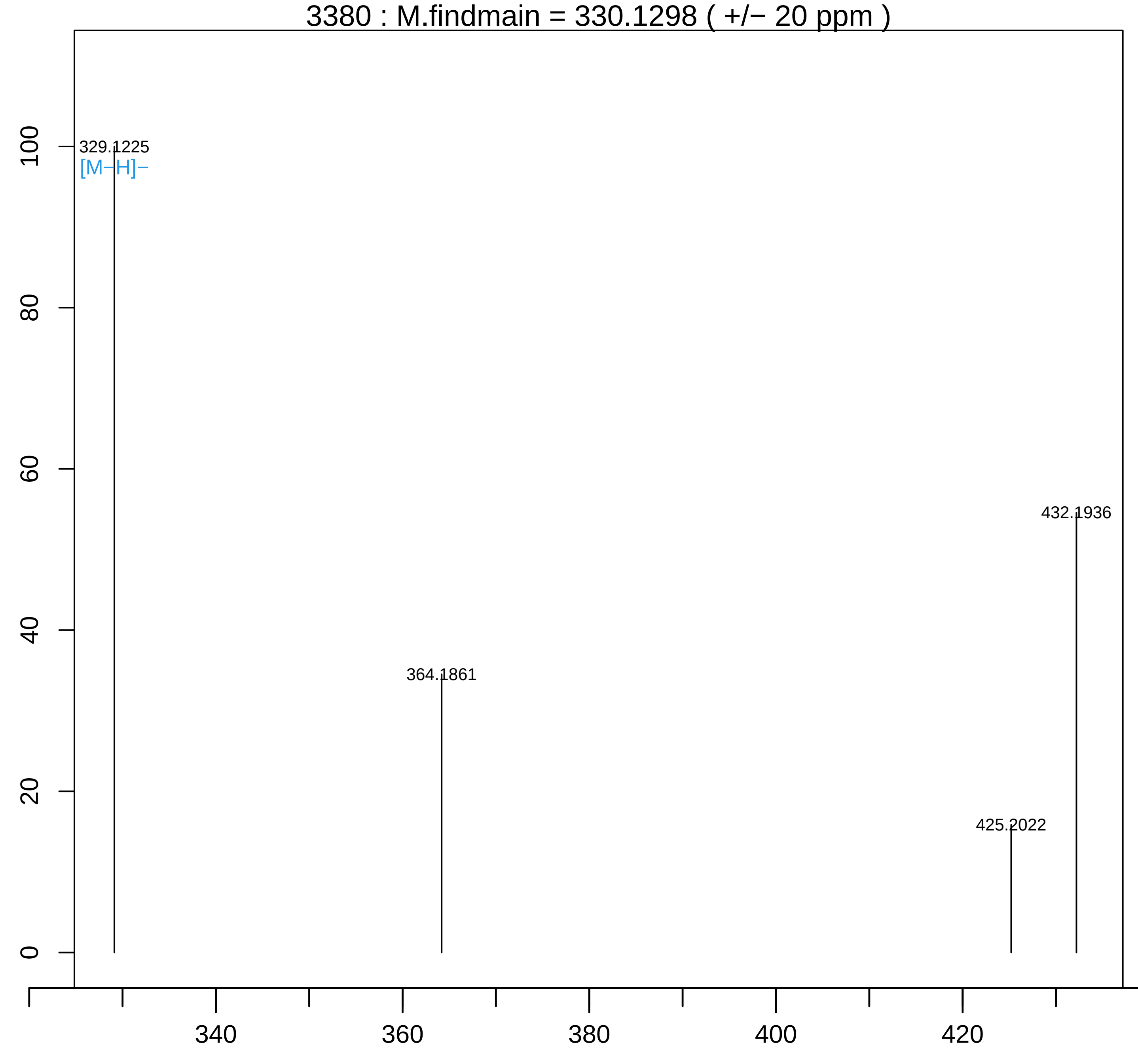
3380 : M.findmain = 330.1298 (+/− 20 ppm)
329.1225
[M−H]−
364.1861
425.2022
432.1936
0
20
40
60
80
100
340
360
380
400
420

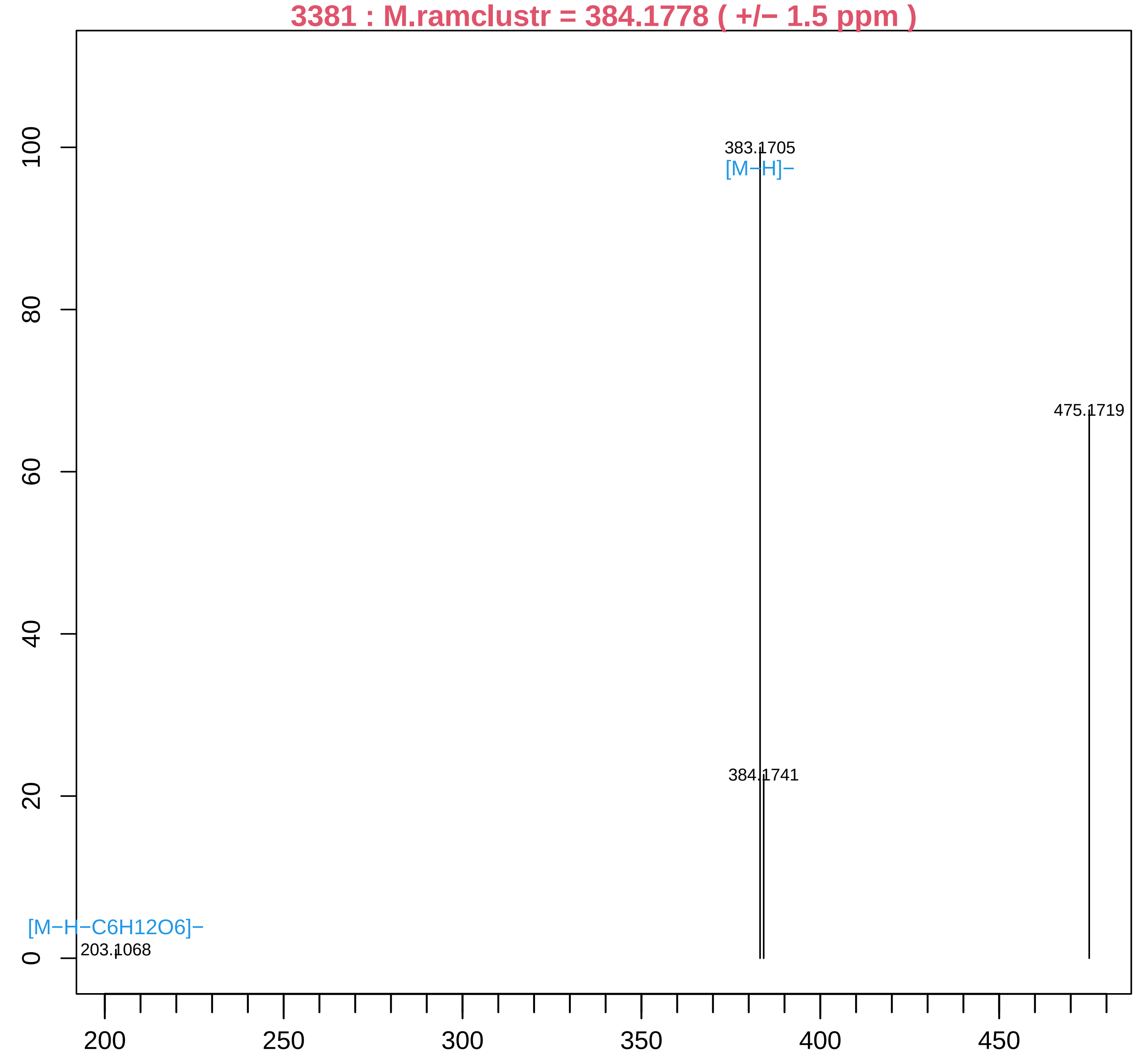
3381 : M.ramclustr = 384.1778 (+/− 1.5 ppm)
383.1705
[M−H]−
475.1719
384.1741
[M−H−C6H12O6]−
203.1068
0
20
40
60
80
100
200
250
300
350
400
450

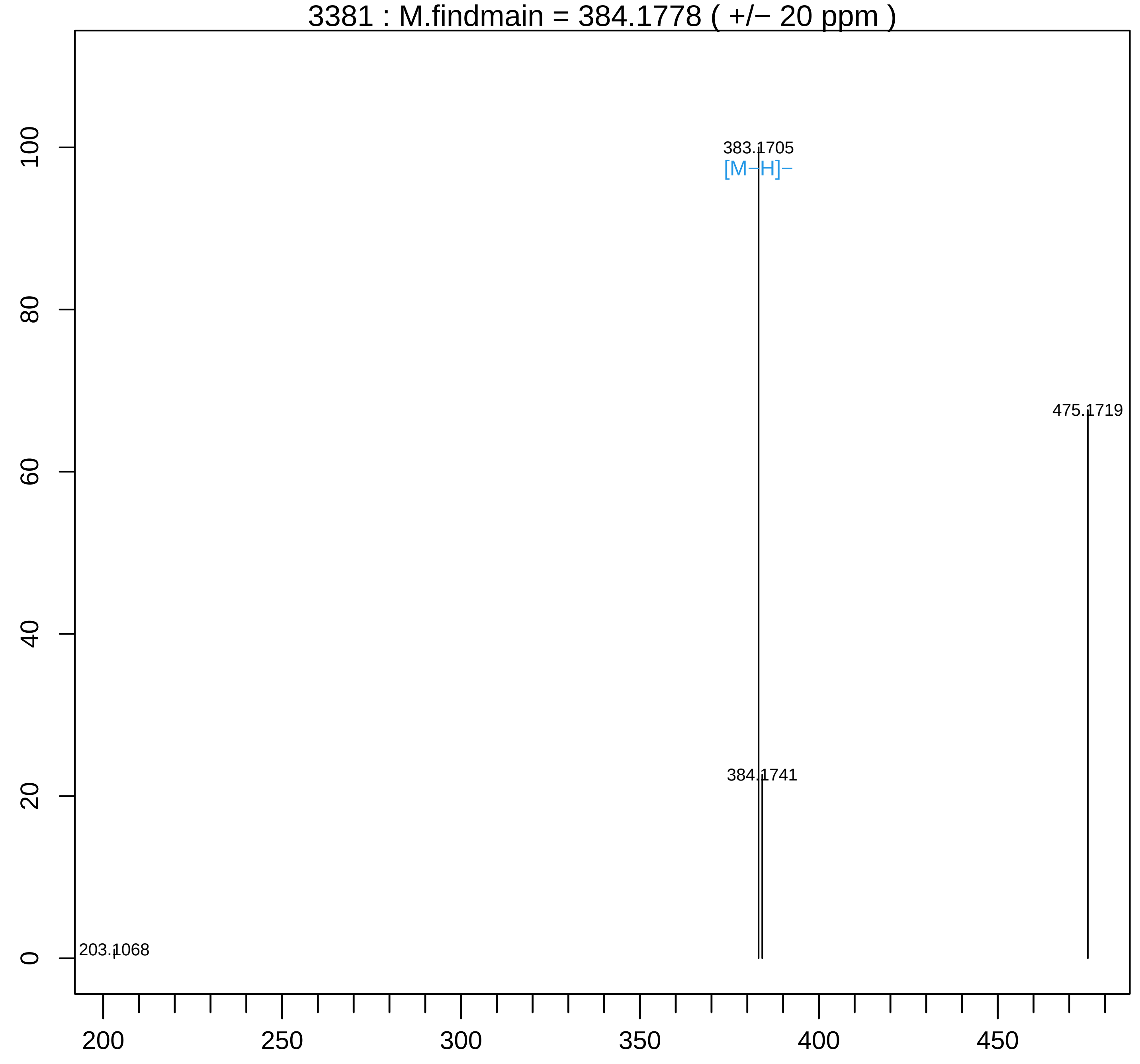
3381 : M.findmain = 384.1778 (+/− 20 ppm)
383.1705
[M−H]−
475.1719
384.1741
203.1068
0
20
40
60
80
100
200
250
300
350
400
450

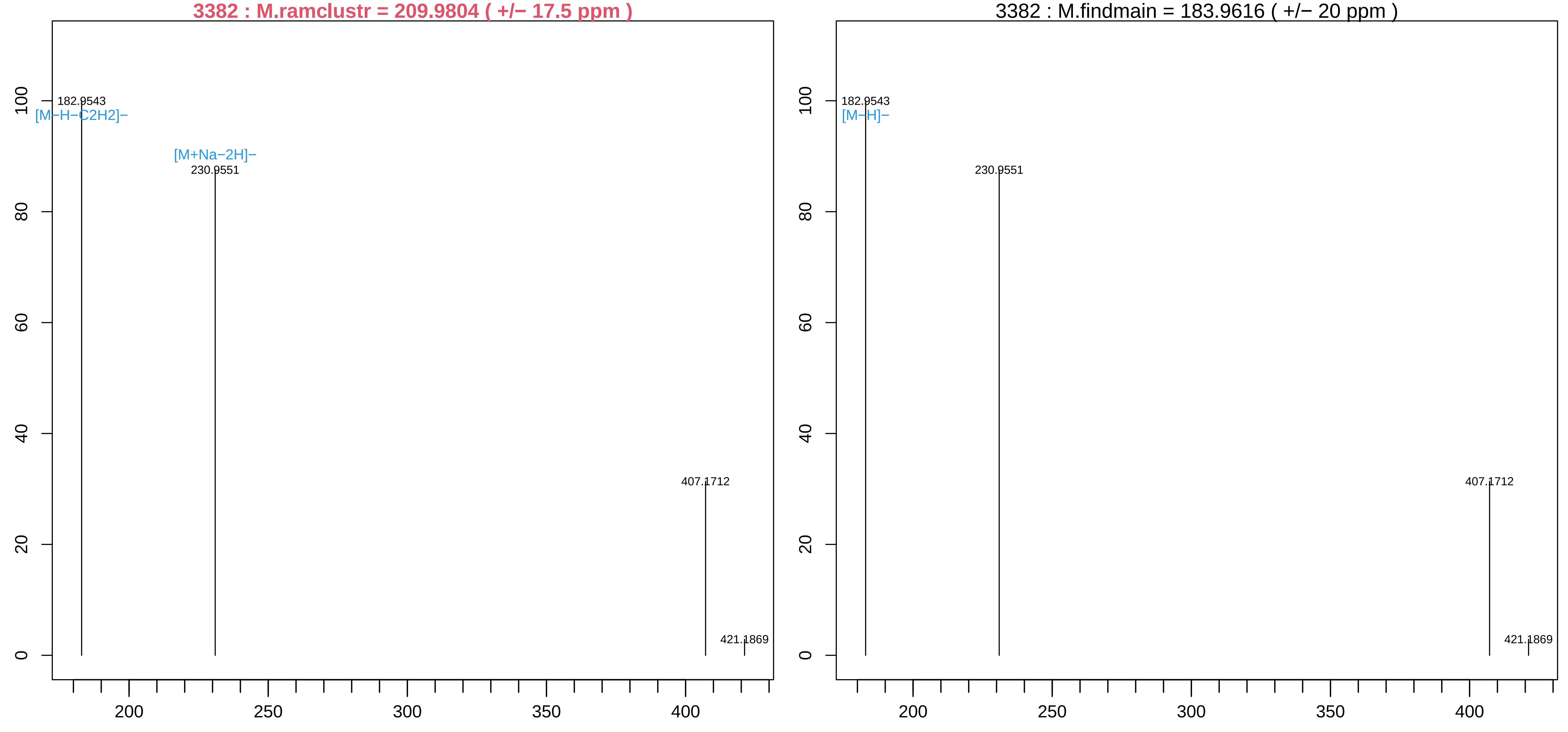
3382 : M.ramclustr = 209.9804 (+/− 17.5 ppm)
182.9543
[M−H−C2H2]−
[M+Na−2H]−
230.9551
407.1712
421.1869
0
20
40
60
80
100
200
250
300
350
400
3382 : M.findmain = 183.9616 (+/− 20 ppm)
182.9543
[M−H]−
230.9551
407.1712
421.1869

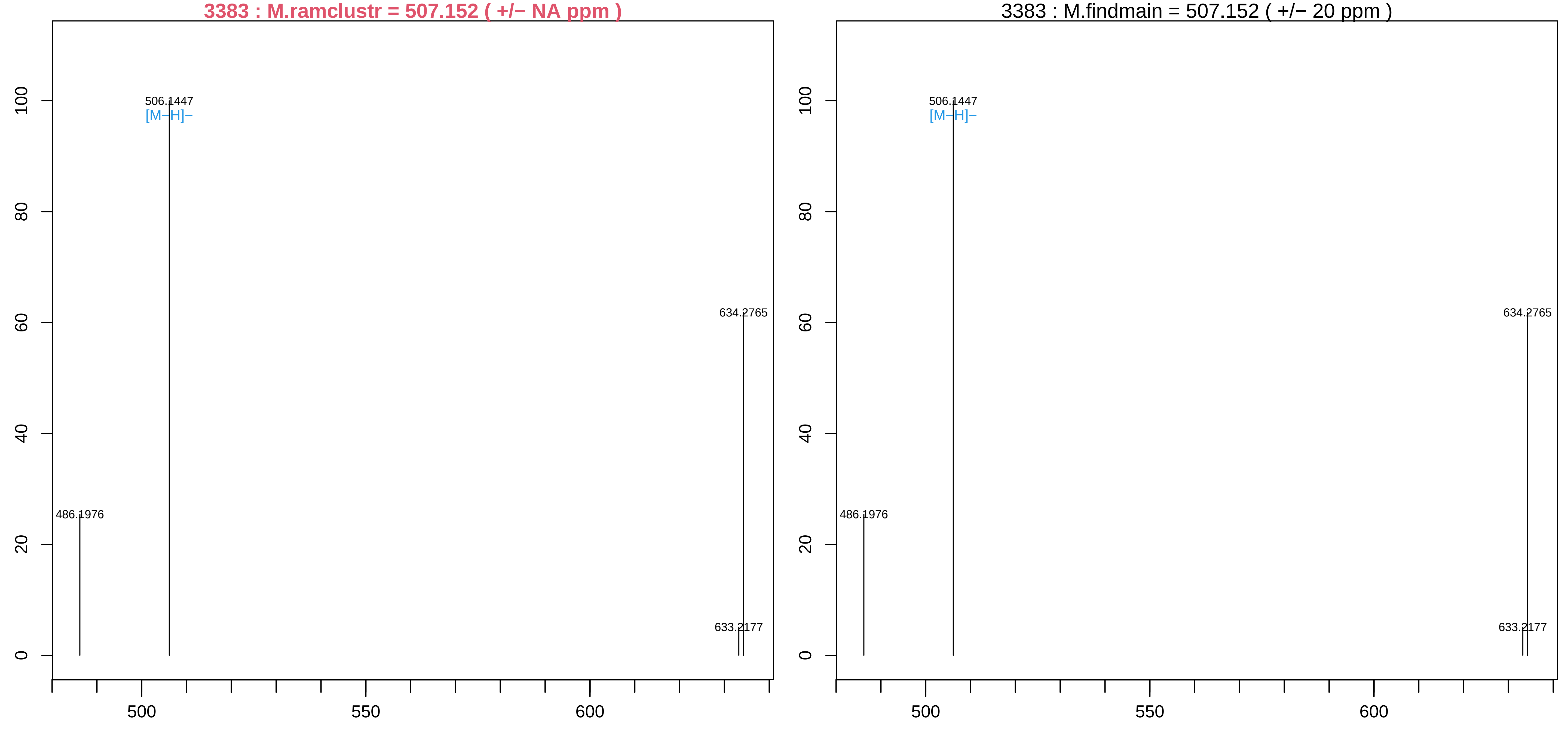

3383 : M.ramclustr = 507.152 (+/− NA ppm)
506.1447
[M−H]−
634.2765
486.1976
633.2177
3383 : M.findmain = 507.152 (+/− 20 ppm)
506.1447
[M−H]−
634.2765
486.1976
633.2177

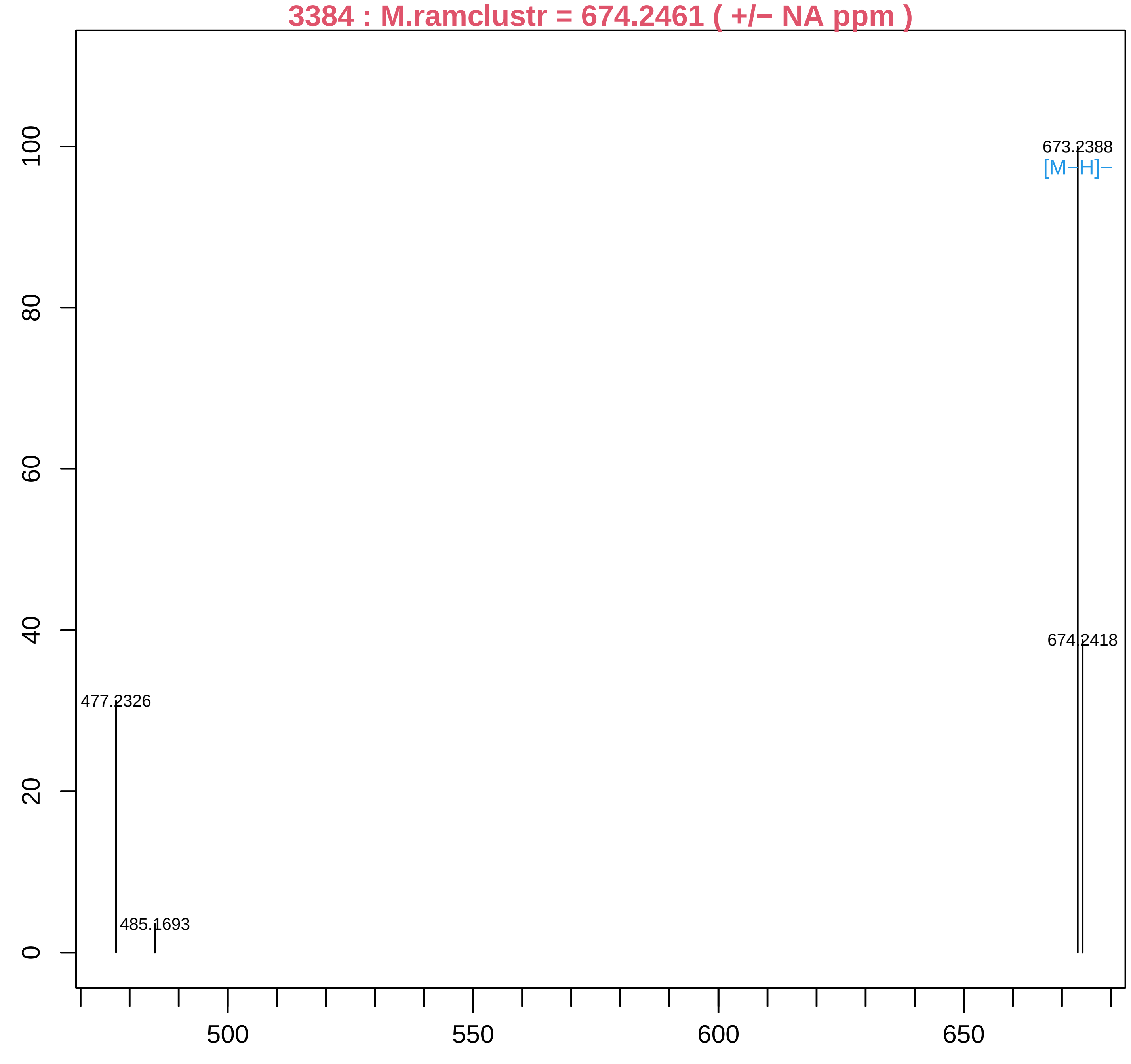
3384 : M.ramclustr = 674.2461 (+/− NA ppm)
673.2388
[M−H]−
674.2418
477.2326
485.1693
0
20
40
60
80
100
500
550
600
650
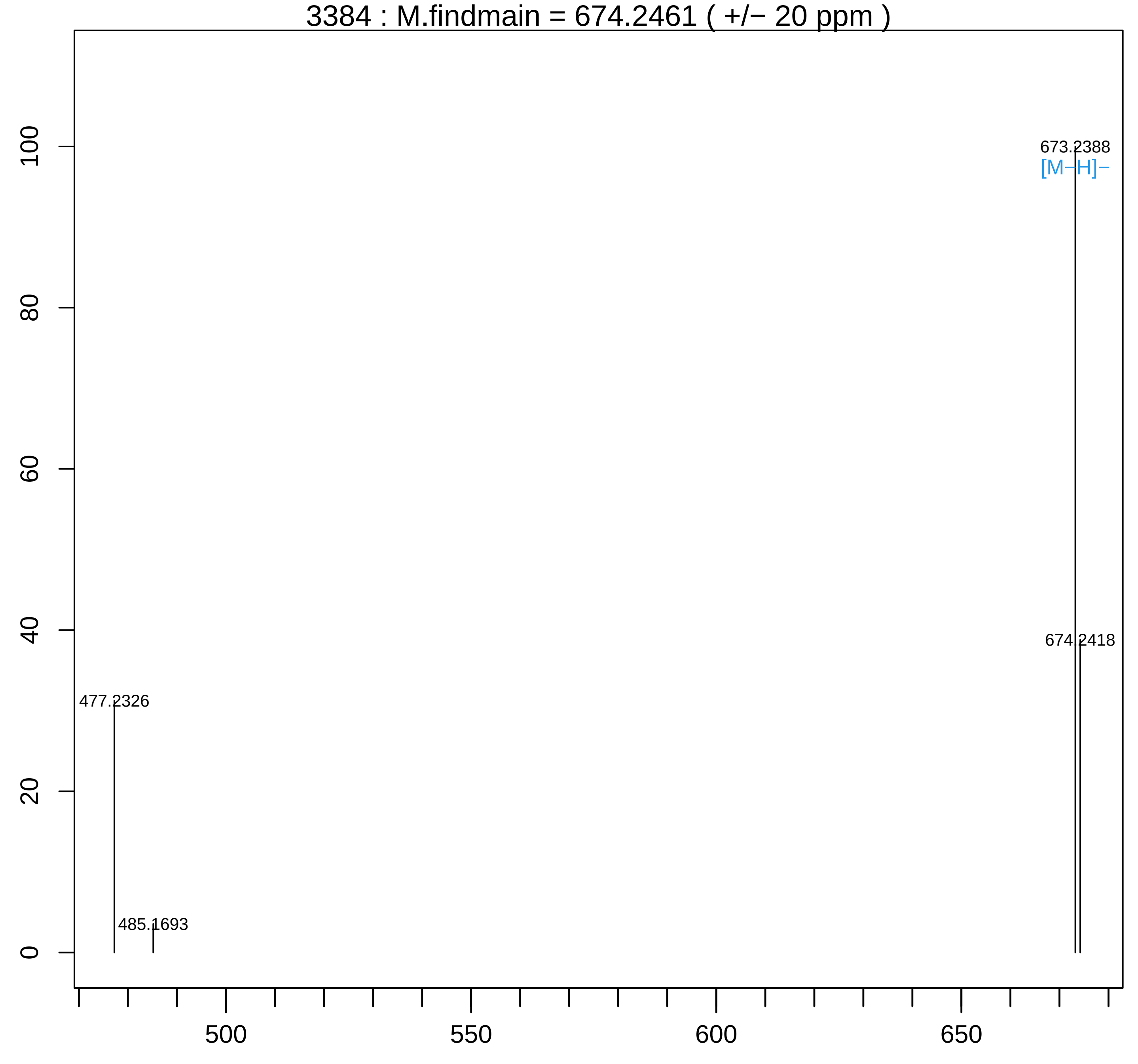
3384 : M.findmain = 674.2461 (+/− 20 ppm)
673.2388
[M−H]−
674.2418
477.2326
485.1693
0
20
40
60
80
100
500
550
600
650

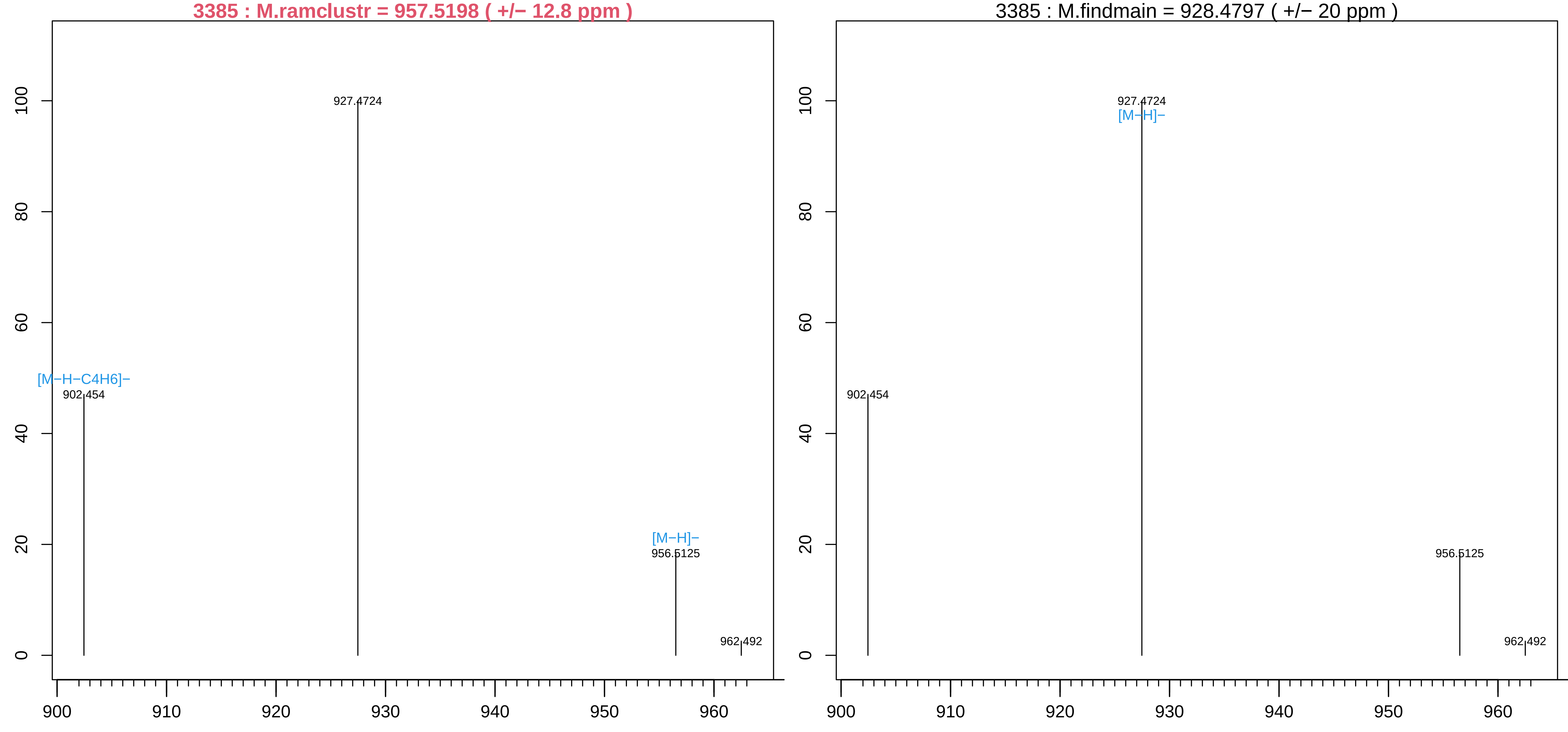
3385 : M.ramclustr = 957.5198 (+/− 12.8 ppm)
[M−H−C4H6]−
902.454
927.4724
[M−H]−
956.5125
962.492
3385 : M.findmain = 928.4797 (+/− 20 ppm)
902.454
927.4724
[M−H]−
956.5125
962.492
0
20
40
60
80
100
900
910
920
930
940
950
960

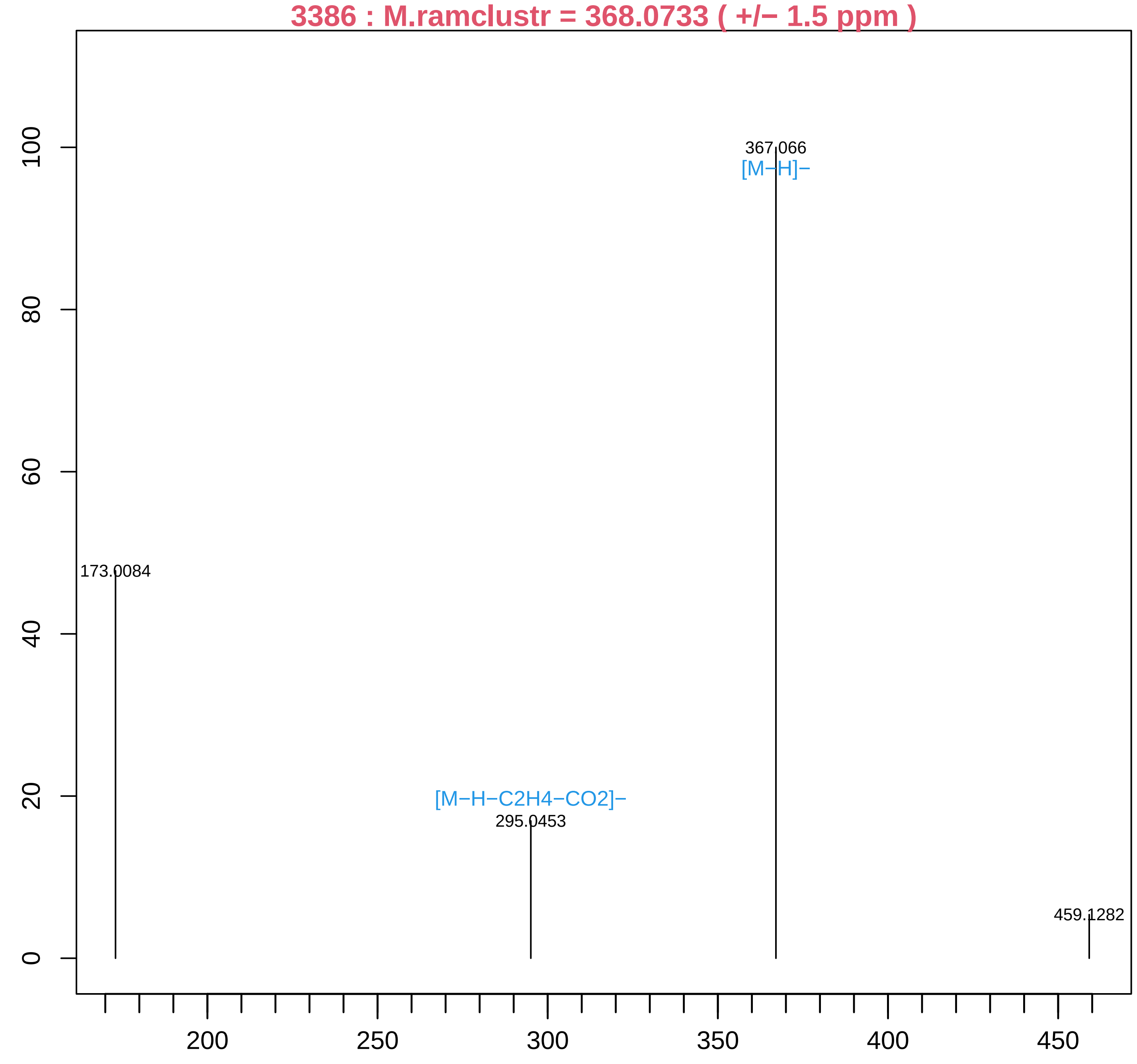

3386 : M.ramclustr = 368.0733 (+/− 1.5 ppm)
367.066
[M−H]−
173.0084
[M−H−C2H4−CO2]−
295.0453
459.1282

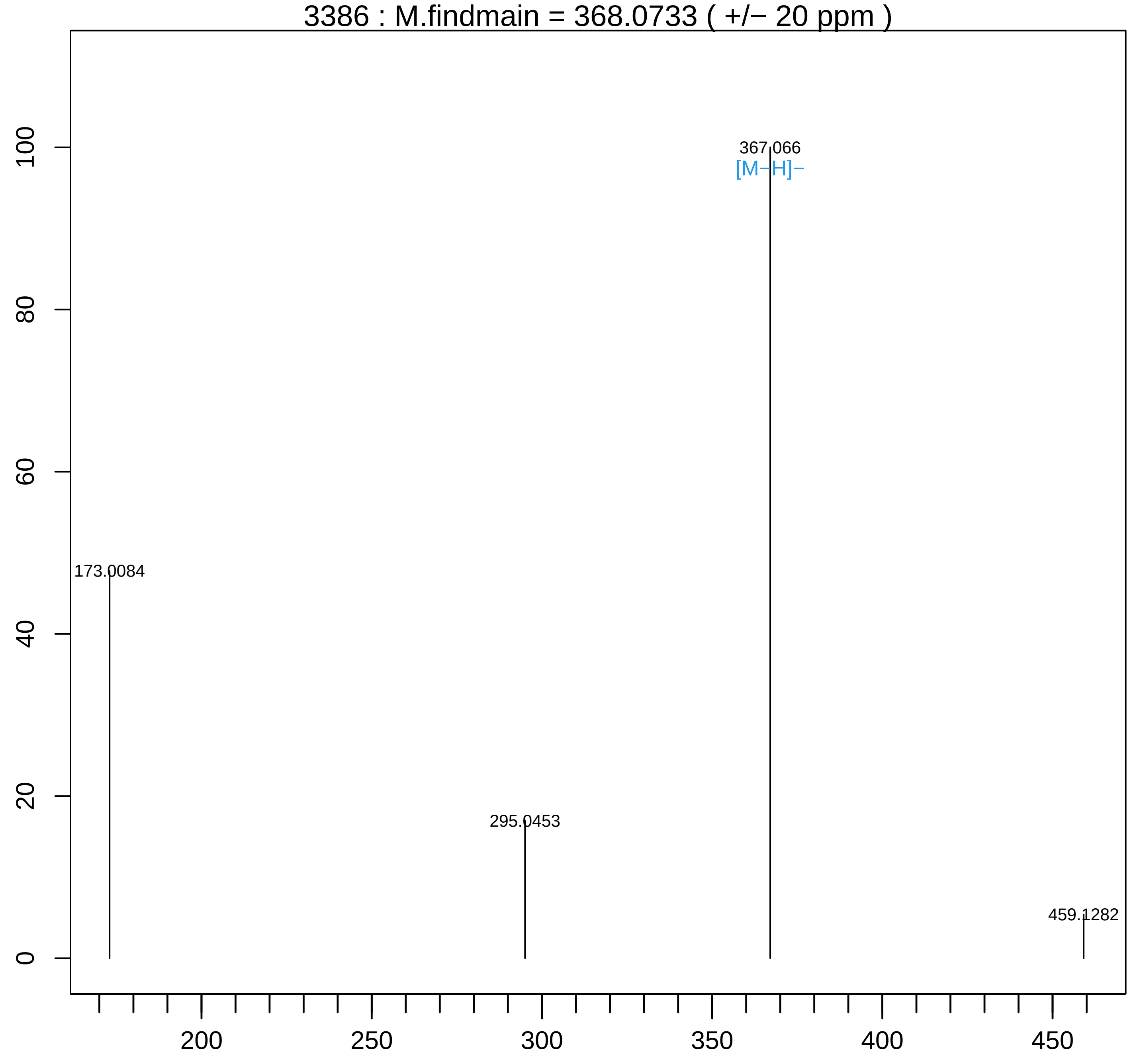

3386 : M.findmain = 368.0733 (+/− 20 ppm)
367.066
[M−H]−
173.0084
295.0453
459.1282

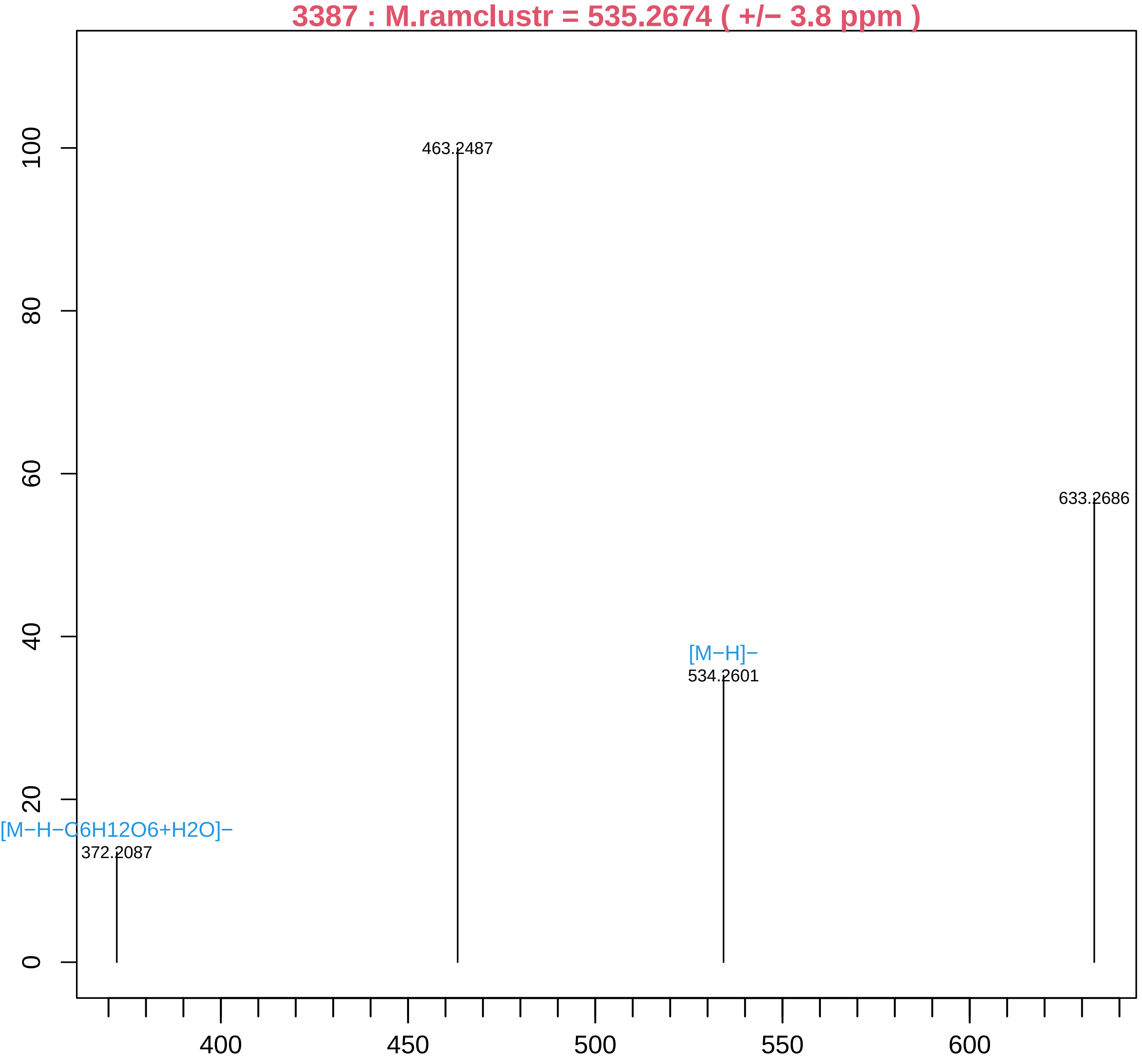
3387 : M.ramclustr = 535.2674 (+/− 3.8 ppm)
463.2487
633.2686
[M−H]−
534.2601
[M−H−C6H12O6+H2O]−
372.2087
0
20
40
60
80
100
400
450
500
550
600

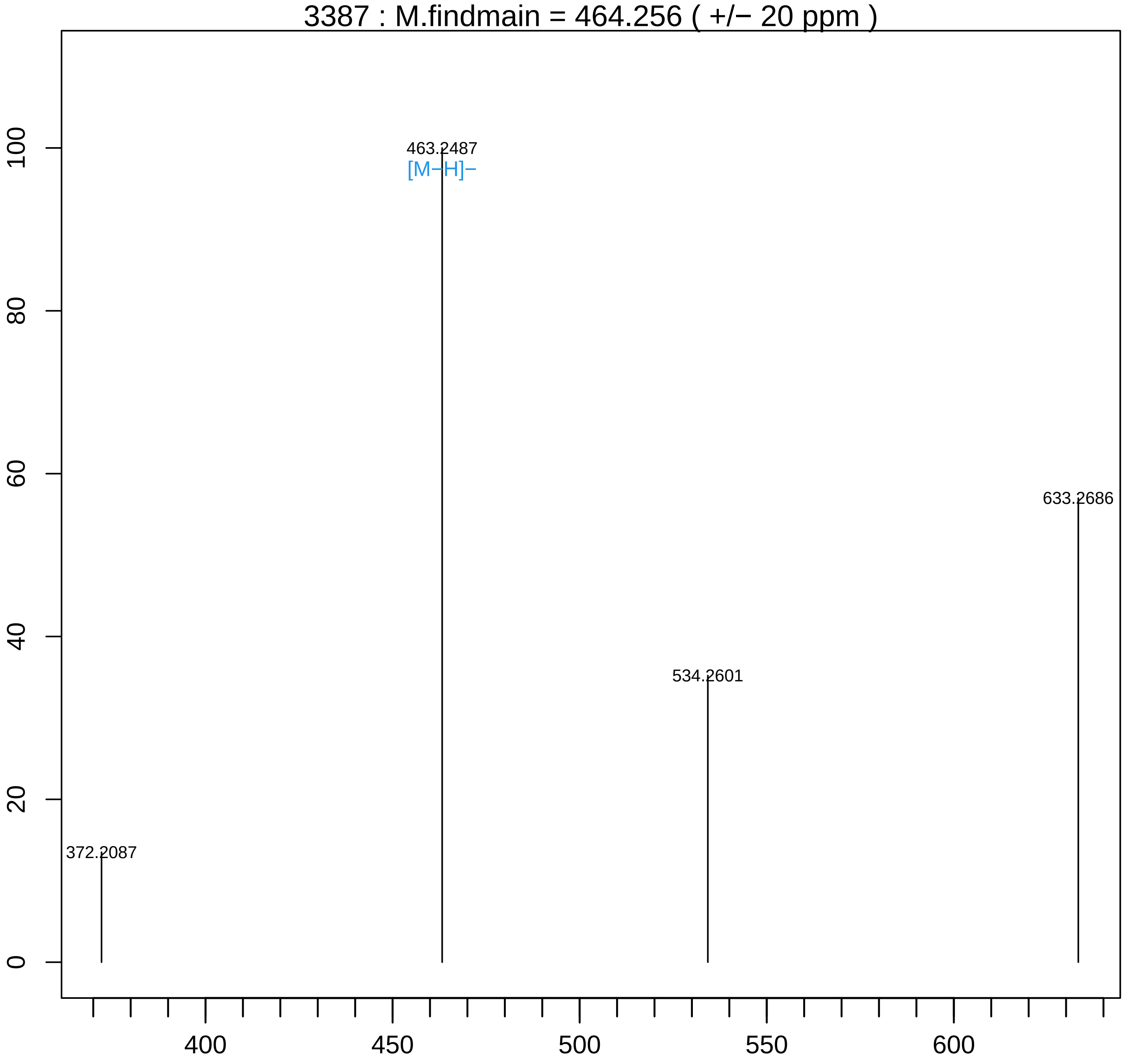
3387 : M.findmain = 464.256 (+/− 20 ppm)
463.2487
[M−H]−
633.2686
534.2601
372.2087
0
20
40
60
80
100
400
450
500
550
600

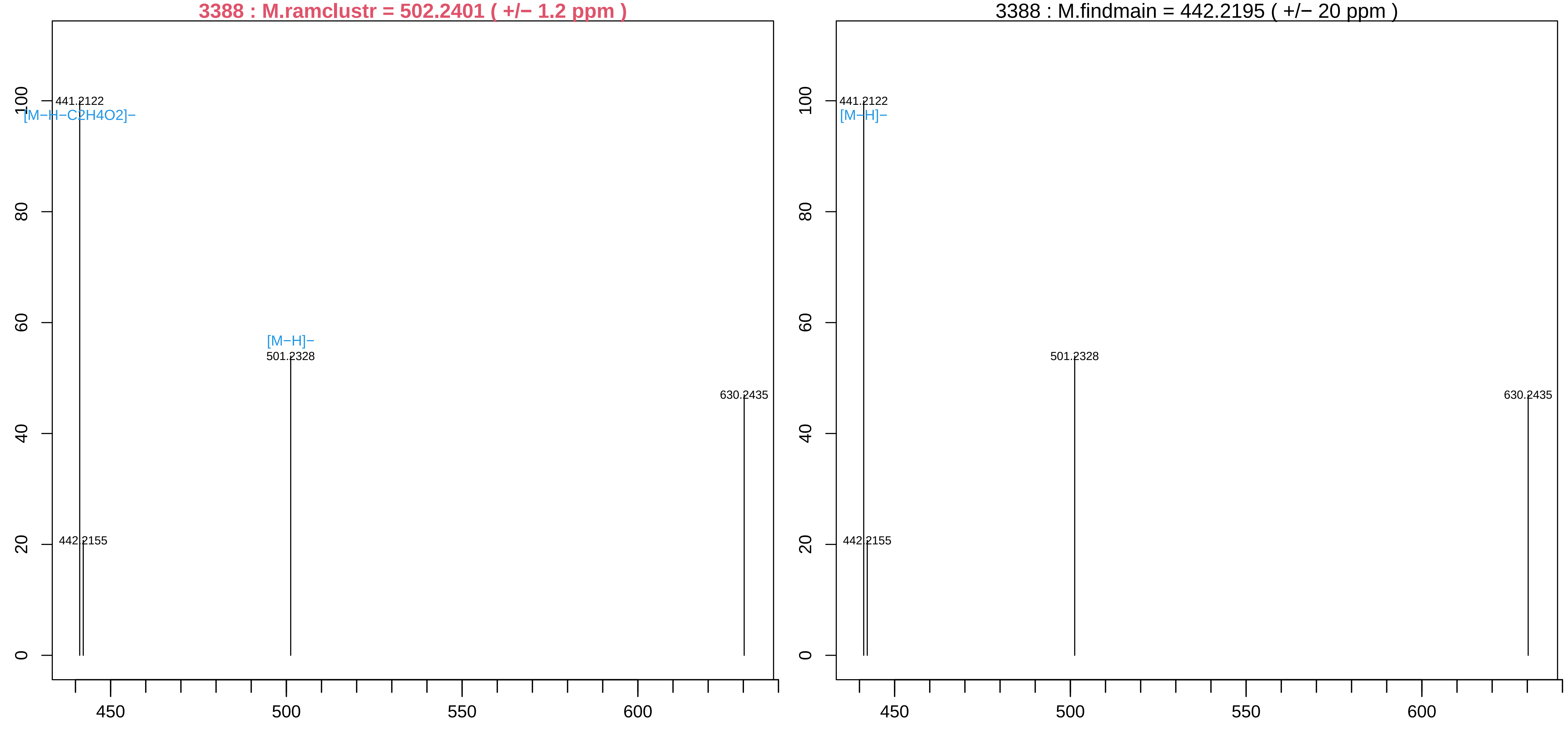
3388 : M.ramclustr = 502.2401 (+/− 1.2 ppm)
441.2122
[M−H−C2H4O2]−
[M−H]−
501.2328
630.2435
442.2155
3388 : M.findmain = 442.2195 (+/− 20 ppm)
441.2122
[M−H]−
501.2328
630.2435
442.2155

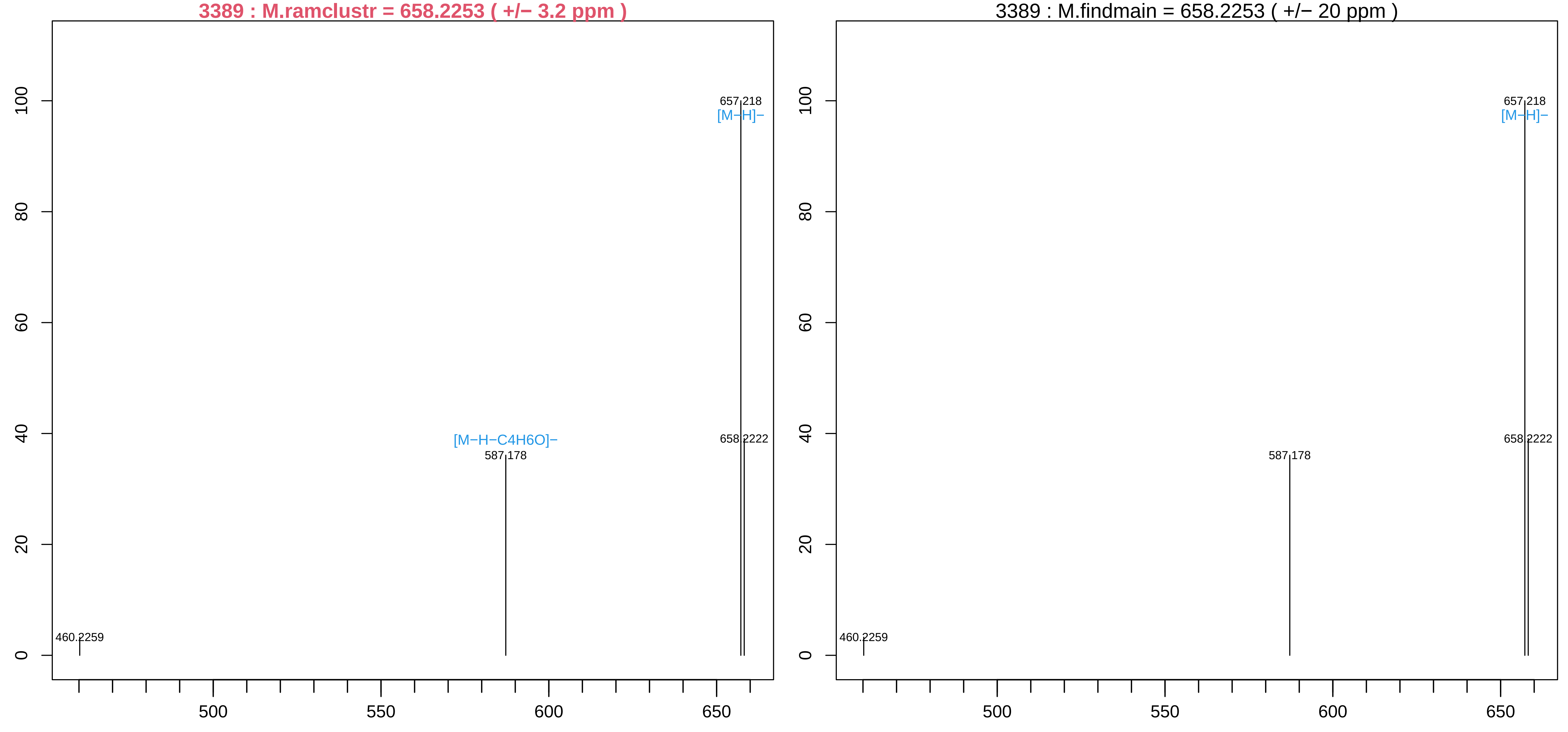
3389 : M.ramclustr = 658.2253 (+/− 3.2 ppm)
3389 : M.findmain = 658.2253 (+/− 20 ppm)
657.218
[M−H]−
658.2222
[M−H−C4H6O]−
587.178
460.2259
0
20
40
60
80
100
500
550
600
650

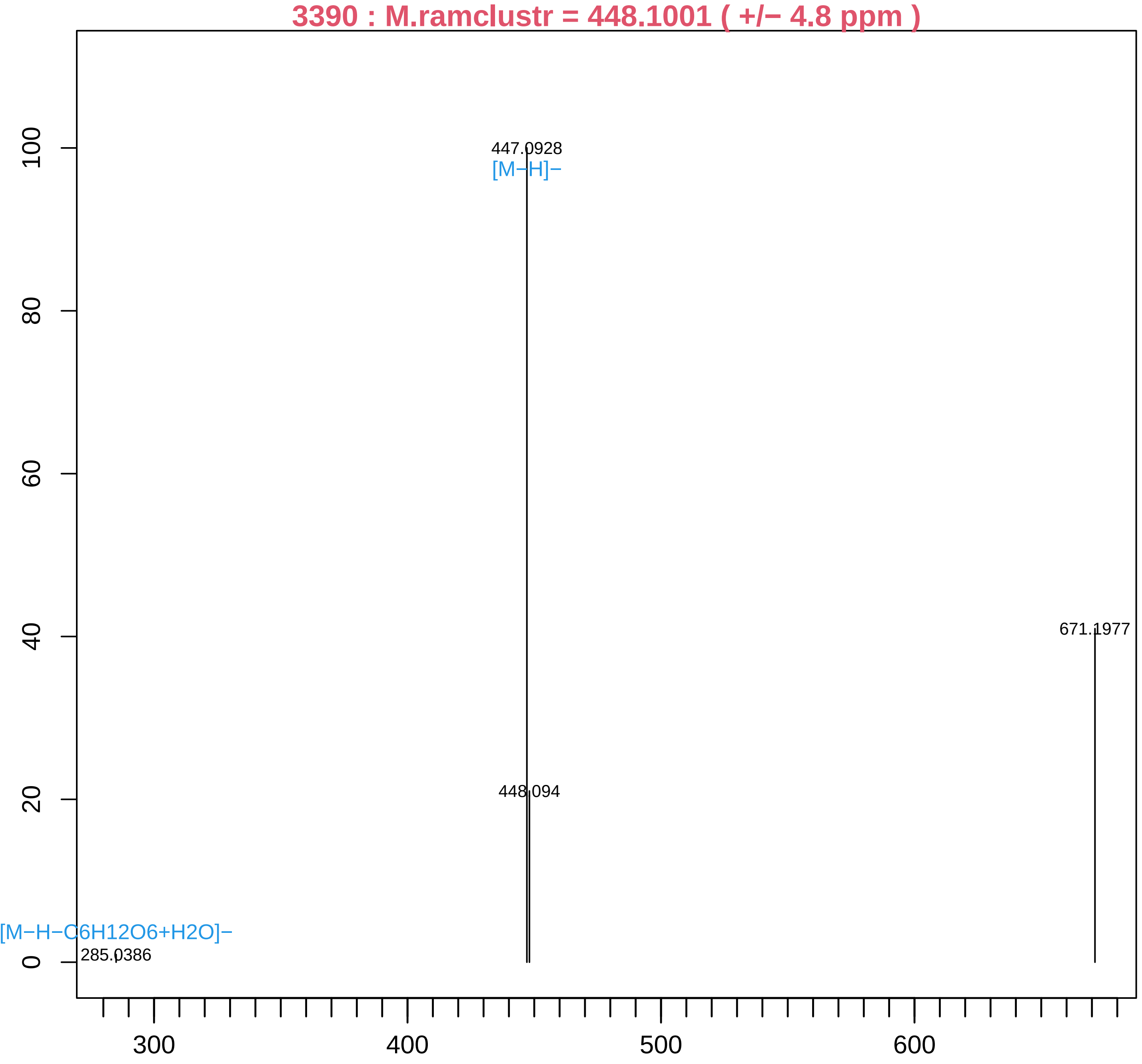

3390 : M.ramclustr = 448.1001 (+/− 4.8 ppm)
447.0928
[M−H]−
671.1977
448.094
[M−H−C6H12O6+H2O]−
285.0386
0
20
40
60
80
100
300
400
500
600

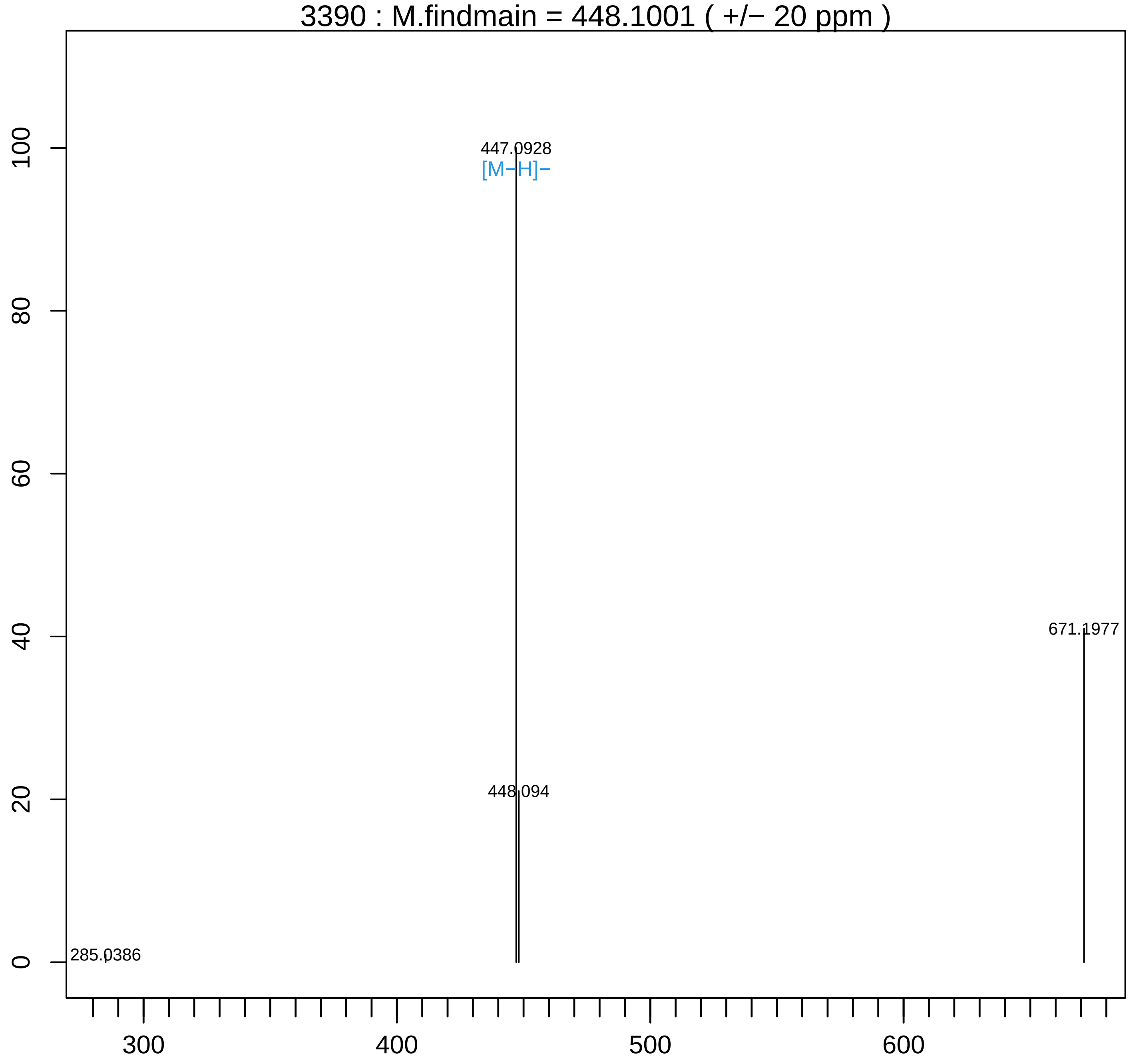

3390 : M.findmain = 448.1001 (+/− 20 ppm)
447.0928
[M−H]−
671.1977
448.094
285.0386
0
20
40
60
80
100
300
400
500
600

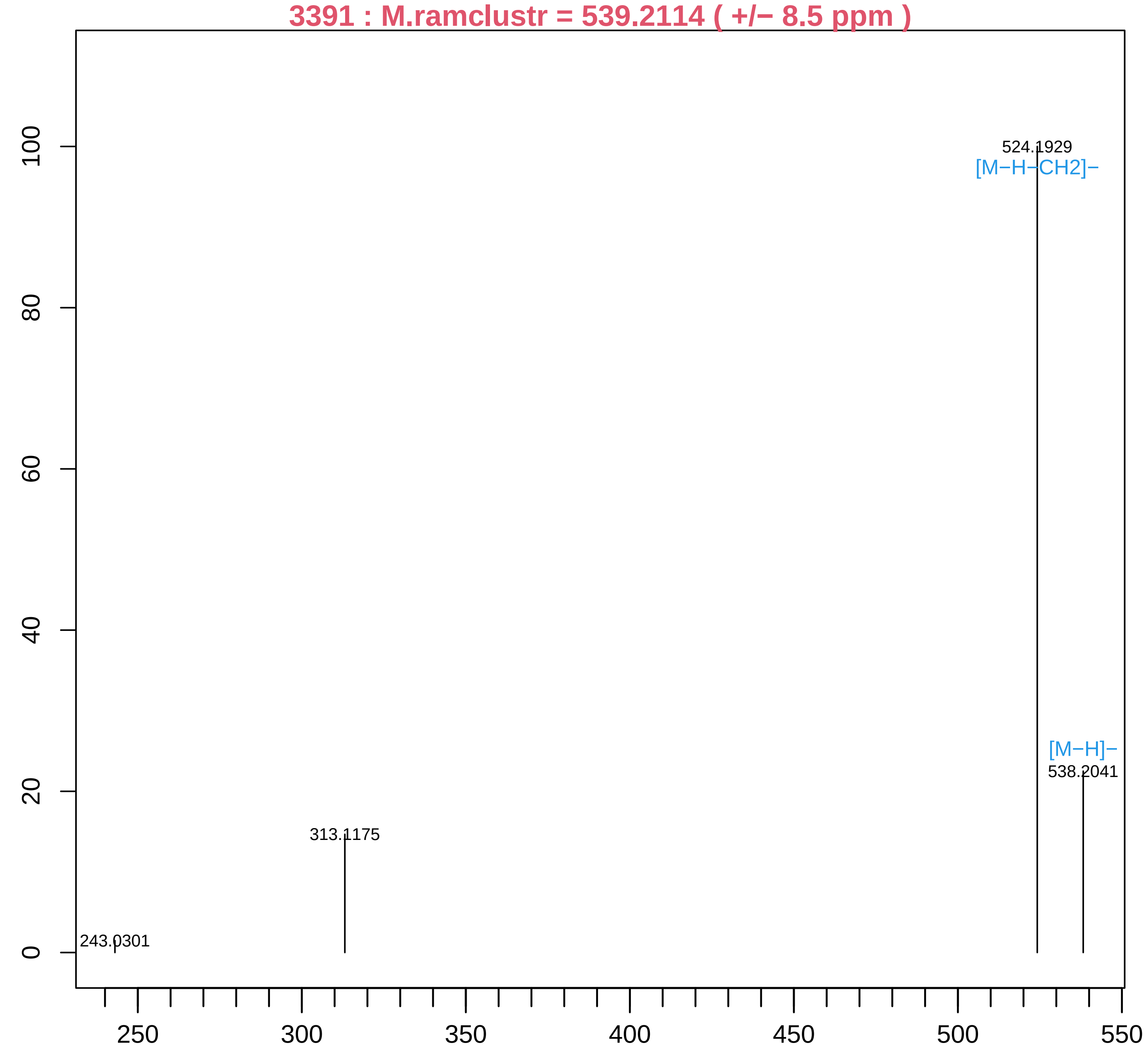
3391 : M.ramclustr = 539.2114 (+/− 8.5 ppm)
524.1929
[M−H−CH2]−
[M−H]−
538.2041
313.1175
243.0301
0
20
40
60
80
100
250
300
350
400
450
500
550

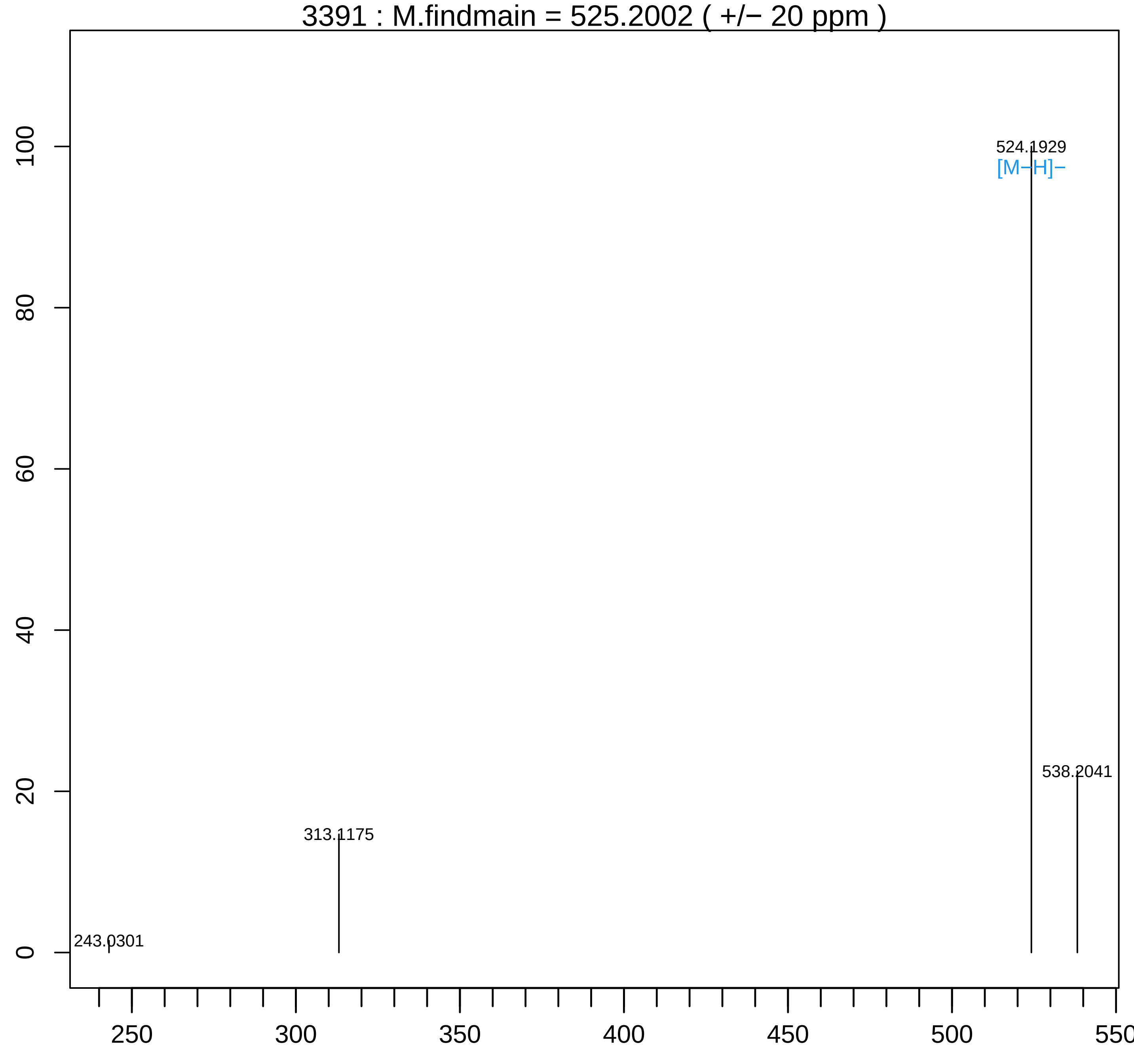
3391 : M.findmain = 525.2002 (+/− 20 ppm)
524.1929
[M−H]−
538.2041
313.1175
243.0301
0
20
40
60
80
100
250
300
350
400
450
500
550

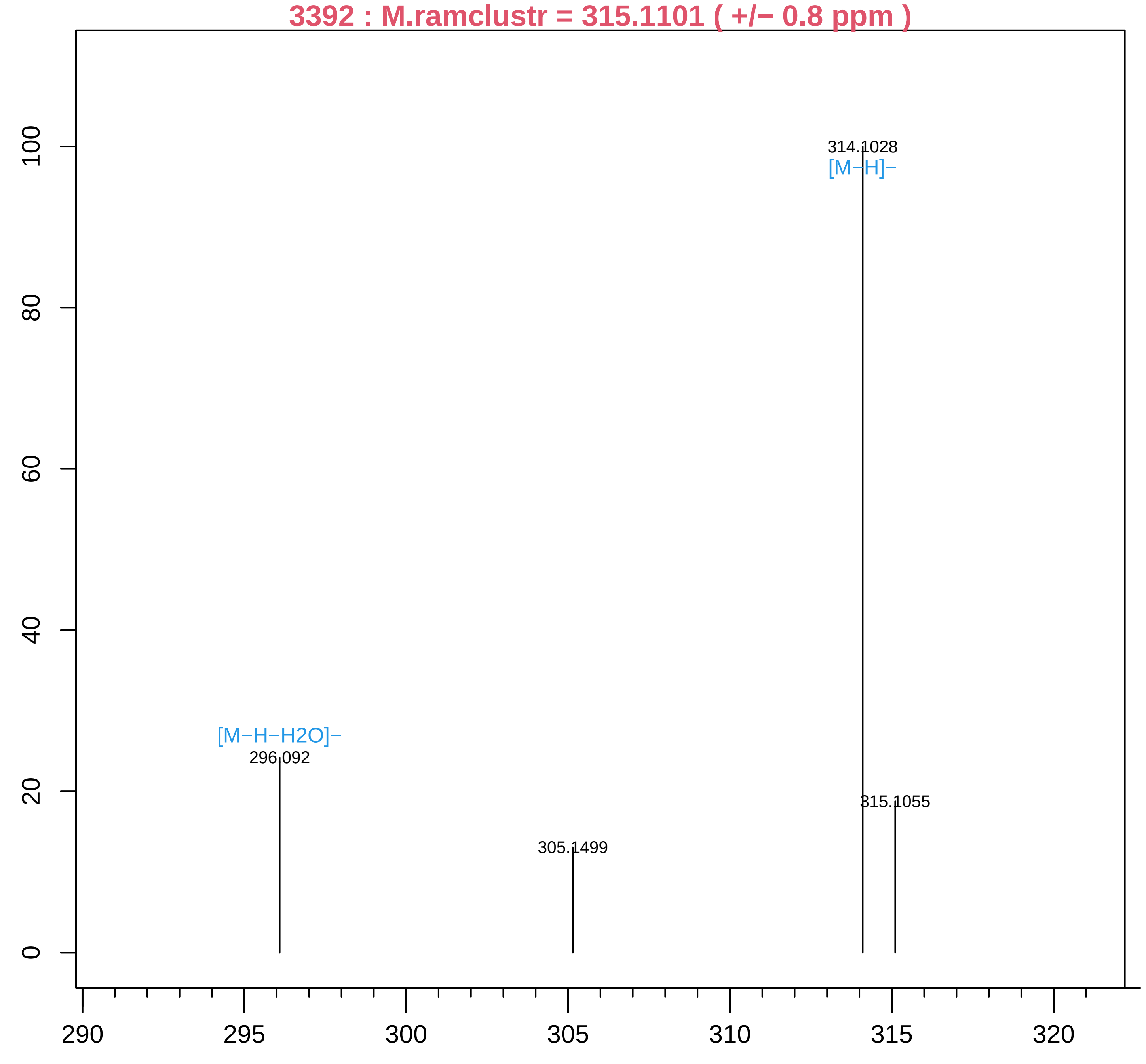
3392 : M.ramclustr = 315.1101 (+/− 0.8 ppm)
314.1028
[M−H]−
[M−H−H2O]−
296.092
315.1055
305.1499
0
20
40
60
80
100
290
295
300
305
310
315
320

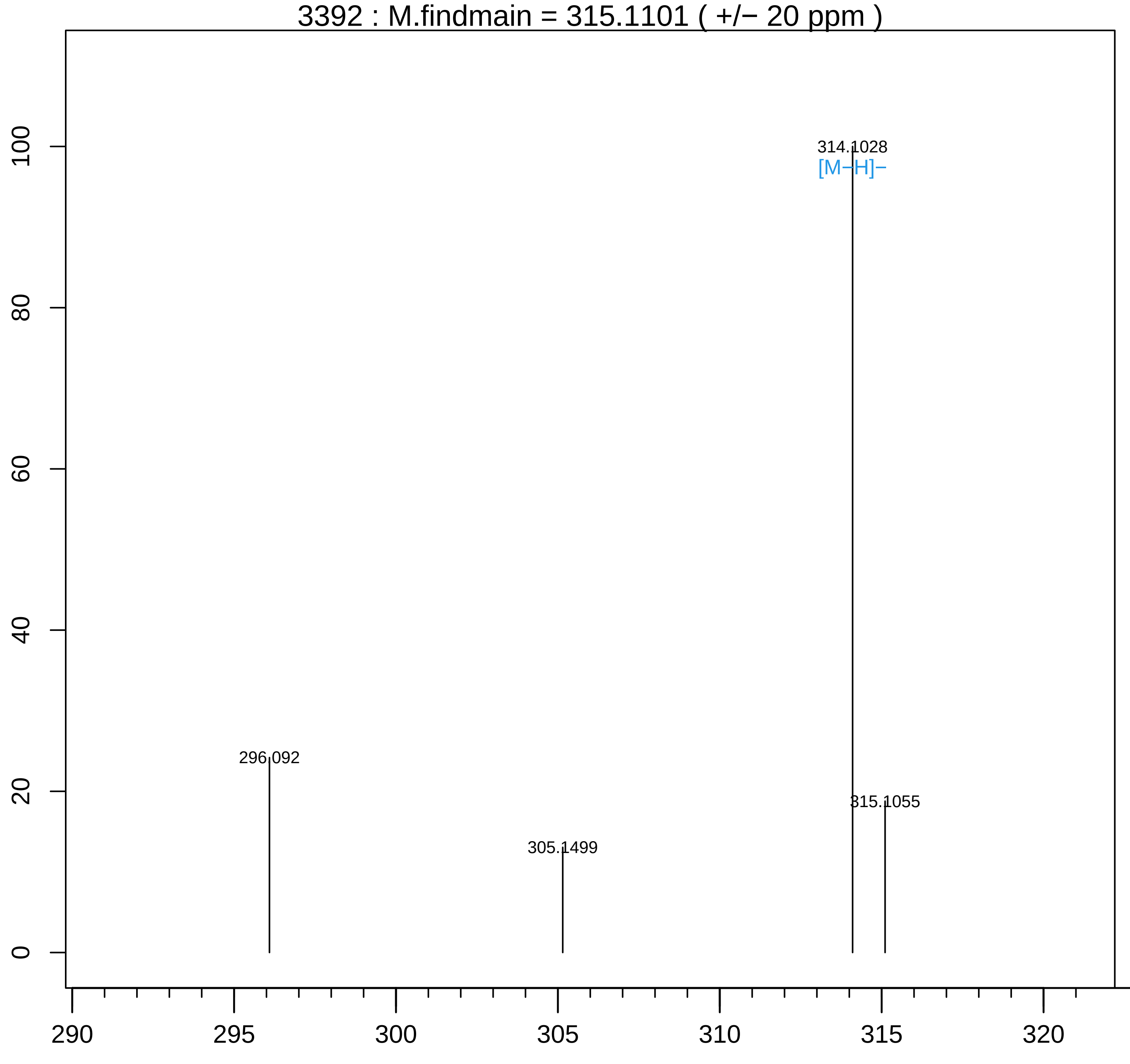
3392 : M.findmain = 315.1101 (+/− 20 ppm)
314.1028
[M−H]−
296.092
315.1055
305.1499
0
20
40
60
80
100
290
295
300
305
310
315
320

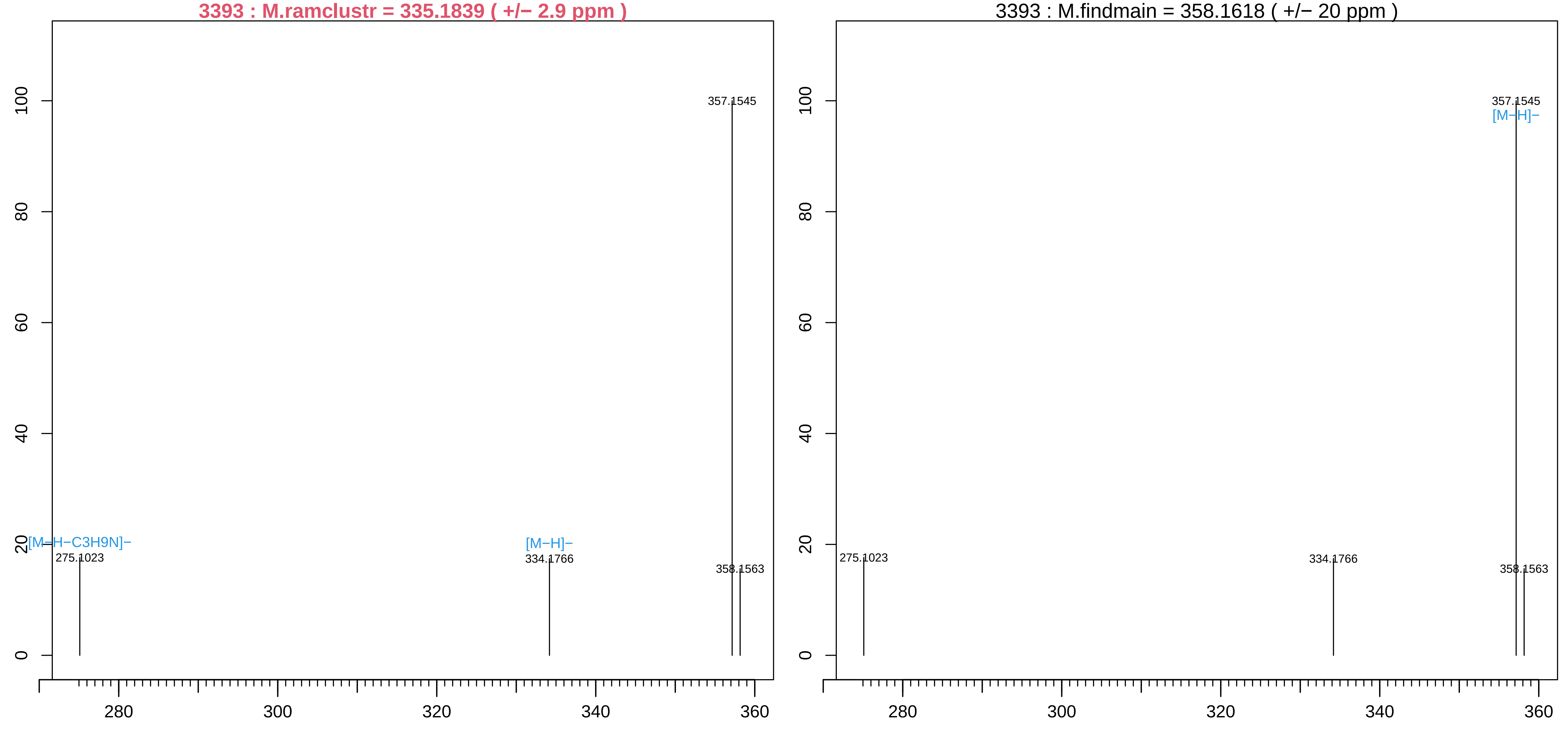
3393 : M.ramclustr = 335.1839 (+/− 2.9 ppm)
3393 : M.findmain = 358.1618 (+/− 20 ppm)
357.1545
[M−H]−
[M−H−C3H9N]−
275.1023
334.1766
358.1563
0
20
40
60
80
100
280
300
320
340
360

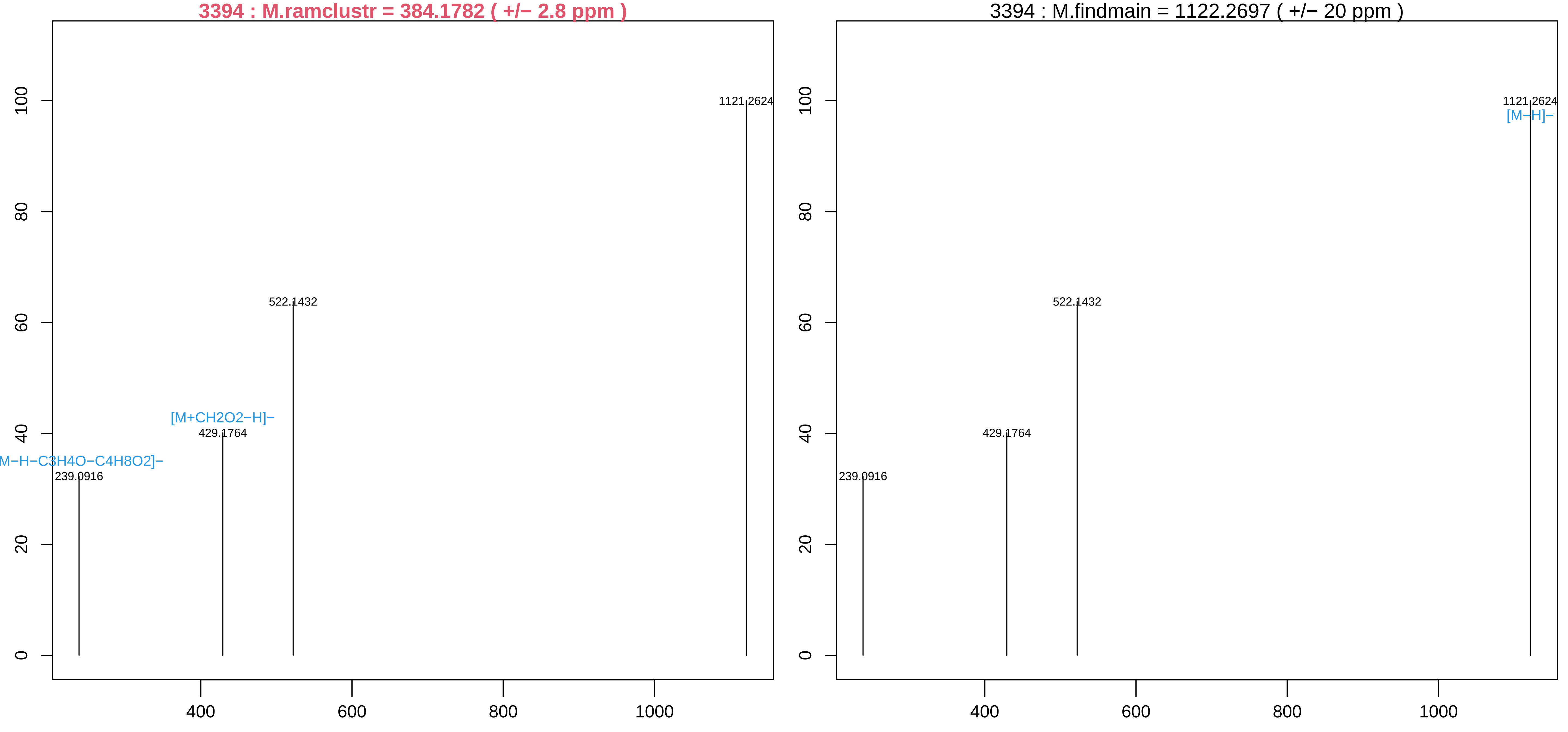

3394 : M.ramclustr = 384.1782 (+/− 2.8 ppm)
1121.2624
522.1432
[M+CH2O2−H]−
429.1764
M−H−C3H4O−C4H8O2]−
239.0916
0
20
40
60
80
100
400
600
800
1000
3394 : M.findmain = 1122.2697 (+/− 20 ppm)
1121.2624
[M−H]−
522.1432
429.1764
239.0916
0
20
40
60
80
100
400
600
800
1000

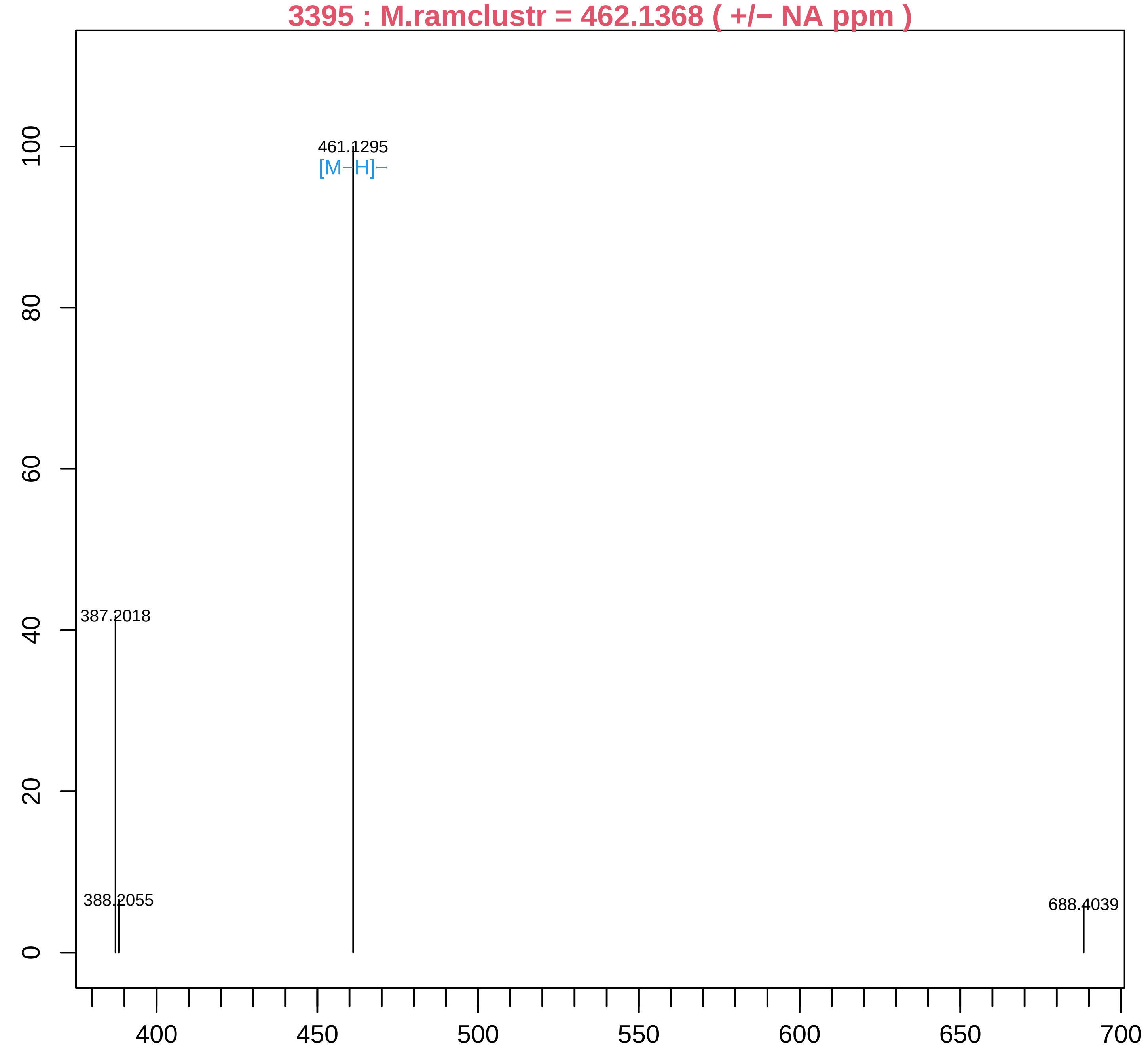
3395 : M.ramclustr = 462.1368 (+/− NA ppm)
461.1295
[M−H]−
387.2018
388.2055
688.4039
0
20
40
60
80
100
400
450
500
550
600
650
700

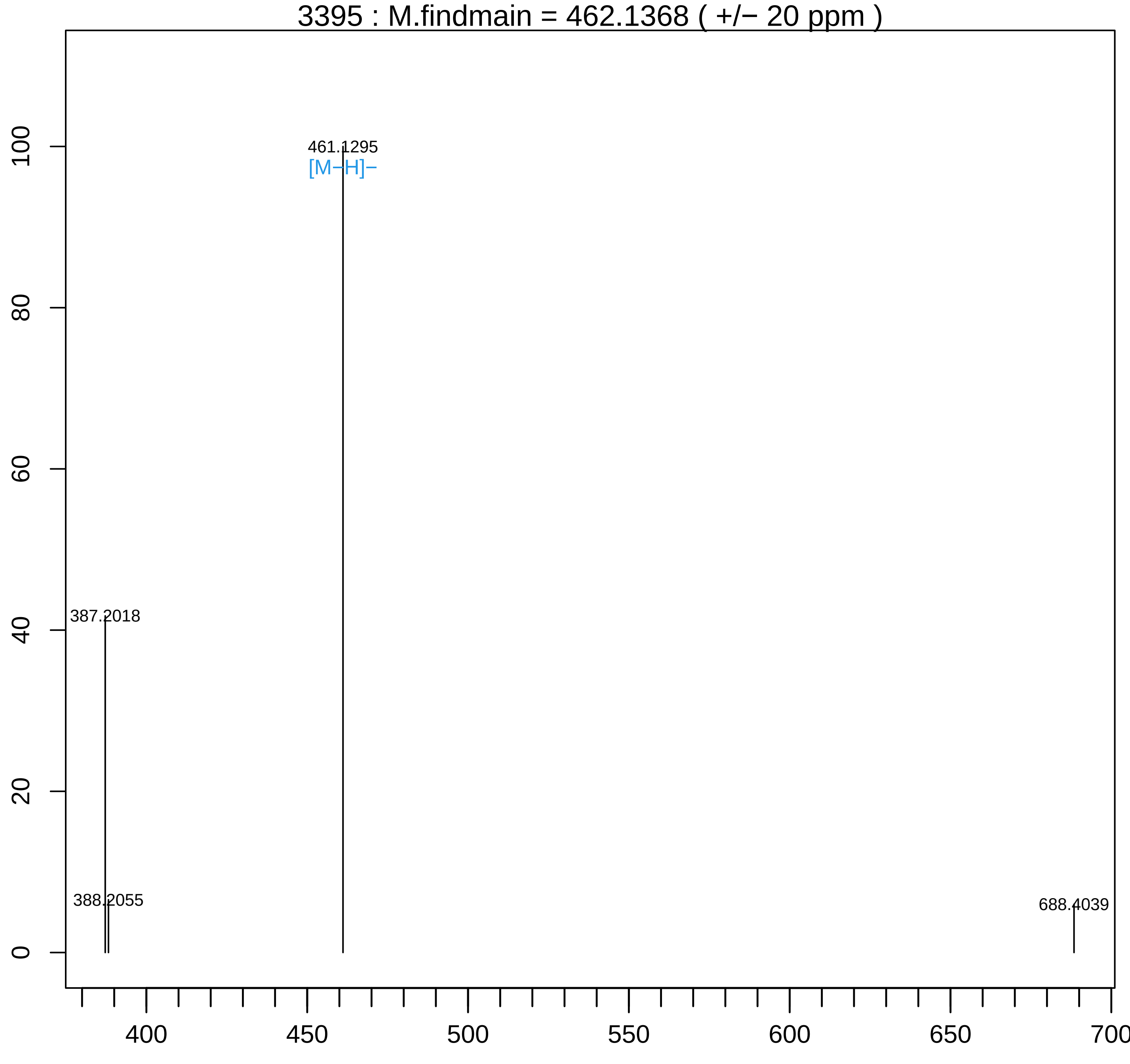
3395 : M.findmain = 462.1368 (+/− 20 ppm)
461.1295
[M−H]−
387.2018
388.2055
688.4039
0
20
40
60
80
100
400
450
500
550
600
650
700

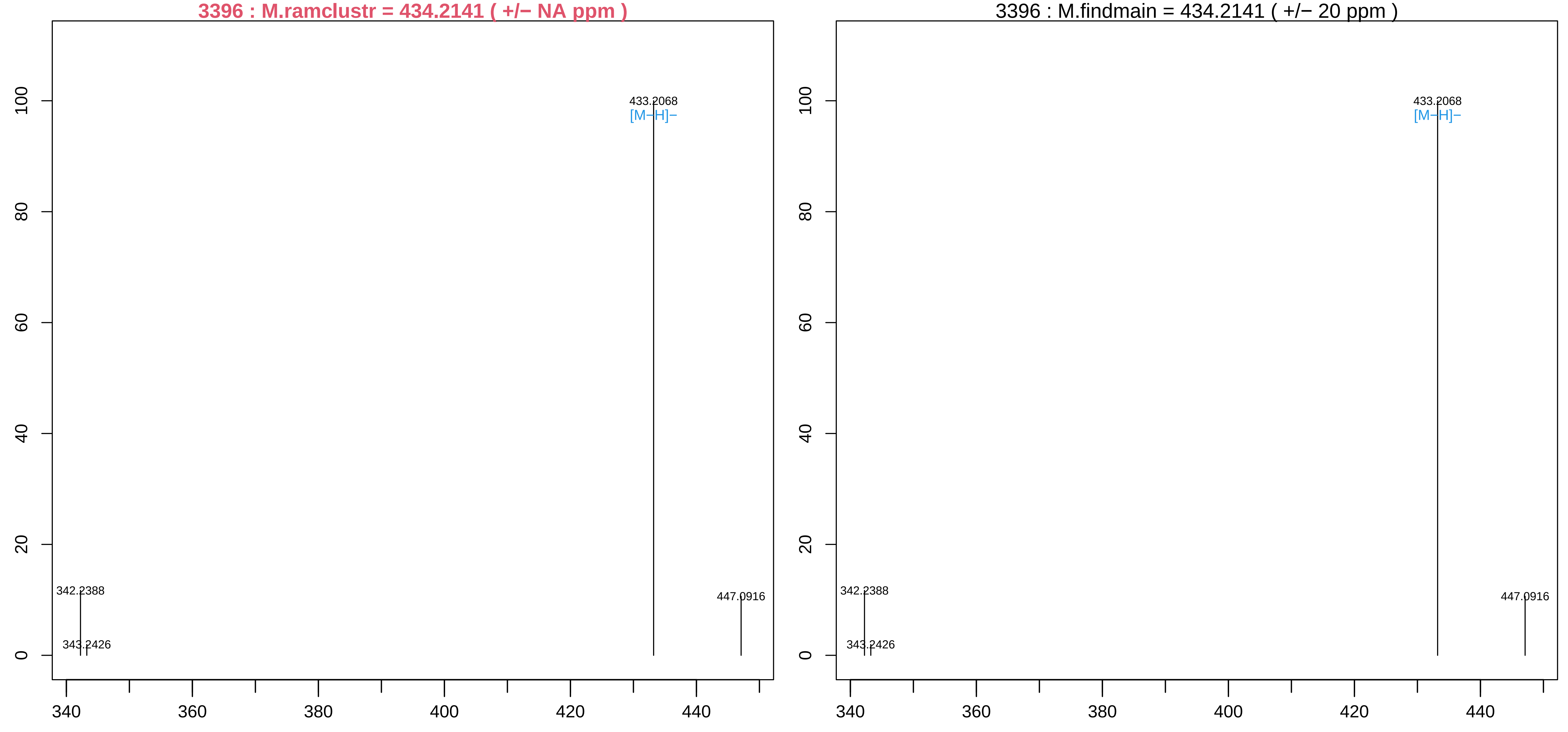

3396 : M.ramclustr = 434.2141 (+/− NA ppm)
3396 : M.findmain = 434.2141 (+/− 20 ppm)
433.2068
[M−H]−
342.2388
343.2426
447.0916
433.2068
[M−H]−
342.2388
343.2426
447.0916

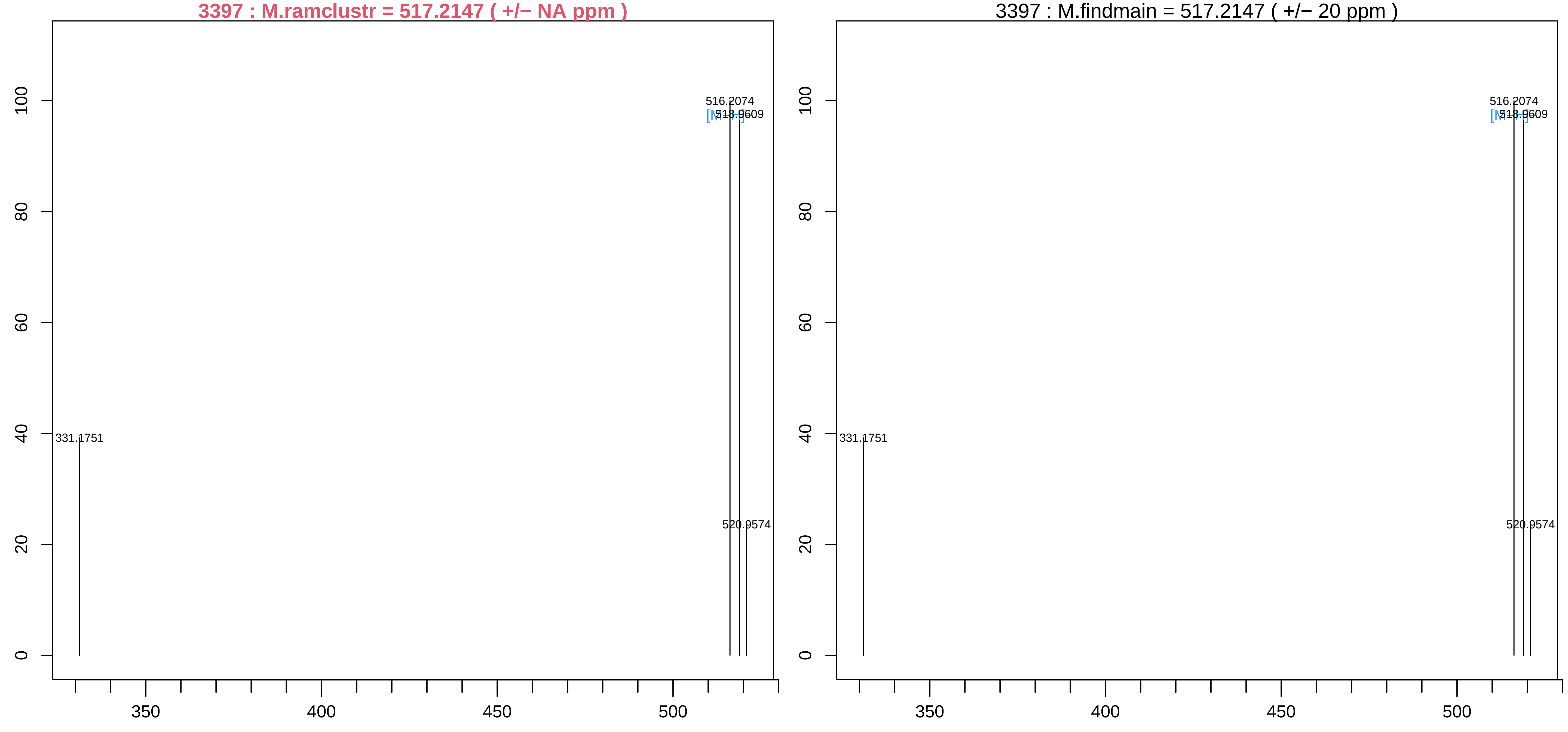

3397 : M.ramclustr = 517.2147 (+/− NA ppm)
3397 : M.findmain = 517.2147 (+/− 20 ppm)
516.2074
518.9609
331.1751
520.9574
0
20
40
60
80
100
350
400
450
500

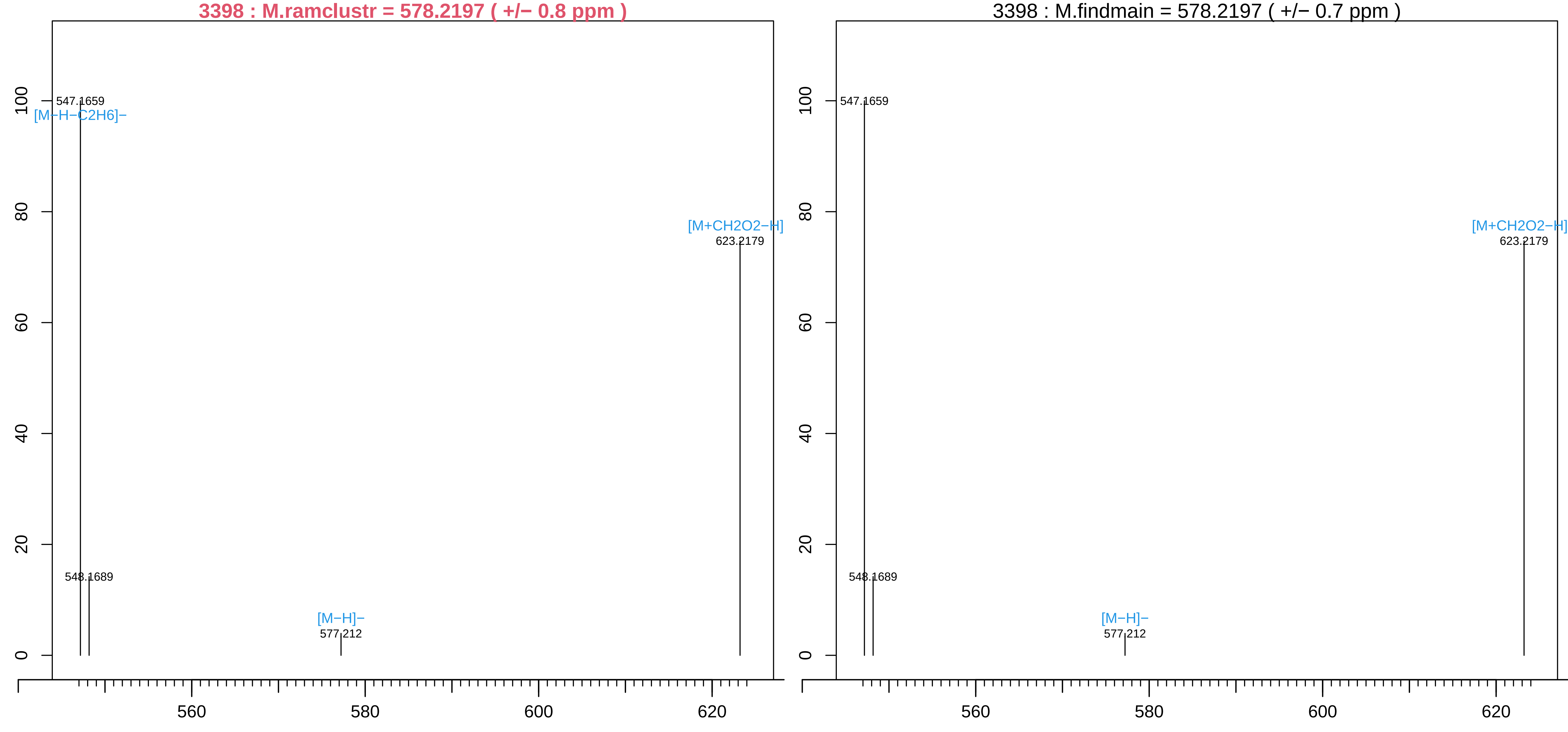
3398 : M.ramclustr = 578.2197 (+/− 0.8 ppm)
547.1659
[M−H−C2H6]−
[M+CH2O2−H]
623.2179
548.1689
[M−H]−
577.212
0
20
40
60
80
100
560
580
600
620
3398 : M.findmain = 578.2197 (+/− 0.7 ppm)
547.1659
[M+CH2O2−H]
623.2179
548.1689
[M−H]−
577.212
0
20
40
60
80
100
560
580
600
620

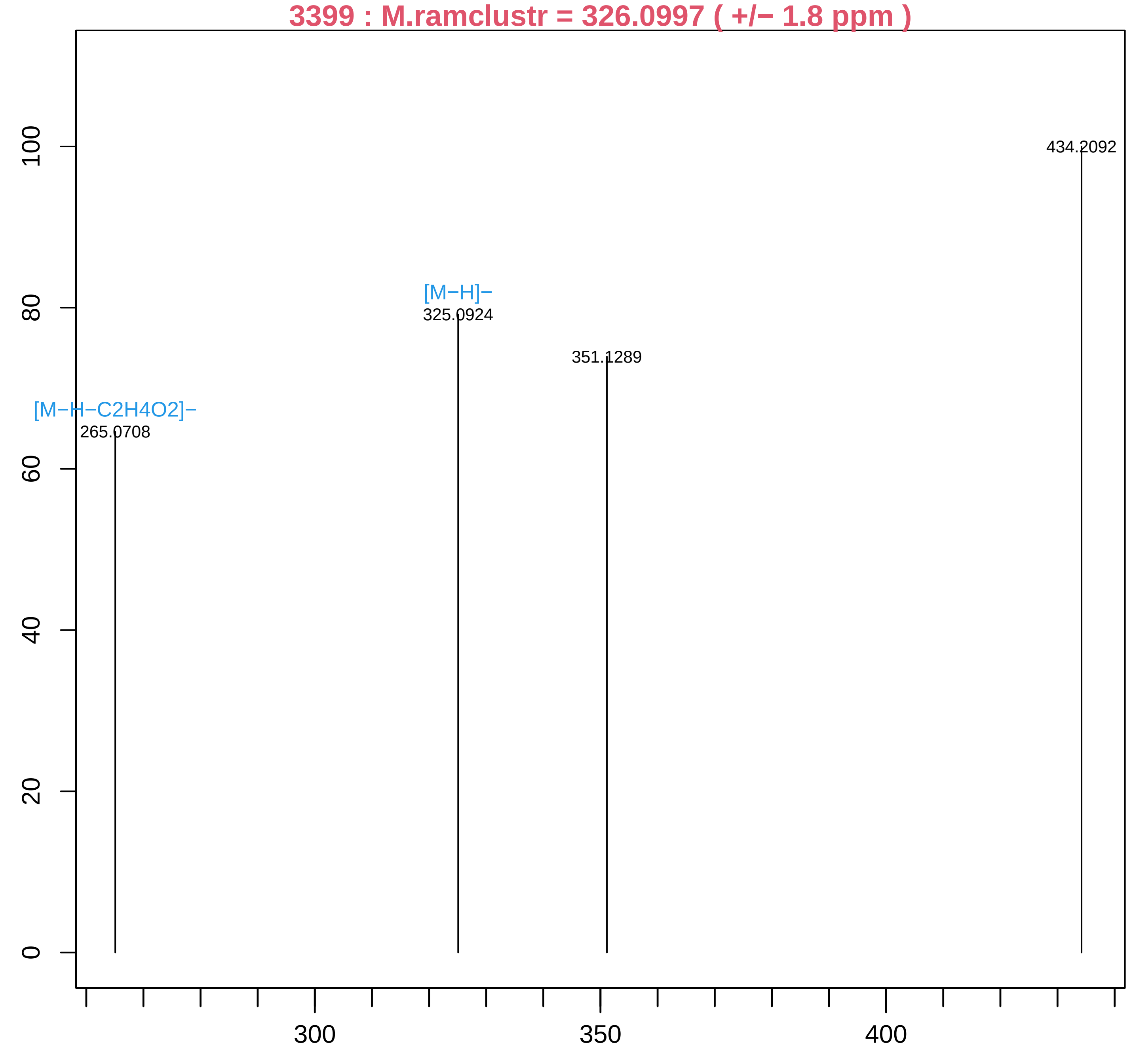

3399 : M.ramclustr = 326.0997 (+/− 1.8 ppm)
[M−H−C2H4O2]−
265.0708
[M−H]−
325.0924
351.1289
434.2092
0
20
40
60
80
100
300
350
400

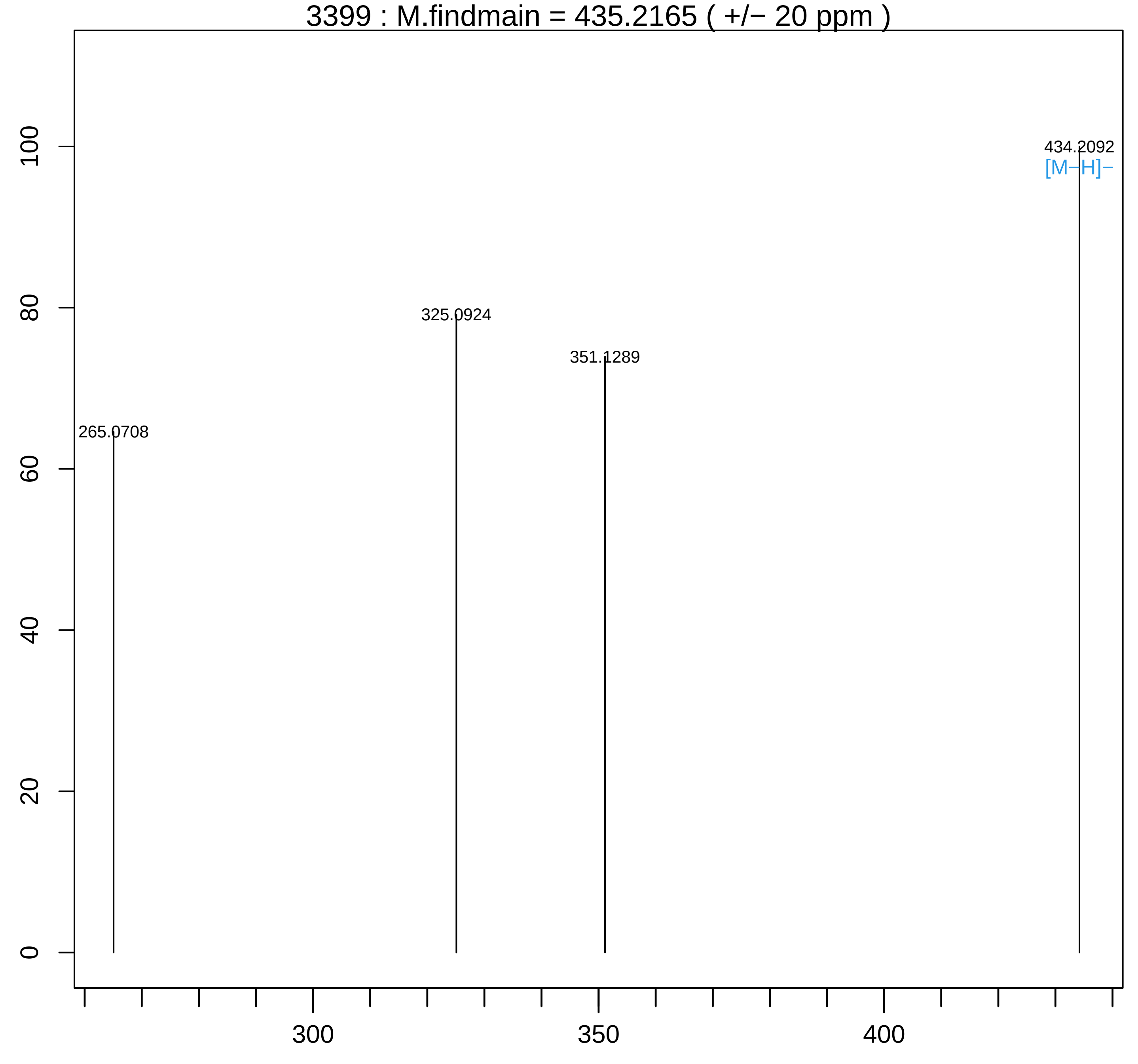

3399 : M.findmain = 435.2165 (+/− 20 ppm)
265.0708
325.0924
351.1289
434.2092
[M−H]−
0
20
40
60
80
100
300
350
400

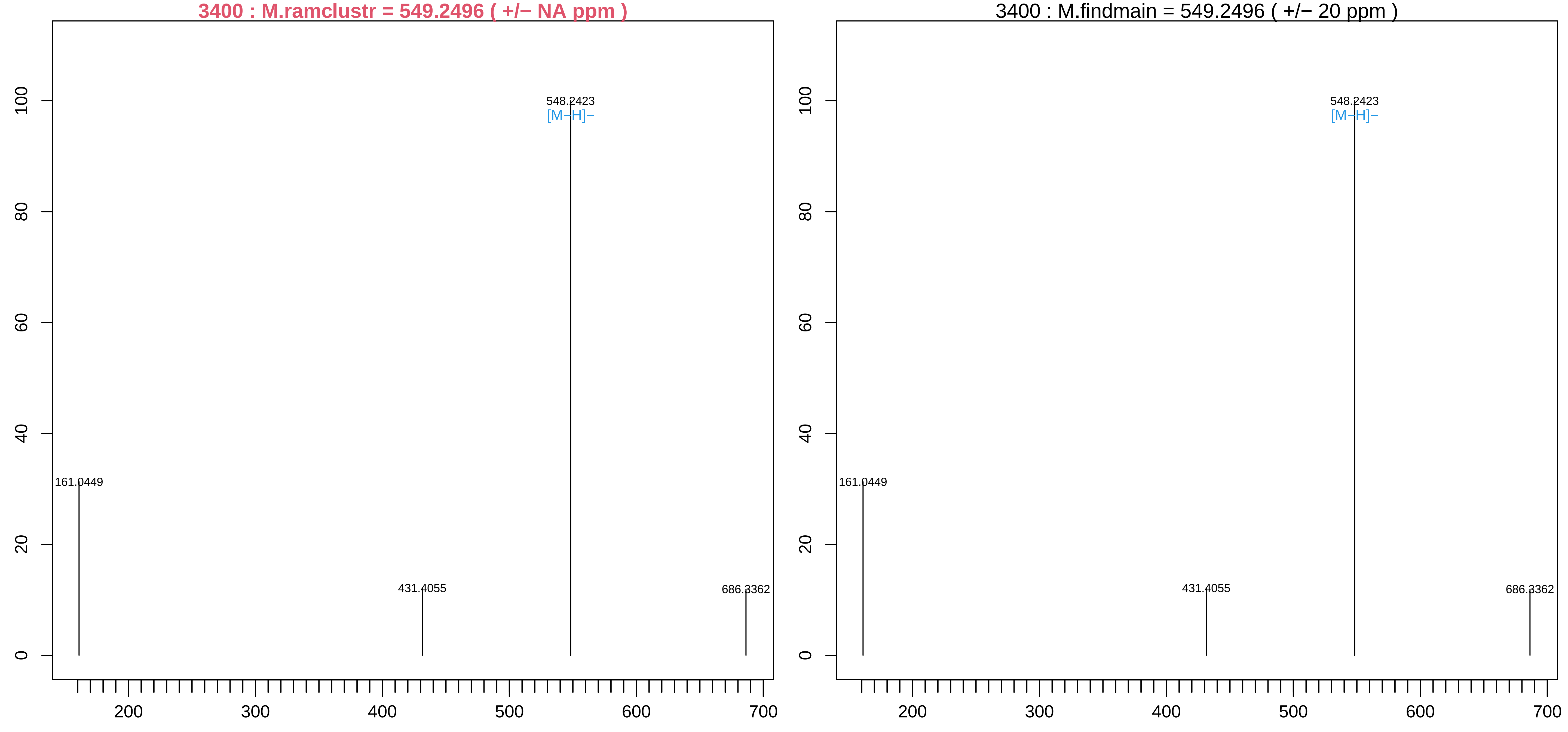
3400 : M.ramclustr = 549.2496 (+/− NA ppm)
548.2423
[M−H]−
161.0449
431.4055
686.3362
3400 : M.findmain = 549.2496 (+/− 20 ppm)
548.2423
[M−H]−
161.0449
431.4055
686.3362

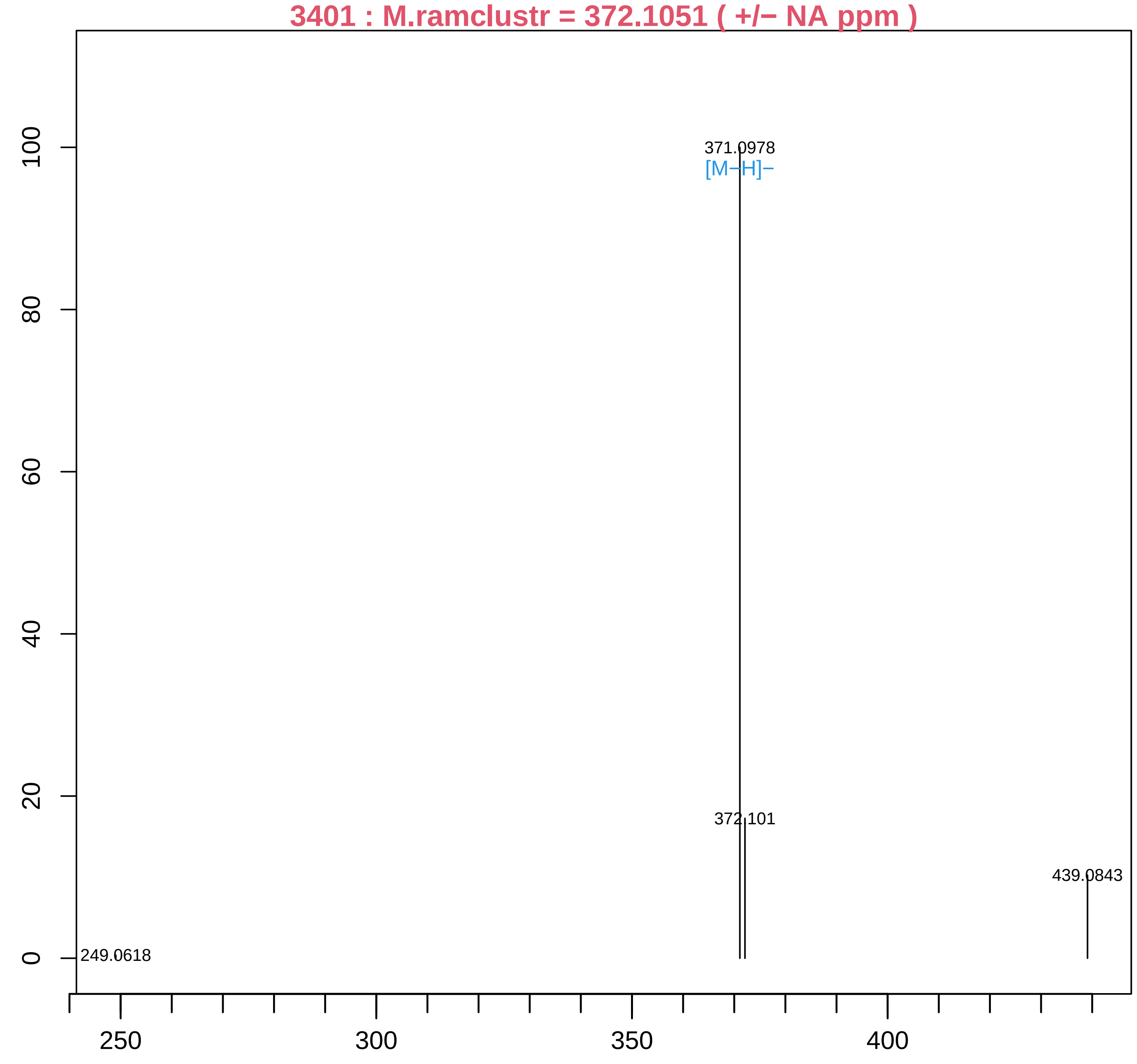
3401 : M.ramclustr = 372.1051 (+/− NA ppm)
371.0978
[M−H]−
372.101
439.0843
249.0618
0
20
40
60
80
100
250
300
350
400

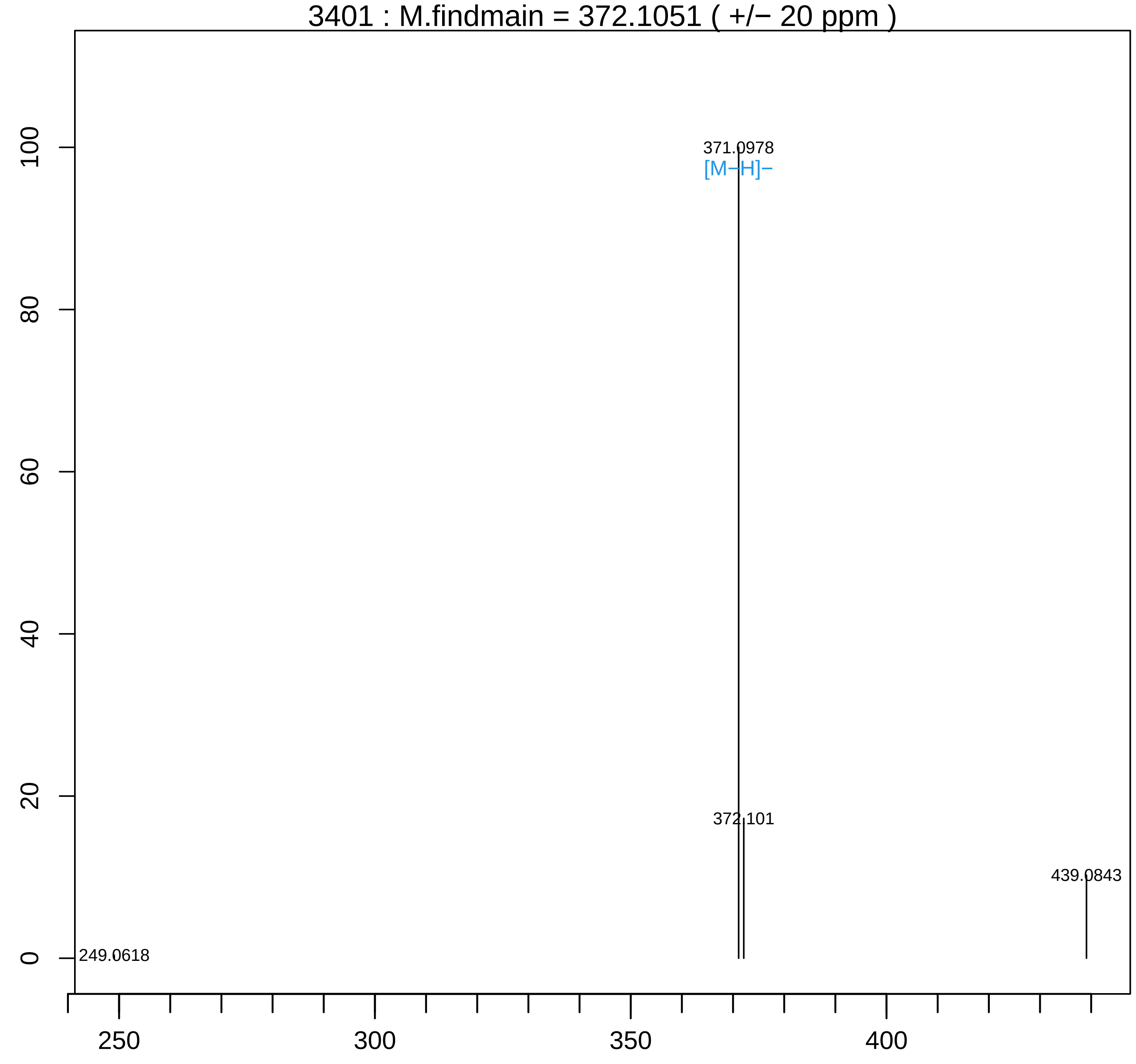
3401 : M.findmain = 372.1051 (+/− 20 ppm)
371.0978
[M−H]−
372.101
439.0843
249.0618
0
20
40
60
80
100
250
300
350
400

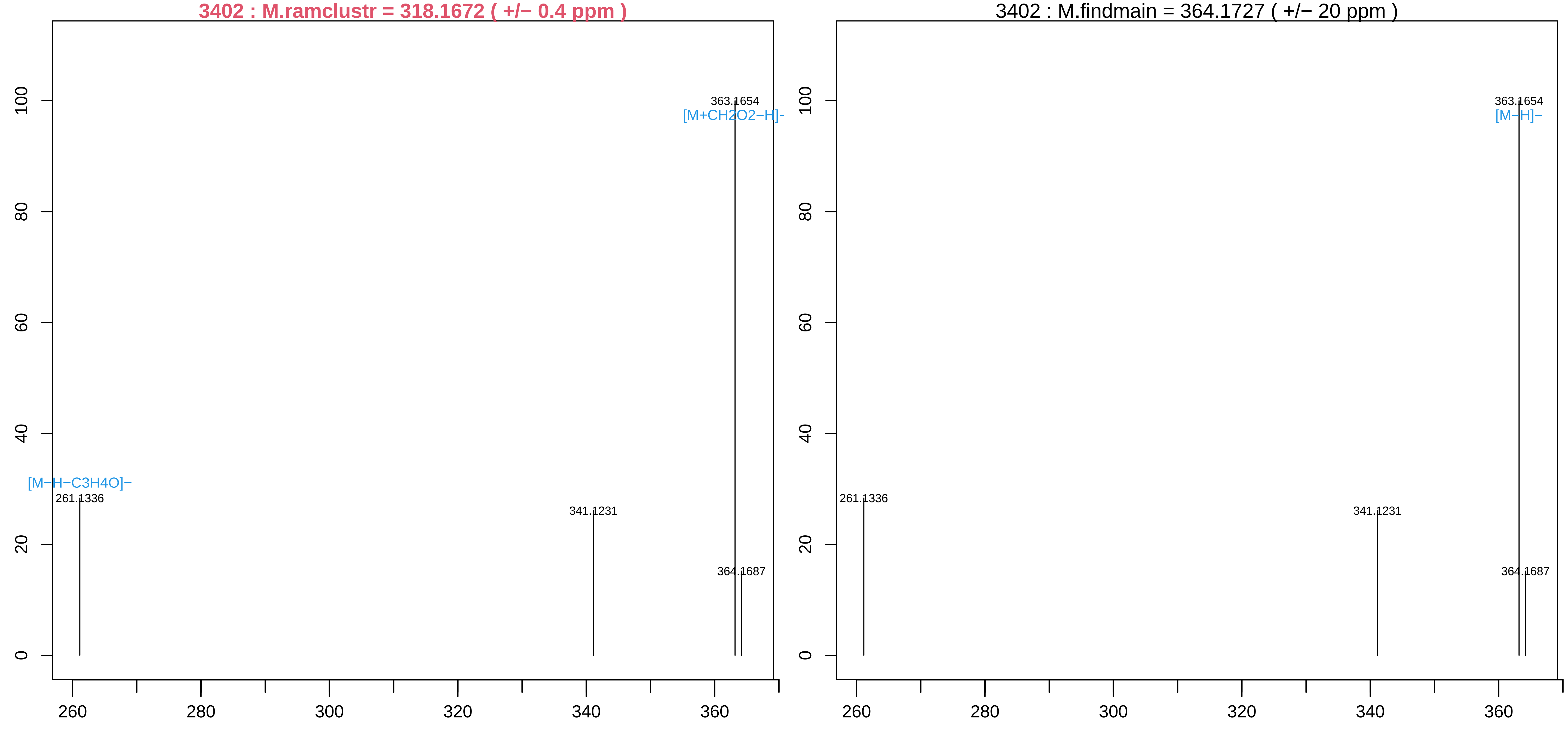

3402 : M.ramclustr = 318.1672 (+/− 0.4 ppm)
363.1654
[M+CH2O2−H]-
[M−H−C3H4O]−
261.1336
341.1231
364.1687
3402 : M.findmain = 364.1727 (+/− 20 ppm)
363.1654
[M−H]−
261.1336
341.1231
364.1687
0
20
40
60
80
100
260
280
300
320
340
360

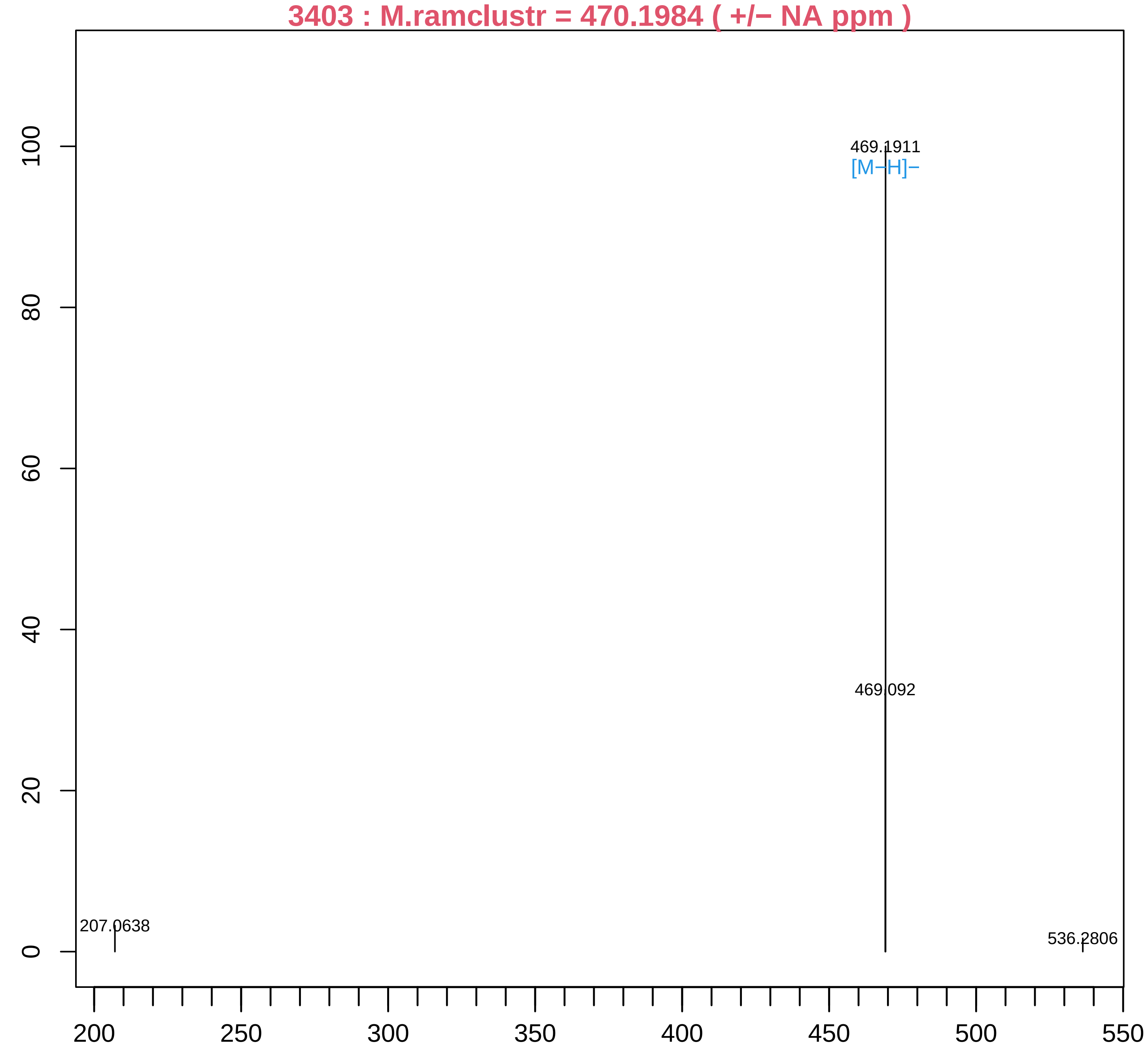
3403 : M.ramclustr = 470.1984 (+/− NA ppm)
469.1911
[M−H]−
469.092
207.0638
536.2806

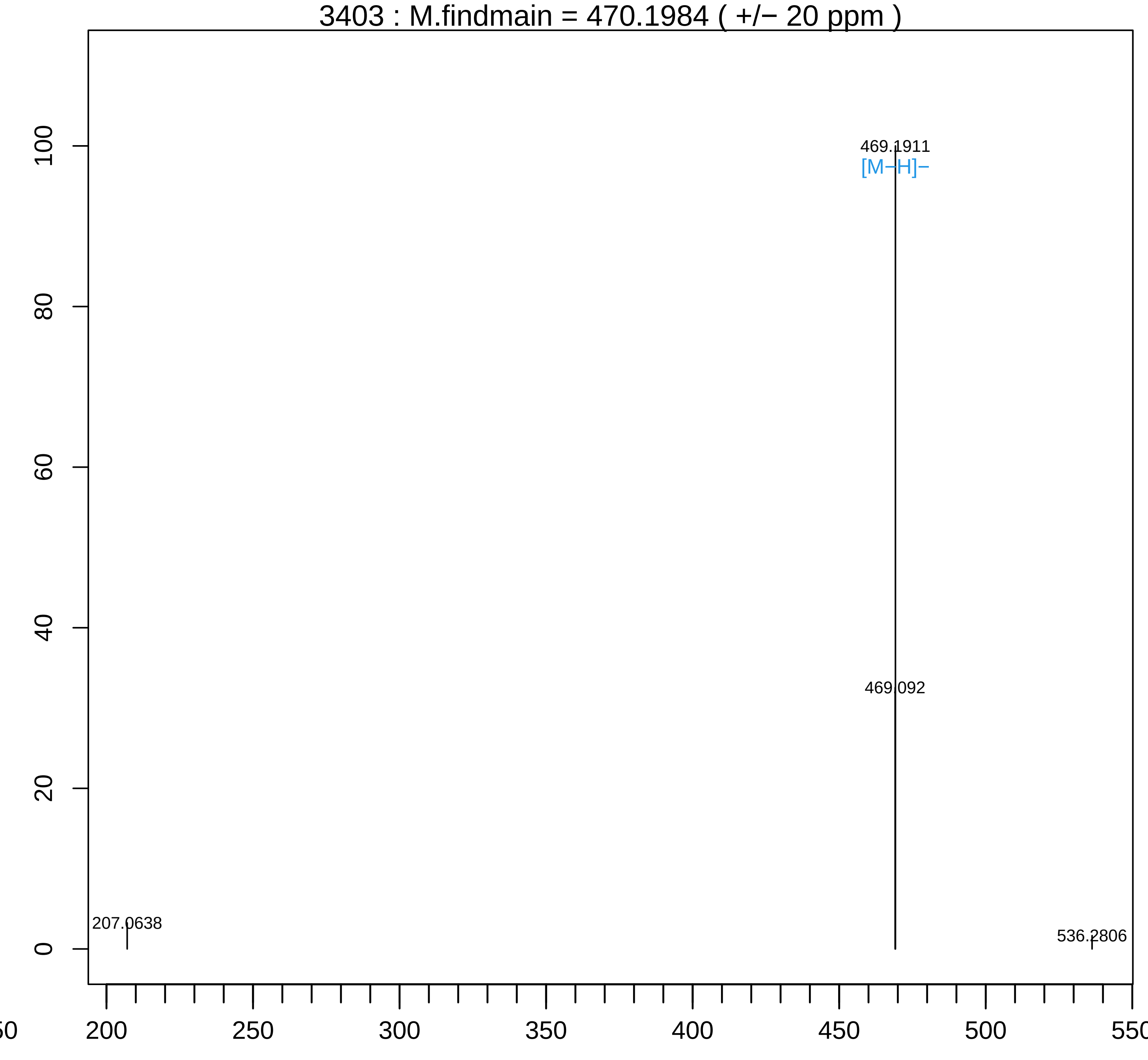
3403 : M.findmain = 470.1984 (+/− 20 ppm)
469.1911
[M−H]−
469.092
207.0638
536.2806

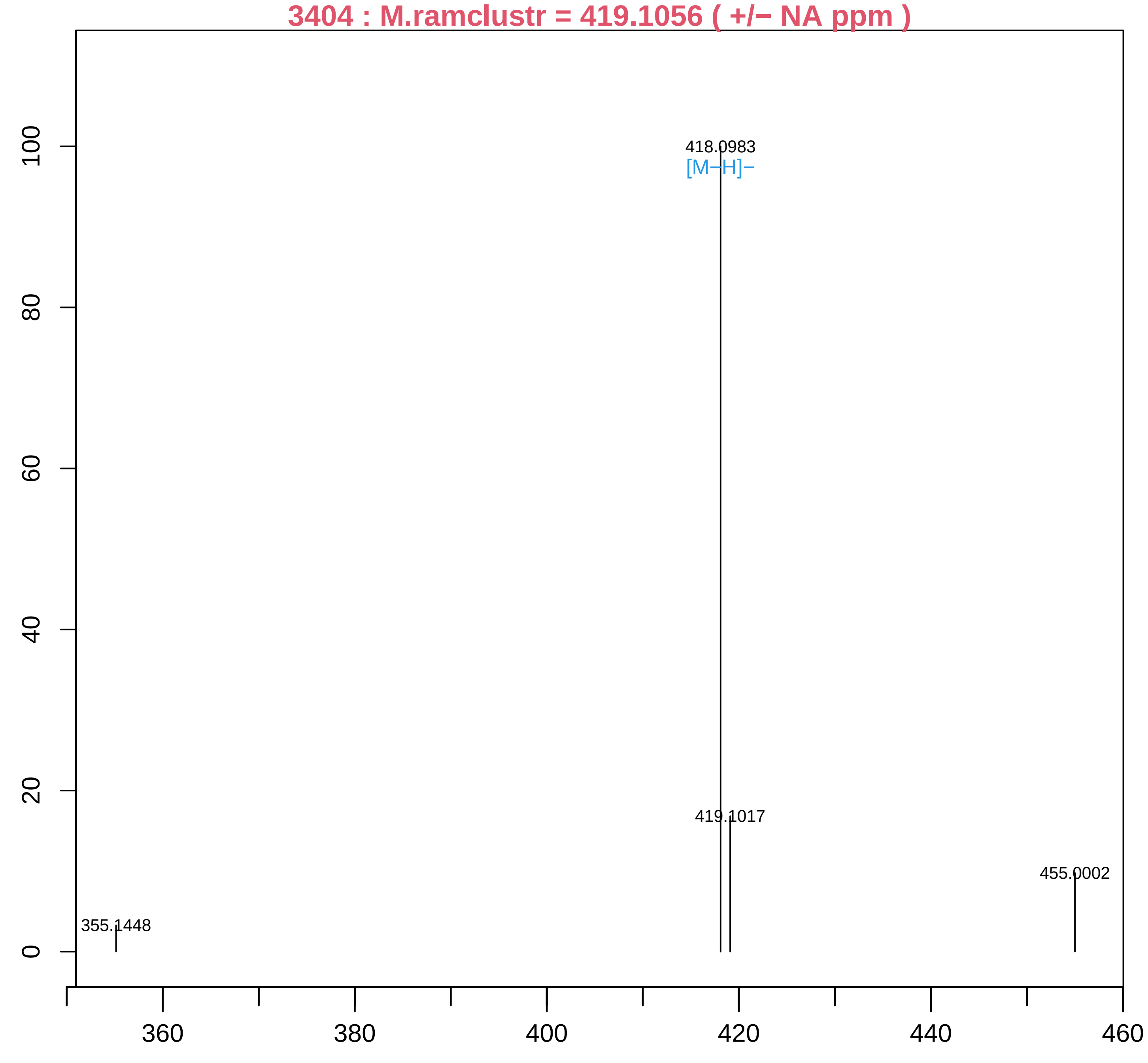
3404 : M.ramclustr = 419.1056 (+/− NA ppm)
418.0983
[M−H]−
419.1017
455.0002
355.1448
0
20
40
60
80
100
360
380
400
420
440
460

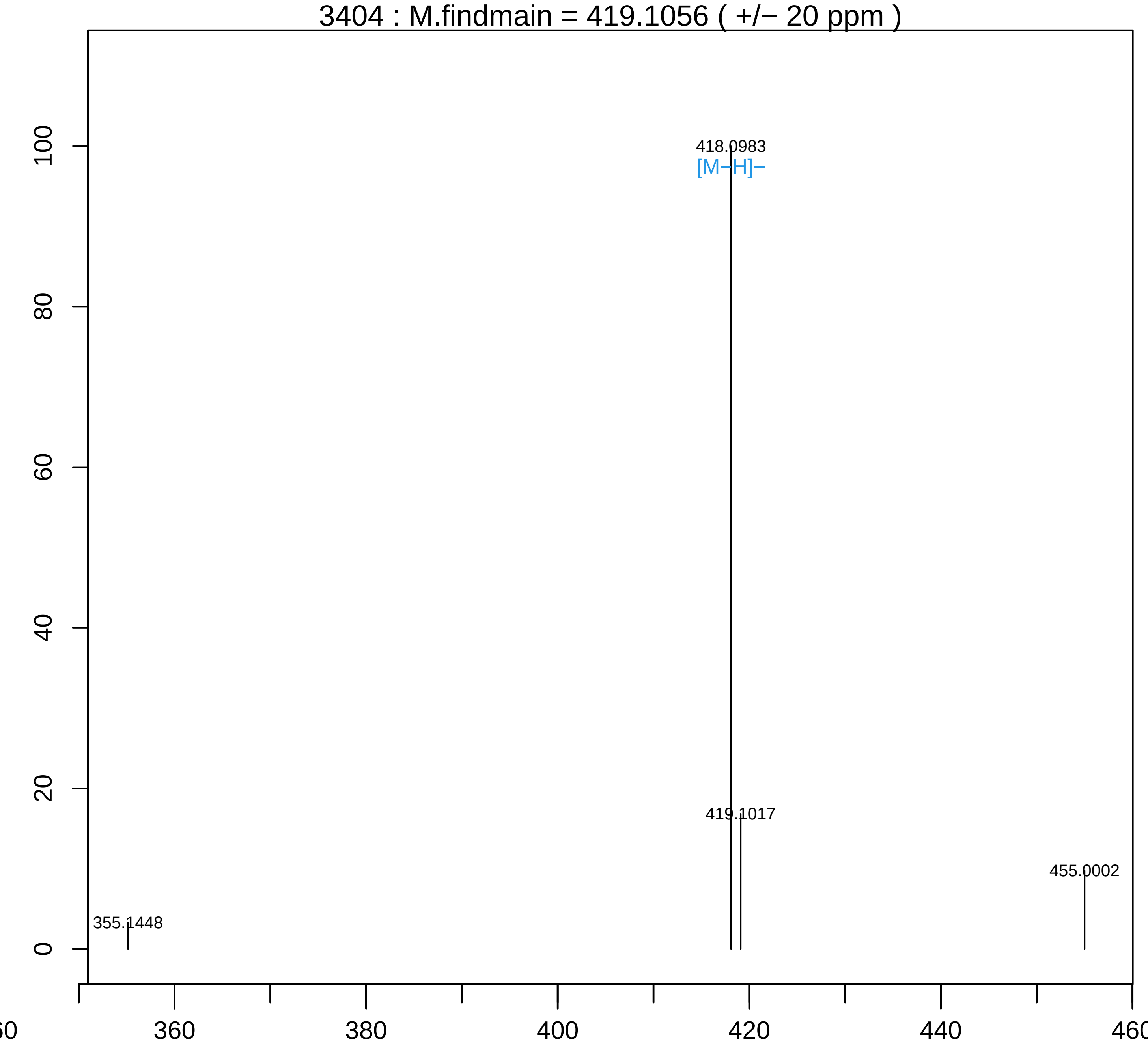
3404 : M.findmain = 419.1056 (+/− 20 ppm)
418.0983
[M−H]−
419.1017
455.0002
355.1448
0
20
40
60
80
100
360
380
400
420
440
460

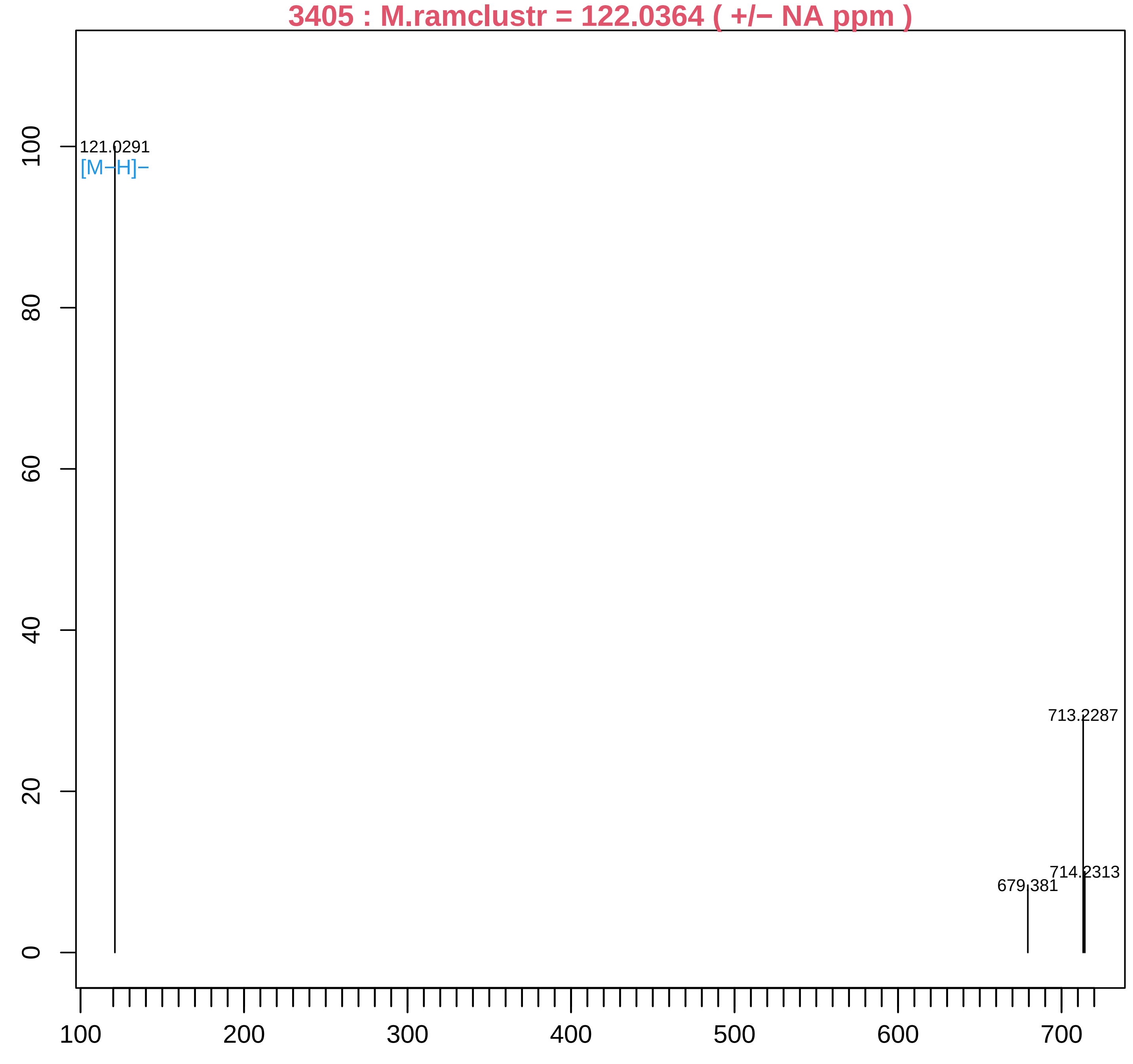
3405 : M.ramclustr = 122.0364 (+/− NA ppm)
121.0291
[M−H]−
713.2287
714.2313
679.381
0
20
40
60
80
100
100
200
300
400
500
600
700

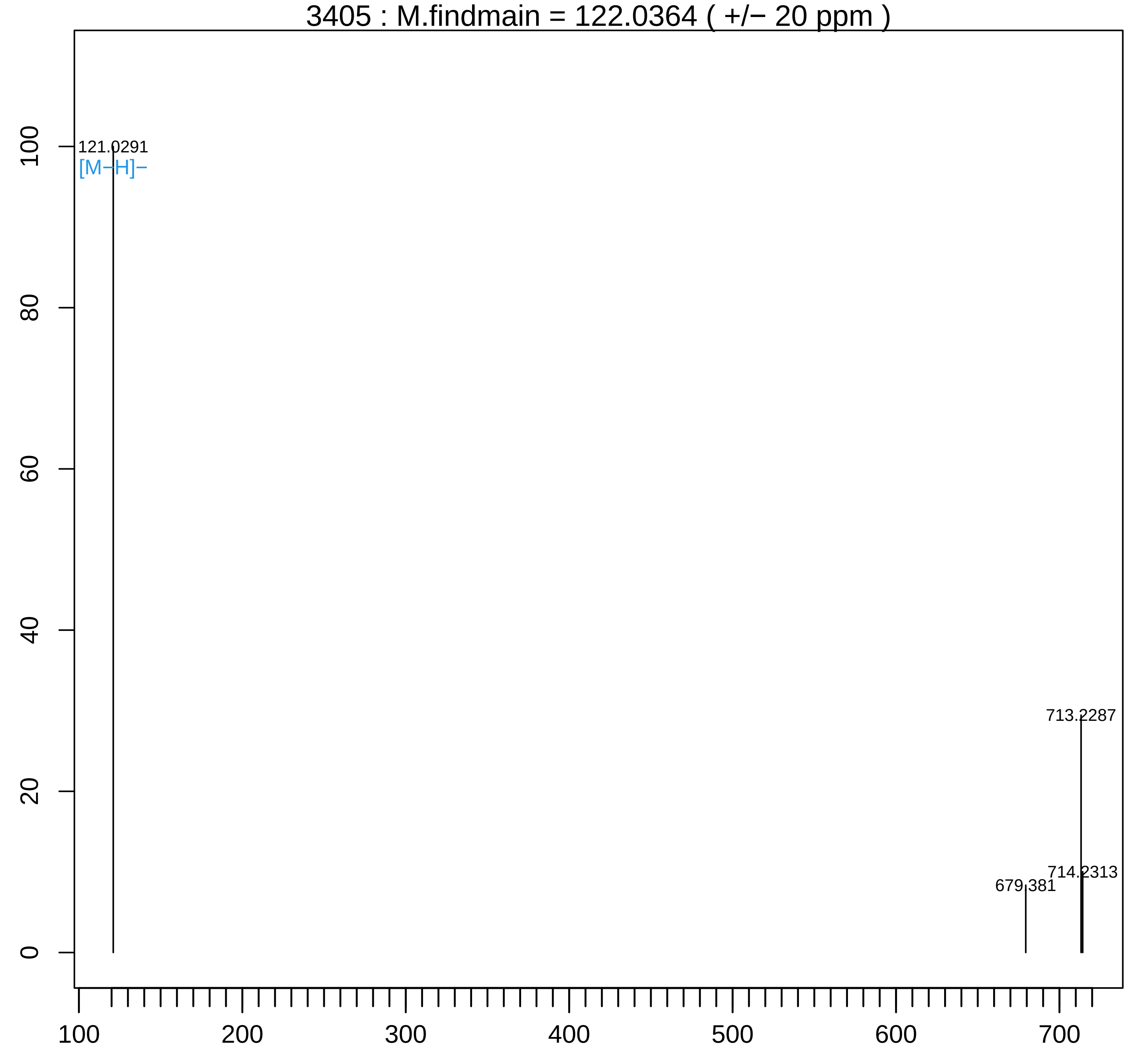
3405 : M.findmain = 122.0364 (+/− 20 ppm)
121.0291
[M−H]−
713.2287
714.2313
679.381
0
20
40
60
80
100
100
200
300
400
500
600
700

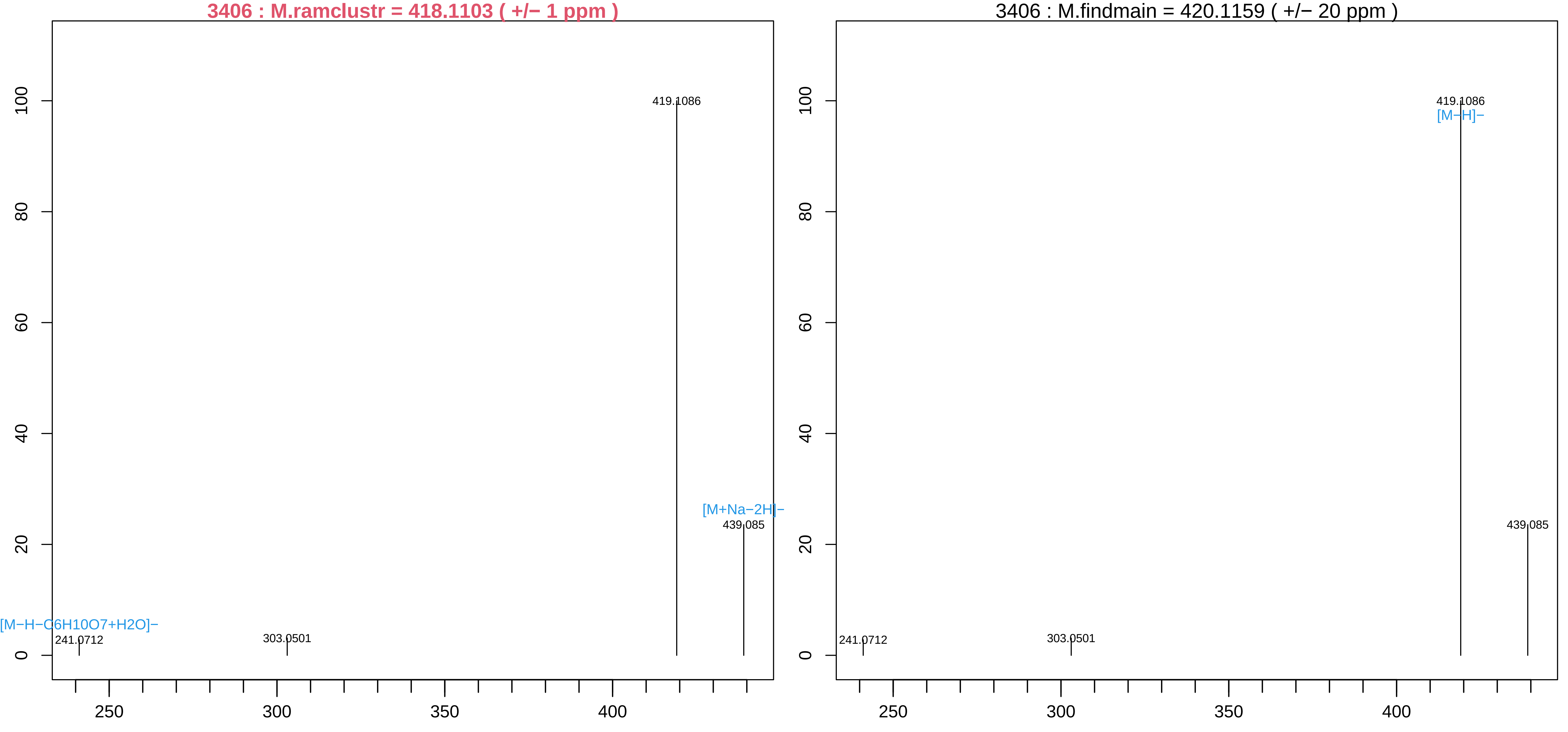

3406 : M.ramclustr = 418.1103 (+/− 1 ppm)
419.1086
[M+Na−2H]−
439.085
[M−H−C6H10O7+H2O]−
241.0712
303.0501
3406 : M.findmain = 420.1159 (+/− 20 ppm)
419.1086
[M−H]−
439.085
241.0712
303.0501
0
20
40
60
80
100
250
300
350
400

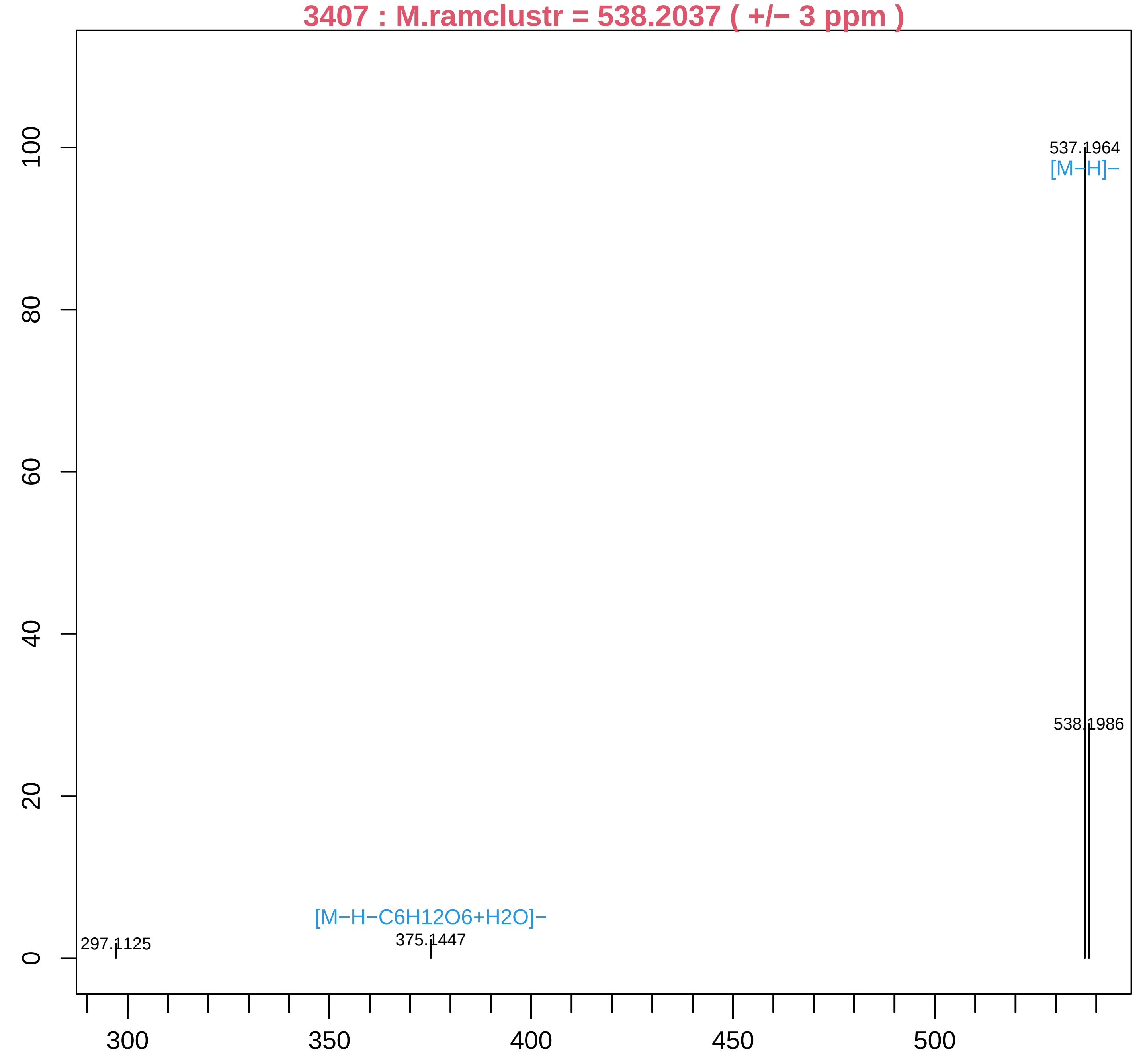

3407 : M.ramclustr = 538.2037 (+/− 3 ppm)
537.1964
[M−H]−
538.1986
[M−H−C6H12O6+H2O]−
375.1447
297.1125
0
20
40
60
80
100
300
350
400
450
500

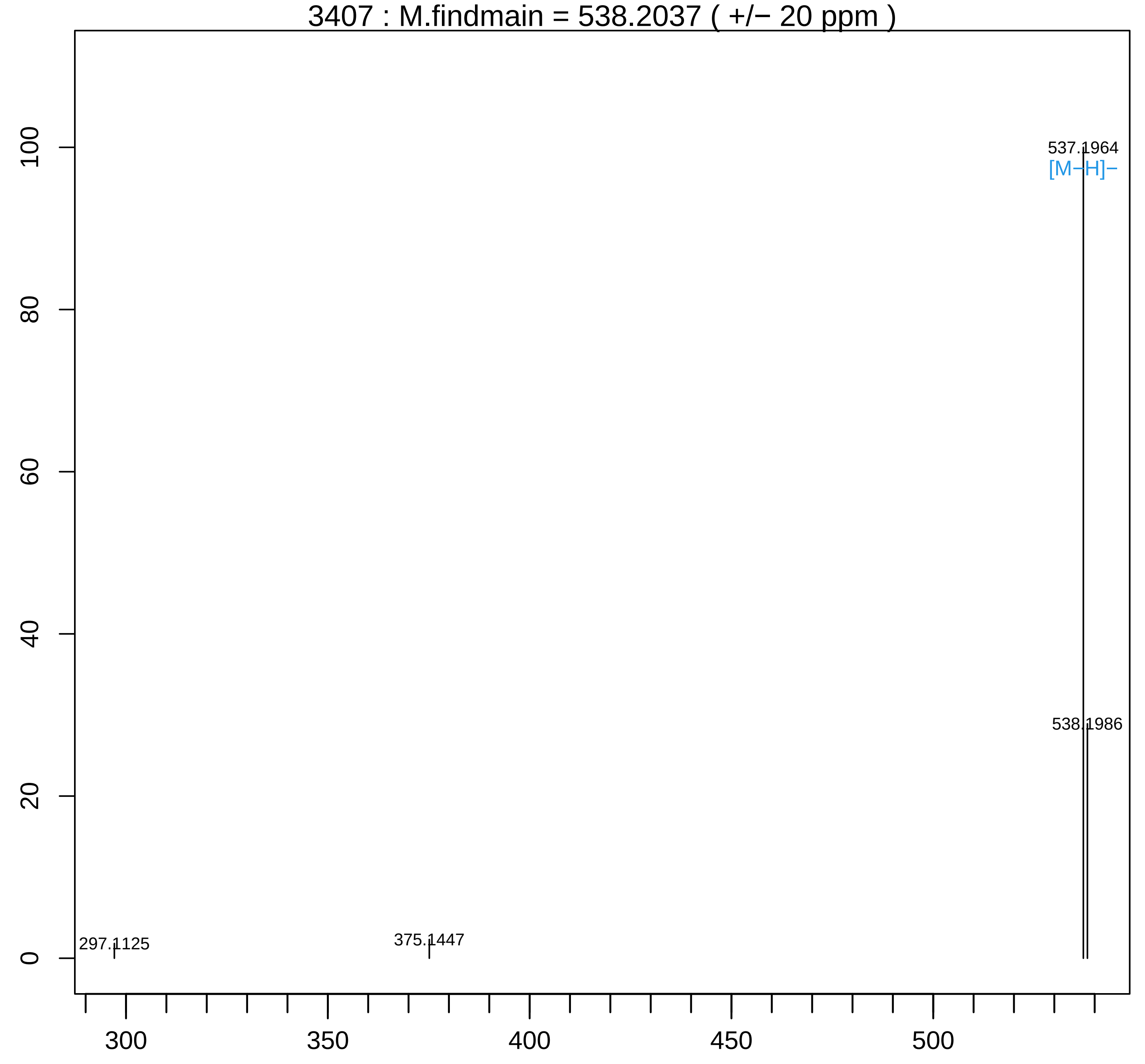

3407 : M.findmain = 538.2037 (+/− 20 ppm)
537.1964
[M−H]−
538.1986
375.1447
297.1125
0
20
40
60
80
100
300
350
400
450
500

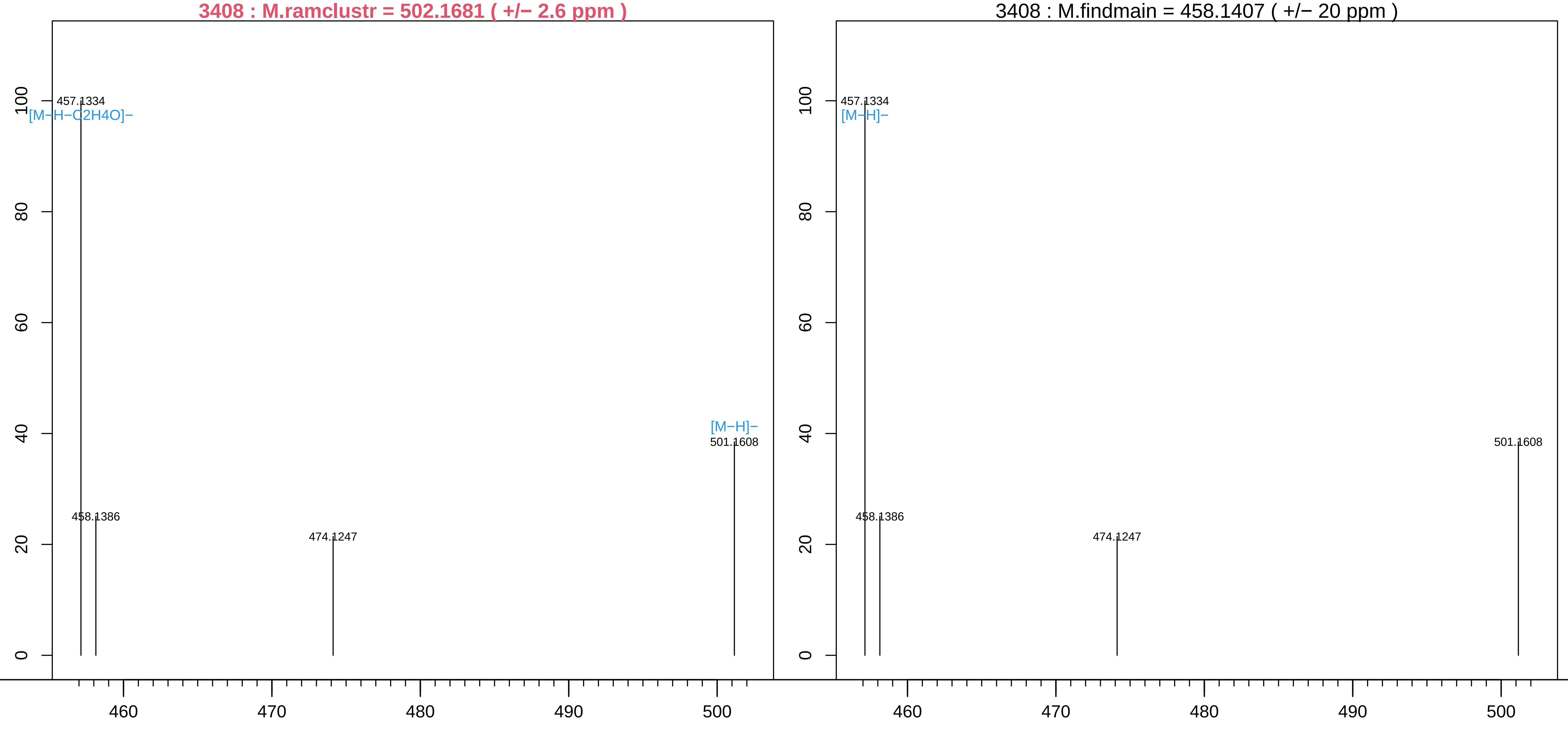
3408 : M.ramclustr = 502.1681 (+/− 2.6 ppm)
457.1334
[M−H−C2H4O]−
458.1386
474.1247
[M−H]−
501.1608
3408 : M.findmain = 458.1407 (+/− 20 ppm)
457.1334
[M−H]−
458.1386
474.1247
501.1608
0
20
40
60
80
100
460
470
480
490
500

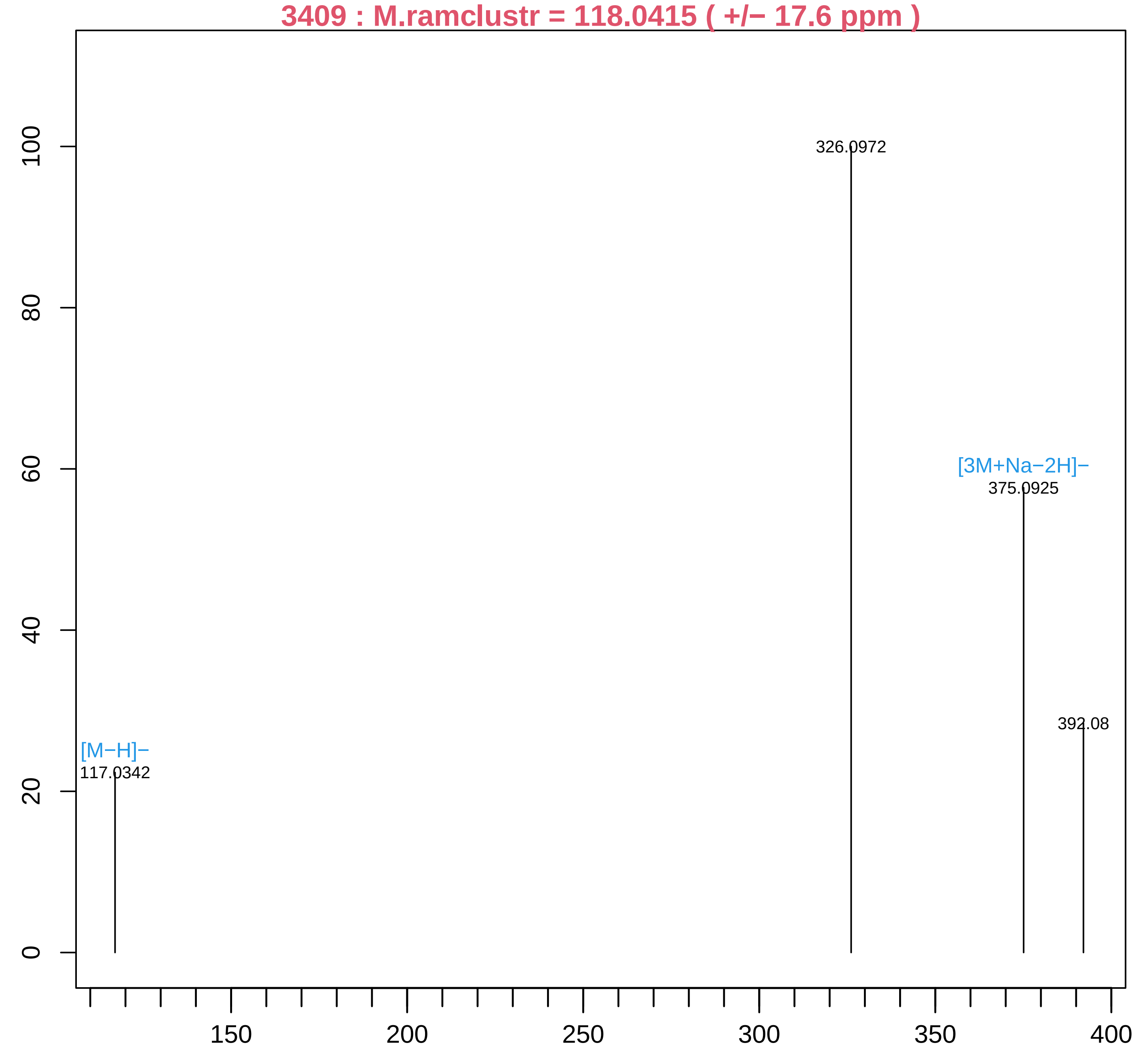
3409 : M.ramclustr = 118.0415 (+/− 17.6 ppm)
326.0972
[3M+Na−2H]−
375.0925
392.08
[M−H]−
117.0342
0
20
40
60
80
100
150
200
250
300
350
400

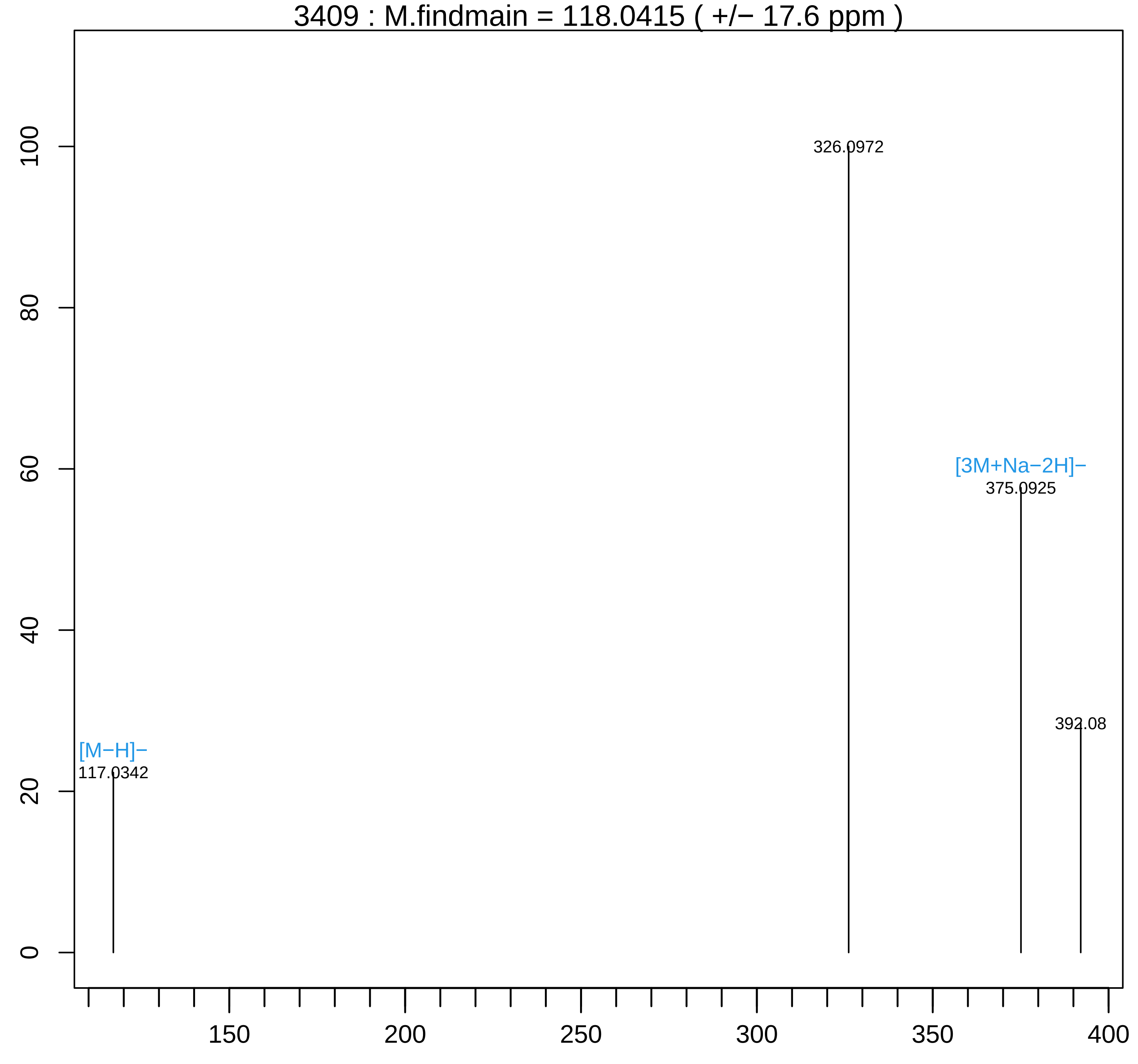
3409 : M.findmain = 118.0415 (+/− 17.6 ppm)
326.0972
[3M+Na−2H]−
375.0925
392.08
[M−H]−
117.0342
0
20
40
60
80
100
150
200
250
300
350
400

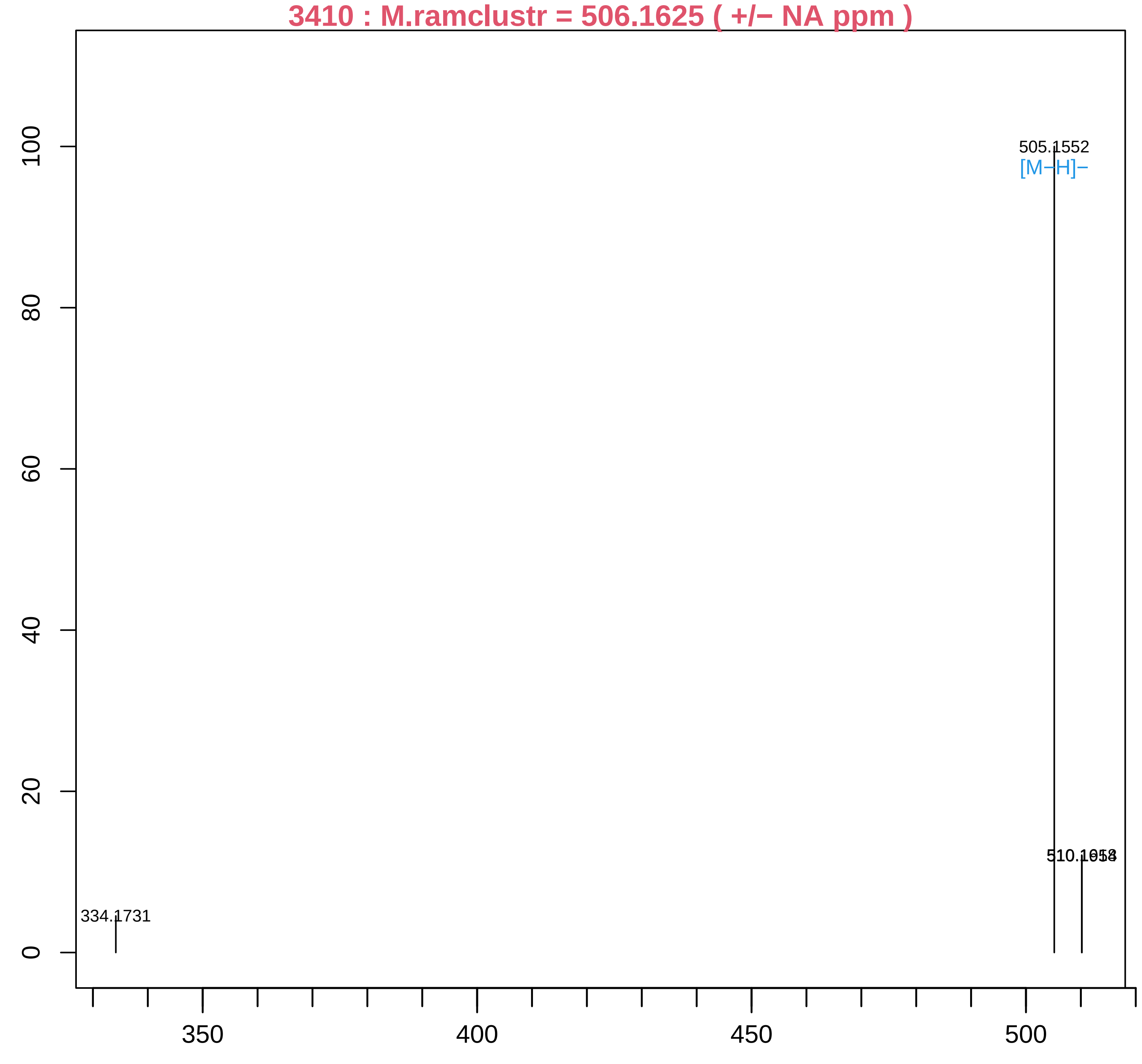
3410 : M.ramclustr = 506.1625 (+/− NA ppm)
505.1552
[M−H]−
334.1731
0
20
40
60
80
100
350
400
450
500

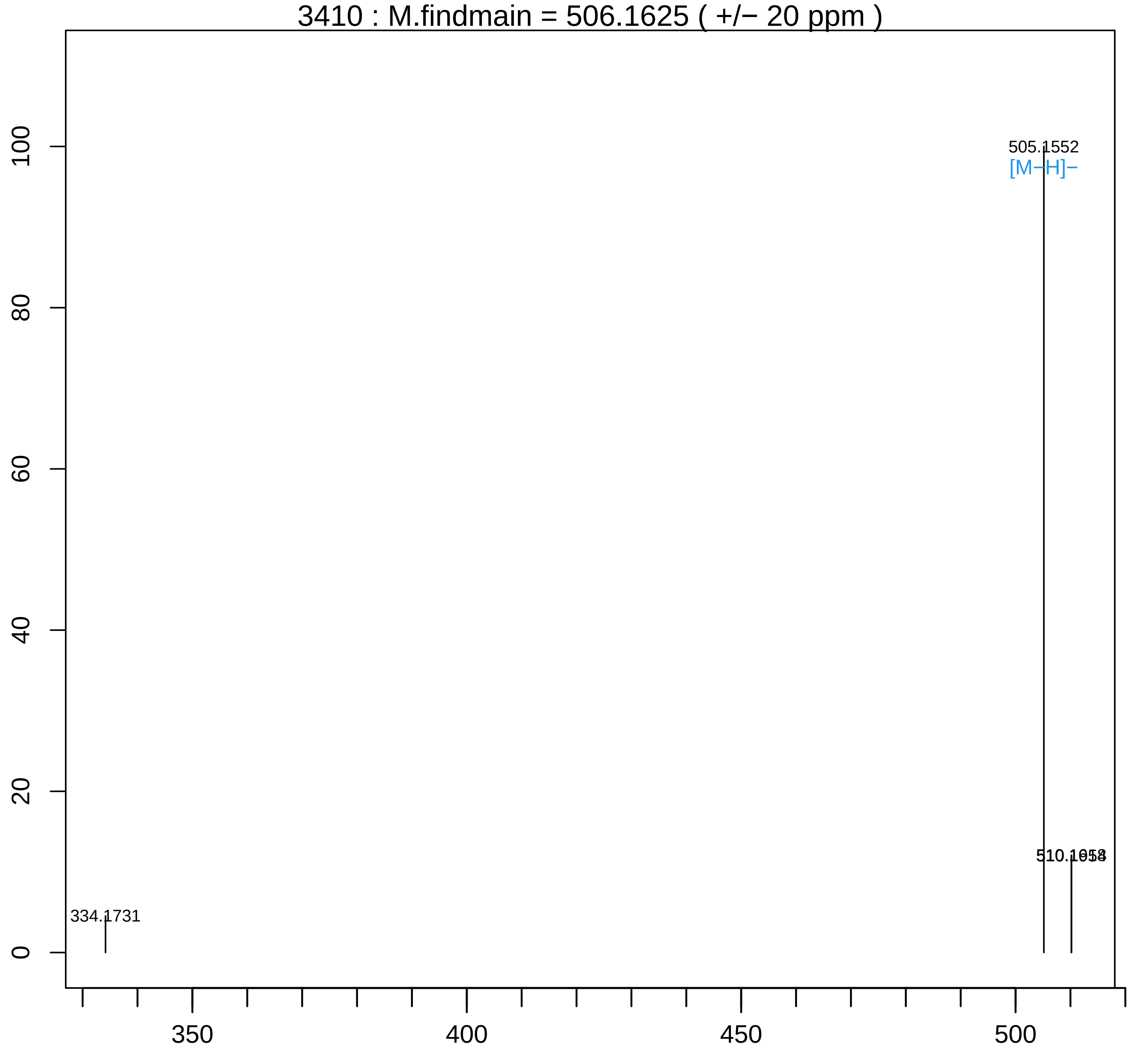
3410 : M.findmain = 506.1625 (+/− 20 ppm)
505.1552
[M−H]−
334.1731
0
20
40
60
80
100
350
400
450
500

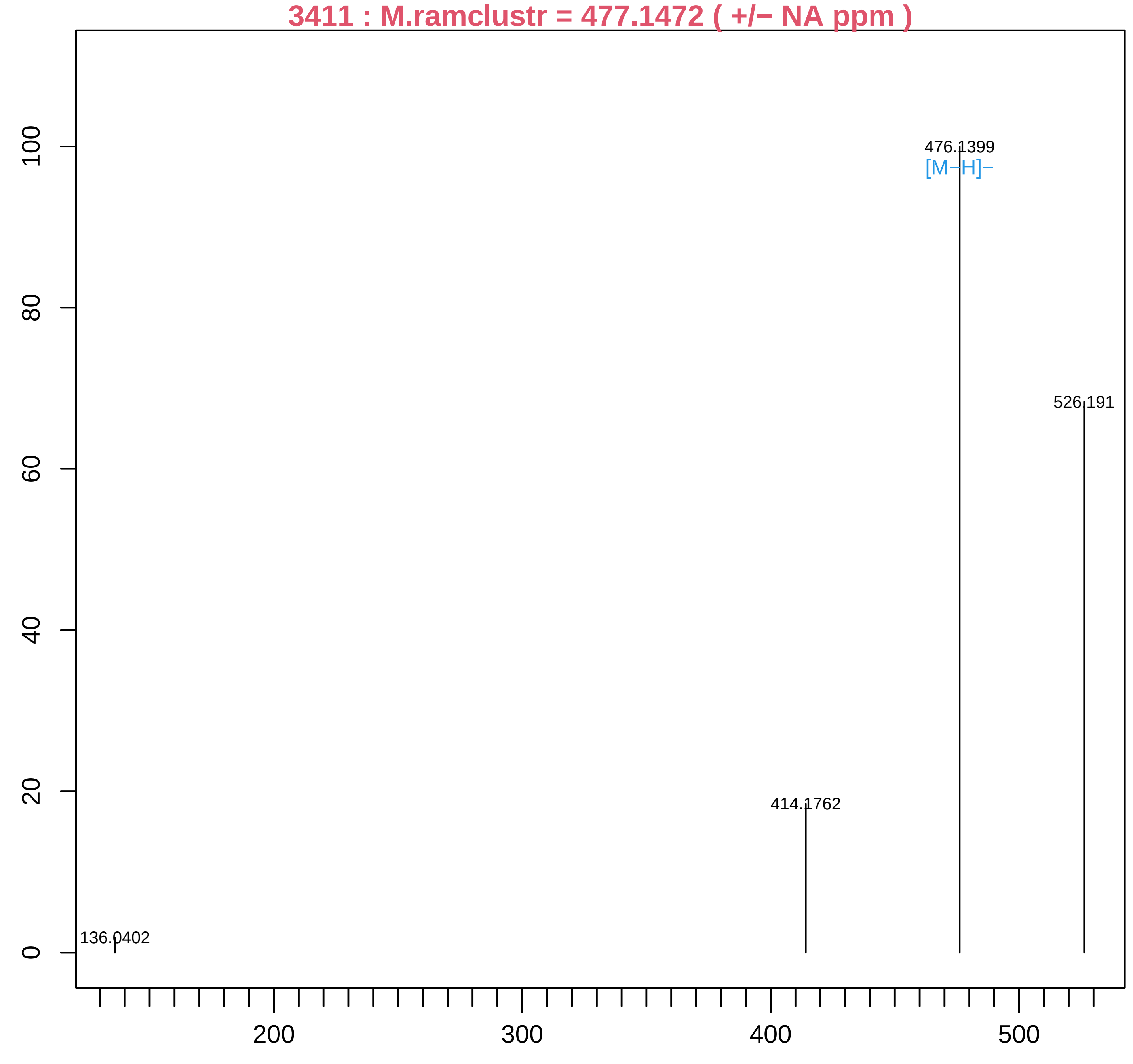
3411 : M.ramclustr = 477.1472 (+/− NA ppm)
476.1399
[M−H]−
526.191
414.1762
136.0402
0
20
40
60
80
100
200
300
400
500

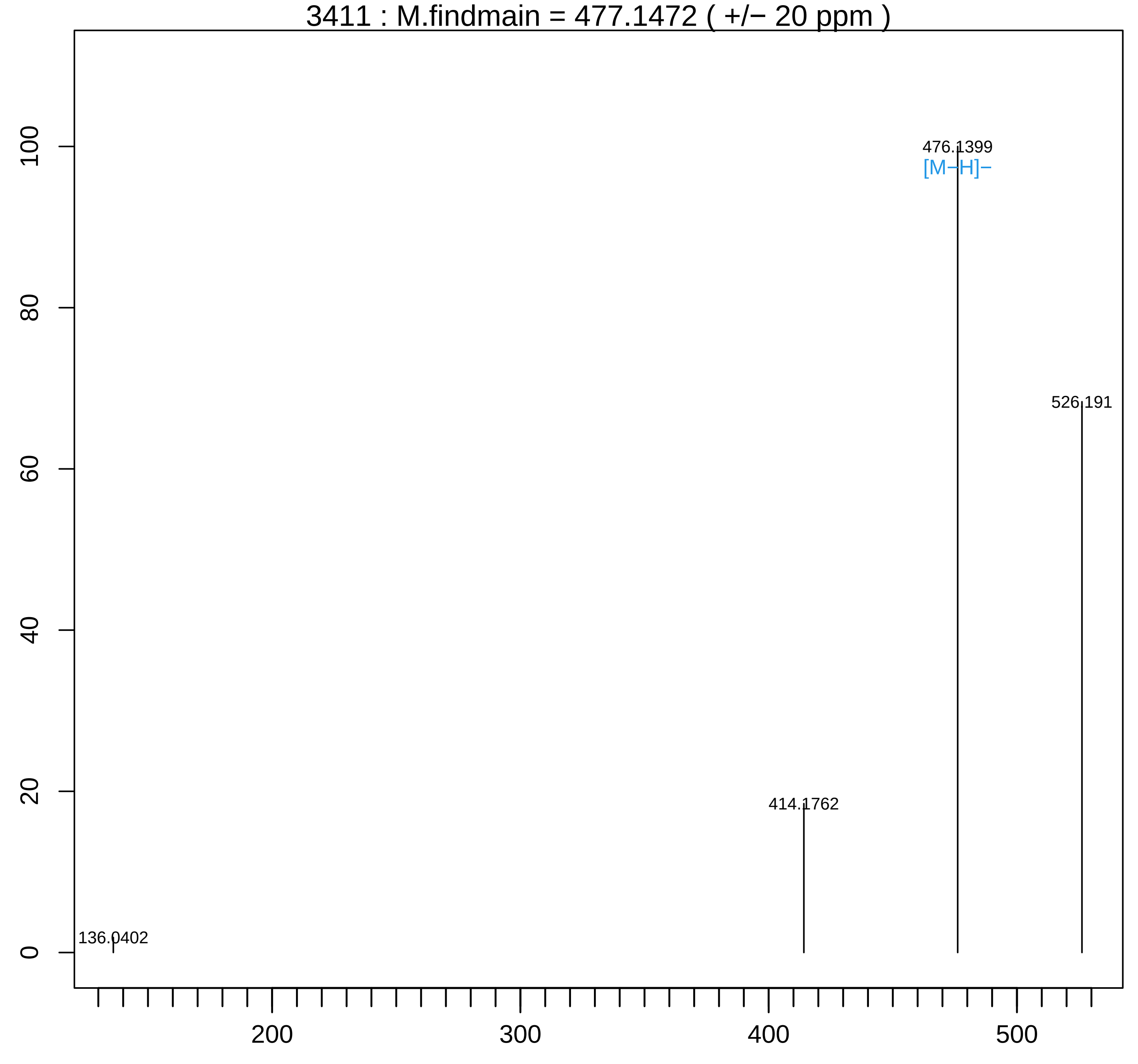
3411 : M.findmain = 477.1472 (+/− 20 ppm)
476.1399
[M−H]−
526.191
414.1762
136.0402
0
20
40
60
80
100
200
300
400
500

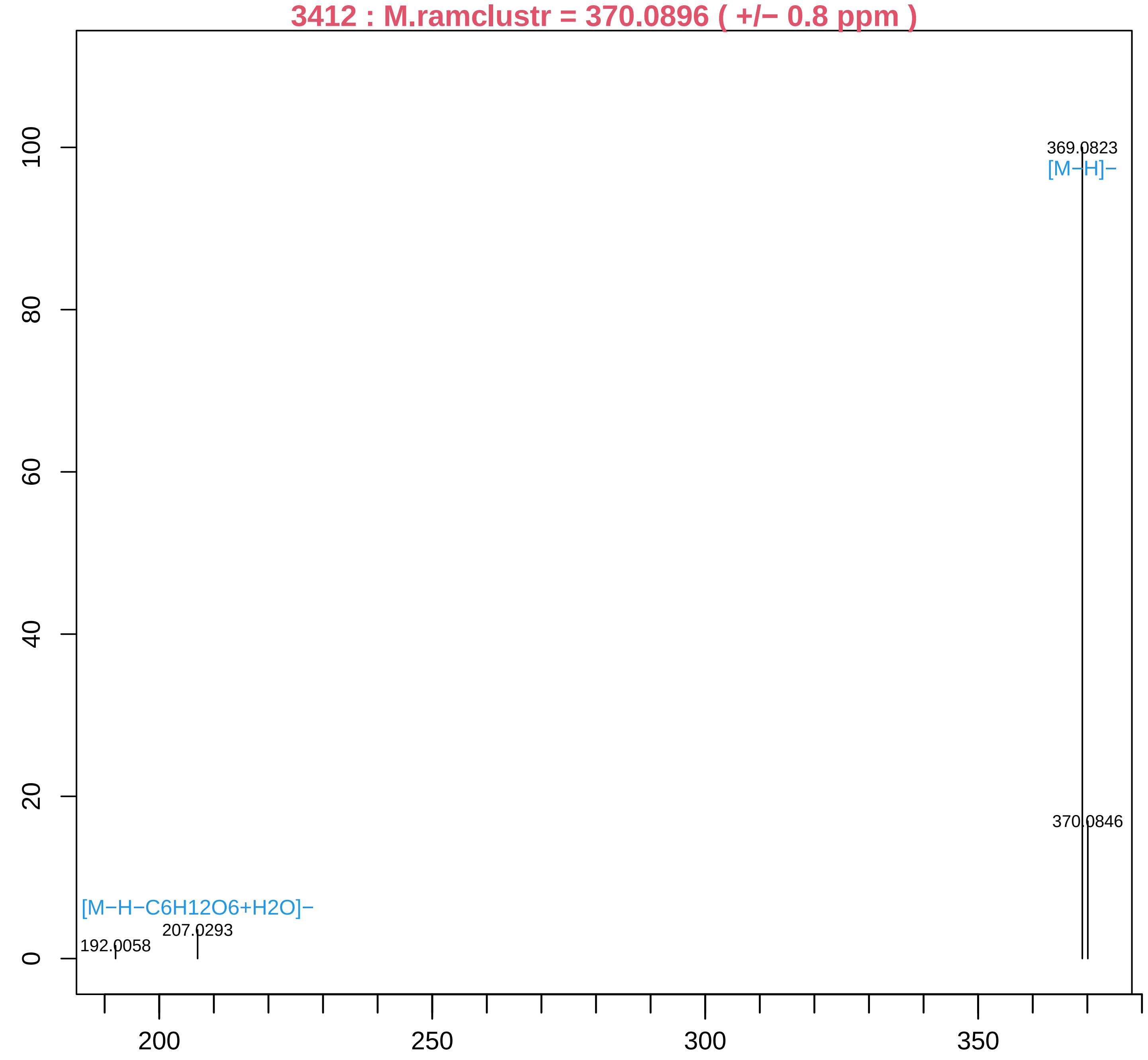
3412 : M.ramclustr = 370.0896 (+/− 0.8 ppm)
369.0823
[M−H]−
370.0846
[M−H−C6H12O6+H2O]−
207.0293
192.0058
0
20
40
60
80
100
200
250
300
350

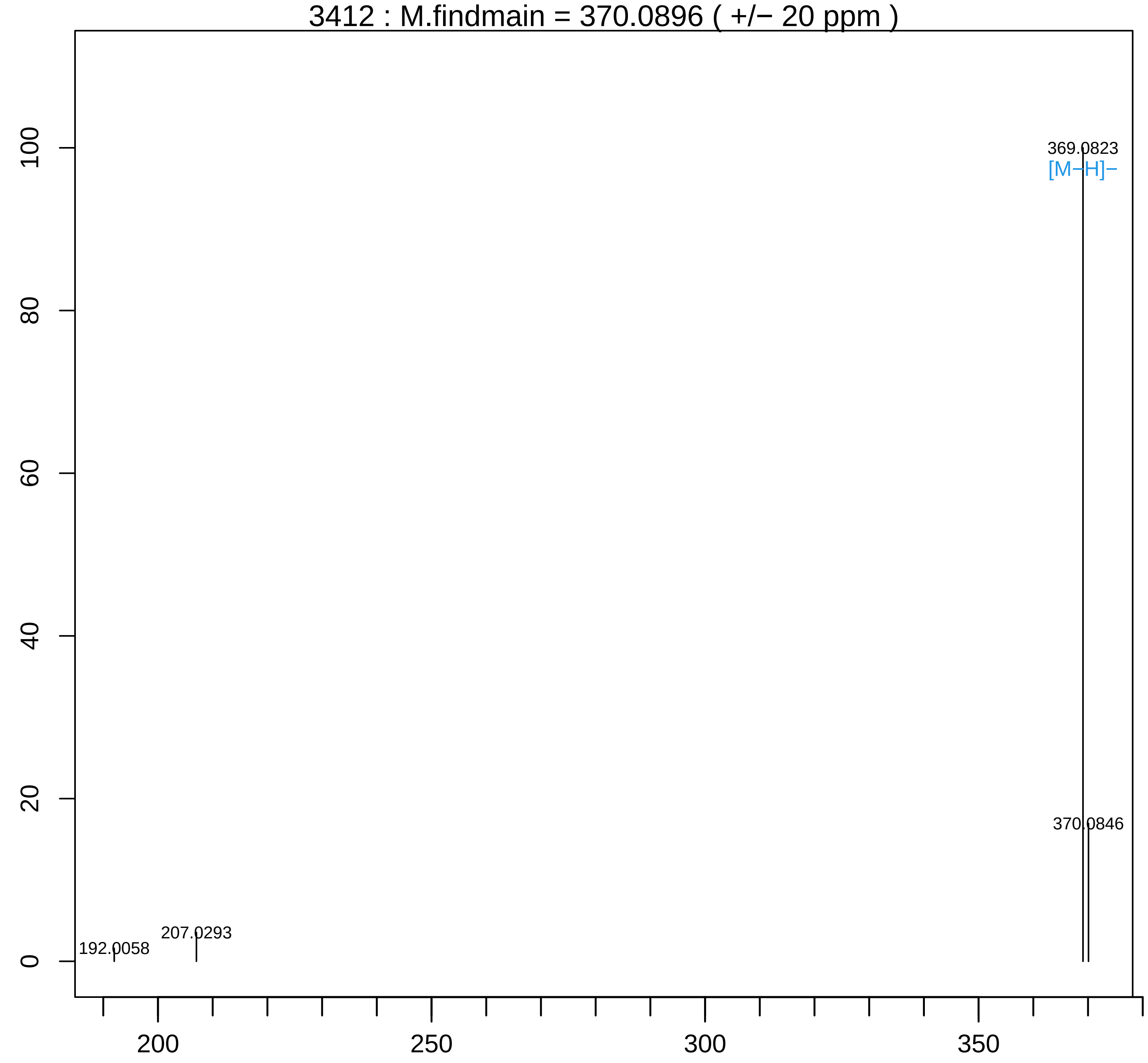
3412 : M.findmain = 370.0896 (+/− 20 ppm)
369.0823
[M−H]−
370.0846
207.0293
192.0058
0
20
40
60
80
100
200
250
300
350

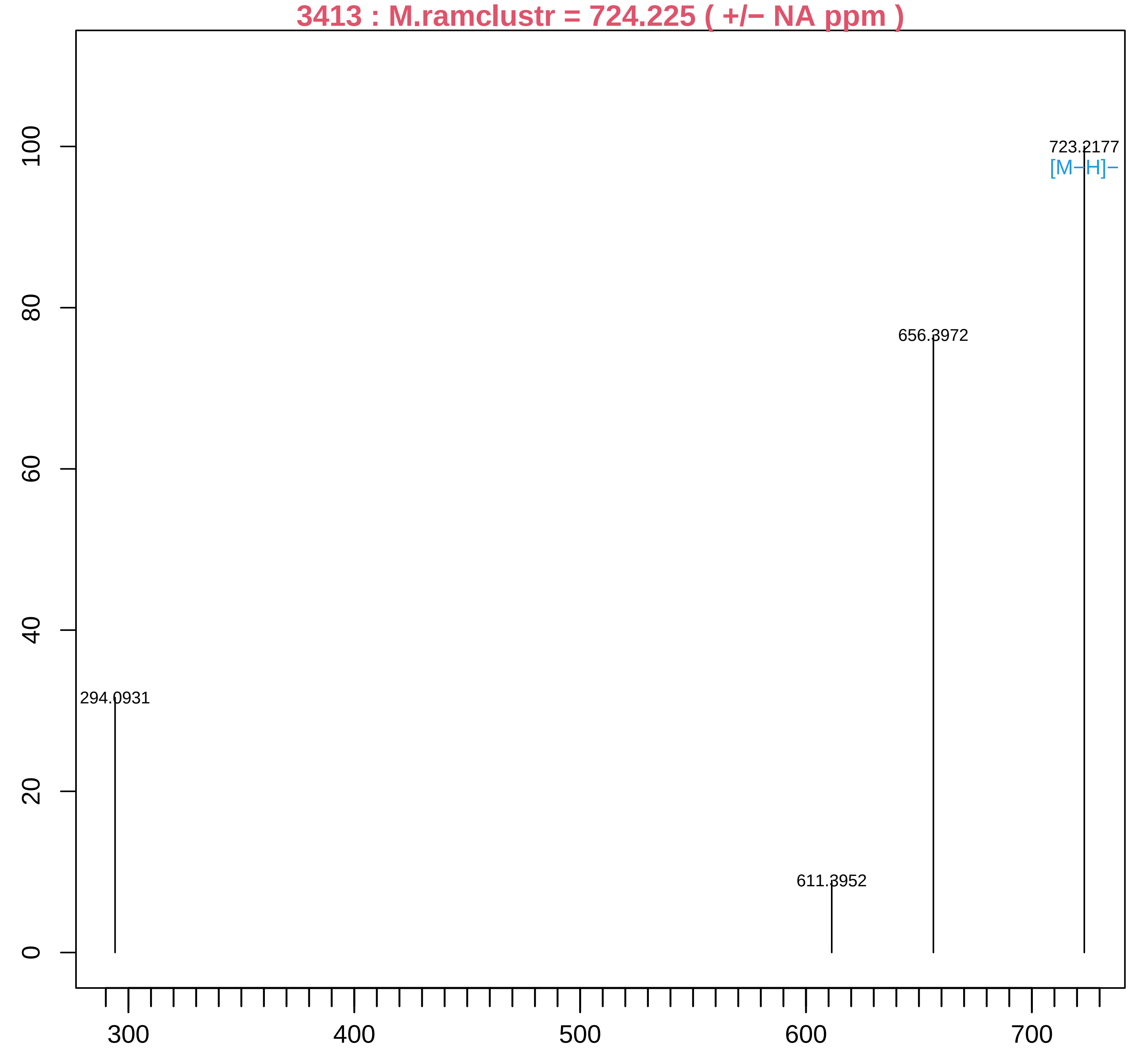
3413 : M.ramclustr = 724.225 (+/− NA ppm)
723.2177
[M−H]−
656.3972
611.3952
294.0931
0
20
40
60
80
100
300
400
500
600
700

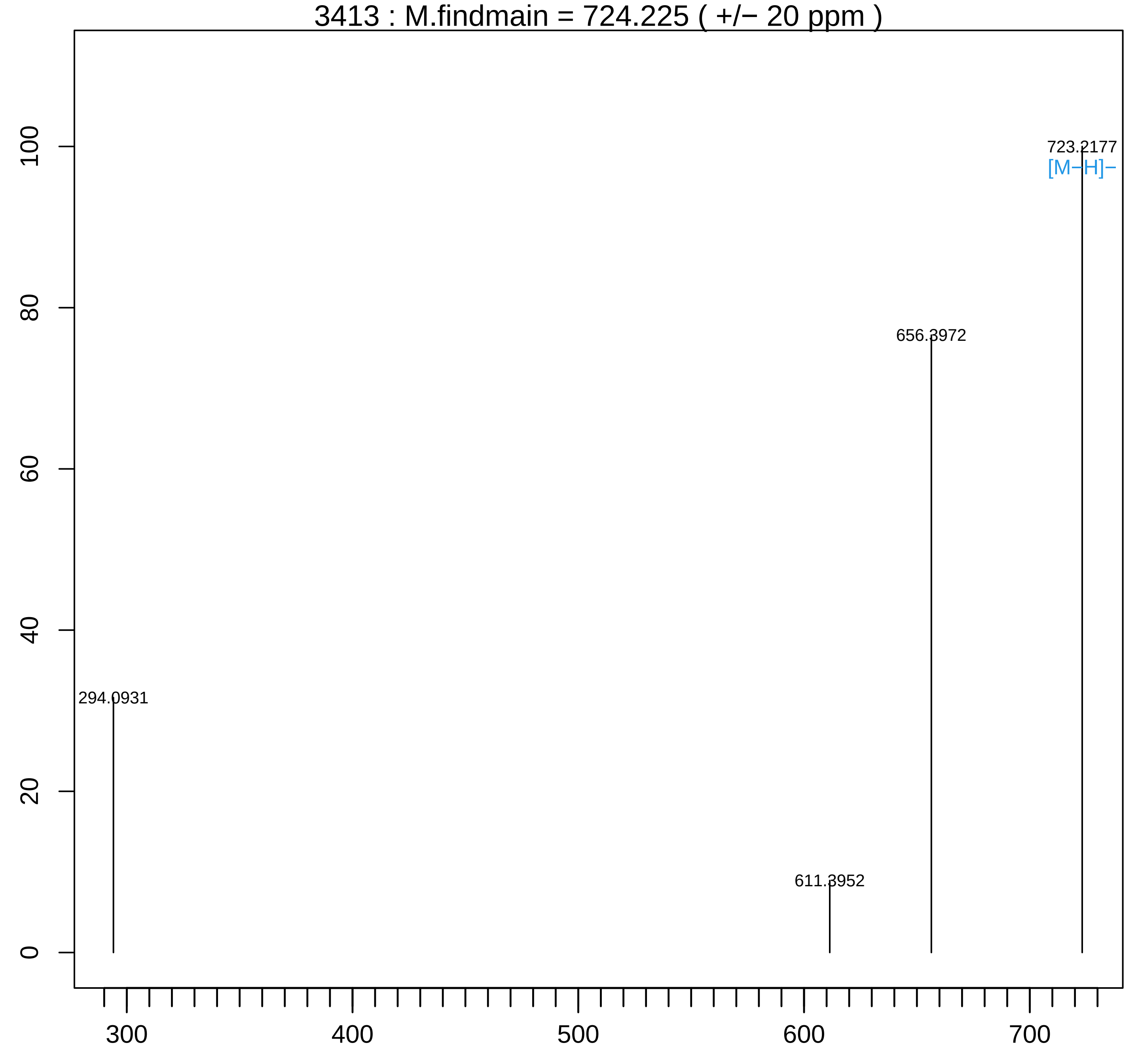
3413 : M.findmain = 724.225 (+/− 20 ppm)
723.2177
[M−H]−
656.3972
611.3952
294.0931
0
20
40
60
80
100
300
400
500
600
700

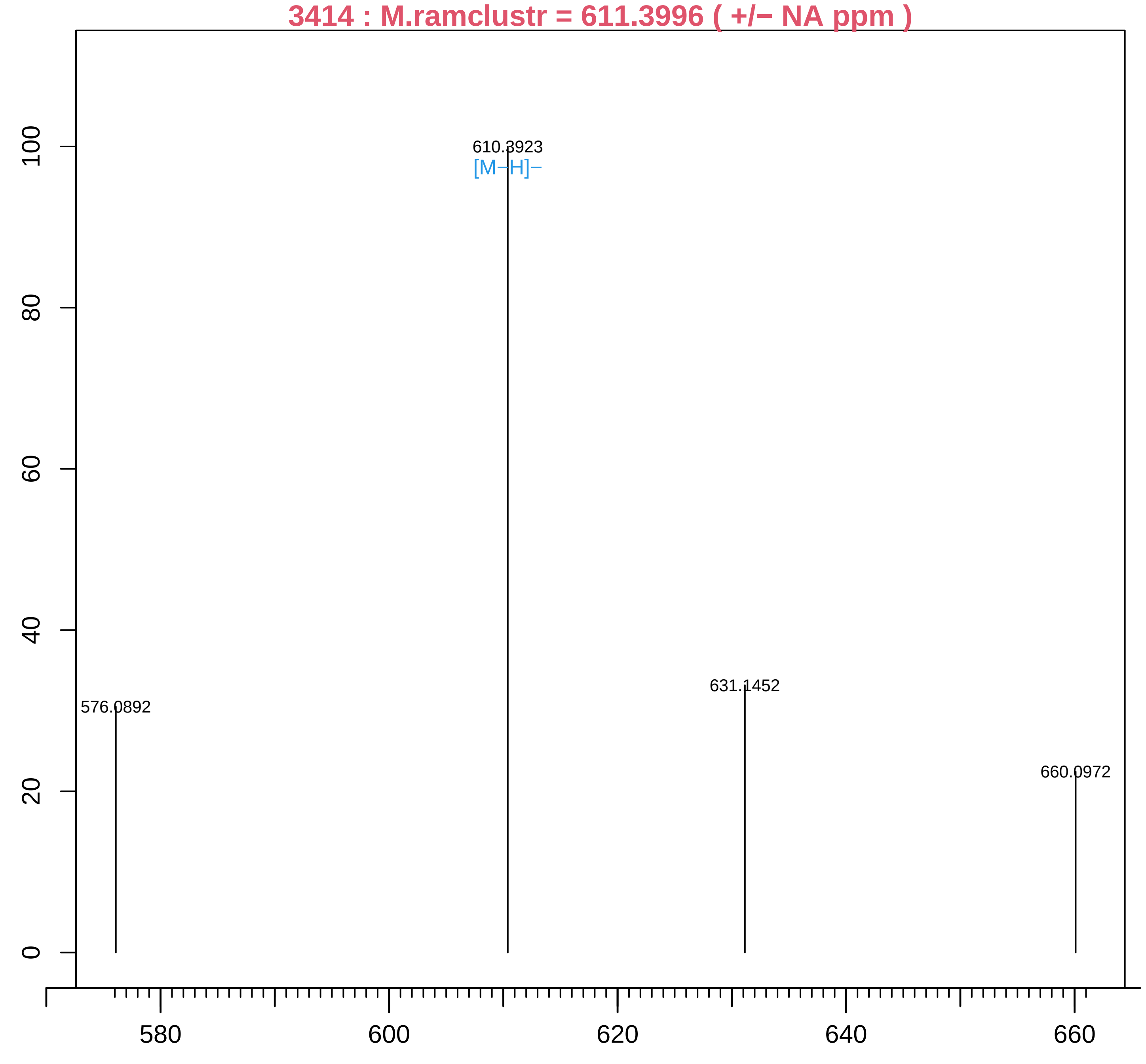
3414 : M.ramclustr = 611.3996 (+/− NA ppm)
100
80
60
40
20
0
610.3923
[M−H]−
576.0892
631.1452
660.0972
580
600
620
640
660

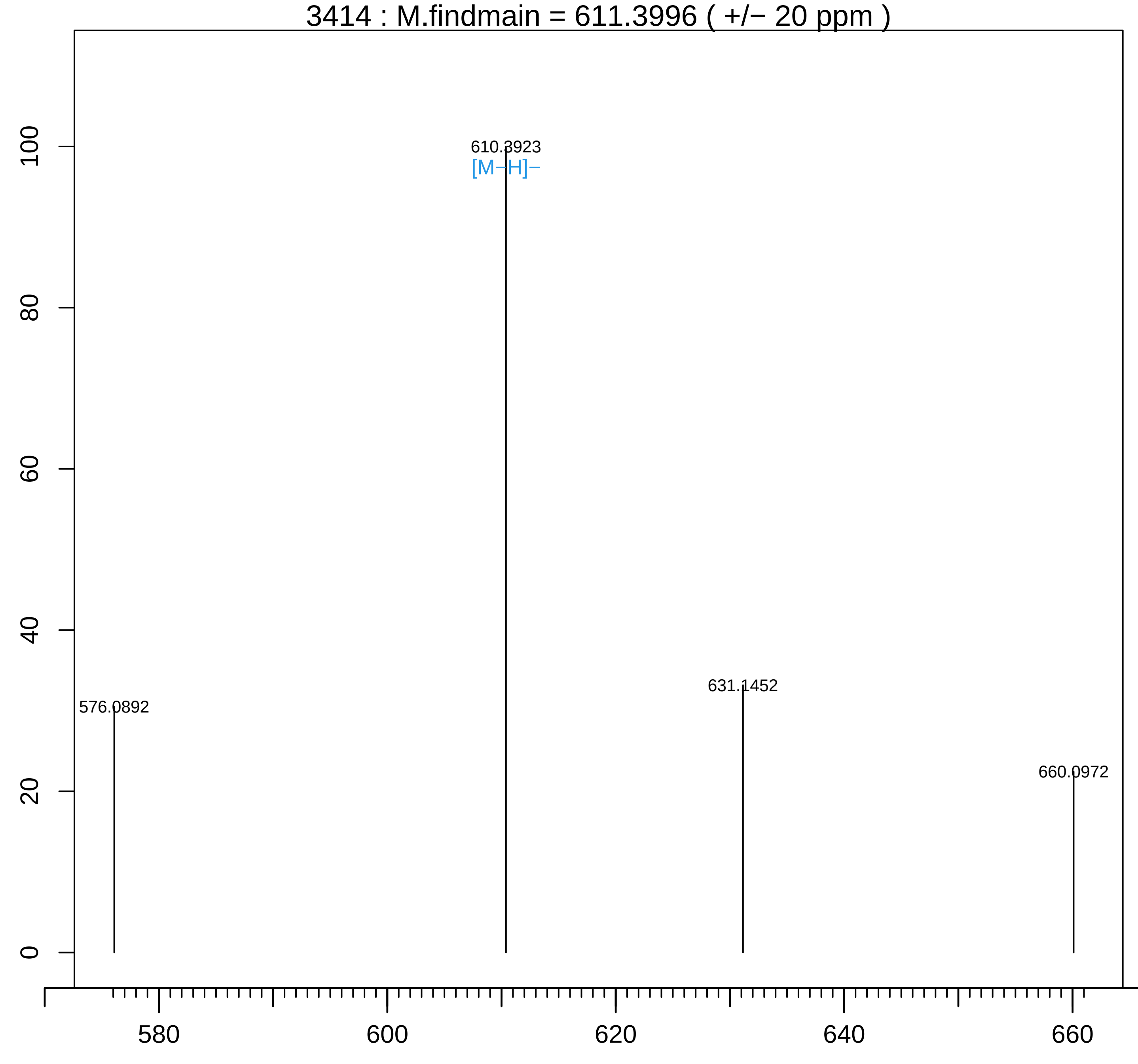
3414 : M.findmain = 611.3996 (+/− 20 ppm)
100
80
60
40
20
0
610.3923
[M−H]−
576.0892
631.1452
660.0972
580
600
620
640
660

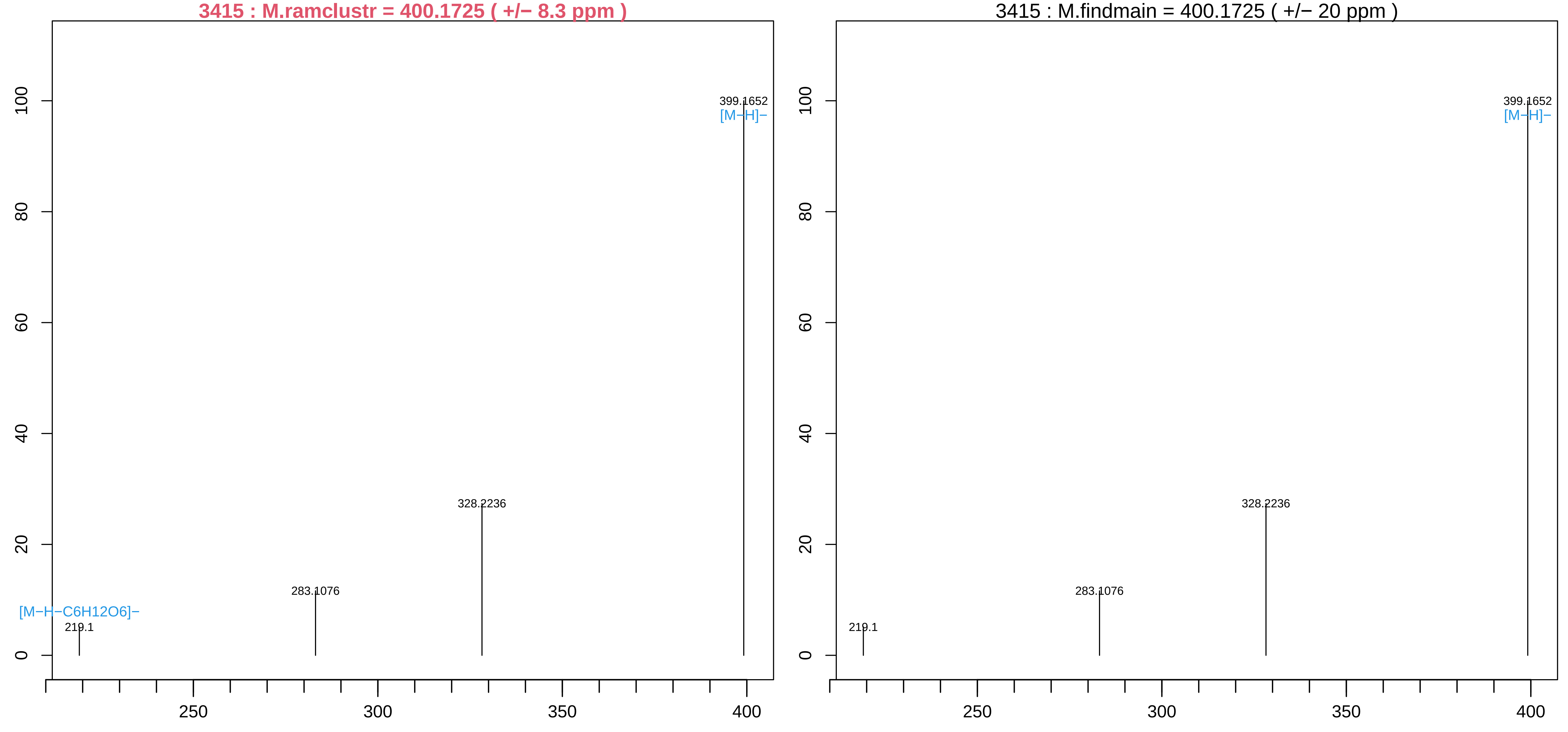

3415 : M.ramclustr = 400.1725 (+/− 8.3 ppm)
399.1652
[M−H]−
328.2236
283.1076
[M−H−C6H12O6]−
219.1
3415 : M.findmain = 400.1725 (+/− 20 ppm)
399.1652
[M−H]−
328.2236
283.1076
219.1
0
20
40
60
80
100
250
300
350
400

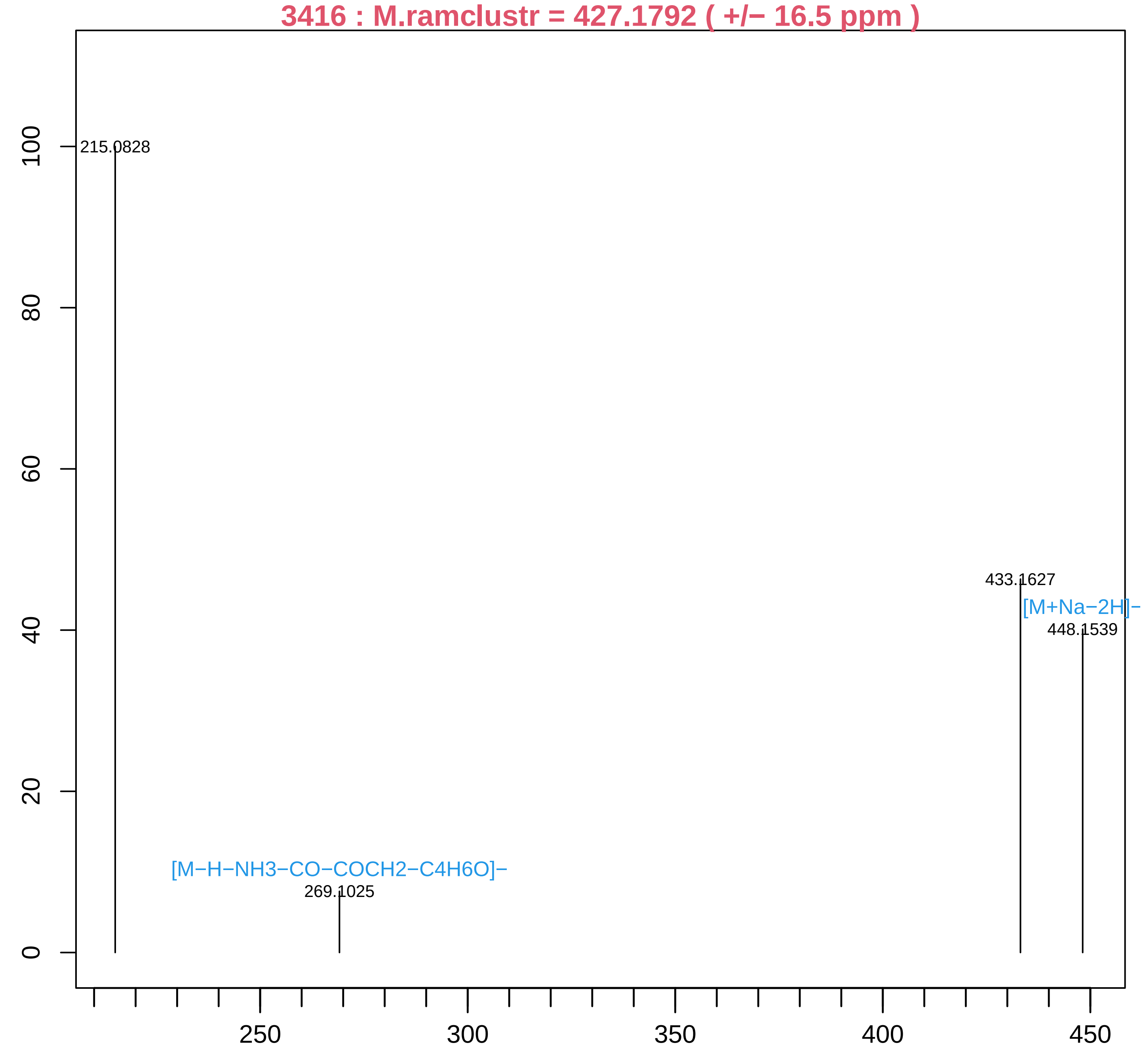
3416 : M.ramclustr = 427.1792 (+/− 16.5 ppm)
215.0828
[M−H−NH3−CO−COCH2−C4H6O]−
269.1025
433.1627
[M+Na−2H]−
448.1539
0
20
40
60
80
100
250
300
350
400
450

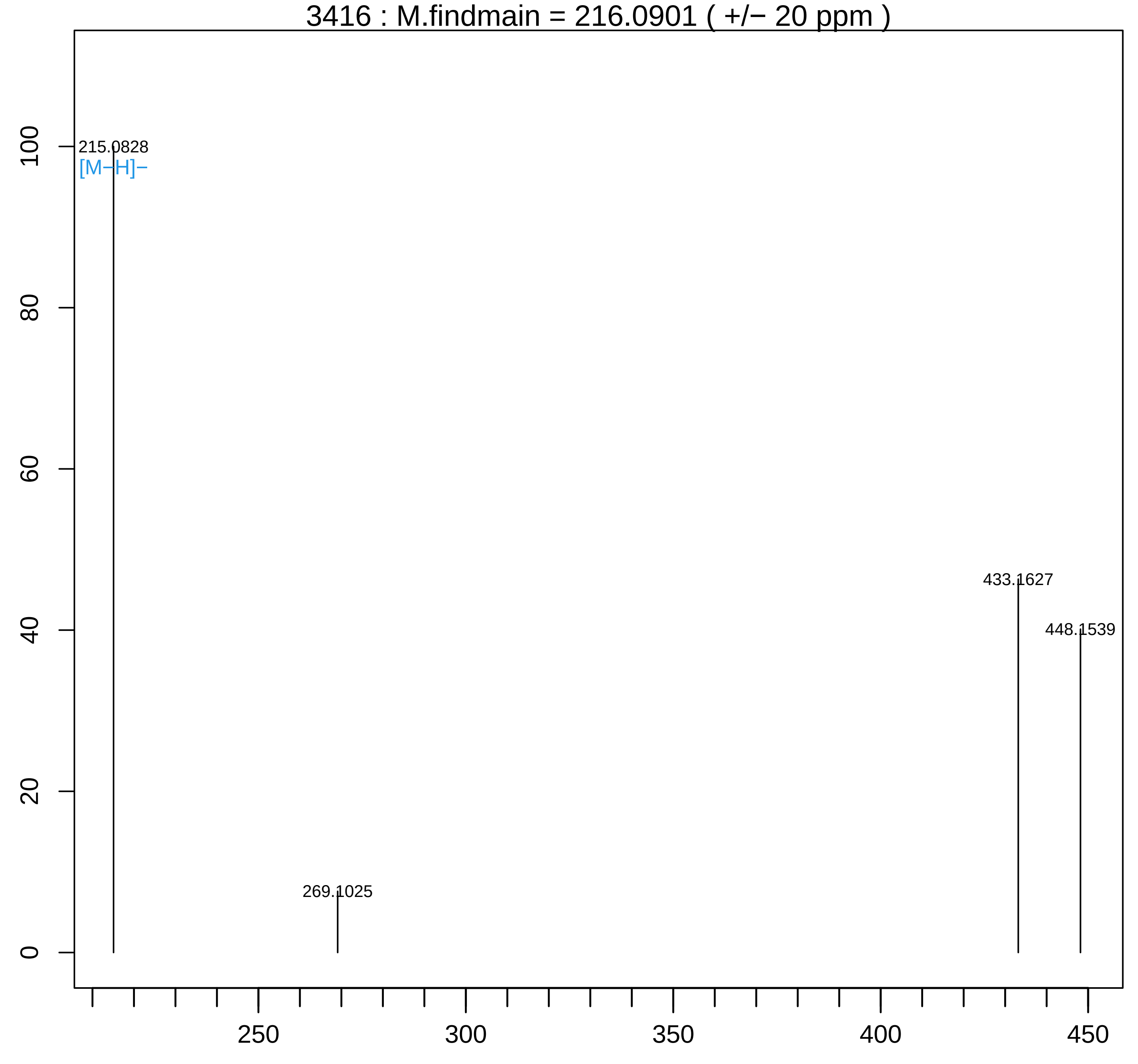
3416 : M.findmain = 216.0901 (+/− 20 ppm)
215.0828
[M−H]−
269.1025
433.1627
448.1539
0
20
40
60
80
100
250
300
350
400
450

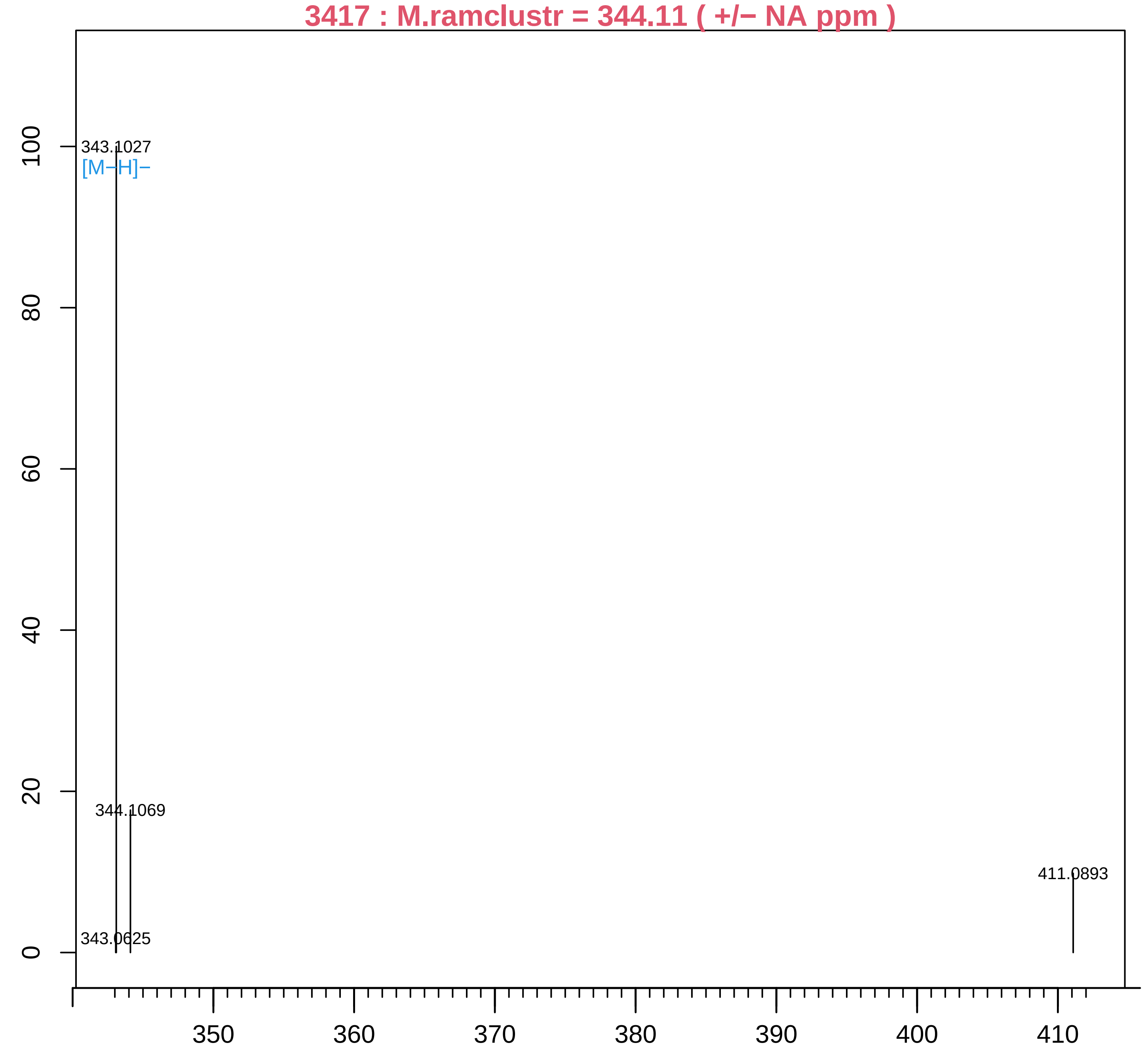

3417 : M.ramclustr = 344.11 (+/− NA ppm)
343.1027
[M−H]−
344.1069
411.0893
343.0625
0
20
40
60
80
100
350
360
370
380
390
400
410

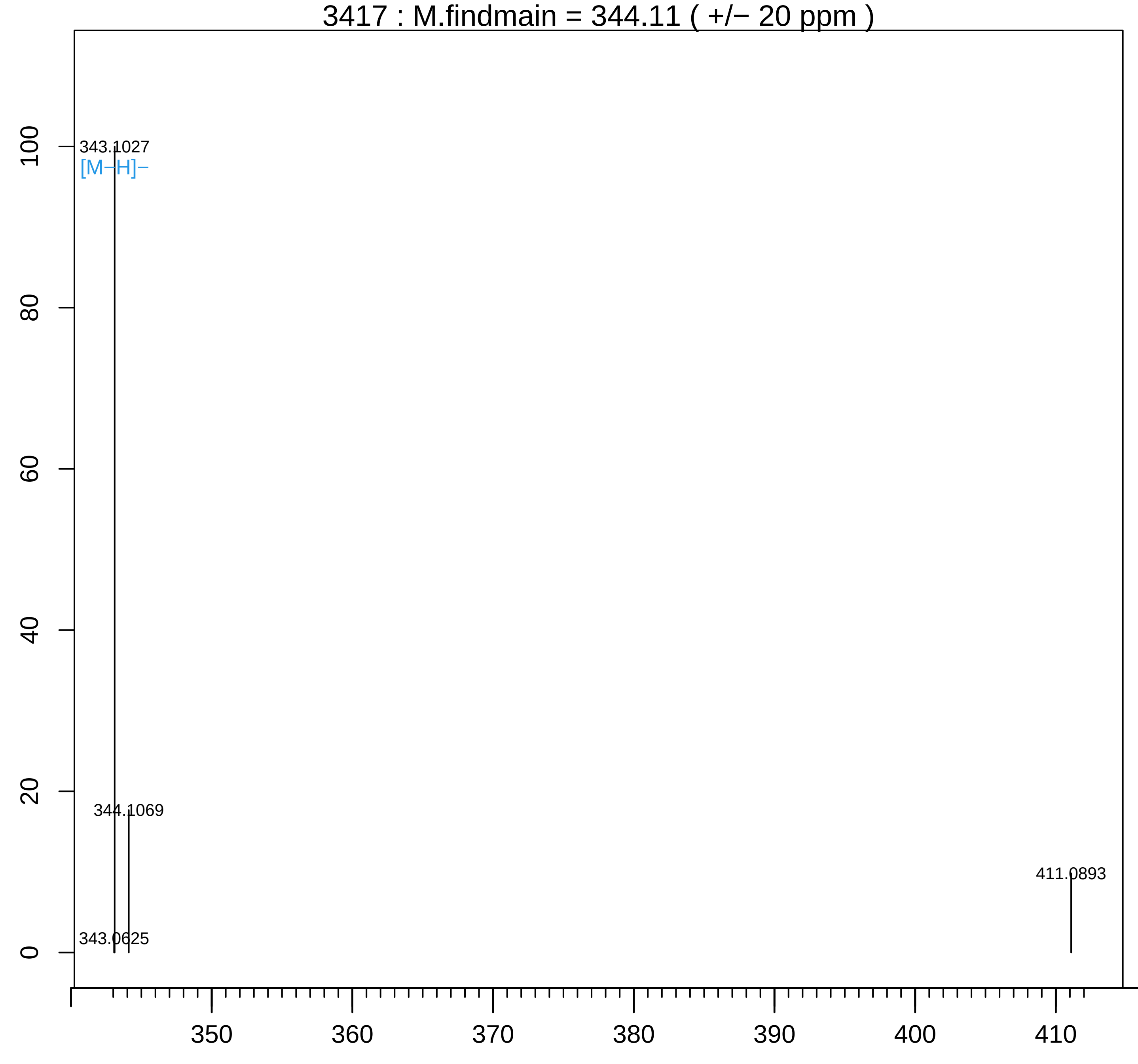

3417 : M.findmain = 344.11 (+/− 20 ppm)
343.1027
[M−H]−
344.1069
411.0893
343.0625
0
20
40
60
80
100
350
360
370
380
390
400
410

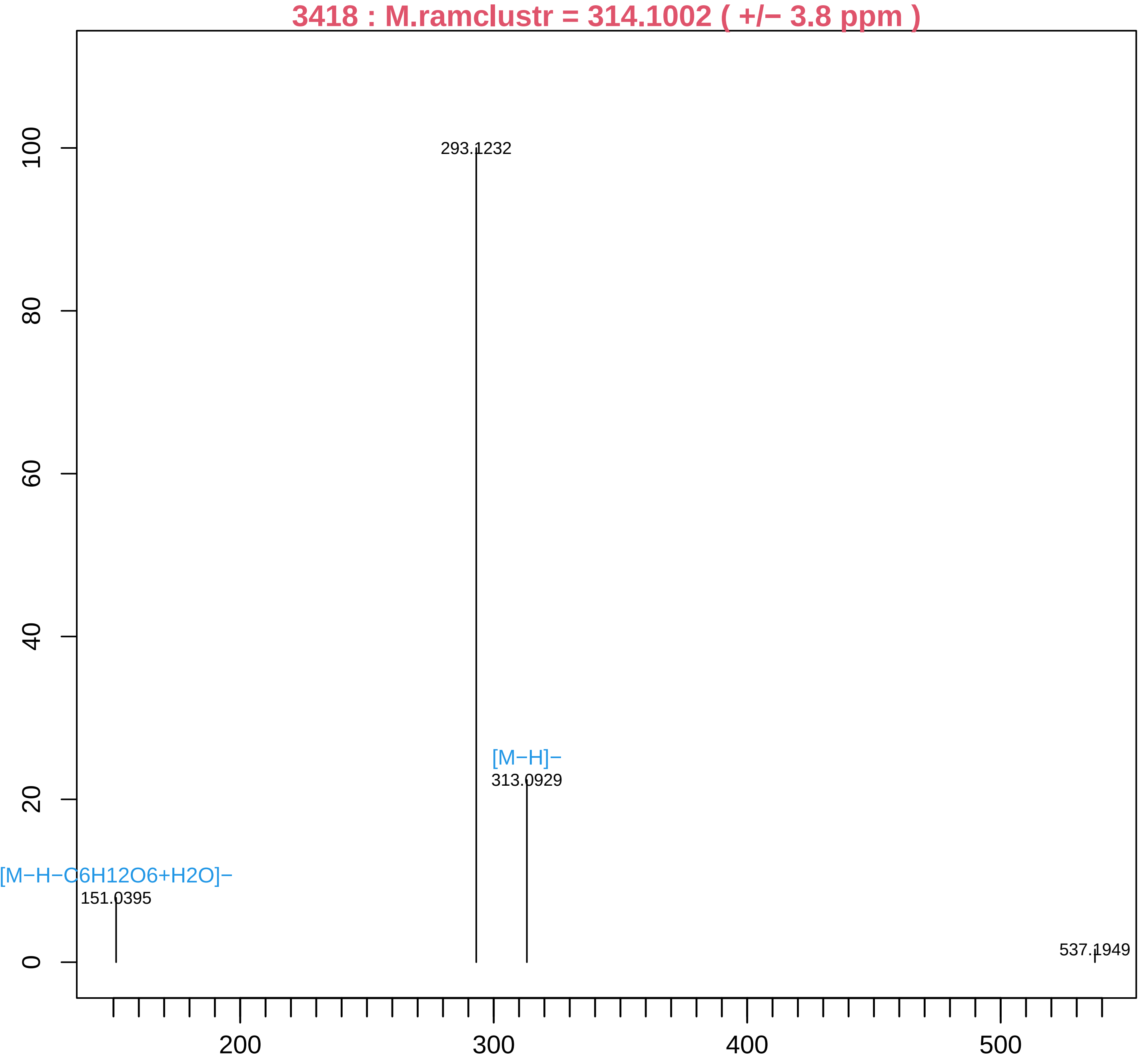

3418 : M.ramclustr = 314.1002 (+/− 3.8 ppm)
293.1232
[M−H]−
313.0929
[M−H−C6H12O6+H2O]−
151.0395
537.1949
0
20
40
60
80
100
200
300
400
500

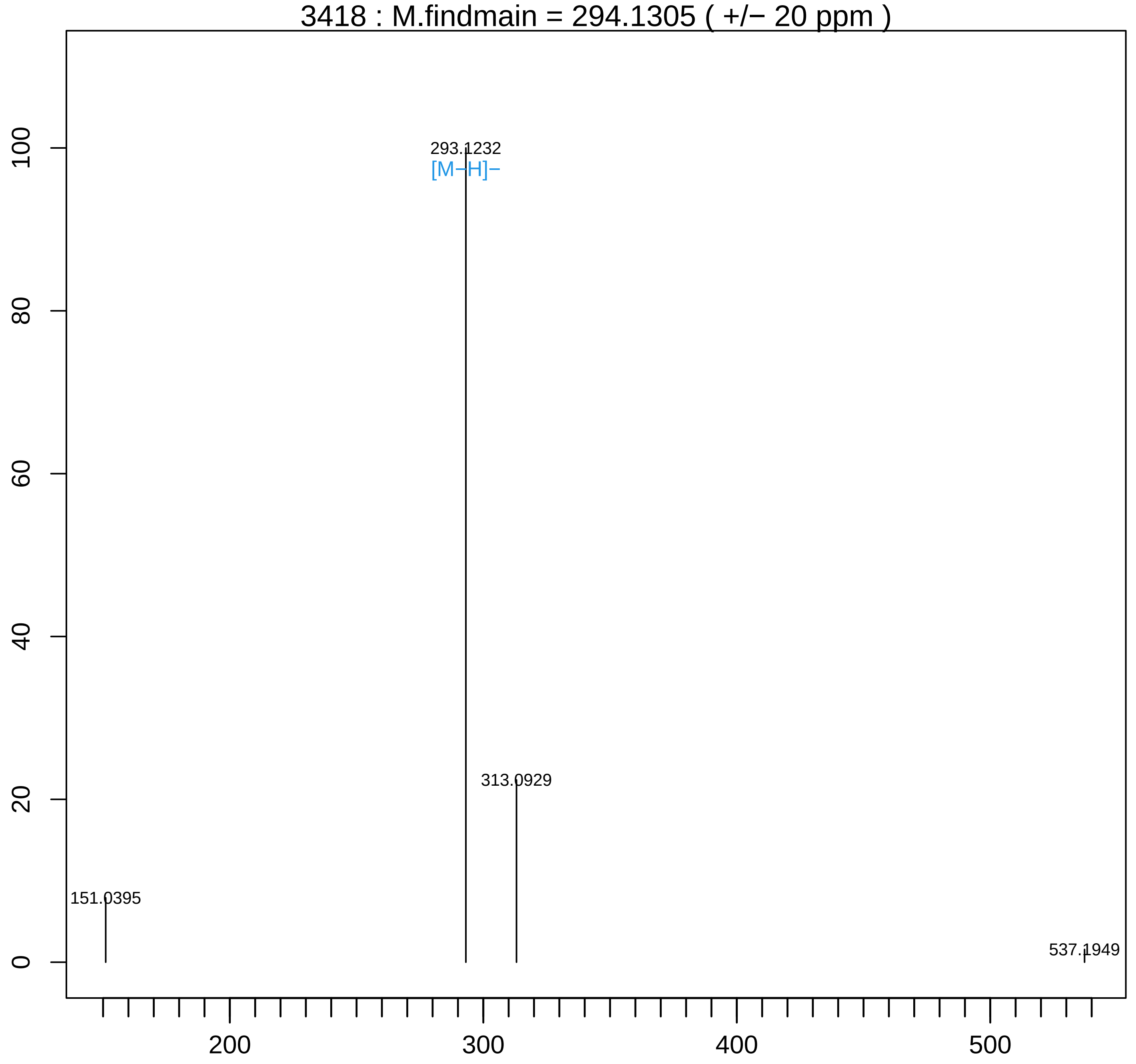

3418 : M.findmain = 294.1305 (+/− 20 ppm)
293.1232
[M−H]−
313.0929
151.0395
537.1949
0
20
40
60
80
100
200
300
400
500

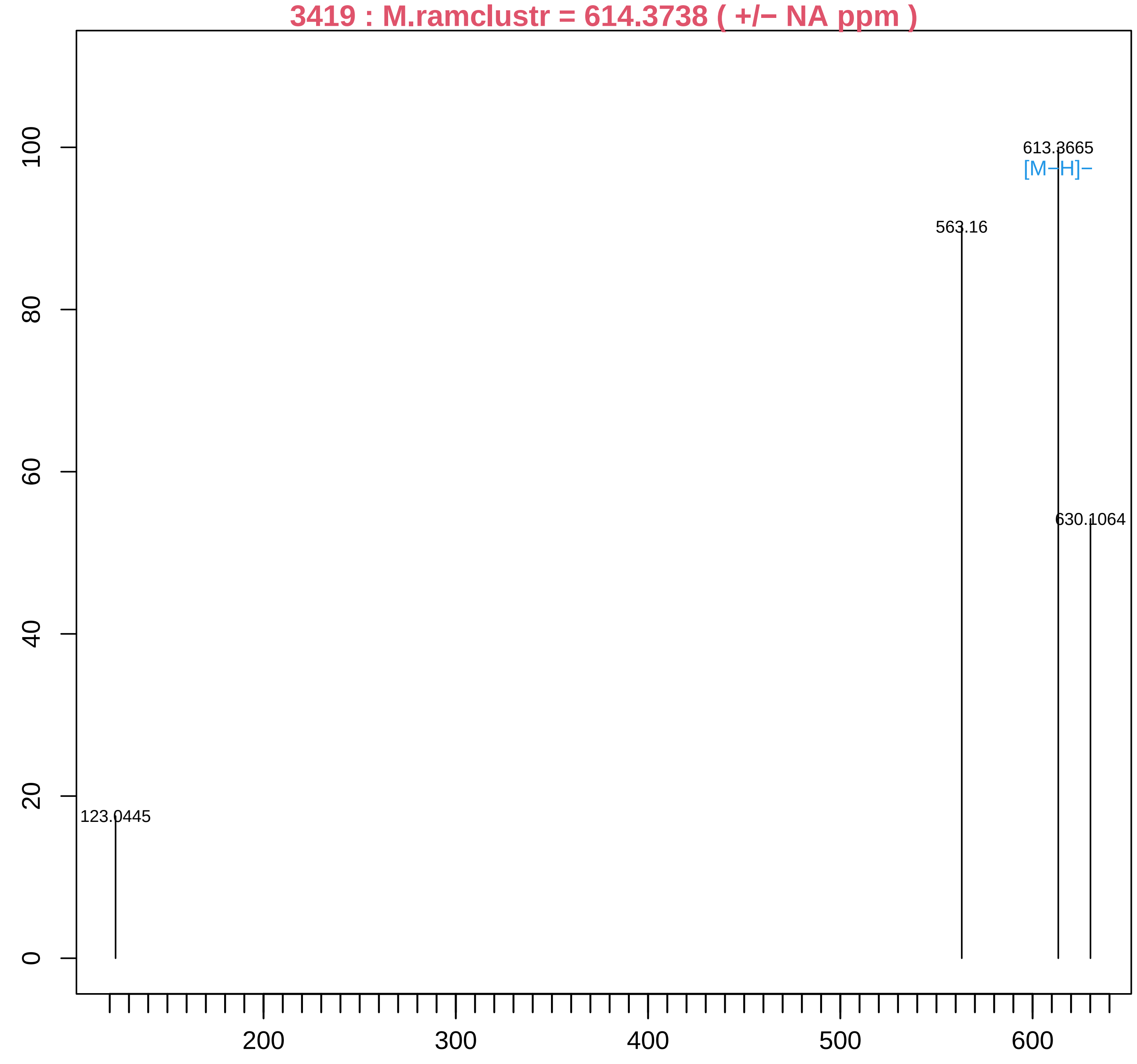
3419 : M.ramclustr = 614.3738 (+/− NA ppm)
613.3665
[M−H]−
563.16
630.1064
123.0445
0
20
40
60
80
100
200
300
400
500
600

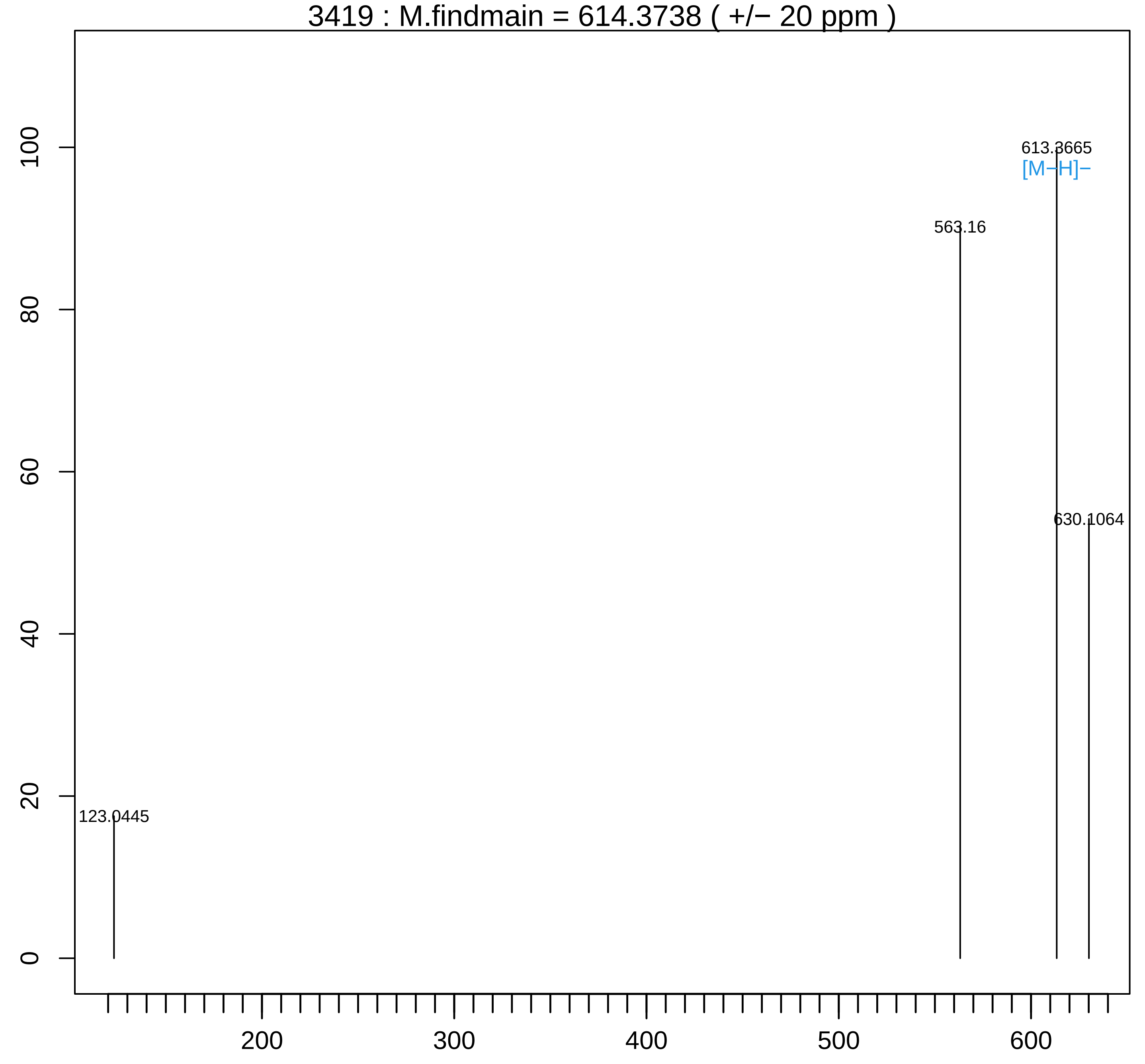
3419 : M.findmain = 614.3738 (+/− 20 ppm)
613.3665
[M−H]−
563.16
630.1064
123.0445
0
20
40
60
80
100
200
300
400
500
600

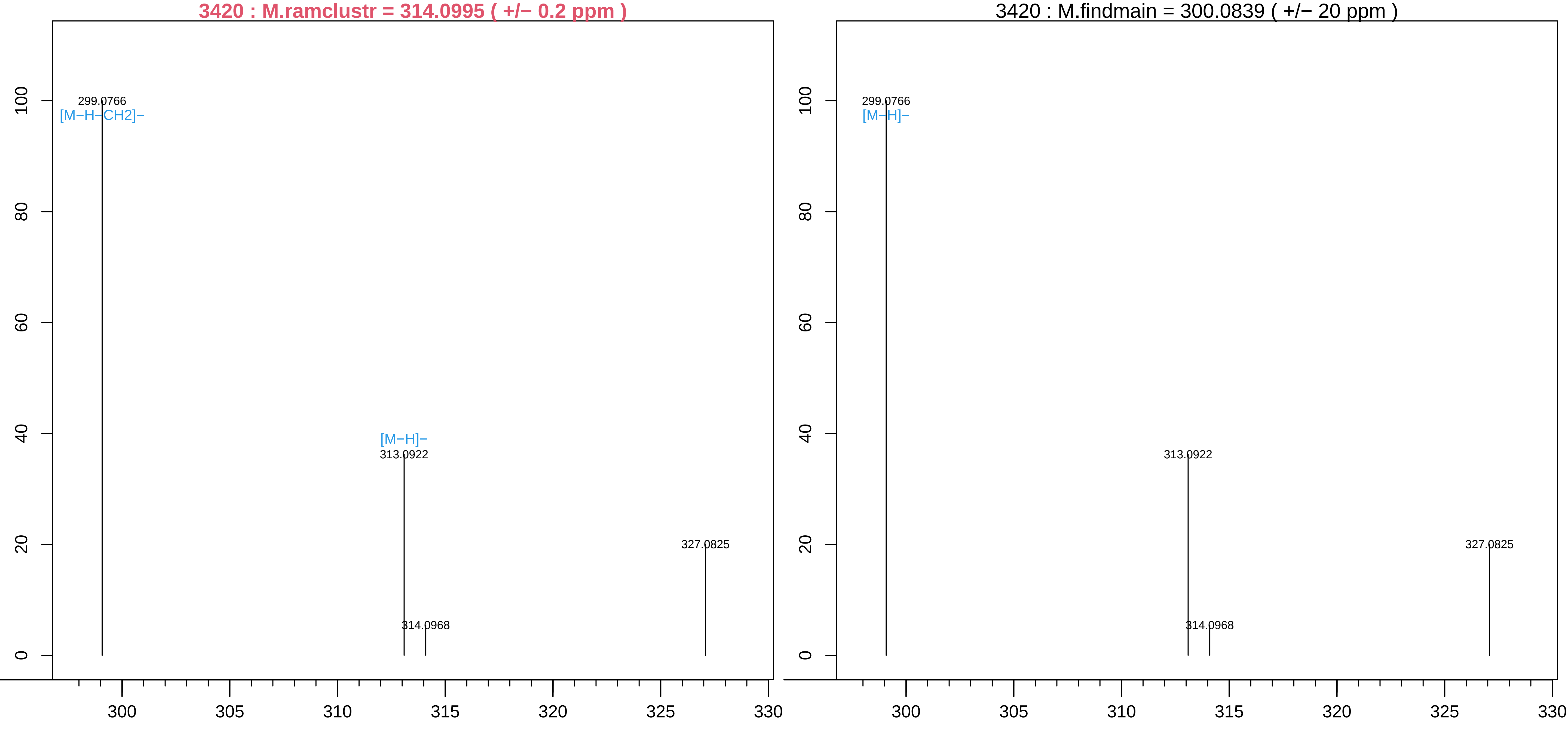
3420 : M.ramclustr = 314.0995 (+/− 0.2 ppm)
299.0766
[M−H−CH2]−
[M−H]−
313.0922
314.0968
327.0825
3420 : M.findmain = 300.0839 (+/− 20 ppm)
299.0766
[M−H]−
313.0922
314.0968
327.0825

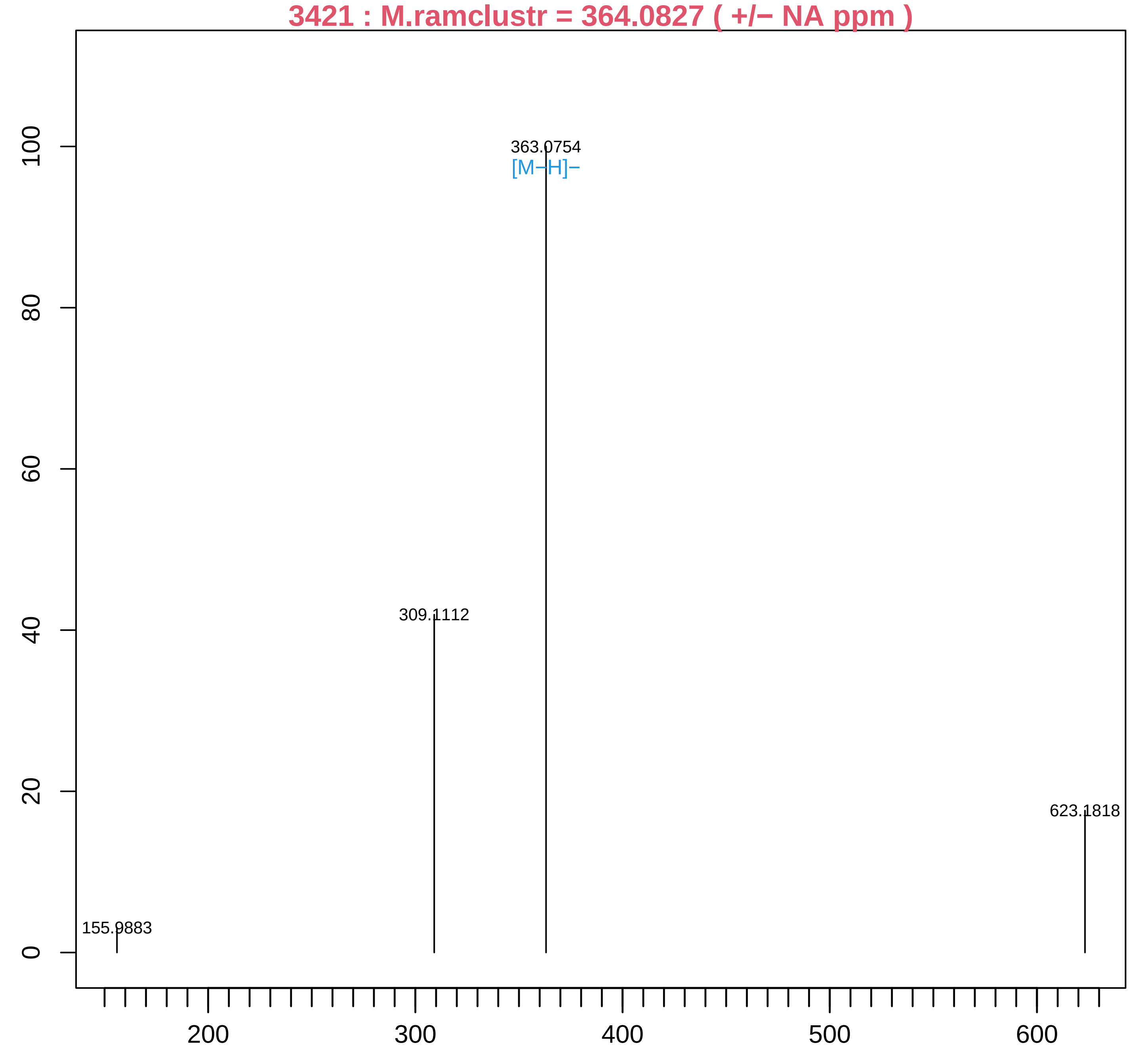

3421 : M.ramclustr = 364.0827 (+/− NA ppm)
363.0754
[M−H]−
309.1112
623.1818
155.9883
0
20
40
60
80
100
200
300
400
500
600

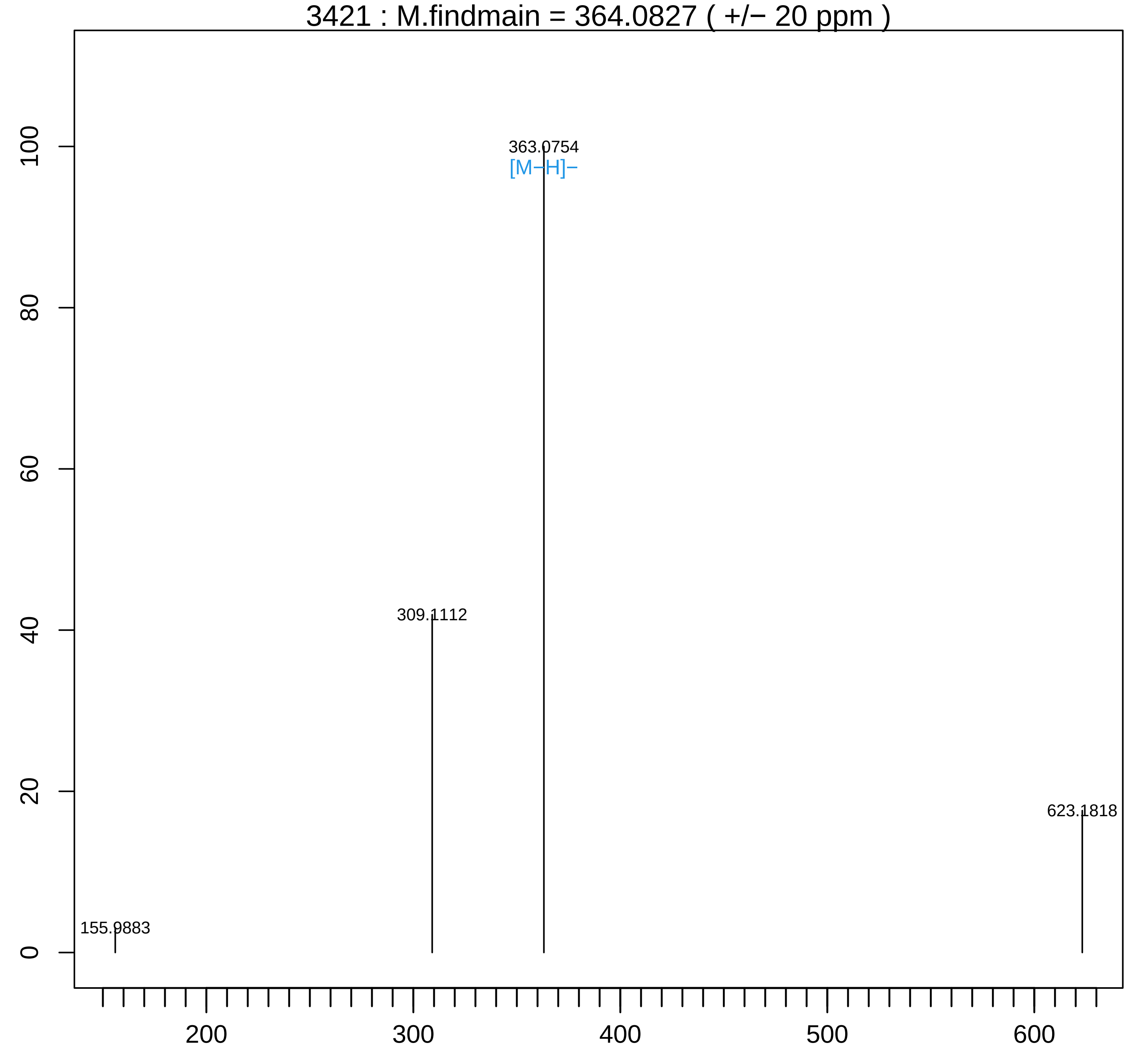

3421 : M.findmain = 364.0827 (+/− 20 ppm)
363.0754
[M−H]−
309.1112
623.1818
155.9883
0
20
40
60
80
100
200
300
400
500
600

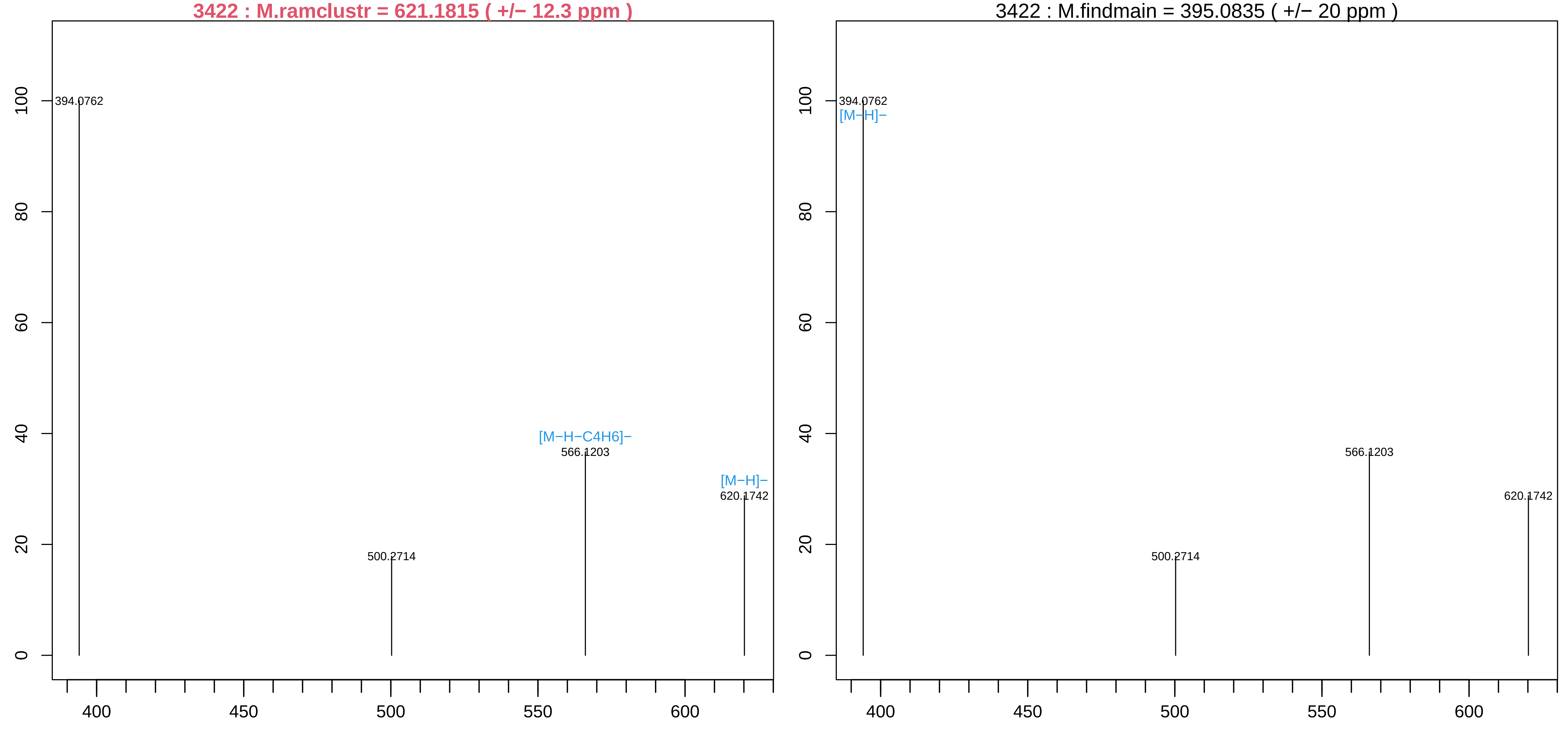
3422 : M.ramclustr = 621.1815 (+/− 12.3 ppm)
394.0762
[M−H−C4H6]−
566.1203
[M−H]−
620.1742
500.2714
3422 : M.findmain = 395.0835 (+/− 20 ppm)
394.0762
[M−H]−
566.1203
620.1742
500.2714
0
20
40
60
80
100
400
450
500
550
600

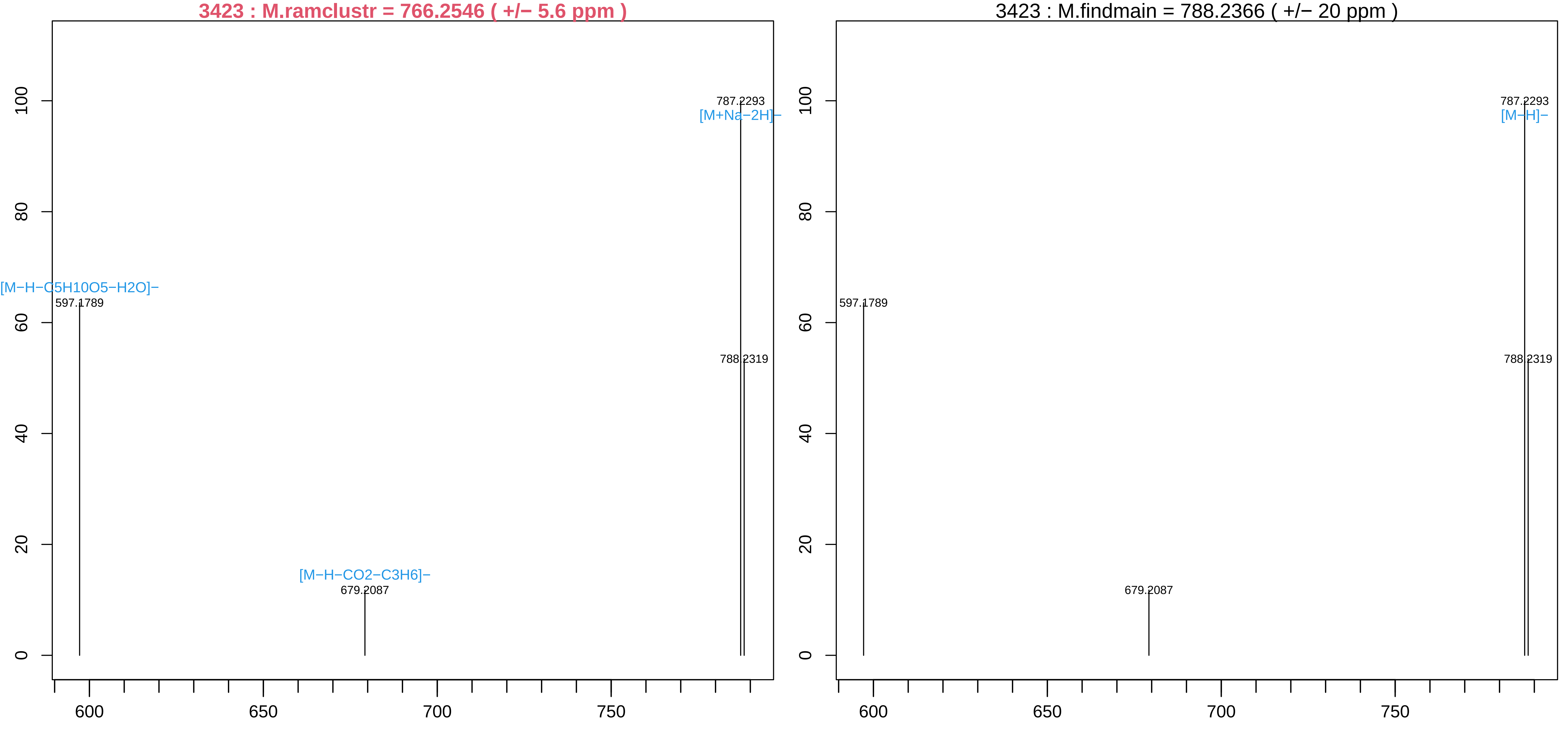
3423 : M.ramclustr = 766.2546 (+/− 5.6 ppm)
787.2293
[M+Na−2H]−
[M−H−C5H10O5−H2O]−
597.1789
788.2319
[M−H−CO2−C3H6]−
679.2087
0
20
40
60
80
100
600
650
700
750
3423 : M.findmain = 788.2366 (+/− 20 ppm)
787.2293
[M−H]−
597.1789
788.2319
679.2087
0
20
40
60
80
100
600
650
700
750

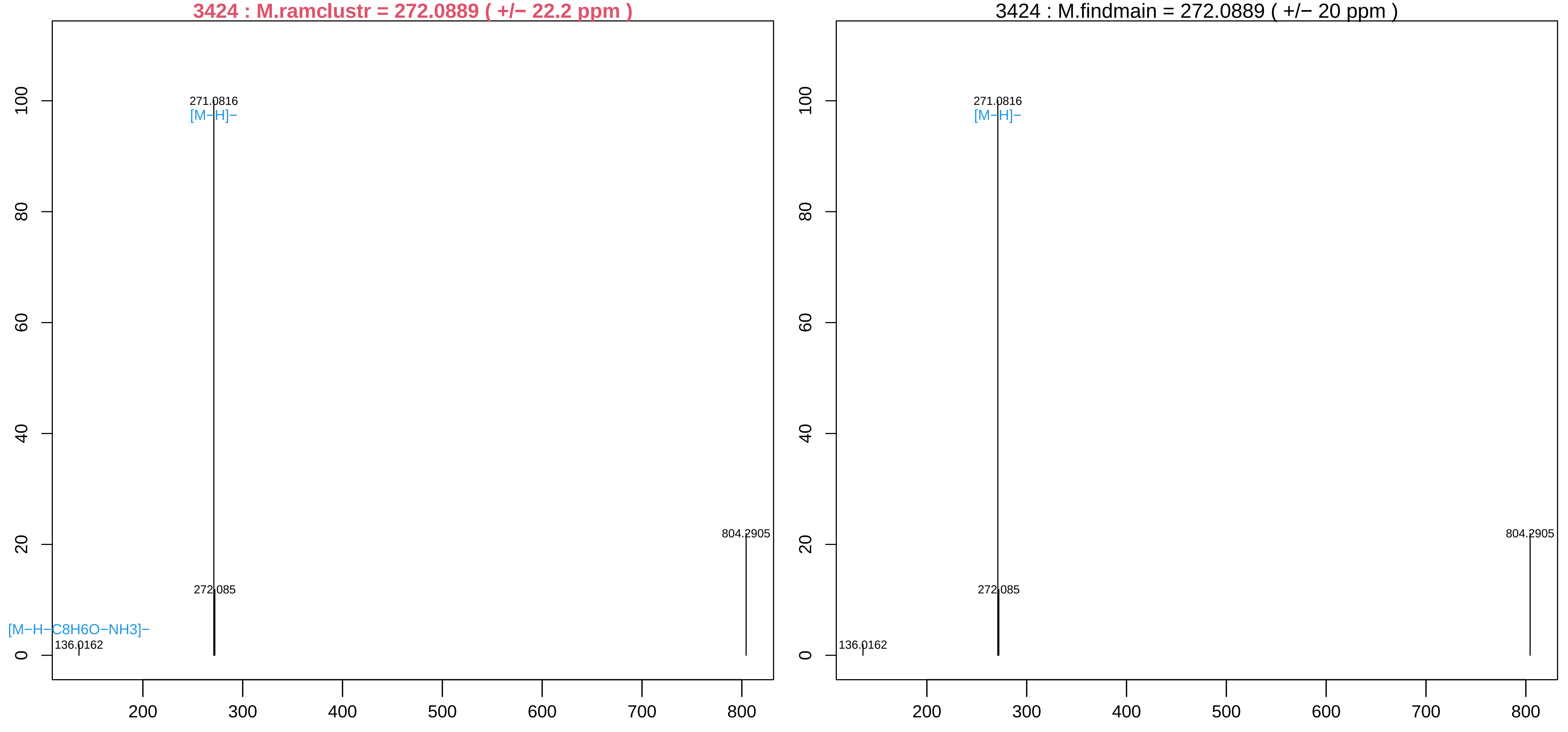
3424 : M.ramclustr = 272.0889 (+/− 22.2 ppm)
271.0816
[M−H]−
804.2905
272.085
[M−H−C8H6O−NH3]−
136.0162
3424 : M.findmain = 272.0889 (+/− 20 ppm)
271.0816
[M−H]−
804.2905
272.085
136.0162
0
20
40
60
80
100
200
300
400
500
600
700
800

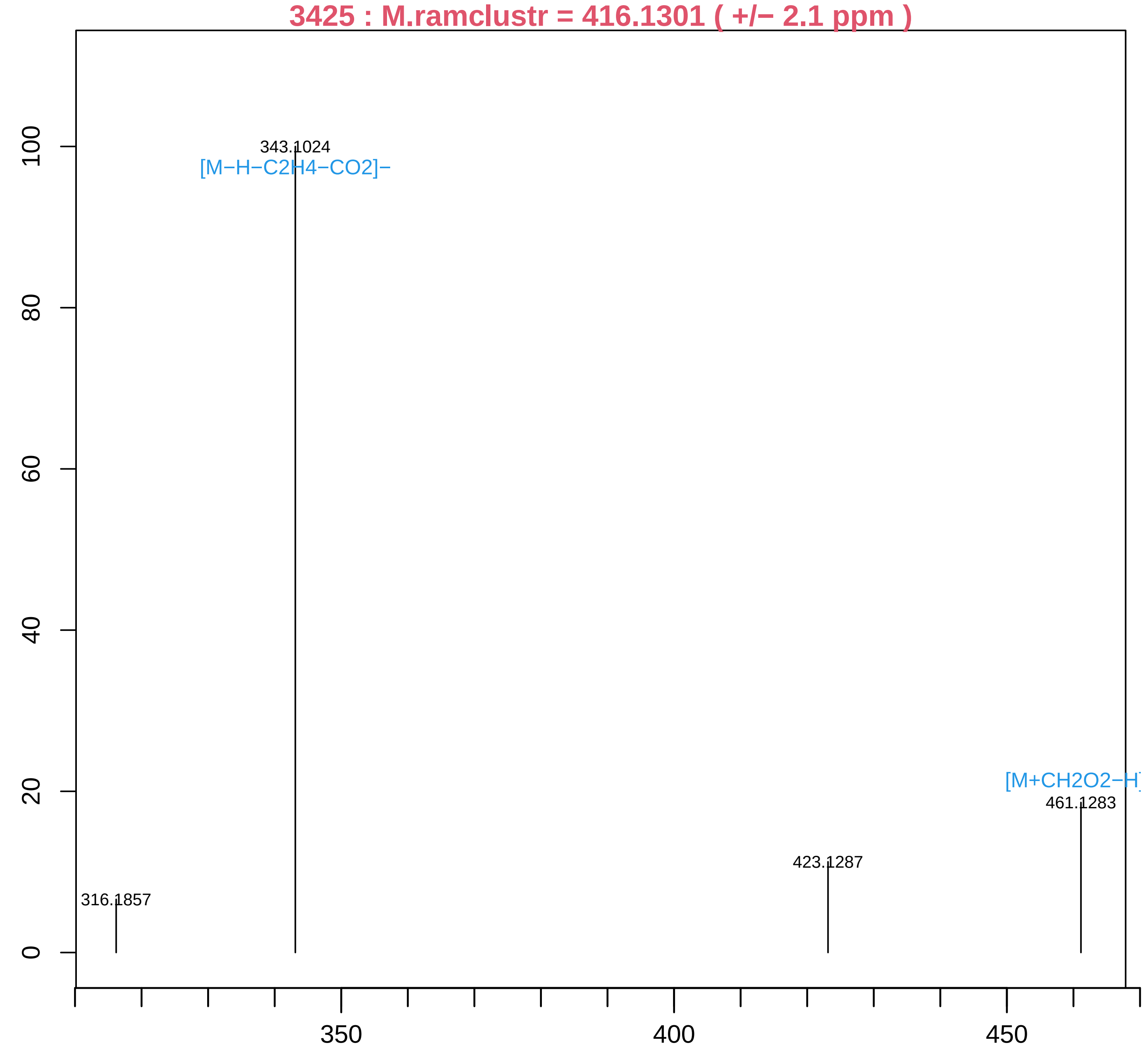

3425 : M.ramclustr = 416.1301 (+/− 2.1 ppm)
343.1024
[M−H−C2H4−CO2]−
[M+CH2O2−H]
461.1283
423.1287
316.1857
0
20
40
60
80
100
350
400
450

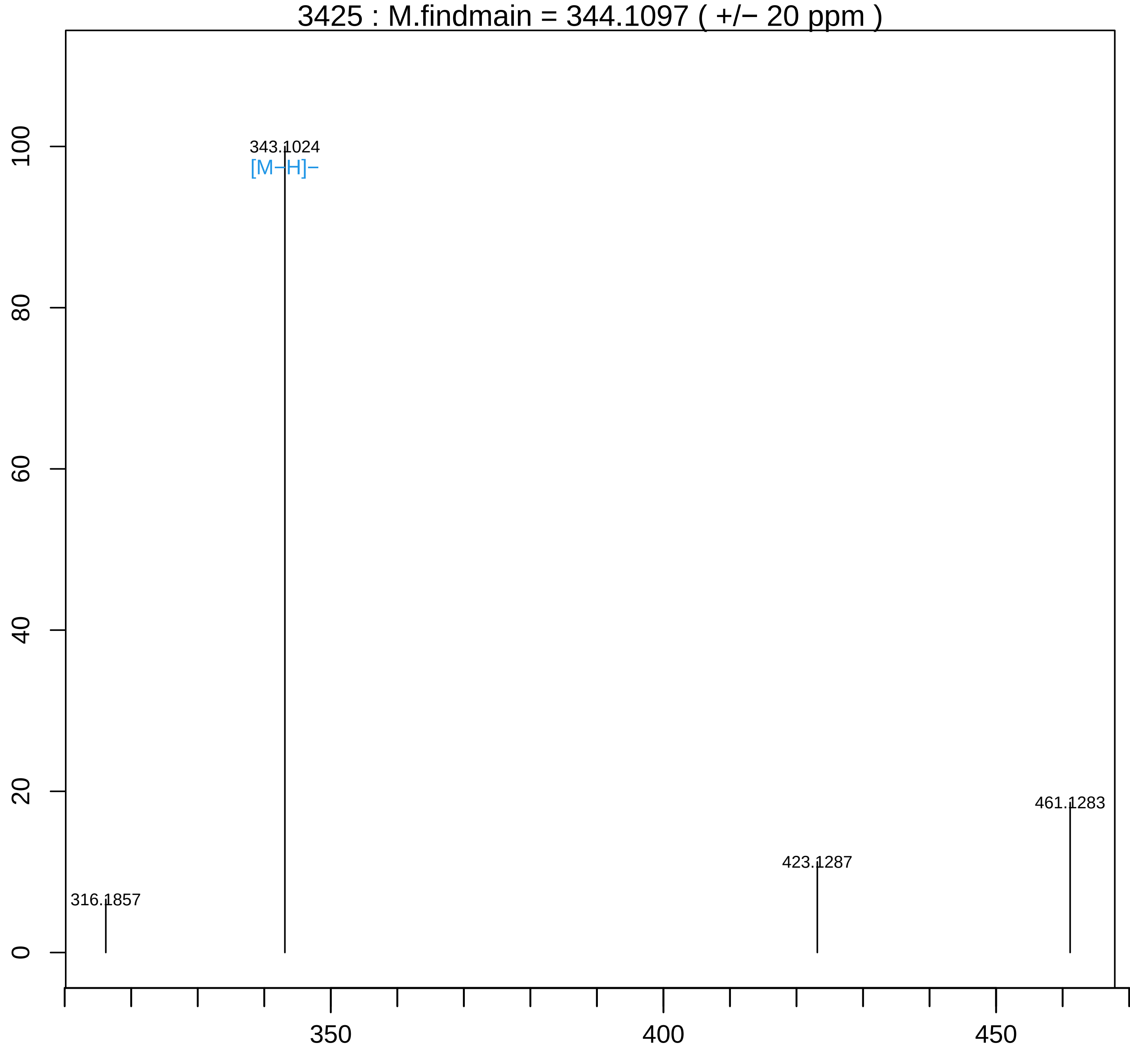

3425 : M.findmain = 344.1097 (+/− 20 ppm)
343.1024
[M−H]−
461.1283
423.1287
316.1857
0
20
40
60
80
100
350
400
450

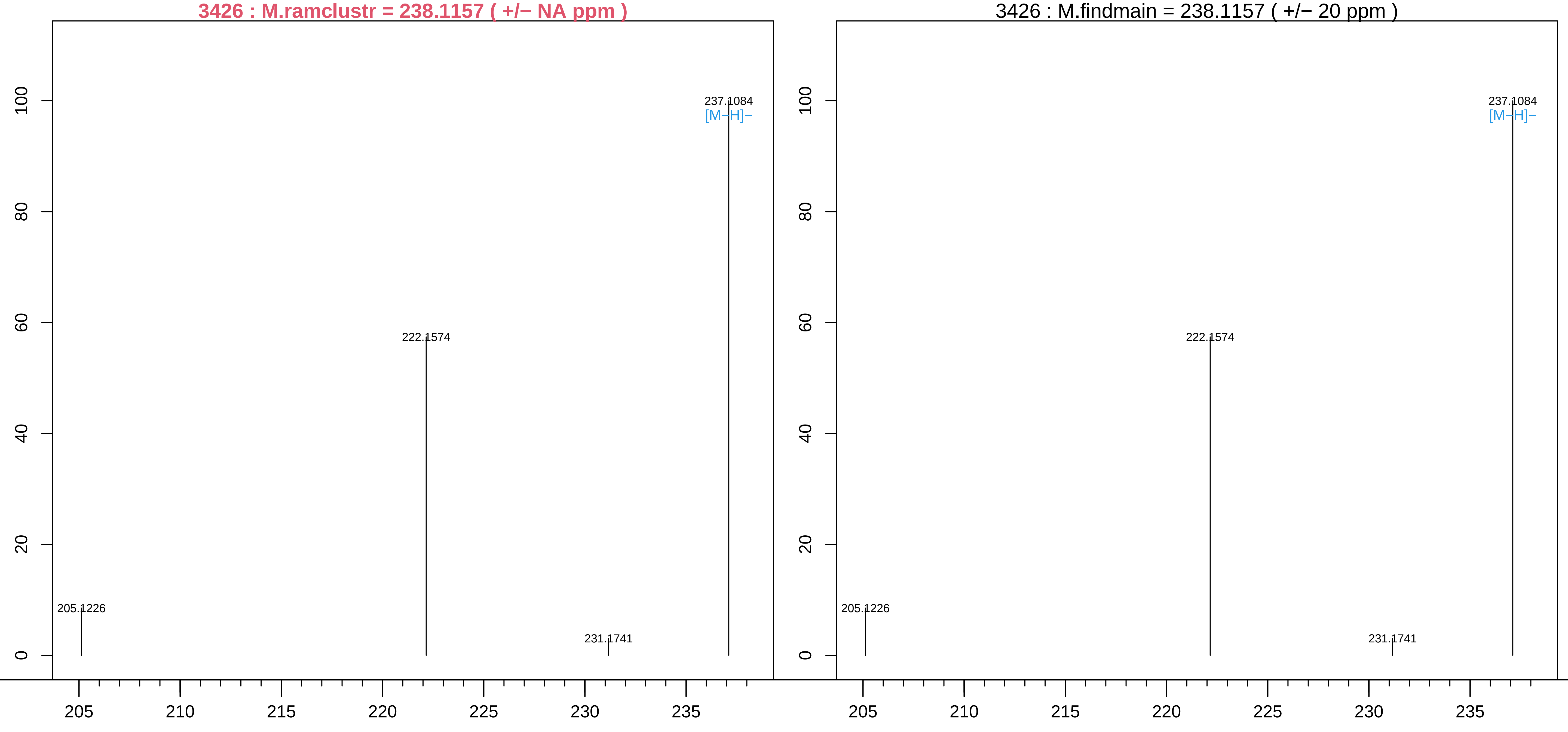

3426 : M.ramclustr = 238.1157 (+/− NA ppm)
3426 : M.findmain = 238.1157 (+/− 20 ppm)
237.1084
[M−H]−
222.1574
205.1226
231.1741
0
20
40
60
80
100
205
210
215
220
225
230
235

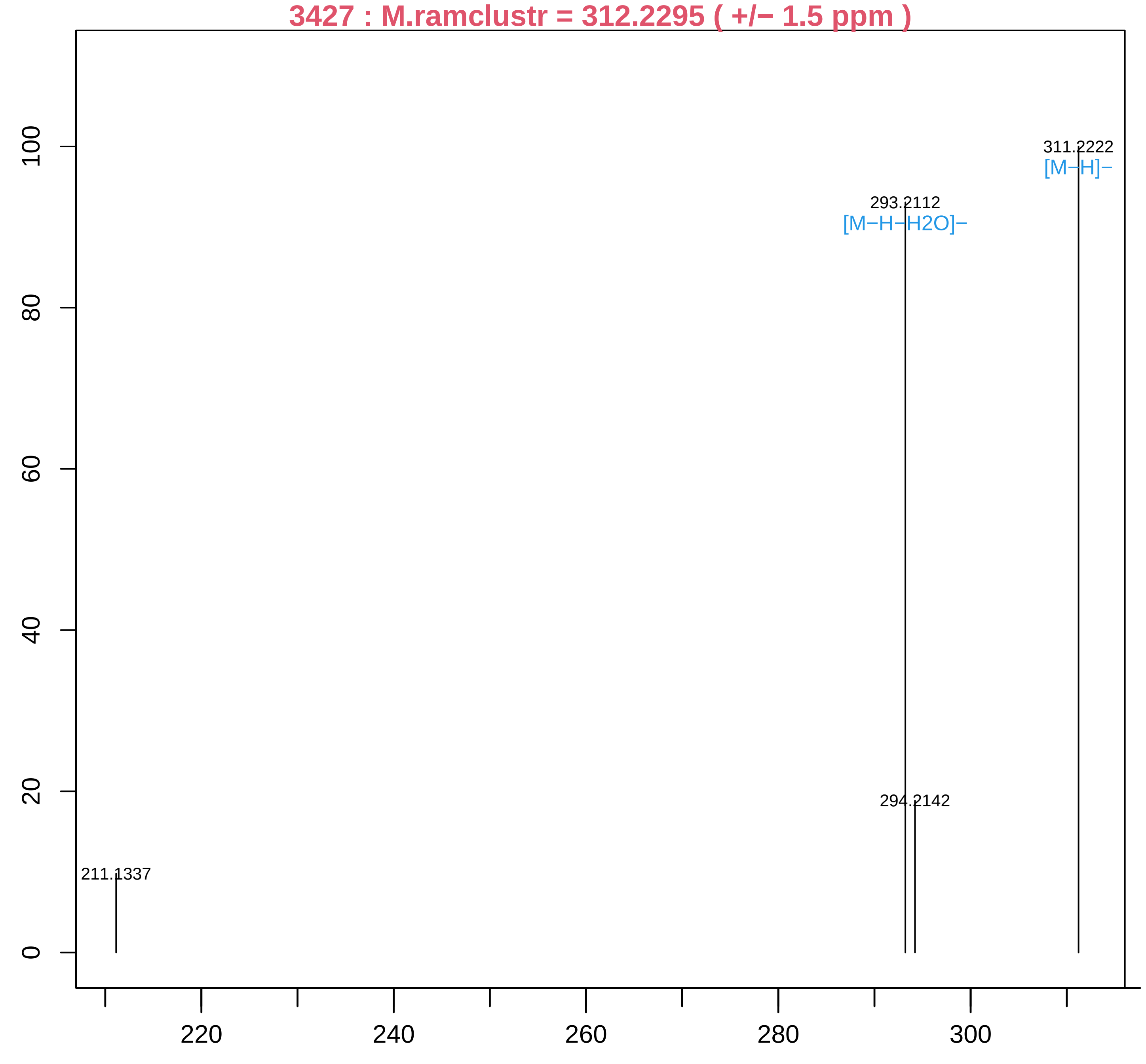

3427 : M.ramclustr = 312.2295 (+/− 1.5 ppm)
311.2222
[M−H]−
293.2112
[M−H−H2O]−
294.2142
211.1337

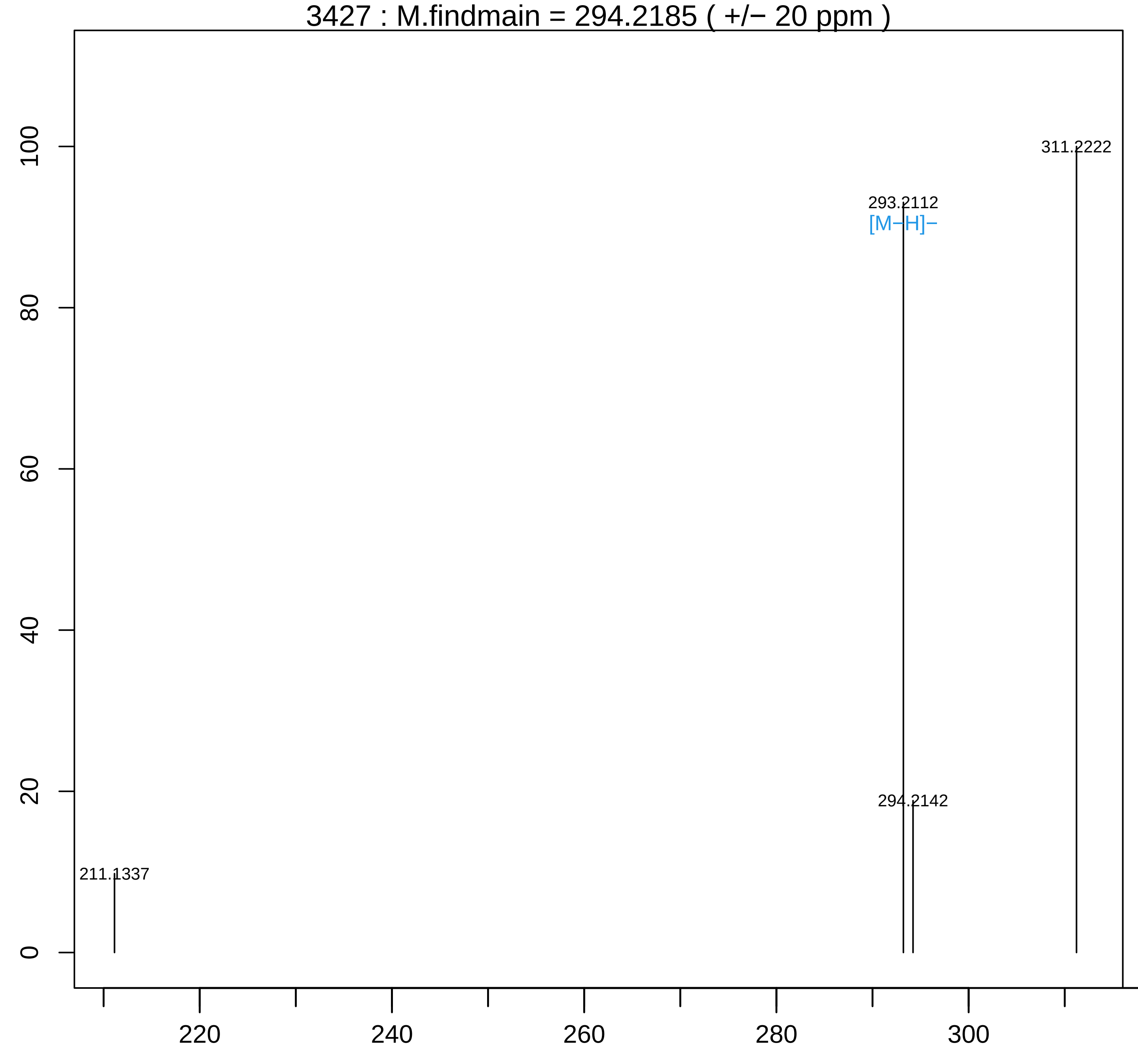

3427 : M.findmain = 294.2185 (+/− 20 ppm)
311.2222
293.2112
[M−H]−
294.2142
211.1337

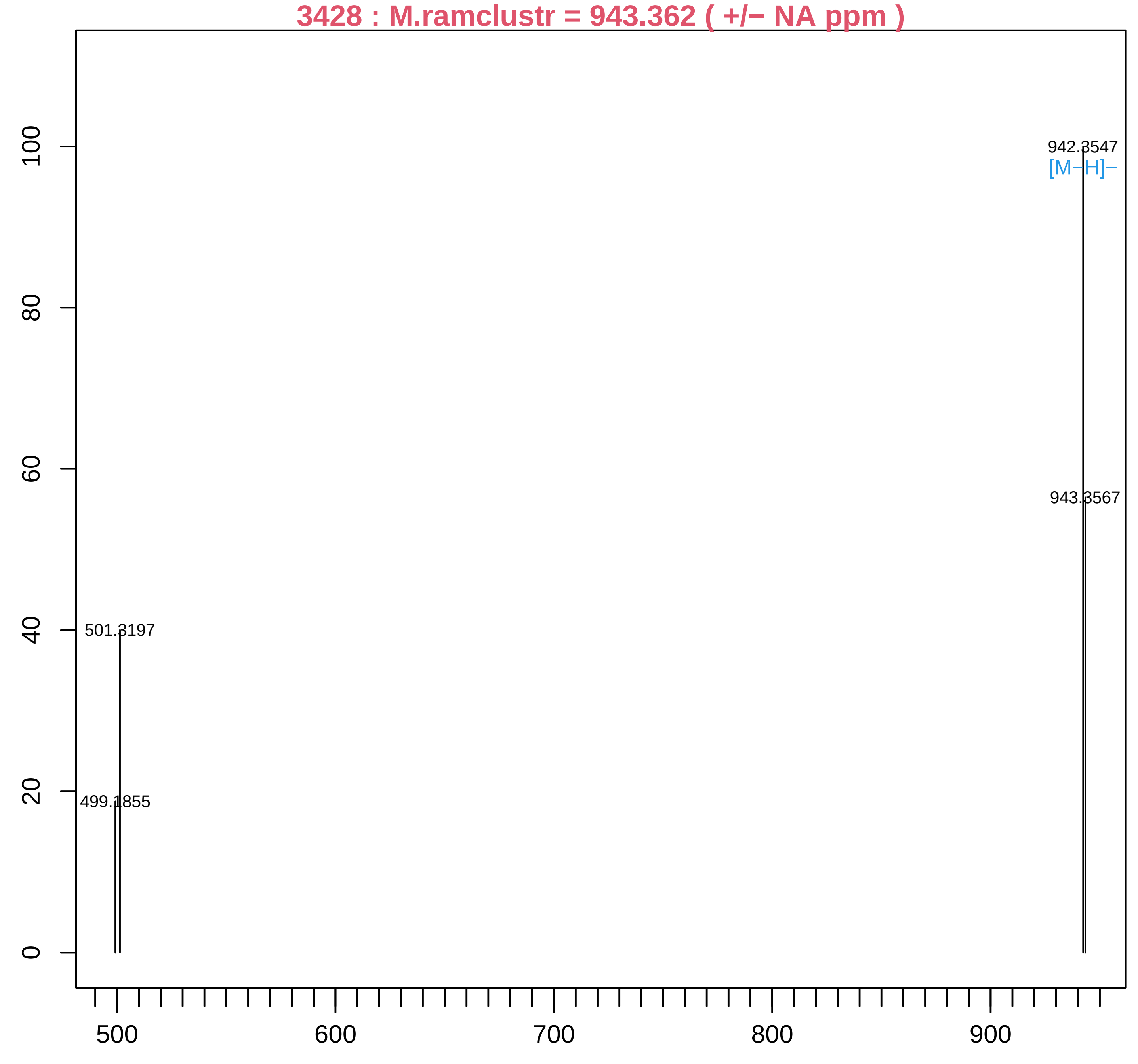
3428 : M.ramclustr = 943.362 (+/− NA ppm)
942.3547
[M−H]−
943.3567
501.3197
499.1855
0
20
40
60
80
100
500
600
700
800
900

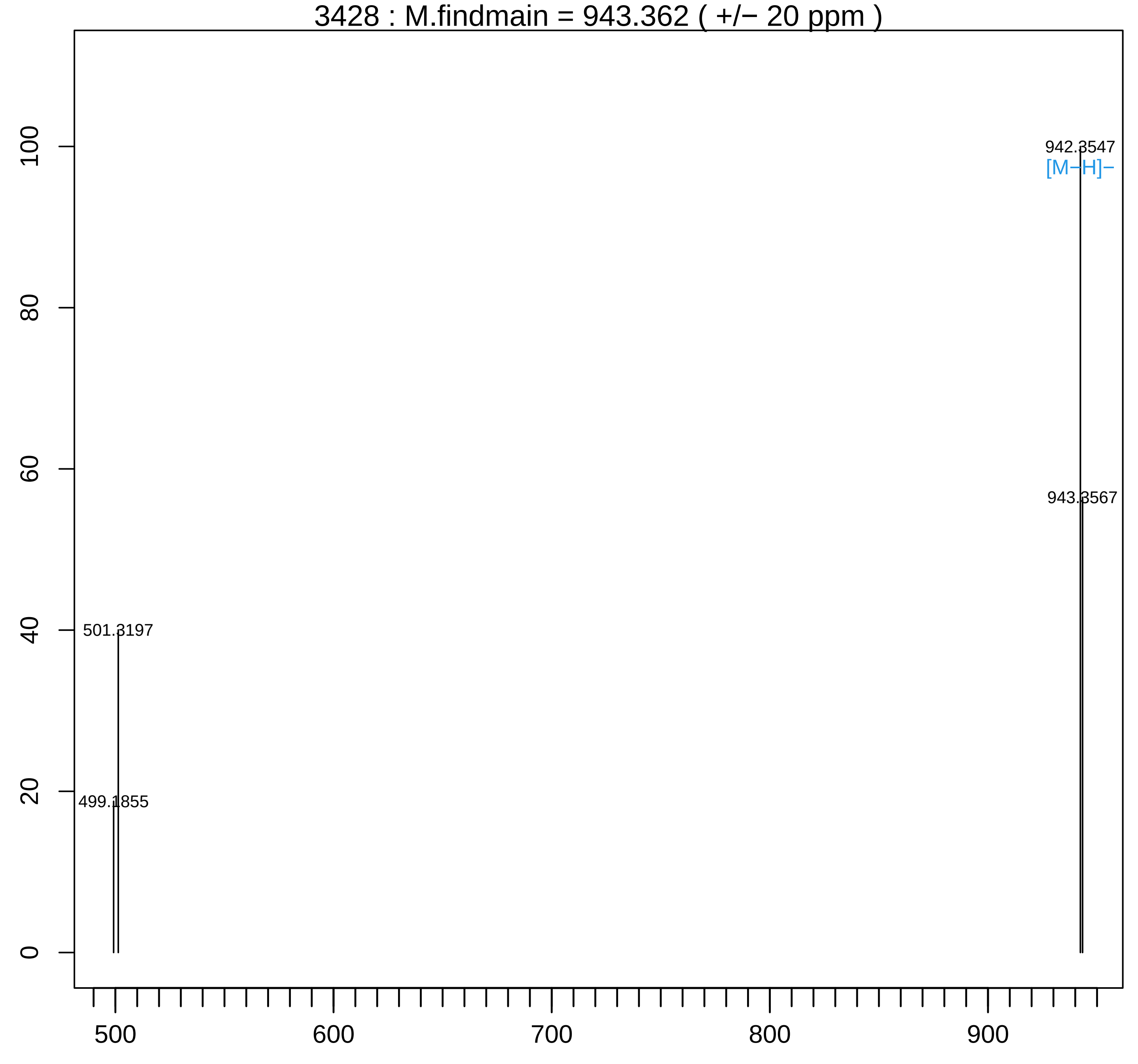
3428 : M.findmain = 943.362 (+/− 20 ppm)
942.3547
[M−H]−
943.3567
501.3197
499.1855
0
20
40
60
80
100
500
600
700
800
900

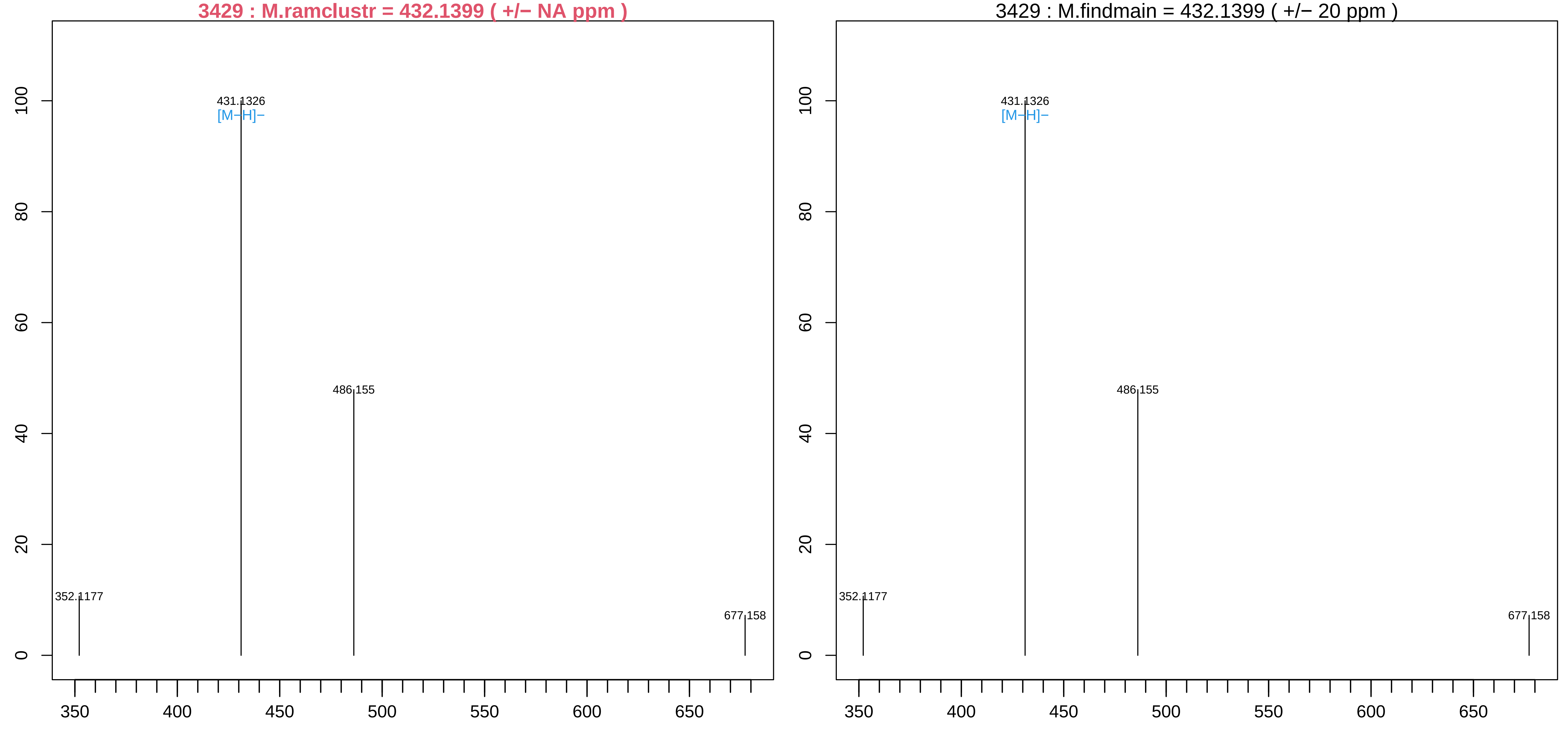
3429 : M.ramclustr = 432.1399 (+/− NA ppm)
431.1326
[M−H]−
486.155
352.1177
677.158
3429 : M.findmain = 432.1399 (+/− 20 ppm)
431.1326
[M−H]−
486.155
352.1177
677.158
0
20
40
60
80
100
350
400
450
500
550
600
650

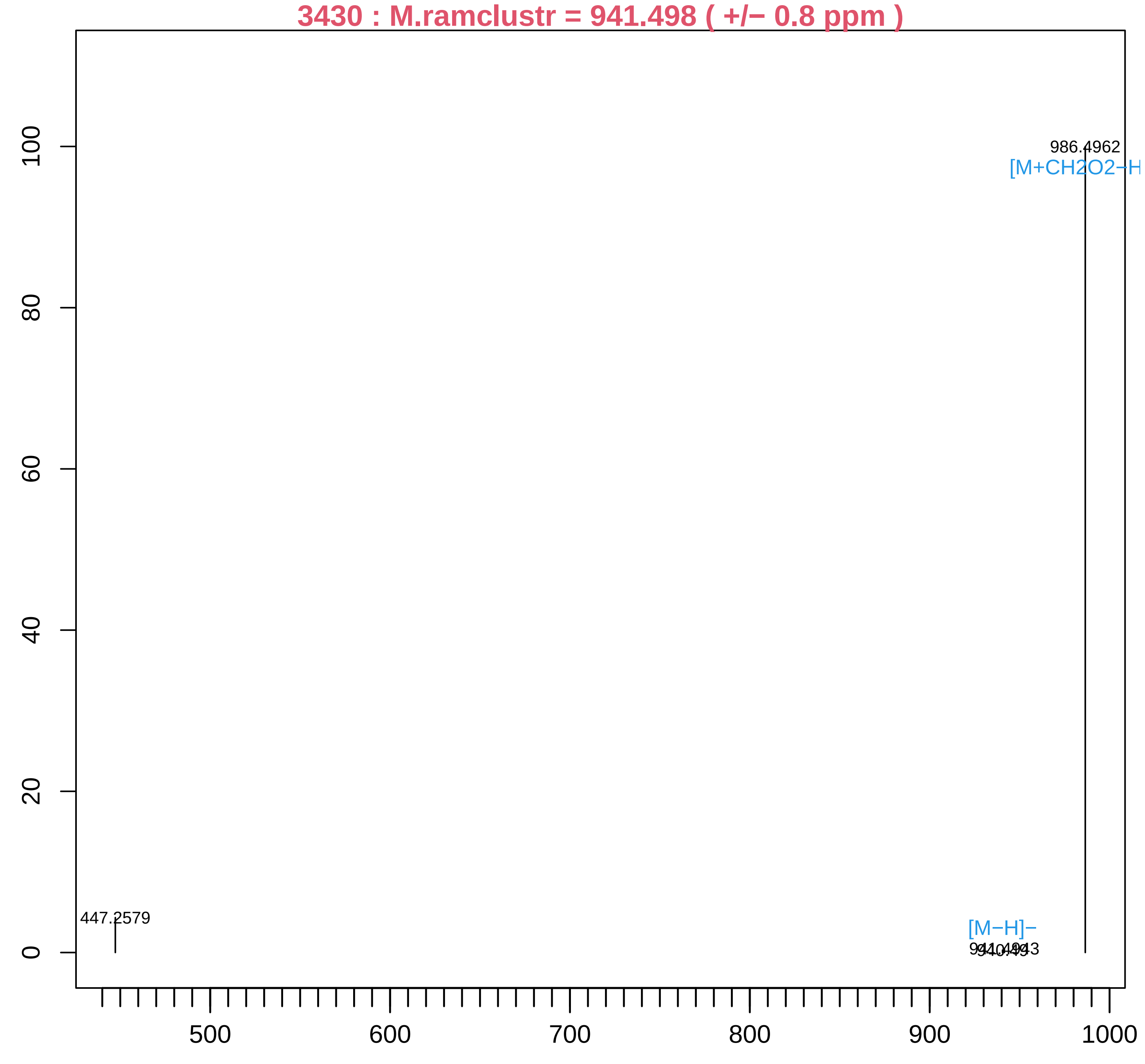
3430 : M.ramclustr = 941.498 (+/− 0.8 ppm)
986.4962
[M+CH2O2−H
447.2579
[M−H]−
0
20
40
60
80
100
500
600
700
800
900
1000

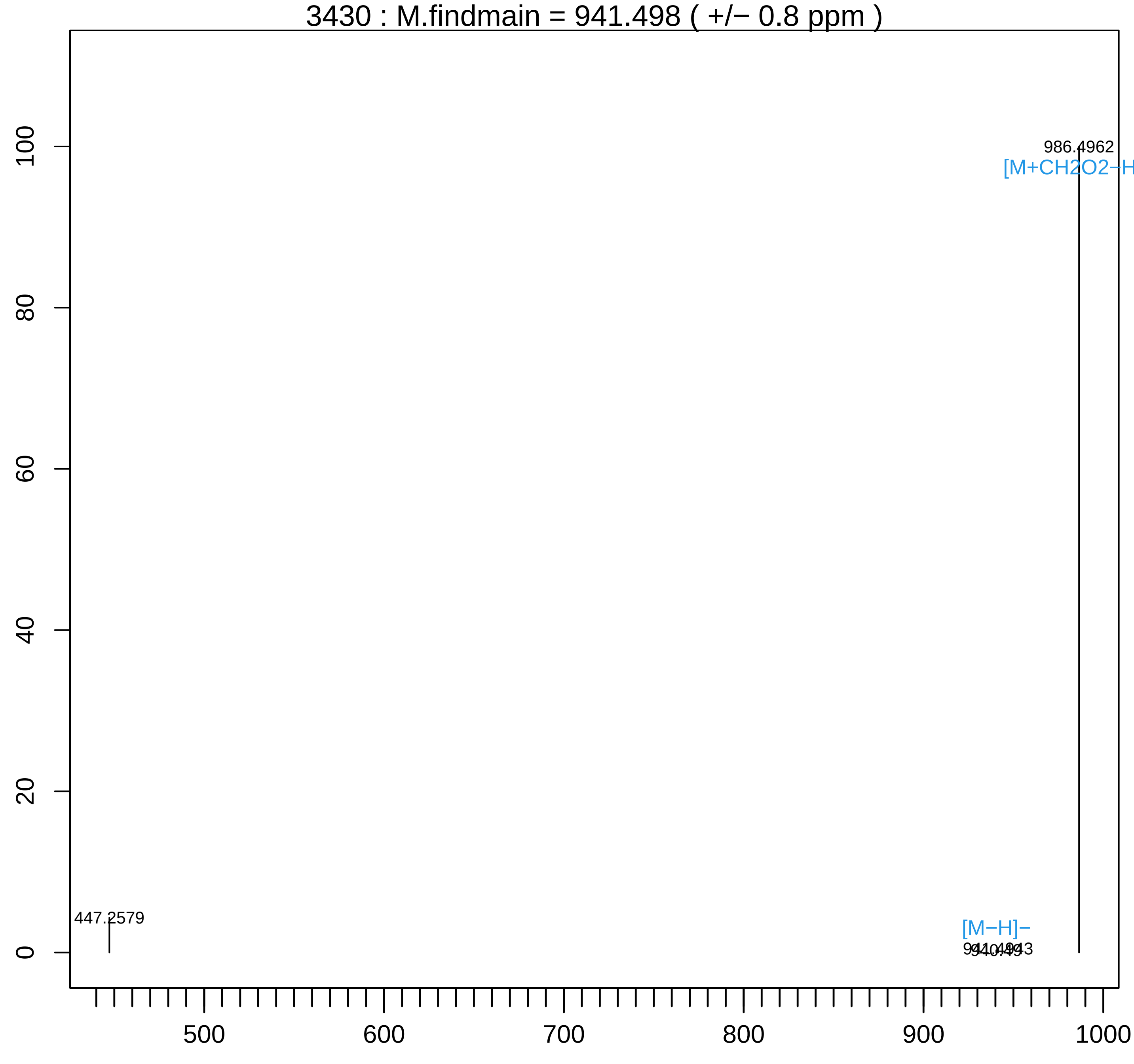
3430 : M.findmain = 941.498 (+/− 0.8 ppm)
986.4962
[M+CH2O2−H
447.2579
[M−H]−
0
20
40
60
80
100
500
600
700
800
900
1000

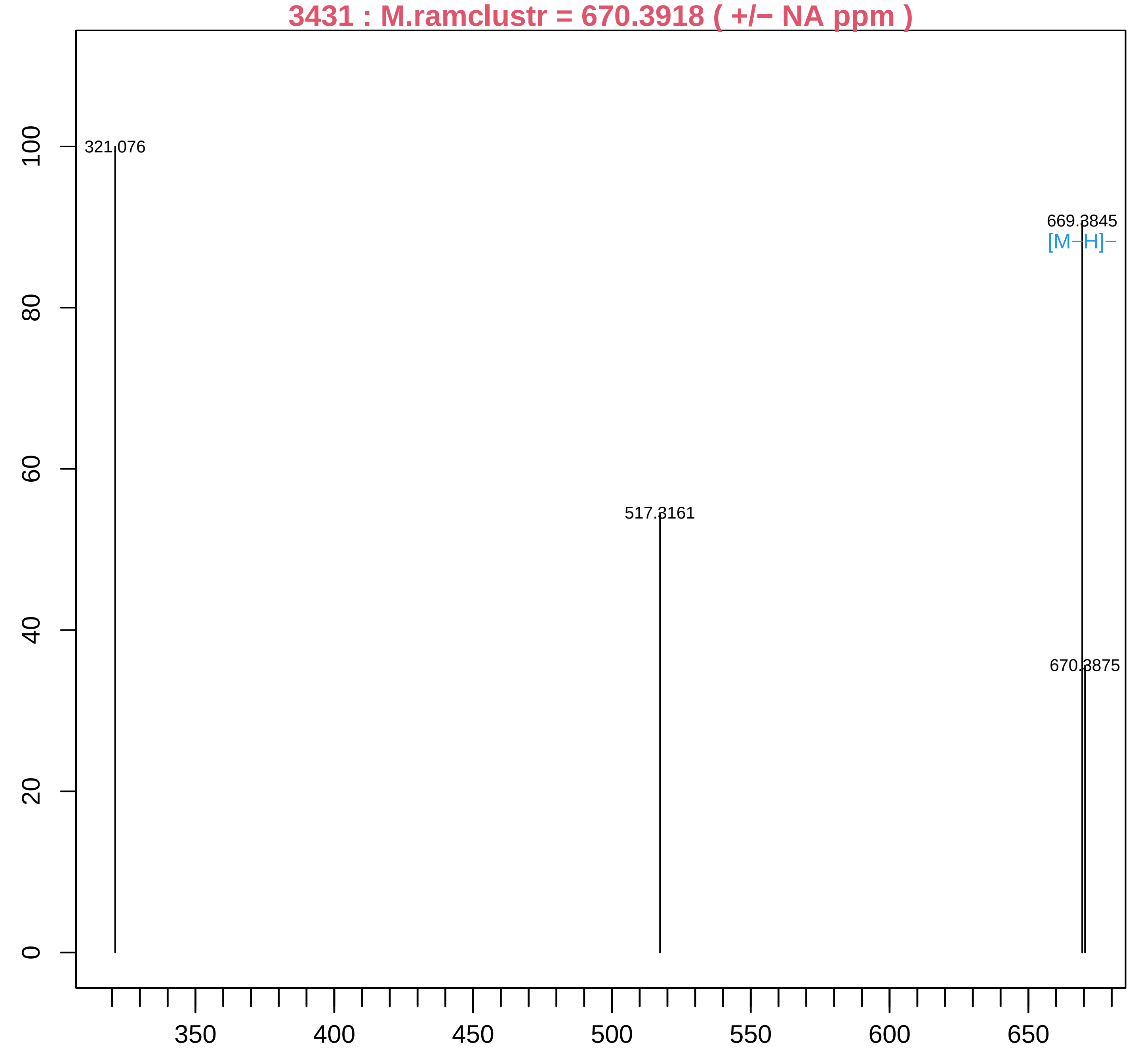
3431 : M.ramclustr = 670.3918 (+/− NA ppm)
321.076
517.3161
669.3845
[M−H]−
670.3875
0
20
40
60
80
100
350
400
450
500
550
600
650

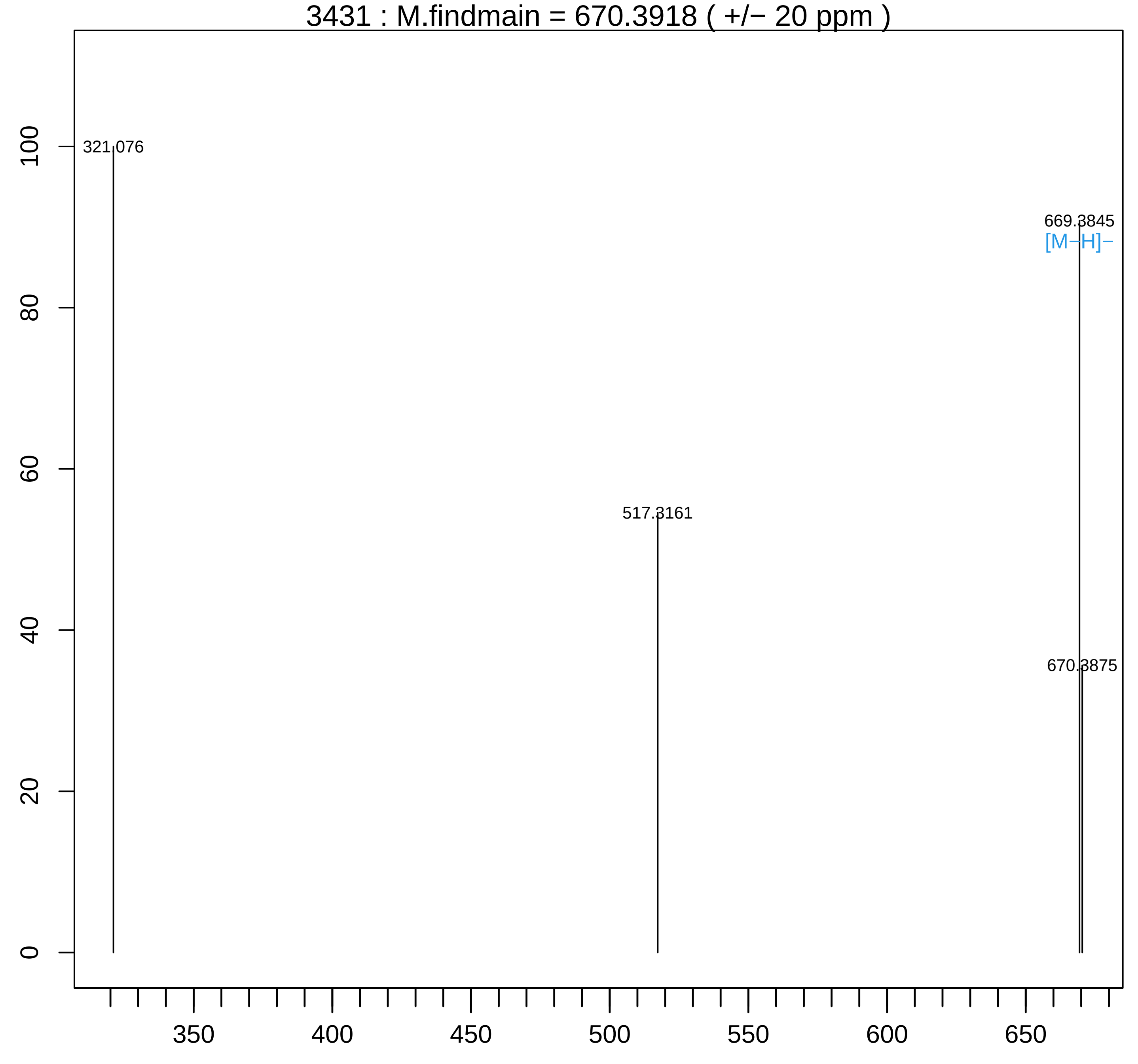
3431 : M.findmain = 670.3918 (+/− 20 ppm)
321.076
517.3161
669.3845
[M−H]−
670.3875
0
20
40
60
80
100
350
400
450
500
550
600
650

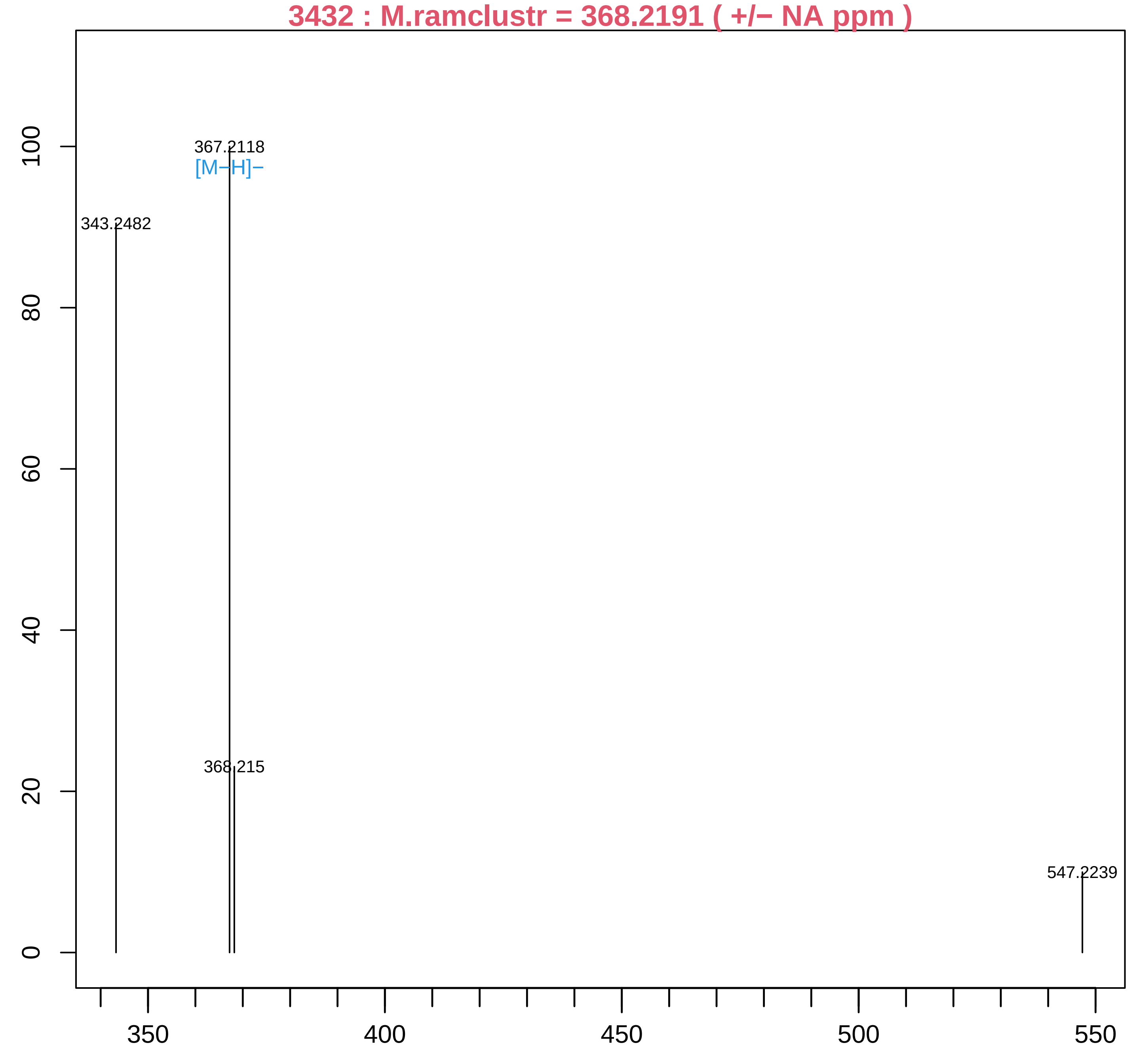
3432 : M.ramclustr = 368.2191 (+/− NA ppm)
367.2118
[M−H]−
343.2482
368.215
547.2239
0
20
40
60
80
100
350
400
450
500
550

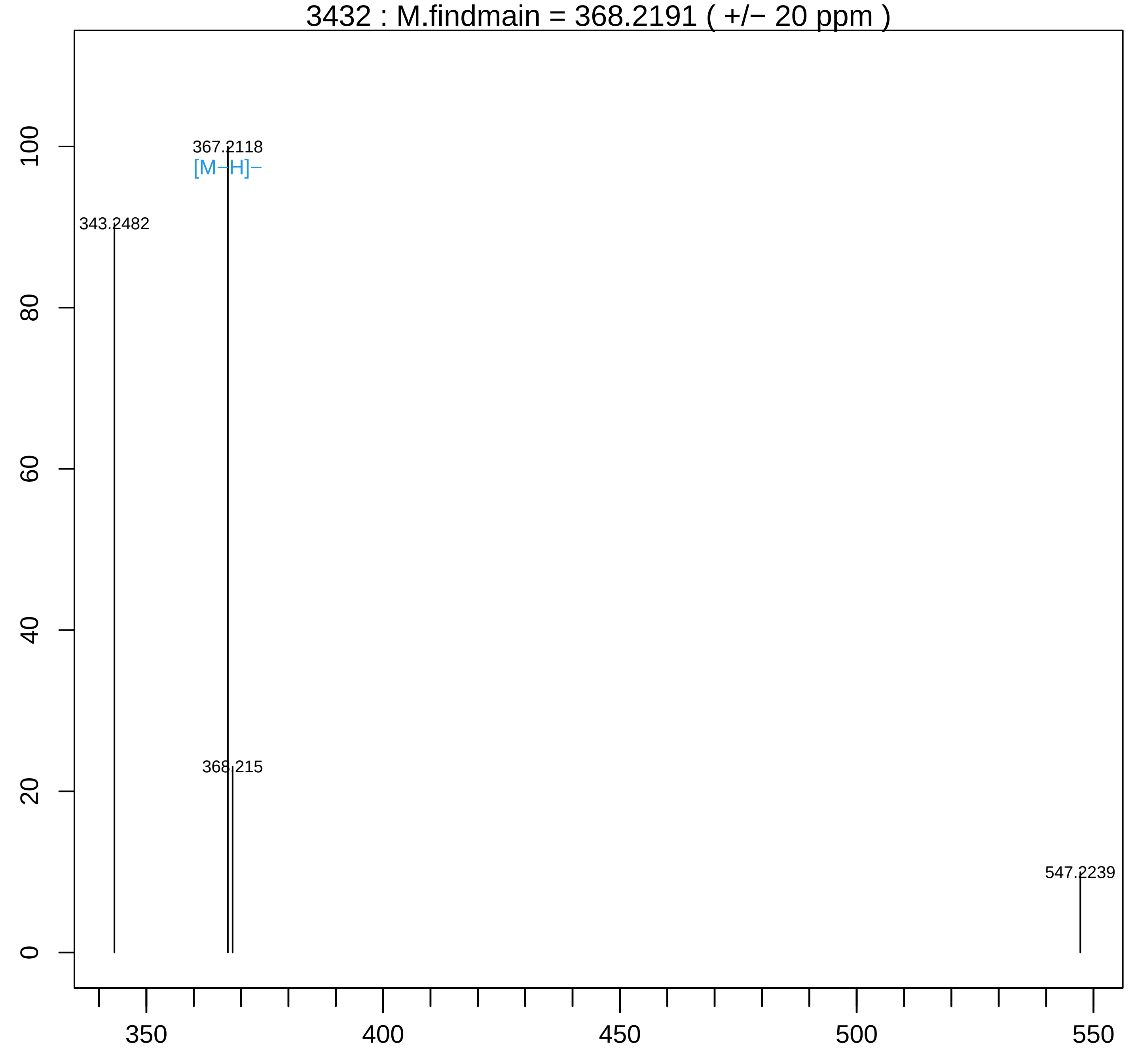
3432 : M.findmain = 368.2191 (+/− 20 ppm)
367.2118
[M−H]−
343.2482
368.215
547.2239
0
20
40
60
80
100
350
400
450
500
550

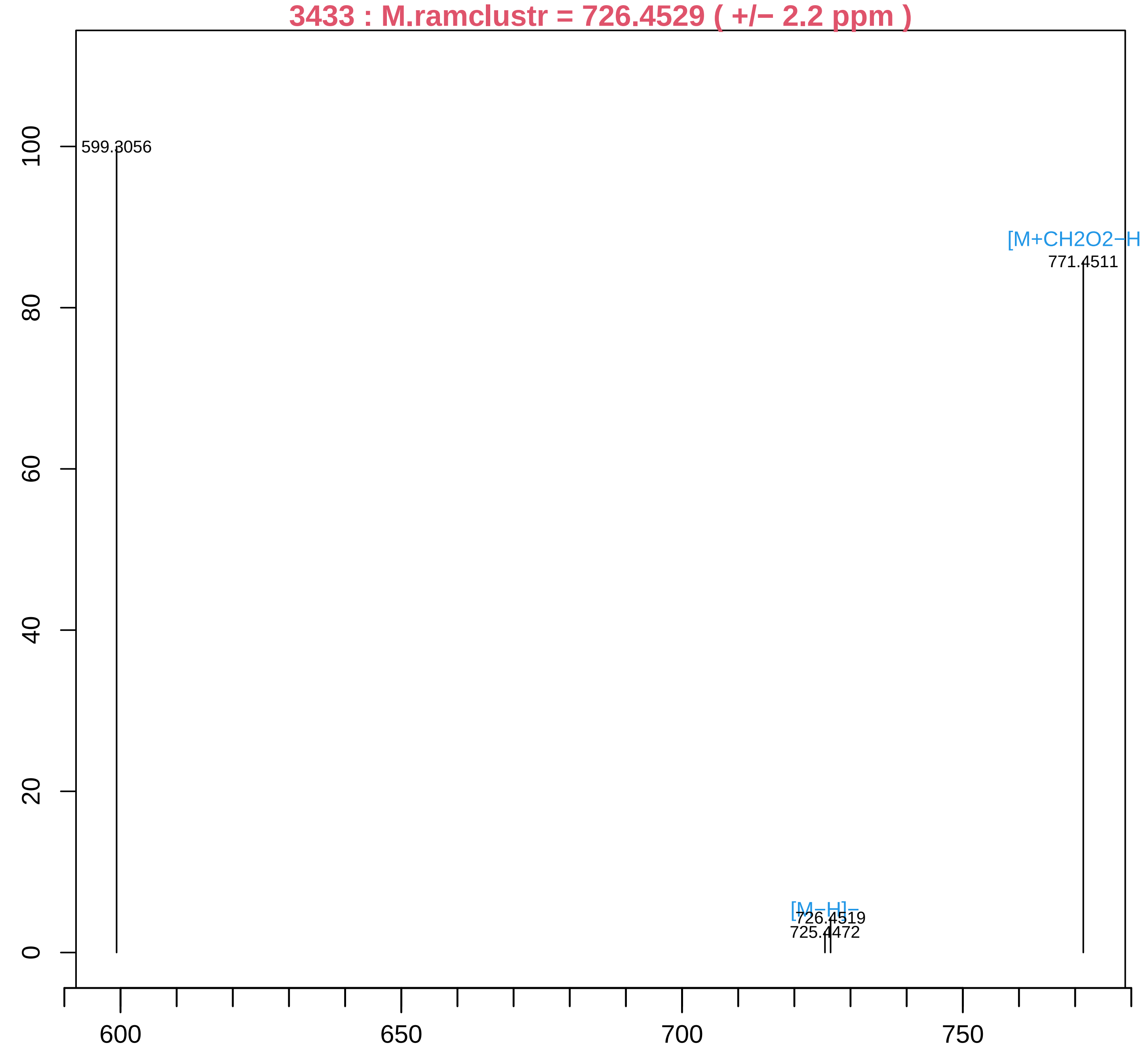
3433 : M.ramclustr = 726.4529 (+/− 2.2 ppm)
599.3056
[M+CH2O2−H
771.4511
[M−H]−
726.4519
725.4472
0
20
40
60
80
100
600
650
700
750

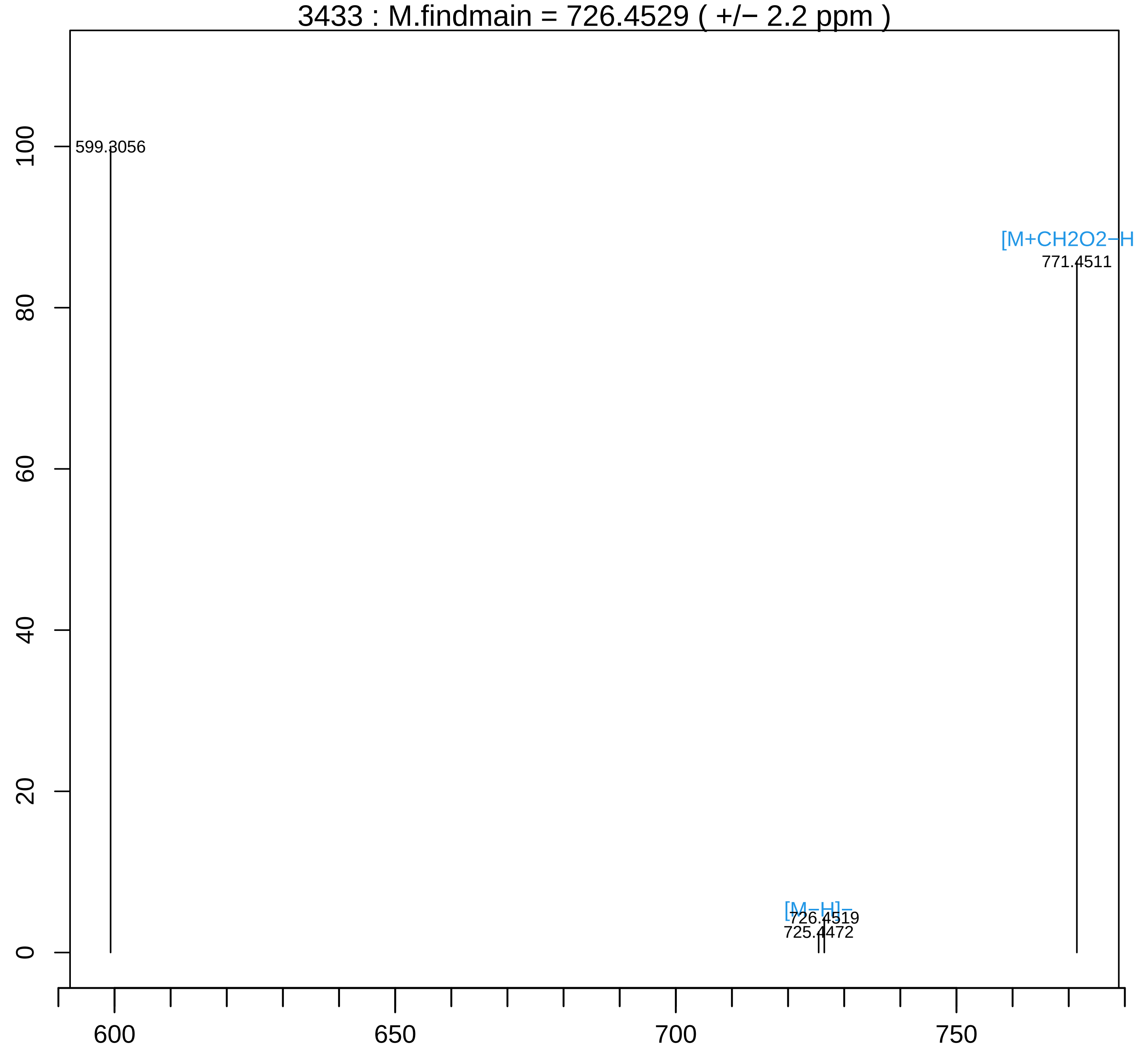
3433 : M.findmain = 726.4529 (+/− 2.2 ppm)
599.3056
[M+CH2O2−H
771.4511
[M−H]−
726.4519
725.4472
0
20
40
60
80
100
600
650
700
750

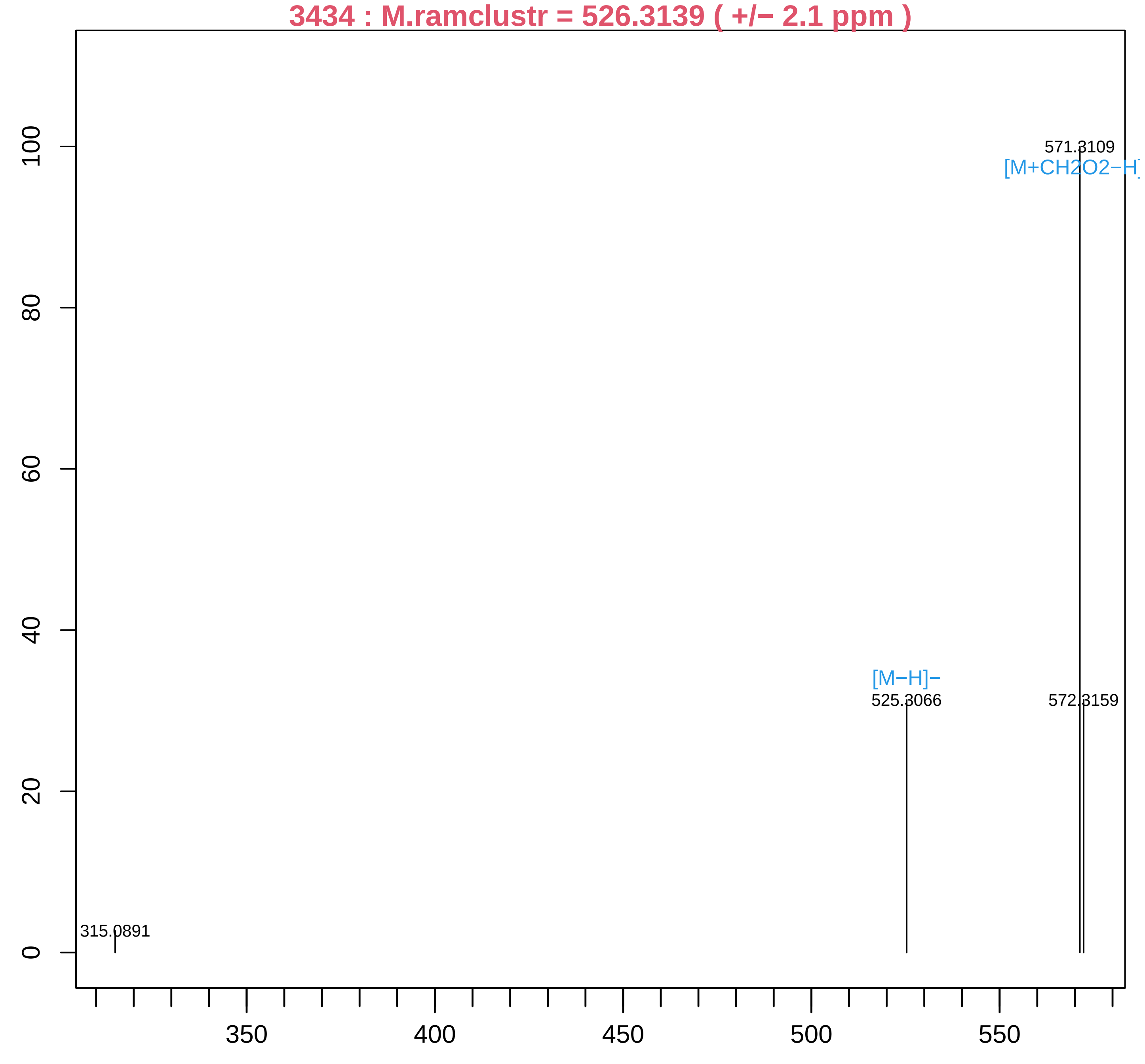
3434 : M.ramclustr = 526.3139 (+/− 2.1 ppm)
571.3109
[M+CH2O2−H]
[M−H]−
525.3066
572.3159
315.0891
0
20
40
60
80
100
350
400
450
500
550

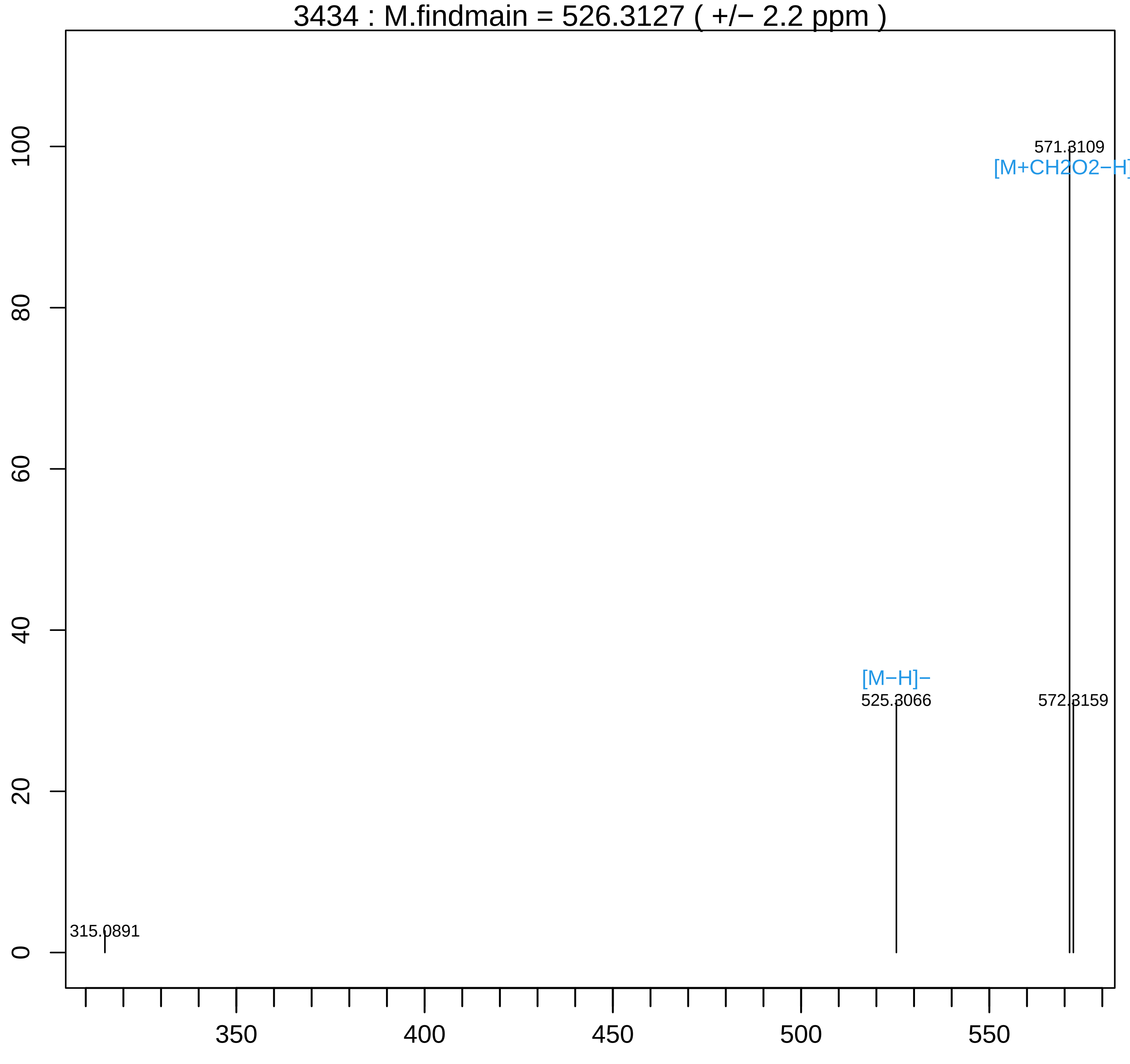
3434 : M.findmain = 526.3127 (+/− 2.2 ppm)
571.3109
[M+CH2O2−H]
[M−H]−
525.3066
572.3159
315.0891
0
20
40
60
80
100
350
400
450
500
550

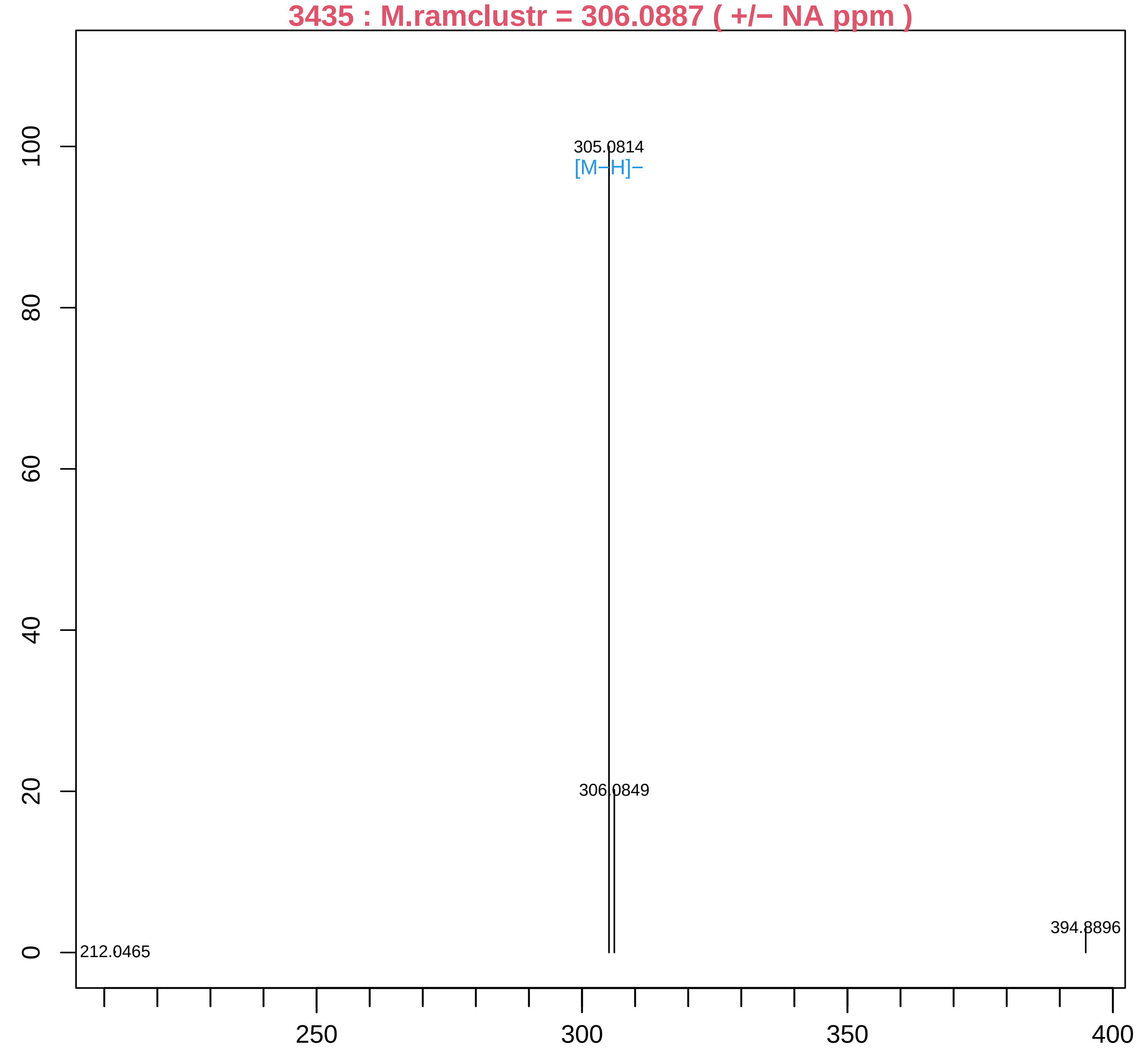

3435 : M.ramclustr = 306.0887 (+/− NA ppm)
305.0814
[M−H]−
306.0849
212.0465
394.8896
0
20
40
60
80
100
250
300
350
400

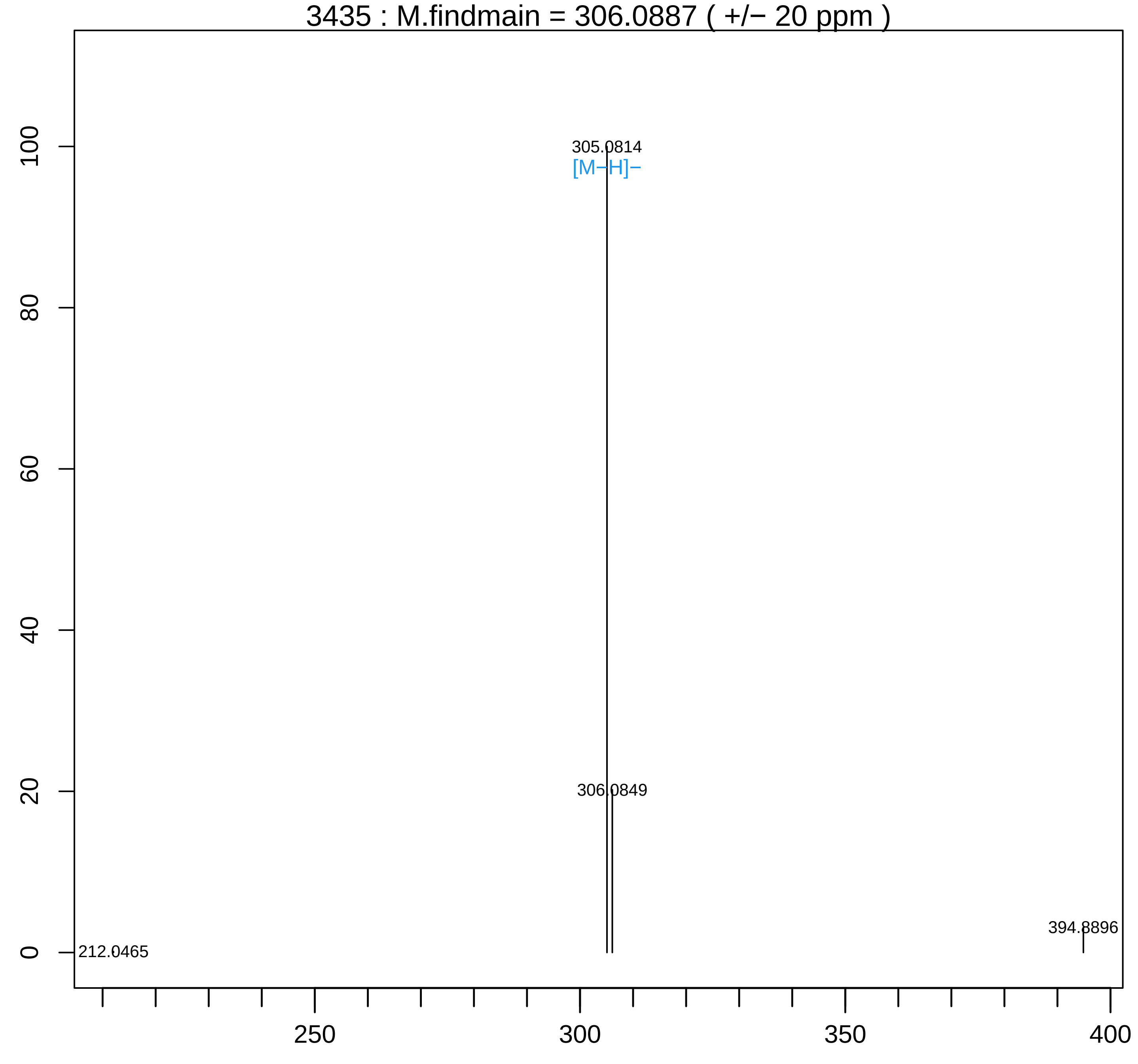

3435 : M.findmain = 306.0887 (+/− 20 ppm)
305.0814
[M−H]−
306.0849
212.0465
394.8896
0
20
40
60
80
100
250
300
350
400

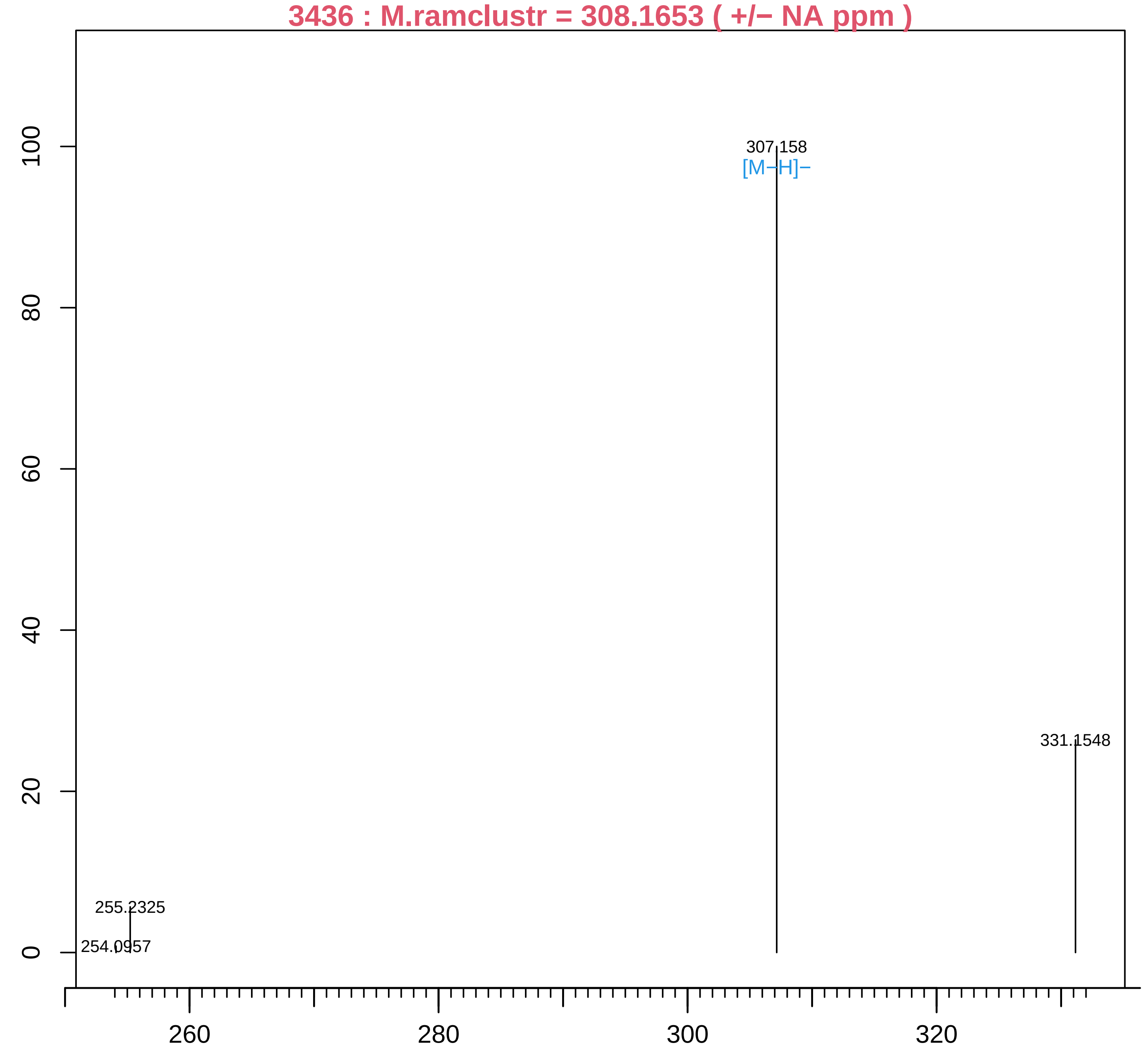
3436 : M.ramclustr = 308.1653 (+/− NA ppm)
100
80
60
40
20
0
260
280
300
320
307.158
[M−H]−
331.1548
255.2325
254.0957

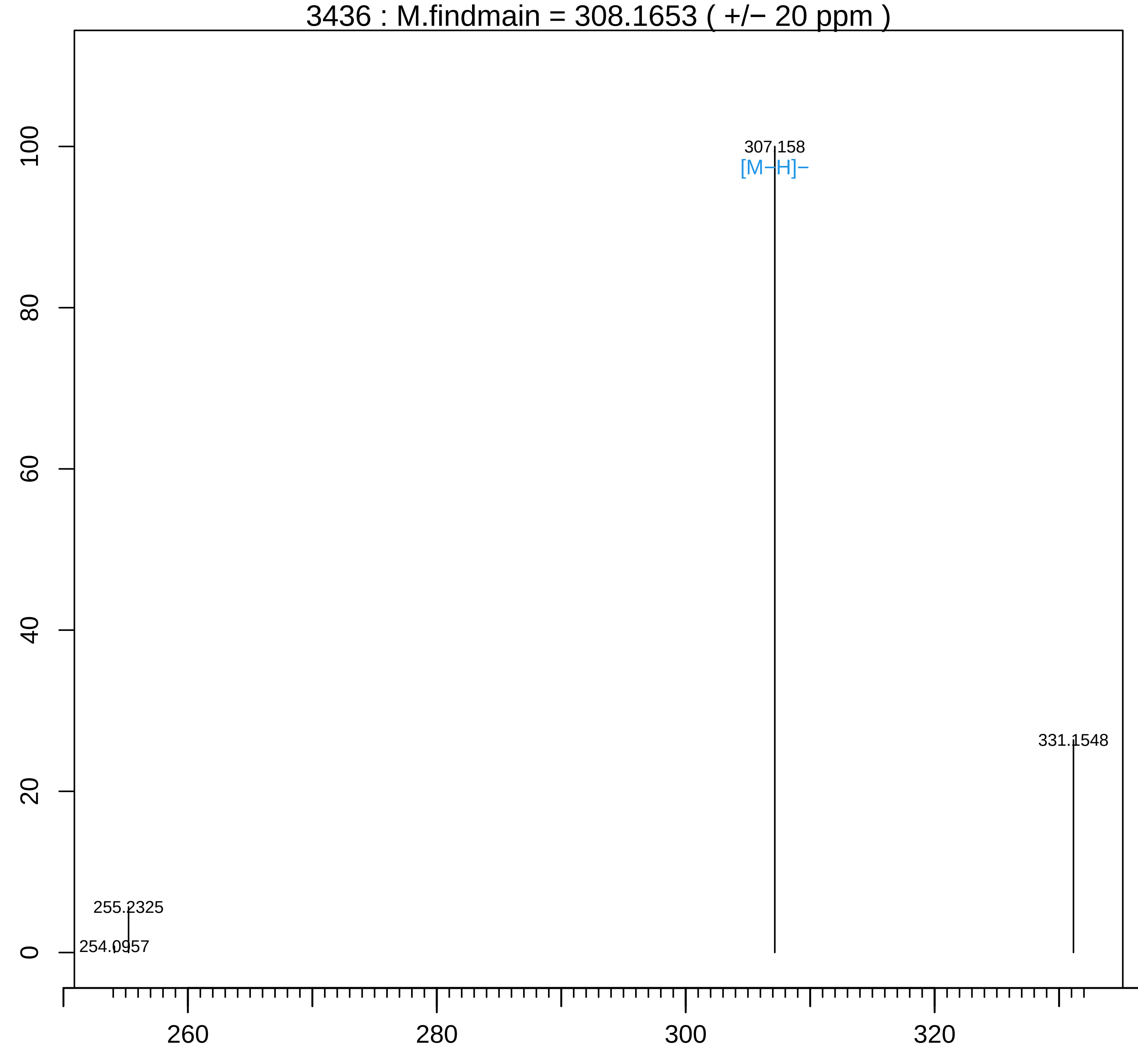
3436 : M.findmain = 308.1653 (+/− 20 ppm)
100
80
60
40
20
0
260
280
300
320
307.158
[M−H]−
331.1548
255.2325
254.0957

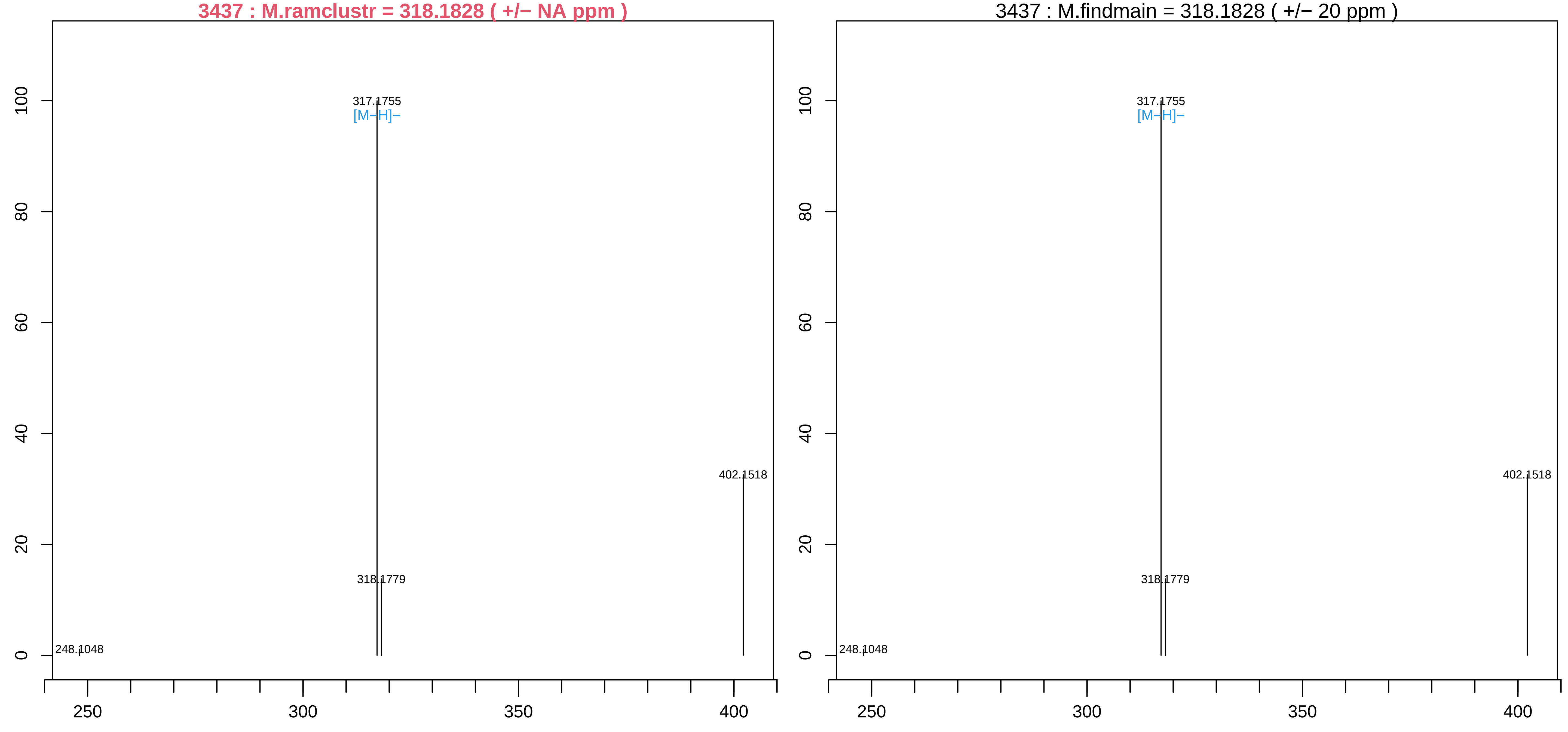

3437 : M.ramclustr = 318.1828 (+/− NA ppm)
317.1755
[M−H]−
318.1779
402.1518
248.1048
3437 : M.findmain = 318.1828 (+/− 20 ppm)
317.1755
[M−H]−
318.1779
402.1518
248.1048

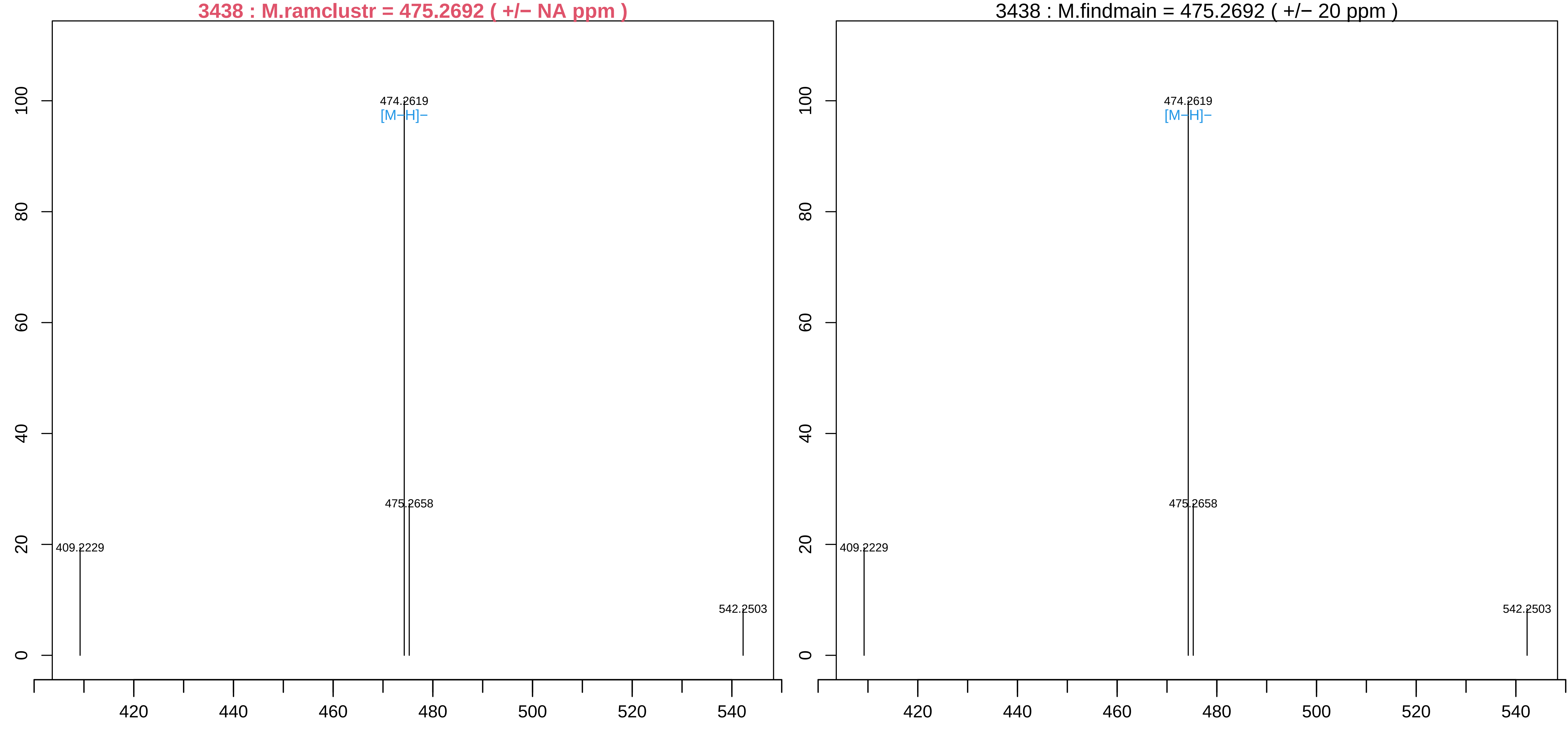

3438 : M.ramclustr = 475.2692 (+/− NA ppm)
474.2619
[M−H]−
475.2658
409.2229
542.2503
3438 : M.findmain = 475.2692 (+/− 20 ppm)
474.2619
[M−H]−
475.2658
409.2229
542.2503

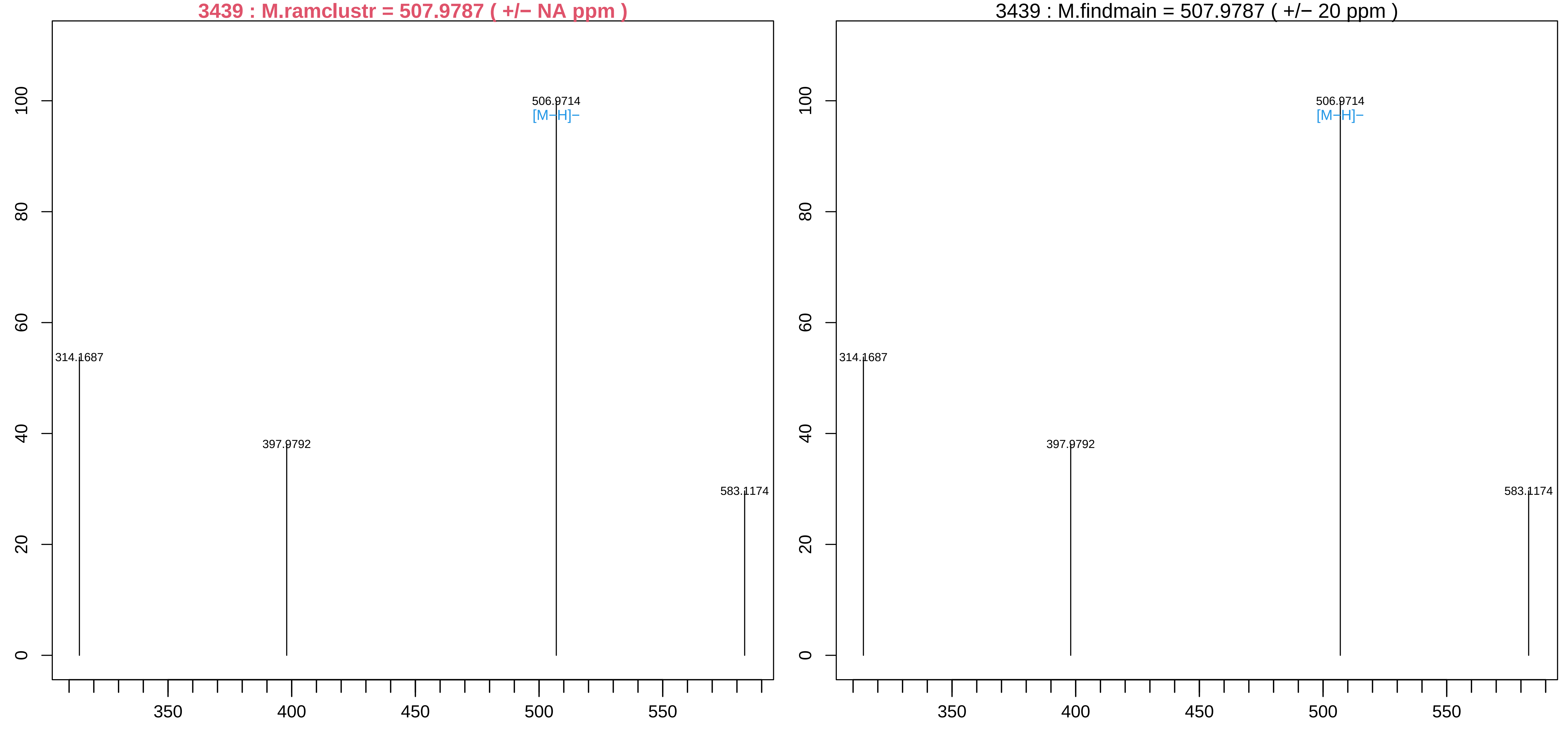
3439 : M.ramclustr = 507.9787 (+/− NA ppm)
3439 : M.findmain = 507.9787 (+/− 20 ppm)
506.9714
[M−H]−
314.1687
397.9792
583.1174
0
20
40
60
80
100
350
400
450
500
550

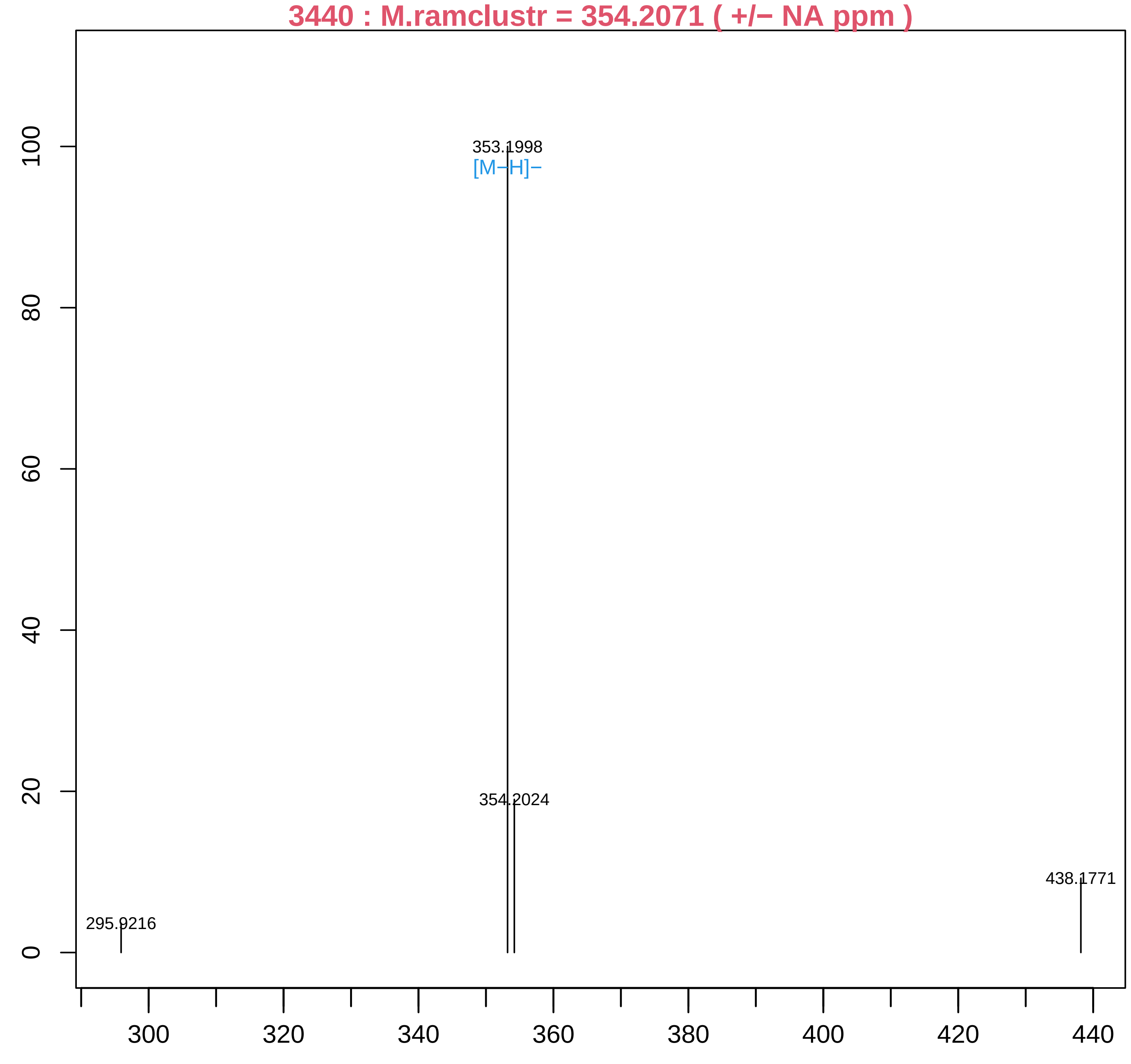

3440 : M.ramclustr = 354.2071 (+/− NA ppm)
353.1998
[M−H]−
354.2024
438.1771
295.9216
0
20
40
60
80
100
300
320
340
360
380
400
420
440

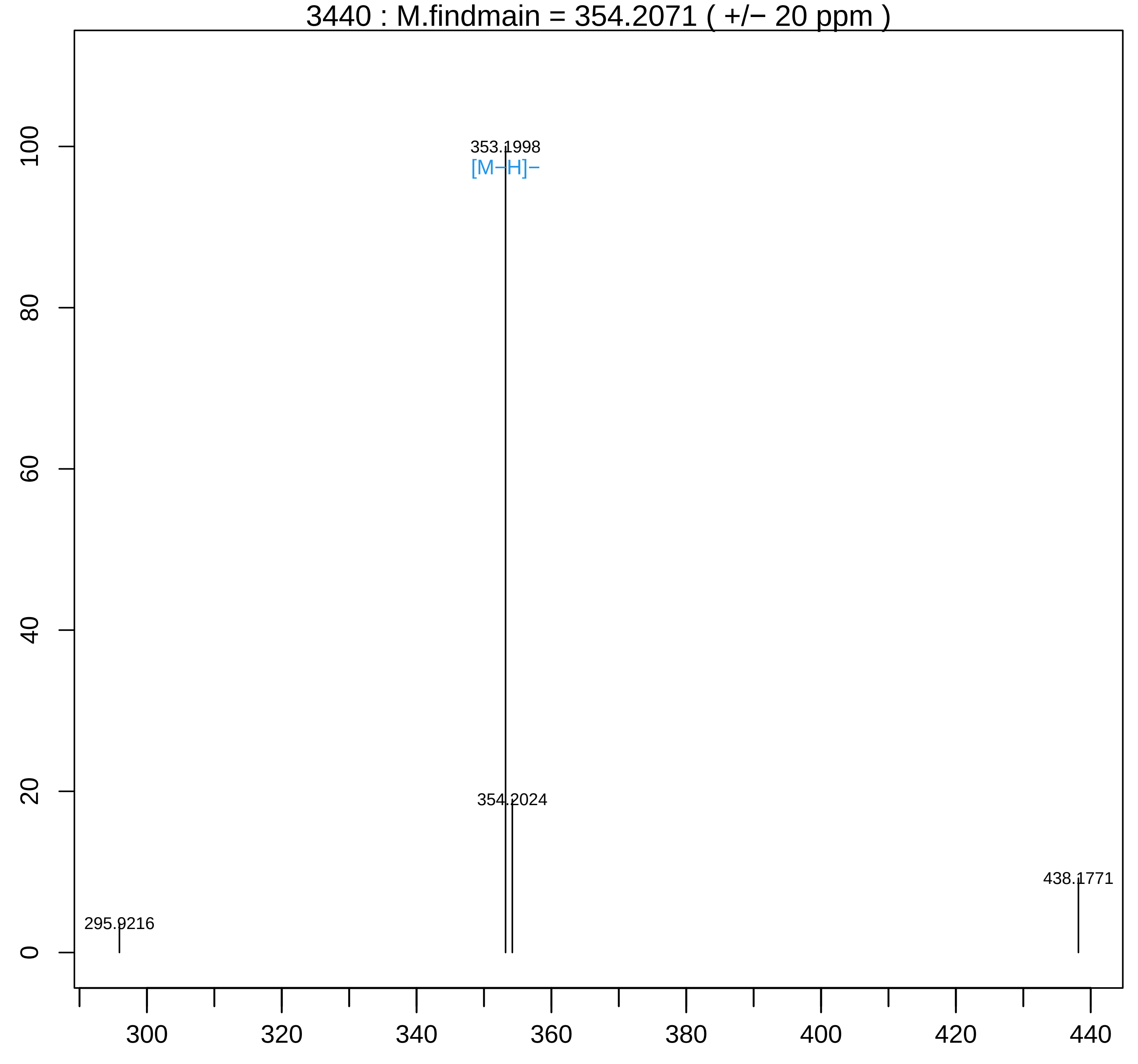

3440 : M.findmain = 354.2071 (+/− 20 ppm)
353.1998
[M−H]−
354.2024
438.1771
295.9216
0
20
40
60
80
100
300
320
340
360
380
400
420
440

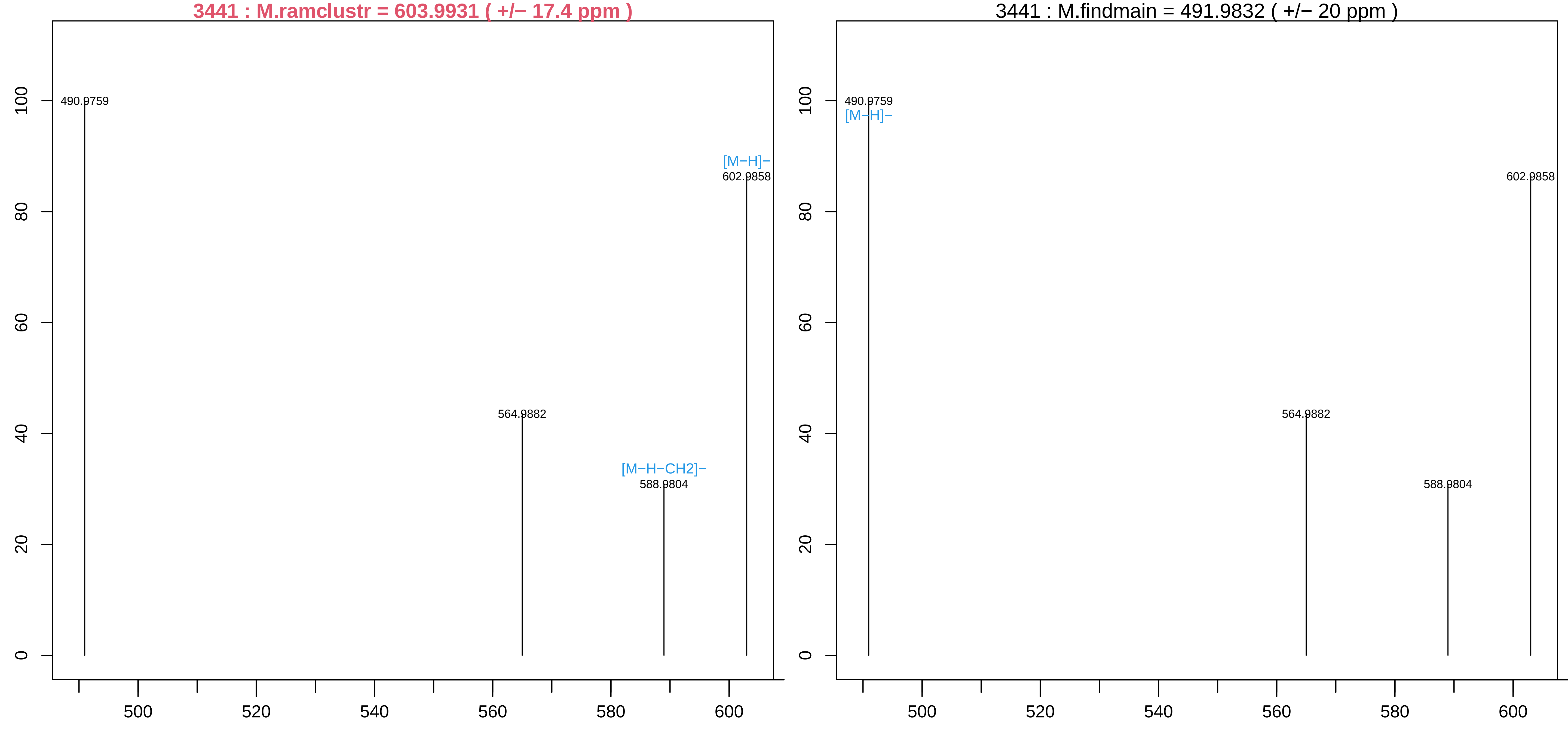
3441 : M.ramclustr = 603.9931 (+/− 17.4 ppm)
490.9759
[M−H]−
602.9858
564.9882
[M−H−CH2]−
588.9804
0
20
40
60
80
100
500
520
540
560
580
600
3441 : M.findmain = 491.9832 (+/− 20 ppm)
490.9759
[M−H]−
602.9858
564.9882
588.9804
0
20
40
60
80
100
500
520
540
560
580
600

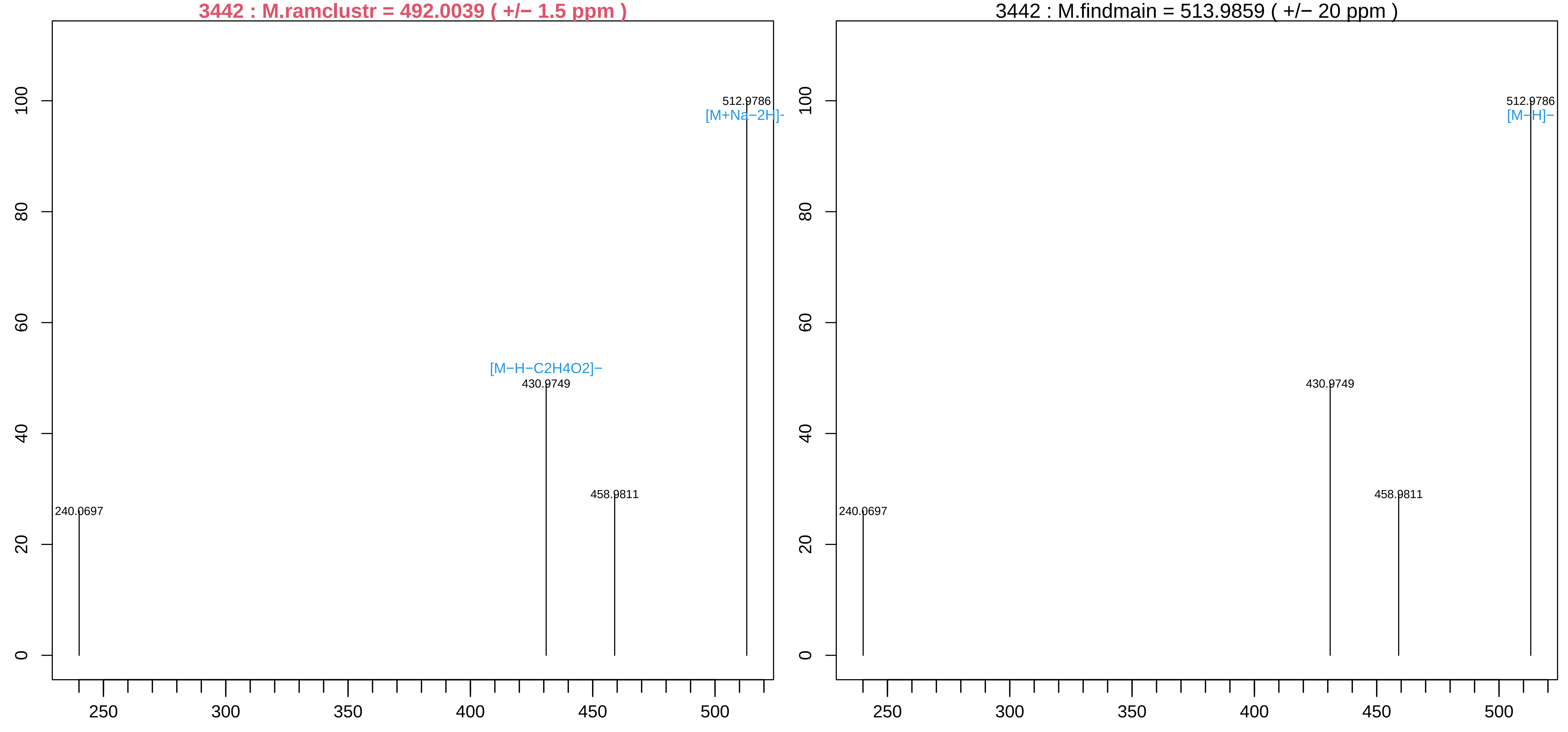
3442 : M.ramclustr = 492.0039 (+/− 1.5 ppm)
512.9786
[M+Na−2H]-
[M−H−C2H4O2]−
430.9749
458.9811
240.0697
3442 : M.findmain = 513.9859 (+/− 20 ppm)
512.9786
[M−H]−
430.9749
458.9811
240.0697
0
20
40
60
80
100
250
300
350
400
450
500

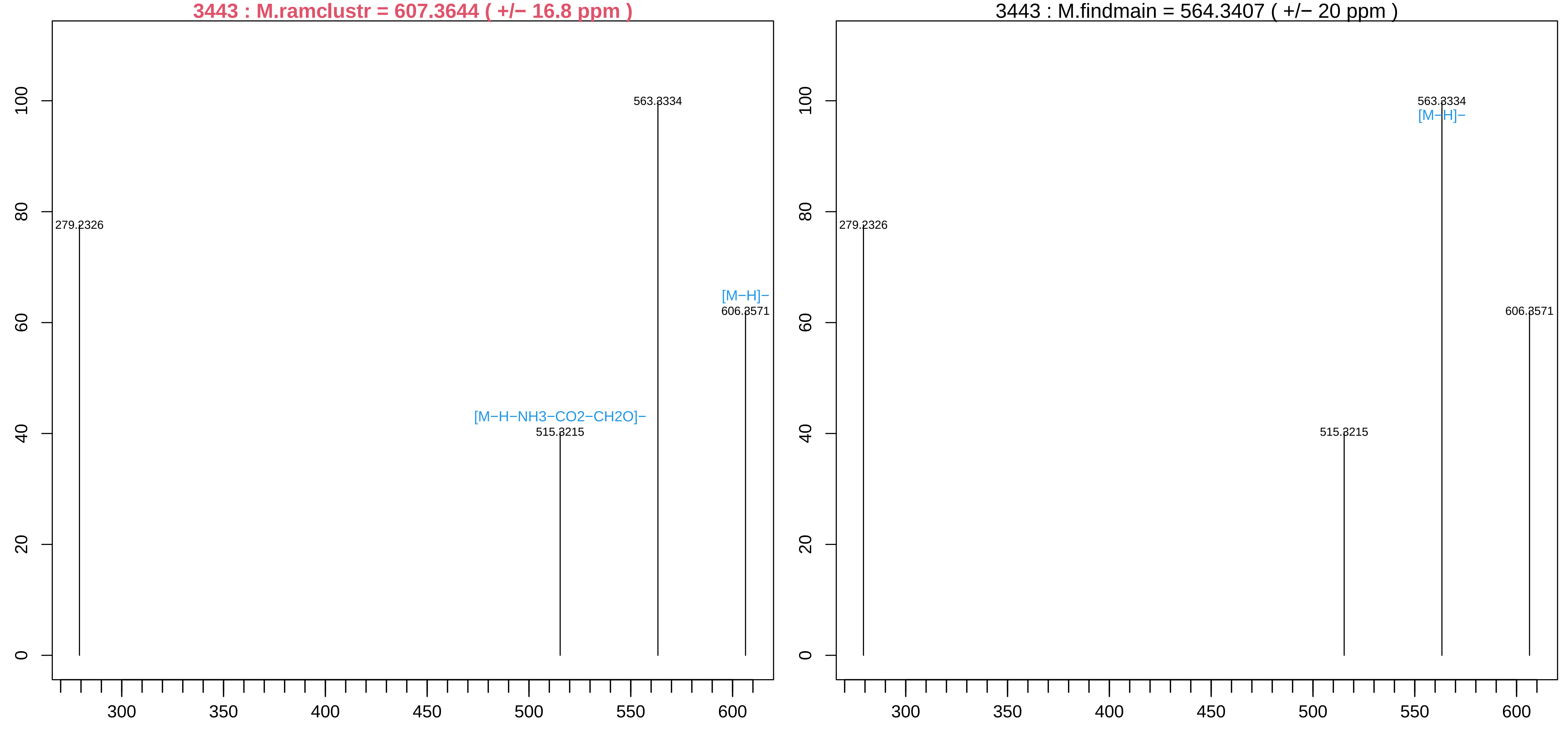
3443 : M.ramclustr = 607.3644 (+/− 16.8 ppm)
279.2326
[M−H−NH3−CO2−CH2O]−
515.3215
563.3334
[M−H]−
606.3571
0
20
40
60
80
100
300
350
400
450
500
550
600
3443 : M.findmain = 564.3407 (+/− 20 ppm)
279.2326
515.3215
563.3334
[M−H]−
606.3571
0
20
40
60
80
100
300
350
400
450
500
550
600

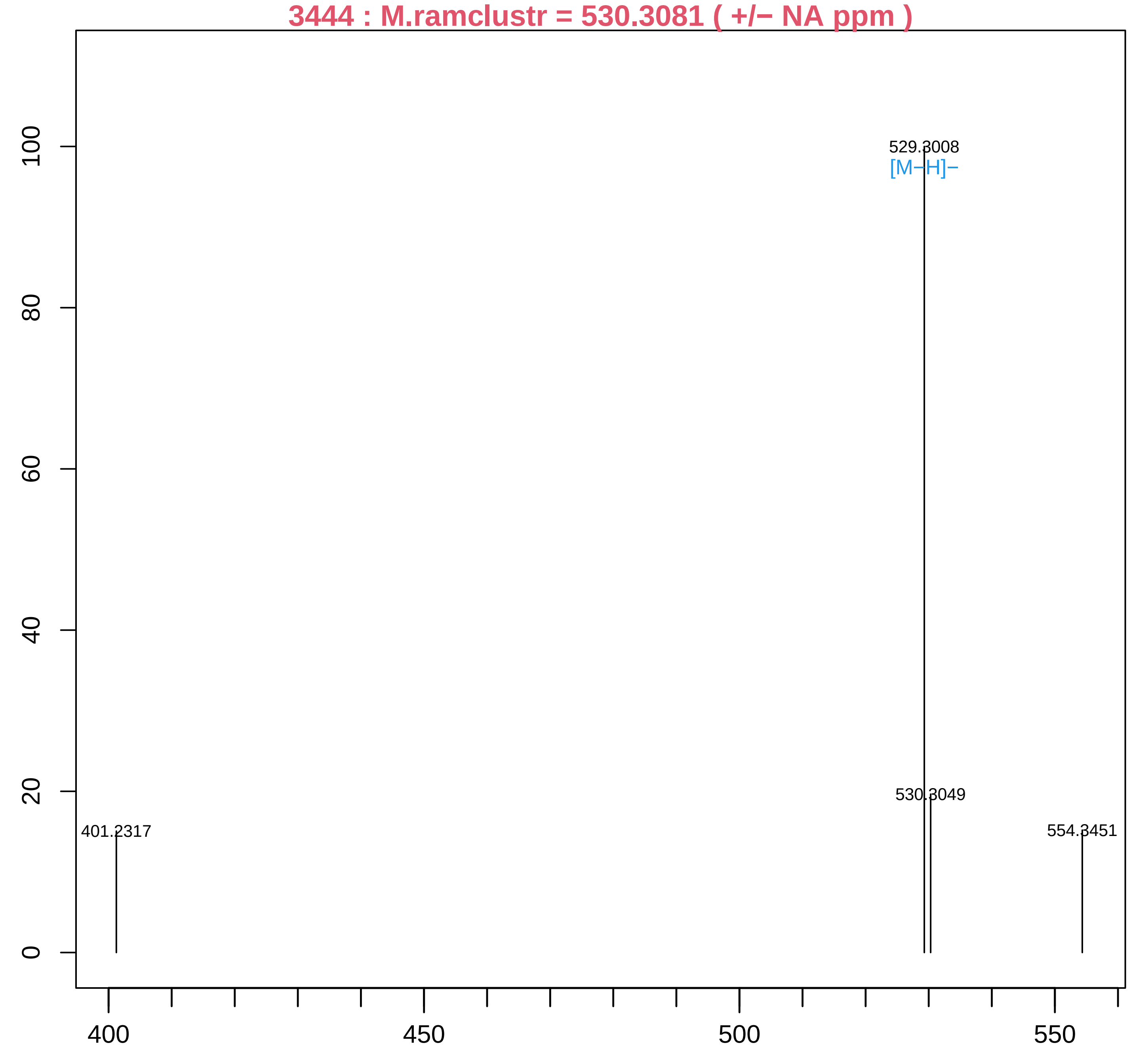
3444 : M.ramclustr = 530.3081 (+/− NA ppm)
529.3008
[M−H]−
530.3049
401.2317
554.3451
0
20
40
60
80
100
400
450
500
550

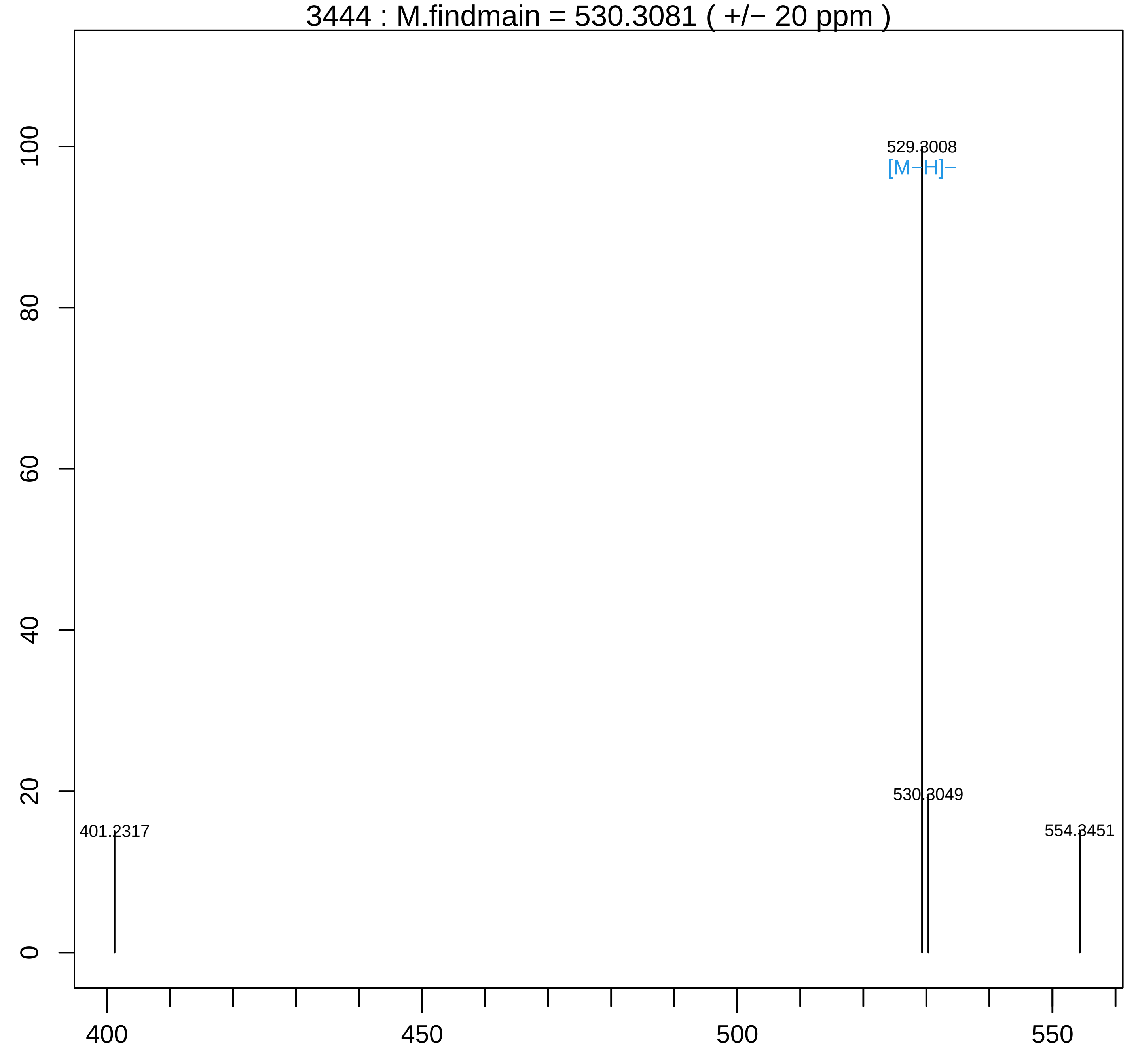
3444 : M.findmain = 530.3081 (+/− 20 ppm)
529.3008
[M−H]−
530.3049
401.2317
554.3451
0
20
40
60
80
100
400
450
500
550

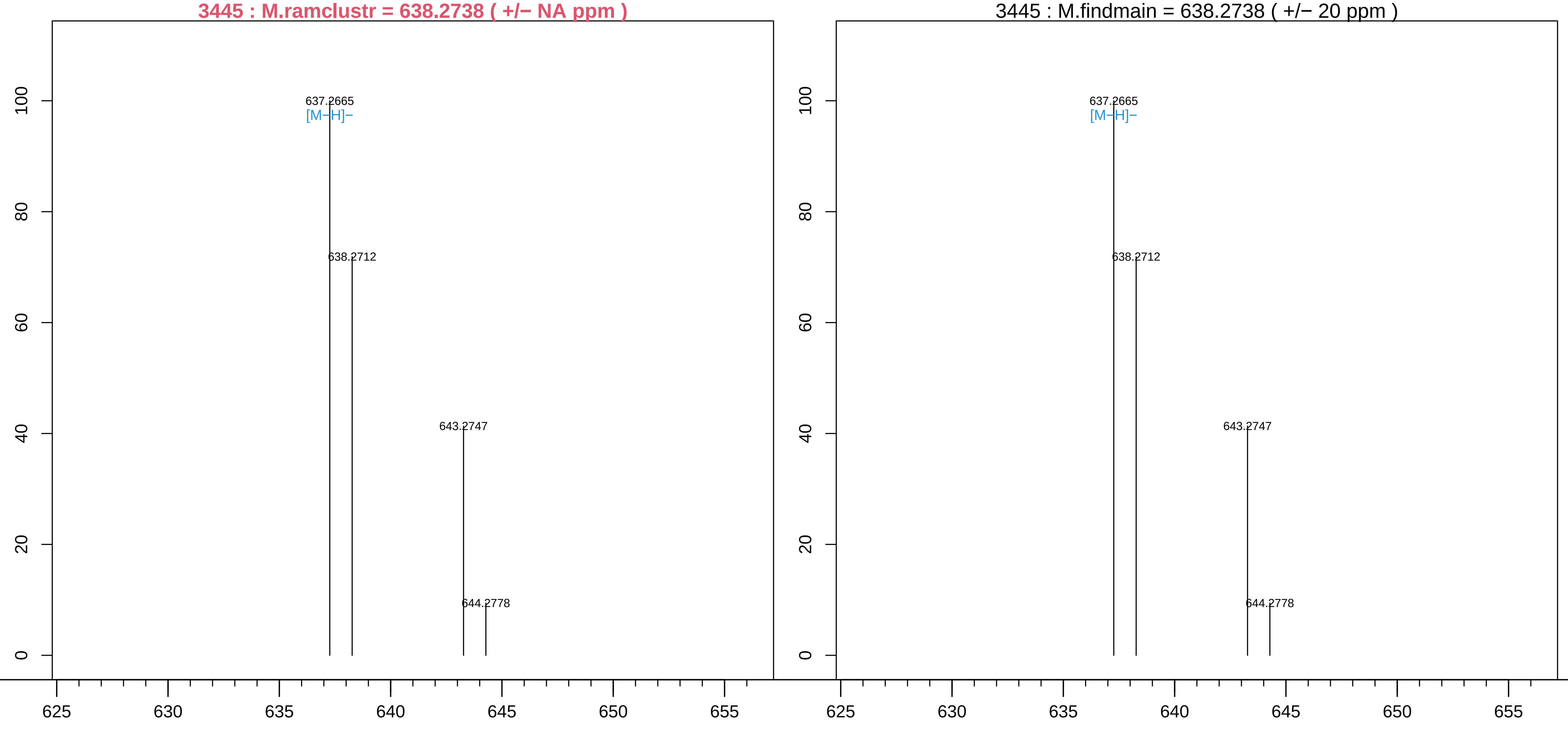
3445 : M.ramclustr = 638.2738 (+/− NA ppm)
3445 : M.findmain = 638.2738 (+/− 20 ppm)
637.2665
[M−H]−
638.2712
643.2747
644.2778
0
20
40
60
80
100
625
630
635
640
645
650
655

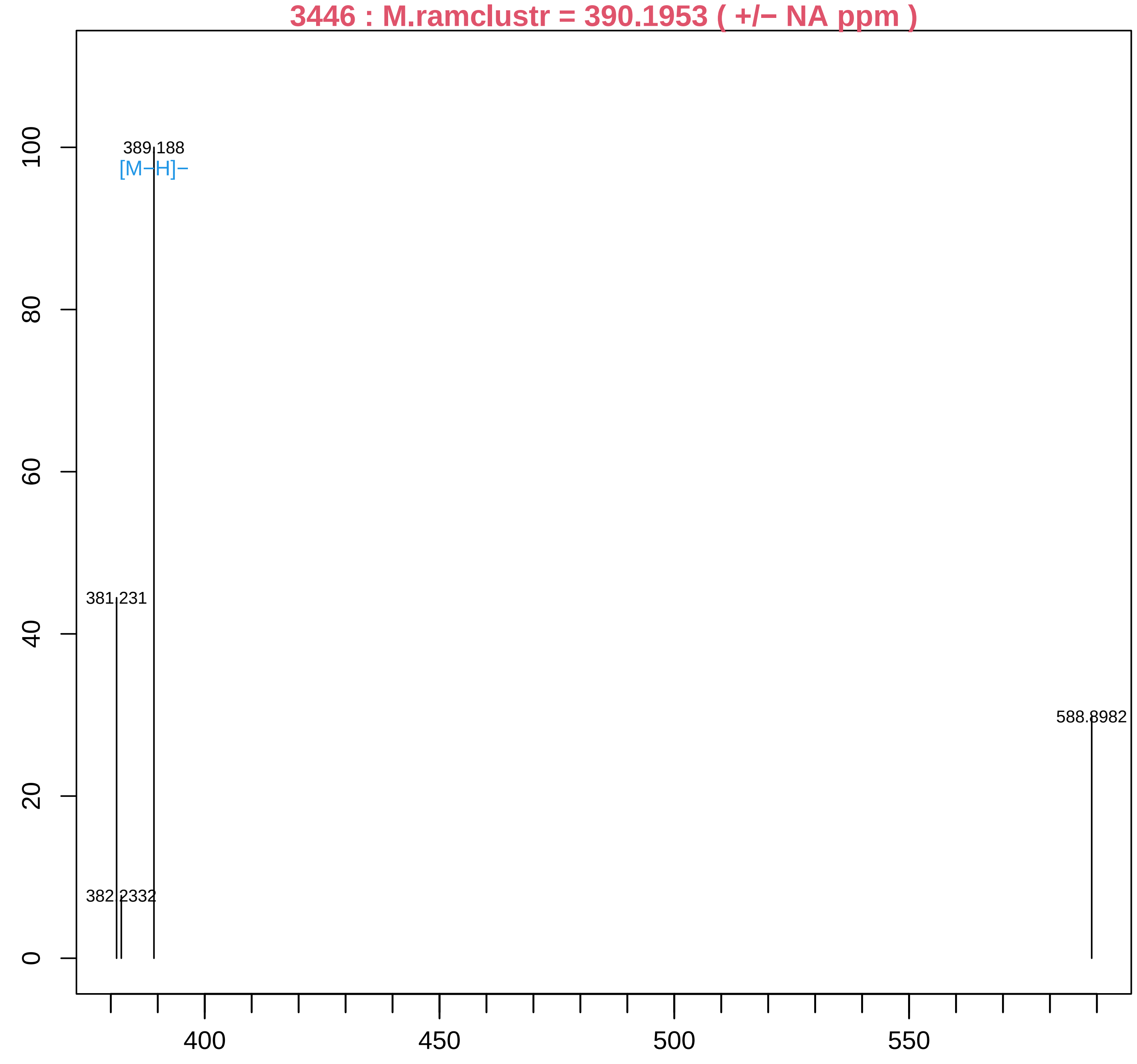
3446 : M.ramclustr = 390.1953 (+/− NA ppm)
389.188
[M−H]−
381.231
382.2332
588.8982
0
20
40
60
80
100
400
450
500
550

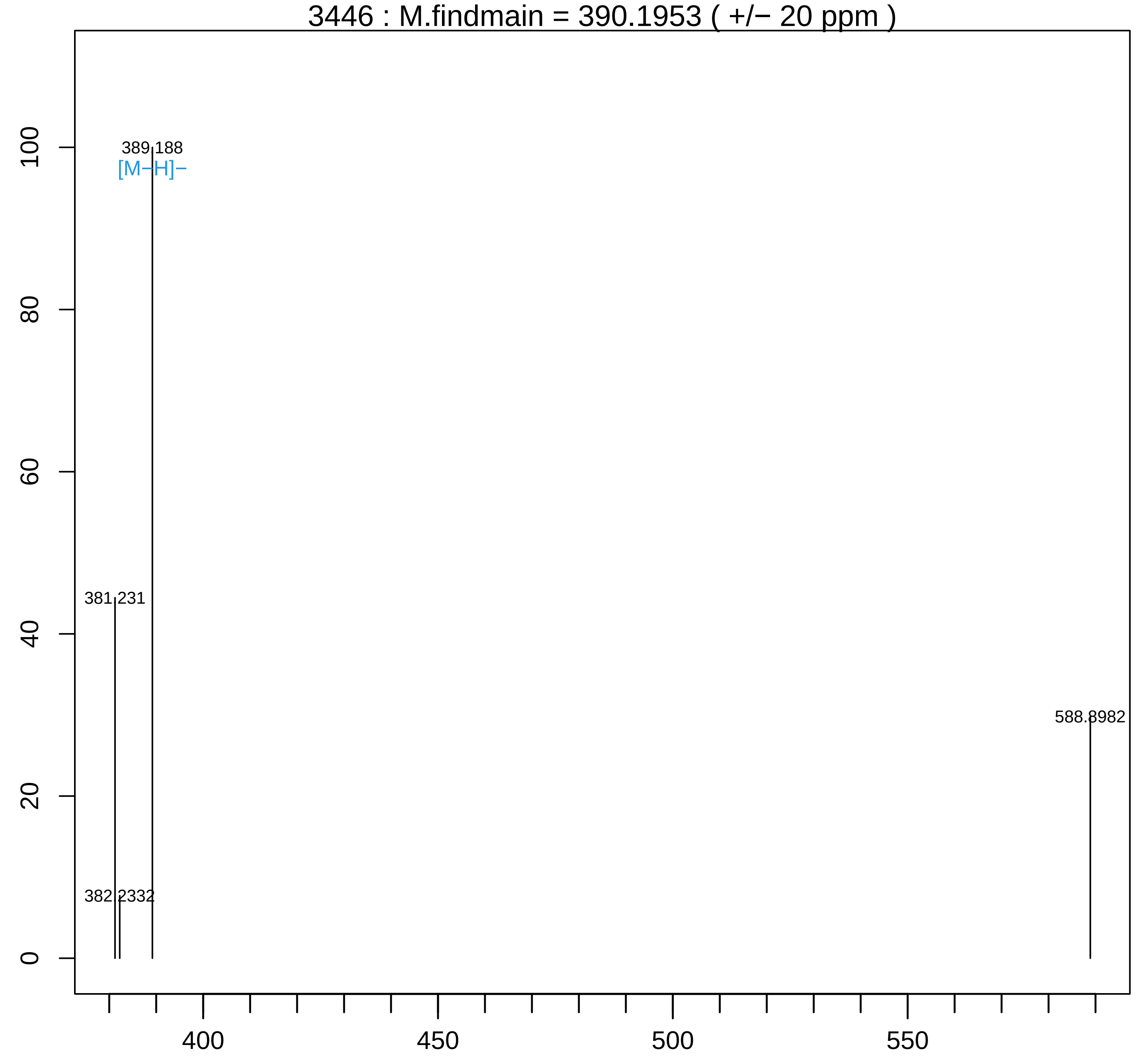
3446 : M.findmain = 390.1953 (+/− 20 ppm)
389.188
[M−H]−
381.231
382.2332
588.8982
0
20
40
60
80
100
400
450
500
550

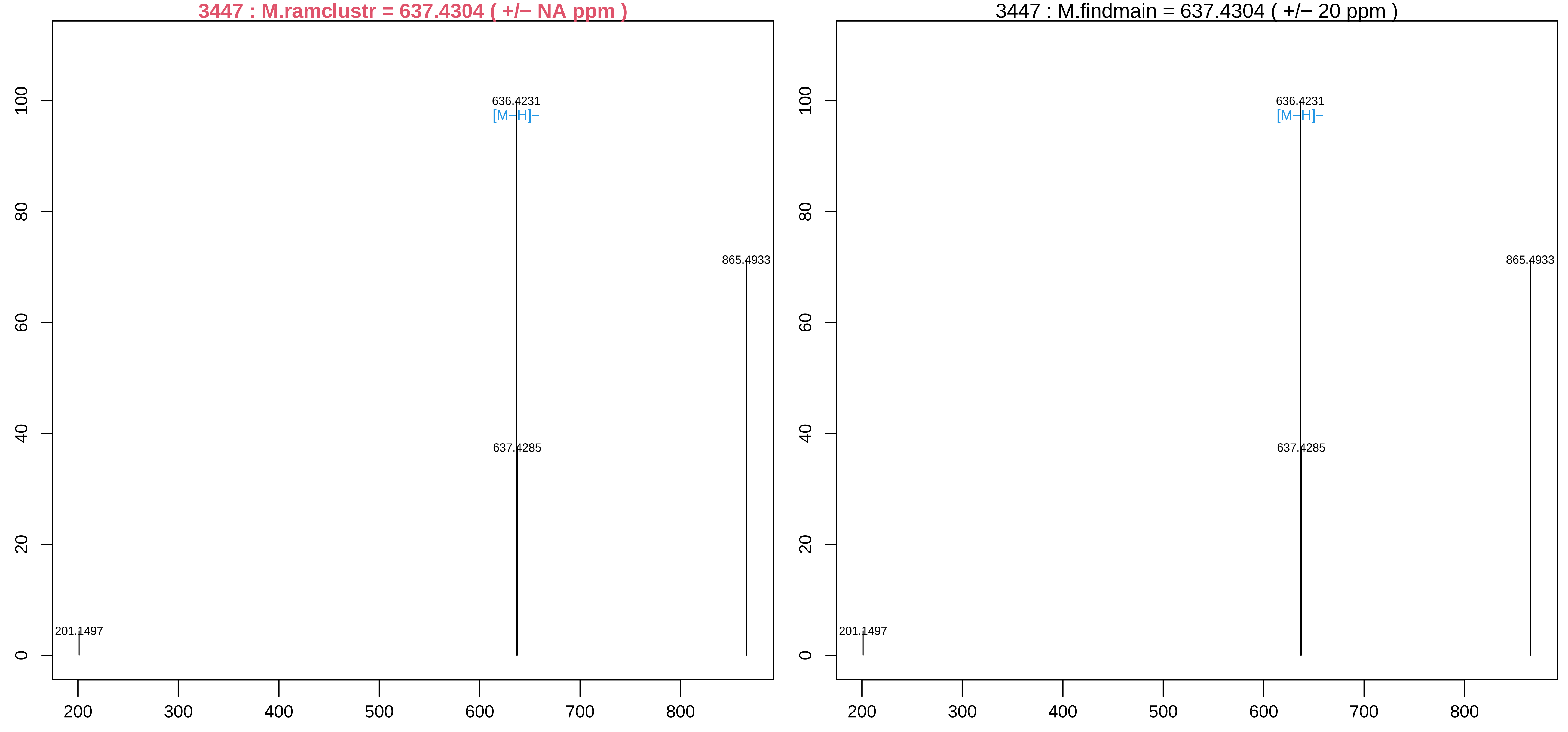
3447 : M.ramclustr = 637.4304 (+/− NA ppm)
636.4231
[M−H]−
865.4933
637.4285
201.1497
3447 : M.findmain = 637.4304 (+/− 20 ppm)
636.4231
[M−H]−
865.4933
637.4285
201.1497
0
20
40
60
80
100
200
300
400
500
600
700
800

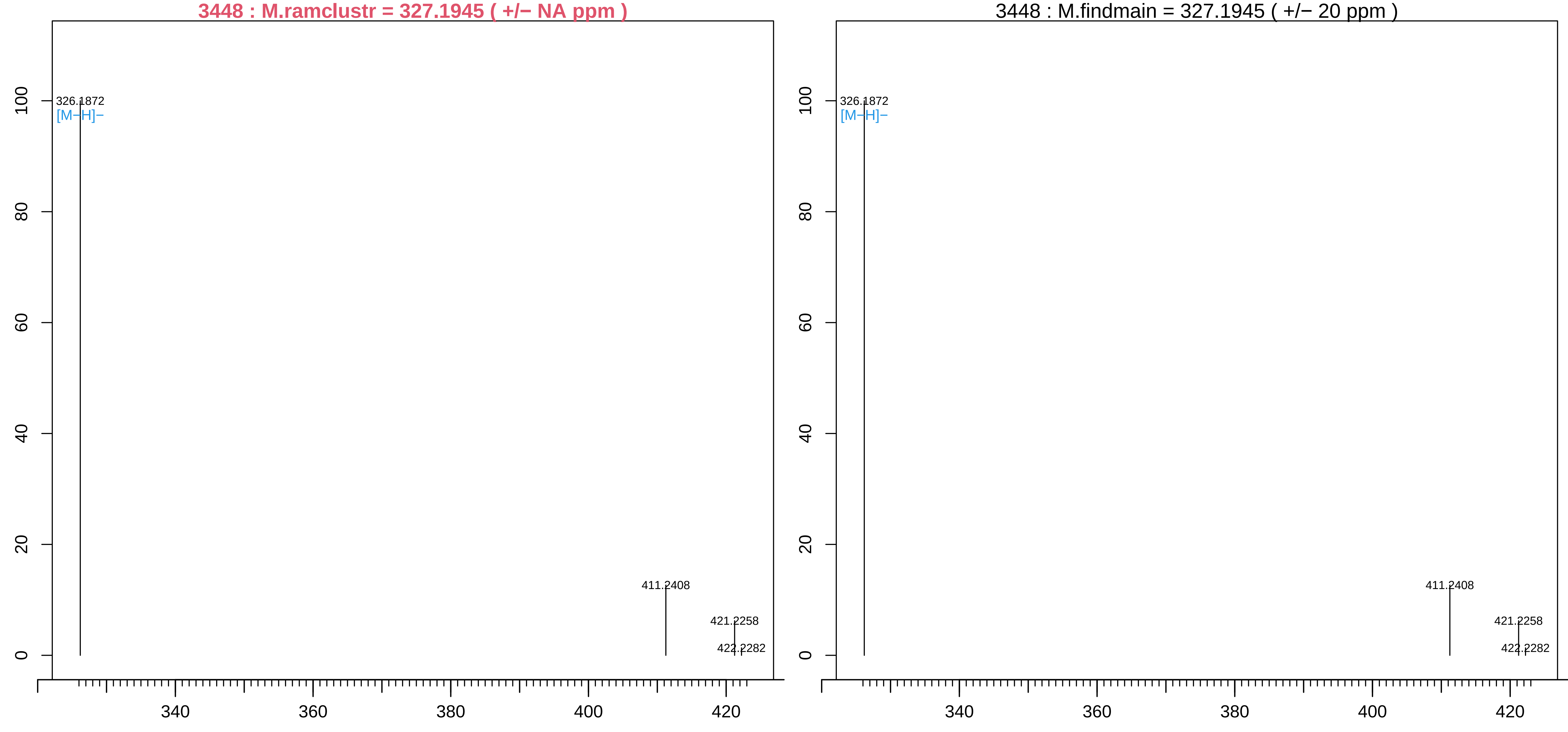
3448 : M.ramclustr = 327.1945 (+/− NA ppm)
326.1872
[M−H]−
411.2408
421.2258
422.2282
0
20
40
60
80
100
340
360
380
400
420
3448 : M.findmain = 327.1945 (+/− 20 ppm)
326.1872
[M−H]−
411.2408
421.2258
422.2282
0
20
40
60
80
100
340
360
380
400
420

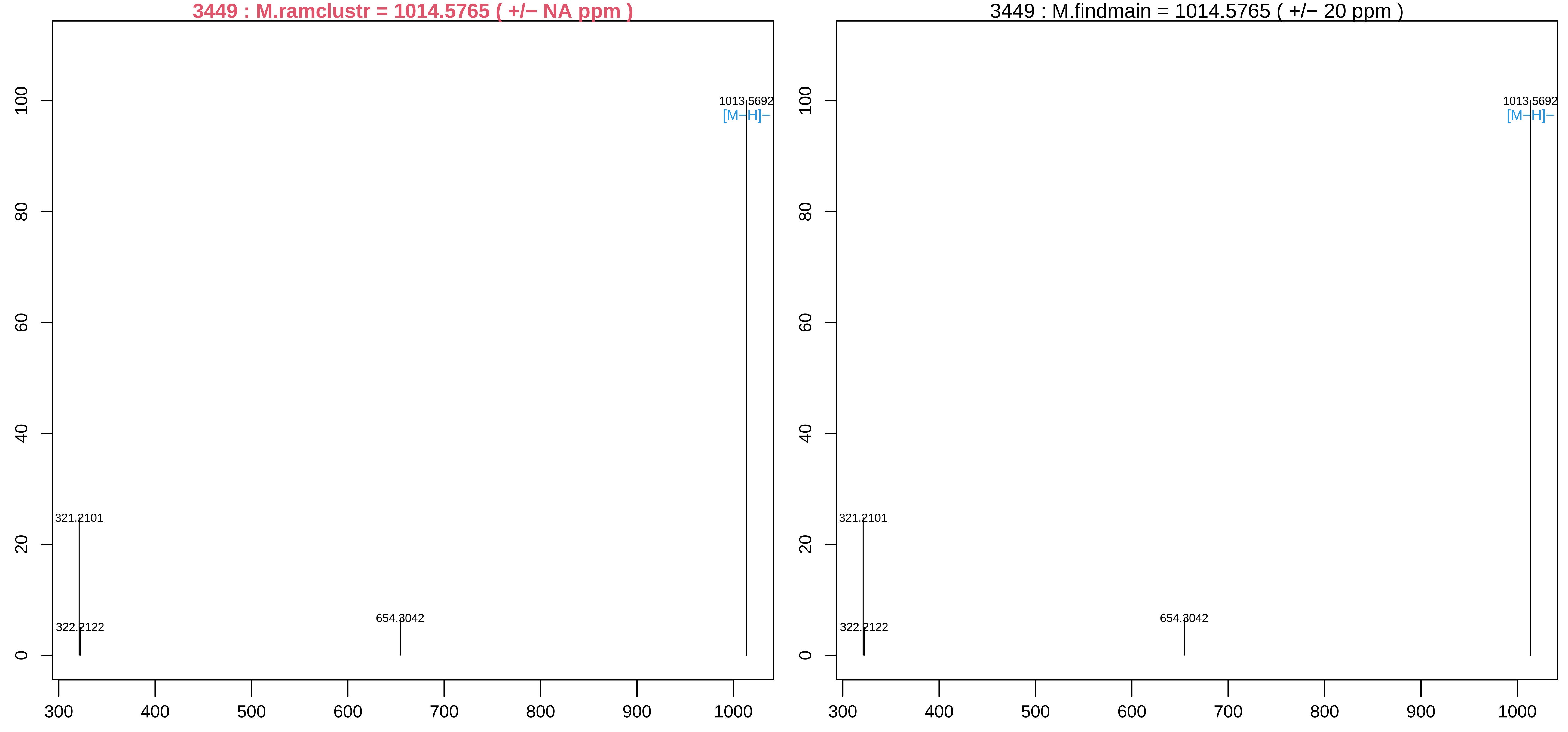
3449 : M.ramclustr = 1014.5765 (+/− NA ppm)
1013.5692
[M−H]−
321.2101
322.2122
654.3042
0
20
40
60
80
100
300
400
500
600
700
800
900
1000
3449 : M.findmain = 1014.5765 (+/− 20 ppm)
1013.5692
[M−H]−
321.2101
322.2122
654.3042
0
20
40
60
80
100
300
400
500
600
700
800
900
1000

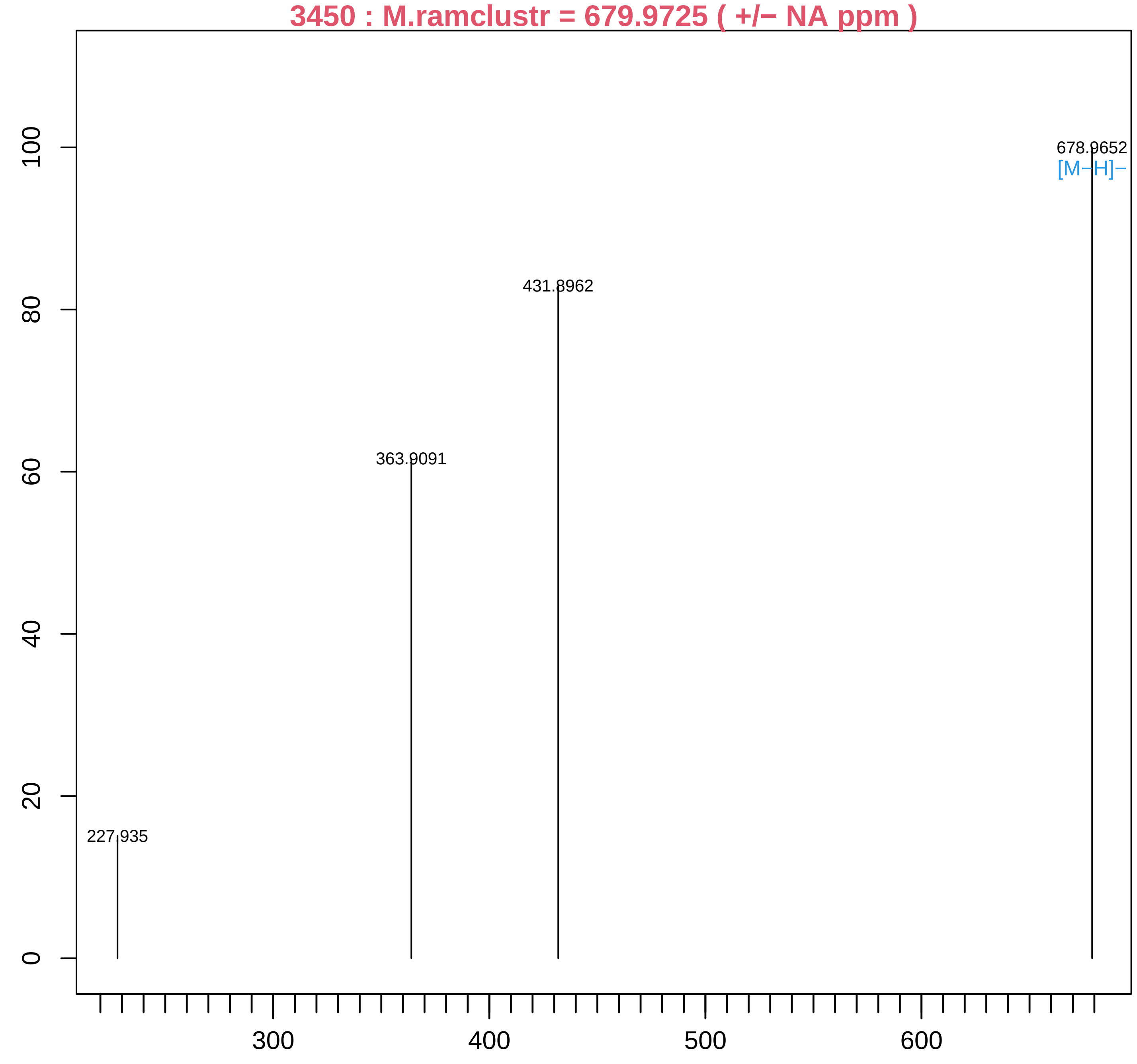

3450 : M.ramclustr = 679.9725 (+/− NA ppm)
678.9652
[M−H]−
431.8962
363.9091
227.935
0
20
40
60
80
100
300
400
500
600

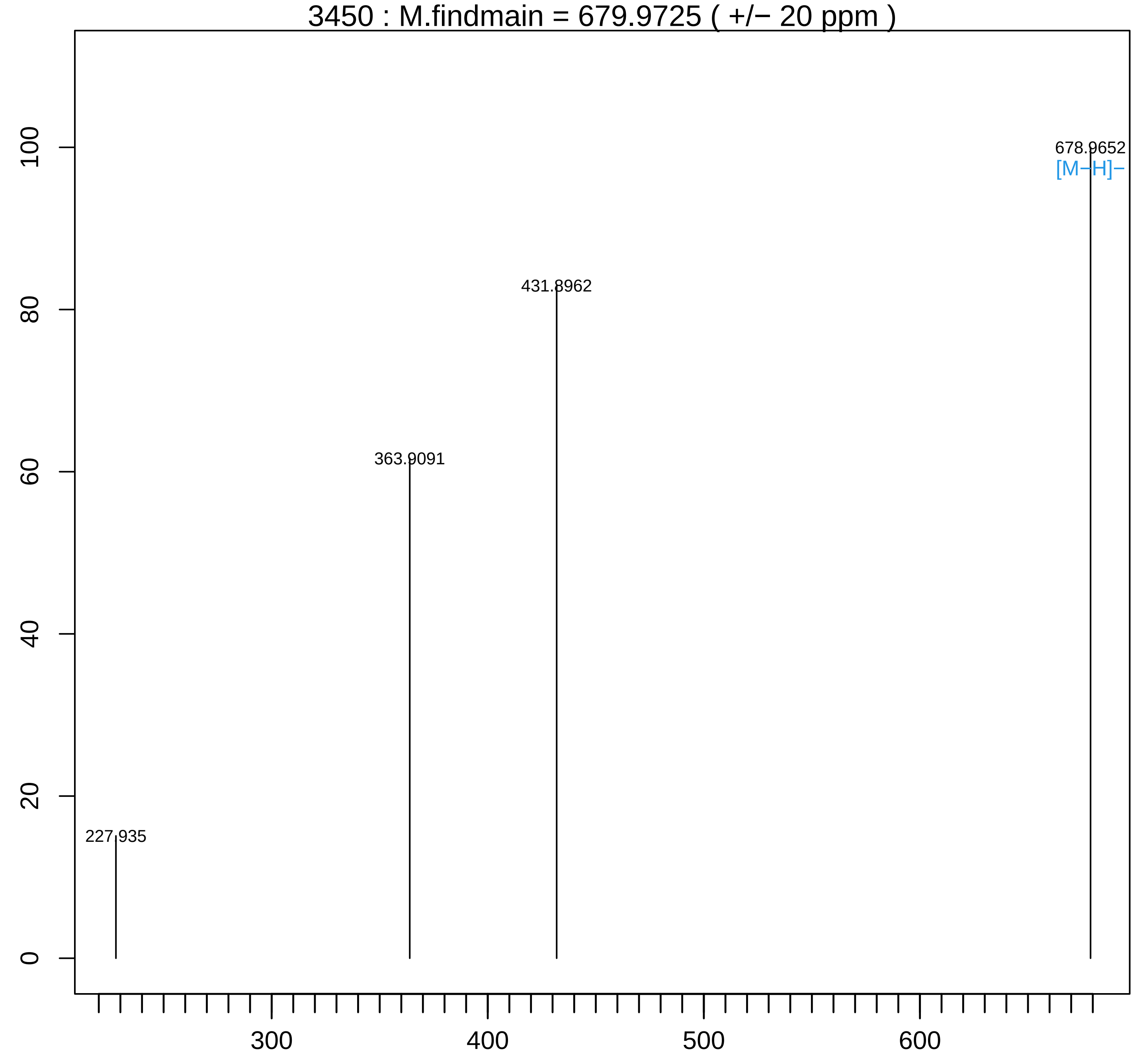

3450 : M.findmain = 679.9725 (+/− 20 ppm)
678.9652
[M−H]−
431.8962
363.9091
227.935
0
20
40
60
80
100
300
400
500
600

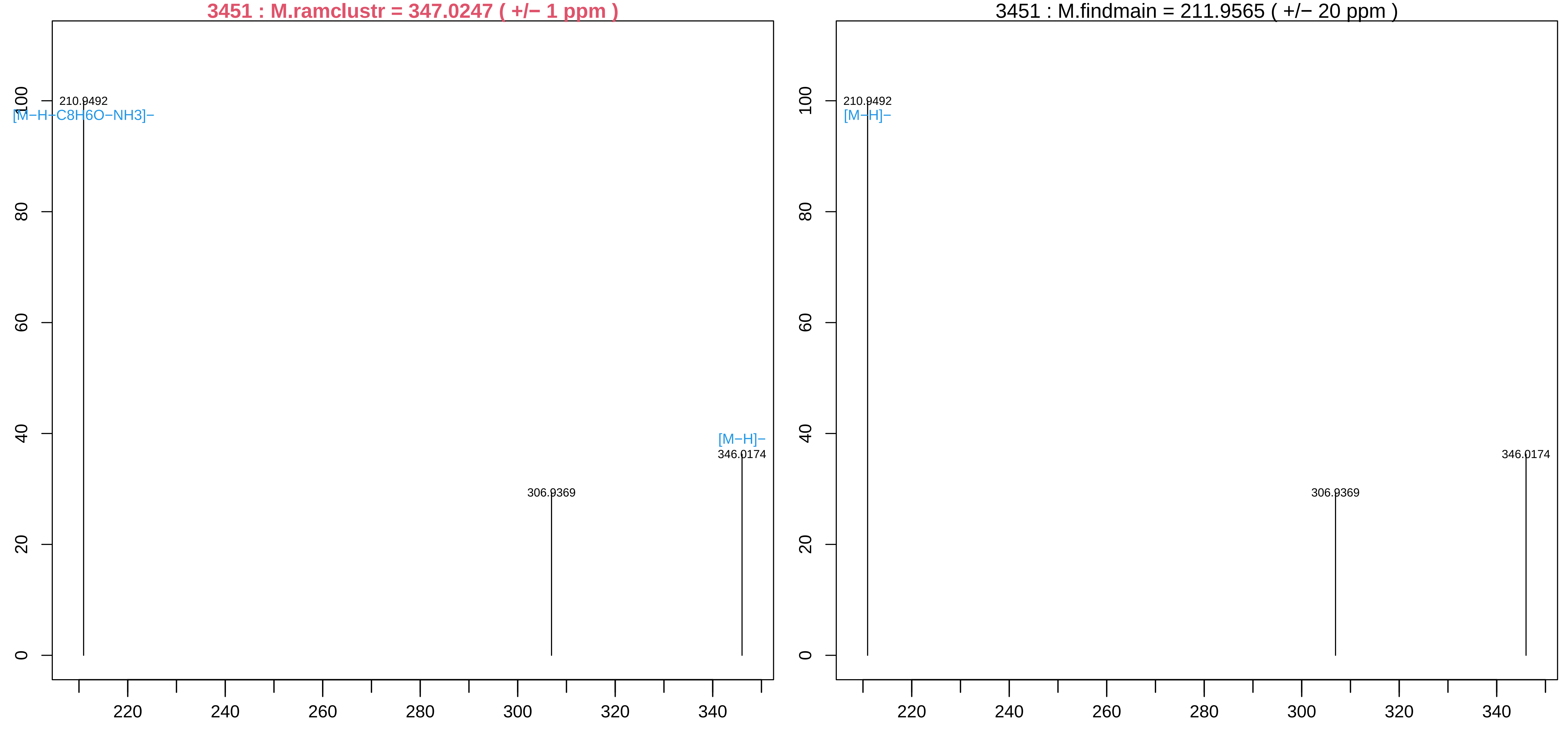
3451 : M.ramclustr = 347.0247 (+/− 1 ppm)
210.9492
[M−H−C8H6O−NH3]−
306.9369
[M−H]−
346.0174
3451 : M.findmain = 211.9565 (+/− 20 ppm)
210.9492
[M−H]−
306.9369
346.0174
0
20
40
60
80
100
220
240
260
280
300
320
340

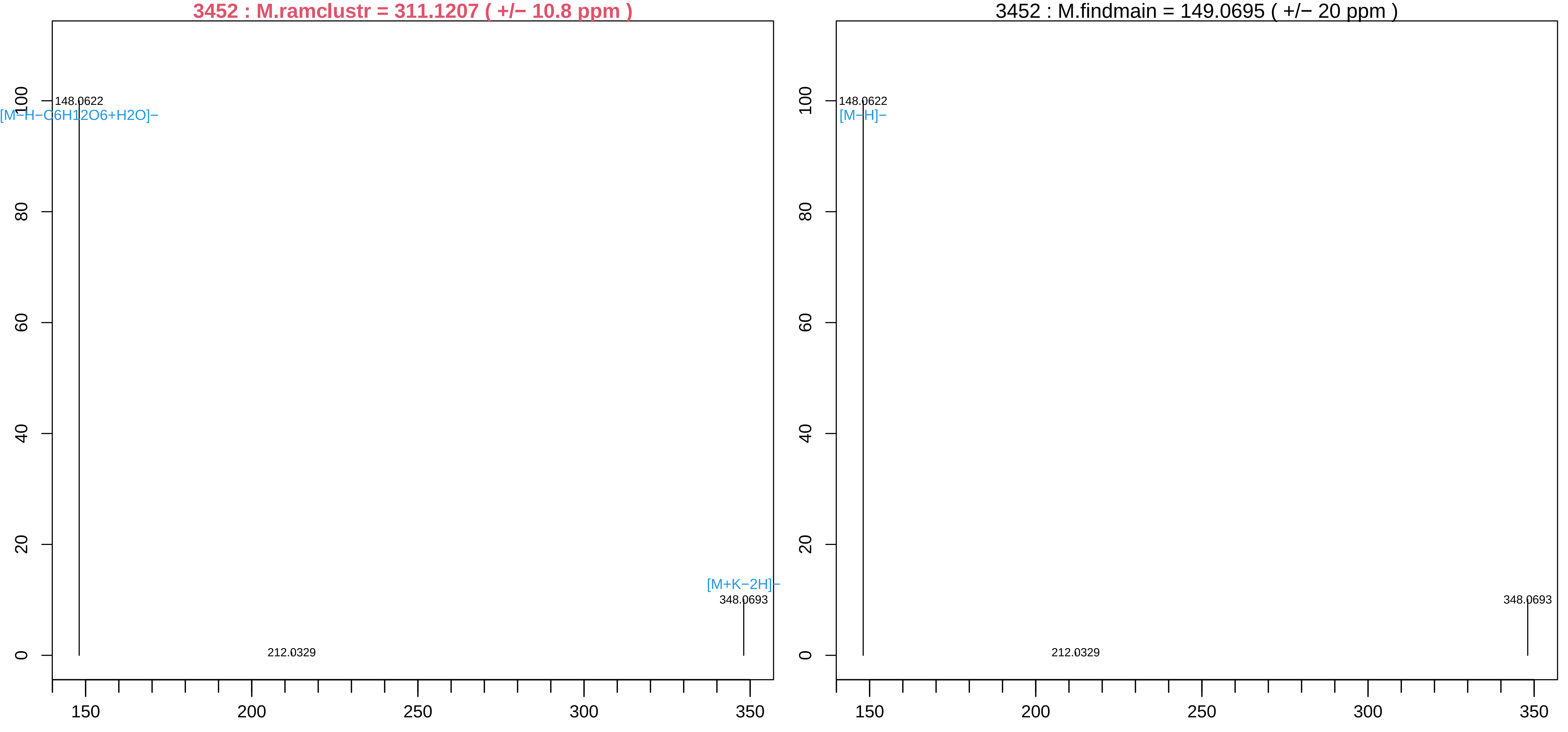
3452 : M.ramclustr = 311.1207 (+/− 10.8 ppm)
148.0622
[M−H−C6H12O6+H2O]−
[M+K−2H]−
348.0693
212.0329
3452 : M.findmain = 149.0695 (+/− 20 ppm)
148.0622
[M−H]−
348.0693
212.0329

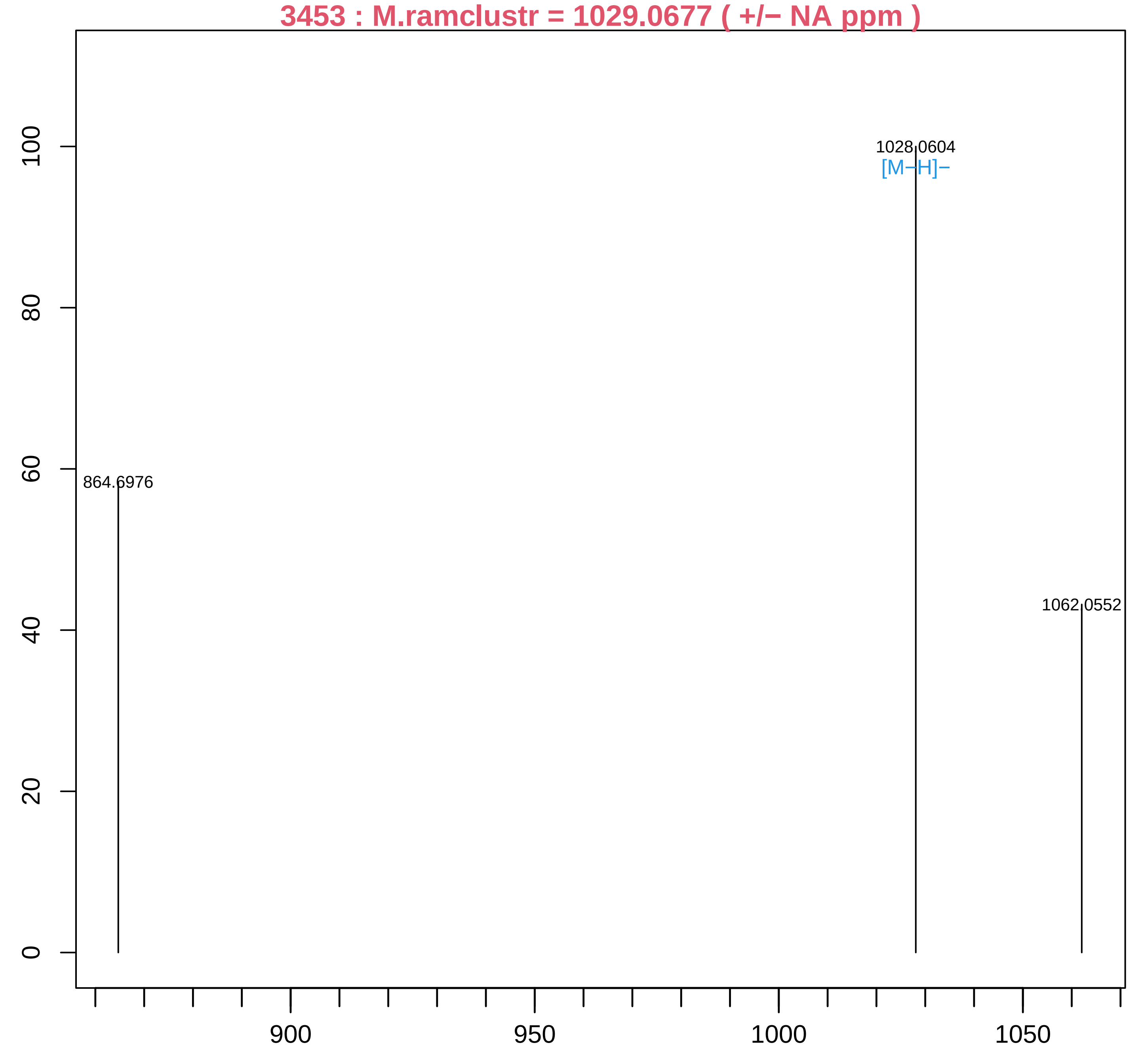
3453 : M.ramclustr = 1029.0677 (+/− NA ppm)
1028.0604
[M−H]−
864.6976
1062.0552
0
20
40
60
80
100
900
950
1000
1050

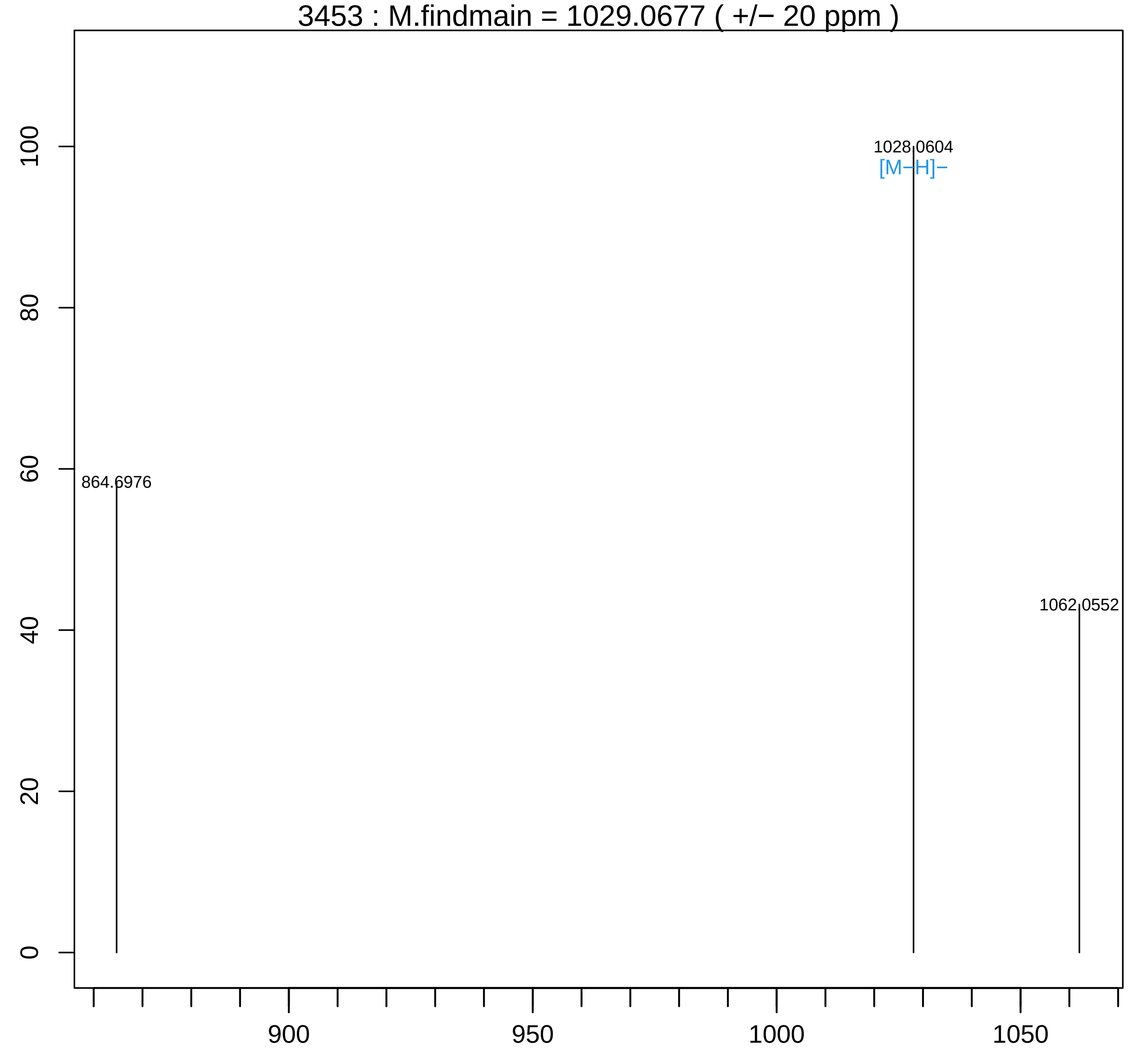
3453 : M.findmain = 1029.0677 (+/− 20 ppm)
1028.0604
[M−H]−
864.6976
1062.0552
0
20
40
60
80
100
900
950
1000
1050

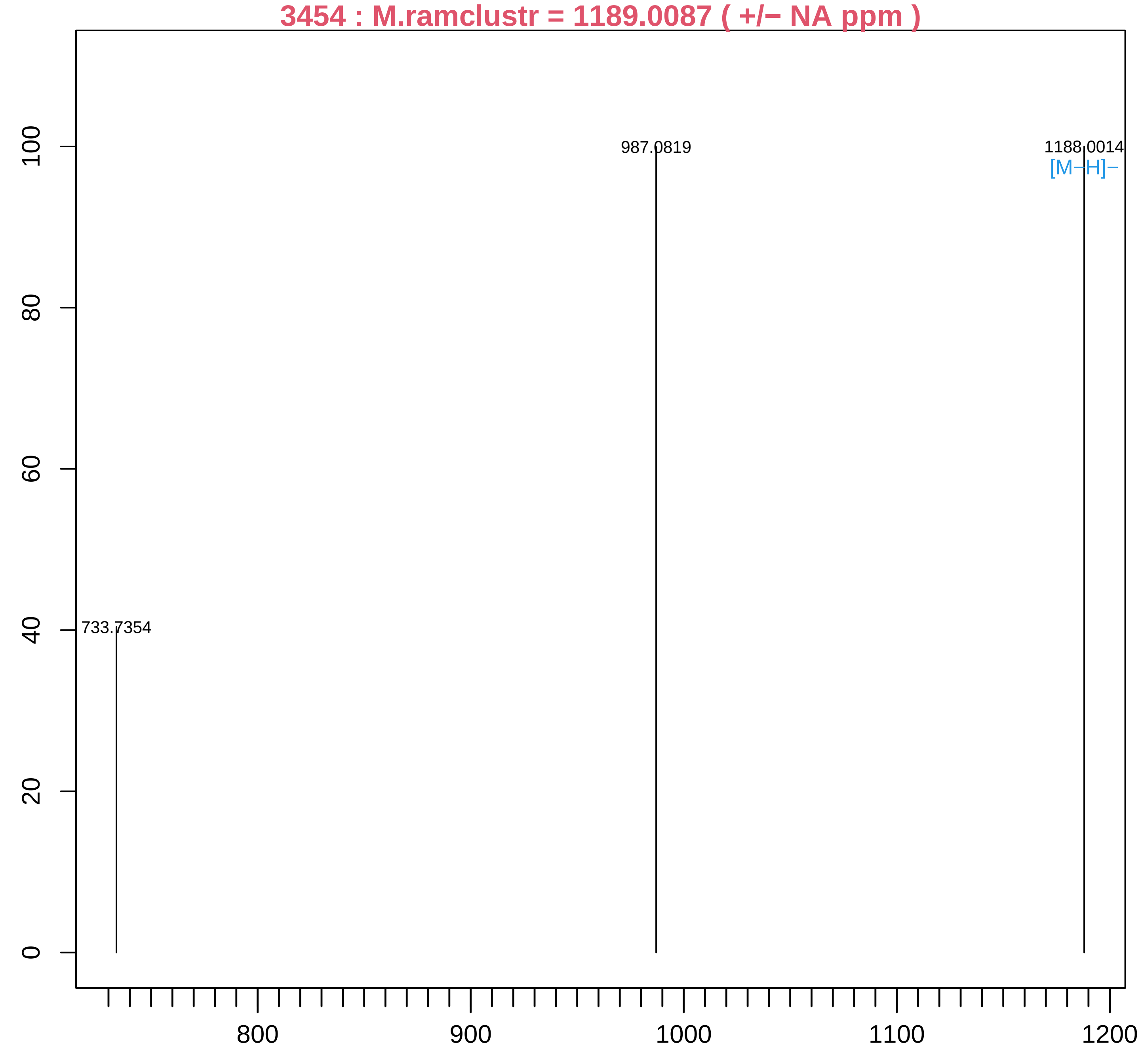
3454 : M.ramclustr = 1189.0087 (+/− NA ppm)
987.0819
1188.0014
[M−H]−
733.7354
0
20
40
60
80
100
800
900
1000
1100
1200

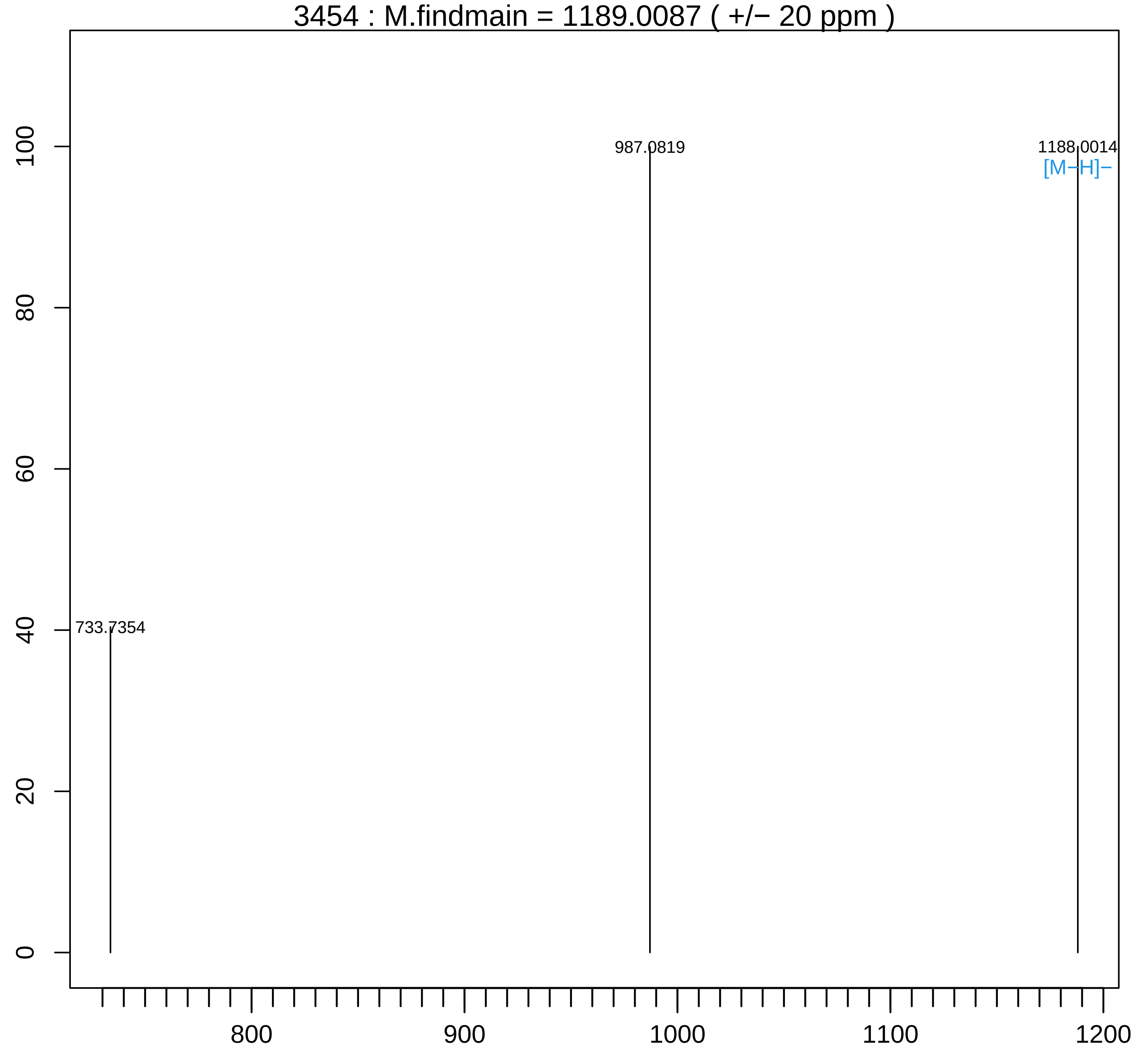
3454 : M.findmain = 1189.0087 (+/− 20 ppm)
987.0819
1188.0014
[M−H]−
733.7354
0
20
40
60
80
100
800
900
1000
1100
1200

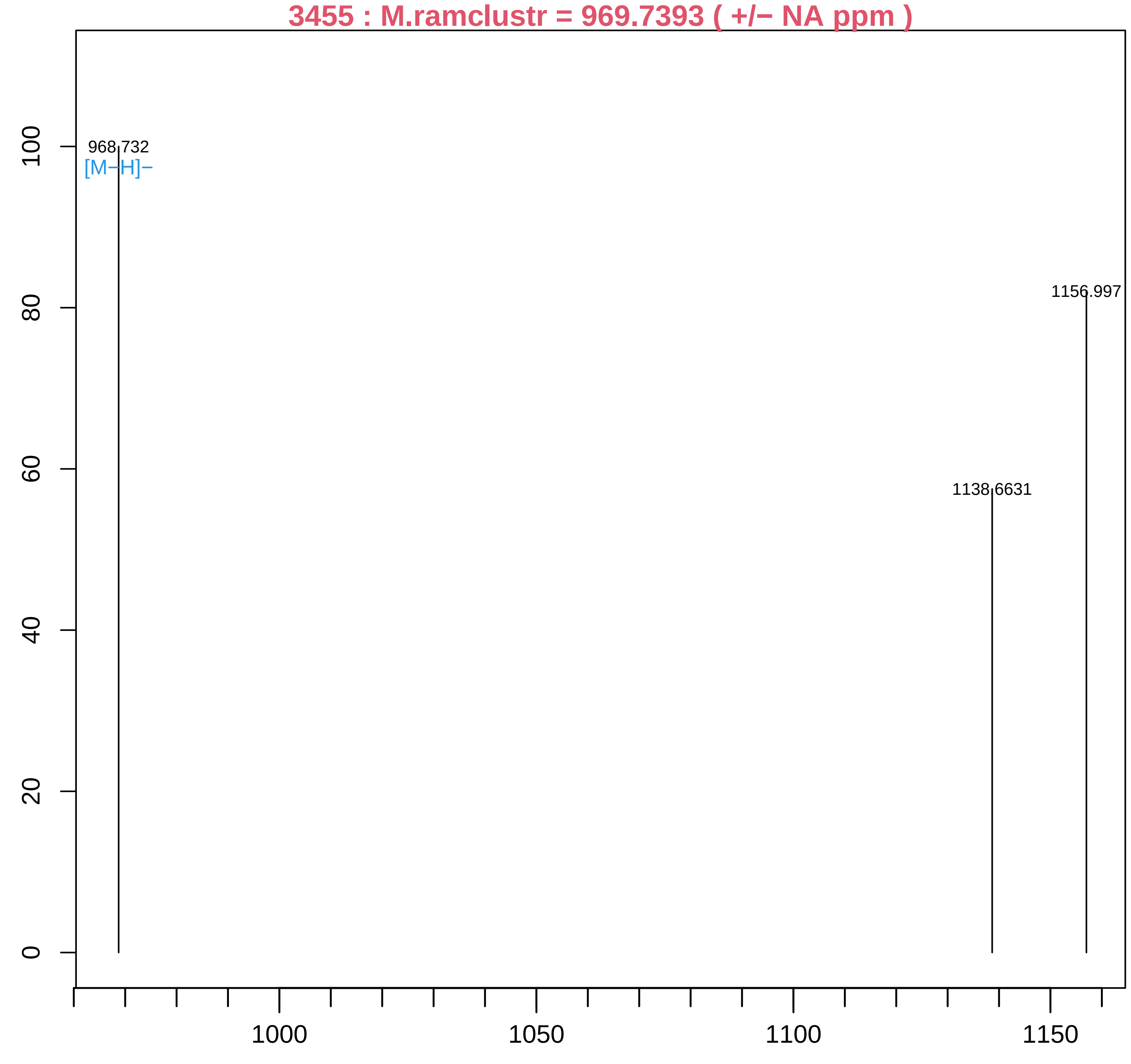
3455 : M.ramclustr = 969.7393 (+/− NA ppm)
968.732
[M−H]−
1138.6631
1156.997
0
20
40
60
80
100
1000
1050
1100
1150

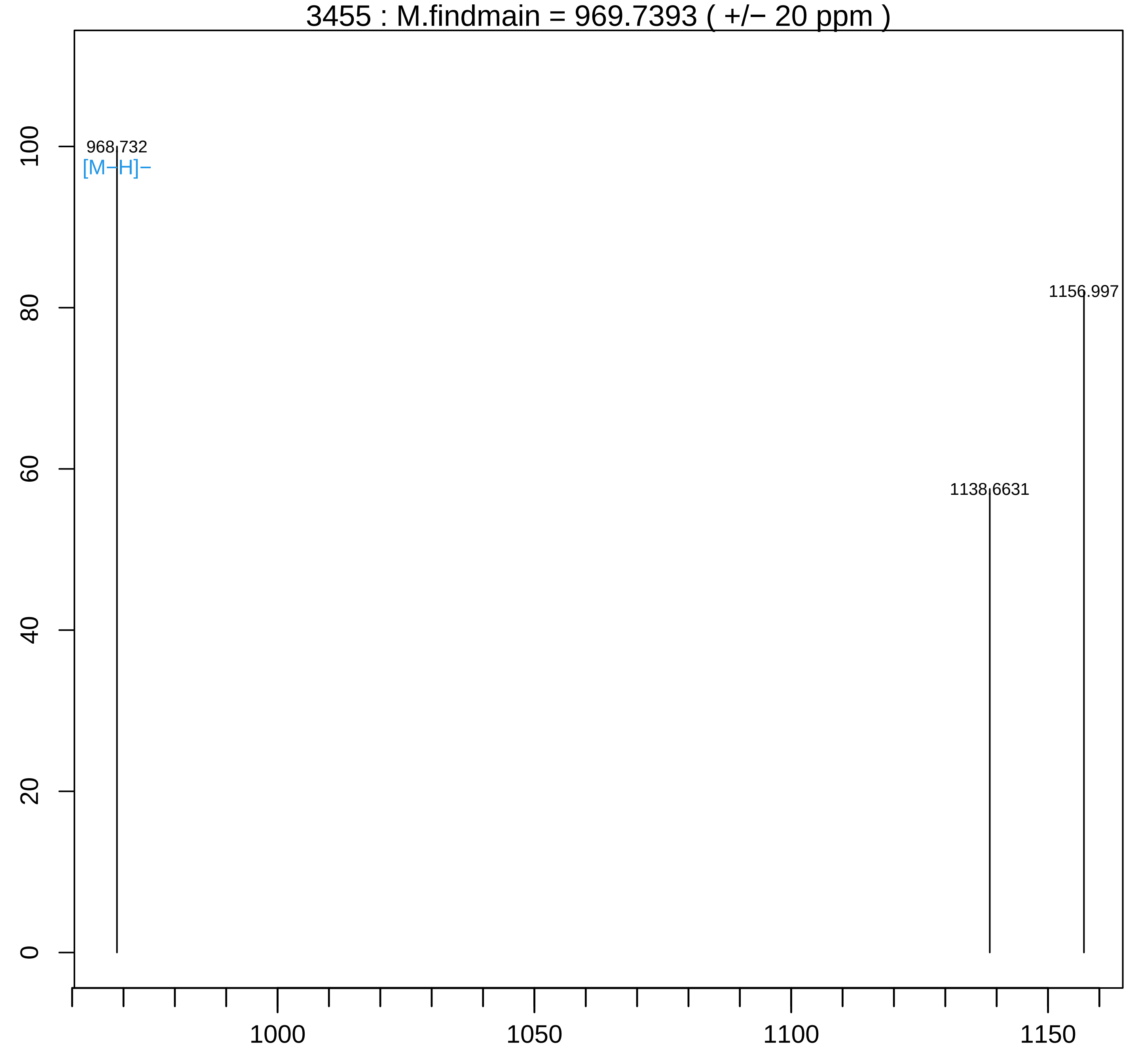
3455 : M.findmain = 969.7393 (+/− 20 ppm)
968.732
[M−H]−
1138.6631
1156.997
0
20
40
60
80
100
1000
1050
1100
1150

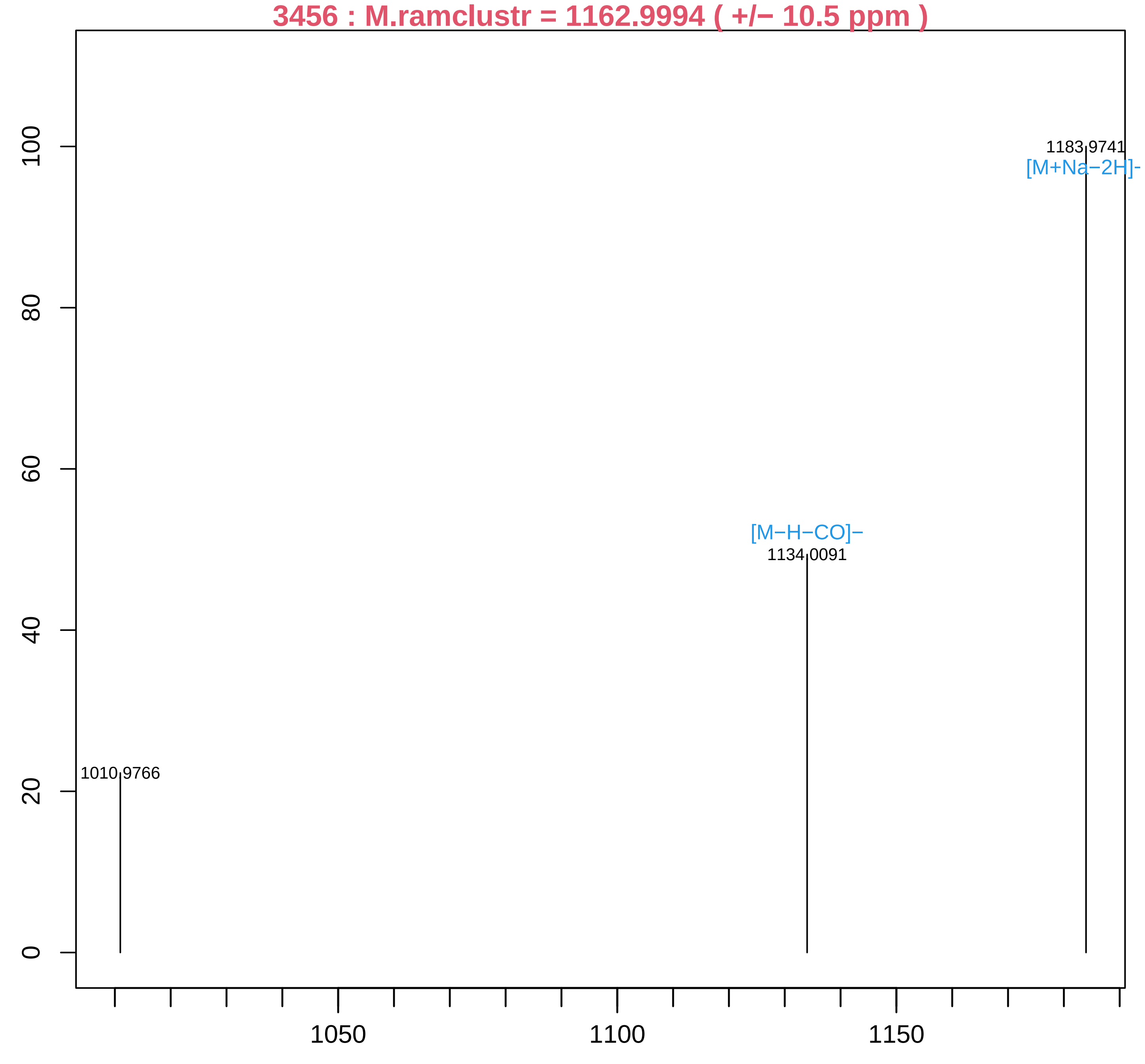
3456 : M.ramclustr = 1162.9994 (+/− 10.5 ppm)
1183.9741
[M+Na−2H]-
[M−H−CO]−
1134.0091
1010.9766
0
20
40
60
80
100
1050
1100
1150

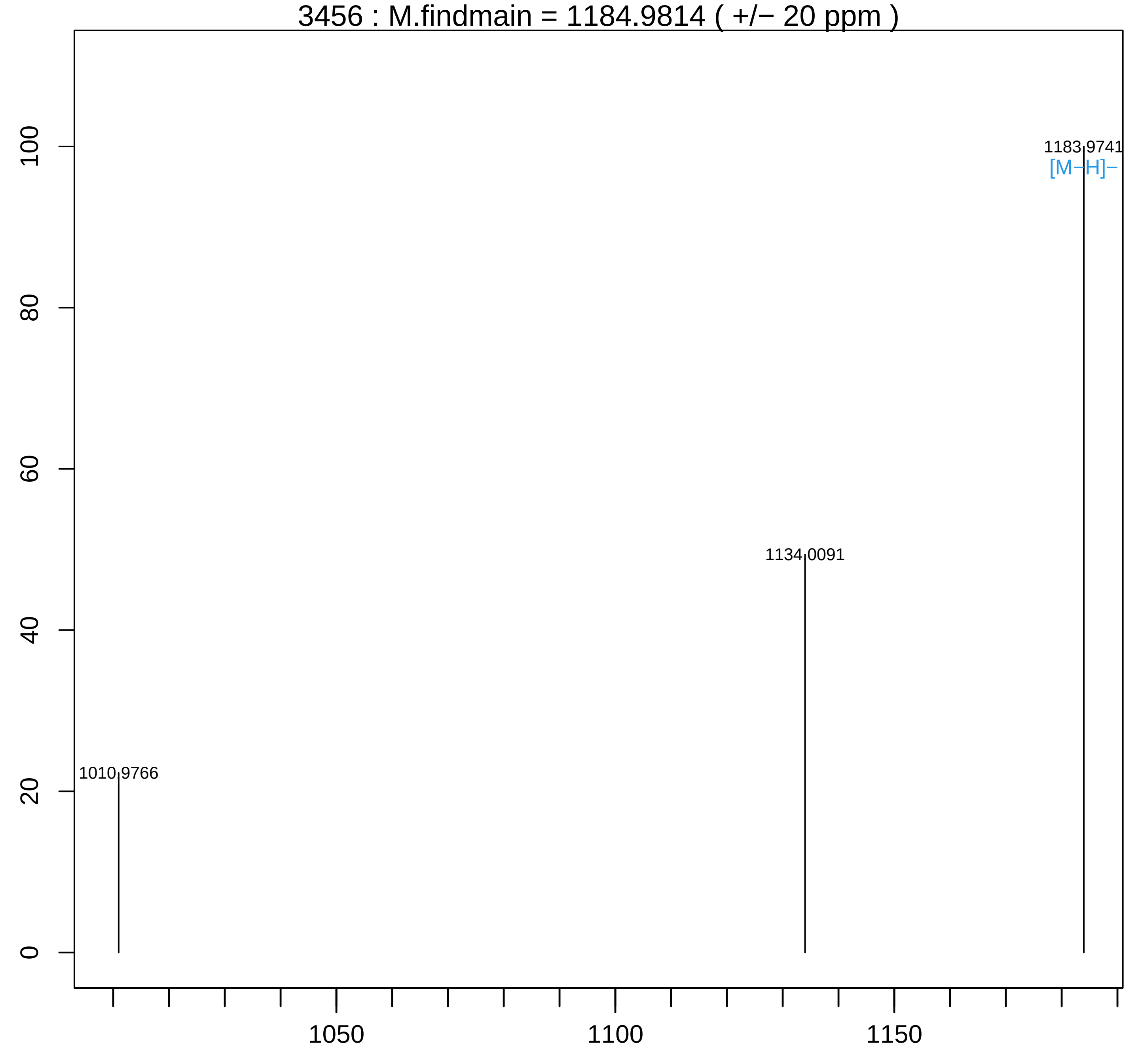
3456 : M.findmain = 1184.9814 (+/− 20 ppm)
1183.9741
[M−H]−
1134.0091
1010.9766
0
20
40
60
80
100
1050
1100
1150

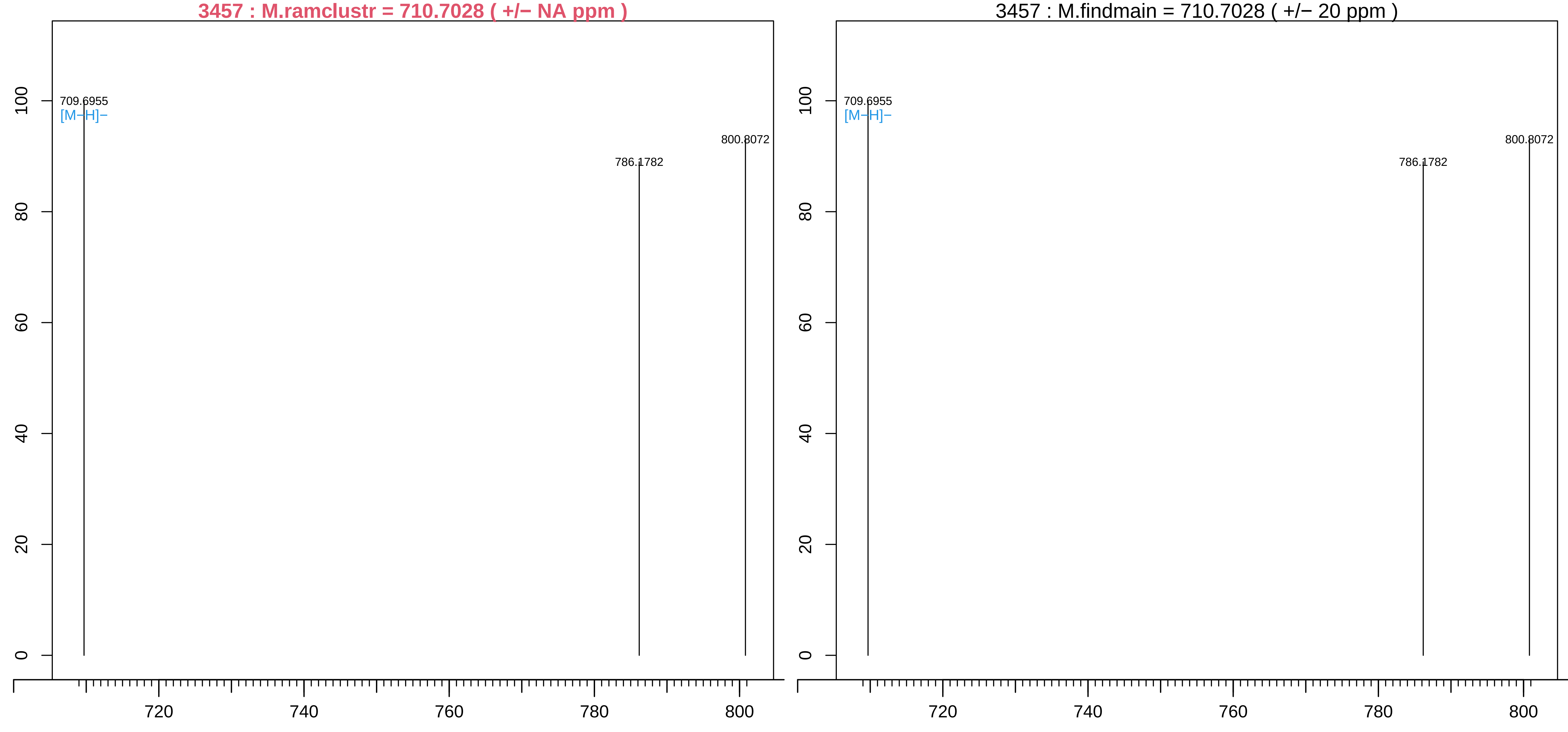
3457 : M.ramclustr = 710.7028 (+/− NA ppm)
709.6955
[M−H]−
786.1782
800.8072
3457 : M.findmain = 710.7028 (+/− 20 ppm)
709.6955
[M−H]−
786.1782
800.8072
0
20
40
60
80
100
720
740
760
780
800

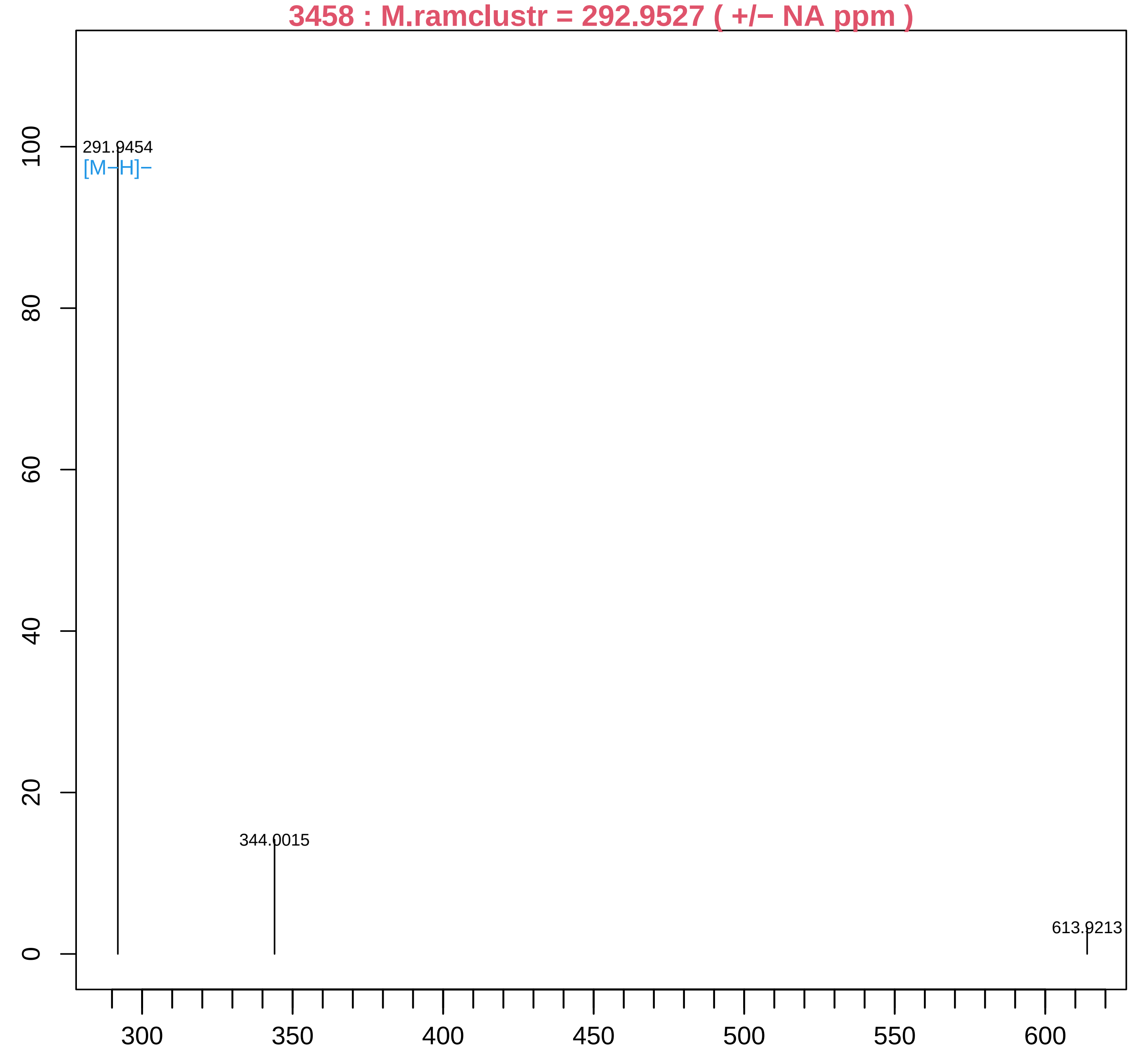
3458 : M.ramclustr = 292.9527 (+/− NA ppm)
291.9454
[M−H]−
344.0015
613.9213
0
20
40
60
80
100
300
350
400
450
500
550
600

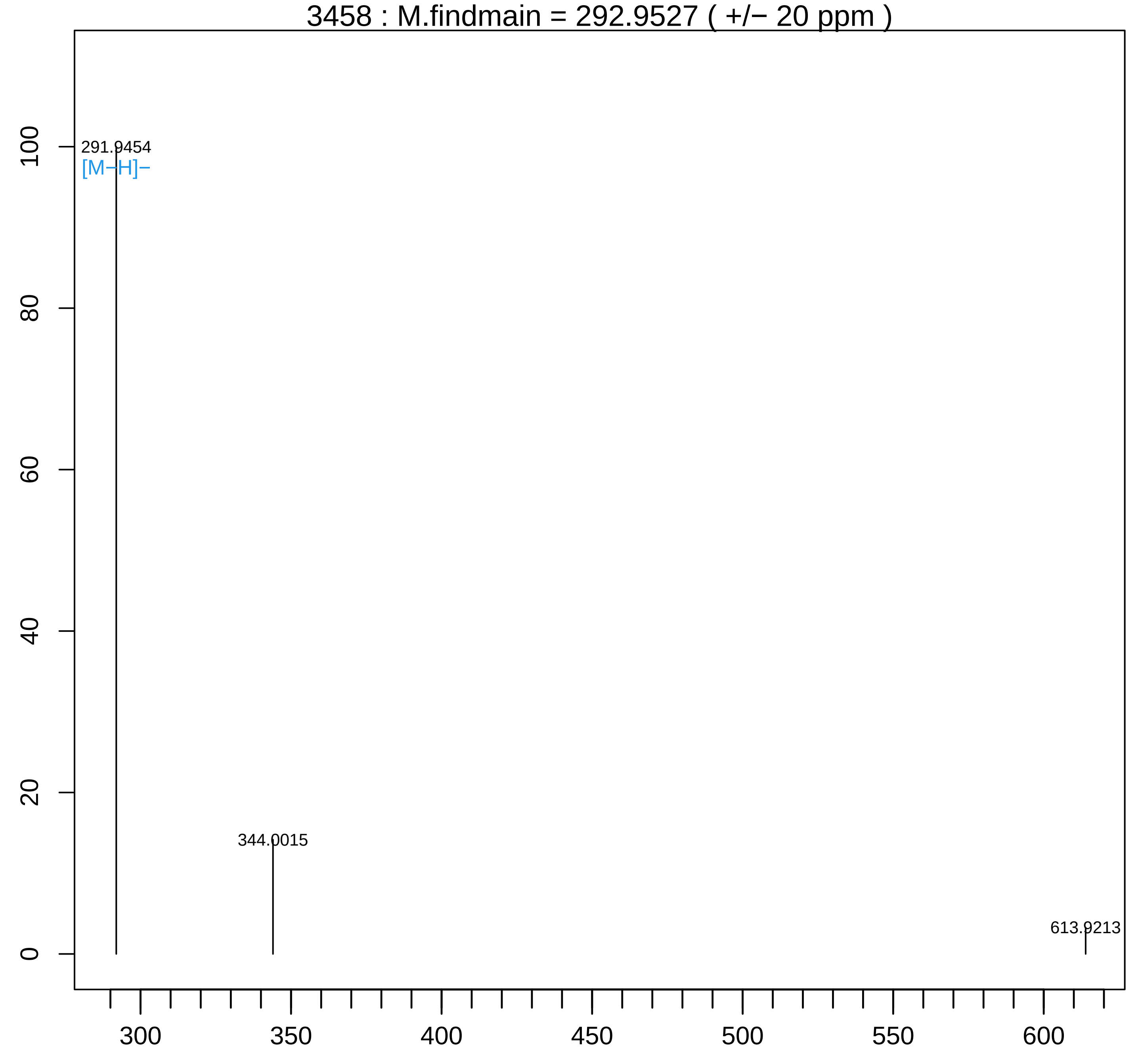
3458 : M.findmain = 292.9527 (+/− 20 ppm)
291.9454
[M−H]−
344.0015
613.9213
0
20
40
60
80
100
300
350
400
450
500
550
600

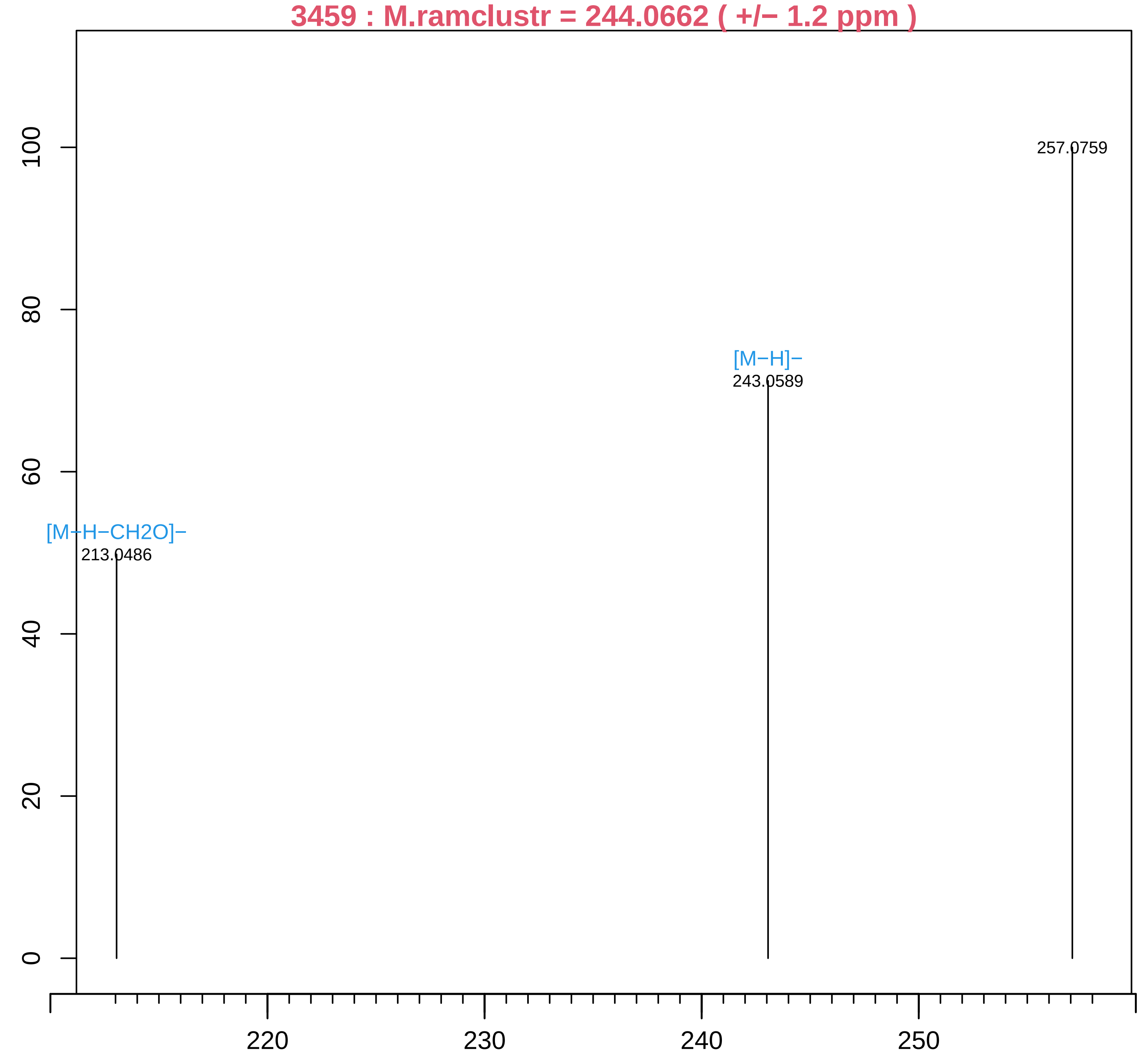
3459 : M.ramclustr = 244.0662 (+/− 1.2 ppm)
257.0759
[M−H]−
243.0589
[M−H−CH2O]−
213.0486
0
20
40
60
80
100
220
230
240
250

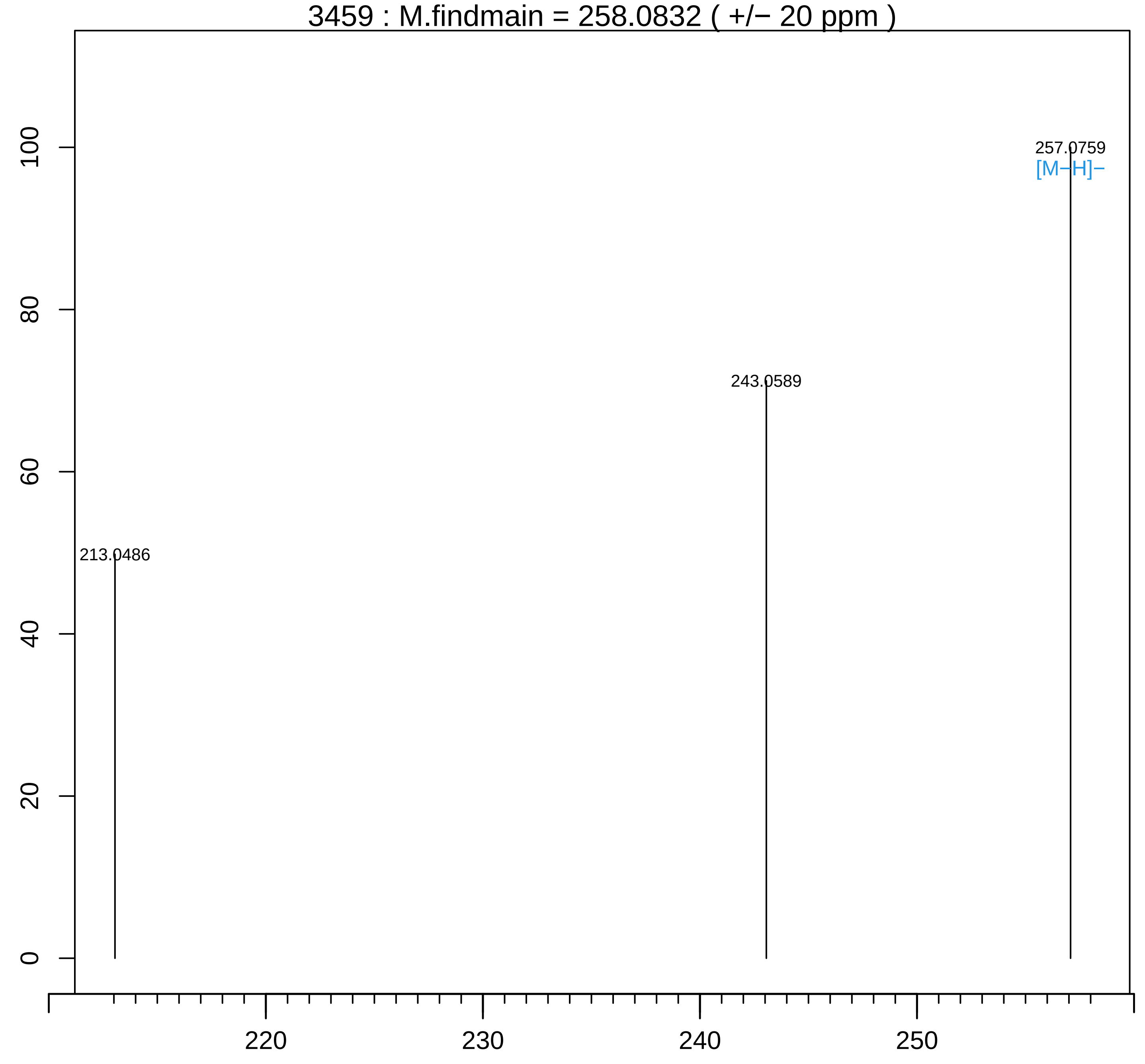
3459 : M.findmain = 258.0832 (+/− 20 ppm)
257.0759
[M−H]−
243.0589
213.0486
0
20
40
60
80
100
220
230
240
250

3460 : M.ramclustr = 676.1309 (+/− NA ppm)

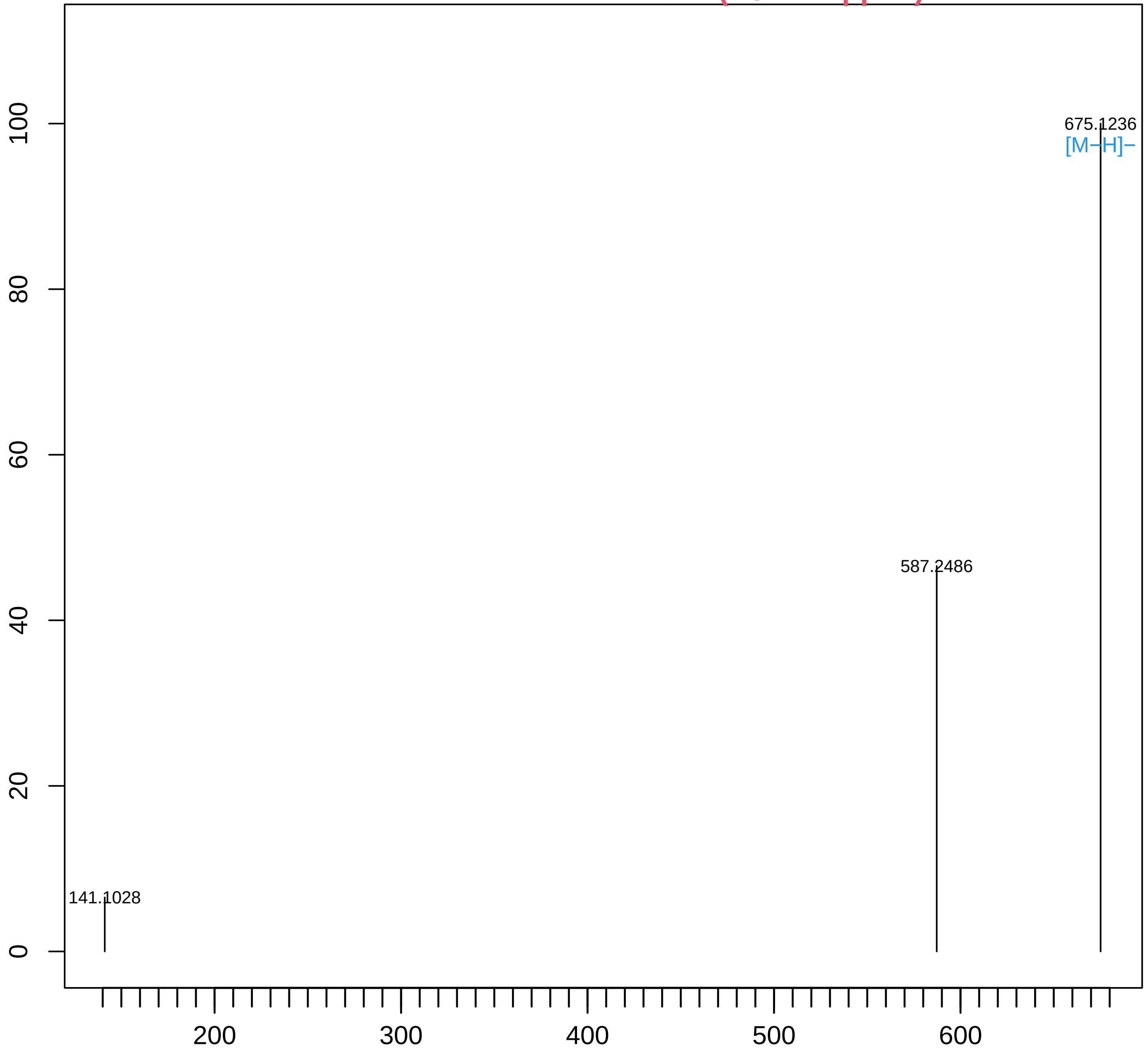

3460 : M.findmain = 676.1309 (+/− 20 ppm)

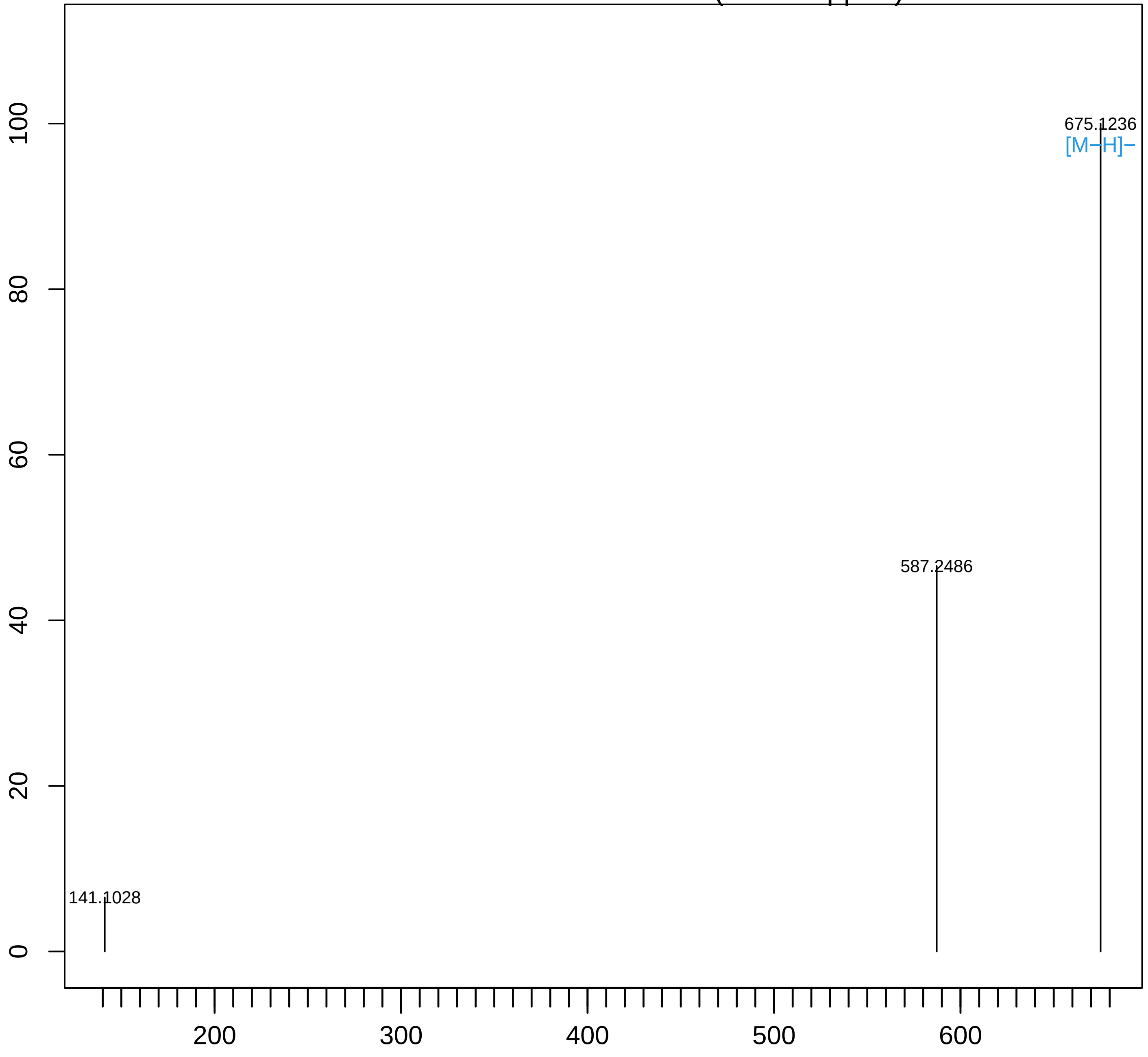

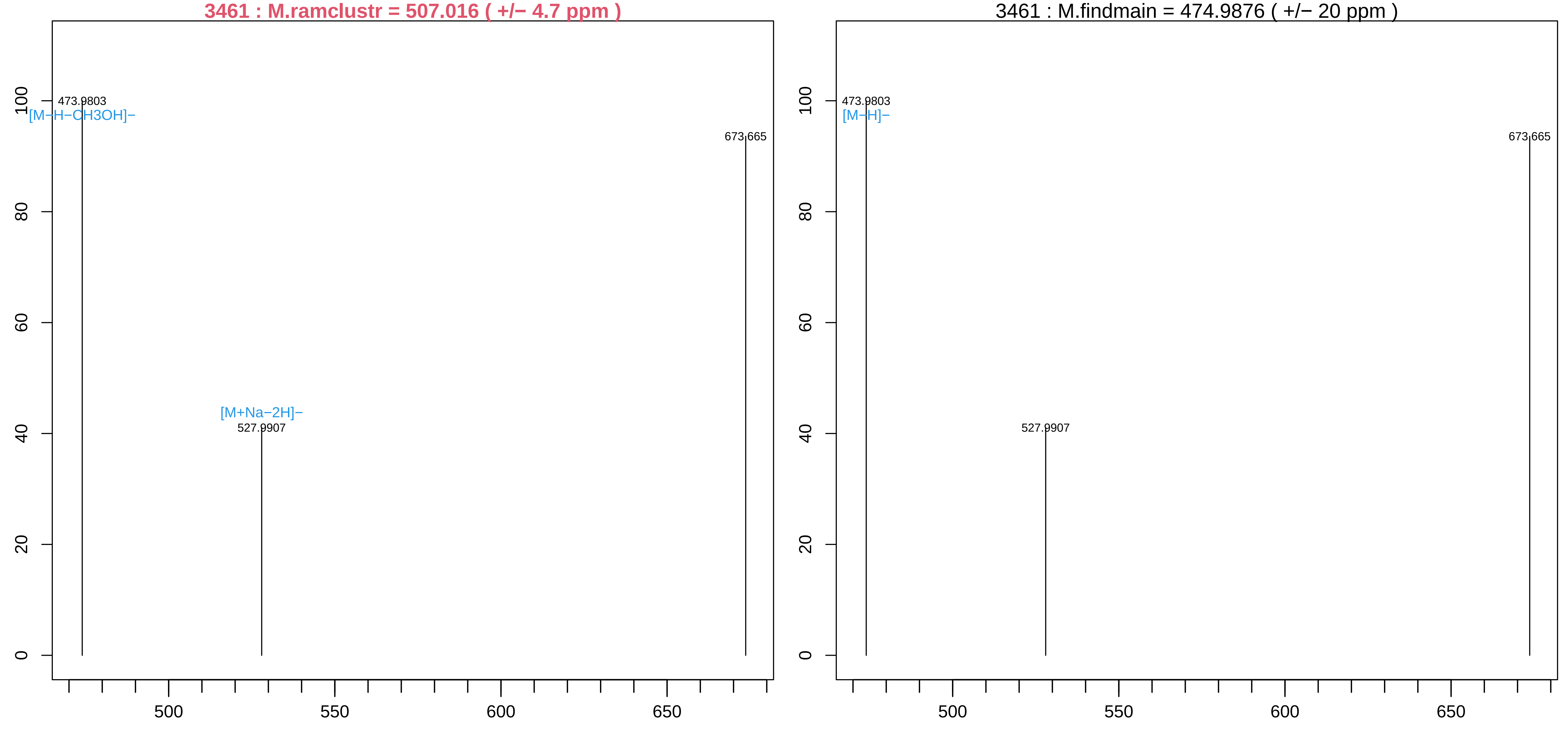
3461 : M.ramclustr = 507.016 (+/− 4.7 ppm)
473.9803
[M−H−CH3OH]−
[M+Na−2H]−
527.9907
673.665
3461 : M.findmain = 474.9876 (+/− 20 ppm)
473.9803
[M−H]−
527.9907
673.665
0
20
40
60
80
100
500
550
600
650

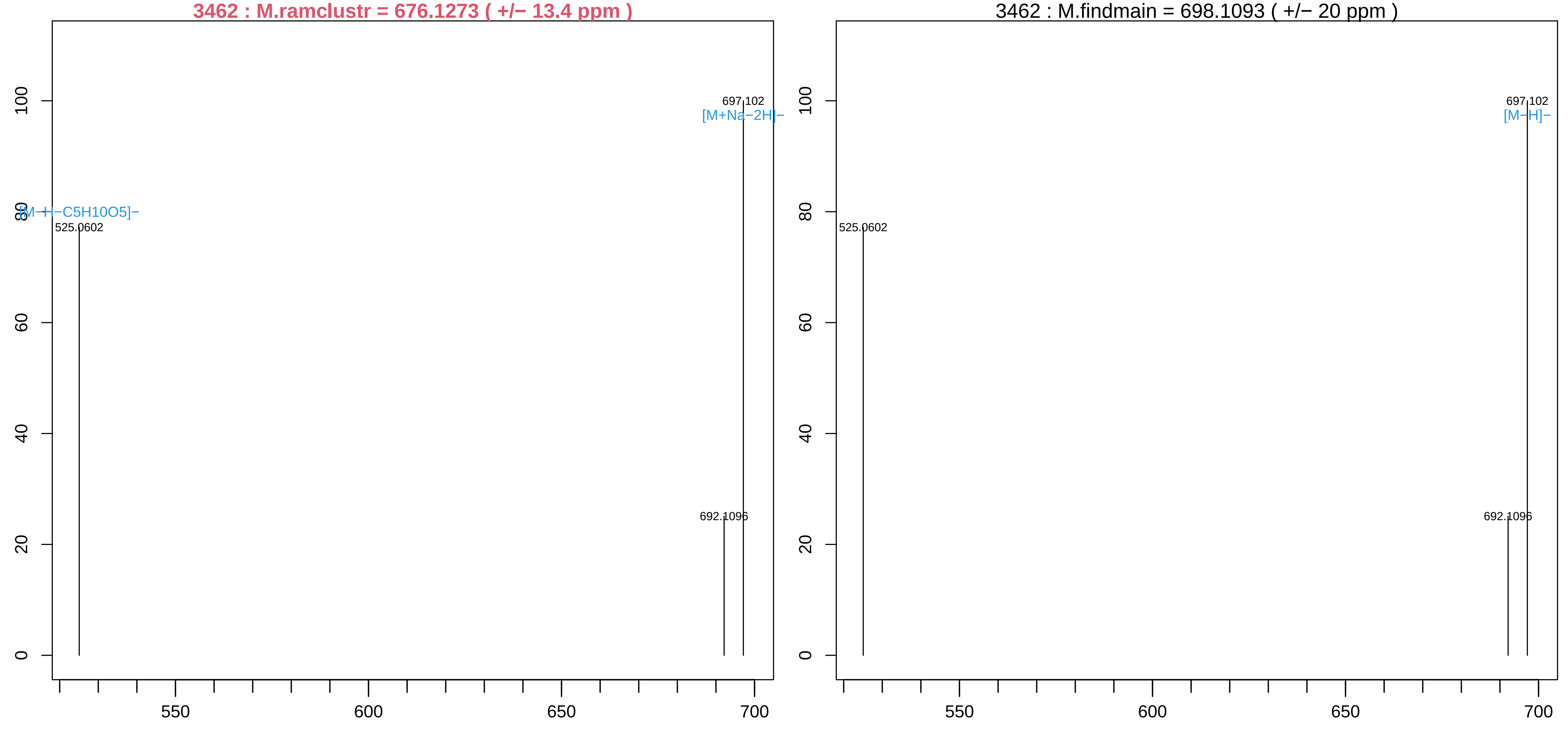
3462 : M.ramclustr = 676.1273 (+/− 13.4 ppm)
697.102
[M+Na−2H]−
[M−H−C5H10O5]−
525.0602
692.1096
3462 : M.findmain = 698.1093 (+/− 20 ppm)
697.102
[M−H]−
525.0602
692.1096
0
20
40
60
80
100
550
600
650
700

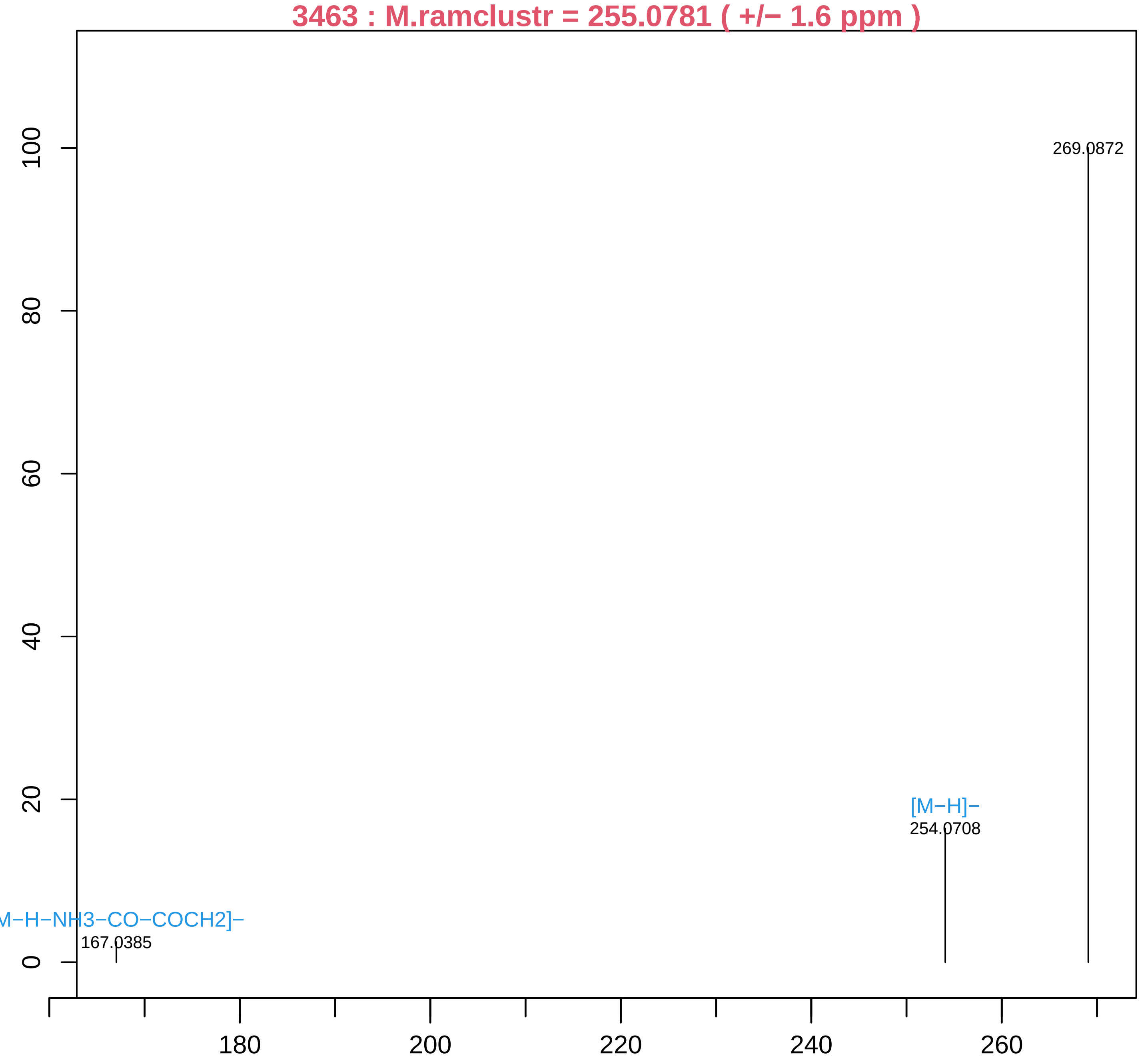
3463 : M.ramclustr = 255.0781 (+/− 1.6 ppm)
269.0872
[M−H]−
254.0708
M−H−NH3−CO−COCH2]−
167.0385
0
20
40
60
80
100
180
200
220
240
260

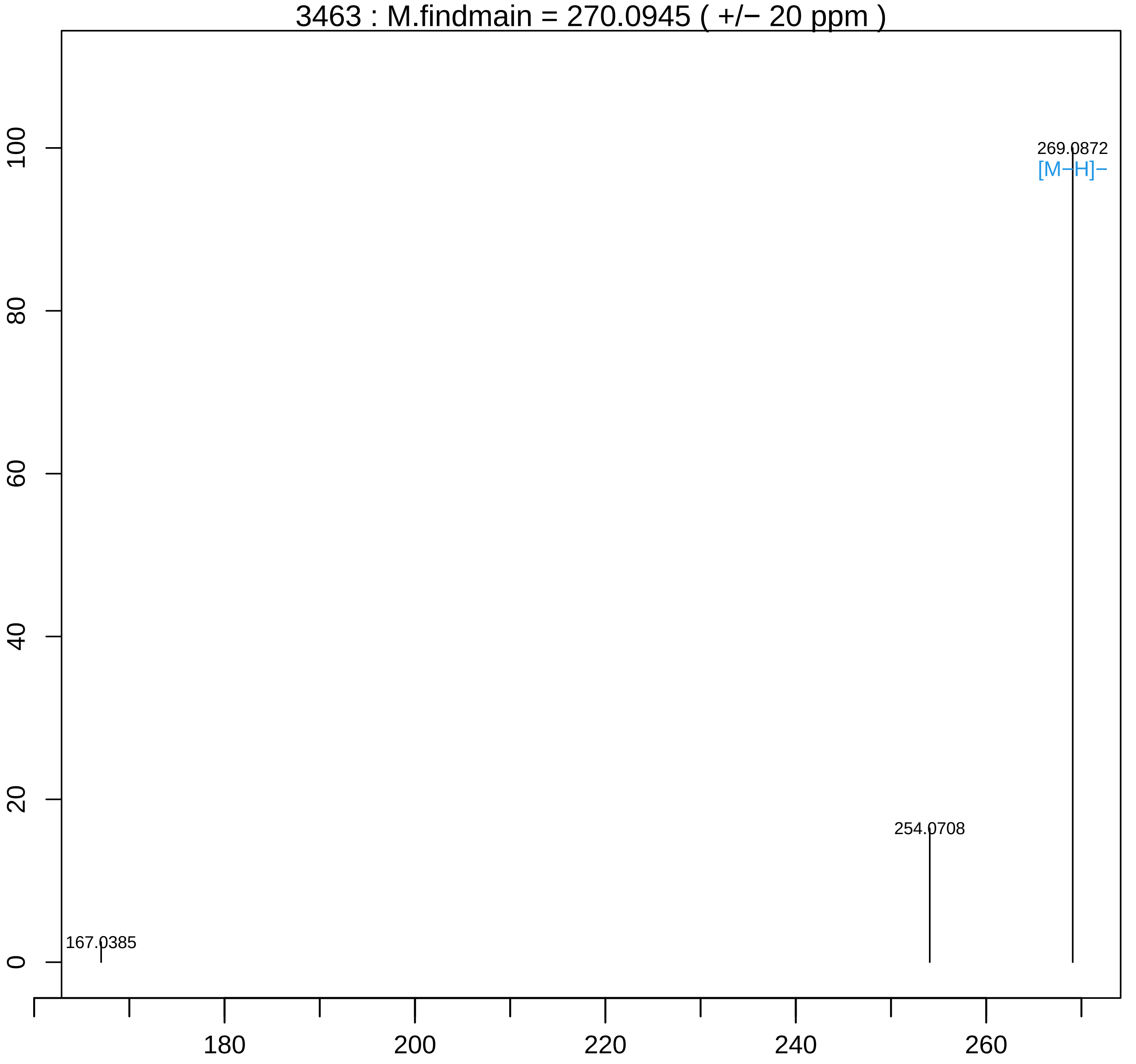
3463 : M.findmain = 270.0945 (+/− 20 ppm)
269.0872
[M−H]−
254.0708
167.0385
0
20
40
60
80
100
180
200
220
240
260

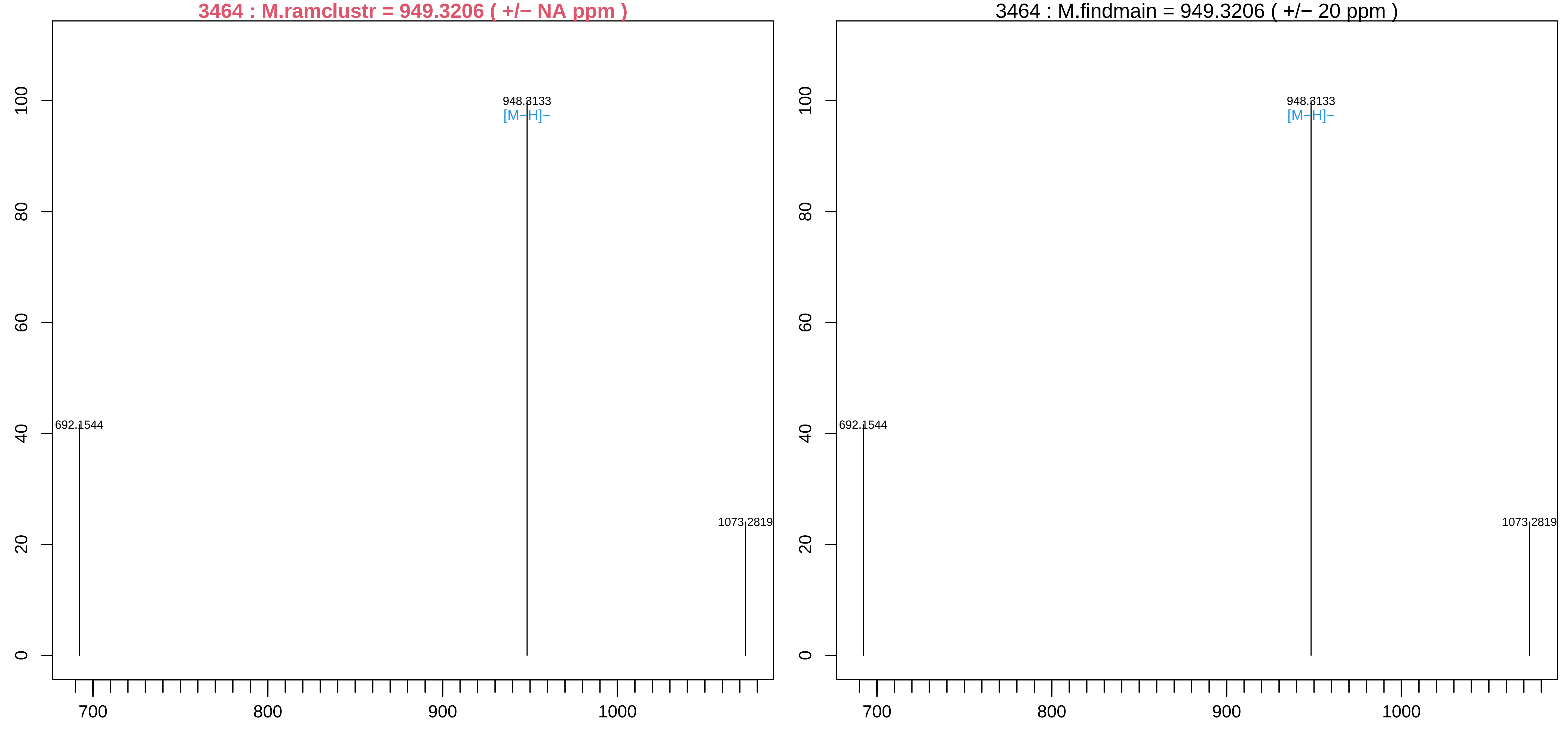

3464 : M.ramclustr = 949.3206 (+/− NA ppm)
948.3133
[M−H]−
692.1544
1073.2819
3464 : M.findmain = 949.3206 (+/− 20 ppm)
948.3133
[M−H]−
692.1544
1073.2819
0
20
40
60
80
100
700
800
900
1000

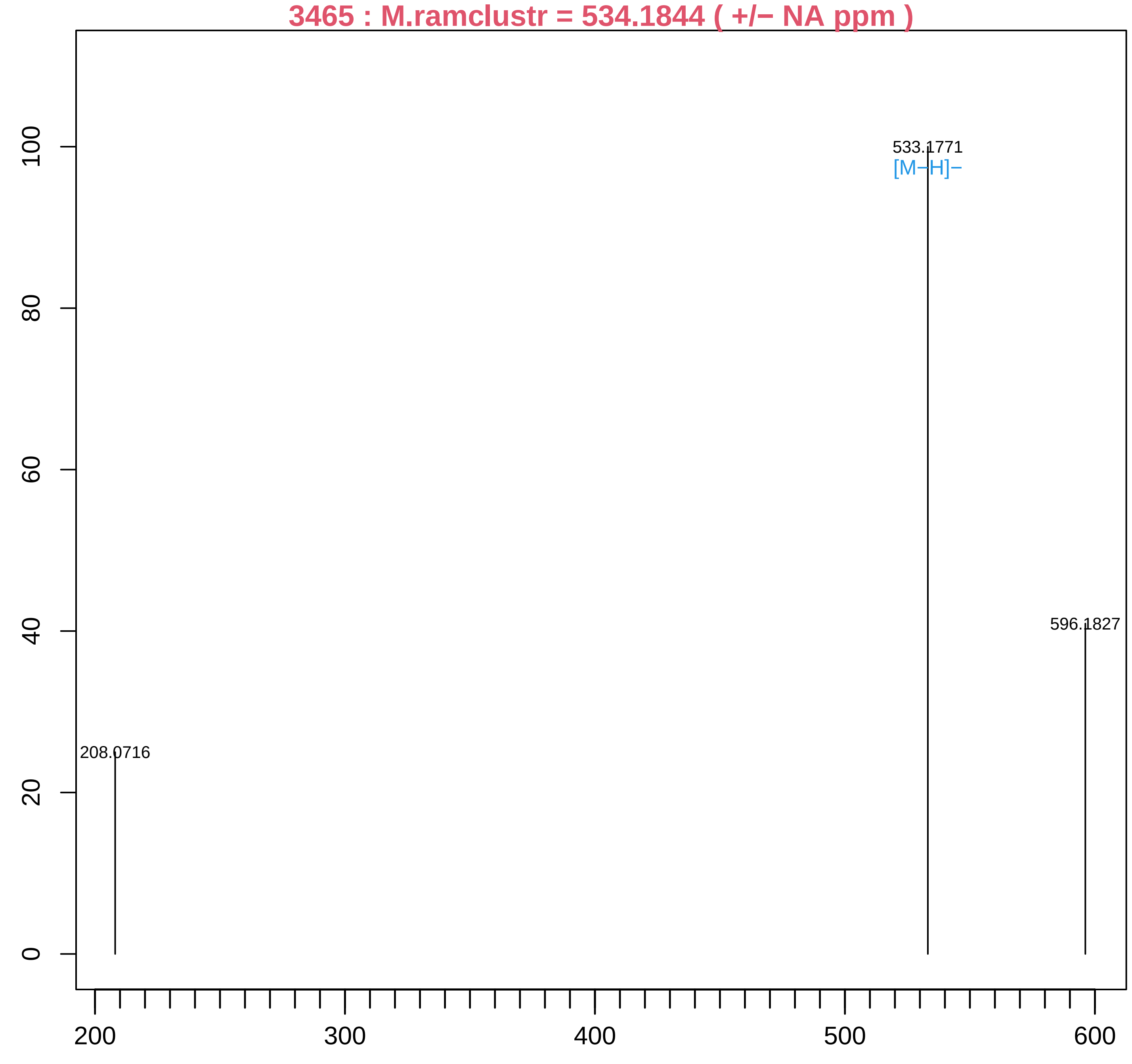
3465 : M.ramclustr = 534.1844 (+/− NA ppm)
533.1771
[M−H]−
596.1827
208.0716
0
20
40
60
80
100
200
300
400
500
600

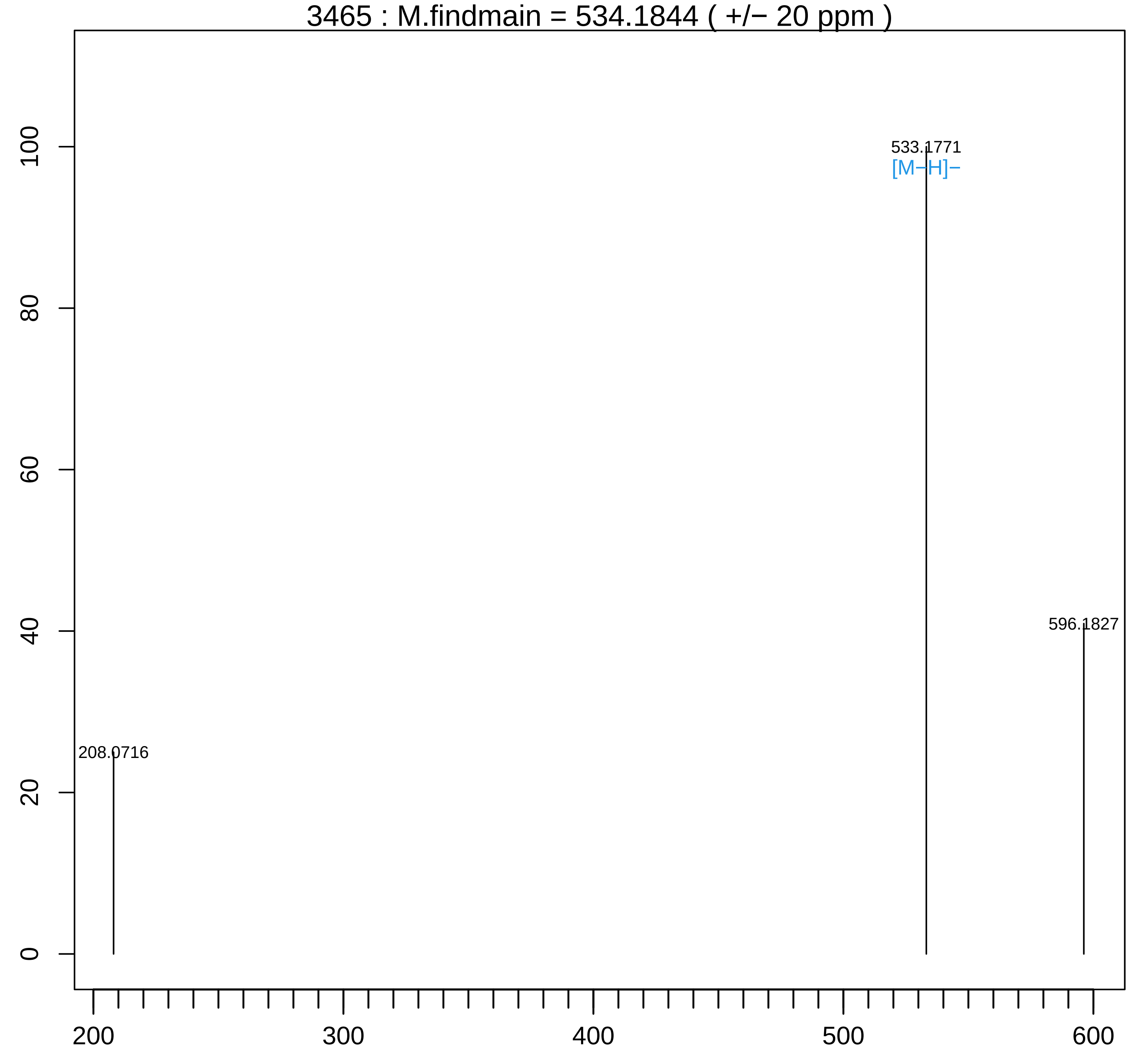
3465 : M.findmain = 534.1844 (+/− 20 ppm)
533.1771
[M−H]−
596.1827
208.0716
0
20
40
60
80
100
200
300
400
500
600

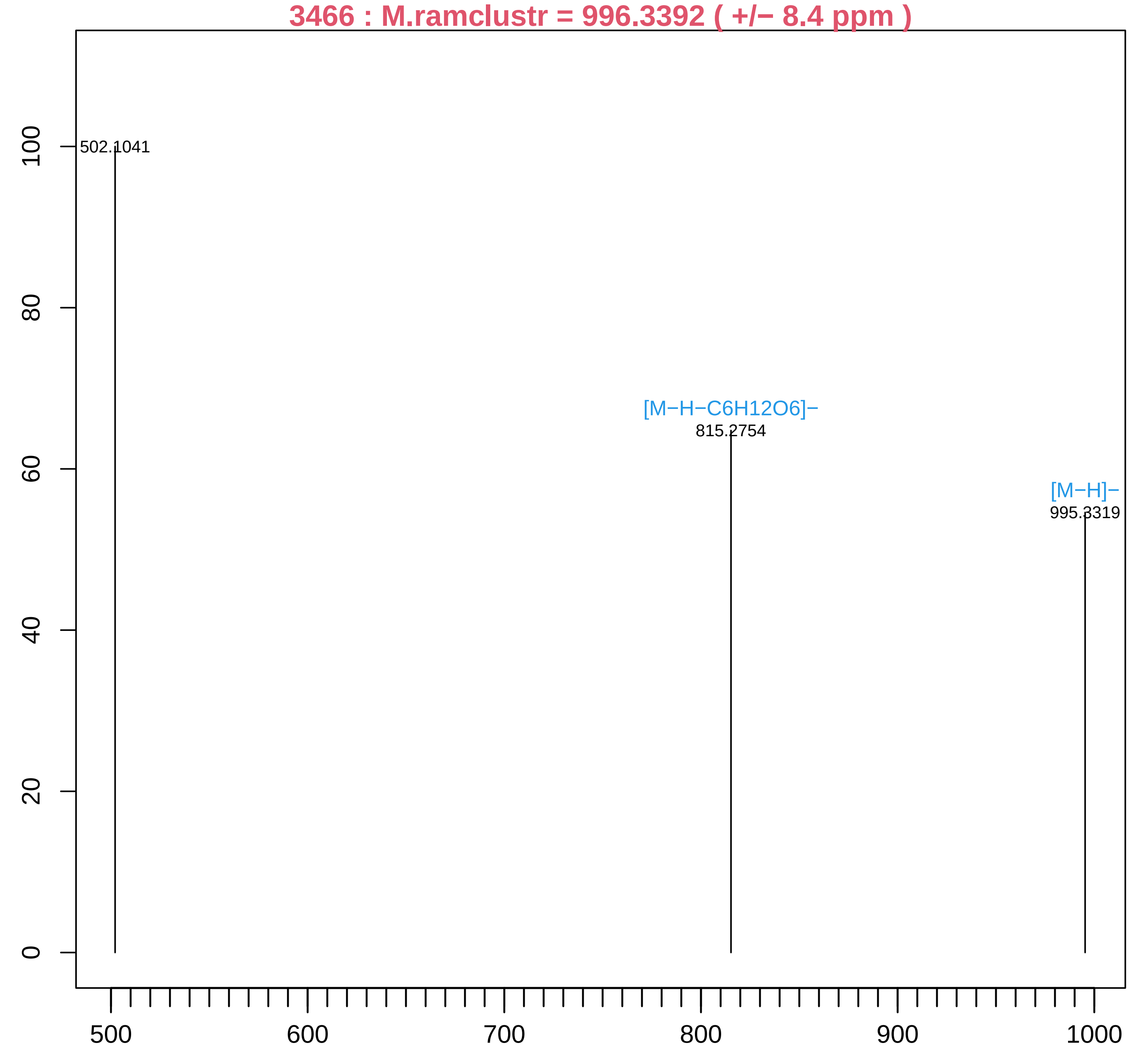
3466 : M.ramclustr = 996.3392 (+/− 8.4 ppm)
502.1041
[M−H−C6H12O6]−
815.2754
[M−H]−
995.3319
0
20
40
60
80
100
500
600
700
800
900
1000

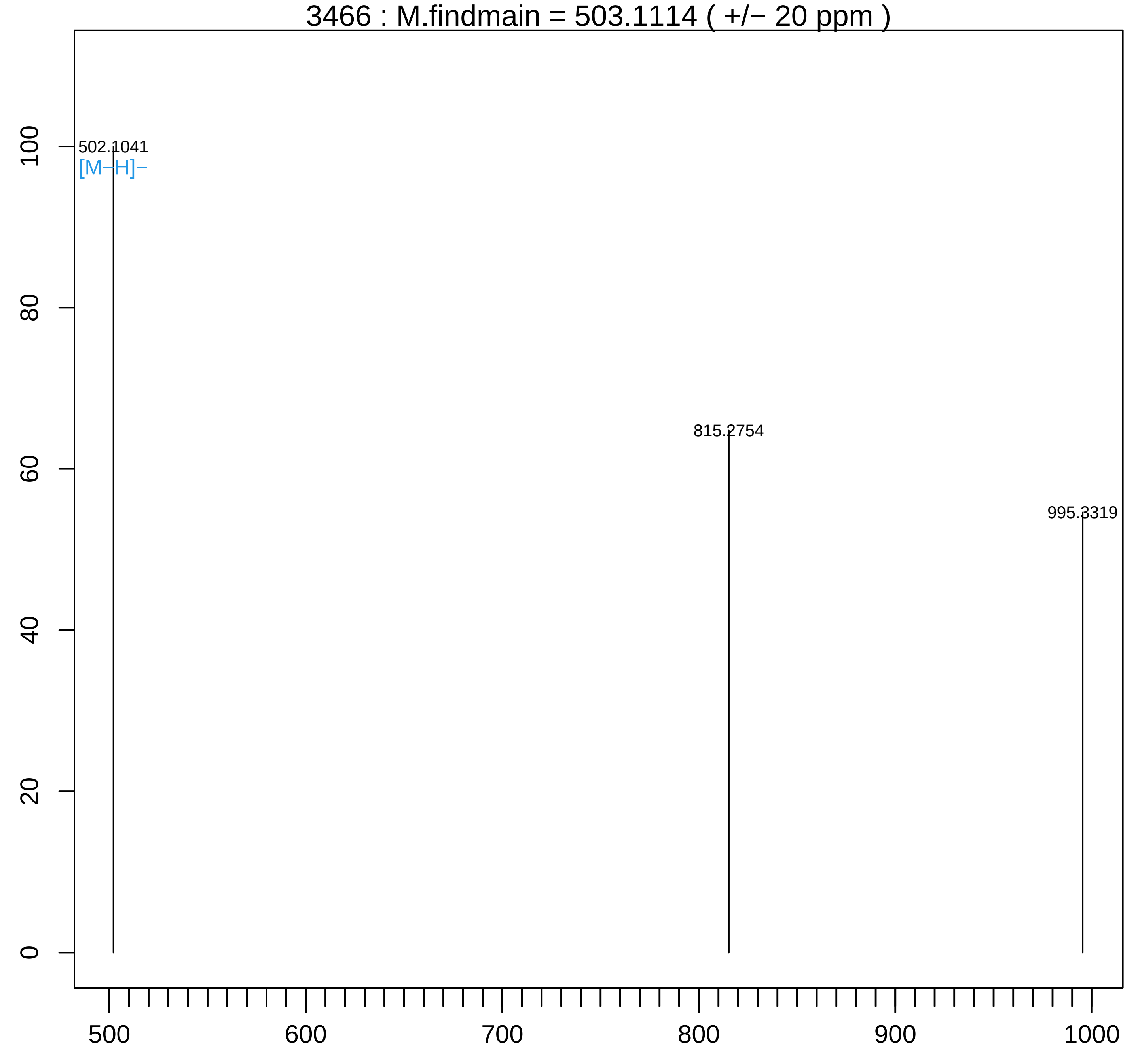
3466 : M.findmain = 503.1114 (+/− 20 ppm)
502.1041
[M−H]−
815.2754
995.3319
0
20
40
60
80
100
500
600
700
800
900
1000

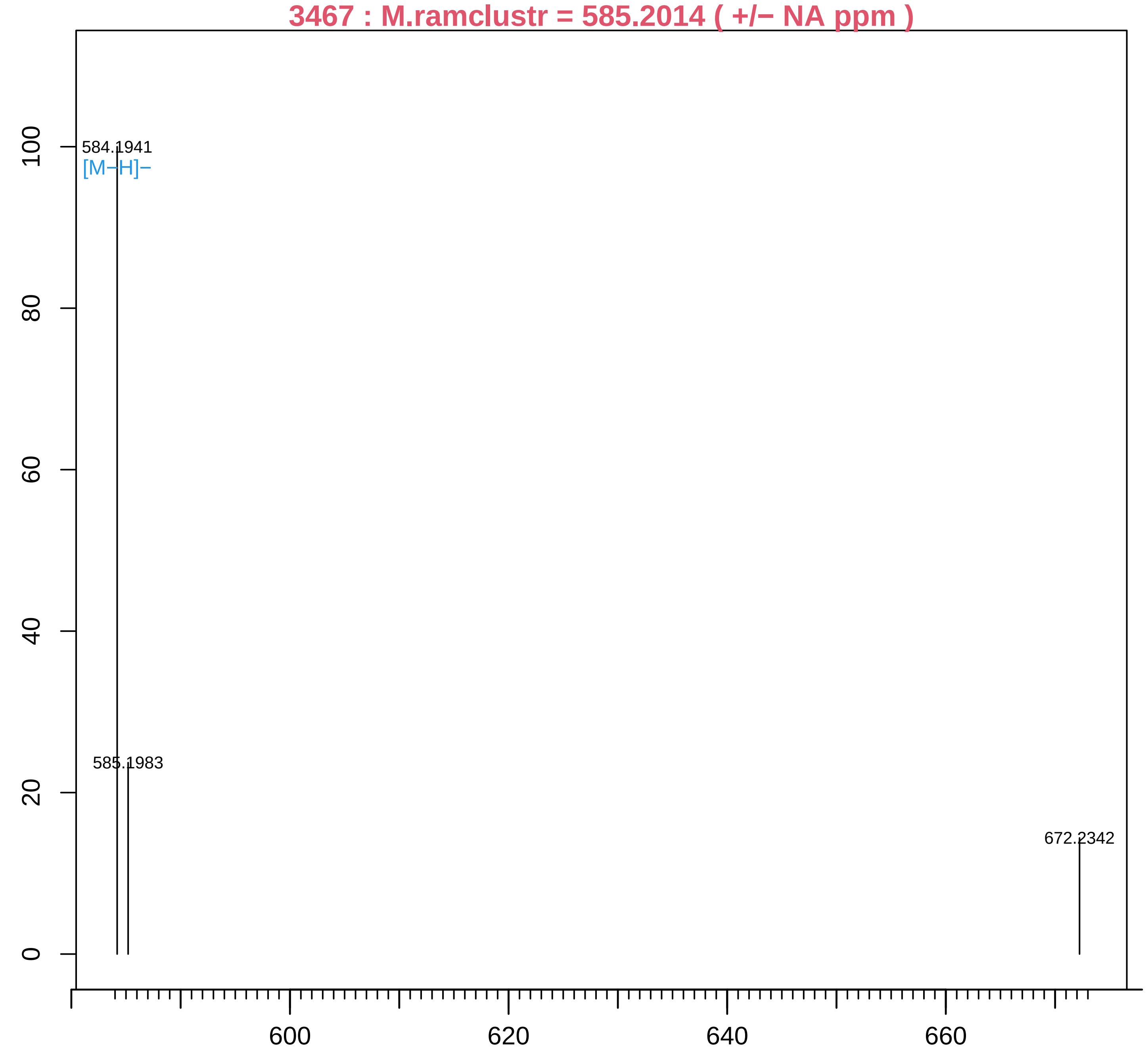
3467 : M.ramclustr = 585.2014 (+/− NA ppm)
584.1941
[M−H]−
585.1983
672.2342
0
20
40
60
80
100
600
620
640
660

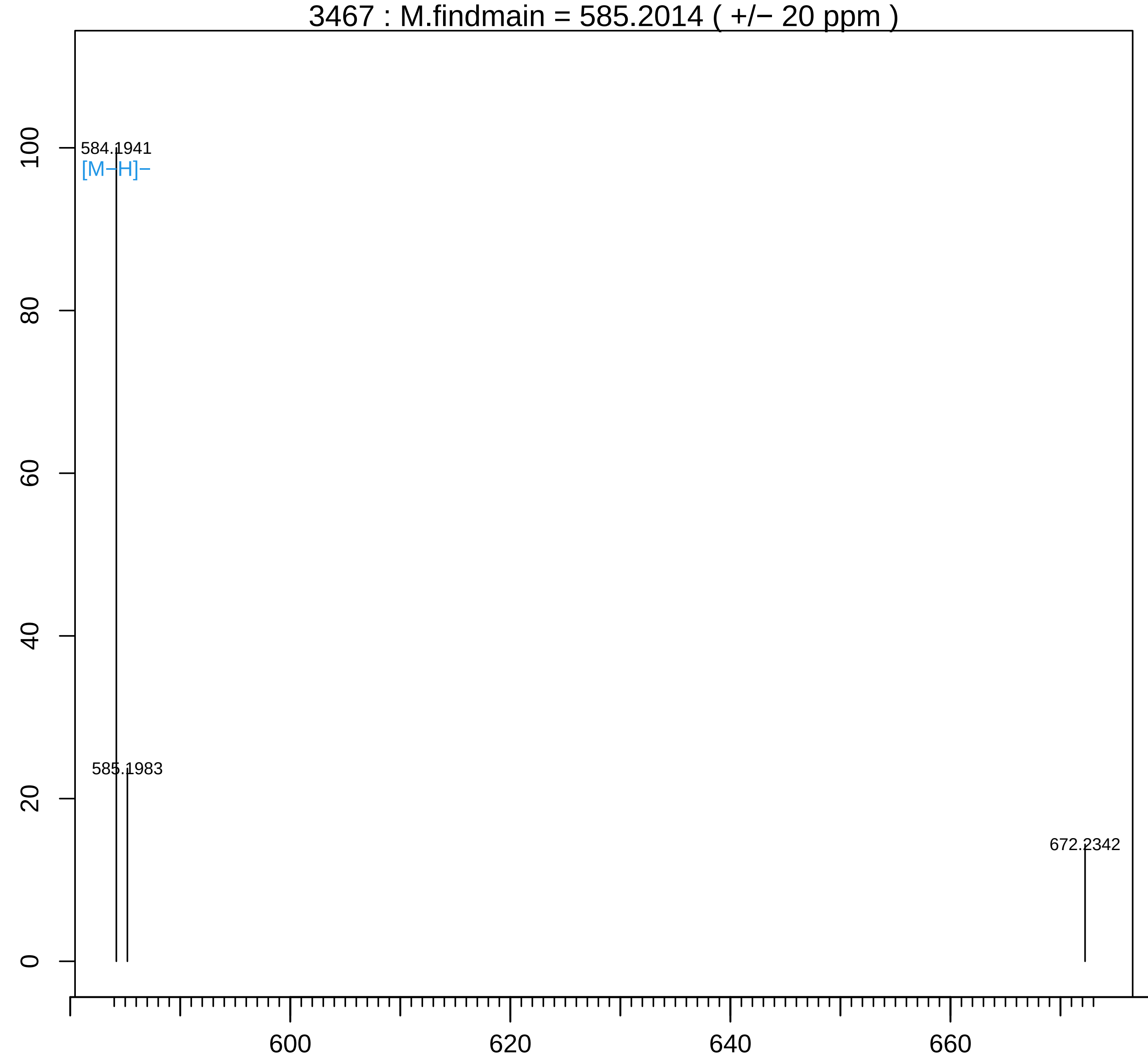
3467 : M.findmain = 585.2014 (+/− 20 ppm)
584.1941
[M−H]−
585.1983
672.2342
0
20
40
60
80
100
600
620
640
660

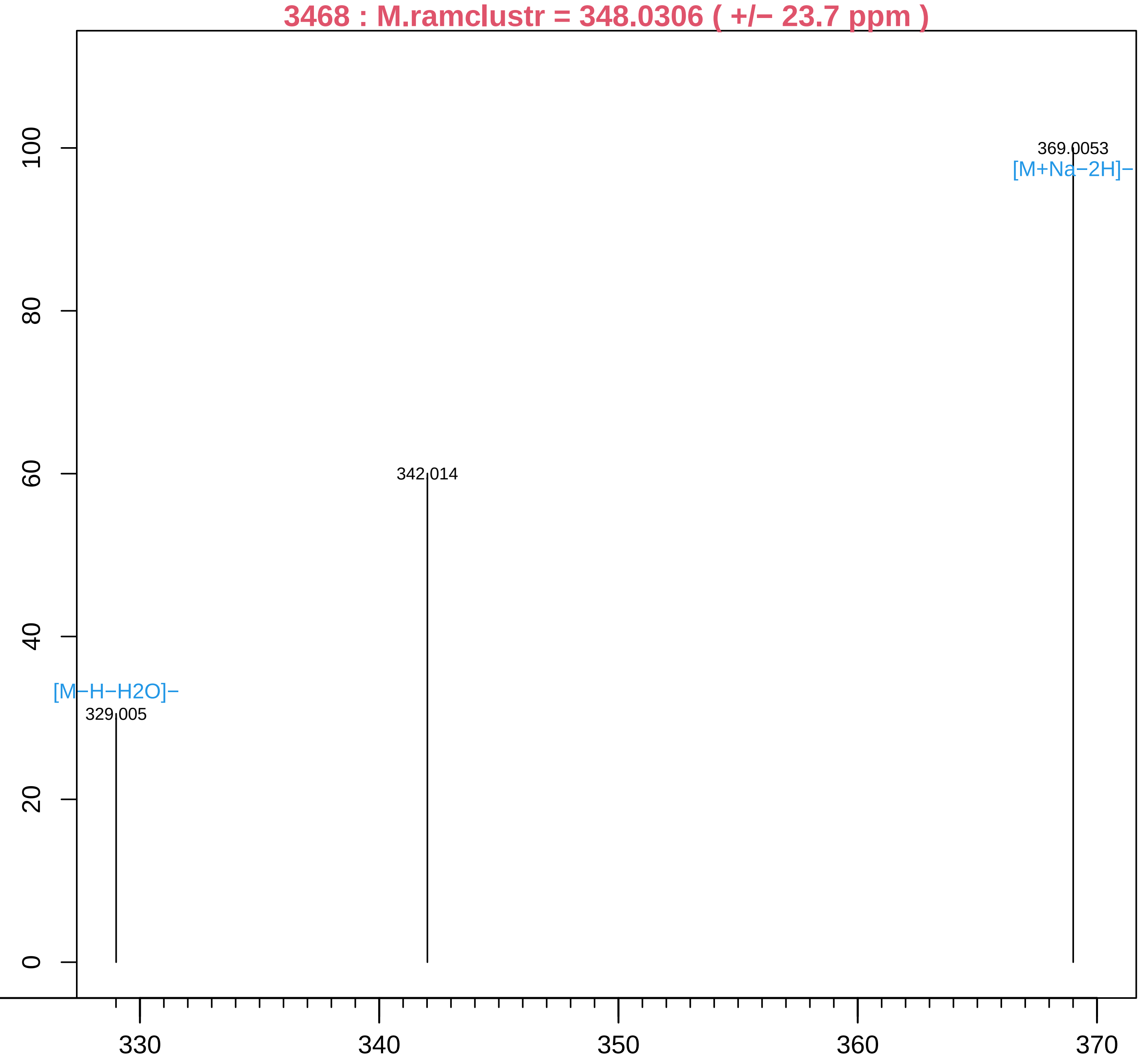

3468 : M.ramclustr = 348.0306 (+/− 23.7 ppm)
369.0053
[M+Na−2H]−
342.014
[M−H−H2O]−
329.005
0
20
40
60
80
100
330
340
350
360
370

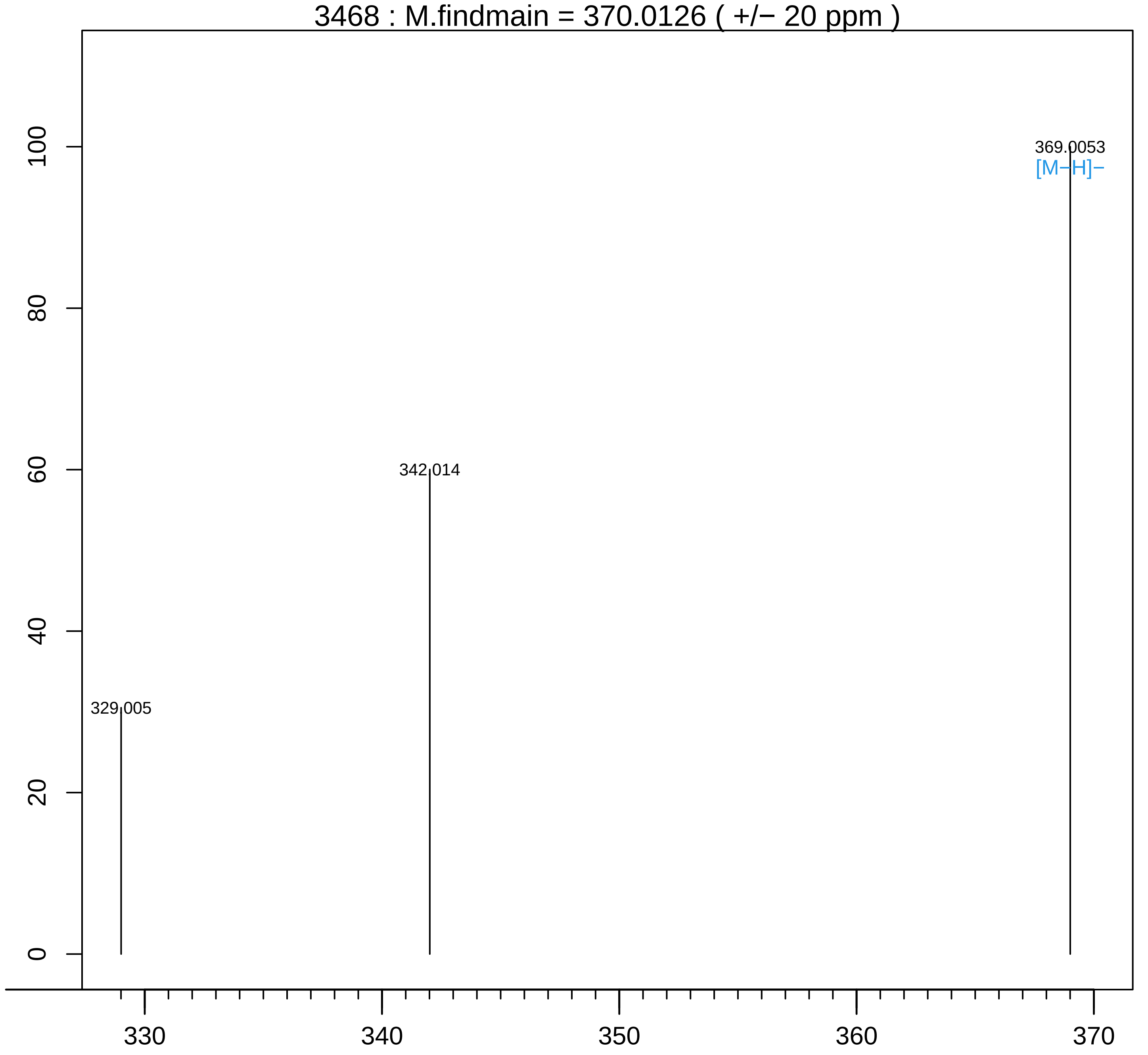

3468 : M.findmain = 370.0126 (+/− 20 ppm)
369.0053
[M−H]−
342.014
329.005
0
20
40
60
80
100
330
340
350
360
370

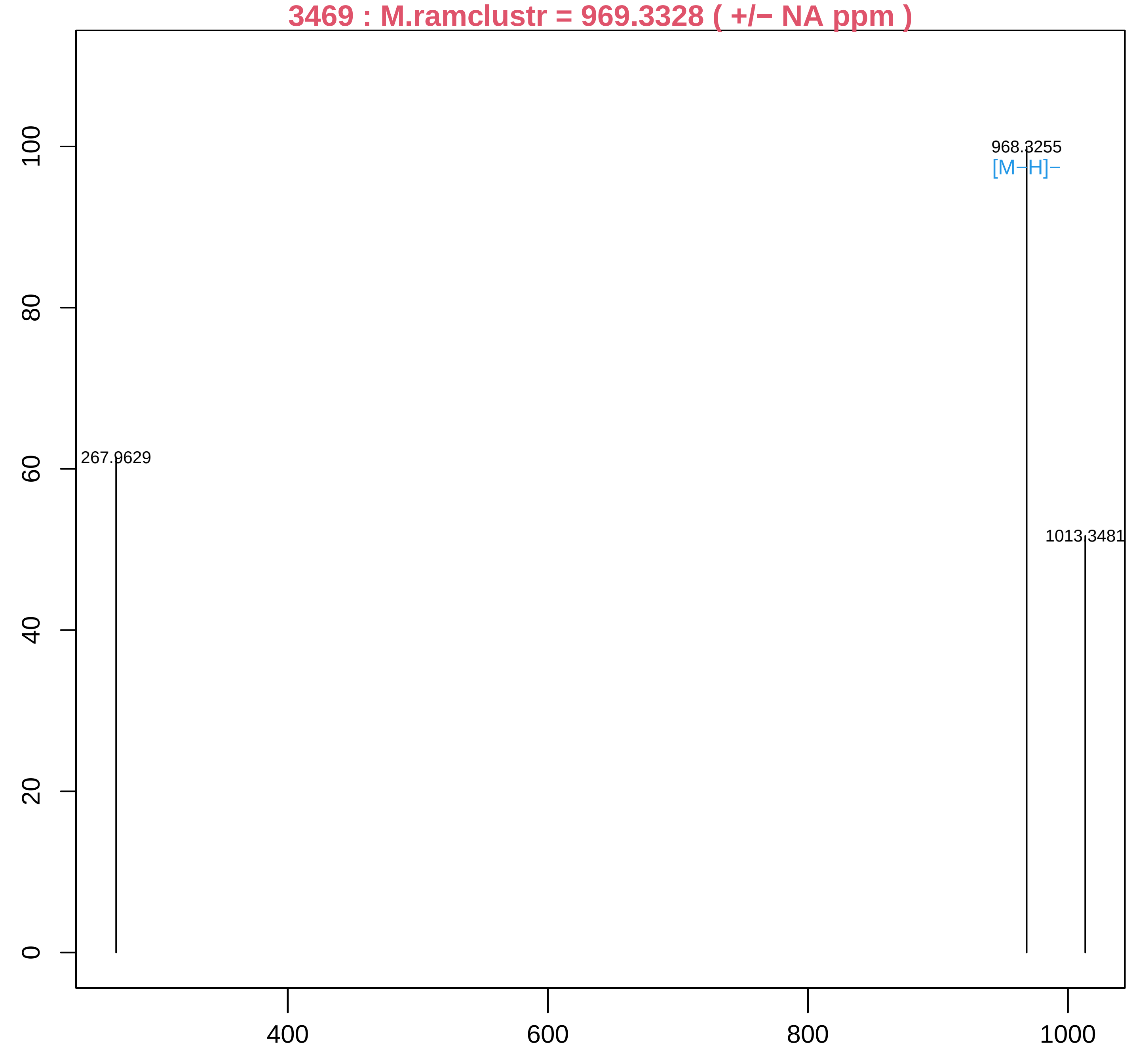
3469 : M.ramclustr = 969.3328 (+/− NA ppm)
968.3255
[M−H]−
267.9629
1013.3481
0
20
40
60
80
100
400
600
800
1000

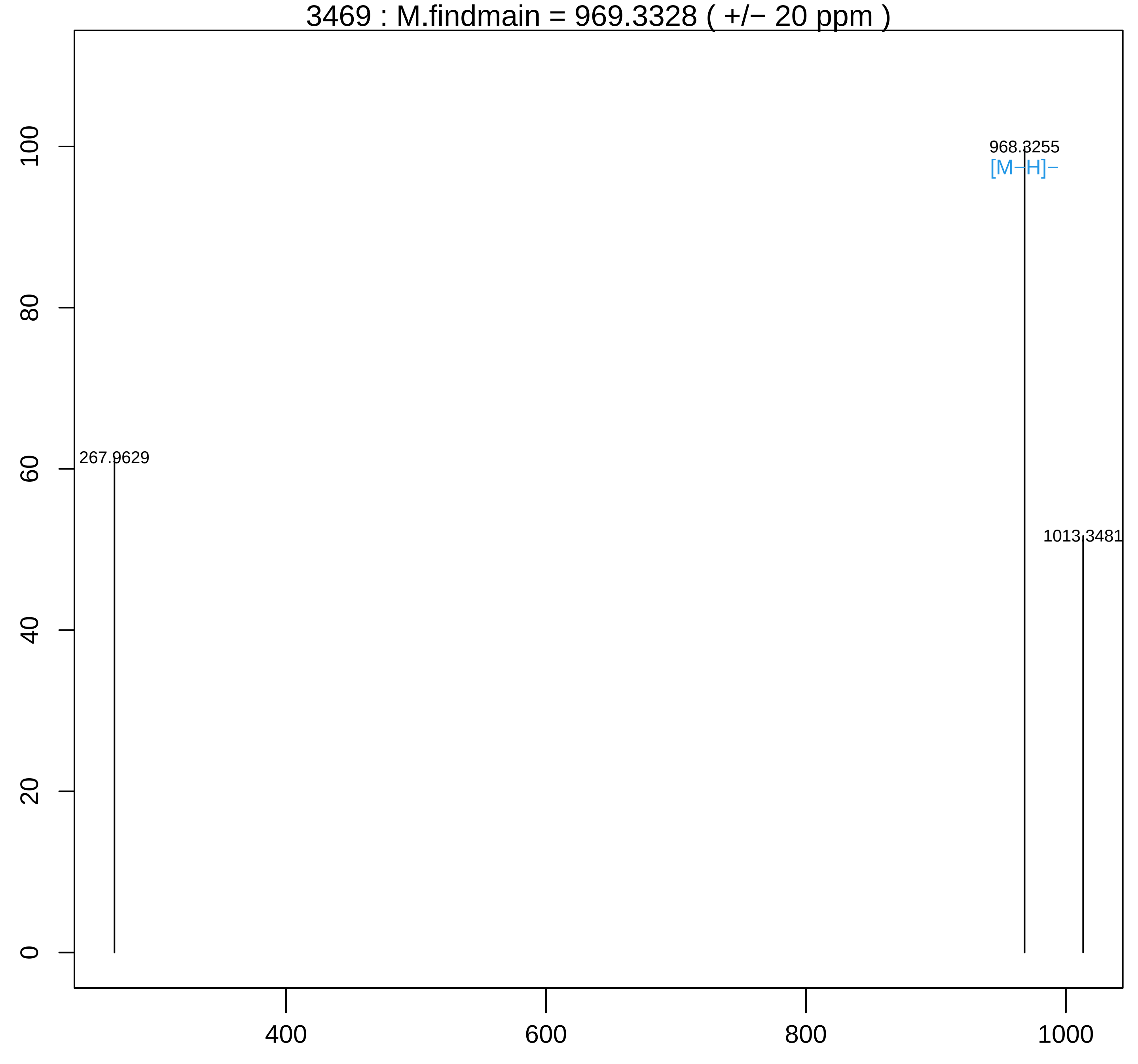
3469 : M.findmain = 969.3328 (+/− 20 ppm)
968.3255
[M−H]−
267.9629
1013.3481
0
20
40
60
80
100
400
600
800
1000

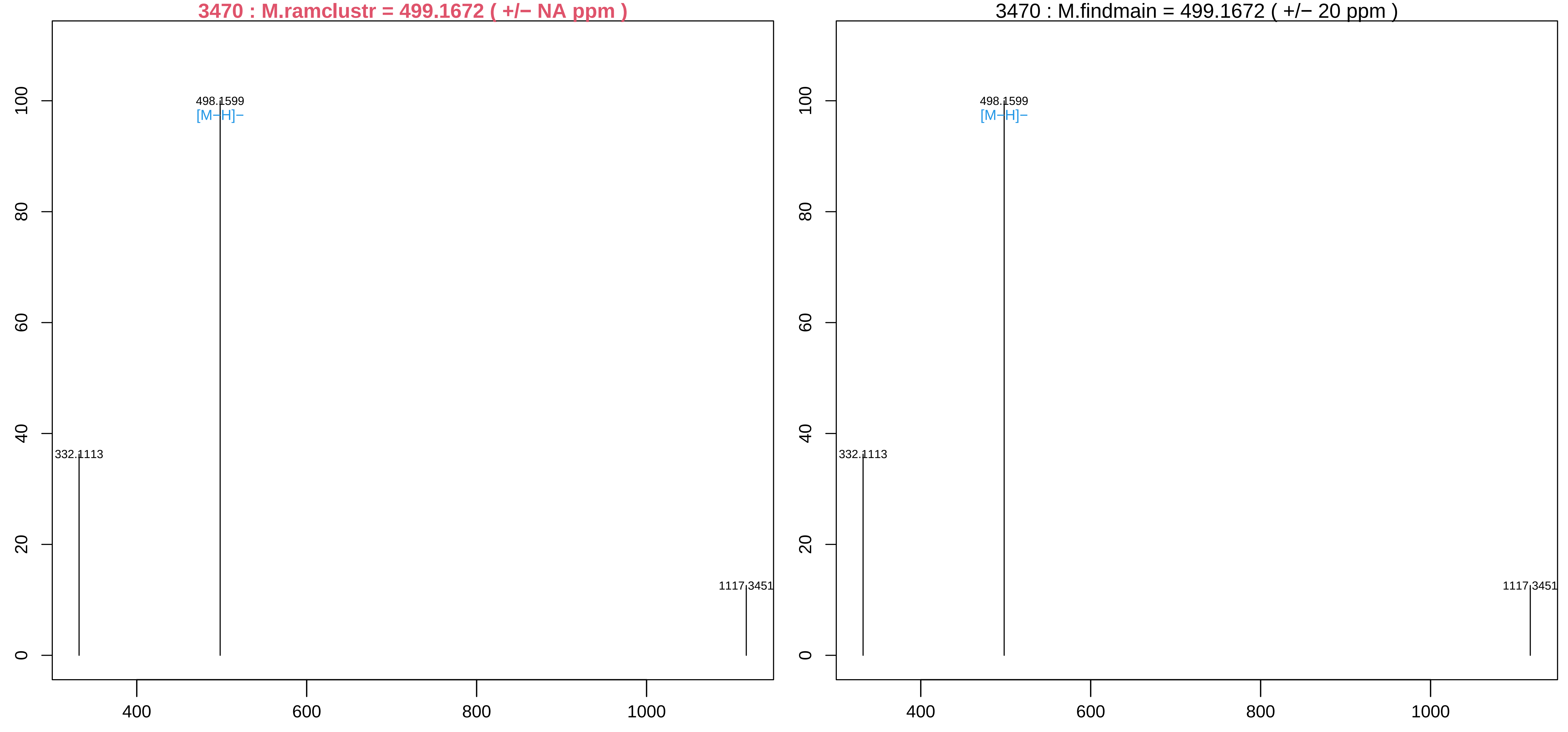

3470 : M.ramclustr = 499.1672 (+/− NA ppm)
498.1599
[M−H]−
332.1113
1117.3451
3470 : M.findmain = 499.1672 (+/− 20 ppm)
498.1599
[M−H]−
332.1113
1117.3451
0
20
40
60
80
100
400
600
800
1000

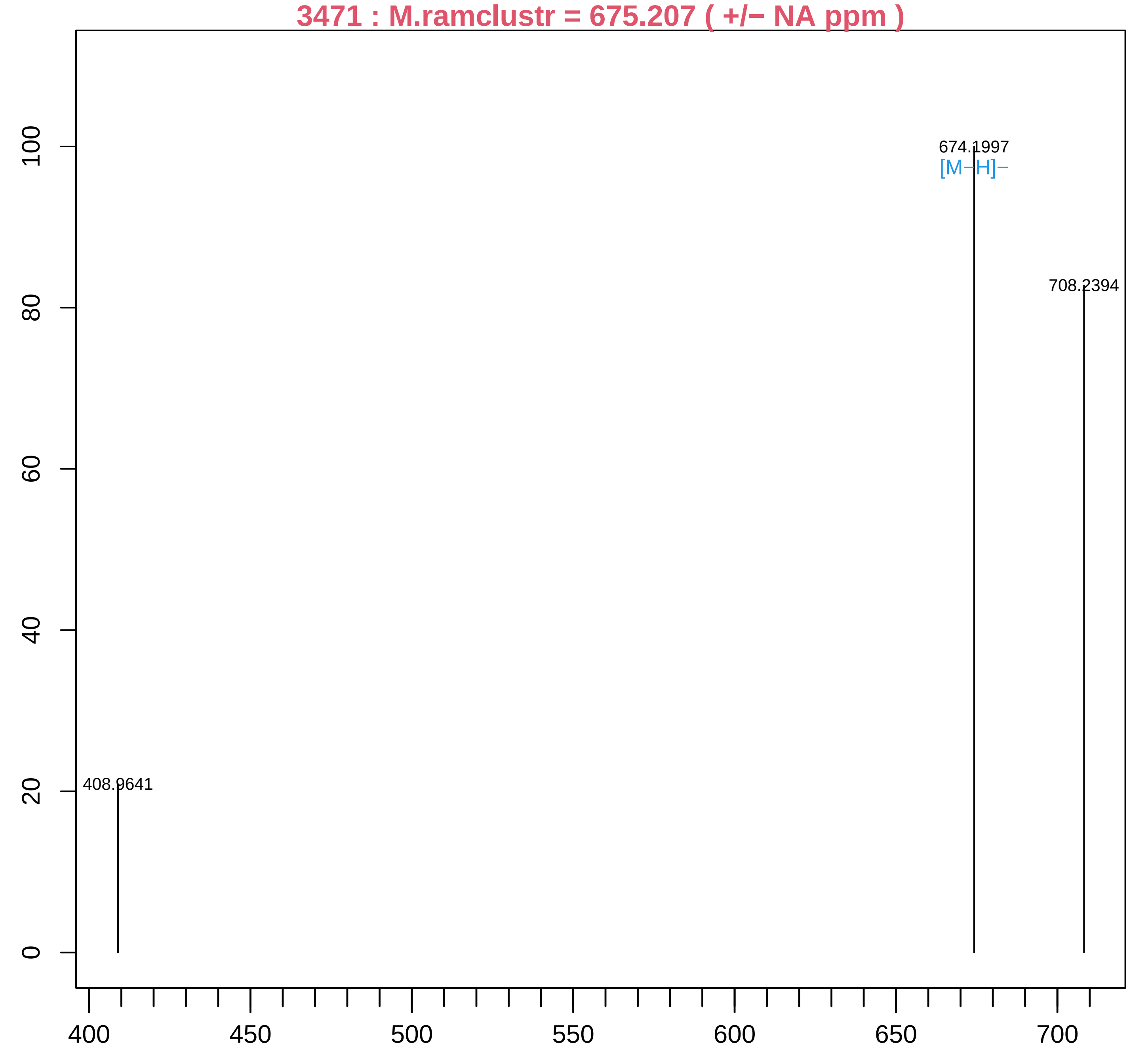
3471 : M.ramclustr = 675.207 (+/− NA ppm)
674.1997
[M−H]−
708.2394
408.9641
0
20
40
60
80
100
400
450
500
550
600
650
700

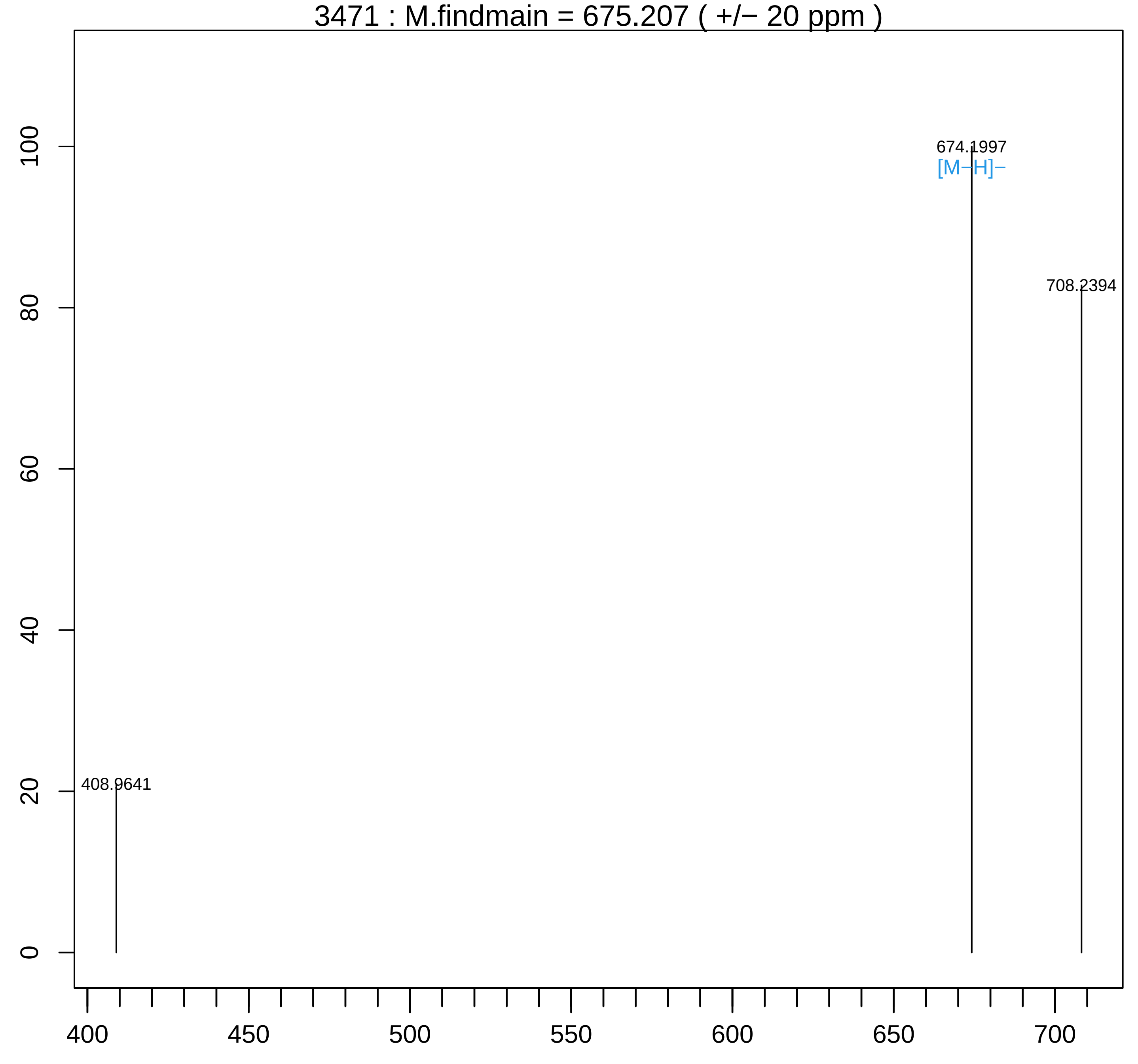
3471 : M.findmain = 675.207 (+/− 20 ppm)
674.1997
[M−H]−
708.2394
408.9641
0
20
40
60
80
100
400
450
500
550
600
650
700

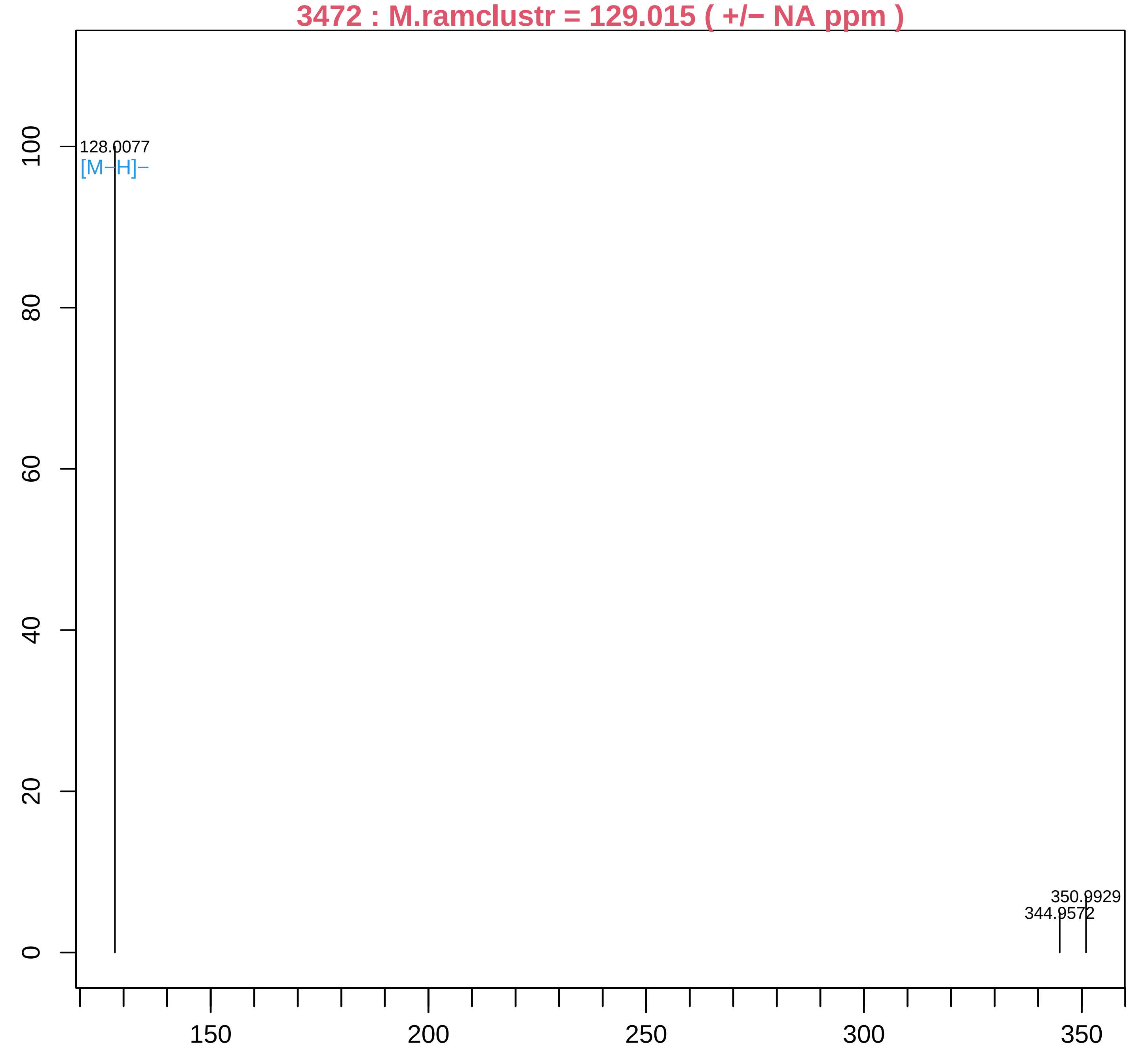
3472 : M.ramclustr = 129.015 (+/− NA ppm)
128.0077
[M−H]−
350.9929
344.9572
0
20
40
60
80
100
150
200
250
300
350

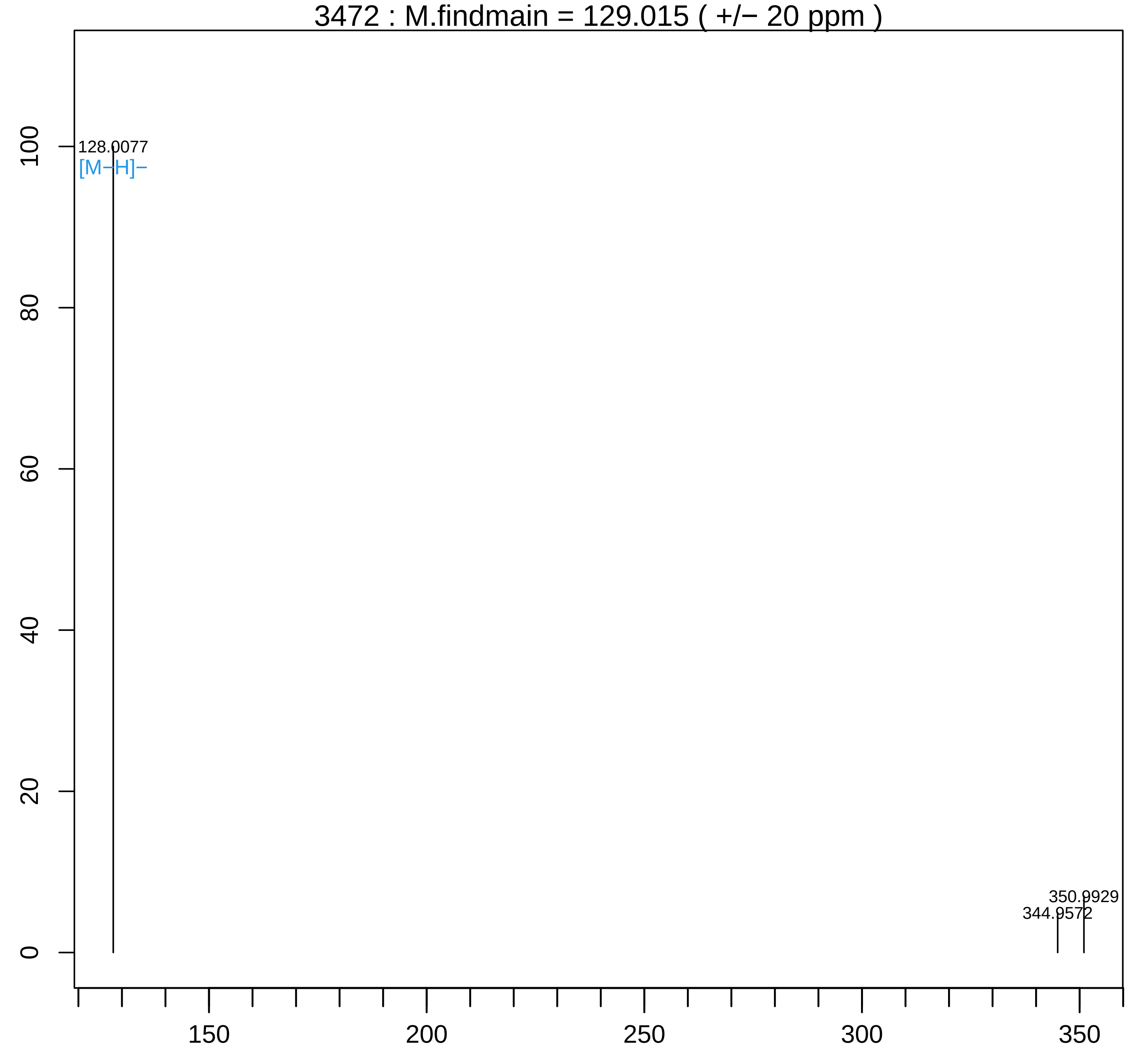
3472 : M.findmain = 129.015 (+/− 20 ppm)
128.0077
[M−H]−
350.9929
344.9572
0
20
40
60
80
100
150
200
250
300
350

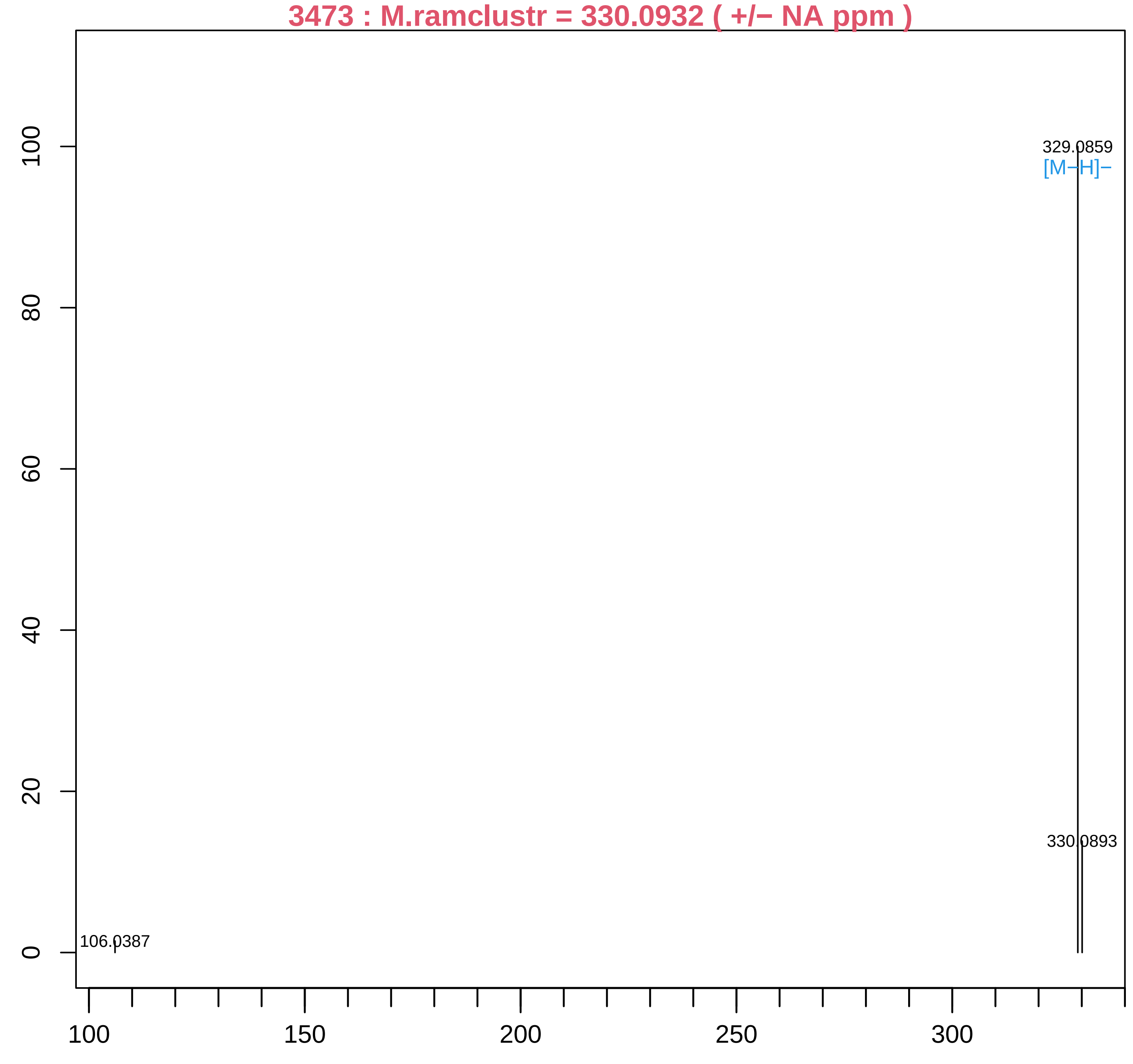

3473 : M.ramclustr = 330.0932 (+/− NA ppm)
329.0859
[M−H]−
330.0893
106.0387
0
20
40
60
80
100
100
150
200
250
300

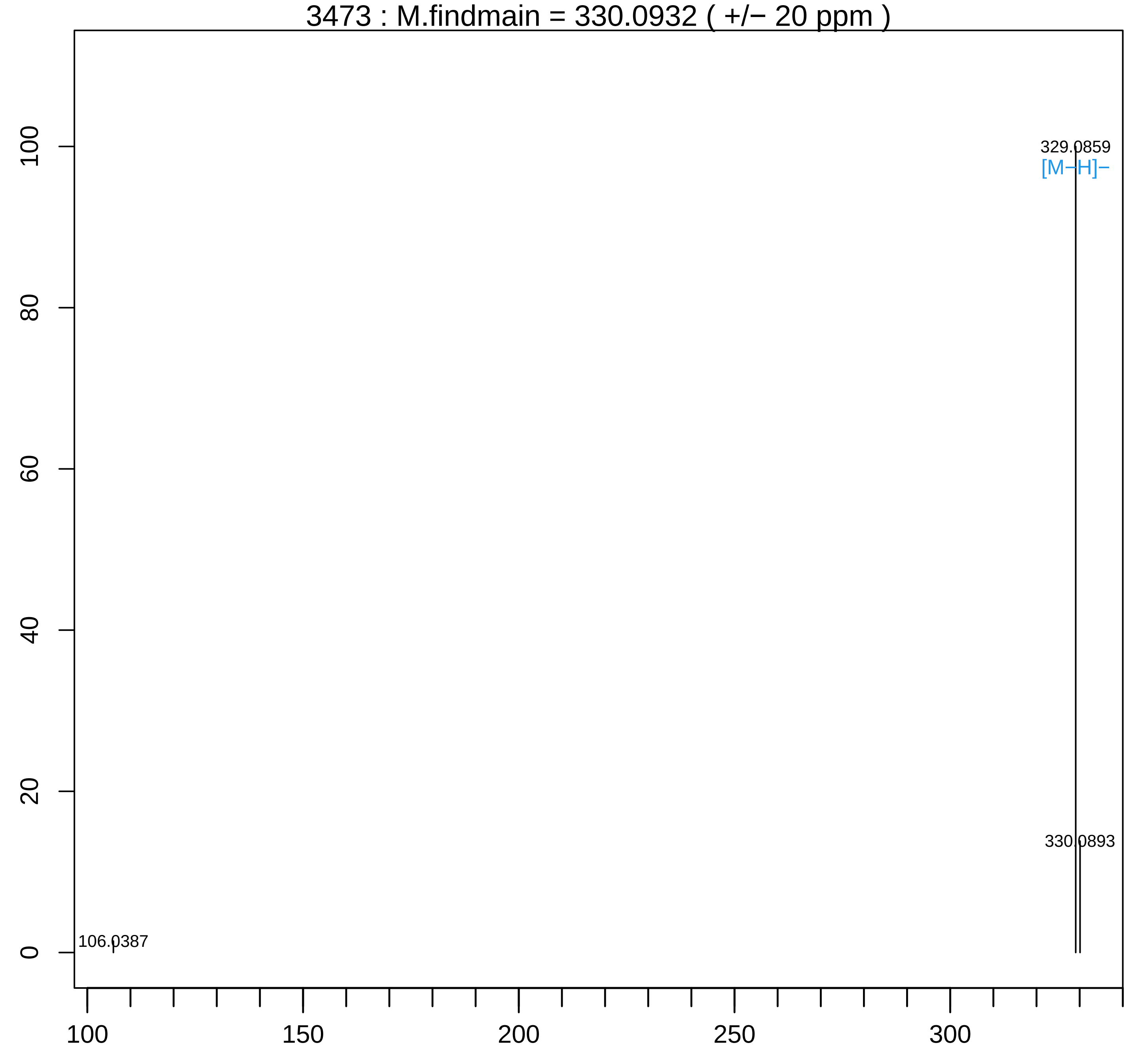

3473 : M.findmain = 330.0932 (+/− 20 ppm)
329.0859
[M−H]−
330.0893
106.0387
0
20
40
60
80
100
100
150
200
250
300

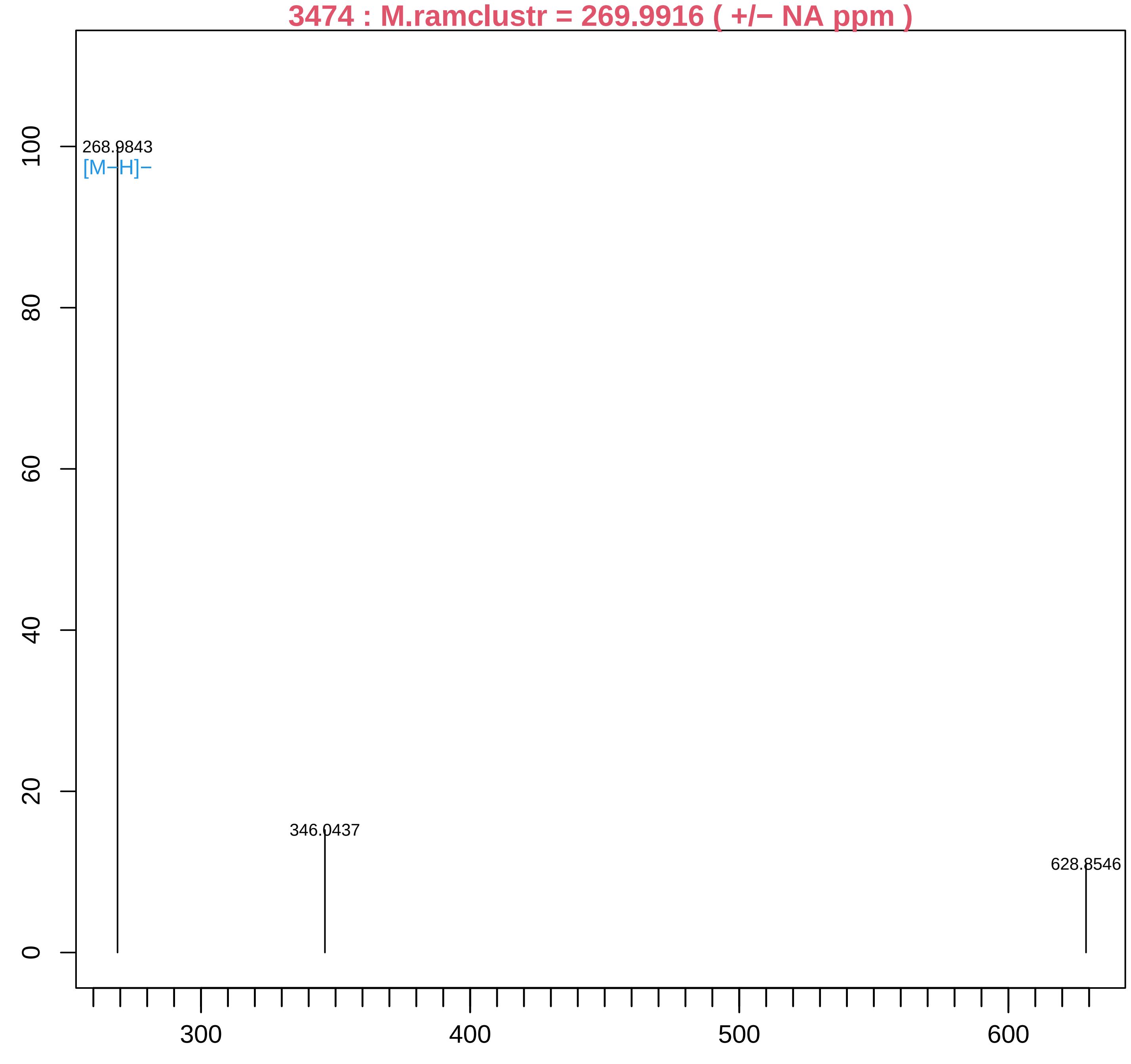
3474 : M.ramclustr = 269.9916 (+/− NA ppm)
268.9843
[M−H]−
346.0437
628.8546
0
20
40
60
80
100
300
400
500
600

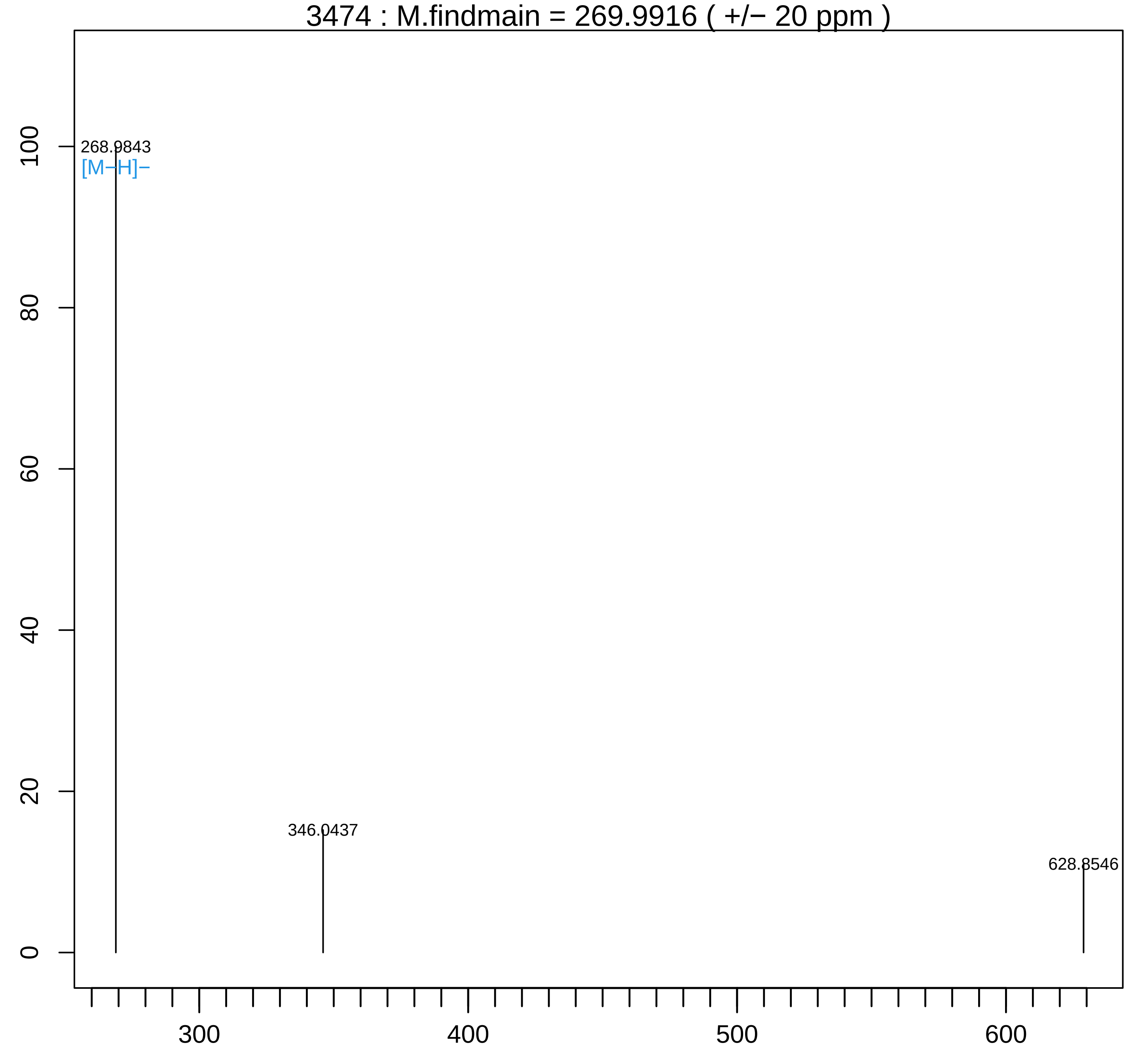
3474 : M.findmain = 269.9916 (+/− 20 ppm)
268.9843
[M−H]−
346.0437
628.8546
0
20
40
60
80
100
300
400
500
600

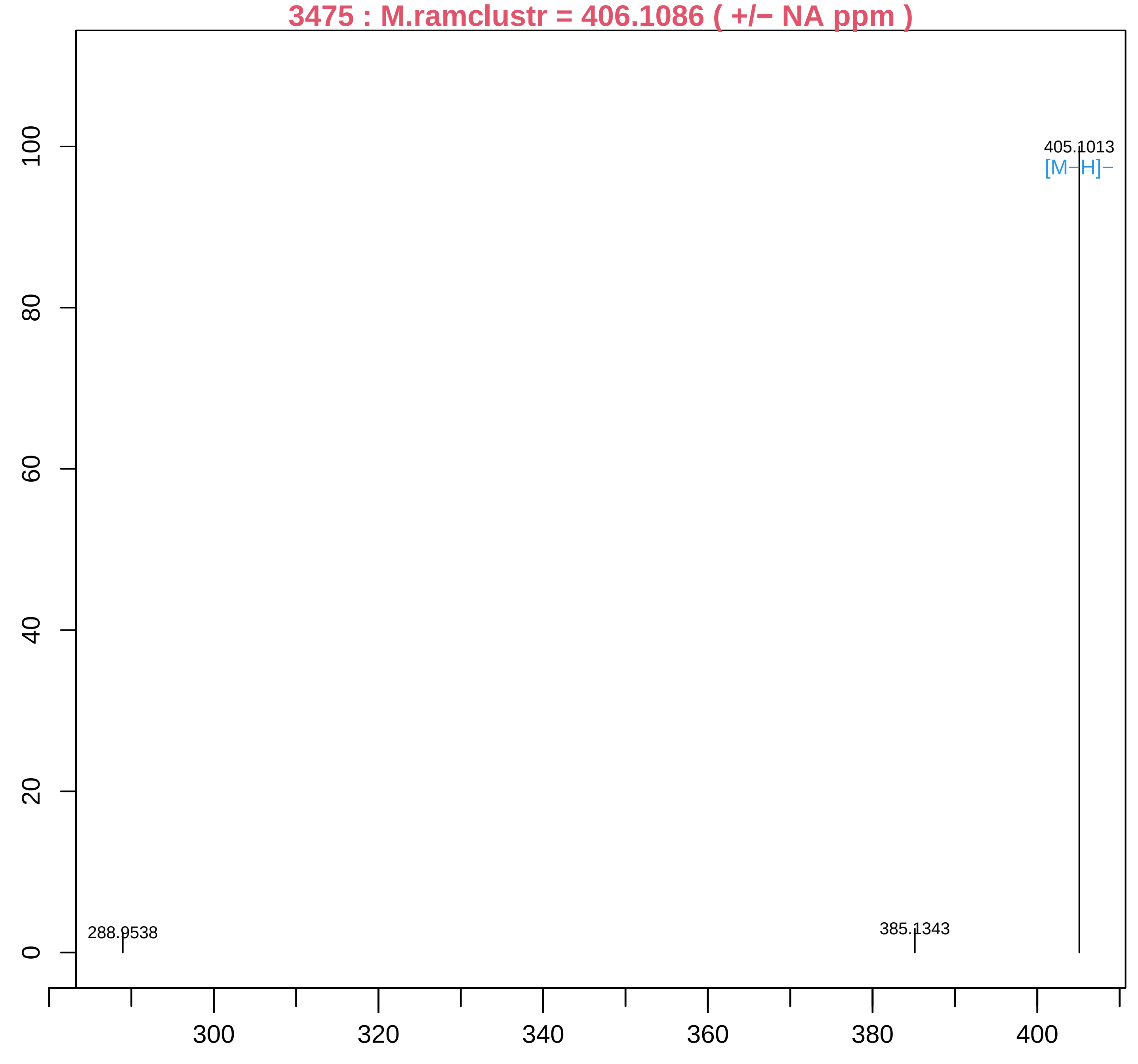

3475 : M.ramclustr = 406.1086 (+/− NA ppm)
405.1013
[M−H]−
288.9538
385.1343
0
20
40
60
80
100
300
320
340
360
380
400

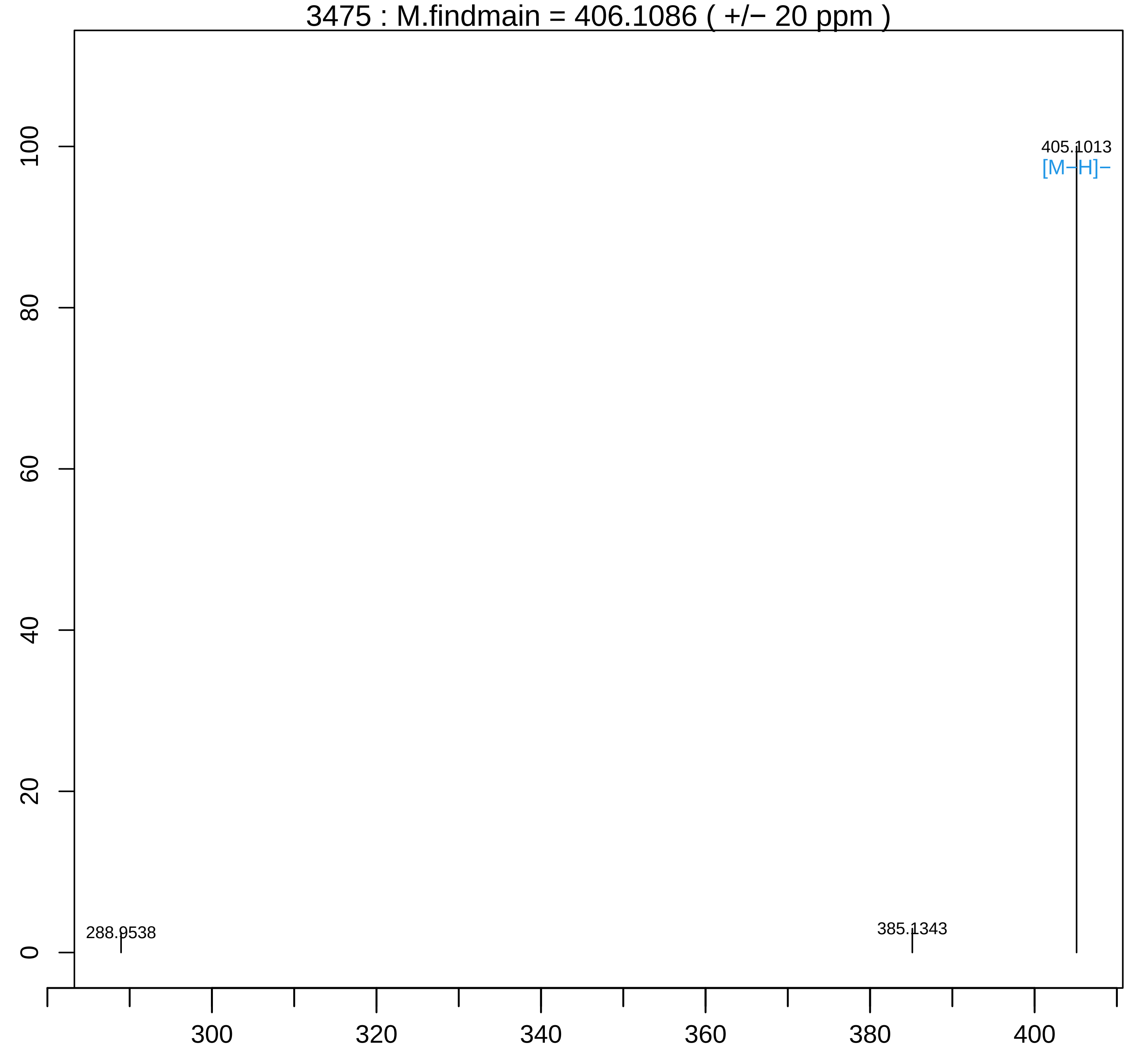

3475 : M.findmain = 406.1086 (+/− 20 ppm)
405.1013
[M−H]−
288.9538
385.1343
0
20
40
60
80
100
300
320
340
360
380
400

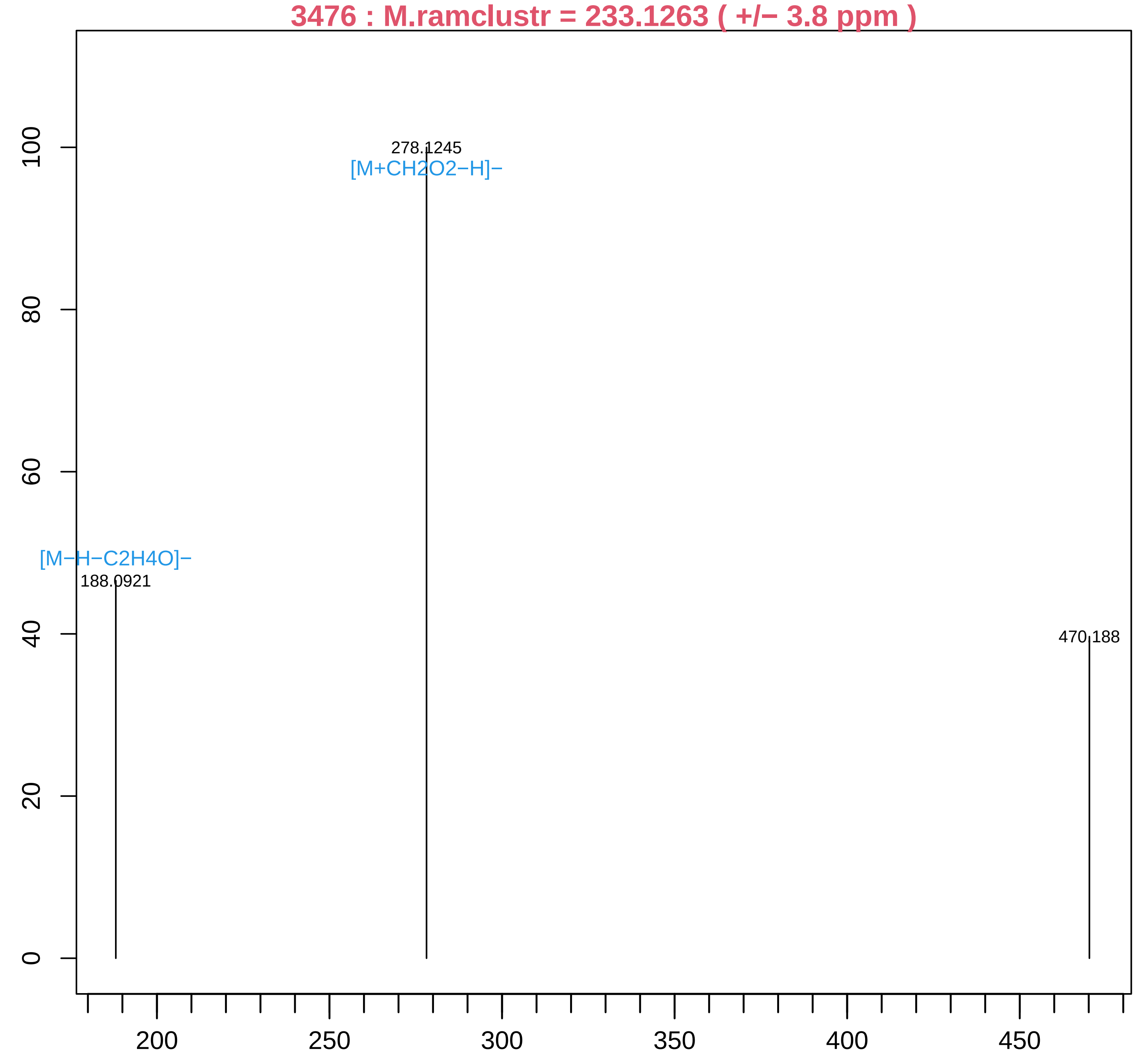
3476 : M.ramclustr = 233.1263 (+/− 3.8 ppm)
278.1245
[M+CH2O2−H]−
[M−H−C2H4O]−
188.0921
470.188
0
20
40
60
80
100
200
250
300
350
400
450

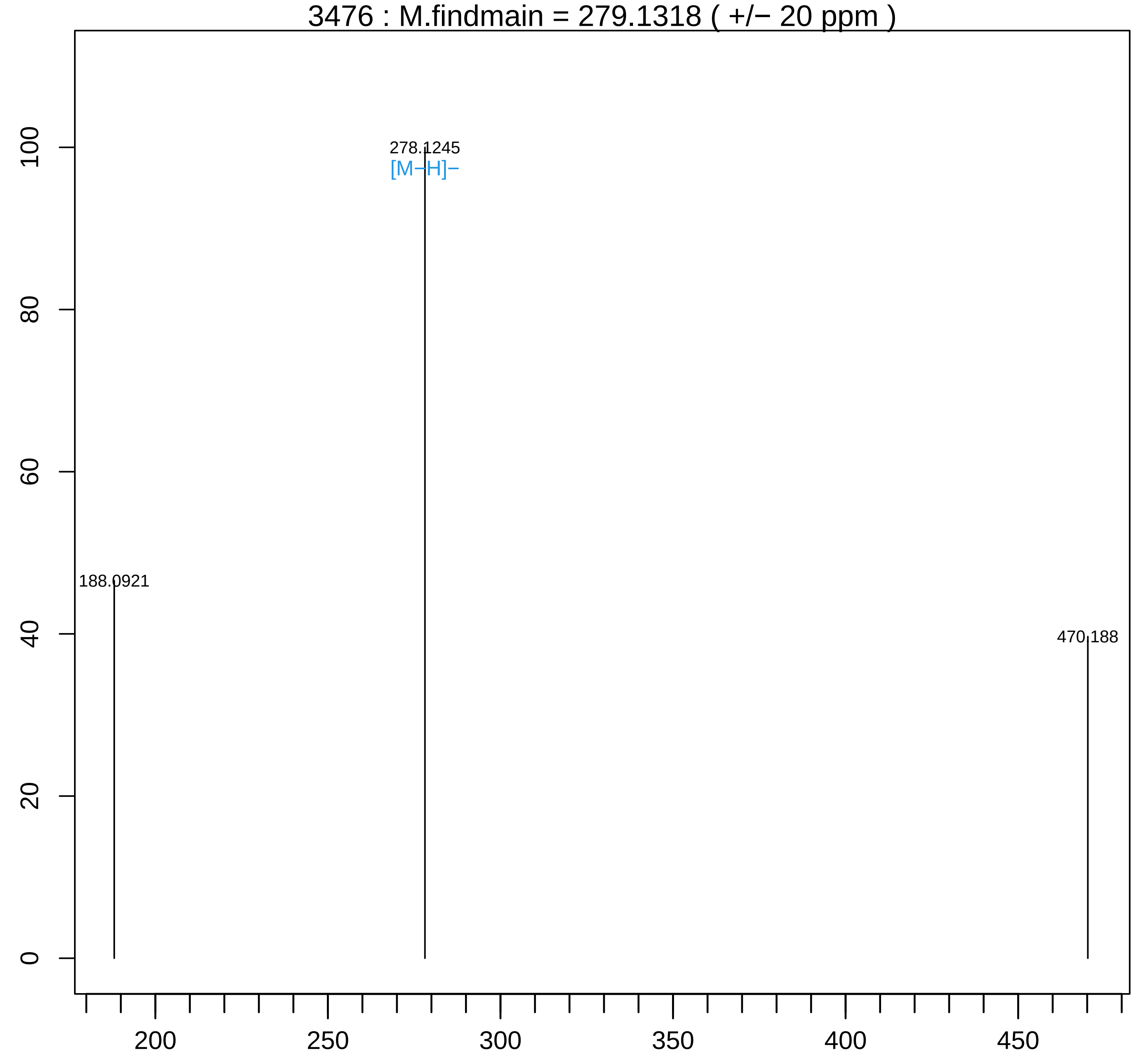
3476 : M.findmain = 279.1318 (+/− 20 ppm)
278.1245
[M−H]−
188.0921
470.188
0
20
40
60
80
100
200
250
300
350
400
450

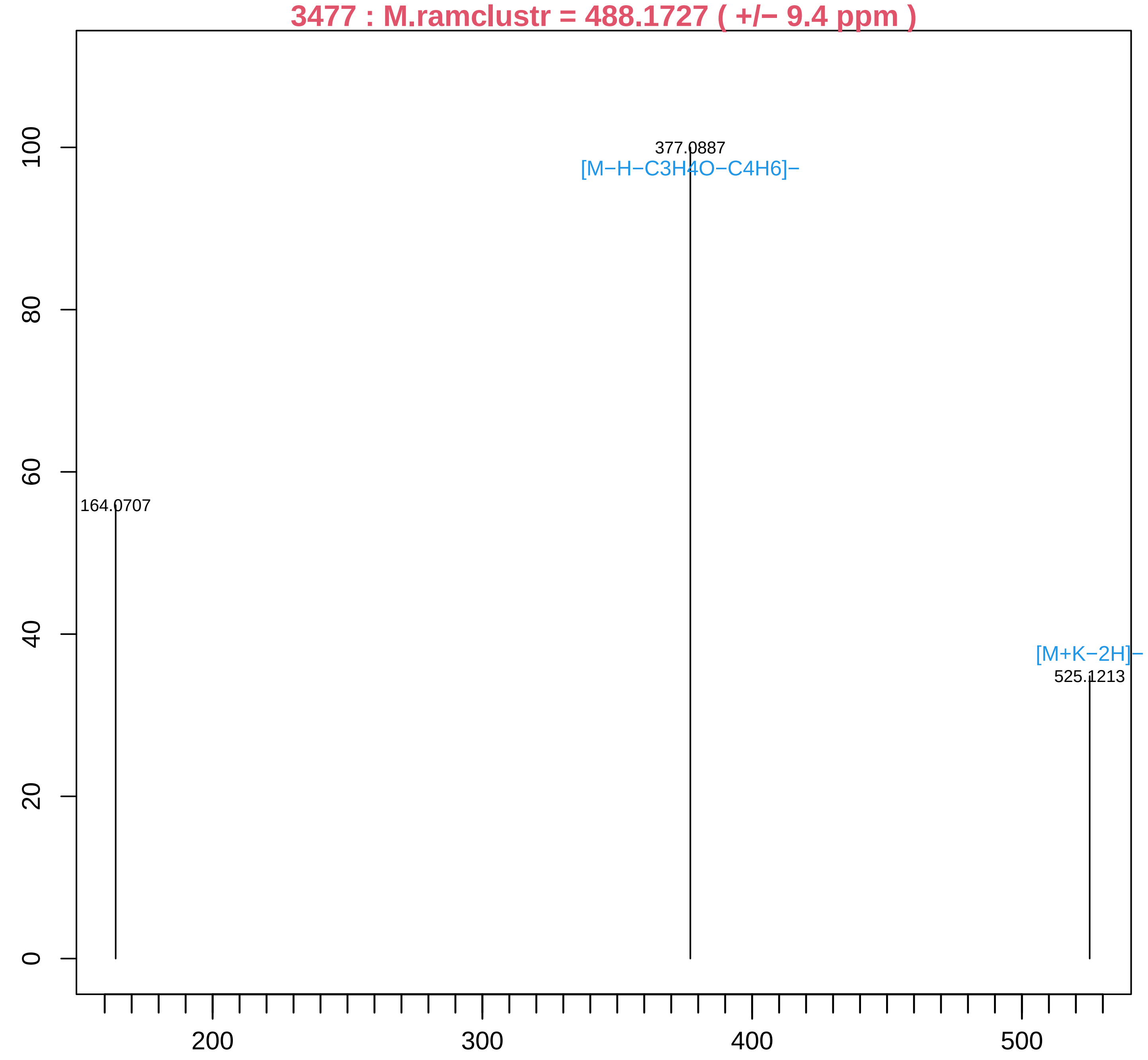
3477 : M.ramclustr = 488.1727 (+/− 9.4 ppm)
377.0887
[M−H−C3H4O−C4H6]−
164.0707
[M+K−2H]−
525.1213
0
20
40
60
80
100
200
300
400
500

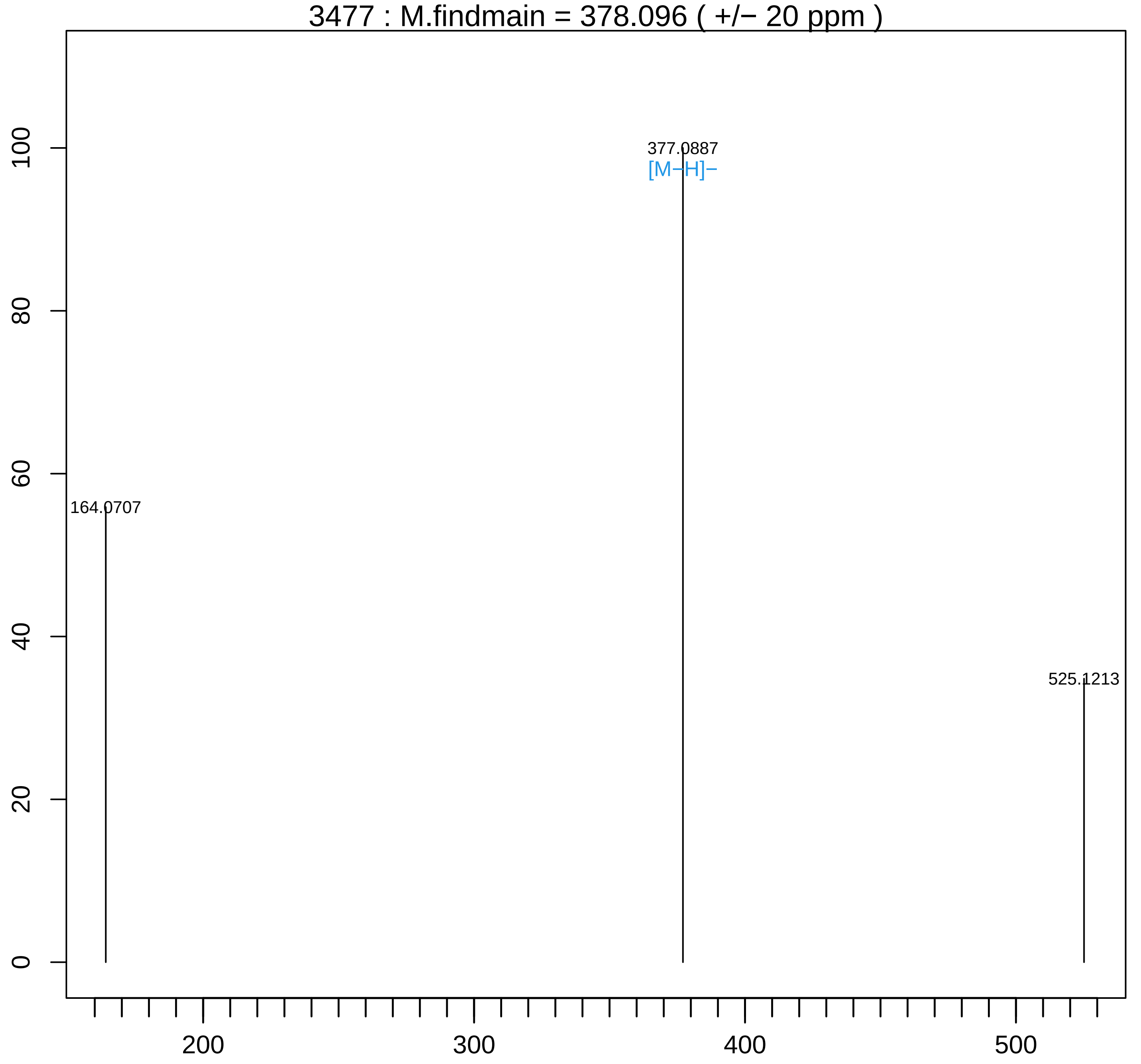
3477 : M.findmain = 378.096 (+/− 20 ppm)
377.0887
[M−H]−
164.0707
525.1213
0
20
40
60
80
100
200
300
400
500

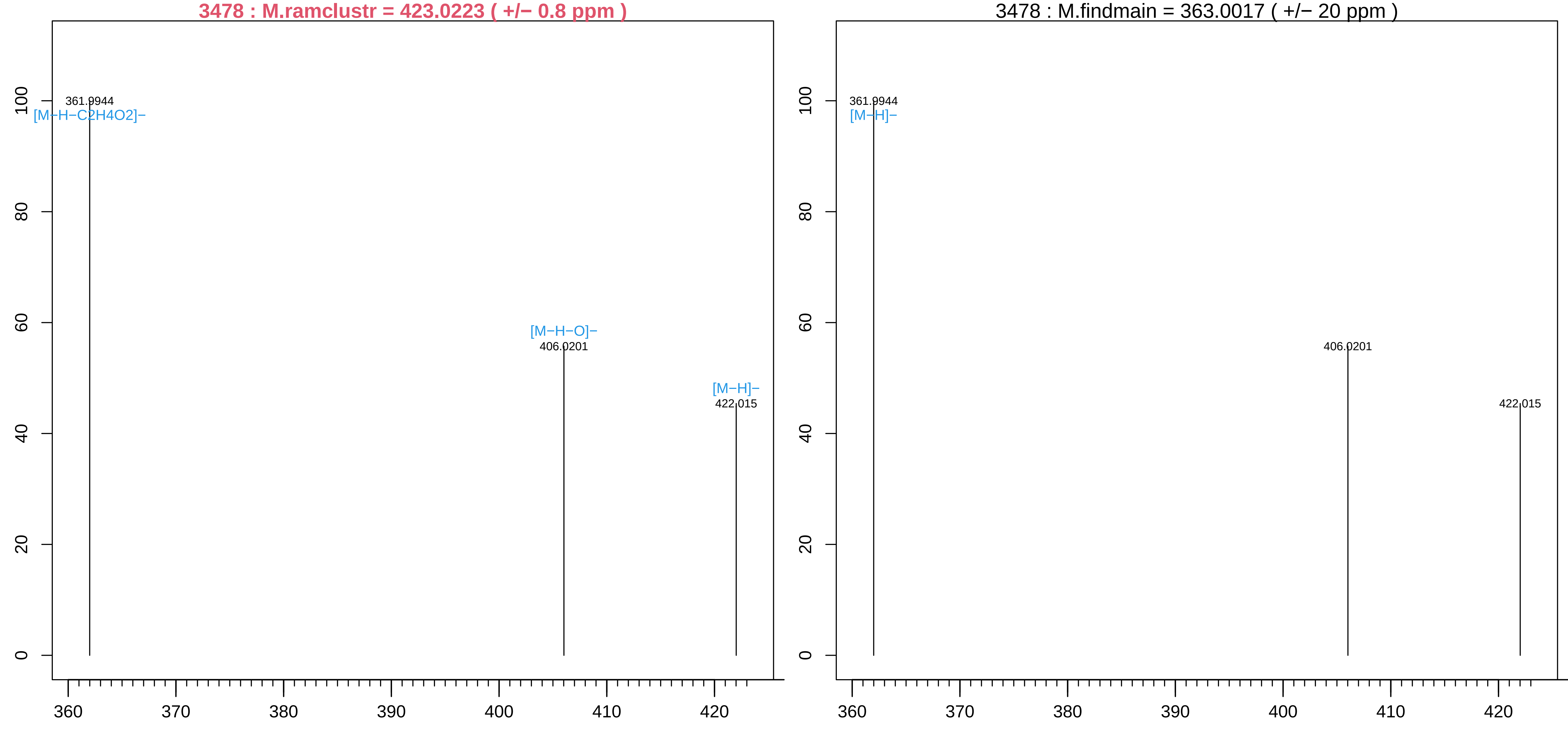
3478 : M.ramclustr = 423.0223 (+/− 0.8 ppm)
361.9944
[M−H−C2H4O2]−
[M−H−O]−
406.0201
[M−H]−
422.015
3478 : M.findmain = 363.0017 (+/− 20 ppm)
361.9944
[M−H]−
406.0201
422.015
0
20
40
60
80
100
360
370
380
390
400
410
420

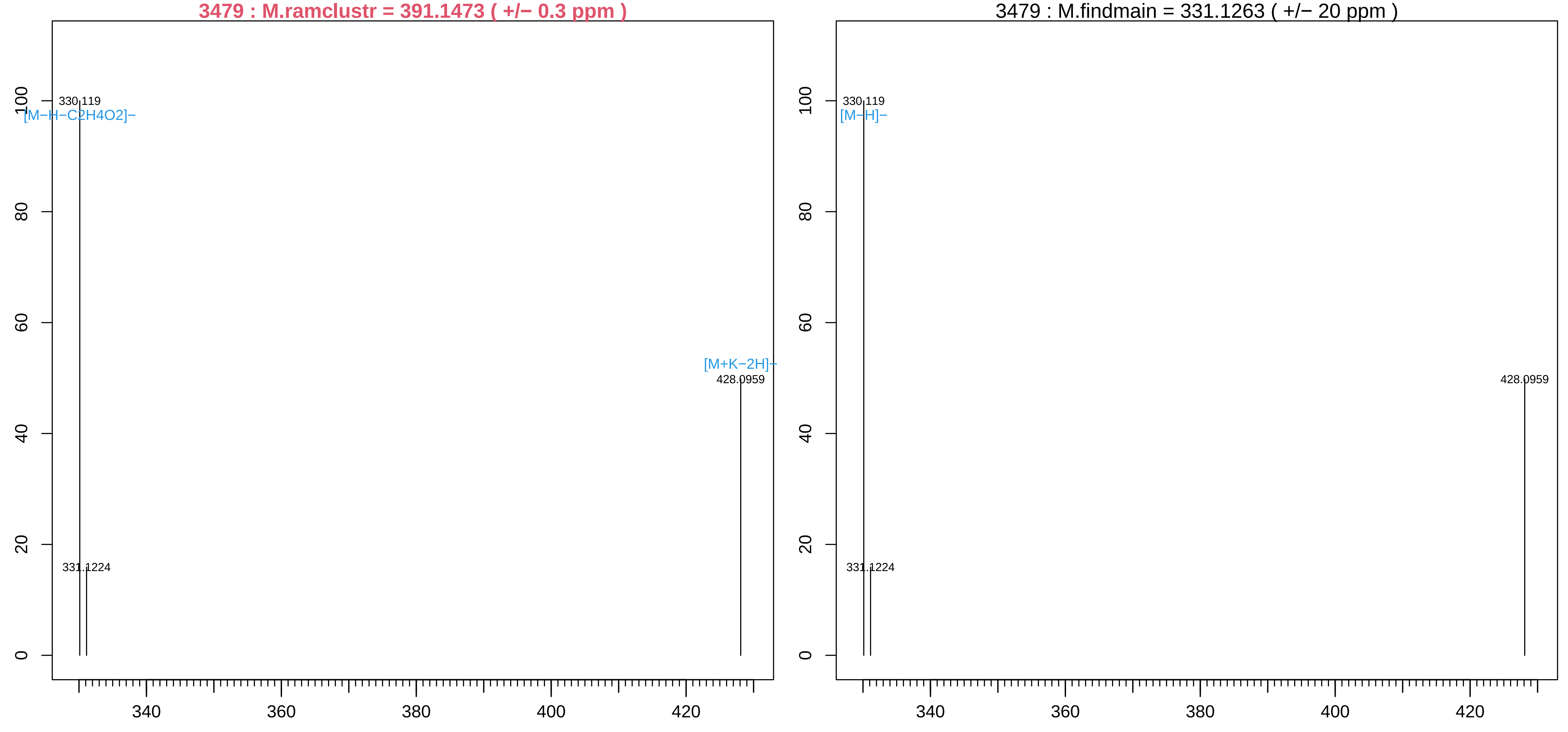
3479 : M.ramclustr = 391.1473 (+/− 0.3 ppm)
330.119
[M−H−C2H4O2]−
331.1224
[M+K−2H]−
428.0959
3479 : M.findmain = 331.1263 (+/− 20 ppm)
330.119
[M−H]−
331.1224
428.0959
0
20
40
60
80
100
340
360
380
400
420

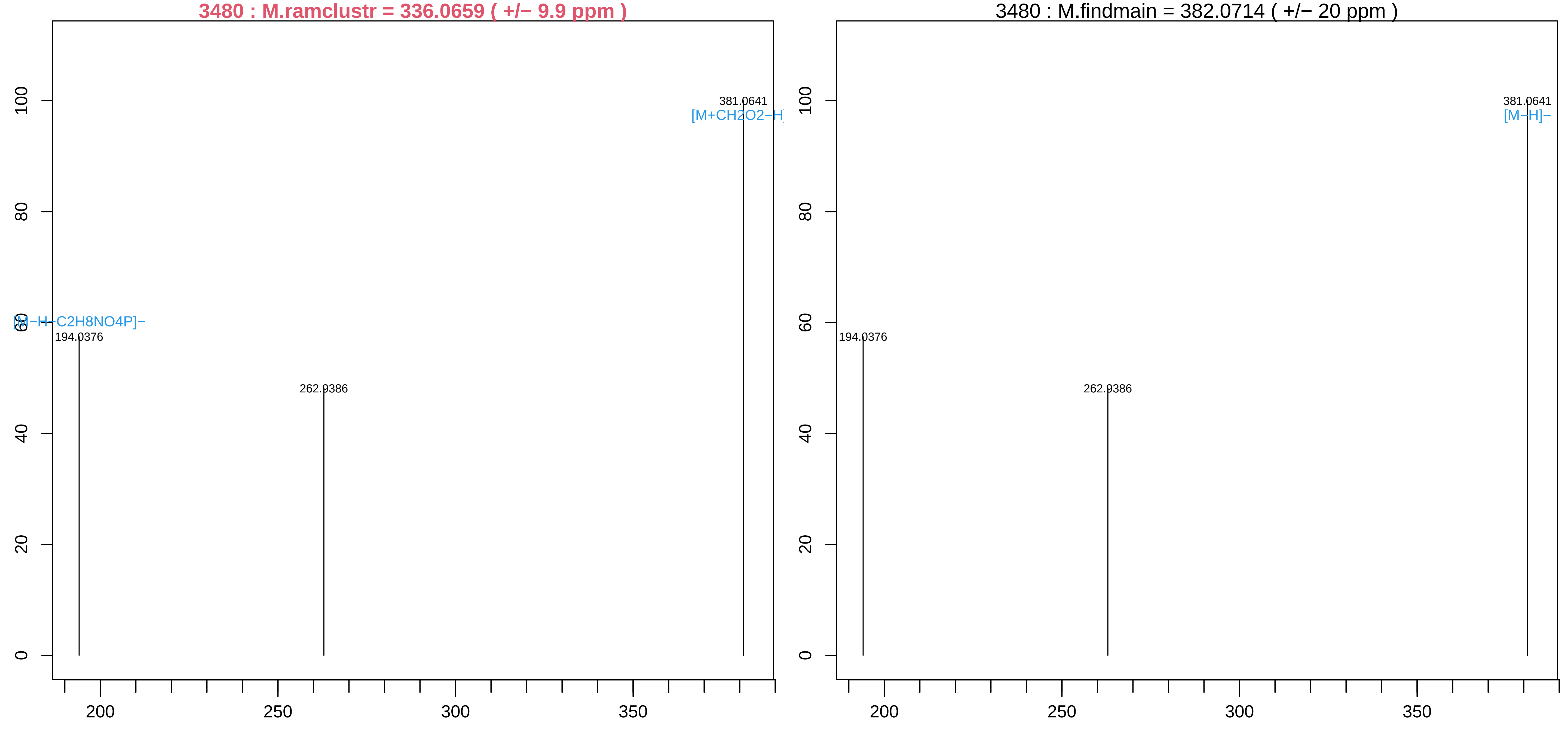
3480 : M.ramclustr = 336.0659 (+/− 9.9 ppm)
381.0641
[M+CH2O2−H]
[M−H−C2H8NO4P]−
194.0376
262.9386
0
20
40
60
80
100
200
250
300
350
3480 : M.findmain = 382.0714 (+/− 20 ppm)
381.0641
[M−H]−
194.0376
262.9386

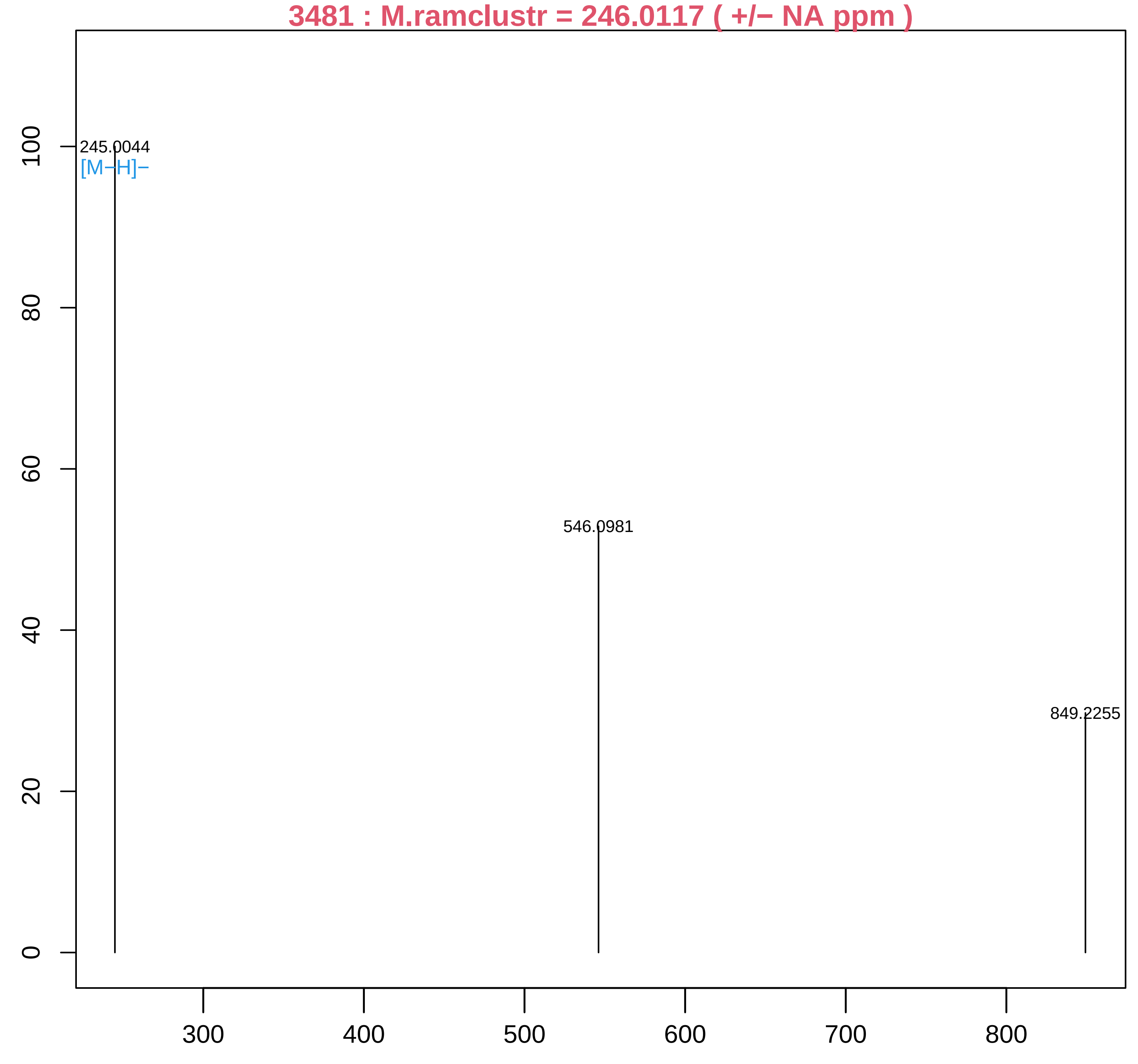
3481 : M.ramclustr = 246.0117 (+/− NA ppm)
245.0044
[M−H]−
546.0981
849.2255
0
20
40
60
80
100
300
400
500
600
700
800

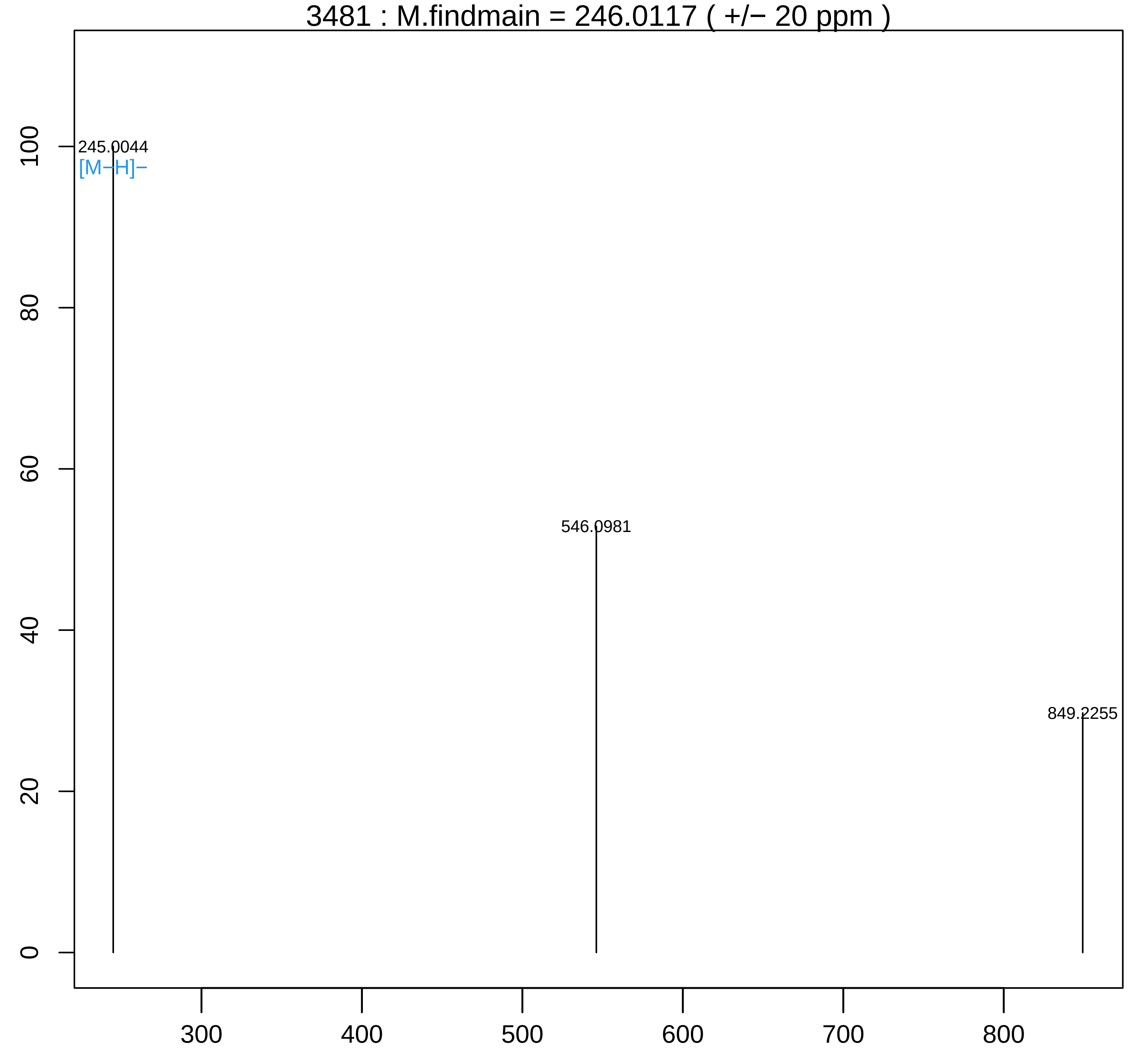
3481 : M.findmain = 246.0117 (+/− 20 ppm)
245.0044
[M−H]−
546.0981
849.2255
0
20
40
60
80
100
300
400
500
600
700
800

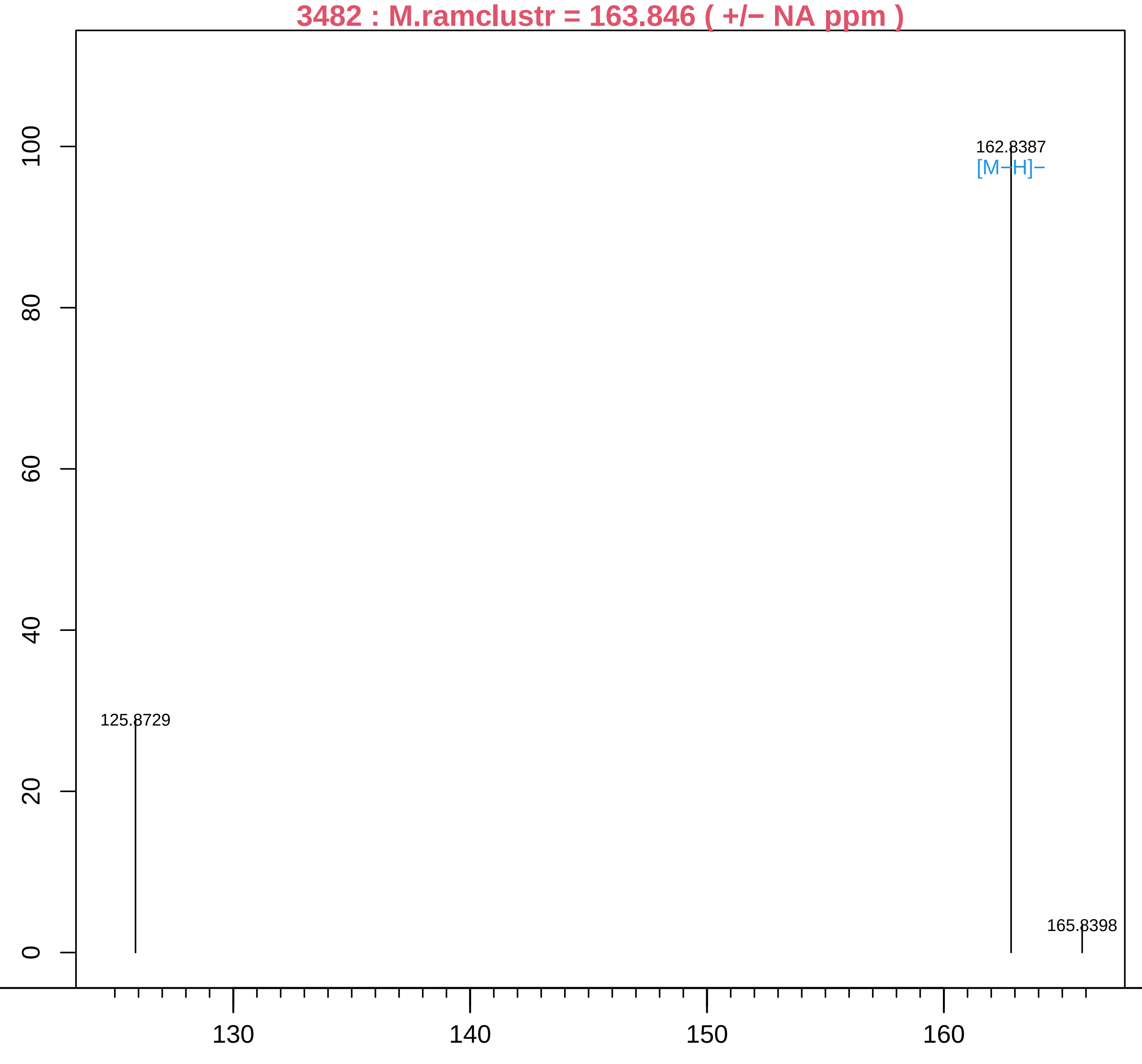
3482 : M.ramclustr = 163.846 (+/− NA ppm)
162.8387
[M−H]−
125.8729
165.8398
0
20
40
60
80
100
130
140
150
160

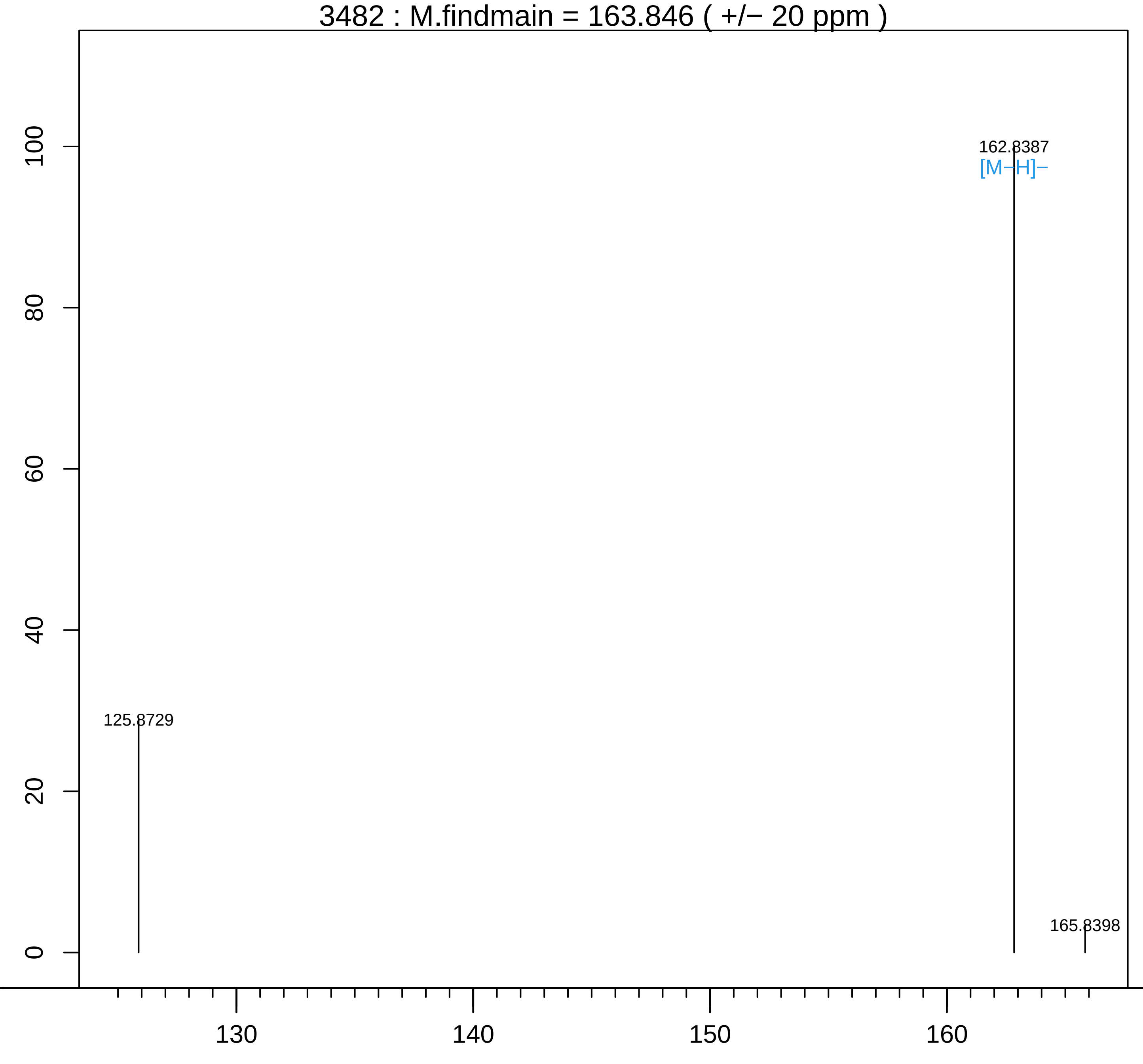
3482 : M.findmain = 163.846 (+/− 20 ppm)
162.8387
[M−H]−
125.8729
165.8398
0
20
40
60
80
100
130
140
150
160

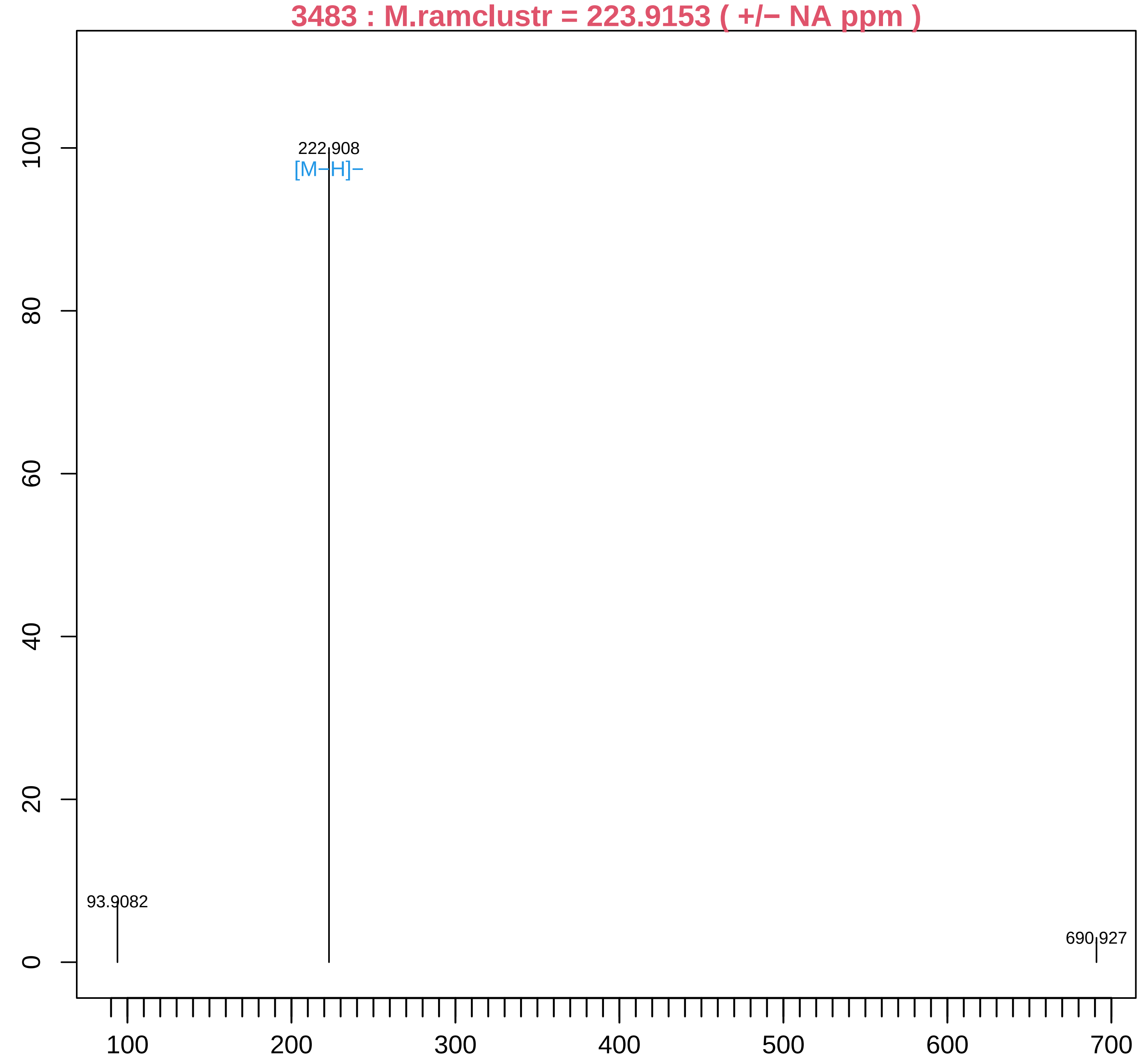
3483 : M.ramclustr = 223.9153 (+/− NA ppm)
222.908
[M−H]−
93.9082
690.927
0
20
40
60
80
100
100
200
300
400
500
600
700

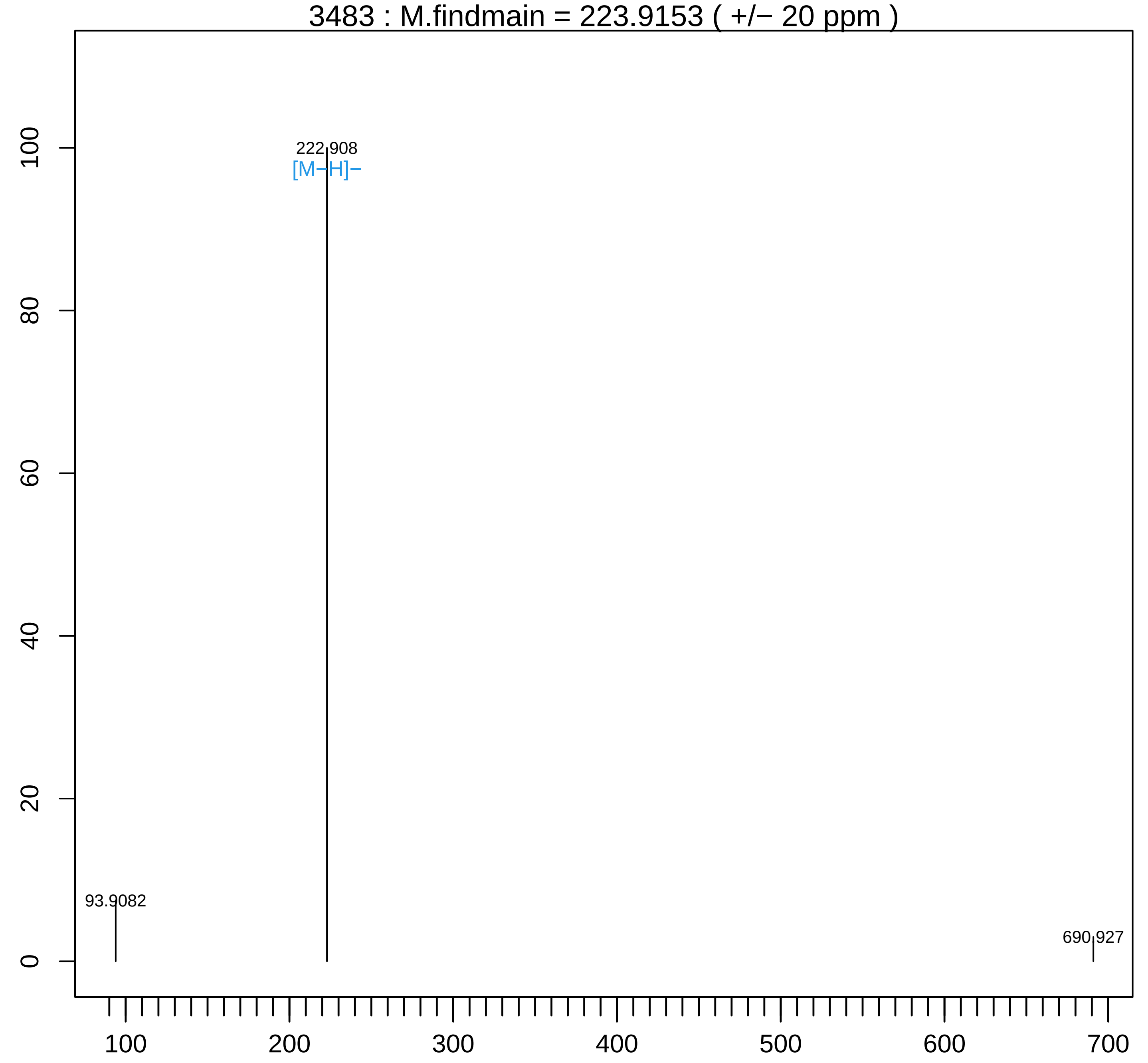
3483 : M.findmain = 223.9153 (+/− 20 ppm)
222.908
[M−H]−
93.9082
690.927
0
20
40
60
80
100
100
200
300
400
500
600
700

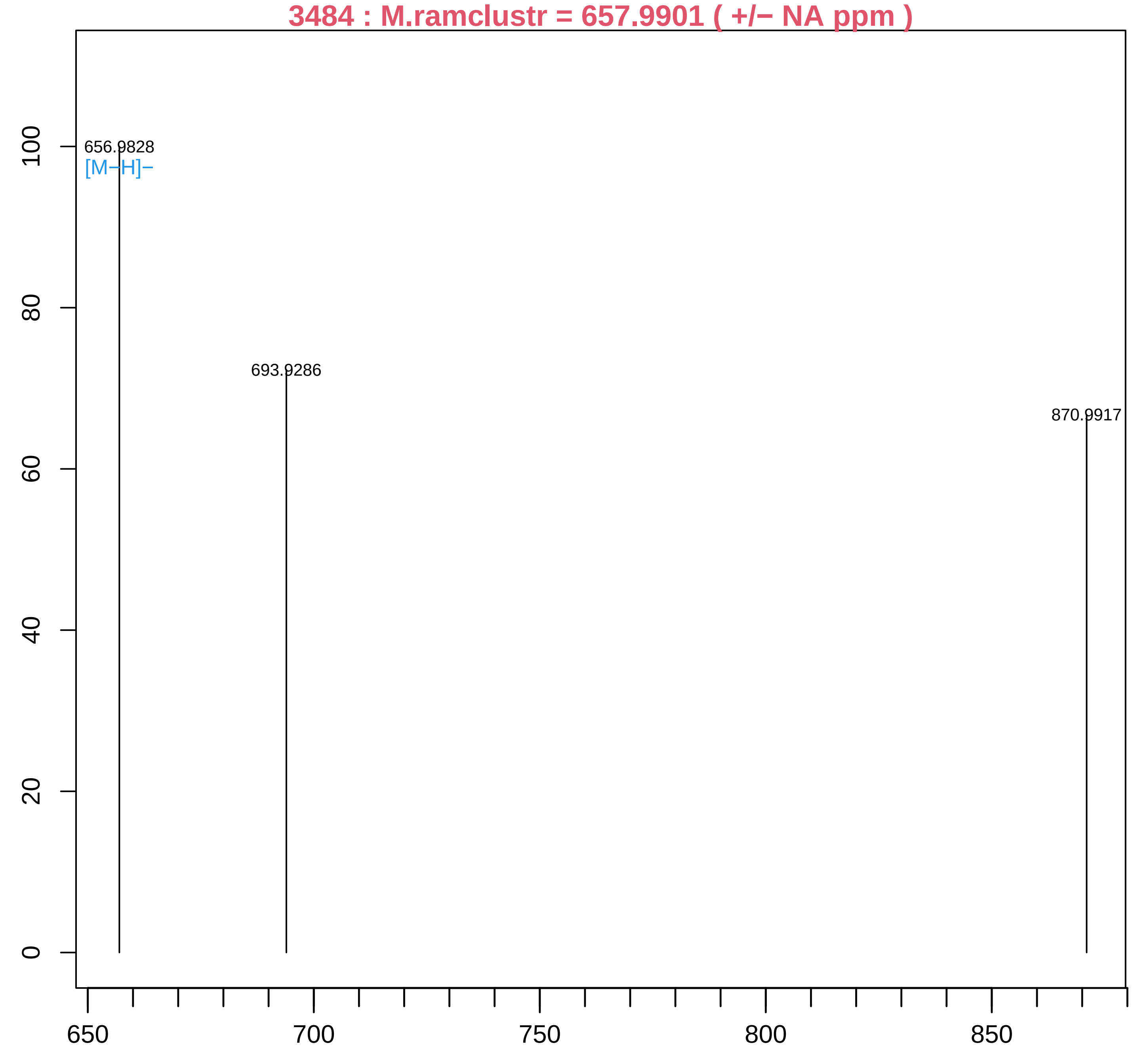
3484 : M.ramclustr = 657.9901 (+/− NA ppm)
656.9828
[M−H]−
693.9286
870.9917
0
20
40
60
80
100
650
700
750
800
850

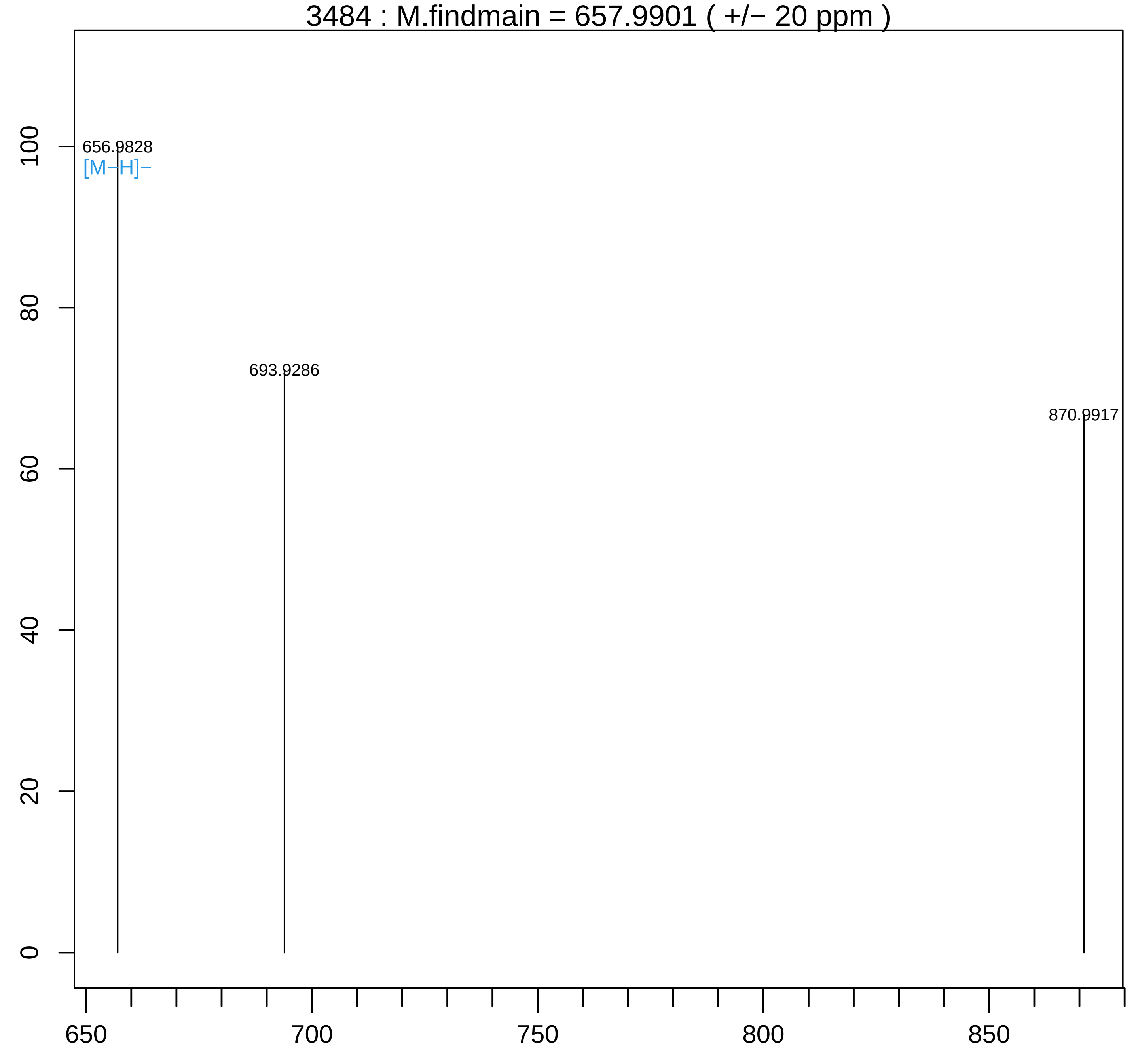
3484 : M.findmain = 657.9901 (+/− 20 ppm)
656.9828
[M−H]−
693.9286
870.9917
0
20
40
60
80
100
650
700
750
800
850

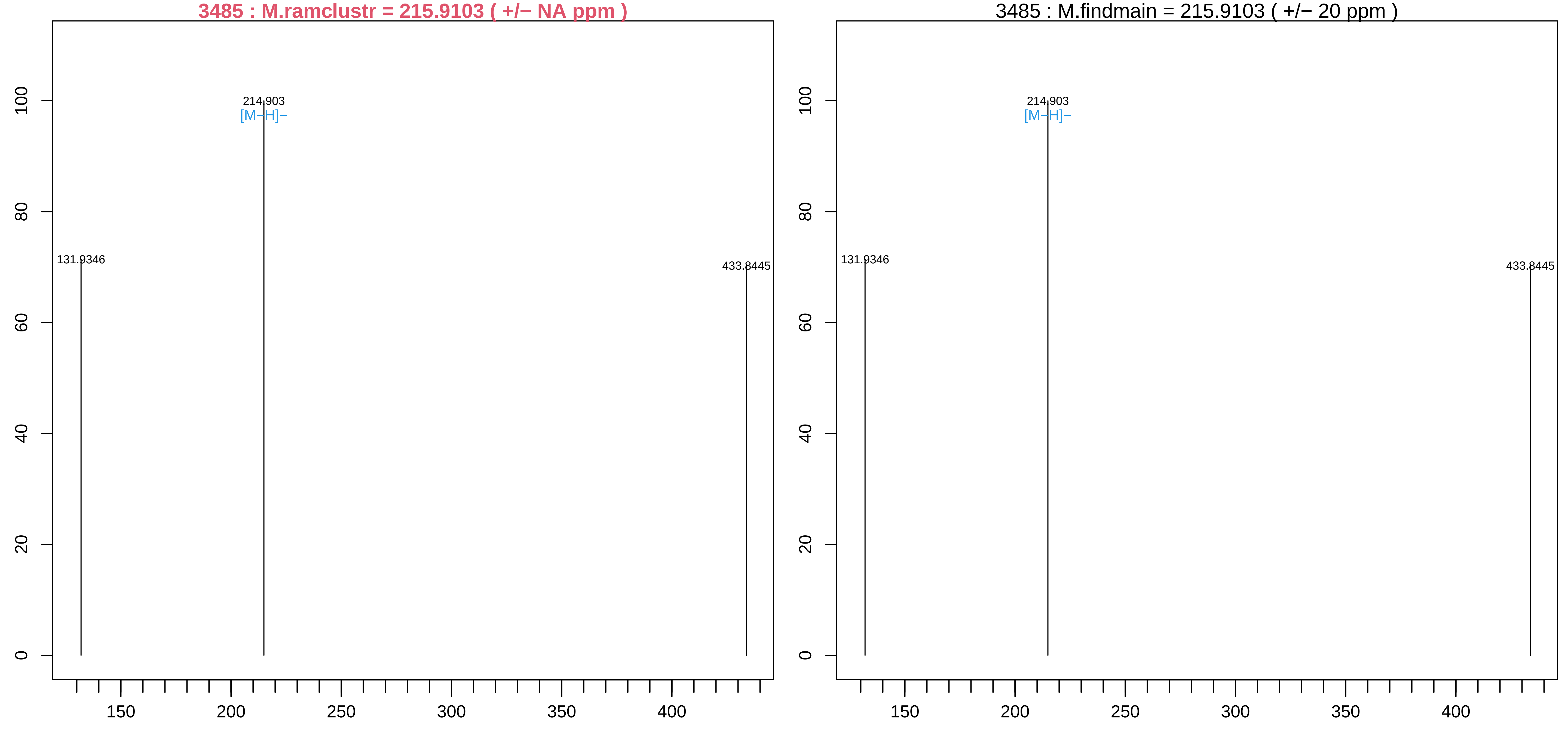

3485 : M.ramclustr = 215.9103 (+/− NA ppm)
131.9346
214.903
[M−H]−
433.8445
0
20
40
60
80
100
150
200
250
300
350
400
3485 : M.findmain = 215.9103 (+/− 20 ppm)
131.9346
214.903
[M−H]−
433.8445
0
20
40
60
80
100
150
200
250
300
350
400

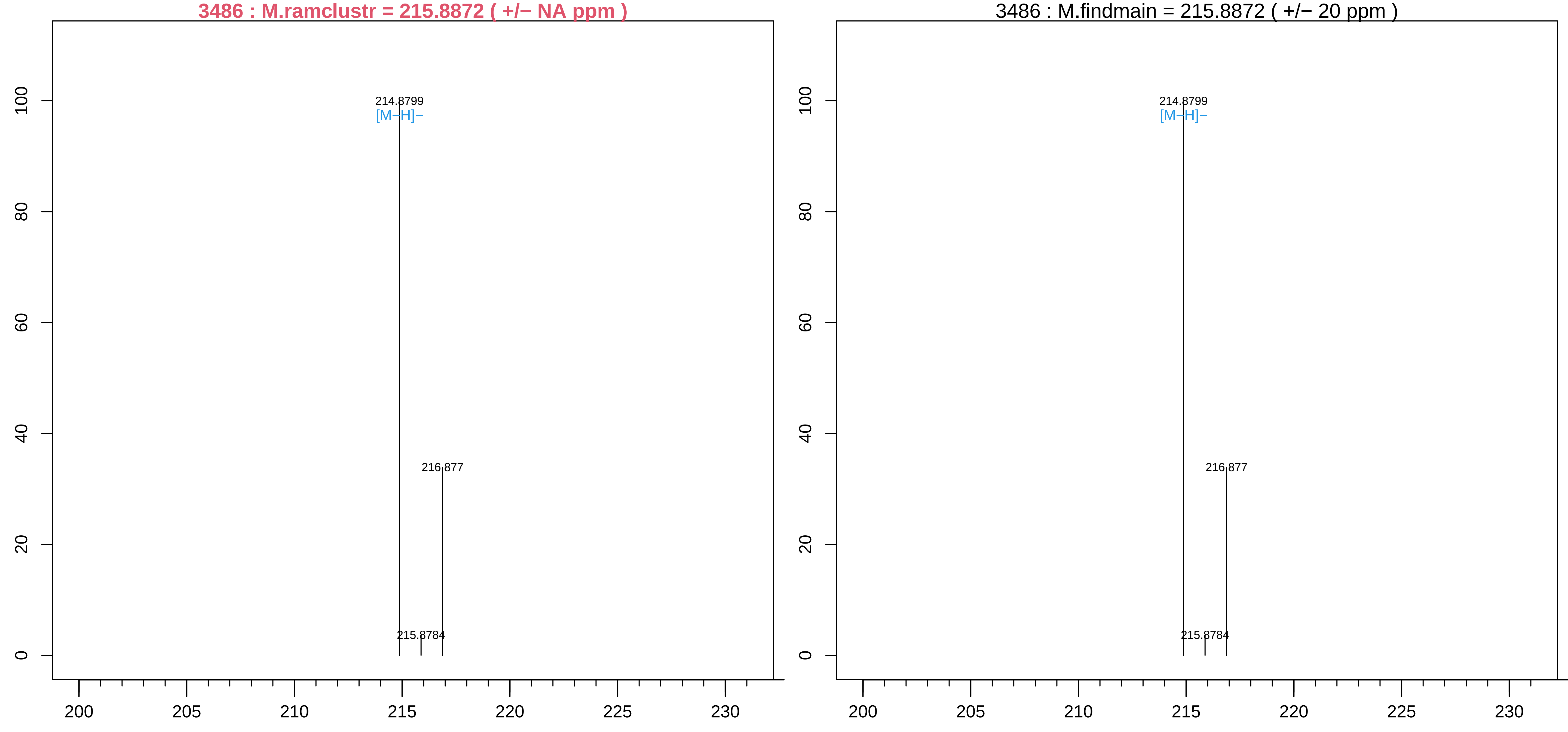

3486 : M.ramclustr = 215.8872 (+/− NA ppm)
214.8799
[M−H]−
216.877
215.8784
3486 : M.findmain = 215.8872 (+/− 20 ppm)
214.8799
[M−H]−
216.877
215.8784

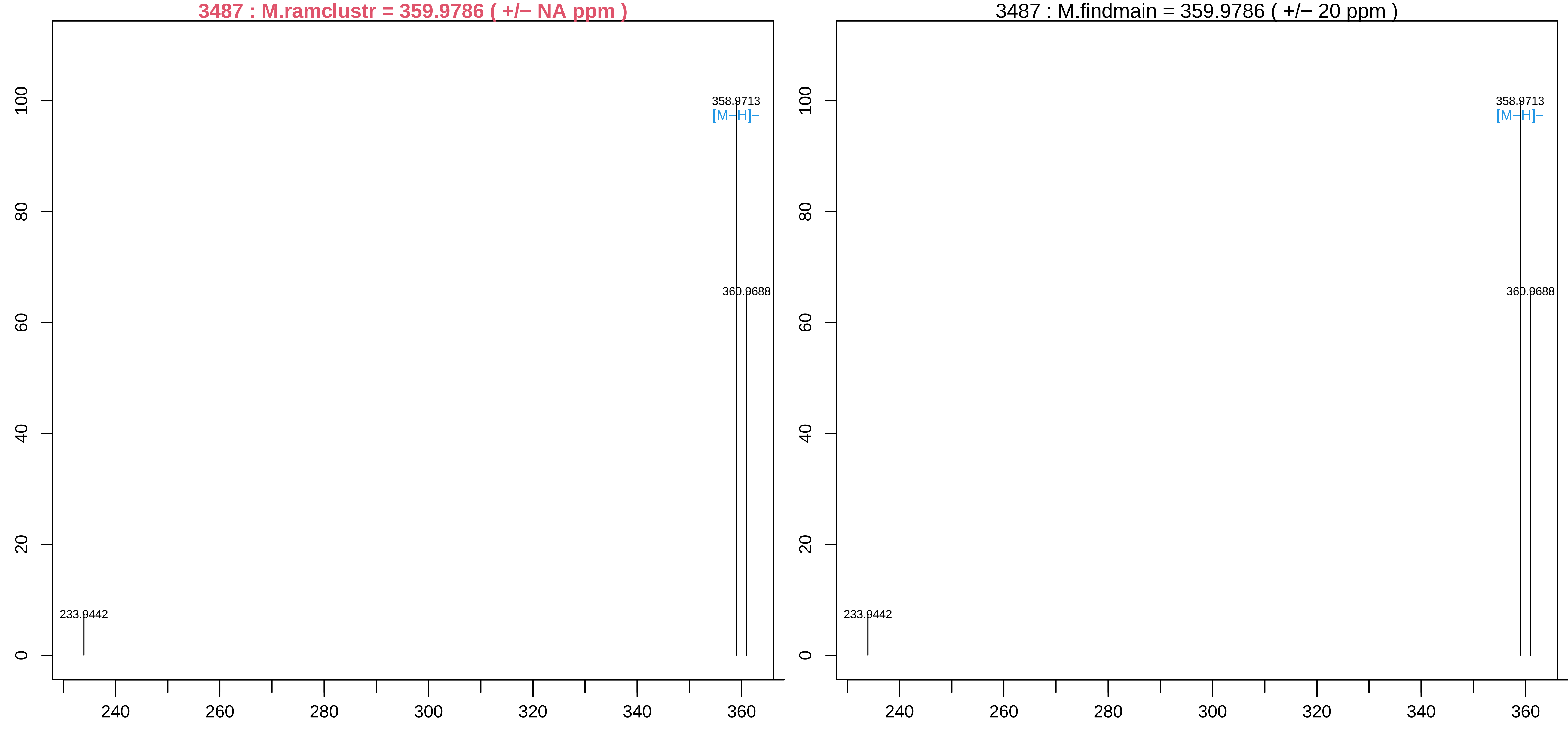
3487 : M.ramclustr = 359.9786 (+/− NA ppm)
358.9713
[M−H]−
360.9688
233.9442
3487 : M.findmain = 359.9786 (+/− 20 ppm)
358.9713
[M−H]−
360.9688
233.9442
0
20
40
60
80
100
240
260
280
300
320
340
360

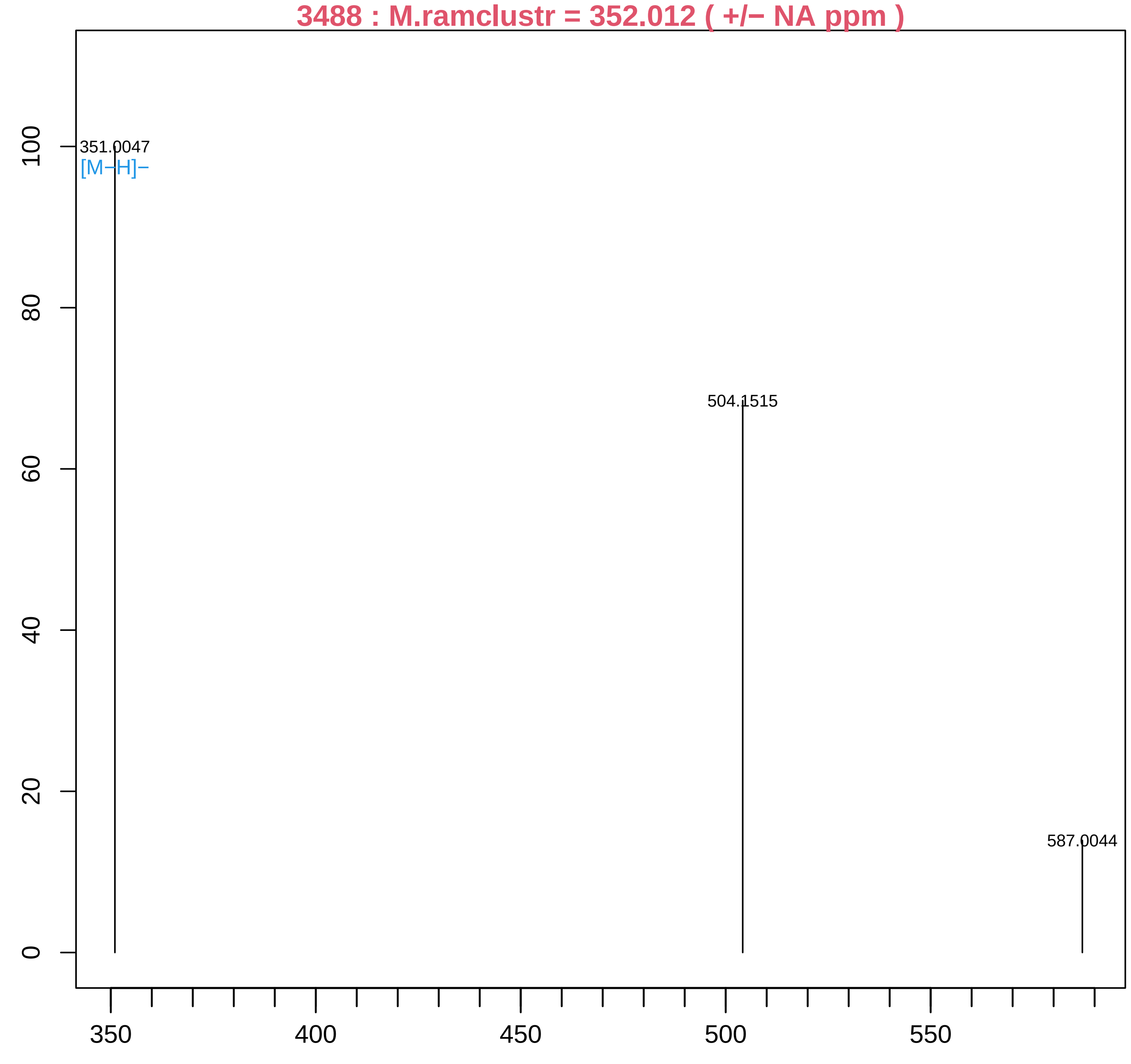
3488 : M.ramclustr = 352.012 (+/− NA ppm)
351.0047
[M−H]−
504.1515
587.0044
0
20
40
60
80
100
350
400
450
500
550

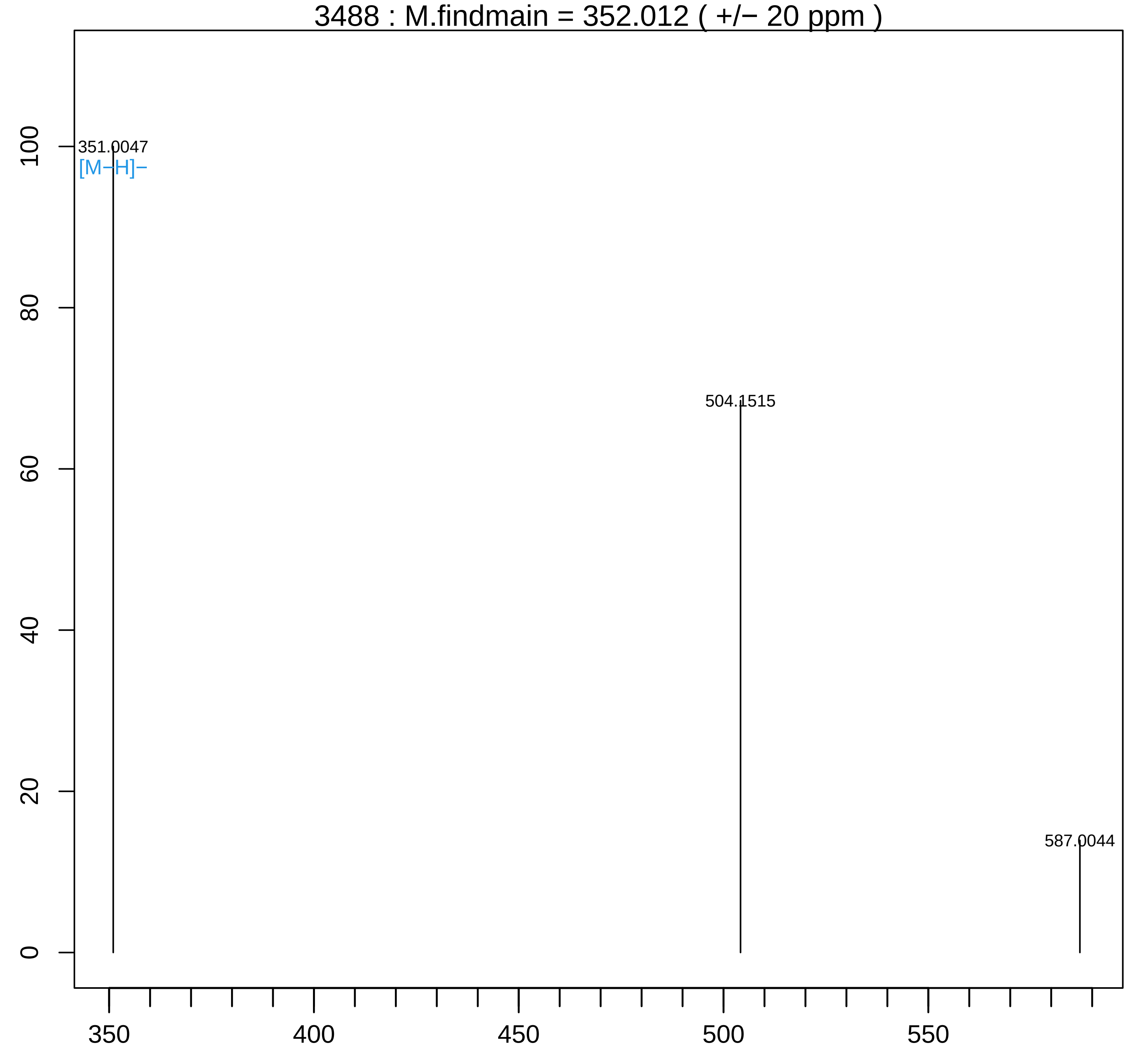
3488 : M.findmain = 352.012 (+/− 20 ppm)
351.0047
[M−H]−
504.1515
587.0044
0
20
40
60
80
100
350
400
450
500
550

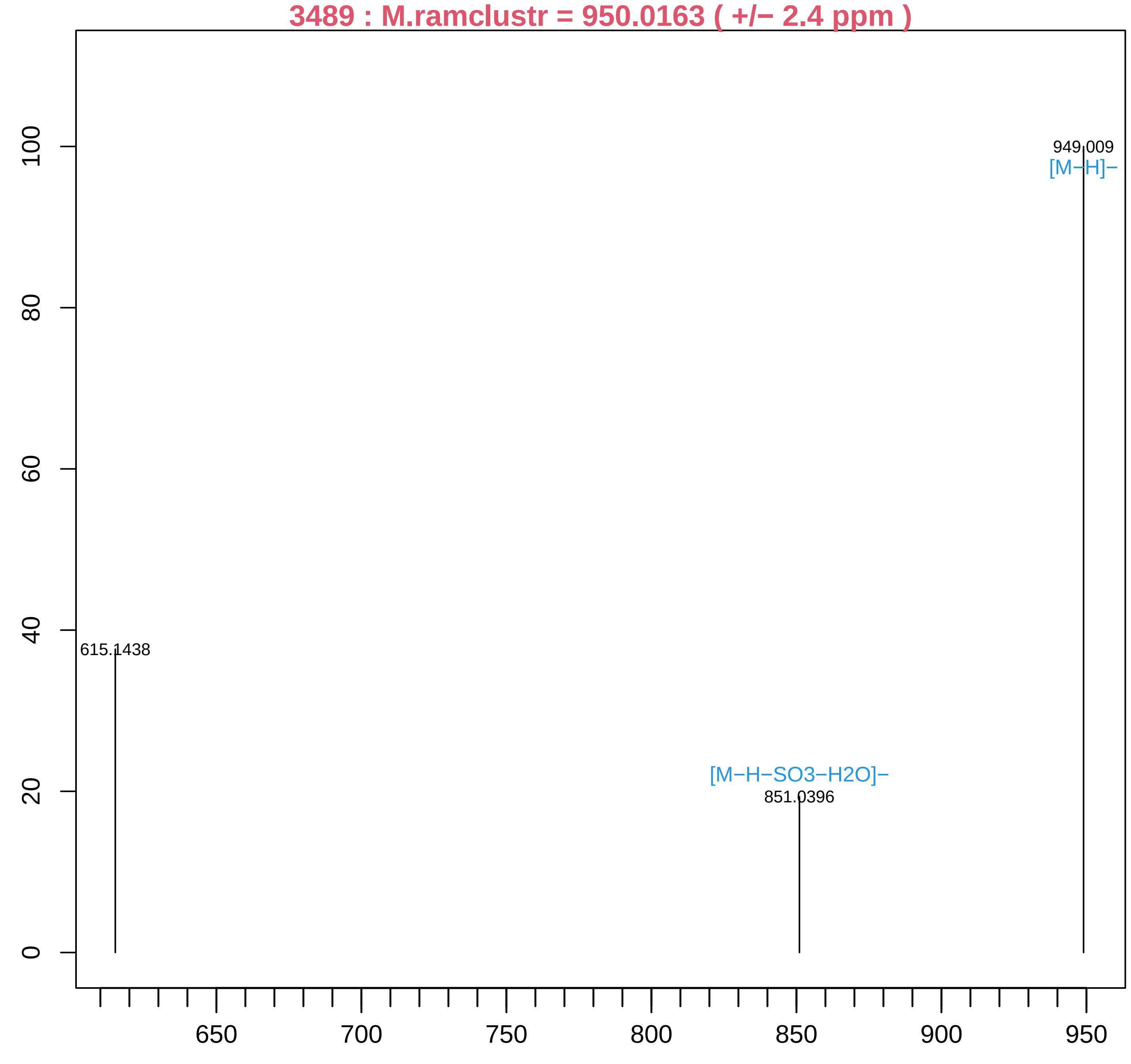
3489 : M.ramclustr = 950.0163 (+/− 2.4 ppm)
949.009
[M−H]−
615.1438
[M−H−SO3−H2O]−
851.0396
0
20
40
60
80
100
650
700
750
800
850
900
950

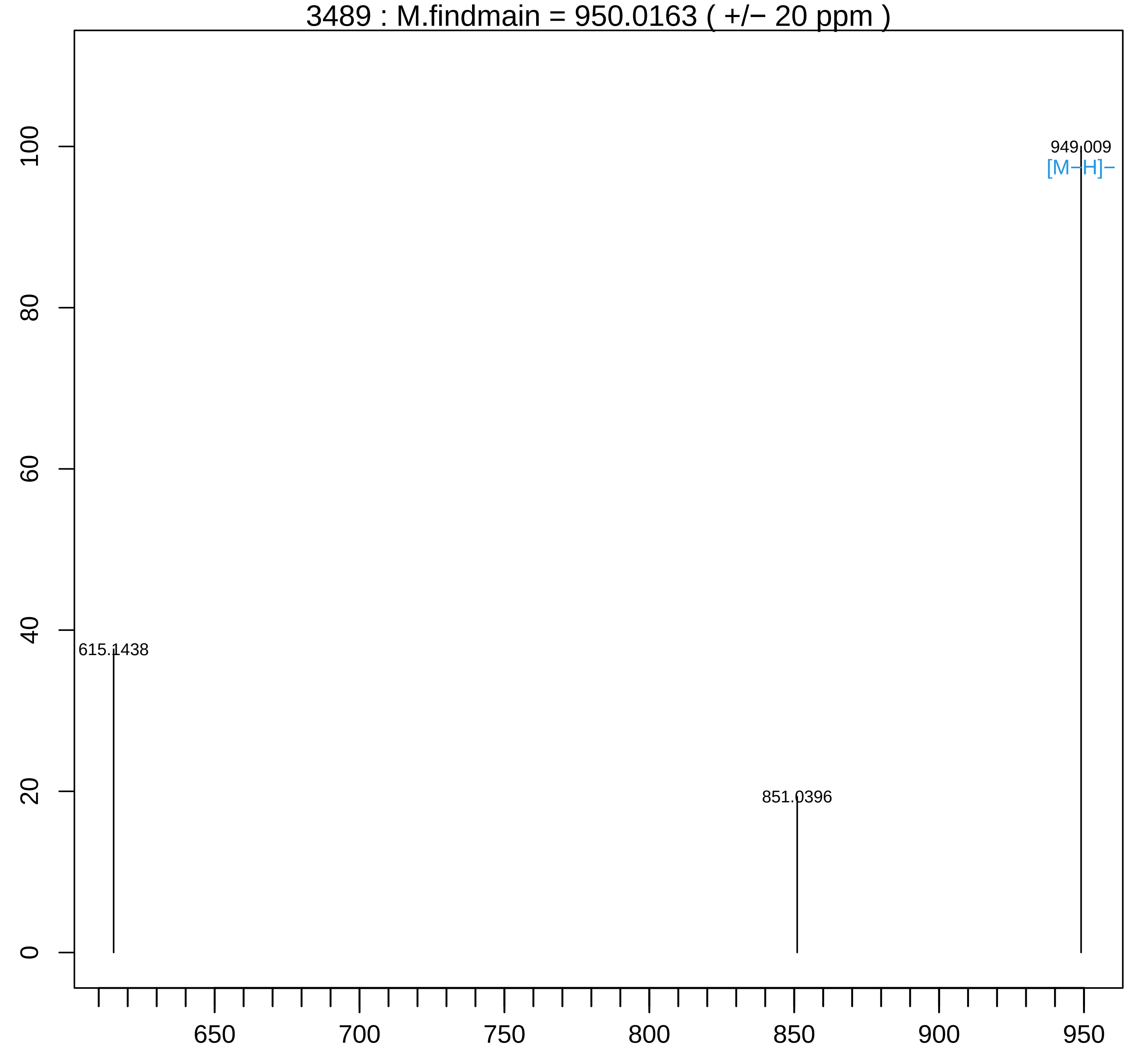
3489 : M.findmain = 950.0163 (+/− 20 ppm)
949.009
[M−H]−
615.1438
851.0396
0
20
40
60
80
100
650
700
750
800
850
900
950

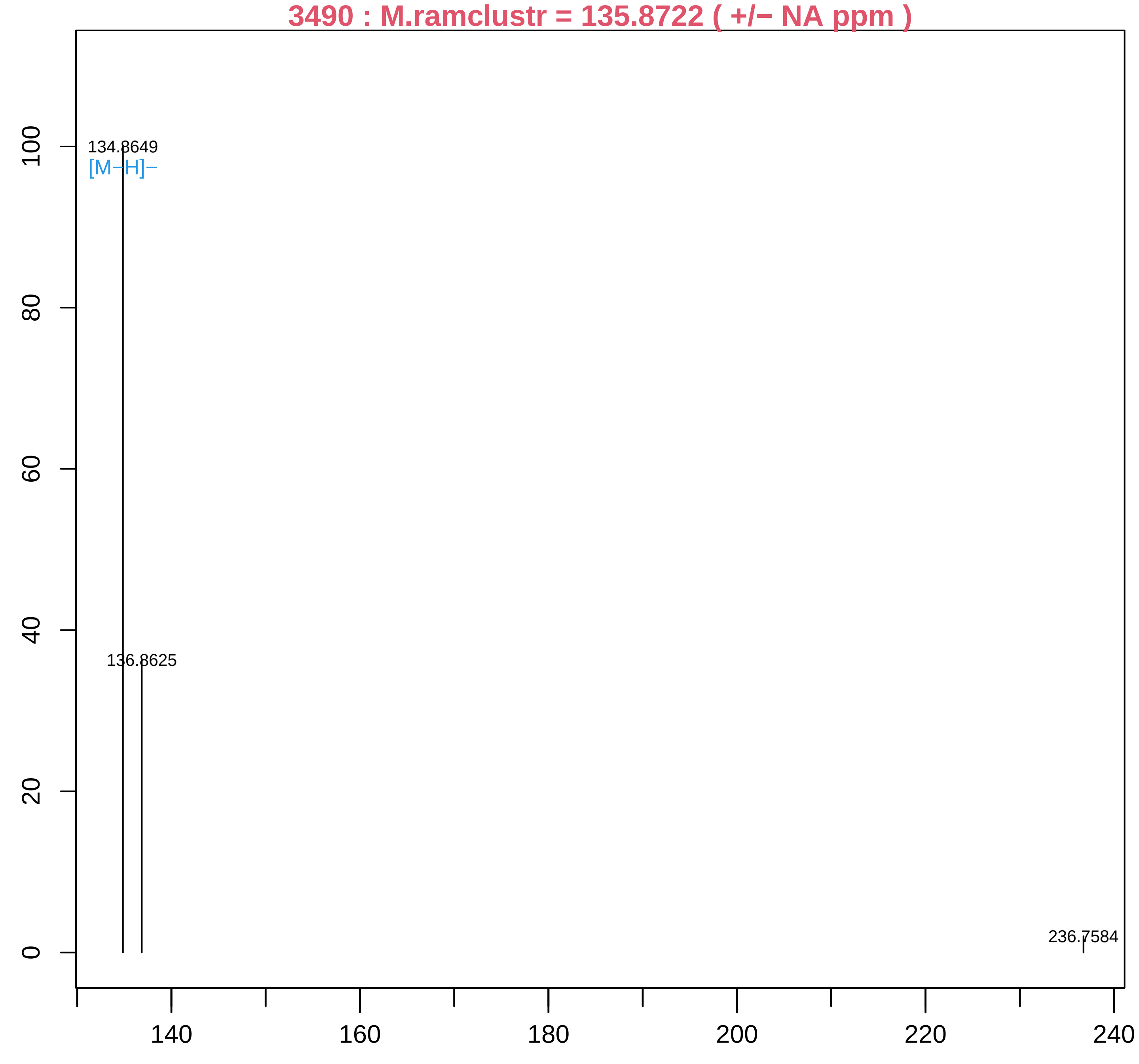

3490 : M.ramclustr = 135.8722 (+/− NA ppm)
134.8649
[M−H]−
136.8625
236.7584
0
20
40
60
80
100
140
160
180
200
220
240

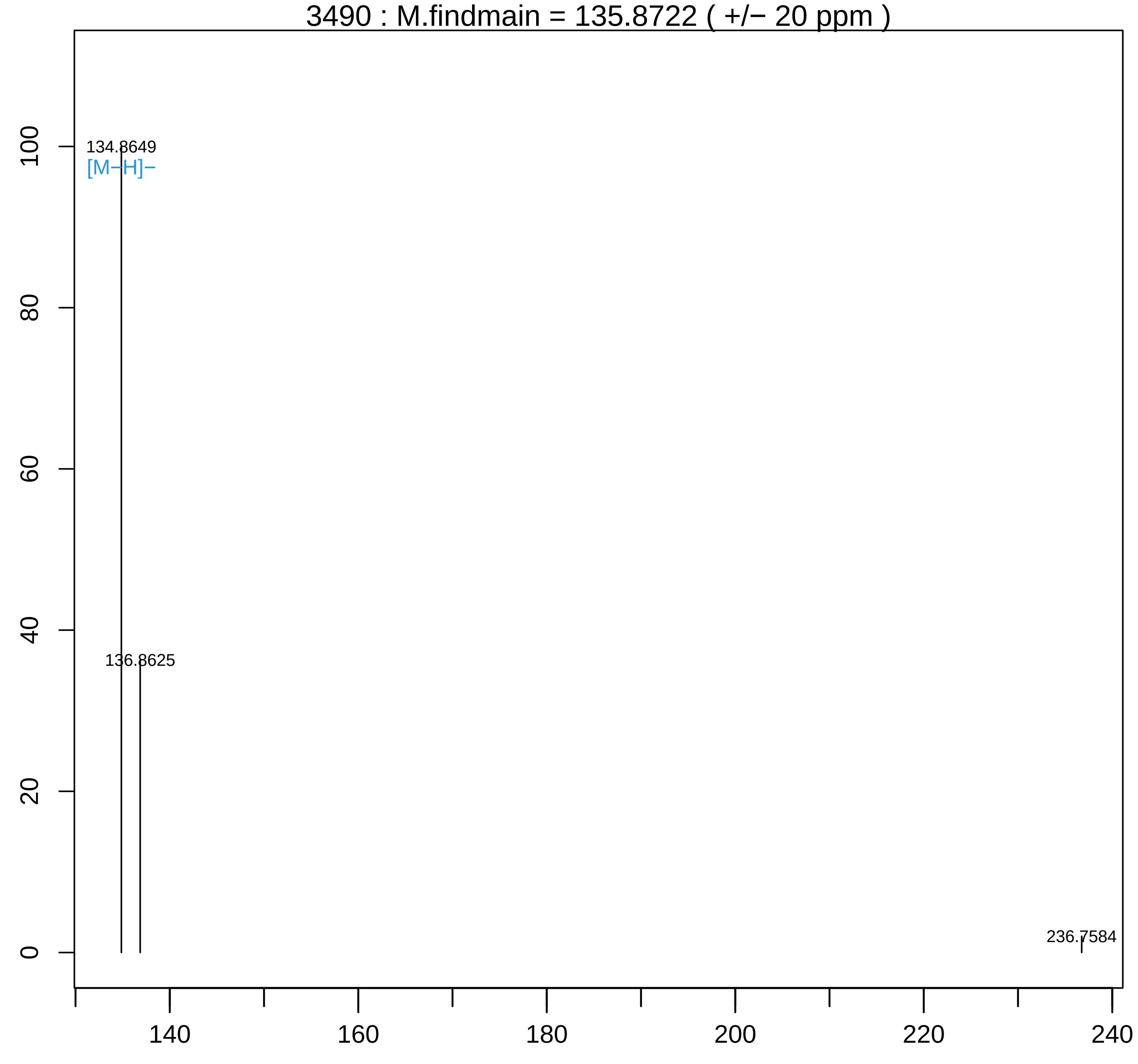

3490 : M.findmain = 135.8722 (+/− 20 ppm)
134.8649
[M−H]−
136.8625
236.7584
0
20
40
60
80
100
140
160
180
200
220
240

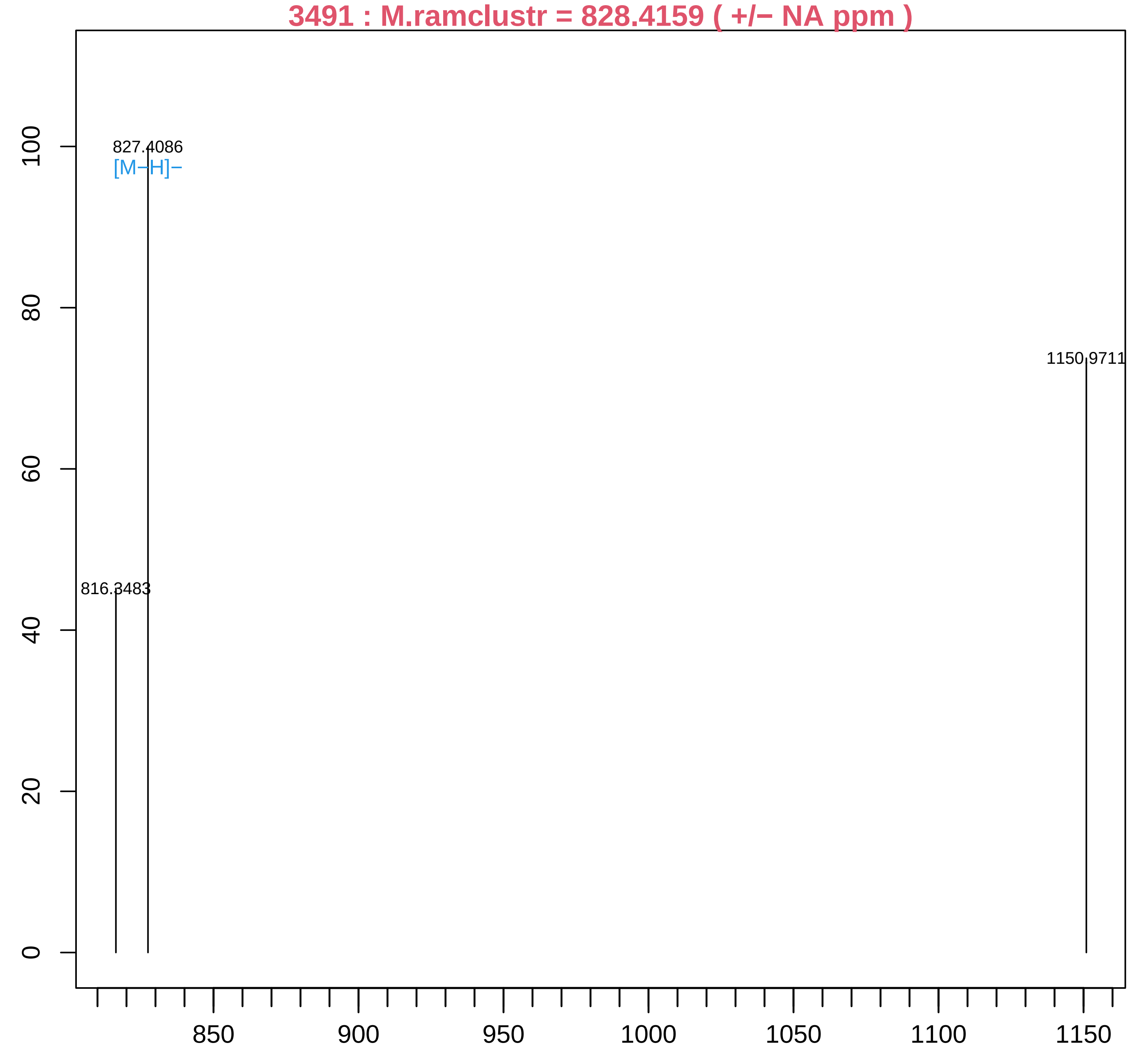
3491 : M.ramclustr = 828.4159 (+/− NA ppm)
827.4086
[M−H]−
816.3483
1150.9711
0
20
40
60
80
100
850
900
950
1000
1050
1100
1150

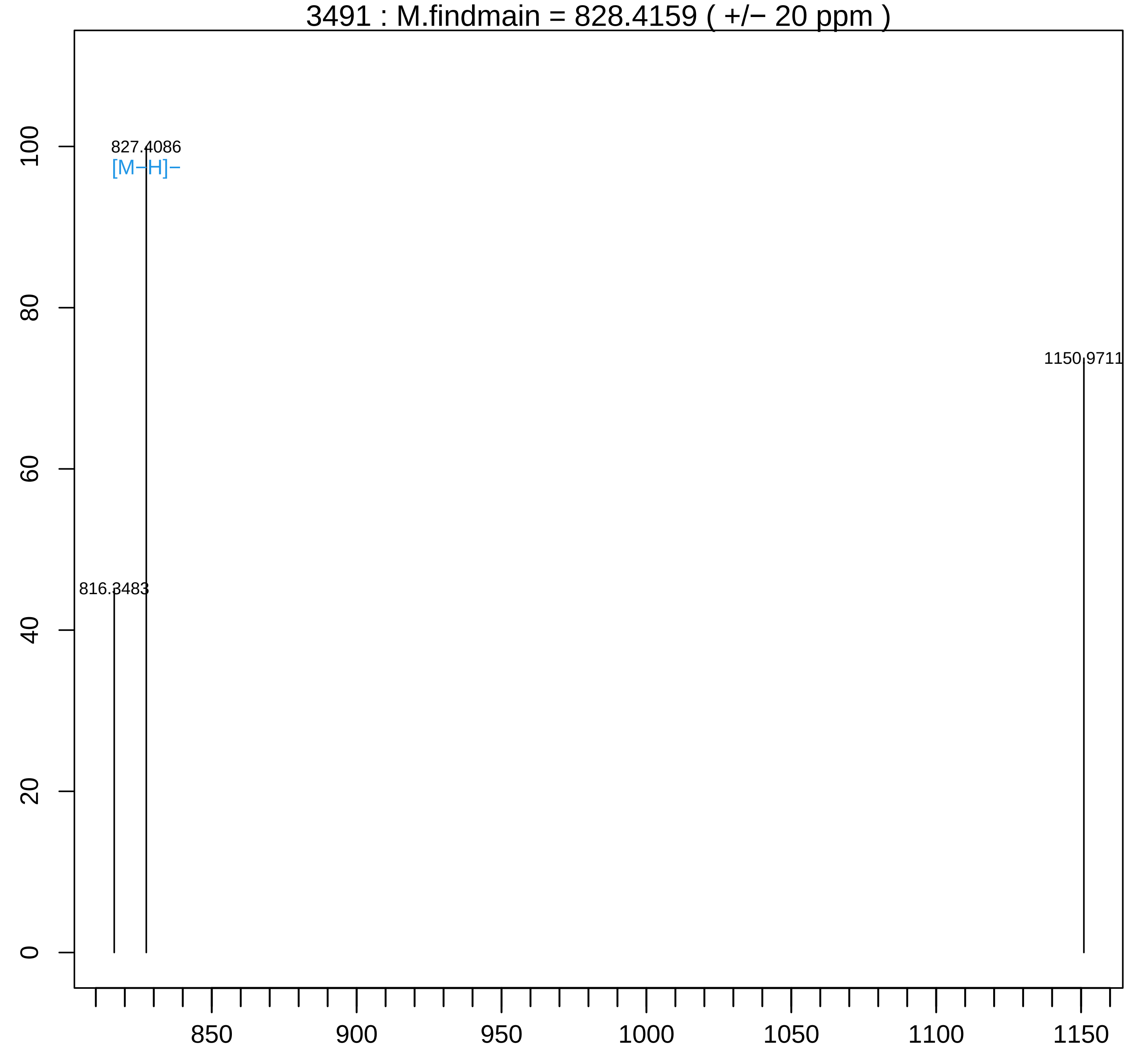
3491 : M.findmain = 828.4159 (+/− 20 ppm)
827.4086
[M−H]−
816.3483
1150.9711
0
20
40
60
80
100
850
900
950
1000
1050
1100
1150

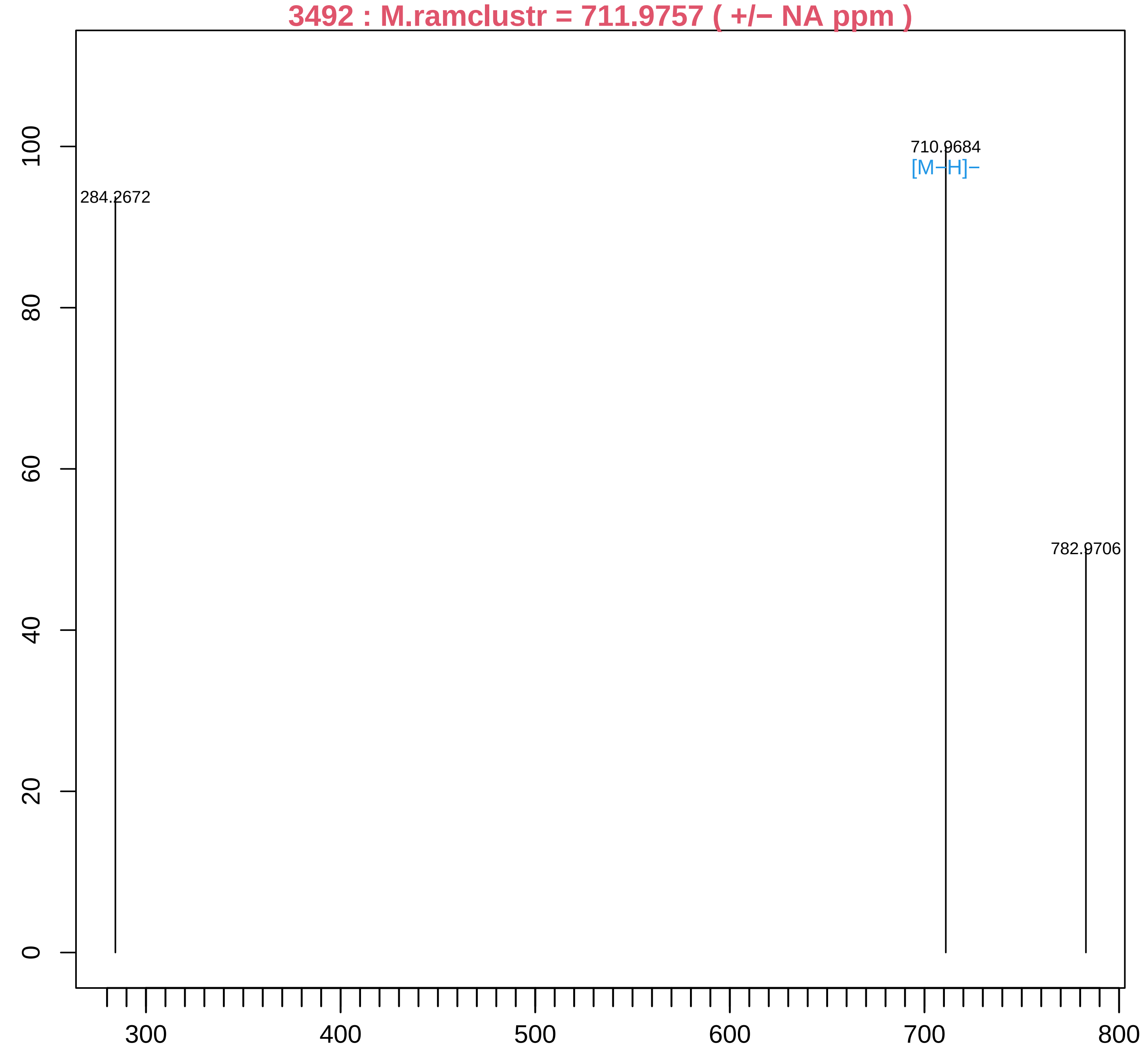

3492 : M.ramclustr = 711.9757 (+/− NA ppm)
284.2672
710.9684
[M−H]−
782.9706
0
20
40
60
80
100
300
400
500
600
700
800

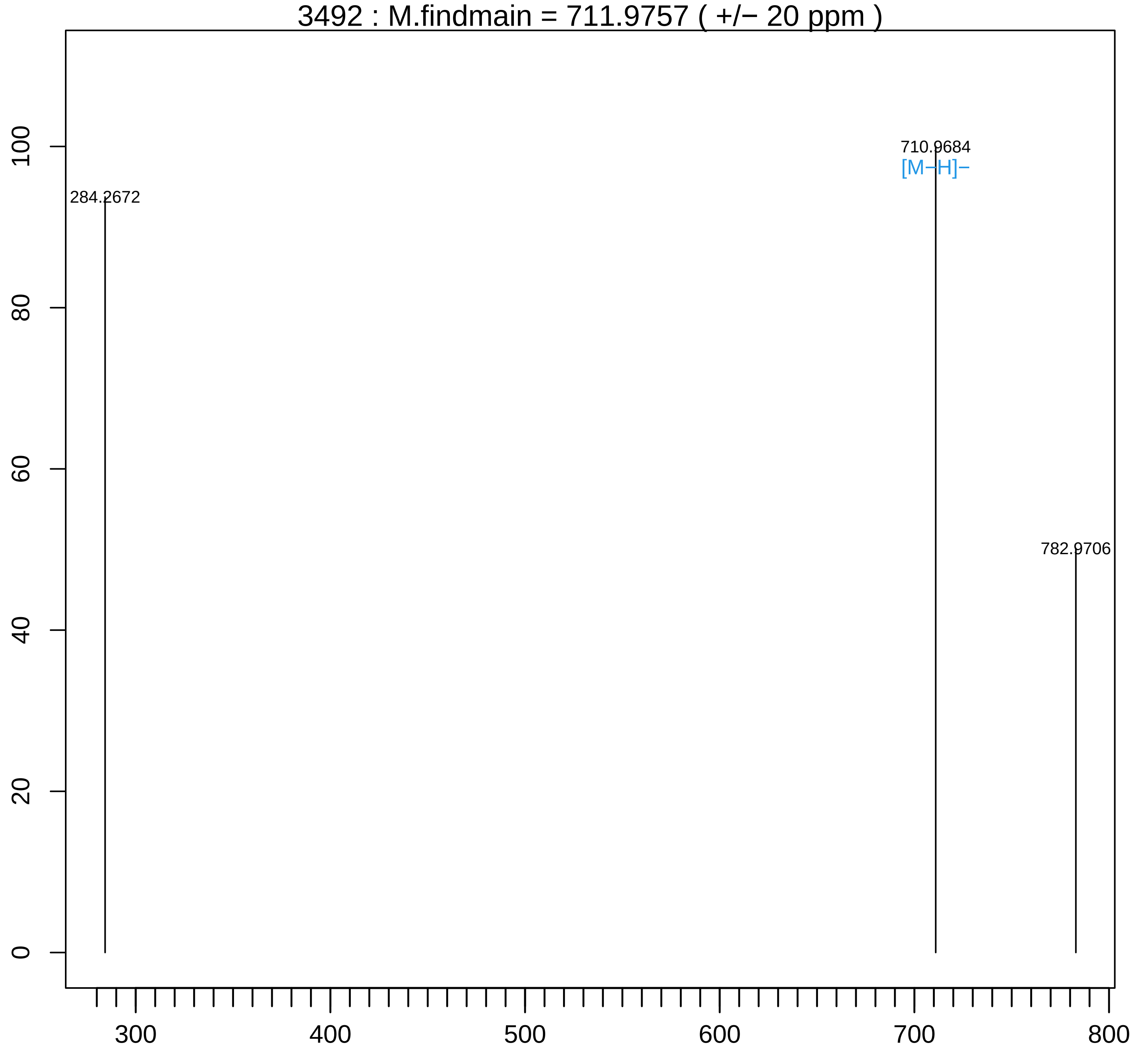

3492 : M.findmain = 711.9757 (+/− 20 ppm)
284.2672
710.9684
[M−H]−
782.9706
0
20
40
60
80
100
300
400
500
600
700
800

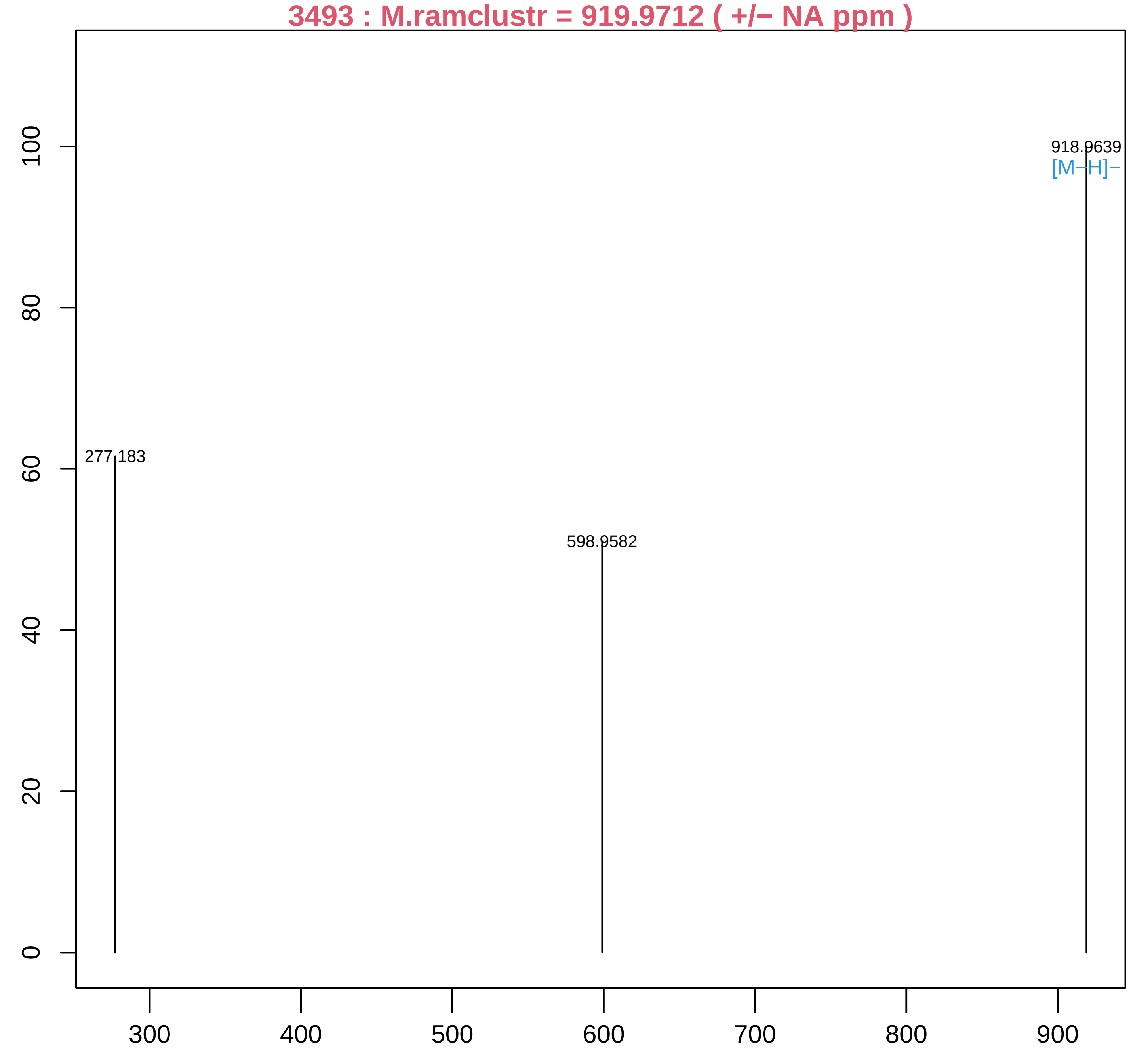
3493 : M.ramclustr = 919.9712 (+/− NA ppm)
918.9639
[M−H]−
277.183
598.9582
0
20
40
60
80
100
300
400
500
600
700
800
900

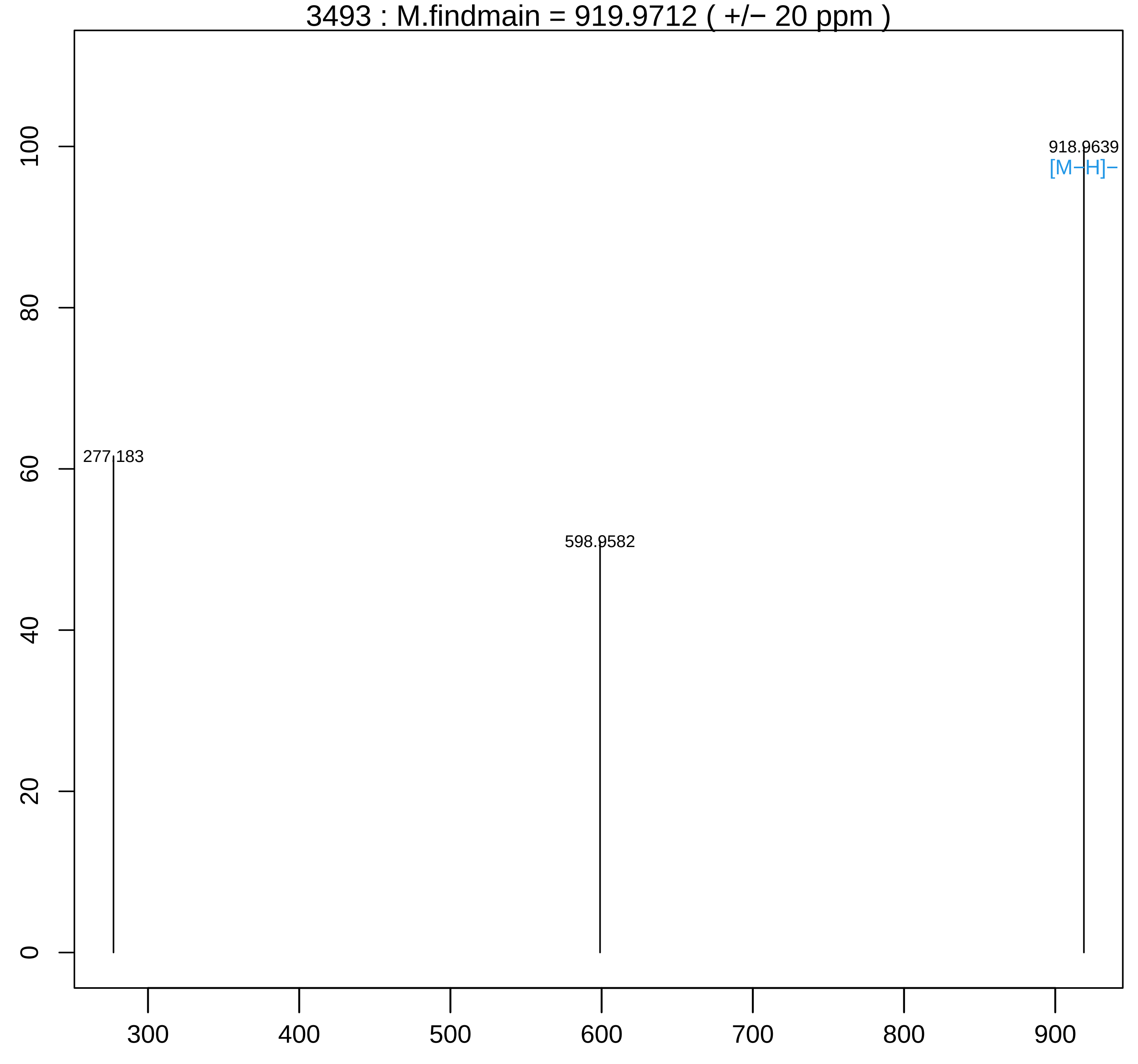
3493 : M.findmain = 919.9712 (+/− 20 ppm)
918.9639
[M−H]−
277.183
598.9582
0
20
40
60
80
100
300
400
500
600
700
800
900

3494 : M.ramclustr = 597.9724 (+/− NA ppm)

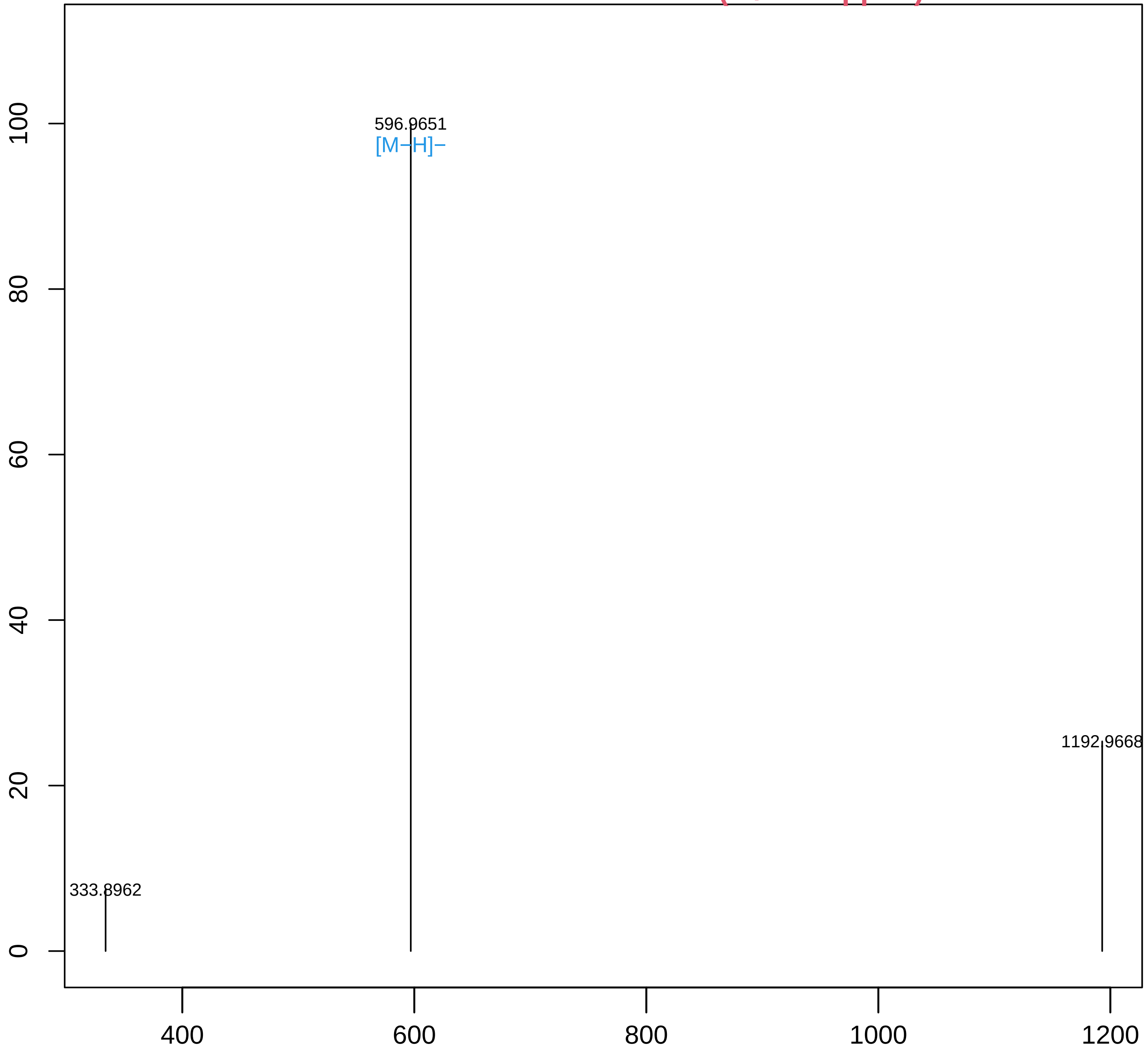

3494 : M.findmain = 597.9724 (+/− 20 ppm)

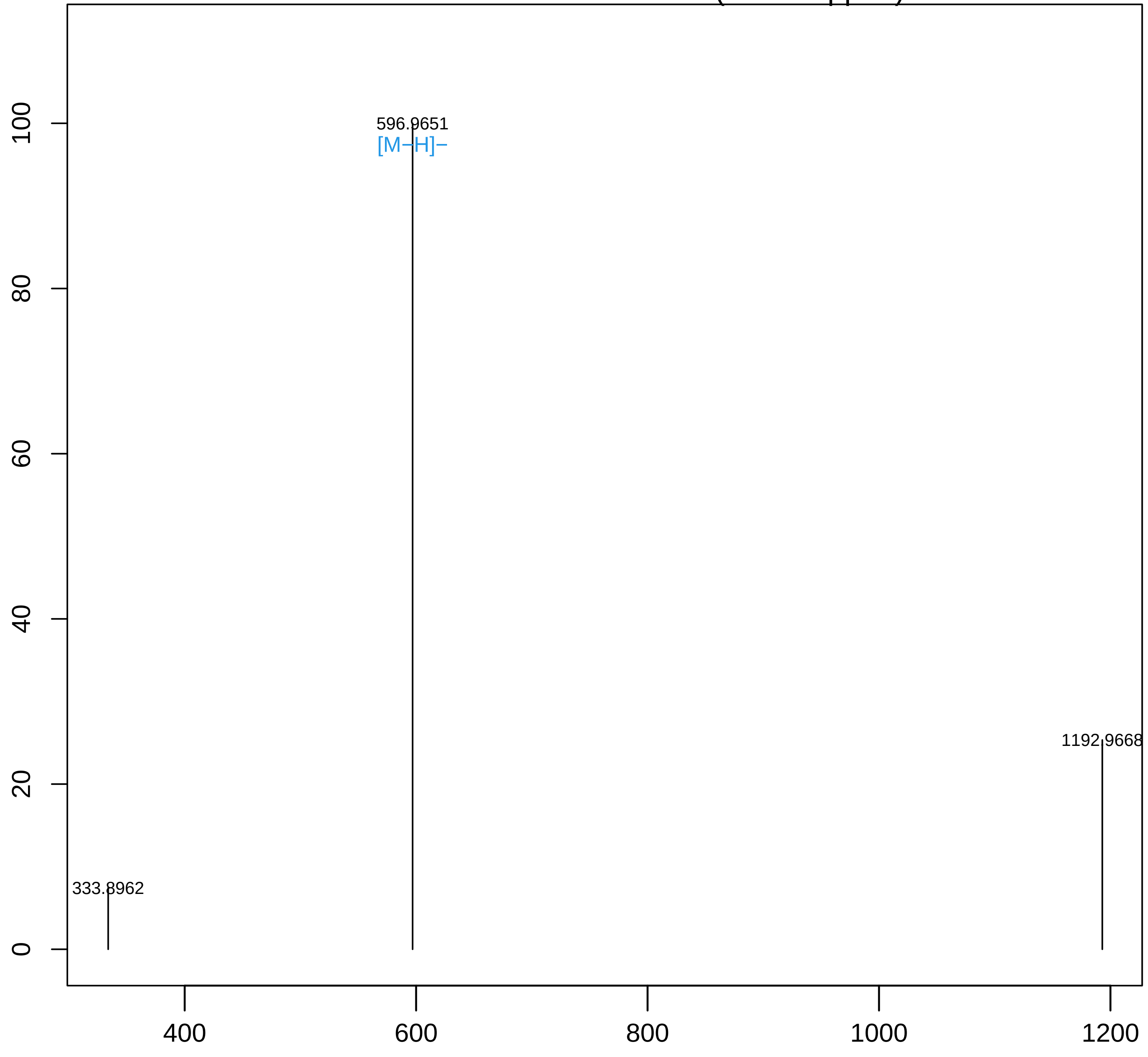

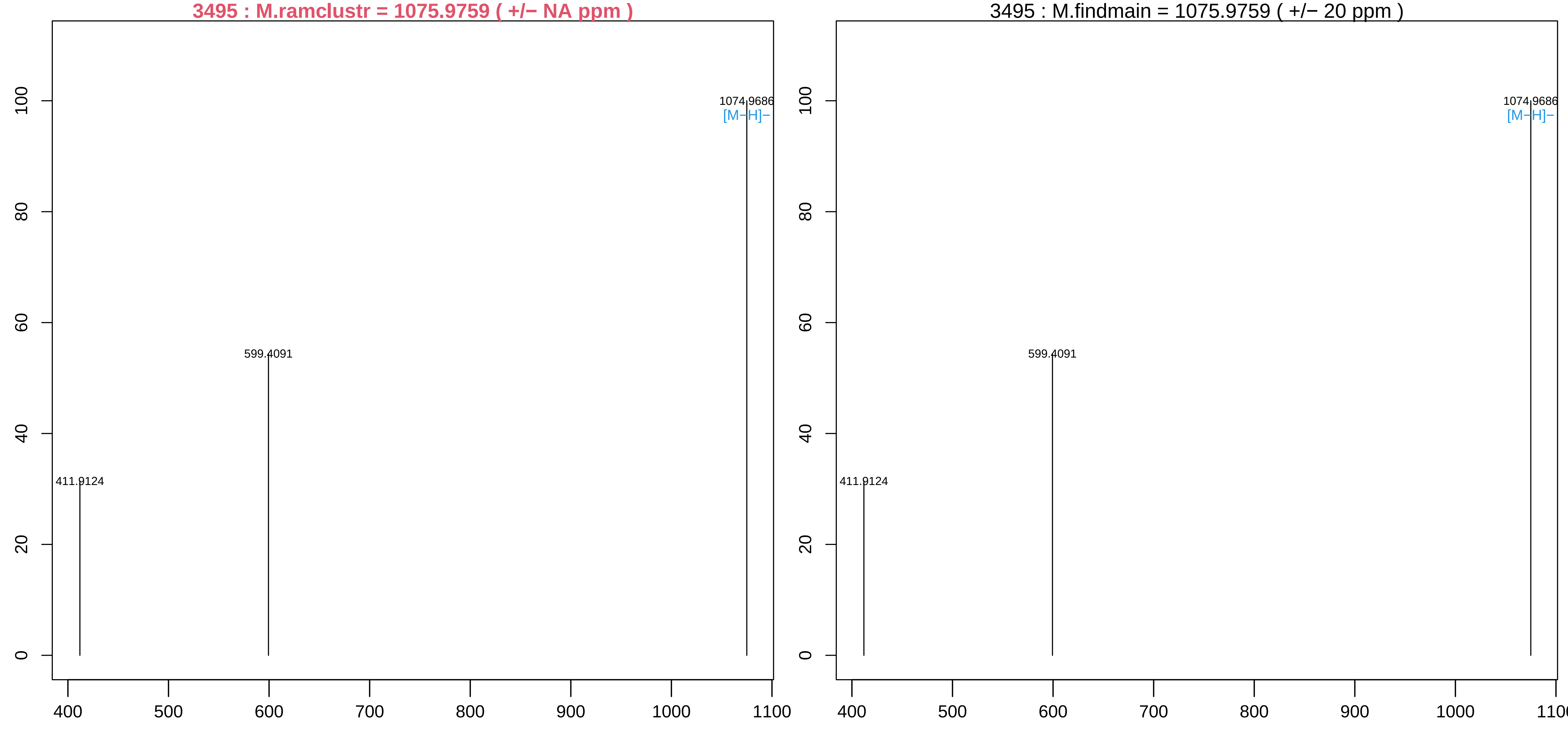

3495 : M.ramclustr = 1075.9759 (+/− NA ppm)
1074.9686
[M−H]−
599.4091
411.9124
0
20
40
60
80
100
400
500
600
700
800
900
1000
1100
3495 : M.findmain = 1075.9759 (+/− 20 ppm)
1074.9686
[M−H]−
599.4091
411.9124

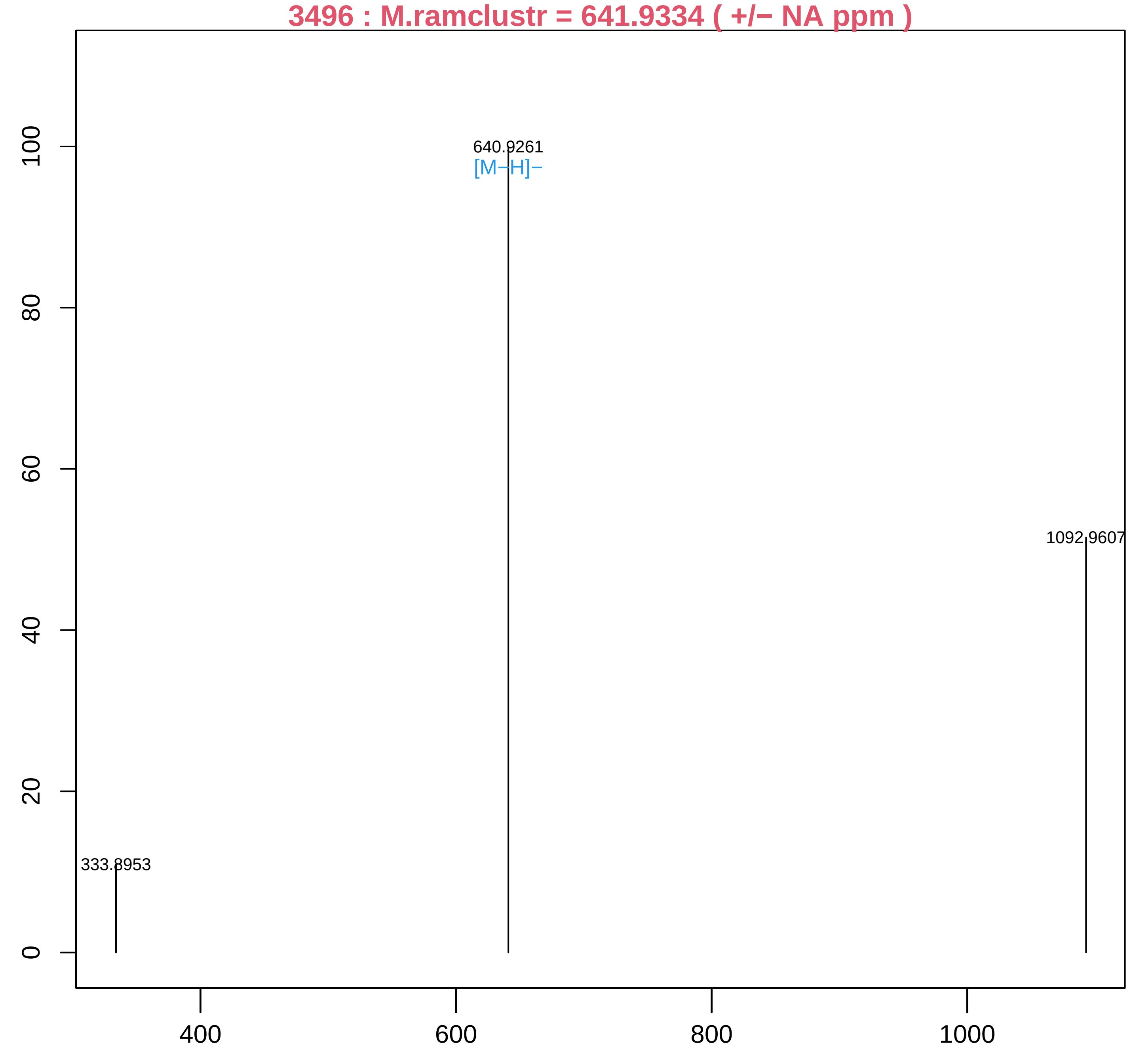
3496 : M.ramclustr = 641.9334 (+/− NA ppm)
640.9261
[M−H]−
1092.9607
333.8953
0
20
40
60
80
100
400
600
800
1000

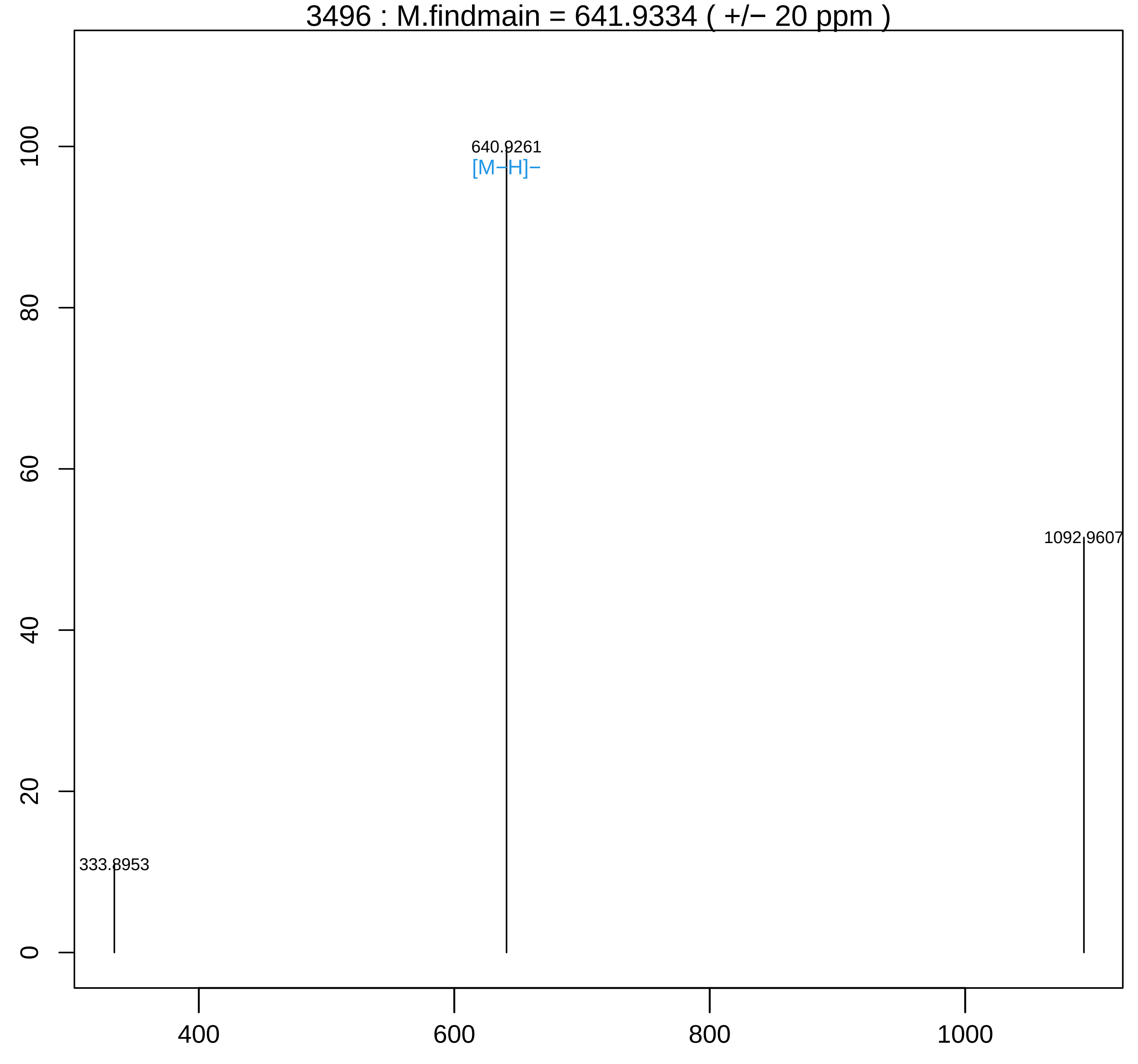
3496 : M.findmain = 641.9334 (+/− 20 ppm)
640.9261
[M−H]−
1092.9607
333.8953
0
20
40
60
80
100
400
600
800
1000

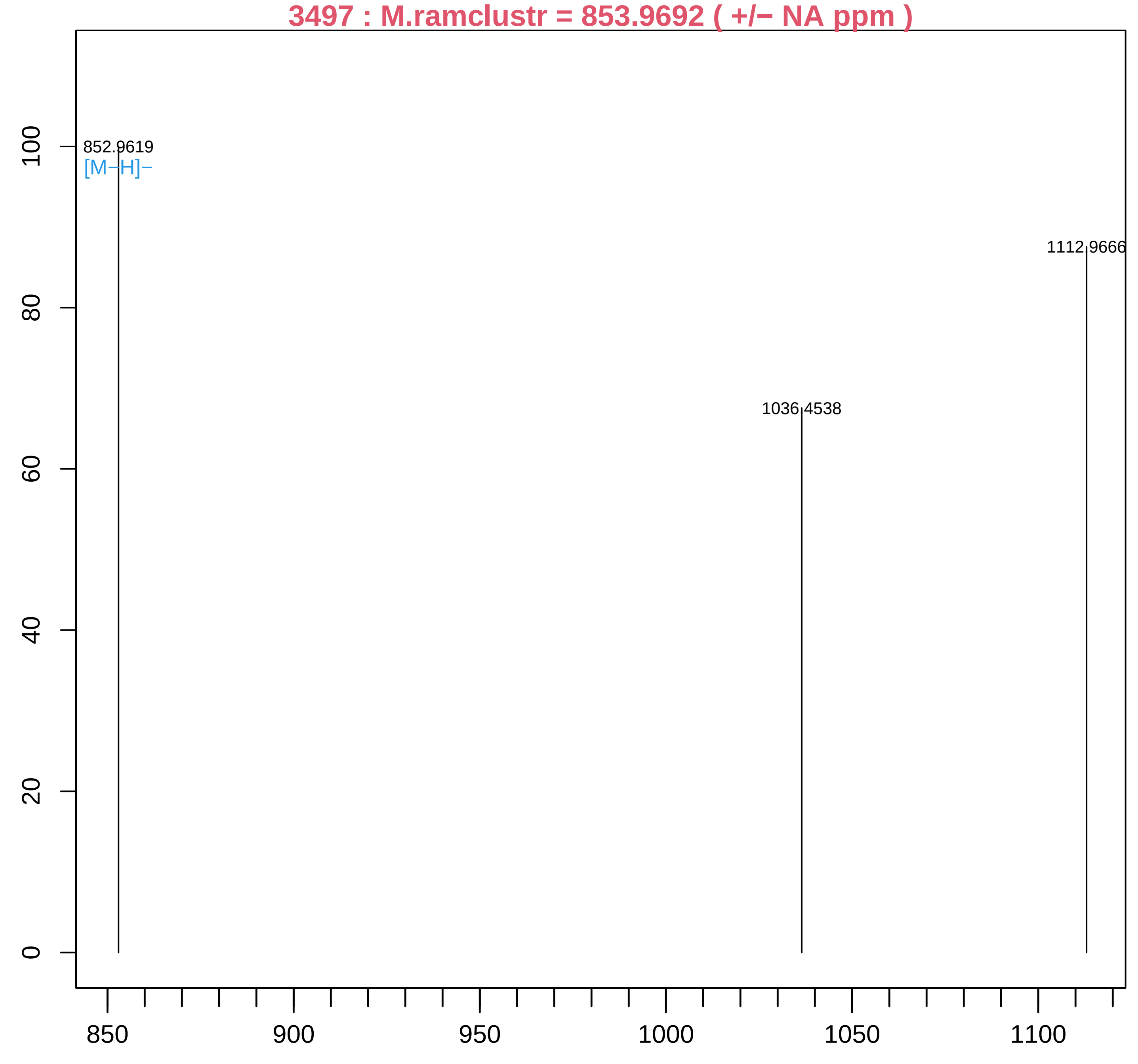
3497 : M.ramclustr = 853.9692 (+/− NA ppm)
852.9619
[M−H]−
1036.4538
1112.9666
0
20
40
60
80
100
850
900
950
1000
1050
1100

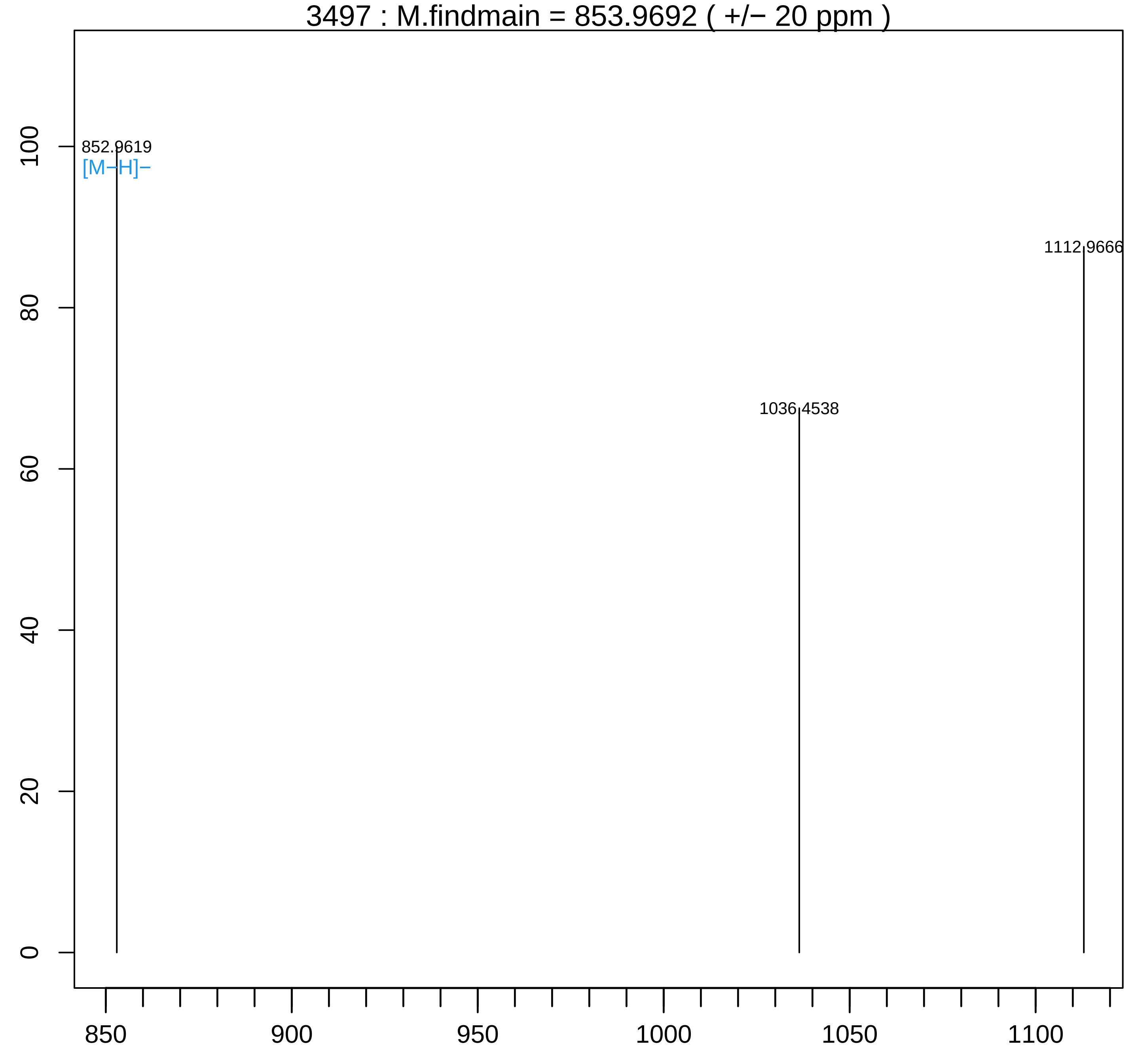
3497 : M.findmain = 853.9692 (+/− 20 ppm)
852.9619
[M−H]−
1036.4538
1112.9666
0
20
40
60
80
100
850
900
950
1000
1050
1100

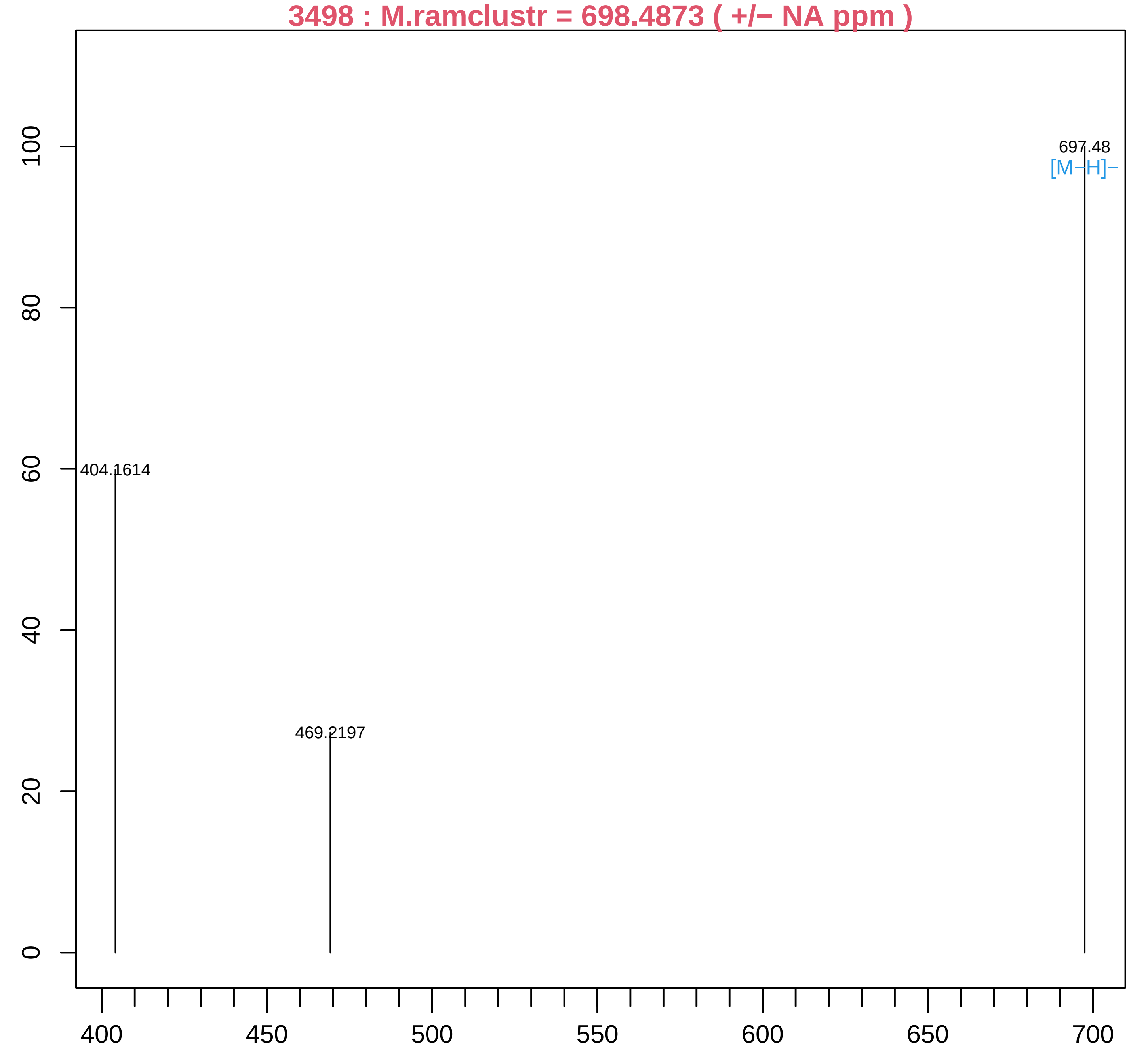
3498 : M.ramclustr = 698.4873 (+/− NA ppm)
404.1614
469.2197
697.48
[M−H]−
0
20
40
60
80
100
400
450
500
550
600
650
700

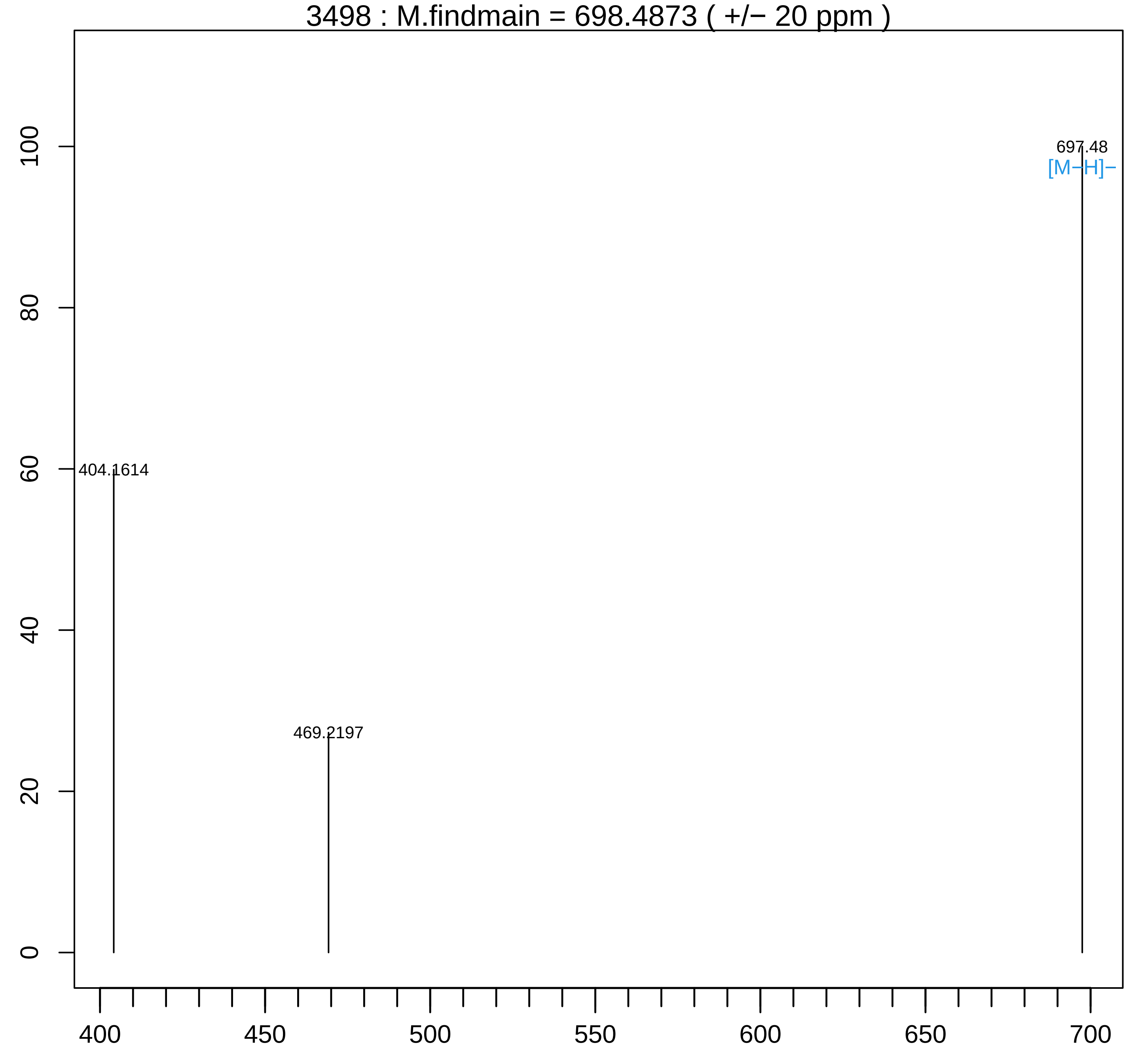
3498 : M.findmain = 698.4873 (+/− 20 ppm)
404.1614
469.2197
697.48
[M−H]−
0
20
40
60
80
100
400
450
500
550
600
650
700

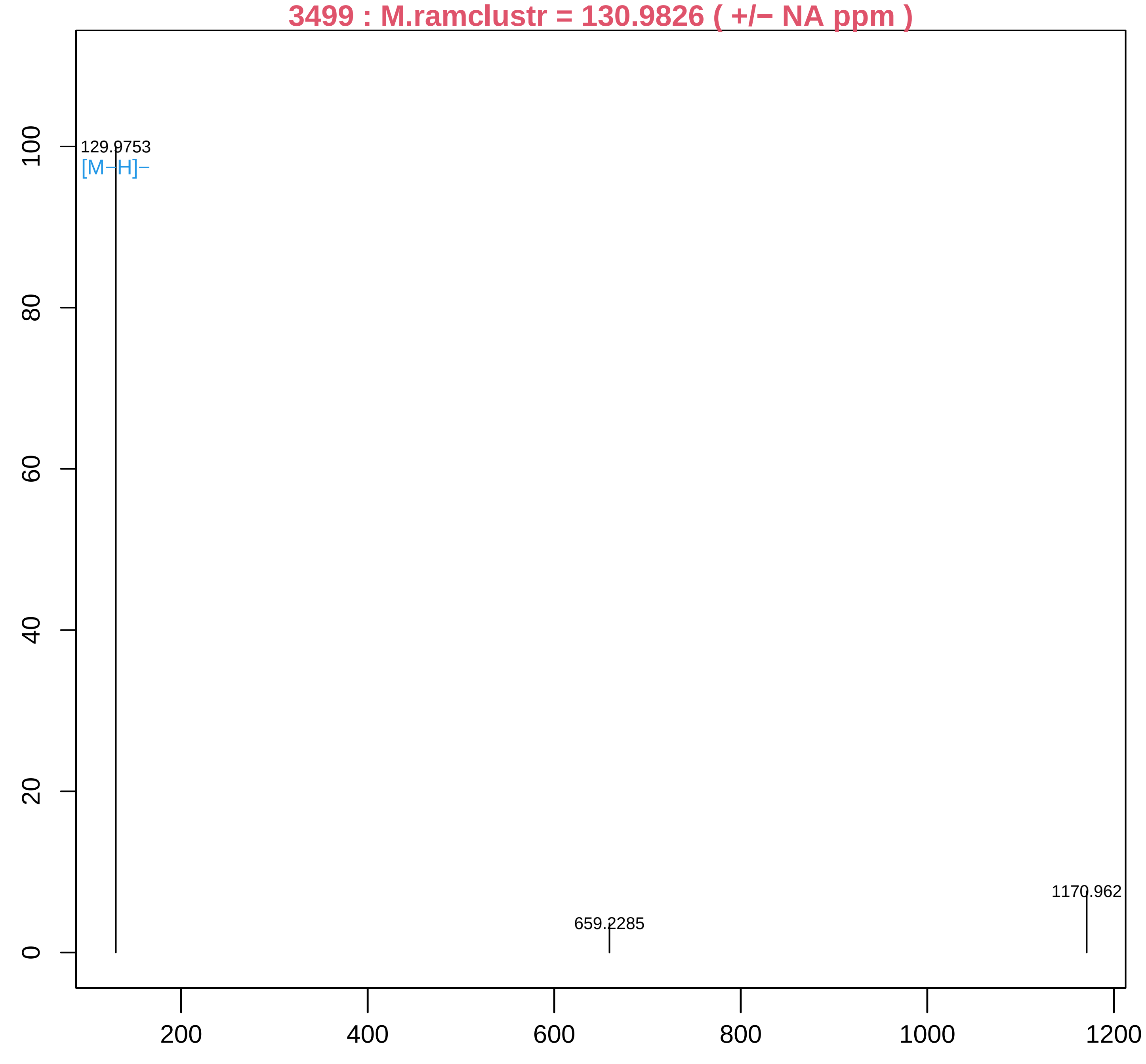
3499 : M.ramclustr = 130.9826 (+/− NA ppm)
129.9753
[M−H]−
659.2285
1170.962
0
20
40
60
80
100
200
400
600
800
1000
1200

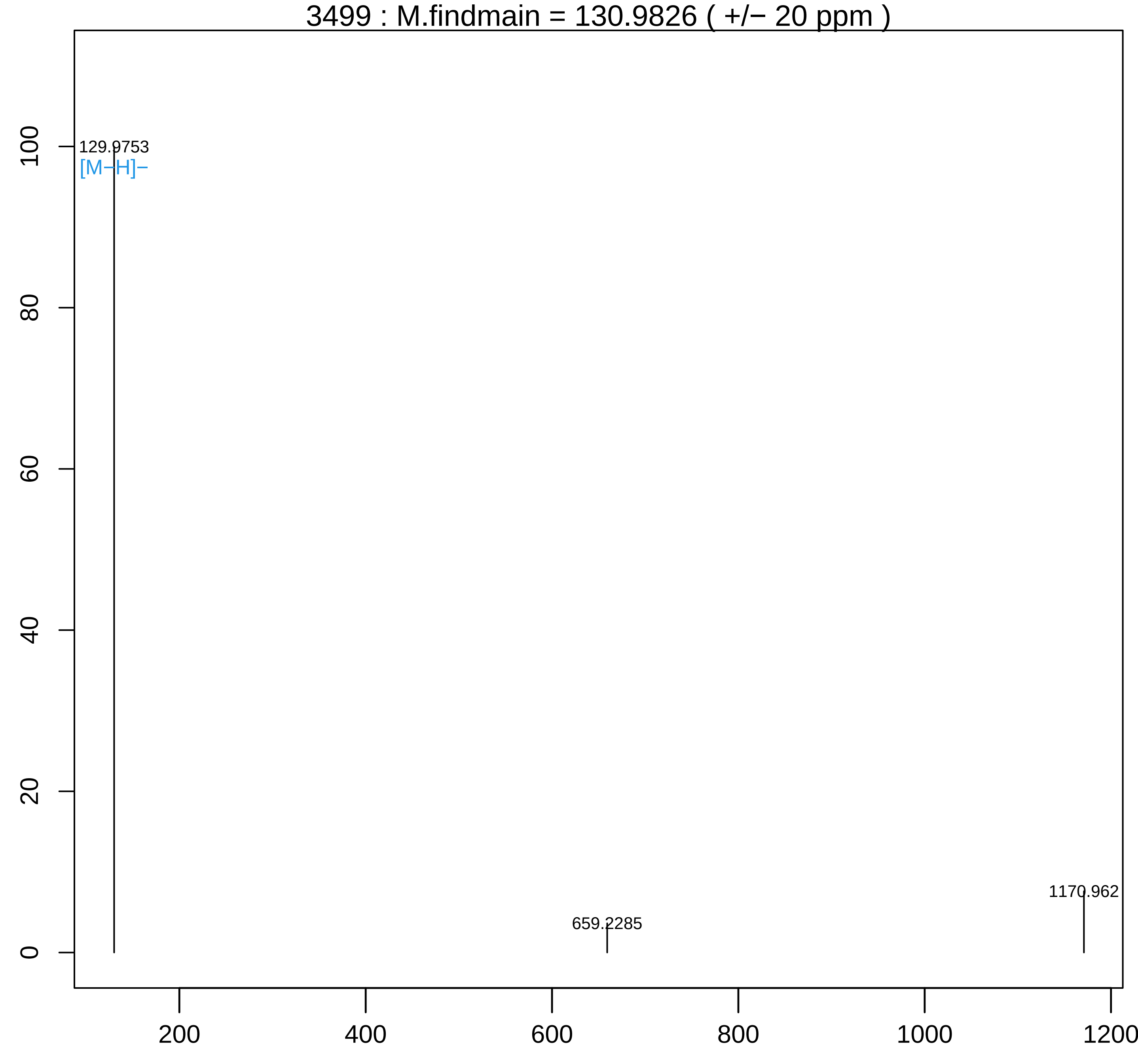
3499 : M.findmain = 130.9826 (+/− 20 ppm)
129.9753
[M−H]−
659.2285
1170.962
0
20
40
60
80
100
200
400
600
800
1000
1200

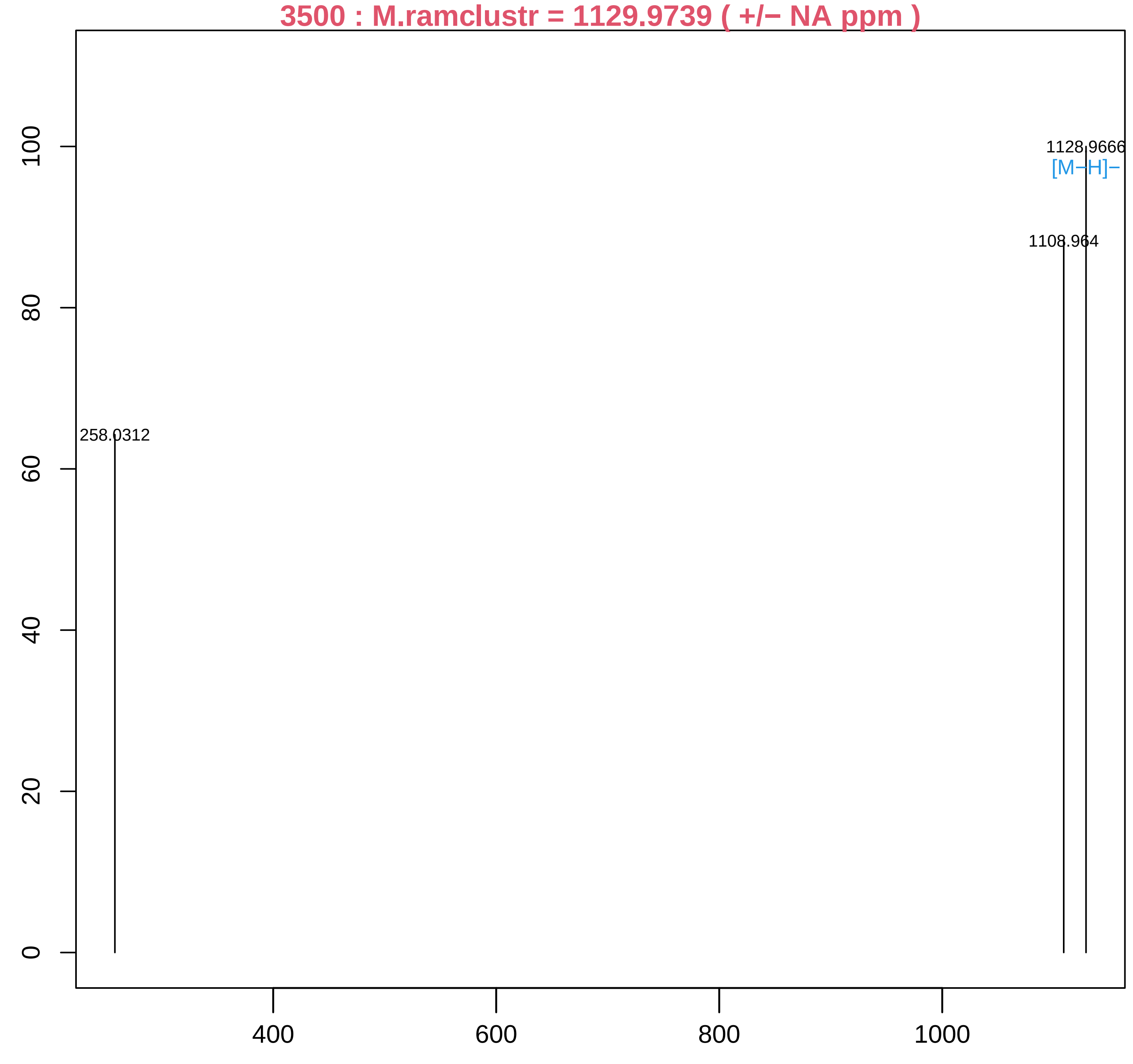
3500 : M.ramclustr = 1129.9739 (+/− NA ppm)
1128.9666
[M−H]−
1108.964
258.0312
0
20
40
60
80
100
400
600
800
1000

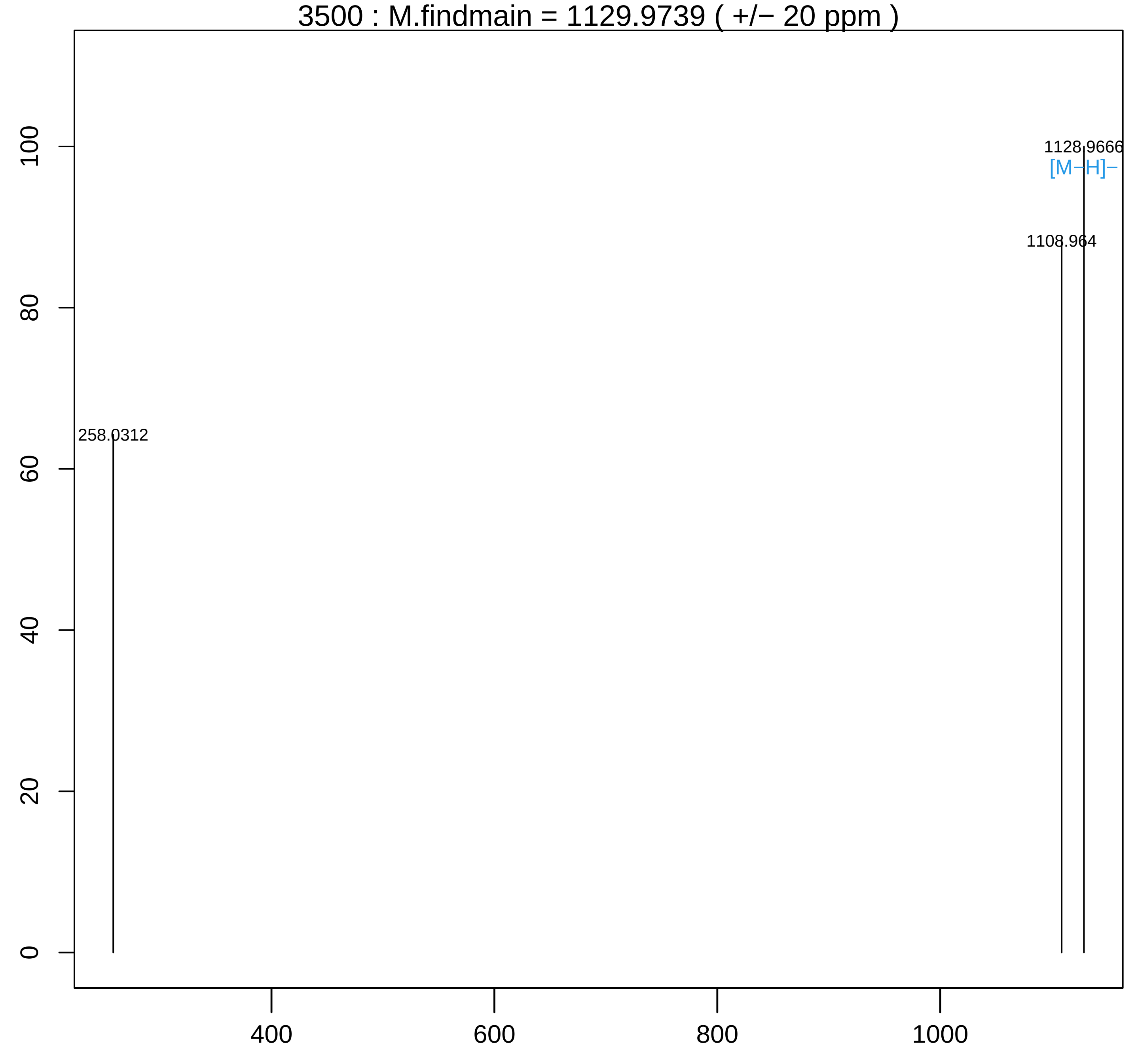
3500 : M.findmain = 1129.9739 (+/− 20 ppm)
1128.9666
[M−H]−
1108.964
258.0312
0
20
40
60
80
100
400
600
800
1000

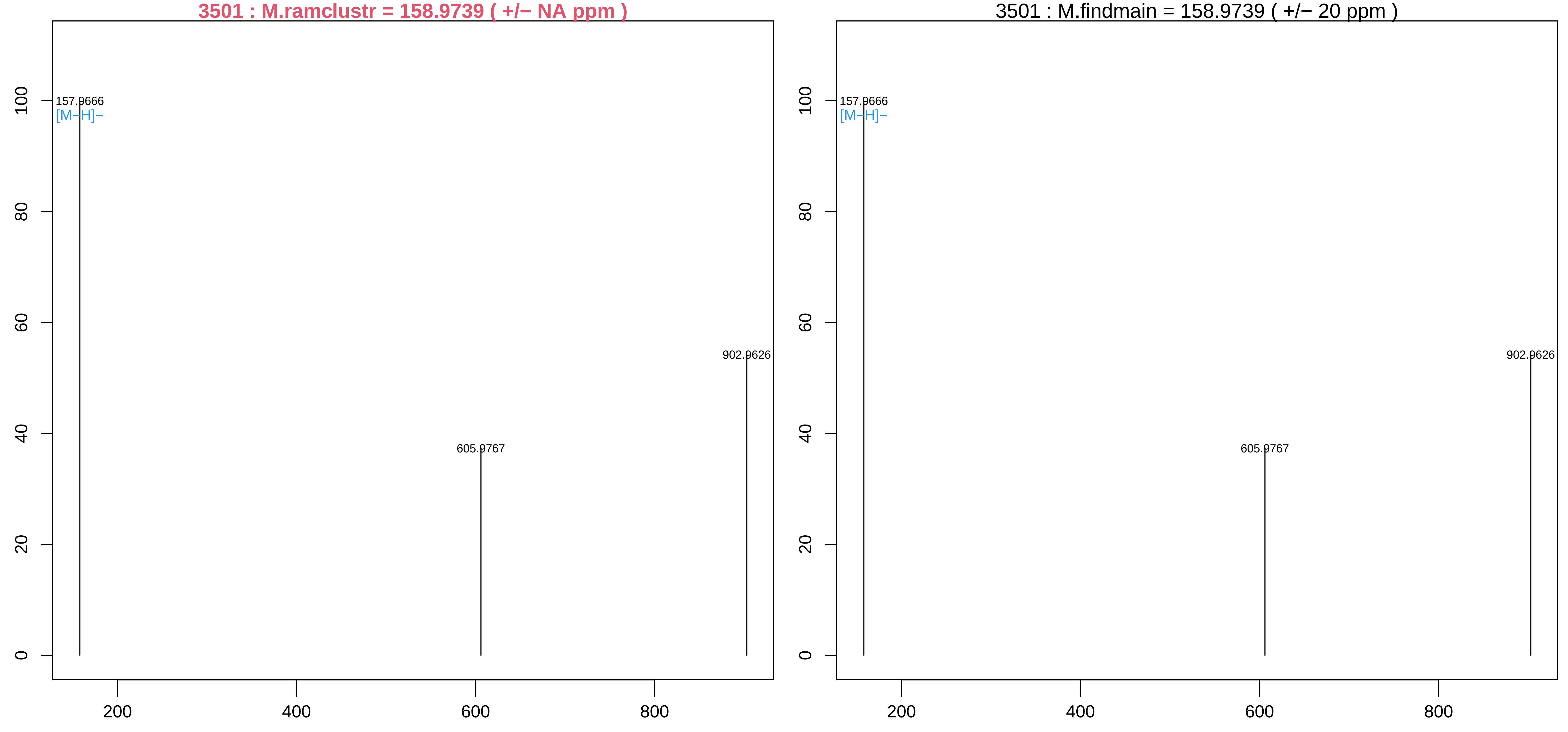
3501 : M.ramclustr = 158.9739 (+/− NA ppm)
157.9666
[M−H]−
605.9767
902.9626
0
20
40
60
80
100
200
400
600
800
3501 : M.findmain = 158.9739 (+/− 20 ppm)
157.9666
[M−H]−
605.9767
902.9626
0
20
40
60
80
100
200
400
600
800

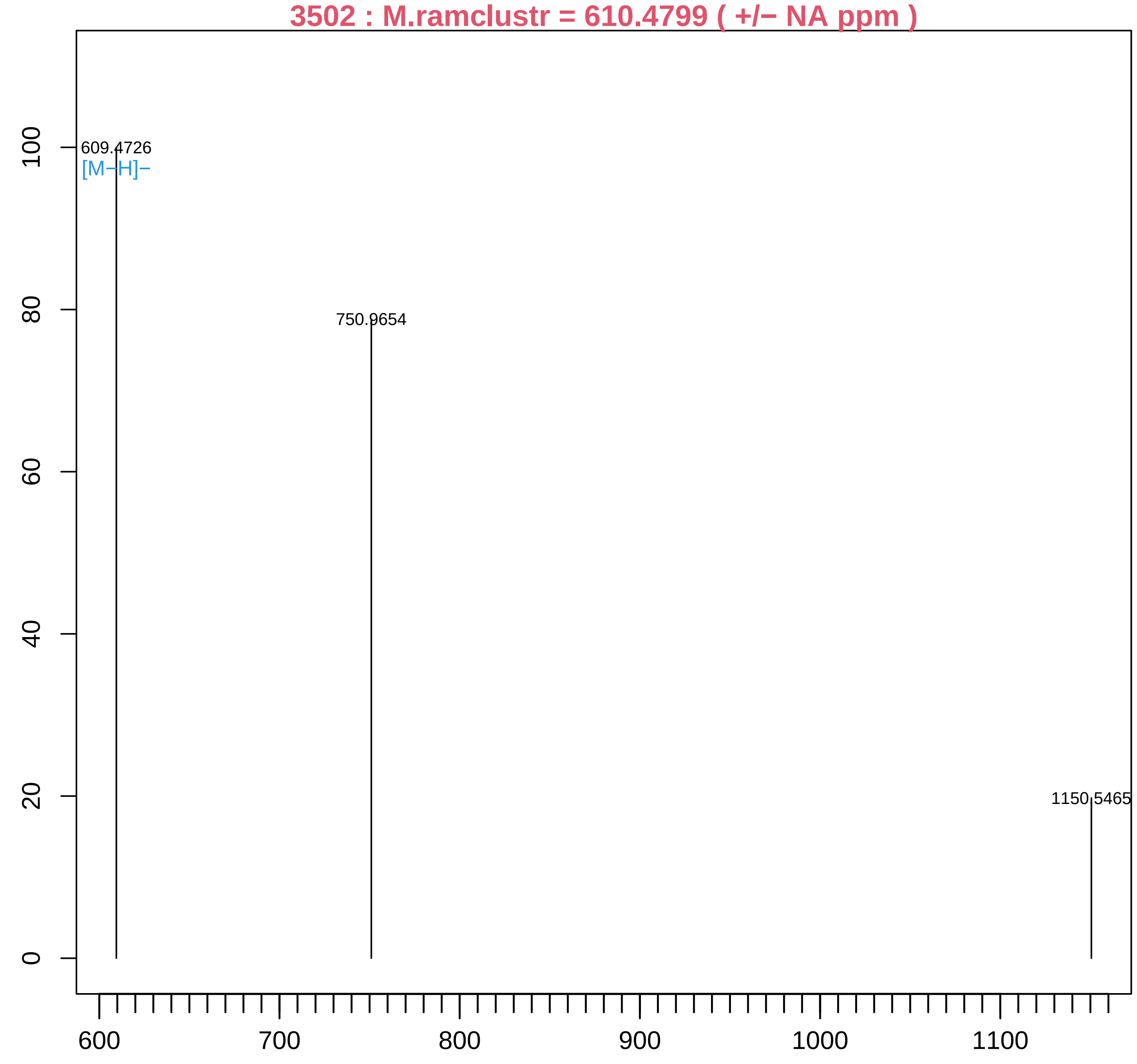
3502 : M.ramclustr = 610.4799 (+/− NA ppm)
609.4726
[M−H]−
750.9654
1150.5465
0
20
40
60
80
100
600
700
800
900
1000
1100

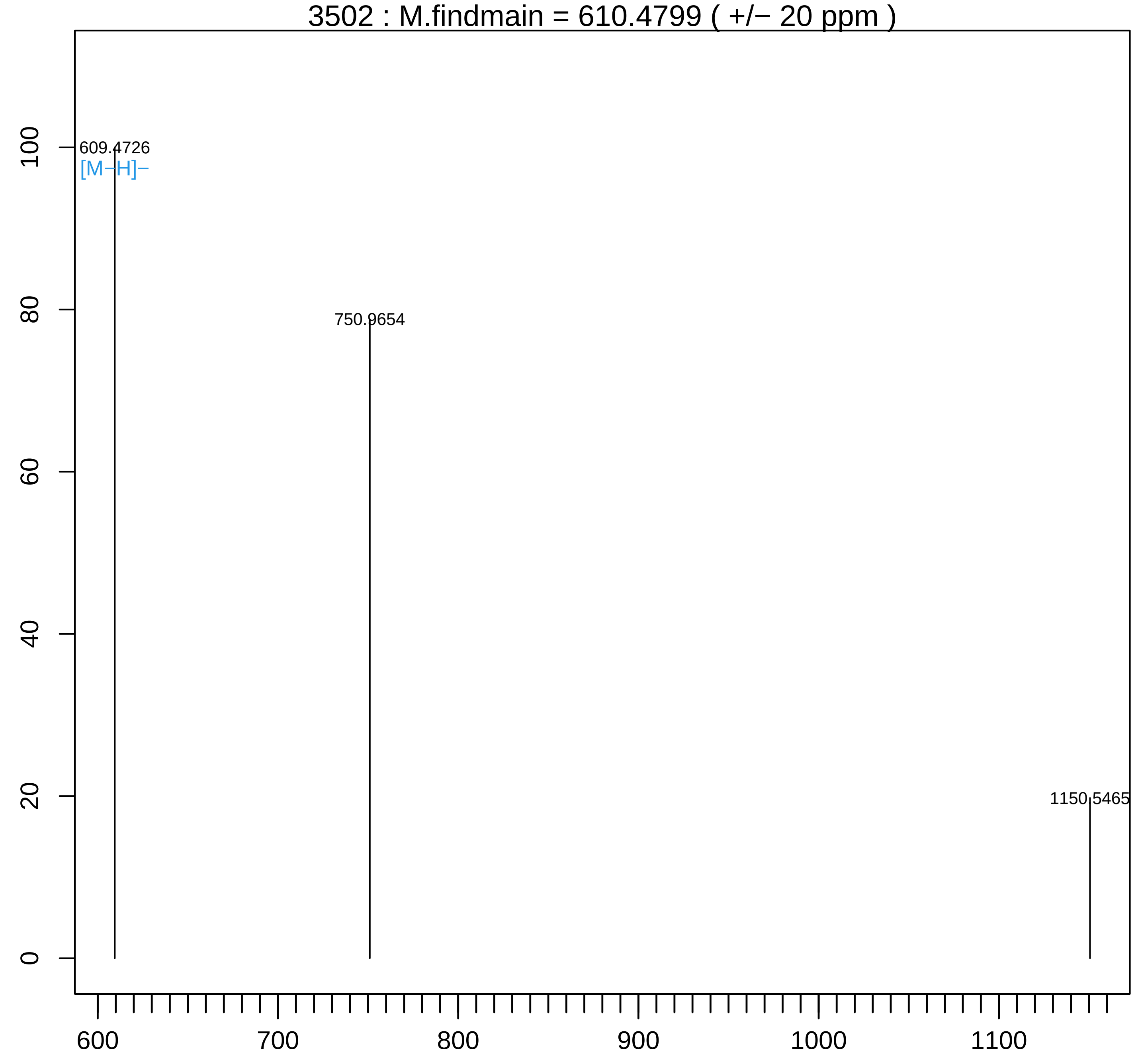
3502 : M.findmain = 610.4799 (+/− 20 ppm)
609.4726
[M−H]−
750.9654
1150.5465
0
20
40
60
80
100
600
700
800
900
1000
1100

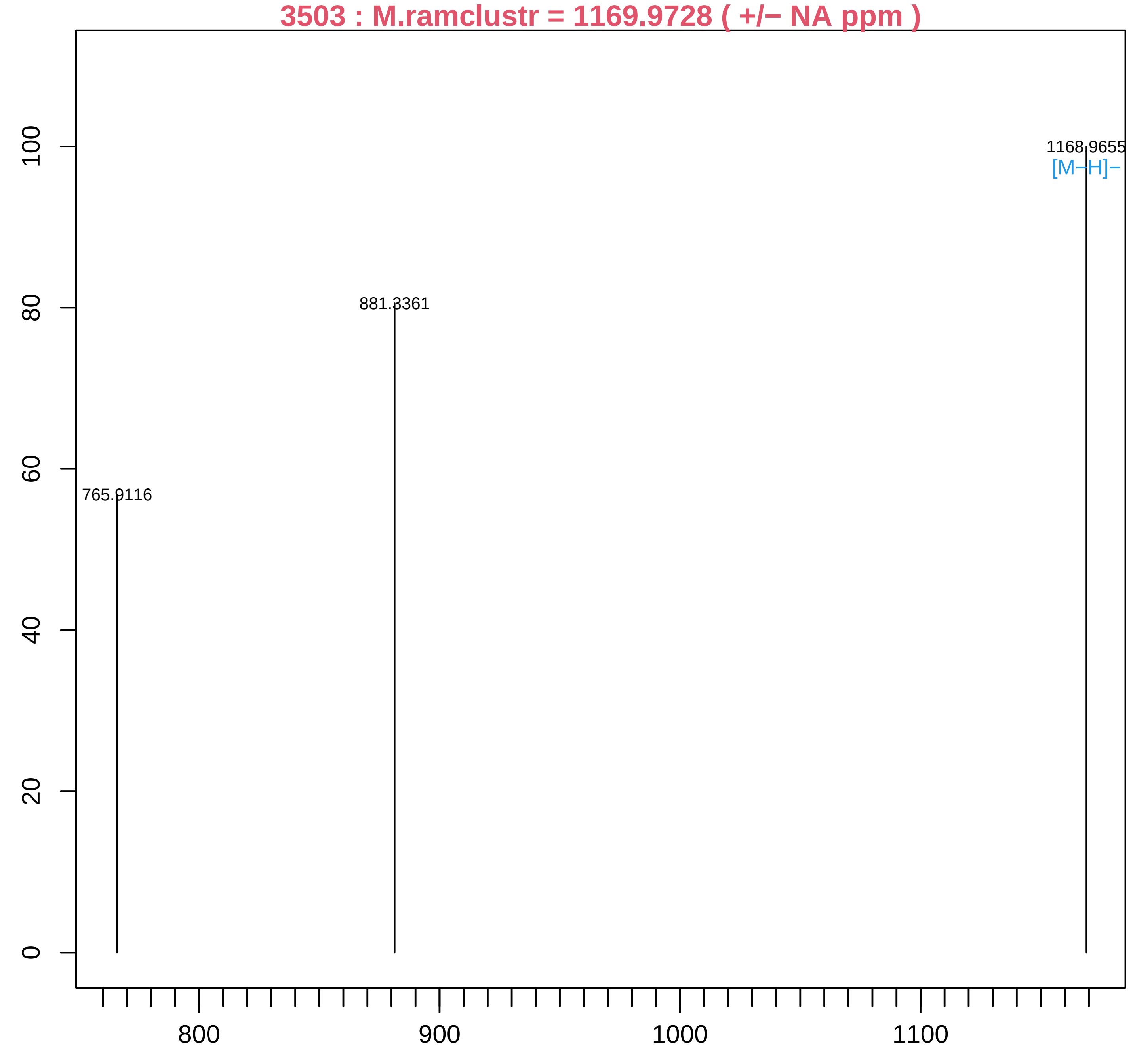
3503 : M.ramclustr = 1169.9728 (+/− NA ppm)
1168.9655
[M−H]−
881.3361
765.9116
0
20
40
60
80
100
800
900
1000
1100
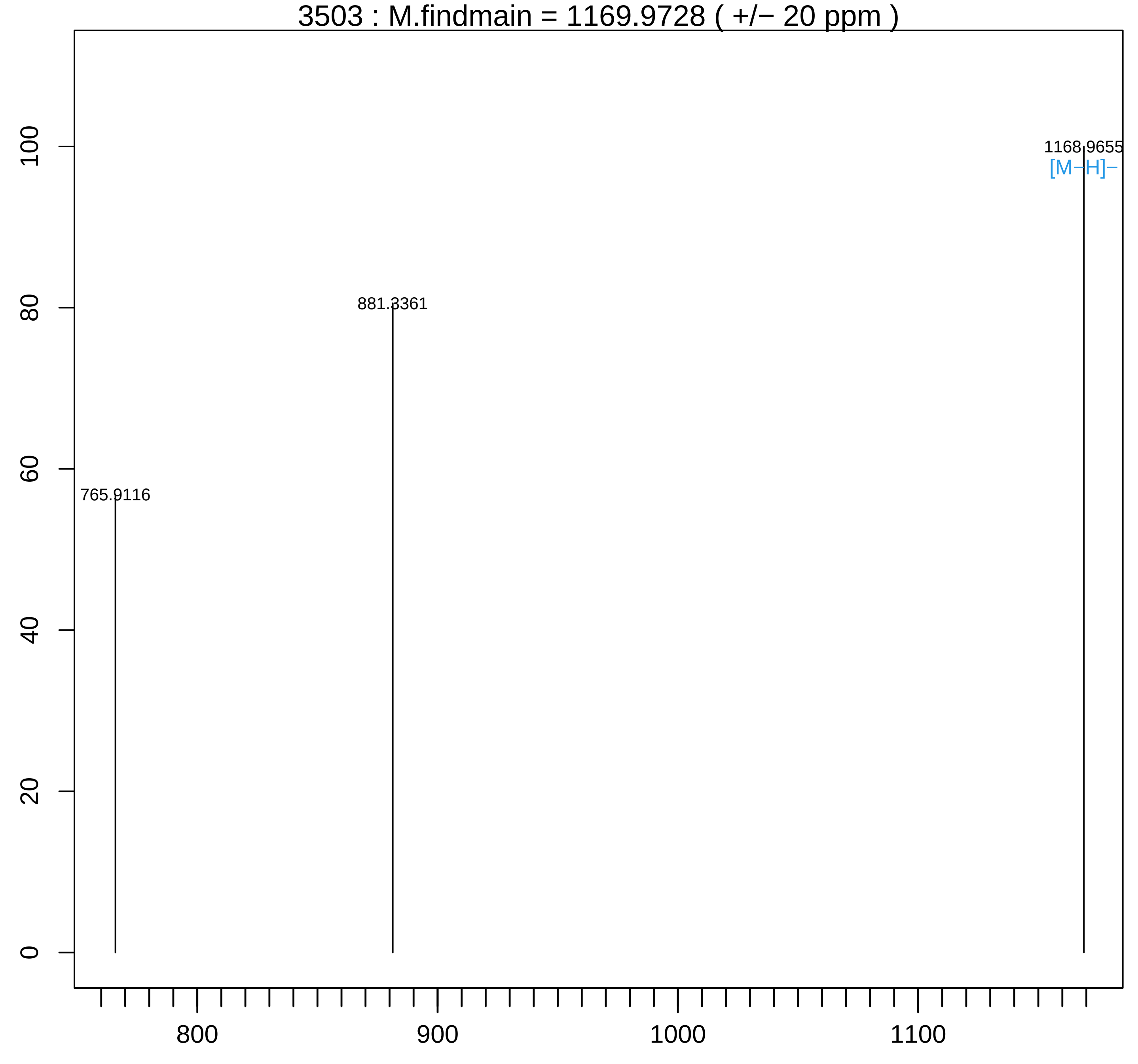
3503 : M.findmain = 1169.9728 (+/− 20 ppm)
1168.9655
[M−H]−
881.3361
765.9116
0
20
40
60
80
100
800
900
1000
1100

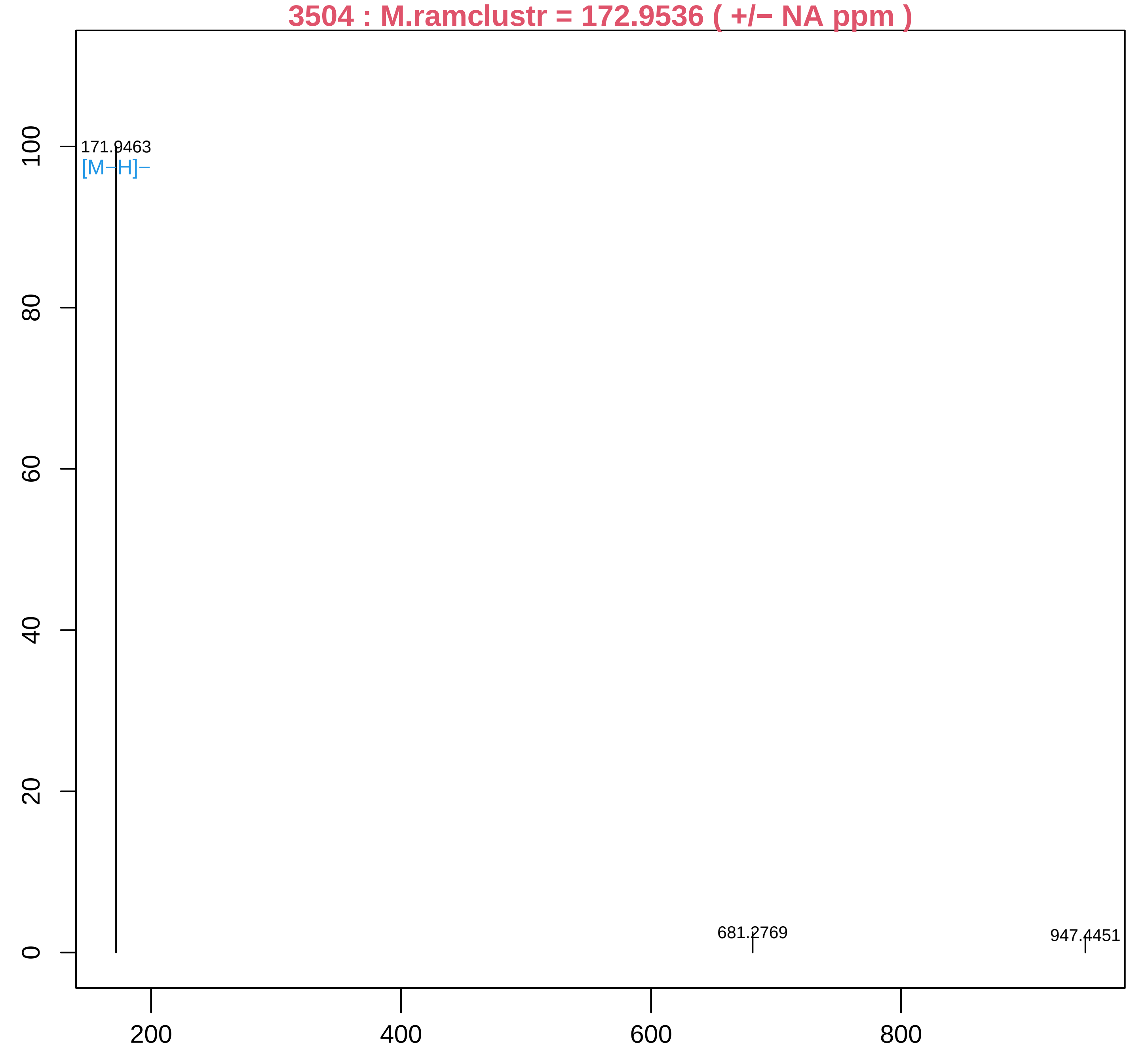

3504 : M.ramclustr = 172.9536 (+/− NA ppm)
171.9463
[M−H]−
681.2769
947.4451
0
20
40
60
80
100
200
400
600
800

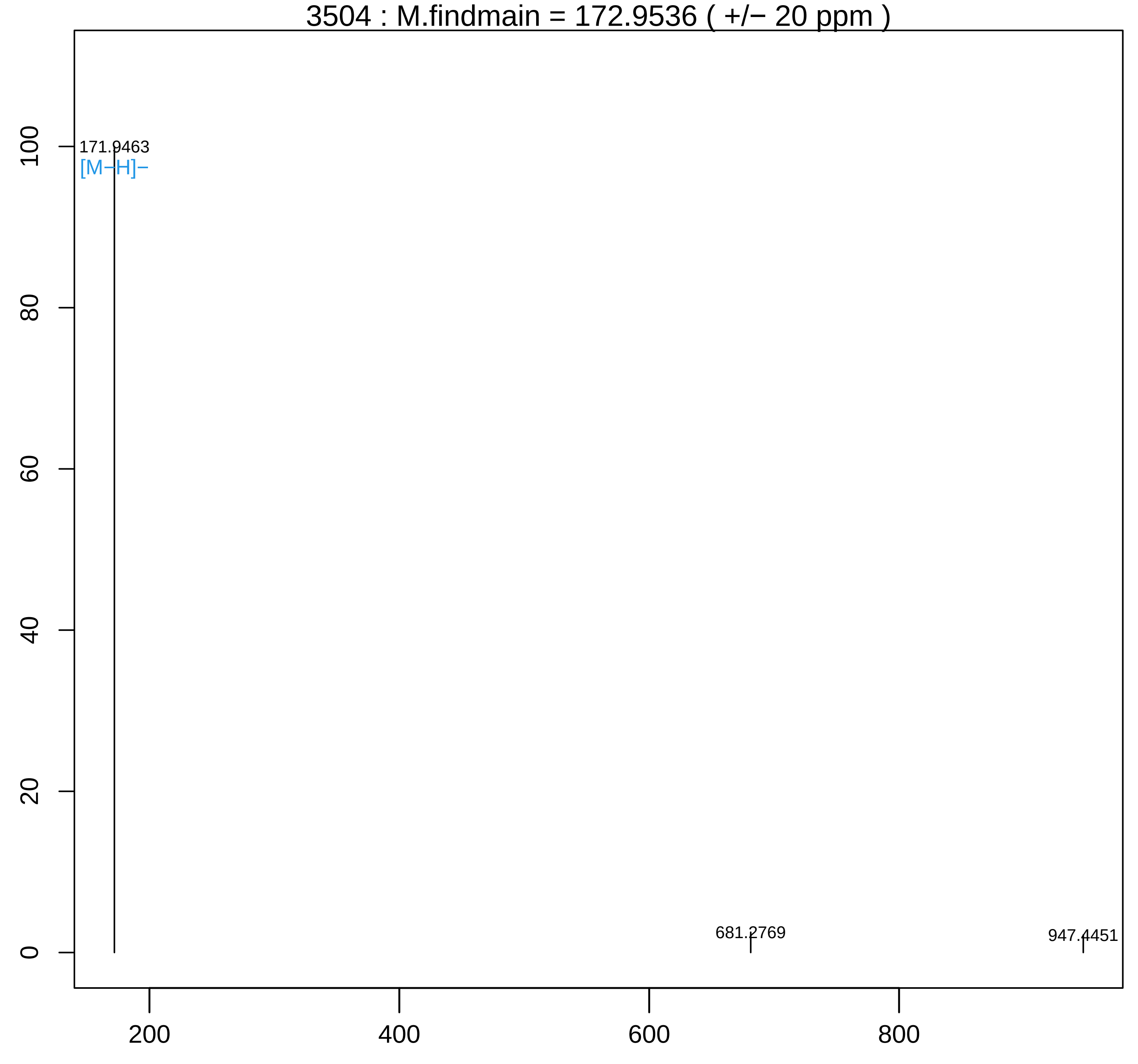

3504 : M.findmain = 172.9536 (+/− 20 ppm)
171.9463
[M−H]−
681.2769
947.4451
0
20
40
60
80
100
200
400
600
800

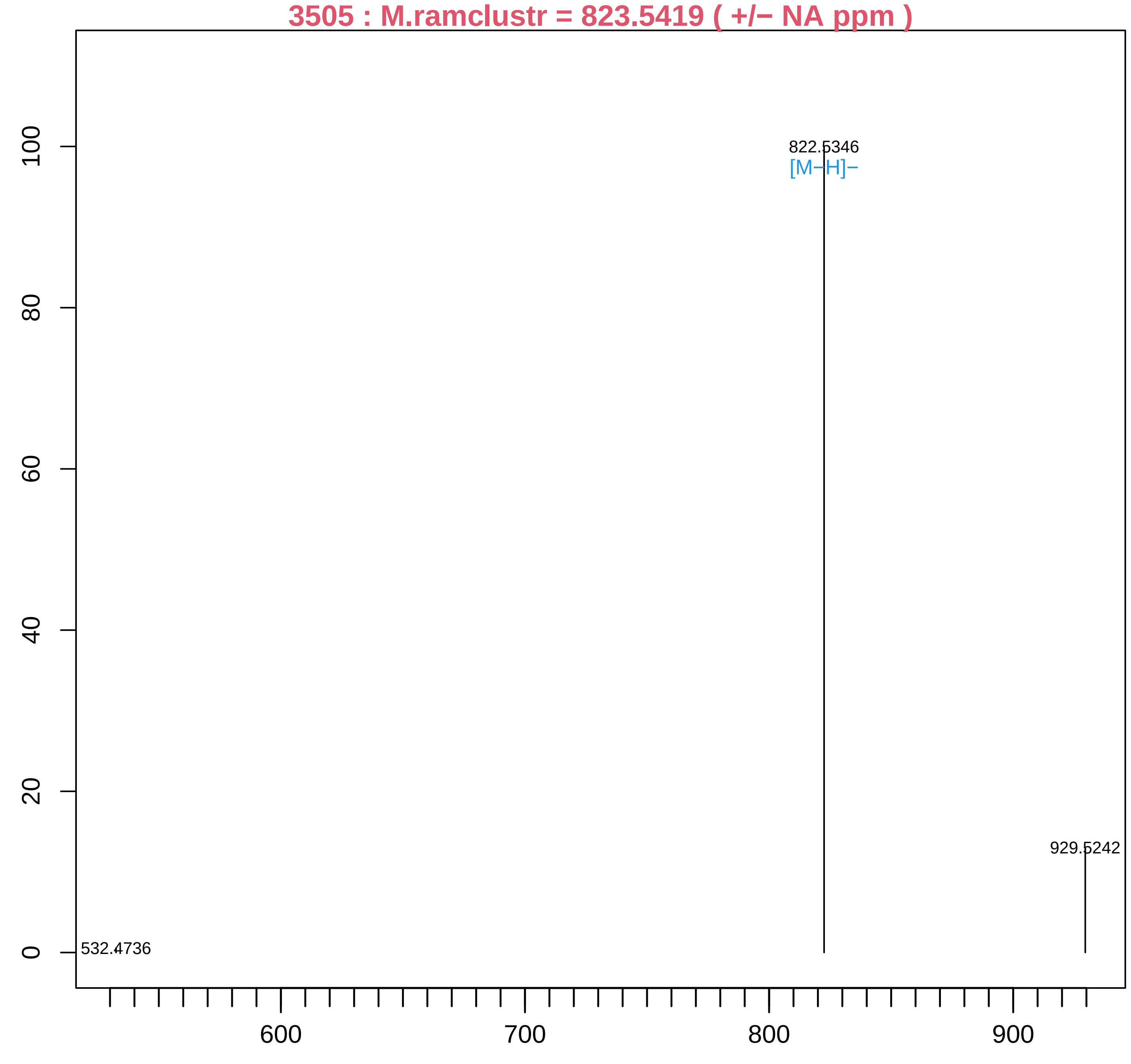
3505 : M.ramclustr = 823.5419 (+/− NA ppm)
822.5346
[M−H]−
929.5242
532.4736
0
20
40
60
80
100
600
700
800
900
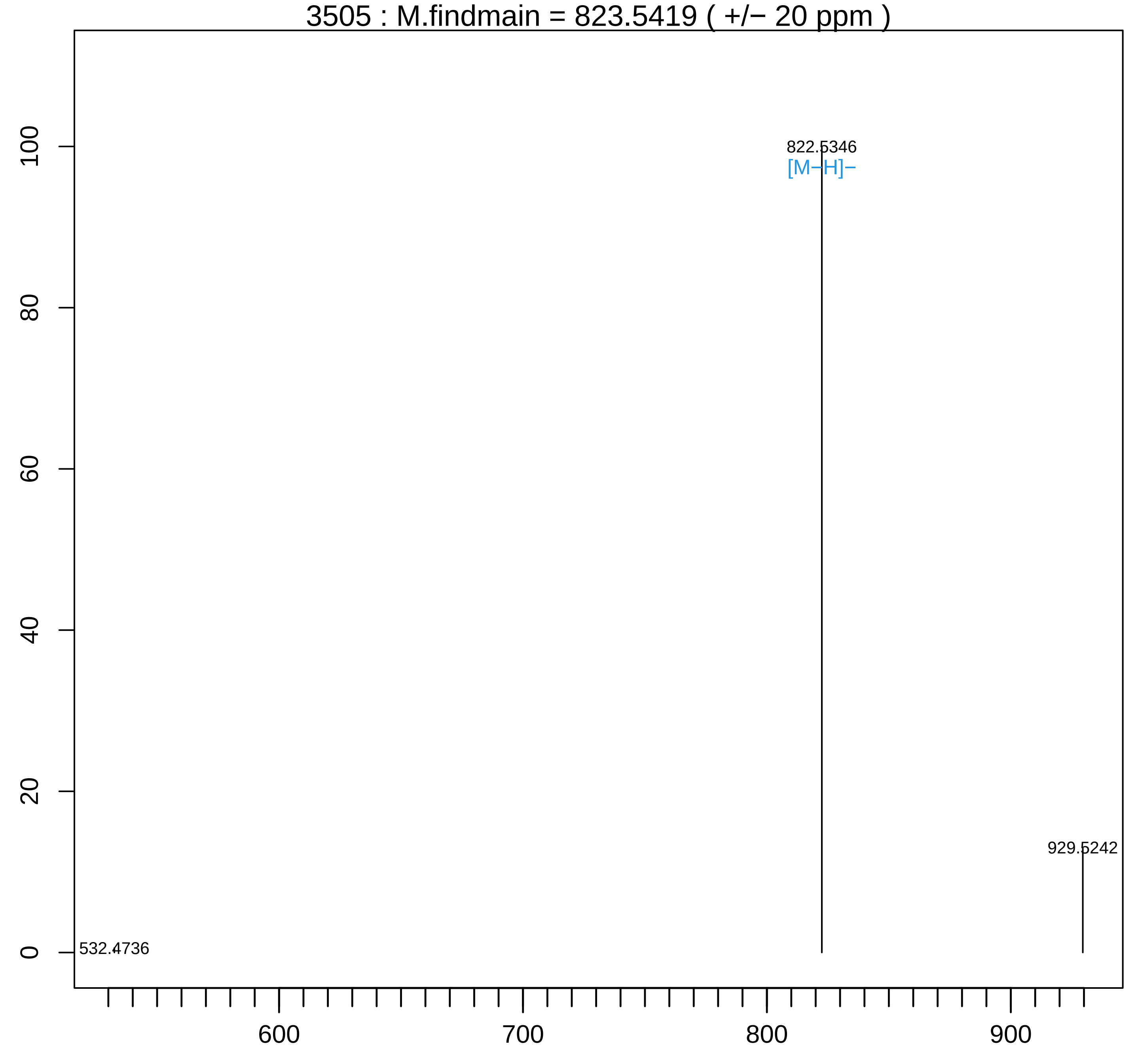
3505 : M.findmain = 823.5419 (+/− 20 ppm)
822.5346
[M−H]−
929.5242
532.4736
0
20
40
60
80
100
600
700
800
900

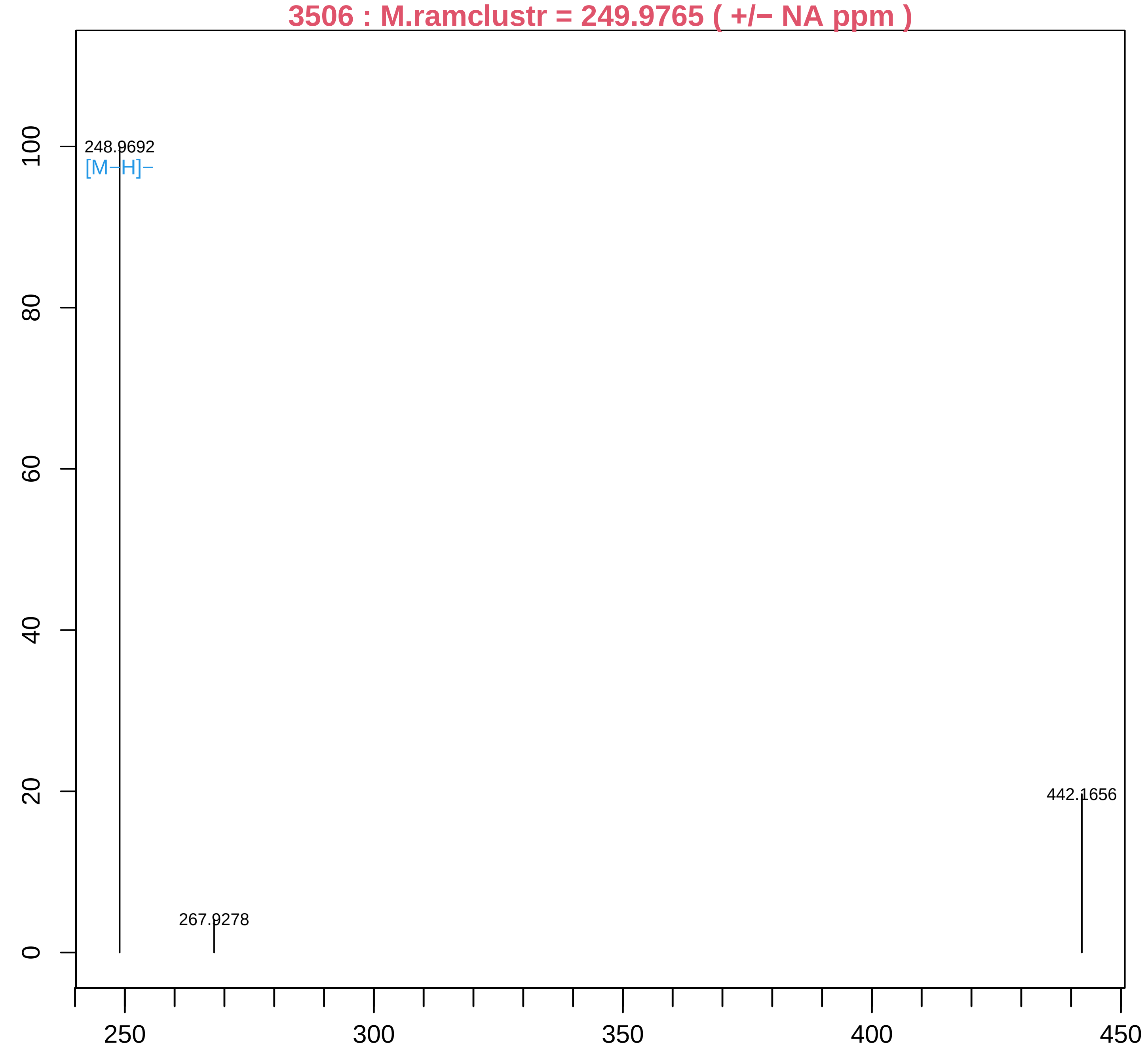
3506 : M.ramclustr = 249.9765 (+/− NA ppm)
248.9692
[M−H]−
267.9278
442.1656
0
20
40
60
80
100
250
300
350
400
450

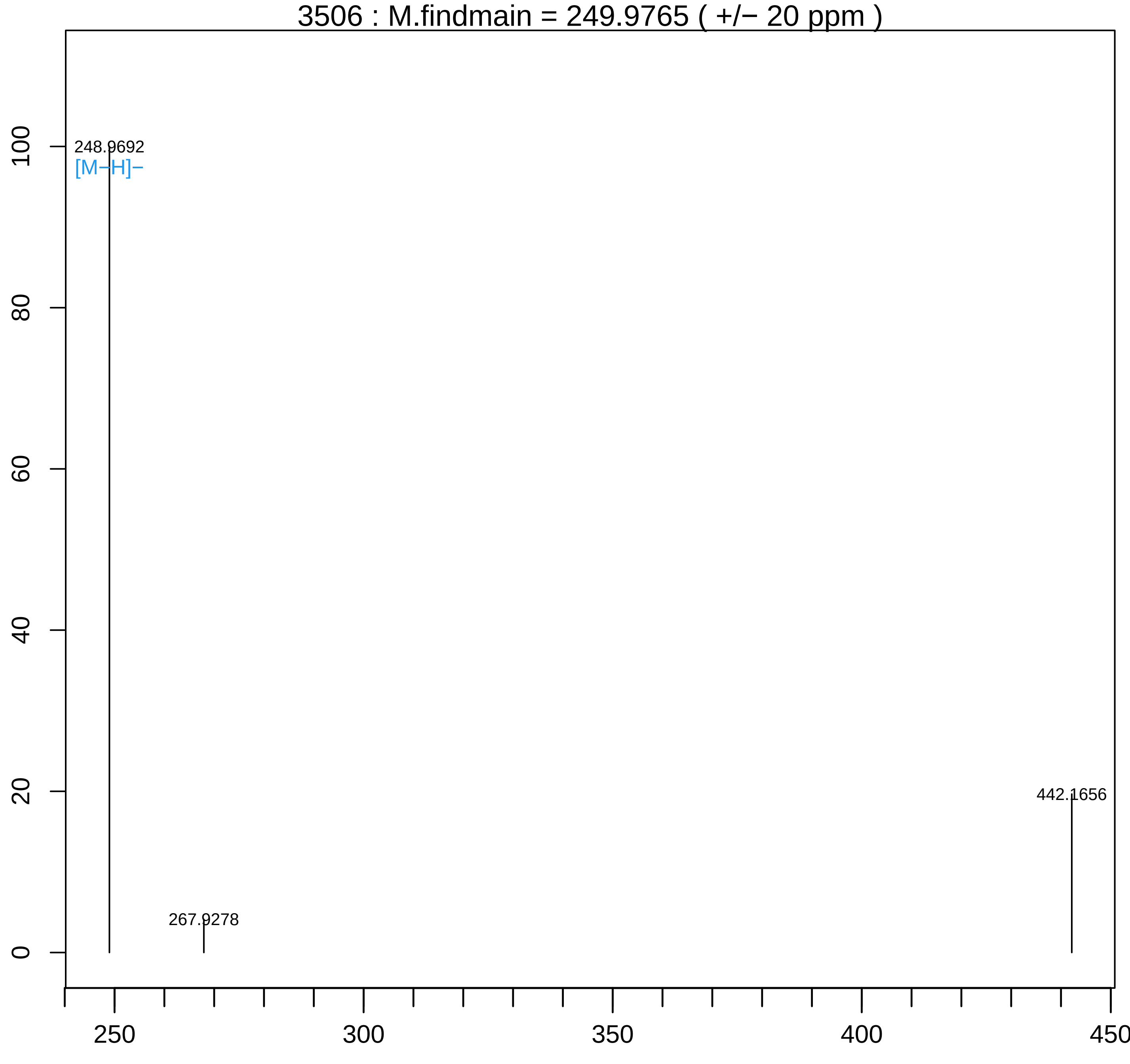
3506 : M.findmain = 249.9765 (+/− 20 ppm)
248.9692
[M−H]−
267.9278
442.1656
0
20
40
60
80
100
250
300
350
400
450

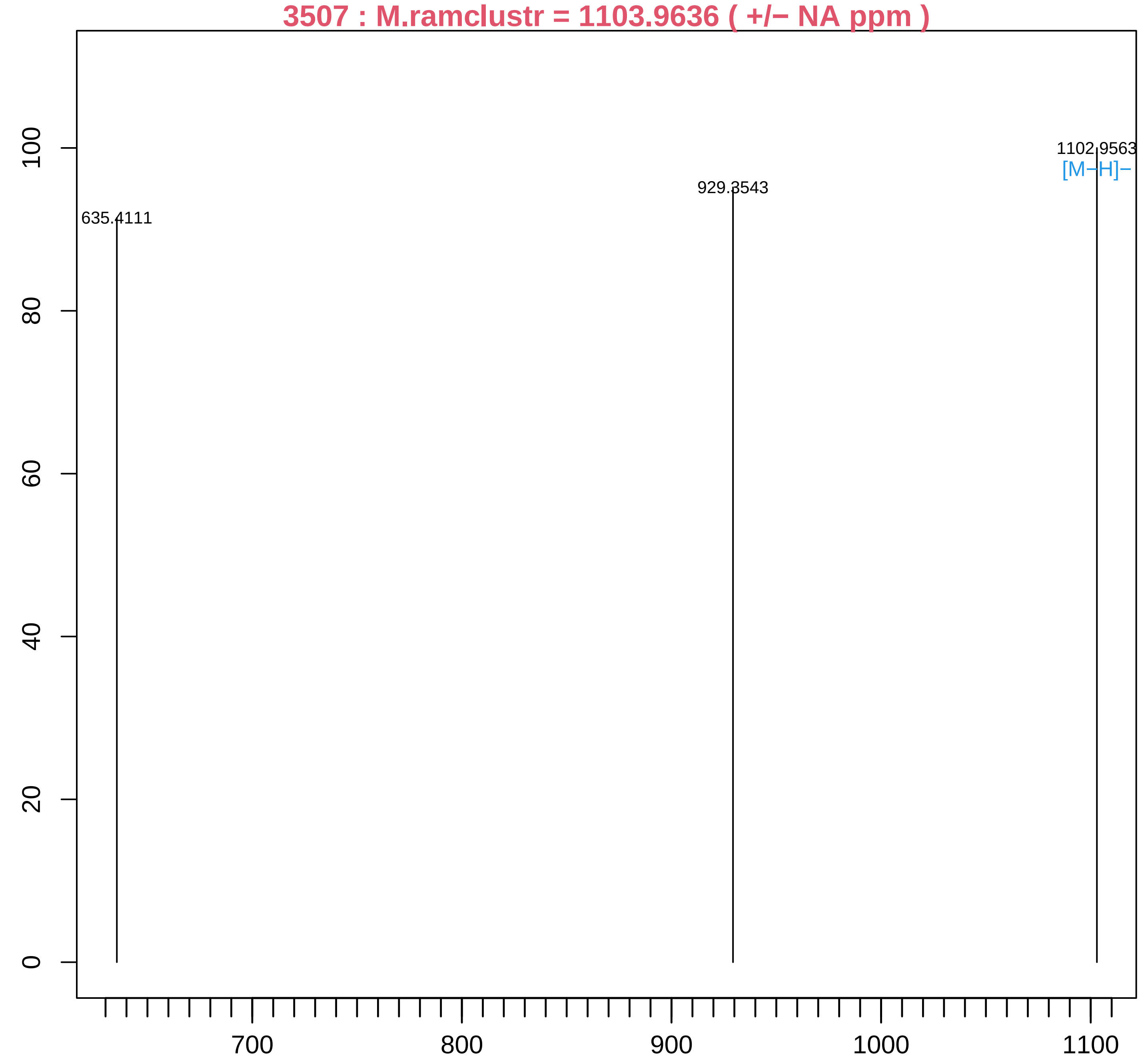
3507 : M.ramclustr = 1103.9636 (+/− NA ppm)
1102.9563
[M−H]−
929.3543
635.4111
0
20
40
60
80
100
700
800
900
1000
1100

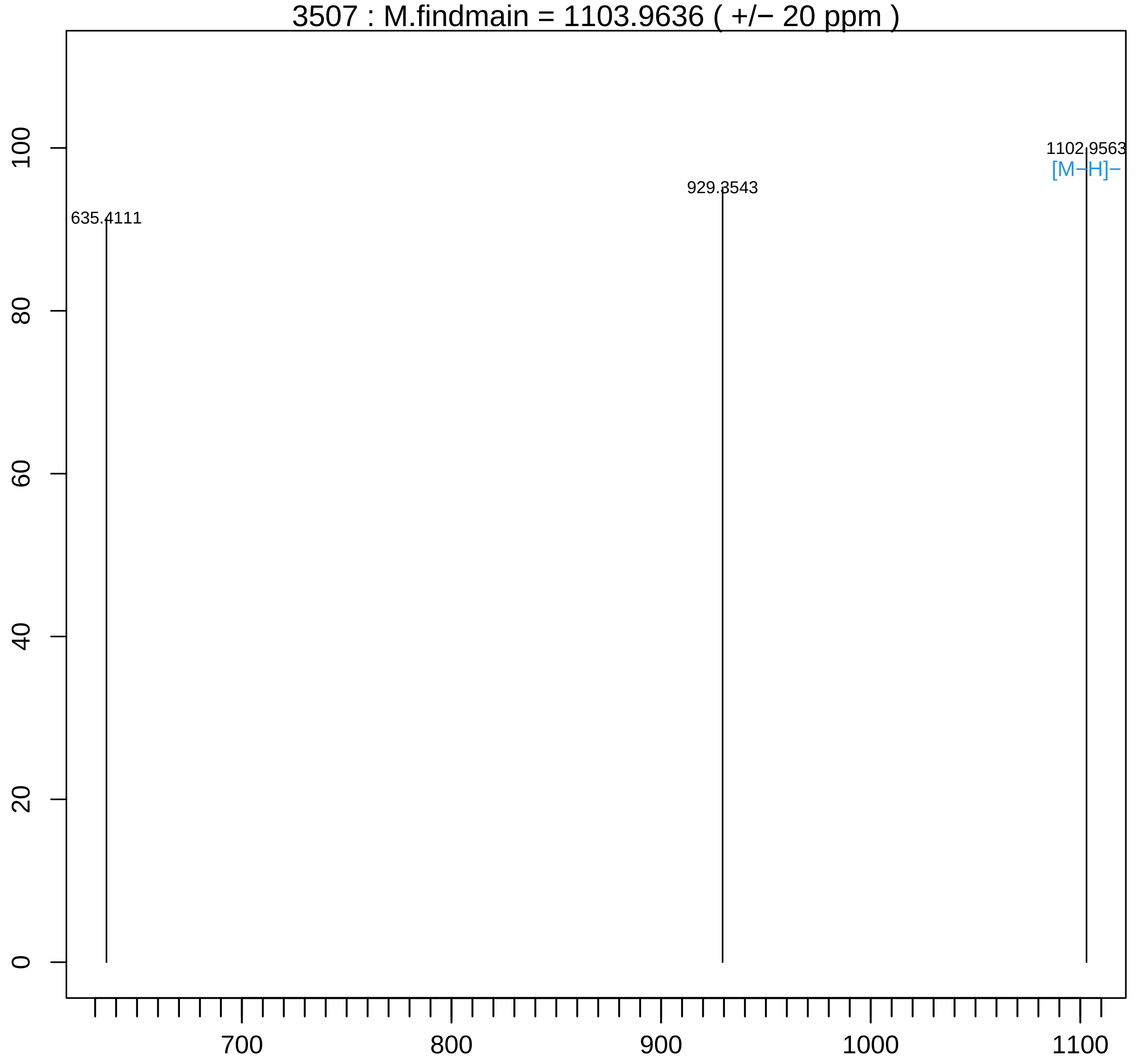
3507 : M.findmain = 1103.9636 (+/− 20 ppm)
1102.9563
[M−H]−
929.3543
635.4111
0
20
40
60
80
100
700
800
900
1000
1100

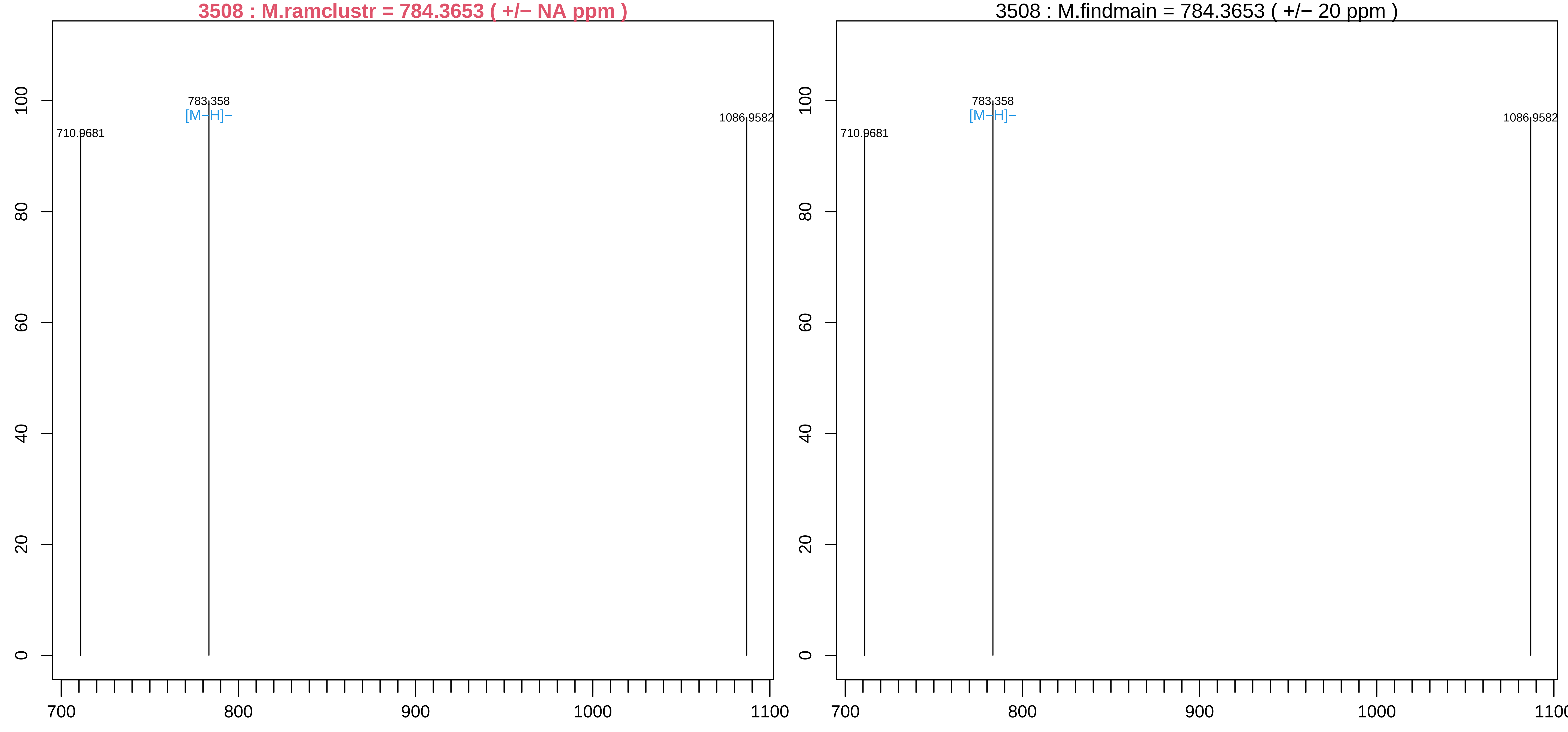

3508 : M.ramclustr = 784.3653 (+/− NA ppm)
710.9681
783.358
[M−H]−
1086.9582
0
20
40
60
80
100
700
800
900
1000
1100
3508 : M.findmain = 784.3653 (+/− 20 ppm)
710.9681
783.358
[M−H]−
1086.9582
0
20
40
60
80
100
700
800
900
1000
1100

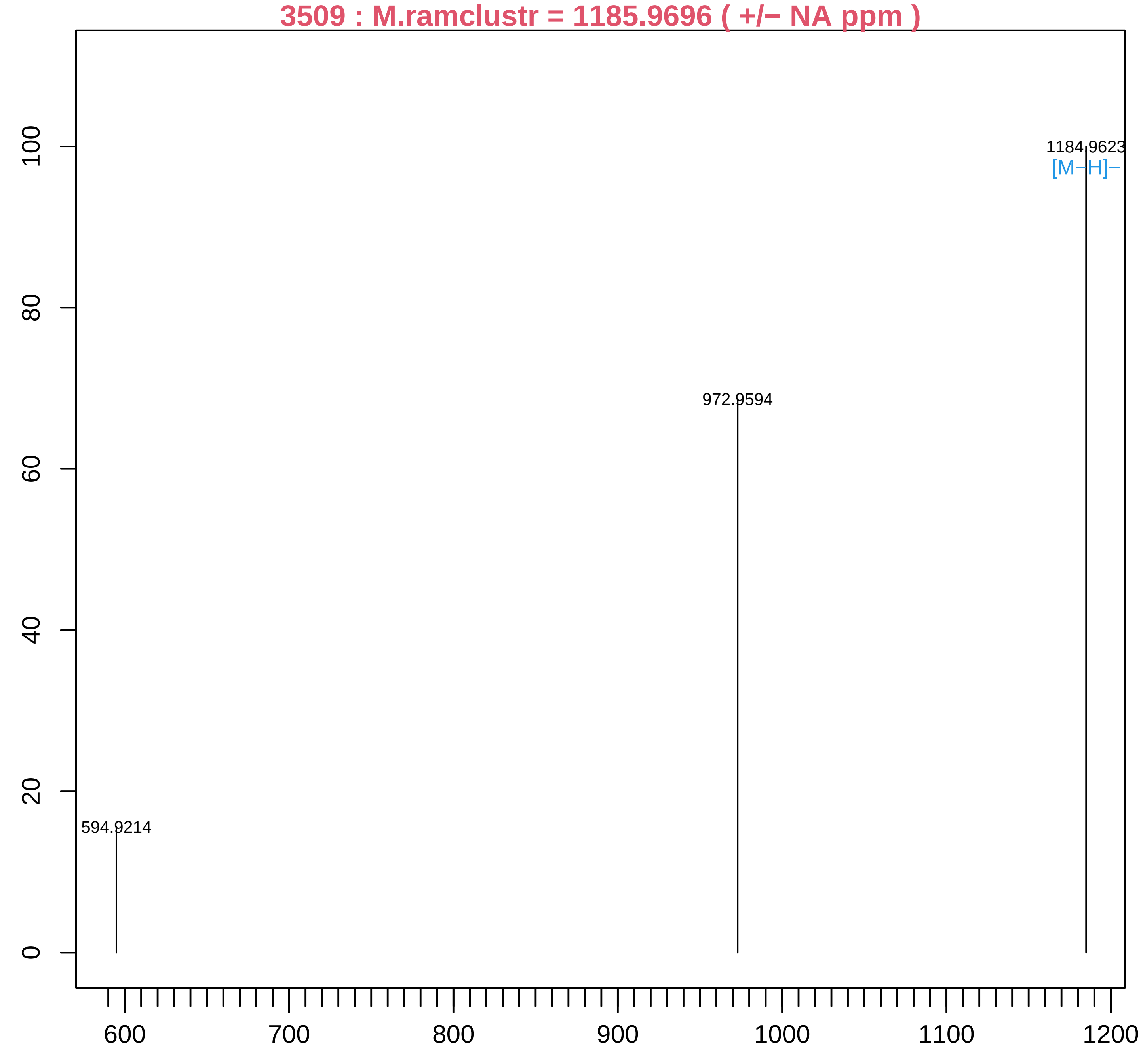

3509 : M.ramclustr = 1185.9696 (+/− NA ppm)
1184.9623
[M−H]−
972.9594
594.9214
0
20
40
60
80
100
600
700
800
900
1000
1100
1200

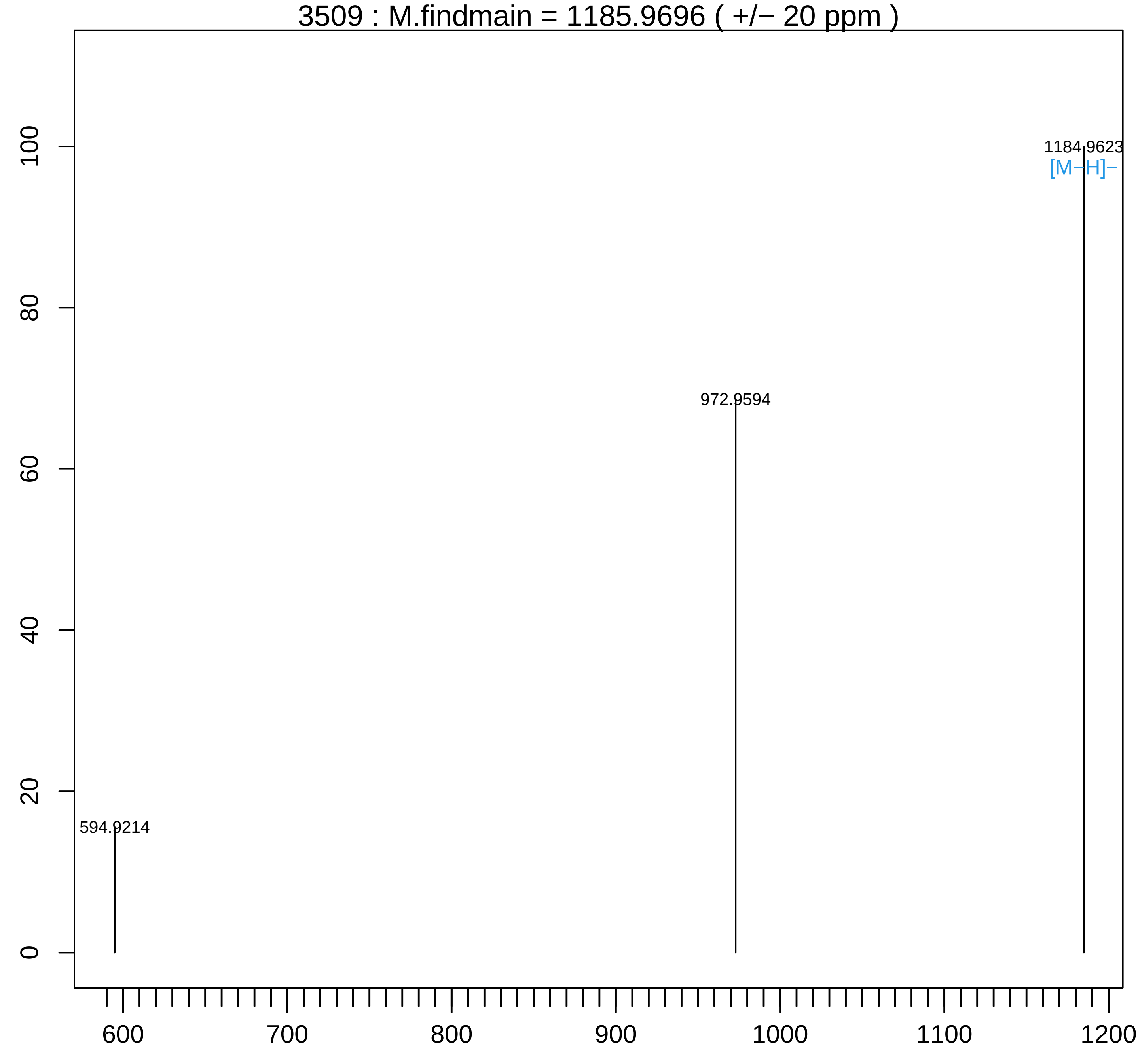

3509 : M.findmain = 1185.9696 (+/− 20 ppm)
1184.9623
[M−H]−
972.9594
594.9214
0
20
40
60
80
100
600
700
800
900
1000
1100
1200

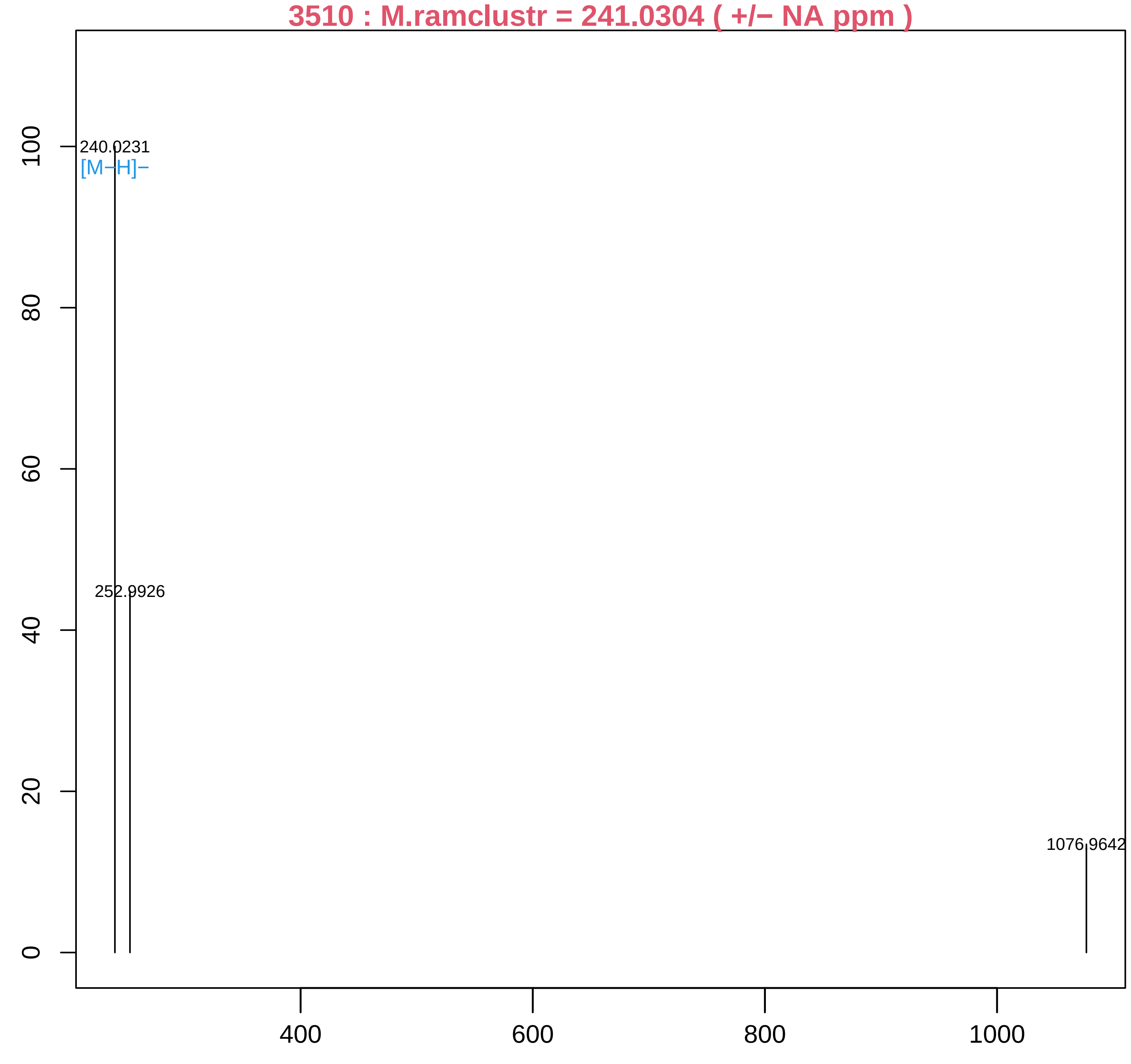
3510 : M.ramclustr = 241.0304 (+/− NA ppm)
240.0231
[M−H]−
252.9926
1076.9642
0
20
40
60
80
100
400
600
800
1000

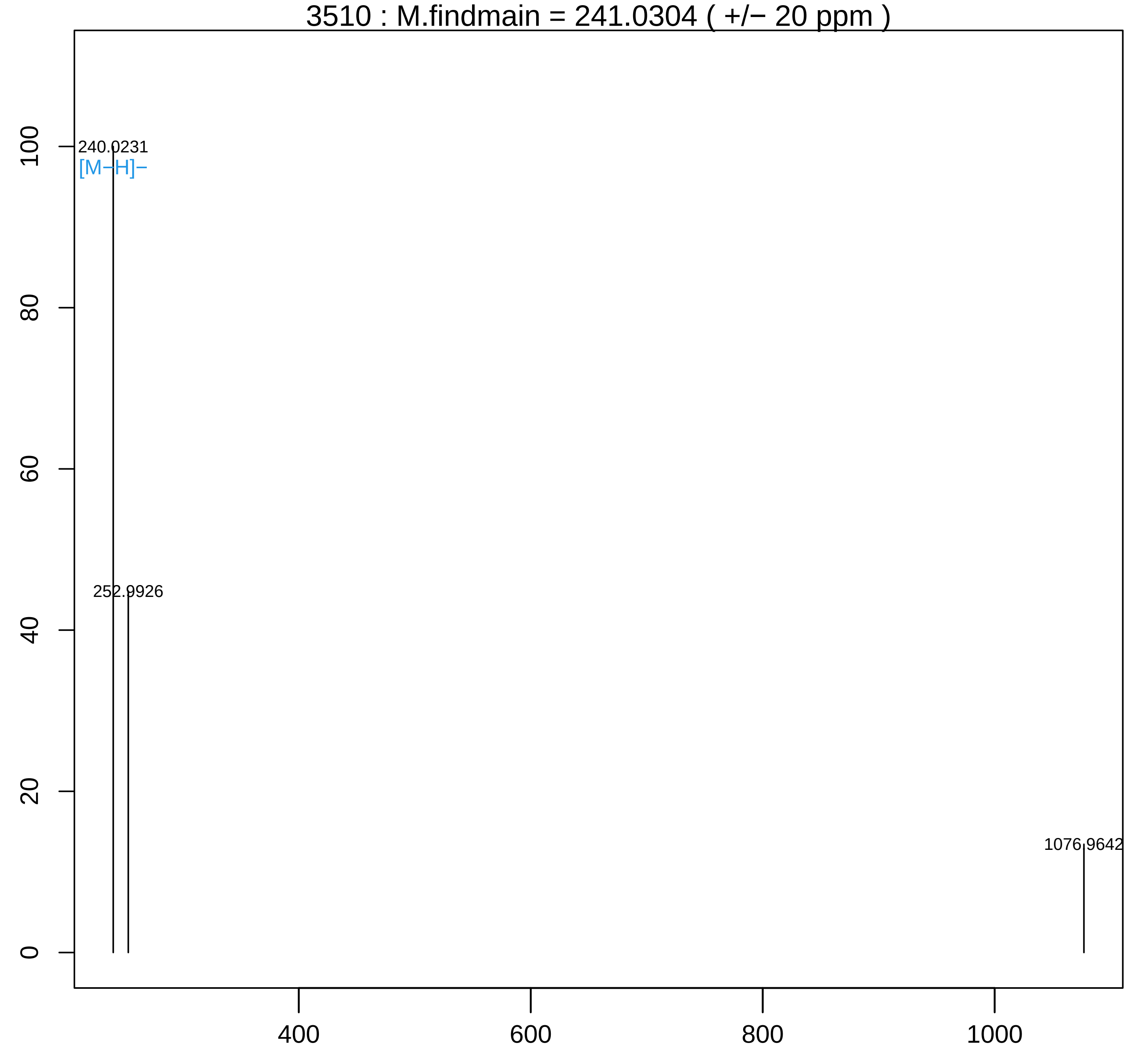
3510 : M.findmain = 241.0304 (+/− 20 ppm)
240.0231
[M−H]−
252.9926
1076.9642
0
20
40
60
80
100
400
600
800
1000

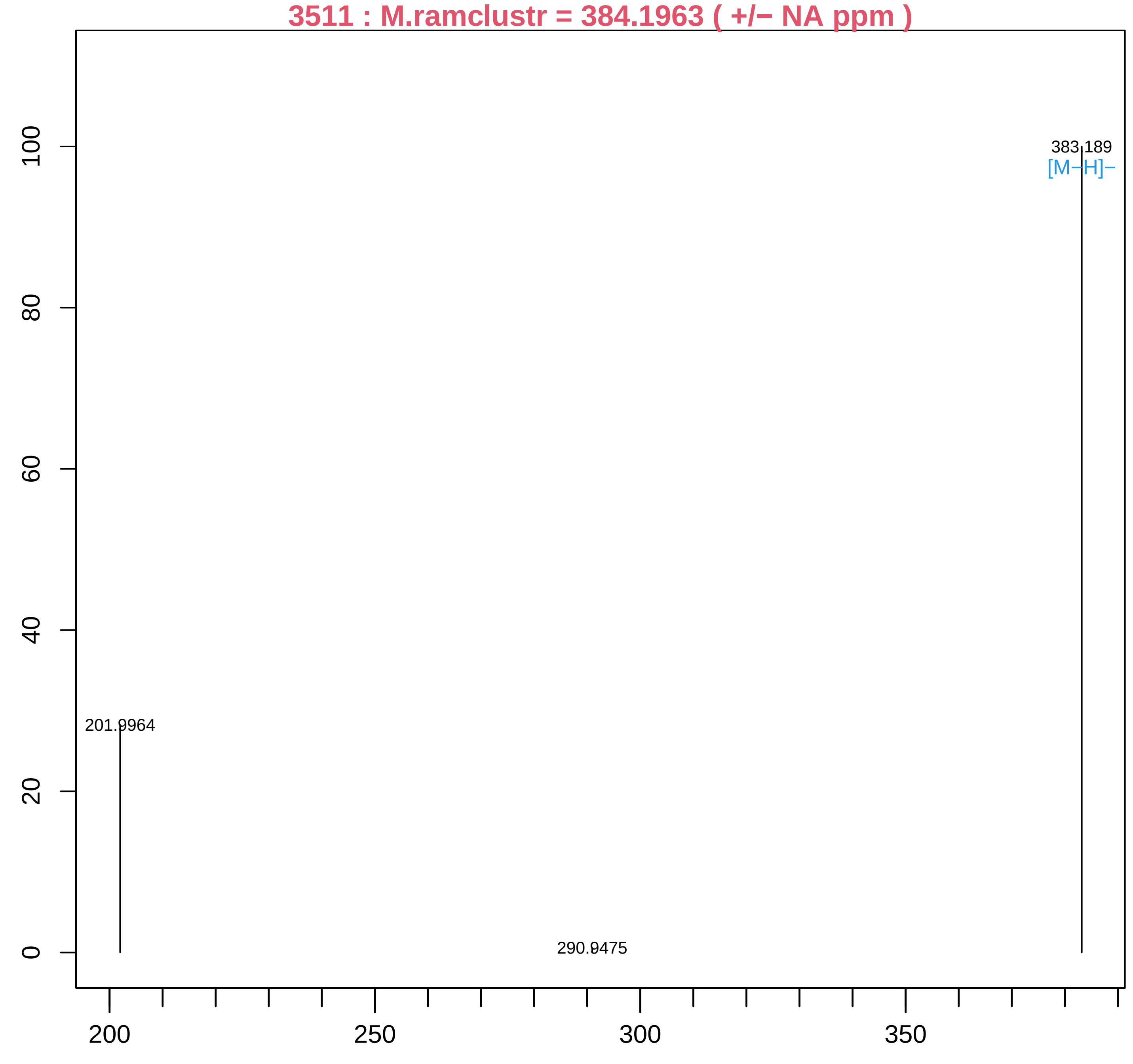
3511 : M.ramclustr = 384.1963 (+/− NA ppm)
383.189
[M−H]−
201.9964
290.9475
0
20
40
60
80
100
200
250
300
350

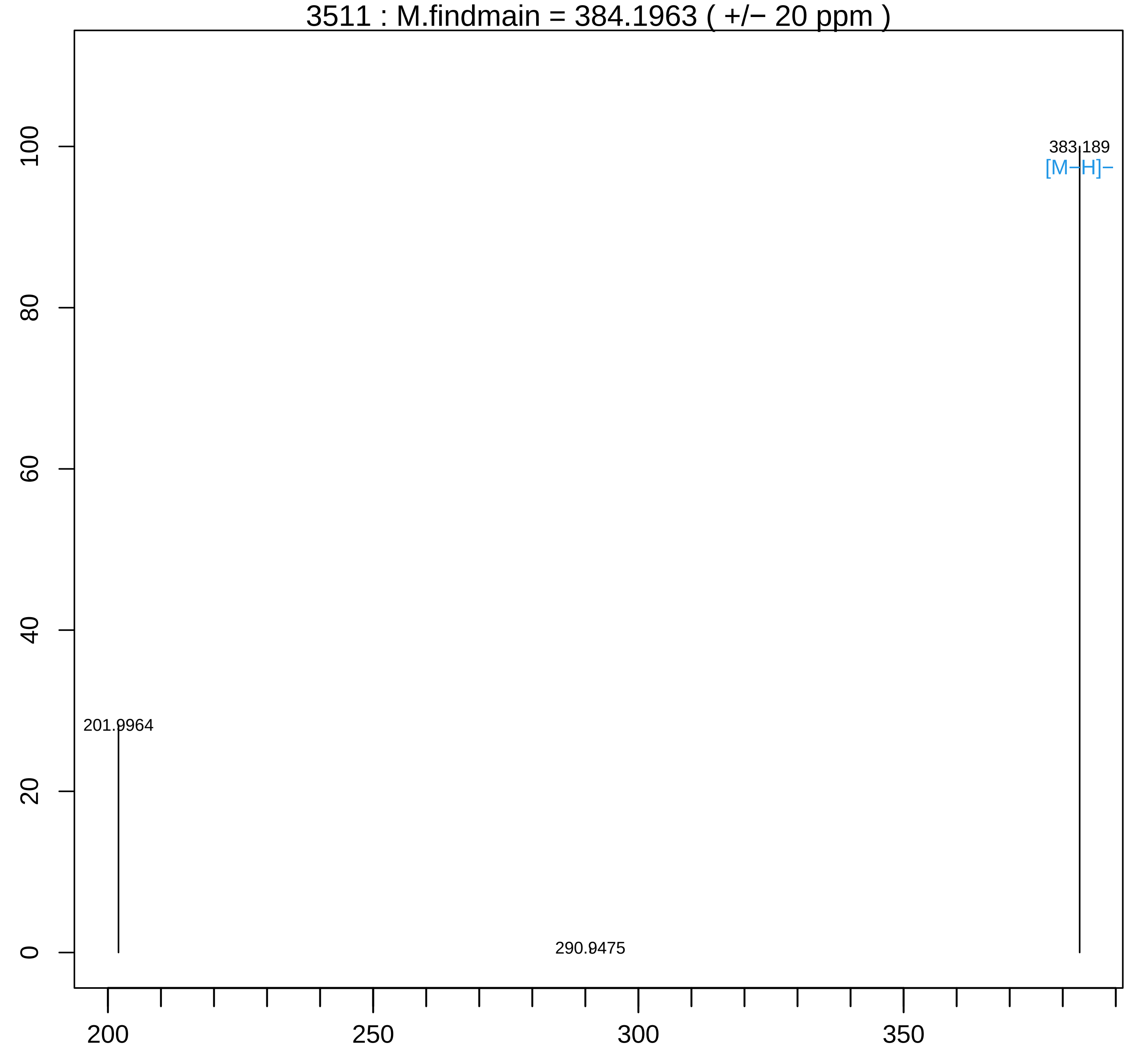
3511 : M.findmain = 384.1963 (+/− 20 ppm)
383.189
[M−H]−
201.9964
290.9475
0
20
40
60
80
100
200
250
300
350

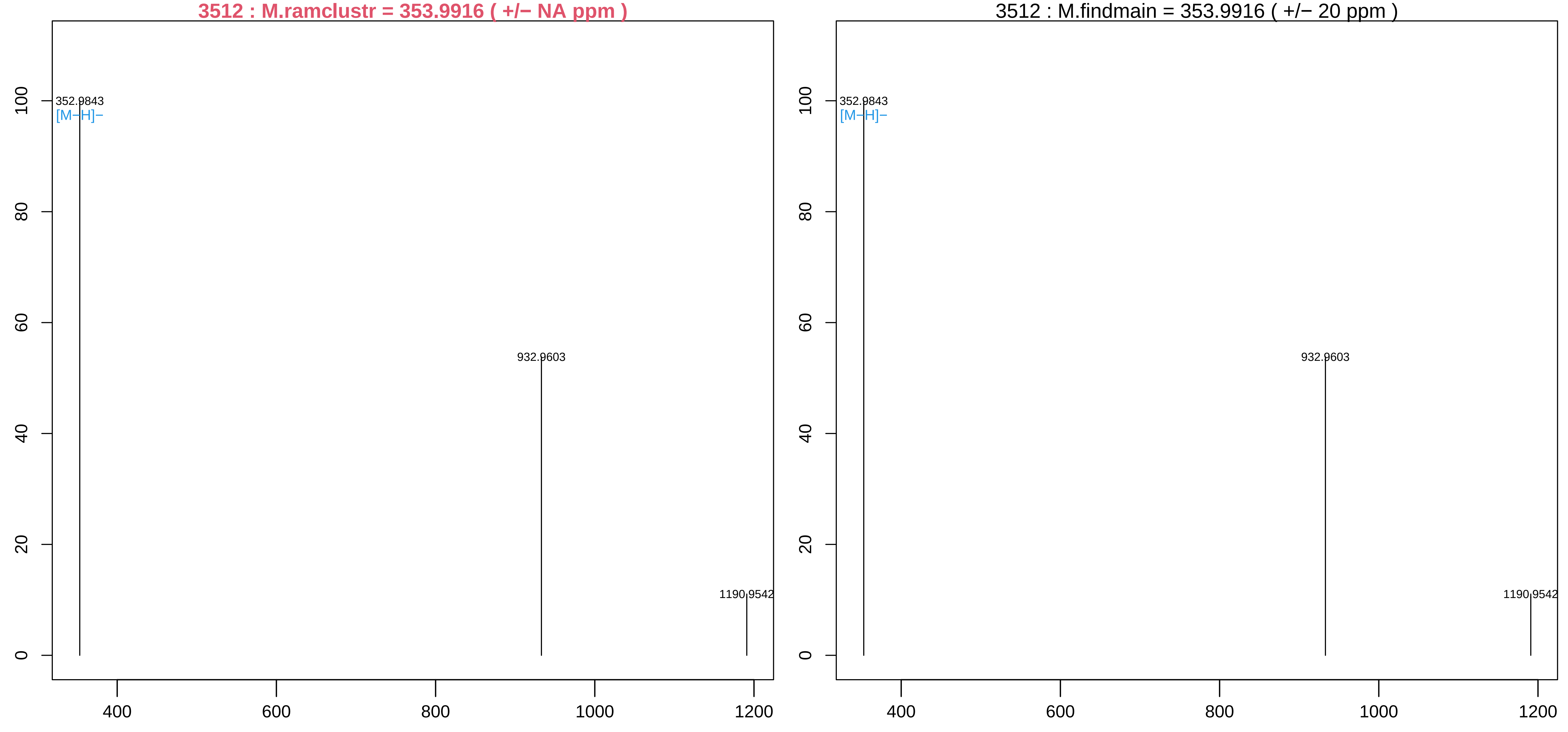
3512 : M.ramclustr = 353.9916 (+/− NA ppm)
3512 : M.findmain = 353.9916 (+/− 20 ppm)
352.9843
[M−H]−
932.9603
1190.9542
0
20
40
60
80
100
400
600
800
1000
1200

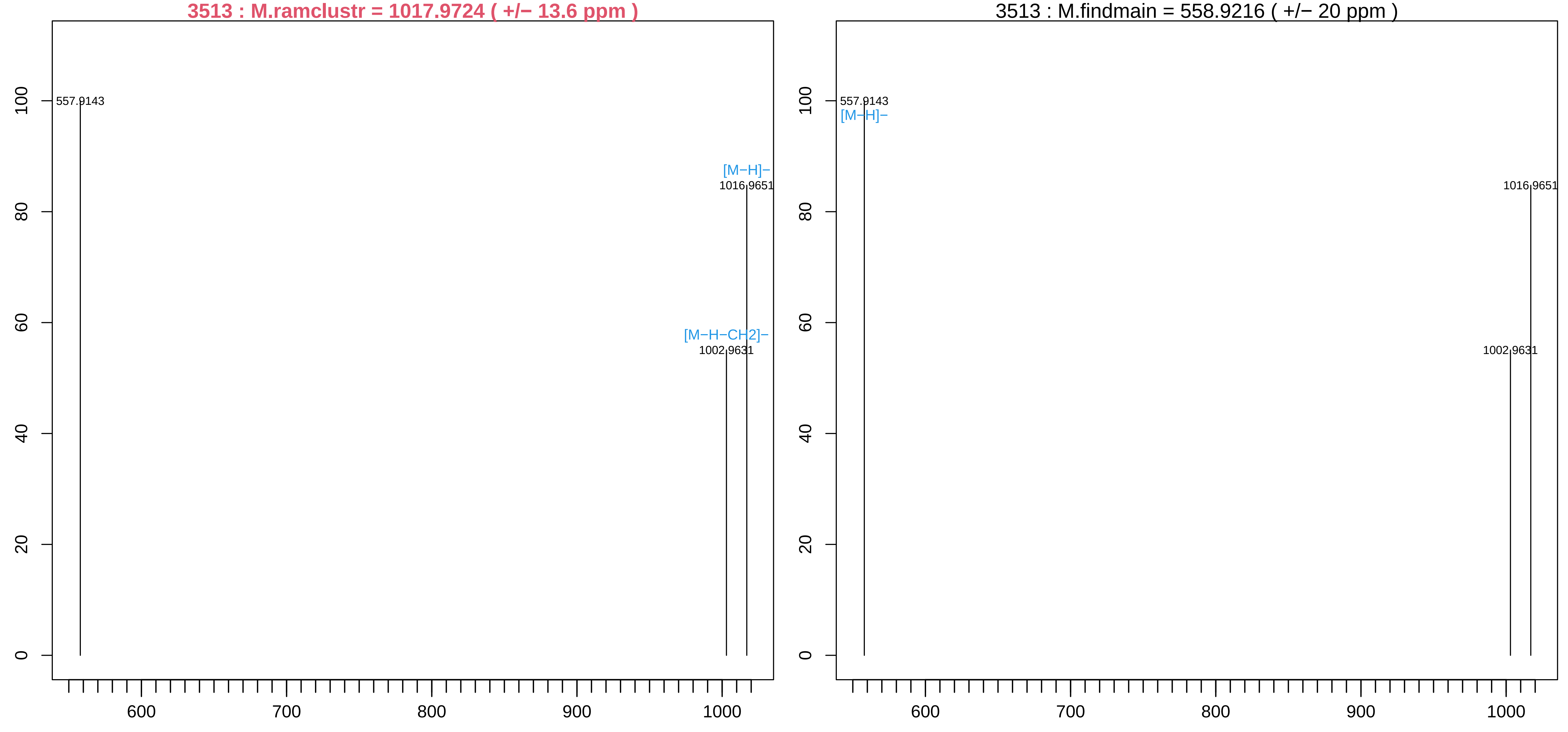
3513 : M.ramclustr = 1017.9724 (+/− 13.6 ppm)
557.9143
[M−H]−
1016.9651
[M−H−CH2]−
1002.9631
0
20
40
60
80
100
600
700
800
900
1000
3513 : M.findmain = 558.9216 (+/− 20 ppm)
557.9143
[M−H]−
1016.9651
1002.9631
0
20
40
60
80
100
600
700
800
900
1000

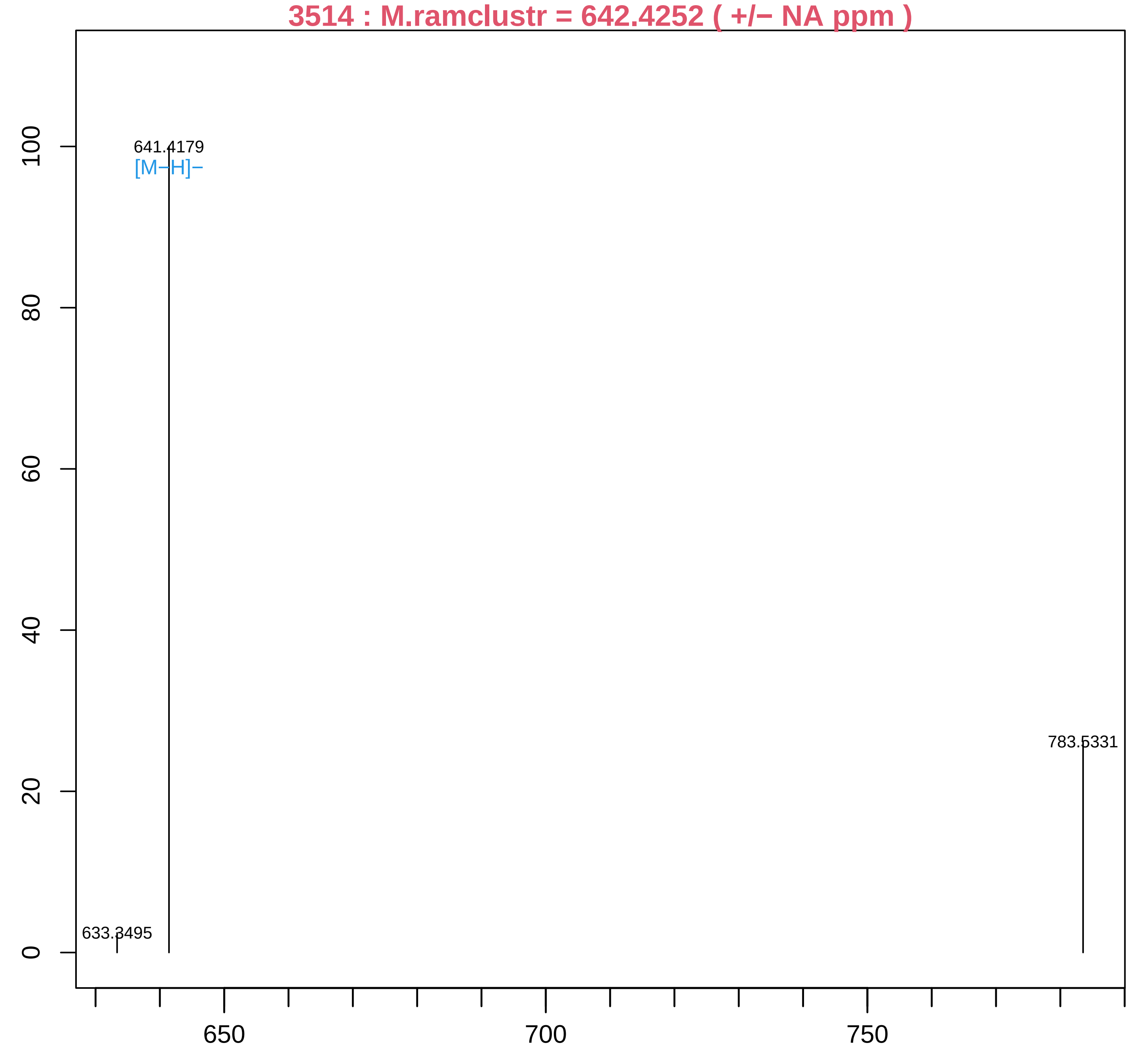
3514 : M.ramclustr = 642.4252 (+/− NA ppm)
641.4179
[M−H]−
633.3495
783.5331
0
20
40
60
80
100
650
700
750

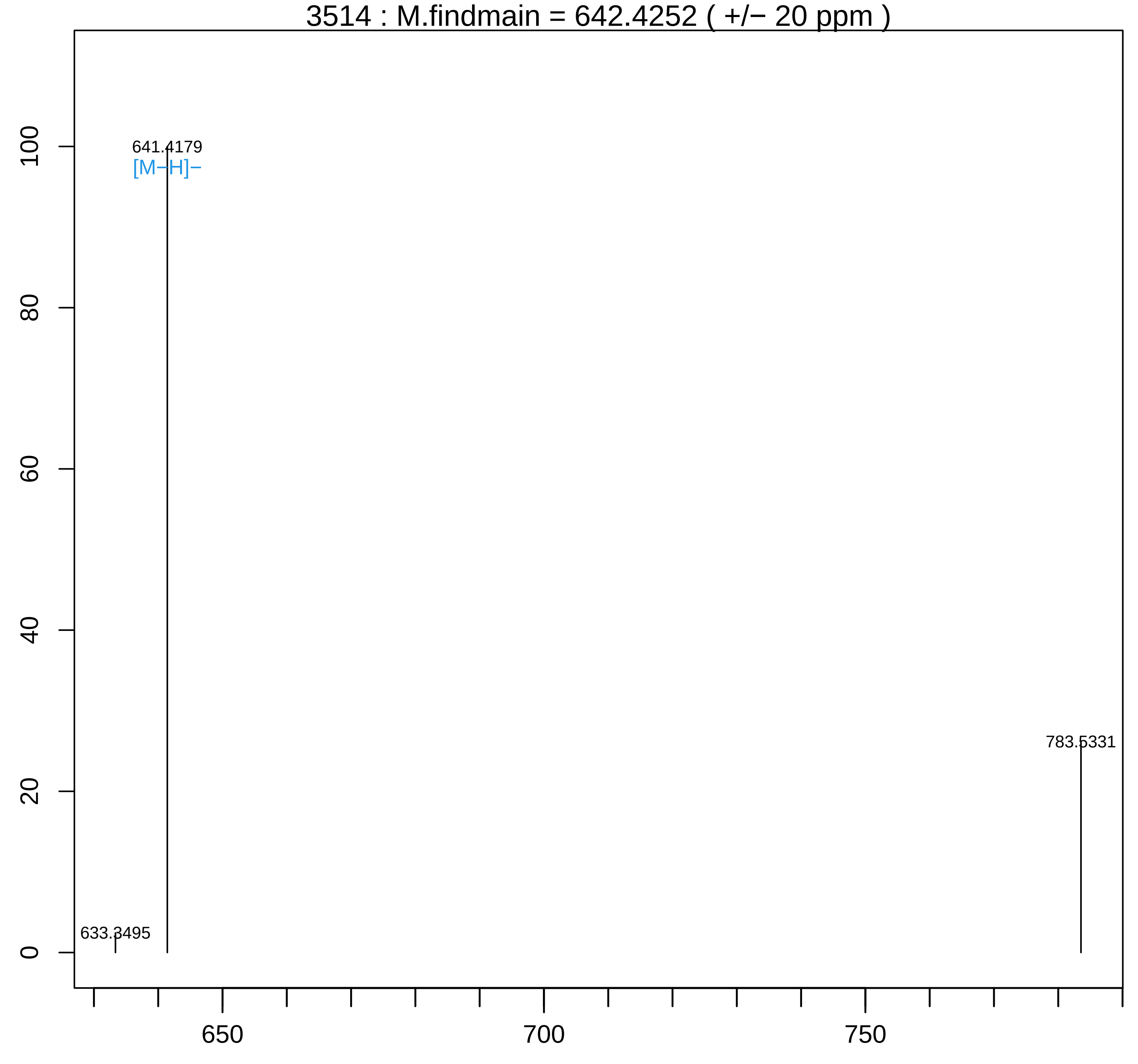
3514 : M.findmain = 642.4252 (+/− 20 ppm)
641.4179
[M−H]−
633.3495
783.5331
0
20
40
60
80
100
650
700
750

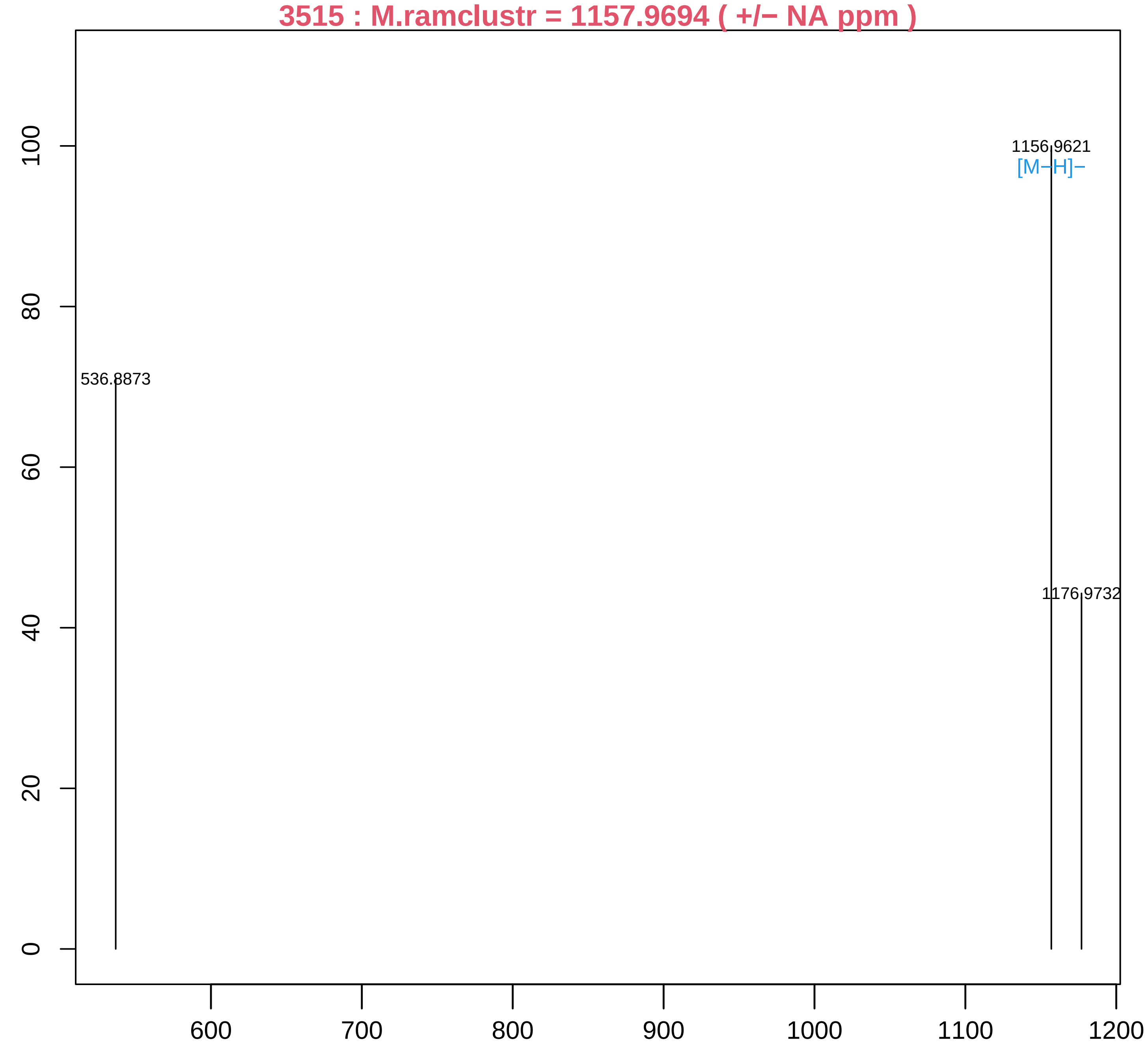

3515 : M.ramclustr = 1157.9694 (+/− NA ppm)
536.8873
1156.9621
[M−H]−
1176.9732
0
20
40
60
80
100
600
700
800
900
1000
1100
1200

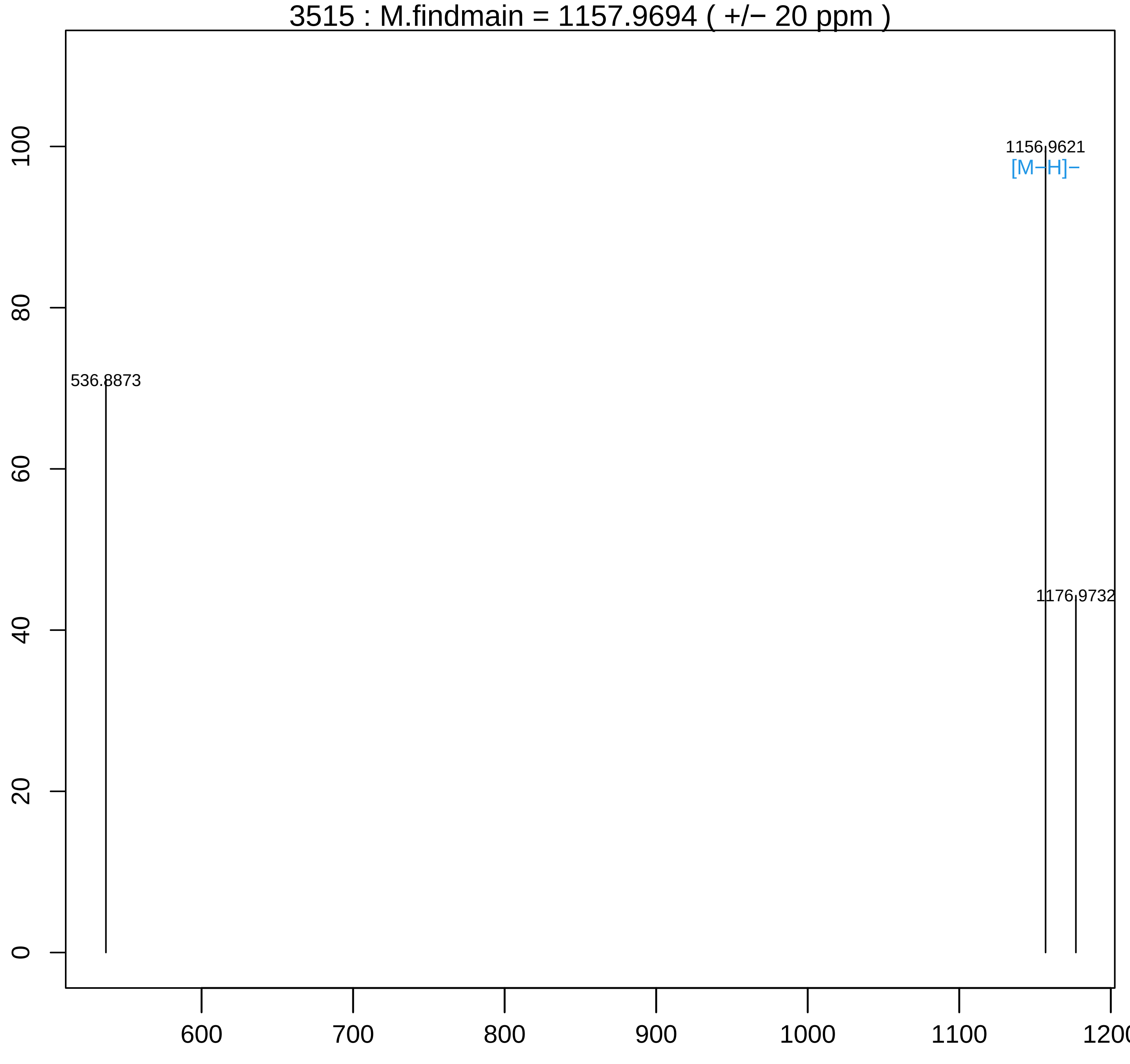

3515 : M.findmain = 1157.9694 (+/− 20 ppm)
536.8873
1156.9621
[M−H]−
1176.9732
0
20
40
60
80
100
600
700
800
900
1000
1100
1200

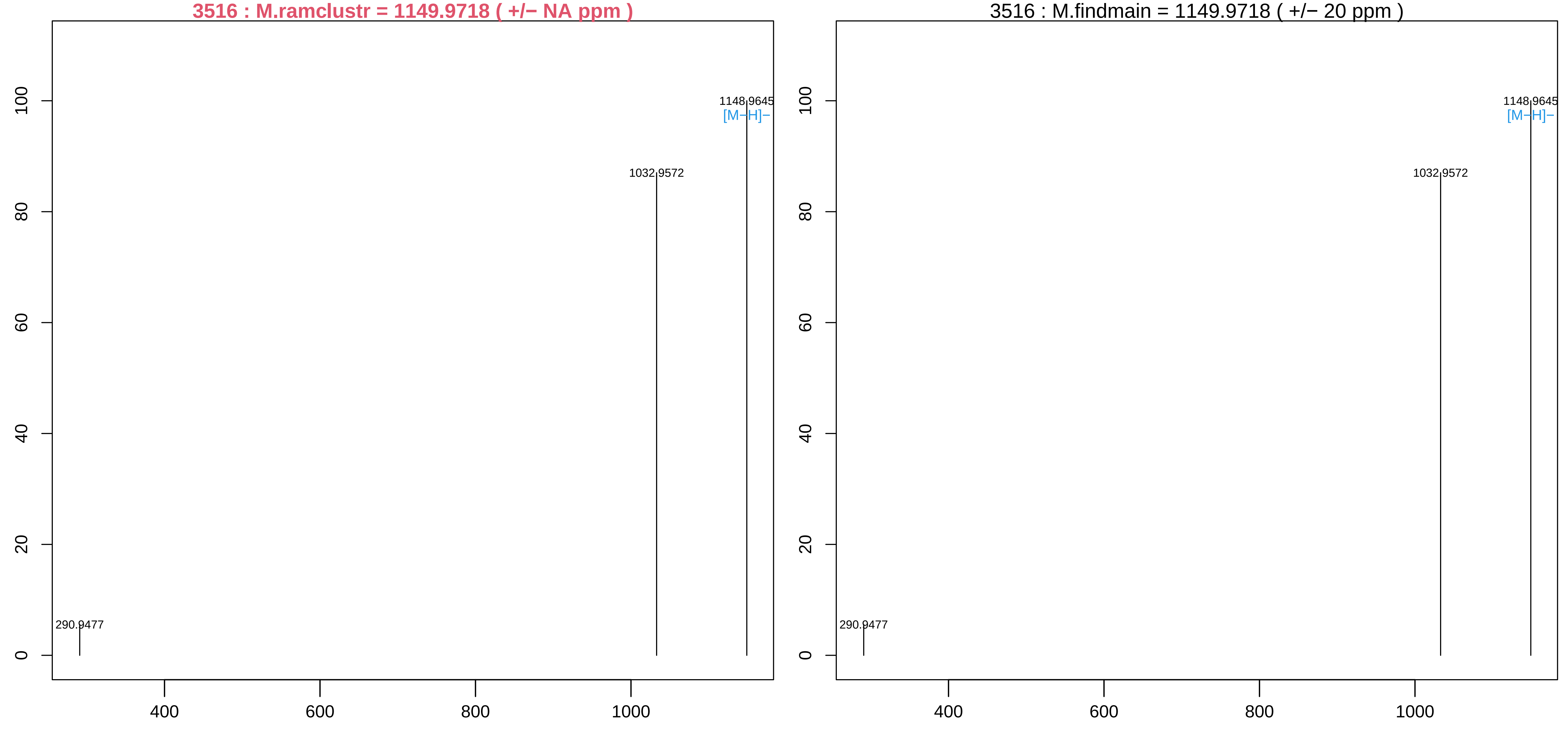
3516 : M.ramclustr = 1149.9718 (+/− NA ppm)
3516 : M.findmain = 1149.9718 (+/− 20 ppm)
1148.9645
[M−H]−
1032.9572
290.9477
0
20
40
60
80
100
400
600
800
1000

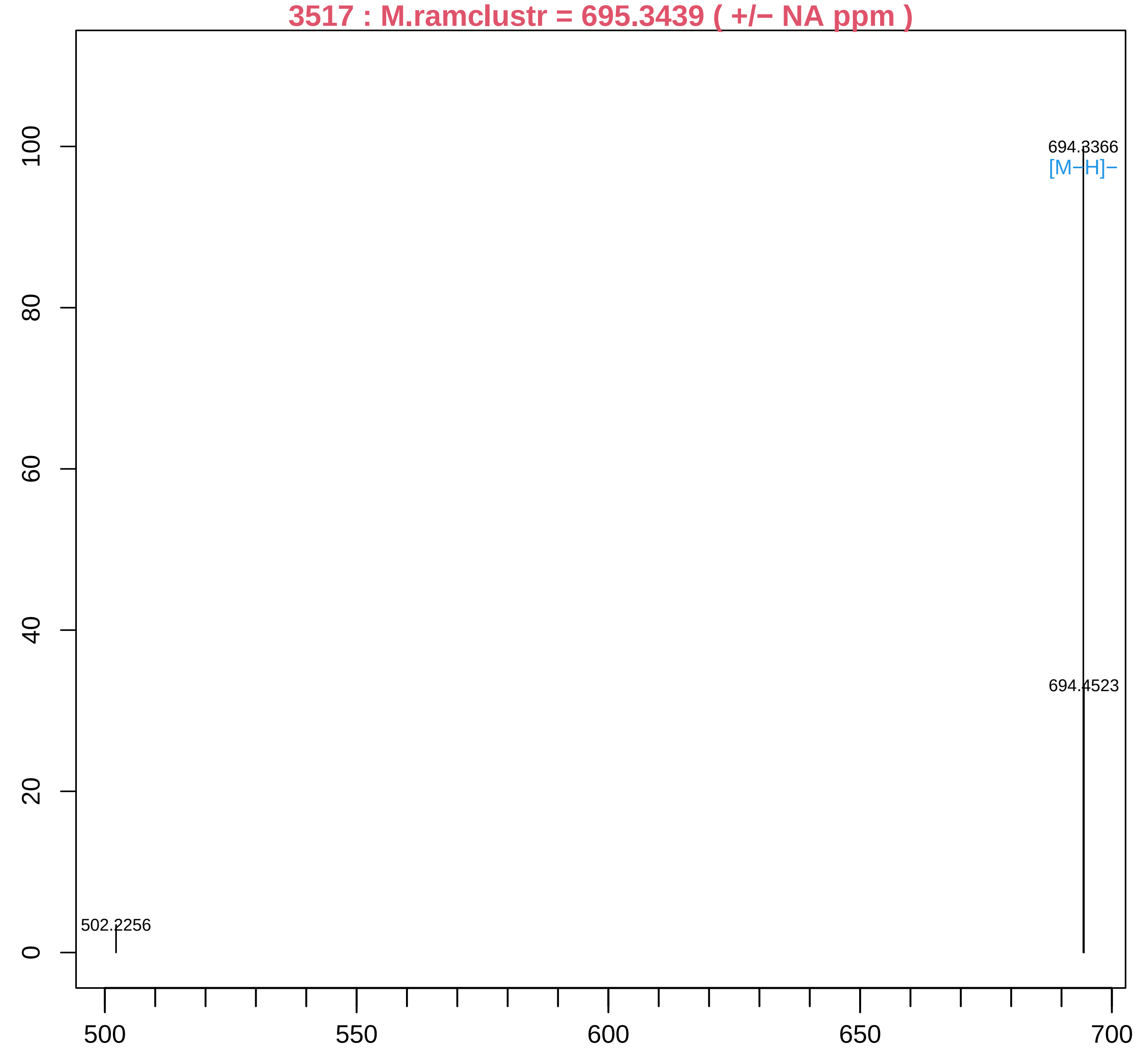

3517 : M.ramclustr = 695.3439 (+/− NA ppm)
694.3366
[M−H]−
694.4523
502.2256
0
20
40
60
80
100
500
550
600
650
700

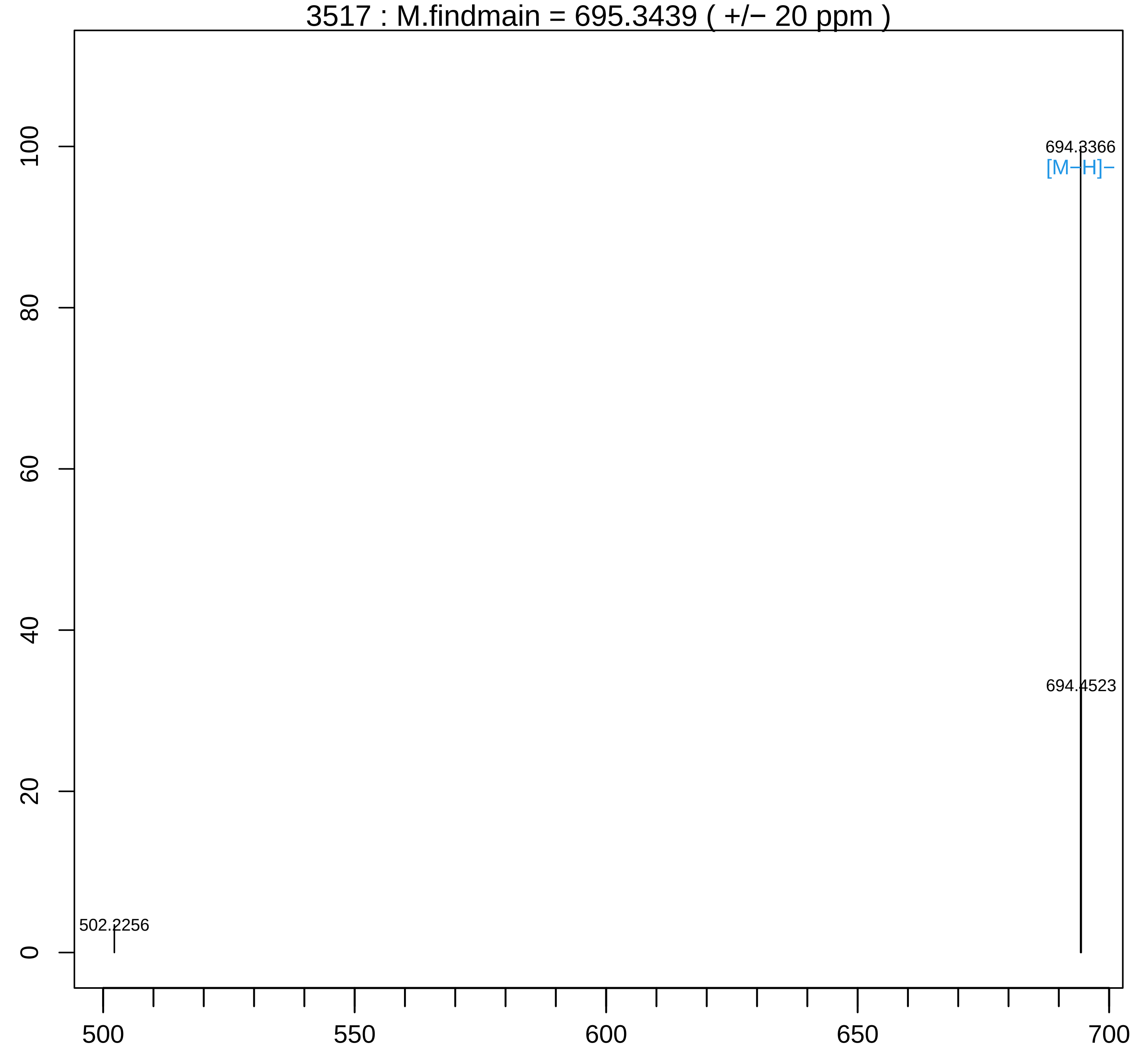

3517 : M.findmain = 695.3439 (+/− 20 ppm)
694.3366
[M−H]−
694.4523
502.2256
0
20
40
60
80
100
500
550
600
650
700

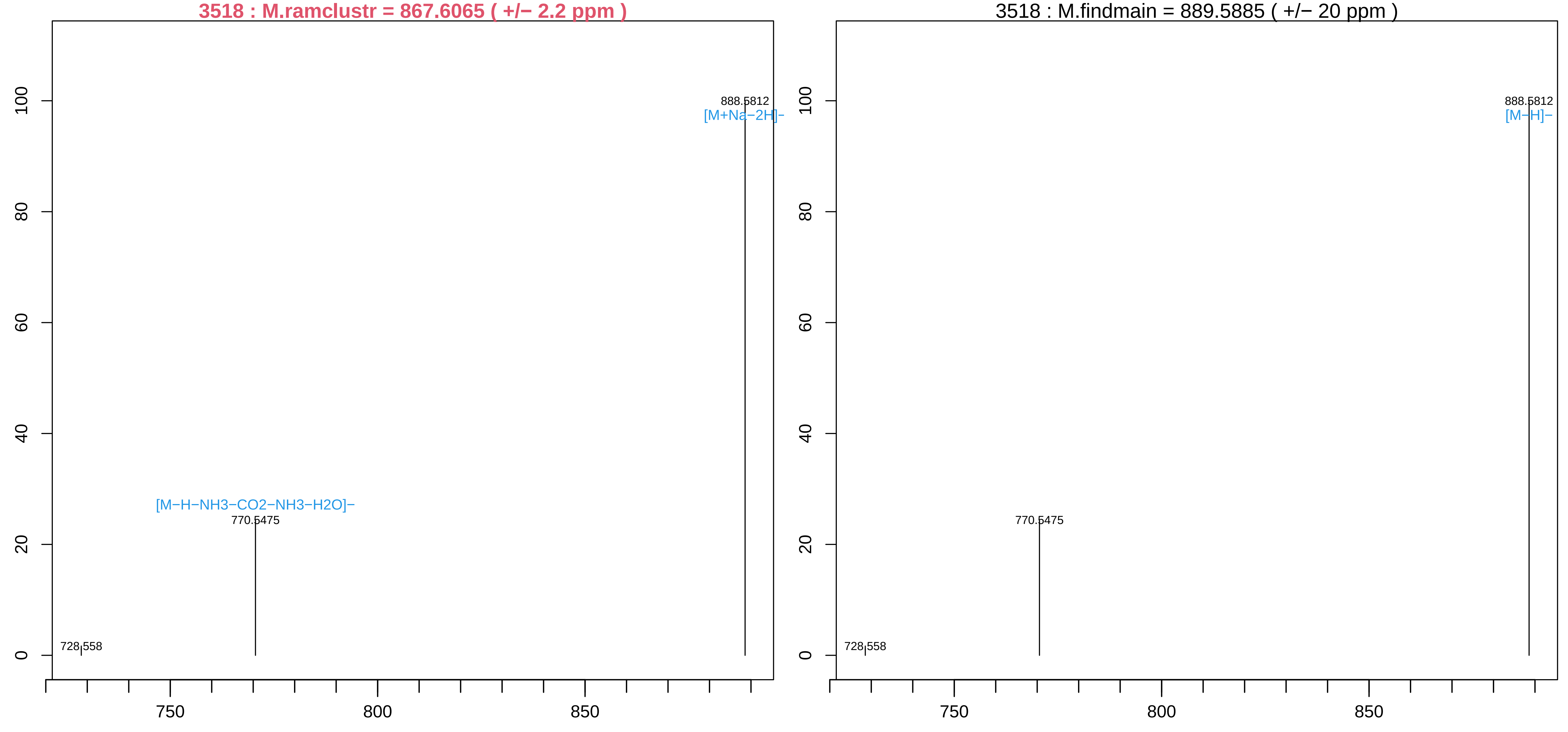

3518 : M.ramclustr = 867.6065 (+/− 2.2 ppm)
888.5812
[M+Na−2H]−
[M−H−NH3−CO2−NH3−H2O]−
770.5475
728.558
0
20
40
60
80
100
750
800
850
3518 : M.findmain = 889.5885 (+/− 20 ppm)
888.5812
[M−H]−
770.5475
728.558

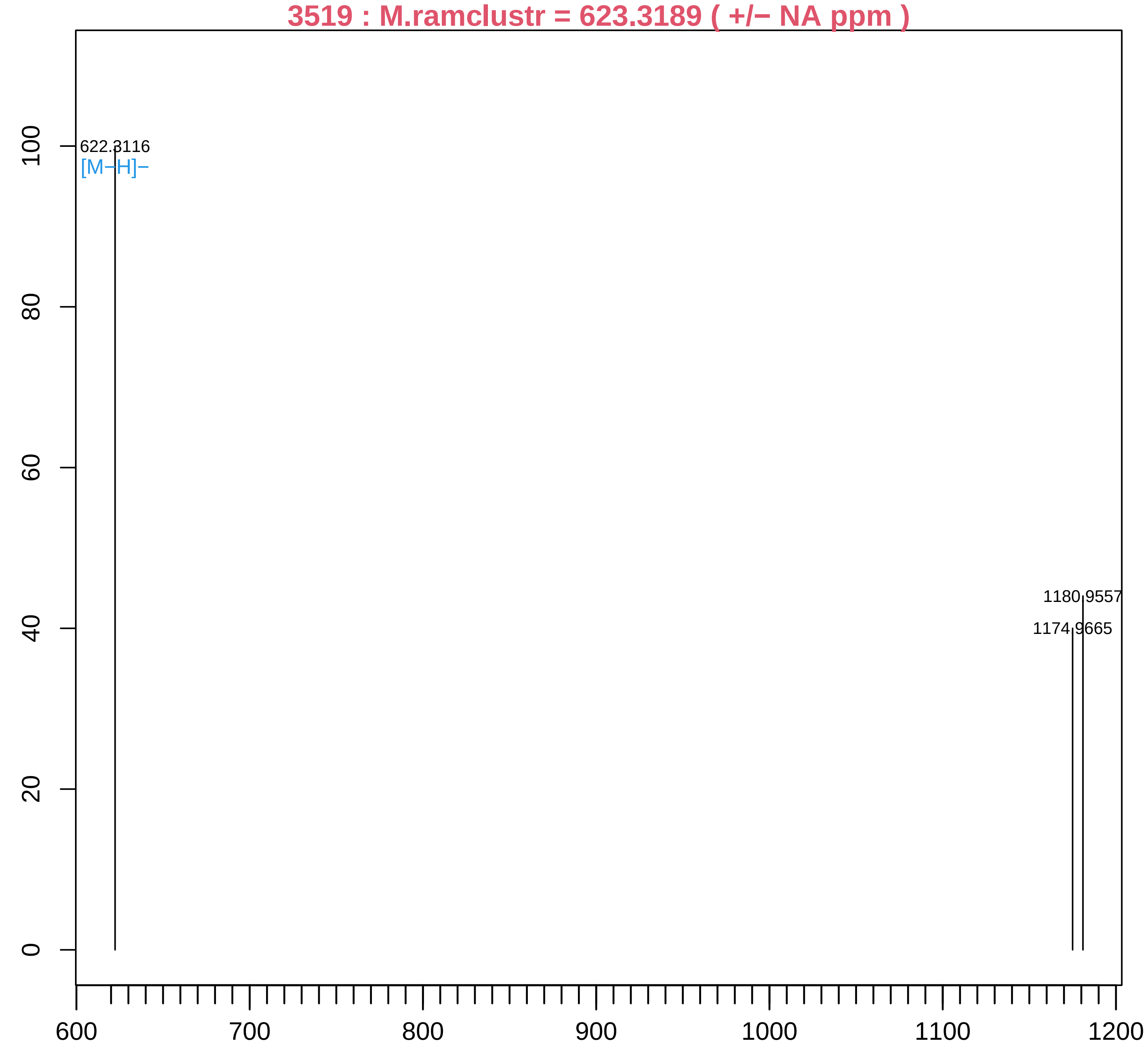
3519 : M.ramclustr = 623.3189 (+/− NA ppm)
622.3116
[M−H]−
1180.9557
1174.9665
0
20
40
60
80
100
600
700
800
900
1000
1100
1200

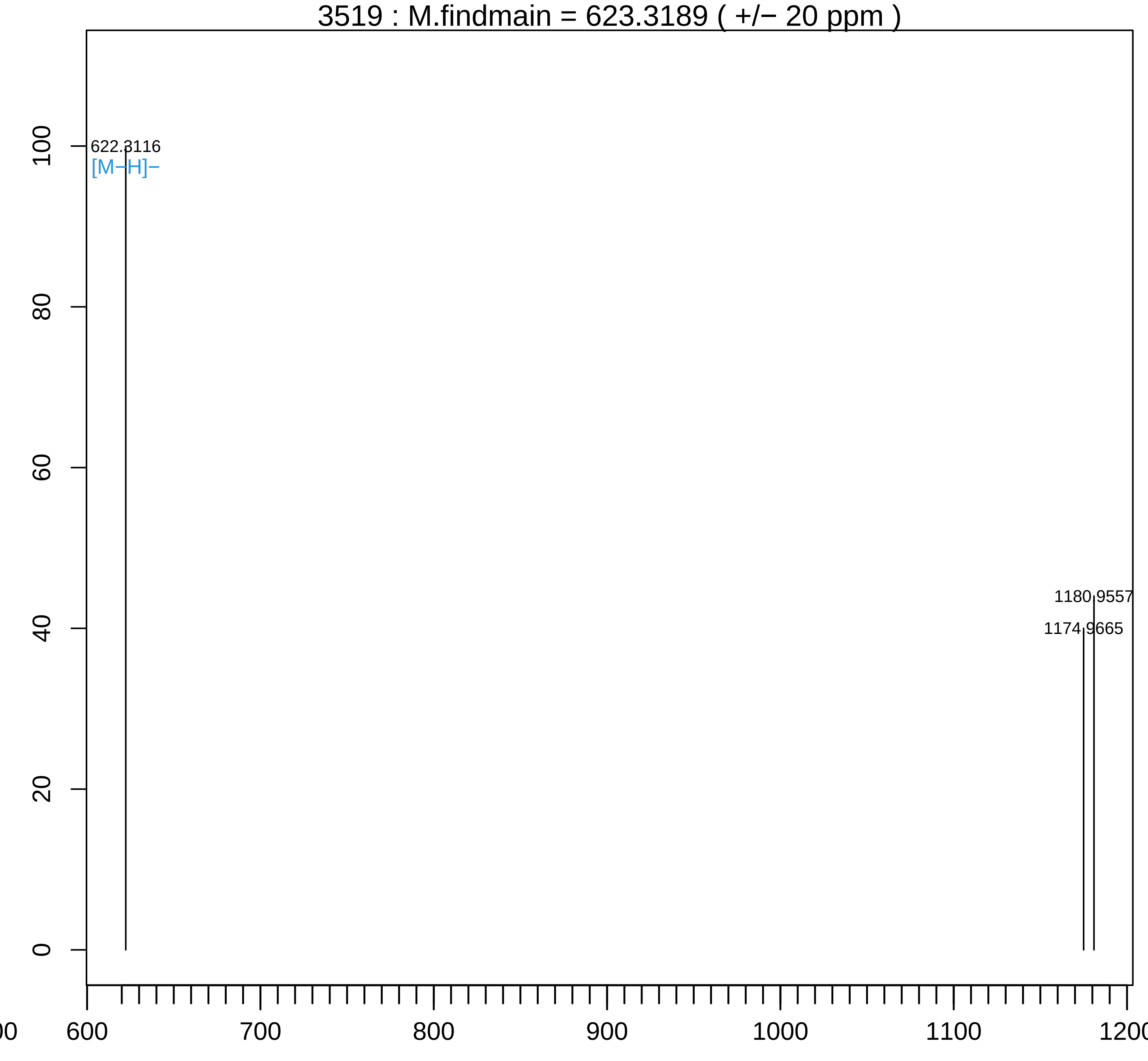
3519 : M.findmain = 623.3189 (+/− 20 ppm)
622.3116
[M−H]−
1180.9557
1174.9665
0
20
40
60
80
100
600
700
800
900
1000
1100
1200

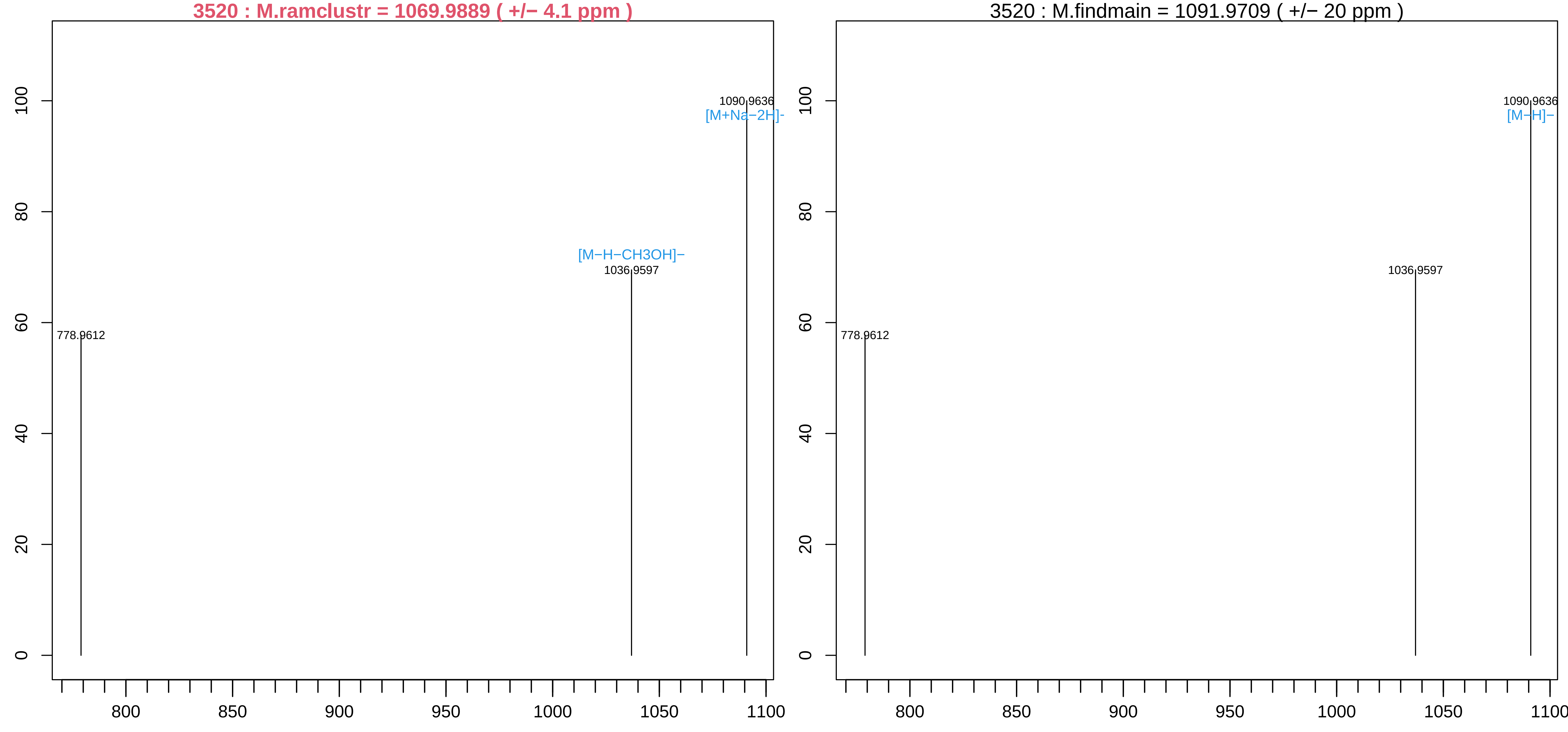
3520 : M.ramclustr = 1069.9889 (+/− 4.1 ppm)
1090.9636
[M+Na−2H]-
[M−H−CH3OH]−
1036.9597
778.9612
0
20
40
60
80
100
800
850
900
950
1000
1050
1100
3520 : M.findmain = 1091.9709 (+/− 20 ppm)
1090.9636
[M−H]−
1036.9597
778.9612

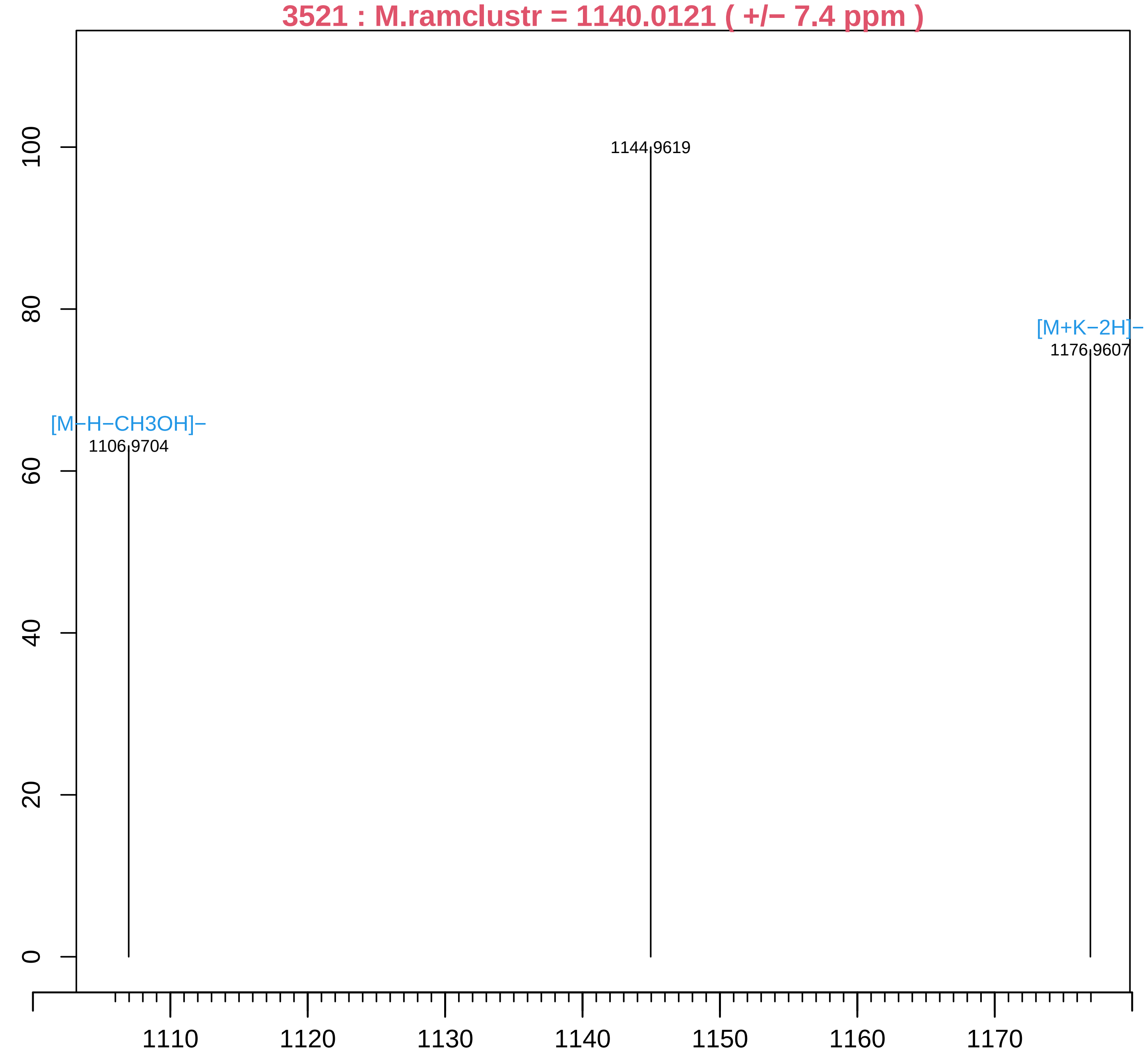
3521 : M.ramclustr = 1140.0121 (+/− 7.4 ppm)
1144.9619
[M+K−2H]−
1176.9607
[M−H−CH3OH]−
1106.9704
0
20
40
60
80
100
1110
1120
1130
1140
1150
1160
1170

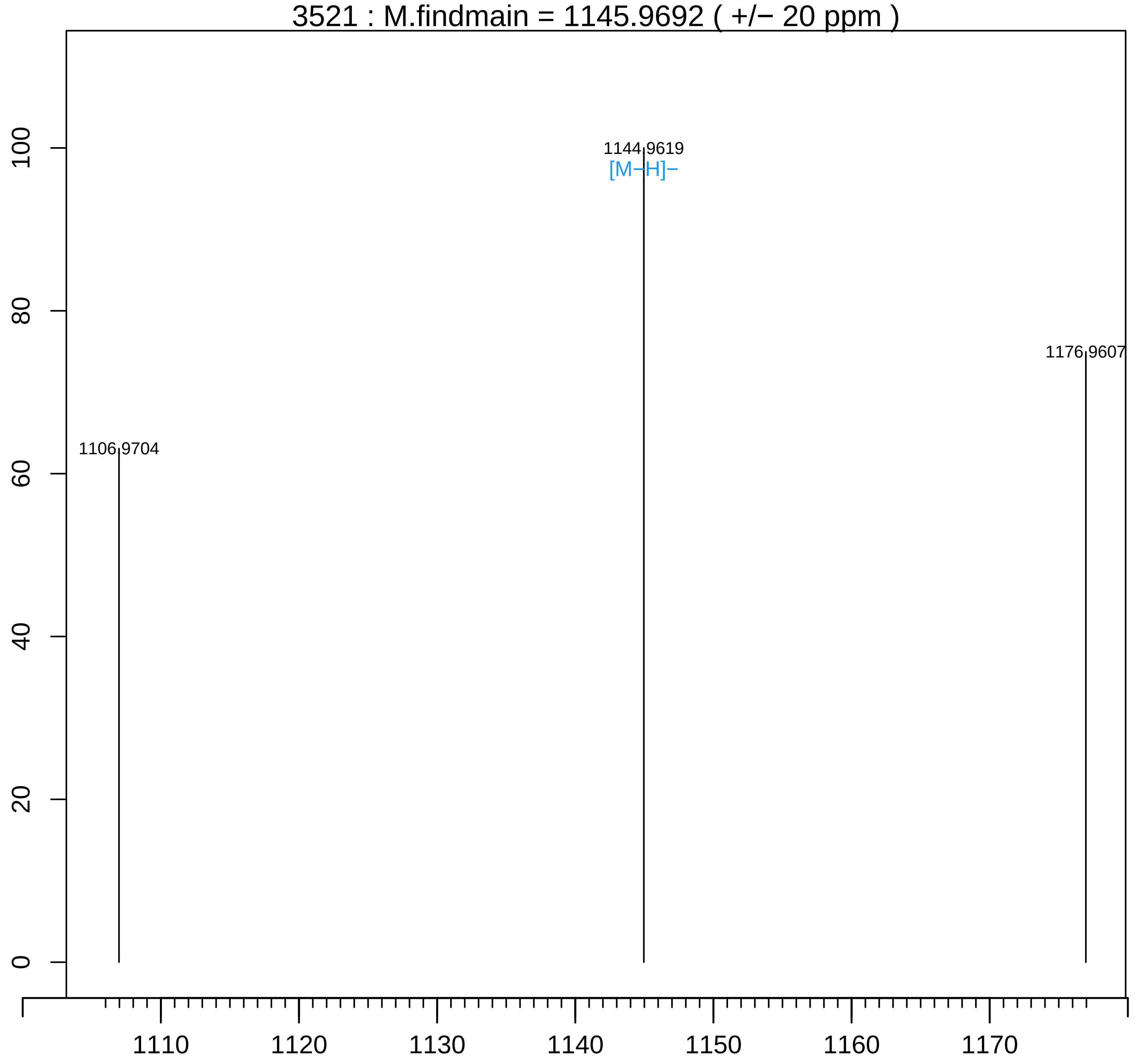
3521 : M.findmain = 1145.9692 (+/− 20 ppm)
1144.9619
[M−H]−
1176.9607
1106.9704
0
20
40
60
80
100
1110
1120
1130
1140
1150
1160
1170

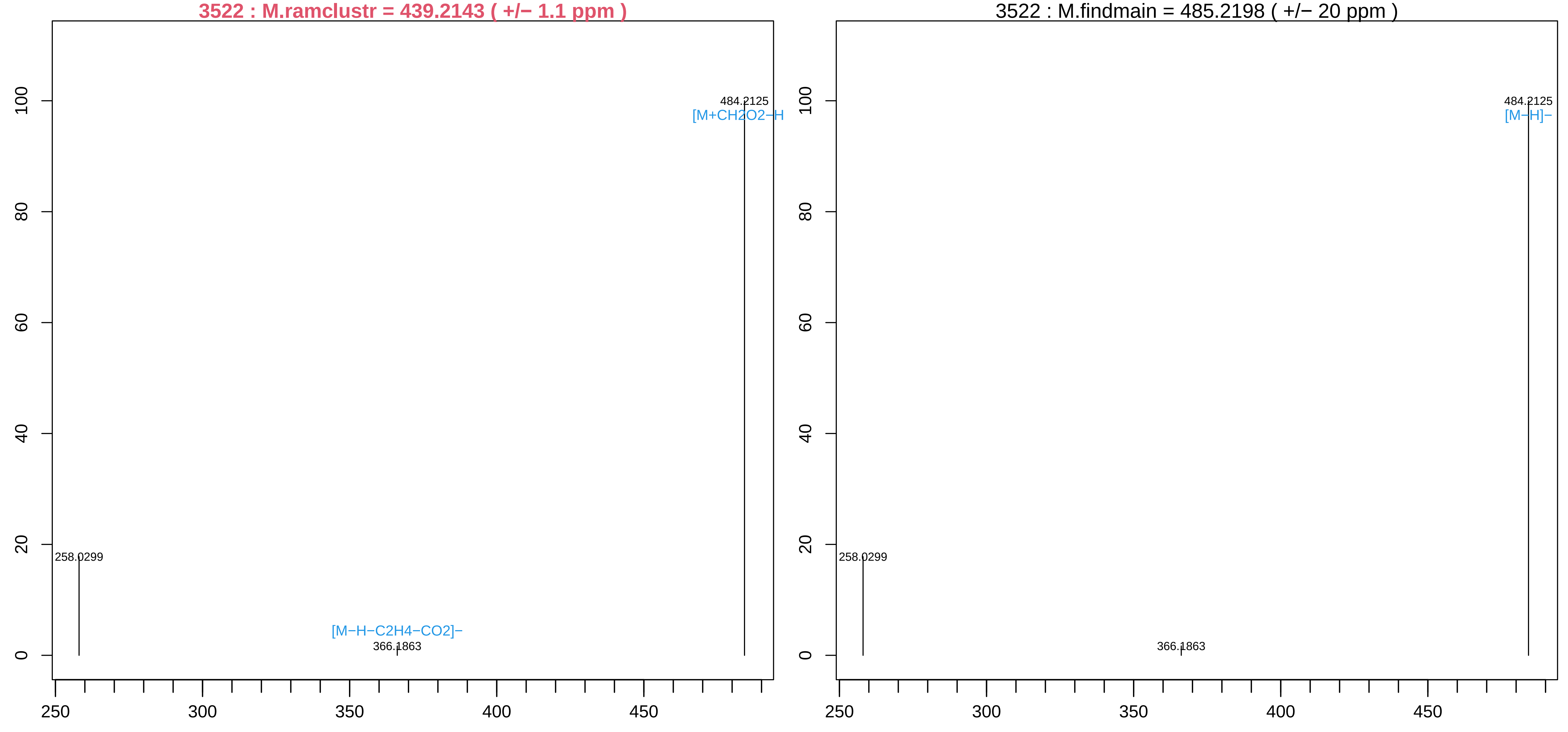
3522 : M.ramclustr = 439.2143 (+/− 1.1 ppm)
484.2125
[M+CH2O2−H
258.0299
[M−H−C2H4−CO2]−
366.1863
0
20
40
60
80
100
250
300
350
400
450
3522 : M.findmain = 485.2198 (+/− 20 ppm)
484.2125
[M−H]−
258.0299
366.1863
0
20
40
60
80
100
250
300
350
400
450

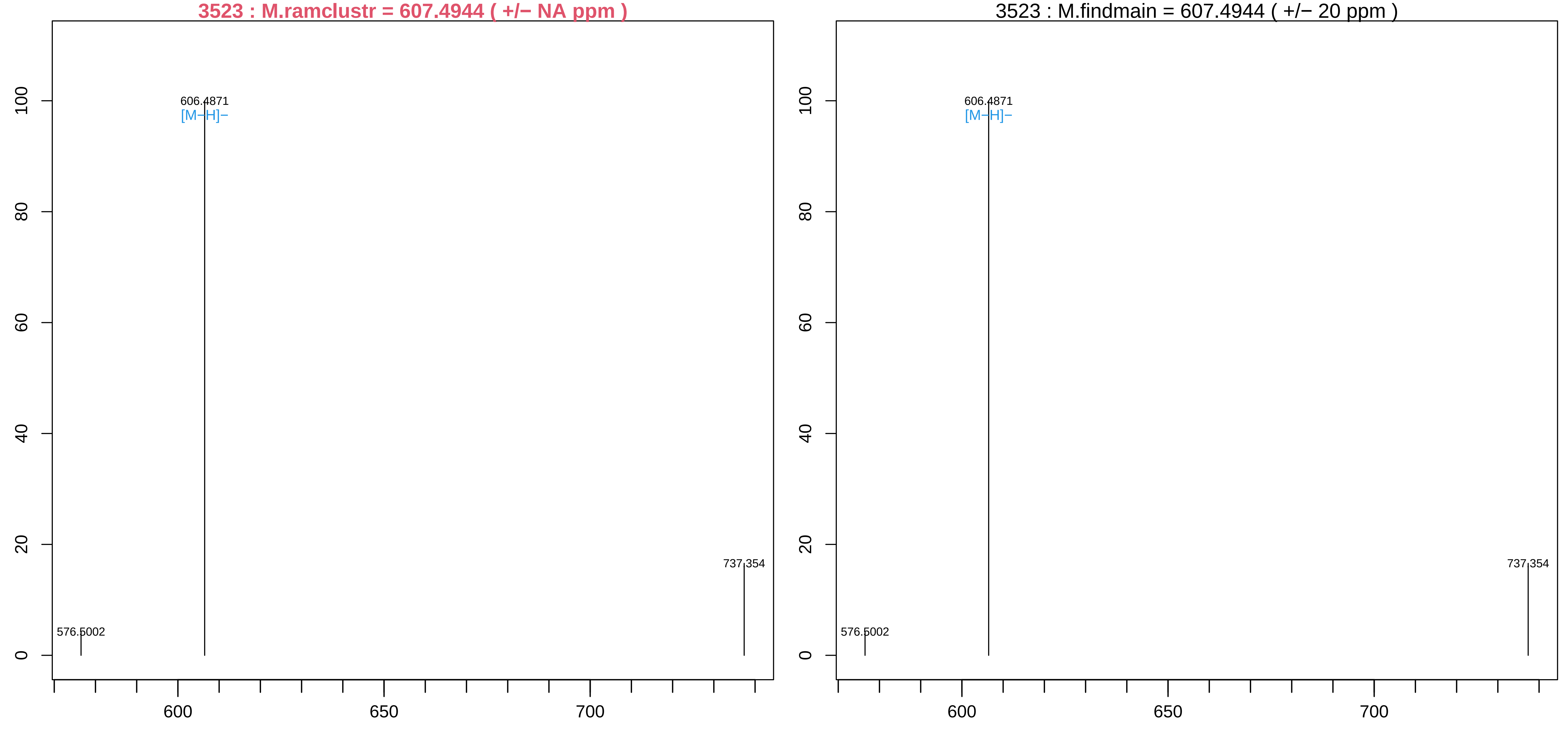
3523 : M.ramclustr = 607.4944 (+/− NA ppm)
606.4871
[M−H]−
737.354
576.5002
0
20
40
60
80
100
600
650
700
3523 : M.findmain = 607.4944 (+/− 20 ppm)
606.4871
[M−H]−
737.354
576.5002
0
20
40
60
80
100
600
650
700

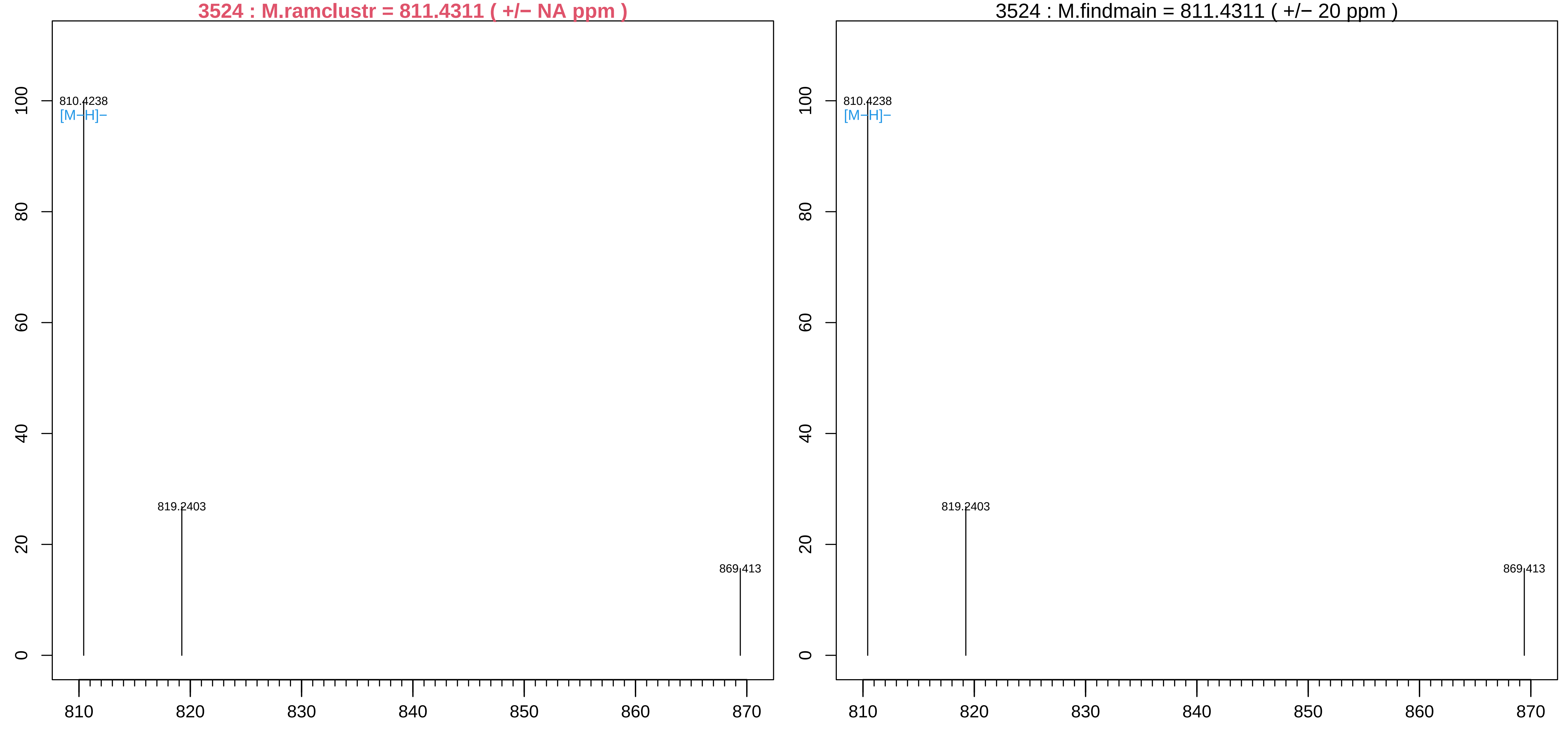

3524 : M.ramclustr = 811.4311 (+/− NA ppm)
810.4238
[M−H]−
819.2403
869.413
3524 : M.findmain = 811.4311 (+/− 20 ppm)
810.4238
[M−H]−
819.2403
869.413

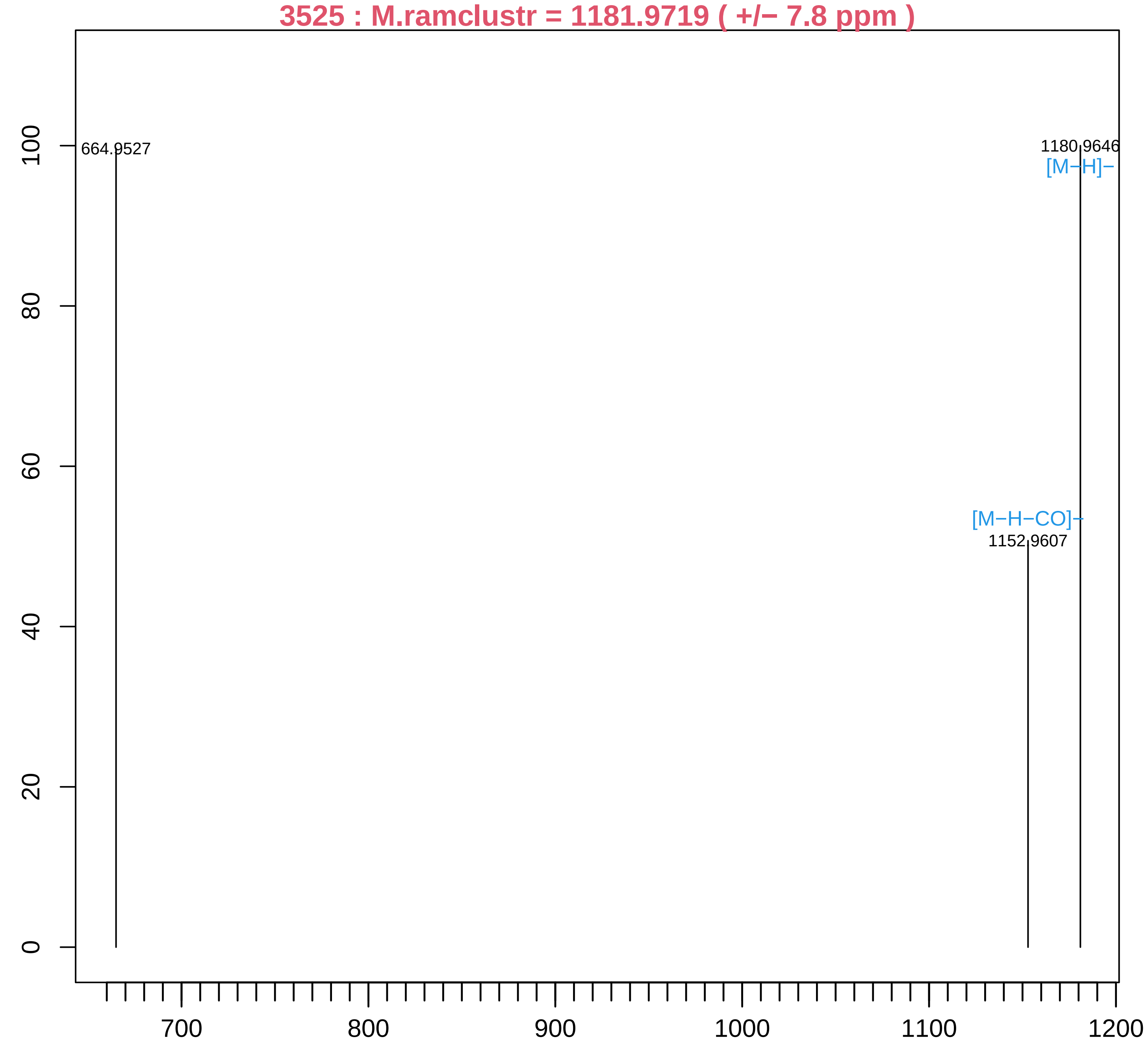
3525 : M.ramclustr = 1181.9719 (+/− 7.8 ppm)
664.9527
1180.9646
[M−H]−
[M−H−CO]−
1152.9607
0
20
40
60
80
100
700
800
900
1000
1100
1200

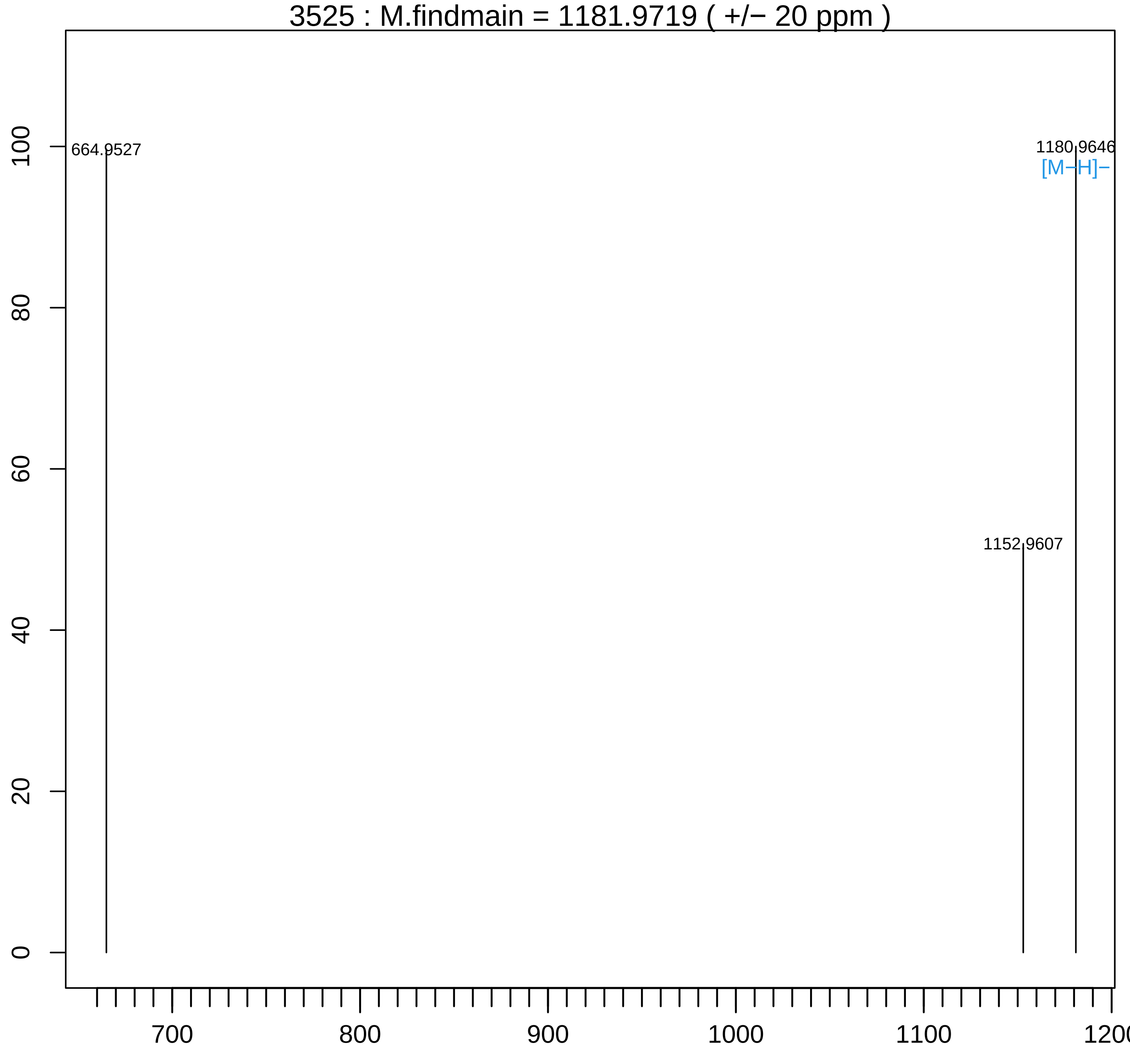
3525 : M.findmain = 1181.9719 (+/− 20 ppm)
664.9527
1180.9646
[M−H]−
1152.9607
0
20
40
60
80
100
700
800
900
1000
1100
1200

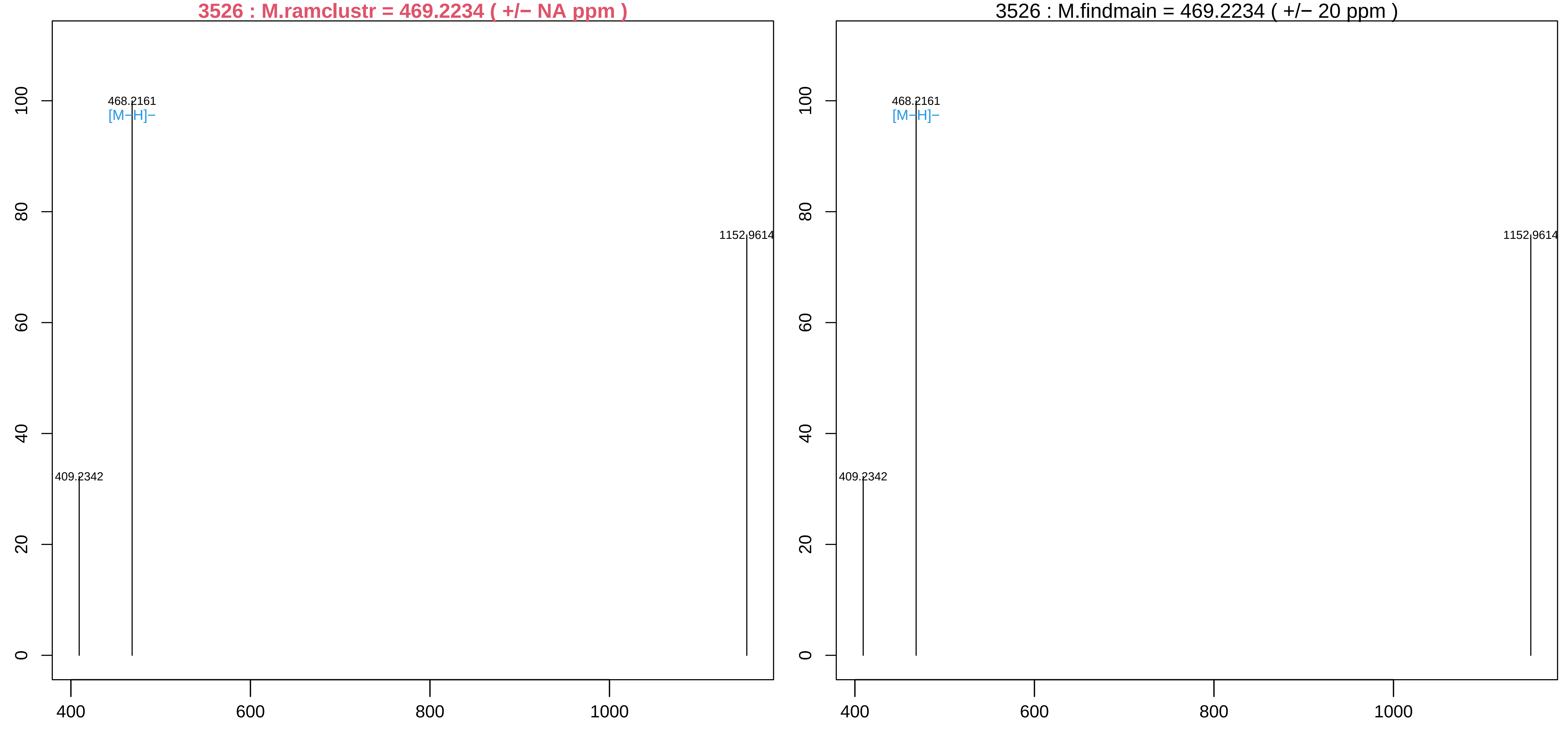
3526 : M.ramclustr = 469.2234 (+/− NA ppm)
468.2161
[M−H]−
409.2342
1152.9614
3526 : M.findmain = 469.2234 (+/− 20 ppm)
468.2161
[M−H]−
409.2342
1152.9614
0
20
40
60
80
100
400
600
800
1000

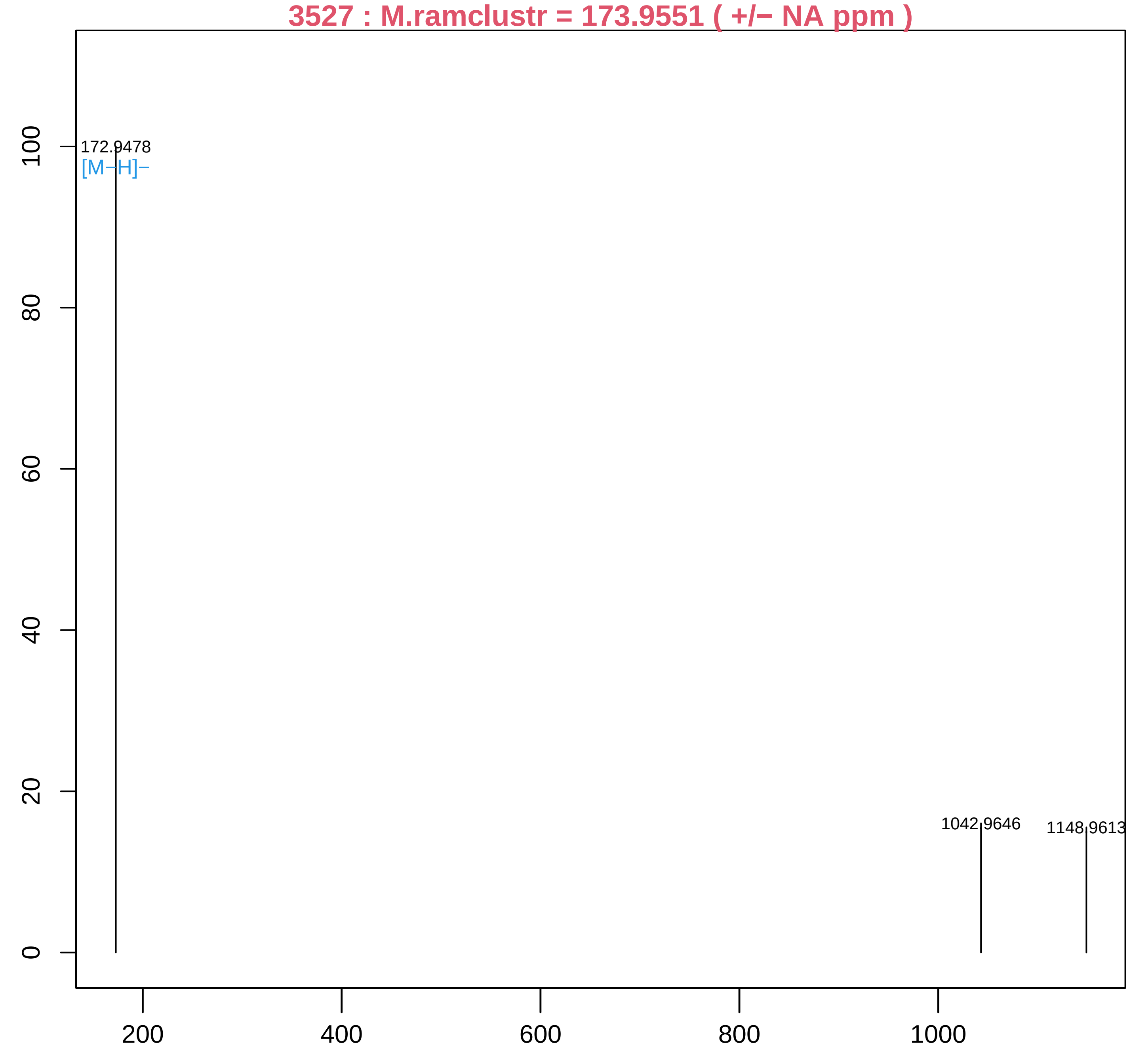

3527 : M.ramclustr = 173.9551 (+/− NA ppm)
172.9478
[M−H]−
1042.9646
1148.9613
0
20
40
60
80
100
200
400
600
800
1000

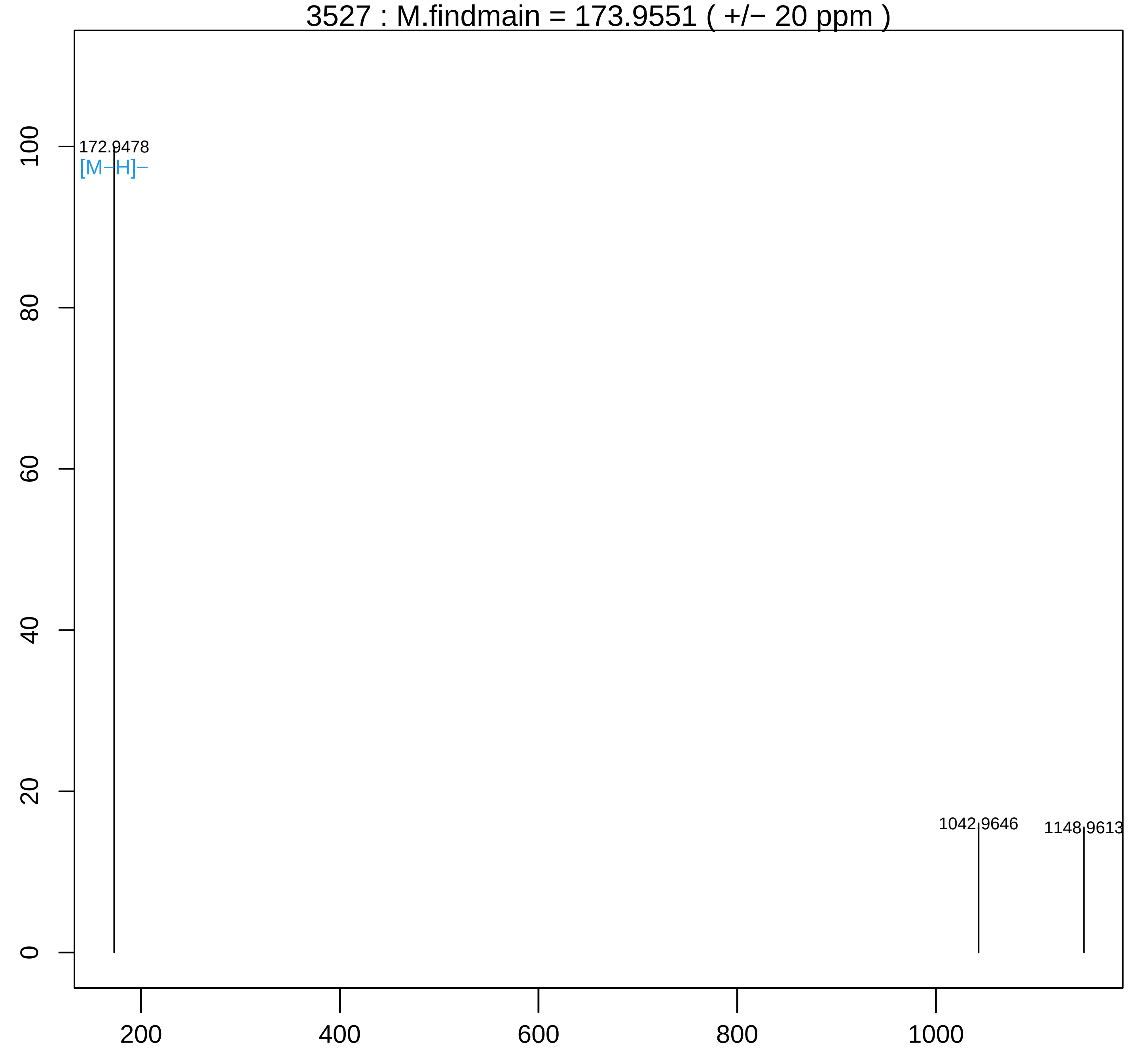

3527 : M.findmain = 173.9551 (+/− 20 ppm)
172.9478
[M−H]−
1042.9646
1148.9613
0
20
40
60
80
100
200
400
600
800
1000

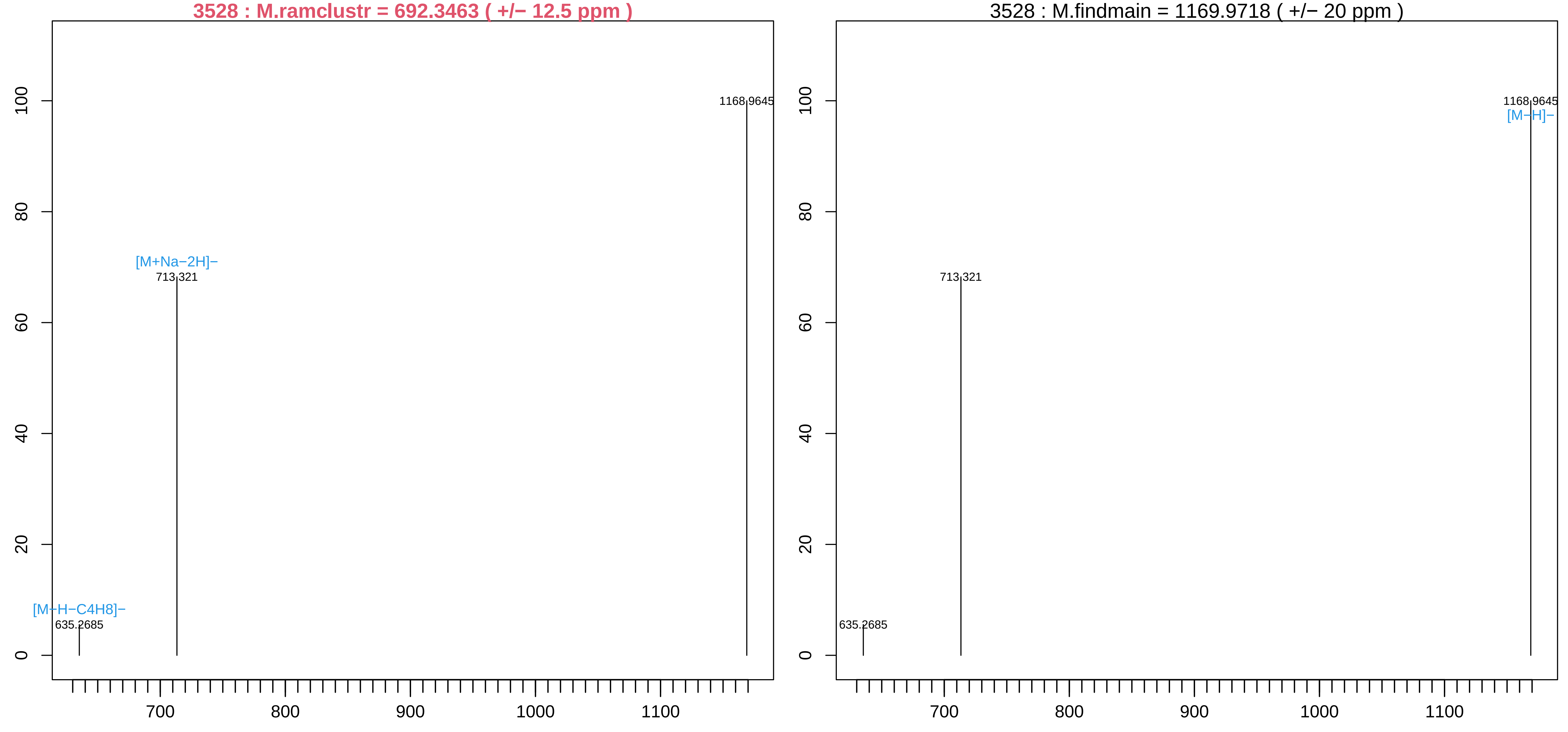
3528 : M.ramclustr = 692.3463 (+/− 12.5 ppm)
1168.9645
[M+Na−2H]−
713.321
[M−H−C4H8]−
635.2685
3528 : M.findmain = 1169.9718 (+/− 20 ppm)
1168.9645
[M−H]−
713.321
635.2685
0
20
40
60
80
100
700
800
900
1000
1100

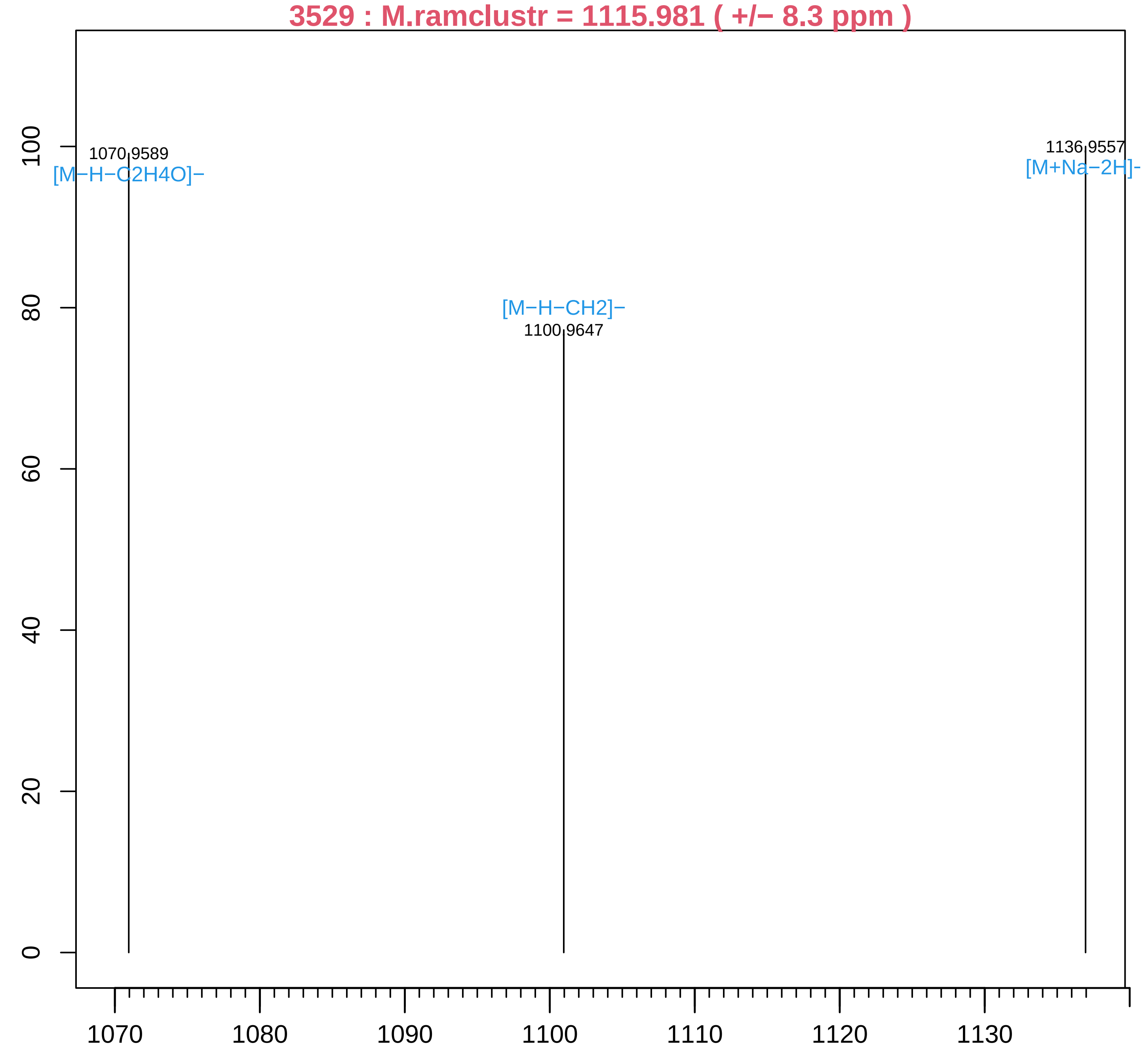
3529 : M.ramclustr = 1115.981 (+/− 8.3 ppm)
1070.9589
[M−H−C2H4O]−
[M−H−CH2]−
1100.9647
1136.9557
[M+Na−2H]-
0
20
40
60
80
100
1070
1080
1090
1100
1110
1120
1130

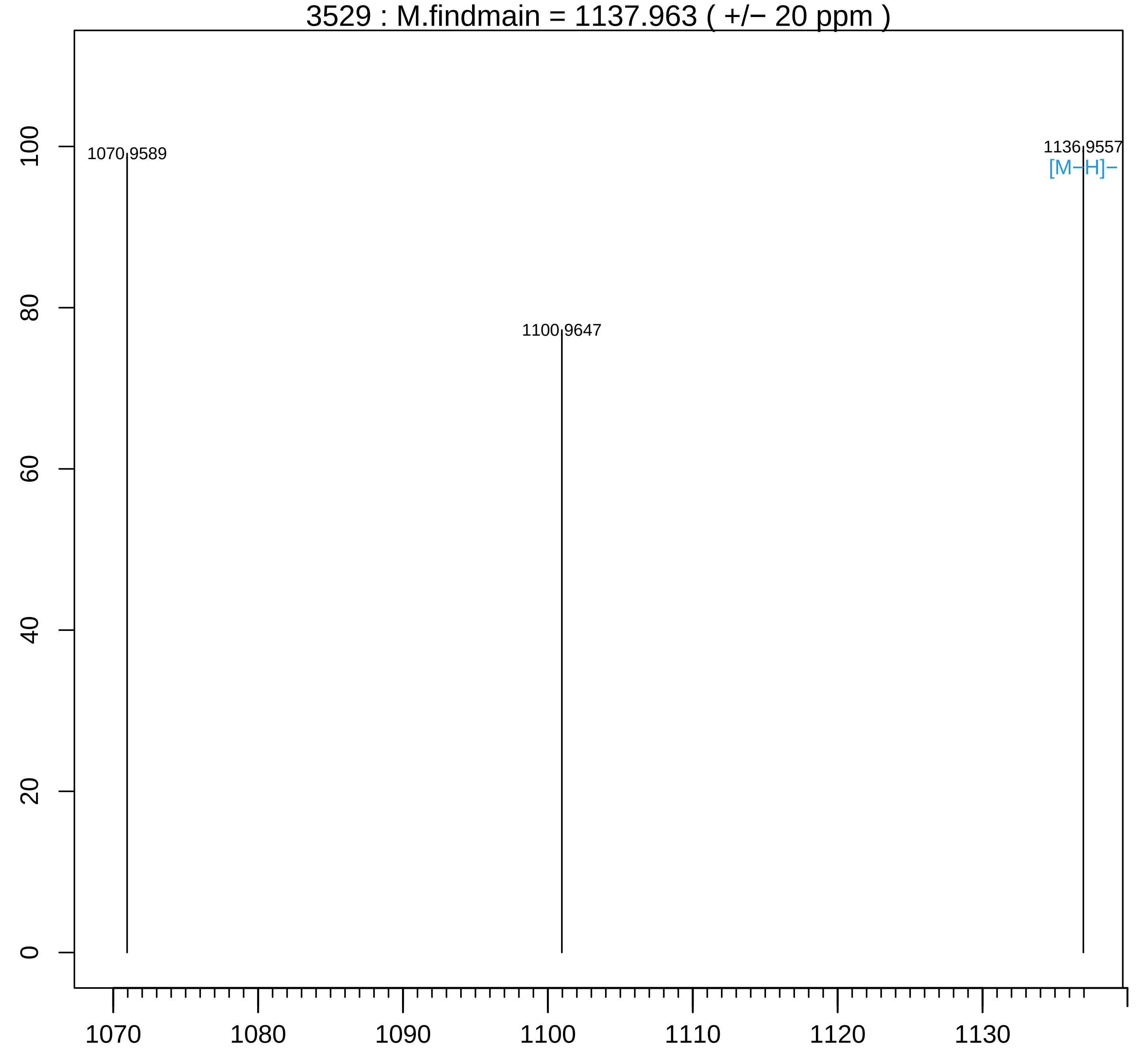
3529 : M.findmain = 1137.963 (+/− 20 ppm)
1070.9589
1100.9647
1136.9557
[M−H]−
0
20
40
60
80
100
1070
1080
1090
1100
1110
1120
1130

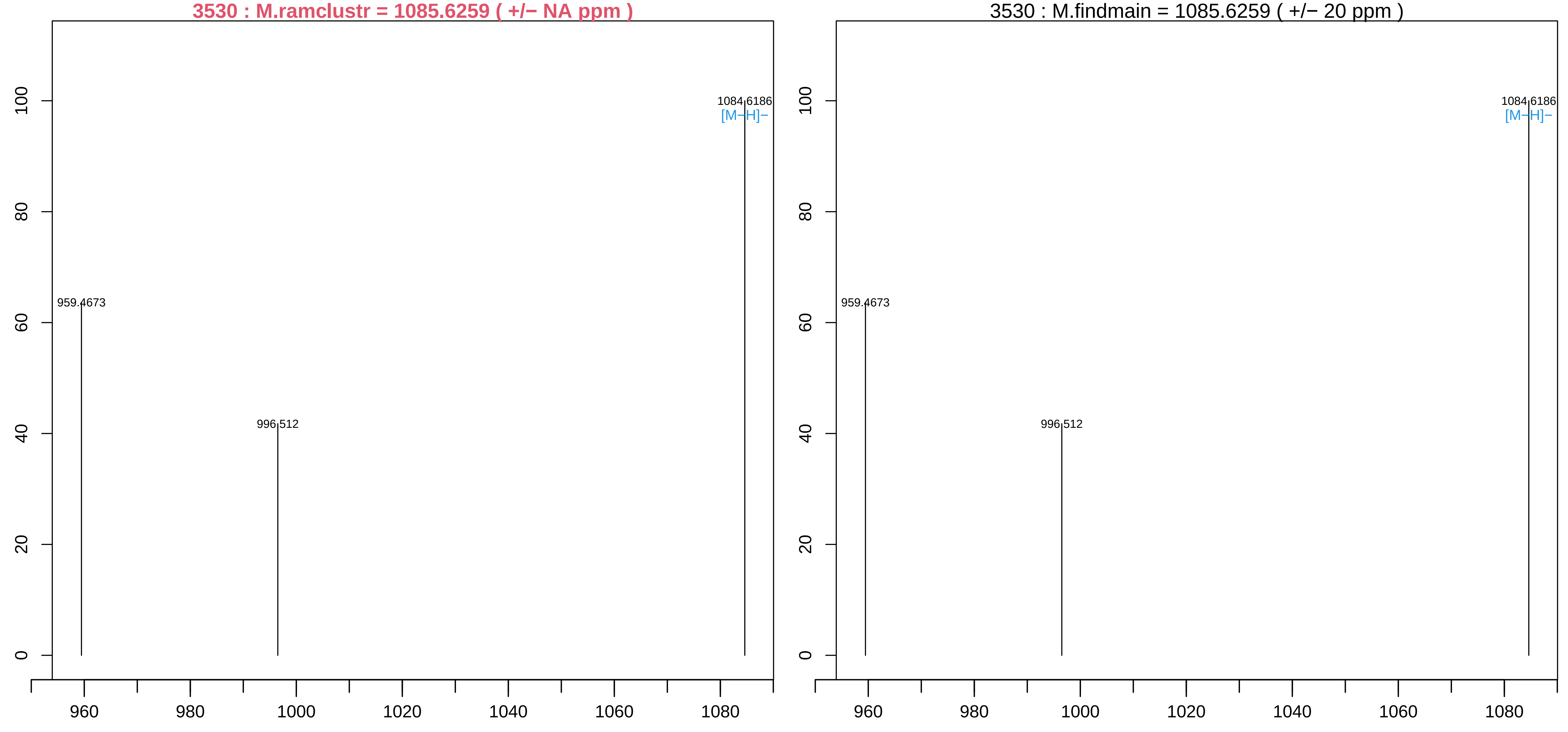
3530 : M.ramclustr = 1085.6259 (+/− NA ppm)
1084.6186
[M−H]−
959.4673
996.512
3530 : M.findmain = 1085.6259 (+/− 20 ppm)
1084.6186
[M−H]−
959.4673
996.512

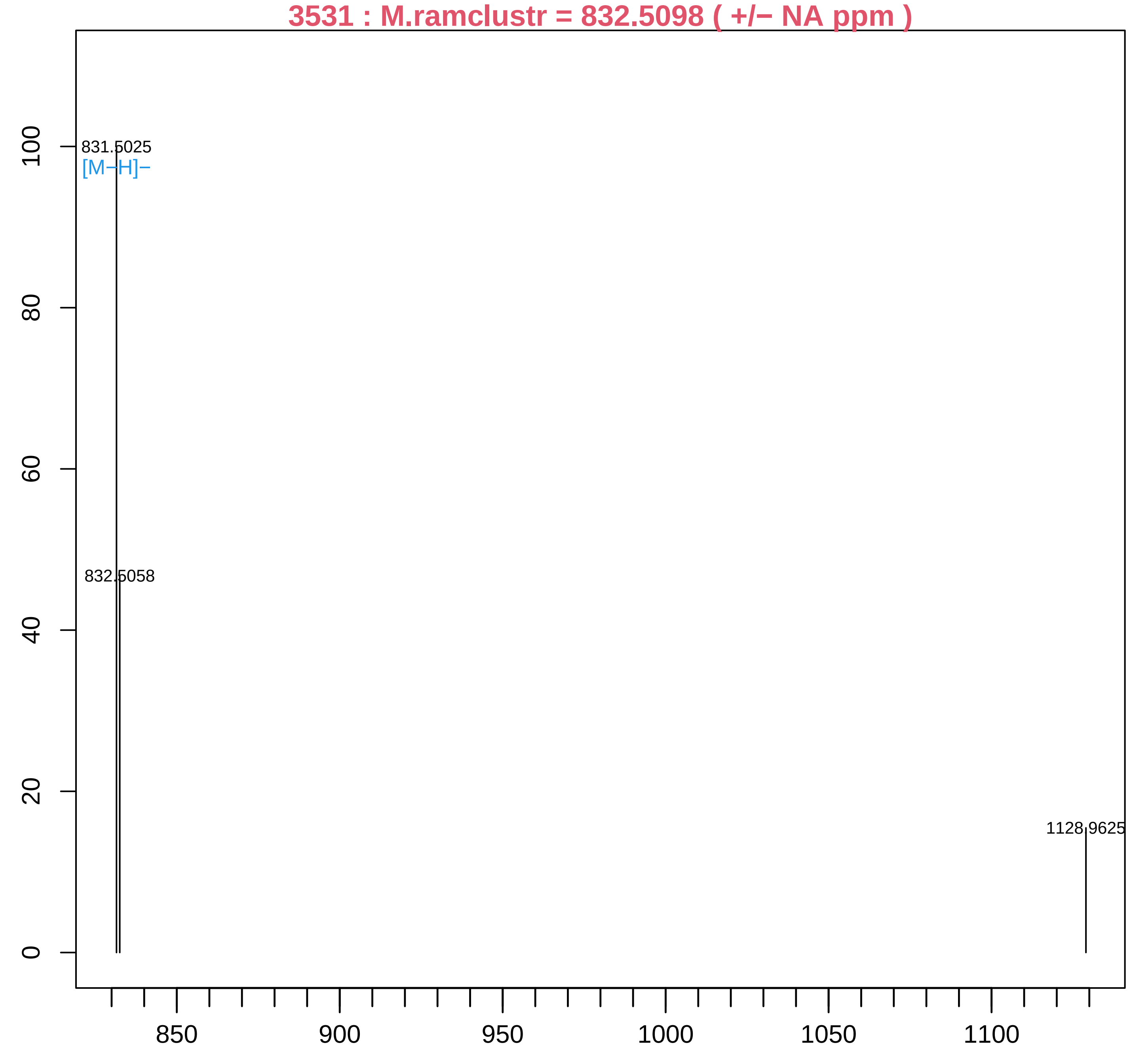

3531 : M.ramclustr = 832.5098 (+/− NA ppm)
831.5025
[M−H]−
832.5058
1128.9625
0
20
40
60
80
100
850
900
950
1000
1050
1100

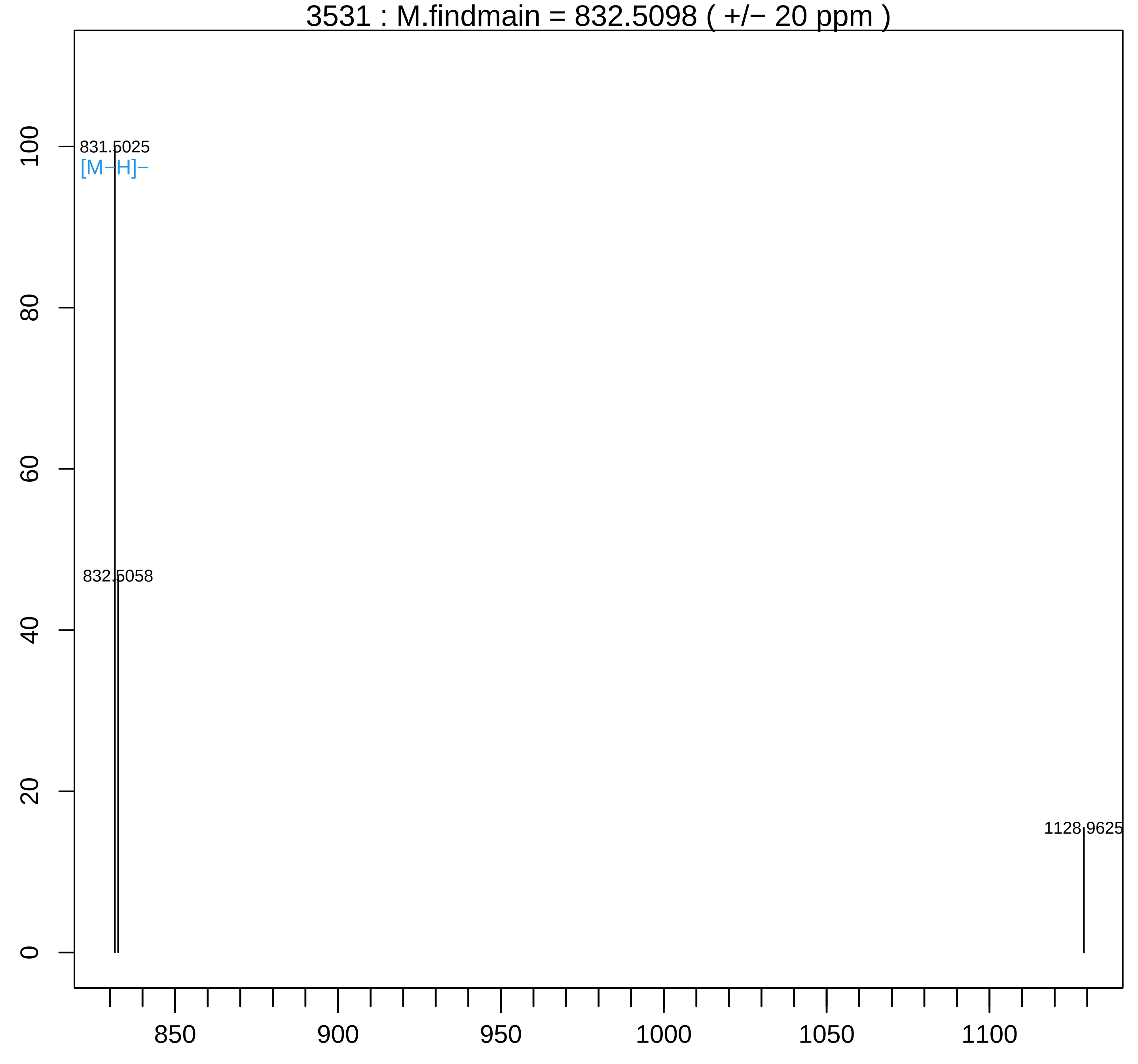

3531 : M.findmain = 832.5098 (+/− 20 ppm)
831.5025
[M−H]−
832.5058
1128.9625
0
20
40
60
80
100
850
900
950
1000
1050
1100

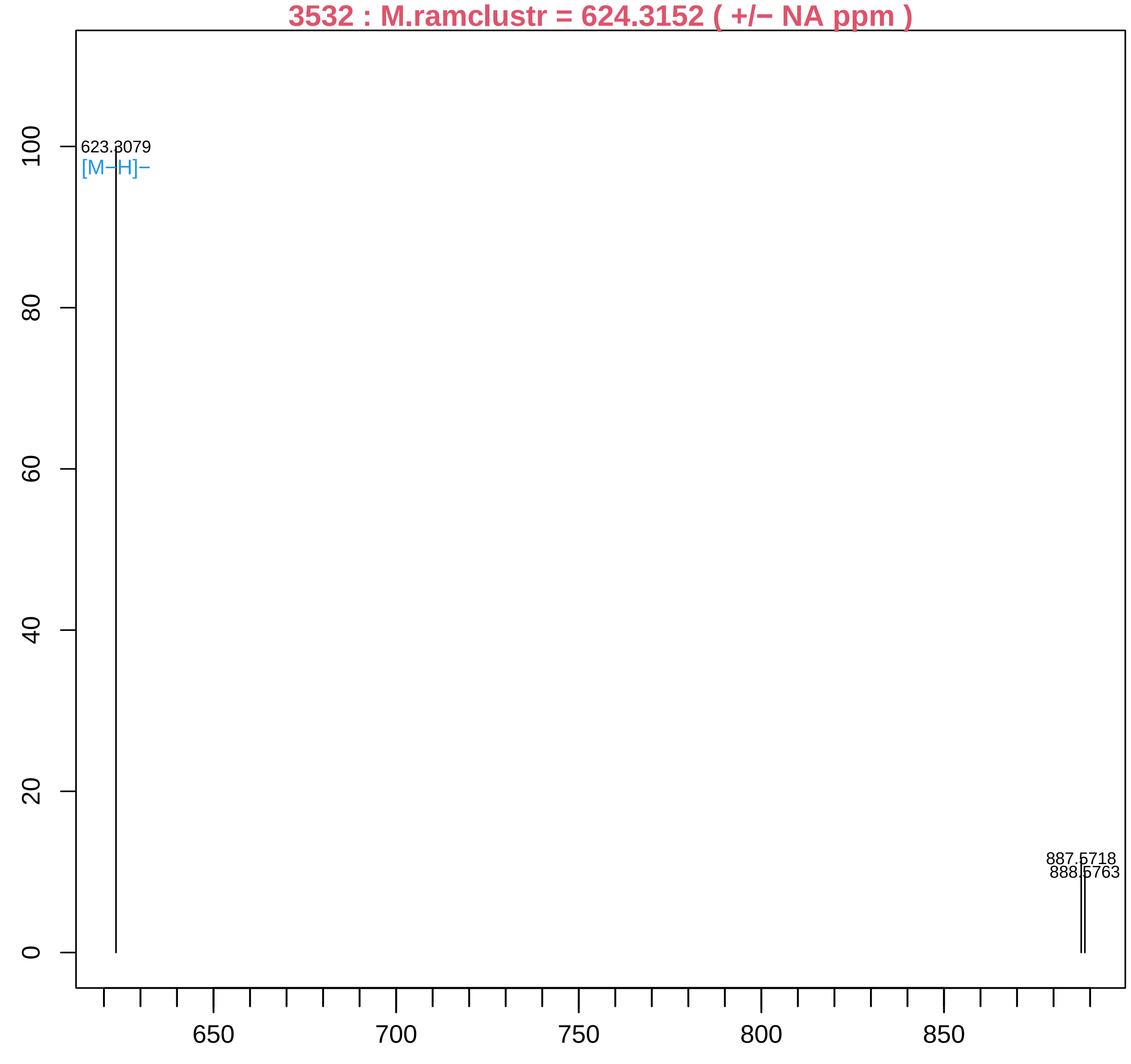
3532 : M.ramclustr = 624.3152 (+/− NA ppm)
623.3079
[M−H]−
887.5718
888.5763
0
20
40
60
80
100
650
700
750
800
850

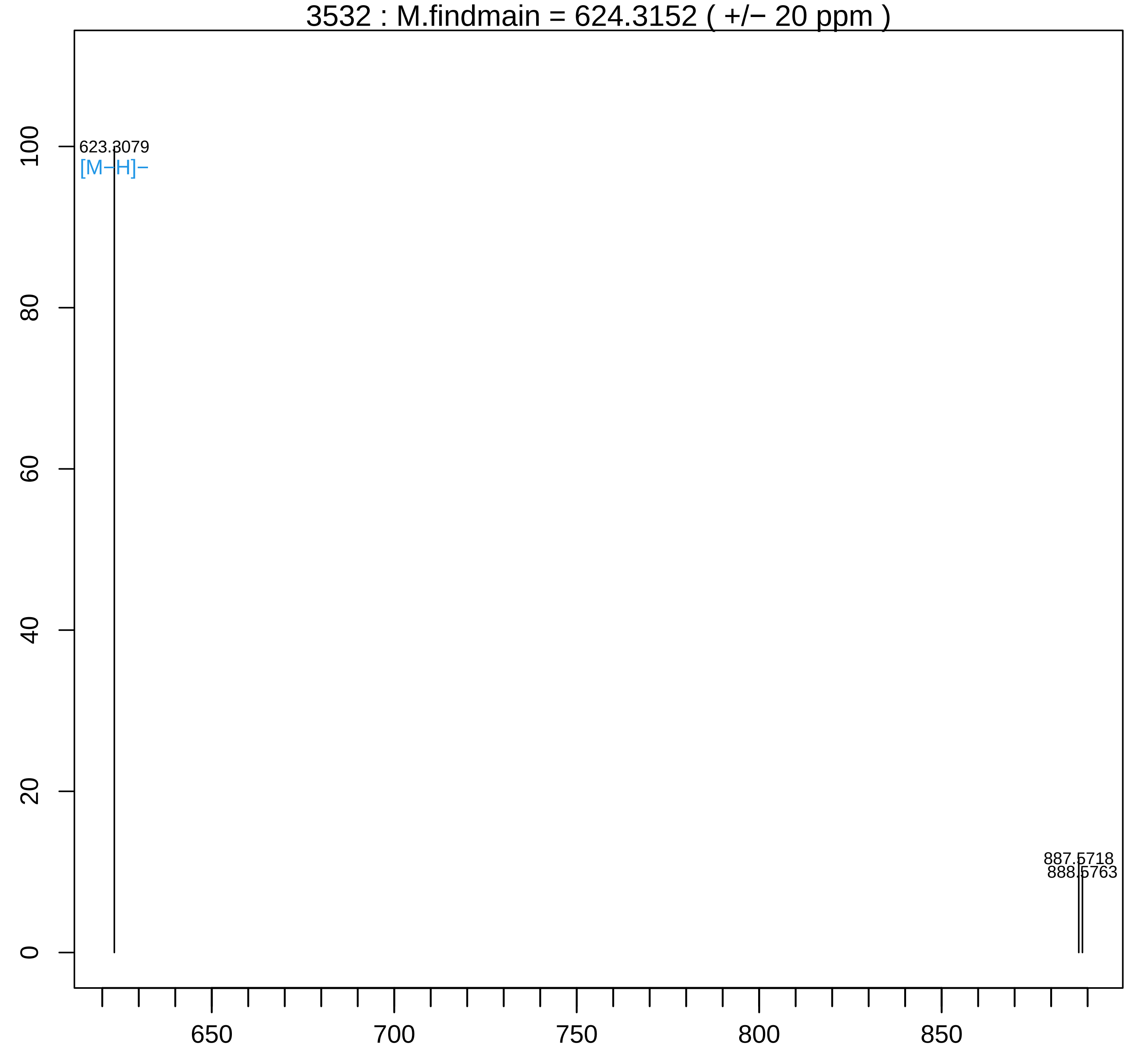
3532 : M.findmain = 624.3152 (+/− 20 ppm)
623.3079
[M−H]−
887.5718
888.5763
0
20
40
60
80
100
650
700
750
800
850

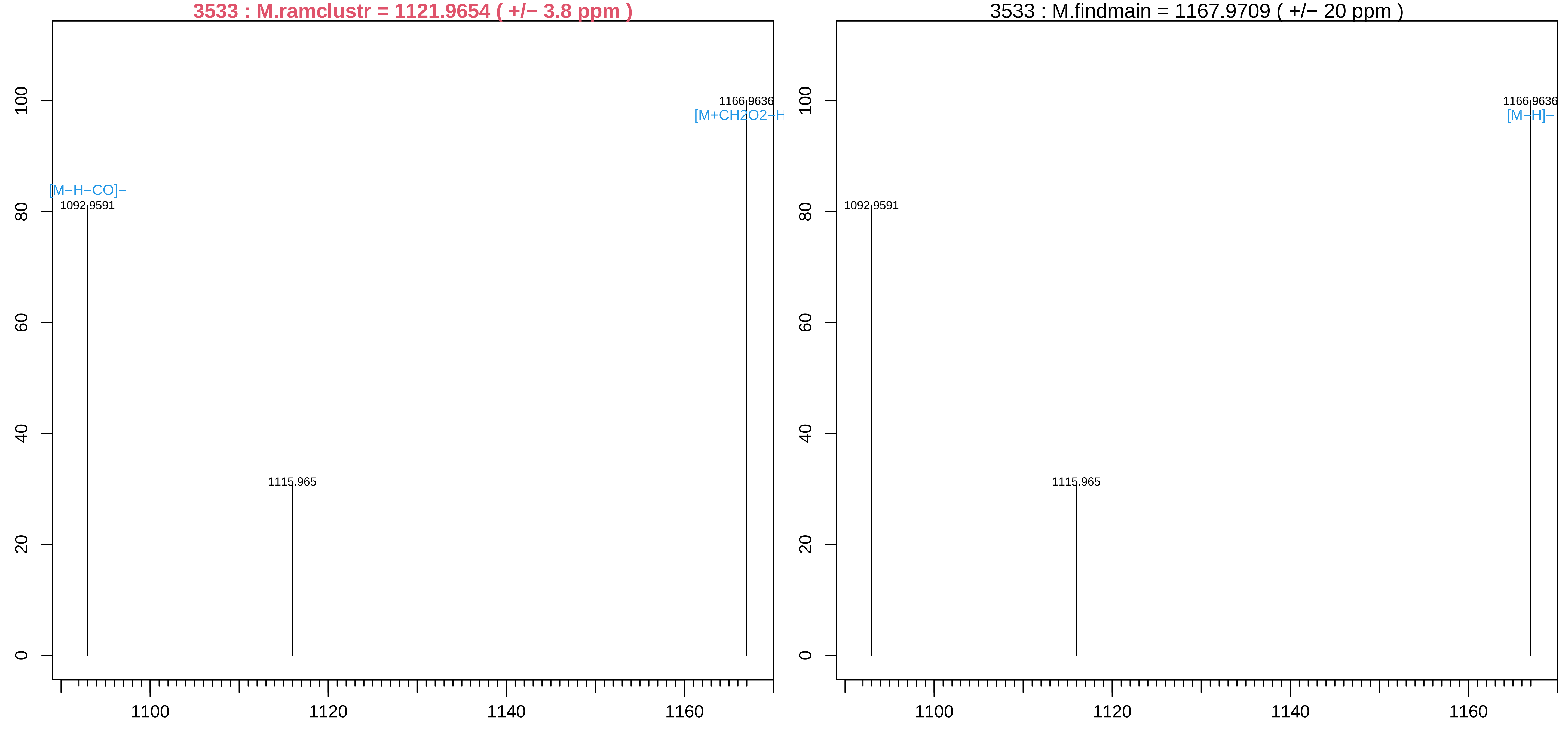

3533 : M.ramclustr = 1121.9654 (+/− 3.8 ppm)
1166.9636
[M+CH2O2−H
[M−H−CO]−
1092.9591
1115.965
3533 : M.findmain = 1167.9709 (+/− 20 ppm)
1166.9636
[M−H]−
1092.9591
1115.965

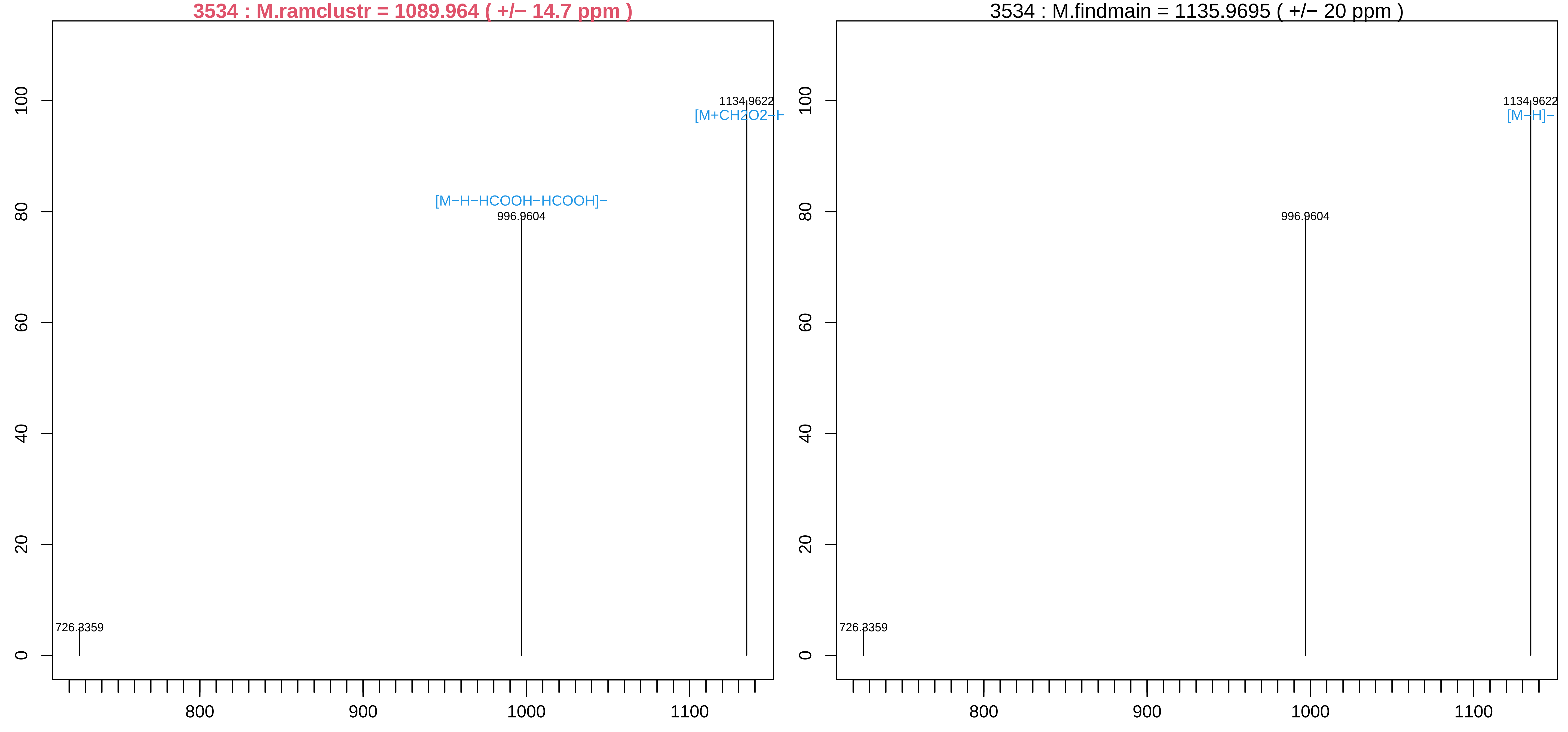

3534 : M.ramclustr = 1089.964 (+/− 14.7 ppm)
1134.9622
[M+CH2O2−H
[M−H−HCOOH−HCOOH]−
996.9604
726.3359
0
20
40
60
80
100
800
900
1000
1100
3534 : M.findmain = 1135.9695 (+/− 20 ppm)
1134.9622
[M−H]−
996.9604
726.3359
0
20
40
60
80
100
800
900
1000
1100

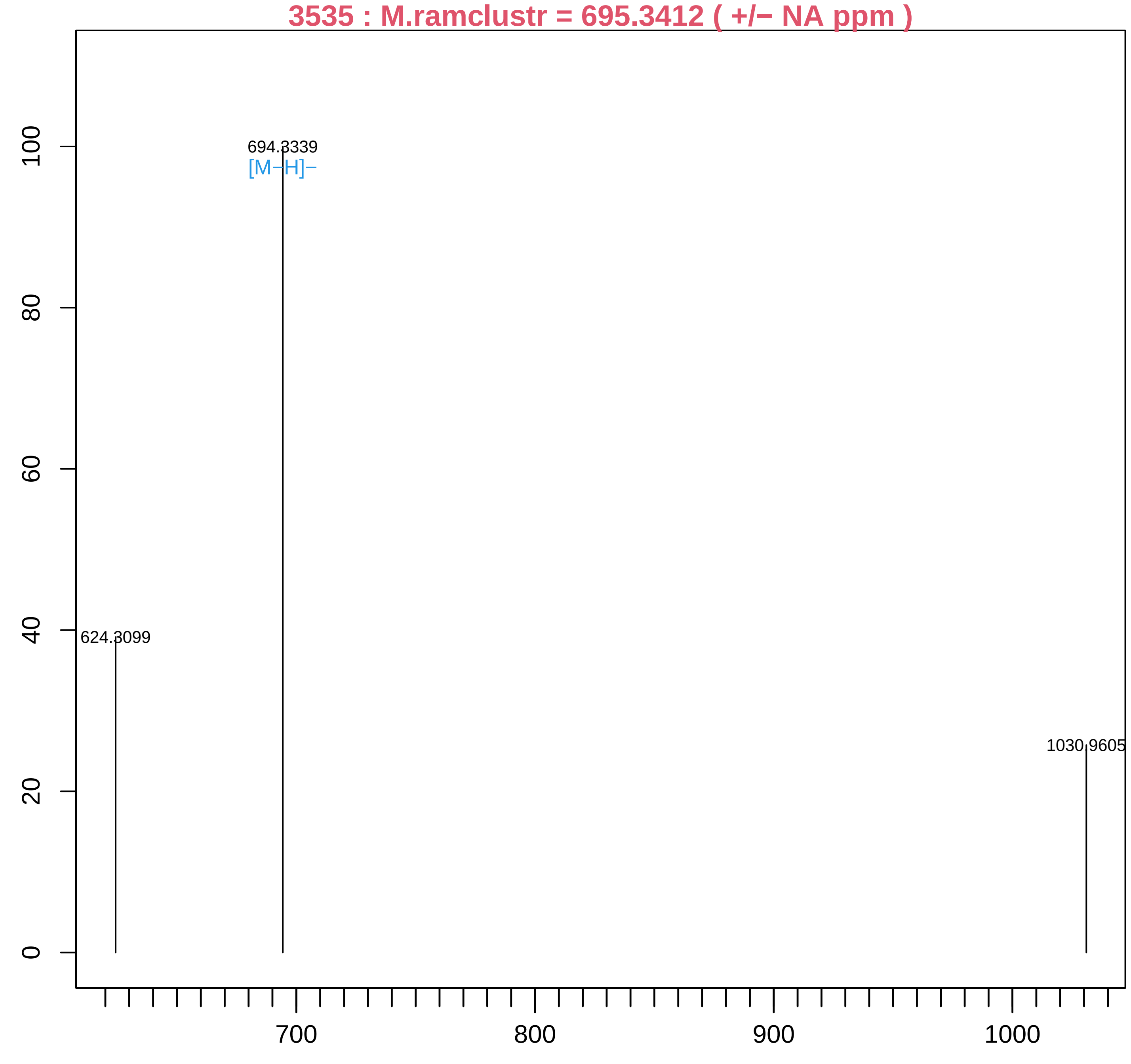

3535 : M.ramclustr = 695.3412 (+/− NA ppm)
694.3339
[M−H]−
624.3099
1030.9605
0
20
40
60
80
100
700
800
900
1000

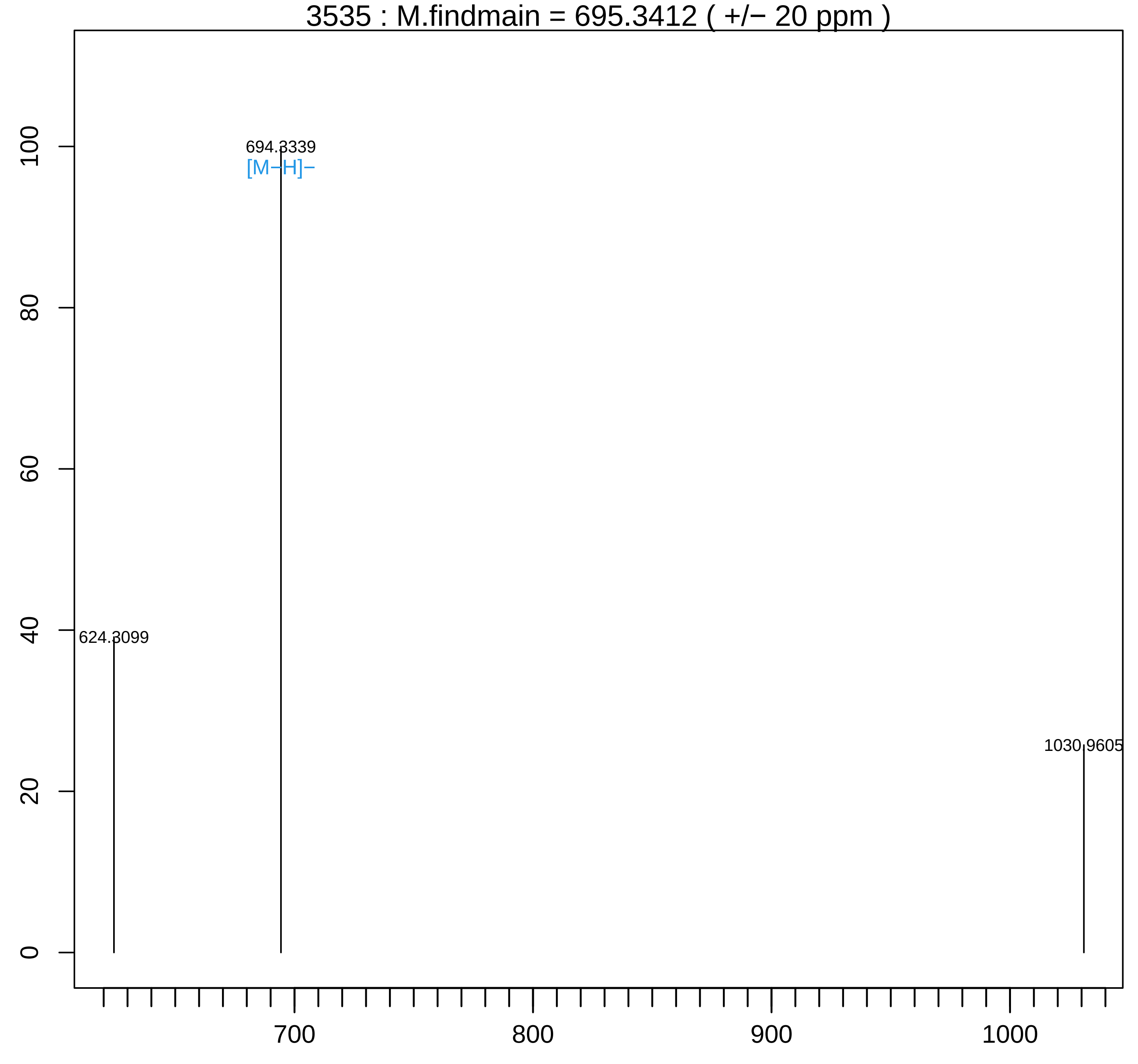

3535 : M.findmain = 695.3412 (+/− 20 ppm)
694.3339
[M−H]−
624.3099
1030.9605
0
20
40
60
80
100
700
800
900
1000

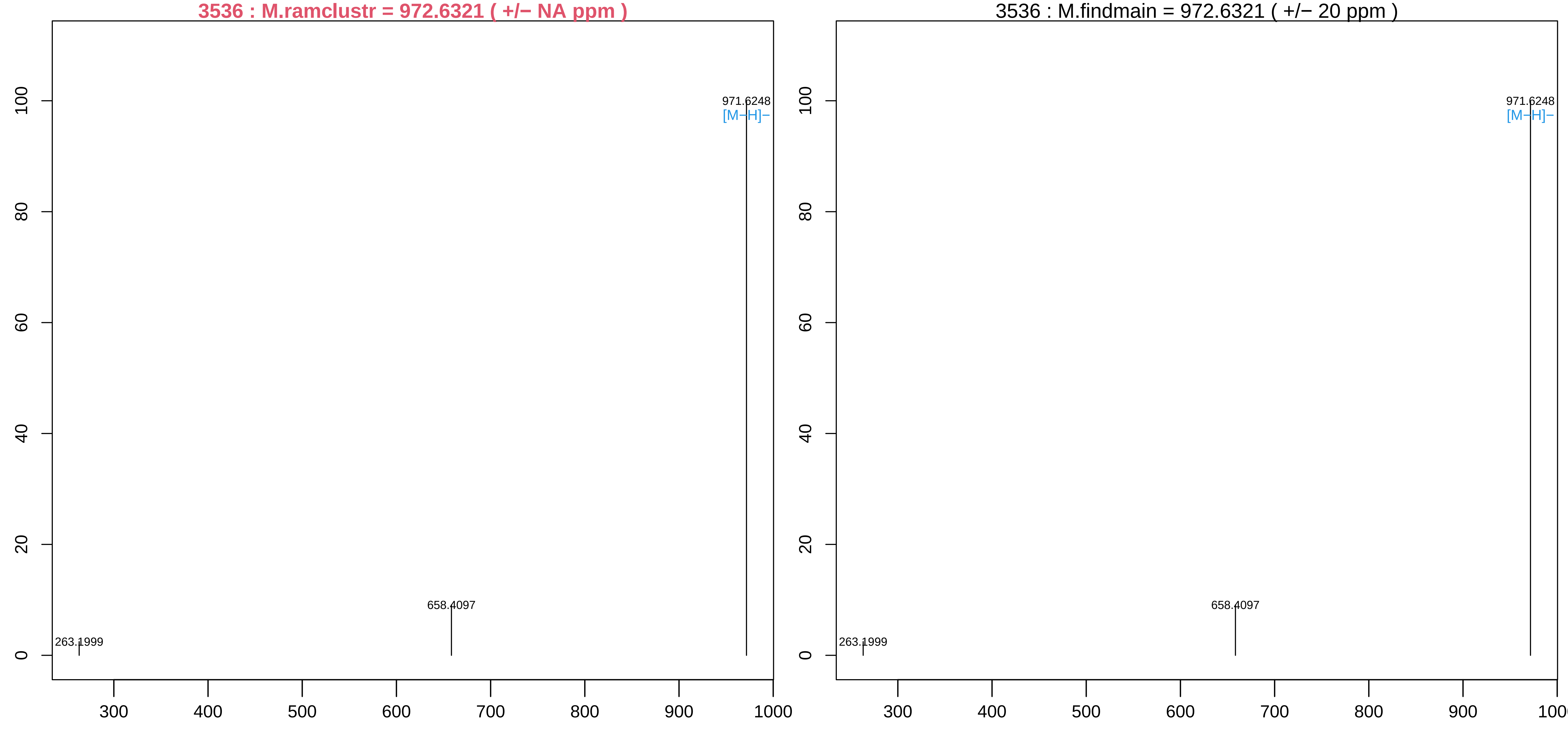
3536 : M.ramclustr = 972.6321 (+/− NA ppm)
971.6248
[M−H]−
658.4097
263.1999
3536 : M.findmain = 972.6321 (+/− 20 ppm)
971.6248
[M−H]−
658.4097
263.1999
0
20
40
60
80
100
300
400
500
600
700
800
900
1000

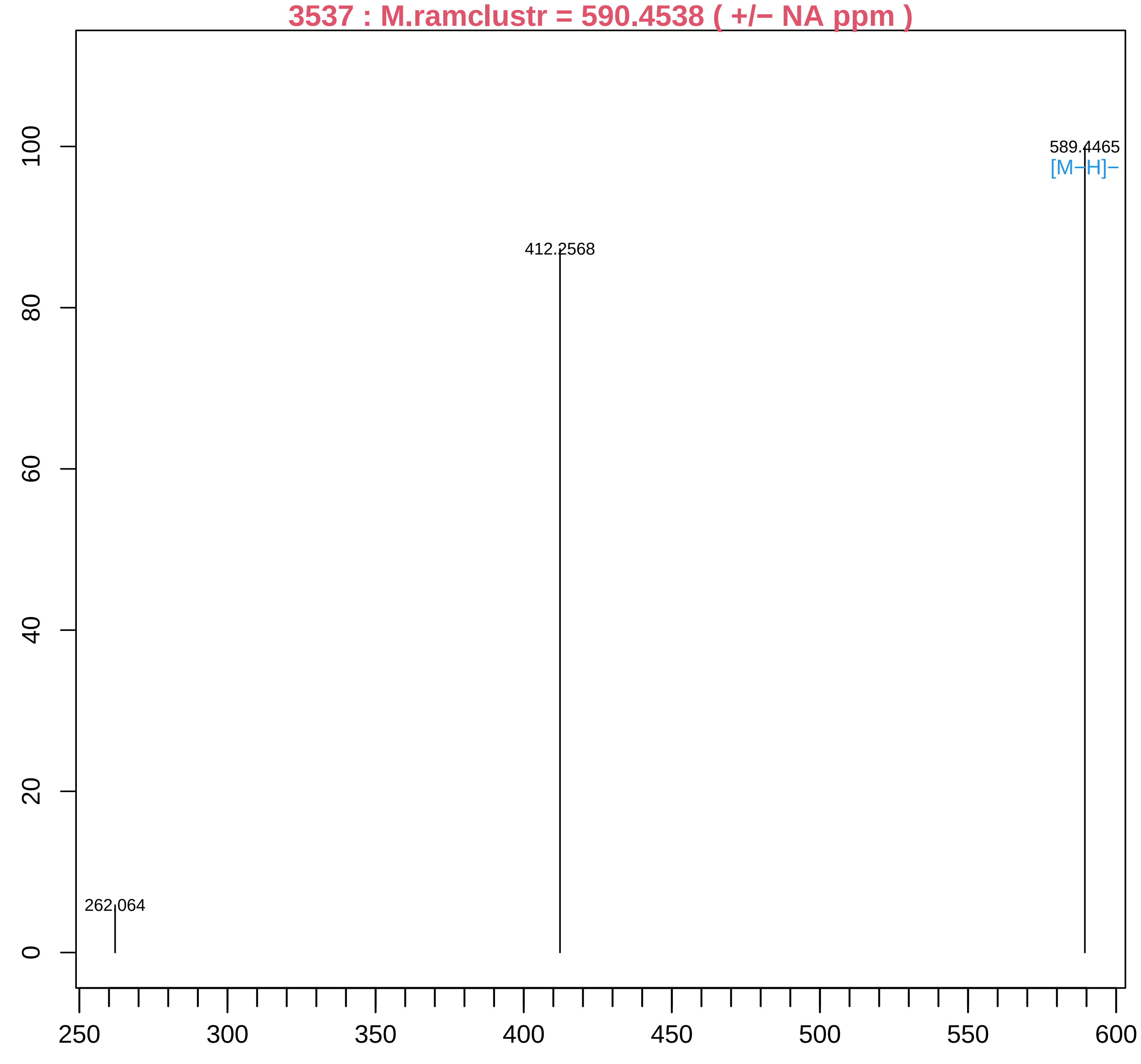
3537 : M.ramclustr = 590.4538 (+/− NA ppm)
589.4465
[M−H]−
412.2568
262.064
0
20
40
60
80
100
250
300
350
400
450
500
550
600

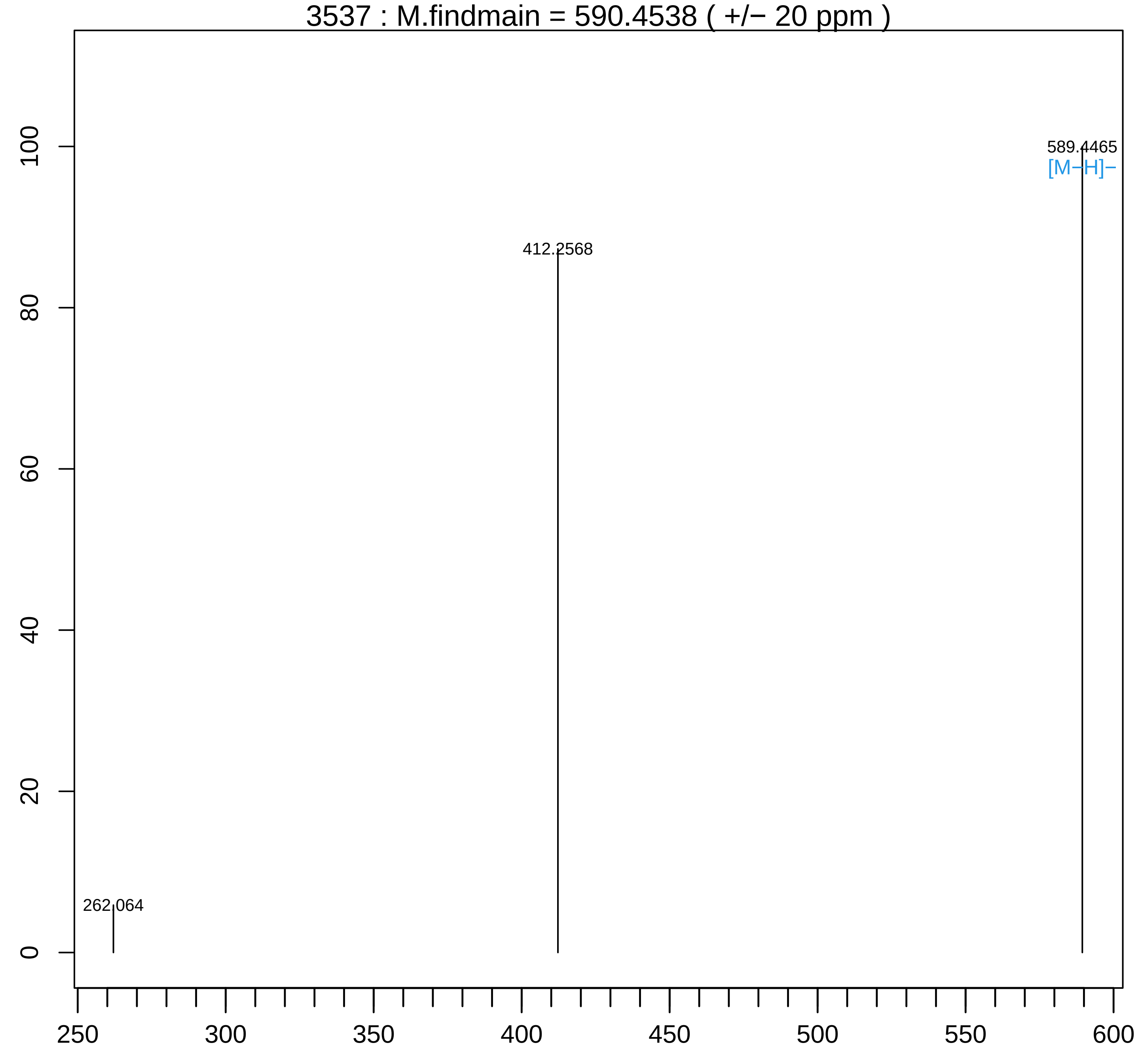
3537 : M.findmain = 590.4538 (+/− 20 ppm)
589.4465
[M−H]−
412.2568
262.064
0
20
40
60
80
100
250
300
350
400
450
500
550
600

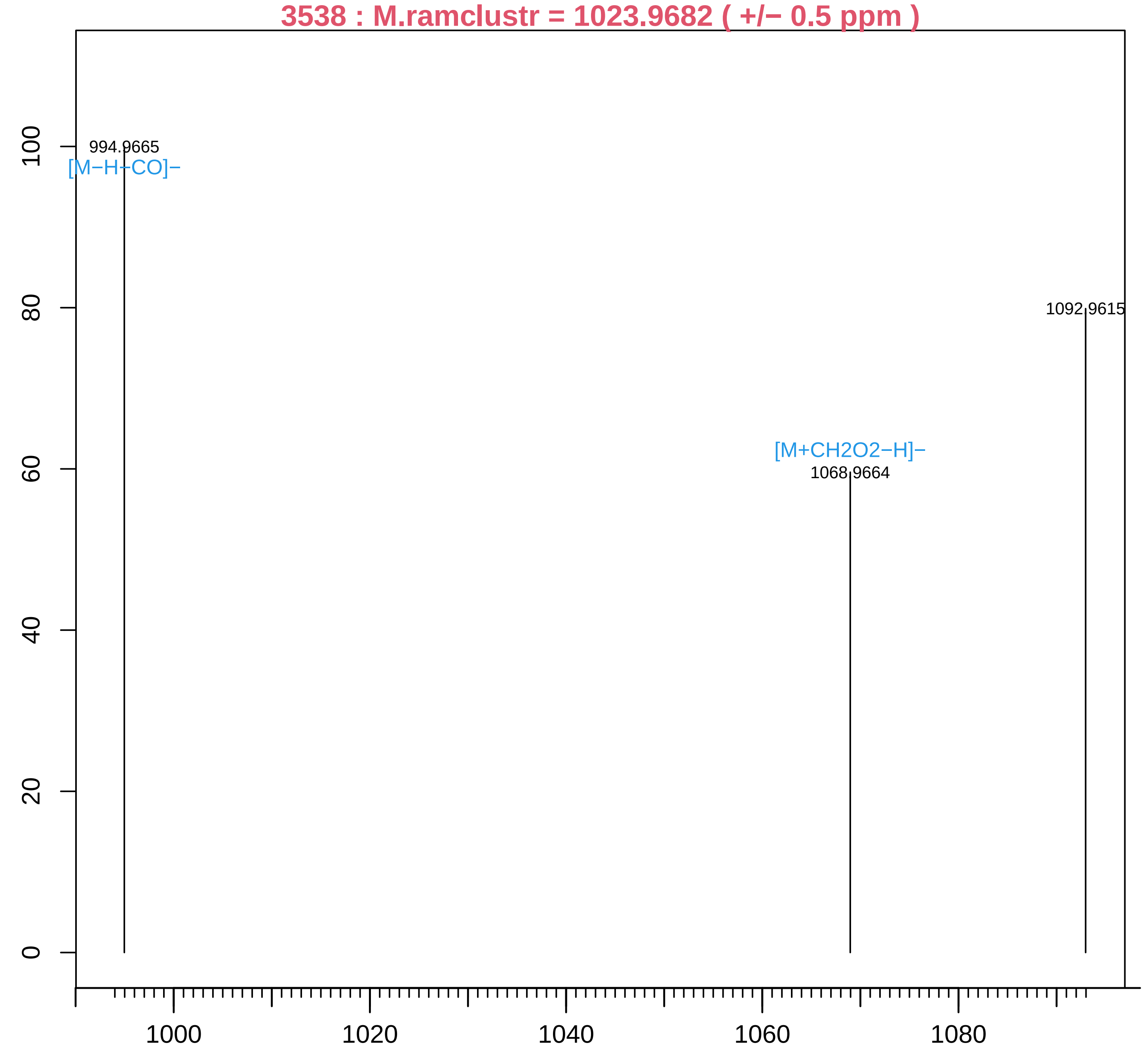
3538 : M.ramclustr = 1023.9682 (+/− 0.5 ppm)
994.9665
[M−H−CO]−
1092.9615
[M+CH2O2−H]−
1068.9664
0
20
40
60
80
100
1000
1020
1040
1060
1080

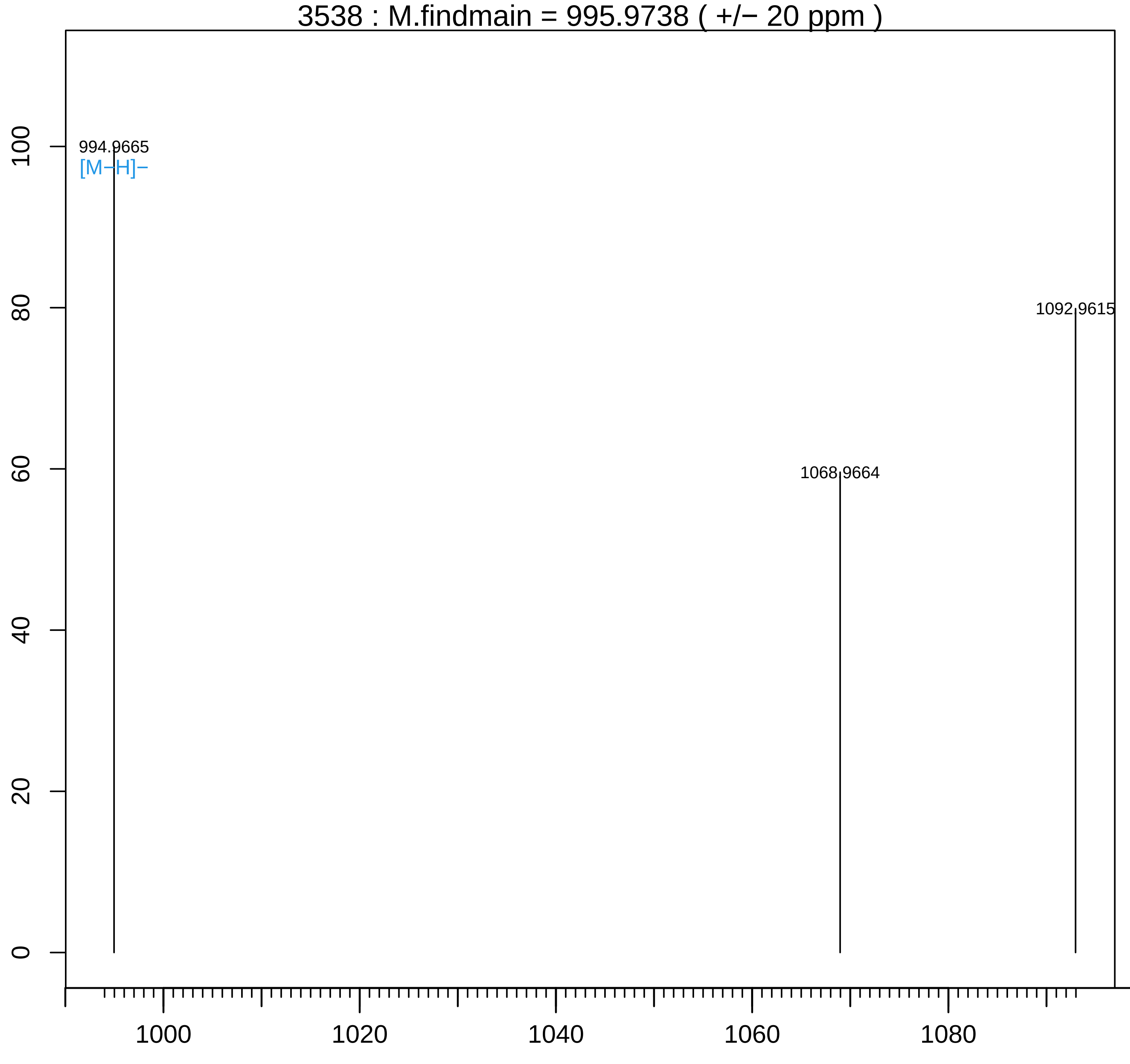
3538 : M.findmain = 995.9738 (+/− 20 ppm)
994.9665
[M−H]−
1092.9615
1068.9664
0
20
40
60
80
100
1000
1020
1040
1060
1080

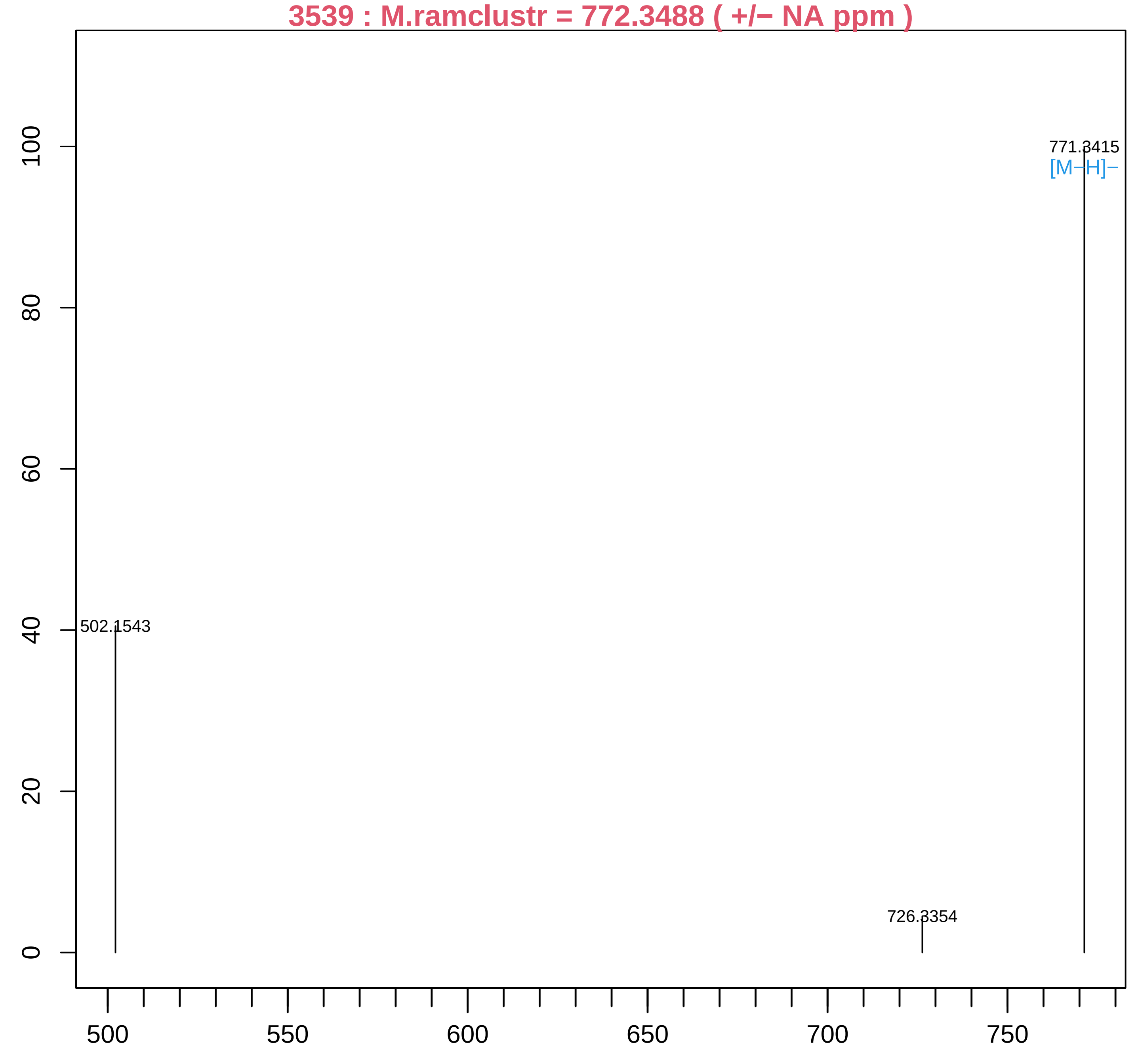
3539 : M.ramclustr = 772.3488 (+/− NA ppm)
771.3415
[M−H]−
502.1543
726.3354
0
20
40
60
80
100
500
550
600
650
700
750

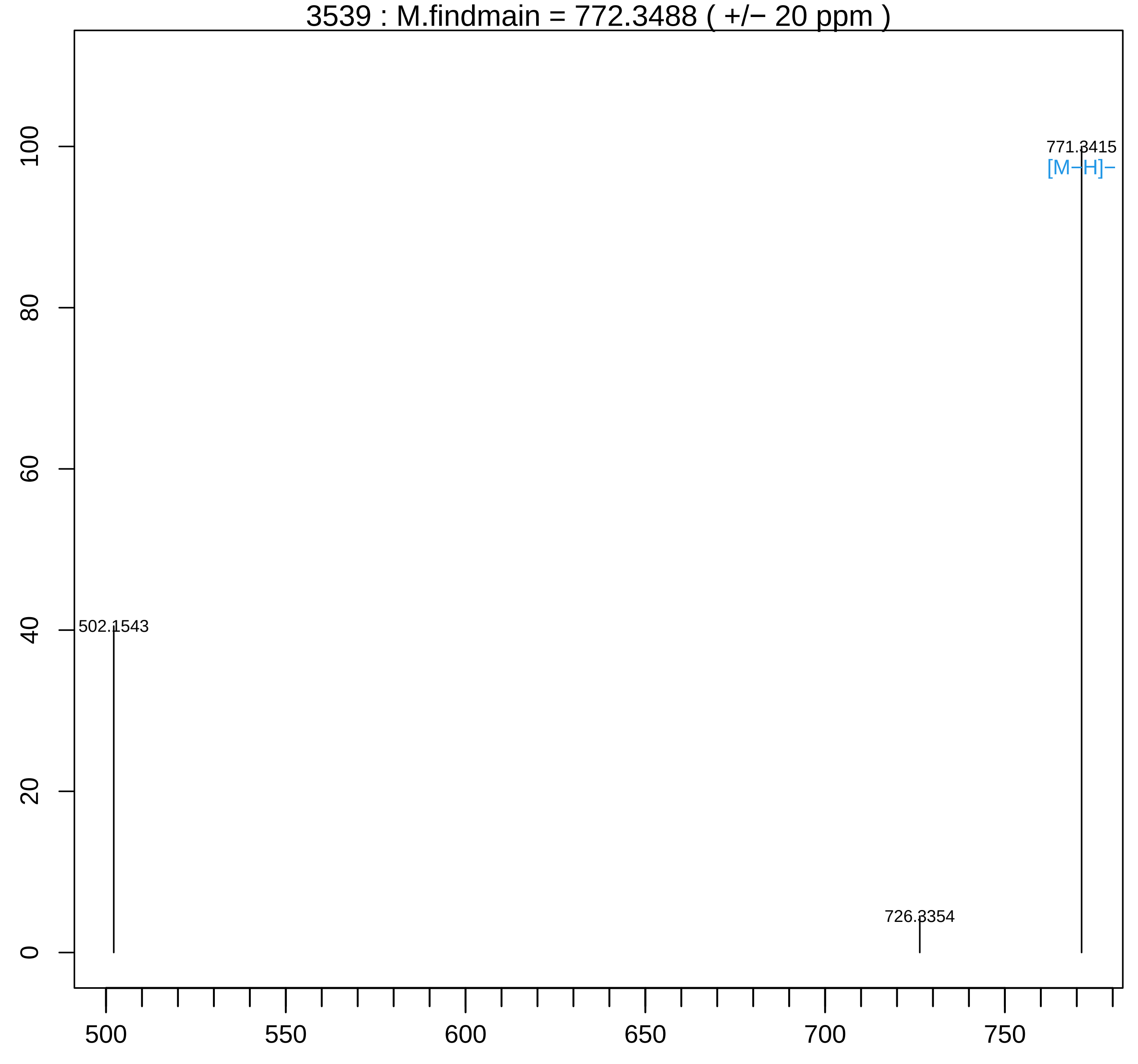
3539 : M.findmain = 772.3488 (+/− 20 ppm)
771.3415
[M−H]−
502.1543
726.3354
0
20
40
60
80
100
500
550
600
650
700
750

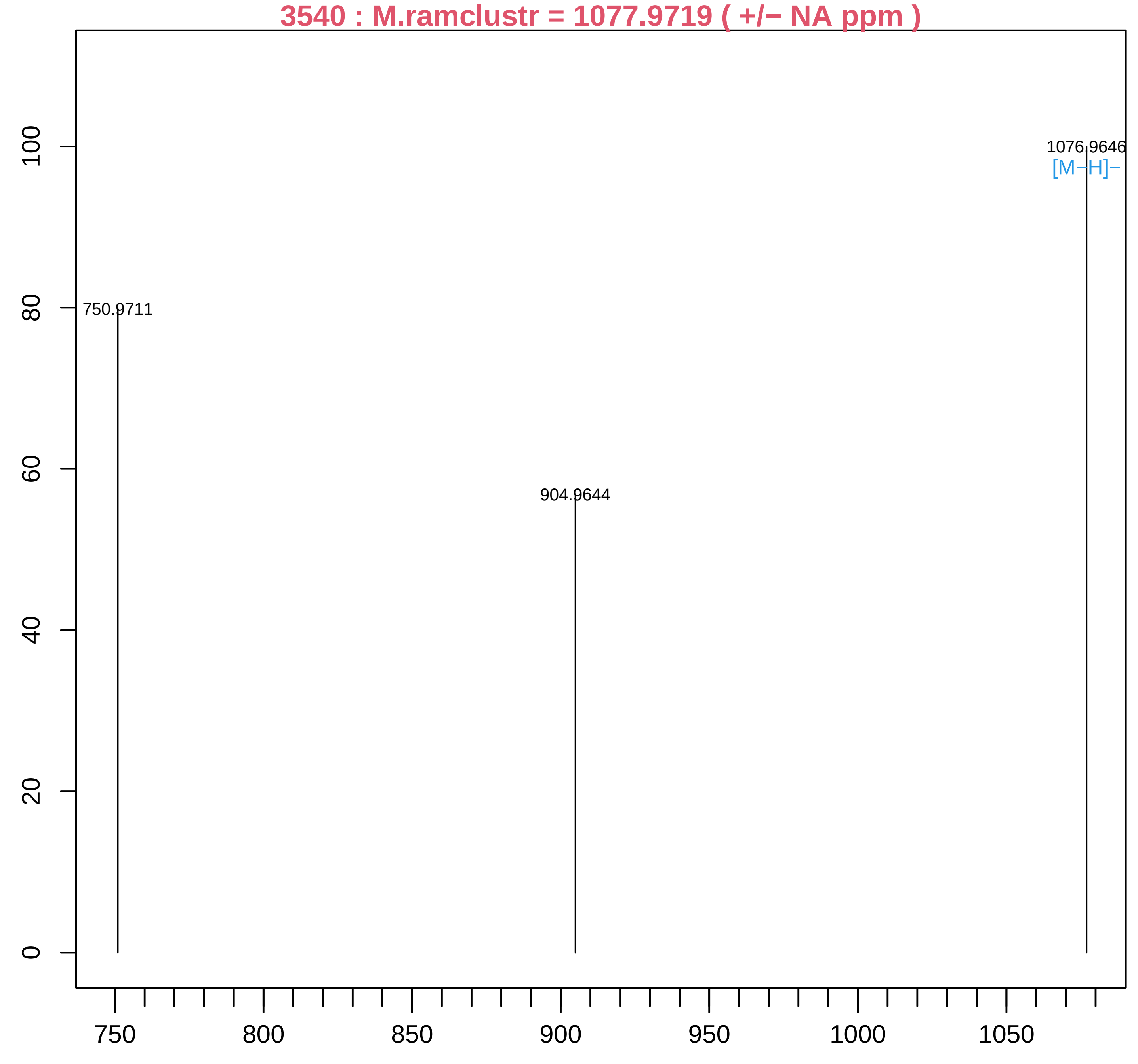
3540 : M.ramclustr = 1077.9719 (+/− NA ppm)
1076.9646
[M−H]−
750.9711
904.9644
0
20
40
60
80
100
750
800
850
900
950
1000
1050

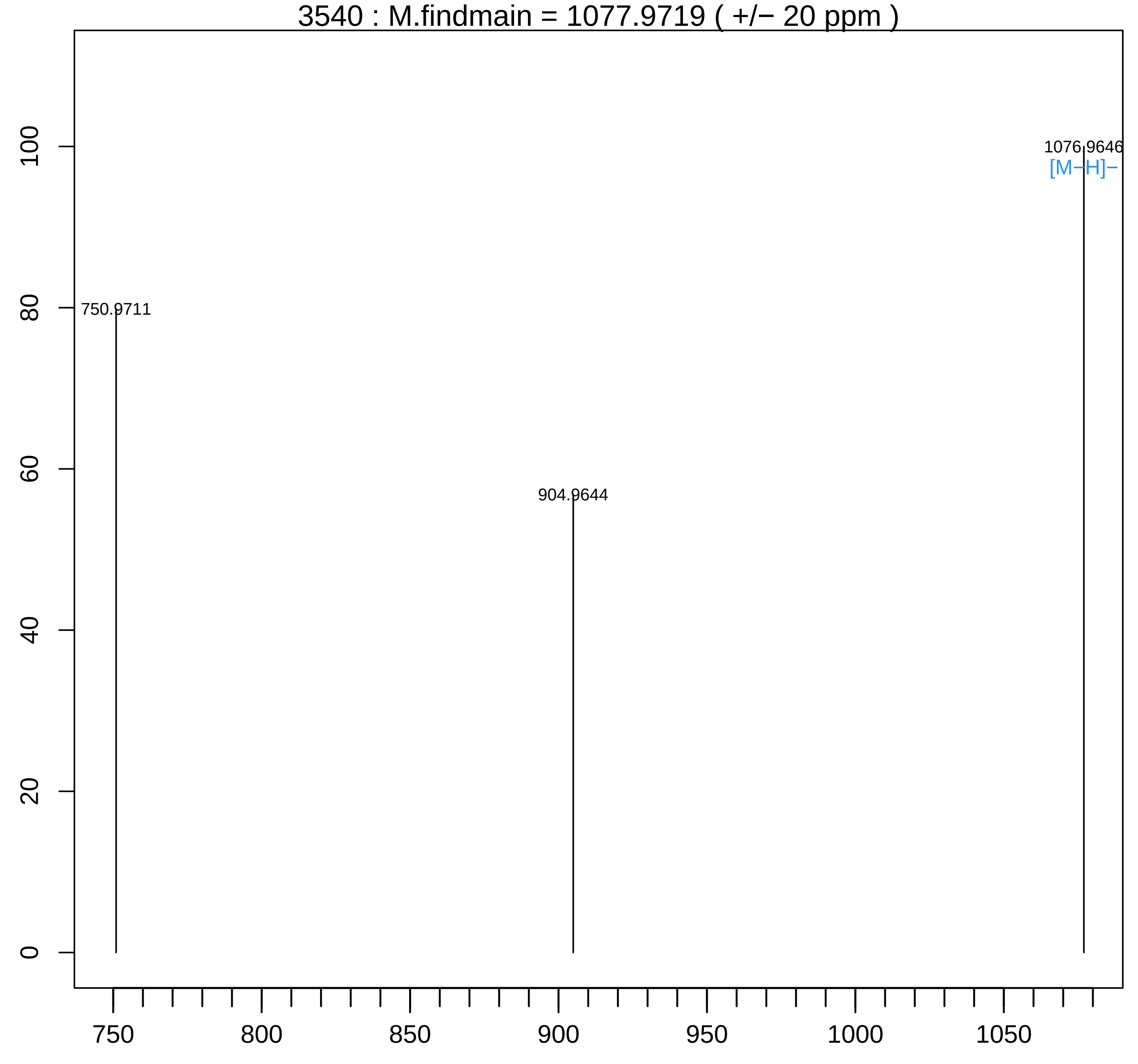
3540 : M.findmain = 1077.9719 (+/− 20 ppm)
1076.9646
[M−H]−
750.9711
904.9644
0
20
40
60
80
100
750
800
850
900
950
1000
1050

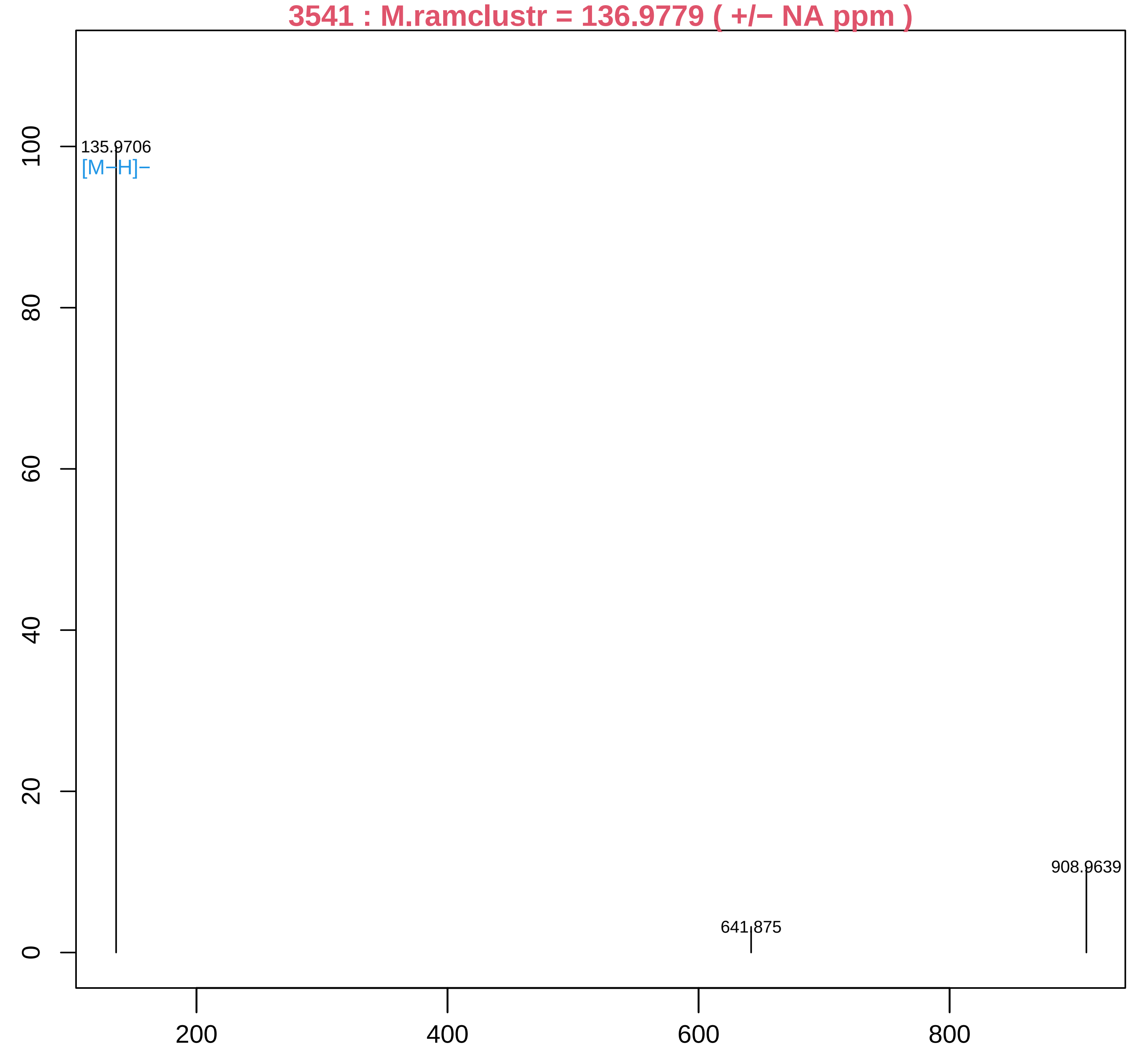
3541 : M.ramclustr = 136.9779 (+/− NA ppm)
135.9706
[M−H]−
641.875
908.9639
0
20
40
60
80
100
200
400
600
800

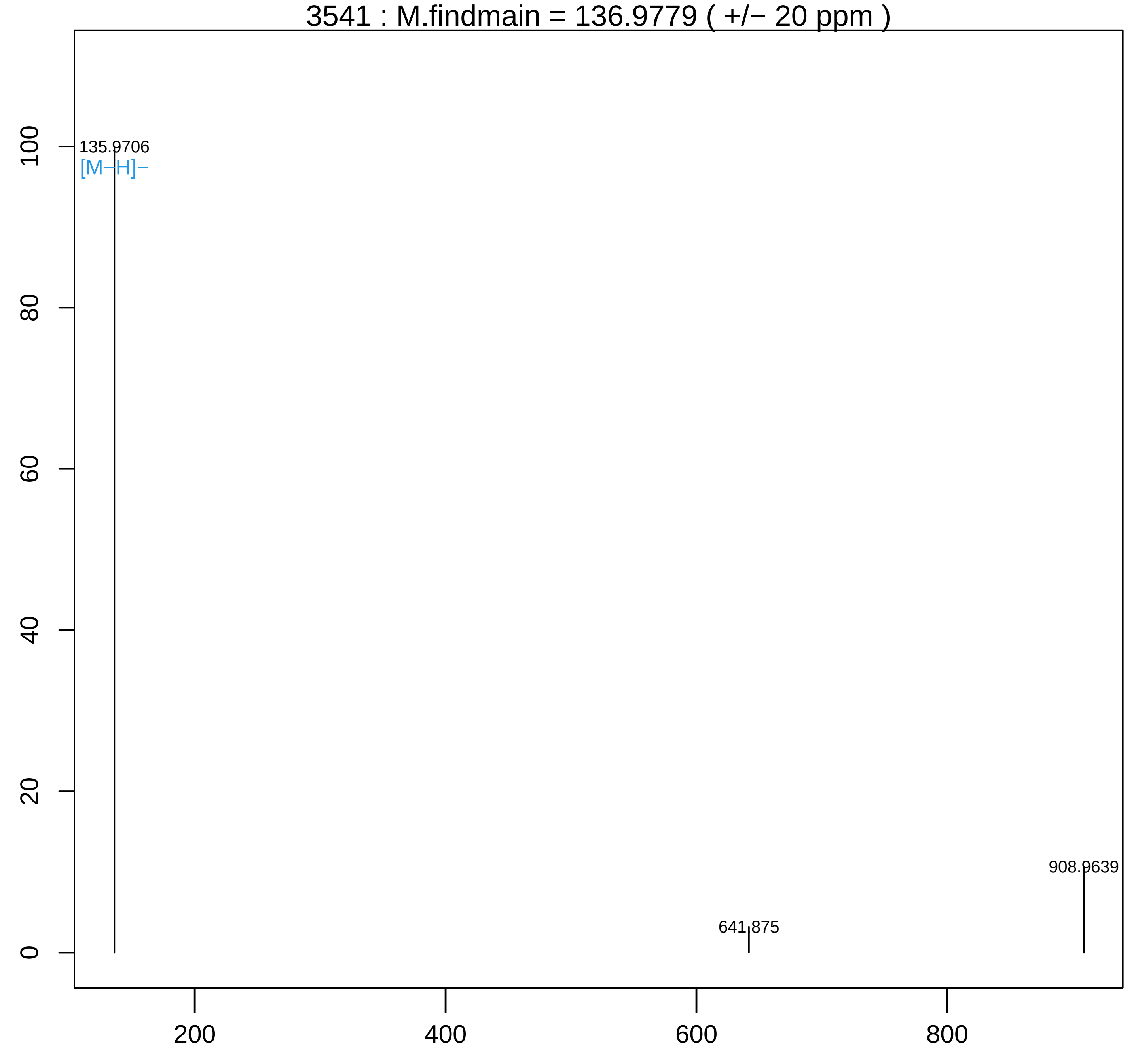
3541 : M.findmain = 136.9779 (+/− 20 ppm)
135.9706
[M−H]−
641.875
908.9639
0
20
40
60
80
100
200
400
600
800

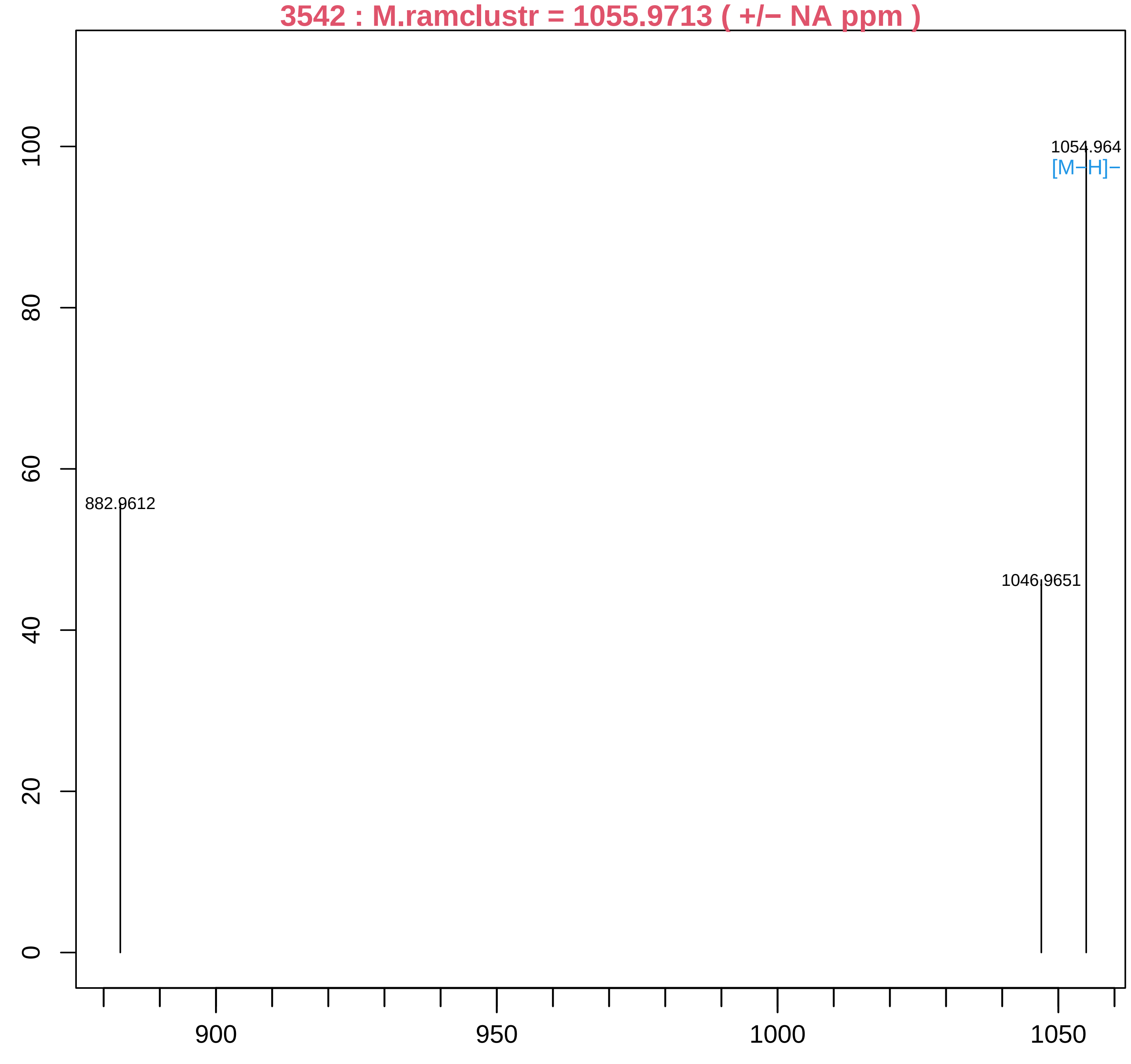
3542 : M.ramclustr = 1055.9713 (+/− NA ppm)
1054.964
[M−H]−
882.9612
1046.9651
0
20
40
60
80
100
900
950
1000
1050

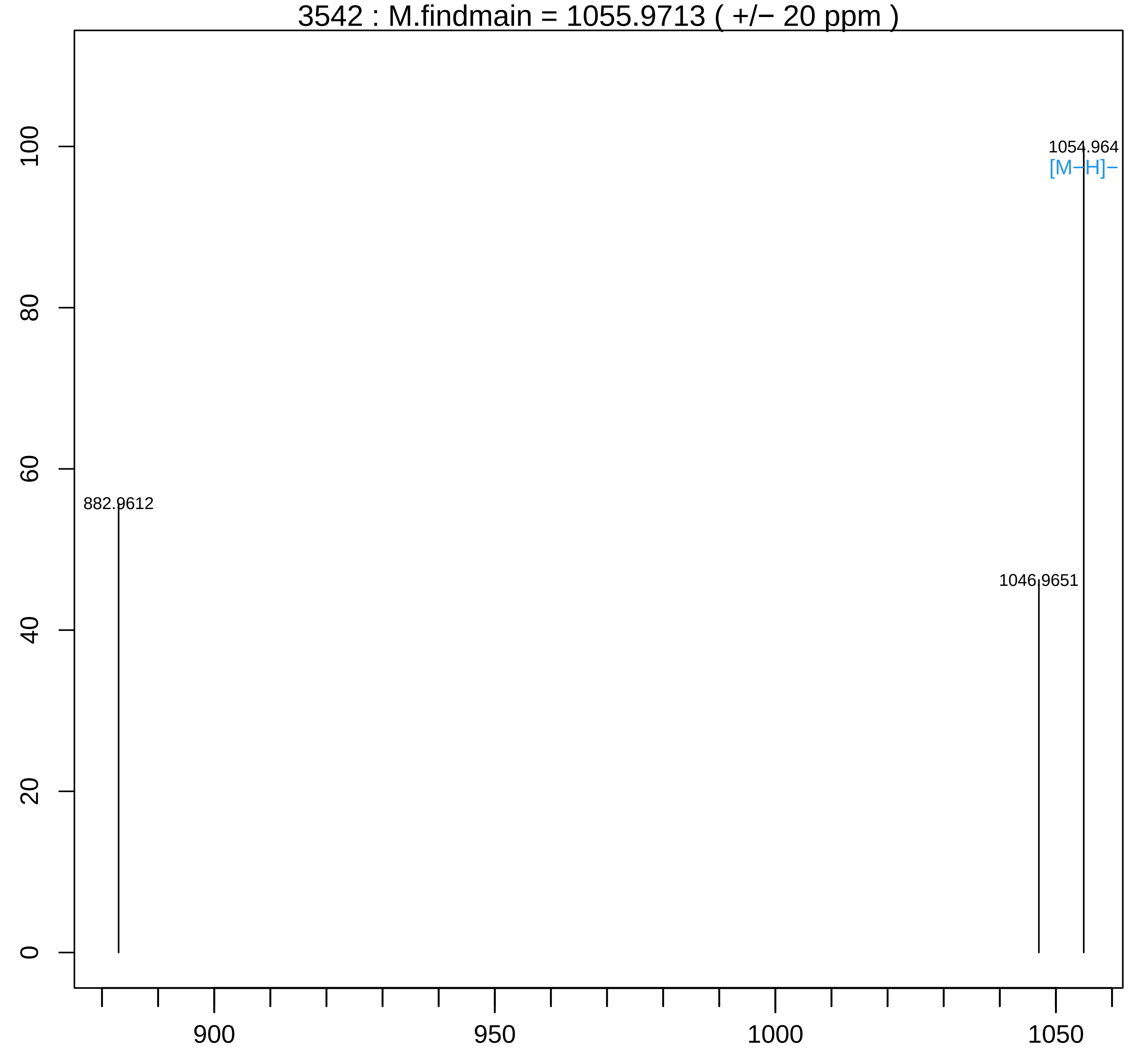
3542 : M.findmain = 1055.9713 (+/− 20 ppm)
1054.964
[M−H]−
882.9612
1046.9651
0
20
40
60
80
100
900
950
1000
1050

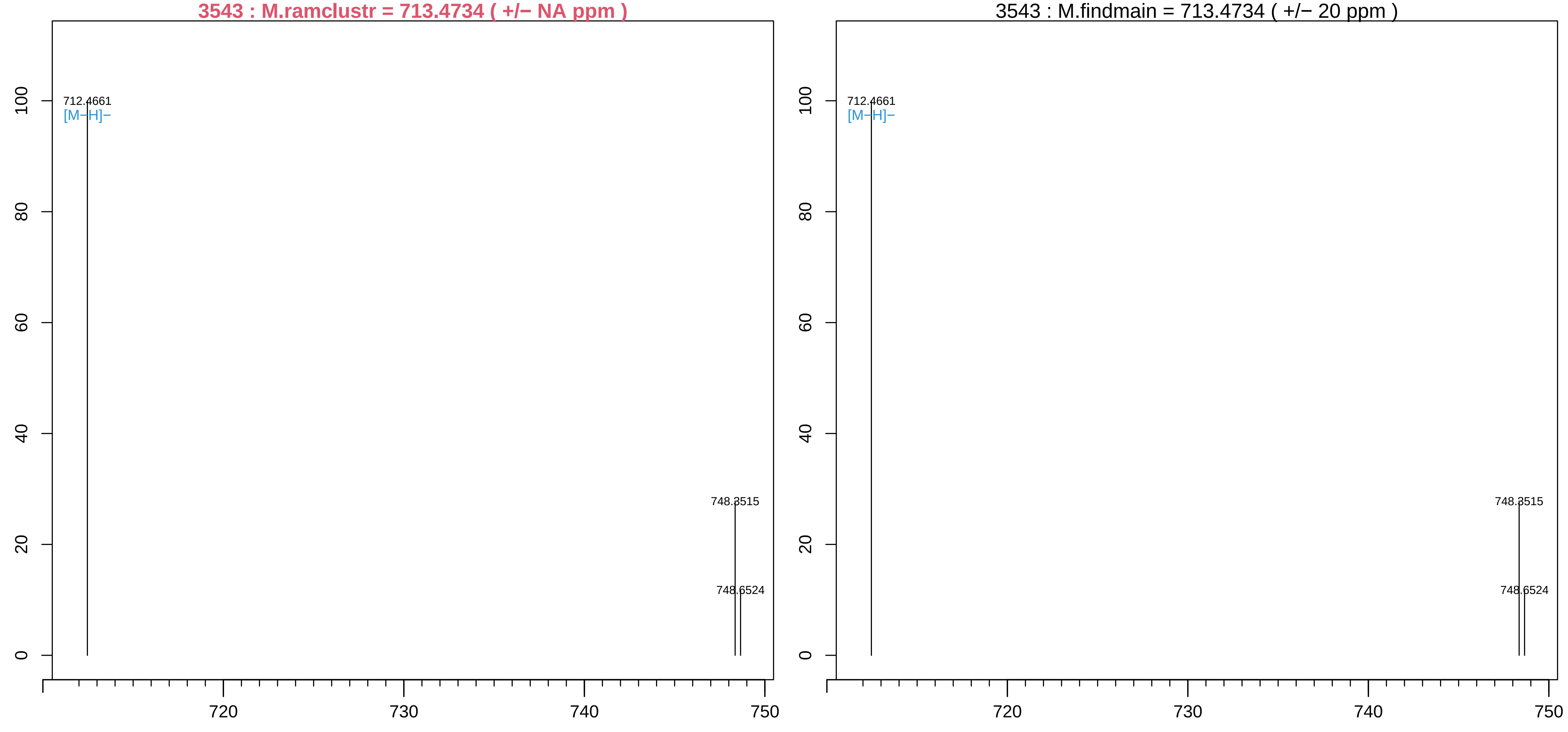

3543 : M.ramclustr = 713.4734 (+/− NA ppm)
712.4661
[M−H]−
748.3515
748.6524
3543 : M.findmain = 713.4734 (+/− 20 ppm)
712.4661
[M−H]−
748.3515
748.6524
0
20
40
60
80
100
720
730
740
750

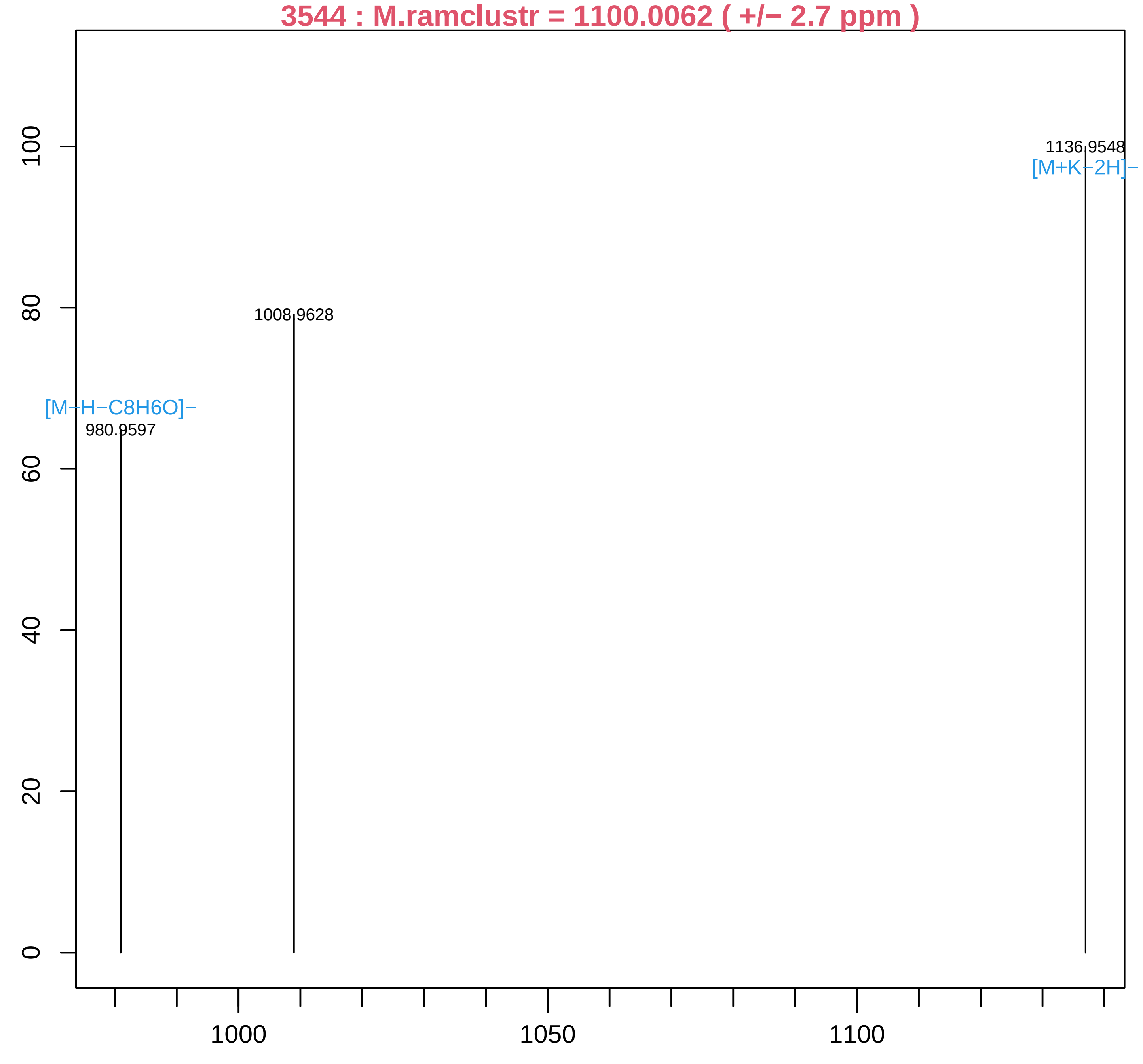
3544 : M.ramclustr = 1100.0062 (+/− 2.7 ppm)
1136.9548
[M+K−2H]−
1008.9628
[M−H−C8H6O]−
980.9597
0
20
40
60
80
100
1000
1050
1100

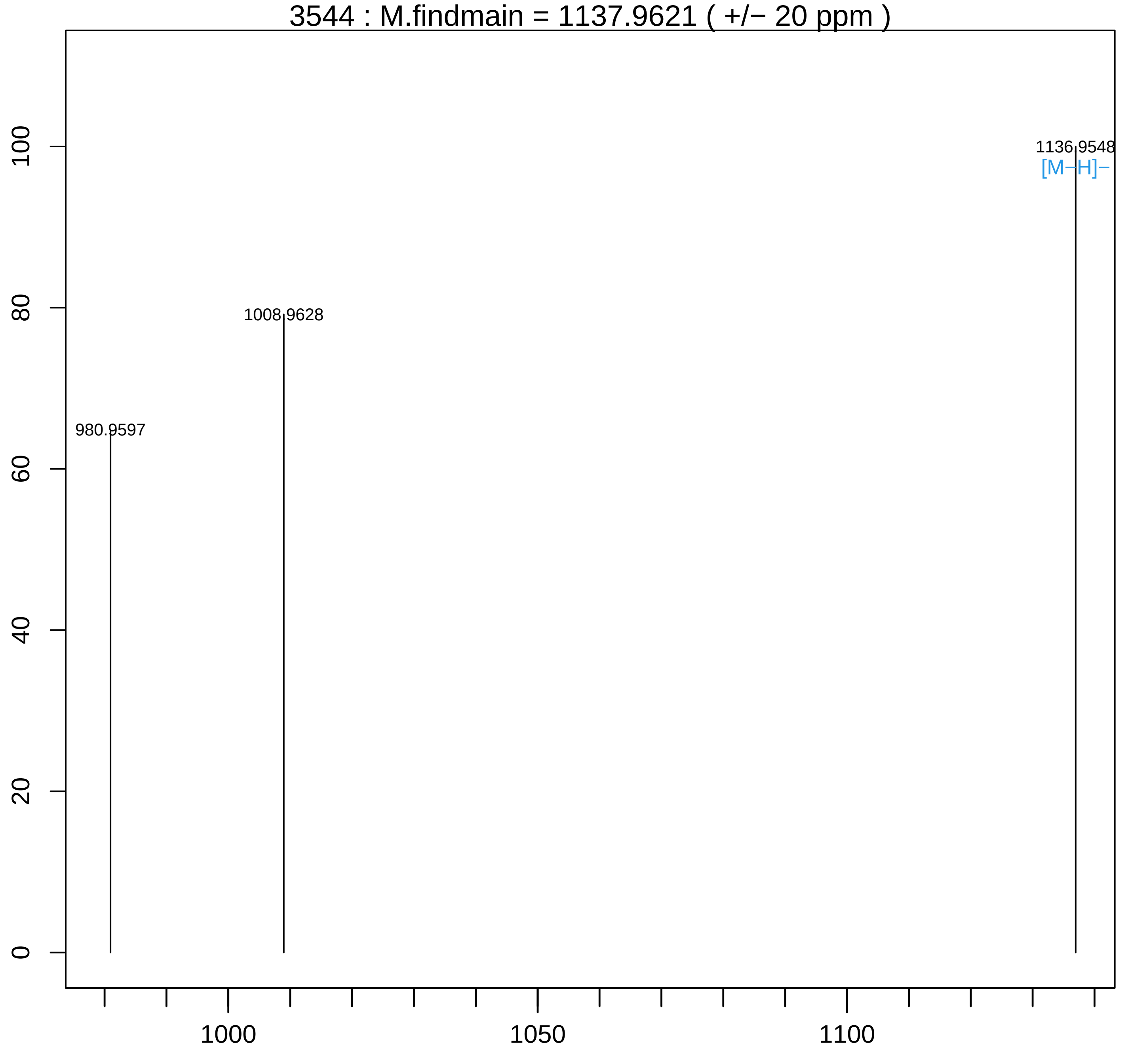
3544 : M.findmain = 1137.9621 (+/− 20 ppm)
1136.9548
[M−H]−
1008.9628
980.9597
0
20
40
60
80
100
1000
1050
1100

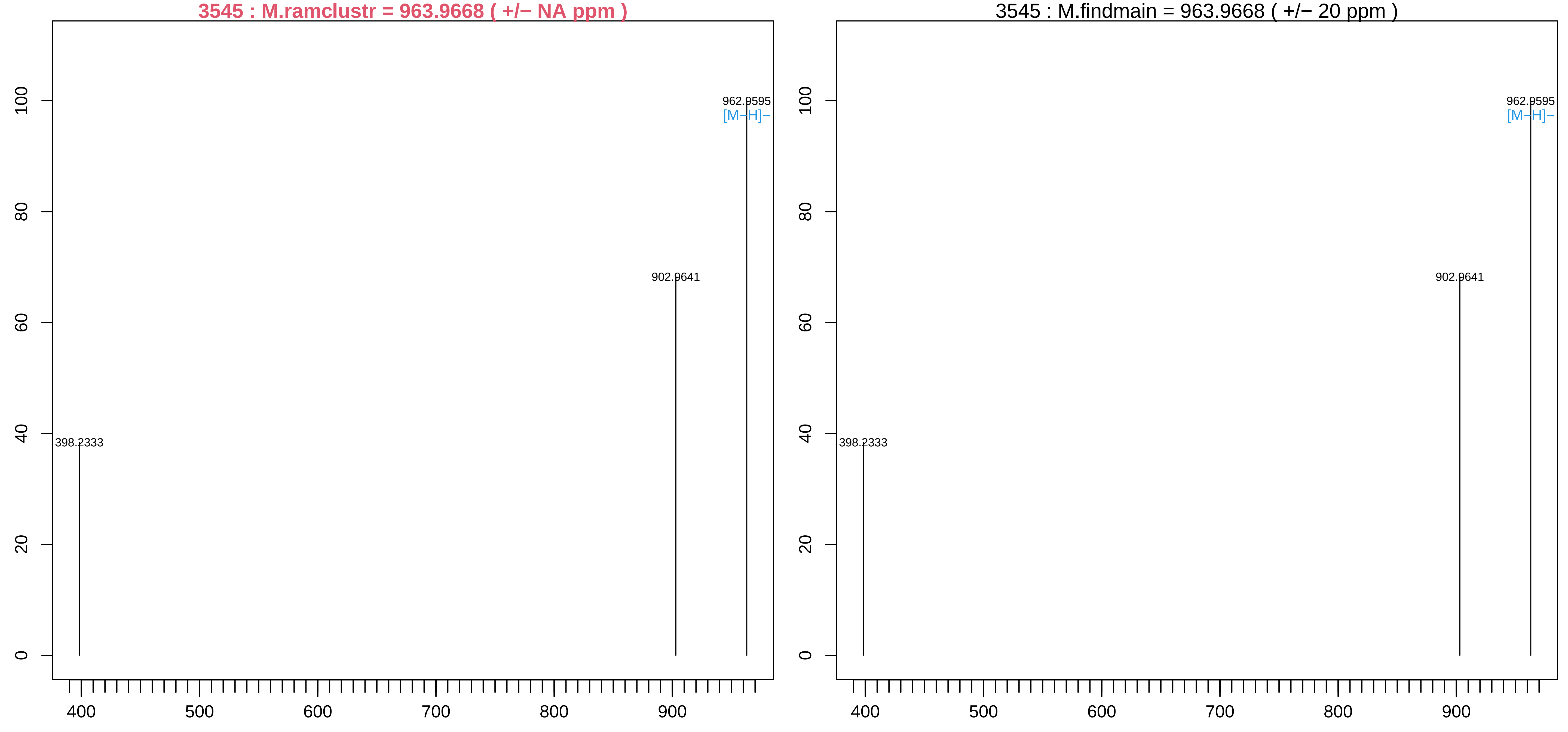

3545 : M.ramclustr = 963.9668 (+/− NA ppm)
962.9595
[M−H]−
902.9641
398.2333
0
20
40
60
80
100
400
500
600
700
800
900
3545 : M.findmain = 963.9668 (+/− 20 ppm)
962.9595
[M−H]−
902.9641
398.2333
0
20
40
60
80
100
400
500
600
700
800
900

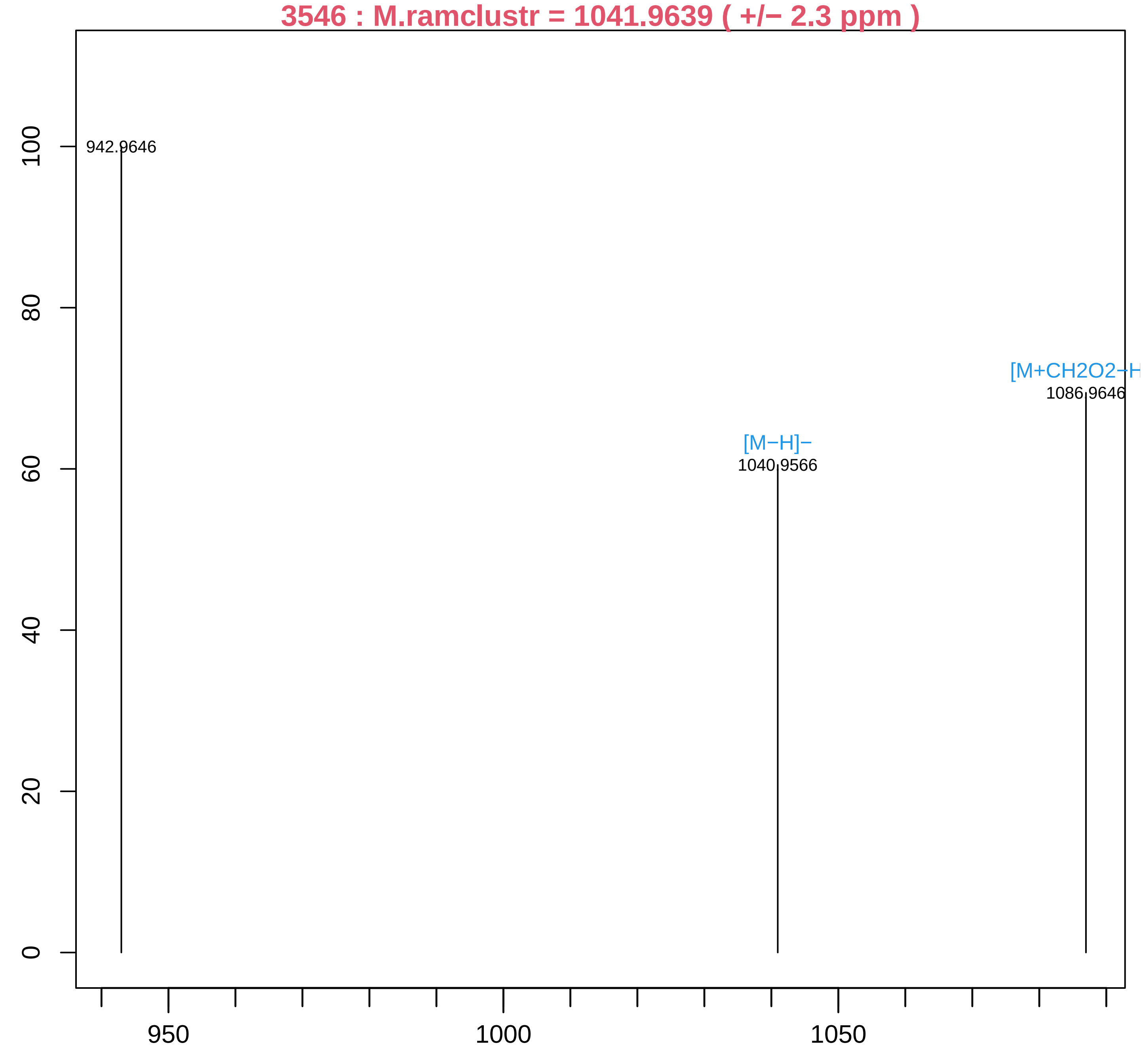

3546 : M.ramclustr = 1041.9639 (+/− 2.3 ppm)
942.9646
[M−H]−
1040.9566
[M+CH2O2−H
1086.9646
0
20
40
60
80
100
950
1000
1050

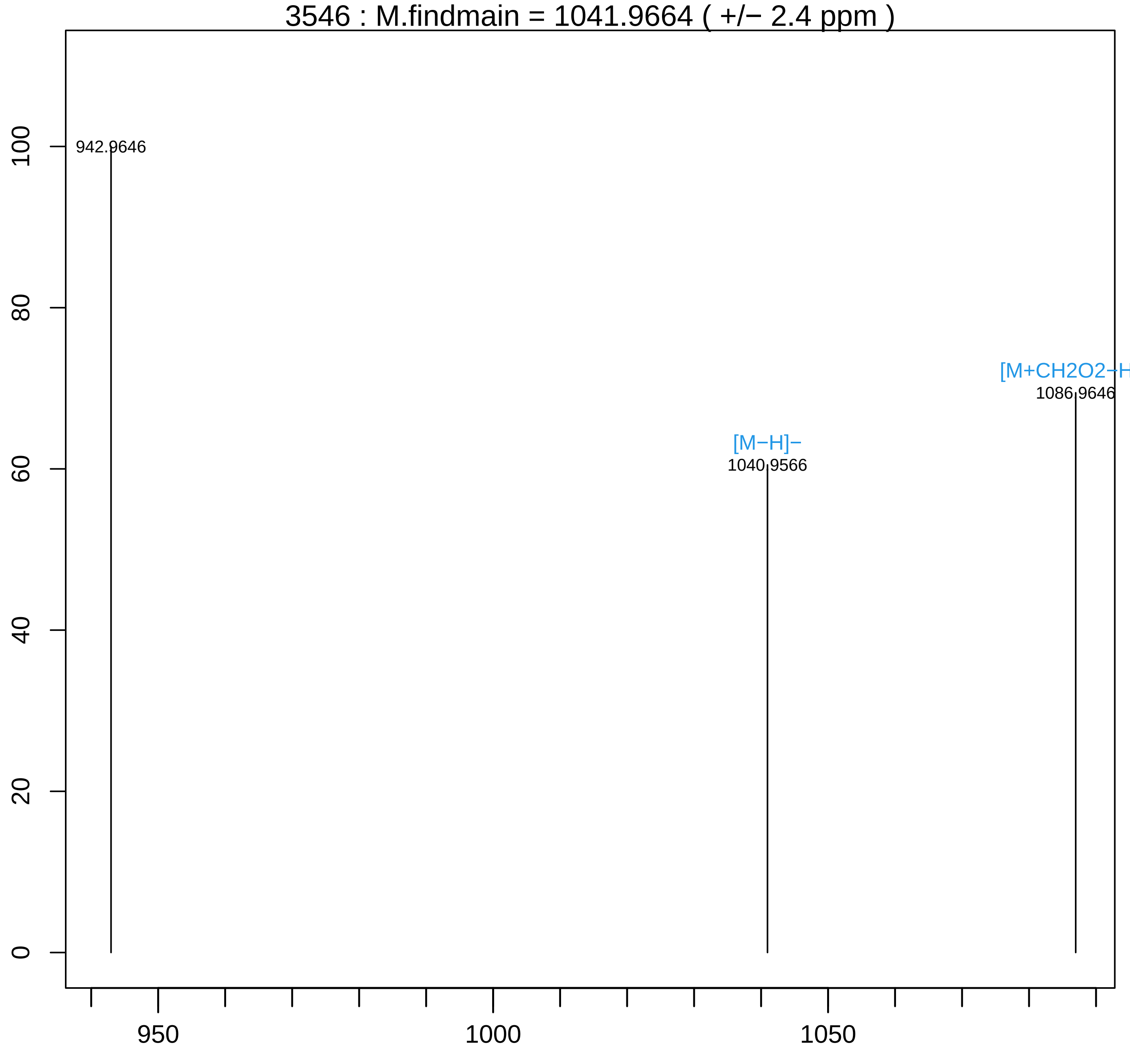

3546 : M.findmain = 1041.9664 (+/− 2.4 ppm)
942.9646
[M−H]−
1040.9566
[M+CH2O2−H
1086.9646
0
20
40
60
80
100
950
1000
1050

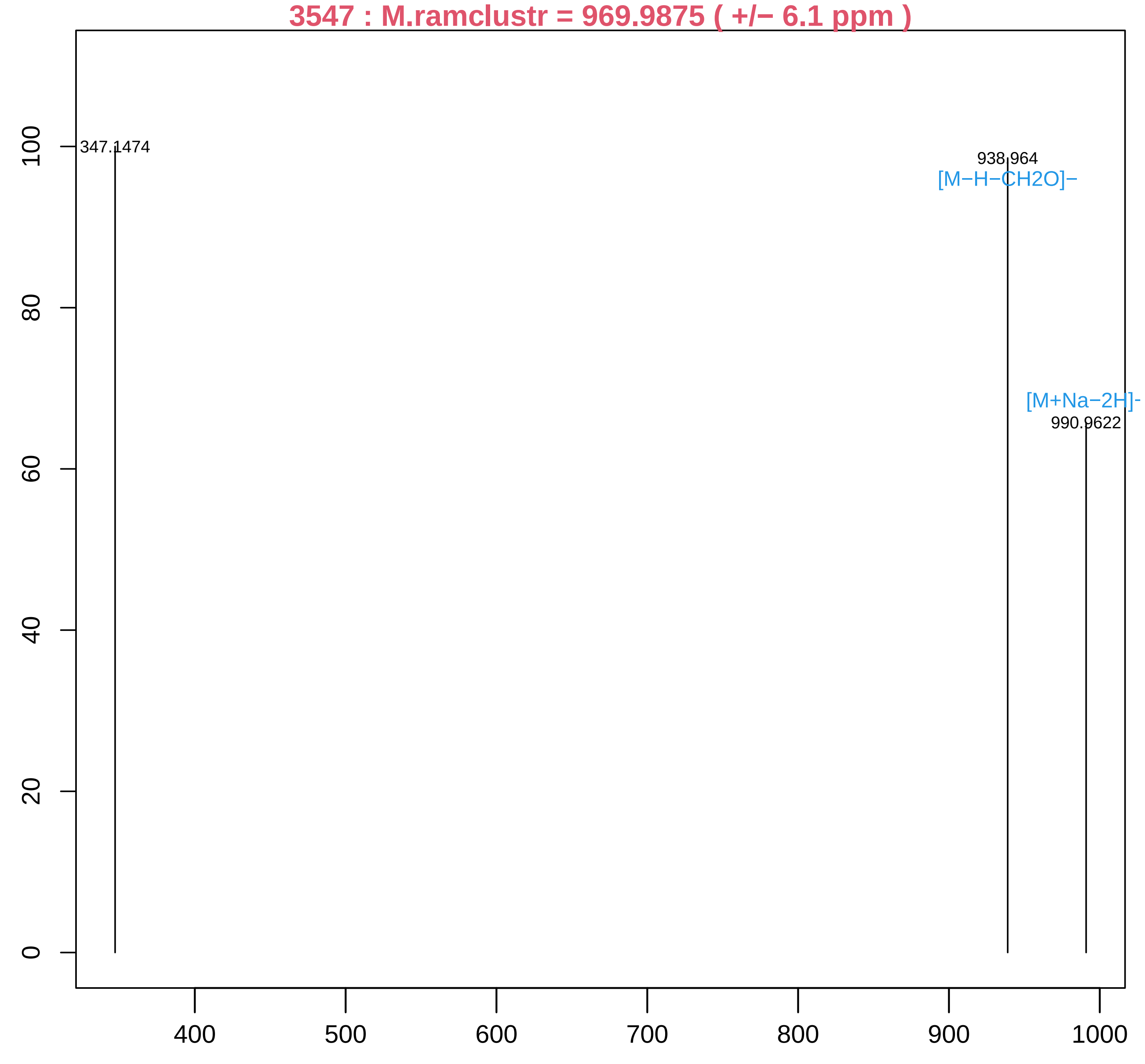

3547 : M.ramclustr = 969.9875 (+/− 6.1 ppm)
347.1474
938.964
[M−H−CH2O]−
[M+Na−2H]-
990.9622
0
20
40
60
80
100
400
500
600
700
800
900
1000

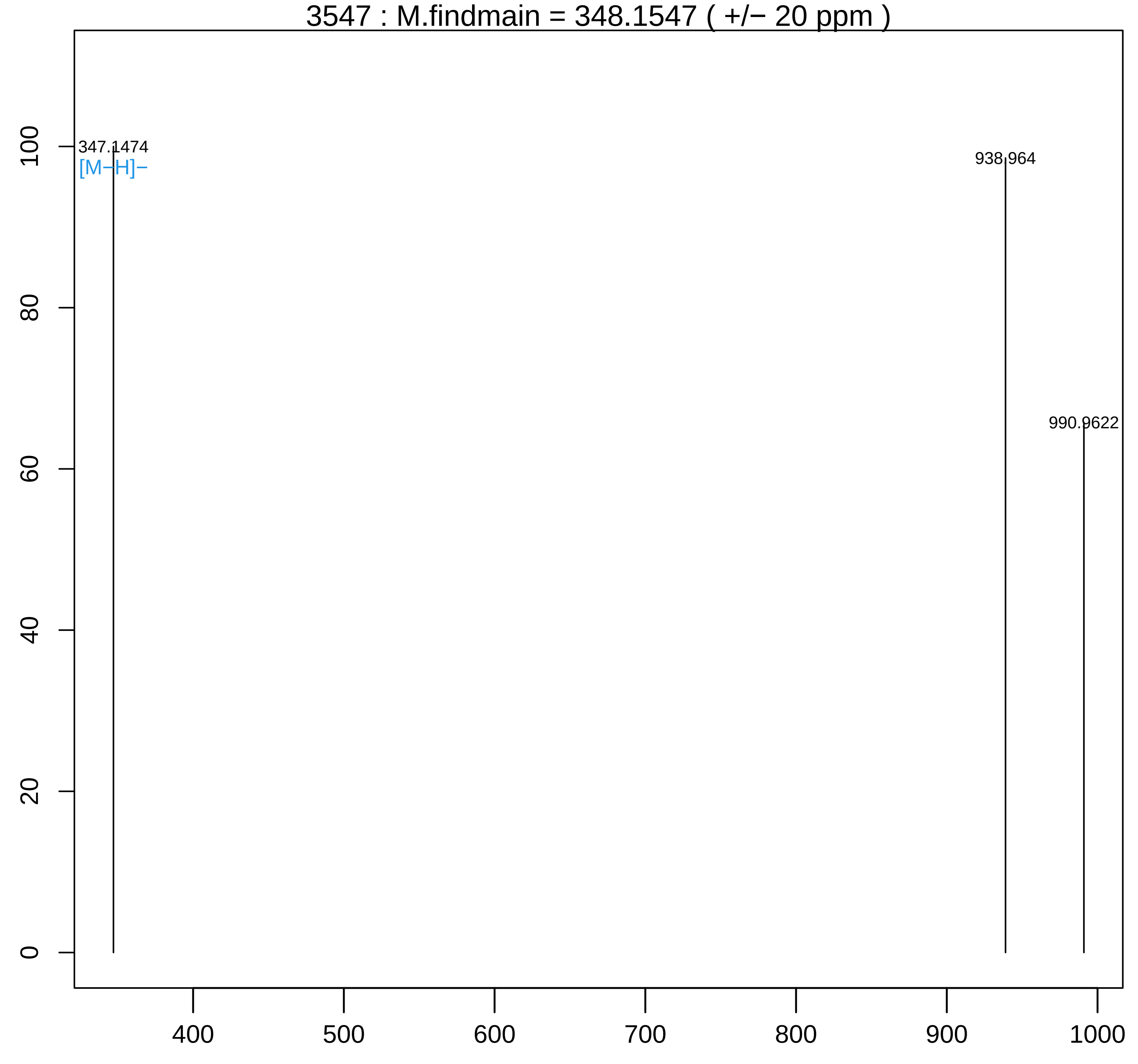

3547 : M.findmain = 348.1547 (+/− 20 ppm)
347.1474
[M−H]−
938.964
990.9622
0
20
40
60
80
100
400
500
600
700
800
900
1000

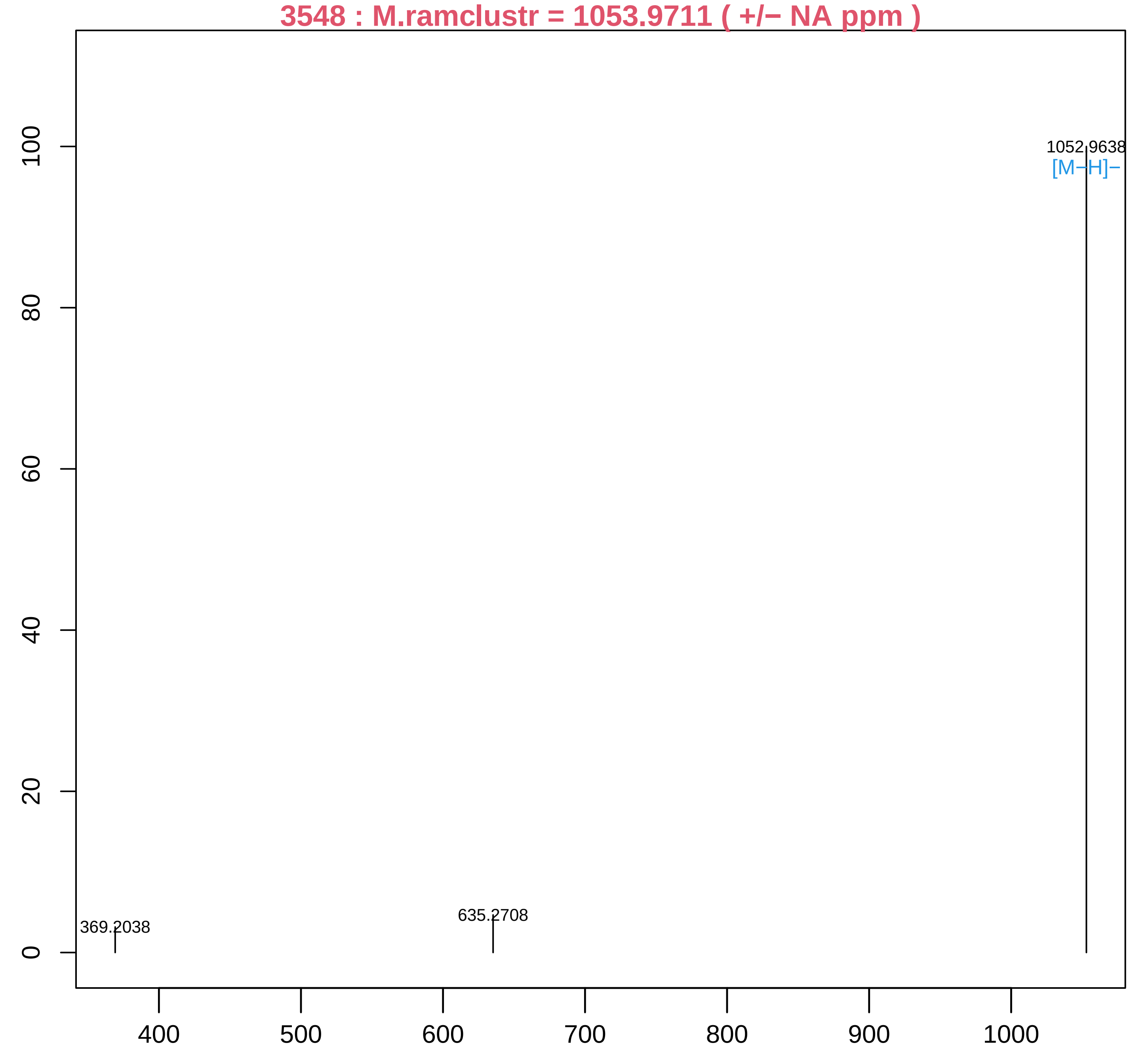
3548 : M.ramclustr = 1053.9711 (+/− NA ppm)
1052.9638
[M−H]−
635.2708
369.2038
0
20
40
60
80
100
400
500
600
700
800
900
1000
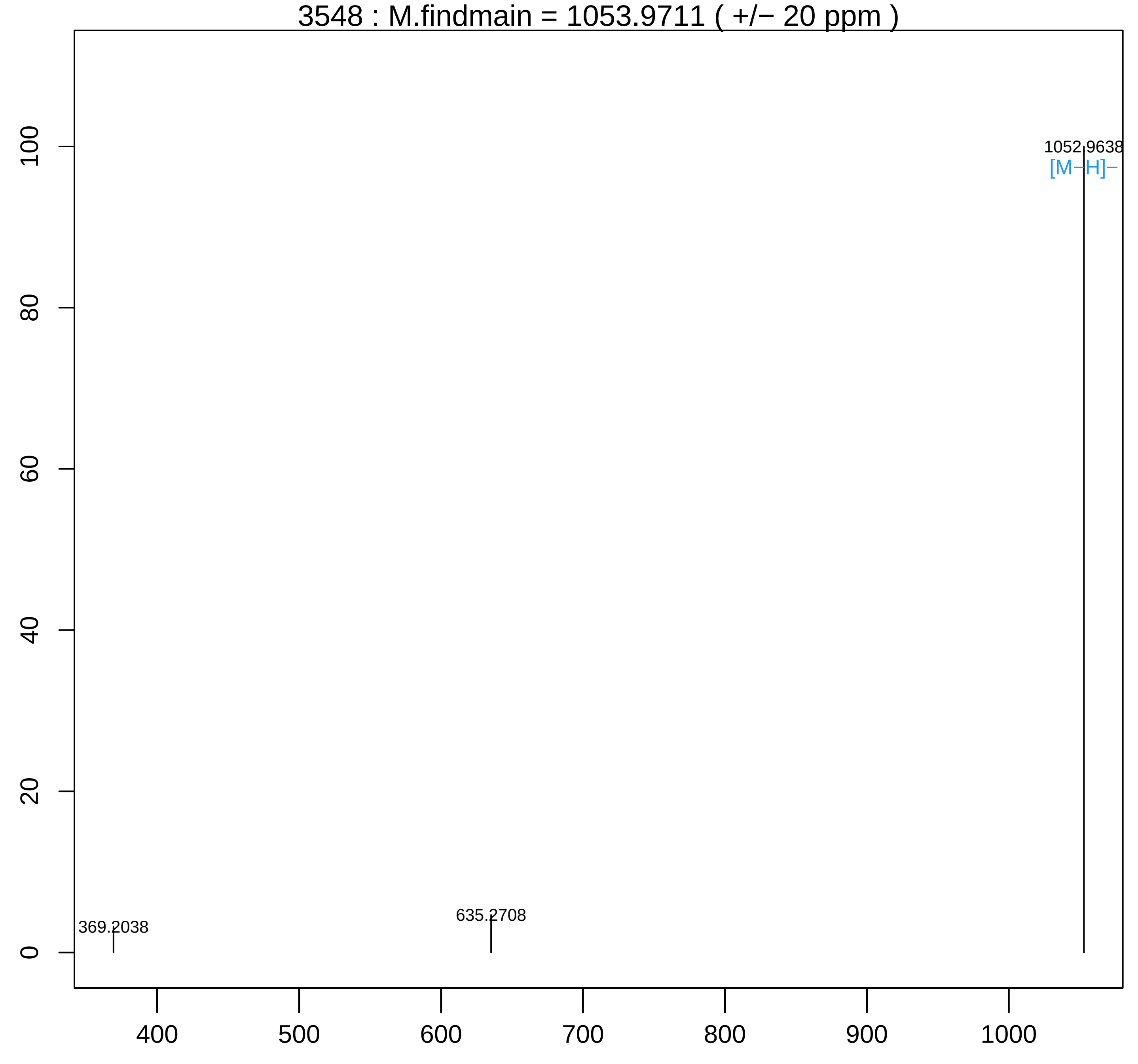
3548 : M.findmain = 1053.9711 (+/− 20 ppm)
1052.9638
[M−H]−
635.2708
369.2038
0
20
40
60
80
100
400
500
600
700
800
900
1000

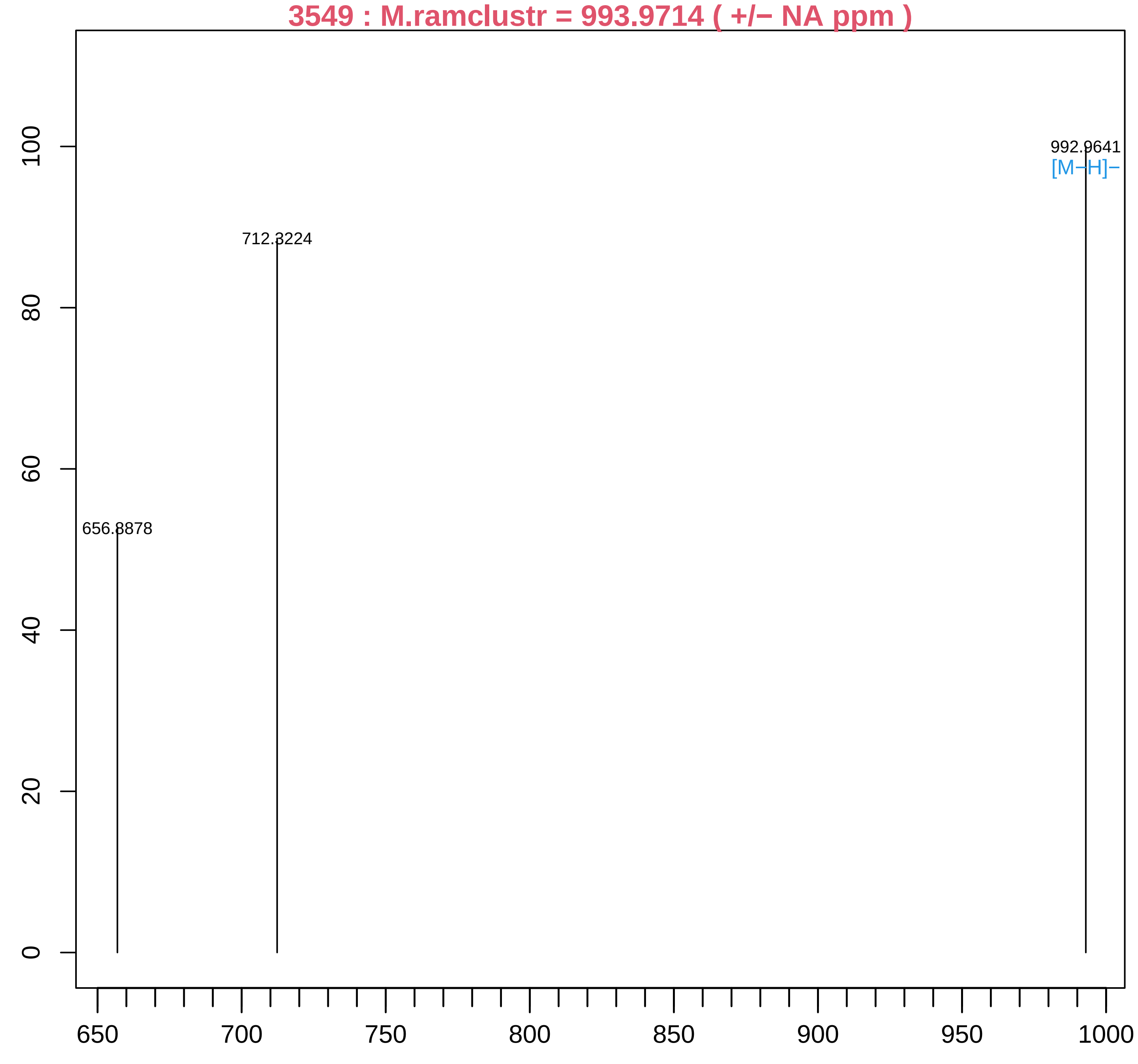
3549 : M.ramclustr = 993.9714 (+/− NA ppm)
992.9641
[M−H]−
712.3224
656.8878
0
20
40
60
80
100
650
700
750
800
850
900
950
1000

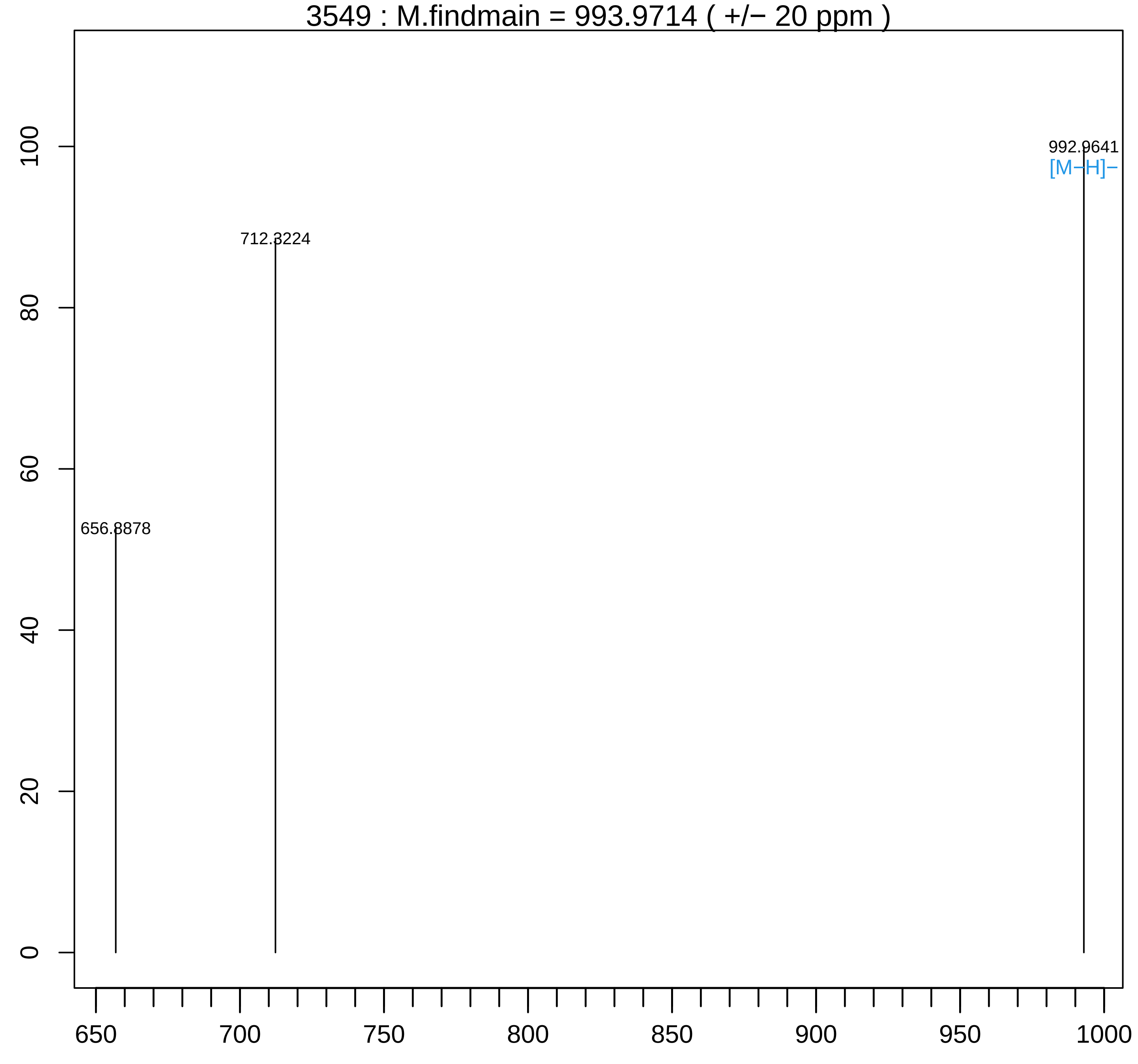
3549 : M.findmain = 993.9714 (+/− 20 ppm)
992.9641
[M−H]−
712.3224
656.8878
0
20
40
60
80
100
650
700
750
800
850
900
950
1000

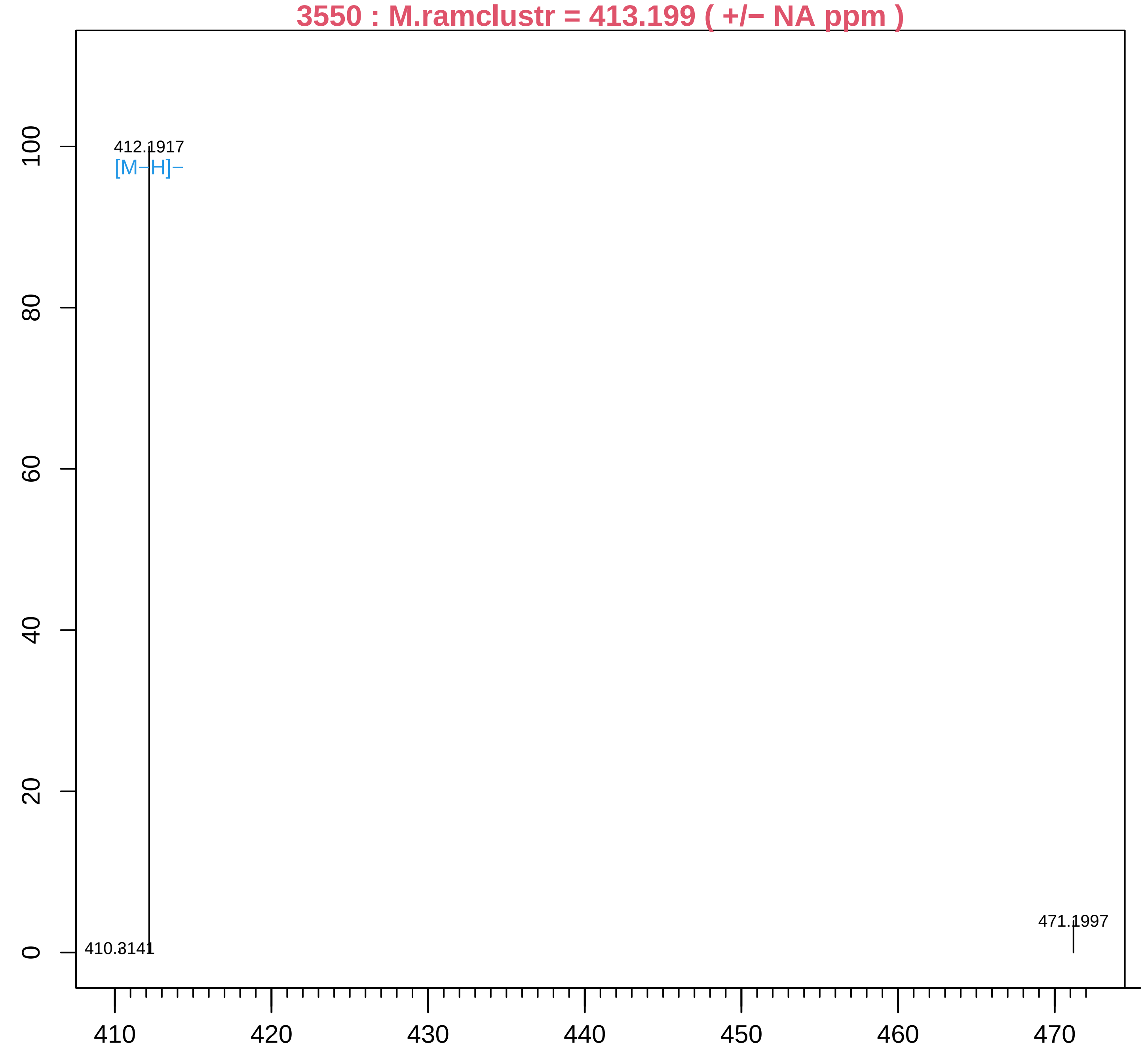
3550 : M.ramclustr = 413.199 (+/− NA ppm)
412.1917
[M−H]−
410.8141
471.1997
0
20
40
60
80
100
410
420
430
440
450
460
470

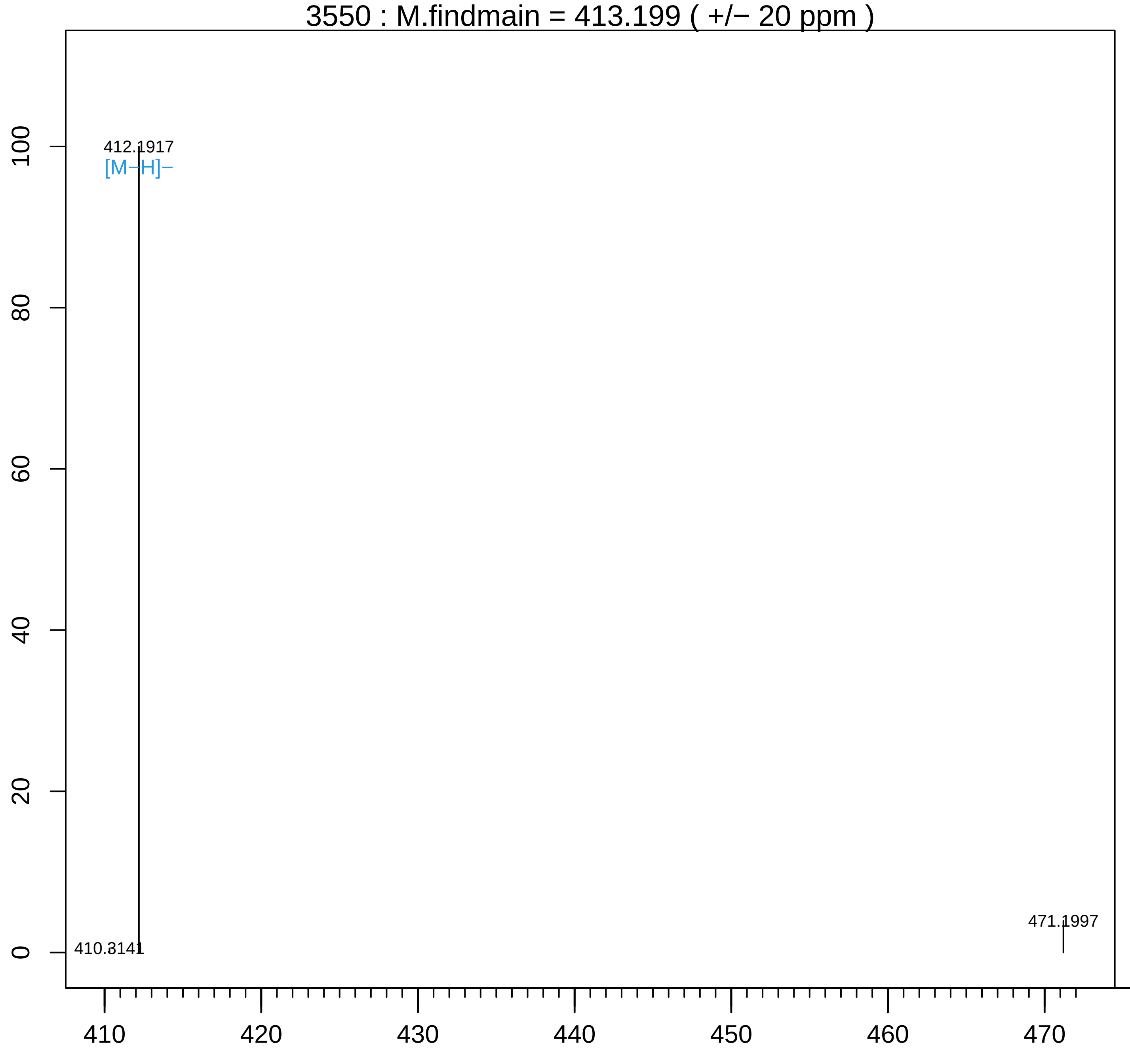
3550 : M.findmain = 413.199 (+/− 20 ppm)
412.1917
[M−H]−
410.8141
471.1997
0
20
40
60
80
100
410
420
430
440
450
460
470

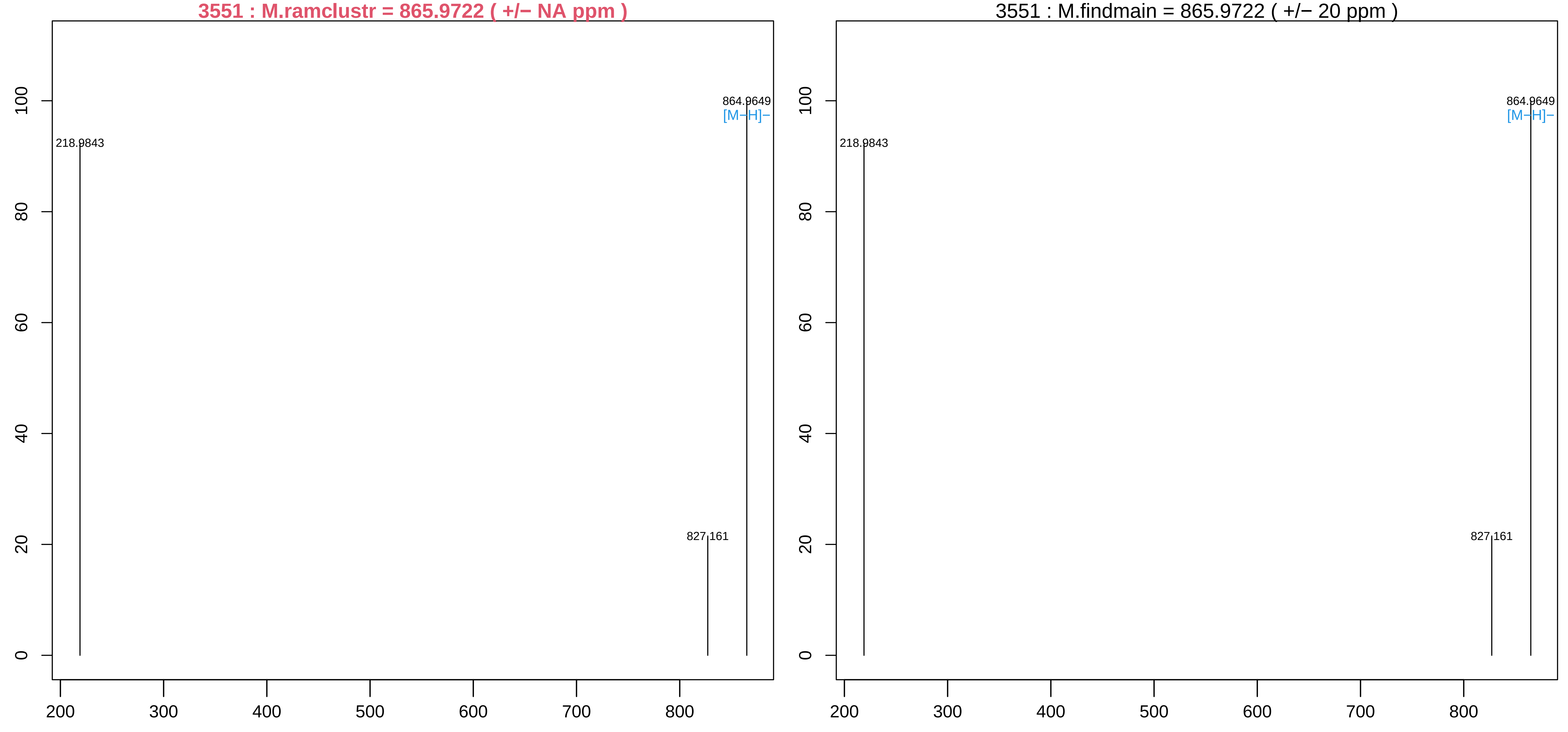
3551 : M.ramclustr = 865.9722 (+/− NA ppm)
864.9649
[M−H]−
218.9843
827.161
3551 : M.findmain = 865.9722 (+/− 20 ppm)
864.9649
[M−H]−
218.9843
827.161
0
20
40
60
80
100
200
300
400
500
600
700
800

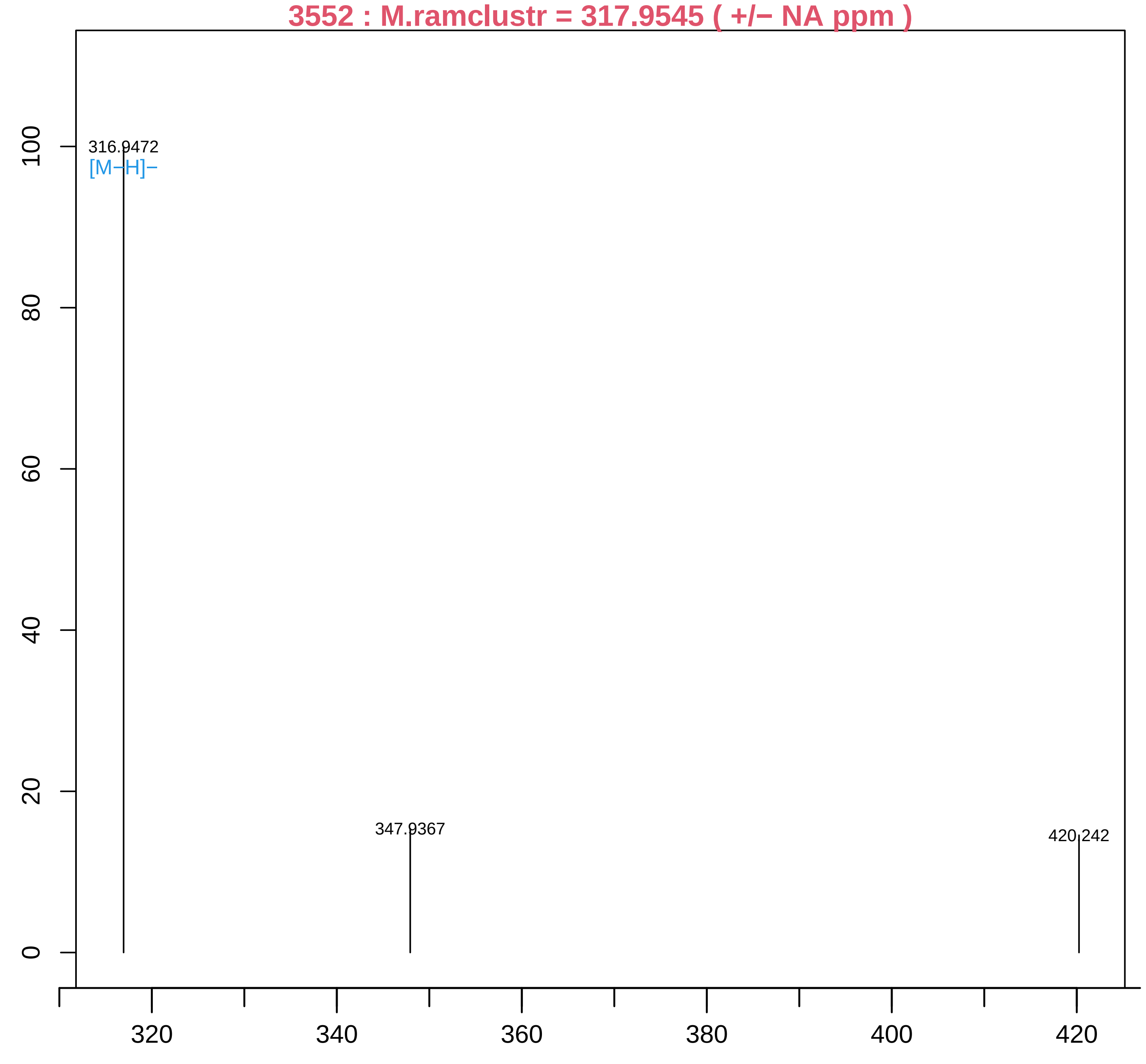
3552 : M.ramclustr = 317.9545 (+/− NA ppm)
316.9472
[M−H]−
347.9367
420.242
0
20
40
60
80
100
320
340
360
380
400
420

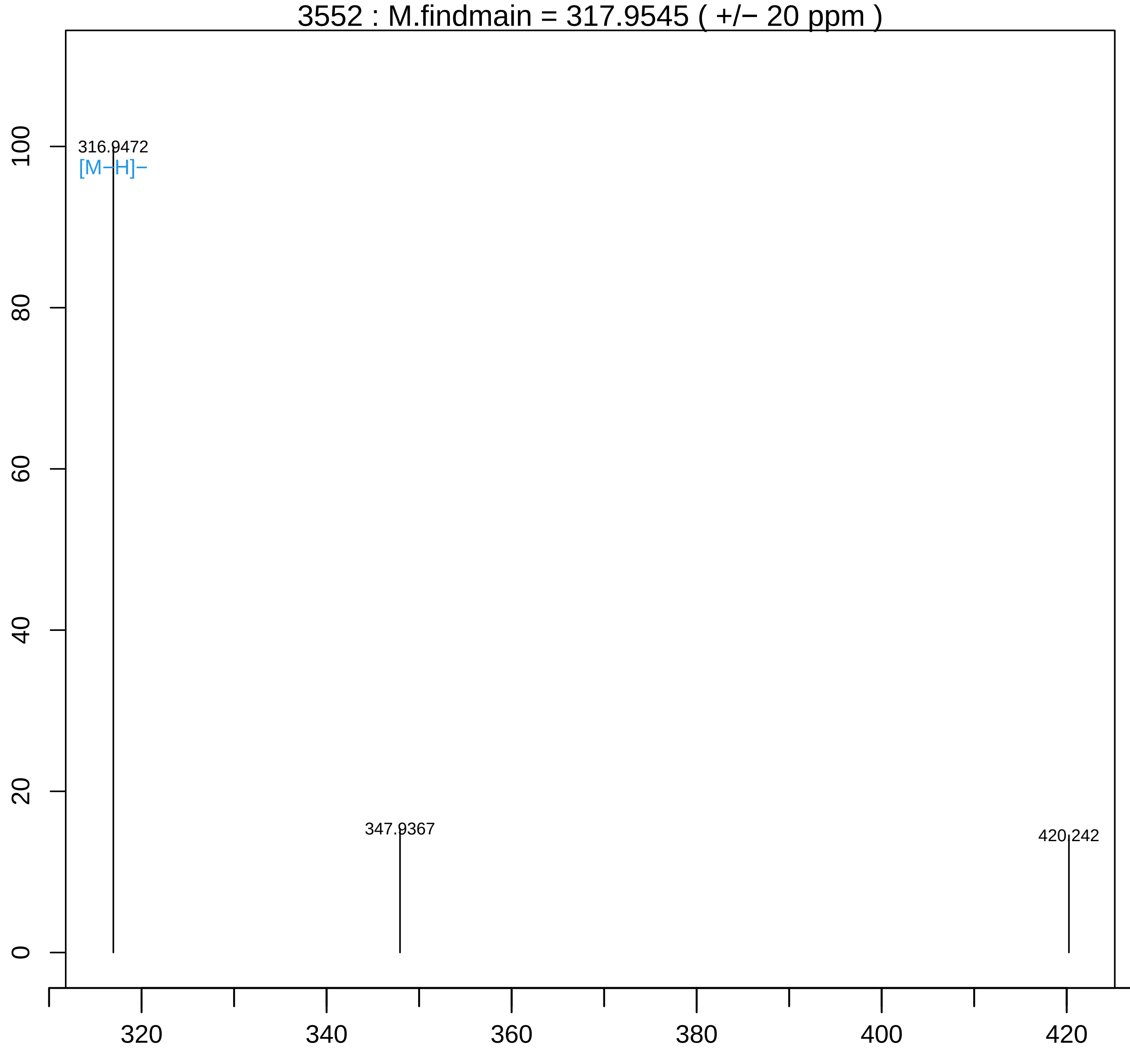
3552 : M.findmain = 317.9545 (+/− 20 ppm)
316.9472
[M−H]−
347.9367
420.242
0
20
40
60
80
100
320
340
360
380
400
420

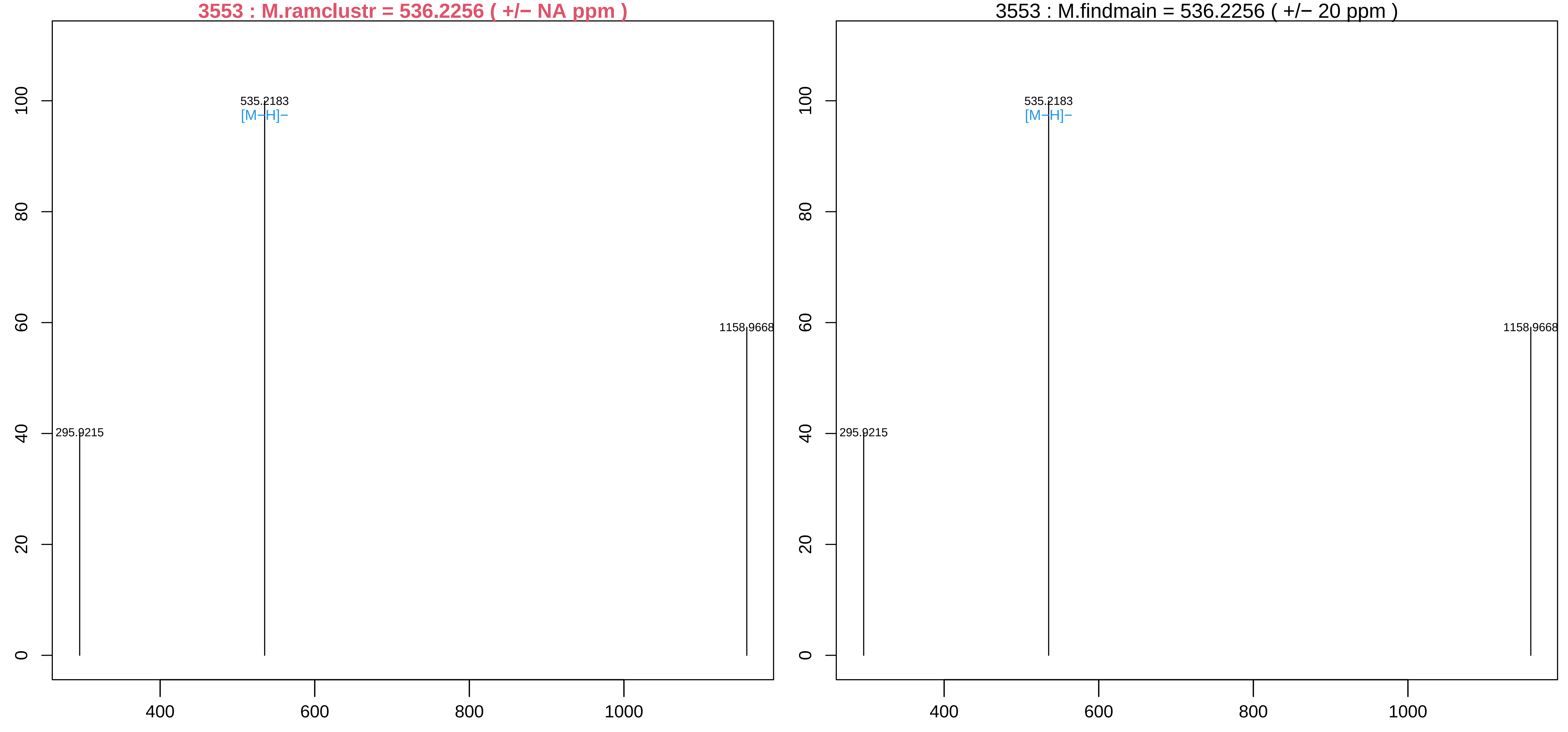
3553 : M.ramclustr = 536.2256 (+/− NA ppm)
3553 : M.findmain = 536.2256 (+/− 20 ppm)
535.2183
[M−H]−
295.9215
1158.9668
0
20
40
60
80
100
400
600
800
1000

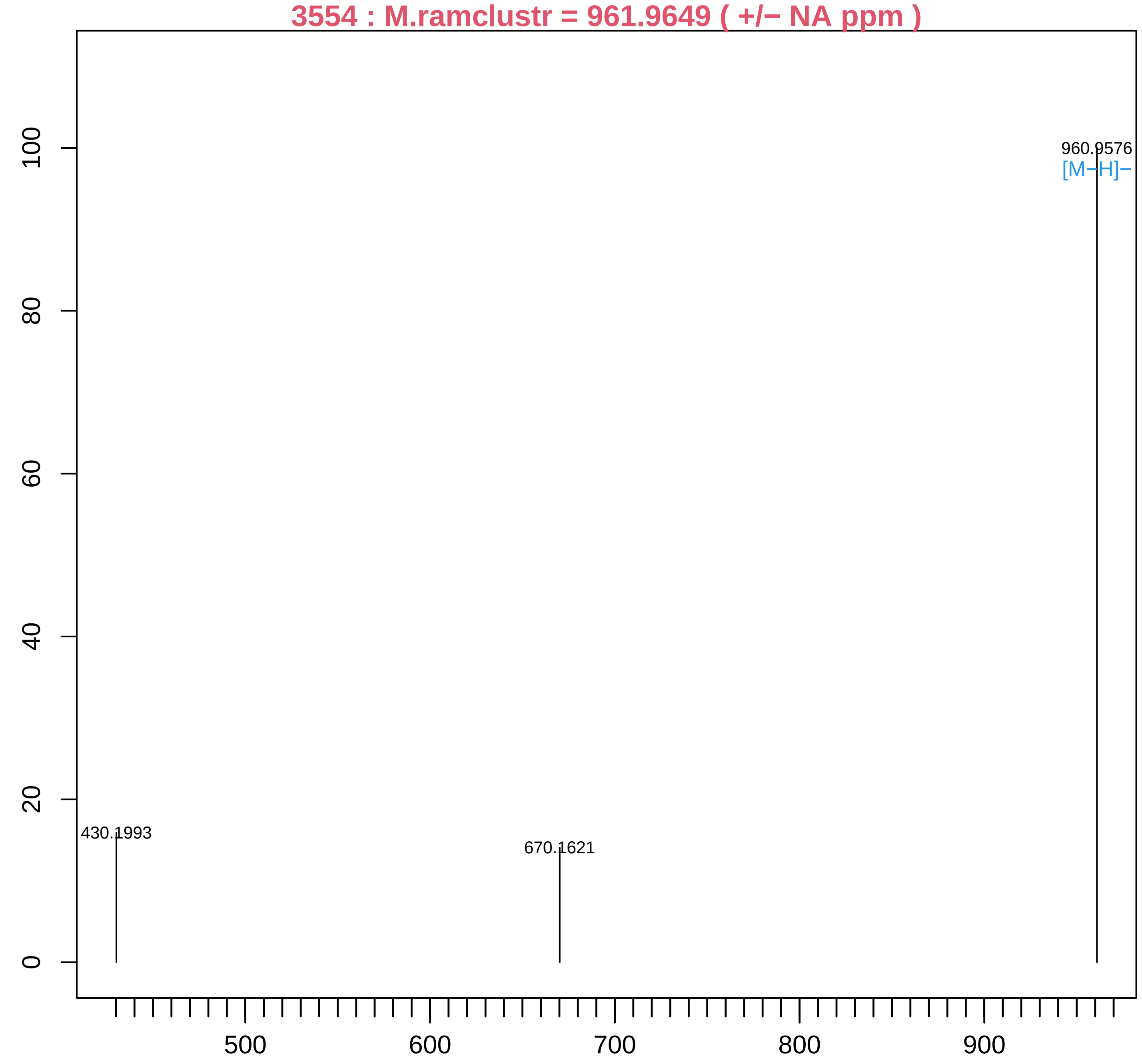
3554 : M.ramclustr = 961.9649 (+/− NA ppm)
960.9576
[M−H]−
430.1993
670.1621
0
20
40
60
80
100
500
600
700
800
900

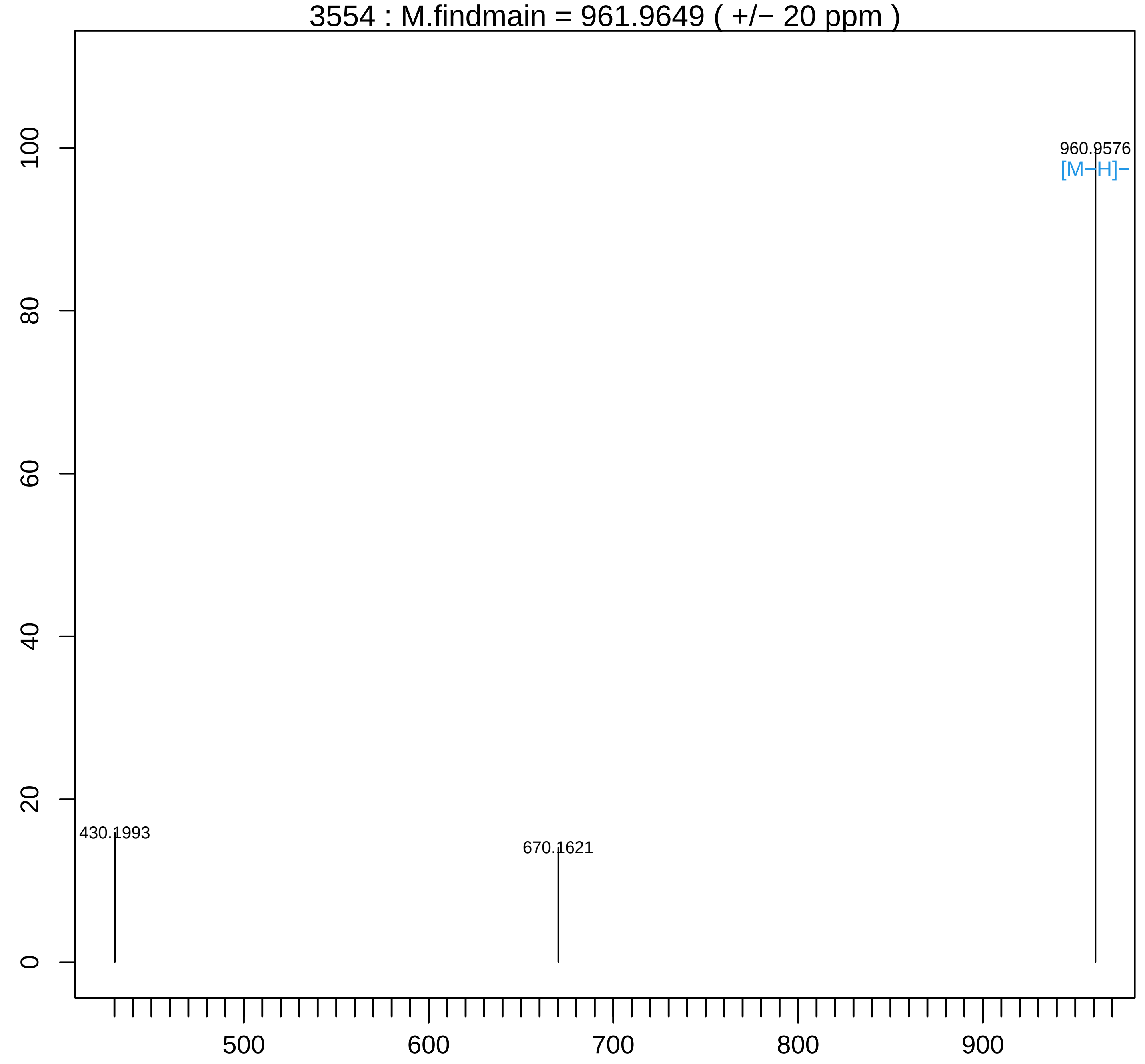
3554 : M.findmain = 961.9649 (+/− 20 ppm)
960.9576
[M−H]−
430.1993
670.1621
0
20
40
60
80
100
500
600
700
800
900

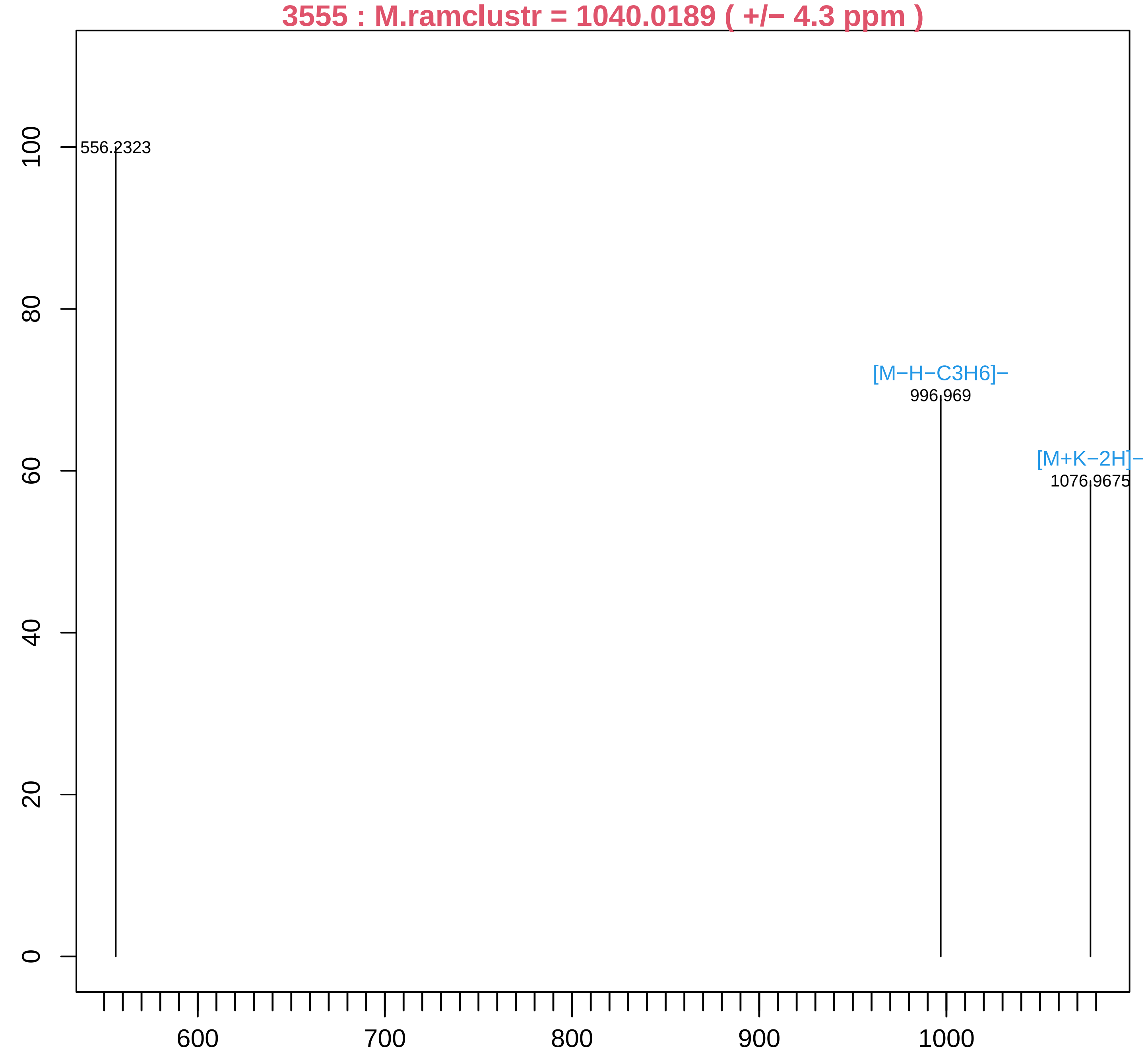
3555 : M.ramclustr = 1040.0189 (+/− 4.3 ppm)
556.2323
[M−H−C3H6]−
996.969
[M+K−2H]−
1076.9675
0
20
40
60
80
100
600
700
800
900
1000

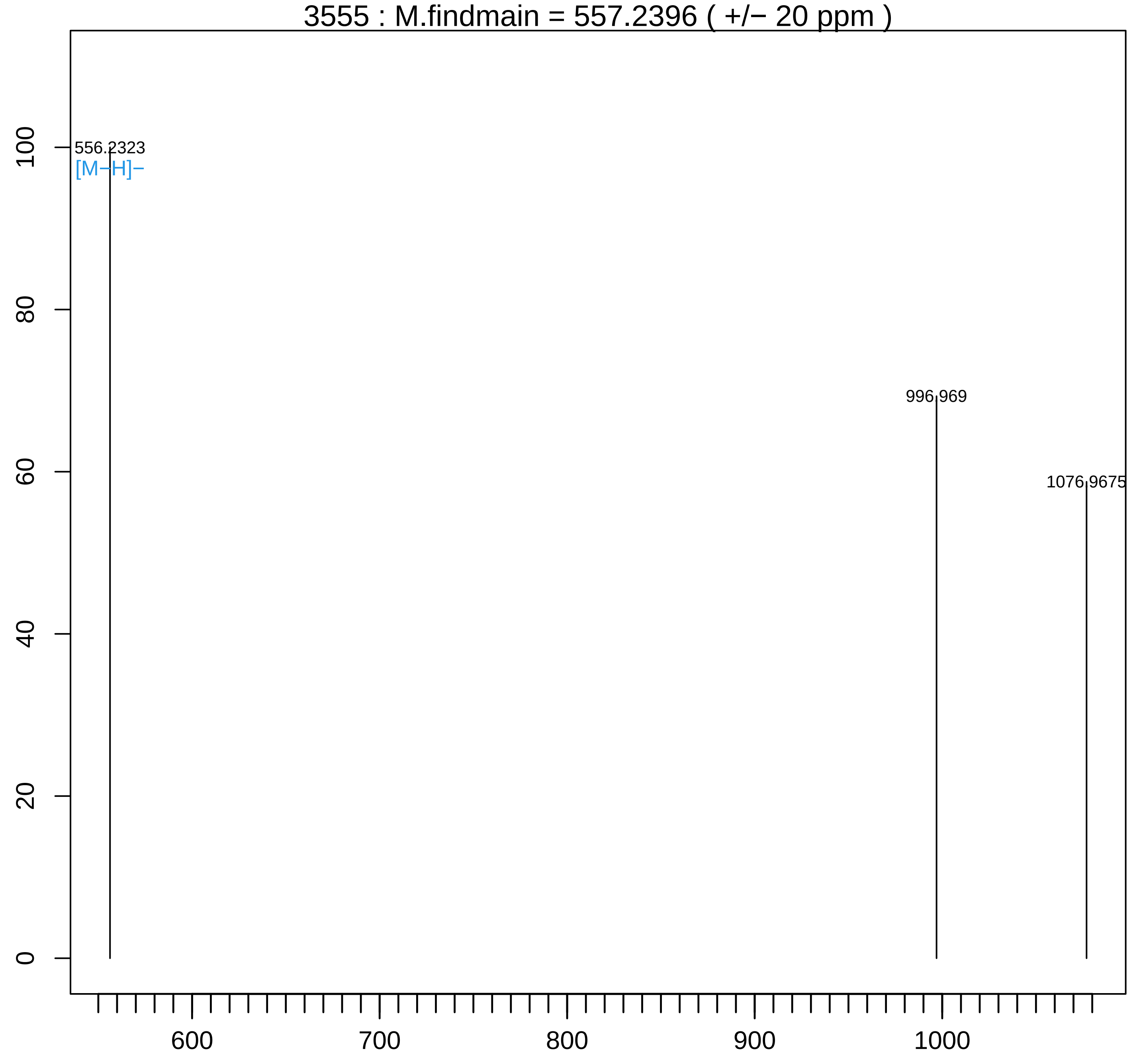
3555 : M.findmain = 557.2396 (+/− 20 ppm)
556.2323
[M−H]−
996.969
1076.9675
0
20
40
60
80
100
600
700
800
900
1000

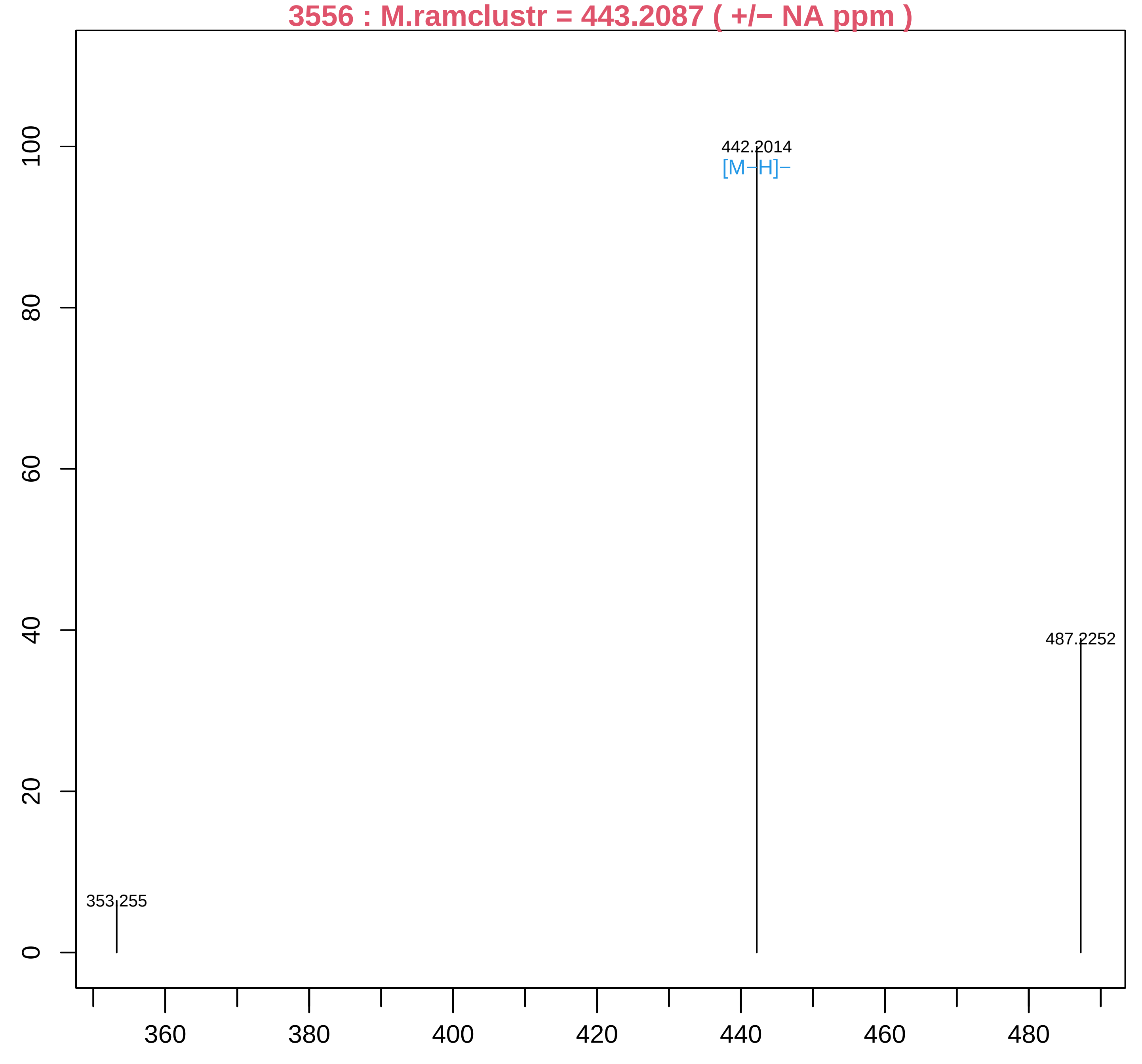
3556 : M.ramclustr = 443.2087 (+/− NA ppm)
442.2014
[M−H]−
487.2252
353.255
0
20
40
60
80
100
360
380
400
420
440
460
480

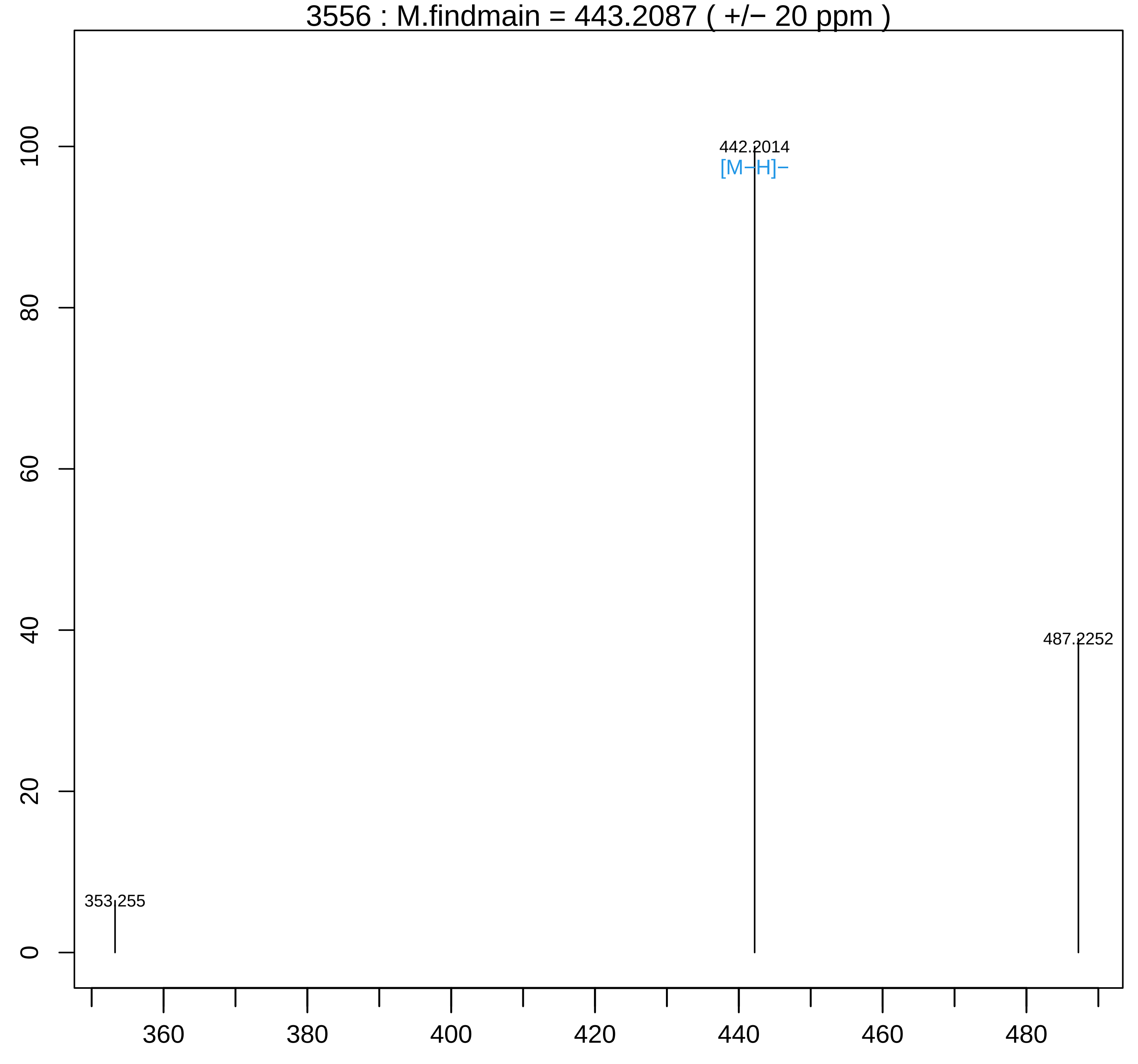
3556 : M.findmain = 443.2087 (+/− 20 ppm)
442.2014
[M−H]−
487.2252
353.255
0
20
40
60
80
100
360
380
400
420
440
460
480

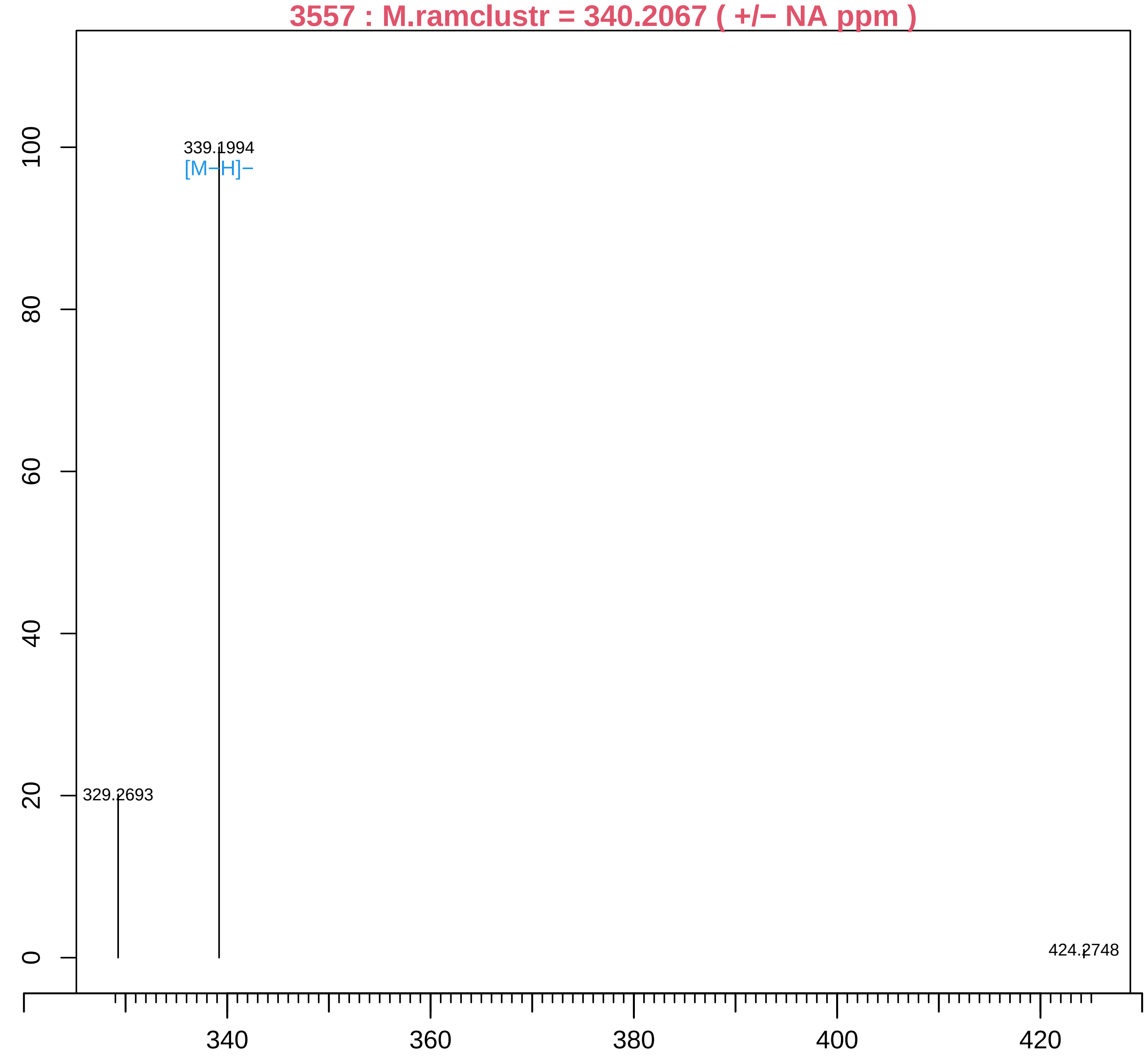
3557 : M.ramclustr = 340.2067 (+/− NA ppm)
339.1994
[M−H]−
329.2693
424.2748
0
20
40
60
80
100
340
360
380
400
420

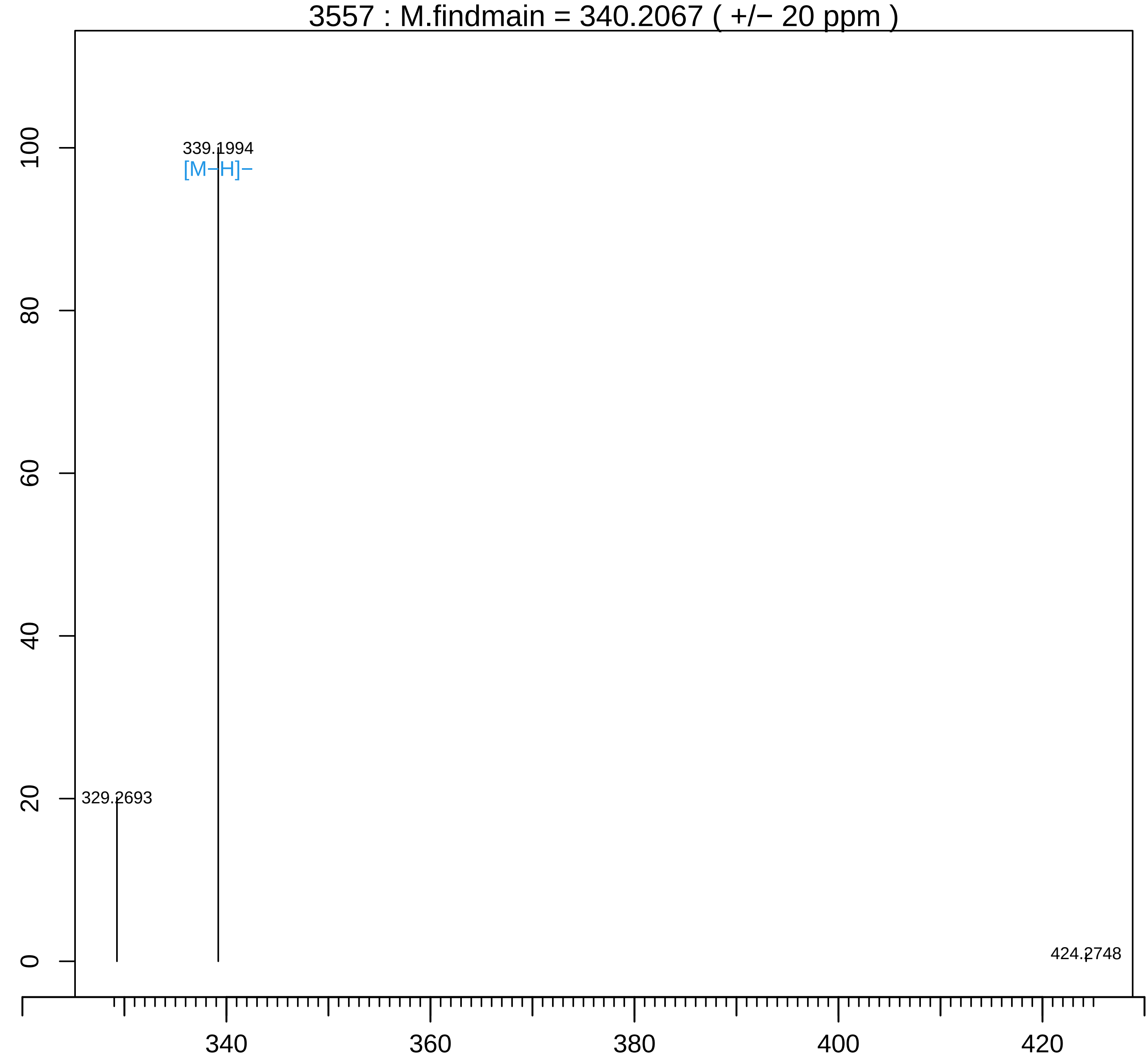
3557 : M.findmain = 340.2067 (+/− 20 ppm)
339.1994
[M−H]−
329.2693
424.2748
0
20
40
60
80
100
340
360
380
400
420

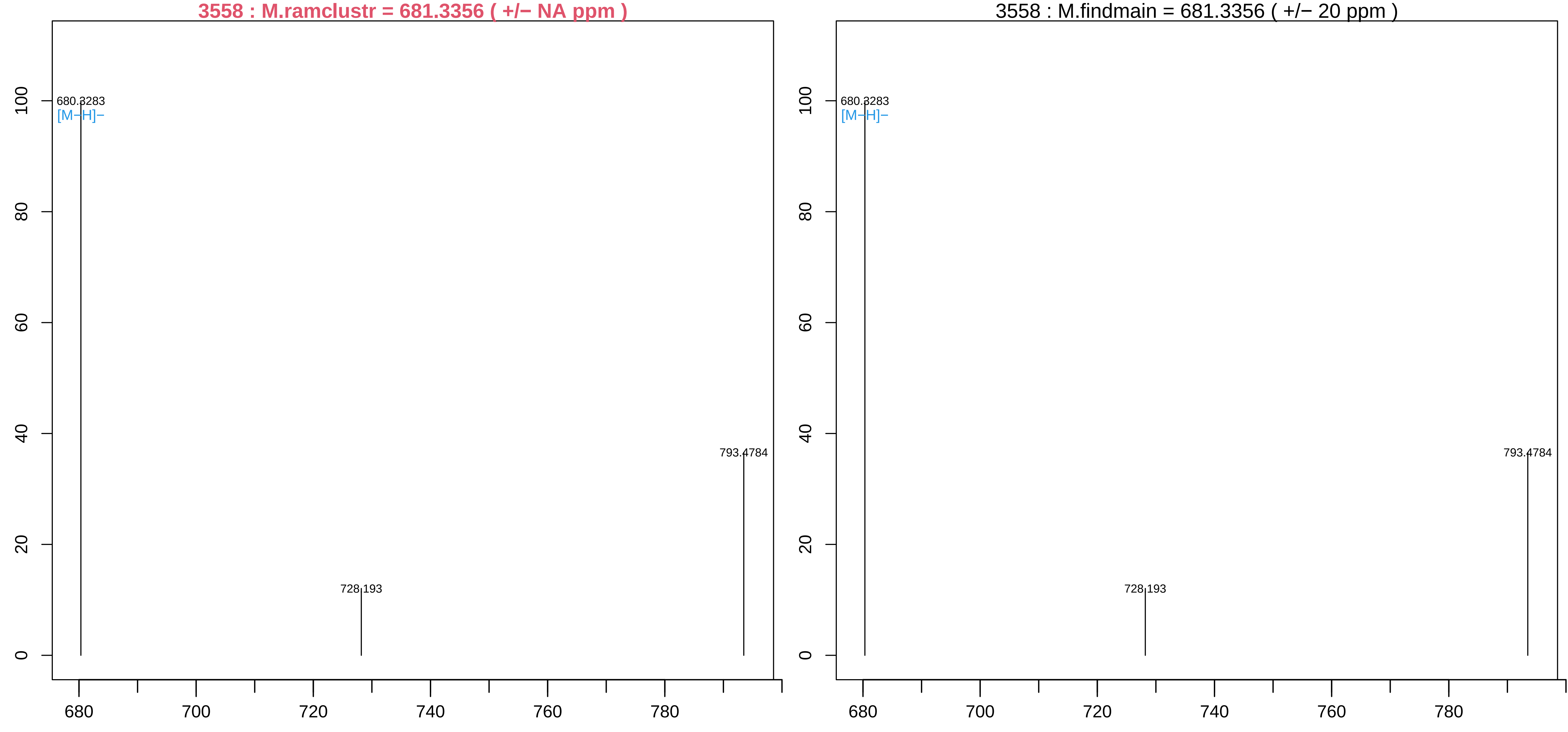

3558 : M.ramclustr = 681.3356 (+/− NA ppm)
680.3283
[M−H]−
728.193
793.4784
3558 : M.findmain = 681.3356 (+/− 20 ppm)
680.3283
[M−H]−
728.193
793.4784
0
20
40
60
80
100
680
700
720
740
760
780

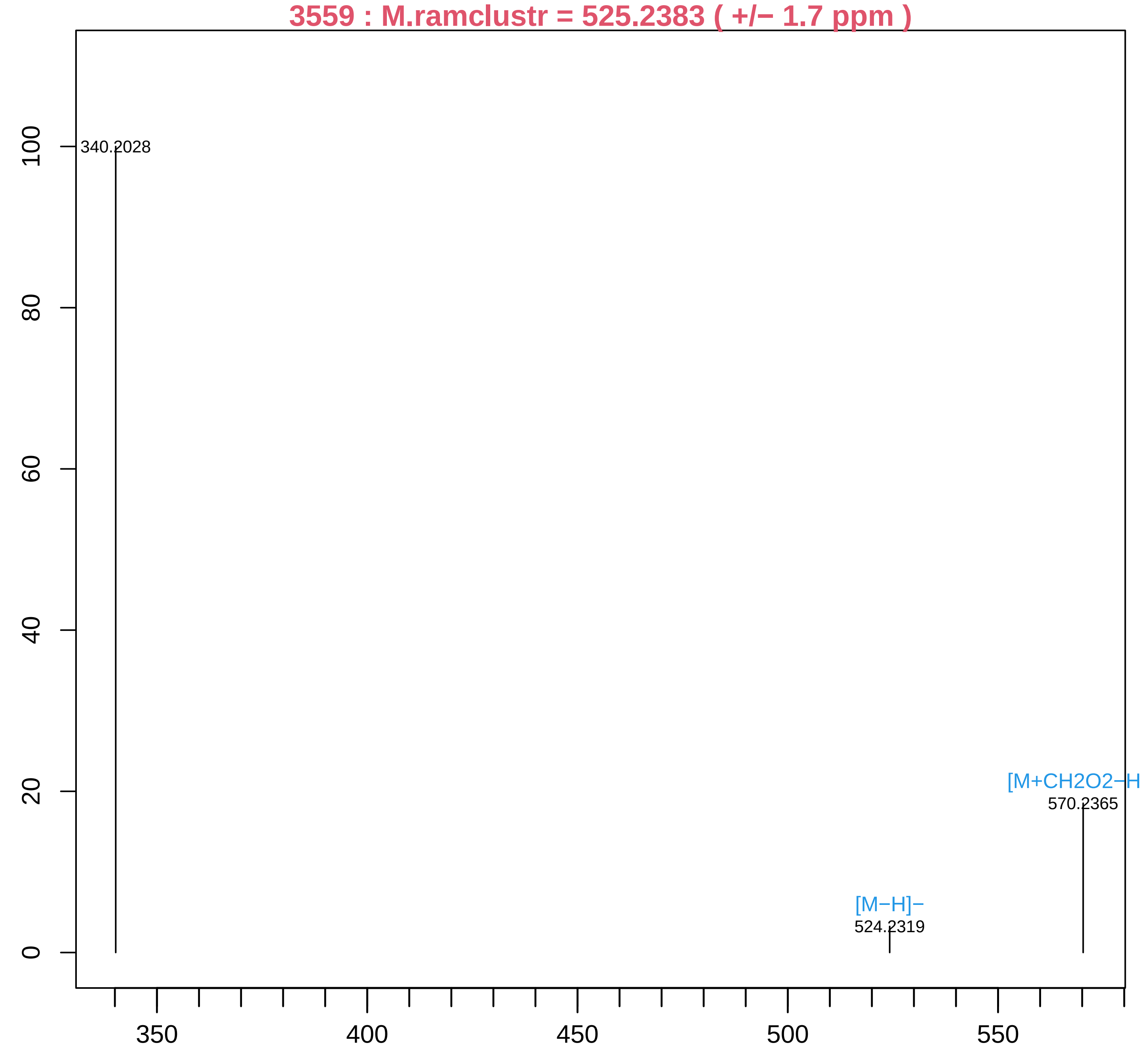
3559 : M.ramclustr = 525.2383 (+/− 1.7 ppm)
340.2028
[M−H]−
524.2319
[M+CH2O2−H
570.2365
0
20
40
60
80
100
350
400
450
500
550

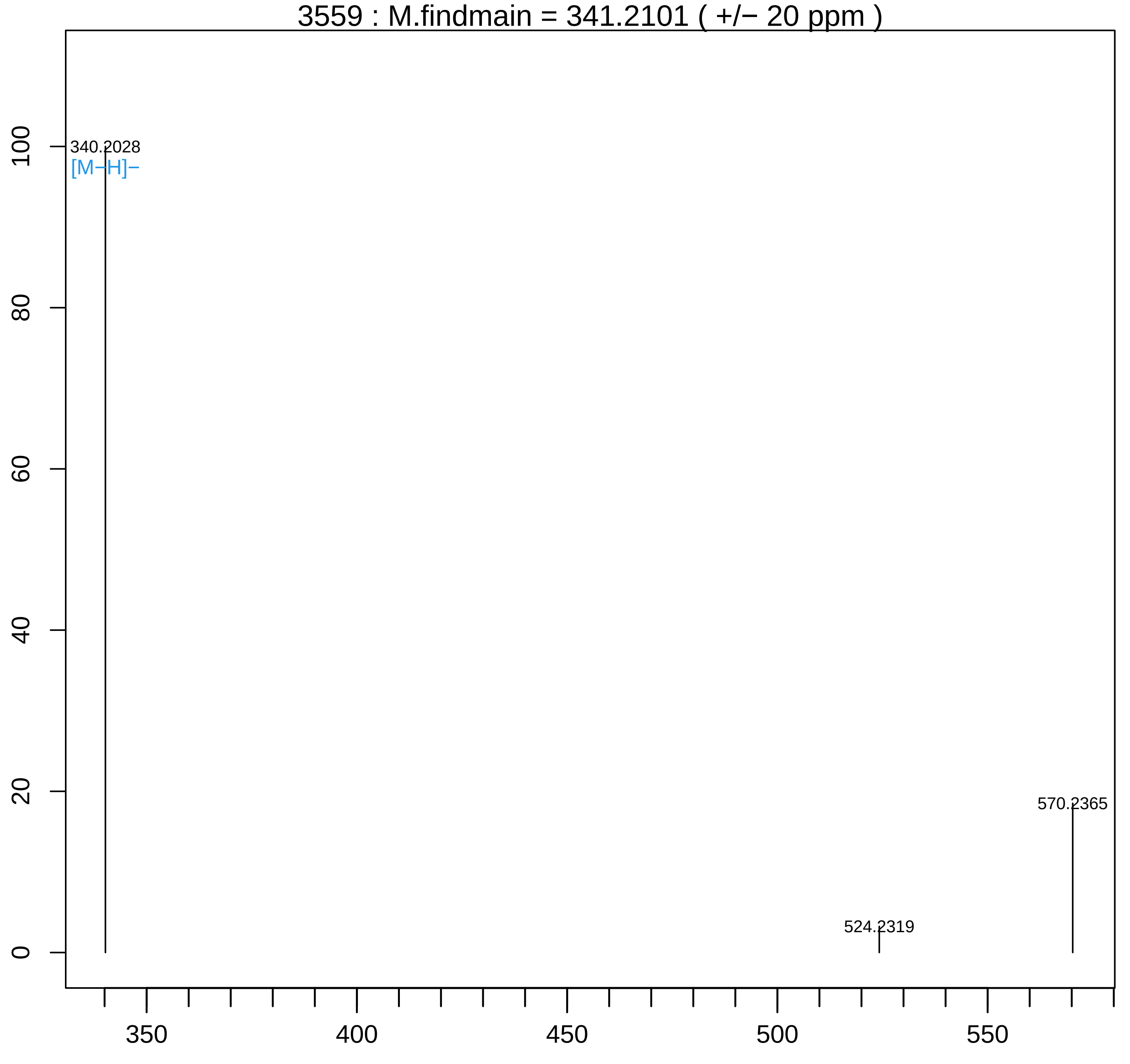
3559 : M.findmain = 341.2101 (+/− 20 ppm)
340.2028
[M−H]−
524.2319
570.2365
0
20
40
60
80
100
350
400
450
500
550

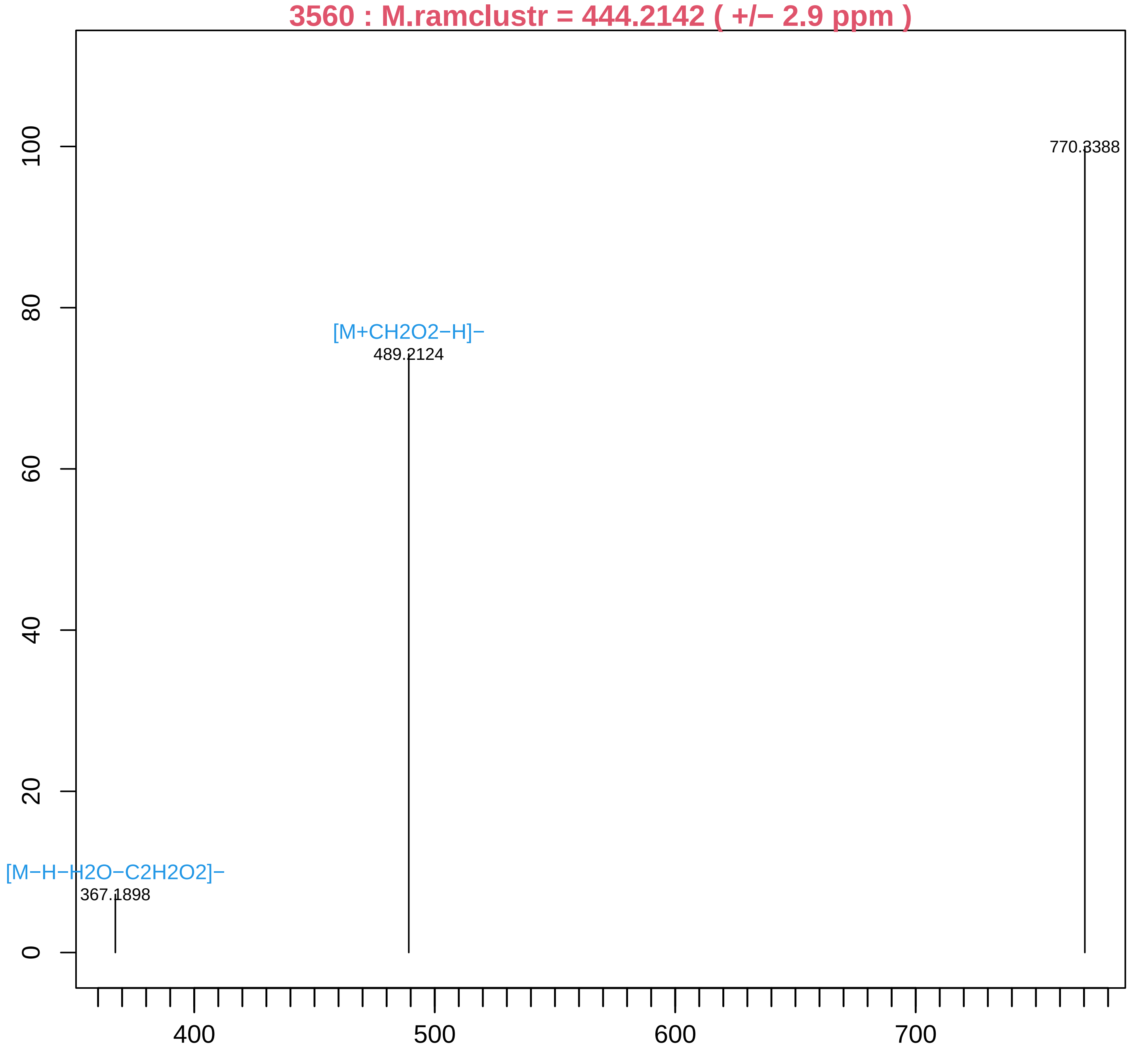
3560 : M.ramclustr = 444.2142 (+/− 2.9 ppm)
770.3388
[M+CH2O2−H]−
489.2124
[M−H−H2O−C2H2O2]−
367.1898
0
20
40
60
80
100
400
500
600
700

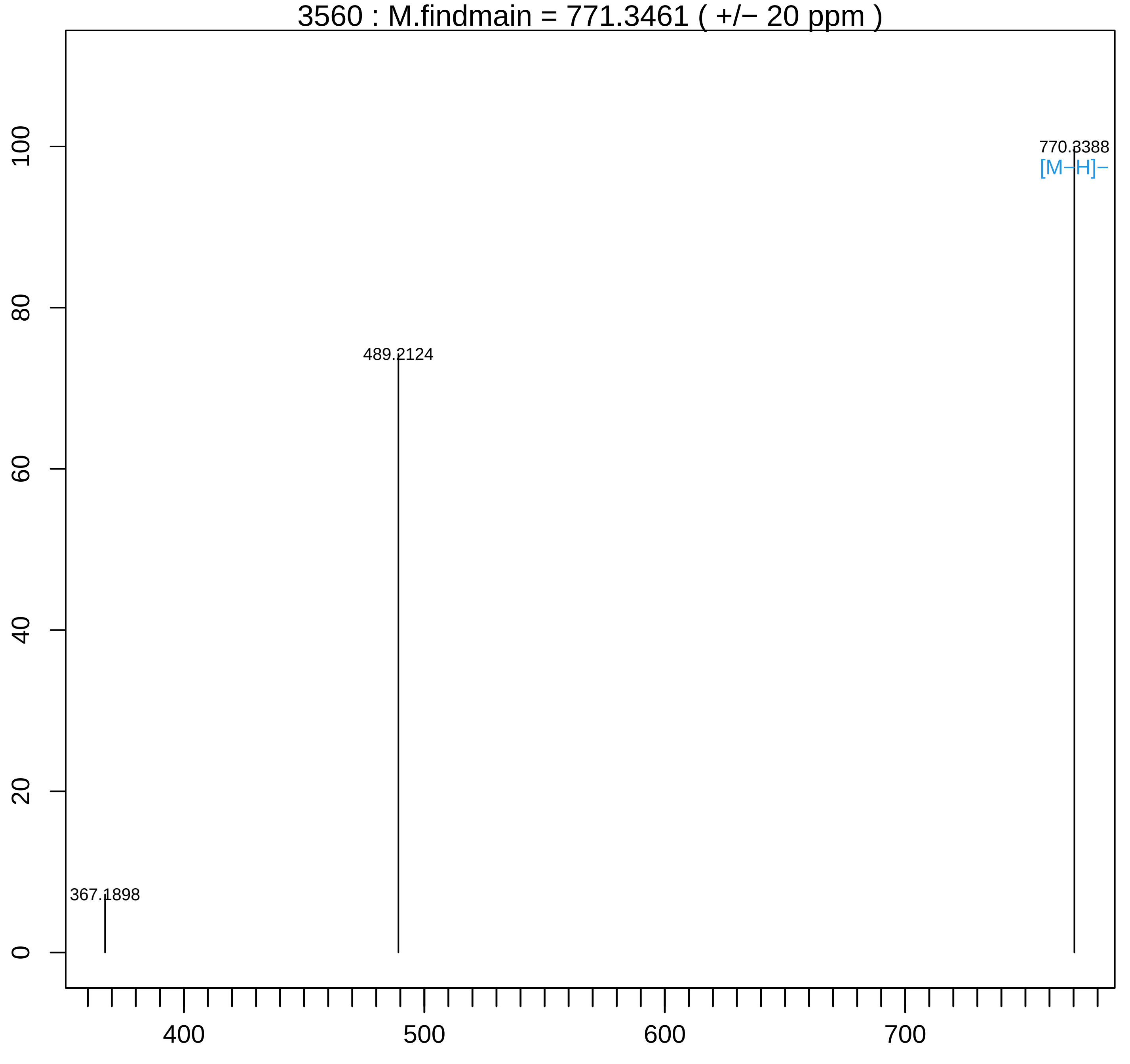
3560 : M.findmain = 771.3461 (+/− 20 ppm)
770.3388
[M−H]−
489.2124
367.1898
0
20
40
60
80
100
400
500
600
700

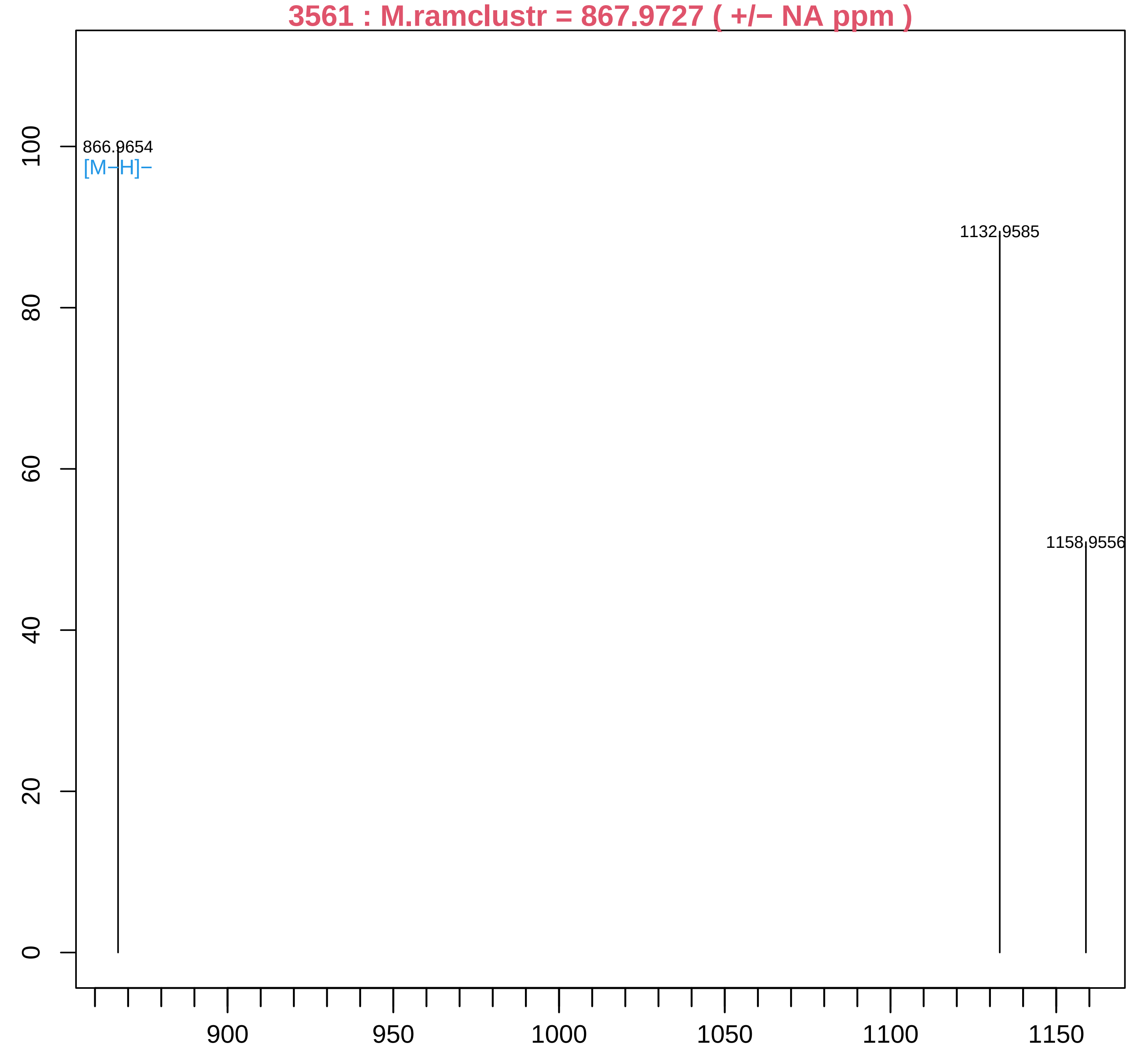

3561 : M.ramclustr = 867.9727 (+/− NA ppm)
866.9654
[M−H]−
1132.9585
1158.9556
0
20
40
60
80
100
900
950
1000
1050
1100
1150

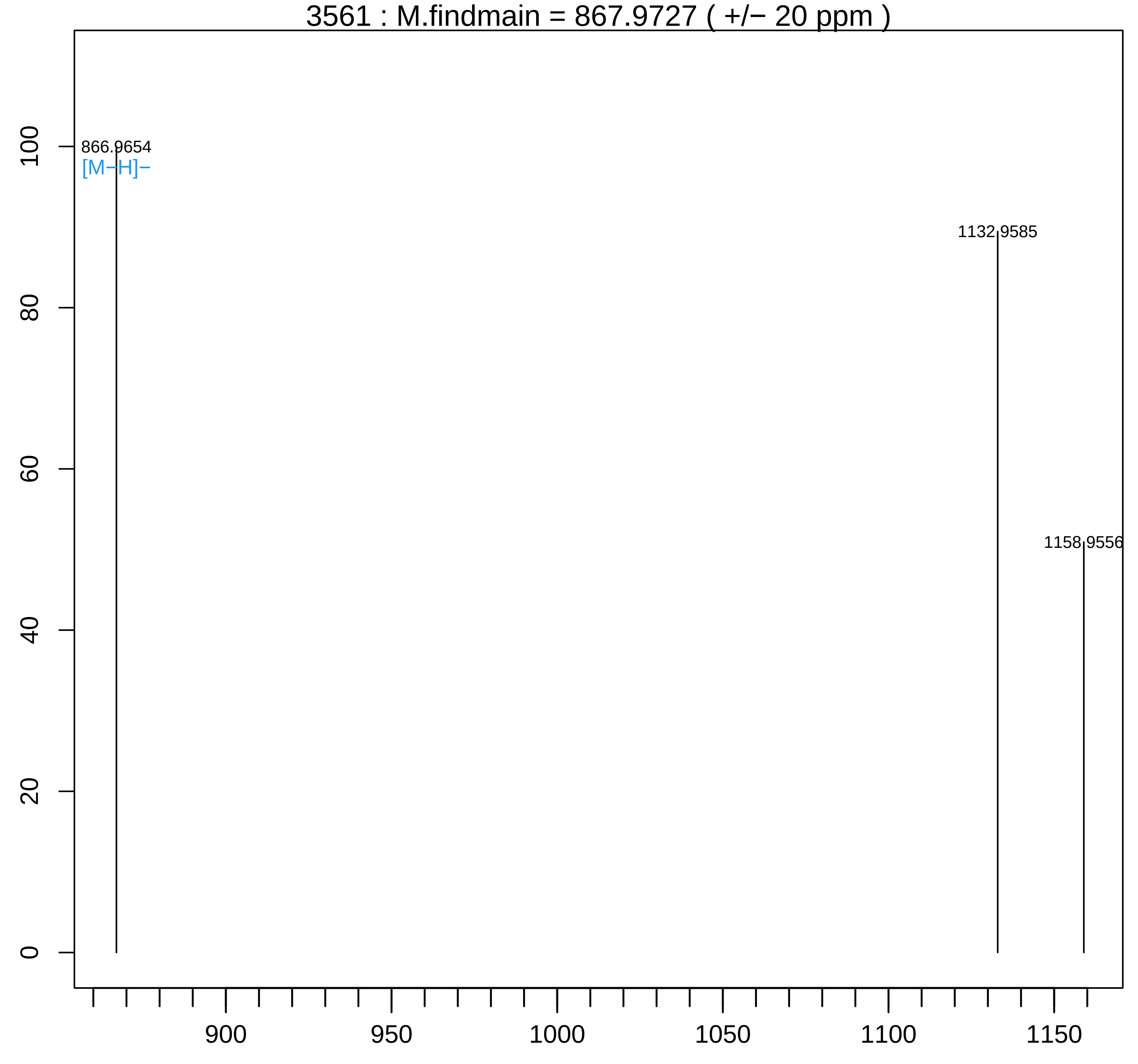

3561 : M.findmain = 867.9727 (+/− 20 ppm)
866.9654
[M−H]−
1132.9585
1158.9556
0
20
40
60
80
100
900
950
1000
1050
1100
1150

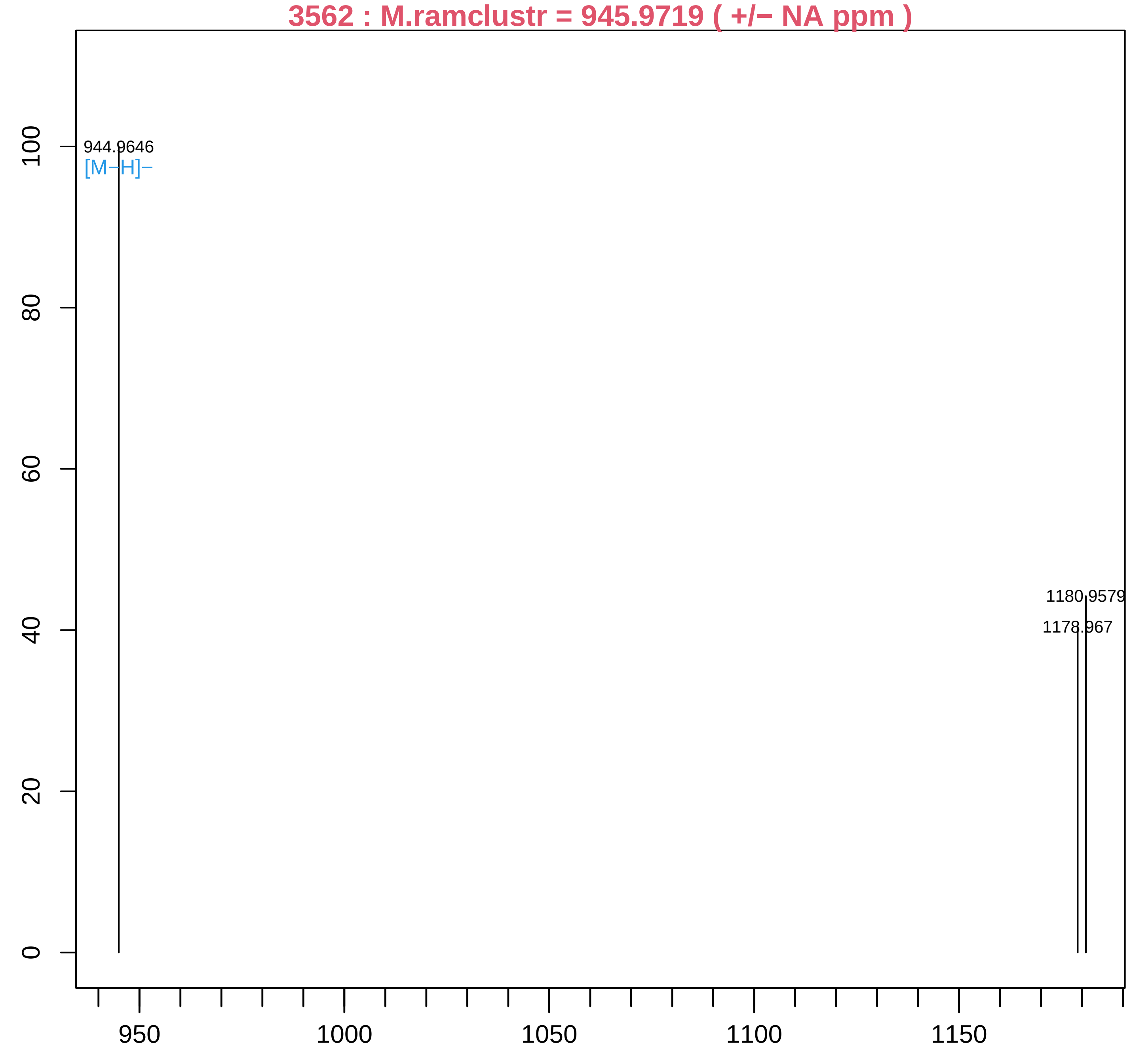
3562 : M.ramclustr = 945.9719 (+/− NA ppm)
944.9646
[M−H]−
1180.9579
1178.967
0
20
40
60
80
100
950
1000
1050
1100
1150

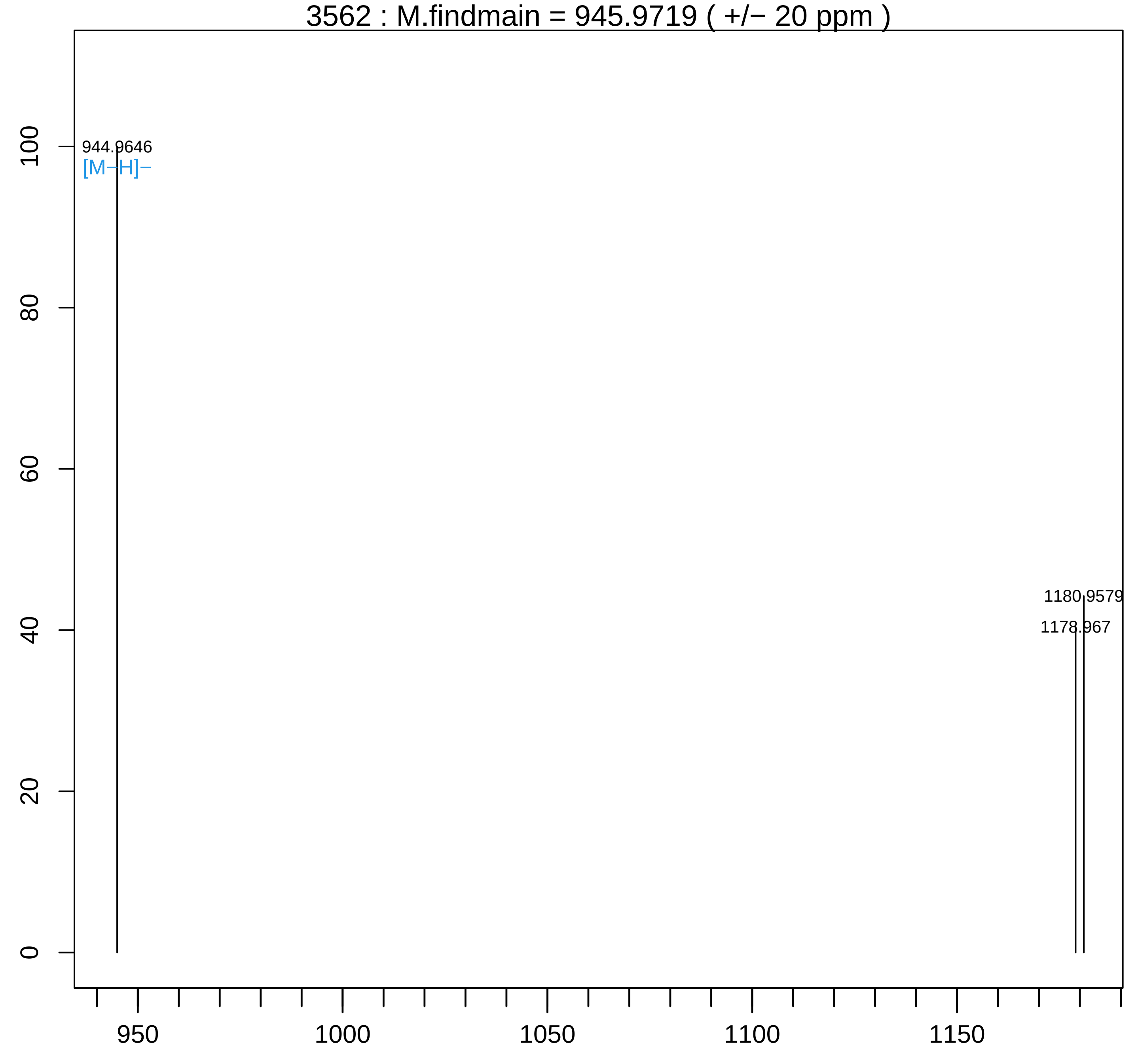
3562 : M.findmain = 945.9719 (+/− 20 ppm)
944.9646
[M−H]−
1180.9579
1178.967
0
20
40
60
80
100
950
1000
1050
1100
1150

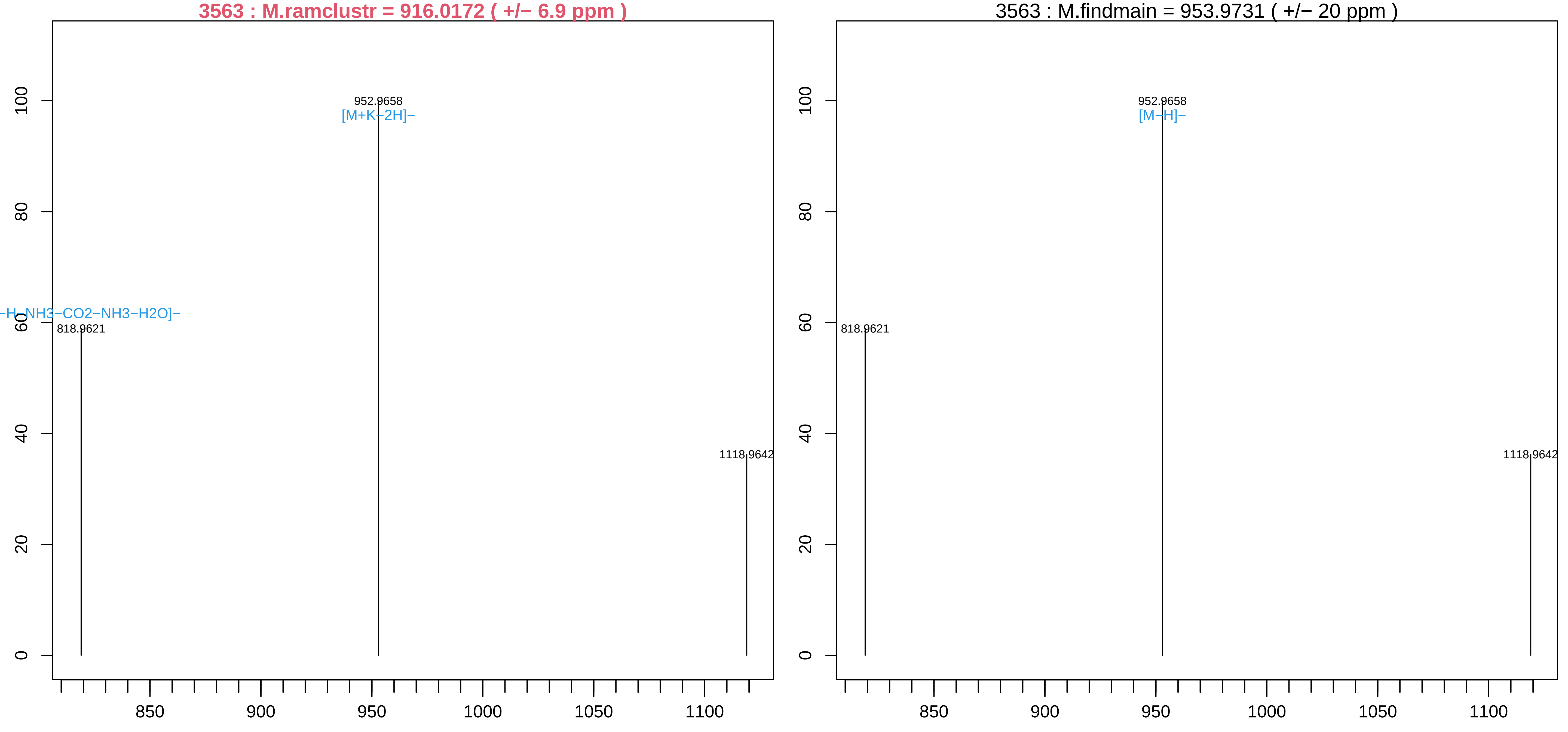
3563 : M.ramclustr = 916.0172 (+/− 6.9 ppm)
952.9658
[M+K−2H]−
−H−NH3−CO2−NH3−H2O]−
818.9621
1118.9642
3563 : M.findmain = 953.9731 (+/− 20 ppm)
952.9658
[M−H]−
818.9621
1118.9642

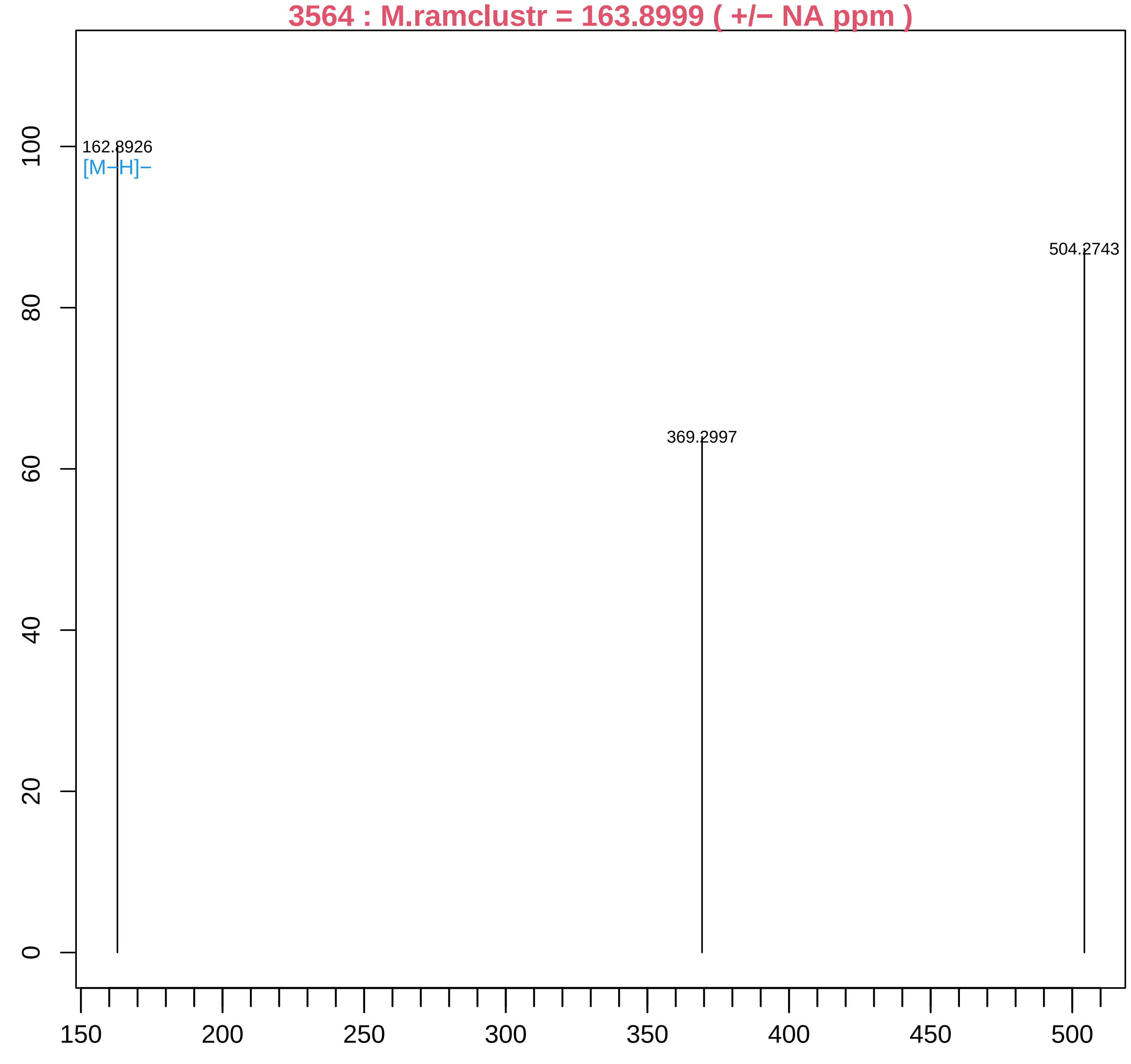
3564 : M.ramclustr = 163.8999 (+/− NA ppm)
162.8926
[M−H]−
369.2997
504.2743
0
20
40
60
80
100
150
200
250
300
350
400
450
500

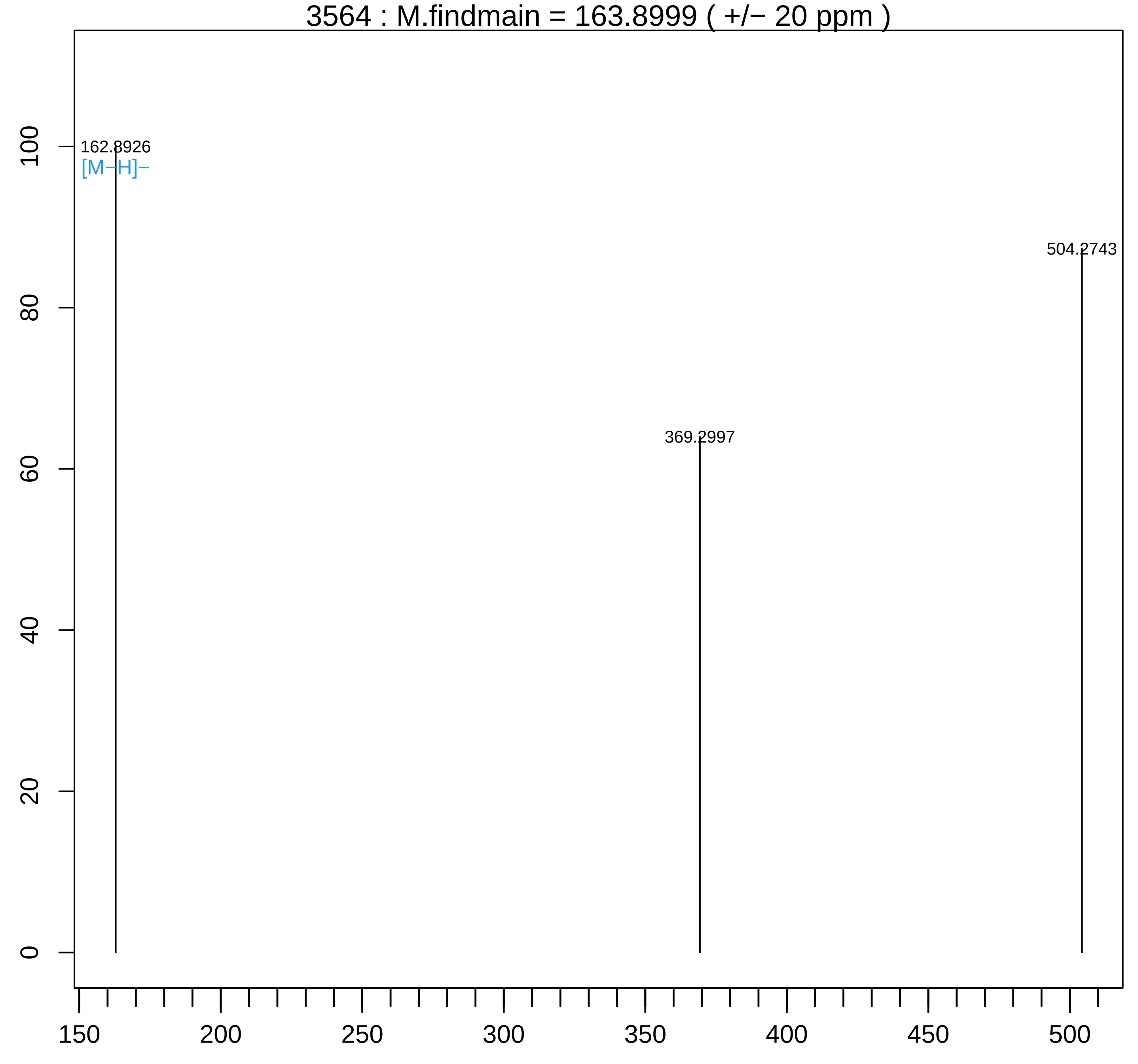
3564 : M.findmain = 163.8999 (+/− 20 ppm)
162.8926
[M−H]−
369.2997
504.2743
0
20
40
60
80
100
150
200
250
300
350
400
450
500

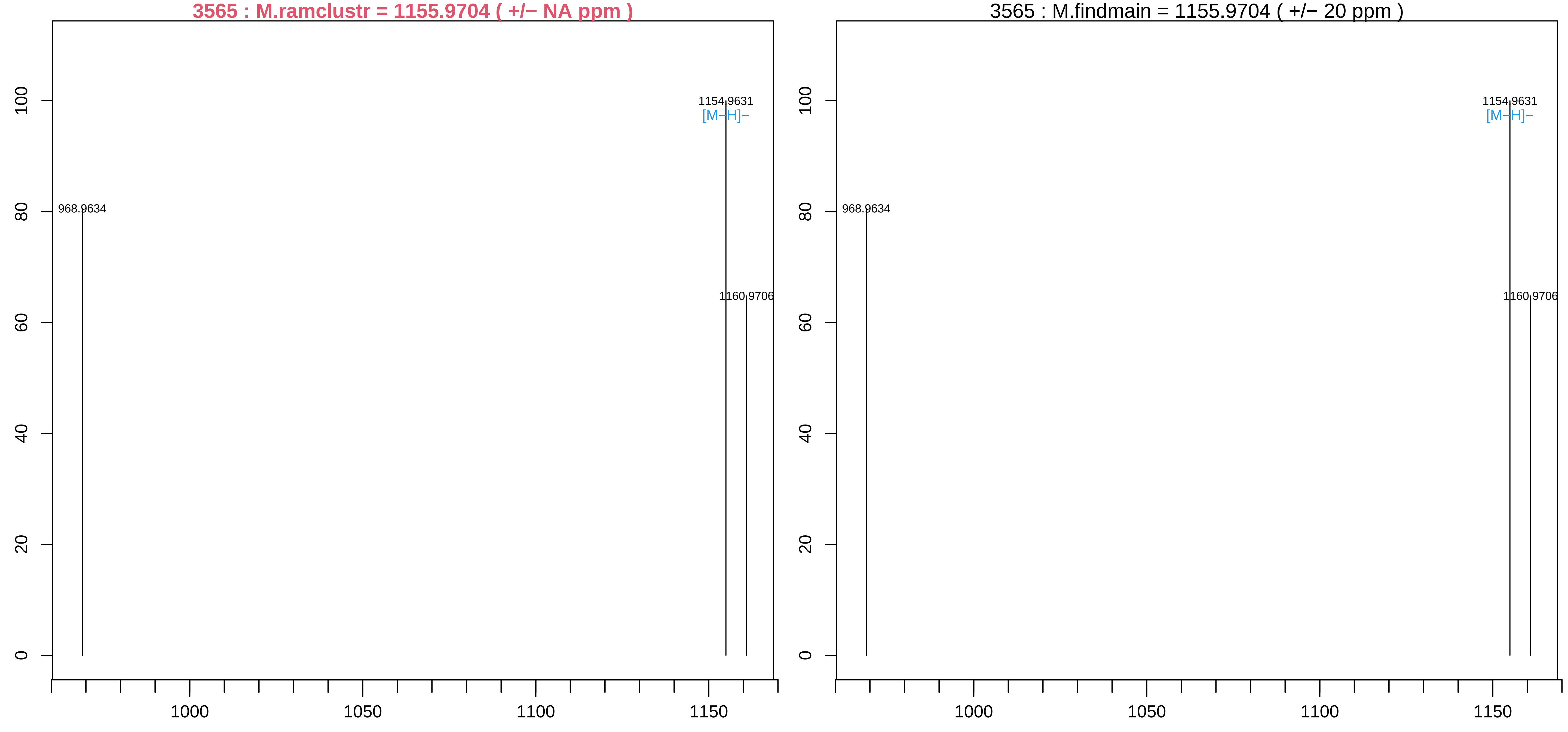

3565 : M.ramclustr = 1155.9704 (+/− NA ppm)
968.9634
1154.9631
[M−H]−
1160.9706
3565 : M.findmain = 1155.9704 (+/− 20 ppm)
968.9634
1154.9631
[M−H]−
1160.9706
0
20
40
60
80
100
1000
1050
1100
1150

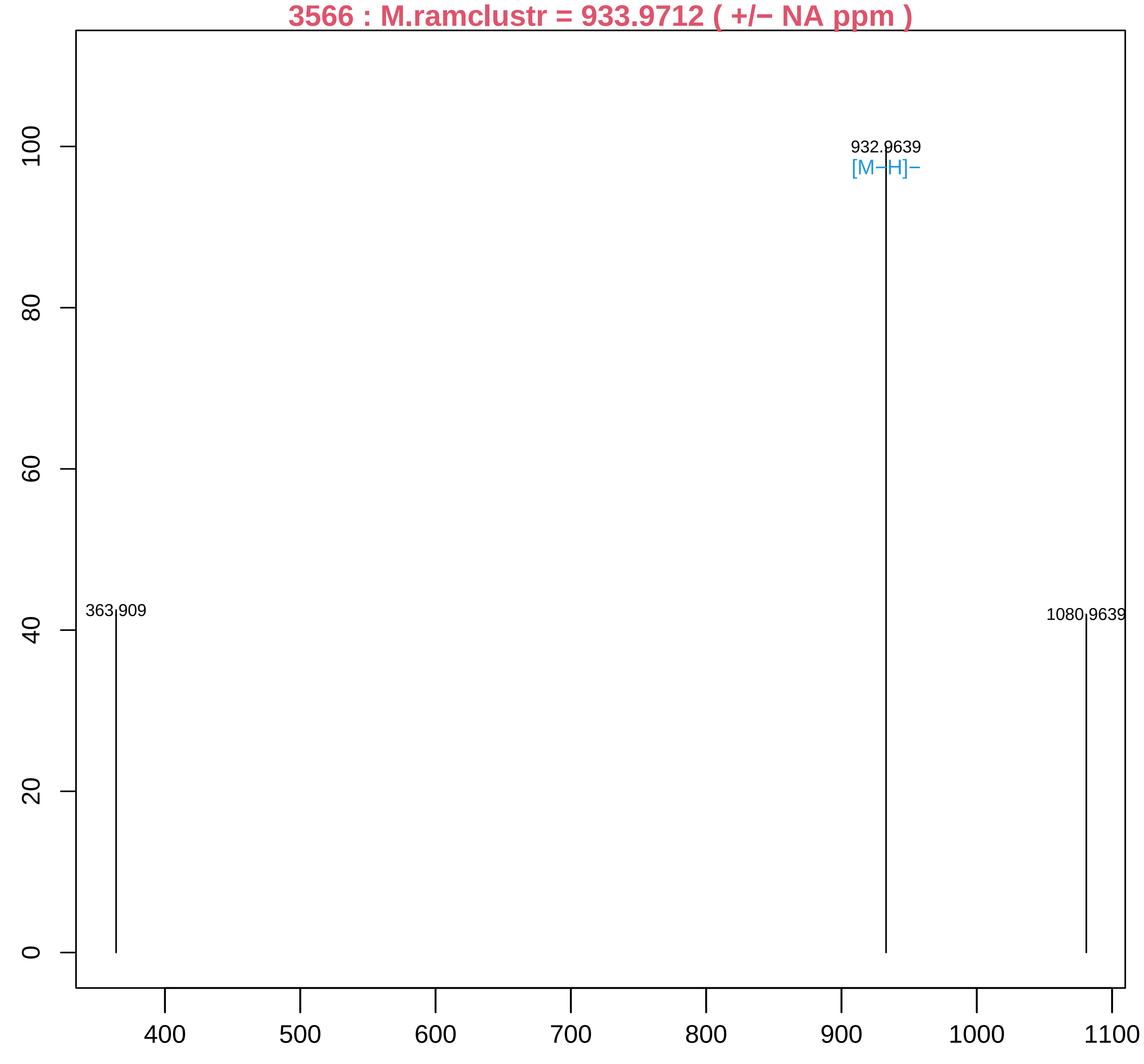

3566 : M.ramclustr = 933.9712 (+/− NA ppm)
932.9639
[M−H]−
363.909
1080.9639
100
80
60
40
20
0
400
500
600
700
800
900
1000
1100

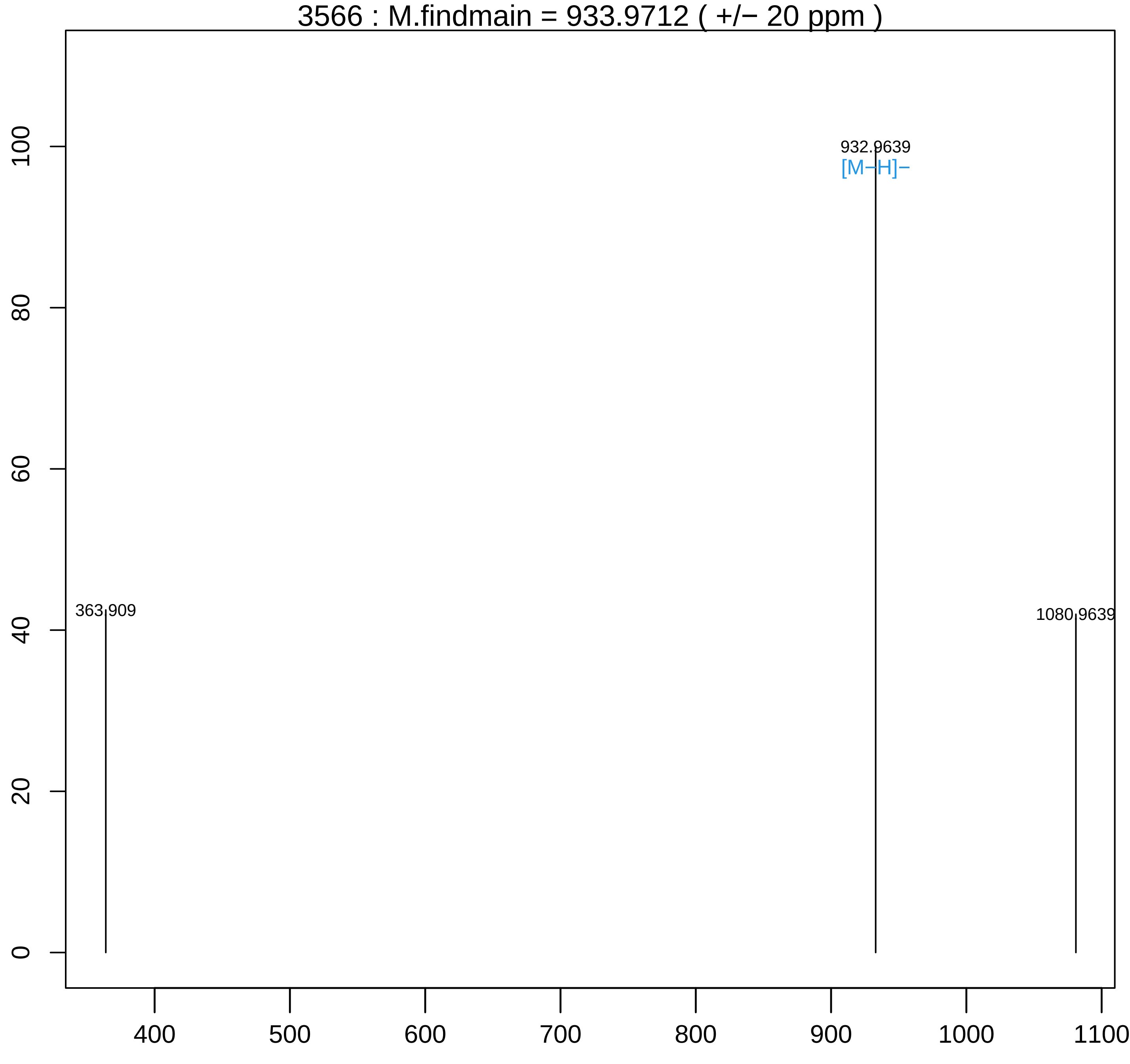

3566 : M.findmain = 933.9712 (+/− 20 ppm)
932.9639
[M−H]−
363.909
1080.9639
100
80
60
40
20
0
400
500
600
700
800
900
1000
1100

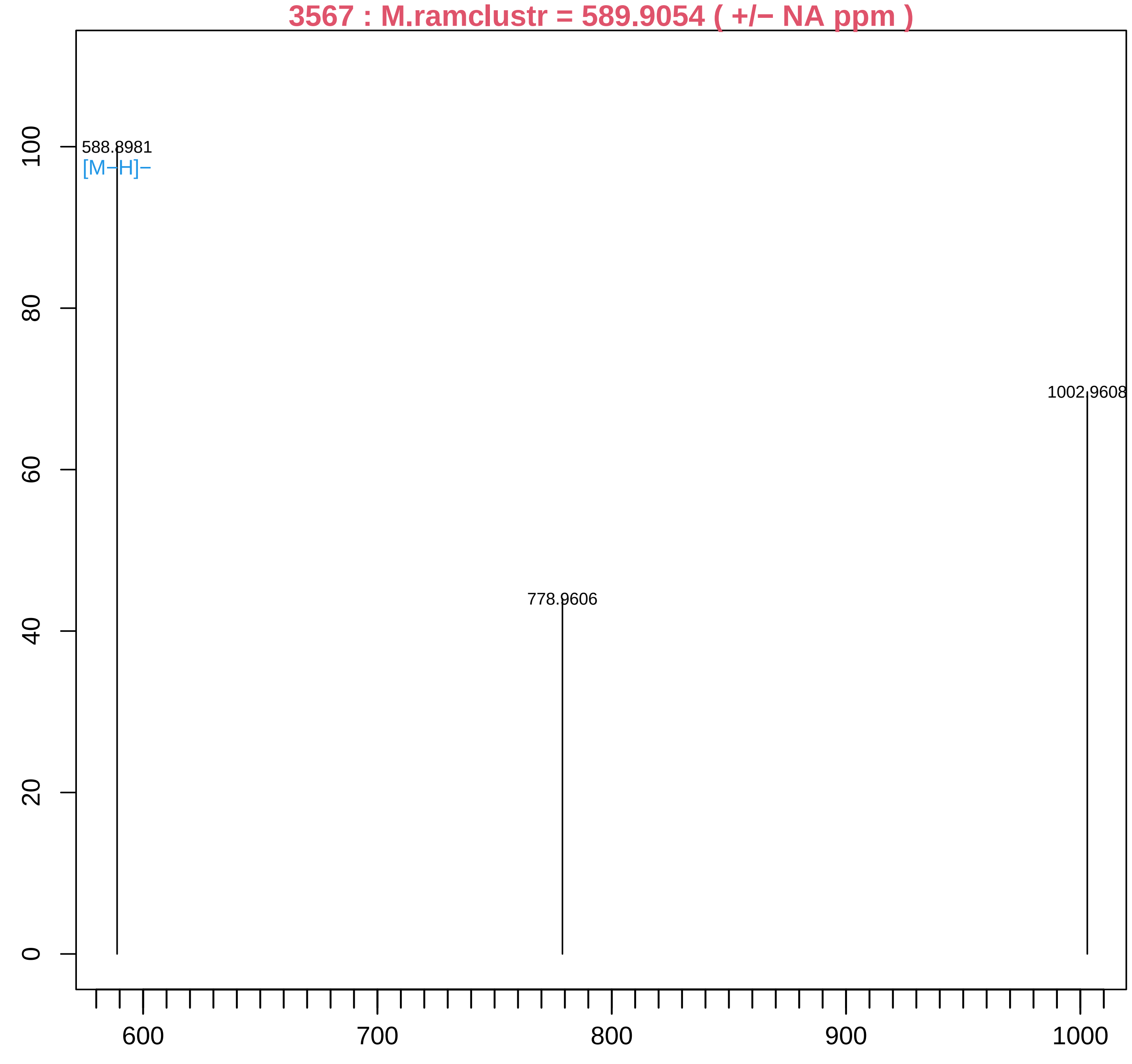
3567 : M.ramclustr = 589.9054 (+/− NA ppm)
588.8981
[M−H]−
778.9606
1002.9608
0
20
40
60
80
100
600
700
800
900
1000

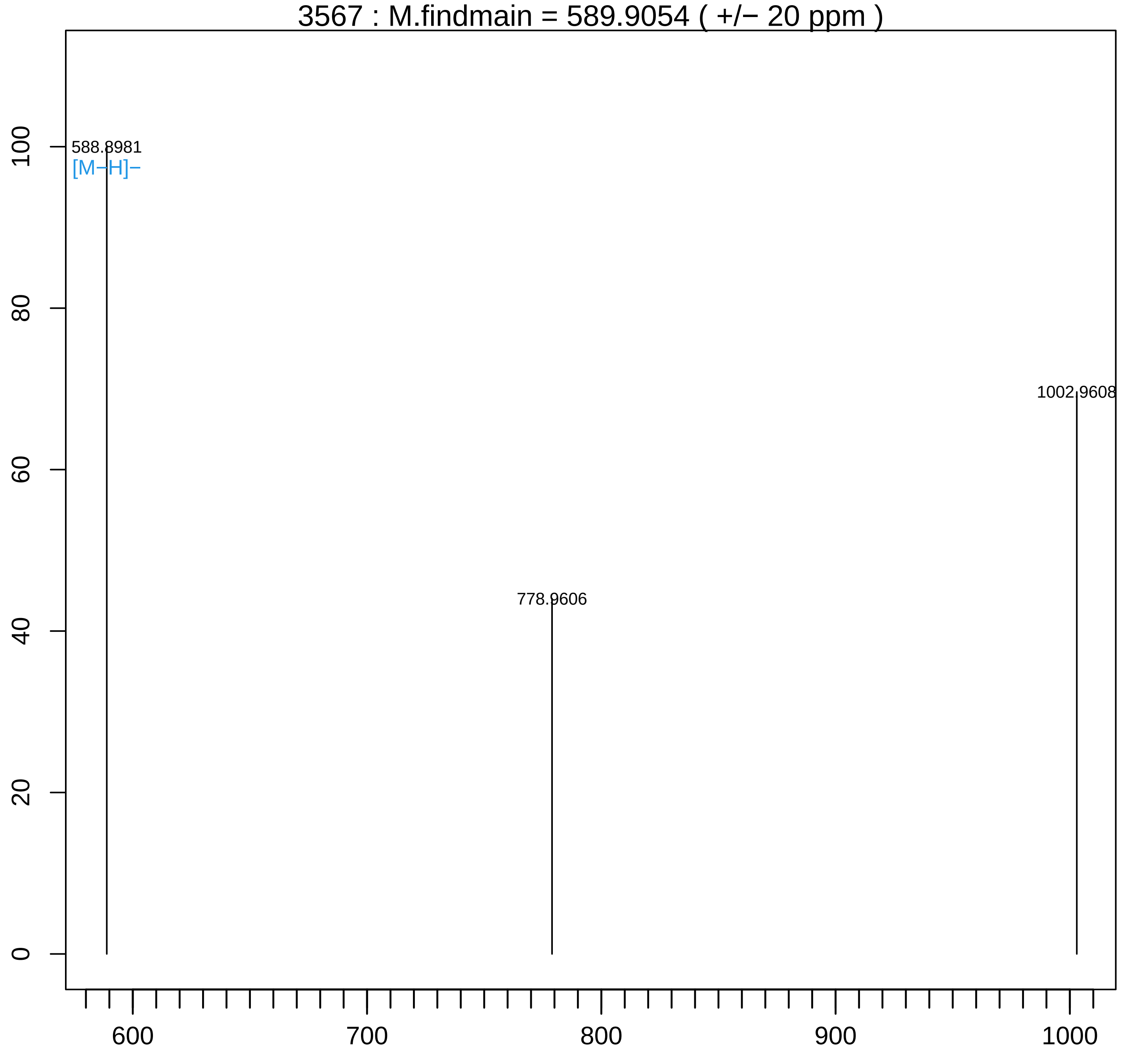
3567 : M.findmain = 589.9054 (+/− 20 ppm)
588.8981
[M−H]−
778.9606
1002.9608
0
20
40
60
80
100
600
700
800
900
1000

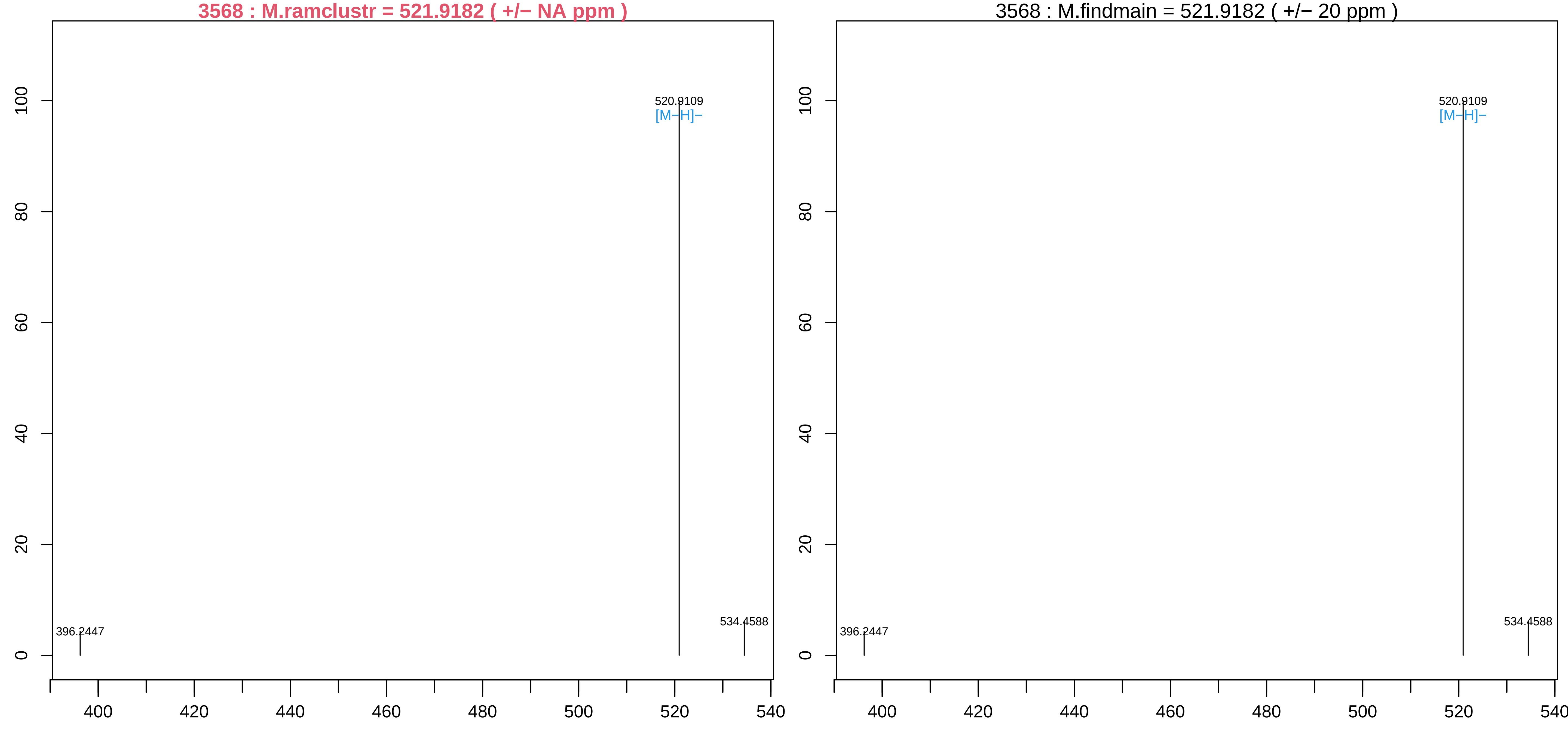
3568 : M.ramclustr = 521.9182 (+/− NA ppm)
3568 : M.findmain = 521.9182 (+/− 20 ppm)
520.9109
[M−H]−
396.2447
534.4588
0
20
40
60
80
100
400
420
440
460
480
500
520
540

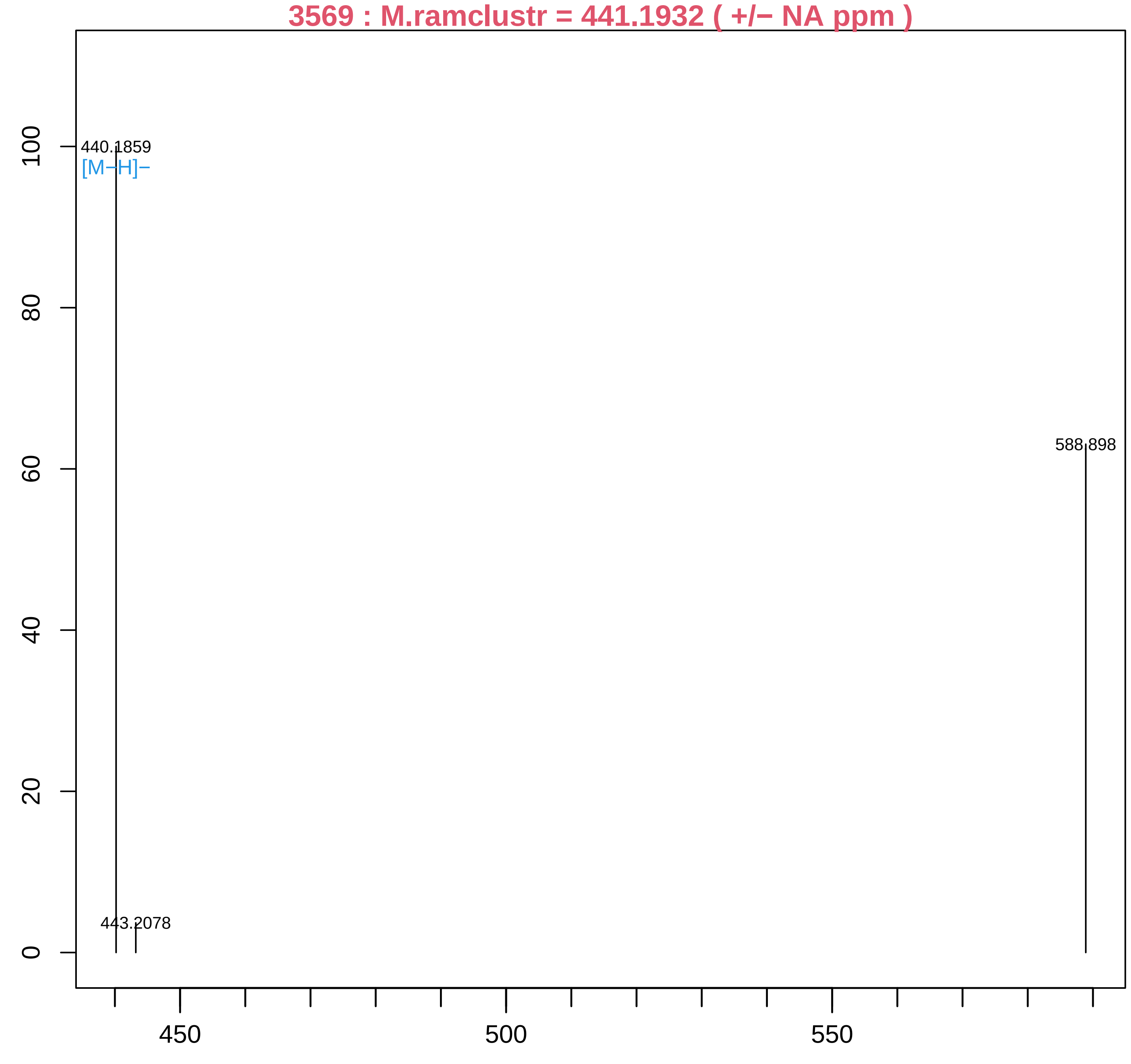
3569 : M.ramclustr = 441.1932 (+/− NA ppm)
440.1859
[M−H]−
588.898
443.2078
0
20
40
60
80
100
450
500
550

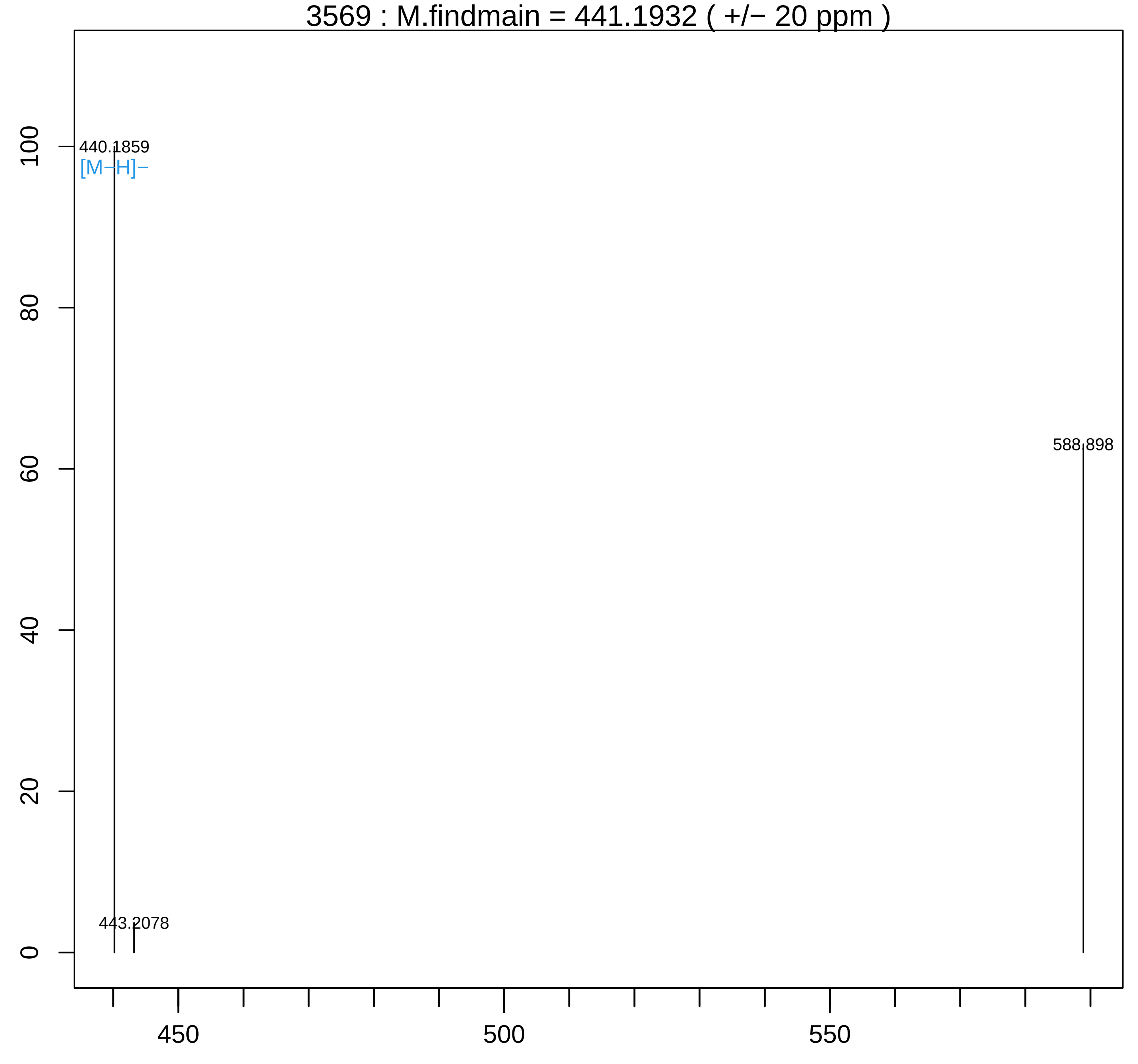
3569 : M.findmain = 441.1932 (+/− 20 ppm)
440.1859
[M−H]−
588.898
443.2078
0
20
40
60
80
100
450
500
550

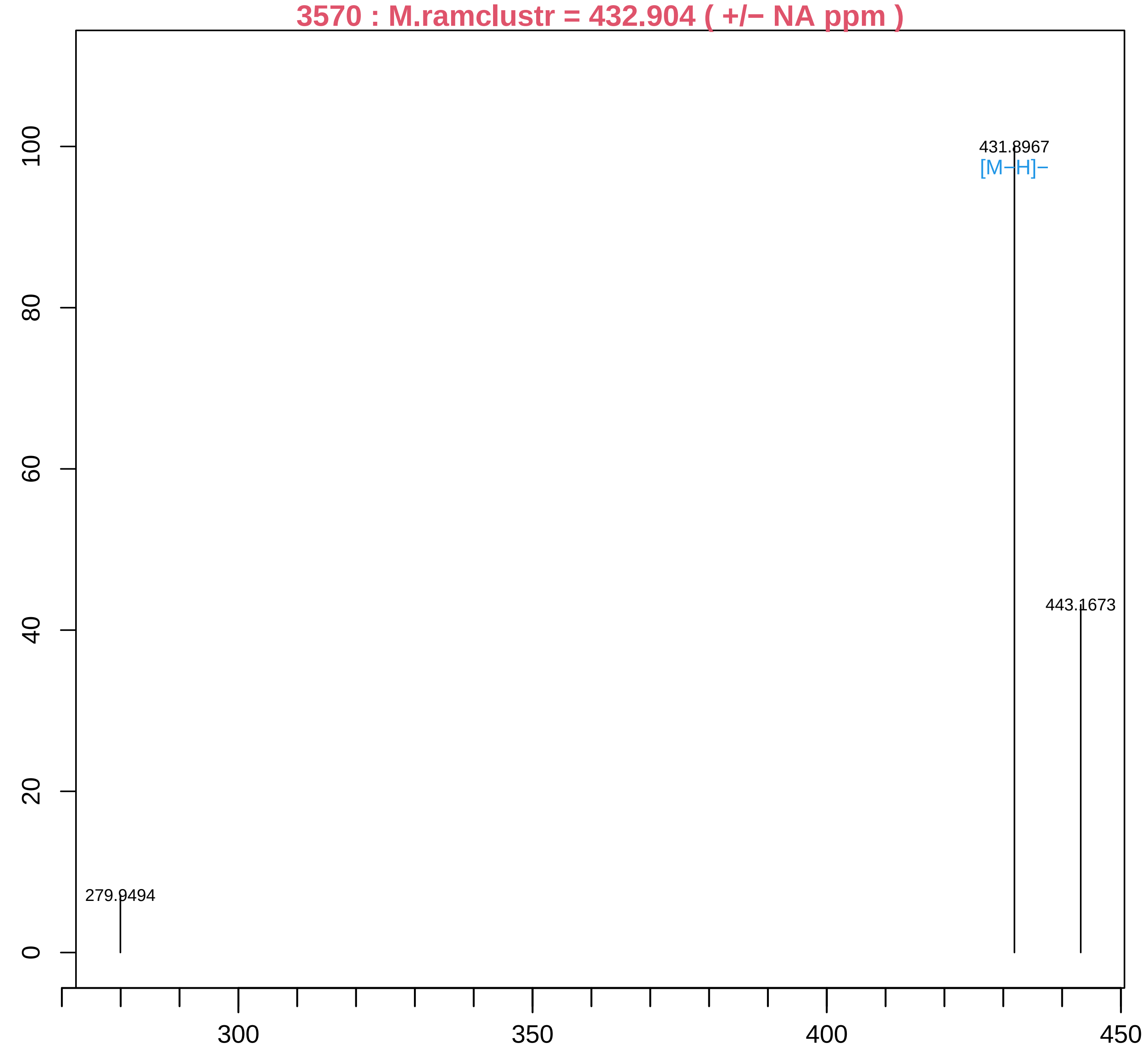
3570 : M.ramclustr = 432.904 (+/− NA ppm)
431.8967
[M−H]−
443.1673
279.9494
0
20
40
60
80
100
300
350
400
450

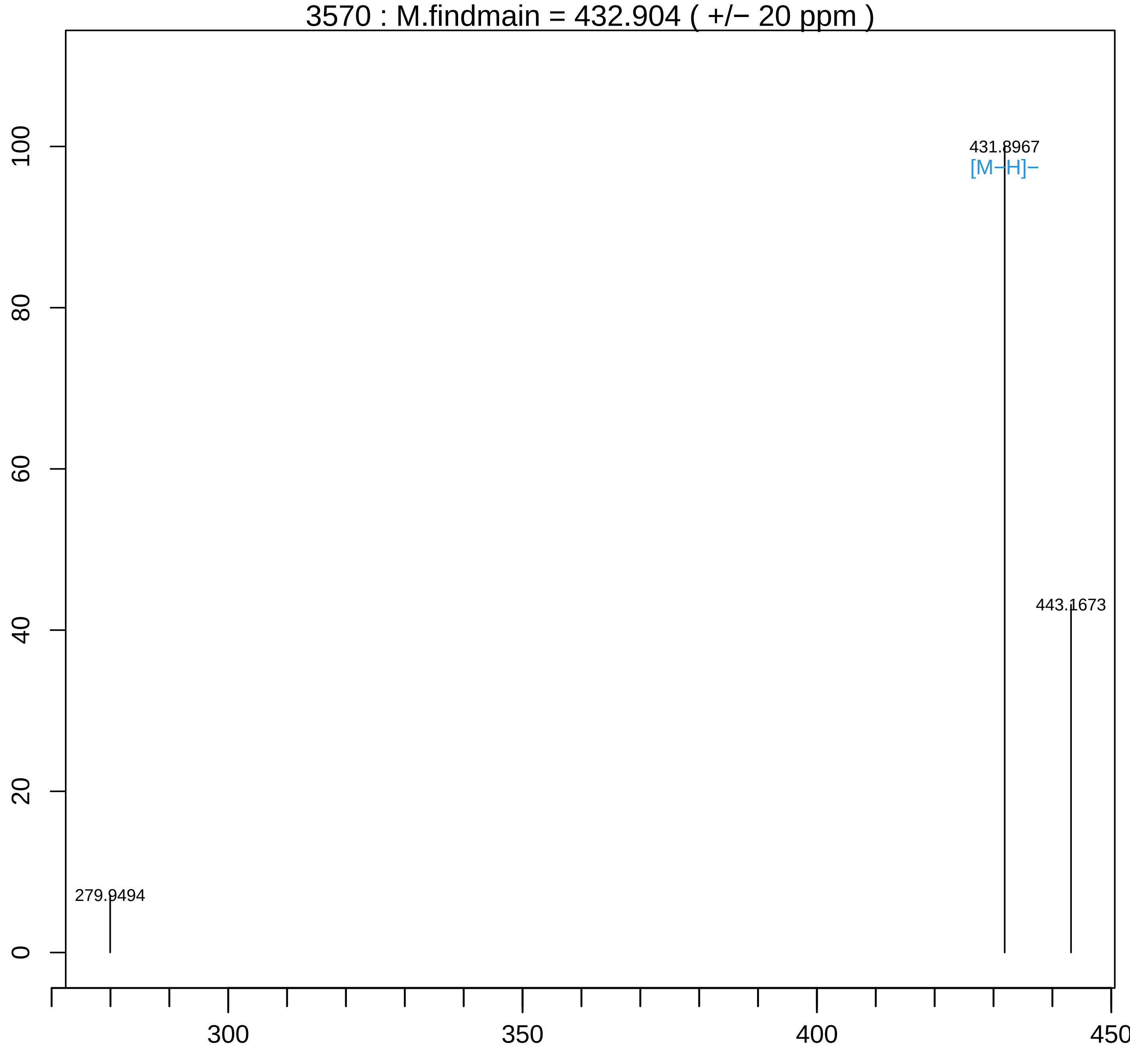
3570 : M.findmain = 432.904 (+/− 20 ppm)
431.8967
[M−H]−
443.1673
279.9494
0
20
40
60
80
100
300
350
400
450

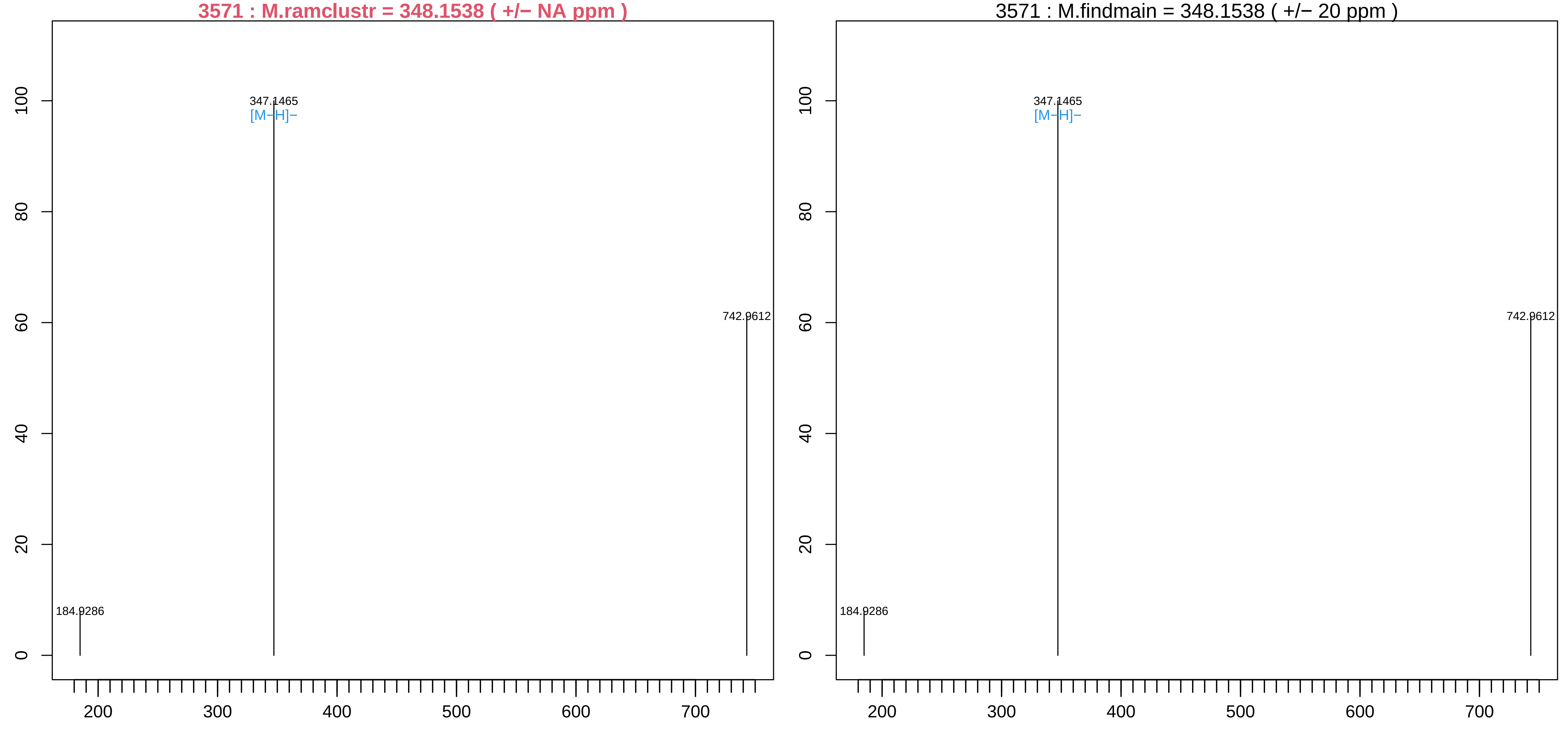
3571 : M.ramclustr = 348.1538 (+/− NA ppm)
347.1465
[M−H]−
742.9612
184.9286
3571 : M.findmain = 348.1538 (+/− 20 ppm)
347.1465
[M−H]−
742.9612
184.9286

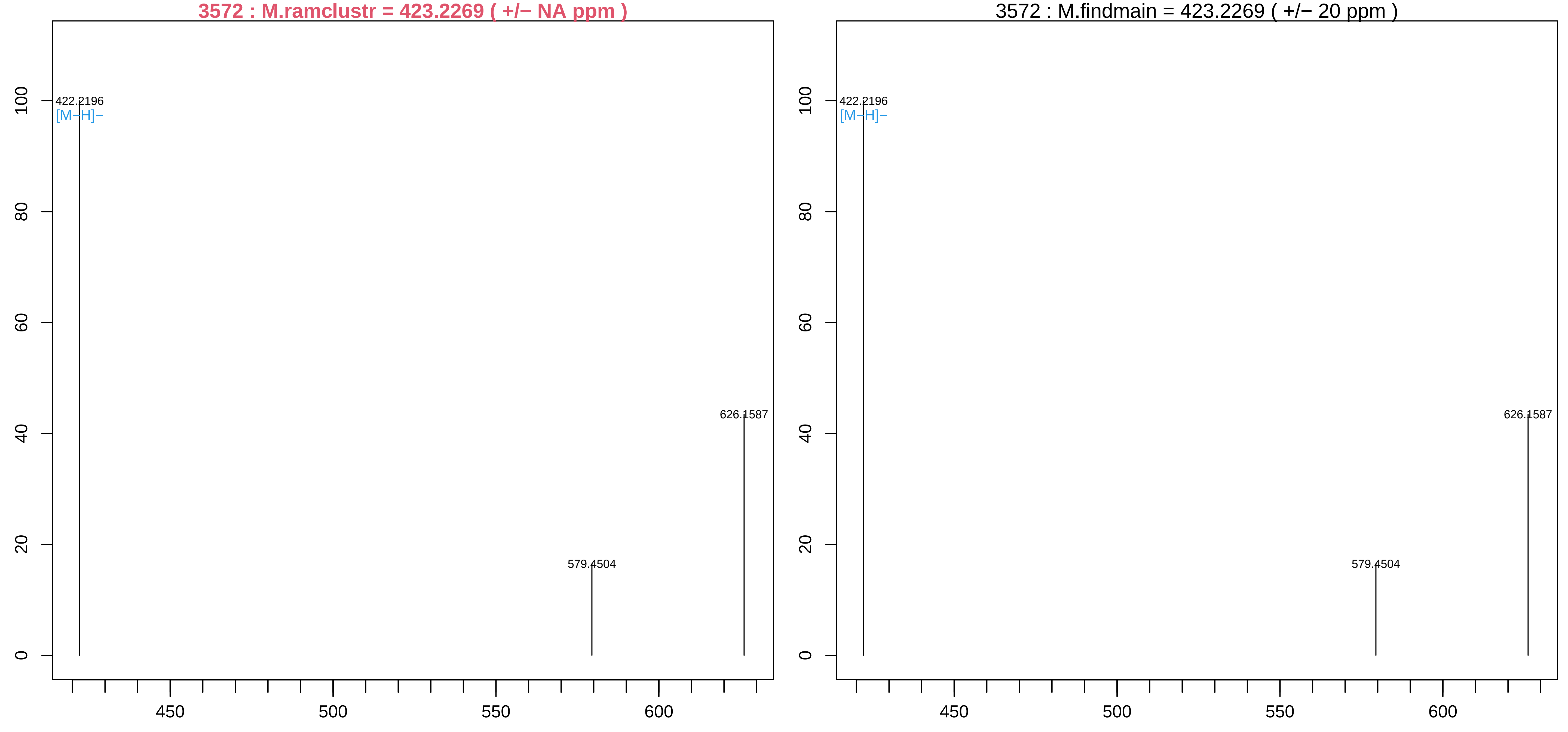

3572 : M.ramclustr = 423.2269 (+/− NA ppm)
422.2196
[M−H]−
579.4504
626.1587
3572 : M.findmain = 423.2269 (+/− 20 ppm)
422.2196
[M−H]−
579.4504
626.1587
0
20
40
60
80
100
450
500
550
600

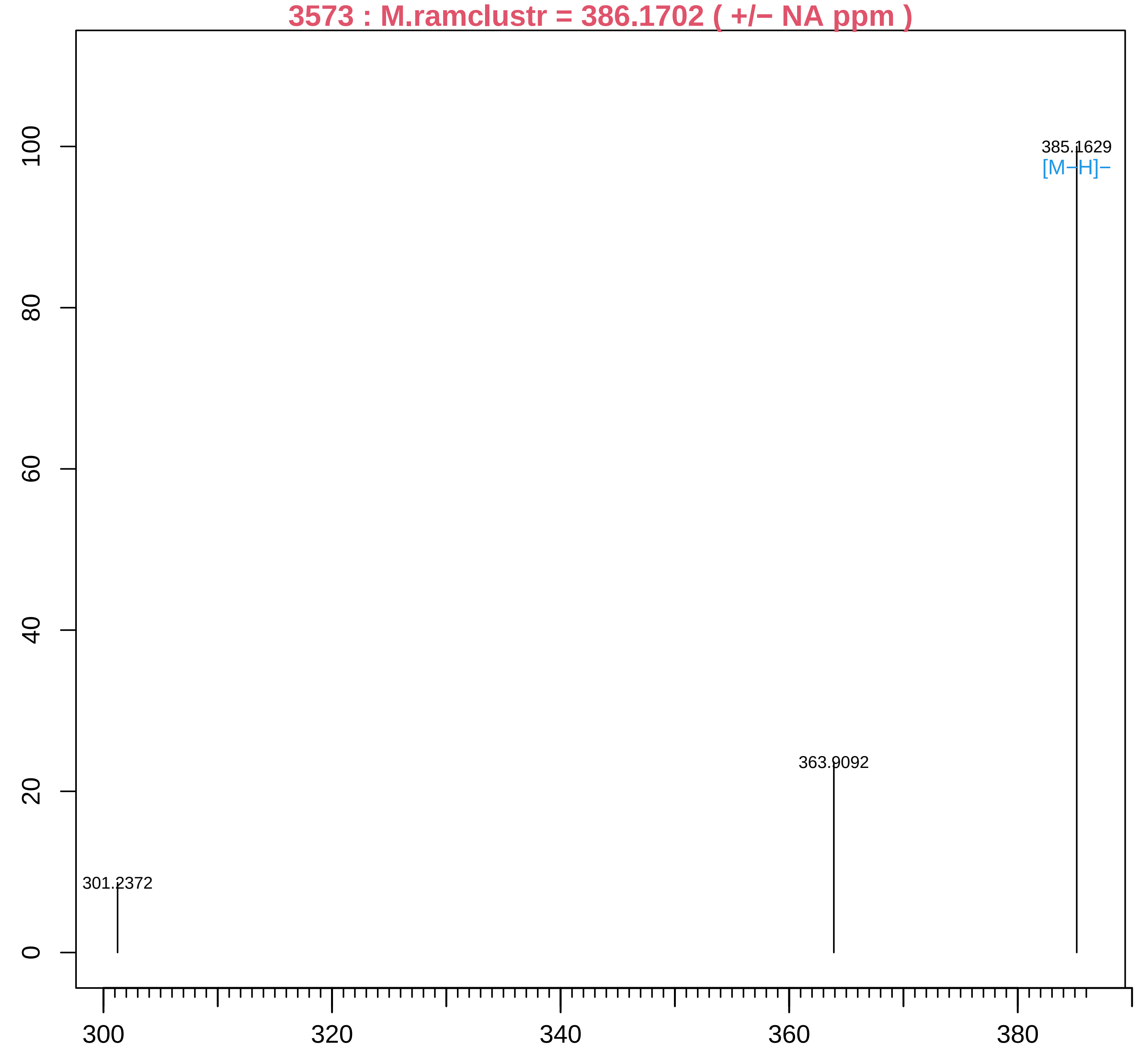
3573 : M.ramclustr = 386.1702 (+/− NA ppm)
385.1629
[M−H]−
363.9092
301.2372
0
20
40
60
80
100
300
320
340
360
380

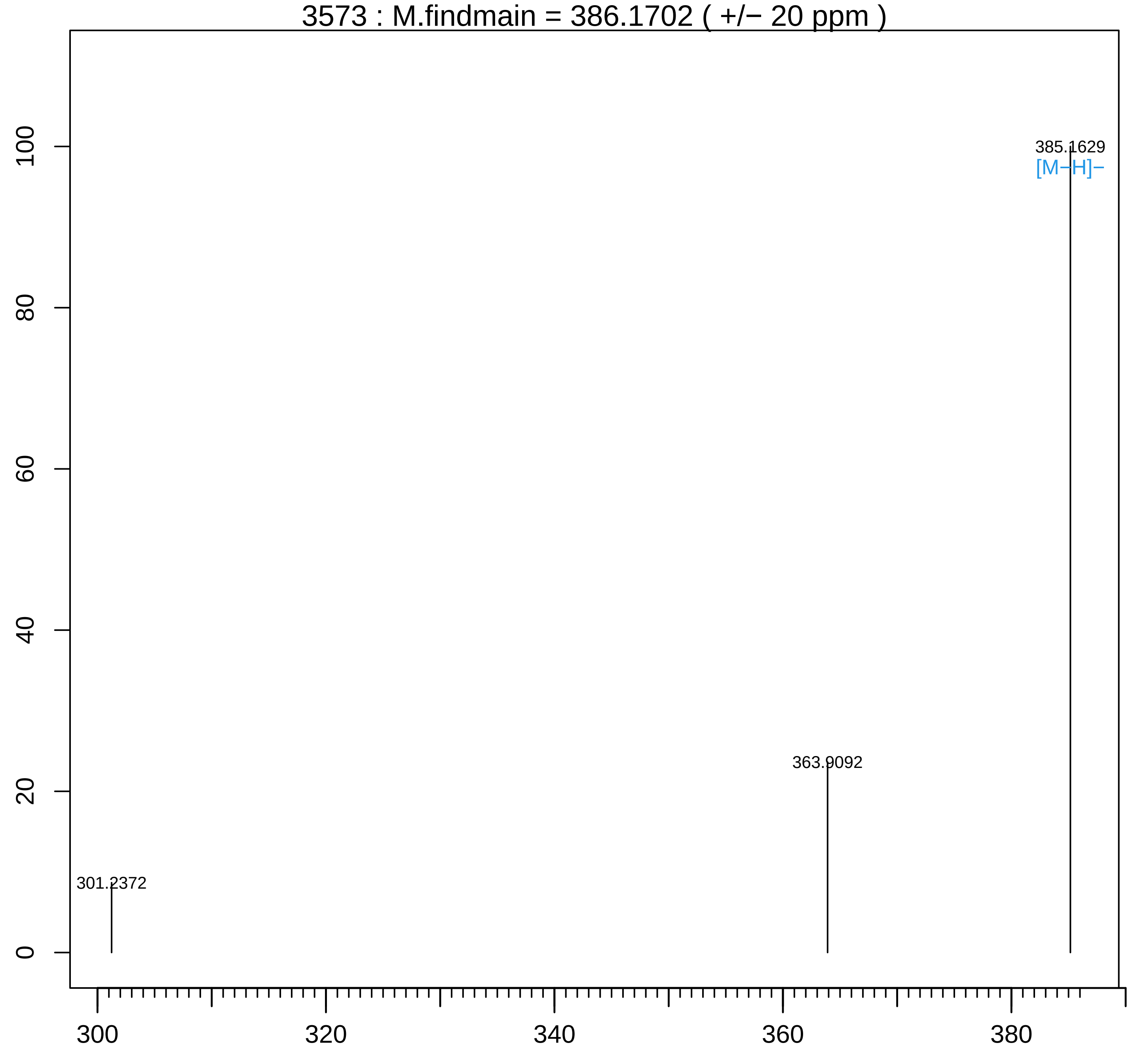
3573 : M.findmain = 386.1702 (+/− 20 ppm)
385.1629
[M−H]−
363.9092
301.2372
0
20
40
60
80
100
300
320
340
360
380

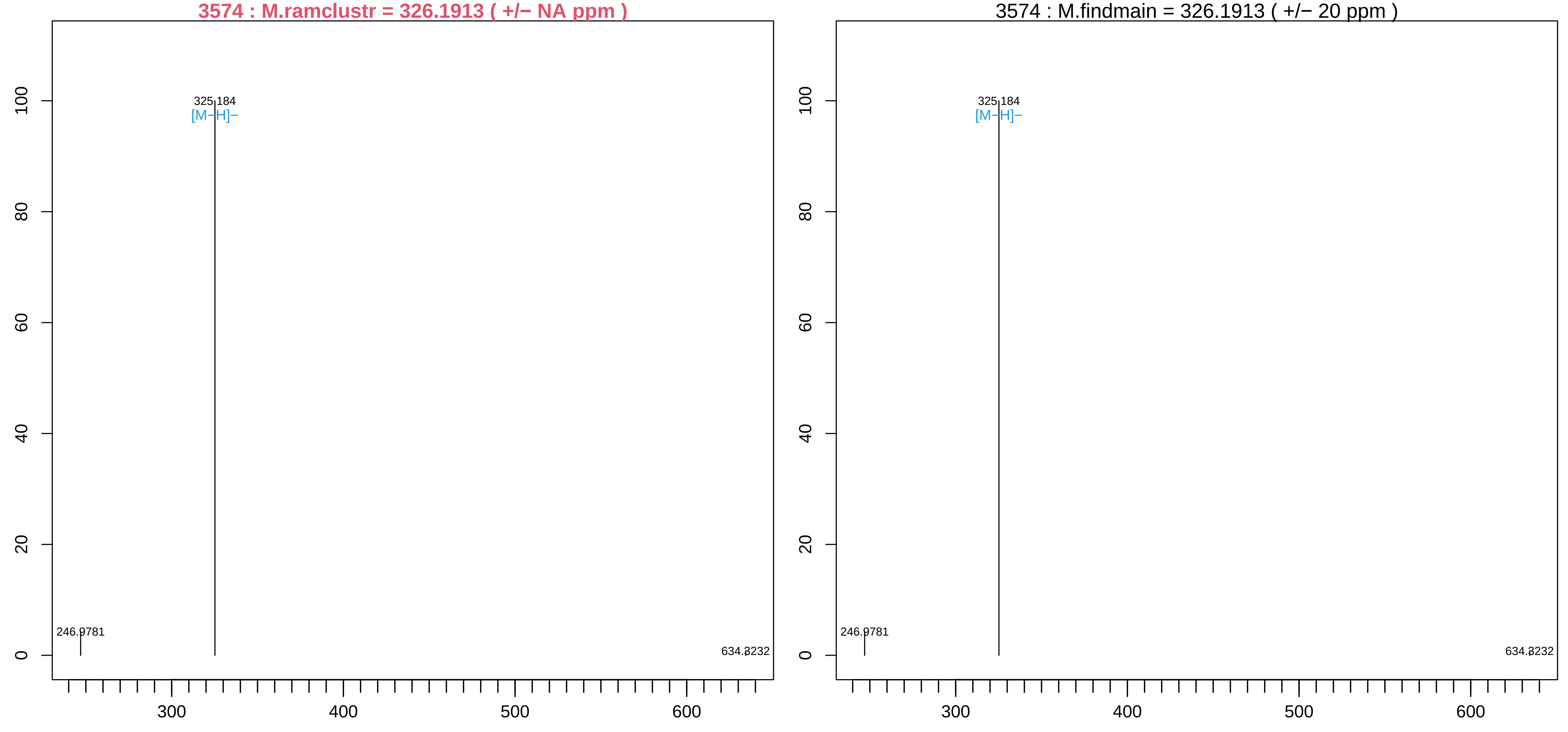
3574 : M.ramclustr = 326.1913 (+/− NA ppm)
325.184
[M−H]−
246.9781
634.3232
3574 : M.findmain = 326.1913 (+/− 20 ppm)
325.184
[M−H]−
246.9781
634.3232
0
20
40
60
80
100
300
400
500
600

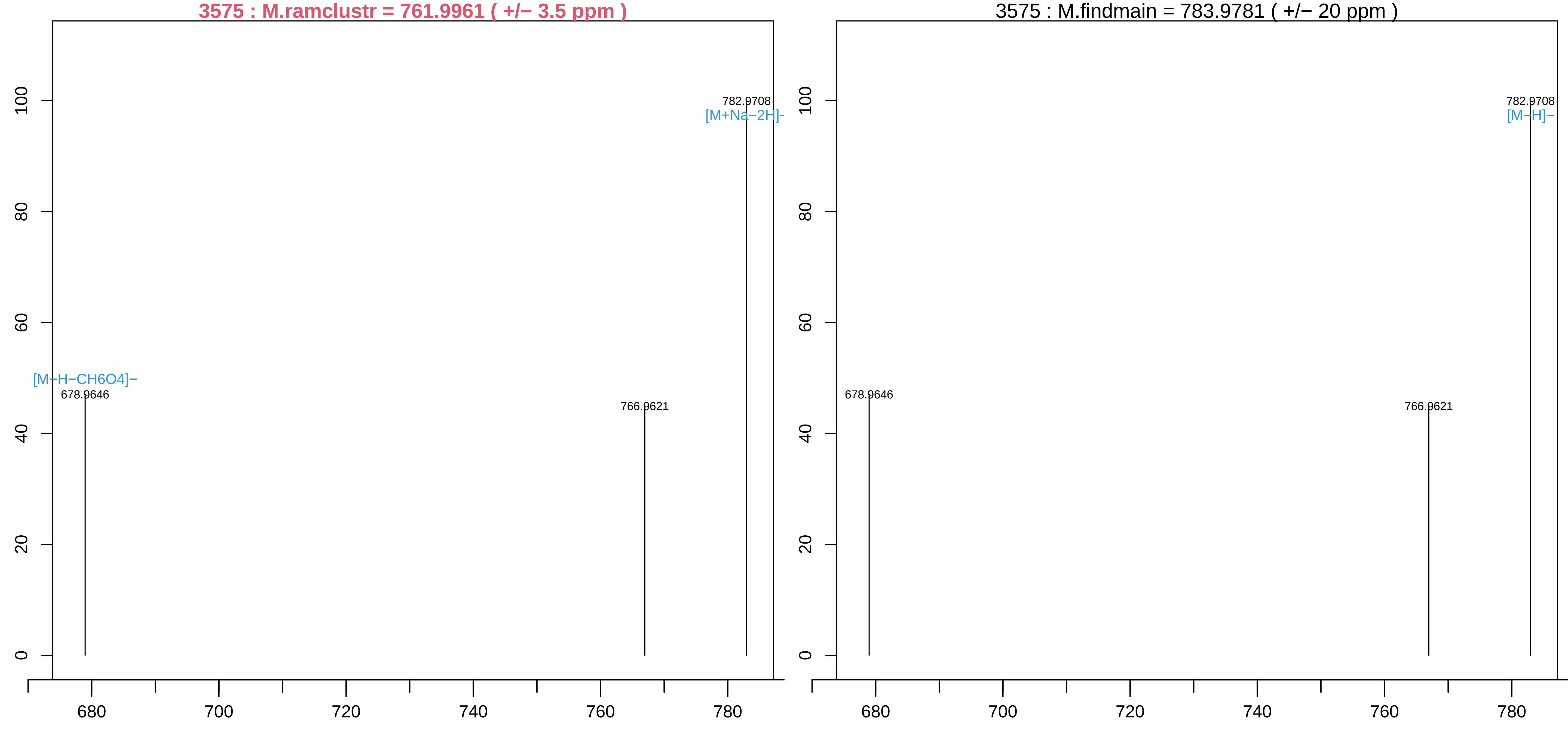
3575 : M.ramclustr = 761.9961 (+/− 3.5 ppm)
[M−H−CH6O4]−
678.9646
766.9621
782.9708
[M+Na−2H]−
0
20
40
60
80
100
680
700
720
740
760
780
3575 : M.findmain = 783.9781 (+/− 20 ppm)
678.9646
766.9621
782.9708
[M−H]−

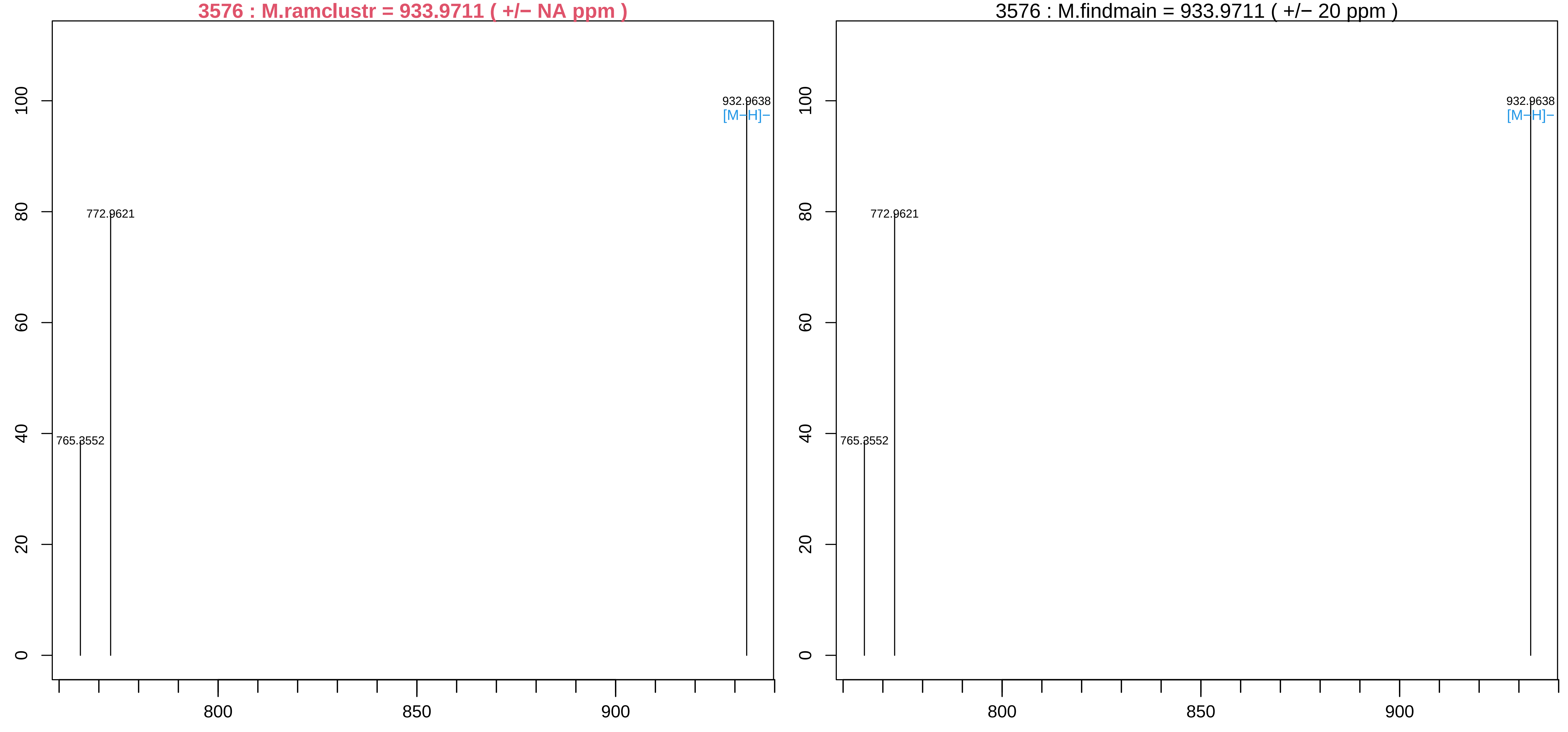
3576 : M.ramclustr = 933.9711 (+/− NA ppm)
932.9638
[M−H]−
772.9621
765.3552
3576 : M.findmain = 933.9711 (+/− 20 ppm)
932.9638
[M−H]−
772.9621
765.3552
0
20
40
60
80
100
800
850
900

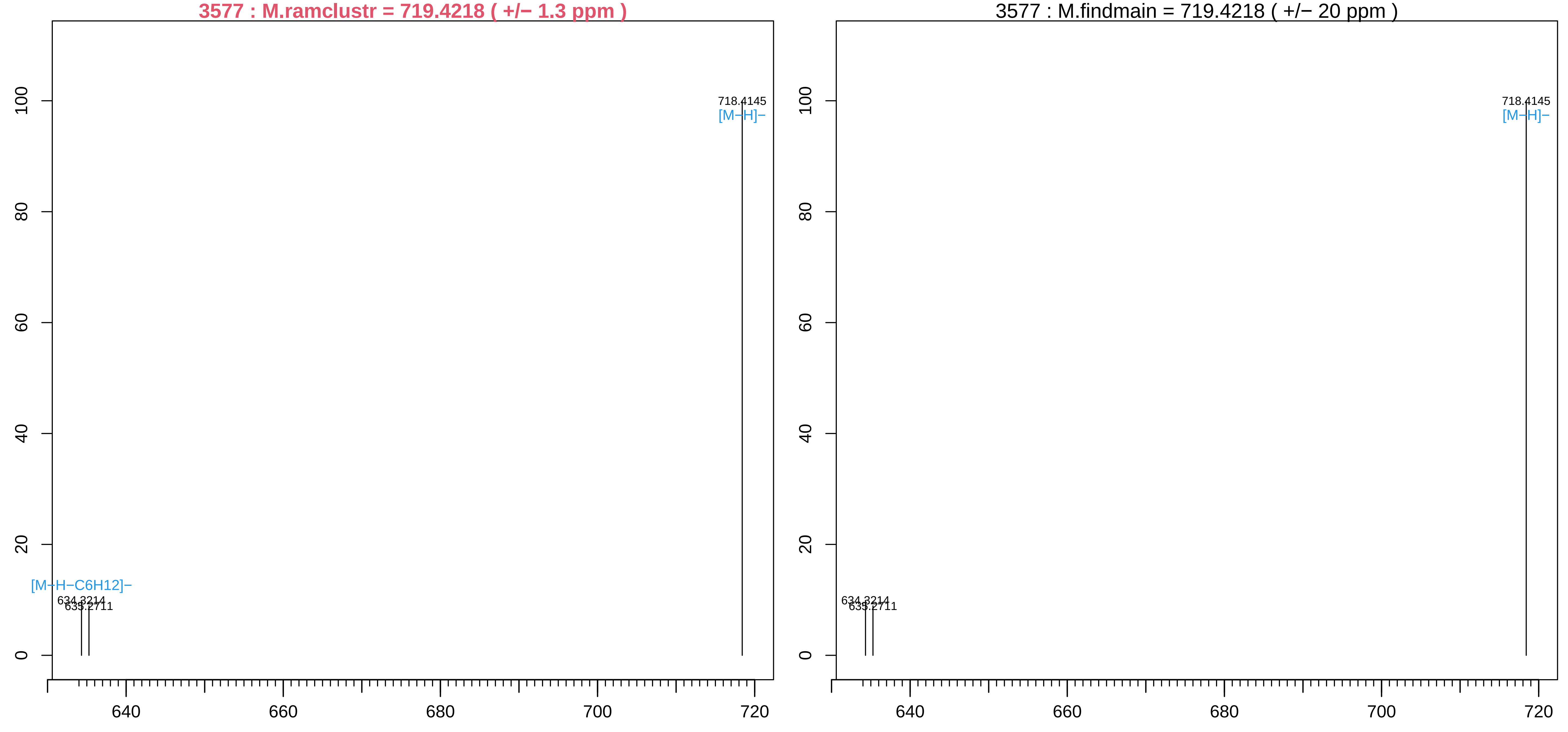
3577 : M.ramclustr = 719.4218 (+/− 1.3 ppm)
718.4145
[M−H]−
[M−H−C6H12]−
634.3214
635.2711
0
20
40
60
80
100
640
660
680
700
720
3577 : M.findmain = 719.4218 (+/− 20 ppm)
718.4145
[M−H]−
634.3214
635.2711

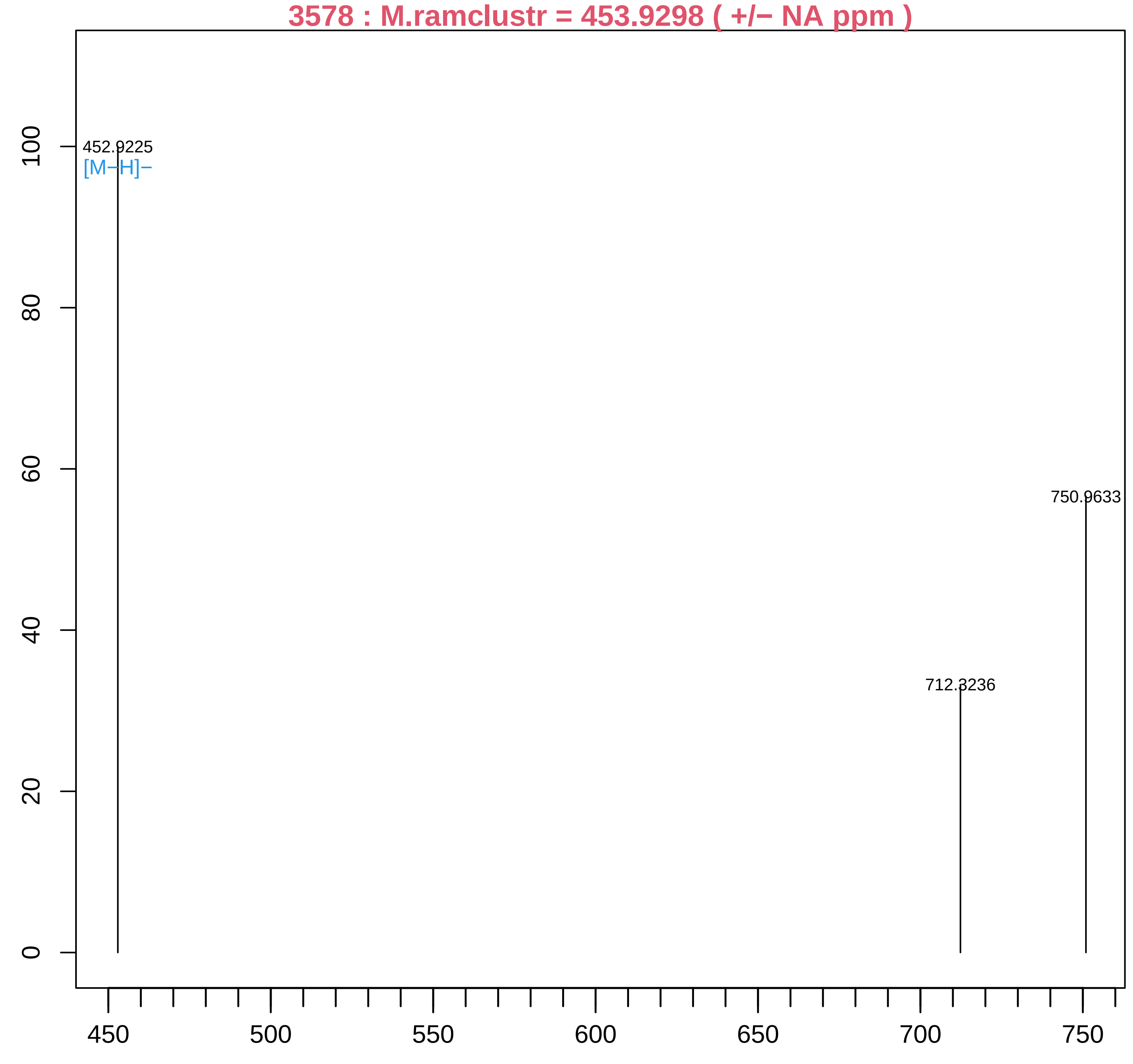
3578 : M.ramclustr = 453.9298 (+/− NA ppm)
452.9225
[M−H]−
712.3236
750.9633
0
20
40
60
80
100
450
500
550
600
650
700
750

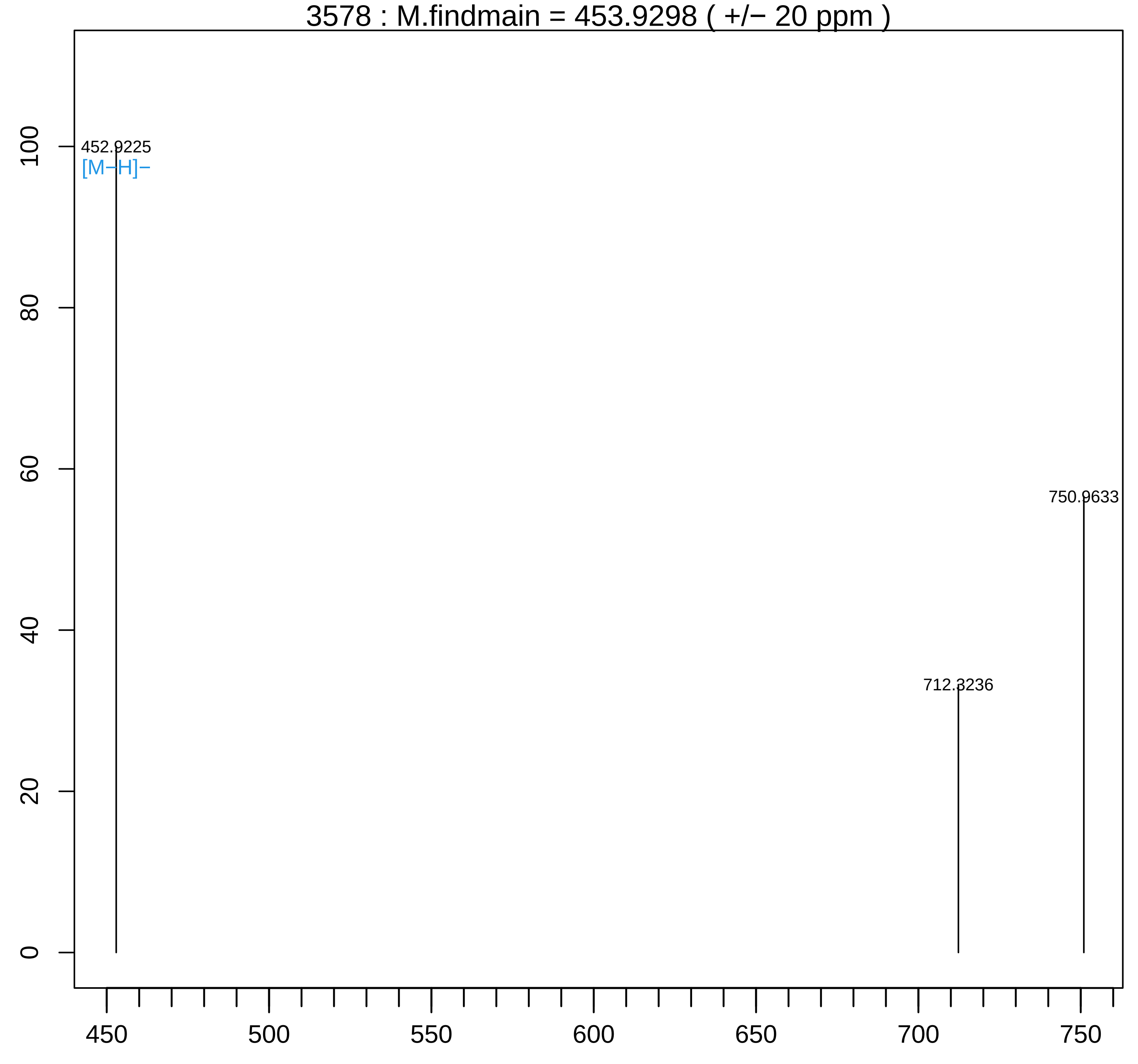
3578 : M.findmain = 453.9298 (+/− 20 ppm)
452.9225
[M−H]−
712.3236
750.9633
0
20
40
60
80
100
450
500
550
600
650
700
750

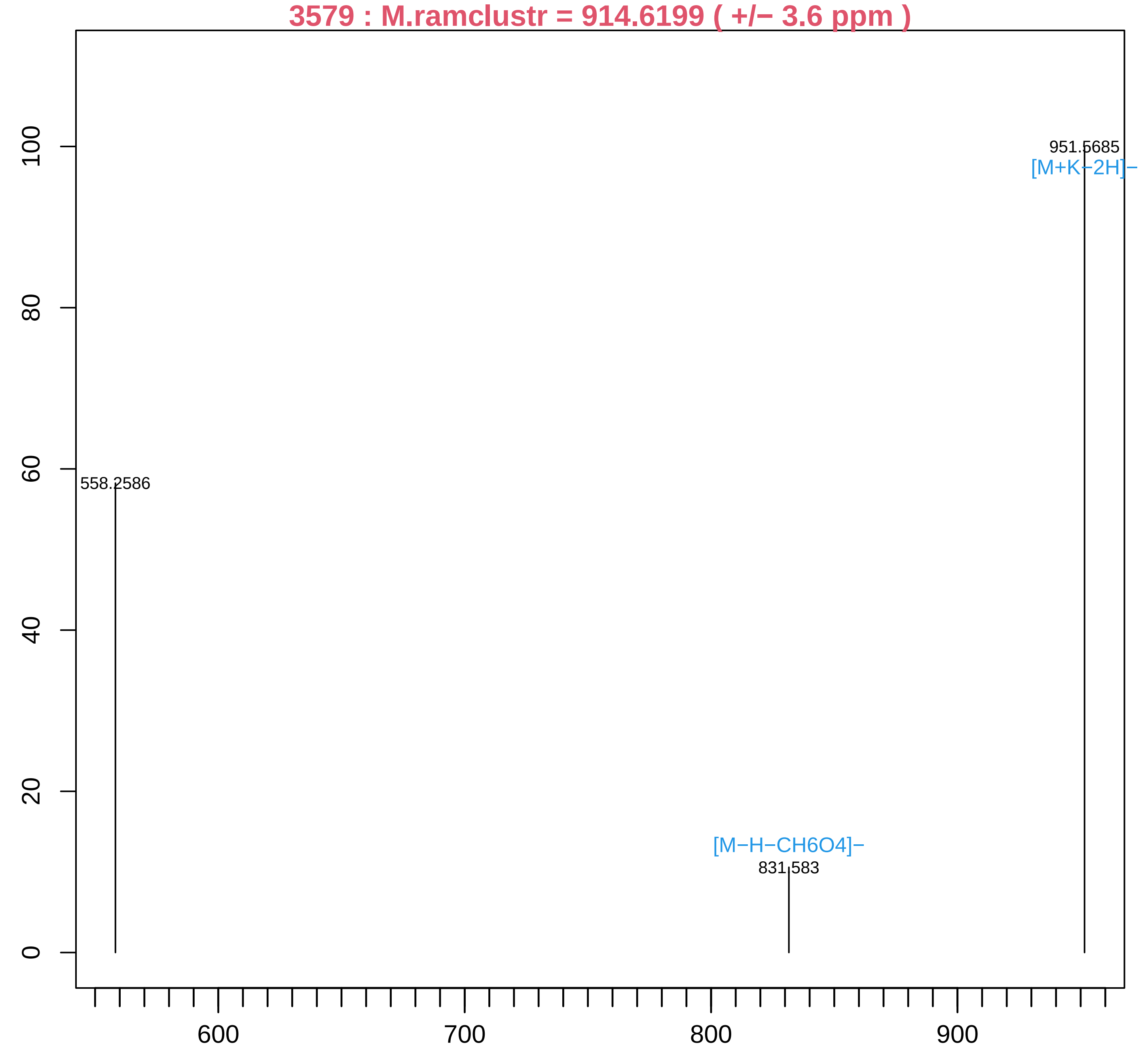
3579 : M.ramclustr = 914.6199 (+/− 3.6 ppm)
951.5685
[M+K−2H]−
558.2586
[M−H−CH6O4]−
831.583
0
20
40
60
80
100
600
700
800
900

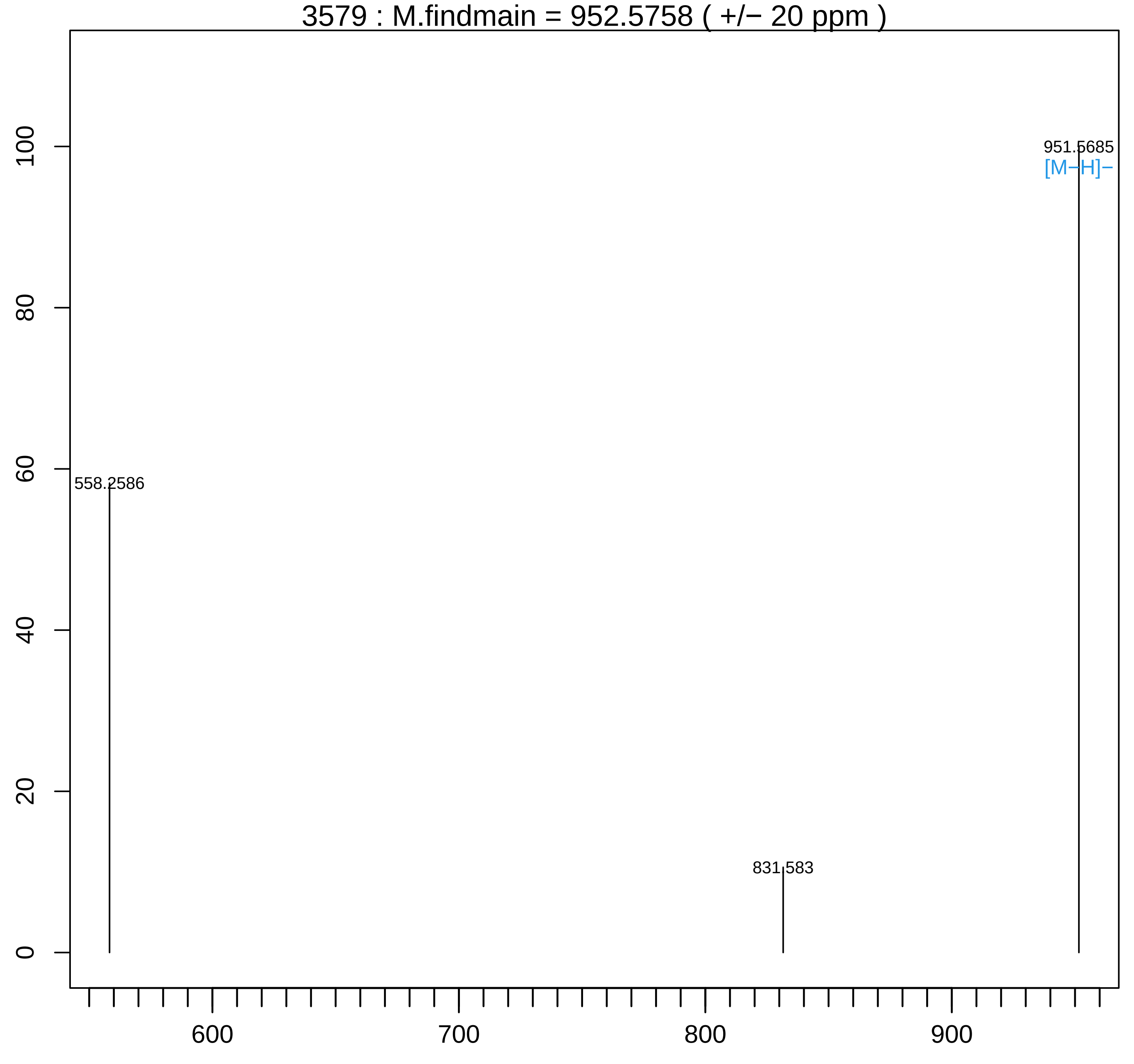
3579 : M.findmain = 952.5758 (+/− 20 ppm)
951.5685
[M−H]−
558.2586
831.583
0
20
40
60
80
100
600
700
800
900

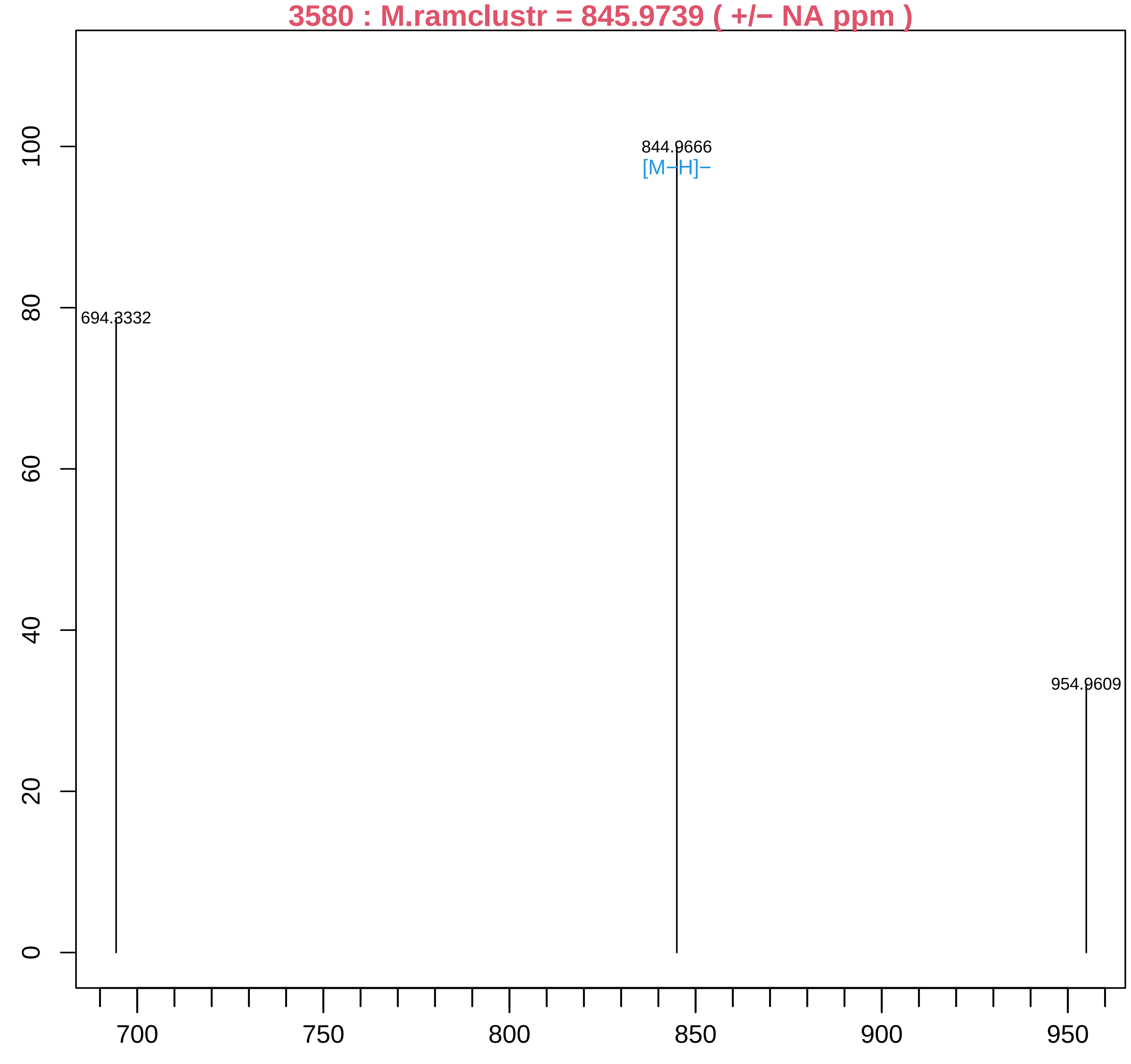
3580 : M.ramclustr = 845.9739 (+/− NA ppm)
694.3332
844.9666
[M−H]−
954.9609
0
20
40
60
80
100
700
750
800
850
900
950
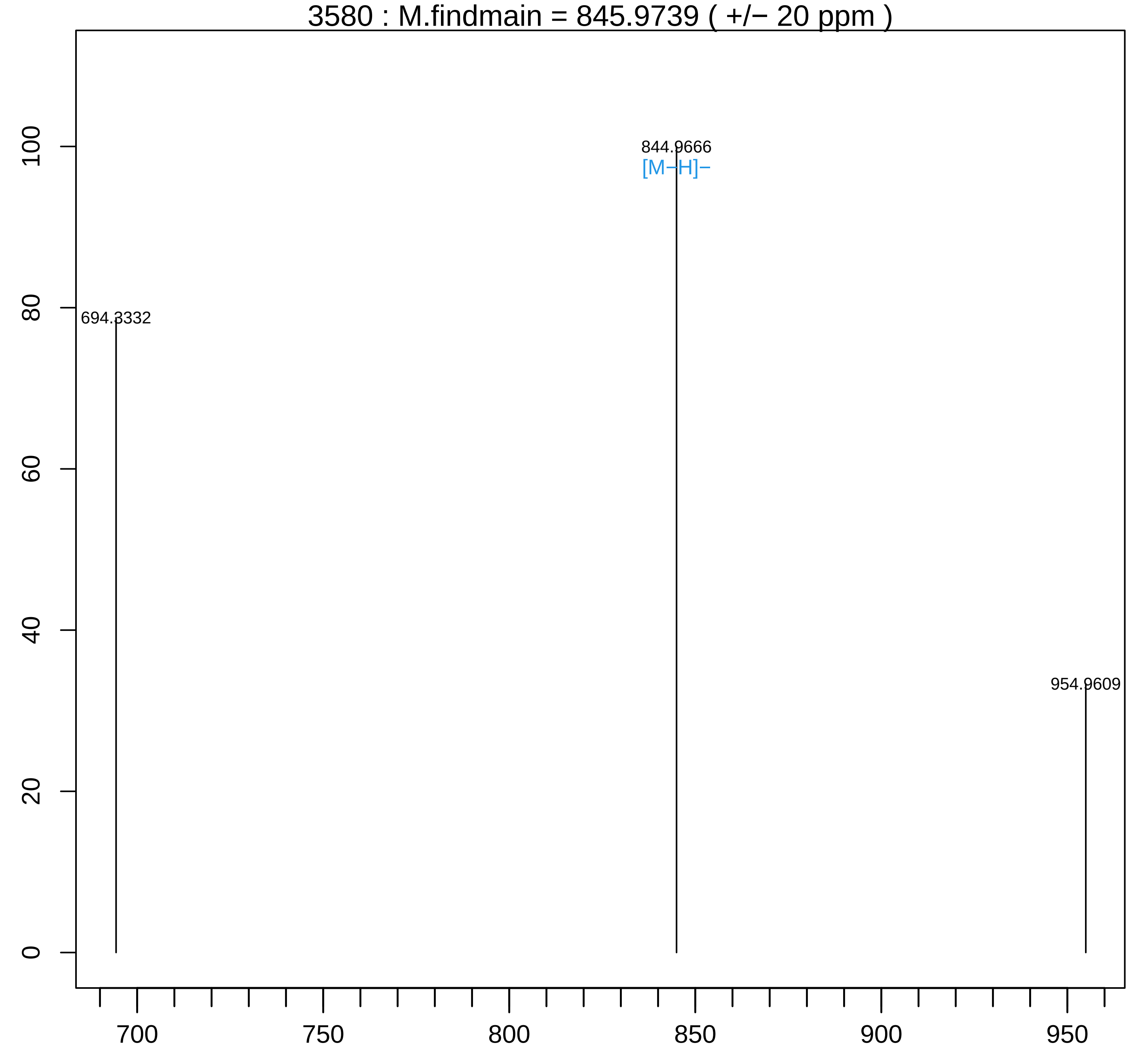
3580 : M.findmain = 845.9739 (+/− 20 ppm)
694.3332
844.9666
[M−H]−
954.9609
0
20
40
60
80
100
700
750
800
850
900
950

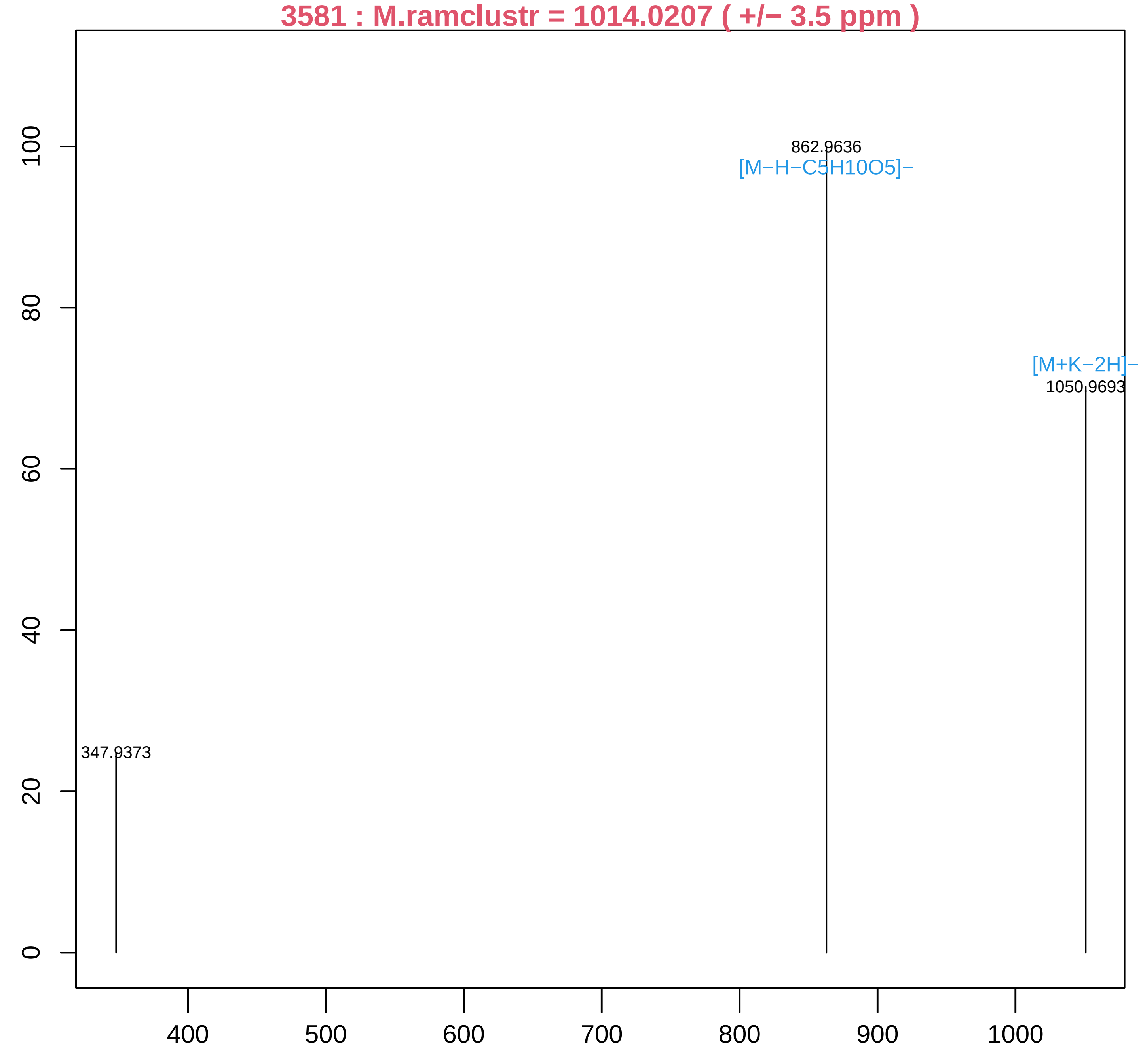
3581 : M.ramclustr = 1014.0207 (+/− 3.5 ppm)
862.9636
[M−H−C5H10O5]−
[M+K−2H]−
1050.9693
347.9373
0
20
40
60
80
100
400
500
600
700
800
900
1000

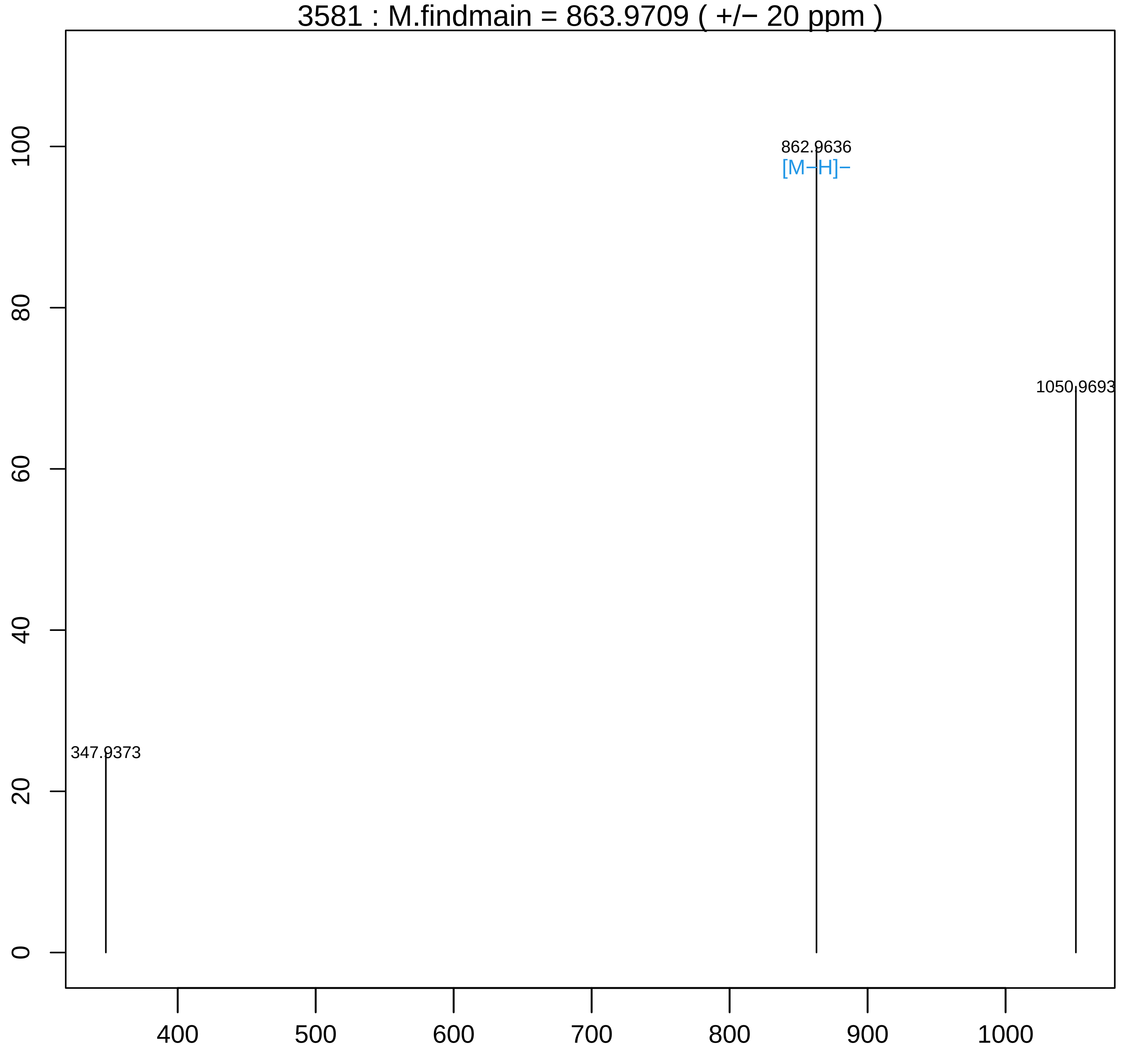
3581 : M.findmain = 863.9709 (+/− 20 ppm)
862.9636
[M−H]−
1050.9693
347.9373
0
20
40
60
80
100
400
500
600
700
800
900
1000

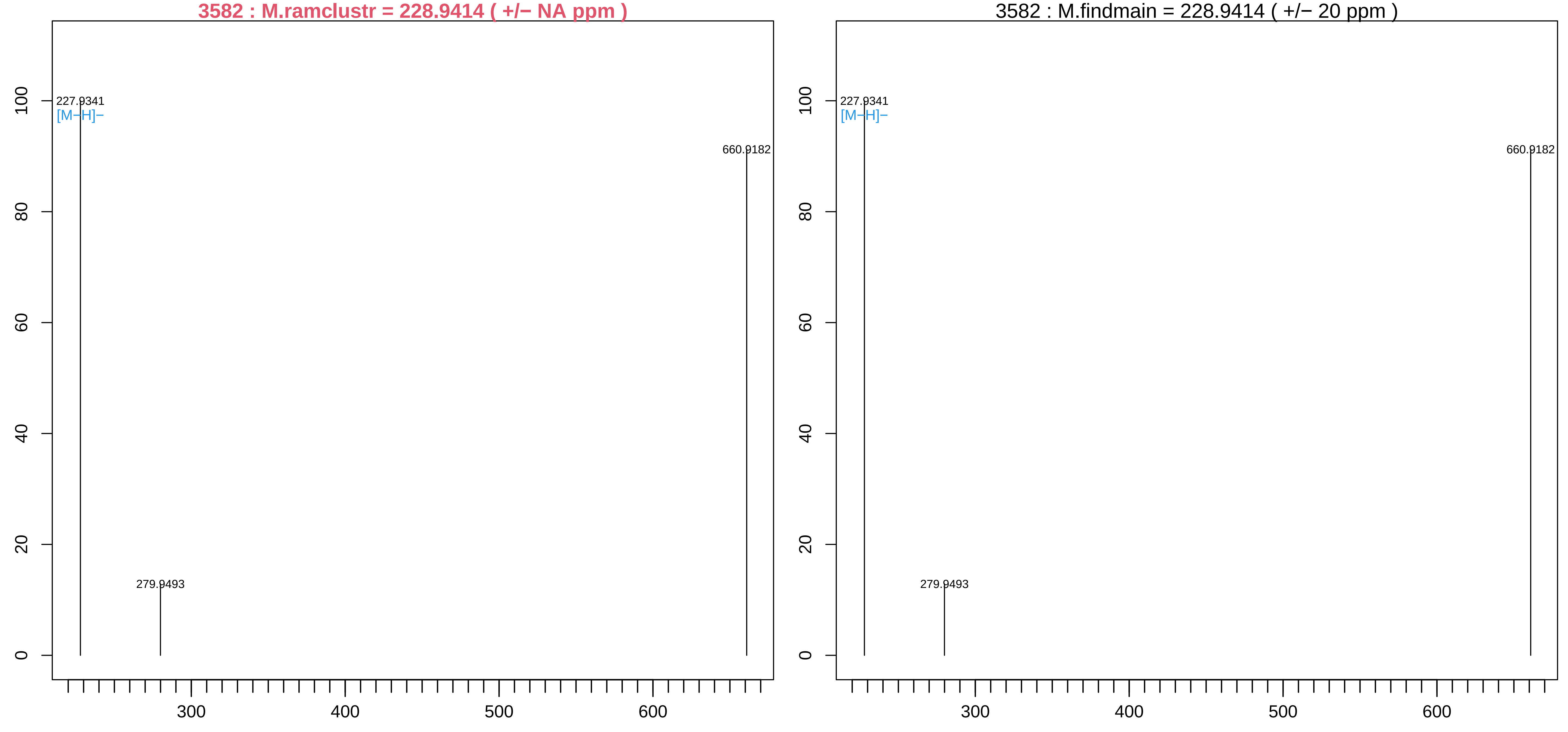
3582 : M.ramclustr = 228.9414 (+/− NA ppm)
227.9341
[M−H]−
279.9493
660.9182
0
20
40
60
80
100
300
400
500
600
3582 : M.findmain = 228.9414 (+/− 20 ppm)
227.9341
[M−H]−
279.9493
660.9182
0
20
40
60
80
100
300
400
500
600

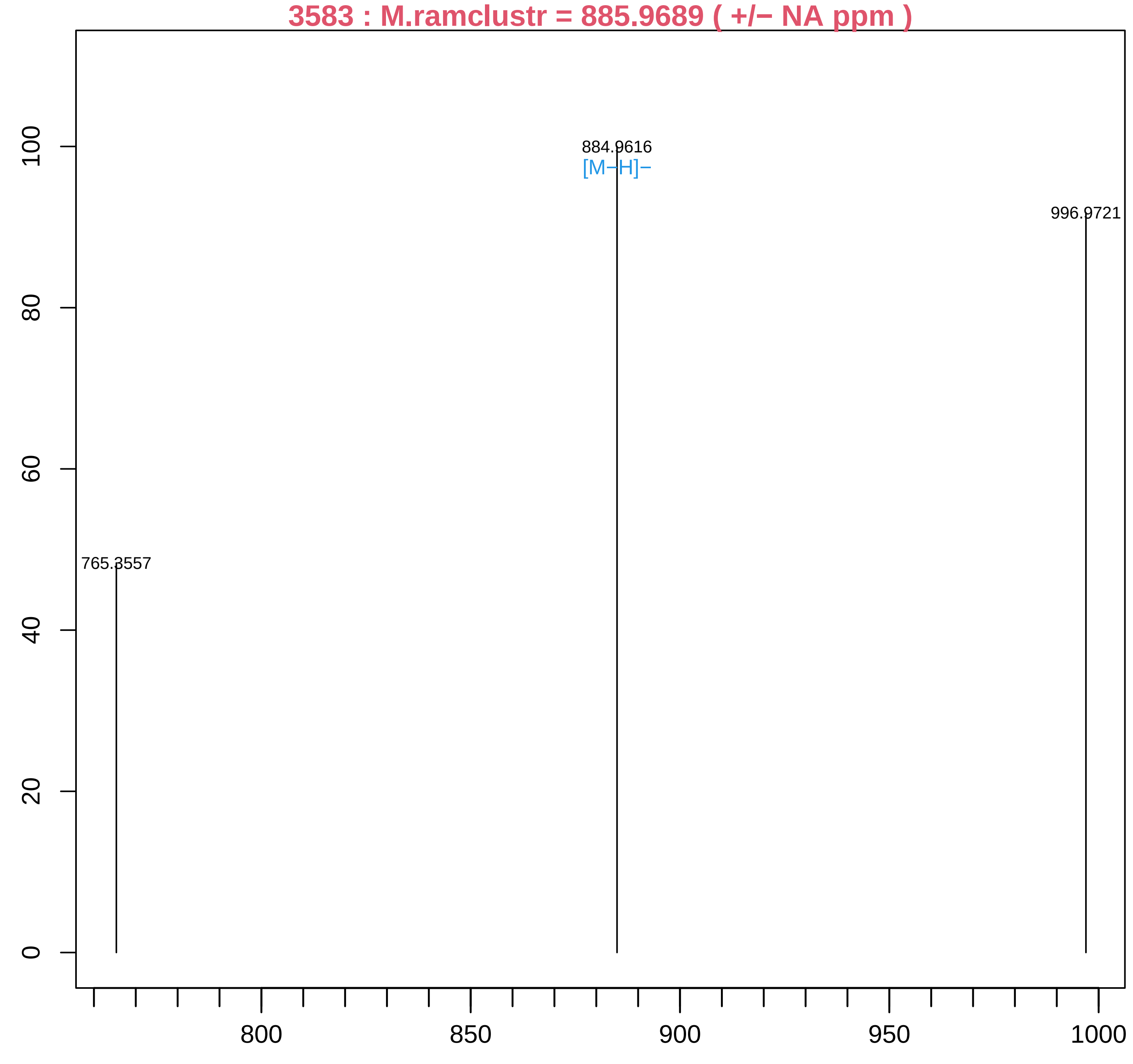
3583 : M.ramclustr = 885.9689 (+/− NA ppm)
884.9616
[M−H]−
996.9721
765.3557
0
20
40
60
80
100
800
850
900
950
1000

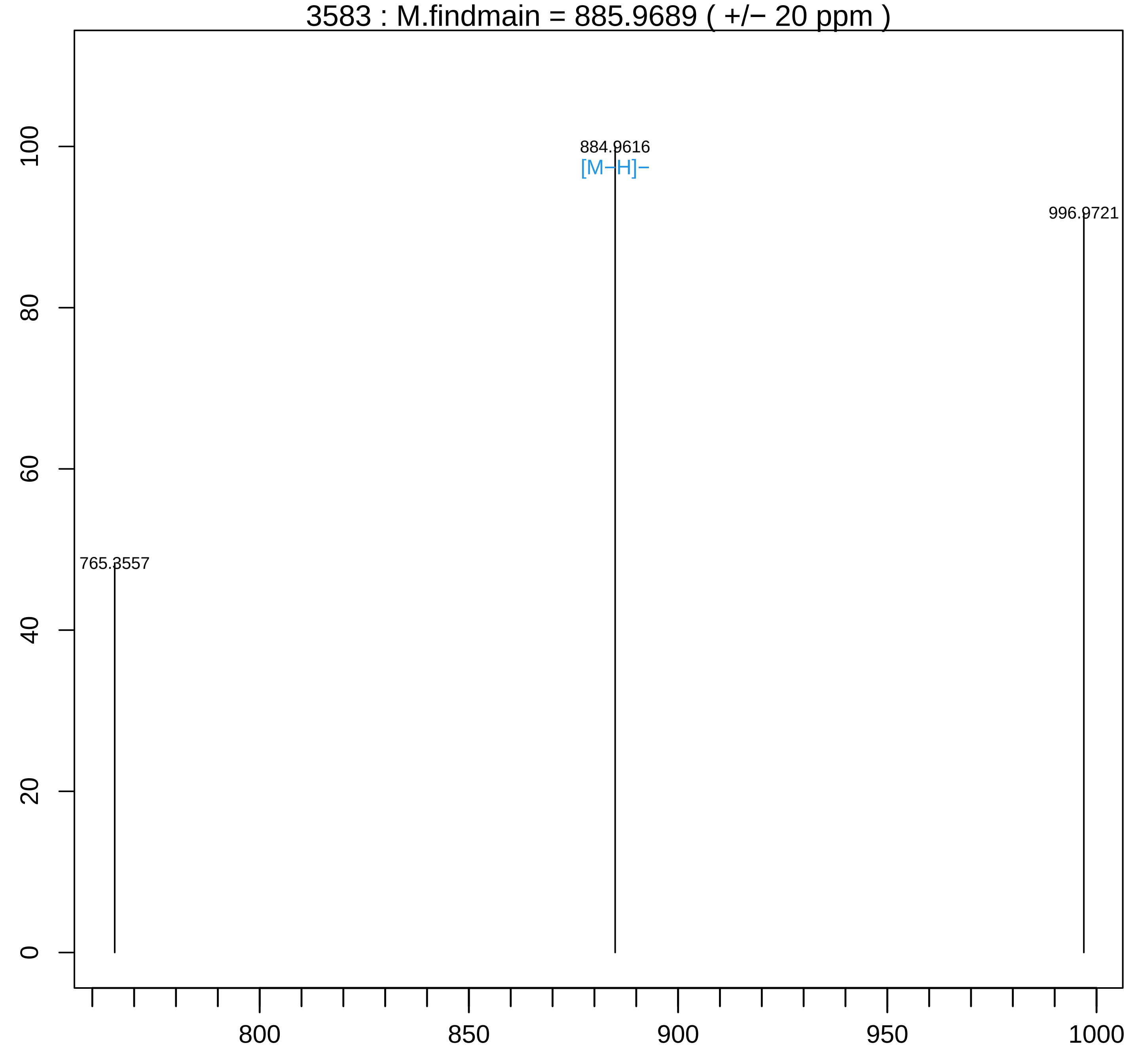
3583 : M.findmain = 885.9689 (+/− 20 ppm)
884.9616
[M−H]−
996.9721
765.3557
0
20
40
60
80
100
800
850
900
950
1000

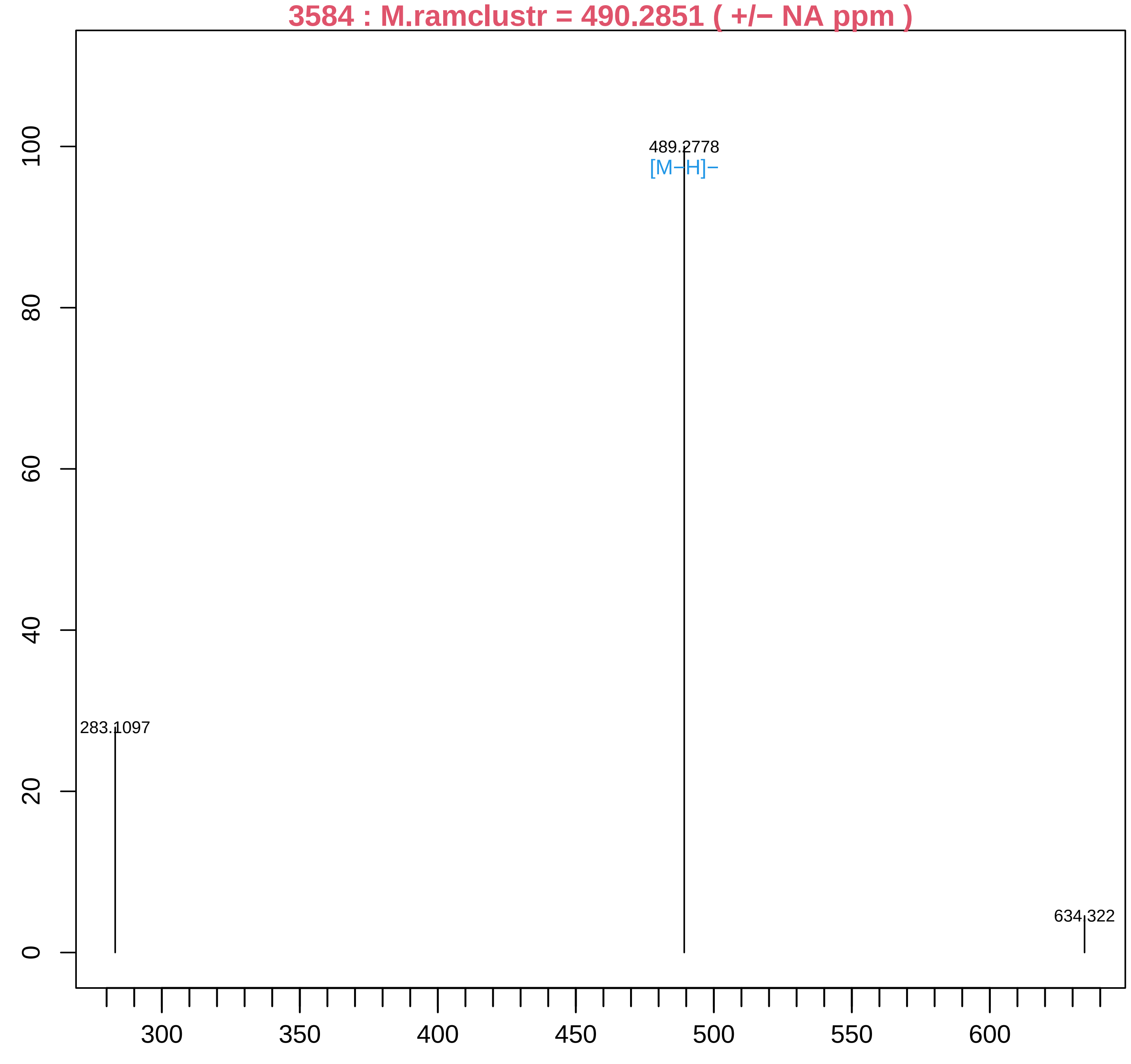
3584 : M.ramclustr = 490.2851 (+/− NA ppm)
489.2778
[M−H]−
283.1097
634.322
0
20
40
60
80
100
300
350
400
450
500
550
600

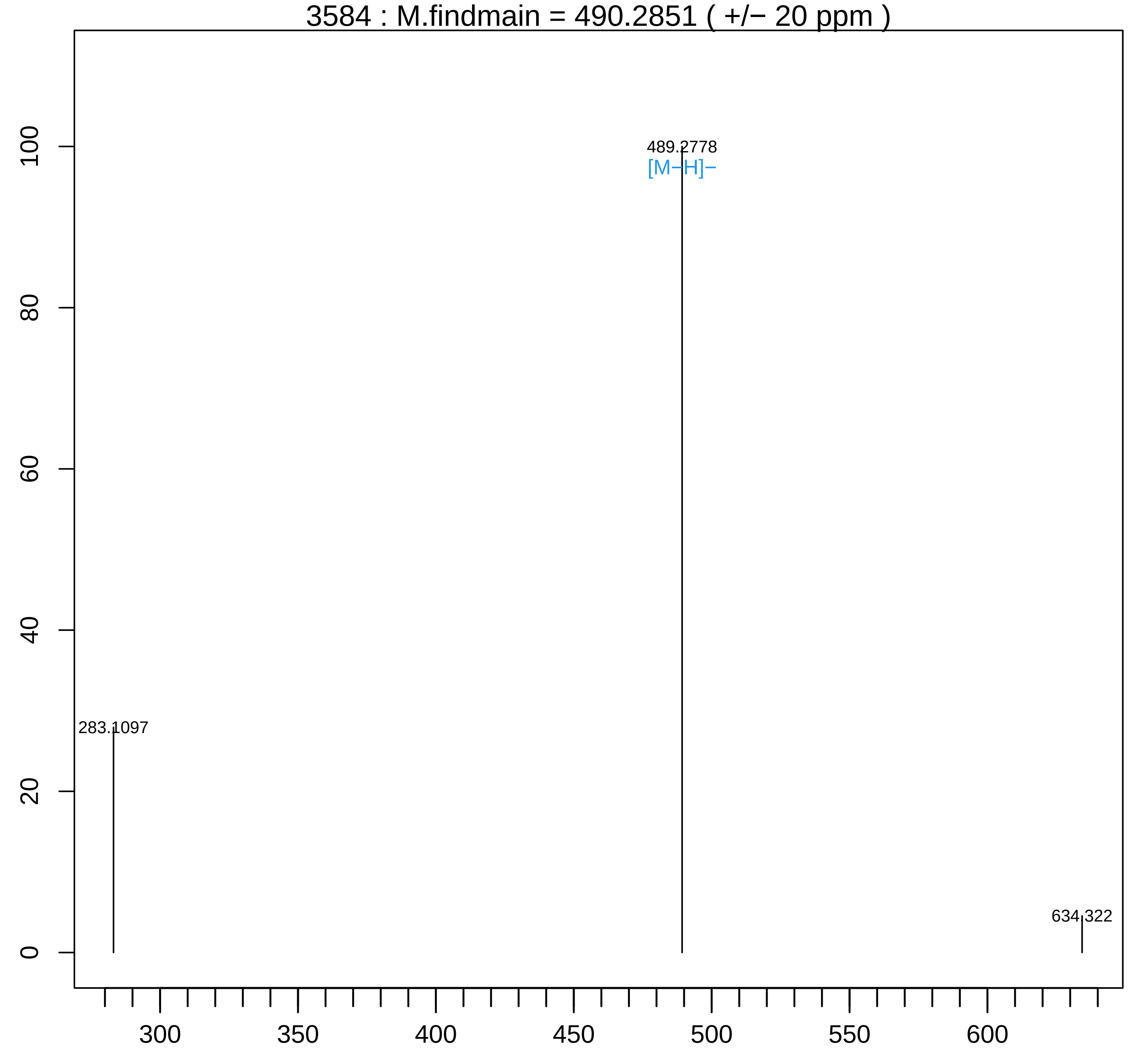
3584 : M.findmain = 490.2851 (+/− 20 ppm)
489.2778
[M−H]−
283.1097
634.322
0
20
40
60
80
100
300
350
400
450
500
550
600

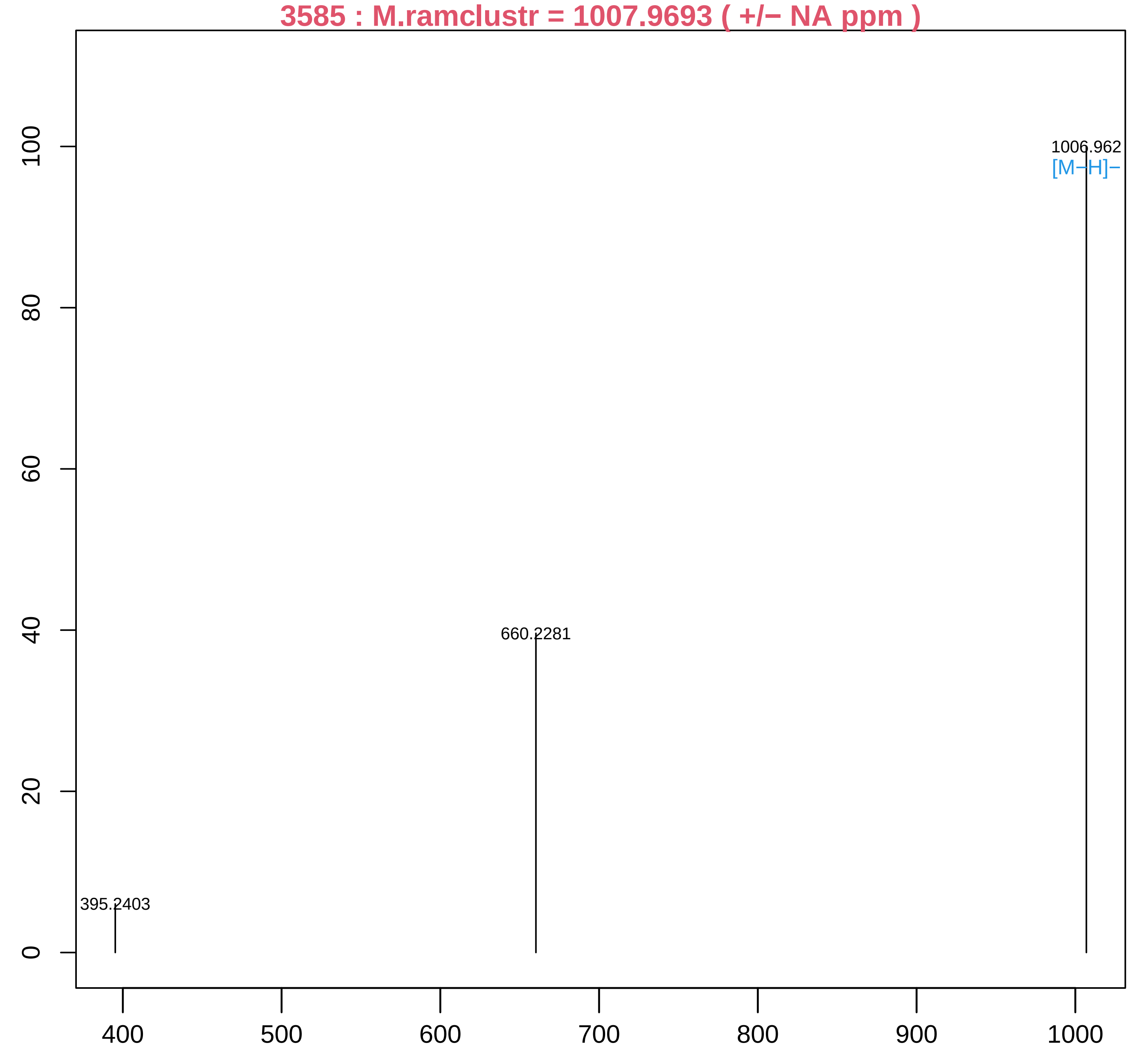
3585 : M.ramclustr = 1007.9693 (+/− NA ppm)
1006.962
[M−H]−
660.2281
395.2403
0
20
40
60
80
100
400
500
600
700
800
900
1000

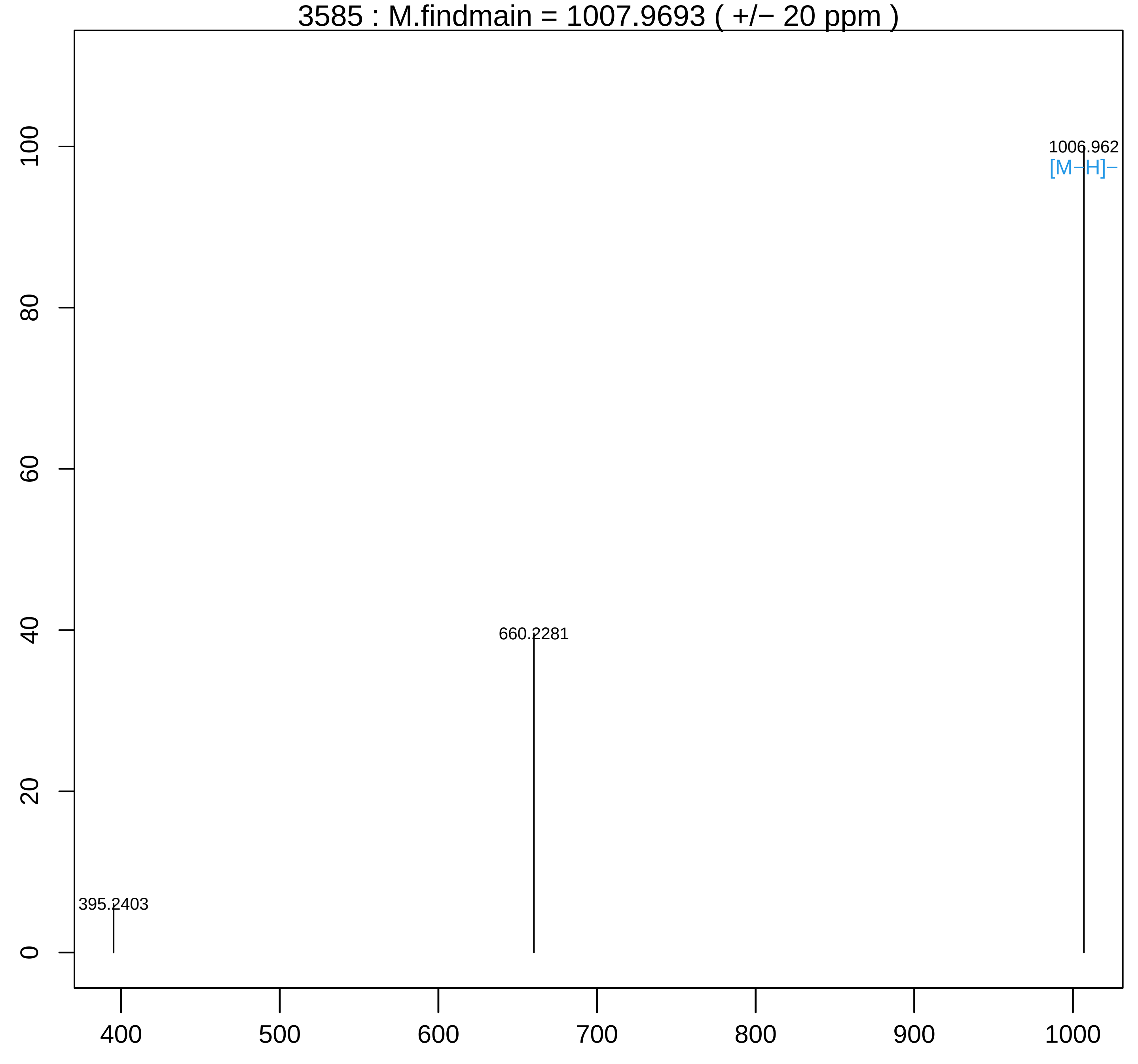
3585 : M.findmain = 1007.9693 (+/− 20 ppm)
1006.962
[M−H]−
660.2281
395.2403
0
20
40
60
80
100
400
500
600
700
800
900
1000

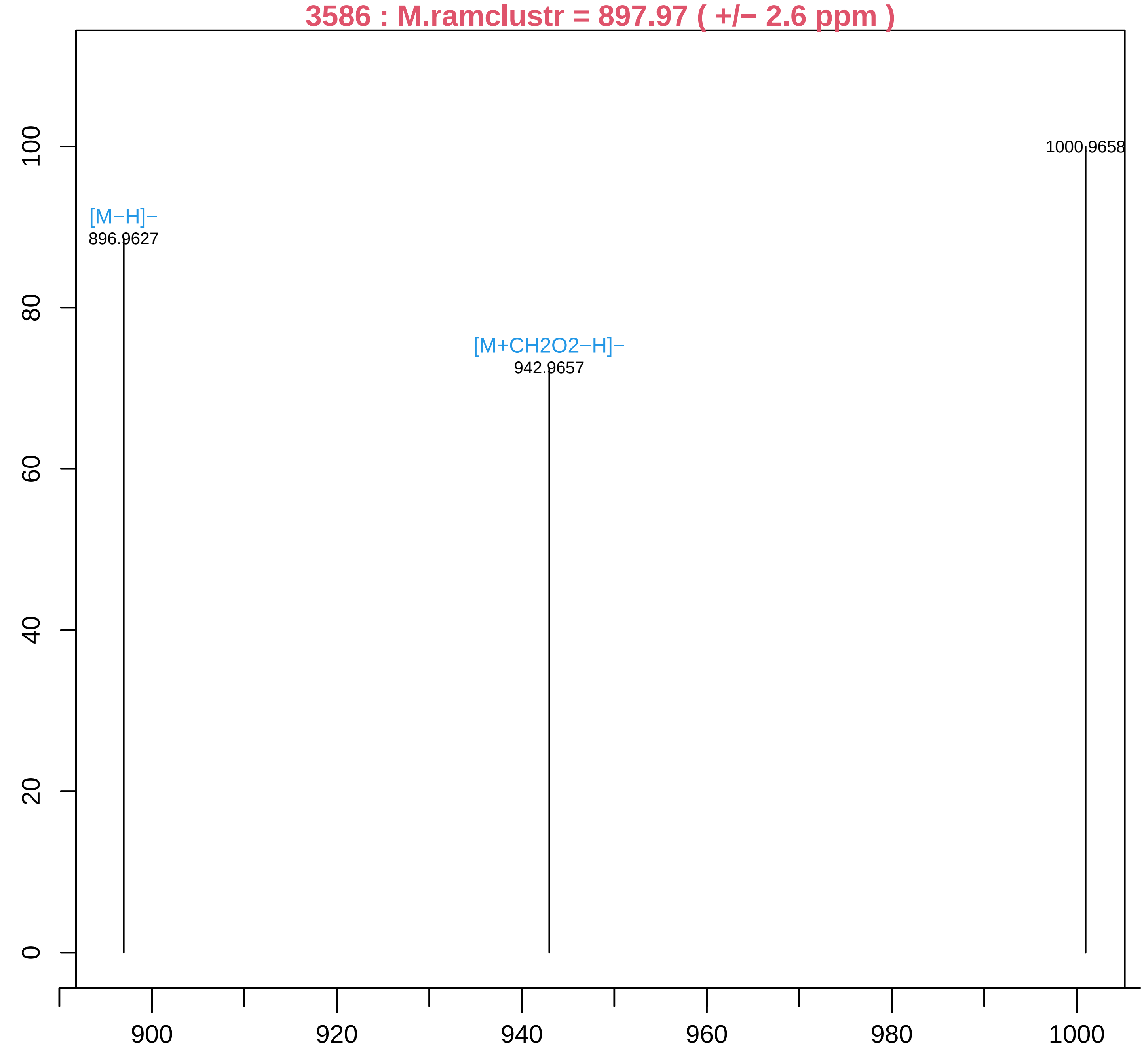
3586 : M.ramclustr = 897.97 (+/− 2.6 ppm)
[M−H]−
896.9627
[M+CH2O2−H]−
942.9657
1000.9658
0
20
40
60
80
100
900
920
940
960
980
1000

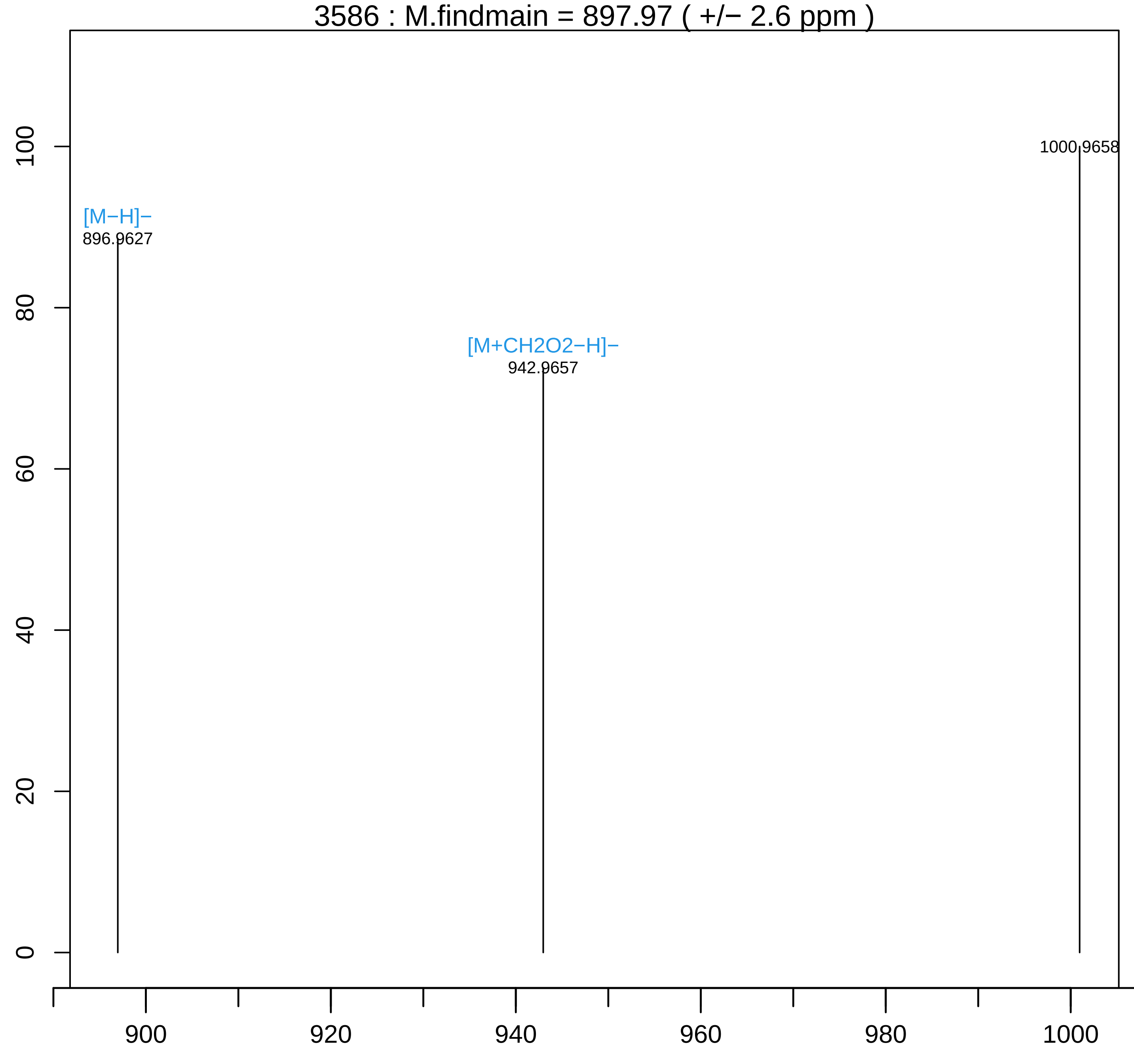
3586 : M.findmain = 897.97 (+/− 2.6 ppm)
[M−H]−
896.9627
[M+CH2O2−H]−
942.9657
1000.9658
0
20
40
60
80
100
900
920
940
960
980
1000

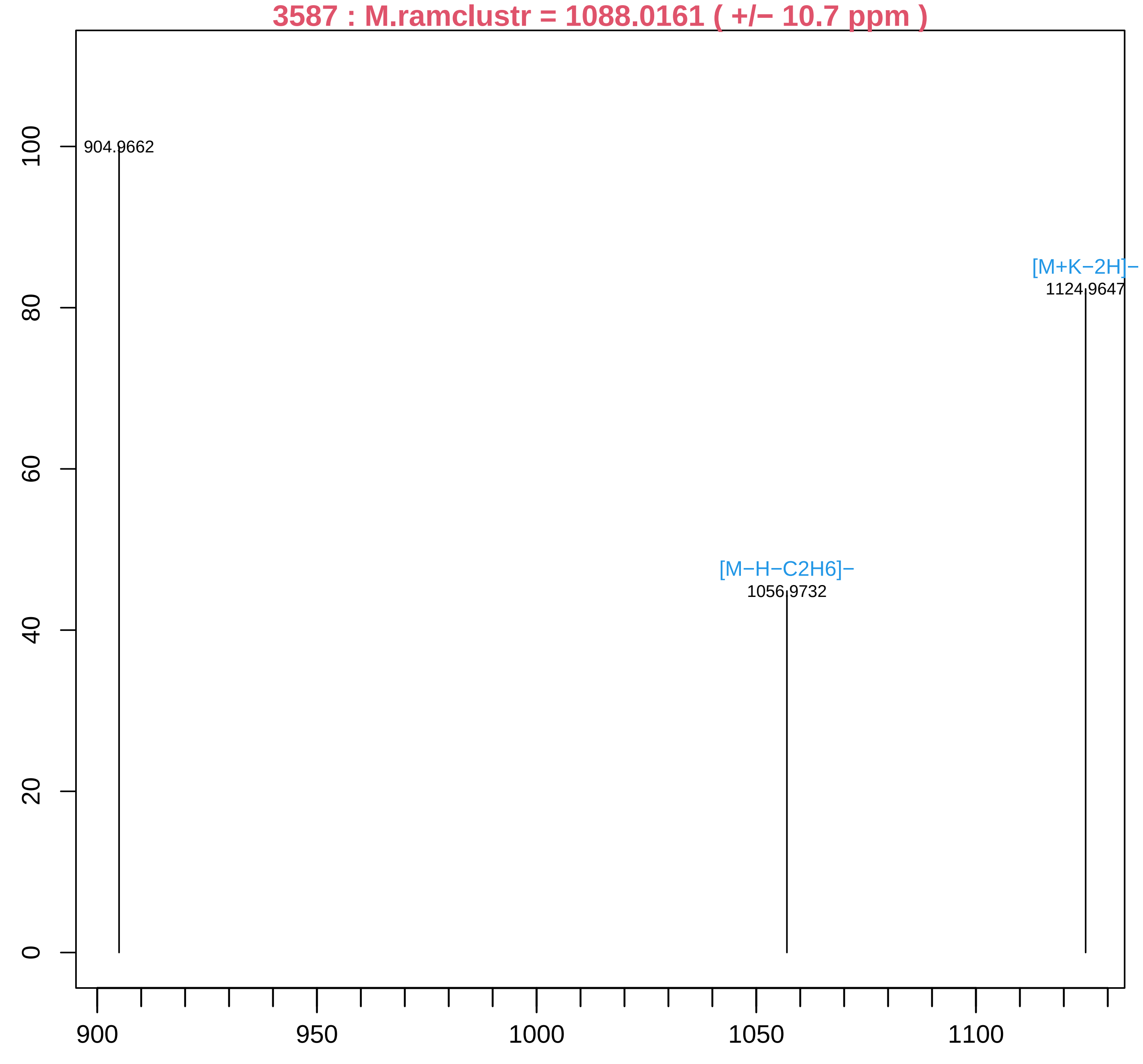

3587 : M.ramclustr = 1088.0161 (+/− 10.7 ppm)
904.9662
[M−H−C2H6]−
1056.9732
[M+K−2H]−
1124.9647
0
20
40
60
80
100
900
950
1000
1050
1100

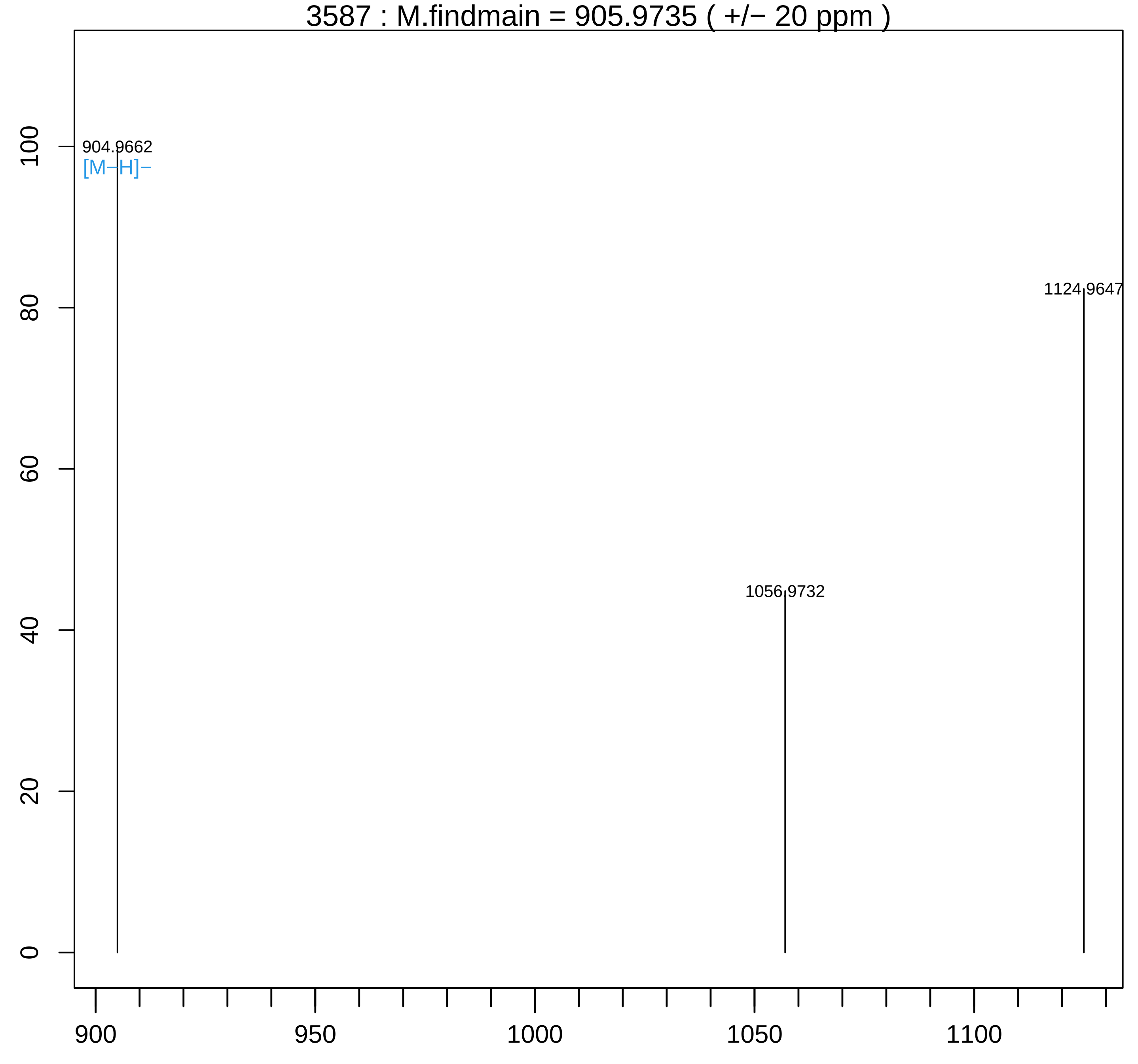

3587 : M.findmain = 905.9735 (+/− 20 ppm)
904.9662
[M−H]−
1056.9732
1124.9647
0
20
40
60
80
100
900
950
1000
1050
1100

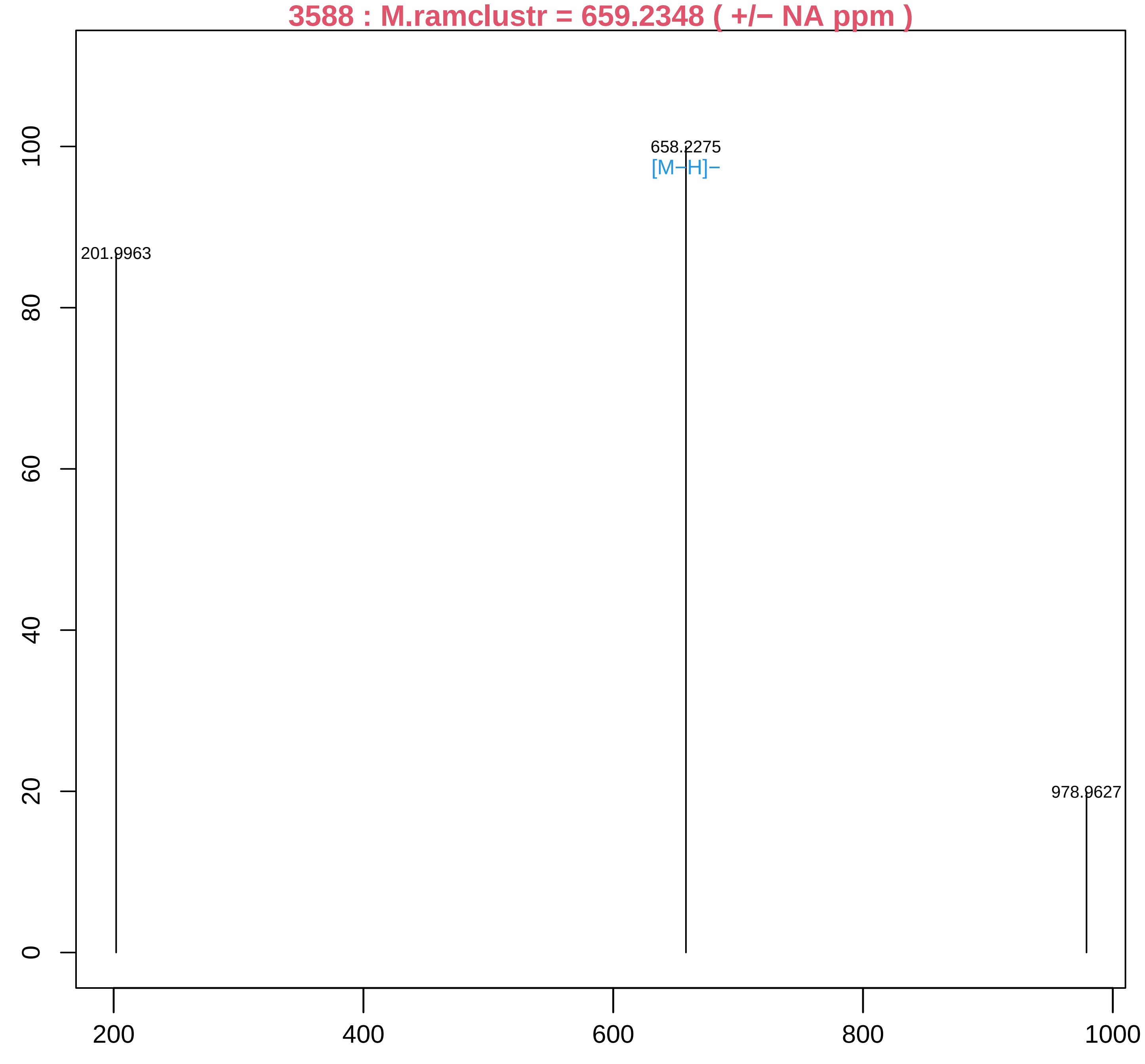
3588 : M.ramclustr = 659.2348 (+/− NA ppm)
658.2275
[M−H]−
201.9963
978.9627
0
20
40
60
80
100
200
400
600
800
1000

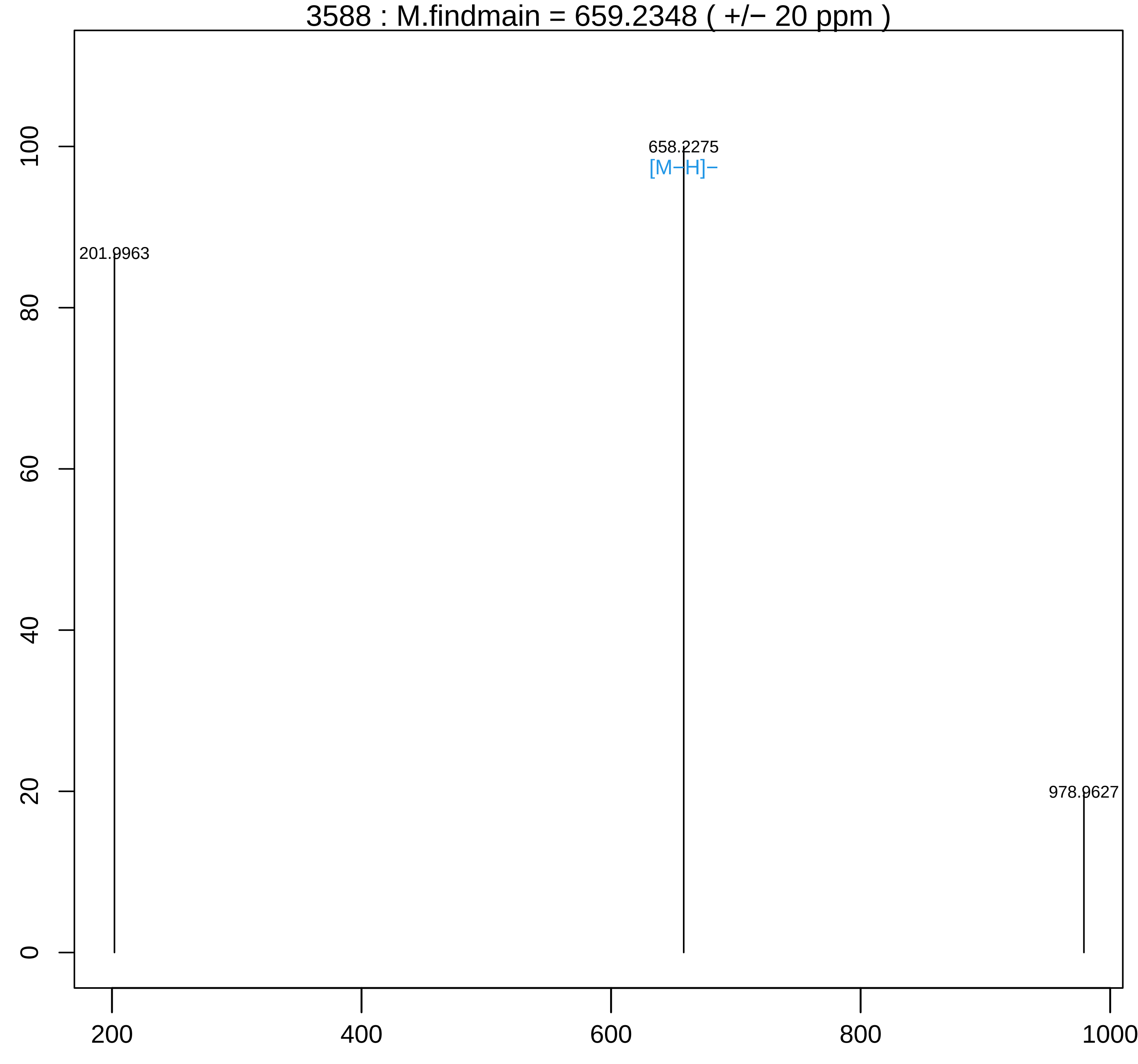
3588 : M.findmain = 659.2348 (+/− 20 ppm)
658.2275
[M−H]−
201.9963
978.9627
0
20
40
60
80
100
200
400
600
800
1000

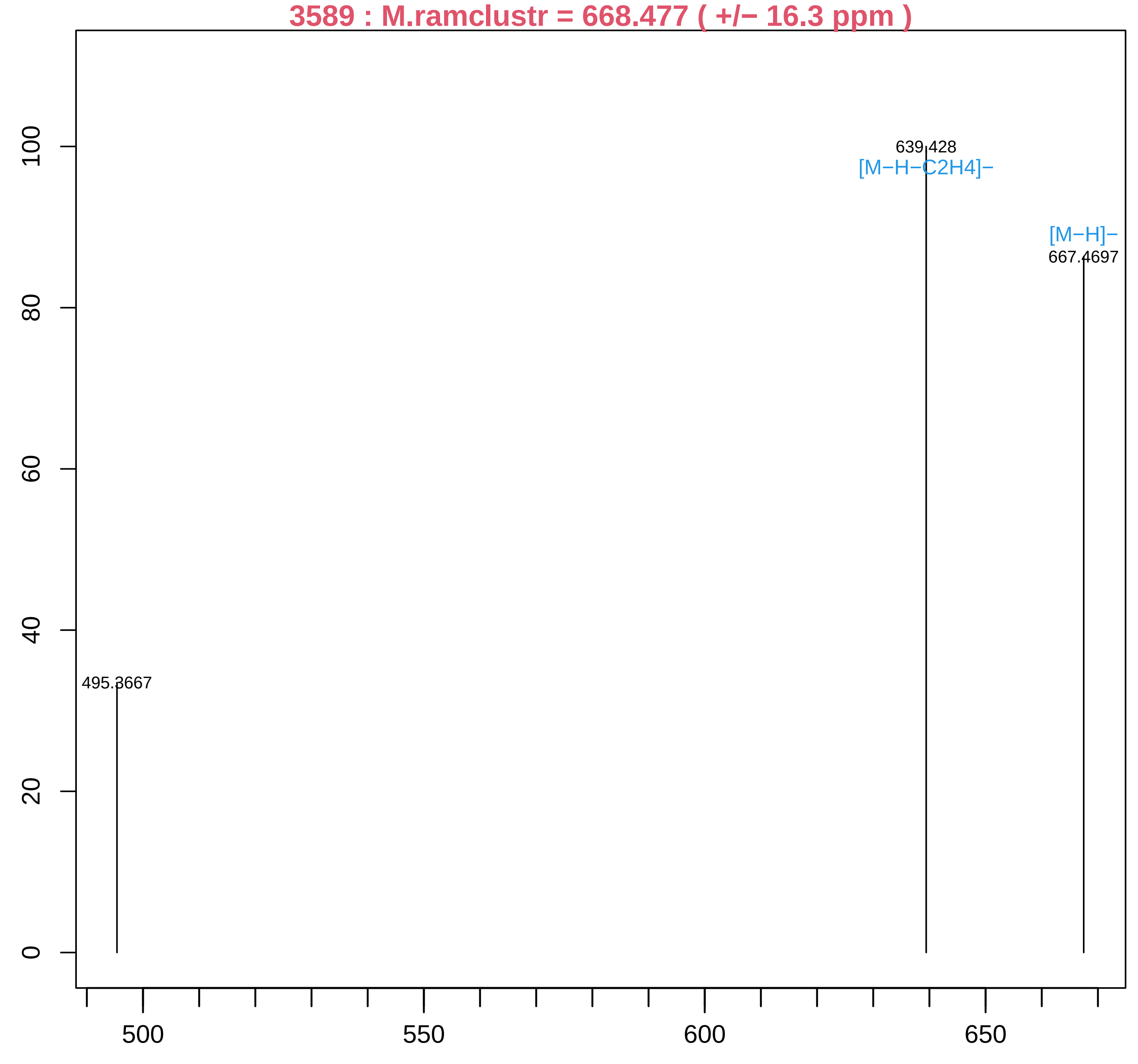
3589 : M.ramclustr = 668.477 (+/− 16.3 ppm)
639.428
[M−H−C2H4]−
[M−H]−
667.4697
495.3667
0
20
40
60
80
100
500
550
600
650

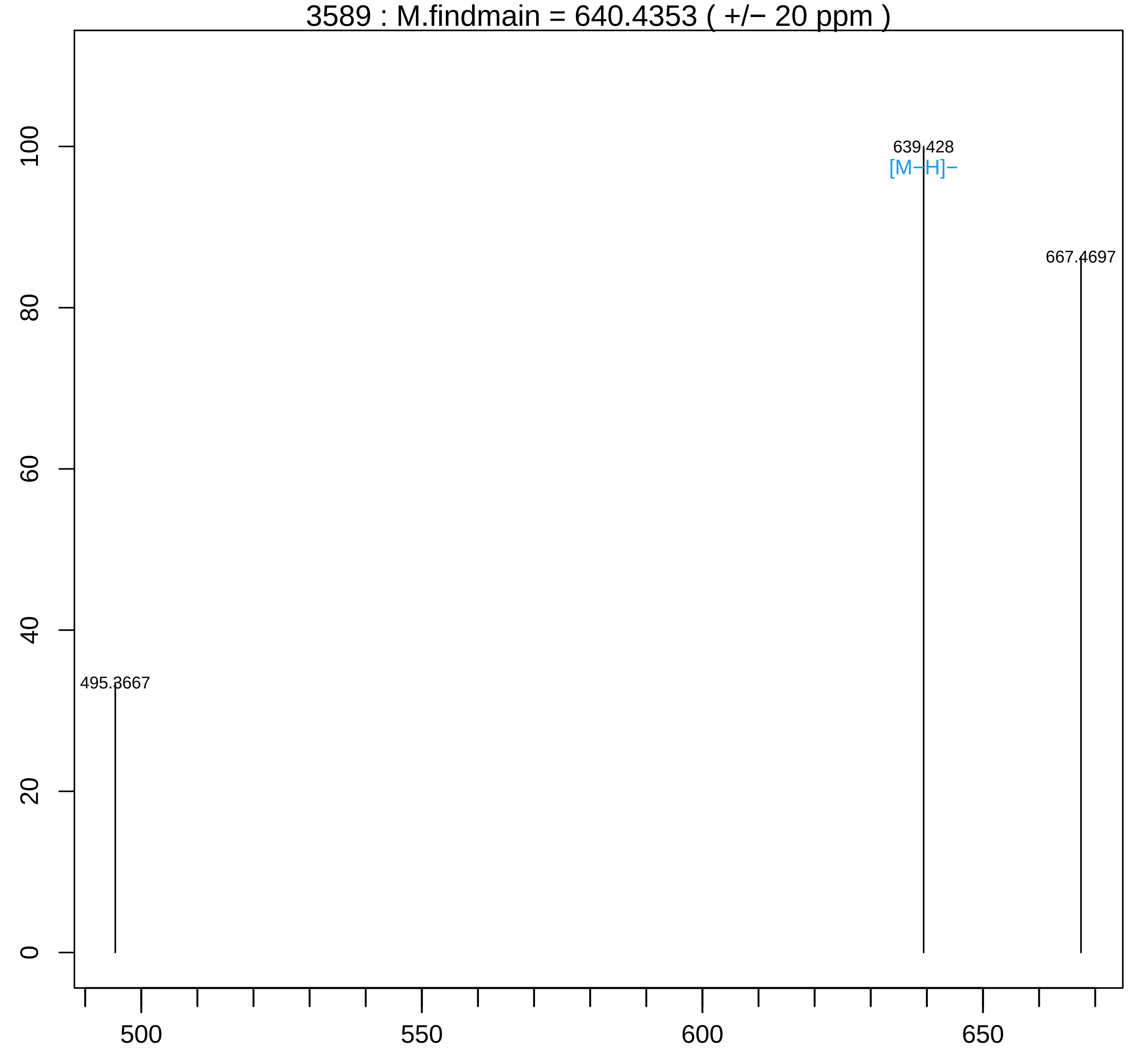
3589 : M.findmain = 640.4353 (+/− 20 ppm)
639.428
[M−H]−
667.4697
495.3667
0
20
40
60
80
100
500
550
600
650

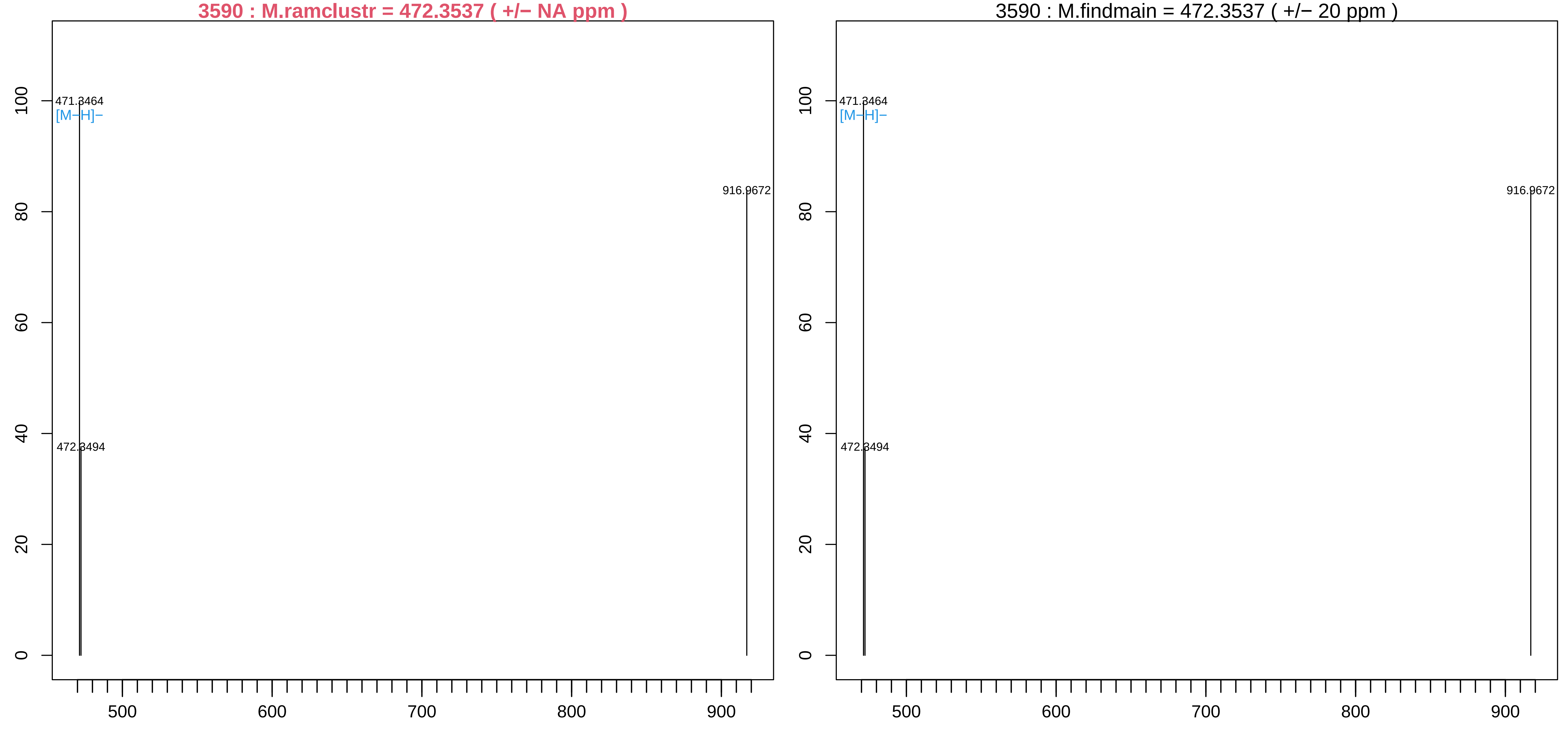
3590 : M.ramclustr = 472.3537 (+/− NA ppm)
471.3464
[M−H]−
472.3494
916.9672
0
20
40
60
80
100
500
600
700
800
900
3590 : M.findmain = 472.3537 (+/− 20 ppm)
471.3464
[M−H]−
472.3494
916.9672
0
20
40
60
80
100
500
600
700
800
900

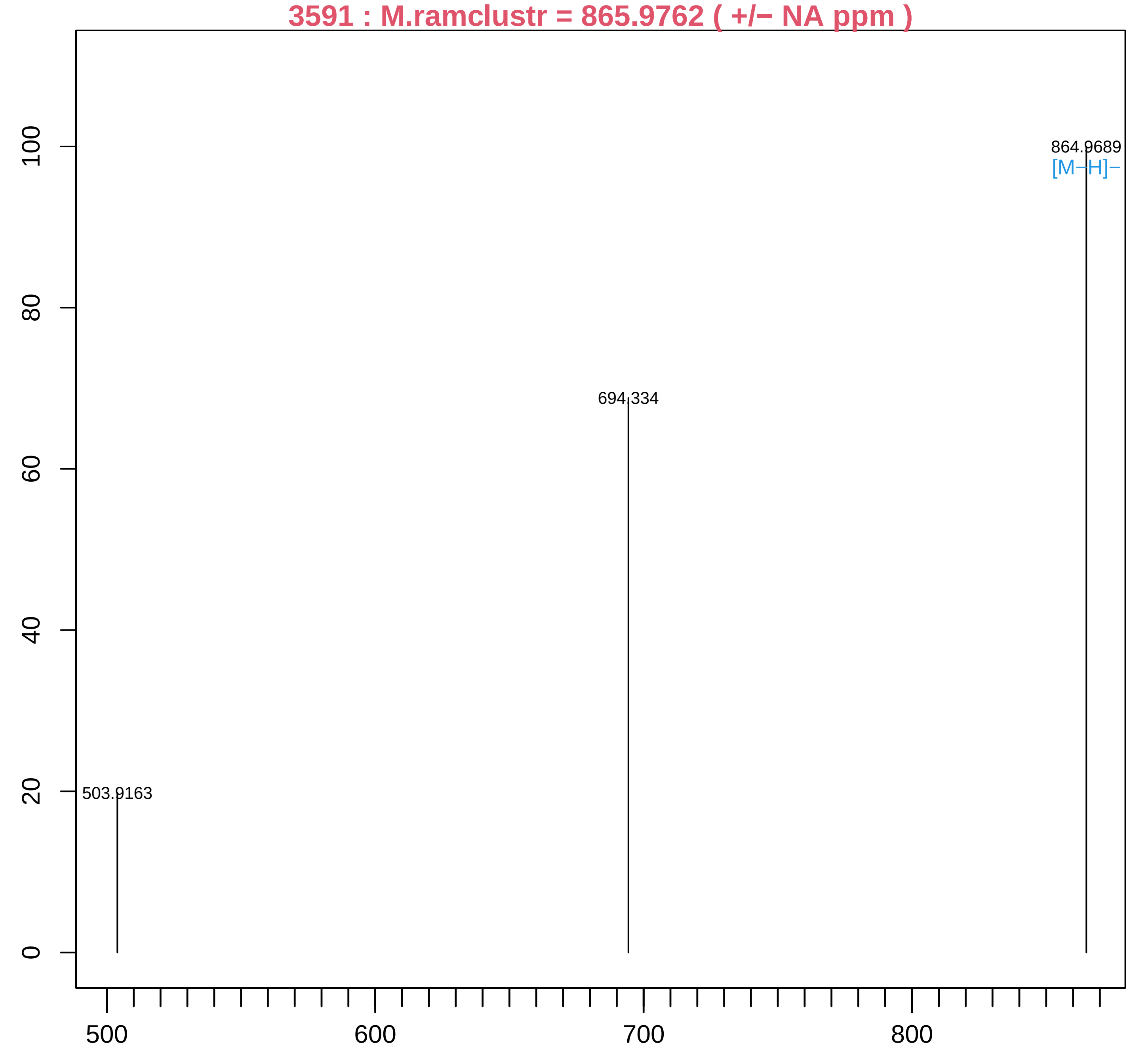
3591 : M.ramclustr = 865.9762 (+/− NA ppm)
864.9689
[M−H]−
694.334
503.9163
0
20
40
60
80
100
500
600
700
800

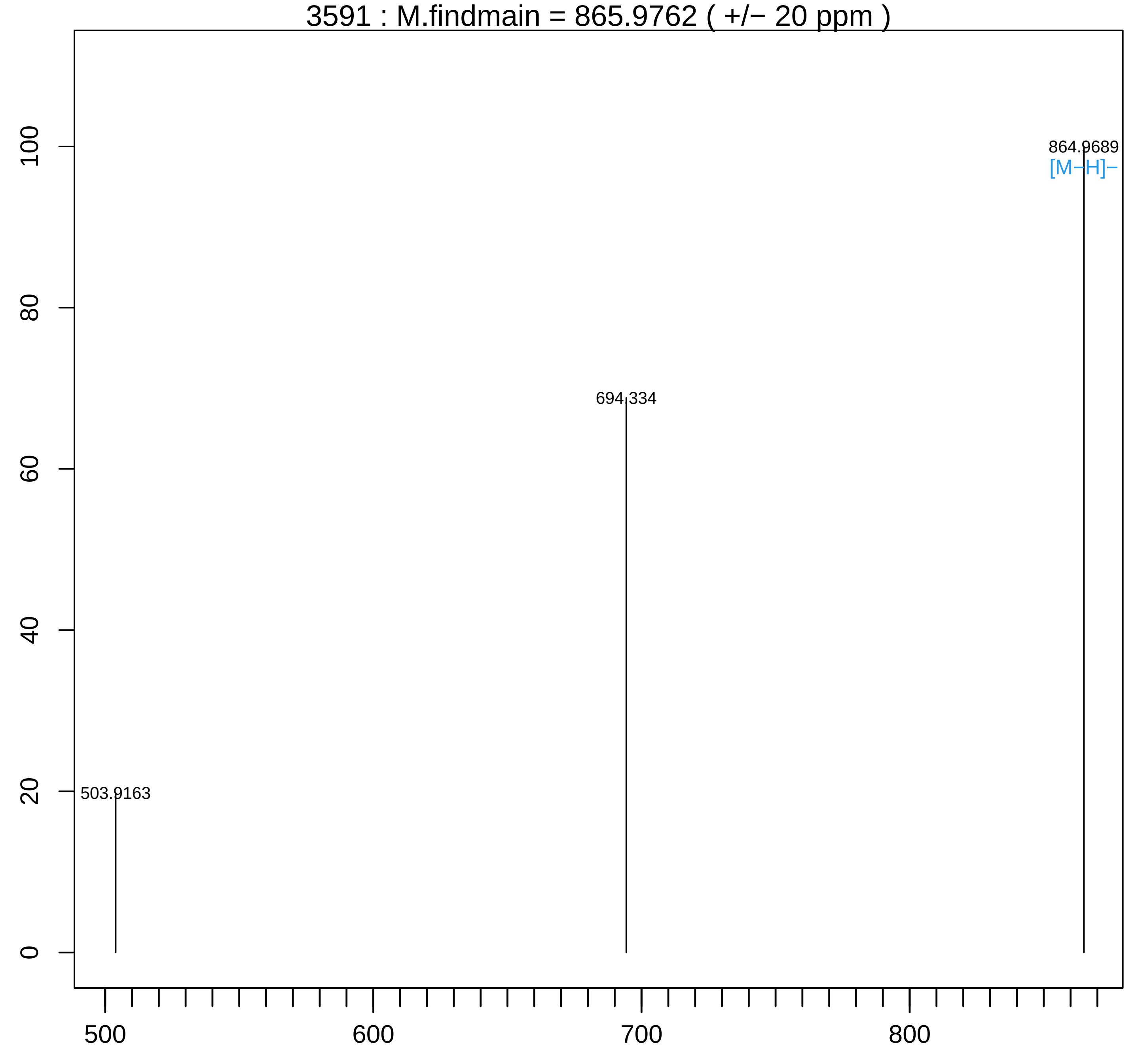
3591 : M.findmain = 865.9762 (+/− 20 ppm)
864.9689
[M−H]−
694.334
503.9163
0
20
40
60
80
100
500
600
700
800

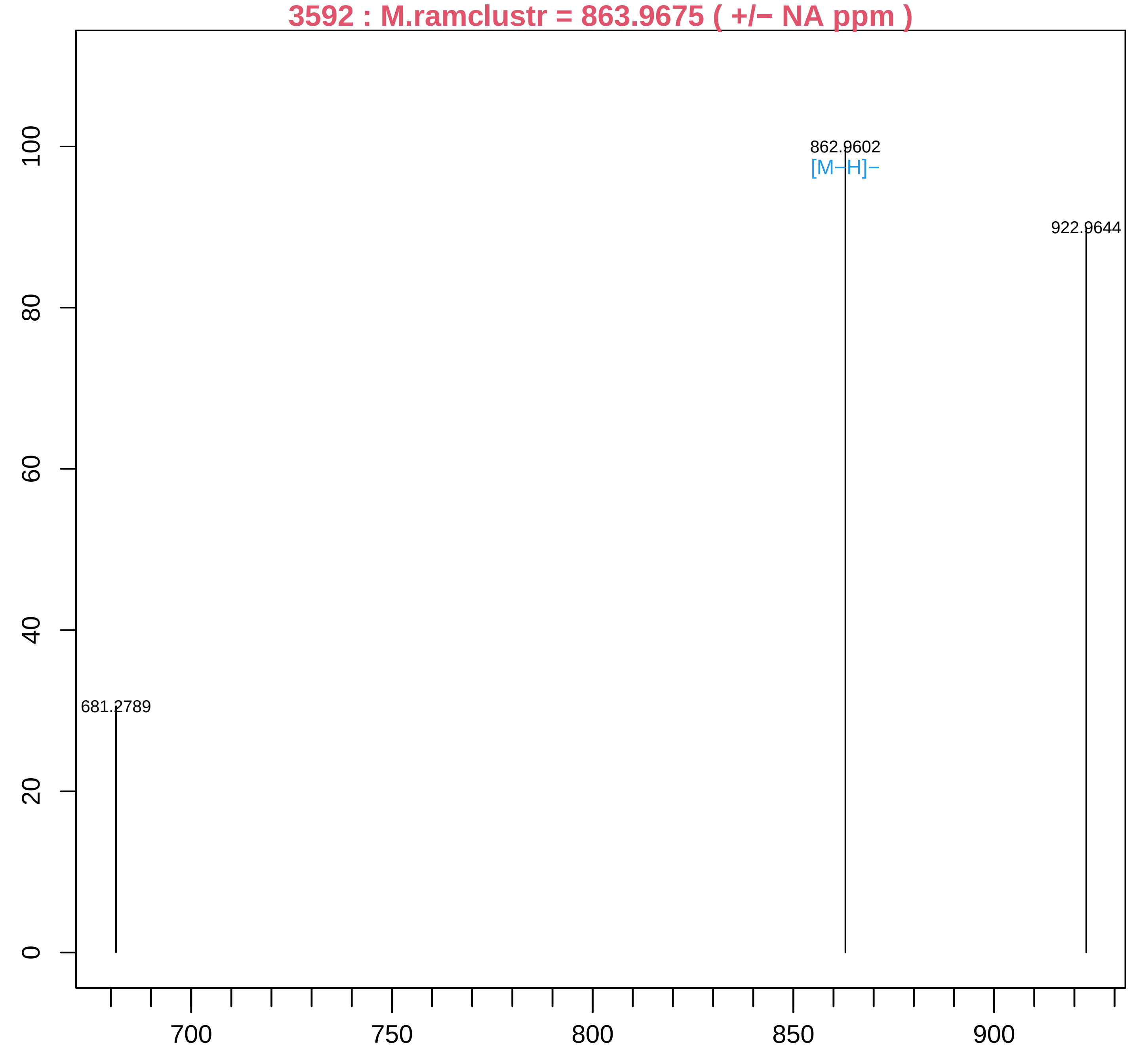
3592 : M.ramclustr = 863.9675 (+/− NA ppm)
862.9602
[M−H]−
922.9644
681.2789
0
20
40
60
80
100
700
750
800
850
900

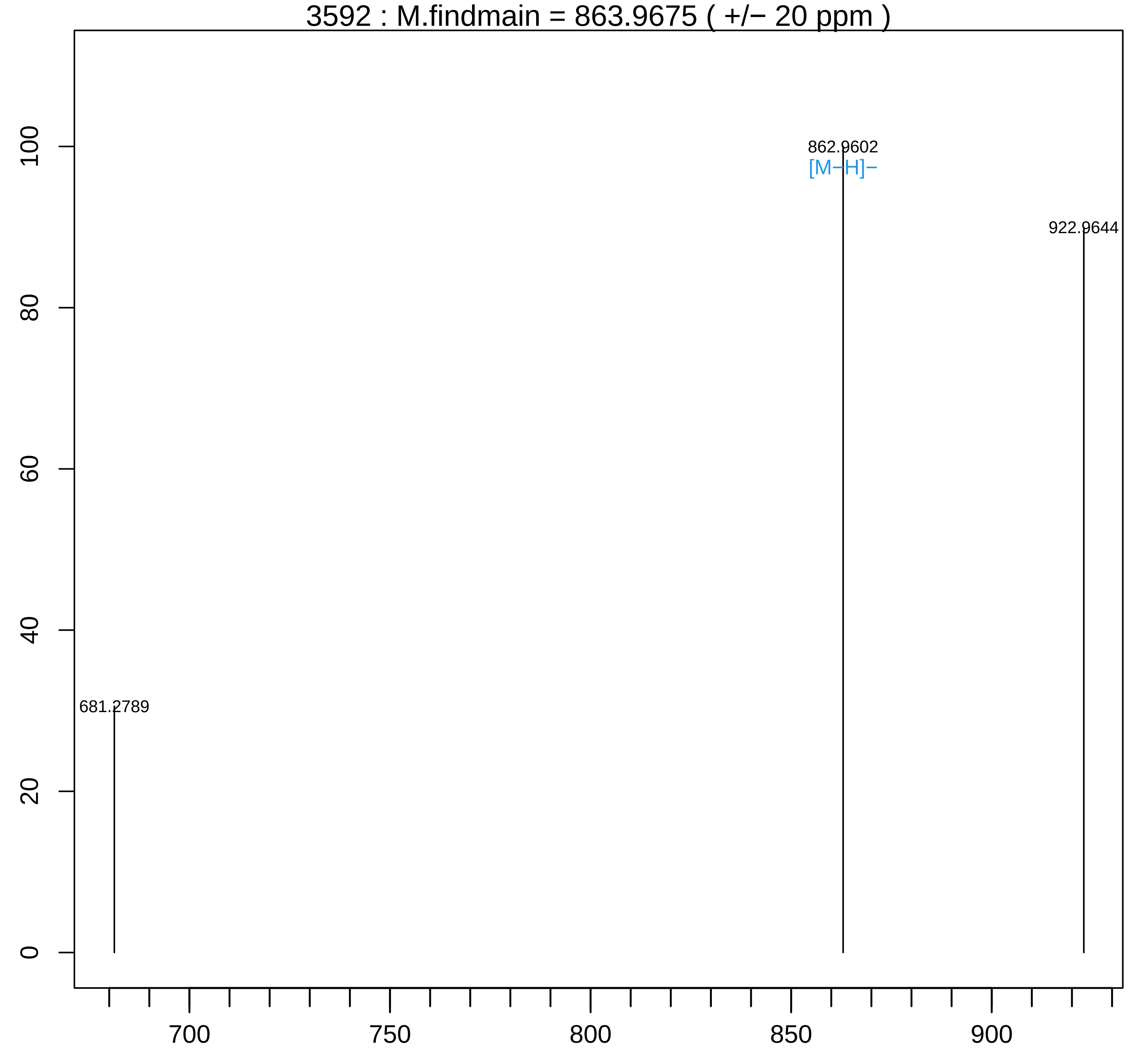
3592 : M.findmain = 863.9675 (+/− 20 ppm)
862.9602
[M−H]−
922.9644
681.2789
0
20
40
60
80
100
700
750
800
850
900

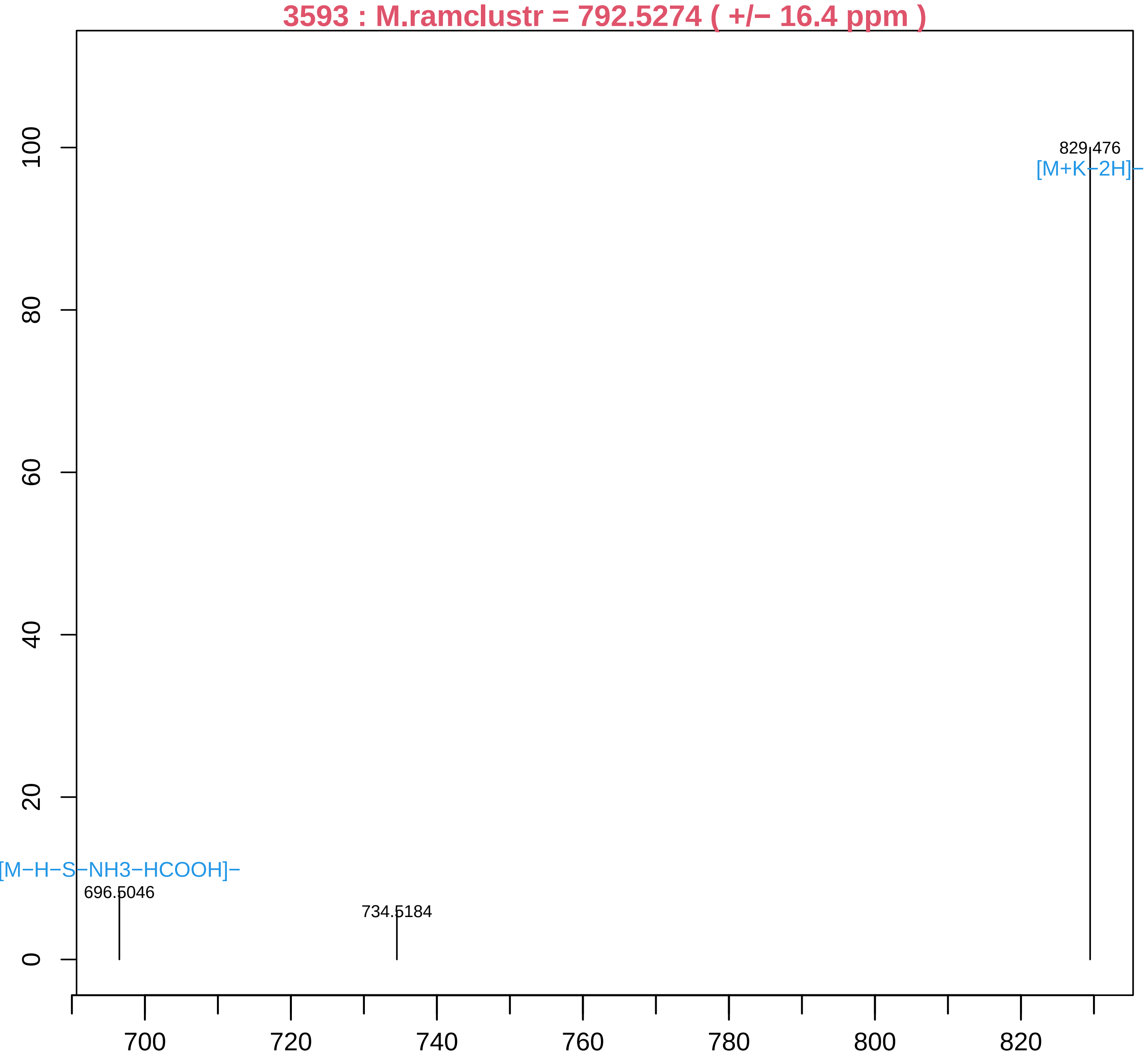
3593 : M.ramclustr = 792.5274 (+/− 16.4 ppm)
829.476
[M+K−2H]−
[M−H−S−NH3−HCOOH]−
696.5046
734.5184
0
20
40
60
80
100
700
720
740
760
780
800
820

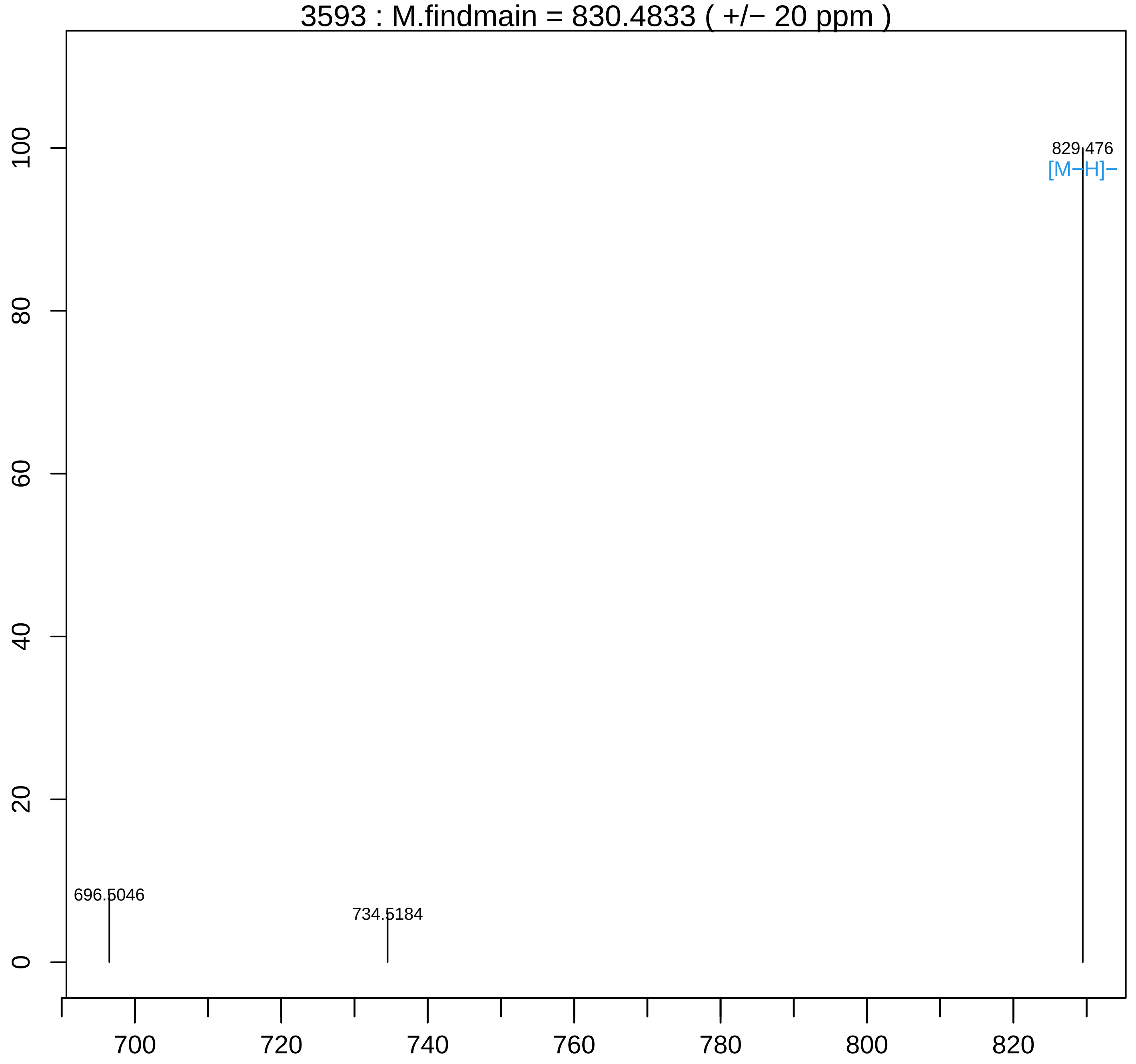
3593 : M.findmain = 830.4833 (+/− 20 ppm)
829.476
[M−H]−
696.5046
734.5184
0
20
40
60
80
100
700
720
740
760
780
800
820

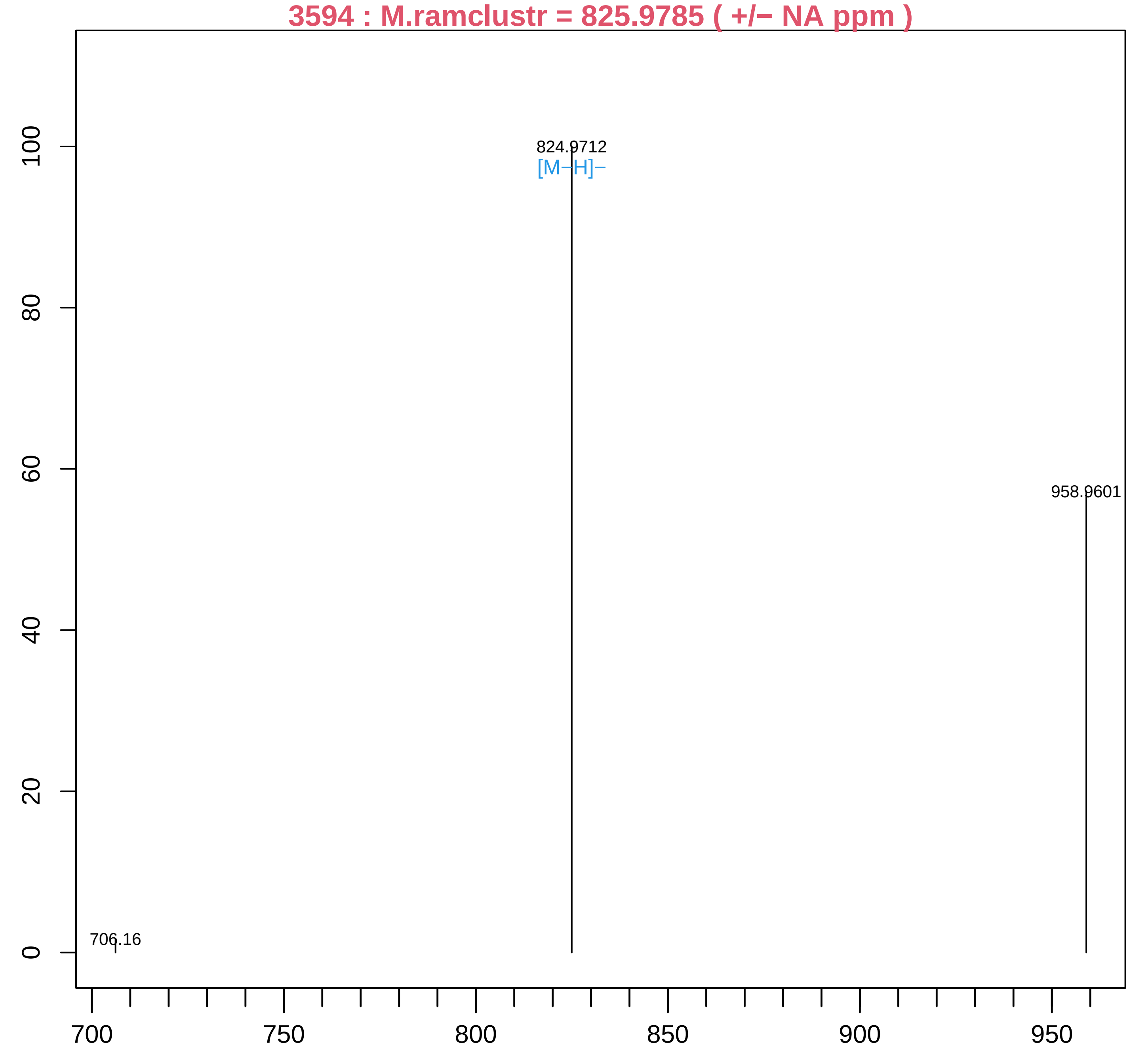
3594 : M.ramclustr = 825.9785 (+/− NA ppm)
824.9712
[M−H]−
958.9601
706.16
0
20
40
60
80
100
700
750
800
850
900
950

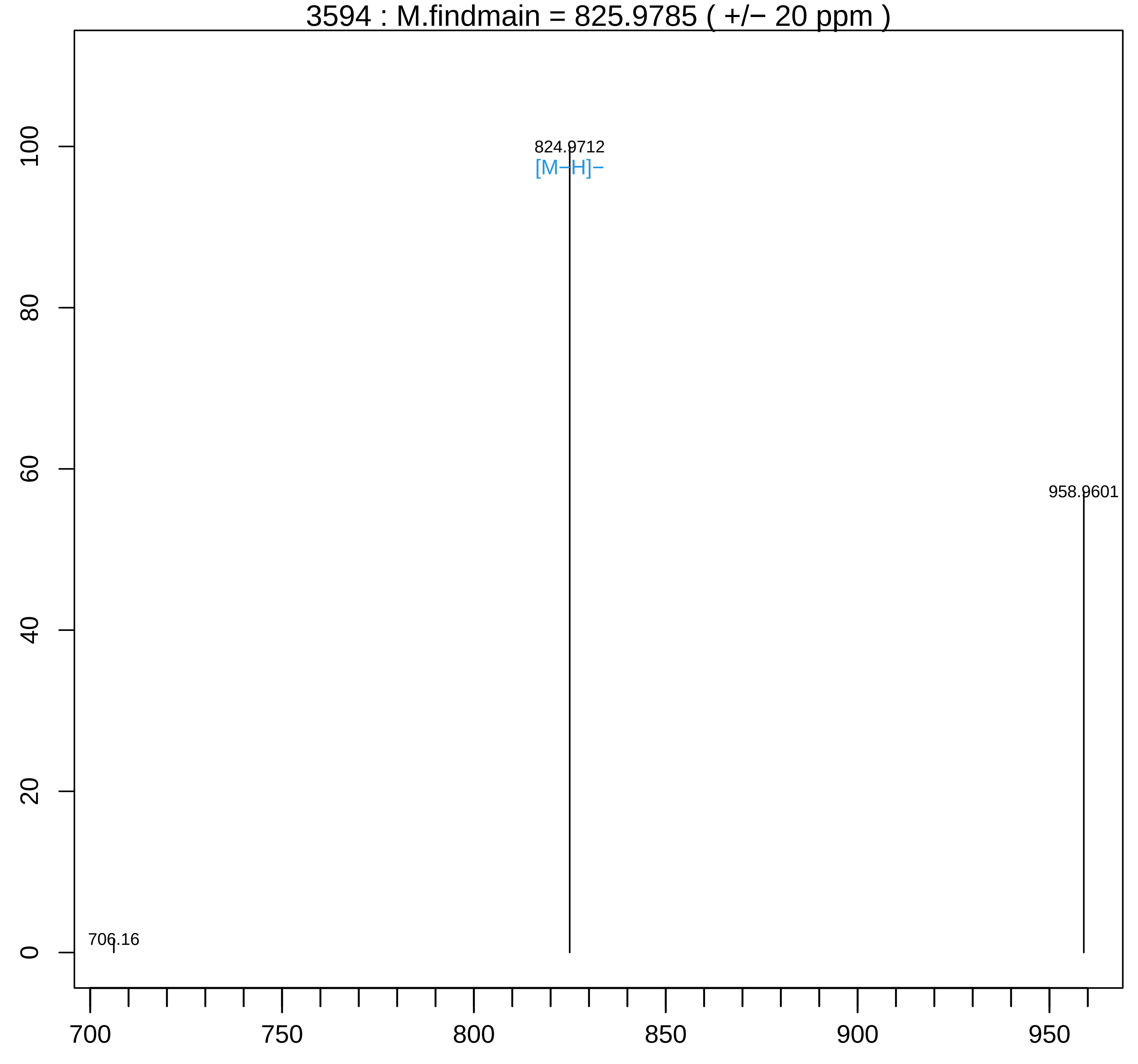
3594 : M.findmain = 825.9785 (+/− 20 ppm)
824.9712
[M−H]−
958.9601
706.16
0
20
40
60
80
100
700
750
800
850
900
950

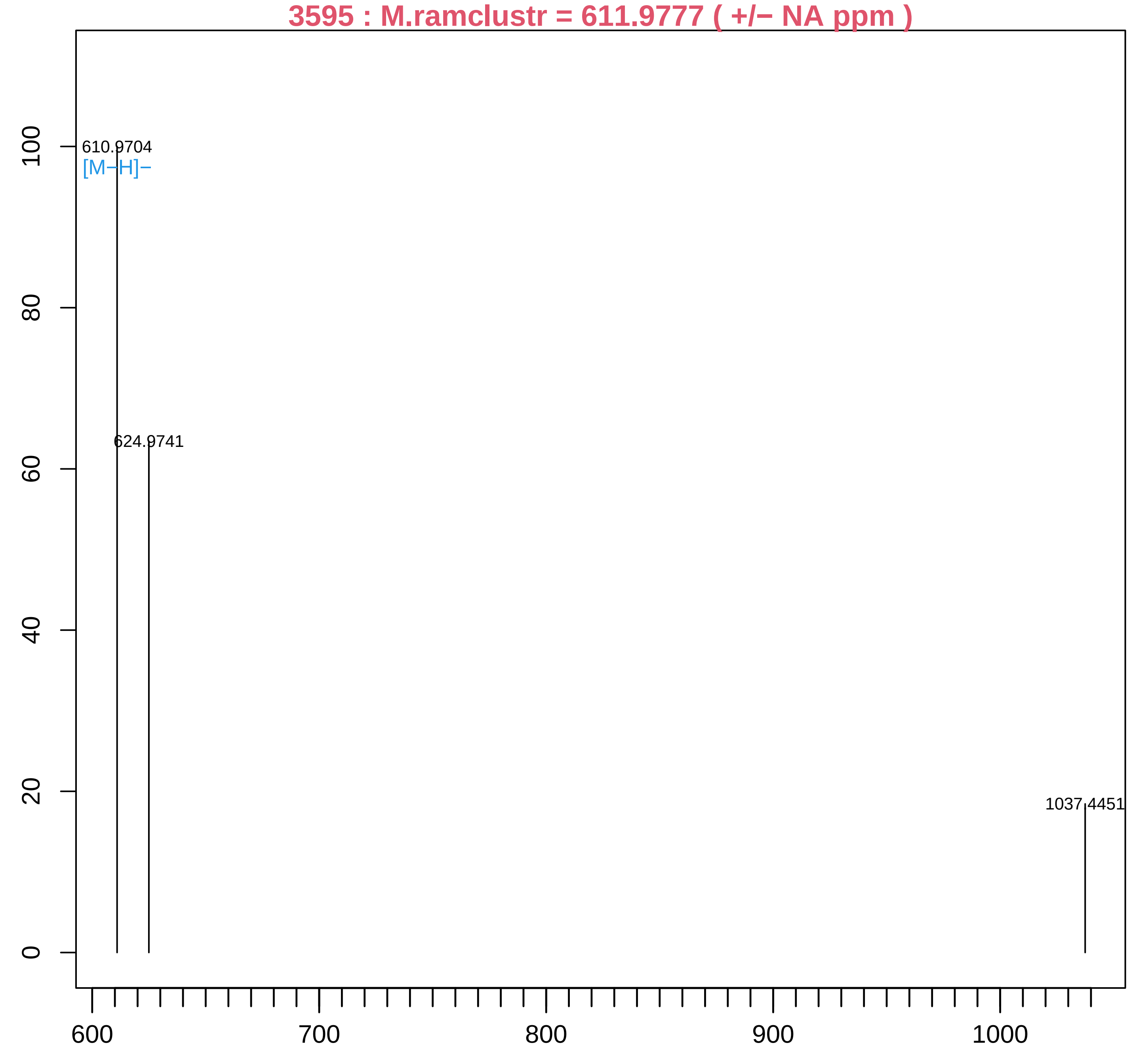
3595 : M.ramclustr = 611.9777 (+/− NA ppm)
610.9704
[M−H]−
624.9741
1037.4451
0
20
40
60
80
100
600
700
800
900
1000

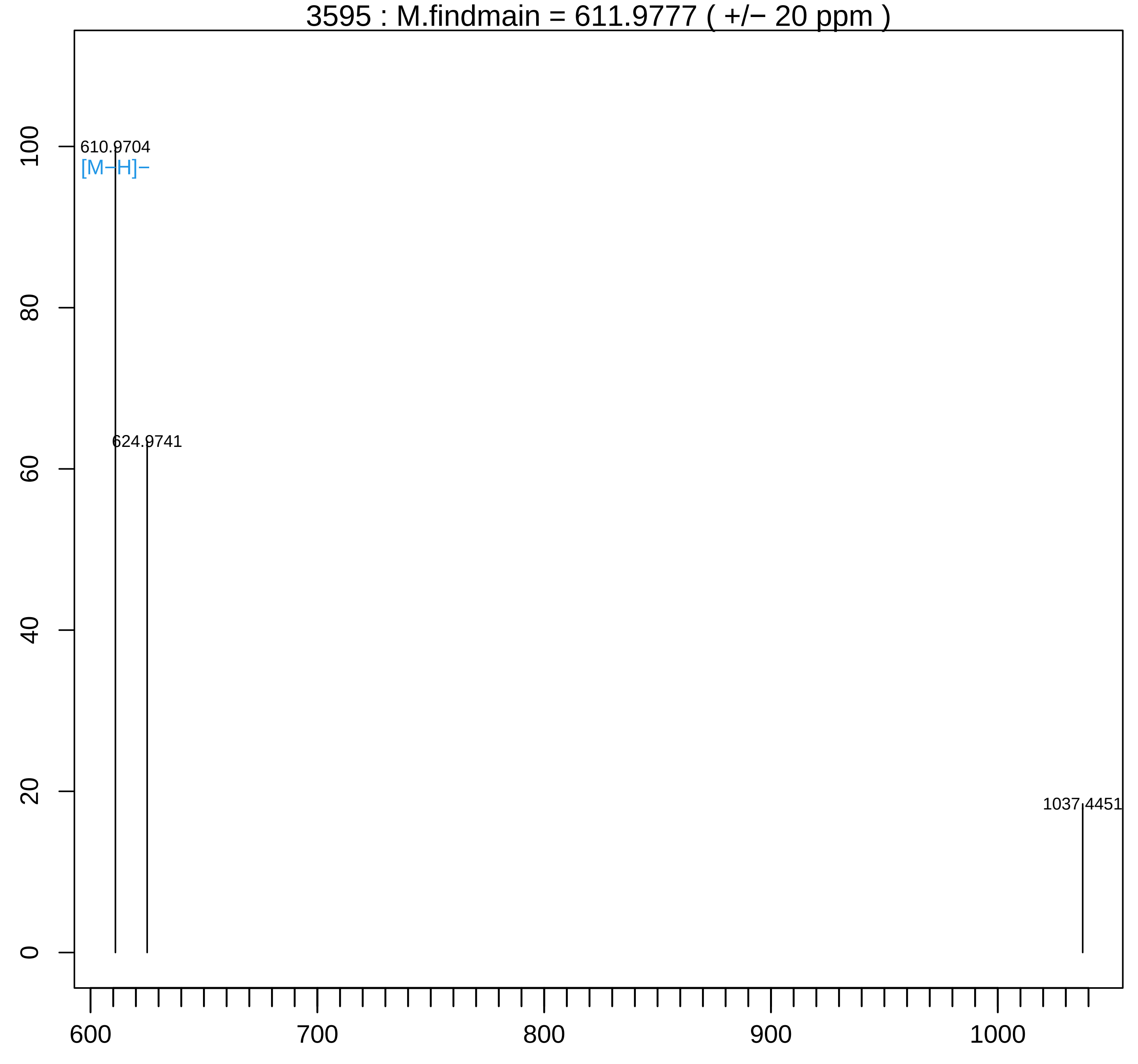
3595 : M.findmain = 611.9777 (+/− 20 ppm)
610.9704
[M−H]−
624.9741
1037.4451
0
20
40
60
80
100
600
700
800
900
1000

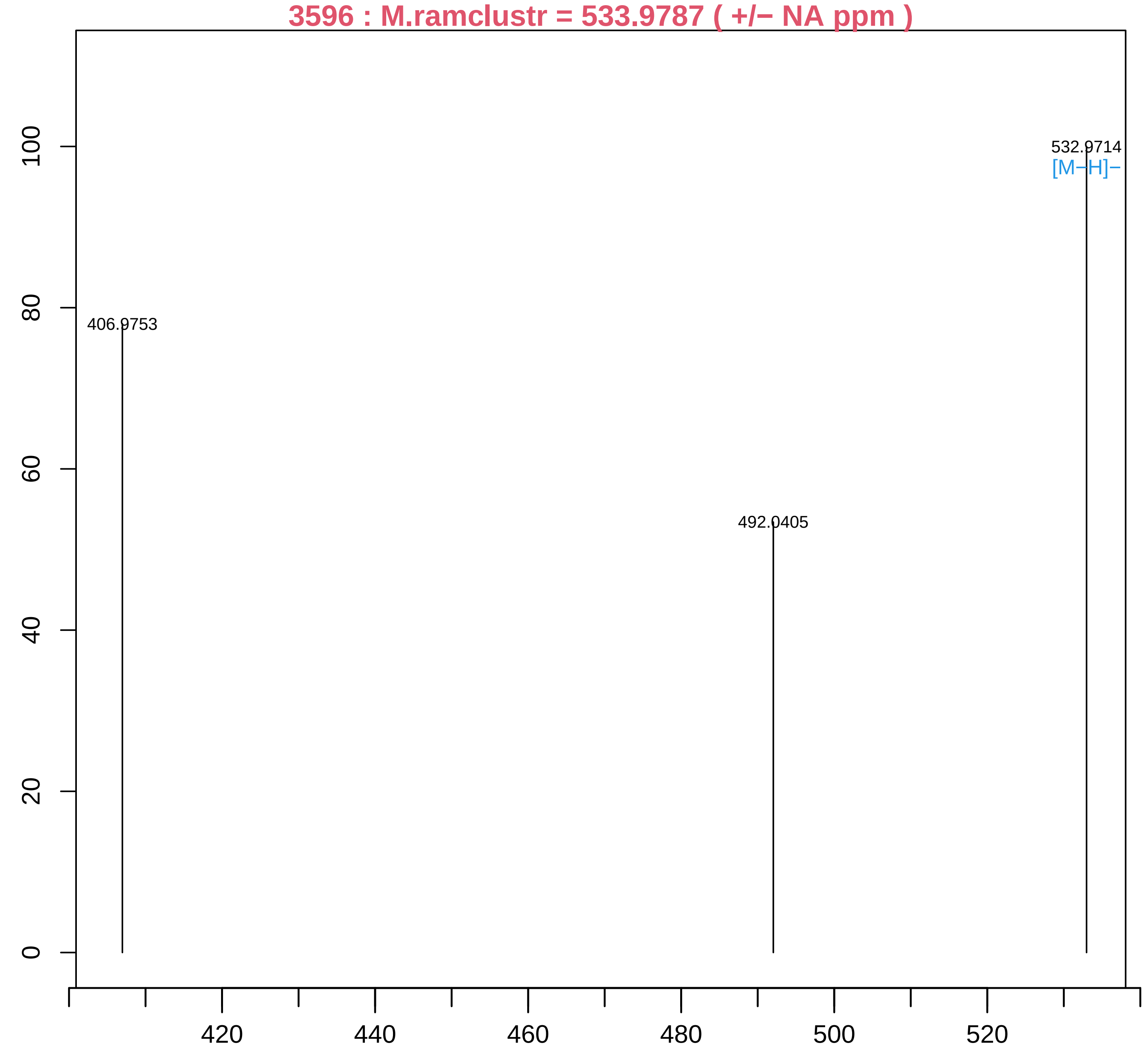
3596 : M.ramclustr = 533.9787 (+/− NA ppm)
406.9753
492.0405
532.9714
[M−H]−
0
20
40
60
80
100
420
440
460
480
500
520

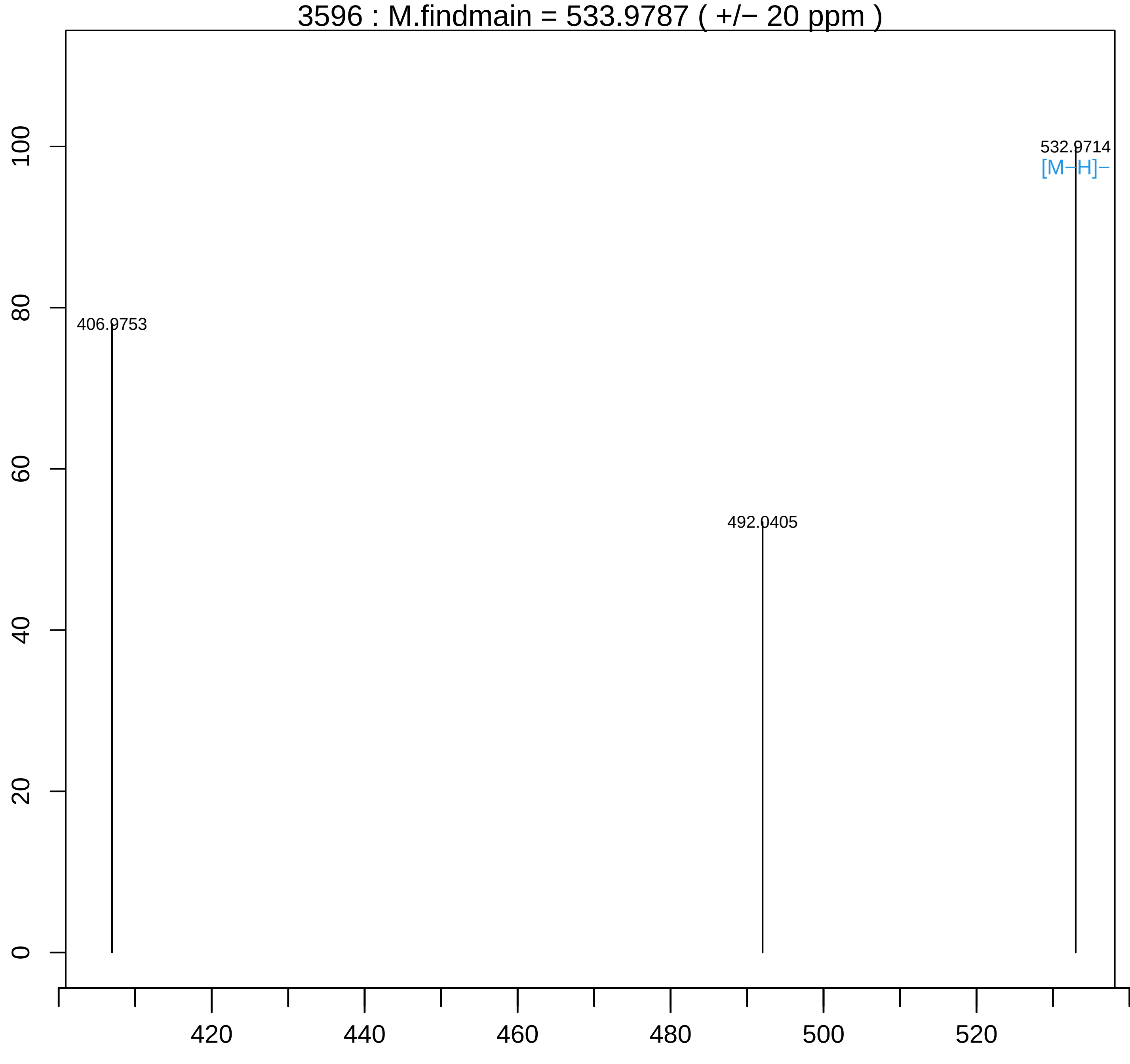
3596 : M.findmain = 533.9787 (+/− 20 ppm)
406.9753
492.0405
532.9714
[M−H]−
0
20
40
60
80
100
420
440
460
480
500
520

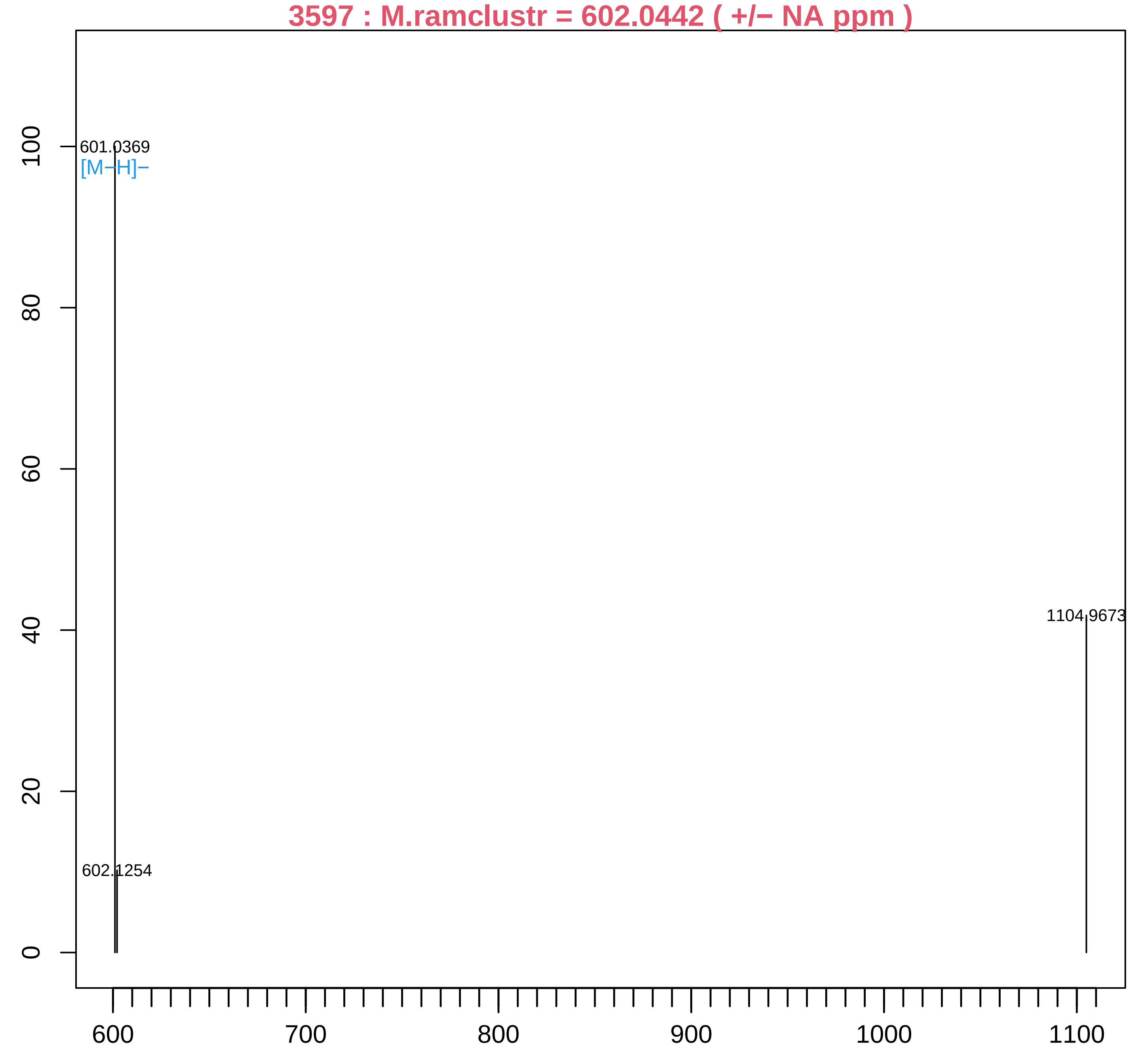

3597 : M.ramclustr = 602.0442 (+/− NA ppm)
601.0369
[M−H]−
602.1254
1104.9673
0
20
40
60
80
100
600
700
800
900
1000
1100

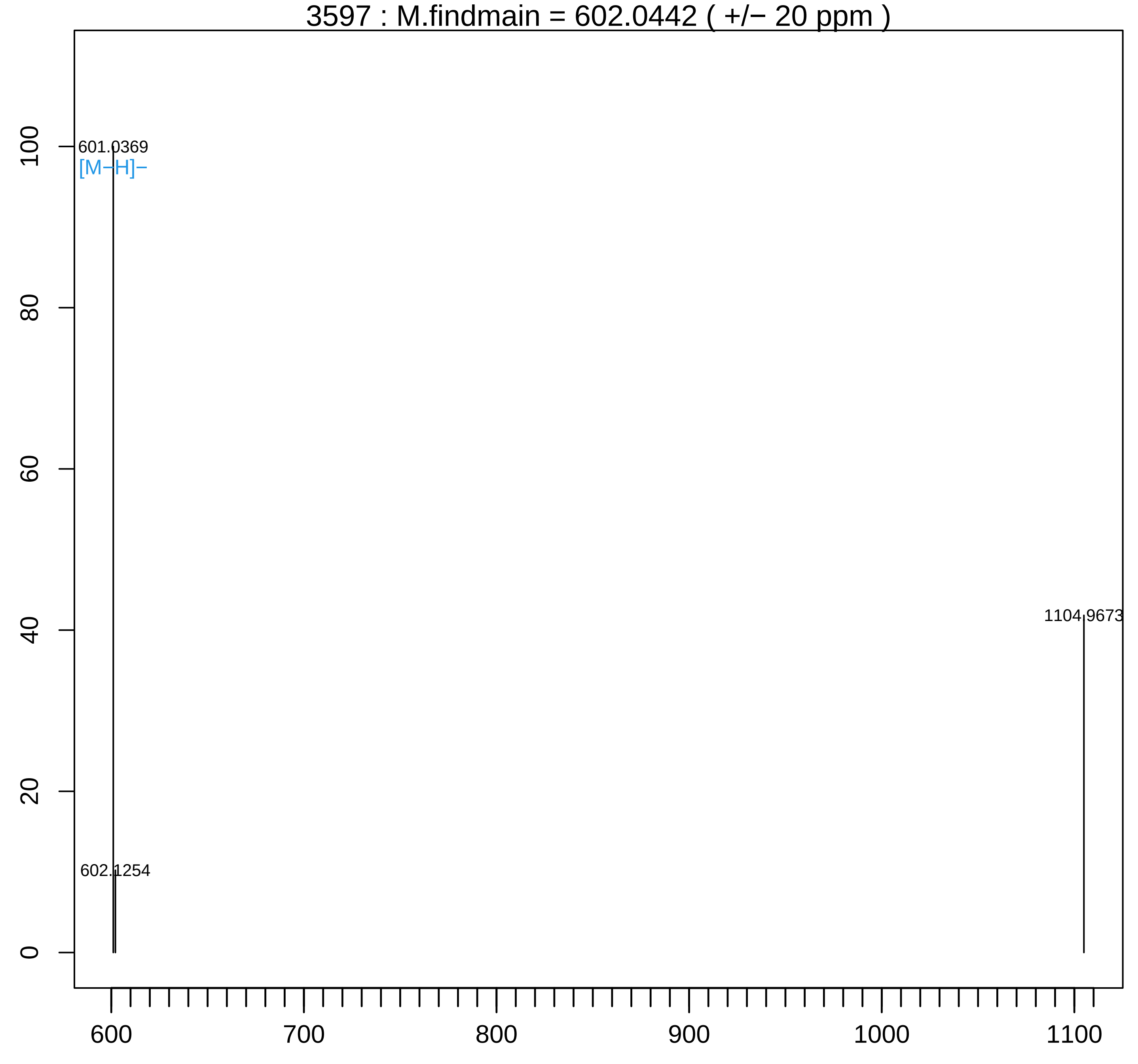

3597 : M.findmain = 602.0442 (+/− 20 ppm)
601.0369
[M−H]−
602.1254
1104.9673
0
20
40
60
80
100
600
700
800
900
1000
1100

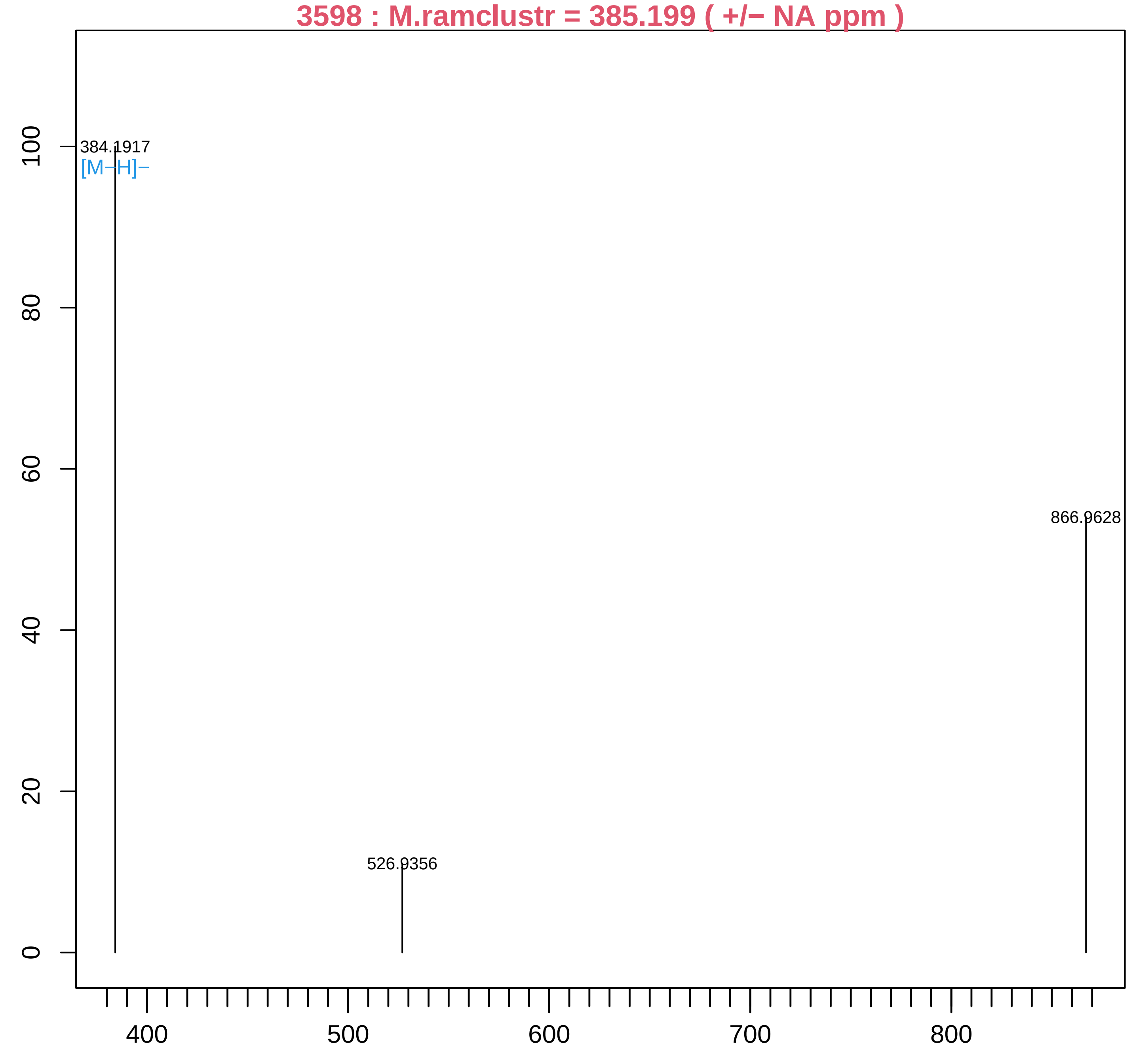

3598 : M.ramclustr = 385.199 (+/− NA ppm)
384.1917
[M−H]−
526.9356
866.9628
0
20
40
60
80
100
400
500
600
700
800

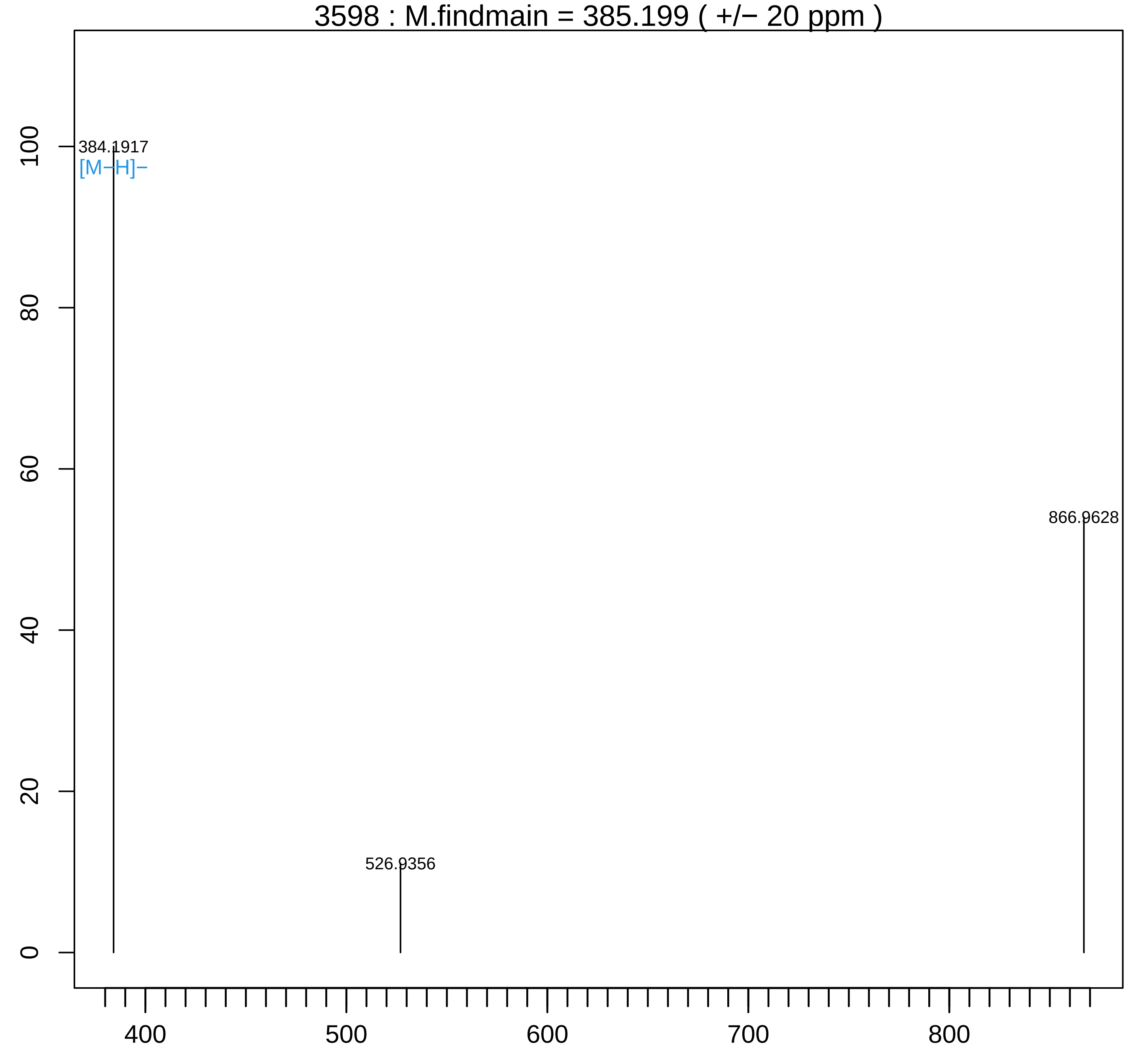

3598 : M.findmain = 385.199 (+/− 20 ppm)
384.1917
[M−H]−
526.9356
866.9628
0
20
40
60
80
100
400
500
600
700
800

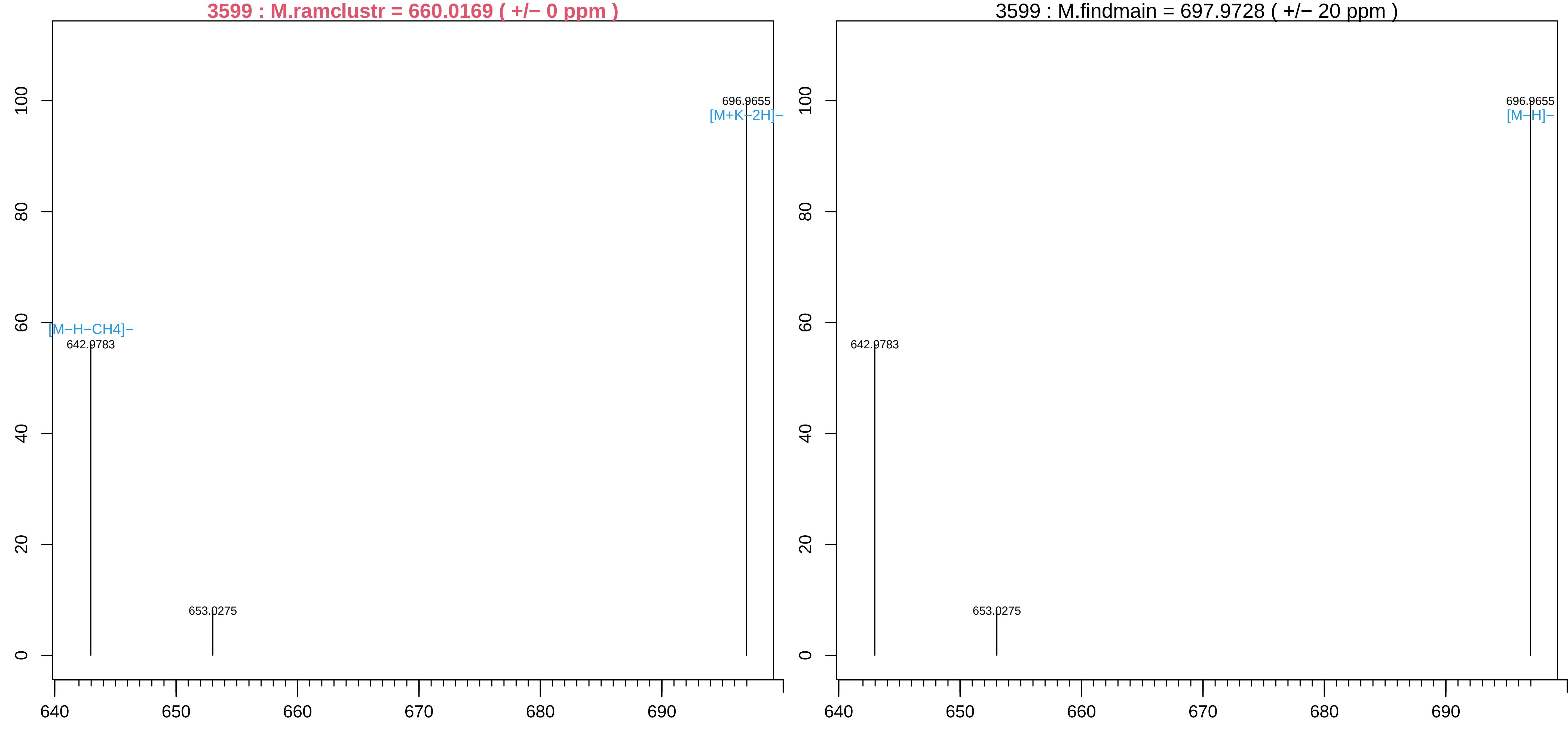
3599 : M.ramclustr = 660.0169 (+/− 0 ppm)
696.9655
[M+K−2H]−
[M−H−CH4]−
642.9783
653.0275
3599 : M.findmain = 697.9728 (+/− 20 ppm)
696.9655
[M−H]−
642.9783
653.0275
0
20
40
60
80
100
640
650
660
670
680
690

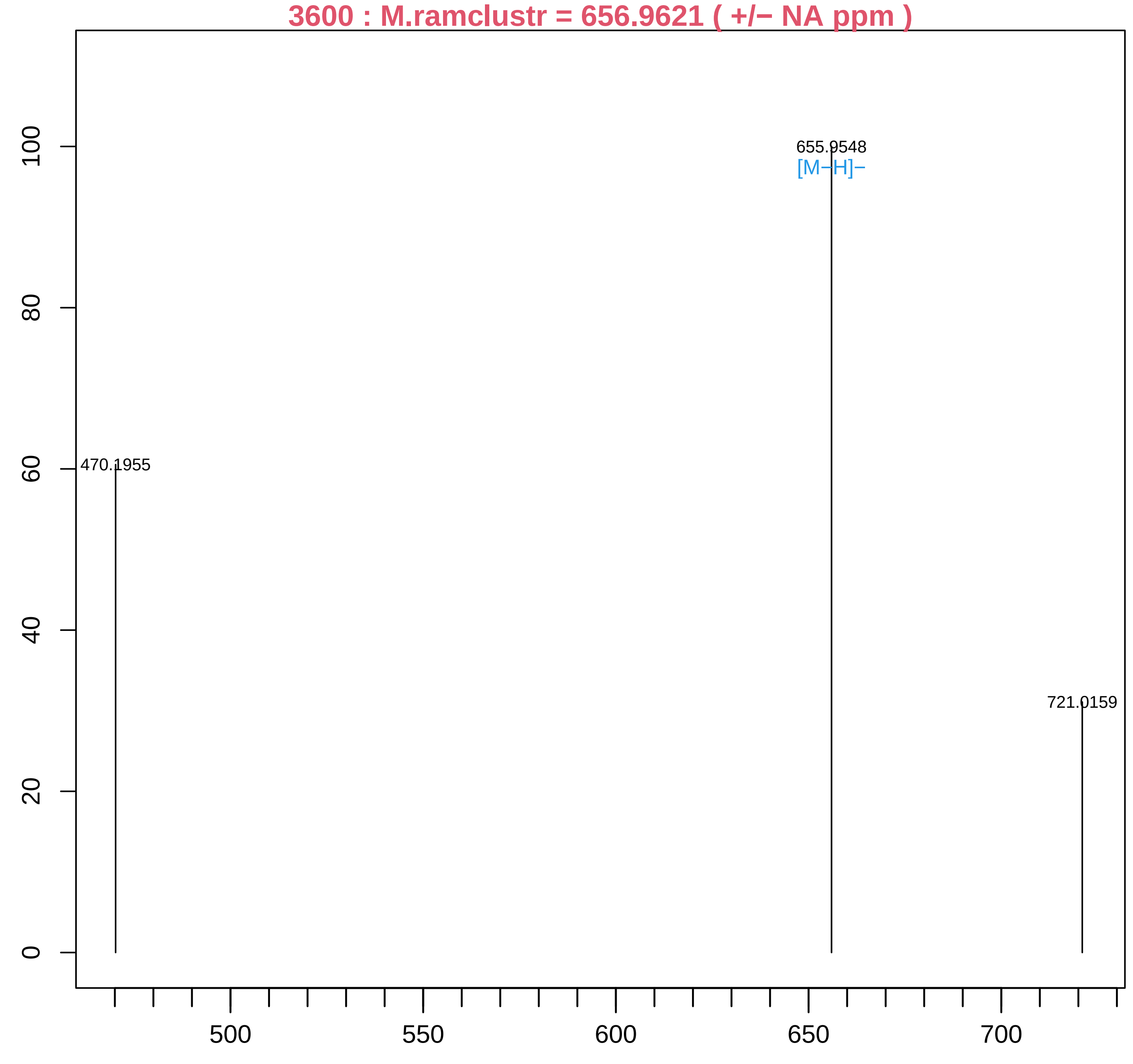
3600 : M.ramclustr = 656.9621 (+/− NA ppm)
655.9548
[M−H]−
470.1955
721.0159
0
20
40
60
80
100
500
550
600
650
700

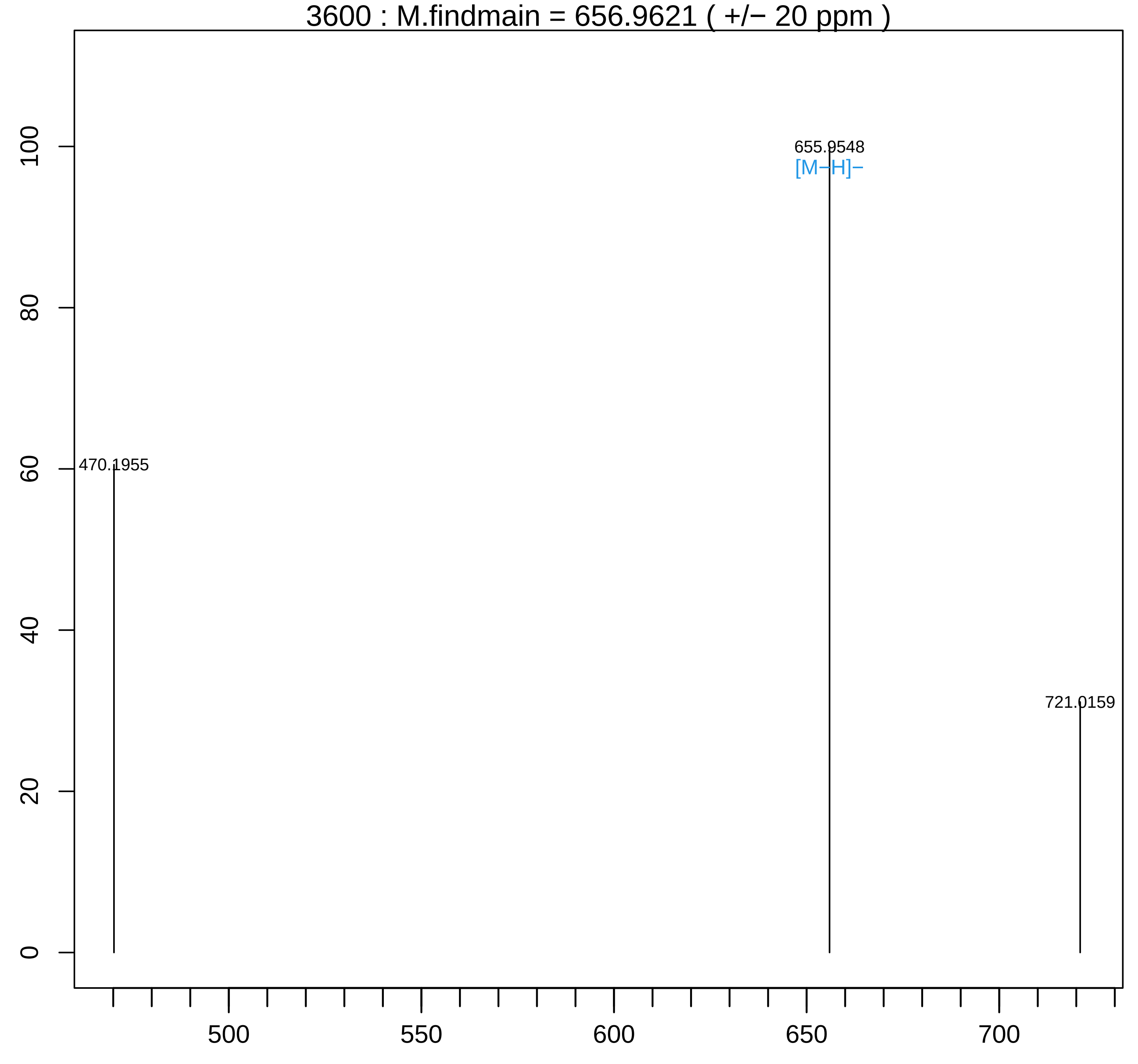
3600 : M.findmain = 656.9621 (+/− 20 ppm)
655.9548
[M−H]−
470.1955
721.0159
0
20
40
60
80
100
500
550
600
650
700

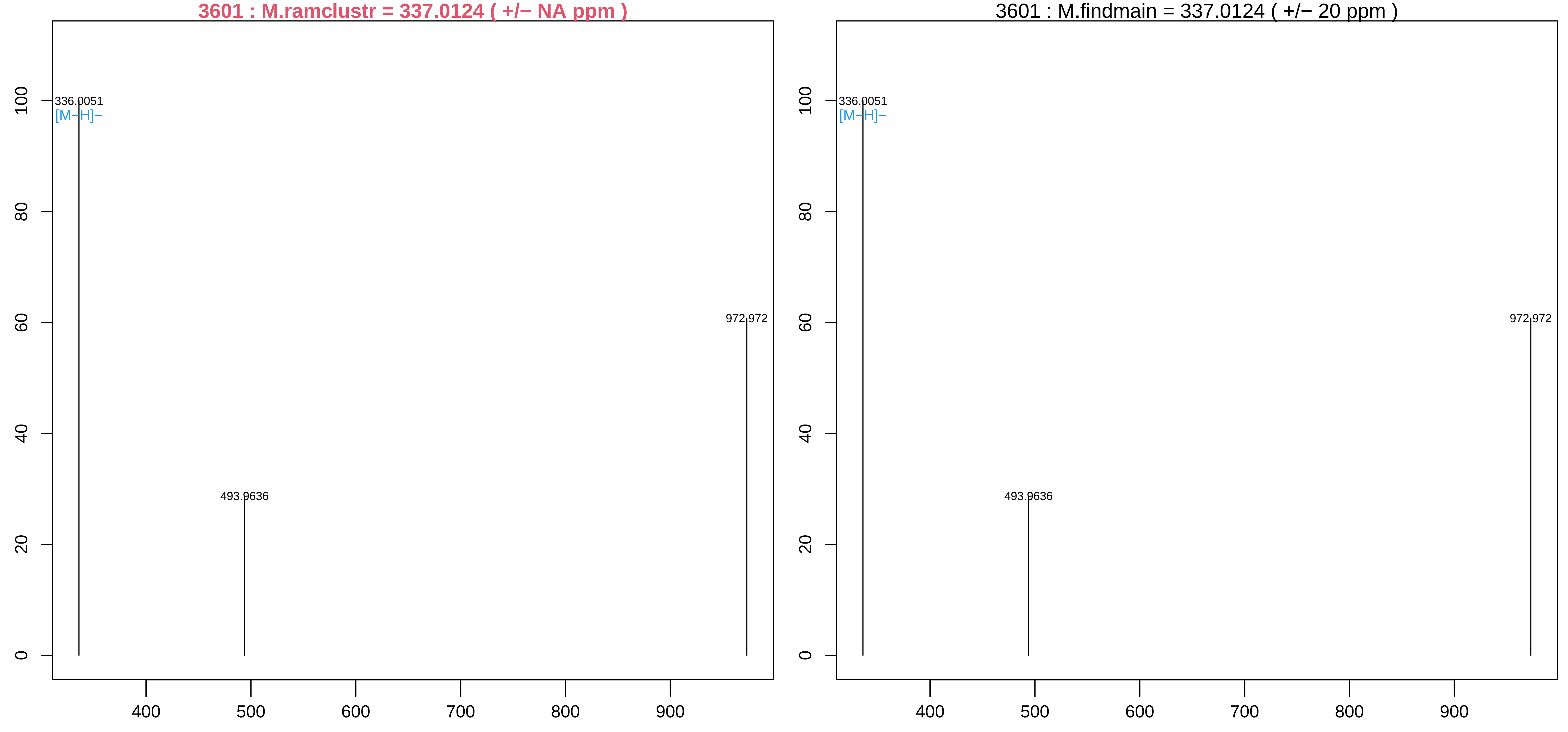
3601 : M.ramclustr = 337.0124 (+/− NA ppm)
336.0051
[M−H]−
493.9636
972.972
3601 : M.findmain = 337.0124 (+/− 20 ppm)
336.0051
[M−H]−
493.9636
972.972
0
20
40
60
80
100
400
500
600
700
800
900

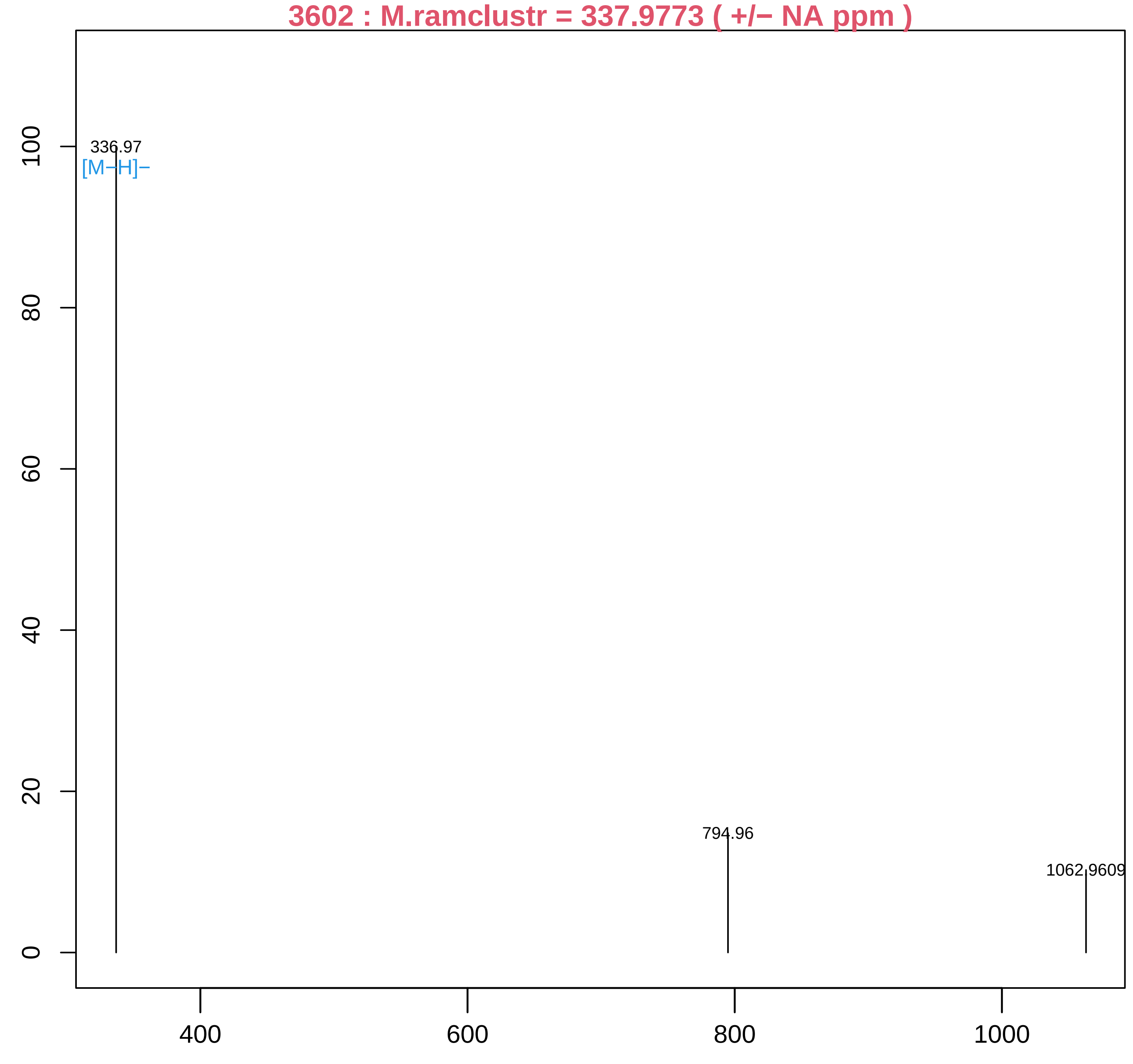
3602 : M.ramclustr = 337.9773 (+/− NA ppm)
336.97
[M−H]−
794.96
1062.9609
0
20
40
60
80
100
400
600
800
1000

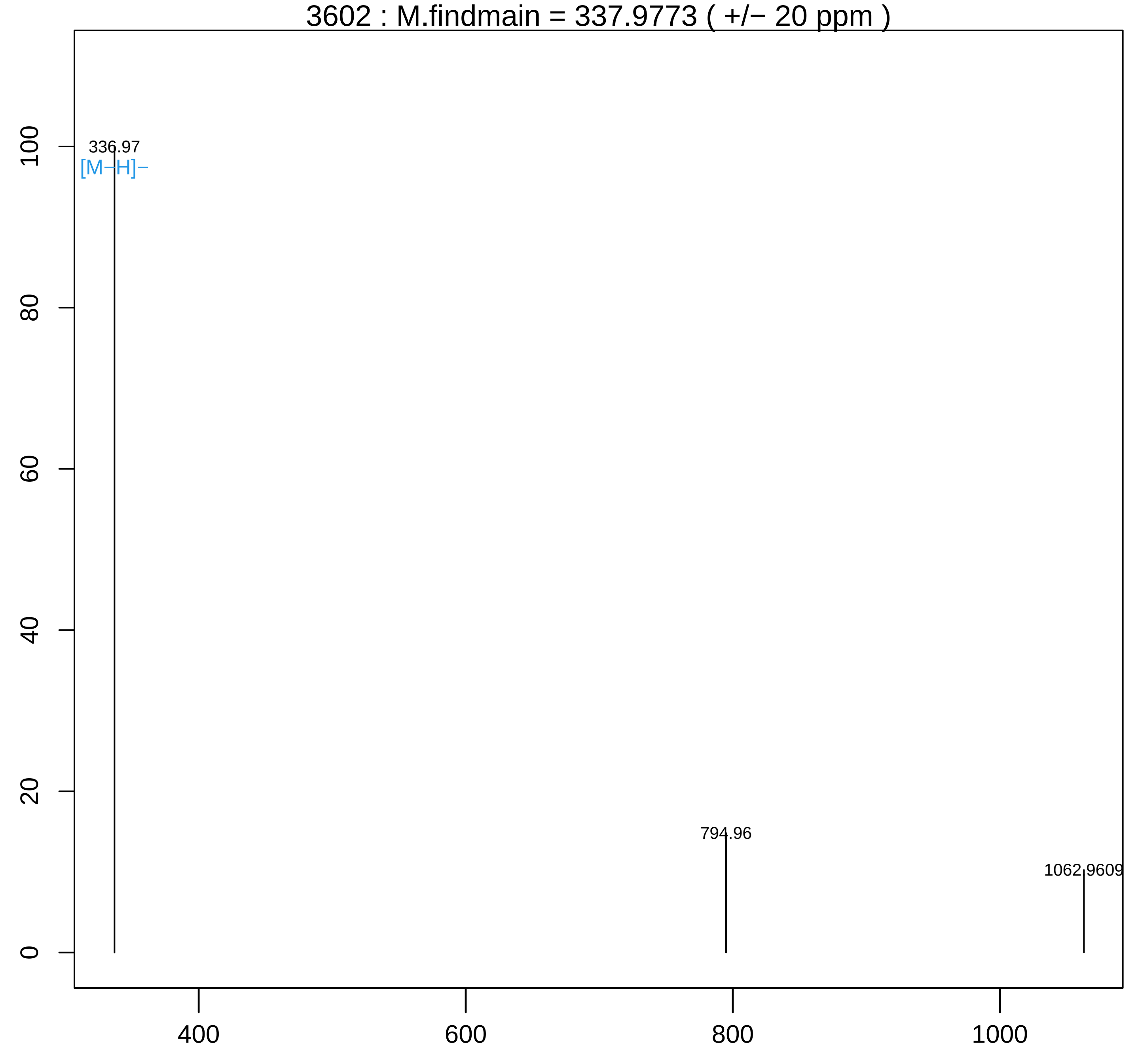
3602 : M.findmain = 337.9773 (+/− 20 ppm)
336.97
[M−H]−
794.96
1062.9609
0
20
40
60
80
100
400
600
800
1000

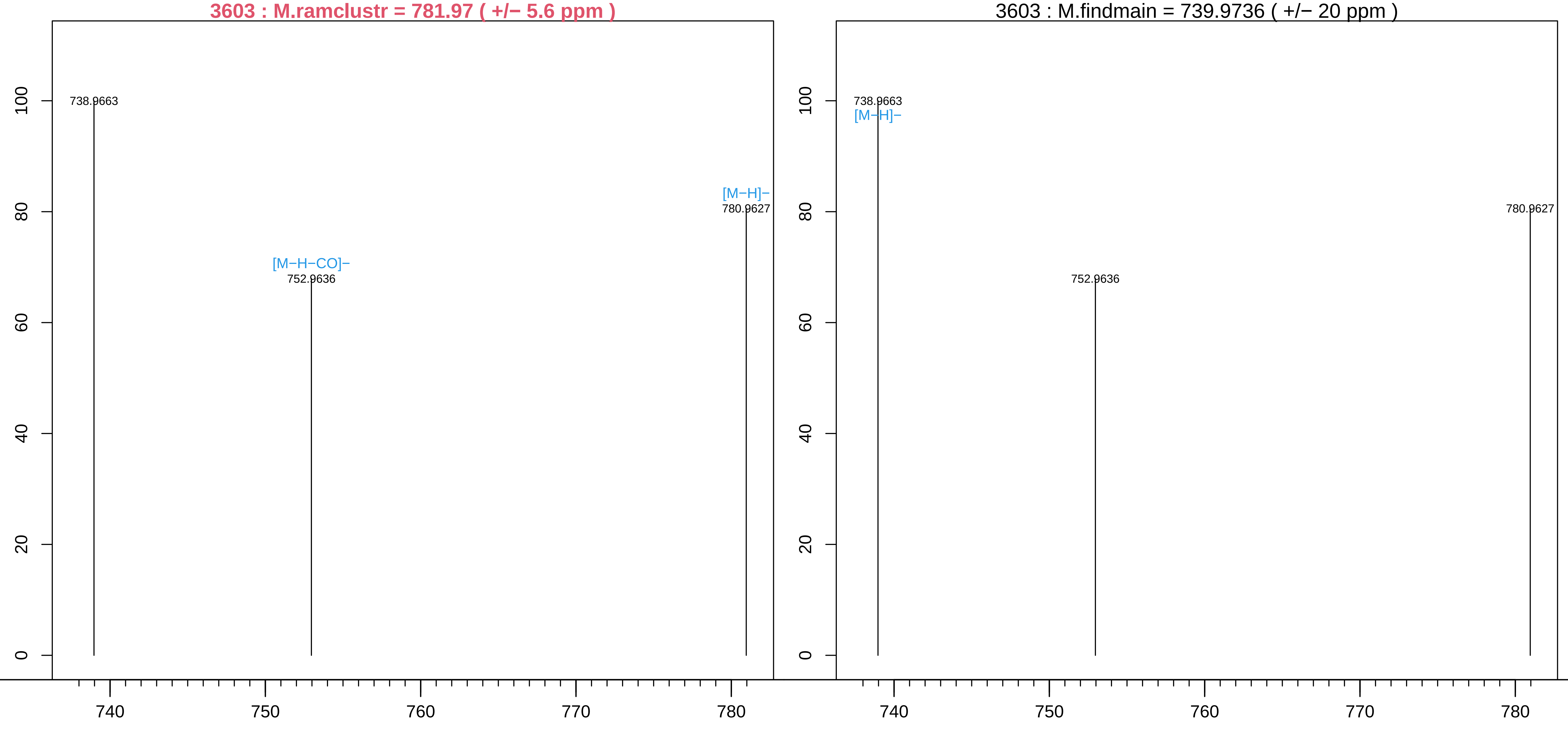
3603 : M.ramclustr = 781.97 (+/− 5.6 ppm)
738.9663
[M−H−CO]−
752.9636
[M−H]−
780.9627
3603 : M.findmain = 739.9736 (+/− 20 ppm)
738.9663
[M−H]−
752.9636
780.9627
0
20
40
60
80
100
740
750
760
770
780

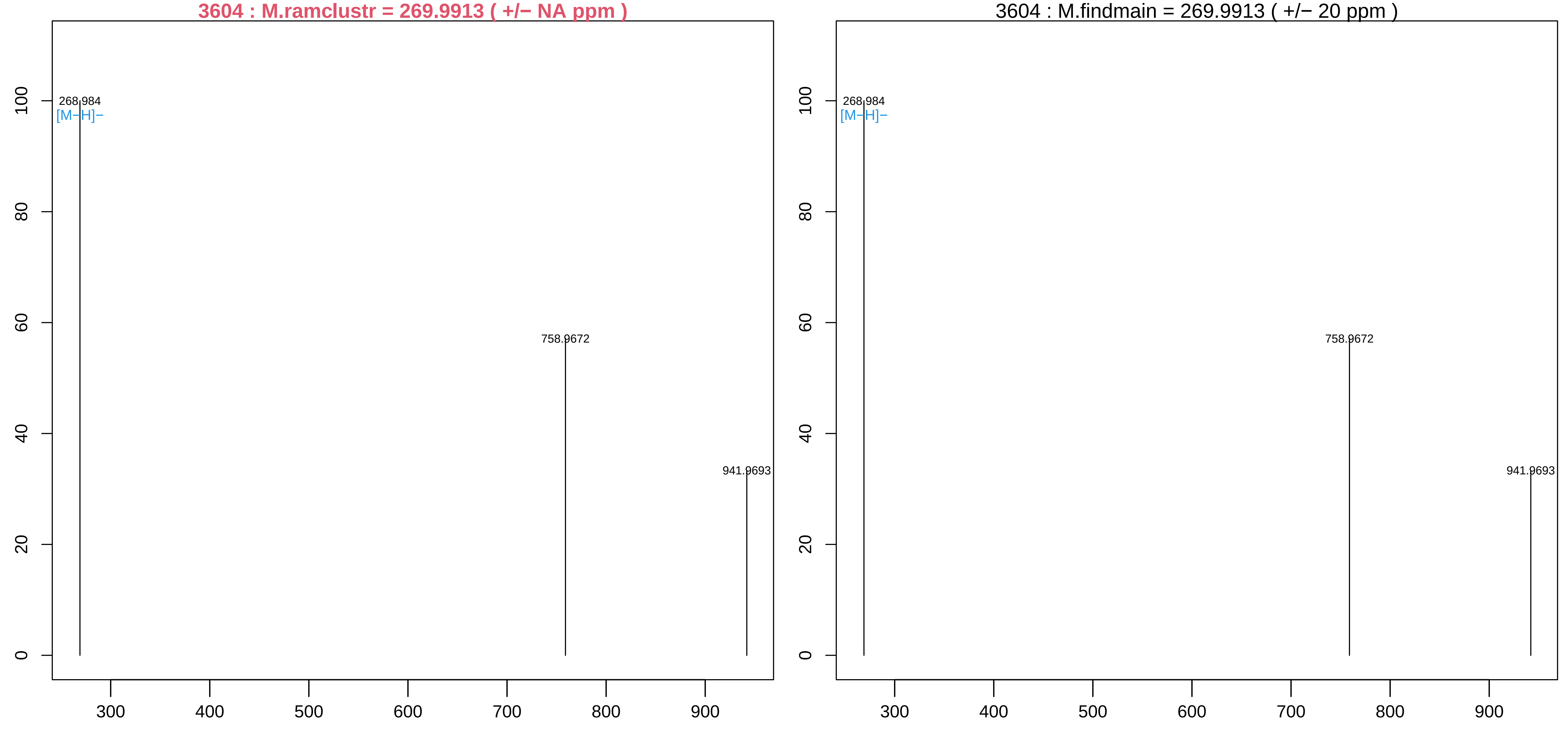

3604 : M.ramclustr = 269.9913 (+/− NA ppm)
268.984
[M−H]−
758.9672
941.9693
3604 : M.findmain = 269.9913 (+/− 20 ppm)
268.984
[M−H]−
758.9672
941.9693
0
20
40
60
80
100
300
400
500
600
700
800
900

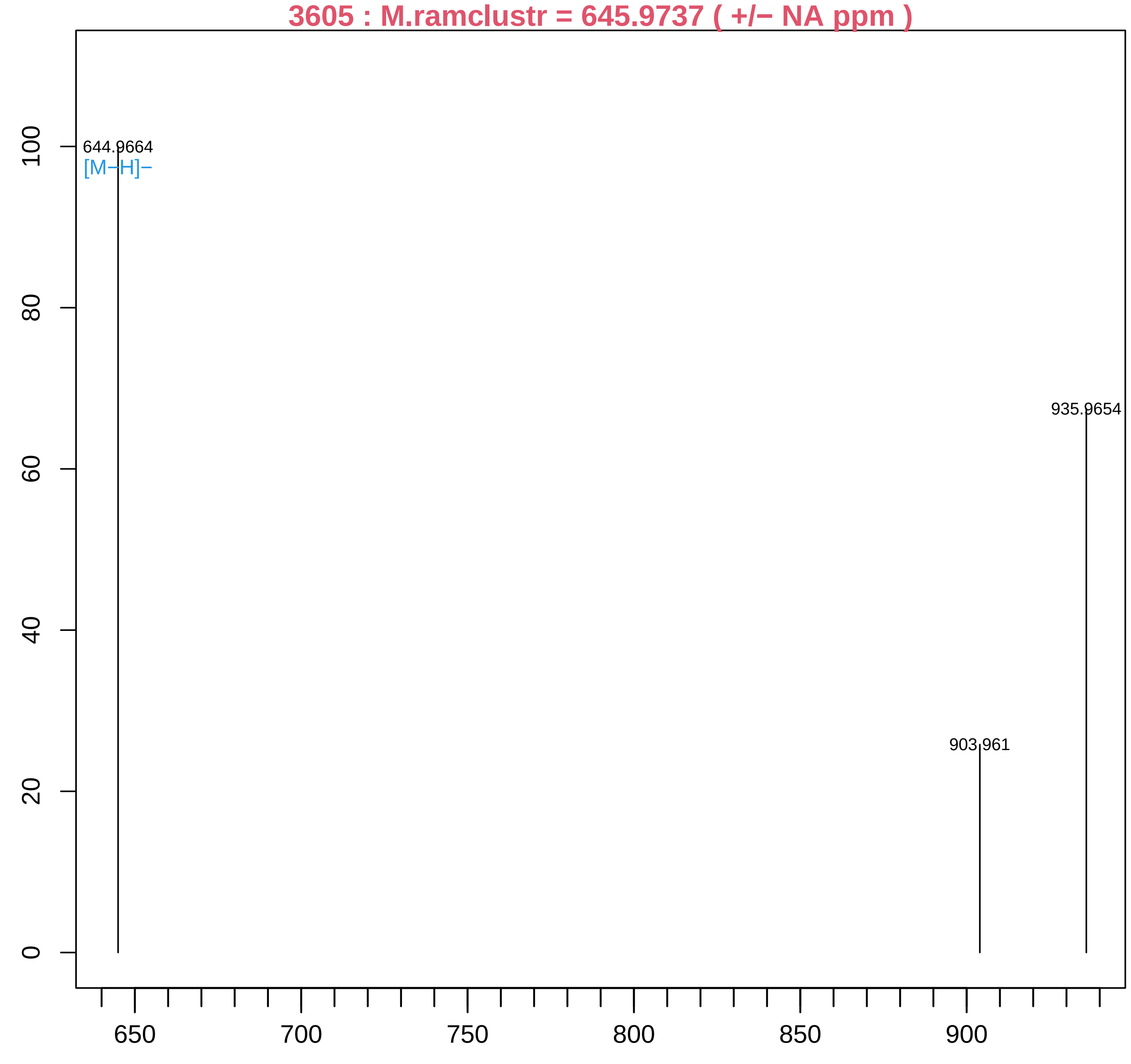
3605 : M.ramclustr = 645.9737 (+/− NA ppm)
644.9664
[M−H]−
935.9654
903.961
0
20
40
60
80
100
650
700
750
800
850
900

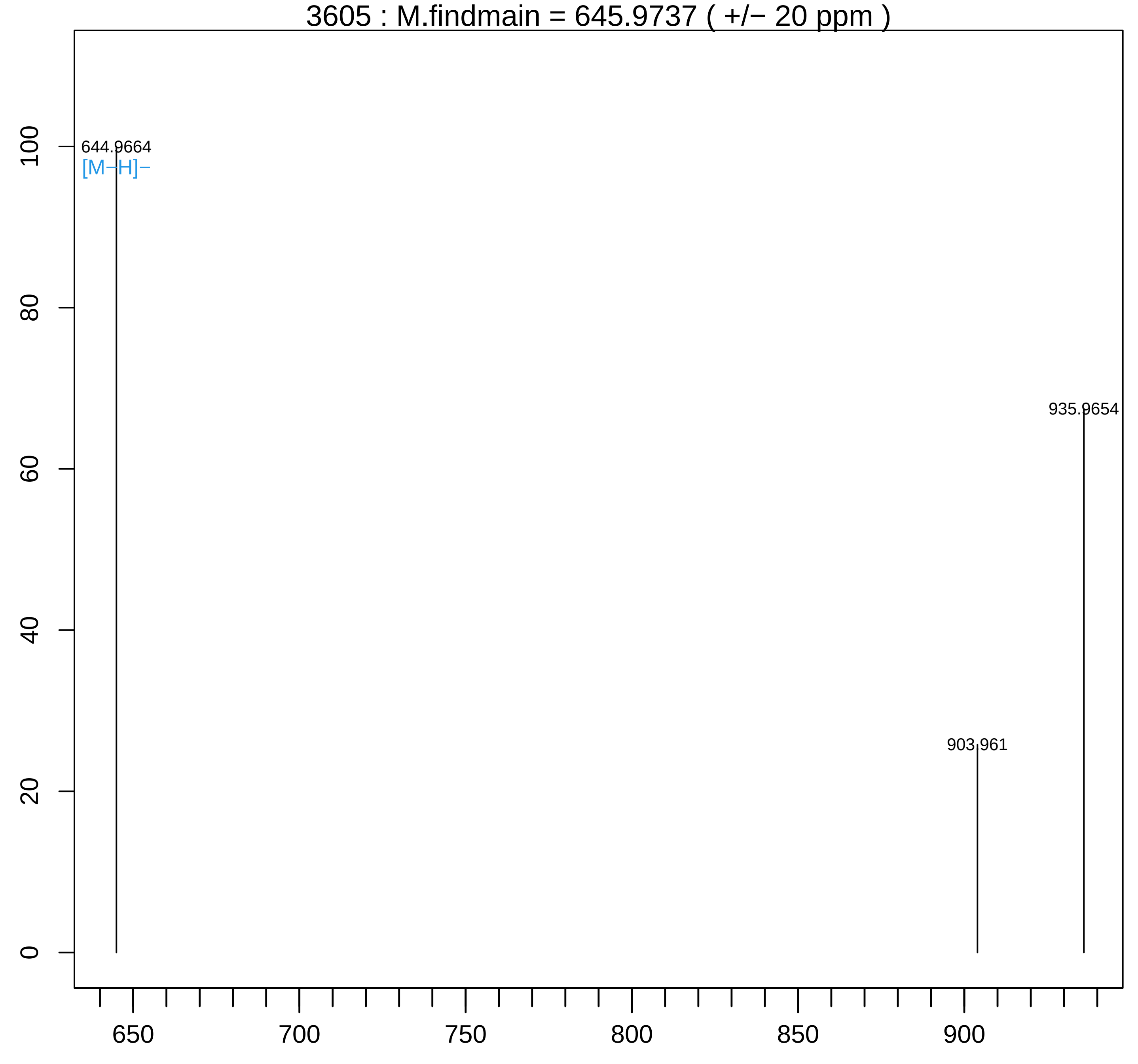
3605 : M.findmain = 645.9737 (+/− 20 ppm)
644.9664
[M−H]−
935.9654
903.961
0
20
40
60
80
100
650
700
750
800
850
900

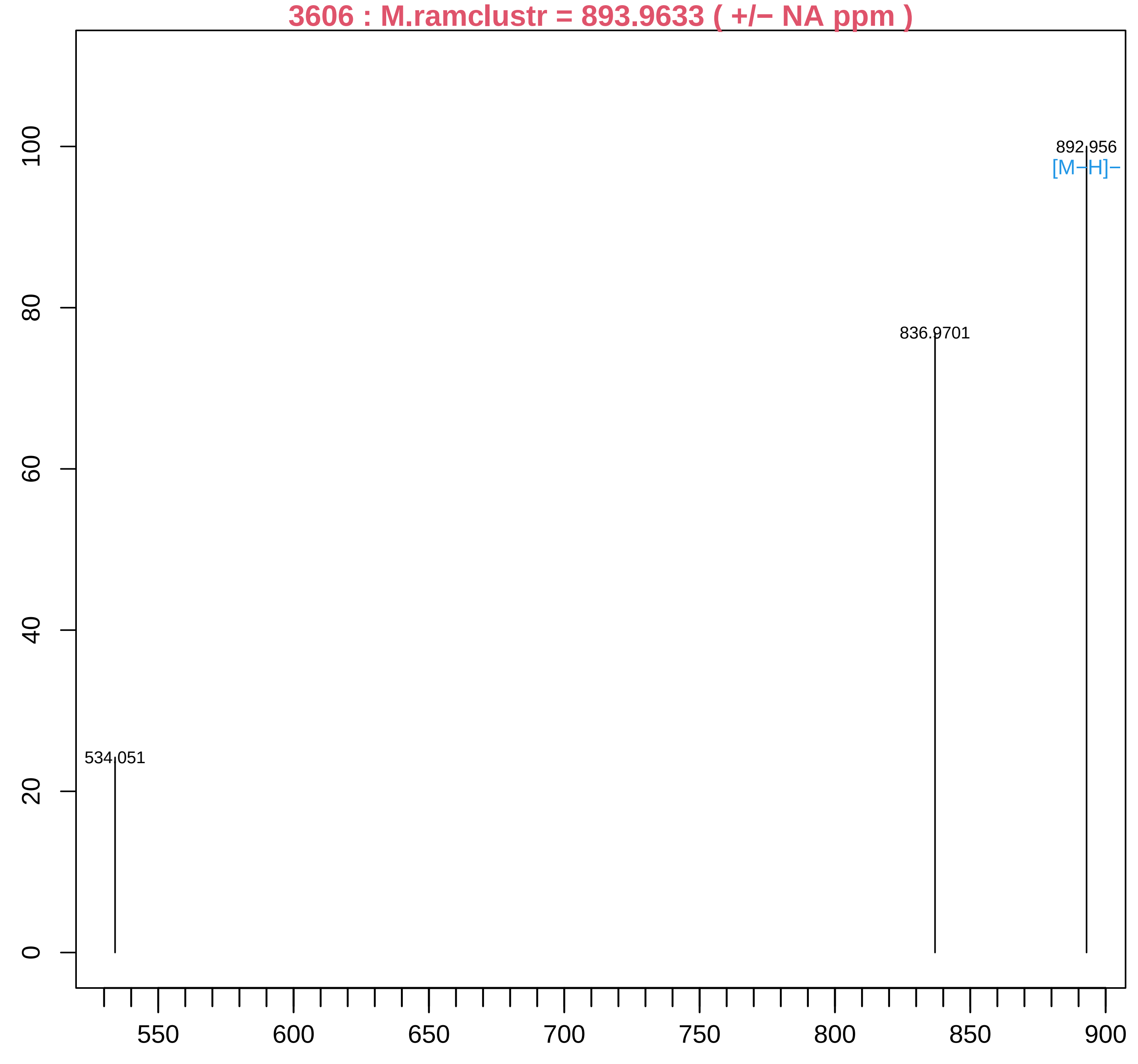
3606 : M.ramclustr = 893.9633 (+/− NA ppm)
892.956
[M−H]−
836.9701
534.051
0
20
40
60
80
100
550
600
650
700
750
800
850
900

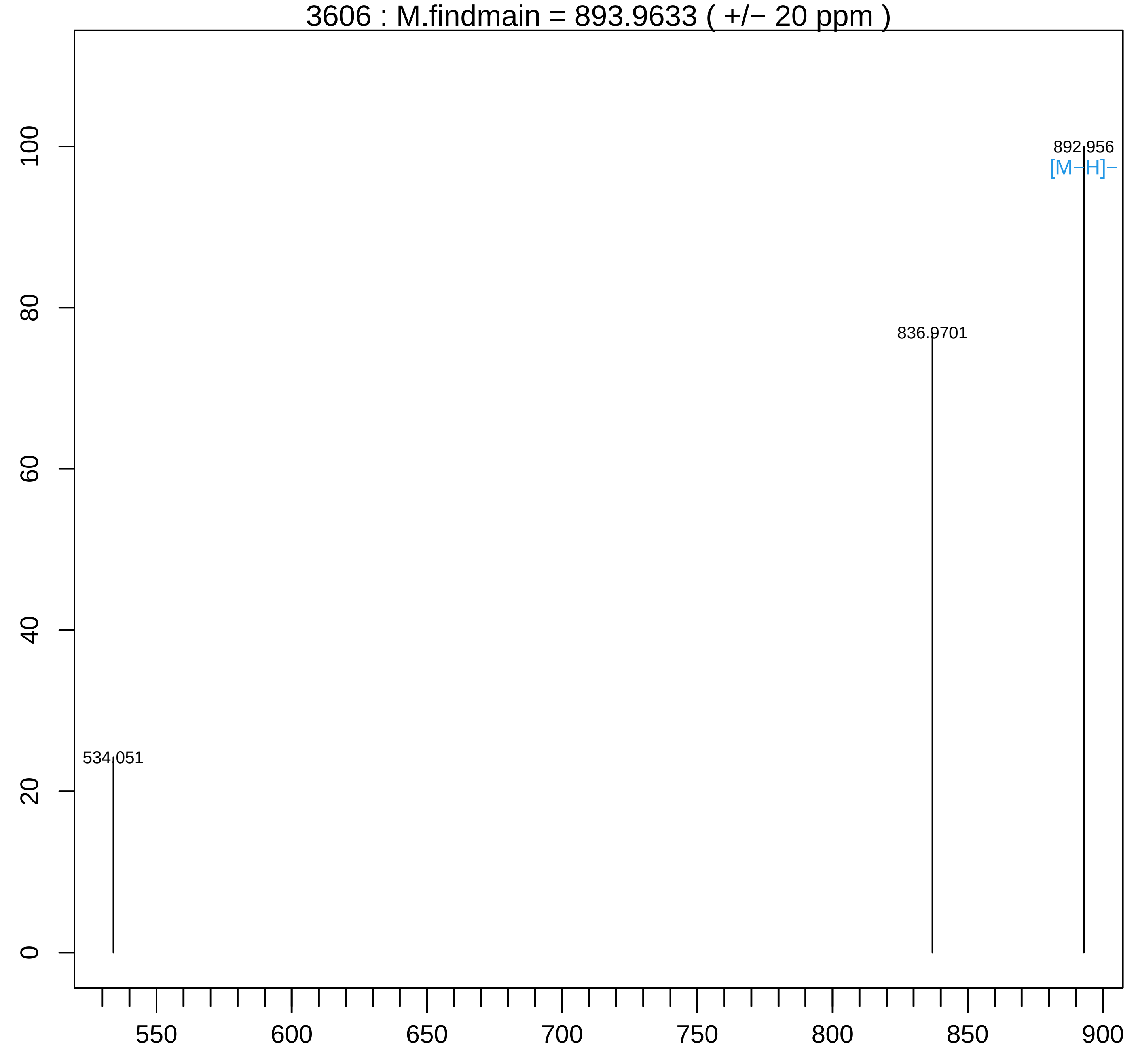
3606 : M.findmain = 893.9633 (+/− 20 ppm)
892.956
[M−H]−
836.9701
534.051
0
20
40
60
80
100
550
600
650
700
750
800
850
900

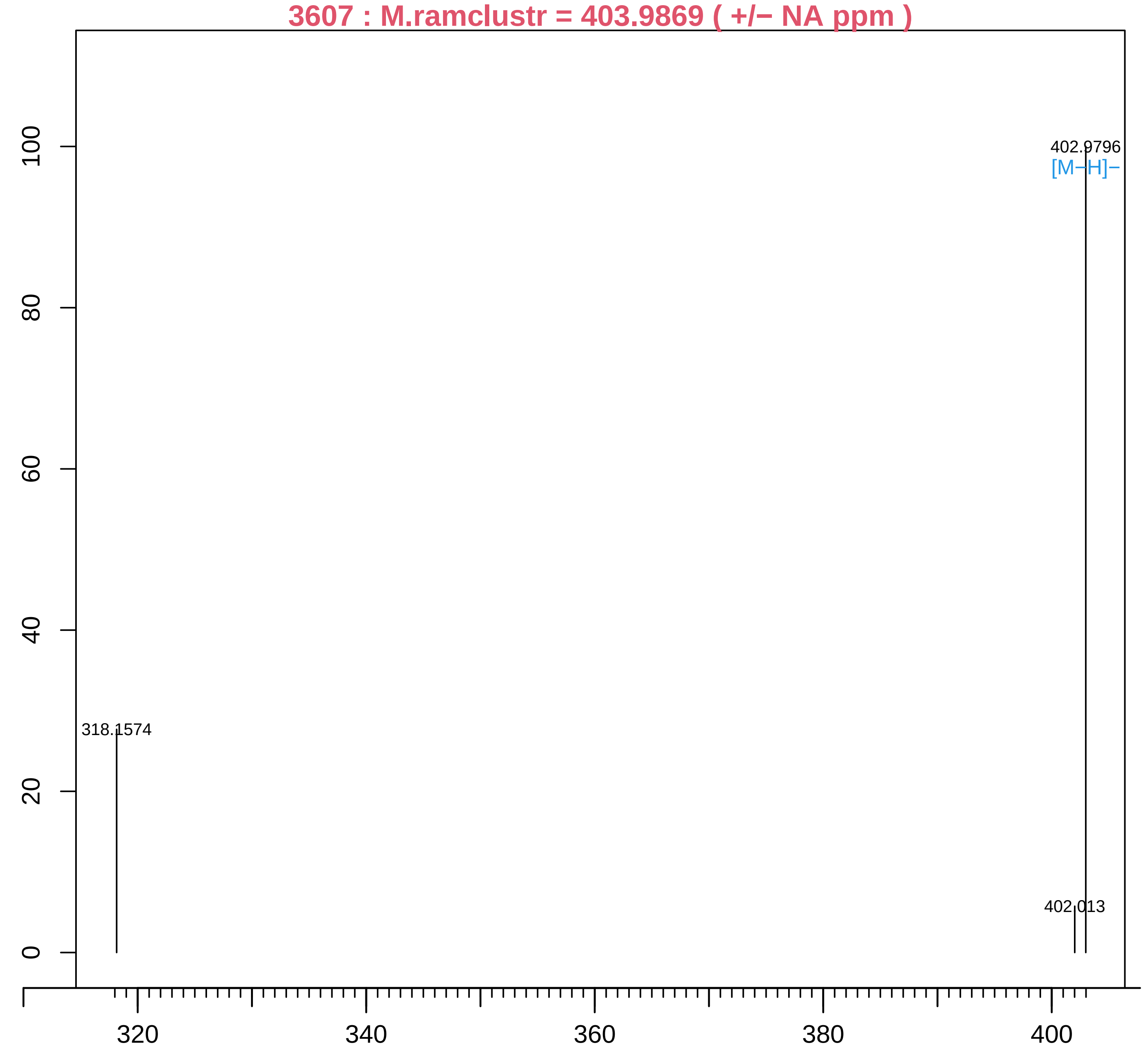
3607 : M.ramclustr = 403.9869 (+/− NA ppm)
402.9796
[M−H]−
318.1574
402.013
0
20
40
60
80
100
320
340
360
380
400

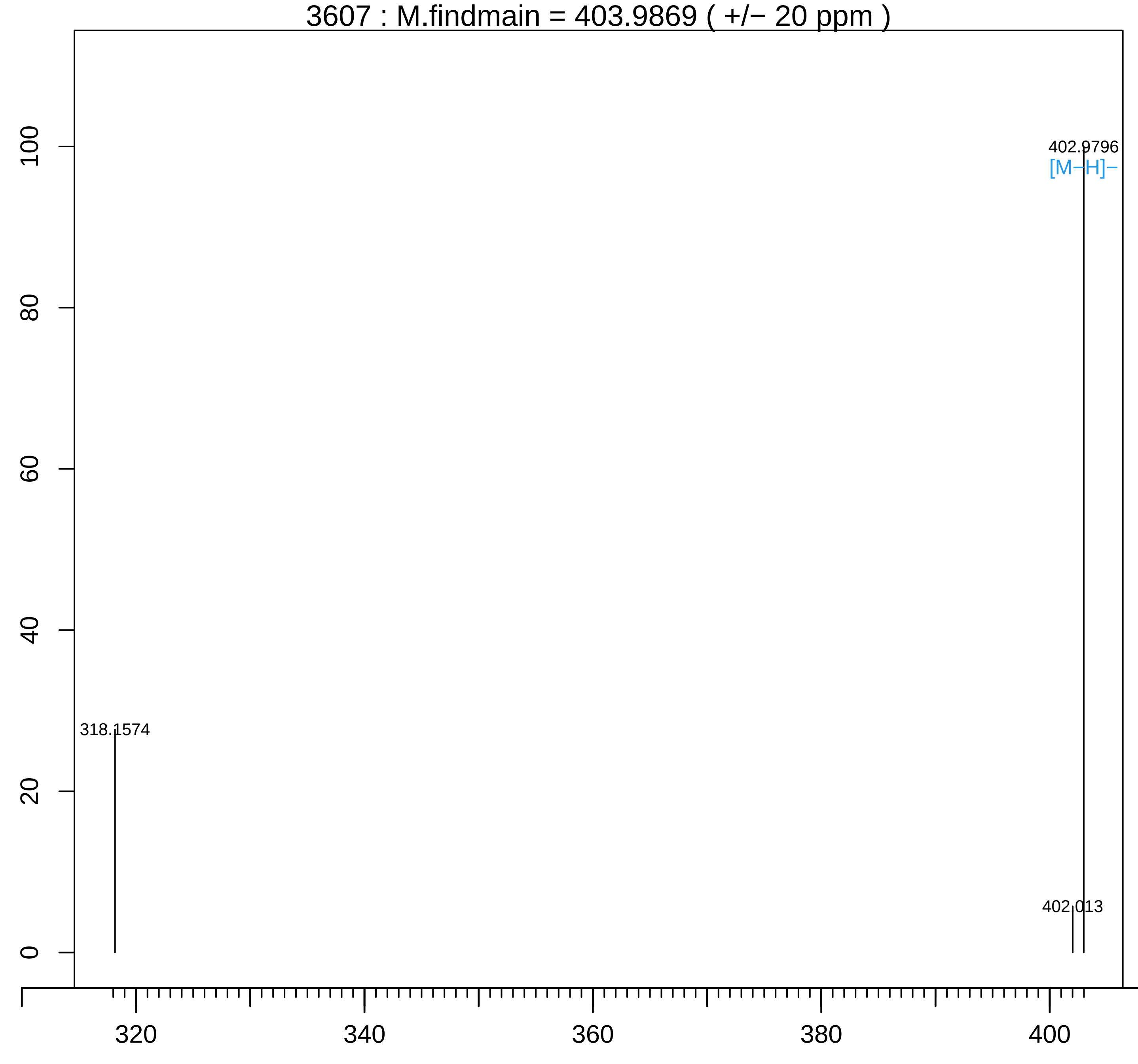
3607 : M.findmain = 403.9869 (+/− 20 ppm)
402.9796
[M−H]−
318.1574
402.013
0
20
40
60
80
100
320
340
360
380
400

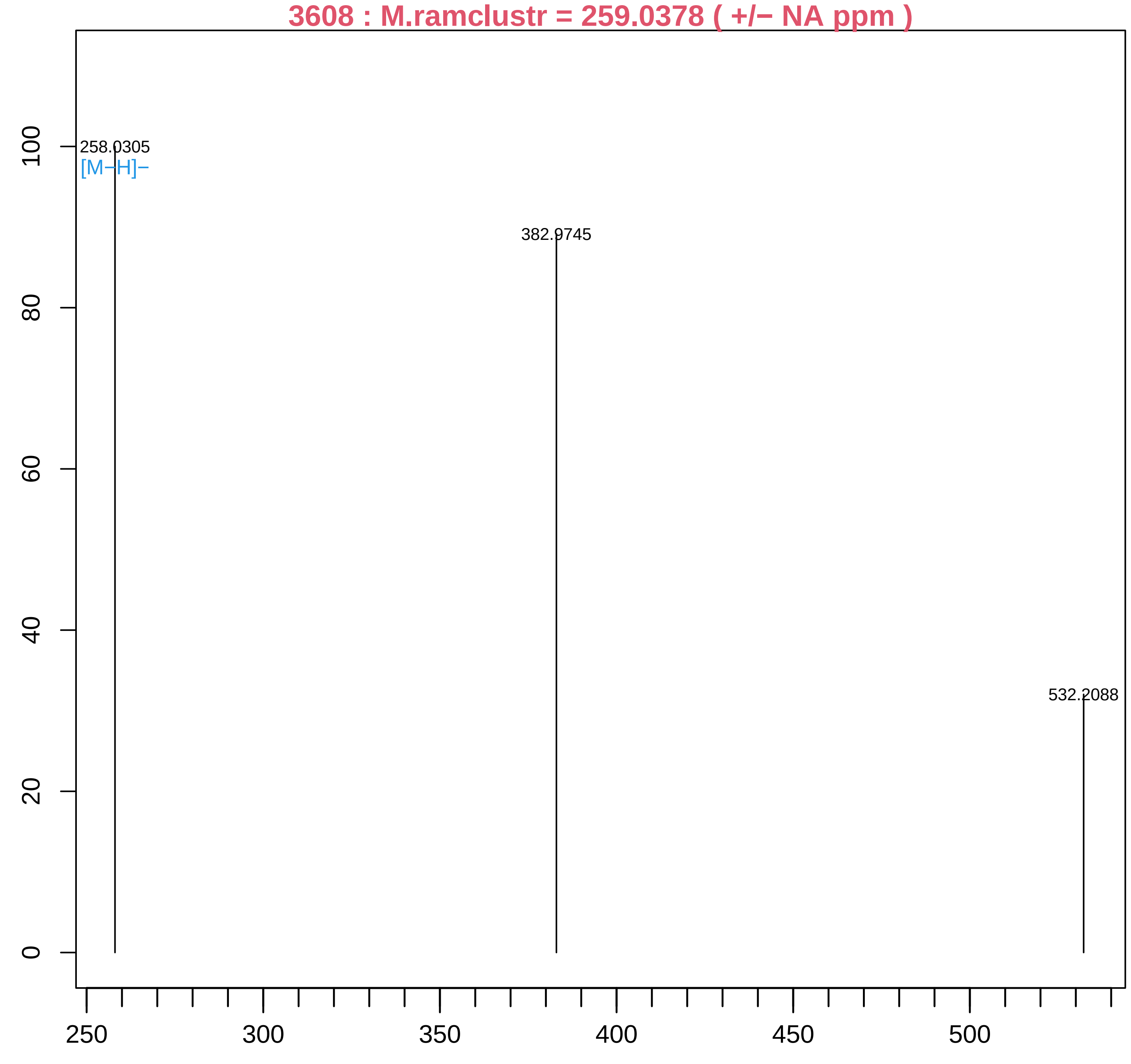
3608 : M.ramclustr = 259.0378 (+/− NA ppm)
258.0305
[M−H]−
382.9745
532.2088
0
20
40
60
80
100
250
300
350
400
450
500

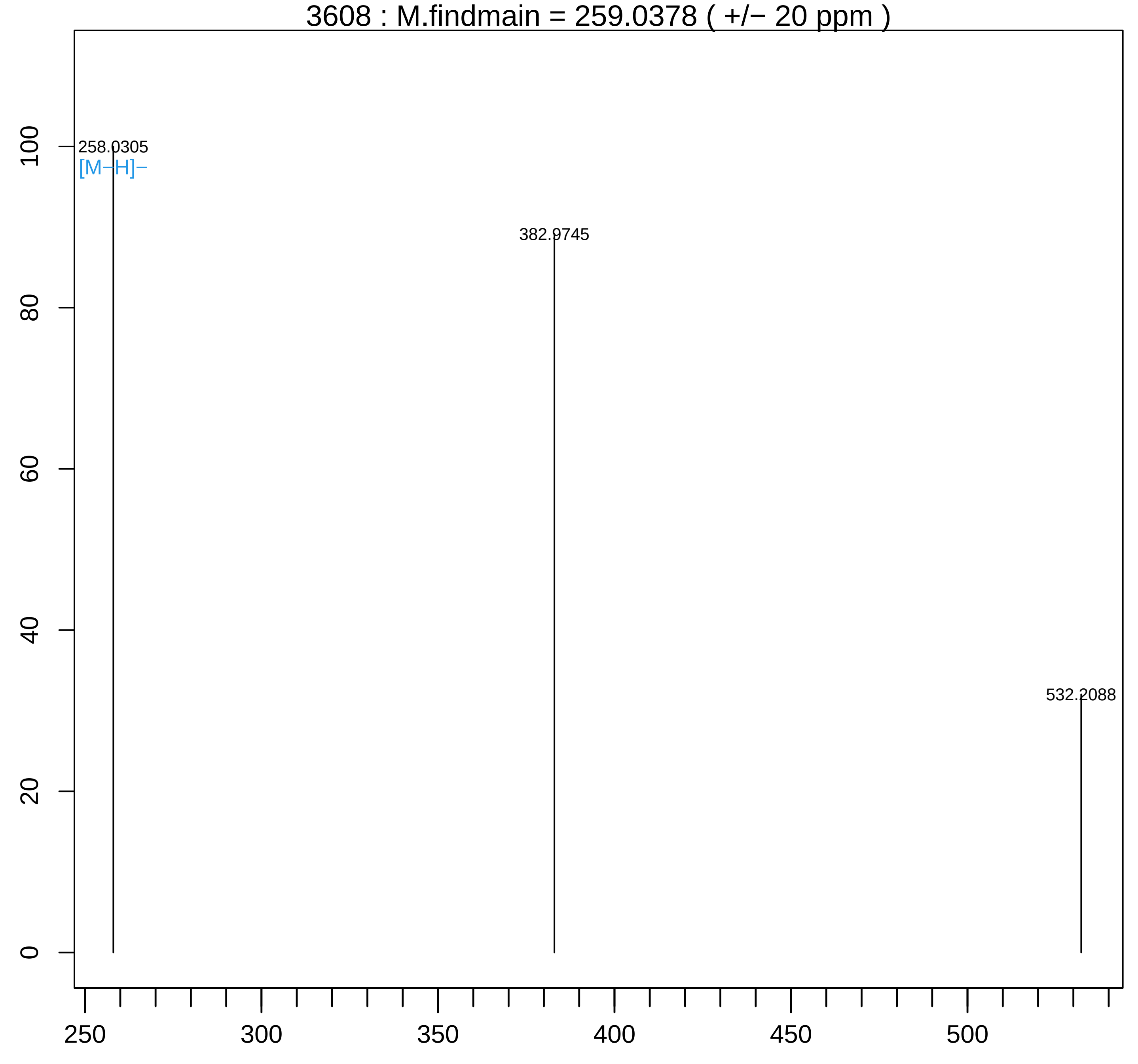
3608 : M.findmain = 259.0378 (+/− 20 ppm)
258.0305
[M−H]−
382.9745
532.2088
0
20
40
60
80
100
250
300
350
400
450
500

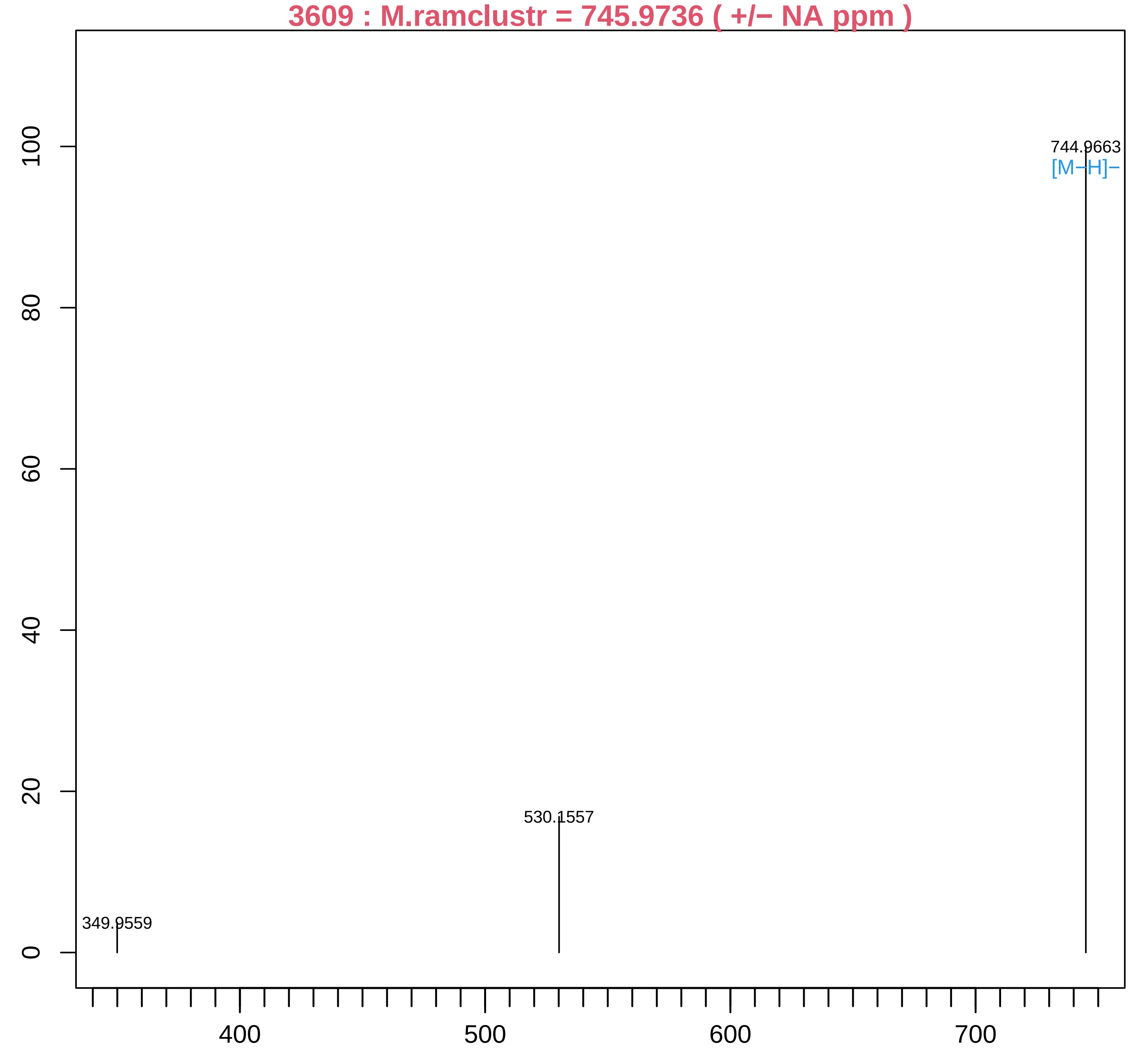
3609 : M.ramclustr = 745.9736 (+/− NA ppm)
744.9663
[M−H]−
530.1557
349.9559
0
20
40
60
80
100
400
500
600
700

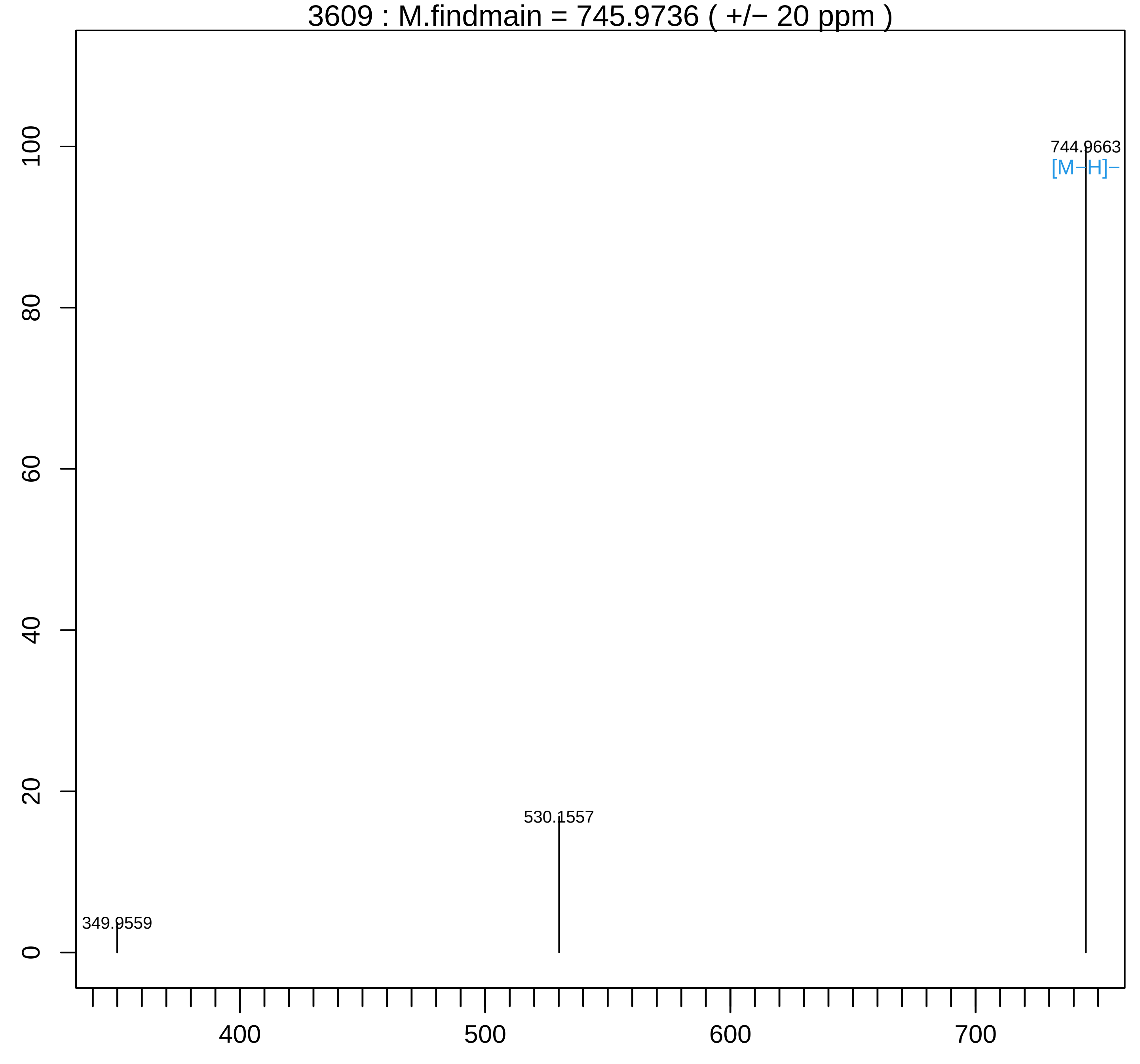
3609 : M.findmain = 745.9736 (+/− 20 ppm)
744.9663
[M−H]−
530.1557
349.9559
0
20
40
60
80
100
400
500
600
700

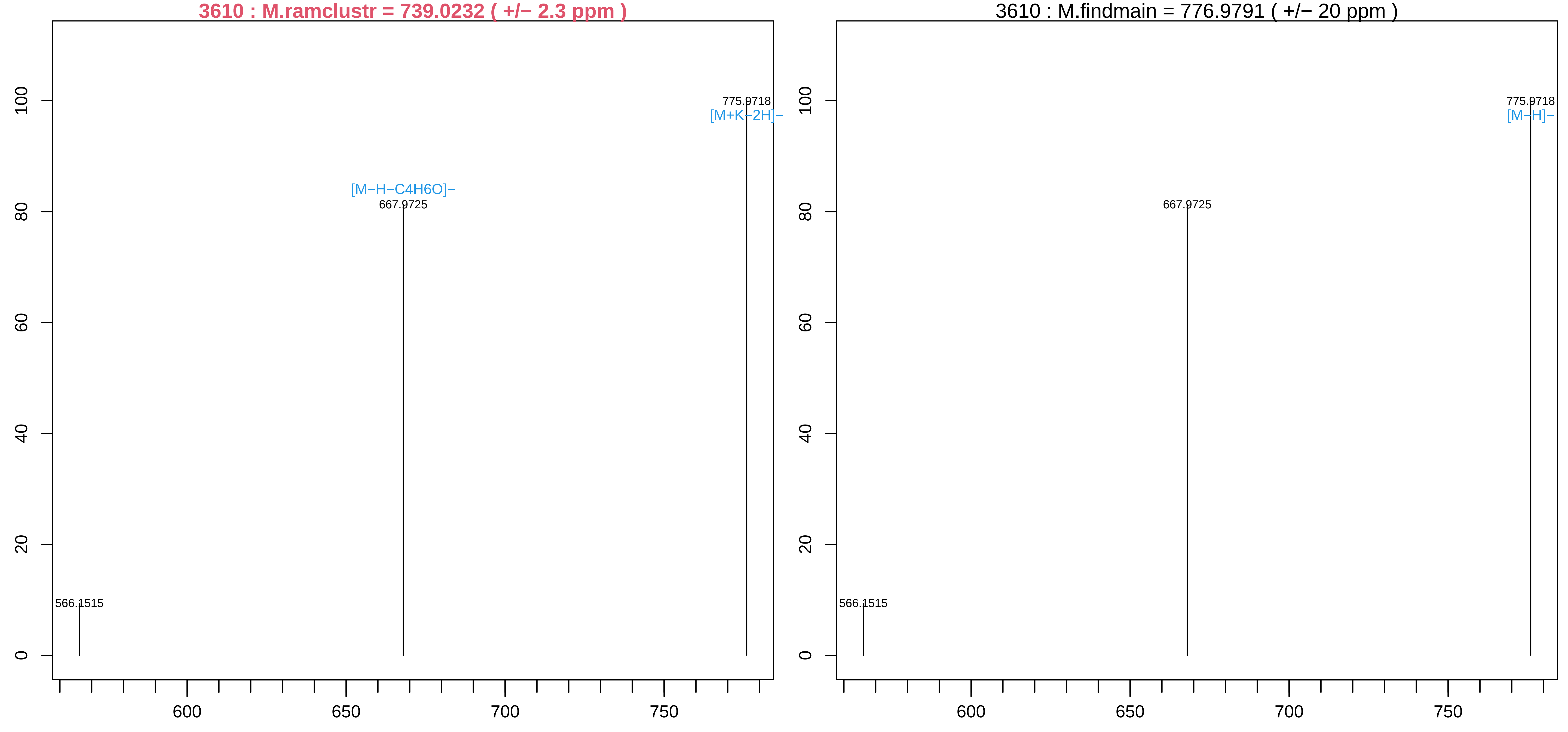
3610 : M.ramclustr = 739.0232 (+/− 2.3 ppm)
775.9718
[M+K−2H]−
[M−H−C4H6O]−
667.9725
566.1515
0
20
40
60
80
100
600
650
700
750
3610 : M.findmain = 776.9791 (+/− 20 ppm)
775.9718
[M−H]−
667.9725
566.1515
0
20
40
60
80
100
600
650
700
750

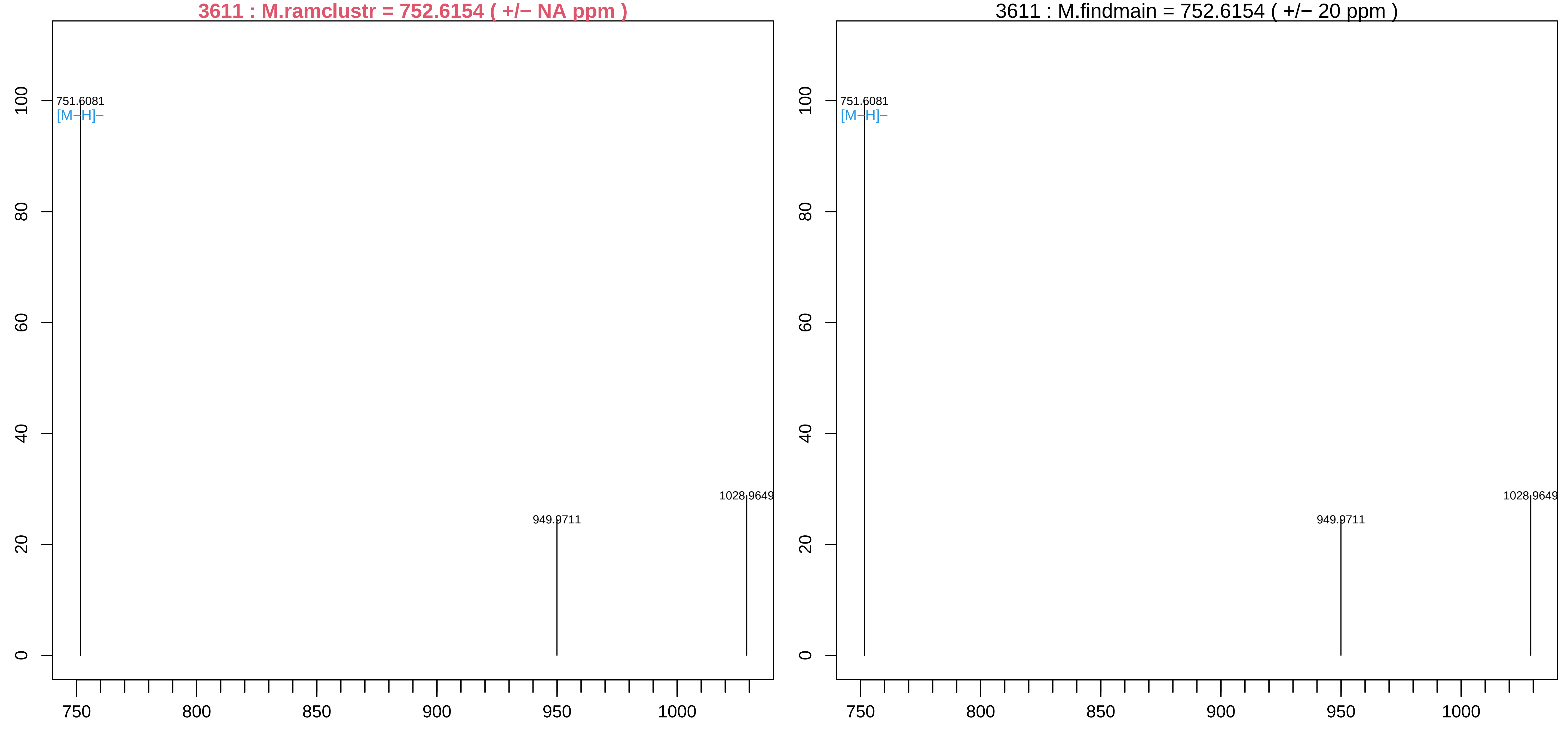
3611 : M.ramclustr = 752.6154 (+/− NA ppm)
751.6081
[M−H]−
949.9711
1028.9649
3611 : M.findmain = 752.6154 (+/− 20 ppm)
751.6081
[M−H]−
949.9711
1028.9649

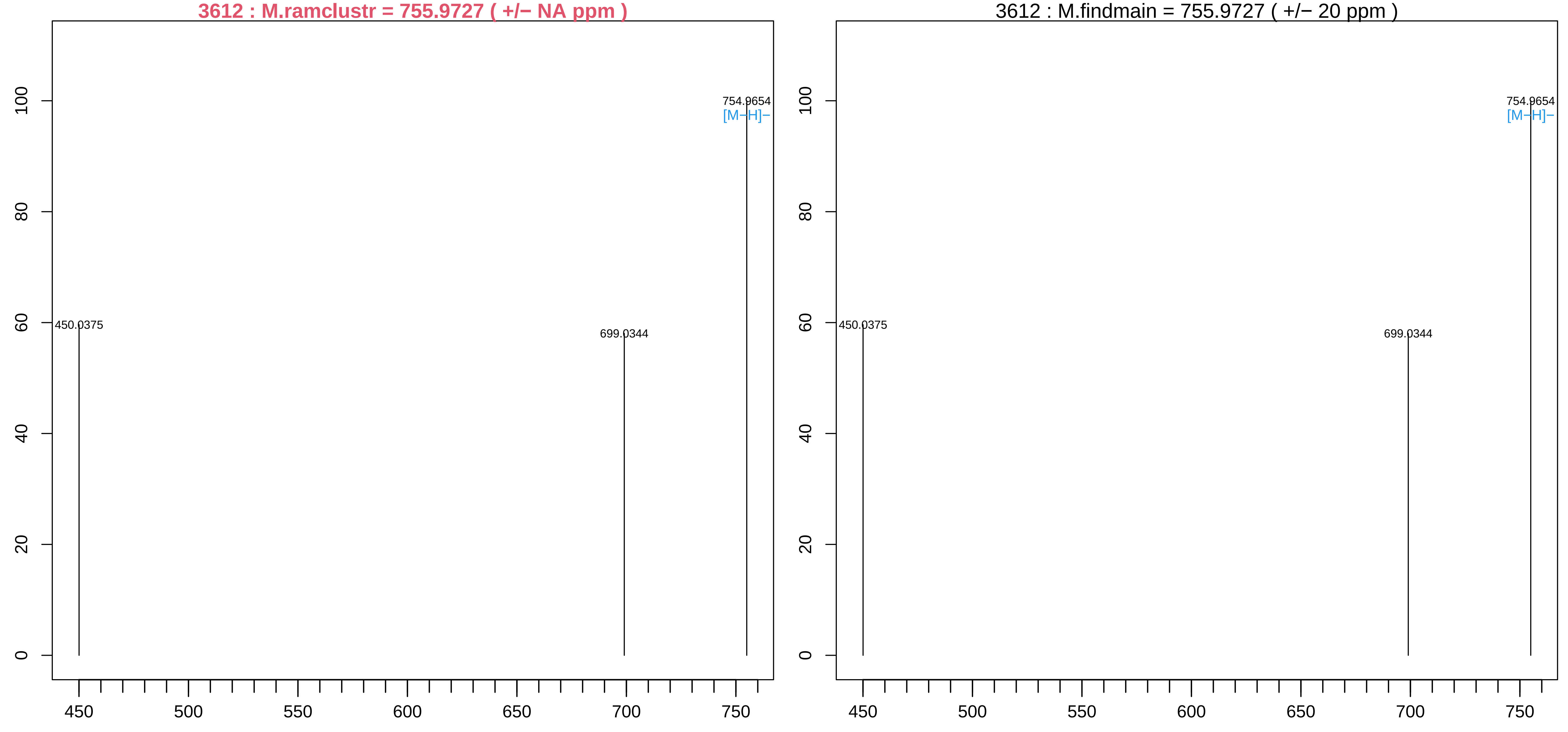
3612 : M.ramclustr = 755.9727 (+/− NA ppm)
3612 : M.findmain = 755.9727 (+/− 20 ppm)
754.9654
[M−H]−
450.0375
699.0344
0
20
40
60
80
100
450
500
550
600
650
700
750

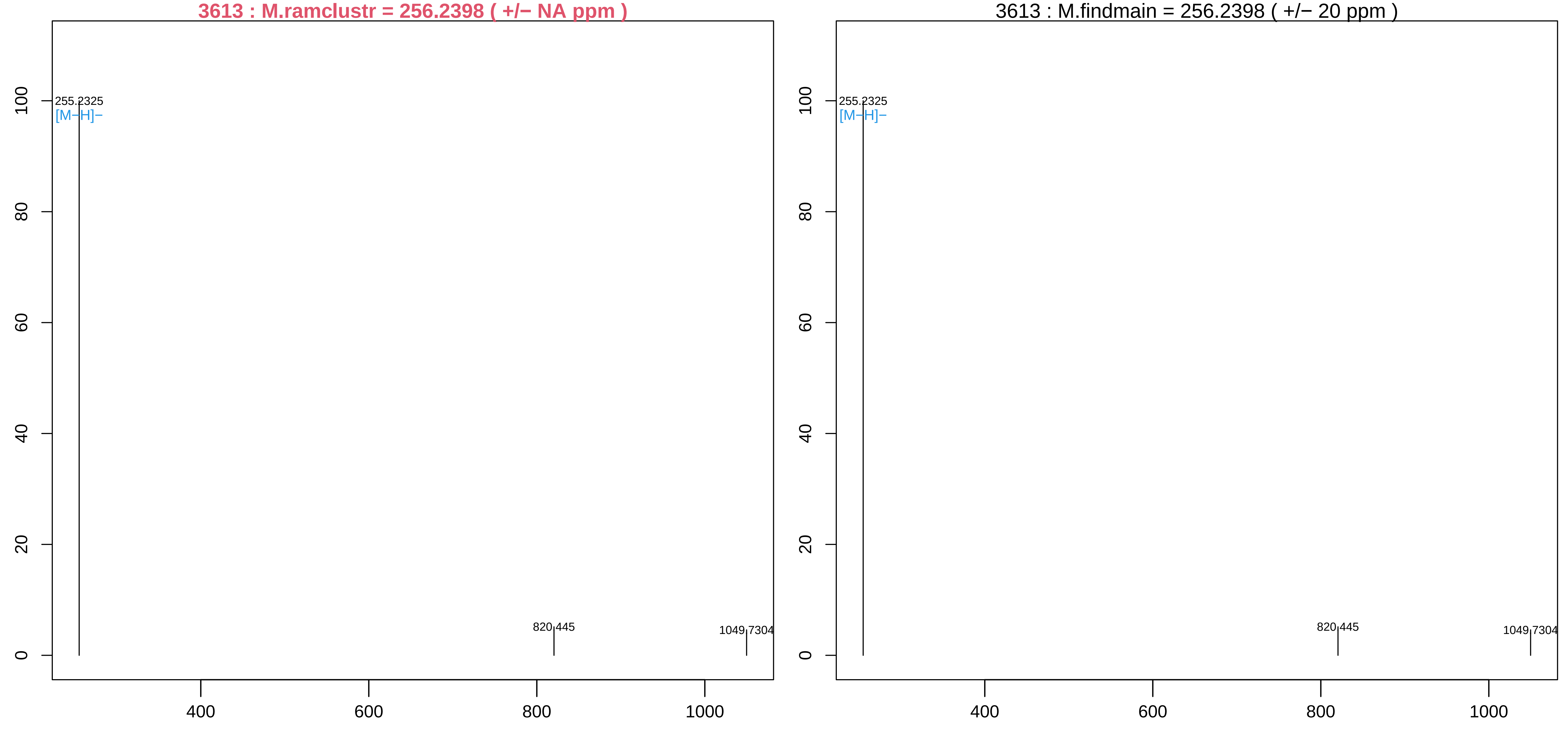

3613 : M.ramclustr = 256.2398 (+/− NA ppm)
255.2325
[M−H]−
820.445
1049.7304
3613 : M.findmain = 256.2398 (+/− 20 ppm)
255.2325
[M−H]−
820.445
1049.7304
0
20
40
60
80
100
400
600
800
1000

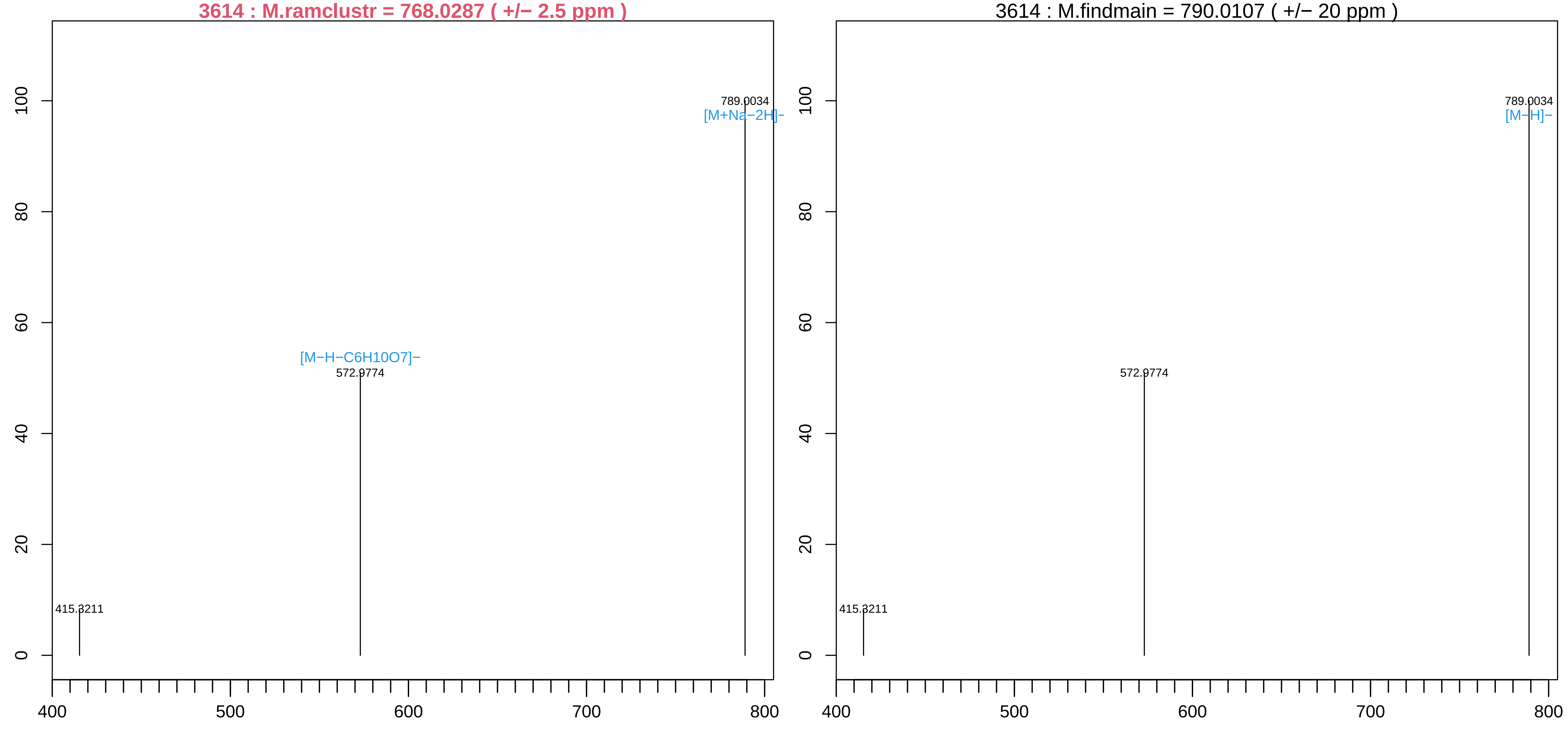

3614 : M.ramclustr = 768.0287 (+/− 2.5 ppm)
789.0034
[M+Na−2H]−
[M−H−C6H10O7]−
572.9774
415.3211
0
20
40
60
80
100
400
500
600
700
800
3614 : M.findmain = 790.0107 (+/− 20 ppm)
789.0034
[M−H]−
572.9774
415.3211
0
20
40
60
80
100
400
500
600
700
800

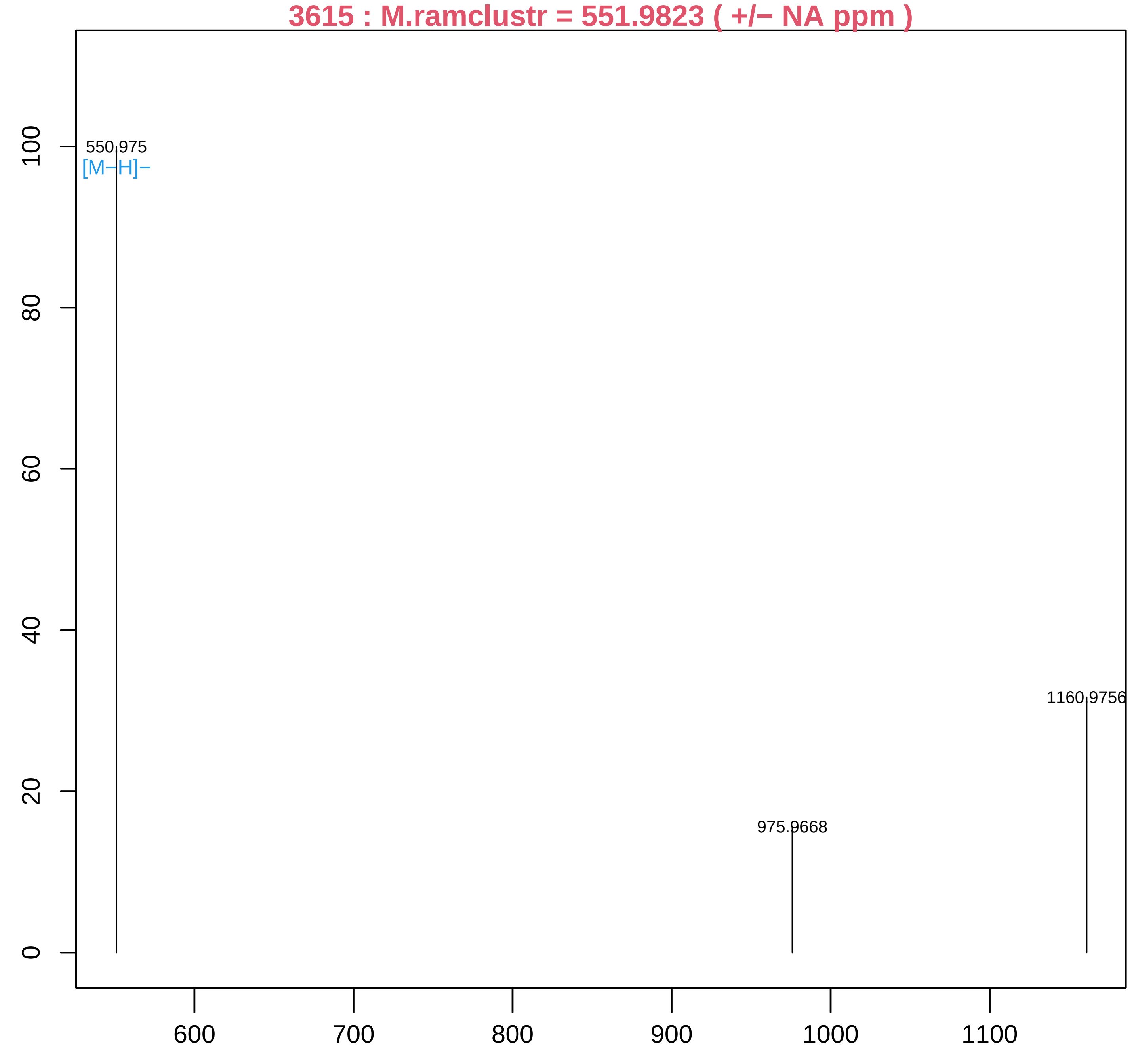
3615 : M.ramclustr = 551.9823 (+/− NA ppm)
550.975
[M−H]−
975.9668
1160.9756
0
20
40
60
80
100
600
700
800
900
1000
1100

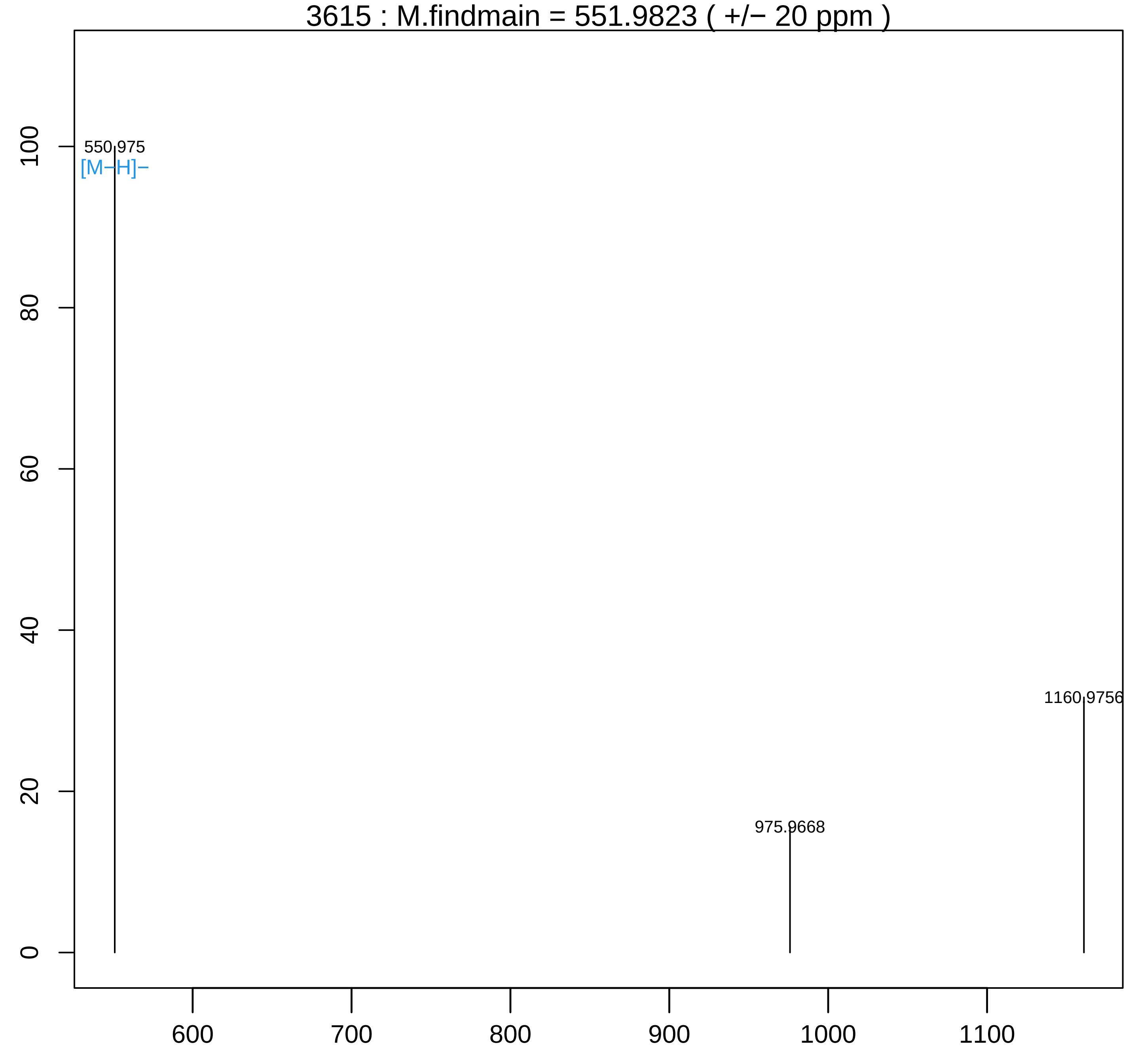
3615 : M.findmain = 551.9823 (+/− 20 ppm)
550.975
[M−H]−
975.9668
1160.9756
0
20
40
60
80
100
600
700
800
900
1000
1100

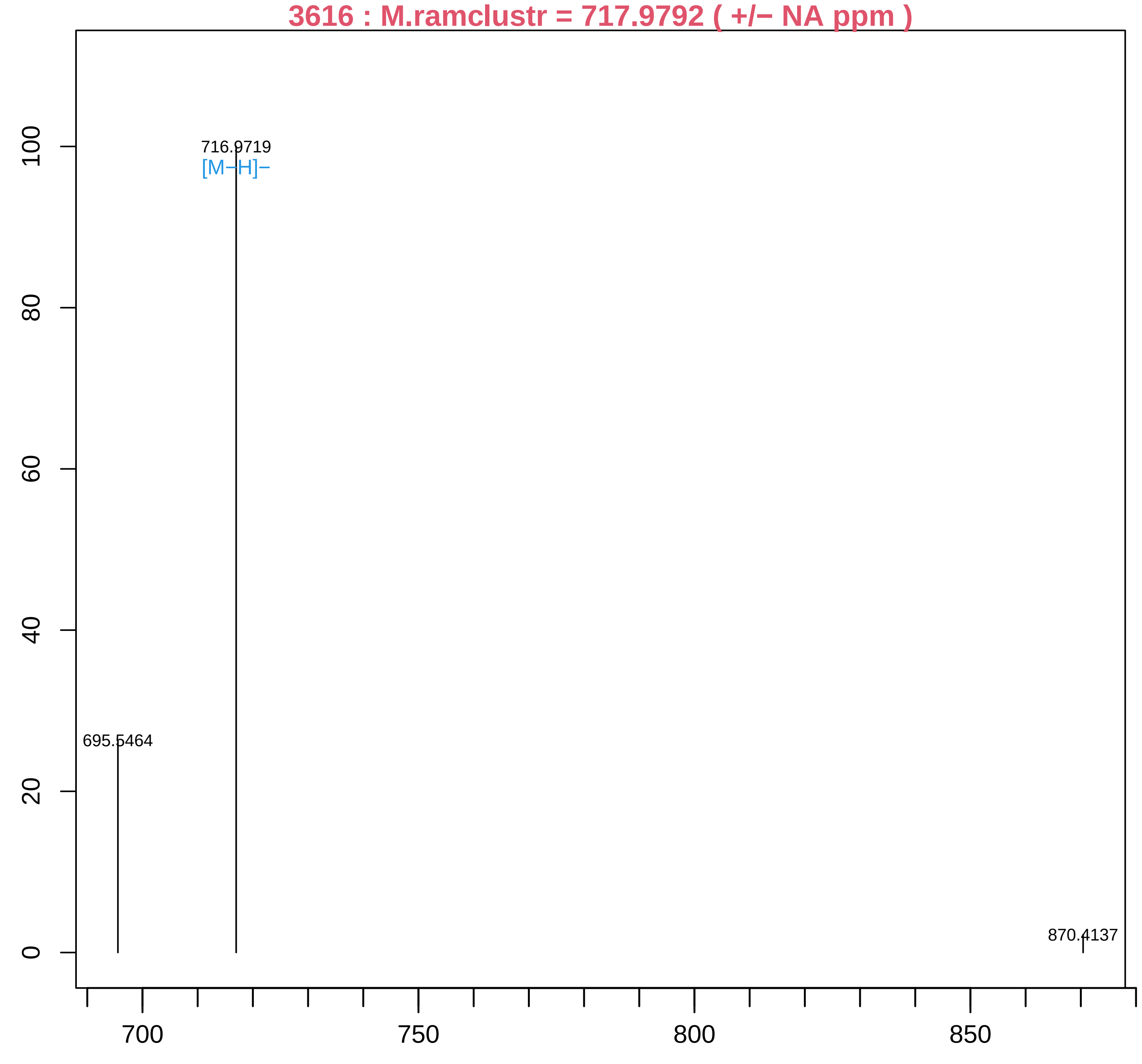
3616 : M.ramclustr = 717.9792 (+/− NA ppm)
716.9719
[M−H]−
695.5464
870.4137
0
20
40
60
80
100
700
750
800
850

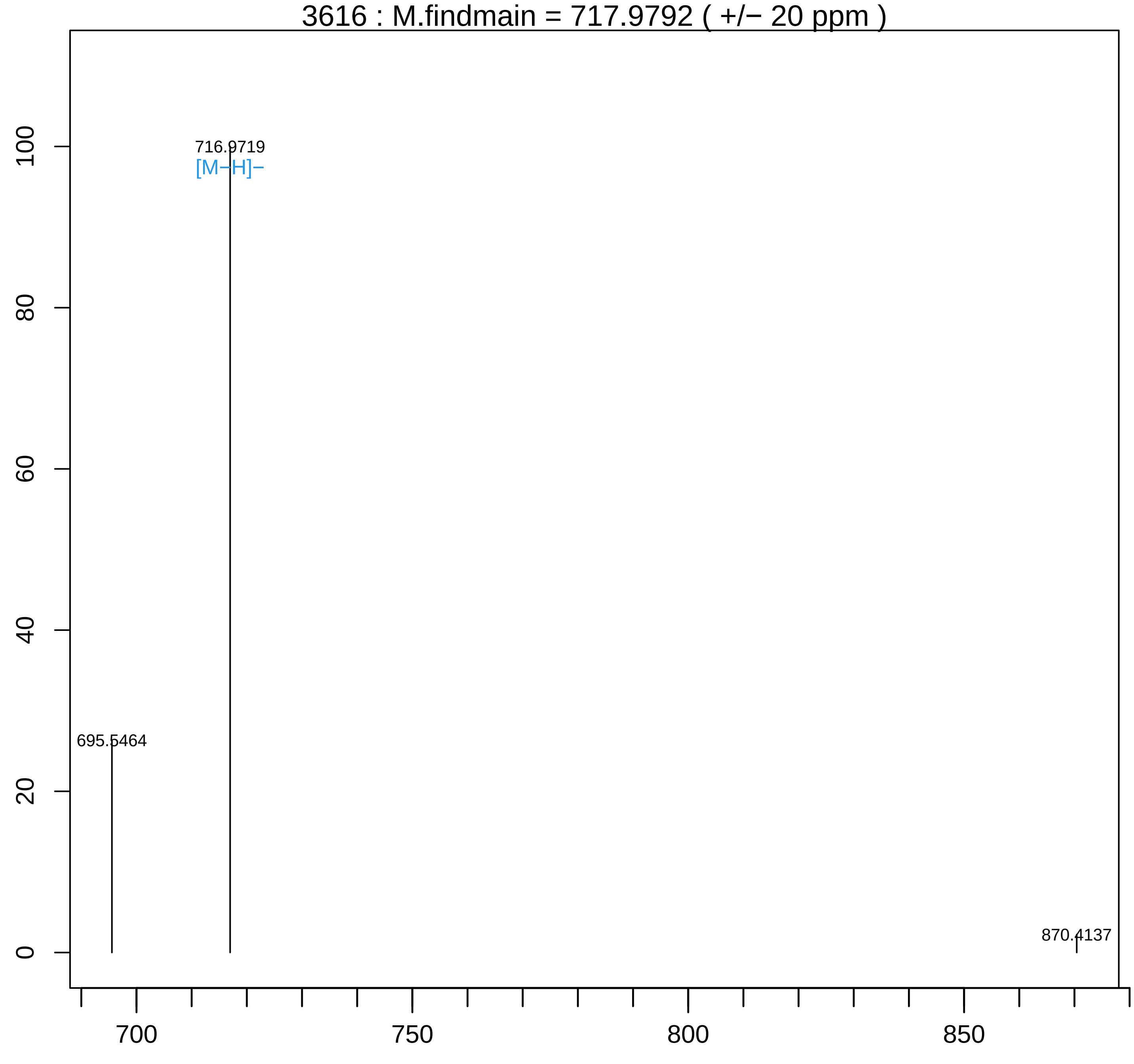
3616 : M.findmain = 717.9792 (+/− 20 ppm)
716.9719
[M−H]−
695.5464
870.4137
0
20
40
60
80
100
700
750
800
850

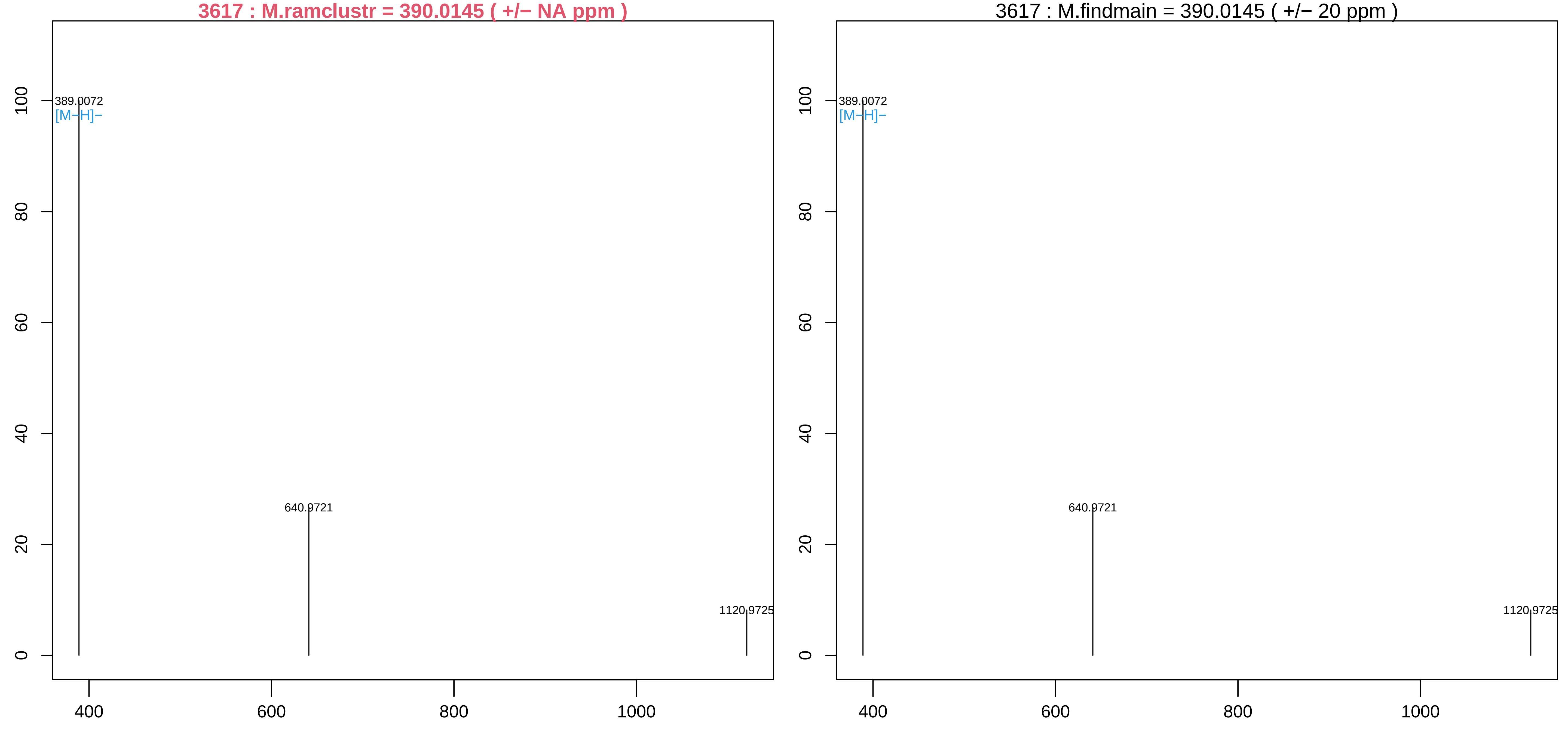
3617 : M.ramclustr = 390.0145 (+/− NA ppm)
389.0072
[M−H]−
640.9721
1120.9725
3617 : M.findmain = 390.0145 (+/− 20 ppm)
389.0072
[M−H]−
640.9721
1120.9725
0
20
40
60
80
100
400
600
800
1000

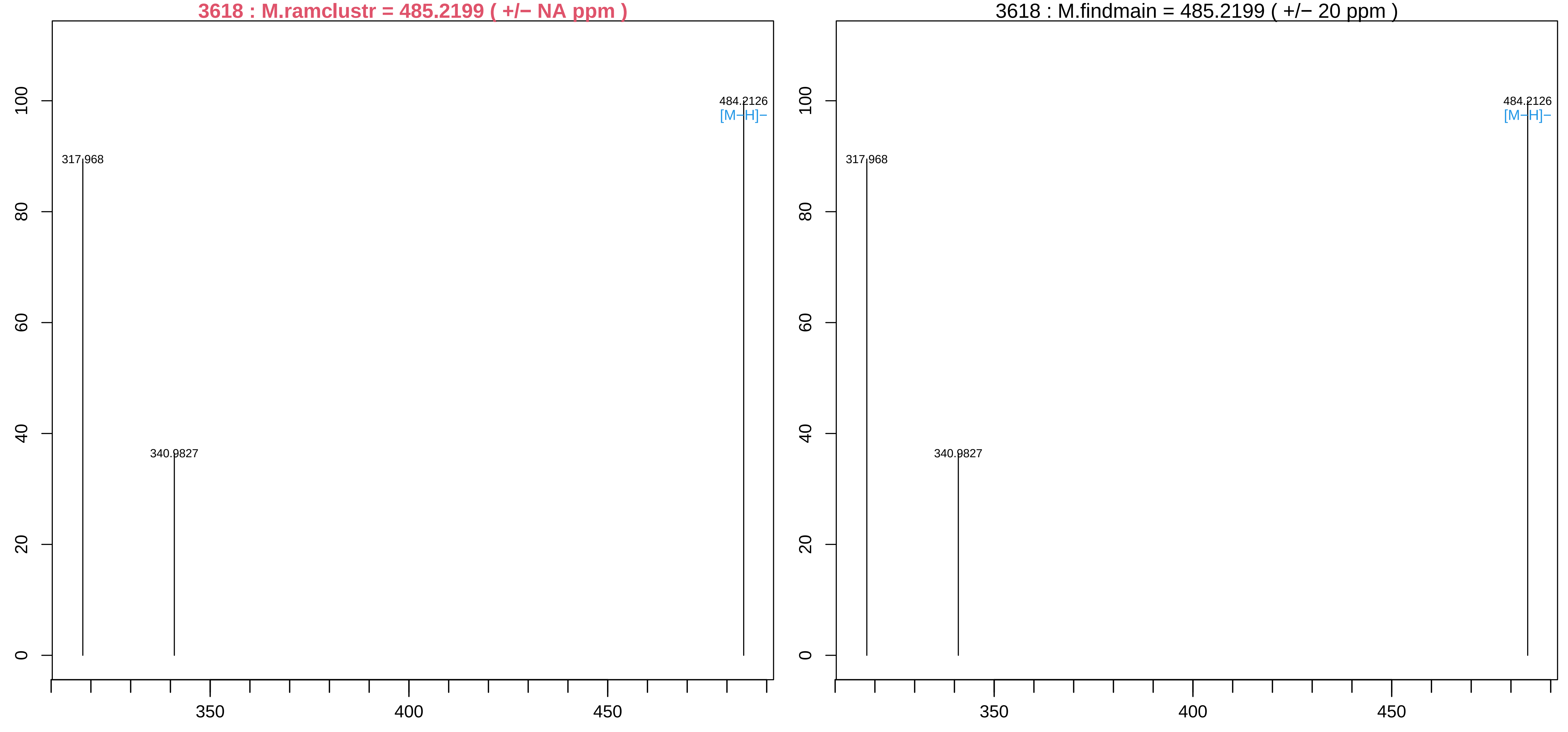
3618 : M.ramclustr = 485.2199 (+/− NA ppm)
3618 : M.findmain = 485.2199 (+/− 20 ppm)
484.2126
[M−H]−
317.968
340.9827
0
20
40
60
80
100
350
400
450

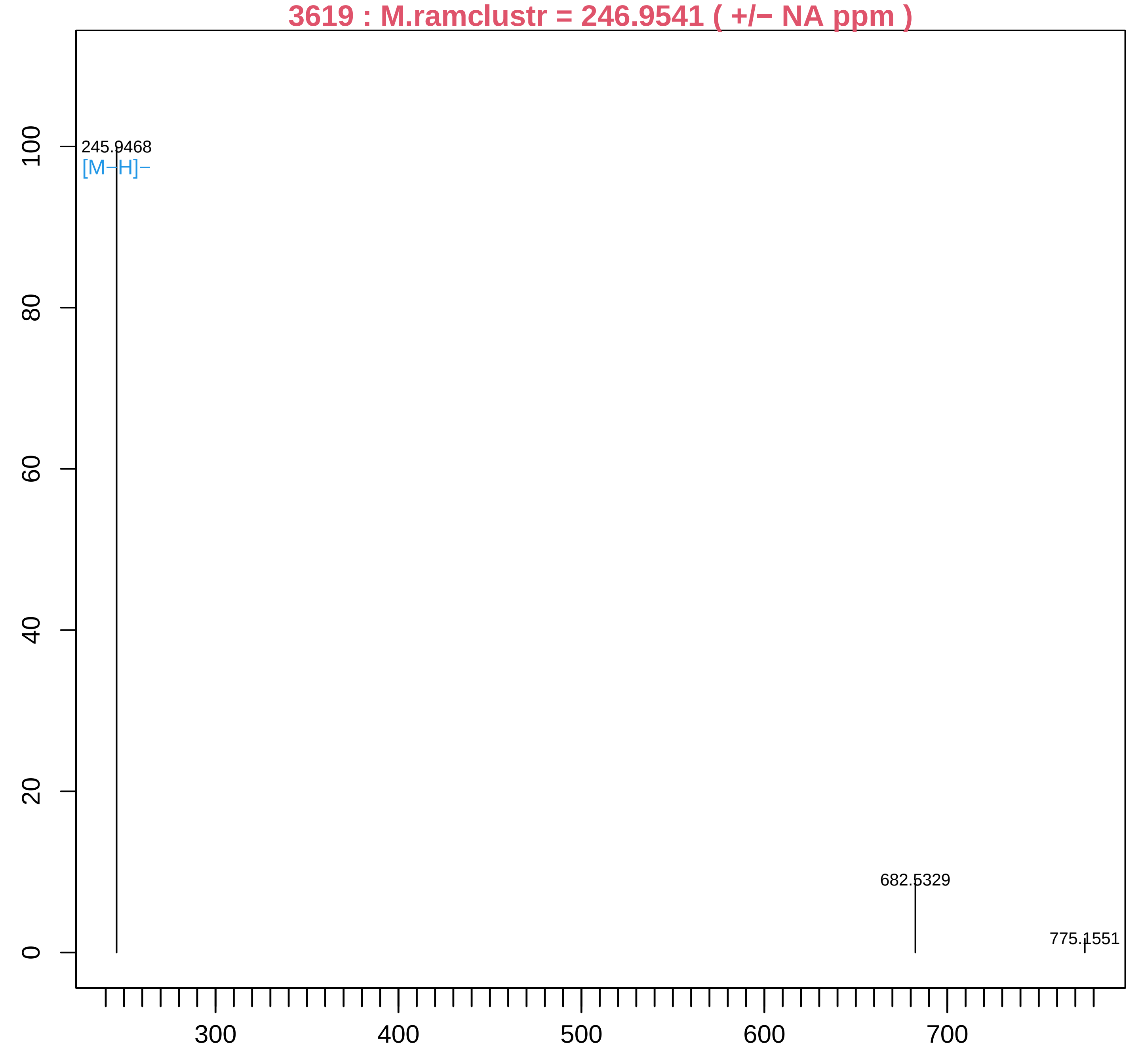
3619 : M.ramclustr = 246.9541 (+/− NA ppm)
245.9468
[M−H]−
682.5329
775.1551
0
20
40
60
80
100
300
400
500
600
700

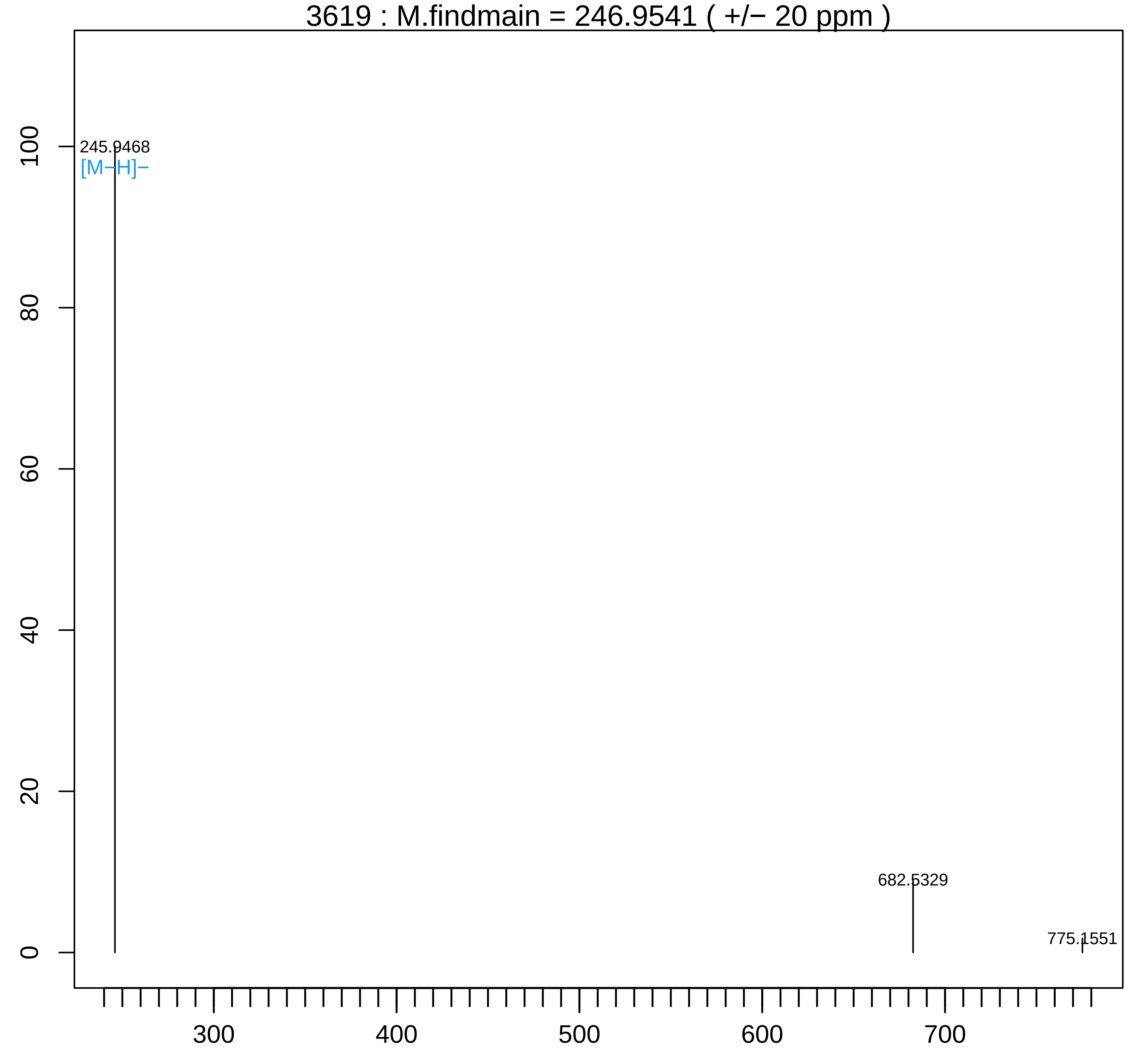
3619 : M.findmain = 246.9541 (+/− 20 ppm)
245.9468
[M−H]−
682.5329
775.1551
0
20
40
60
80
100
300
400
500
600
700

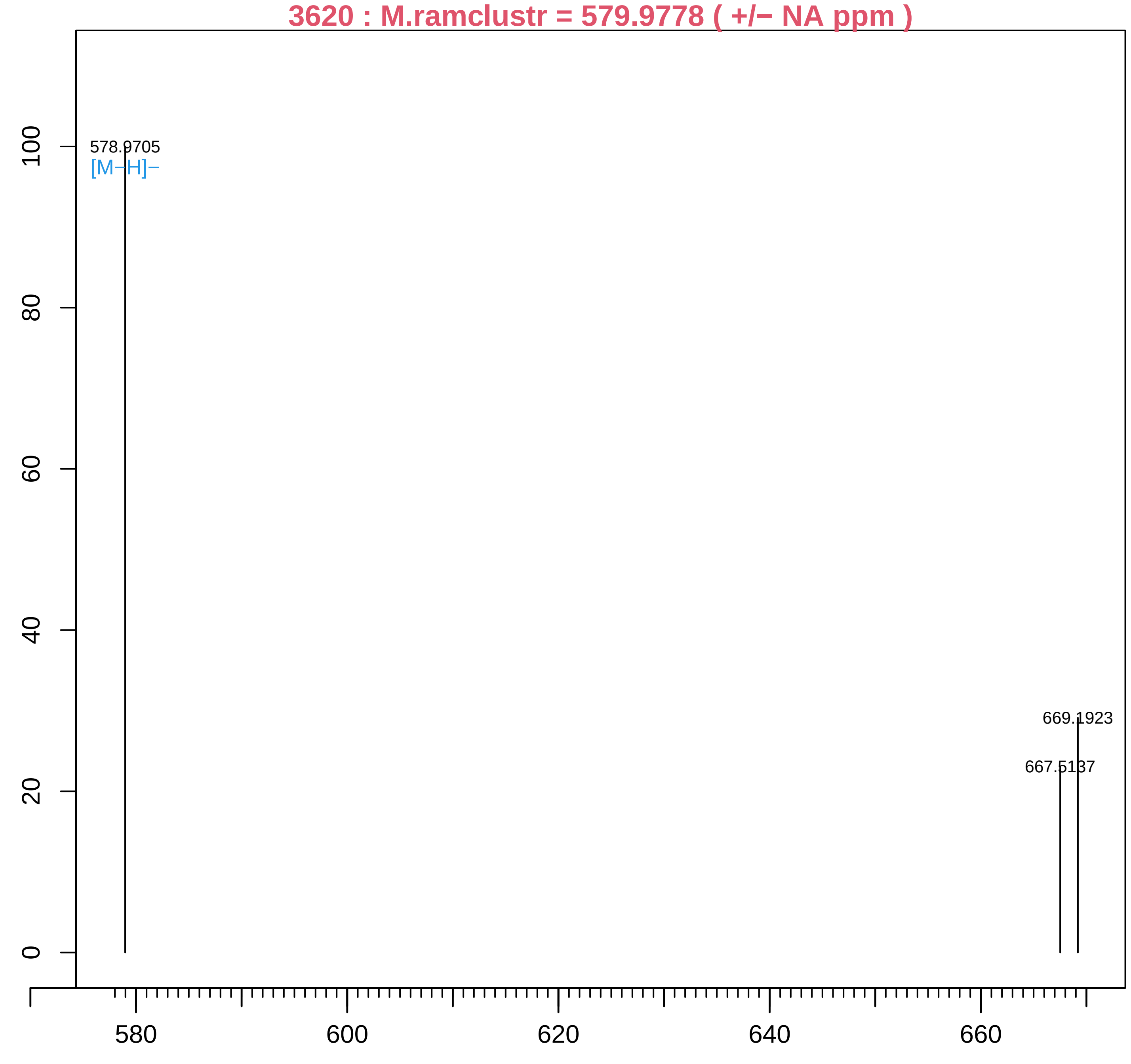
3620 : M.ramclustr = 579.9778 (+/− NA ppm)
578.9705
[M−H]−
669.1923
667.5137
0
20
40
60
80
100
580
600
620
640
660

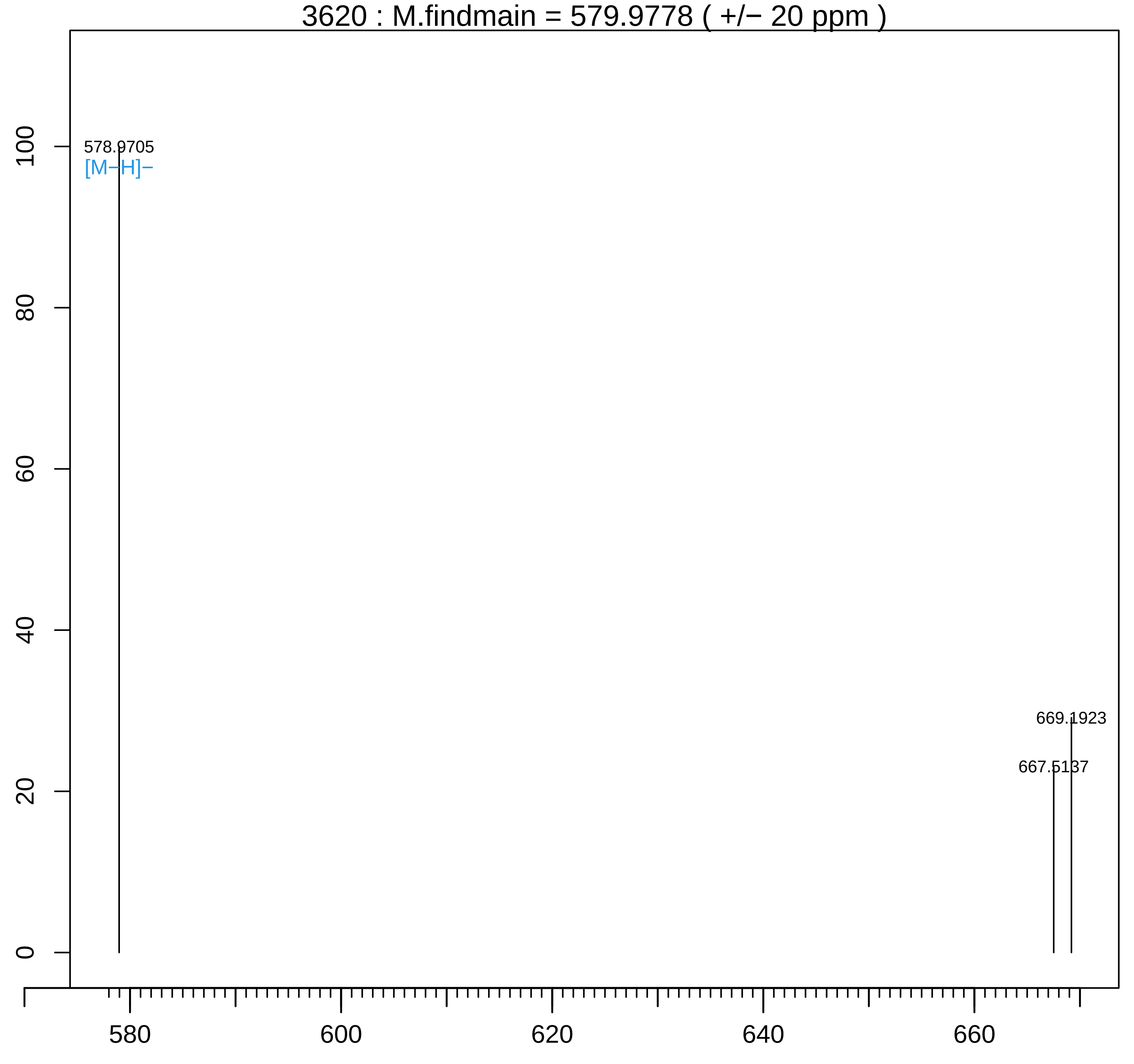
3620 : M.findmain = 579.9778 (+/− 20 ppm)
578.9705
[M−H]−
669.1923
667.5137
0
20
40
60
80
100
580
600
620
640
660

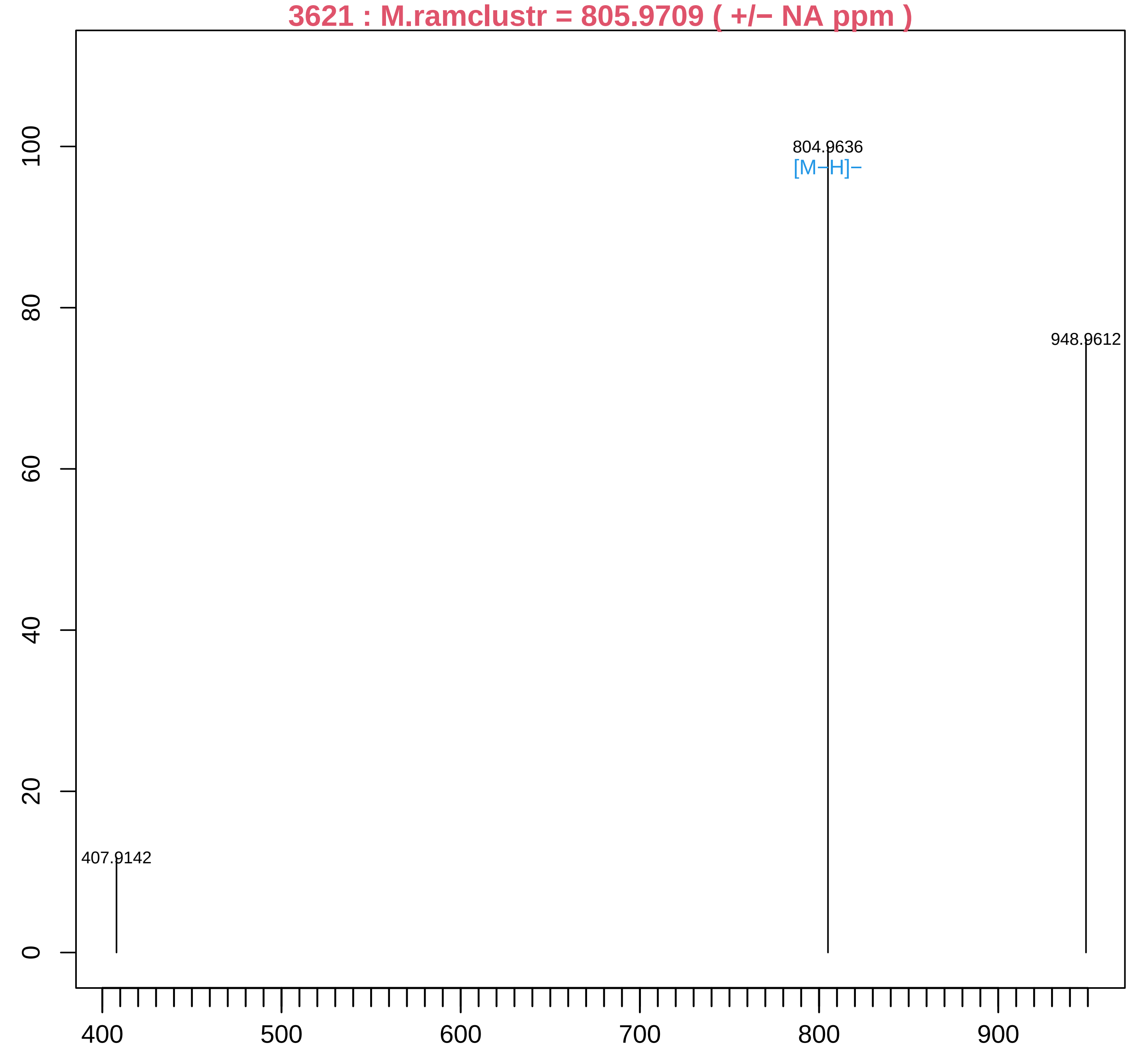
3621 : M.ramclustr = 805.9709 (+/− NA ppm)
804.9636
[M−H]−
948.9612
407.9142
0
20
40
60
80
100
400
500
600
700
800
900

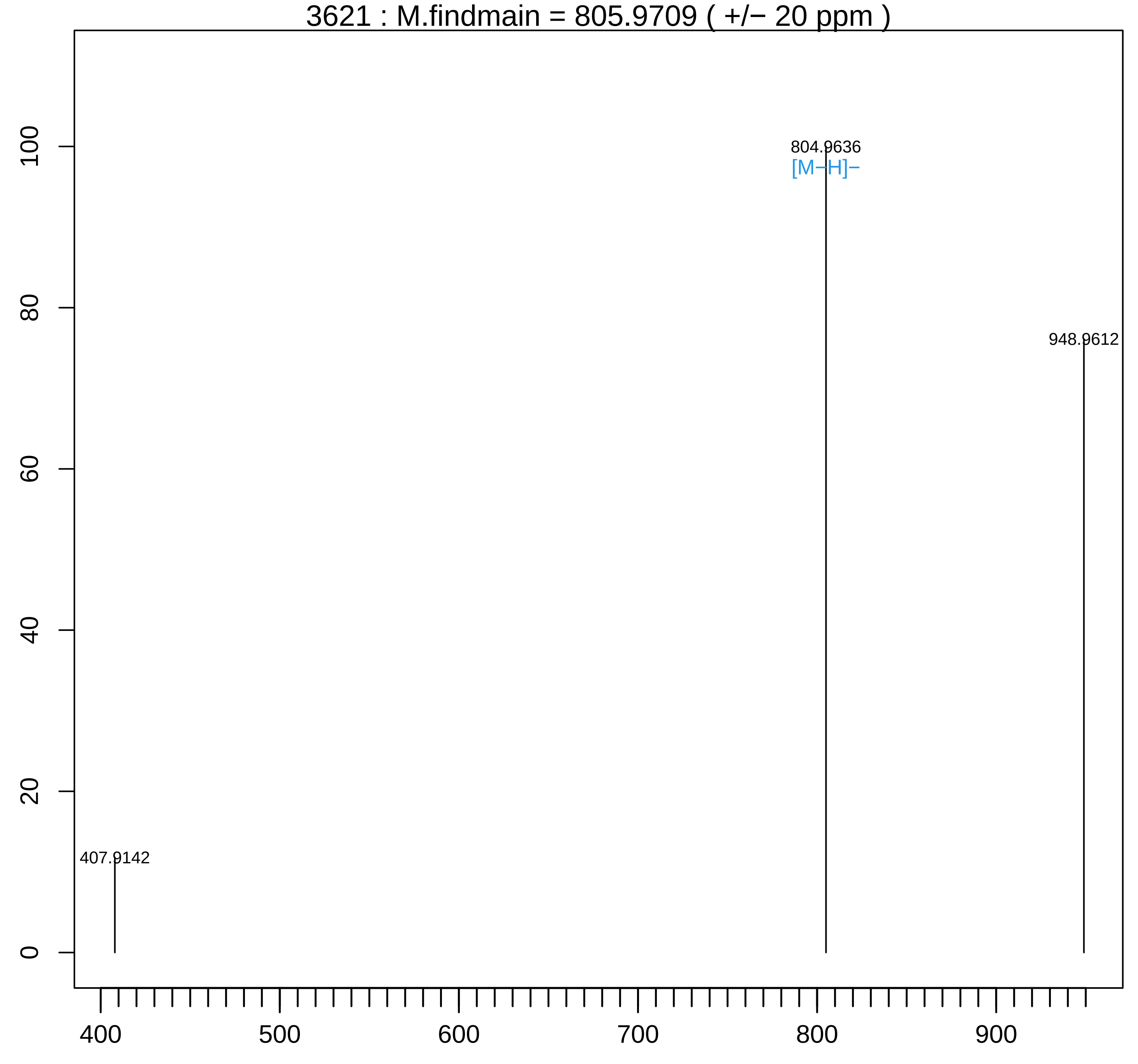
3621 : M.findmain = 805.9709 (+/− 20 ppm)
804.9636
[M−H]−
948.9612
407.9142
0
20
40
60
80
100
400
500
600
700
800
900

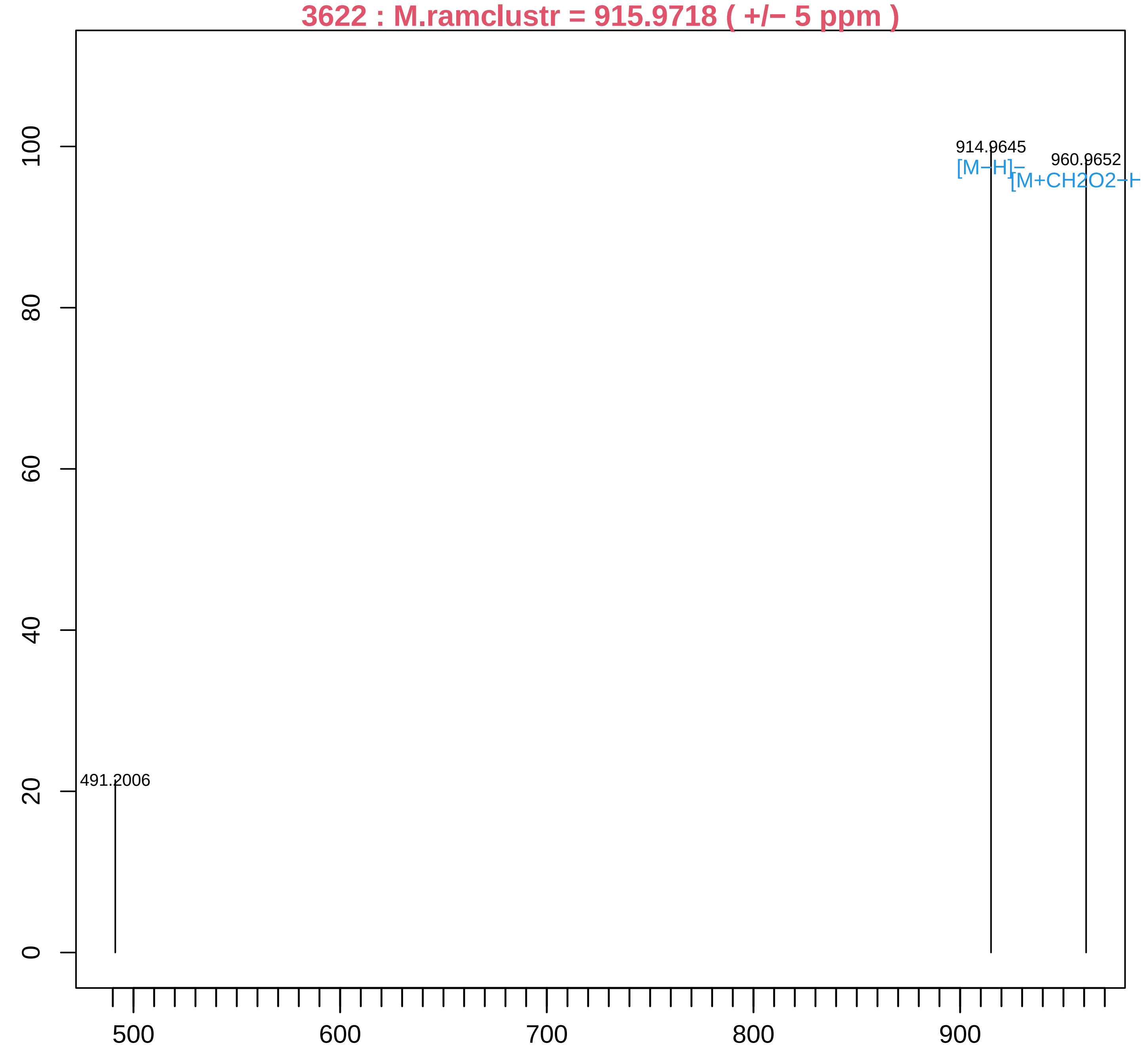

3622 : M.ramclustr = 915.9718 (+/− 5 ppm)
914.9645
[M−H]−
960.9652
[M+CH2O2-H
491.2006
0
20
40
60
80
100
500
600
700
800
900

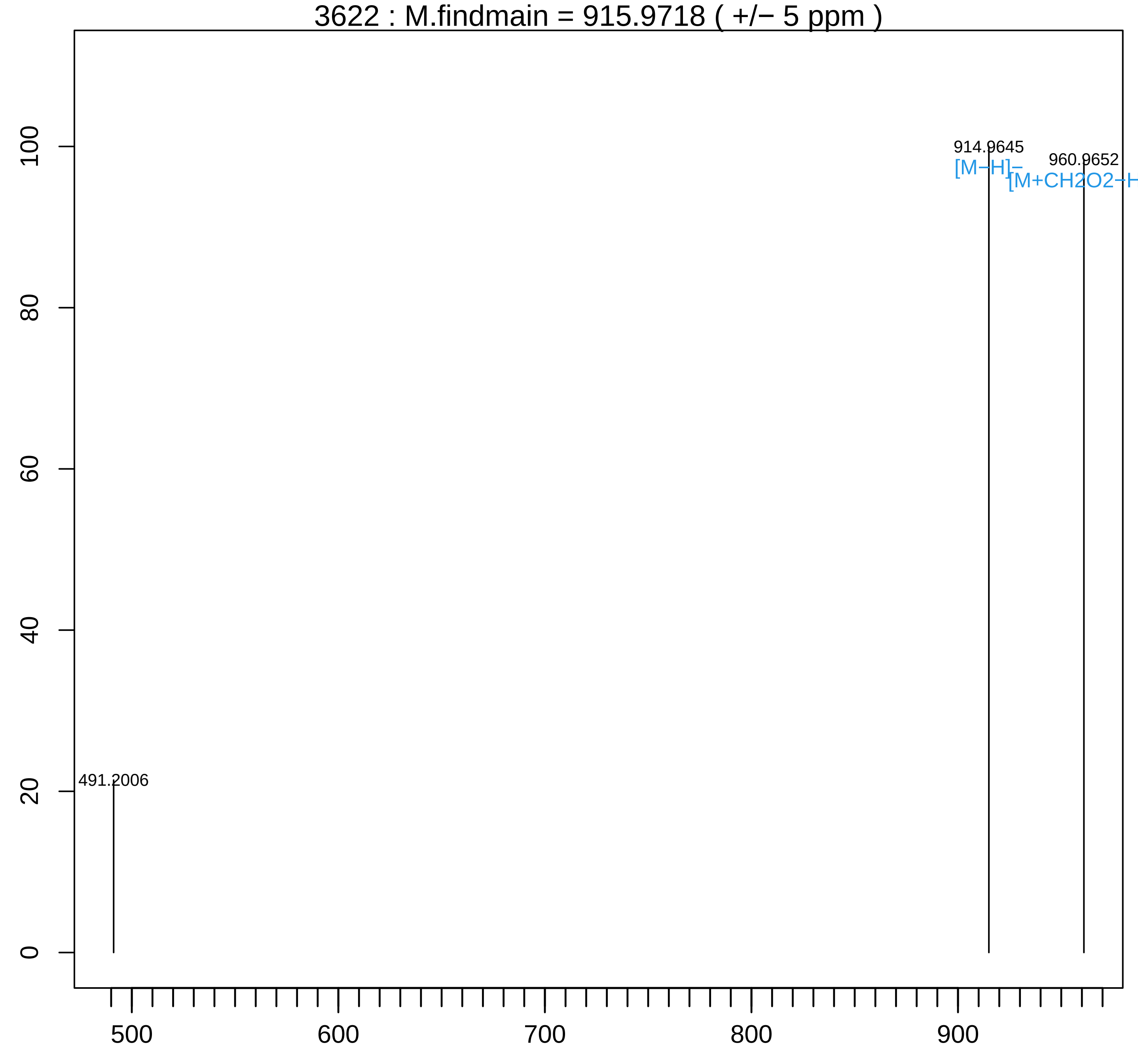

3622 : M.findmain = 915.9718 (+/− 5 ppm)
914.9645
[M−H]−
960.9652
[M+CH2O2-H
491.2006
0
20
40
60
80
100
500
600
700
800
900

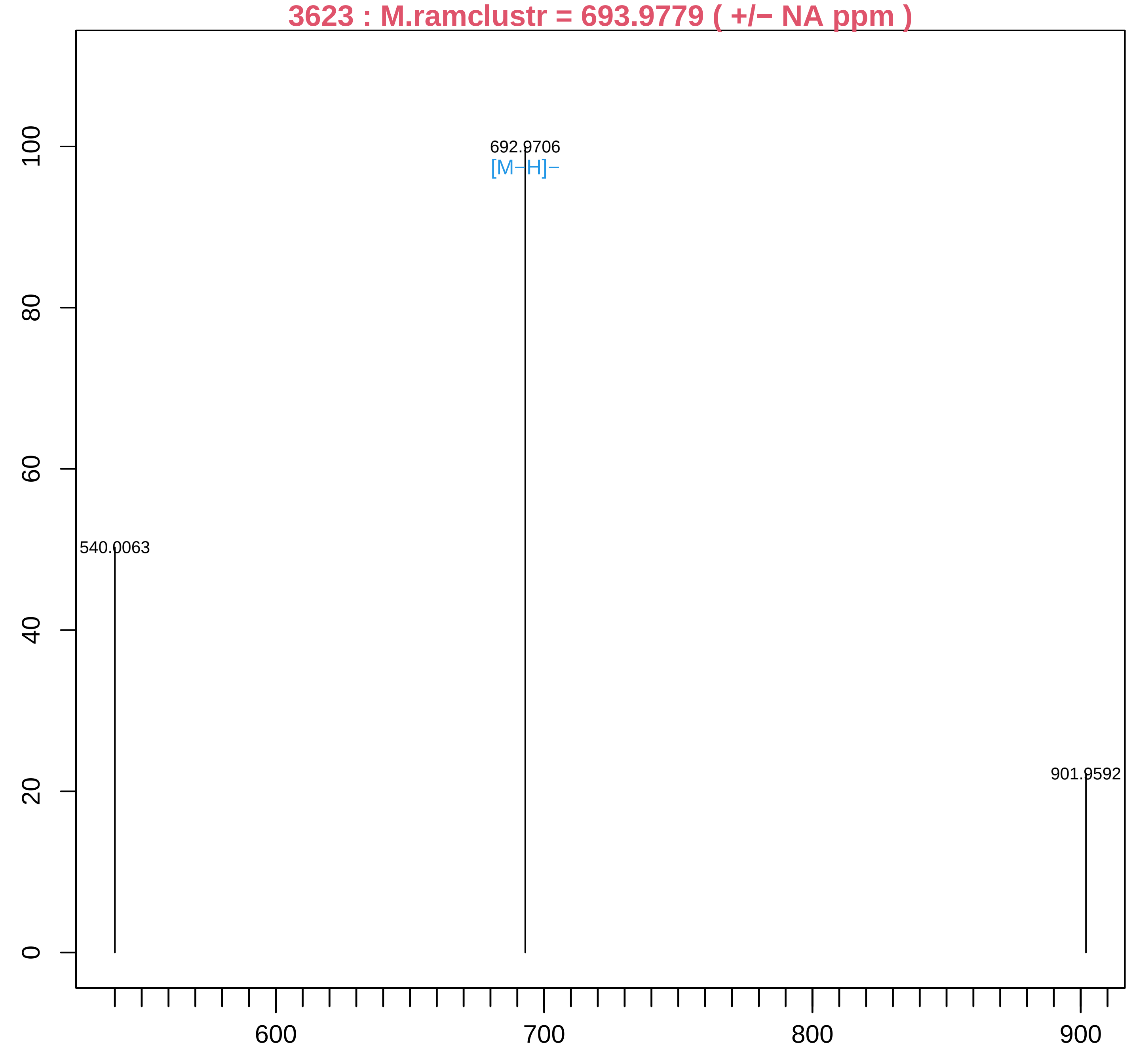
3623 : M.ramclustr = 693.9779 (+/− NA ppm)
692.9706
[M−H]−
540.0063
901.9592
0
20
40
60
80
100
600
700
800
900

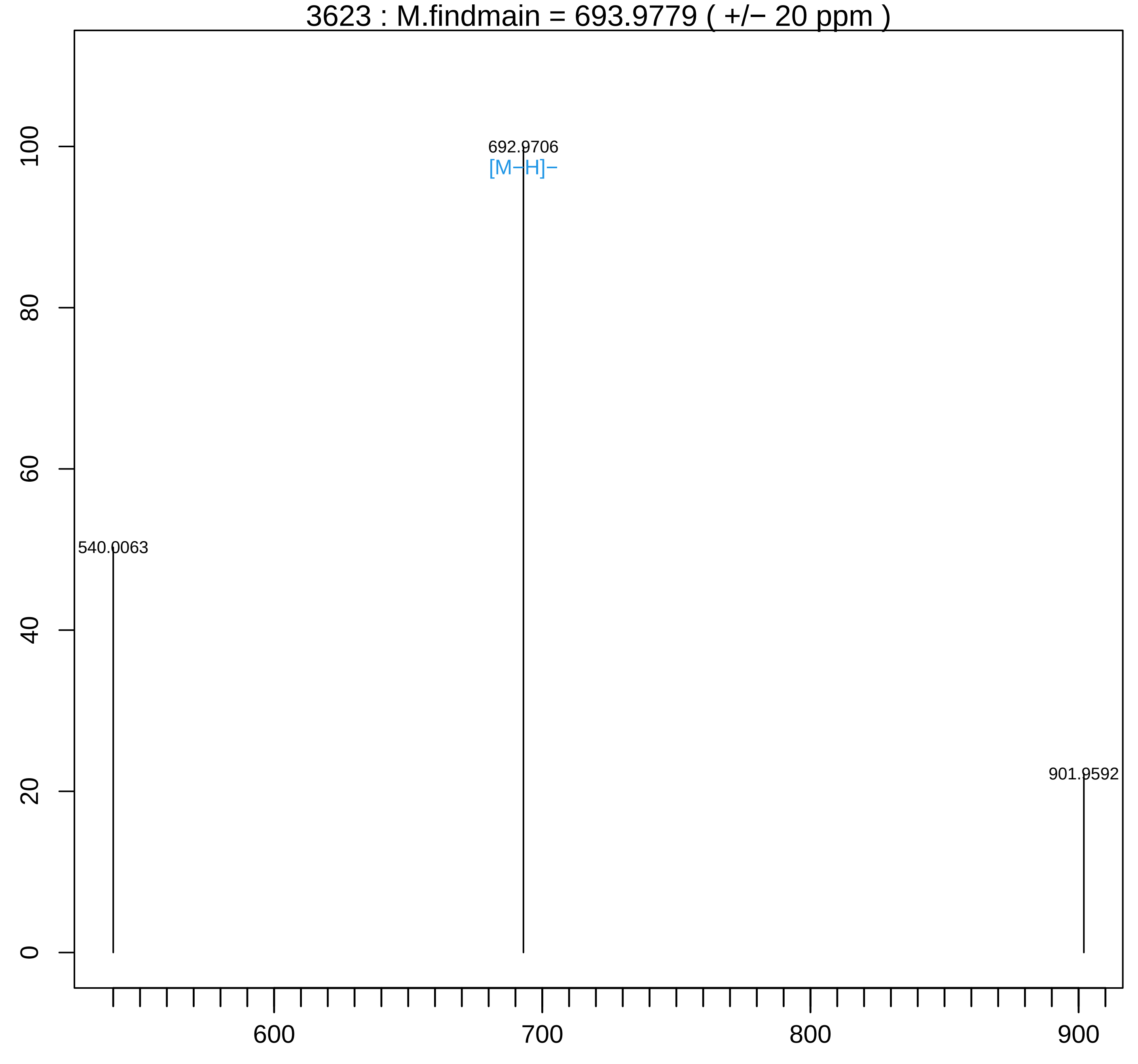
3623 : M.findmain = 693.9779 (+/− 20 ppm)
692.9706
[M−H]−
540.0063
901.9592
0
20
40
60
80
100
600
700
800
900

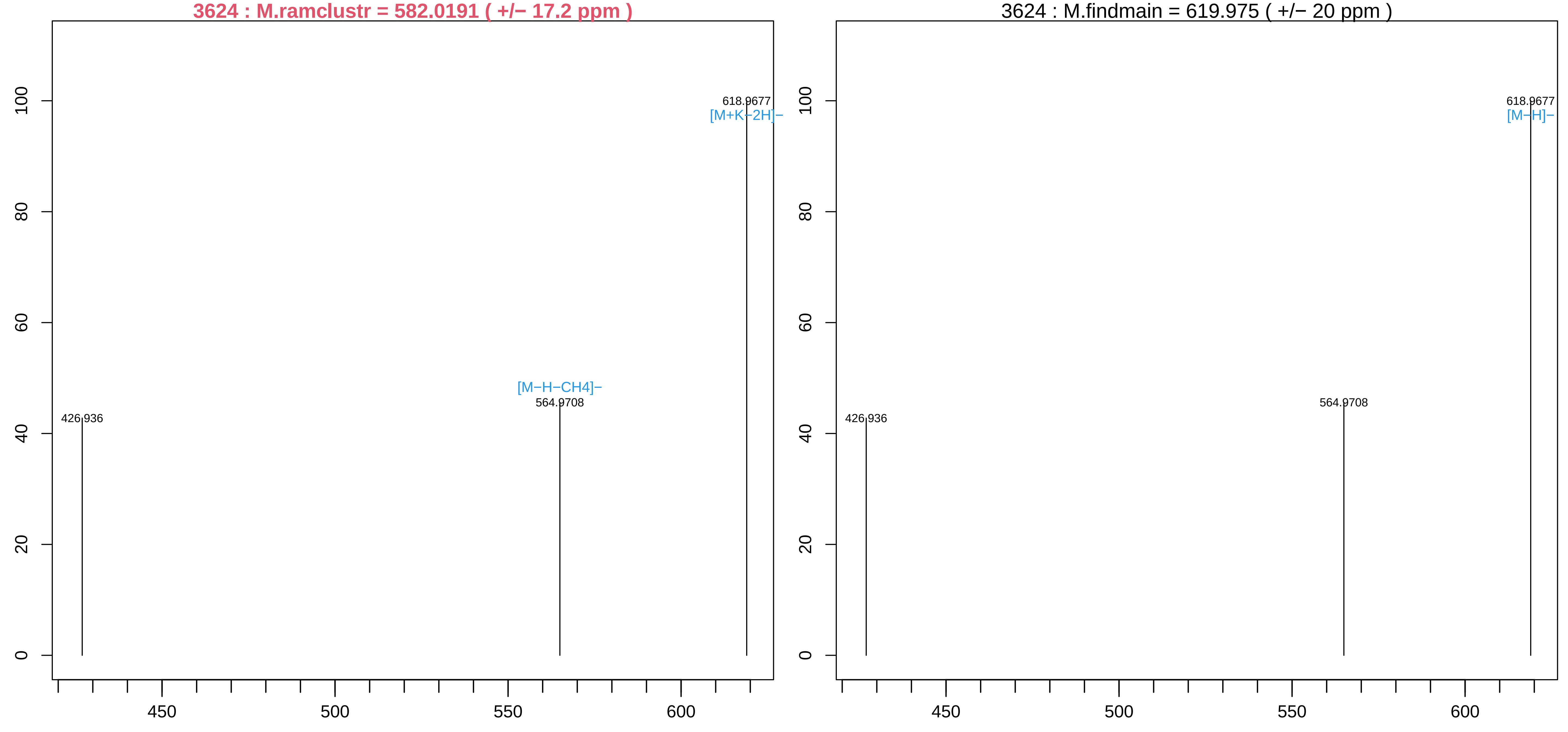
3624 : M.ramclustr = 582.0191 (+/− 17.2 ppm)
618.9677
[M+K−2H]−
[M−H−CH4]−
564.9708
426.936
3624 : M.findmain = 619.975 (+/− 20 ppm)
618.9677
[M−H]−
564.9708
426.936

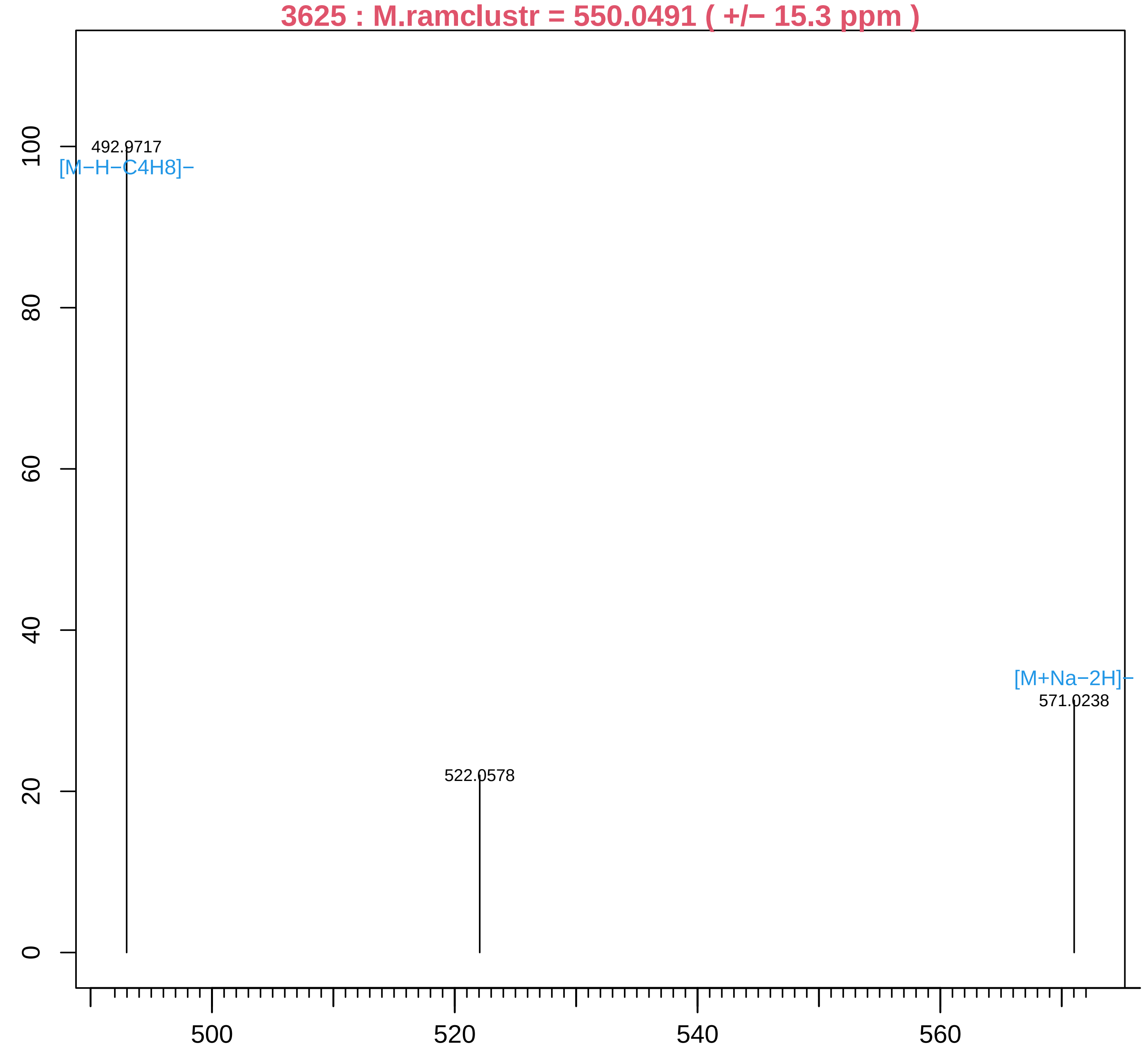
3625 : M.ramclustr = 550.0491 (+/− 15.3 ppm)
492.9717
[M−H−C4H8]−
522.0578
[M+Na−2H]−
571.0238
0
20
40
60
80
100
500
520
540
560

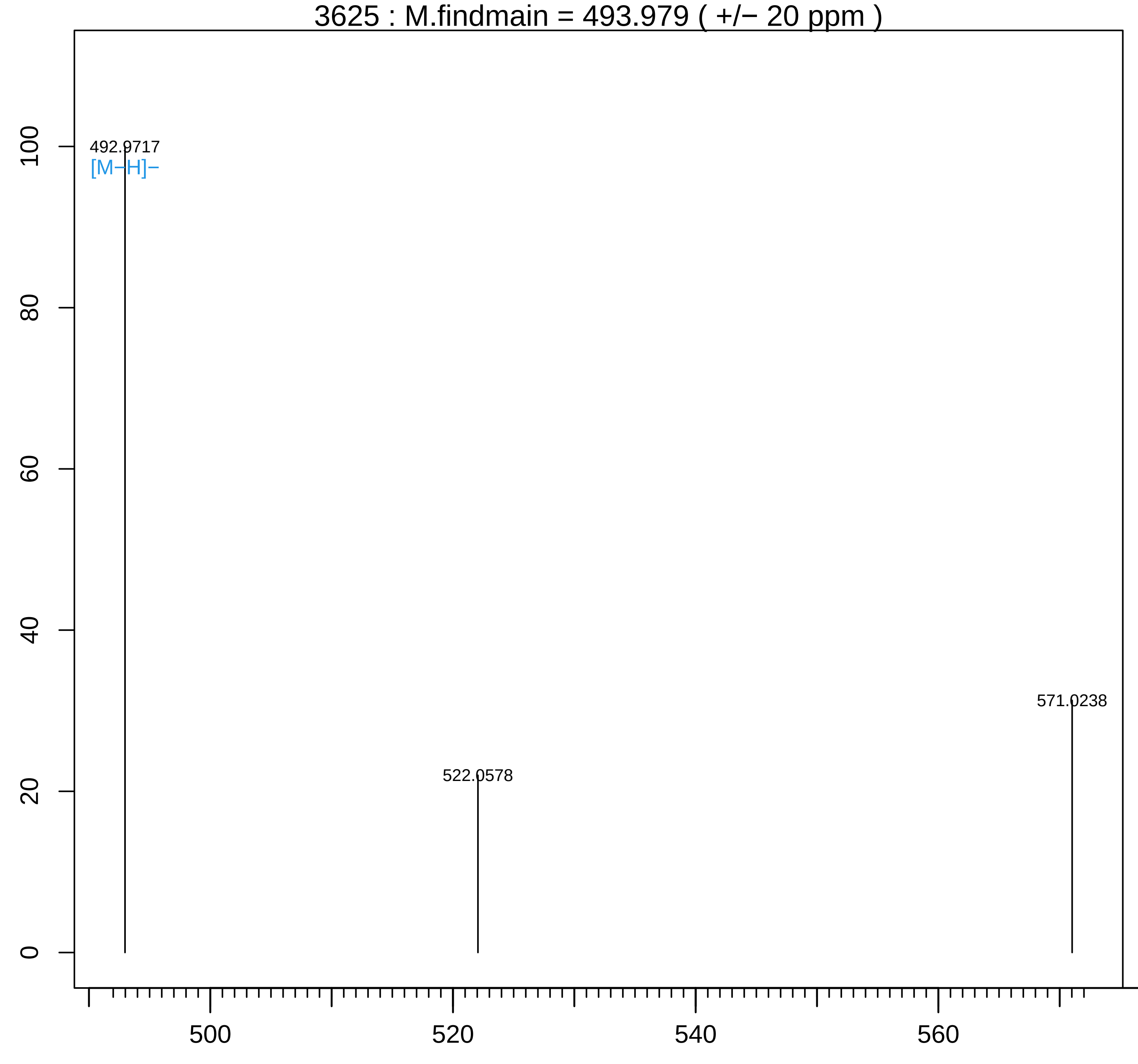
3625 : M.findmain = 493.979 (+/− 20 ppm)
492.9717
[M−H]−
522.0578
571.0238
0
20
40
60
80
100
500
520
540
560

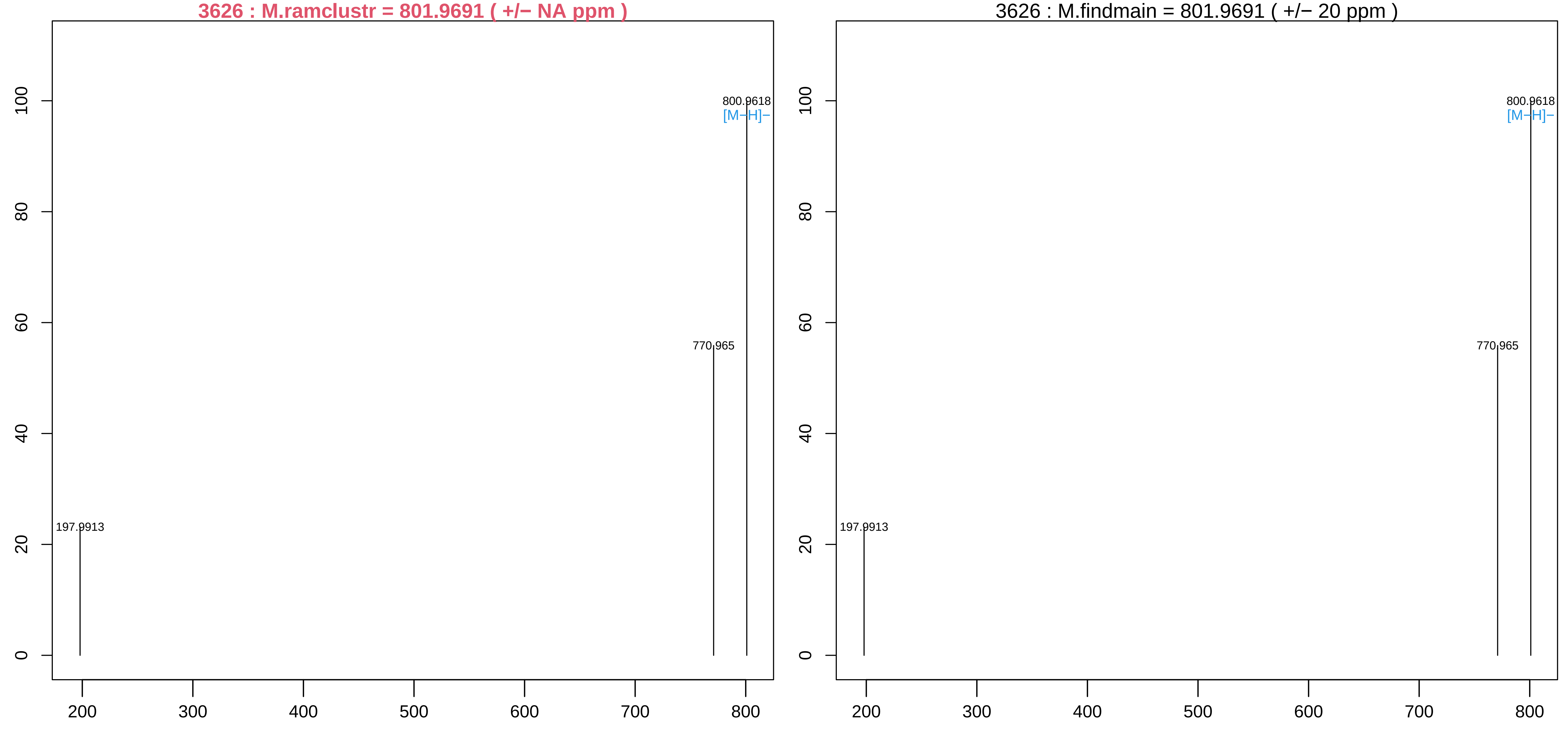

3626 : M.ramclustr = 801.9691 (+/− NA ppm)
800.9618
[M−H]−
770.965
197.9913
3626 : M.findmain = 801.9691 (+/− 20 ppm)
800.9618
[M−H]−
770.965
197.9913

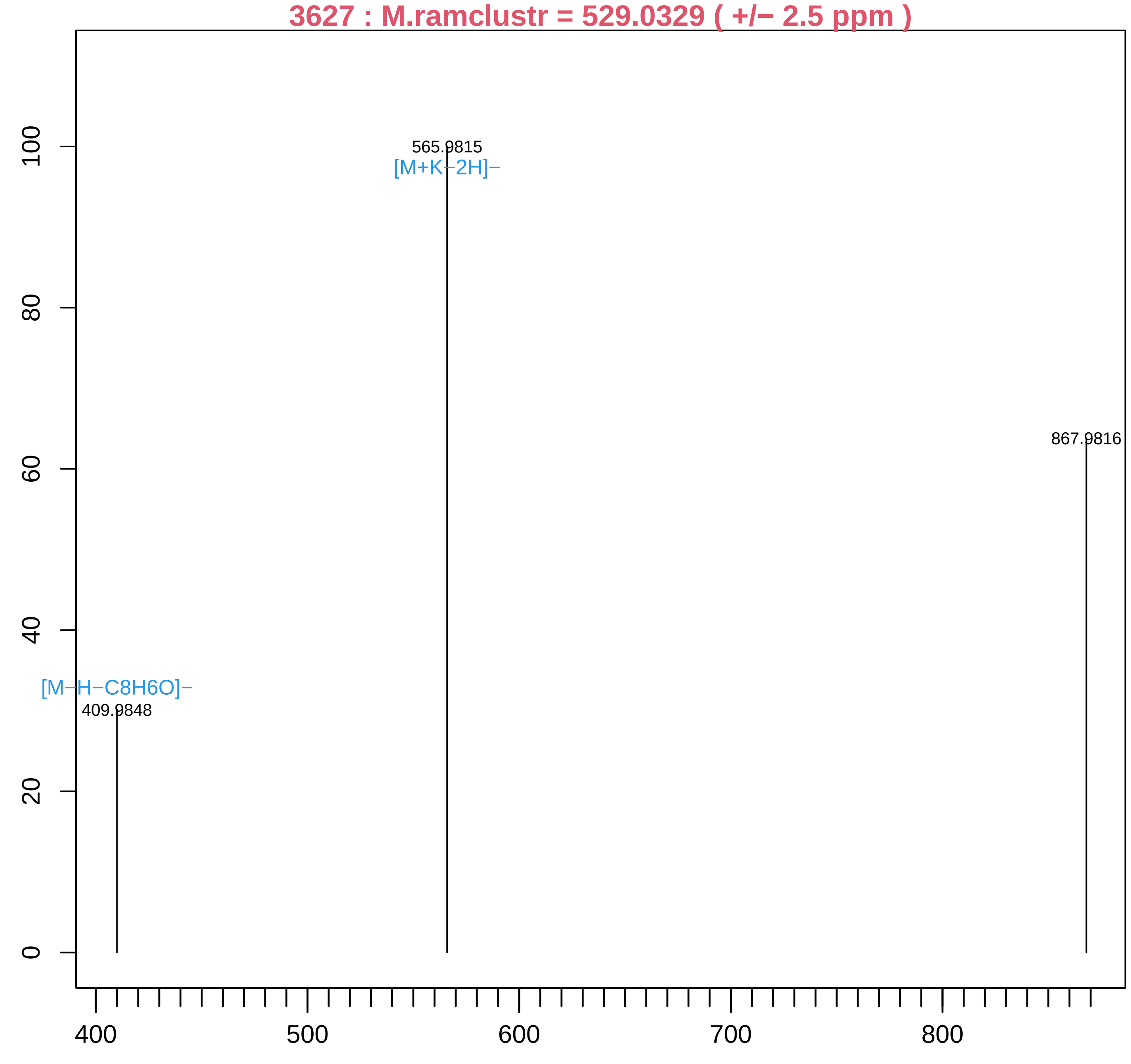
3627 : M.ramclustr = 529.0329 (+/− 2.5 ppm)
565.9815
[M+K−2H]−
867.9816
[M−H−C8H6O]−
409.9848
0
20
40
60
80
100
400
500
600
700
800

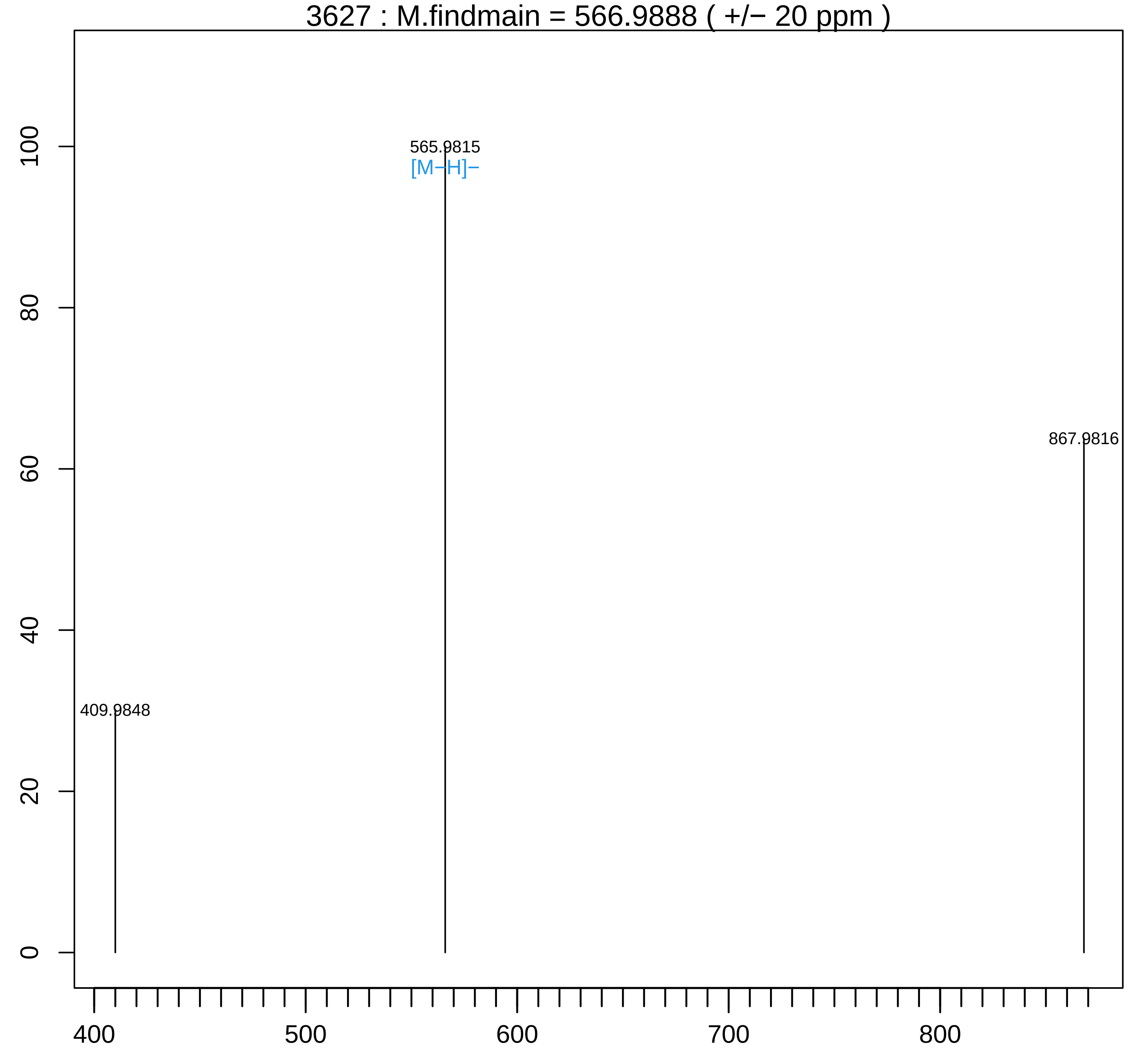
3627 : M.findmain = 566.9888 (+/− 20 ppm)
565.9815
[M−H]−
867.9816
409.9848
0
20
40
60
80
100
400
500
600
700
800

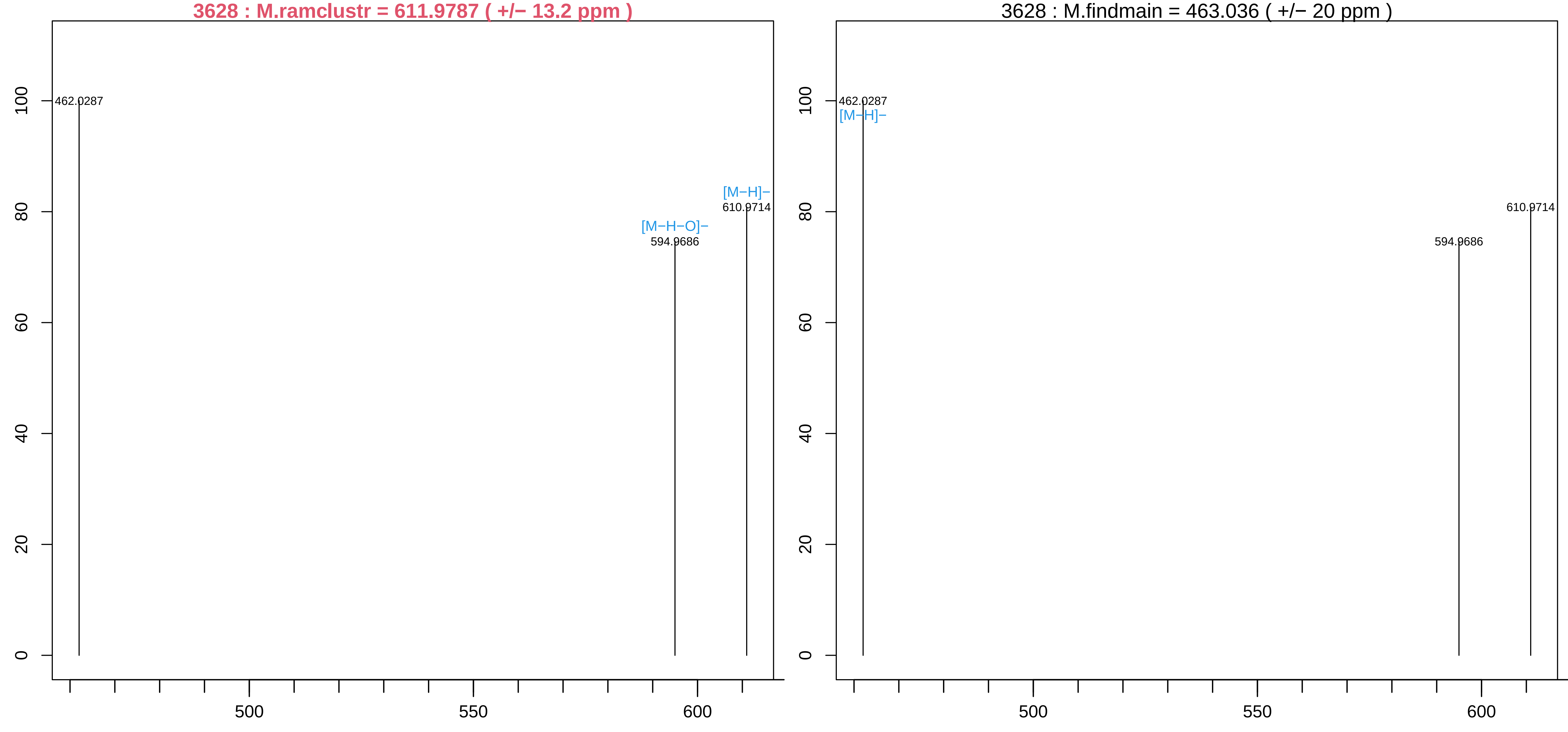
3628 : M.ramclustr = 611.9787 (+/− 13.2 ppm)
462.0287
[M−H−O]−
594.9686
[M−H]−
610.9714
100
80
60
40
20
0
500
550
600
3628 : M.findmain = 463.036 (+/− 20 ppm)
462.0287
[M−H]−
594.9686
610.9714
100
80
60
40
20
0
500
550
600

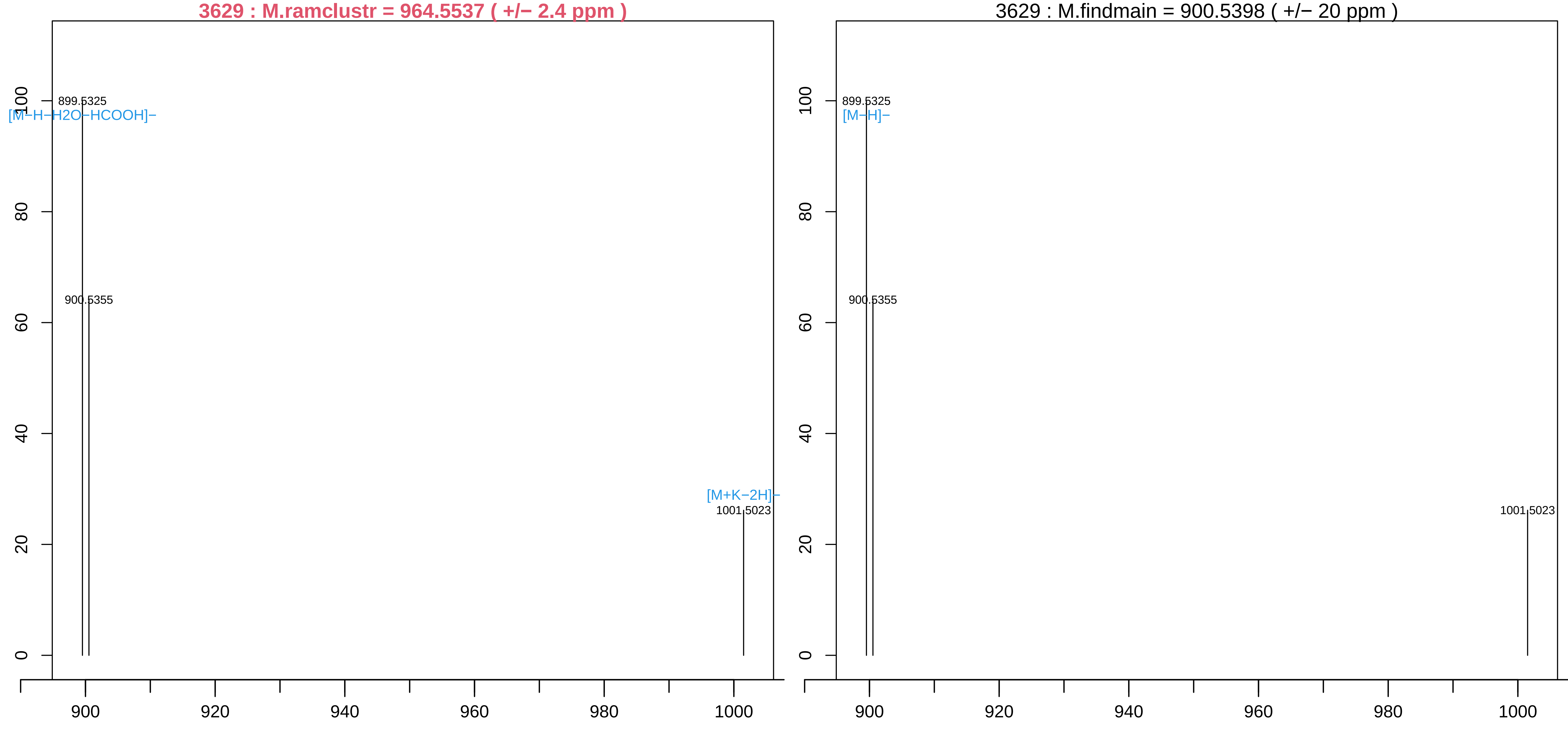
3629 : M.ramclustr = 964.5537 (+/− 2.4 ppm)
899.5325
[M−H−H2O−HCOOH]−
900.5355
[M+K−2H]−
1001.5023
3629 : M.findmain = 900.5398 (+/− 20 ppm)
899.5325
[M−H]−
900.5355
1001.5023
0
20
40
60
80
100
900
920
940
960
980
1000

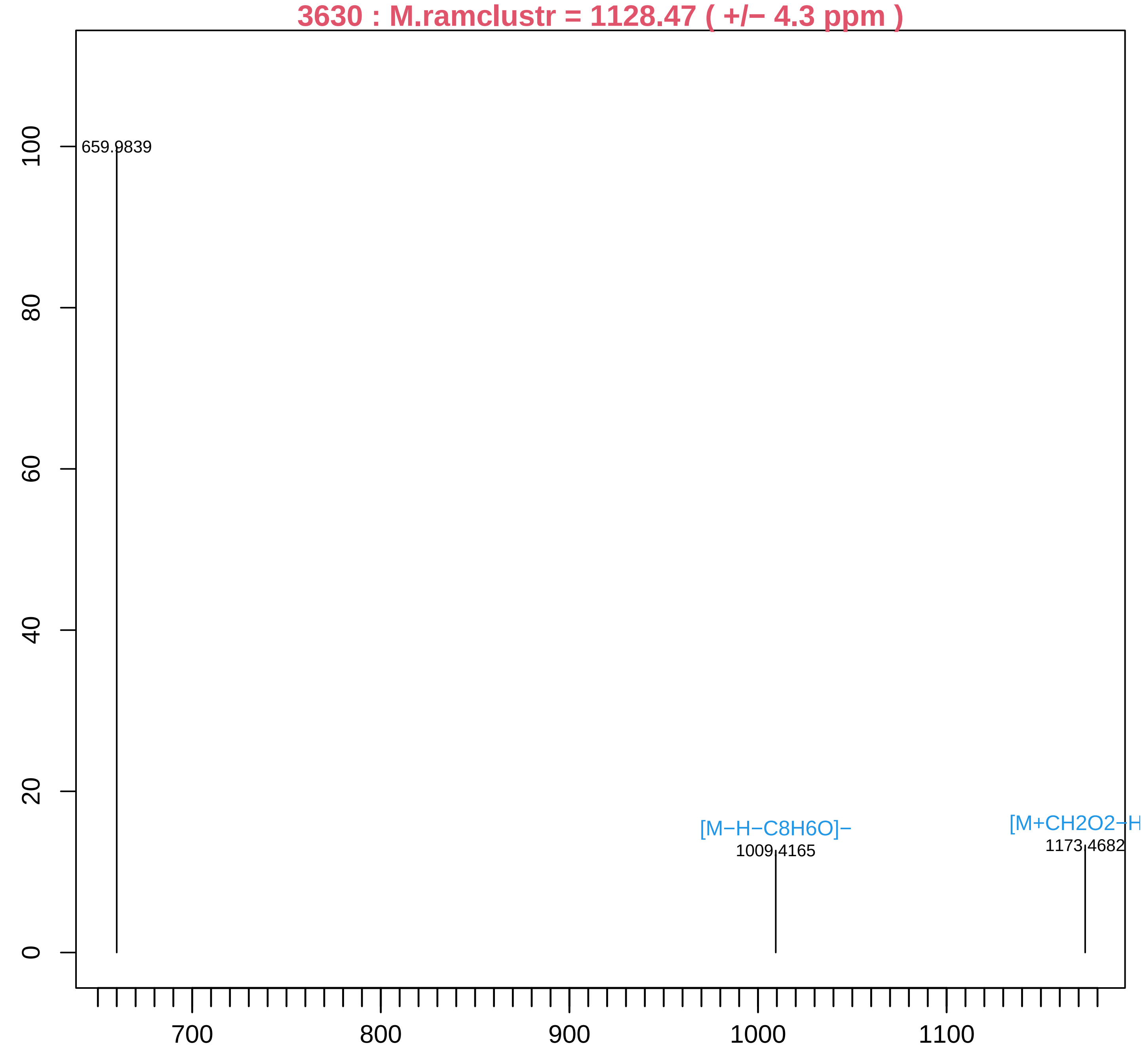
3630 : M.ramclustr = 1128.47 (+/− 4.3 ppm)
659.9839
[M−H−C8H6O]−
1009.4165
[M+CH2O2−H
1173.4682
0
20
40
60
80
100
700
800
900
1000
1100

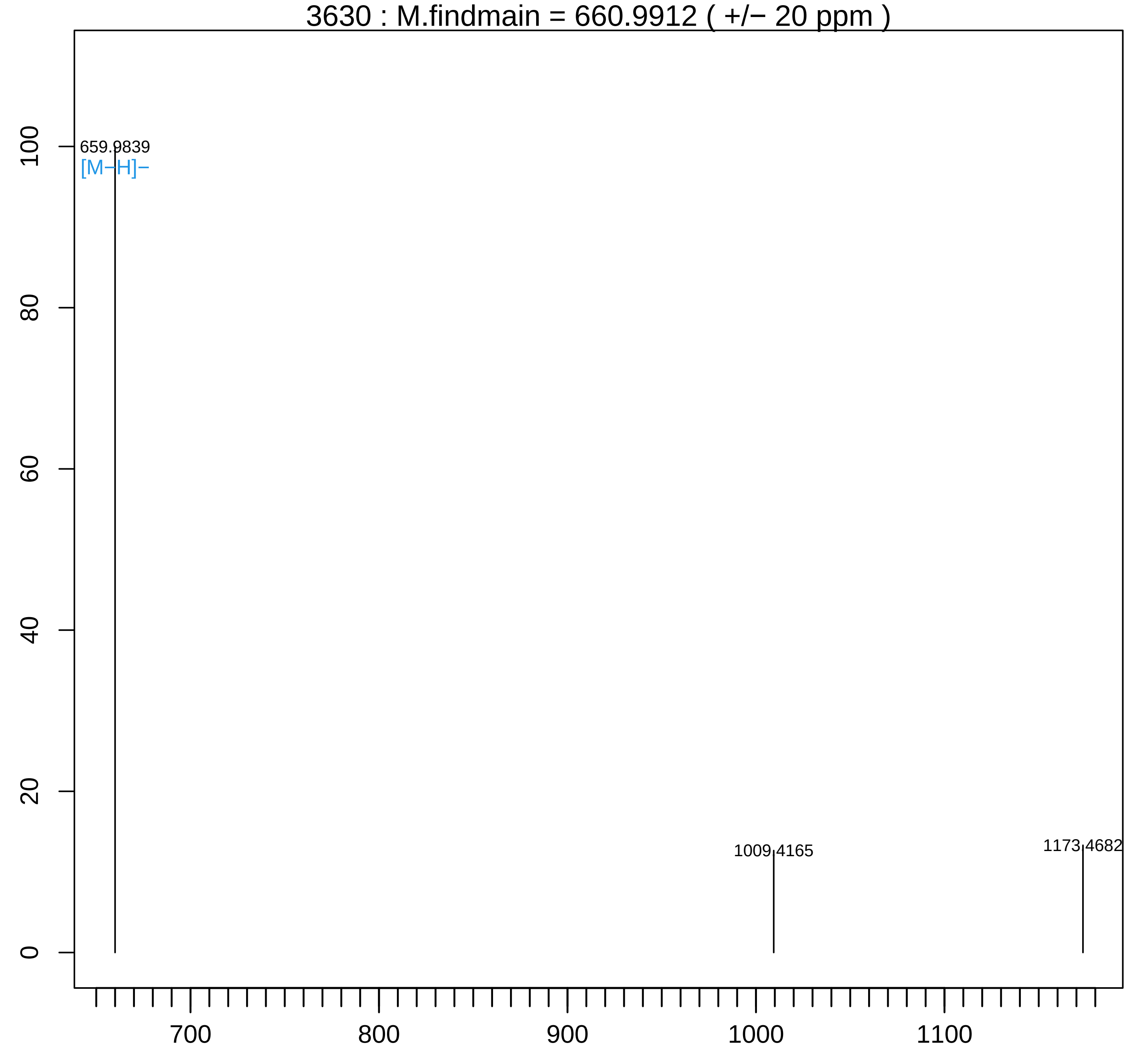
3630 : M.findmain = 660.9912 (+/− 20 ppm)
659.9839
[M−H]−
1009.4165
1173.4682
0
20
40
60
80
100
700
800
900
1000
1100

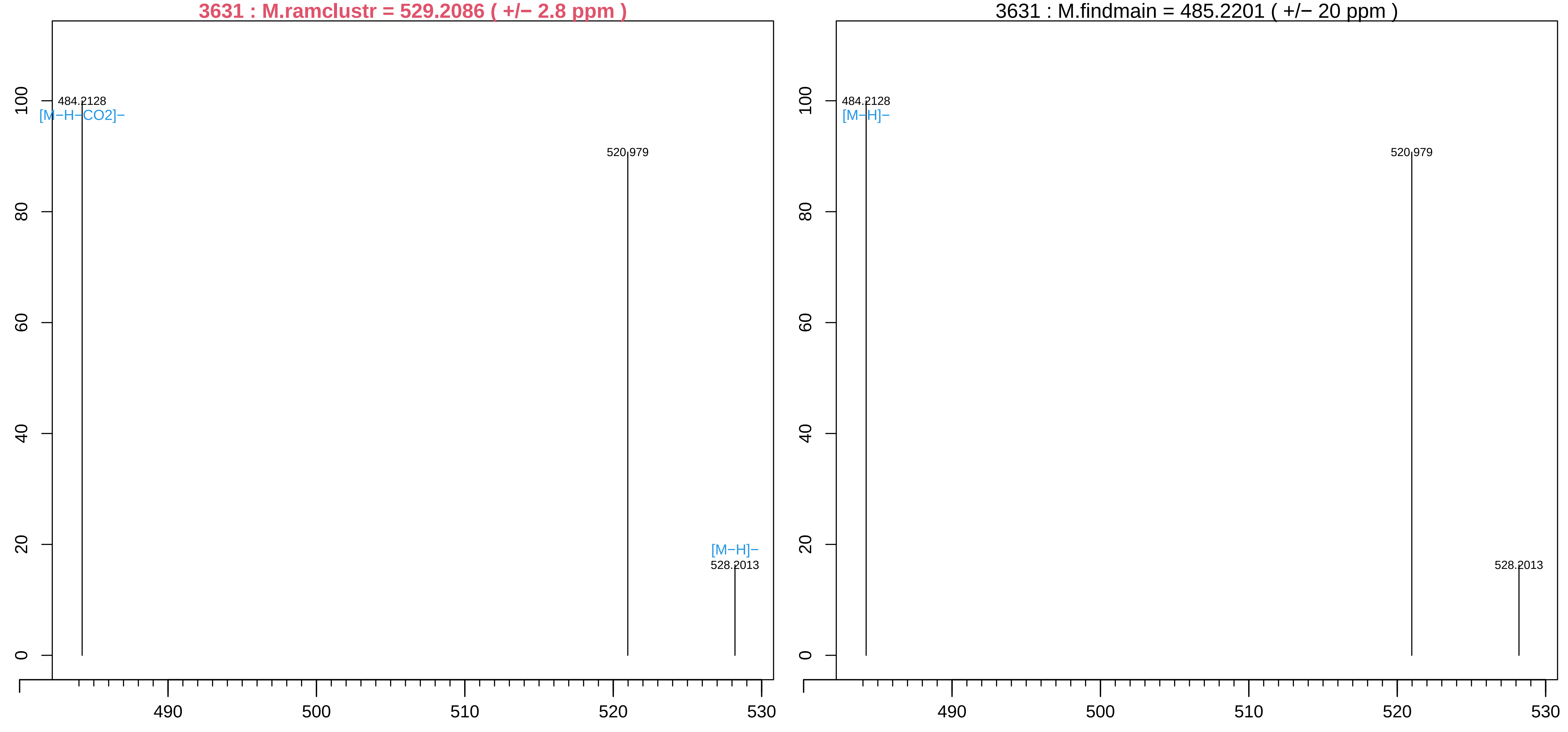
3631 : M.ramclustr = 529.2086 (+/− 2.8 ppm)
484.2128
[M−H−CO2]−
520.979
[M−H]−
528.2013
3631 : M.findmain = 485.2201 (+/− 20 ppm)
484.2128
[M−H]−
520.979
528.2013

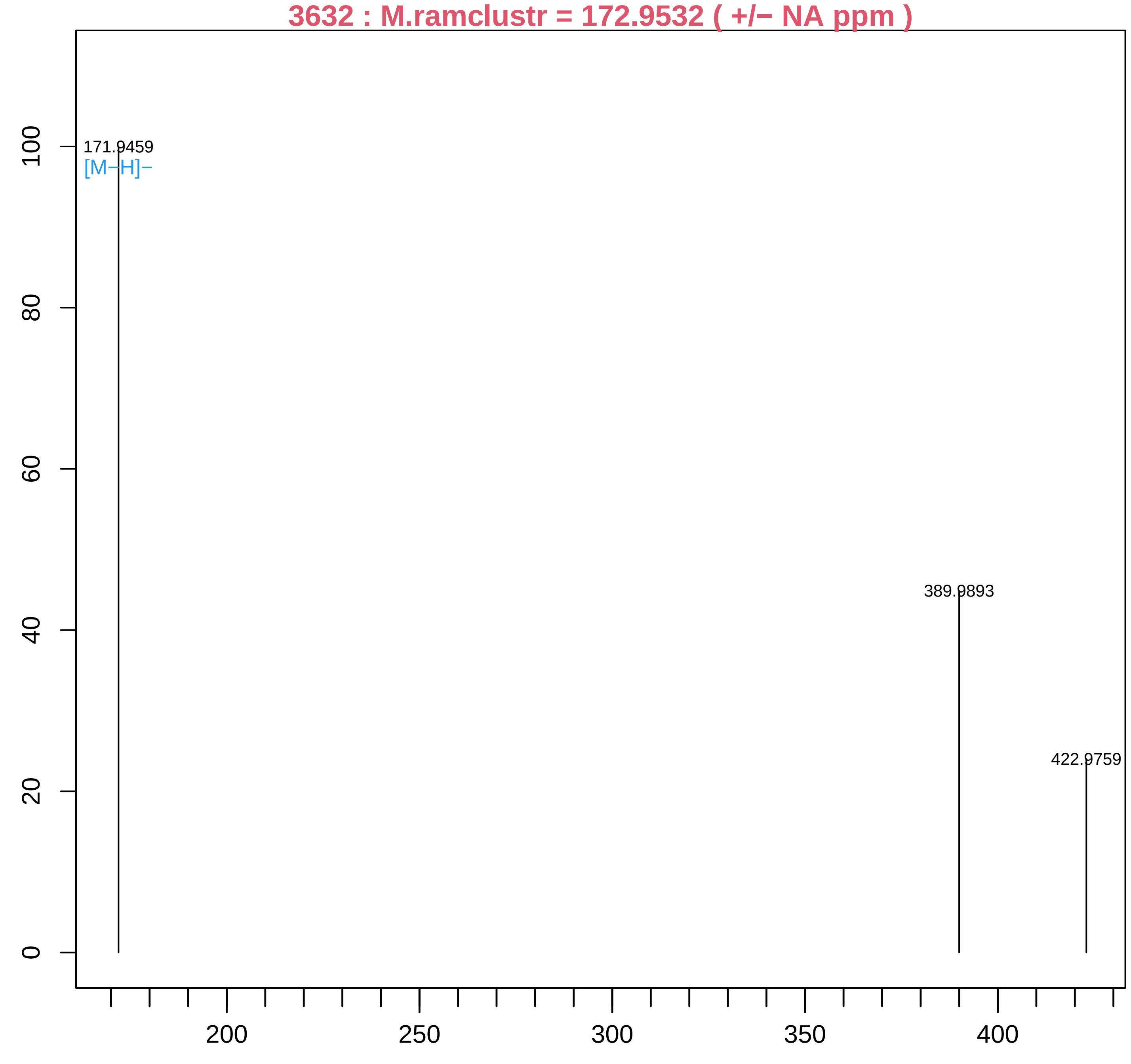
3632 : M.ramclustr = 172.9532 (+/− NA ppm)
171.9459
[M−H]−
389.9893
422.9759
0
20
40
60
80
100
200
250
300
350
400

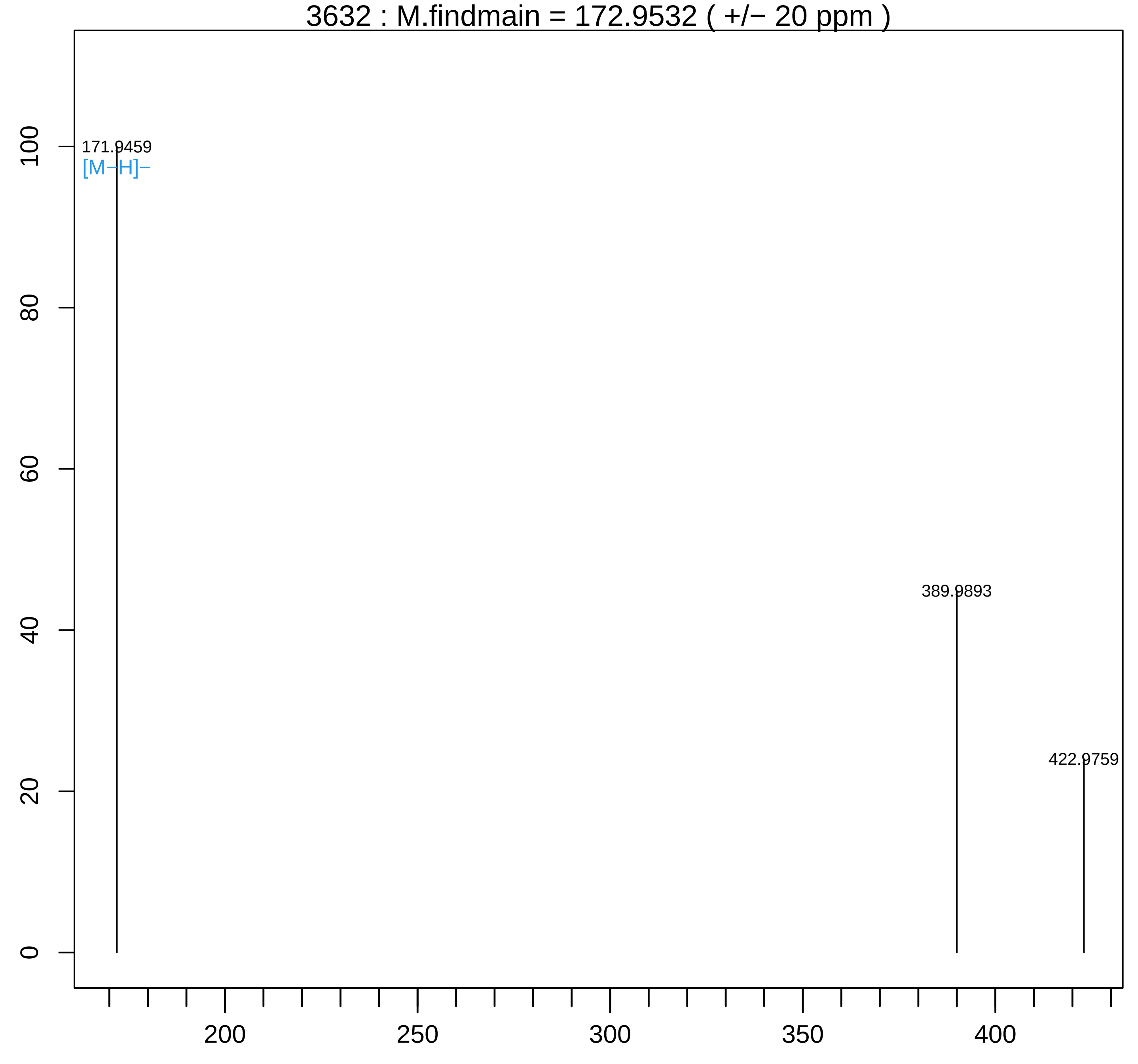
3632 : M.findmain = 172.9532 (+/− 20 ppm)
171.9459
[M−H]−
389.9893
422.9759
0
20
40
60
80
100
200
250
300
350
400

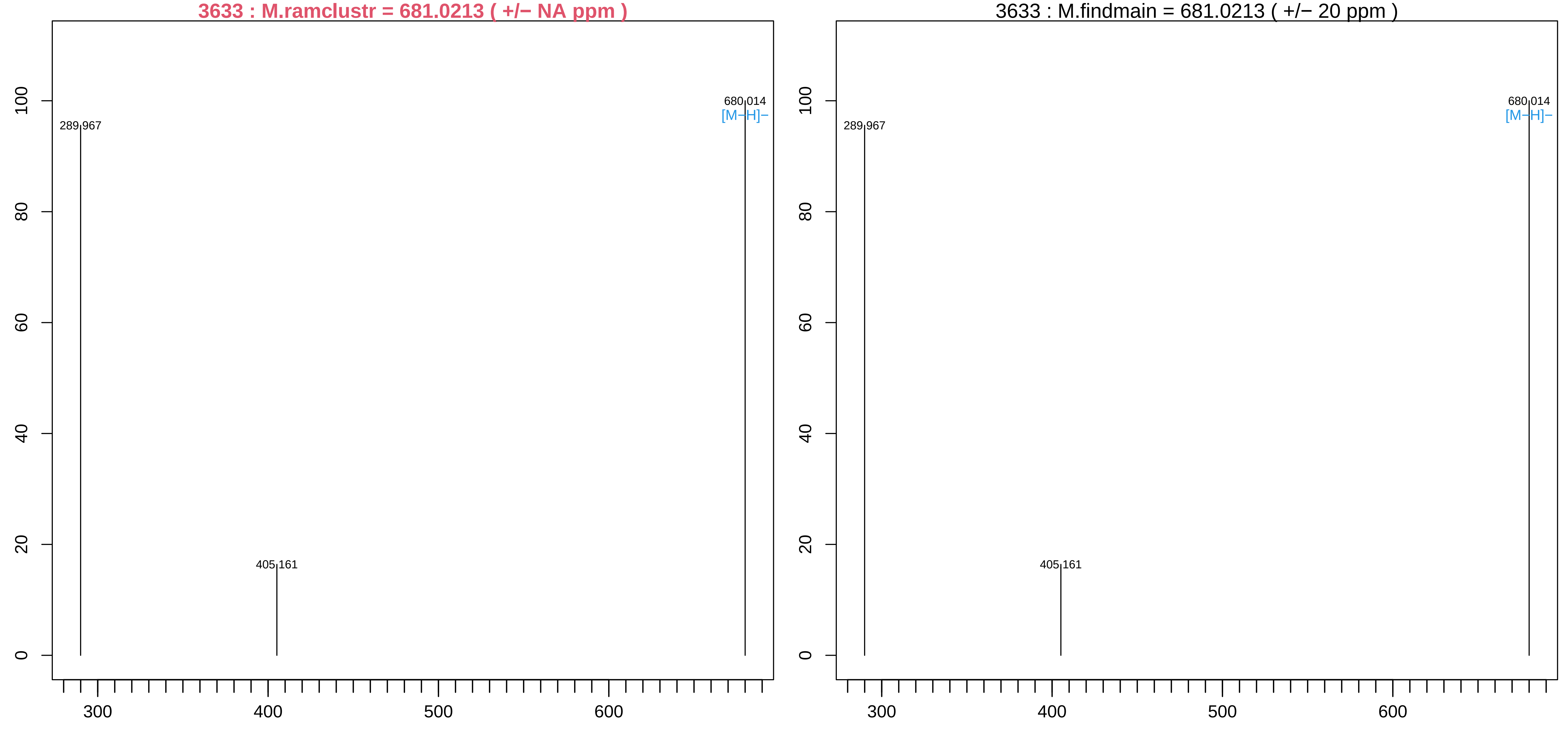
3633 : M.ramclustr = 681.0213 (+/− NA ppm)
3633 : M.findmain = 681.0213 (+/− 20 ppm)
289.967
405.161
680.014
[M−H]−
0
20
40
60
80
100
300
400
500
600

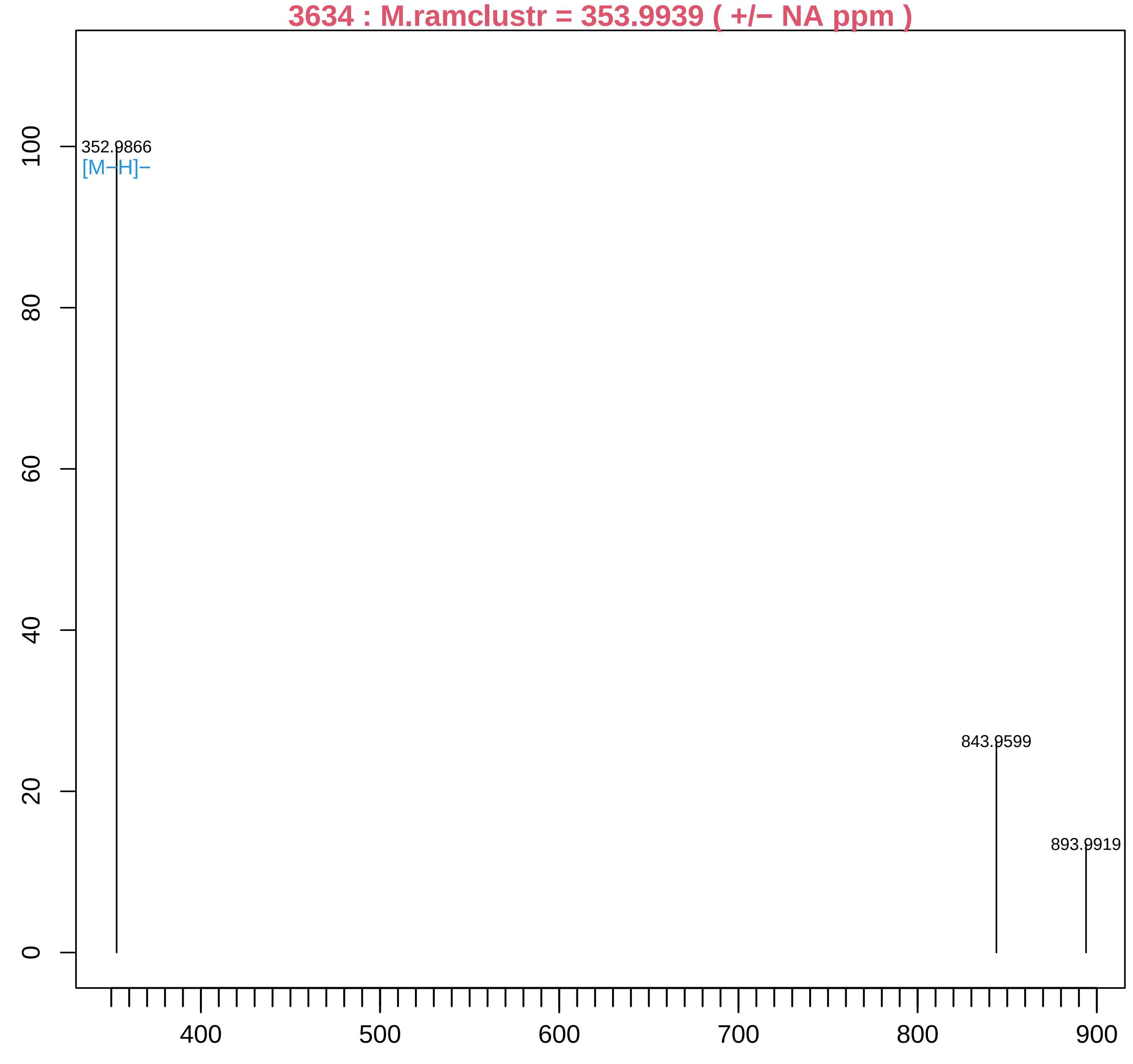
3634 : M.ramclustr = 353.9939 (+/− NA ppm)
352.9866
[M−H]−
843.9599
893.9919
0
20
40
60
80
100
400
500
600
700
800
900

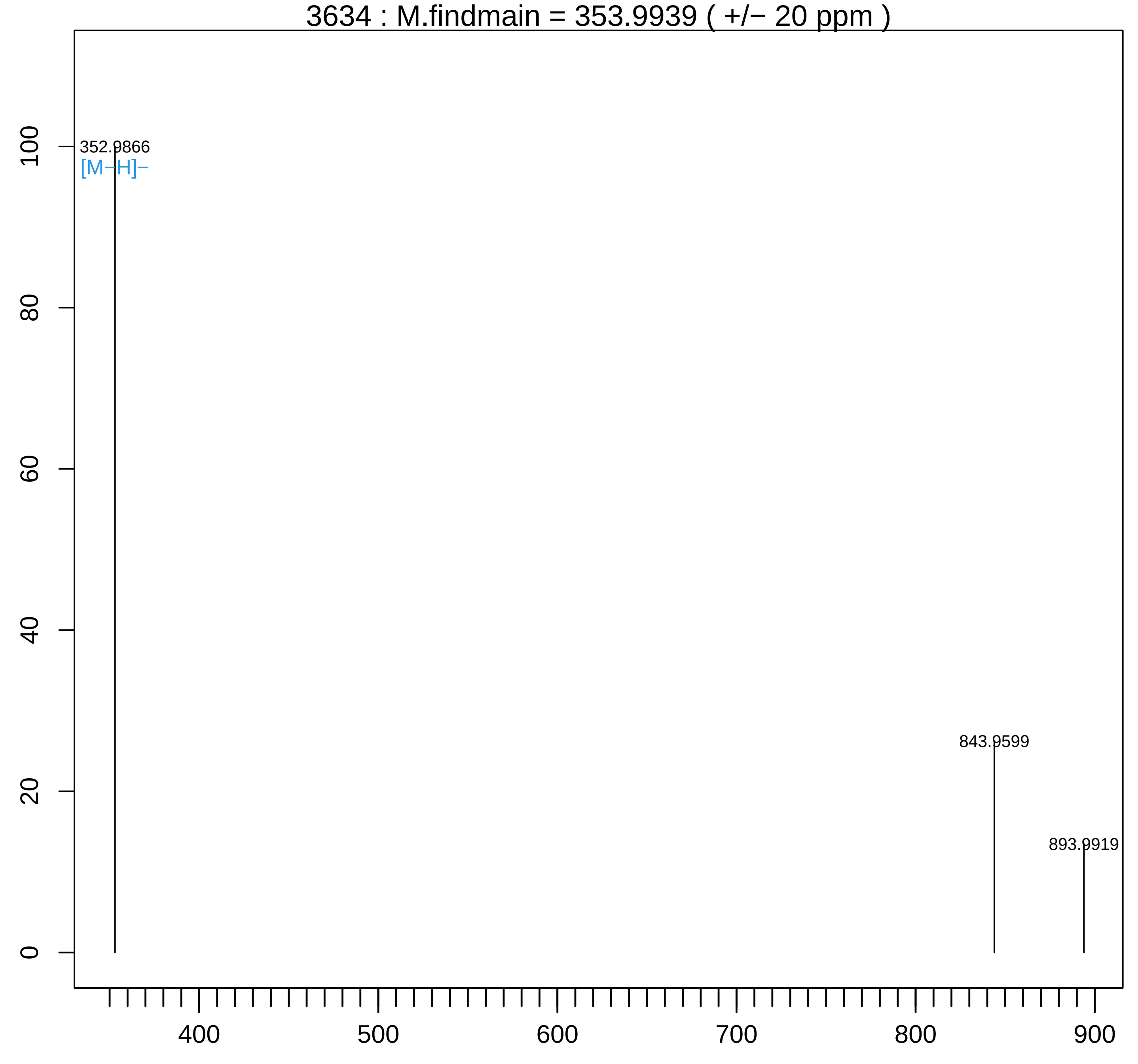
3634 : M.findmain = 353.9939 (+/− 20 ppm)
352.9866
[M−H]−
843.9599
893.9919
0
20
40
60
80
100
400
500
600
700
800
900

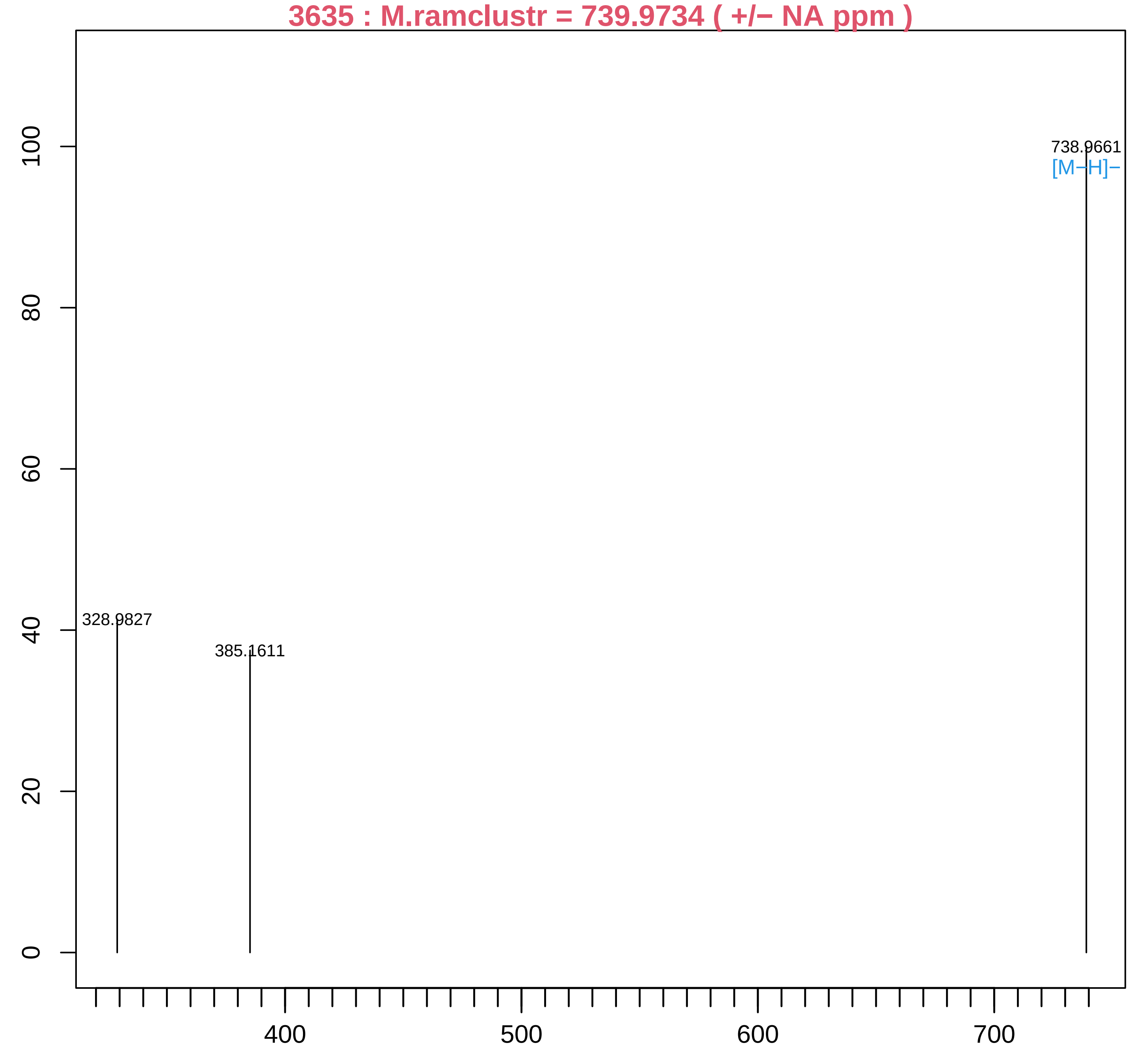
3635 : M.ramclustr = 739.9734 (+/− NA ppm)
738.9661
[M−H]−
328.9827
385.1611
0
20
40
60
80
100
400
500
600
700

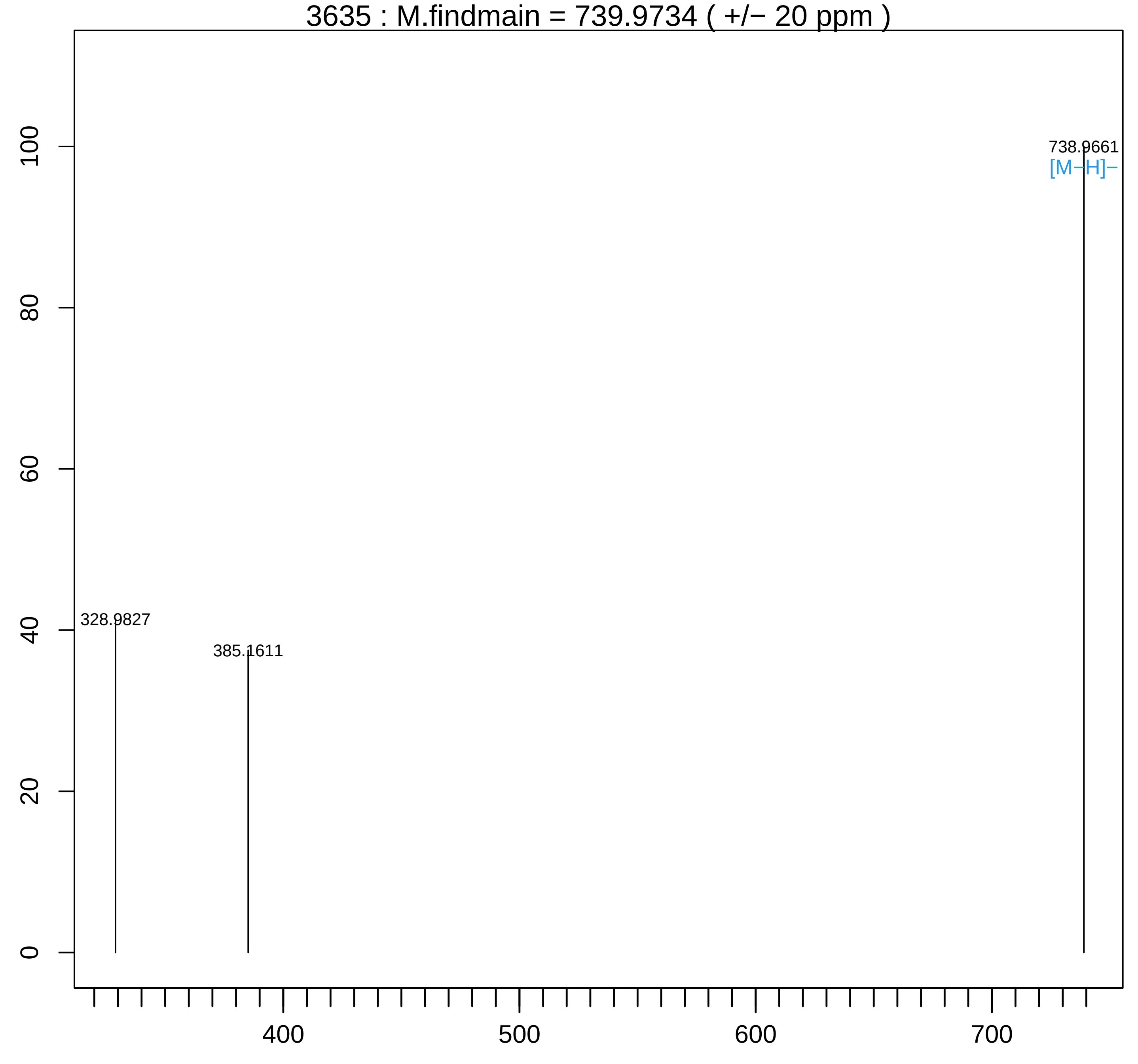
3635 : M.findmain = 739.9734 (+/− 20 ppm)
738.9661
[M−H]−
328.9827
385.1611
0
20
40
60
80
100
400
500
600
700

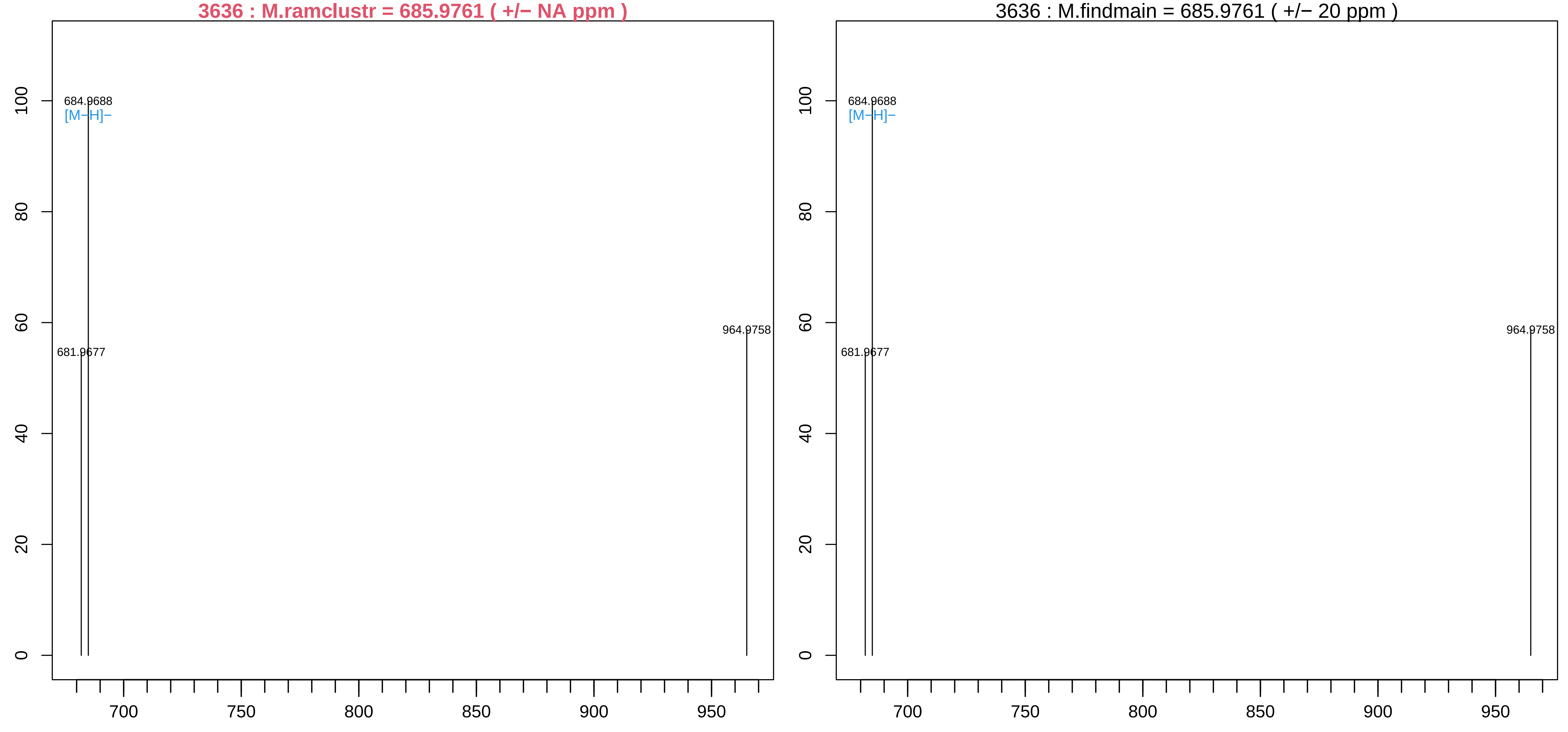

3636 : M.ramclustr = 685.9761 (+/− NA ppm)
684.9688
[M−H]−
681.9677
964.9758
3636 : M.findmain = 685.9761 (+/− 20 ppm)
684.9688
[M−H]−
681.9677
964.9758

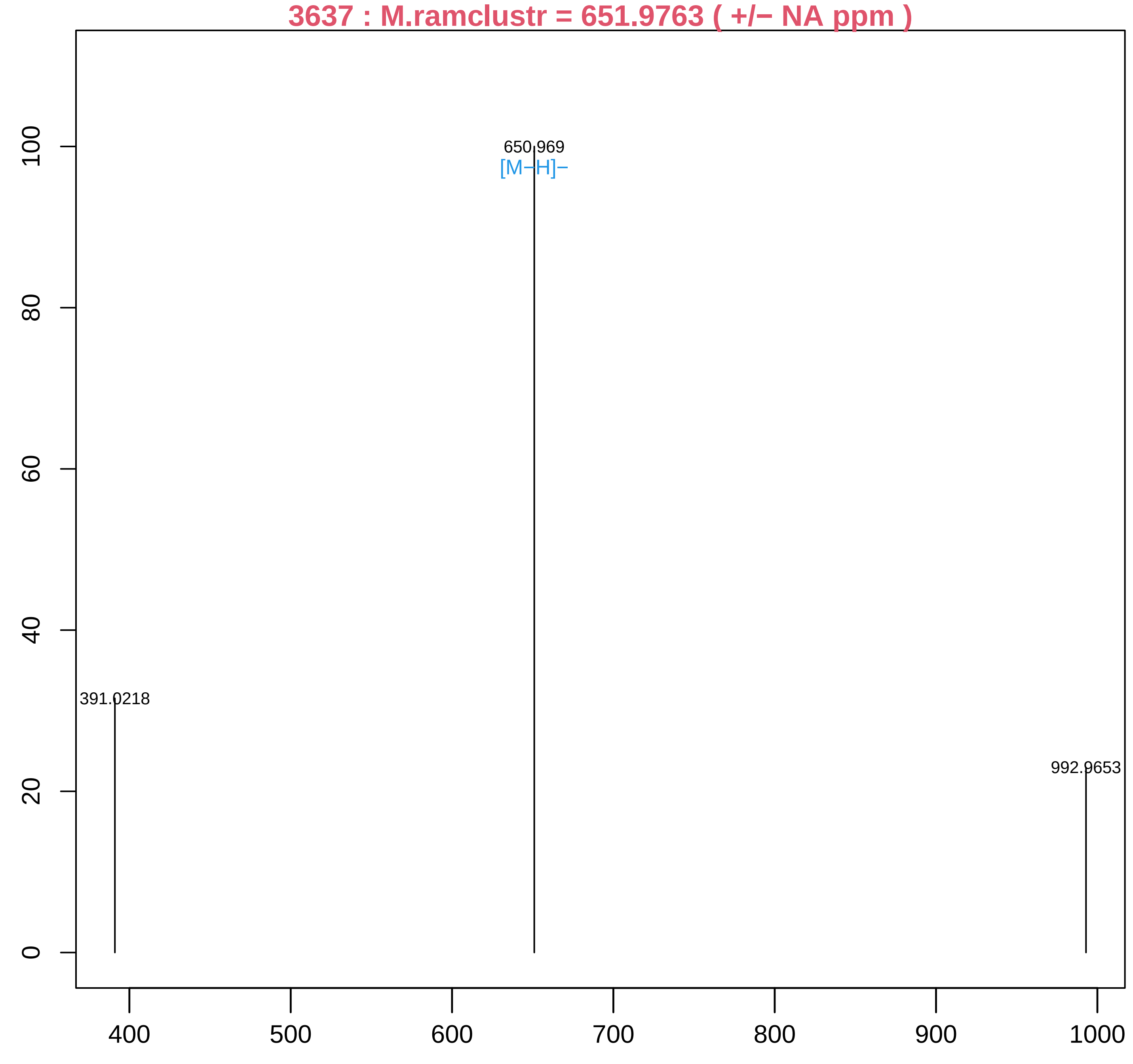
3637 : M.ramclustr = 651.9763 (+/− NA ppm)
650.969
[M−H]−
391.0218
992.9653
0
20
40
60
80
100
400
500
600
700
800
900
1000

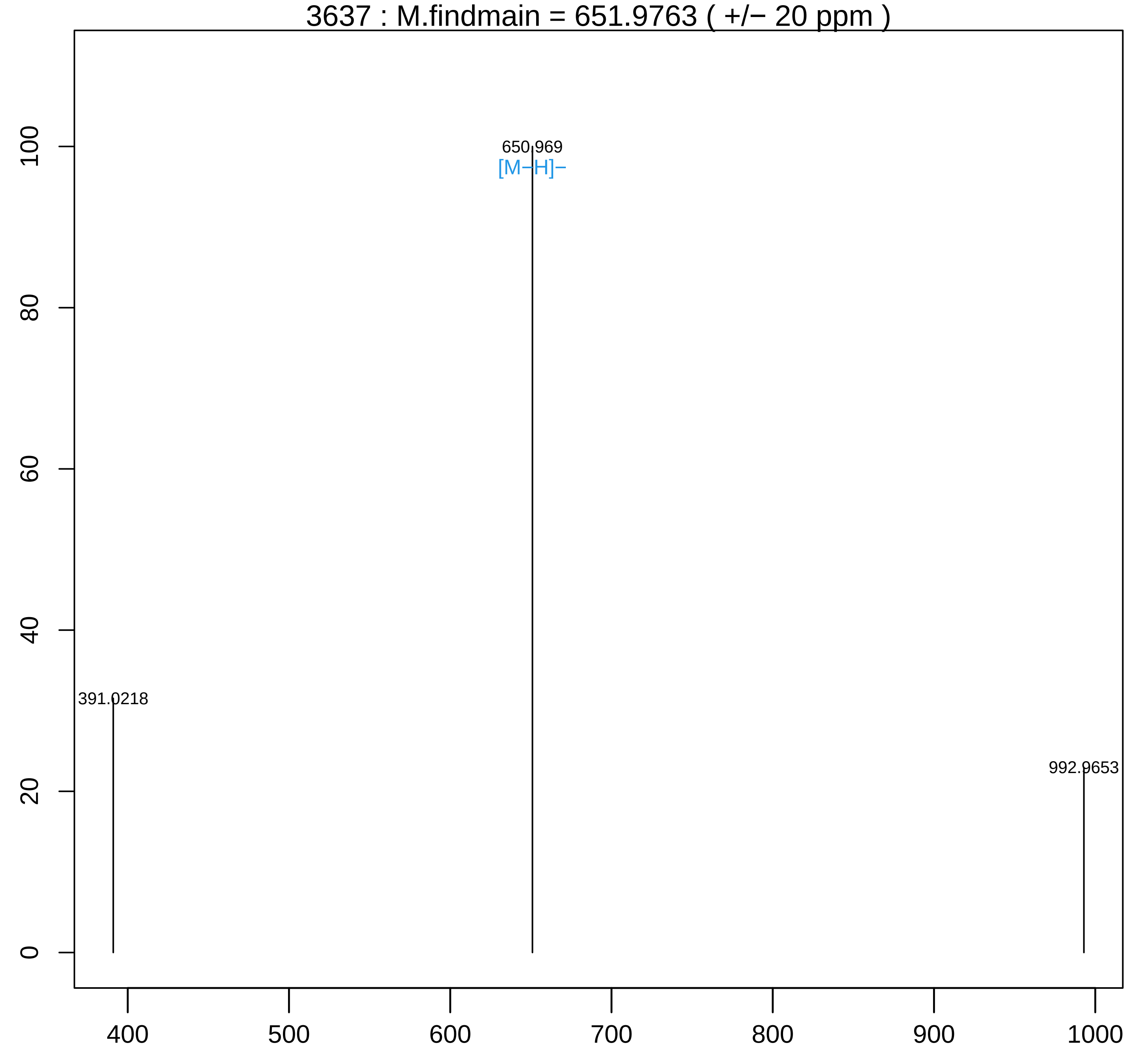
3637 : M.findmain = 651.9763 (+/− 20 ppm)
650.969
[M−H]−
391.0218
992.9653
0
20
40
60
80
100
400
500
600
700
800
900
1000

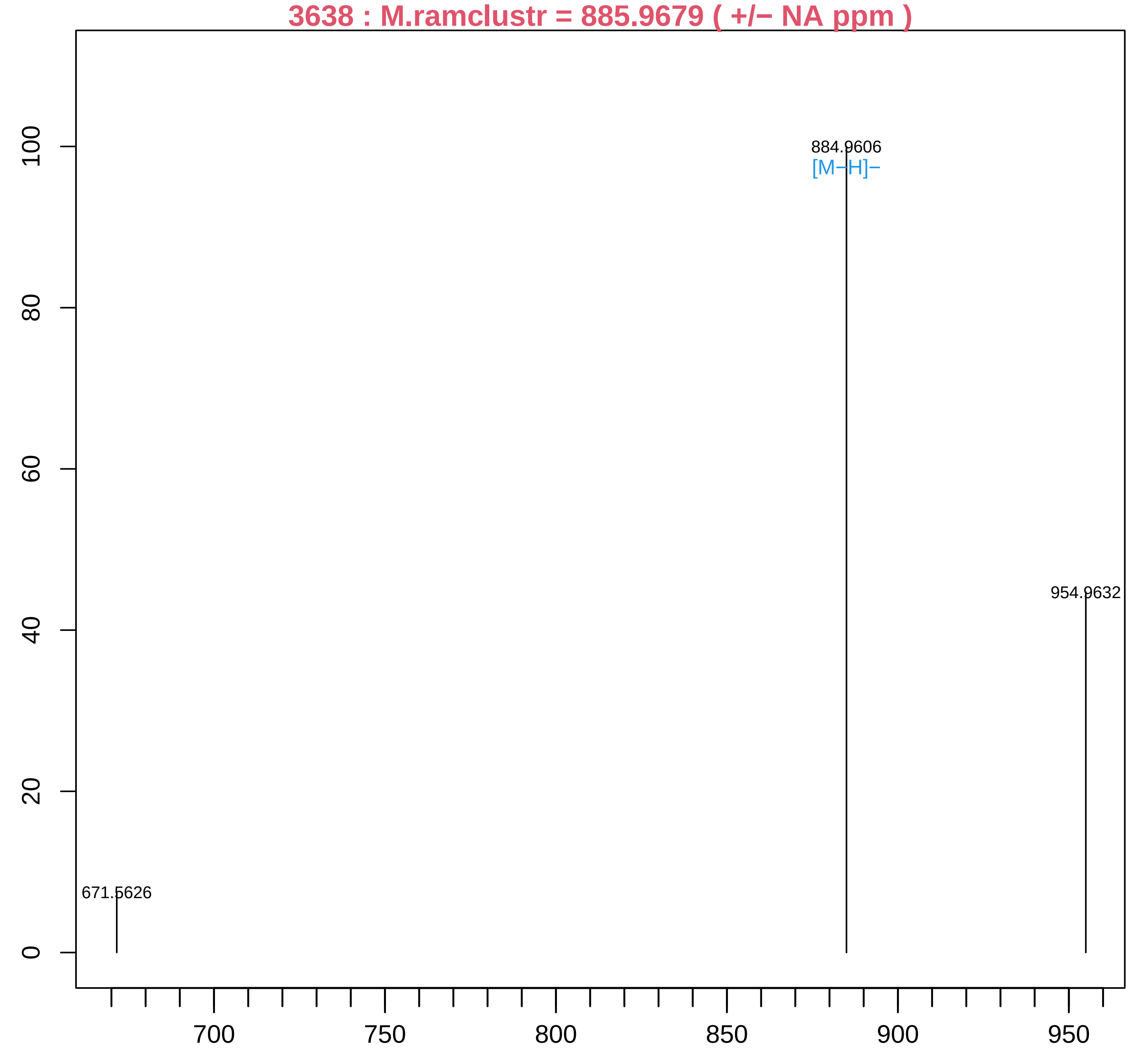

3638 : M.ramclustr = 885.9679 (+/− NA ppm)
884.9606
[M−H]−
954.9632
671.5626
0
20
40
60
80
100
700
750
800
850
900
950

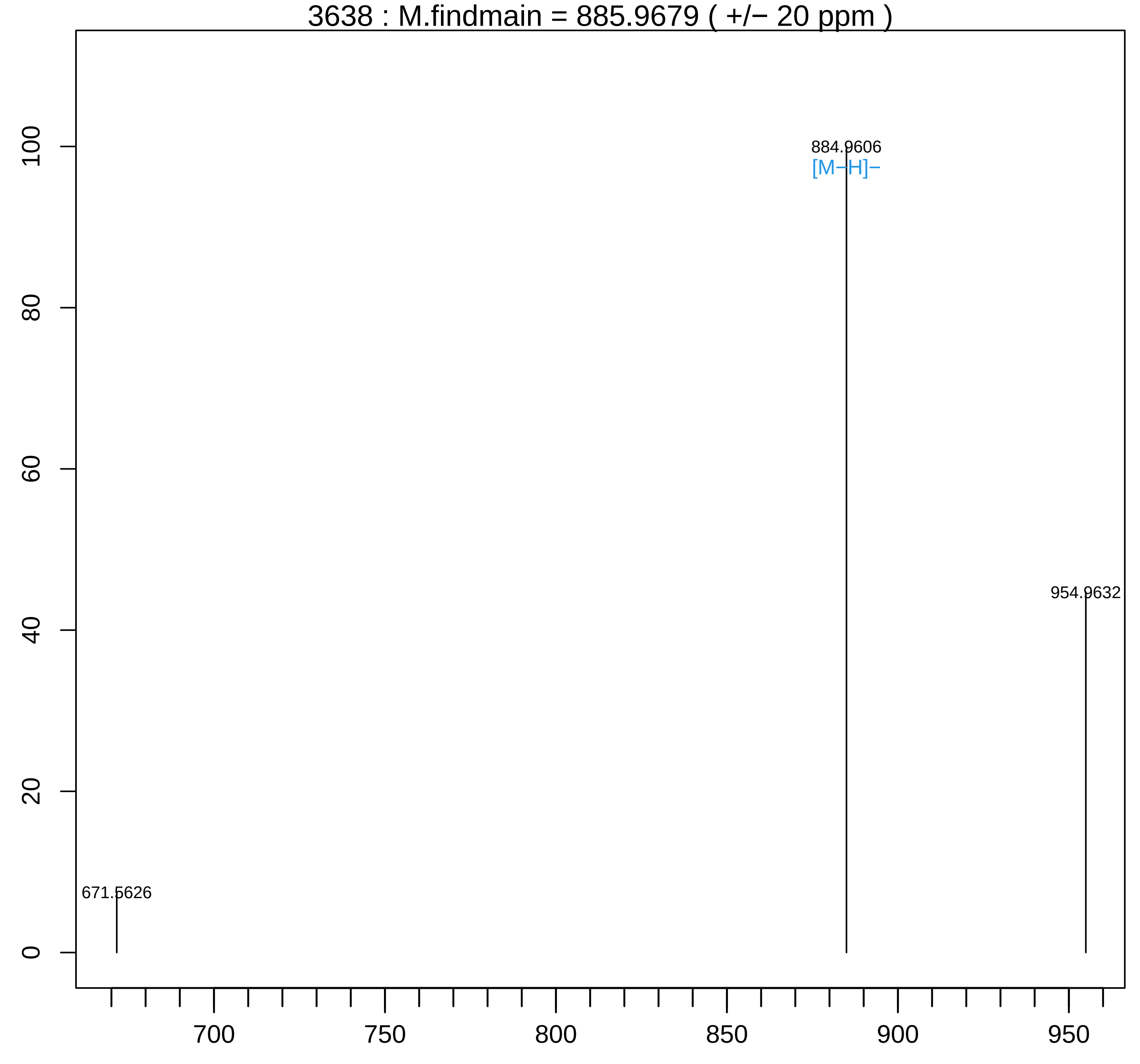

3638 : M.findmain = 885.9679 (+/− 20 ppm)
884.9606
[M−H]−
954.9632
671.5626
0
20
40
60
80
100
700
750
800
850
900
950

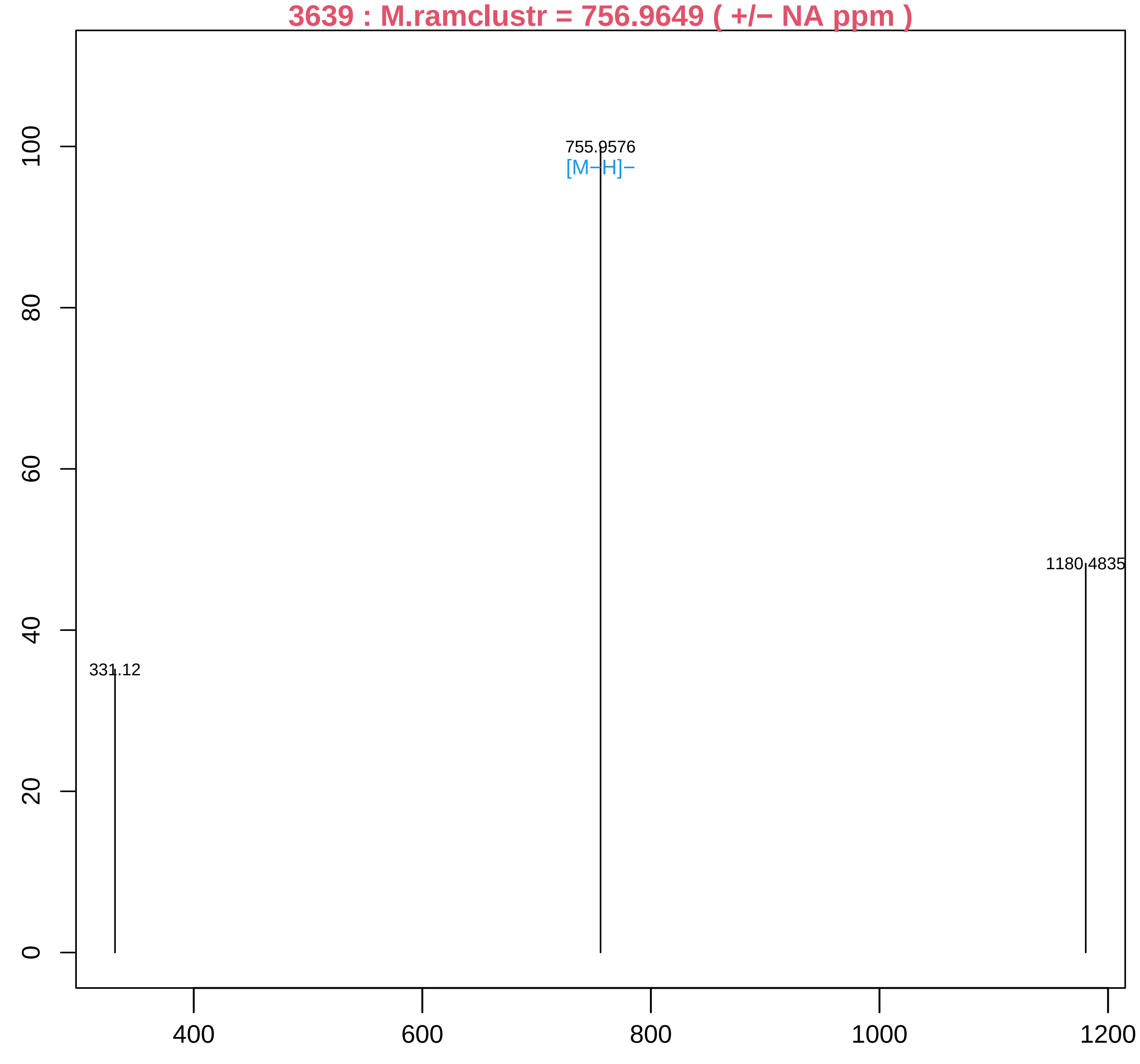
3639 : M.ramclustr = 756.9649 (+/− NA ppm)
755.9576
[M−H]−
1180.4835
331.12
0
20
40
60
80
100
400
600
800
1000
1200

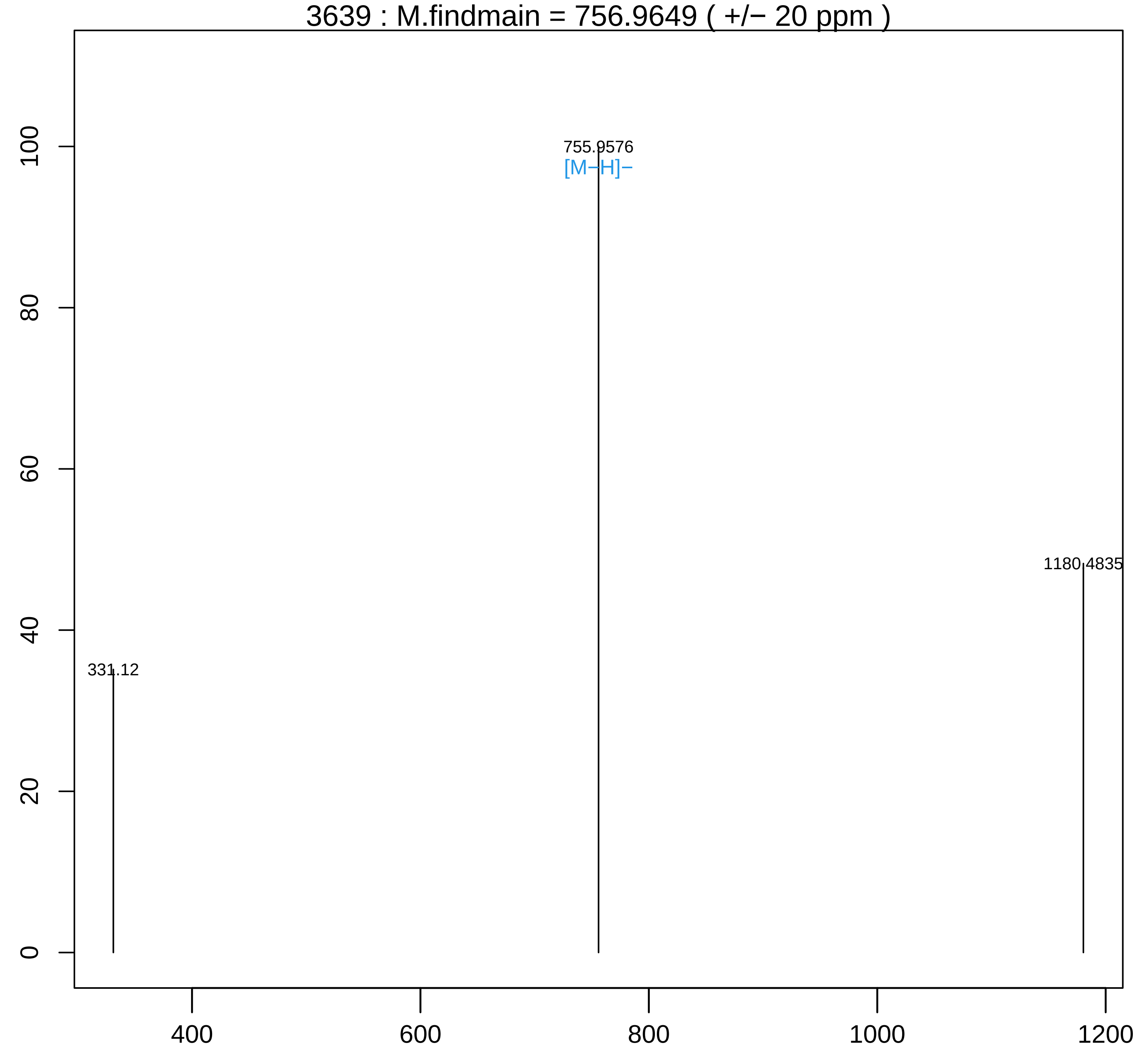
3639 : M.findmain = 756.9649 (+/− 20 ppm)
755.9576
[M−H]−
1180.4835
331.12
0
20
40
60
80
100
400
600
800
1000
1200

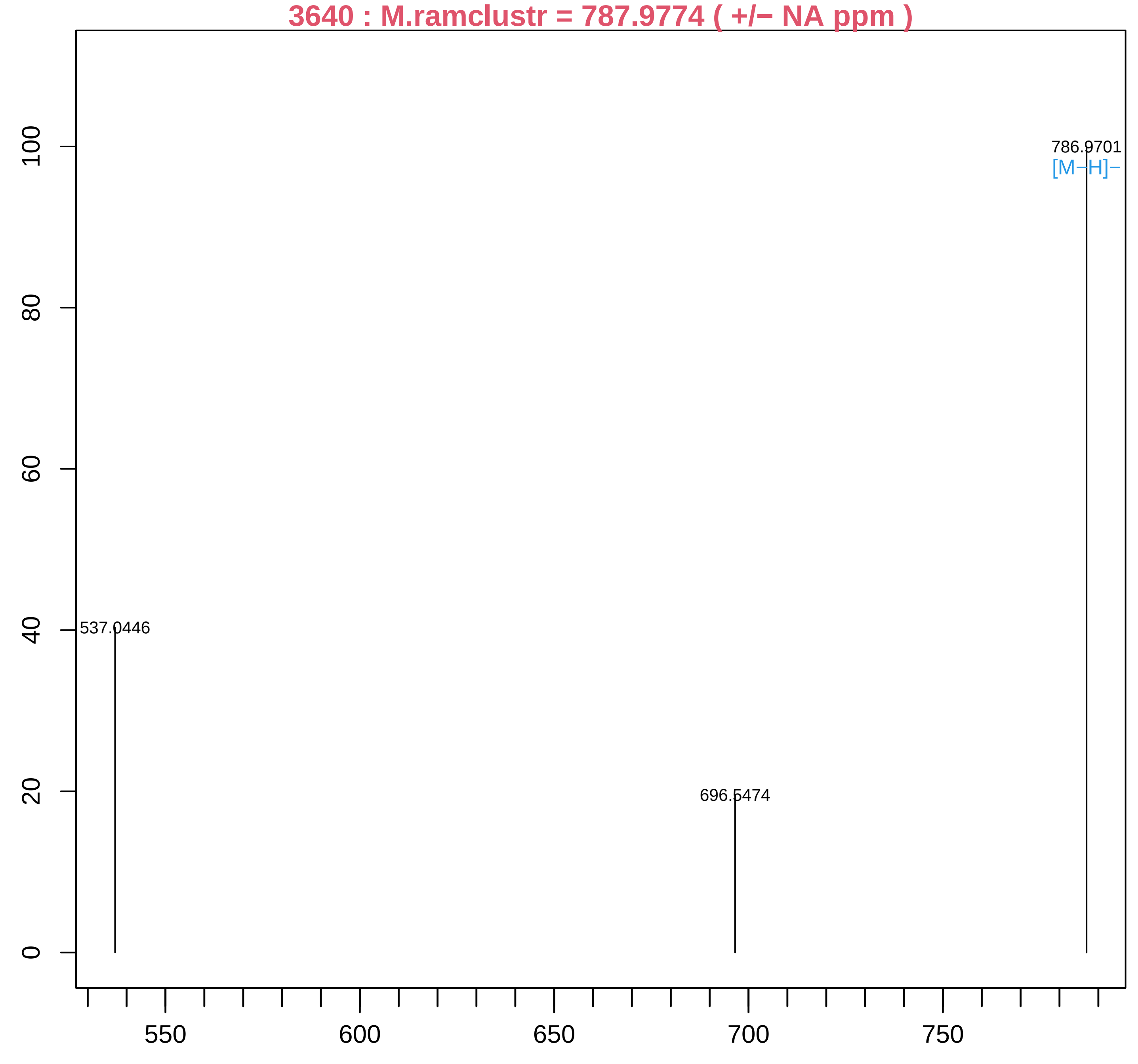
3640 : M.ramclustr = 787.9774 (+/− NA ppm)
786.9701
[M−H]−
537.0446
696.5474
0
20
40
60
80
100
550
600
650
700
750

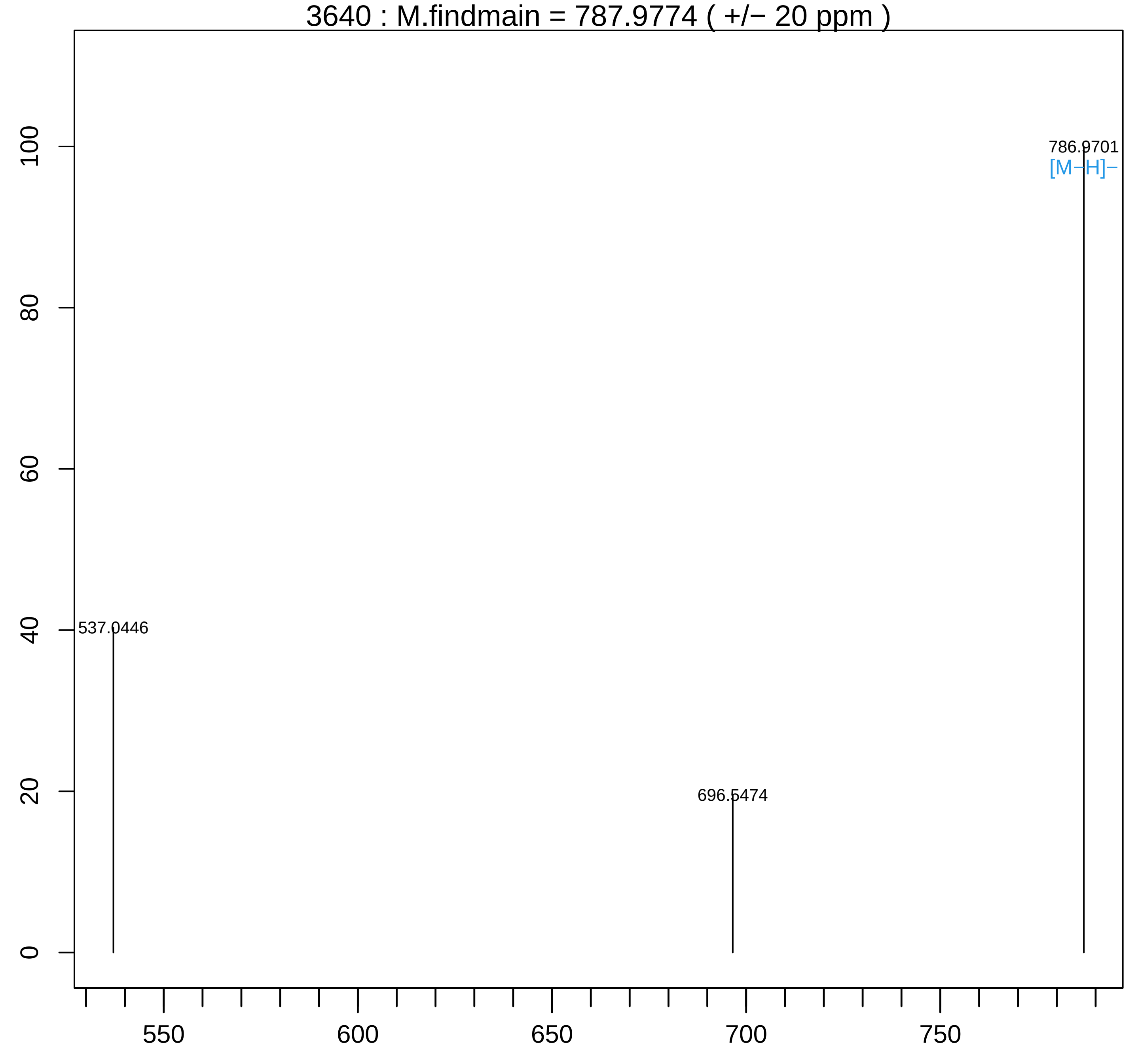
3640 : M.findmain = 787.9774 (+/− 20 ppm)
786.9701
[M−H]−
537.0446
696.5474
0
20
40
60
80
100
550
600
650
700
750

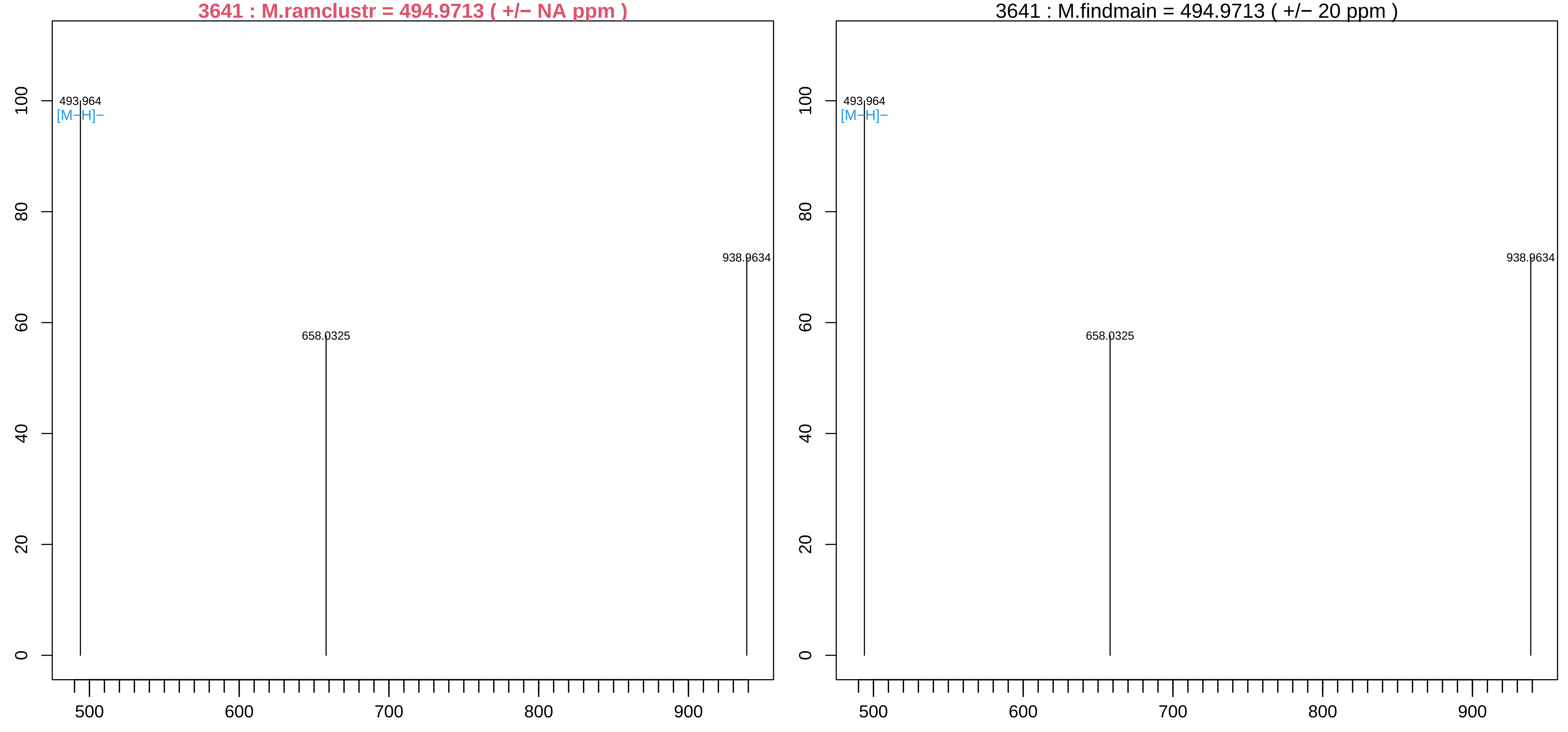

3641 : M.ramclustr = 494.9713 (+/− NA ppm)
493.964
[M−H]−
658.0325
938.9634
0
20
40
60
80
100
500
600
700
800
900
3641 : M.findmain = 494.9713 (+/− 20 ppm)
493.964
[M−H]−
658.0325
938.9634
0
20
40
60
80
100
500
600
700
800
900

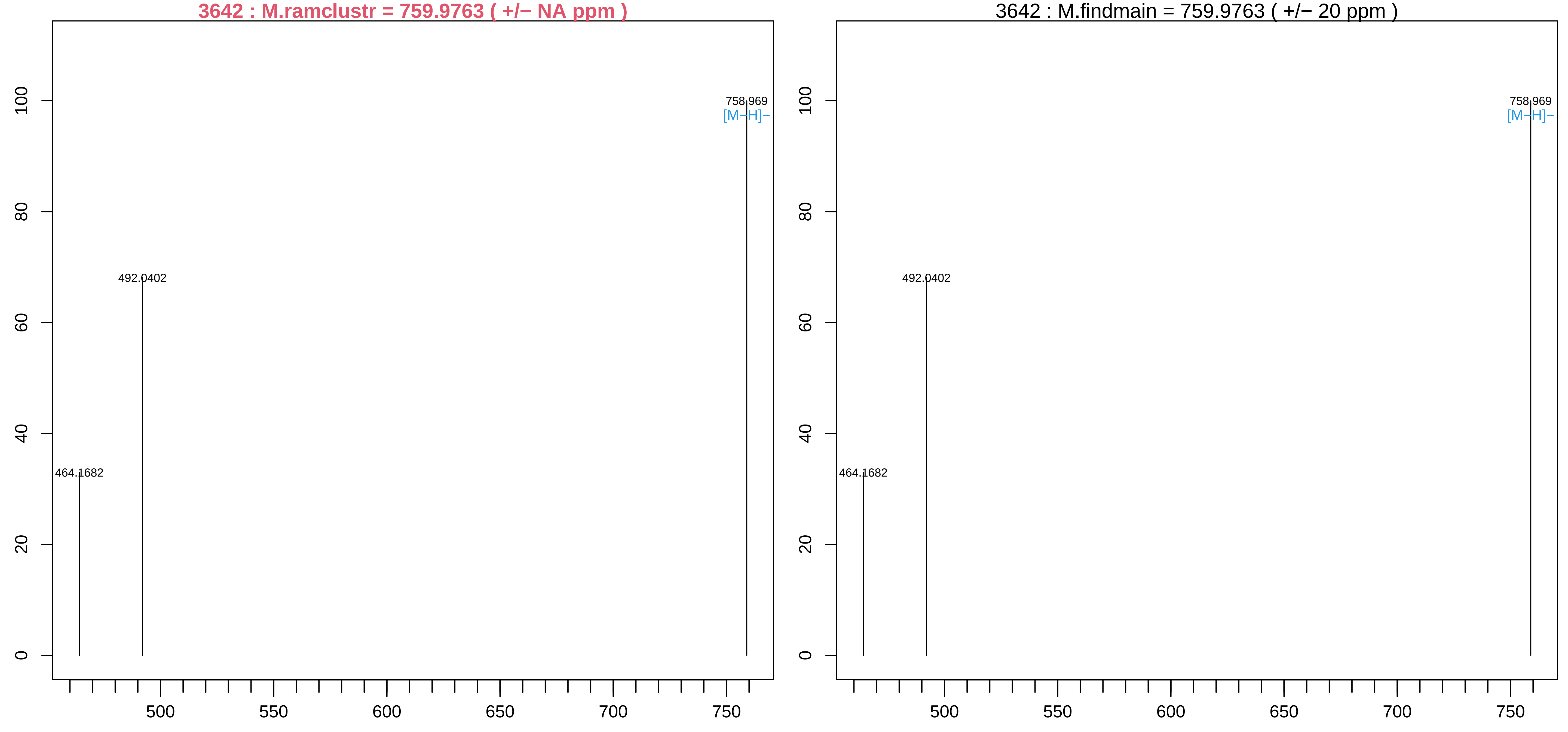
3642 : M.ramclustr = 759.9763 (+/− NA ppm)
758.969
[M−H]−
492.0402
464.1682
3642 : M.findmain = 759.9763 (+/− 20 ppm)
758.969
[M−H]−
492.0402
464.1682

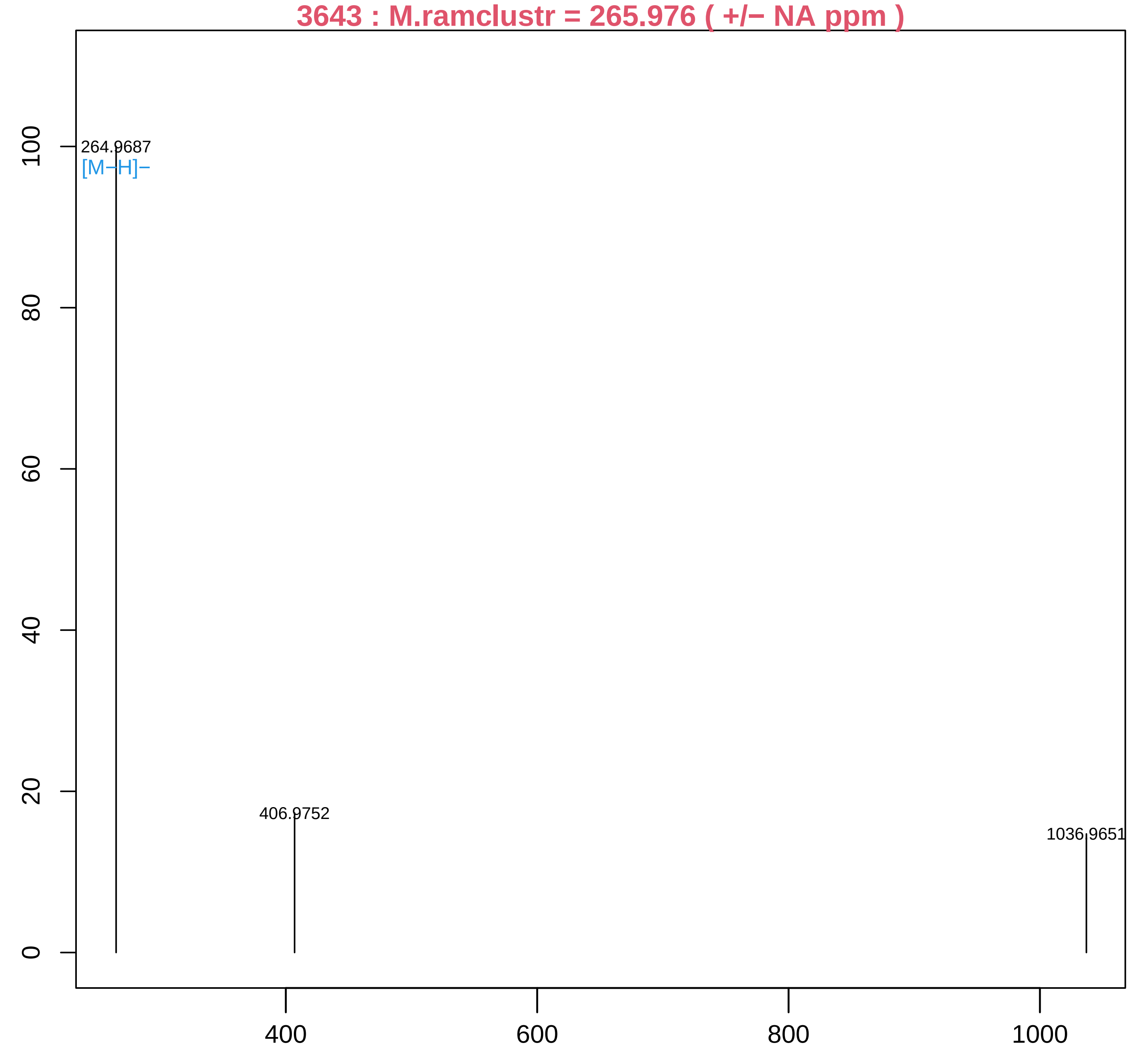
3643 : M.ramclustr = 265.976 (+/− NA ppm)
264.9687
[M−H]−
406.9752
1036.9651
0
20
40
60
80
100
400
600
800
1000

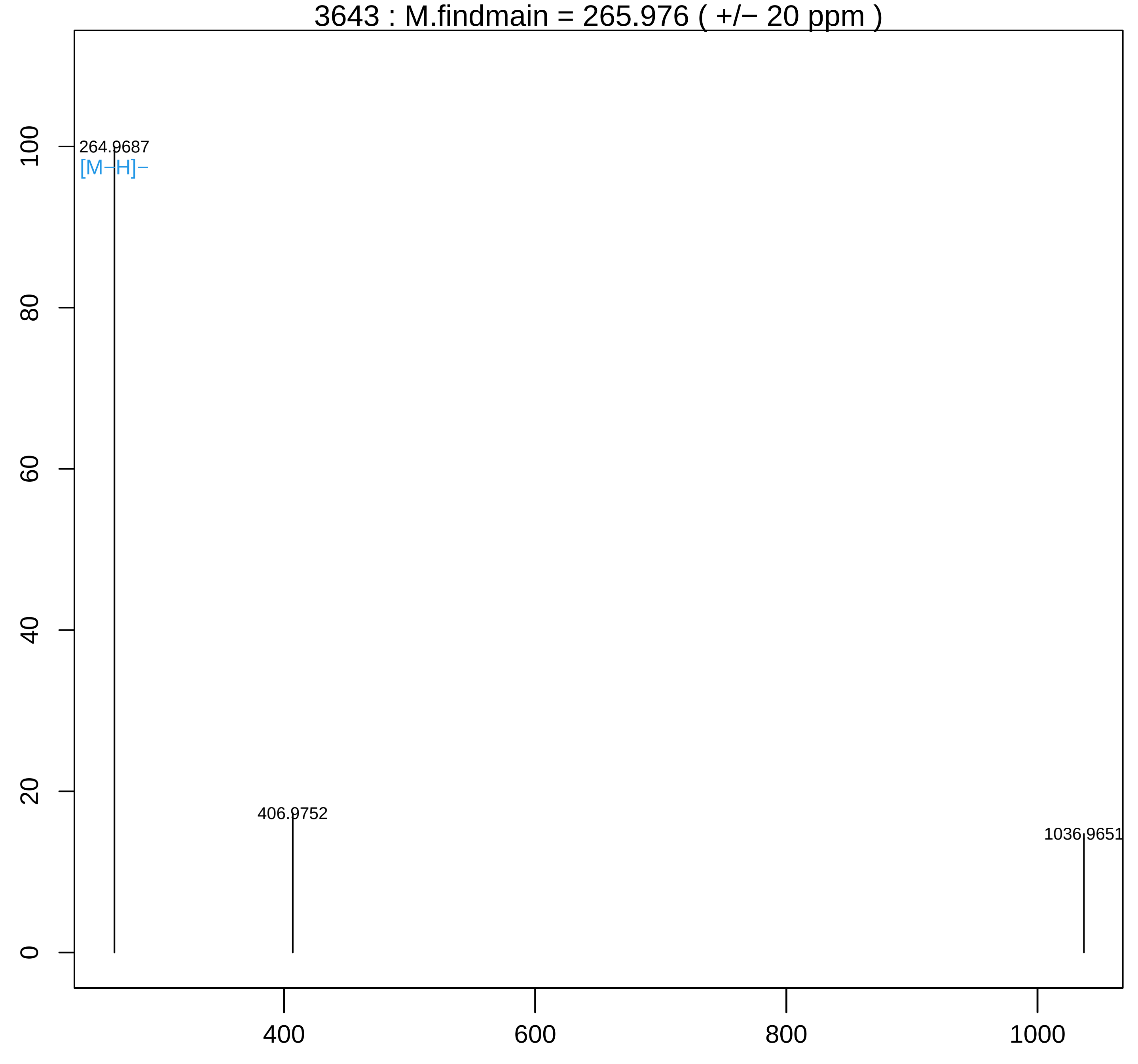
3643 : M.findmain = 265.976 (+/− 20 ppm)
264.9687
[M−H]−
406.9752
1036.9651
0
20
40
60
80
100
400
600
800
1000

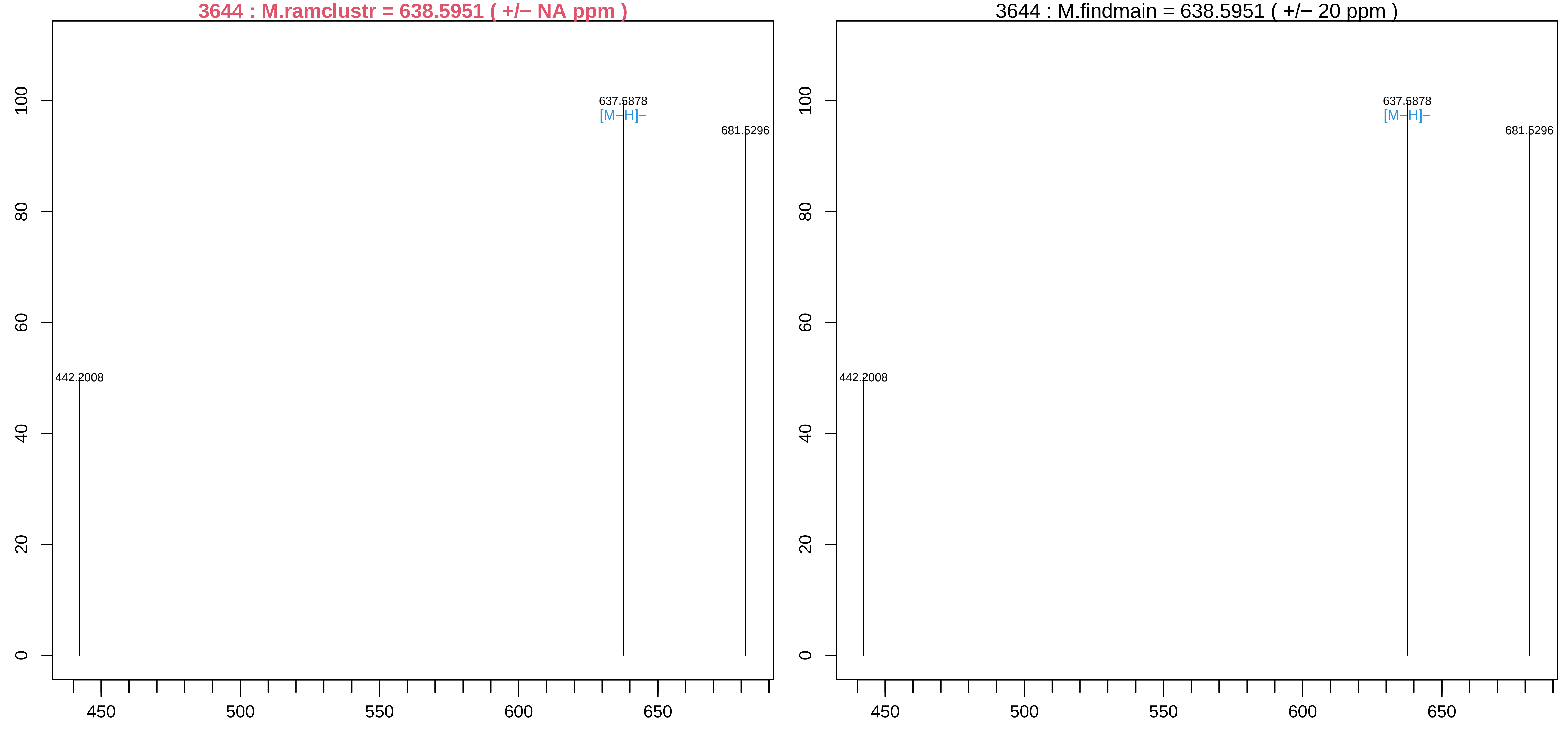
3644 : M.ramclustr = 638.5951 (+/− NA ppm)
637.5878
[M−H]−
681.5296
442.2008
3644 : M.findmain = 638.5951 (+/− 20 ppm)
637.5878
[M−H]−
681.5296
442.2008
0
20
40
60
80
100
450
500
550
600
650

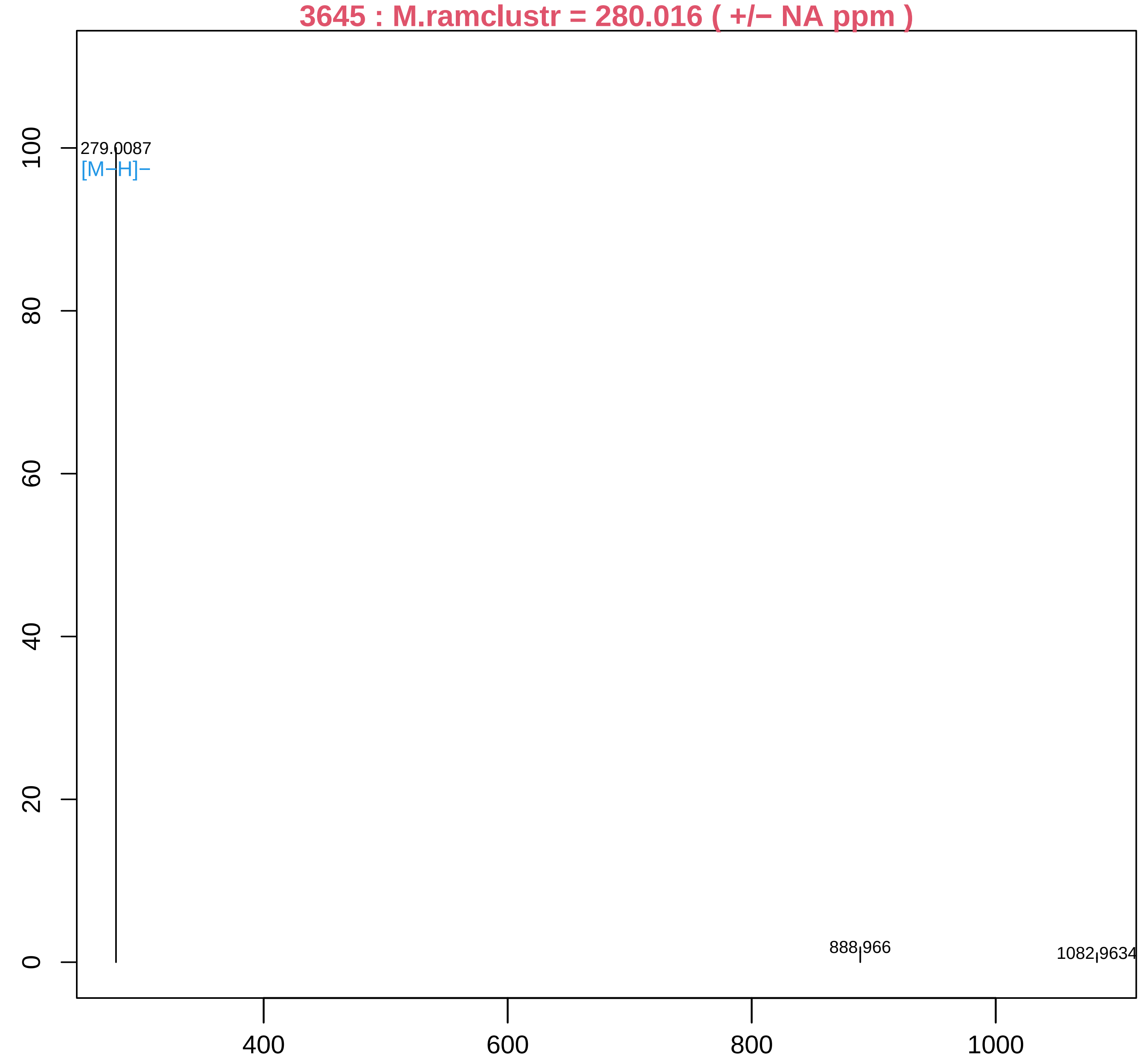

3645 : M.ramclustr = 280.016 (+/− NA ppm)
279.0087
[M−H]−
888.966
1082.9634
0
20
40
60
80
100
400
600
800
1000

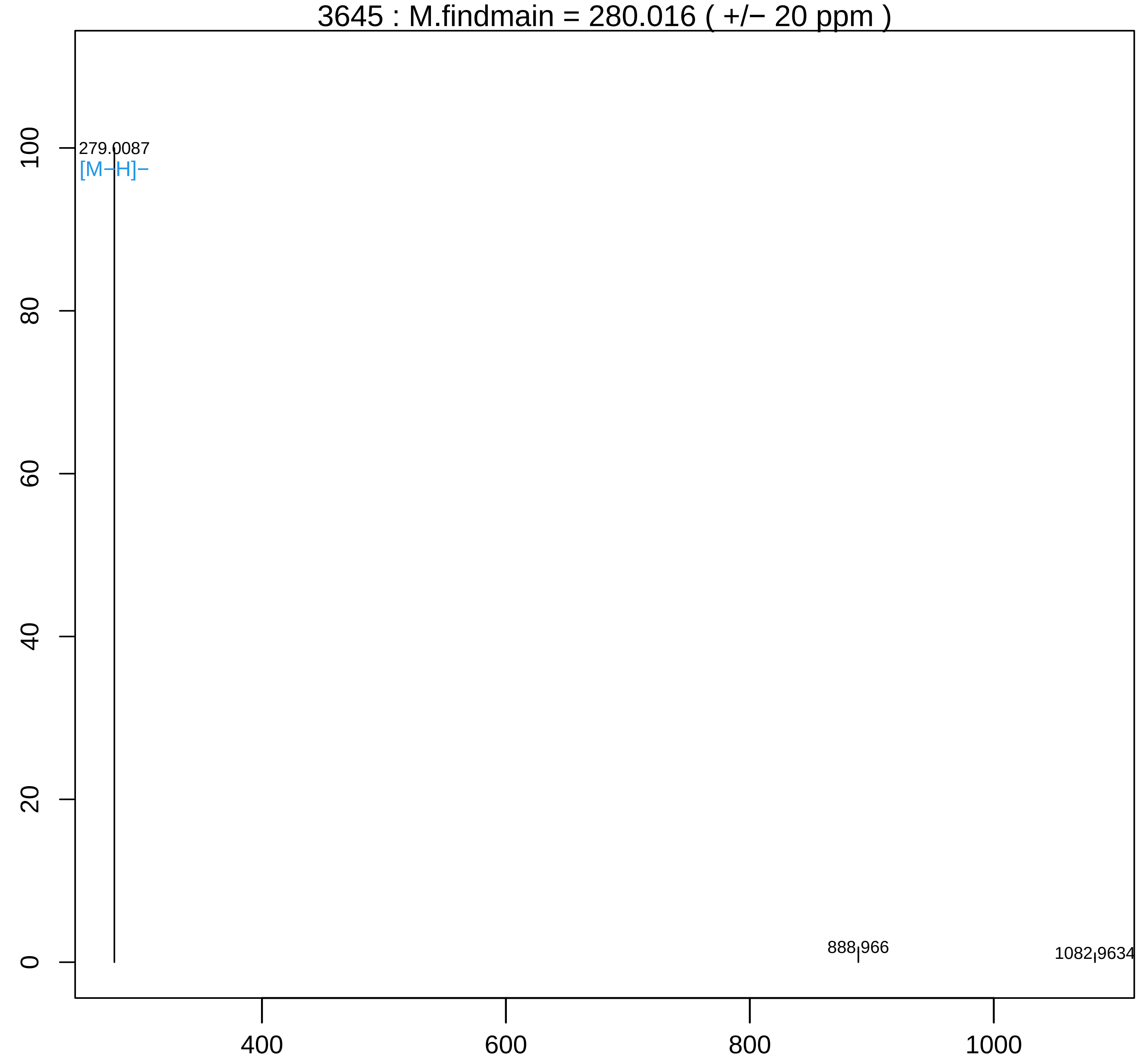

3645 : M.findmain = 280.016 (+/− 20 ppm)
279.0087
[M−H]−
888.966
1082.9634
0
20
40
60
80
100
400
600
800
1000

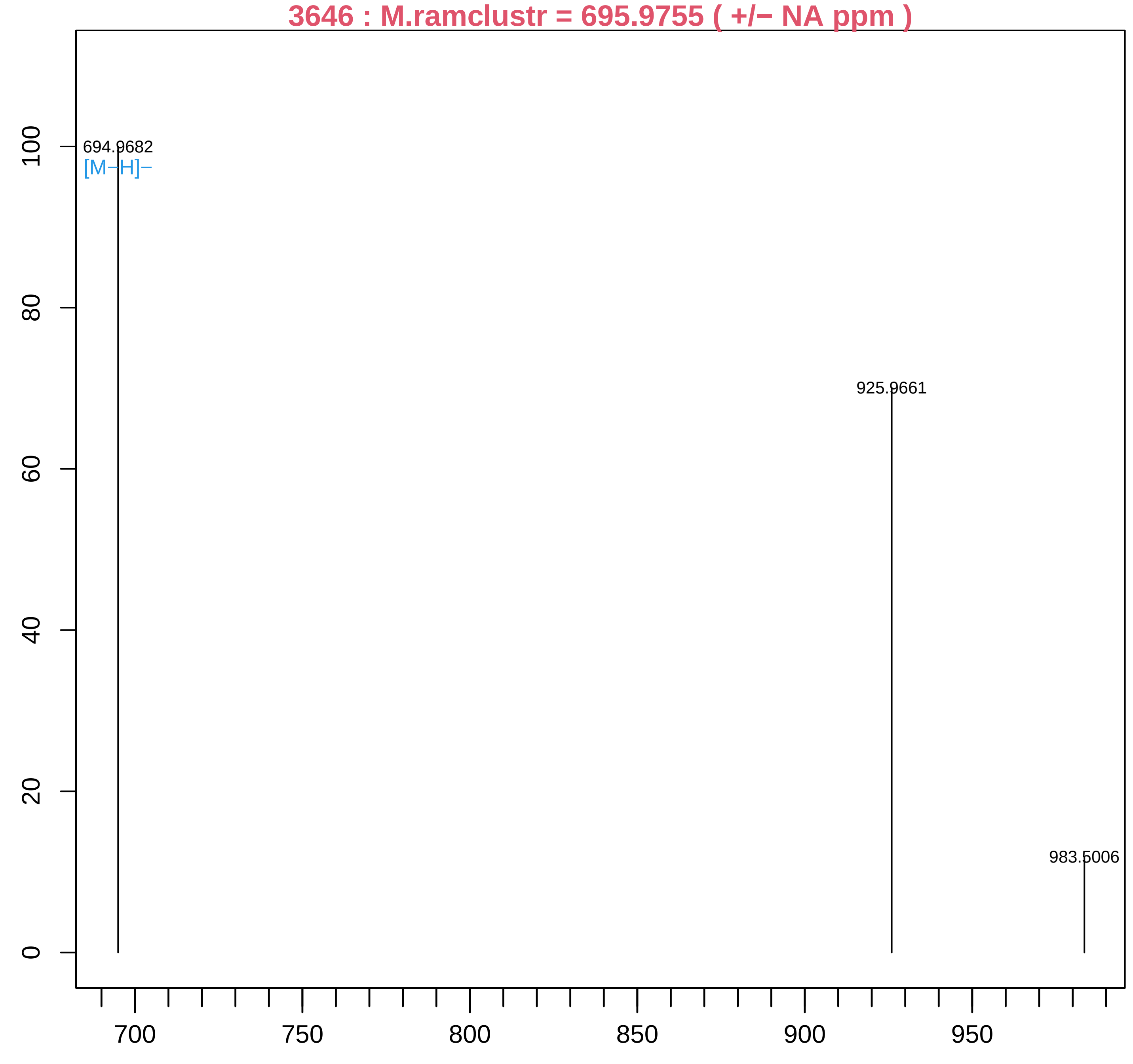
3646 : M.ramclustr = 695.9755 (+/− NA ppm)
694.9682
[M−H]−
925.9661
983.5006
0
20
40
60
80
100
700
750
800
850
900
950
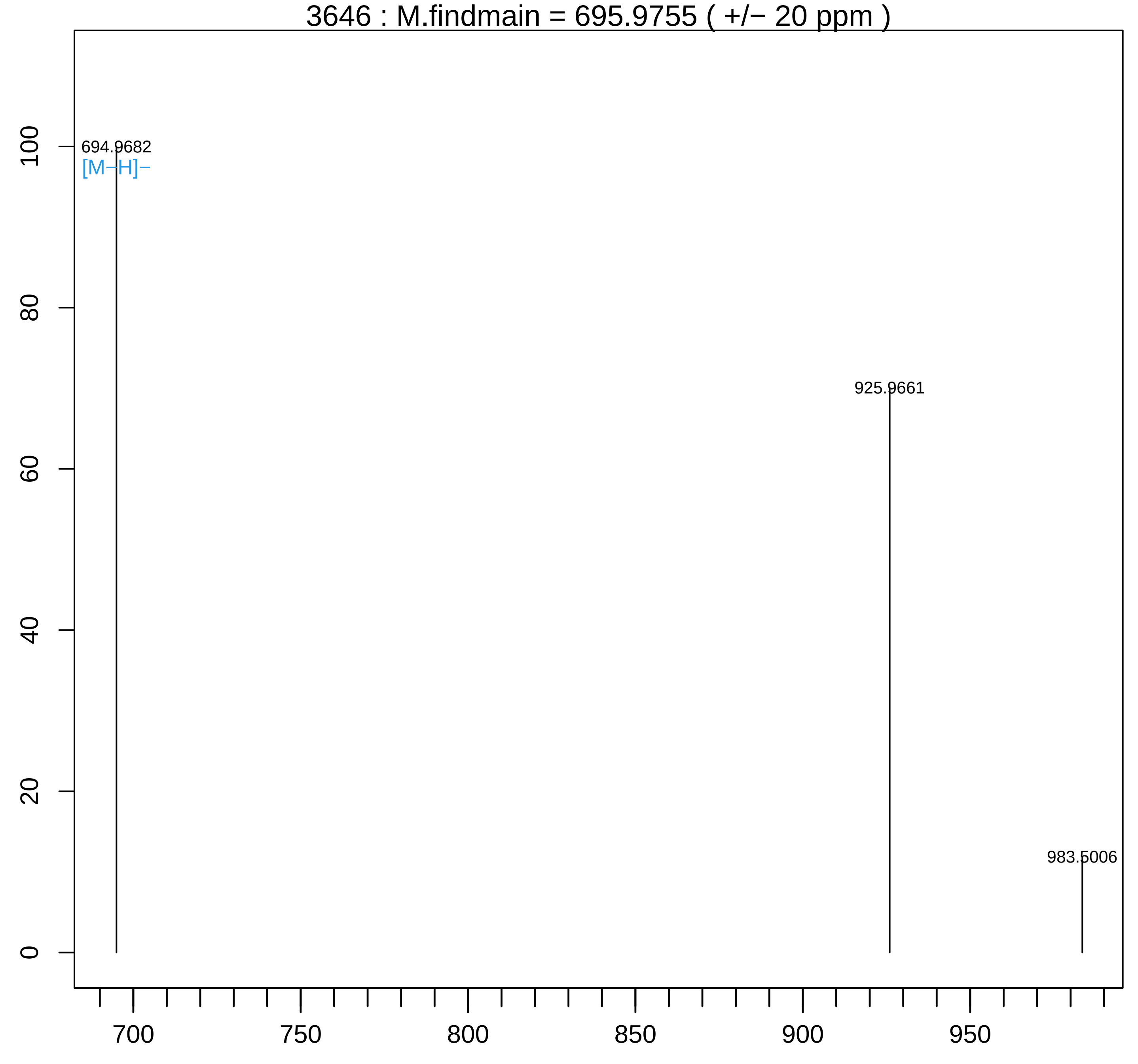
3646 : M.findmain = 695.9755 (+/− 20 ppm)
694.9682
[M−H]−
925.9661
983.5006
0
20
40
60
80
100
700
750
800
850
900
950

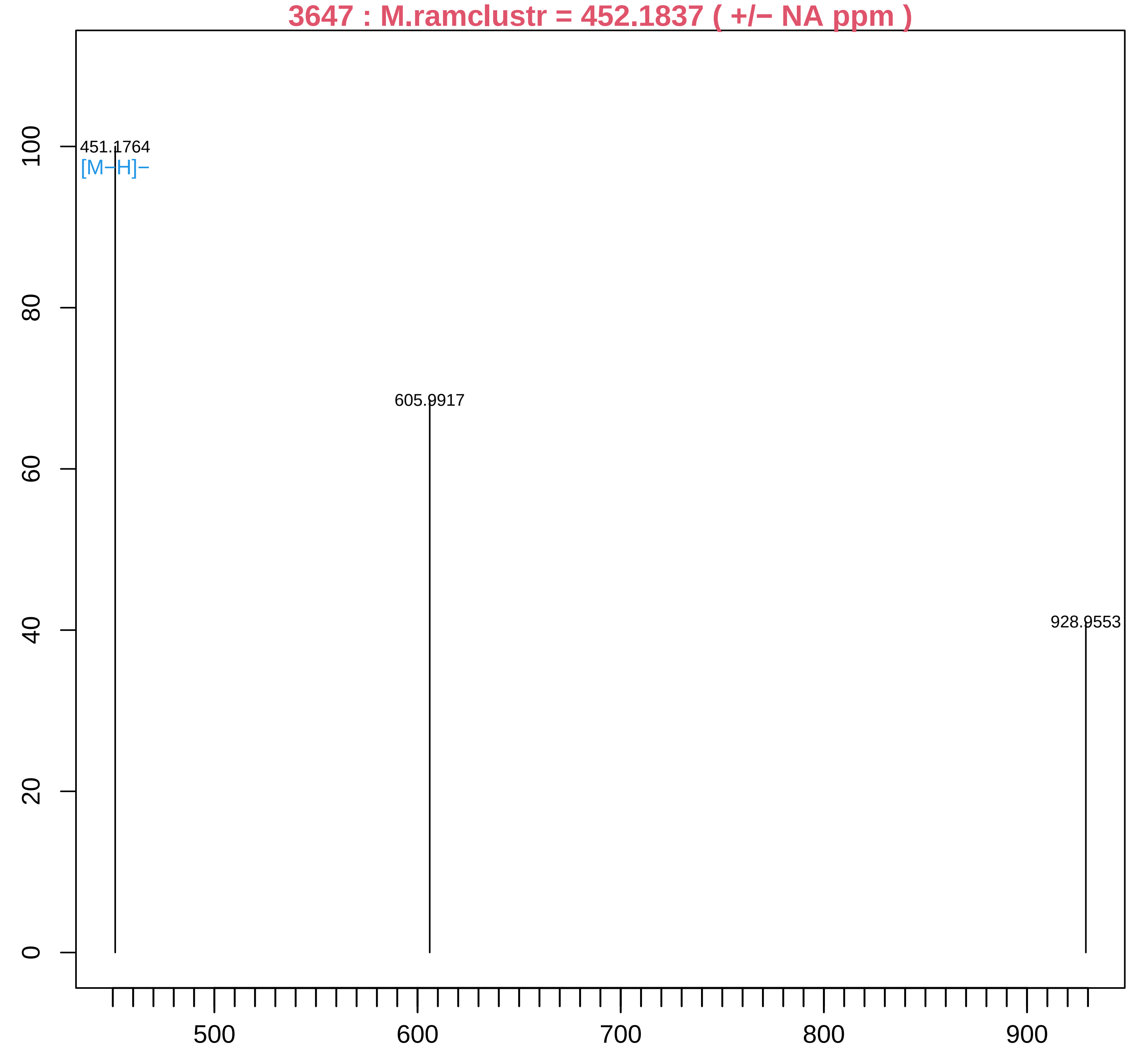
3647 : M.ramclustr = 452.1837 (+/− NA ppm)
451.1764
[M−H]−
605.9917
928.9553
0
20
40
60
80
100
500
600
700
800
900

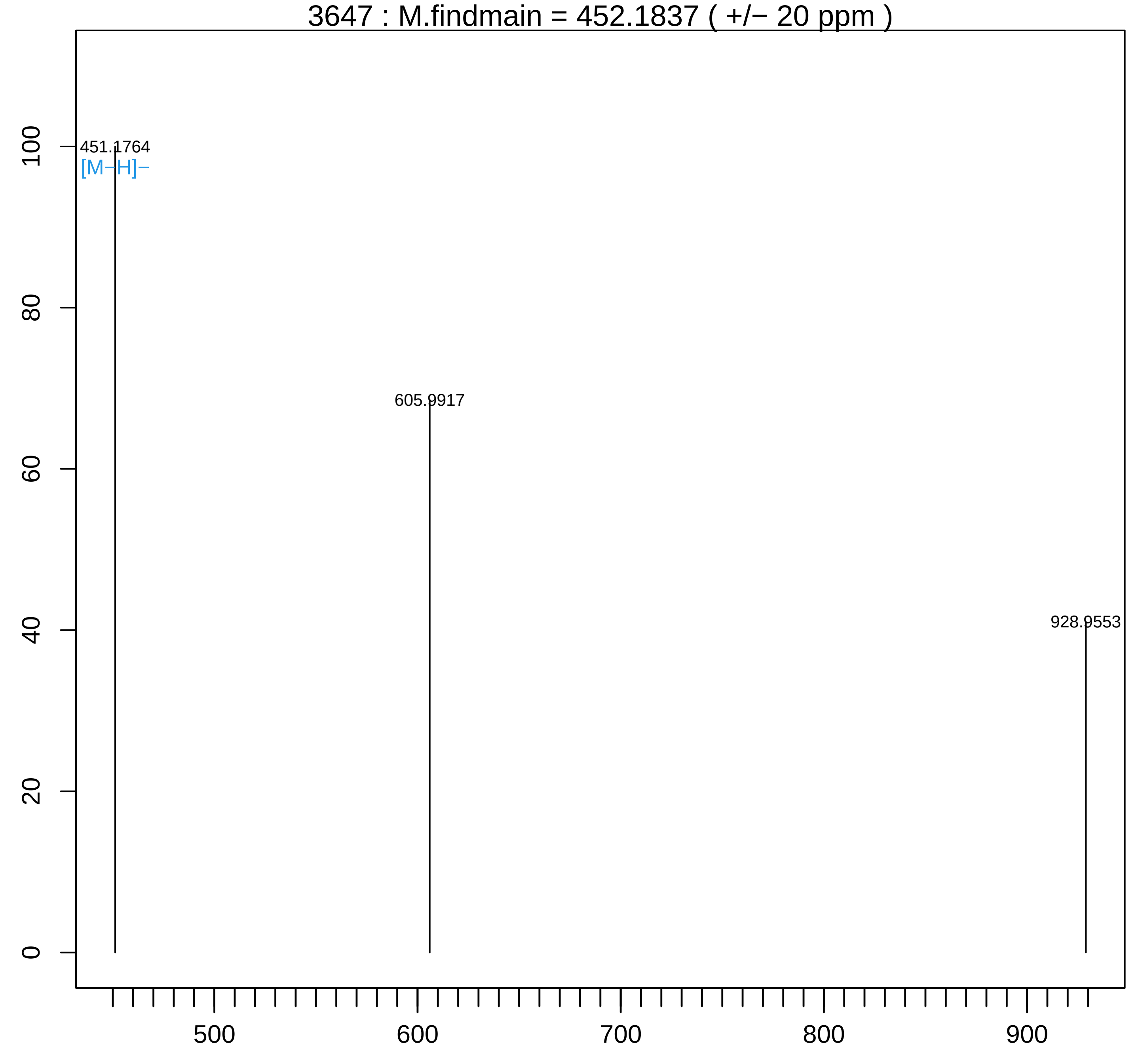
3647 : M.findmain = 452.1837 (+/− 20 ppm)
451.1764
[M−H]−
605.9917
928.9553
0
20
40
60
80
100
500
600
700
800
900

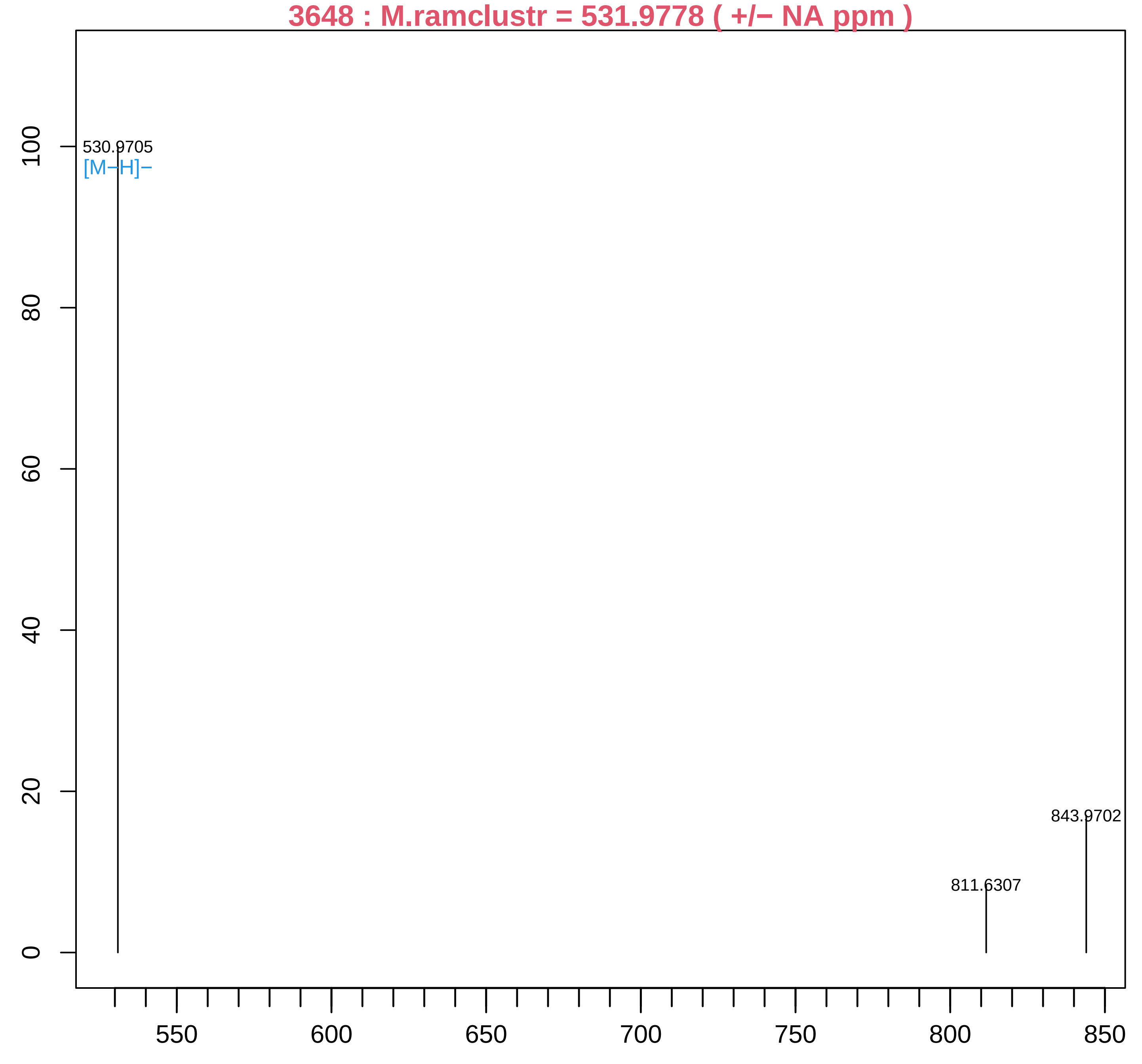
3648 : M.ramclustr = 531.9778 (+/− NA ppm)
530.9705
[M−H]−
843.9702
811.6307
0
20
40
60
80
100
550
600
650
700
750
800
850

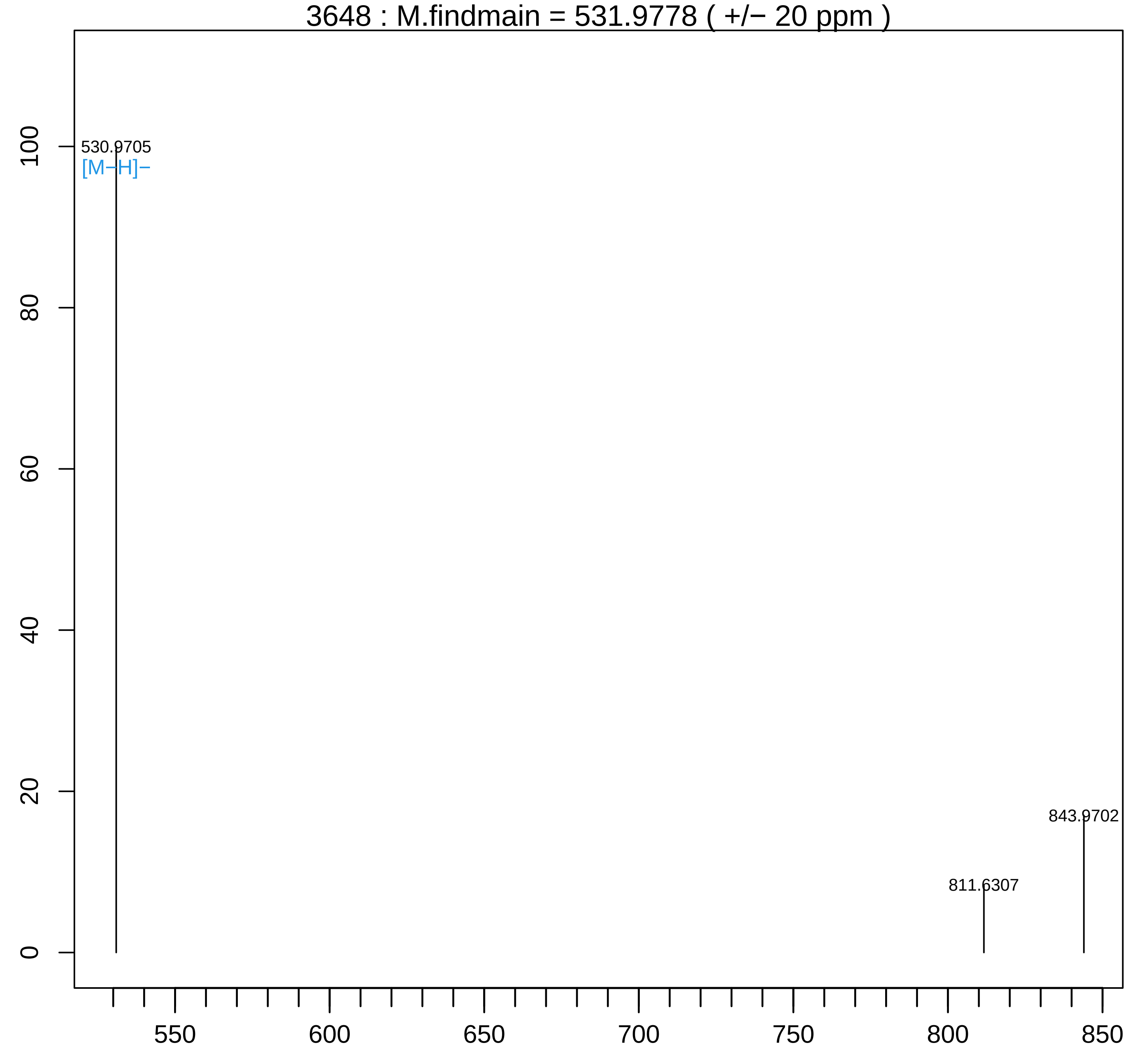
3648 : M.findmain = 531.9778 (+/− 20 ppm)
530.9705
[M−H]−
843.9702
811.6307
0
20
40
60
80
100
550
600
650
700
750
800
850

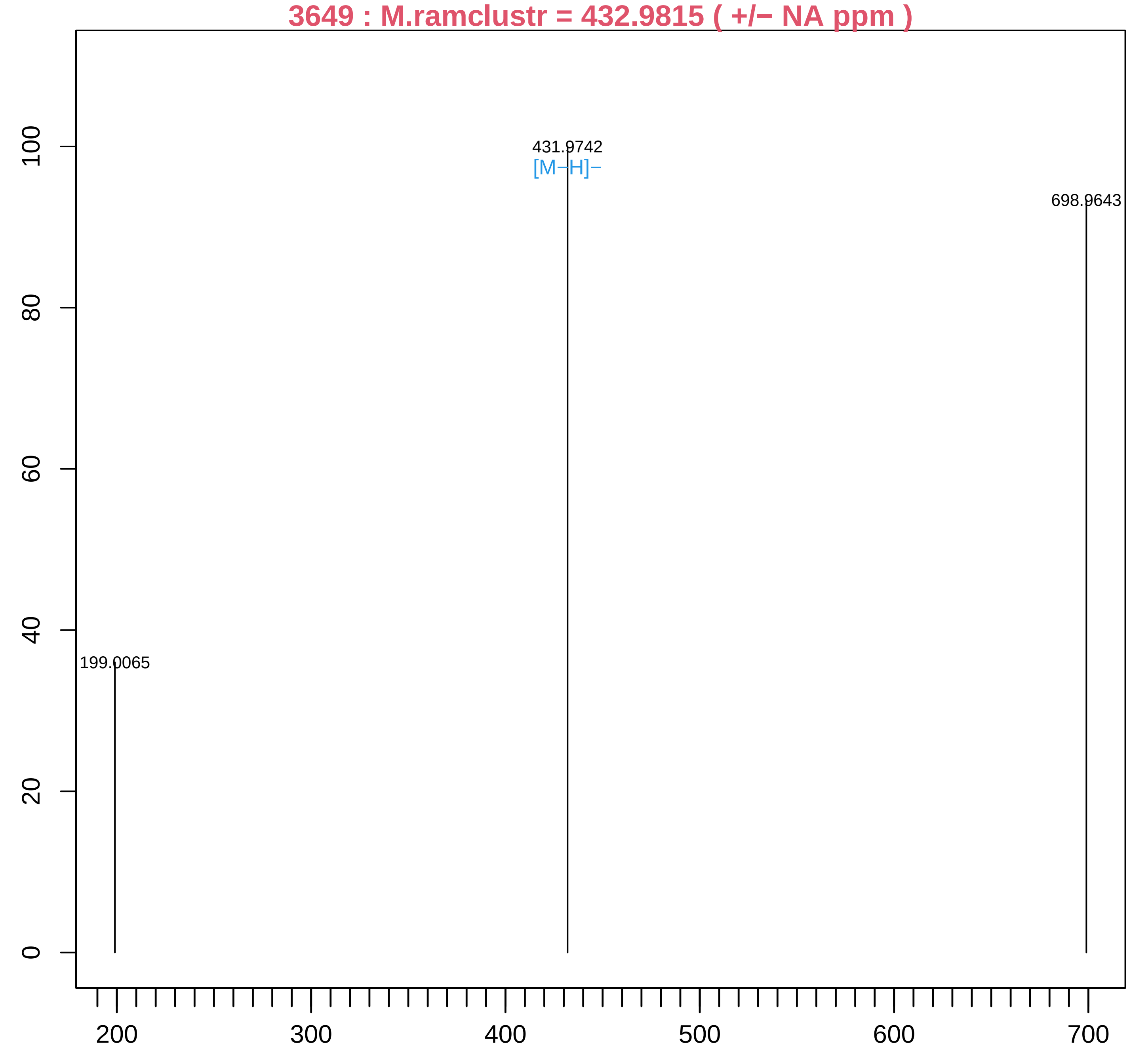
3649 : M.ramclustr = 432.9815 (+/− NA ppm)
431.9742
[M−H]−
698.9643
199.0065
0
20
40
60
80
100
200
300
400
500
600
700

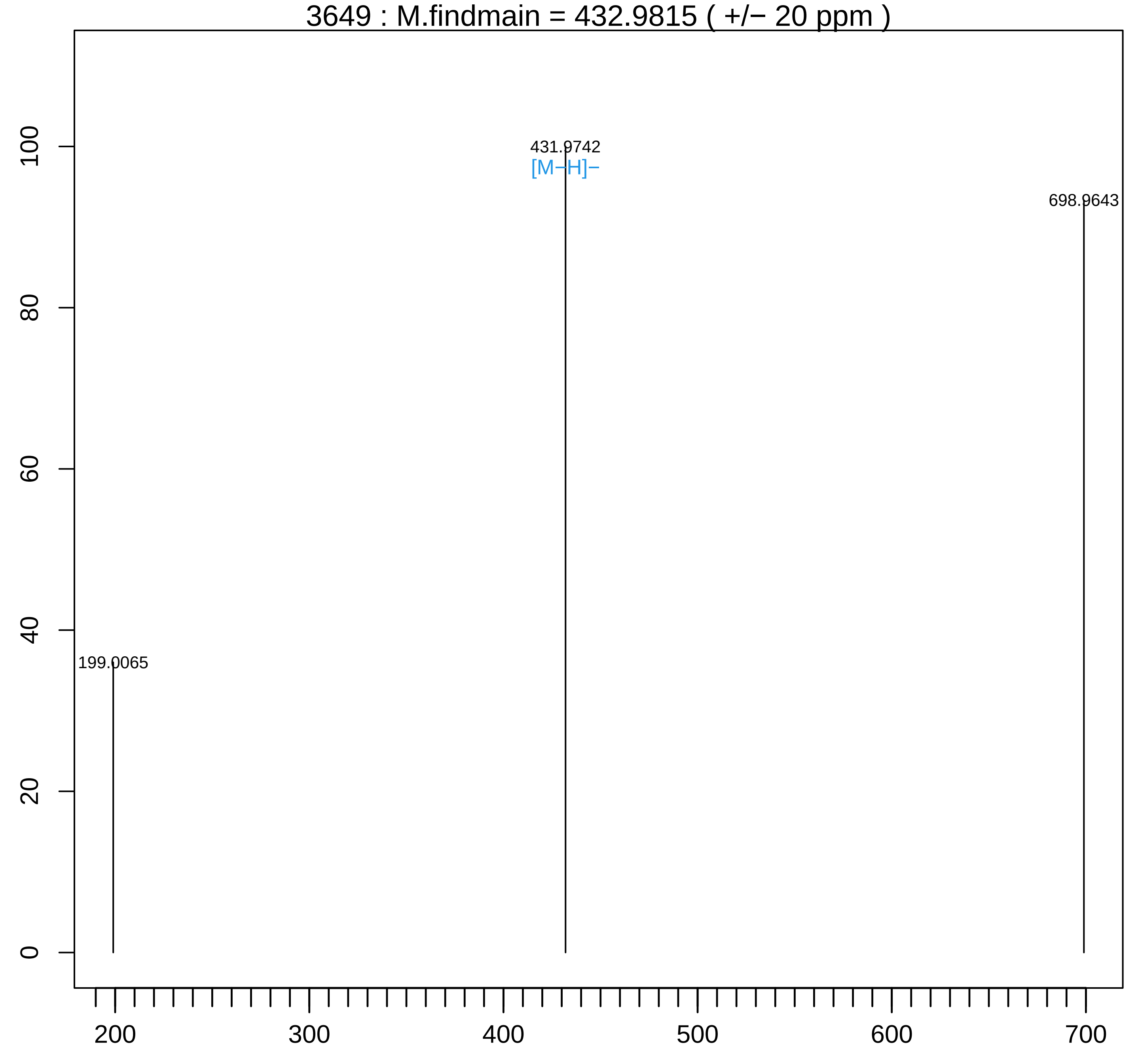
3649 : M.findmain = 432.9815 (+/− 20 ppm)
431.9742
[M−H]−
698.9643
199.0065
0
20
40
60
80
100
200
300
400
500
600
700

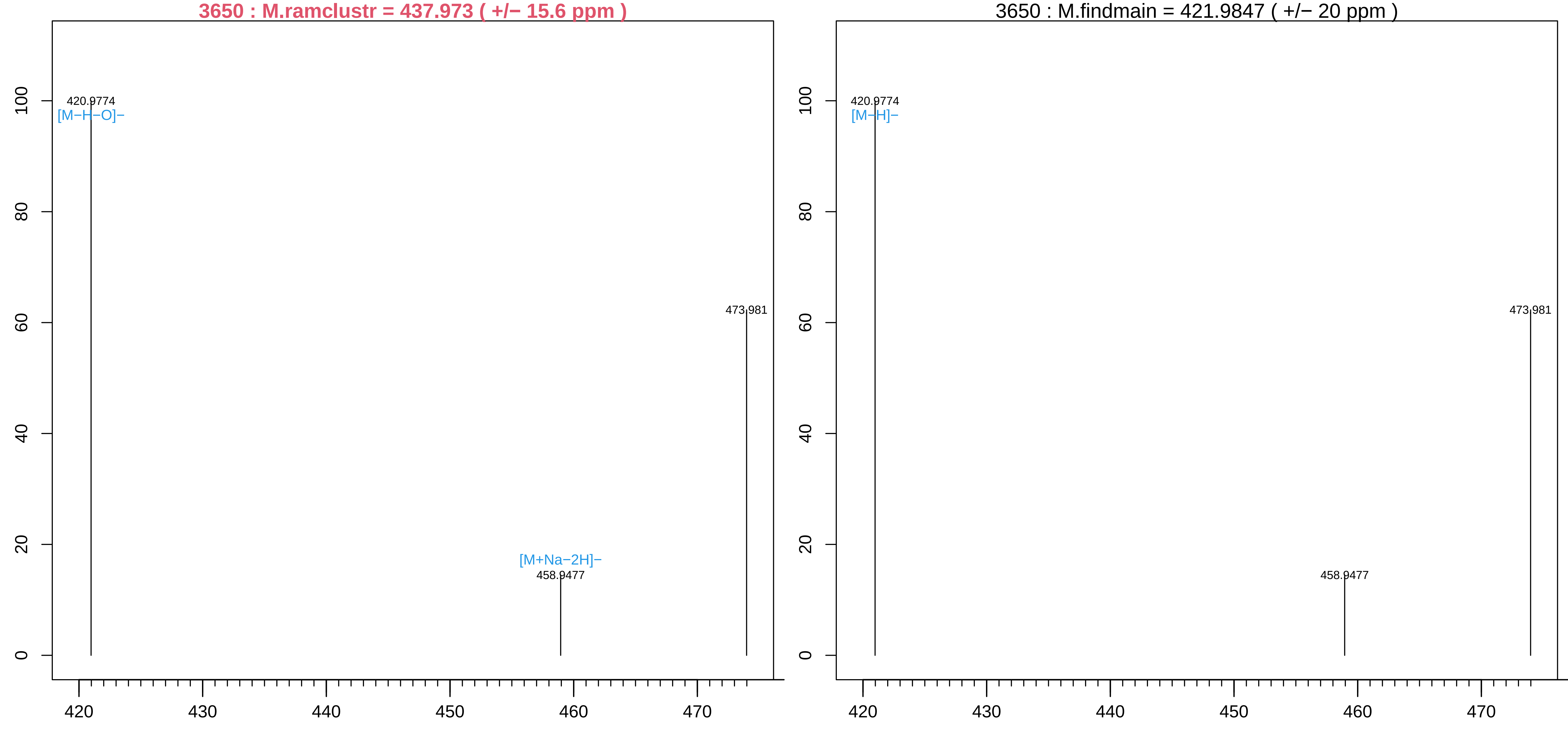
3650 : M.ramclustr = 437.973 (+/− 15.6 ppm)
420.9774
[M−H−O]−
[M+Na−2H]−
458.9477
473.981
3650 : M.findmain = 421.9847 (+/− 20 ppm)
420.9774
[M−H]−
458.9477
473.981

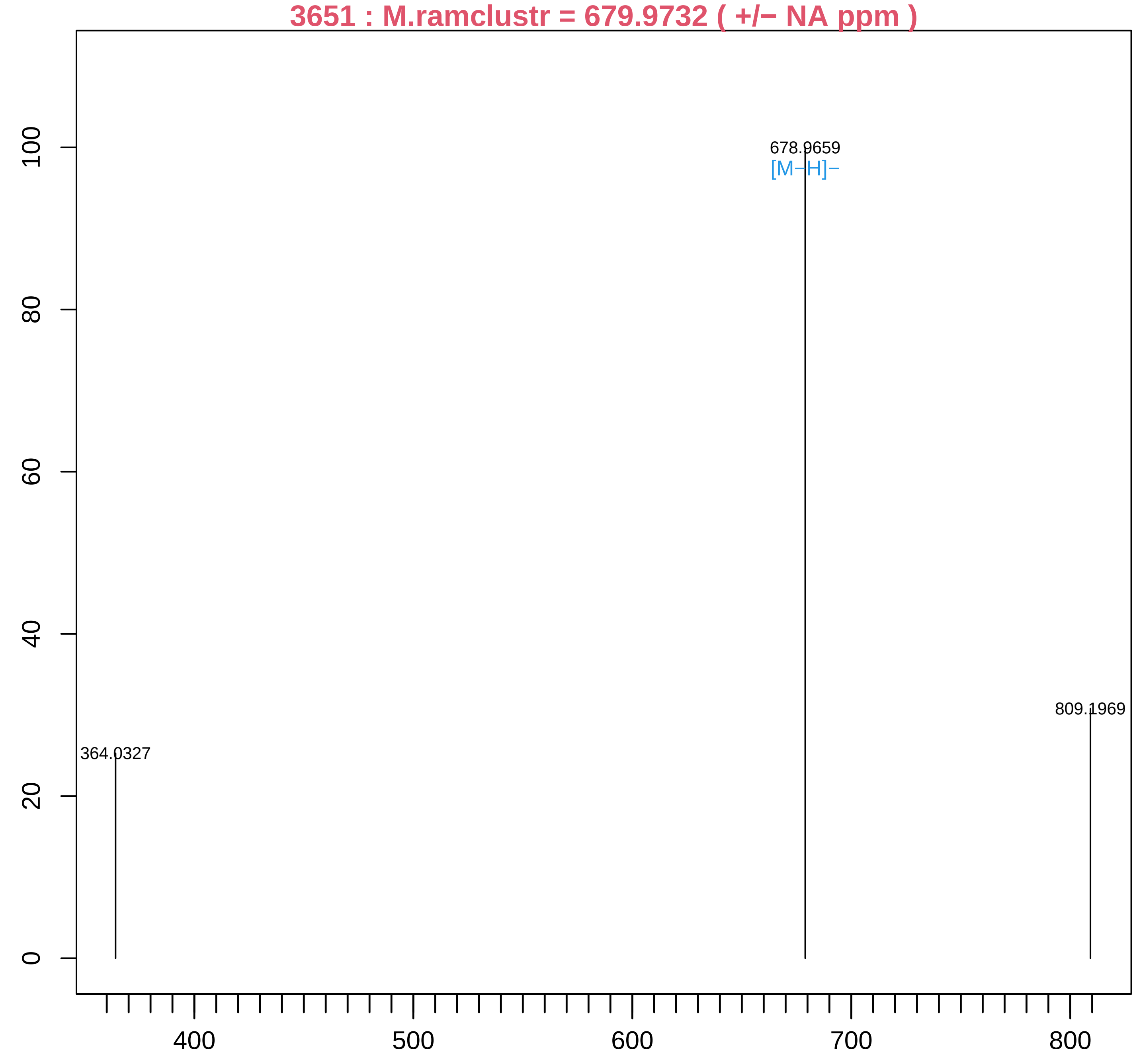
3651 : M.ramclustr = 679.9732 (+/− NA ppm)
678.9659
[M−H]−
364.0327
809.1969
0
20
40
60
80
100
400
500
600
700
800

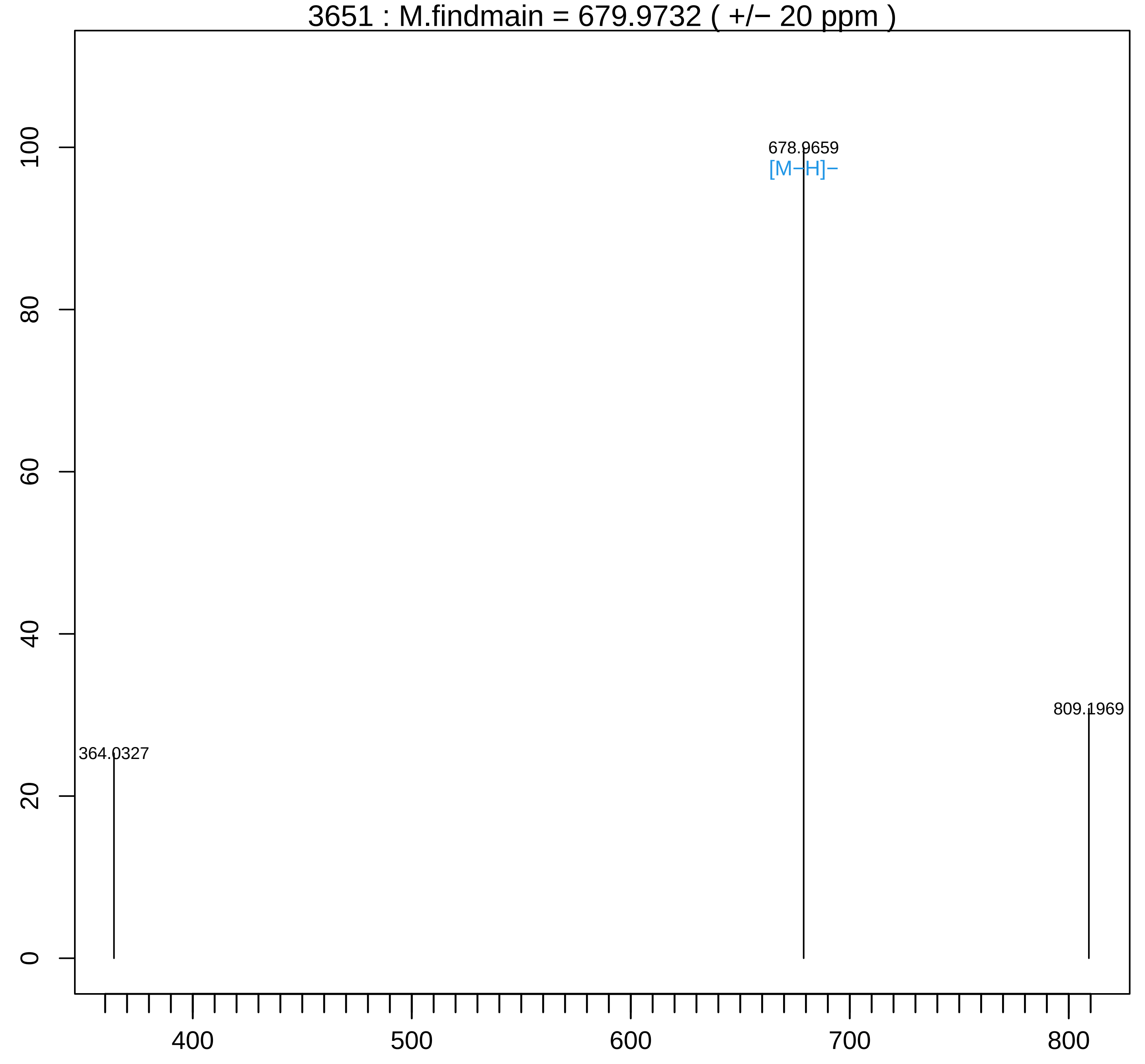
3651 : M.findmain = 679.9732 (+/− 20 ppm)
678.9659
[M−H]−
364.0327
809.1969
0
20
40
60
80
100
400
500
600
700
800

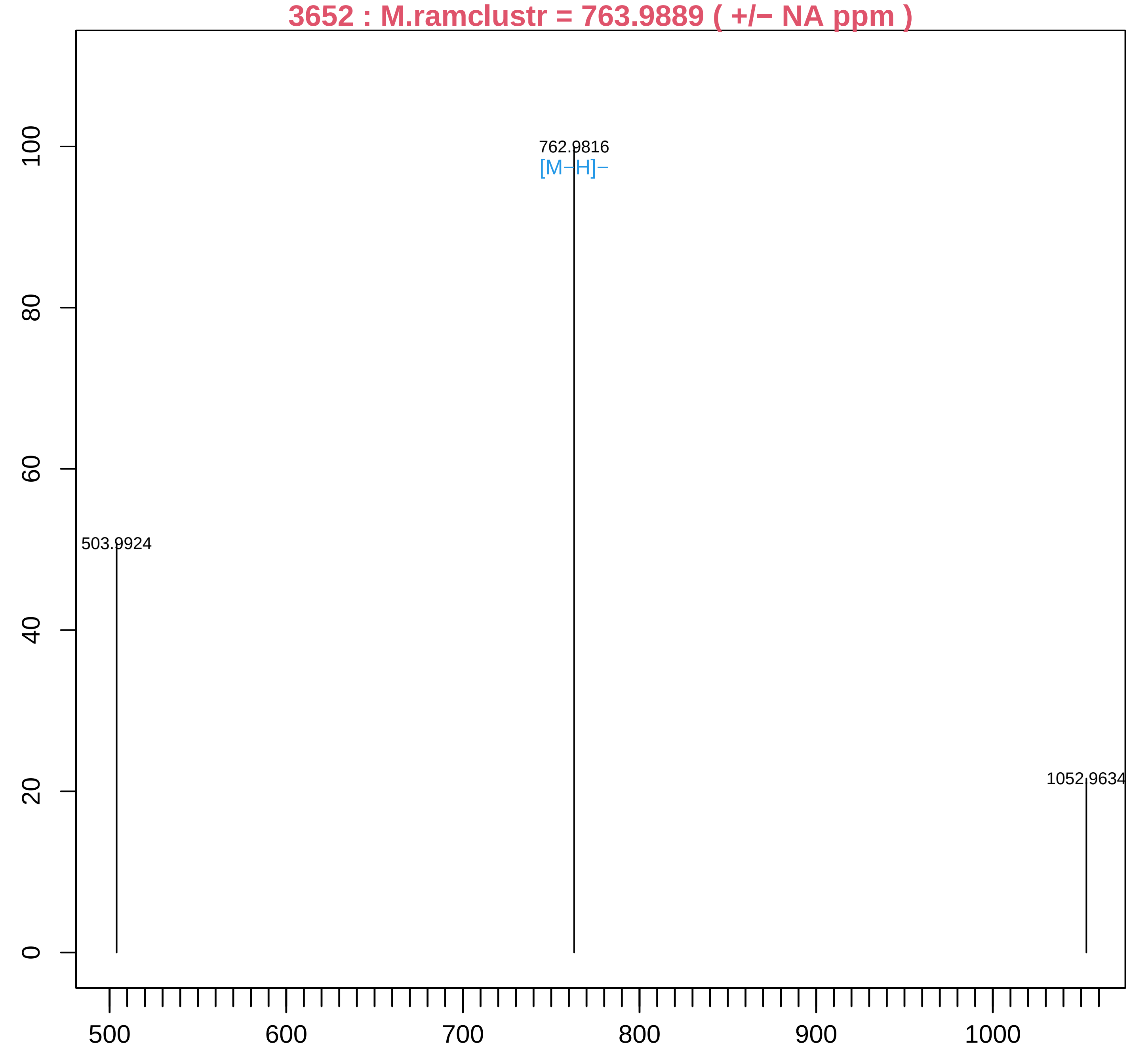
3652 : M.ramclustr = 763.9889 (+/− NA ppm)
762.9816
[M−H]−
503.9924
1052.9634
0
20
40
60
80
100
500
600
700
800
900
1000

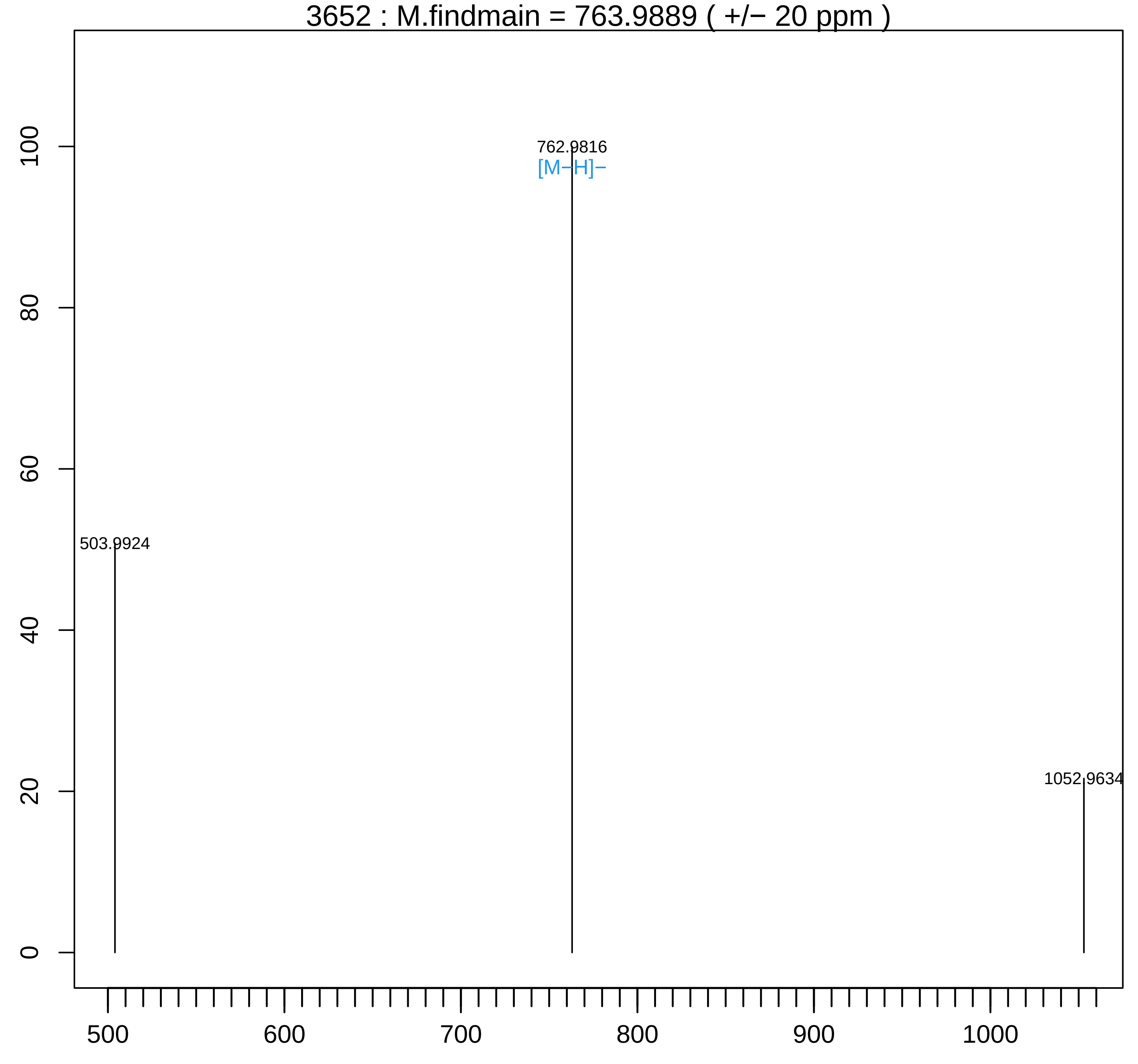
3652 : M.findmain = 763.9889 (+/− 20 ppm)
762.9816
[M−H]−
503.9924
1052.9634
0
20
40
60
80
100
500
600
700
800
900
1000

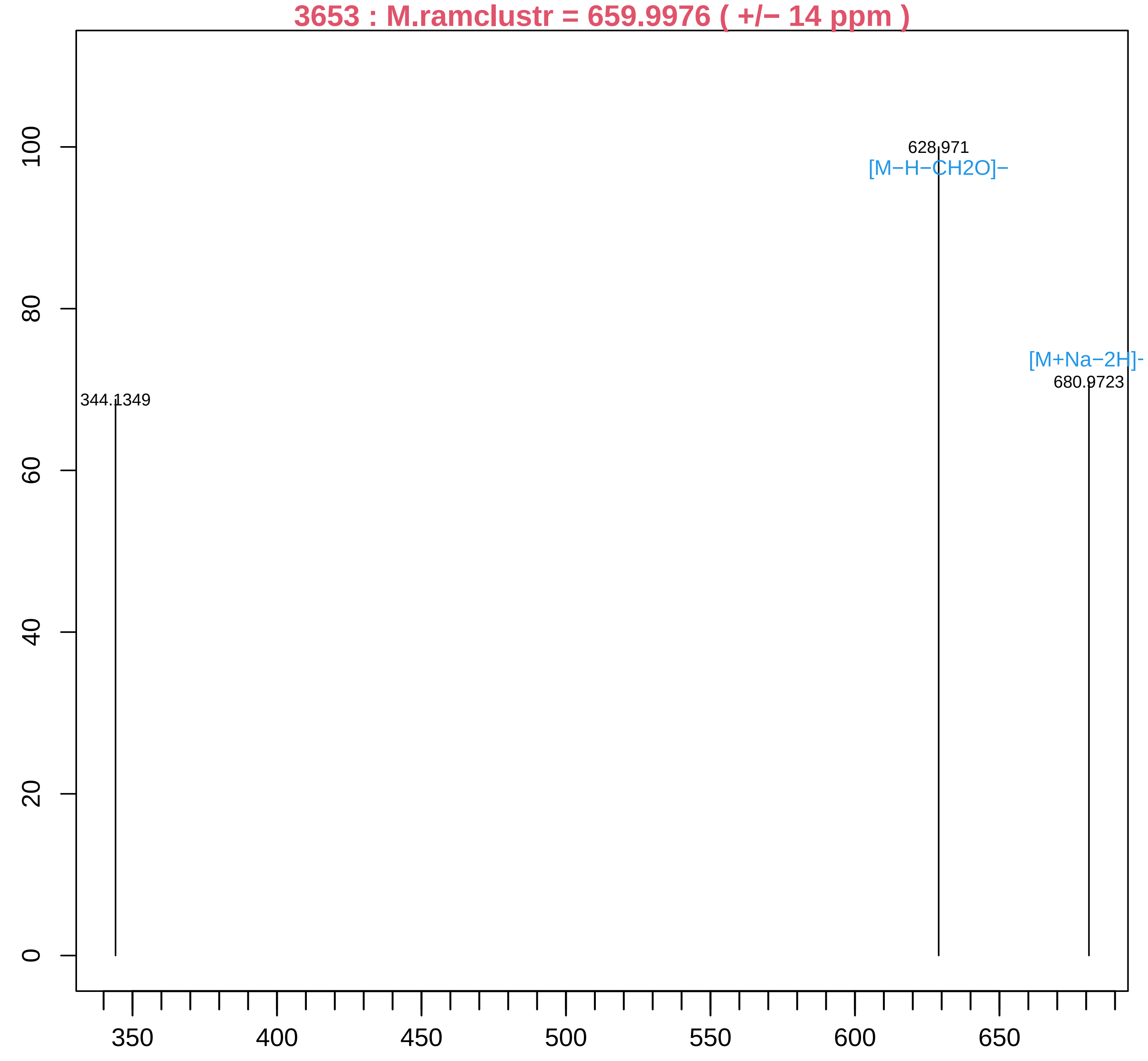
3653 : M.ramclustr = 659.9976 (+/− 14 ppm)
628.971
[M−H−CH2O]−
[M+Na−2H]-
680.9723
344.1349
0
20
40
60
80
100
350
400
450
500
550
600
650

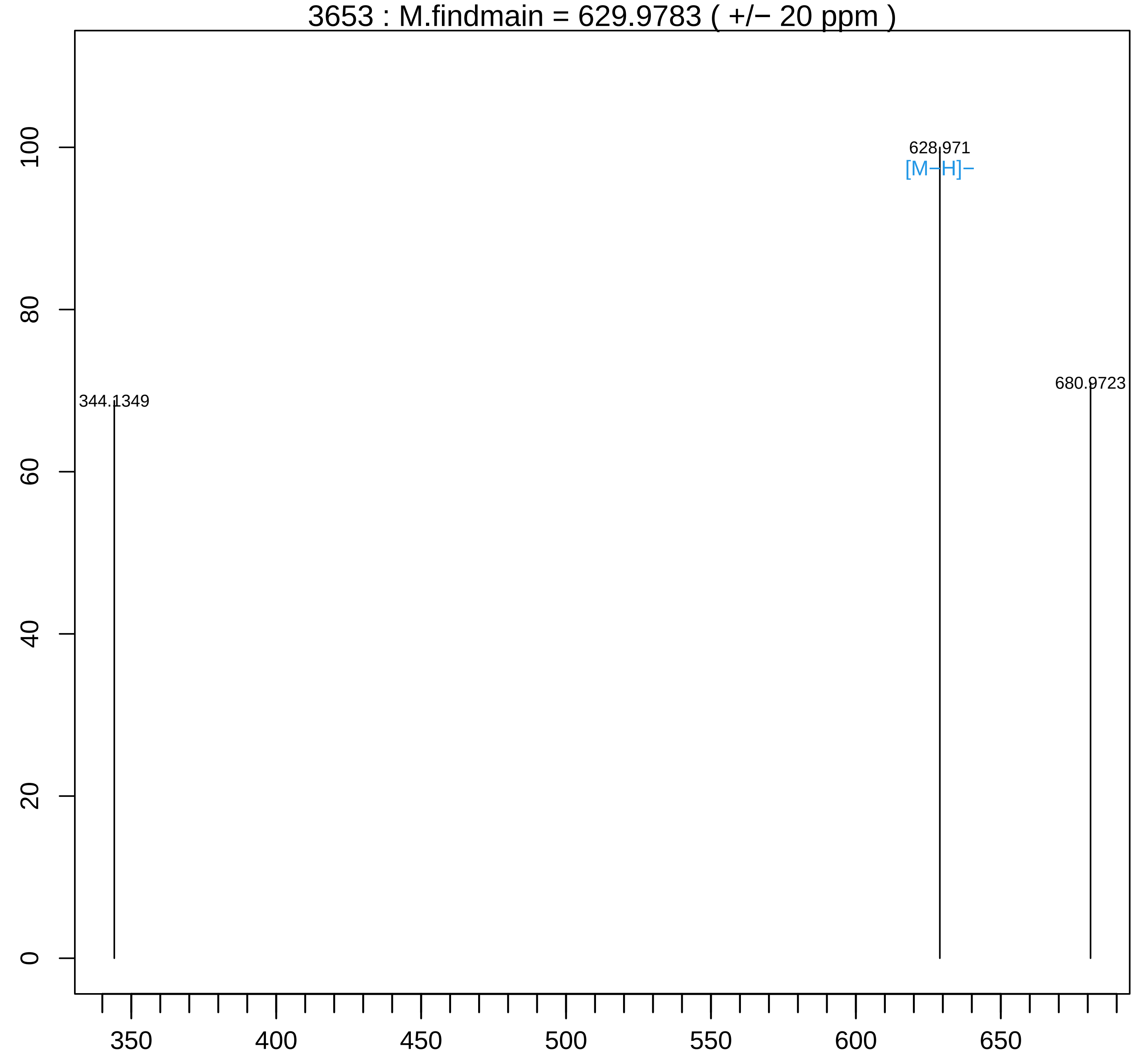
3653 : M.findmain = 629.9783 (+/− 20 ppm)
628.971
[M−H]−
680.9723
344.1349
0
20
40
60
80
100
350
400
450
500
550
600
650

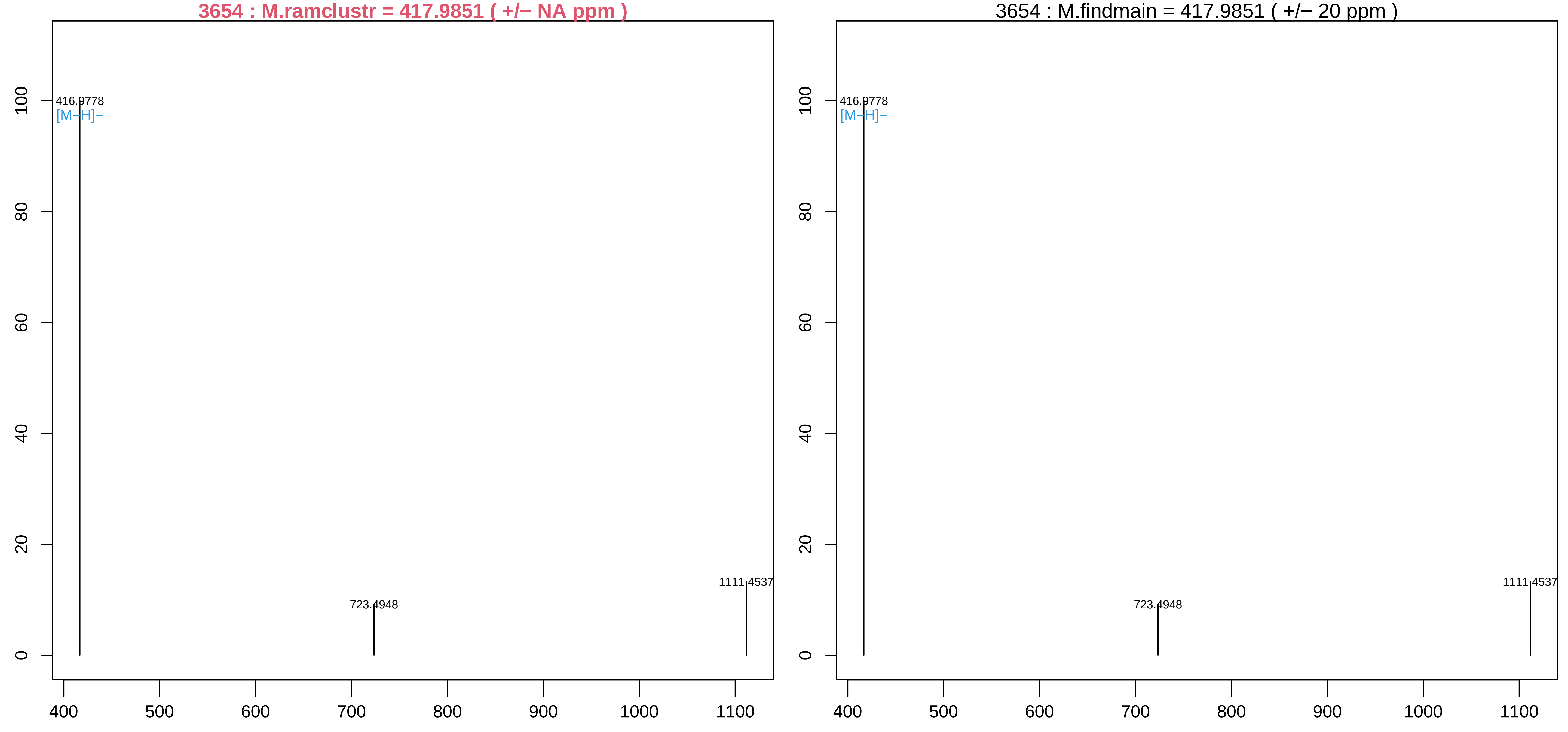
3654 : M.ramclustr = 417.9851 (+/− NA ppm)
416.9778
[M−H]−
723.4948
1111.4537
3654 : M.findmain = 417.9851 (+/− 20 ppm)
416.9778
[M−H]−
723.4948
1111.4537

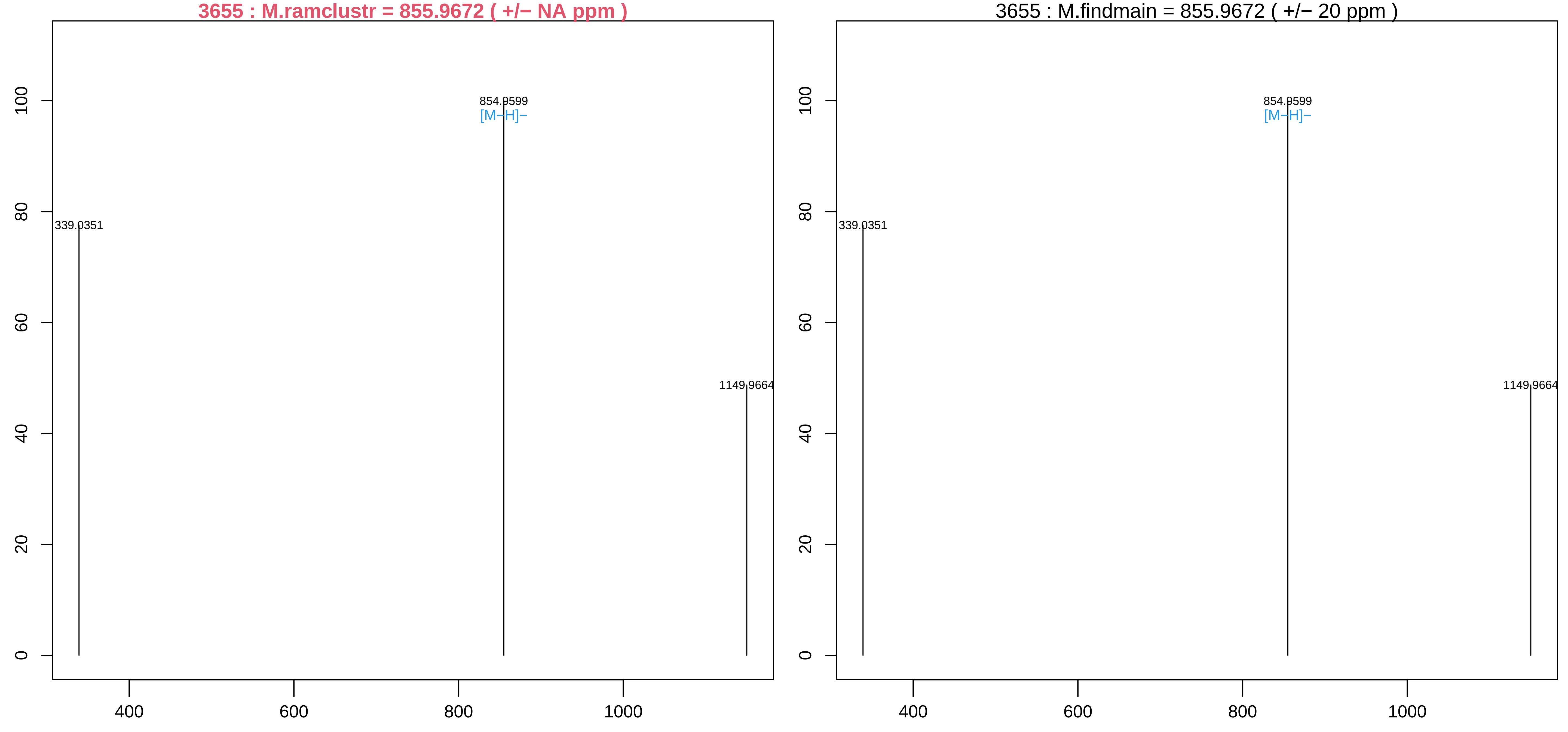
3655 : M.ramclustr = 855.9672 (+/− NA ppm)
339.0351
854.9599
[M−H]−
1149.9664
0
20
40
60
80
100
400
600
800
1000
3655 : M.findmain = 855.9672 (+/− 20 ppm)
339.0351
854.9599
[M−H]−
1149.9664
0
20
40
60
80
100
400
600
800
1000

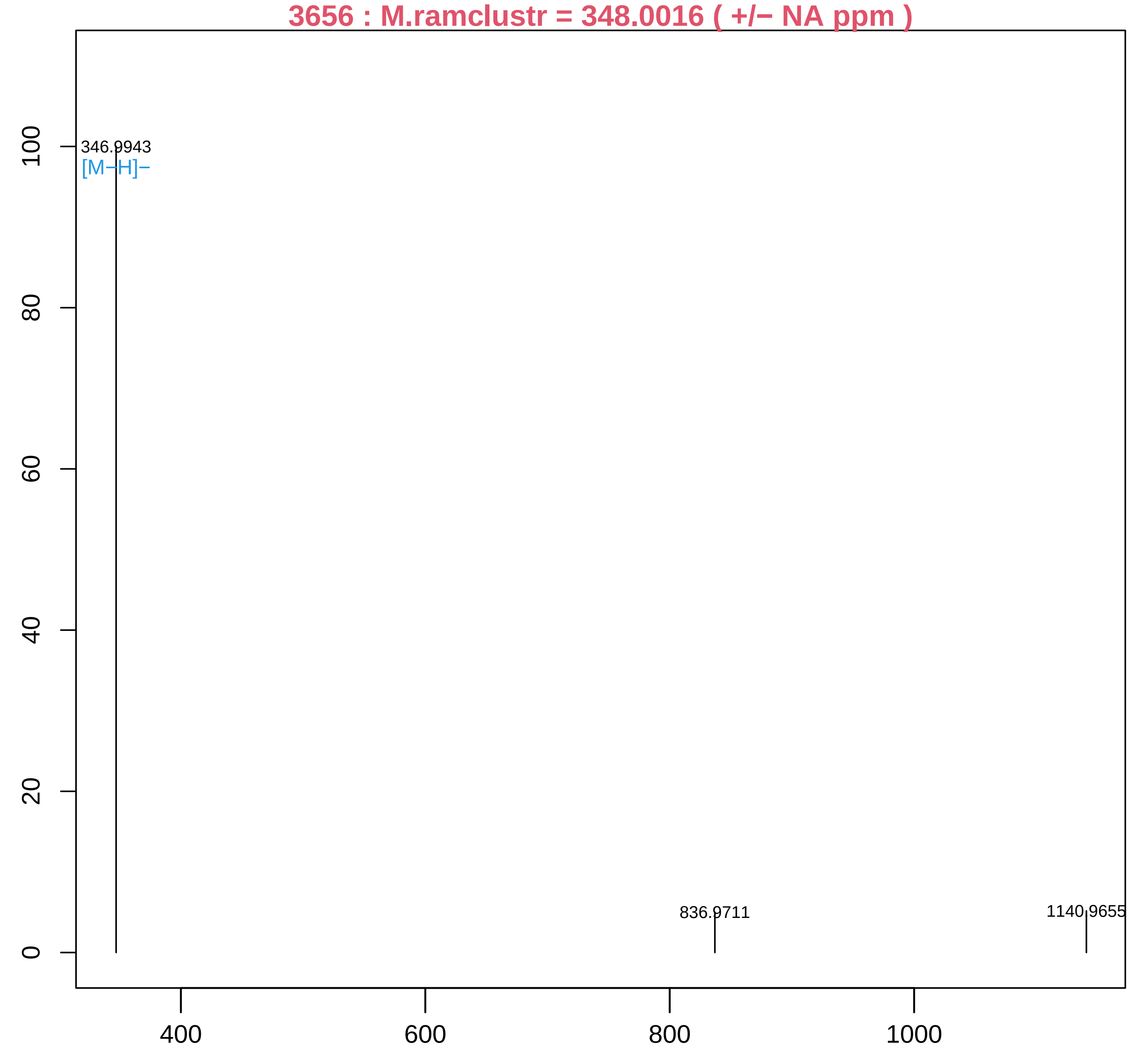
3656 : M.ramclustr = 348.0016 (+/− NA ppm)
346.9943
[M−H]−
836.9711
1140.9655
0
20
40
60
80
100
400
600
800
1000
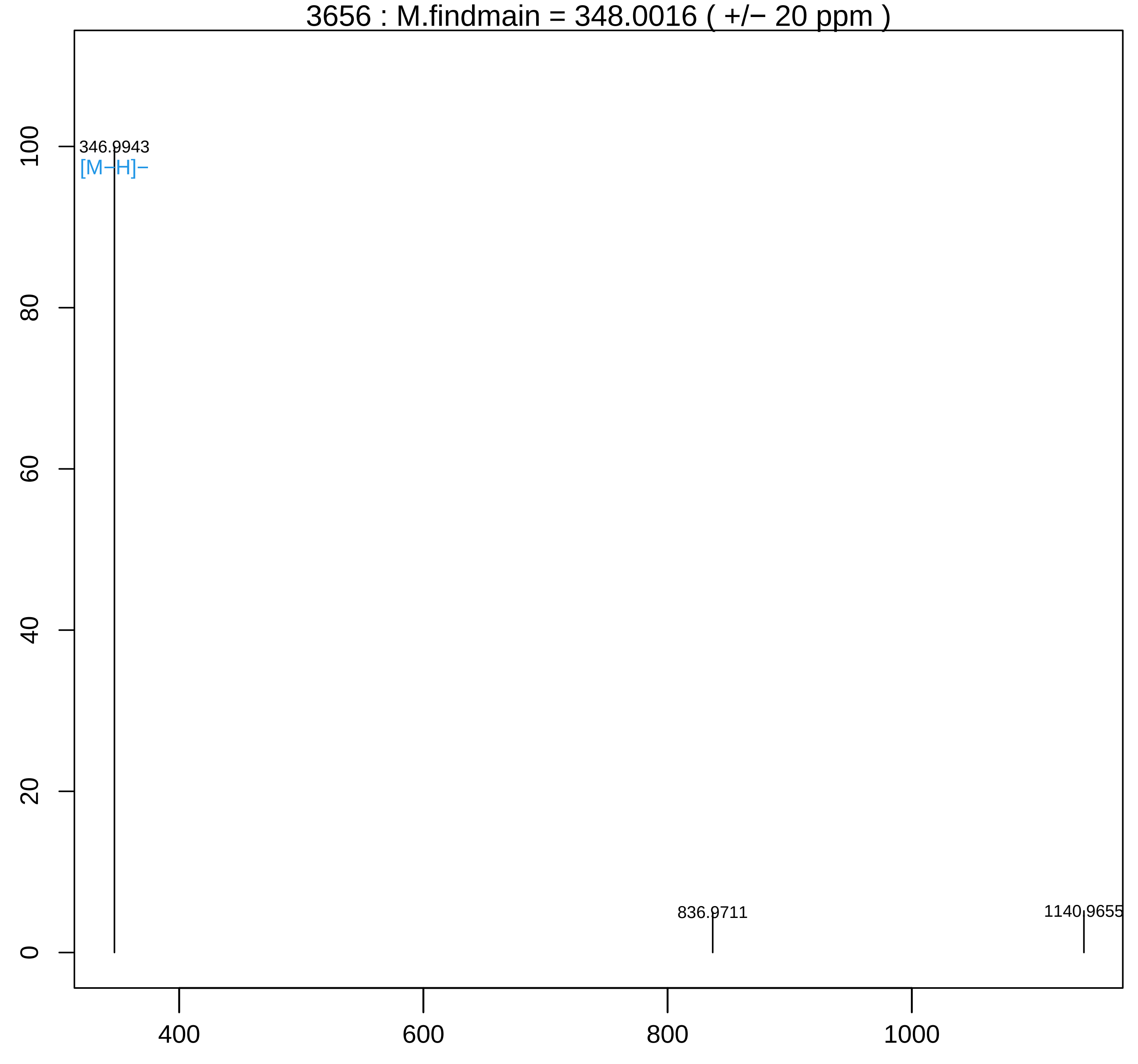
3656 : M.findmain = 348.0016 (+/− 20 ppm)
346.9943
[M−H]−
836.9711
1140.9655
0
20
40
60
80
100
400
600
800
1000

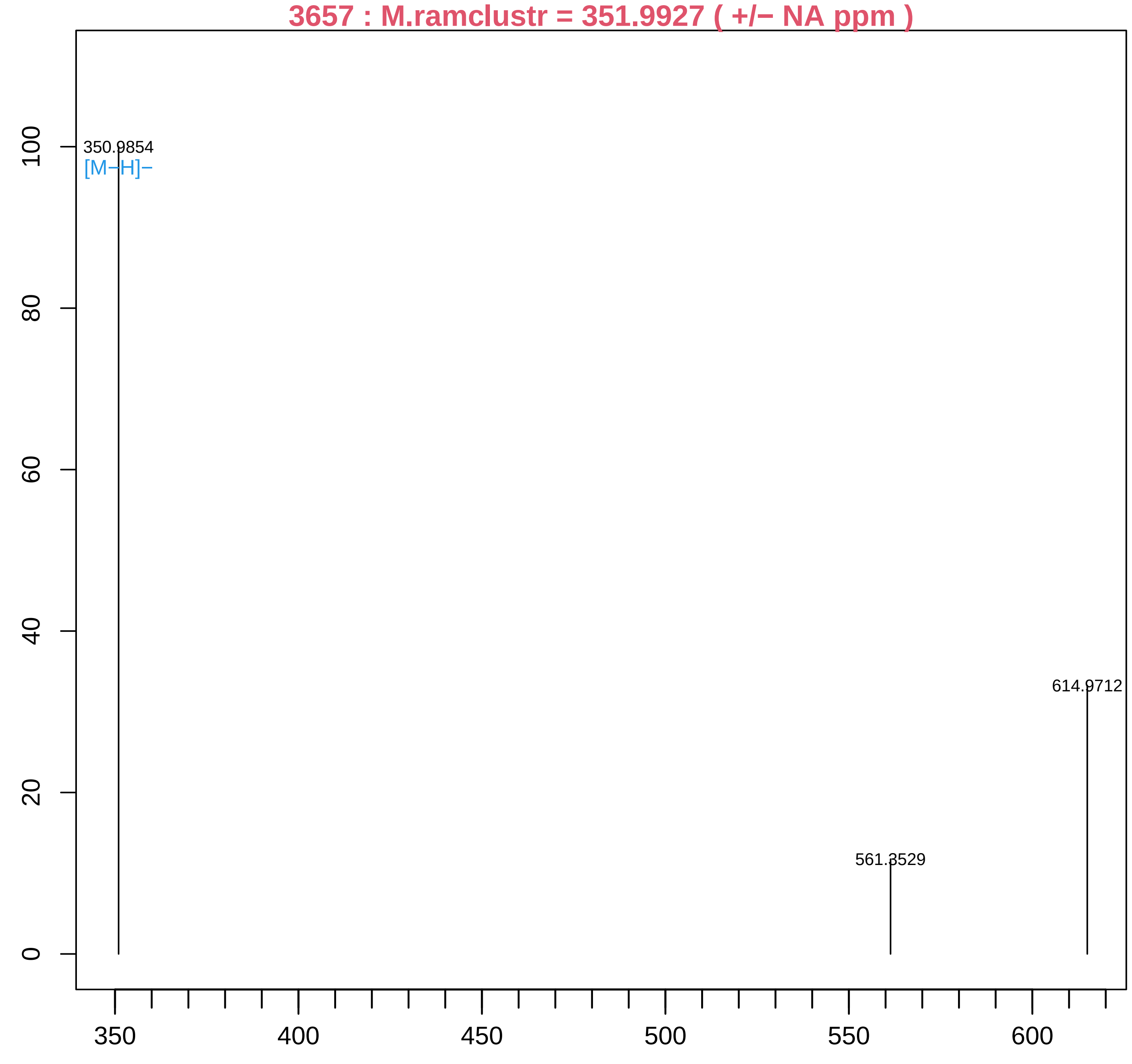
3657 : M.ramclustr = 351.9927 (+/− NA ppm)
350.9854
[M−H]−
614.9712
561.3529
0
20
40
60
80
100
350
400
450
500
550
600

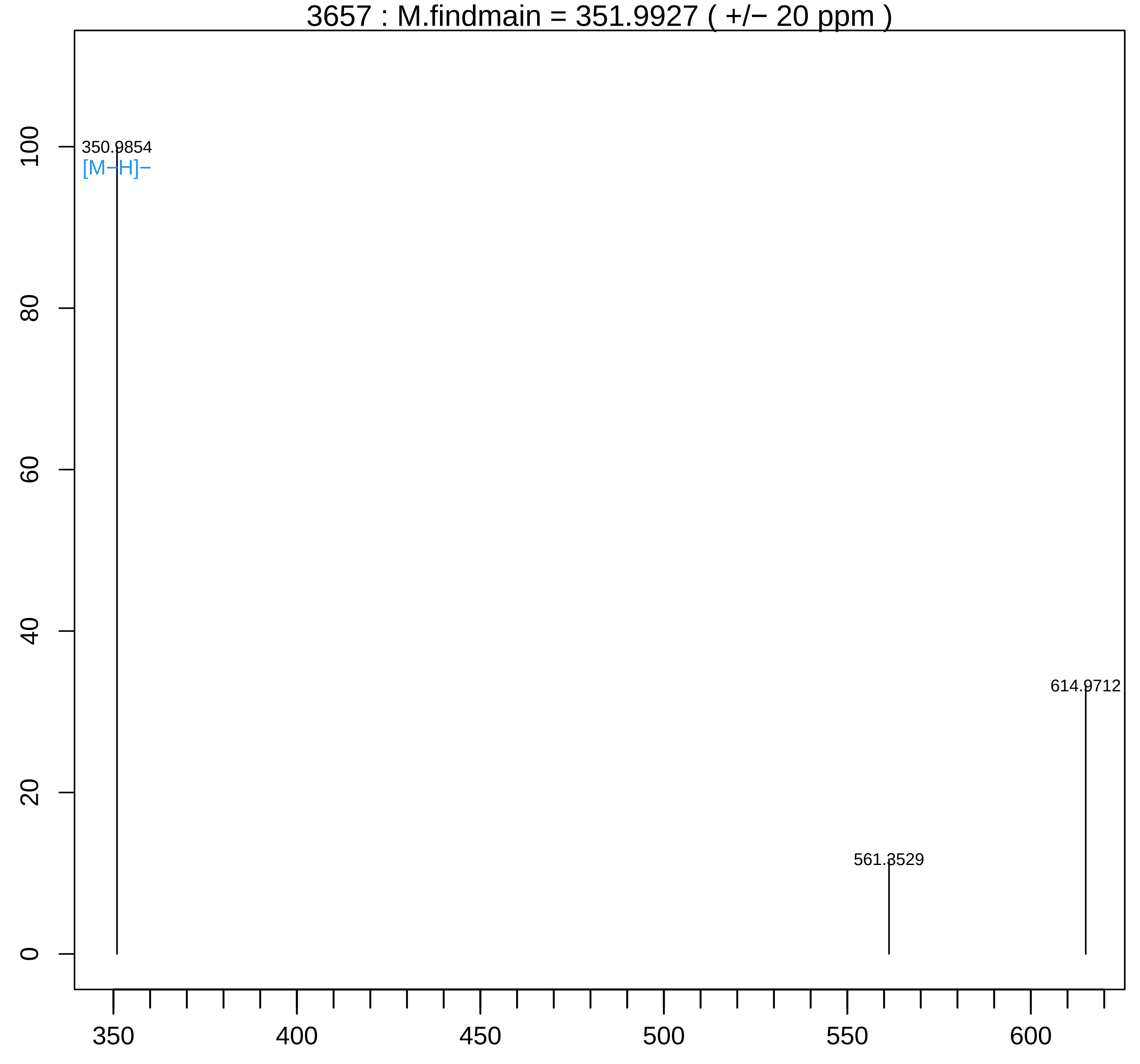
3657 : M.findmain = 351.9927 (+/− 20 ppm)
350.9854
[M−H]−
614.9712
561.3529
0
20
40
60
80
100
350
400
450
500
550
600

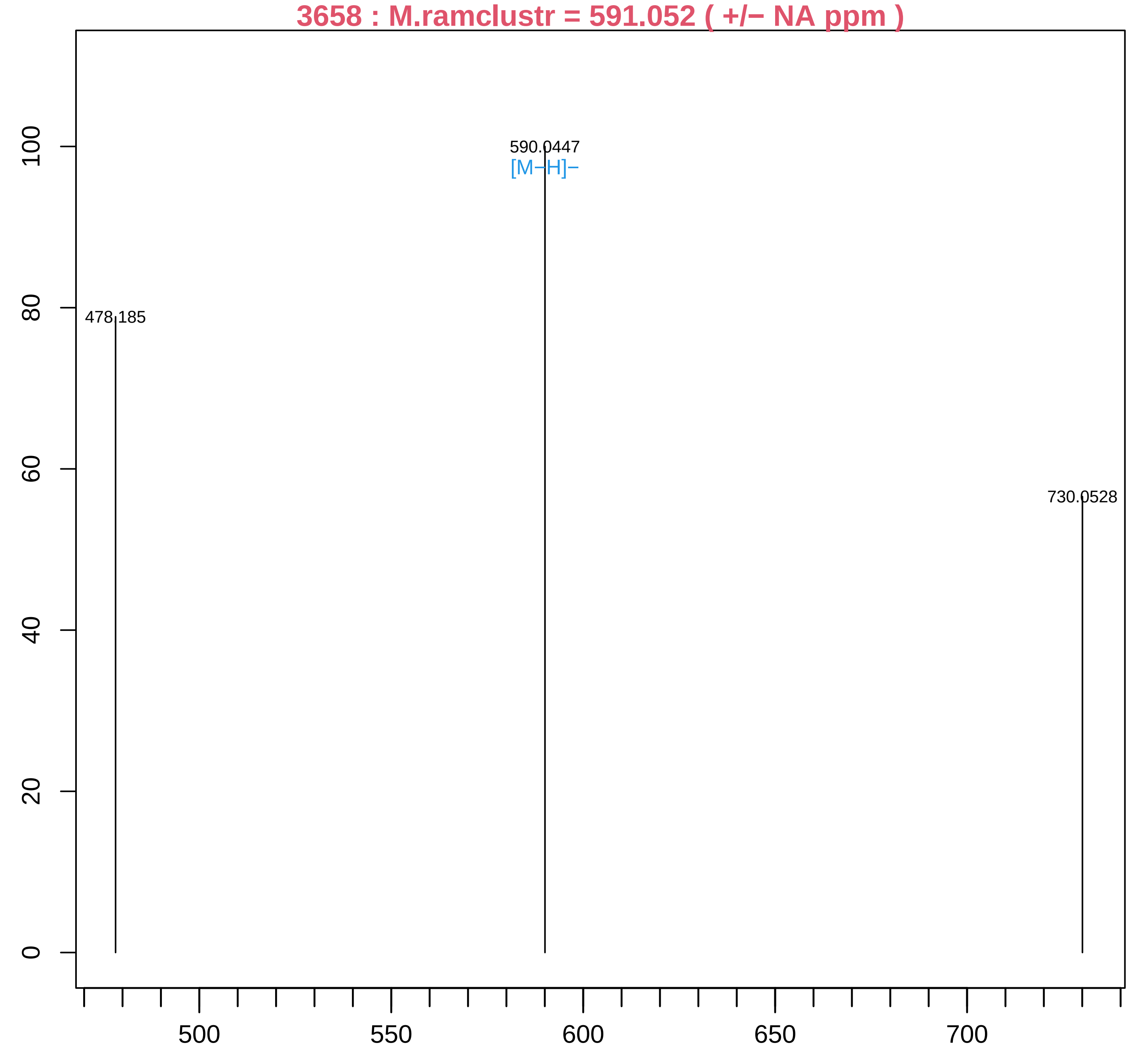

3658 : M.ramclustr = 591.052 (+/− NA ppm)
590.0447
[M−H]−
478.185
730.0528
0
20
40
60
80
100
500
550
600
650
700

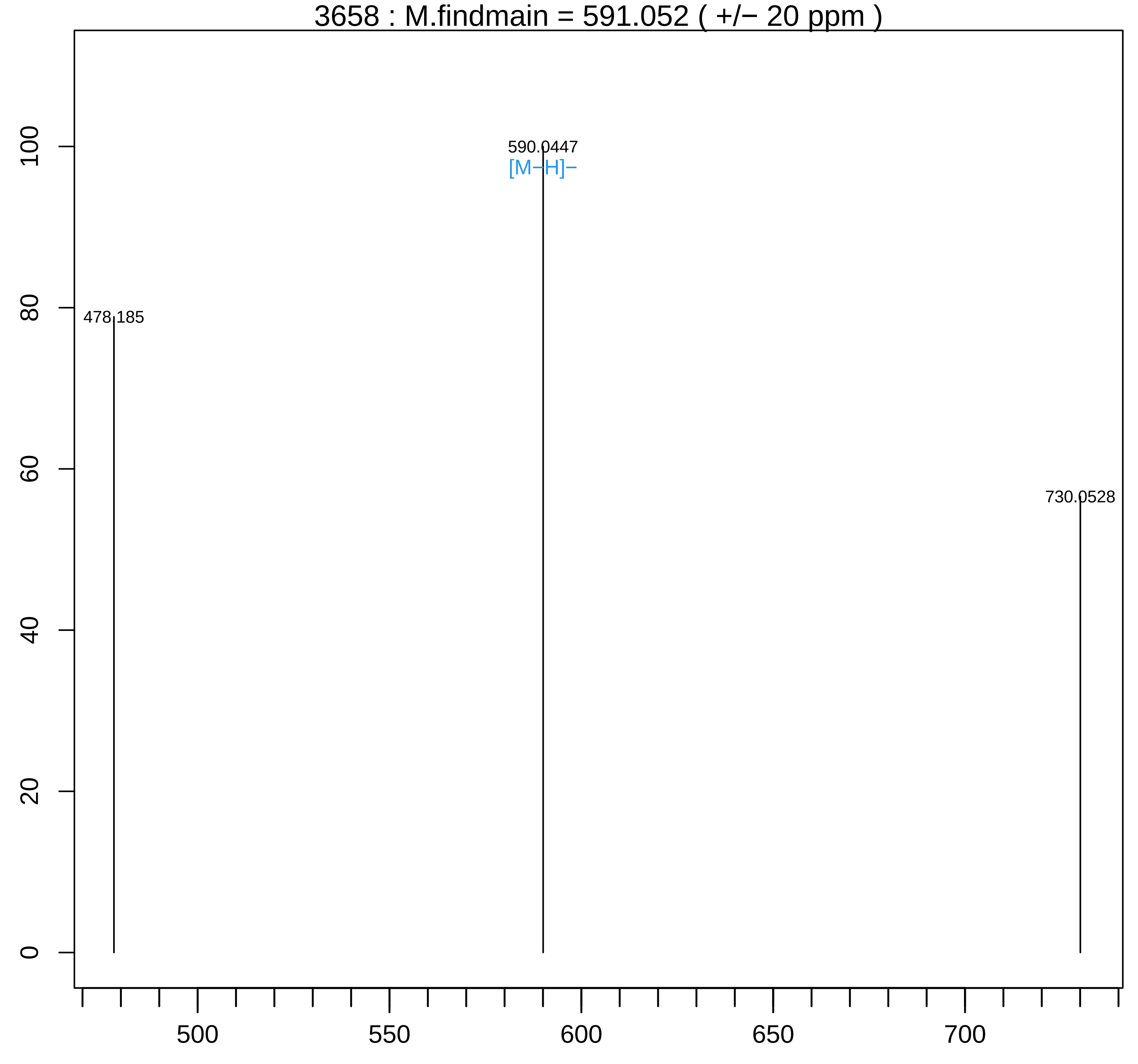

3658 : M.findmain = 591.052 (+/− 20 ppm)
590.0447
[M−H]−
478.185
730.0528
0
20
40
60
80
100
500
550
600
650
700

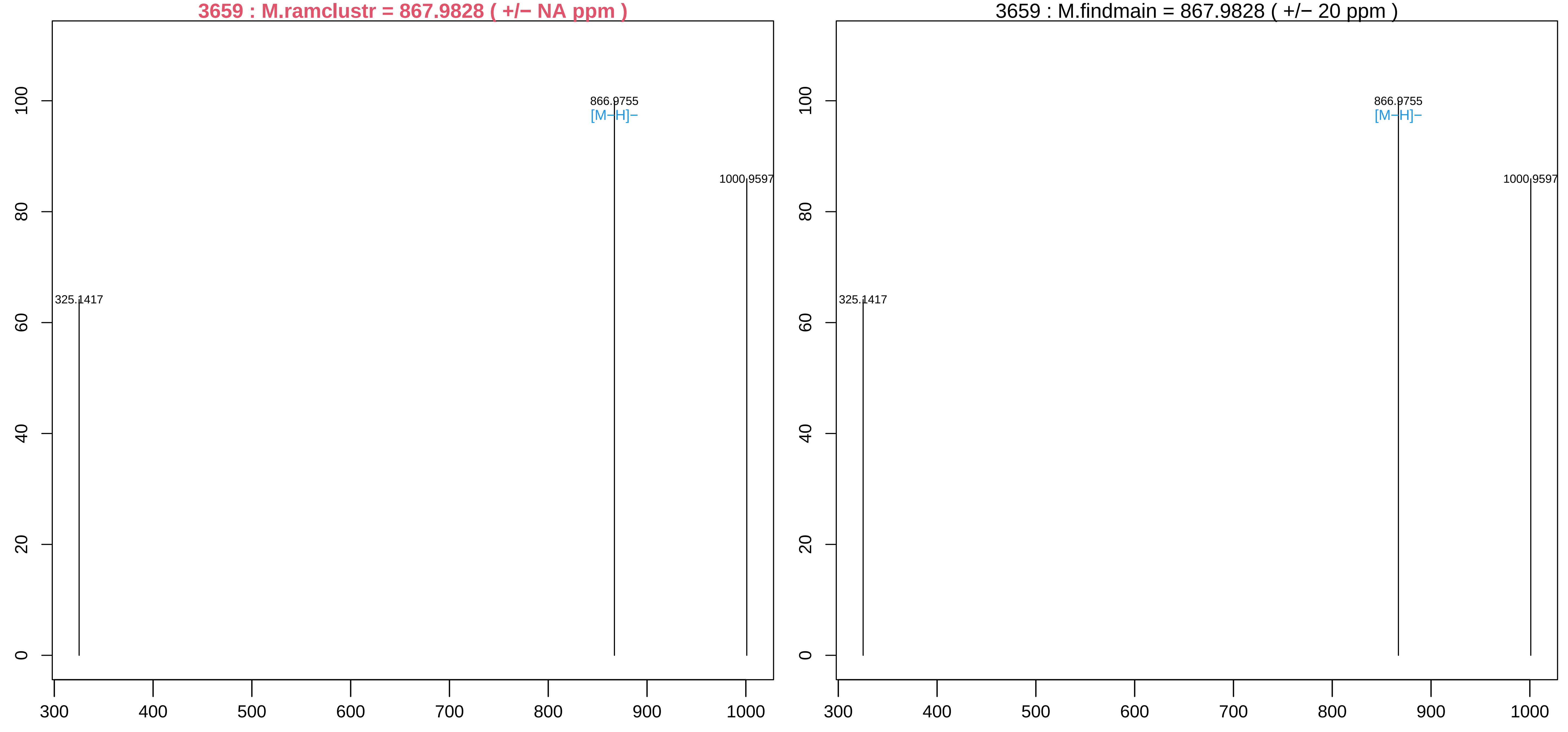
3659 : M.ramclustr = 867.9828 (+/− NA ppm)
866.9755
[M−H]−
1000.9597
325.1417
0
20
40
60
80
100
300
400
500
600
700
800
900
1000
3659 : M.findmain = 867.9828 (+/− 20 ppm)
866.9755
[M−H]−
1000.9597
325.1417
0
20
40
60
80
100
300
400
500
600
700
800
900
1000

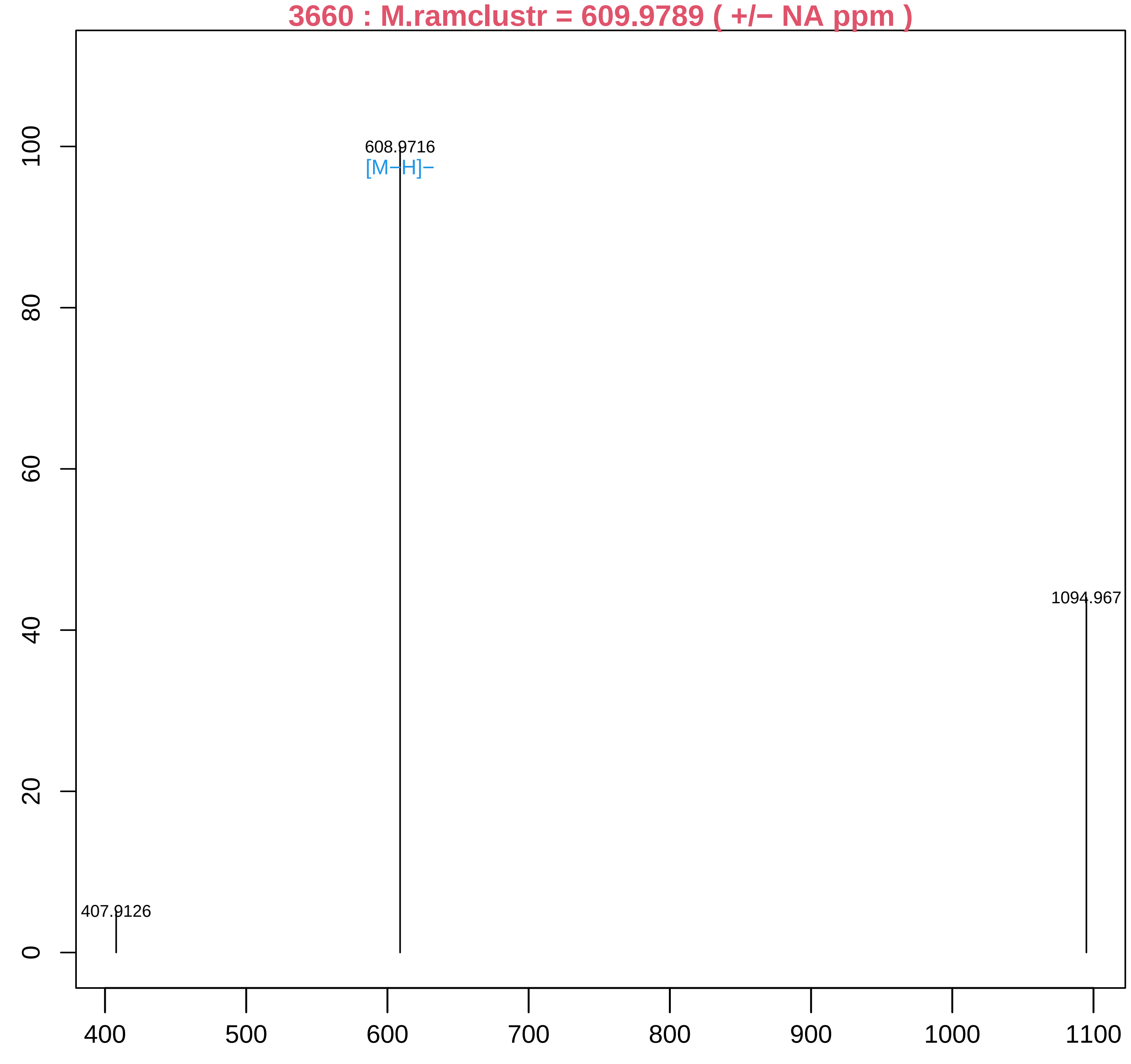
3660 : M.ramclustr = 609.9789 (+/− NA ppm)
608.9716
[M−H]−
1094.967
407.9126
0
20
40
60
80
100
400
500
600
700
800
900
1000
1100

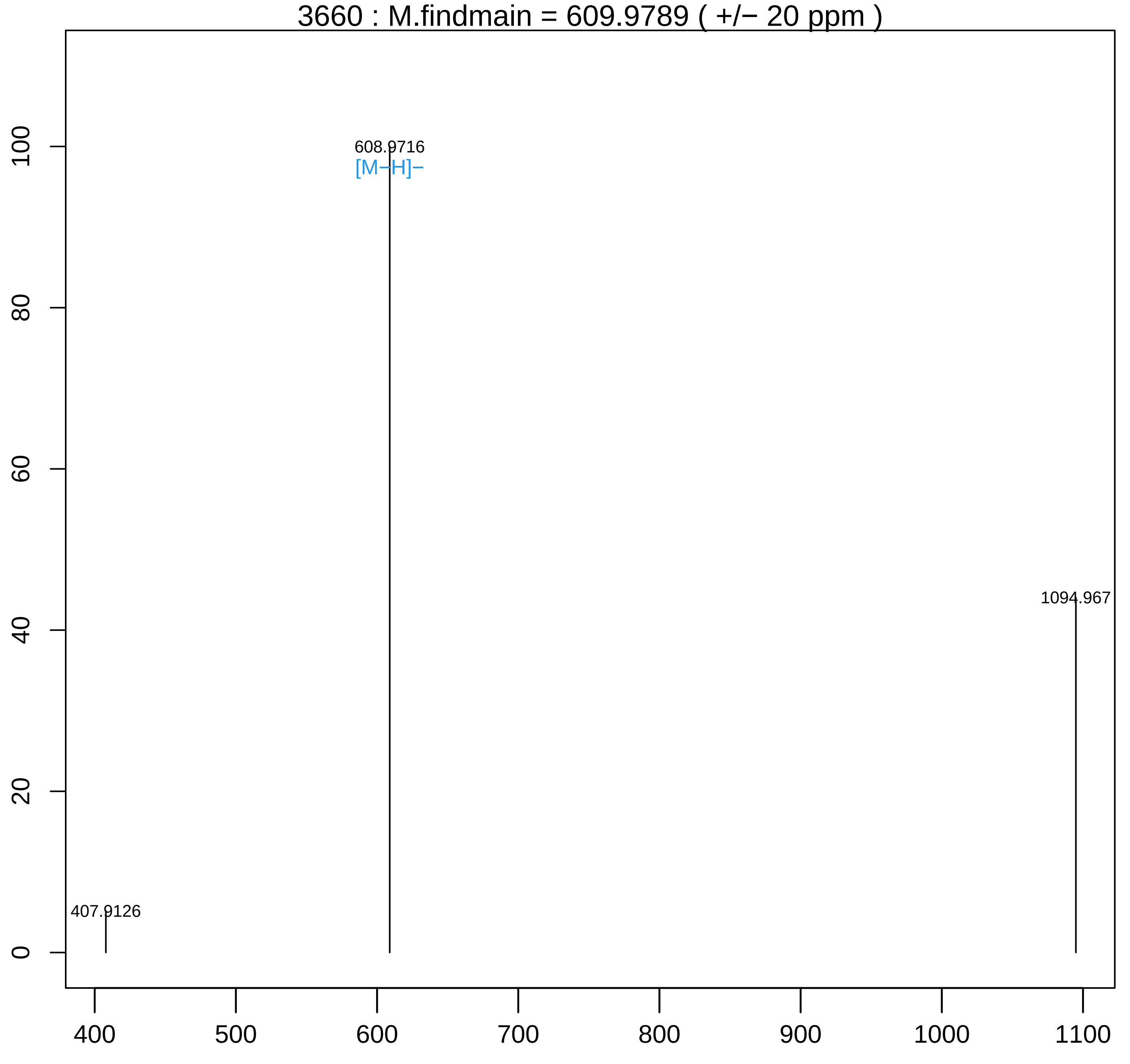
3660 : M.findmain = 609.9789 (+/− 20 ppm)
608.9716
[M−H]−
1094.967
407.9126
0
20
40
60
80
100
400
500
600
700
800
900
1000
1100

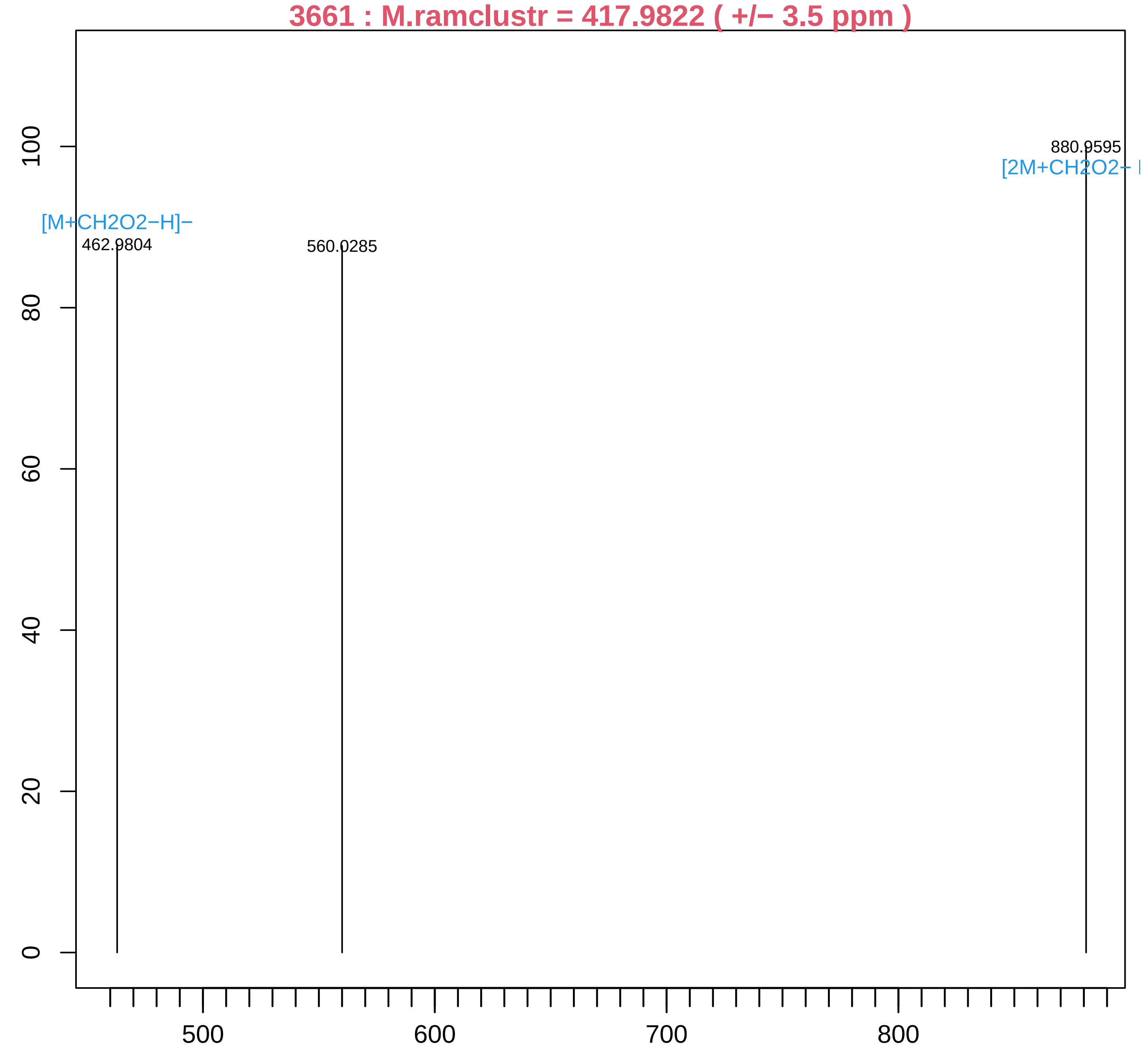
3661 : M.ramclustr = 417.9822 (+/− 3.5 ppm)
880.9595
[2M+CH2O2−
[M+CH2O2−H]−
462.9804
560.0285
0
20
40
60
80
100
500
600
700
800

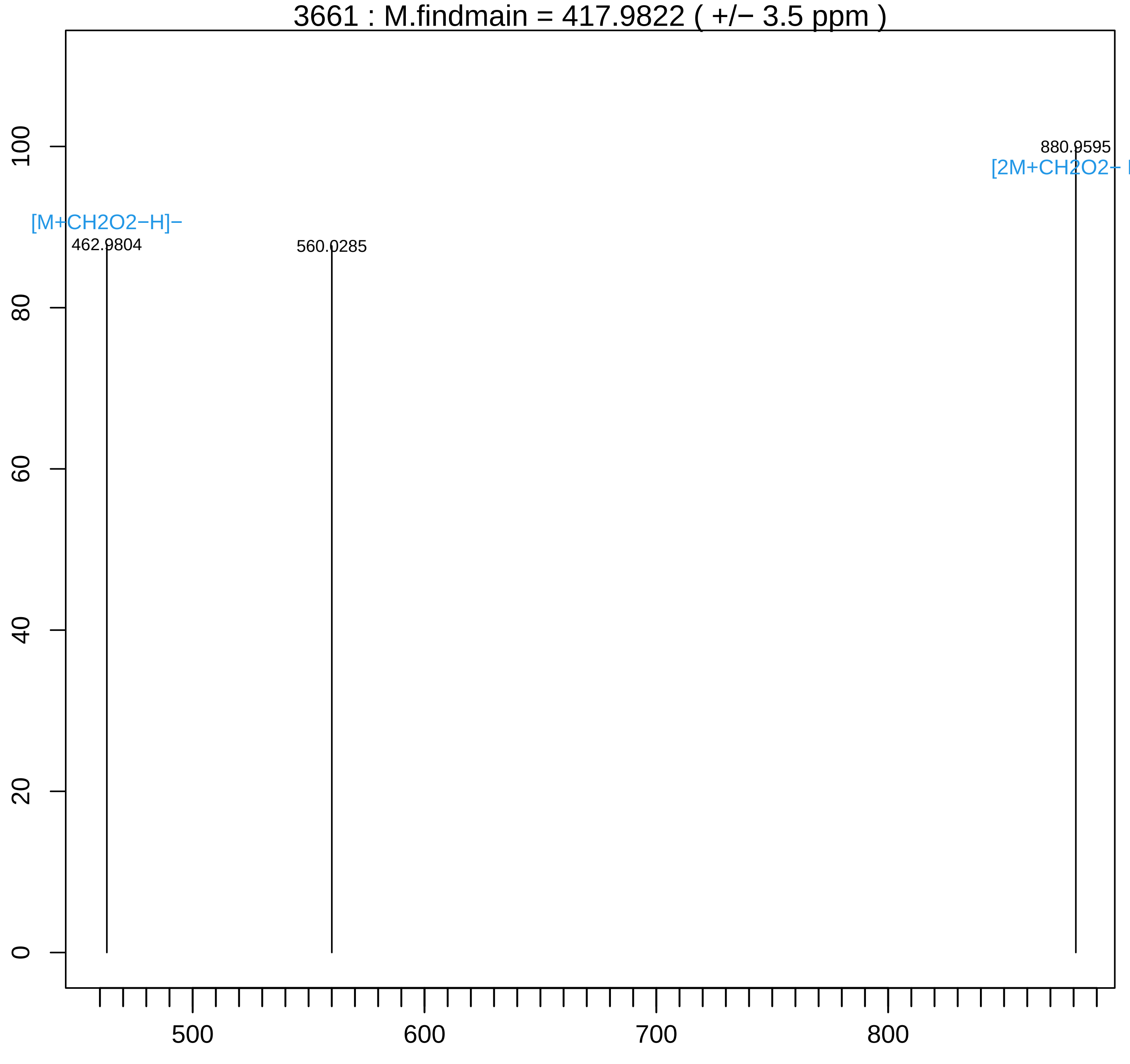
3661 : M.findmain = 417.9822 (+/− 3.5 ppm)
880.9595
[2M+CH2O2−
[M+CH2O2−H]−
462.9804
560.0285
0
20
40
60
80
100
500
600
700
800

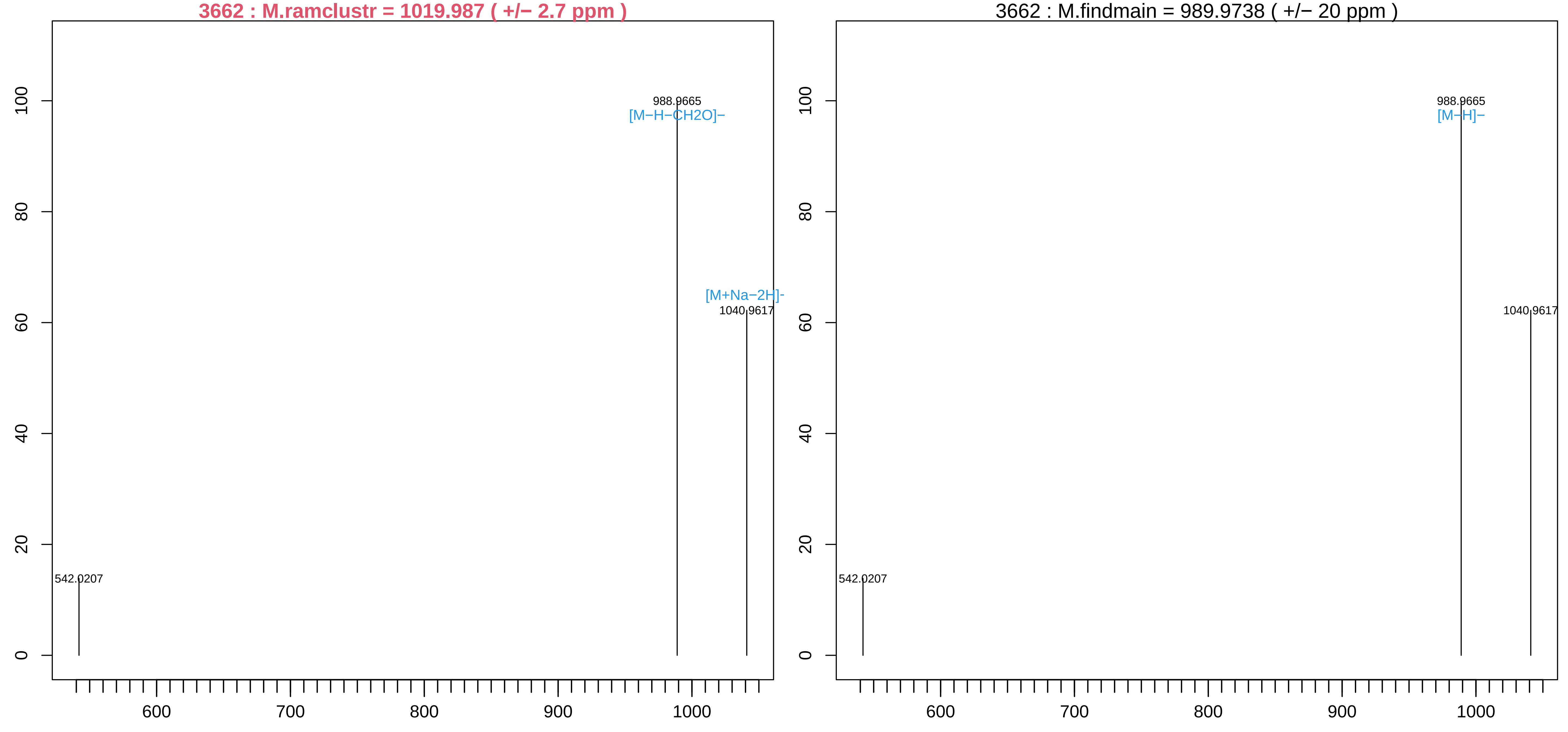
3662 : M.ramclustr = 1019.987 (+/− 2.7 ppm)
988.9665
[M−H−CH2O]−
[M+Na−2H]-
1040.9617
542.0207
3662 : M.findmain = 989.9738 (+/− 20 ppm)
988.9665
[M−H]−
1040.9617
542.0207
0
20
40
60
80
100
600
700
800
900
1000

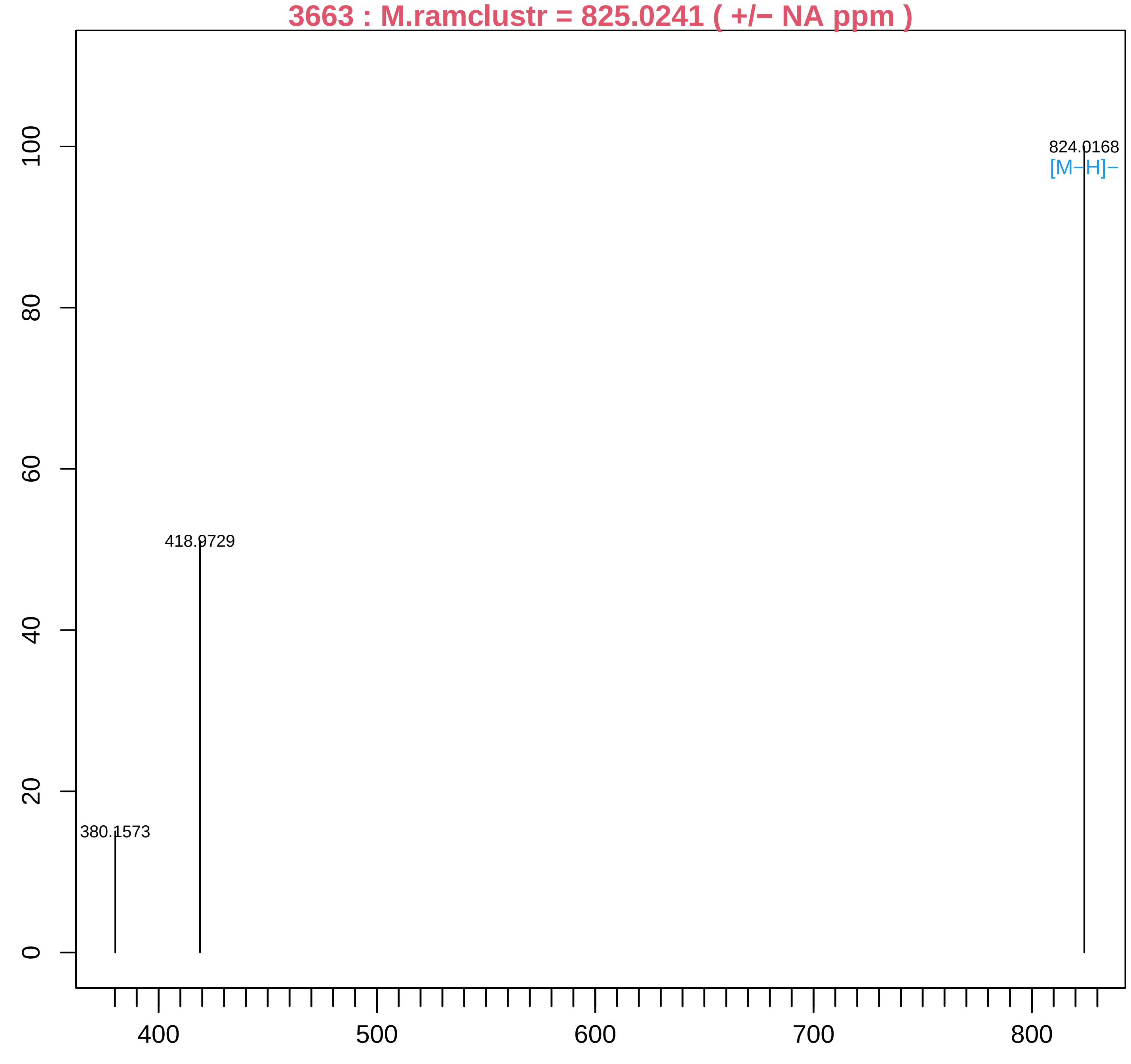
3663 : M.ramclustr = 825.0241 (+/− NA ppm)
824.0168
[M−H]−
418.9729
380.1573
0
20
40
60
80
100
400
500
600
700
800

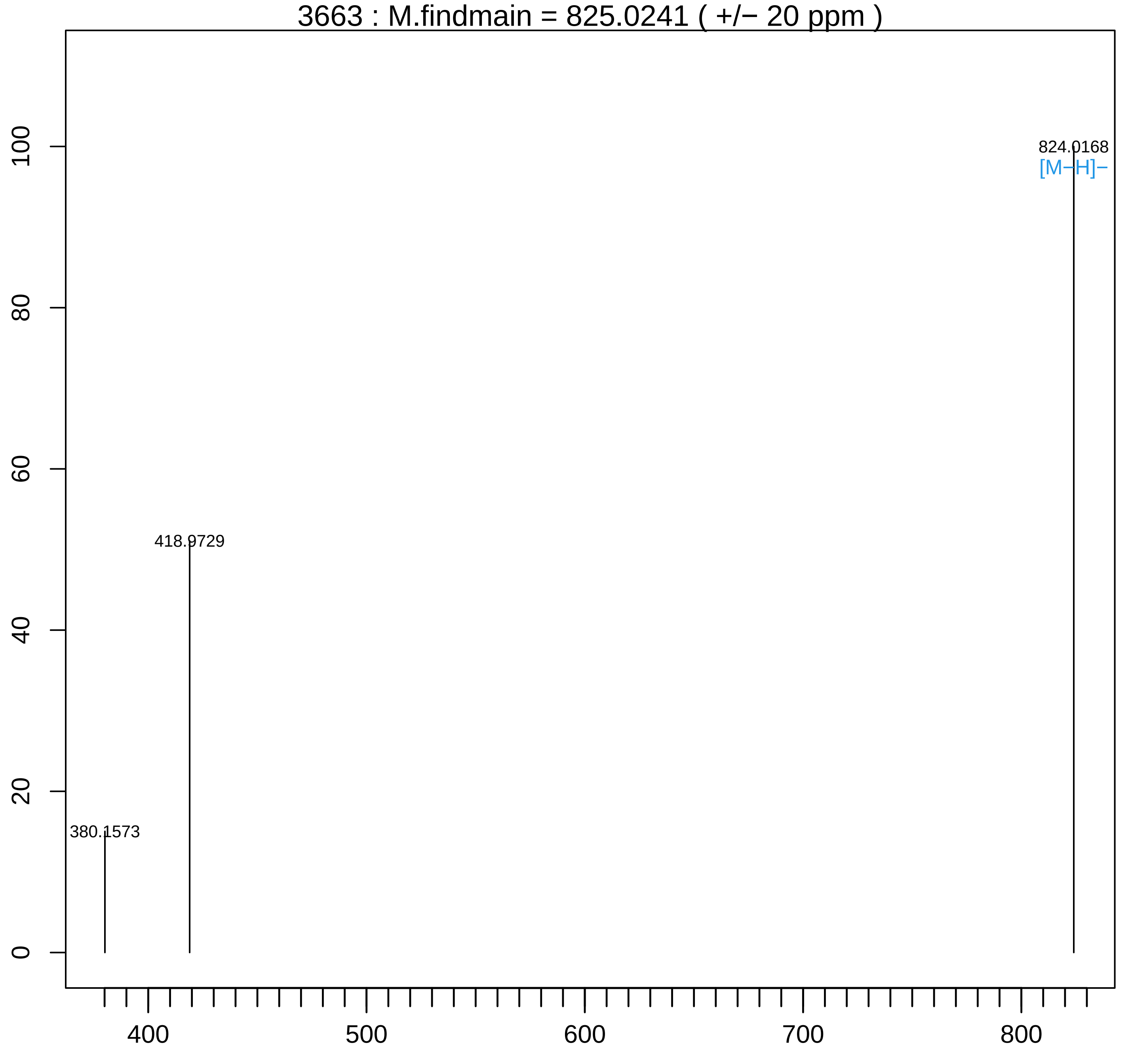
3663 : M.findmain = 825.0241 (+/− 20 ppm)
824.0168
[M−H]−
418.9729
380.1573
0
20
40
60
80
100
400
500
600
700
800

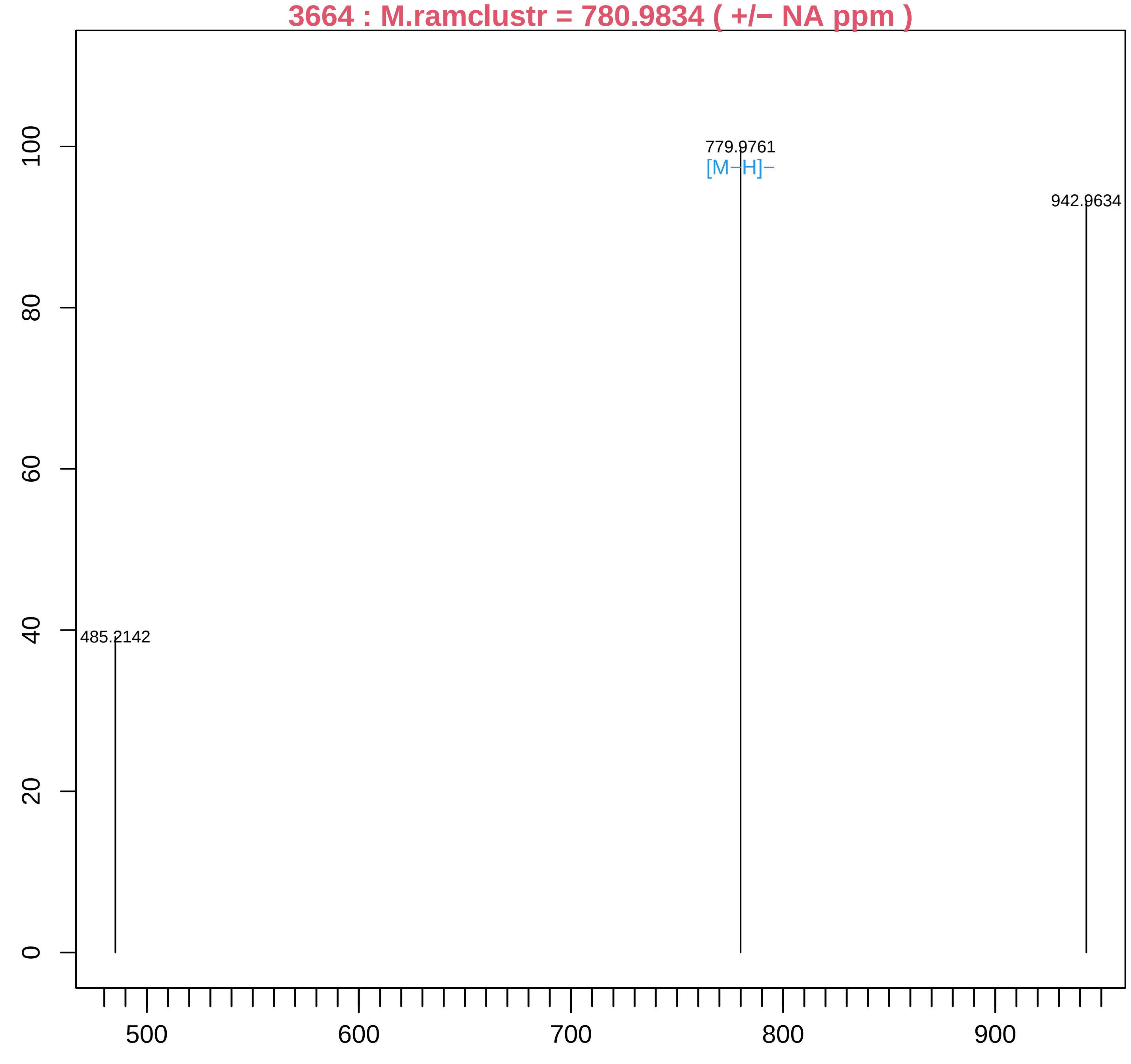

3664 : M.ramclustr = 780.9834 (+/− NA ppm)
779.9761
[M−H]−
942.9634
485.2142
0
20
40
60
80
100
500
600
700
800
900

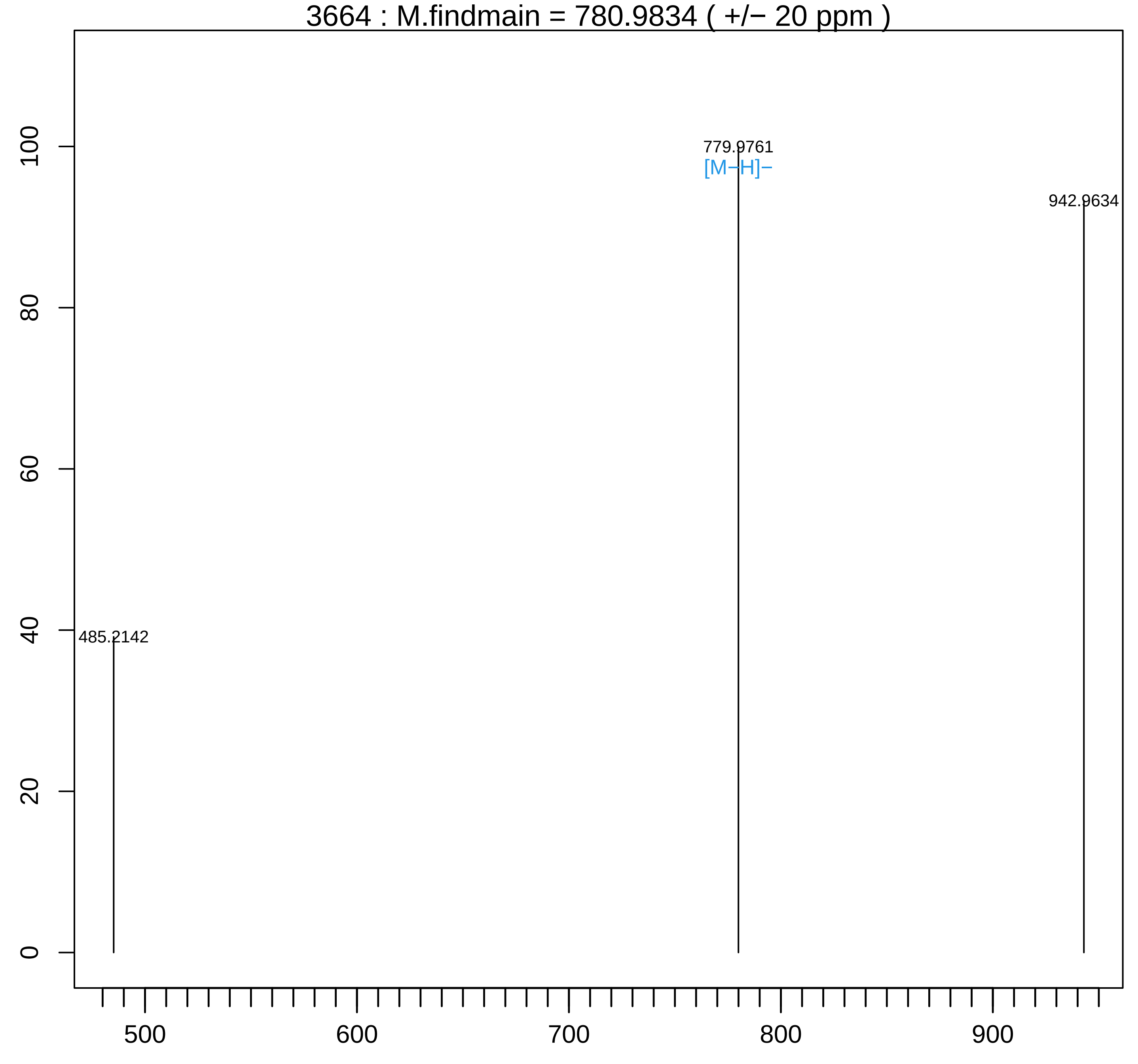

3664 : M.findmain = 780.9834 (+/− 20 ppm)
779.9761
[M−H]−
942.9634
485.2142
0
20
40
60
80
100
500
600
700
800
900

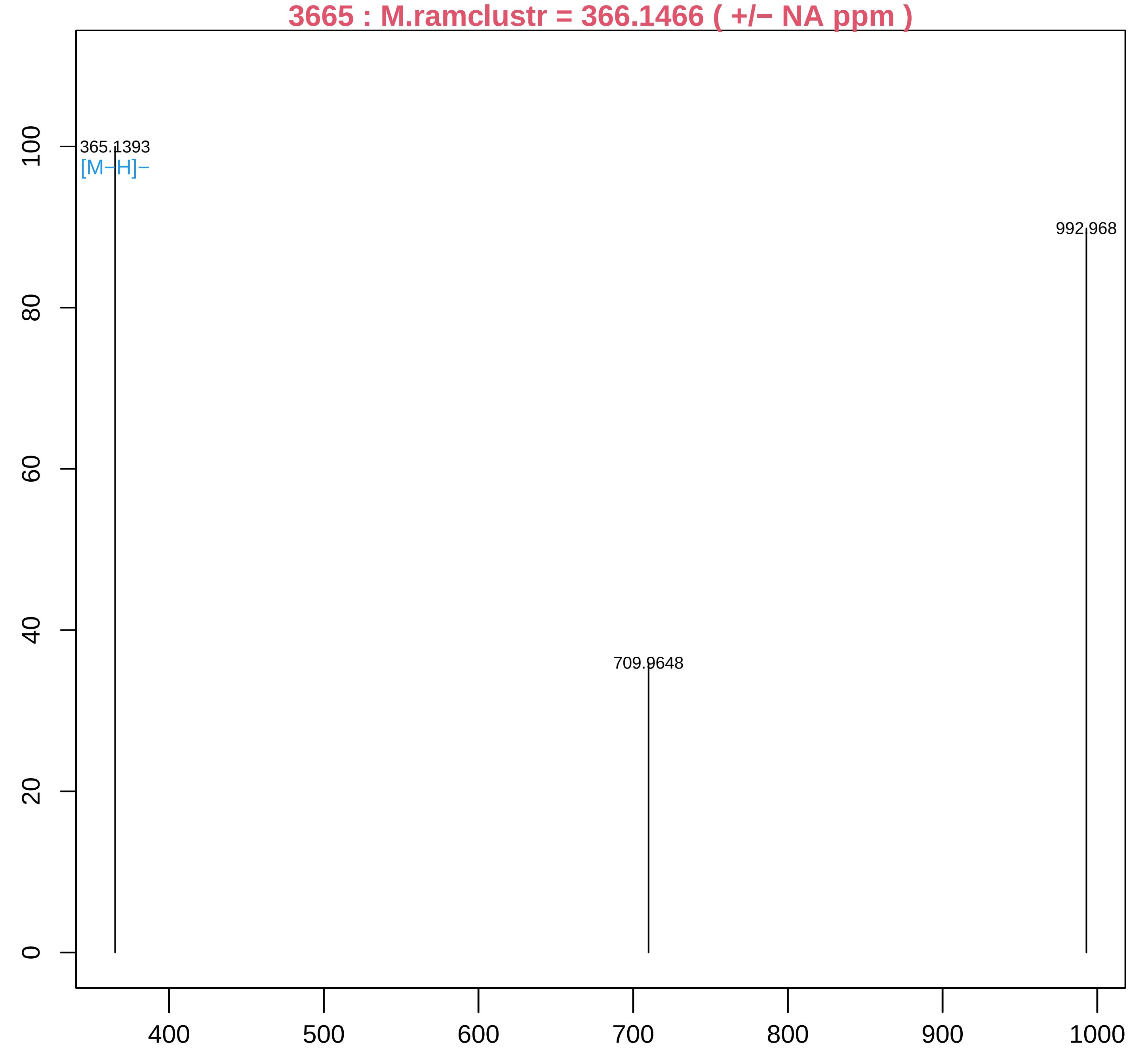
3665 : M.ramclustr = 366.1466 (+/− NA ppm)
365.1393
[M−H]−
709.9648
992.968
0
20
40
60
80
100
400
500
600
700
800
900
1000

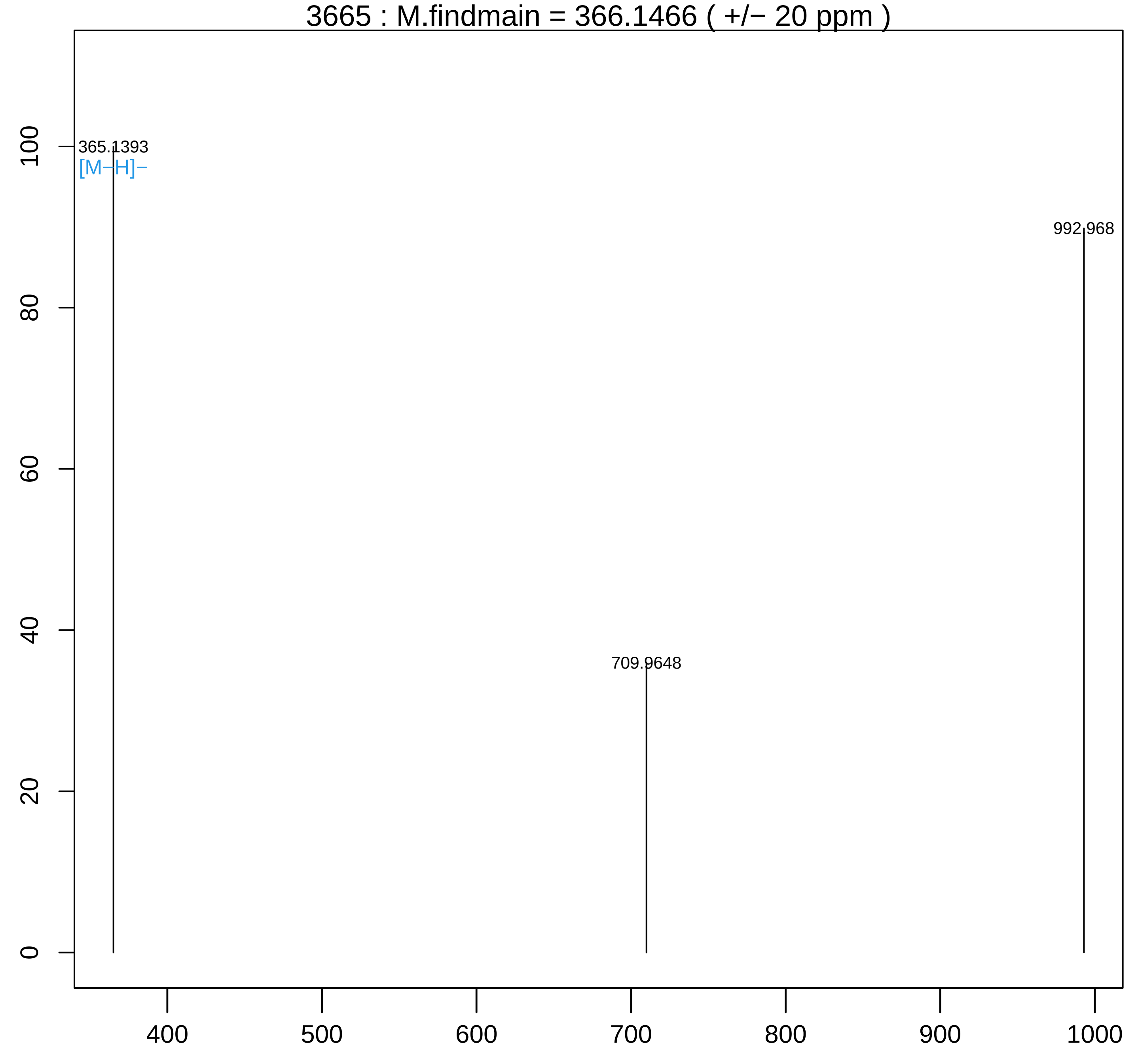
3665 : M.findmain = 366.1466 (+/− 20 ppm)
365.1393
[M−H]−
709.9648
992.968
0
20
40
60
80
100
400
500
600
700
800
900
1000

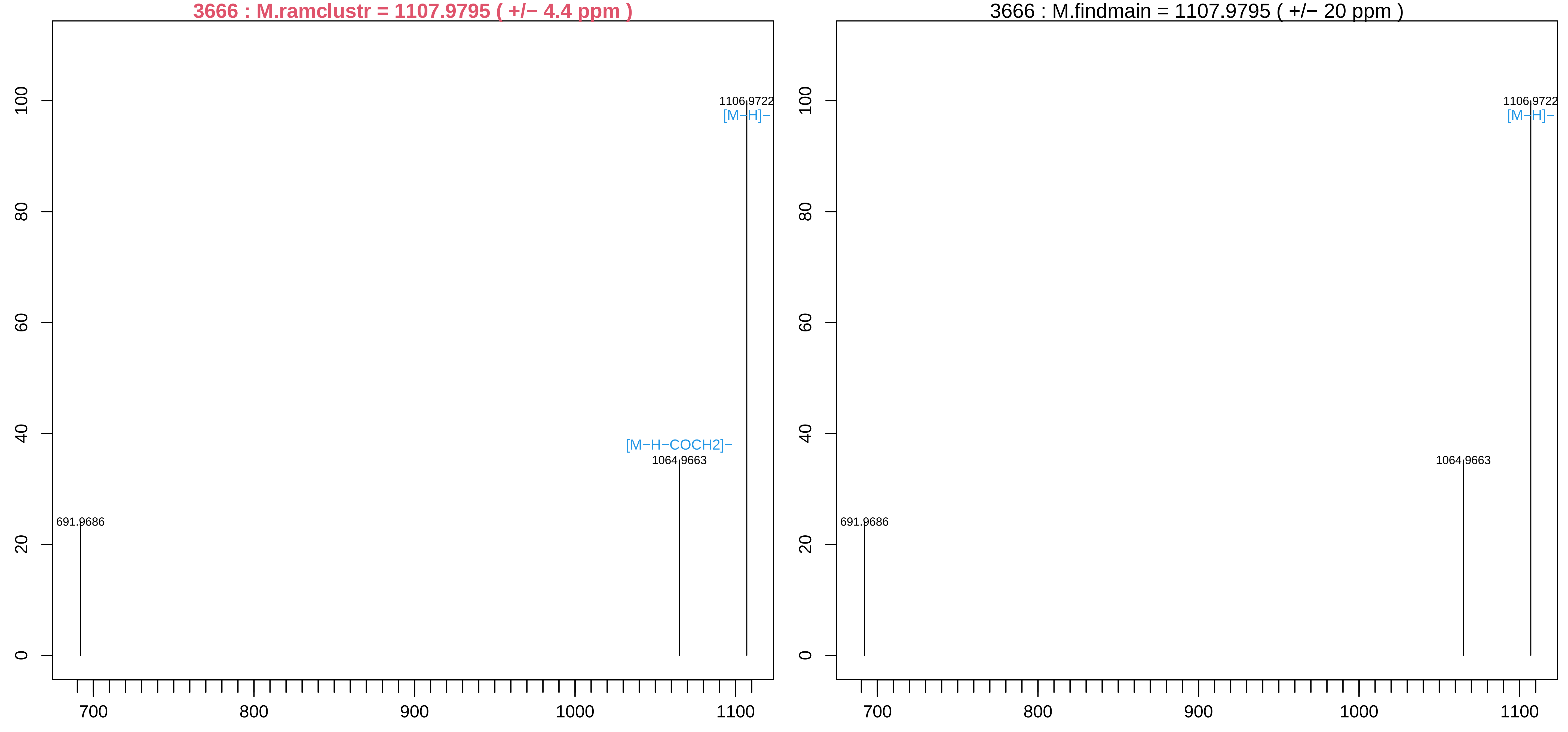

3666 : M.ramclustr = 1107.9795 (+/− 4.4 ppm)
1106.9722
[M−H]−
[M−H−COCH2]−
1064.9663
691.9686
0
20
40
60
80
100
700
800
900
1000
1100
3666 : M.findmain = 1107.9795 (+/− 20 ppm)
1106.9722
[M−H]−
1064.9663
691.9686
0
20
40
60
80
100
700
800
900
1000
1100

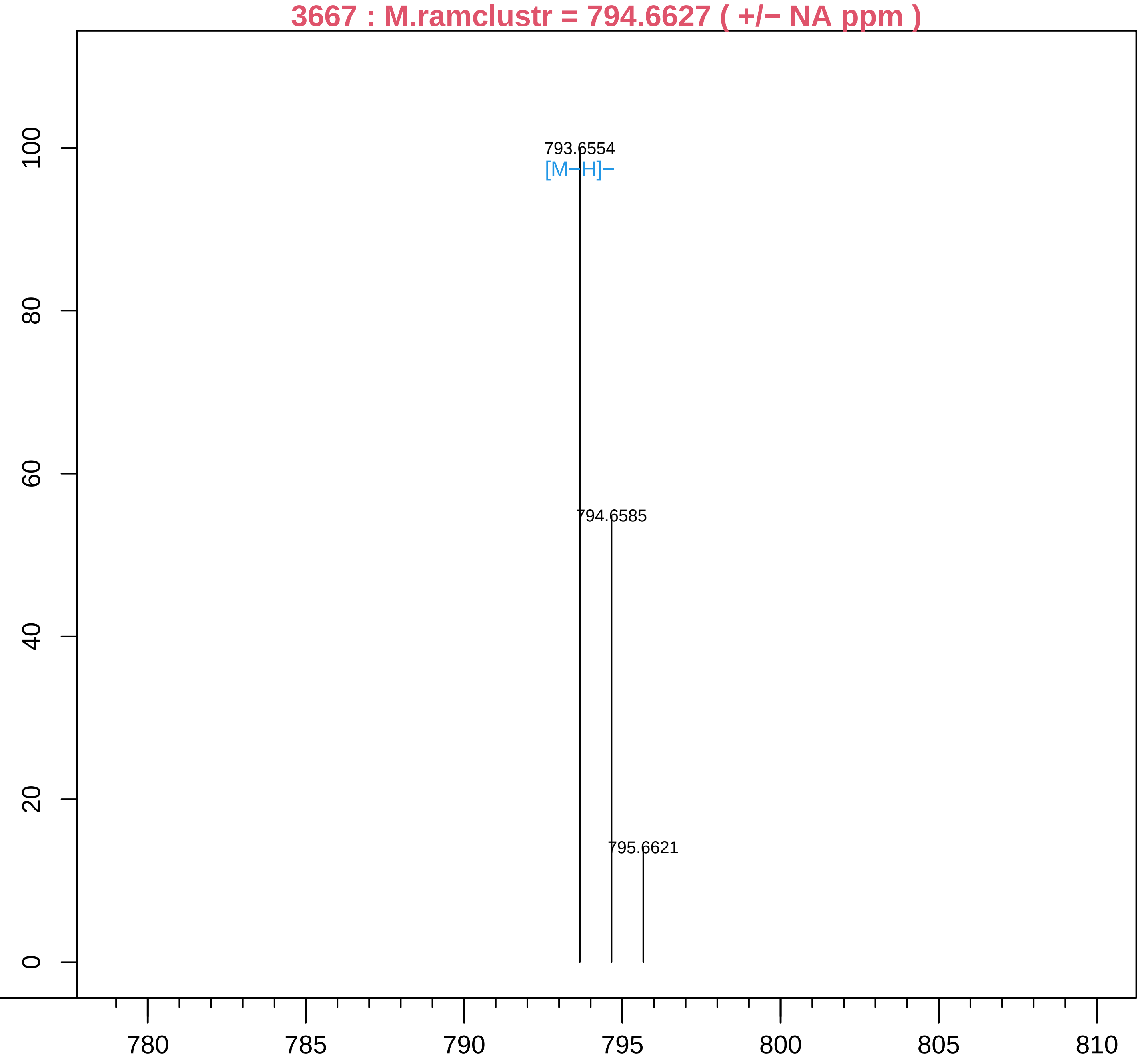
3667 : M.ramclustr = 794.6627 (+/− NA ppm)
793.6554
[M−H]−
794.6585
795.6621
0
20
40
60
80
100
780
785
790
795
800
805
810

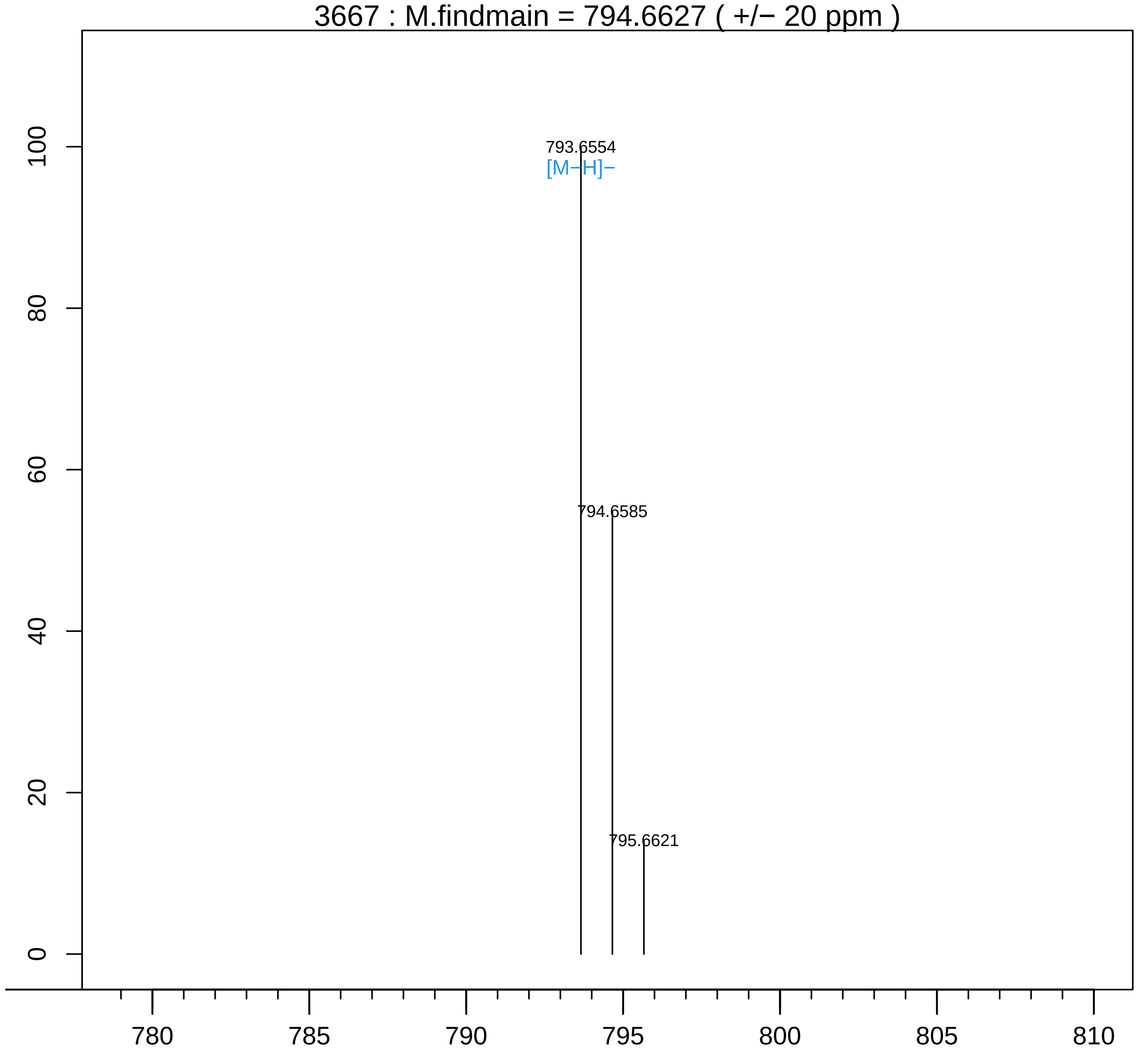
3667 : M.findmain = 794.6627 (+/− 20 ppm)
793.6554
[M−H]−
794.6585
795.6621
0
20
40
60
80
100
780
785
790
795
800
805
810

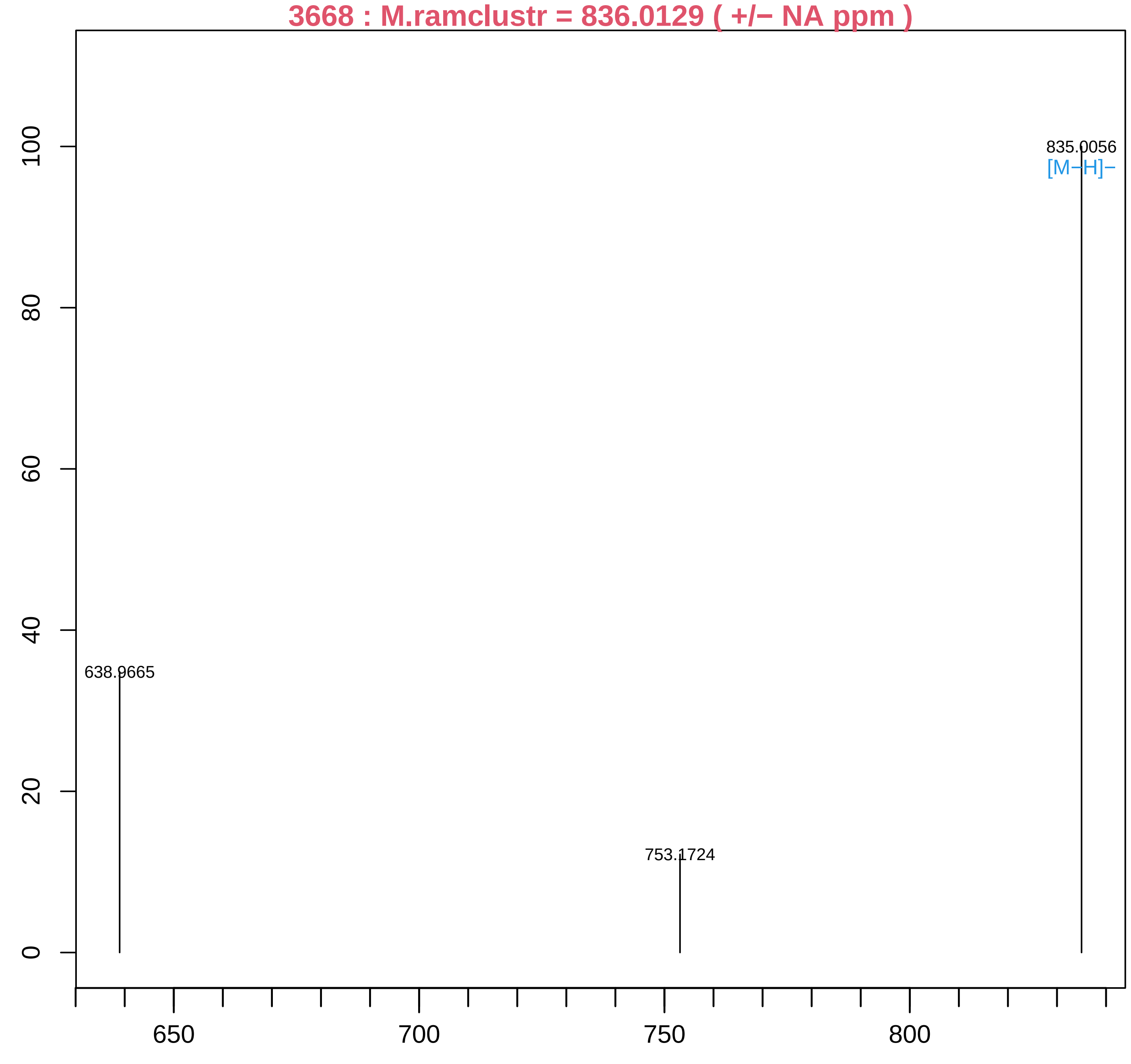
3668 : M.ramclustr = 836.0129 (+/− NA ppm)
835.0056
[M−H]−
638.9665
753.1724
0
20
40
60
80
100
650
700
750
800

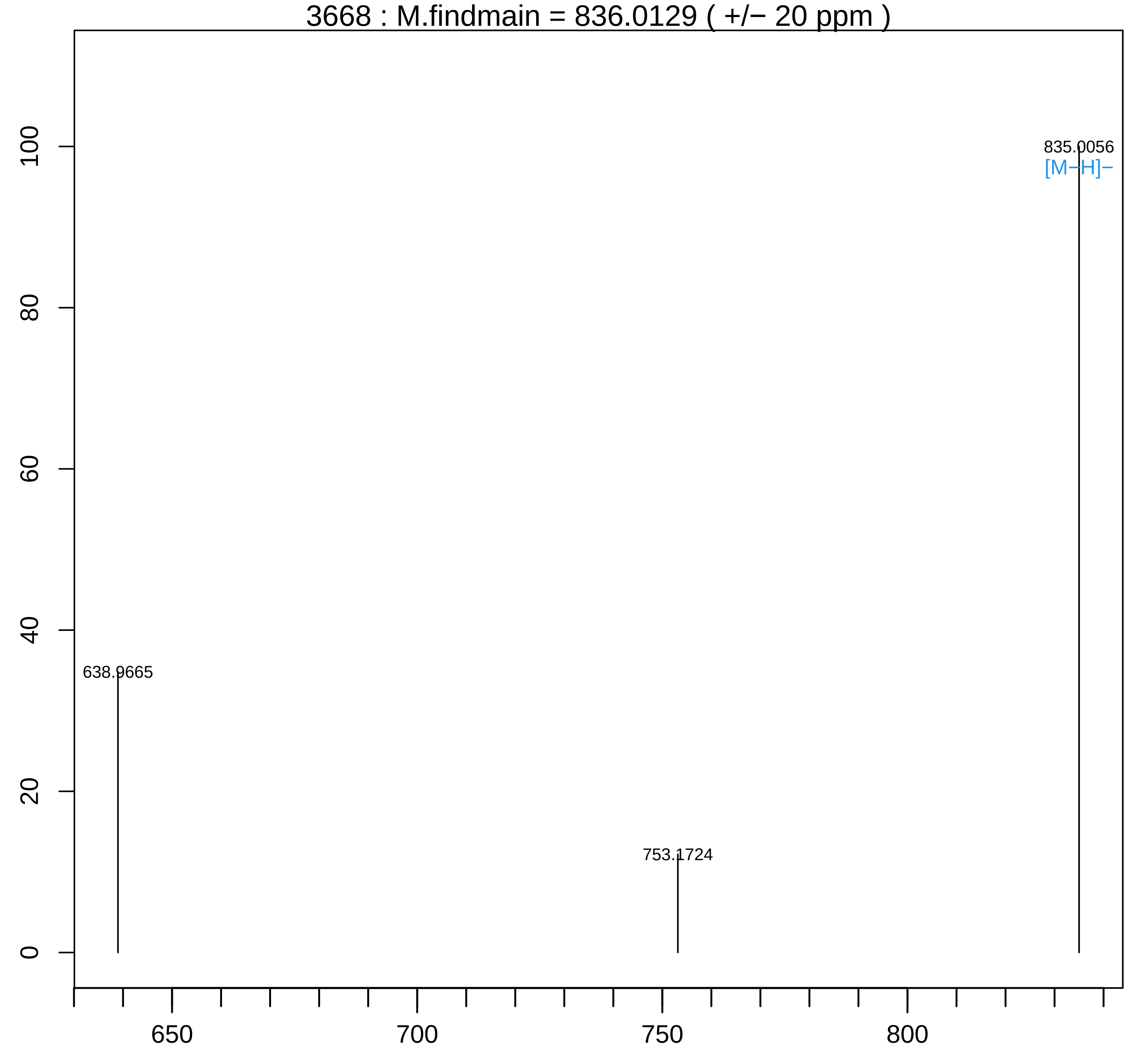
3668 : M.findmain = 836.0129 (+/− 20 ppm)
835.0056
[M−H]−
638.9665
753.1724
0
20
40
60
80
100
650
700
750
800

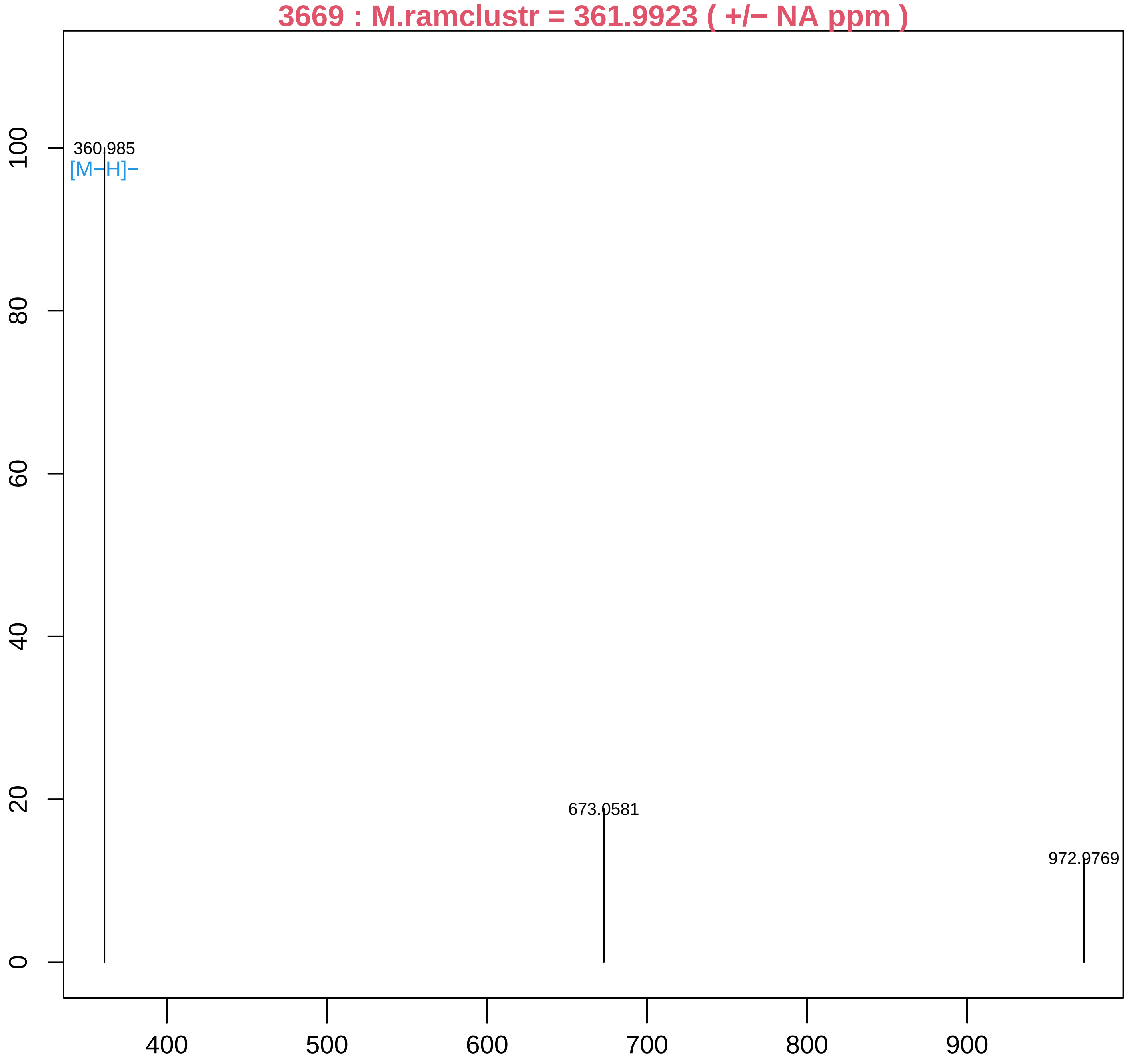
3669 : M.ramclustr = 361.9923 (+/− NA ppm)
360.985
[M−H]−
673.0581
972.9769
0
20
40
60
80
100
400
500
600
700
800
900

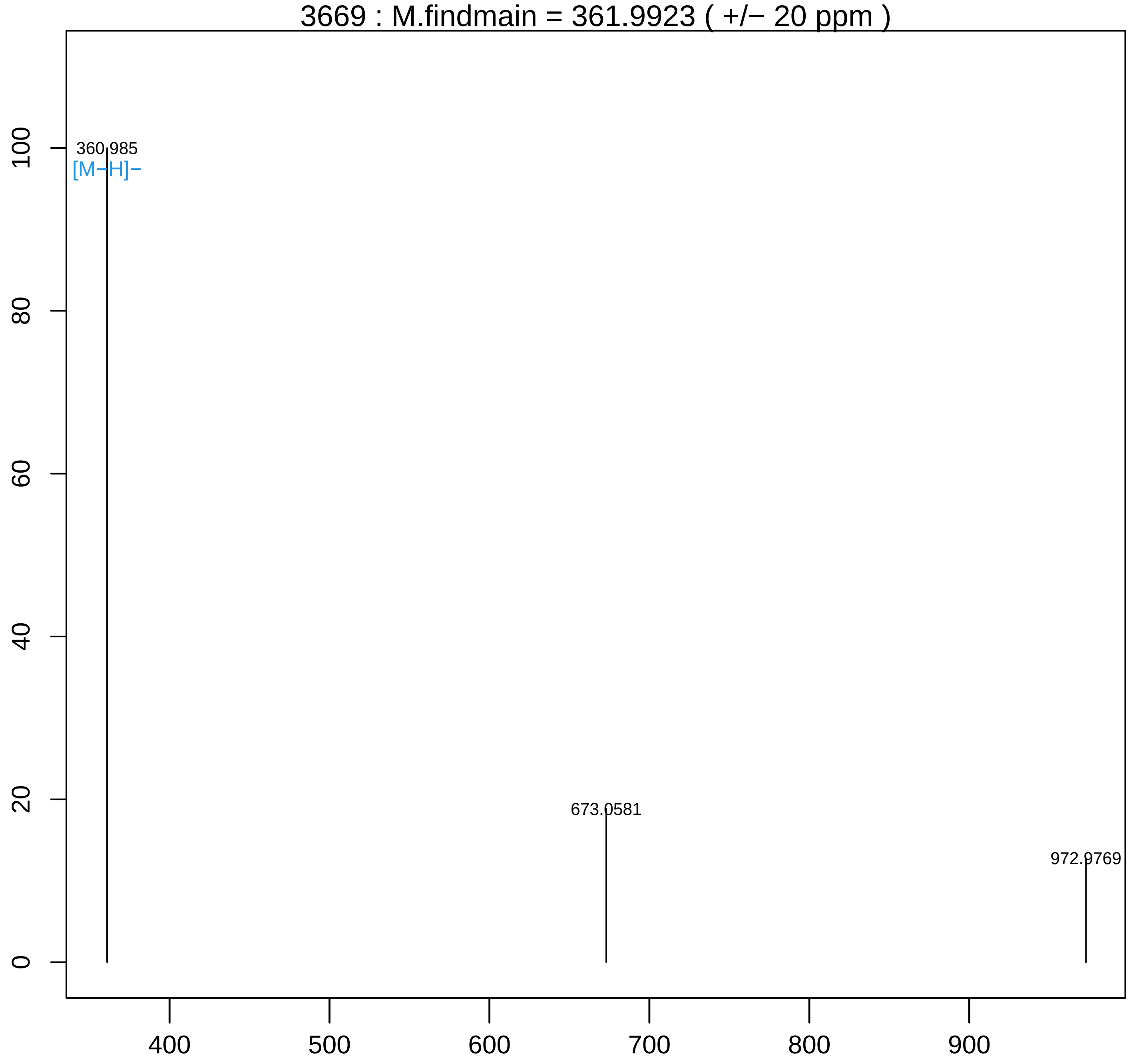
3669 : M.findmain = 361.9923 (+/− 20 ppm)
360.985
[M−H]−
673.0581
972.9769
0
20
40
60
80
100
400
500
600
700
800
900

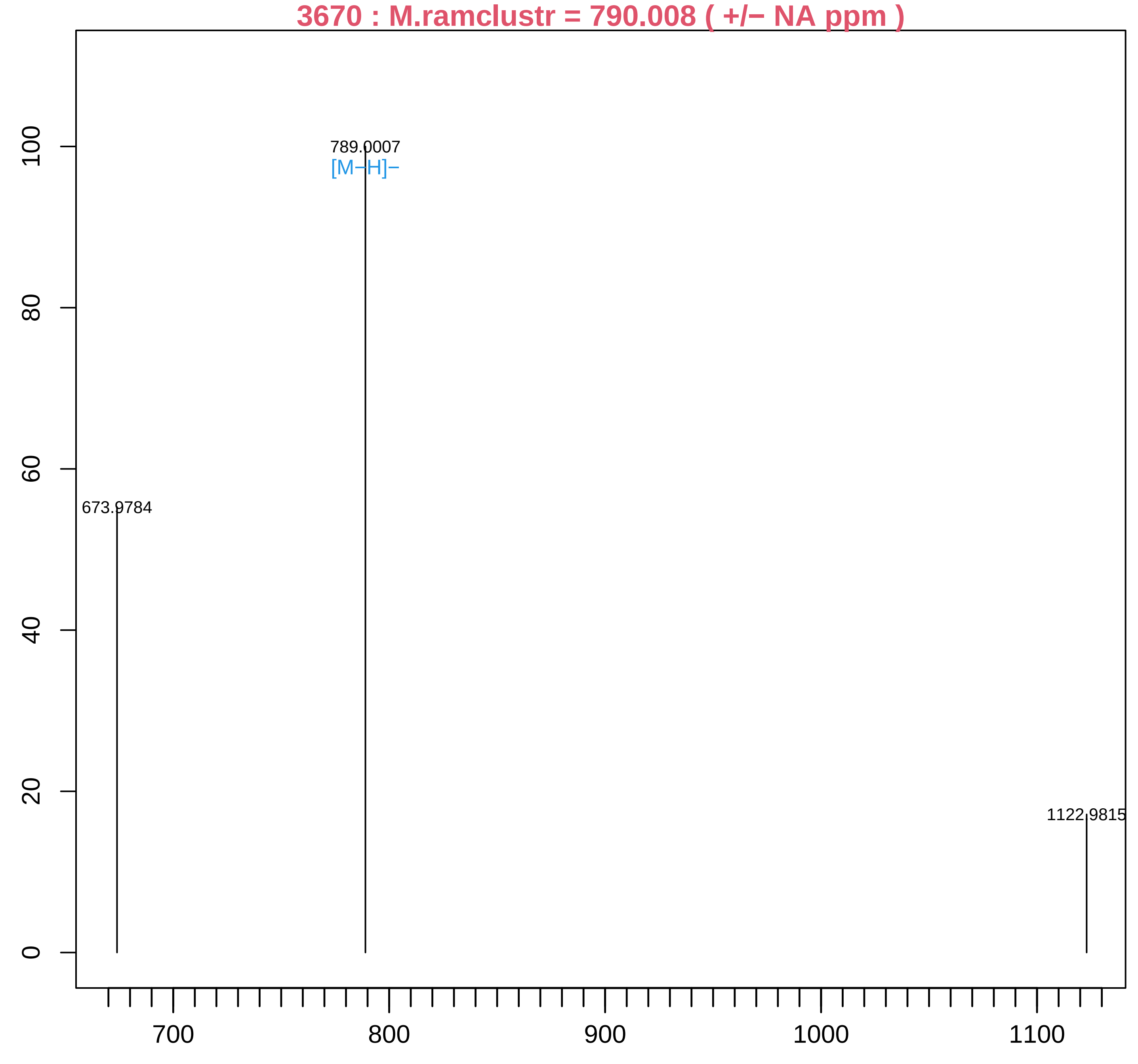

3670 : M.ramclustr = 790.008 (+/− NA ppm)
789.0007
[M−H]−
673.9784
1122.9815
0
20
40
60
80
100
700
800
900
1000
1100

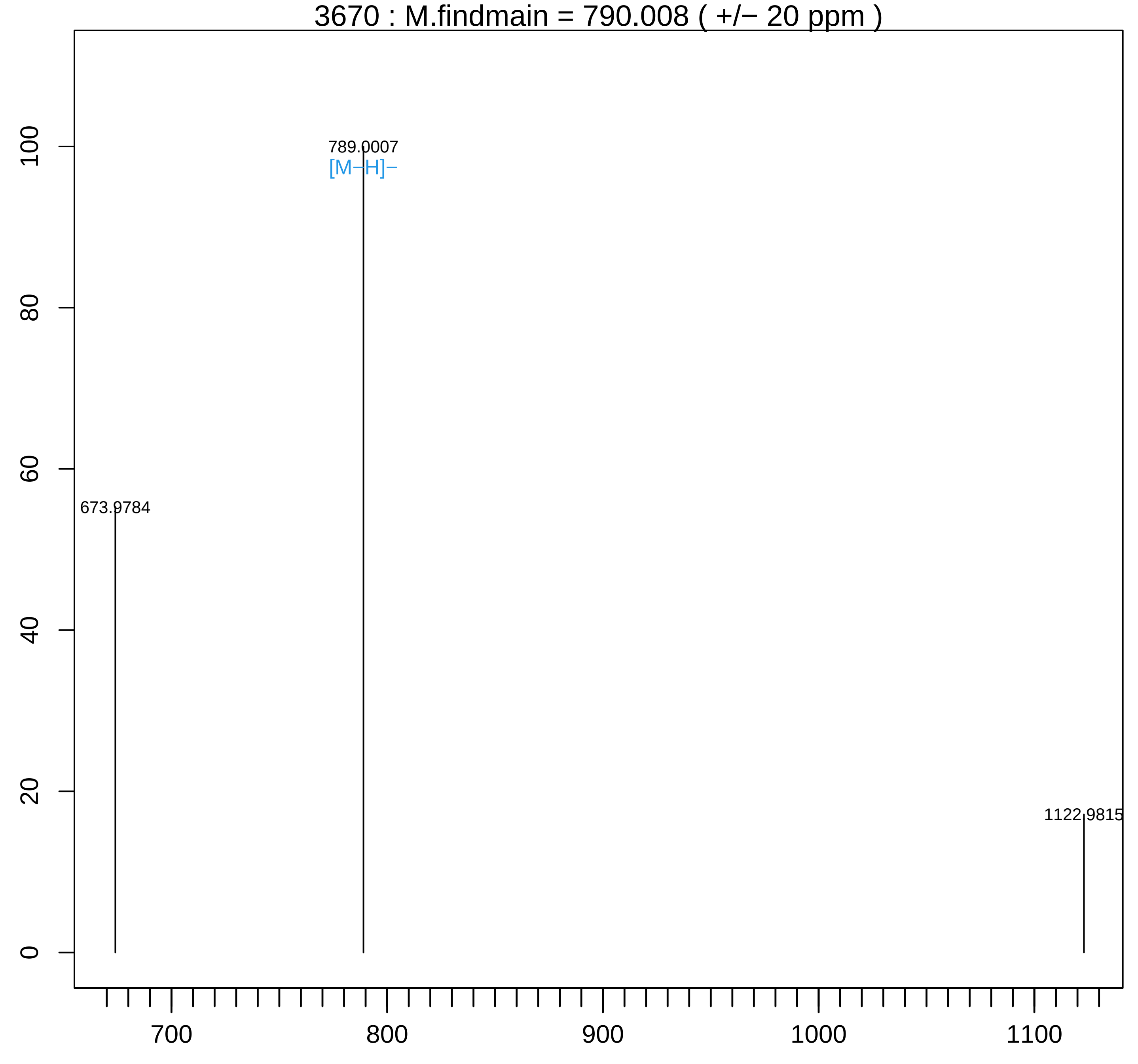

3670 : M.findmain = 790.008 (+/− 20 ppm)
789.0007
[M−H]−
673.9784
1122.9815
0
20
40
60
80
100
700
800
900
1000
1100

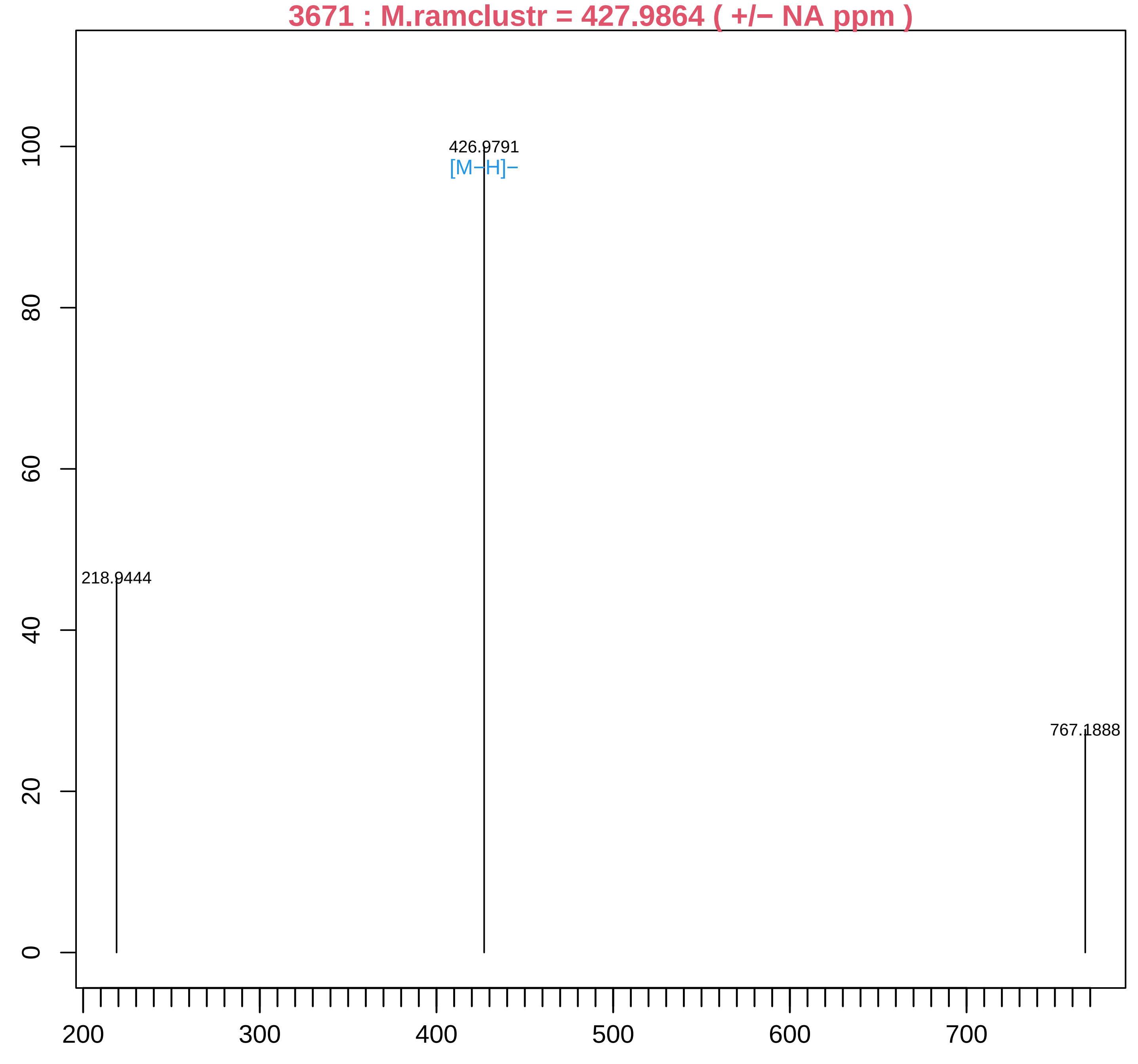
3671 : M.ramclustr = 427.9864 (+/− NA ppm)
426.9791
[M−H]−
218.9444
767.1888
0
20
40
60
80
100
200
300
400
500
600
700

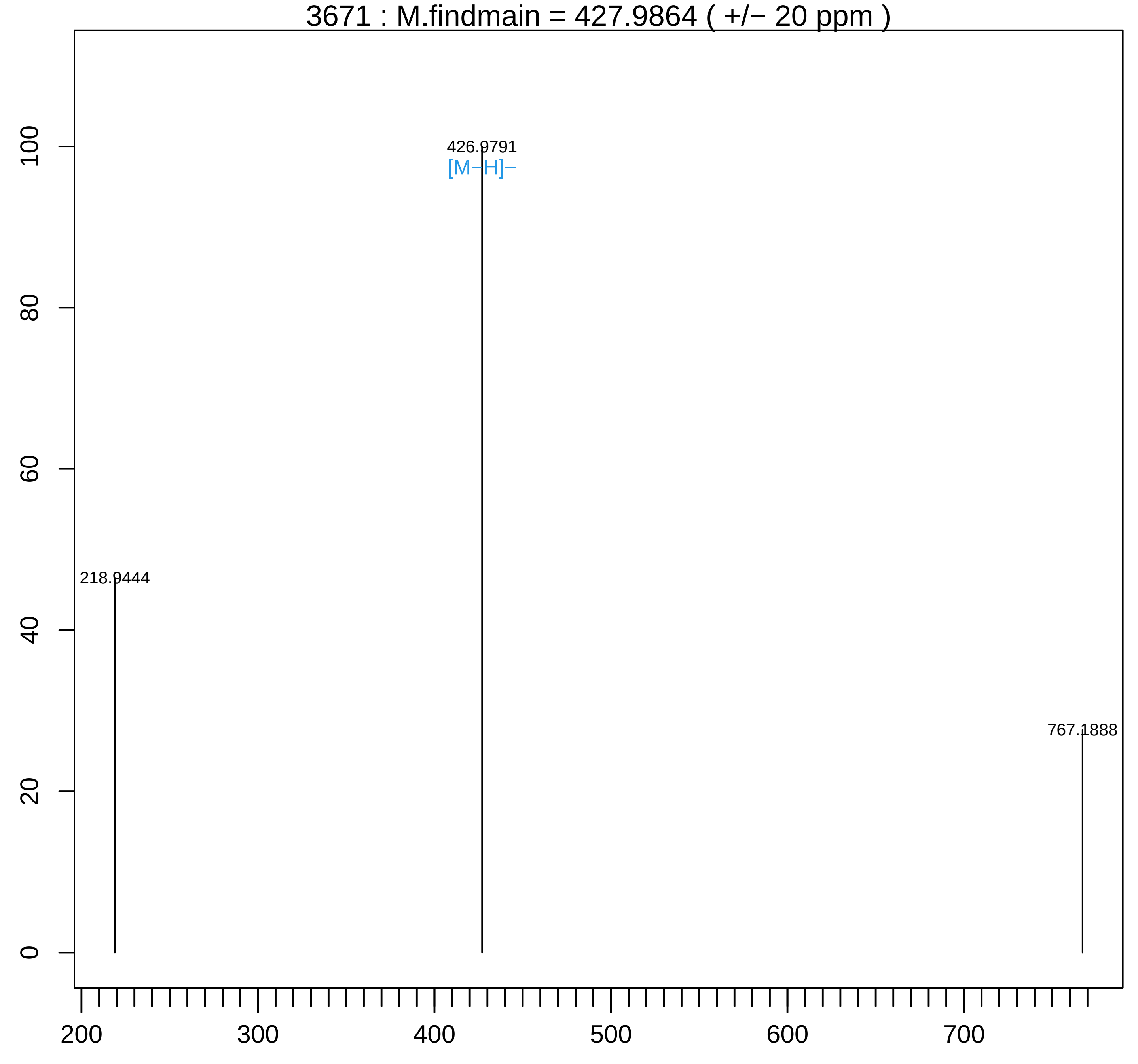
3671 : M.findmain = 427.9864 (+/− 20 ppm)
426.9791
[M−H]−
218.9444
767.1888
0
20
40
60
80
100
200
300
400
500
600
700

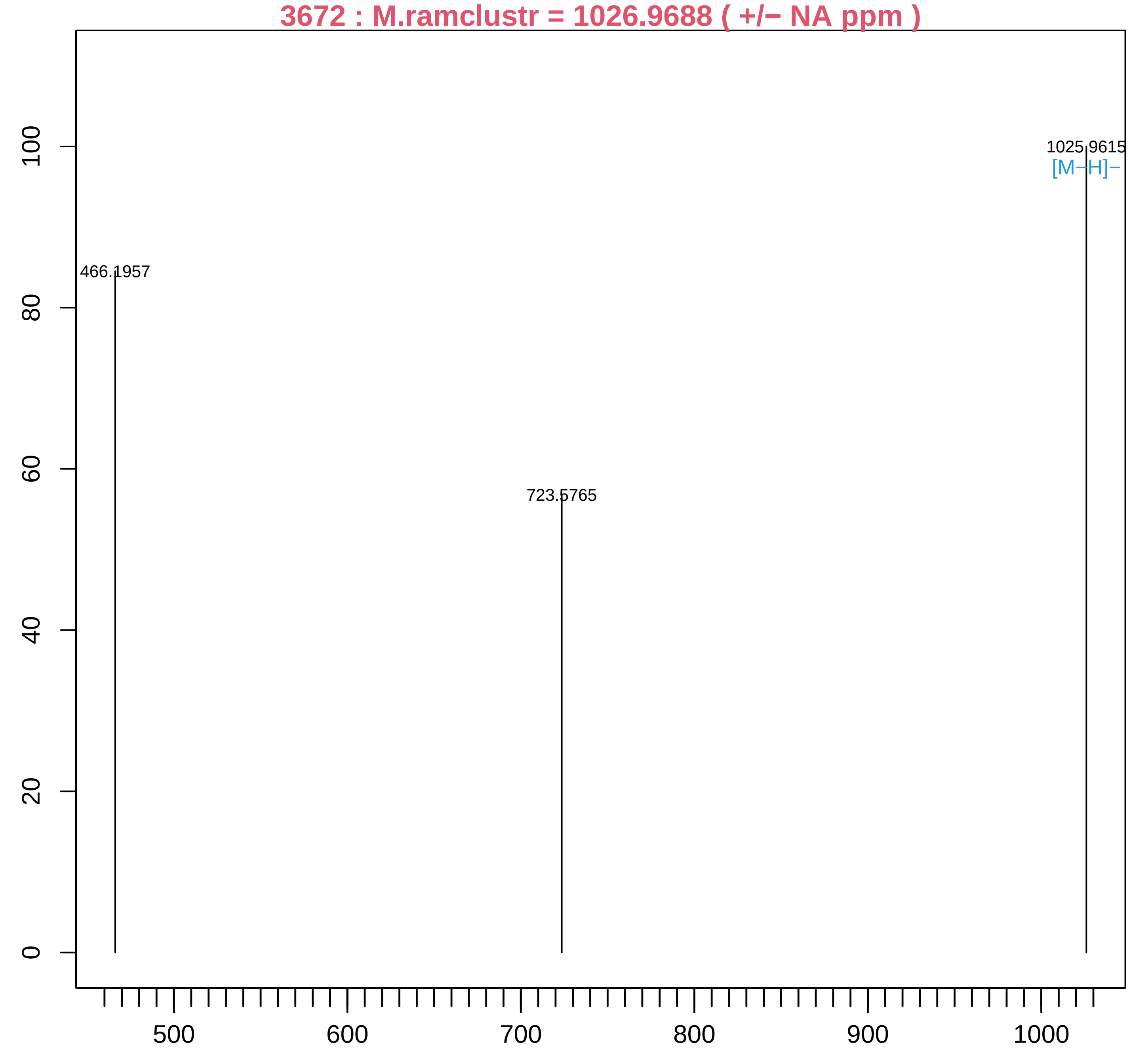

3672 : M.ramclustr = 1026.9688 (+/− NA ppm)
466.1957
723.5765
1025.9615
[M−H]−
0
20
40
60
80
100
500
600
700
800
900
1000

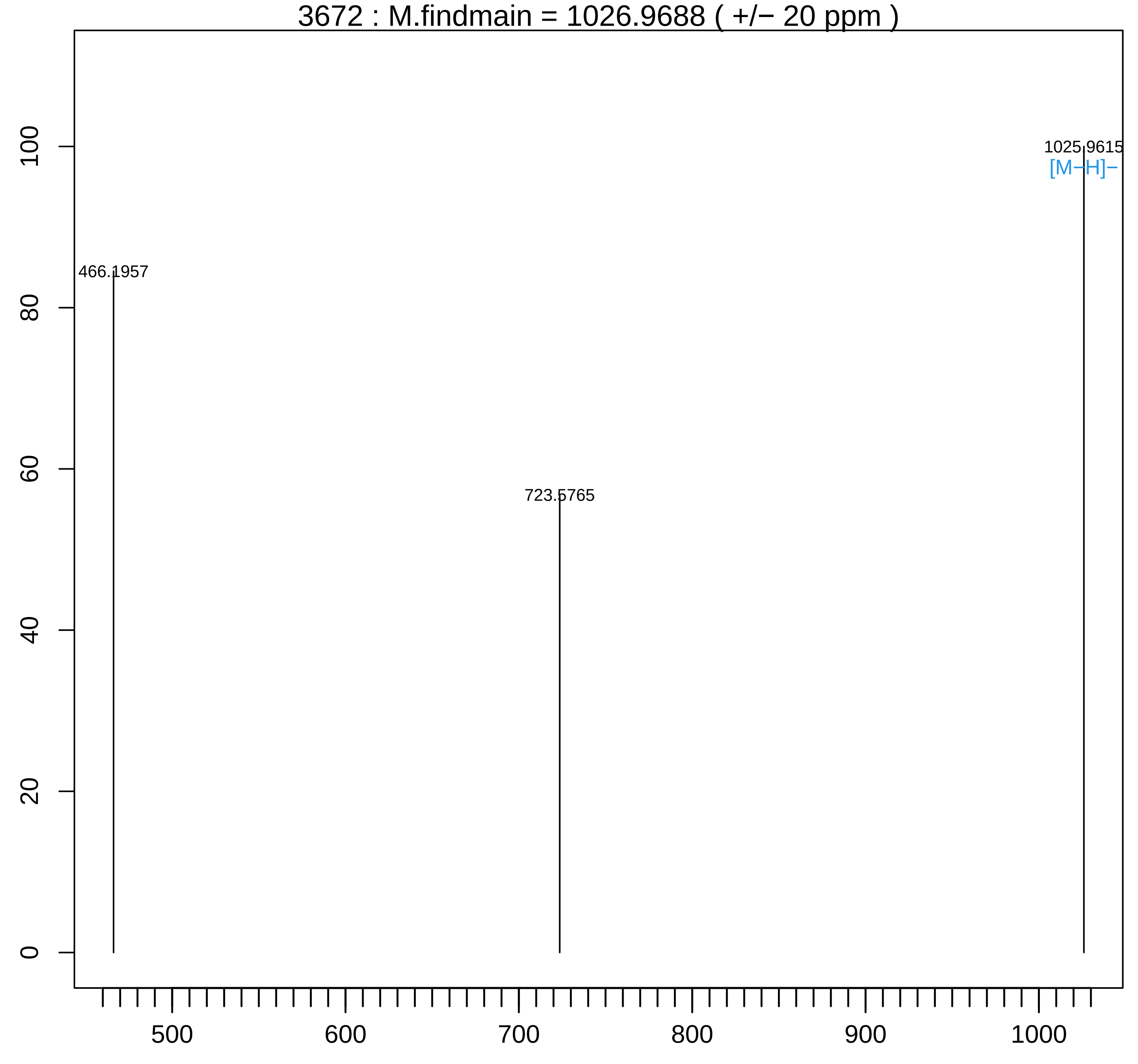

3672 : M.findmain = 1026.9688 (+/− 20 ppm)
466.1957
723.5765
1025.9615
[M−H]−
0
20
40
60
80
100
500
600
700
800
900
1000

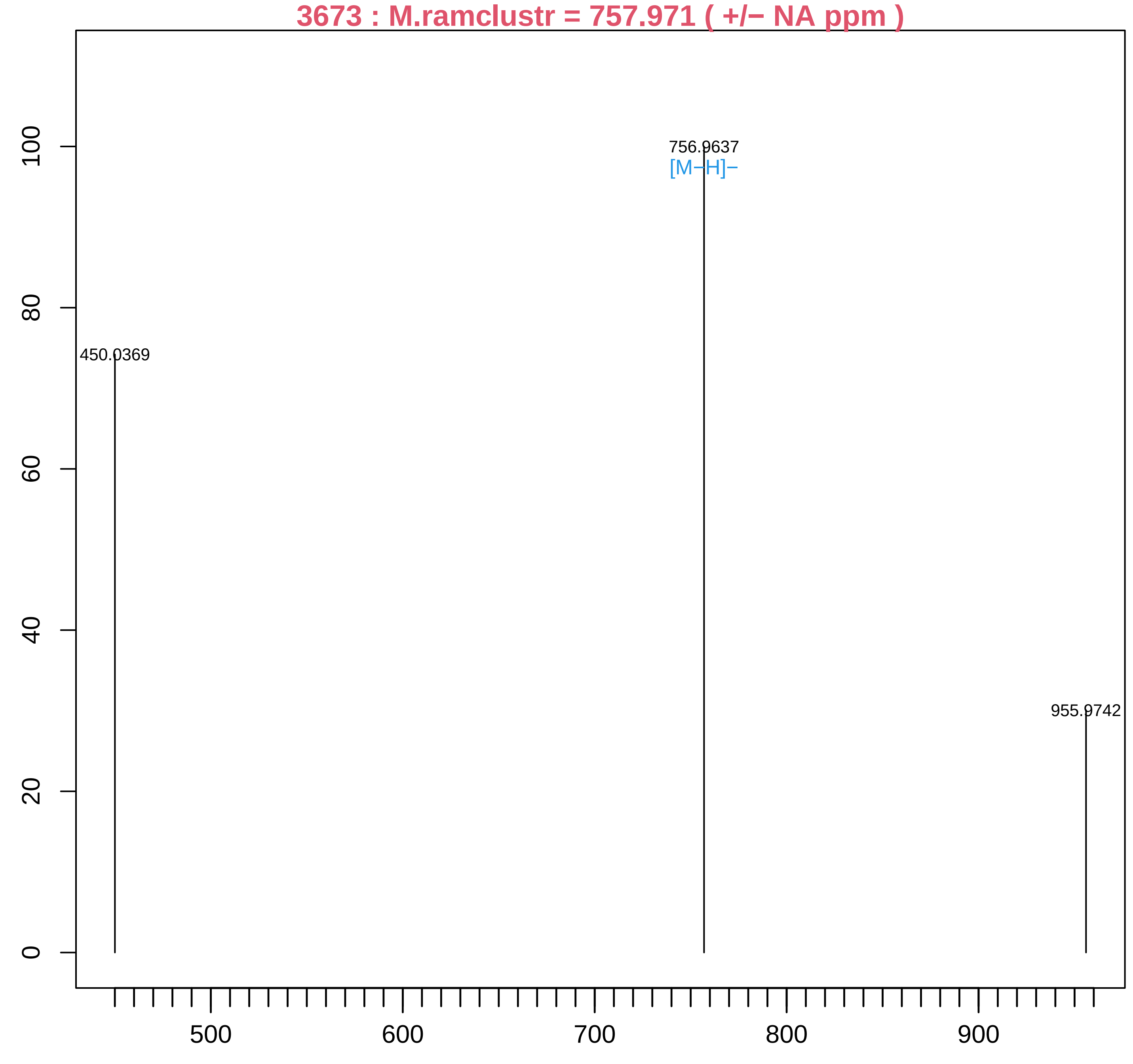
3673 : M.ramclustr = 757.971 (+/− NA ppm)
756.9637
[M−H]−
450.0369
955.9742
0
20
40
60
80
100
500
600
700
800
900

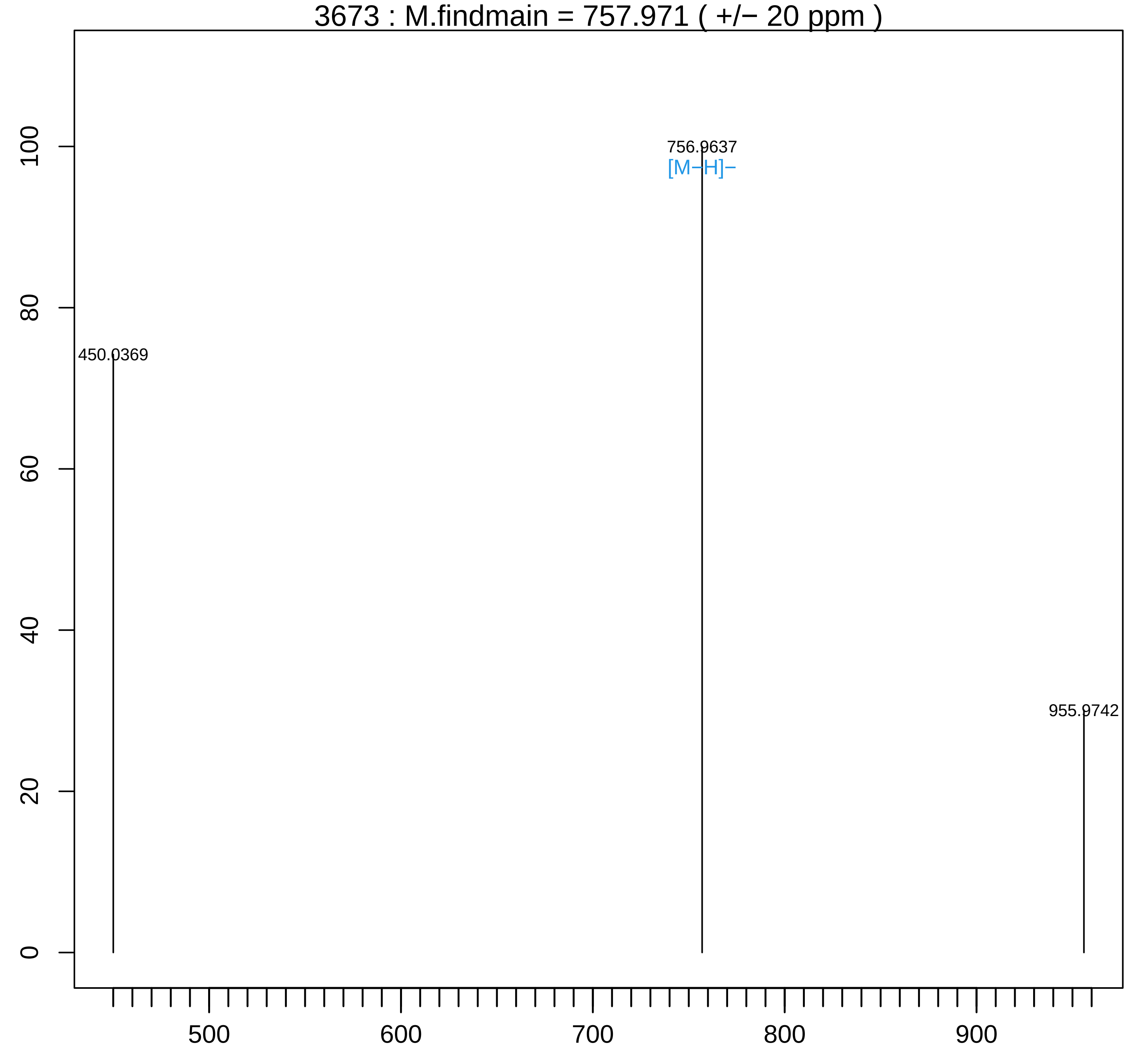
3673 : M.findmain = 757.971 (+/− 20 ppm)
756.9637
[M−H]−
450.0369
955.9742
0
20
40
60
80
100
500
600
700
800
900

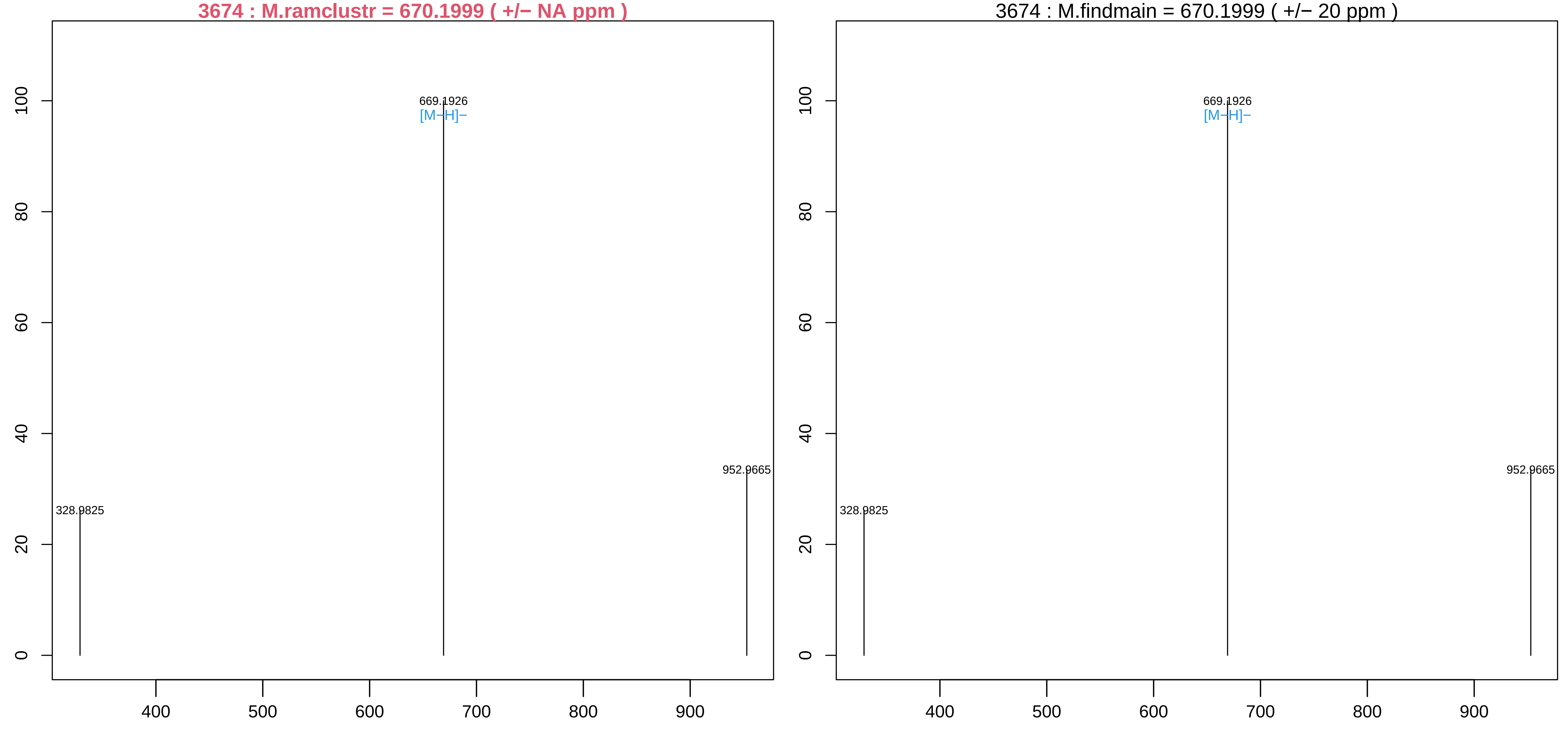
3674 : M.ramclustr = 670.1999 (+/− NA ppm)
669.1926
[M−H]−
952.9665
328.9825
3674 : M.findmain = 670.1999 (+/− 20 ppm)
669.1926
[M−H]−
952.9665
328.9825
0
20
40
60
80
100
400
500
600
700
800
900

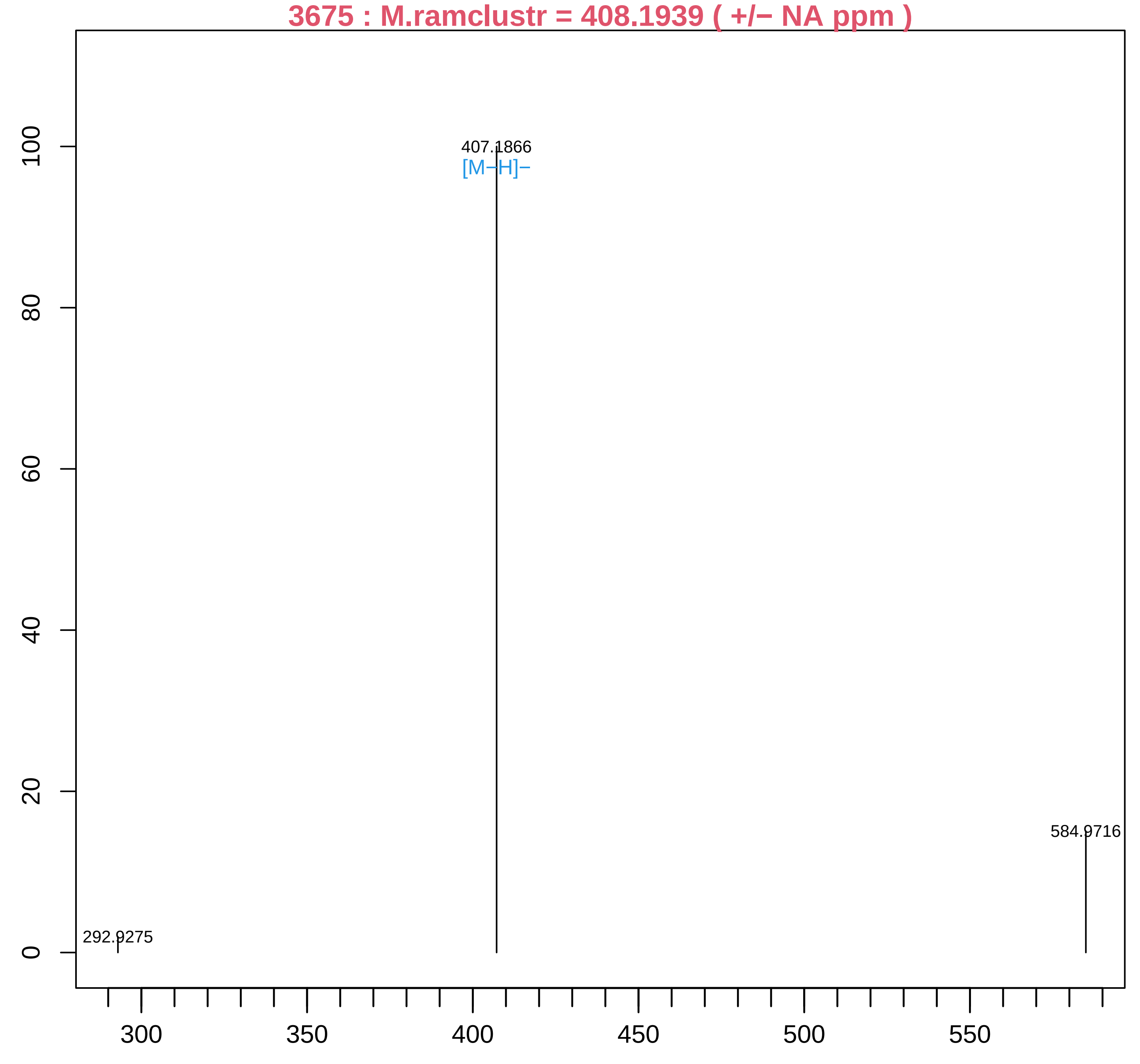
3675 : M.ramclustr = 408.1939 (+/− NA ppm)
407.1866
[M−H]−
584.9716
292.9275
0
20
40
60
80
100
300
350
400
450
500
550
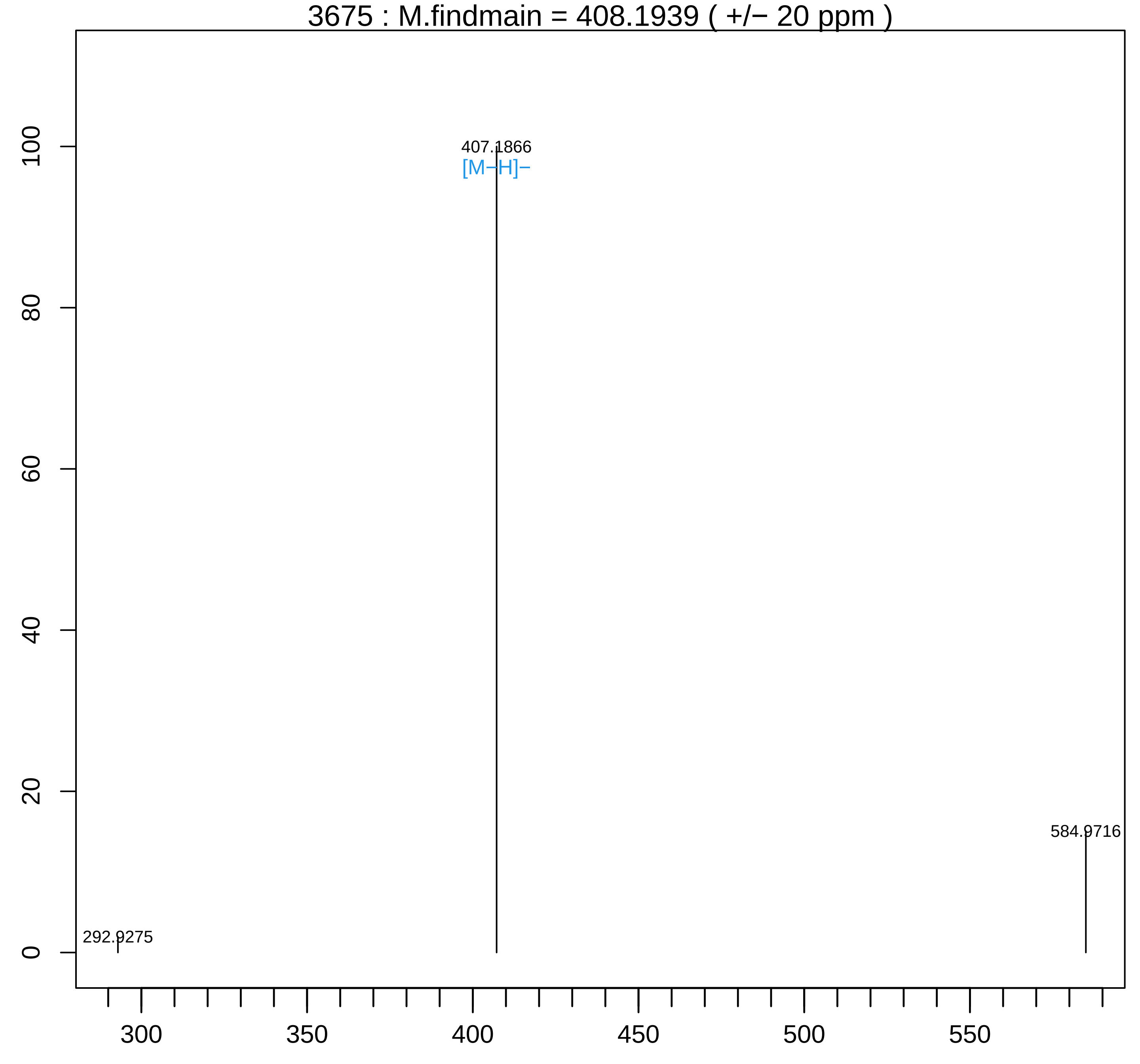
3675 : M.findmain = 408.1939 (+/− 20 ppm)
407.1866
[M−H]−
584.9716
292.9275
0
20
40
60
80
100
300
350
400
450
500
550

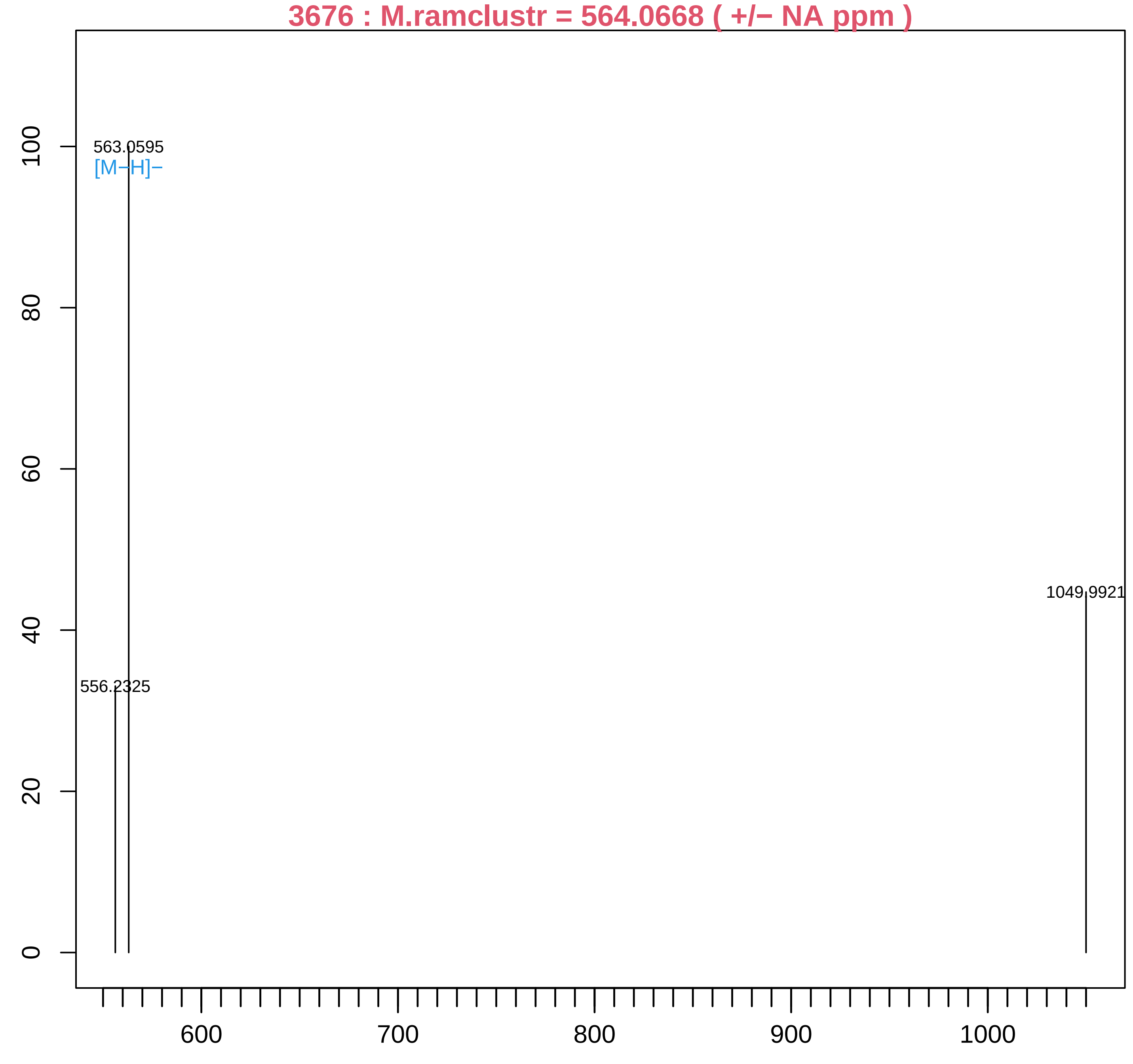

3676 : M.ramclustr = 564.0668 (+/− NA ppm)
563.0595
[M−H]−
556.2325
1049.9921
0
20
40
60
80
100
600
700
800
900
1000

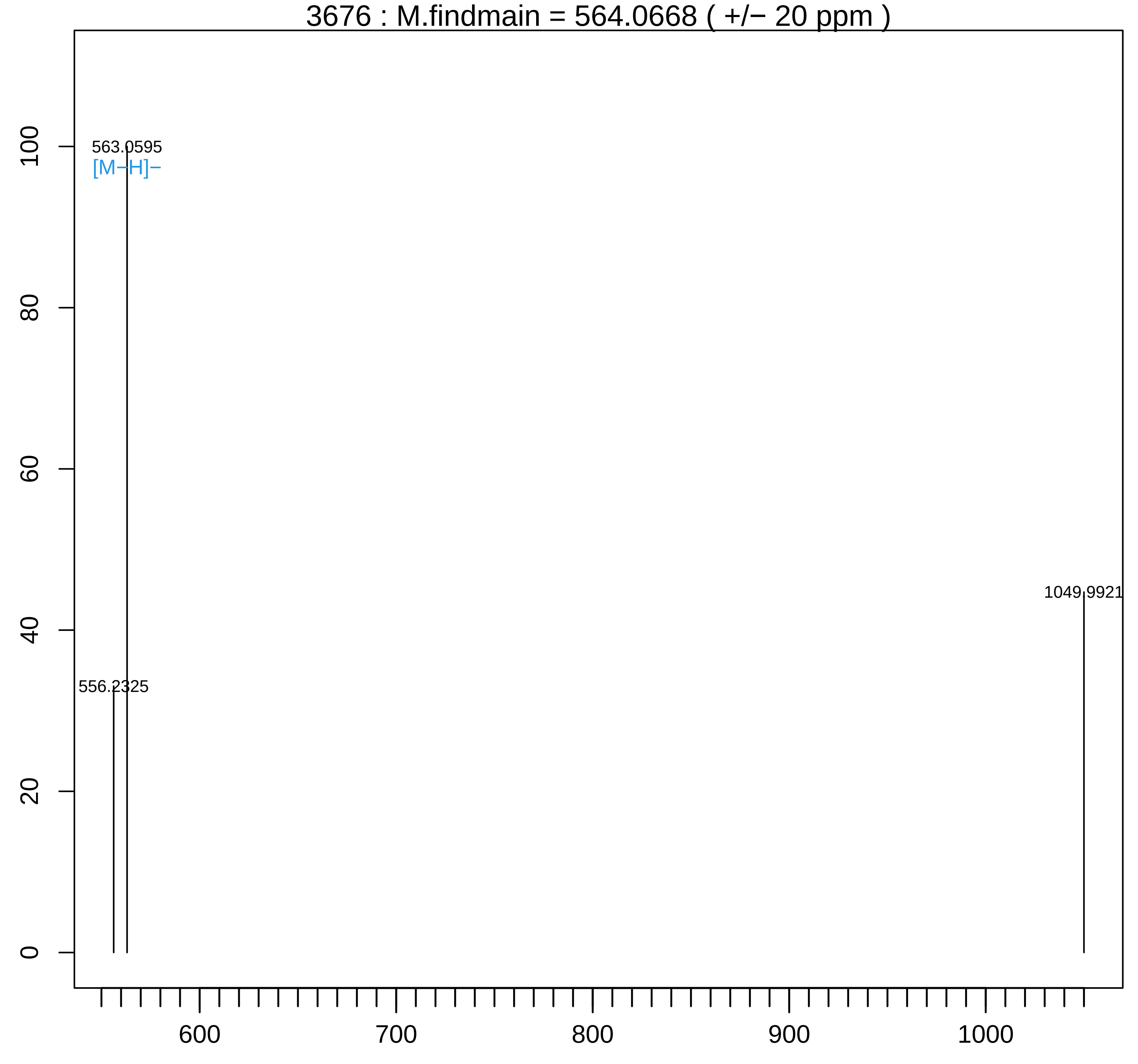

3676 : M.findmain = 564.0668 (+/− 20 ppm)
563.0595
[M−H]−
556.2325
1049.9921
0
20
40
60
80
100
600
700
800
900
1000

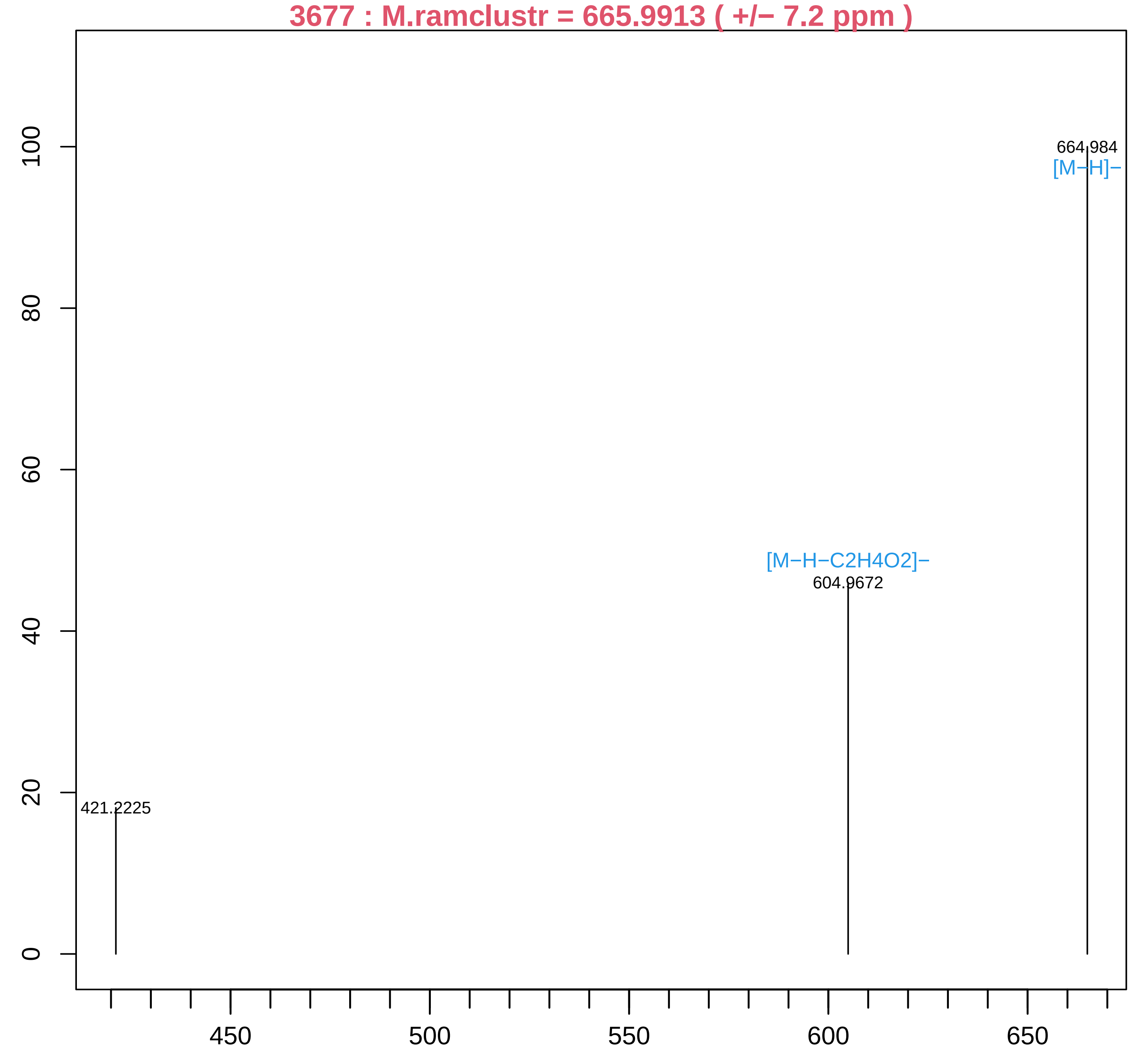
3677 : M.ramclustr = 665.9913 (+/− 7.2 ppm)
664.984
[M−H]−
[M−H−C2H4O2]−
604.9672
421.2225
0
20
40
60
80
100
450
500
550
600
650

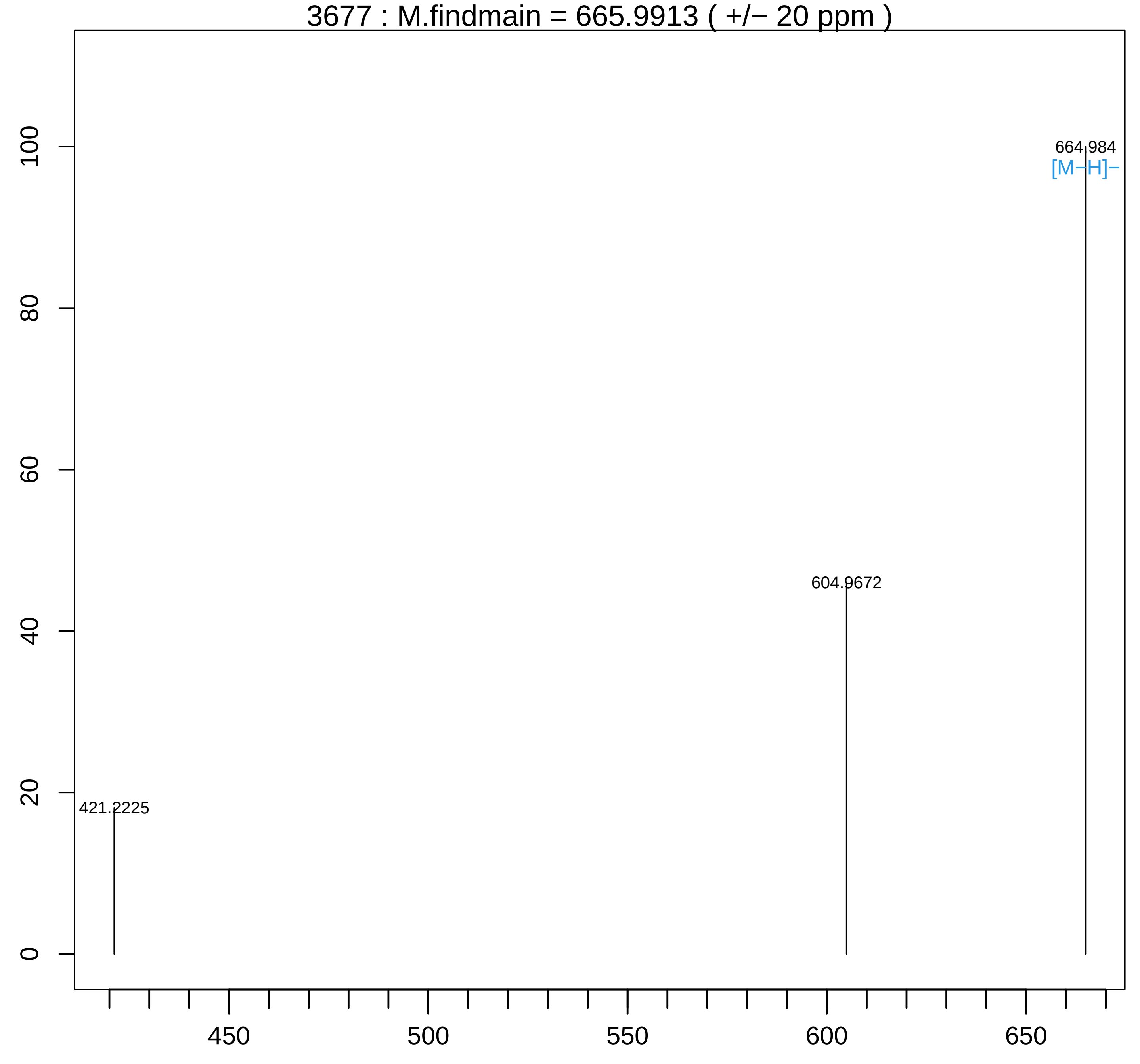
3677 : M.findmain = 665.9913 (+/− 20 ppm)
664.984
[M−H]−
604.9672
421.2225
0
20
40
60
80
100
450
500
550
600
650

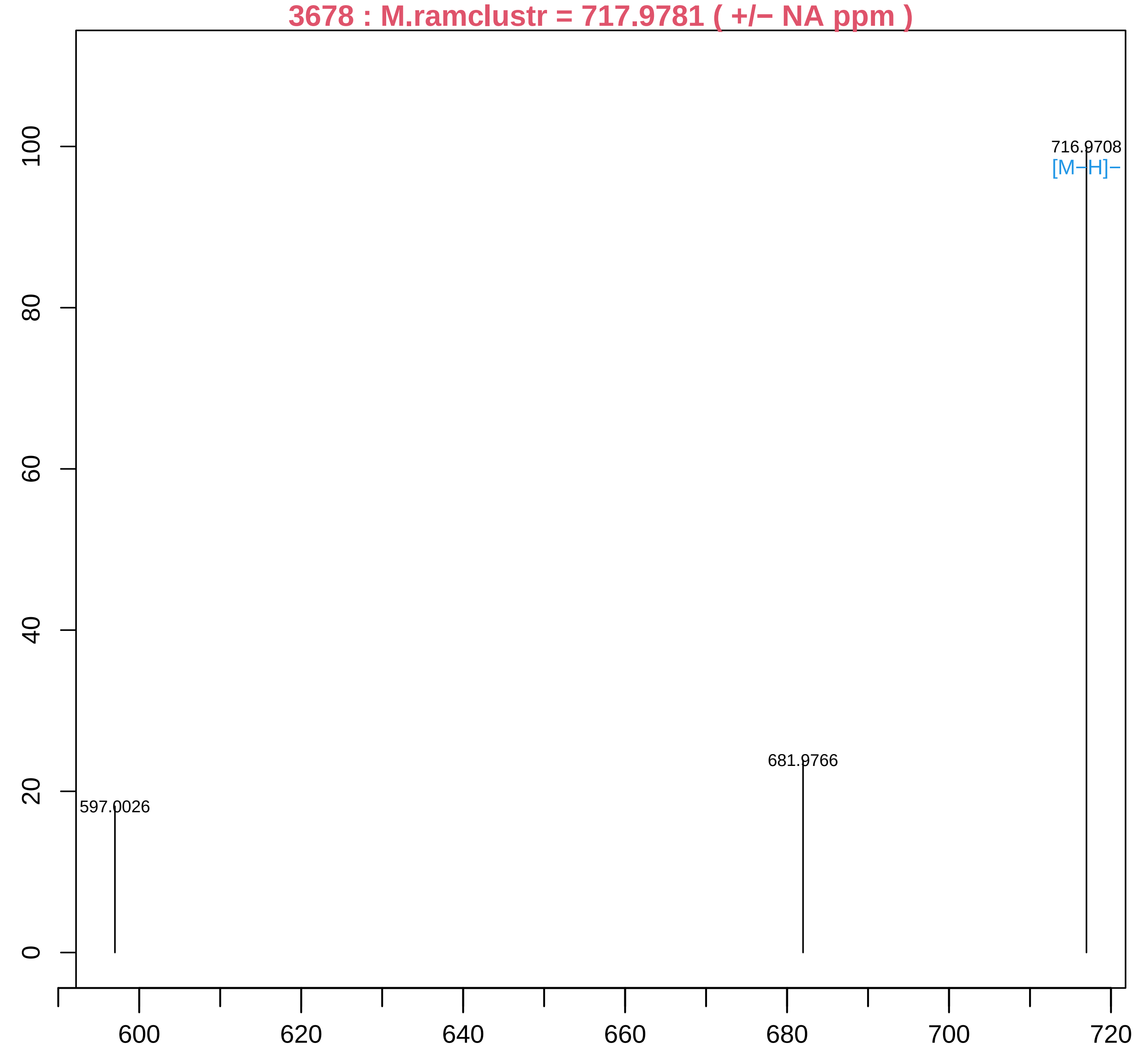
3678 : M.ramclustr = 717.9781 (+/− NA ppm)
716.9708
[M−H]−
681.9766
597.0026
0
20
40
60
80
100
600
620
640
660
680
700
720

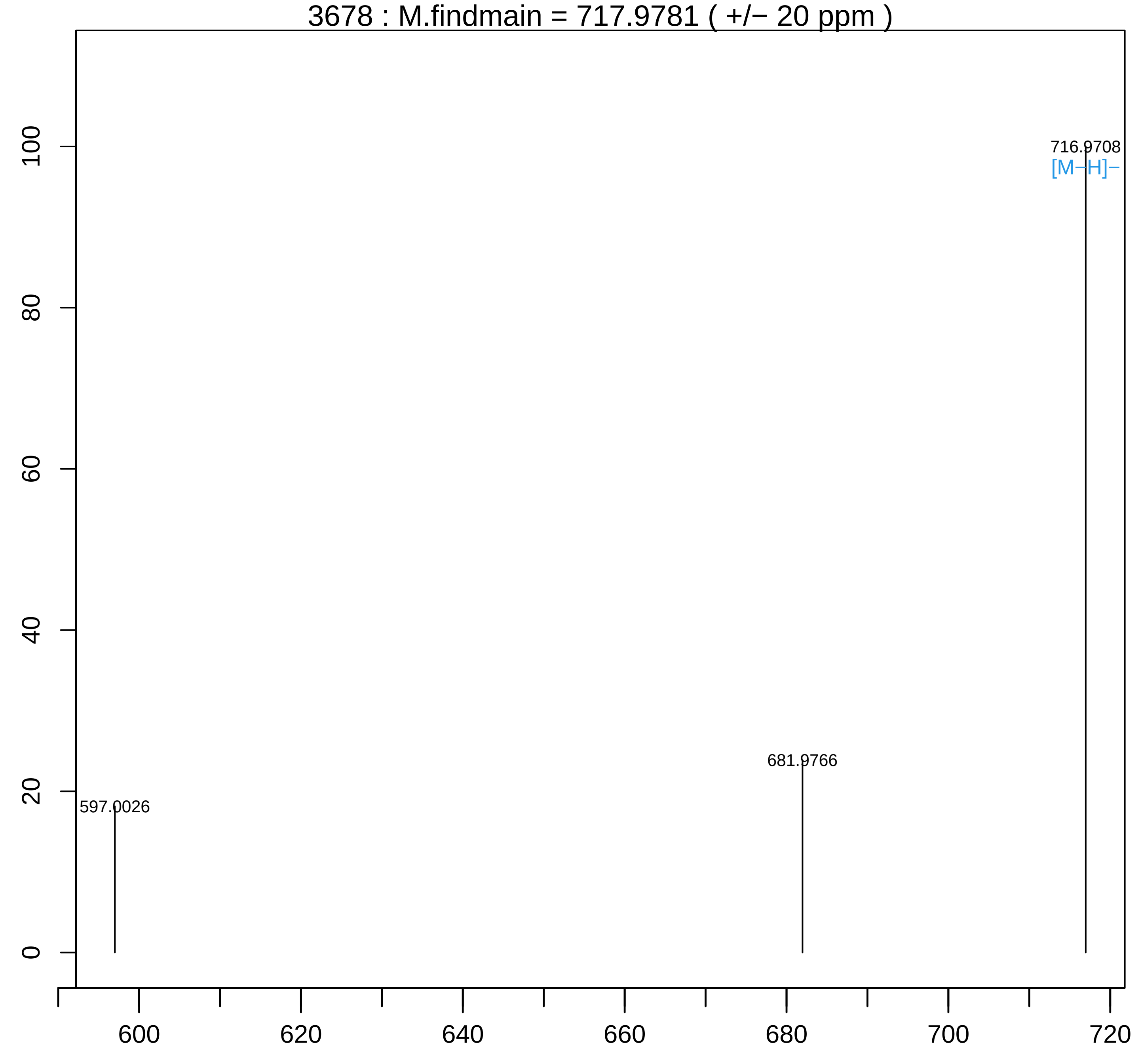
3678 : M.findmain = 717.9781 (+/− 20 ppm)
716.9708
[M−H]−
681.9766
597.0026
0
20
40
60
80
100
600
620
640
660
680
700
720

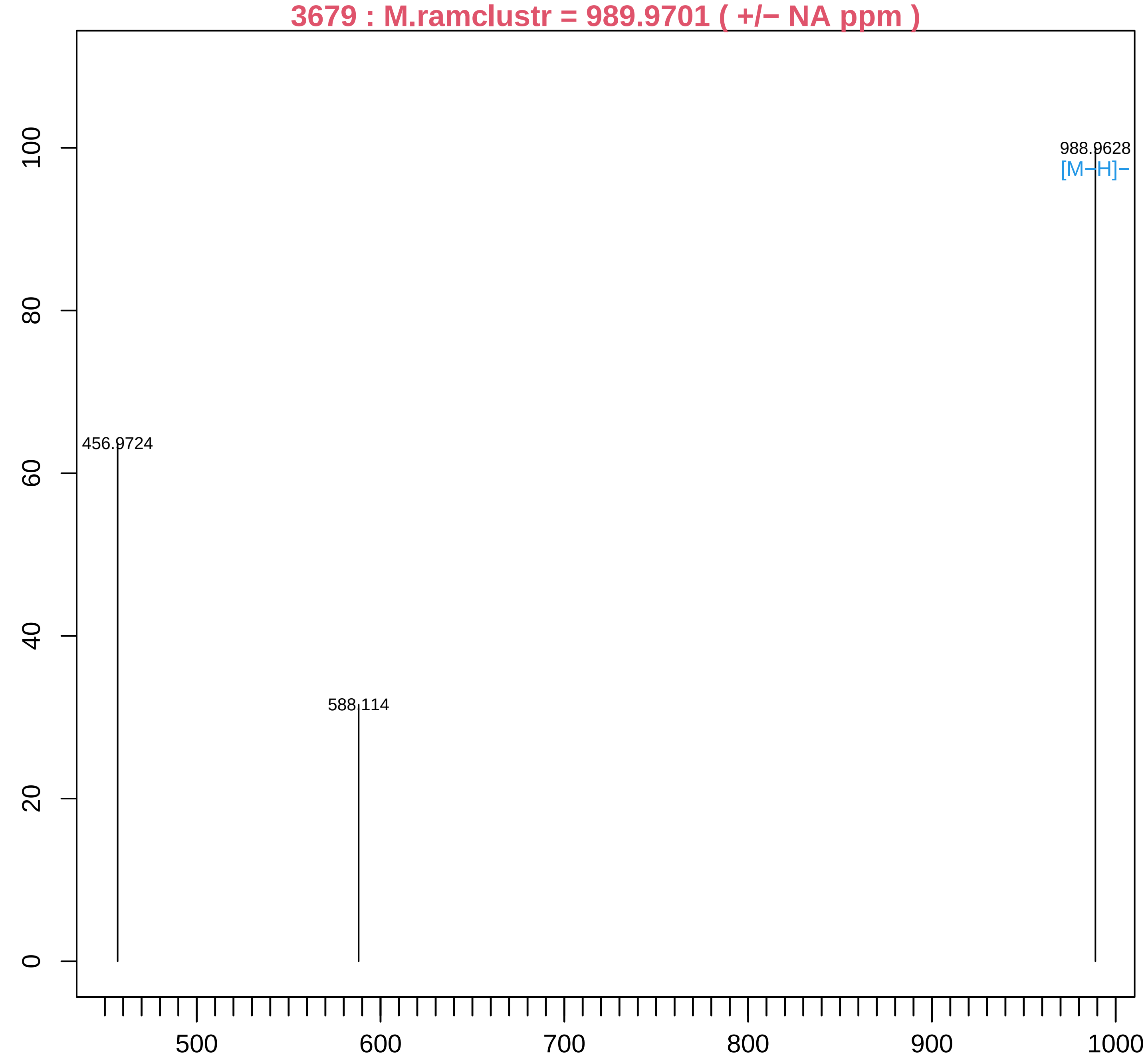
3679 : M.ramclustr = 989.9701 (+/− NA ppm)
988.9628
[M−H]−
456.9724
588.114
0
20
40
60
80
100
500
600
700
800
900
1000

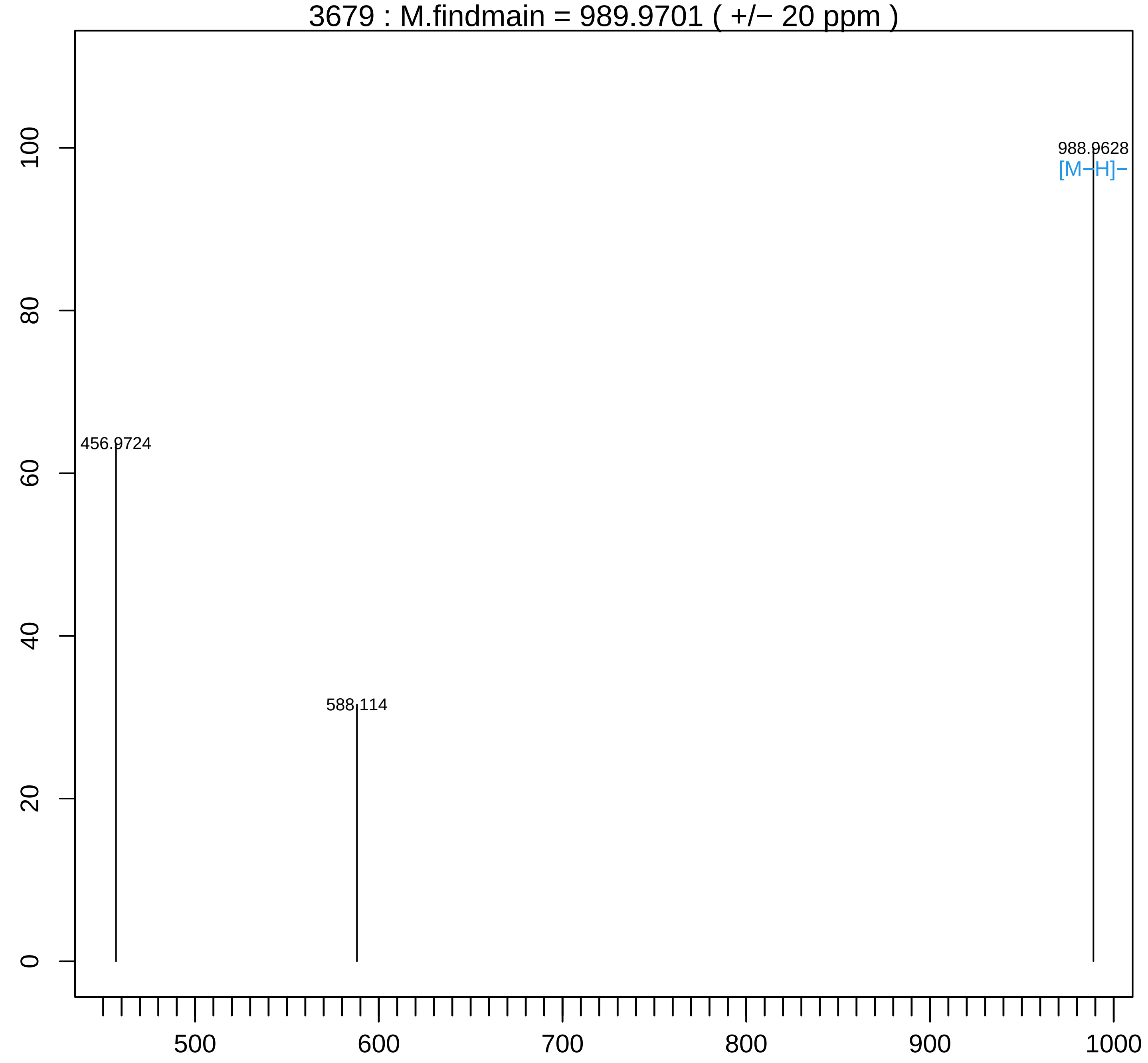
3679 : M.findmain = 989.9701 (+/− 20 ppm)
988.9628
[M−H]−
456.9724
588.114
0
20
40
60
80
100
500
600
700
800
900
1000

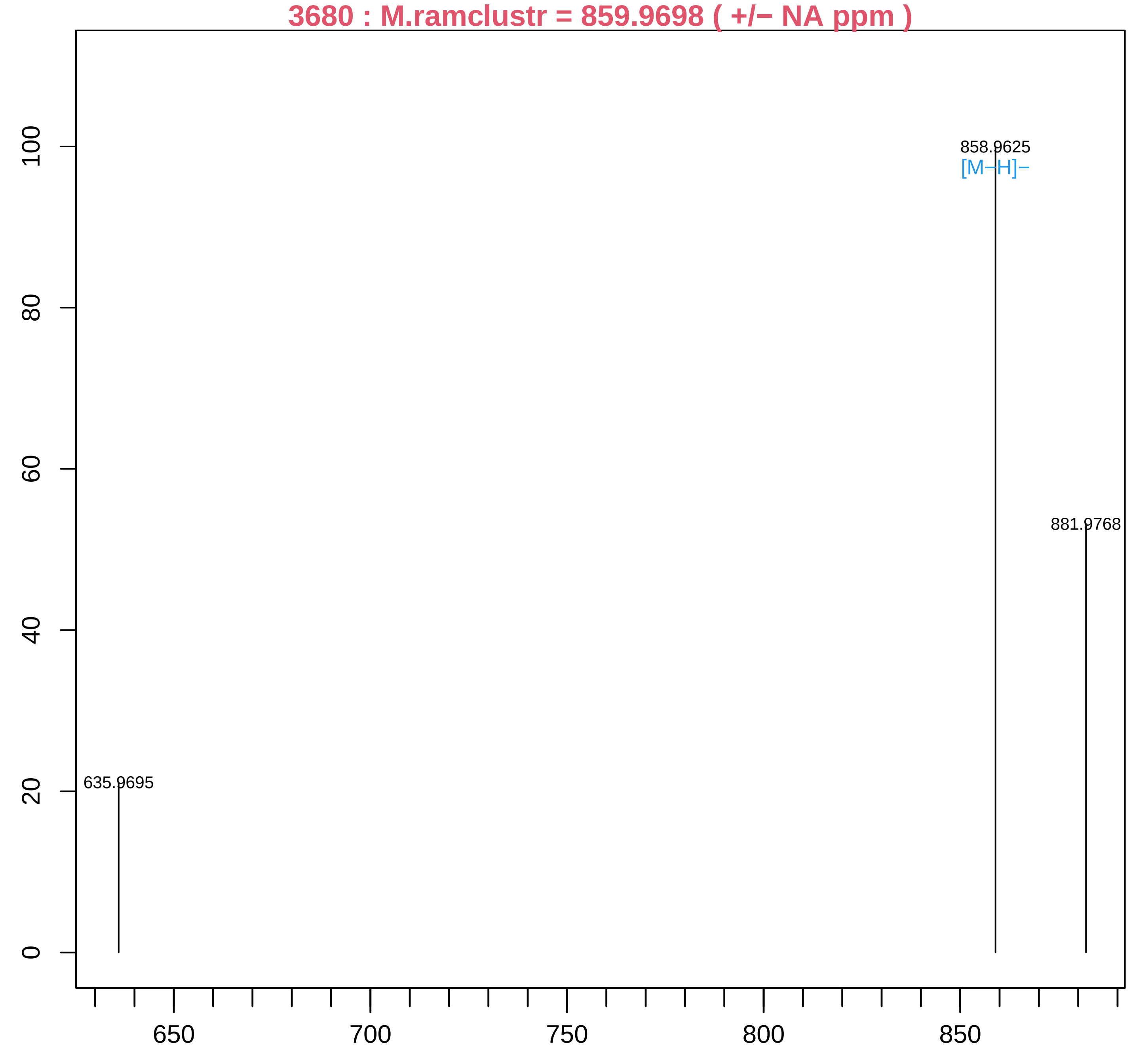

3680 : M.ramclustr = 859.9698 (+/− NA ppm)
858.9625
[M−H]−
881.9768
635.9695
0
20
40
60
80
100
650
700
750
800
850

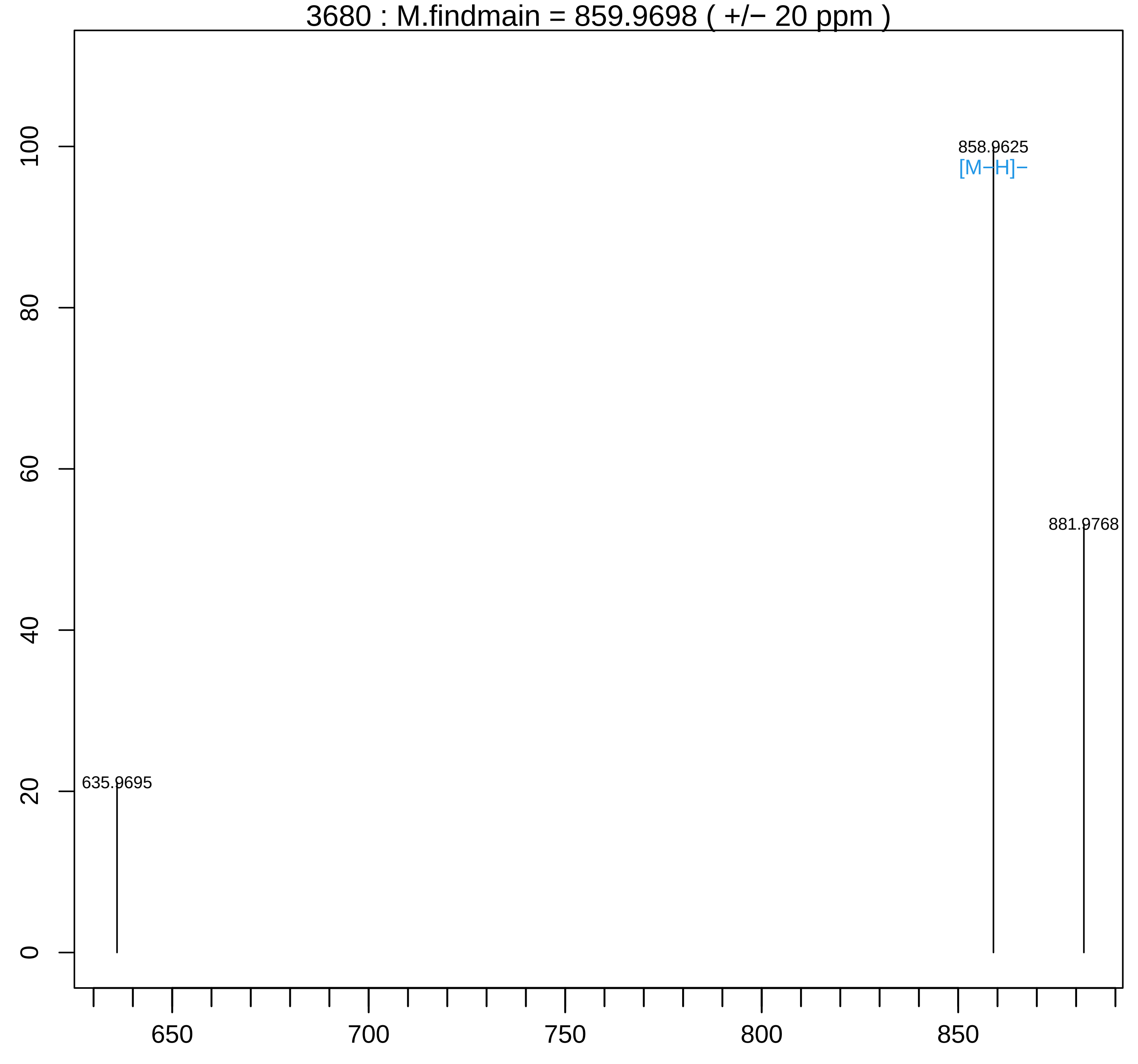

3680 : M.findmain = 859.9698 (+/− 20 ppm)
858.9625
[M−H]−
881.9768
635.9695
0
20
40
60
80
100
650
700
750
800
850

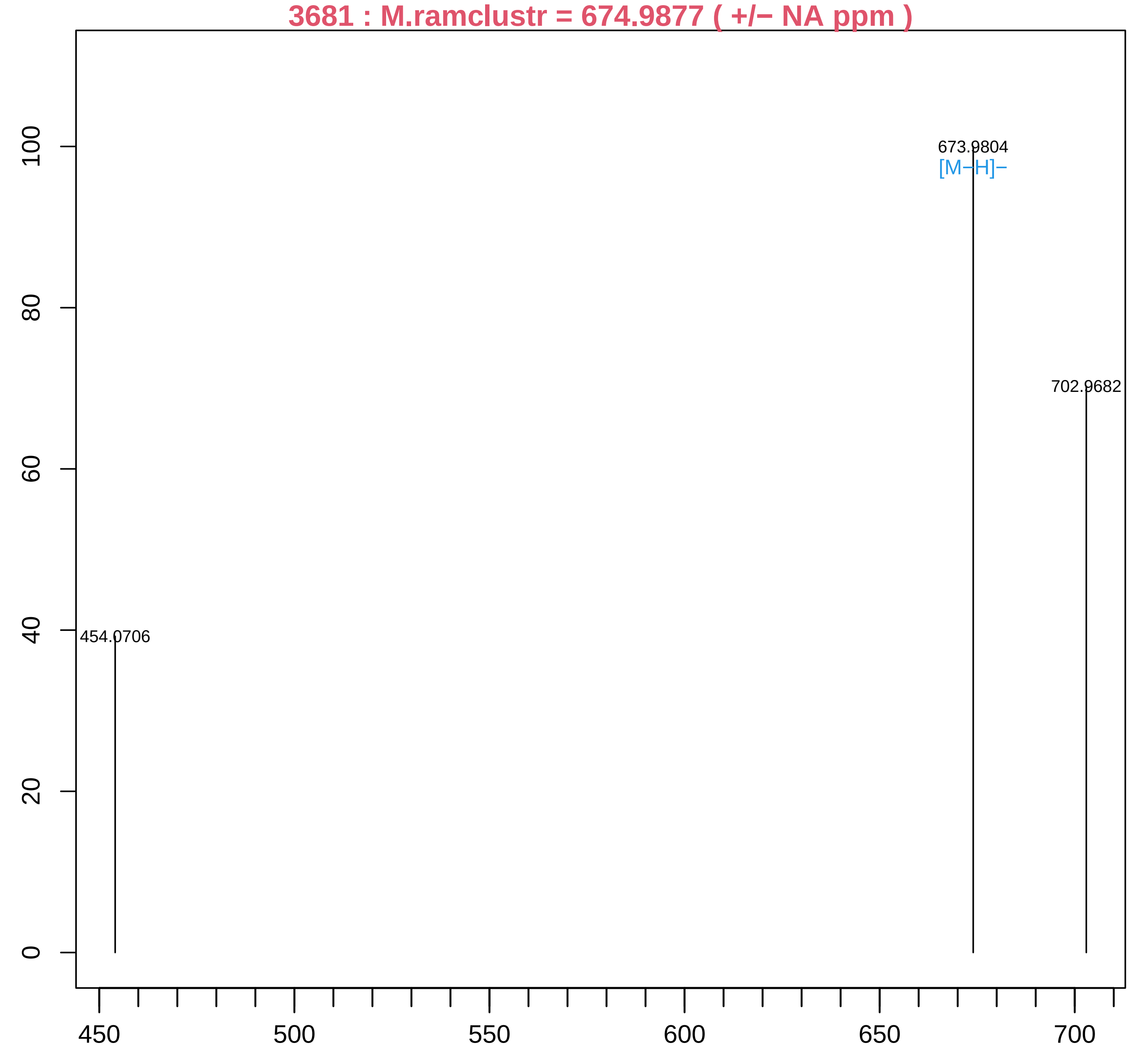
3681 : M.ramclustr = 674.9877 (+/− NA ppm)
673.9804
[M−H]−
702.9682
454.0706
0
20
40
60
80
100
450
500
550
600
650
700

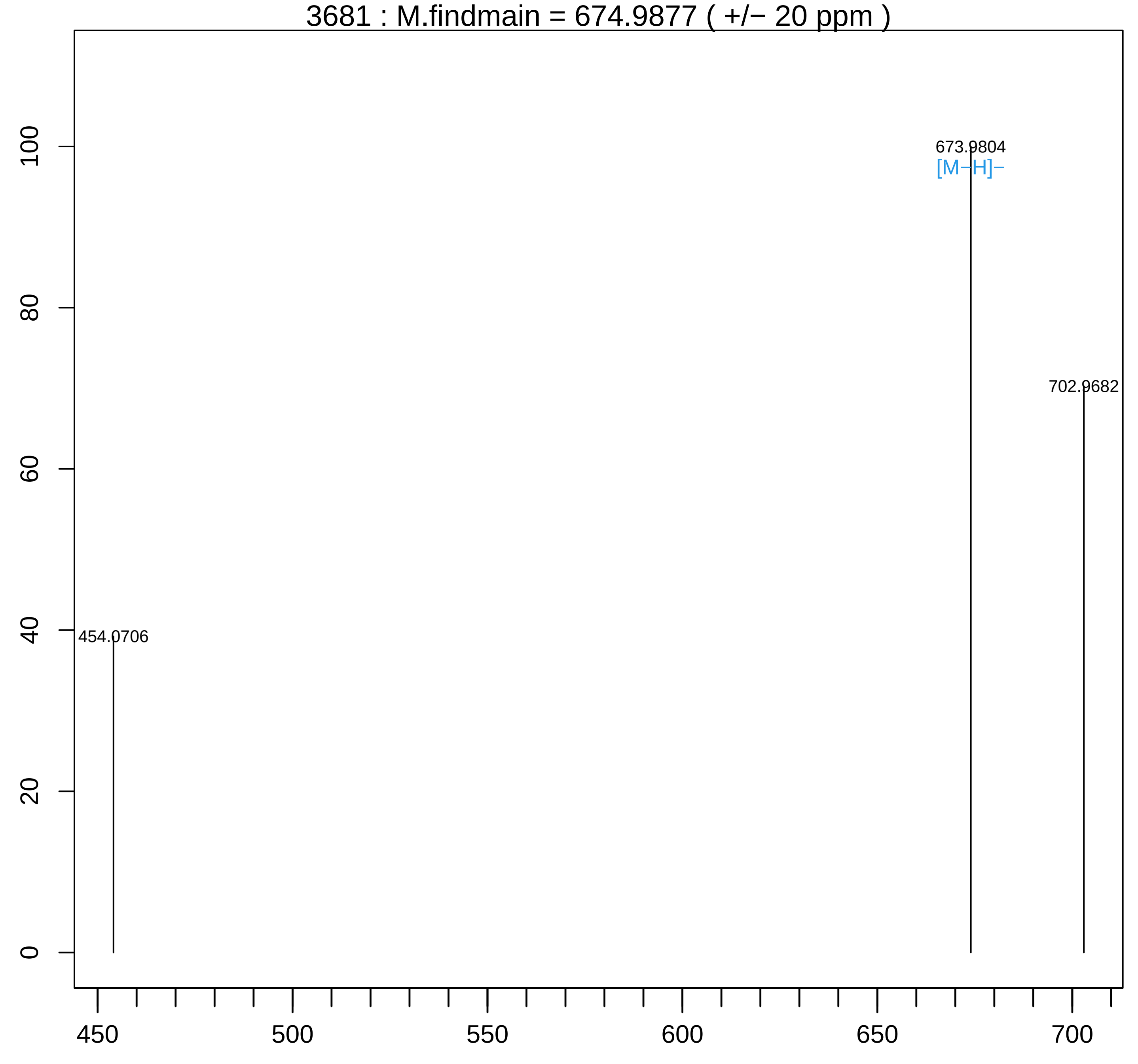
3681 : M.findmain = 674.9877 (+/− 20 ppm)
673.9804
[M−H]−
702.9682
454.0706
0
20
40
60
80
100
450
500
550
600
650
700

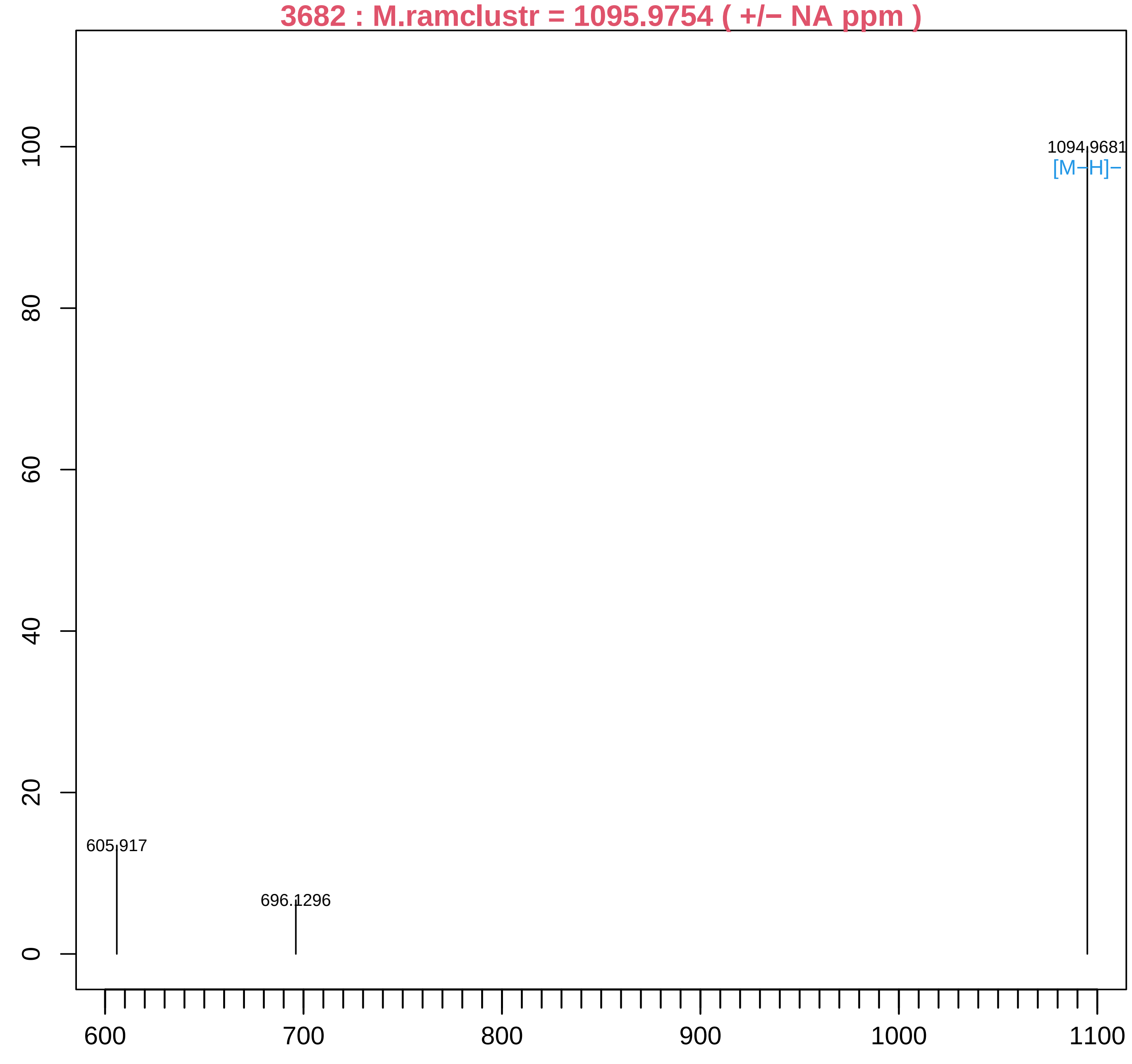
3682 : M.ramclustr = 1095.9754 (+/− NA ppm)
1094.9681
[M−H]−
605.917
696.1296
0
20
40
60
80
100
600
700
800
900
1000
1100

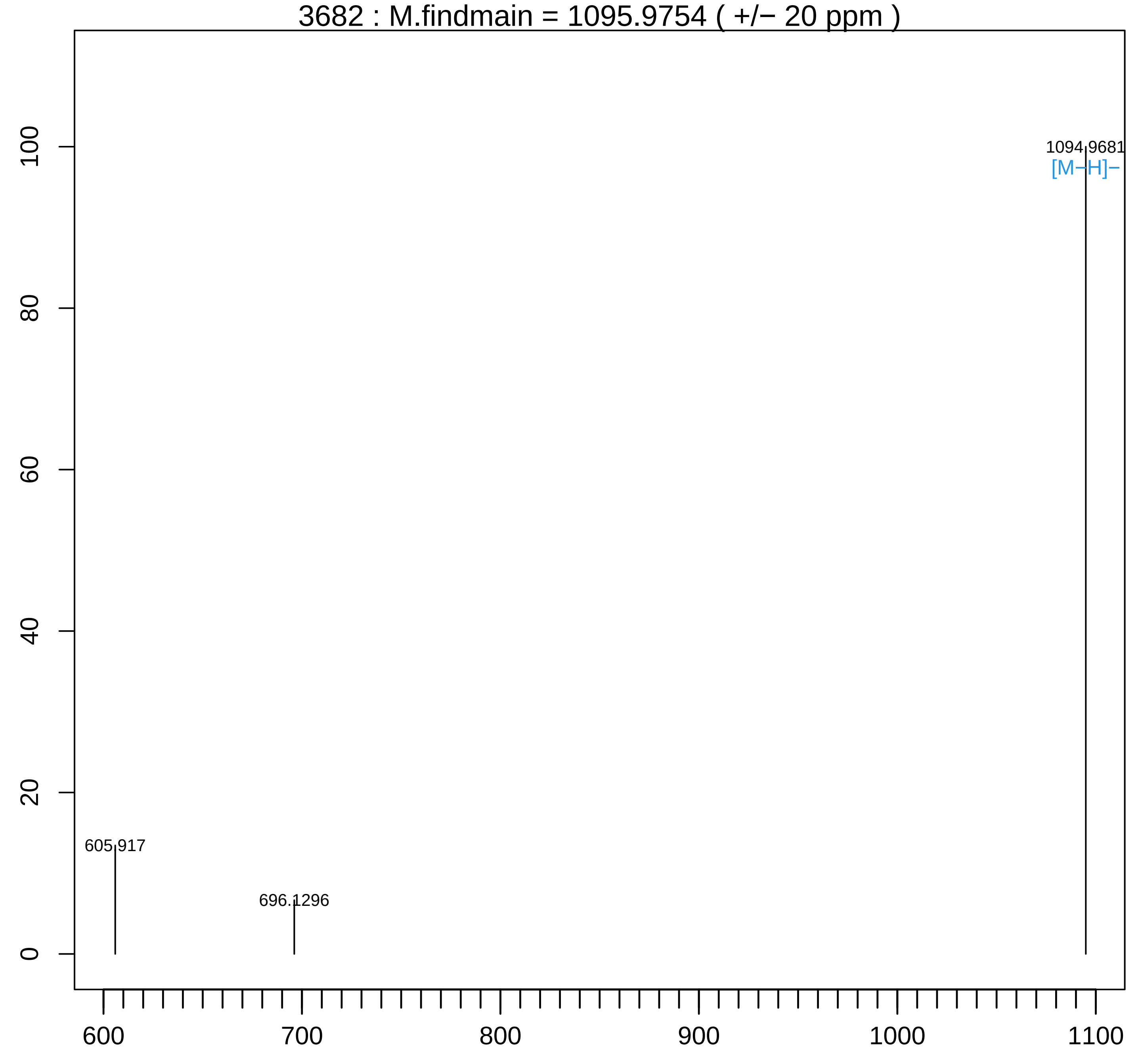
3682 : M.findmain = 1095.9754 (+/− 20 ppm)
1094.9681
[M−H]−
605.917
696.1296
0
20
40
60
80
100
600
700
800
900
1000
1100

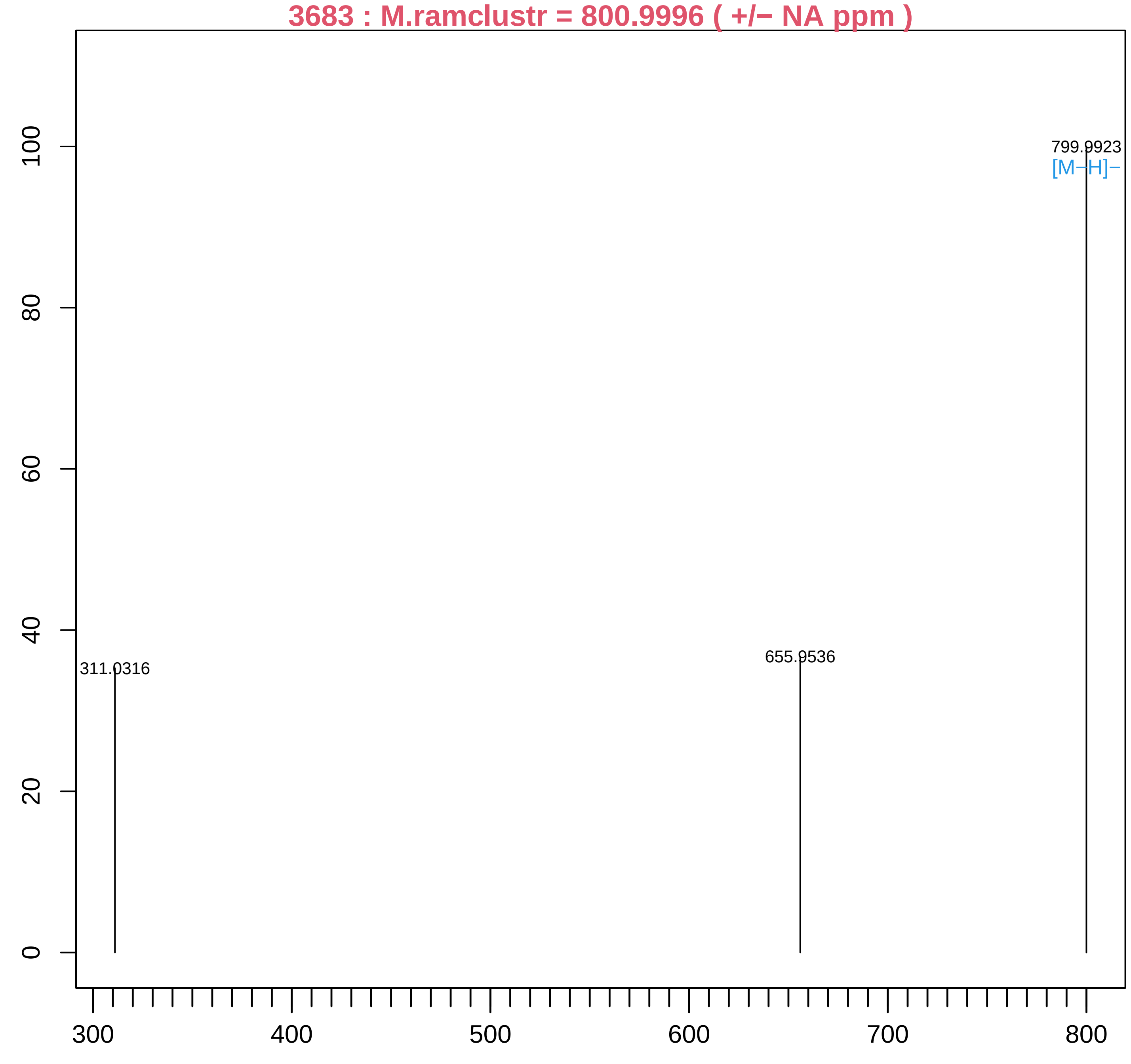
3683 : M.ramclustr = 800.9996 (+/− NA ppm)
799.9923
[M−H]−
311.0316
655.9536
0
20
40
60
80
100
300
400
500
600
700
800

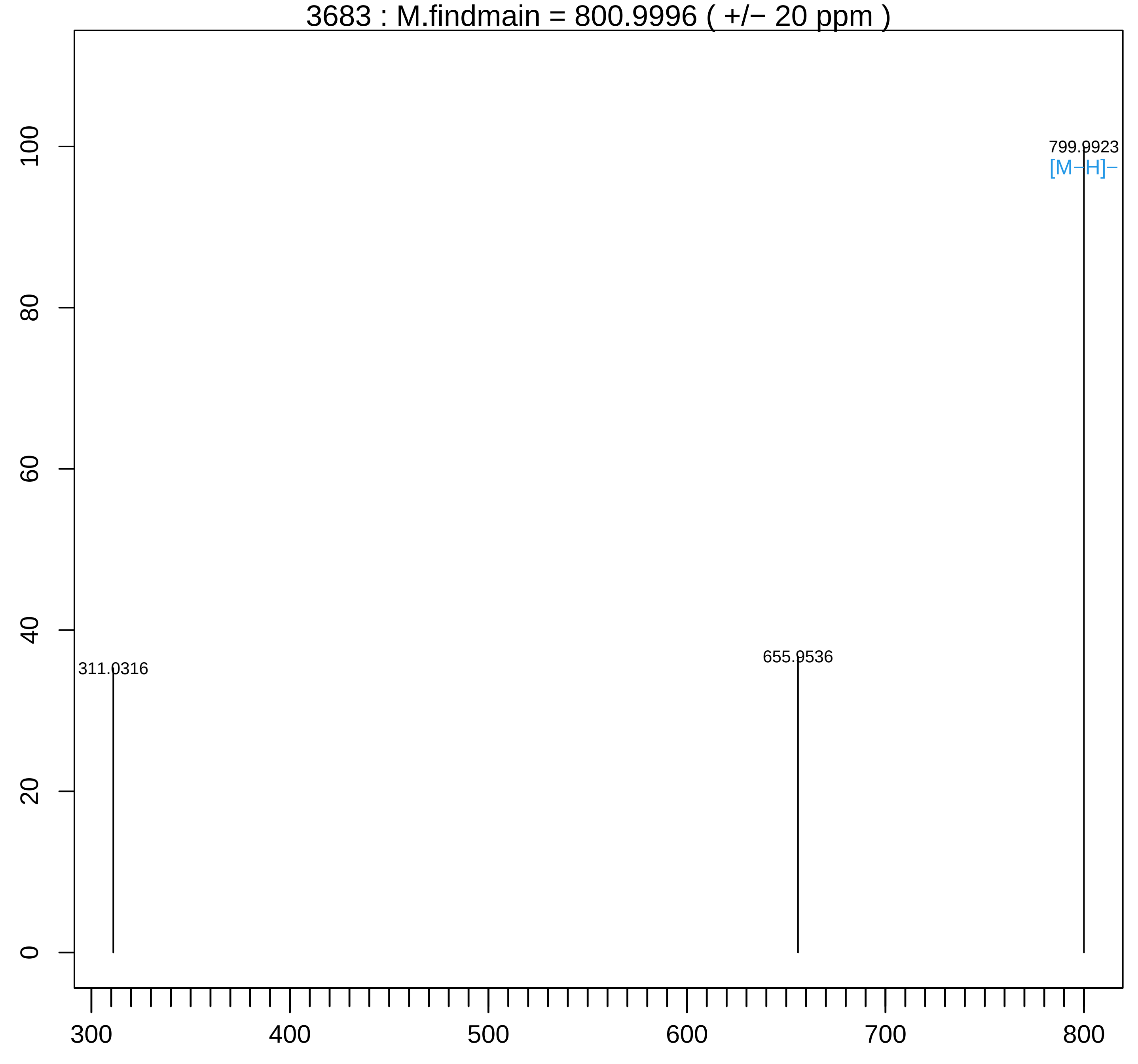
3683 : M.findmain = 800.9996 (+/− 20 ppm)
799.9923
[M−H]−
311.0316
655.9536
0
20
40
60
80
100
300
400
500
600
700
800

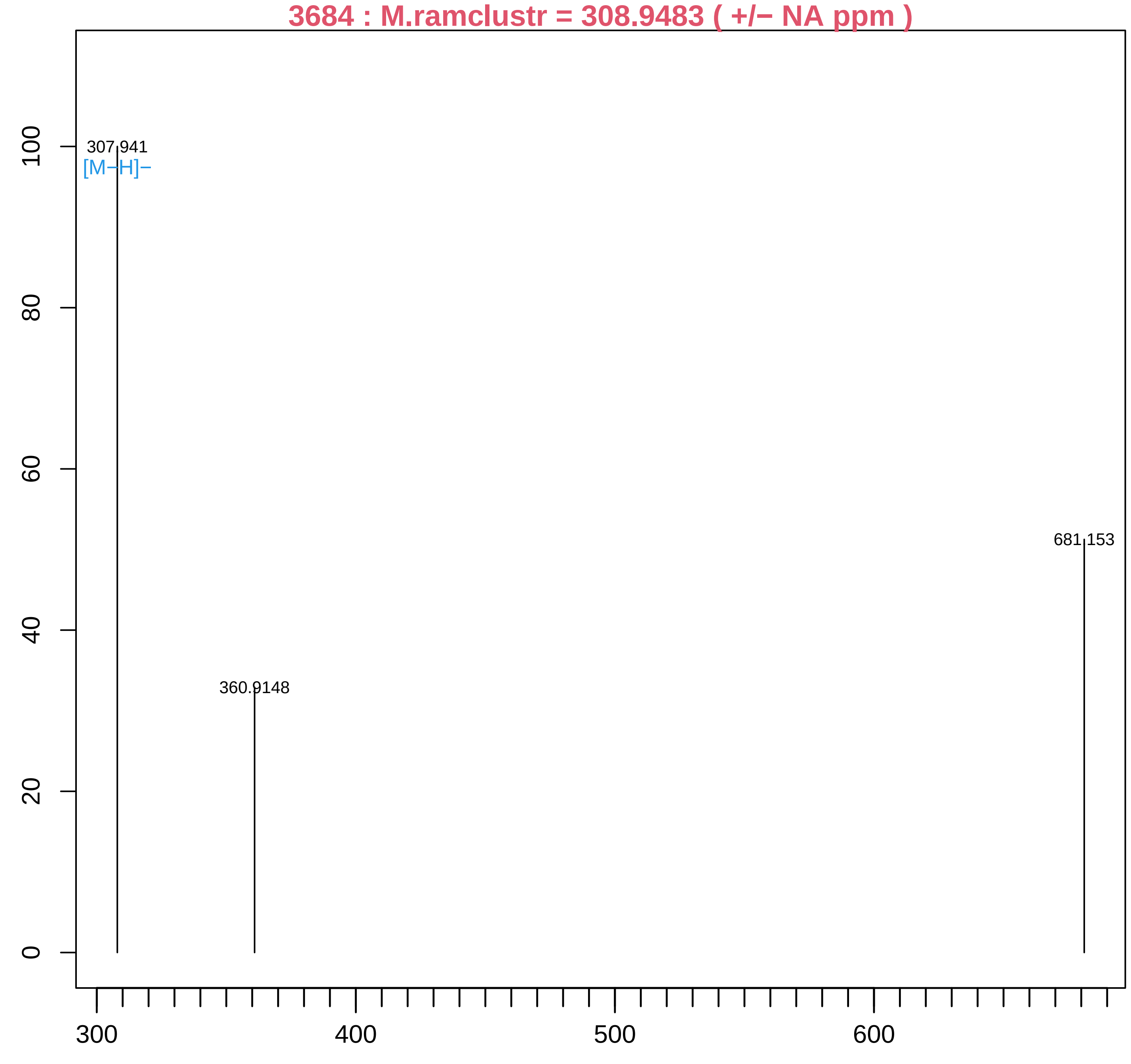
3684 : M.ramclustr = 308.9483 (+/− NA ppm)
307.941
[M−H]−
360.9148
681.153
0
20
40
60
80
100
300
400
500
600

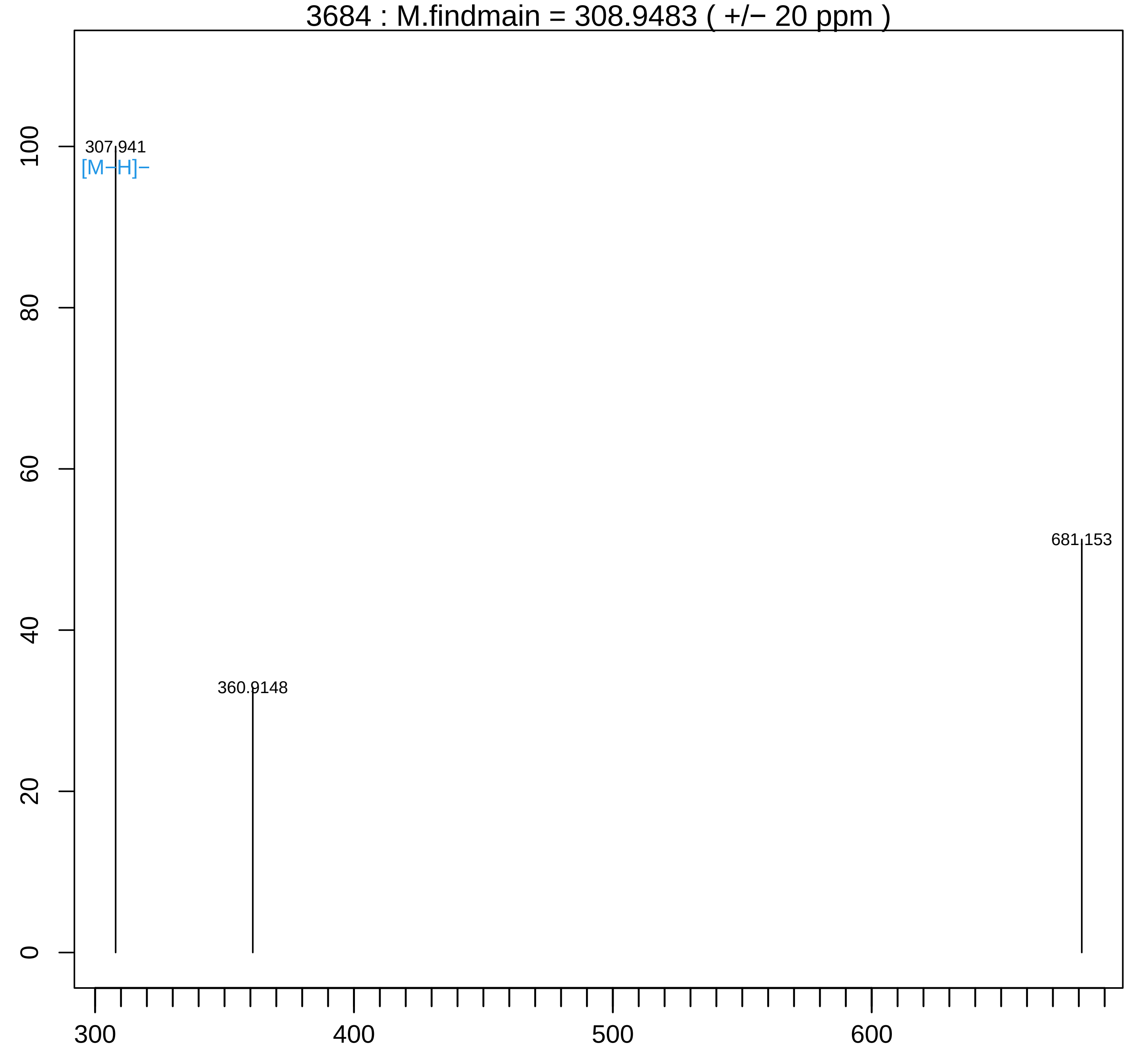
3684 : M.findmain = 308.9483 (+/− 20 ppm)
307.941
[M−H]−
360.9148
681.153
0
20
40
60
80
100
300
400
500
600

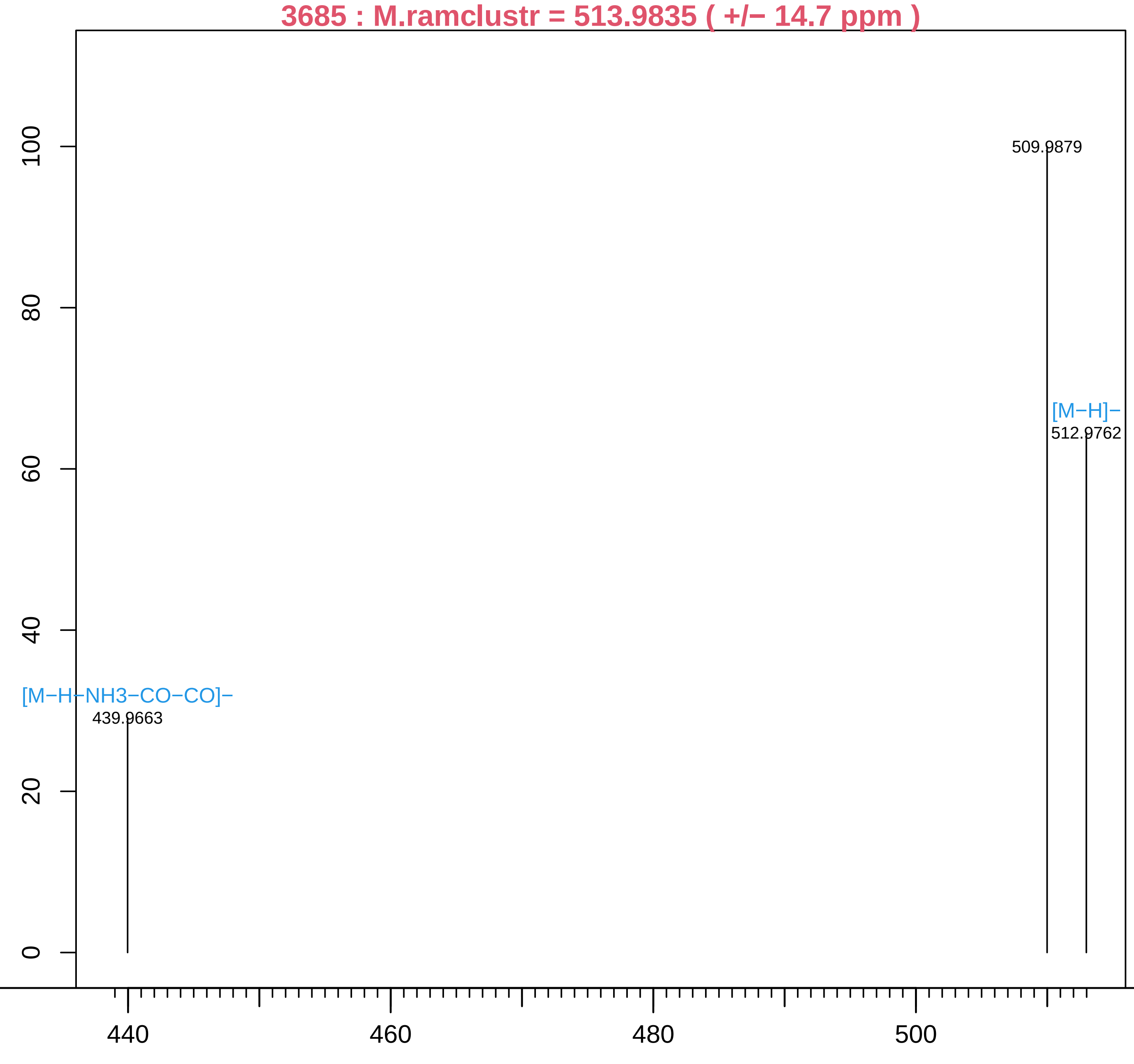
3685 : M.ramclustr = 513.9835 (+/− 14.7 ppm)
509.9879
[M−H]−
512.9762
[M−H−NH3−CO−CO]−
439.9663
0
20
40
60
80
100
440
460
480
500

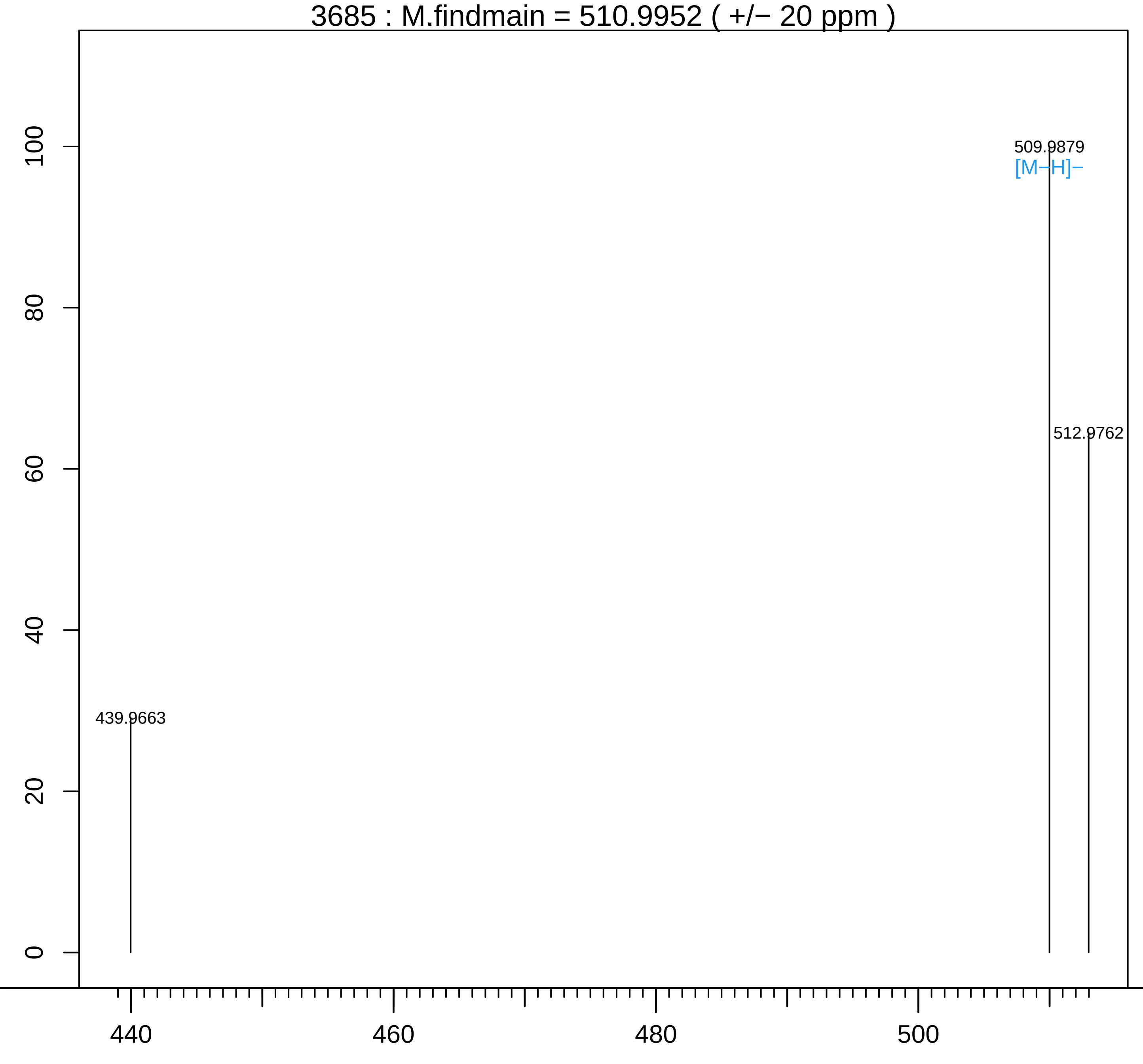
3685 : M.findmain = 510.9952 (+/− 20 ppm)
509.9879
[M−H]−
512.9762
439.9663
0
20
40
60
80
100
440
460
480
500

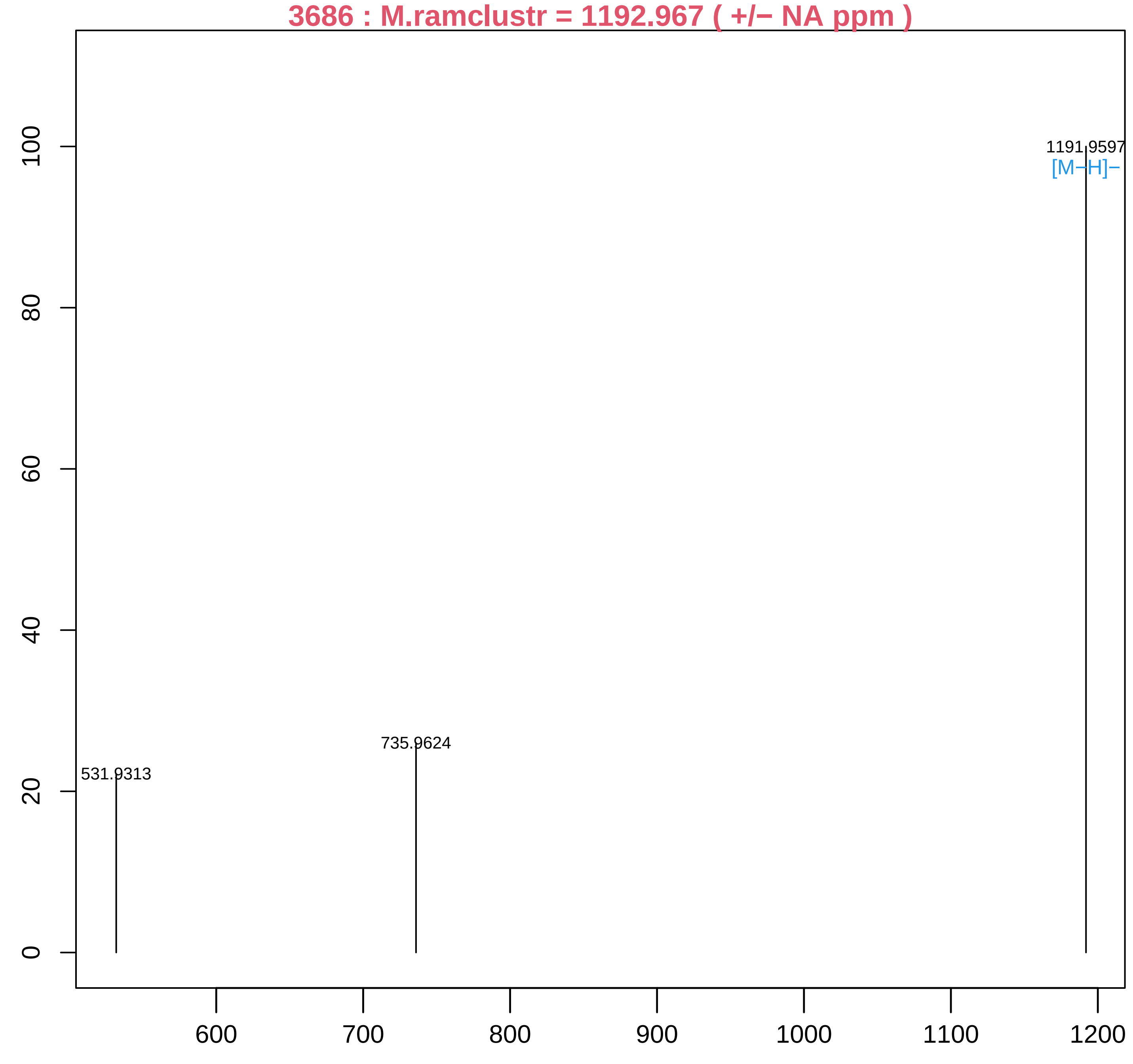
3686 : M.ramclustr = 1192.967 (+/− NA ppm)
1191.9597
[M−H]−
735.9624
531.9313
0
20
40
60
80
100
600
700
800
900
1000
1100
1200

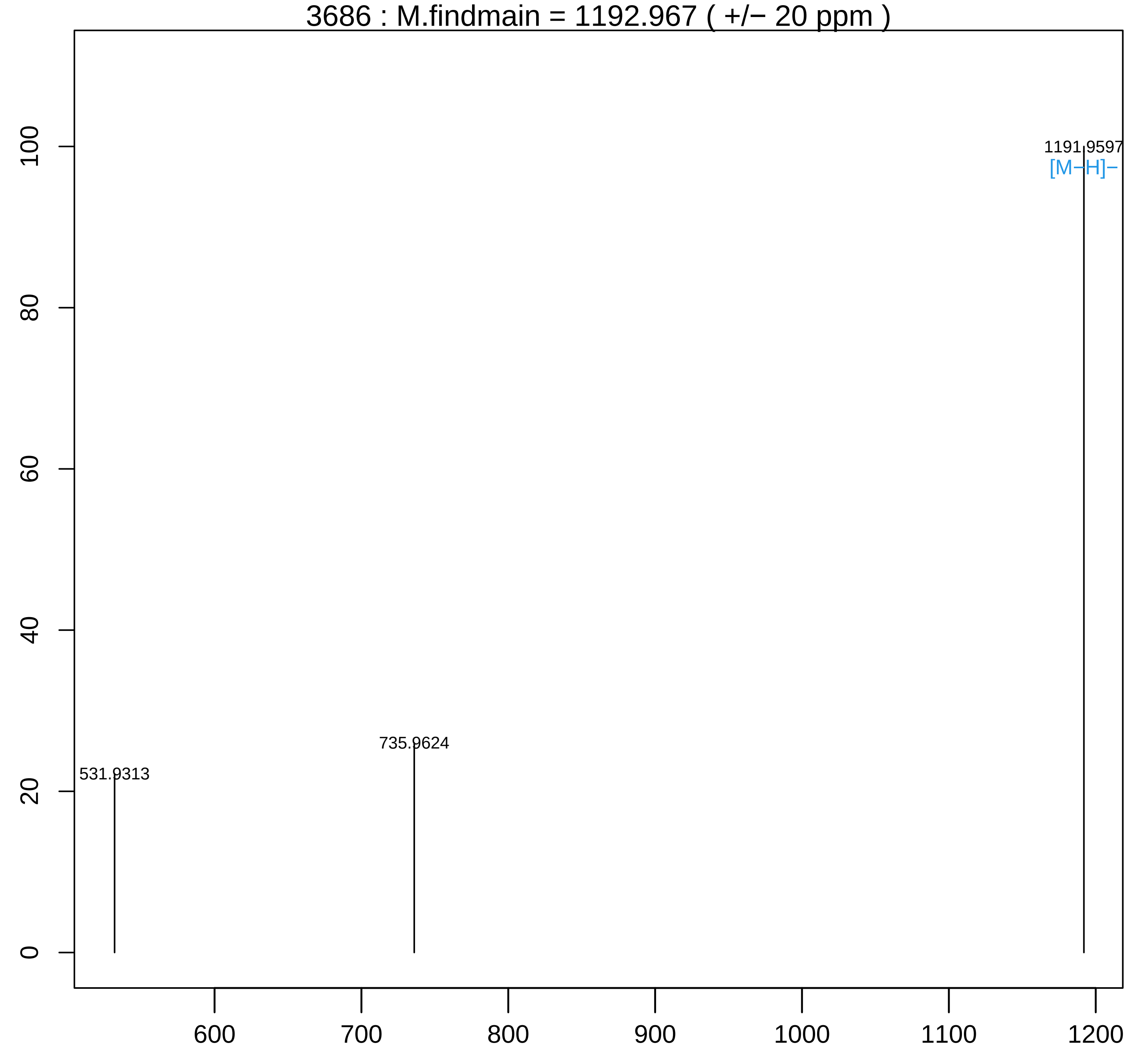
3686 : M.findmain = 1192.967 (+/− 20 ppm)
1191.9597
[M−H]−
735.9624
531.9313
0
20
40
60
80
100
600
700
800
900
1000
1100
1200

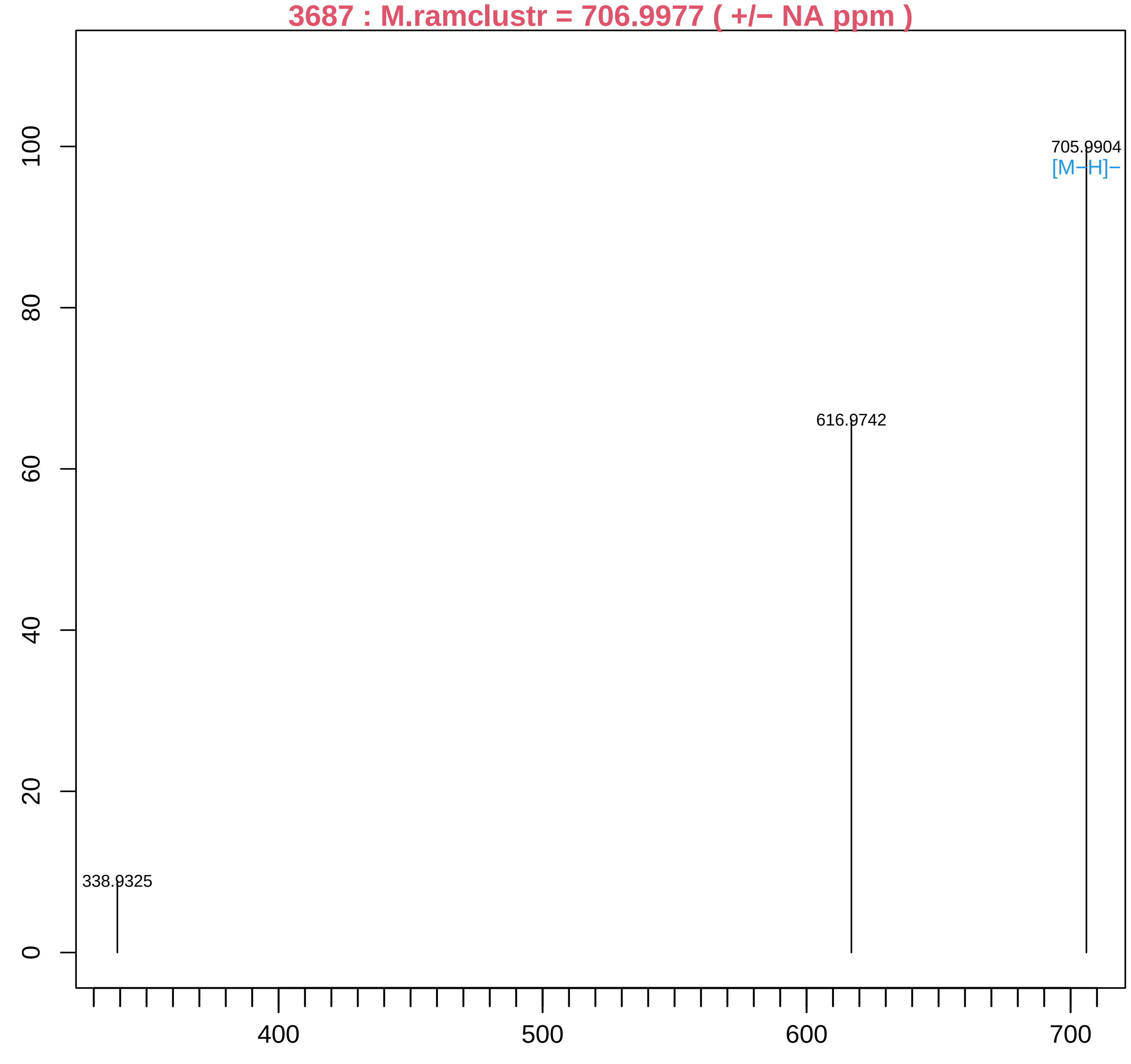
3687 : M.ramclustr = 706.9977 (+/− NA ppm)
705.9904
[M−H]−
616.9742
338.9325
0
20
40
60
80
100
400
500
600
700

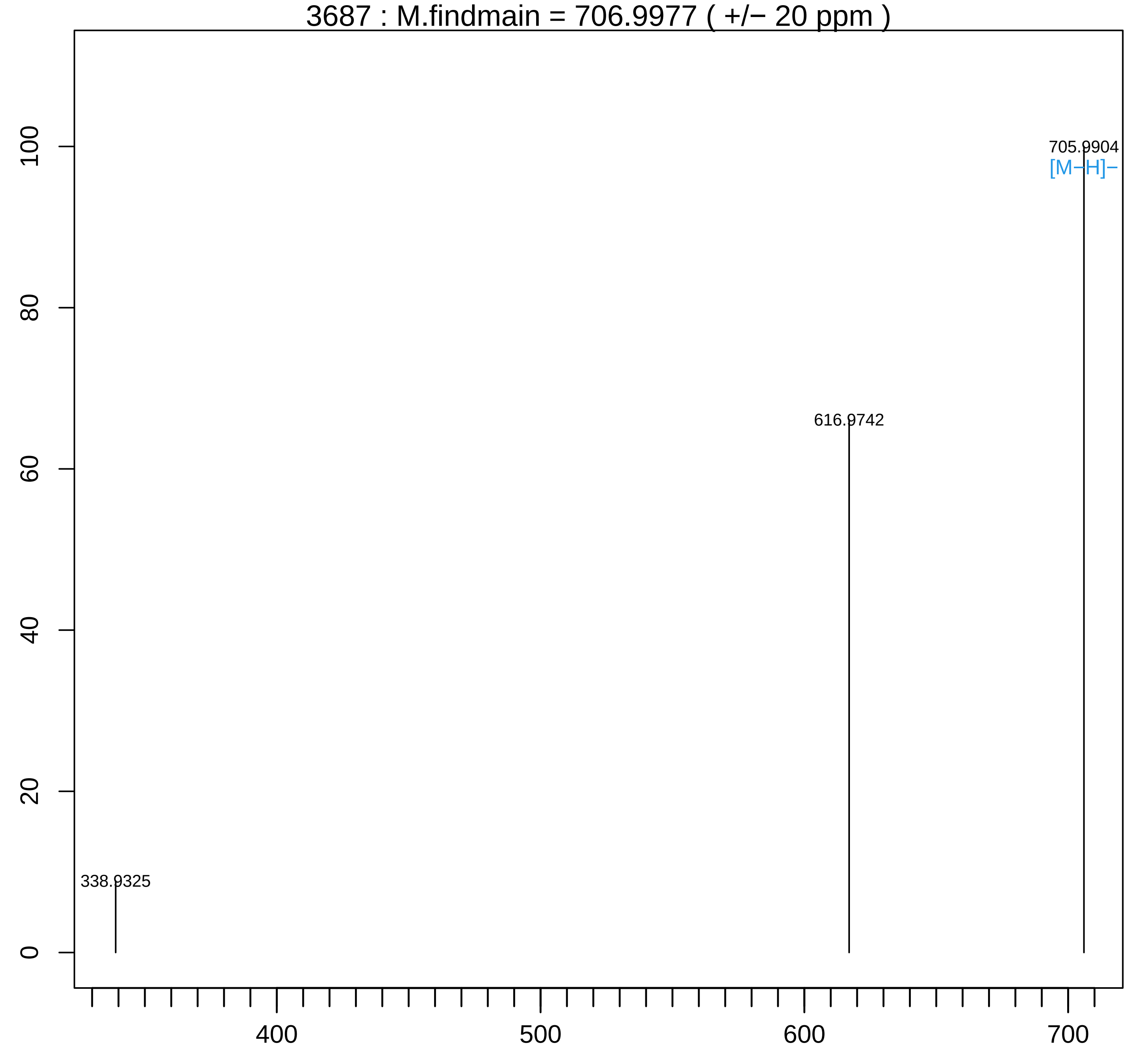
3687 : M.findmain = 706.9977 (+/− 20 ppm)
705.9904
[M−H]−
616.9742
338.9325
0
20
40
60
80
100
400
500
600
700

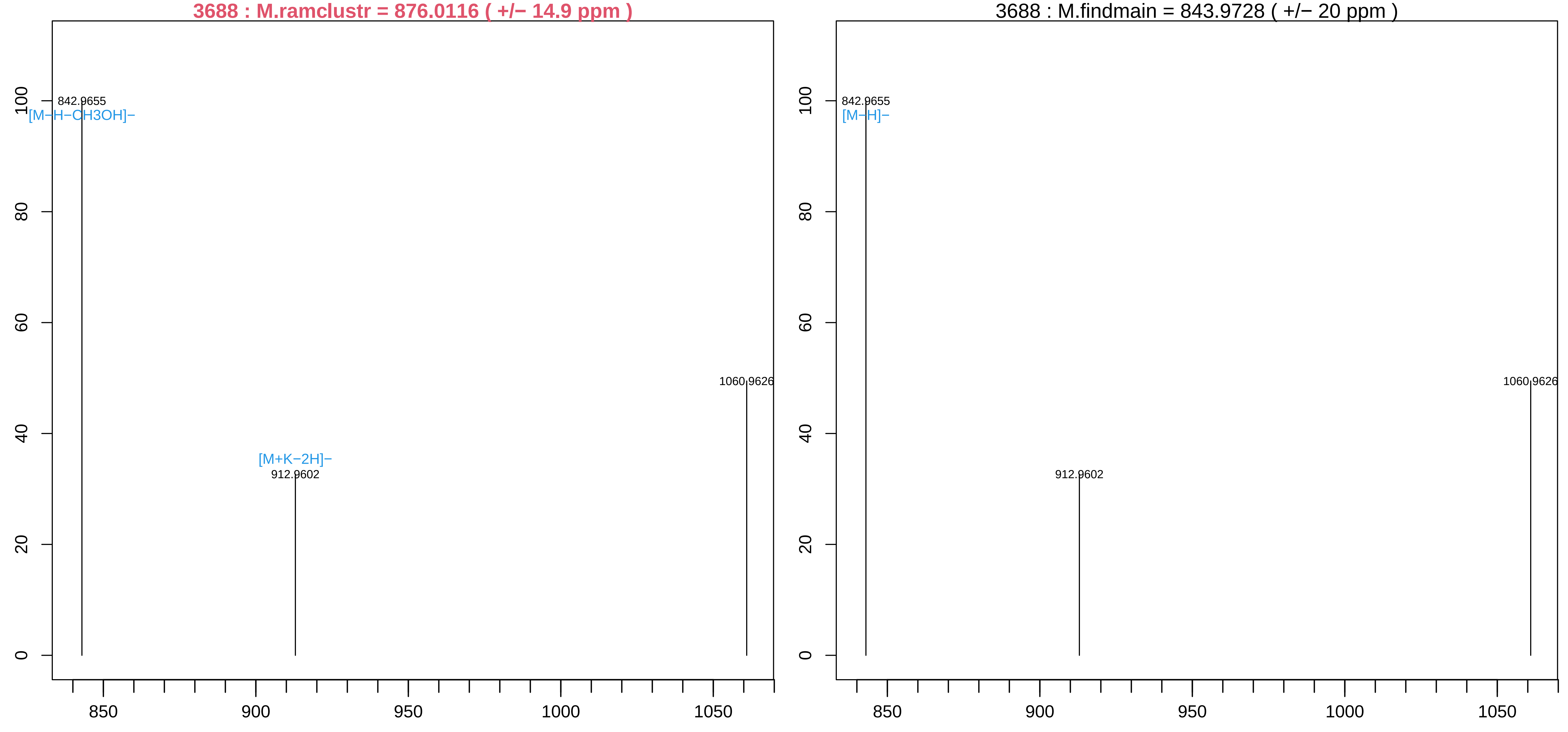
3688 : M.ramclustr = 876.0116 (+/− 14.9 ppm)
842.9655
[M−H−CH3OH]−
[M+K−2H]−
912.9602
1060.9626
3688 : M.findmain = 843.9728 (+/− 20 ppm)
842.9655
[M−H]−
912.9602
1060.9626

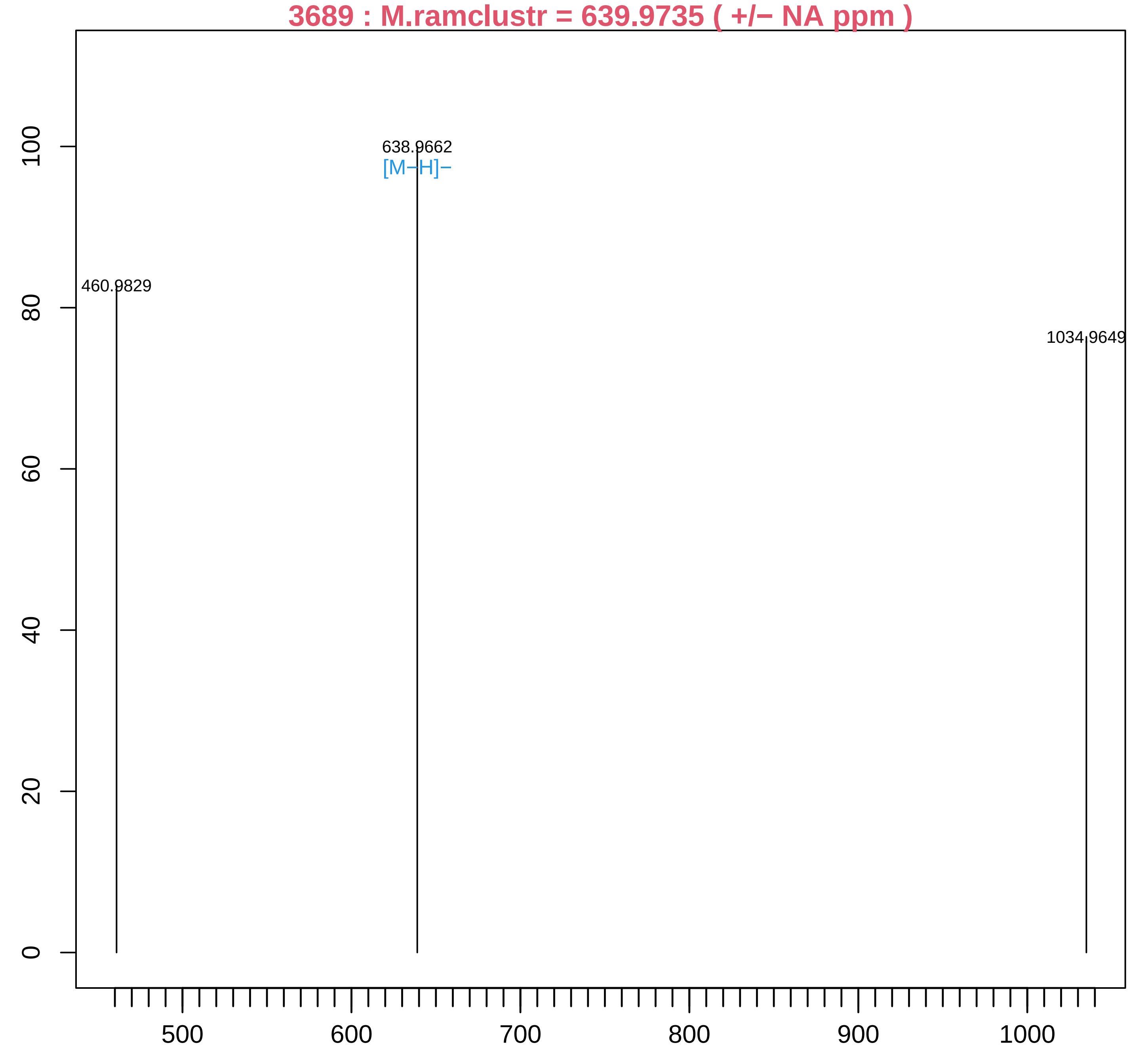
3689 : M.ramclustr = 639.9735 (+/− NA ppm)
638.9662
[M−H]−
460.9829
1034.9649
0
20
40
60
80
100
500
600
700
800
900
1000

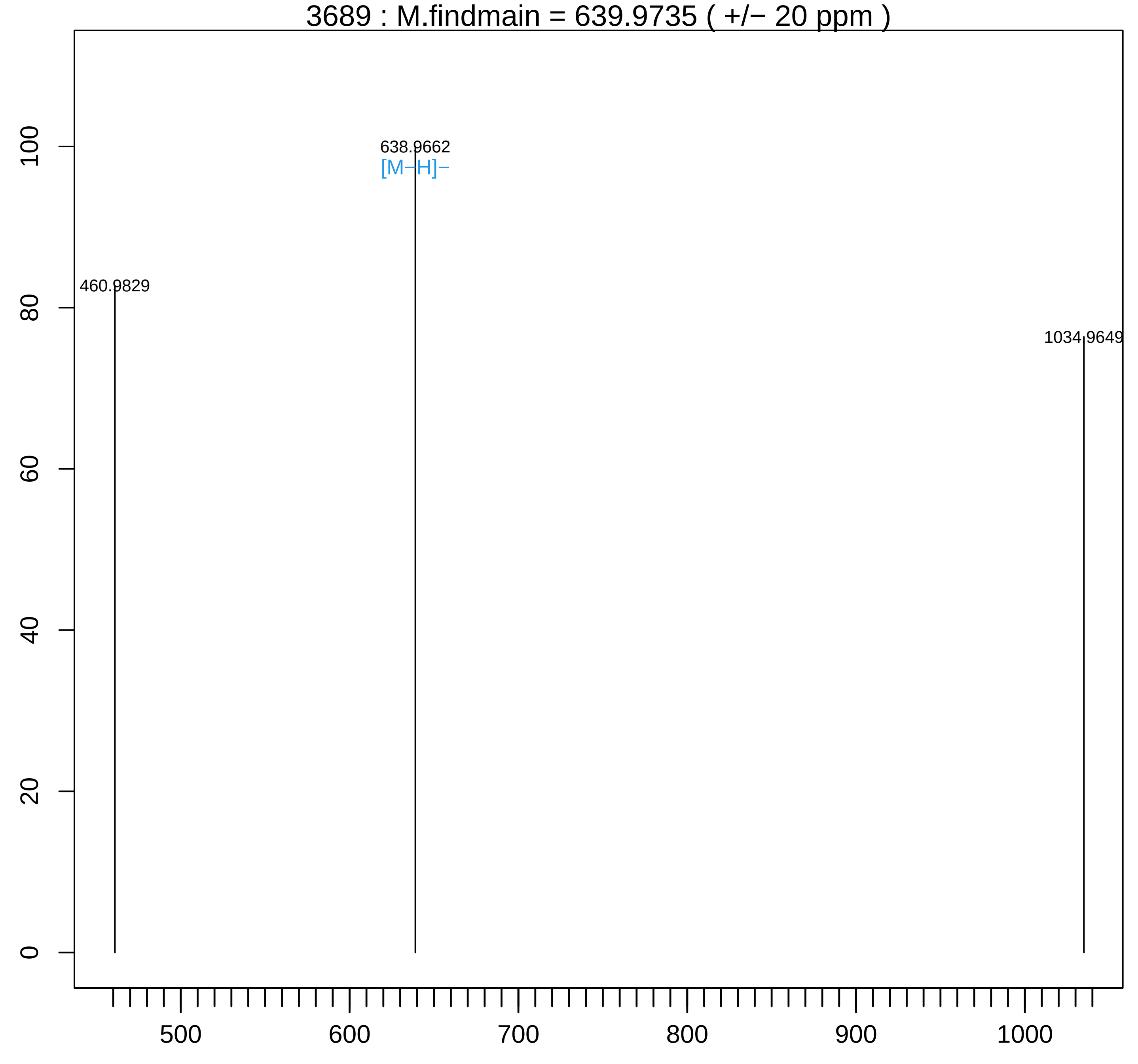
3689 : M.findmain = 639.9735 (+/− 20 ppm)
638.9662
[M−H]−
460.9829
1034.9649
0
20
40
60
80
100
500
600
700
800
900
1000

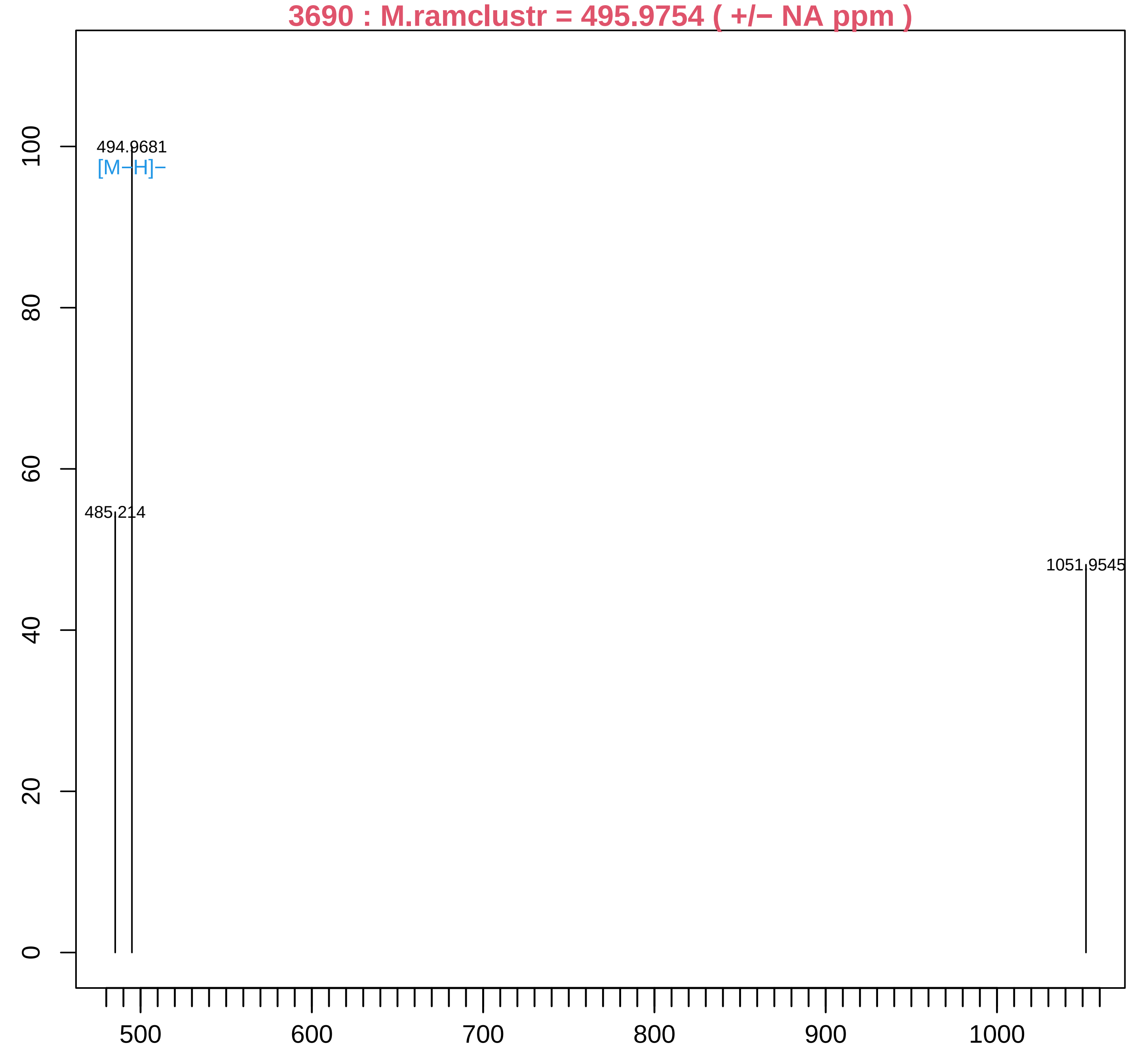
3690 : M.ramclustr = 495.9754 (+/− NA ppm)
494.9681
[M−H]−
485.214
1051.9545
0
20
40
60
80
100
500
600
700
800
900
1000

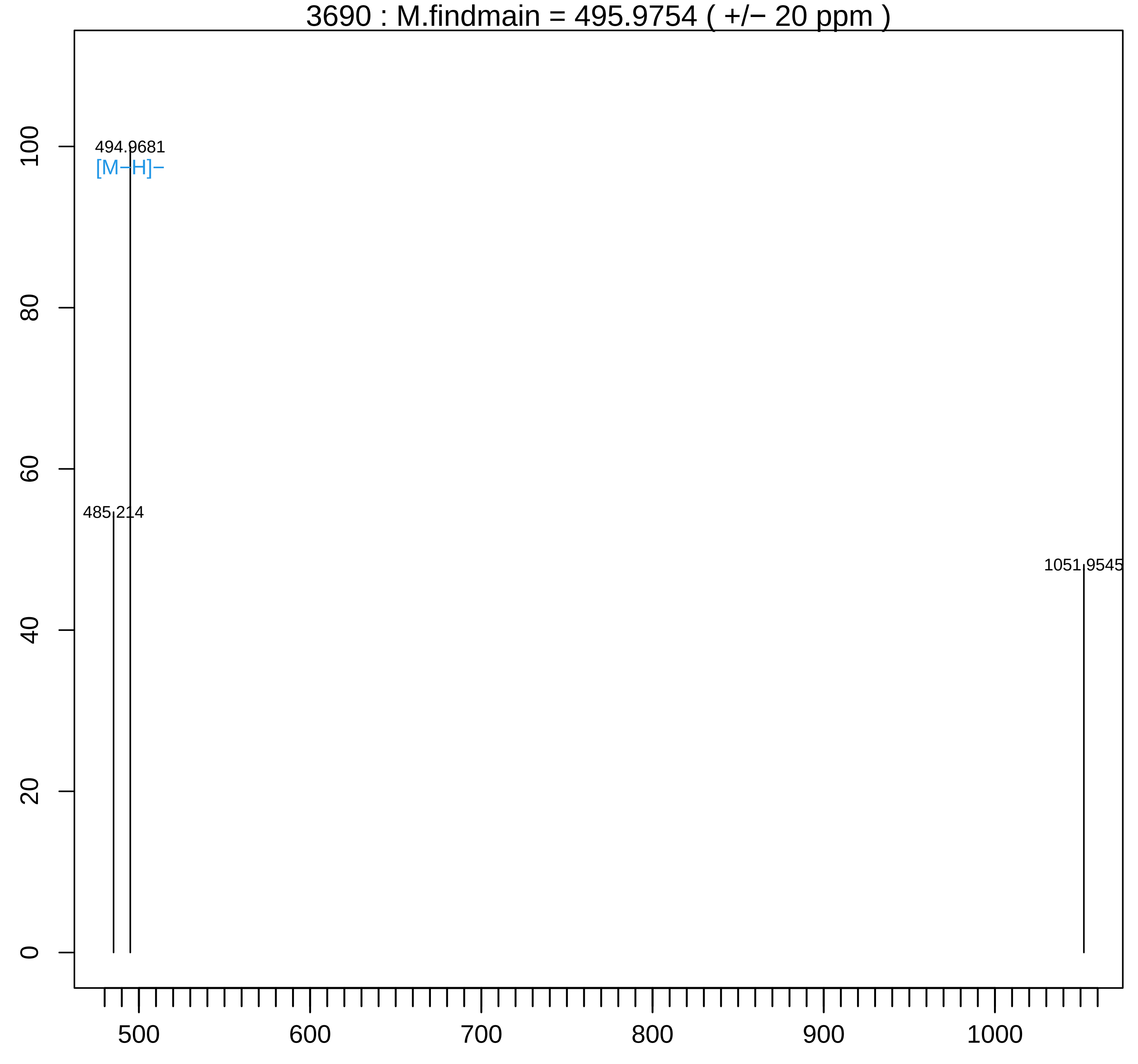
3690 : M.findmain = 495.9754 (+/− 20 ppm)
494.9681
[M−H]−
485.214
1051.9545
0
20
40
60
80
100
500
600
700
800
900
1000

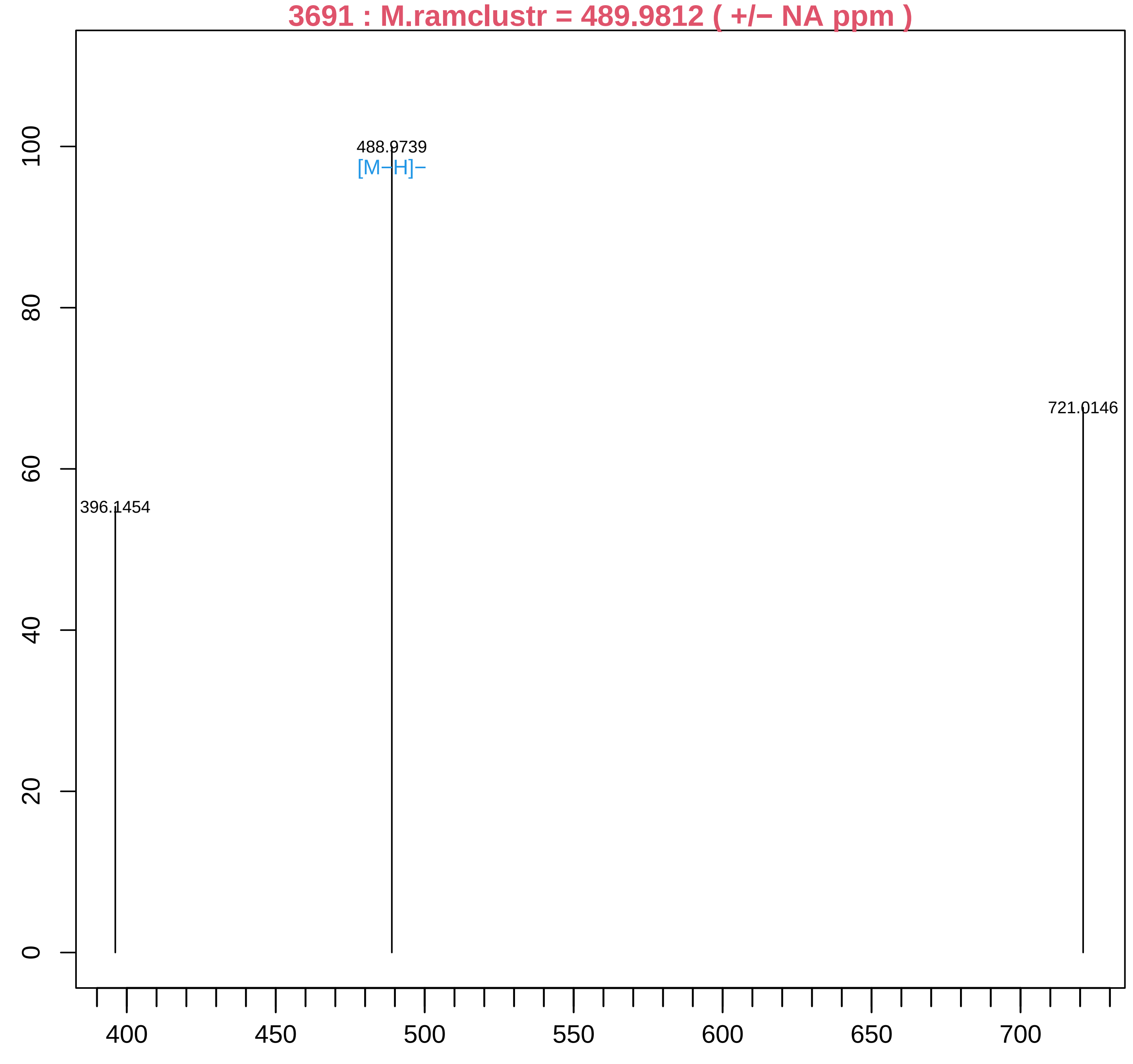
3691 : M.ramclustr = 489.9812 (+/− NA ppm)
488.9739
[M−H]−
721.0146
396.1454
0
20
40
60
80
100
400
450
500
550
600
650
700

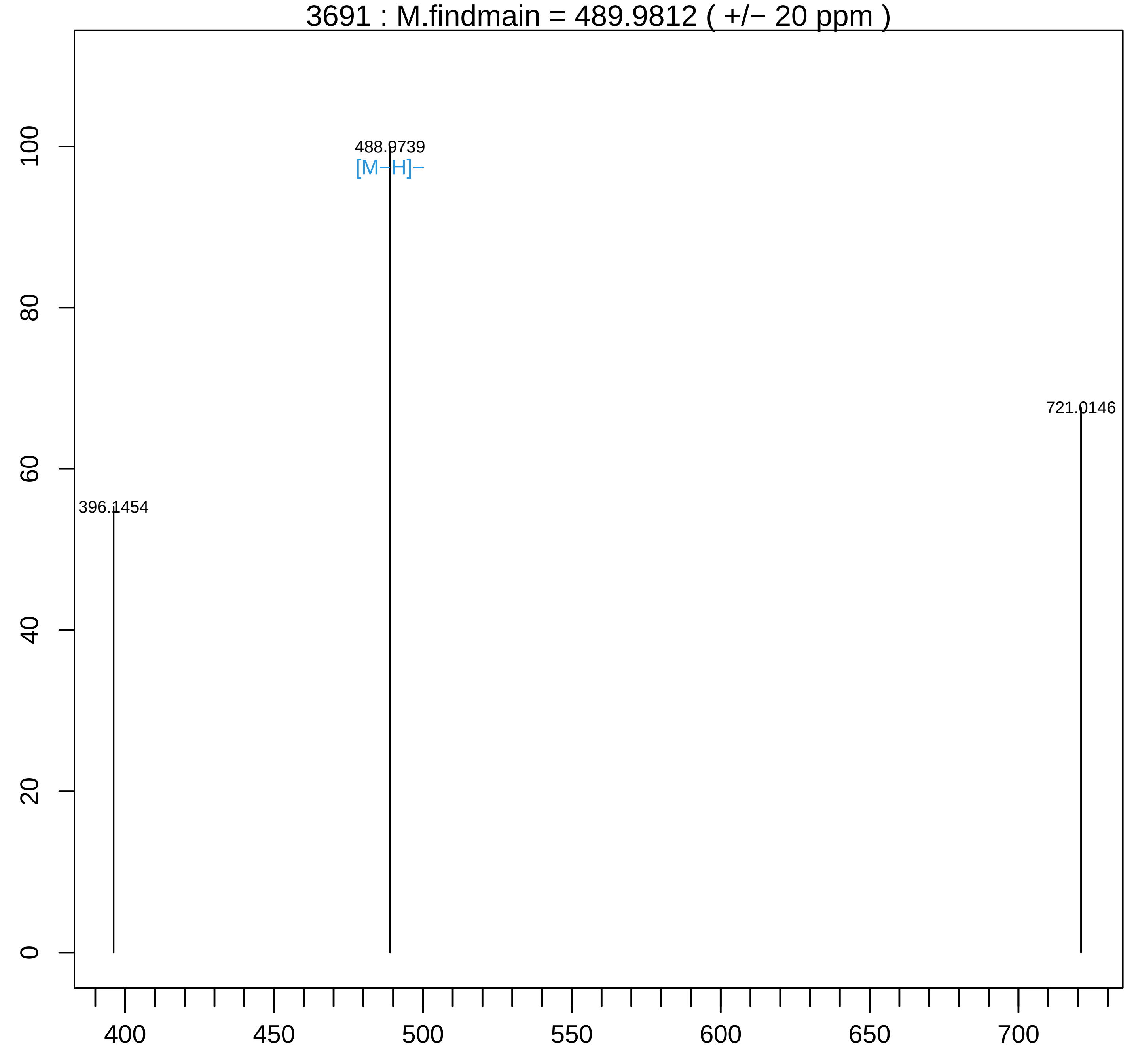
3691 : M.findmain = 489.9812 (+/− 20 ppm)
488.9739
[M−H]−
721.0146
396.1454
0
20
40
60
80
100
400
450
500
550
600
650
700

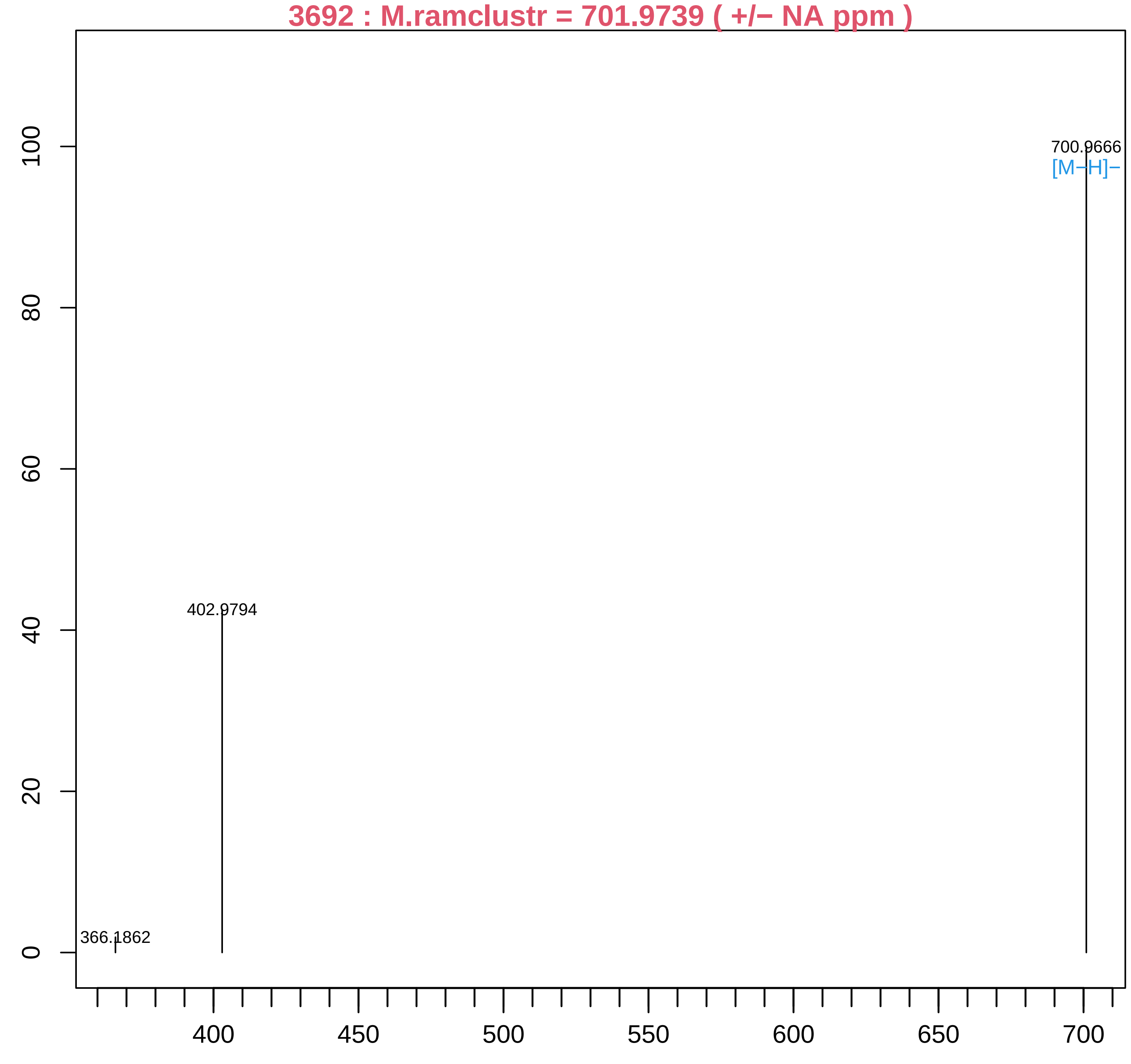
3692 : M.ramclustr = 701.9739 (+/− NA ppm)
700.9666
[M−H]−
402.9794
366.1862
0
20
40
60
80
100
400
450
500
550
600
650
700

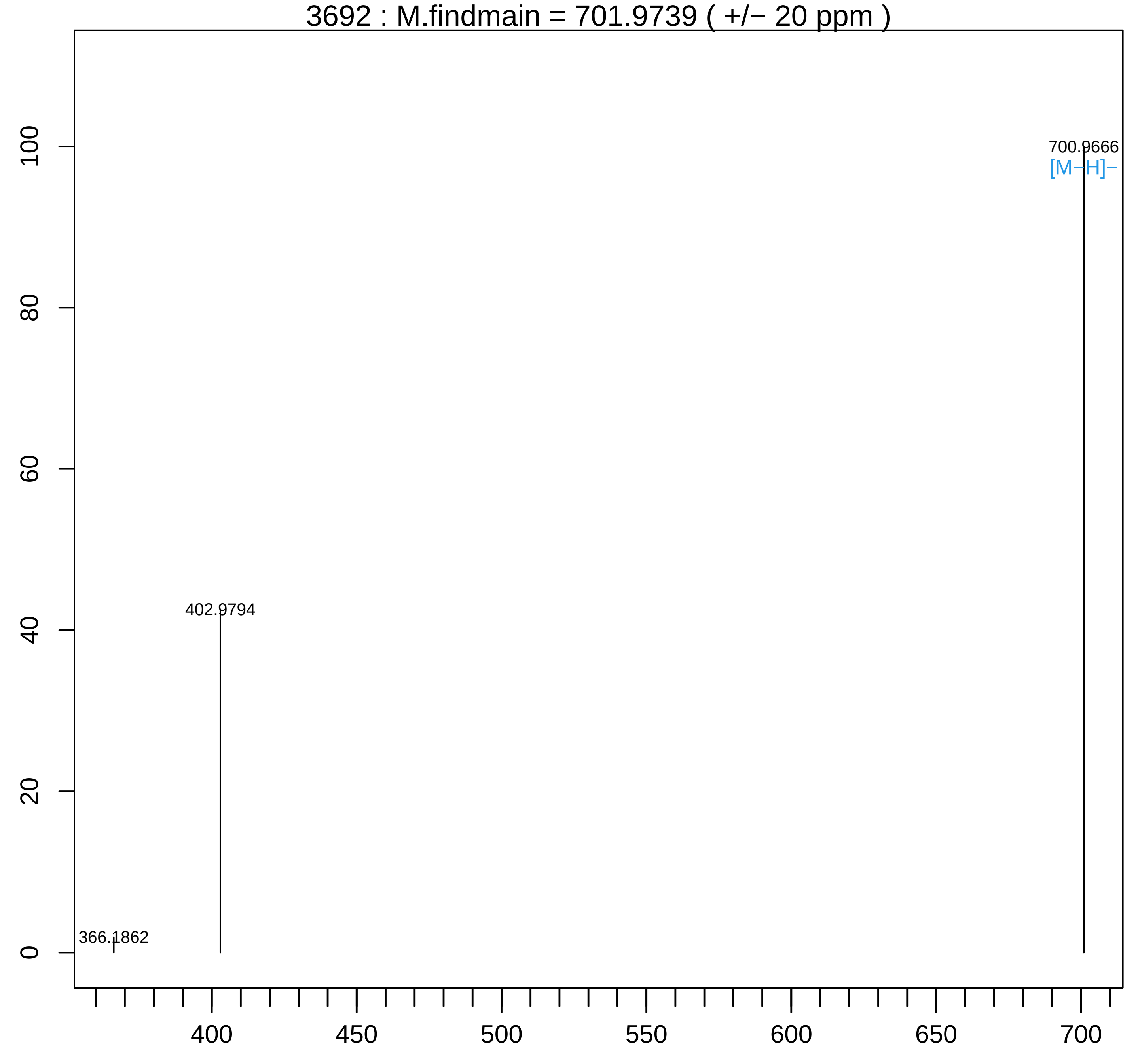
3692 : M.findmain = 701.9739 (+/− 20 ppm)
700.9666
[M−H]−
402.9794
366.1862
0
20
40
60
80
100
400
450
500
550
600
650
700

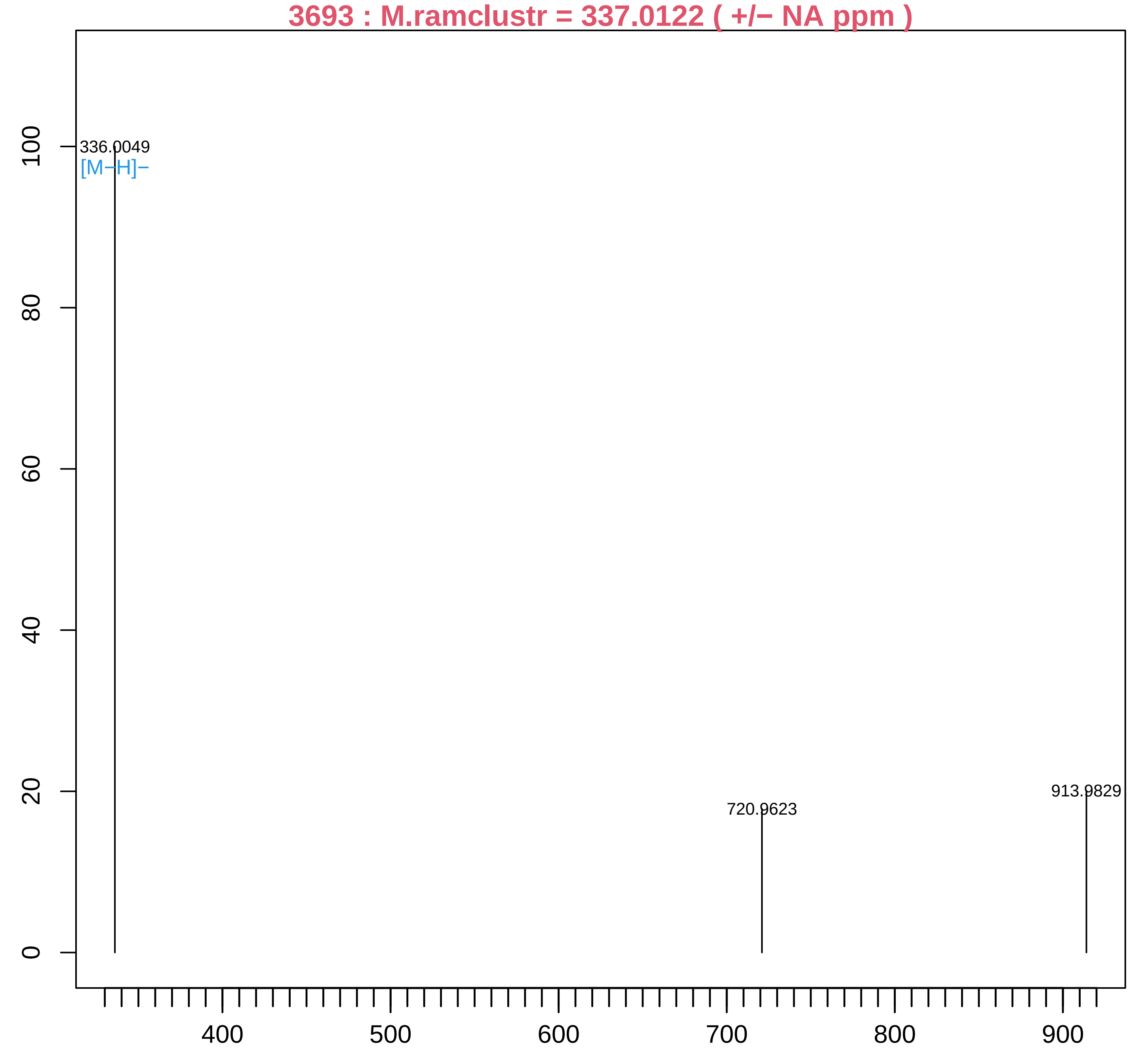
3693 : M.ramclustr = 337.0122 (+/− NA ppm)
336.0049
[M−H]−
720.9623
913.9829
0
20
40
60
80
100
400
500
600
700
800
900

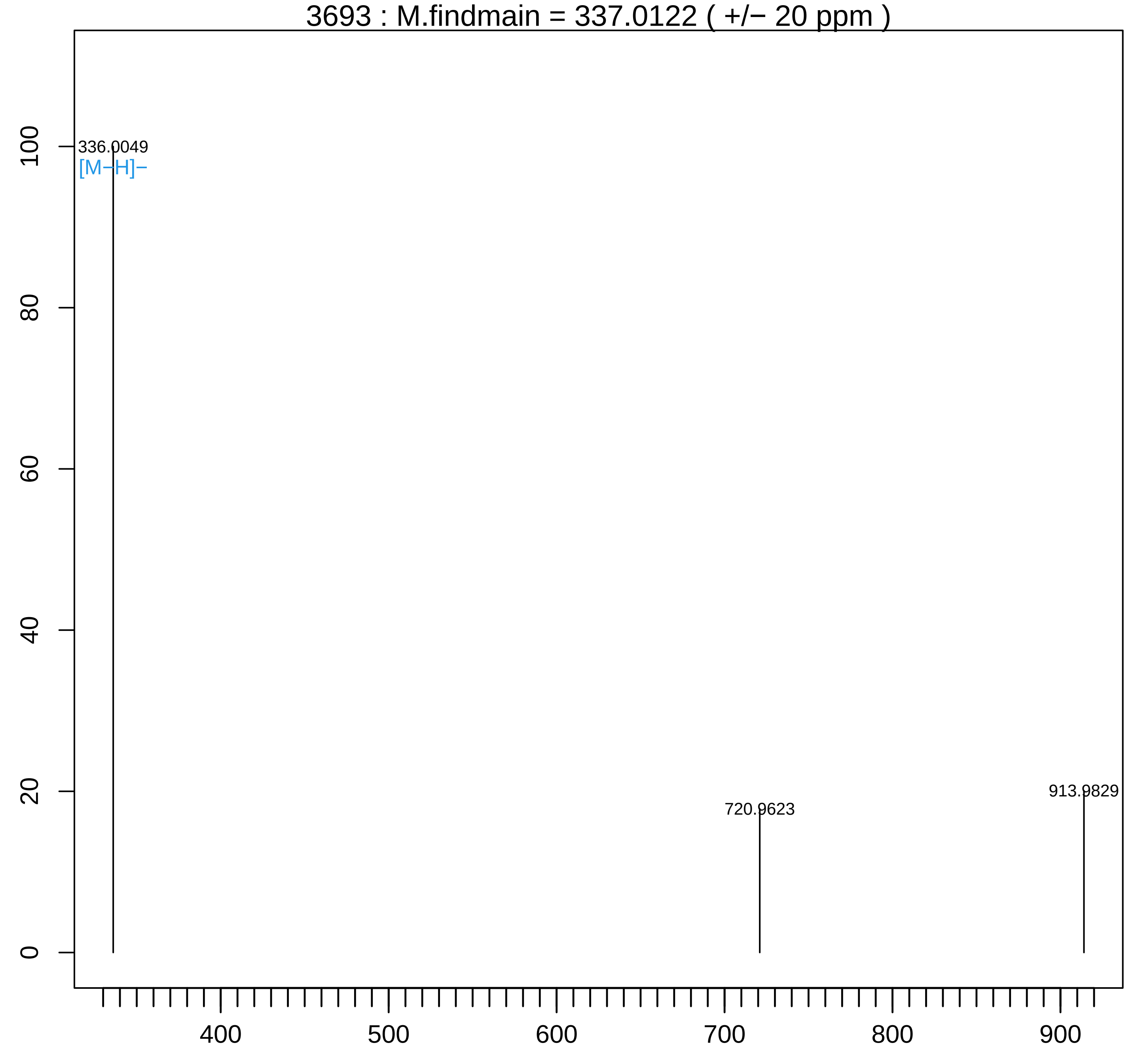
3693 : M.findmain = 337.0122 (+/− 20 ppm)
336.0049
[M−H]−
720.9623
913.9829
0
20
40
60
80
100
400
500
600
700
800
900

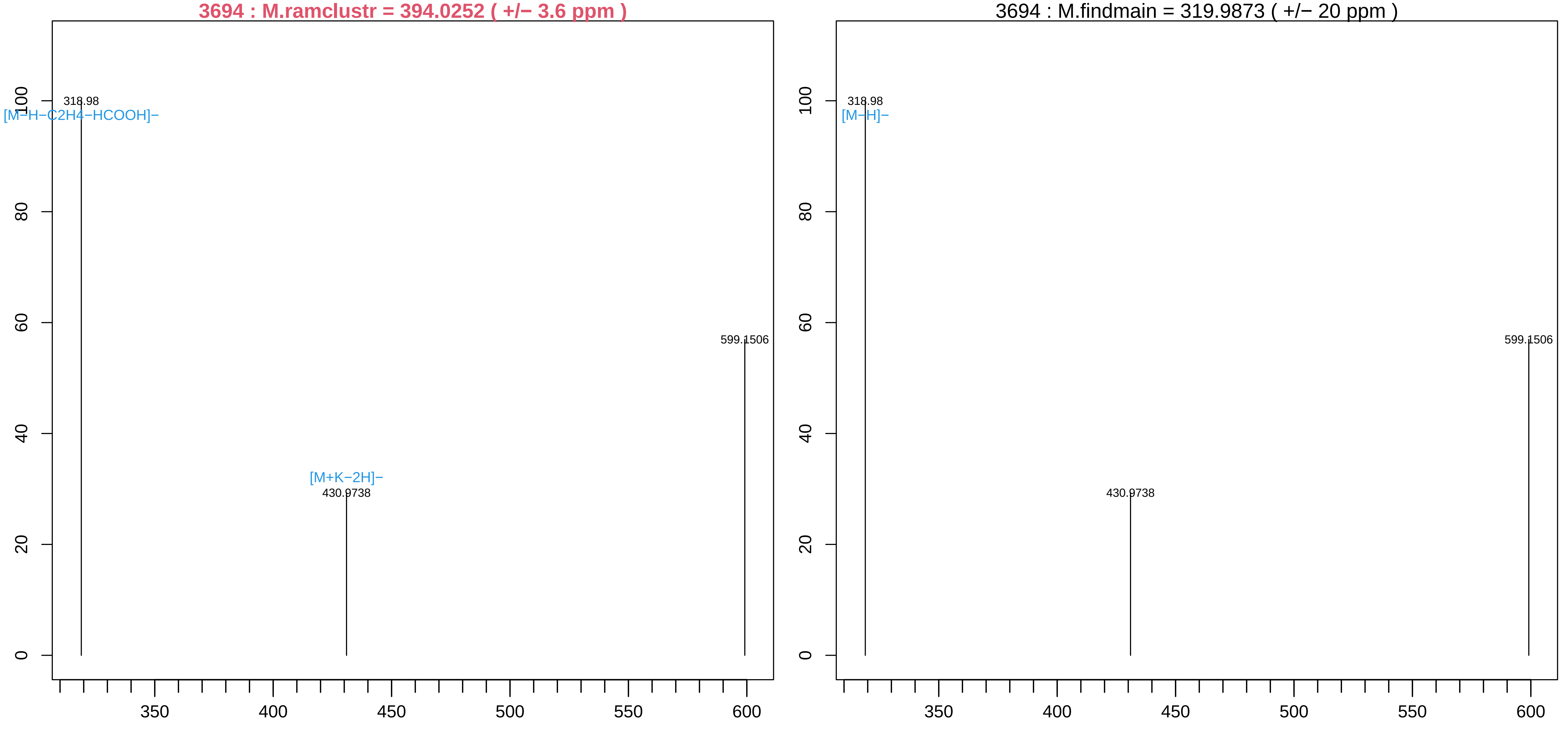
3694 : M.ramclustr = 394.0252 (+/− 3.6 ppm)
318.98
[M−H−C2H4−HCOOH]−
[M+K−2H]−
430.9738
599.1506
3694 : M.findmain = 319.9873 (+/− 20 ppm)
318.98
[M−H]−
430.9738
599.1506

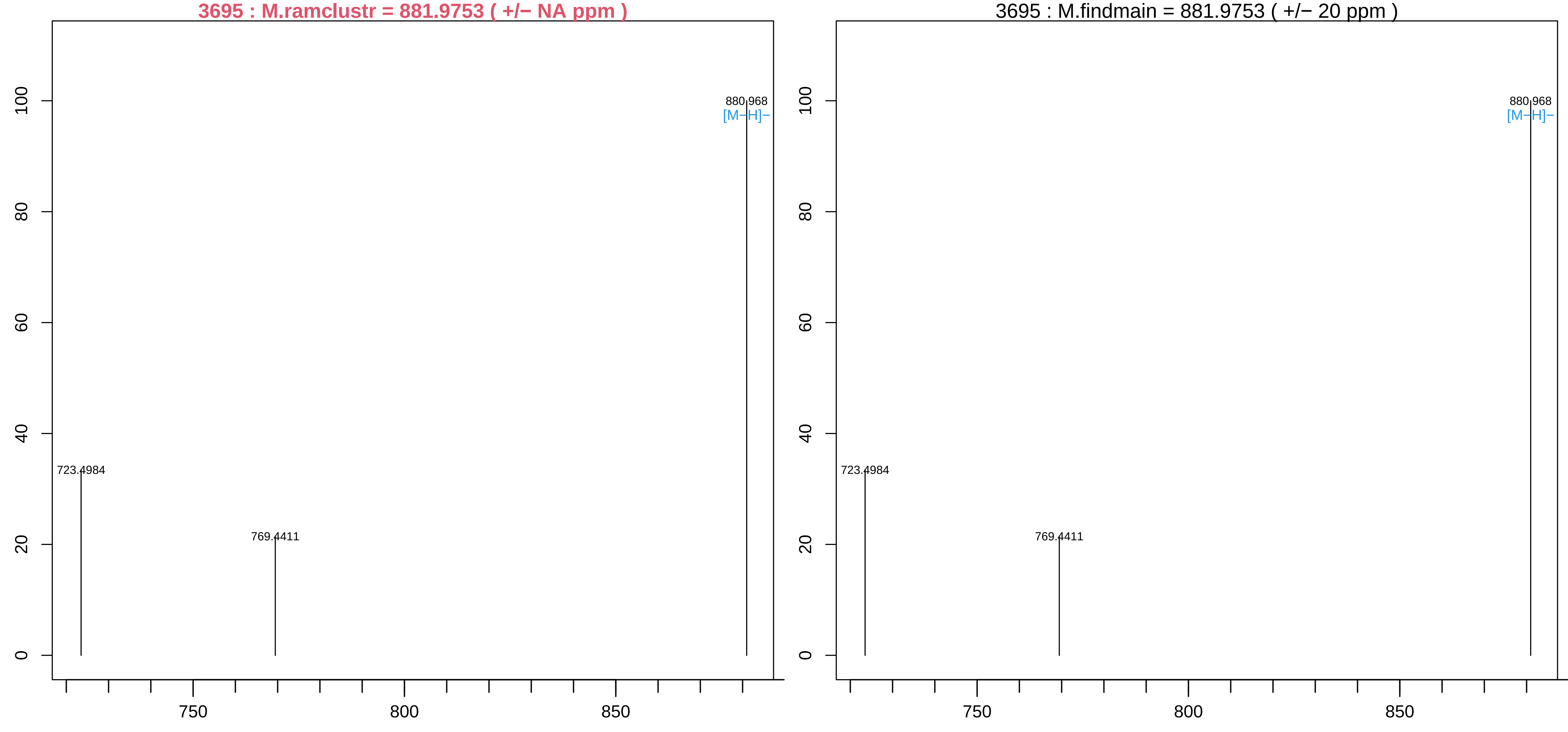

3695 : M.ramclustr = 881.9753 (+/− NA ppm)
880.968
[M−H]−
723.4984
769.4411
0
20
40
60
80
100
750
800
850
3695 : M.findmain = 881.9753 (+/− 20 ppm)
880.968
[M−H]−
723.4984
769.4411
0
20
40
60
80
100
750
800
850

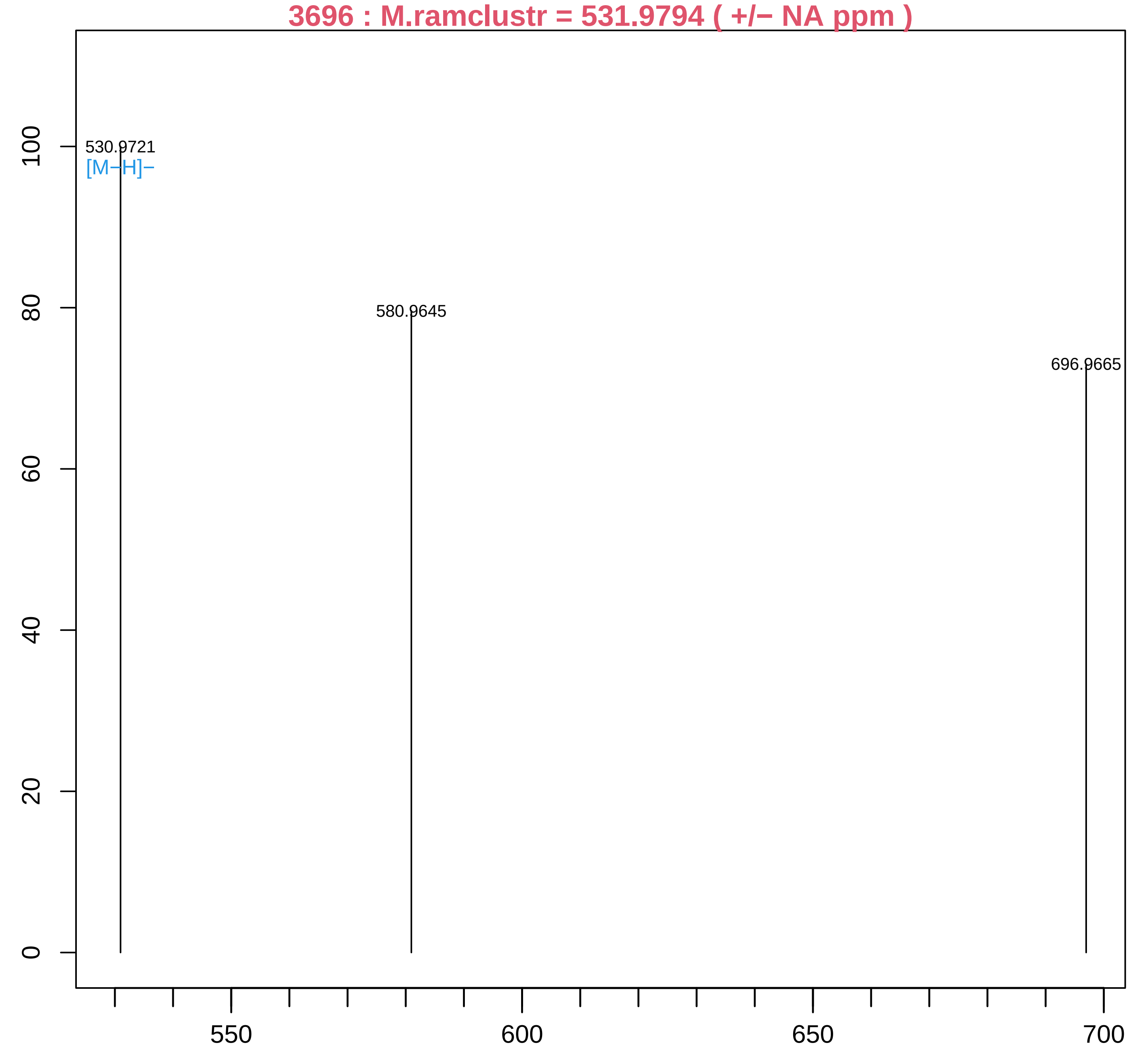
3696 : M.ramclustr = 531.9794 (+/− NA ppm)
530.9721
[M−H]−
580.9645
696.9665
0
20
40
60
80
100
550
600
650
700

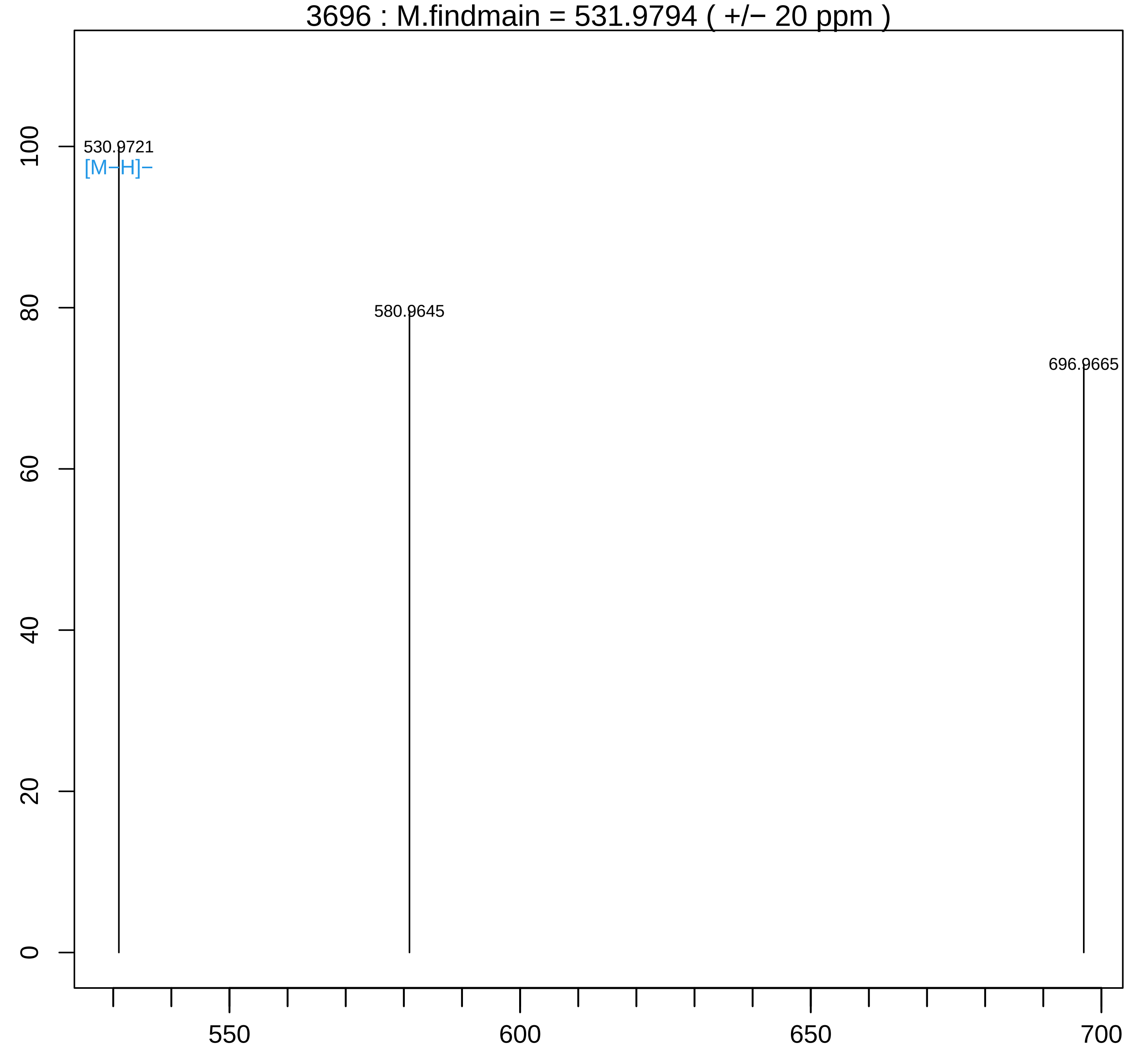
3696 : M.findmain = 531.9794 (+/− 20 ppm)
530.9721
[M−H]−
580.9645
696.9665
0
20
40
60
80
100
550
600
650
700

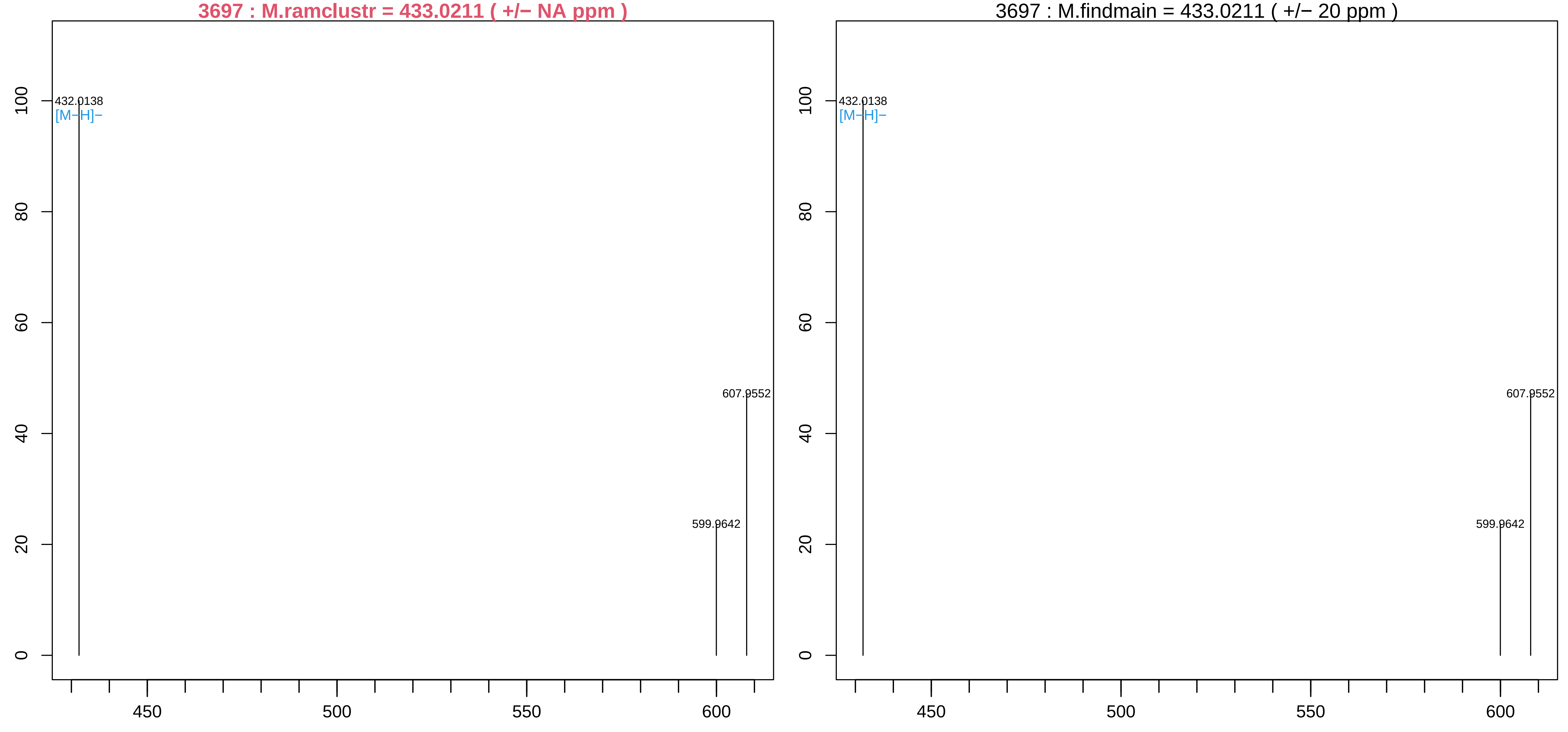
3697 : M.ramclustr = 433.0211 (+/− NA ppm)
432.0138
[M−H]−
607.9552
599.9642
0
20
40
60
80
100
450
500
550
600
3697 : M.findmain = 433.0211 (+/− 20 ppm)
432.0138
[M−H]−
607.9552
599.9642
0
20
40
60
80
100
450
500
550
600

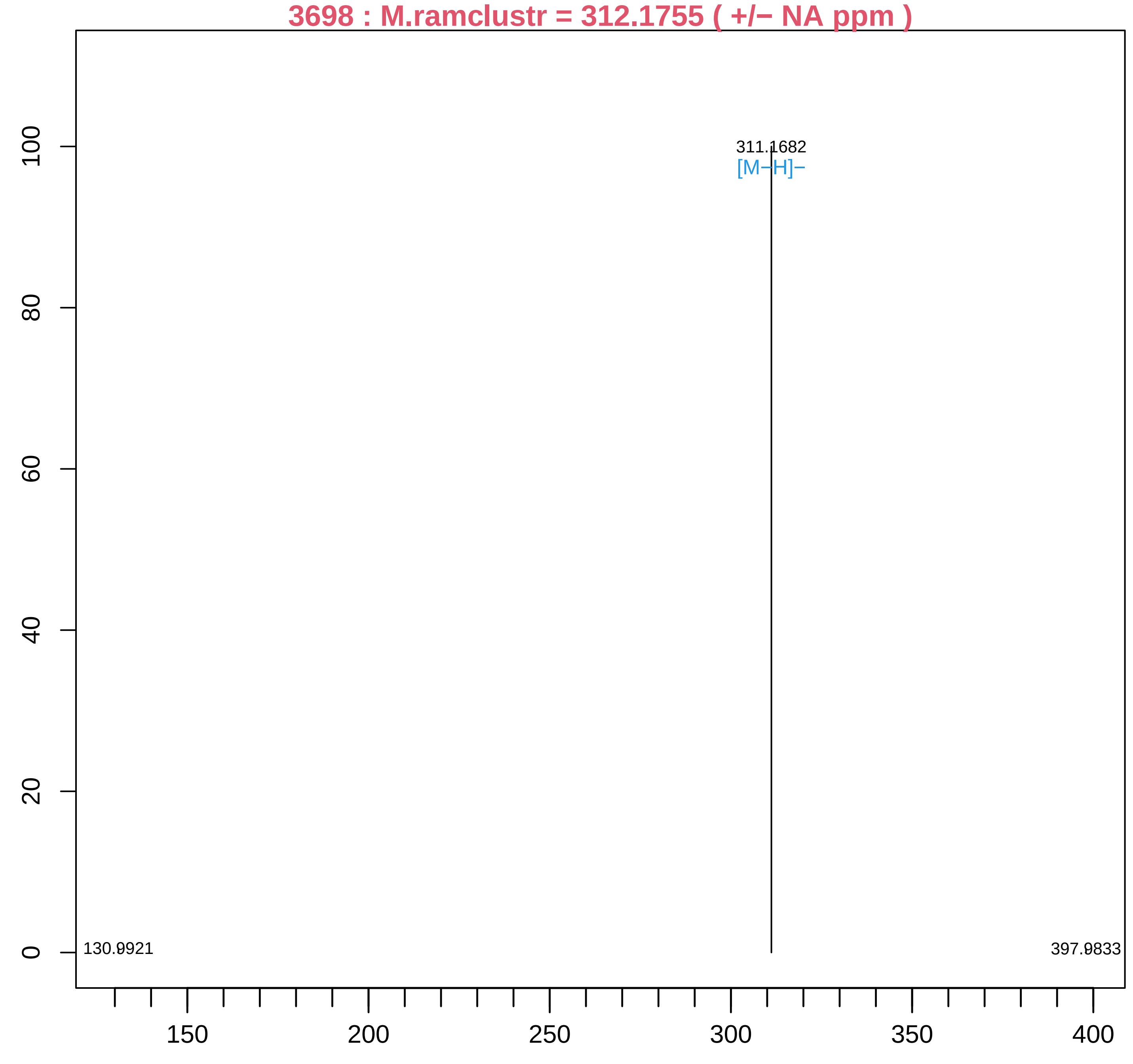
3698 : M.ramclustr = 312.1755 (+/− NA ppm)
311.1682
[M−H]−
130.9921
397.9833
0
20
40
60
80
100
150
200
250
300
350
400

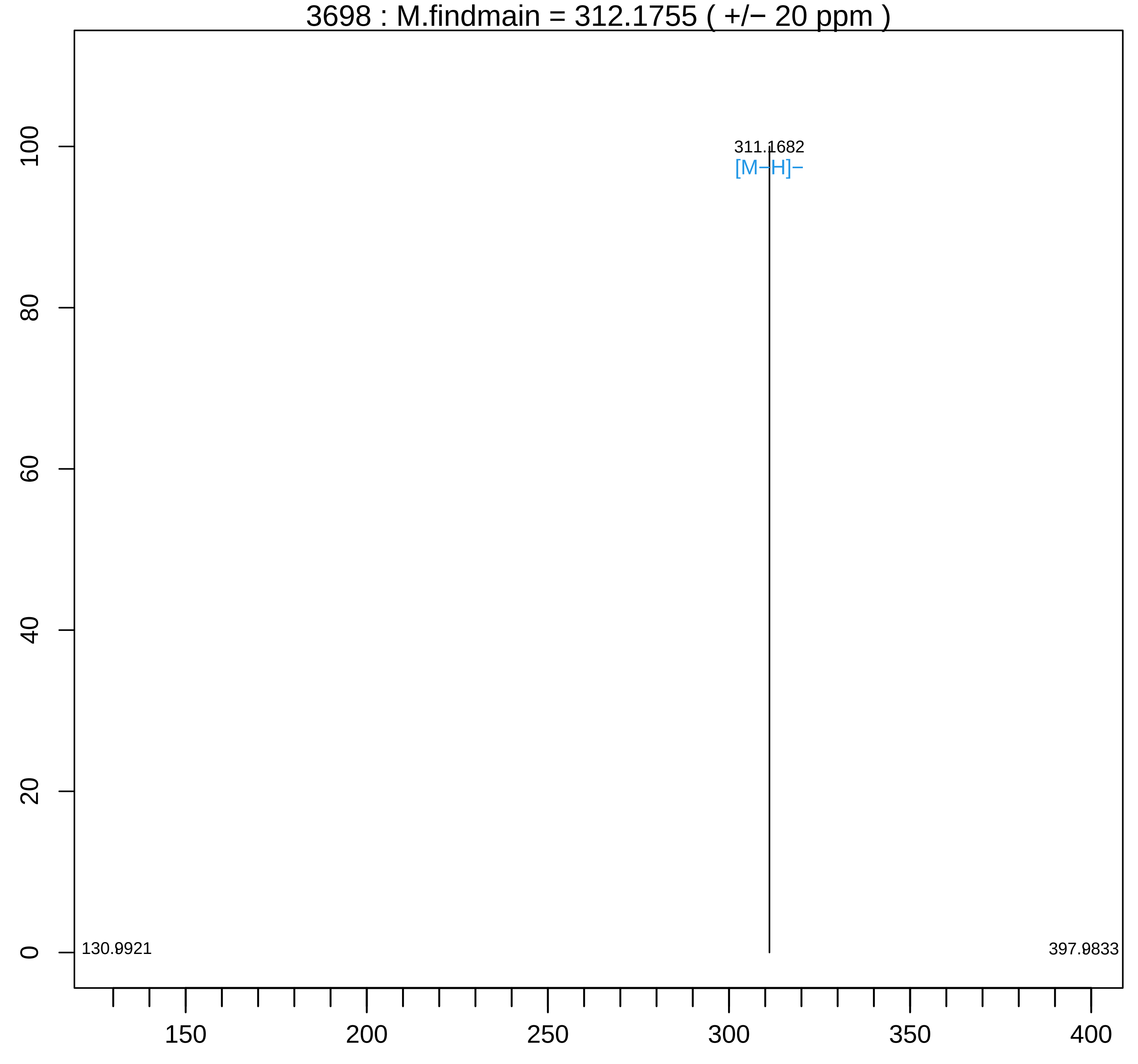
3698 : M.findmain = 312.1755 (+/− 20 ppm)
311.1682
[M−H]−
130.9921
397.9833
0
20
40
60
80
100
150
200
250
300
350
400

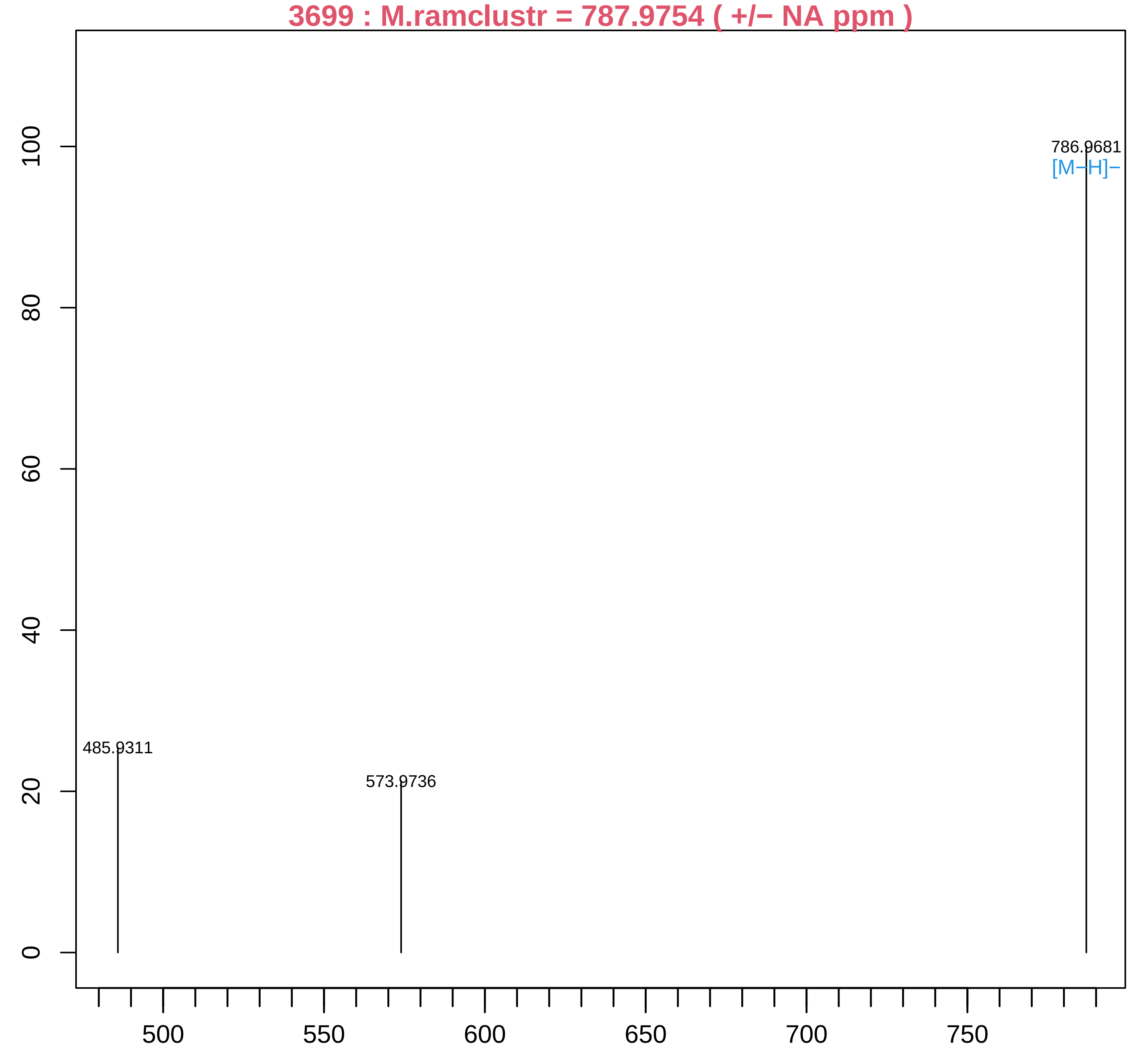
3699 : M.ramclustr = 787.9754 (+/− NA ppm)
786.9681
[M−H]−
485.9311
573.9736
0
20
40
60
80
100
500
550
600
650
700
750

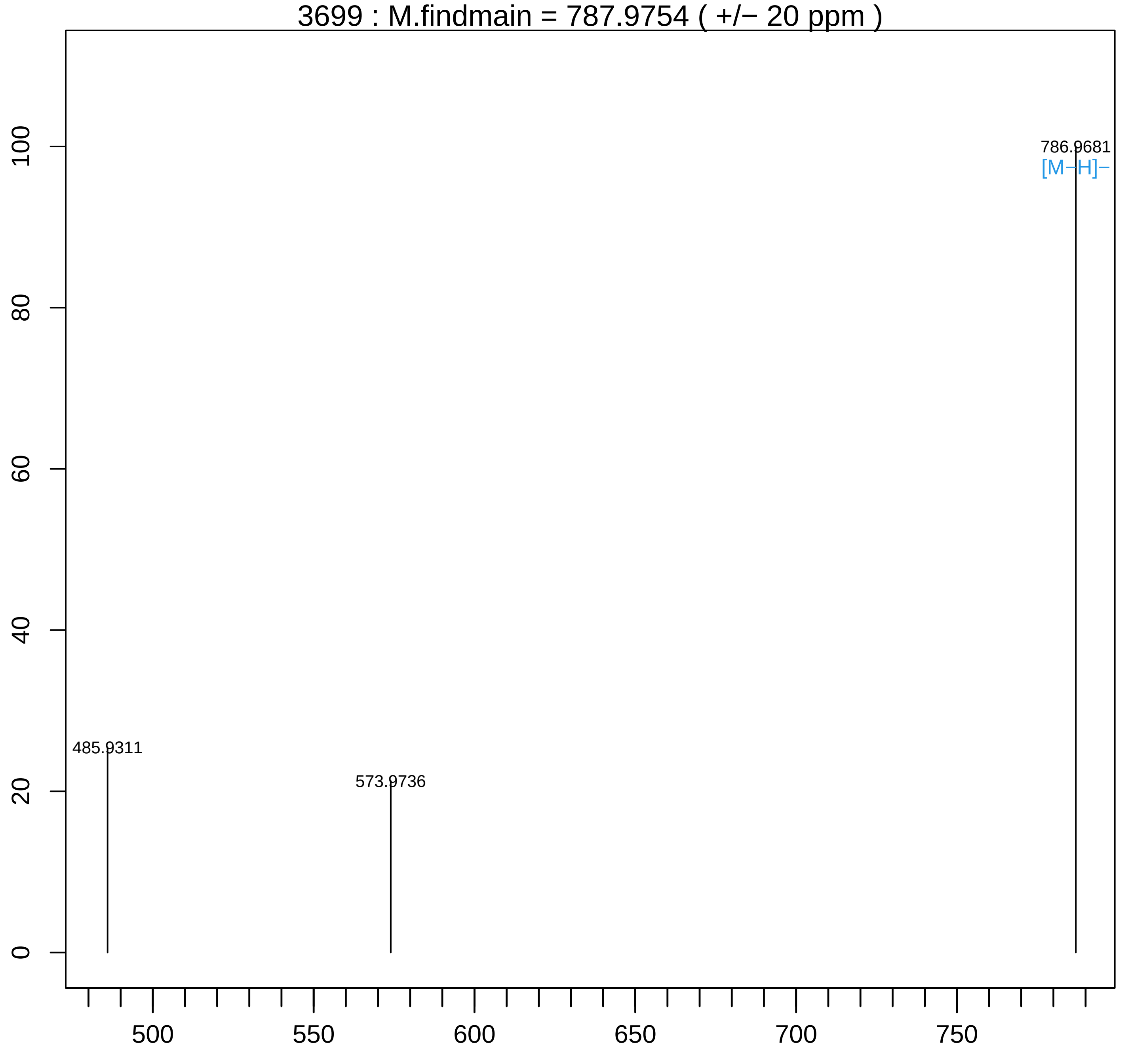
3699 : M.findmain = 787.9754 (+/− 20 ppm)
786.9681
[M−H]−
485.9311
573.9736
0
20
40
60
80
100
500
550
600
650
700
750

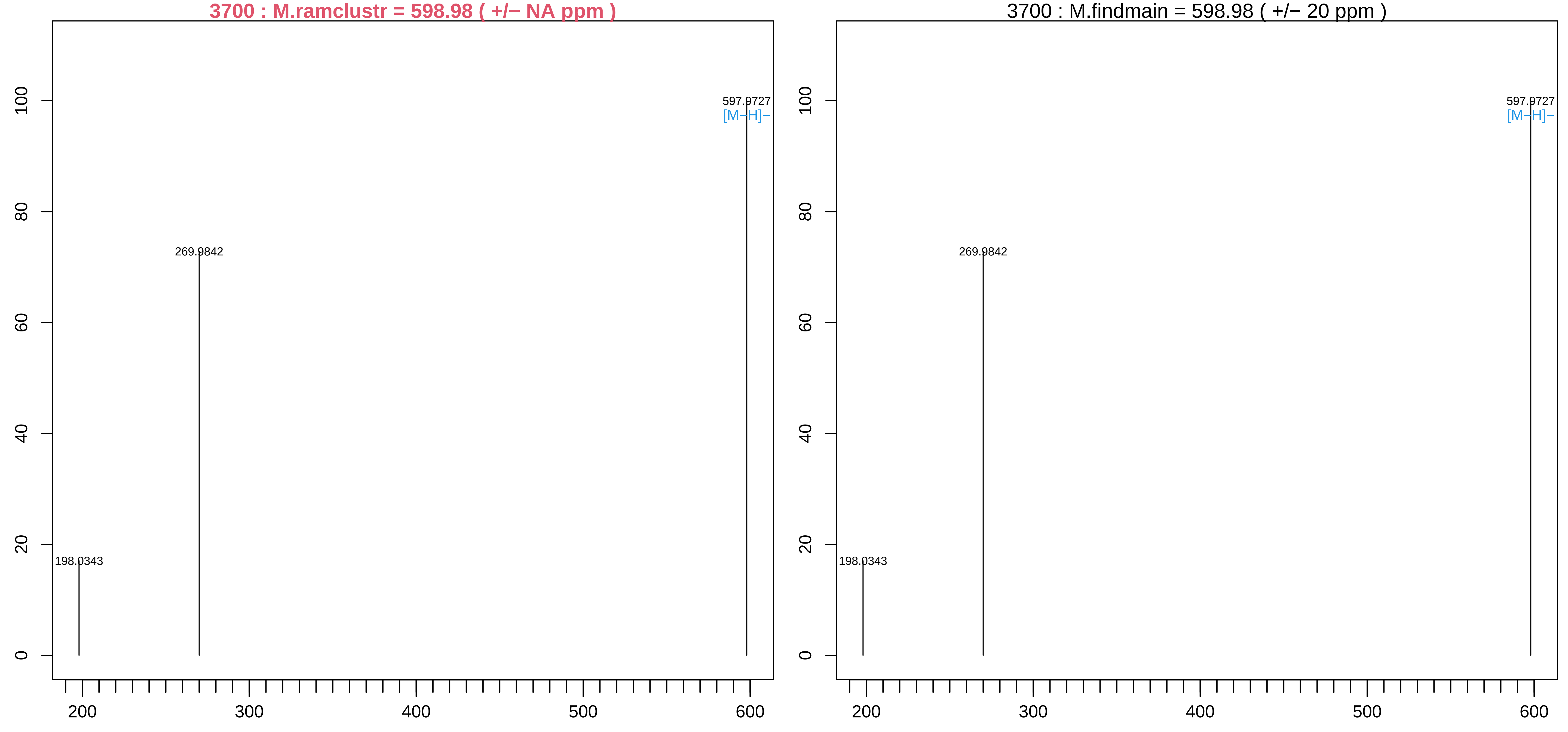
3700 : M.ramclustr = 598.98 (+/− NA ppm)
597.9727
[M−H]−
269.9842
198.0343
0
20
40
60
80
100
200
300
400
500
600
3700 : M.findmain = 598.98 (+/− 20 ppm)
597.9727
[M−H]−
269.9842
198.0343
0
20
40
60
80
100
200
300
400
500
600

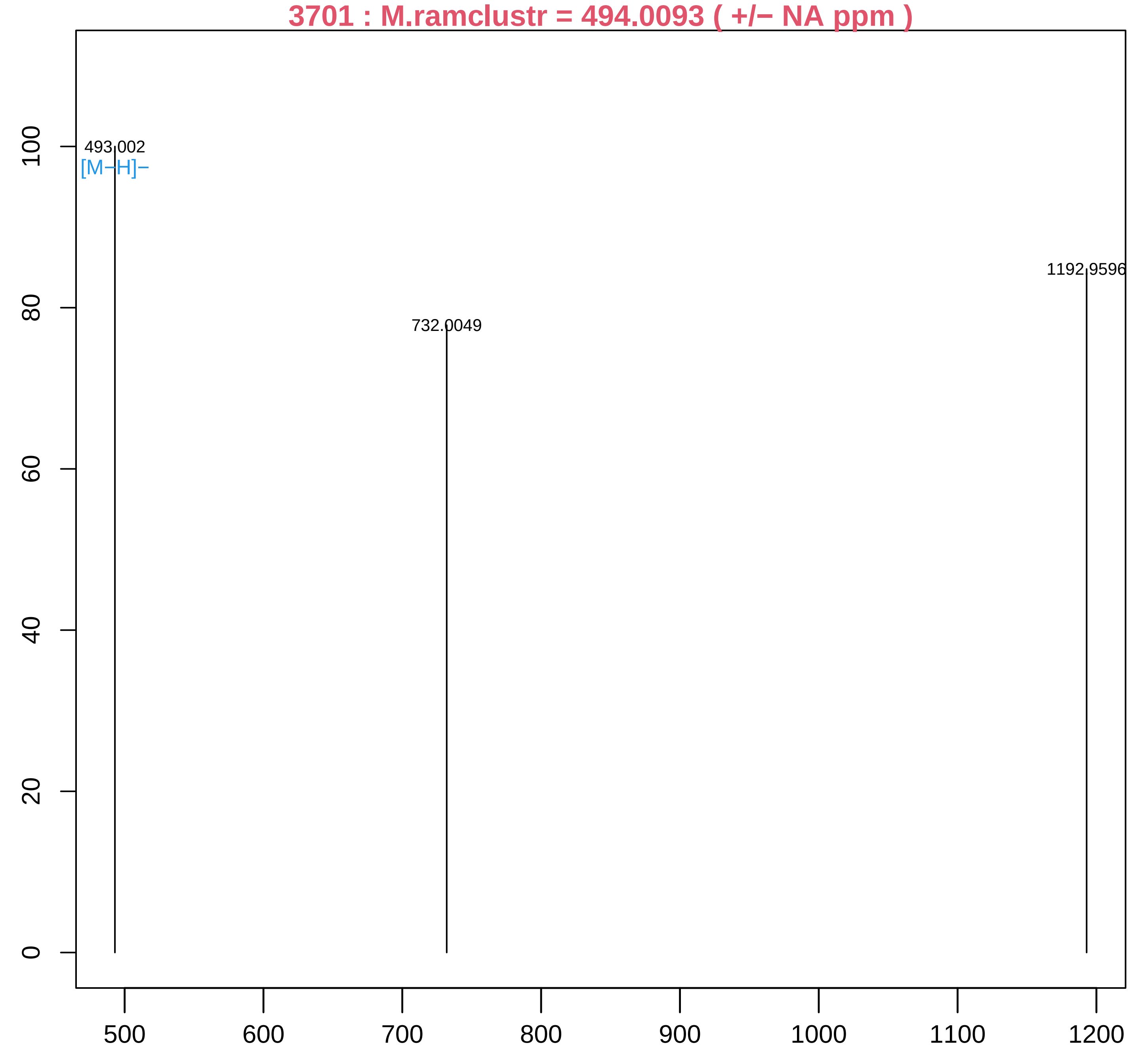

3701 : M.ramclustr = 494.0093 (+/− NA ppm)
493.002
[M−H]−
732.0049
1192.9596
0
20
40
60
80
100
500
600
700
800
900
1000
1100
1200

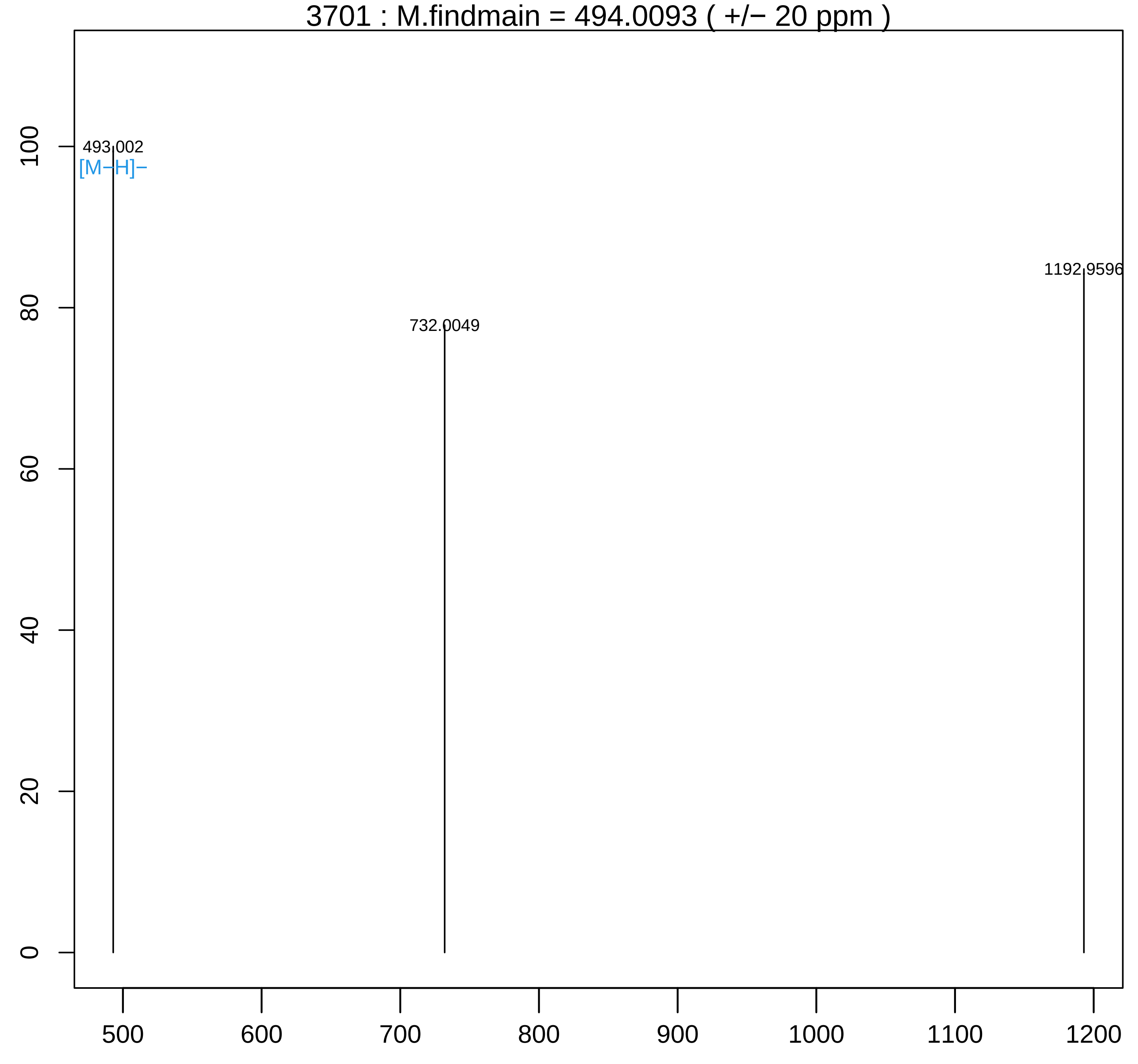

3701 : M.findmain = 494.0093 (+/− 20 ppm)
493.002
[M−H]−
732.0049
1192.9596
0
20
40
60
80
100
500
600
700
800
900
1000
1100
1200

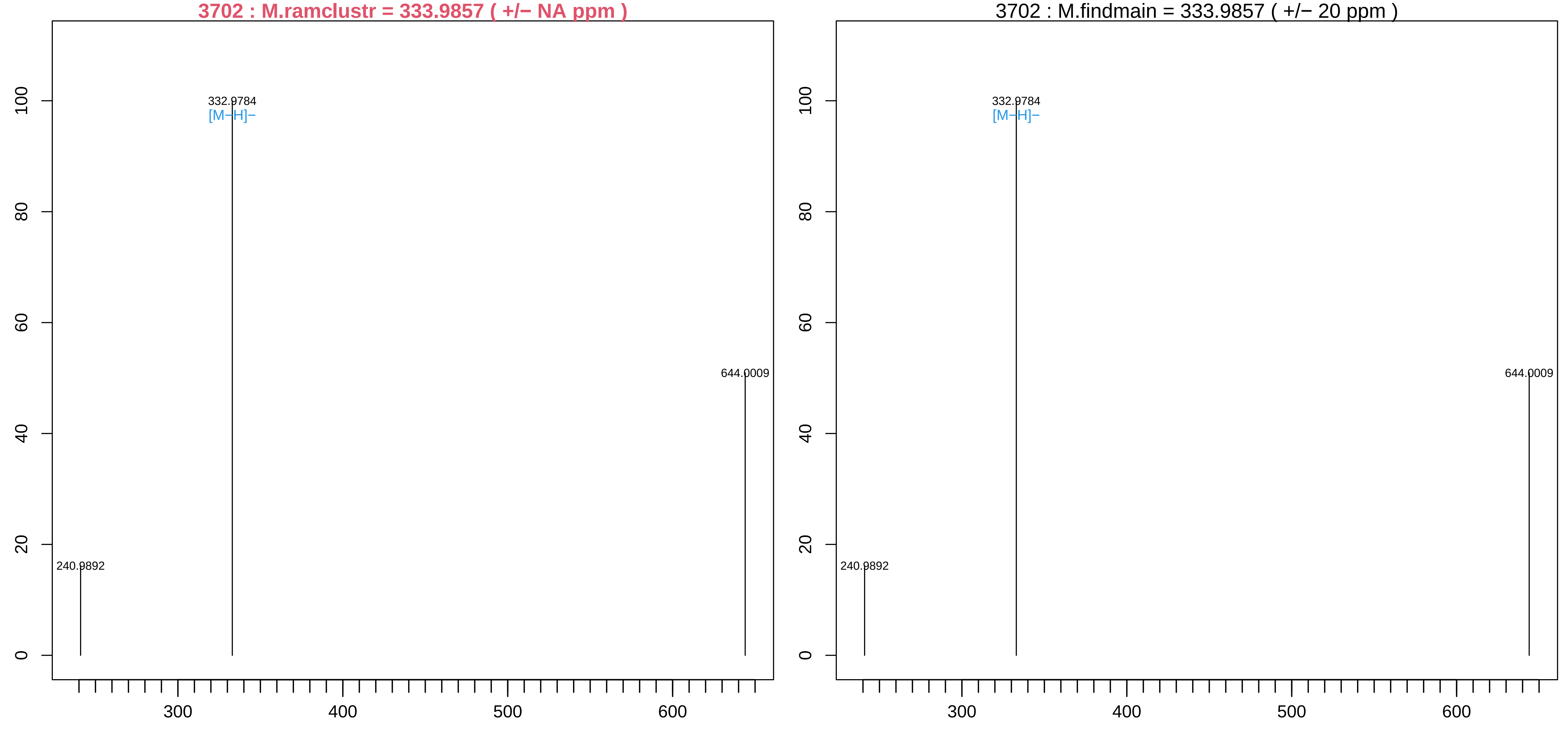
3702 : M.ramclustr = 333.9857 (+/− NA ppm)
332.9784
[M−H]−
644.0009
240.9892
3702 : M.findmain = 333.9857 (+/− 20 ppm)
332.9784
[M−H]−
644.0009
240.9892
0
20
40
60
80
100
300
400
500
600

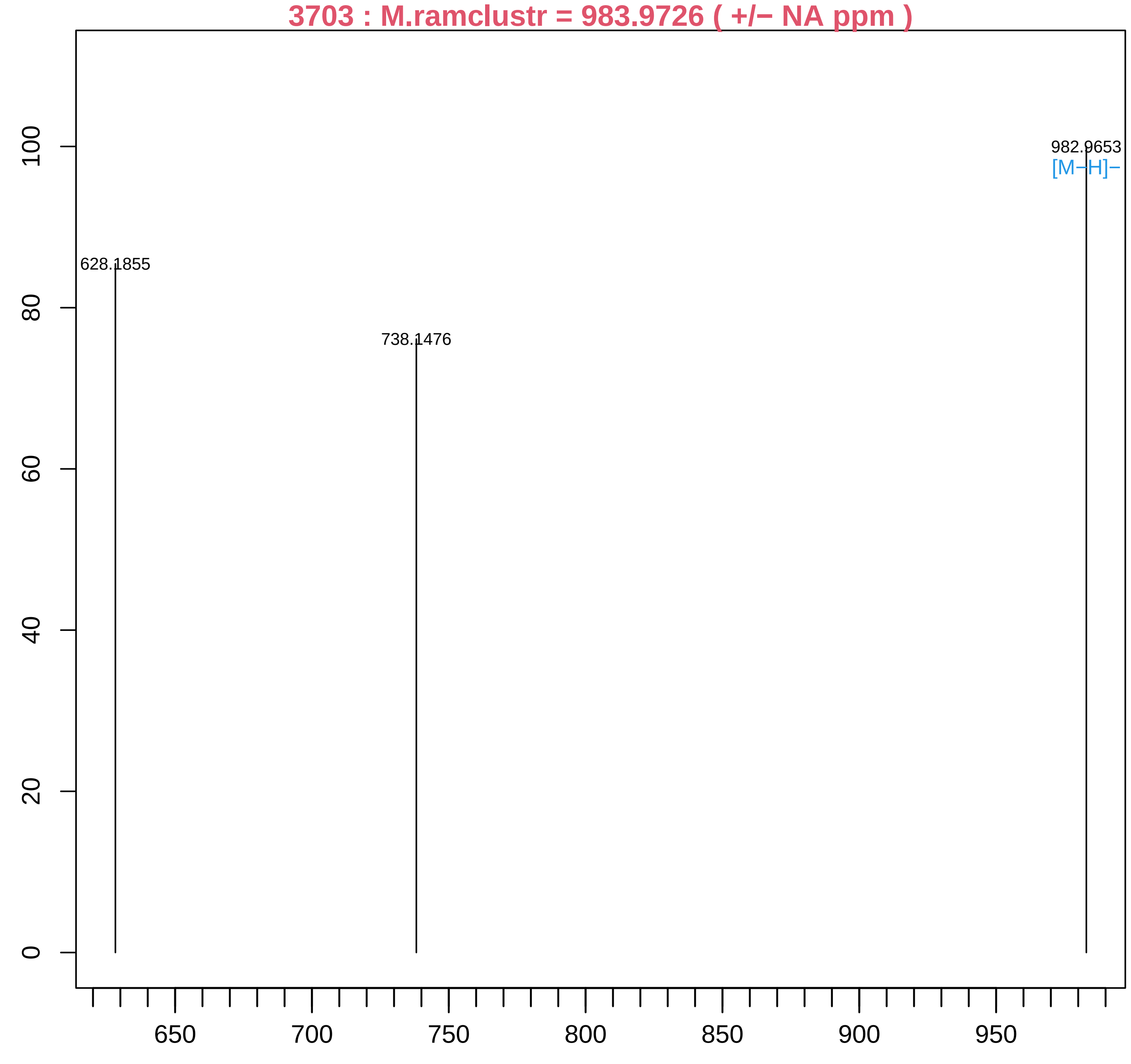
3703 : M.ramclustr = 983.9726 (+/− NA ppm)
982.9653
[M−H]−
628.1855
738.1476
0
20
40
60
80
100
650
700
750
800
850
900
950

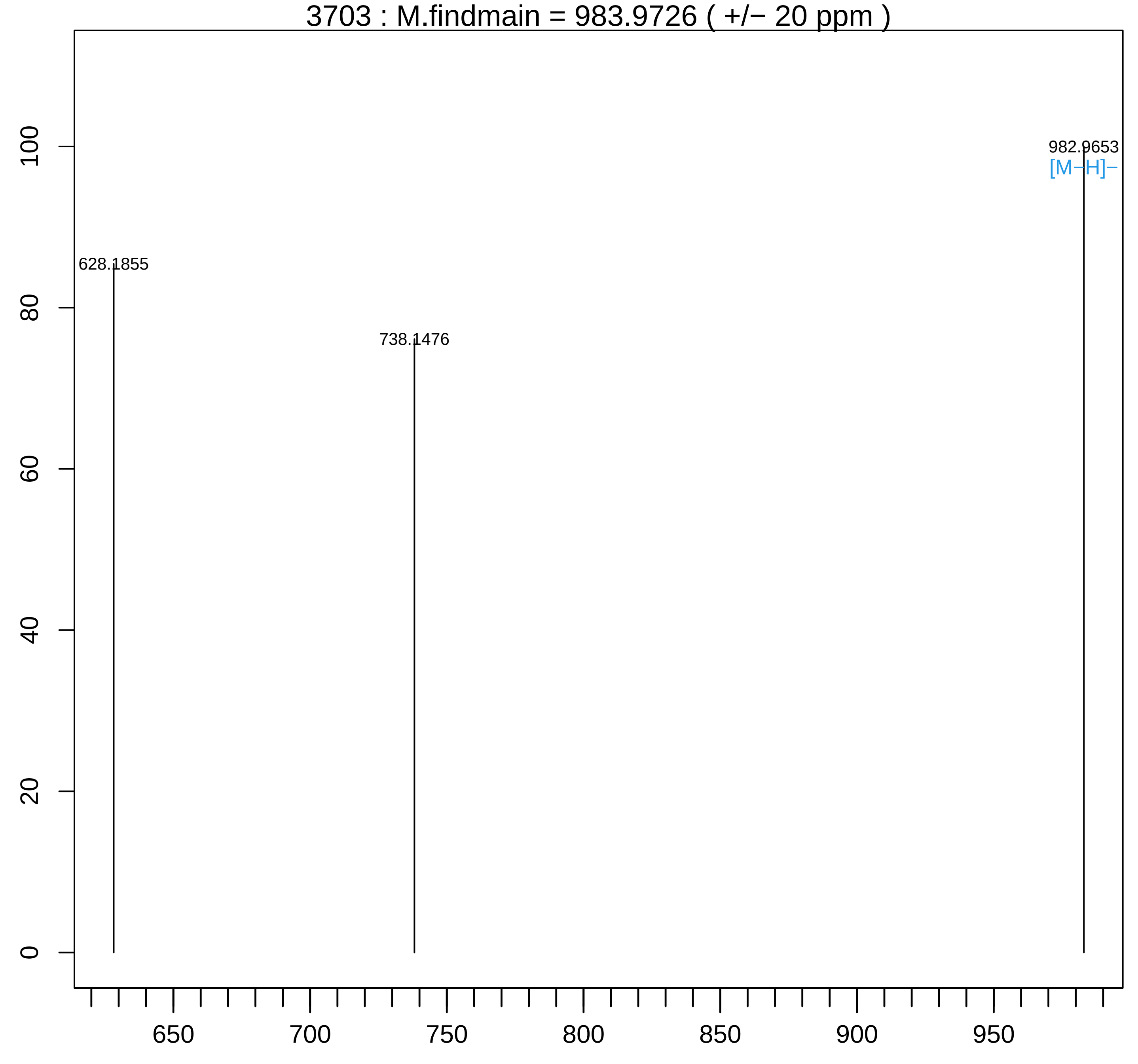
3703 : M.findmain = 983.9726 (+/− 20 ppm)
982.9653
[M−H]−
628.1855
738.1476
0
20
40
60
80
100
650
700
750
800
850
900
950

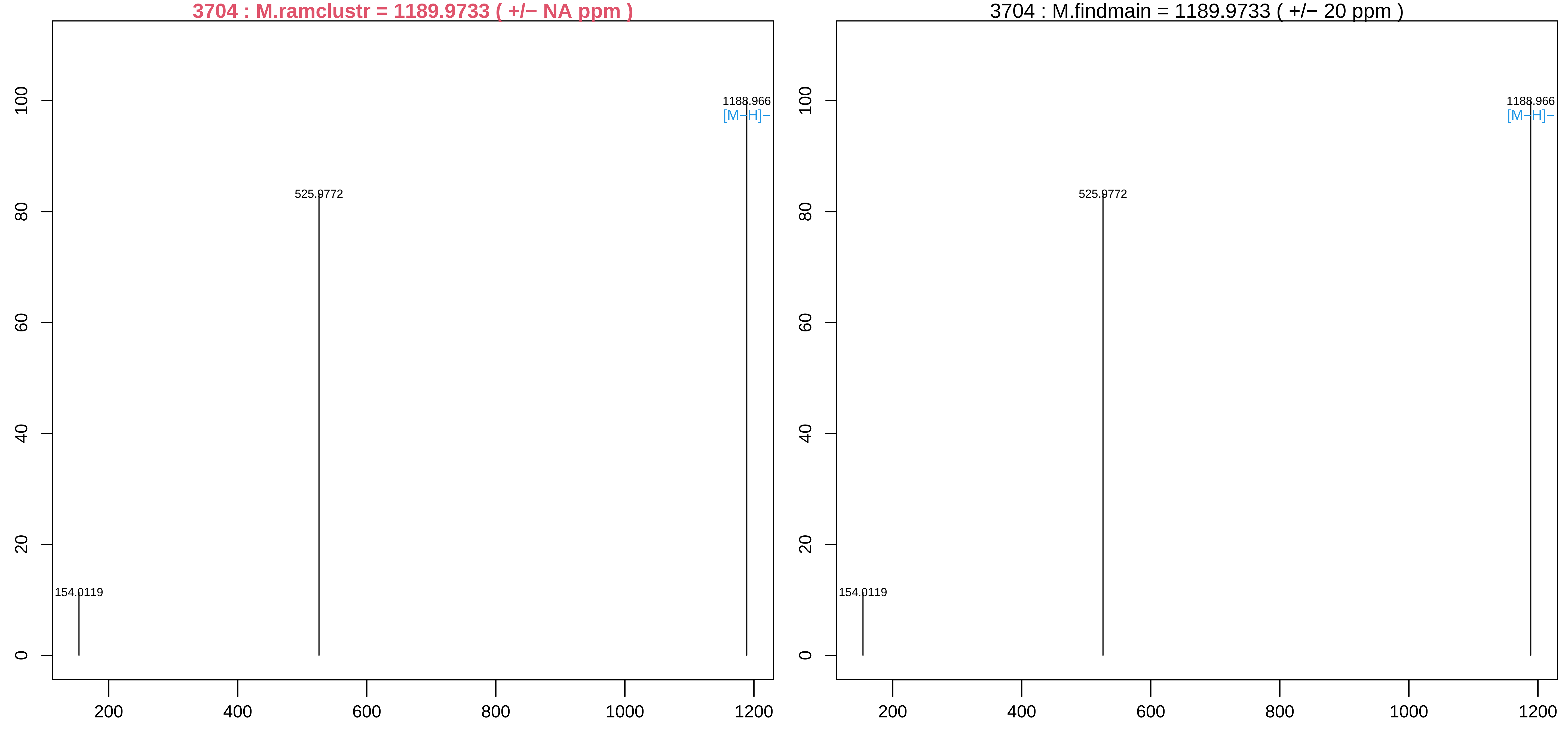
3704 : M.ramclustr = 1189.9733 (+/− NA ppm)
3704 : M.findmain = 1189.9733 (+/− 20 ppm)
1188.966
[M−H]−
525.9772
154.0119
0
20
40
60
80
100
200
400
600
800
1000
1200

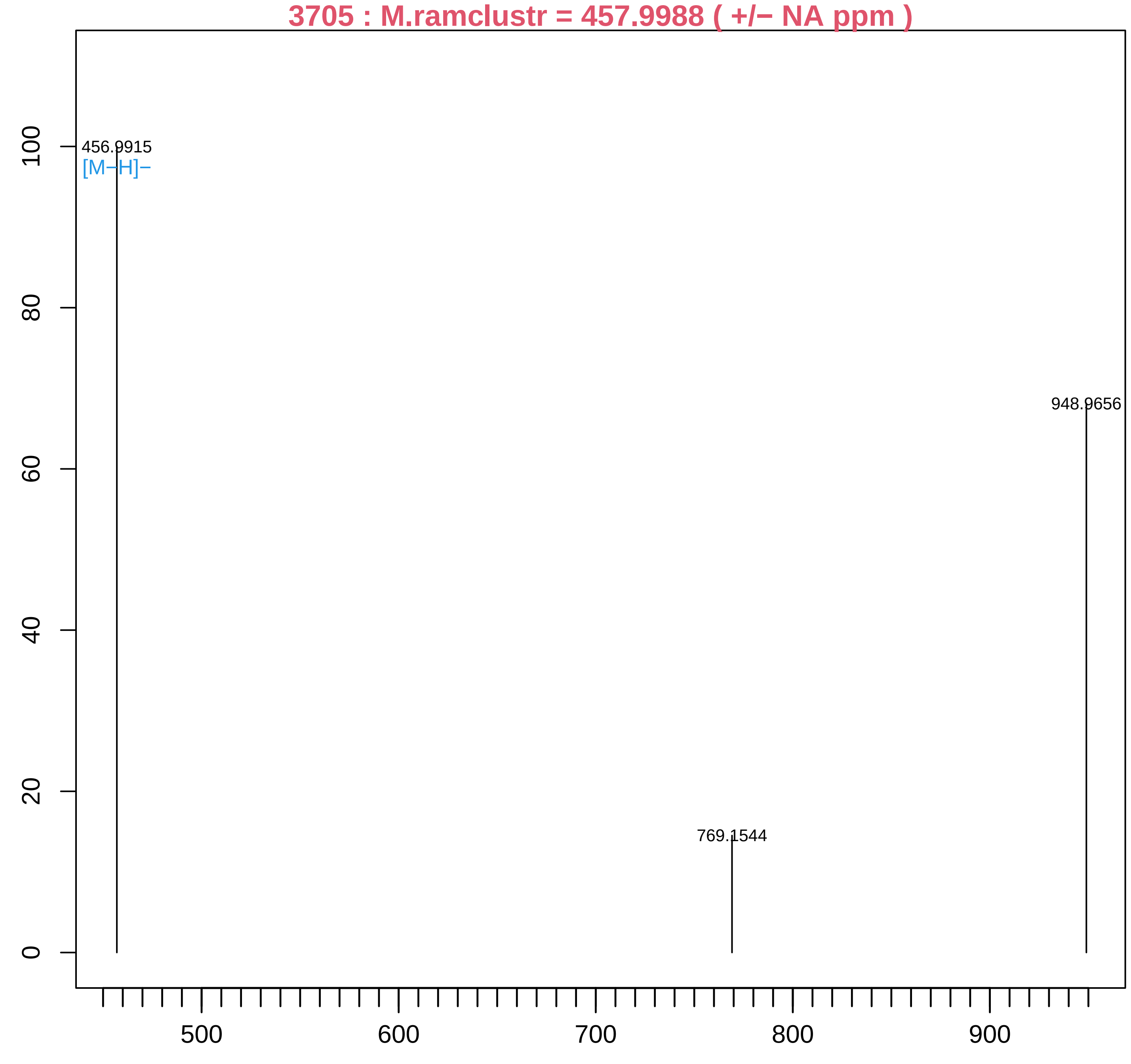
3705 : M.ramclustr = 457.9988 (+/− NA ppm)
456.9915
[M−H]−
948.9656
769.1544
0
20
40
60
80
100
500
600
700
800
900

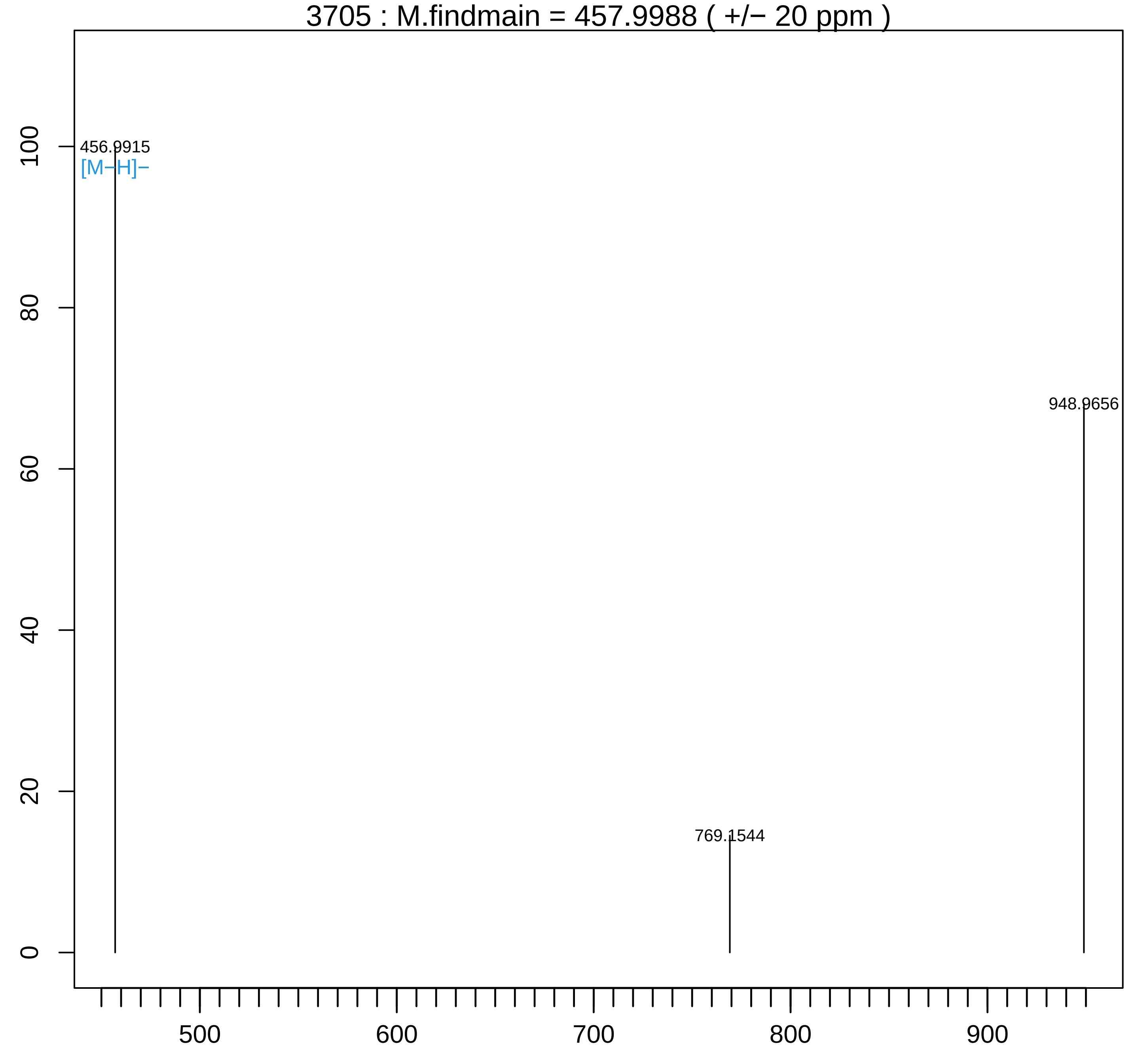
3705 : M.findmain = 457.9988 (+/− 20 ppm)
456.9915
[M−H]−
948.9656
769.1544
0
20
40
60
80
100
500
600
700
800
900

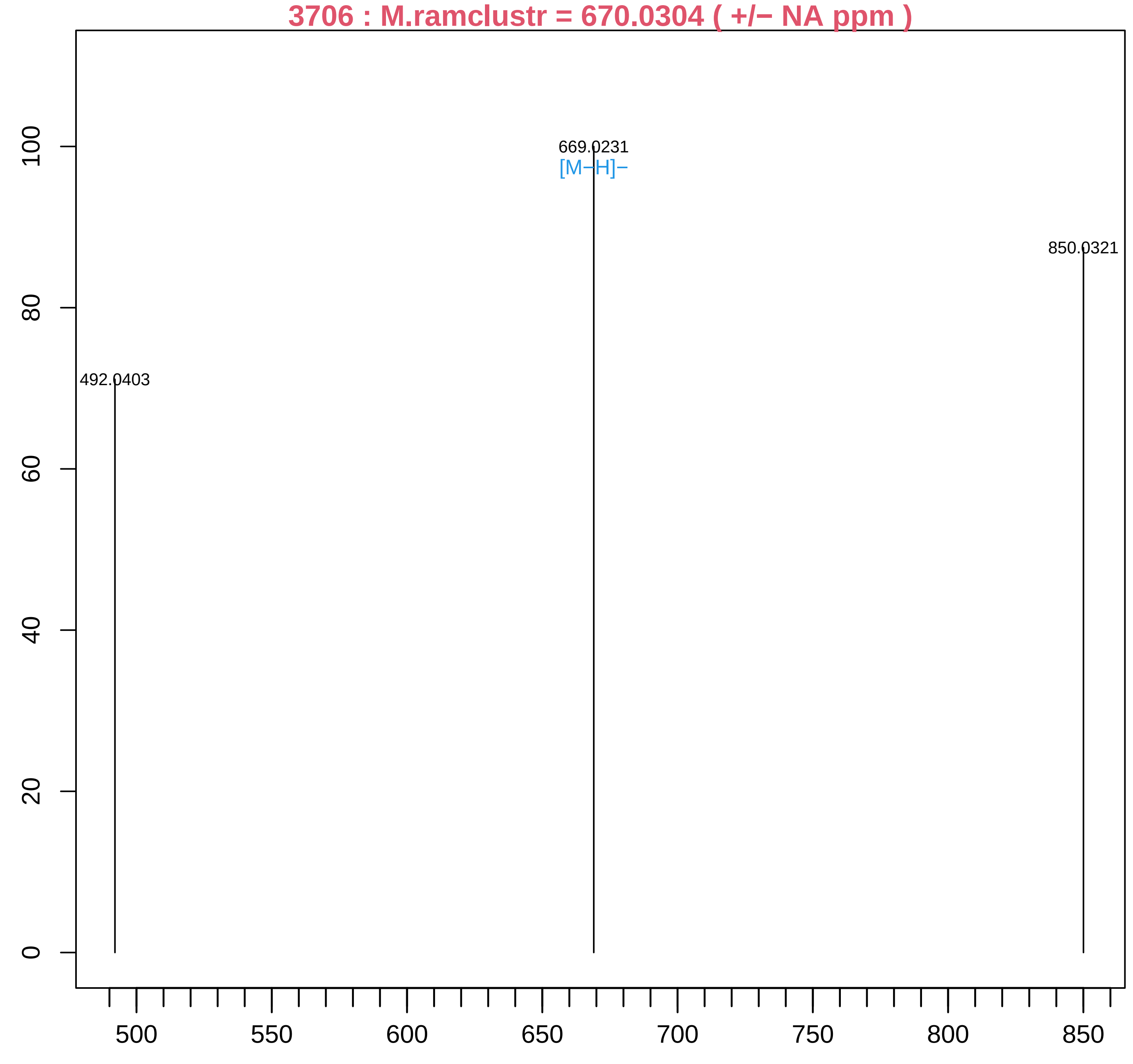
3706 : M.ramclustr = 670.0304 (+/− NA ppm)
669.0231
[M−H]−
850.0321
492.0403
0
20
40
60
80
100
500
550
600
650
700
750
800
850

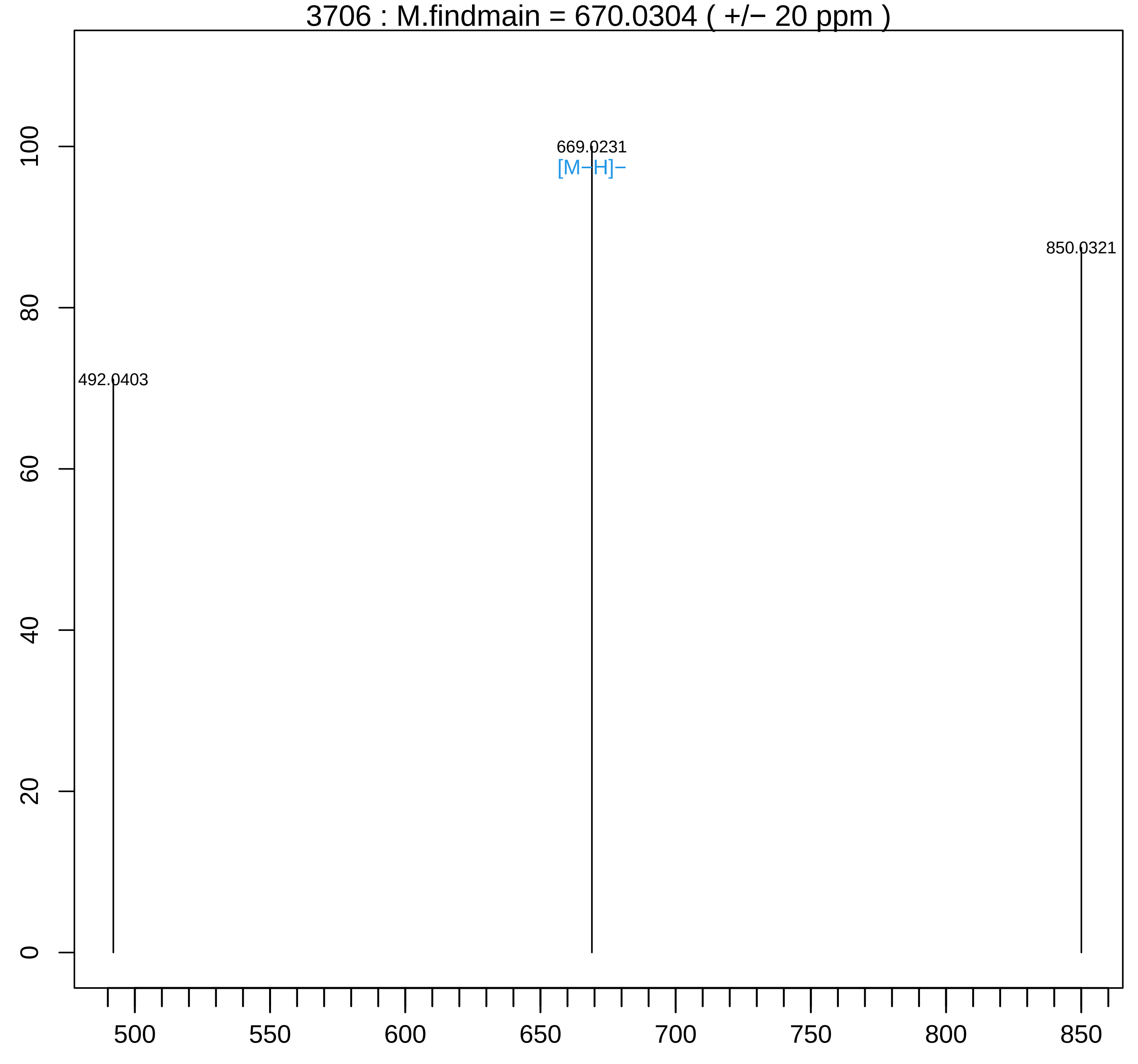
3706 : M.findmain = 670.0304 (+/− 20 ppm)
669.0231
[M−H]−
850.0321
492.0403
0
20
40
60
80
100
500
550
600
650
700
750
800
850

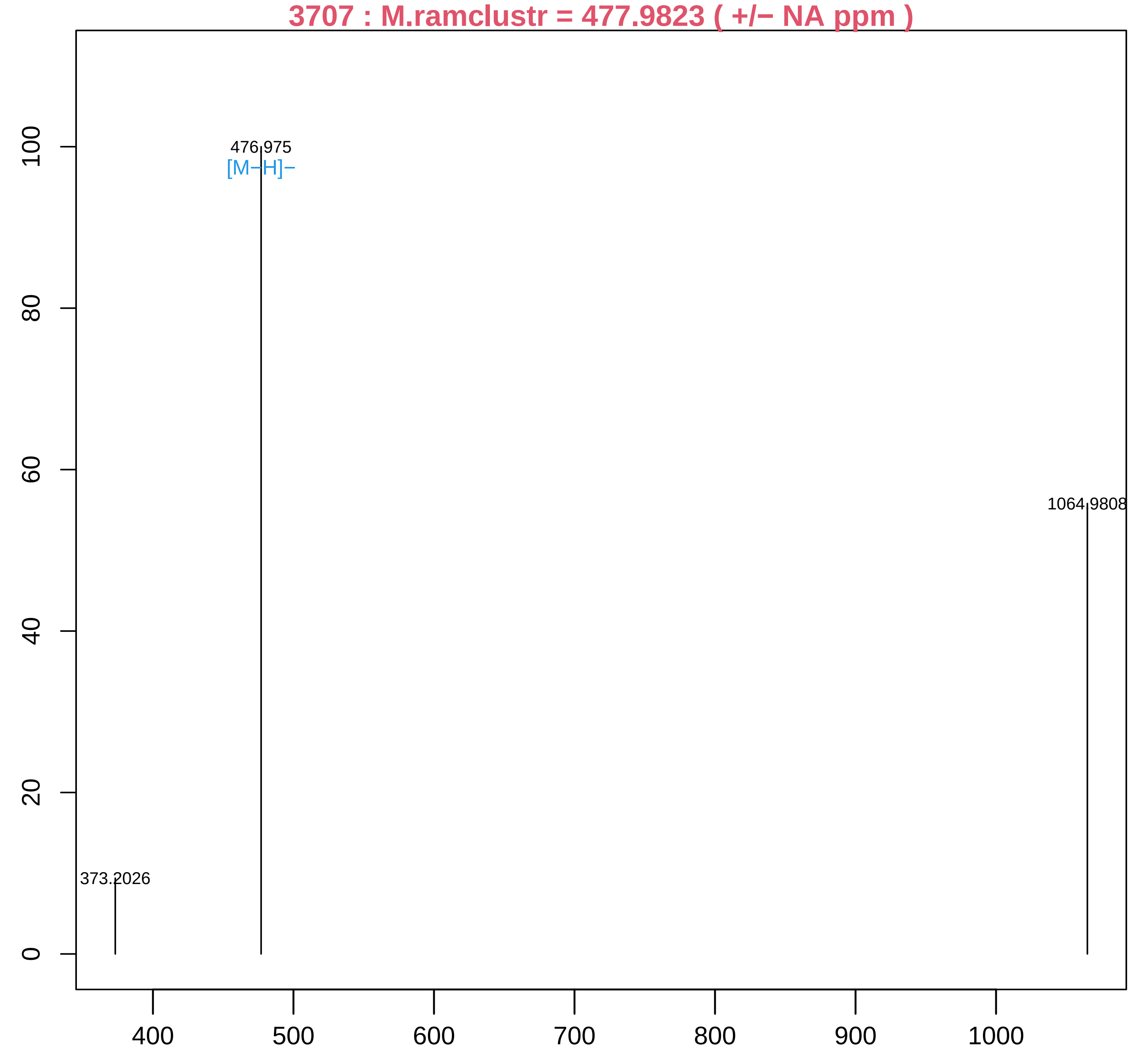
3707 : M.ramclustr = 477.9823 (+/− NA ppm)
476.975
[M−H]−
1064.9808
373.2026
0
20
40
60
80
100
400
500
600
700
800
900
1000

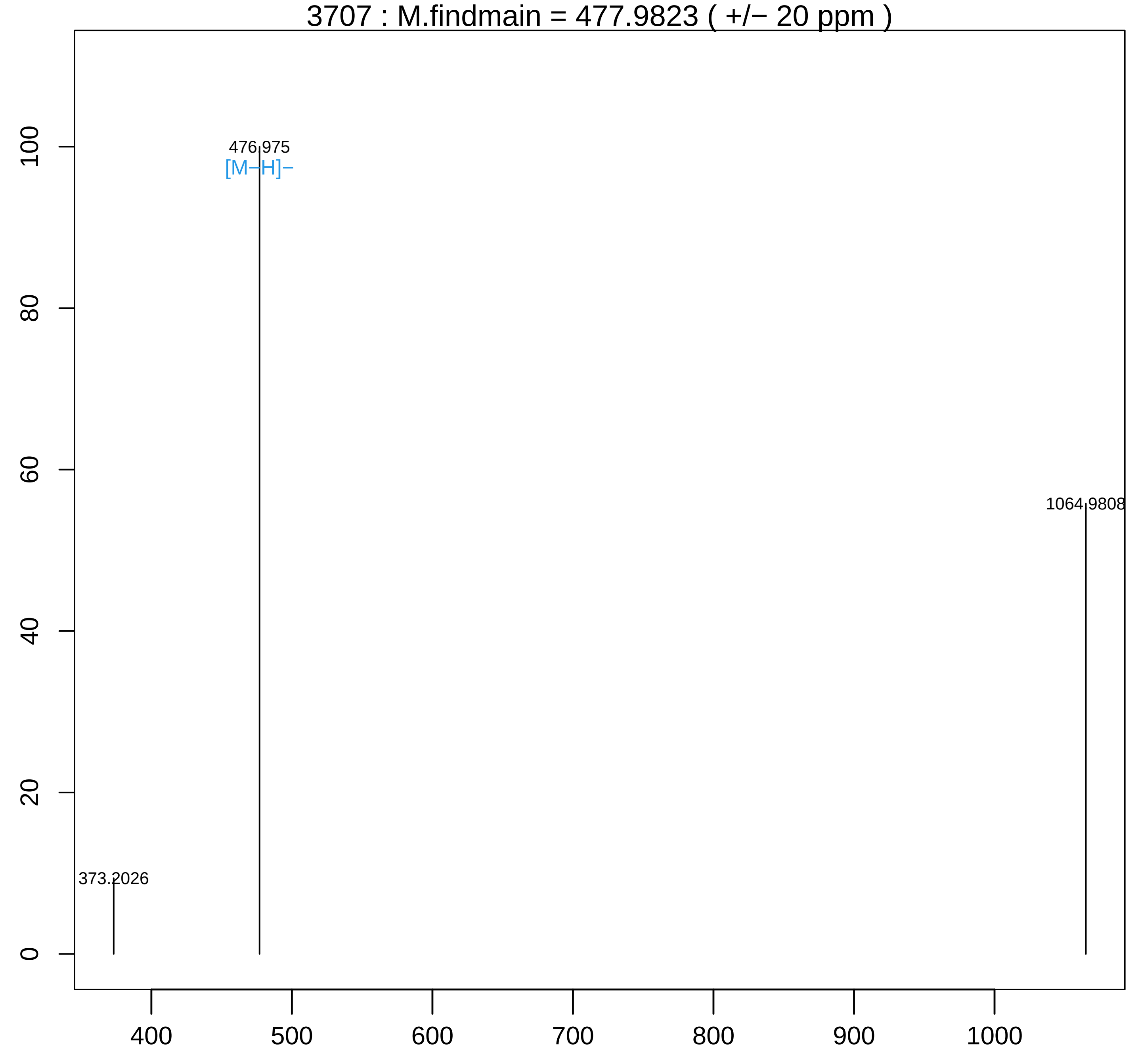
3707 : M.findmain = 477.9823 (+/− 20 ppm)
476.975
[M−H]−
1064.9808
373.2026
0
20
40
60
80
100
400
500
600
700
800
900
1000

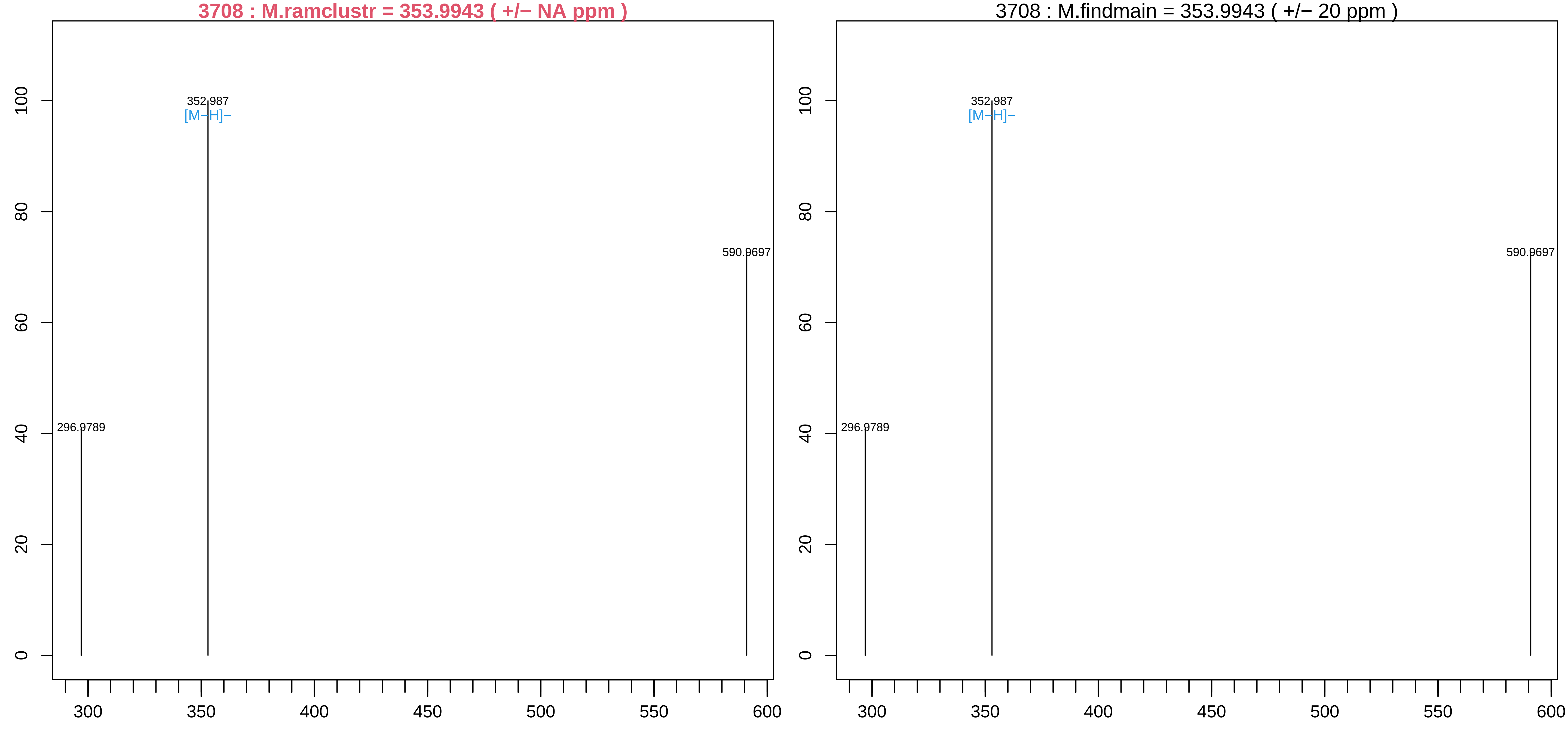
3708 : M.ramclustr = 353.9943 (+/− NA ppm)
352.987
[M−H]−
590.9697
296.9789
3708 : M.findmain = 353.9943 (+/− 20 ppm)
352.987
[M−H]−
590.9697
296.9789
0
20
40
60
80
100
300
350
400
450
500
550
600

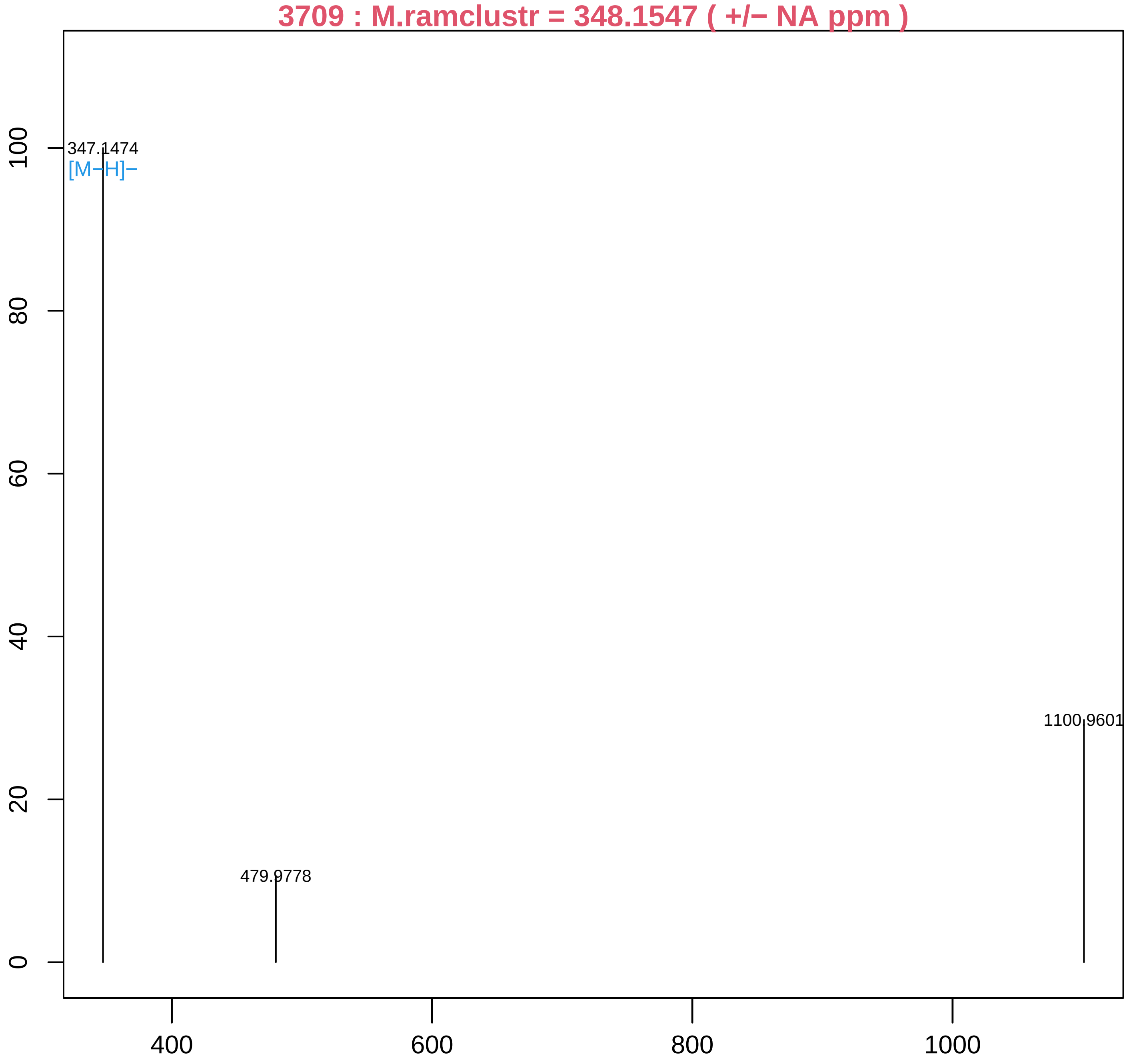
3709 : M.ramclustr = 348.1547 (+/− NA ppm)
347.1474
[M−H]−
479.9778
1100.9601
0
20
40
60
80
100
400
600
800
1000

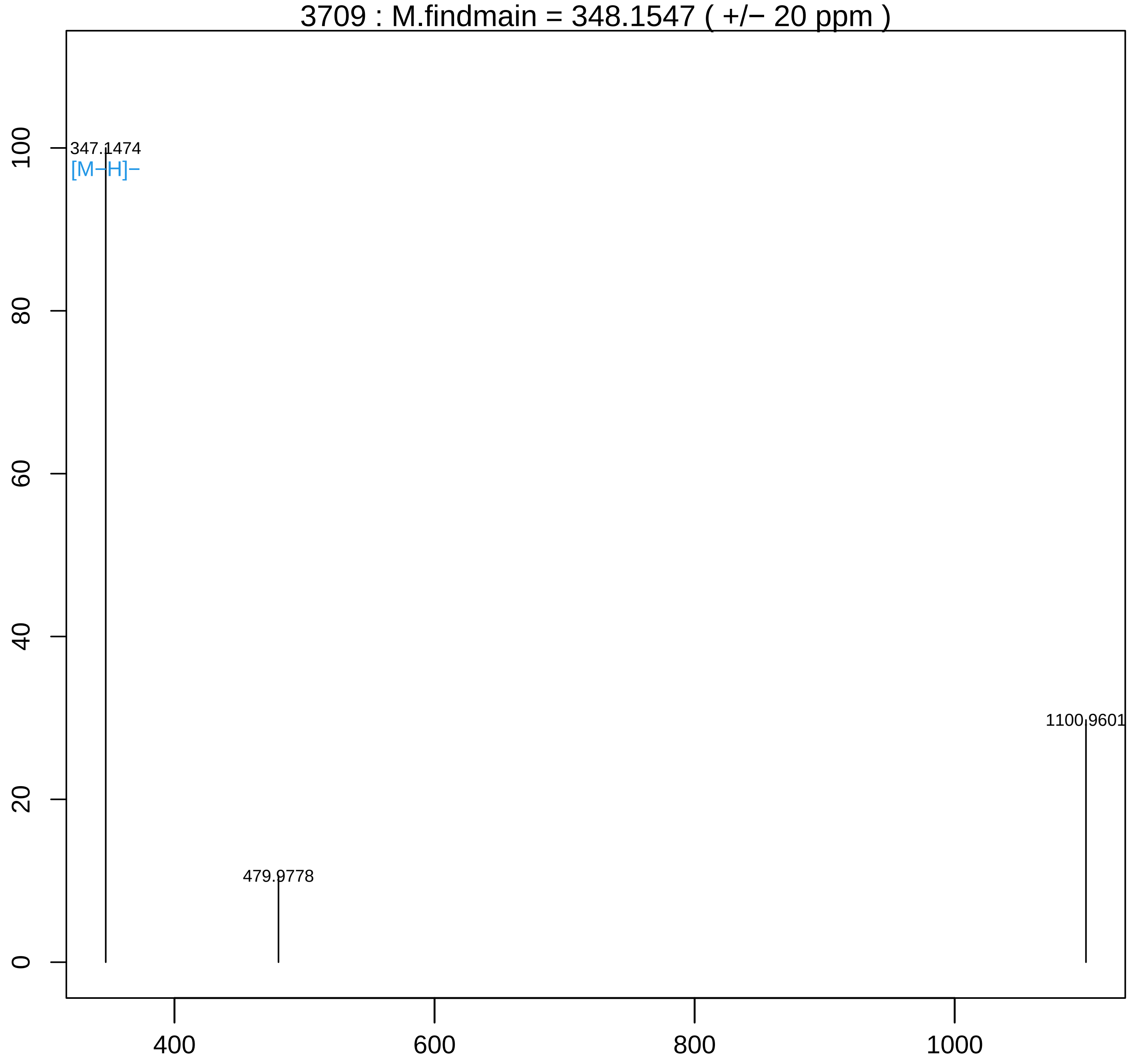
3709 : M.findmain = 348.1547 (+/− 20 ppm)
347.1474
[M−H]−
479.9778
1100.9601
0
20
40
60
80
100
400
600
800
1000

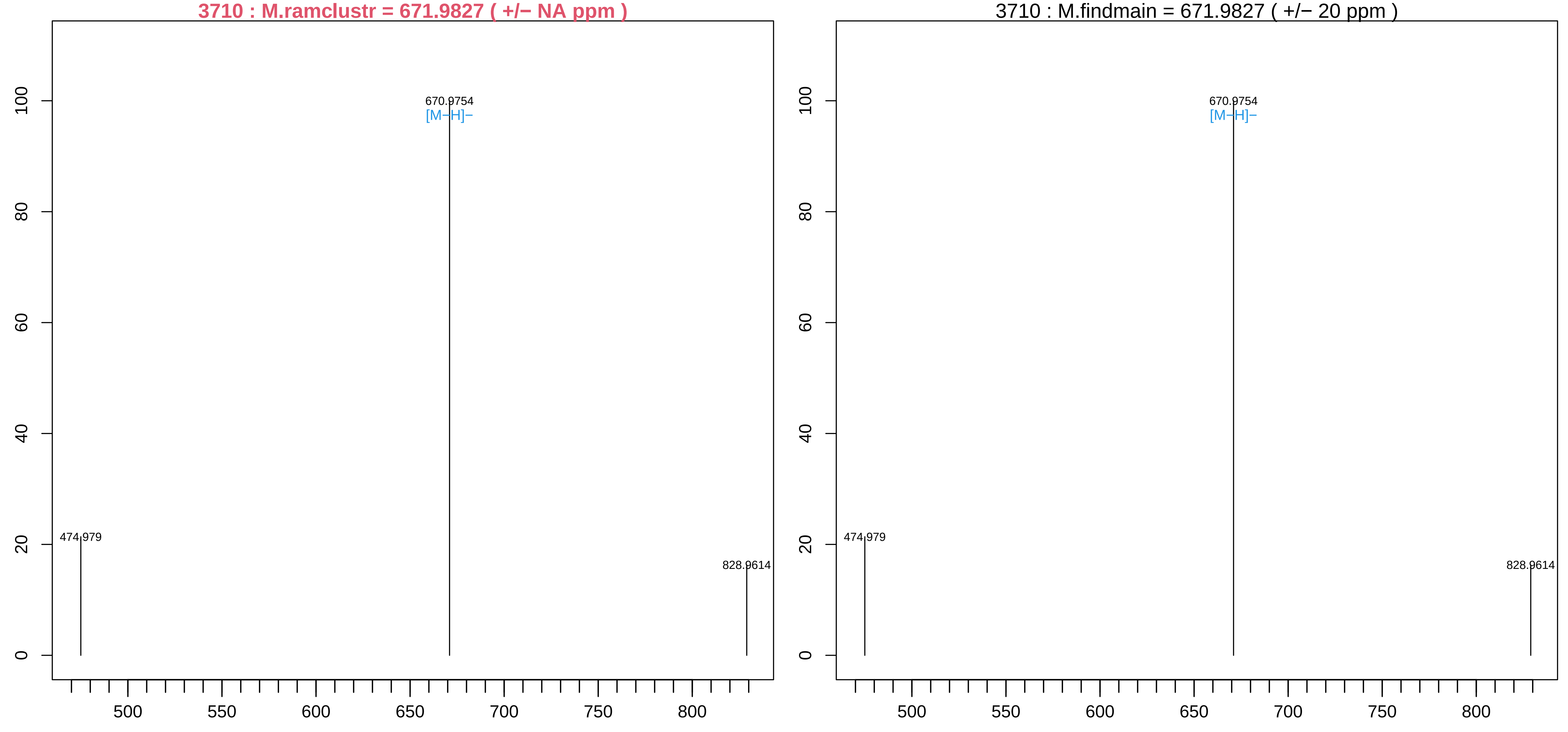
3710 : M.ramclustr = 671.9827 (+/− NA ppm)
670.9754
[M−H]−
474.979
828.9614
3710 : M.findmain = 671.9827 (+/− 20 ppm)
670.9754
[M−H]−
474.979
828.9614

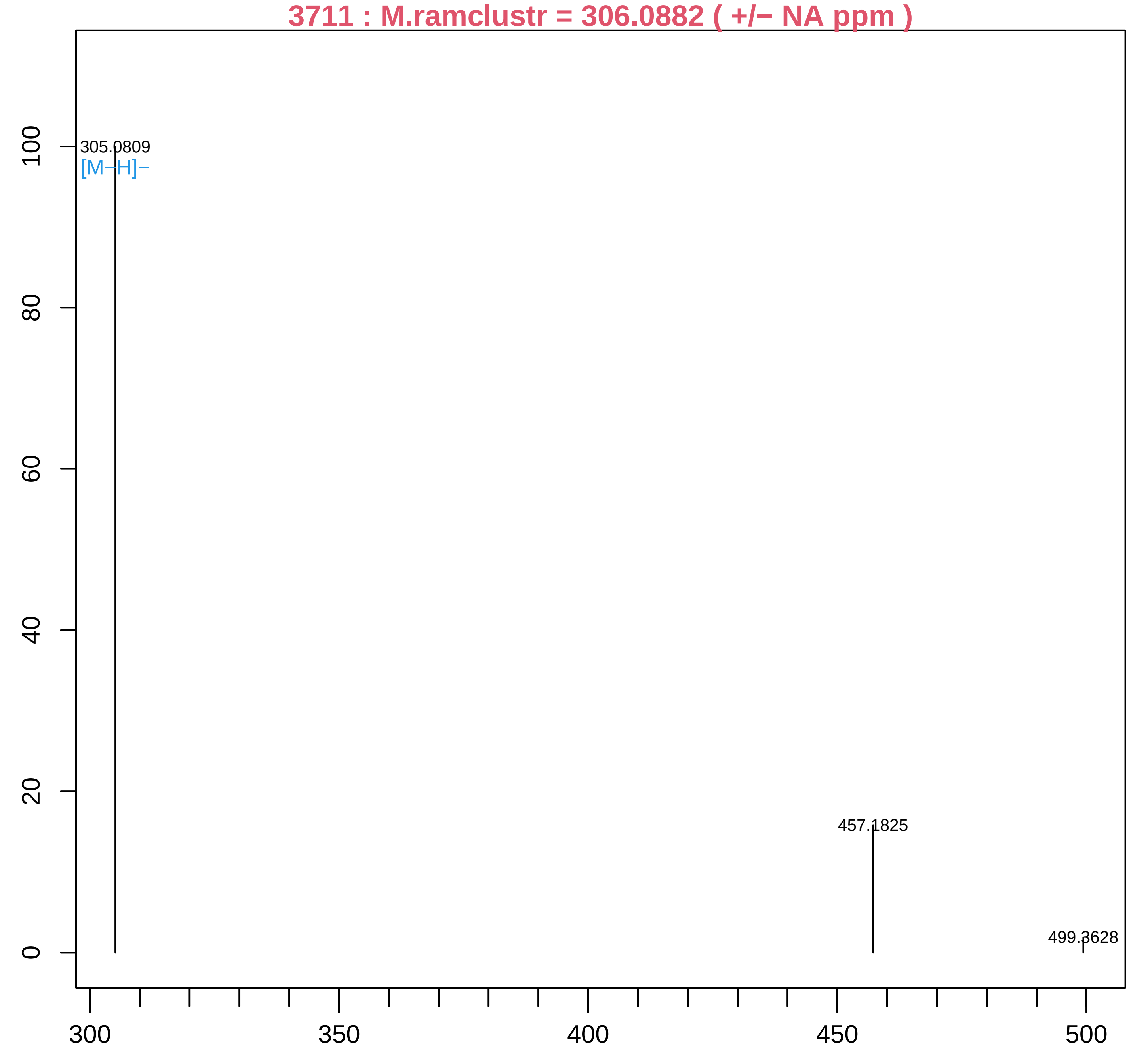
3711 : M.ramclustr = 306.0882 (+/− NA ppm)
305.0809
[M−H]−
457.1825
499.3628
0
20
40
60
80
100
300
350
400
450
500

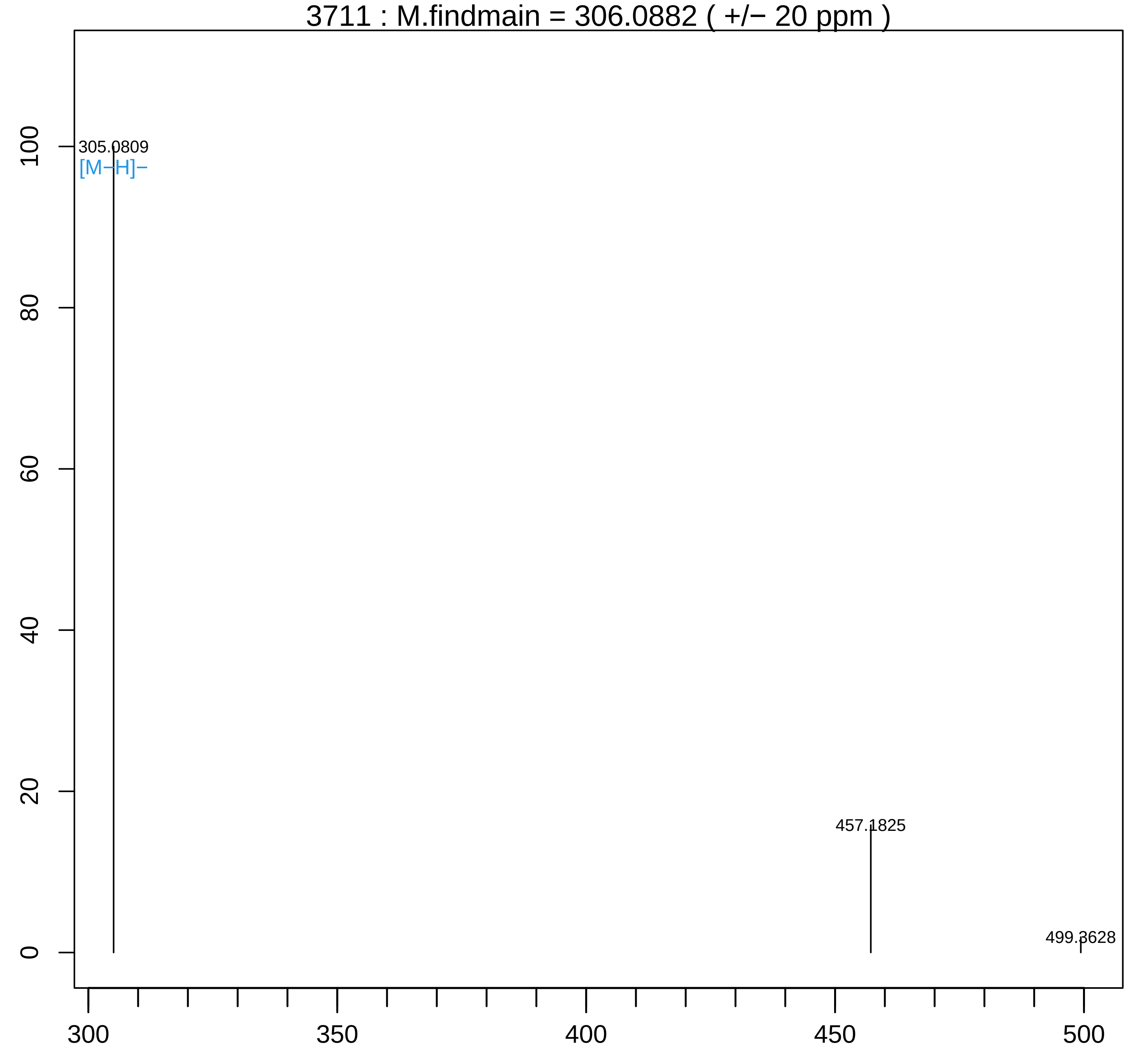
3711 : M.findmain = 306.0882 (+/− 20 ppm)
305.0809
[M−H]−
457.1825
499.3628
0
20
40
60
80
100
300
350
400
450
500

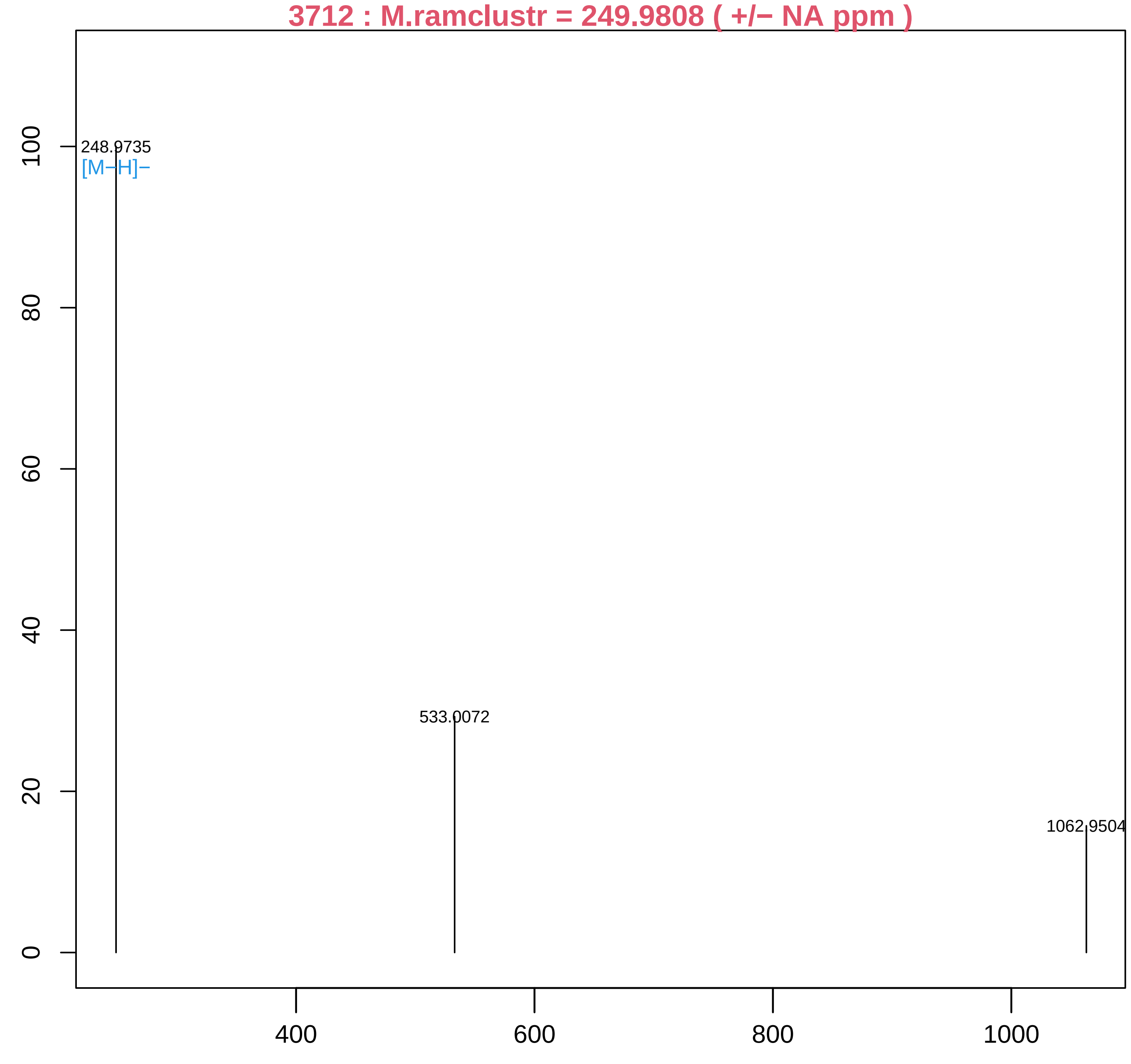
3712 : M.ramclustr = 249.9808 (+/− NA ppm)
248.9735
[M−H]−
533.0072
1062.9504
0
20
40
60
80
100
400
600
800
1000

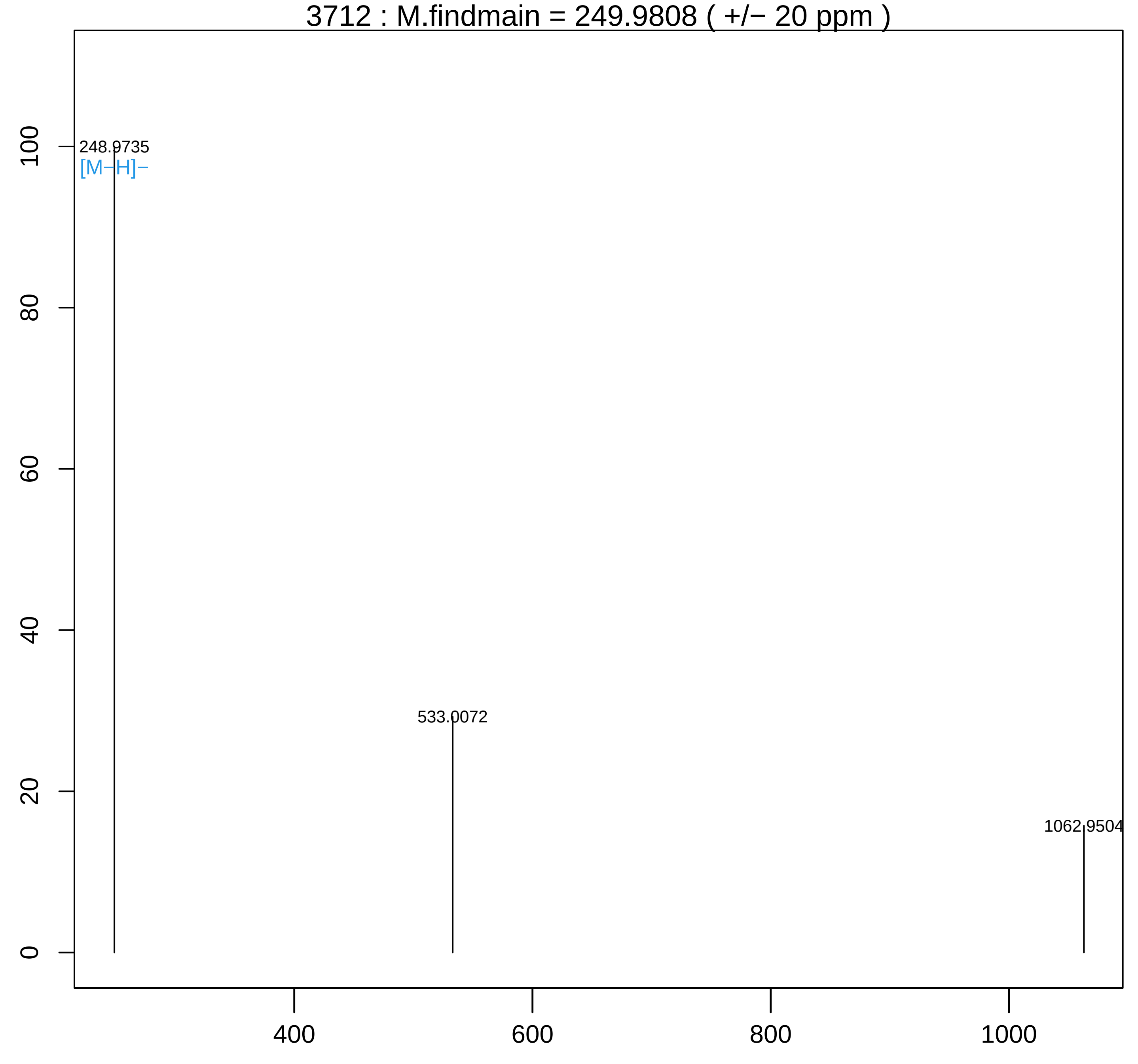
3712 : M.findmain = 249.9808 (+/− 20 ppm)
248.9735
[M−H]−
533.0072
1062.9504
0
20
40
60
80
100
400
600
800
1000

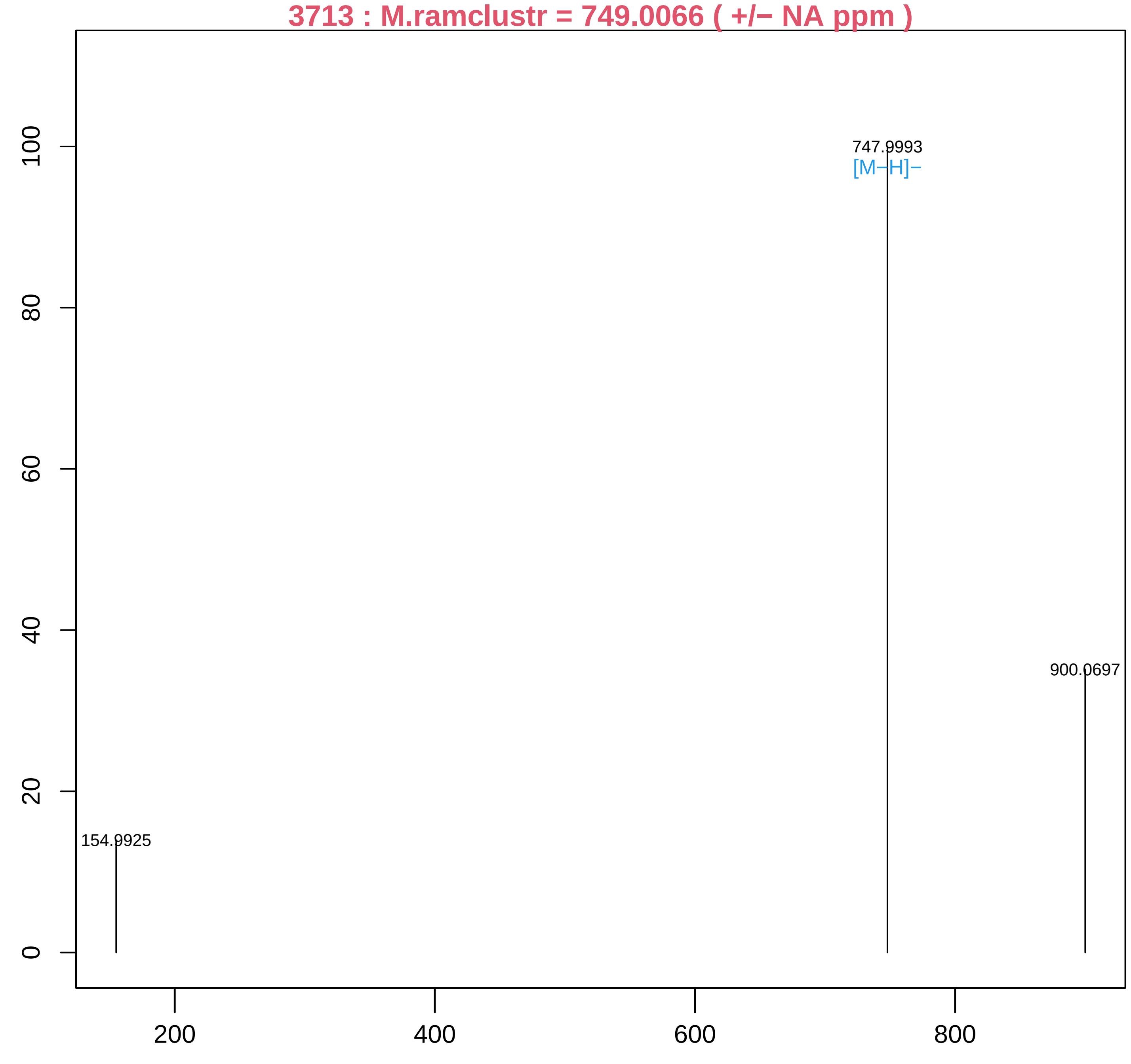
3713 : M.ramclustr = 749.0066 (+/− NA ppm)
747.9993
[M−H]−
900.0697
154.9925
0
20
40
60
80
100
200
400
600
800

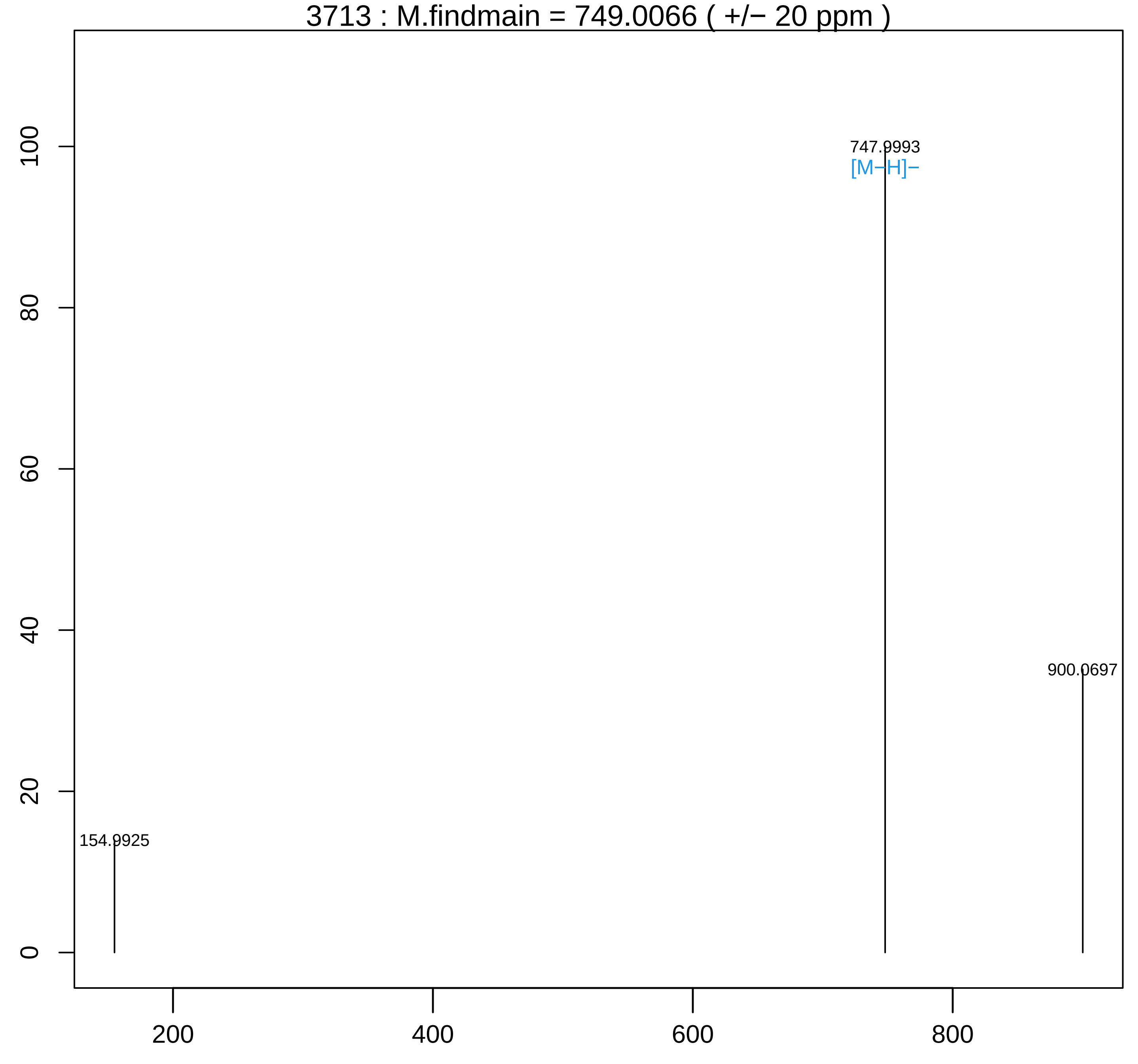
3713 : M.findmain = 749.0066 (+/− 20 ppm)
747.9993
[M−H]−
900.0697
154.9925
0
20
40
60
80
100
200
400
600
800

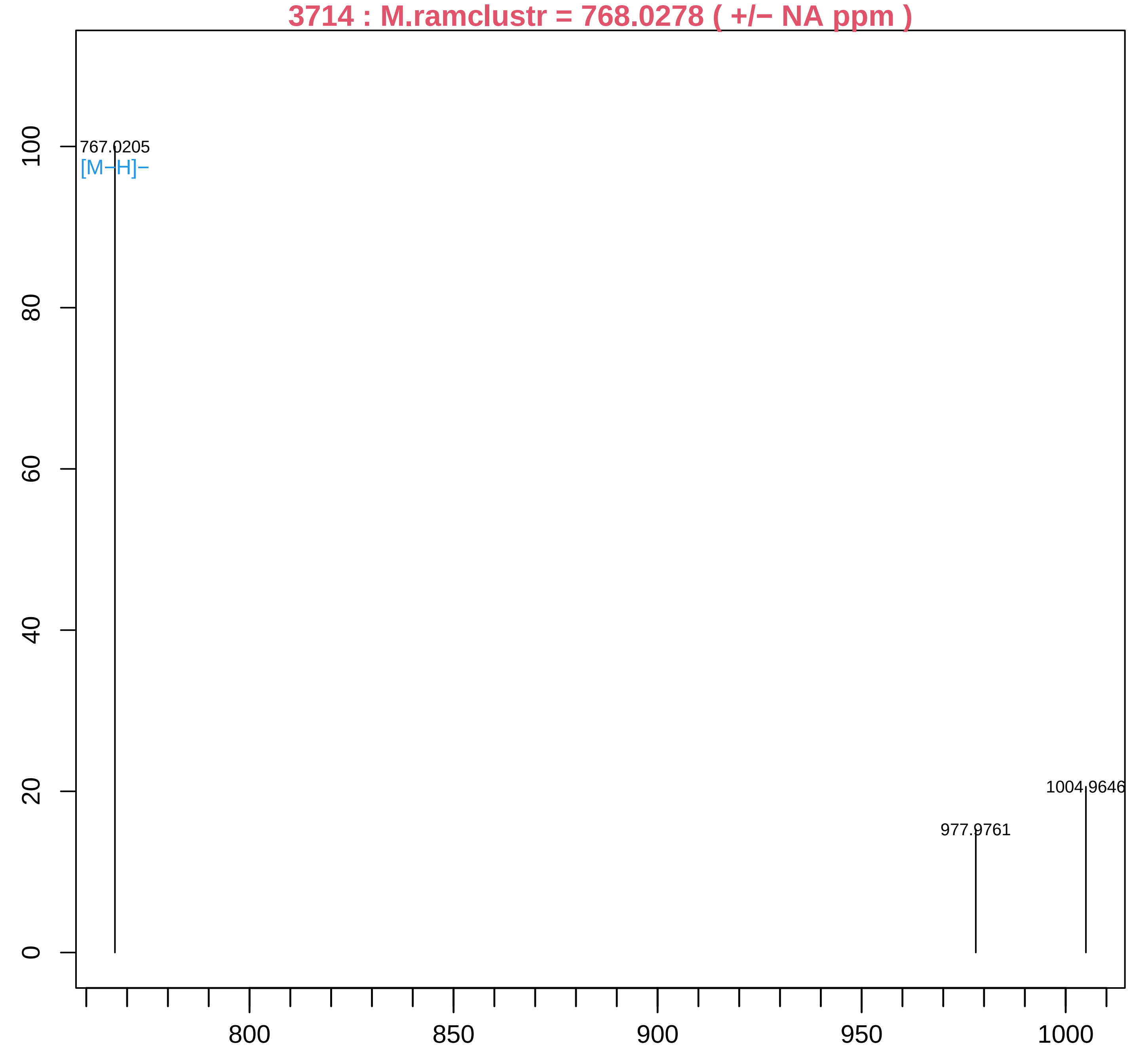
3714 : M.ramclustr = 768.0278 (+/− NA ppm)
767.0205
[M−H]−
977.9761
1004.9646
0
20
40
60
80
100
800
850
900
950
1000

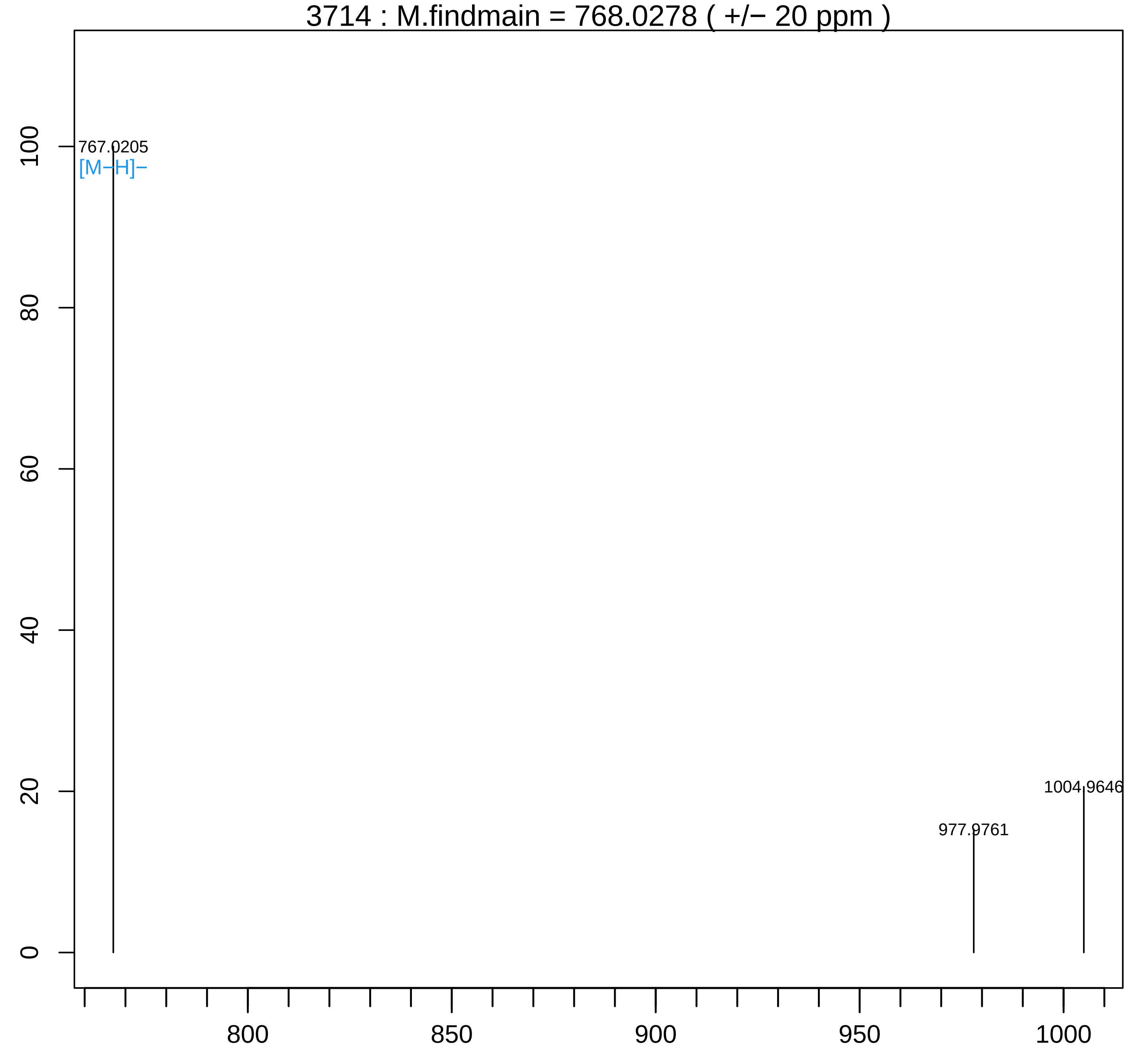
3714 : M.findmain = 768.0278 (+/− 20 ppm)
767.0205
[M−H]−
977.9761
1004.9646
0
20
40
60
80
100
800
850
900
950
1000

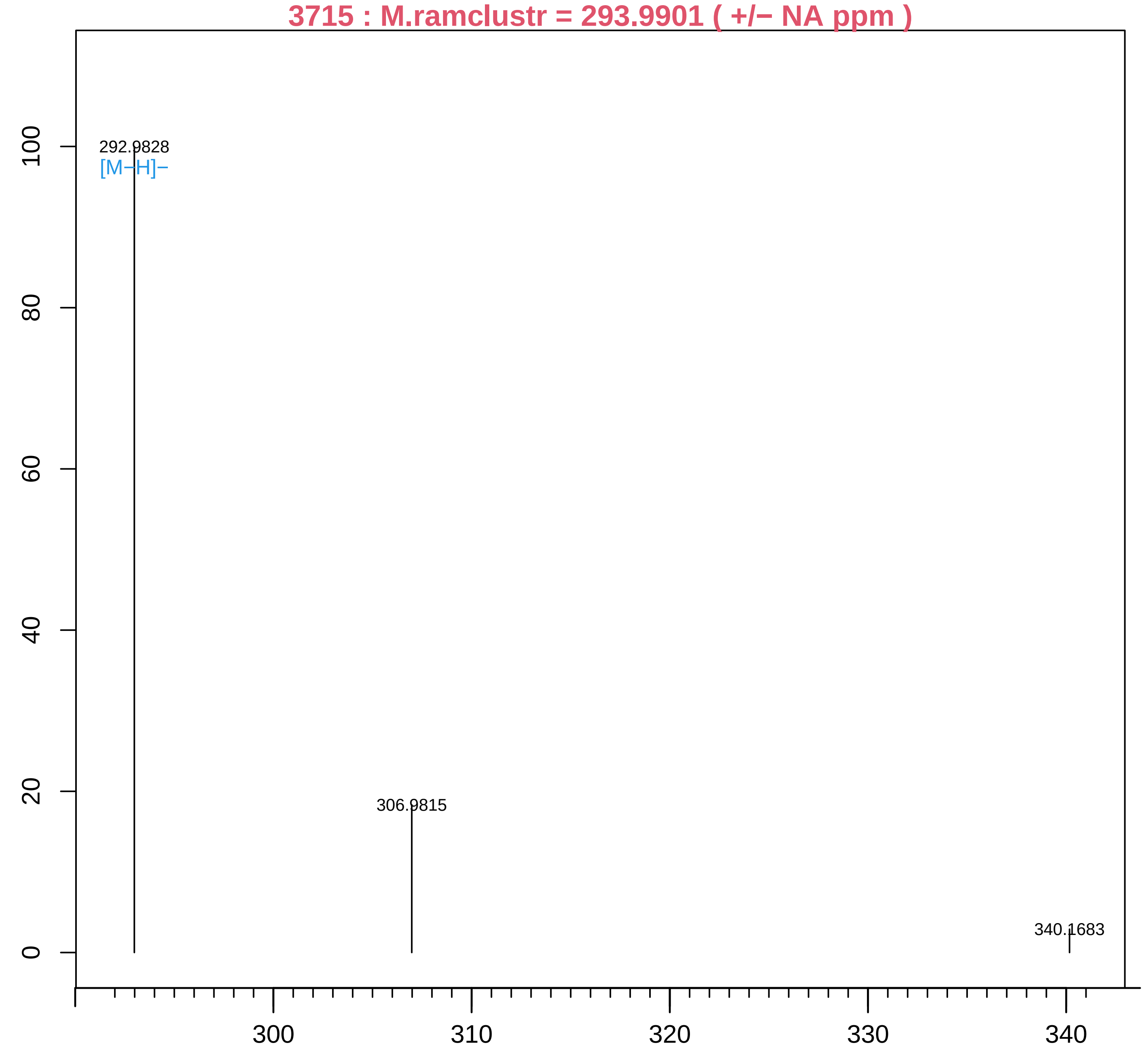
3715 : M.ramclustr = 293.9901 (+/− NA ppm)
292.9828
[M−H]−
306.9815
340.1683
0
20
40
60
80
100
300
310
320
330
340

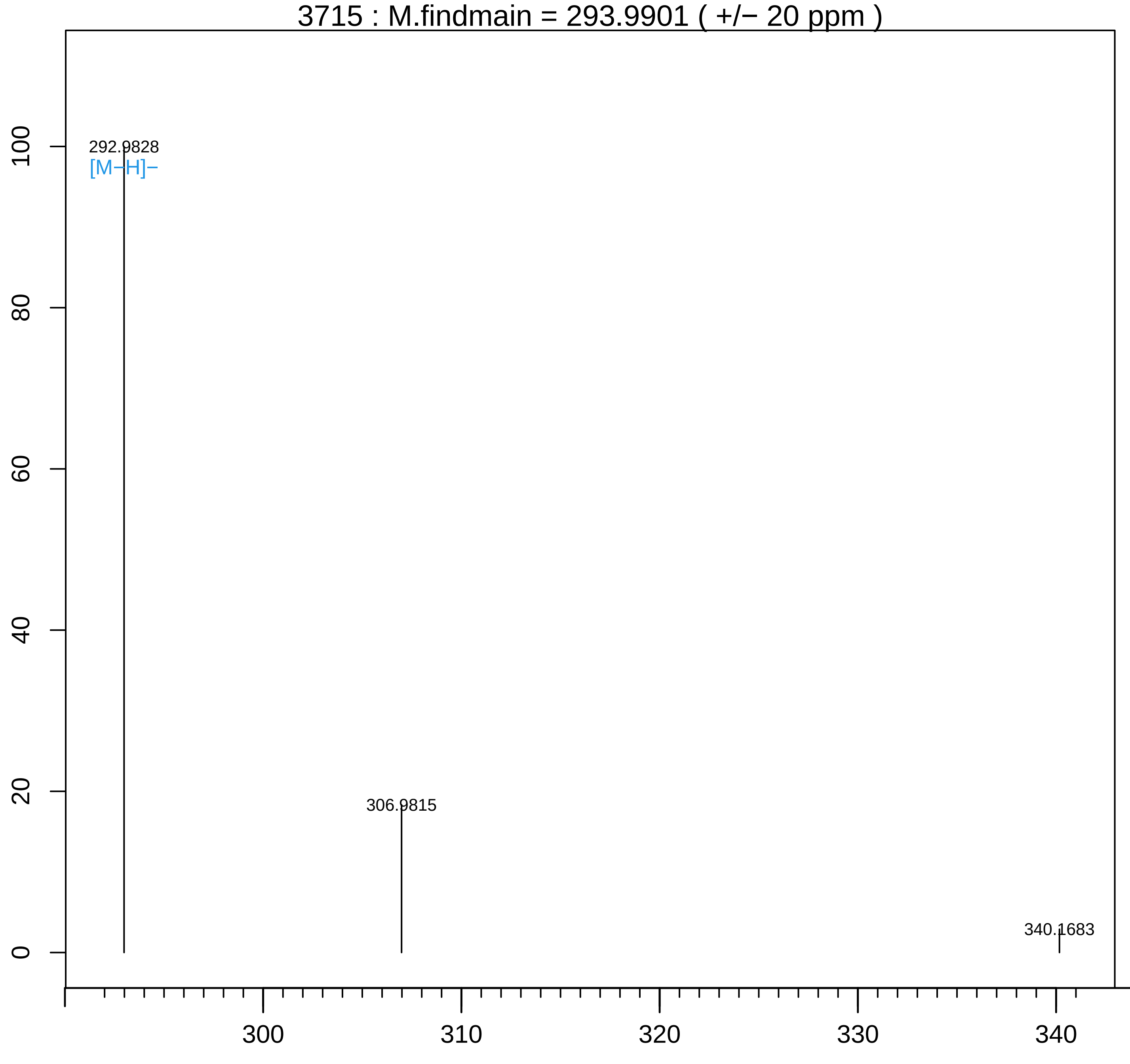
3715 : M.findmain = 293.9901 (+/− 20 ppm)
292.9828
[M−H]−
306.9815
340.1683
0
20
40
60
80
100
300
310
320
330
340

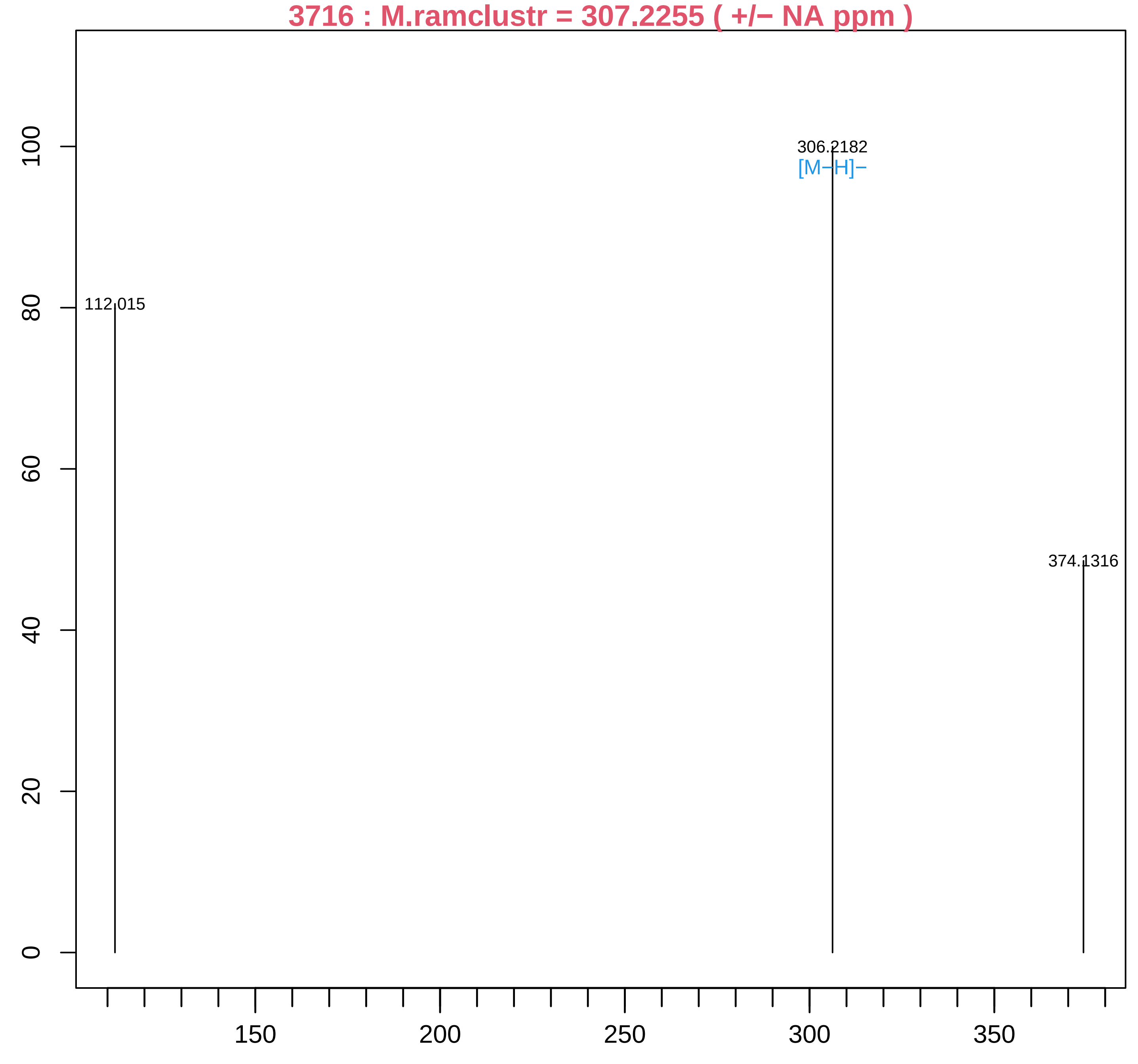
3716 : M.ramclustr = 307.2255 (+/− NA ppm)
100
80
60
40
20
0
150
200
250
300
350
112.015
306.2182
[M−H]−
374.1316

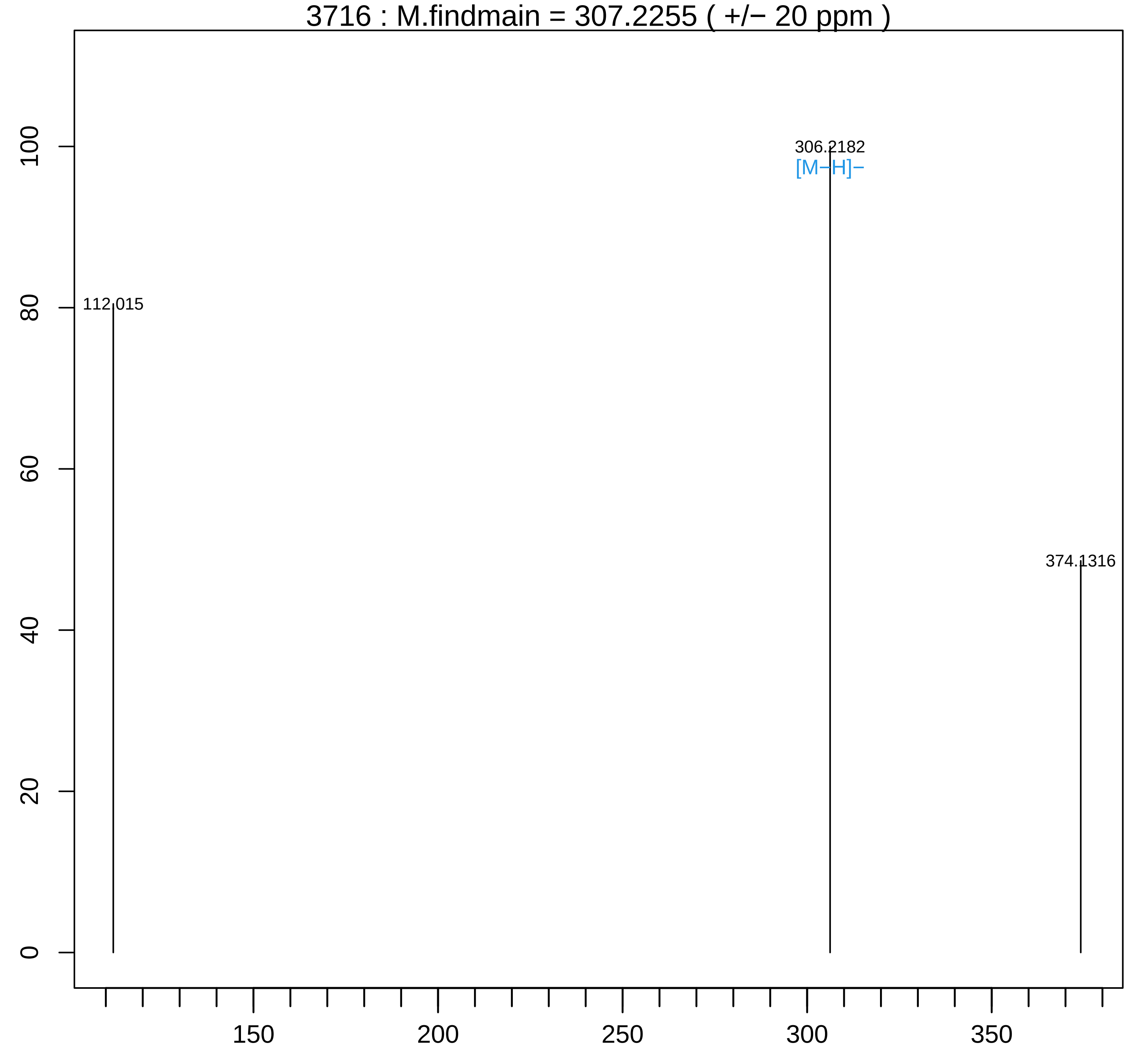
3716 : M.findmain = 307.2255 (+/− 20 ppm)
100
80
60
40
20
0
150
200
250
300
350
112.015
306.2182
[M−H]−
374.1316

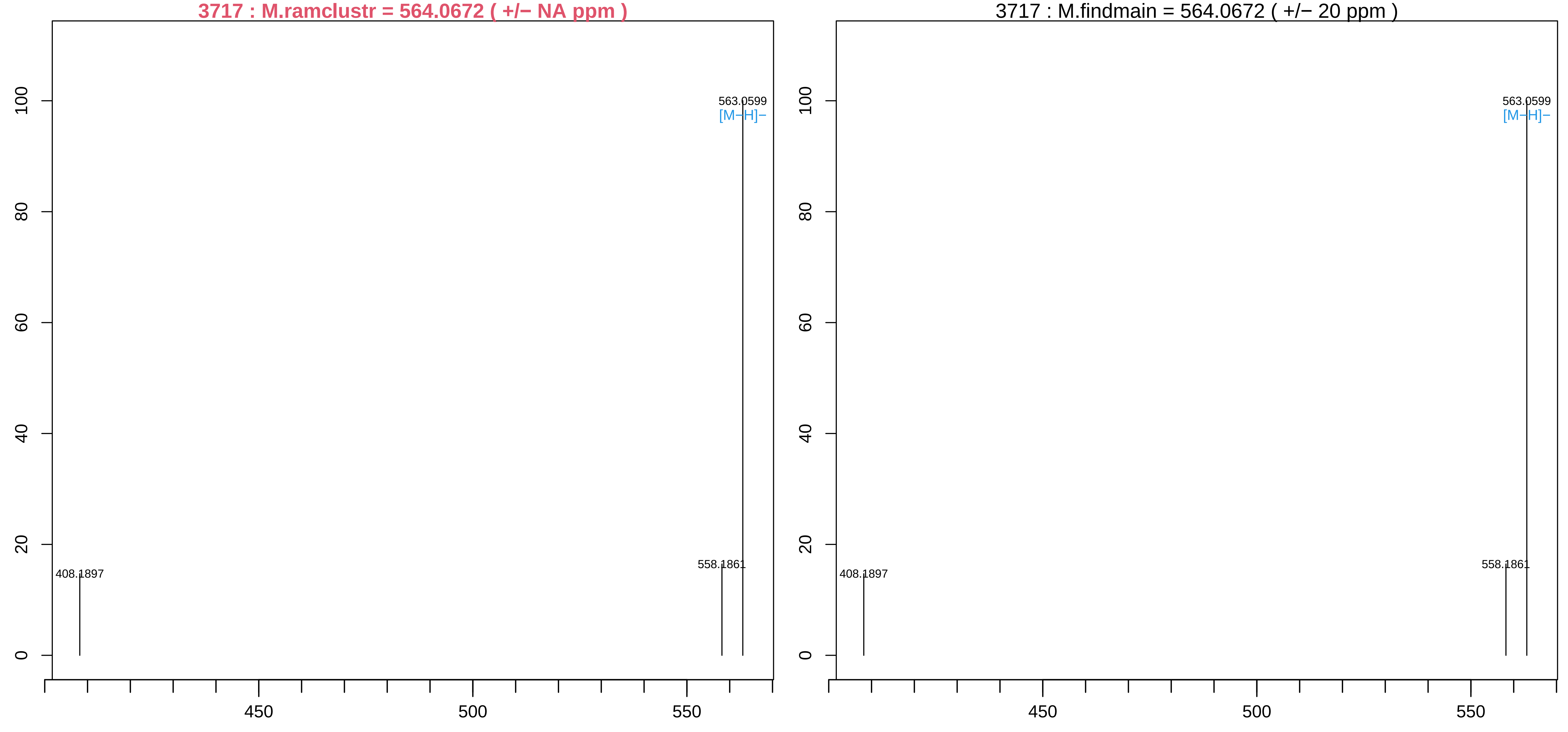
3717 : M.ramclustr = 564.0672 (+/− NA ppm)
563.0599
[M−H]−
558.1861
408.1897
3717 : M.findmain = 564.0672 (+/− 20 ppm)
563.0599
[M−H]−
558.1861
408.1897
0
20
40
60
80
100
450
500
550

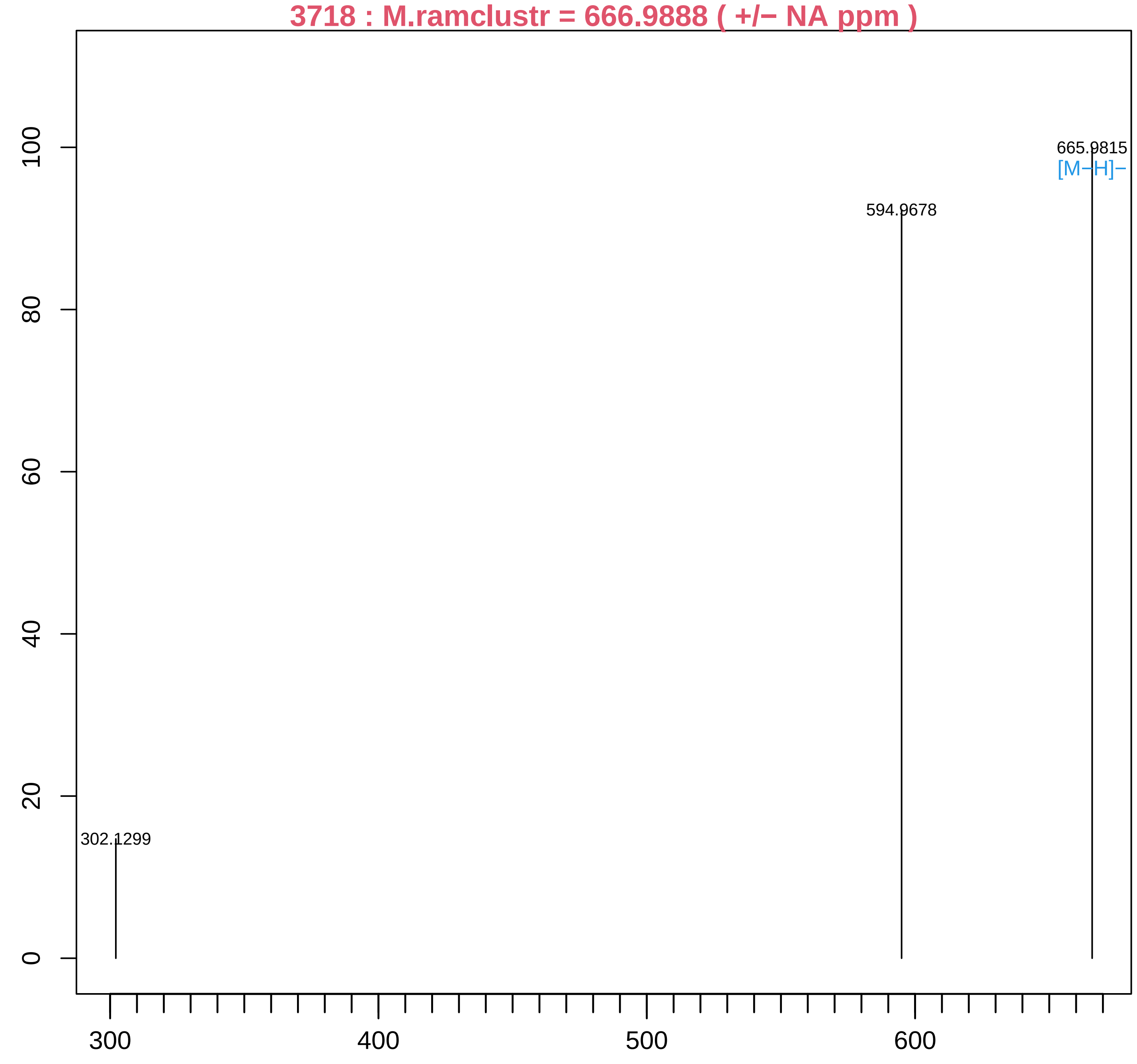
3718 : M.ramclustr = 666.9888 (+/− NA ppm)
665.9815
[M−H]−
594.9678
302.1299
0
20
40
60
80
100
300
400
500
600

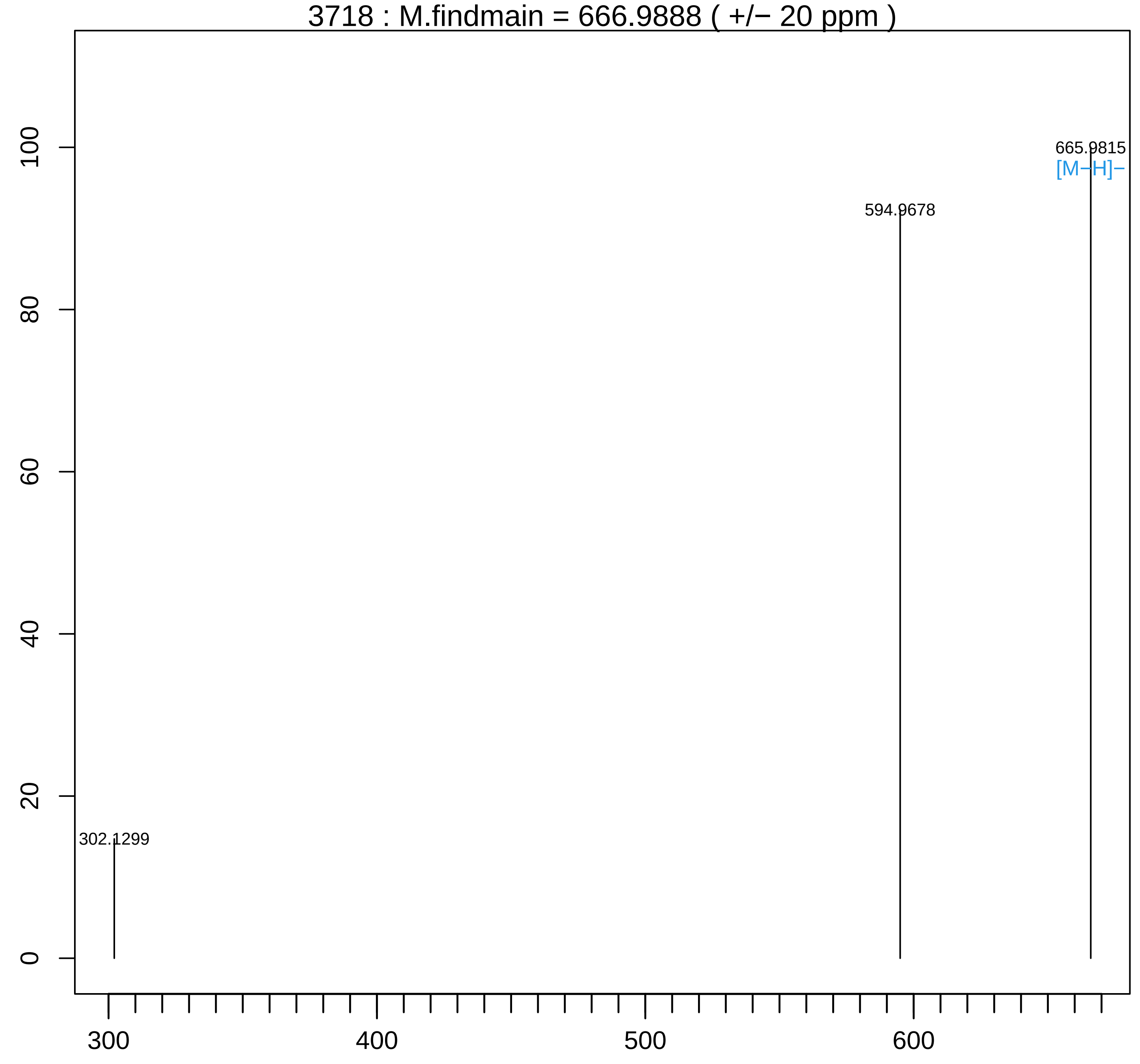
3718 : M.findmain = 666.9888 (+/− 20 ppm)
665.9815
[M−H]−
594.9678
302.1299
0
20
40
60
80
100
300
400
500
600

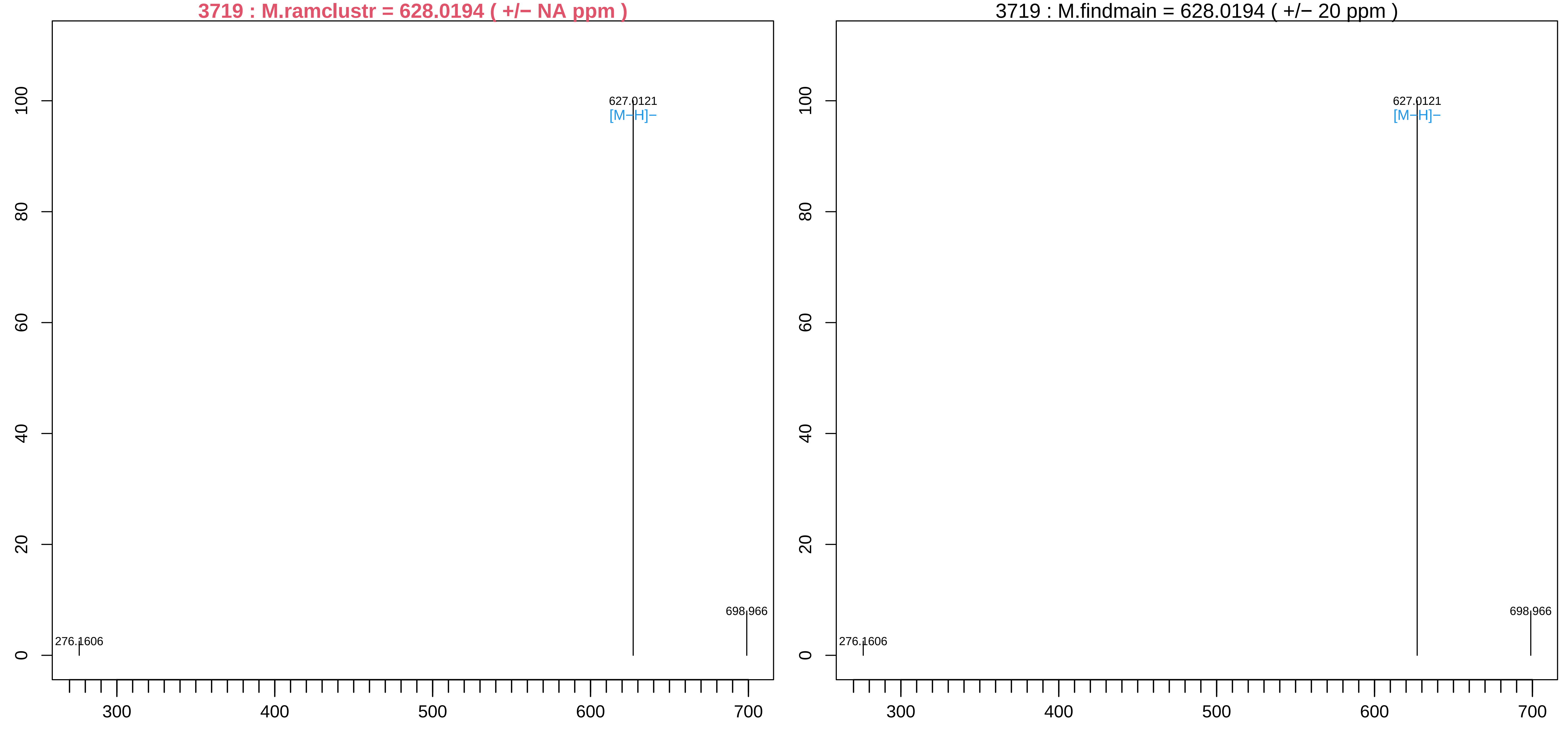
3719 : M.ramclustr = 628.0194 (+/− NA ppm)
3719 : M.findmain = 628.0194 (+/− 20 ppm)
627.0121
[M−H]−
698.966
276.1606
0
20
40
60
80
100
300
400
500
600
700

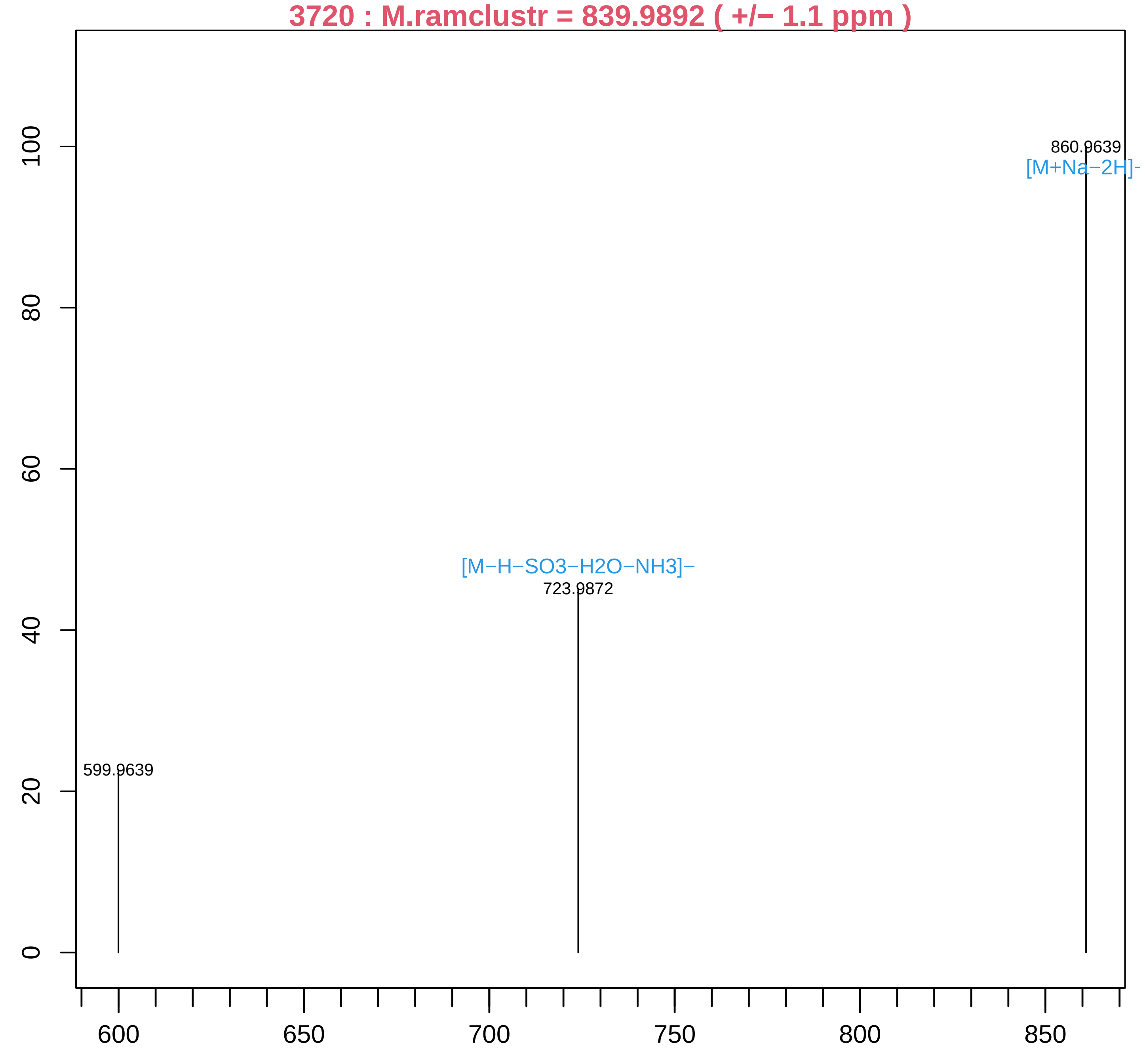
3720 : M.ramclustr = 839.9892 (+/− 1.1 ppm)
860.9639
[M+Na−2H]-
[M−H−SO3−H2O−NH3]−
723.9872
599.9639
0
20
40
60
80
100
600
650
700
750
800
850

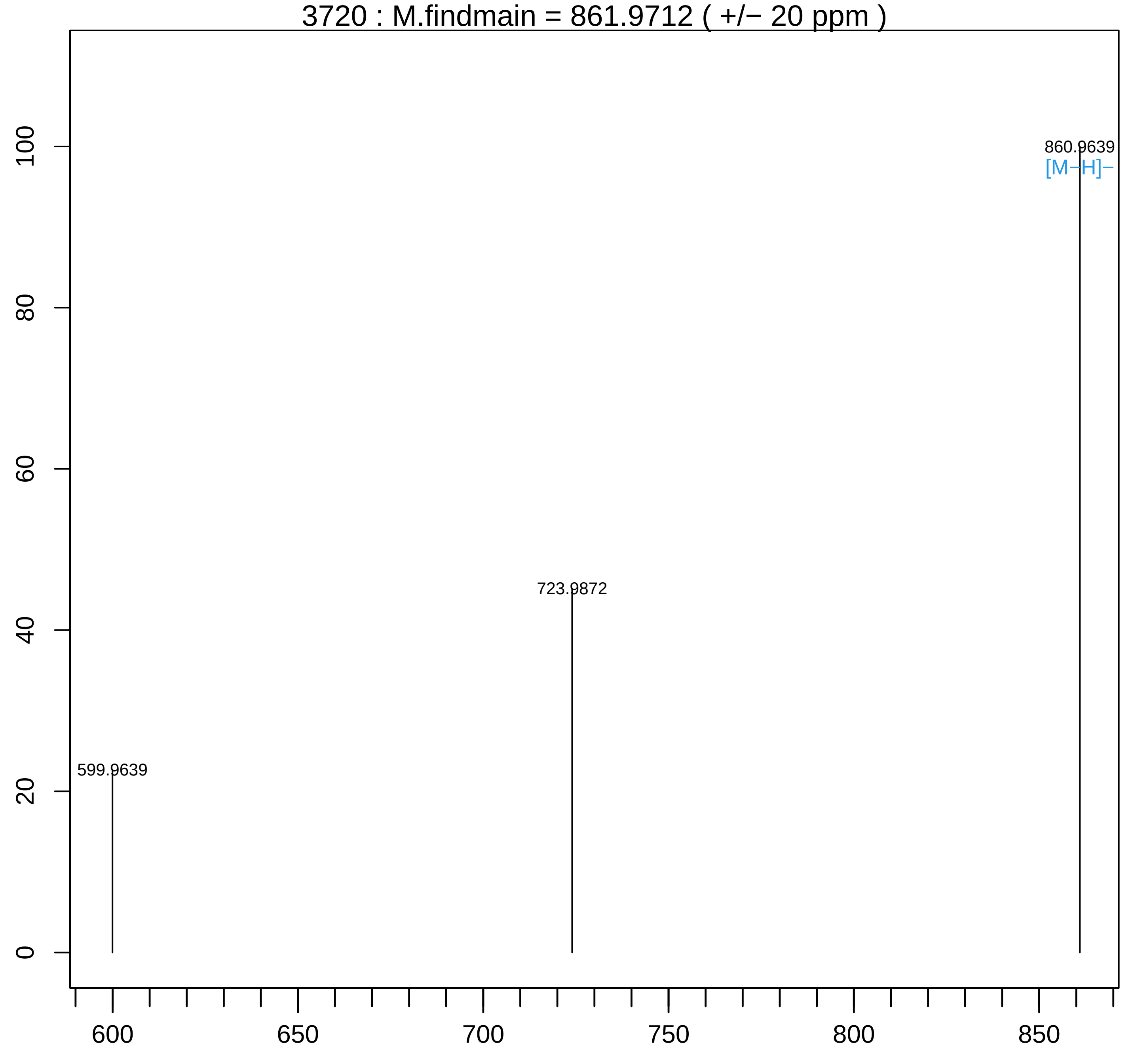
3720 : M.findmain = 861.9712 (+/− 20 ppm)
860.9639
[M−H]−
723.9872
599.9639
0
20
40
60
80
100
600
650
700
750
800
850

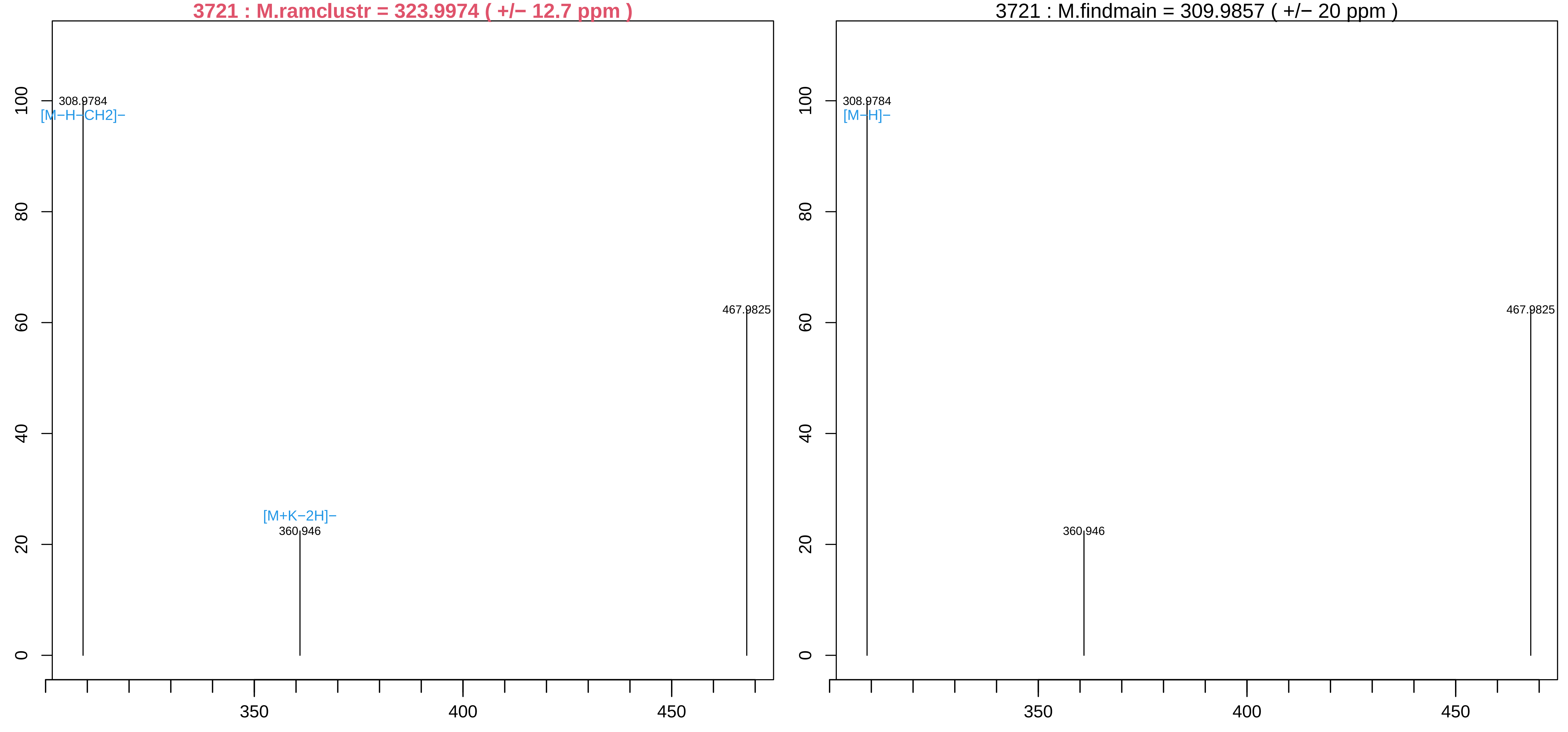
3721 : M.ramclustr = 323.9974 (+/− 12.7 ppm)
308.9784
[M−H−CH2]−
[M+K−2H]−
360.946
467.9825
3721 : M.findmain = 309.9857 (+/− 20 ppm)
308.9784
[M−H]−
360.946
467.9825
0
20
40
60
80
100
350
400
450

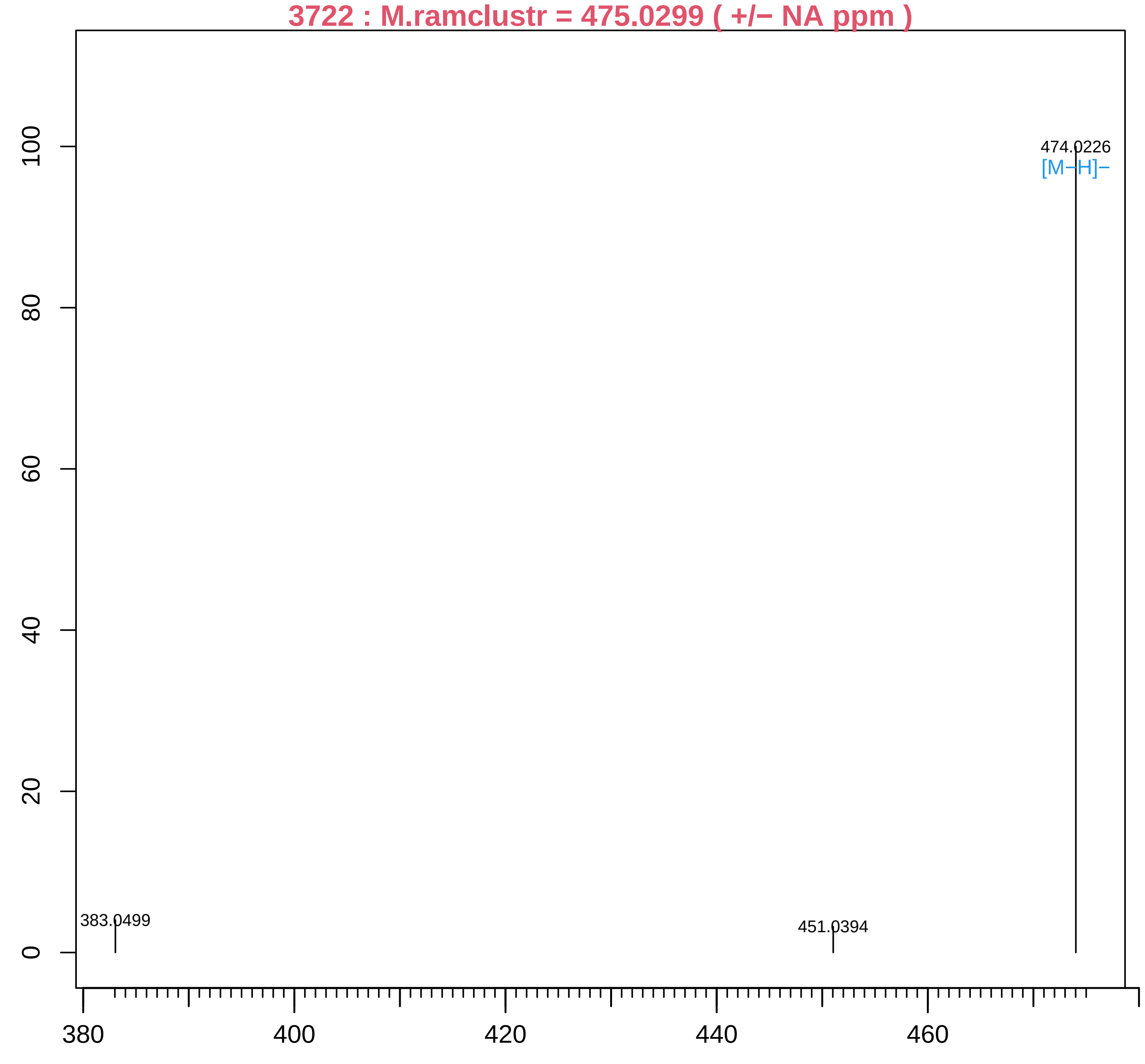
3722 : M.ramclustr = 475.0299 (+/− NA ppm)
474.0226
[M−H]−
383.0499
451.0394

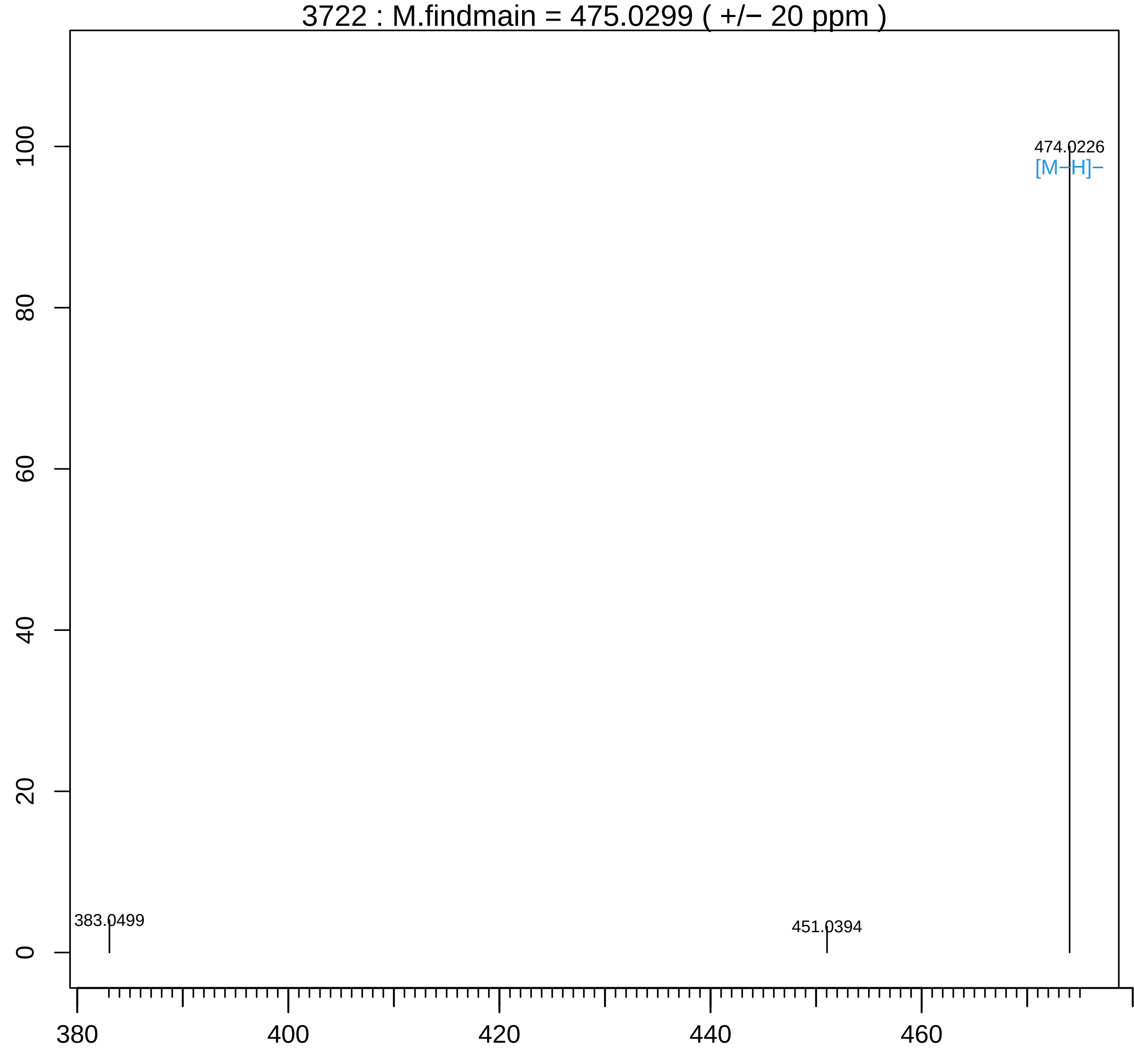
3722 : M.findmain = 475.0299 (+/− 20 ppm)
474.0226
[M−H]−
383.0499
451.0394

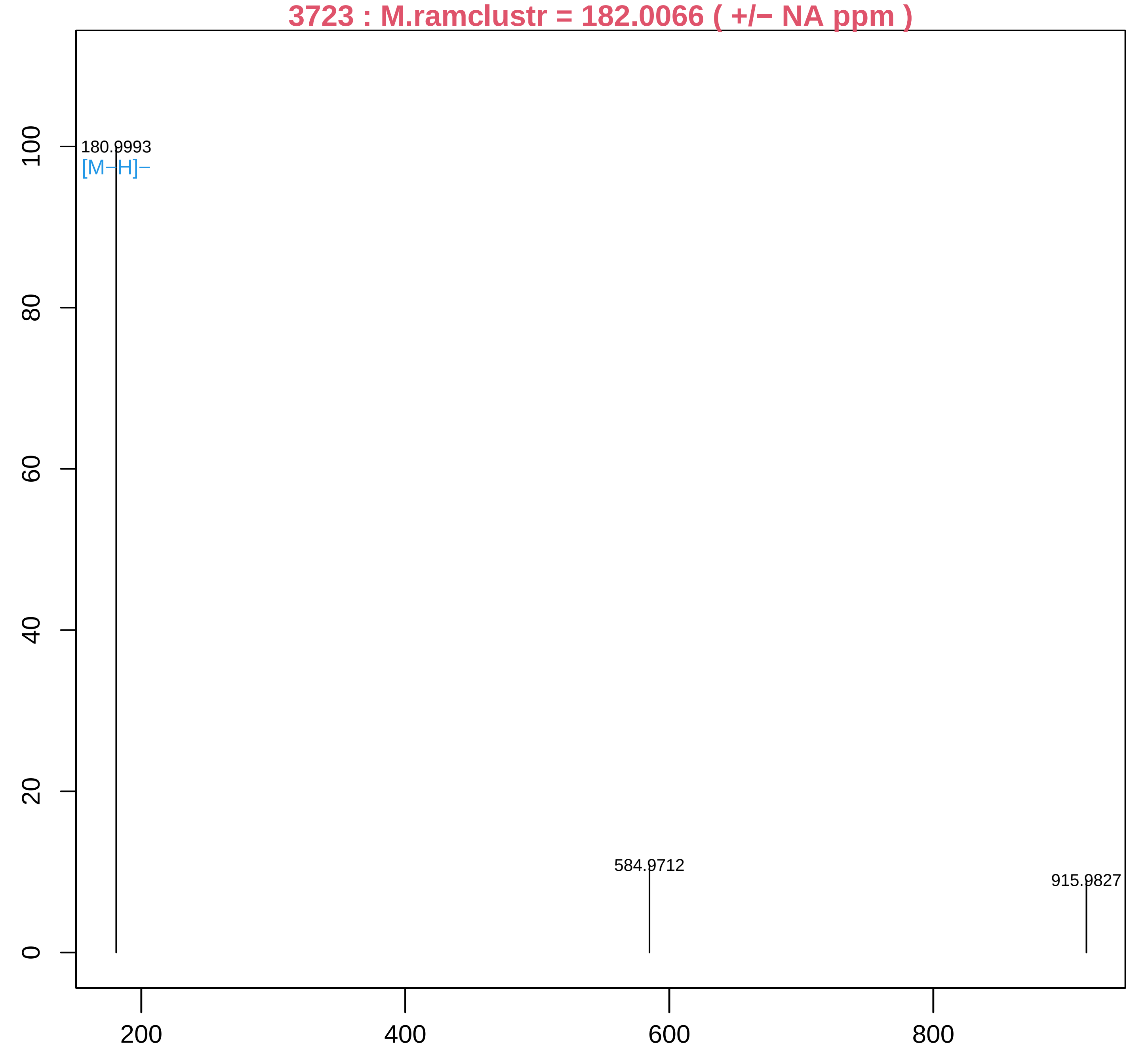
3723 : M.ramclustr = 182.0066 (+/− NA ppm)
180.9993
[M−H]−
584.9712
915.9827
0
20
40
60
80
100
200
400
600
800

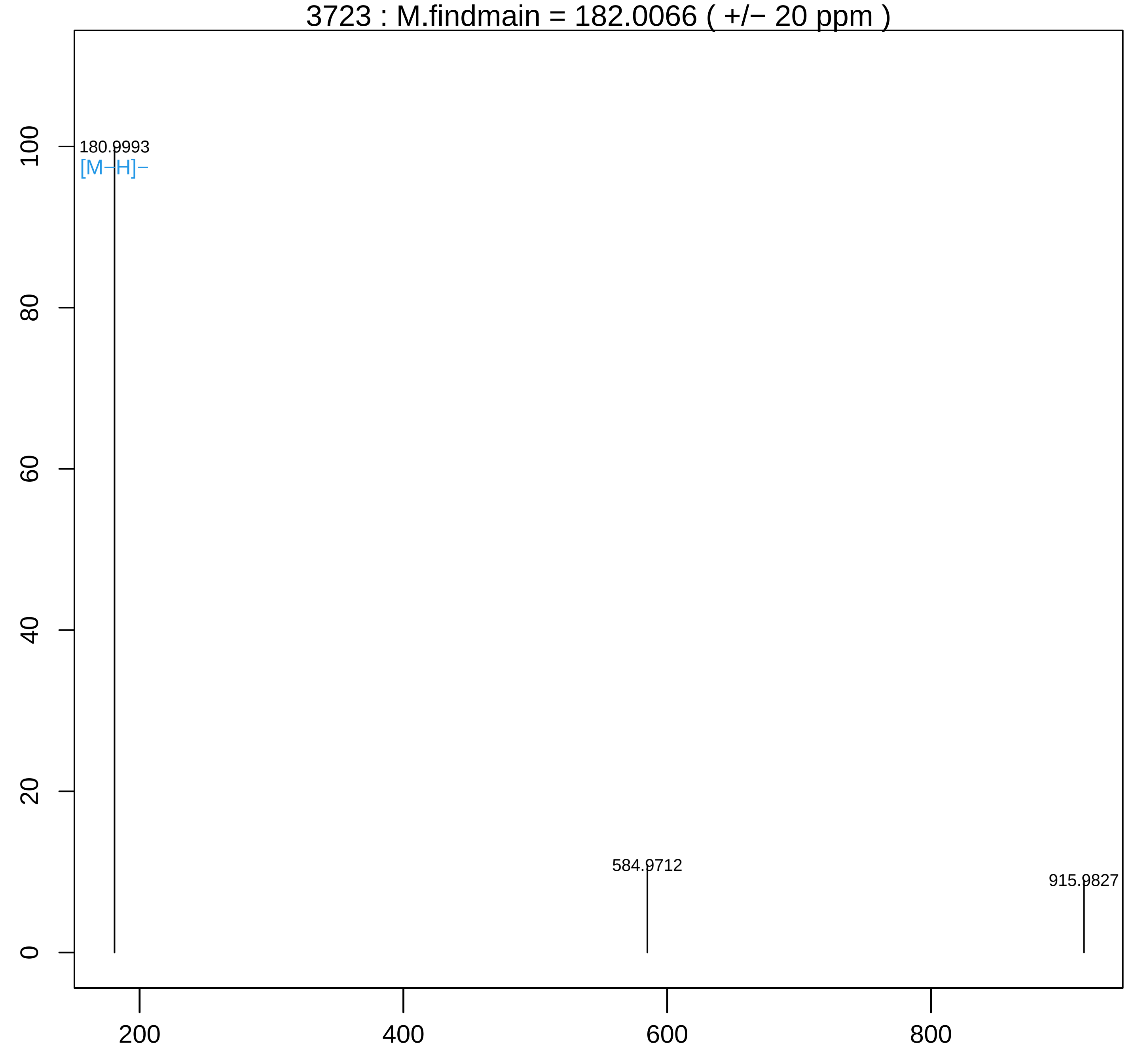
3723 : M.findmain = 182.0066 (+/− 20 ppm)
180.9993
[M−H]−
584.9712
915.9827
0
20
40
60
80
100
200
400
600
800

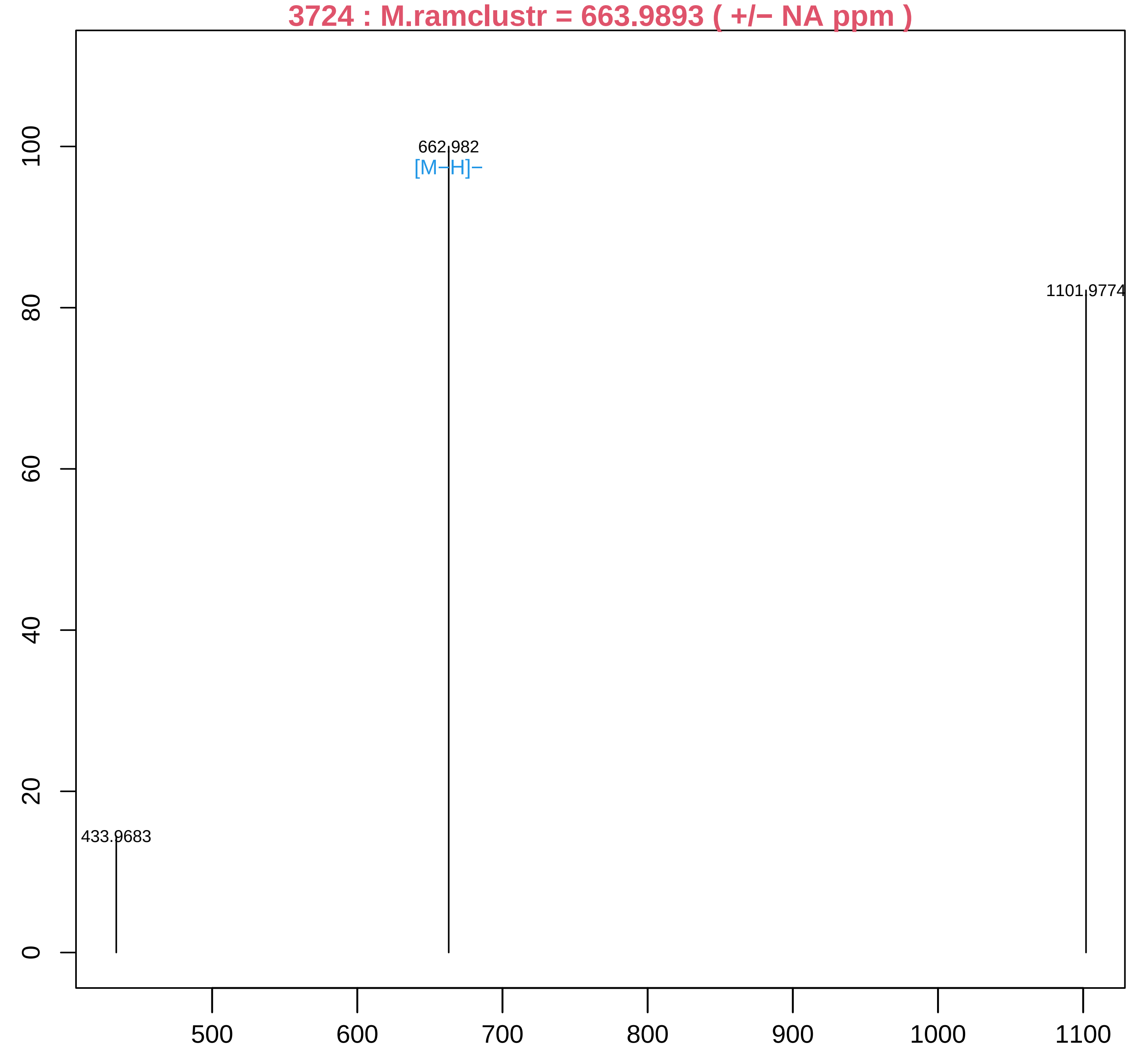
3724 : M.ramclustr = 663.9893 (+/− NA ppm)
662.982
[M−H]−
1101.9774
433.9683
0
20
40
60
80
100
500
600
700
800
900
1000
1100

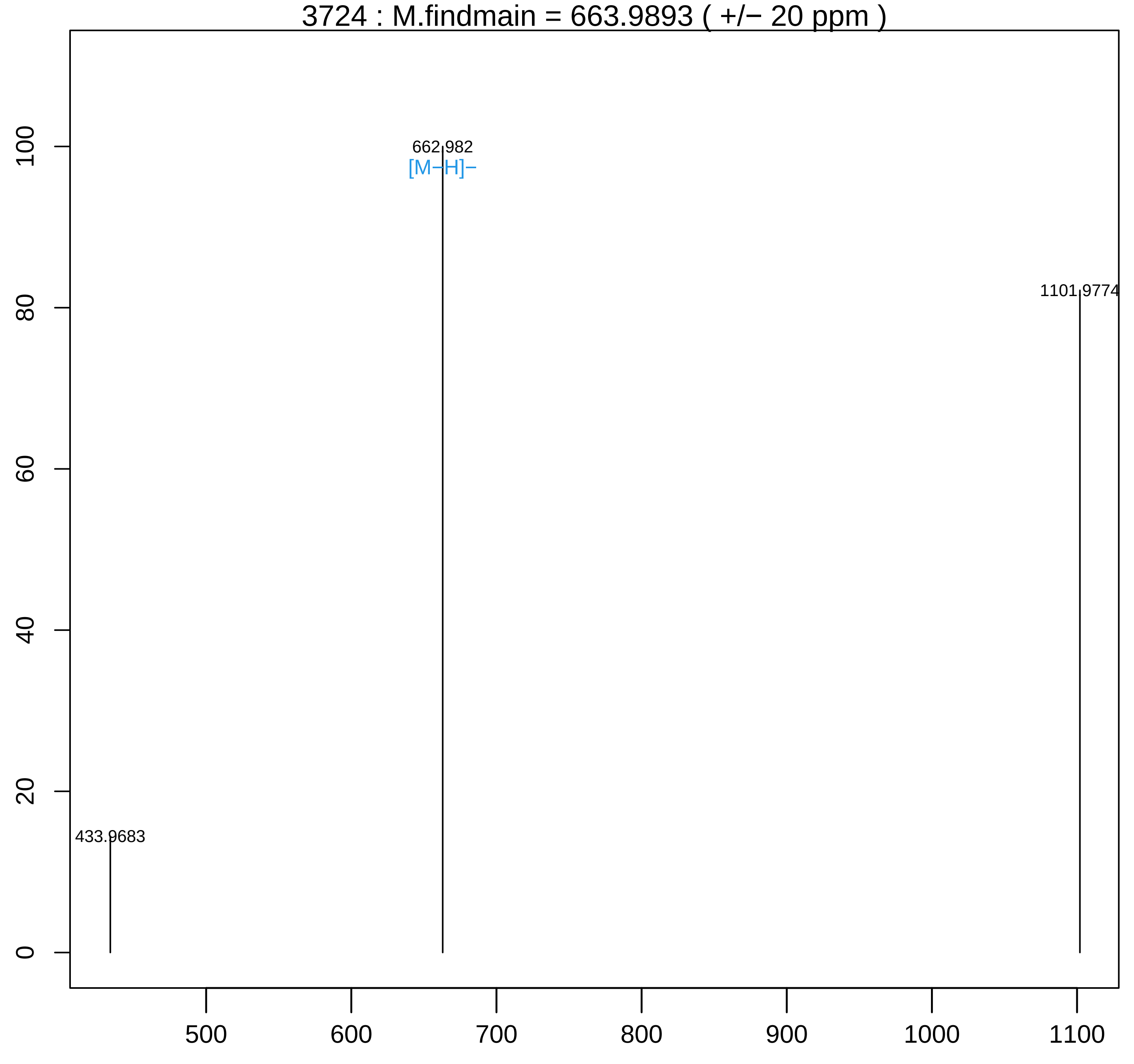
3724 : M.findmain = 663.9893 (+/− 20 ppm)
662.982
[M−H]−
1101.9774
433.9683
0
20
40
60
80
100
500
600
700
800
900
1000
1100

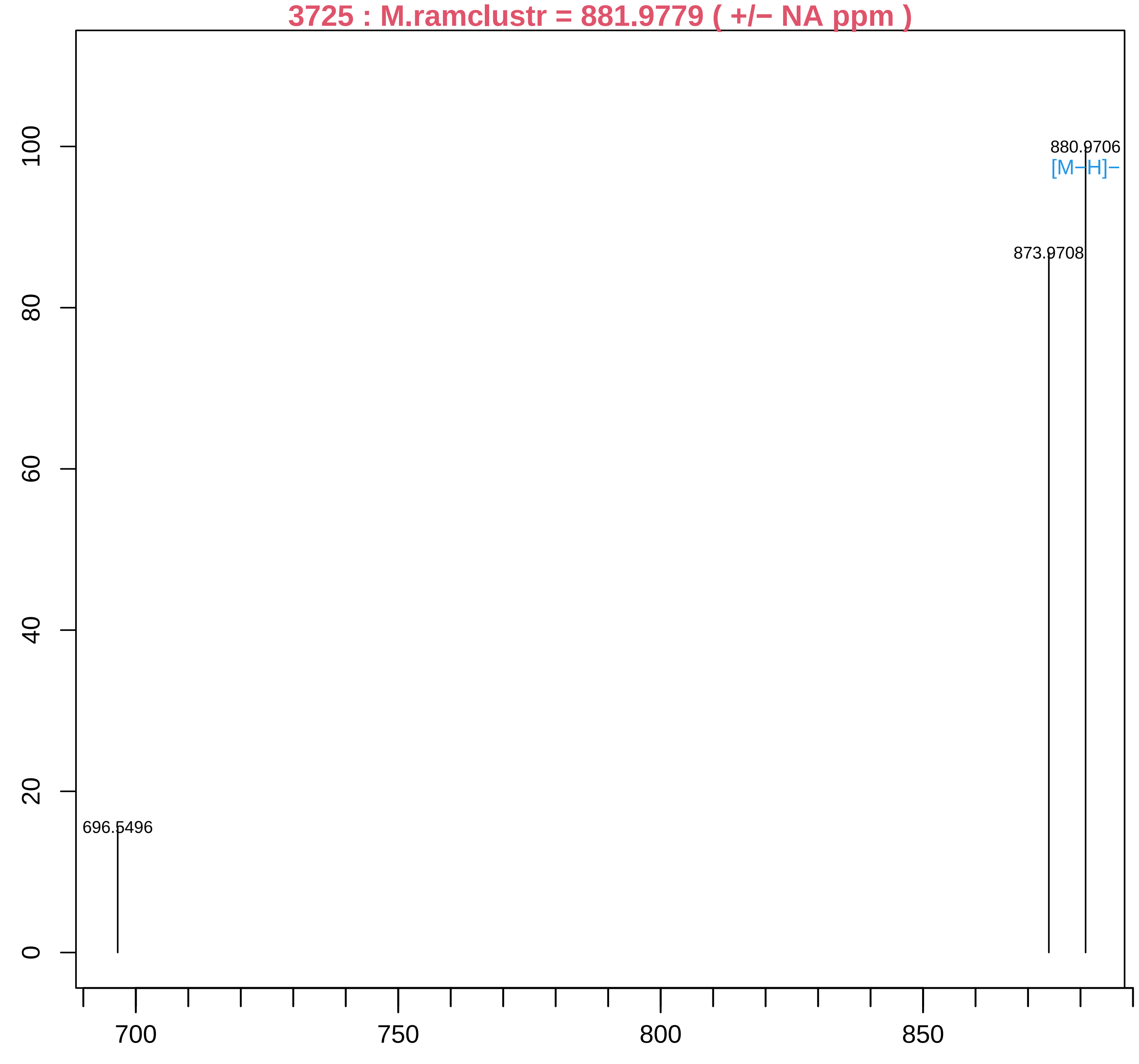
3725 : M.ramclustr = 881.9779 (+/− NA ppm)
880.9706
[M−H]−
873.9708
696.5496
0
20
40
60
80
100
700
750
800
850

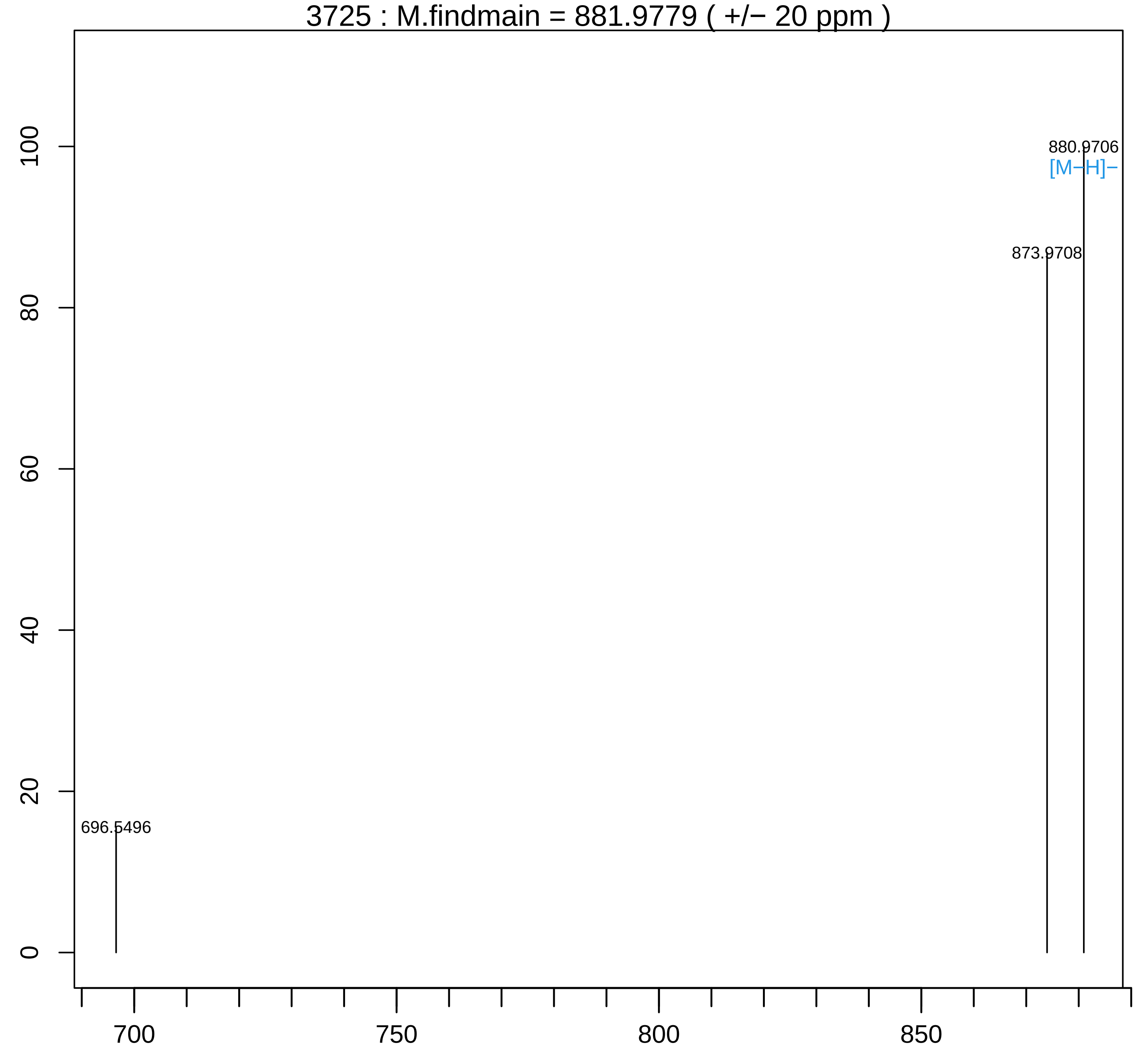
3725 : M.findmain = 881.9779 (+/− 20 ppm)
880.9706
[M−H]−
873.9708
696.5496
0
20
40
60
80
100
700
750
800
850

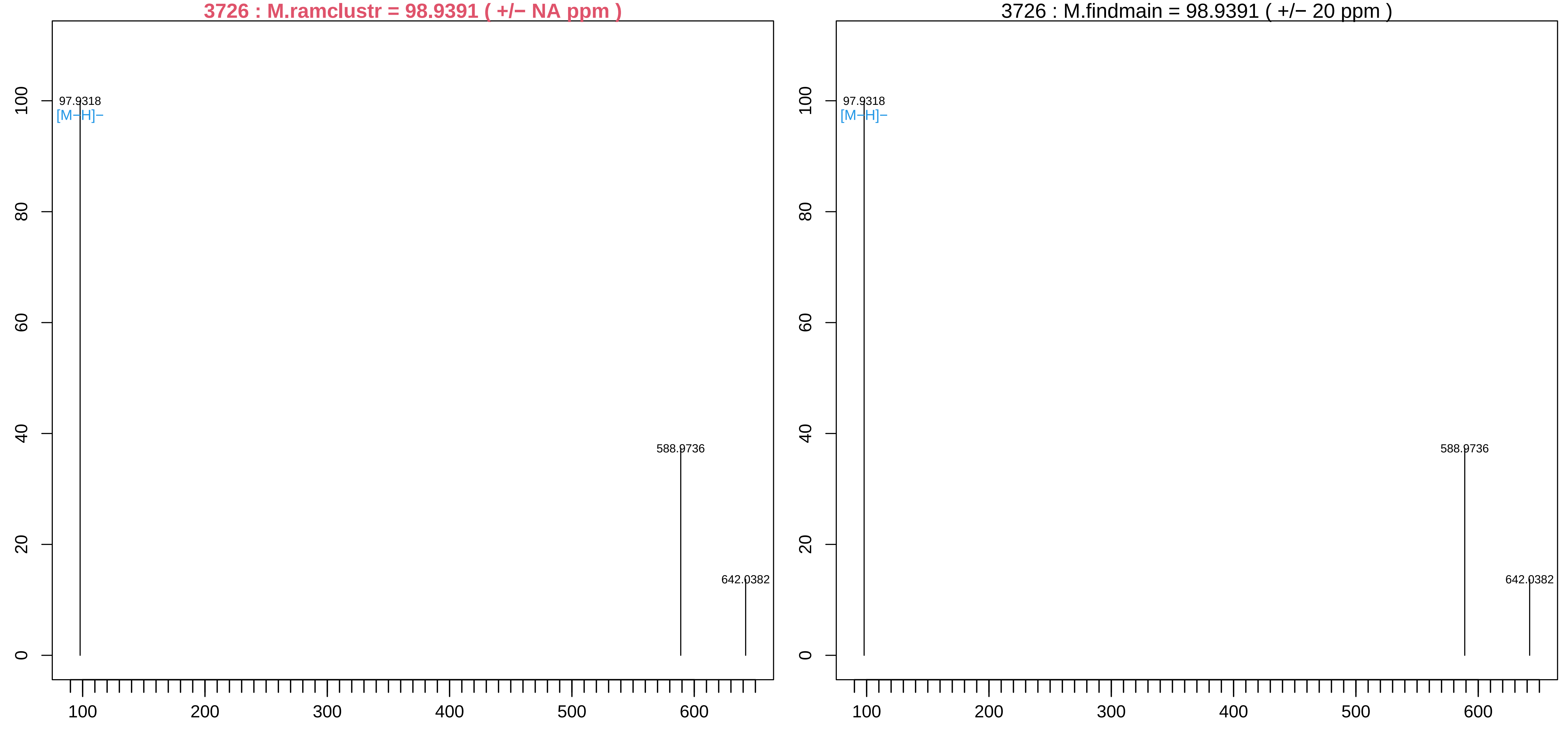
3726 : M.ramclustr = 98.9391 (+/− NA ppm)
3726 : M.findmain = 98.9391 (+/− 20 ppm)
97.9318
[M−H]−
588.9736
642.0382
0
20
40
60
80
100
200
300
400
500
600

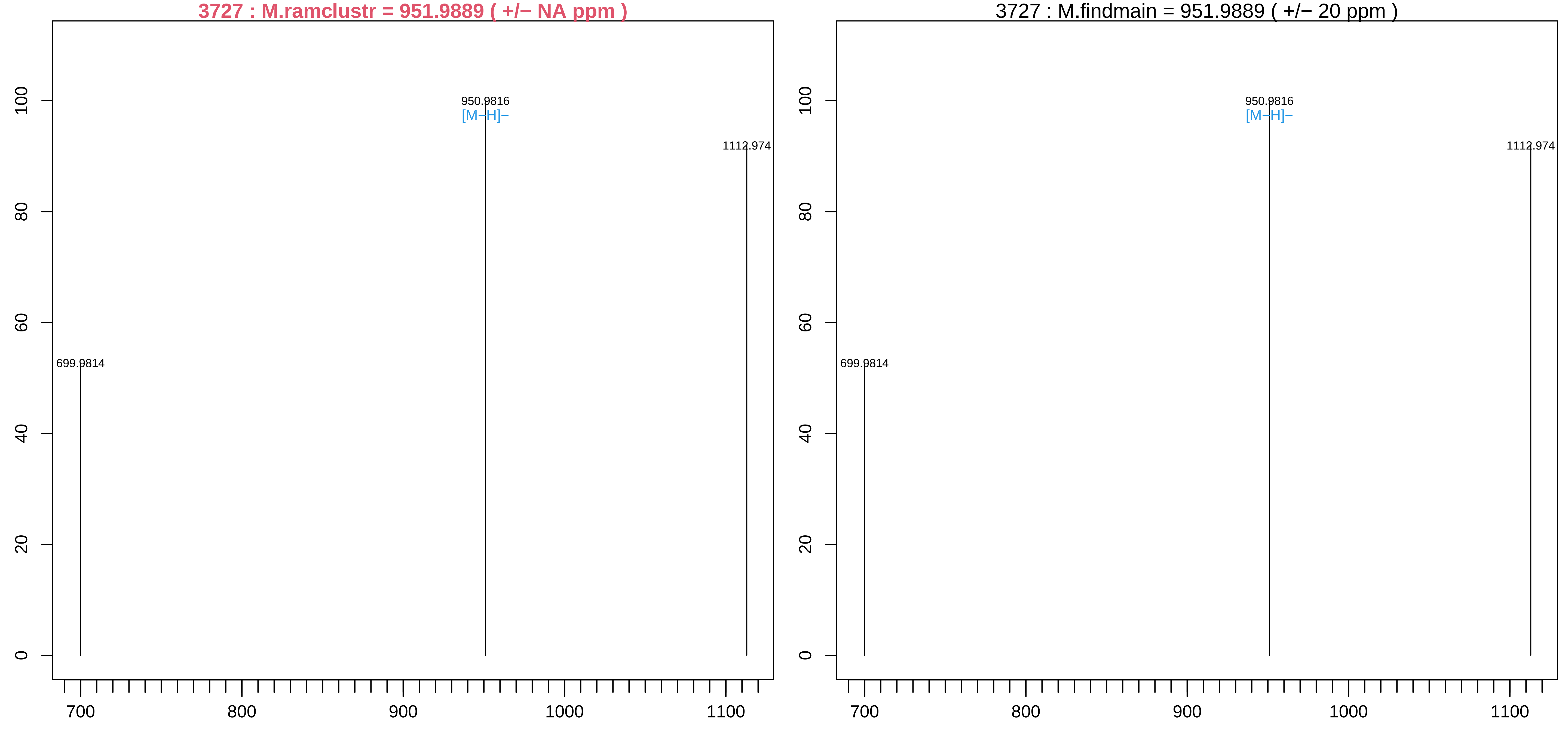
3727 : M.ramclustr = 951.9889 (+/− NA ppm)
950.9816
[M−H]−
1112.974
699.9814
0
20
40
60
80
100
700
800
900
1000
1100
3727 : M.findmain = 951.9889 (+/− 20 ppm)
950.9816
[M−H]−
1112.974
699.9814
0
20
40
60
80
100
700
800
900
1000
1100

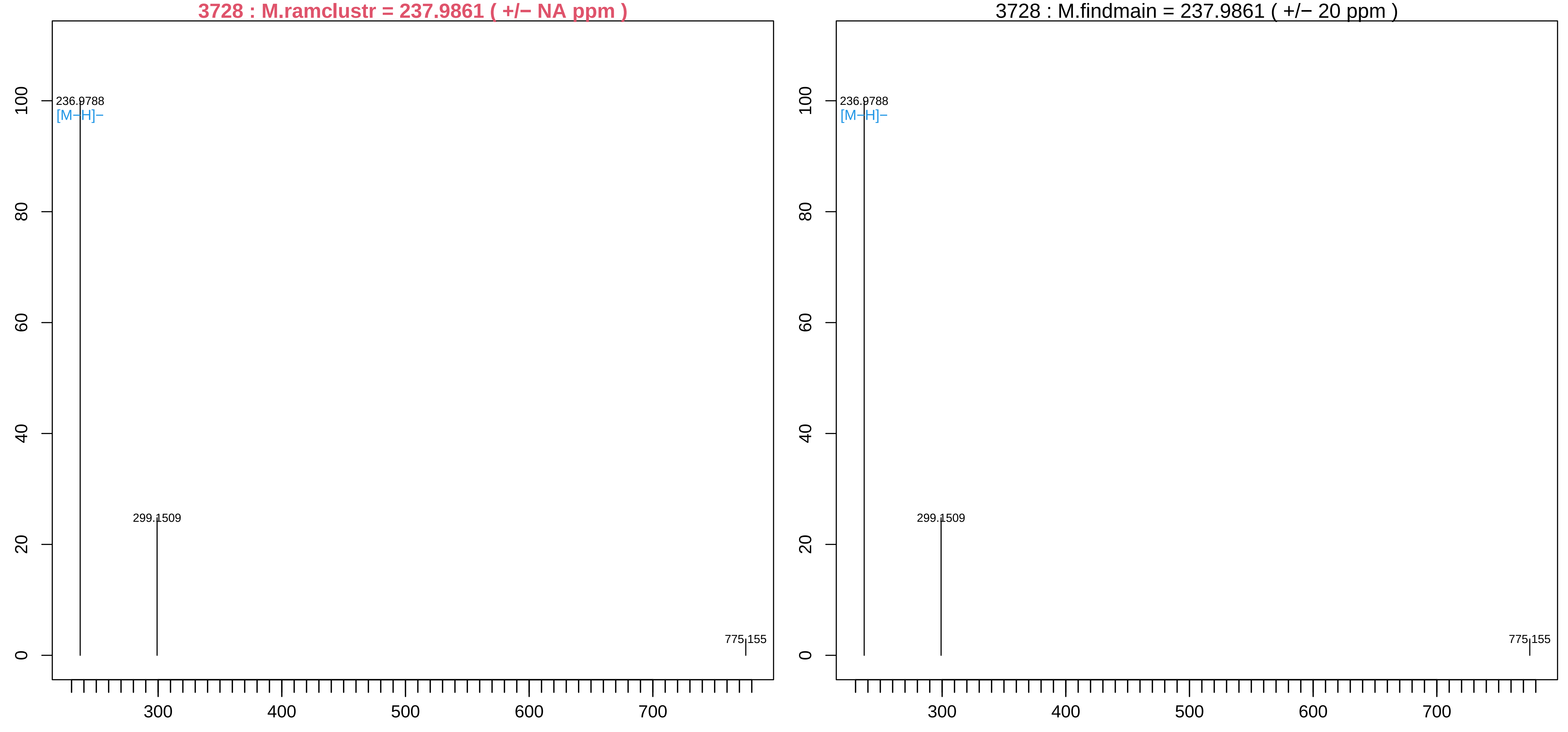
3728 : M.ramclustr = 237.9861 (+/− NA ppm)
236.9788
[M−H]−
299.1509
775.155
3728 : M.findmain = 237.9861 (+/− 20 ppm)
236.9788
[M−H]−
299.1509
775.155
0
20
40
60
80
100
300
400
500
600
700

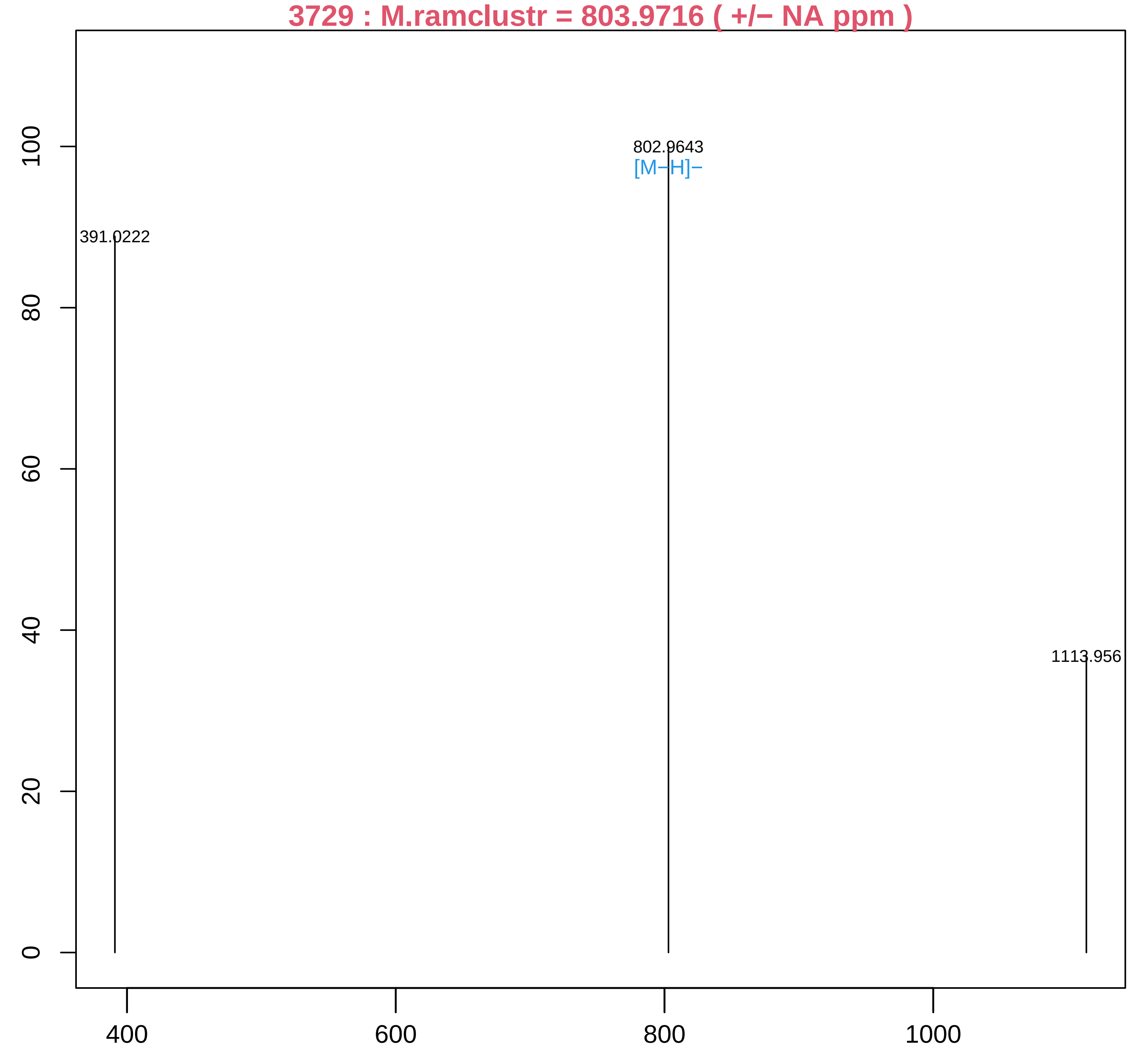
3729 : M.ramclustr = 803.9716 (+/− NA ppm)
391.0222
802.9643
[M−H]−
1113.956
0
20
40
60
80
100
400
600
800
1000

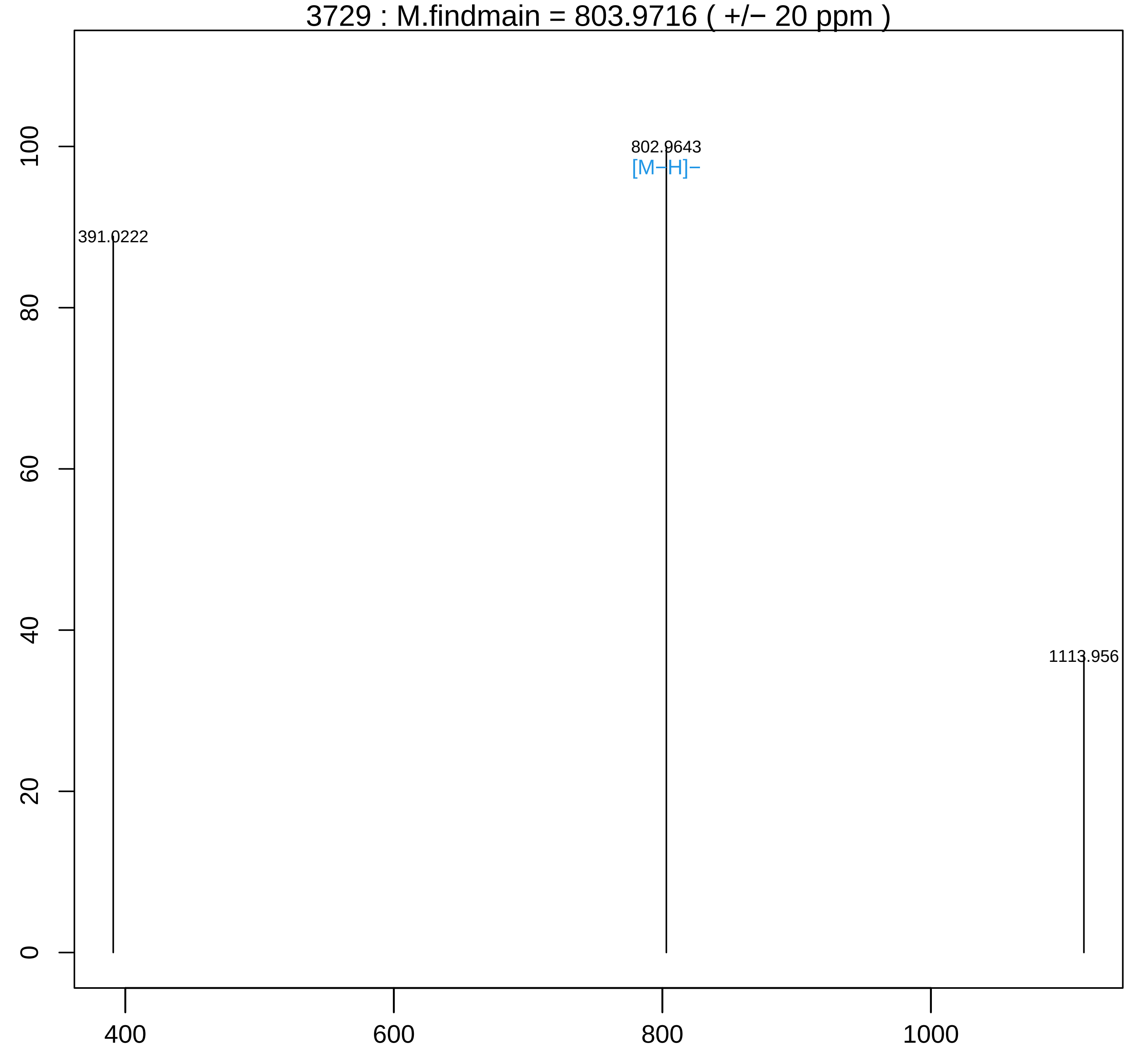
3729 : M.findmain = 803.9716 (+/− 20 ppm)
391.0222
802.9643
[M−H]−
1113.956
0
20
40
60
80
100
400
600
800
1000

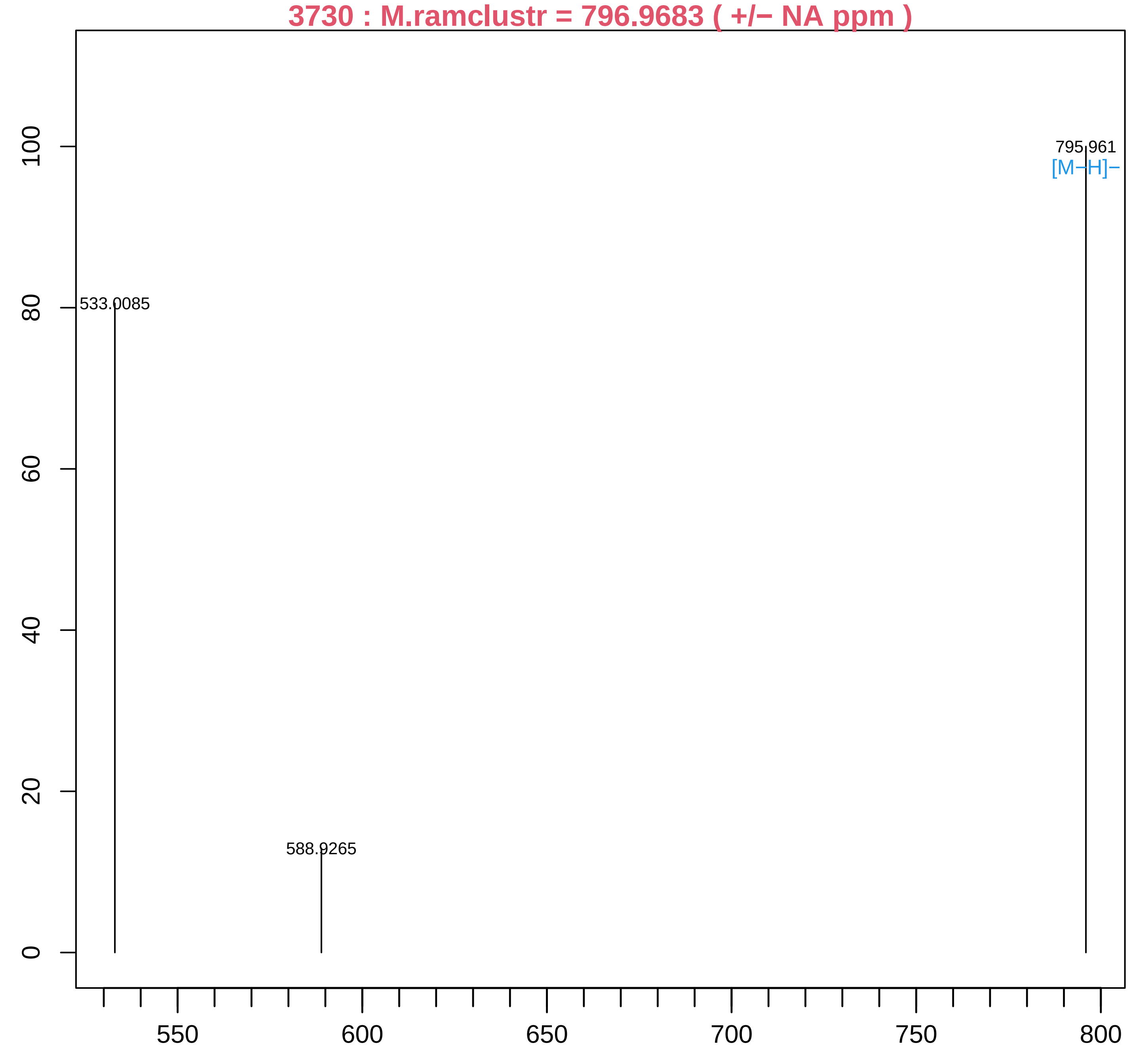
3730 : M.ramclustr = 796.9683 (+/− NA ppm)
795.961
[M−H]−
533.0085
588.9265
0
20
40
60
80
100
550
600
650
700
750
800

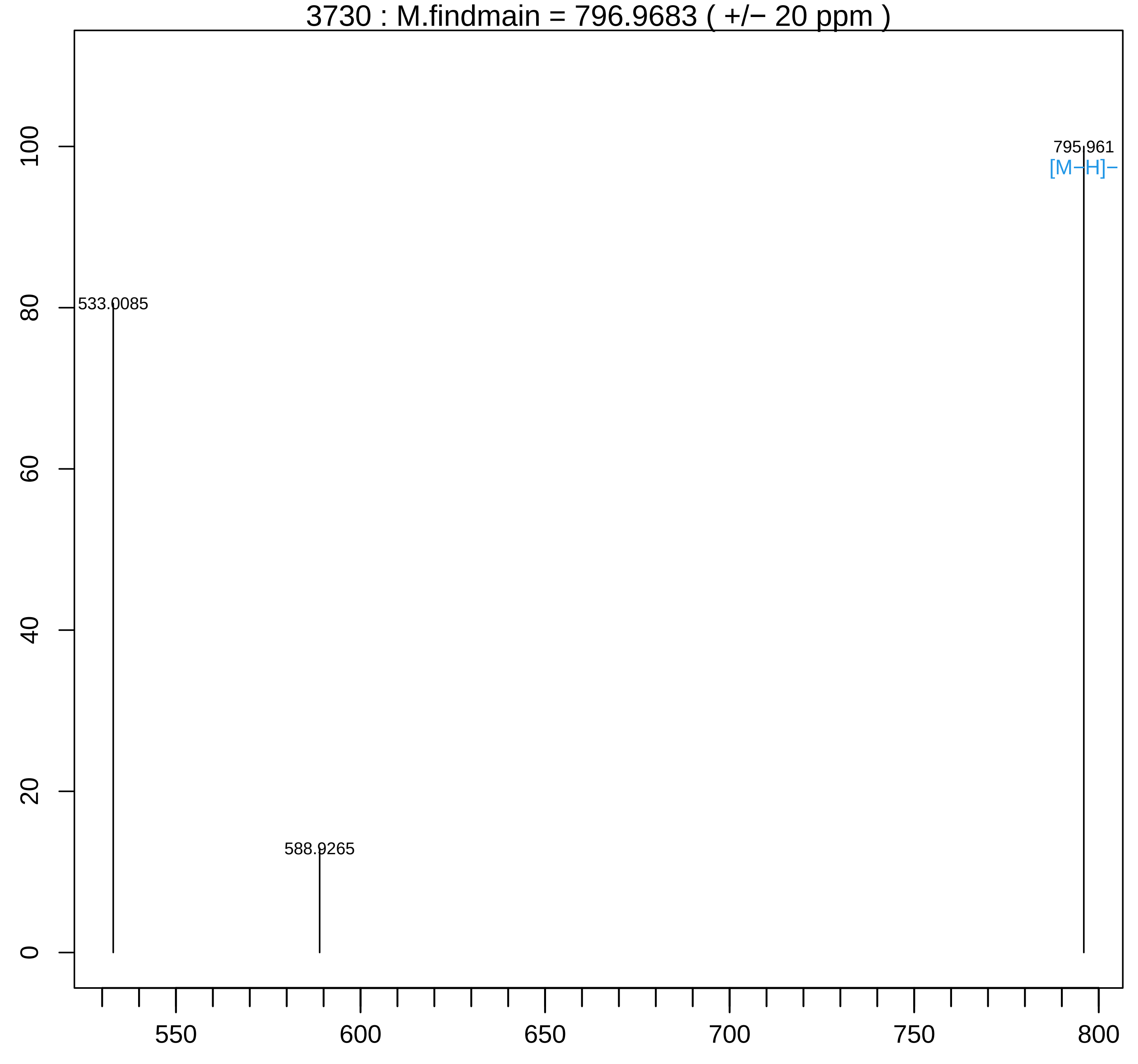
3730 : M.findmain = 796.9683 (+/− 20 ppm)
795.961
[M−H]−
533.0085
588.9265
0
20
40
60
80
100
550
600
650
700
750
800

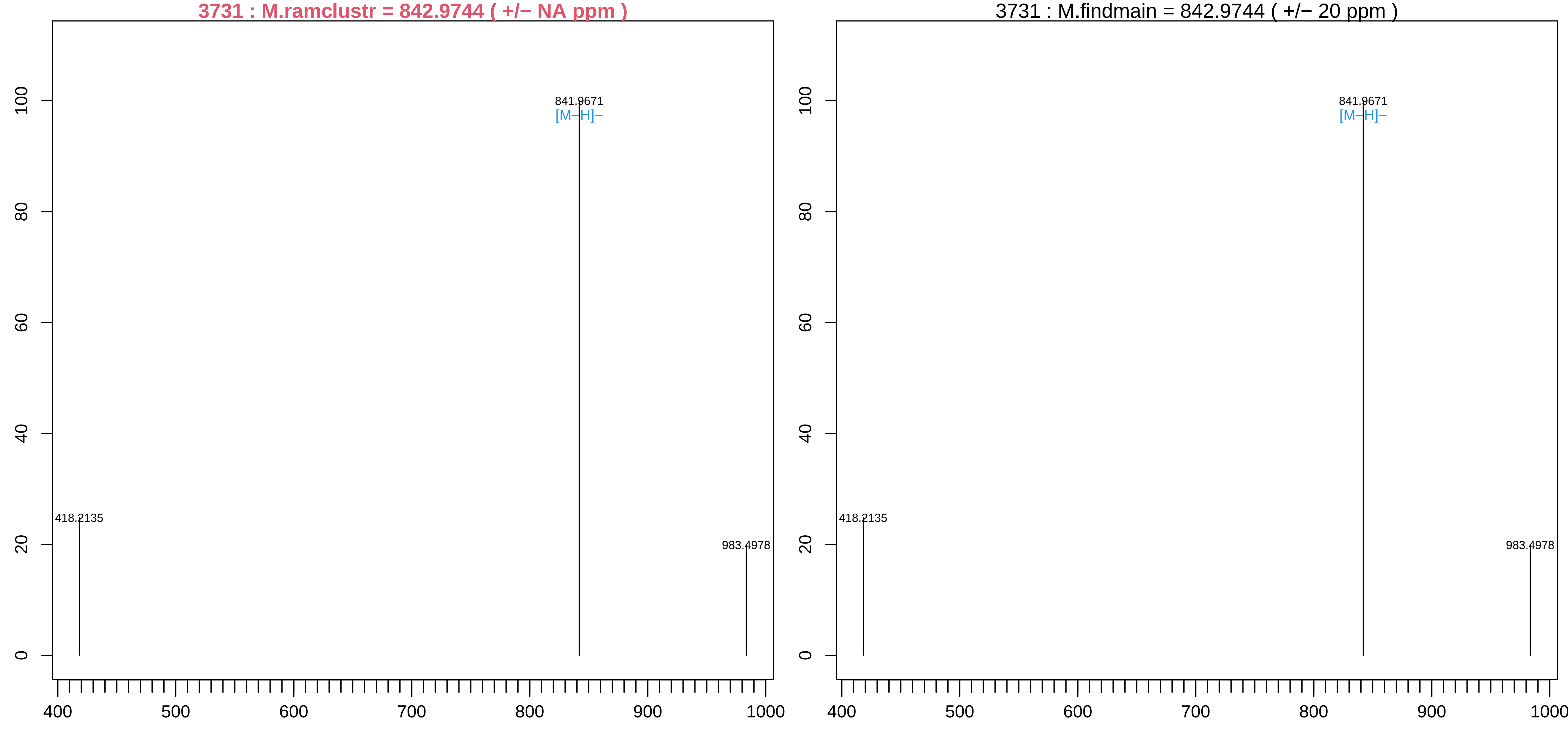
3731 : M.ramclustr = 842.9744 (+/− NA ppm)
841.9671
[M−H]−
418.2135
983.4978
3731 : M.findmain = 842.9744 (+/− 20 ppm)
841.9671
[M−H]−
418.2135
983.4978
0
20
40
60
80
100
400
500
600
700
800
900
1000

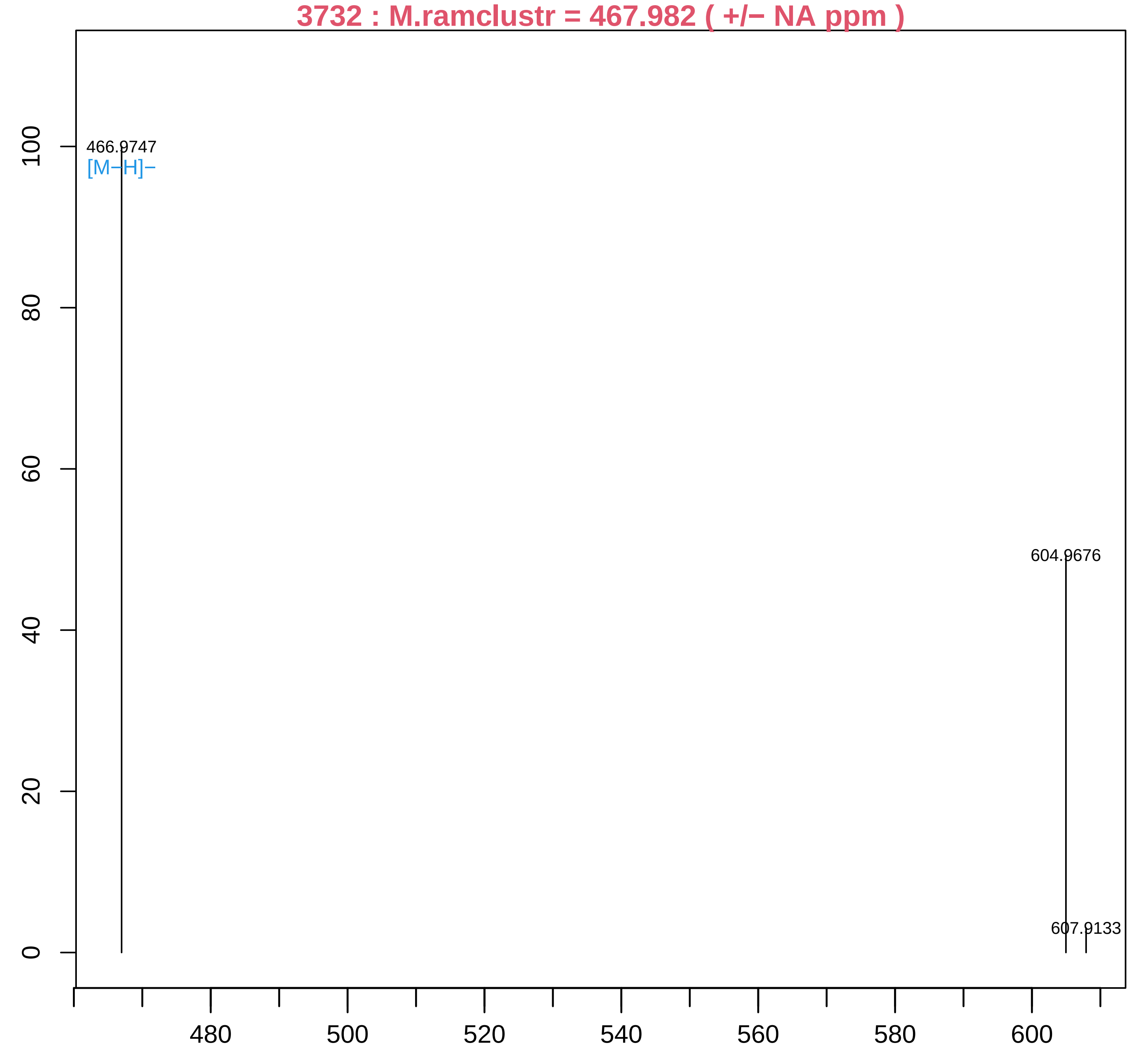

3732 : M.ramclustr = 467.982 (+/− NA ppm)
466.9747
[M−H]−
604.9676
607.9133
0
20
40
60
80
100
480
500
520
540
560
580
600

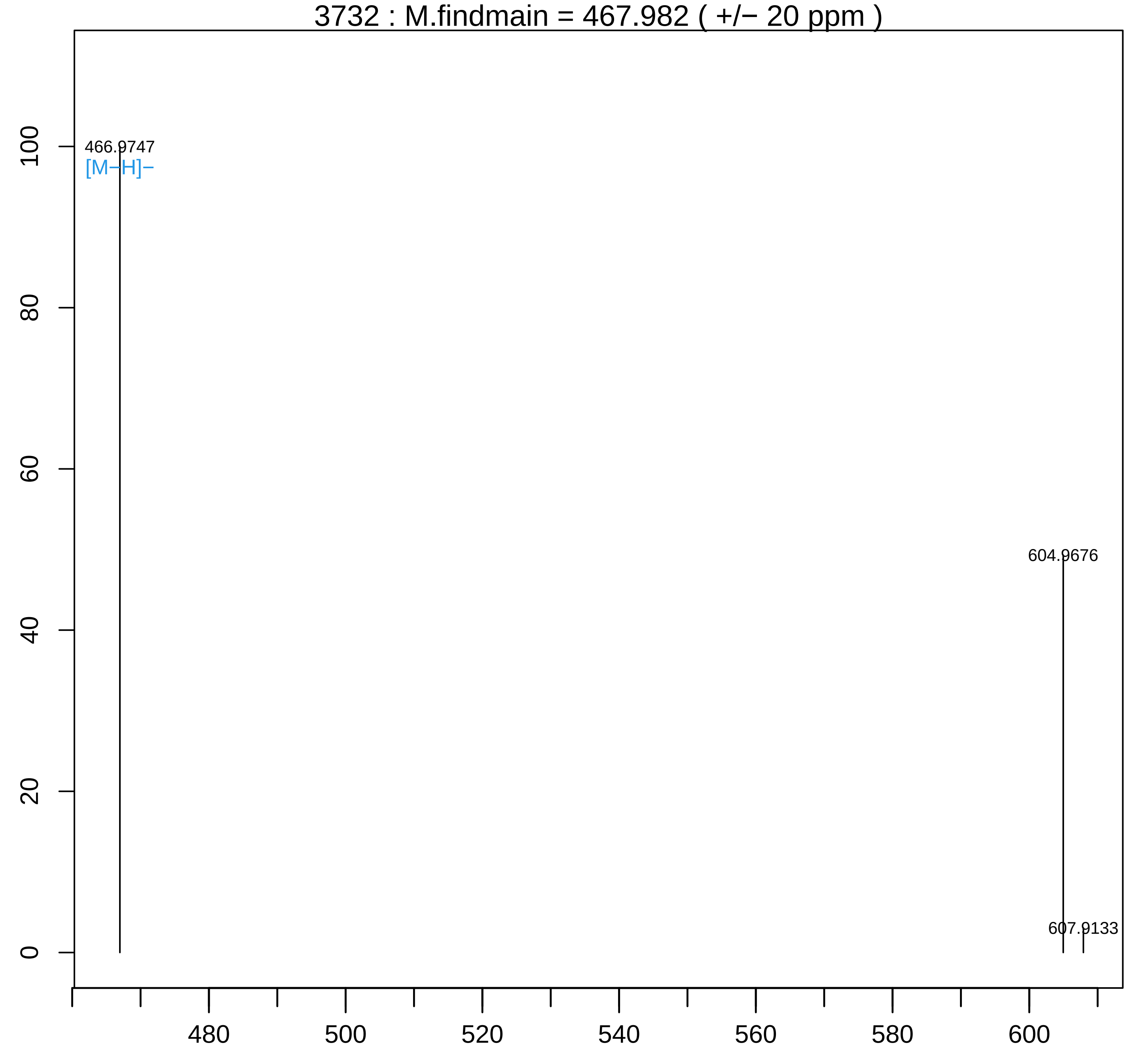

3732 : M.findmain = 467.982 (+/− 20 ppm)
466.9747
[M−H]−
604.9676
607.9133
0
20
40
60
80
100
480
500
520
540
560
580
600

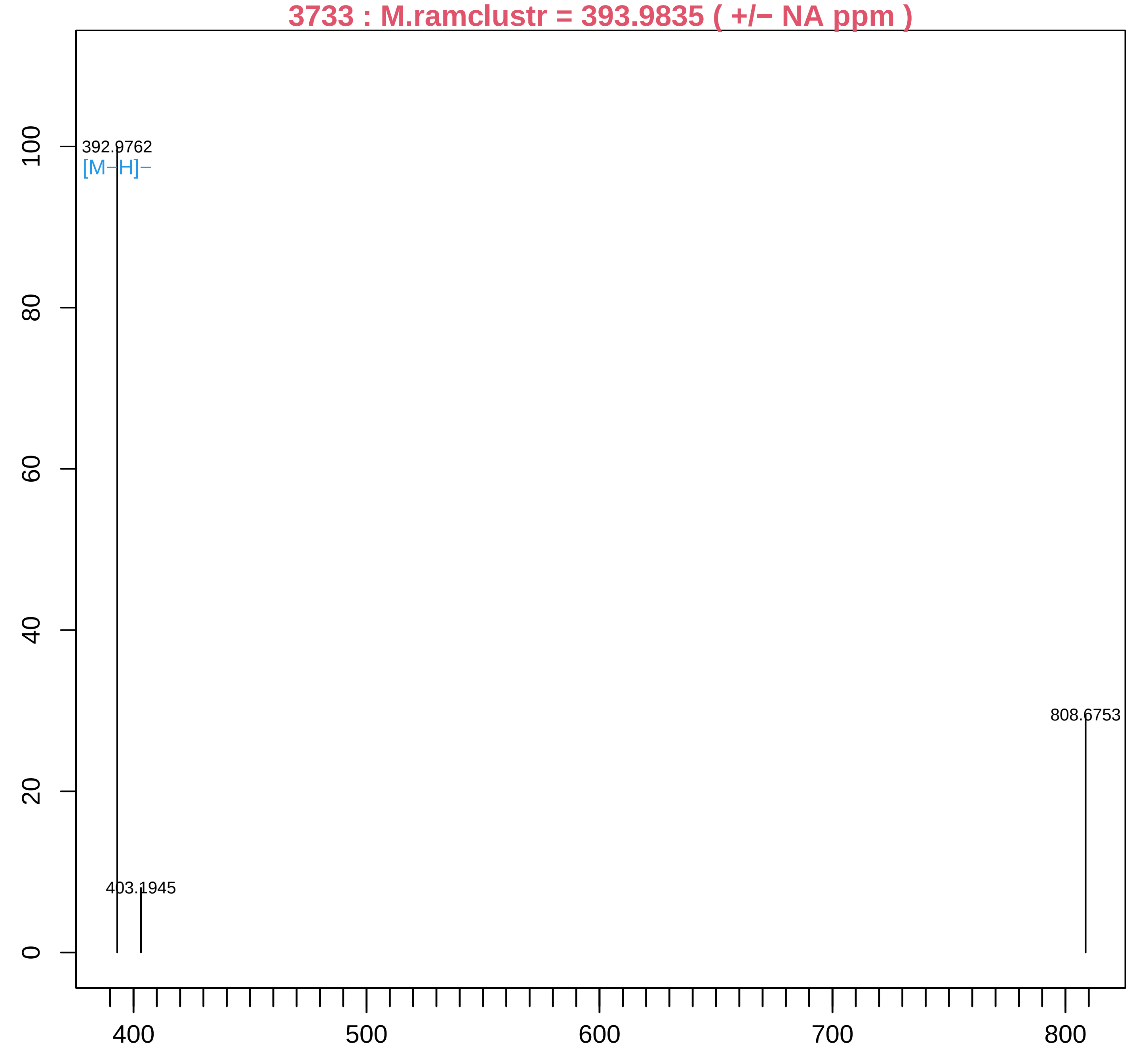
3733 : M.ramclustr = 393.9835 (+/− NA ppm)
392.9762
[M−H]−
403.1945
808.6753
0
20
40
60
80
100
400
500
600
700
800

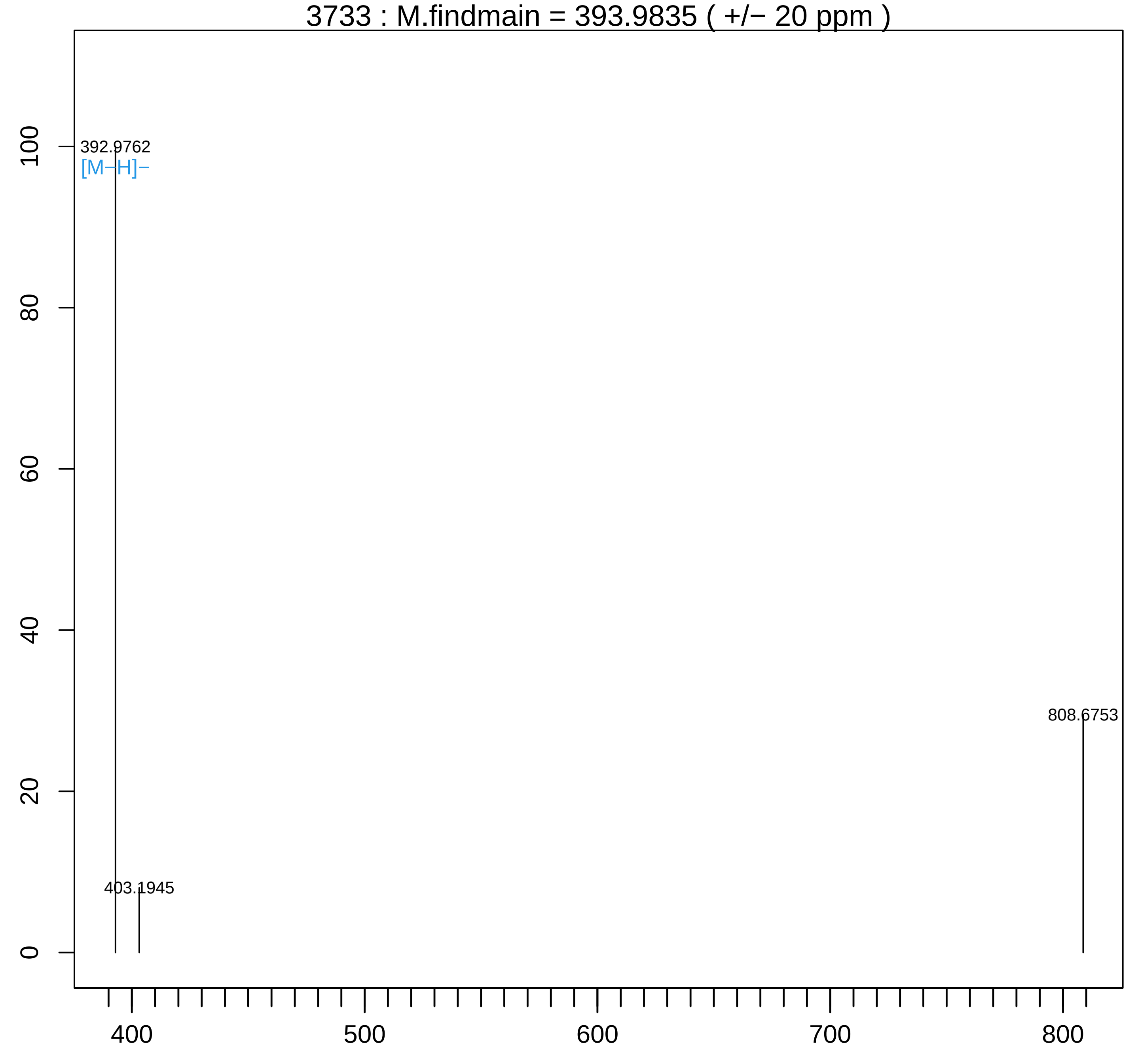
3733 : M.findmain = 393.9835 (+/− 20 ppm)
392.9762
[M−H]−
403.1945
808.6753
0
20
40
60
80
100
400
500
600
700
800

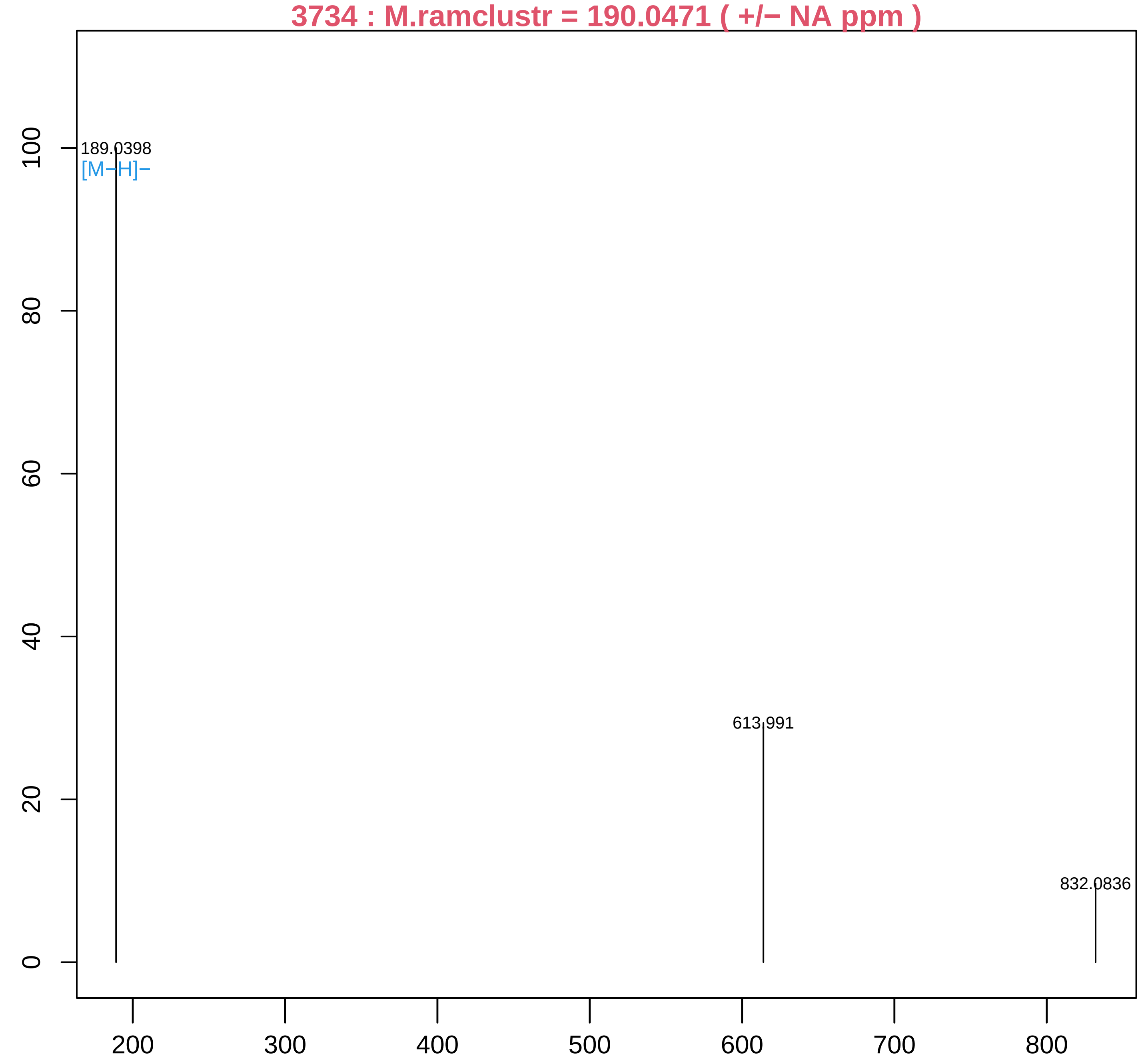
3734 : M.ramclustr = 190.0471 (+/− NA ppm)
189.0398
[M−H]−
613.991
832.0836
0
20
40
60
80
100
200
300
400
500
600
700
800

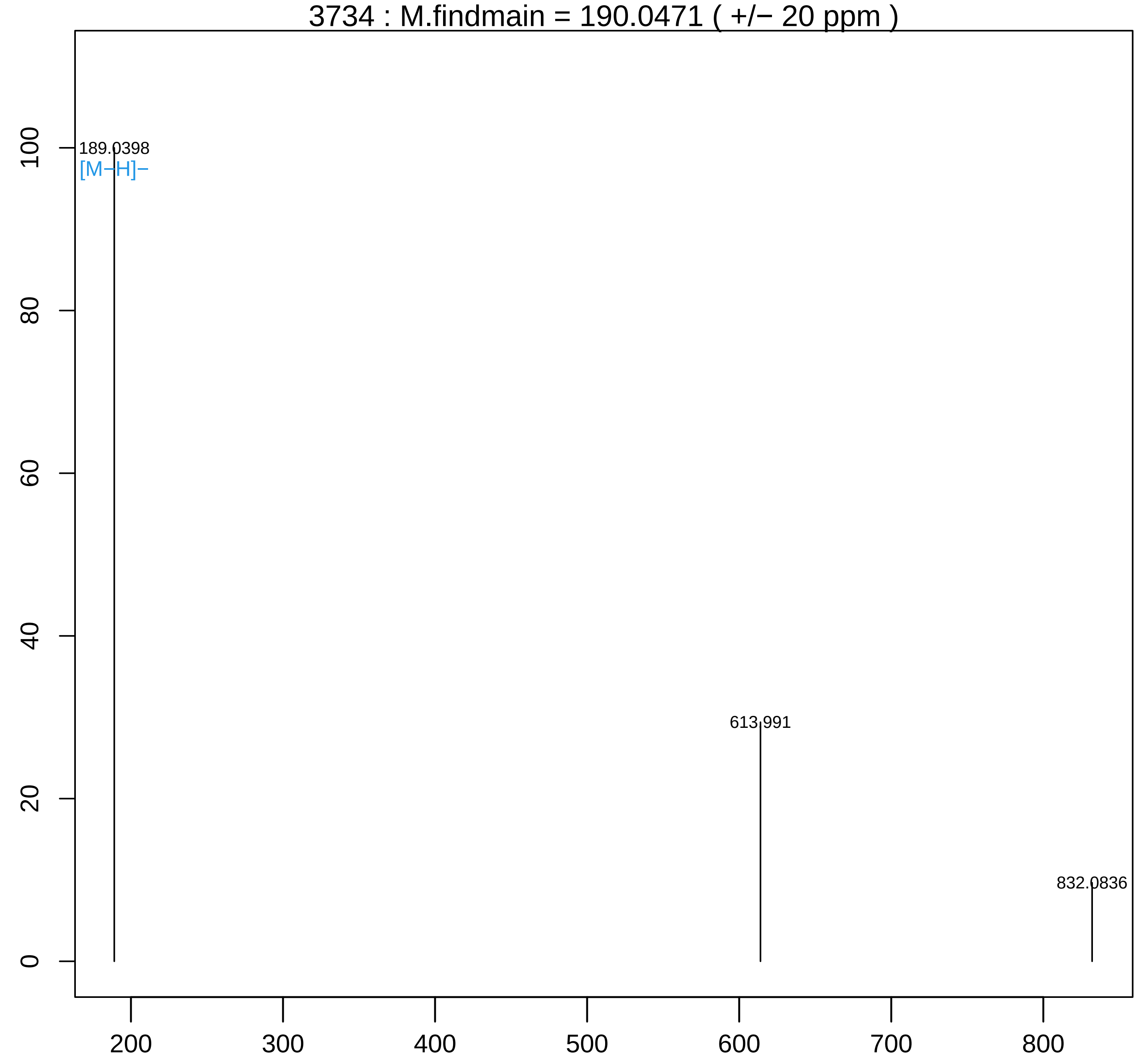
3734 : M.findmain = 190.0471 (+/− 20 ppm)
189.0398
[M−H]−
613.991
832.0836
0
20
40
60
80
100
200
300
400
500
600
700
800

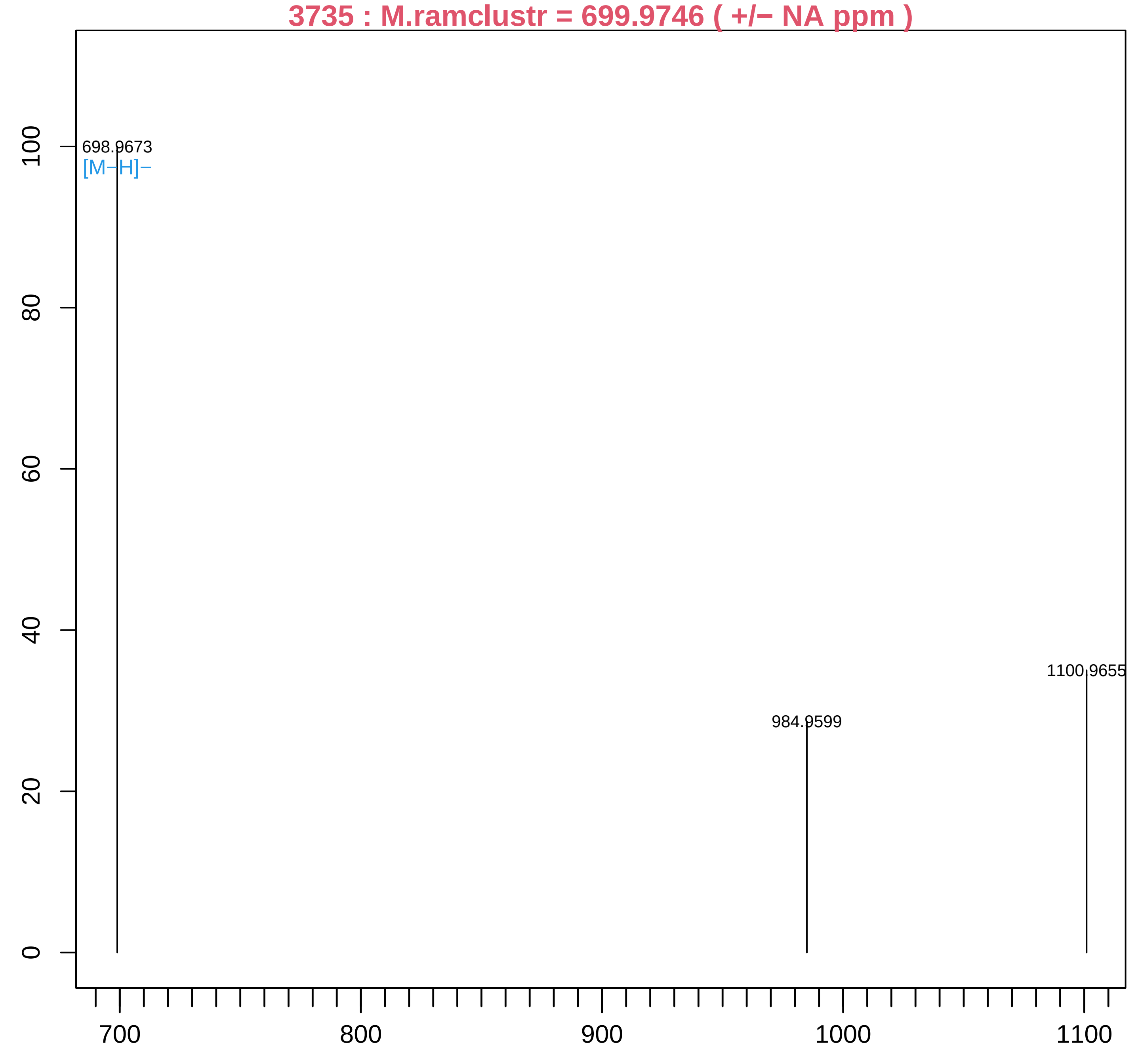
3735 : M.ramclustr = 699.9746 (+/− NA ppm)
698.9673
[M−H]−
984.9599
1100.9655
0
20
40
60
80
100
700
800
900
1000
1100

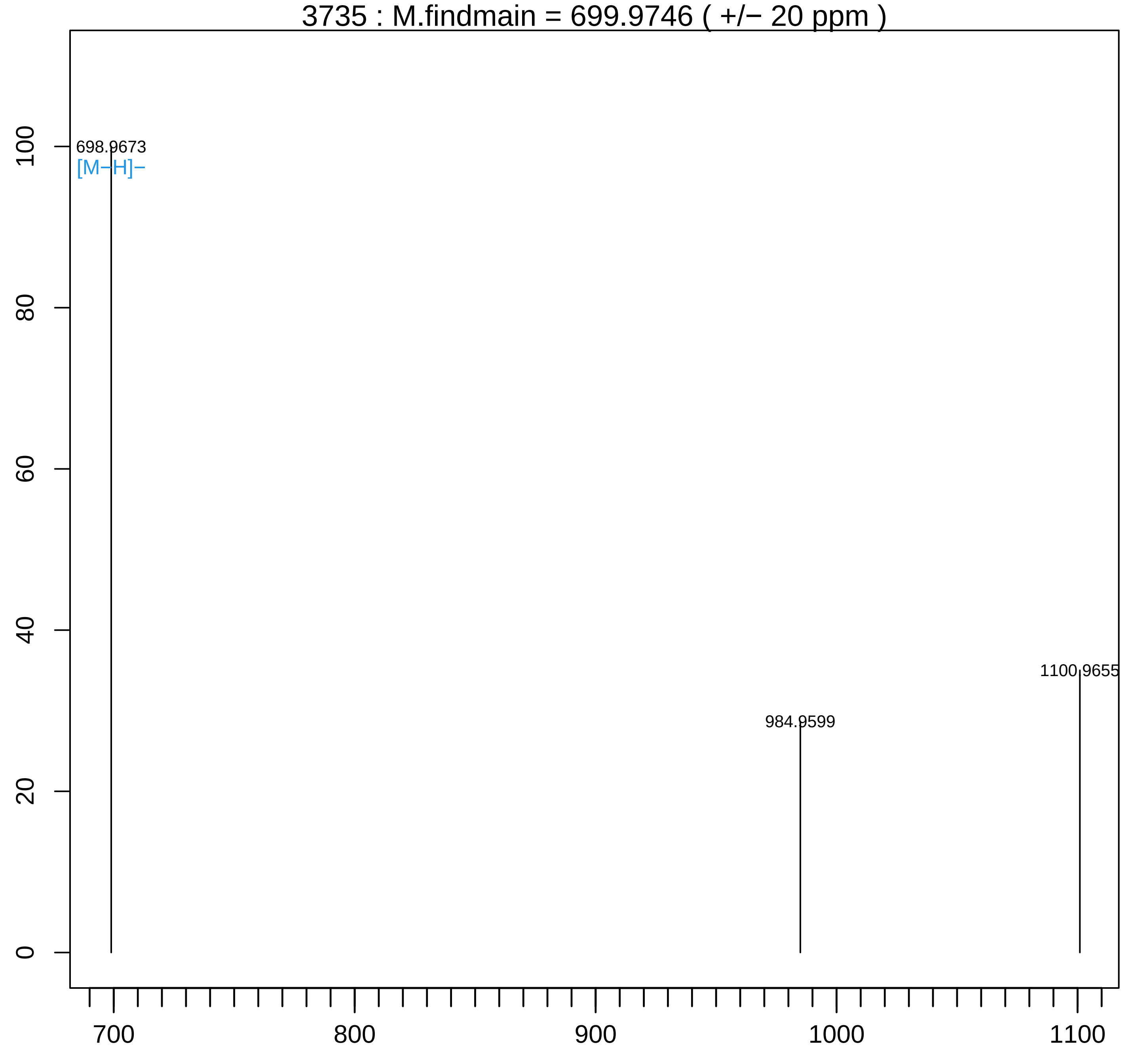
3735 : M.findmain = 699.9746 (+/− 20 ppm)
698.9673
[M−H]−
984.9599
1100.9655
0
20
40
60
80
100
700
800
900
1000
1100

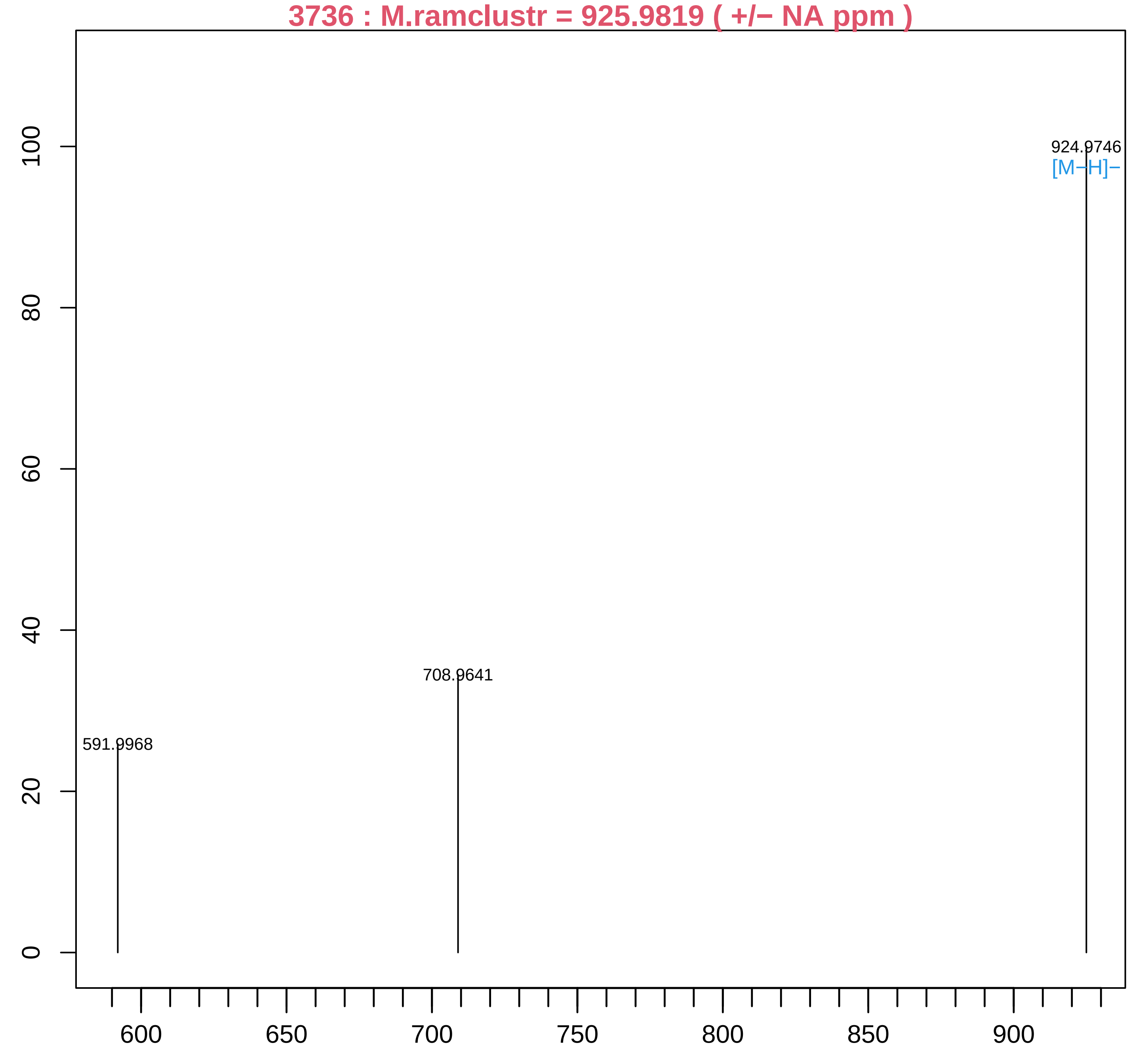
3736 : M.ramclustr = 925.9819 (+/− NA ppm)
924.9746
[M−H]−
708.9641
591.9968
0
20
40
60
80
100
600
650
700
750
800
850
900

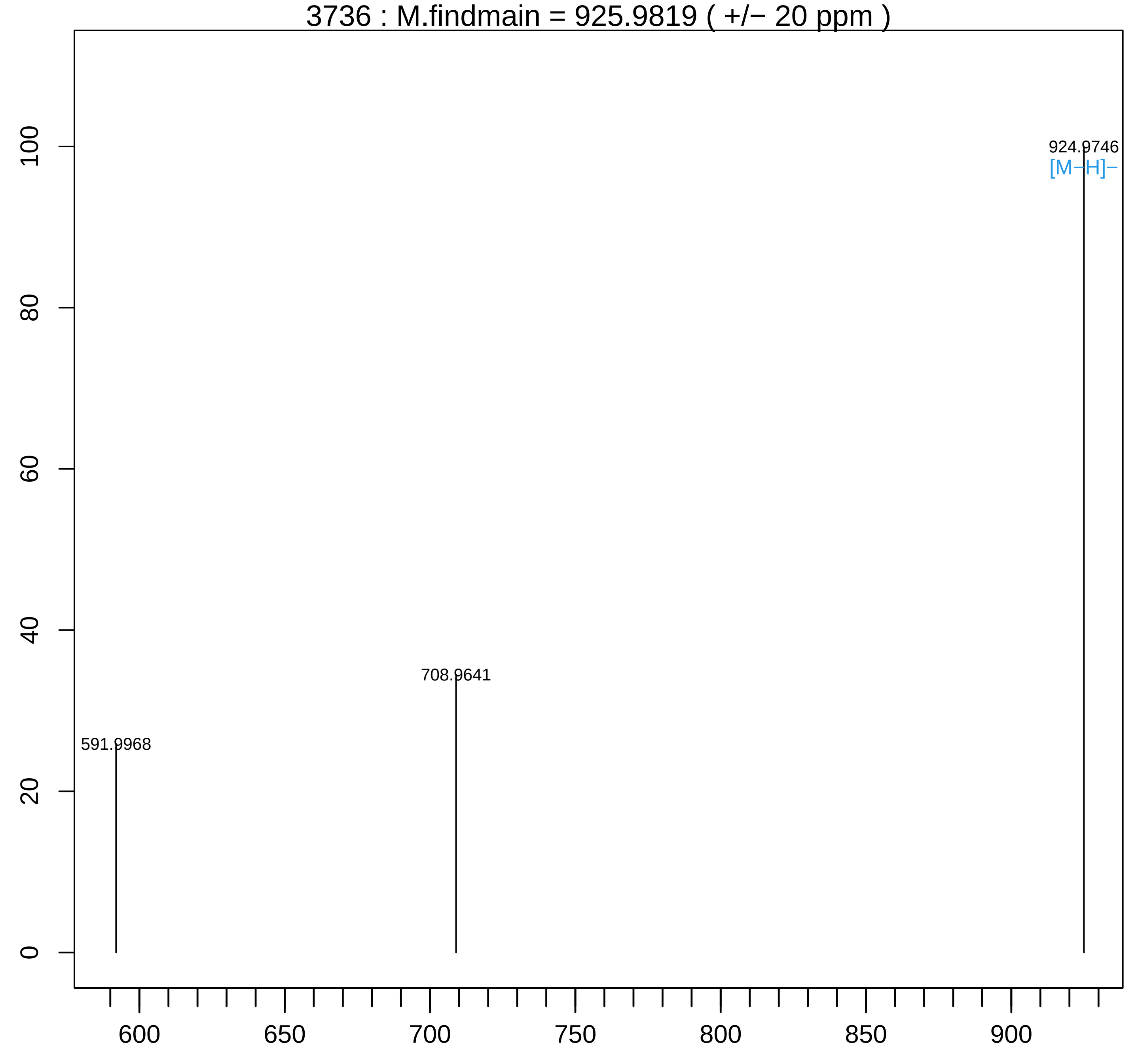
3736 : M.findmain = 925.9819 (+/− 20 ppm)
924.9746
[M−H]−
708.9641
591.9968
0
20
40
60
80
100
600
650
700
750
800
850
900

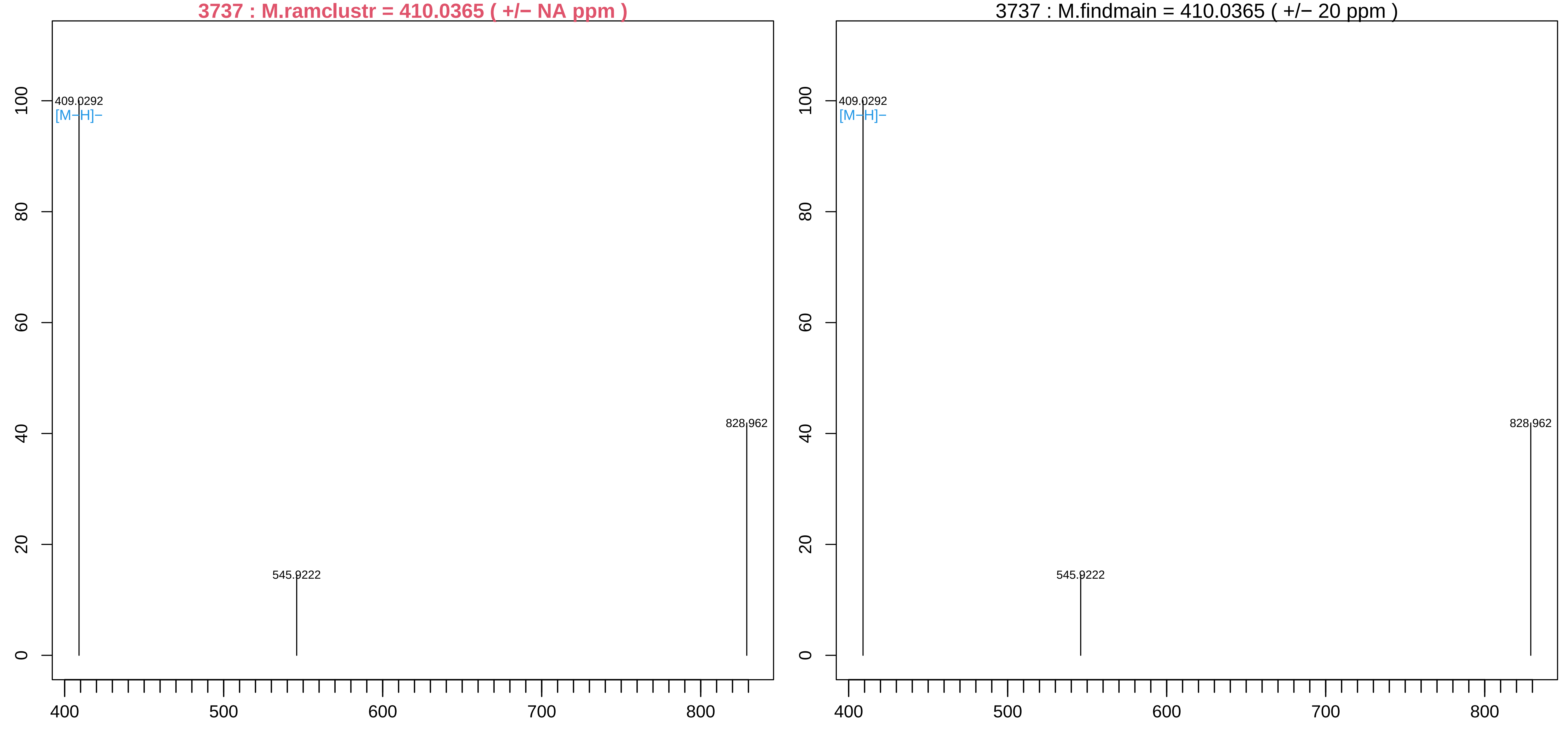

3737 : M.ramclustr = 410.0365 (+/− NA ppm)
409.0292
[M−H]−
545.9222
828.962
3737 : M.findmain = 410.0365 (+/− 20 ppm)
409.0292
[M−H]−
545.9222
828.962

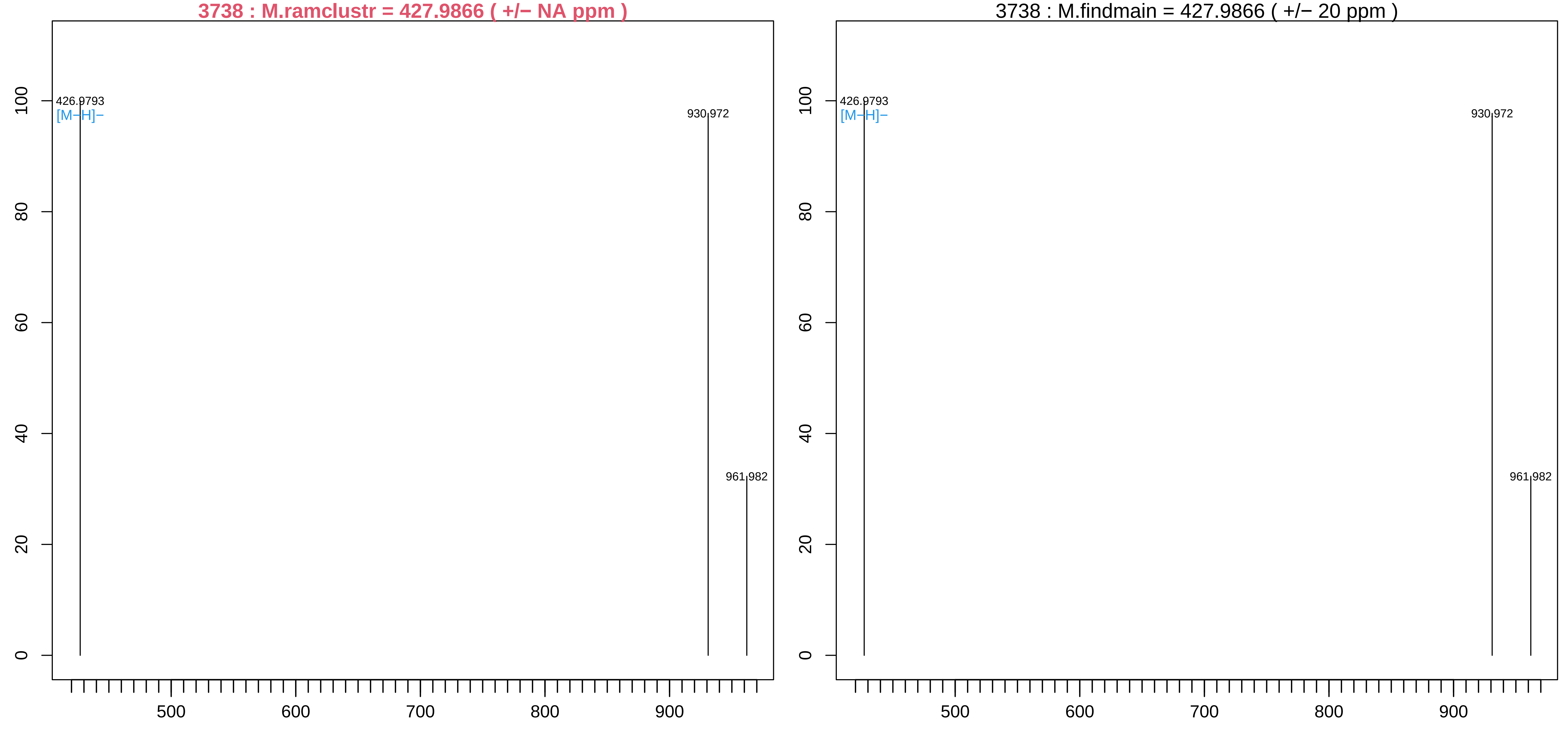

3738 : M.ramclustr = 427.9866 (+/− NA ppm)
426.9793
[M−H]−
930.972
961.982
3738 : M.findmain = 427.9866 (+/− 20 ppm)
426.9793
[M−H]−
930.972
961.982

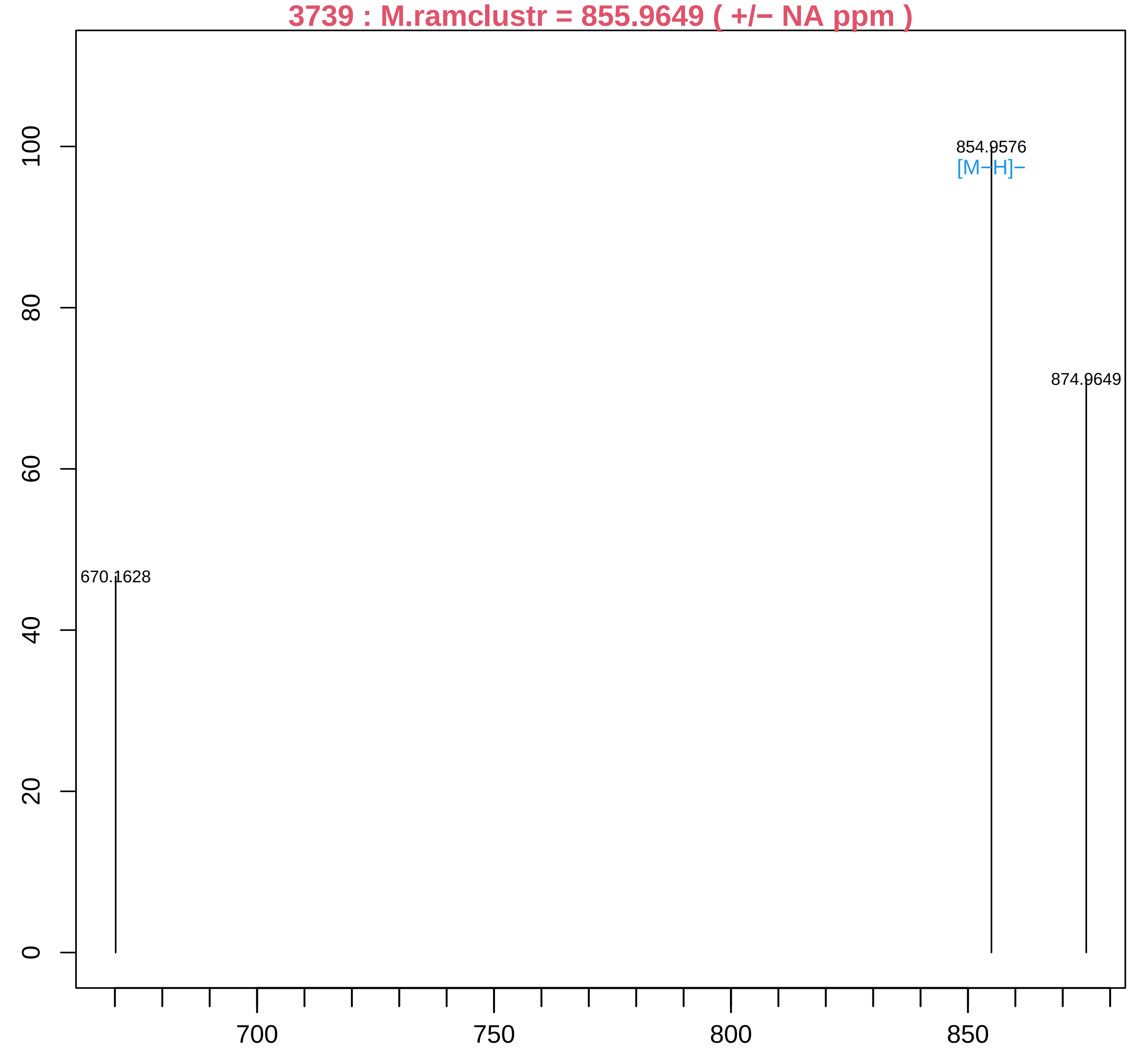
3739 : M.ramclustr = 855.9649 (+/− NA ppm)
854.9576
[M−H]−
874.9649
670.1628
0
20
40
60
80
100
700
750
800
850

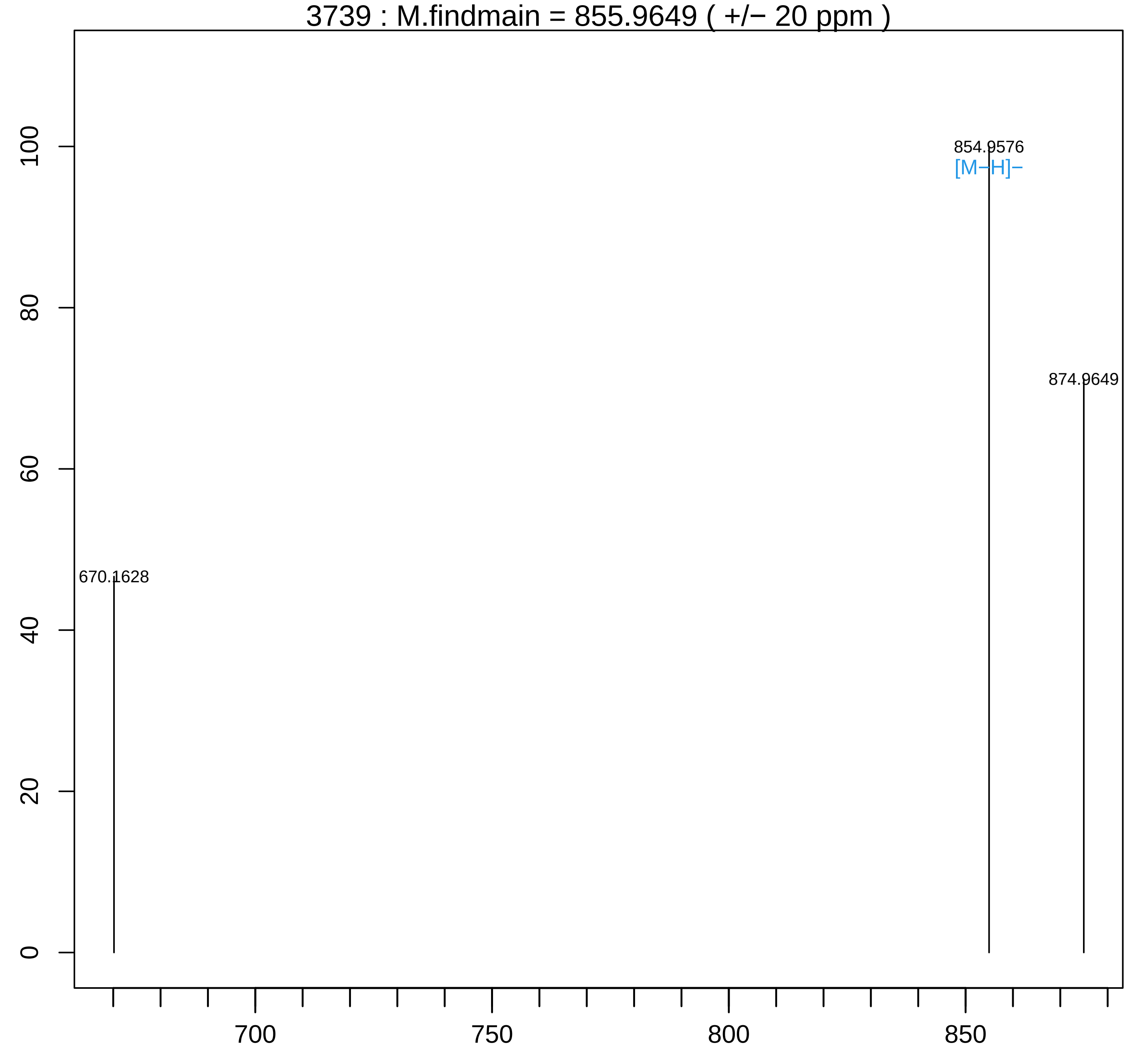
3739 : M.findmain = 855.9649 (+/− 20 ppm)
854.9576
[M−H]−
874.9649
670.1628
0
20
40
60
80
100
700
750
800
850

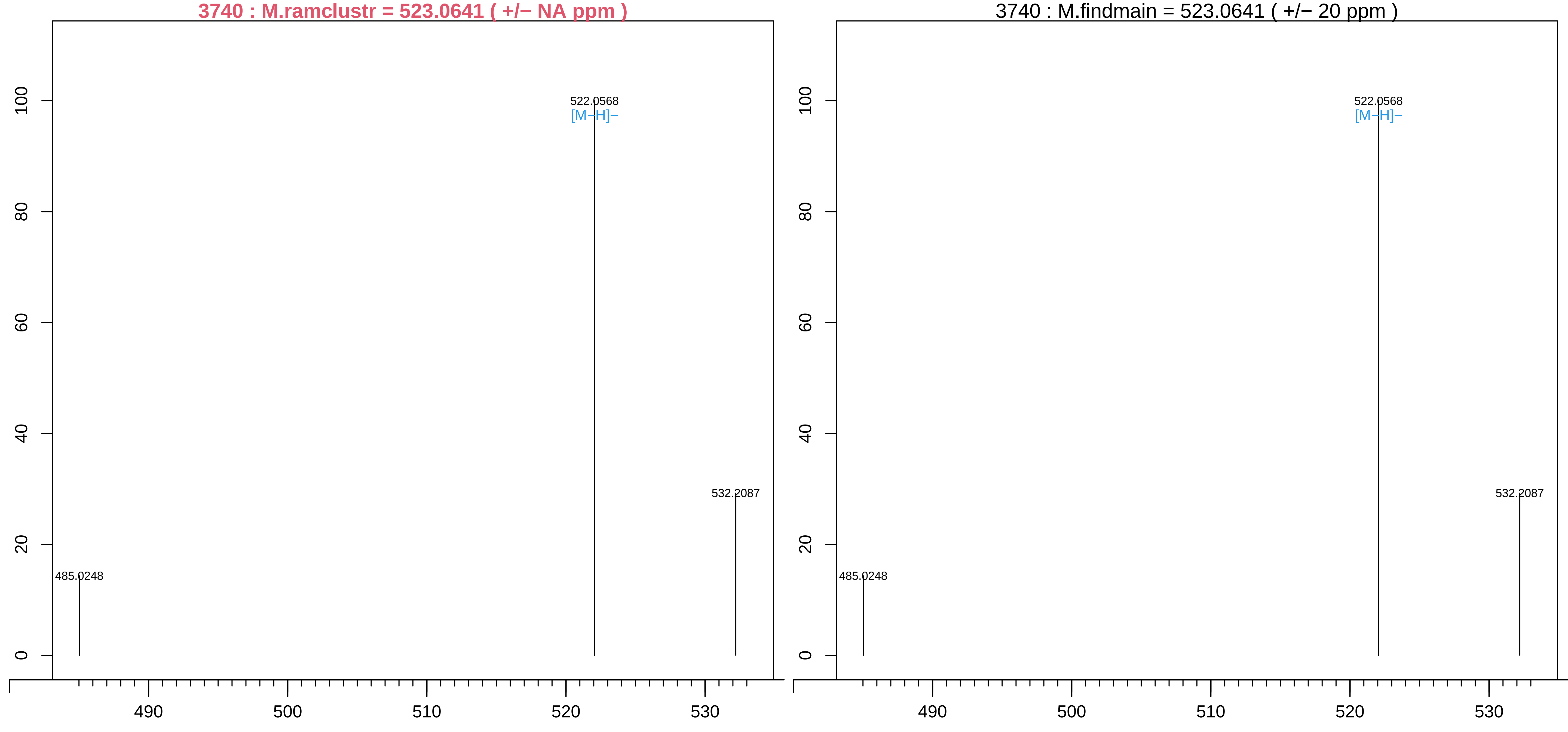

3740 : M.ramclustr = 523.0641 (+/− NA ppm)
3740 : M.findmain = 523.0641 (+/− 20 ppm)
522.0568
[M−H]−
532.2087
485.0248
0
20
40
60
80
100
490
500
510
520
530

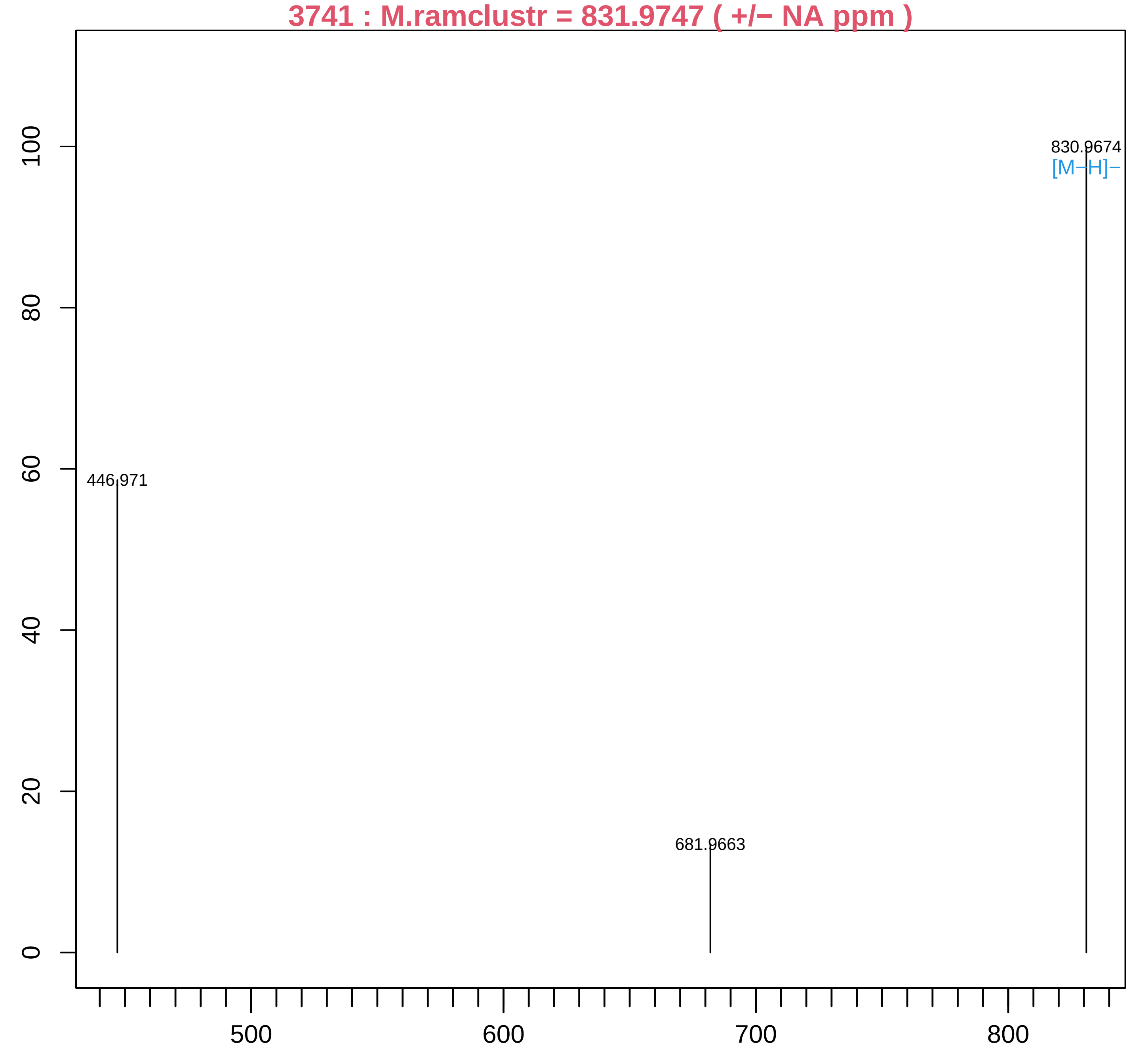
3741 : M.ramclustr = 831.9747 (+/− NA ppm)
830.9674
[M−H]−
446.971
681.9663
0
20
40
60
80
100
500
600
700
800

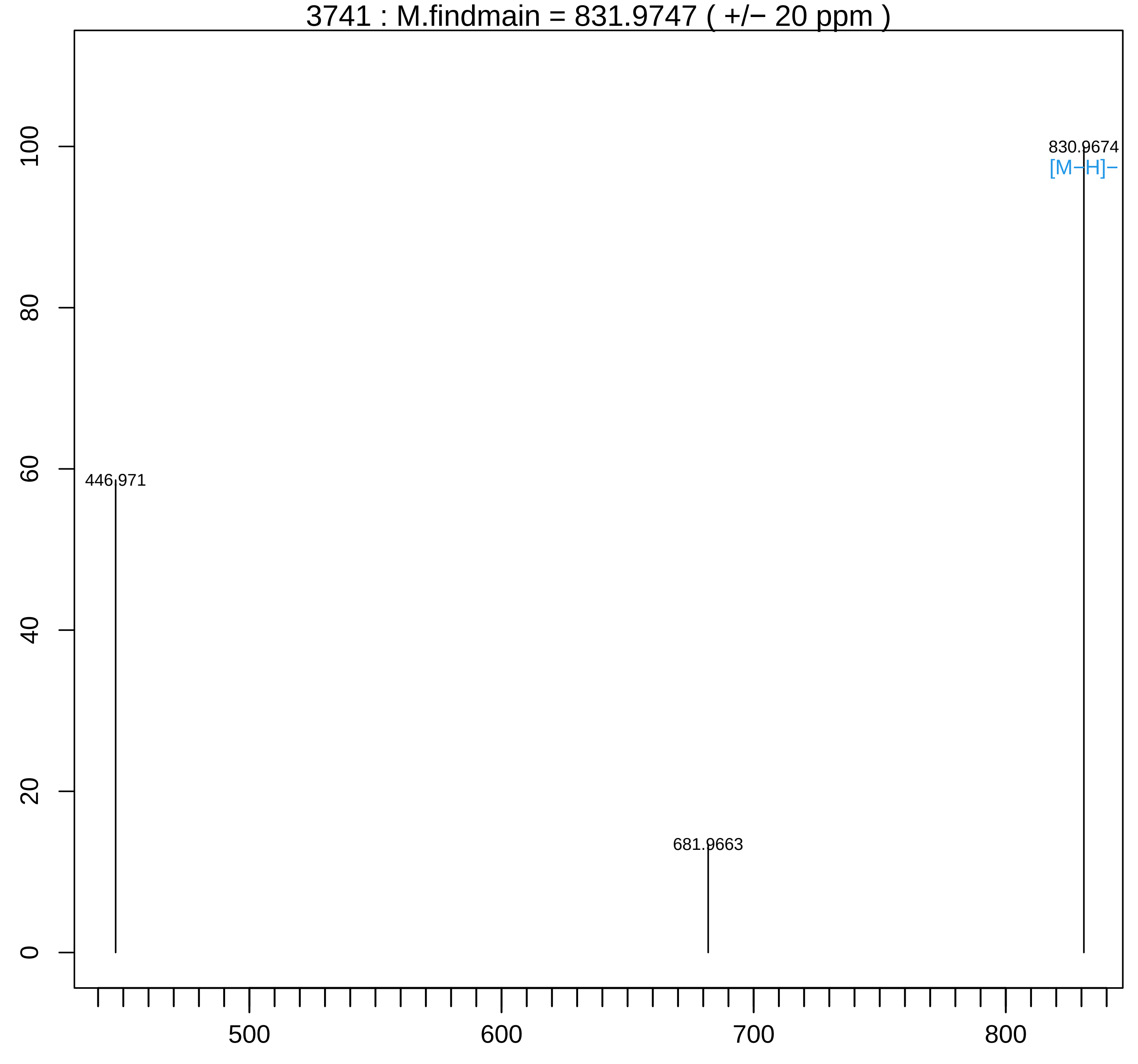
3741 : M.findmain = 831.9747 (+/− 20 ppm)
830.9674
[M−H]−
446.971
681.9663
0
20
40
60
80
100
500
600
700
800

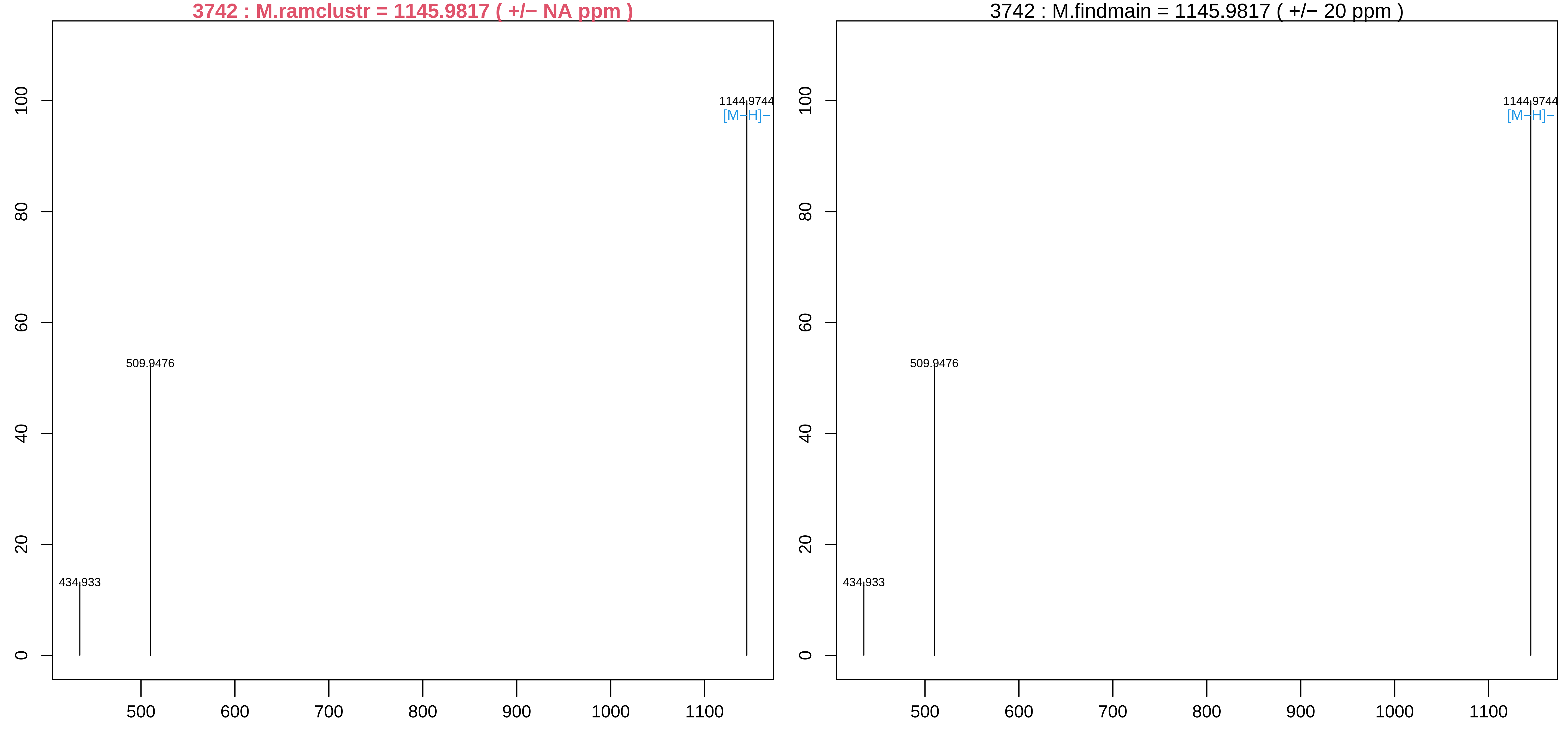
3742 : M.ramclustr = 1145.9817 (+/− NA ppm)
1144.9744
[M−H]−
509.9476
434.933
3742 : M.findmain = 1145.9817 (+/− 20 ppm)
1144.9744
[M−H]−
509.9476
434.933
0
20
40
60
80
100
500
600
700
800
900
1000
1100

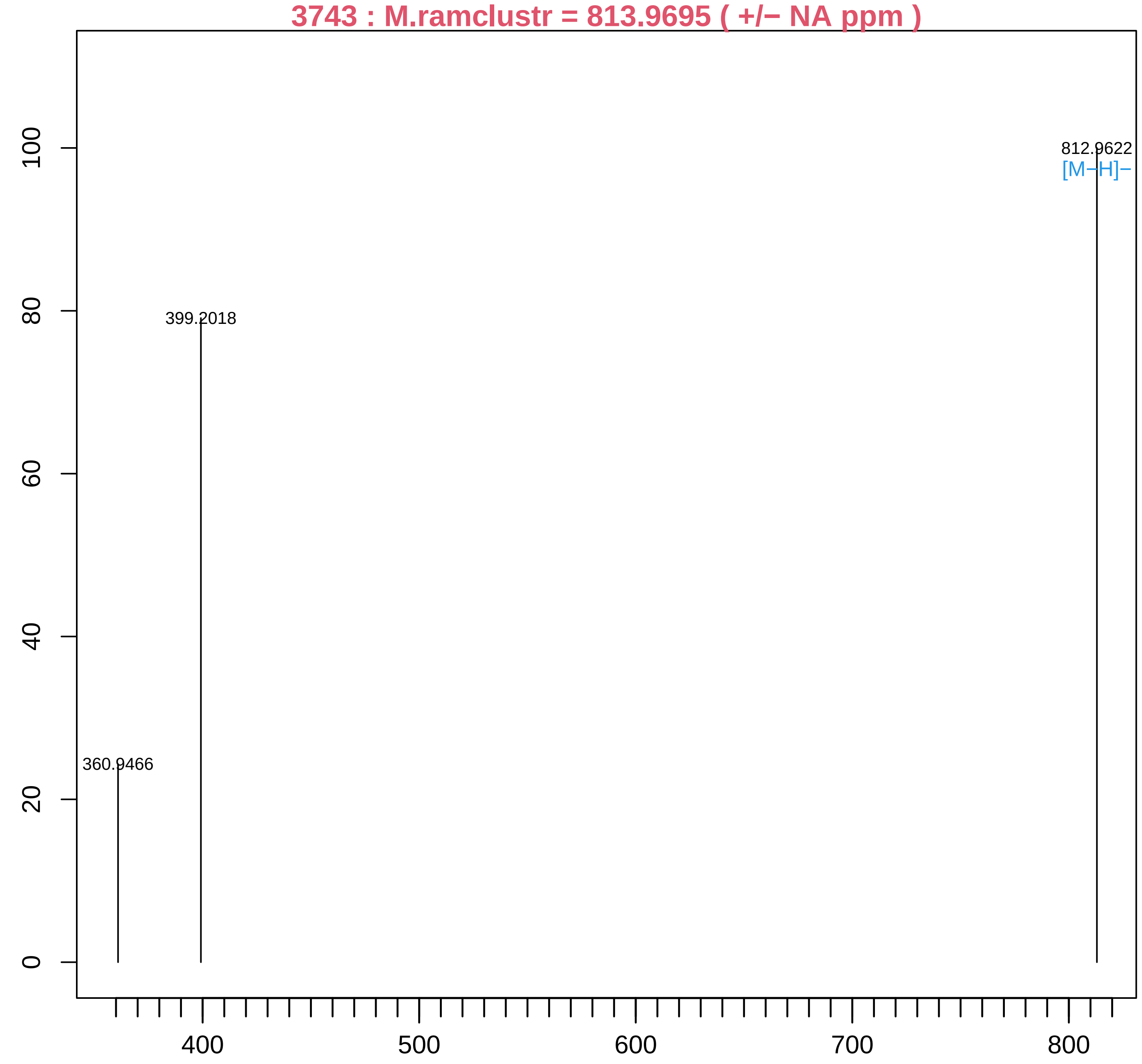

3743 : M.ramclustr = 813.9695 (+/− NA ppm)
100
80
60
40
20
0
400
500
600
700
800
812.9622
[M−H]−
399.2018
360.9466

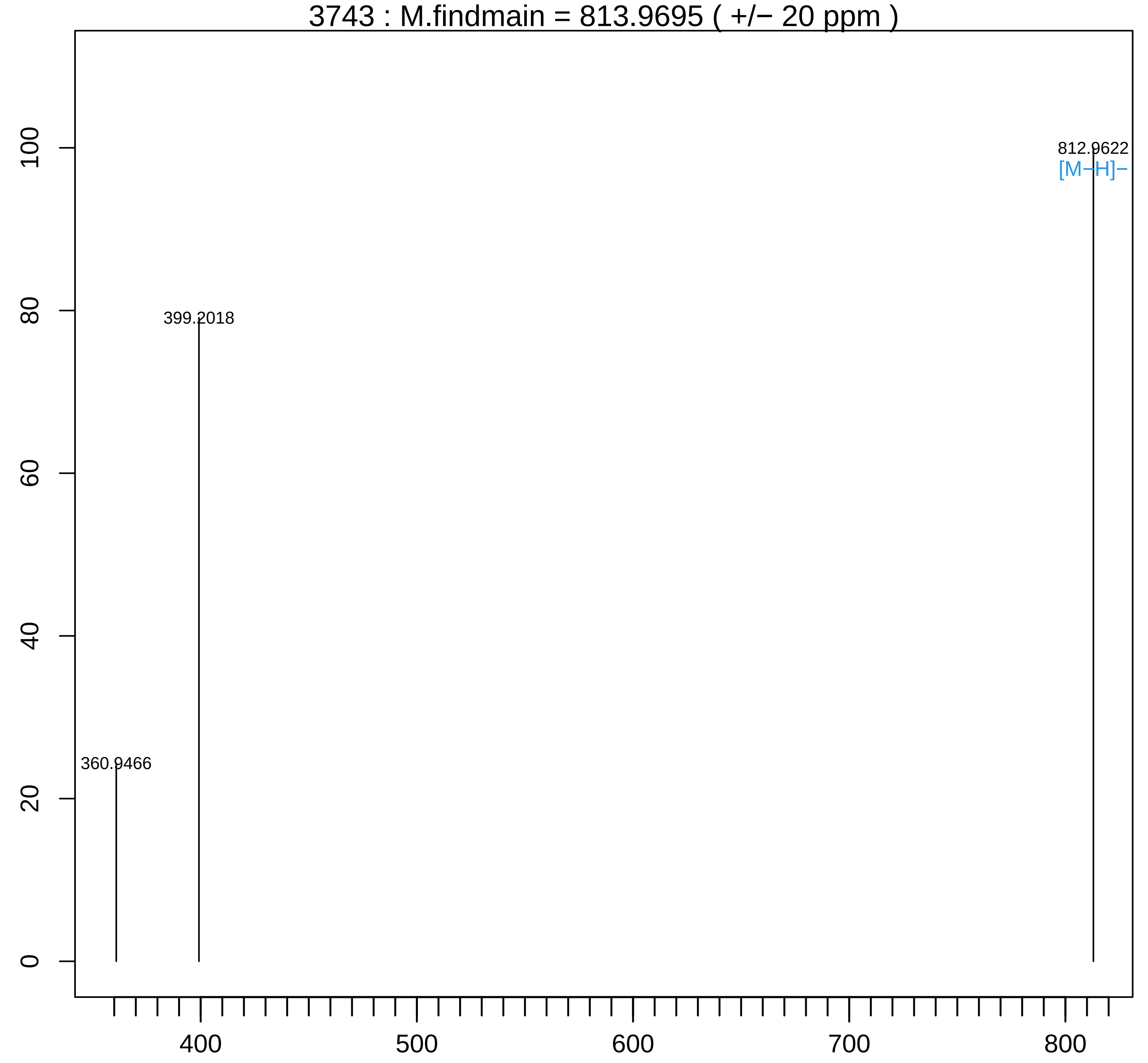

3743 : M.findmain = 813.9695 (+/− 20 ppm)
100
80
60
40
20
0
400
500
600
700
800
812.9622
[M−H]−
399.2018
360.9466

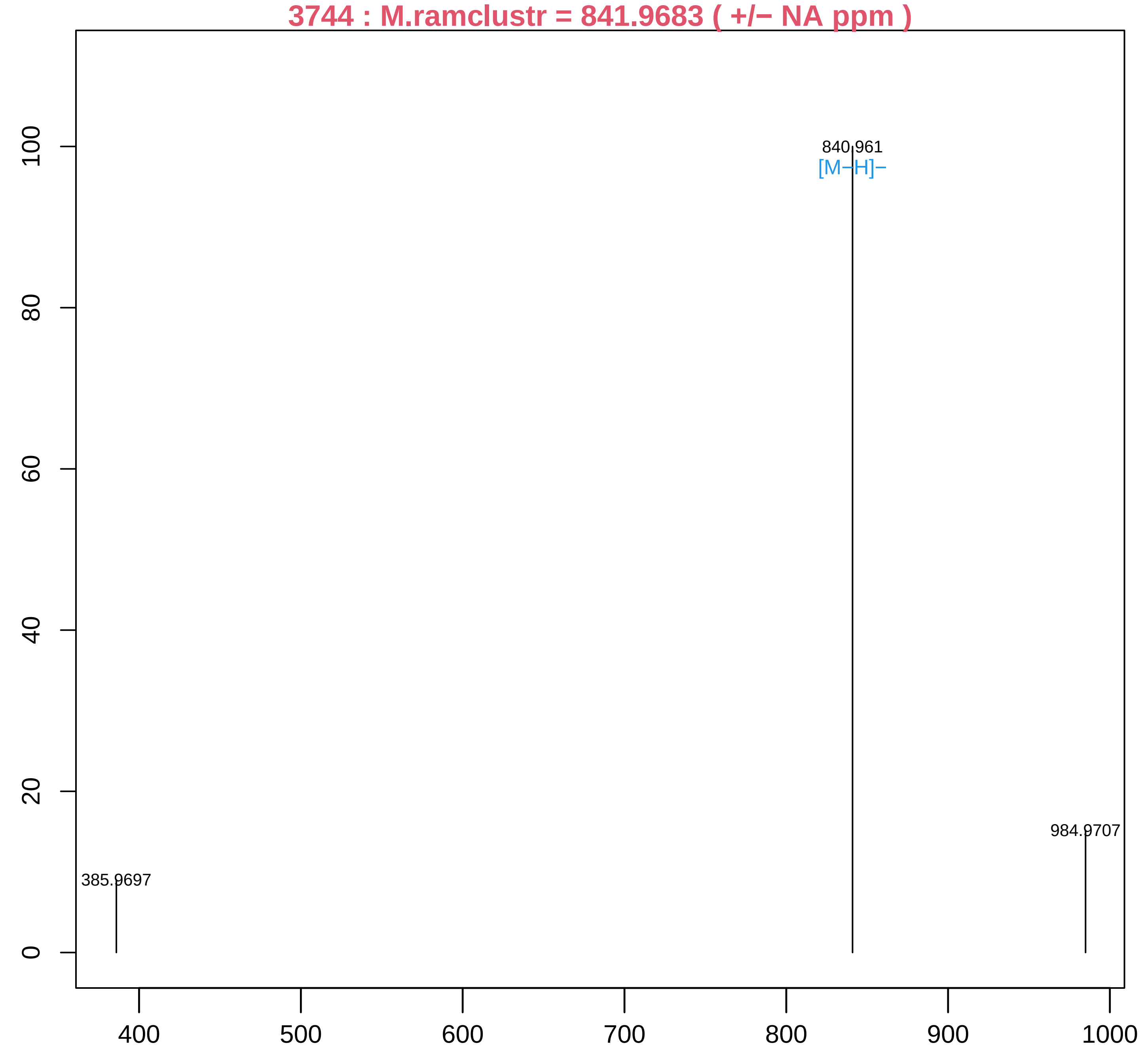

3744 : M.ramclustr = 841.9683 (+/− NA ppm)
840.961
[M−H]−
984.9707
385.9697
0
20
40
60
80
100
400
500
600
700
800
900
1000

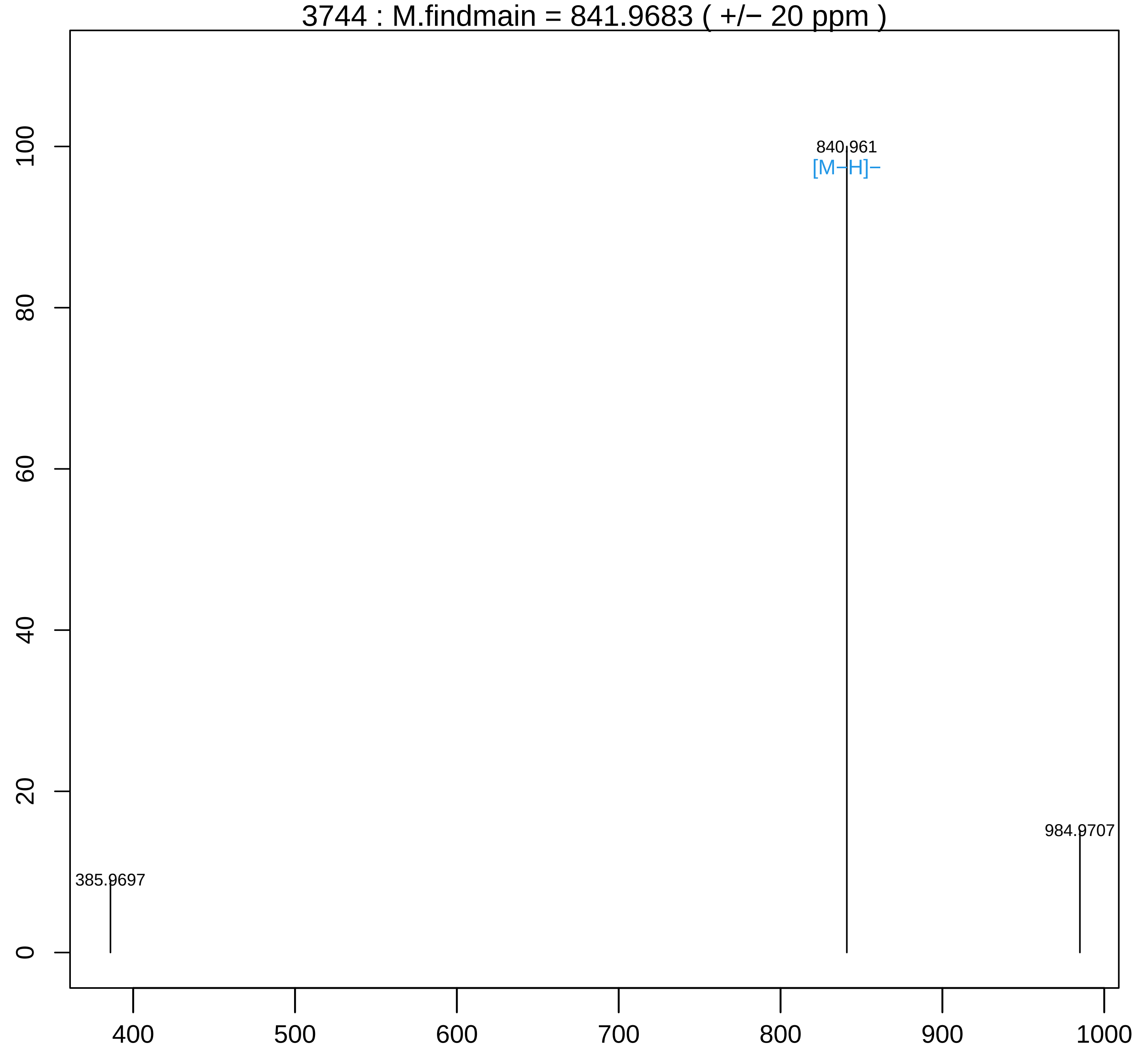

3744 : M.findmain = 841.9683 (+/− 20 ppm)
840.961
[M−H]−
984.9707
385.9697
0
20
40
60
80
100
400
500
600
700
800
900
1000

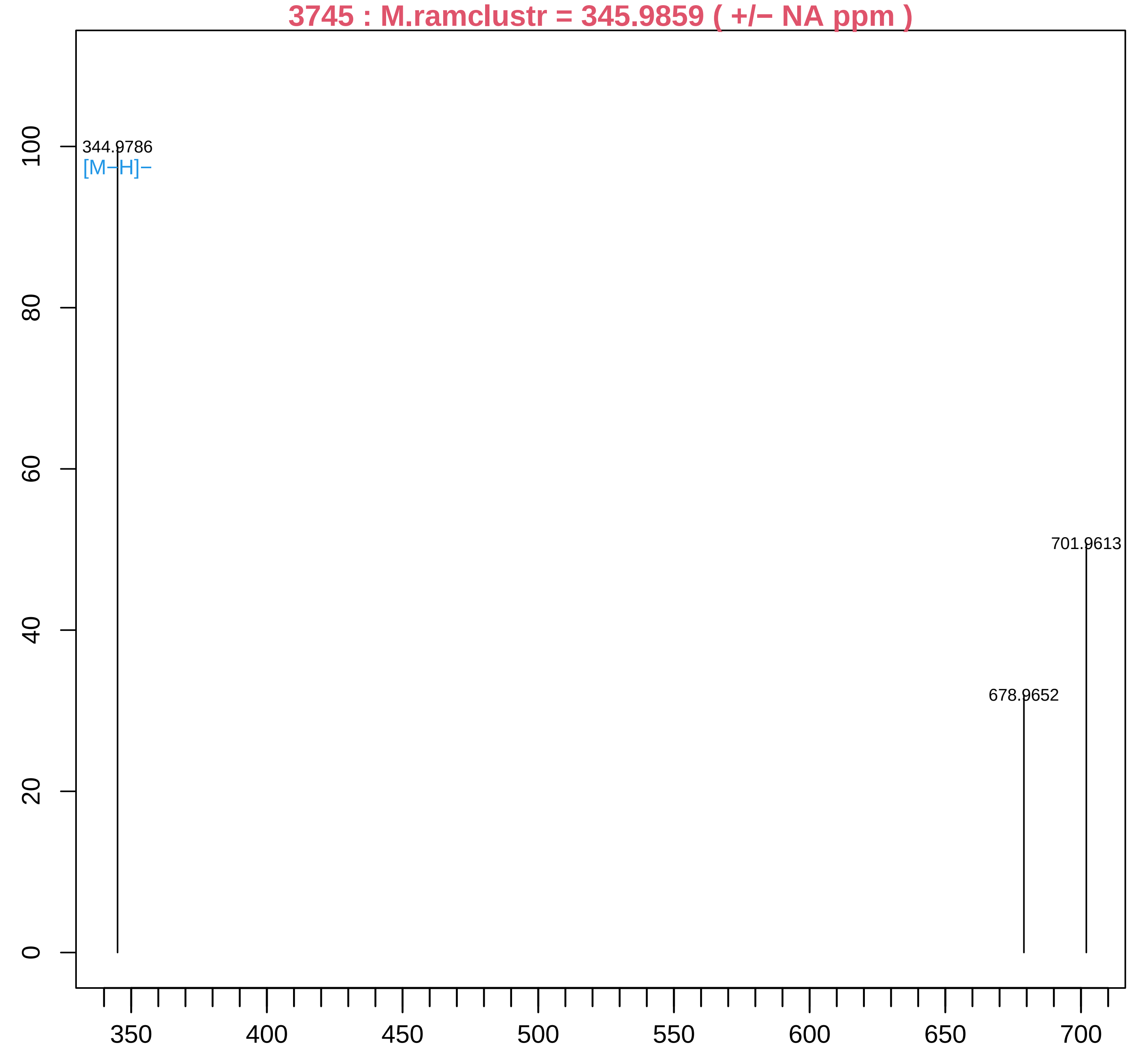
3745 : M.ramclustr = 345.9859 (+/− NA ppm)
344.9786
[M−H]−
701.9613
678.9652
0
20
40
60
80
100
350
400
450
500
550
600
650
700

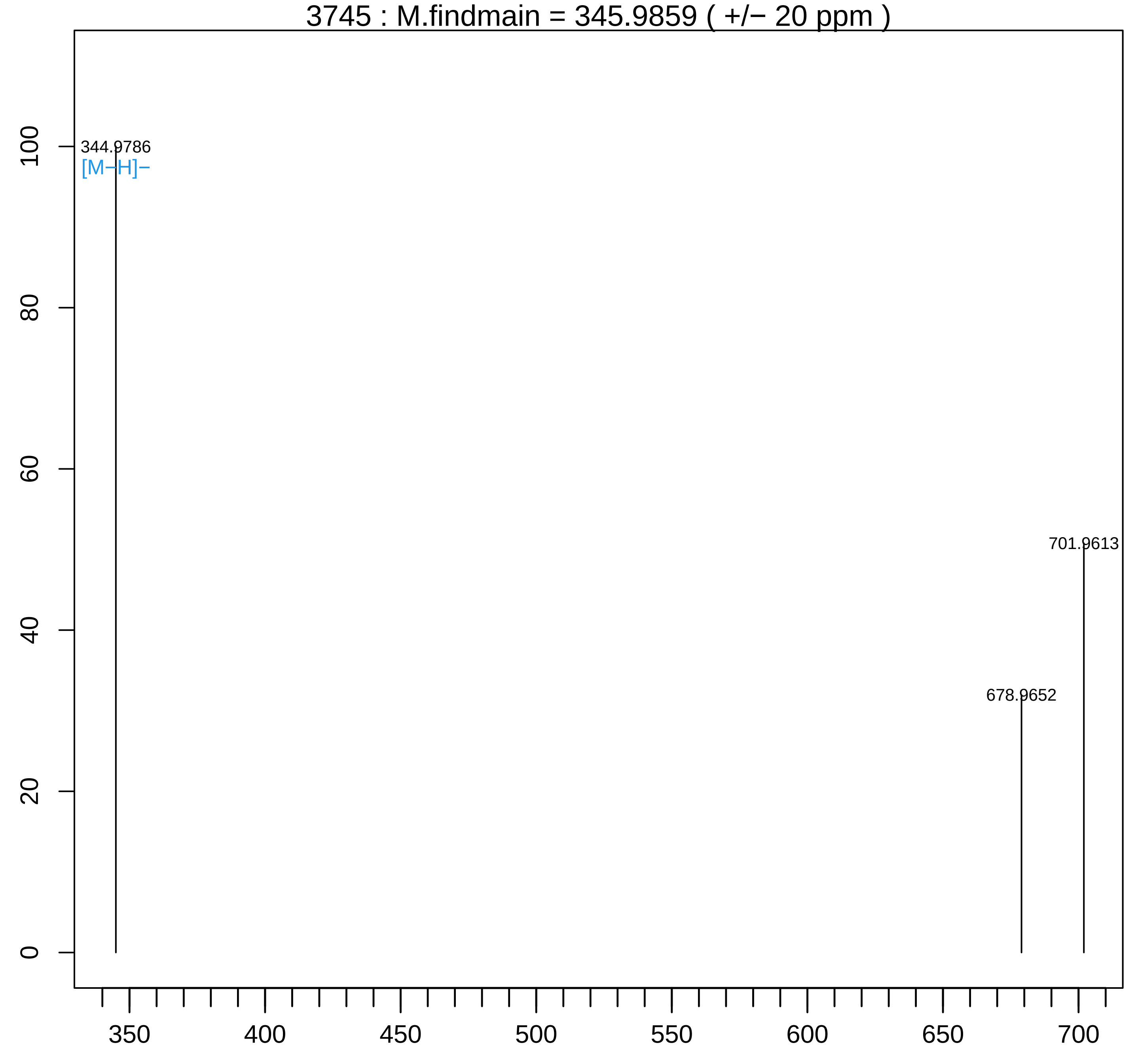
3745 : M.findmain = 345.9859 (+/− 20 ppm)
344.9786
[M−H]−
701.9613
678.9652
0
20
40
60
80
100
350
400
450
500
550
600
650
700

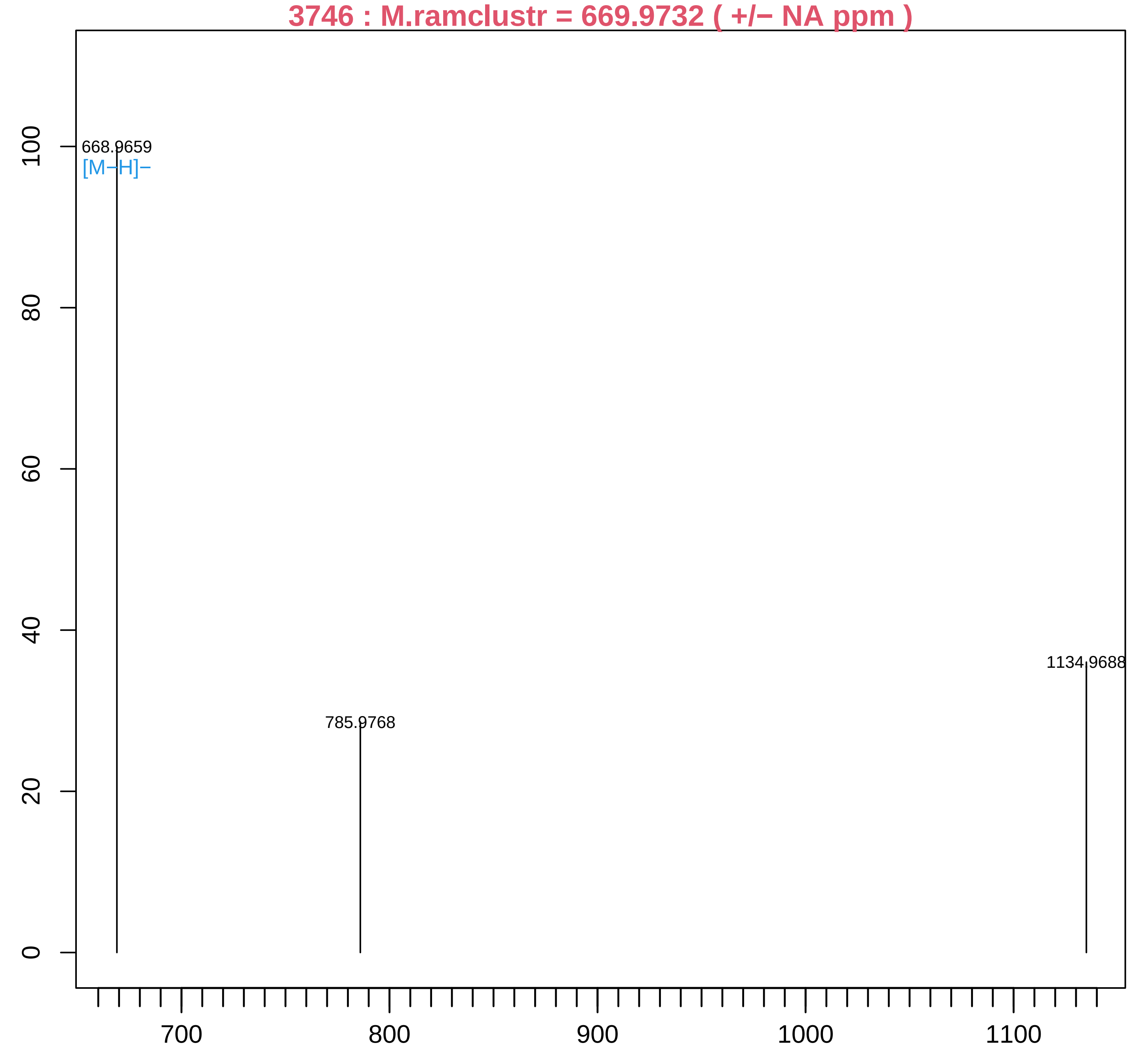
3746 : M.ramclustr = 669.9732 (+/− NA ppm)
668.9659
[M−H]−
785.9768
1134.9688
0
20
40
60
80
100
700
800
900
1000
1100

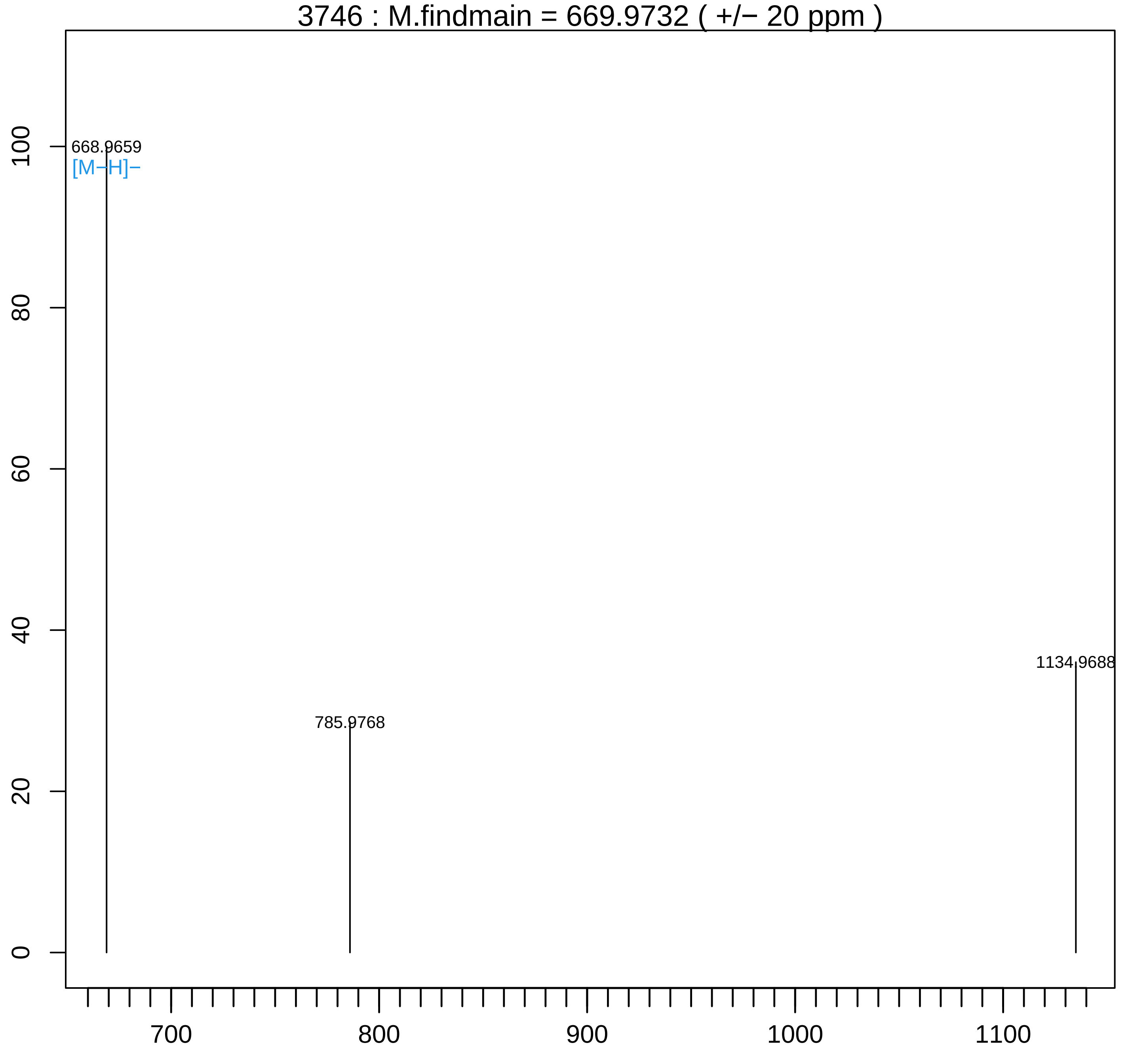
3746 : M.findmain = 669.9732 (+/− 20 ppm)
668.9659
[M−H]−
785.9768
1134.9688
0
20
40
60
80
100
700
800
900
1000
1100

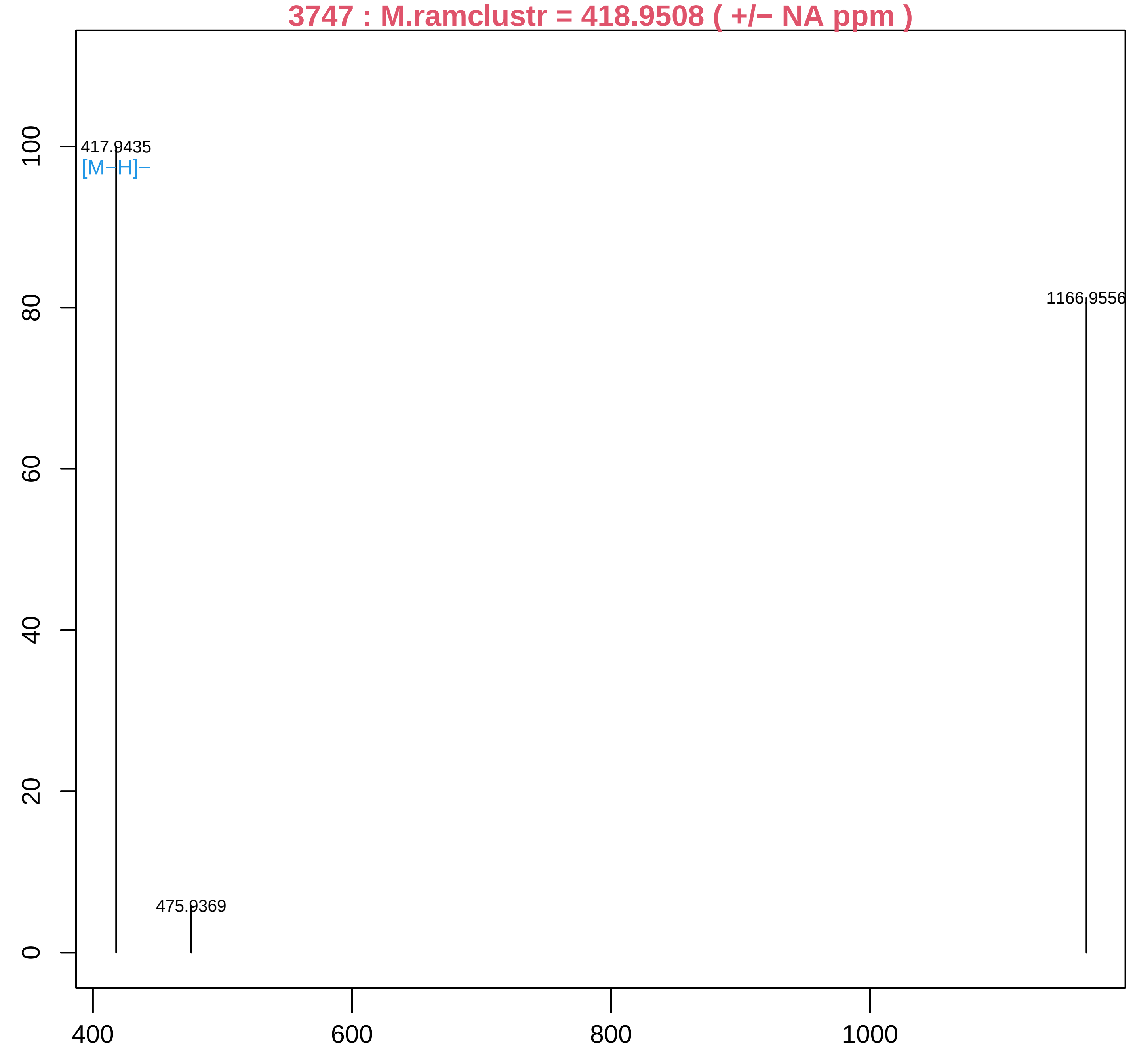
3747 : M.ramclustr = 418.9508 (+/− NA ppm)
417.9435
[M−H]−
1166.9556
475.9369
0
20
40
60
80
100
400
600
800
1000
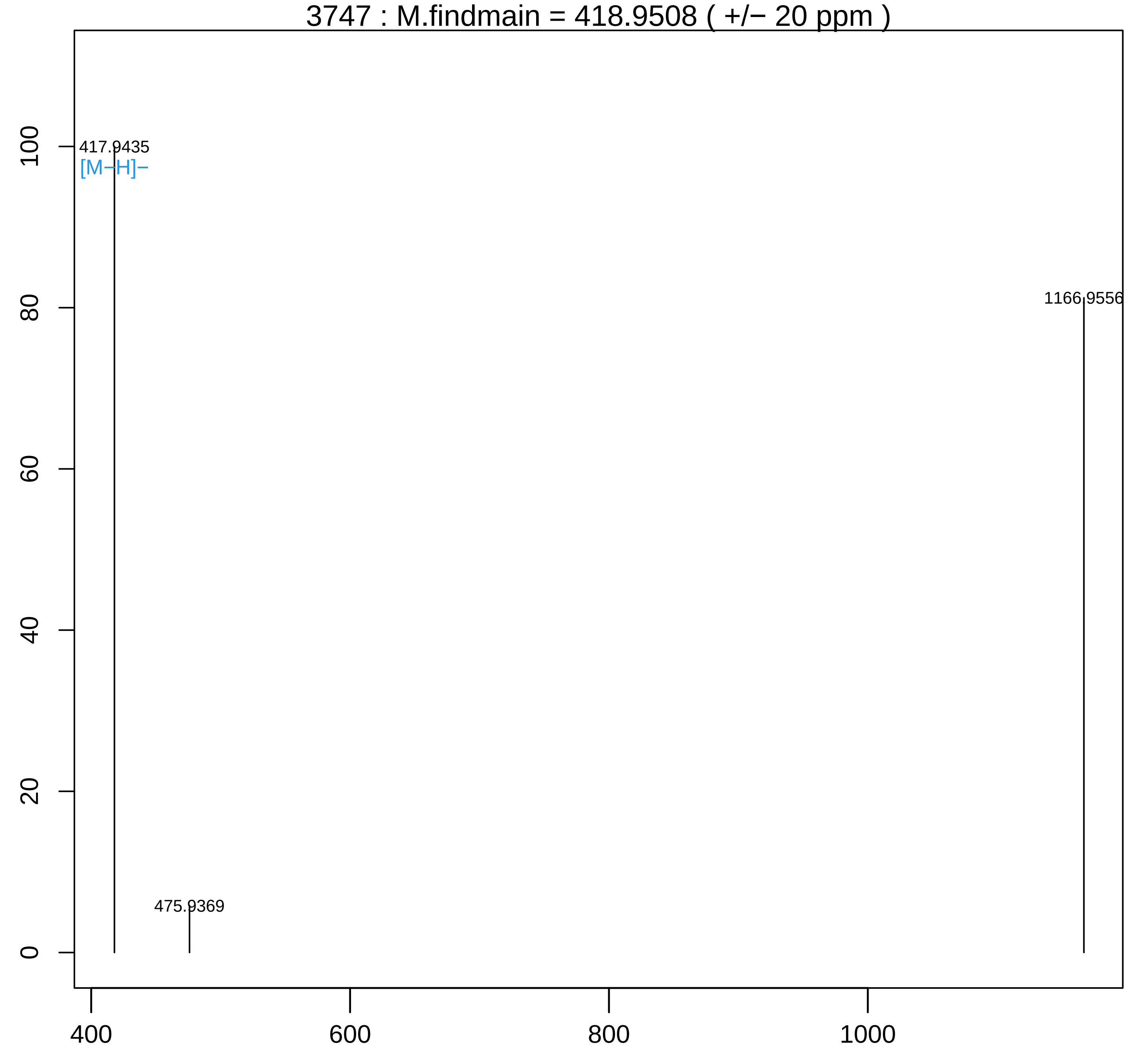
3747 : M.findmain = 418.9508 (+/− 20 ppm)
417.9435
[M−H]−
1166.9556
475.9369
0
20
40
60
80
100
400
600
800
1000

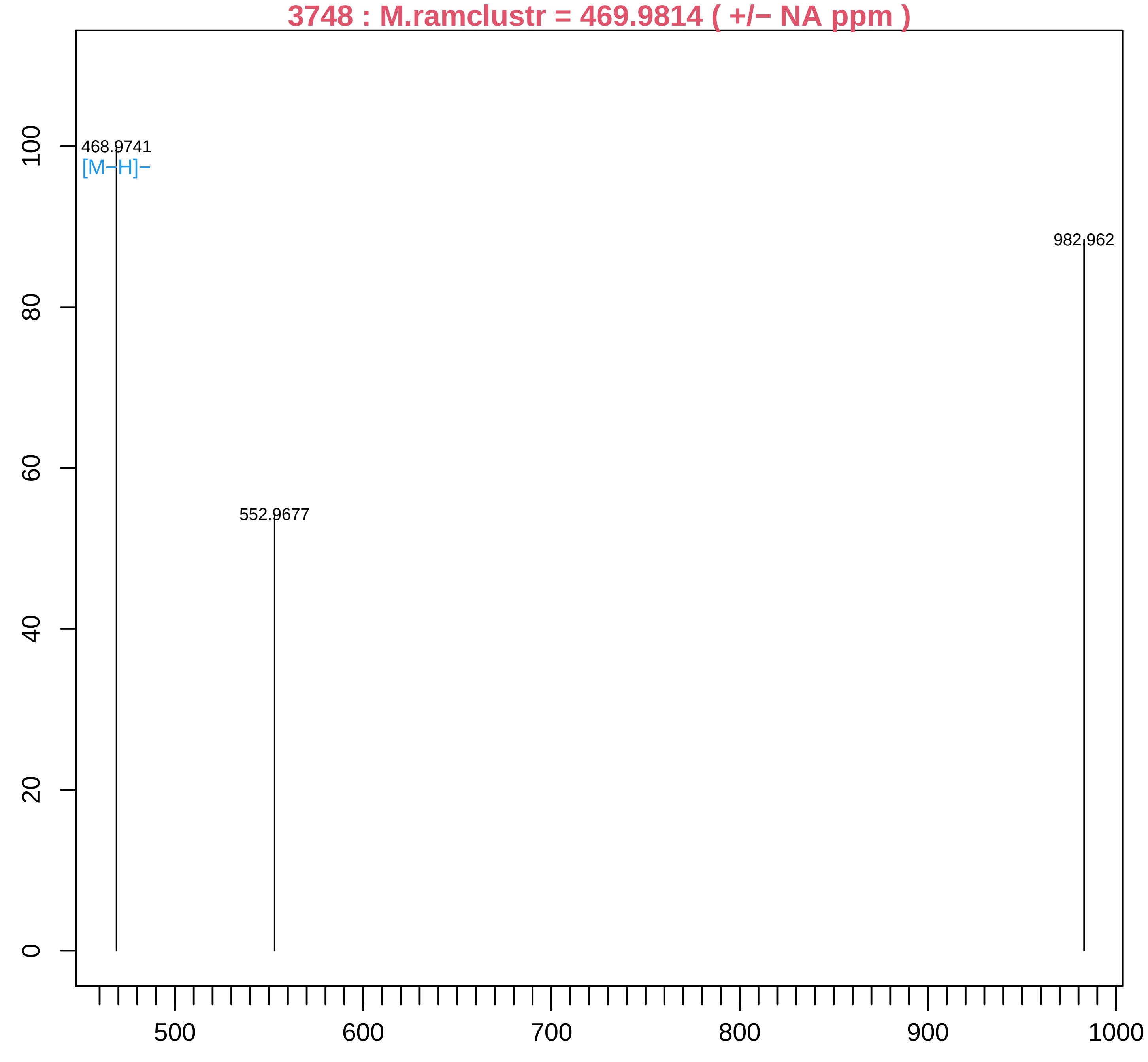
3748 : M.ramclustr = 469.9814 (+/− NA ppm)
468.9741
[M−H]−
552.9677
982.962
0
20
40
60
80
100
500
600
700
800
900
1000

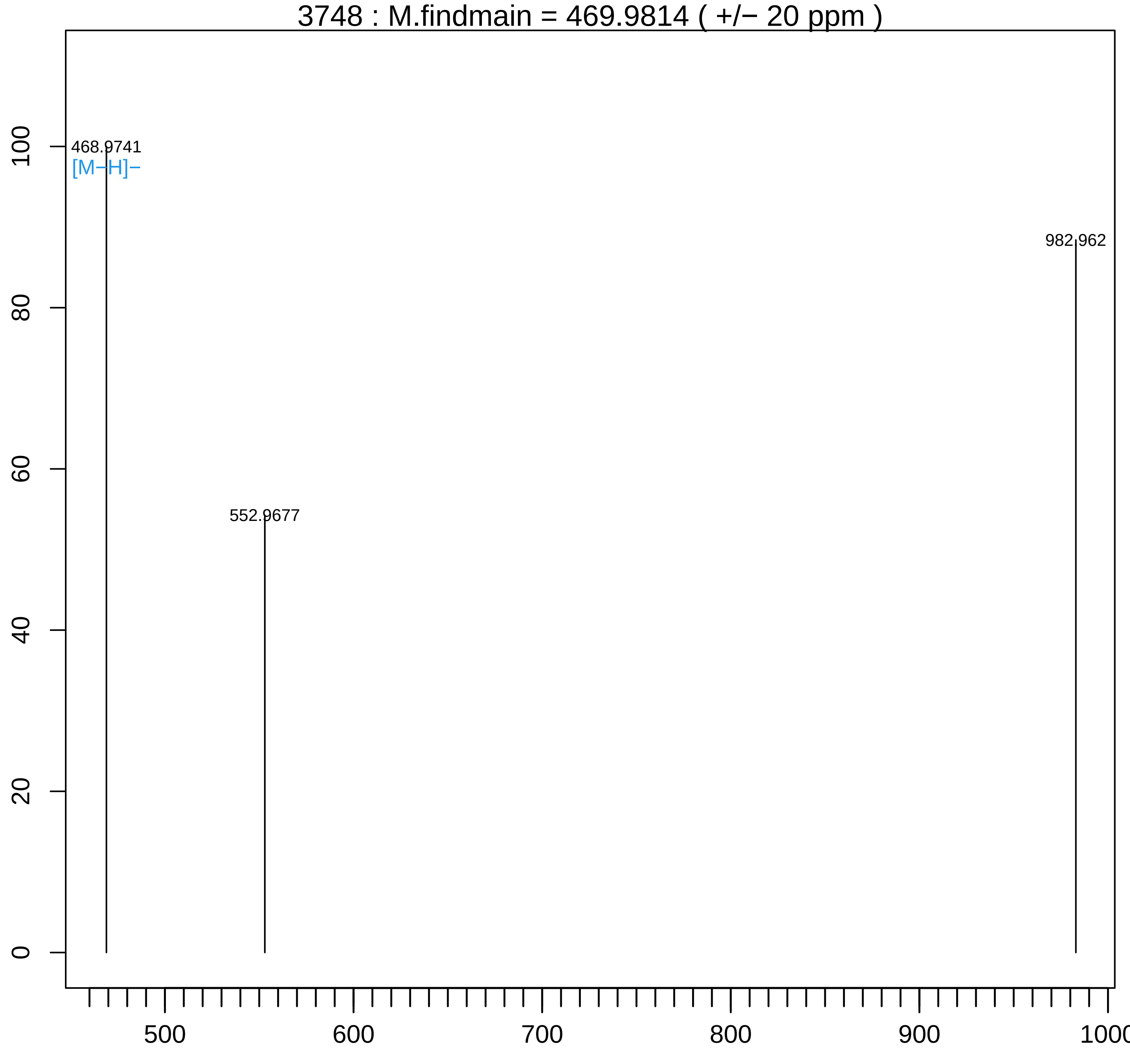
3748 : M.findmain = 469.9814 (+/− 20 ppm)
468.9741
[M−H]−
552.9677
982.962
0
20
40
60
80
100
500
600
700
800
900
1000

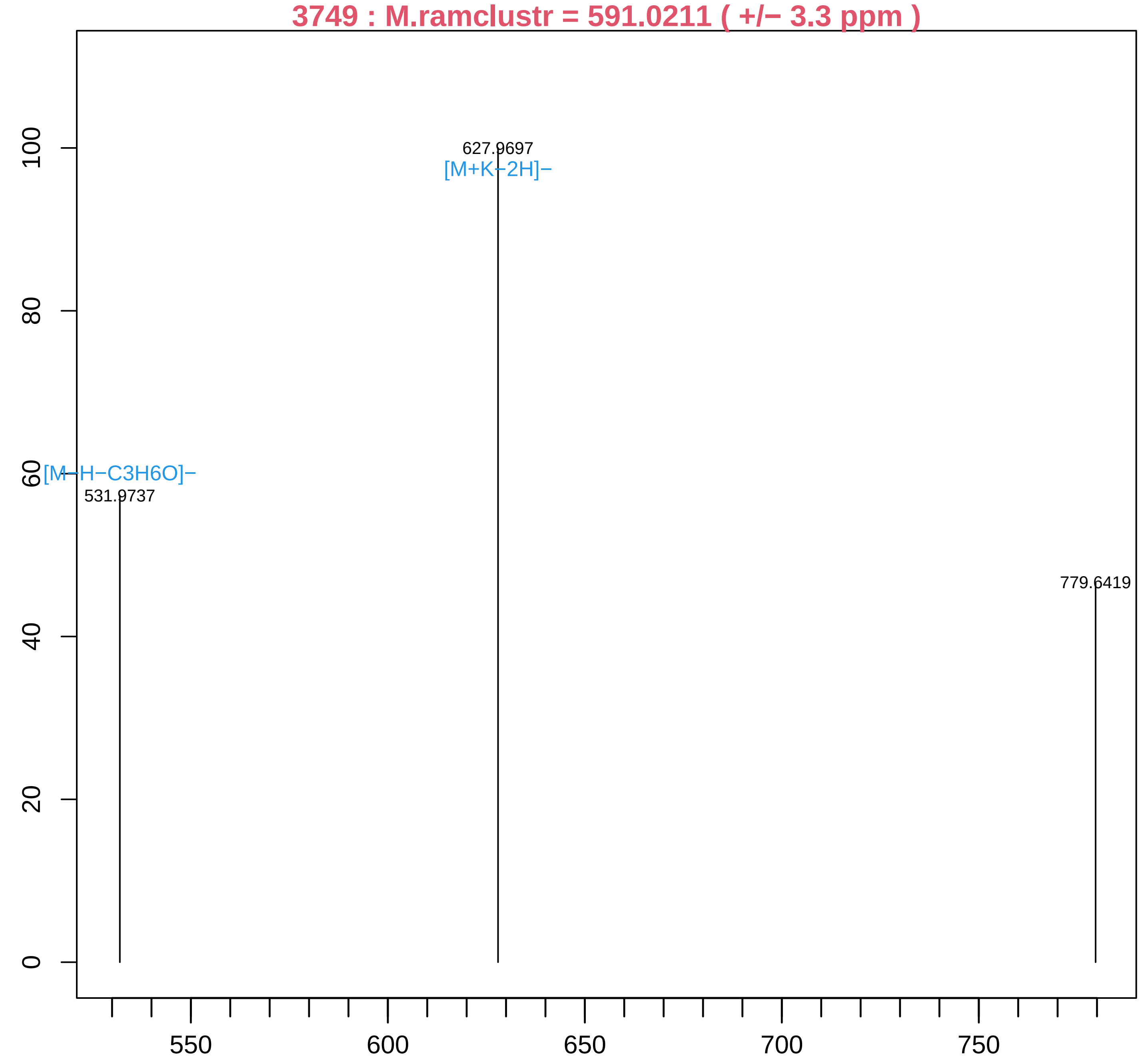

3749 : M.ramclustr = 591.0211 (+/− 3.3 ppm)
627.9697
[M+K−2H]−
[M−H−C3H6O]−
531.9737
779.6419
0
20
40
60
80
100
550
600
650
700
750

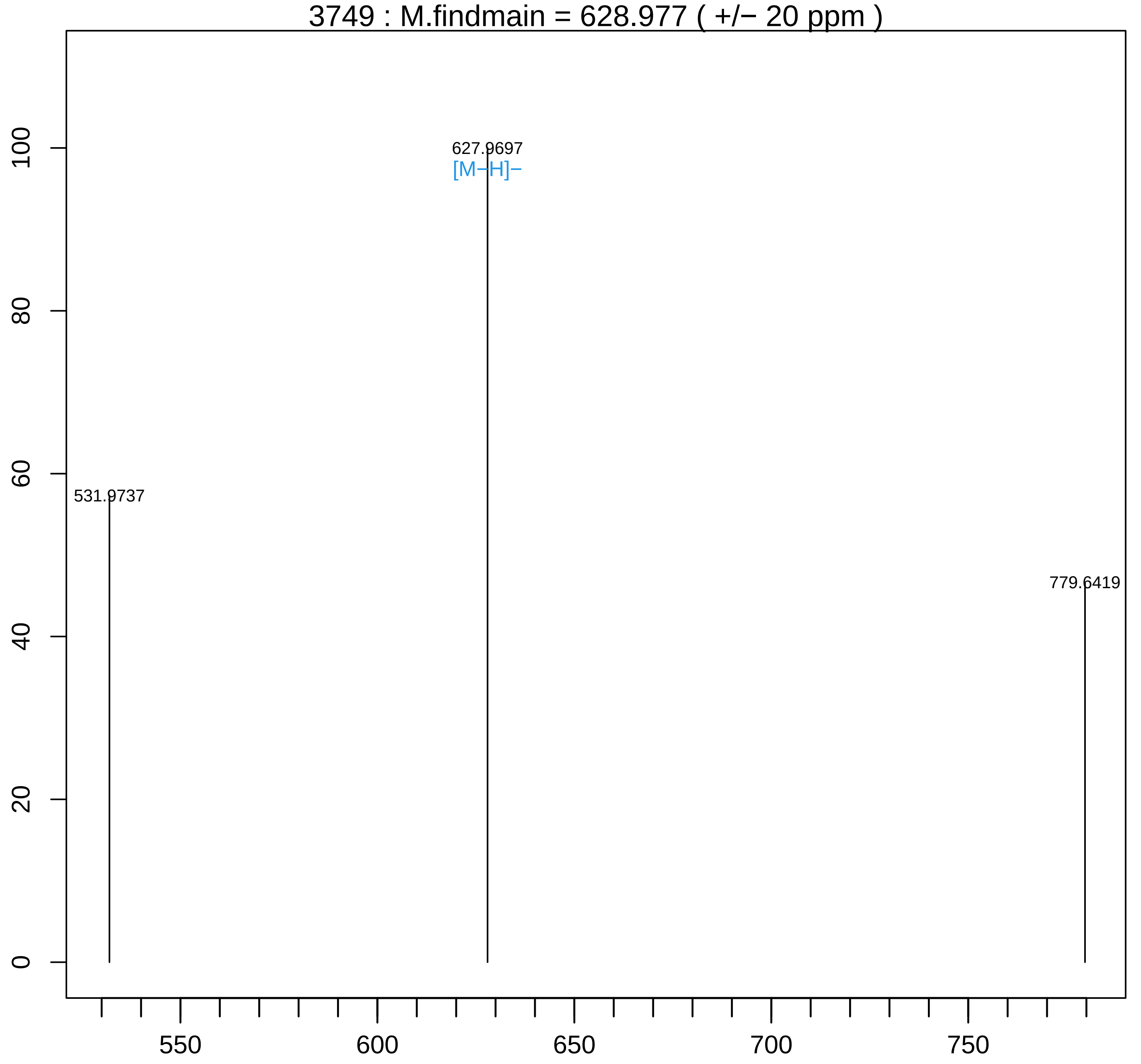

3749 : M.findmain = 628.977 (+/− 20 ppm)
627.9697
[M−H]−
531.9737
779.6419
0
20
40
60
80
100
550
600
650
700
750

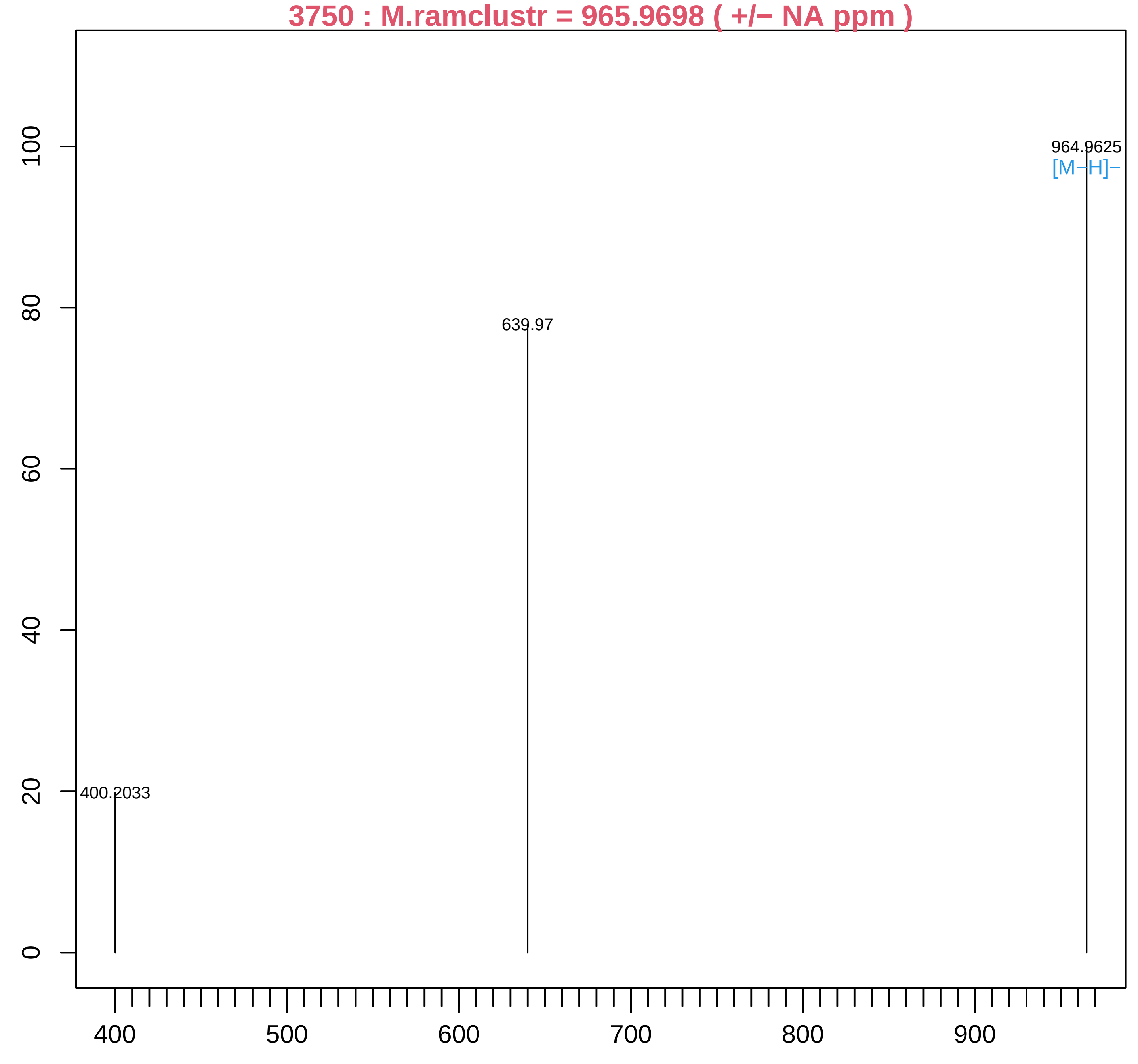
3750 : M.ramclustr = 965.9698 (+/− NA ppm)
964.9625
[M−H]−
639.97
400.2033
0
20
40
60
80
100
400
500
600
700
800
900

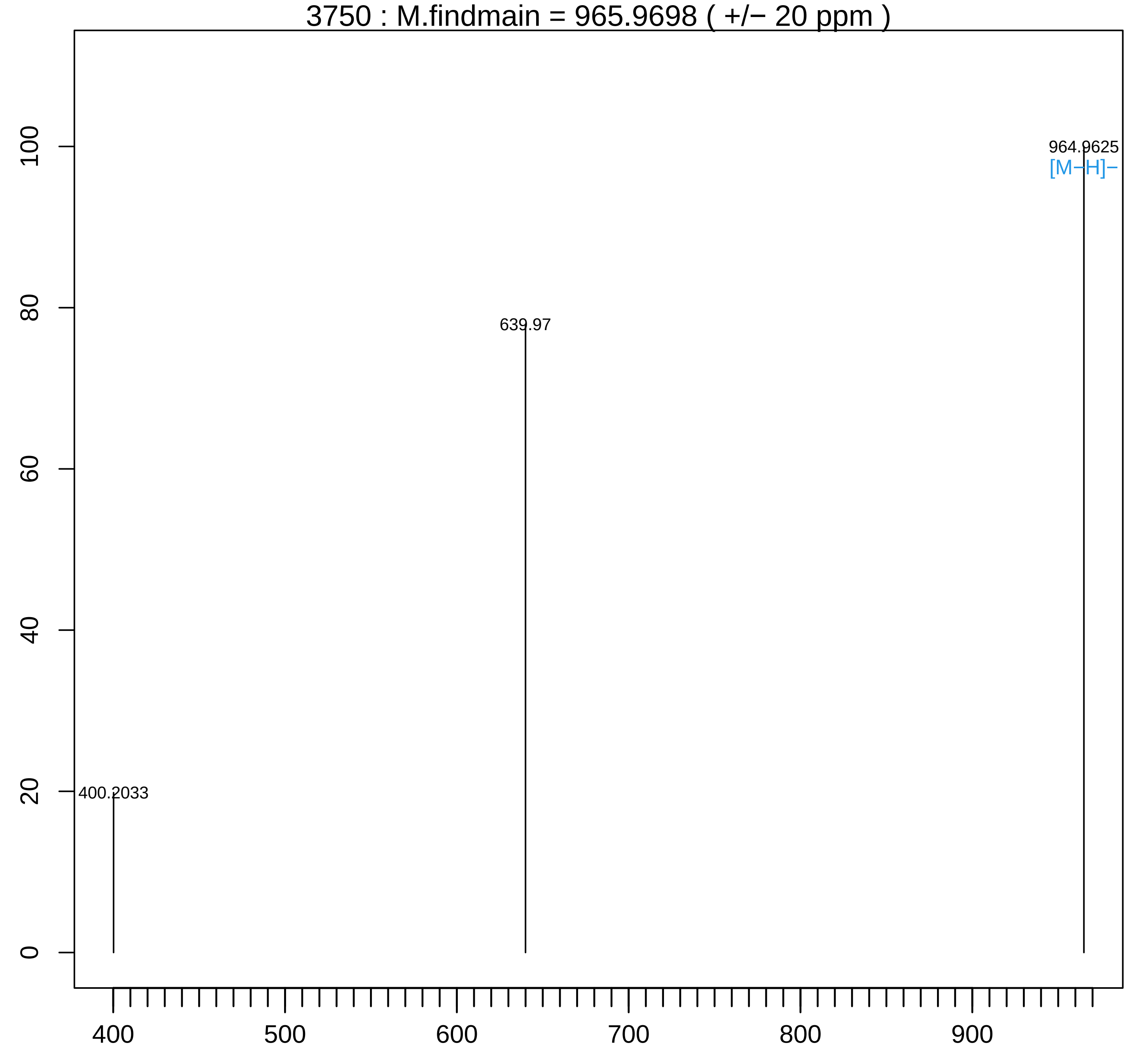
3750 : M.findmain = 965.9698 (+/− 20 ppm)
964.9625
[M−H]−
639.97
400.2033
0
20
40
60
80
100
400
500
600
700
800
900

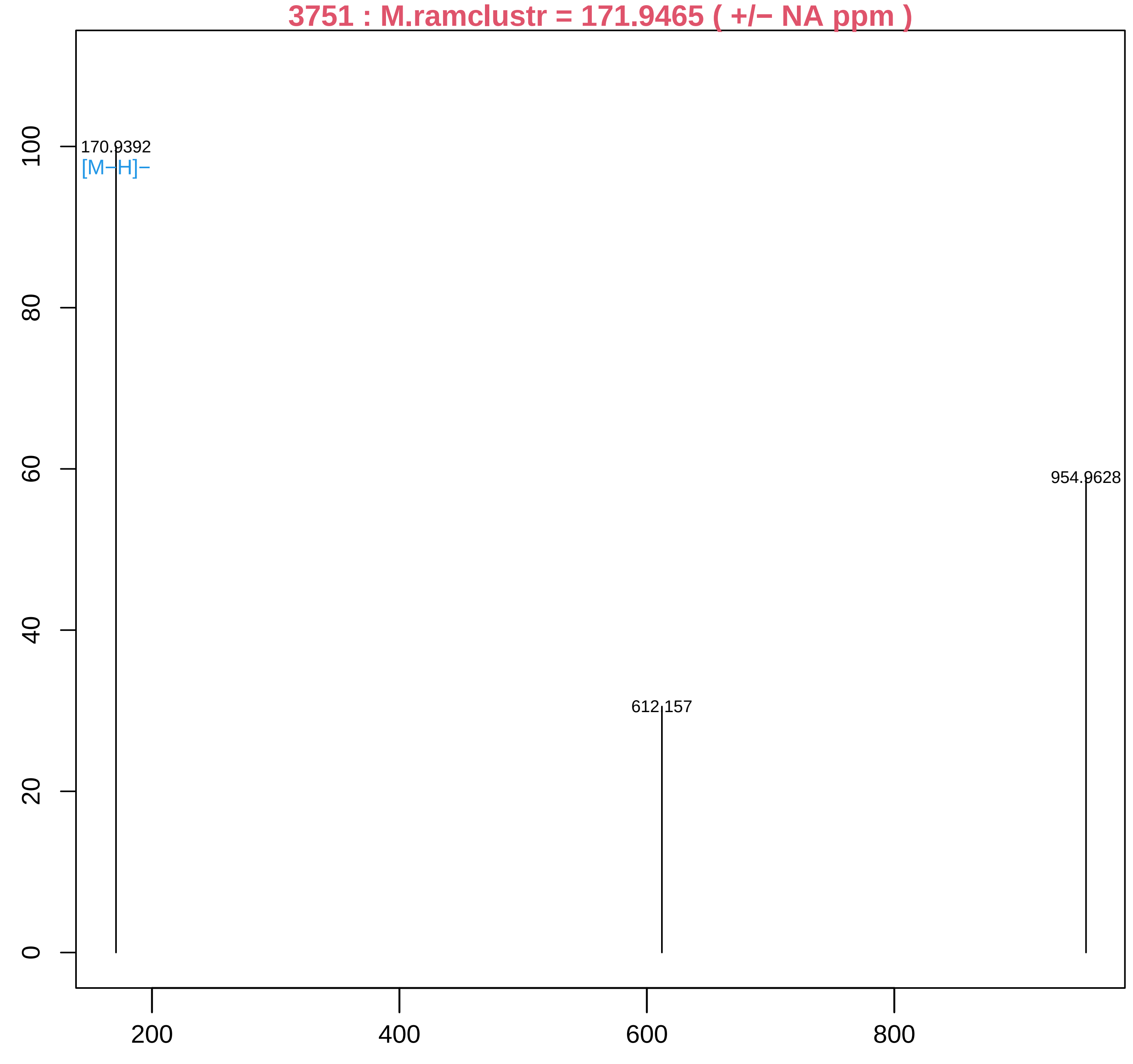
3751 : M.ramclustr = 171.9465 (+/− NA ppm)
170.9392
[M−H]−
612.157
954.9628
0
20
40
60
80
100
200
400
600
800

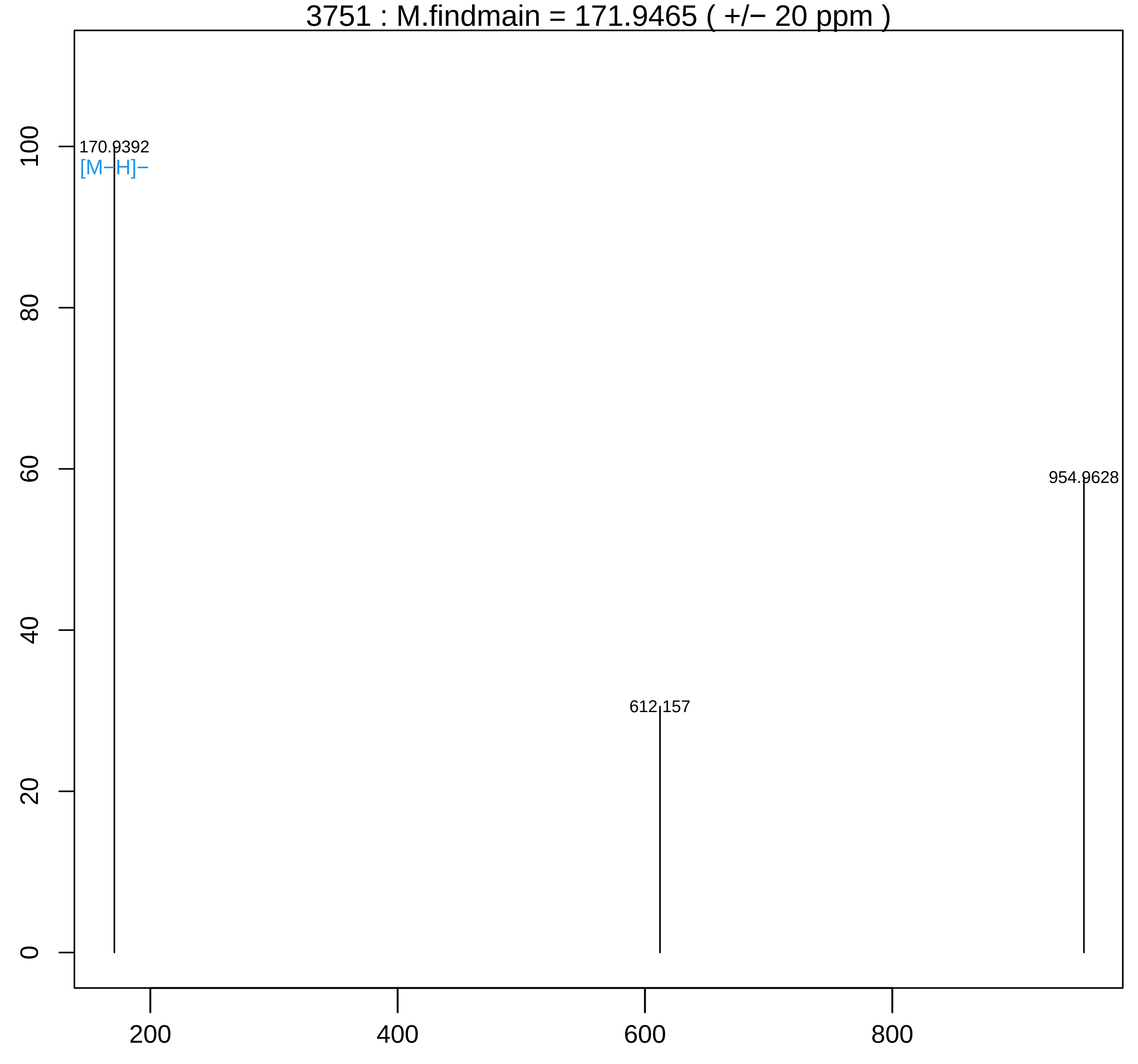
3751 : M.findmain = 171.9465 (+/− 20 ppm)
170.9392
[M−H]−
612.157
954.9628
0
20
40
60
80
100
200
400
600
800

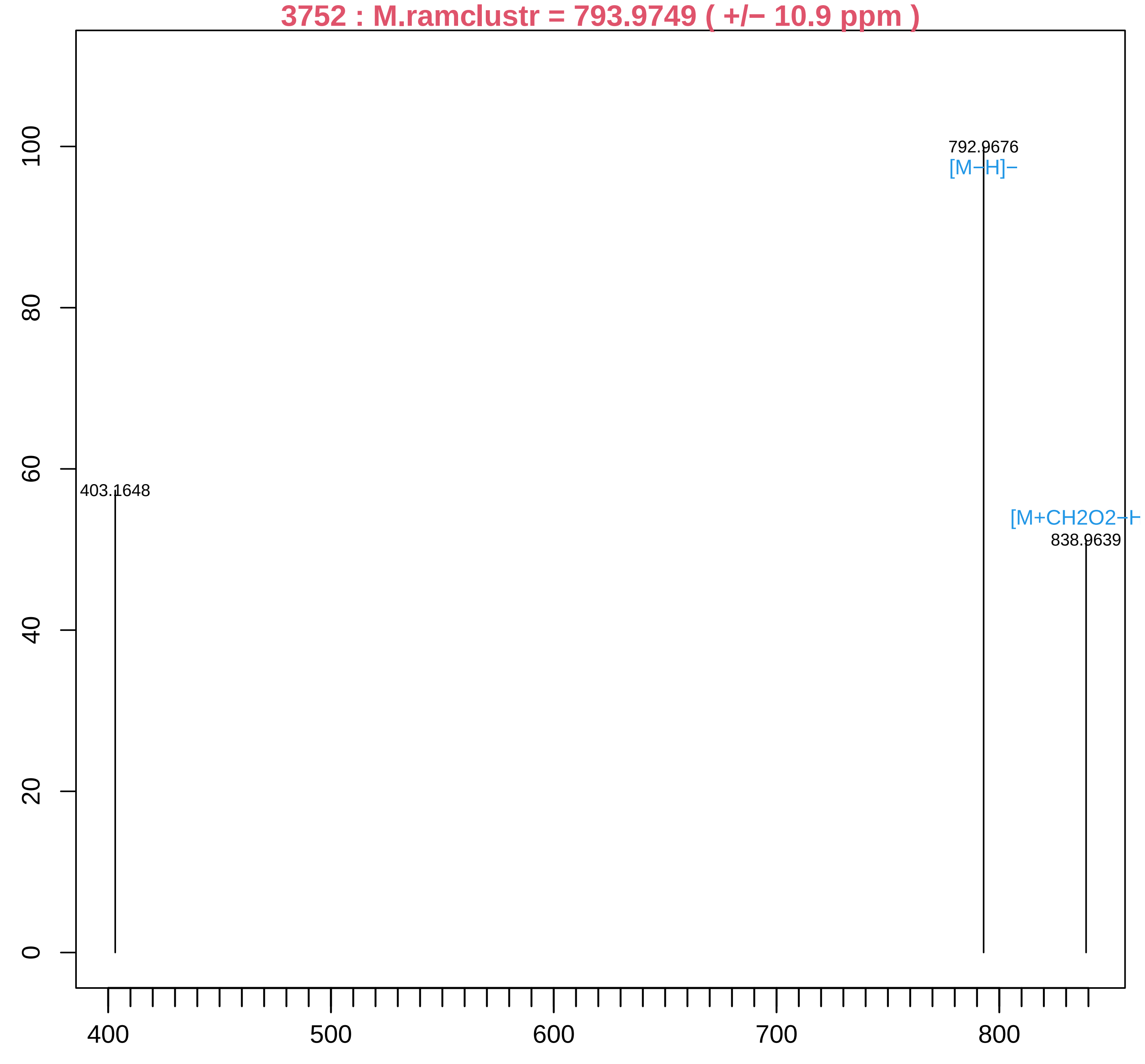
3752 : M.ramclustr = 793.9749 (+/− 10.9 ppm)
792.9676
[M−H]−
403.1648
[M+CH2O2−H
838.9639
0
20
40
60
80
100
400
500
600
700
800

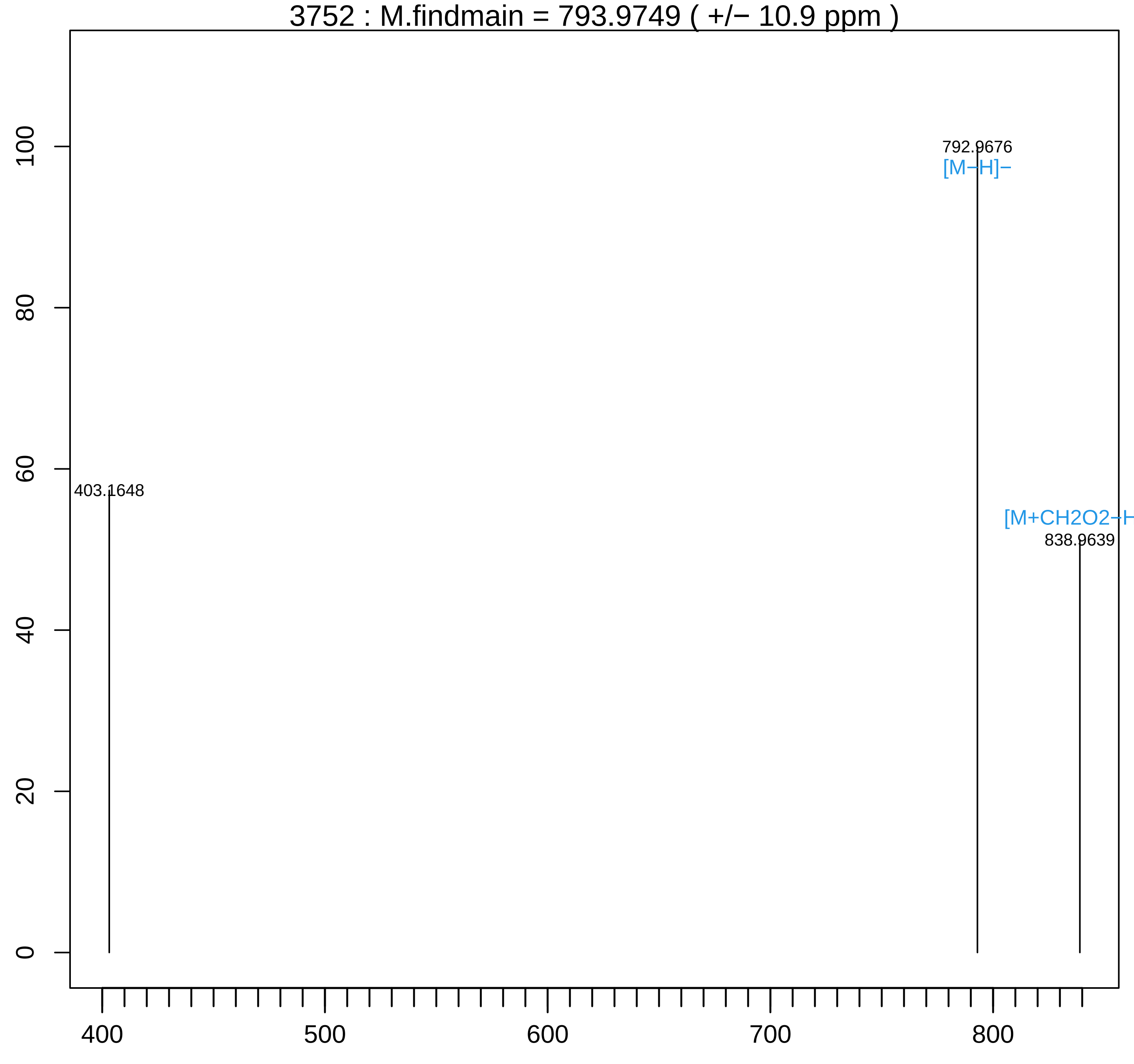
3752 : M.findmain = 793.9749 (+/− 10.9 ppm)
792.9676
[M−H]−
403.1648
[M+CH2O2−H
838.9639
0
20
40
60
80
100
400
500
600
700
800

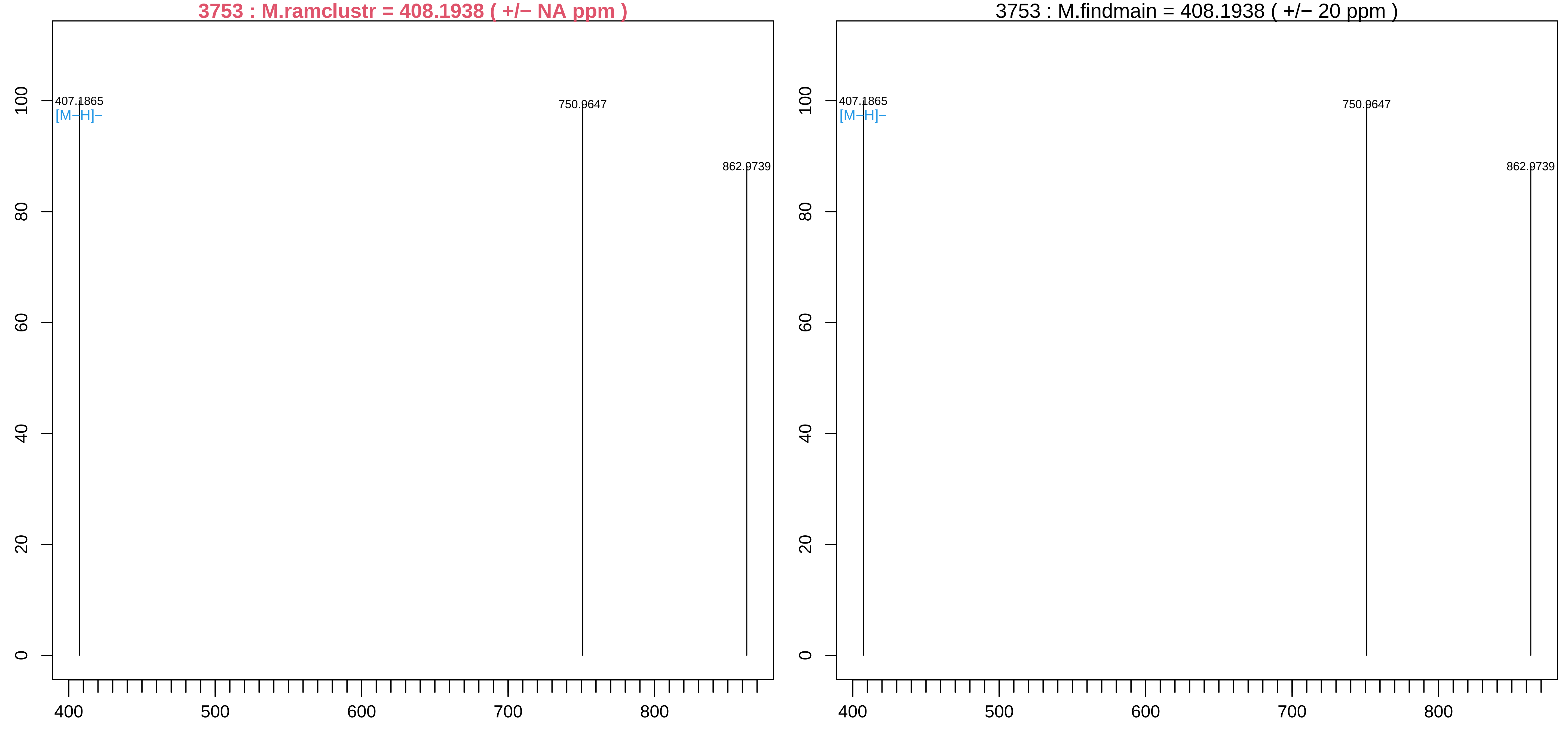

3753 : M.ramclustr = 408.1938 (+/− NA ppm)
407.1865
[M−H]−
750.9647
862.9739
3753 : M.findmain = 408.1938 (+/− 20 ppm)
407.1865
[M−H]−
750.9647
862.9739
0
20
40
60
80
100
400
500
600
700
800

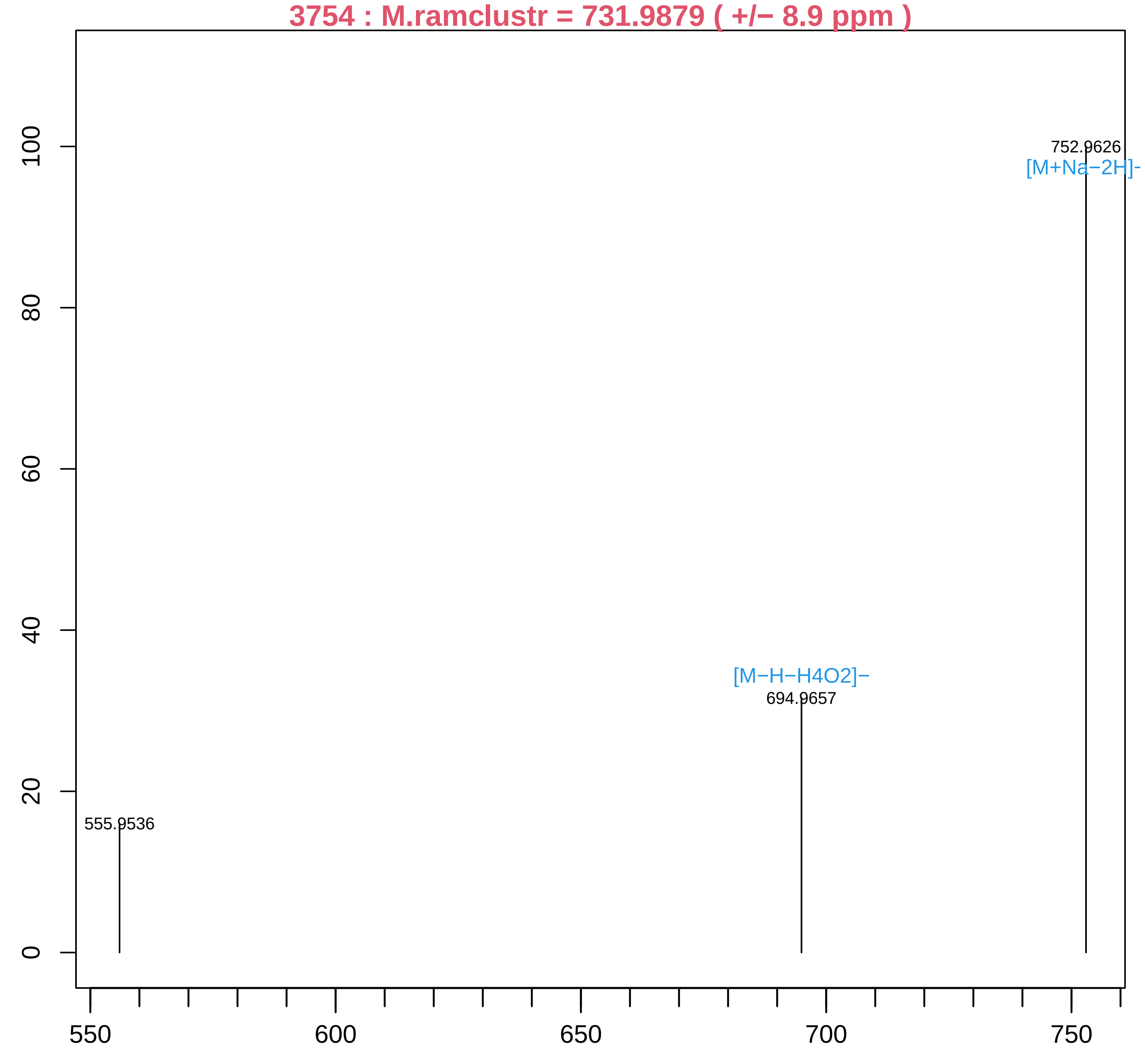

3754 : M.ramclustr = 731.9879 (+/− 8.9 ppm)
752.9626
[M+Na−2H]-
[M−H−H4O2]−
694.9657
555.9536
0
20
40
60
80
100
550
600
650
700
750

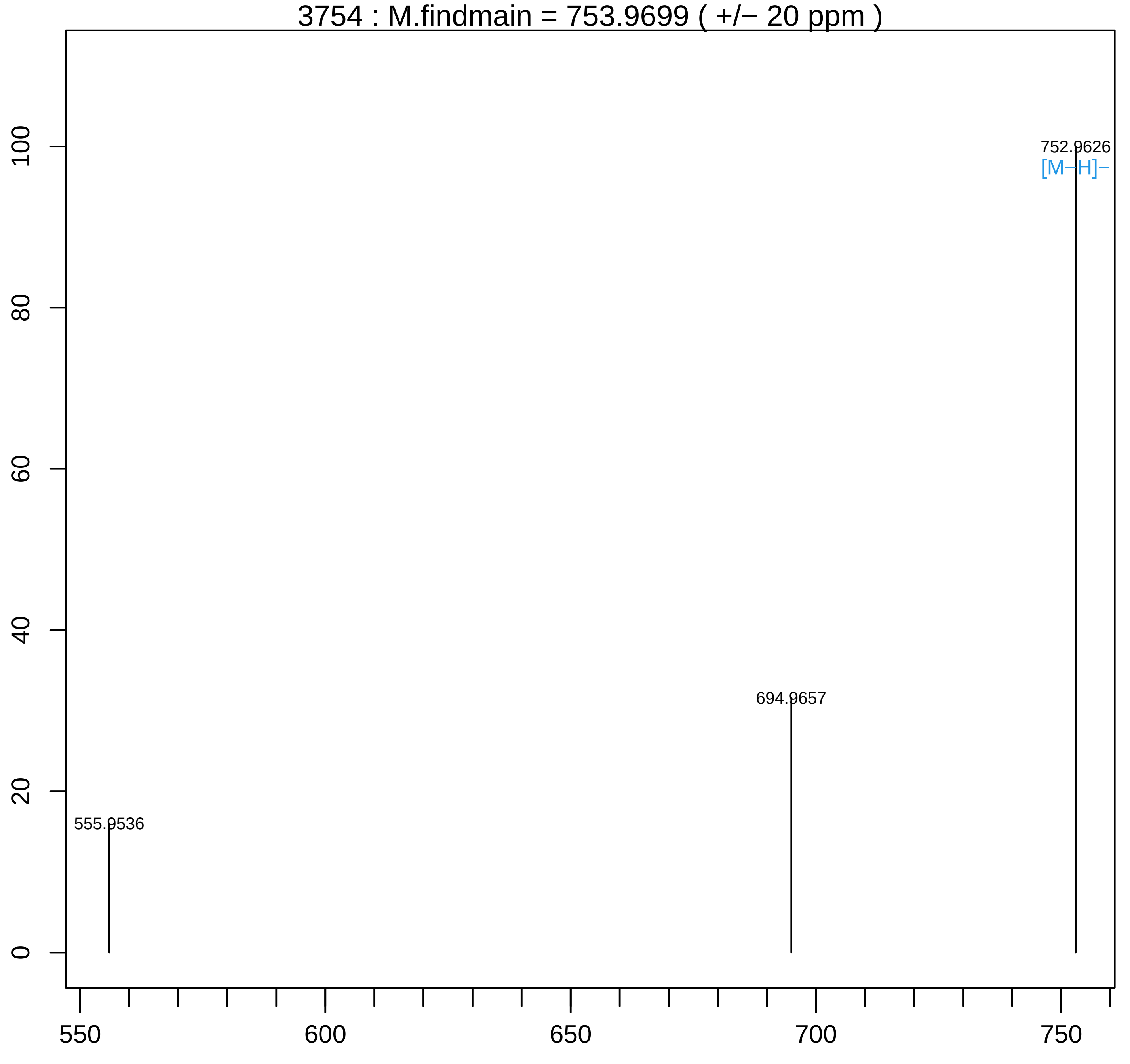

3754 : M.findmain = 753.9699 (+/− 20 ppm)
752.9626
[M−H]−
694.9657
555.9536
0
20
40
60
80
100
550
600
650
700
750

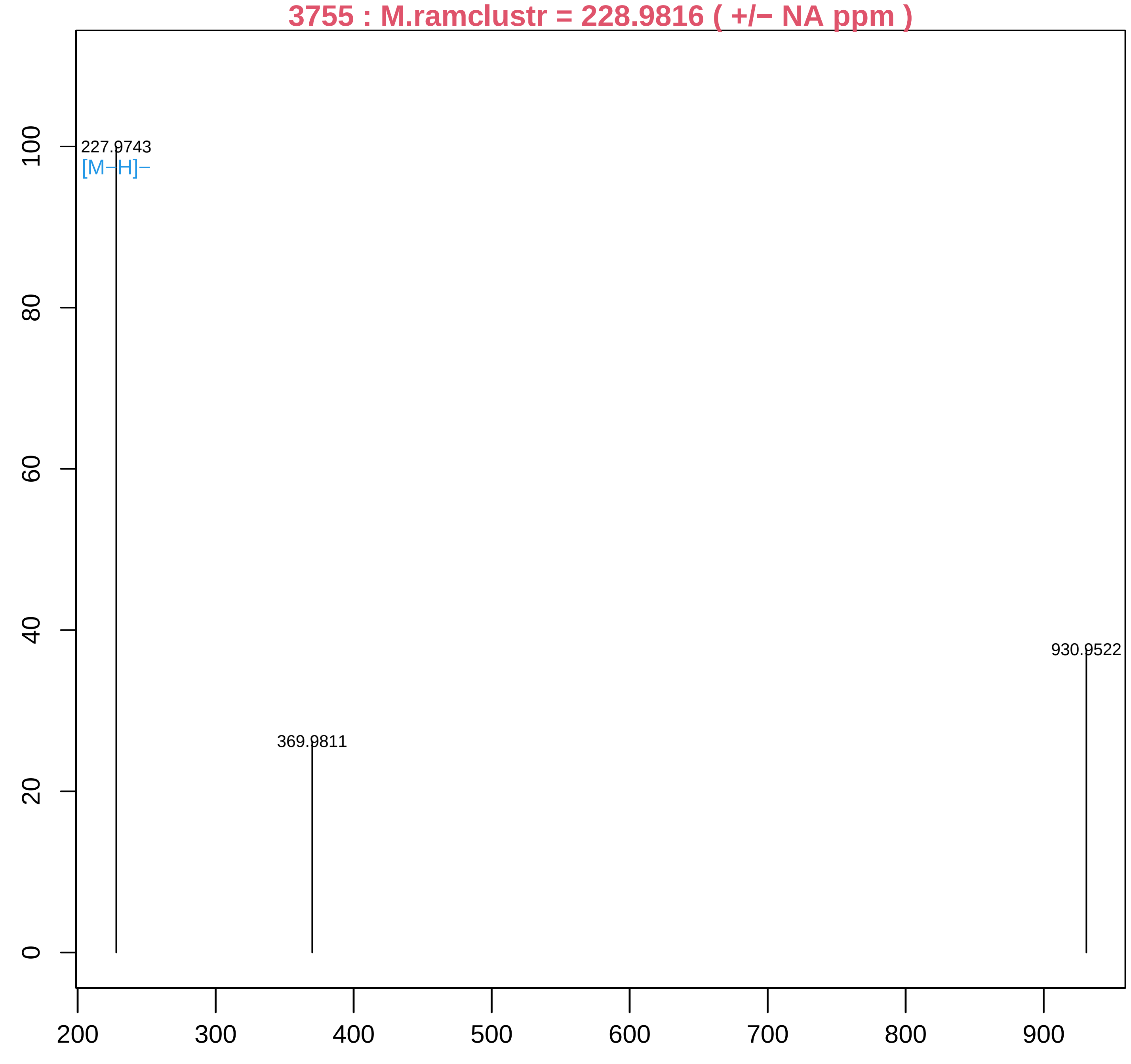
3755 : M.ramclustr = 228.9816 (+/− NA ppm)
227.9743
[M−H]−
369.9811
930.9522
0
20
40
60
80
100
200
300
400
500
600
700
800
900

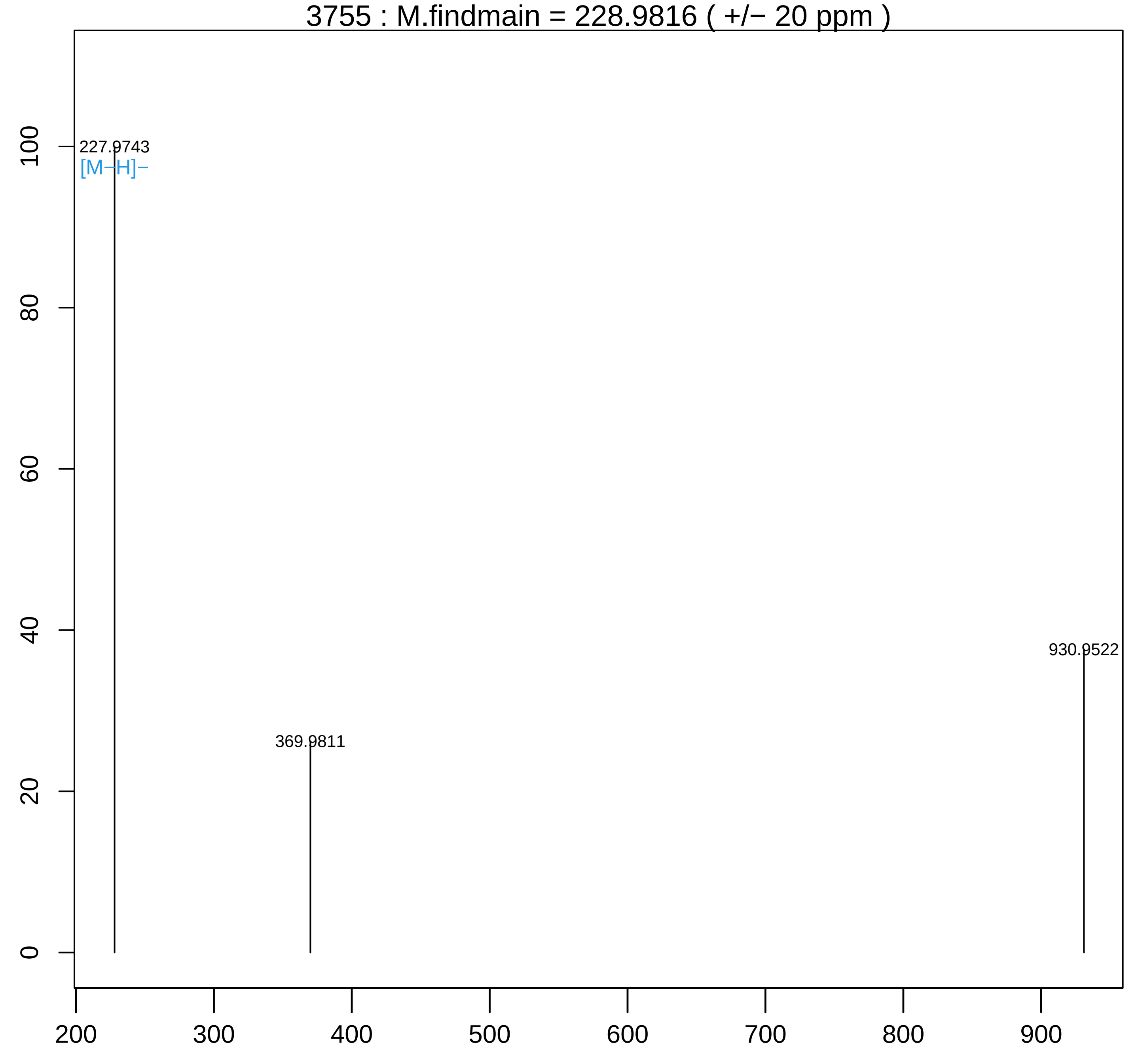
3755 : M.findmain = 228.9816 (+/− 20 ppm)
227.9743
[M−H]−
369.9811
930.9522
0
20
40
60
80
100
200
300
400
500
600
700
800
900

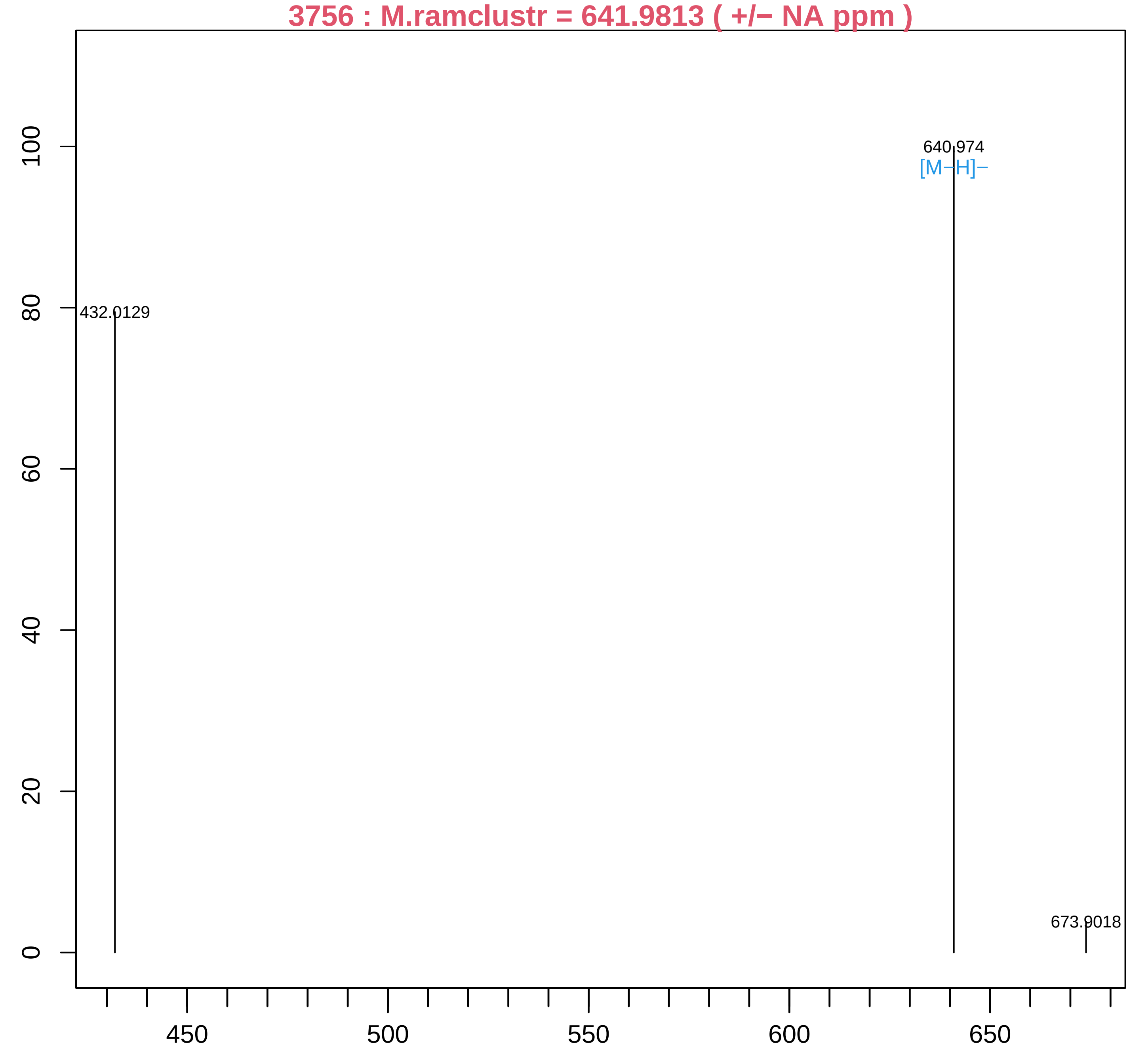
3756 : M.ramclustr = 641.9813 (+/− NA ppm)
640.974
[M−H]−
432.0129
673.9018
0
20
40
60
80
100
450
500
550
600
650

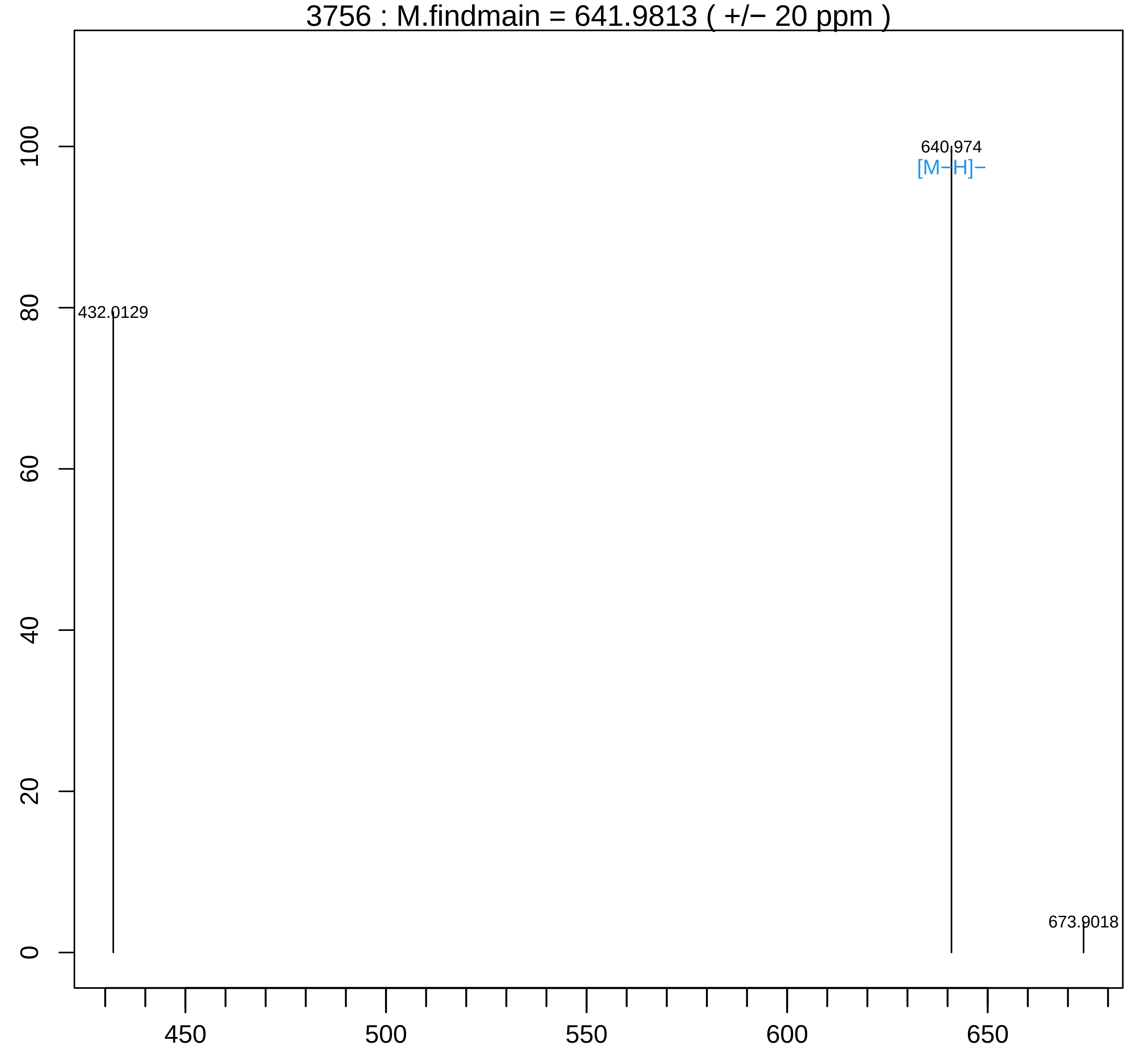
3756 : M.findmain = 641.9813 (+/− 20 ppm)
640.974
[M−H]−
432.0129
673.9018
0
20
40
60
80
100
450
500
550
600
650

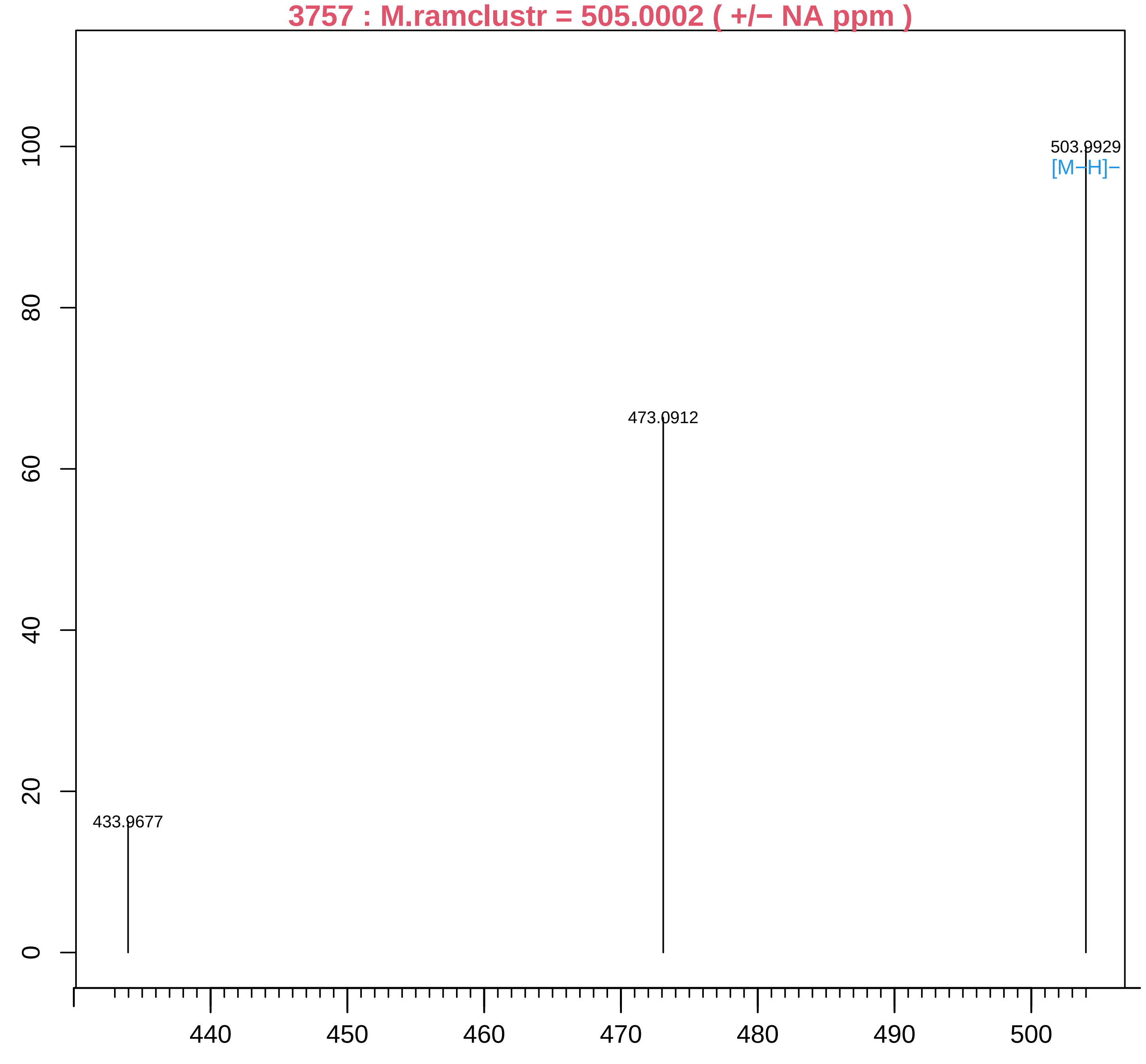
3757 : M.ramclustr = 505.0002 (+/− NA ppm)
503.9929
[M−H]−
473.0912
433.9677
0
20
40
60
80
100
440
450
460
470
480
490
500

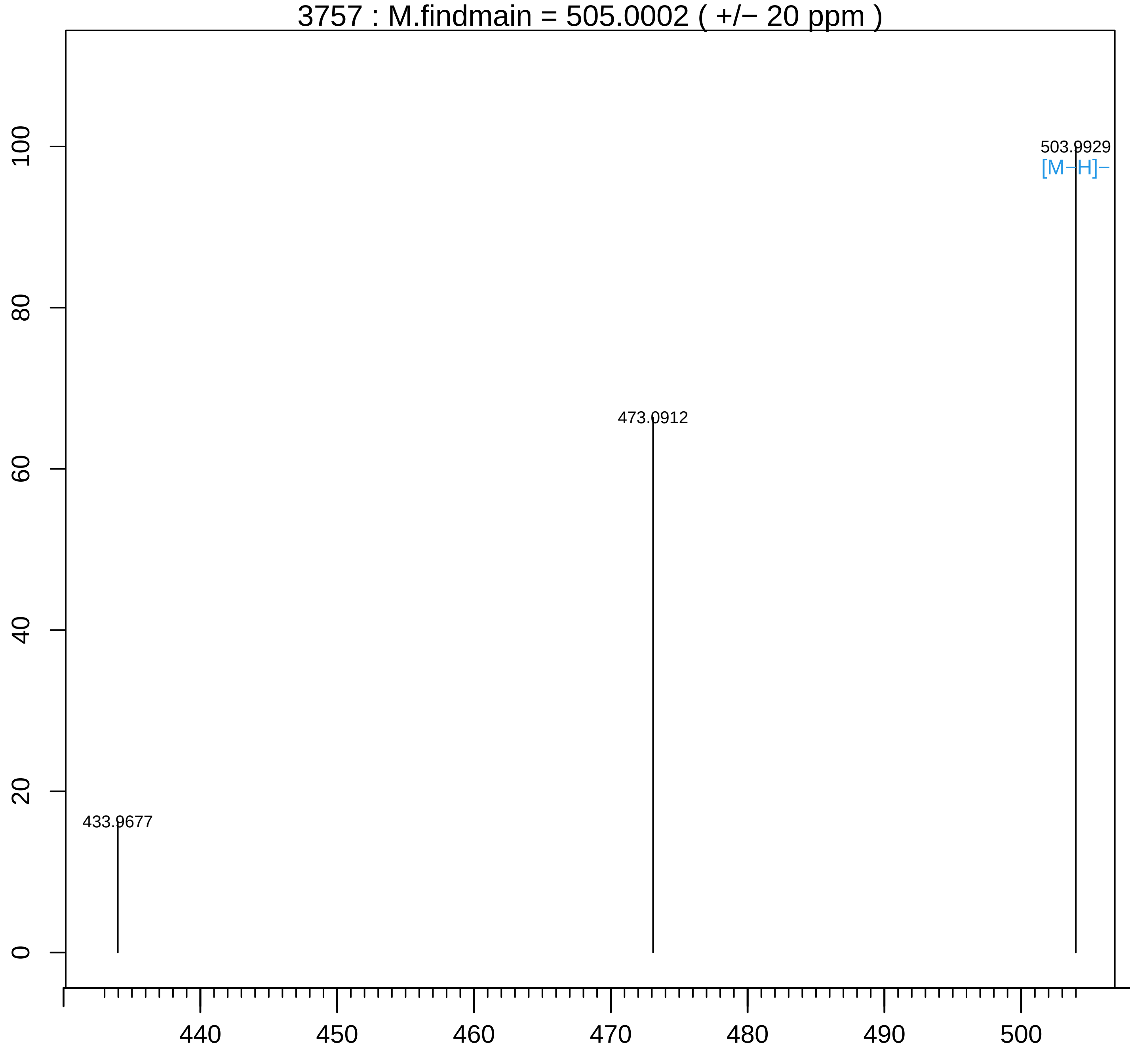
3757 : M.findmain = 505.0002 (+/− 20 ppm)
503.9929
[M−H]−
473.0912
433.9677
0
20
40
60
80
100
440
450
460
470
480
490
500

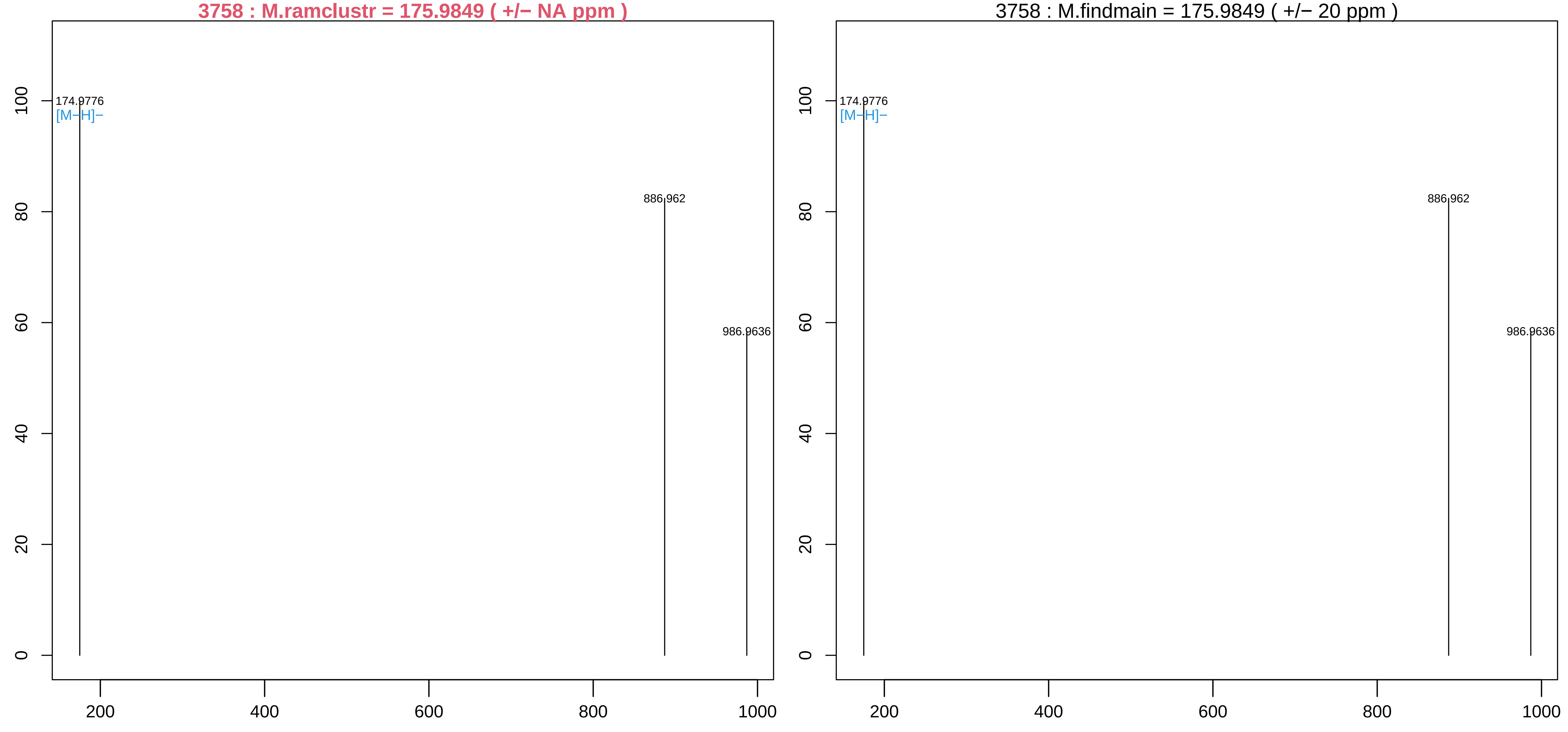
3758 : M.ramclustr = 175.9849 (+/− NA ppm)
174.9776
[M−H]−
886.962
986.9636
3758 : M.findmain = 175.9849 (+/− 20 ppm)
174.9776
[M−H]−
886.962
986.9636
0
20
40
60
80
100
200
400
600
800
1000

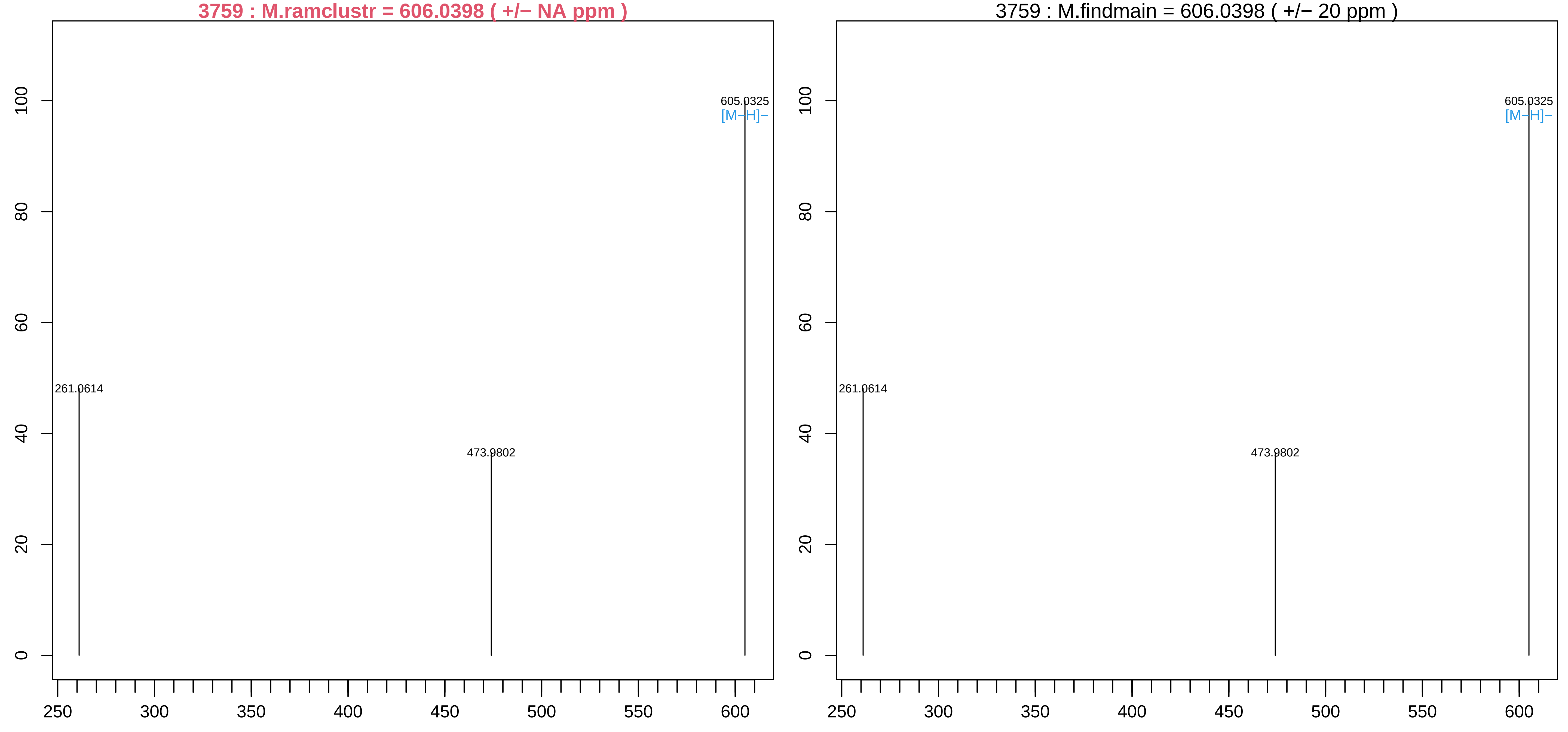
3759 : M.ramclustr = 606.0398 (+/− NA ppm)
3759 : M.findmain = 606.0398 (+/− 20 ppm)
605.0325
[M−H]−
261.0614
473.9802
0
20
40
60
80
100
250
300
350
400
450
500
550
600

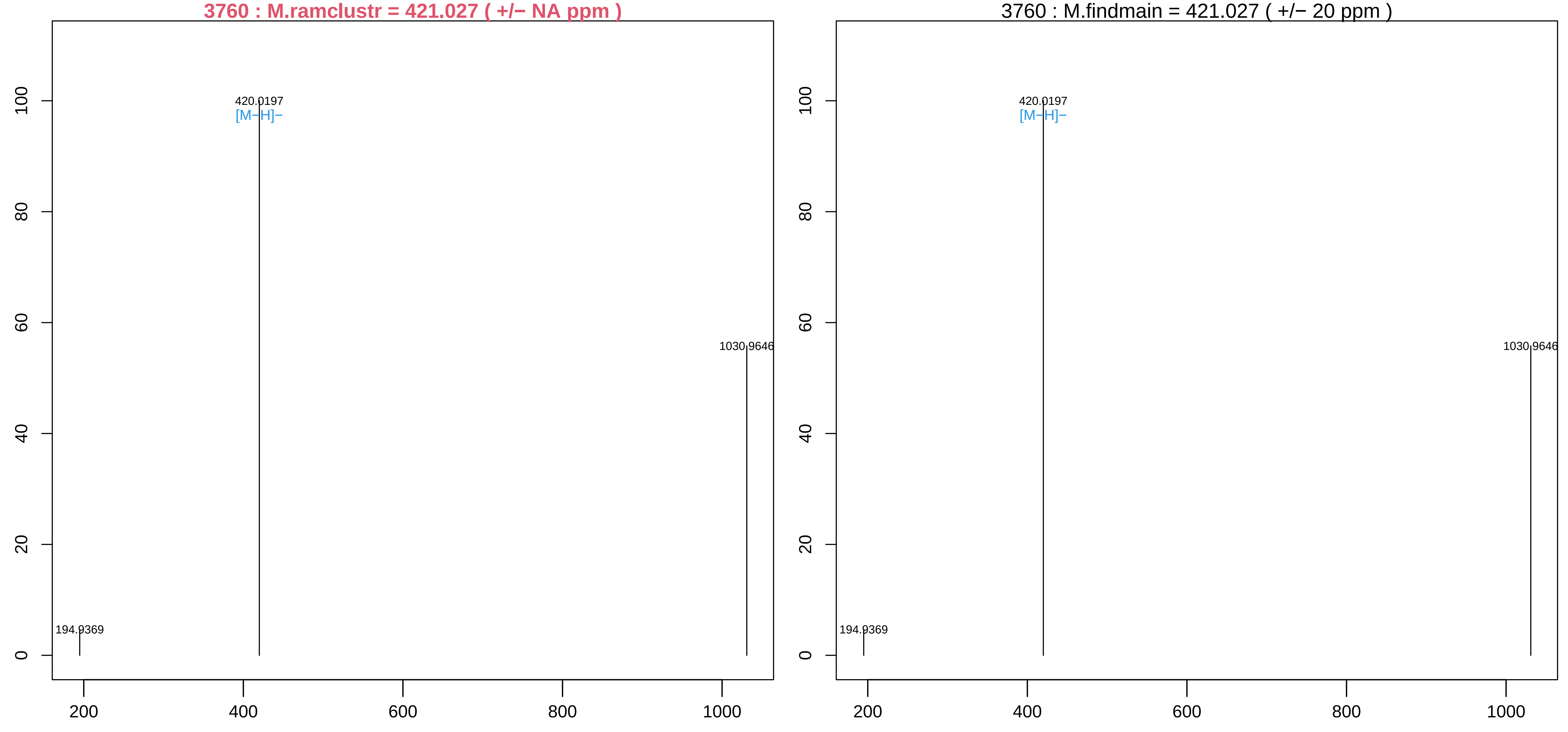
3760 : M.ramclustr = 421.027 (+/− NA ppm)
3760 : M.findmain = 421.027 (+/− 20 ppm)
420.0197
[M−H]−
1030.9646
194.9369
420.0197
[M−H]−
1030.9646
194.9369
0
20
40
60
80
100
200
400
600
800
1000

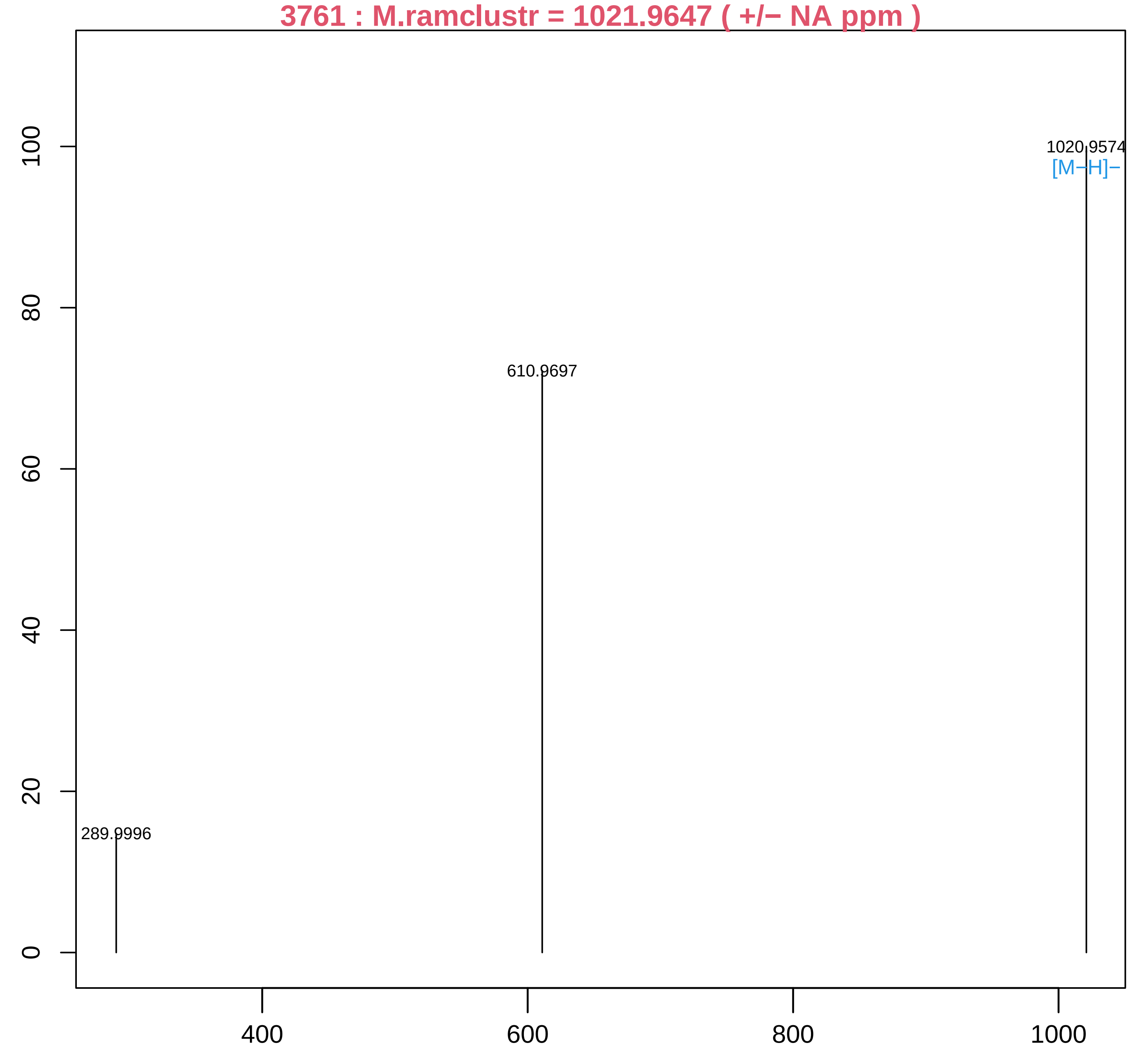

3761 : M.ramclustr = 1021.9647 (+/− NA ppm)
1020.9574
[M−H]−
610.9697
289.9996
0
20
40
60
80
100
400
600
800
1000

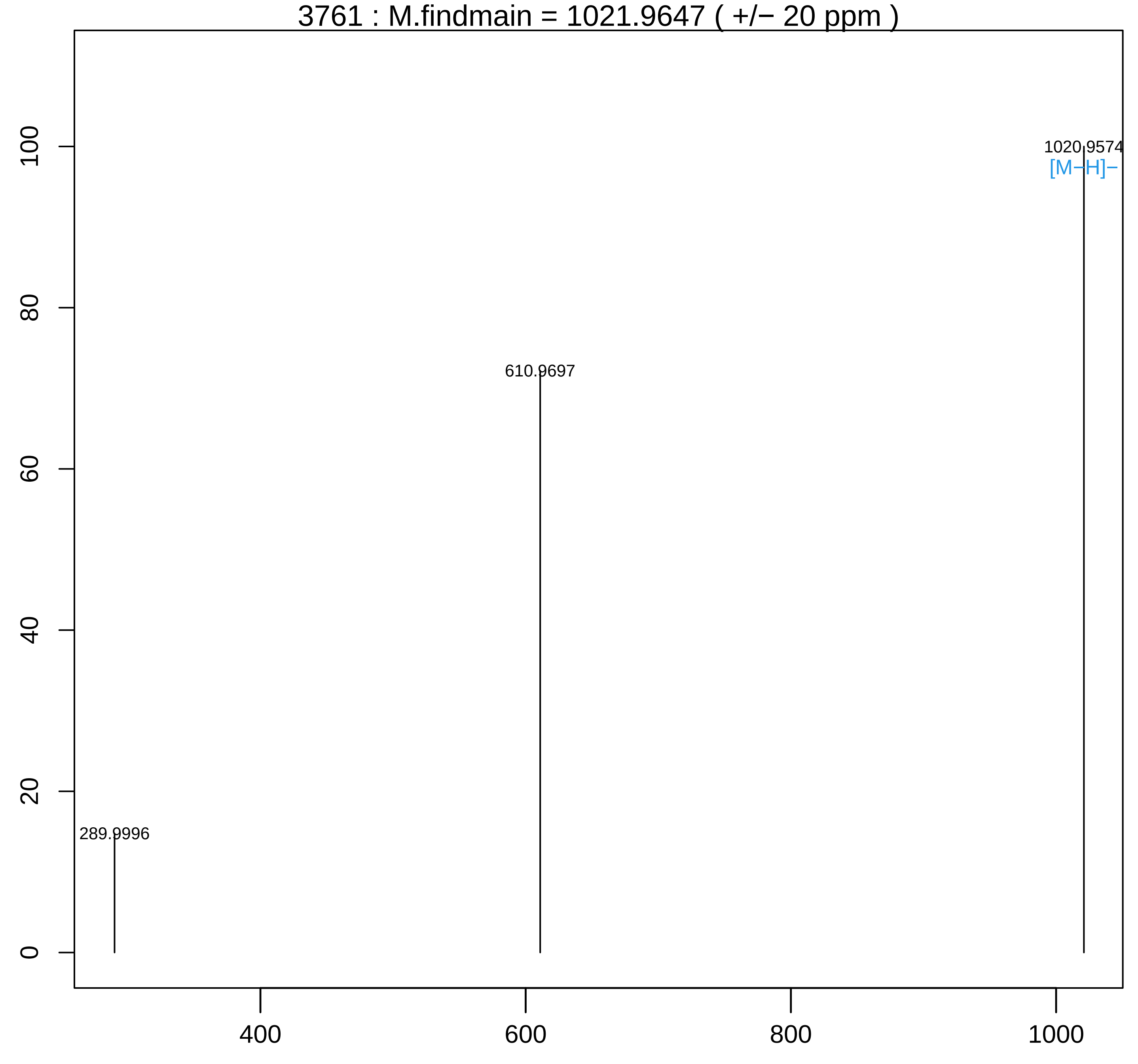

3761 : M.findmain = 1021.9647 (+/− 20 ppm)
1020.9574
[M−H]−
610.9697
289.9996
0
20
40
60
80
100
400
600
800
1000

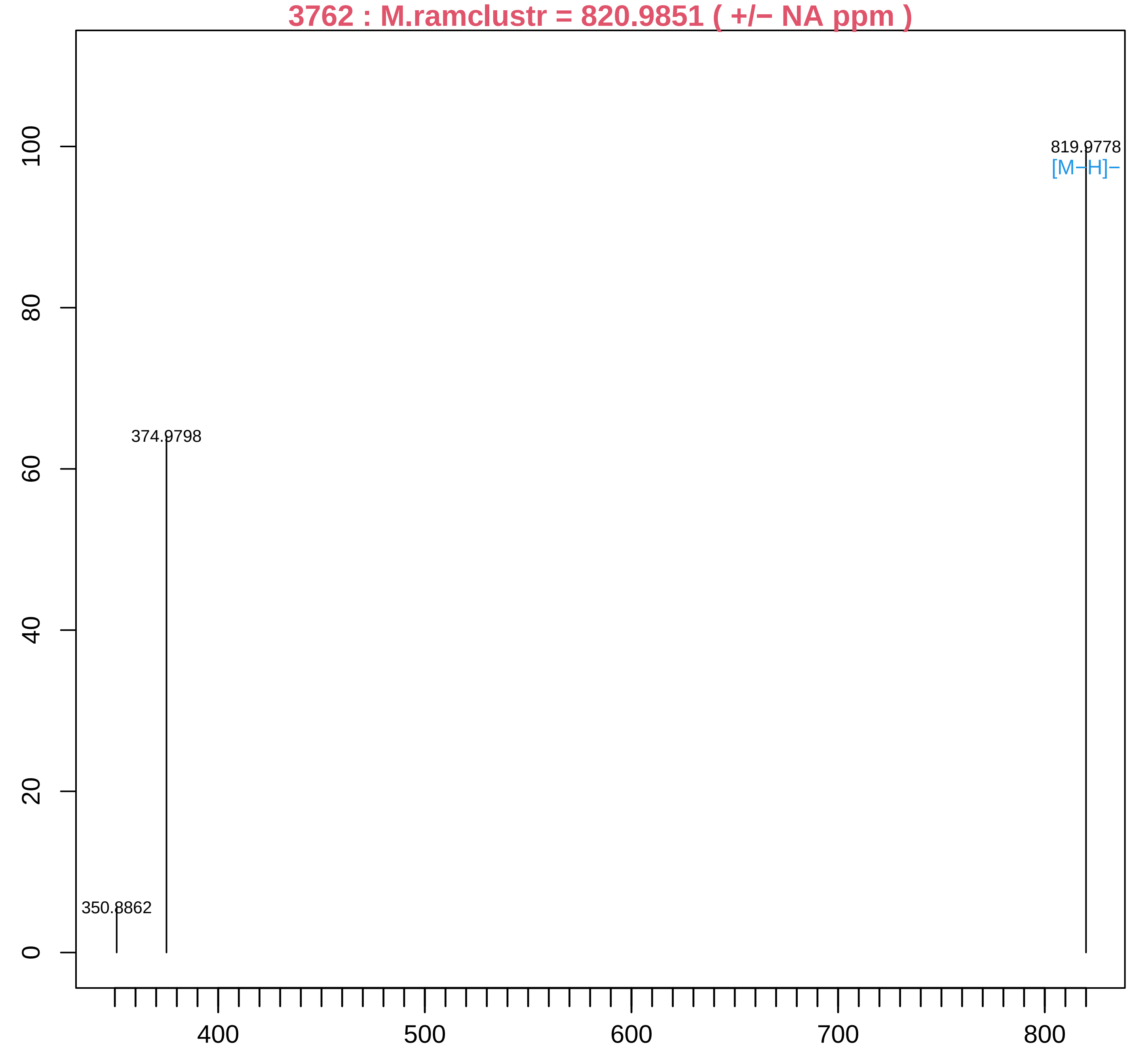
3762 : M.ramclustr = 820.9851 (+/− NA ppm)
819.9778
[M−H]−
374.9798
350.8862
0
20
40
60
80
100
400
500
600
700
800

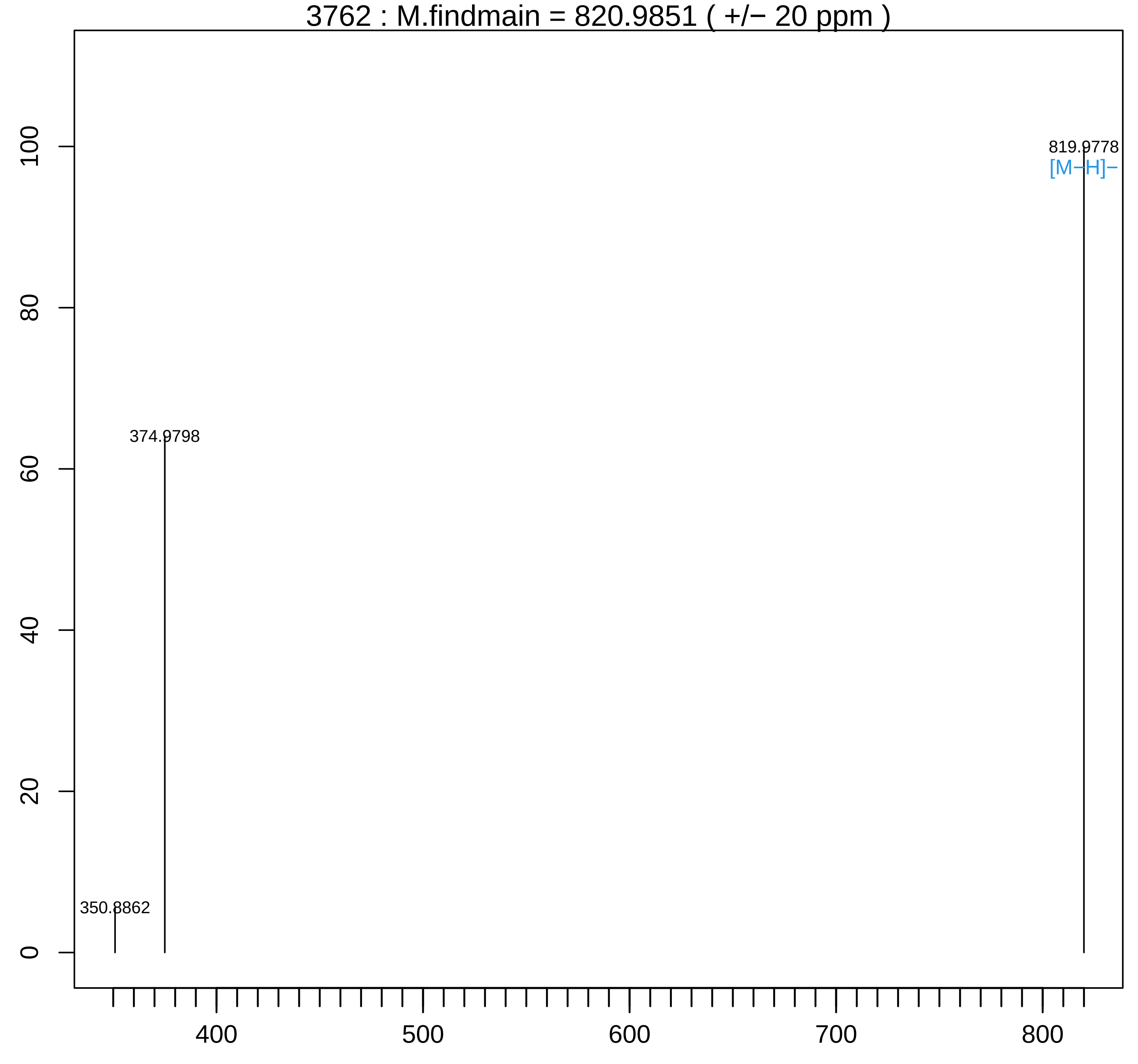
3762 : M.findmain = 820.9851 (+/− 20 ppm)
819.9778
[M−H]−
374.9798
350.8862
0
20
40
60
80
100
400
500
600
700
800

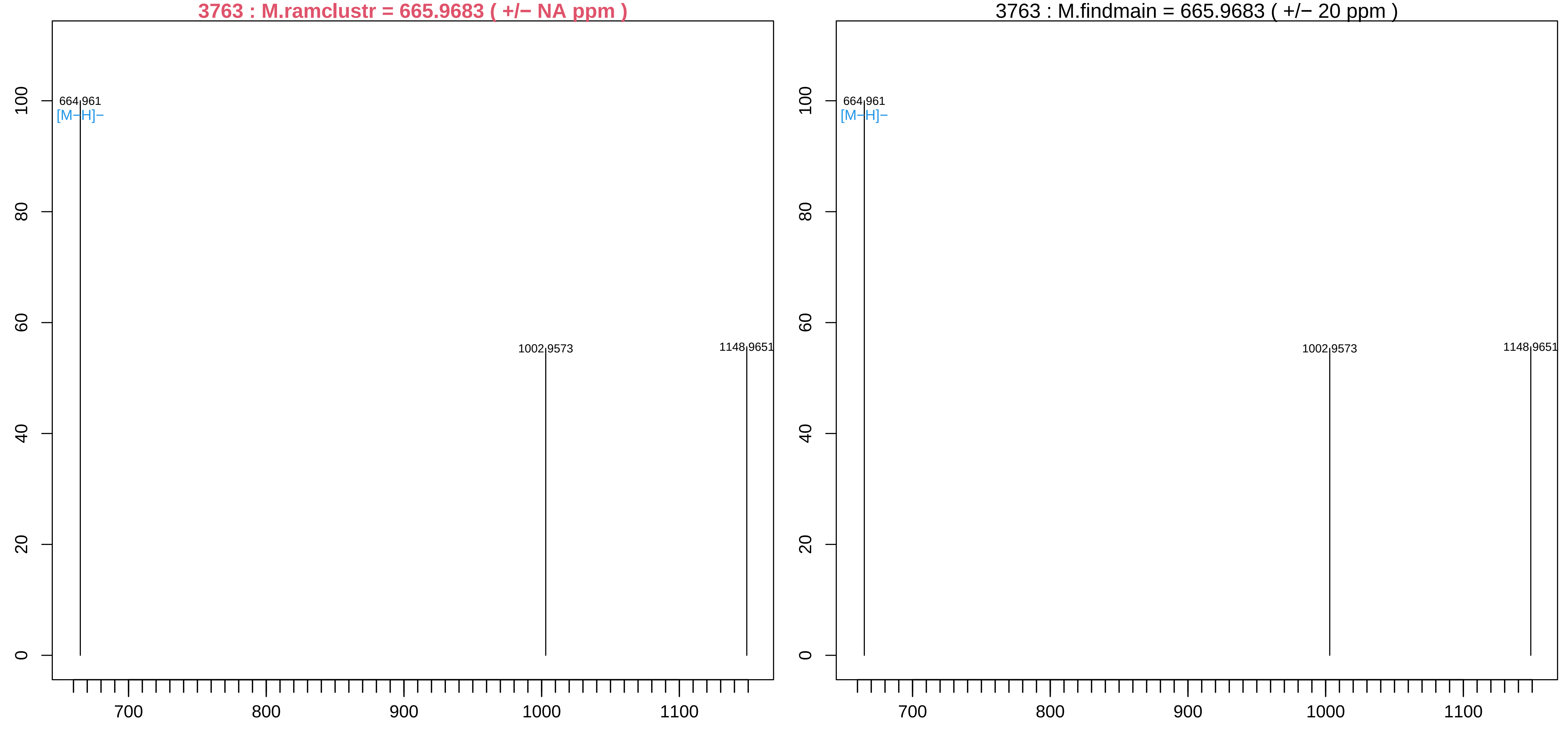

3763 : M.ramclustr = 665.9683 (+/− NA ppm)
664.961
[M−H]−
1002.9573
1148.9651
3763 : M.findmain = 665.9683 (+/− 20 ppm)
664.961
[M−H]−
1002.9573
1148.9651
0
20
40
60
80
100
700
800
900
1000
1100

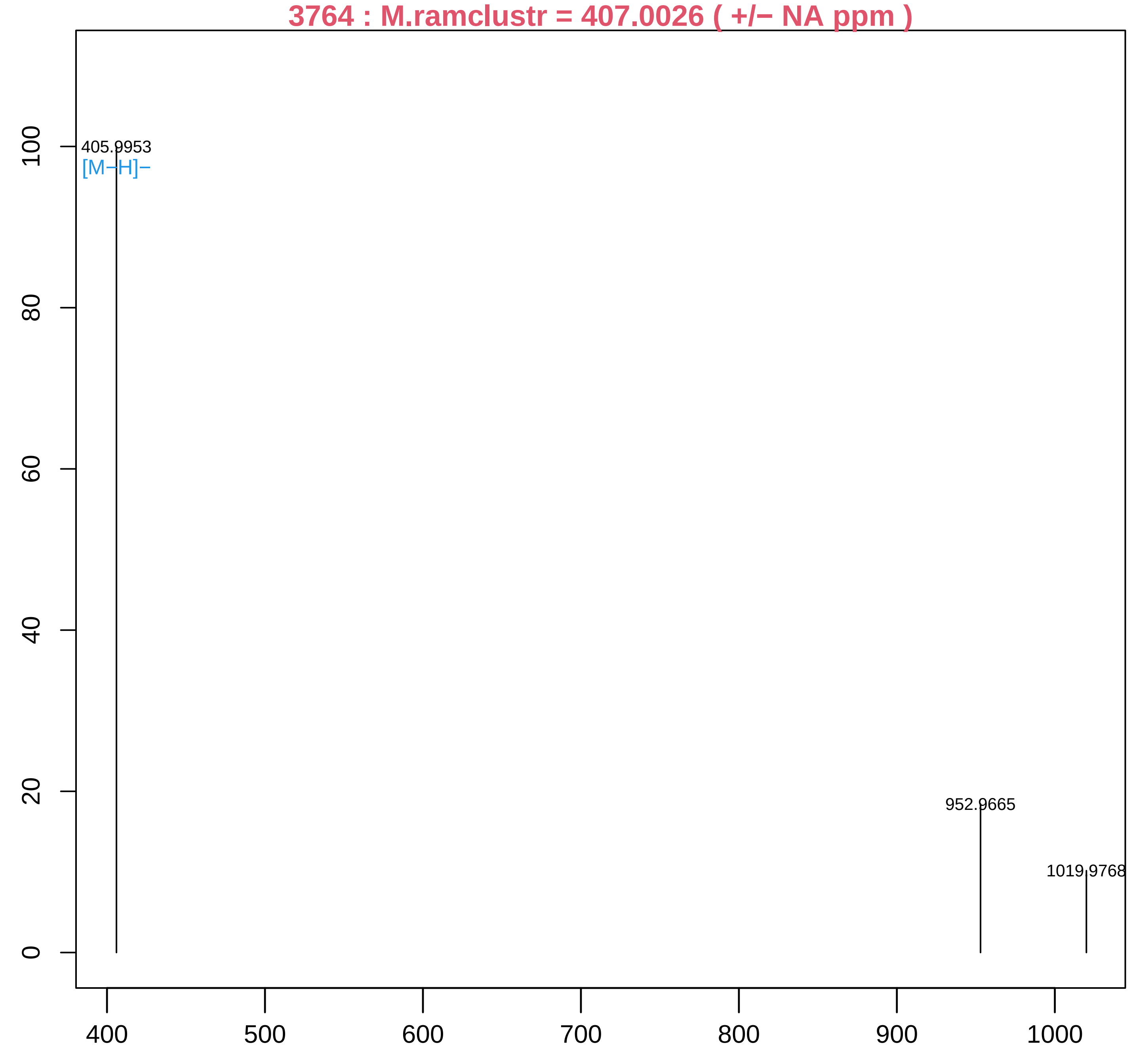
3764 : M.ramclustr = 407.0026 (+/− NA ppm)
405.9953
[M−H]−
952.9665
1019.9768
0
20
40
60
80
100
400
500
600
700
800
900
1000

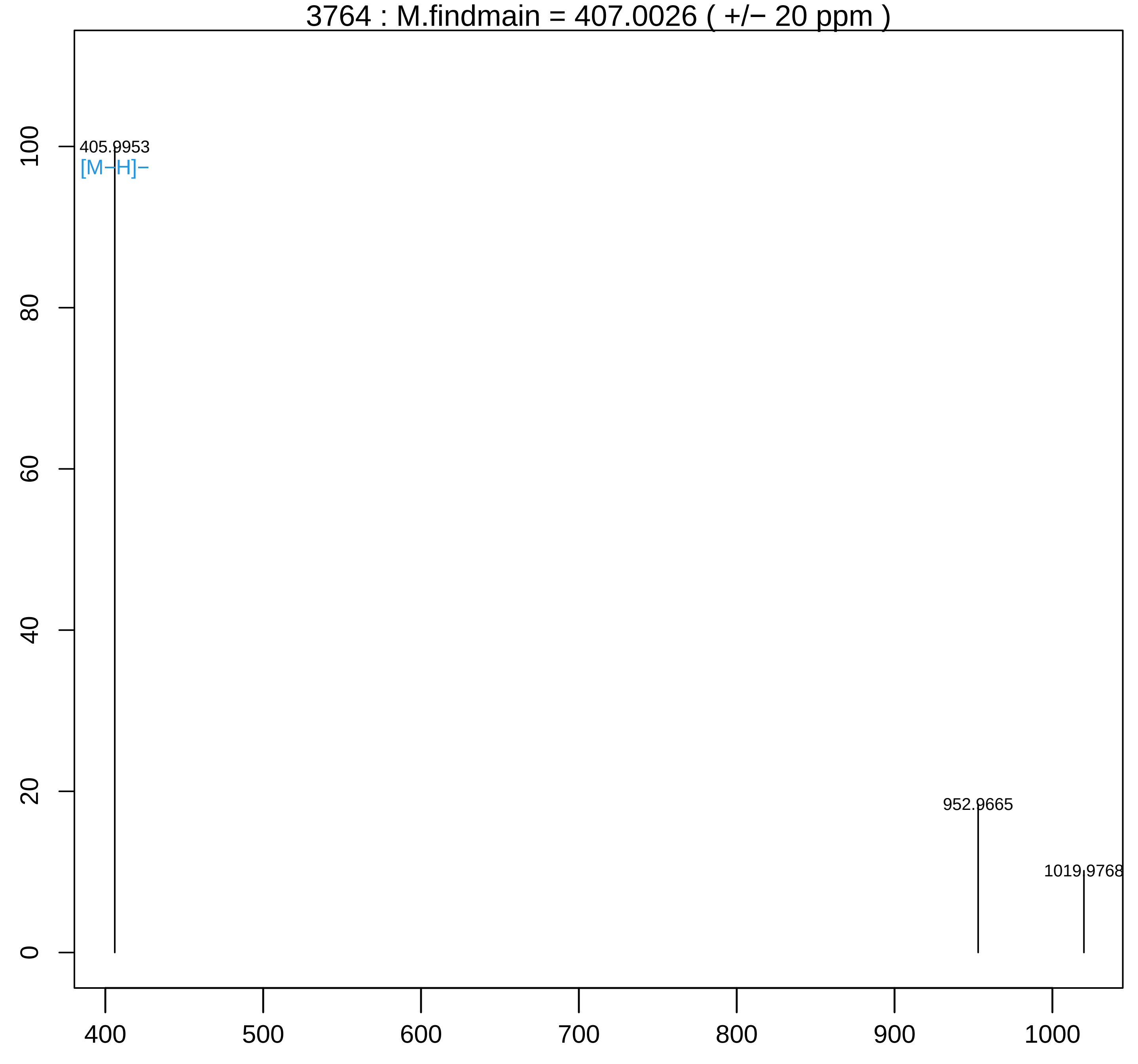
3764 : M.findmain = 407.0026 (+/− 20 ppm)
405.9953
[M−H]−
952.9665
1019.9768
0
20
40
60
80
100
400
500
600
700
800
900
1000

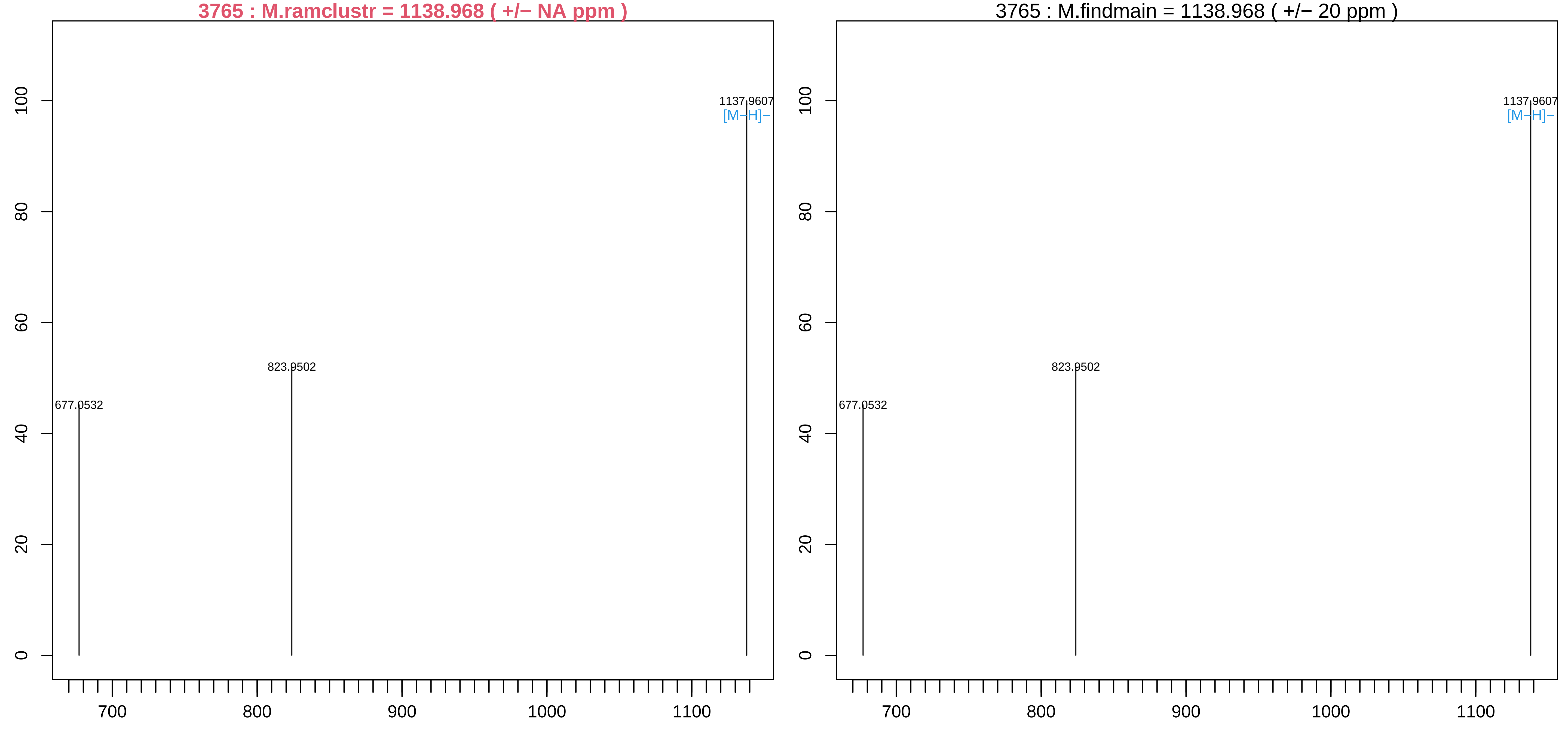

3765 : M.ramclustr = 1138.968 (+/− NA ppm)
1137.9607
[M−H]−
823.9502
677.0532
3765 : M.findmain = 1138.968 (+/− 20 ppm)
1137.9607
[M−H]−
823.9502
677.0532

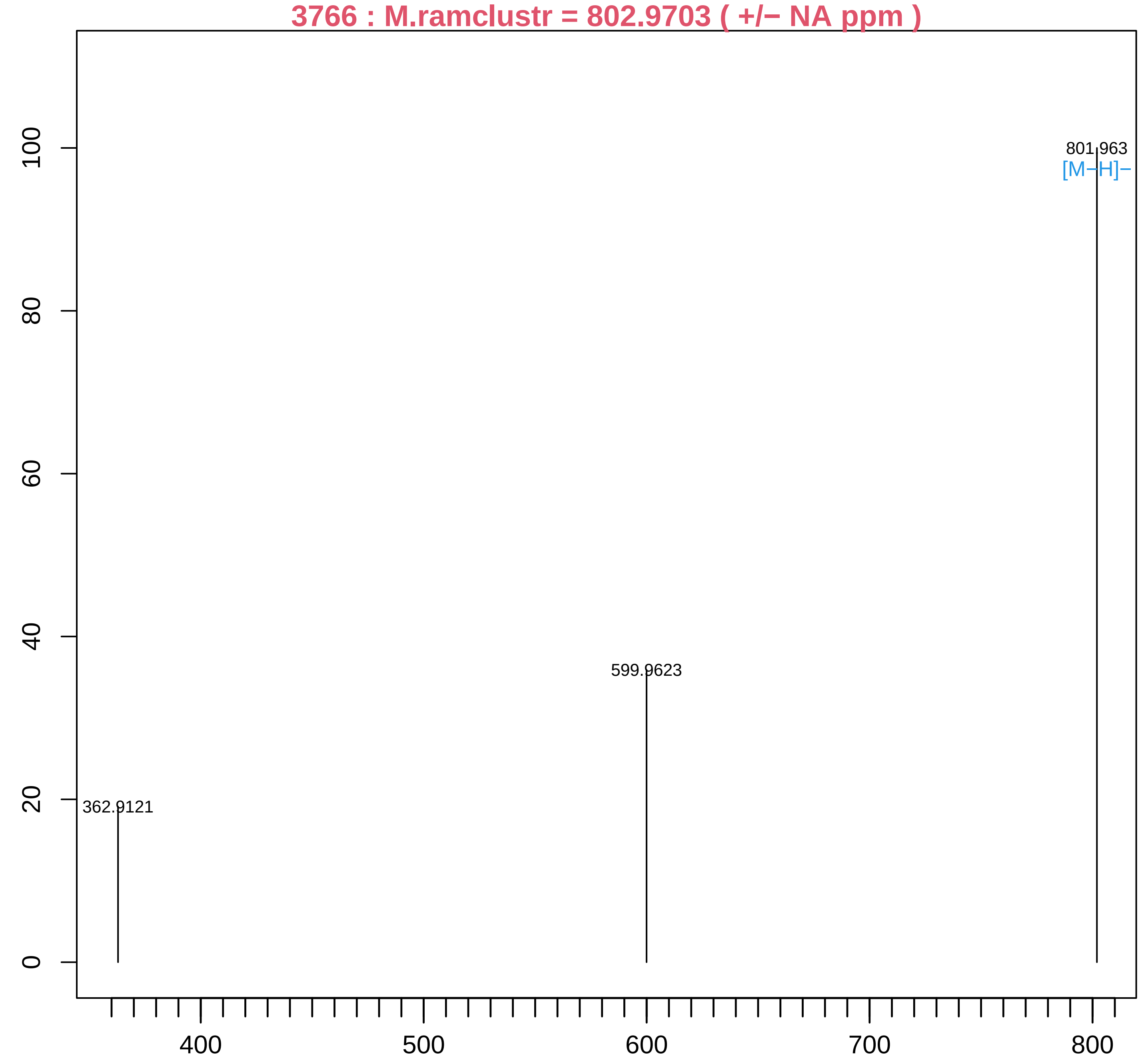
3766 : M.ramclustr = 802.9703 (+/− NA ppm)
801.963
[M−H]−
599.9623
362.9121
0
20
40
60
80
100
400
500
600
700
800

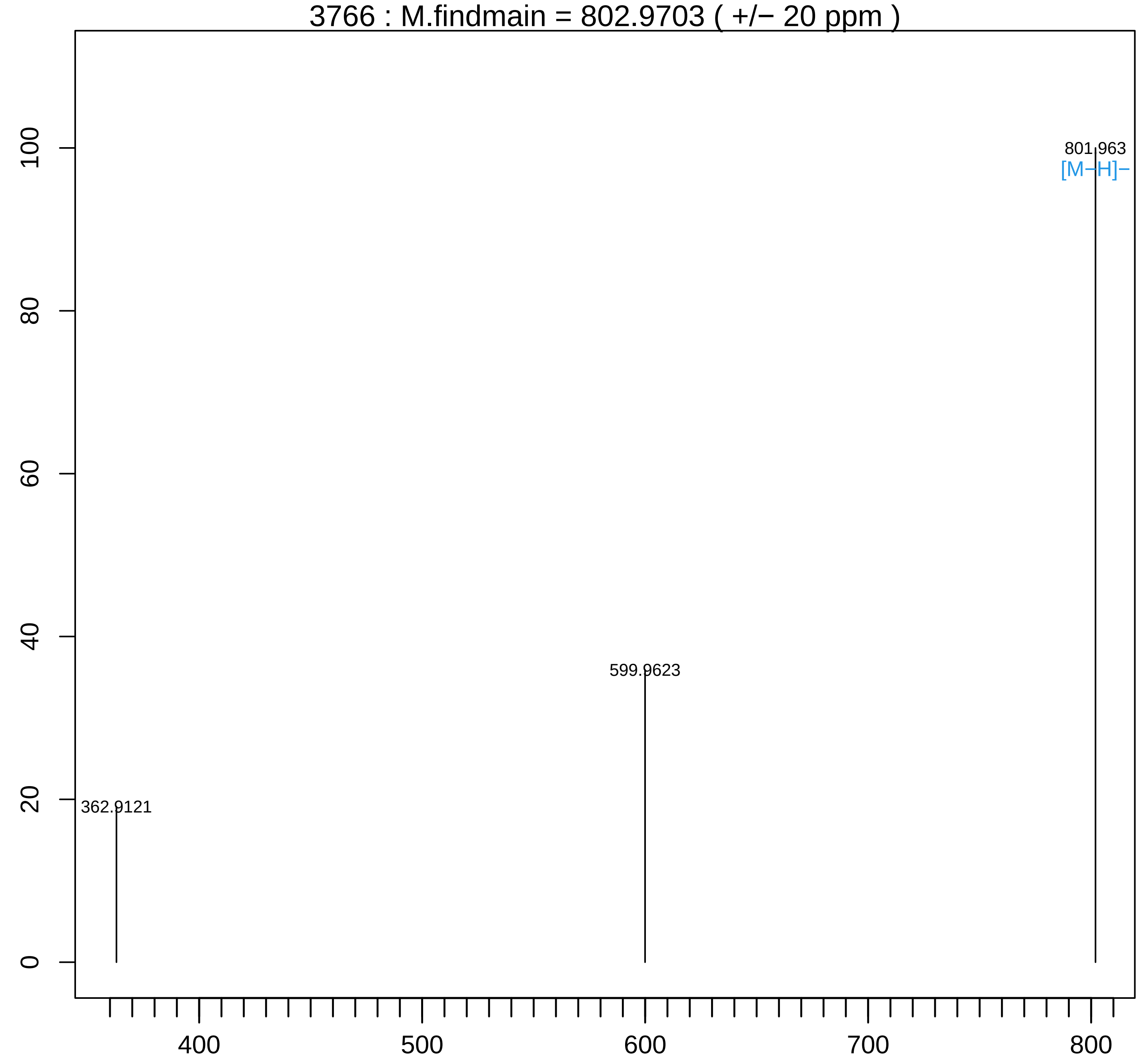
3766 : M.findmain = 802.9703 (+/− 20 ppm)
801.963
[M−H]−
599.9623
362.9121
0
20
40
60
80
100
400
500
600
700
800

3767 : M.ramclustr = 403.1701 (+/− NA ppm)

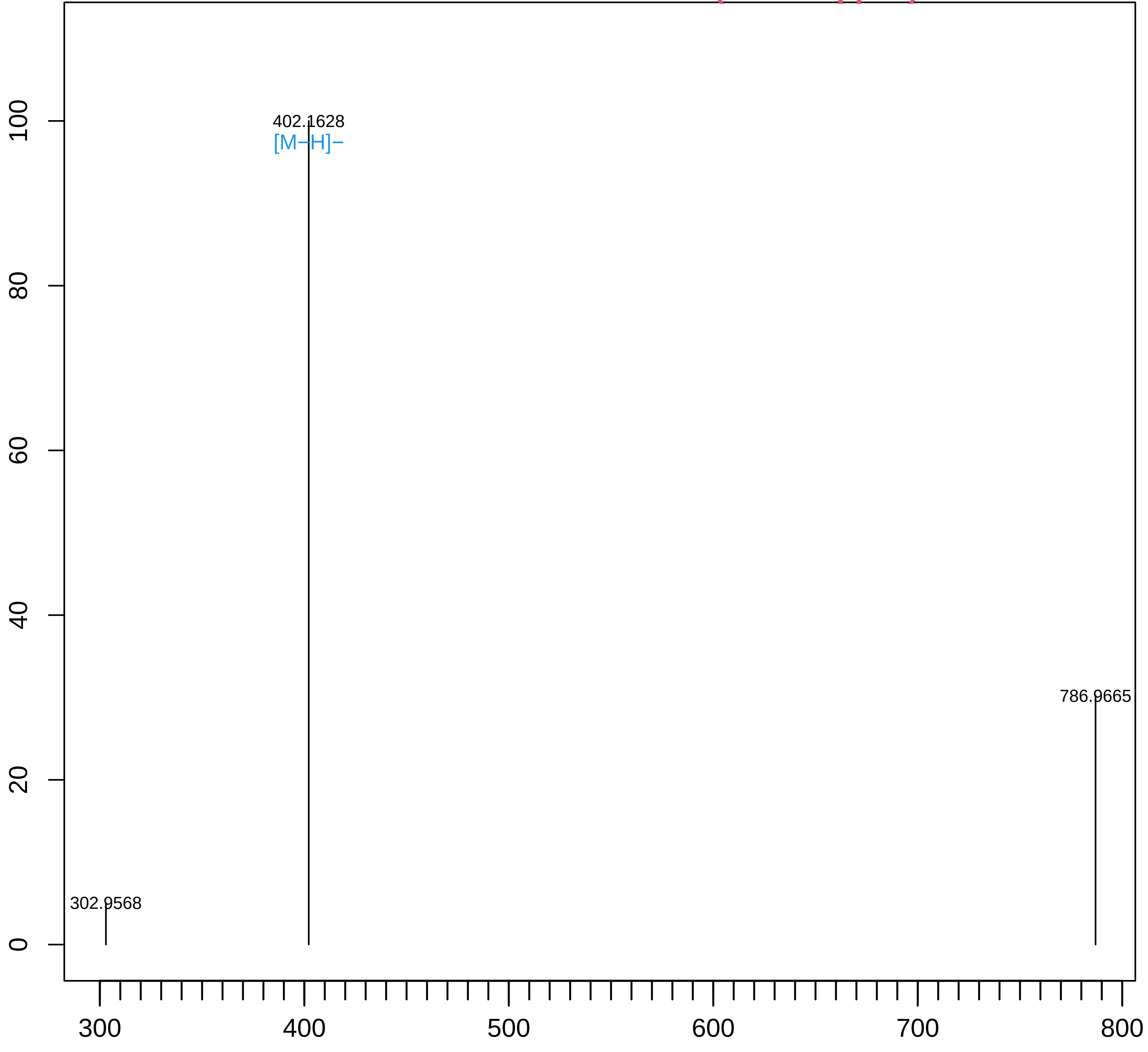

3767 : M.findmain = 403.1701 (+/− 20 ppm)

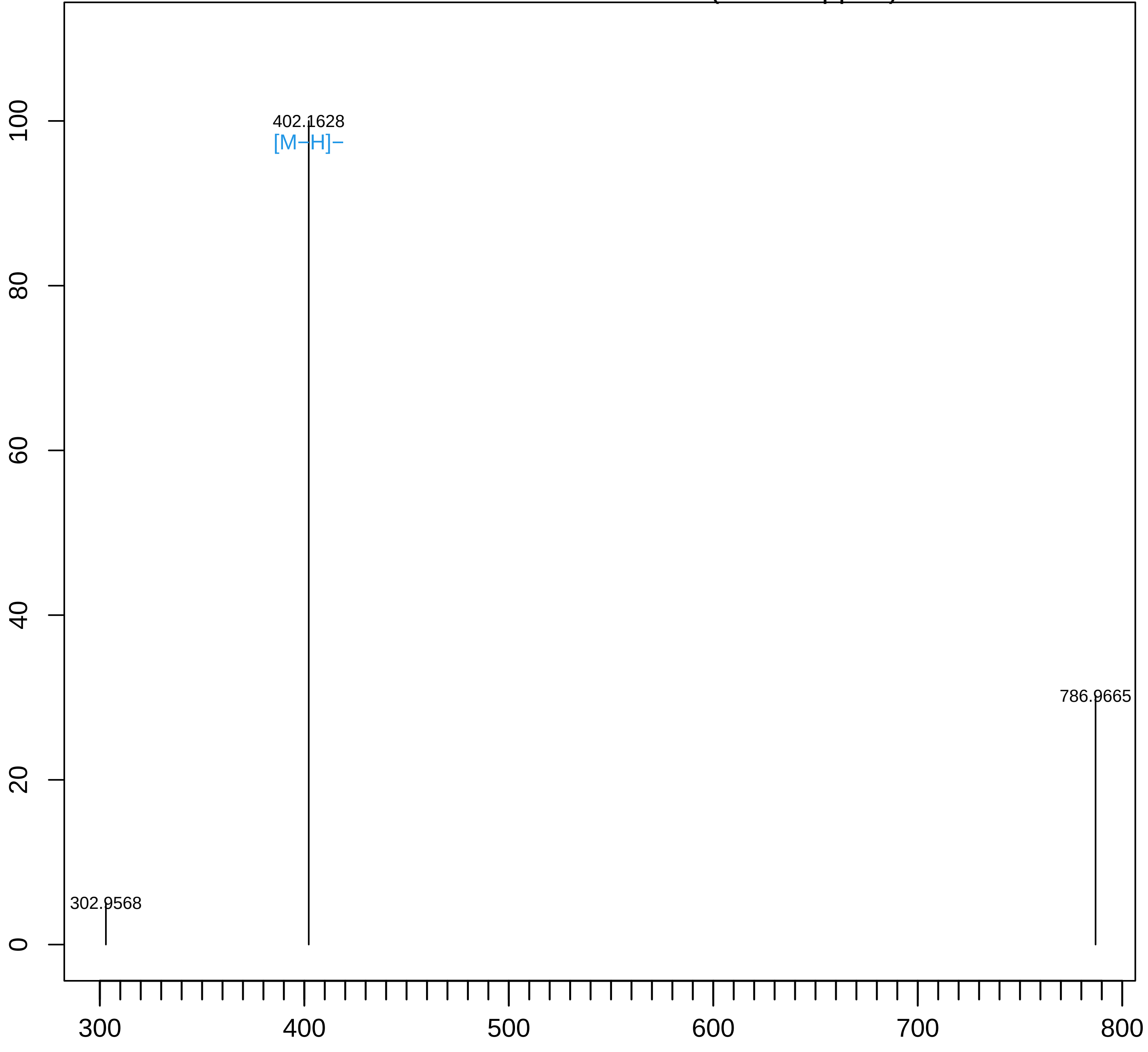

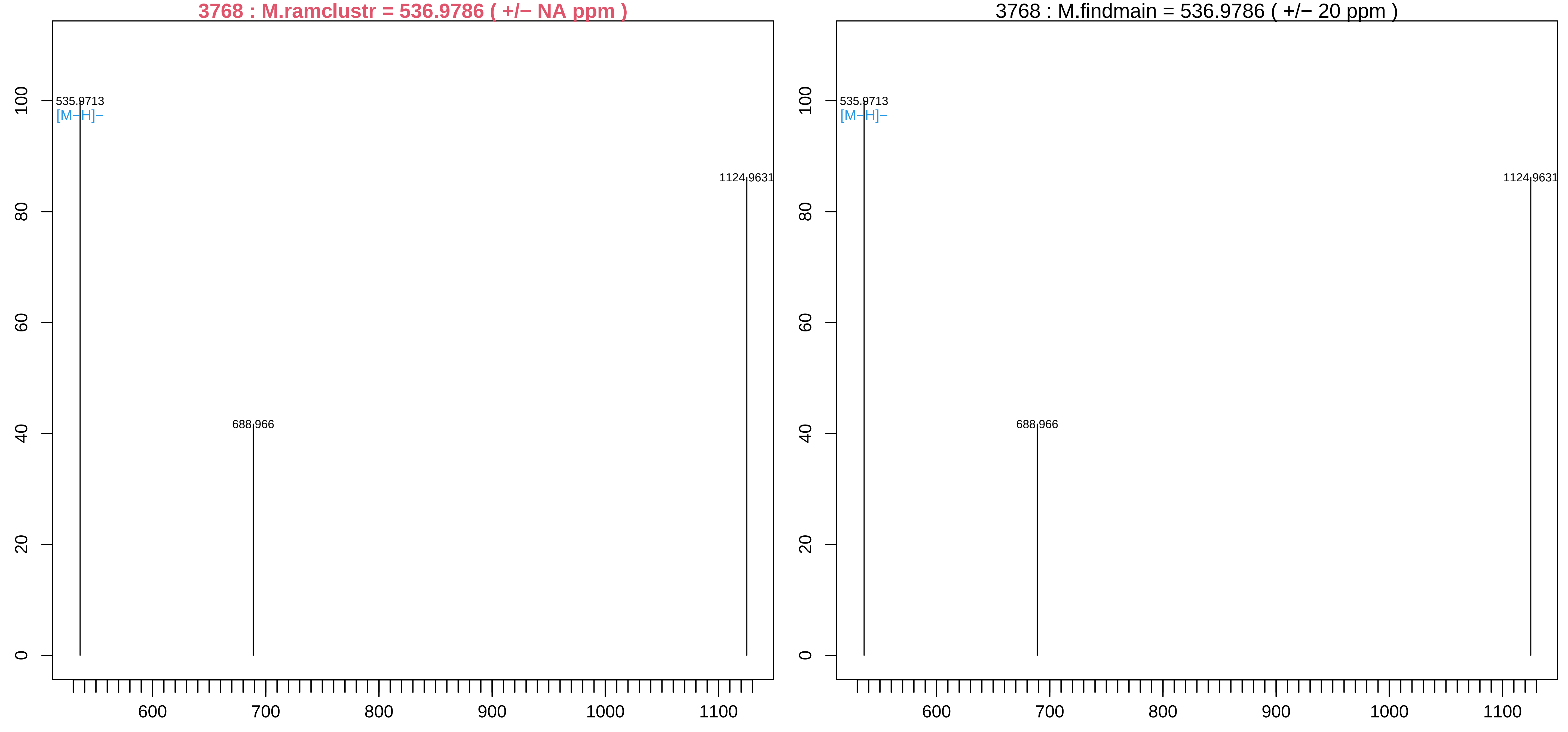
3768 : M.ramclustr = 536.9786 (+/− NA ppm)
535.9713
[M−H]−
688.966
1124.9631
0
20
40
60
80
100
600
700
800
900
1000
1100
3768 : M.findmain = 536.9786 (+/− 20 ppm)
535.9713
[M−H]−
688.966
1124.9631
0
20
40
60
80
100
600
700
800
900
1000
1100

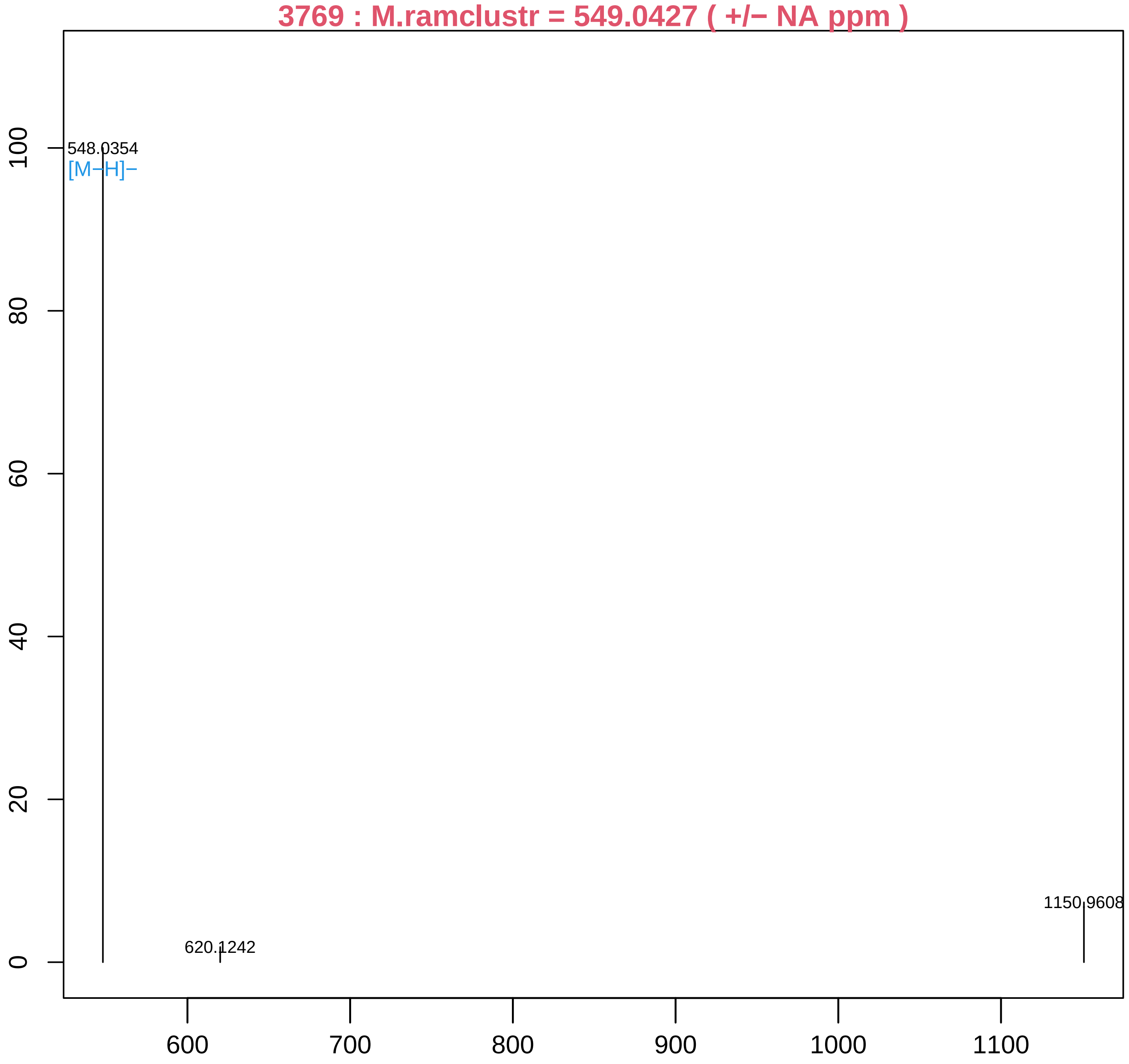
3769 : M.ramclustr = 549.0427 (+/− NA ppm)
548.0354
[M−H]−
620.1242
1150.9608
0
20
40
60
80
100
600
700
800
900
1000
1100

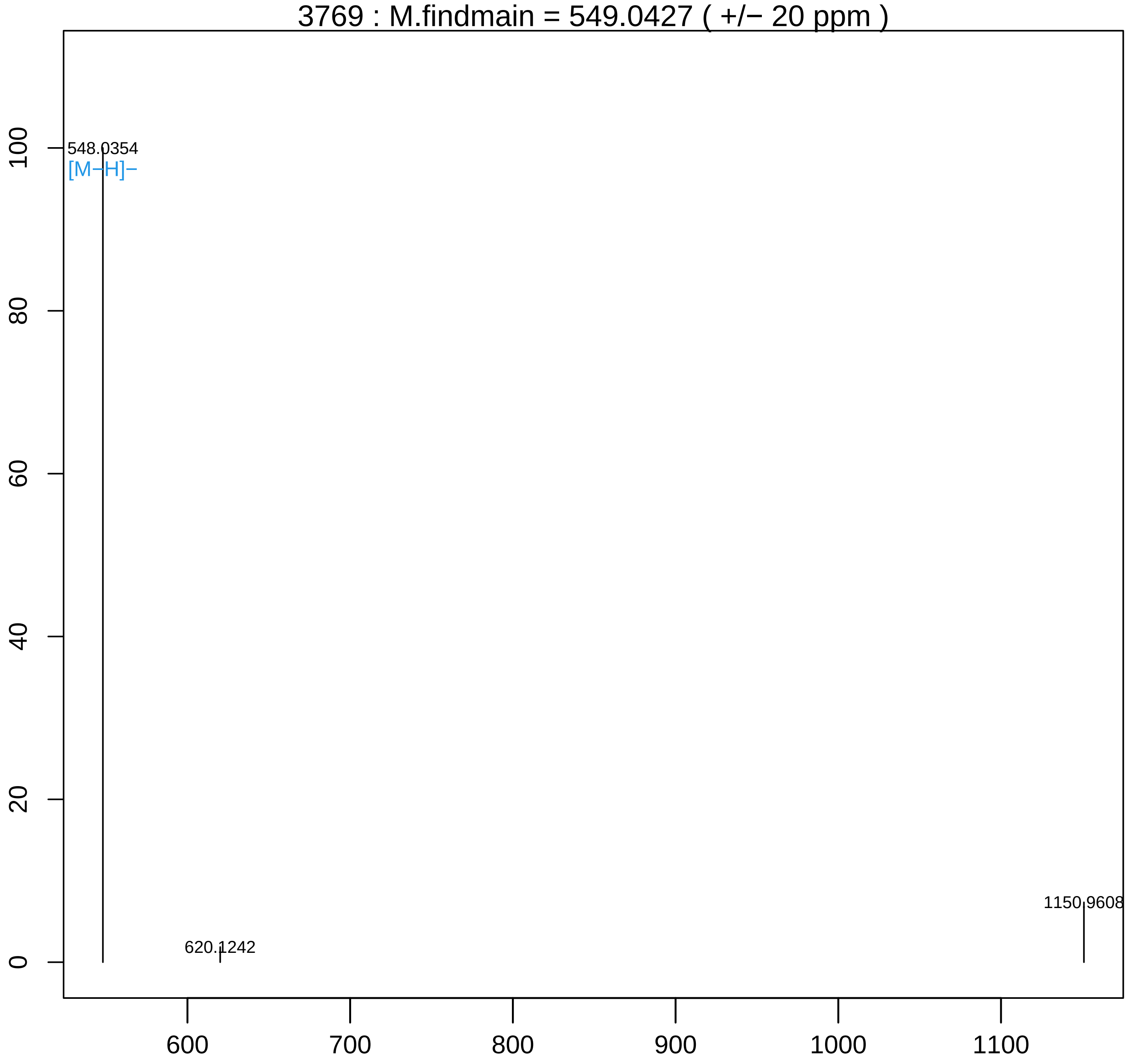
3769 : M.findmain = 549.0427 (+/− 20 ppm)
548.0354
[M−H]−
620.1242
1150.9608
0
20
40
60
80
100
600
700
800
900
1000
1100

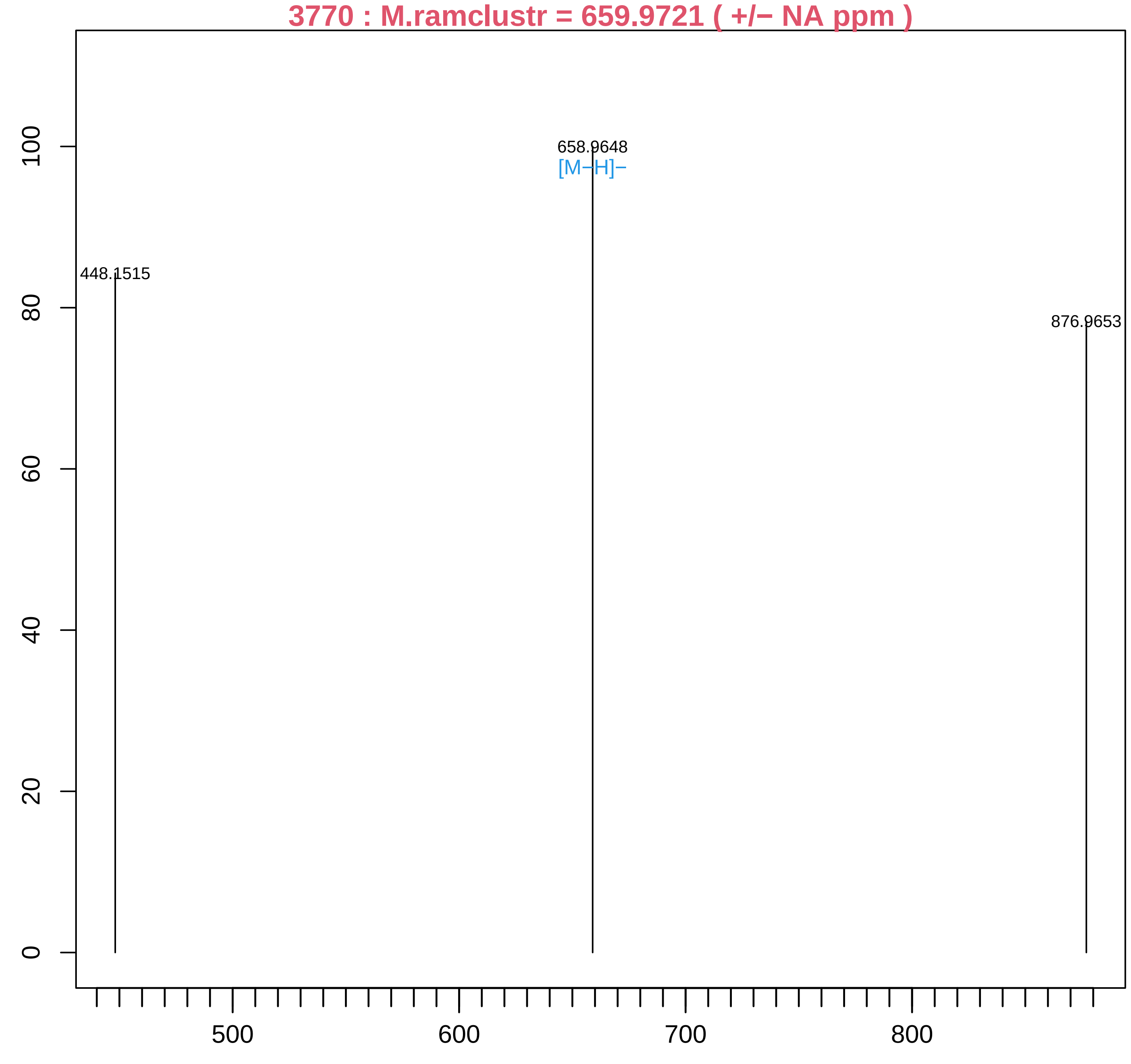
3770 : M.ramclustr = 659.9721 (+/− NA ppm)
658.9648
[M−H]−
448.1515
876.9653
0
20
40
60
80
100
500
600
700
800

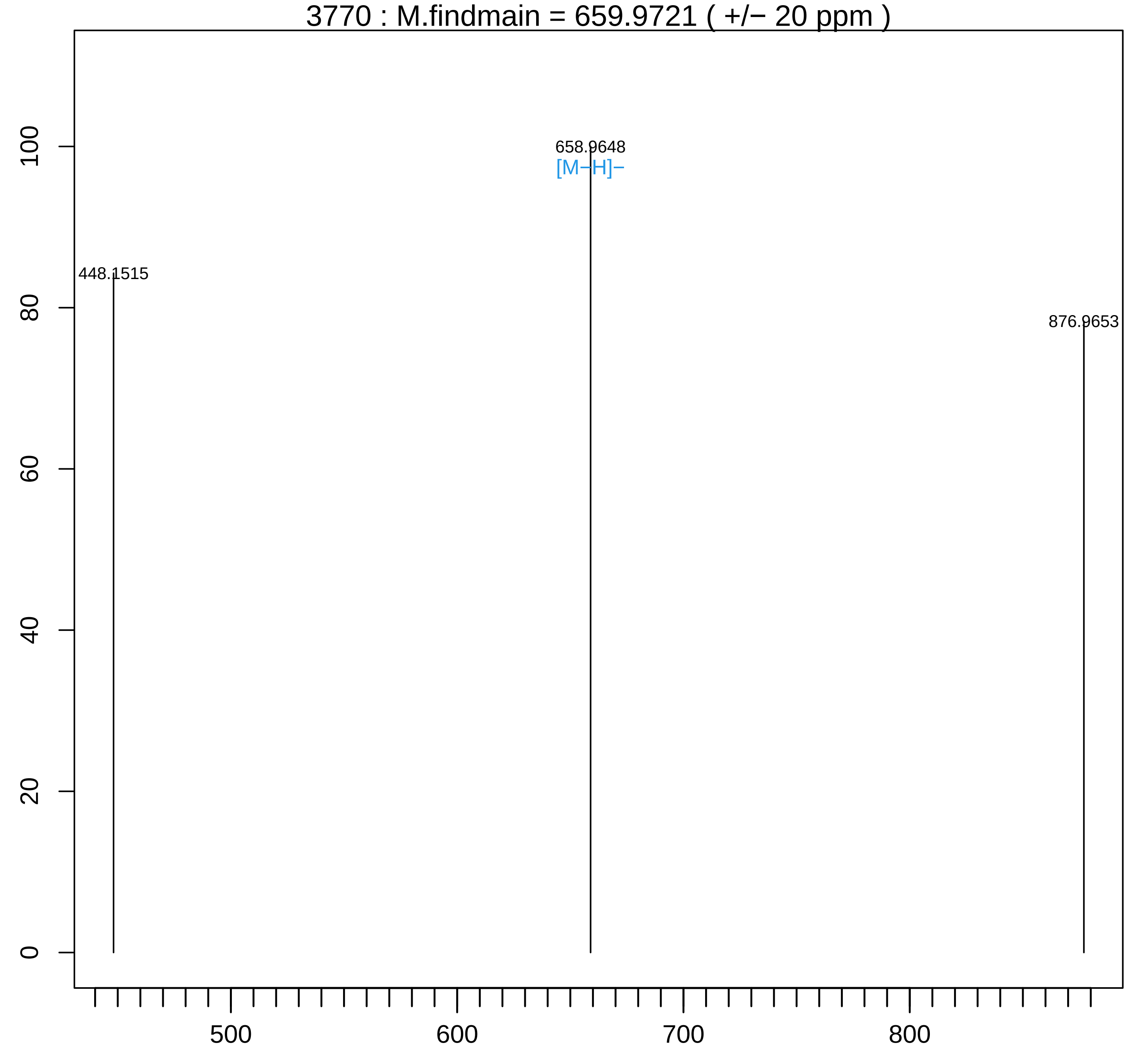
3770 : M.findmain = 659.9721 (+/− 20 ppm)
658.9648
[M−H]−
448.1515
876.9653
0
20
40
60
80
100
500
600
700
800

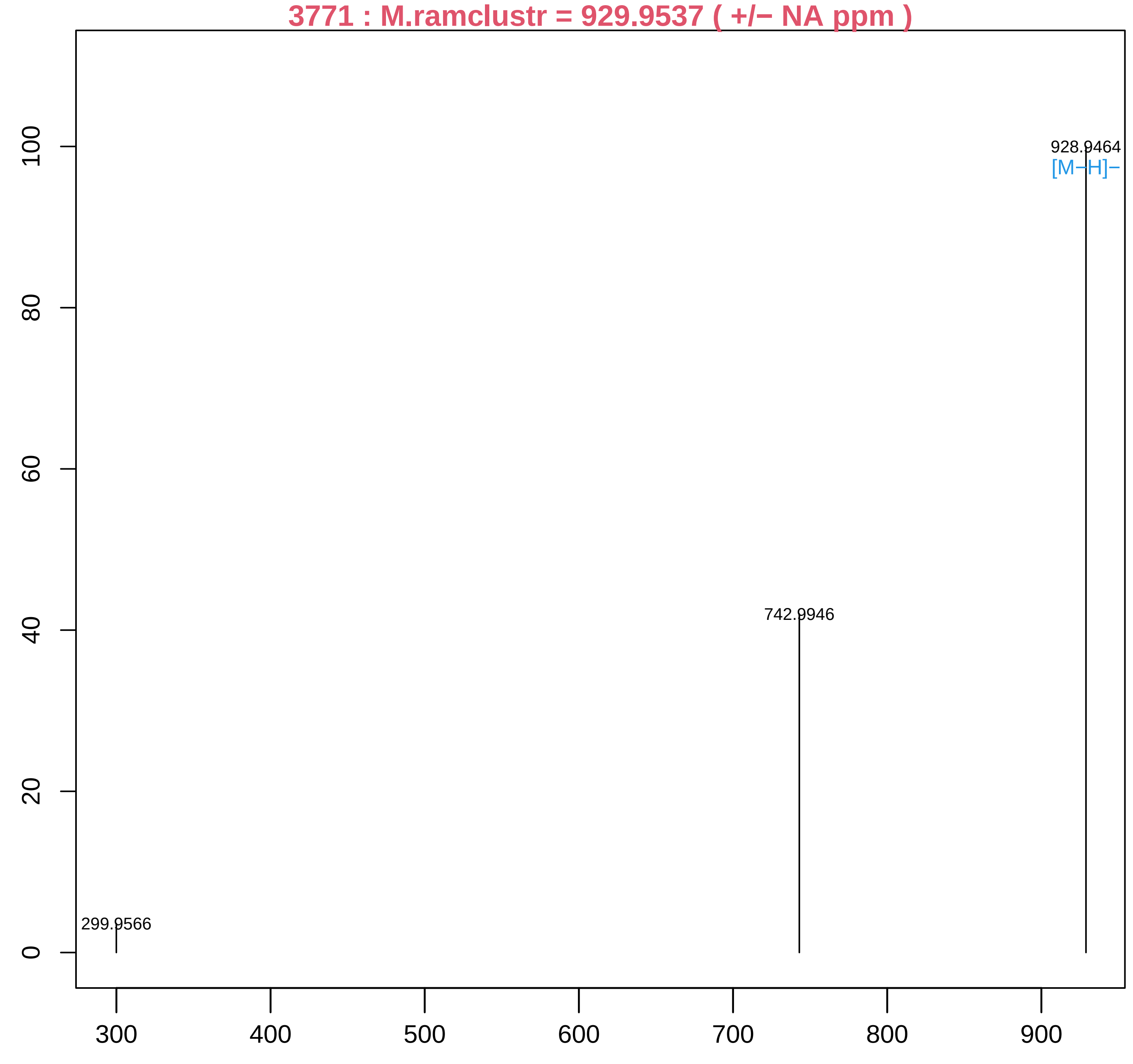

3771 : M.ramclustr = 929.9537 (+/− NA ppm)
928.9464
[M−H]−
742.9946
299.9566
0
20
40
60
80
100
300
400
500
600
700
800
900

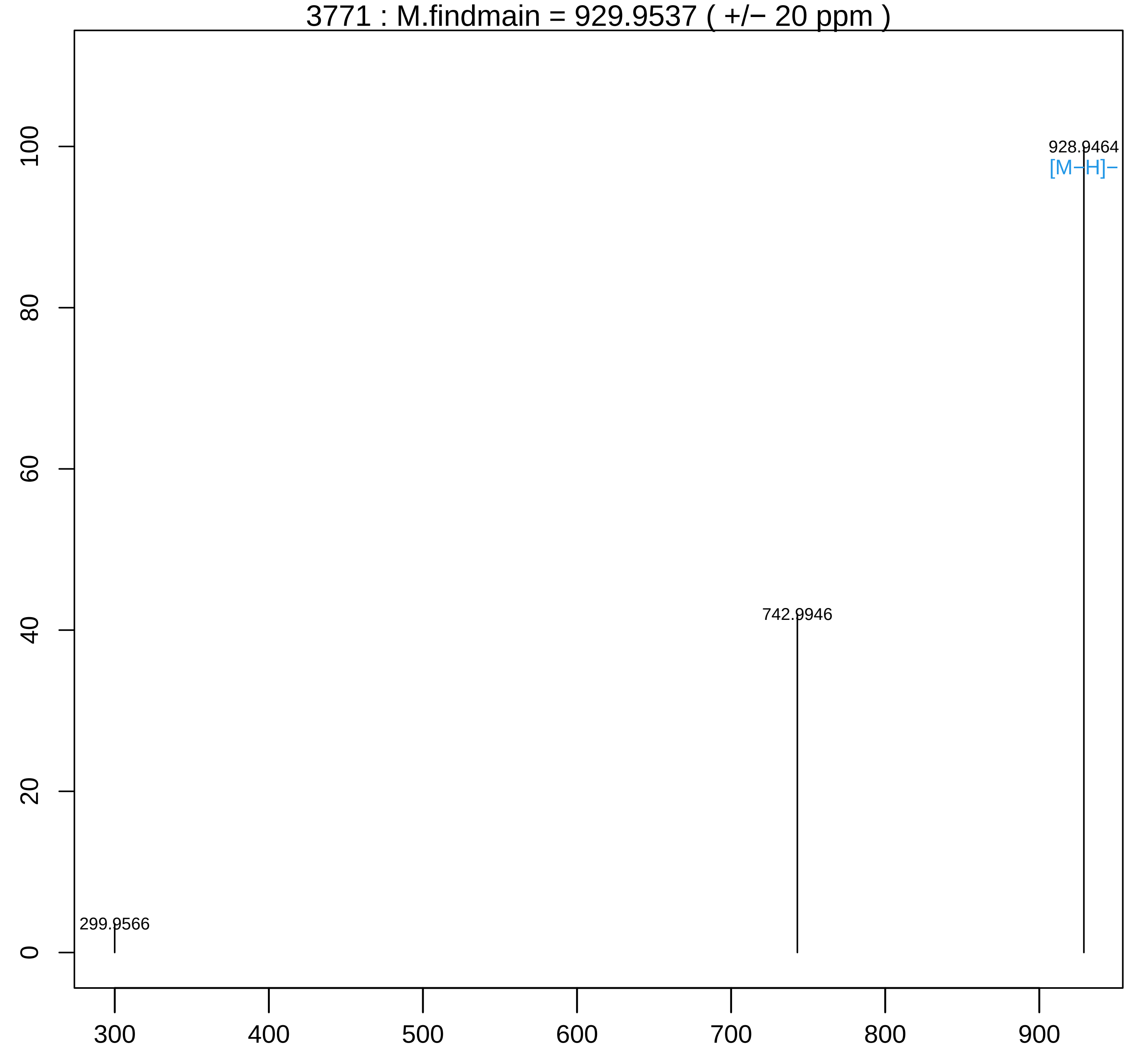

3771 : M.findmain = 929.9537 (+/− 20 ppm)
928.9464
[M−H]−
742.9946
299.9566
0
20
40
60
80
100
300
400
500
600
700
800
900

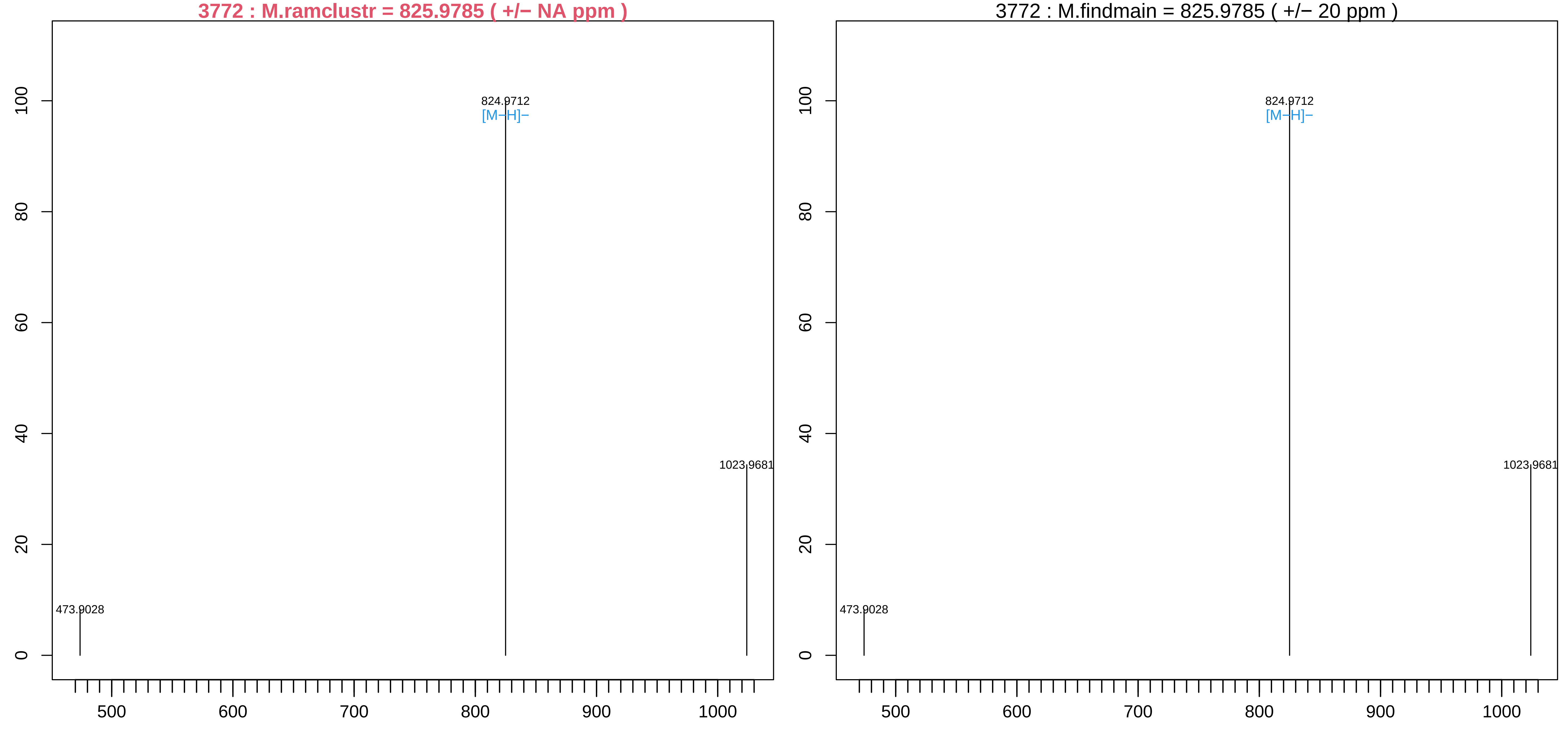
3772 : M.ramclustr = 825.9785 (+/− NA ppm)
824.9712
[M−H]−
1023.9681
473.9028
3772 : M.findmain = 825.9785 (+/− 20 ppm)
824.9712
[M−H]−
1023.9681
473.9028
0
20
40
60
80
100
500
600
700
800
900
1000

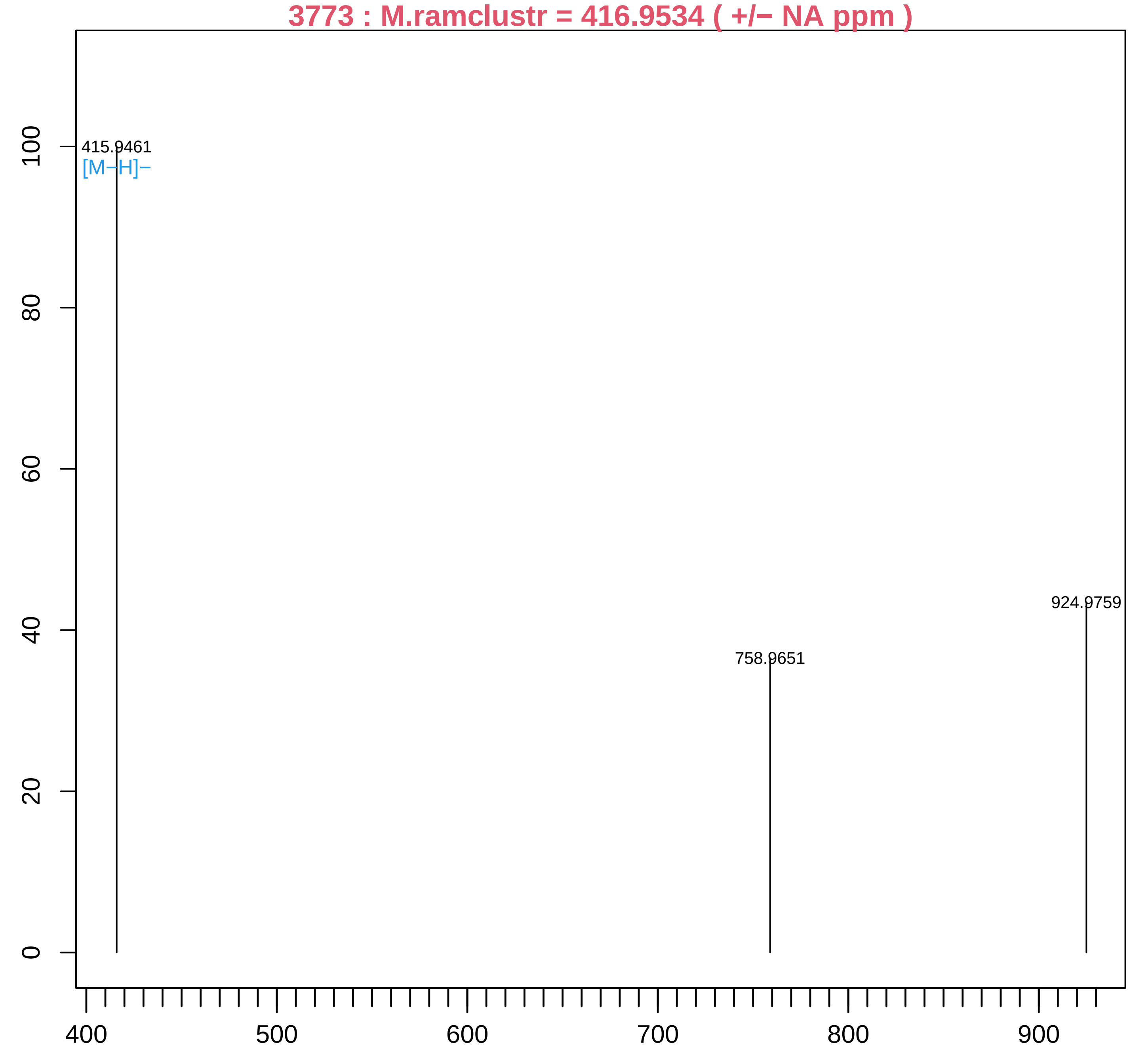
3773 : M.ramclustr = 416.9534 (+/− NA ppm)
415.9461
[M−H]−
758.9651
924.9759
0
20
40
60
80
100
400
500
600
700
800
900
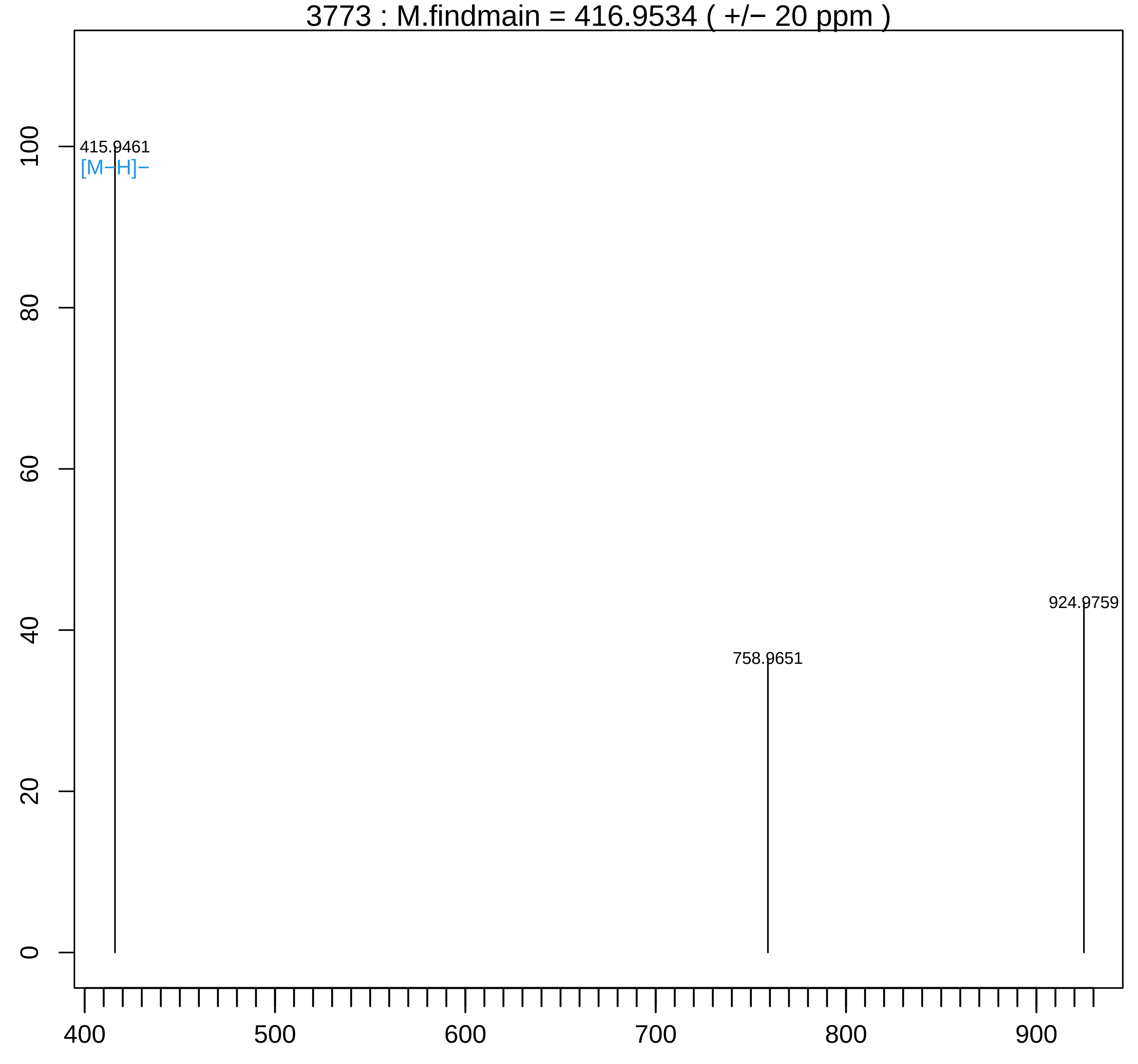
3773 : M.findmain = 416.9534 (+/− 20 ppm)
415.9461
[M−H]−
758.9651
924.9759
0
20
40
60
80
100
400
500
600
700
800
900

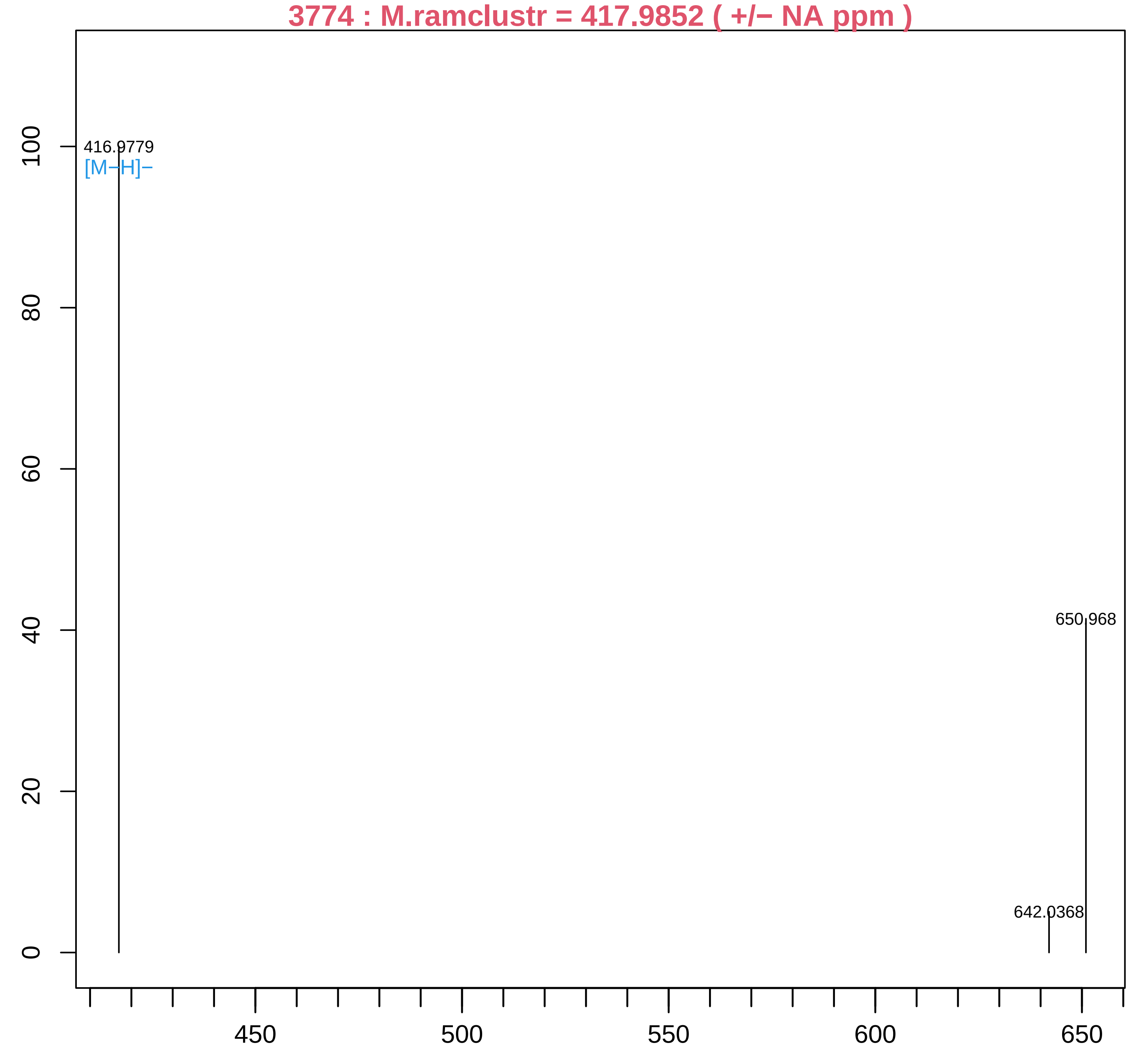
3774 : M.ramclustr = 417.9852 (+/− NA ppm)
416.9779
[M−H]−
650.968
642.0368
0
20
40
60
80
100
450
500
550
600
650

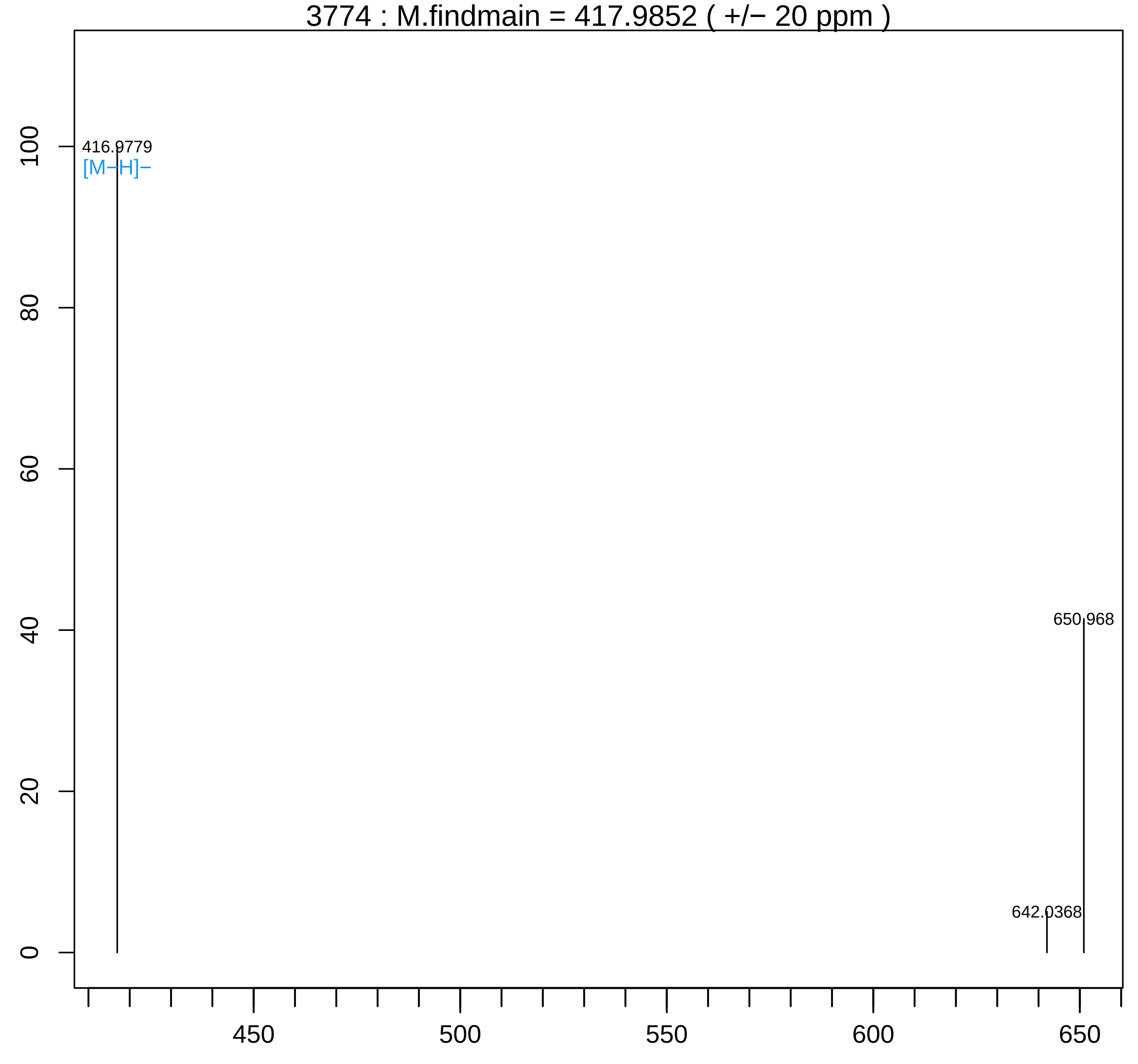
3774 : M.findmain = 417.9852 (+/− 20 ppm)
416.9779
[M−H]−
650.968
642.0368
0
20
40
60
80
100
450
500
550
600
650

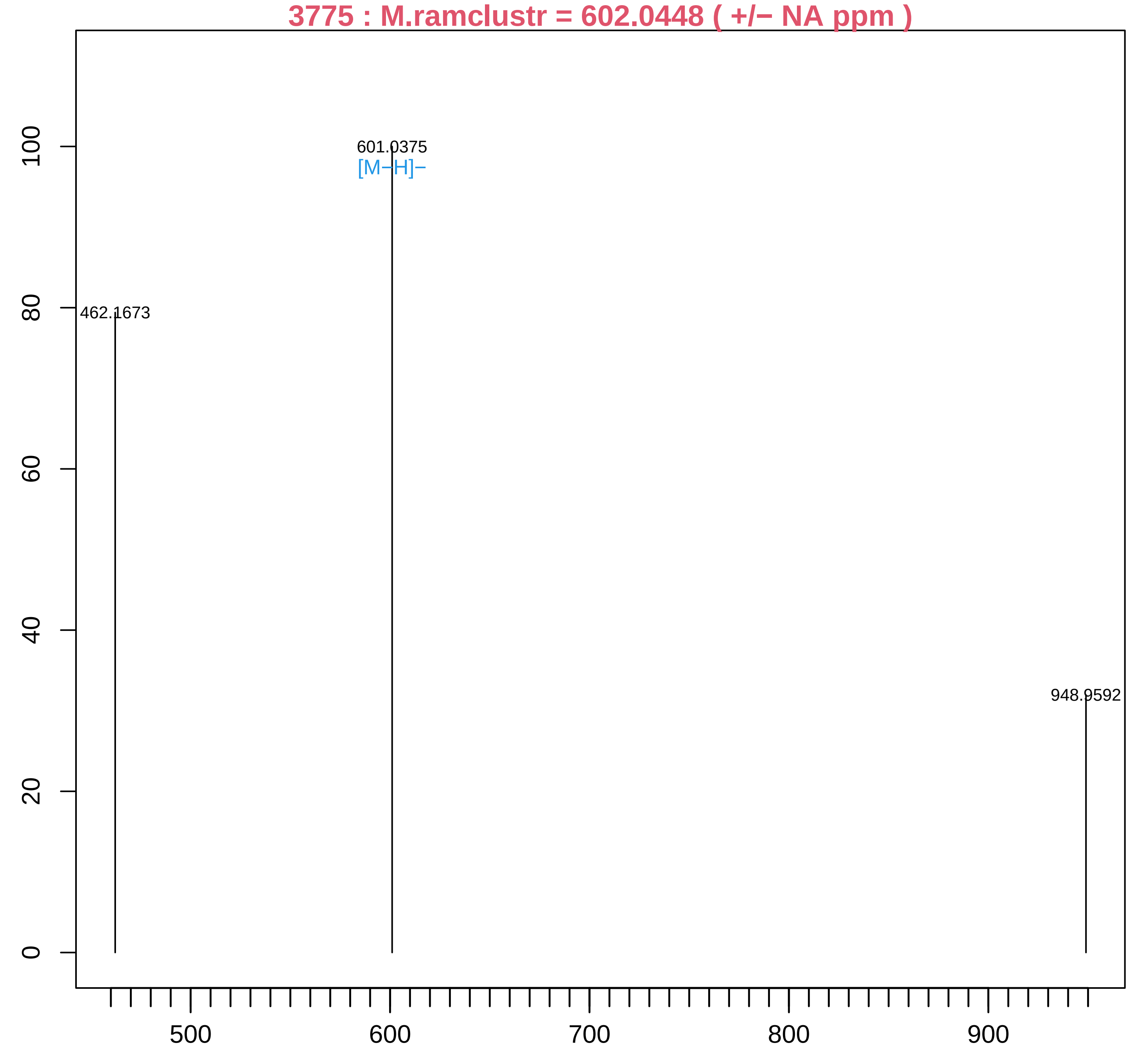

3775 : M.ramclustr = 602.0448 (+/− NA ppm)
601.0375
[M−H]−
462.1673
948.9592
0
20
40
60
80
100
500
600
700
800
900

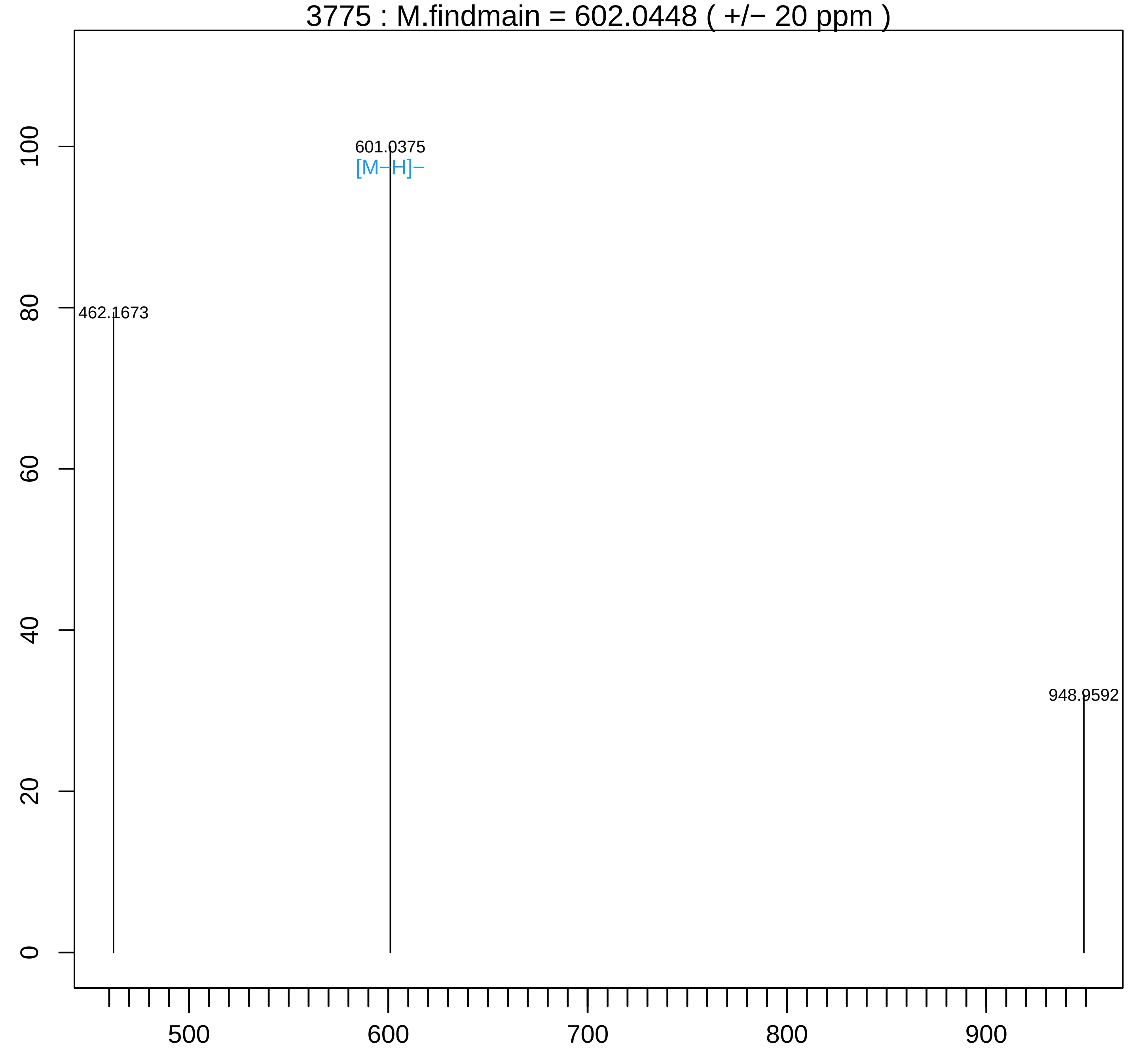

3775 : M.findmain = 602.0448 (+/− 20 ppm)
601.0375
[M−H]−
462.1673
948.9592
0
20
40
60
80
100
500
600
700
800
900

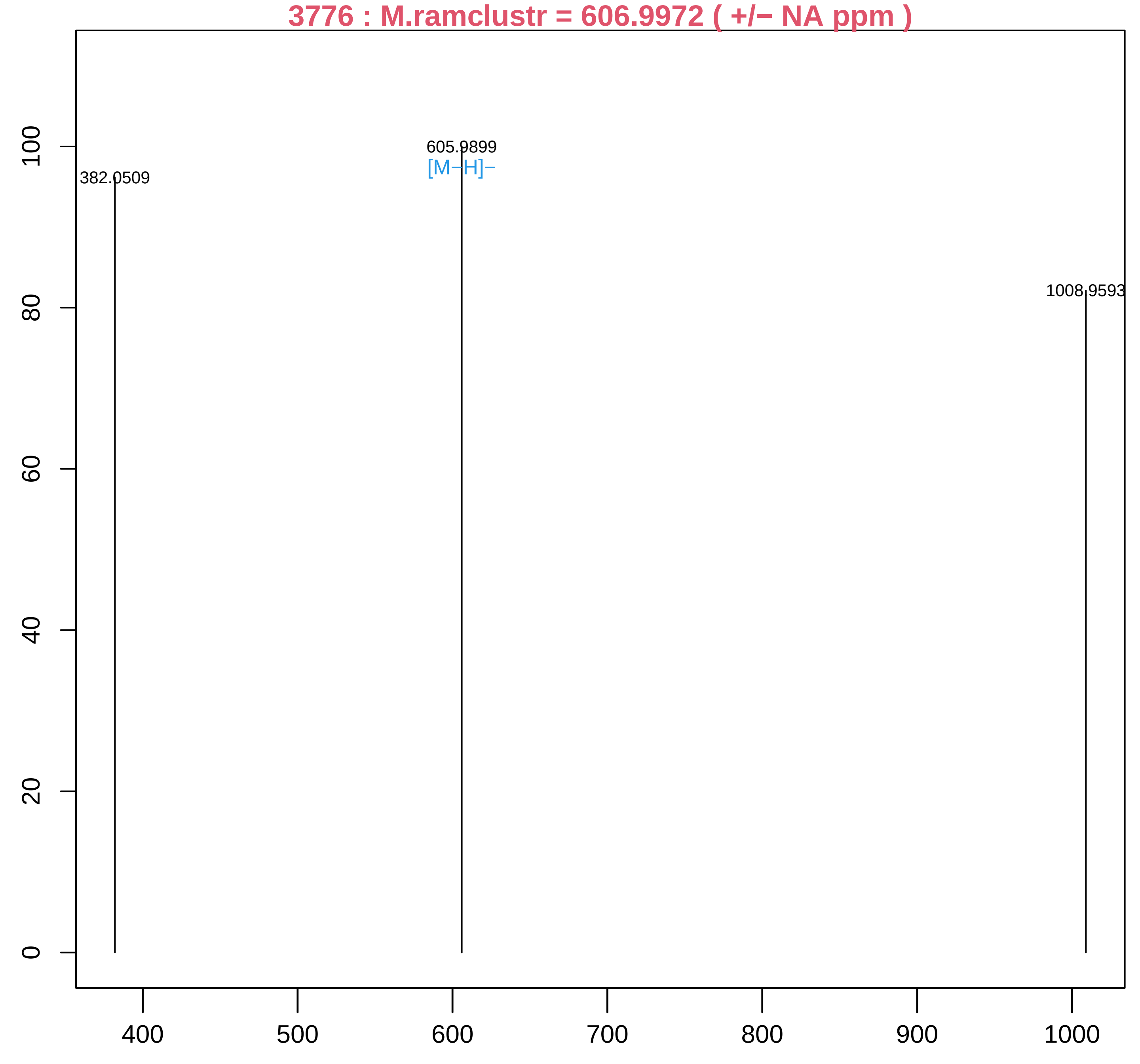
3776 : M.ramclustr = 606.9972 (+/− NA ppm)
382.0509
605.9899
[M−H]−
1008.9593
0
20
40
60
80
100
400
500
600
700
800
900
1000
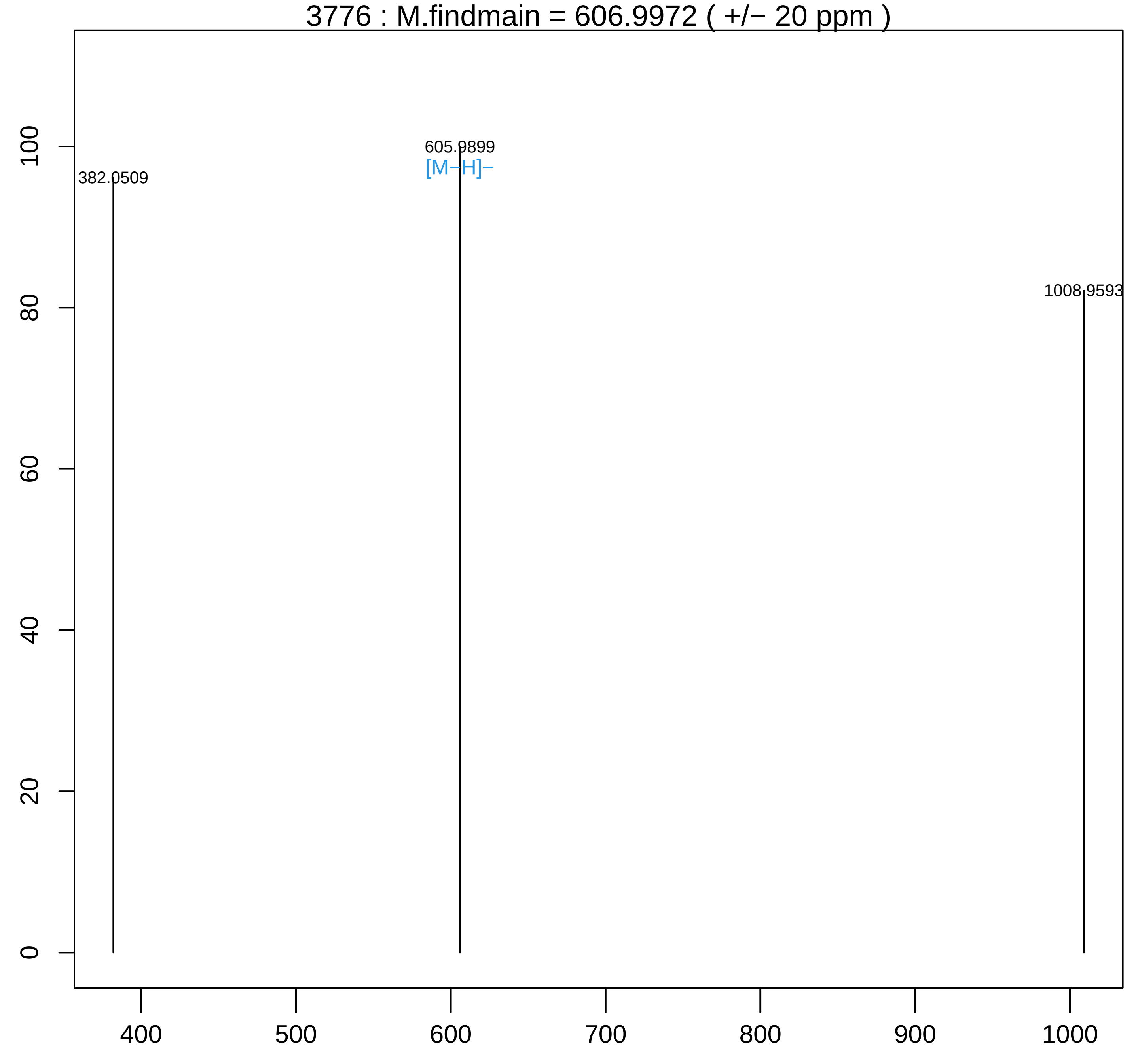
3776 : M.findmain = 606.9972 (+/− 20 ppm)
382.0509
605.9899
[M−H]−
1008.9593
0
20
40
60
80
100
400
500
600
700
800
900
1000

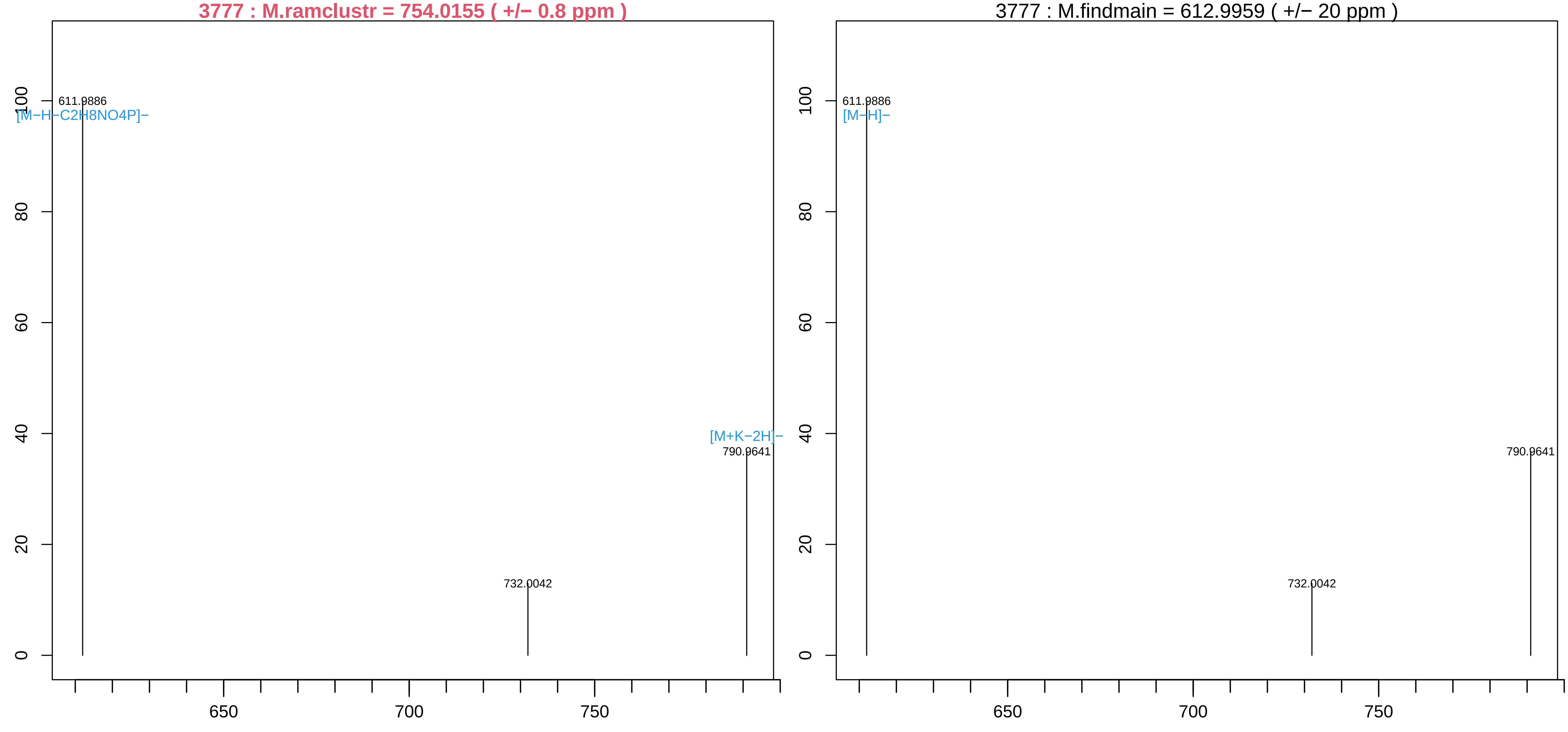

3777 : M.ramclustr = 754.0155 (+/− 0.8 ppm)
611.9886
[M−H−C2H8NO4P]−
732.0042
[M+K−2H]−
790.9641
3777 : M.findmain = 612.9959 (+/− 20 ppm)
611.9886
[M−H]−
732.0042
790.9641

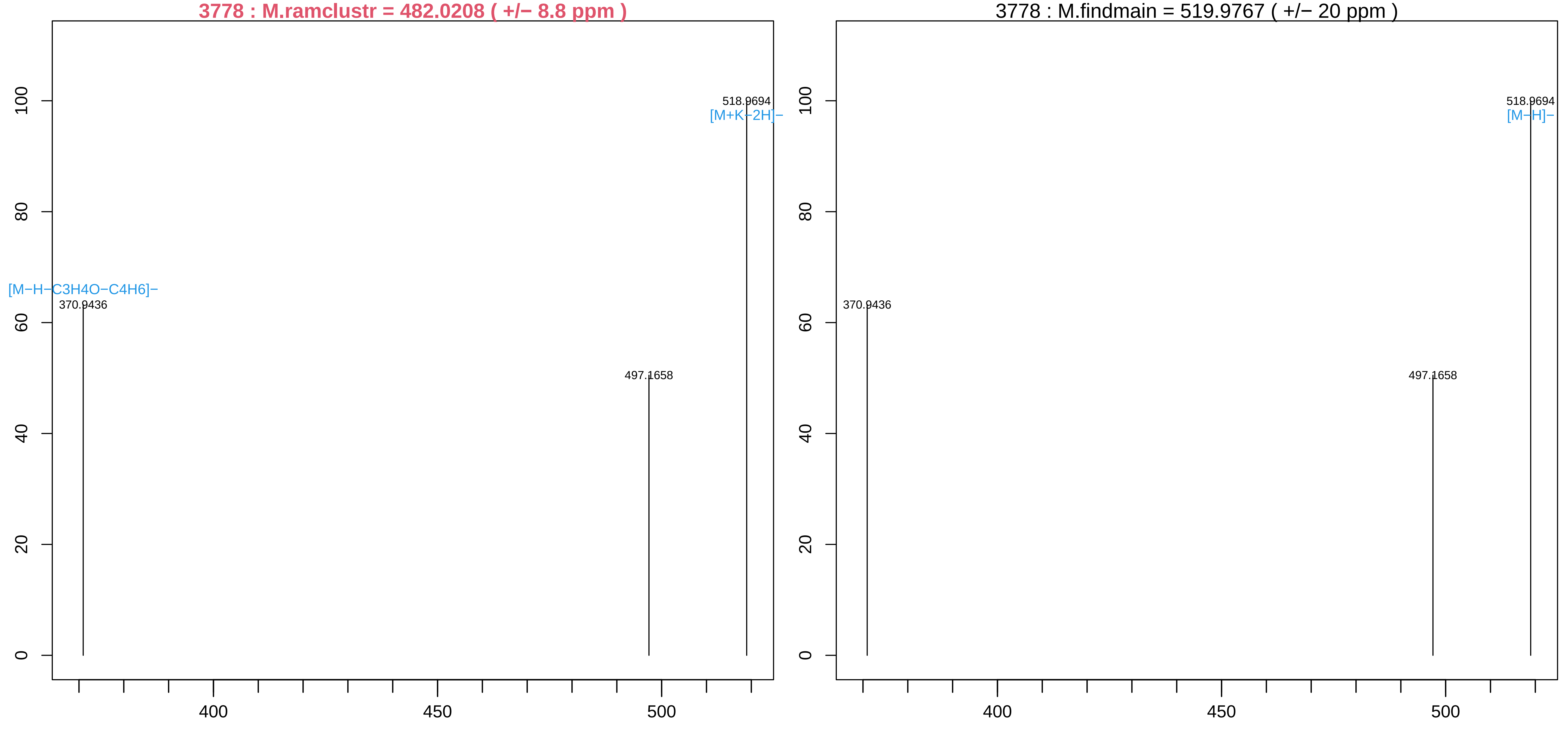

3778 : M.ramclustr = 482.0208 (+/− 8.8 ppm)
518.9694
[M+K−2H]−
[M−H−C3H4O−C4H6]−
370.9436
497.1658
3778 : M.findmain = 519.9767 (+/− 20 ppm)
518.9694
[M−H]−
370.9436
497.1658
0
20
40
60
80
100
400
450
500

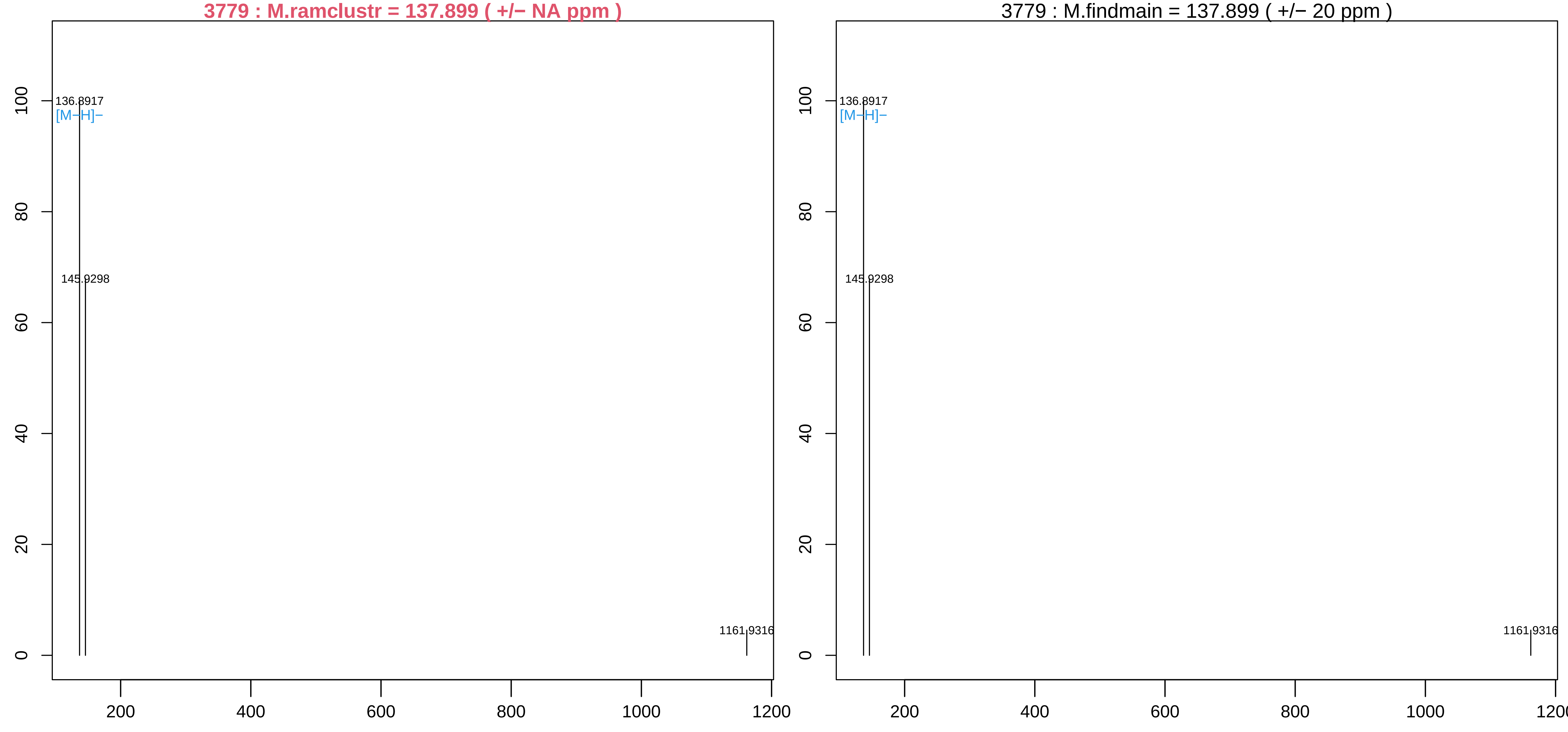
3779 : M.ramclustr = 137.899 (+/− NA ppm)
136.8917
[M−H]−
145.9298
1161.9316
3779 : M.findmain = 137.899 (+/− 20 ppm)
136.8917
[M−H]−
145.9298
1161.9316
0
20
40
60
80
100
200
400
600
800
1000
1200

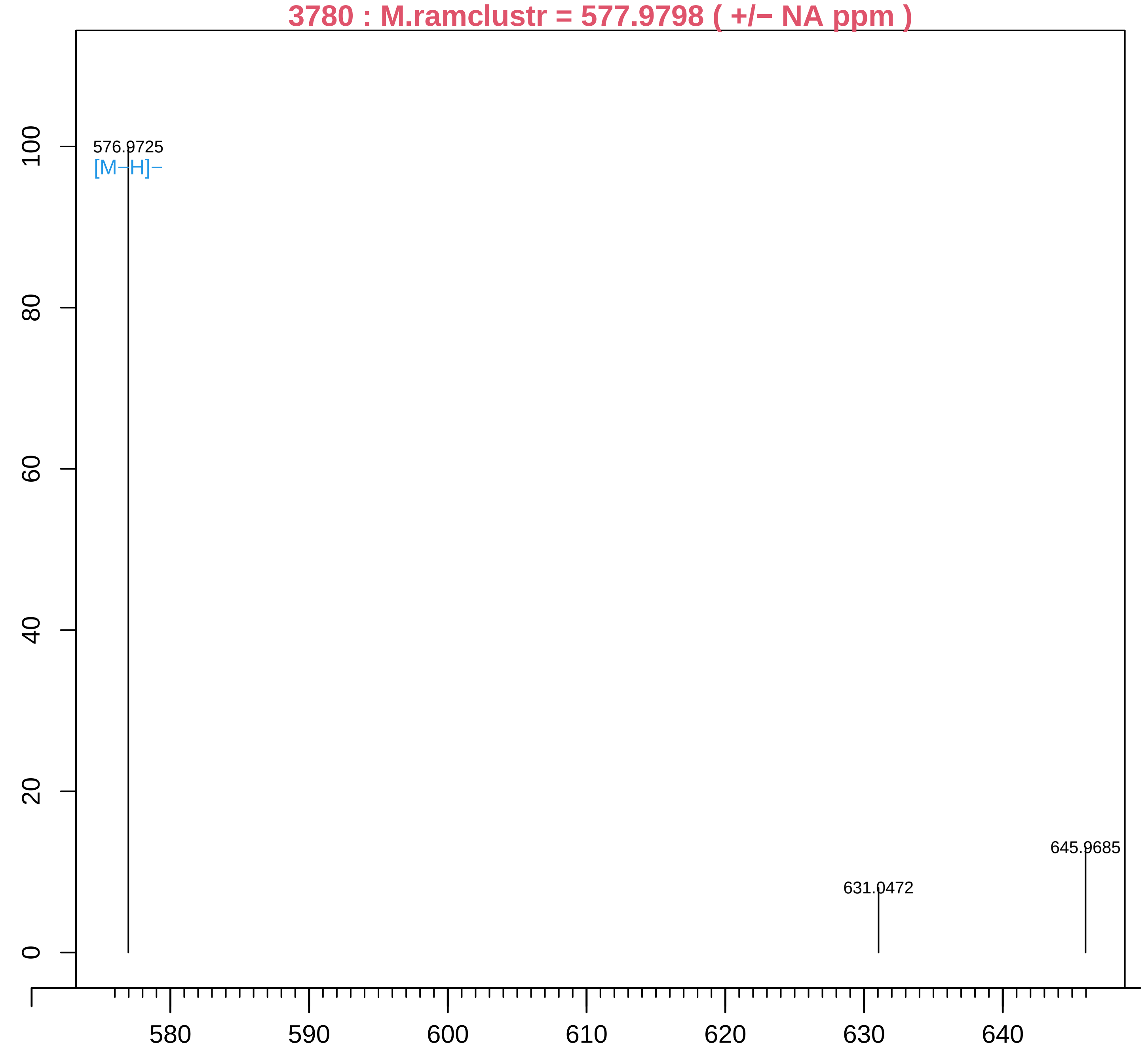
3780 : M.ramclustr = 577.9798 (+/− NA ppm)
576.9725
[M−H]−
631.0472
645.9685
0
20
40
60
80
100
580
590
600
610
620
630
640

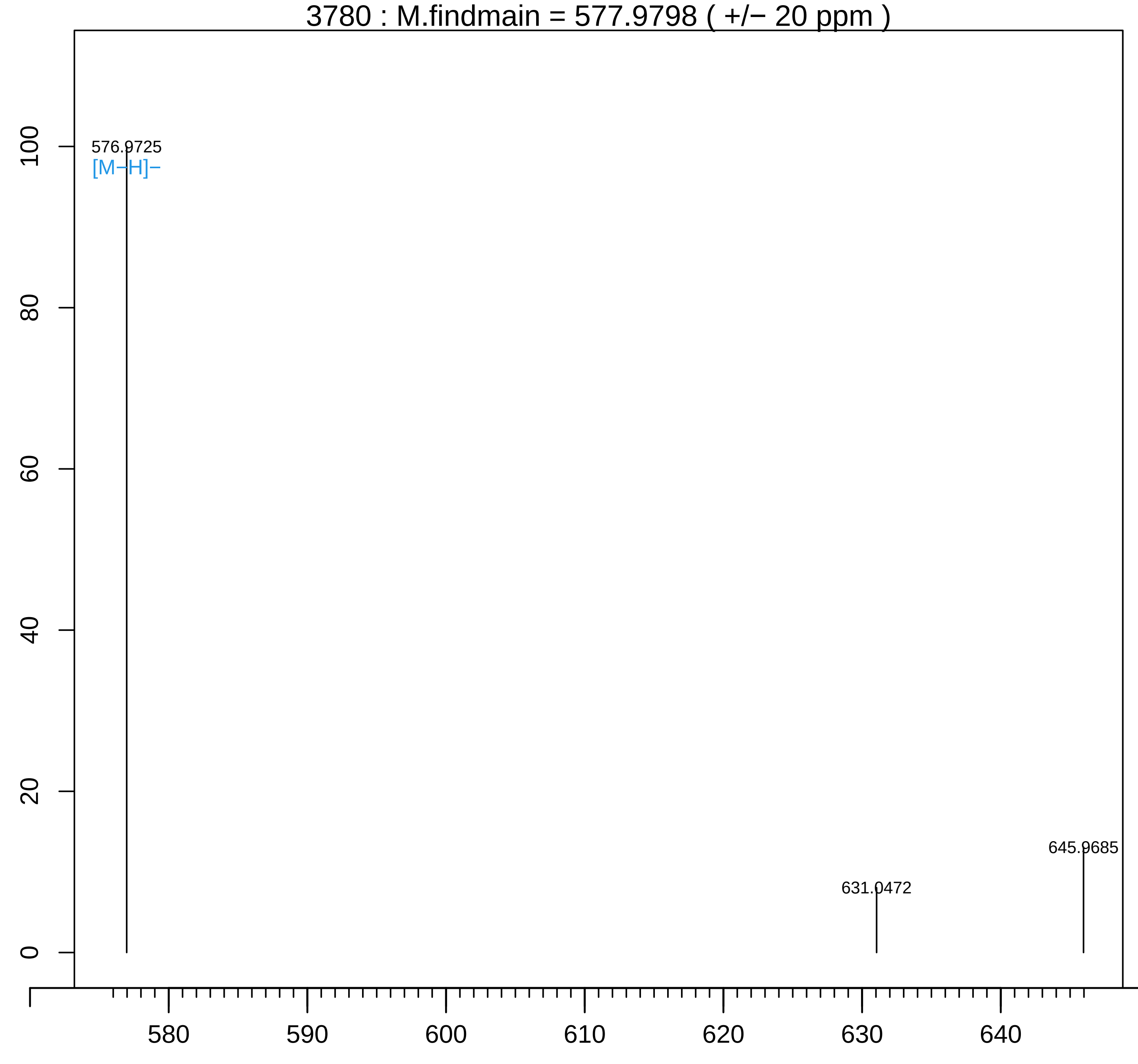
3780 : M.findmain = 577.9798 (+/− 20 ppm)
576.9725
[M−H]−
631.0472
645.9685
0
20
40
60
80
100
580
590
600
610
620
630
640

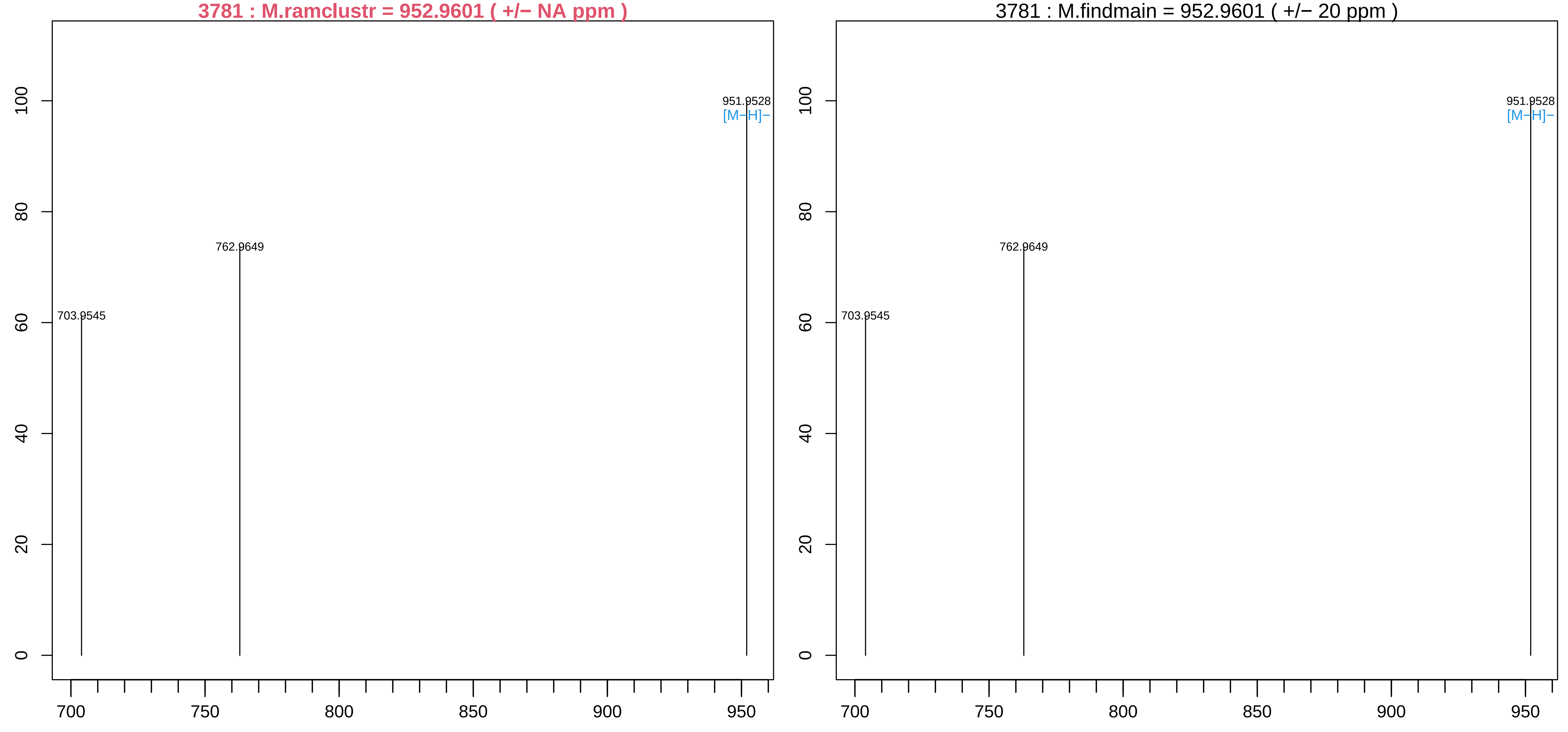
3781 : M.ramclustr = 952.9601 (+/− NA ppm)
3781 : M.findmain = 952.9601 (+/− 20 ppm)
703.9545
762.9649
951.9528
[M−H]−
0
20
40
60
80
100
700
750
800
850
900
950

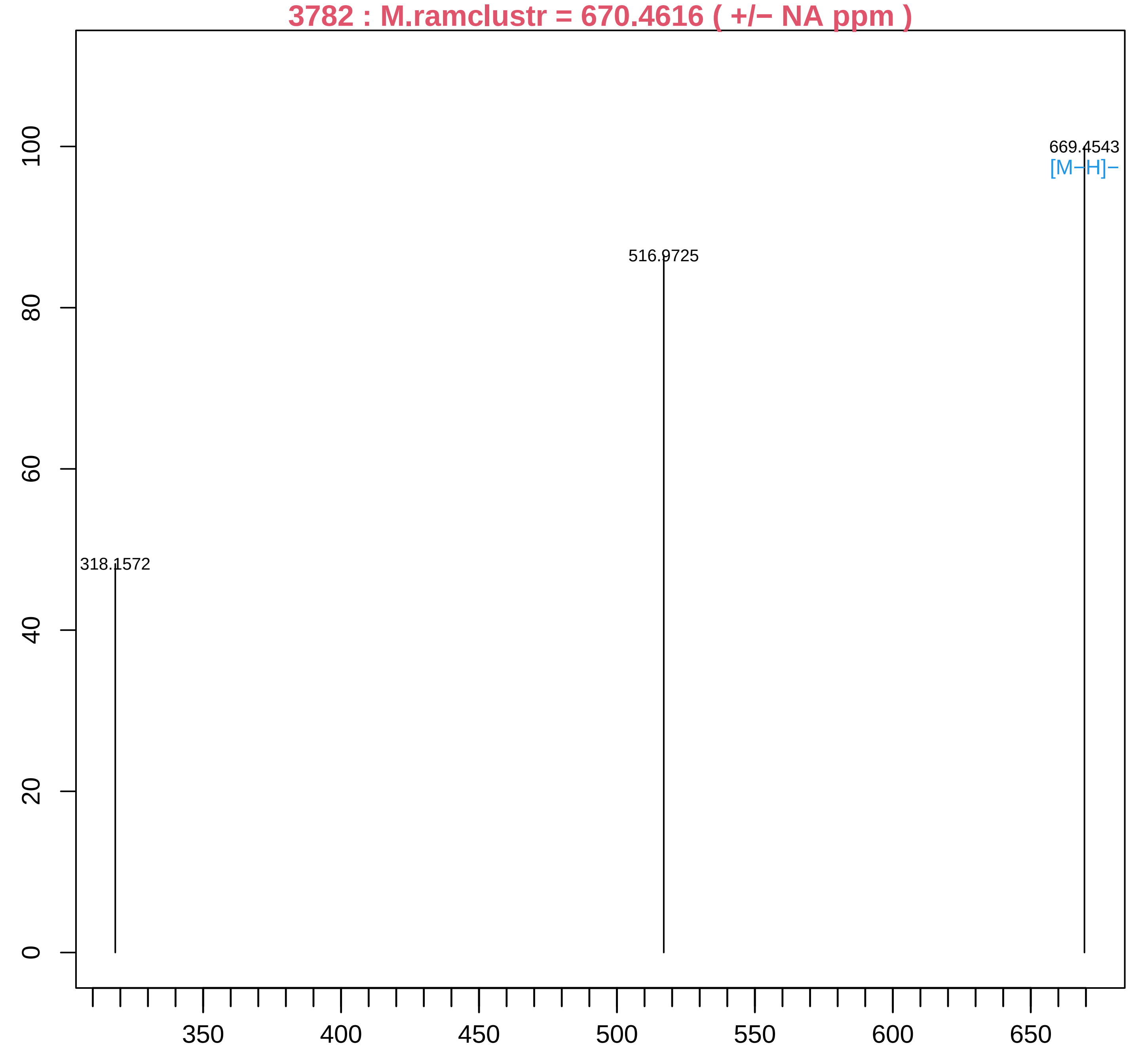

3782 : M.ramclustr = 670.4616 (+/− NA ppm)
669.4543
[M−H]−
516.9725
318.1572
0
20
40
60
80
100
350
400
450
500
550
600
650

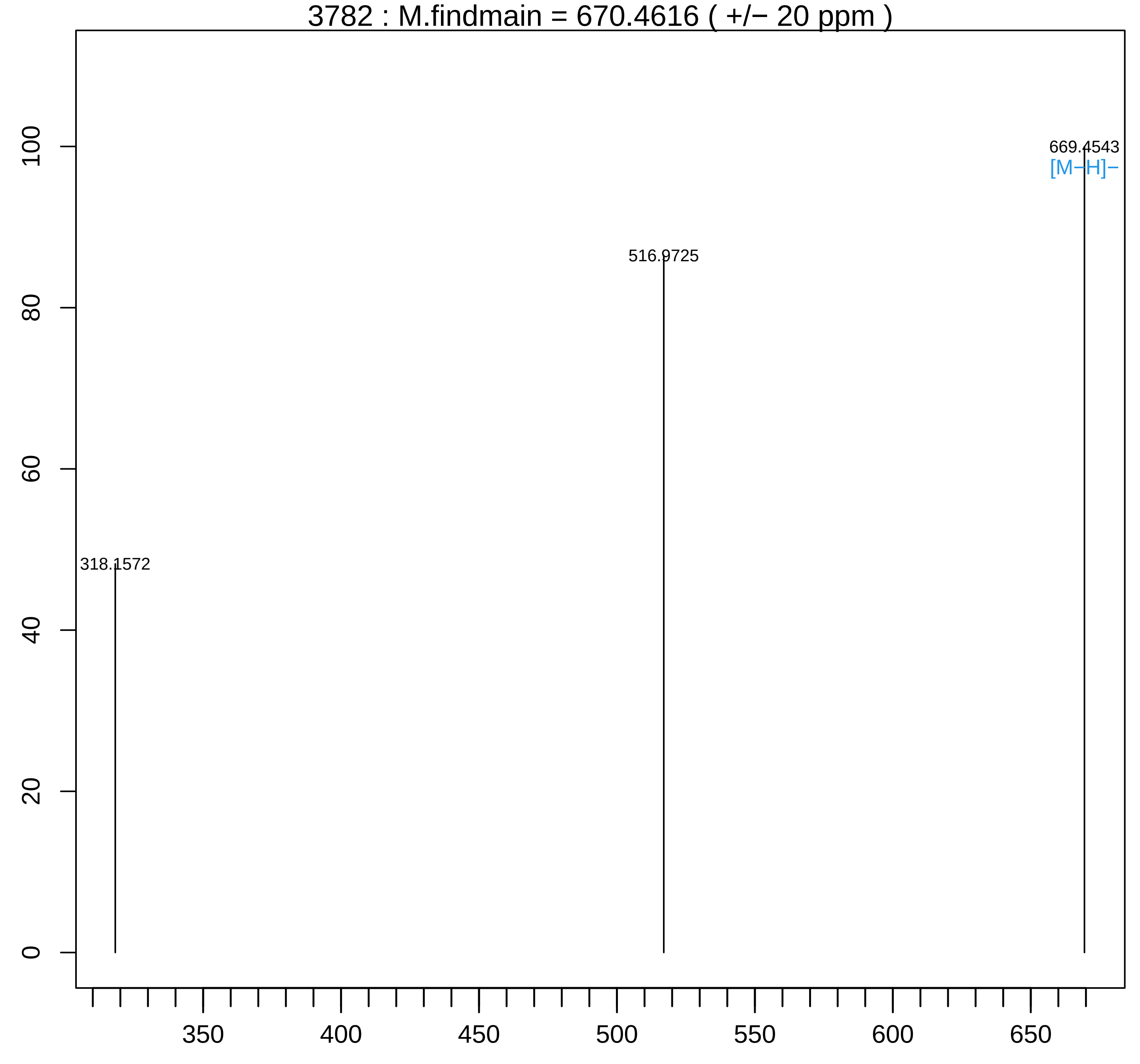

3782 : M.findmain = 670.4616 (+/− 20 ppm)
669.4543
[M−H]−
516.9725
318.1572
0
20
40
60
80
100
350
400
450
500
550
600
650

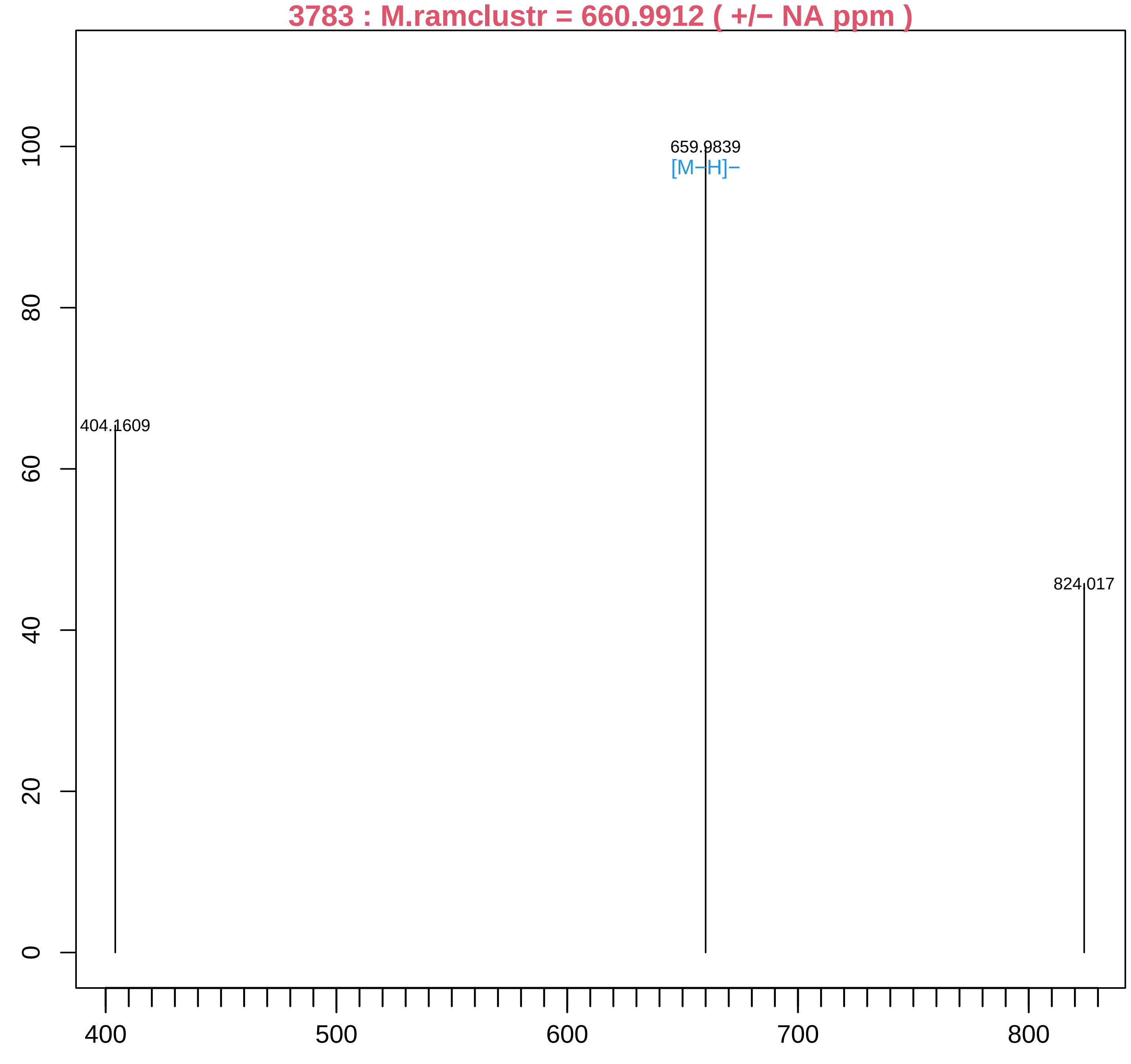
3783 : M.ramclustr = 660.9912 (+/− NA ppm)
659.9839
[M−H]−
404.1609
824.017
0
20
40
60
80
100
400
500
600
700
800

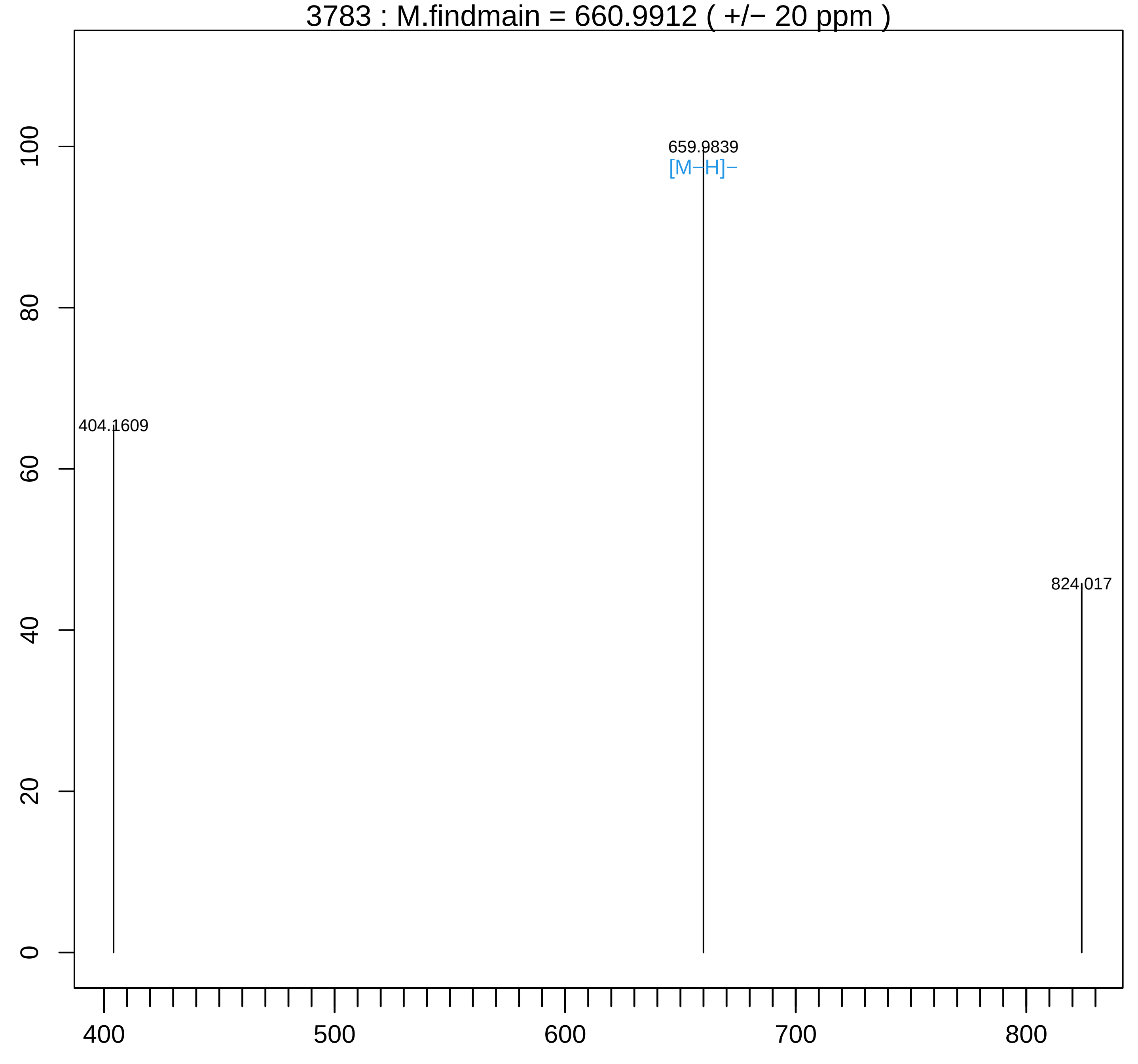
3783 : M.findmain = 660.9912 (+/− 20 ppm)
659.9839
[M−H]−
404.1609
824.017
0
20
40
60
80
100
400
500
600
700
800

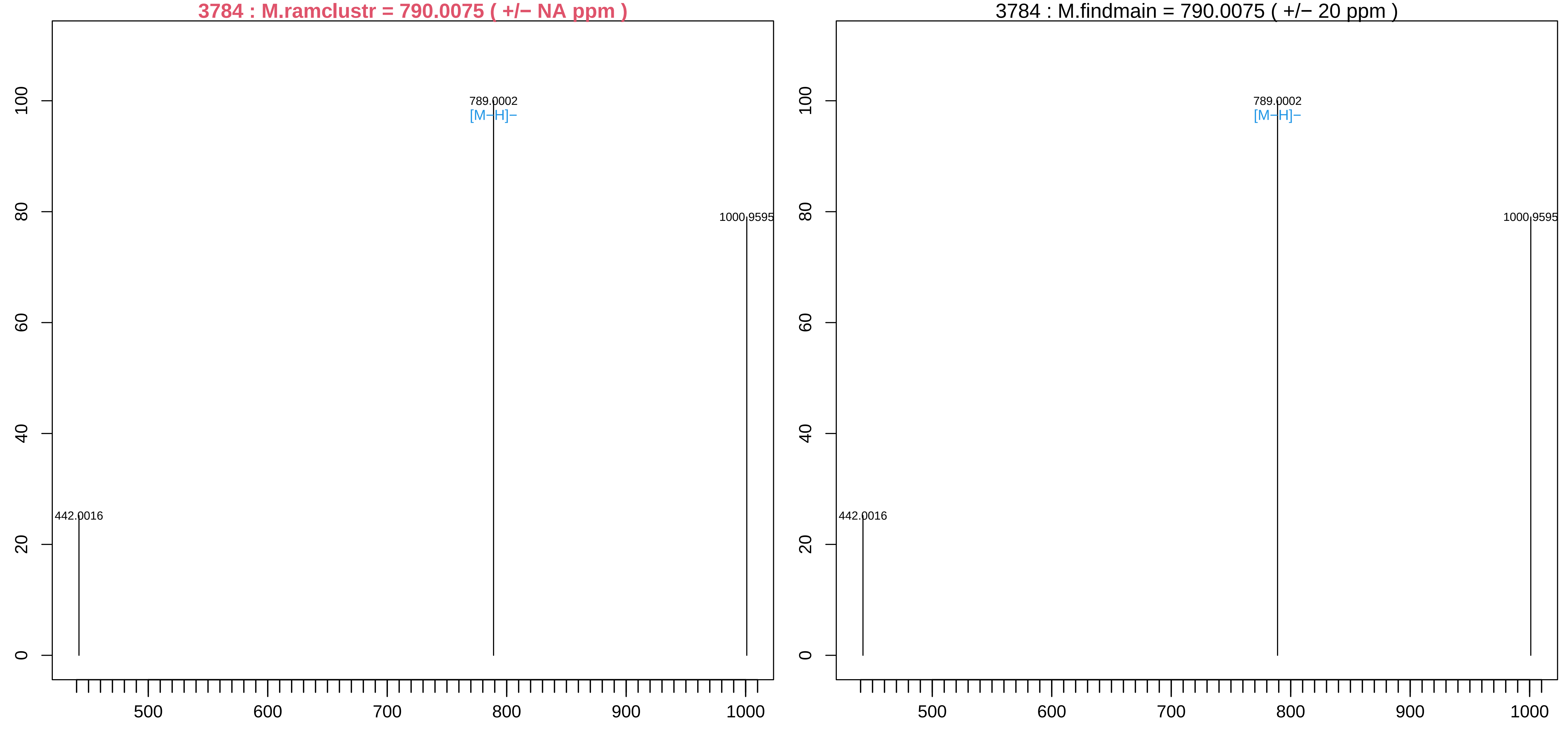
3784 : M.ramclustr = 790.0075 (+/− NA ppm)
3784 : M.findmain = 790.0075 (+/− 20 ppm)
789.0002
[M−H]−
1000.9595
442.0016
0
20
40
60
80
100
500
600
700
800
900
1000

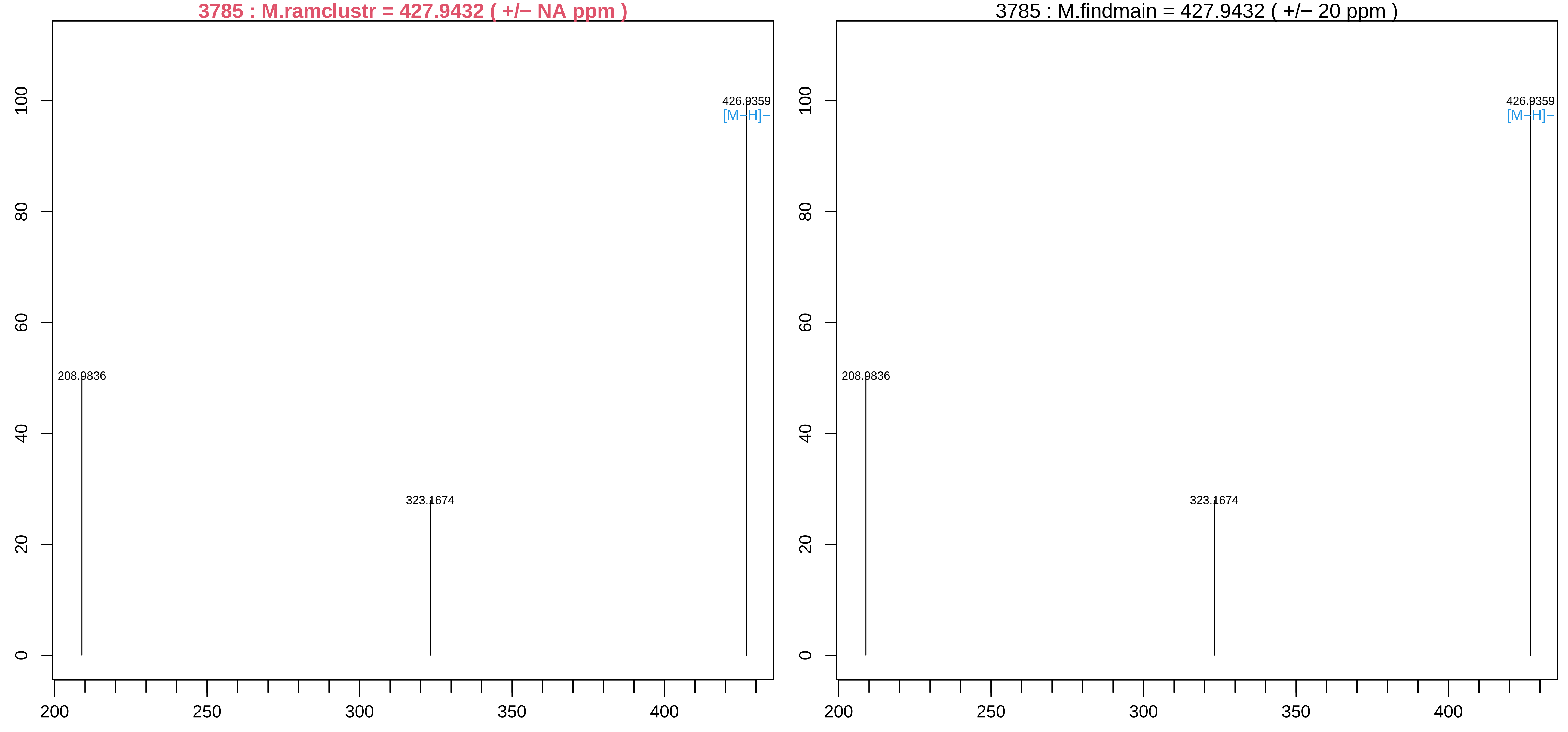
3785 : M.ramclustr = 427.9432 (+/− NA ppm)
426.9359
[M−H]−
208.9836
323.1674
3785 : M.findmain = 427.9432 (+/− 20 ppm)
426.9359
[M−H]−
208.9836
323.1674

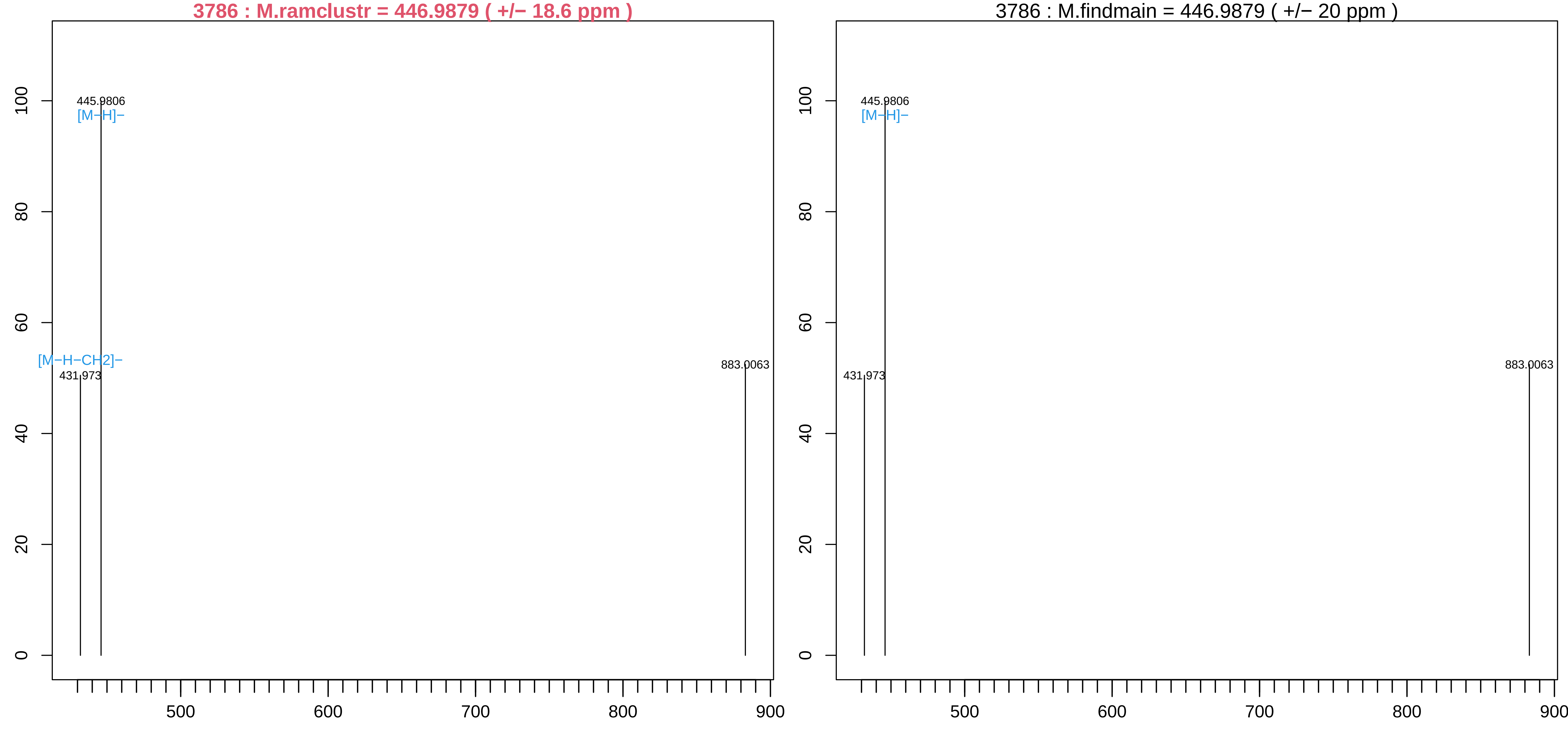
3786 : M.ramclustr = 446.9879 (+/− 18.6 ppm)
445.9806
[M−H]−
[M−H−CH2]−
431.973
883.0063
3786 : M.findmain = 446.9879 (+/− 20 ppm)
445.9806
[M−H]−
431.973
883.0063
0
20
40
60
80
100
500
600
700
800
900

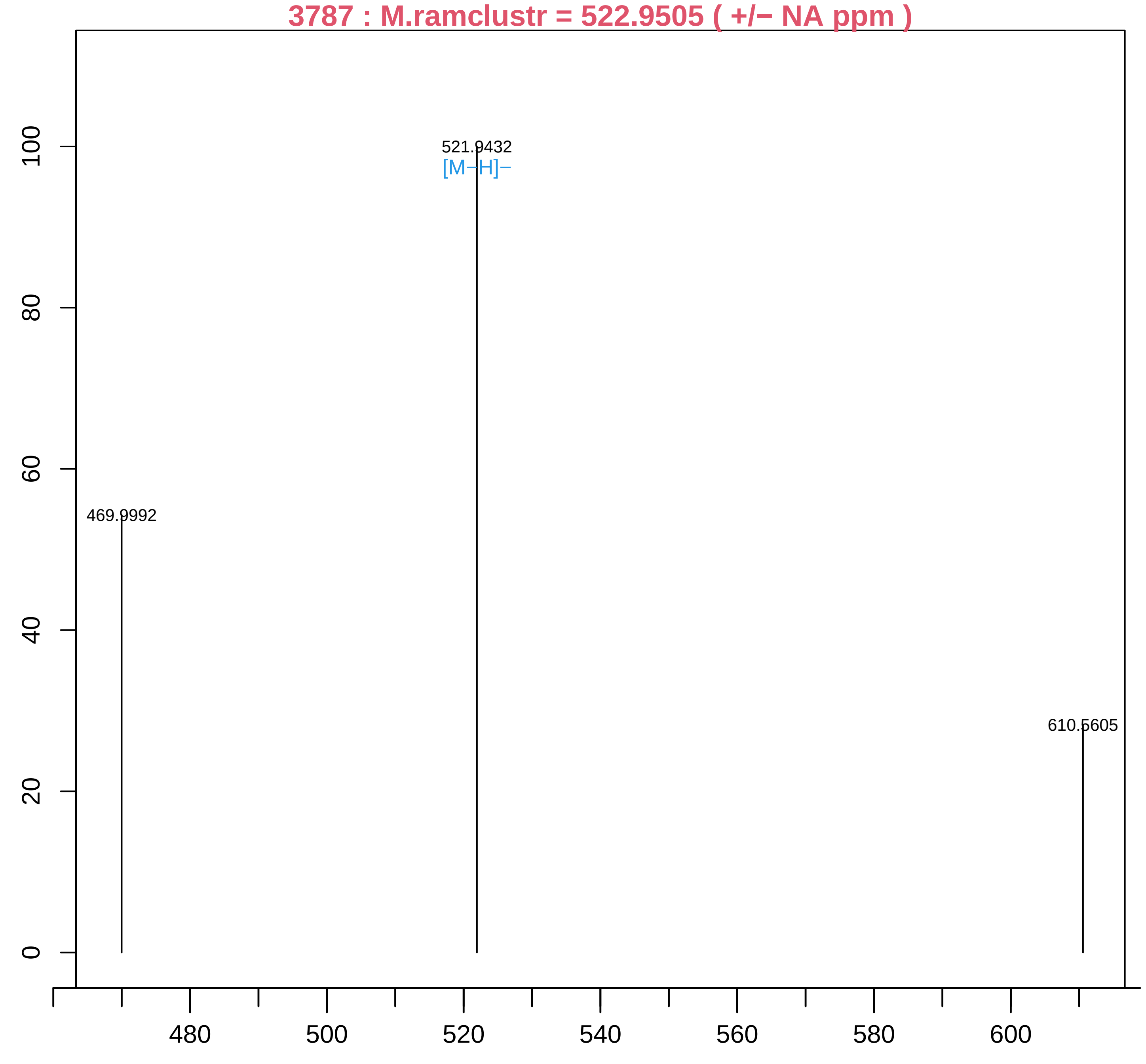
3787 : M.ramclustr = 522.9505 (+/− NA ppm)
521.9432
[M−H]−
469.9992
610.5605
0
20
40
60
80
100
480
500
520
540
560
580
600

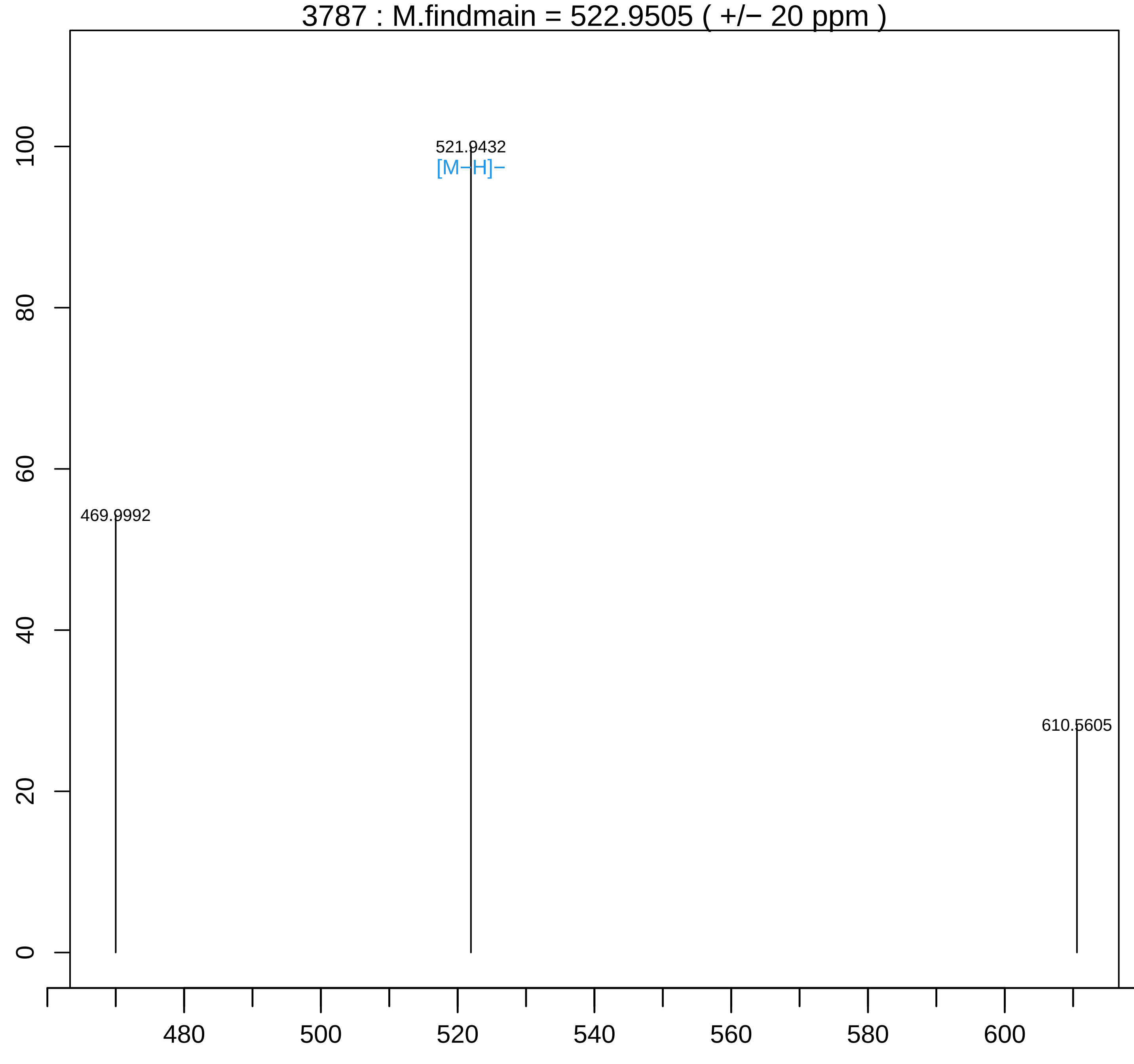
3787 : M.findmain = 522.9505 (+/− 20 ppm)
521.9432
[M−H]−
469.9992
610.5605
0
20
40
60
80
100
480
500
520
540
560
580
600

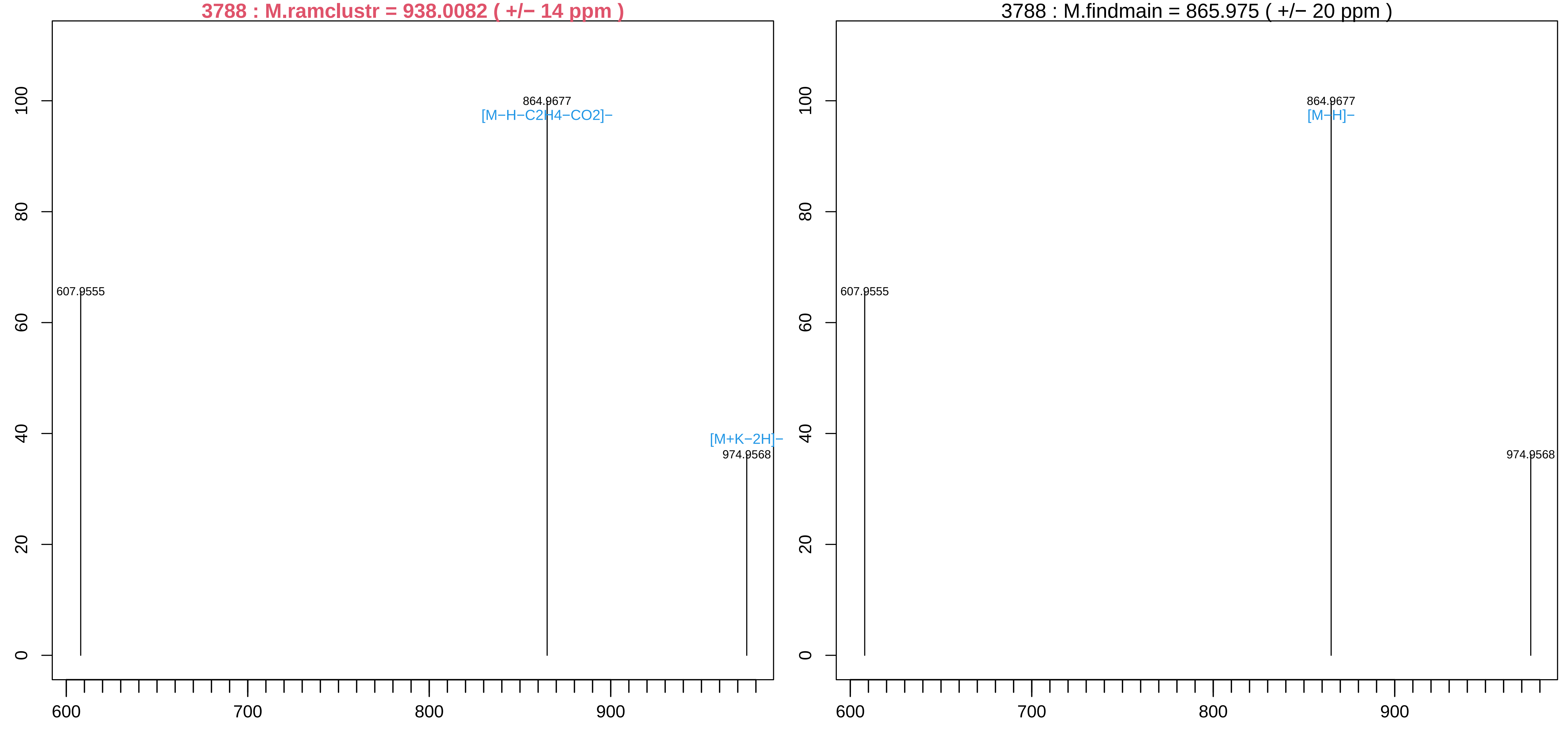

3788 : M.ramclustr = 938.0082 (+/− 14 ppm)
864.9677
[M−H−C2H4−CO2]−
607.9555
[M+K−2H]−
974.9568
3788 : M.findmain = 865.975 (+/− 20 ppm)
864.9677
[M−H]−
607.9555
974.9568
0
20
40
60
80
100
600
700
800
900

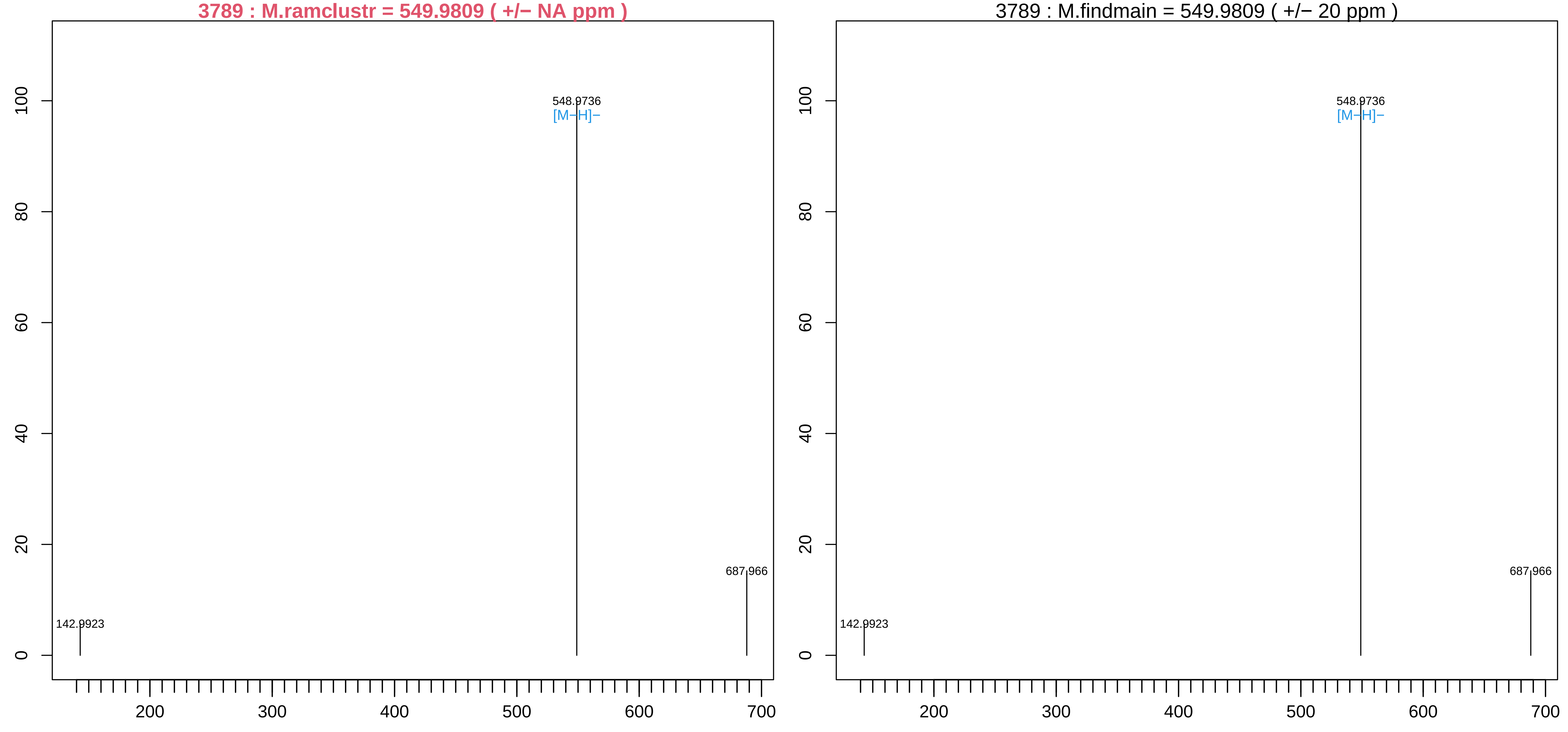
3789 : M.ramclustr = 549.9809 (+/− NA ppm)
3789 : M.findmain = 549.9809 (+/− 20 ppm)
548.9736
[M−H]−
687.966
142.9923
0
20
40
60
80
100
200
300
400
500
600
700

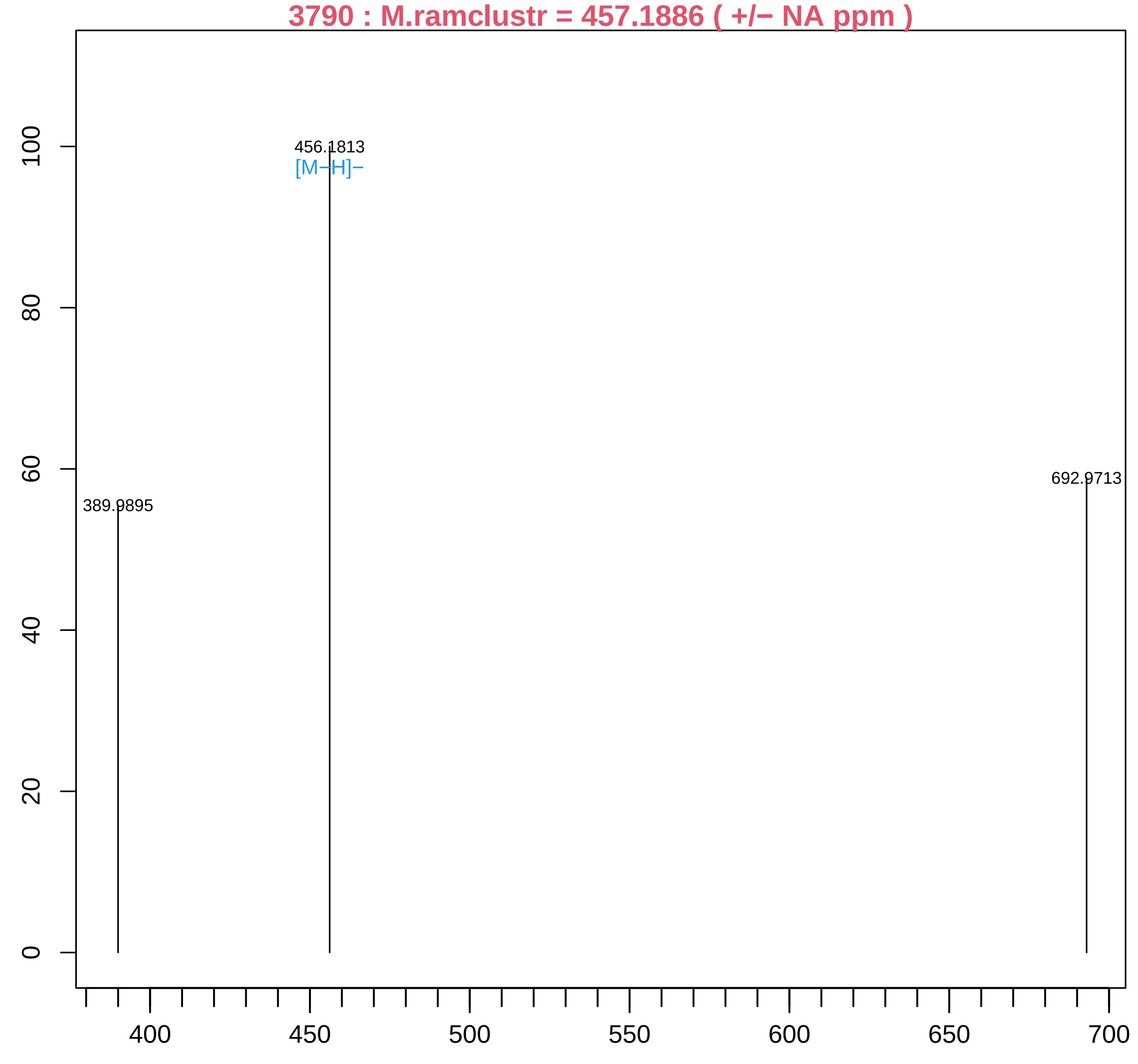
3790 : M.ramclustr = 457.1886 (+/− NA ppm)
456.1813
[M−H]−
389.9895
692.9713
0
20
40
60
80
100
400
450
500
550
600
650
700

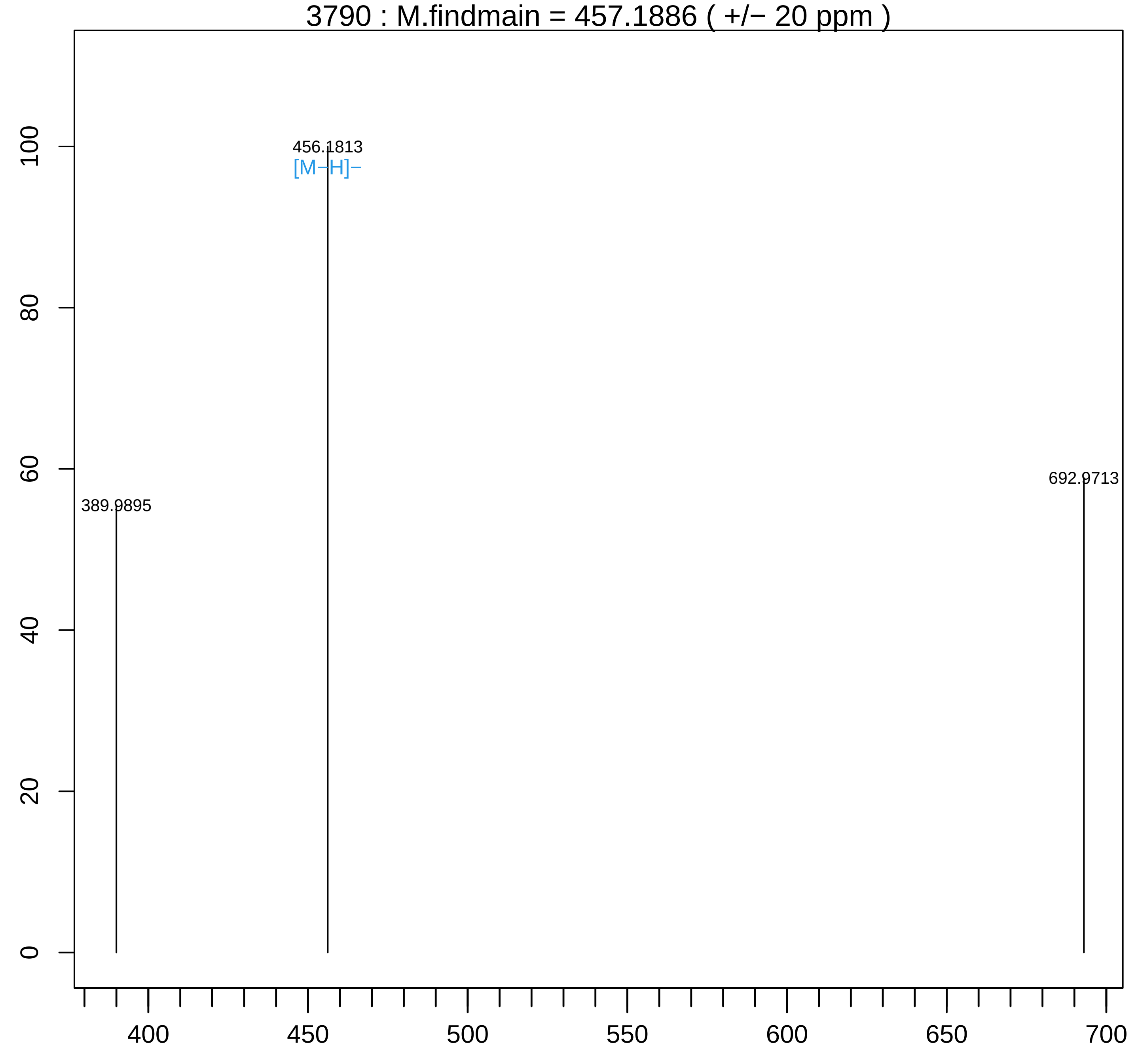
3790 : M.findmain = 457.1886 (+/− 20 ppm)
456.1813
[M−H]−
389.9895
692.9713
0
20
40
60
80
100
400
450
500
550
600
650
700

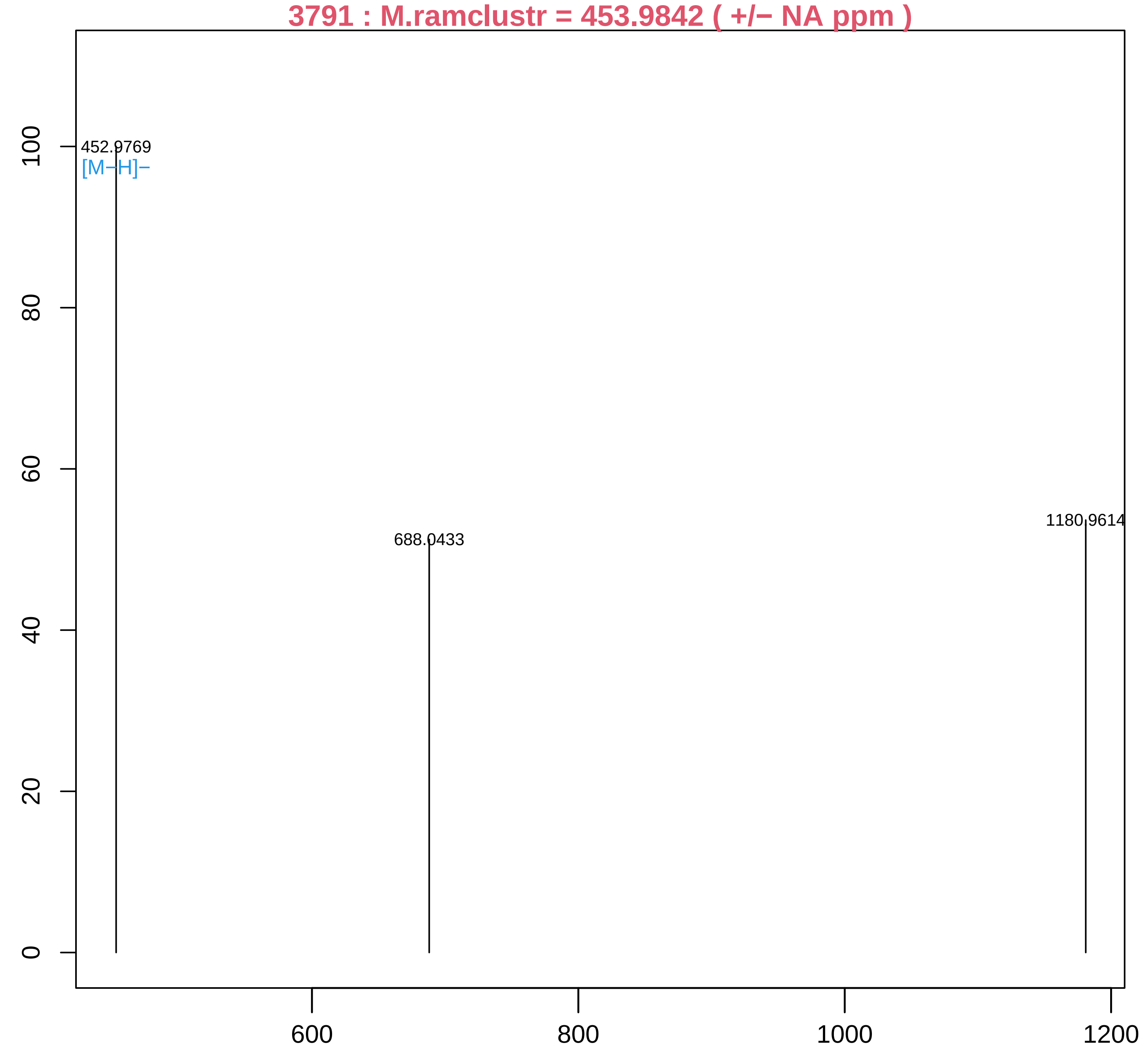
3791 : M.ramclustr = 453.9842 (+/− NA ppm)
452.9769
[M−H]−
688.0433
1180.9614
0
20
40
60
80
100
600
800
1000
1200

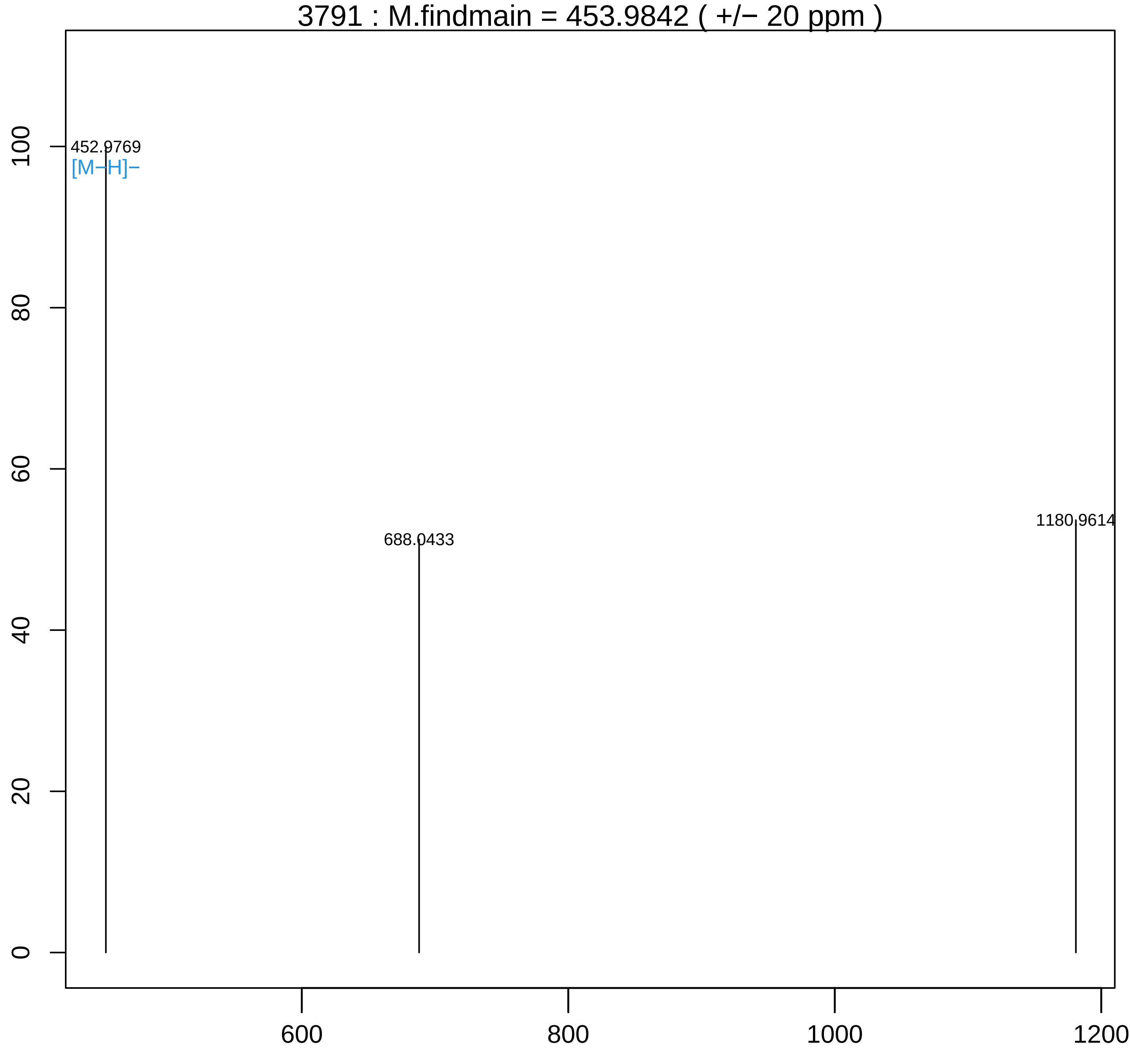
3791 : M.findmain = 453.9842 (+/− 20 ppm)
452.9769
[M−H]−
688.0433
1180.9614
0
20
40
60
80
100
600
800
1000
1200

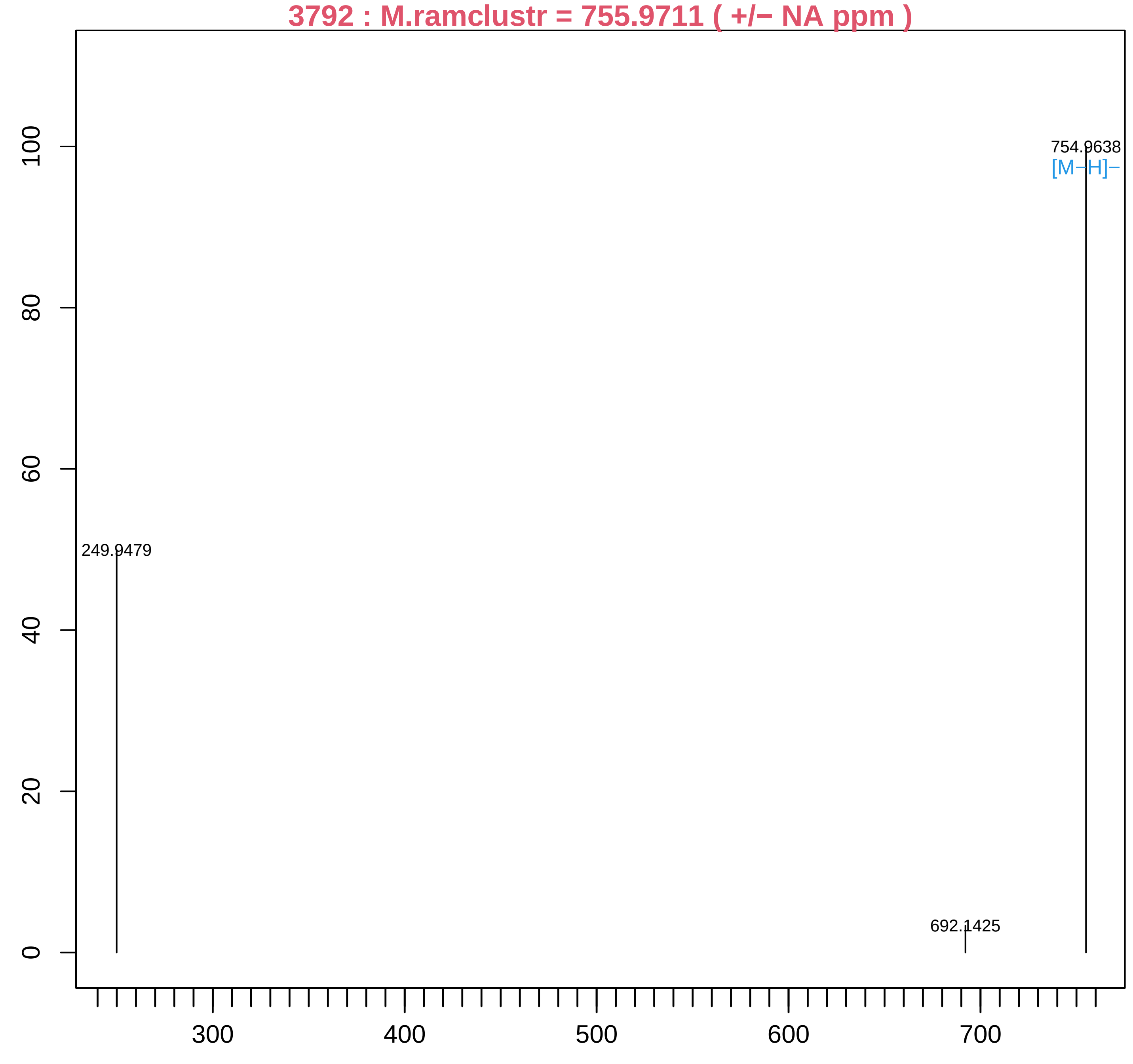

3792 : M.ramclustr = 755.9711 (+/− NA ppm)
754.9638
[M−H]−
249.9479
692.1425
0
20
40
60
80
100
300
400
500
600
700

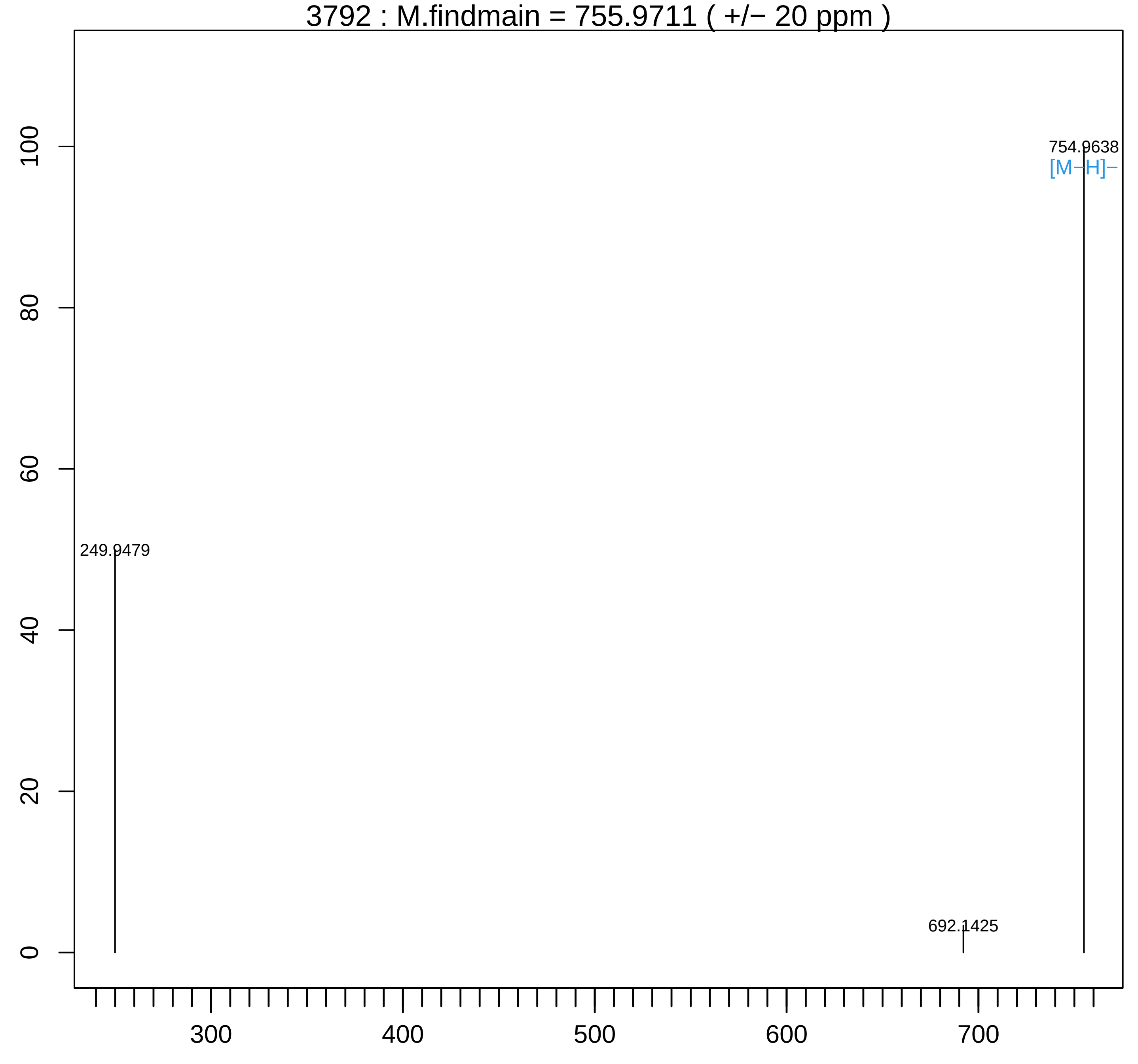

3792 : M.findmain = 755.9711 (+/− 20 ppm)
754.9638
[M−H]−
249.9479
692.1425
0
20
40
60
80
100
300
400
500
600
700

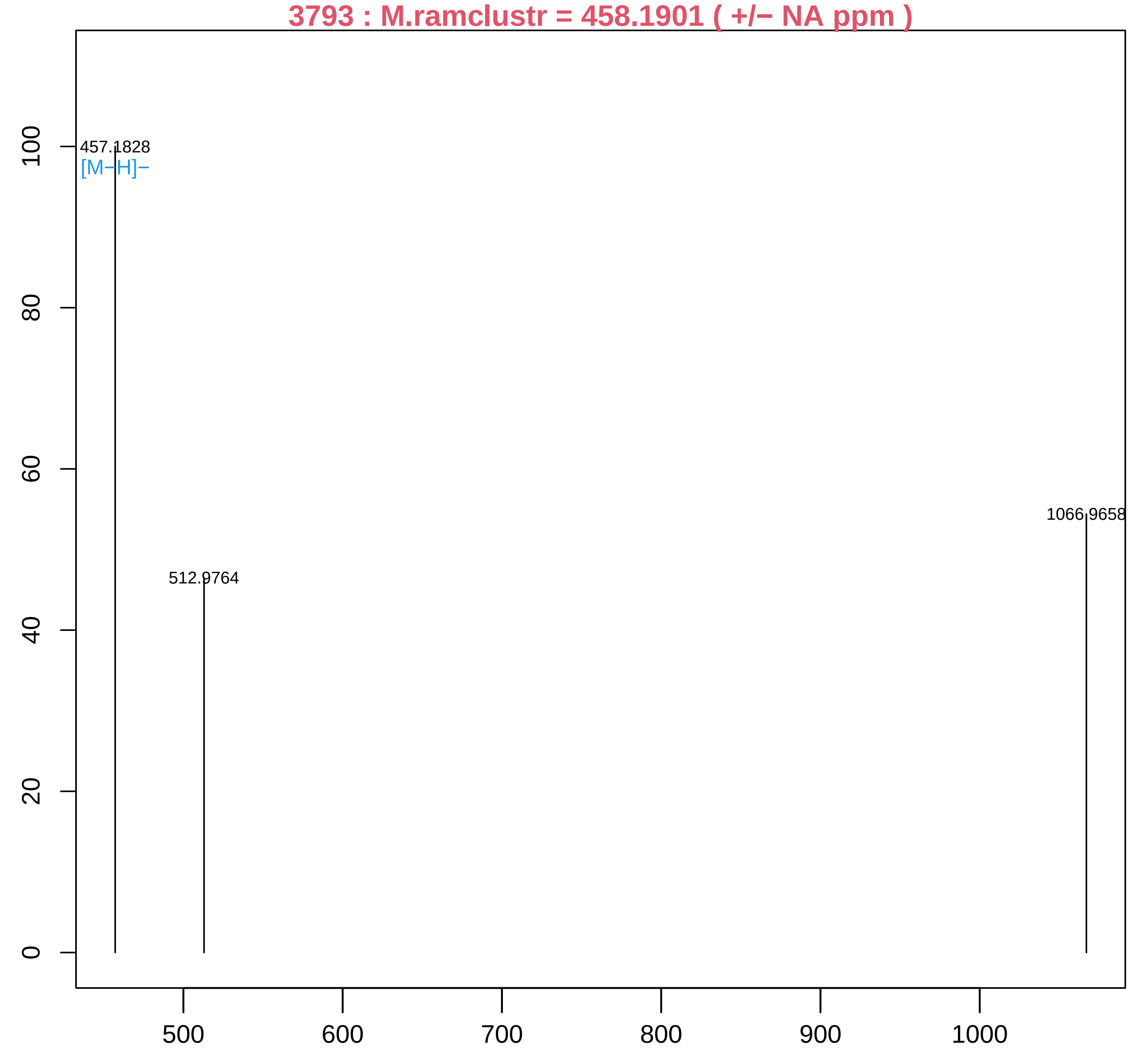

3793 : M.ramclustr = 458.1901 (+/− NA ppm)
457.1828
[M−H]−
512.9764
1066.9658
0
20
40
60
80
100
500
600
700
800
900
1000

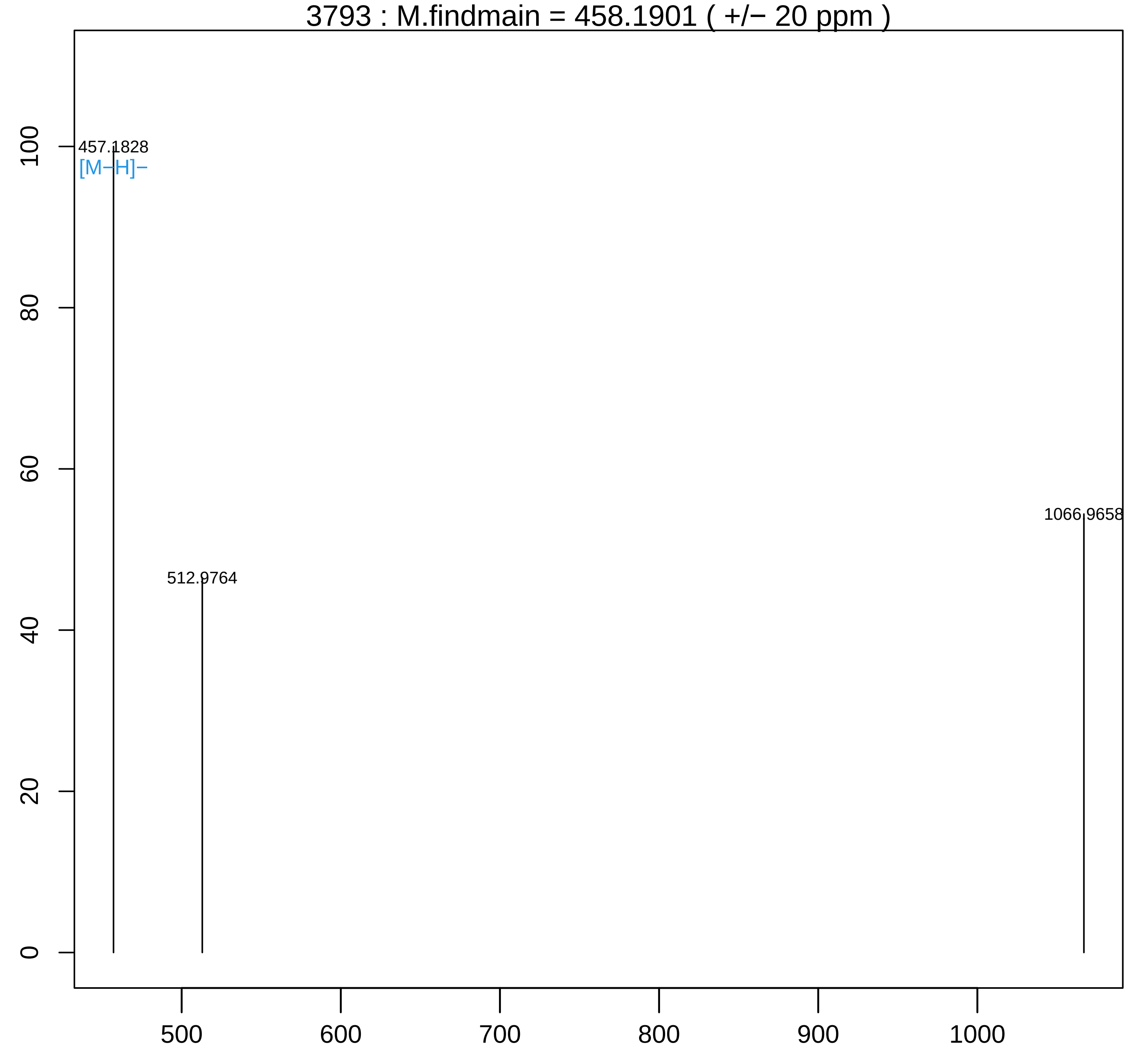

3793 : M.findmain = 458.1901 (+/− 20 ppm)
457.1828
[M−H]−
512.9764
1066.9658
0
20
40
60
80
100
500
600
700
800
900
1000

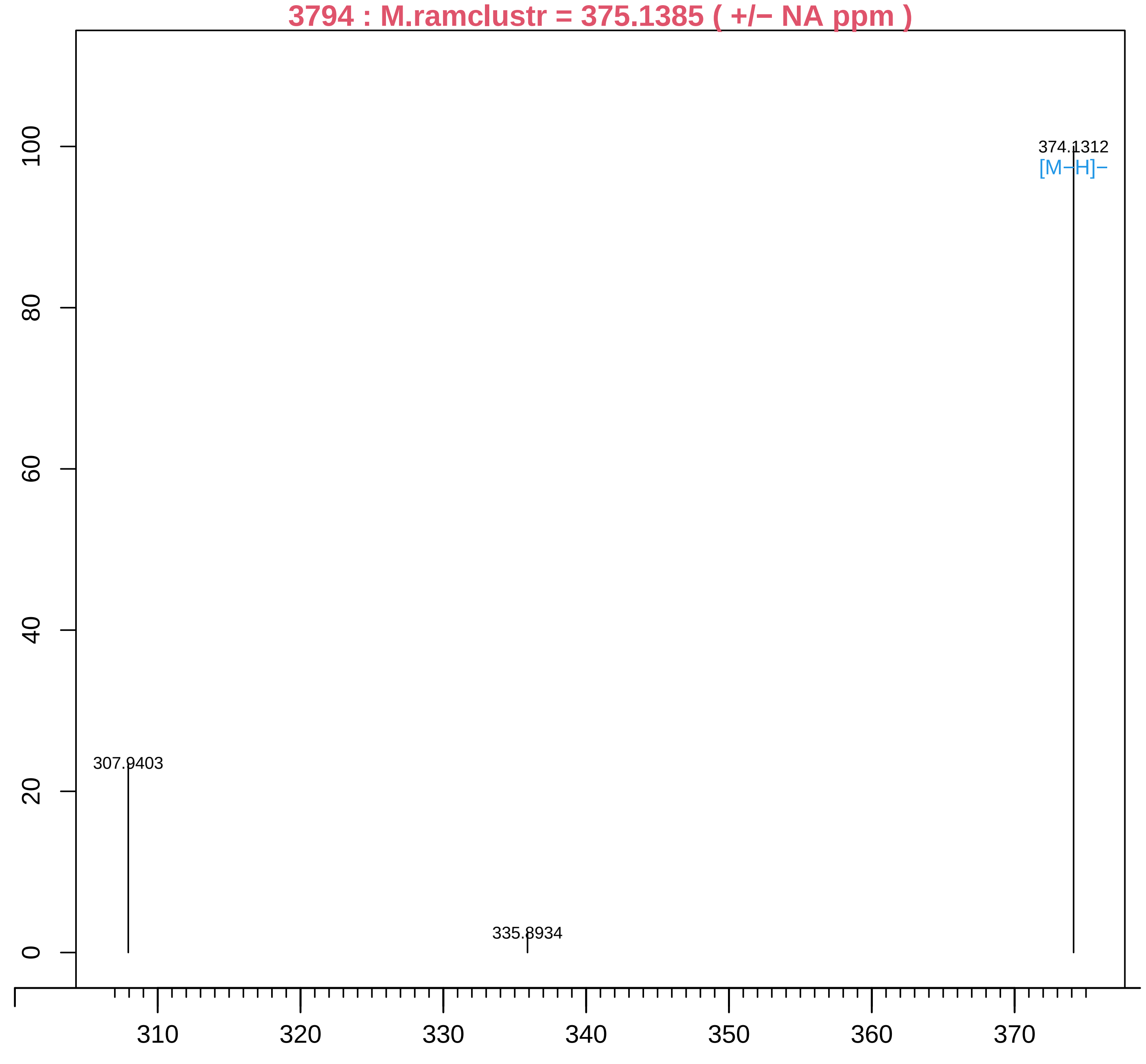
3794 : M.ramclustr = 375.1385 (+/− NA ppm)
374.1312
[M−H]−
307.9403
335.8934
0
20
40
60
80
100
310
320
330
340
350
360
370

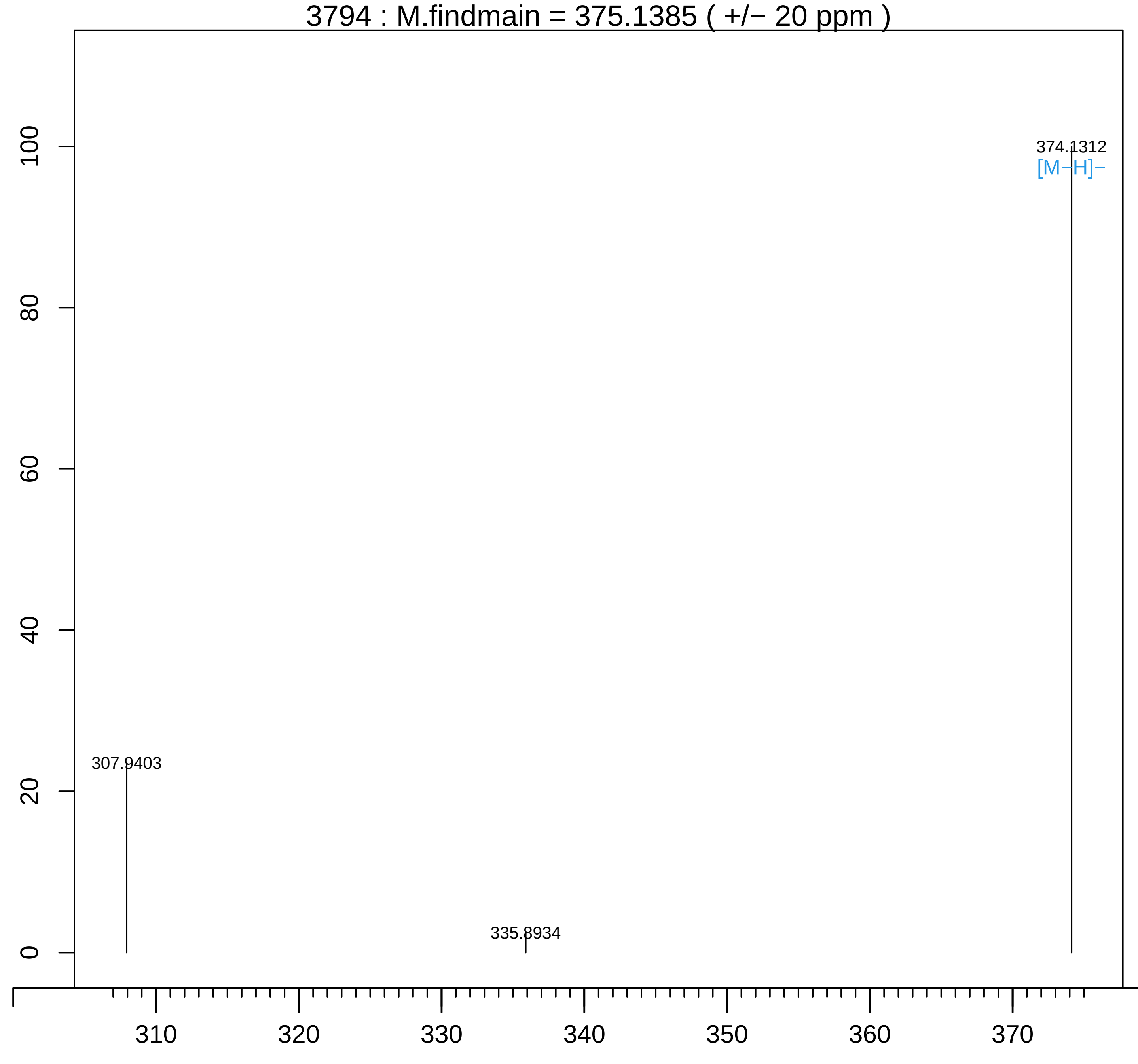
3794 : M.findmain = 375.1385 (+/− 20 ppm)
374.1312
[M−H]−
307.9403
335.8934
0
20
40
60
80
100
310
320
330
340
350
360
370

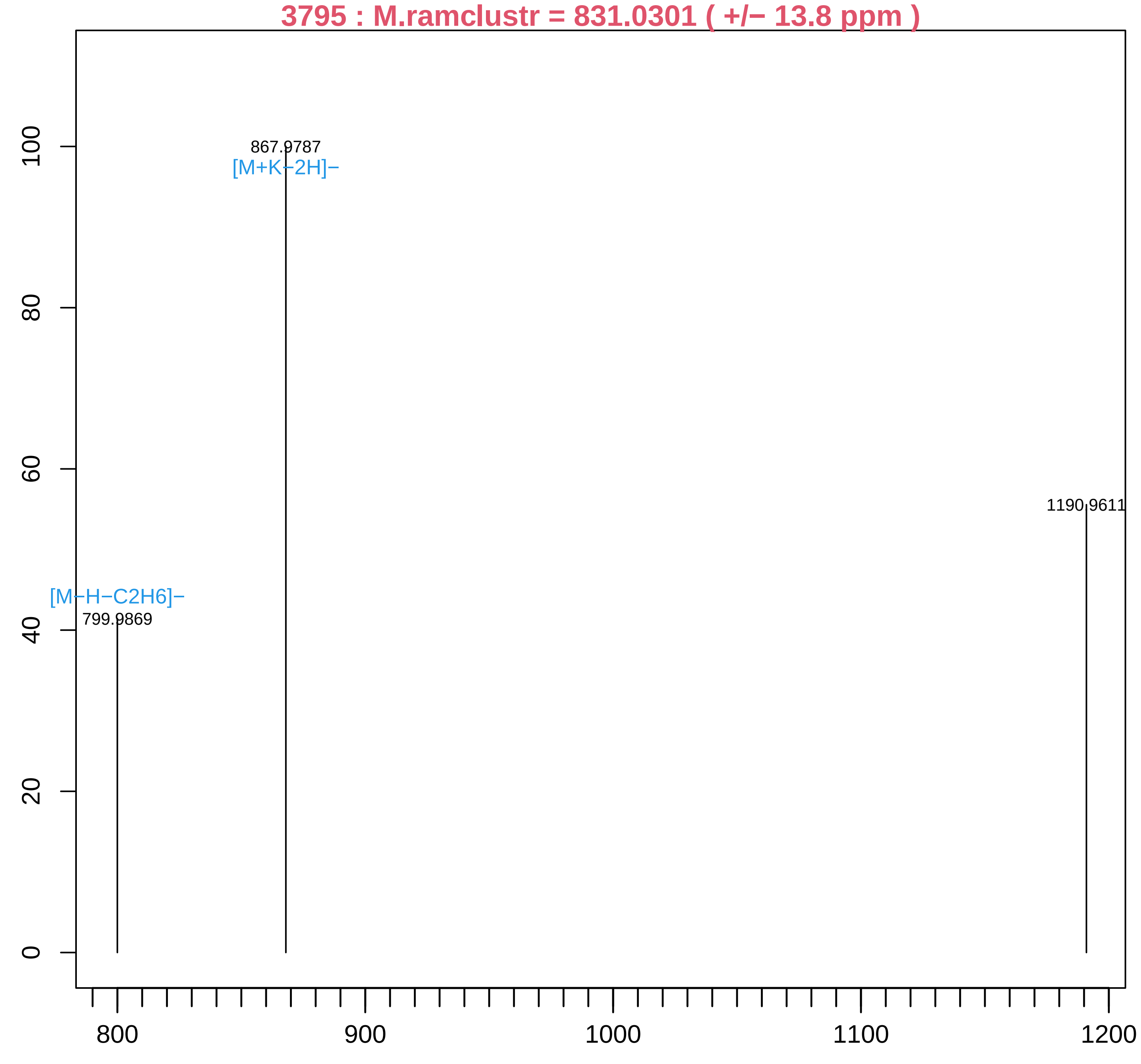
3795 : M.ramclustr = 831.0301 (+/− 13.8 ppm)
867.9787
[M+K−2H]−
[M−H−C2H6]−
799.9869
1190.9611
0
20
40
60
80
100
800
900
1000
1100
1200

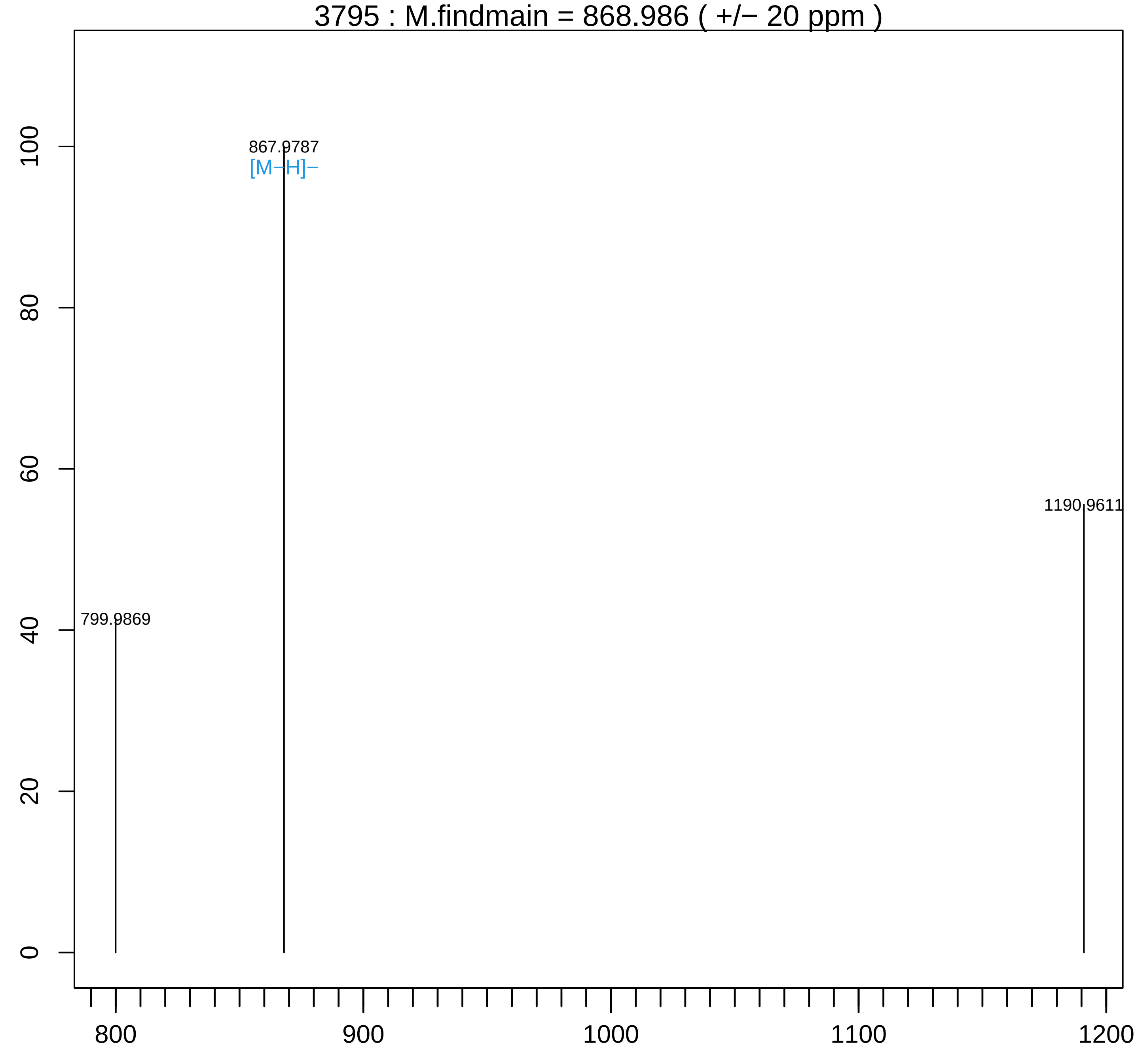
3795 : M.findmain = 868.986 (+/− 20 ppm)
867.9787
[M−H]−
799.9869
1190.9611
0
20
40
60
80
100
800
900
1000
1100
1200

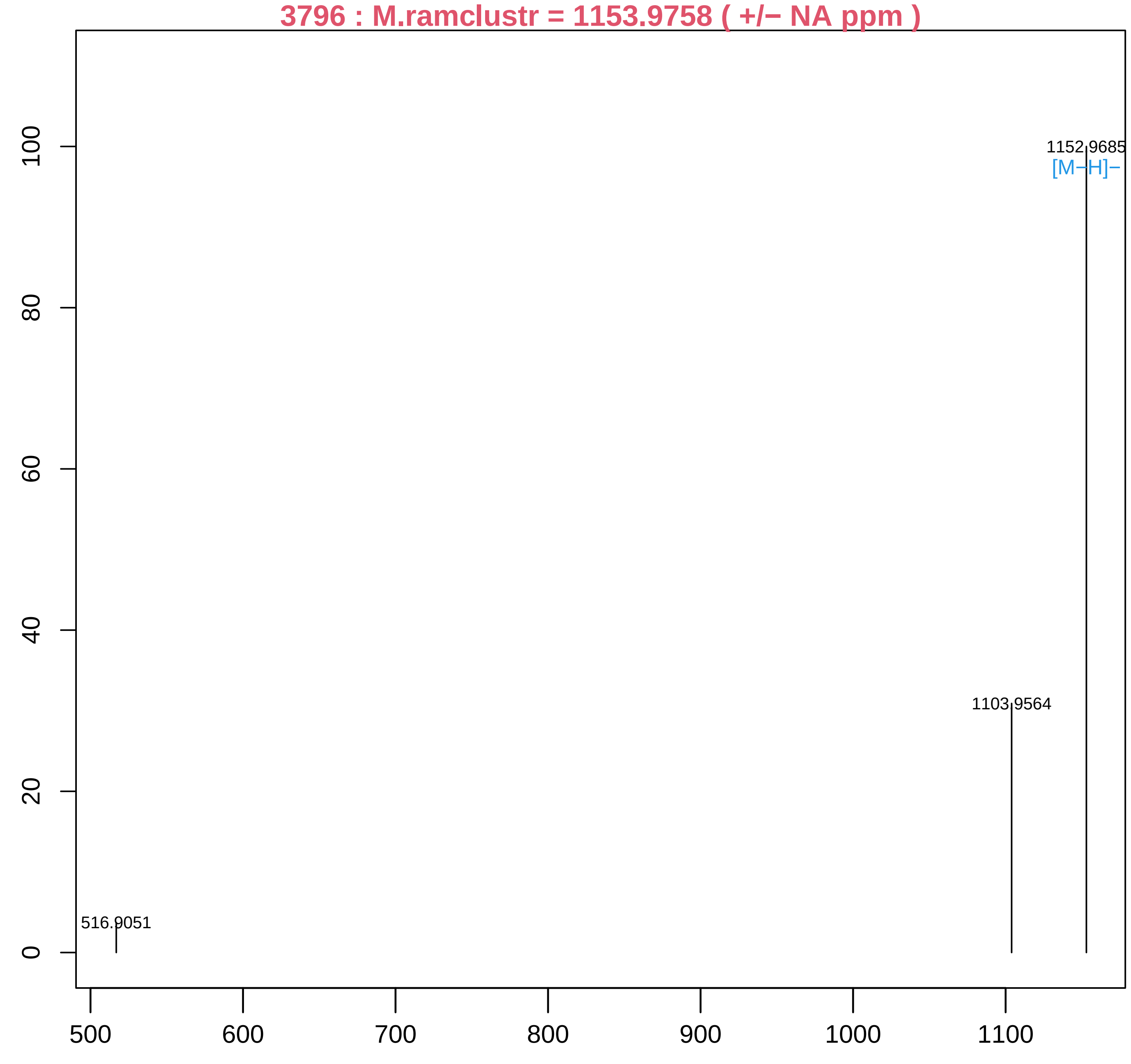
3796 : M.ramclustr = 1153.9758 (+/− NA ppm)
1152.9685
[M−H]−
1103.9564
516.9051
0
20
40
60
80
100
500
600
700
800
900
1000
1100

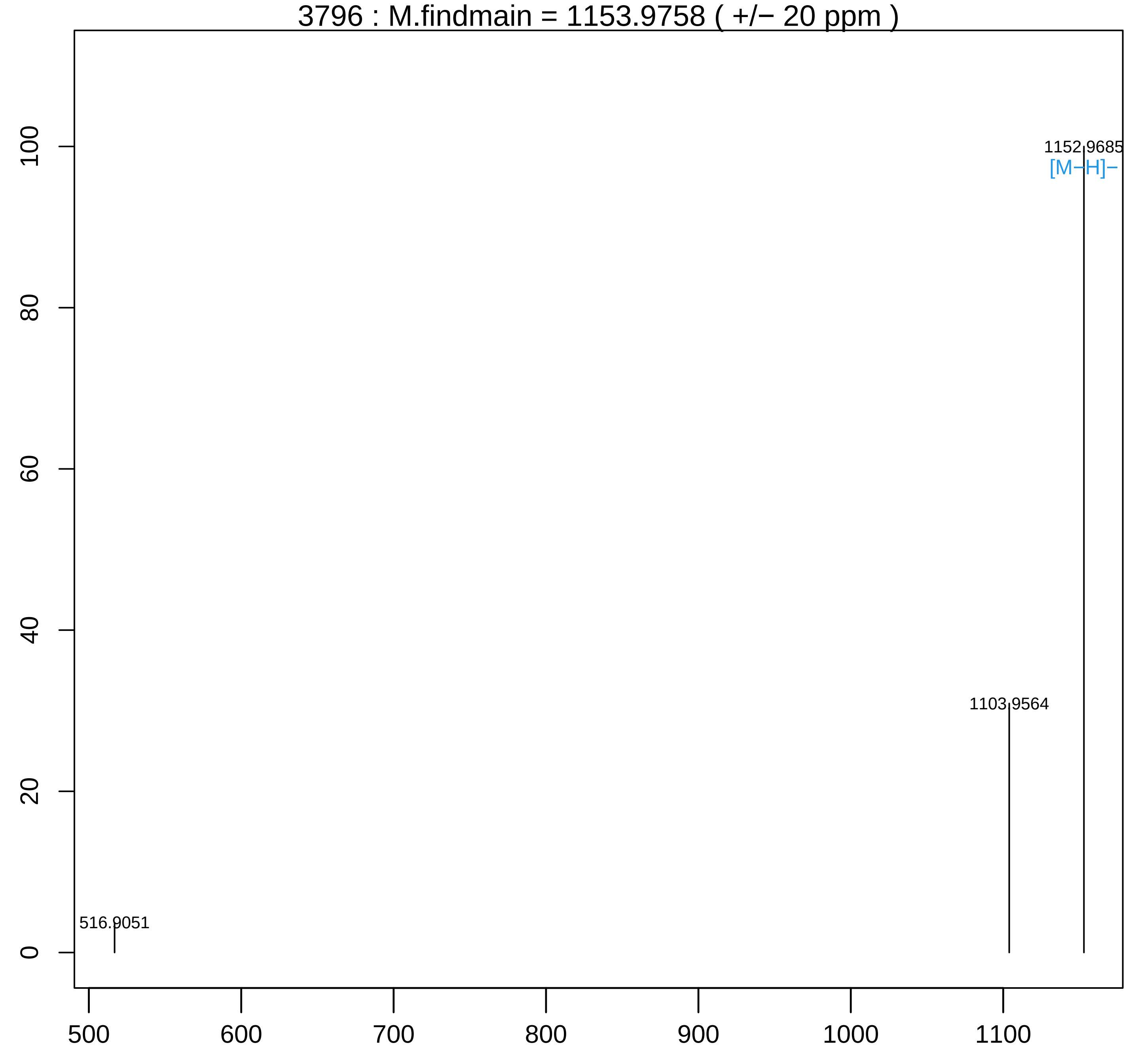
3796 : M.findmain = 1153.9758 (+/− 20 ppm)
1152.9685
[M−H]−
1103.9564
516.9051
0
20
40
60
80
100
500
600
700
800
900
1000
1100

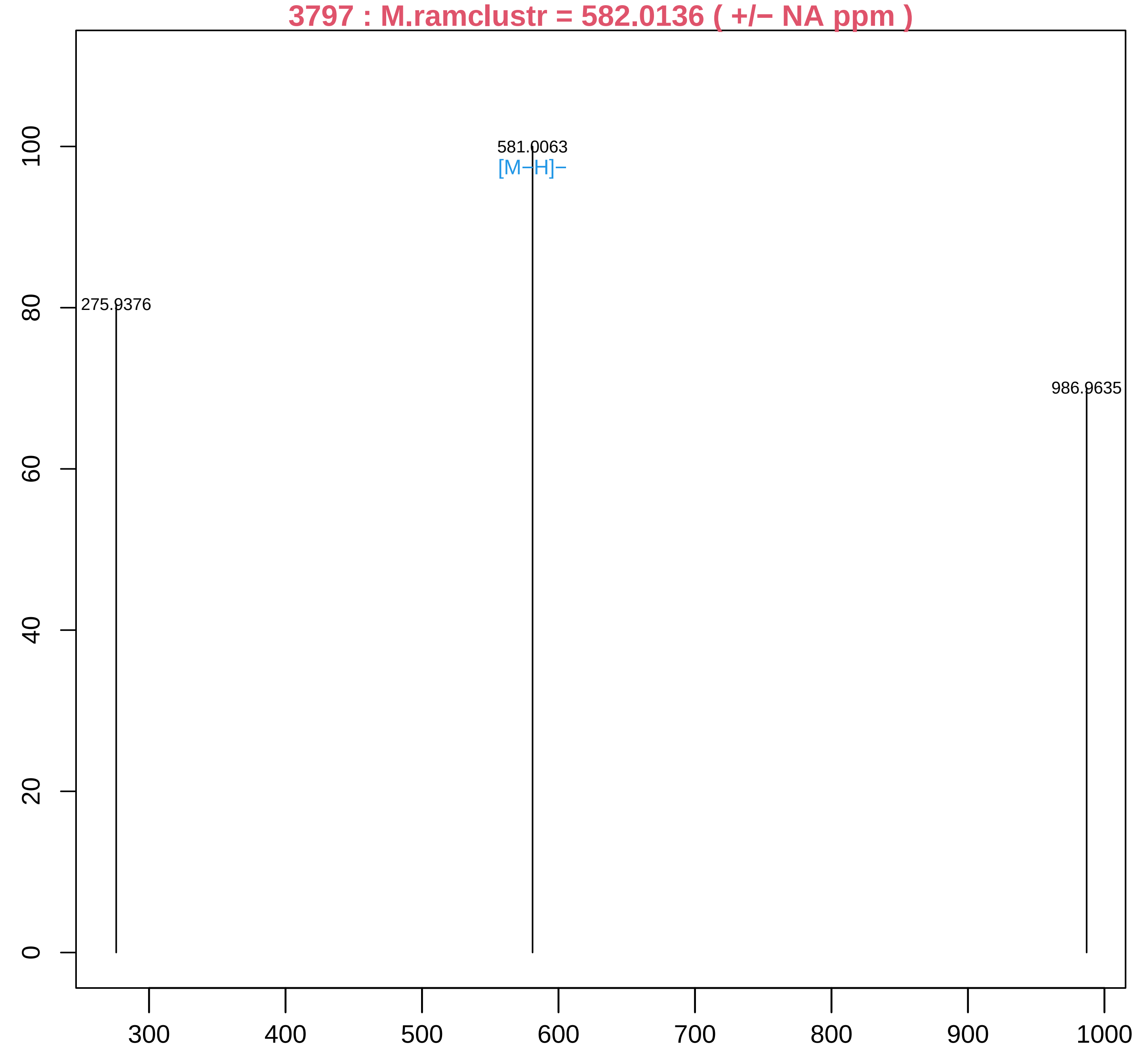
3797 : M.ramclustr = 582.0136 (+/− NA ppm)
581.0063
[M−H]−
275.9376
986.9635
0
20
40
60
80
100
300
400
500
600
700
800
900
1000

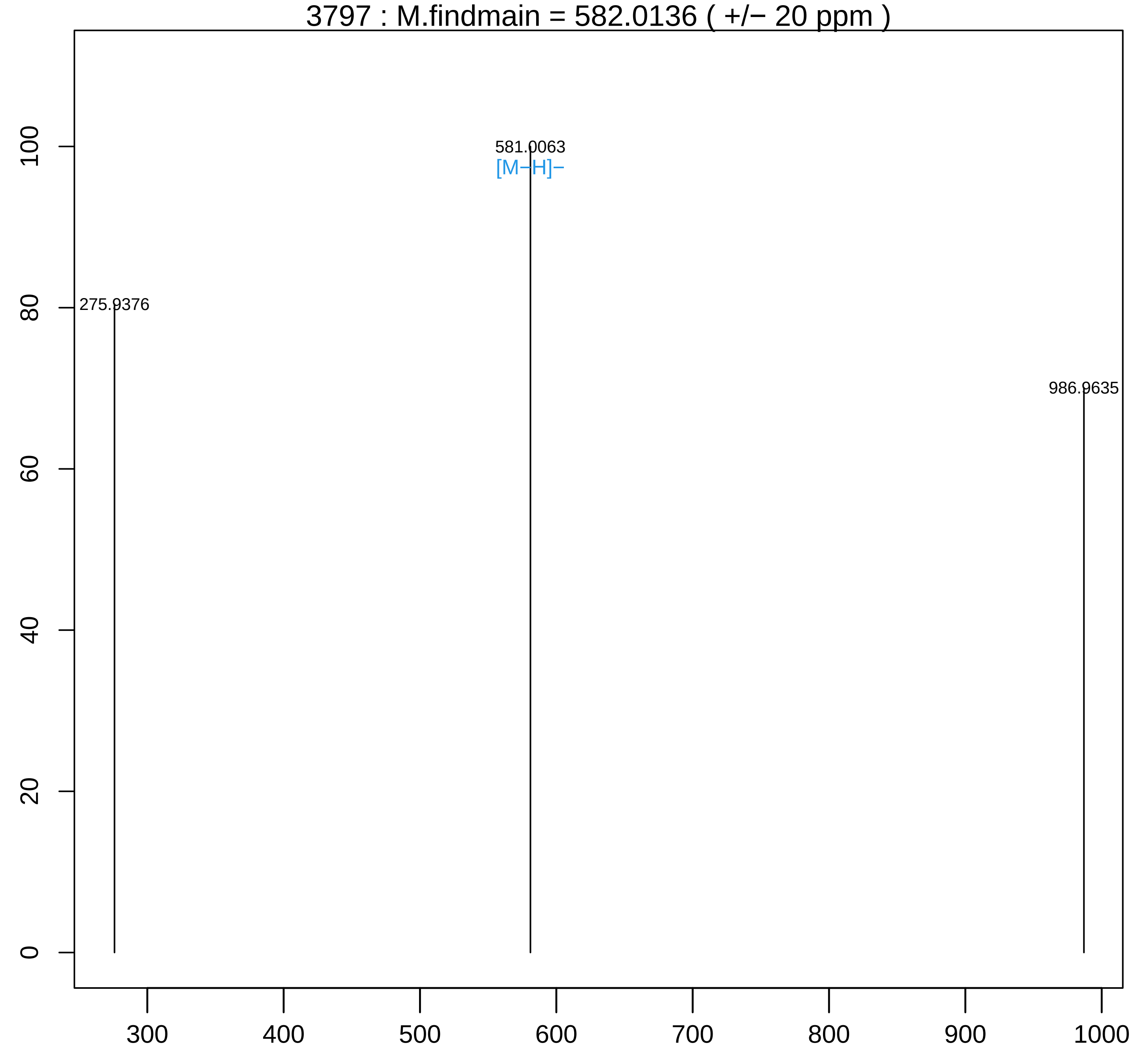
3797 : M.findmain = 582.0136 (+/− 20 ppm)
581.0063
[M−H]−
275.9376
986.9635
0
20
40
60
80
100
300
400
500
600
700
800
900
1000

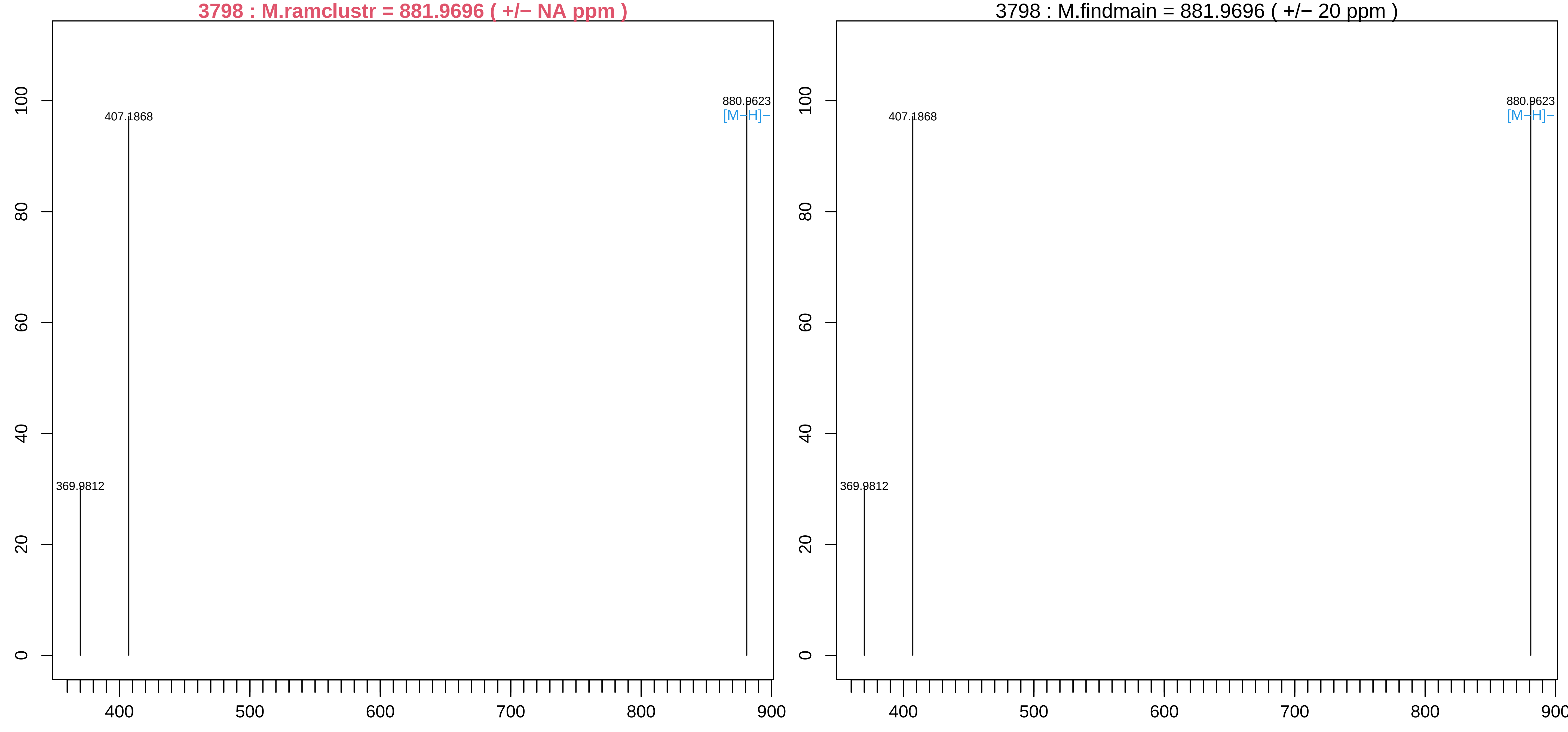
3798 : M.ramclustr = 881.9696 (+/− NA ppm)
3798 : M.findmain = 881.9696 (+/− 20 ppm)
369.9812
407.1868
880.9623
[M−H]−
0
20
40
60
80
100
400
500
600
700
800
900

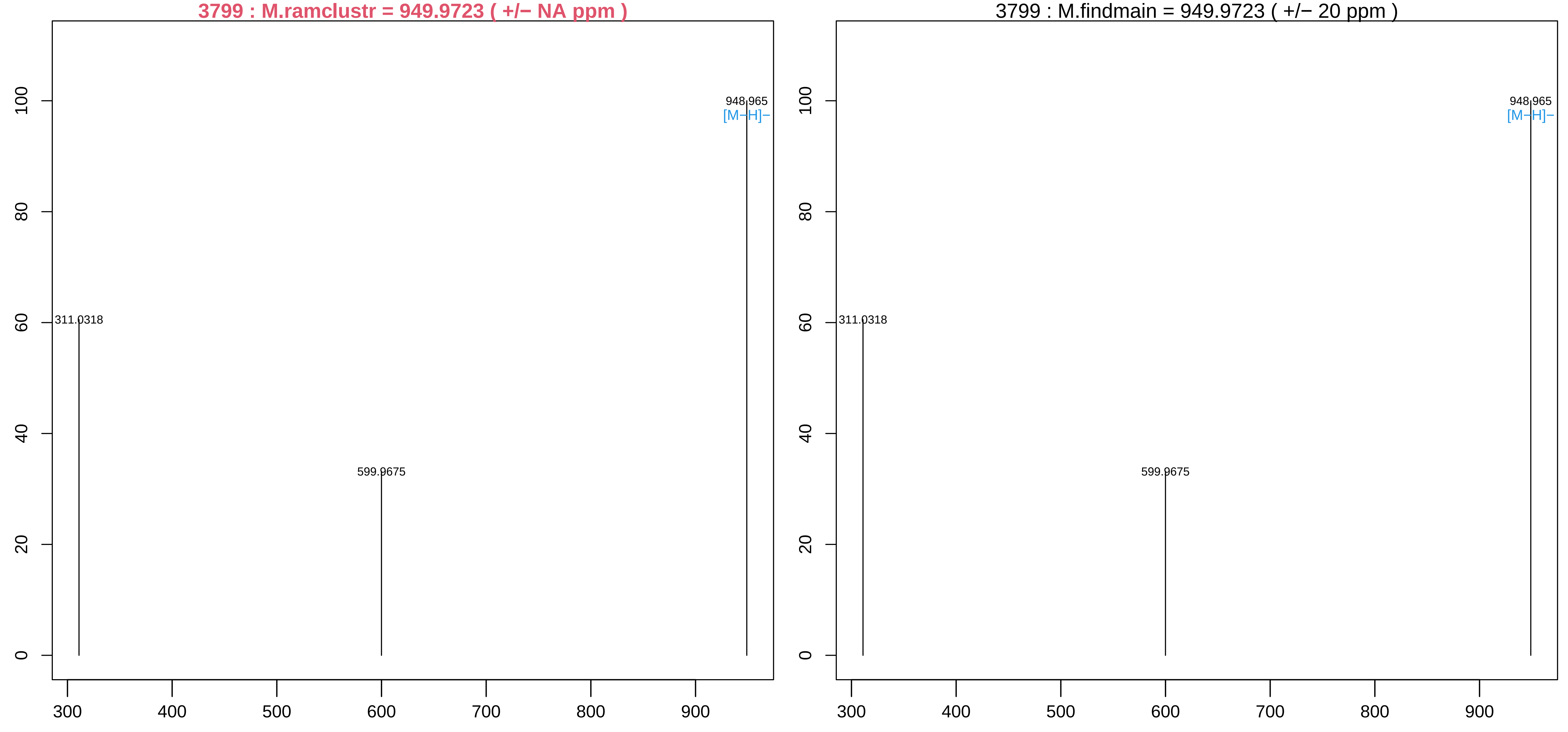

3799 : M.ramclustr = 949.9723 (+/− NA ppm)
948.965
[M−H]−
311.0318
599.9675
3799 : M.findmain = 949.9723 (+/− 20 ppm)
948.965
[M−H]−
311.0318
599.9675

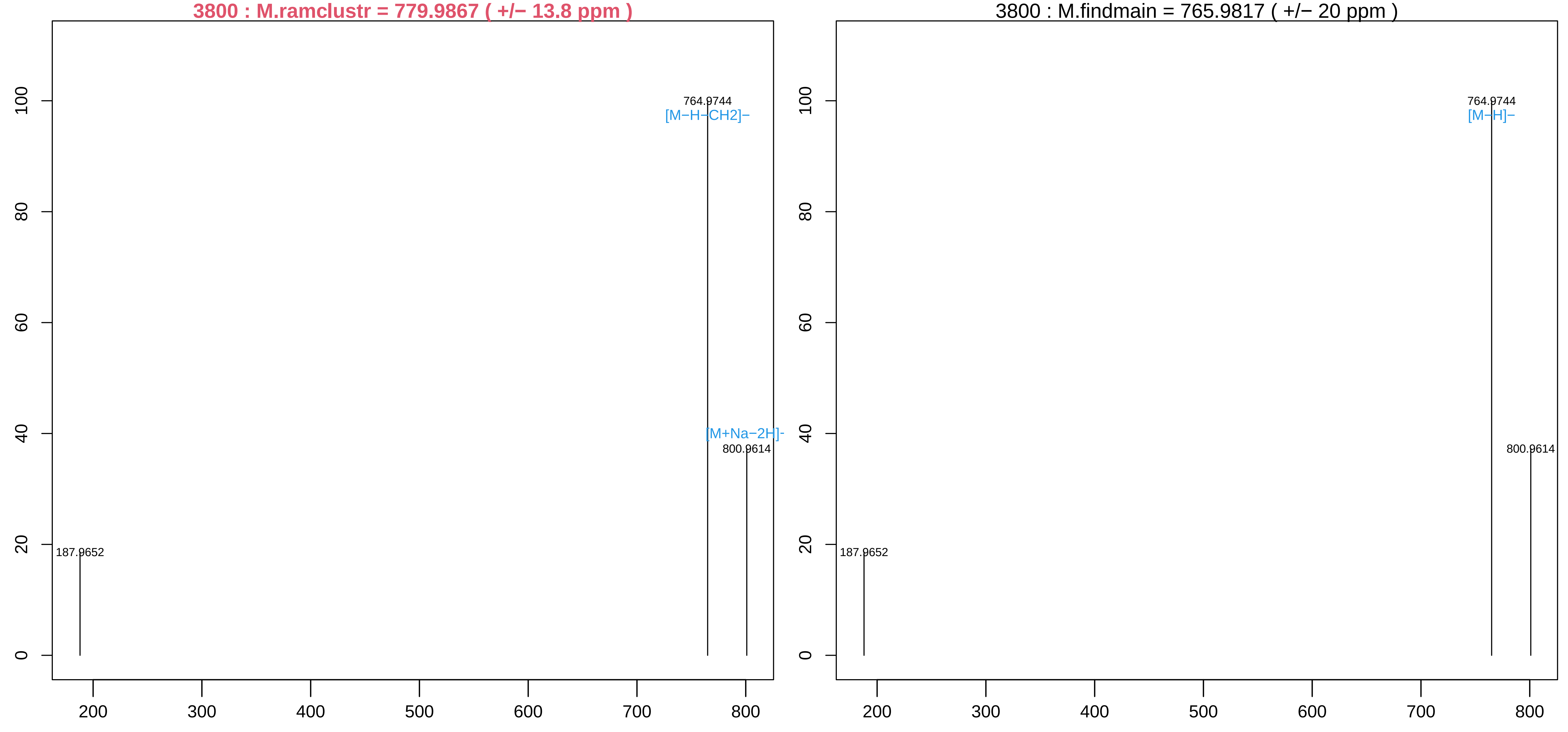
3800 : M.ramclustr = 779.9867 (+/− 13.8 ppm)
764.9744
[M−H−CH2]−
[M+Na−2H]-
800.9614
187.9652
0
20
40
60
80
100
200
300
400
500
600
700
800
3800 : M.findmain = 765.9817 (+/− 20 ppm)
764.9744
[M−H]−
800.9614
187.9652
0
20
40
60
80
100
200
300
400
500
600
700
800

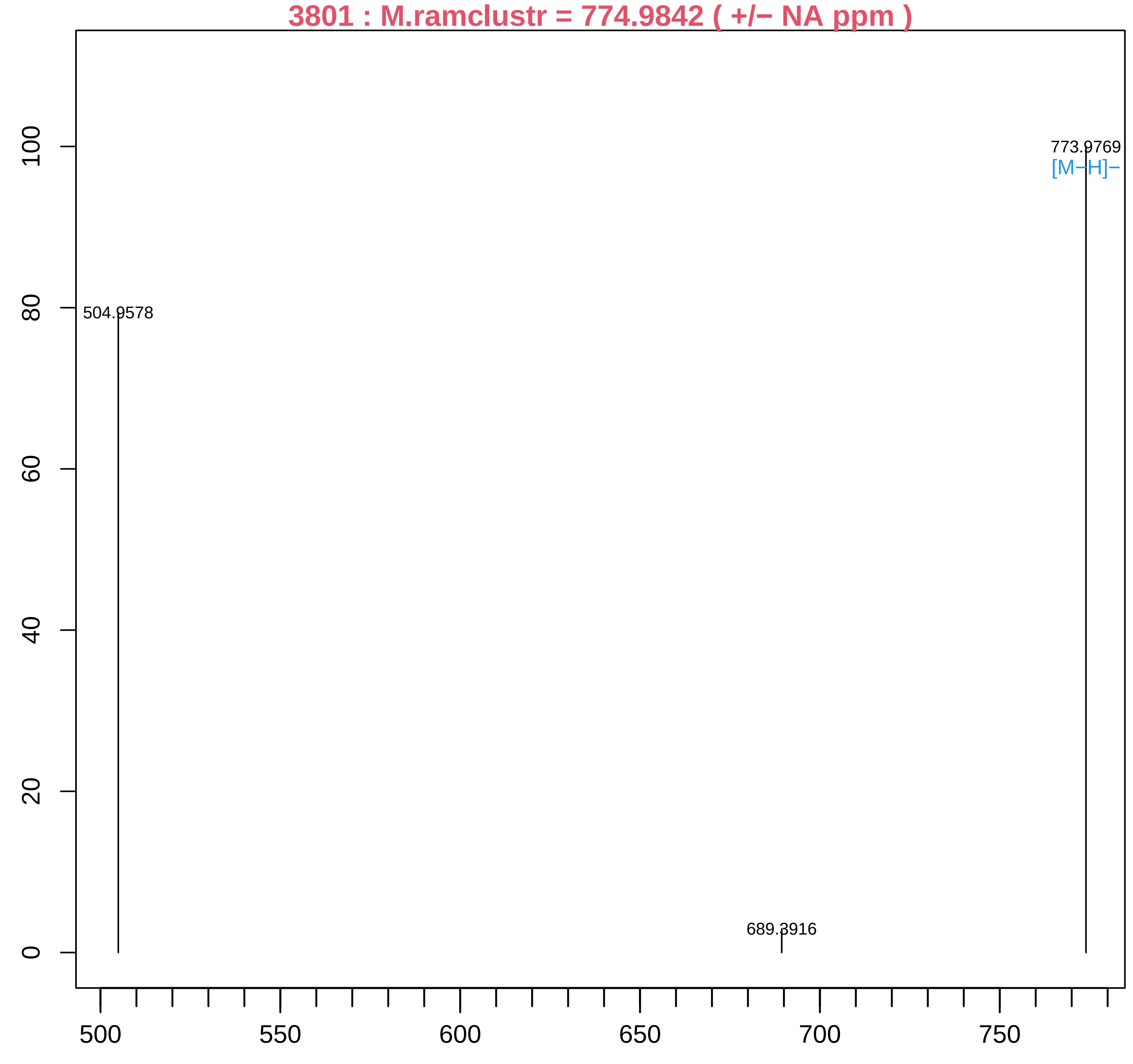
3801 : M.ramclustr = 774.9842 (+/− NA ppm)
773.9769
[M−H]−
504.9578
689.3916
0
20
40
60
80
100
500
550
600
650
700
750

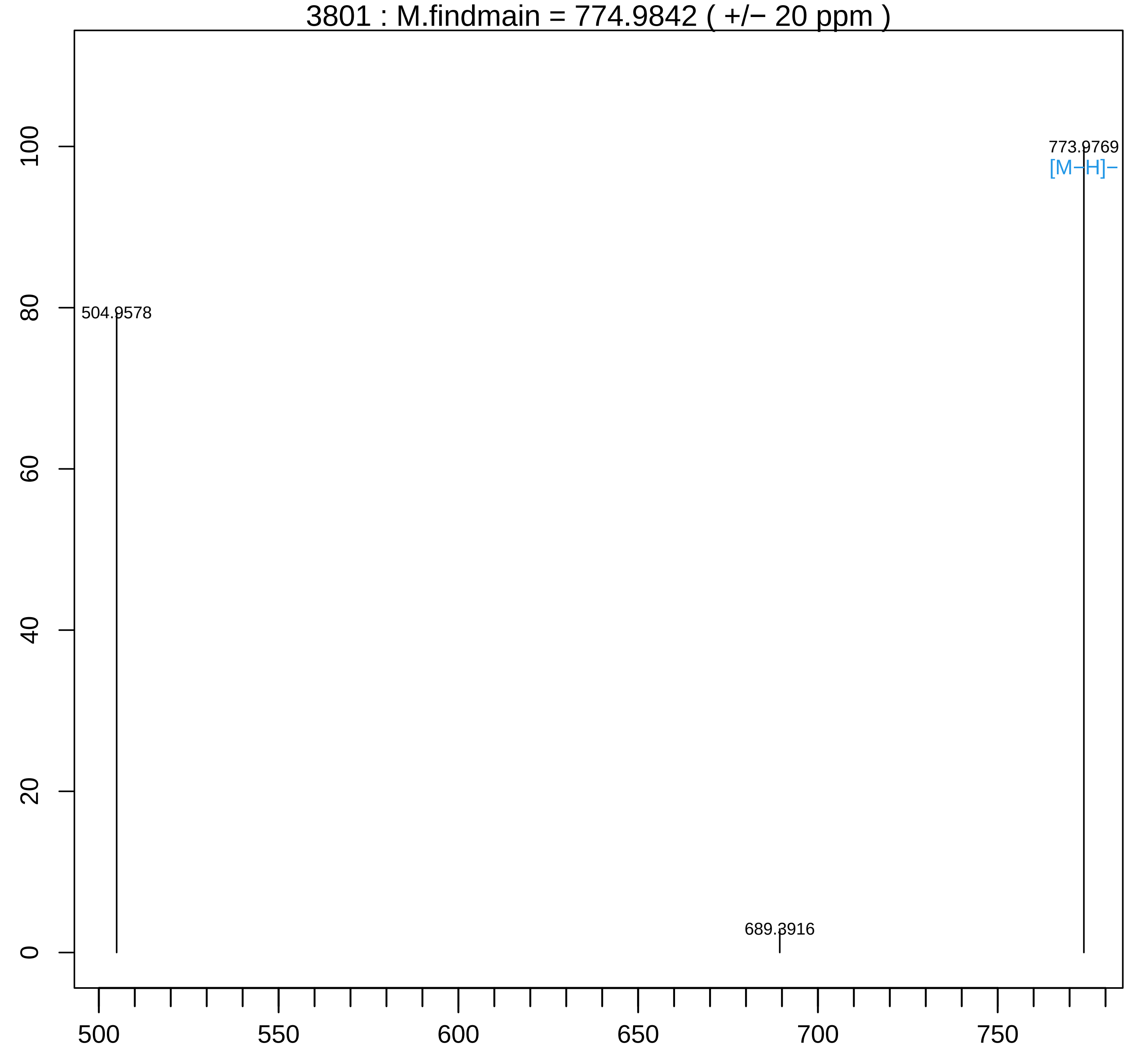
3801 : M.findmain = 774.9842 (+/− 20 ppm)
773.9769
[M−H]−
504.9578
689.3916
0
20
40
60
80
100
500
550
600
650
700
750

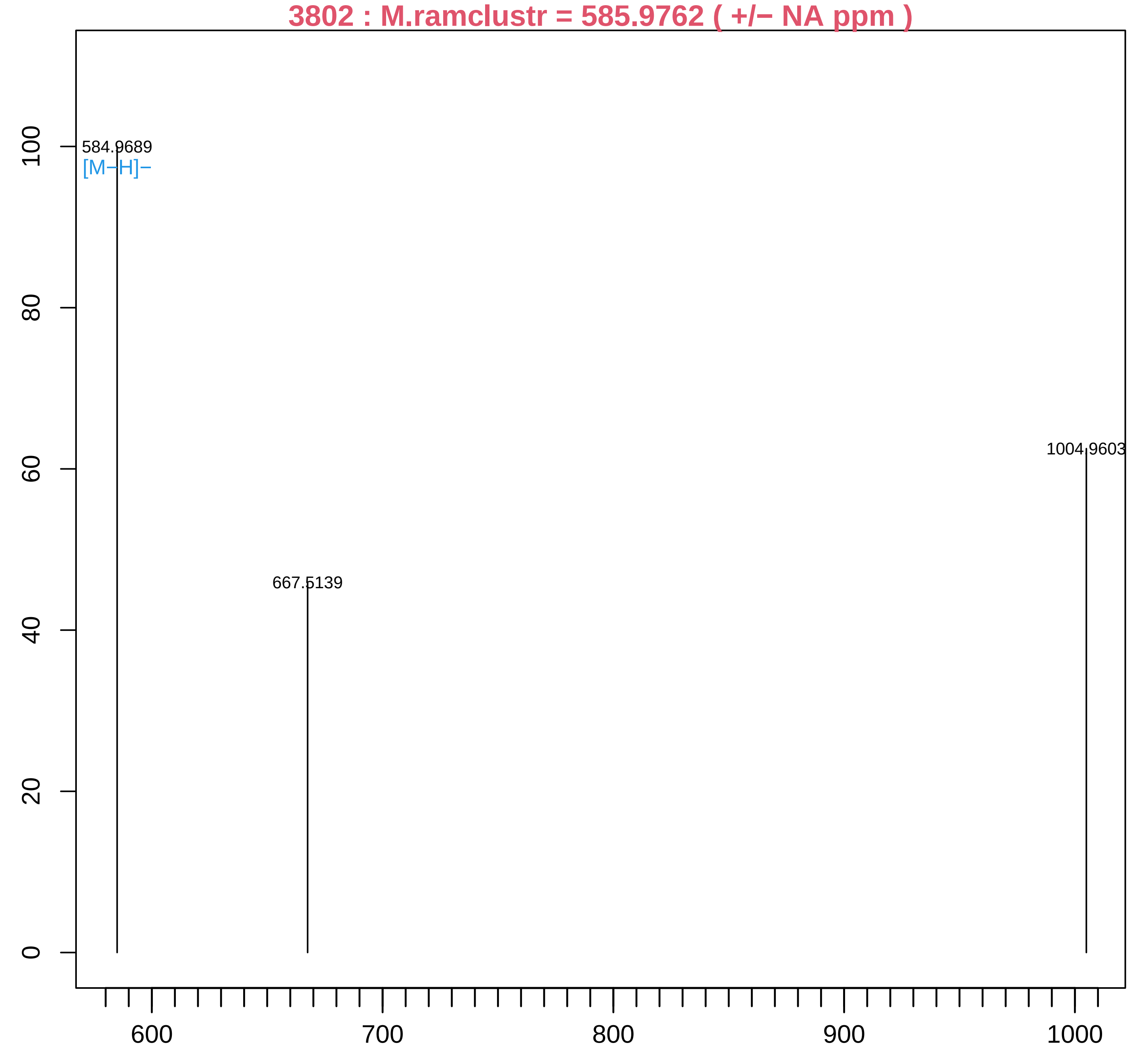
3802 : M.ramclustr = 585.9762 (+/− NA ppm)
584.9689
[M−H]−
667.5139
1004.9603
0
20
40
60
80
100
600
700
800
900
1000

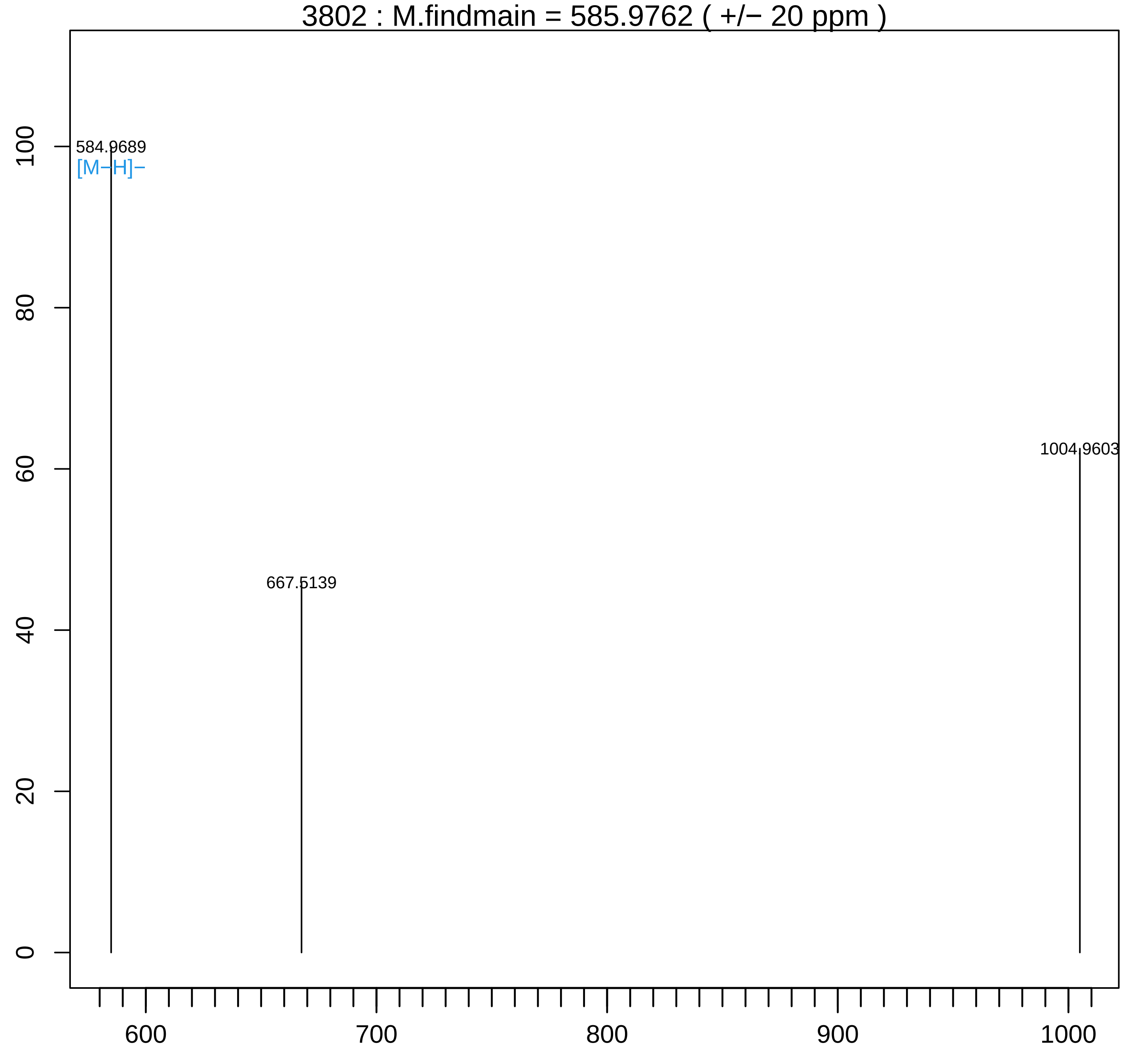
3802 : M.findmain = 585.9762 (+/− 20 ppm)
584.9689
[M−H]−
667.5139
1004.9603
0
20
40
60
80
100
600
700
800
900
1000

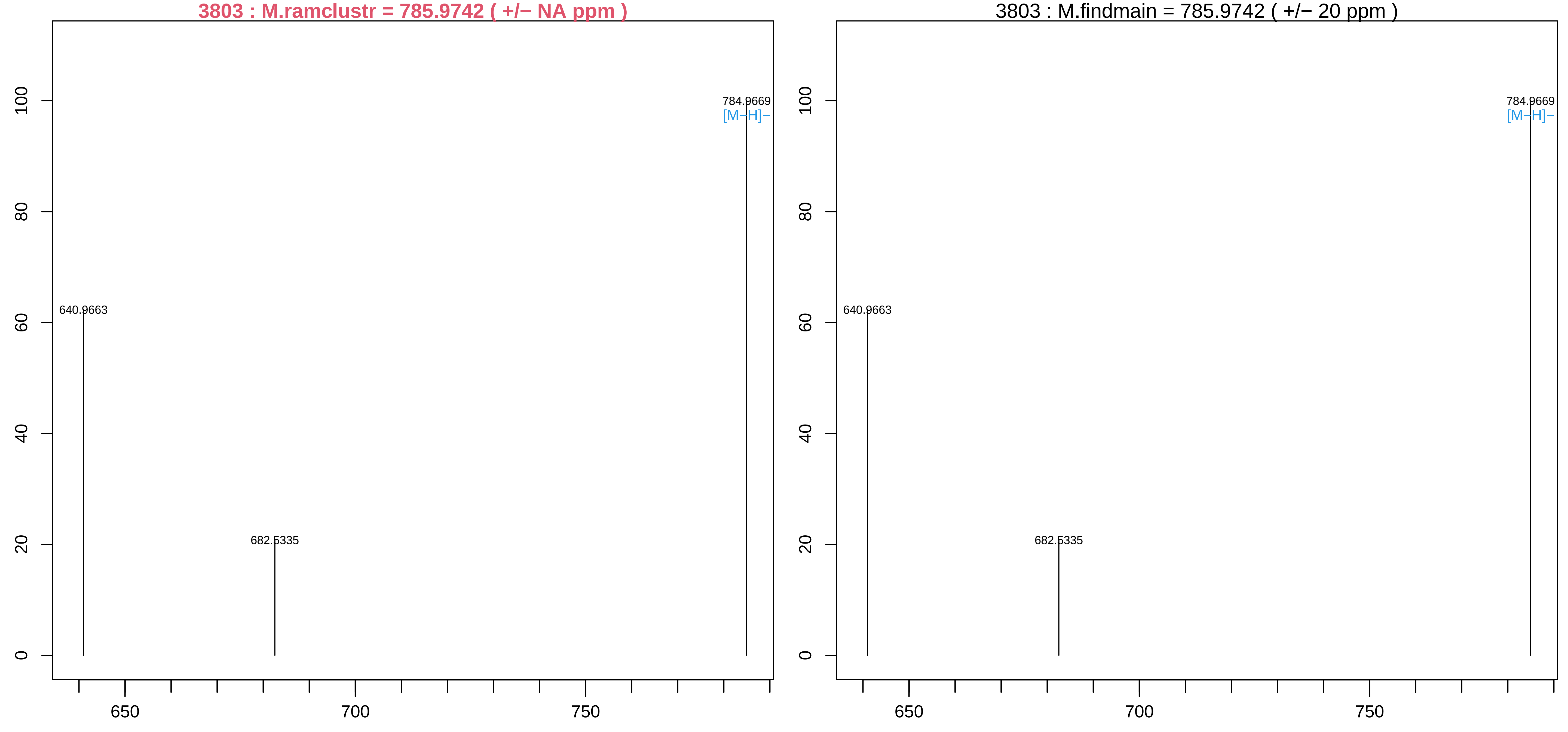
3803 : M.ramclustr = 785.9742 (+/− NA ppm)
784.9669
[M−H]−
640.9663
682.5335
0
20
40
60
80
100
650
700
750
3803 : M.findmain = 785.9742 (+/− 20 ppm)
784.9669
[M−H]−
640.9663
682.5335
0
20
40
60
80
100
650
700
750

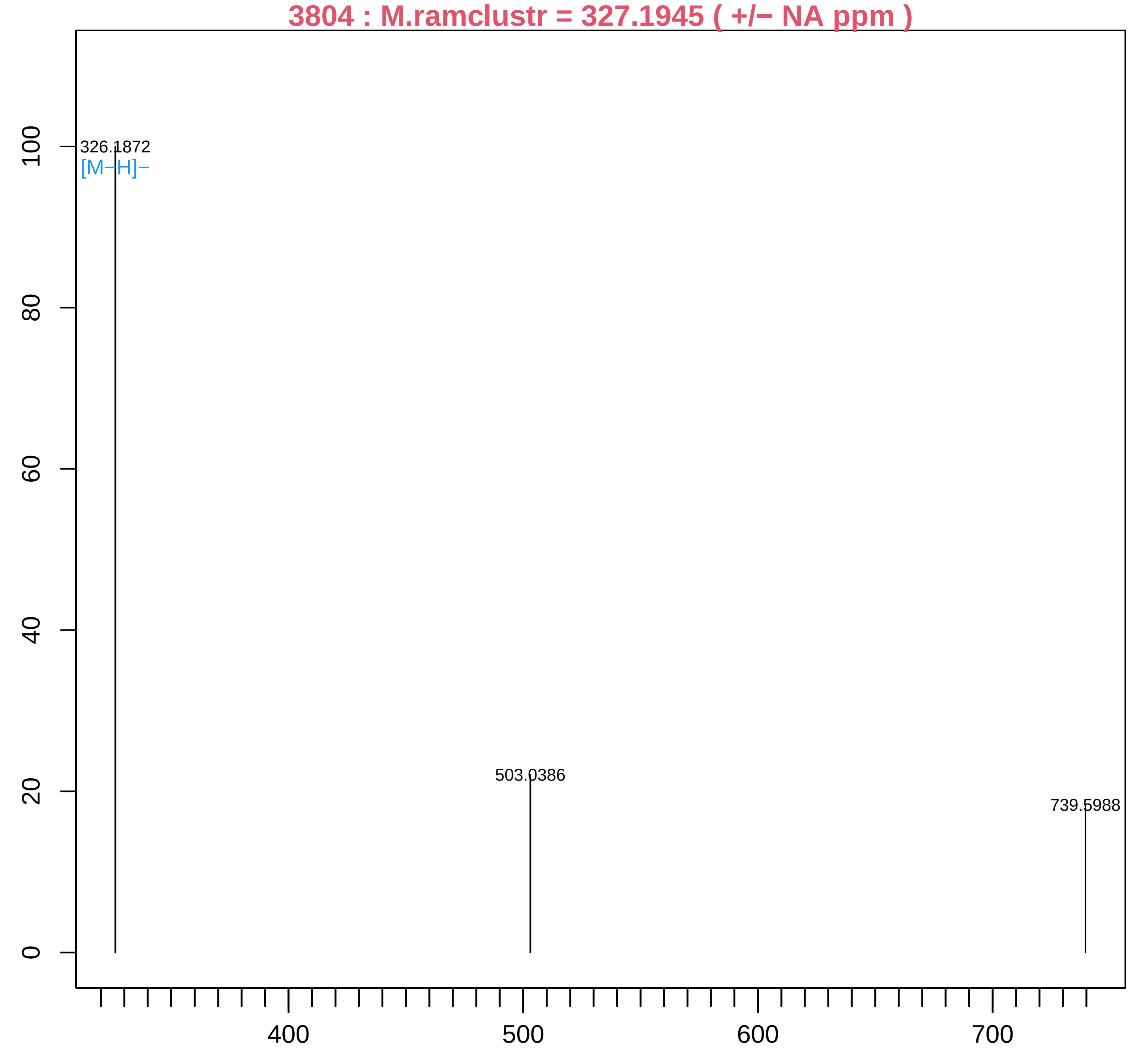
3804 : M.ramclustr = 327.1945 (+/− NA ppm)
326.1872
[M−H]−
503.0386
739.5988
0
20
40
60
80
100
400
500
600
700

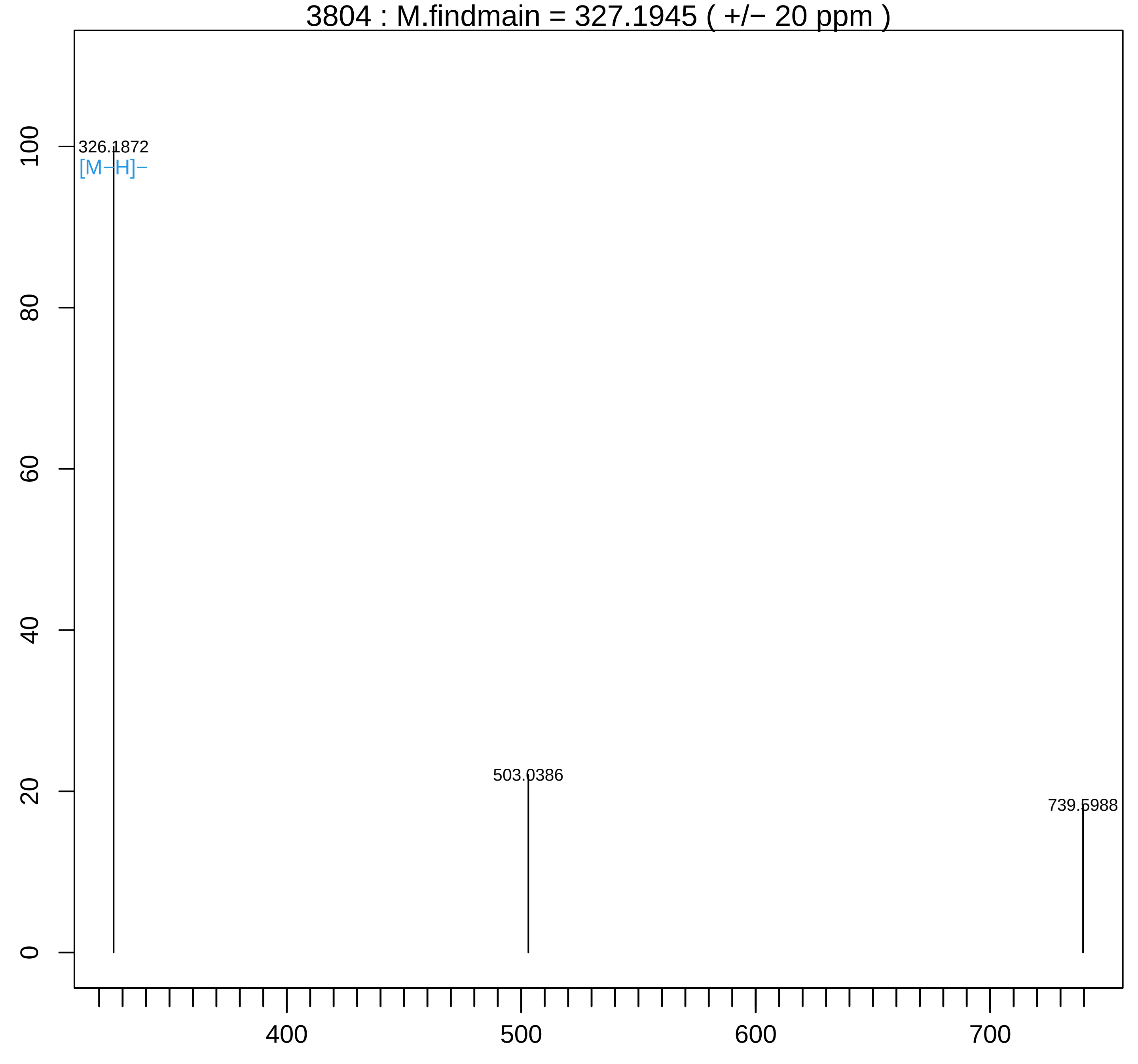
3804 : M.findmain = 327.1945 (+/− 20 ppm)
326.1872
[M−H]−
503.0386
739.5988
0
20
40
60
80
100
400
500
600
700

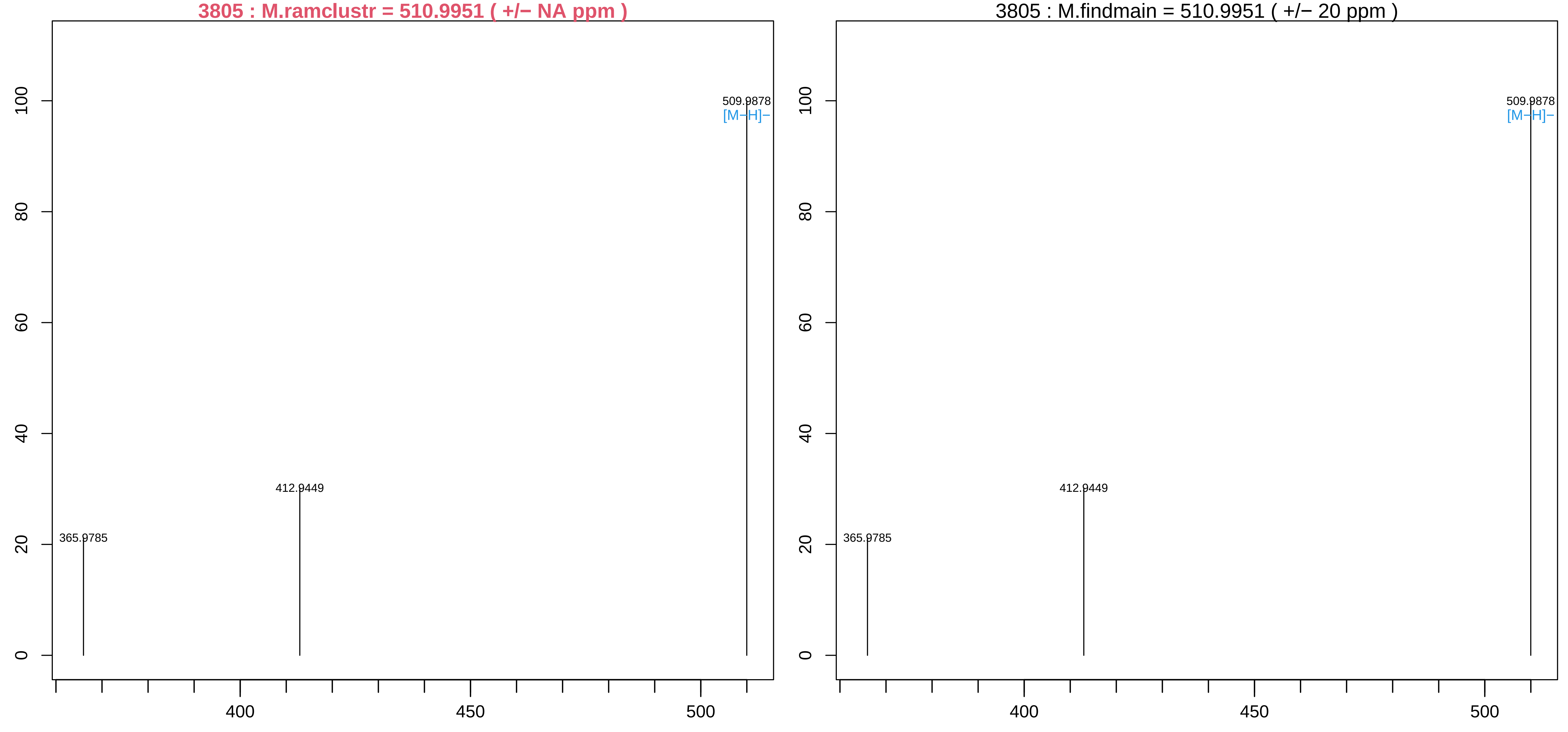
3805 : M.ramclustr = 510.9951 (+/− NA ppm)
3805 : M.findmain = 510.9951 (+/− 20 ppm)
509.9878
[M−H]−
412.9449
365.9785
0
20
40
60
80
100
400
450
500

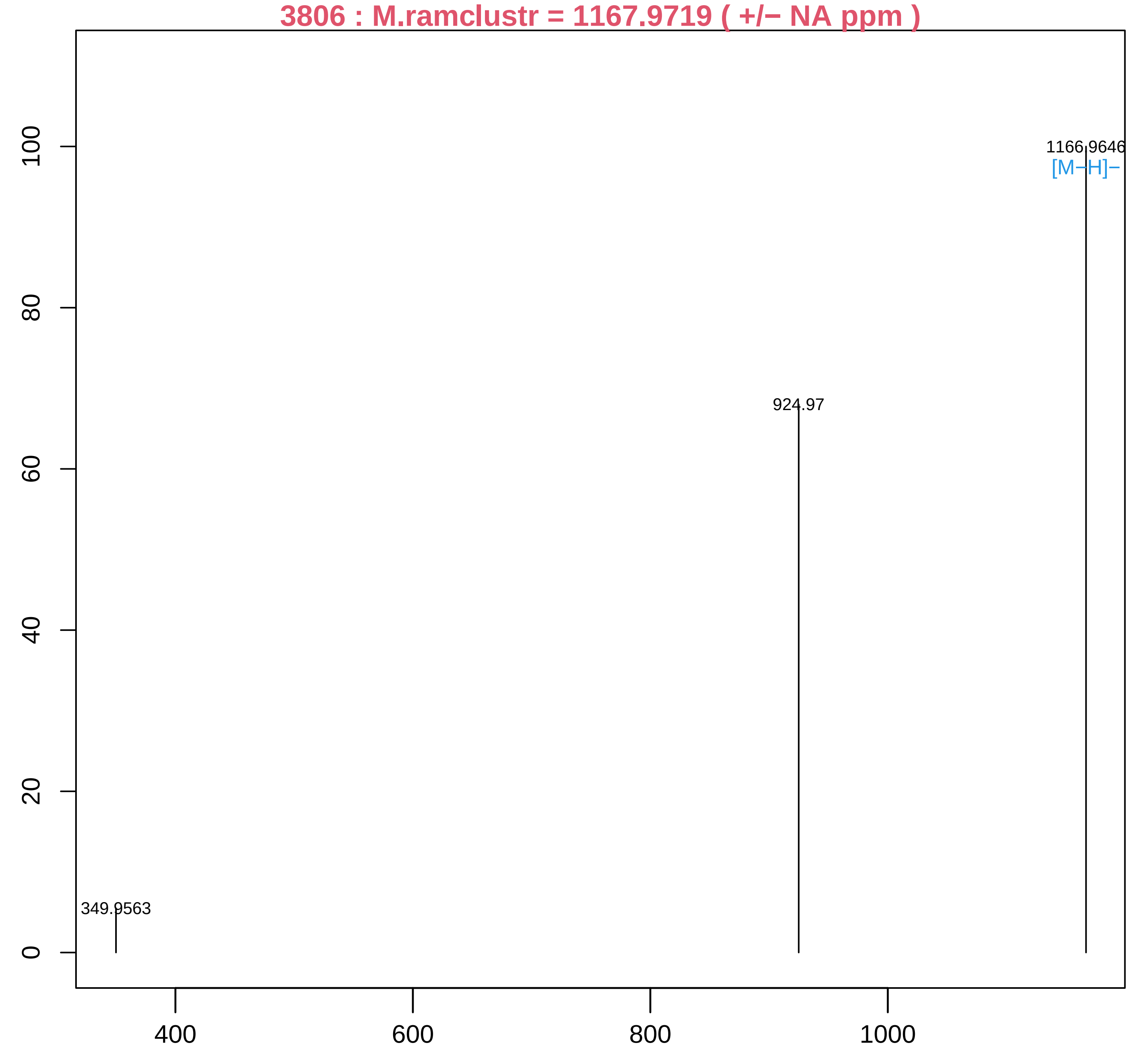
3806 : M.ramclustr = 1167.9719 (+/− NA ppm)
1166.9646
[M−H]−
924.97
349.9563
0
20
40
60
80
100
400
600
800
1000

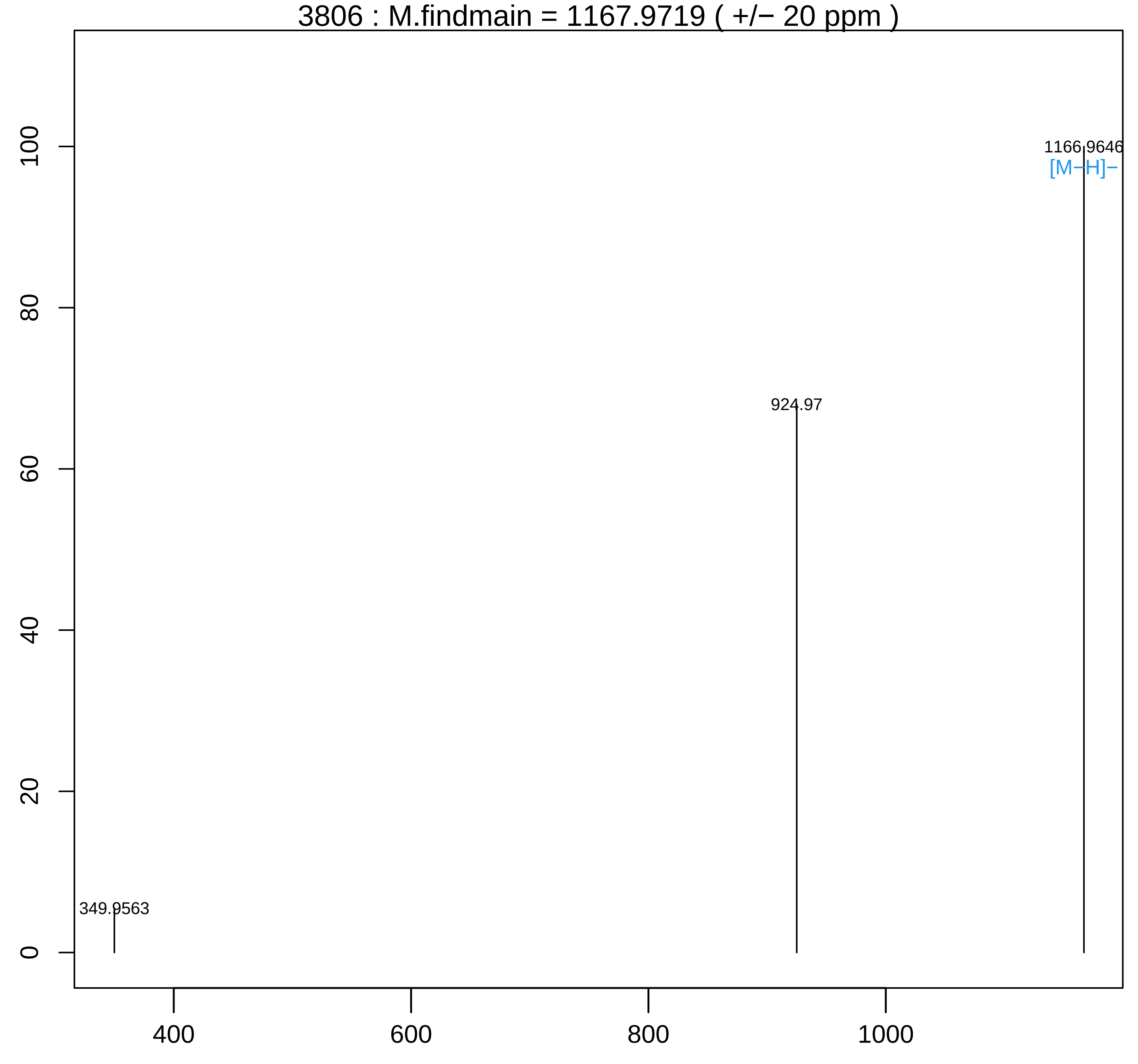
3806 : M.findmain = 1167.9719 (+/− 20 ppm)
1166.9646
[M−H]−
924.97
349.9563
0
20
40
60
80
100
400
600
800
1000

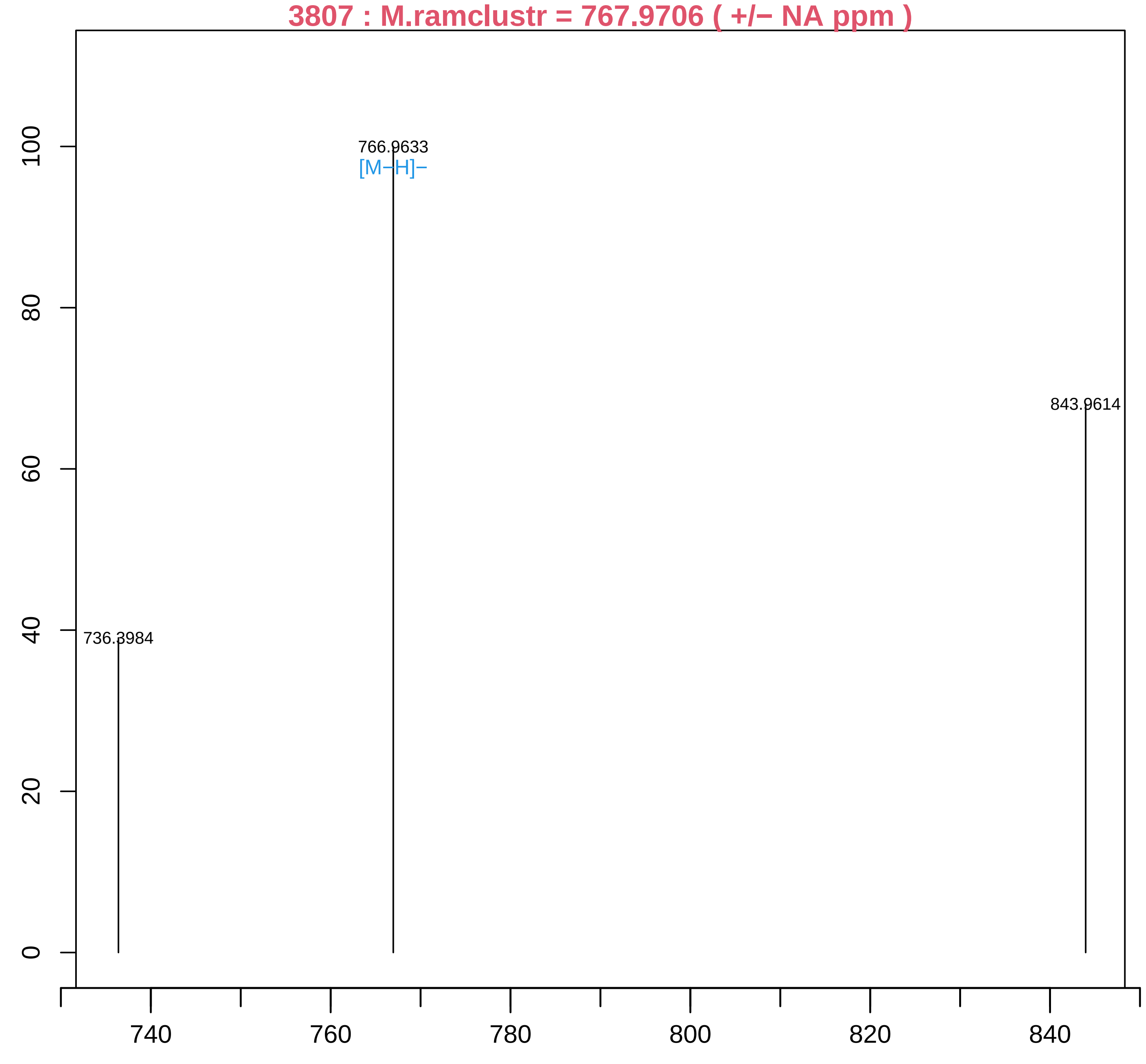
3807 : M.ramclustr = 767.9706 (+/− NA ppm)
766.9633
[M−H]−
843.9614
736.3984
0
20
40
60
80
100
740
760
780
800
820
840
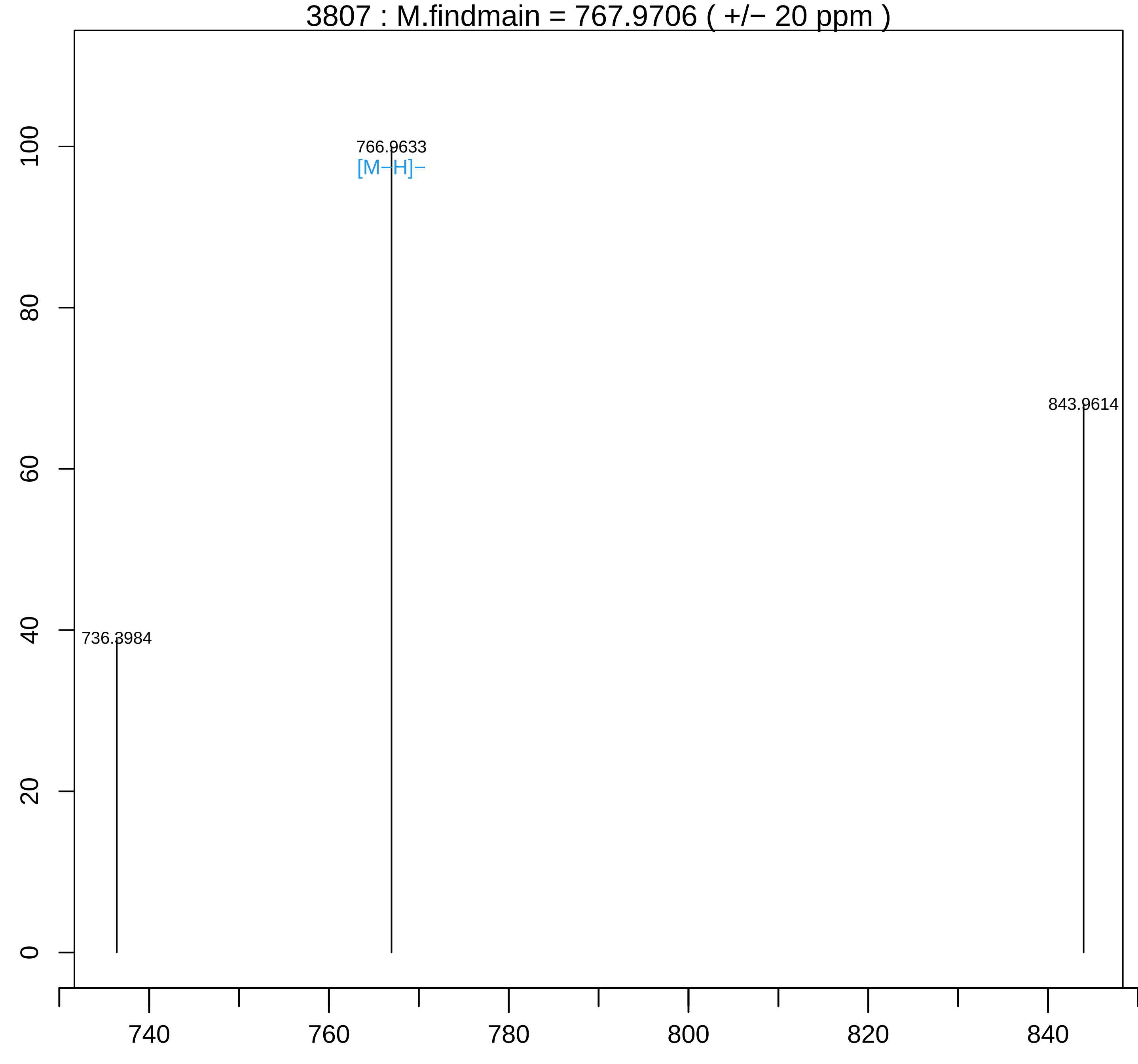
3807 : M.findmain = 767.9706 (+/− 20 ppm)
766.9633
[M−H]−
843.9614
736.3984
0
20
40
60
80
100
740
760
780
800
820
840

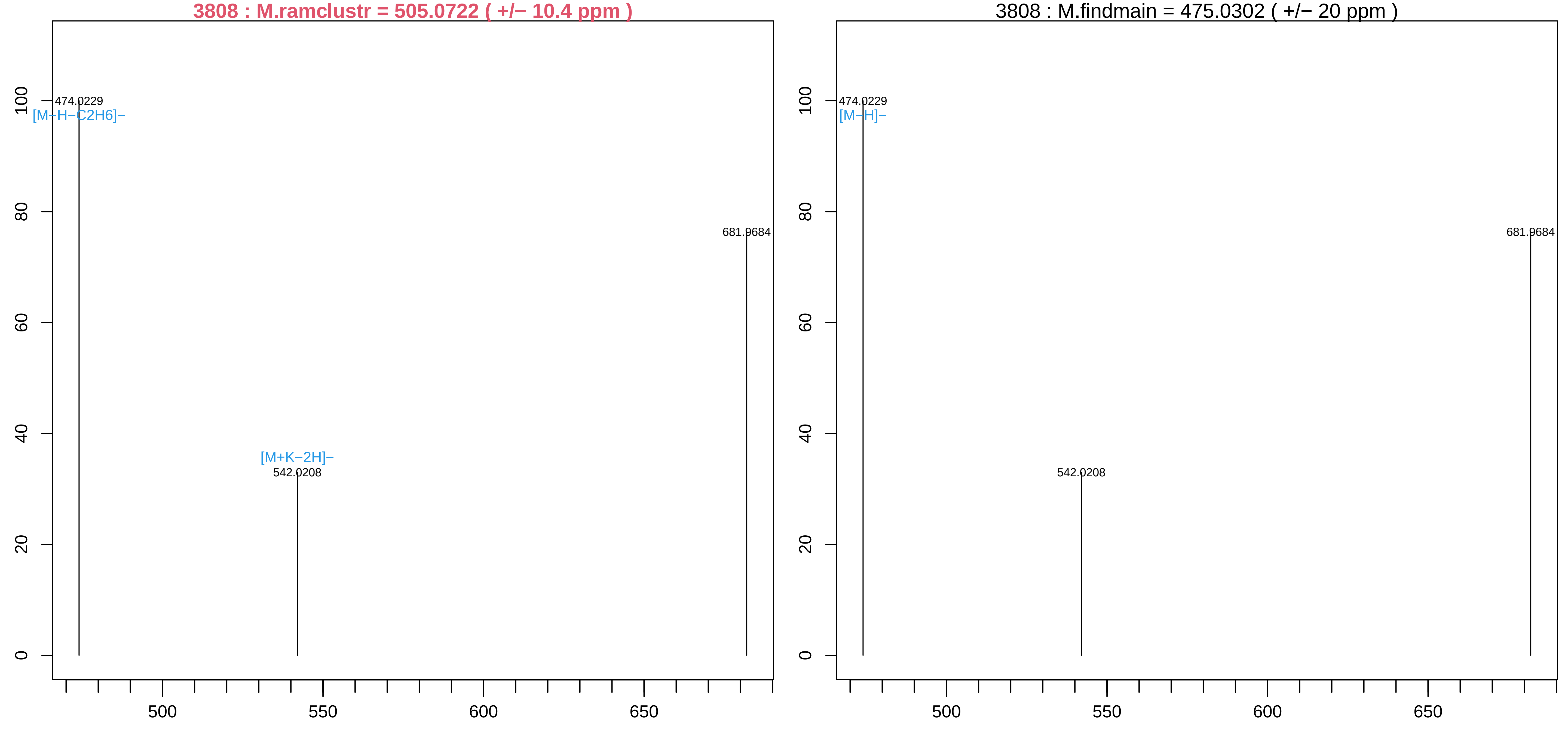
3808 : M.ramclustr = 505.0722 (+/− 10.4 ppm)
474.0229
[M−H−C2H6]−
[M+K−2H]−
542.0208
681.9684
3808 : M.findmain = 475.0302 (+/− 20 ppm)
474.0229
[M−H]−
542.0208
681.9684
0
20
40
60
80
100
500
550
600
650

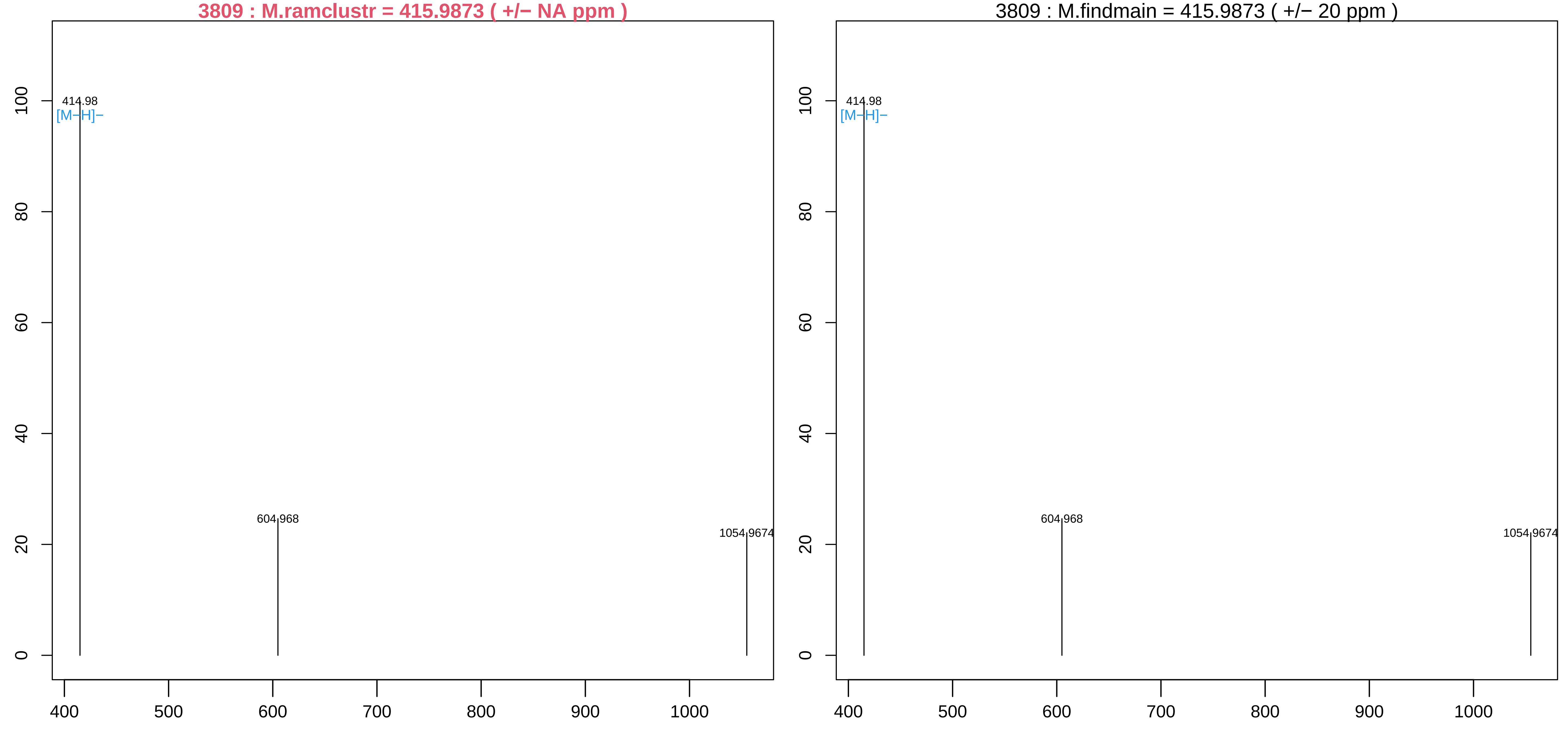
3809 : M.ramclustr = 415.9873 (+/− NA ppm)
414.98
[M−H]−
604.968
1054.9674
3809 : M.findmain = 415.9873 (+/− 20 ppm)
414.98
[M−H]−
604.968
1054.9674

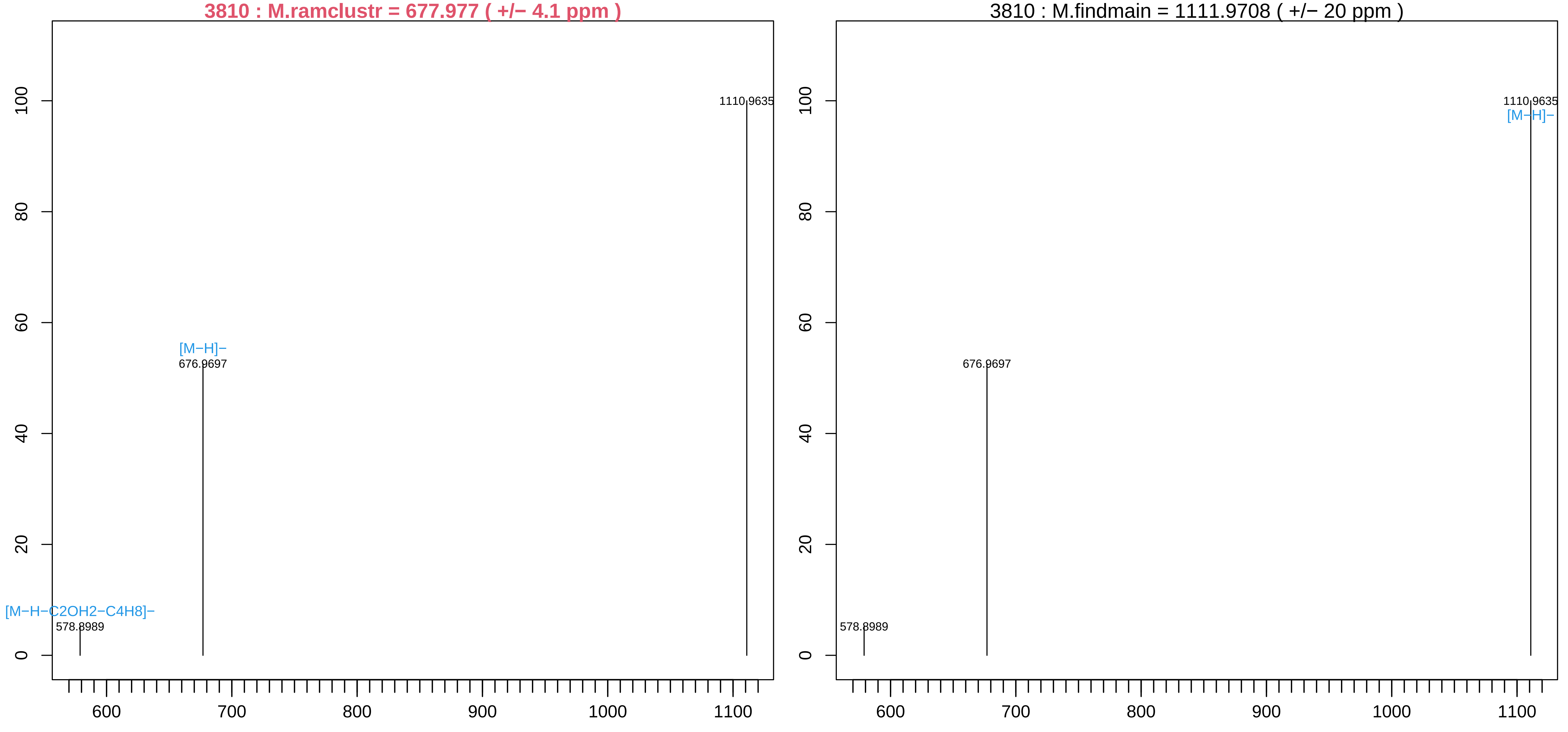
3810 : M.ramclustr = 677.977 (+/− 4.1 ppm)
1110.9635
[M−H]−
676.9697
[M−H−C2OH2−C4H8]−
578.8989
3810 : M.findmain = 1111.9708 (+/− 20 ppm)
1110.9635
[M−H]−
676.9697
578.8989

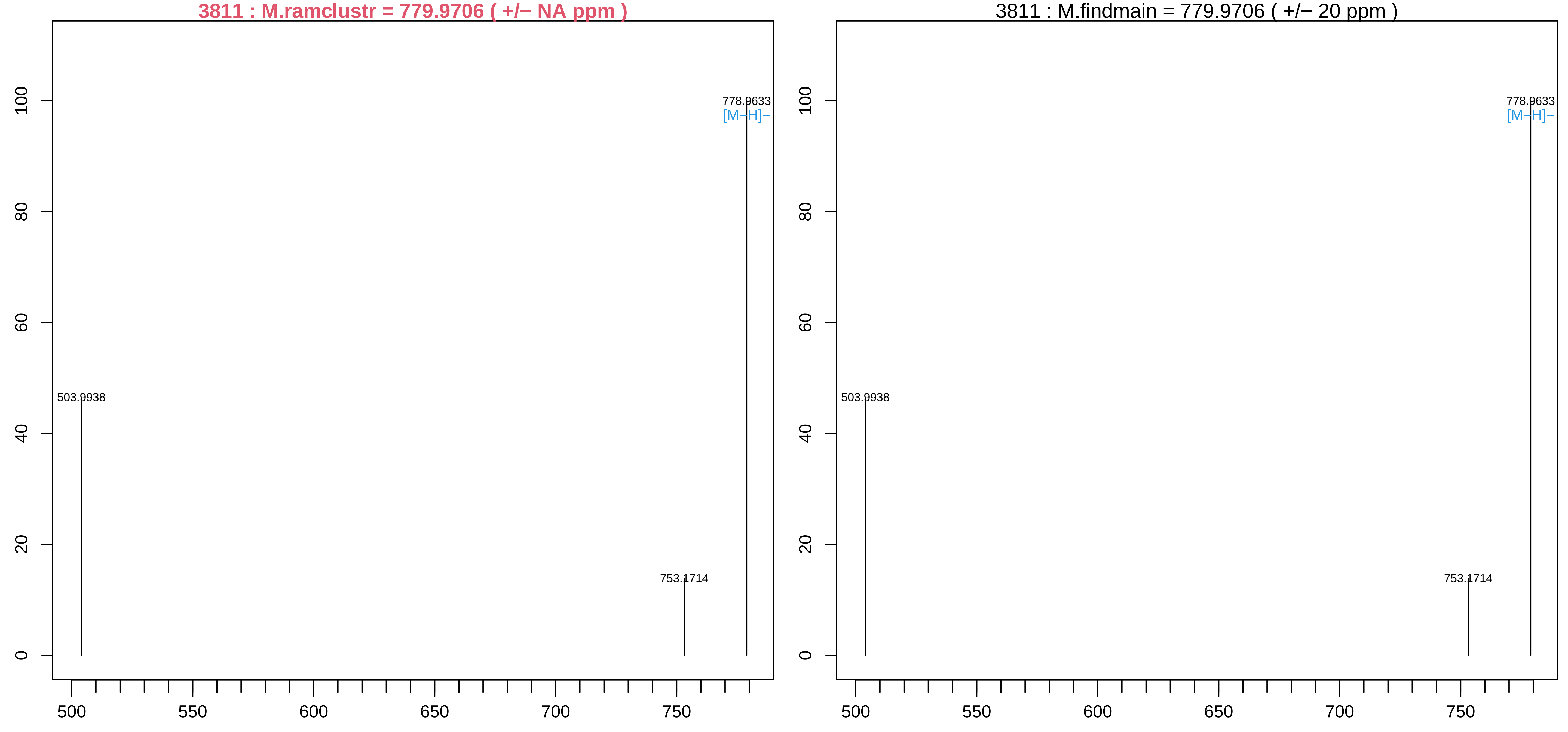
3811 : M.ramclustr = 779.9706 (+/− NA ppm)
3811 : M.findmain = 779.9706 (+/− 20 ppm)
778.9633
[M−H]−
503.9938
753.1714
0
20
40
60
80
100
500
550
600
650
700
750

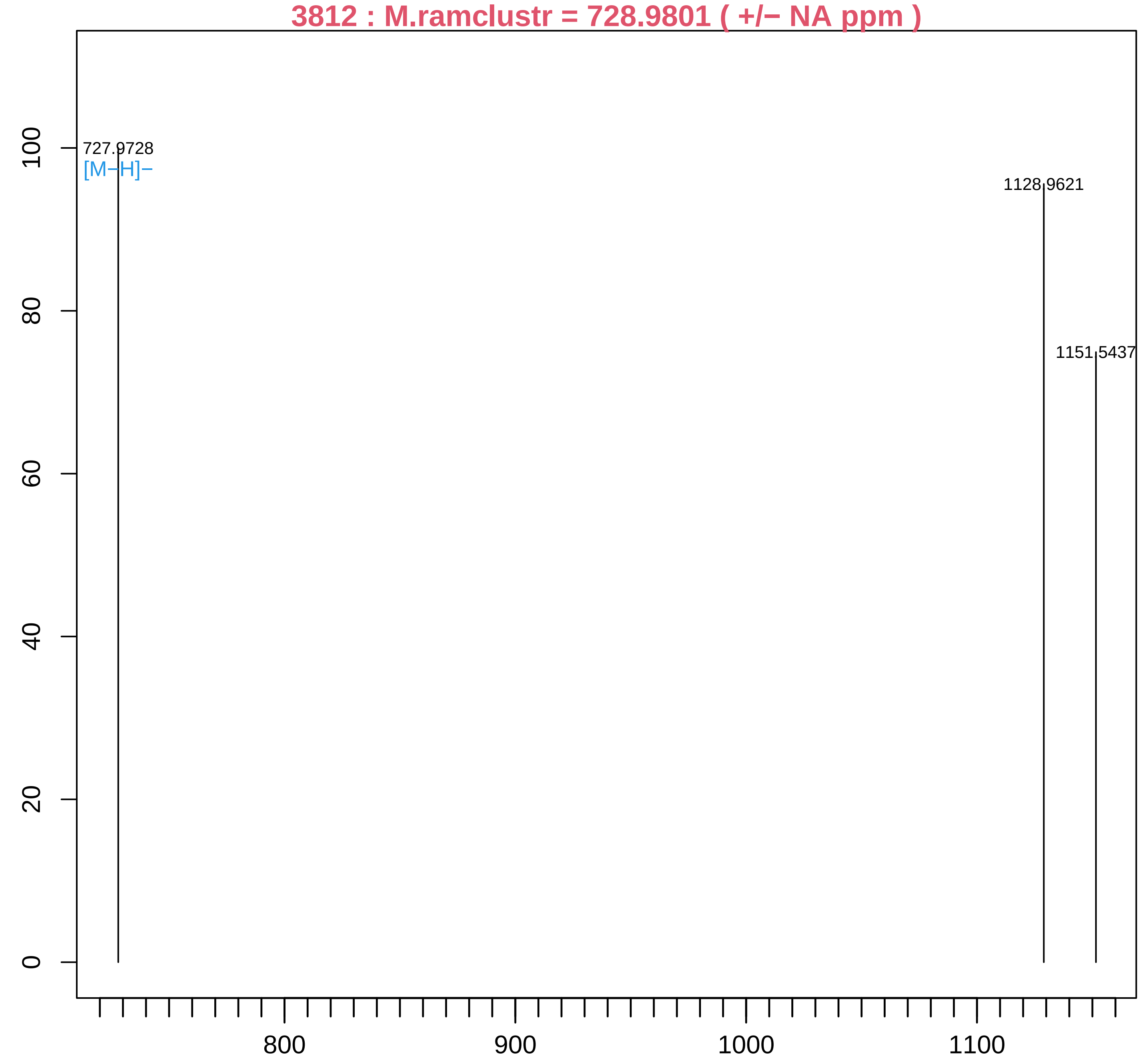
3812 : M.ramclustr = 728.9801 (+/− NA ppm)
727.9728
[M−H]−
1128.9621
1151.5437
0
20
40
60
80
100
800
900
1000
1100

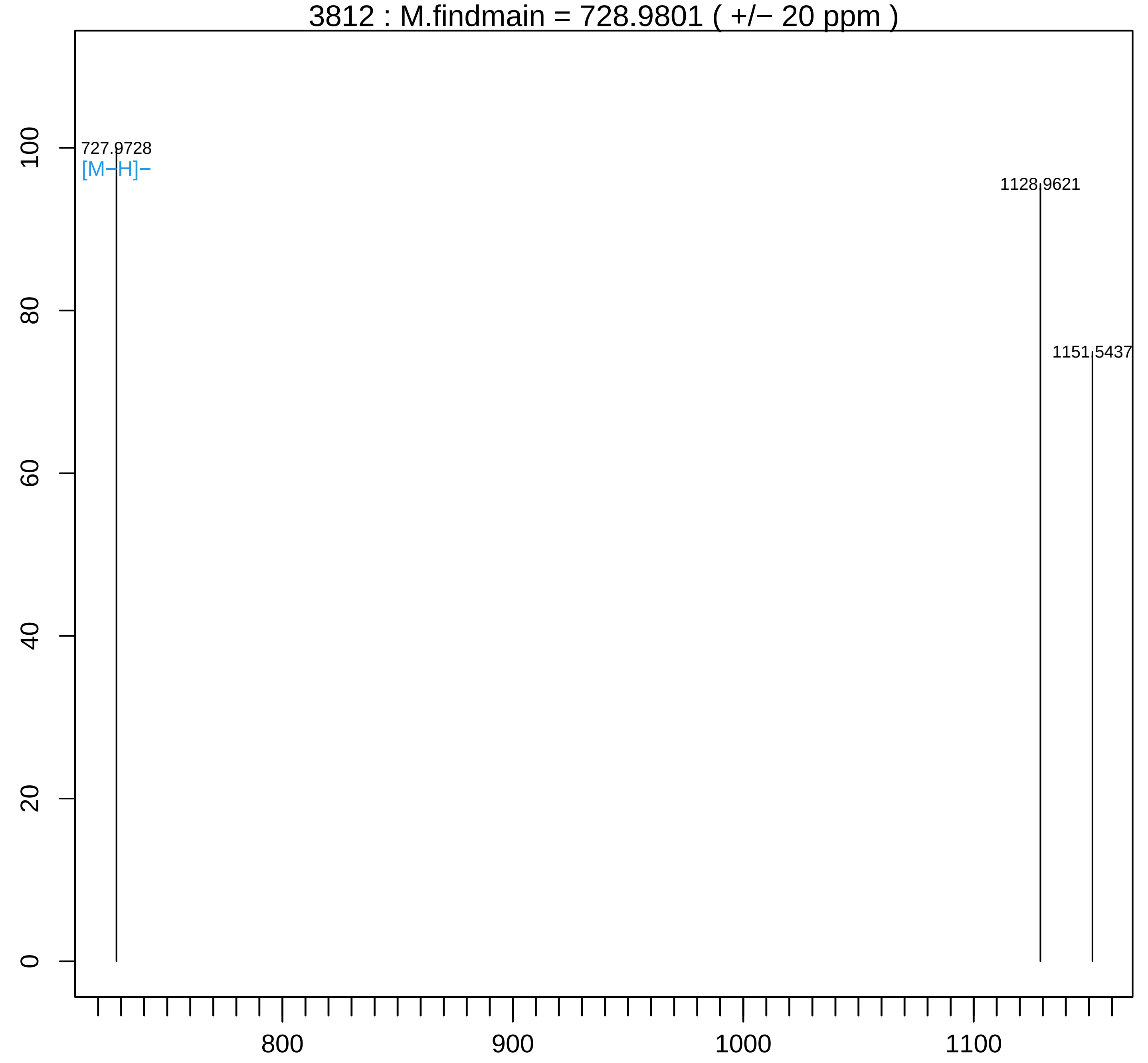
3812 : M.findmain = 728.9801 (+/− 20 ppm)
727.9728
[M−H]−
1128.9621
1151.5437
0
20
40
60
80
100
800
900
1000
1100

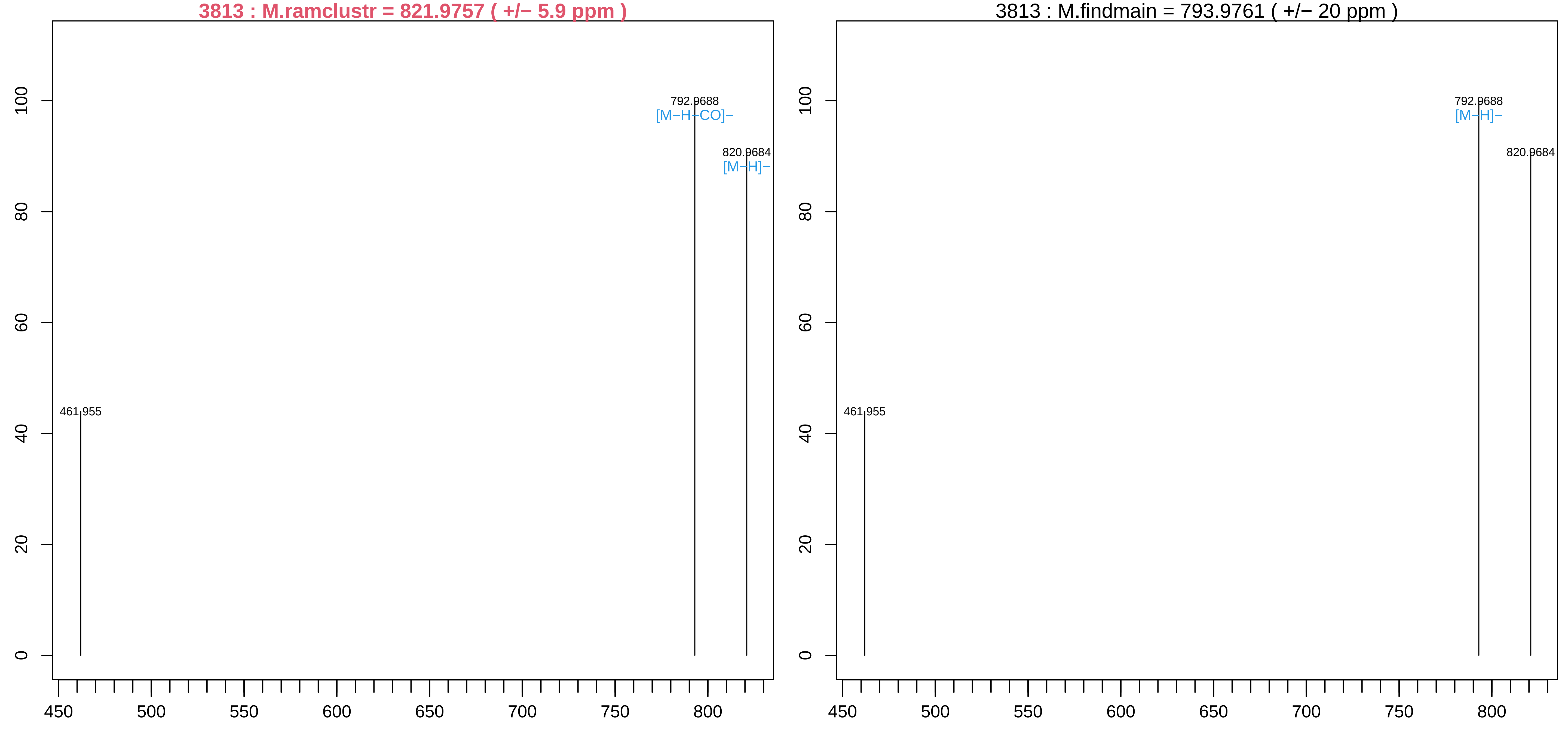
3813 : M.ramclustr = 821.9757 (+/− 5.9 ppm)
792.9688
[M−H−CO]−
820.9684
[M−H]−
461.955
3813 : M.findmain = 793.9761 (+/− 20 ppm)
792.9688
[M−H]−
820.9684
461.955
0
20
40
60
80
100
450
500
550
600
650
700
750
800

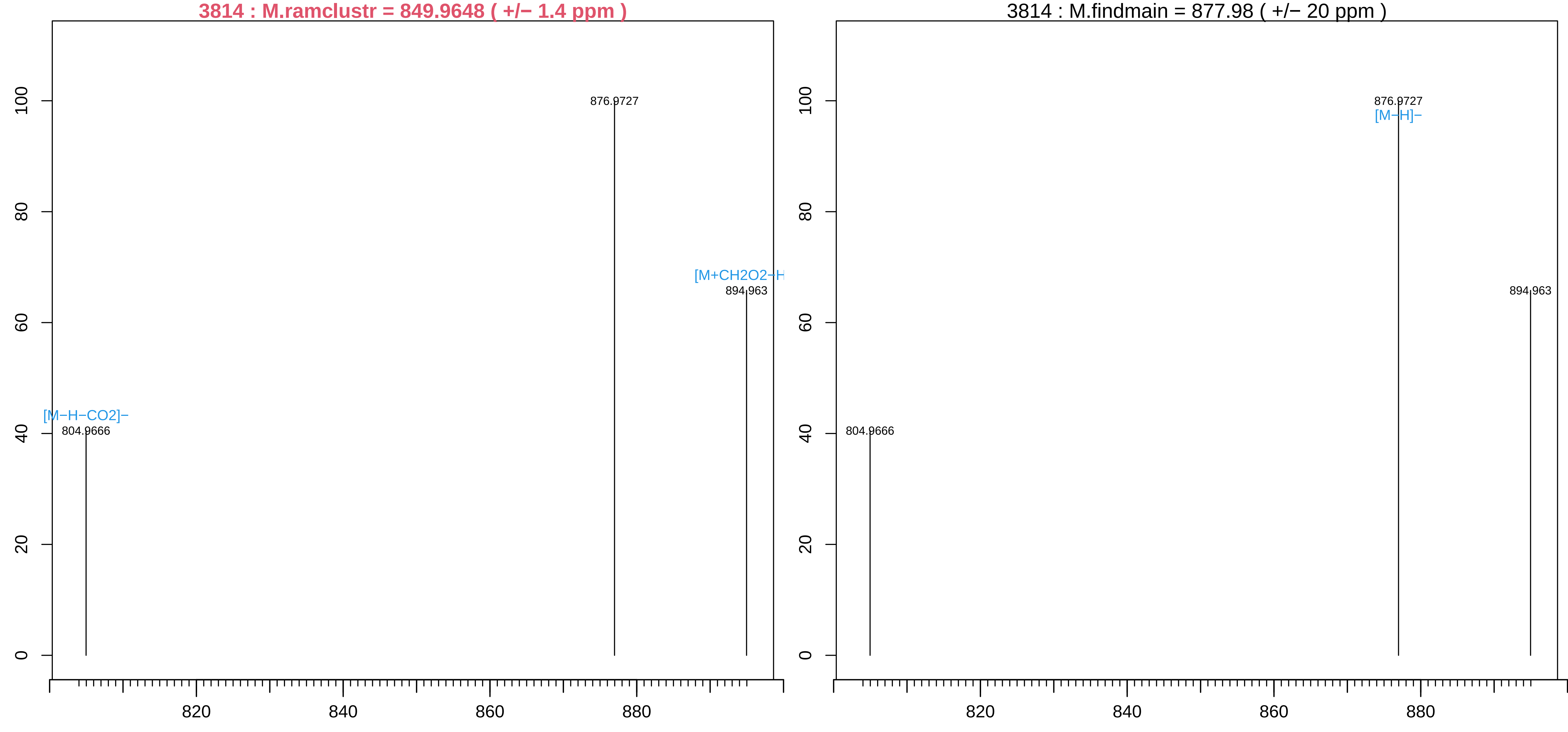
3814 : M.ramclustr = 849.9648 (+/− 1.4 ppm)
876.9727
[M+CH2O2−H
894.963
[M−H−CO2]−
804.9666
3814 : M.findmain = 877.98 (+/− 20 ppm)
876.9727
[M−H]−
894.963
804.9666
0
20
40
60
80
100
820
840
860
880

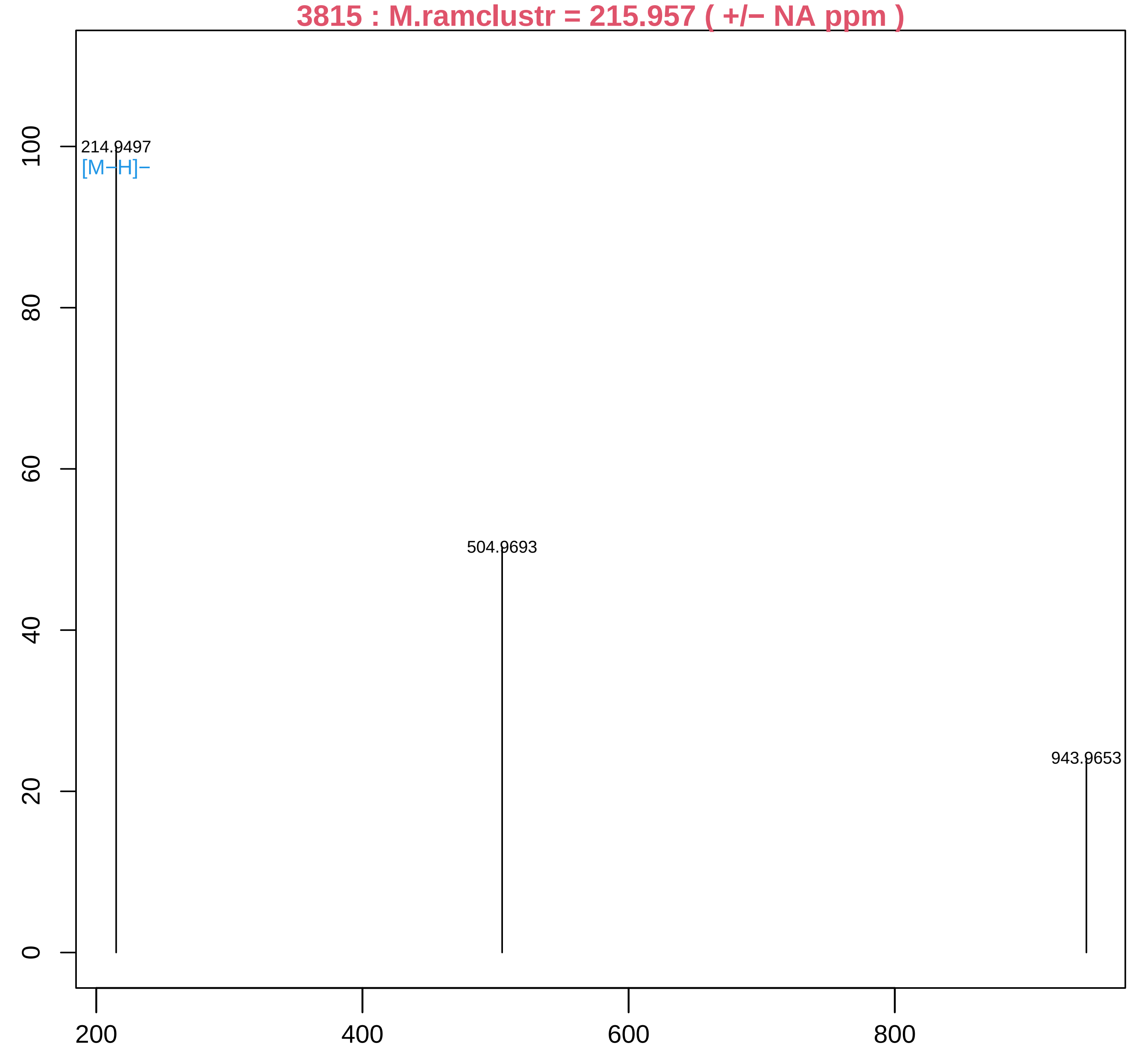
3815 : M.ramclustr = 215.957 (+/− NA ppm)
214.9497
[M−H]−
504.9693
943.9653
0
20
40
60
80
100
200
400
600
800

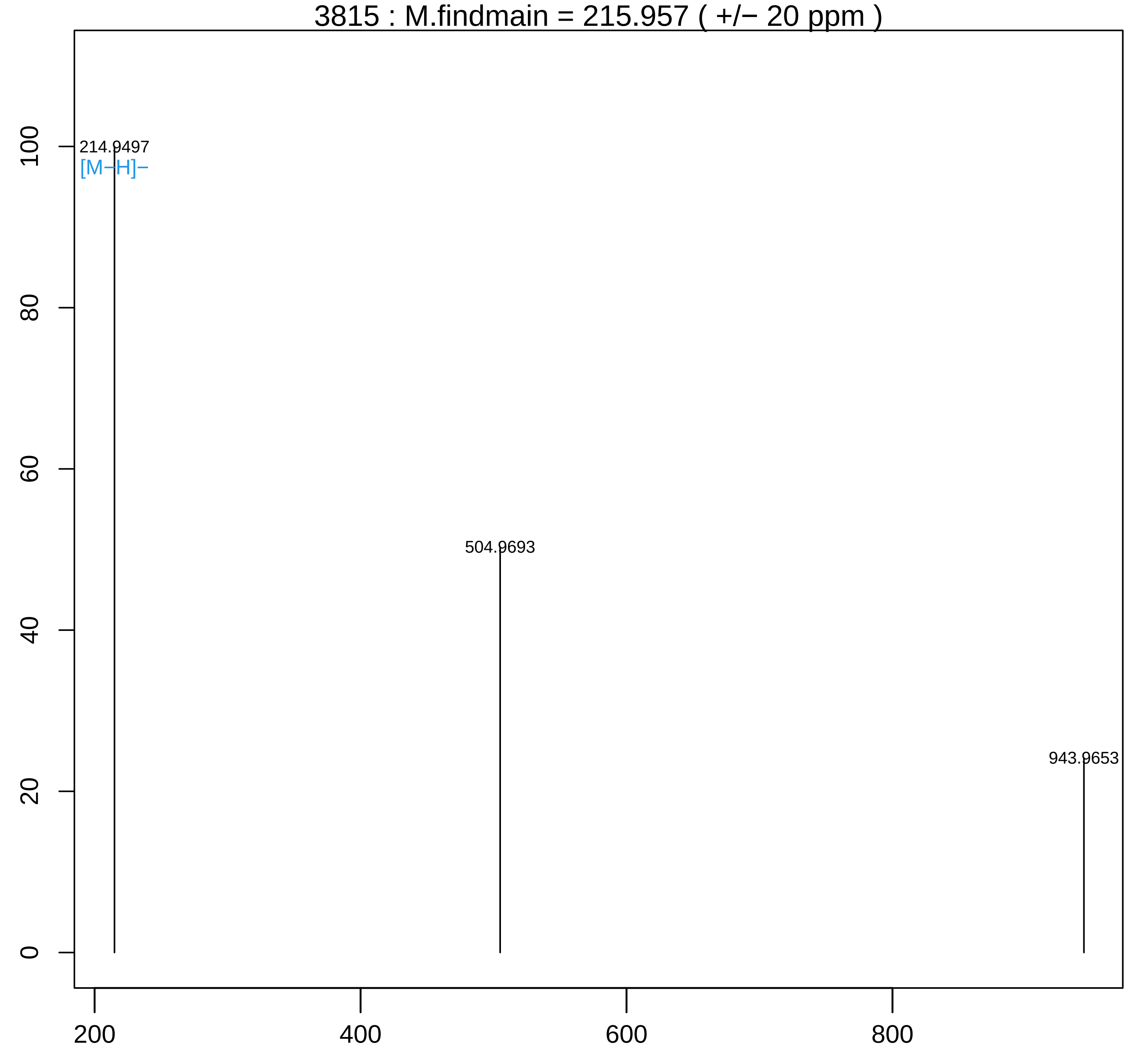
3815 : M.findmain = 215.957 (+/− 20 ppm)
214.9497
[M−H]−
504.9693
943.9653
0
20
40
60
80
100
200
400
600
800

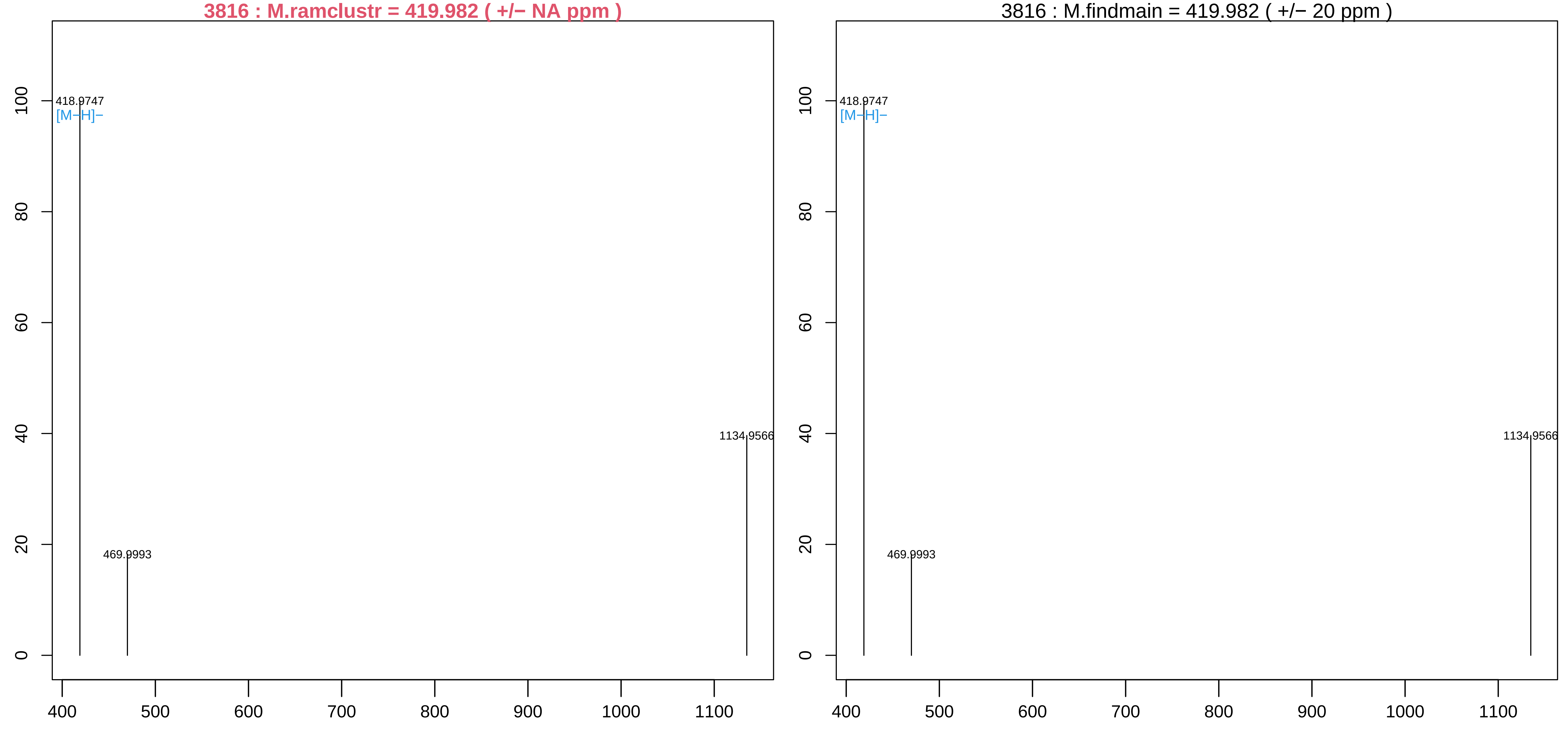
3816 : M.ramclustr = 419.982 (+/− NA ppm)
418.9747
[M−H]−
469.9993
1134.9566
3816 : M.findmain = 419.982 (+/− 20 ppm)
418.9747
[M−H]−
469.9993
1134.9566
0
20
40
60
80
100
400
500
600
700
800
900
1000
1100

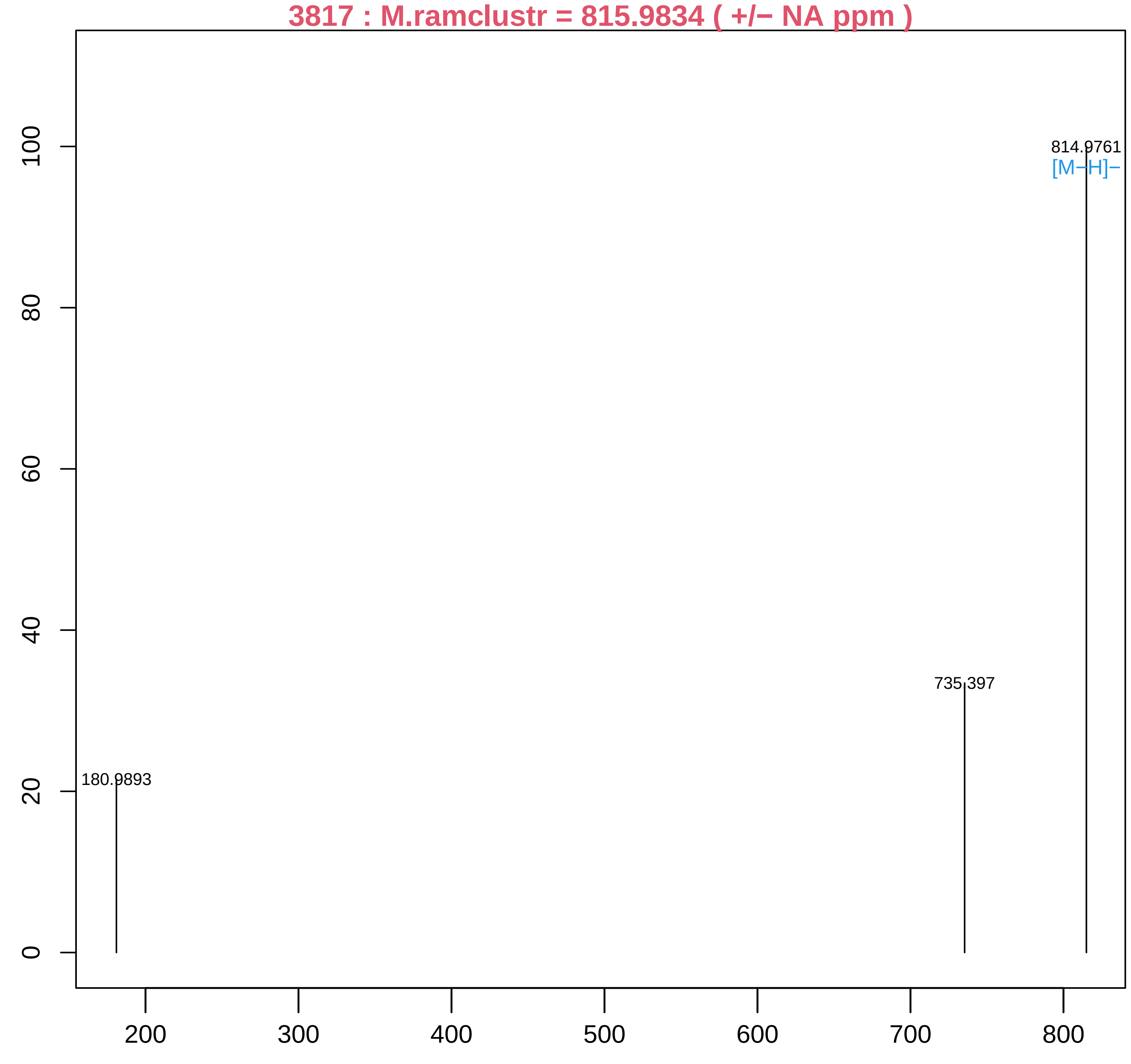
3817 : M.ramclustr = 815.9834 (+/− NA ppm)
814.9761
[M−H]−
735.397
180.9893
0
20
40
60
80
100
200
300
400
500
600
700
800

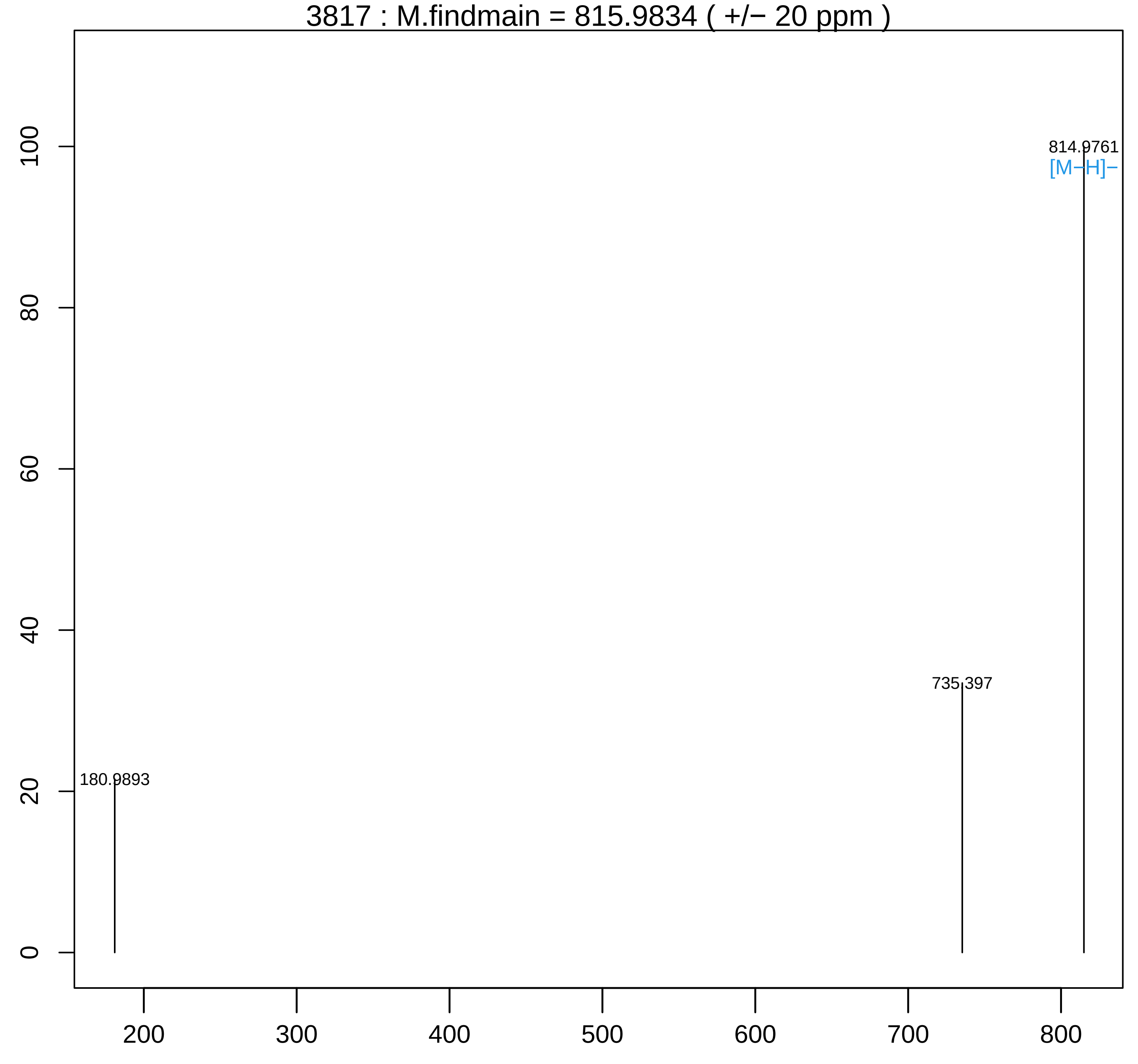
3817 : M.findmain = 815.9834 (+/− 20 ppm)
814.9761
[M−H]−
735.397
180.9893
0
20
40
60
80
100
200
300
400
500
600
700
800

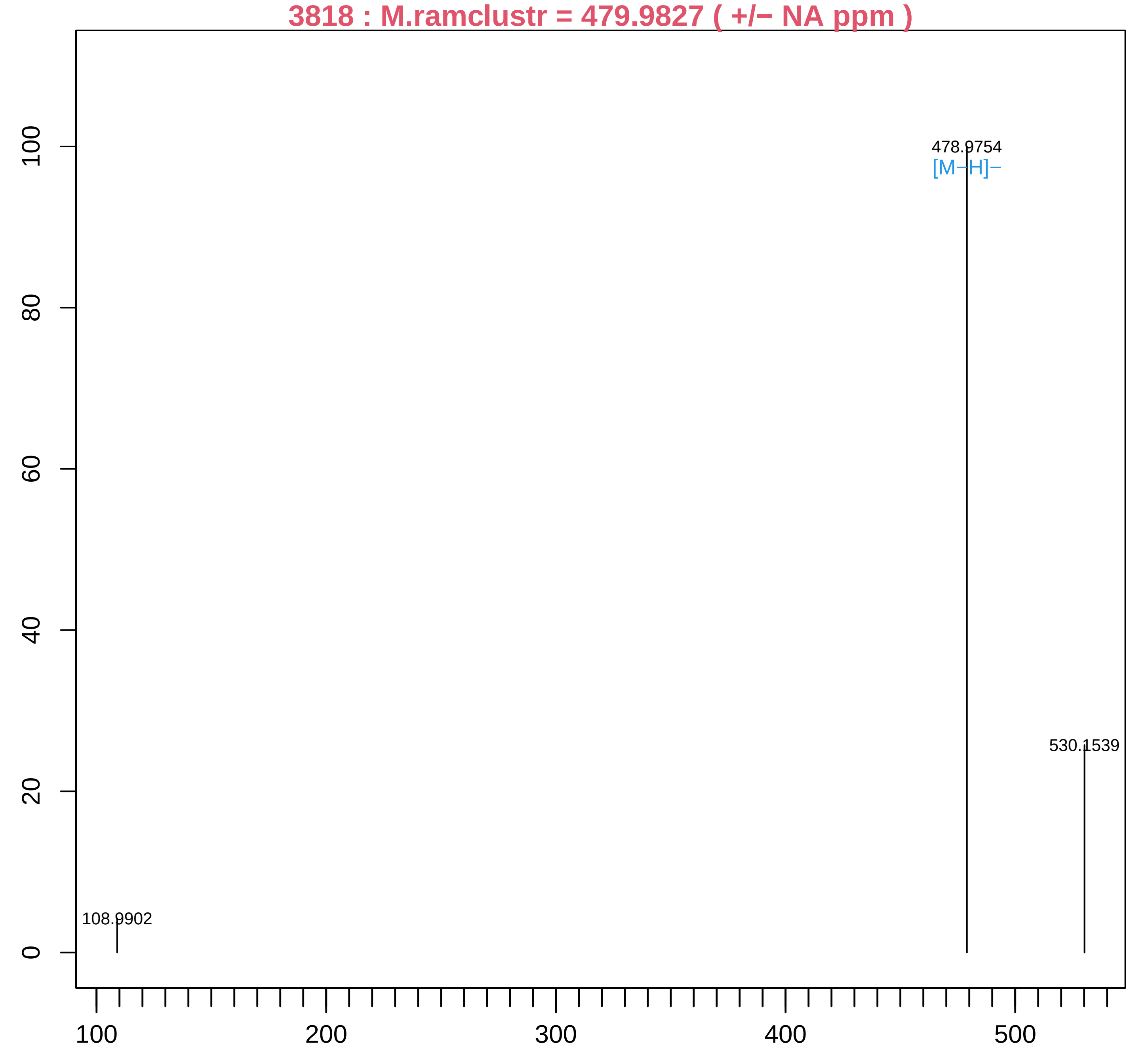
3818 : M.ramclustr = 479.9827 (+/− NA ppm)
478.9754
[M−H]−
530.1539
108.9902
0
20
40
60
80
100
100
200
300
400
500

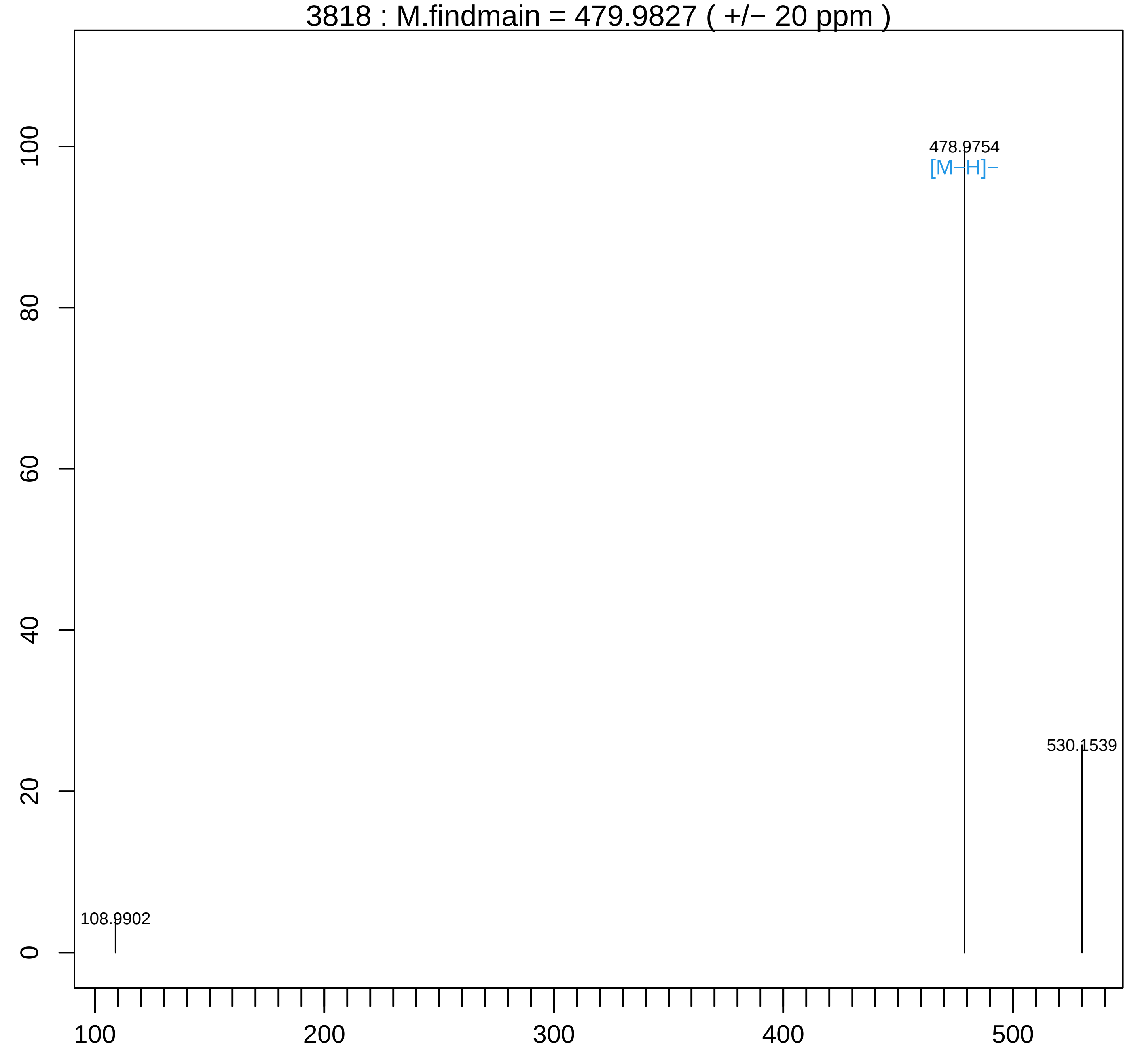
3818 : M.findmain = 479.9827 (+/− 20 ppm)
478.9754
[M−H]−
530.1539
108.9902
0
20
40
60
80
100
100
200
300
400
500

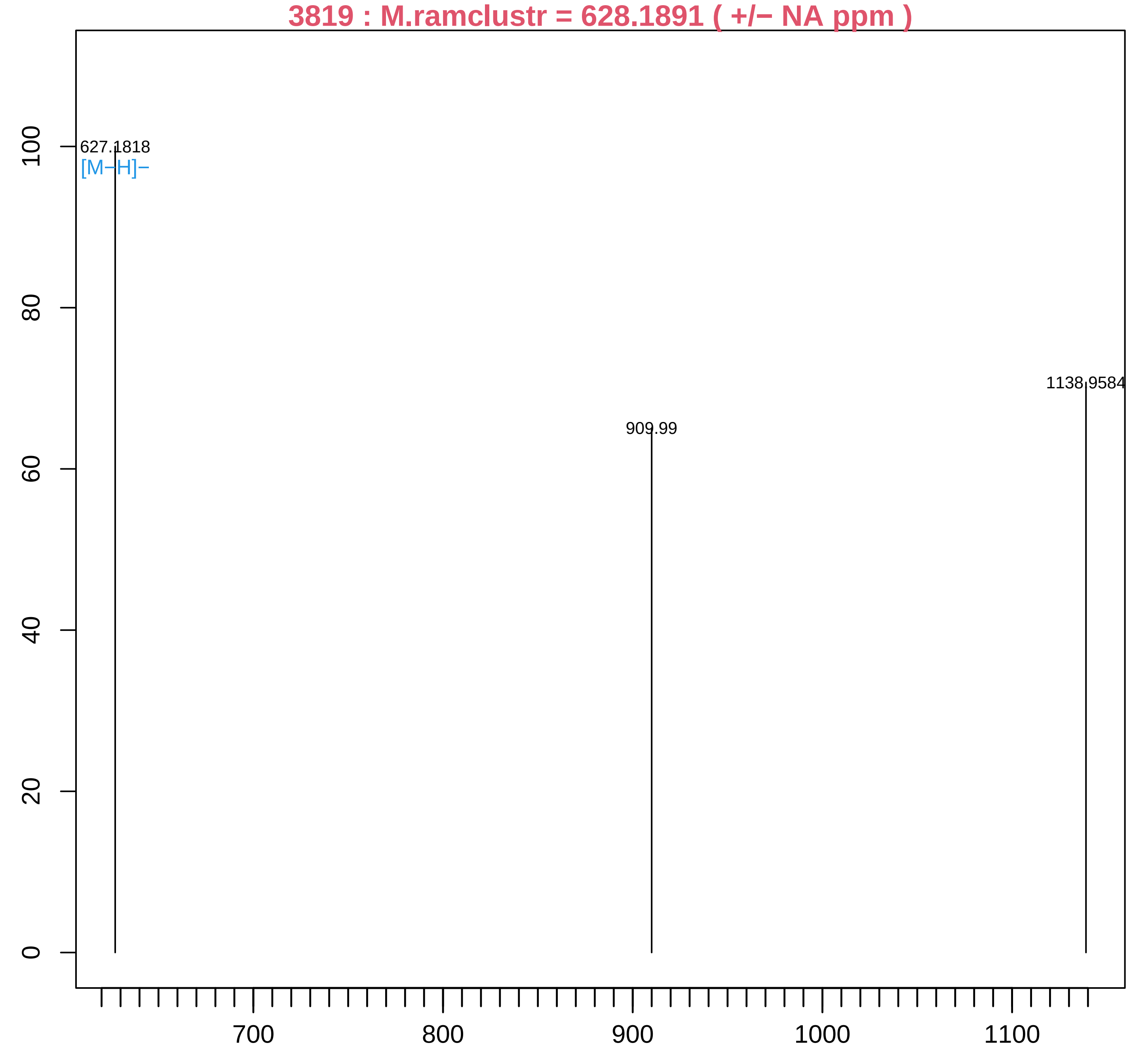
3819 : M.ramclustr = 628.1891 (+/− NA ppm)
627.1818
[M−H]−
909.99
1138.9584
0
20
40
60
80
100
700
800
900
1000
1100

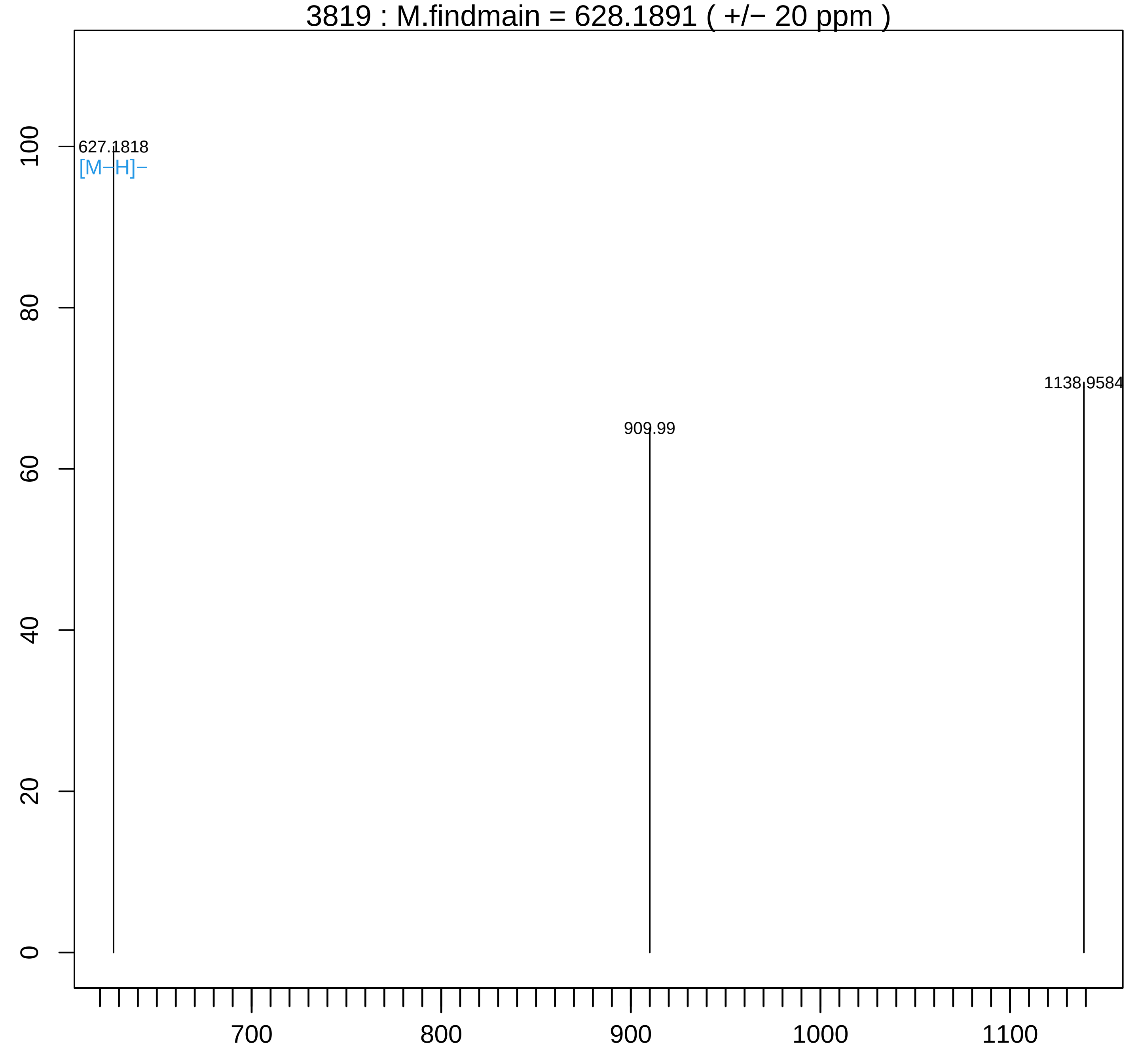
3819 : M.findmain = 628.1891 (+/− 20 ppm)
627.1818
[M−H]−
909.99
1138.9584
0
20
40
60
80
100
700
800
900
1000
1100

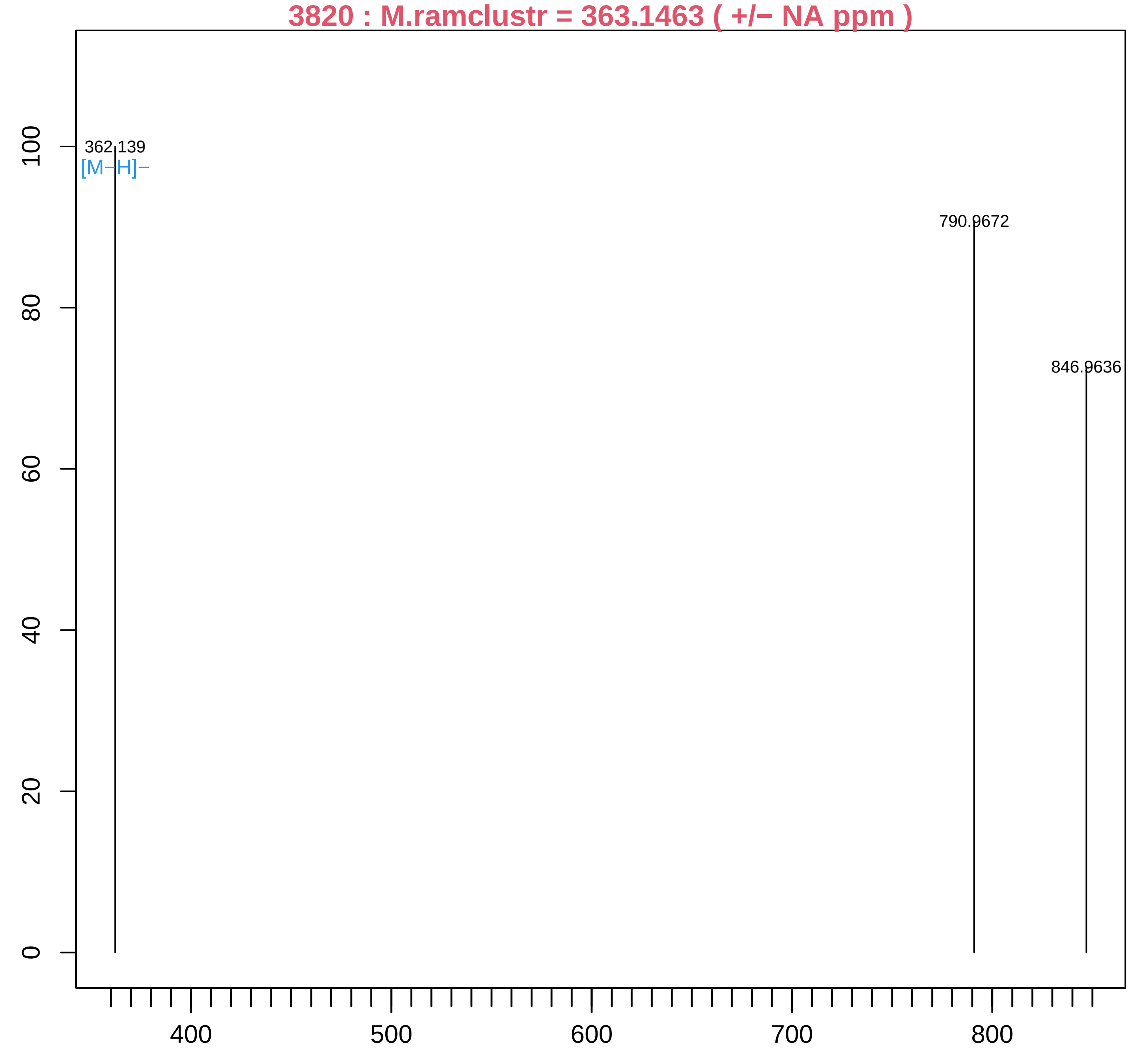
3820 : M.ramclustr = 363.1463 (+/− NA ppm)
362.139
[M−H]−
790.9672
846.9636
0
20
40
60
80
100
400
500
600
700
800

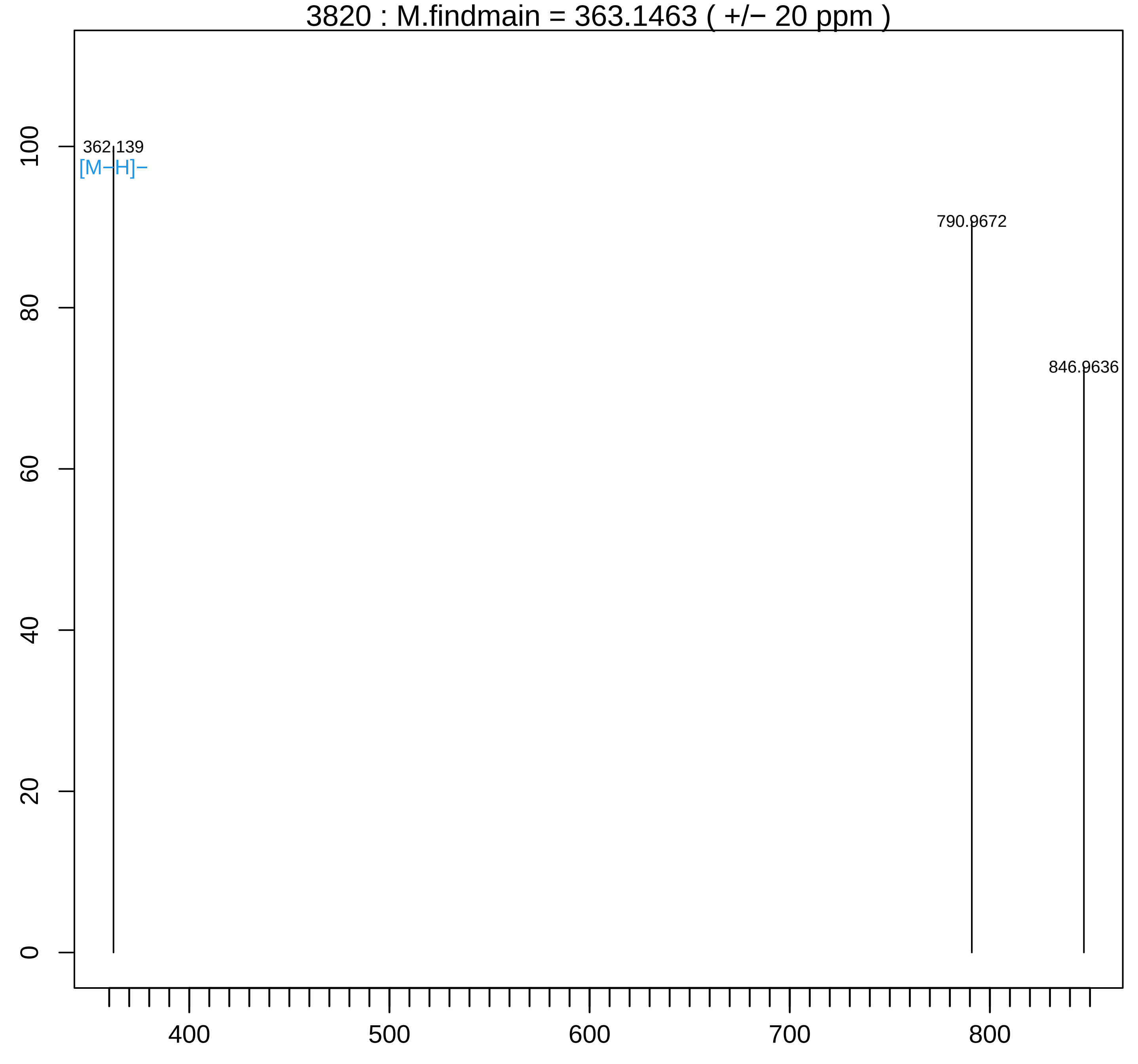
3820 : M.findmain = 363.1463 (+/− 20 ppm)
362.139
[M−H]−
790.9672
846.9636
0
20
40
60
80
100
400
500
600
700
800

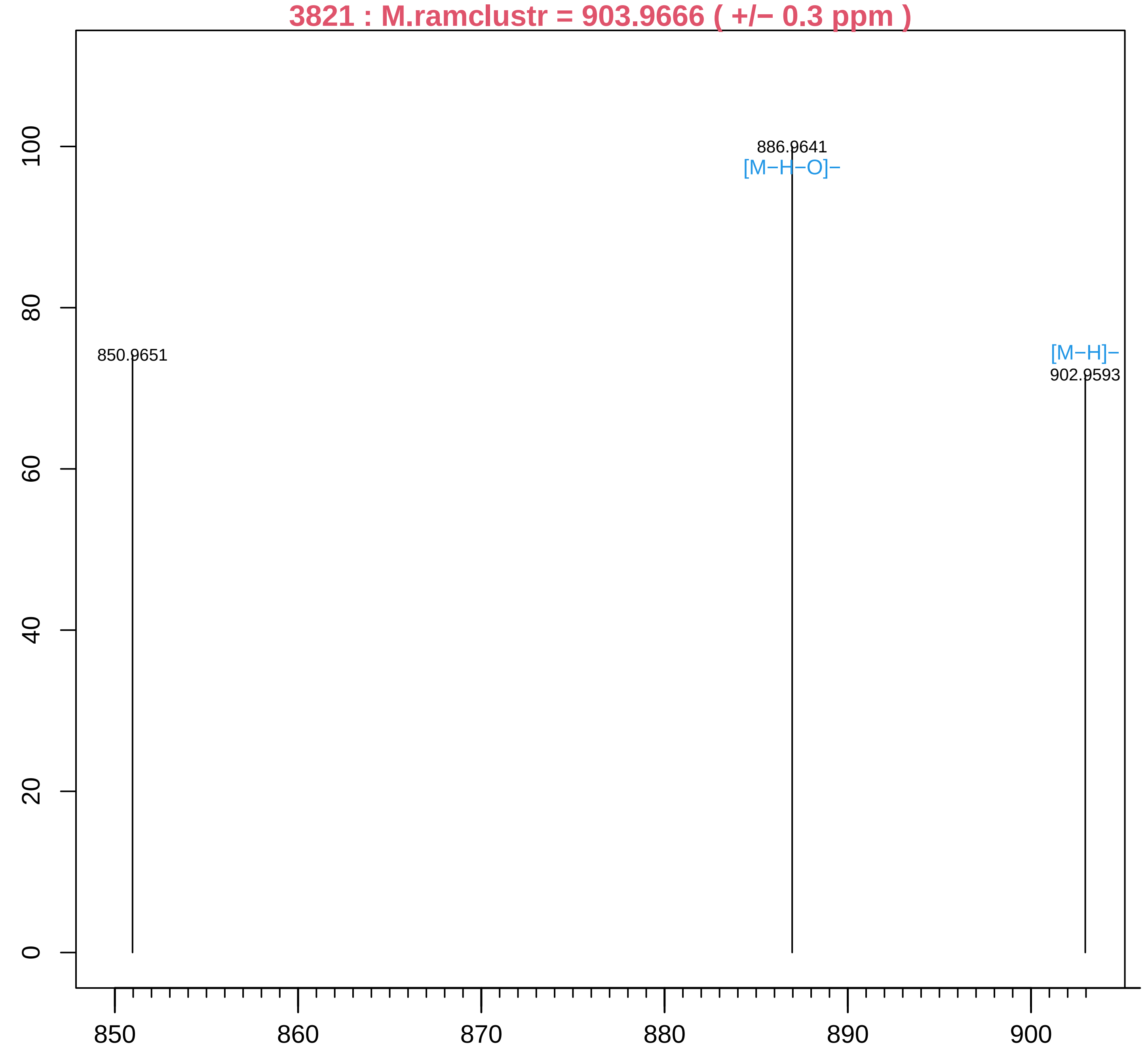
3821 : M.ramclustr = 903.9666 (+/− 0.3 ppm)
886.9641
[M−H−O]−
850.9651
[M−H]−
902.9593
0
20
40
60
80
100
850
860
870
880
890
900

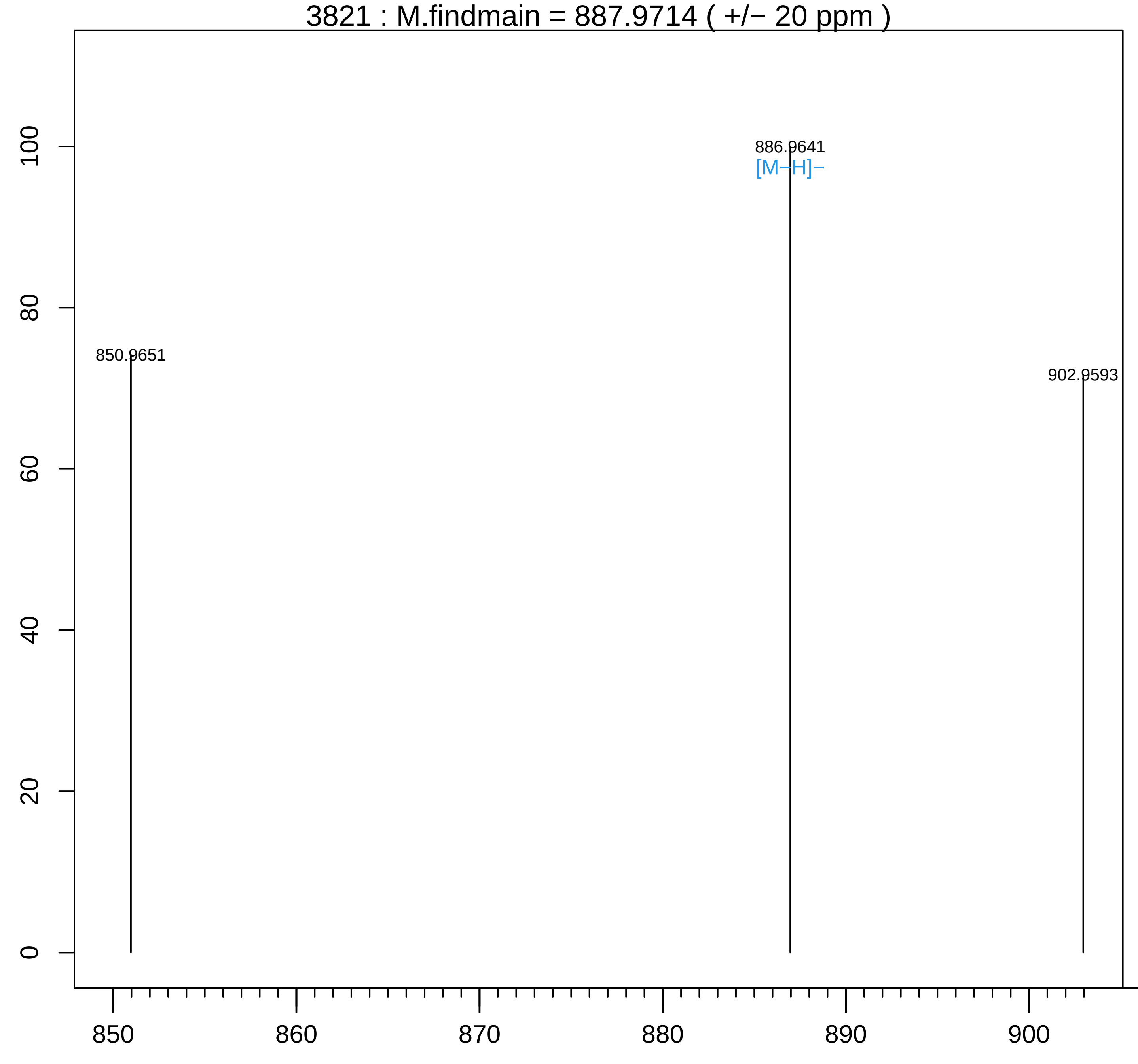
3821 : M.findmain = 887.9714 (+/− 20 ppm)
886.9641
[M−H]−
850.9651
902.9593
0
20
40
60
80
100
850
860
870
880
890
900

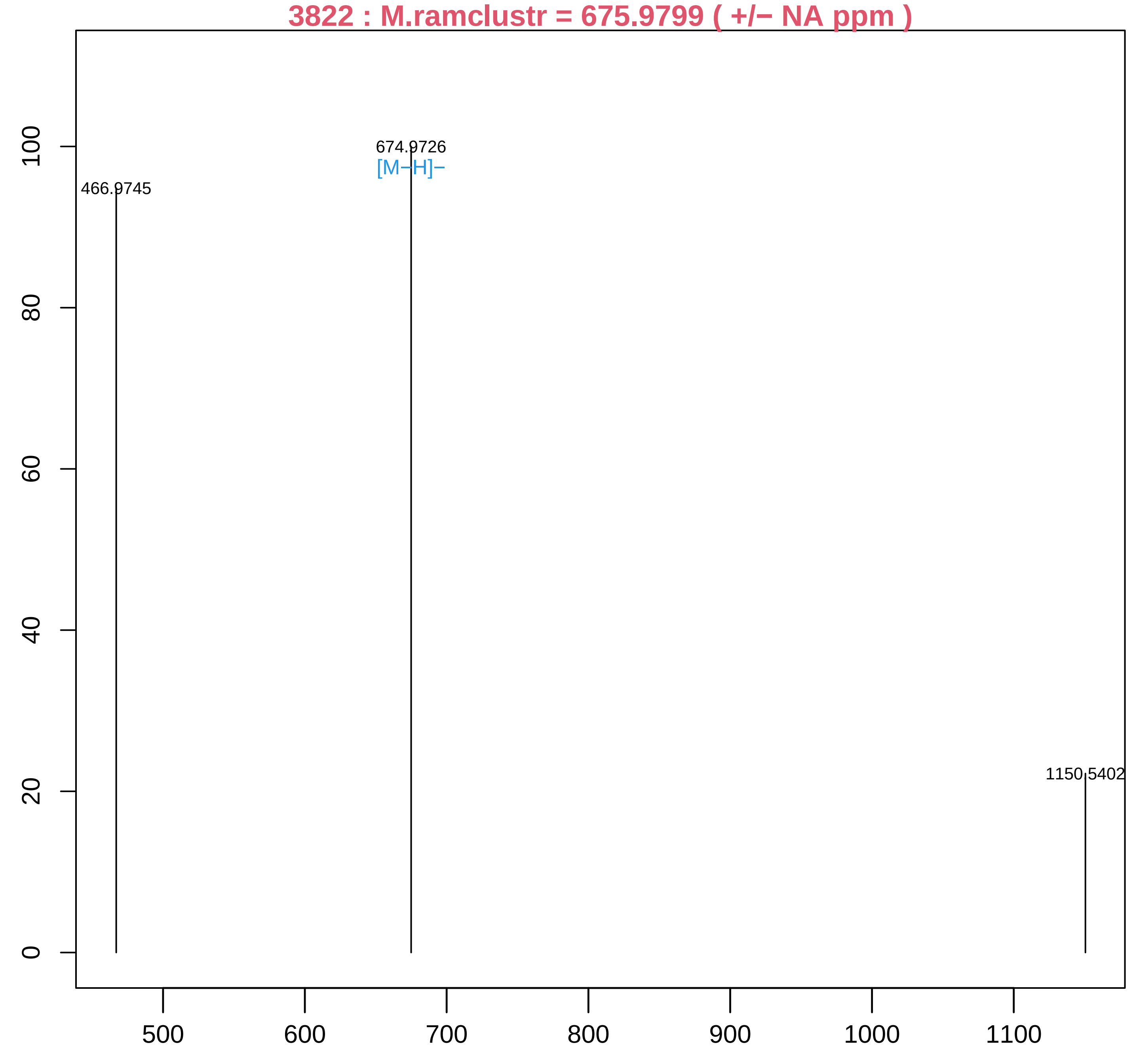
3822 : M.ramclustr = 675.9799 (+/− NA ppm)
674.9726
[M−H]−
466.9745
1150.5402
0
20
40
60
80
100
500
600
700
800
900
1000
1100

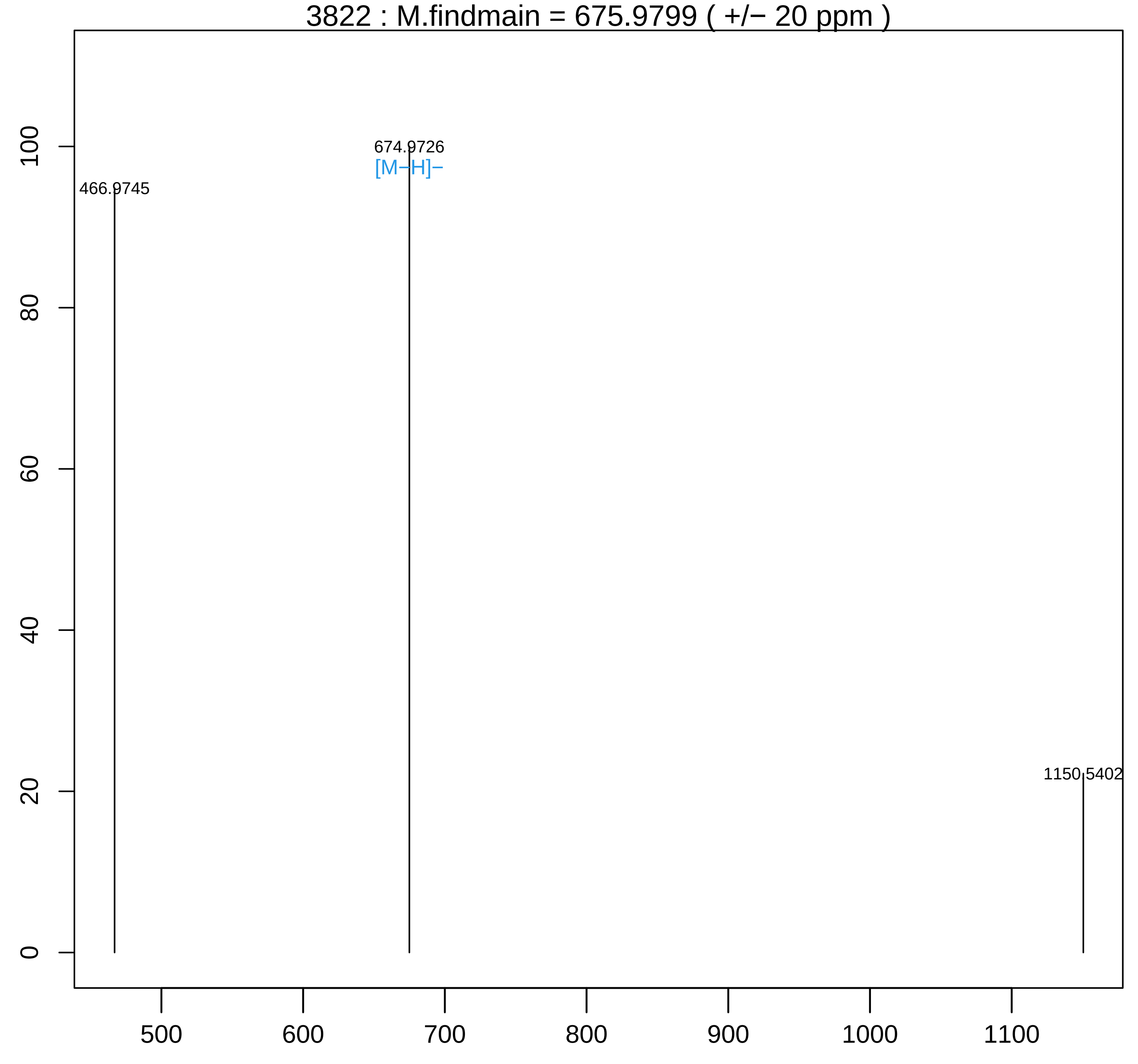
3822 : M.findmain = 675.9799 (+/− 20 ppm)
674.9726
[M−H]−
466.9745
1150.5402
0
20
40
60
80
100
500
600
700
800
900
1000
1100

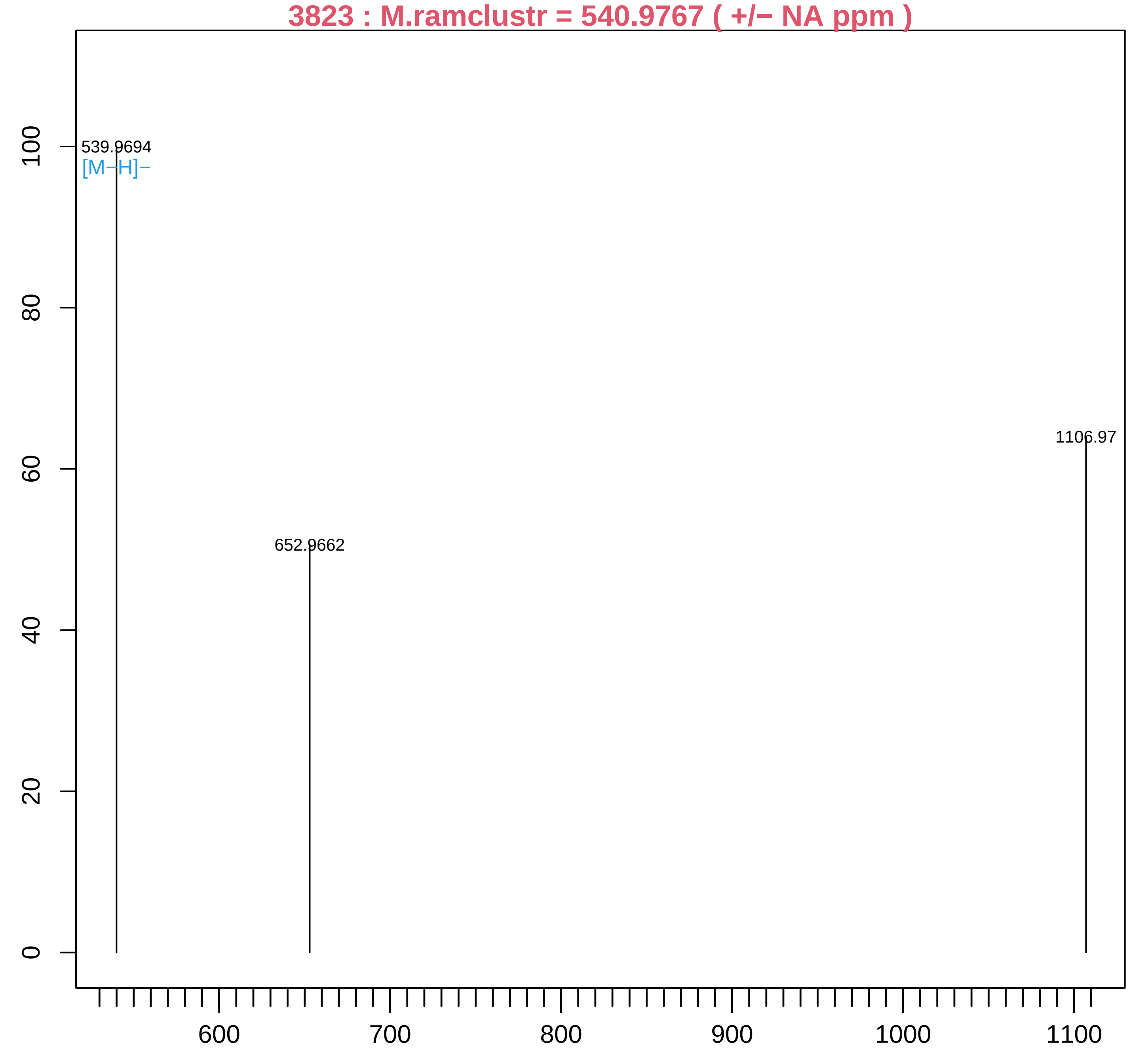
3823 : M.ramclustr = 540.9767 (+/− NA ppm)
539.9694
[M−H]−
652.9662
1106.97
0
20
40
60
80
100
600
700
800
900
1000
1100

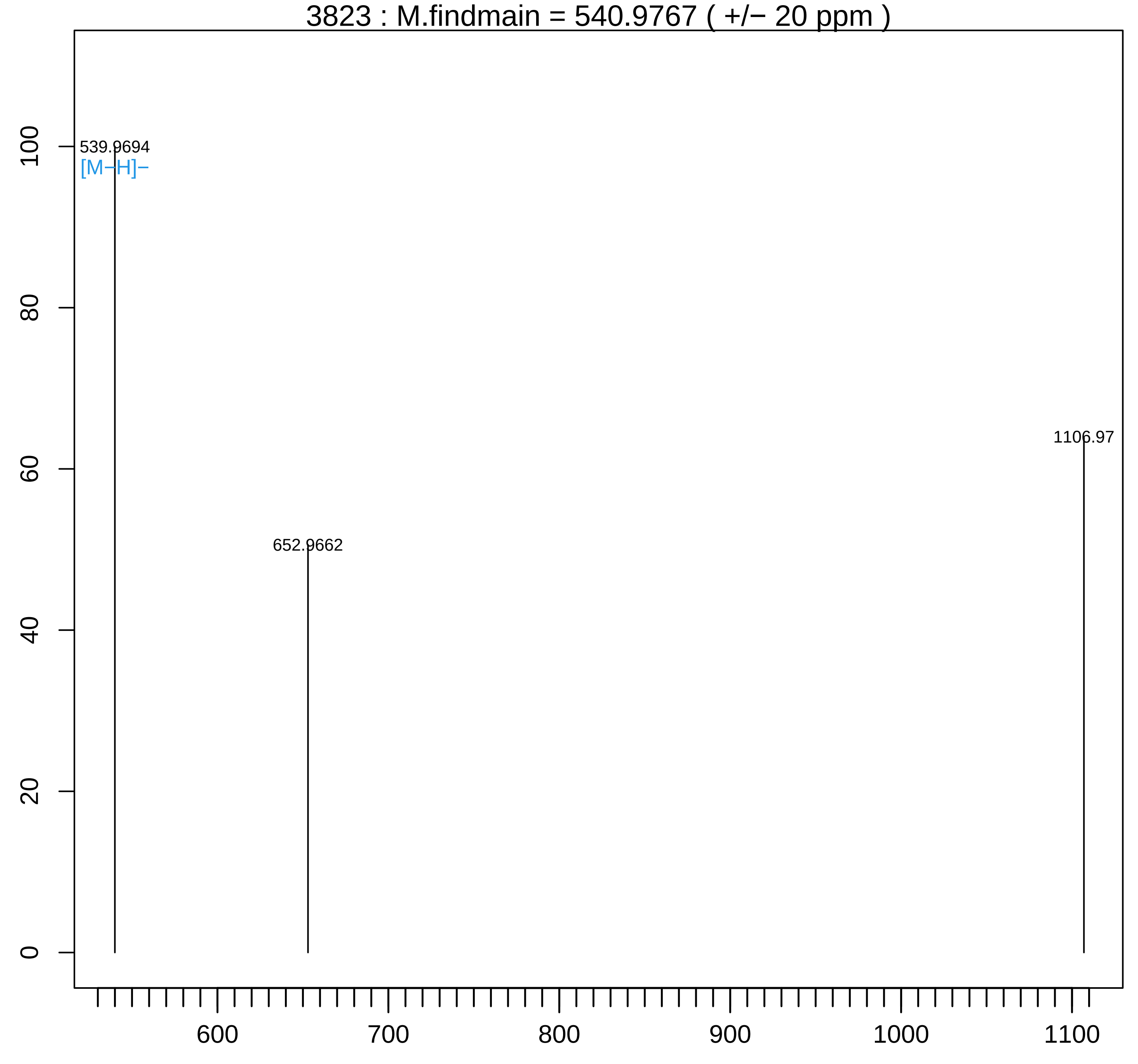
3823 : M.findmain = 540.9767 (+/− 20 ppm)
539.9694
[M−H]−
652.9662
1106.97
0
20
40
60
80
100
600
700
800
900
1000
1100

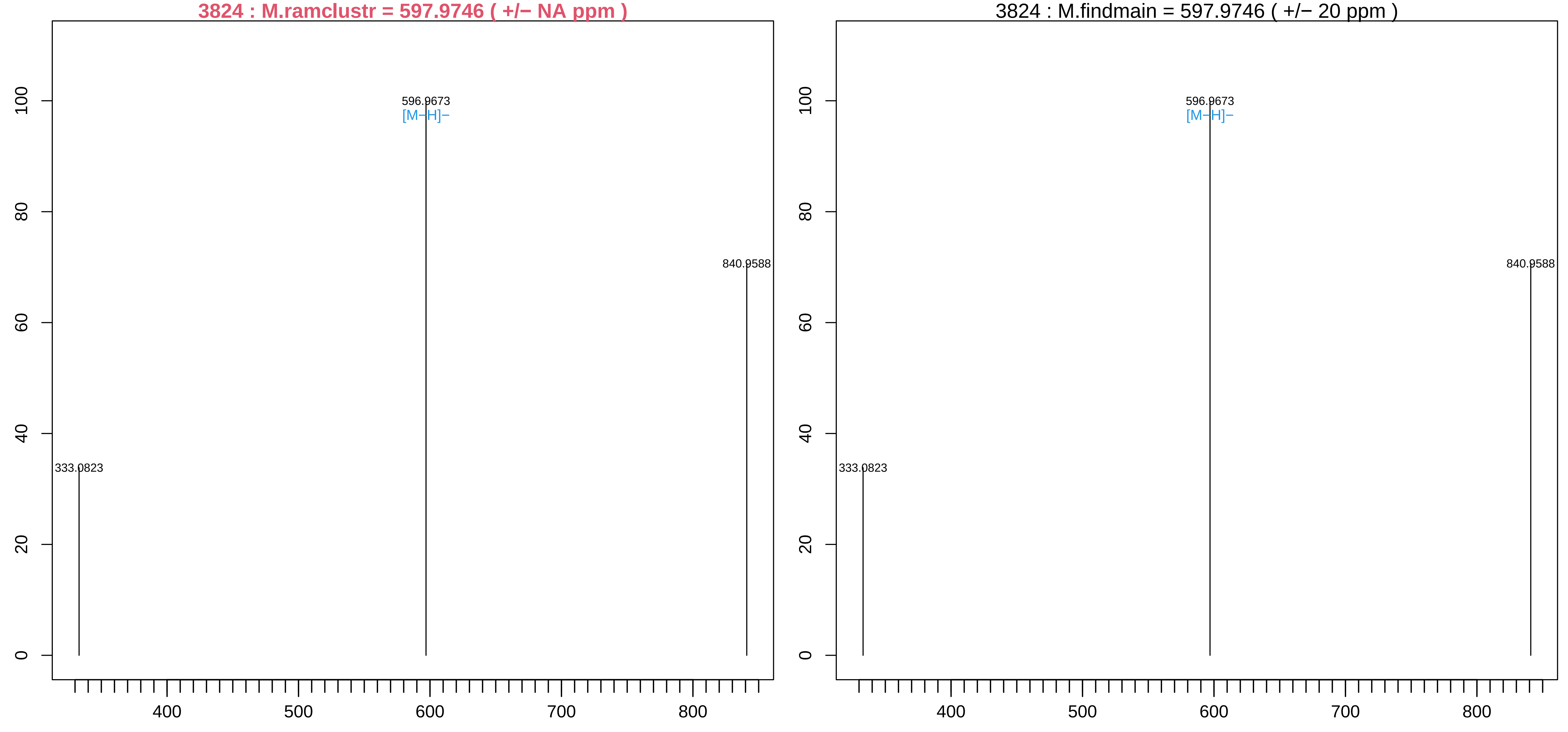
3824 : M.ramclustr = 597.9746 (+/− NA ppm)
3824 : M.findmain = 597.9746 (+/− 20 ppm)
596.9673
[M−H]−
840.9588
333.0823
0
20
40
60
80
100
400
500
600
700
800

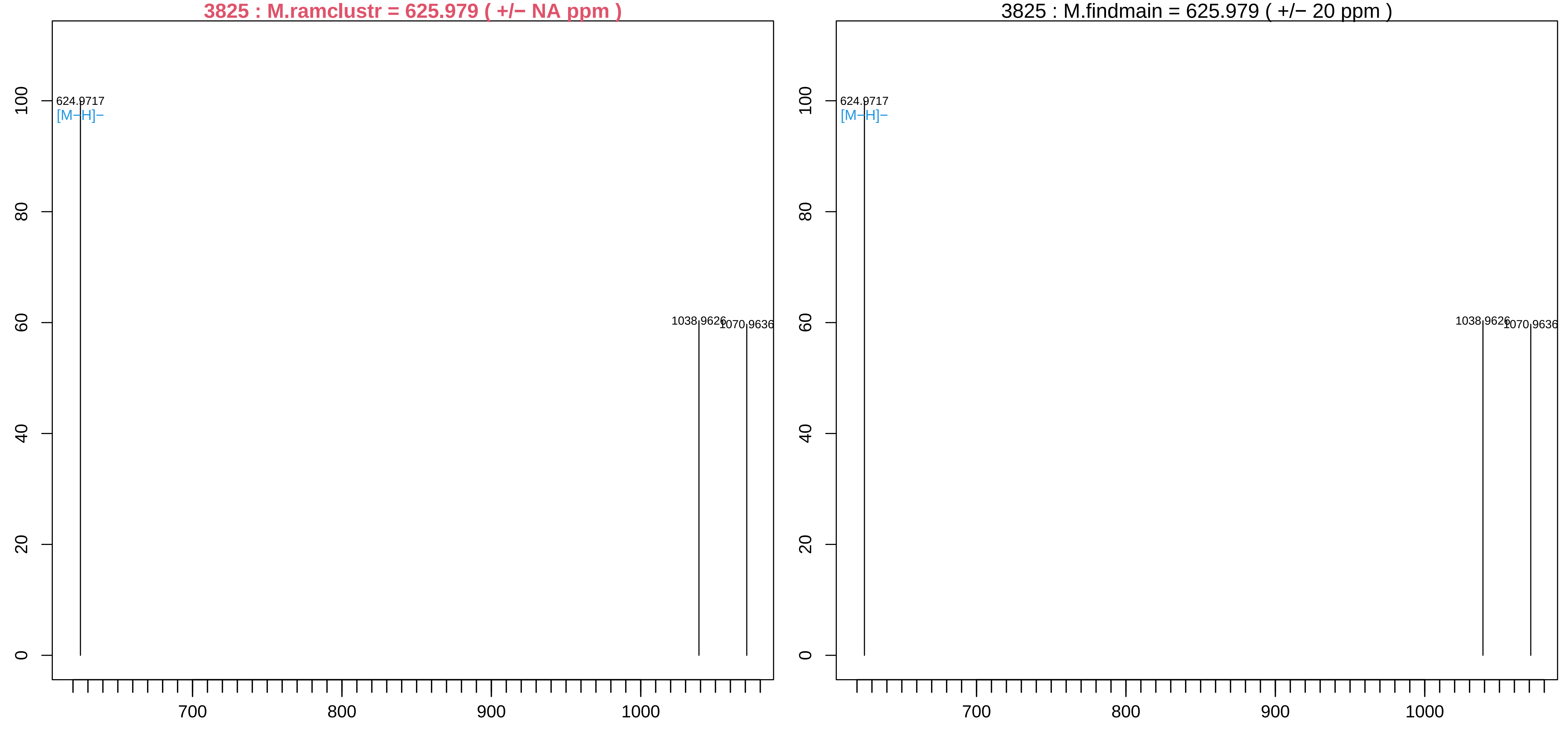

3825 : M.ramclustr = 625.979 (+/− NA ppm)
624.9717
[M−H]−
1038.9626
1070.9636
0
20
40
60
80
100
700
800
900
1000
3825 : M.findmain = 625.979 (+/− 20 ppm)
624.9717
[M−H]−
1038.9626
1070.9636
0
20
40
60
80
100
700
800
900
1000

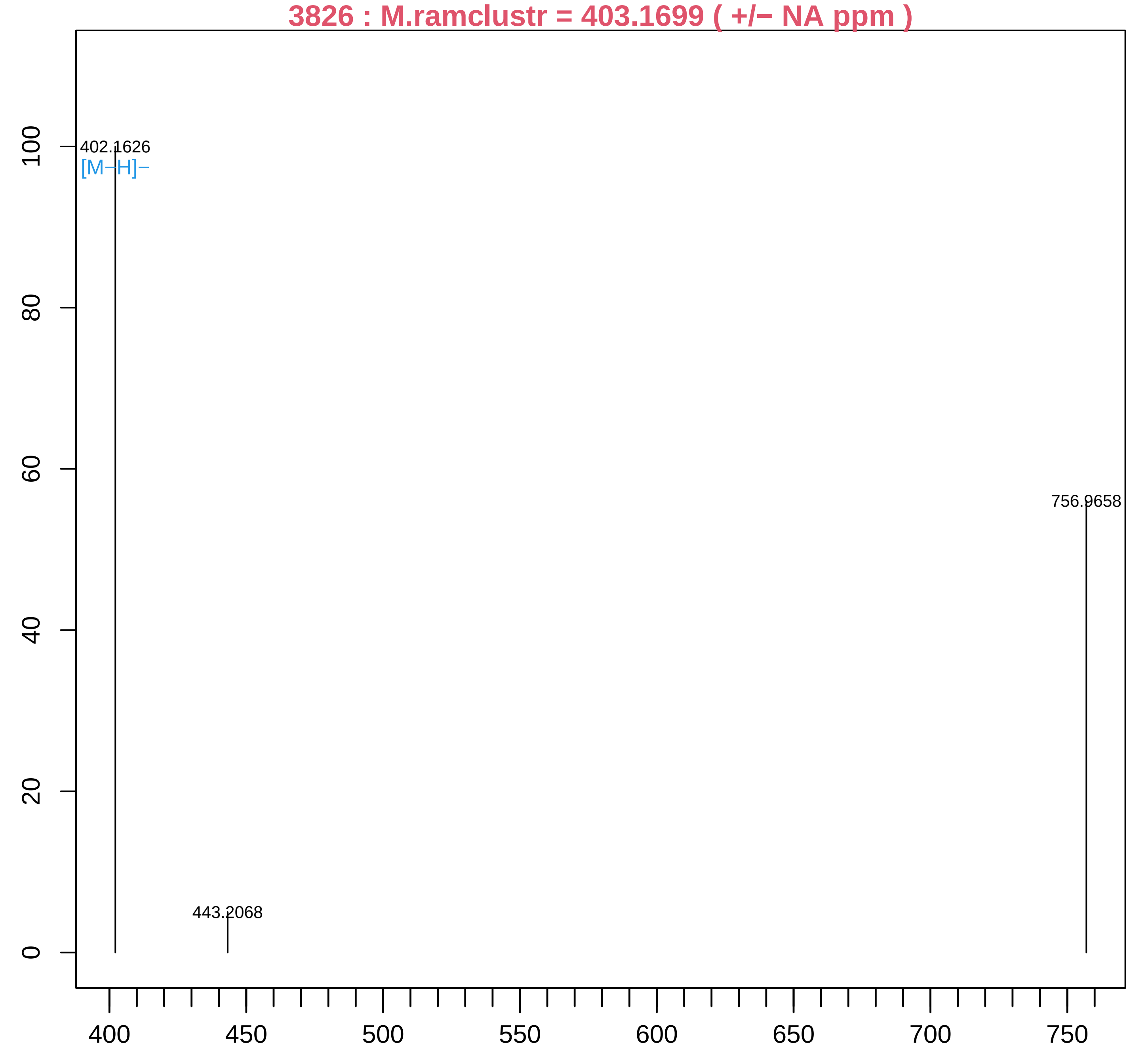
3826 : M.ramclustr = 403.1699 (+/− NA ppm)
402.1626
[M−H]−
443.2068
756.9658
0
20
40
60
80
100
400
450
500
550
600
650
700
750

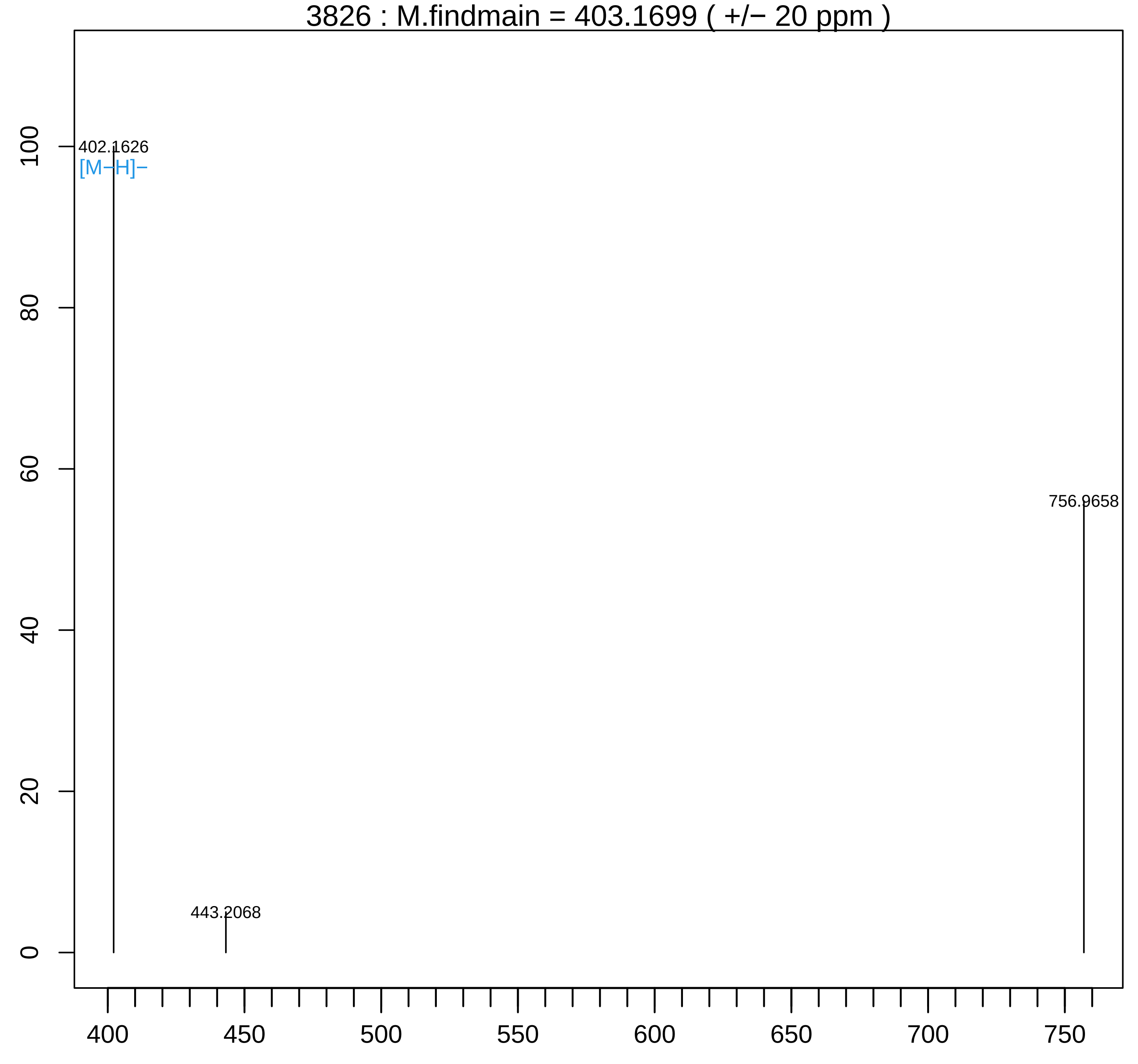
3826 : M.findmain = 403.1699 (+/− 20 ppm)
402.1626
[M−H]−
443.2068
756.9658
0
20
40
60
80
100
400
450
500
550
600
650
700
750

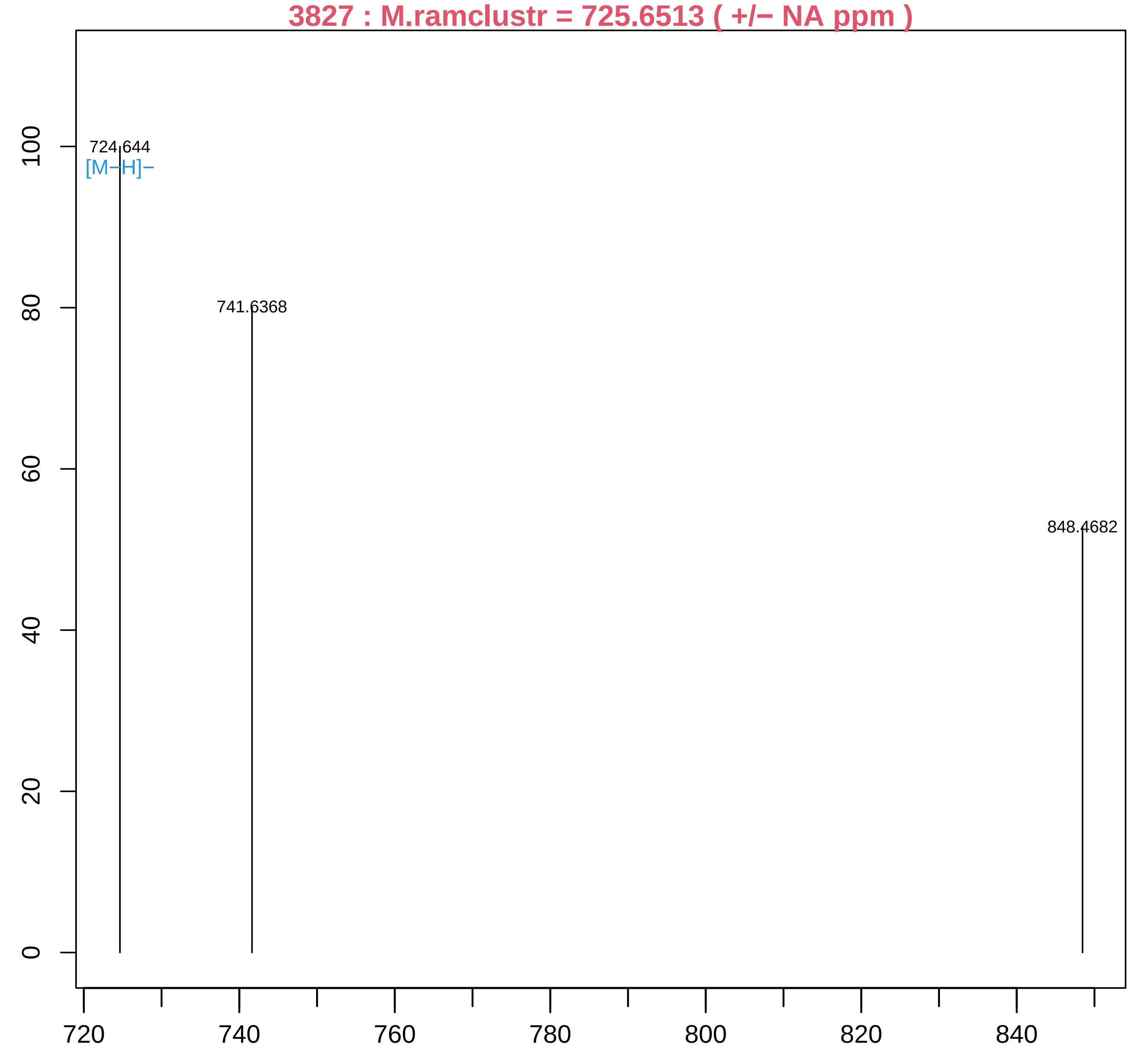
3827 : M.ramclustr = 725.6513 (+/− NA ppm)
724.644
[M−H]−
741.6368
848.4682
0
20
40
60
80
100
720
740
760
780
800
820
840
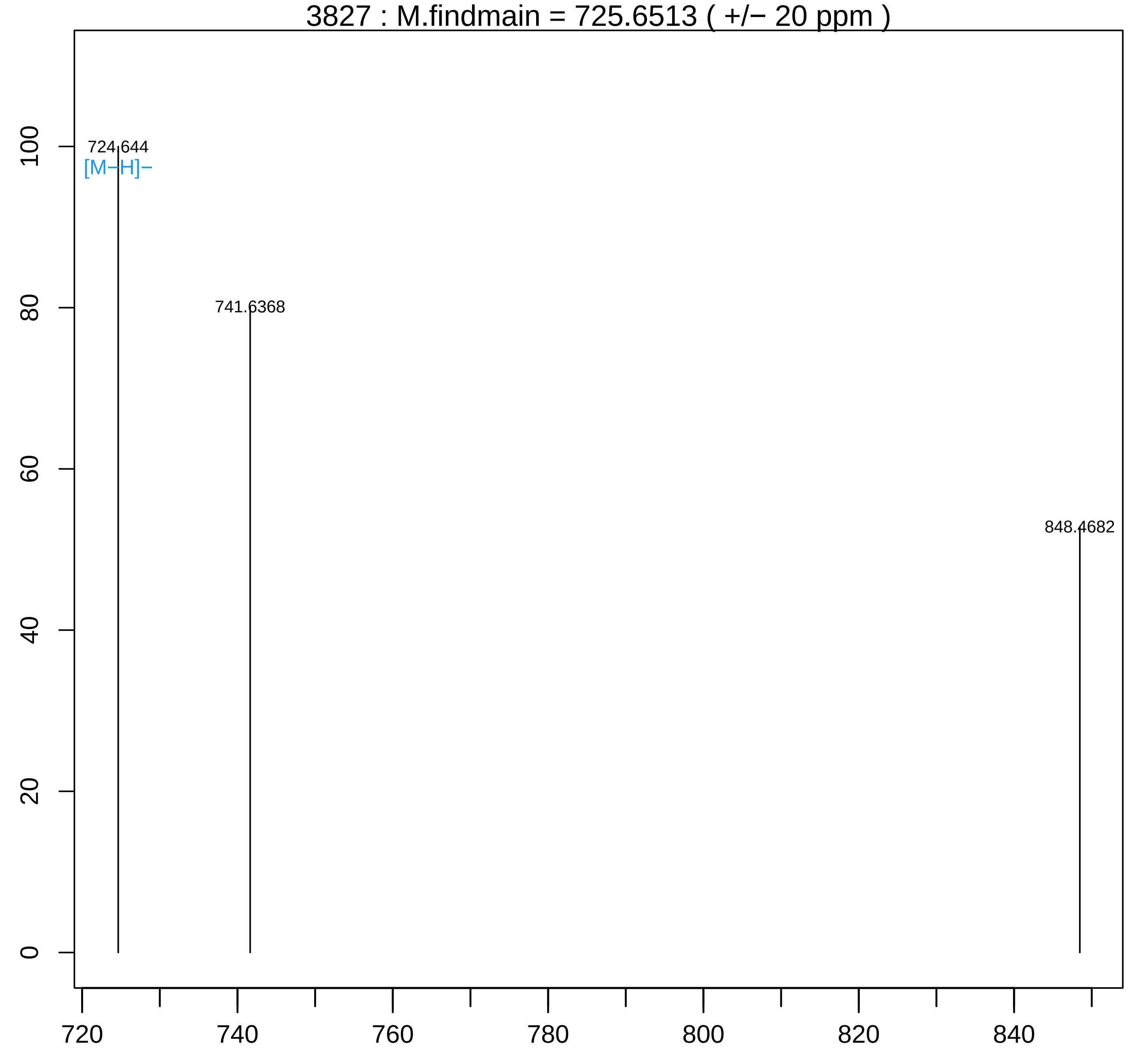
3827 : M.findmain = 725.6513 (+/− 20 ppm)
724.644
[M−H]−
741.6368
848.4682
0
20
40
60
80
100
720
740
760
780
800
820
840

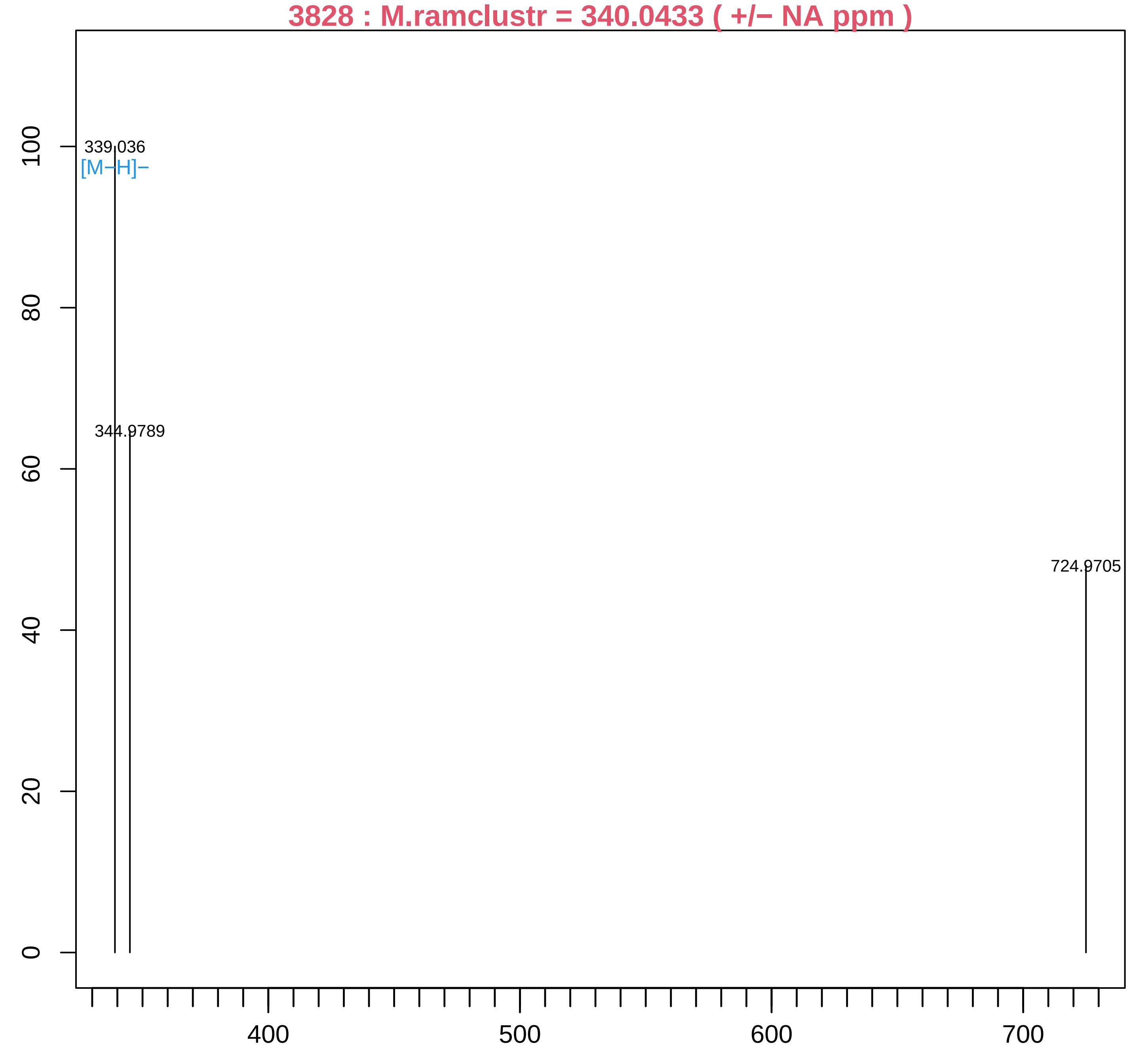
3828 : M.ramclustr = 340.0433 (+/− NA ppm)
339.036
[M−H]−
344.9789
724.9705
0
20
40
60
80
100
400
500
600
700

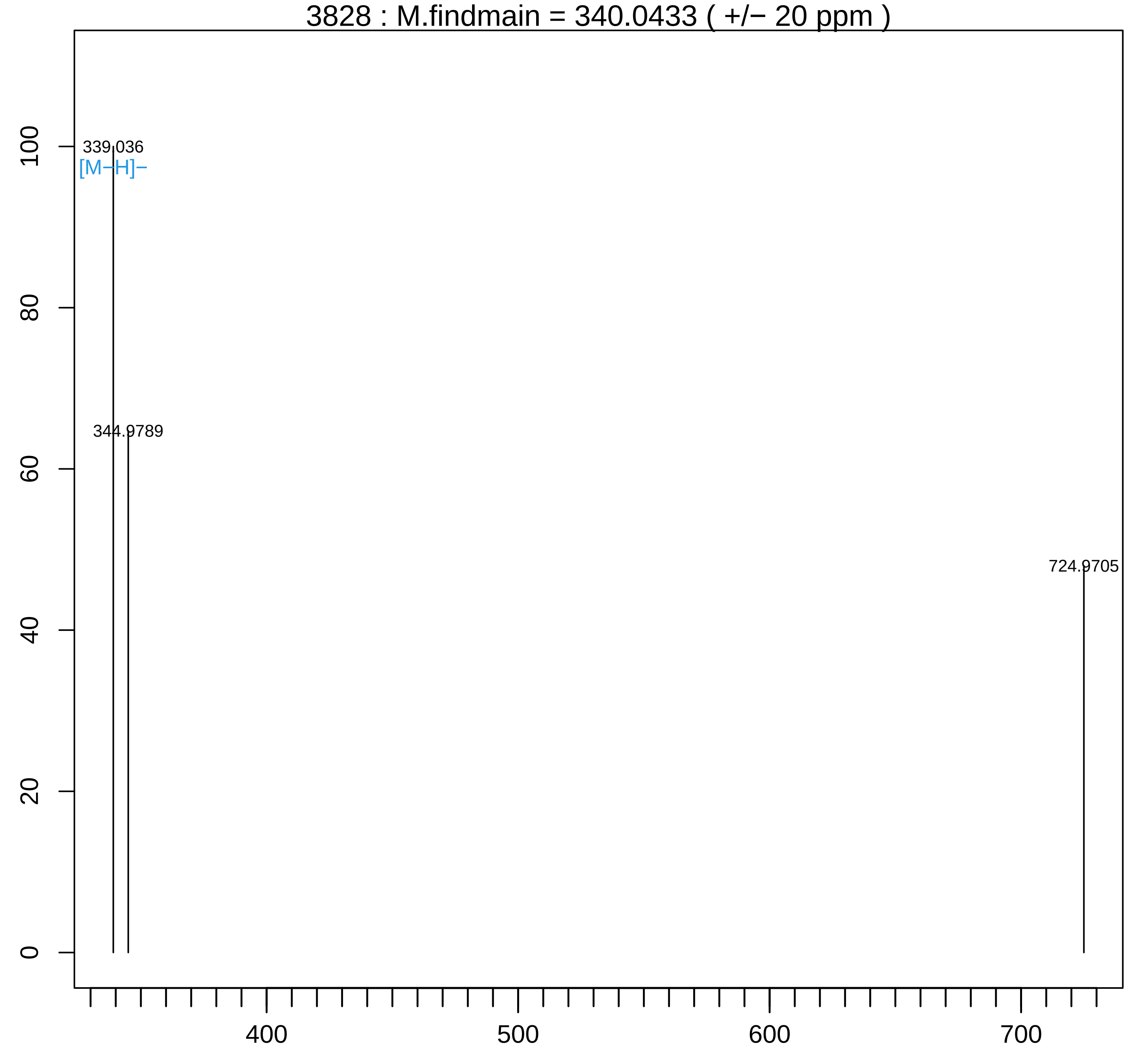
3828 : M.findmain = 340.0433 (+/− 20 ppm)
339.036
[M−H]−
344.9789
724.9705
0
20
40
60
80
100
400
500
600
700

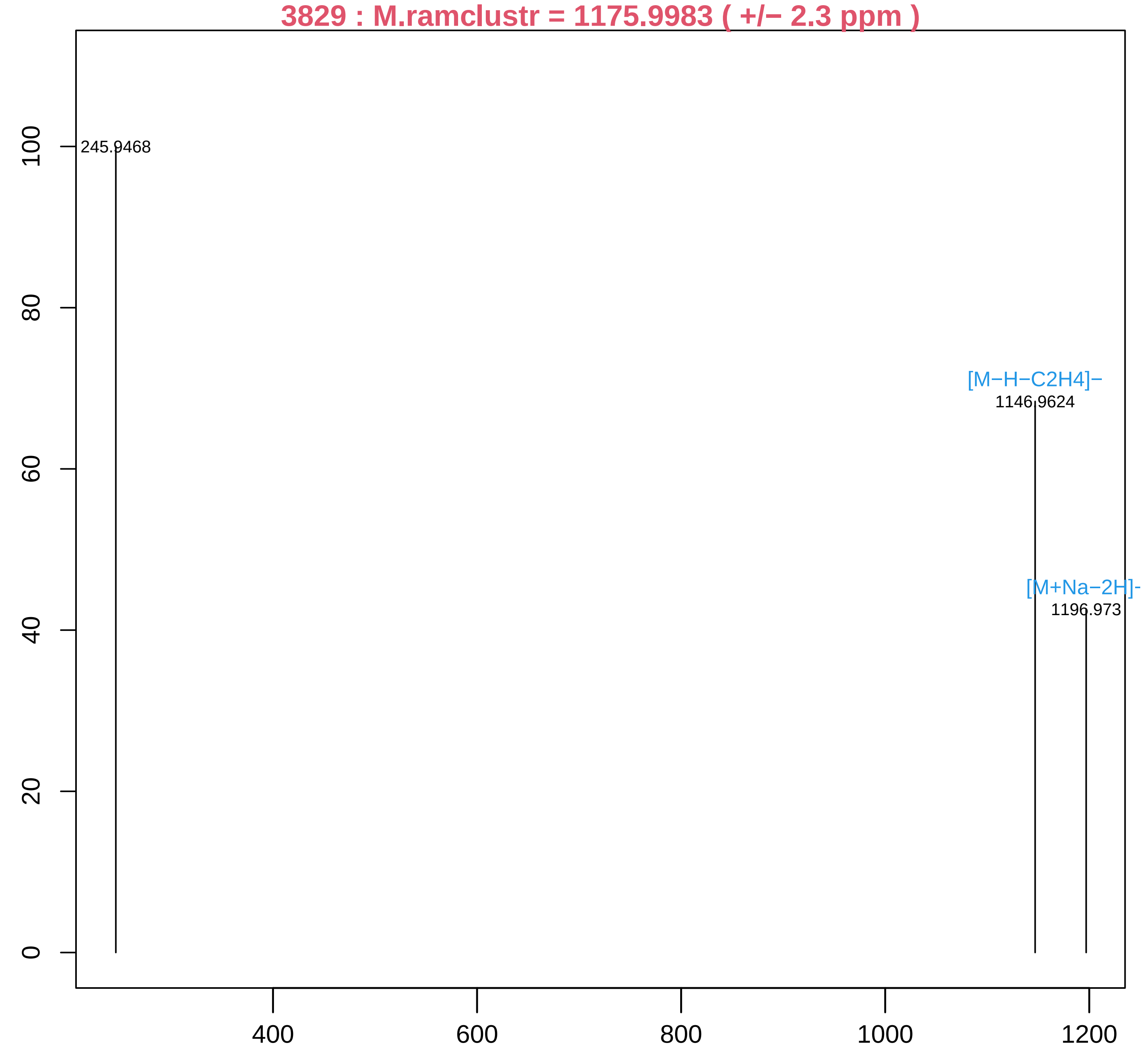
3829 : M.ramclustr = 1175.9983 (+/− 2.3 ppm)
245.9468
[M−H−C2H4]−
1146.9624
[M+Na−2H]-
1196.973
0
20
40
60
80
100
400
600
800
1000
1200

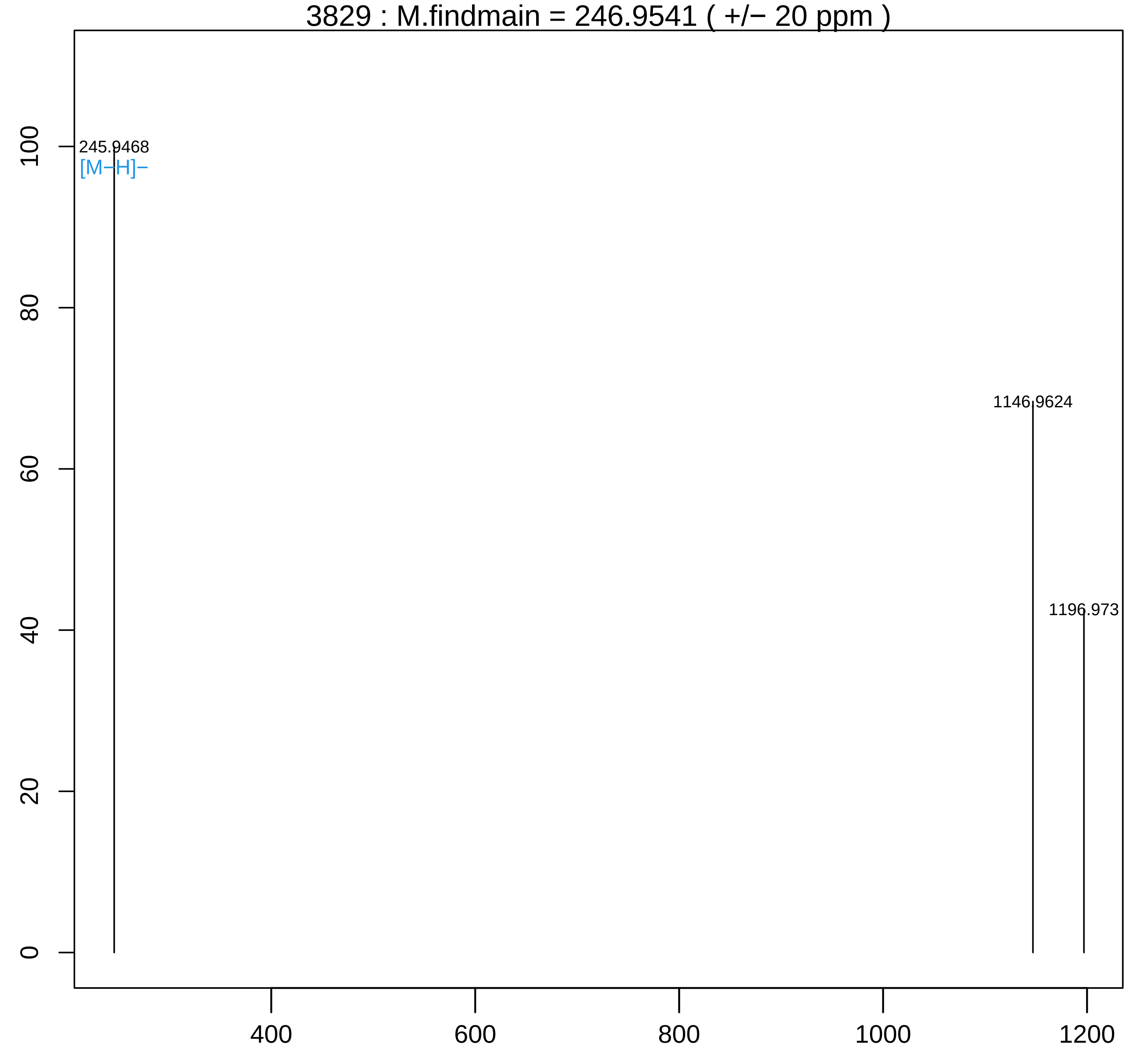
3829 : M.findmain = 246.9541 (+/− 20 ppm)
245.9468
[M−H]−
1146.9624
1196.973
0
20
40
60
80
100
400
600
800
1000
1200

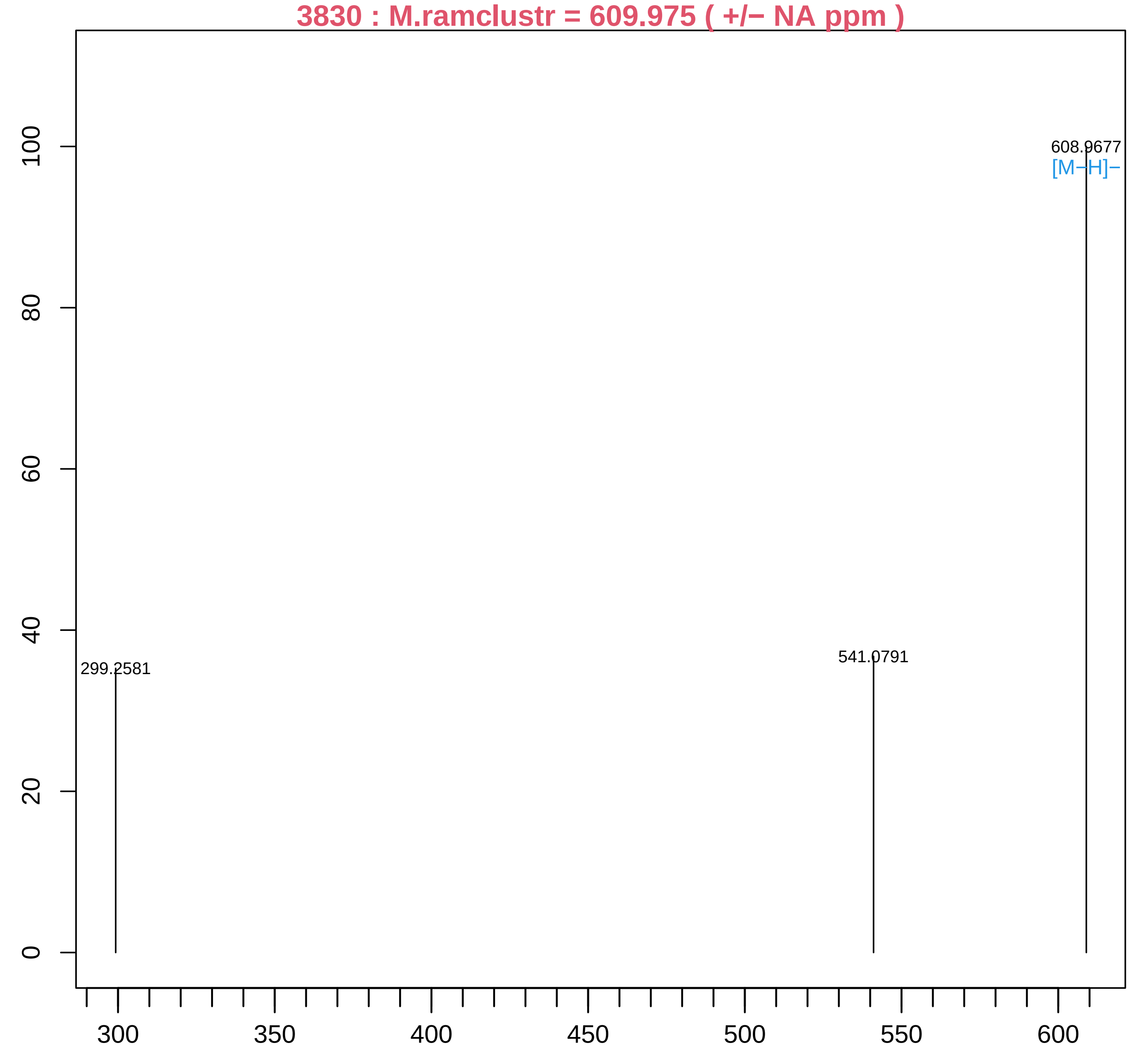
3830 : M.ramclustr = 609.975 (+/− NA ppm)
608.9677
[M−H]−
541.0791
299.2581
0
20
40
60
80
100
300
350
400
450
500
550
600

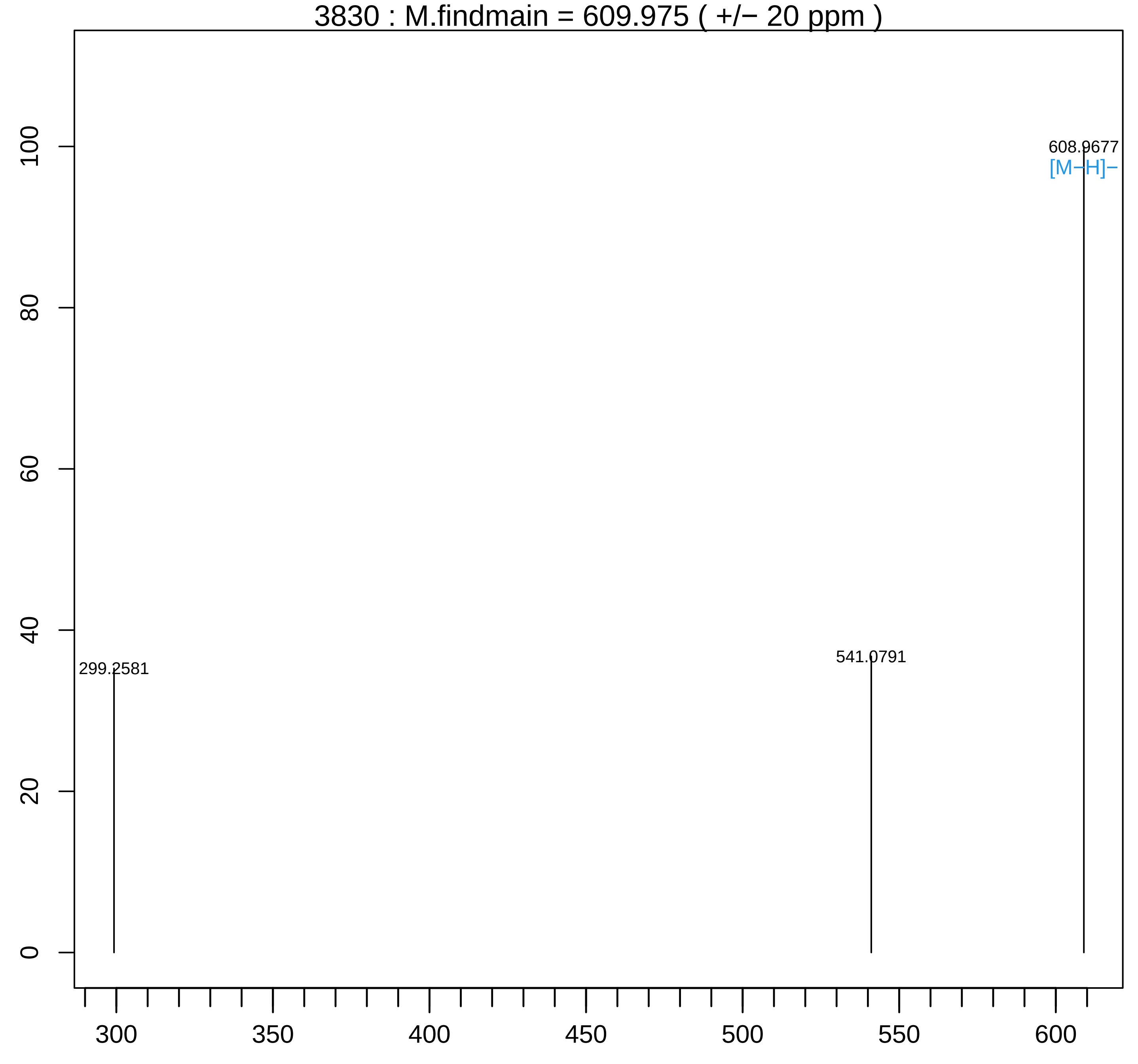
3830 : M.findmain = 609.975 (+/− 20 ppm)
608.9677
[M−H]−
541.0791
299.2581
0
20
40
60
80
100
300
350
400
450
500
550
600

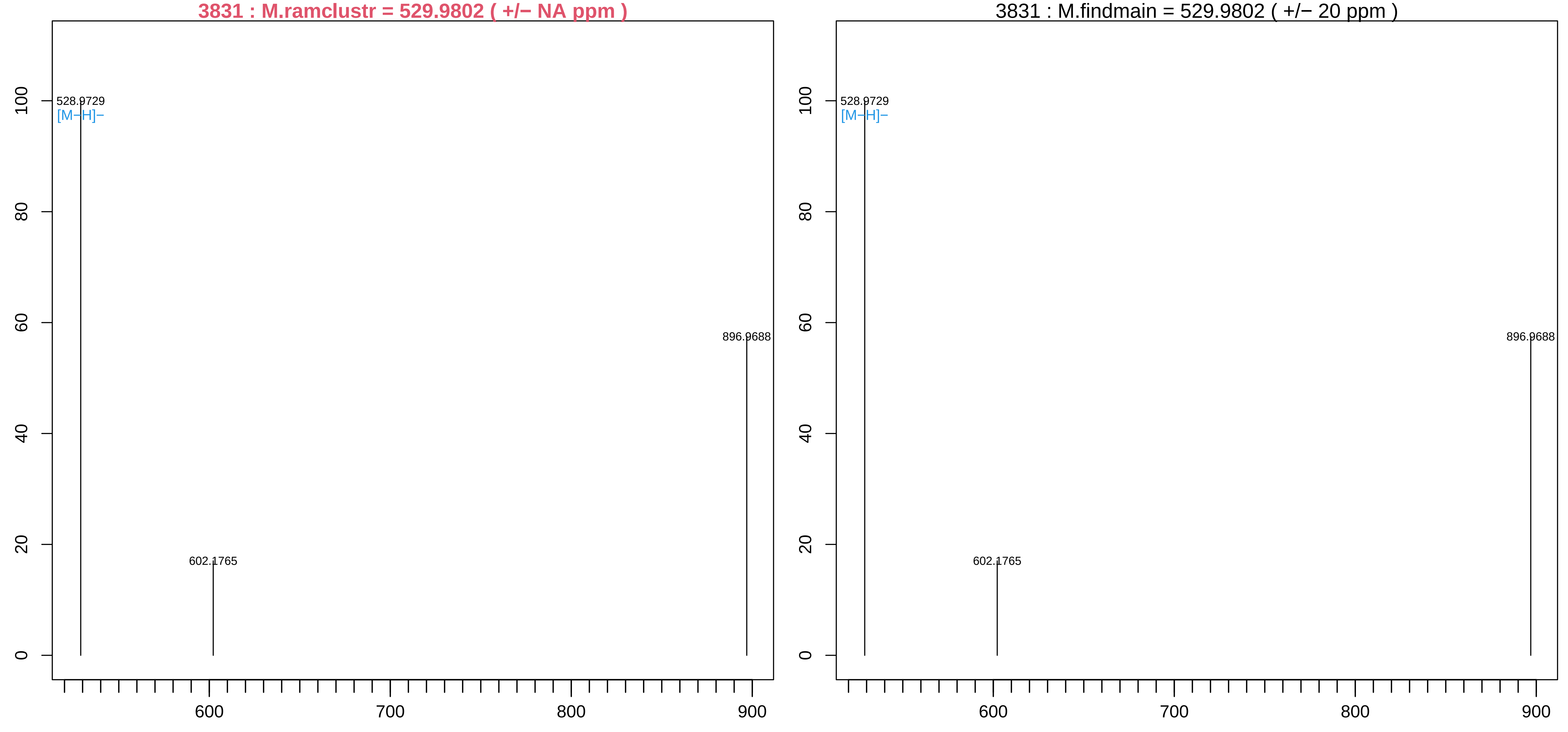
3831 : M.ramclustr = 529.9802 (+/− NA ppm)
3831 : M.findmain = 529.9802 (+/− 20 ppm)
528.9729
[M−H]−
602.1765
896.9688
0
20
40
60
80
100
600
700
800
900

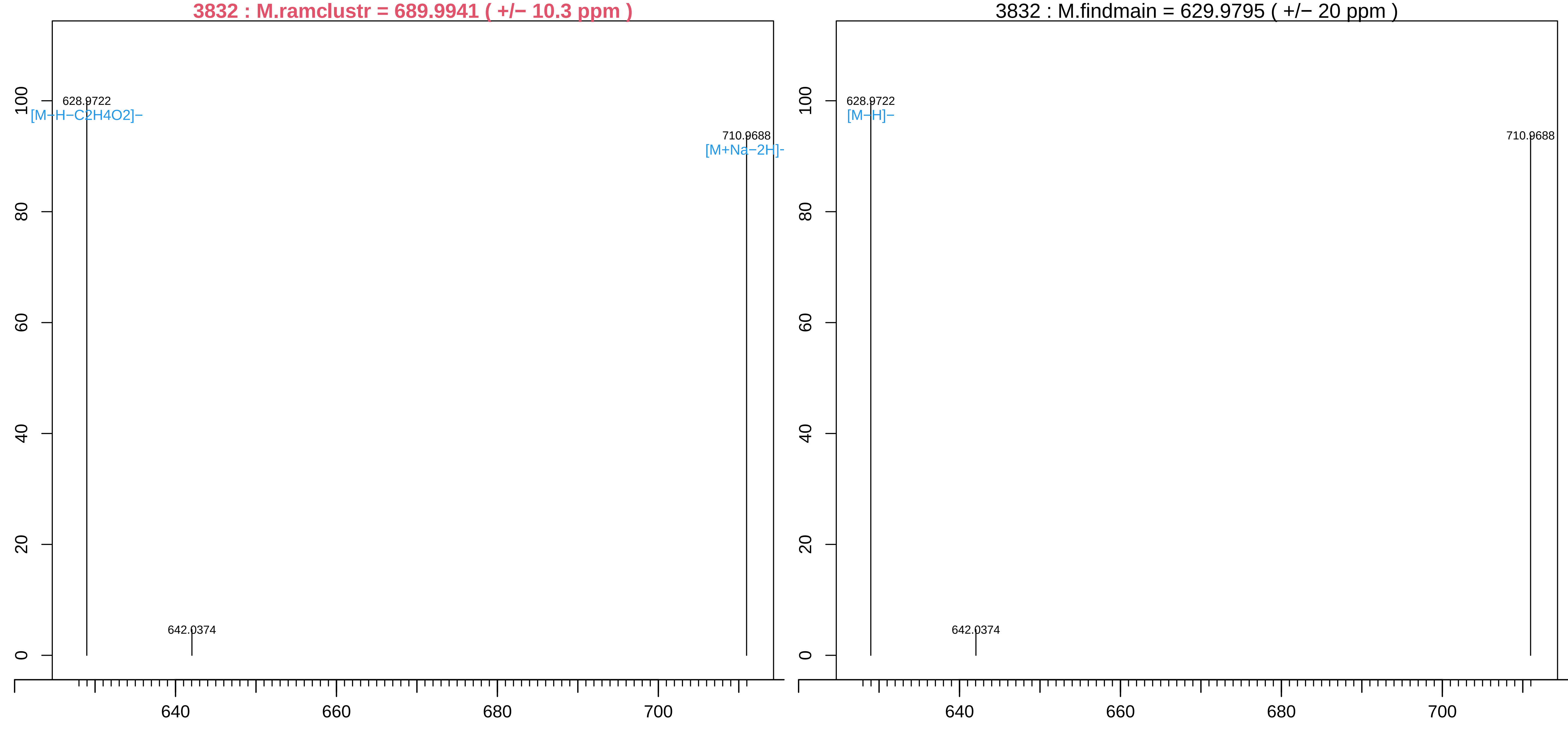
3832 : M.ramclustr = 689.9941 (+/− 10.3 ppm)
628.9722
[M−H−C2H4O2]−
710.9688
[M+Na−2H]-
642.0374
0
20
40
60
80
100
640
660
680
700
3832 : M.findmain = 629.9795 (+/− 20 ppm)
628.9722
[M−H]−
710.9688
642.0374
0
20
40
60
80
100
640
660
680
700

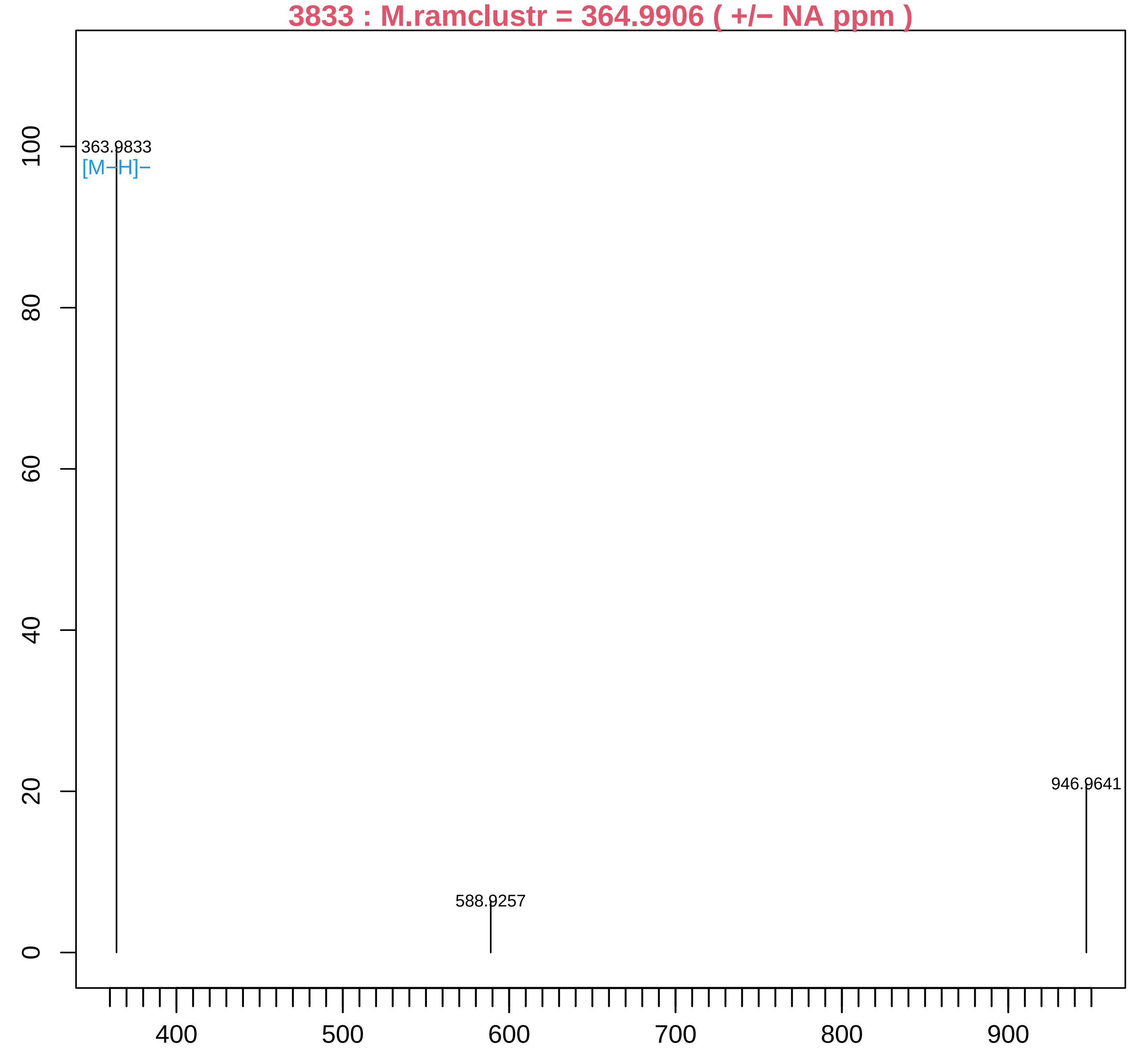
3833 : M.ramclustr = 364.9906 (+/− NA ppm)
363.9833
[M−H]−
588.9257
946.9641
0
20
40
60
80
100
400
500
600
700
800
900
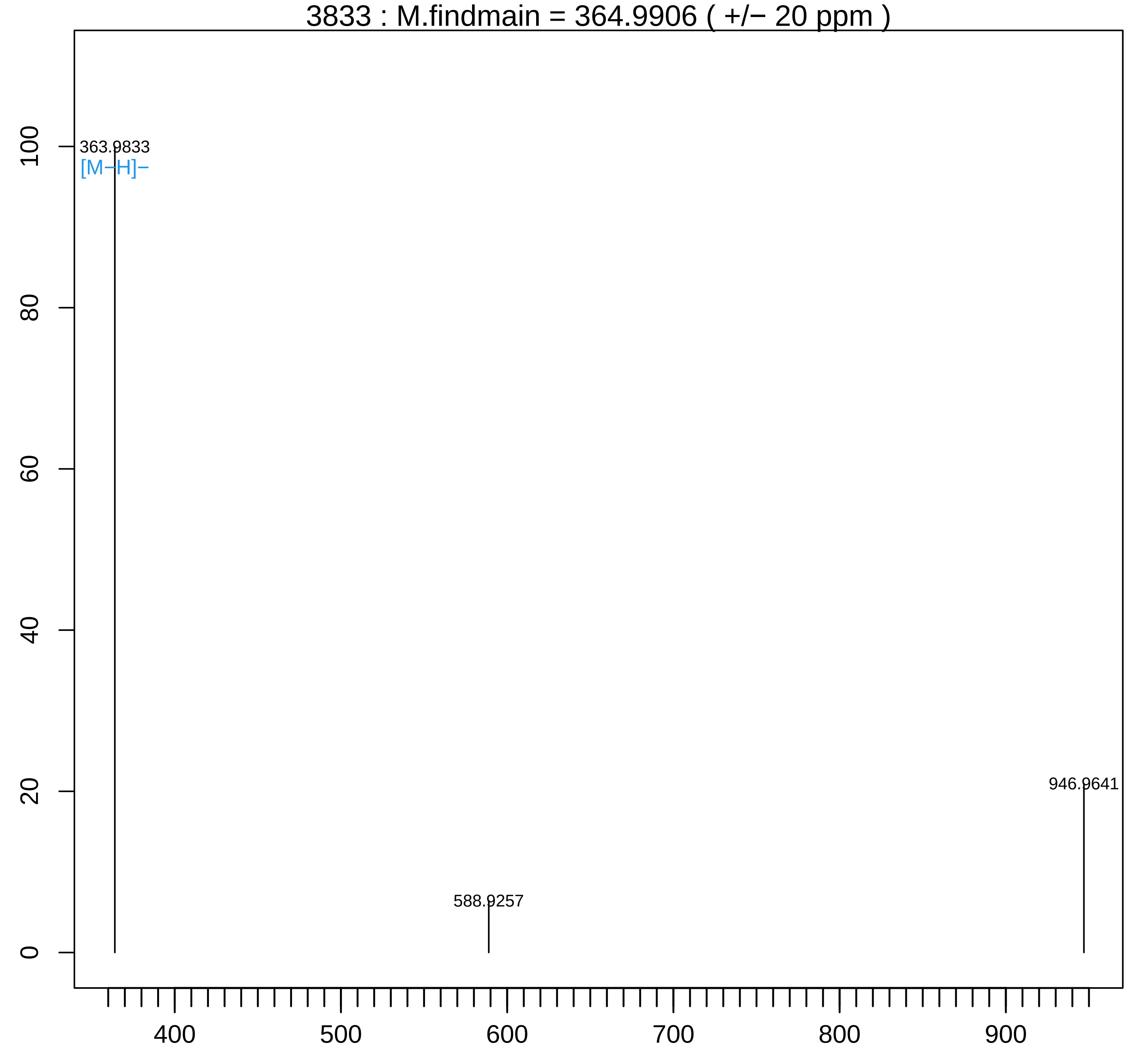
3833 : M.findmain = 364.9906 (+/− 20 ppm)
363.9833
[M−H]−
588.9257
946.9641
0
20
40
60
80
100
400
500
600
700
800
900

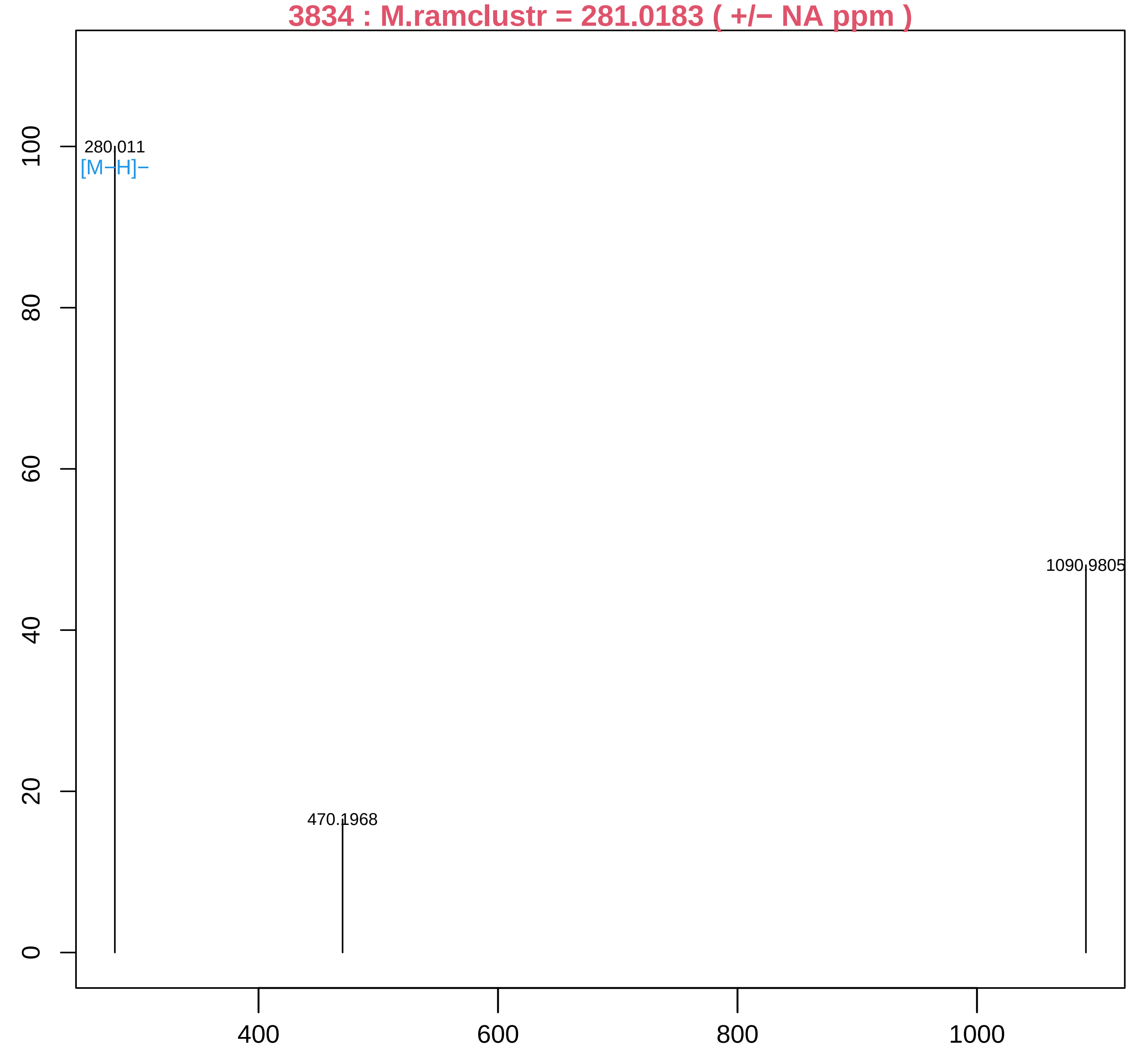
3834 : M.ramclustr = 281.0183 (+/− NA ppm)
280.011
[M−H]−
470.1968
1090.9805
0
20
40
60
80
100
400
600
800
1000

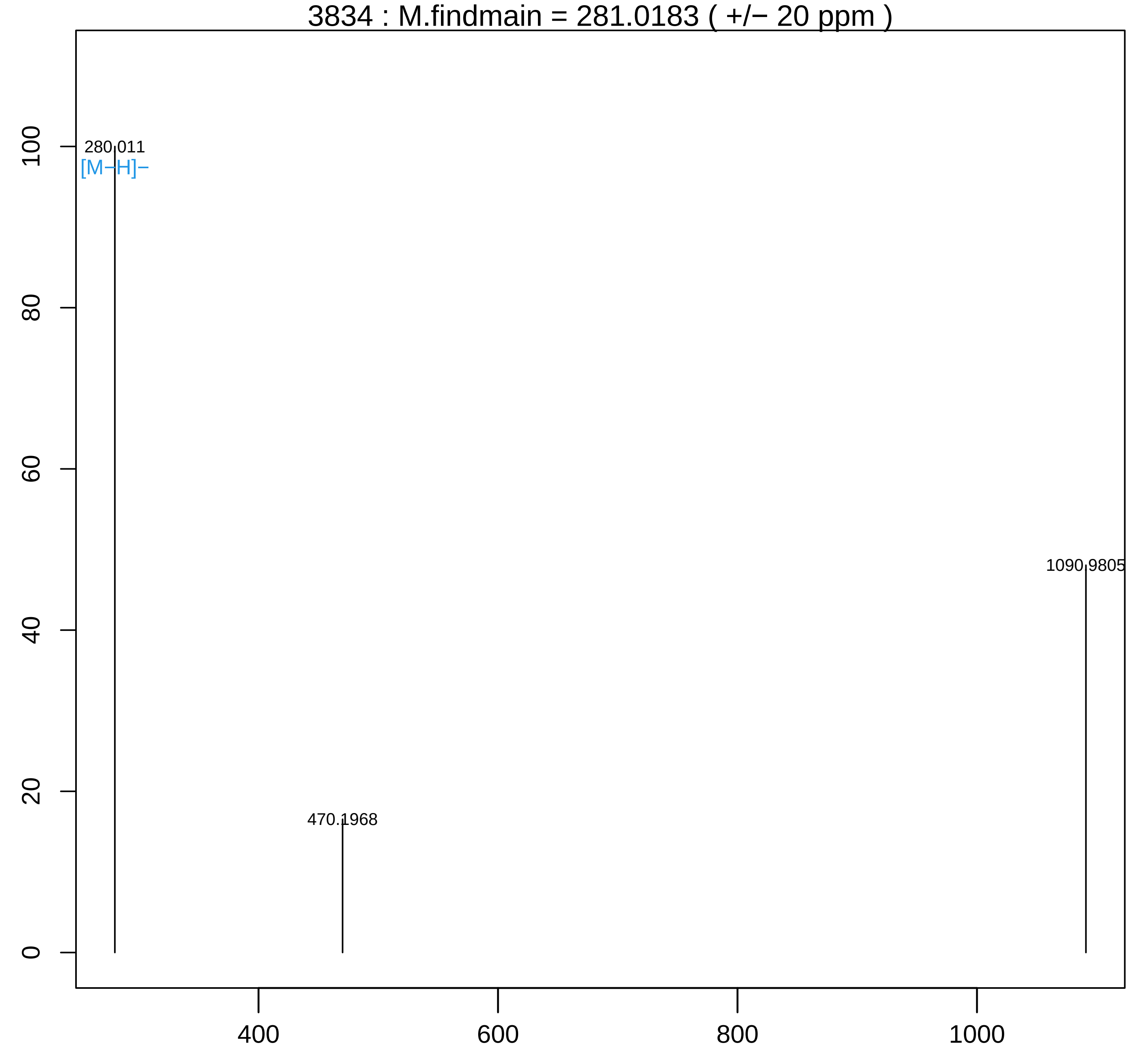
3834 : M.findmain = 281.0183 (+/− 20 ppm)
280.011
[M−H]−
470.1968
1090.9805
0
20
40
60
80
100
400
600
800
1000

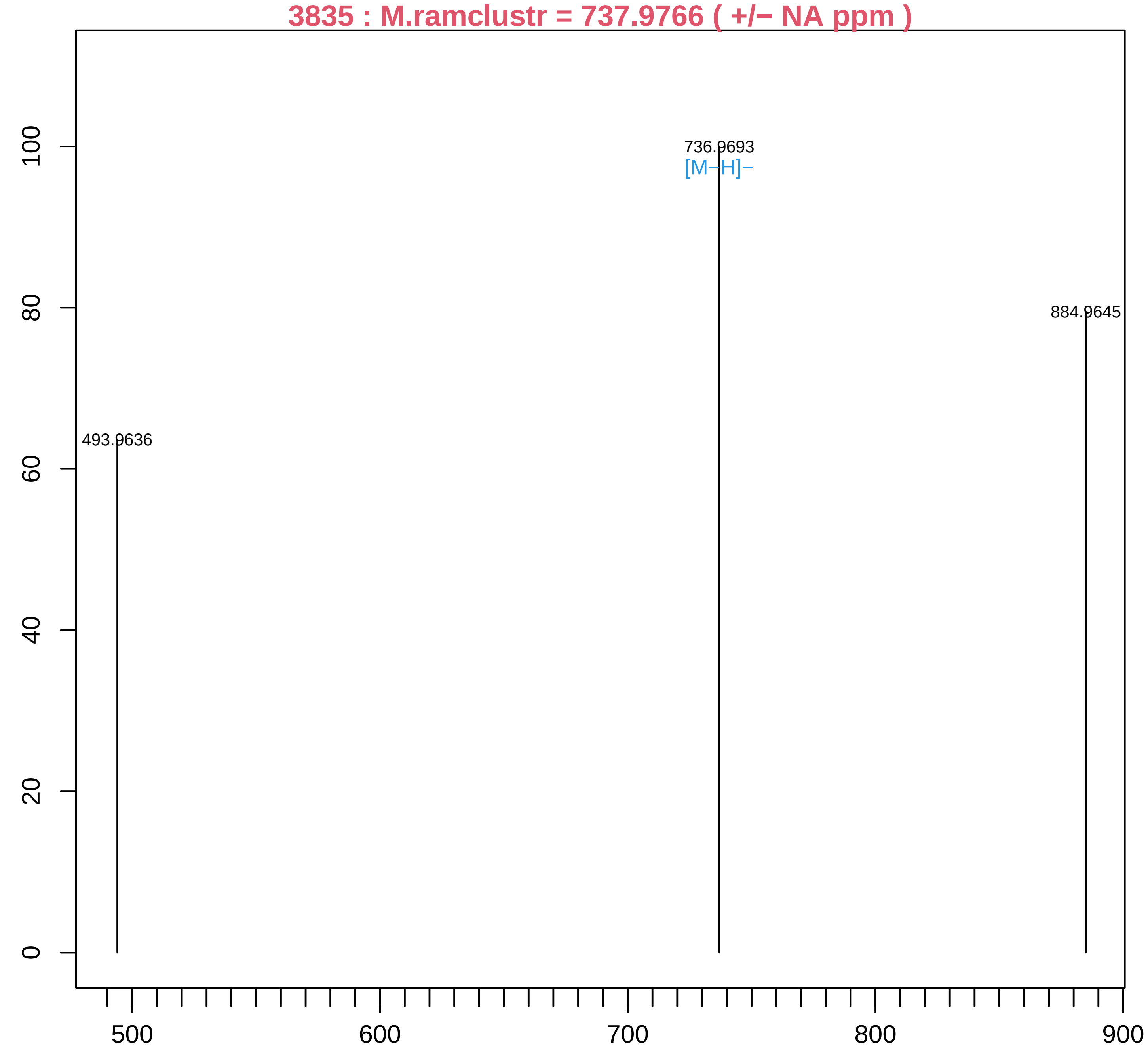

3835 : M.ramclustr = 737.9766 (+/− NA ppm)
736.9693
[M−H]−
884.9645
493.9636
0
20
40
60
80
100
500
600
700
800
900

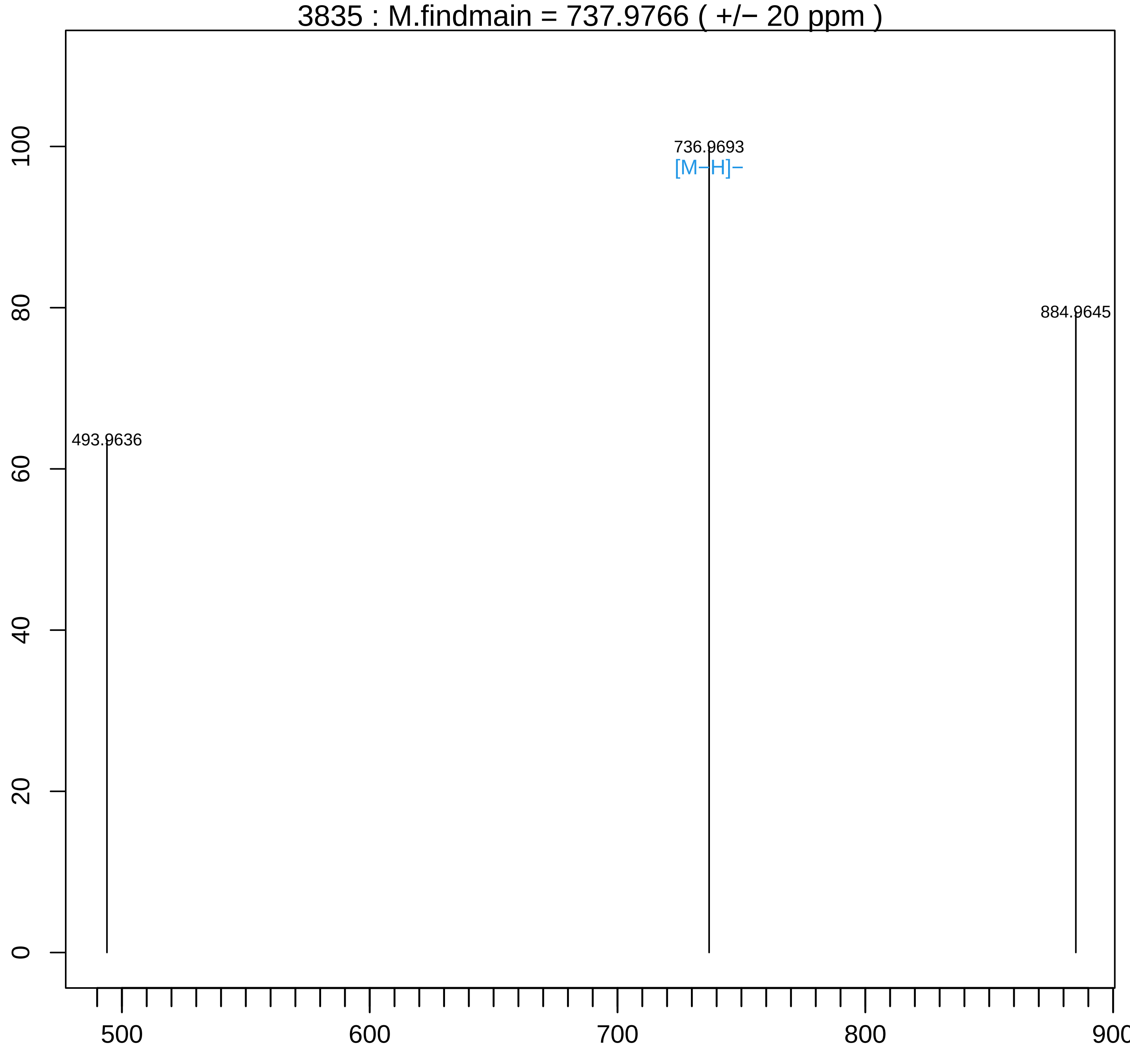

3835 : M.findmain = 737.9766 (+/− 20 ppm)
736.9693
[M−H]−
884.9645
493.9636
0
20
40
60
80
100
500
600
700
800
900

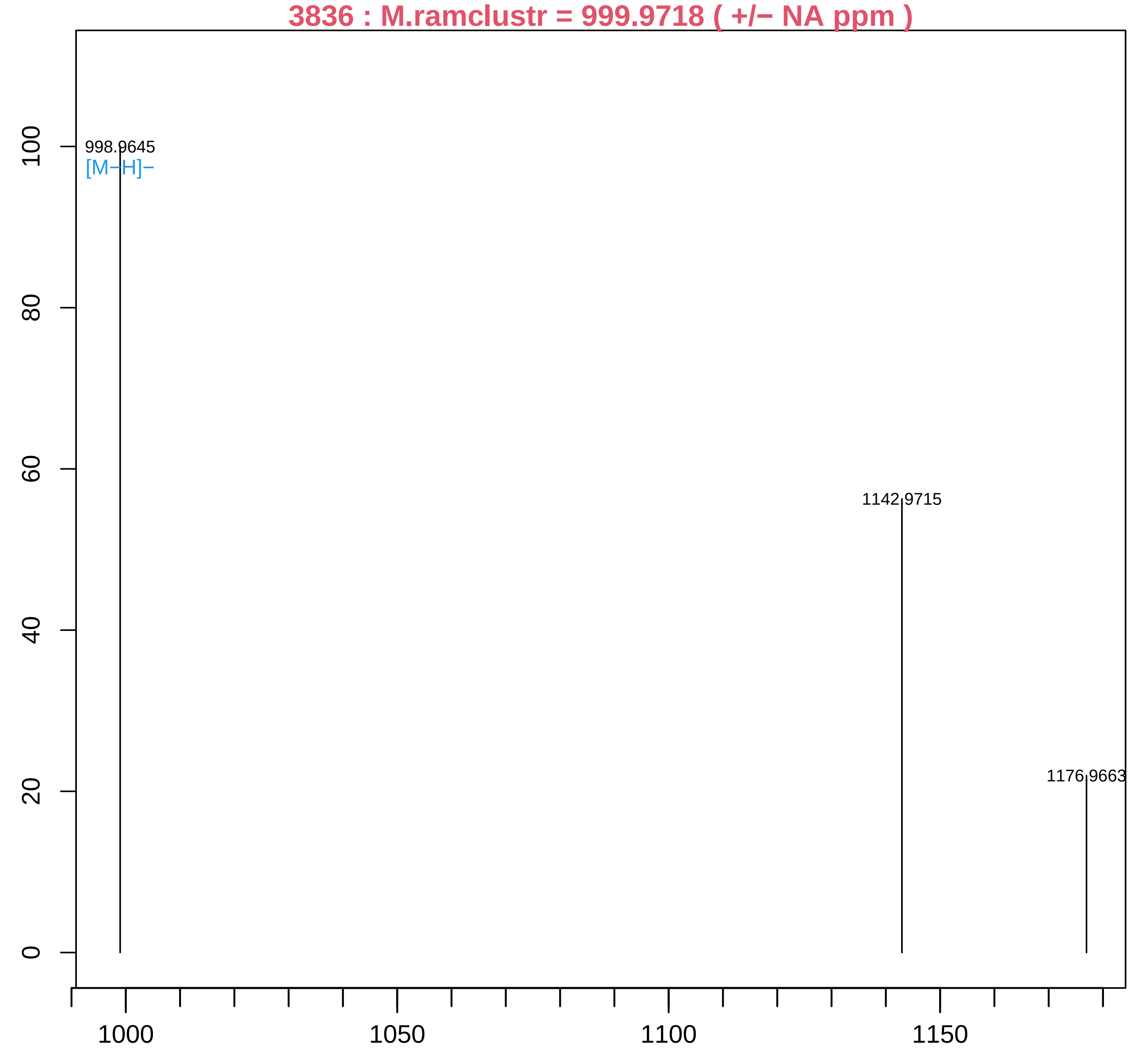
3836 : M.ramclustr = 999.9718 (+/− NA ppm)
998.9645
[M−H]−
1142.9715
1176.9663
0
20
40
60
80
100
1000
1050
1100
1150
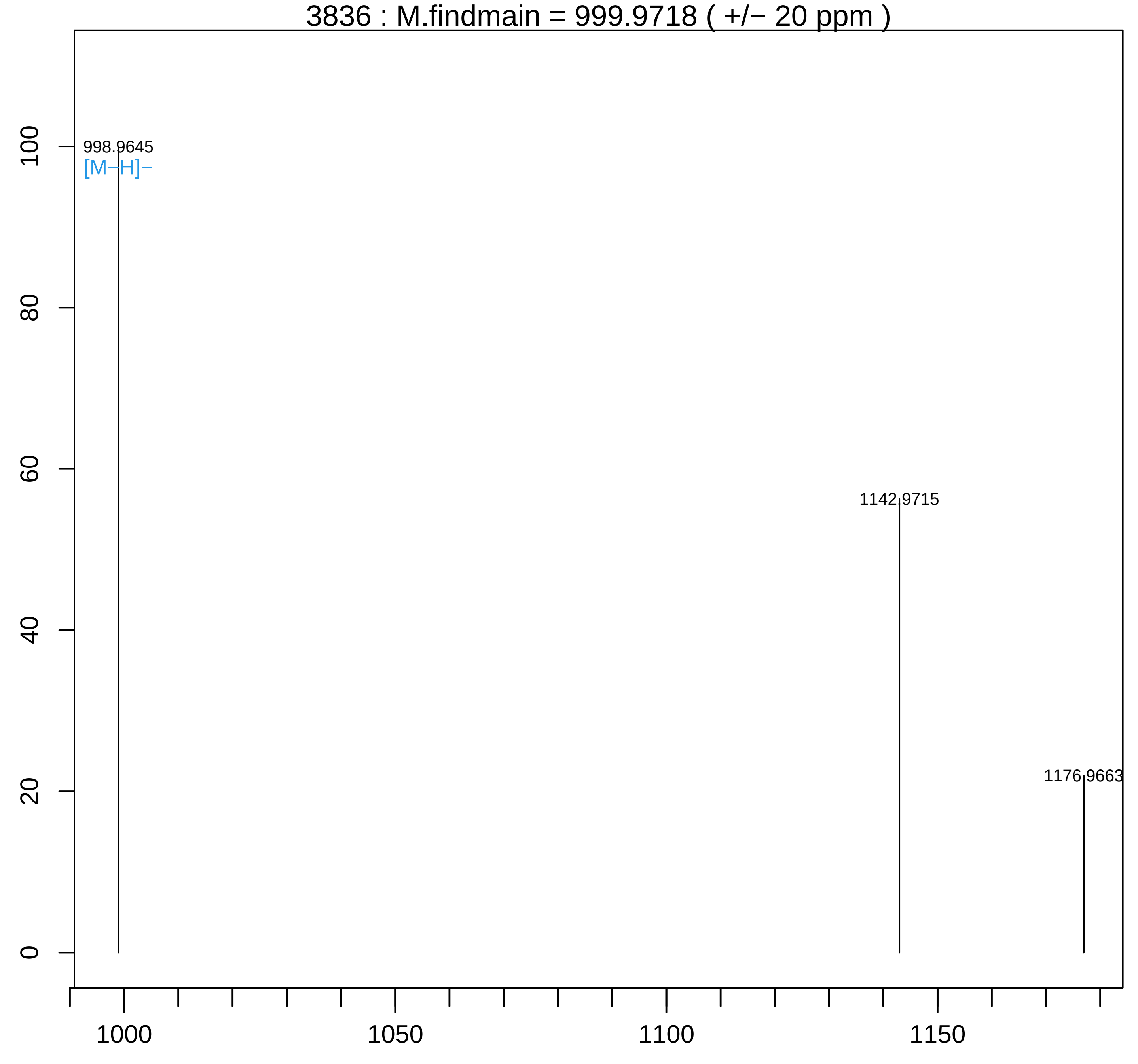
3836 : M.findmain = 999.9718 (+/− 20 ppm)
998.9645
[M−H]−
1142.9715
1176.9663
0
20
40
60
80
100
1000
1050
1100
1150

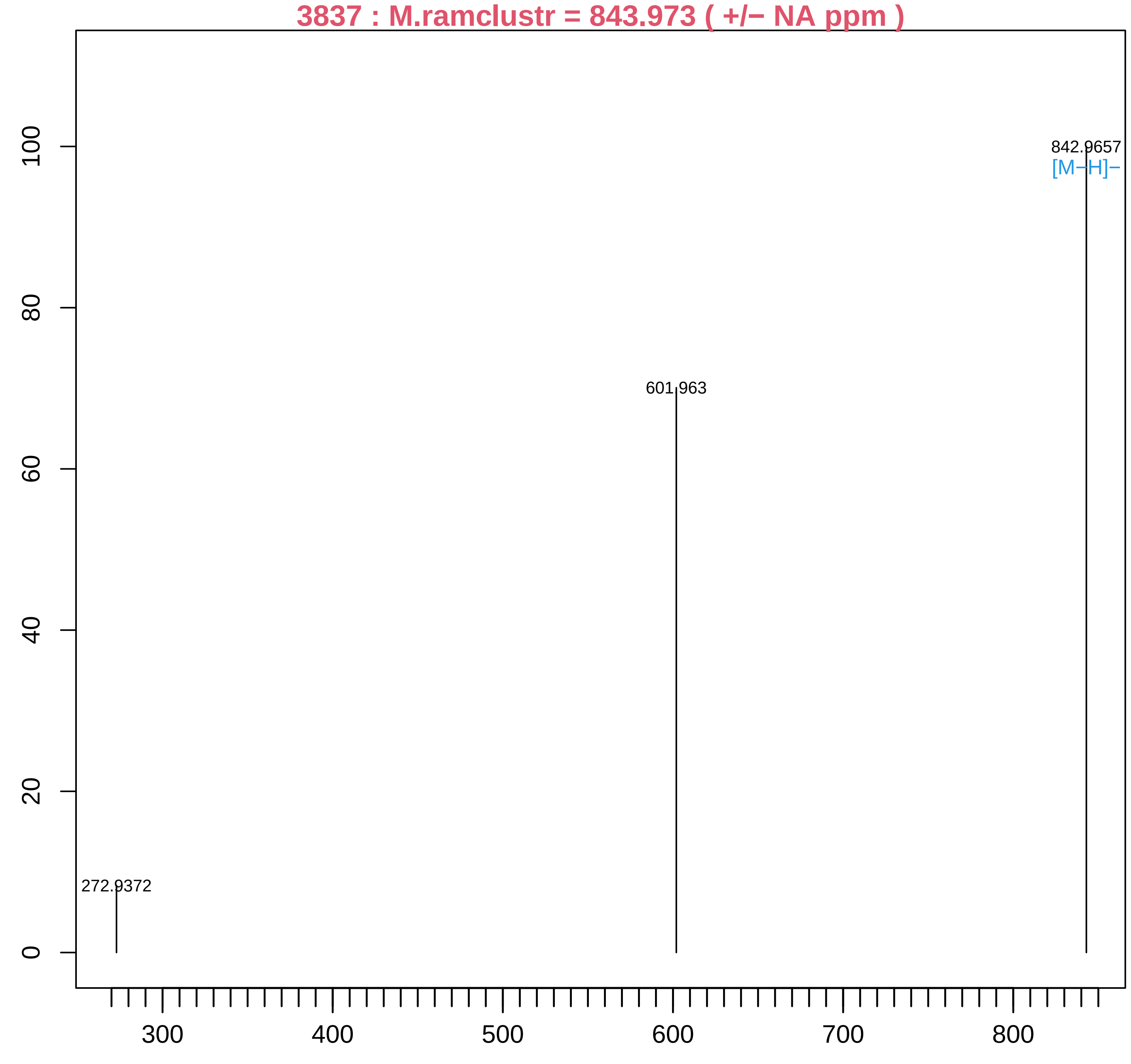
3837 : M.ramclustr = 843.973 (+/− NA ppm)
842.9657
[M−H]−
601.963
272.9372
0
20
40
60
80
100
300
400
500
600
700
800
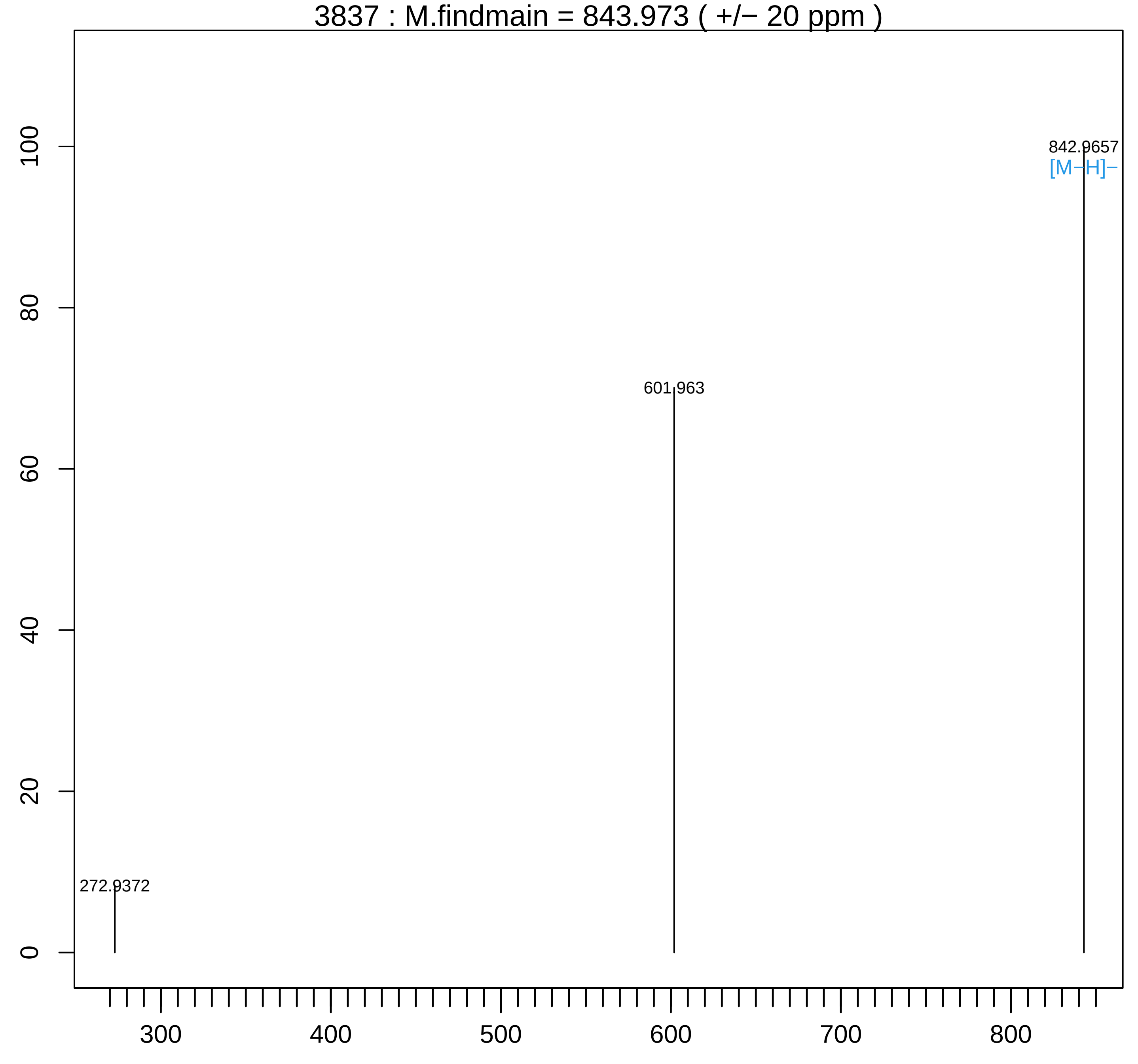
3837 : M.findmain = 843.973 (+/− 20 ppm)
842.9657
[M−H]−
601.963
272.9372
0
20
40
60
80
100
300
400
500
600
700
800

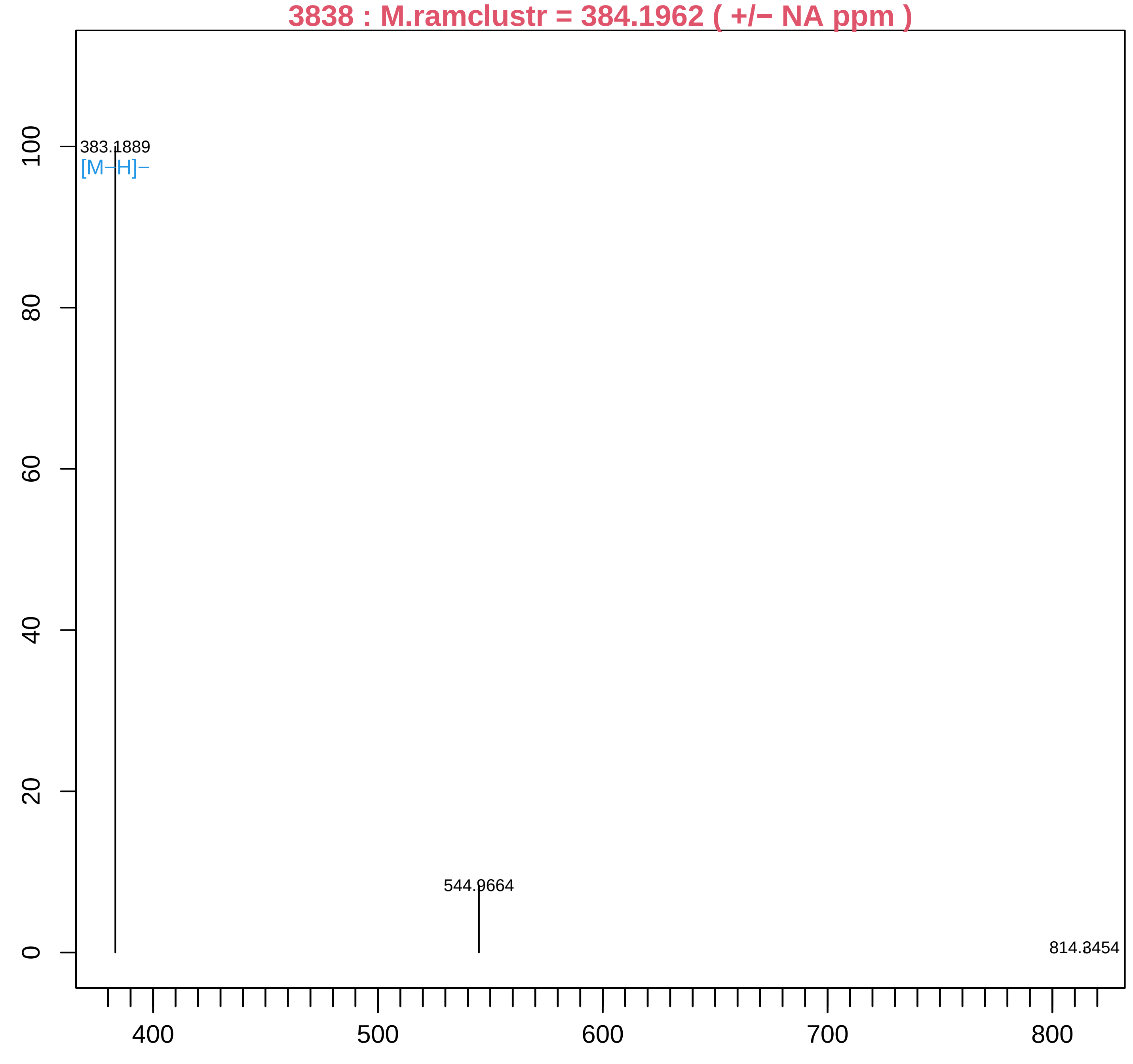
3838 : M.ramclustr = 384.1962 (+/− NA ppm)
383.1889
[M−H]−
544.9664
814.8454
0
20
40
60
80
100
400
500
600
700
800

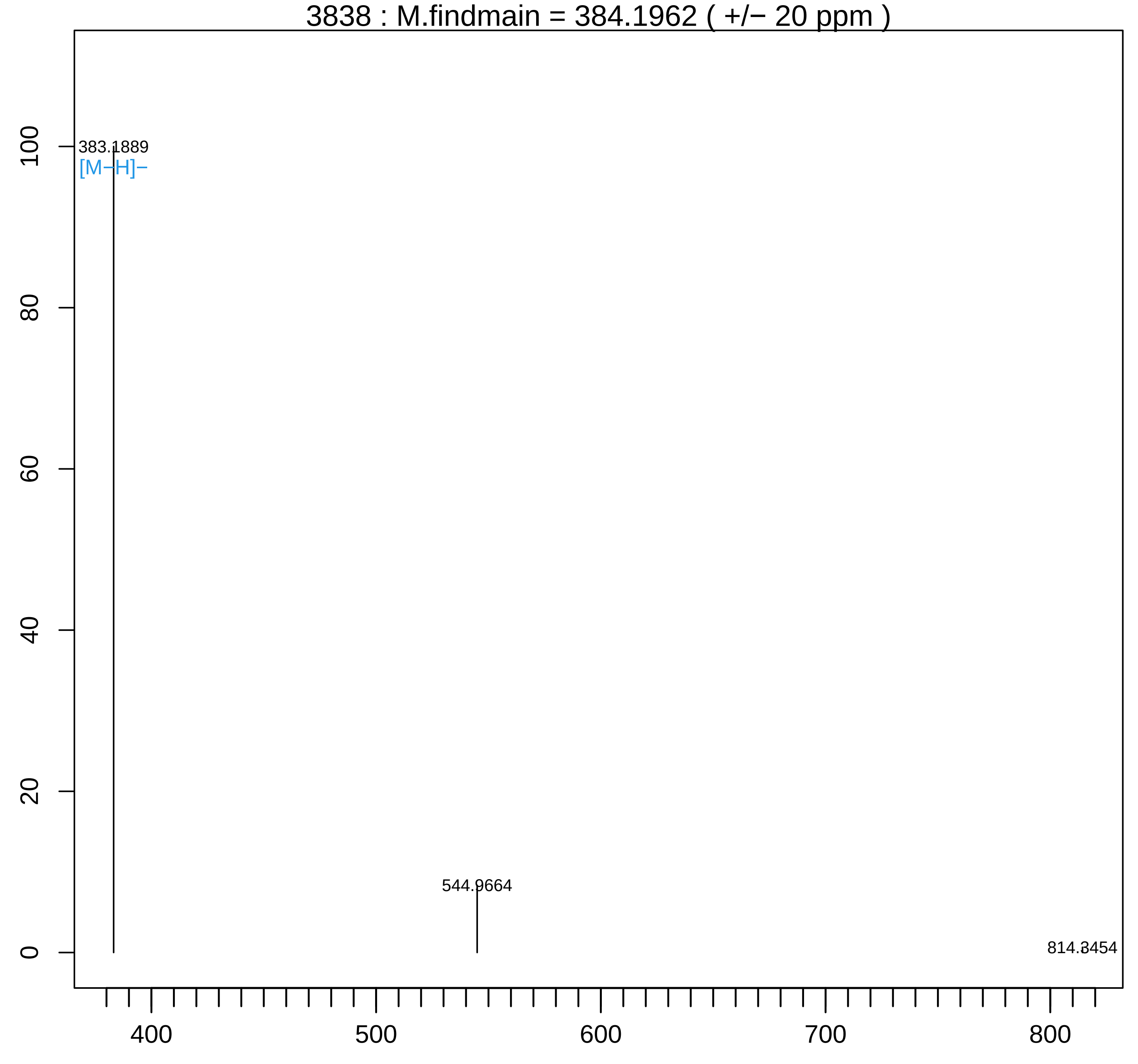
3838 : M.findmain = 384.1962 (+/− 20 ppm)
383.1889
[M−H]−
544.9664
814.8454
0
20
40
60
80
100
400
500
600
700
800

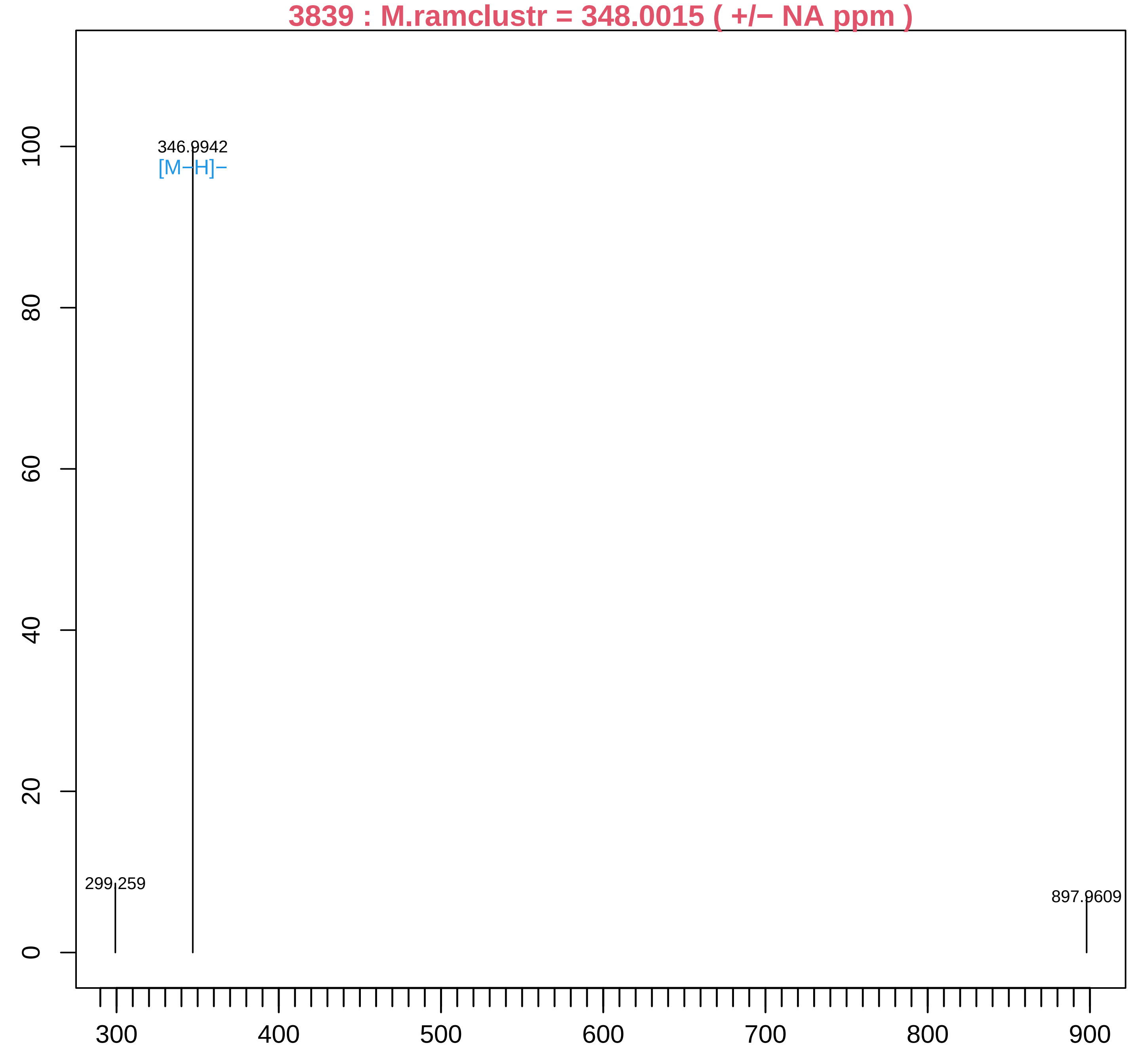

3839 : M.ramclustr = 348.0015 (+/− NA ppm)
346.9942
[M−H]−
299.259
897.9609
0
20
40
60
80
100
300
400
500
600
700
800
900

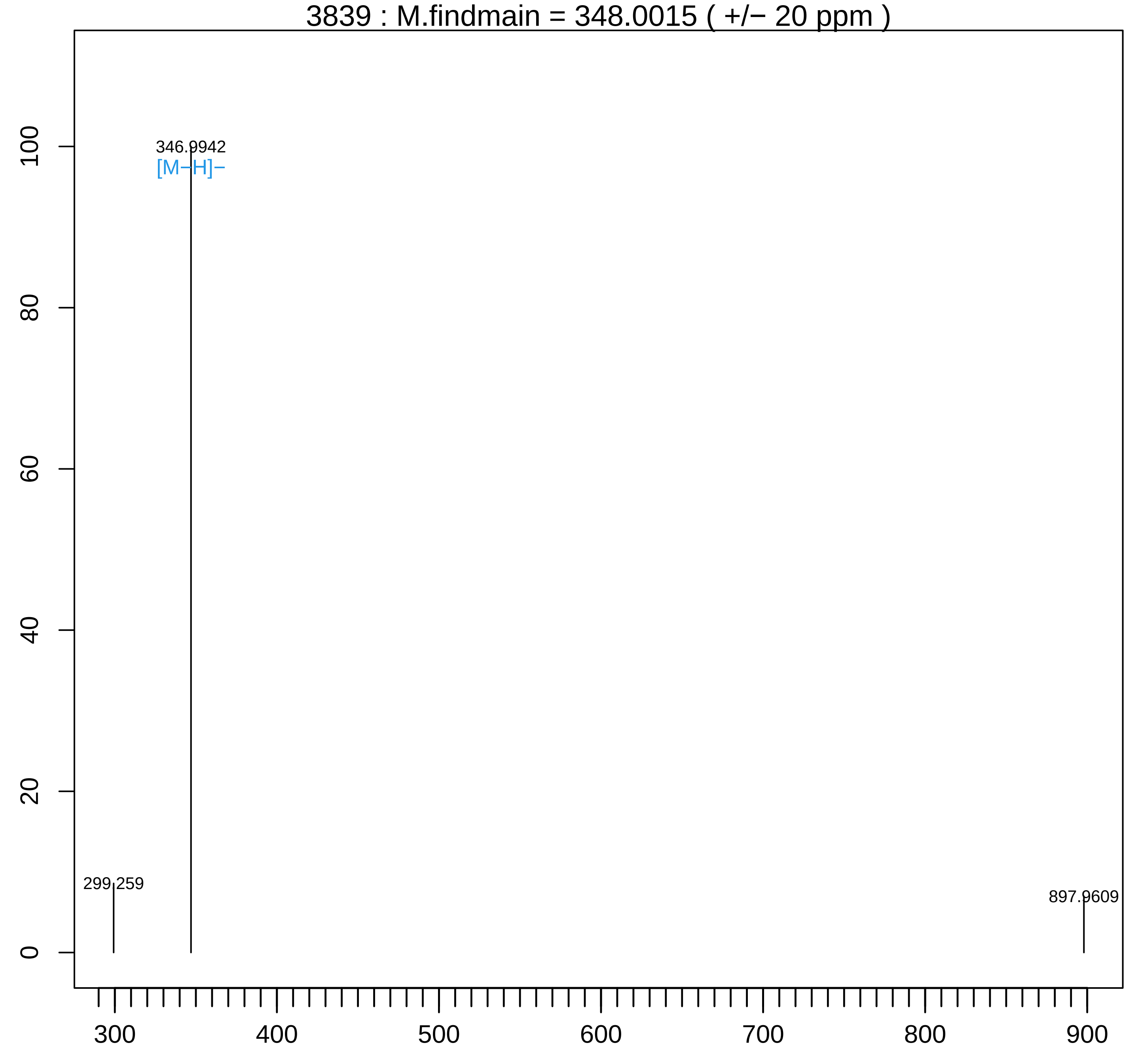

3839 : M.findmain = 348.0015 (+/− 20 ppm)
346.9942
[M−H]−
299.259
897.9609
0
20
40
60
80
100
300
400
500
600
700
800
900

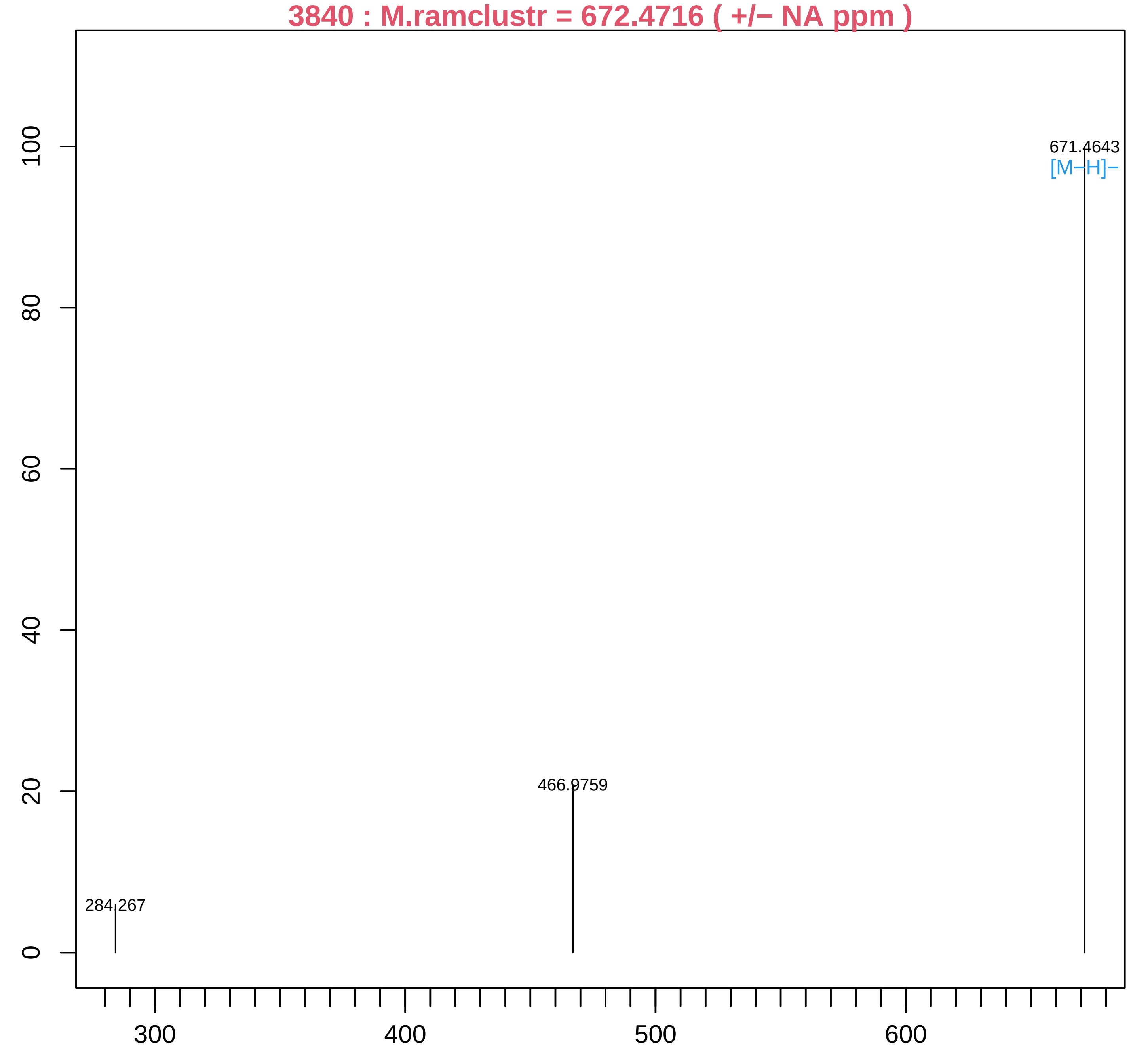
3840 : M.ramclustr = 672.4716 (+/− NA ppm)
671.4643
[M−H]−
466.9759
284.267
0
20
40
60
80
100
300
400
500
600

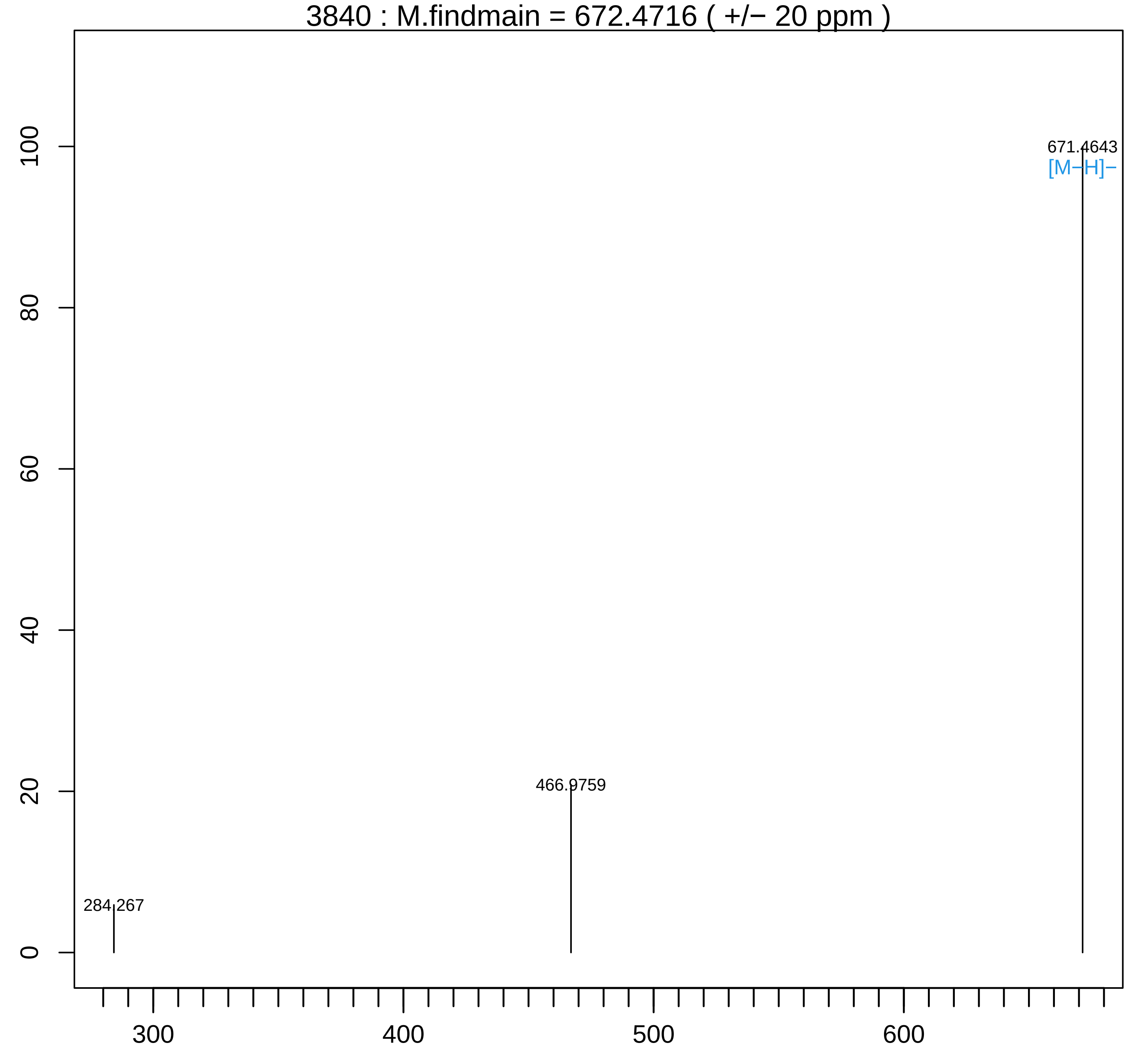
3840 : M.findmain = 672.4716 (+/− 20 ppm)
671.4643
[M−H]−
466.9759
284.267
0
20
40
60
80
100
300
400
500
600

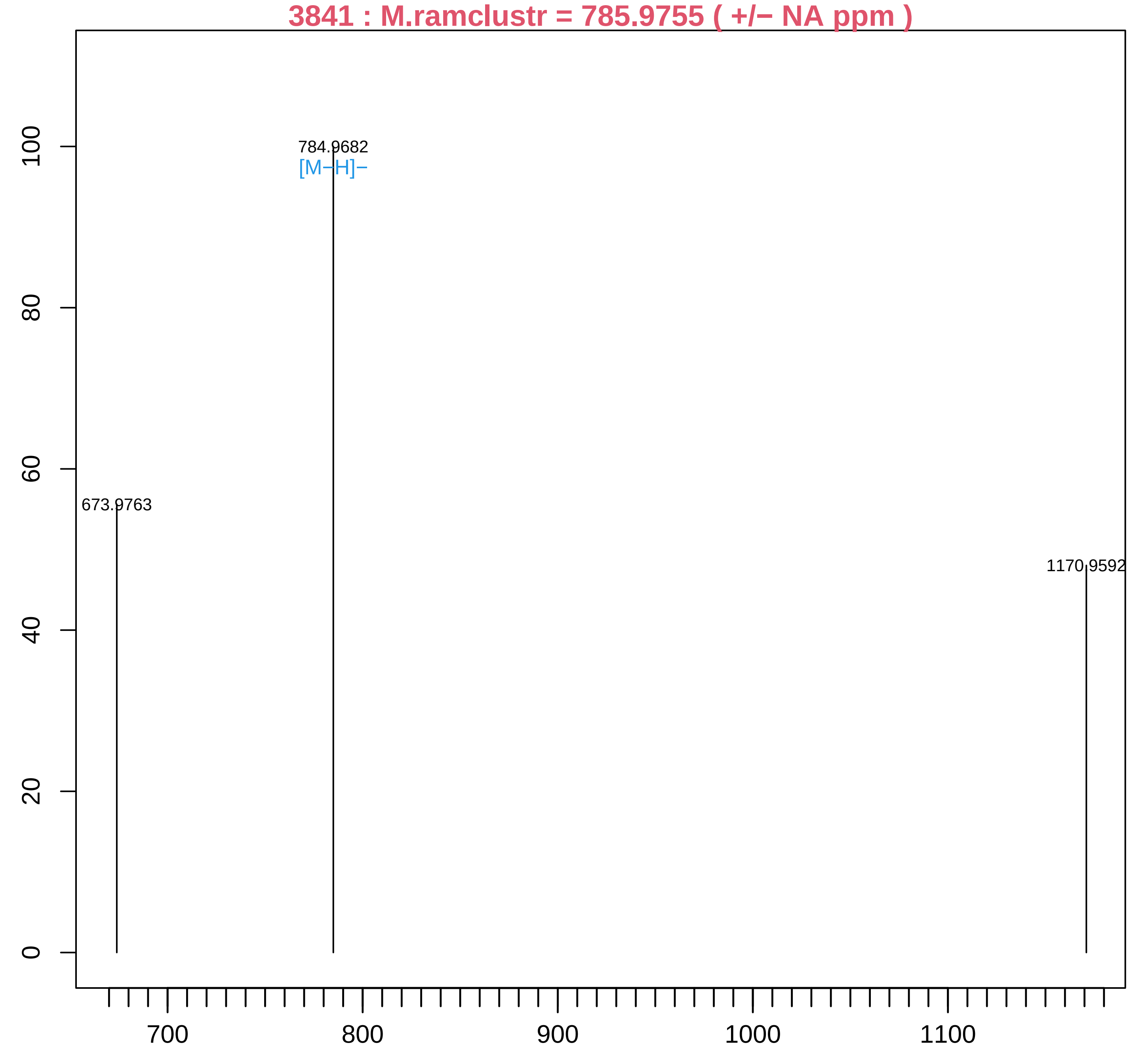
3841 : M.ramclustr = 785.9755 (+/− NA ppm)
784.9682
[M−H]−
673.9763
1170.9592
0
20
40
60
80
100
700
800
900
1000
1100

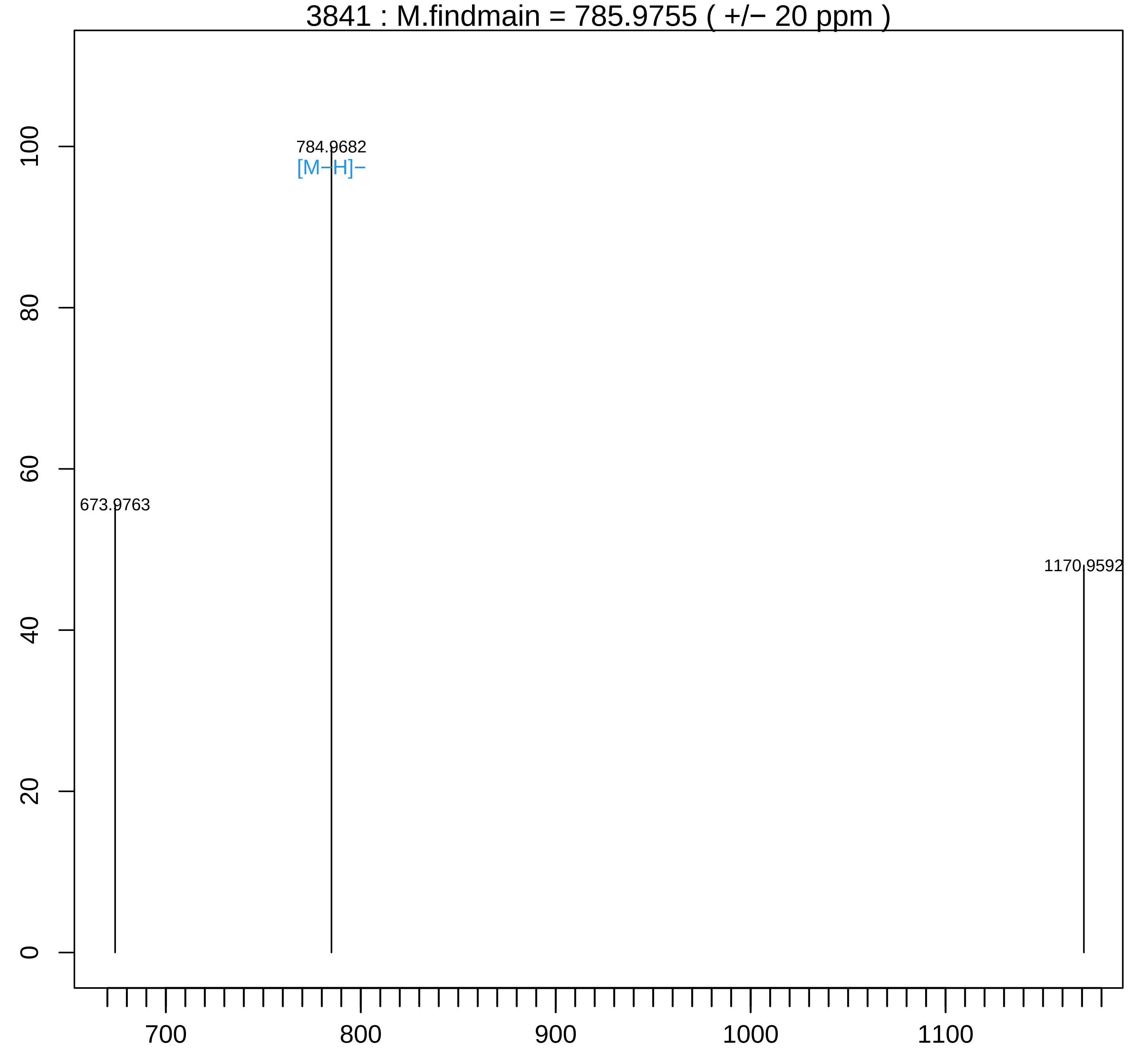
3841 : M.findmain = 785.9755 (+/− 20 ppm)
784.9682
[M−H]−
673.9763
1170.9592
0
20
40
60
80
100
700
800
900
1000
1100

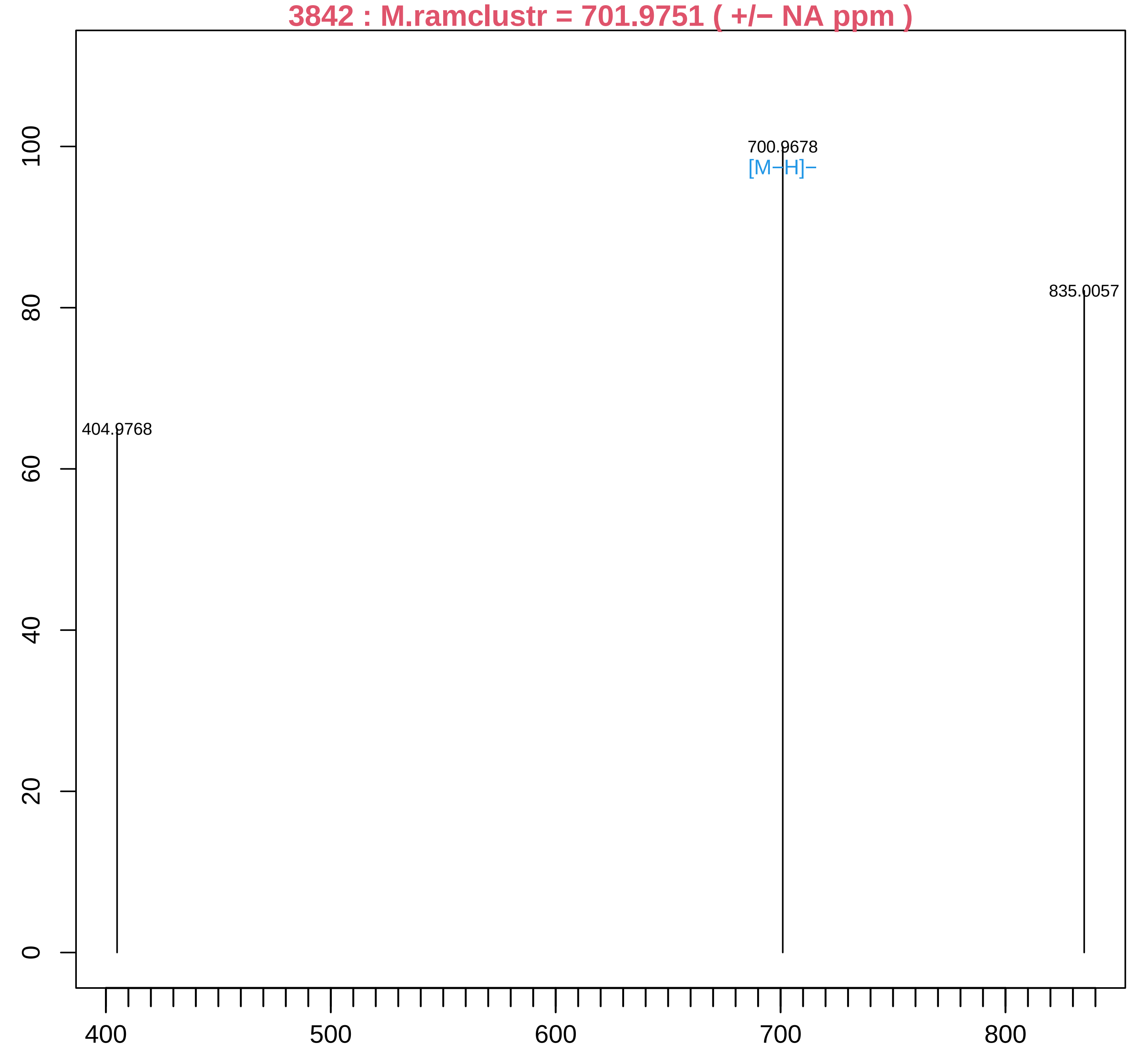
3842 : M.ramclustr = 701.9751 (+/− NA ppm)
700.9678
[M−H]−
835.0057
404.9768
0
20
40
60
80
100
400
500
600
700
800

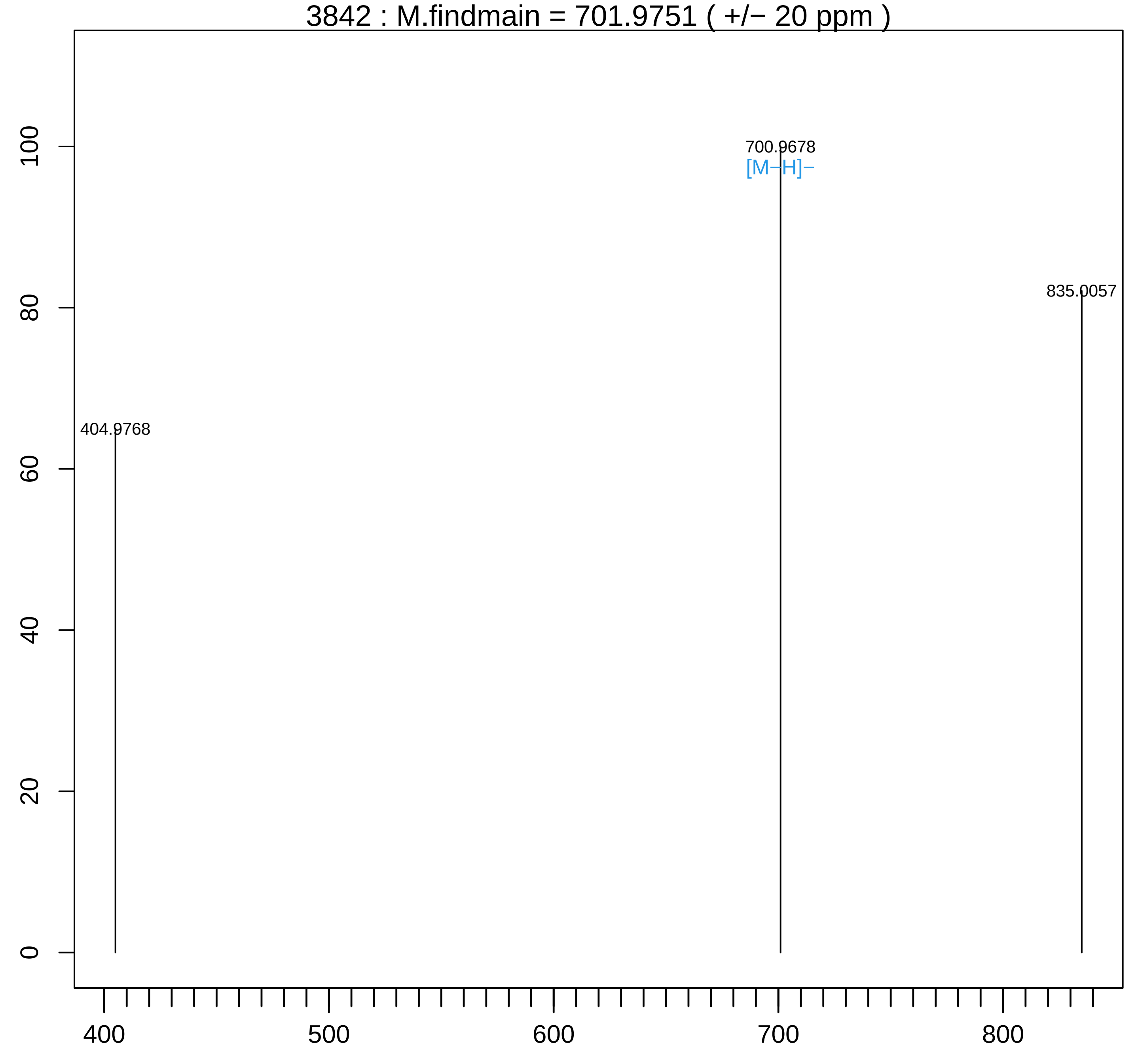
3842 : M.findmain = 701.9751 (+/− 20 ppm)
700.9678
[M−H]−
835.0057
404.9768
0
20
40
60
80
100
400
500
600
700
800

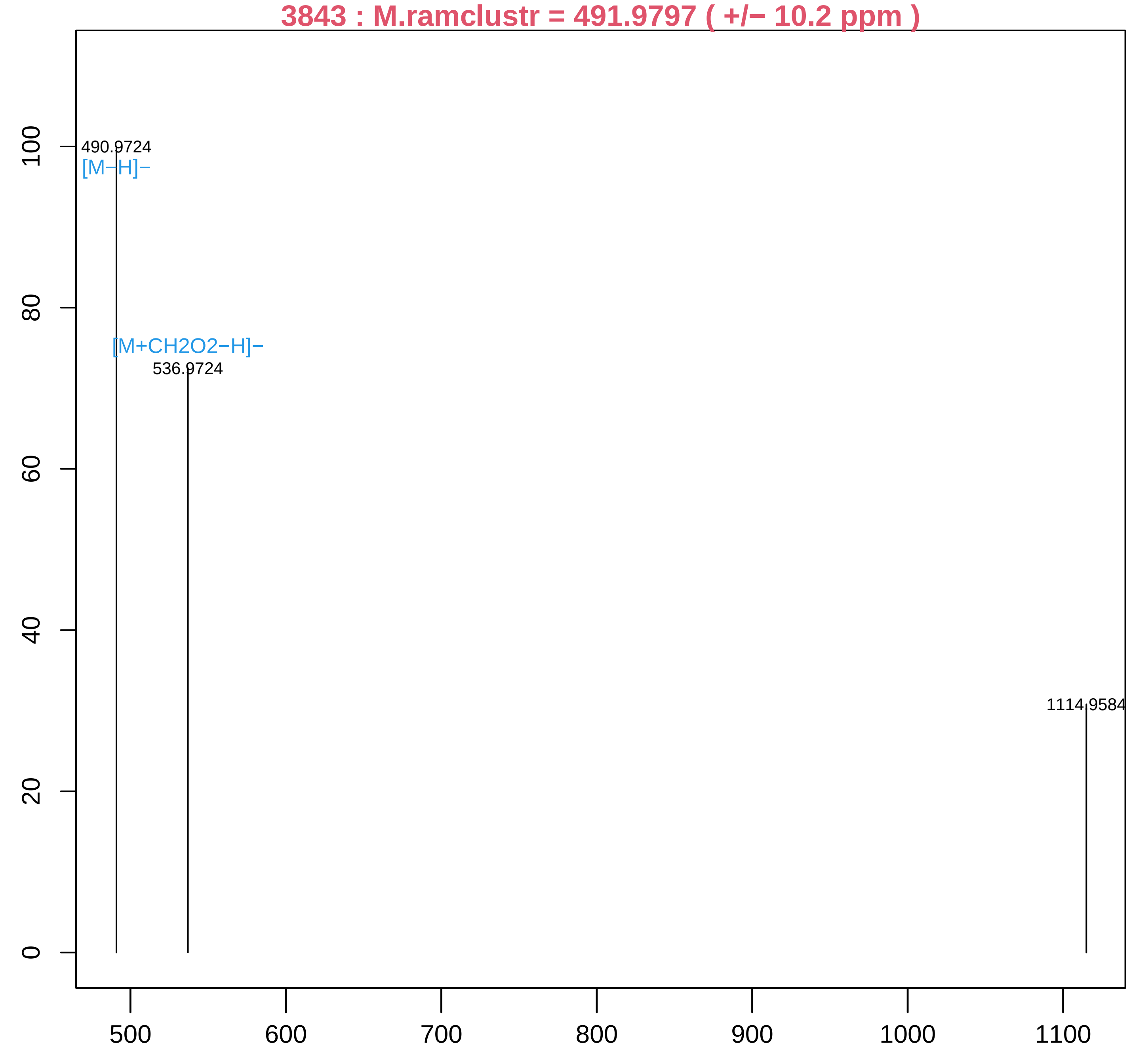
3843 : M.ramclustr = 491.9797 (+/− 10.2 ppm)
490.9724
[M−H]−
[M+CH2O2−H]−
536.9724
1114.9584
0
20
40
60
80
100
500
600
700
800
900
1000
1100

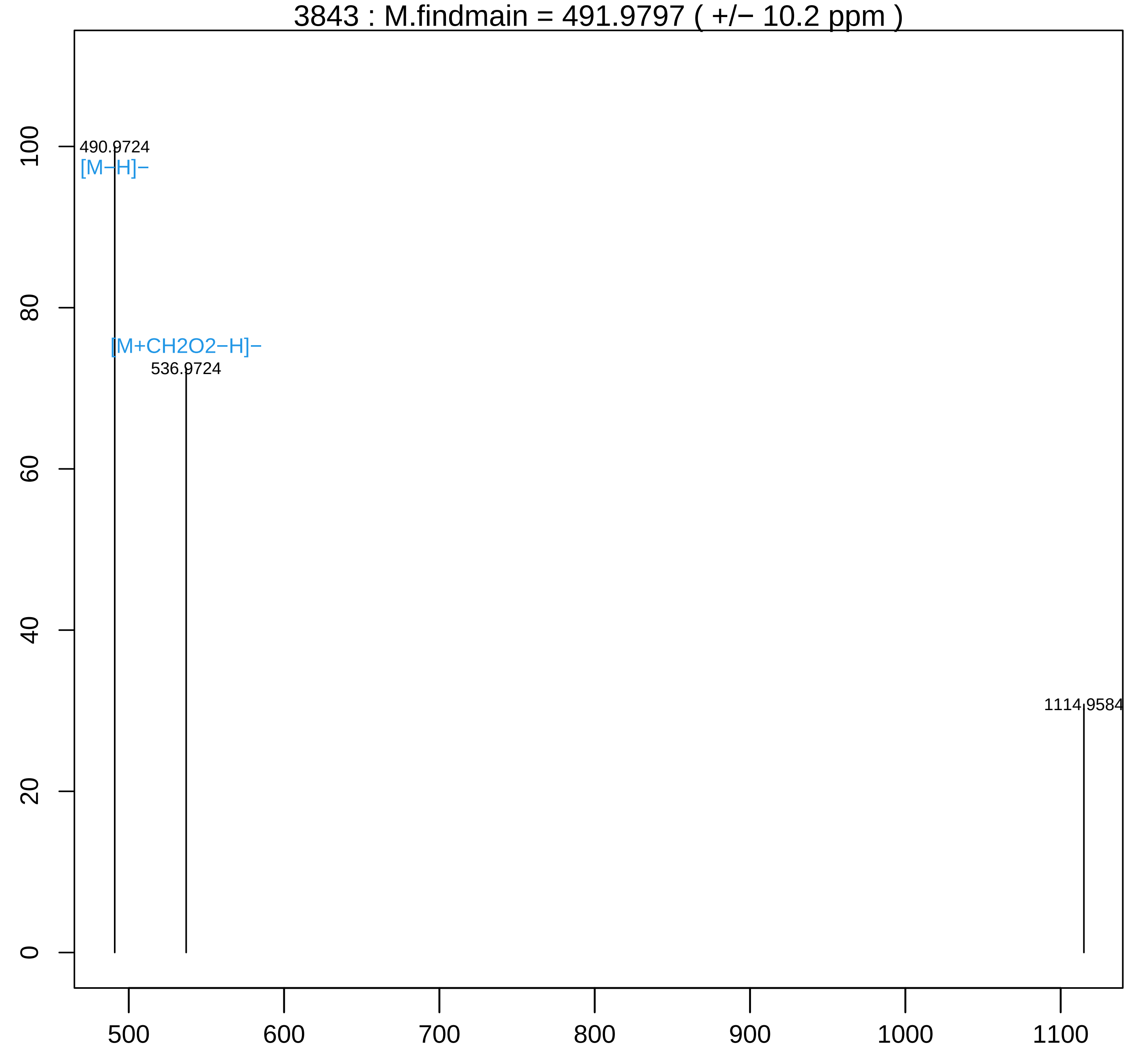
3843 : M.findmain = 491.9797 (+/− 10.2 ppm)
490.9724
[M−H]−
[M+CH2O2−H]−
536.9724
1114.9584
0
20
40
60
80
100
500
600
700
800
900
1000
1100

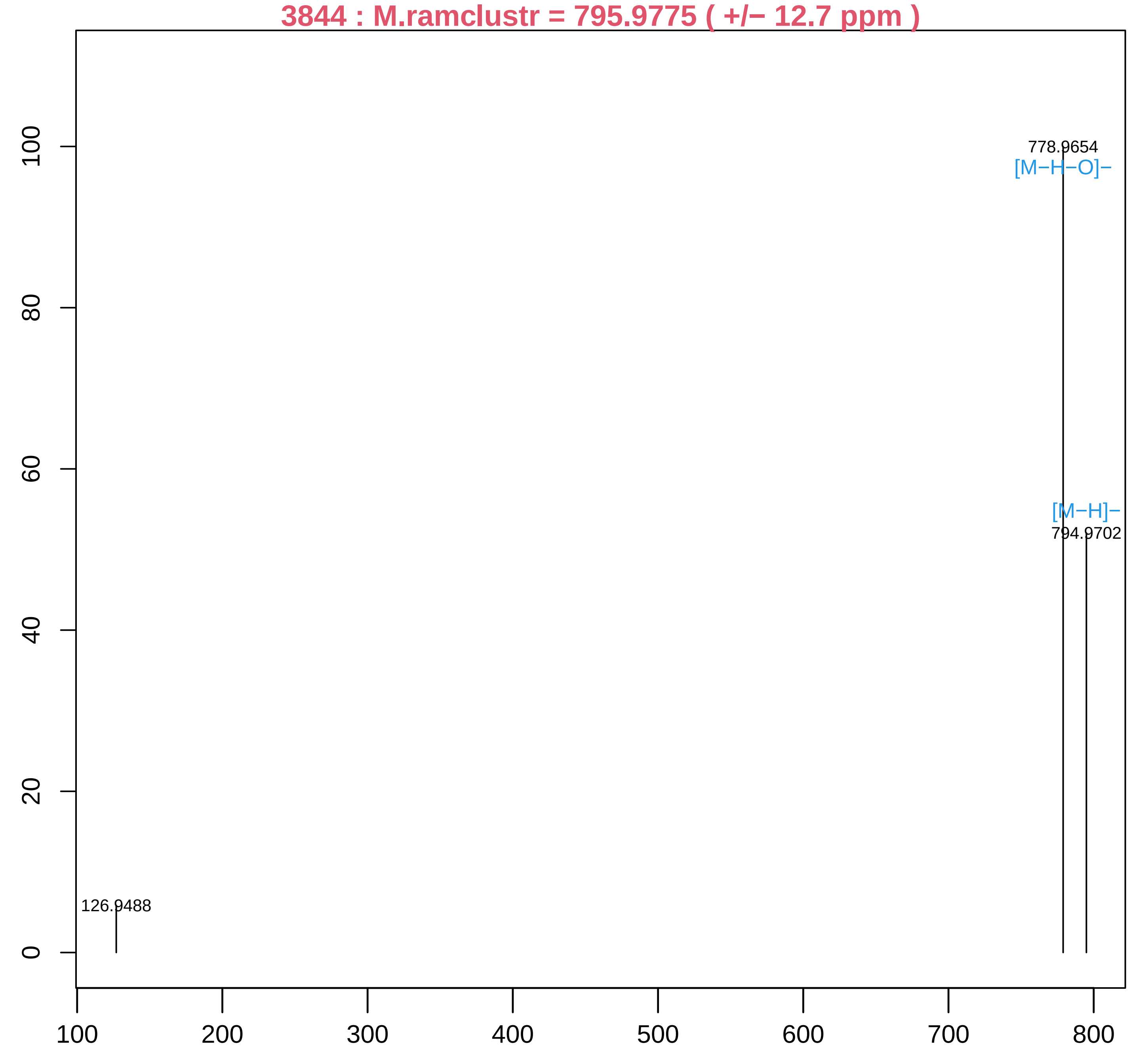
3844 : M.ramclustr = 795.9775 (+/− 12.7 ppm)
778.9654
[M−H−O]−
[M−H]−
794.9702
126.9488
0
20
40
60
80
100
100
200
300
400
500
600
700
800

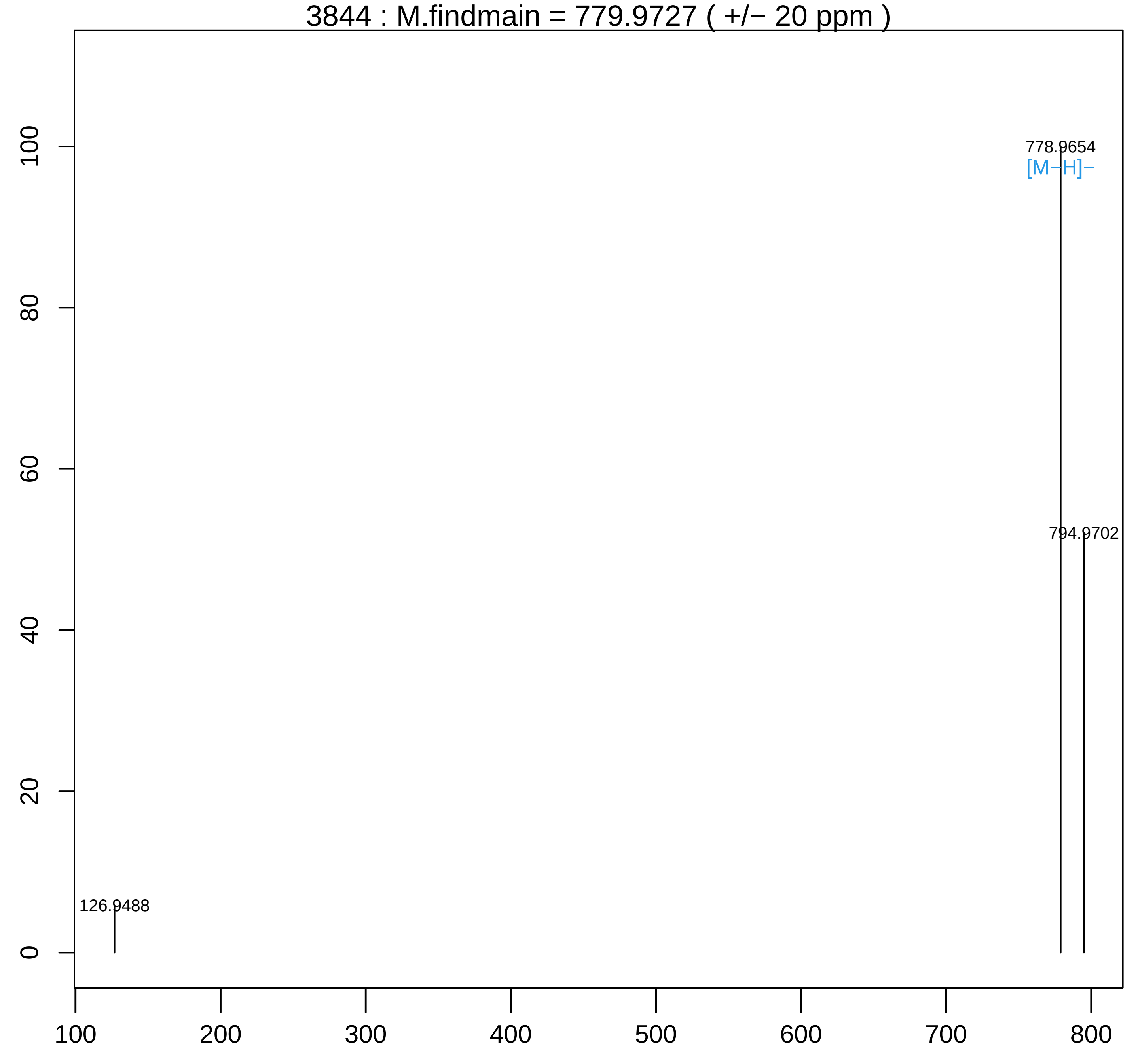
3844 : M.findmain = 779.9727 (+/− 20 ppm)
778.9654
[M−H]−
794.9702
126.9488
0
20
40
60
80
100
100
200
300
400
500
600
700
800

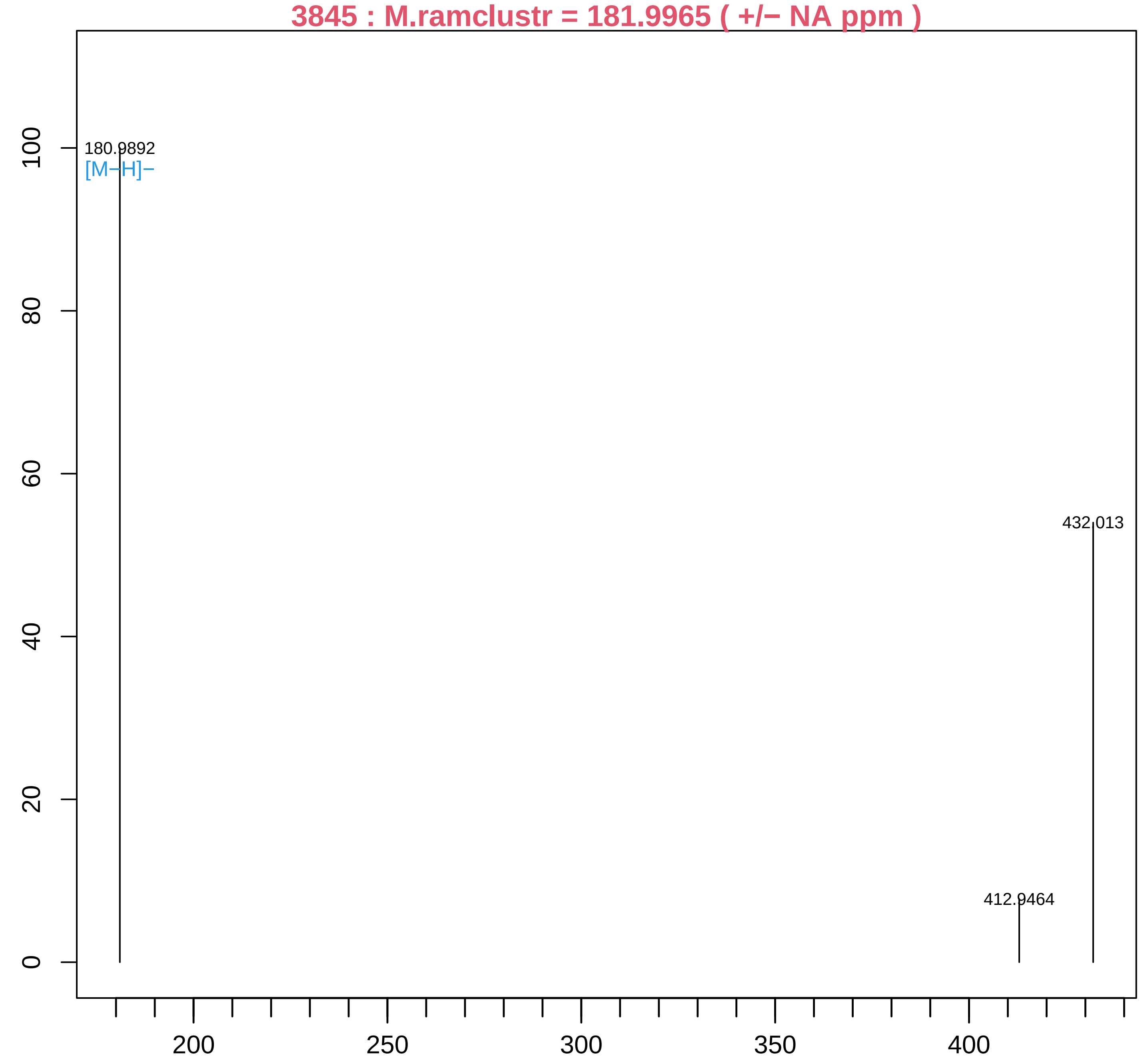
3845 : M.ramclustr = 181.9965 (+/− NA ppm)
180.9892
[M−H]−
432.013
412.9464
0
20
40
60
80
100
200
250
300
350
400

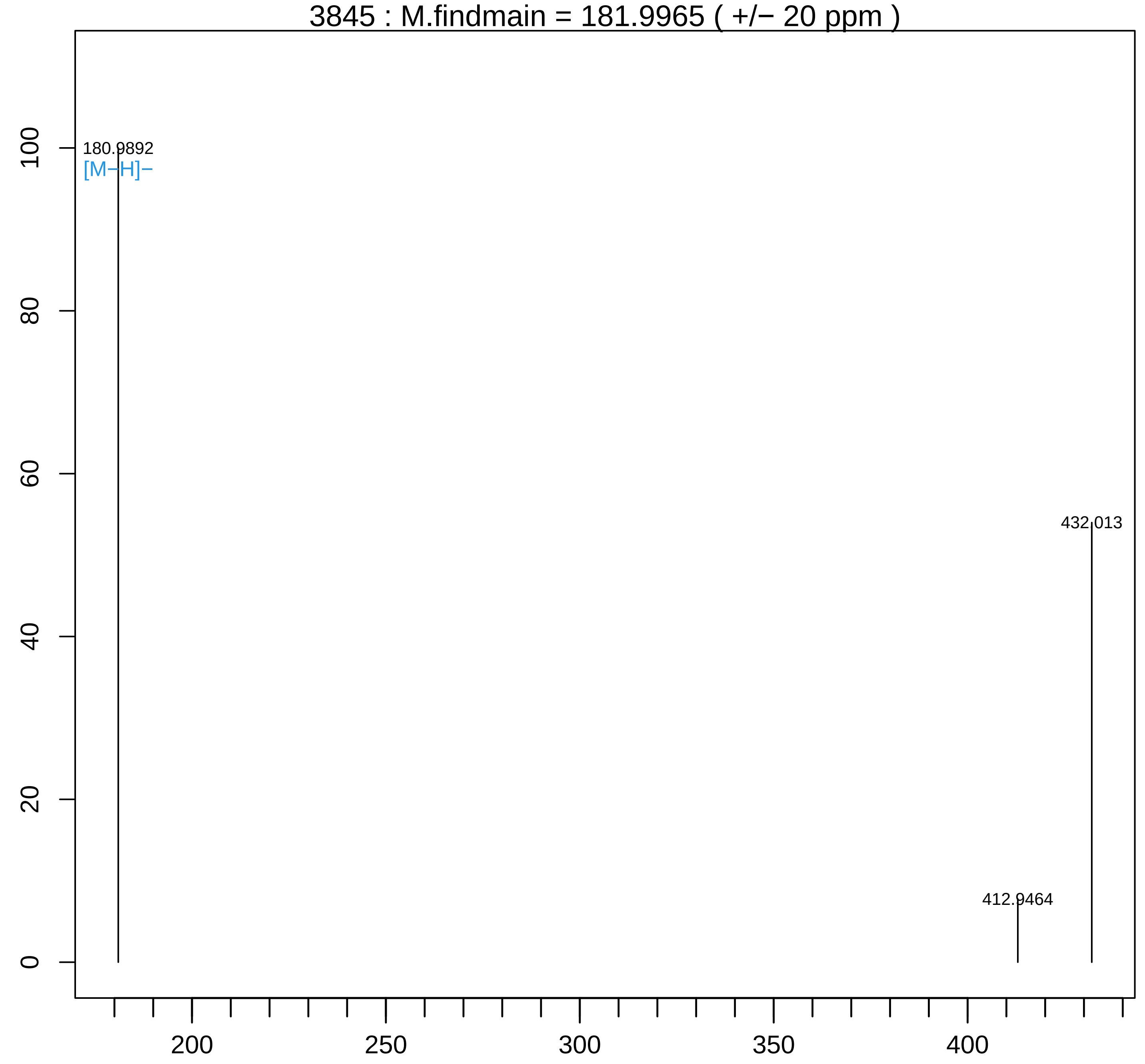
3845 : M.findmain = 181.9965 (+/− 20 ppm)
180.9892
[M−H]−
432.013
412.9464
0
20
40
60
80
100
200
250
300
350
400

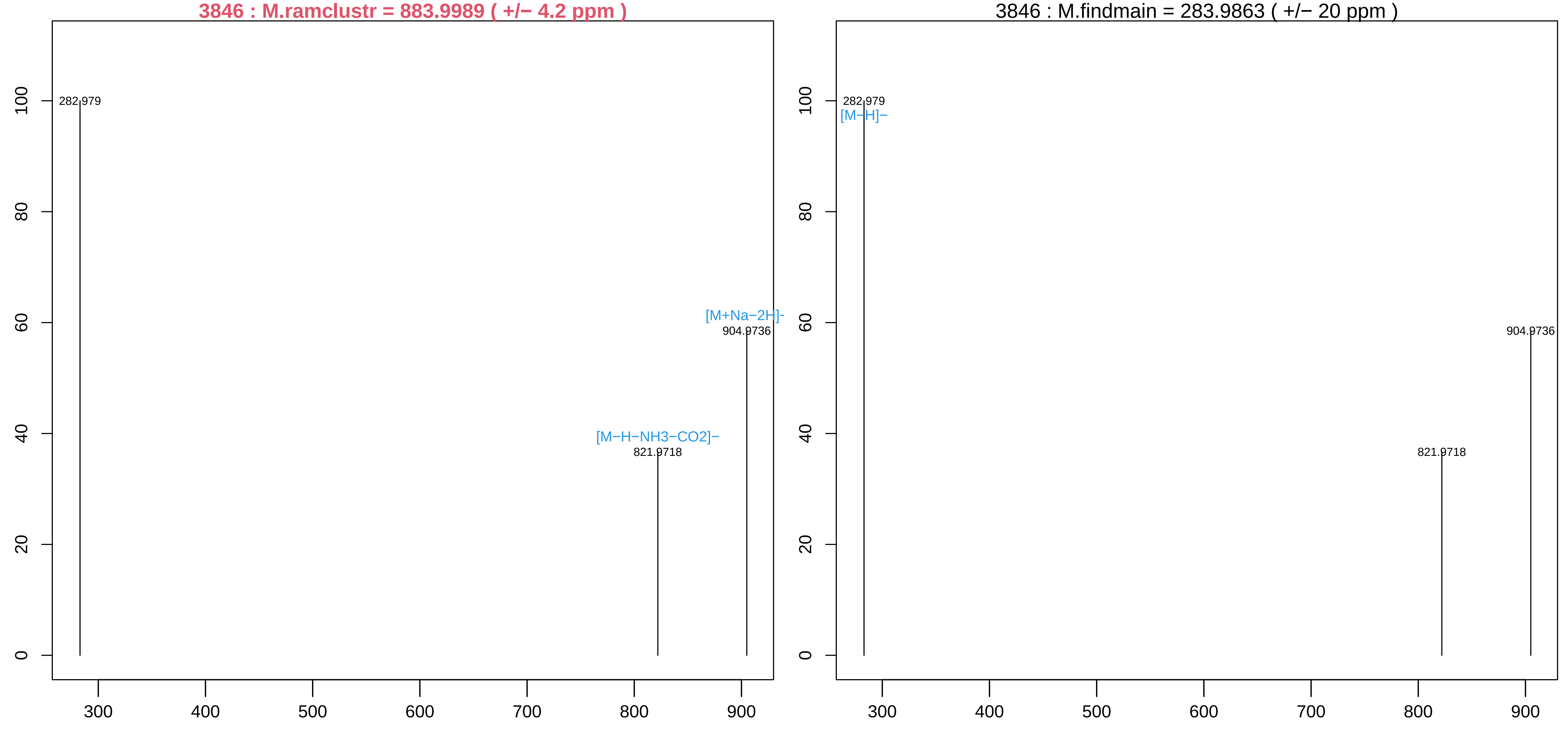

3846 : M.ramclustr = 883.9989 (+/− 4.2 ppm)
282.979
[M−H−NH3−CO2]−
821.9718
[M+Na−2H]-
904.9736
0
20
40
60
80
100
300
400
500
600
700
800
900
3846 : M.findmain = 283.9863 (+/− 20 ppm)
282.979
[M−H]−
821.9718
904.9736
0
20
40
60
80
100
300
400
500
600
700
800
900

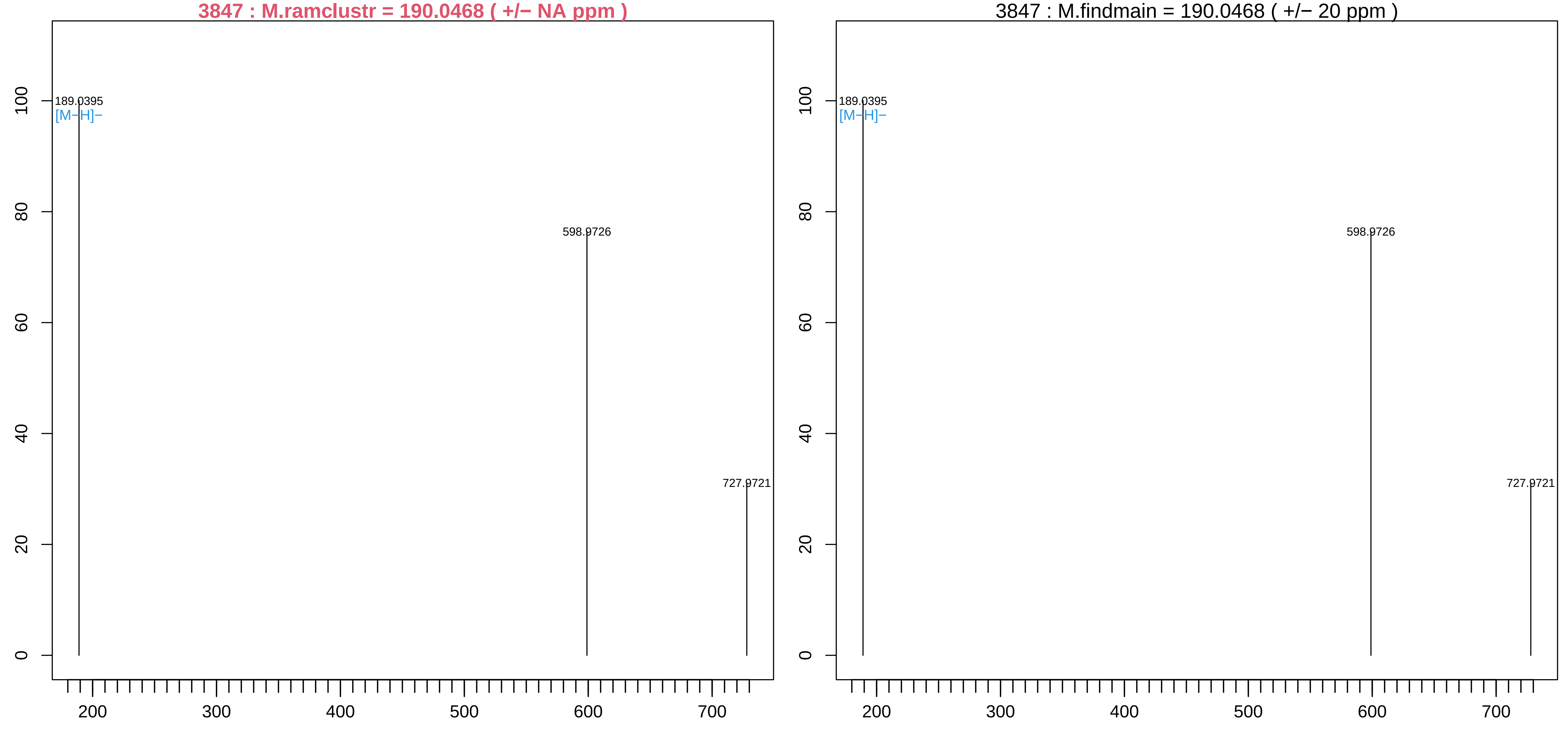
3847 : M.ramclustr = 190.0468 (+/− NA ppm)
189.0395
[M−H]−
598.9726
727.9721
3847 : M.findmain = 190.0468 (+/− 20 ppm)
189.0395
[M−H]−
598.9726
727.9721
0
20
40
60
80
100
200
300
400
500
600
700

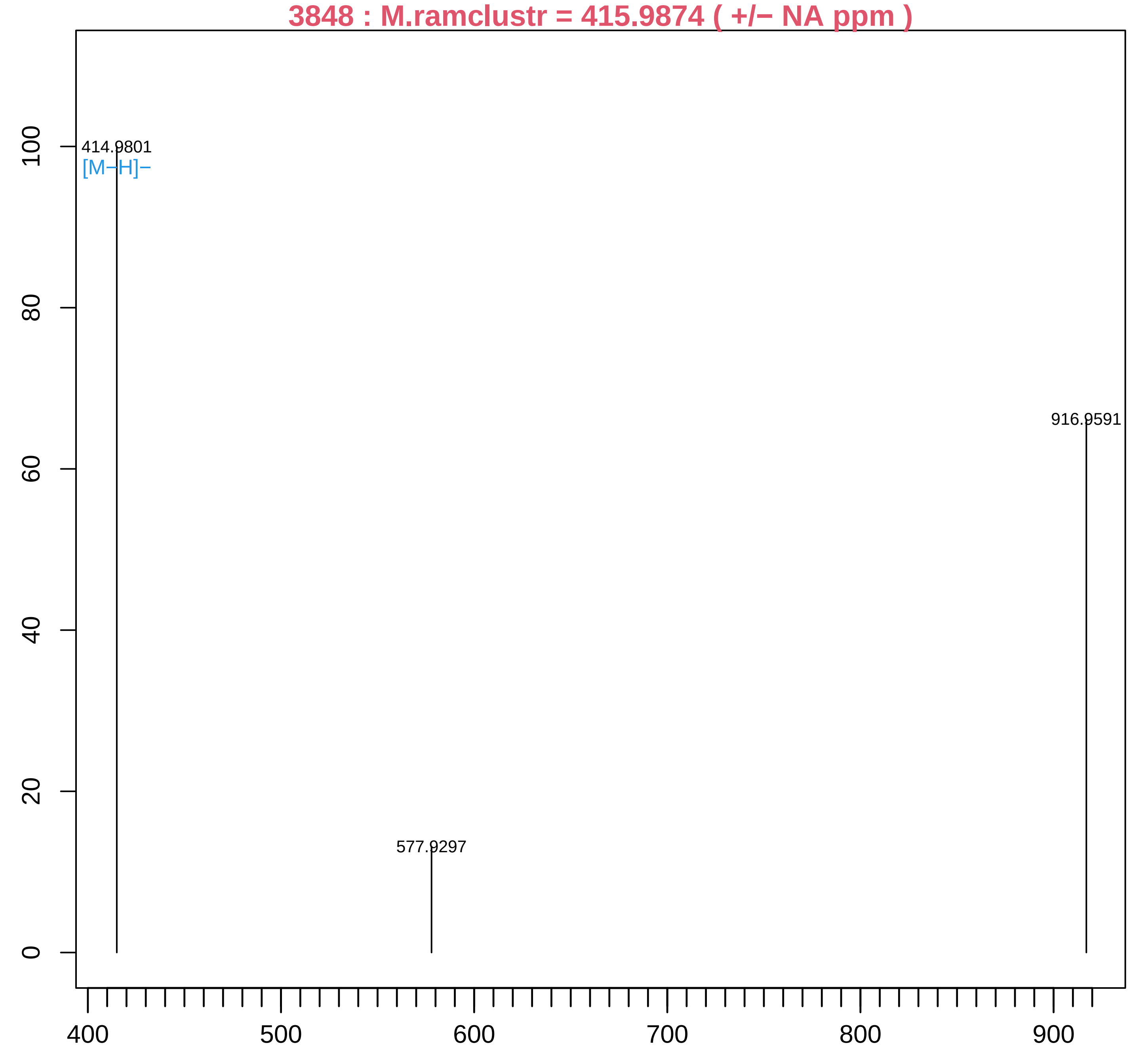
3848 : M.ramclustr = 415.9874 (+/− NA ppm)
414.9801
[M−H]−
577.9297
916.9591
0
20
40
60
80
100
400
500
600
700
800
900

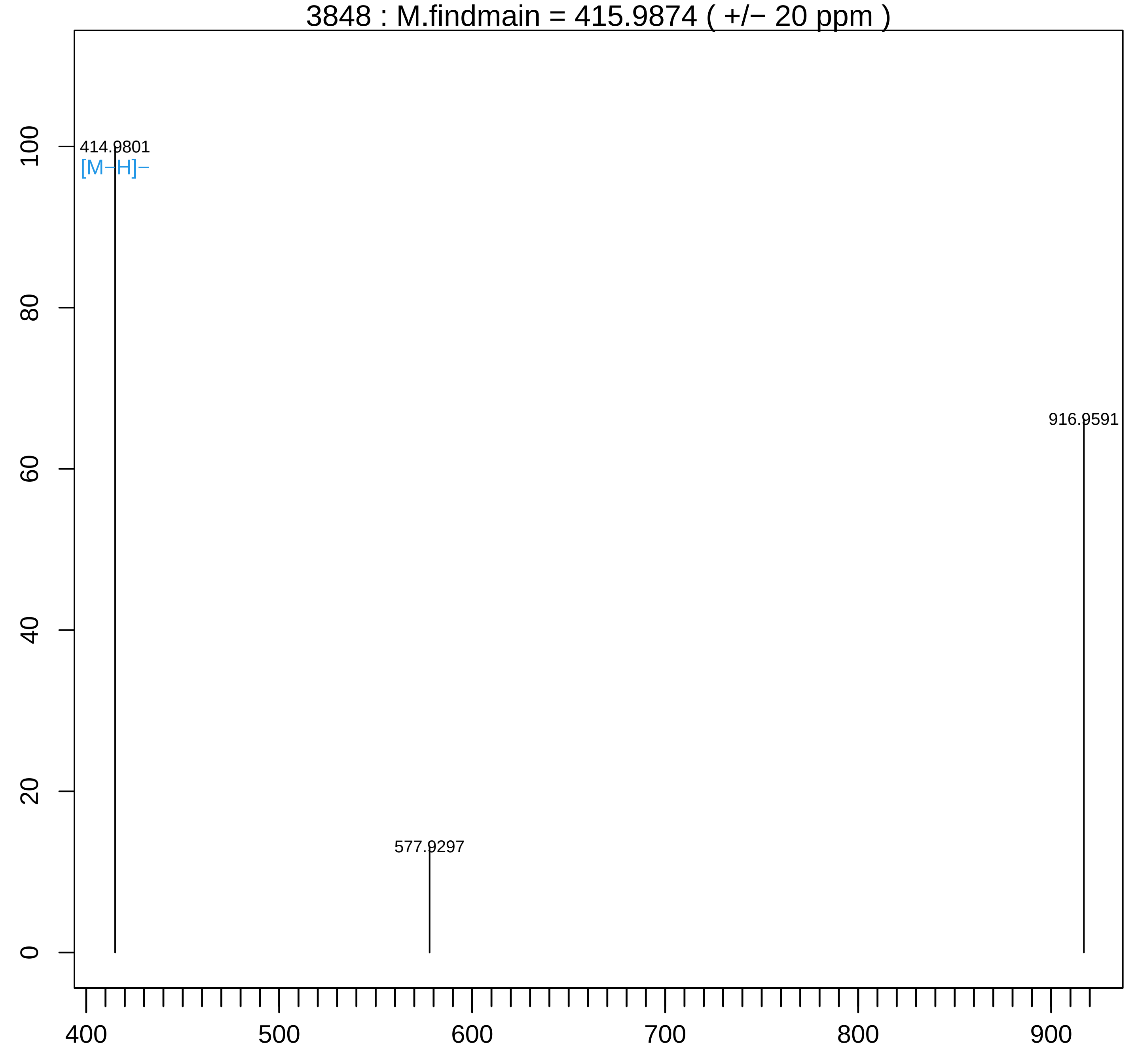
3848 : M.findmain = 415.9874 (+/− 20 ppm)
414.9801
[M−H]−
577.9297
916.9591
0
20
40
60
80
100
400
500
600
700
800
900

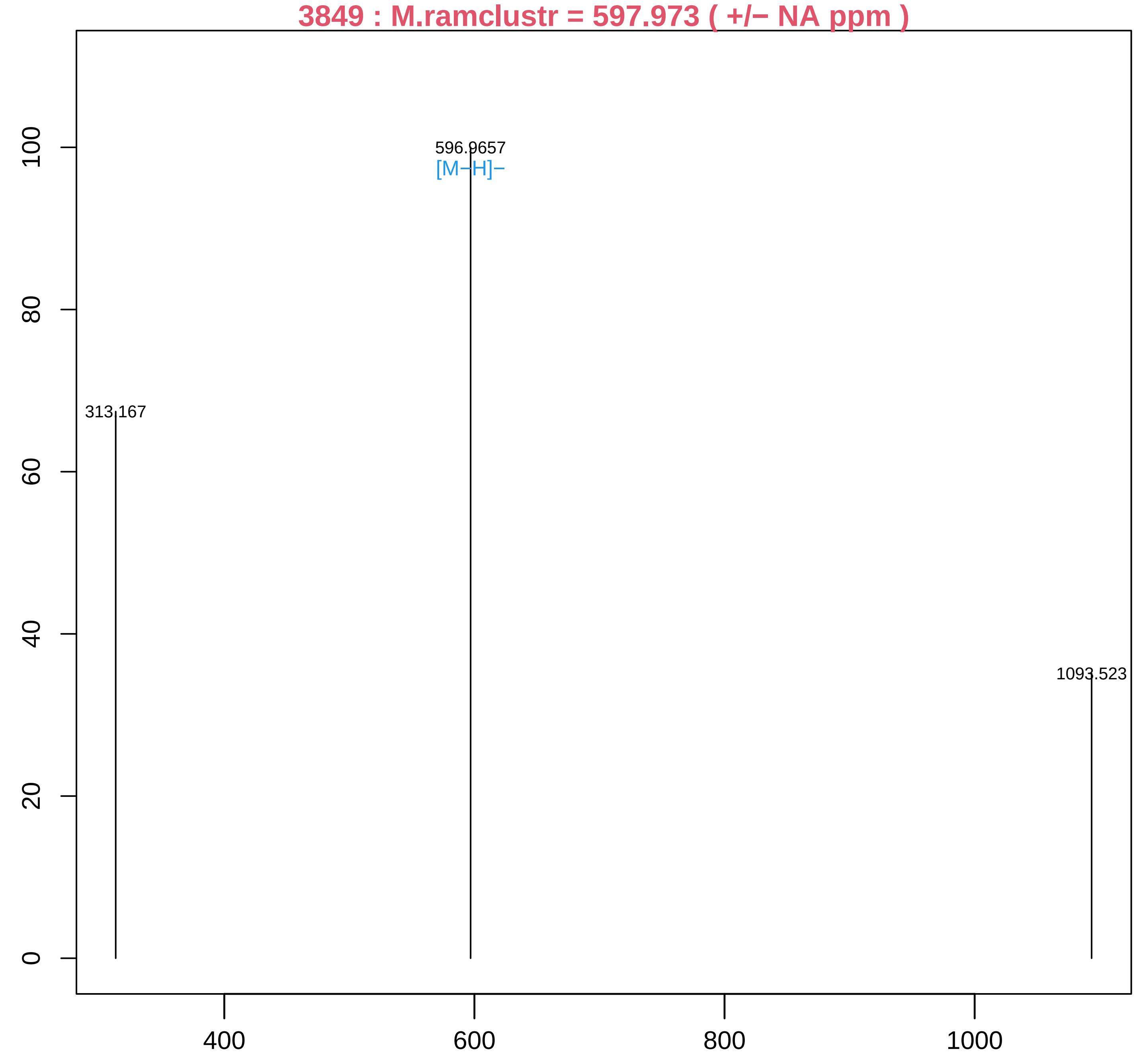
3849 : M.ramclustr = 597.973 (+/− NA ppm)
596.9657
[M−H]−
313.167
1093.523
0
20
40
60
80
100
400
600
800
1000

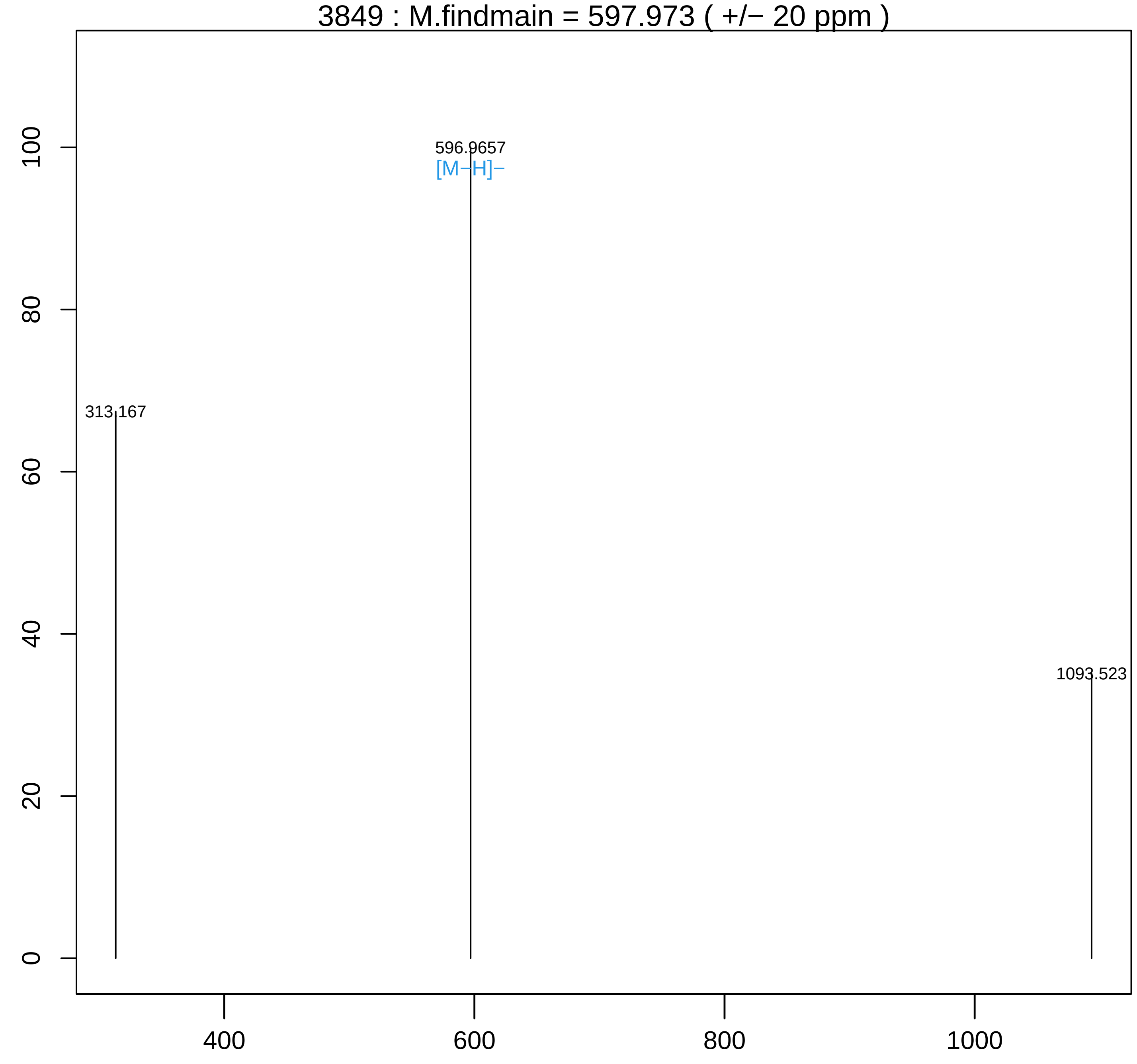
3849 : M.findmain = 597.973 (+/− 20 ppm)
596.9657
[M−H]−
313.167
1093.523
0
20
40
60
80
100
400
600
800
1000

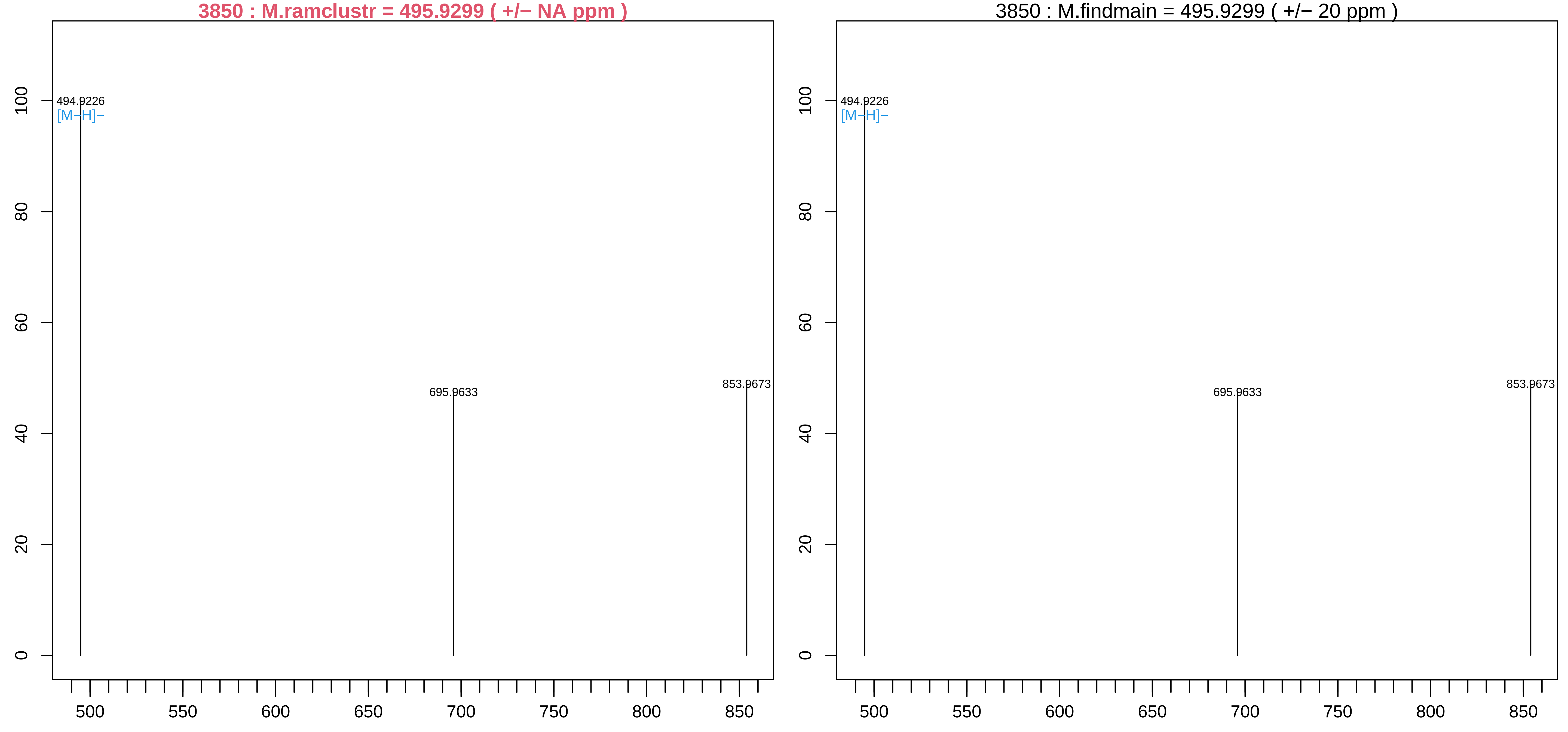

3850 : M.ramclustr = 495.9299 (+/− NA ppm)
494.9226
[M−H]−
695.9633
853.9673
3850 : M.findmain = 495.9299 (+/− 20 ppm)
494.9226
[M−H]−
695.9633
853.9673
0
20
40
60
80
100
500
550
600
650
700
750
800
850

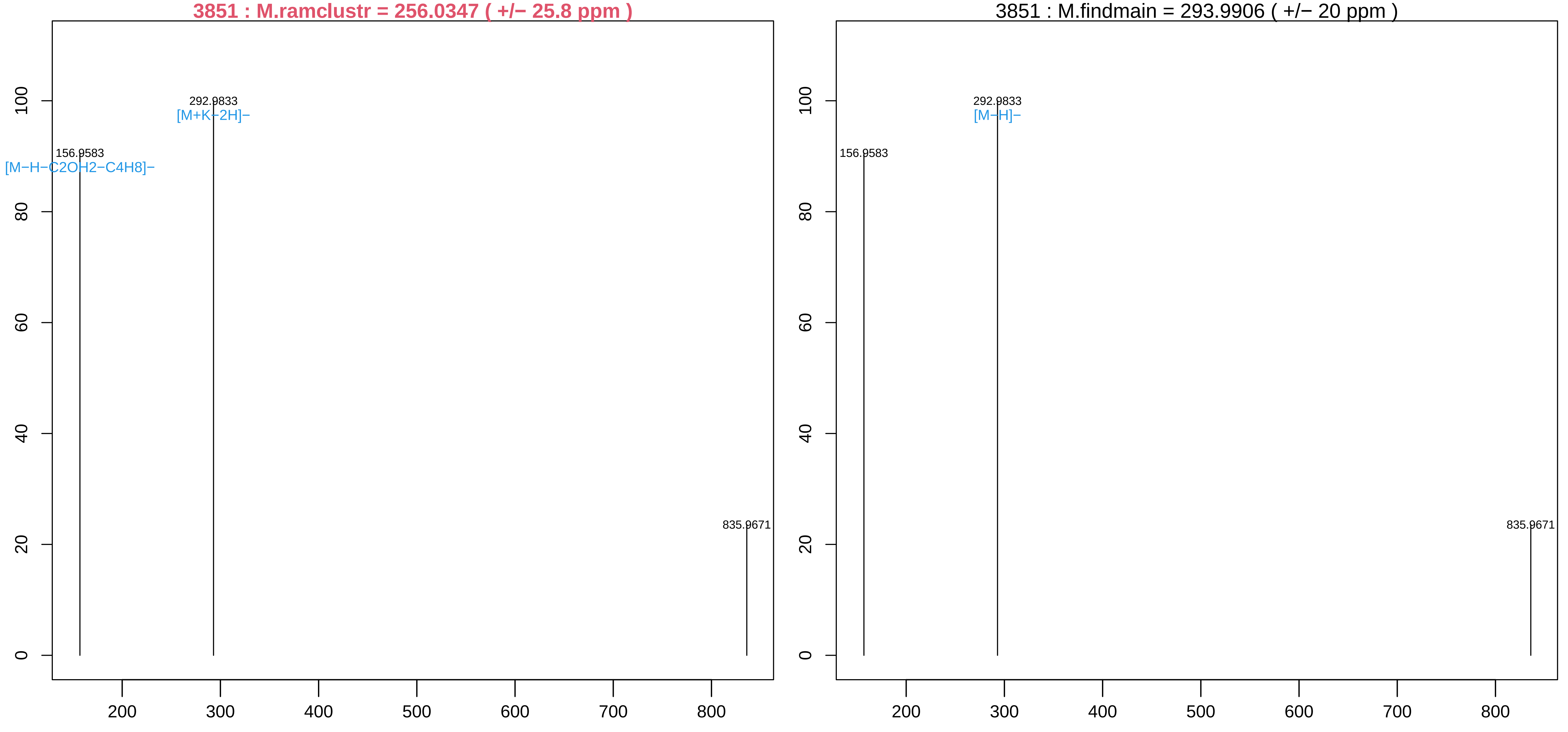
3851 : M.ramclustr = 256.0347 (+/− 25.8 ppm)
292.9833
[M+K−2H]−
156.9583
[M−H−C2OH2−C4H8]−
835.9671
3851 : M.findmain = 293.9906 (+/− 20 ppm)
292.9833
[M−H]−
156.9583
835.9671
0
20
40
60
80
100
200
300
400
500
600
700
800

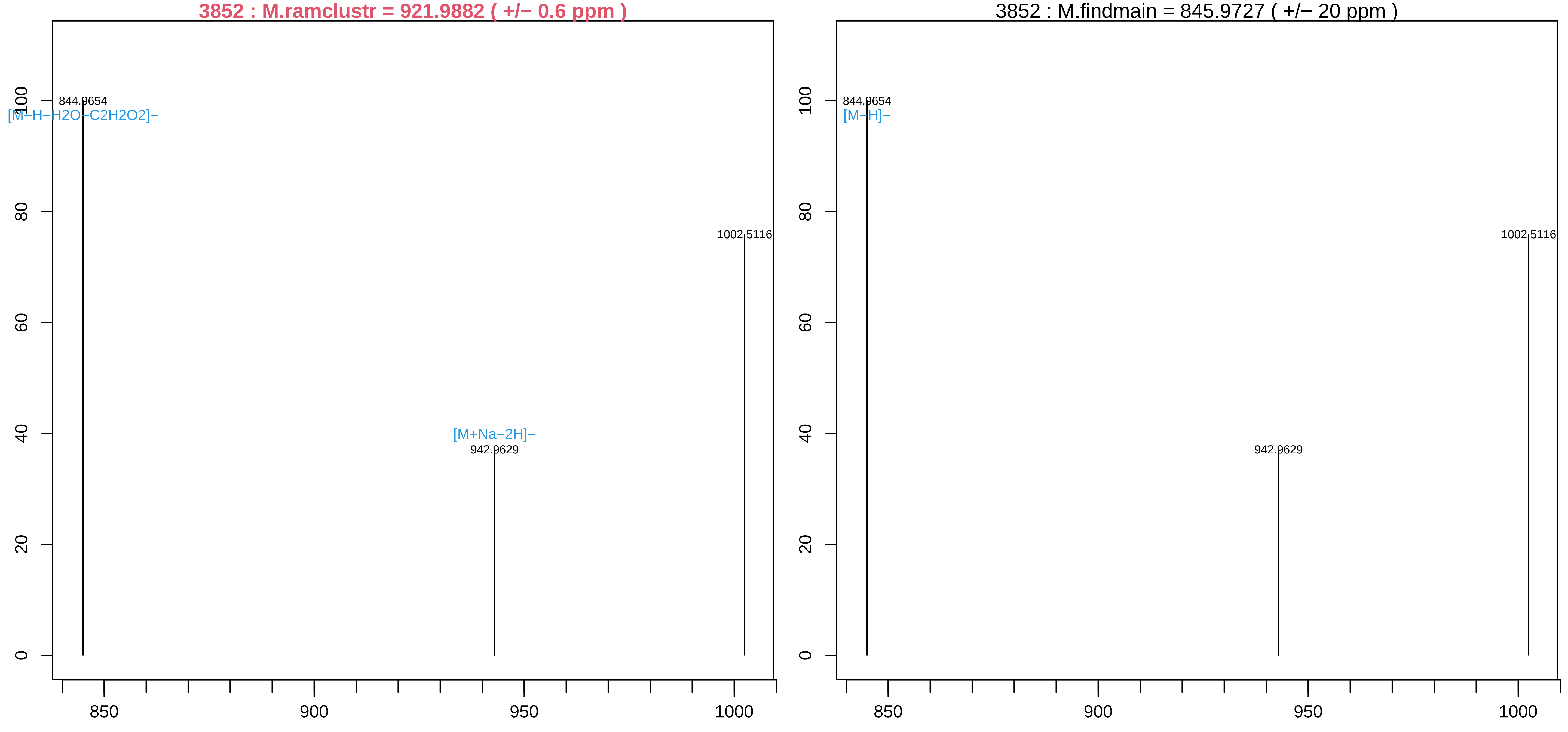
3852 : M.ramclustr = 921.9882 (+/− 0.6 ppm)
844.9654
[M−H−H2O−C2H2O2]−
[M+Na−2H]−
942.9629
1002.5116
0
20
40
60
80
100
850
900
950
1000
3852 : M.findmain = 845.9727 (+/− 20 ppm)
844.9654
[M−H]−
942.9629
1002.5116

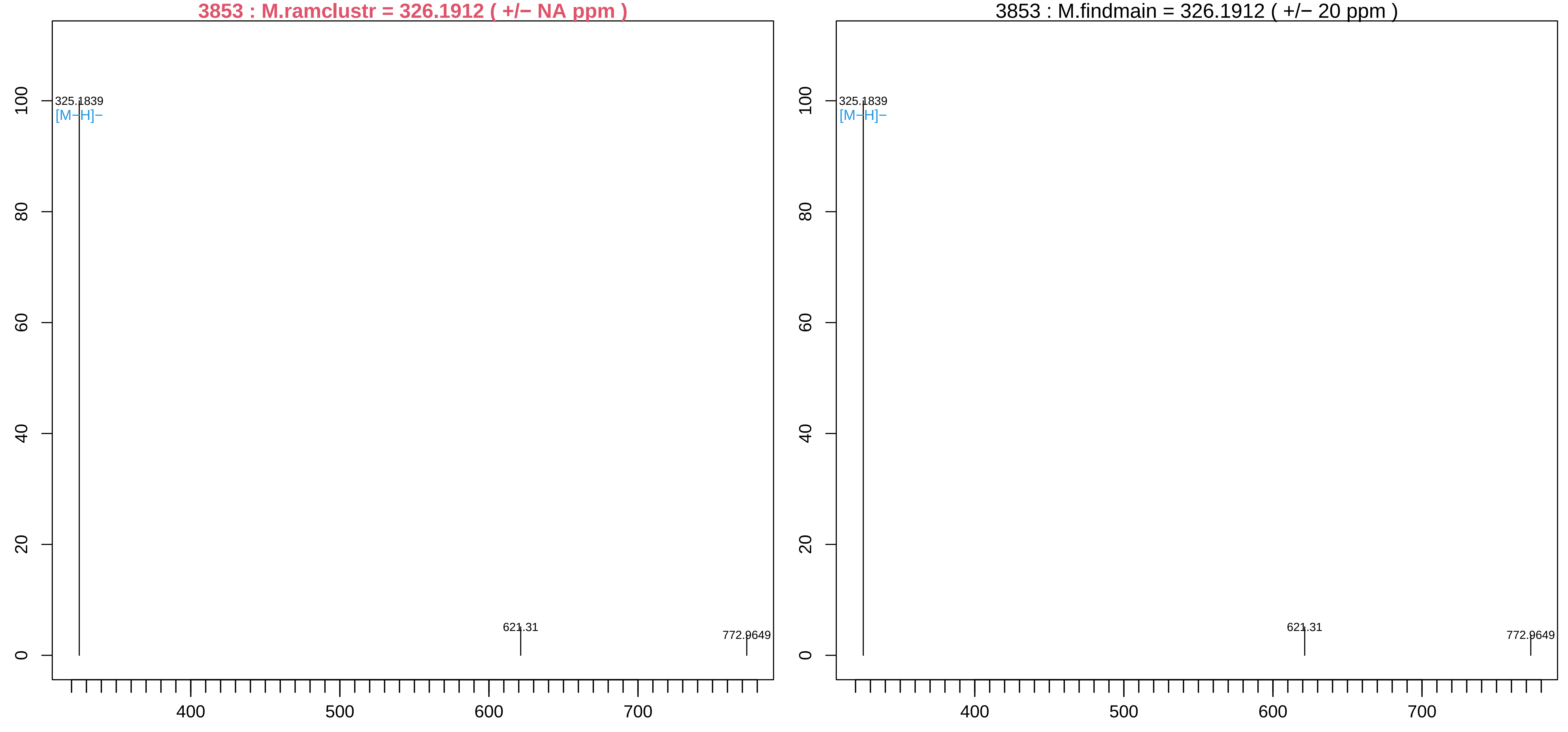

3853 : M.ramclustr = 326.1912 (+/− NA ppm)
325.1839
[M−H]−
621.31
772.9649
3853 : M.findmain = 326.1912 (+/− 20 ppm)
325.1839
[M−H]−
621.31
772.9649
0
20
40
60
80
100
400
500
600
700

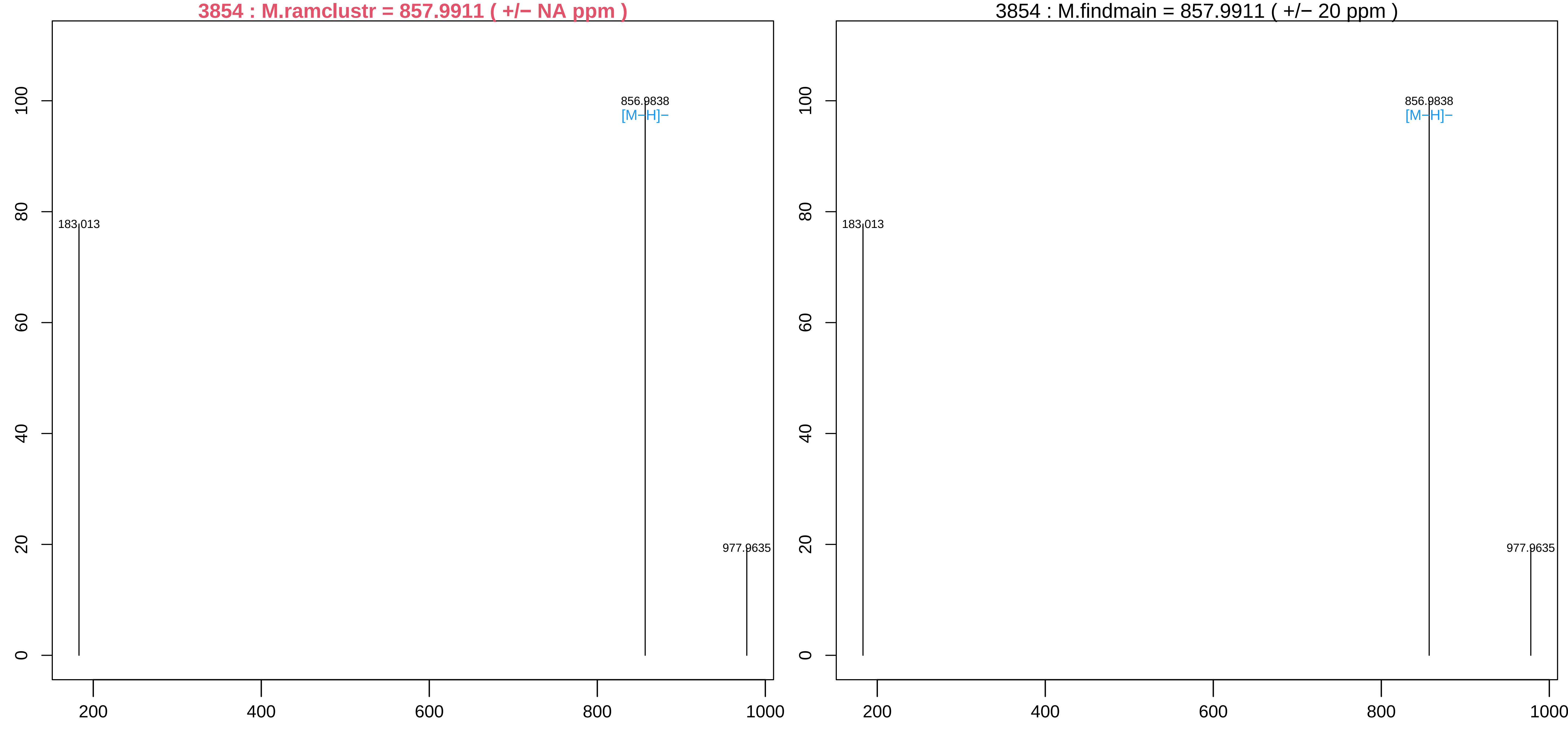
3854 : M.ramclustr = 857.9911 (+/− NA ppm)
183.013
856.9838
[M−H]−
977.9635
0
20
40
60
80
100
200
400
600
800
1000
3854 : M.findmain = 857.9911 (+/− 20 ppm)
183.013
856.9838
[M−H]−
977.9635
0
20
40
60
80
100
200
400
600
800
1000

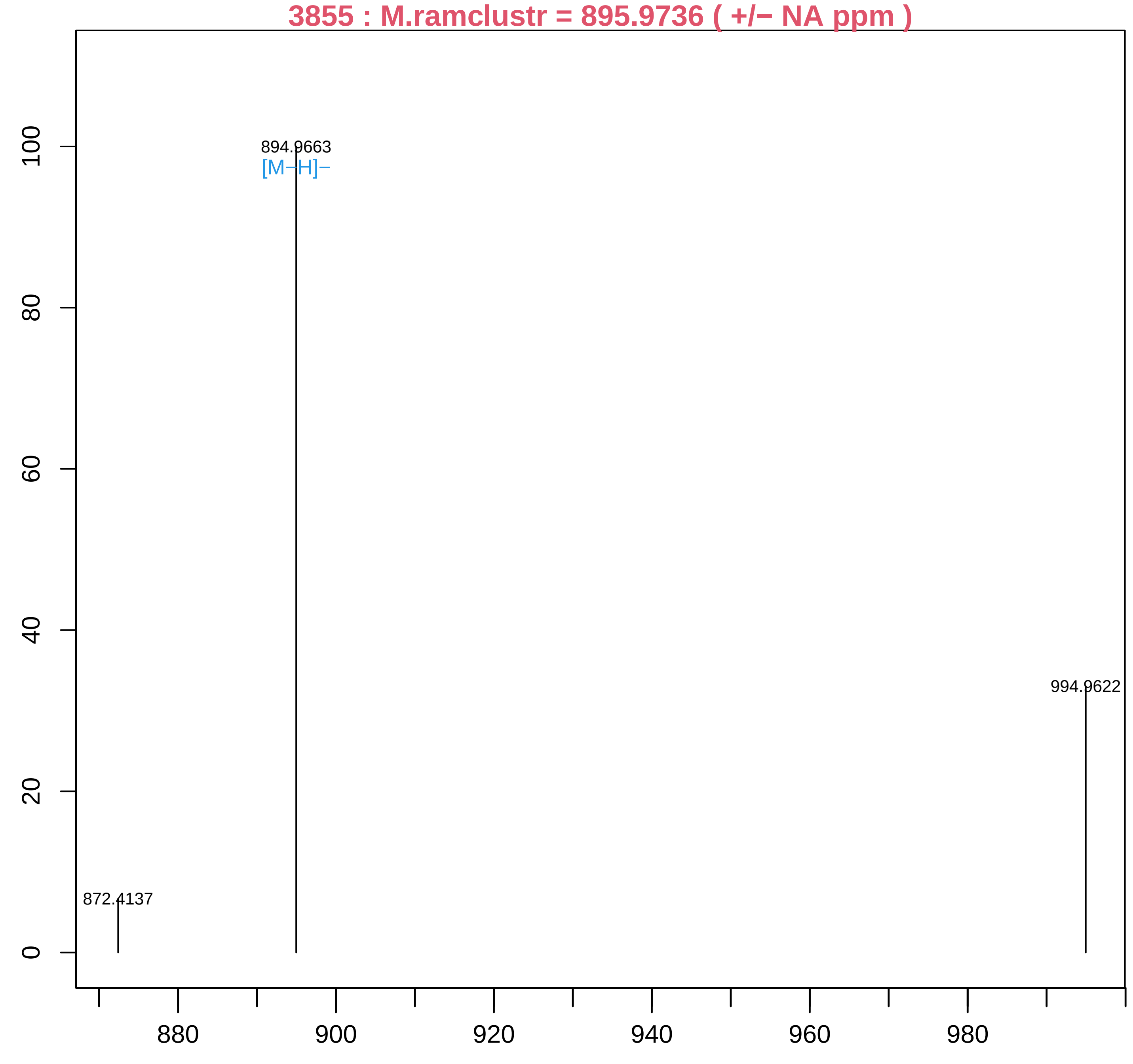
3855 : M.ramclustr = 895.9736 (+/− NA ppm)
894.9663
[M−H]−
872.4137
994.9622
0
20
40
60
80
100
880
900
920
940
960
980
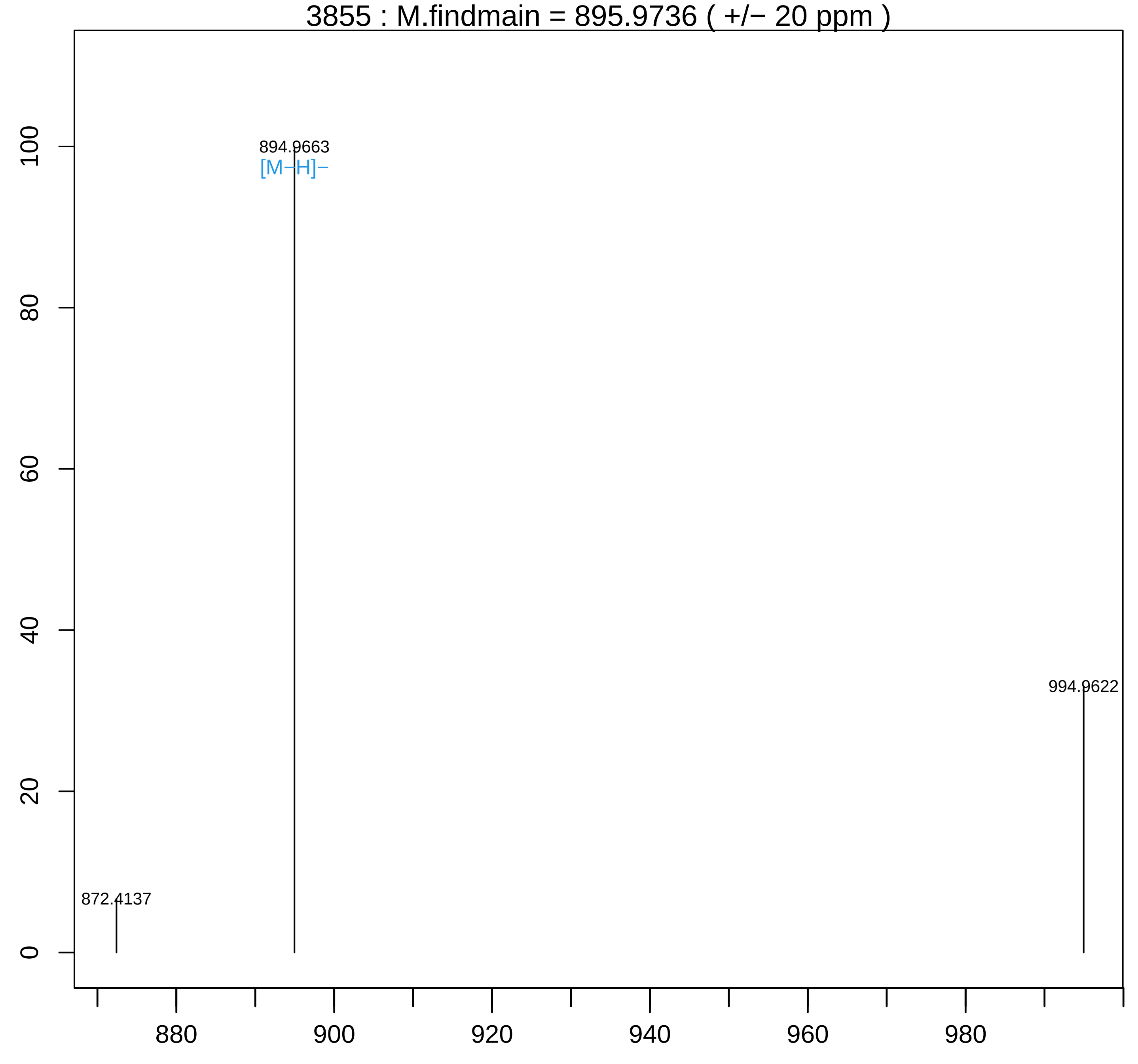
3855 : M.findmain = 895.9736 (+/− 20 ppm)
894.9663
[M−H]−
872.4137
994.9622
0
20
40
60
80
100
880
900
920
940
960
980

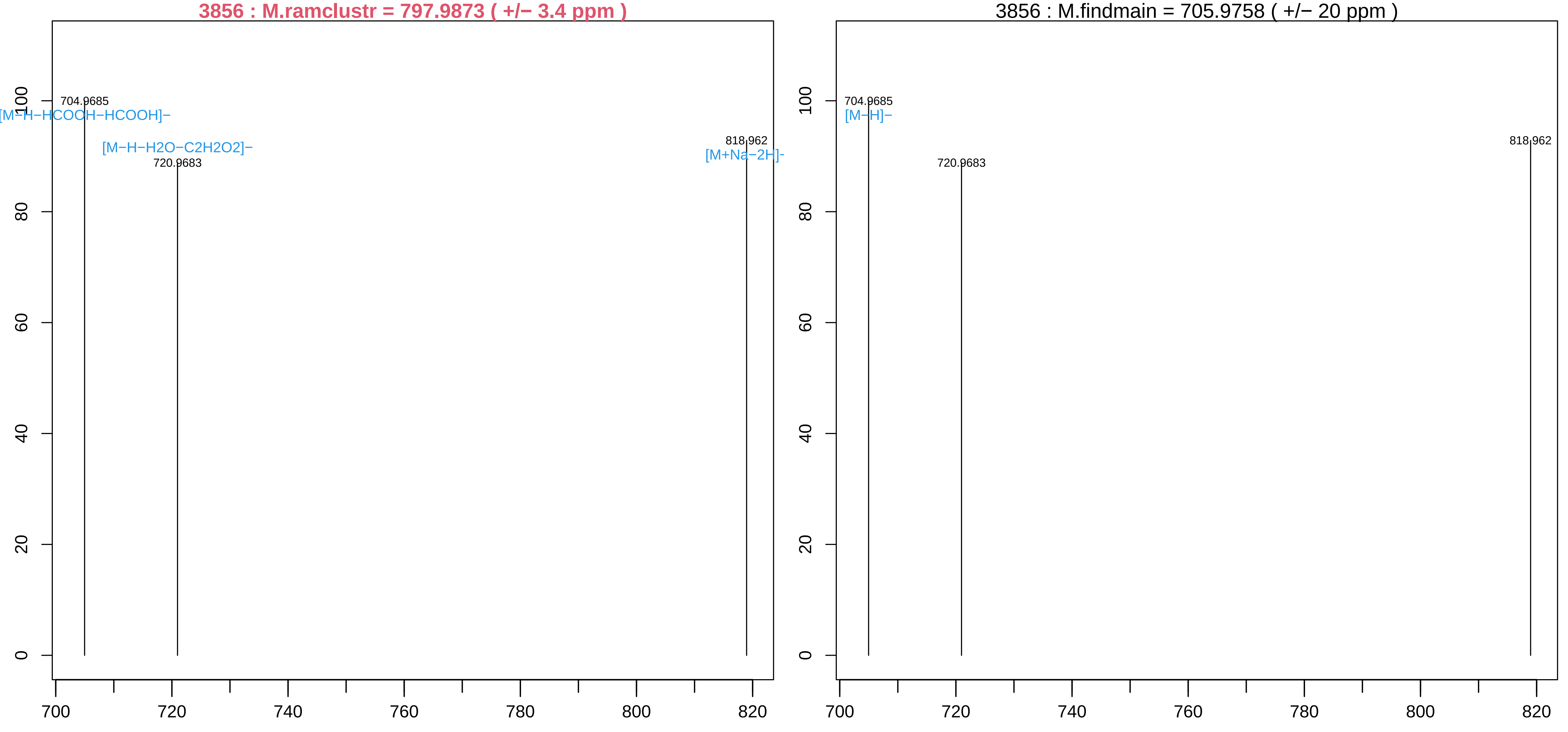
3856 : M.ramclustr = 797.9873 (+/− 3.4 ppm)
704.9685
[M-H−HCOOH−HCOOH]−
[M−H−H2O−C2H2O2]−
720.9683
818.962
[M+Na−2H]-
3856 : M.findmain = 705.9758 (+/− 20 ppm)
704.9685
[M−H]−
720.9683
818.962
0
20
40
60
80
100
700
720
740
760
780
800
820

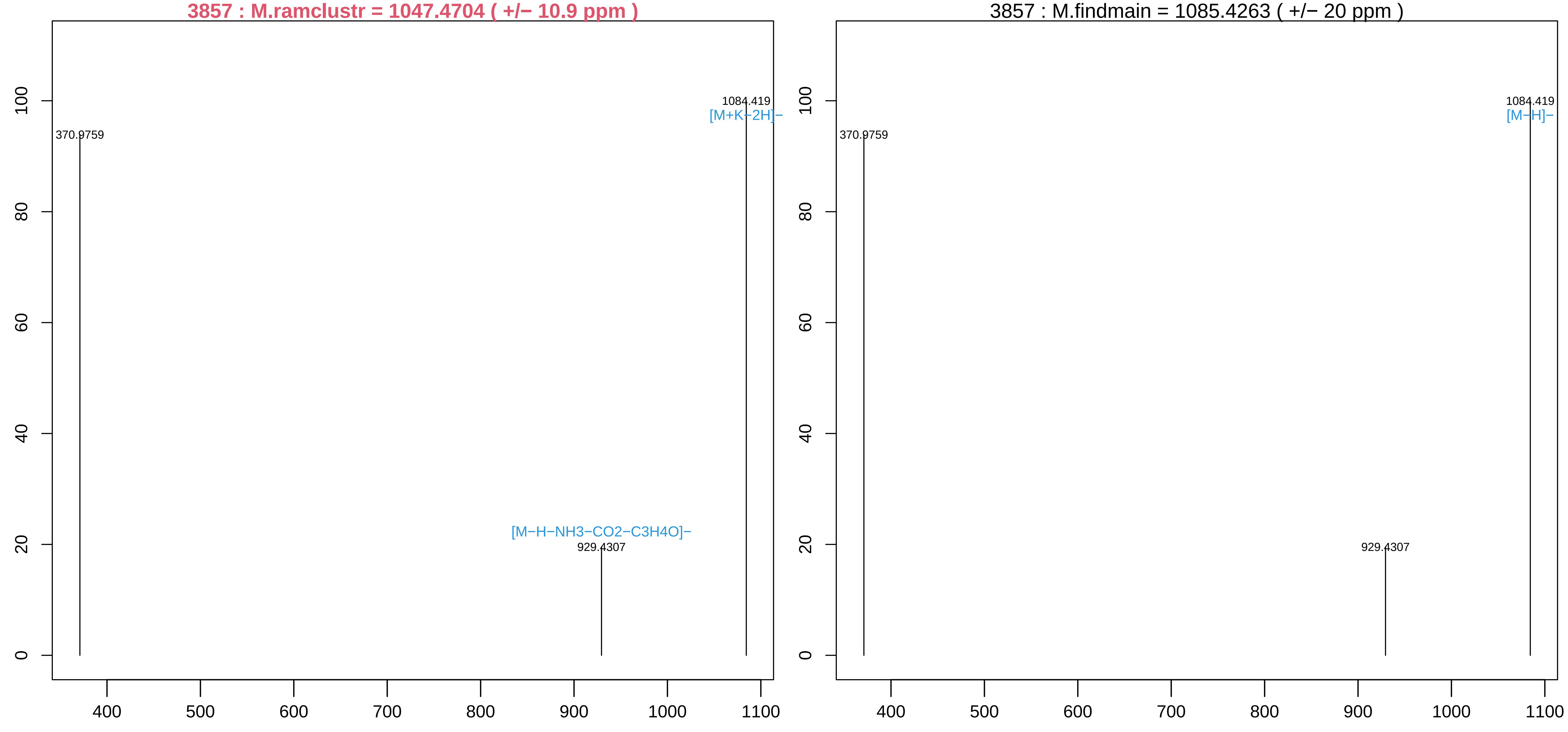
3857 : M.ramclustr = 1047.4704 (+/− 10.9 ppm)
1084.419
[M+K−2H]−
370.9759
[M−H−NH3−CO2−C3H4O]−
929.4307
3857 : M.findmain = 1085.4263 (+/− 20 ppm)
1084.419
[M−H]−
370.9759
929.4307

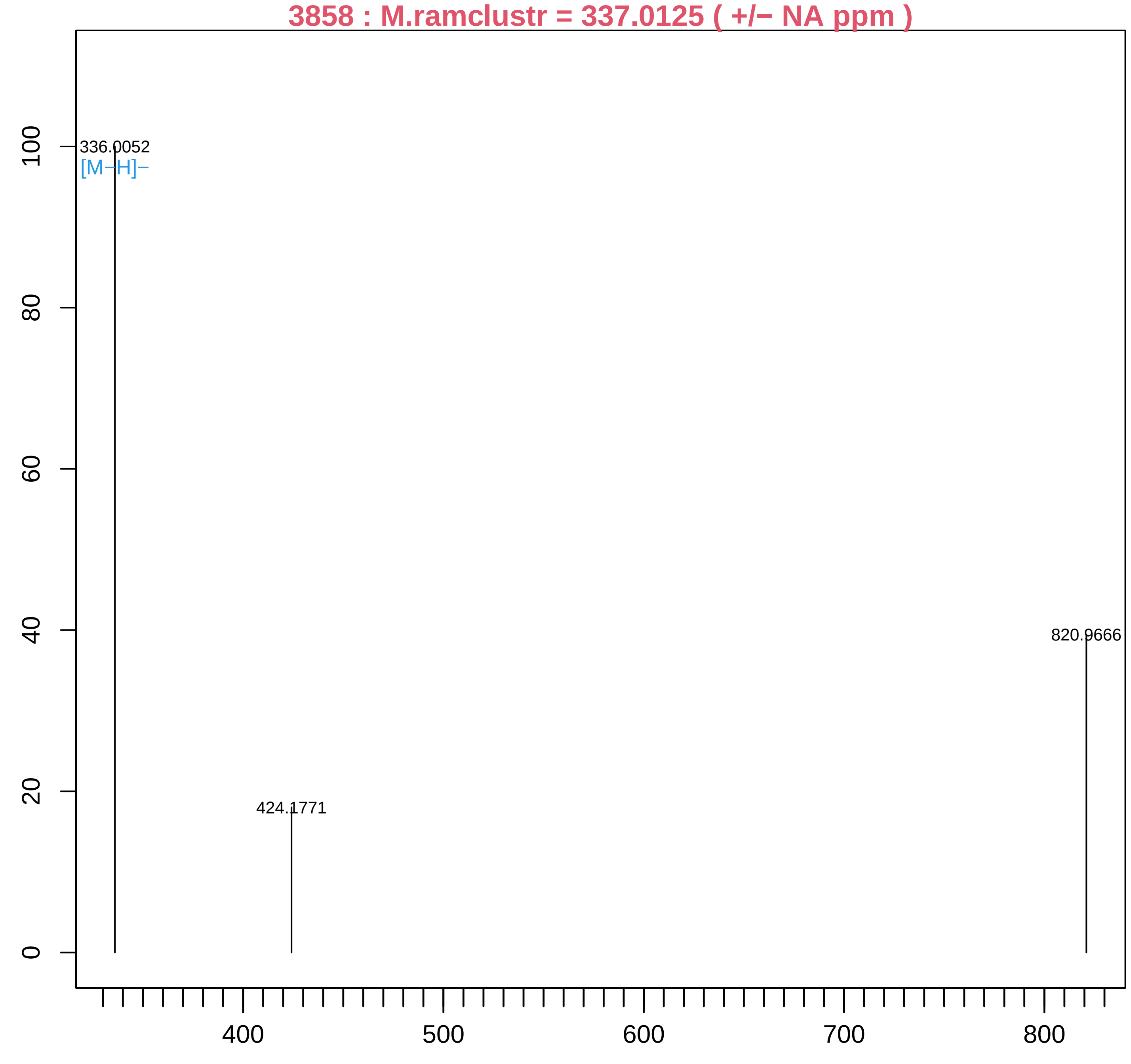
3858 : M.ramclustr = 337.0125 (+/− NA ppm)
336.0052
[M−H]−
424.1771
820.9666
0
20
40
60
80
100
400
500
600
700
800

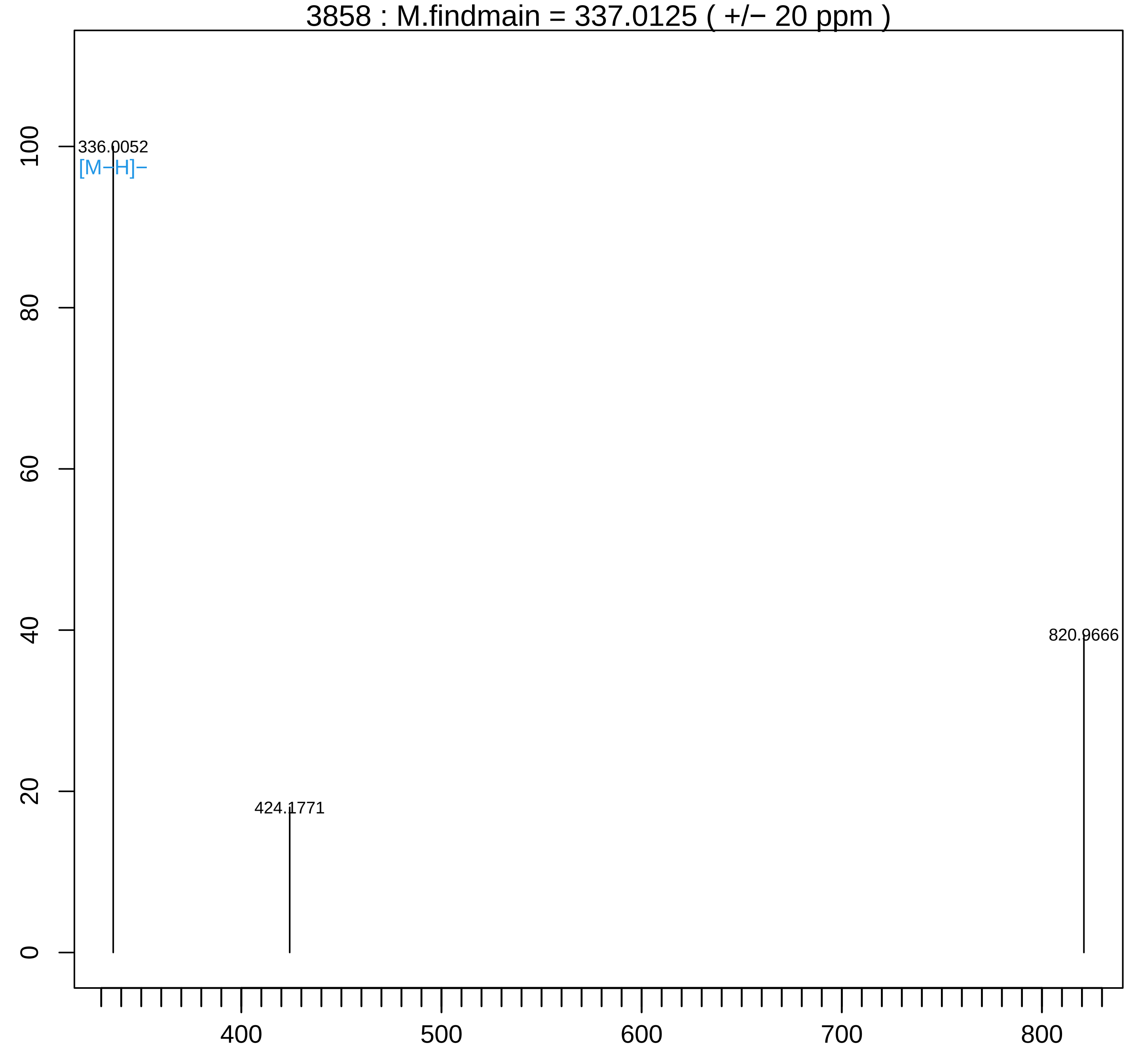
3858 : M.findmain = 337.0125 (+/− 20 ppm)
336.0052
[M−H]−
424.1771
820.9666
0
20
40
60
80
100
400
500
600
700
800

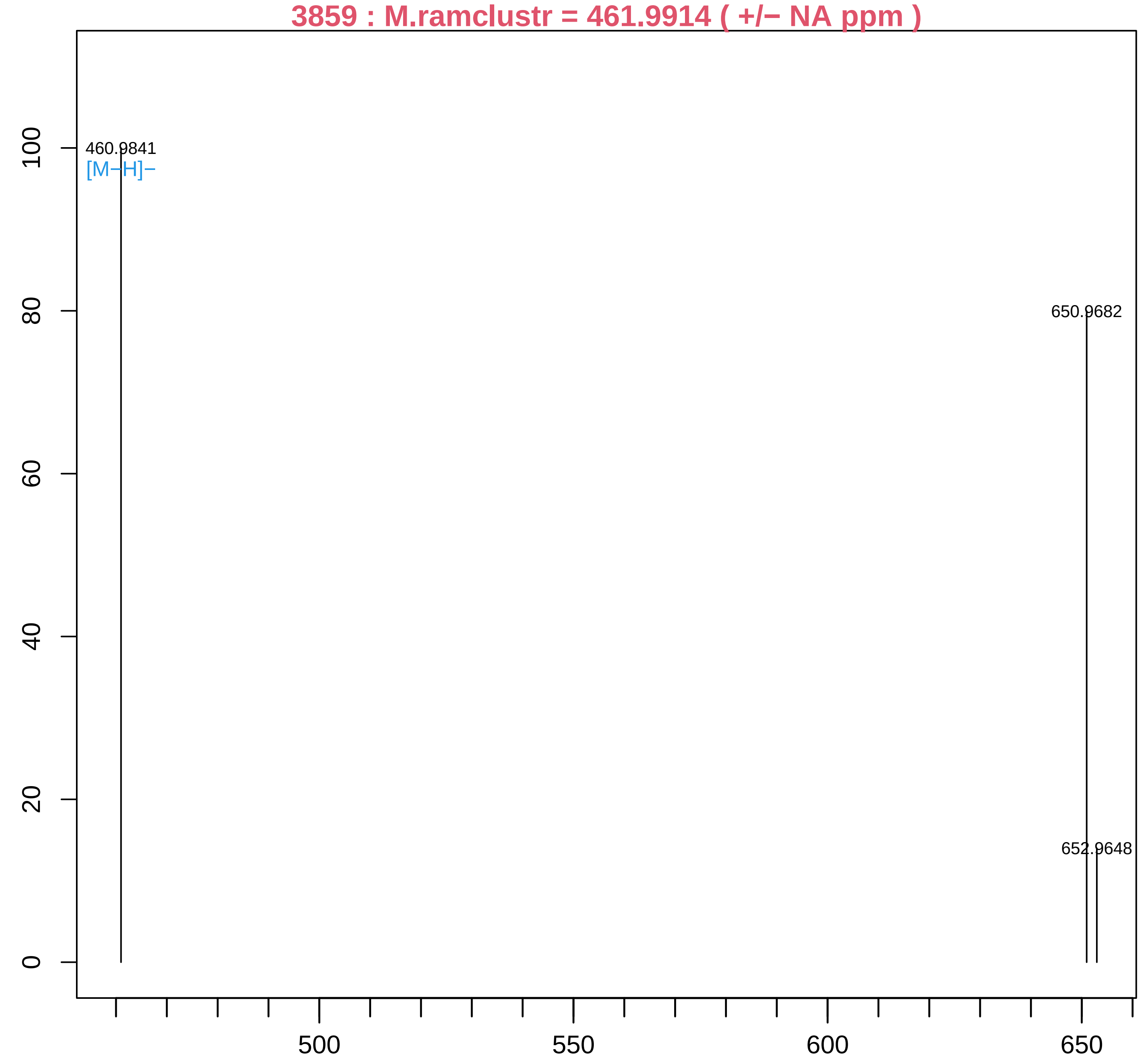
3859 : M.ramclustr = 461.9914 (+/− NA ppm)
460.9841
[M−H]−
650.9682
652.9648
0
20
40
60
80
100
500
550
600
650

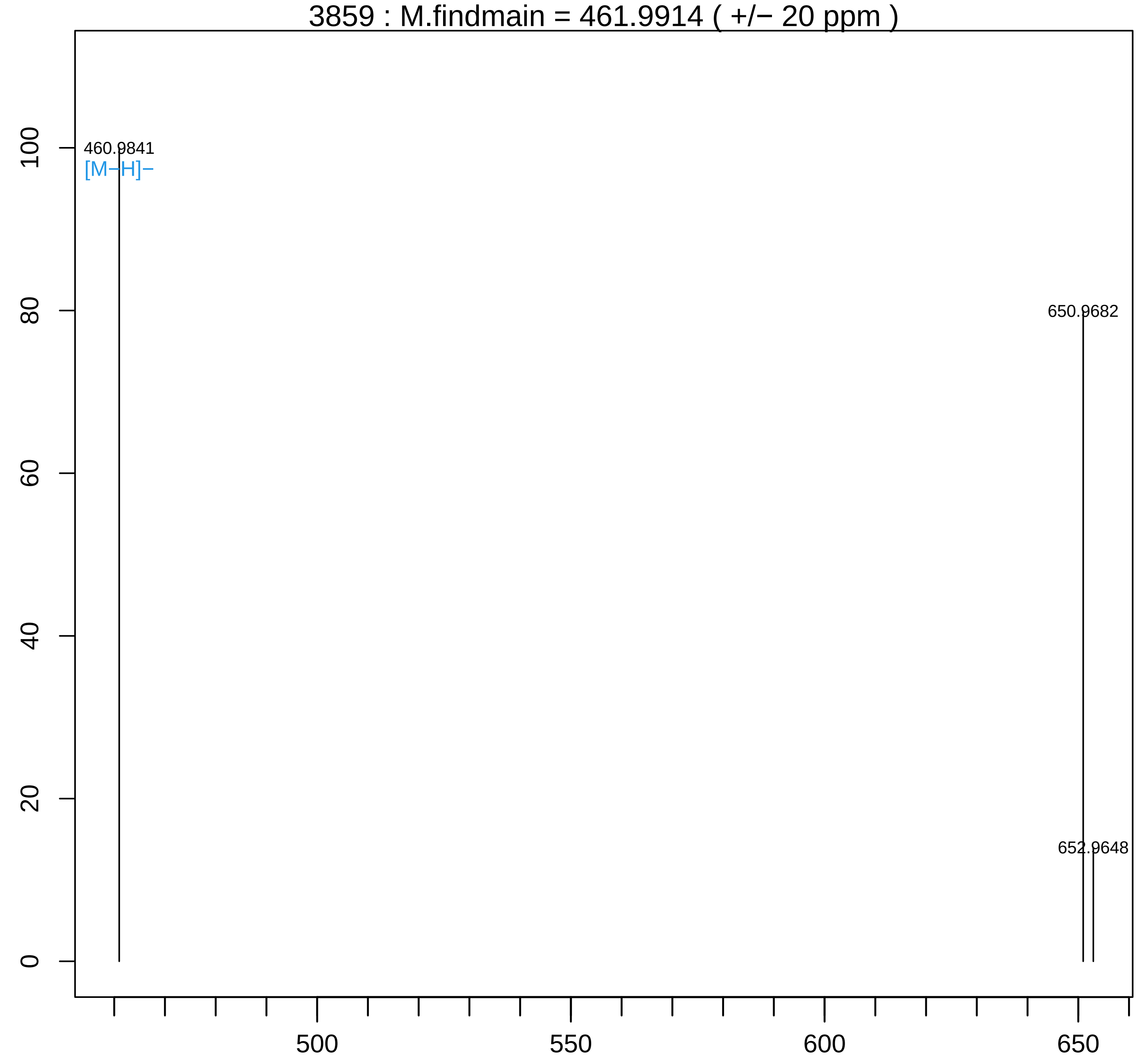
3859 : M.findmain = 461.9914 (+/− 20 ppm)
460.9841
[M−H]−
650.9682
652.9648
0
20
40
60
80
100
500
550
600
650

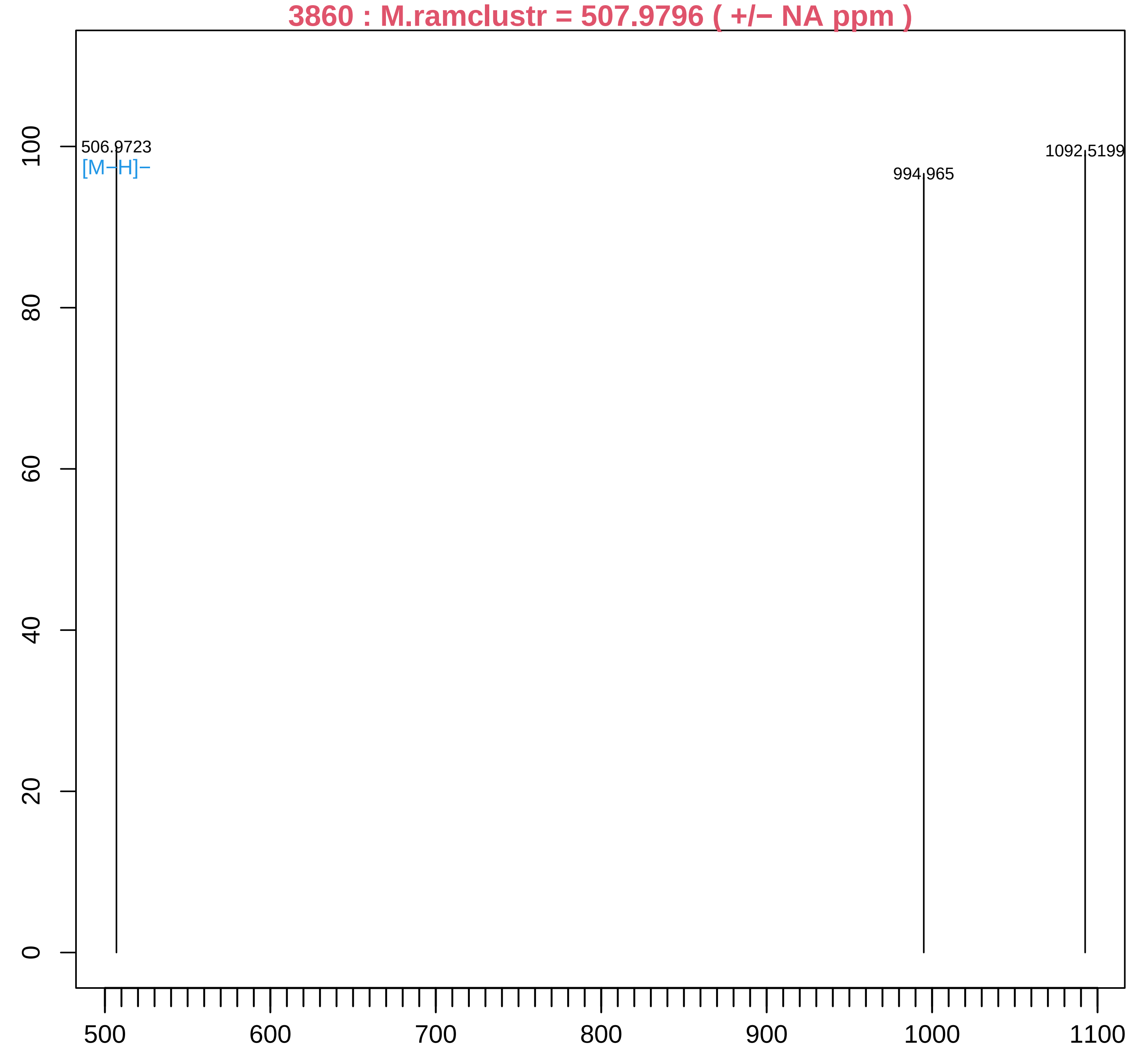
3860 : M.ramclustr = 507.9796 (+/− NA ppm)
506.9723
[M−H]−
994.965
1092.5199
0
20
40
60
80
100
500
600
700
800
900
1000
1100

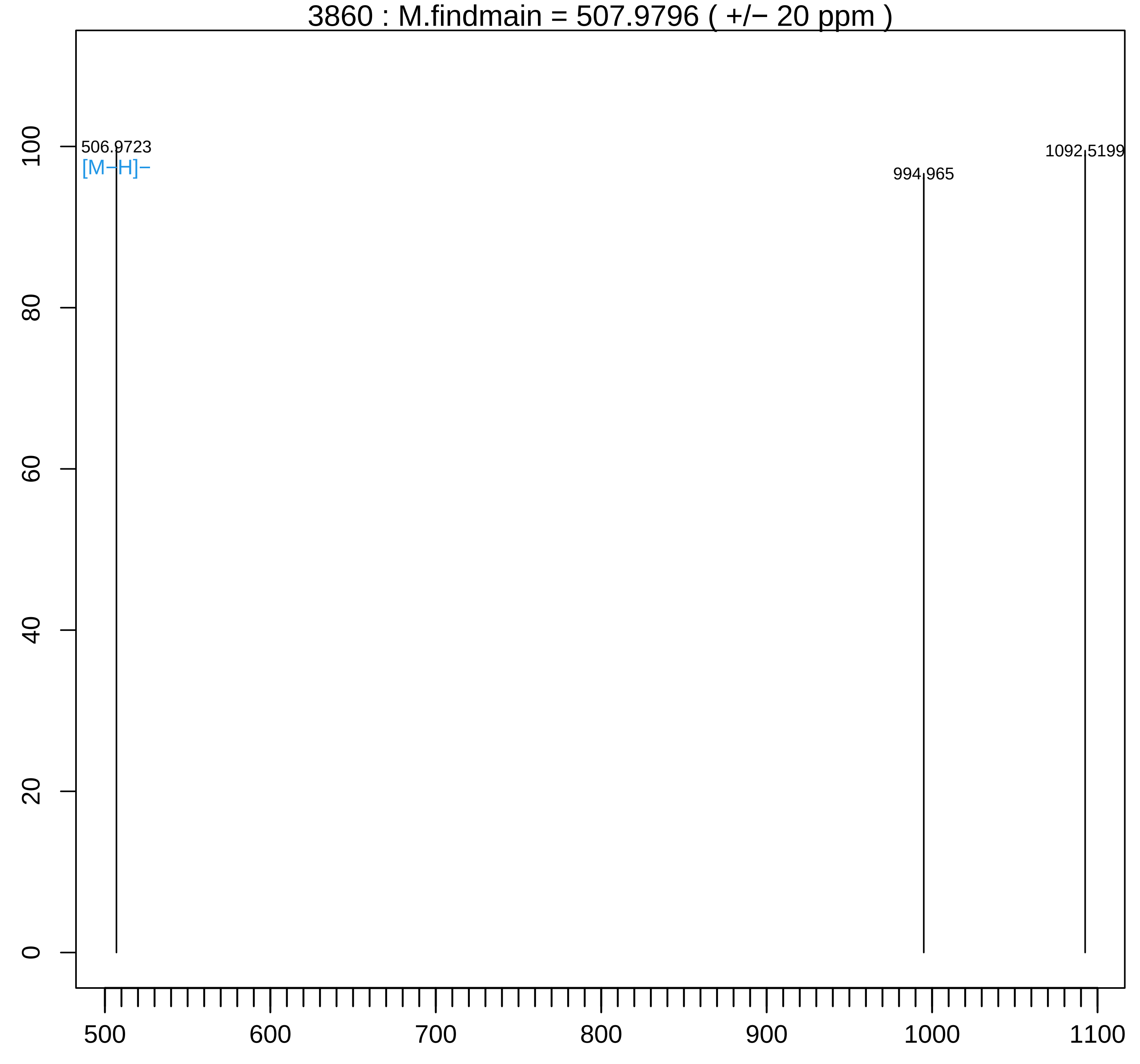
3860 : M.findmain = 507.9796 (+/− 20 ppm)
506.9723
[M−H]−
994.965
1092.5199
0
20
40
60
80
100
500
600
700
800
900
1000
1100

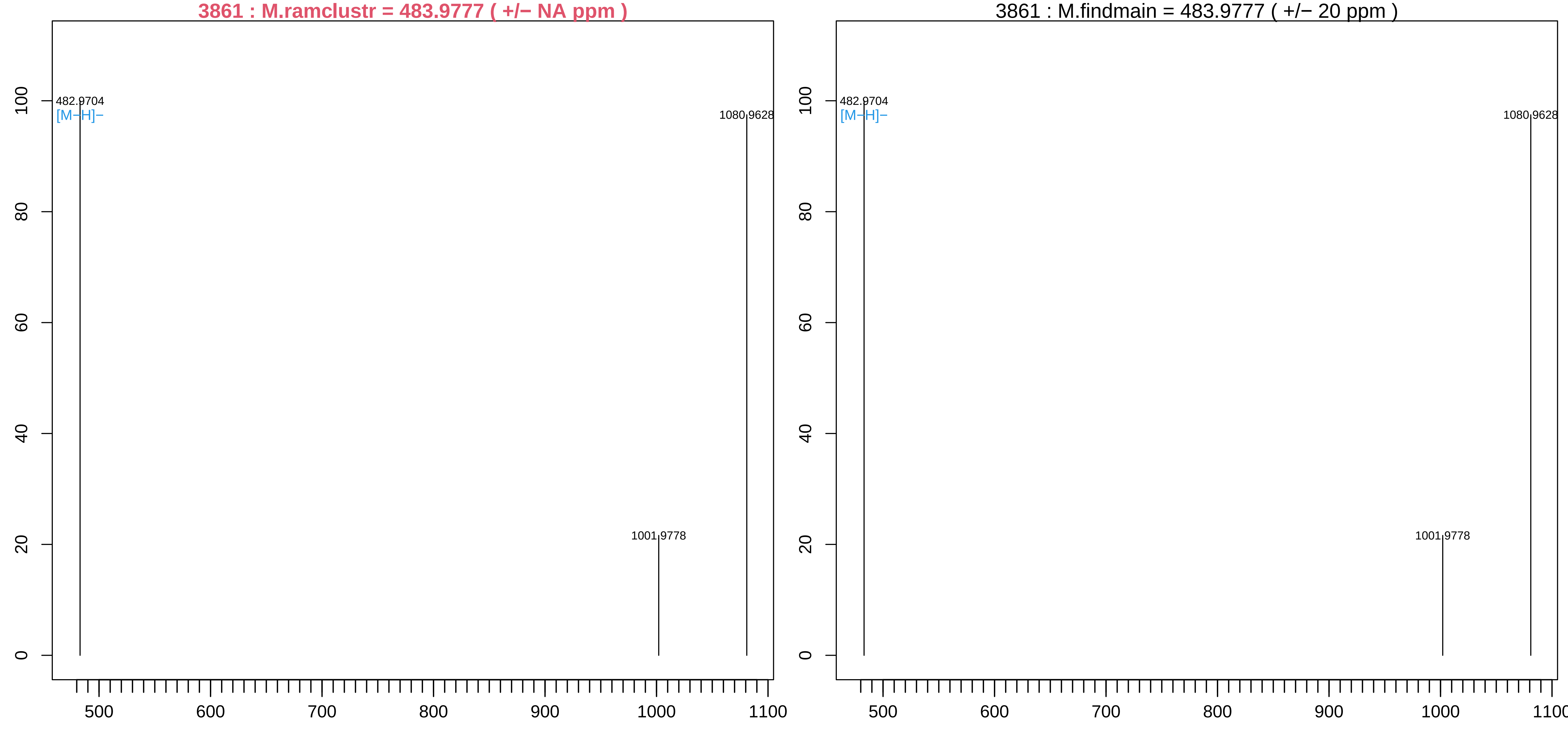

3861 : M.ramclustr = 483.9777 (+/− NA ppm)
482.9704
[M−H]−
1080.9628
1001.9778
3861 : M.findmain = 483.9777 (+/− 20 ppm)
482.9704
[M−H]−
1080.9628
1001.9778

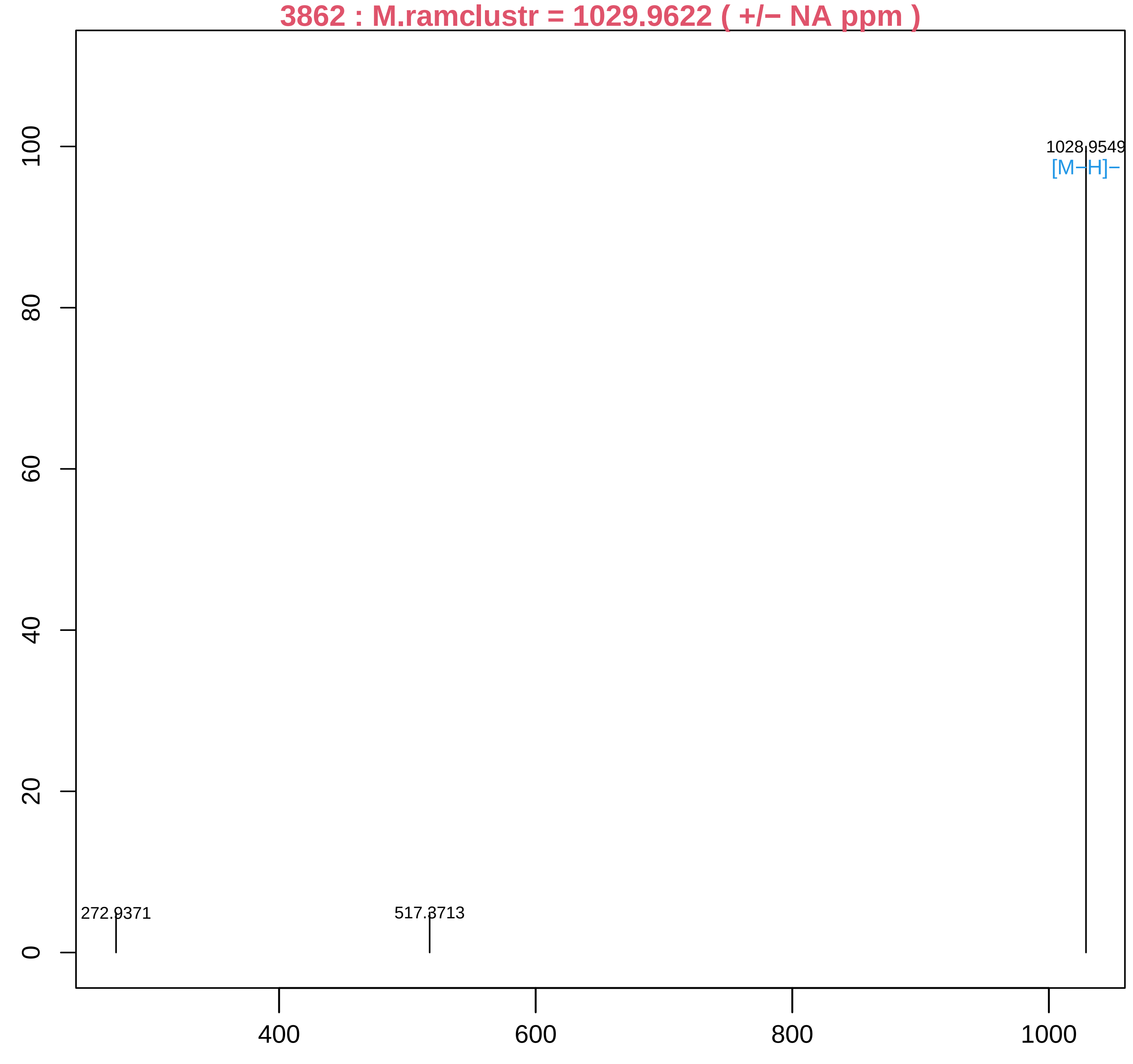
3862 : M.ramclustr = 1029.9622 (+/− NA ppm)
1028.9549
[M−H]−
272.9371
517.3713
0
20
40
60
80
100
400
600
800
1000

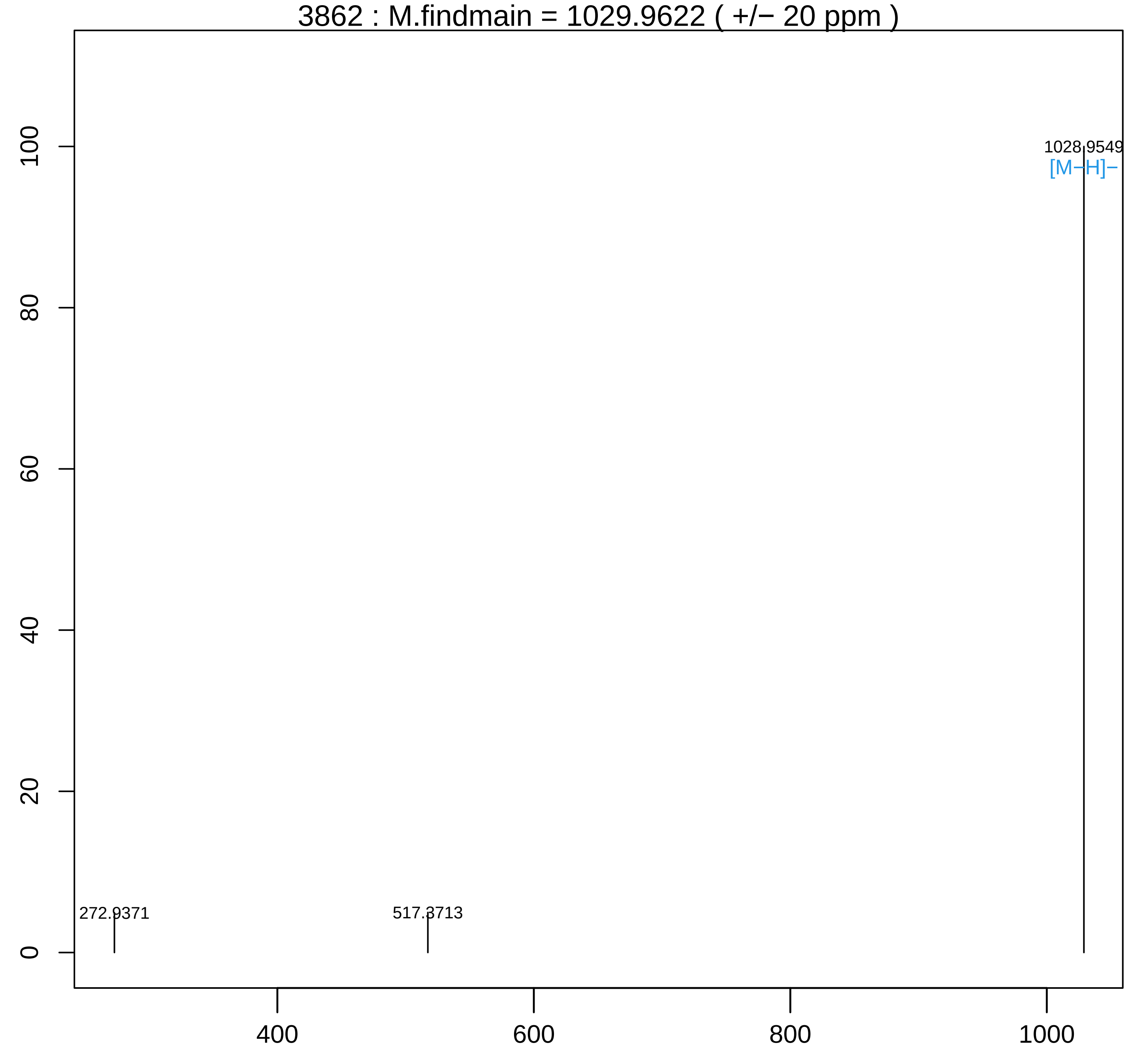
3862 : M.findmain = 1029.9622 (+/− 20 ppm)
1028.9549
[M−H]−
272.9371
517.3713
0
20
40
60
80
100
400
600
800
1000

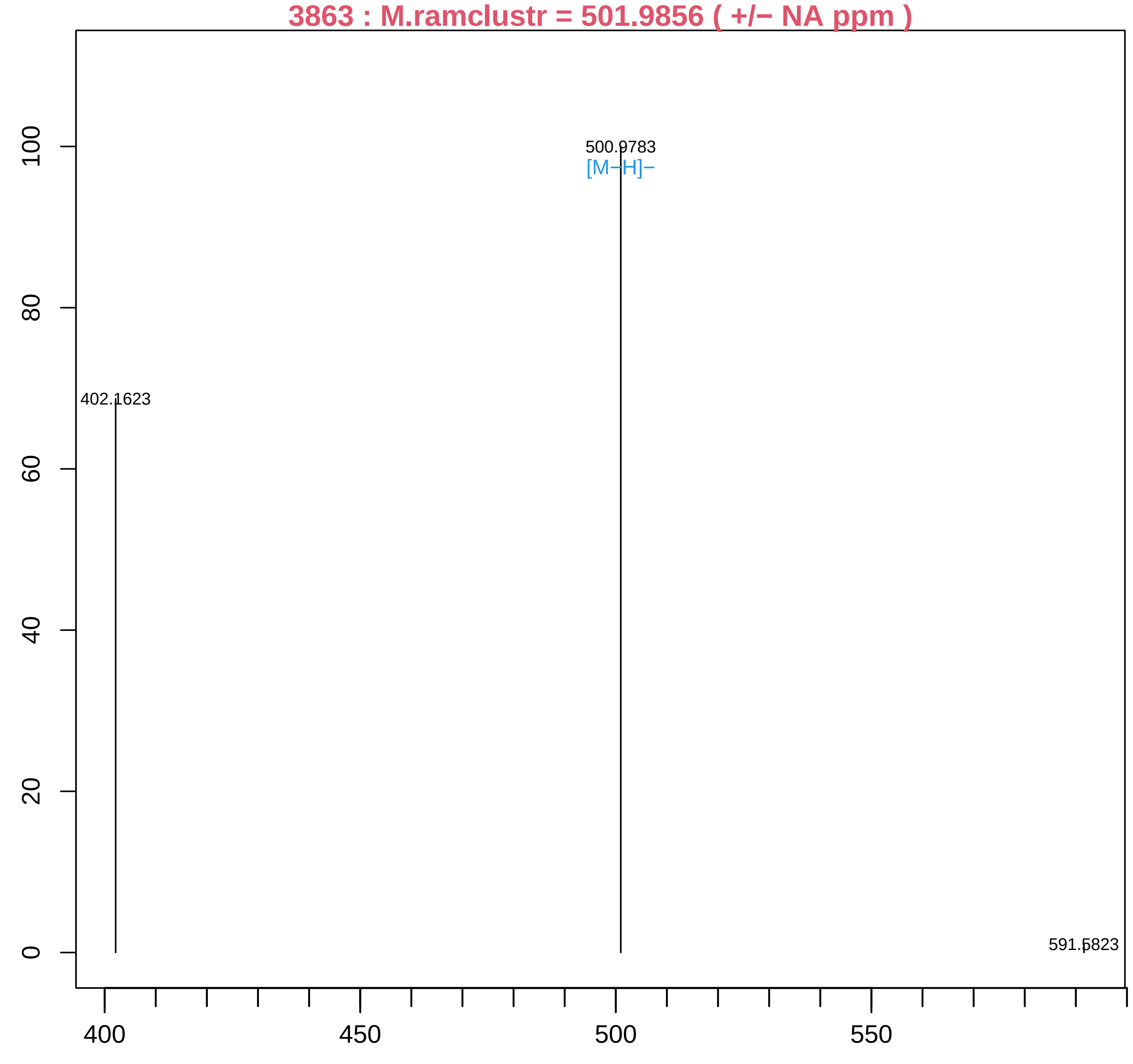
3863 : M.ramclustr = 501.9856 (+/− NA ppm)
500.9783
[M−H]−
402.1623
591.5823
0
20
40
60
80
100
400
450
500
550

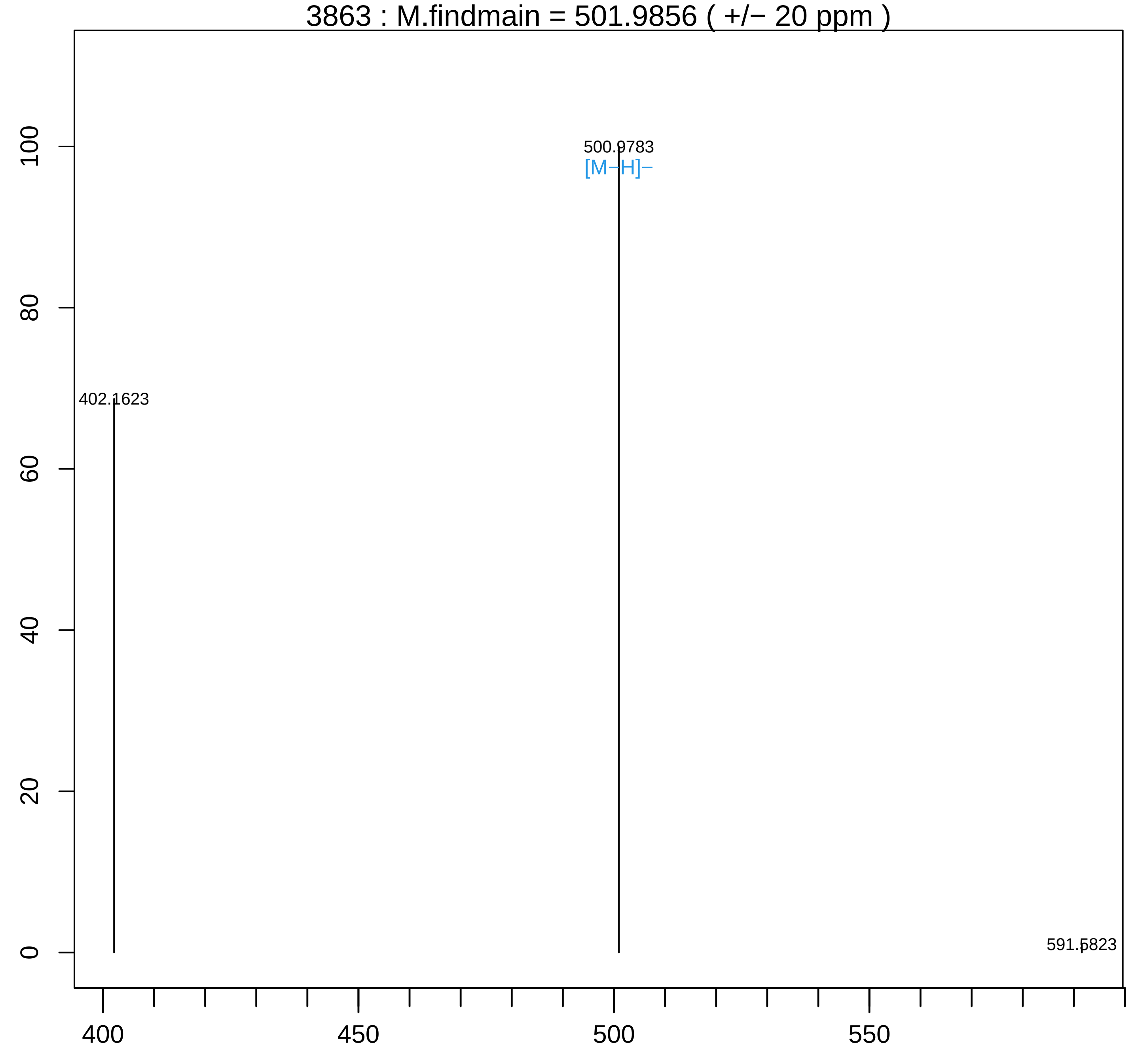
3863 : M.findmain = 501.9856 (+/− 20 ppm)
500.9783
[M−H]−
402.1623
591.5823
0
20
40
60
80
100
400
450
500
550

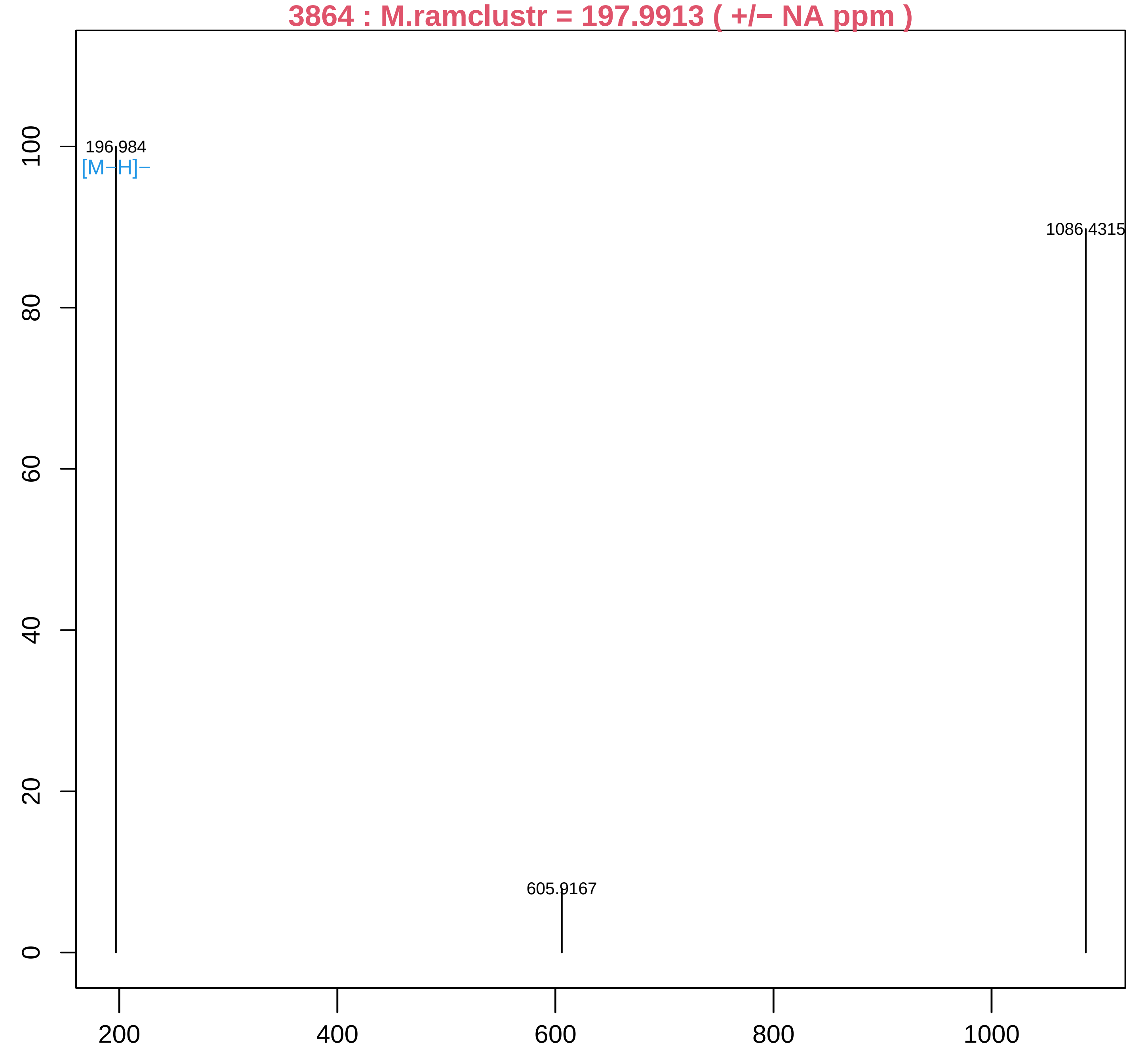
3864 : M.ramclustr = 197.9913 (+/− NA ppm)
196.984
[M−H]−
1086.4315
605.9167
0
20
40
60
80
100
200
400
600
800
1000

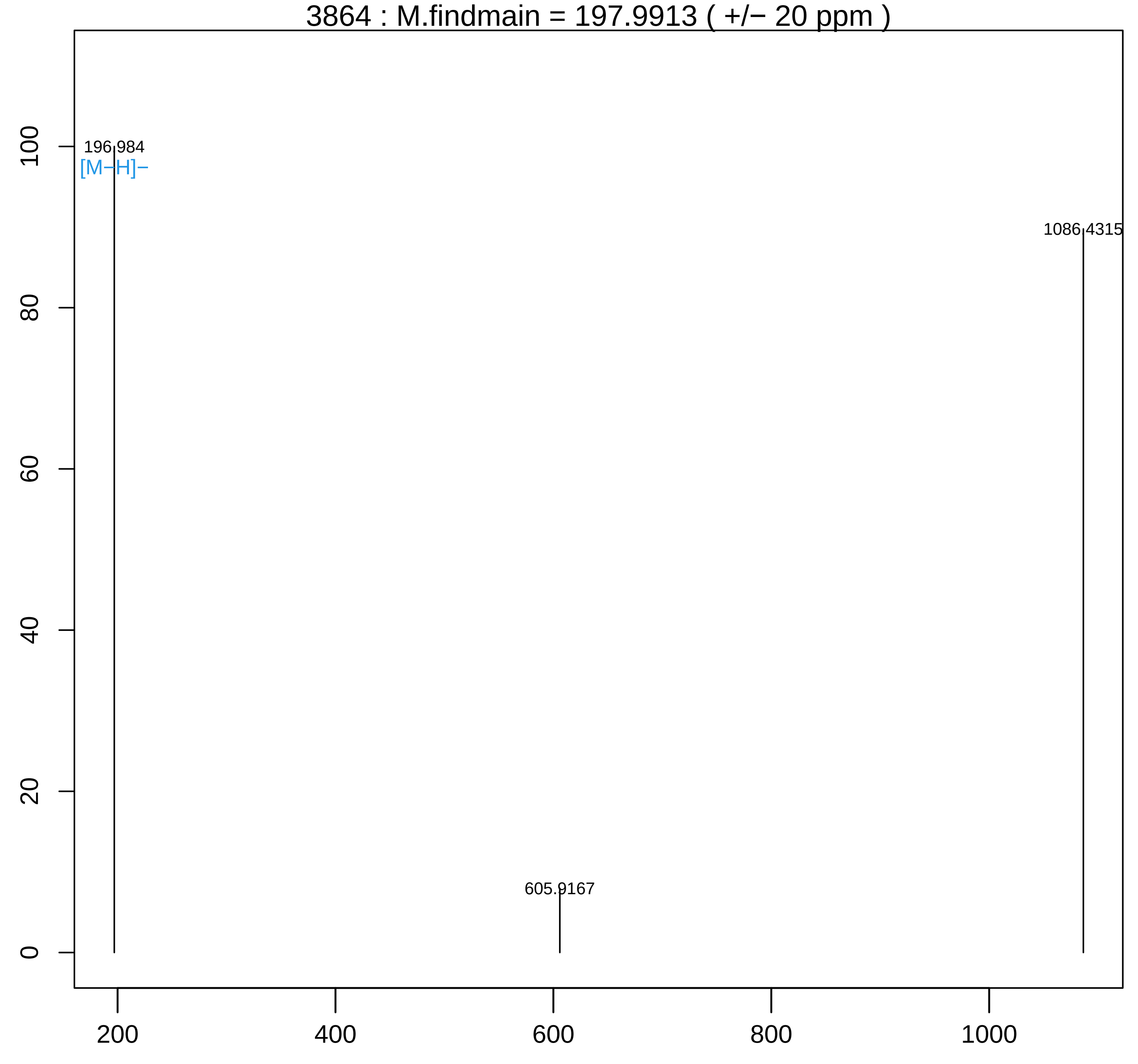
3864 : M.findmain = 197.9913 (+/− 20 ppm)
196.984
[M−H]−
1086.4315
605.9167
0
20
40
60
80
100
200
400
600
800
1000

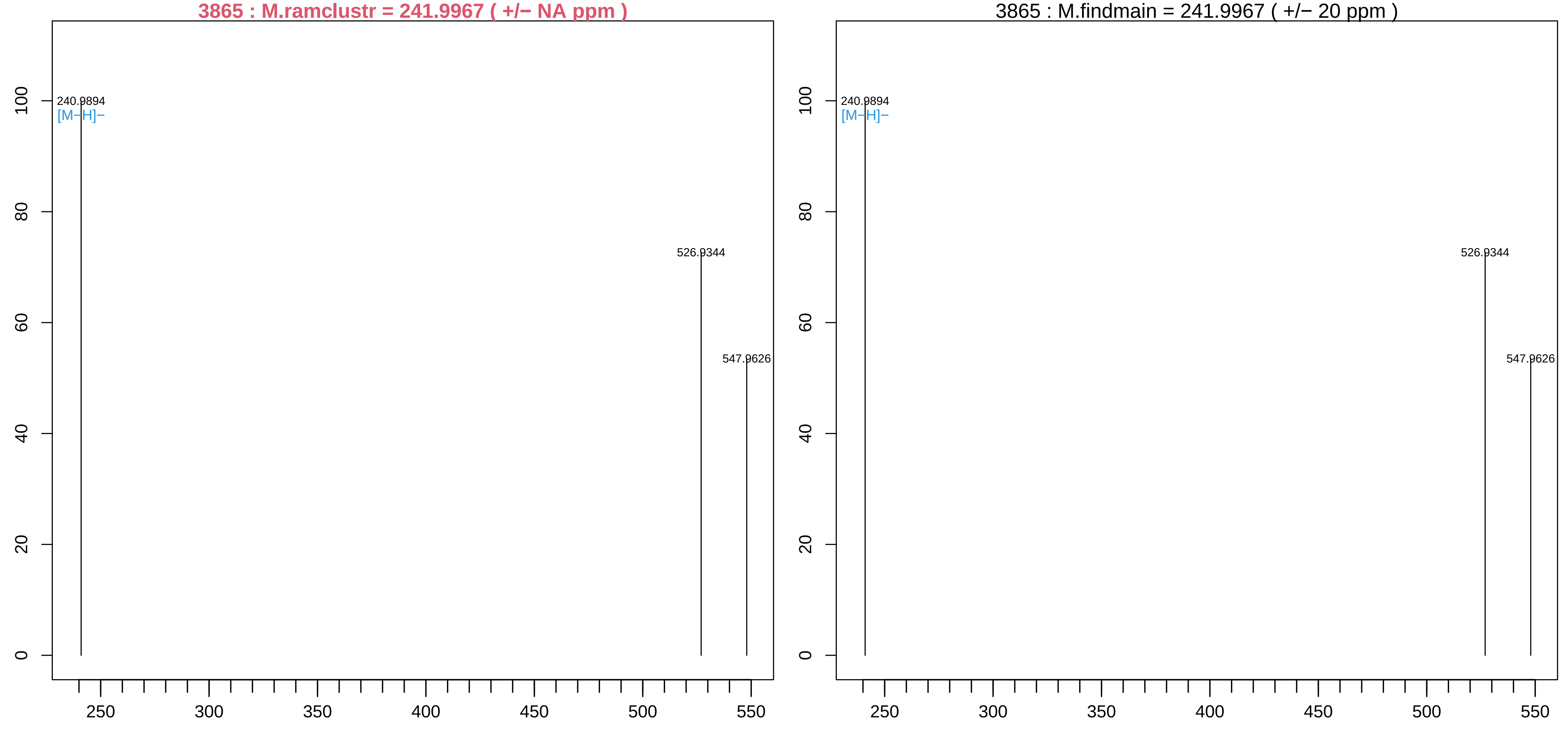
3865 : M.ramclustr = 241.9967 (+/− NA ppm)
3865 : M.findmain = 241.9967 (+/− 20 ppm)
240.9894
[M−H]−
526.9344
547.9626
0
20
40
60
80
100
250
300
350
400
450
500
550

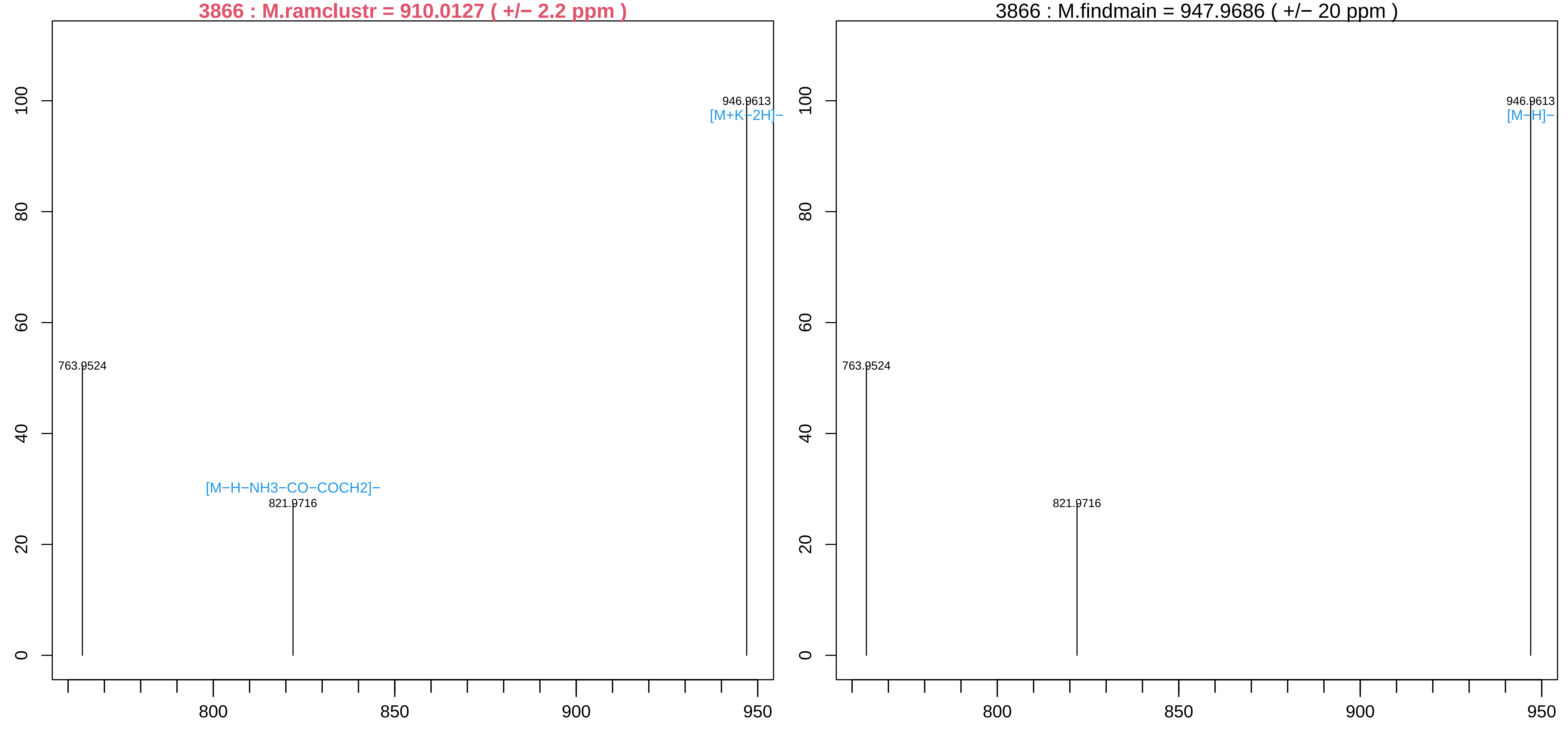
3866 : M.ramclustr = 910.0127 (+/− 2.2 ppm)
946.9613
[M+K−2H]−
763.9524
[M−H−NH3−CO−COCH2]−
821.9716
3866 : M.findmain = 947.9686 (+/− 20 ppm)
946.9613
[M−H]−
763.9524
821.9716
0
20
40
60
80
100
800
850
900
950

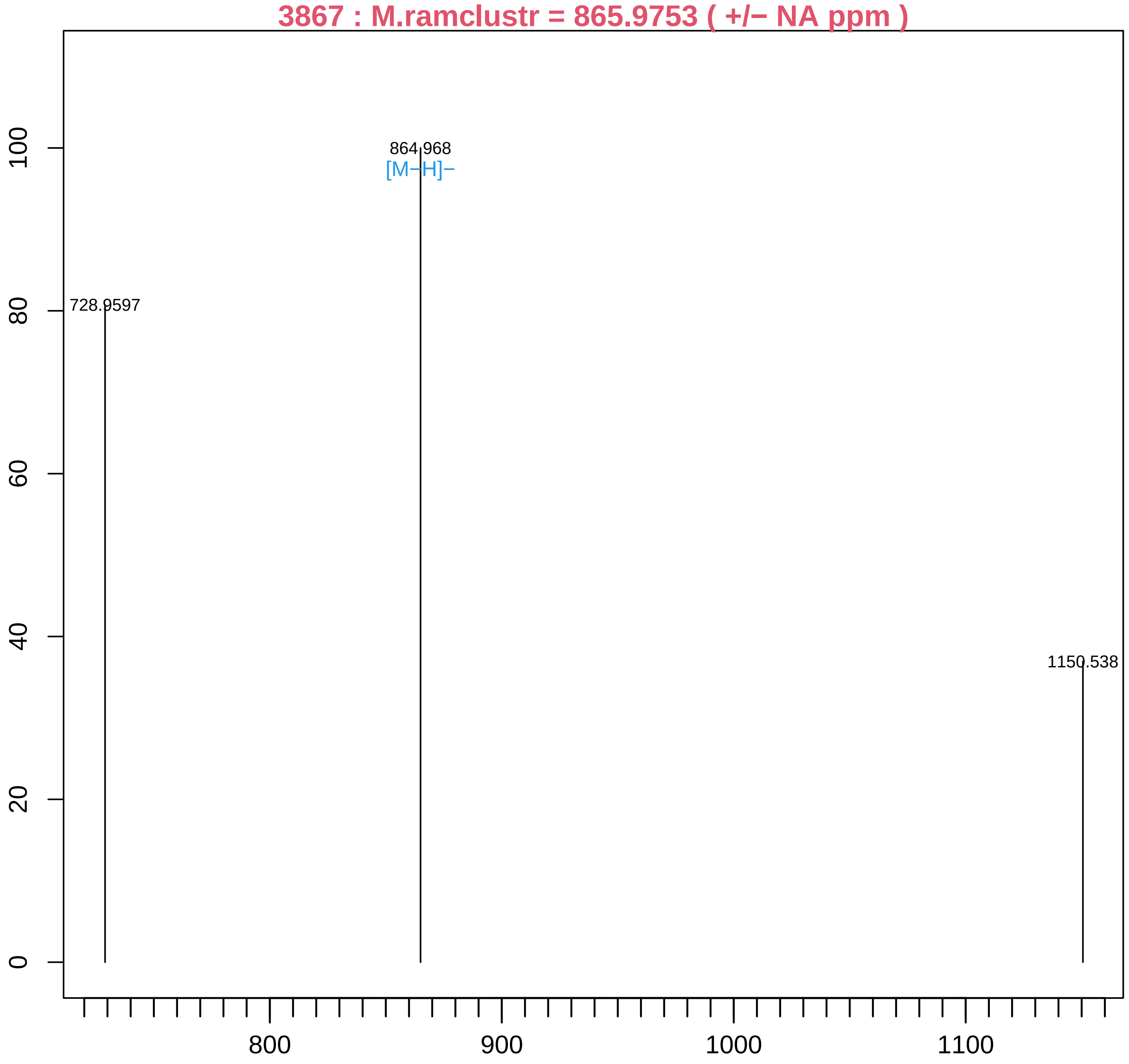
3867 : M.ramclustr = 865.9753 (+/− NA ppm)
864.968
[M−H]−
728.9597
1150.538
0
20
40
60
80
100
800
900
1000
1100

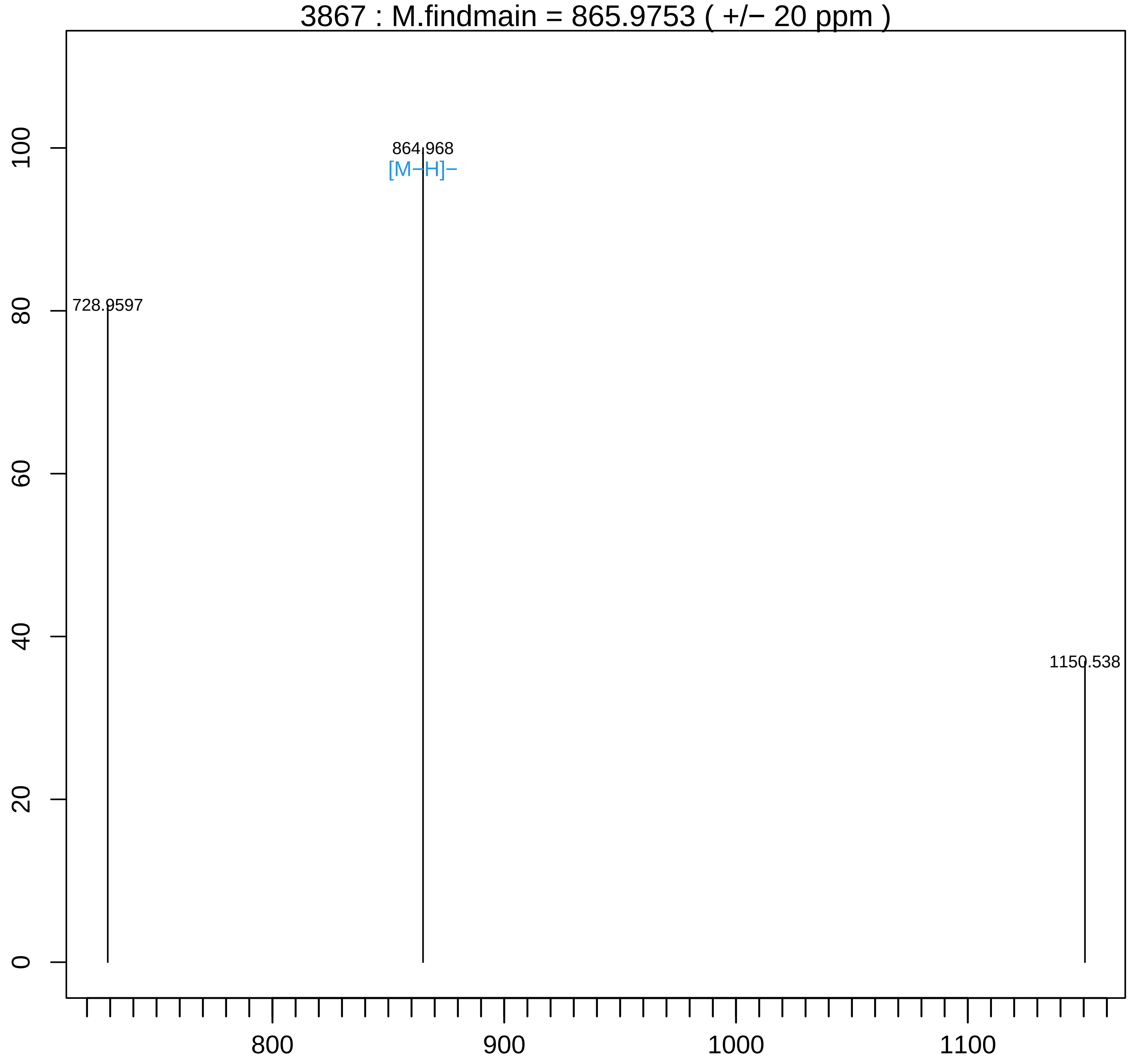
3867 : M.findmain = 865.9753 (+/− 20 ppm)
864.968
[M−H]−
728.9597
1150.538
0
20
40
60
80
100
800
900
1000
1100

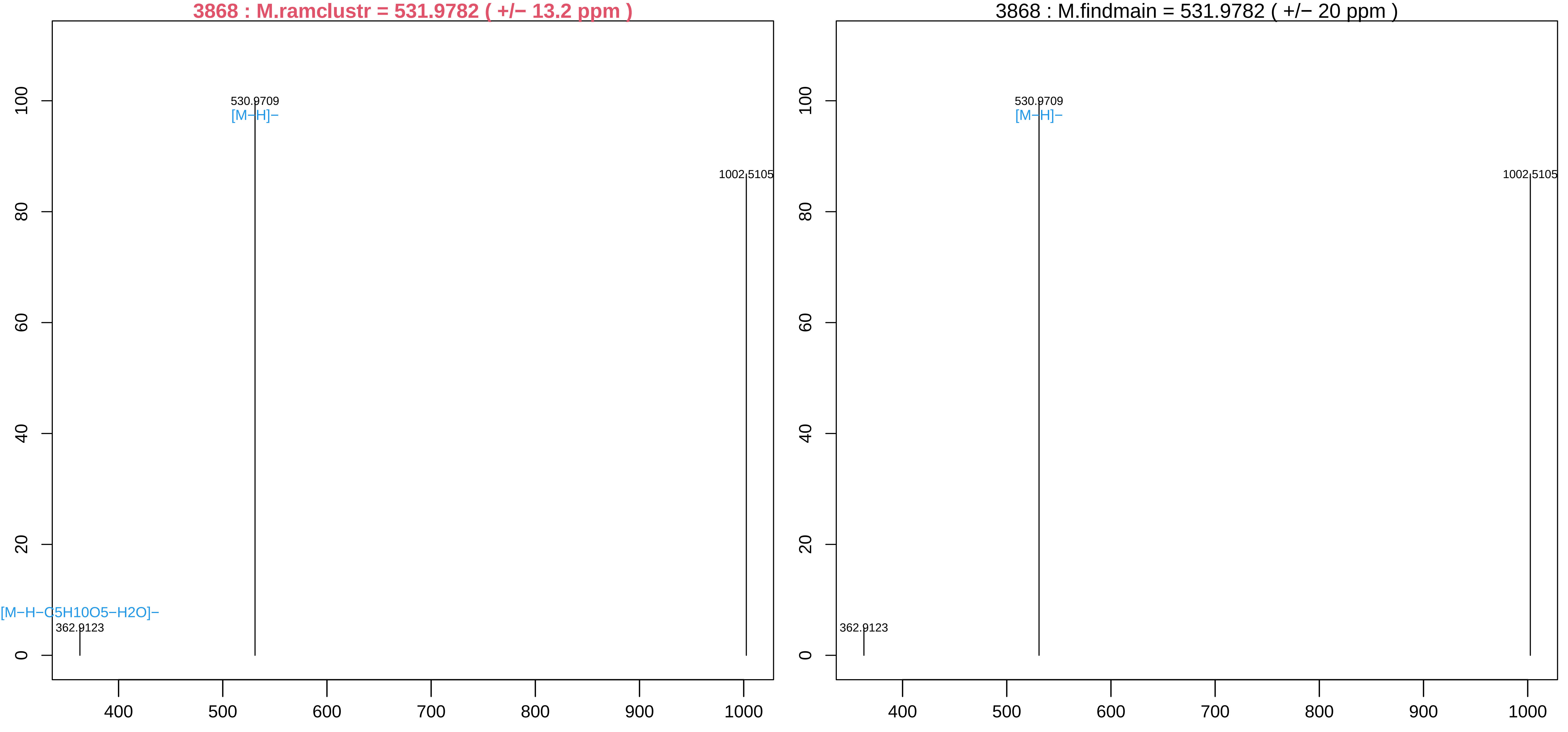
3868 : M.ramclustr = 531.9782 (+/− 13.2 ppm)
530.9709
[M−H]−
1002.5105
[M−H−C5H10O5−H2O]−
362.9123
3868 : M.findmain = 531.9782 (+/− 20 ppm)
530.9709
[M−H]−
1002.5105
362.9123

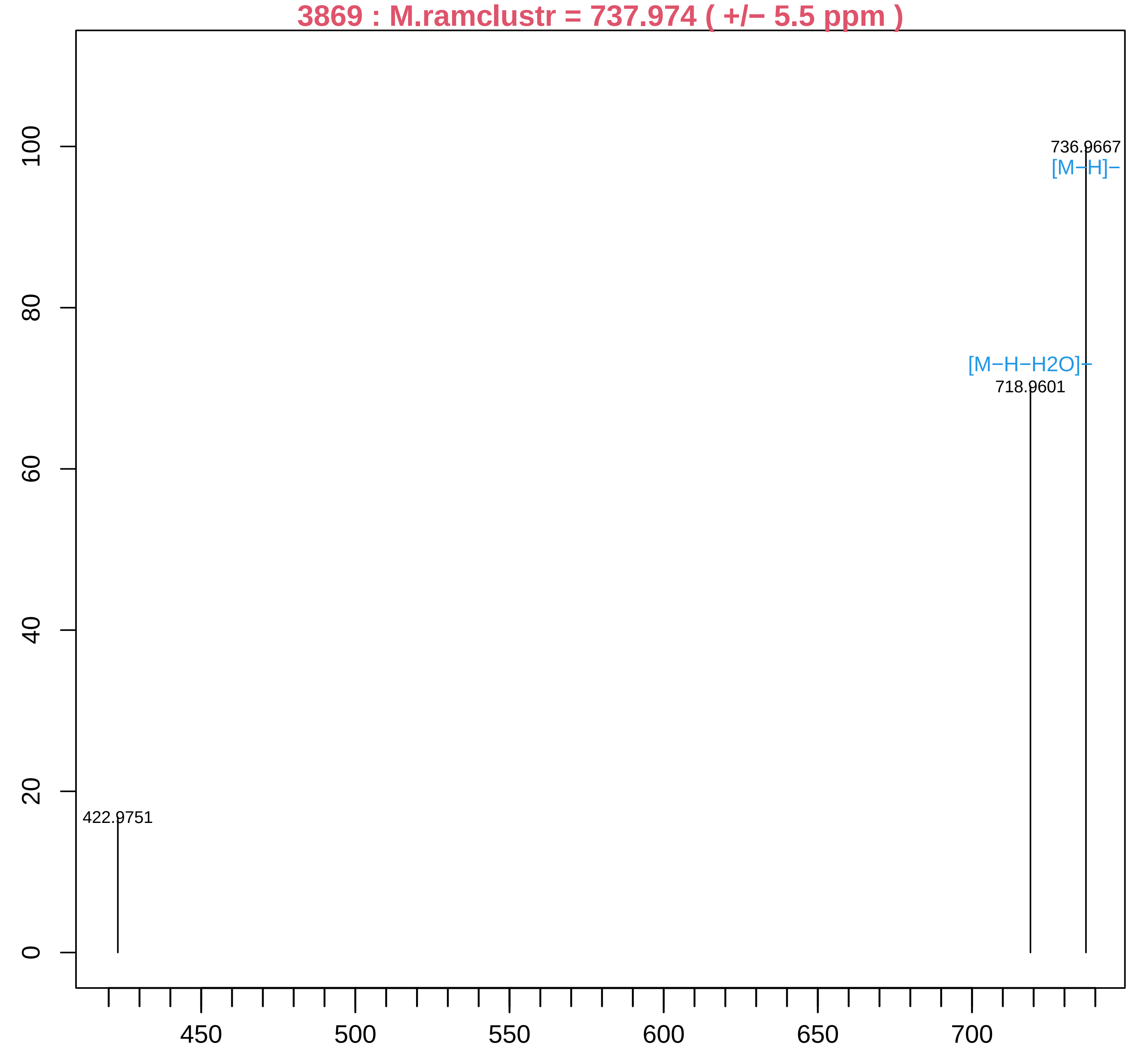
3869 : M.ramclustr = 737.974 (+/− 5.5 ppm)
736.9667
[M−H]−
[M−H−H2O]−
718.9601
422.9751
0
20
40
60
80
100
450
500
550
600
650
700

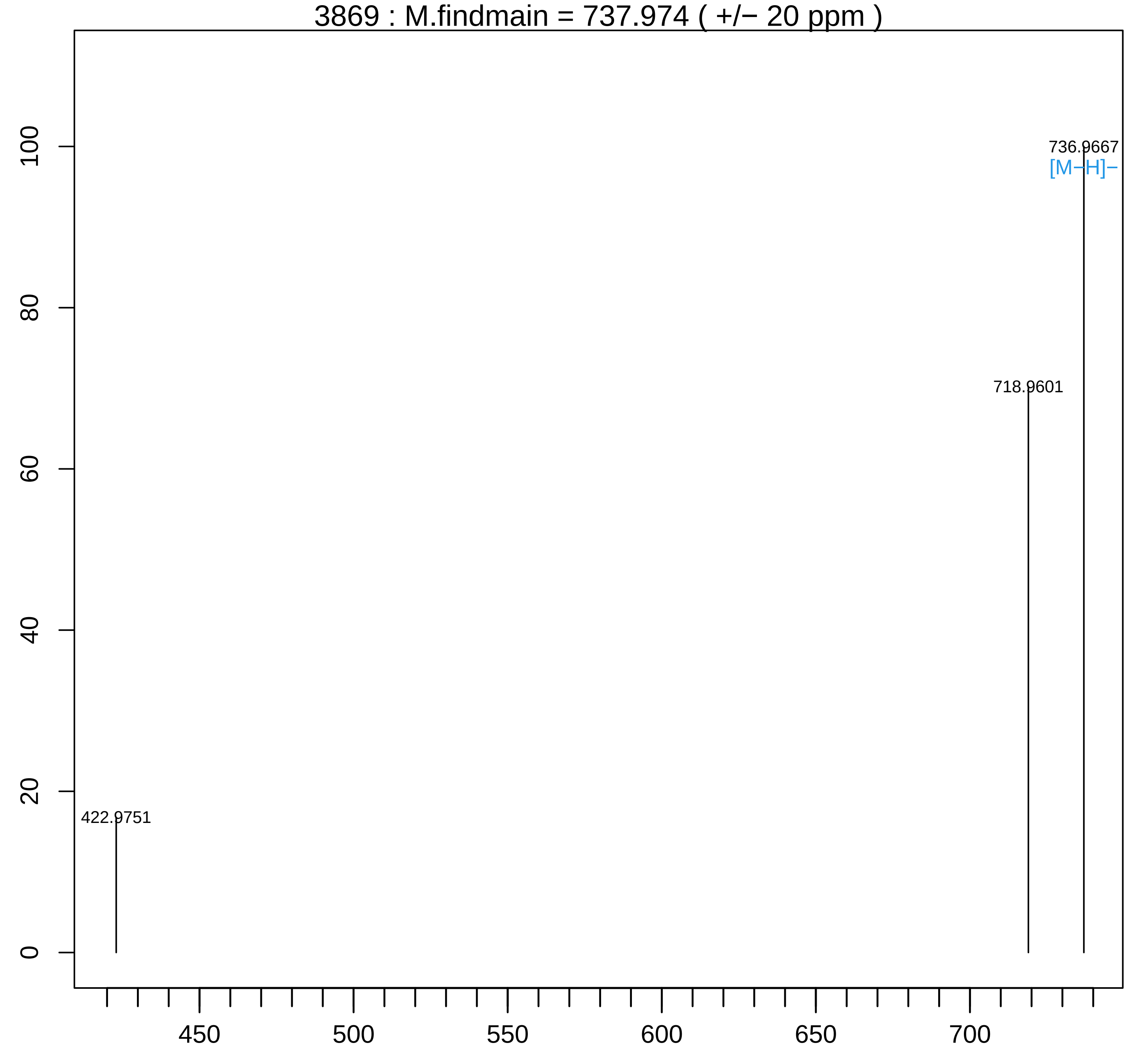
3869 : M.findmain = 737.974 (+/− 20 ppm)
736.9667
[M−H]−
718.9601
422.9751
0
20
40
60
80
100
450
500
550
600
650
700

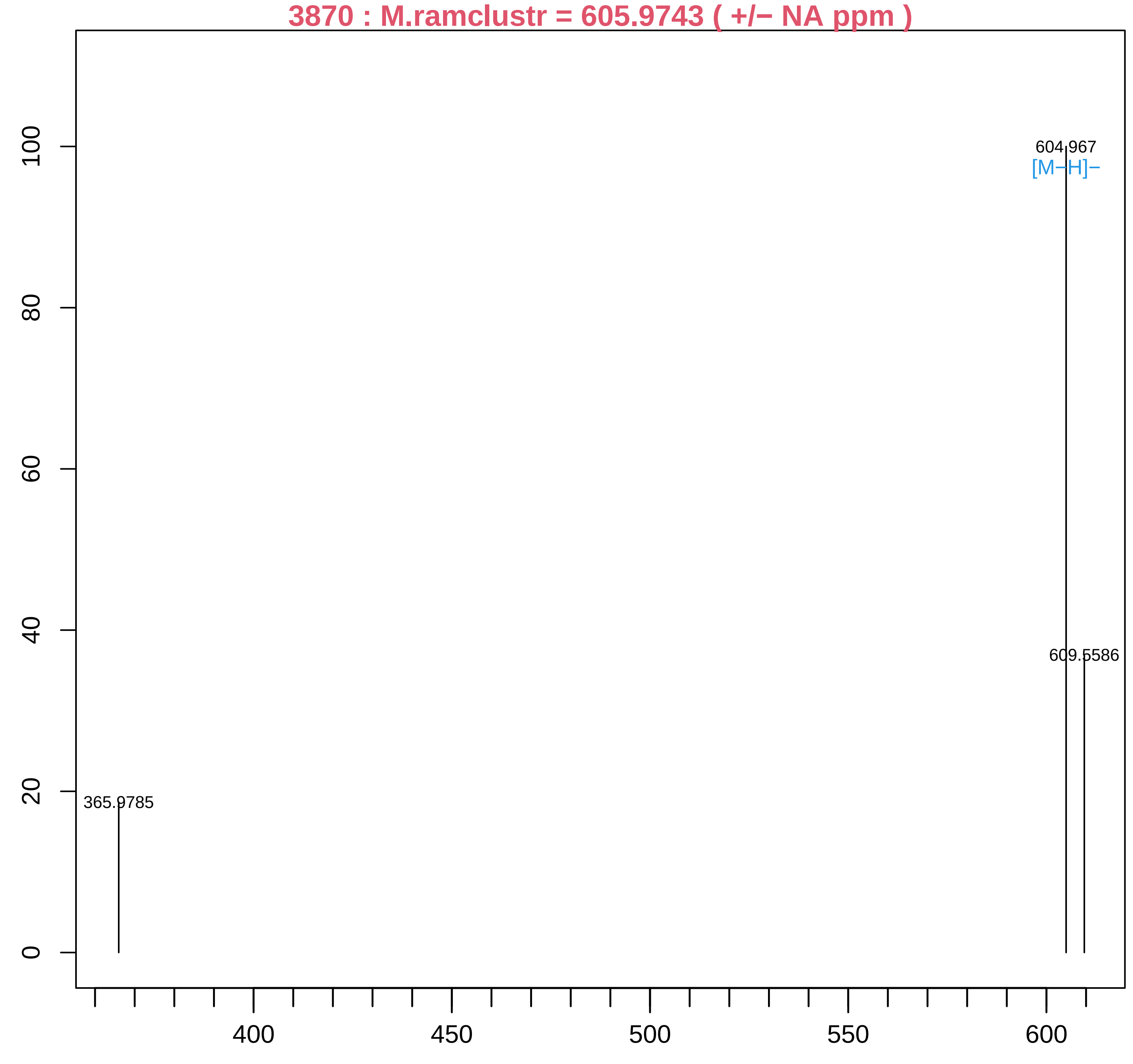
3870 : M.ramclustr = 605.9743 (+/− NA ppm)
604.967
[M−H]−
609.5586
365.9785
0
20
40
60
80
100
400
450
500
550
600

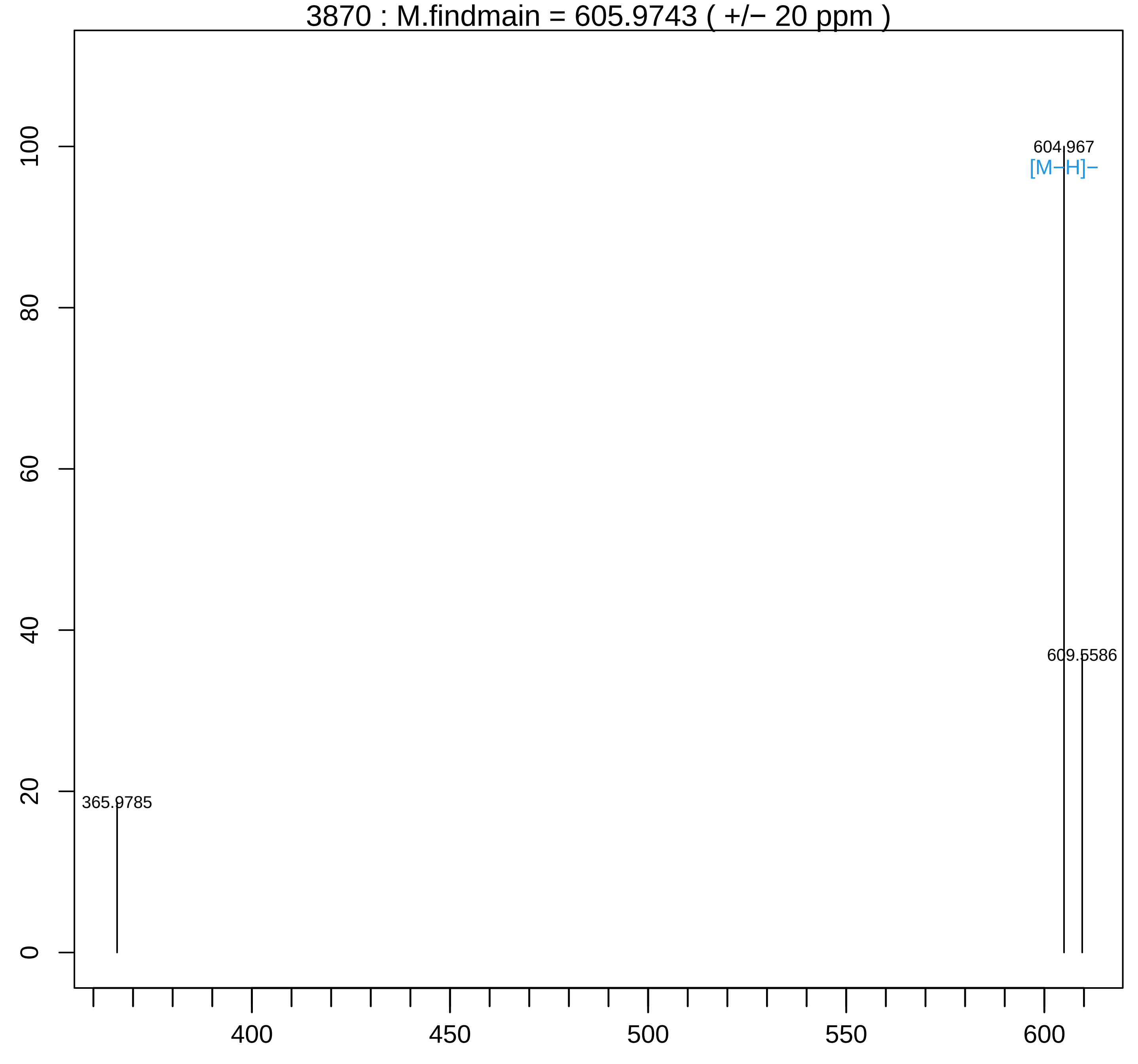
3870 : M.findmain = 605.9743 (+/− 20 ppm)
604.967
[M−H]−
609.5586
365.9785
0
20
40
60
80
100
400
450
500
550
600

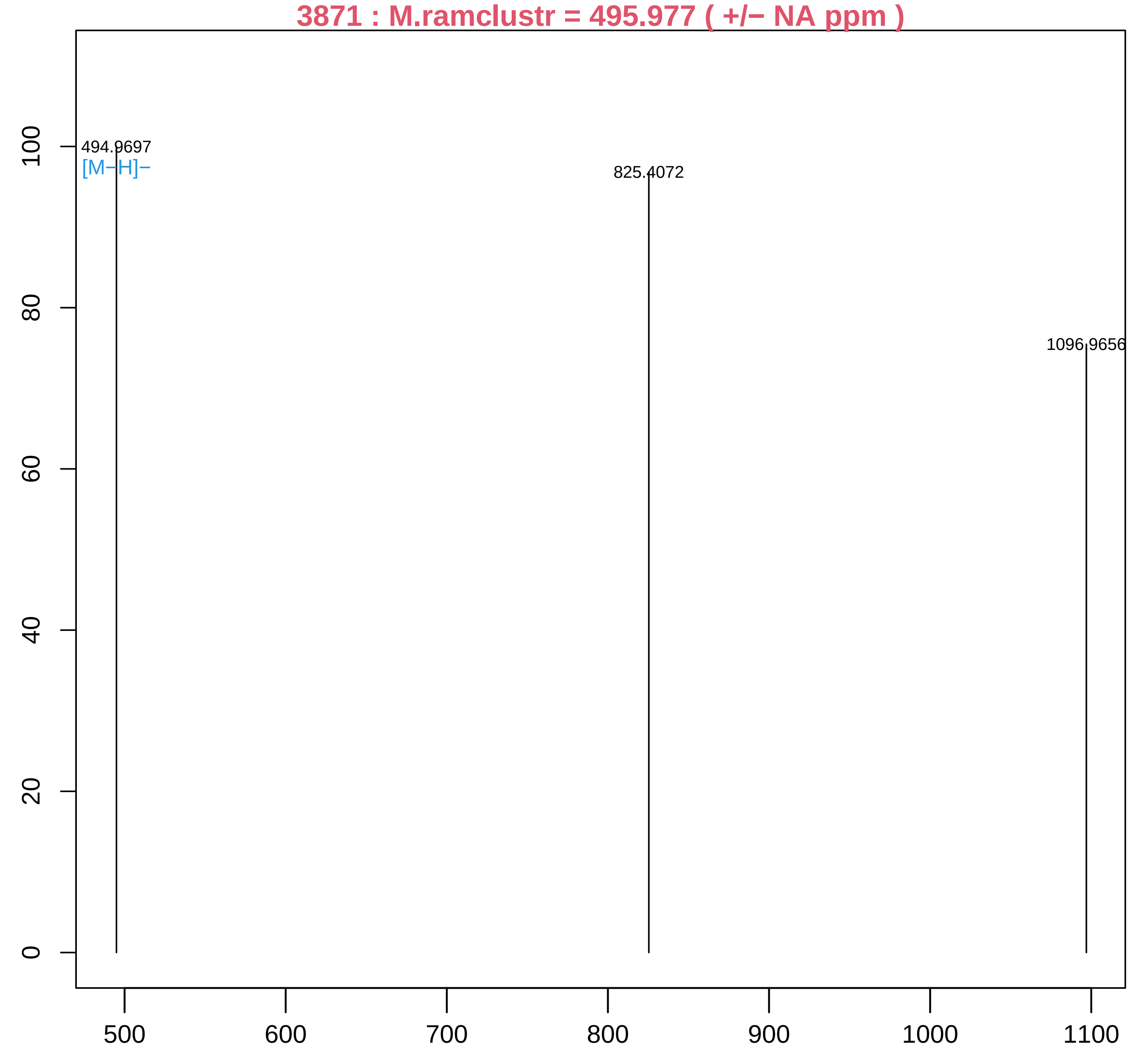
3871 : M.ramclustr = 495.977 (+/− NA ppm)
494.9697
[M−H]−
825.4072
1096.9656
0
20
40
60
80
100
500
600
700
800
900
1000
1100

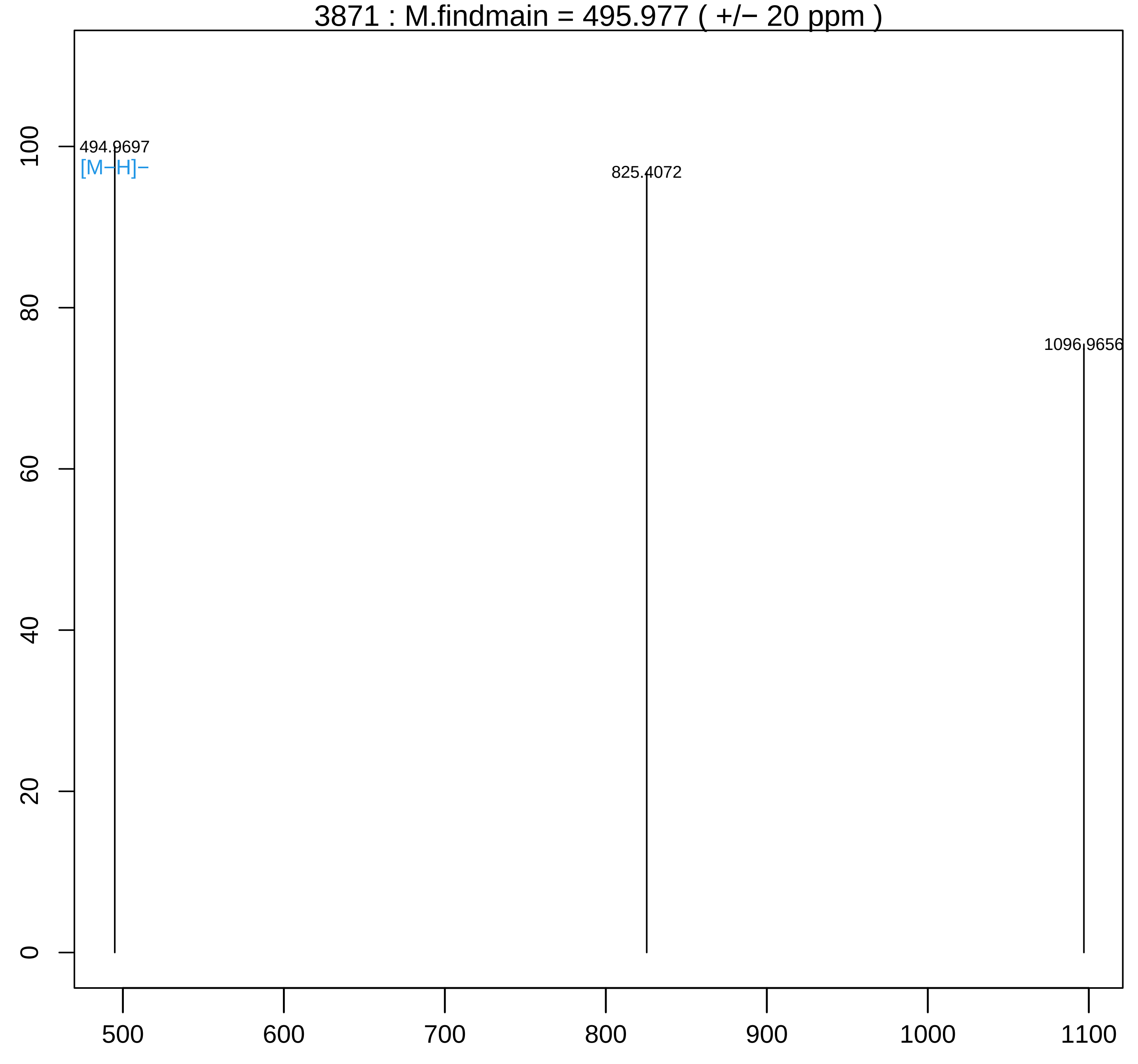
3871 : M.findmain = 495.977 (+/− 20 ppm)
494.9697
[M−H]−
825.4072
1096.9656
0
20
40
60
80
100
500
600
700
800
900
1000
1100

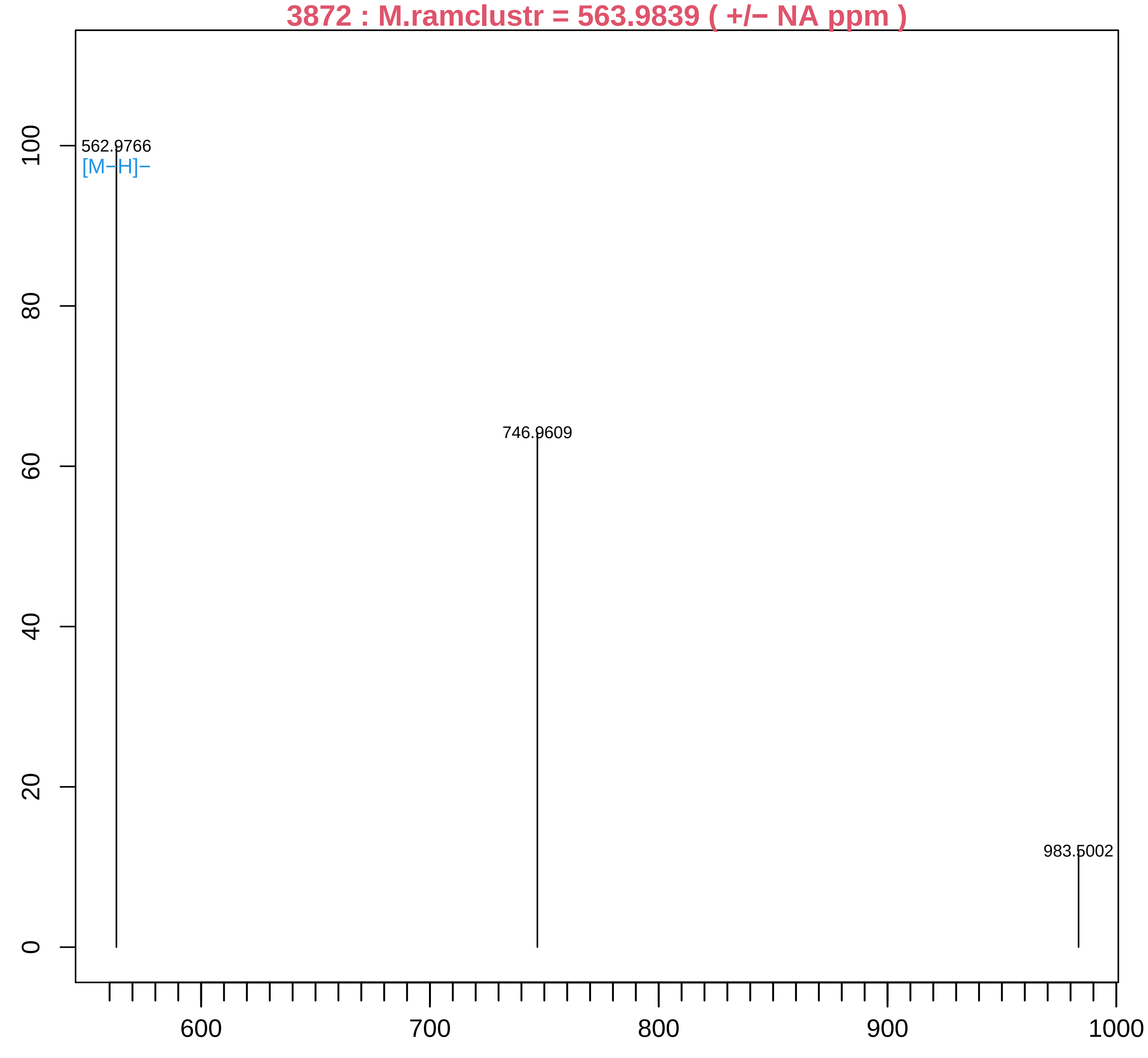
3872 : M.ramclustr = 563.9839 (+/− NA ppm)
562.9766
[M−H]−
746.9609
983.5002
0
20
40
60
80
100
600
700
800
900
1000

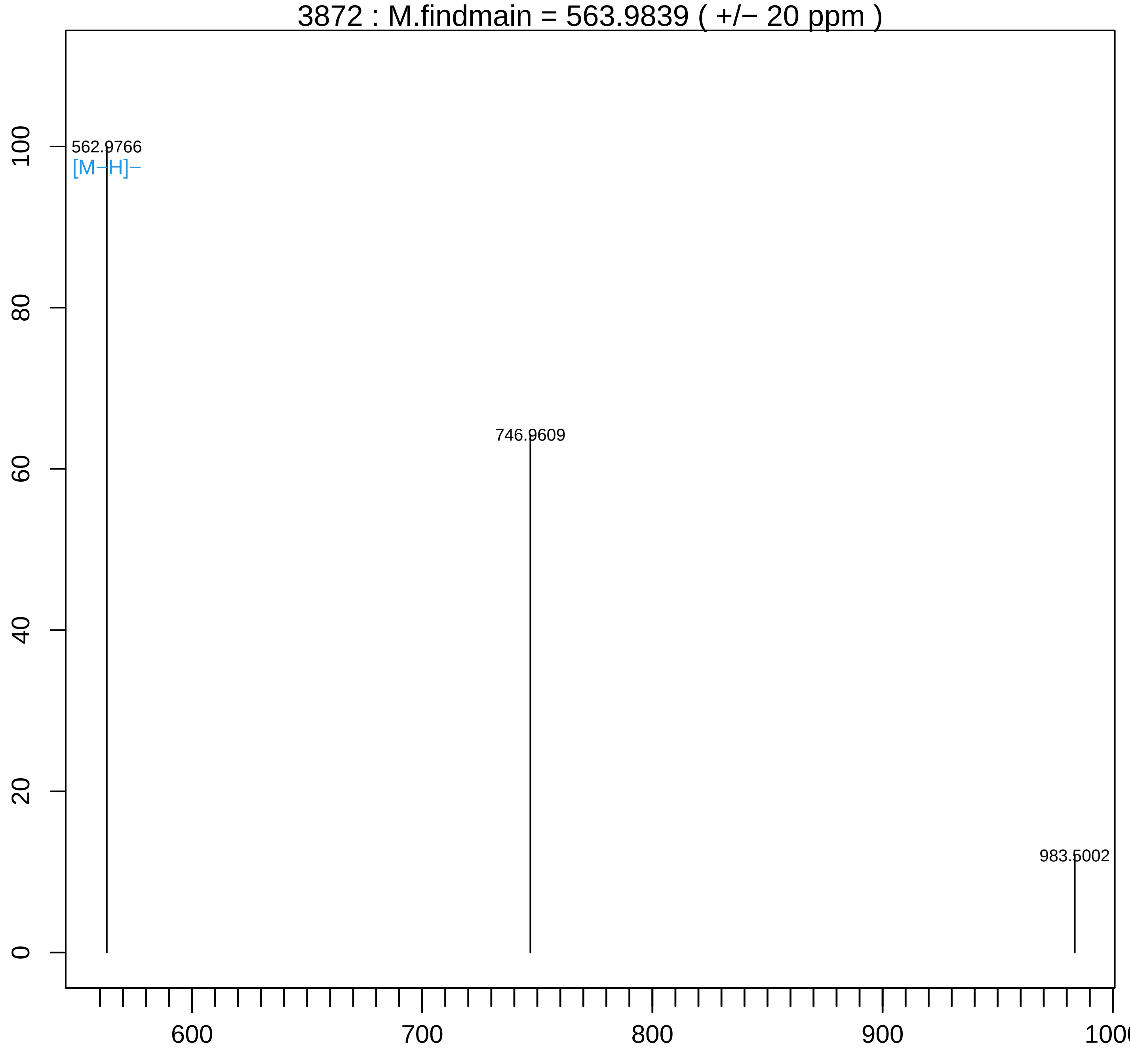
3872 : M.findmain = 563.9839 (+/− 20 ppm)
562.9766
[M−H]−
746.9609
983.5002
0
20
40
60
80
100
600
700
800
900

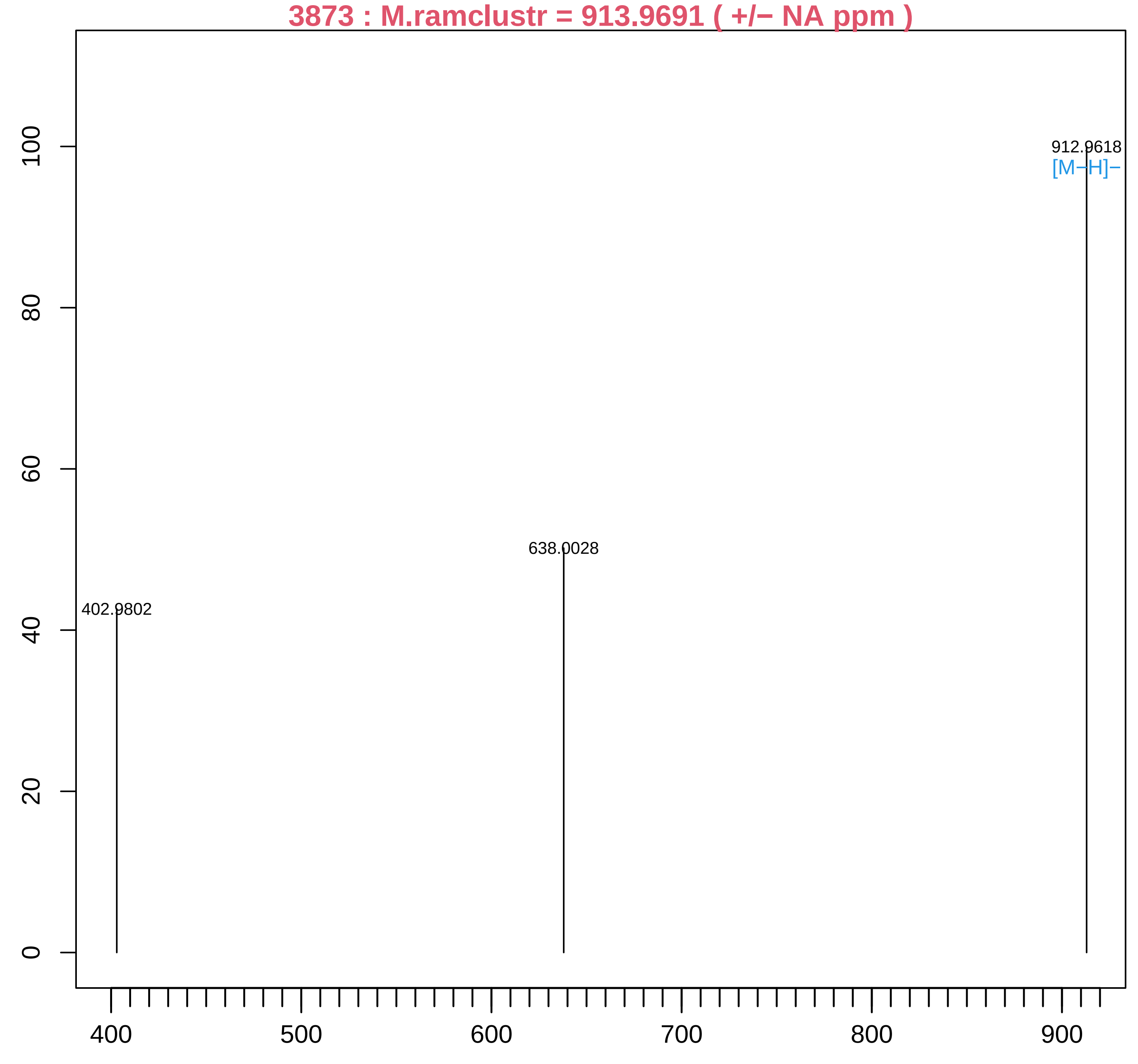
3873 : M.ramclustr = 913.9691 (+/− NA ppm)
402.9802
638.0028
912.9618
[M−H]−

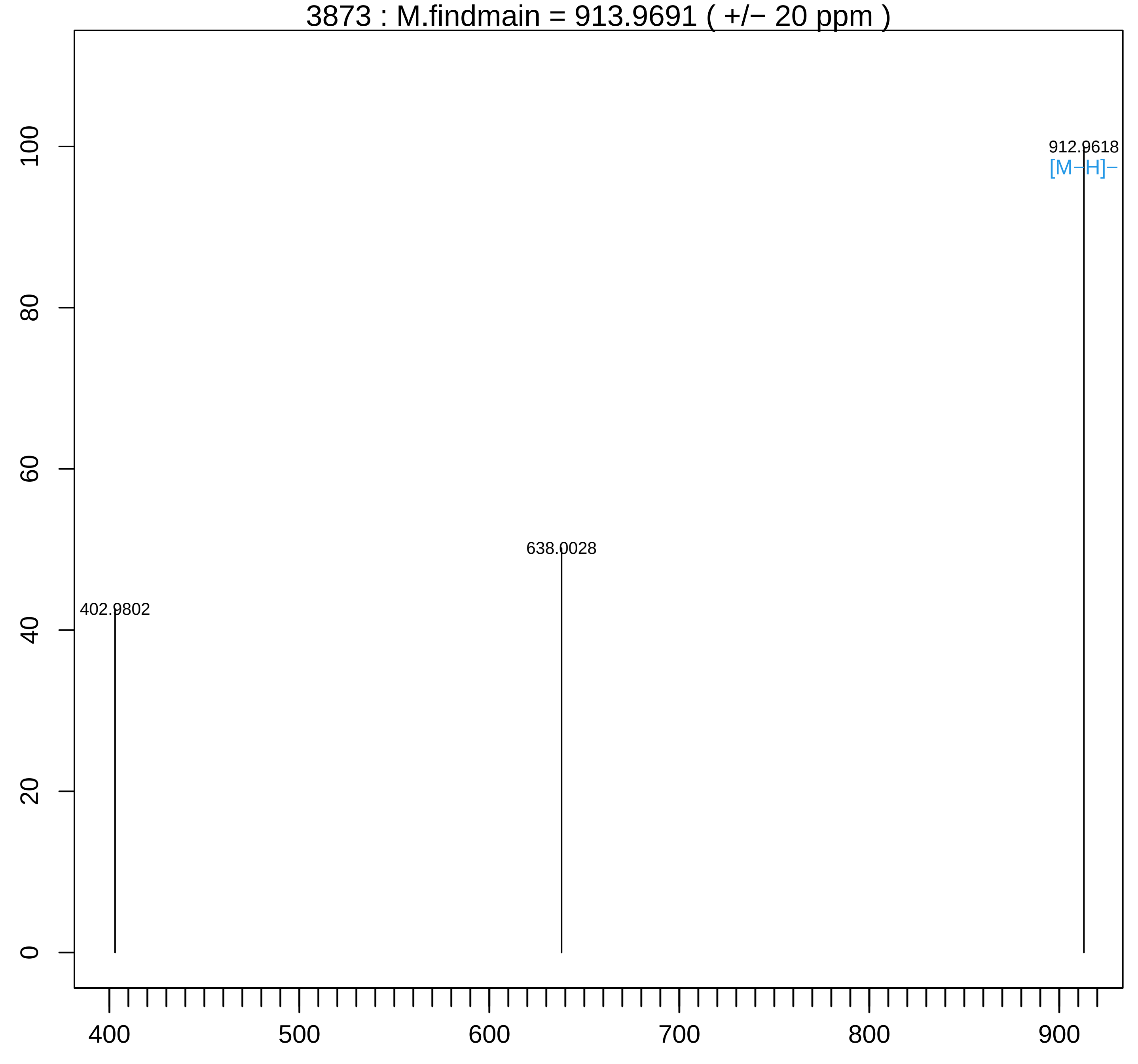
3873 : M.findmain = 913.9691 (+/− 20 ppm)
402.9802
638.0028
912.9618
[M−H]−

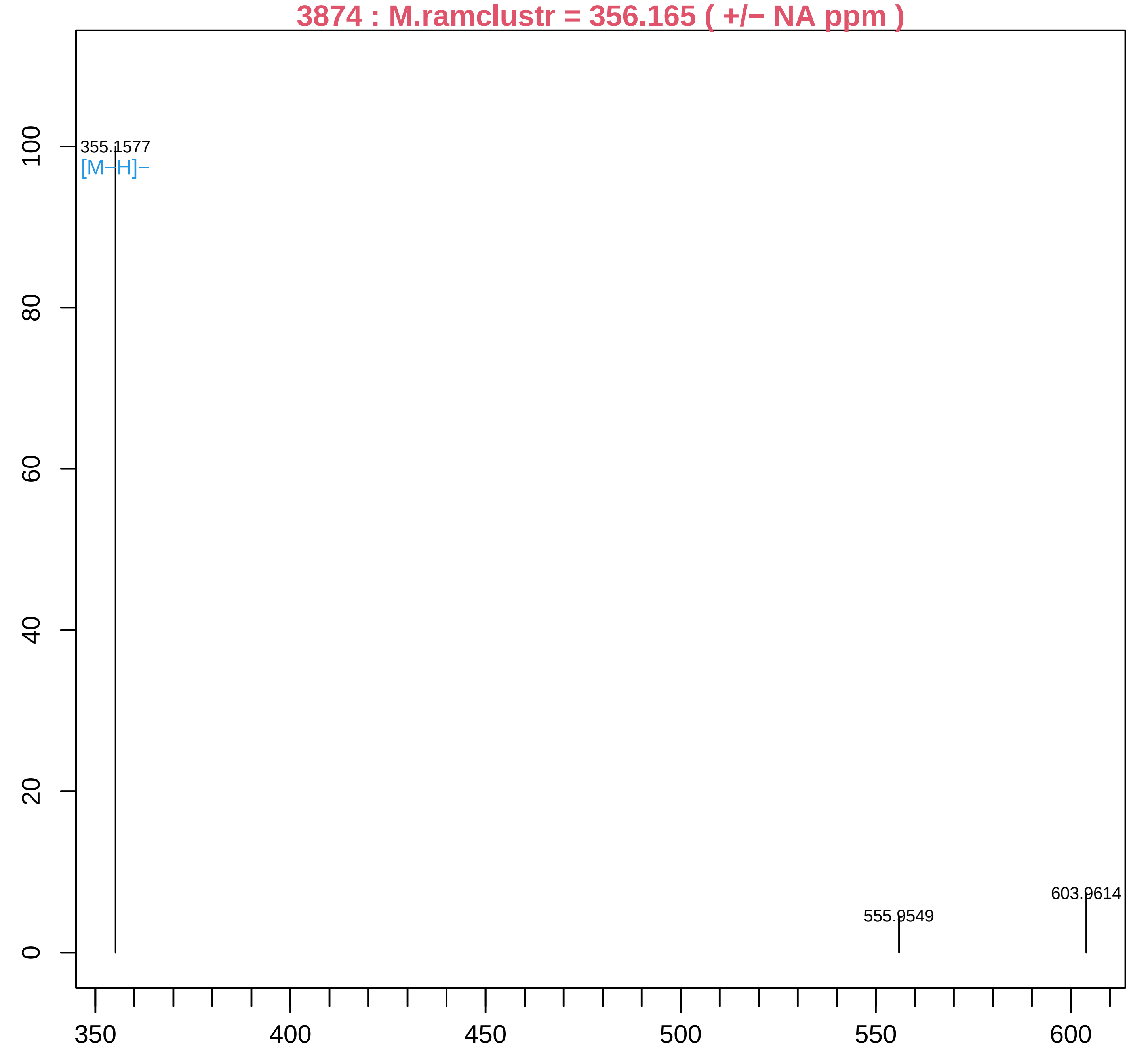
3874 : M.ramclustr = 356.165 (+/− NA ppm)
355.1577
[M−H]−
555.9549
603.9614
0
20
40
60
80
100
350
400
450
500
550
600

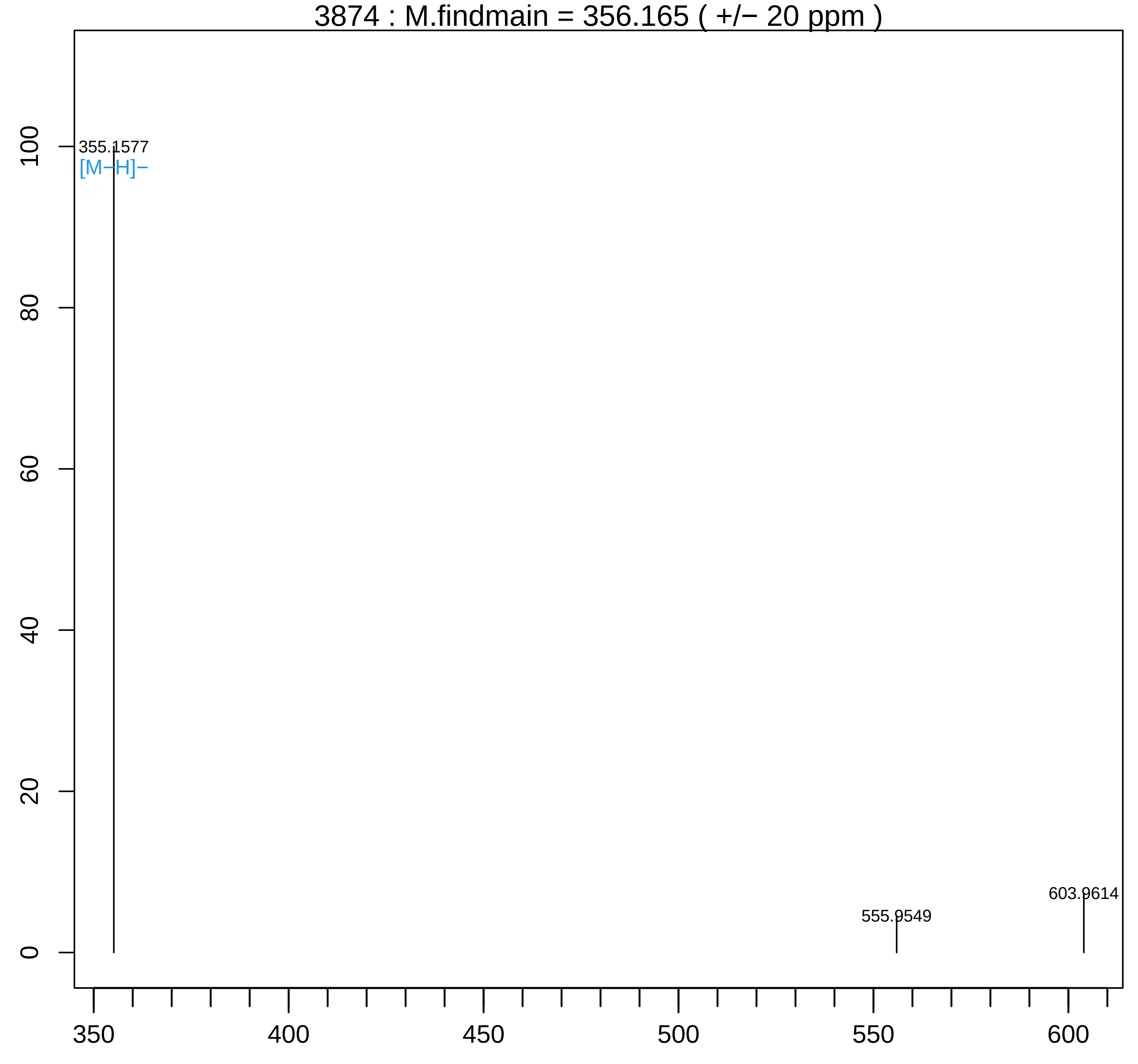
3874 : M.findmain = 356.165 (+/− 20 ppm)
355.1577
[M−H]−
555.9549
603.9614
0
20
40
60
80
100
350
400
450
500
550
600

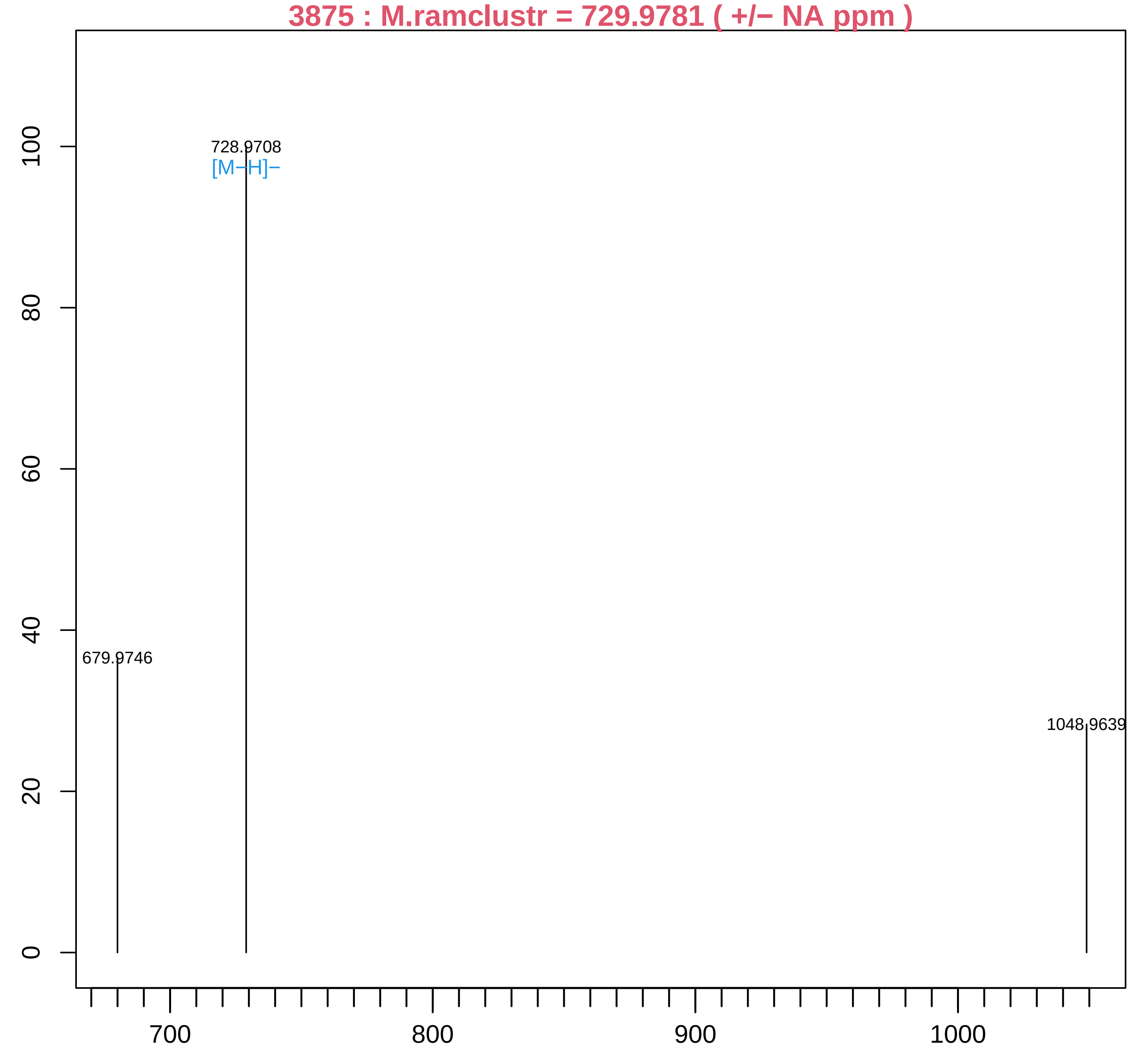
3875 : M.ramclustr = 729.9781 (+/− NA ppm)
728.9708
[M−H]−
679.9746
1048.9639
0
20
40
60
80
100
700
800
900
1000
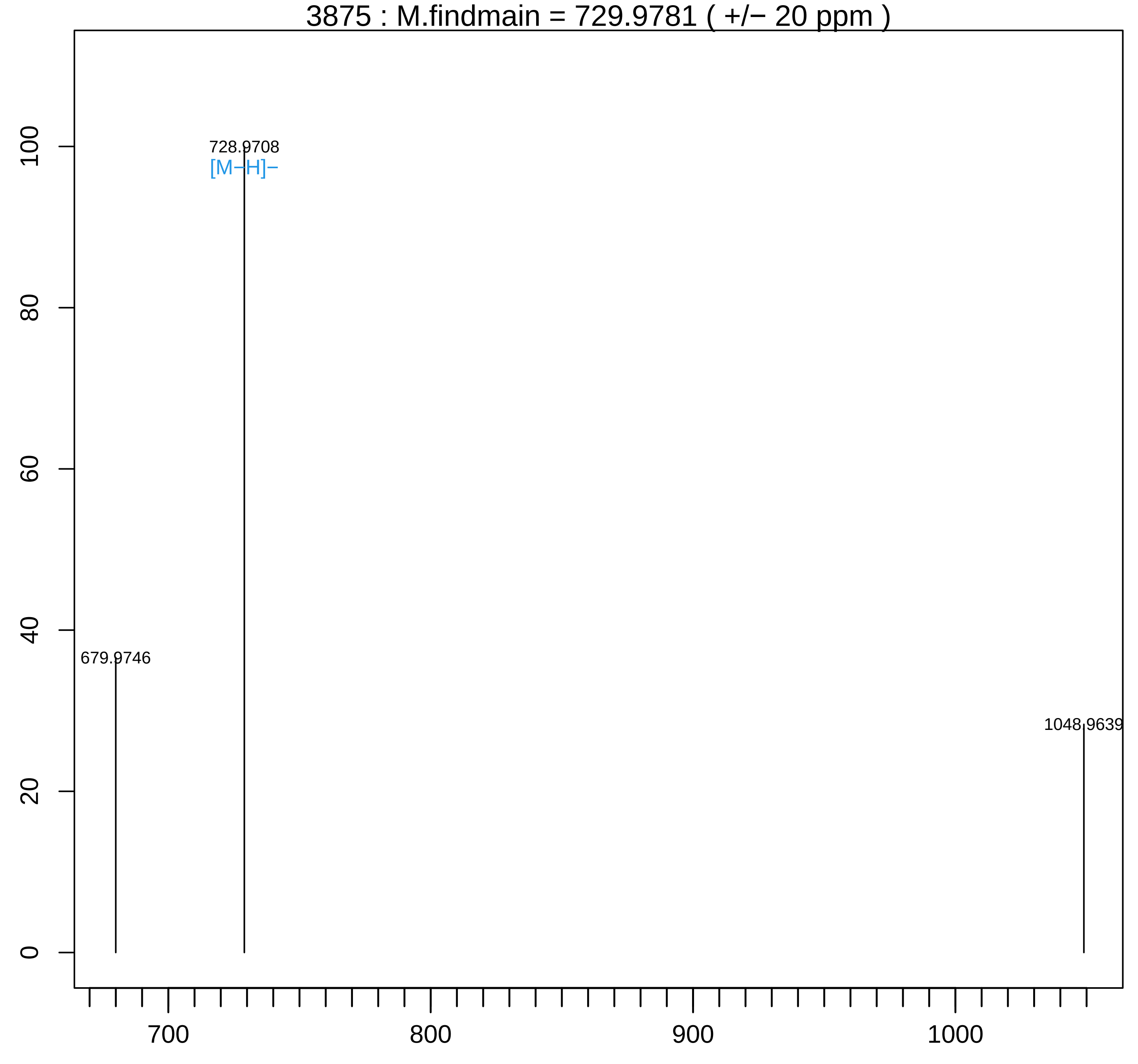
3875 : M.findmain = 729.9781 (+/− 20 ppm)
728.9708
[M−H]−
679.9746
1048.9639
0
20
40
60
80
100
700
800
900
1000

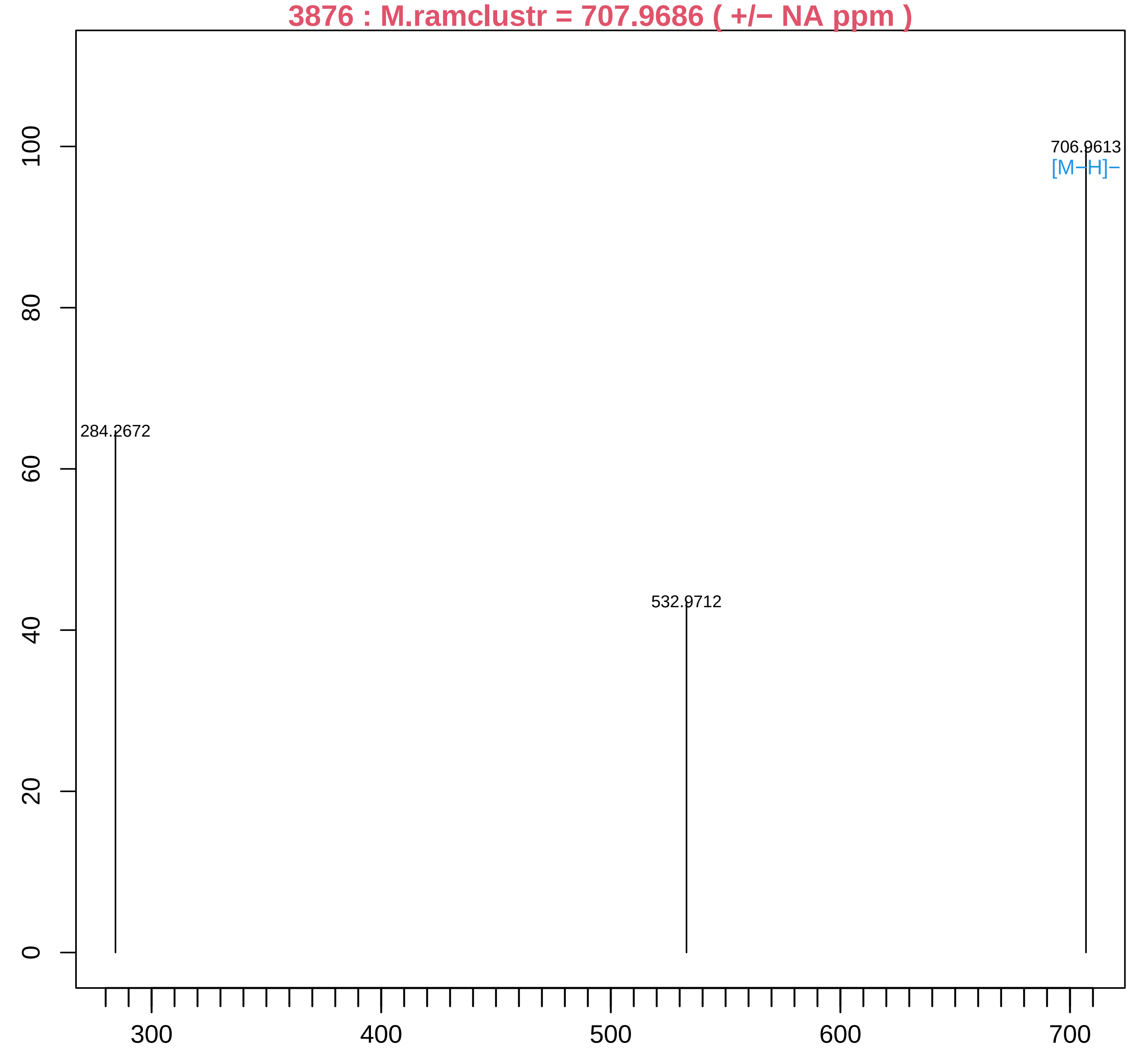
3876 : M.ramclustr = 707.9686 (+/− NA ppm)
706.9613
[M−H]−
284.2672
532.9712
0
20
40
60
80
100
300
400
500
600
700

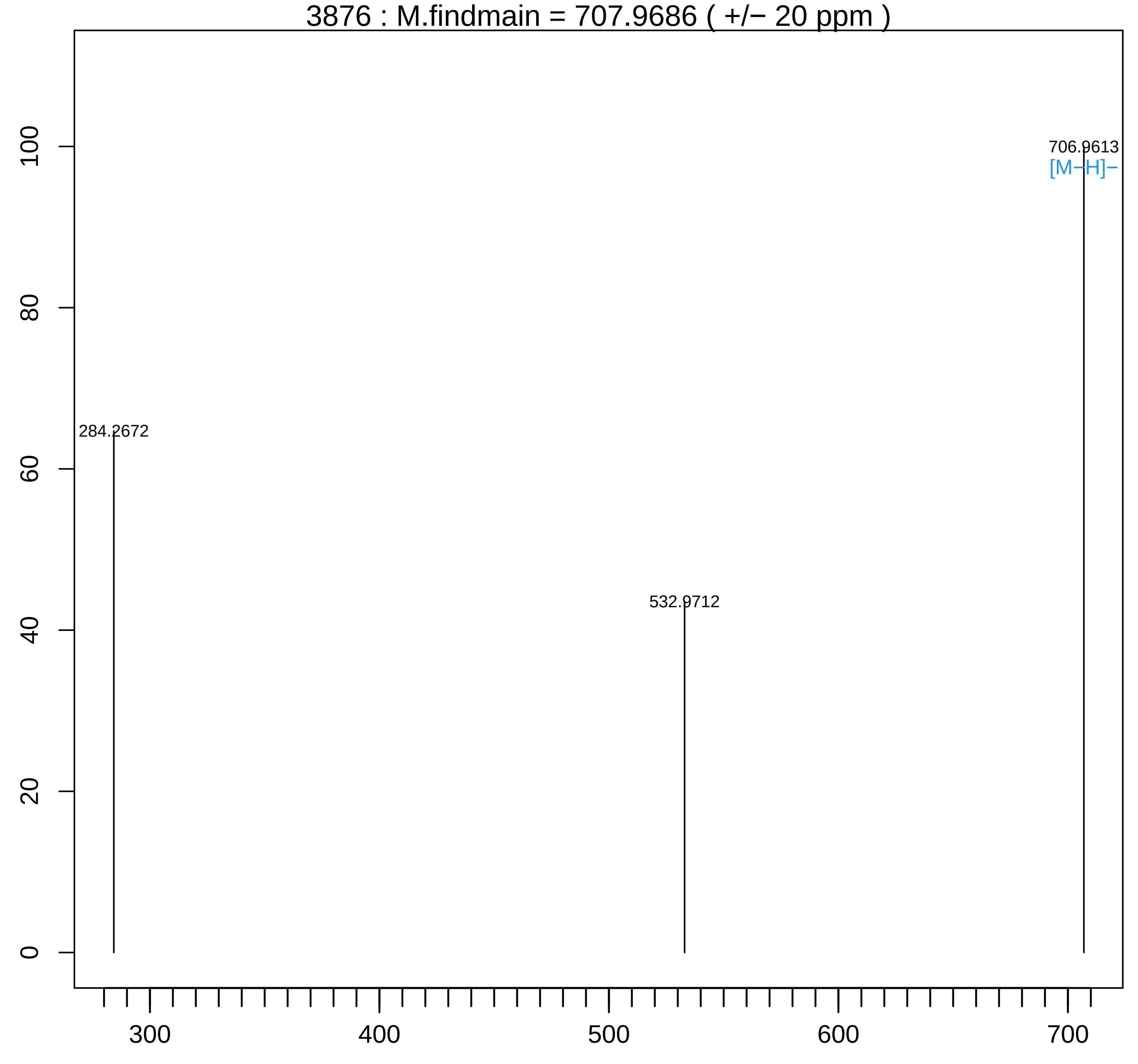
3876 : M.findmain = 707.9686 (+/− 20 ppm)
706.9613
[M−H]−
284.2672
532.9712
0
20
40
60
80
100
300
400
500
600
700

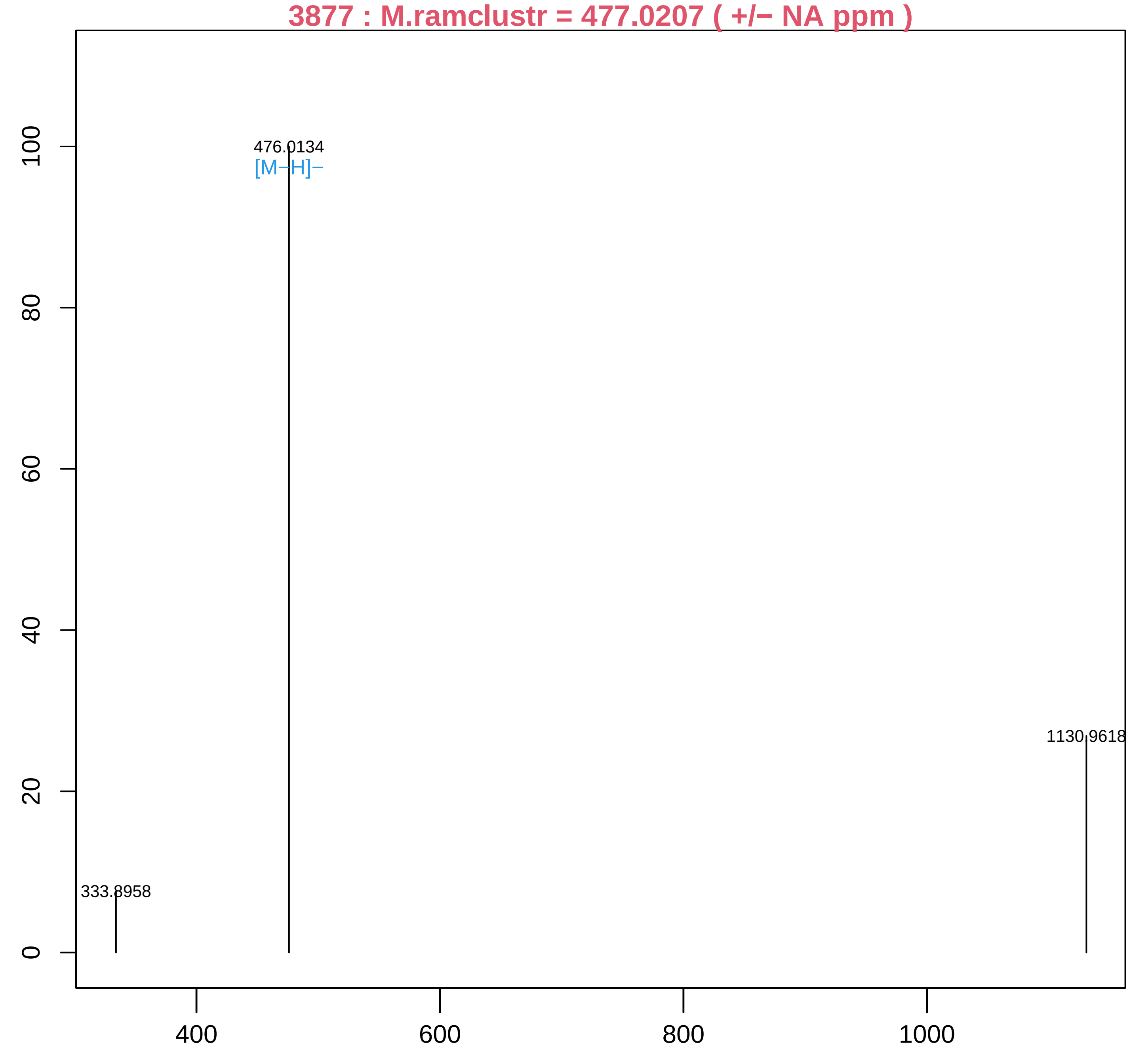
3877 : M.ramclustr = 477.0207 (+/− NA ppm)
476.0134
[M−H]−
333.8958
1130.9618
0
20
40
60
80
100
400
600
800
1000

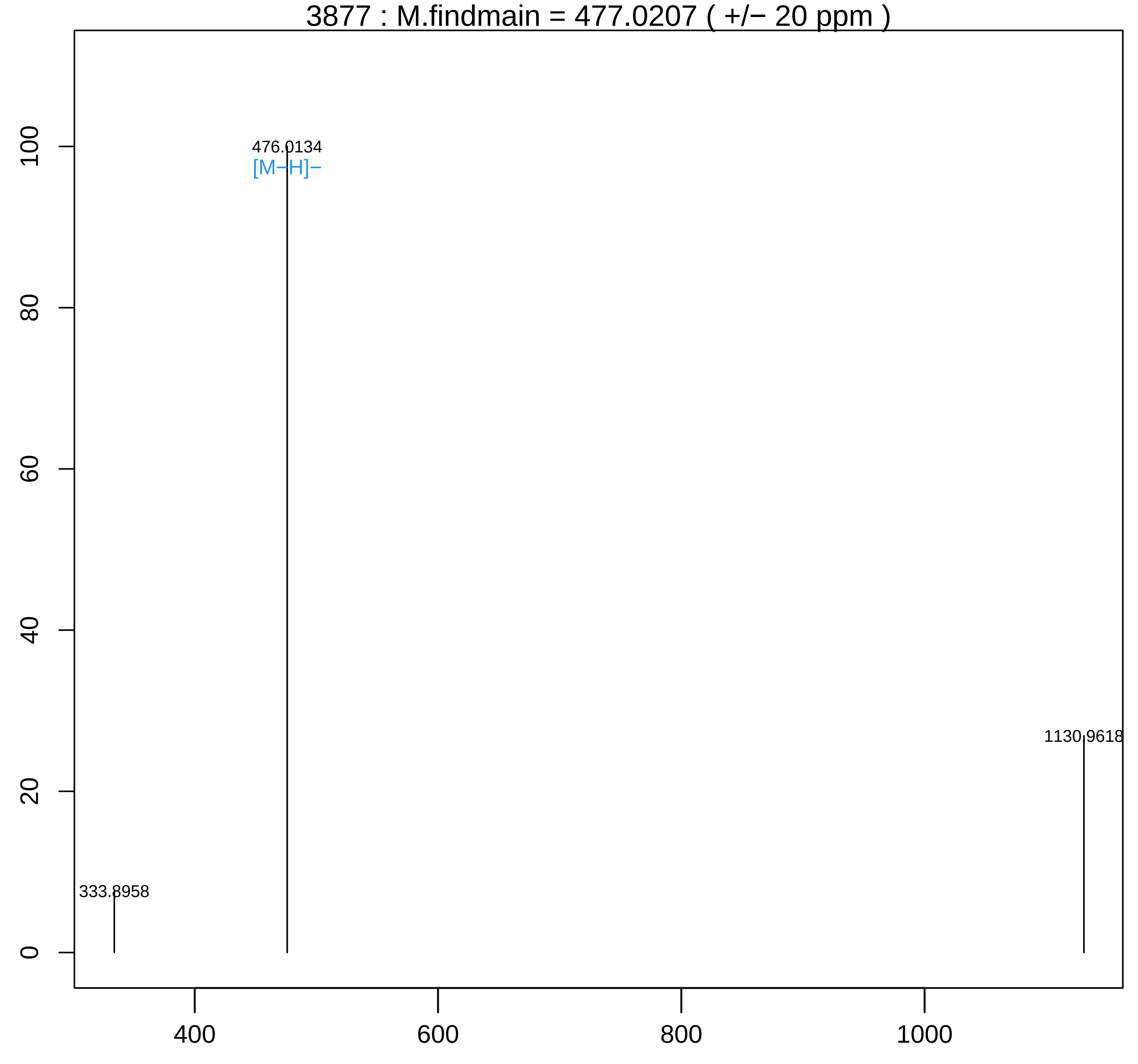
3877 : M.findmain = 477.0207 (+/− 20 ppm)
476.0134
[M−H]−
333.8958
1130.9618
0
20
40
60
80
100
400
600
800
1000

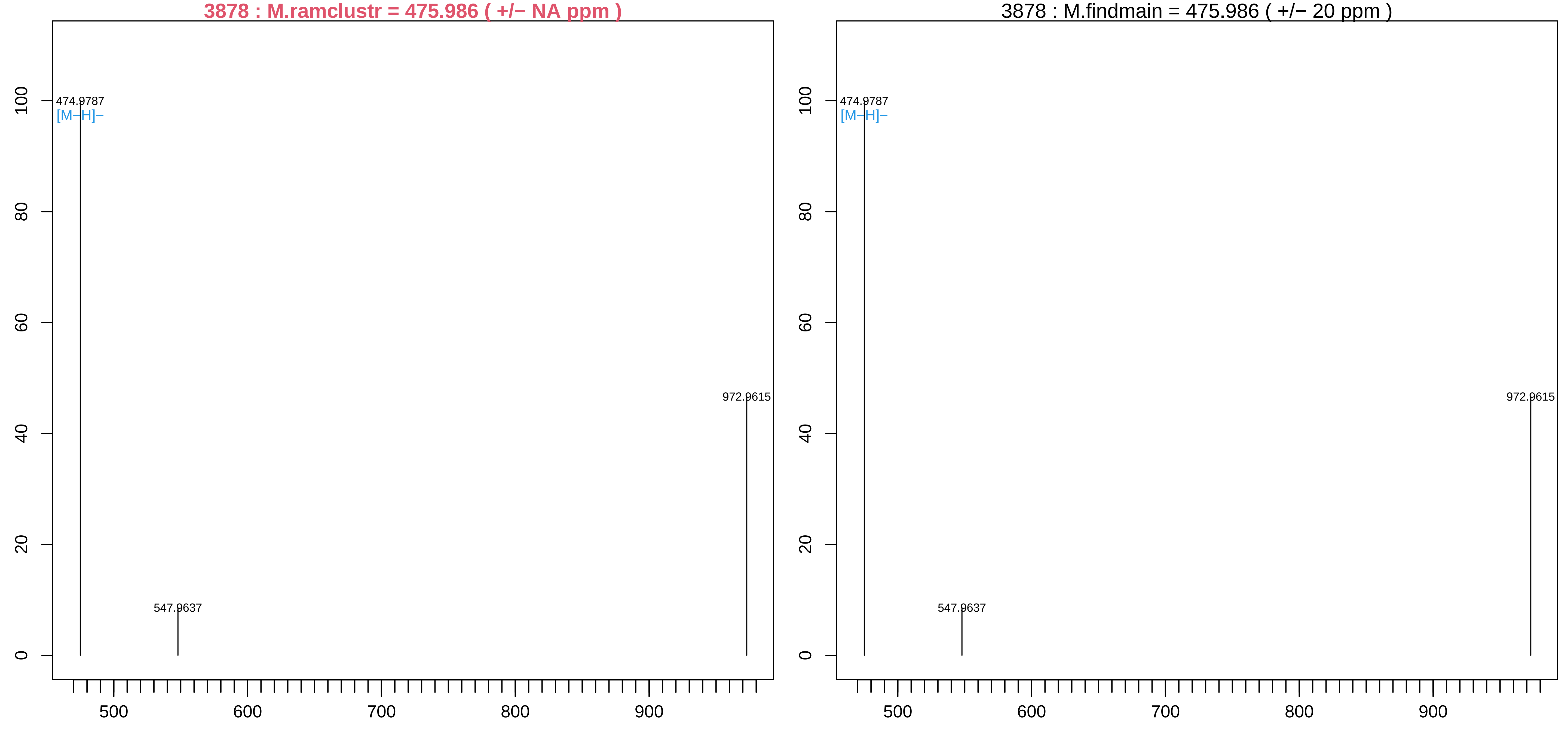
3878 : M.ramclustr = 475.986 (+/− NA ppm)
474.9787
[M−H]−
547.9637
972.9615
3878 : M.findmain = 475.986 (+/− 20 ppm)
474.9787
[M−H]−
547.9637
972.9615
0
20
40
60
80
100
500
600
700
800
900

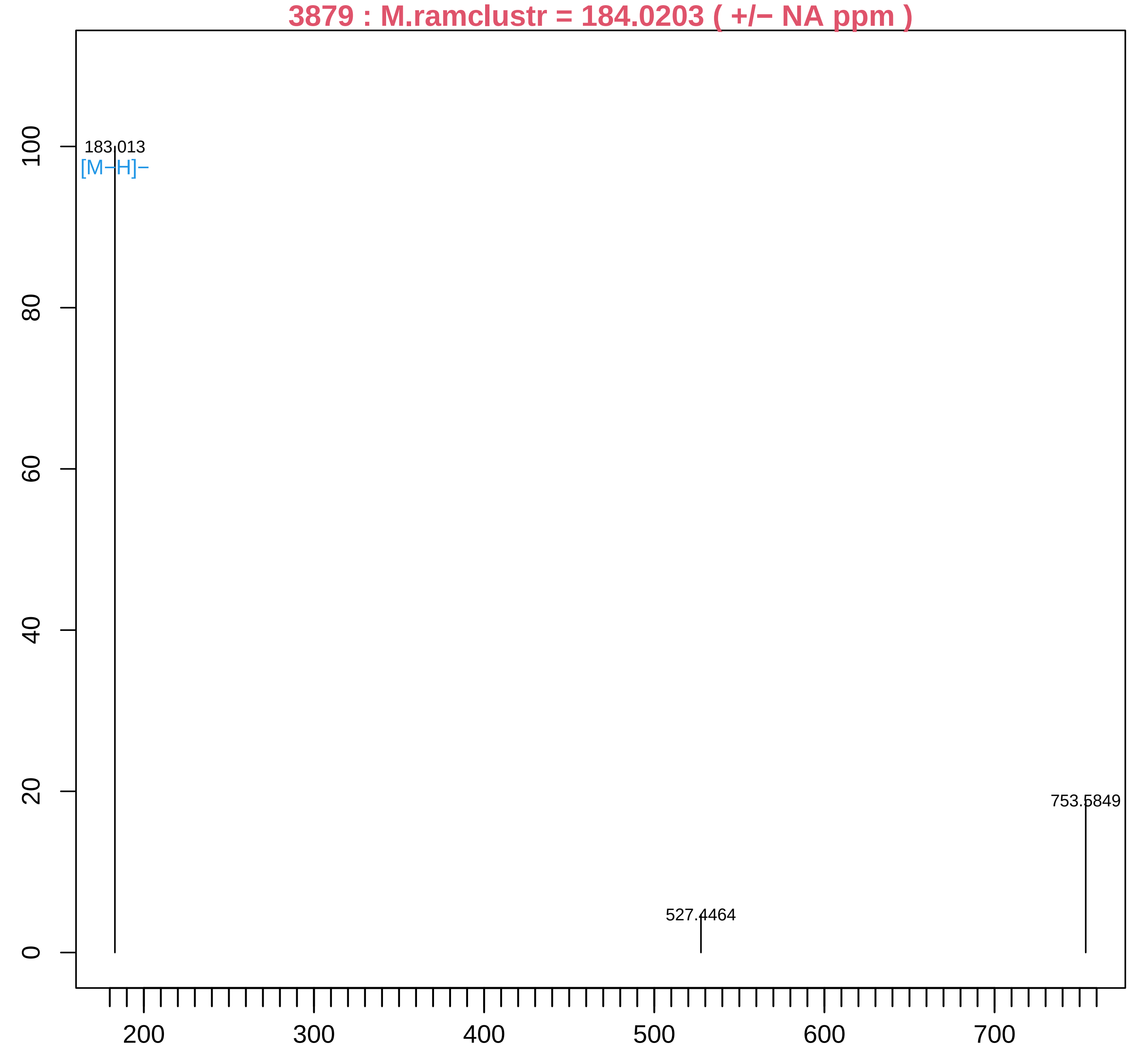
3879 : M.ramclustr = 184.0203 (+/− NA ppm)
183.013
[M−H]−
527.4464
753.5849
0
20
40
60
80
100
200
300
400
500
600
700

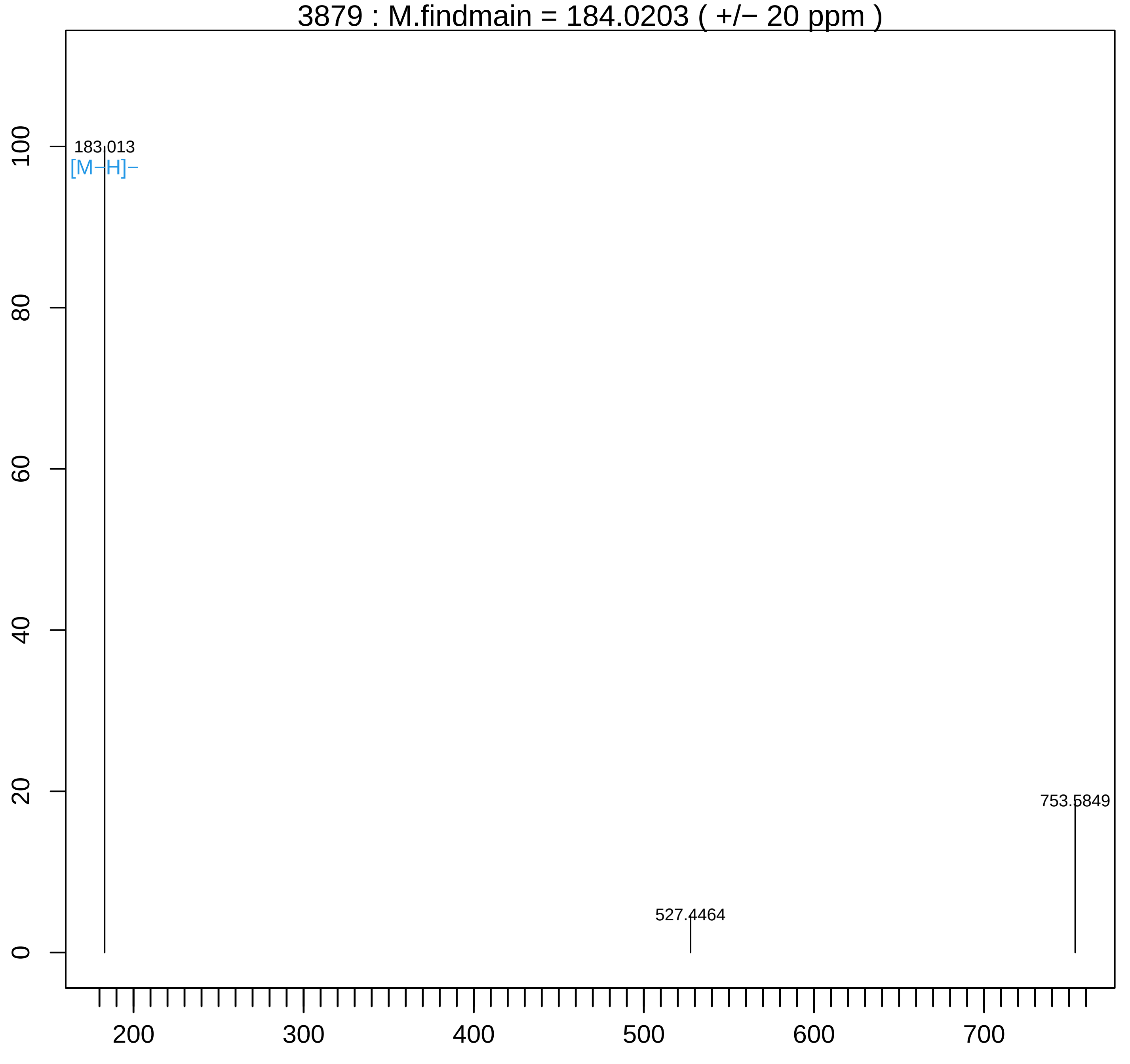
3879 : M.findmain = 184.0203 (+/− 20 ppm)
183.013
[M−H]−
527.4464
753.5849
0
20
40
60
80
100
200
300
400
500
600
700

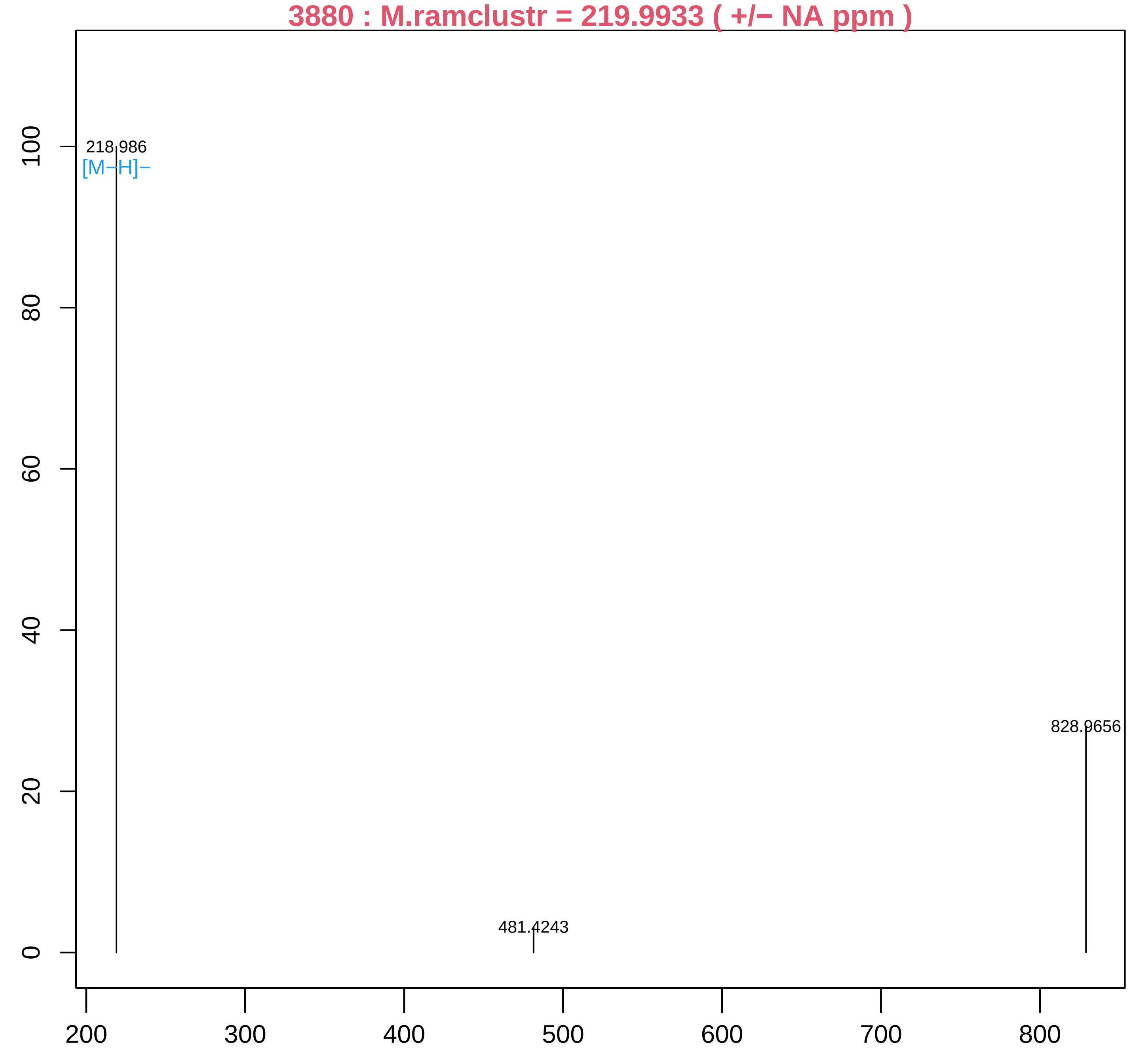
3880 : M.ramclustr = 219.9933 (+/− NA ppm)
218.986
[M−H]−
481.4243
828.9656
0
20
40
60
80
100
200
300
400
500
600
700
800

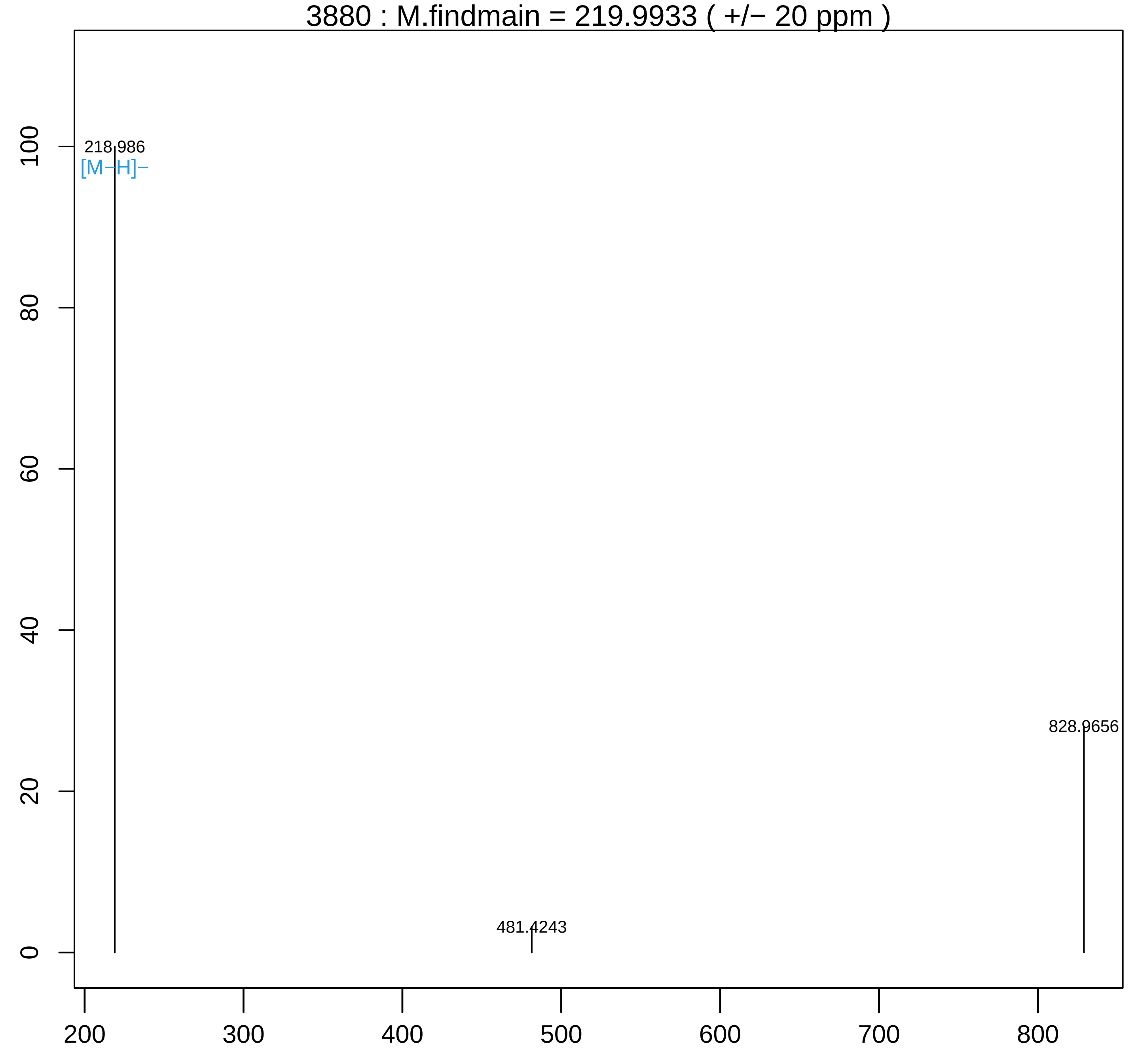
3880 : M.findmain = 219.9933 (+/− 20 ppm)
218.986
[M−H]−
481.4243
828.9656
0
20
40
60
80
100
200
300
400
500
600
700
800

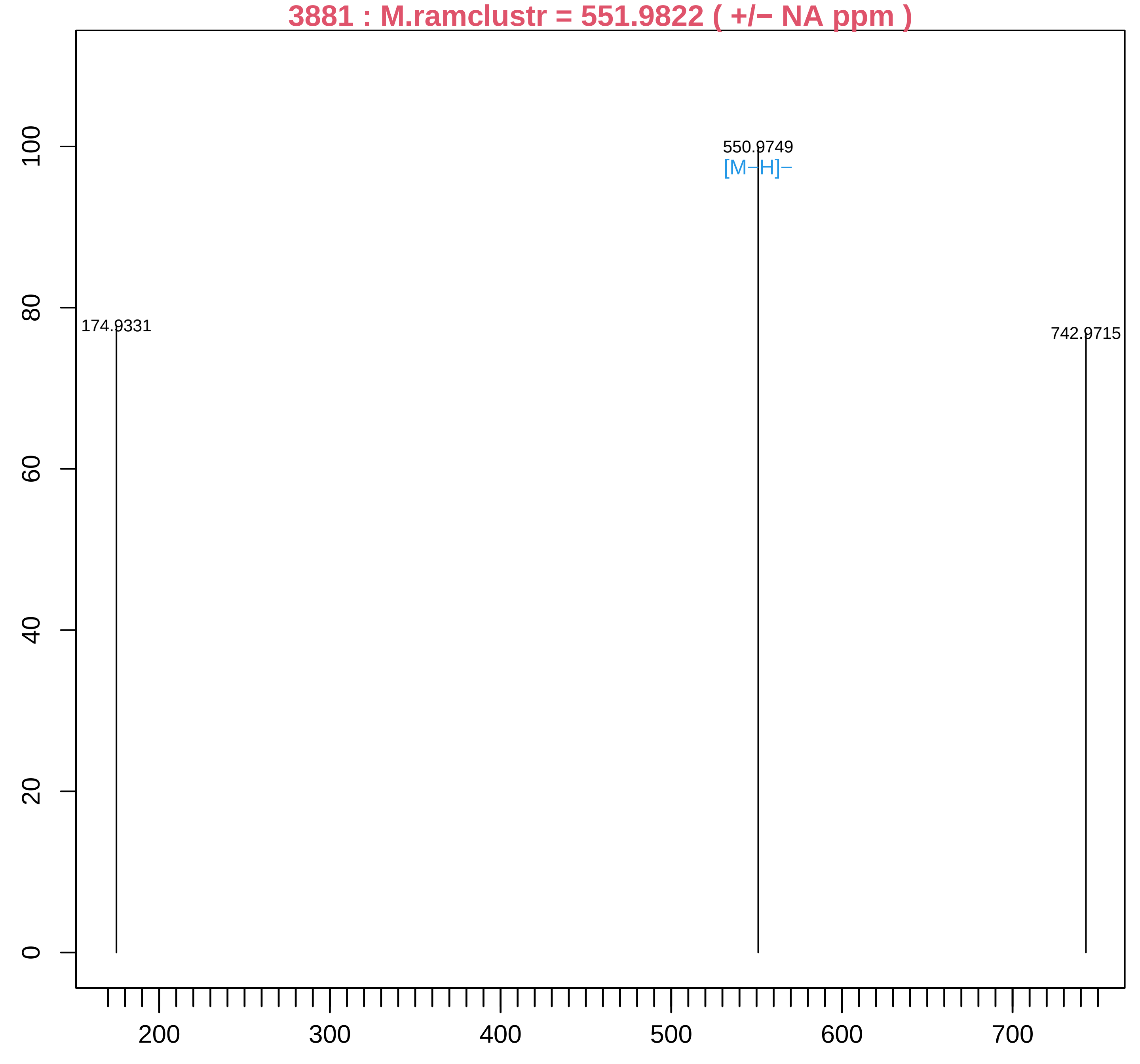
3881 : M.ramclustr = 551.9822 (+/− NA ppm)
550.9749
[M−H]−
174.9331
742.9715
0
20
40
60
80
100
200
300
400
500
600
700
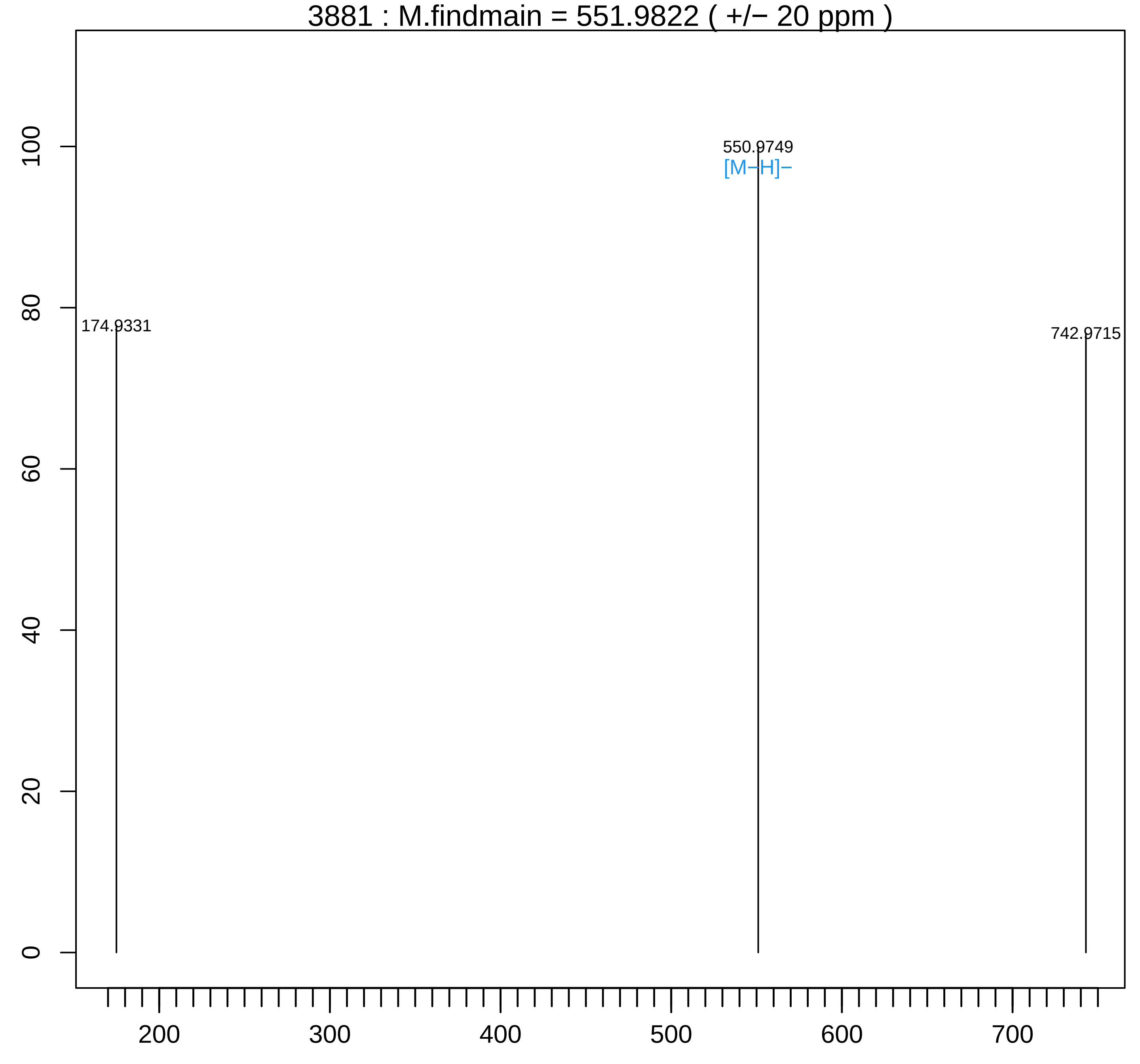
3881 : M.findmain = 551.9822 (+/− 20 ppm)
550.9749
[M−H]−
174.9331
742.9715
0
20
40
60
80
100
200
300
400
500
600
700

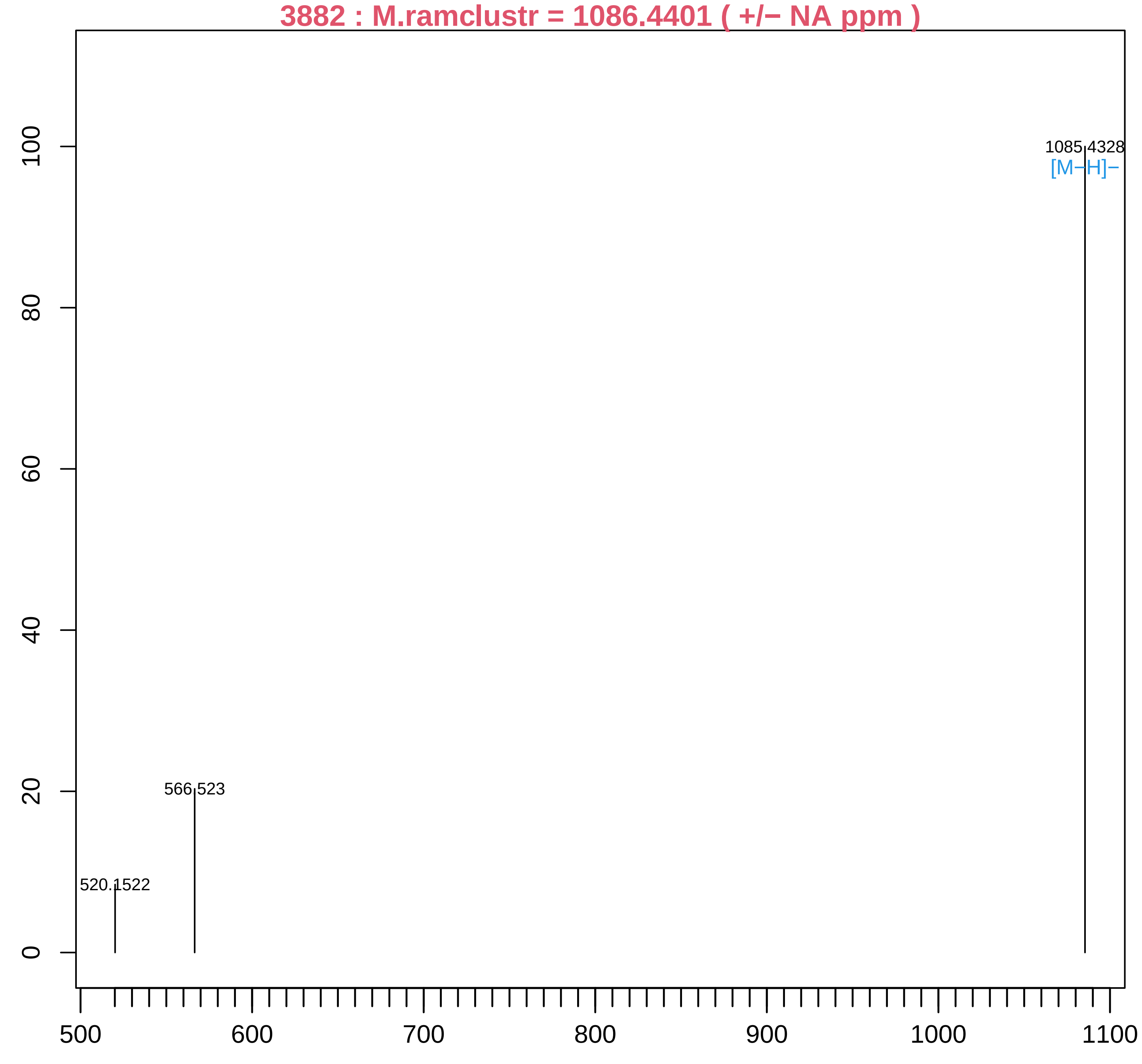
3882 : M.ramclustr = 1086.4401 (+/− NA ppm)
1085.4328
[M−H]−
566.523
520.1522
0
20
40
60
80
100
500
600
700
800
900
1000
1100
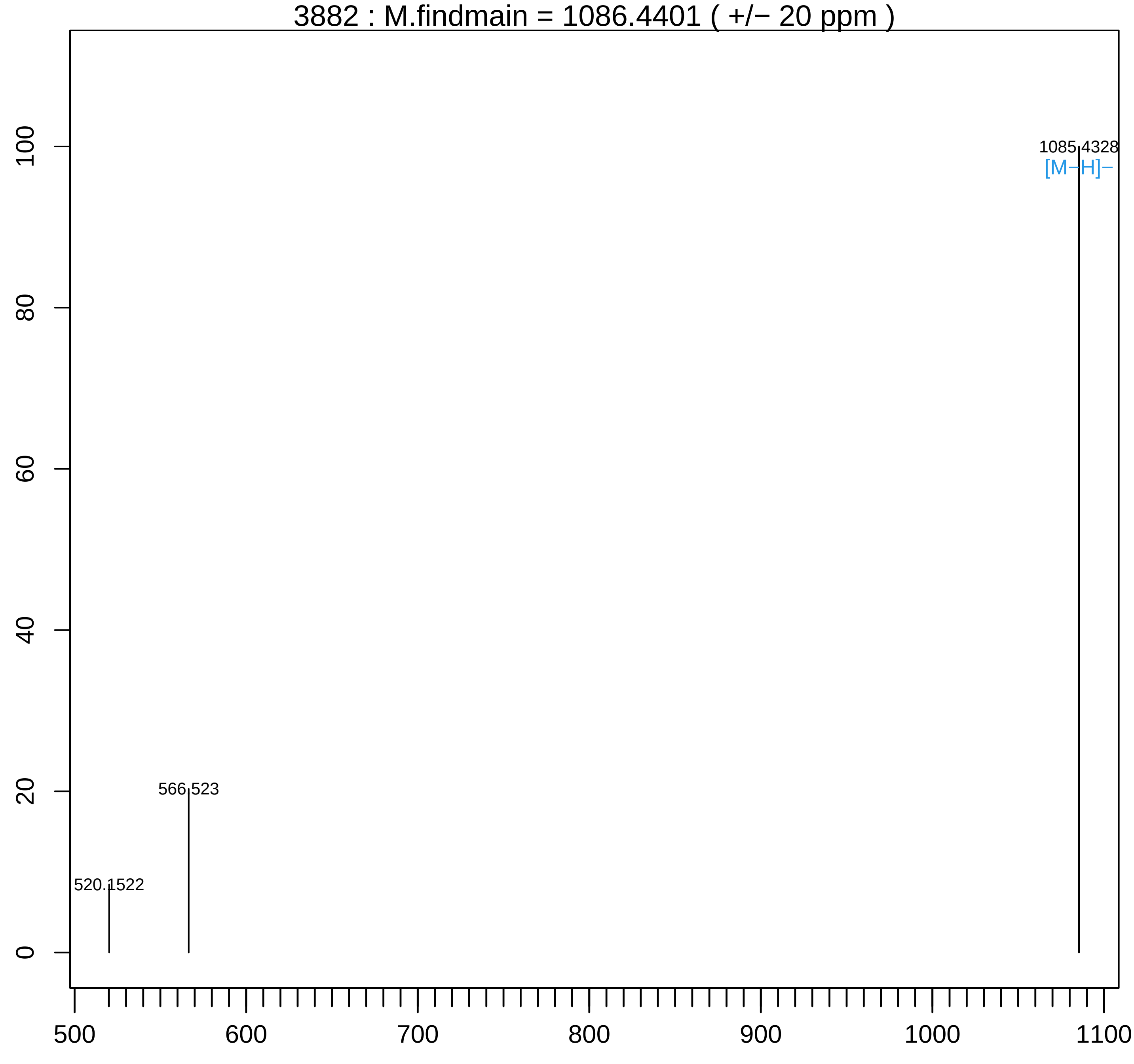
3882 : M.findmain = 1086.4401 (+/− 20 ppm)
1085.4328
[M−H]−
566.523
520.1522
0
20
40
60
80
100
500
600
700
800
900
1000
1100

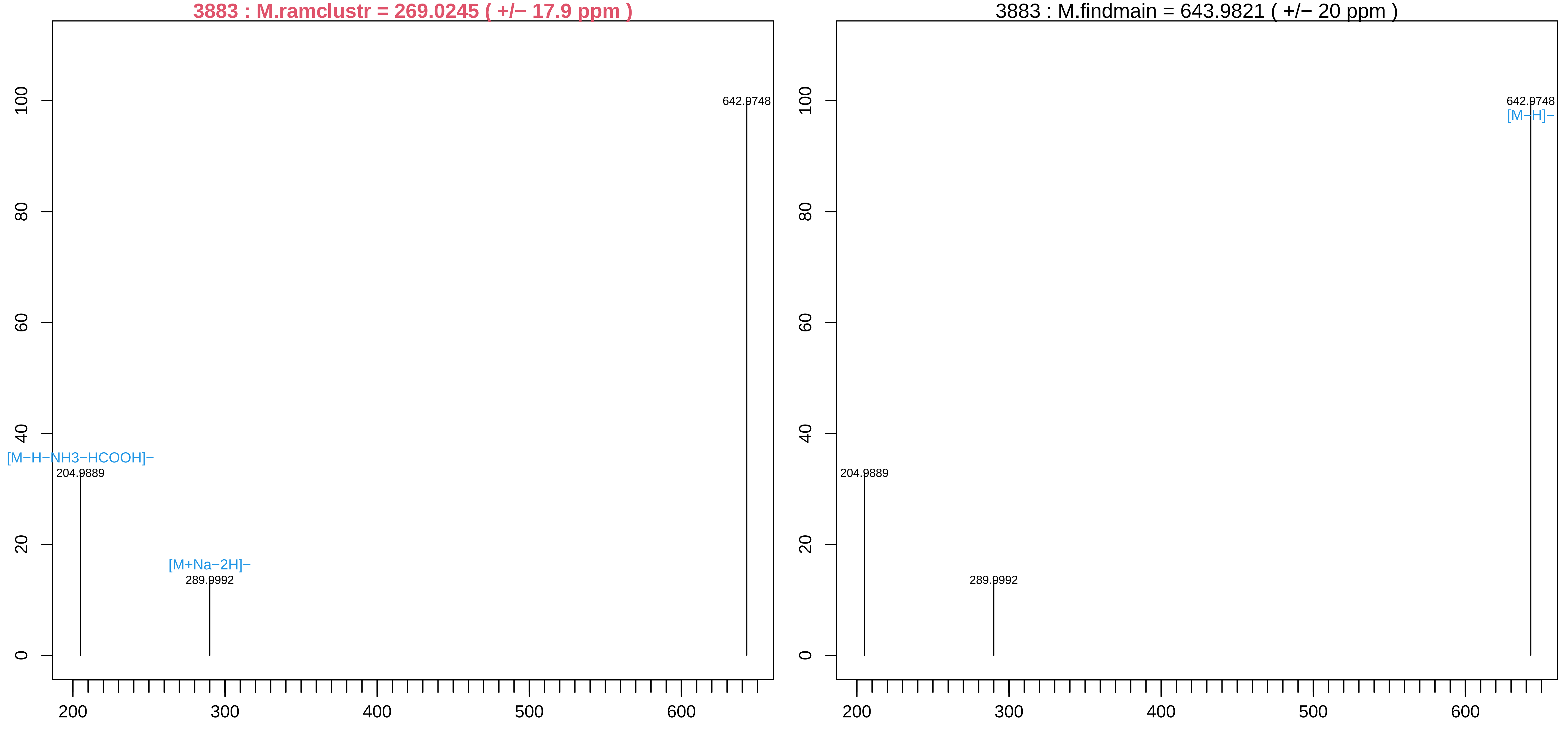
3883 : M.ramclustr = 269.0245 (+/− 17.9 ppm)
642.9748
[M−H−NH3−HCOOH]−
204.9889
[M+Na−2H]−
289.9992
3883 : M.findmain = 643.9821 (+/− 20 ppm)
642.9748
[M−H]−
204.9889
289.9992
0
20
40
60
80
100
200
300
400
500
600

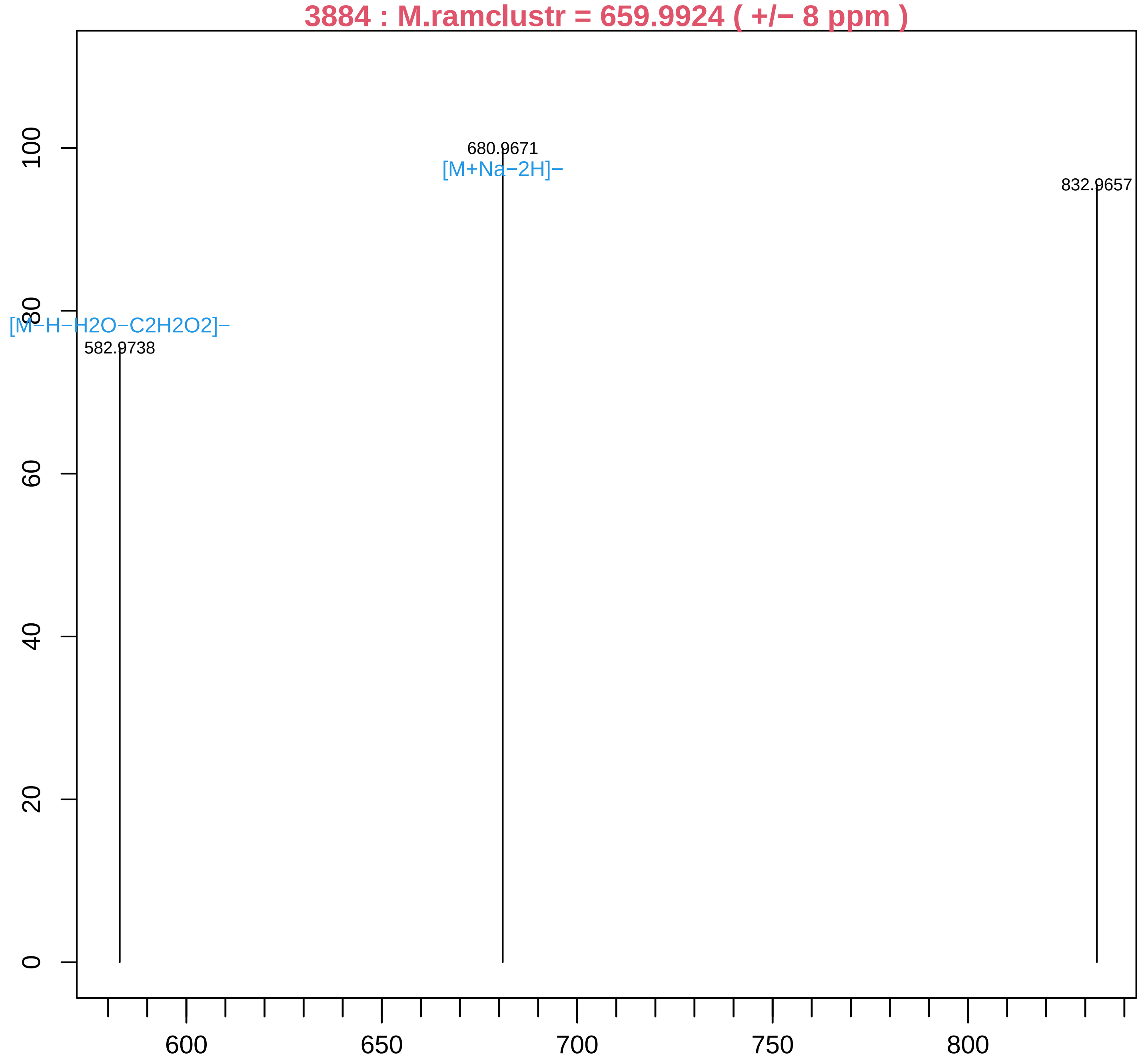
3884 : M.ramclustr = 659.9924 (+/− 8 ppm)
680.9671
[M+Na−2H]−
832.9657
[M−H−H2O−C2H2O2]−
582.9738
0
20
40
60
80
100
600
650
700
750
800

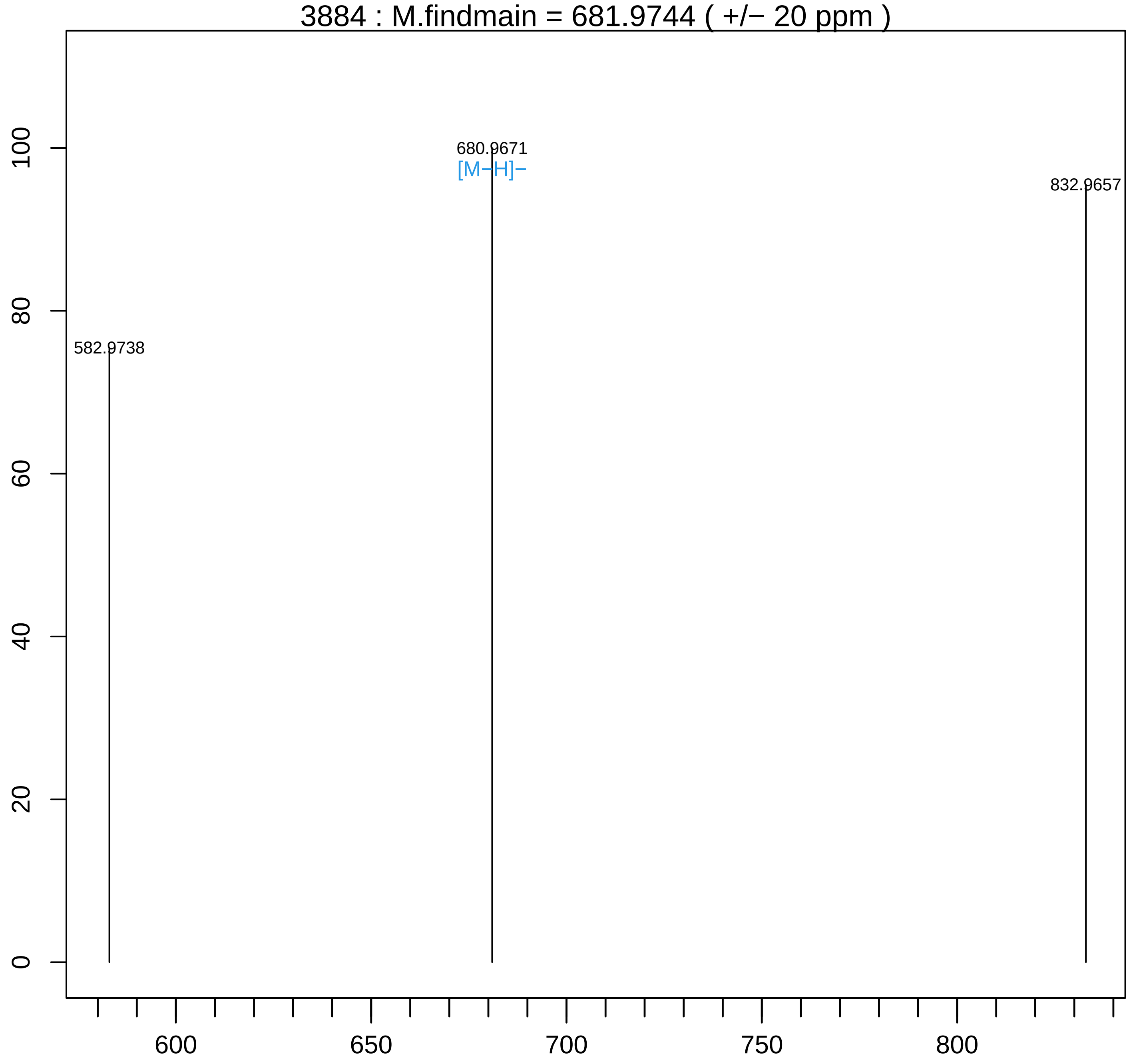
3884 : M.findmain = 681.9744 (+/− 20 ppm)
680.9671
[M−H]−
832.9657
582.9738
0
20
40
60
80
100
600
650
700
750
800

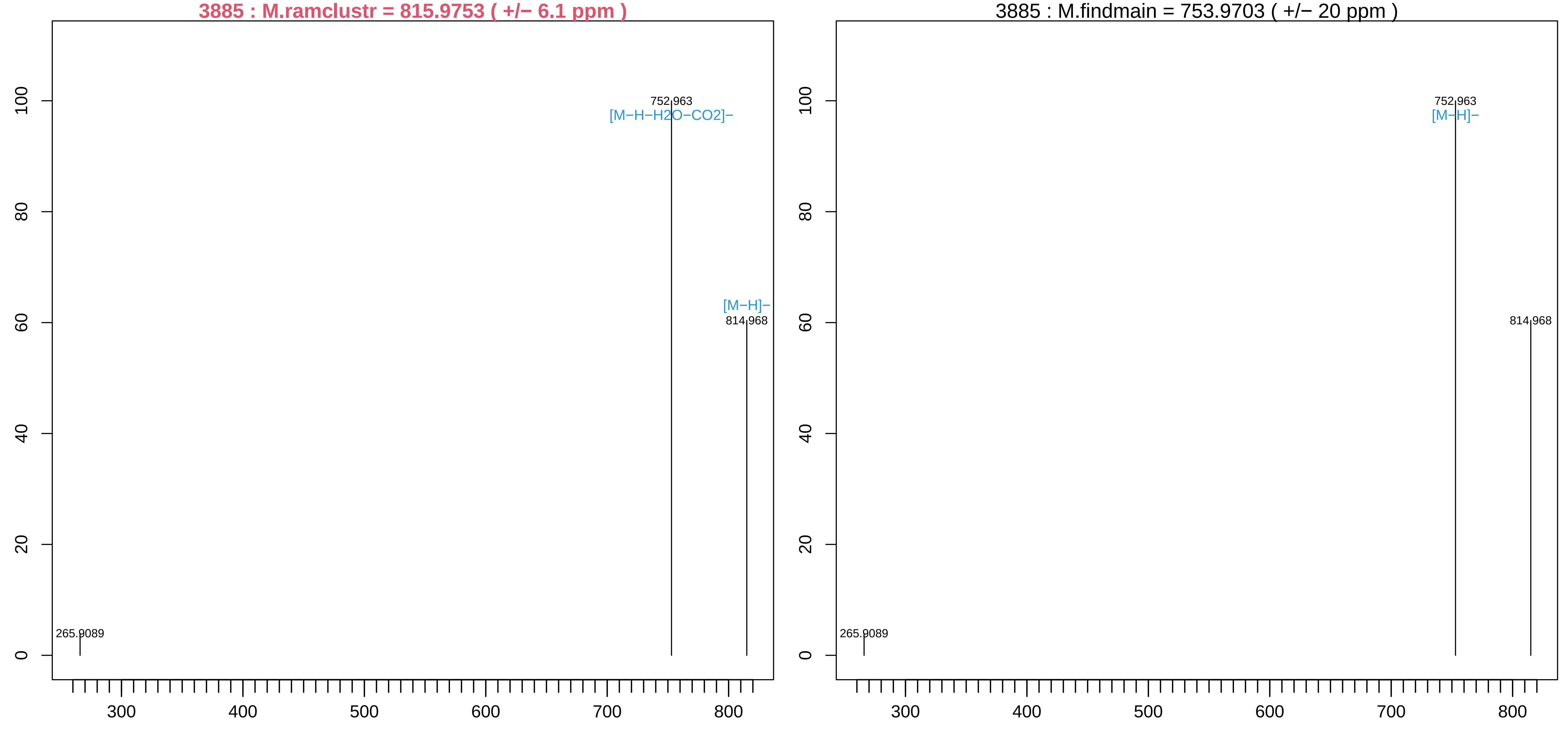

3885 : M.ramclustr = 815.9753 (+/− 6.1 ppm)
752.963
[M−H−H2O−CO2]−
[M−H]−
814.968
265.9089
0
20
40
60
80
100
300
400
500
600
700
800
3885 : M.findmain = 753.9703 (+/− 20 ppm)
752.963
[M−H]−
814.968
265.9089
0
20
40
60
80
100
300
400
500
600
700
800

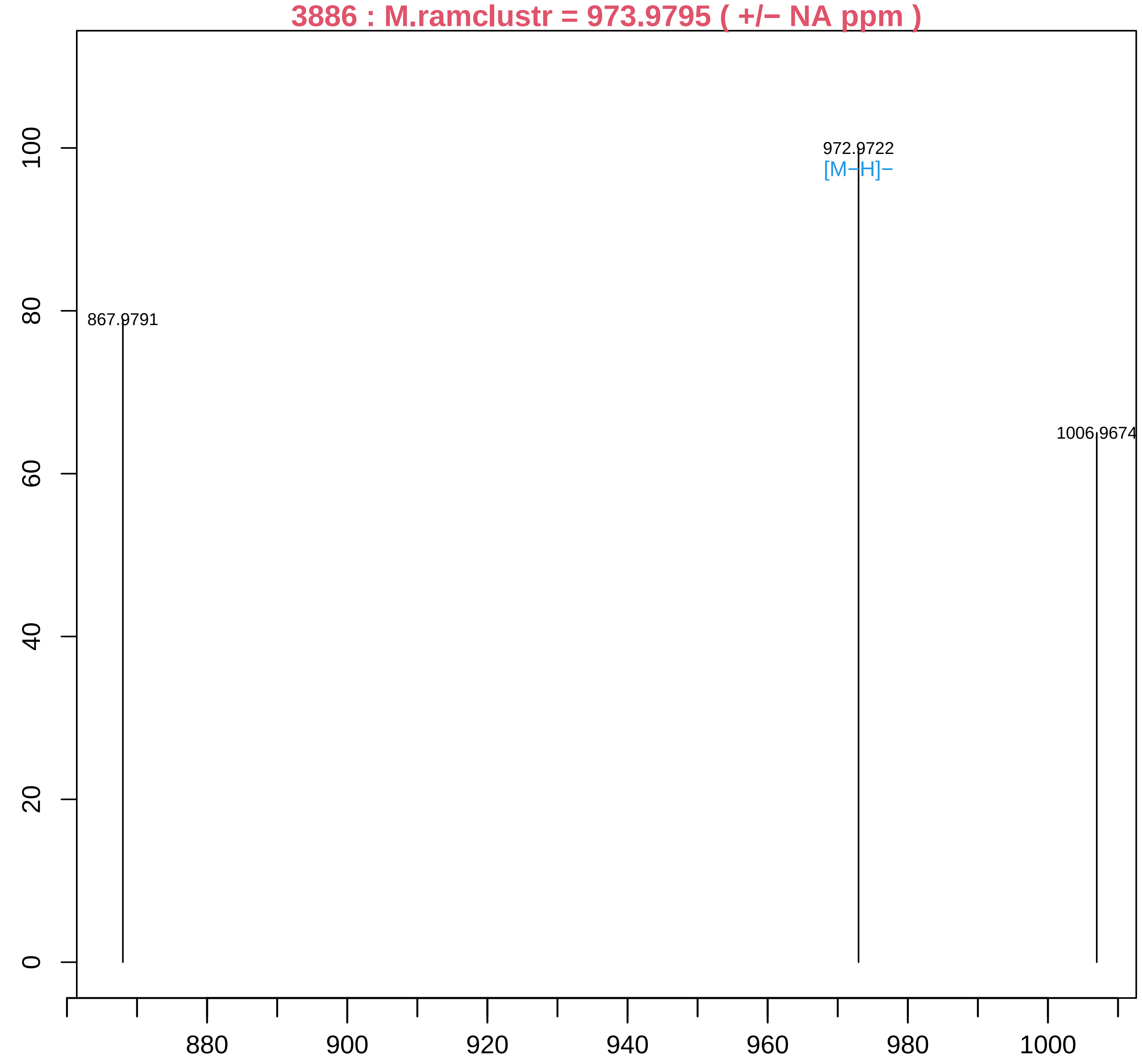
3886 : M.ramclustr = 973.9795 (+/− NA ppm)
972.9722
[M−H]−
867.9791
1006.9674
0
20
40
60
80
100
880
900
920
940
960
980
1000

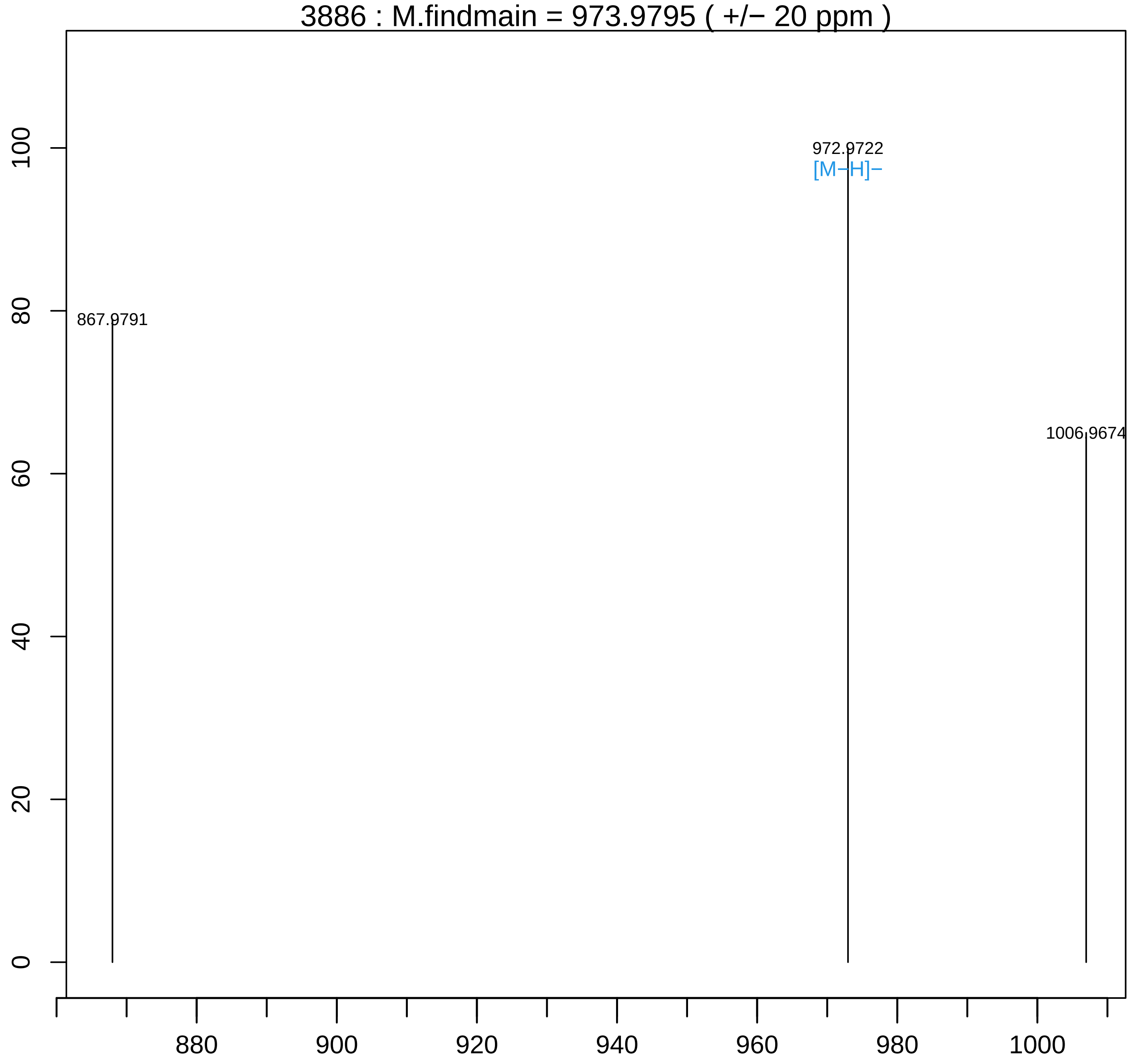
3886 : M.findmain = 973.9795 (+/− 20 ppm)
972.9722
[M−H]−
867.9791
1006.9674
0
20
40
60
80
100
880
900
920
940
960
980
1000

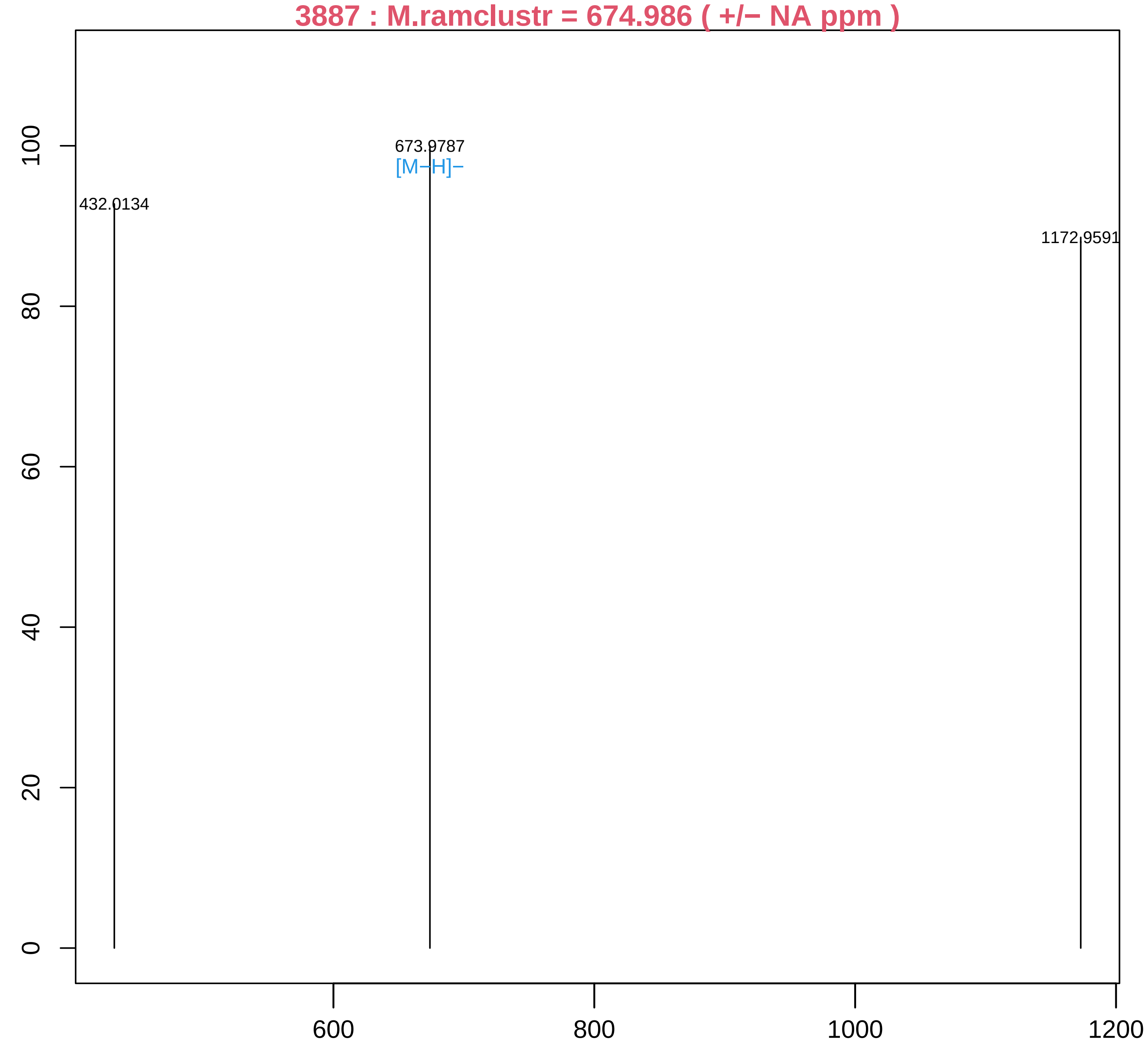
3887 : M.ramclustr = 674.986 (+/− NA ppm)
432.0134
673.9787
[M−H]−
1172.9591
0
20
40
60
80
100
600
800
1000
1200

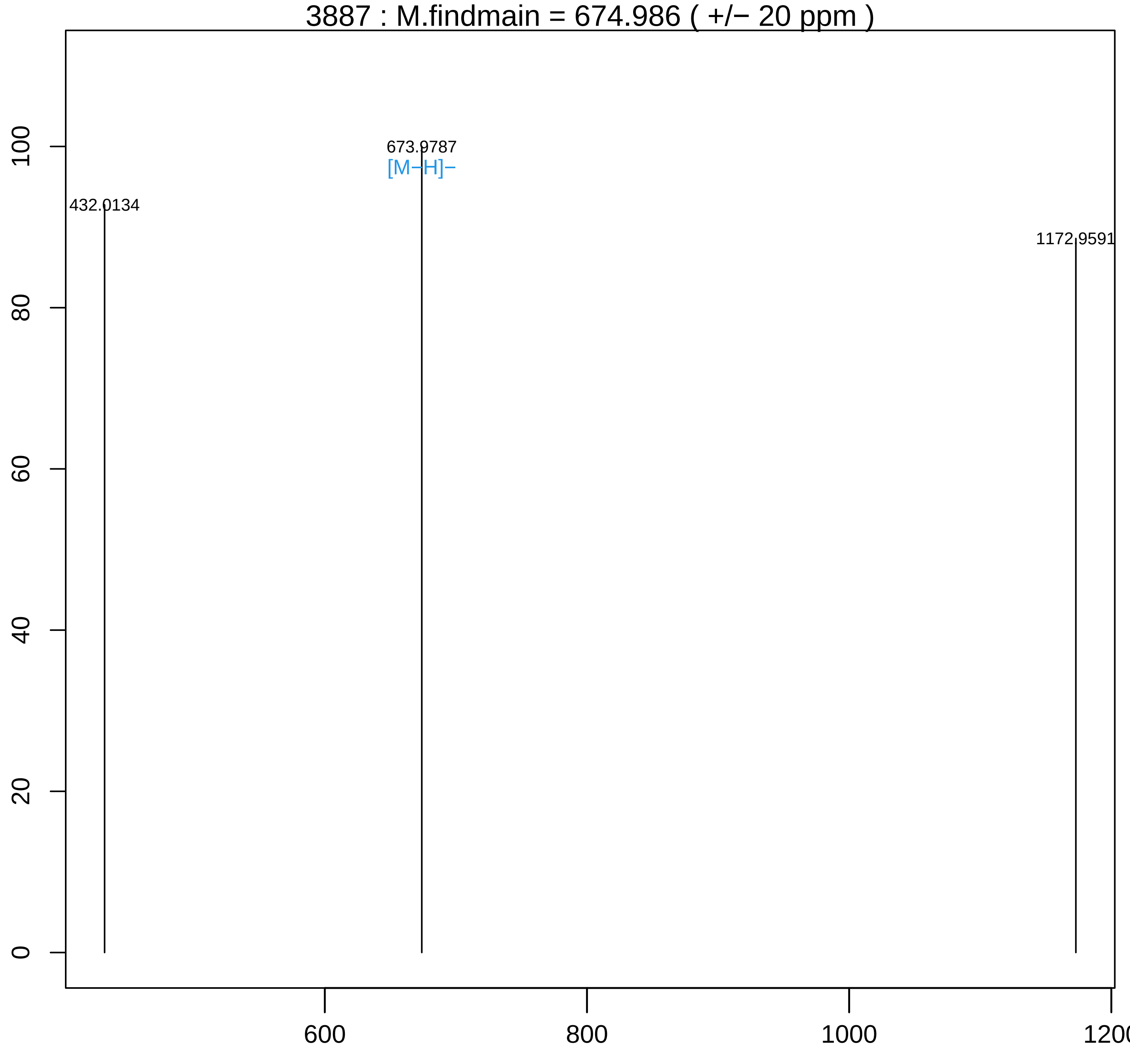
3887 : M.findmain = 674.986 (+/− 20 ppm)
432.0134
673.9787
[M−H]−
1172.9591
0
20
40
60
80
100
600
800
1000

3888 : M.ramclustr = 335.9842 (+/− 4 ppm)

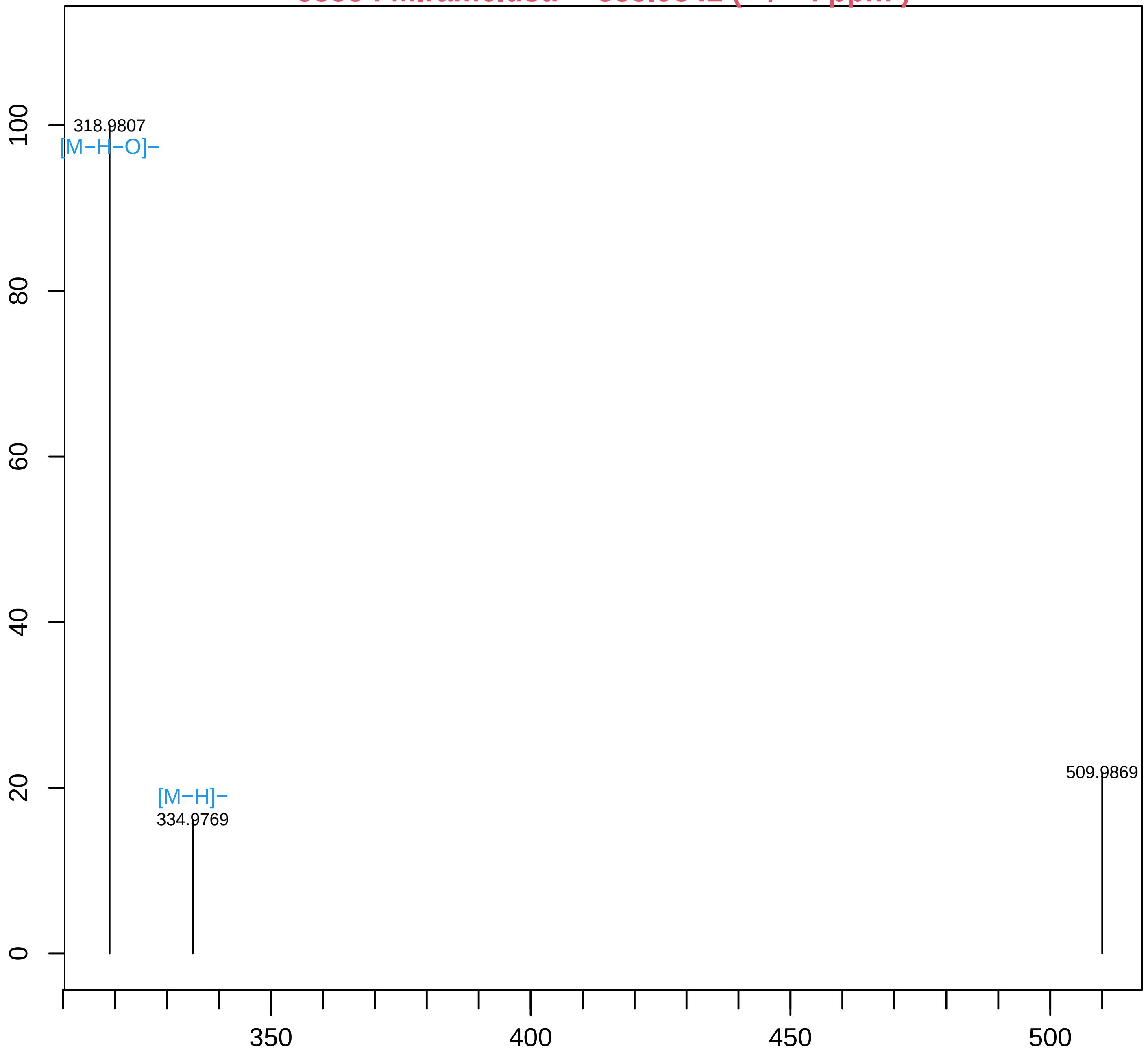

3888 : M.findmain = 319.988 (+/− 20 ppm)

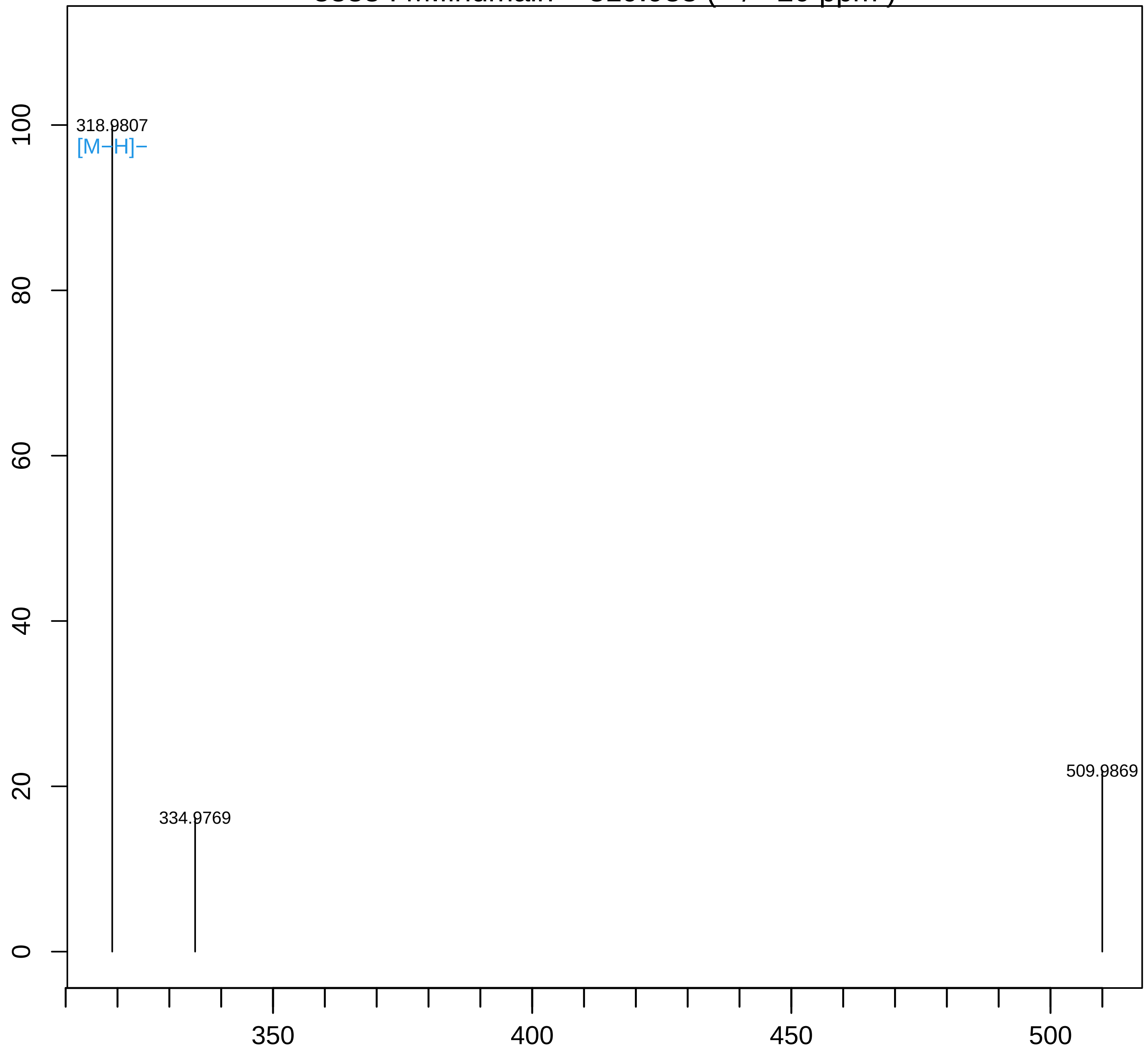

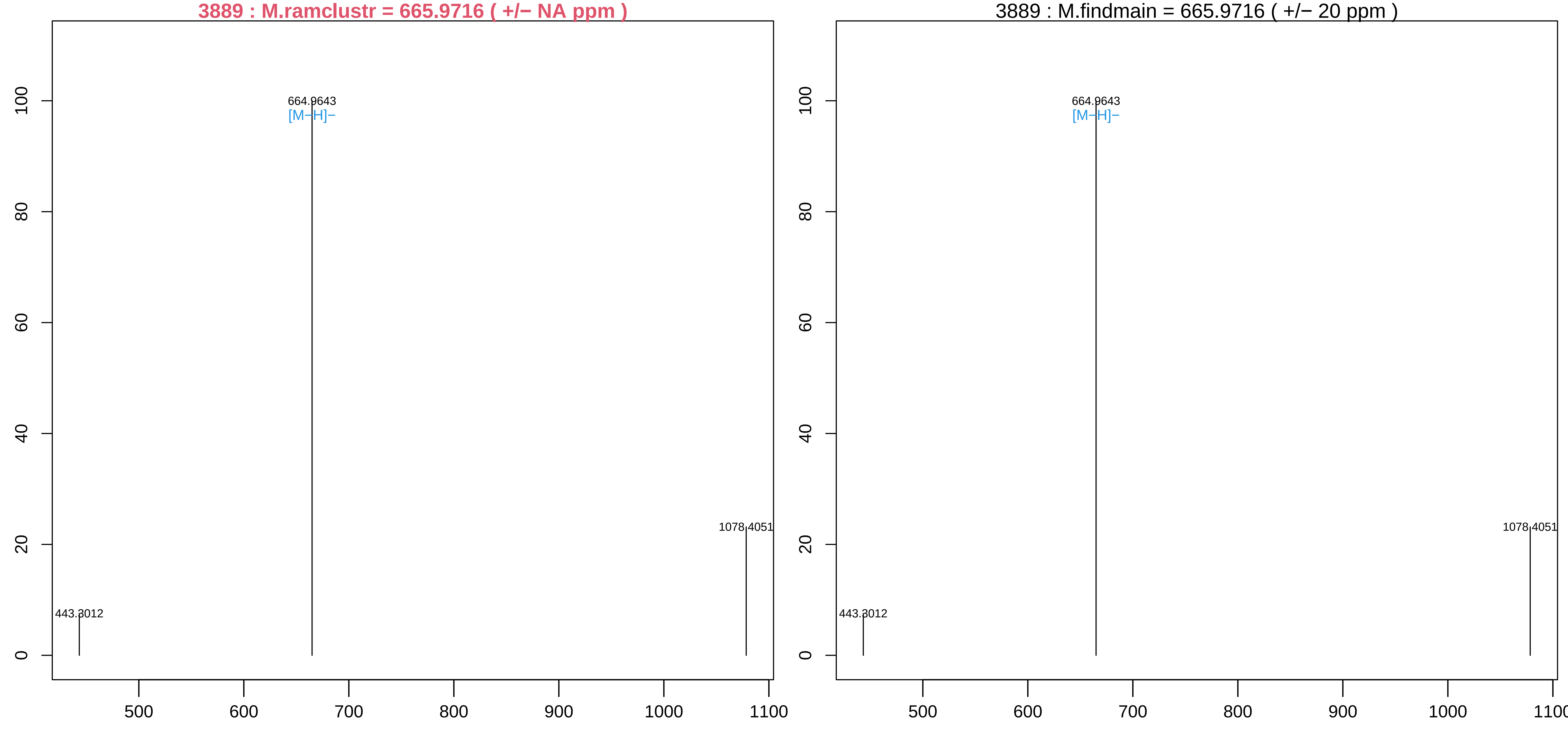
3889 : M.ramclustr = 665.9716 (+/− NA ppm)
664.9643
[M−H]−
1078.4051
443.3012
3889 : M.findmain = 665.9716 (+/− 20 ppm)
664.9643
[M−H]−
1078.4051
443.3012

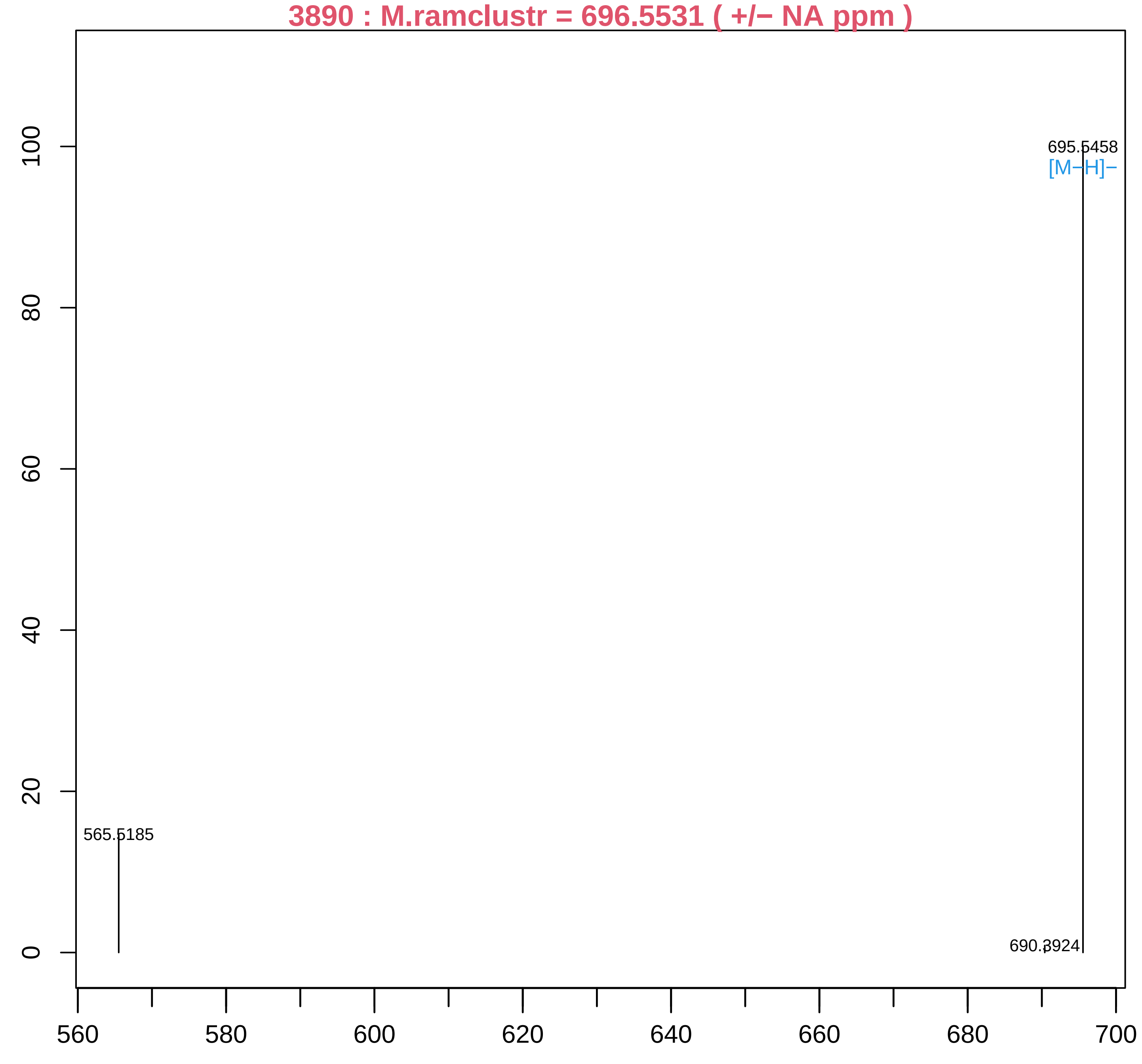
3890 : M.ramclustr = 696.5531 (+/− NA ppm)
695.5458
[M−H]−
565.5185
690.3924
0
20
40
60
80
100
560
580
600
620
640
660
680
700

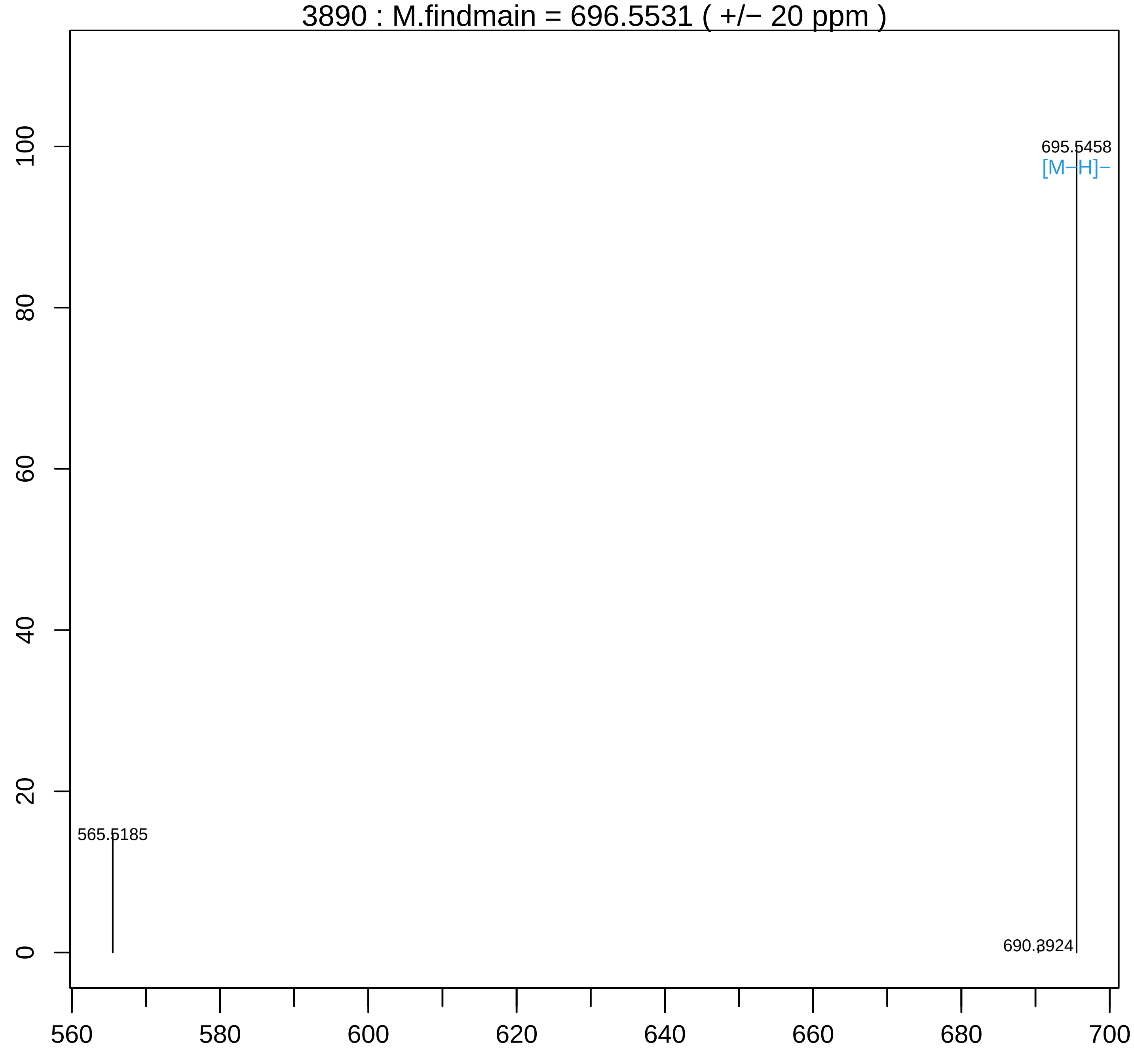
3890 : M.findmain = 696.5531 (+/− 20 ppm)
695.5458
[M−H]−
565.5185
690.3924
0
20
40
60
80
100
560
580
600
620
640
660
680
700

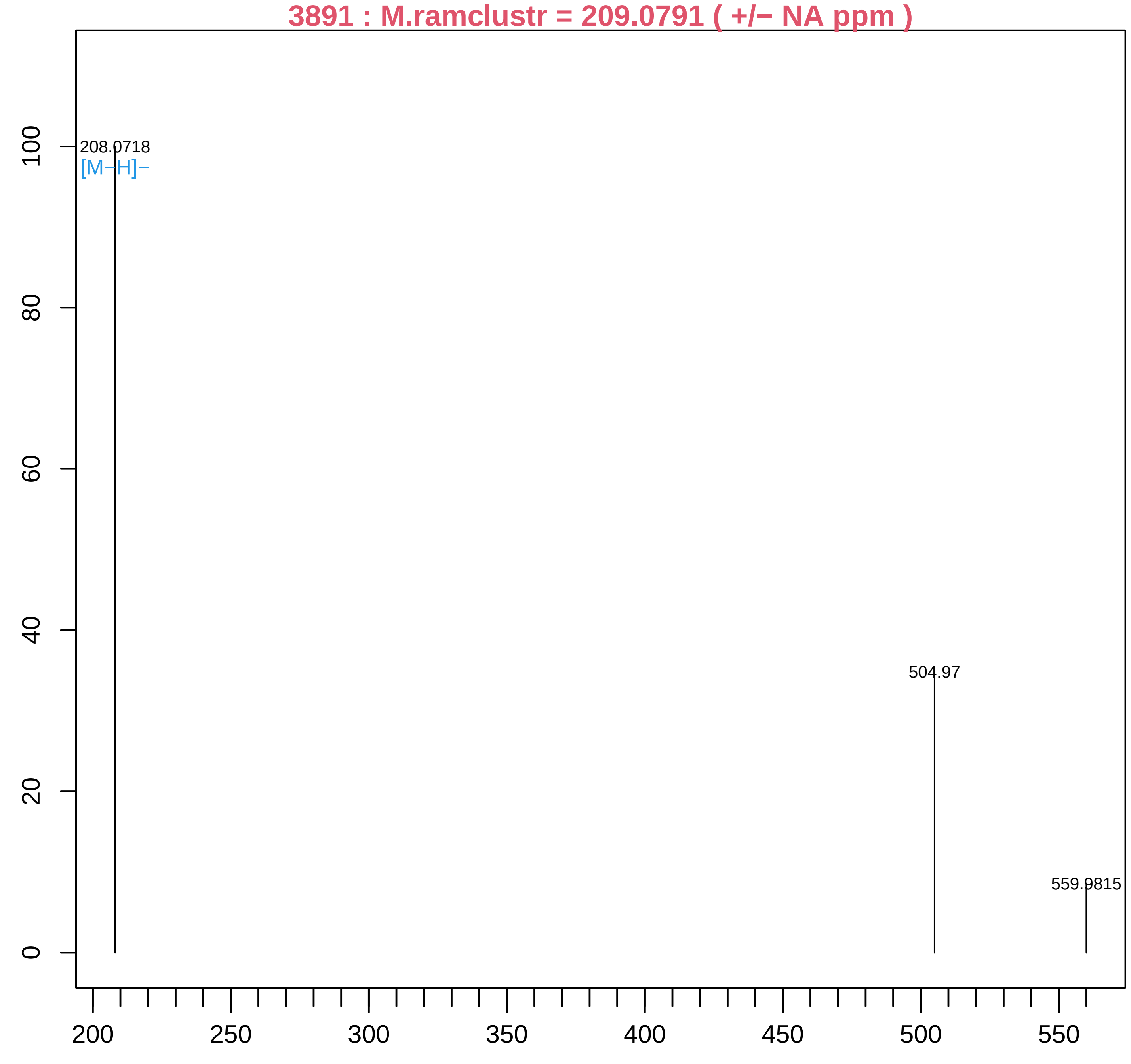
3891 : M.ramclustr = 209.0791 (+/− NA ppm)
208.0718
[M−H]−
504.97
559.9815
0
20
40
60
80
100
200
250
300
350
400
450
500
550

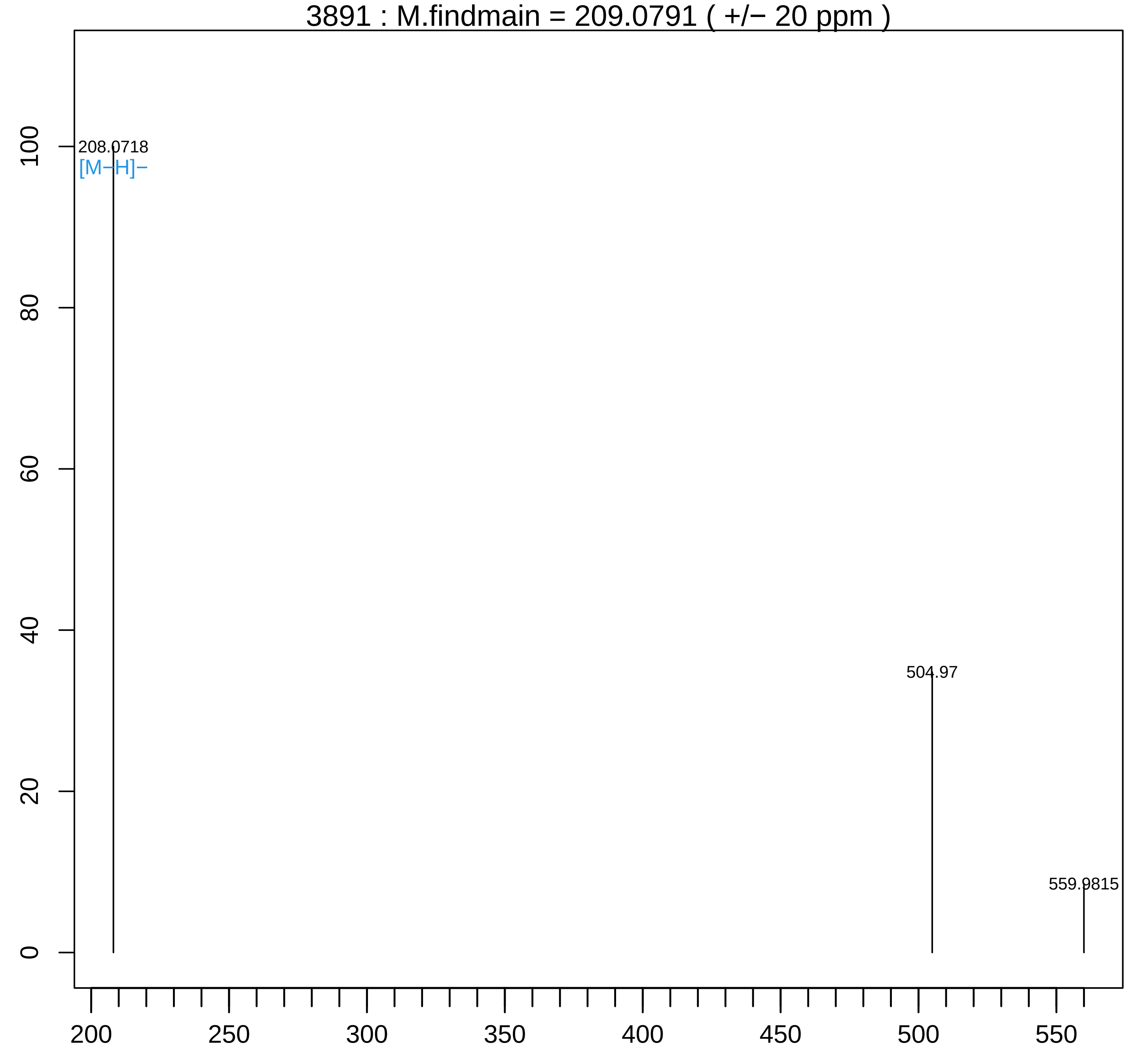
3891 : M.findmain = 209.0791 (+/− 20 ppm)
208.0718
[M−H]−
504.97
559.9815
0
20
40
60
80
100
200
250
300
350
400
450
500
550

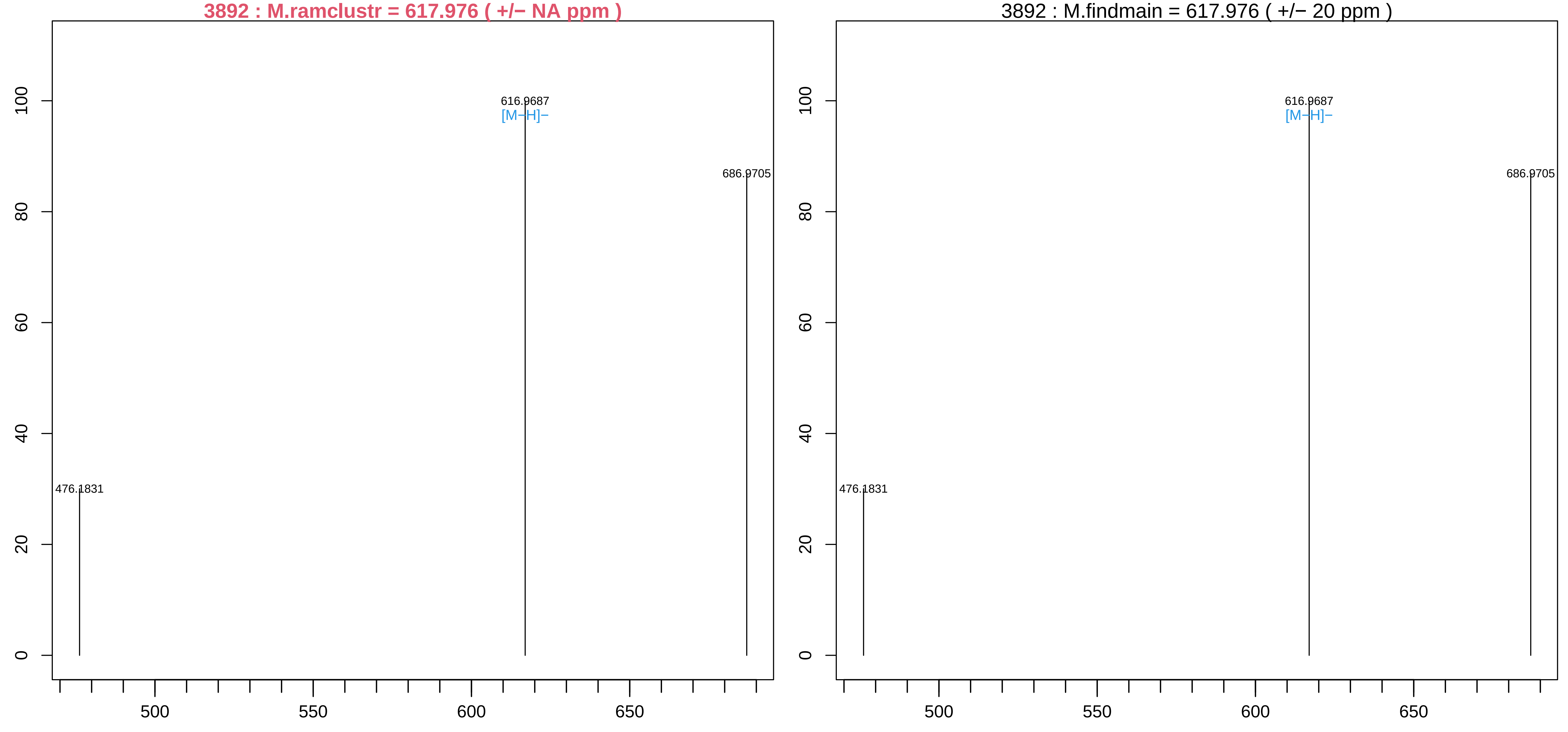
3892 : M.ramclustr = 617.976 (+/− NA ppm)
616.9687
[M−H]−
686.9705
476.1831
0
20
40
60
80
100
500
550
600
650
3892 : M.findmain = 617.976 (+/− 20 ppm)
616.9687
[M−H]−
686.9705
476.1831
0
20
40
60
80
100
500
550
600
650

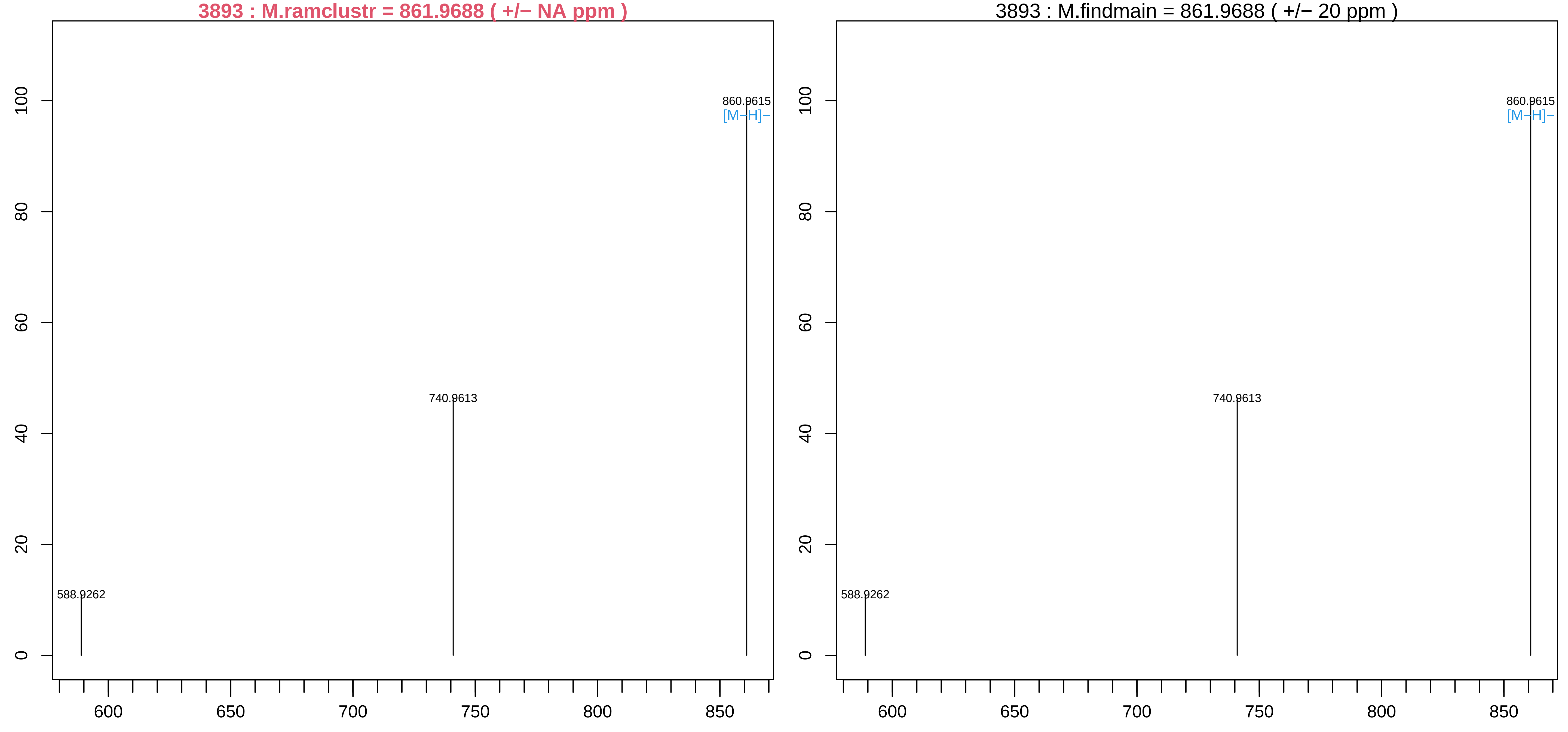
3893 : M.ramclustr = 861.9688 (+/− NA ppm)
860.9615
[M−H]−
740.9613
588.9262
3893 : M.findmain = 861.9688 (+/− 20 ppm)
860.9615
[M−H]−
740.9613
588.9262

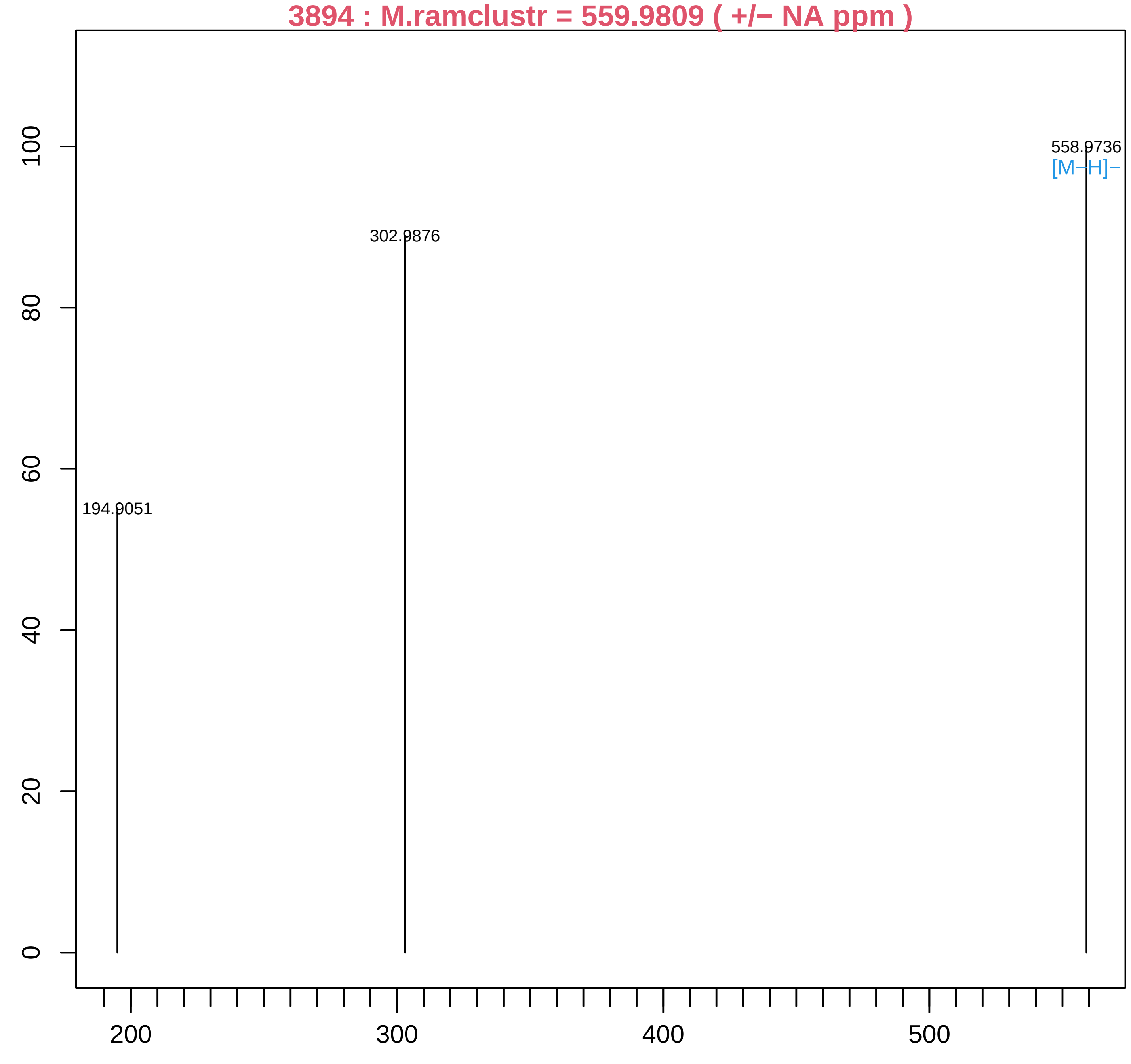

3894 : M.ramclustr = 559.9809 (+/− NA ppm)
558.9736
[M−H]−
302.9876
194.9051
0
20
40
60
80
100
200
300
400
500

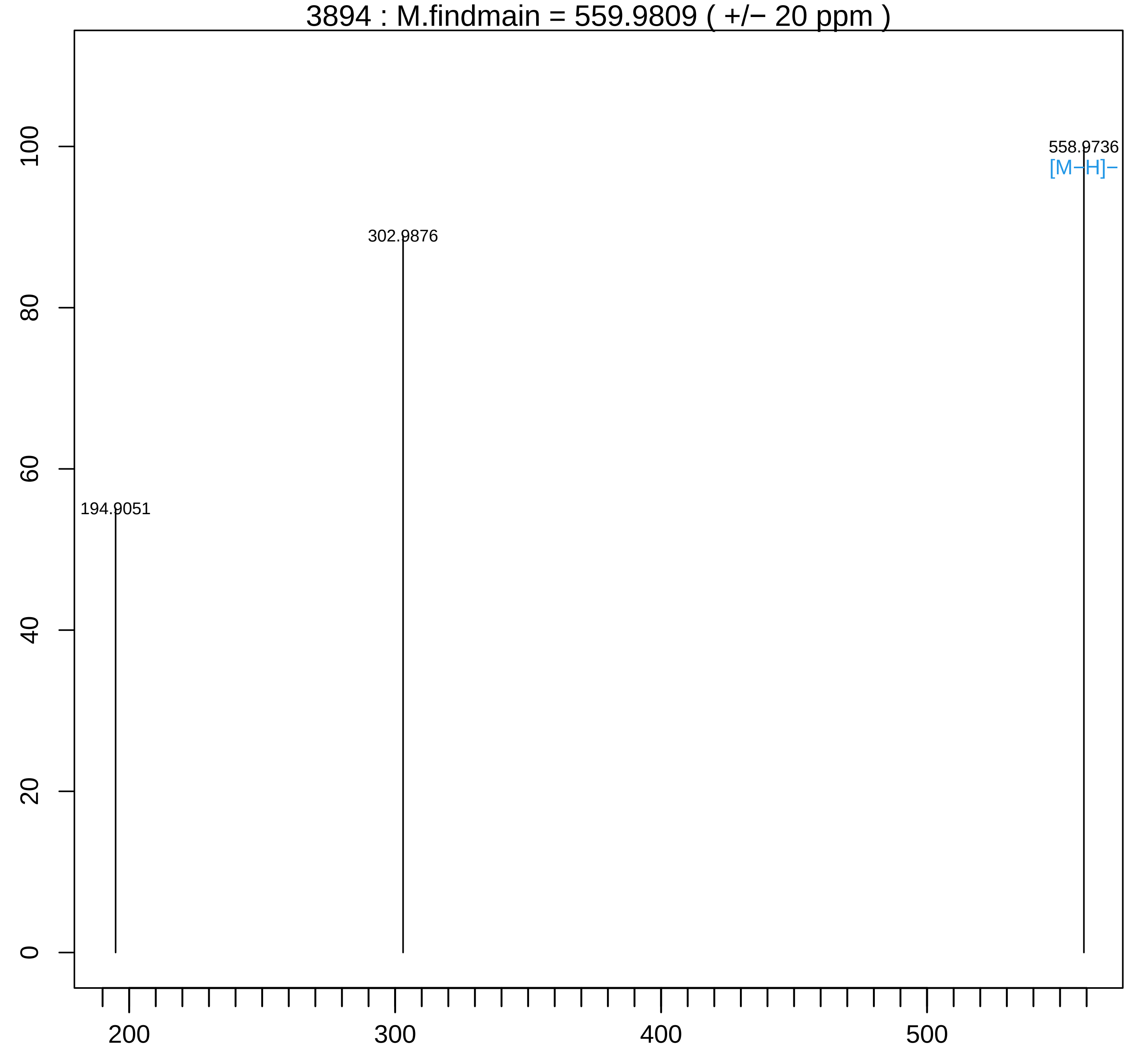

3894 : M.findmain = 559.9809 (+/− 20 ppm)
558.9736
[M−H]−
302.9876
194.9051
0
20
40
60
80
100
200
300
400
500

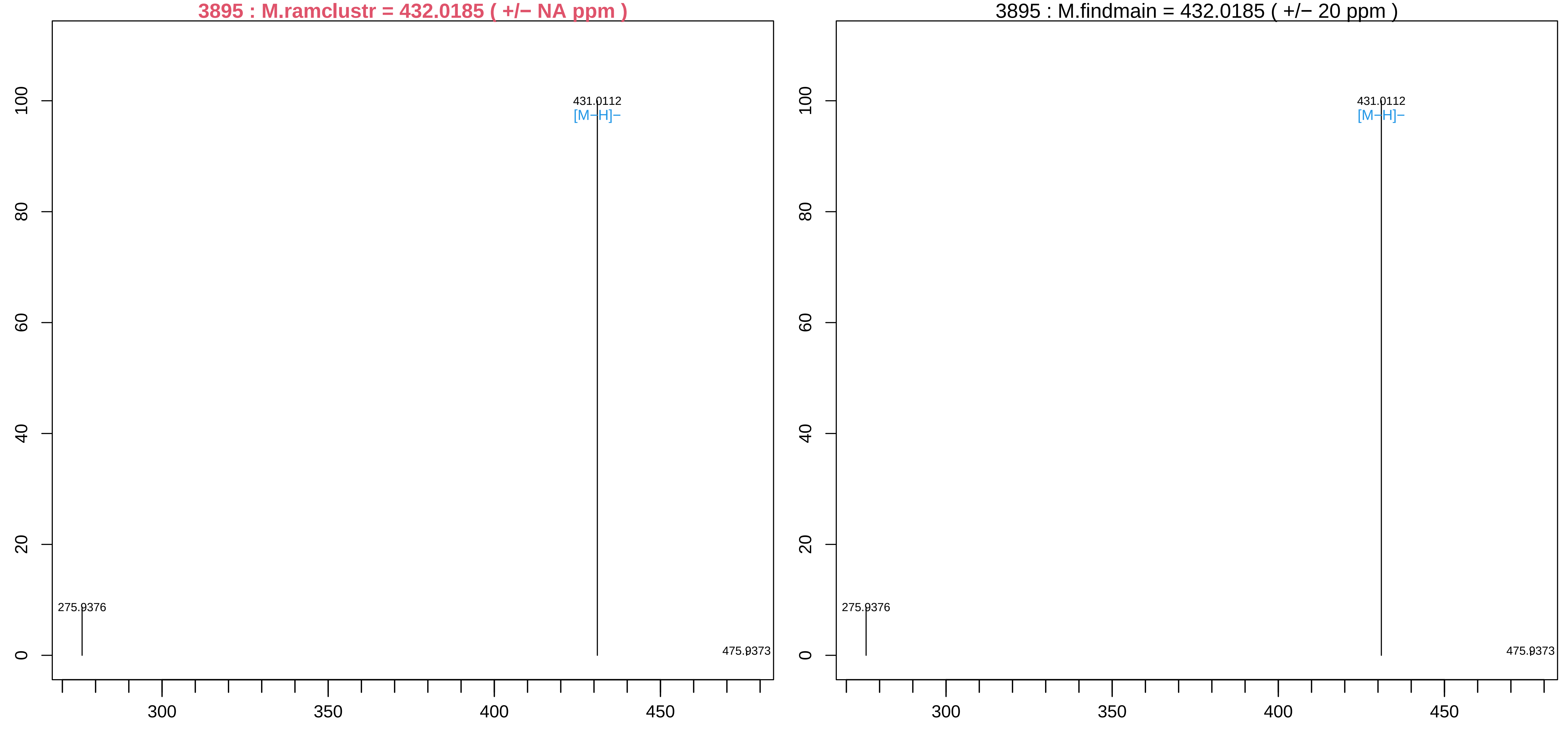

3895 : M.ramclustr = 432.0185 (+/− NA ppm)
431.0112
[M−H]−
275.9376
475.9373
3895 : M.findmain = 432.0185 (+/− 20 ppm)
431.0112
[M−H]−
275.9376
475.9373

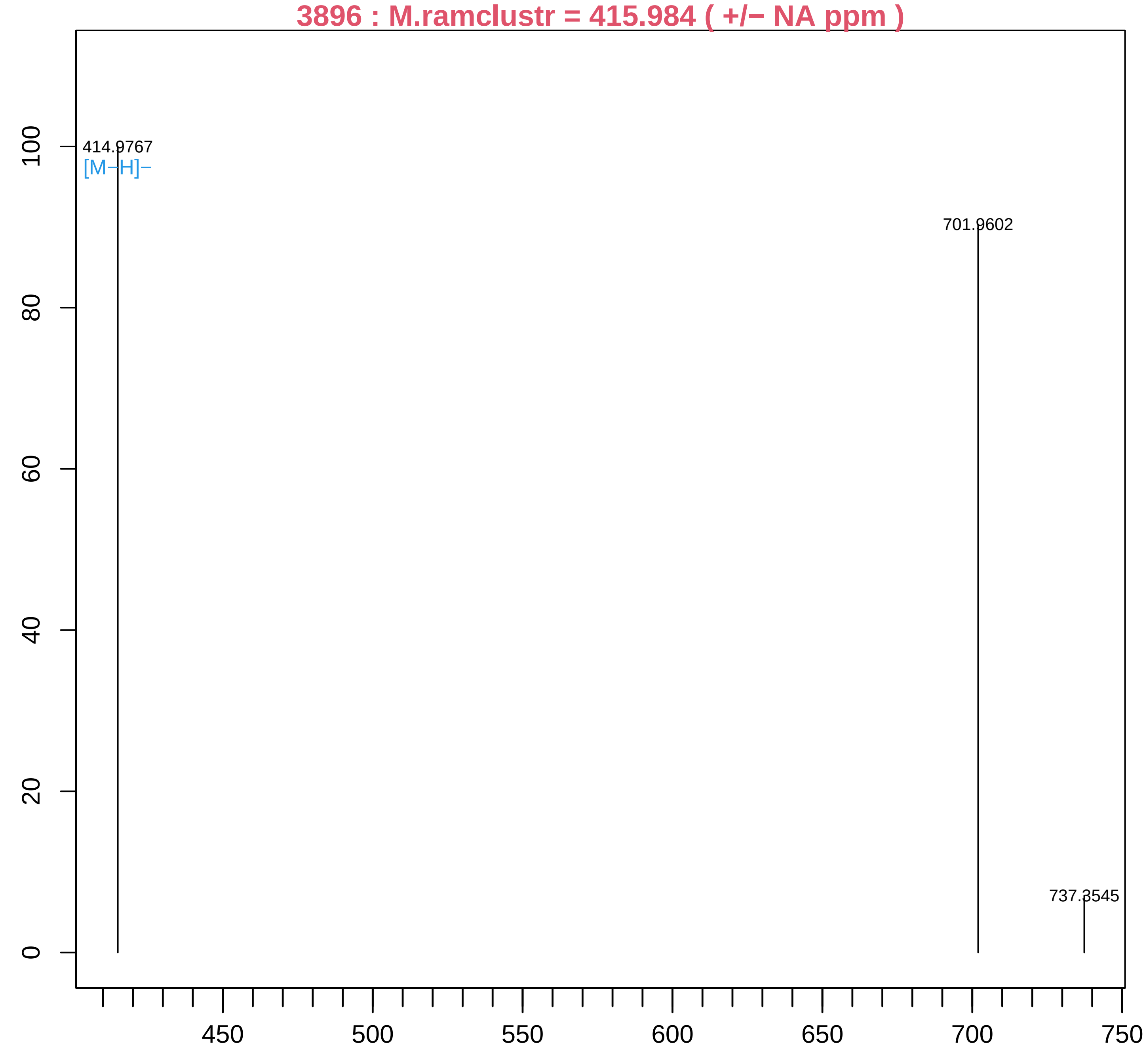
3896 : M.ramclustr = 415.984 (+/− NA ppm)
414.9767
[M−H]−
701.9602
737.3545
0
20
40
60
80
100
450
500
550
600
650
700
750

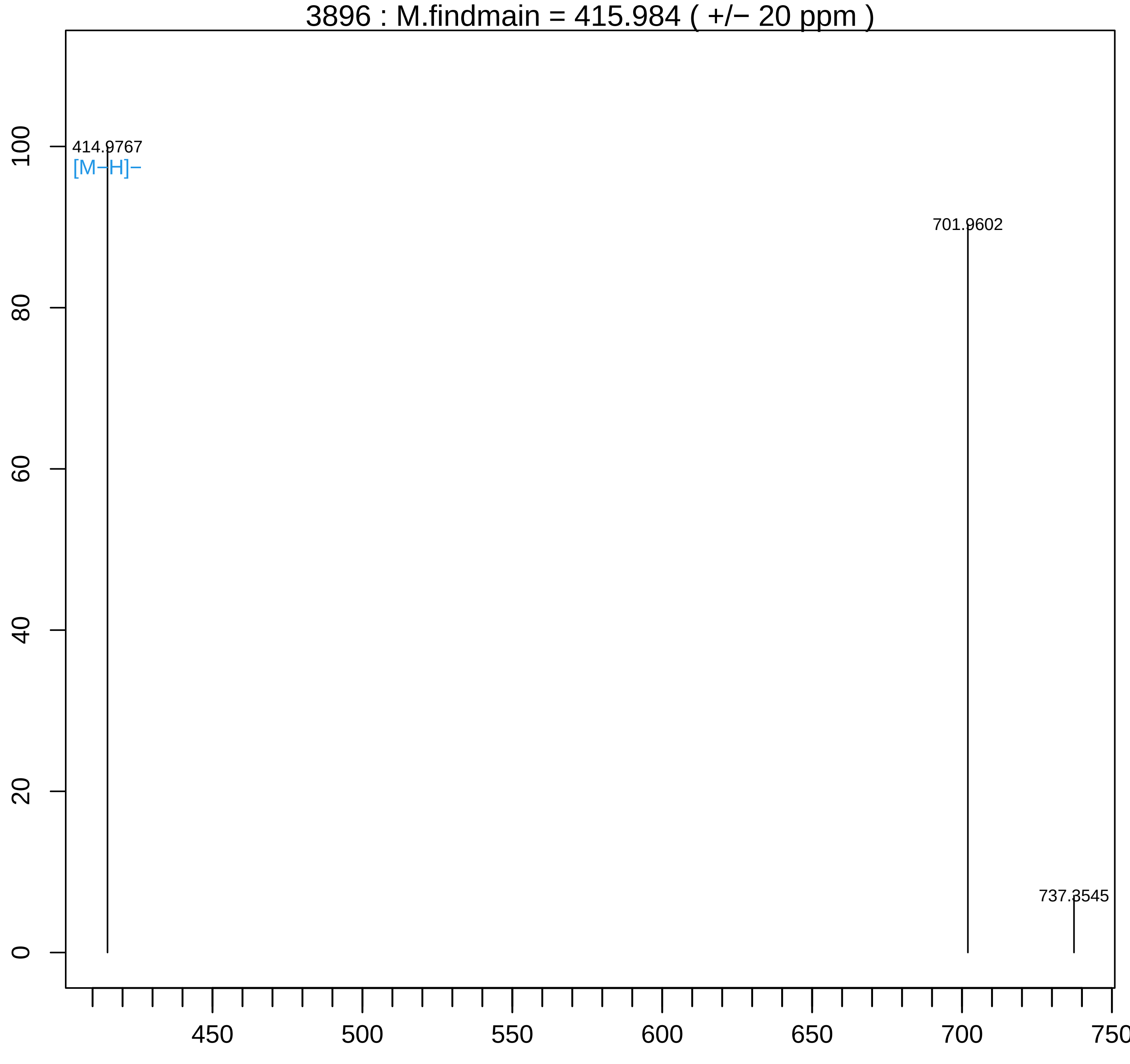
3896 : M.findmain = 415.984 (+/− 20 ppm)
414.9767
[M−H]−
701.9602
737.3545
0
20
40
60
80
100
450
500
550
600
650
700
750

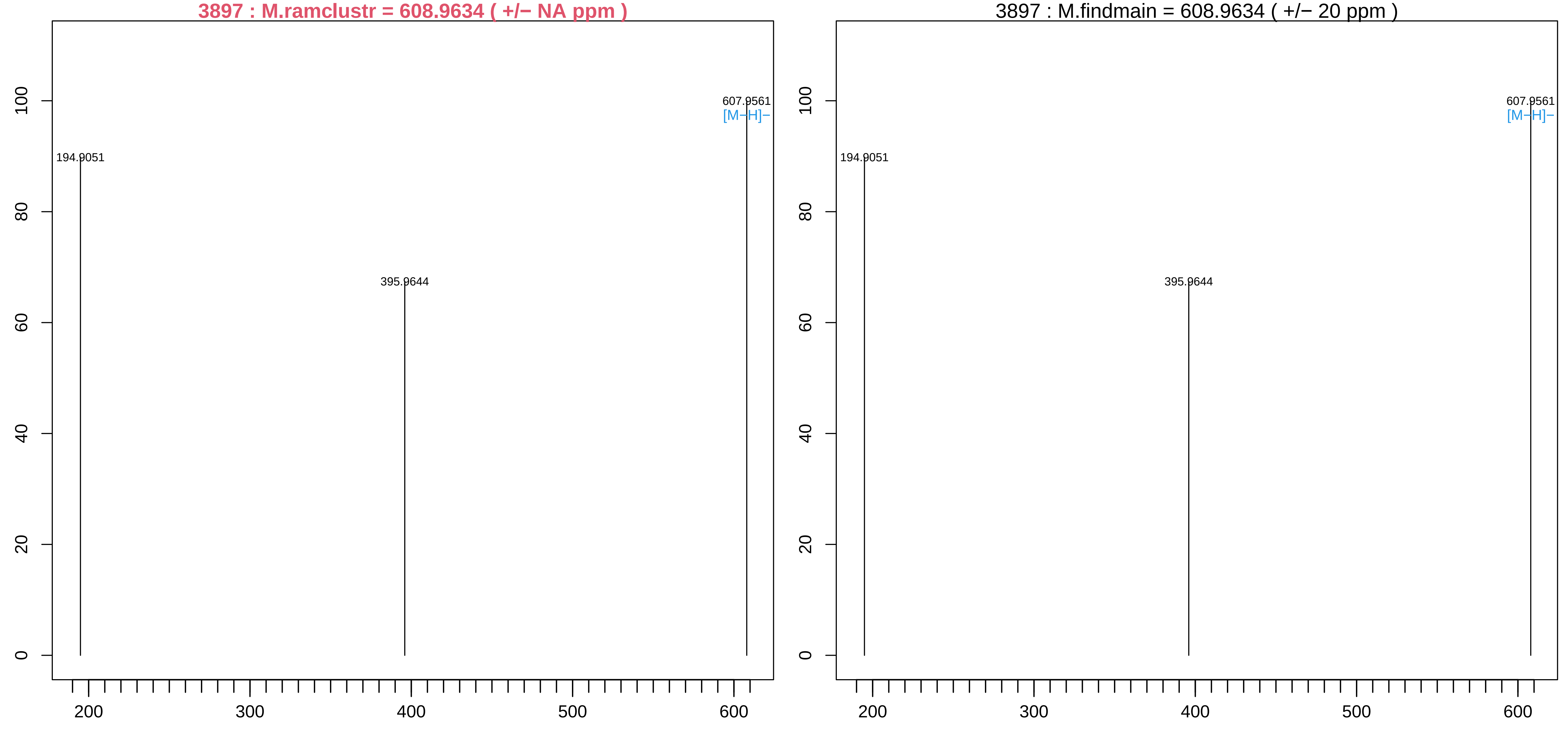
3897 : M.ramclustr = 608.9634 (+/− NA ppm)
607.9561
[M−H]−
194.9051
395.9644
0
20
40
60
80
100
200
300
400
500
600
3897 : M.findmain = 608.9634 (+/− 20 ppm)
607.9561
[M−H]−
194.9051
395.9644
0
20
40
60
80
100
200
300
400
500
600

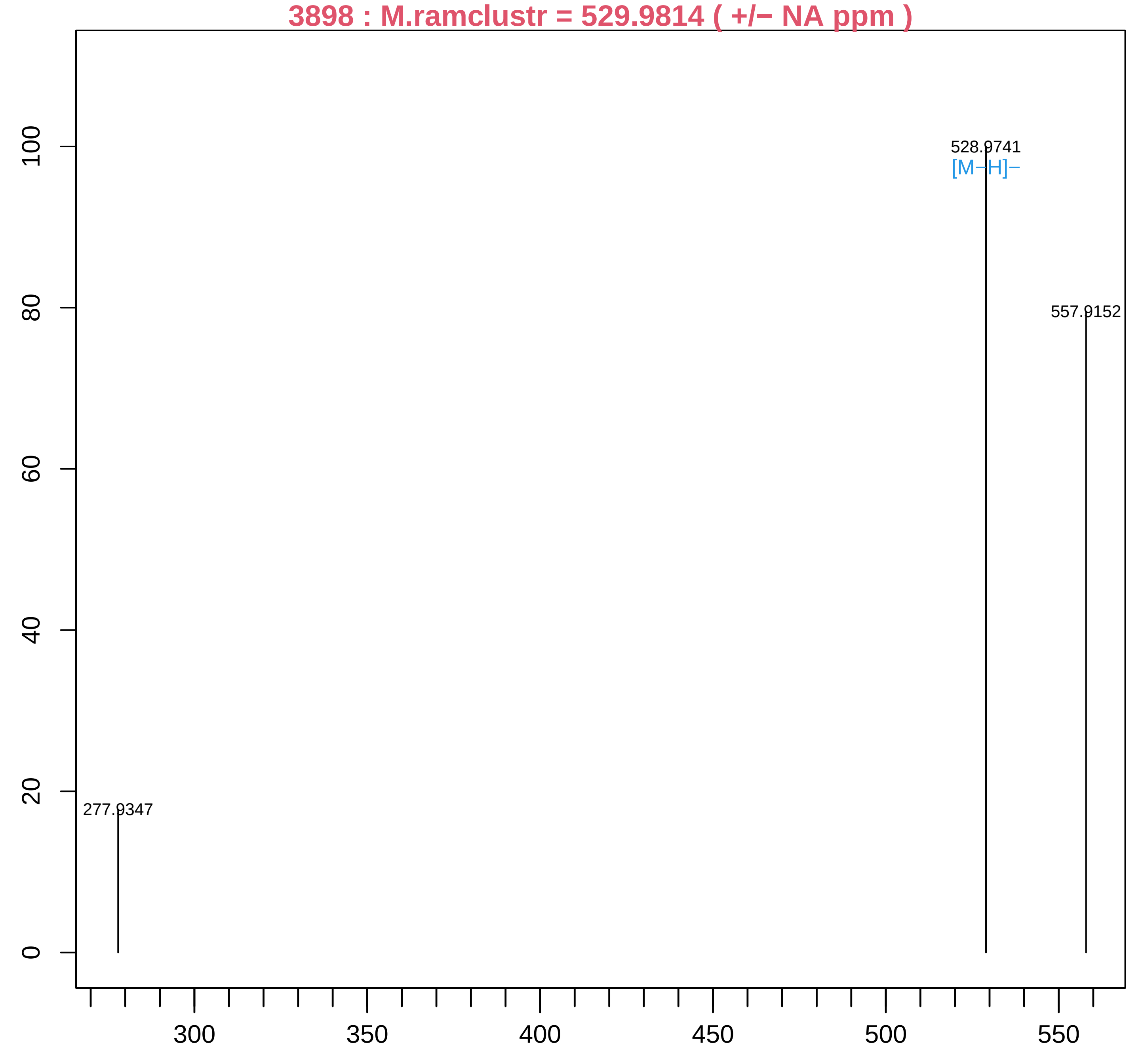
3898 : M.ramclustr = 529.9814 (+/− NA ppm)
528.9741
[M−H]−
557.9152
277.9347
0
20
40
60
80
100
300
350
400
450
500
550

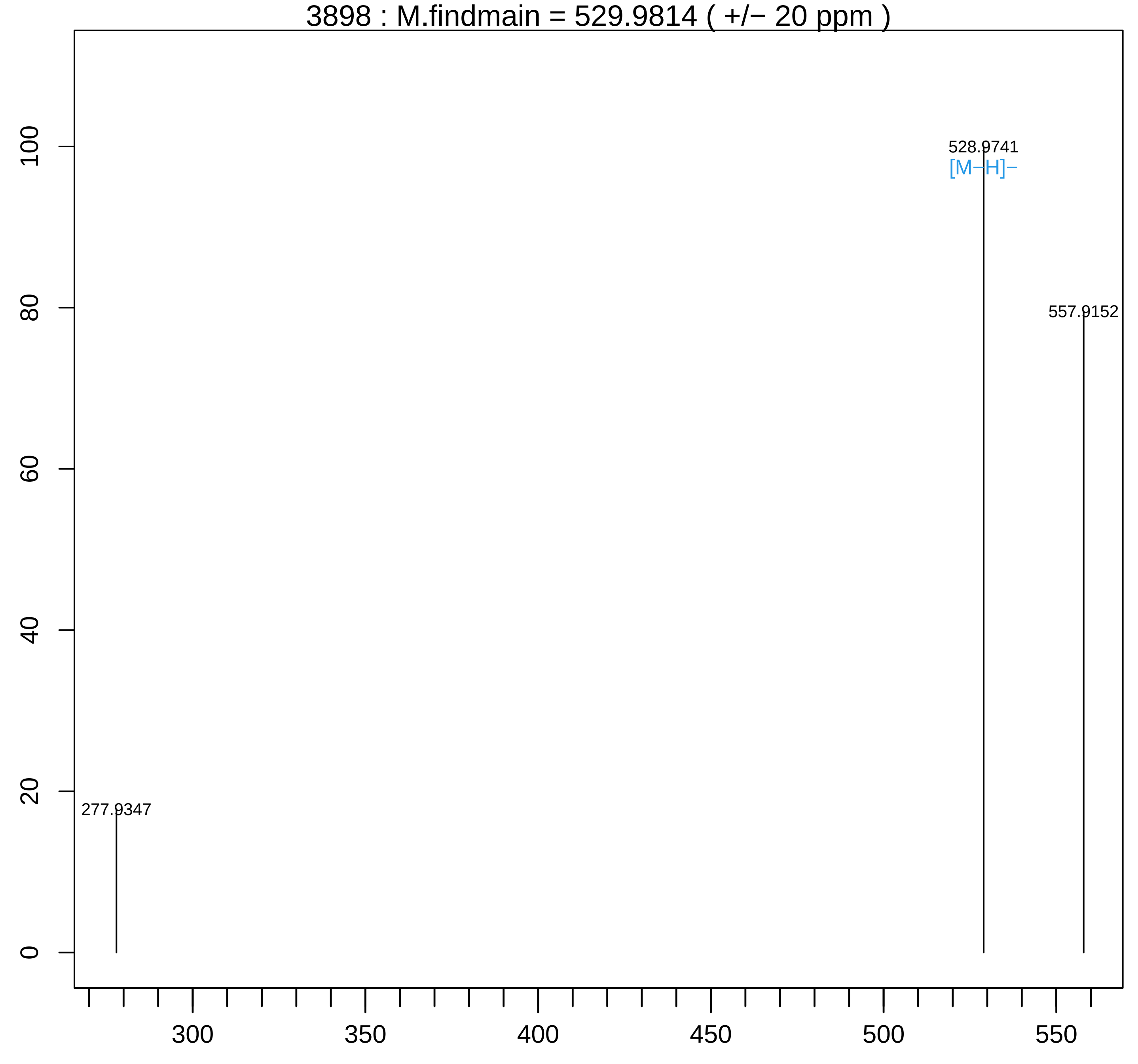
3898 : M.findmain = 529.9814 (+/− 20 ppm)
528.9741
[M−H]−
557.9152
277.9347
0
20
40
60
80
100
300
350
400
450
500
550

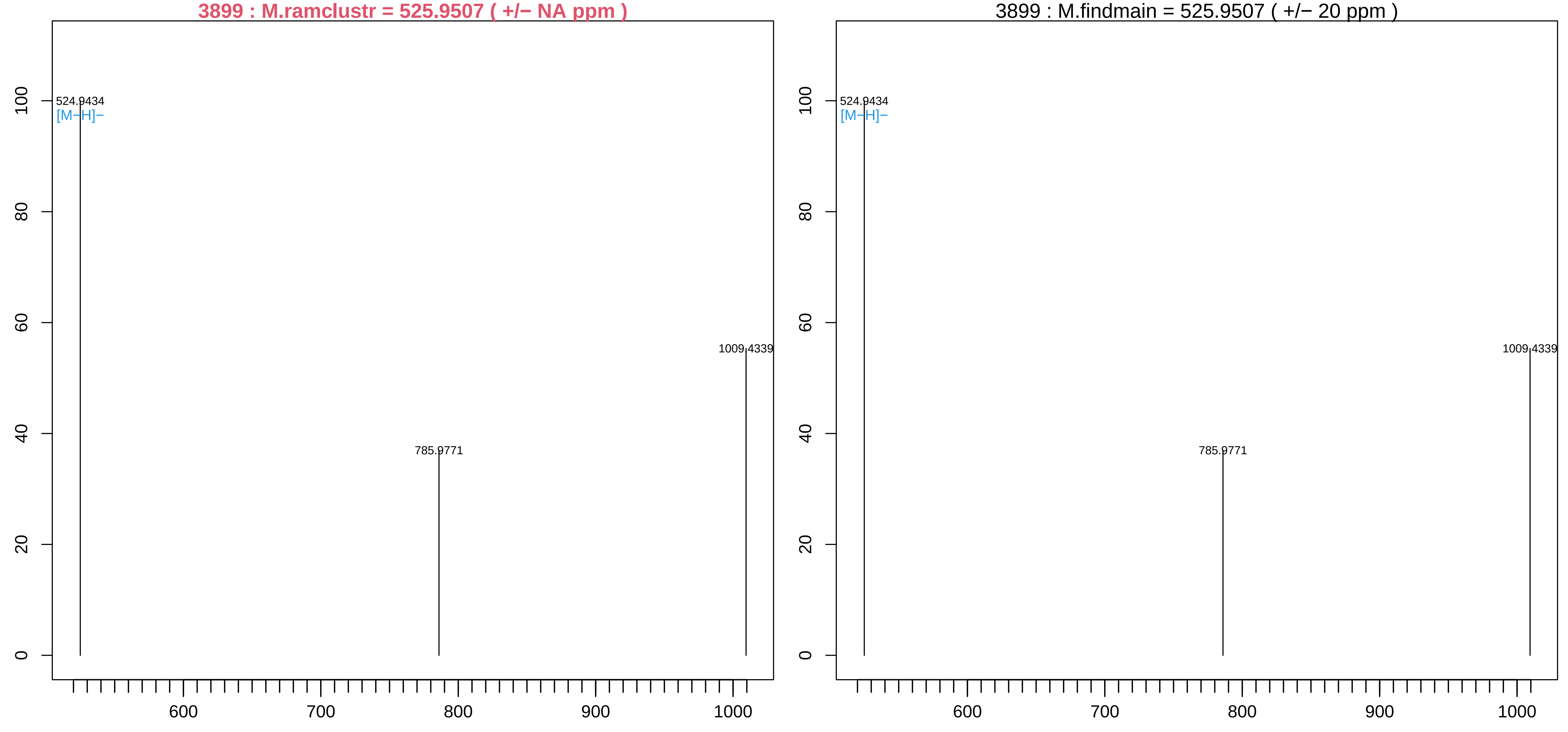
3899 : M.ramclustr = 525.9507 (+/− NA ppm)
524.9434
[M−H]−
785.9771
1009.4339
3899 : M.findmain = 525.9507 (+/− 20 ppm)
524.9434
[M−H]−
785.9771
1009.4339

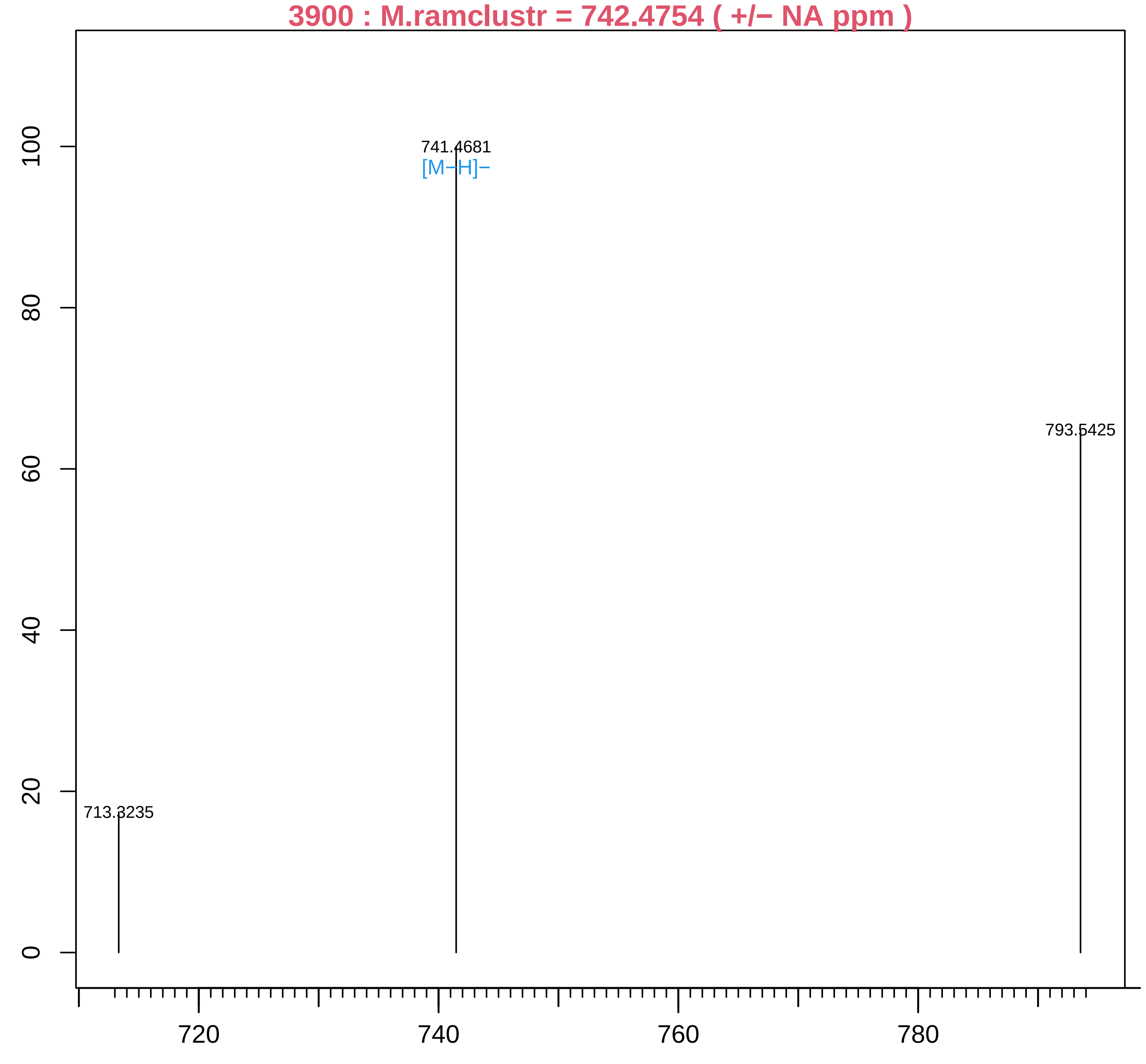
3900 : M.ramclustr = 742.4754 (+/− NA ppm)
741.4681
[M−H]−
793.5425
713.3235
0
20
40
60
80
100
720
740
760
780

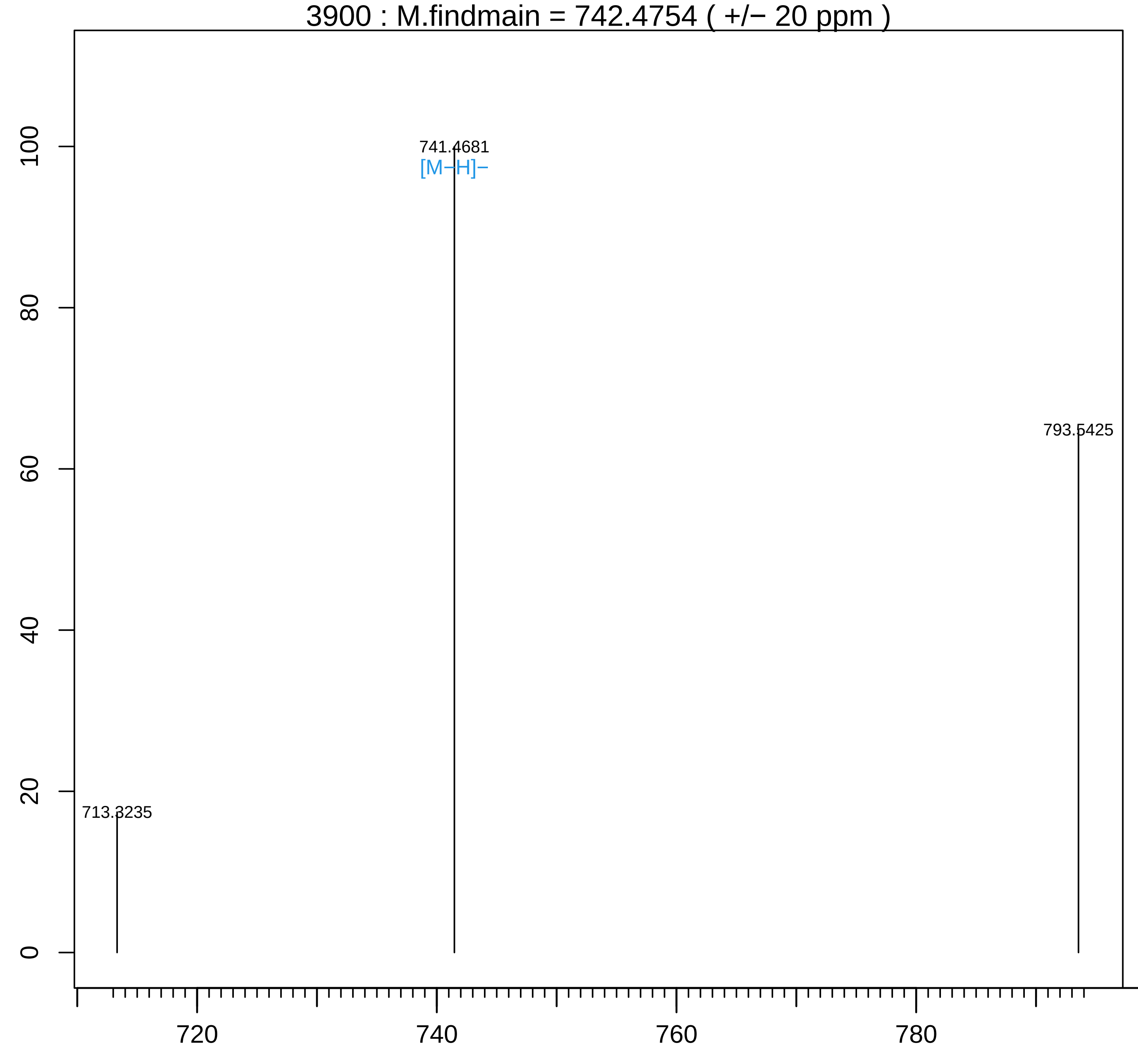
3900 : M.findmain = 742.4754 (+/− 20 ppm)
741.4681
[M−H]−
793.5425
713.3235
0
20
40
60
80
100
720
740
760
780

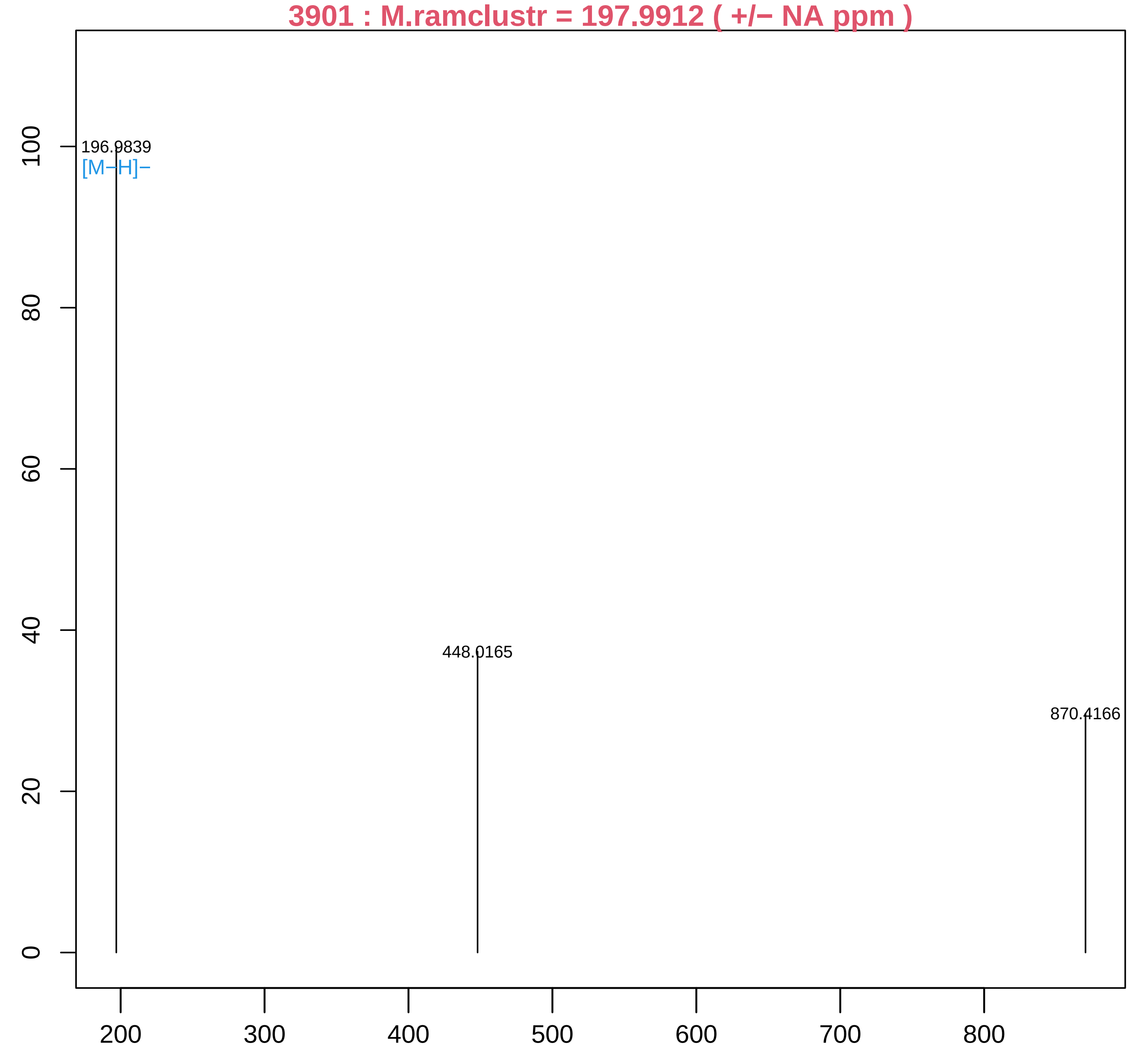
3901 : M.ramclustr = 197.9912 (+/− NA ppm)
196.9839
[M−H]−
448.0165
870.4166
0
20
40
60
80
100
200
300
400
500
600
700
800

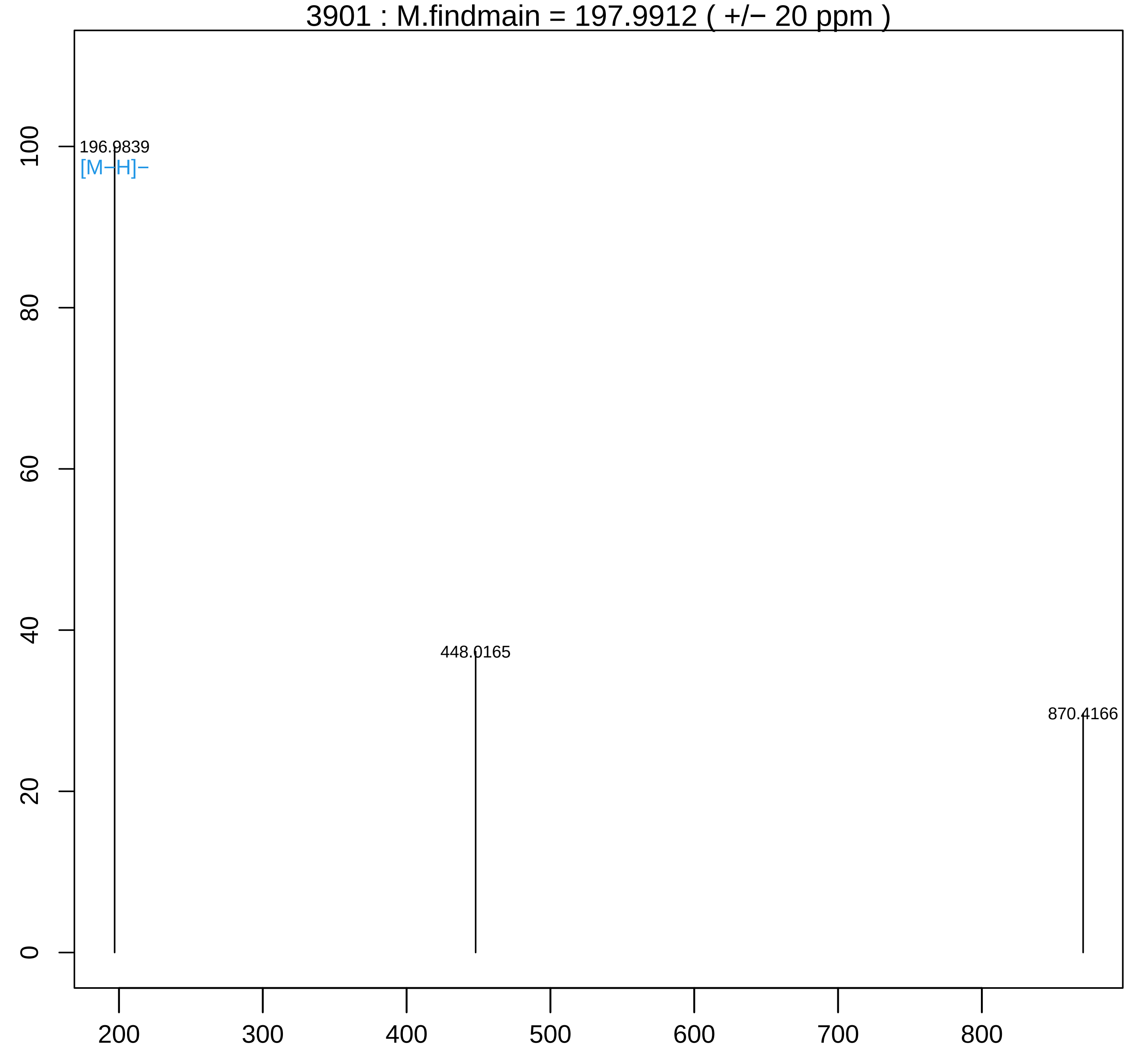
3901 : M.findmain = 197.9912 (+/− 20 ppm)
196.9839
[M−H]−
448.0165
870.4166
0
20
40
60
80
100
200
300
400
500
600
700
800

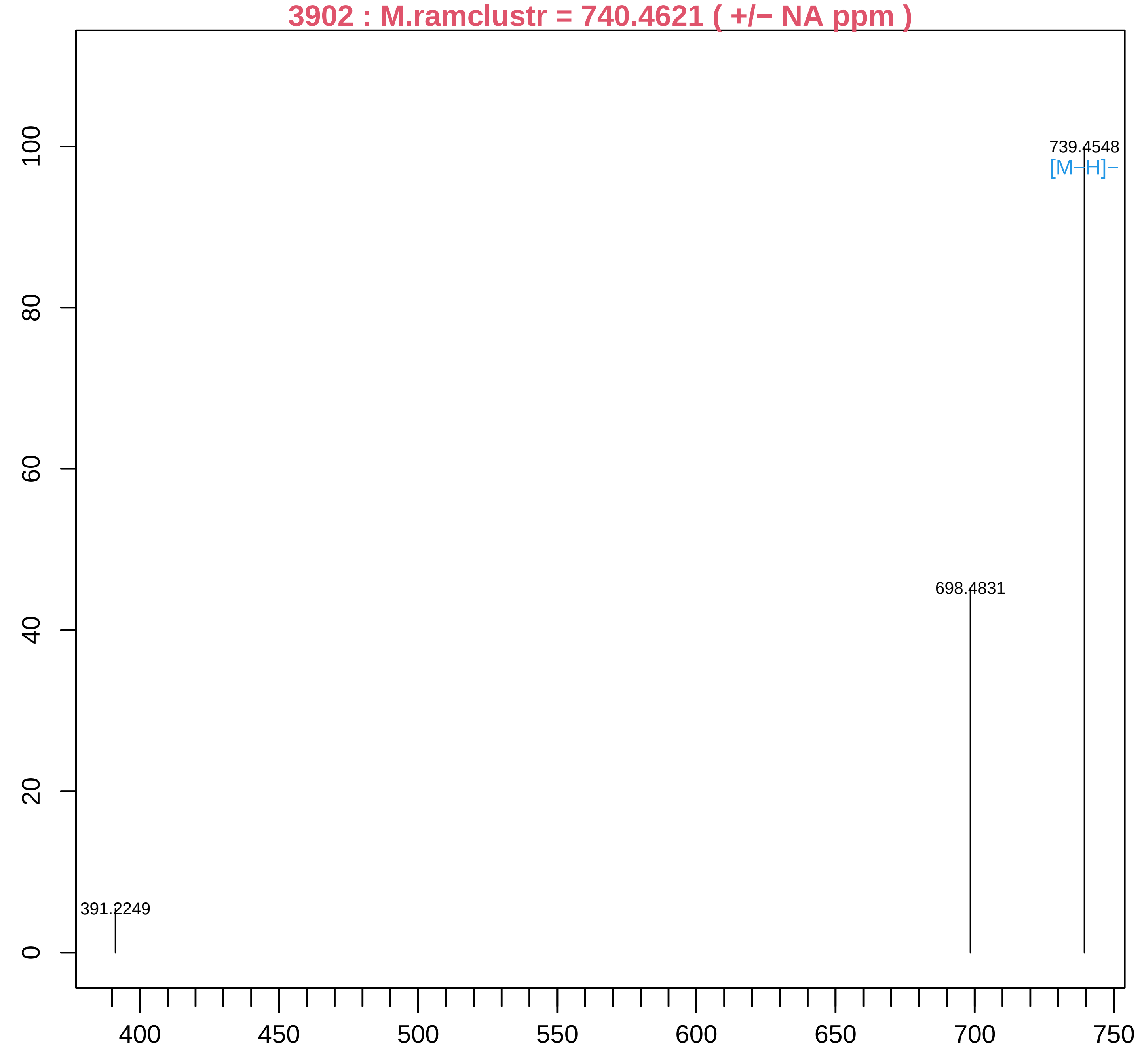
3902 : M.ramclustr = 740.4621 (+/− NA ppm)
739.4548
[M−H]−
698.4831
391.2249
0
20
40
60
80
100
400
450
500
550
600
650
700
750

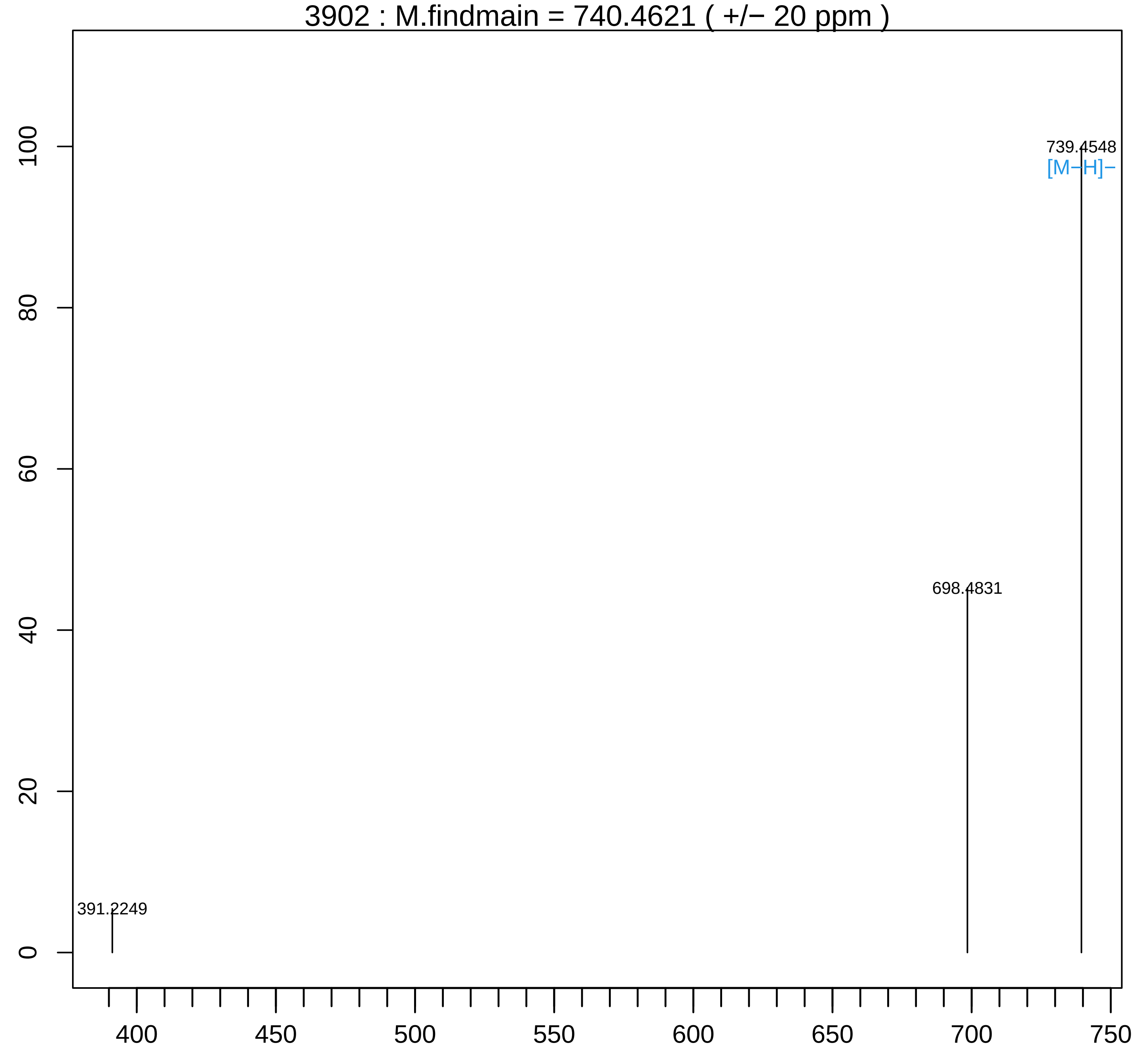
3902 : M.findmain = 740.4621 (+/− 20 ppm)
739.4548
[M−H]−
698.4831
391.2249
0
20
40
60
80
100
400
450
500
550
600
650
700
750

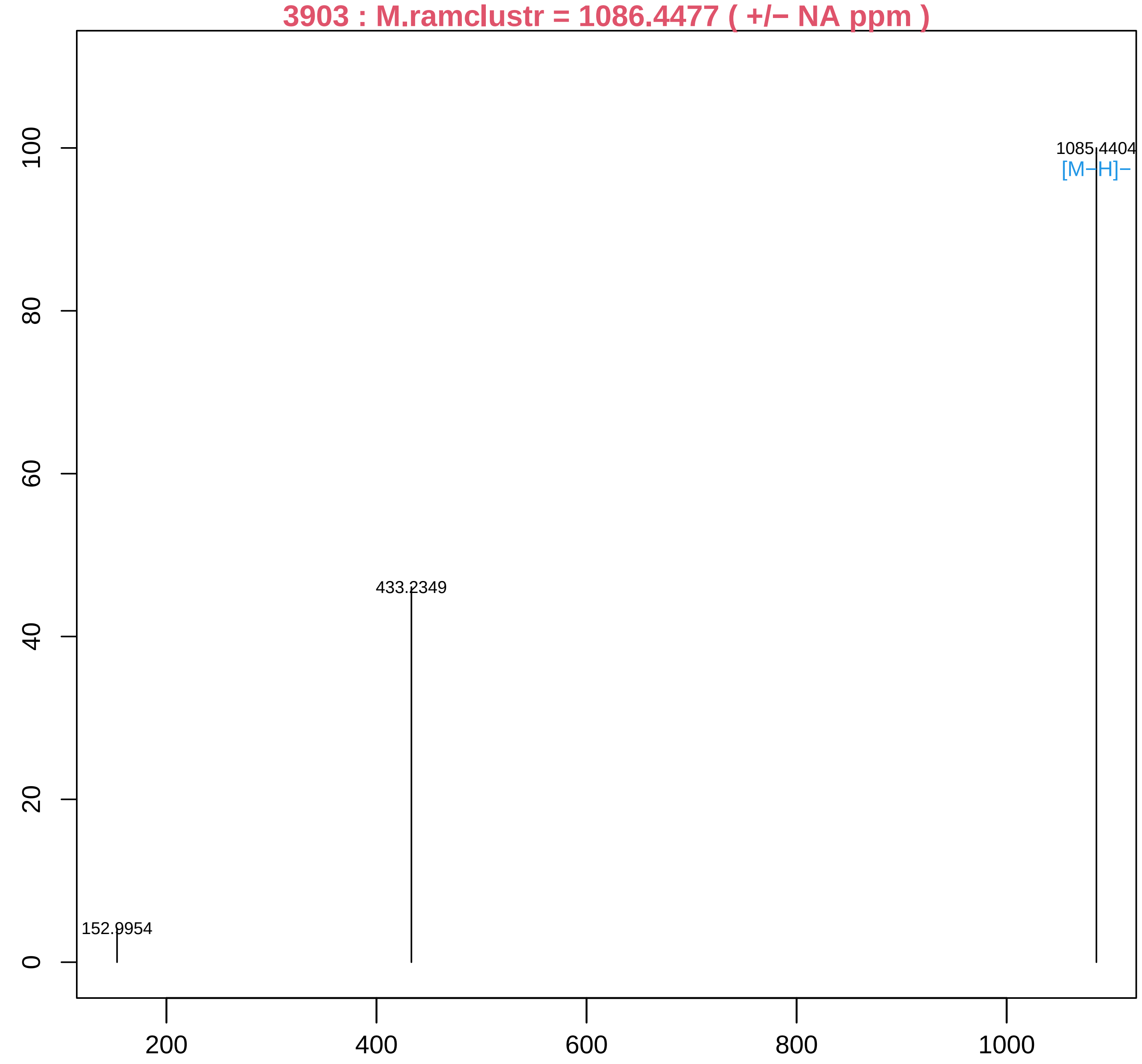

3903 : M.ramclustr = 1086.4477 (+/− NA ppm)
0
20
40
60
80
100
200
400
600
800
1000
152.9954
433.2349
1085.4404
[M−H]−

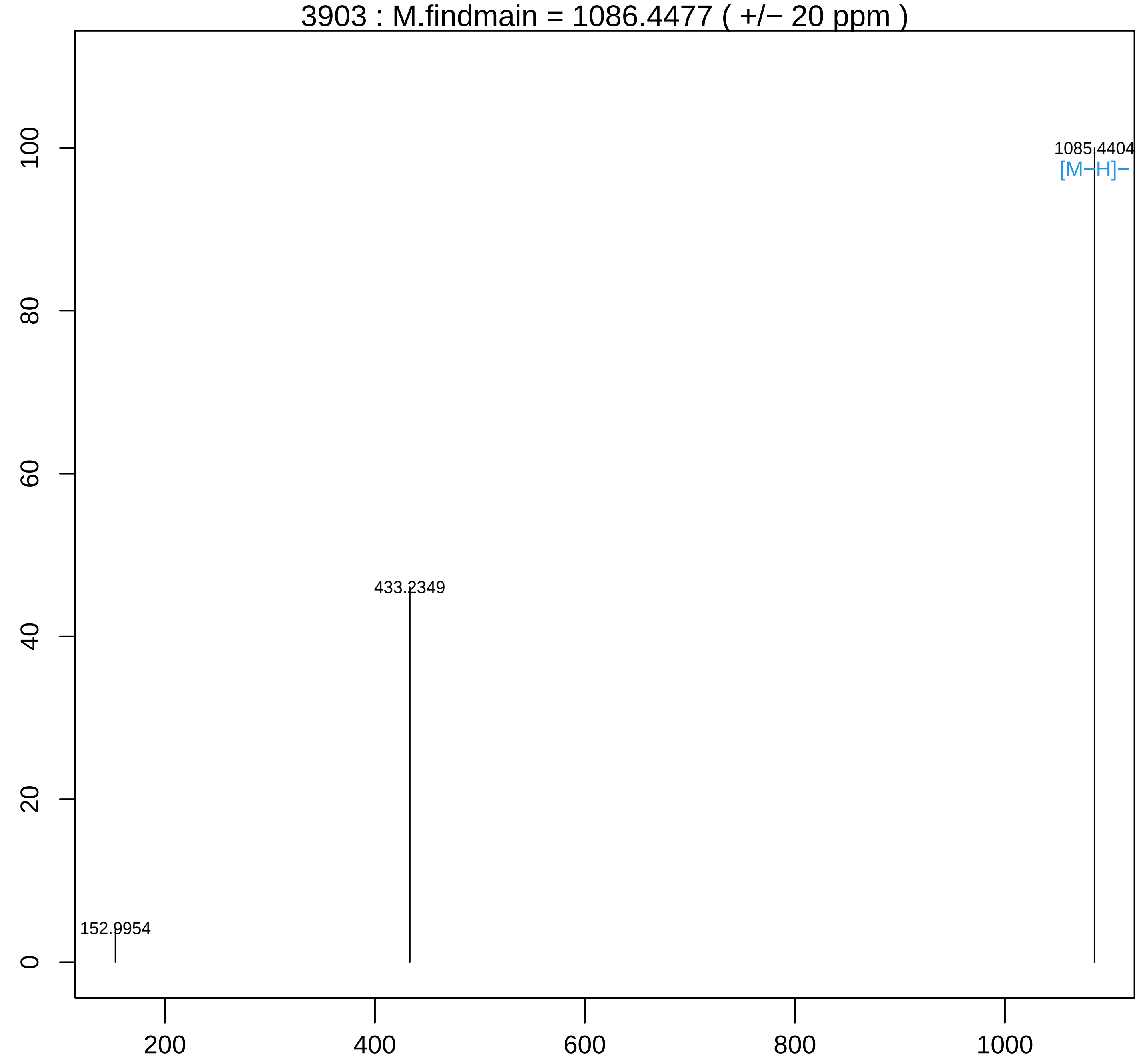

3903 : M.findmain = 1086.4477 (+/− 20 ppm)
0
20
40
60
80
100
200
400
600
800
1000
152.9954
433.2349
1085.4404
[M−H]−

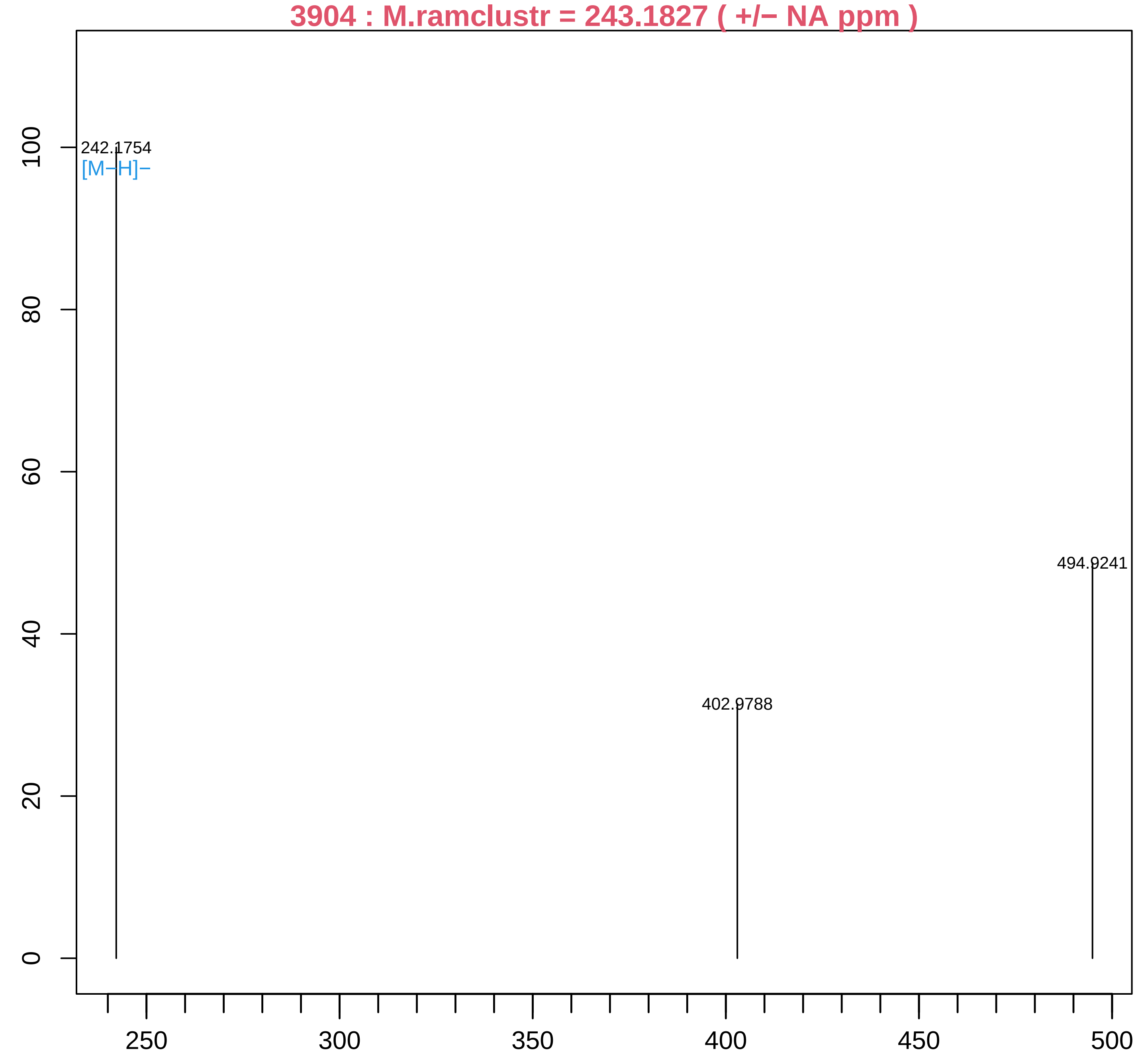
3904 : M.ramclustr = 243.1827 (+/− NA ppm)
242.1754
[M−H]−
402.9788
494.9241
0
20
40
60
80
100
250
300
350
400
450
500
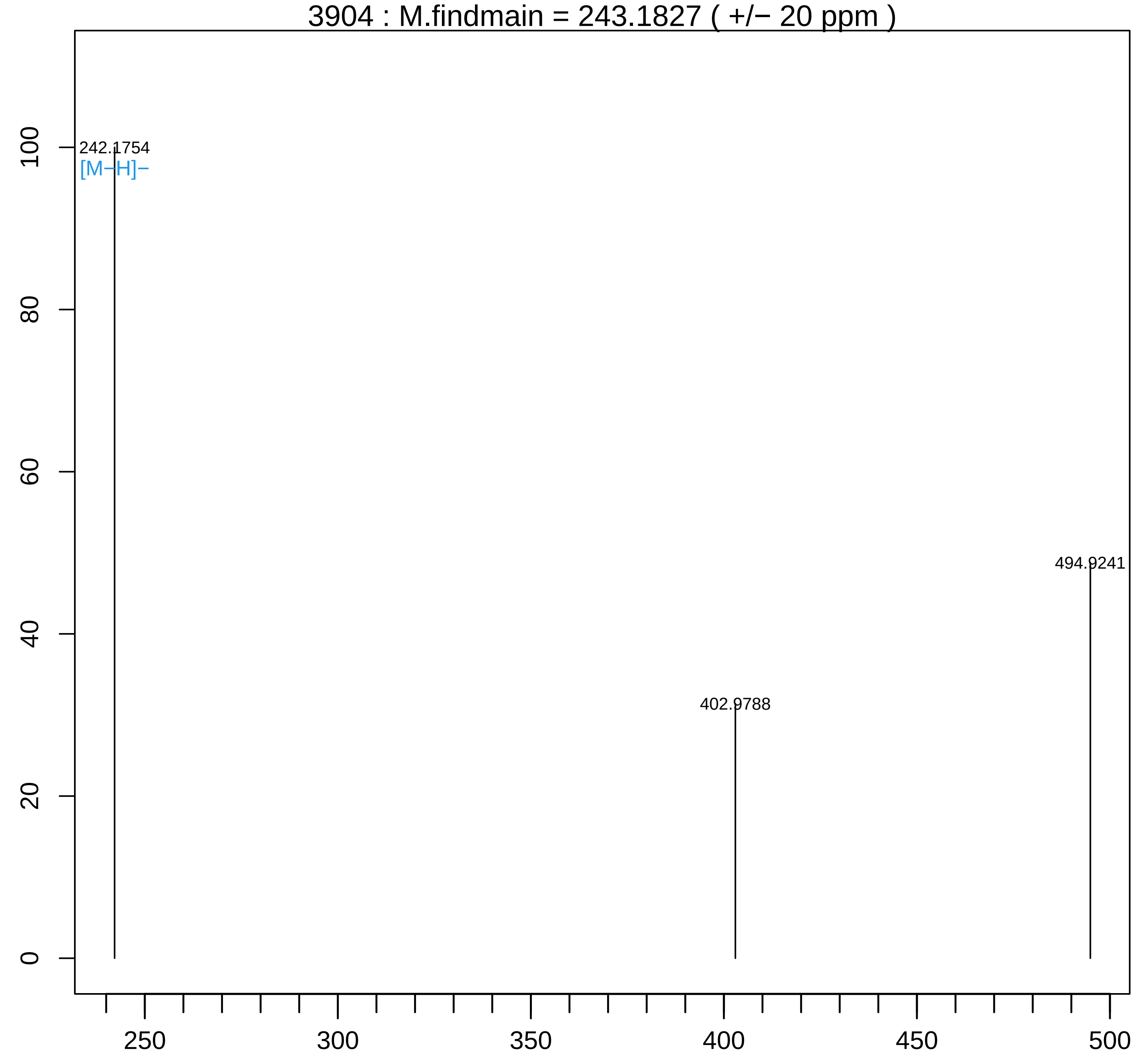
3904 : M.findmain = 243.1827 (+/− 20 ppm)
242.1754
[M−H]−
402.9788
494.9241
0
20
40
60
80
100
250
300
350
400
450
500

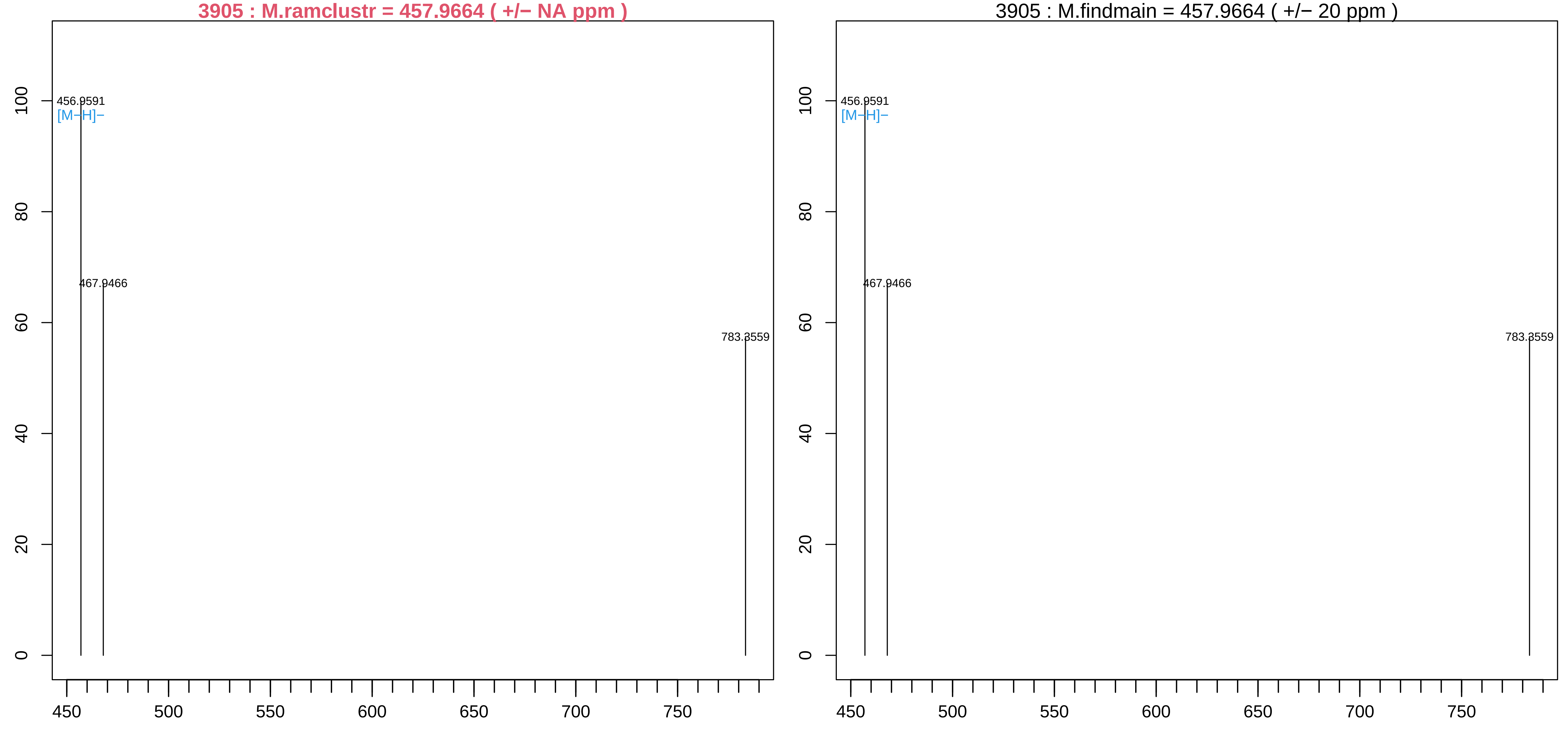
3905 : M.ramclustr = 457.9664 (+/− NA ppm)
456.9591
[M−H]−
467.9466
783.3559
3905 : M.findmain = 457.9664 (+/− 20 ppm)
456.9591
[M−H]−
467.9466
783.3559
0
20
40
60
80
100
450
500
550
600
650
700
750

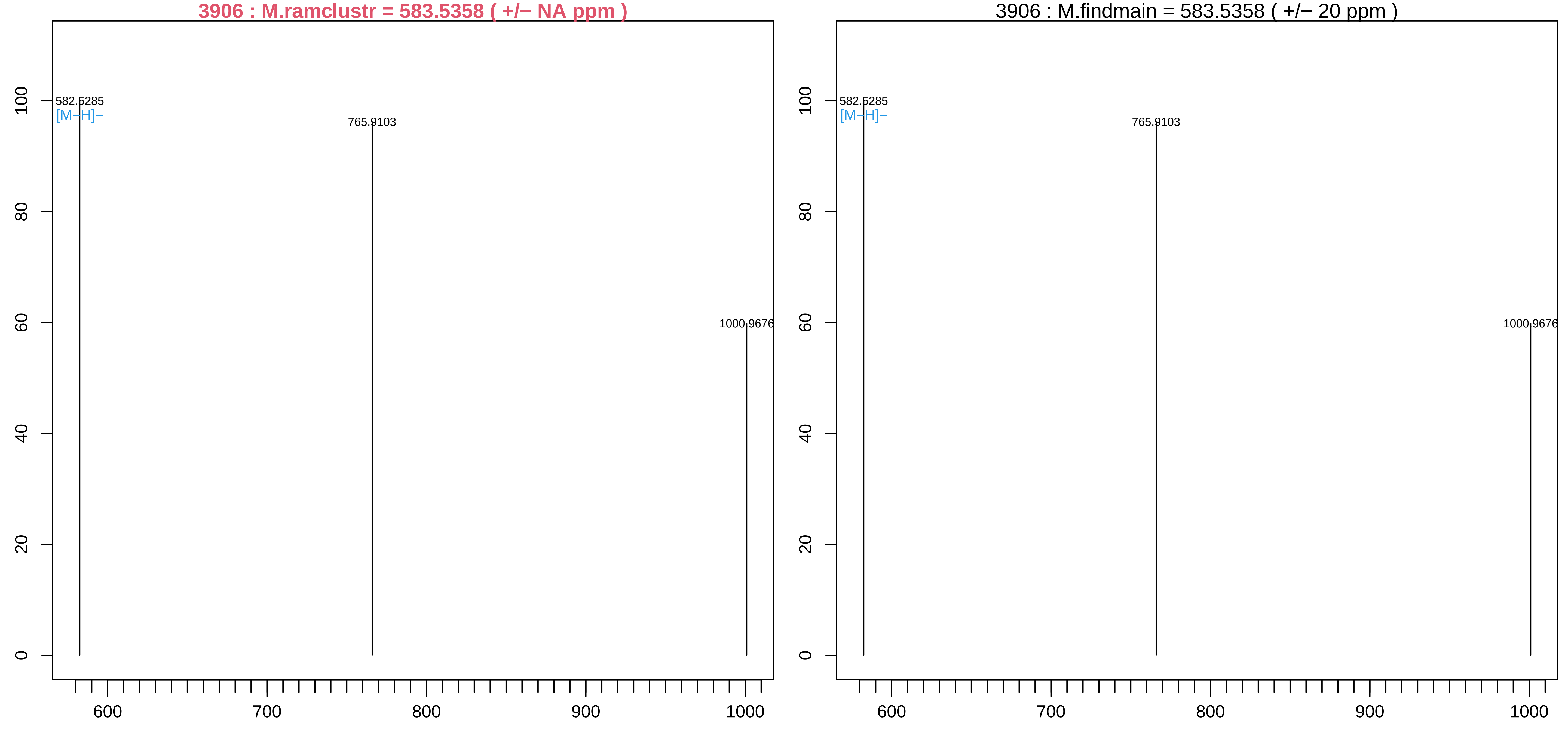

3906 : M.ramclustr = 583.5358 (+/− NA ppm)
582.5285
[M−H]−
765.9103
1000.9676
0
20
40
60
80
100
600
700
800
900
1000
3906 : M.findmain = 583.5358 (+/− 20 ppm)
582.5285
[M−H]−
765.9103
1000.9676
0
20
40
60
80
100
600
700
800
900
1000

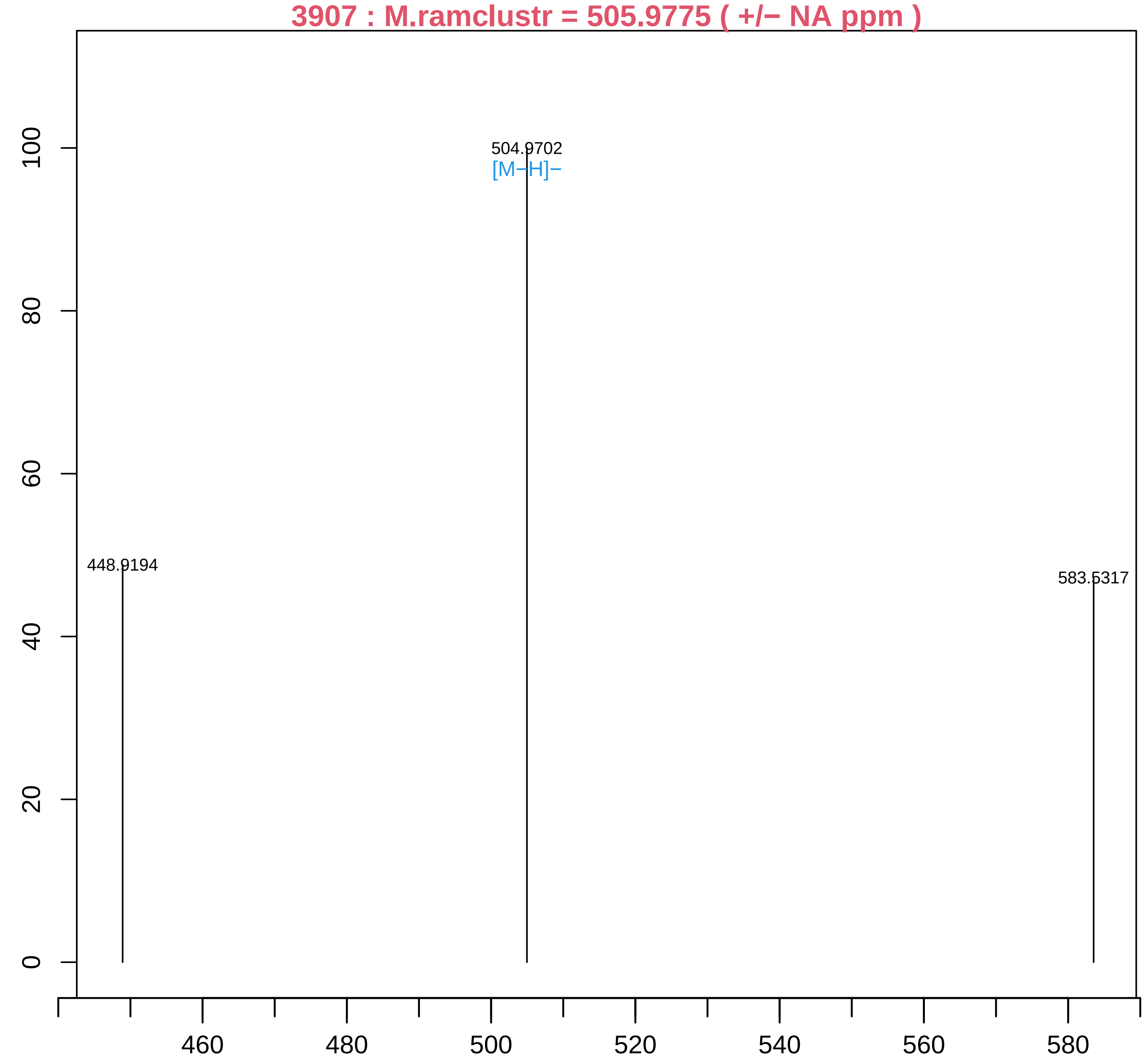
3907 : M.ramclustr = 505.9775 (+/− NA ppm)
504.9702
[M−H]−
448.9194
583.5317
0
20
40
60
80
100
460
480
500
520
540
560
580

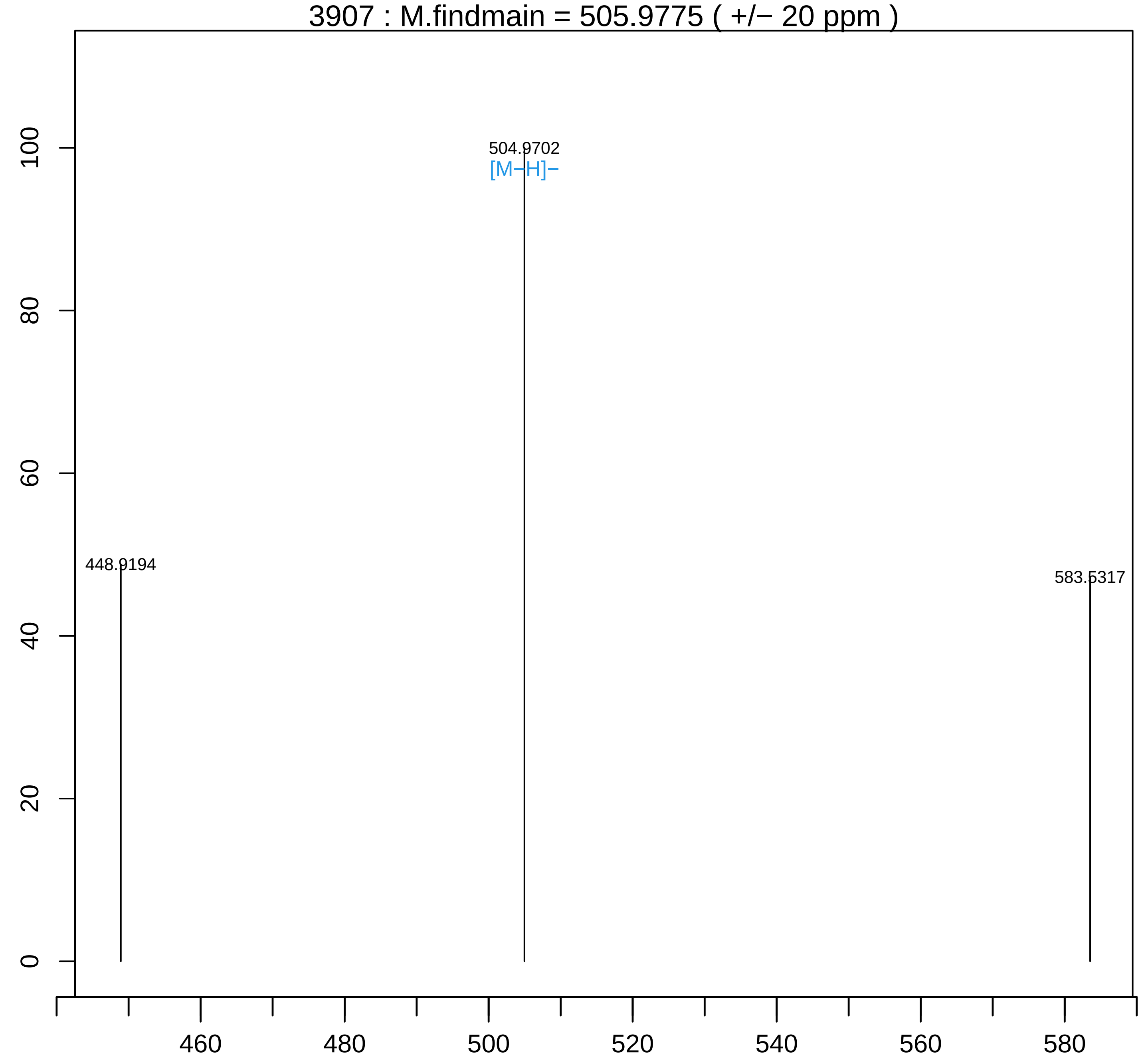
3907 : M.findmain = 505.9775 (+/− 20 ppm)
504.9702
[M−H]−
448.9194
583.5317
0
20
40
60
80
100
460
480
500
520
540
560
580

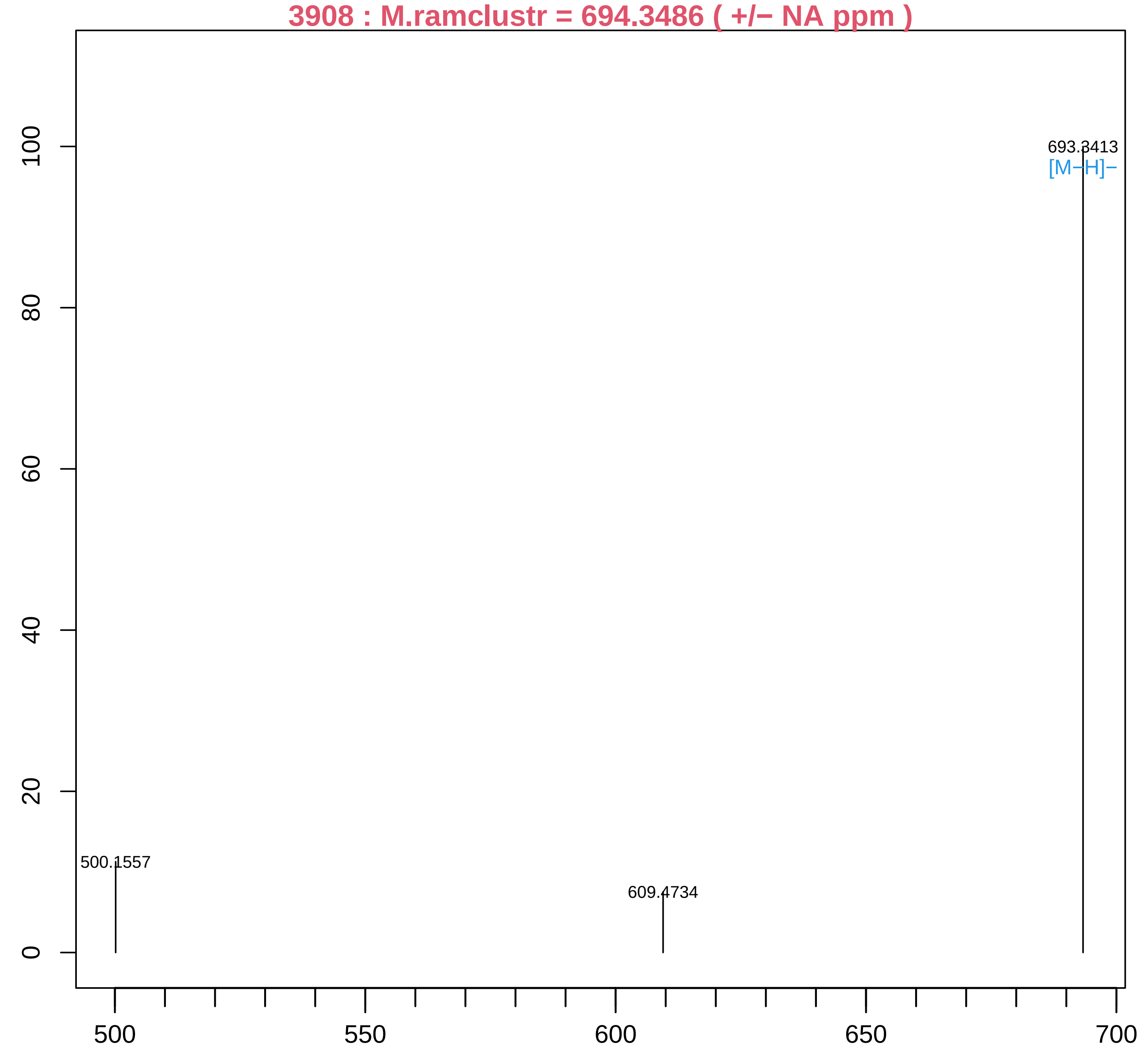

3908 : M.ramclustr = 694.3486 (+/− NA ppm)
693.3413
[M−H]−
500.1557
609.4734
0
20
40
60
80
100
500
550
600
650
700

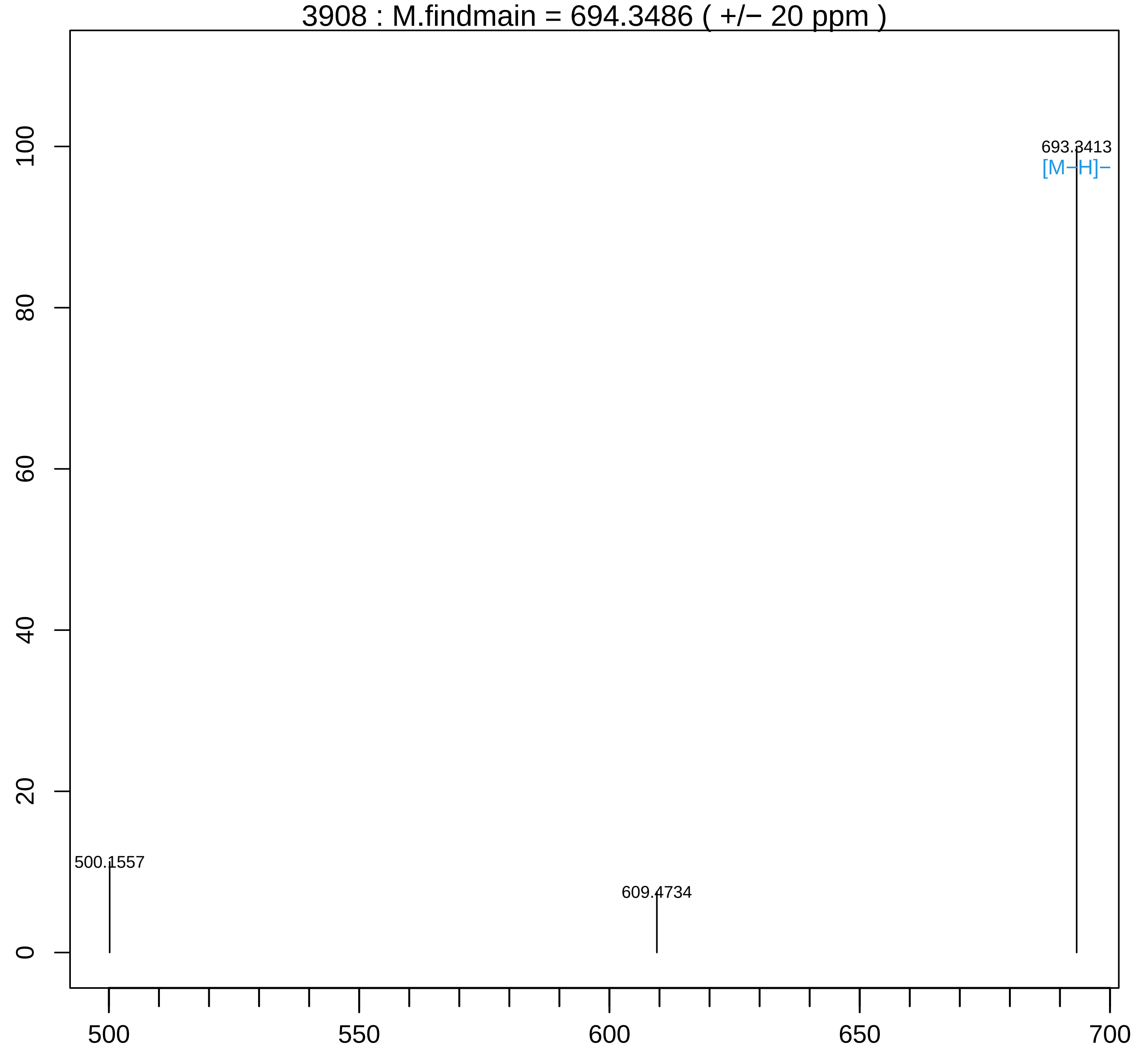

3908 : M.findmain = 694.3486 (+/− 20 ppm)
693.3413
[M−H]−
500.1557
609.4734
0
20
40
60
80
100
500
550
600
650
700

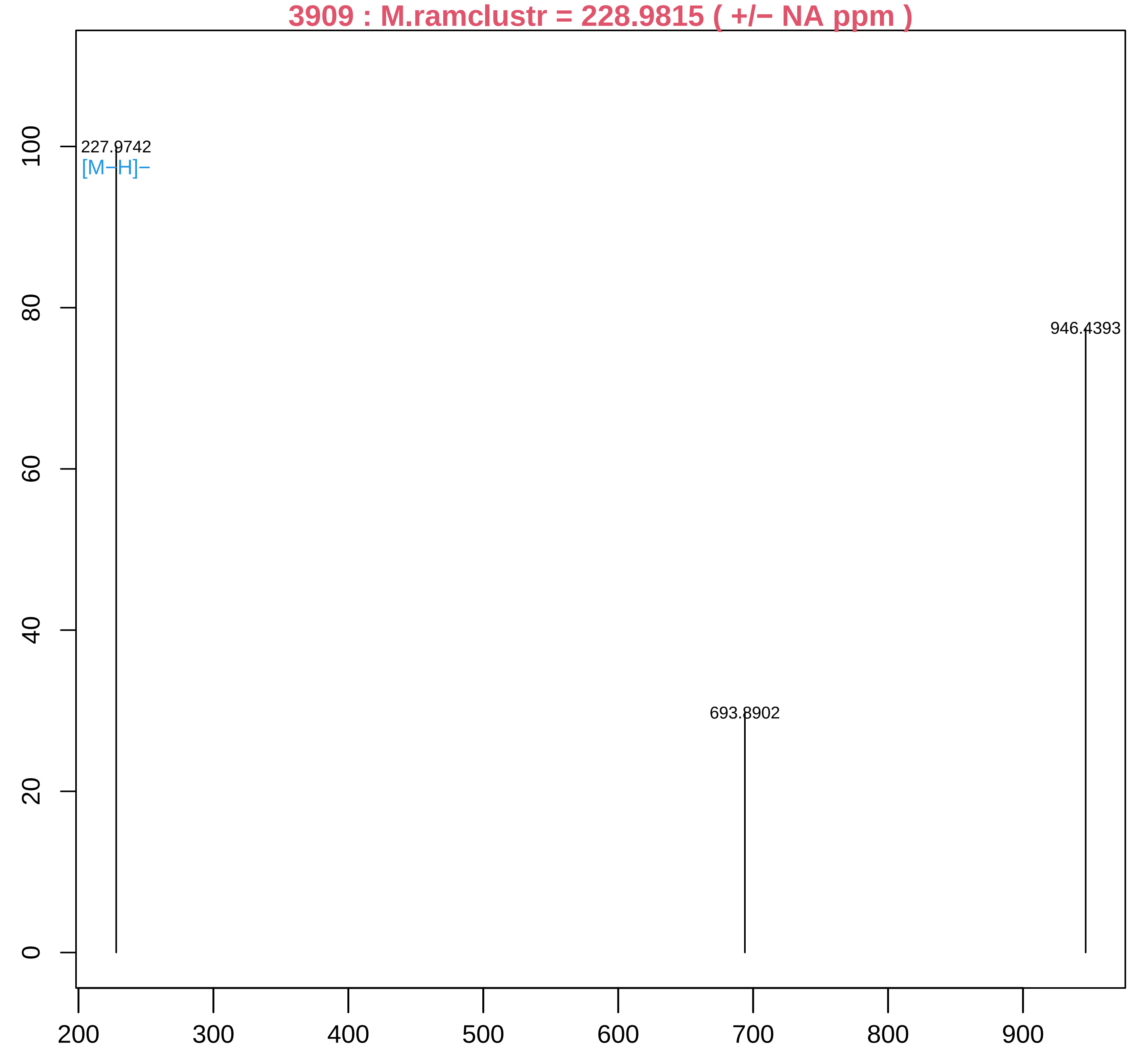
3909 : M.ramclustr = 228.9815 (+/− NA ppm)
227.9742
[M−H]−
946.4393
693.8902
0
20
40
60
80
100
200
300
400
500
600
700
800
900

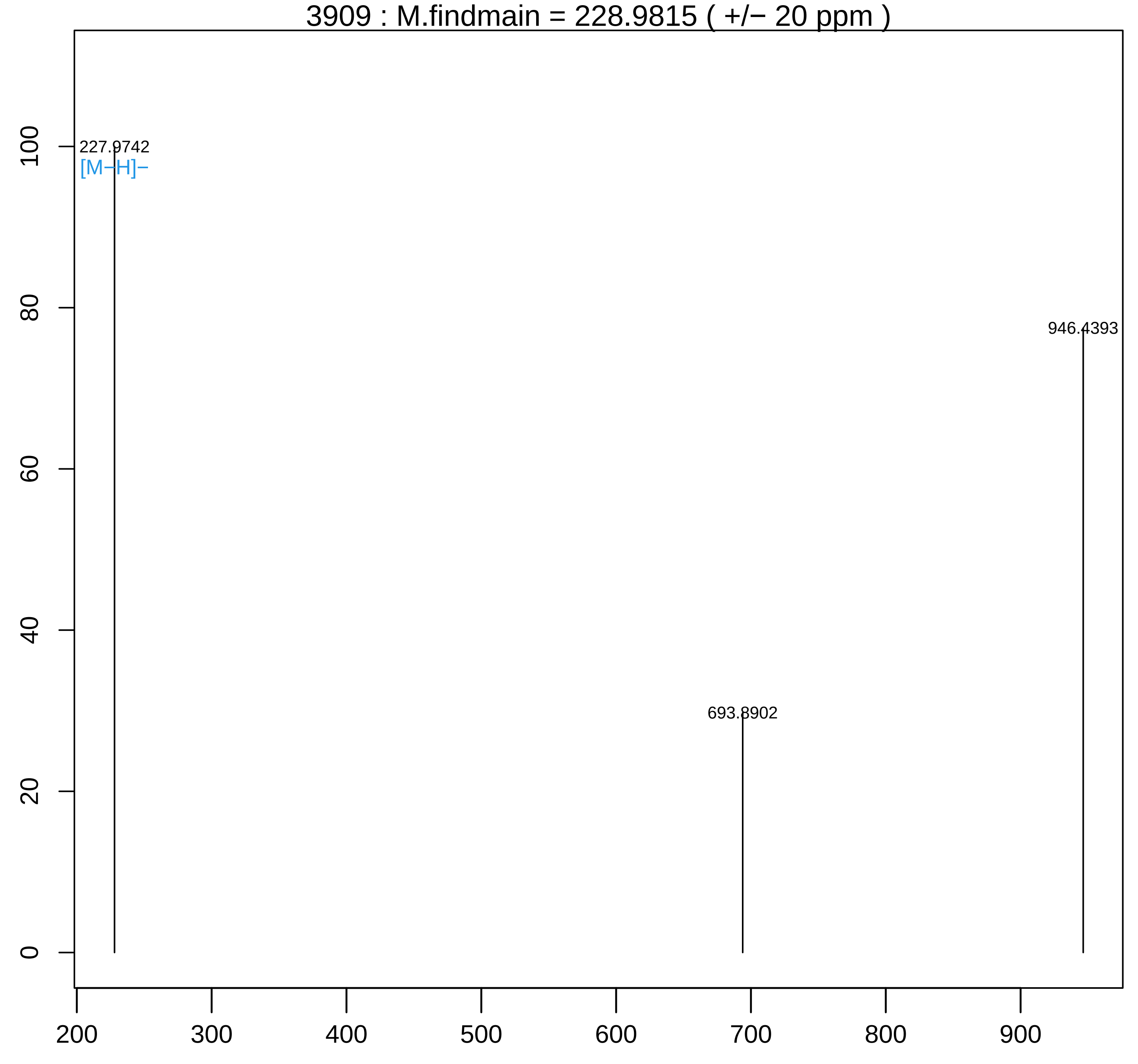
3909 : M.findmain = 228.9815 (+/− 20 ppm)
227.9742
[M−H]−
946.4393
693.8902
0
20
40
60
80
100
200
300
400
500
600
700
800
900

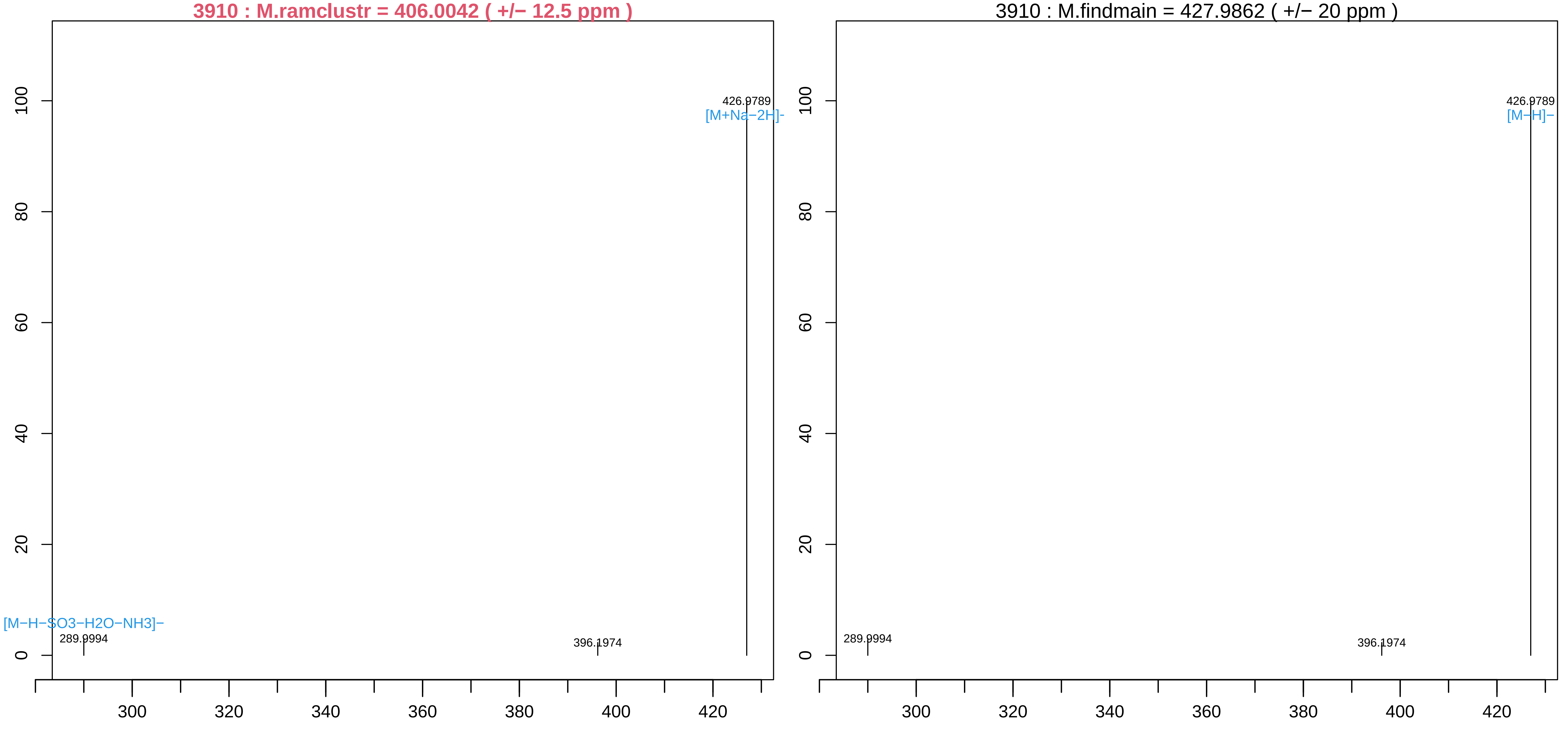
3910 : M.ramclustr = 406.0042 (+/− 12.5 ppm)
426.9789
[M+Na−2H]-
[M−H−SO3−H2O−NH3]−
289.9994
396.1974
0
20
40
60
80
100
300
320
340
360
380
400
420
3910 : M.findmain = 427.9862 (+/− 20 ppm)
426.9789
[M−H]−
289.9994
396.1974

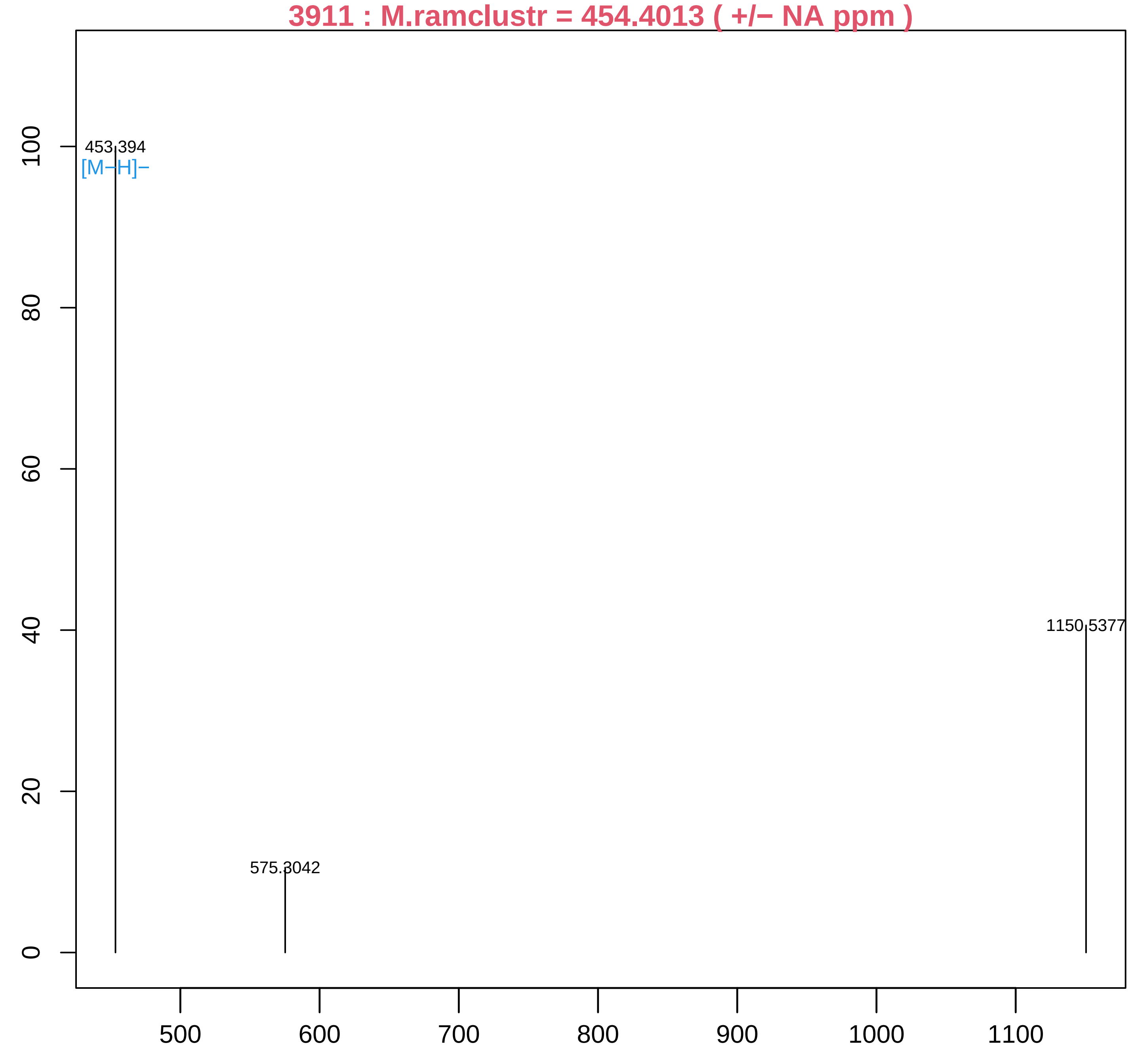
3911 : M.ramclustr = 454.4013 (+/− NA ppm)
453.394
[M−H]−
575.3042
1150.5377
0
20
40
60
80
100
500
600
700
800
900
1000
1100

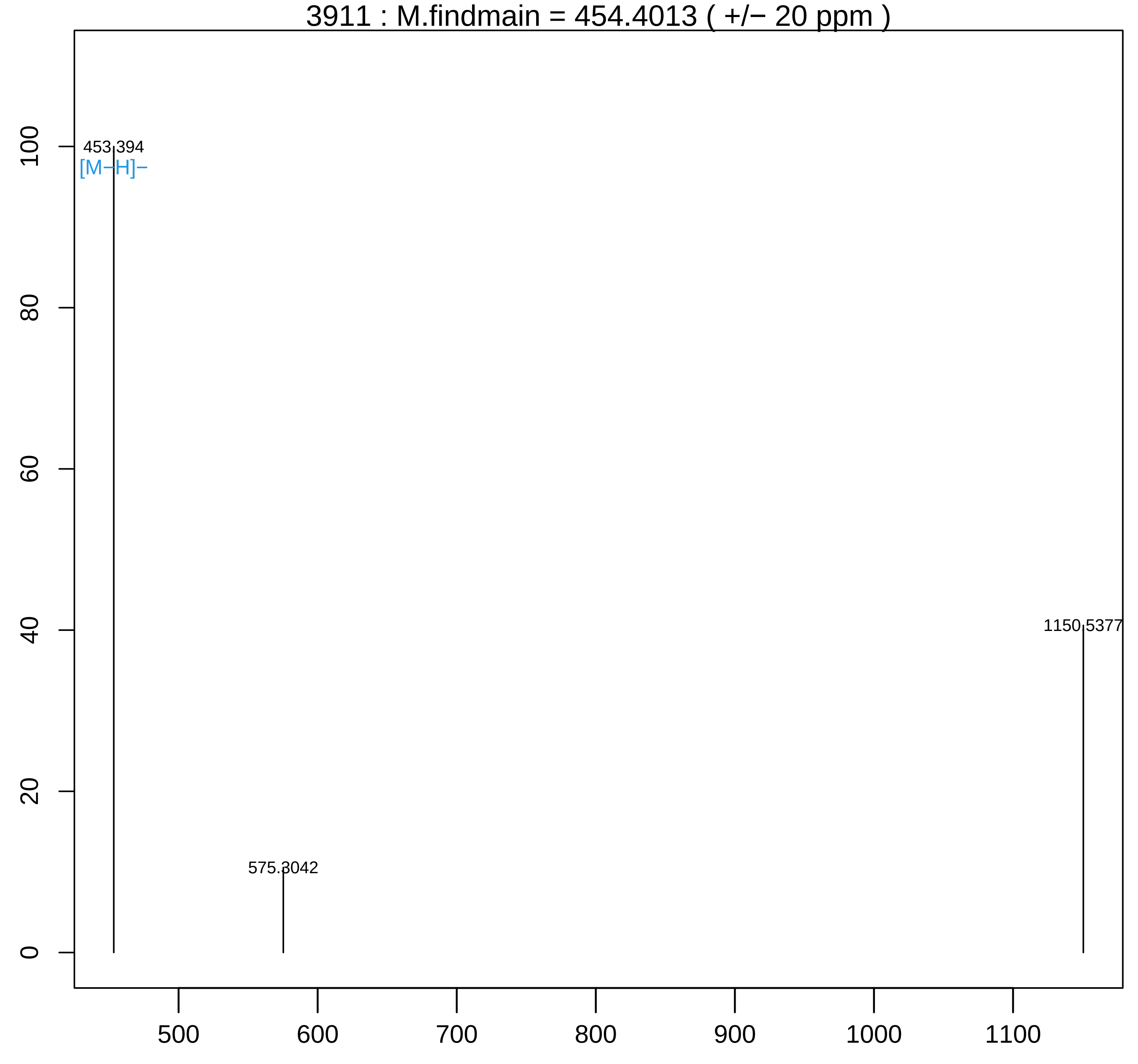
3911 : M.findmain = 454.4013 (+/− 20 ppm)
453.394
[M−H]−
575.3042
1150.5377
0
20
40
60
80
100
500
600
700
800
900
1000
1100

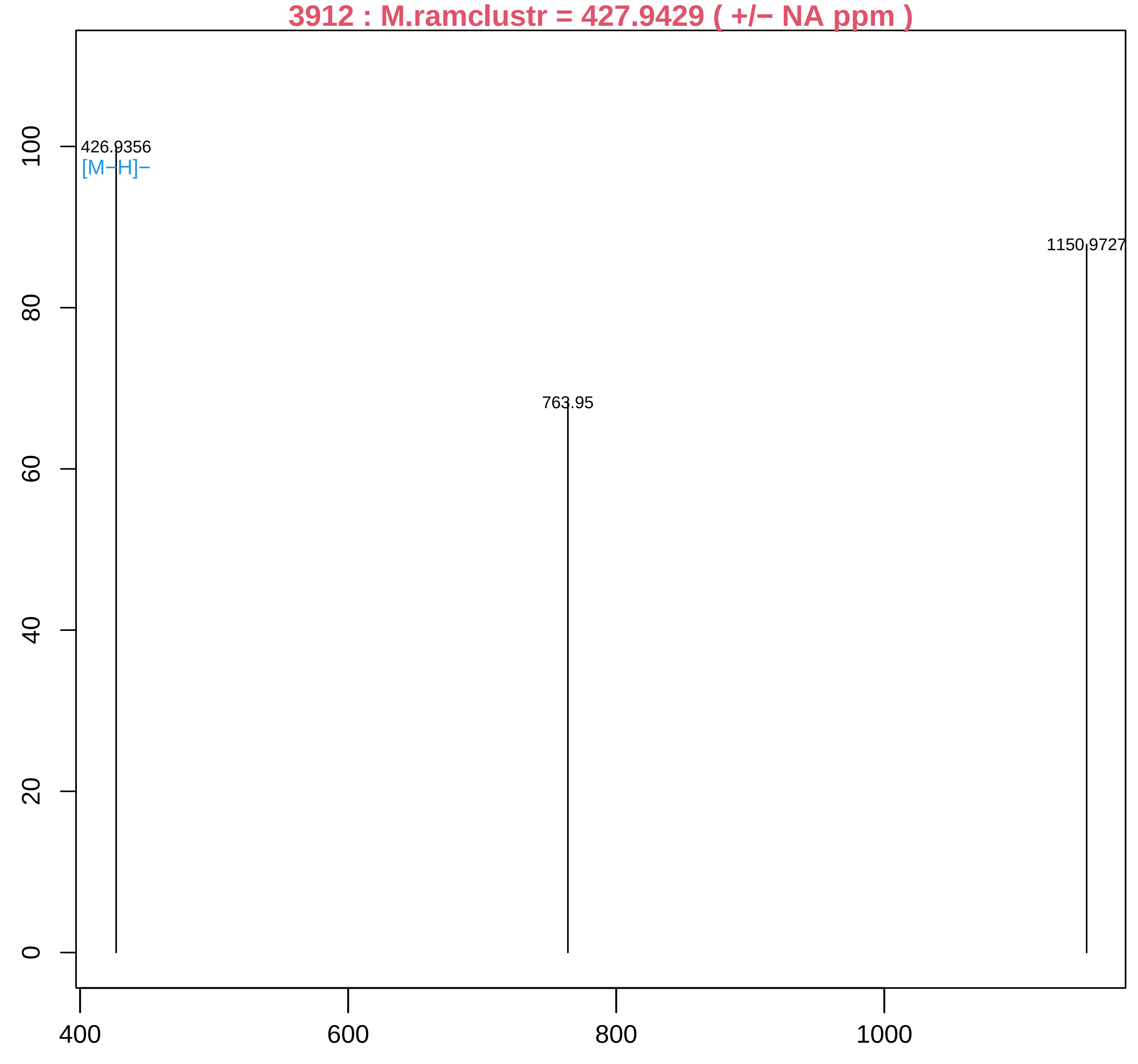
3912 : M.ramclustr = 427.9429 (+/− NA ppm)
426.9356
[M−H]−
763.95
1150.9727
0
20
40
60
80
100
400
600
800
1000

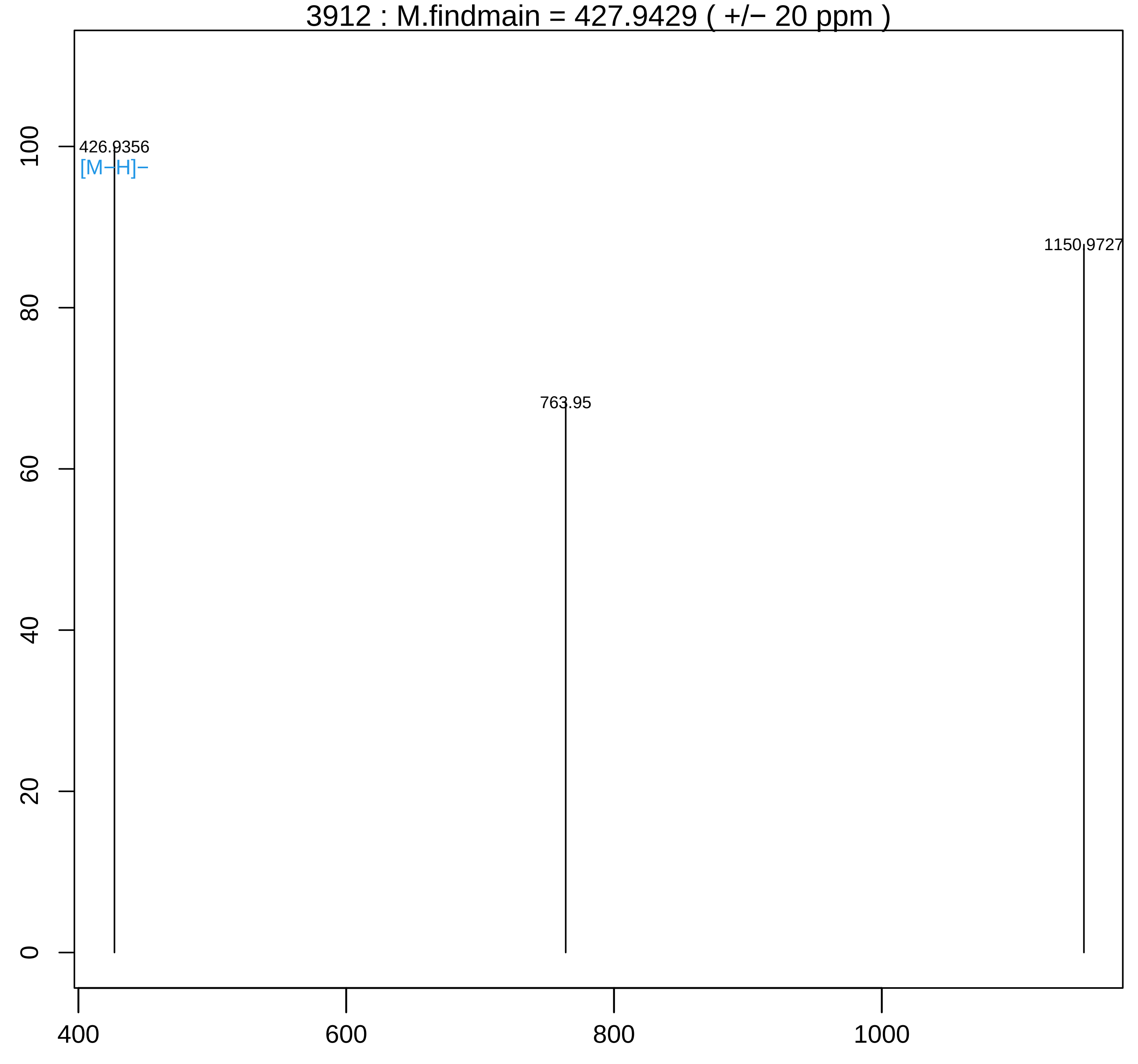
3912 : M.findmain = 427.9429 (+/− 20 ppm)
426.9356
[M−H]−
763.95
1150.9727
0
20
40
60
80
100
400
600
800
1000

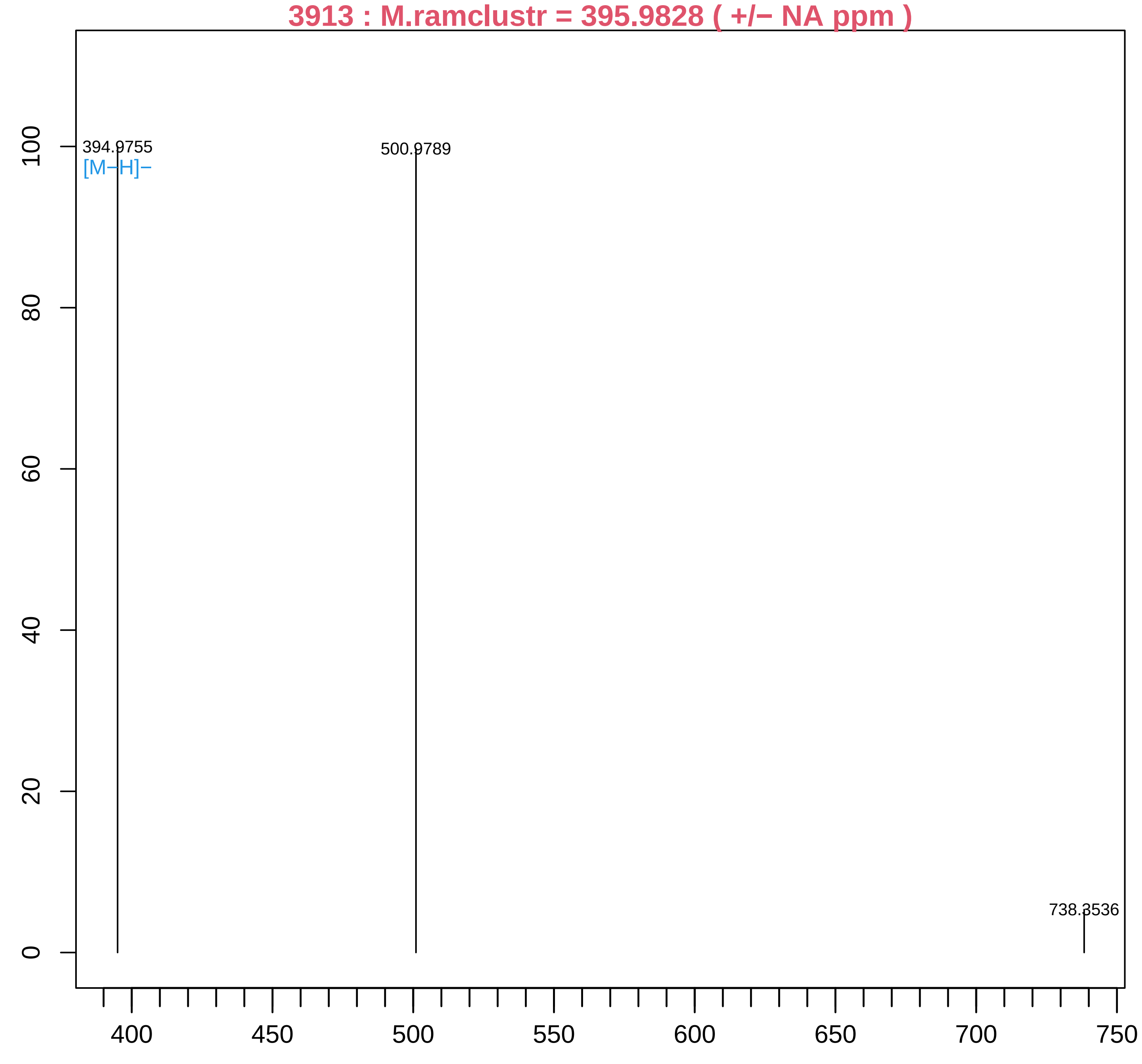

3913 : M.ramclustr = 395.9828 (+/− NA ppm)
394.9755
[M−H]−
500.9789
738.3536
0
20
40
60
80
100
400
450
500
550
600
650
700
750

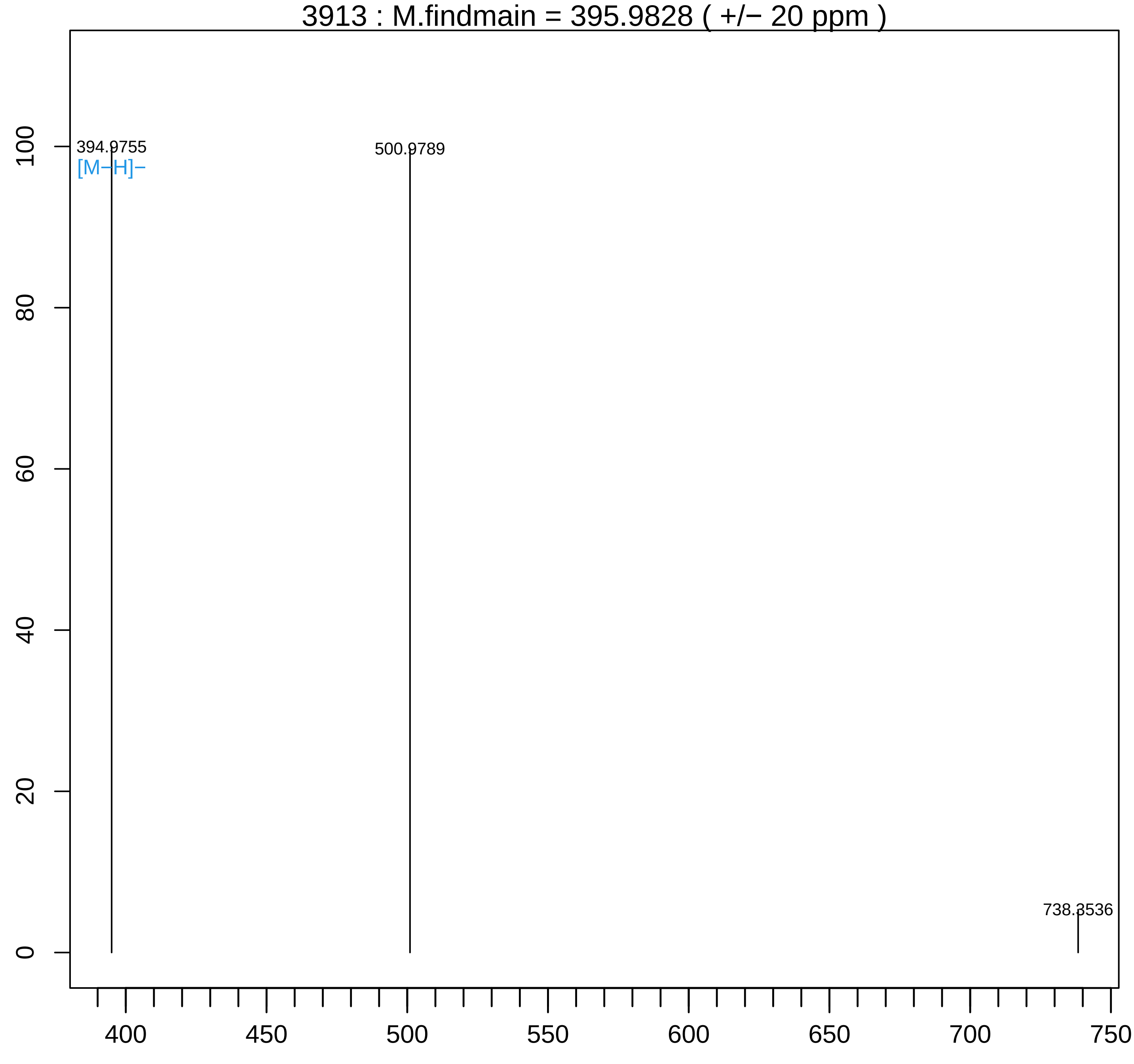

3913 : M.findmain = 395.9828 (+/− 20 ppm)
394.9755
[M−H]−
500.9789
738.3536
0
20
40
60
80
100
400
450
500
550
600
650
700
750

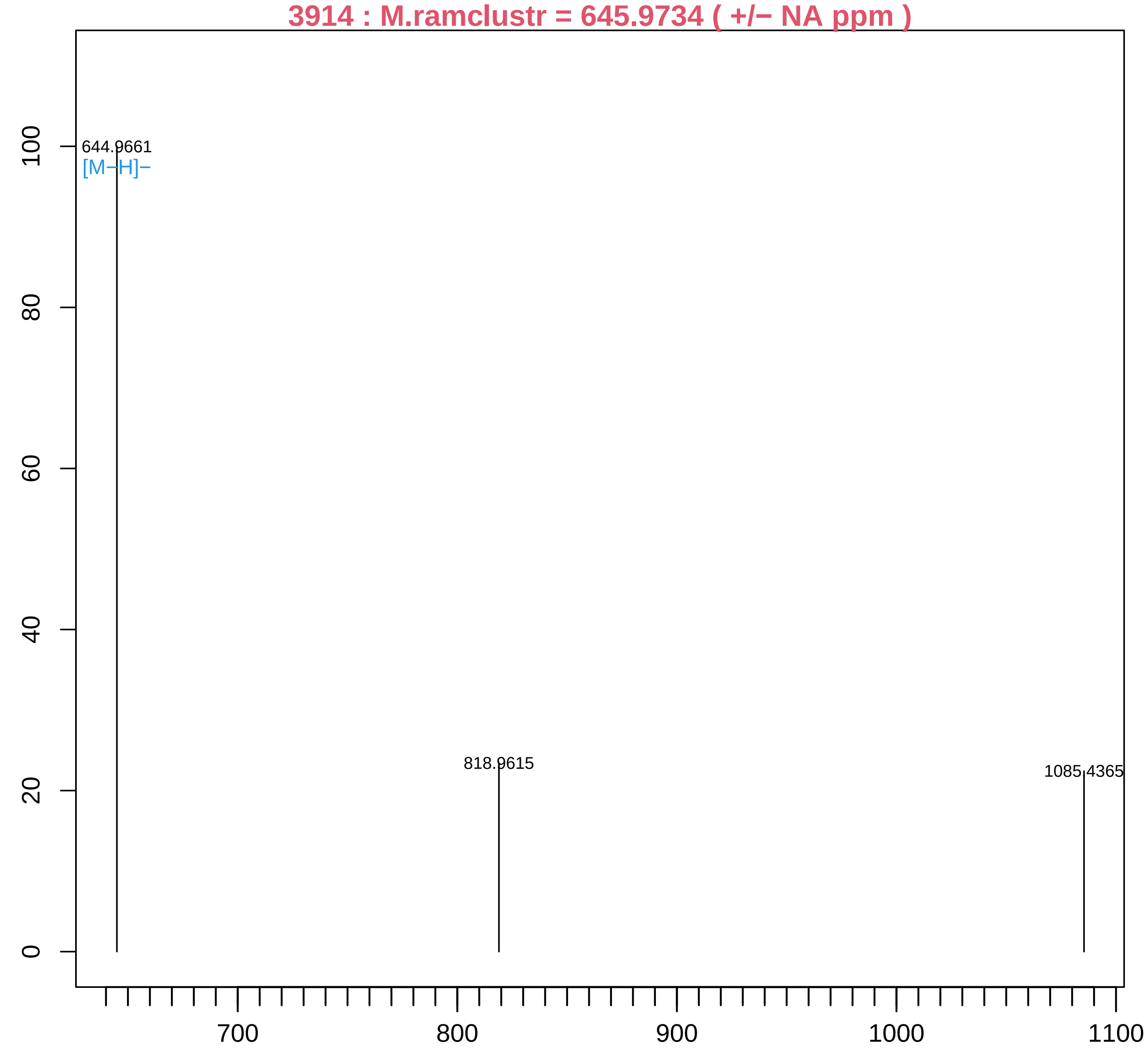
3914 : M.ramclustr = 645.9734 (+/− NA ppm)
644.9661
[M−H]−
818.9615
1085.4365
0
20
40
60
80
100
700
800
900
1000
1100

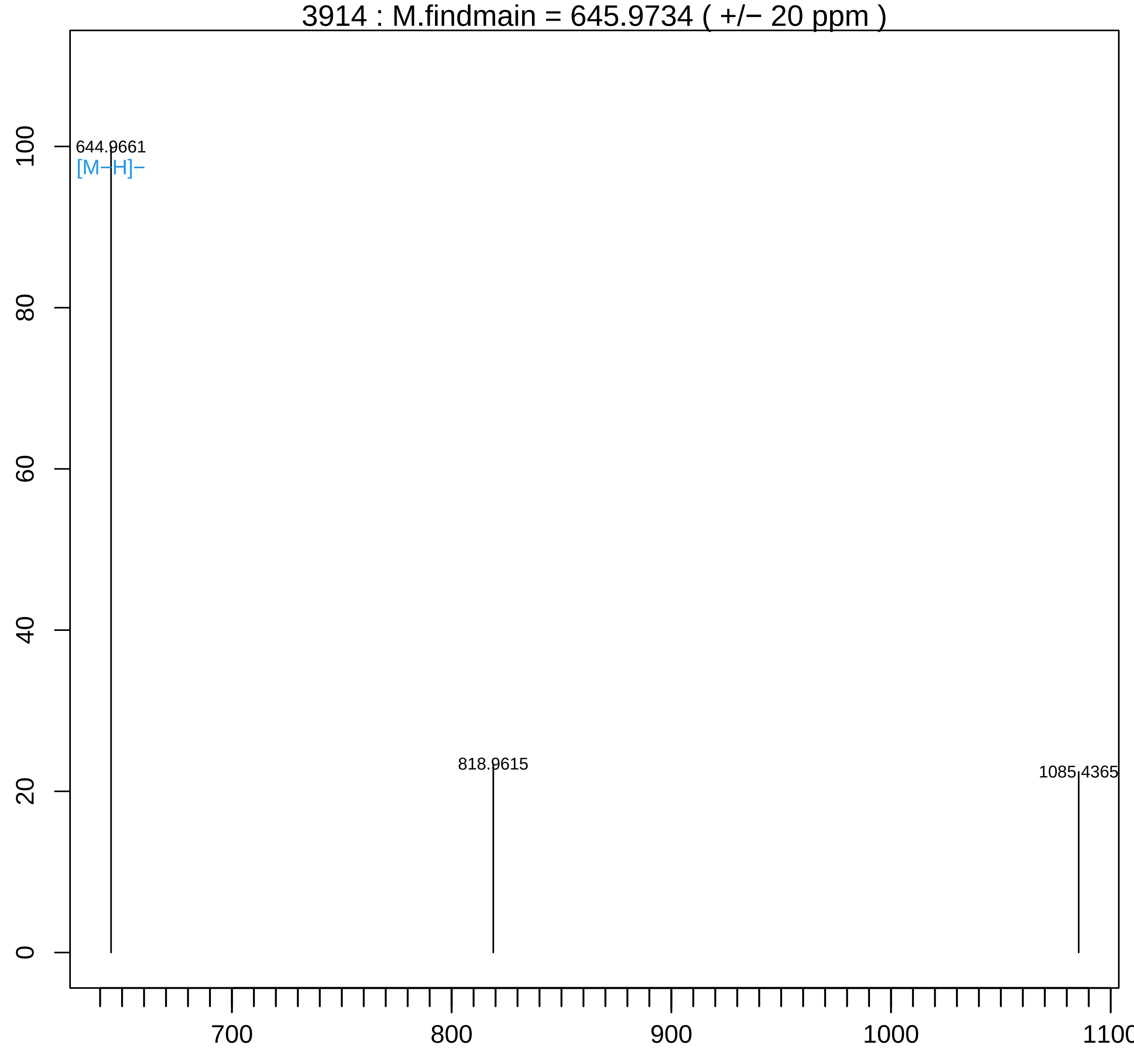
3914 : M.findmain = 645.9734 (+/− 20 ppm)
644.9661
[M−H]−
818.9615
1085.4365
0
20
40
60
80
100
700
800
900
1000
1100

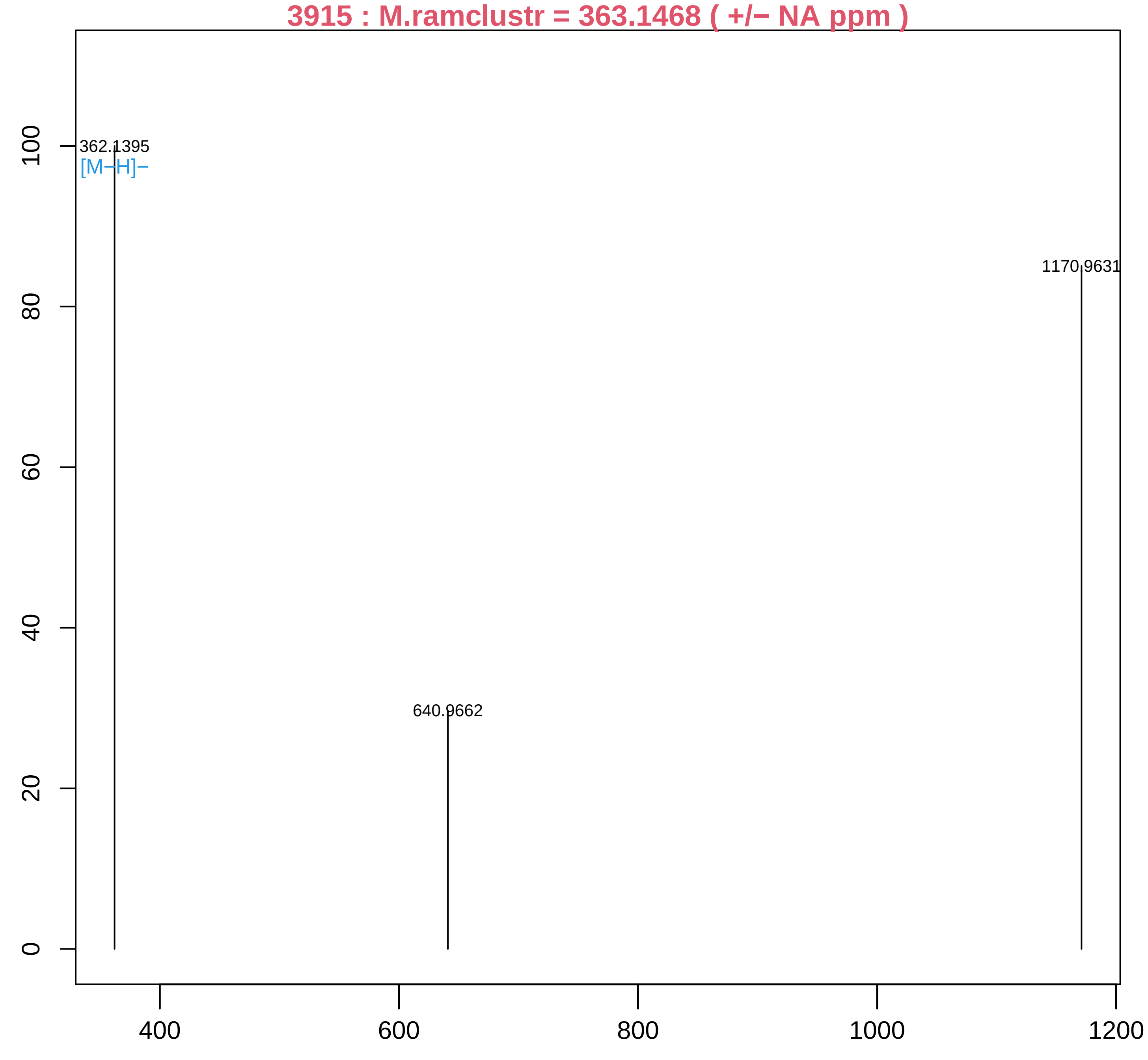
3915 : M.ramclustr = 363.1468 (+/− NA ppm)
362.1395
[M−H]−
640.9662
1170.9631
0
20
40
60
80
100
400
600
800
1000
1200

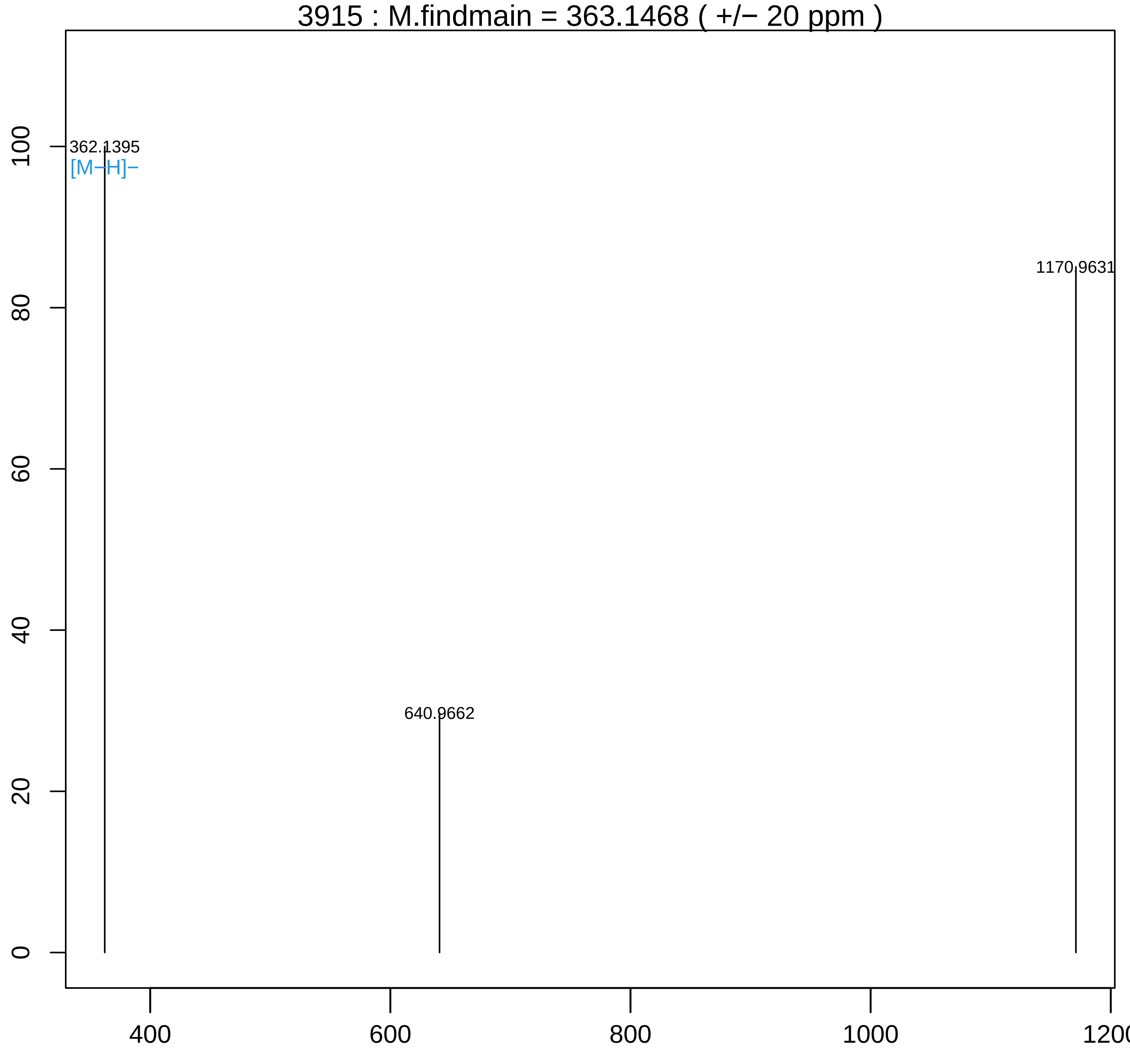
3915 : M.findmain = 363.1468 (+/− 20 ppm)
362.1395
[M−H]−
640.9662
1170.9631
0
20
40
60
80
100
400
600
800
1000
1200

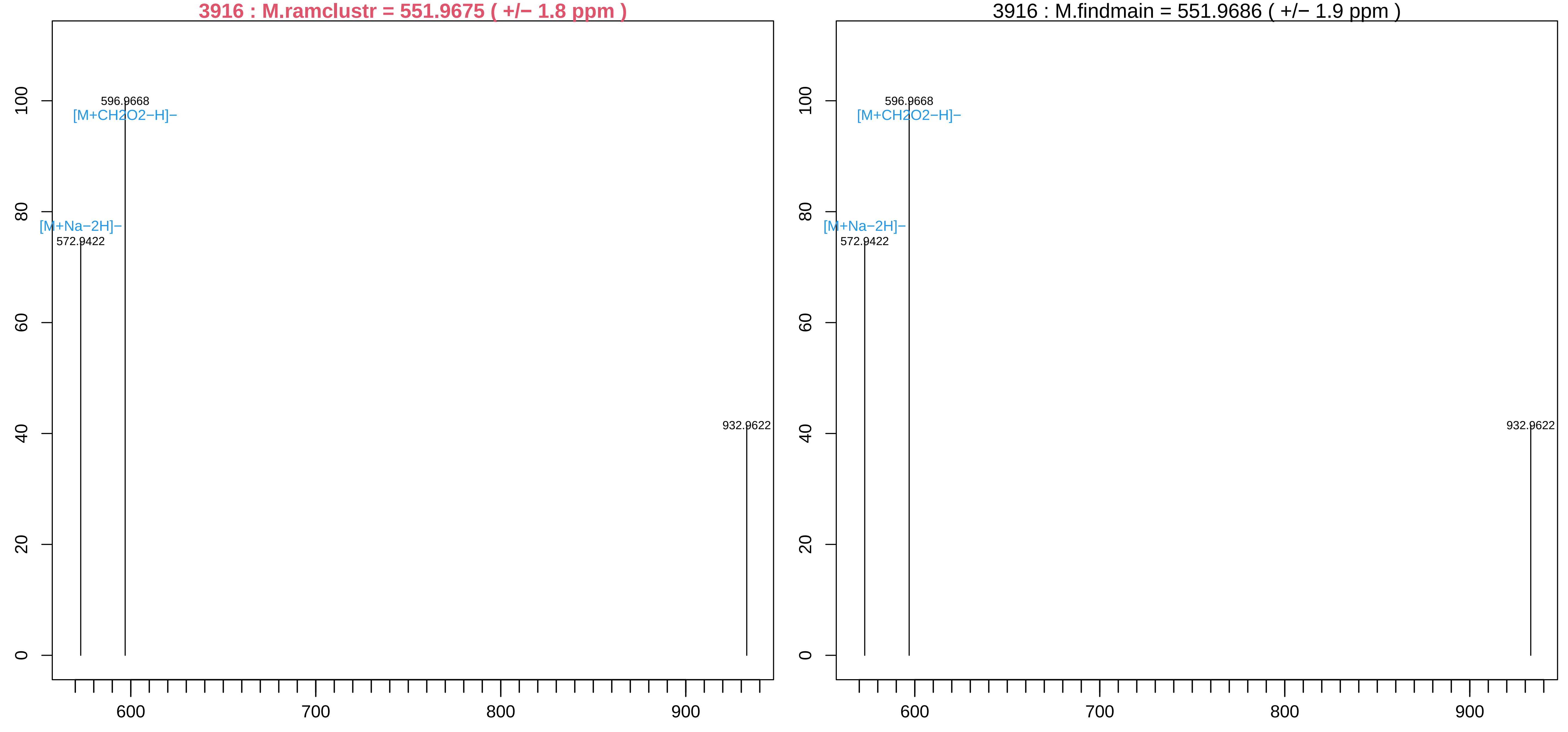
3916 : M.ramclustr = 551.9675 (+/− 1.8 ppm)
596.9668
[M+CH2O2−H]−
[M+Na−2H]−
572.9422
932.9622
3916 : M.findmain = 551.9686 (+/− 1.9 ppm)
596.9668
[M+CH2O2−H]−
[M+Na−2H]−
572.9422
932.9622
0
20
40
60
80
100
600
700
800
900

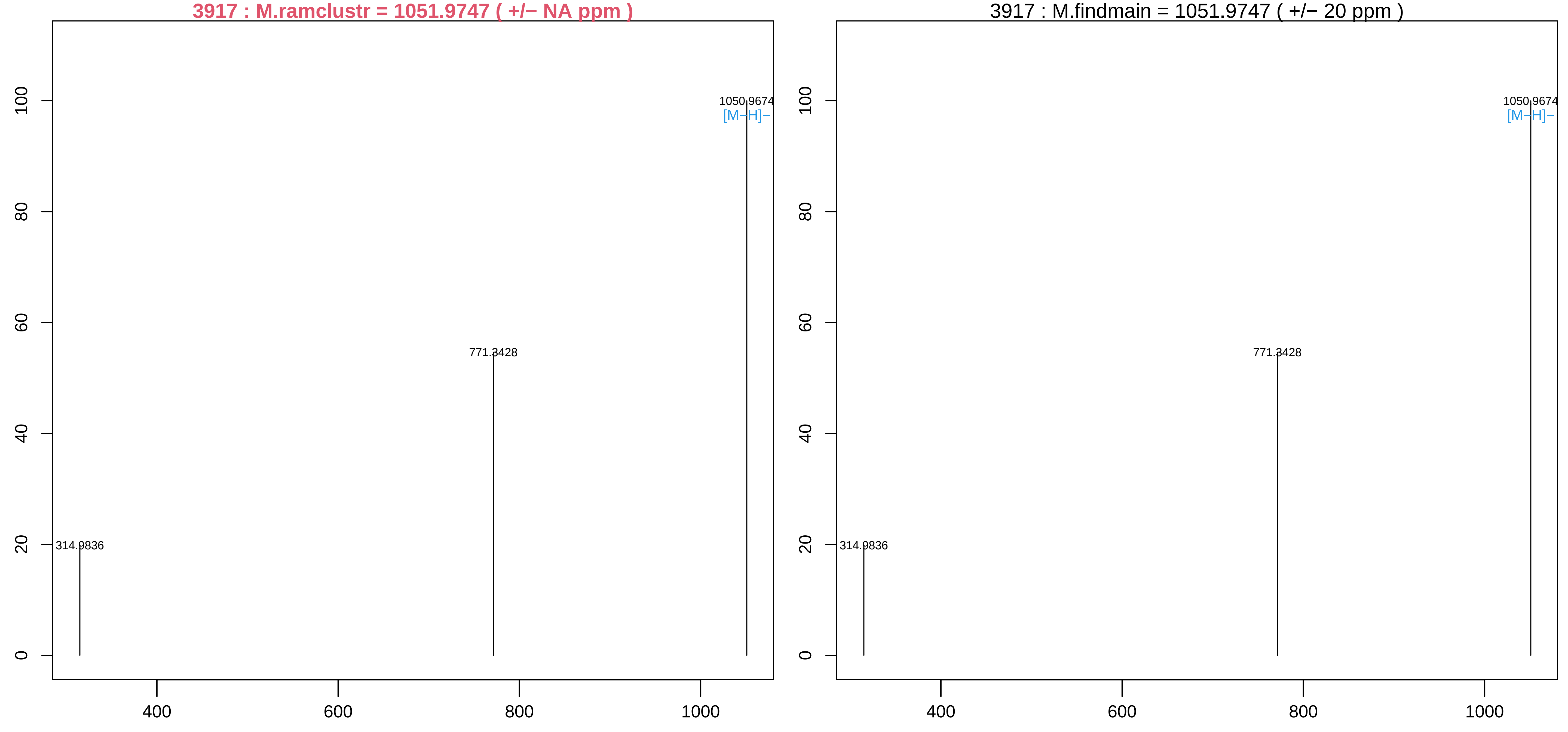

3917 : M.ramclustr = 1051.9747 (+/− NA ppm)
1050.9674
[M−H]−
771.3428
314.9836
0
20
40
60
80
100
400
600
800
1000
3917 : M.findmain = 1051.9747 (+/− 20 ppm)
1050.9674
[M−H]−
771.3428
314.9836
0
20
40
60
80
100
400
600
800
1000

3918 : M.ramclustr = 853.9696 (+/− NA ppm)

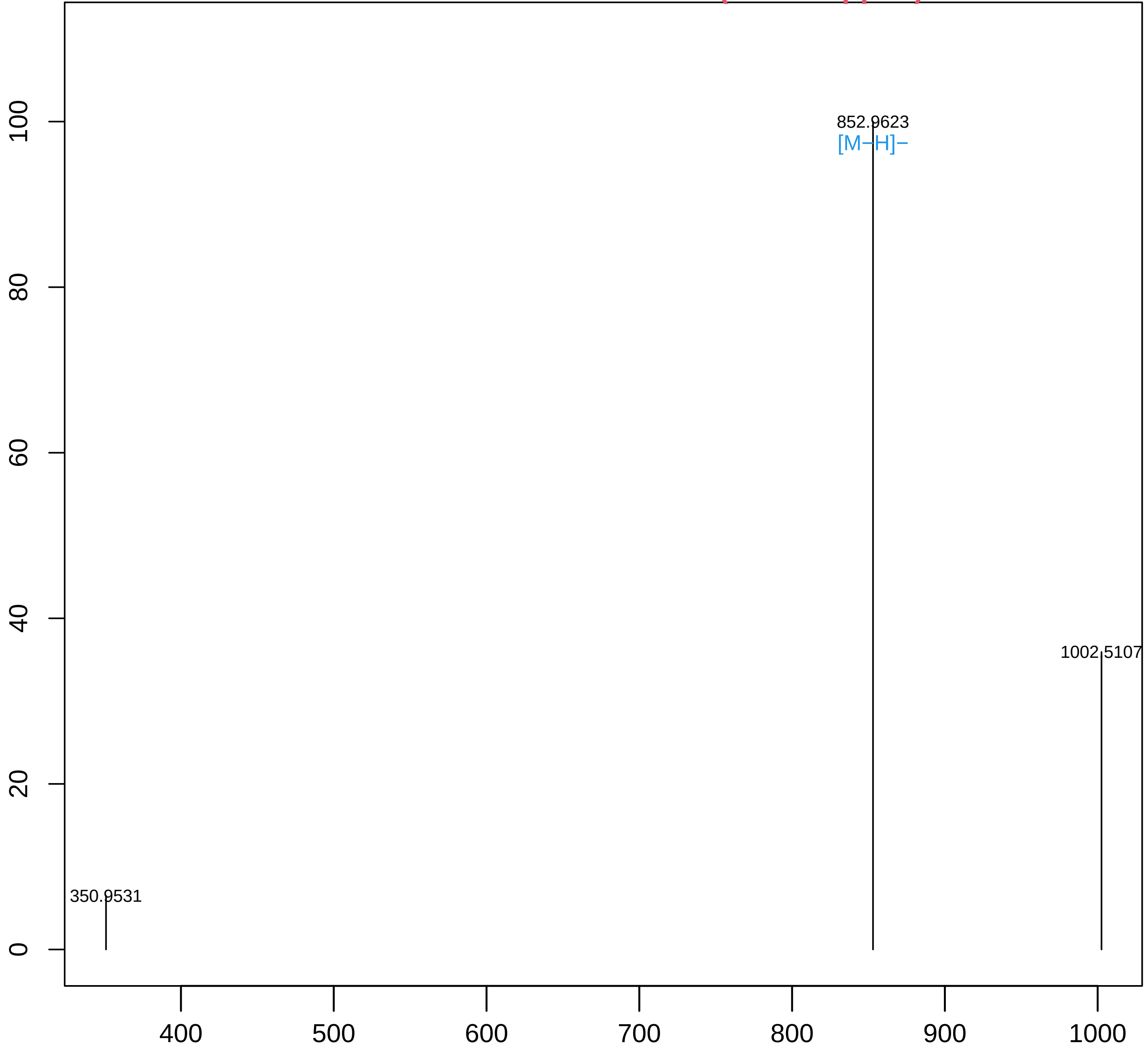

3918 : M.findmain = 853.9696 (+/− 20 ppm)

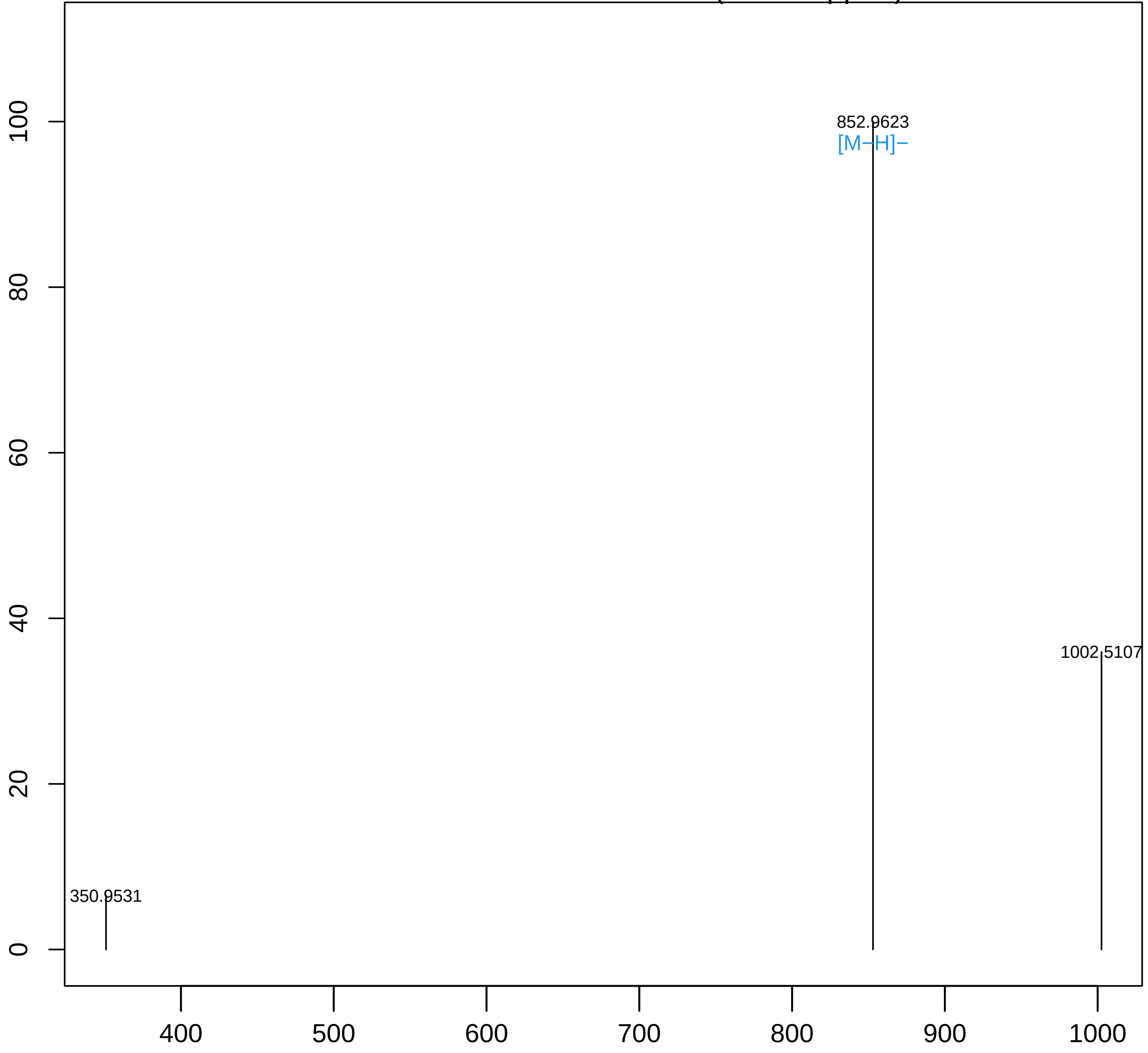

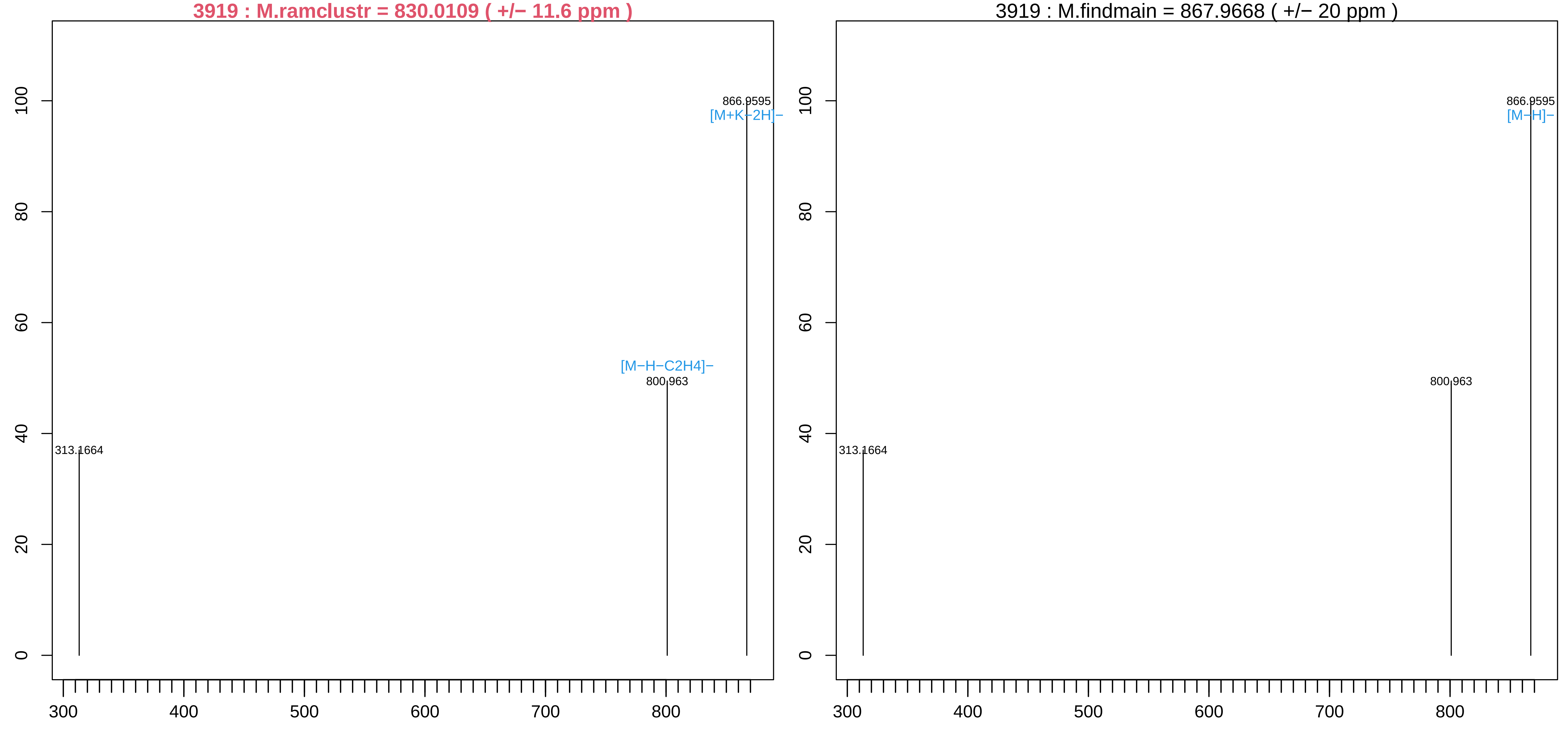

3919 : M.ramclustr = 830.0109 (+/− 11.6 ppm)
866.9595
[M+K−2H]−
[M−H−C2H4]−
800.963
313.1664
3919 : M.findmain = 867.9668 (+/− 20 ppm)
866.9595
[M−H]−
800.963
313.1664

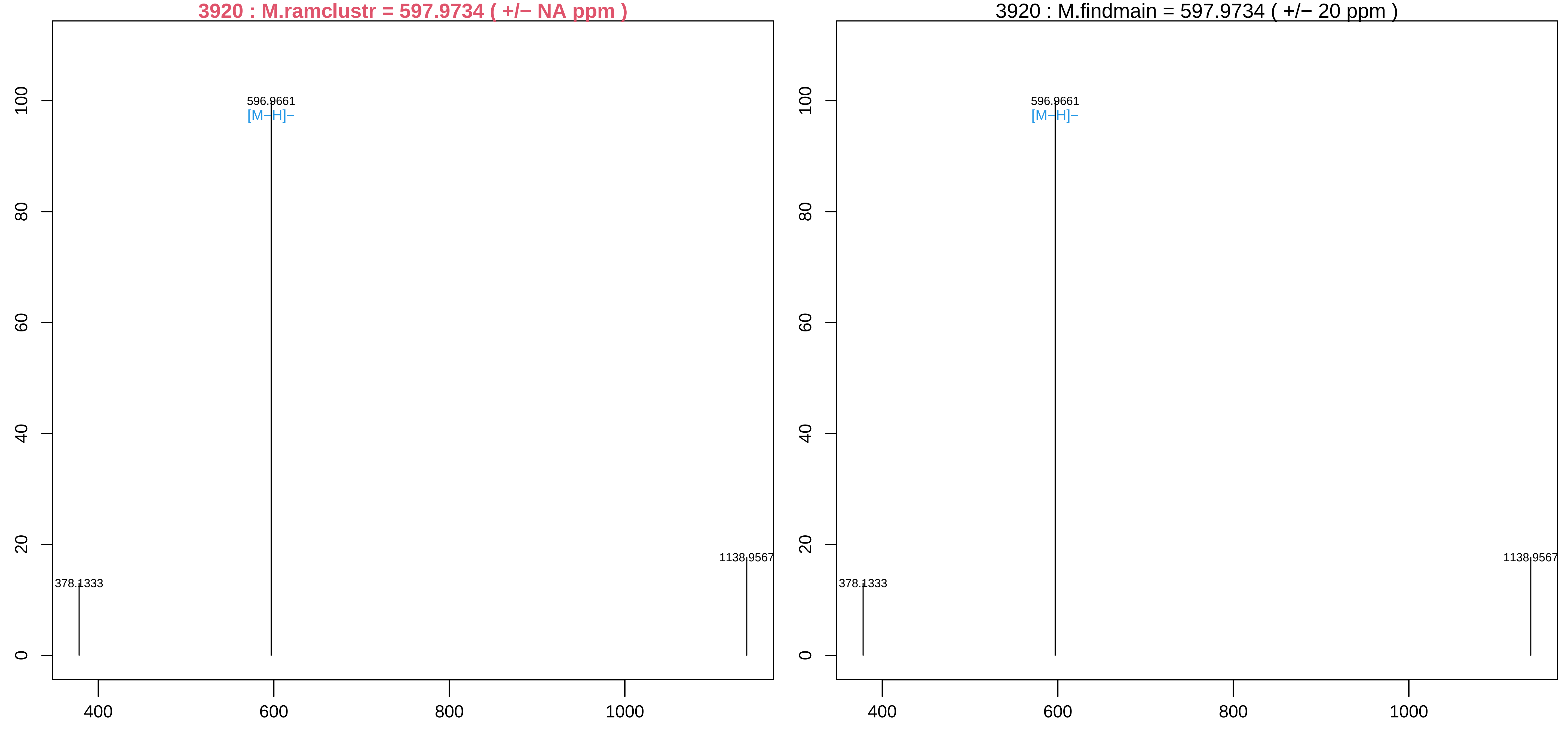
3920 : M.ramclustr = 597.9734 (+/− NA ppm)
596.9661
[M−H]−
378.1333
1138.9567
0
20
40
60
80
100
400
600
800
1000
3920 : M.findmain = 597.9734 (+/− 20 ppm)
596.9661
[M−H]−
378.1333
1138.9567
0
20
40
60
80
100
400
600
800
1000

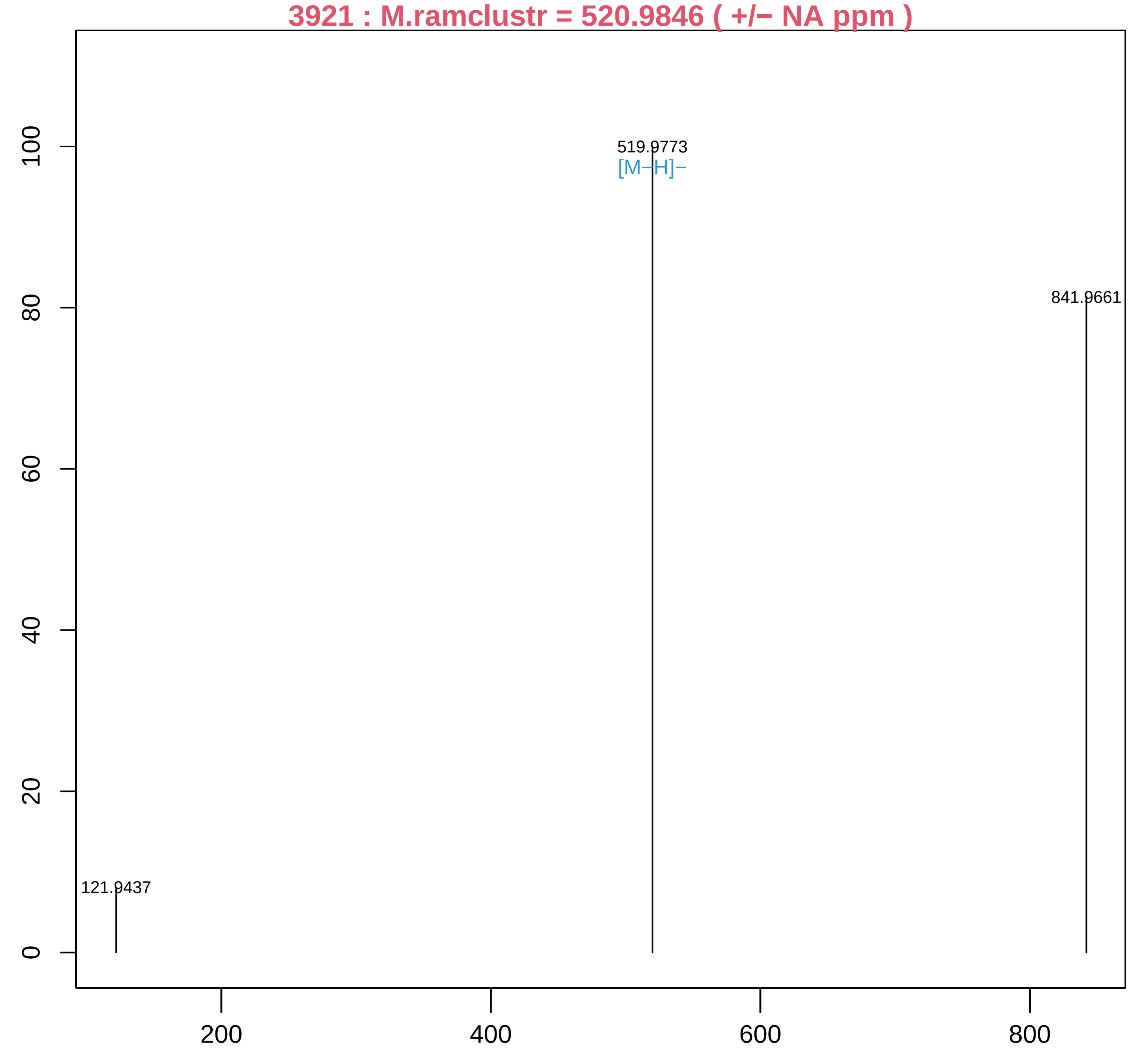
3921 : M.ramclustr = 520.9846 (+/− NA ppm)
519.9773
[M−H]−
841.9661
121.9437
0
20
40
60
80
100
200
400
600
800

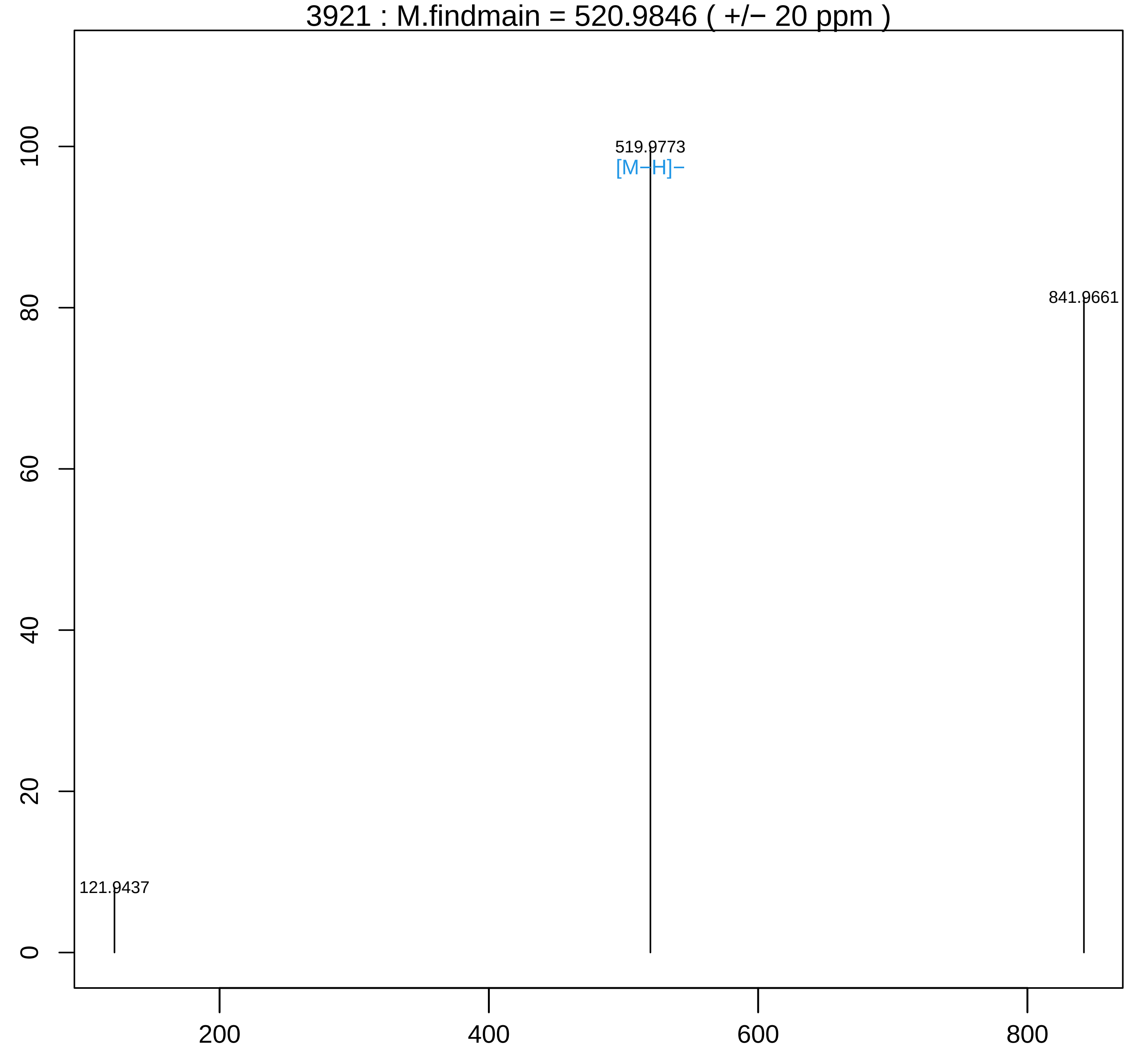
3921 : M.findmain = 520.9846 (+/− 20 ppm)
519.9773
[M−H]−
841.9661
121.9437
0
20
40
60
80
100
200
400
600
800

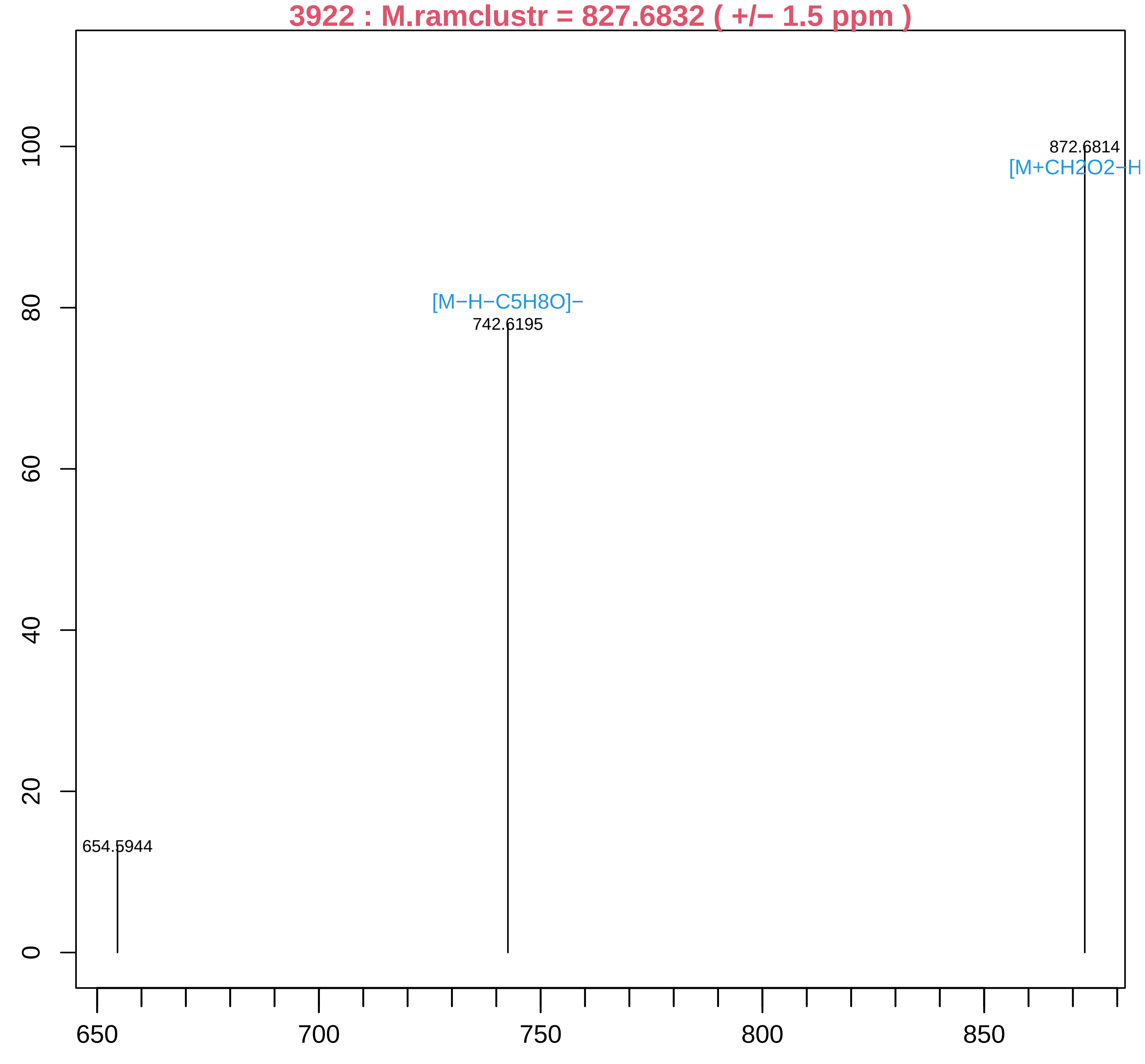

3922 : M.ramclustr = 827.6832 (+/− 1.5 ppm)
872.6814
[M+CH2O2−H
[M−H−C5H8O]−
742.6195
654.5944
0
20
40
60
80
100
650
700
750
800
850

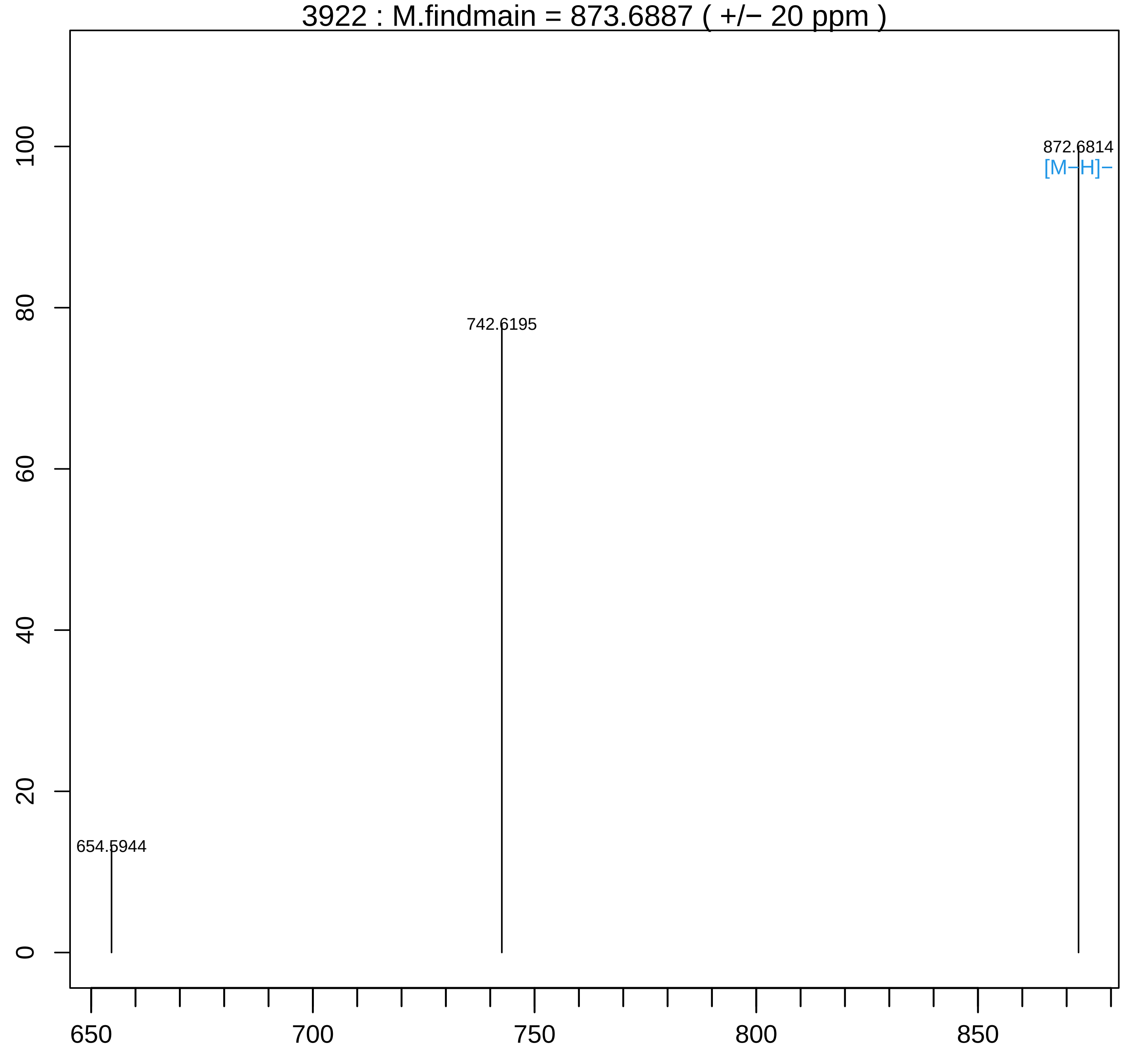

3922 : M.findmain = 873.6887 (+/− 20 ppm)
872.6814
[M−H]−
742.6195
654.5944
0
20
40
60
80
100
650
700
750
800
850

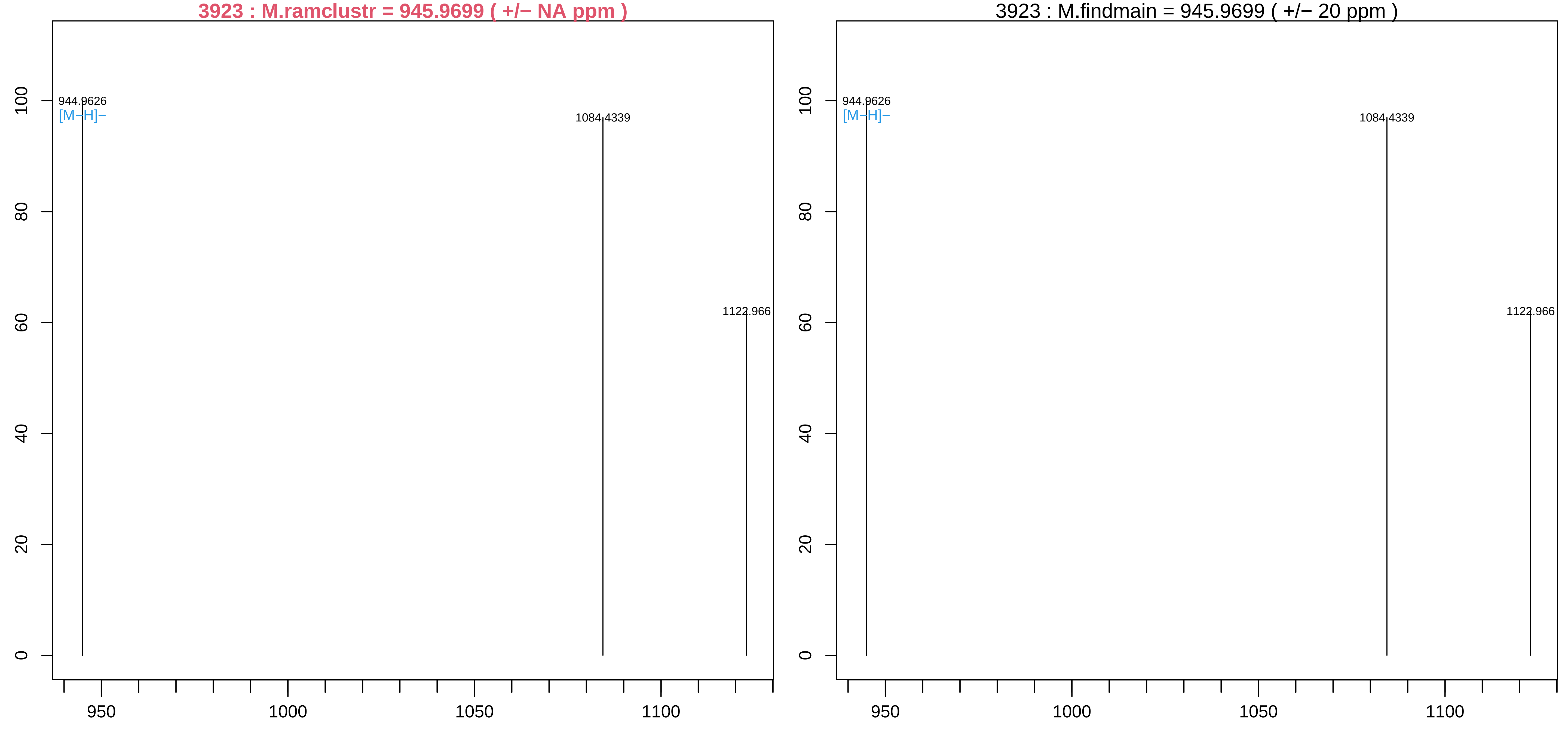
3923 : M.ramclustr = 945.9699 (+/− NA ppm)
944.9626
[M−H]−
1084.4339
1122.966
0
20
40
60
80
100
950
1000
1050
1100
3923 : M.findmain = 945.9699 (+/− 20 ppm)
944.9626
[M−H]−
1084.4339
1122.966
0
20
40
60
80
100
950
1000
1050
1100

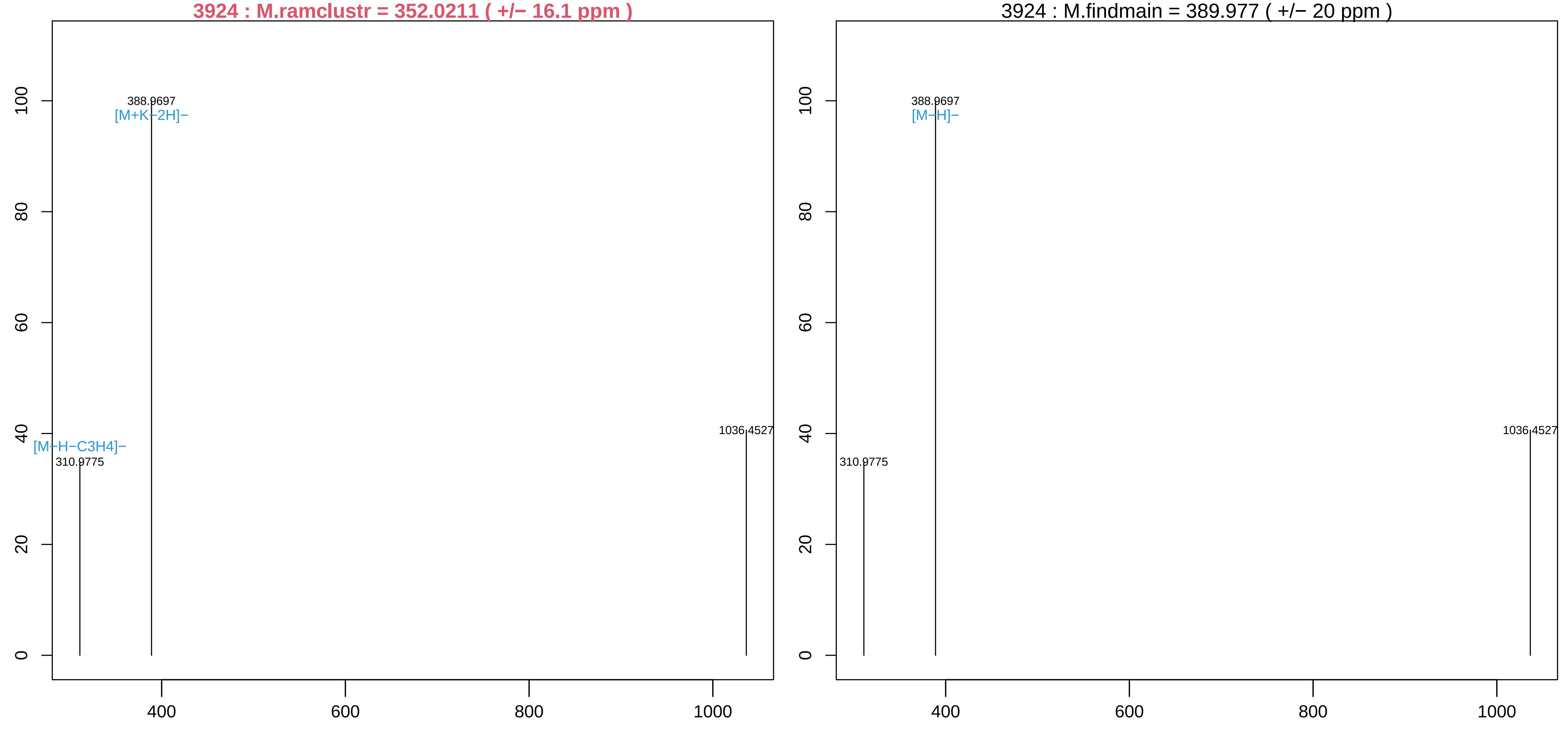

3924 : M.ramclustr = 352.0211 (+/− 16.1 ppm)
388.9697
[M+K−2H]−
[M−H−C3H4]−
310.9775
1036.4527
100
80
60
40
20
0
400
600
800
1000
3924 : M.findmain = 389.977 (+/− 20 ppm)
388.9697
[M−H]−
310.9775
1036.4527
100
80
60
40
20
0
400
600
800
1000

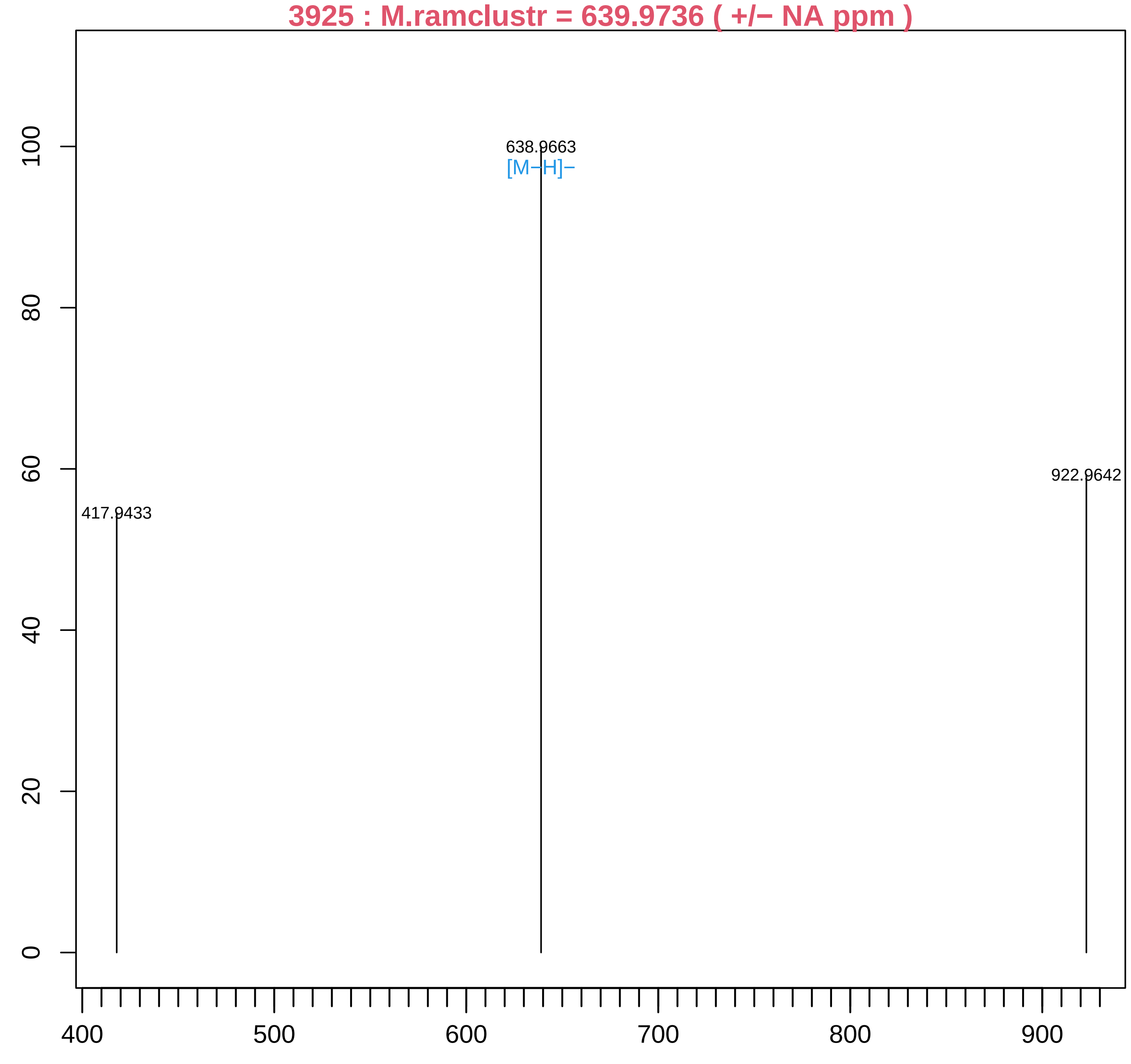
3925 : M.ramclustr = 639.9736 (+/− NA ppm)
638.9663
[M−H]−
922.9642
417.9433
0
20
40
60
80
100
400
500
600
700
800
900

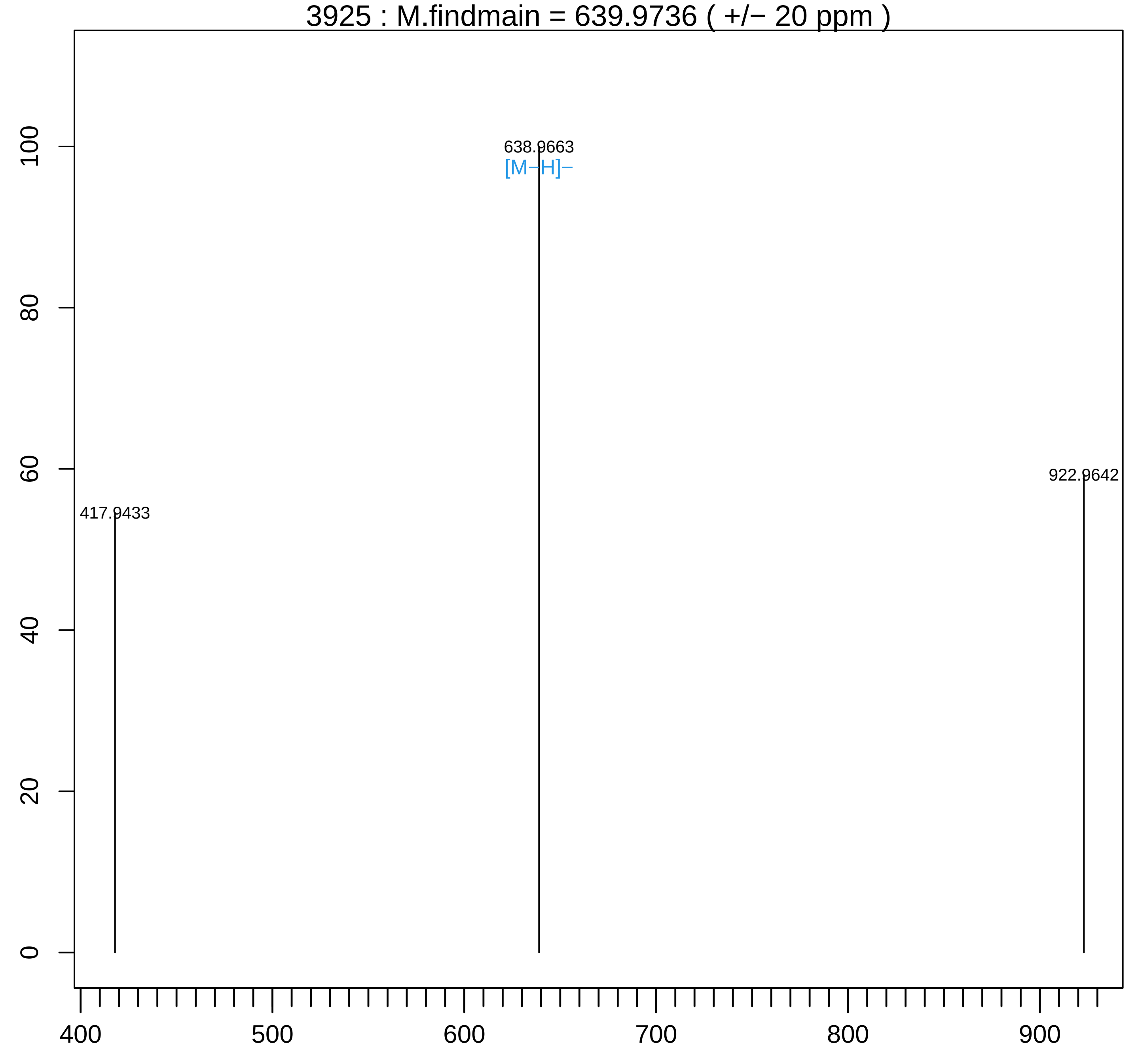
3925 : M.findmain = 639.9736 (+/− 20 ppm)
638.9663
[M−H]−
922.9642
417.9433
0
20
40
60
80
100
400
500
600
700
800
900

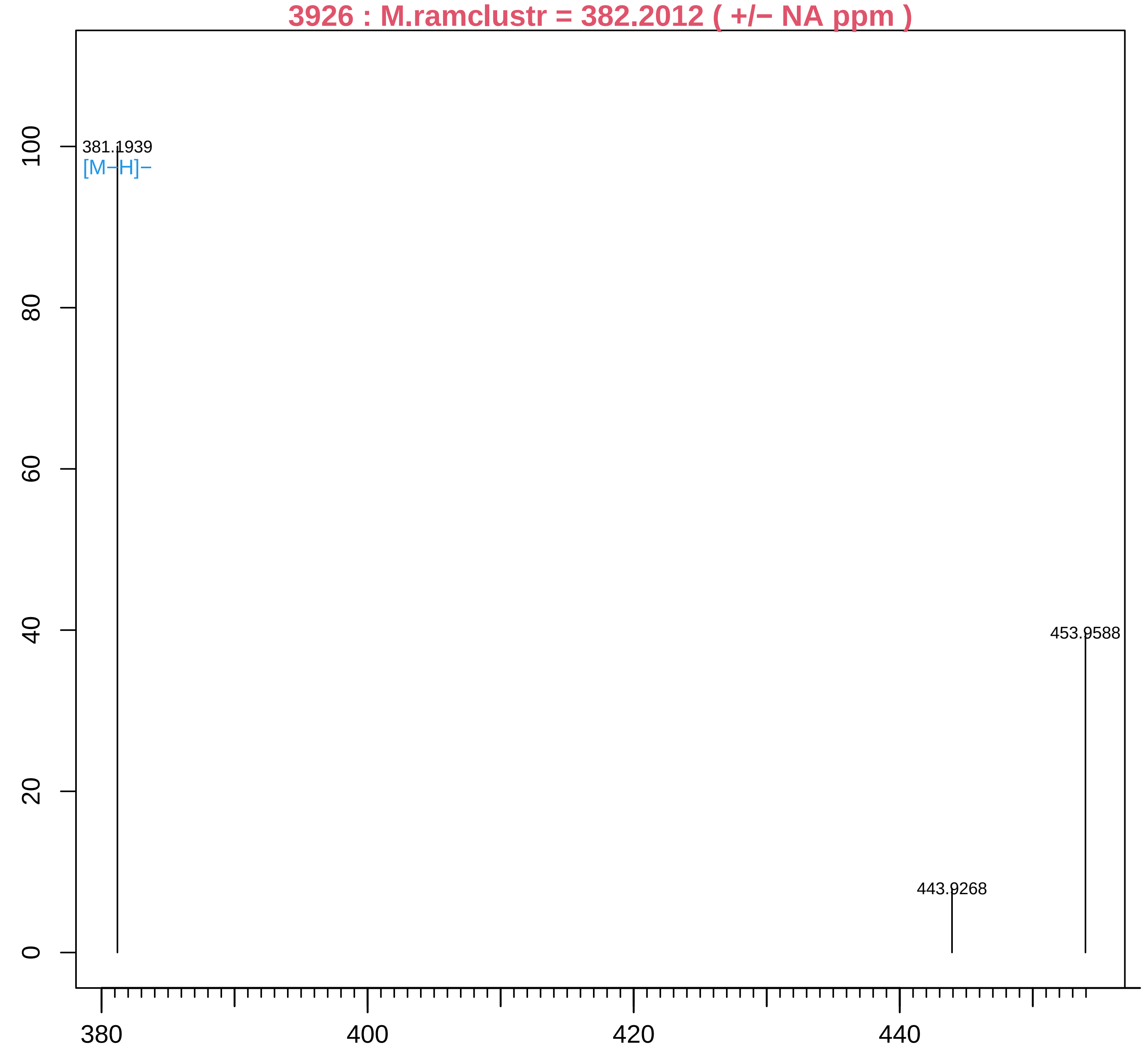
3926 : M.ramclustr = 382.2012 (+/− NA ppm)
381.1939
[M−H]−
443.9268
453.9588
0
20
40
60
80
100
380
400
420
440

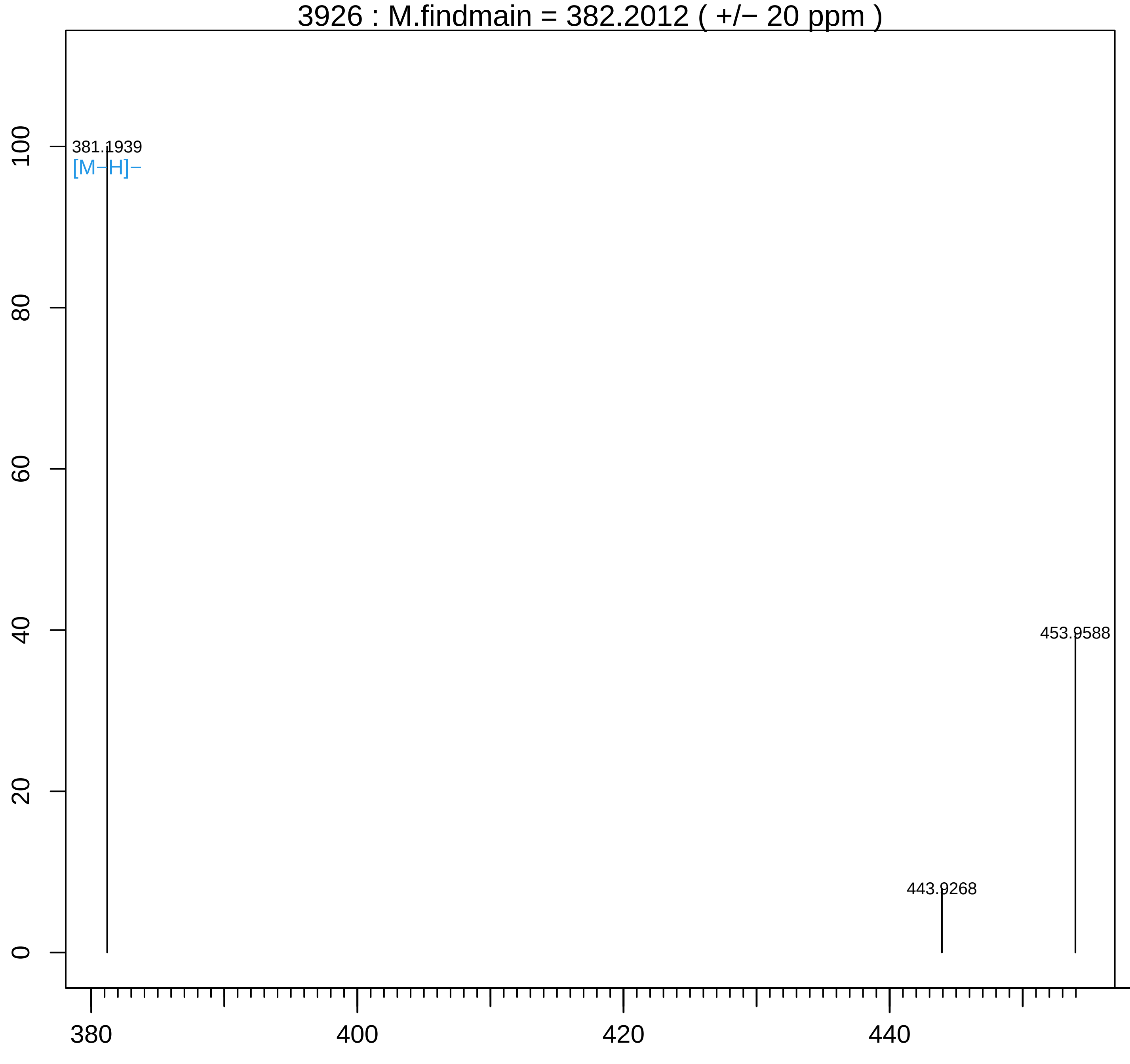
3926 : M.findmain = 382.2012 (+/− 20 ppm)
381.1939
[M−H]−
443.9268
453.9588
0
20
40
60
80
100
380
400
420
440

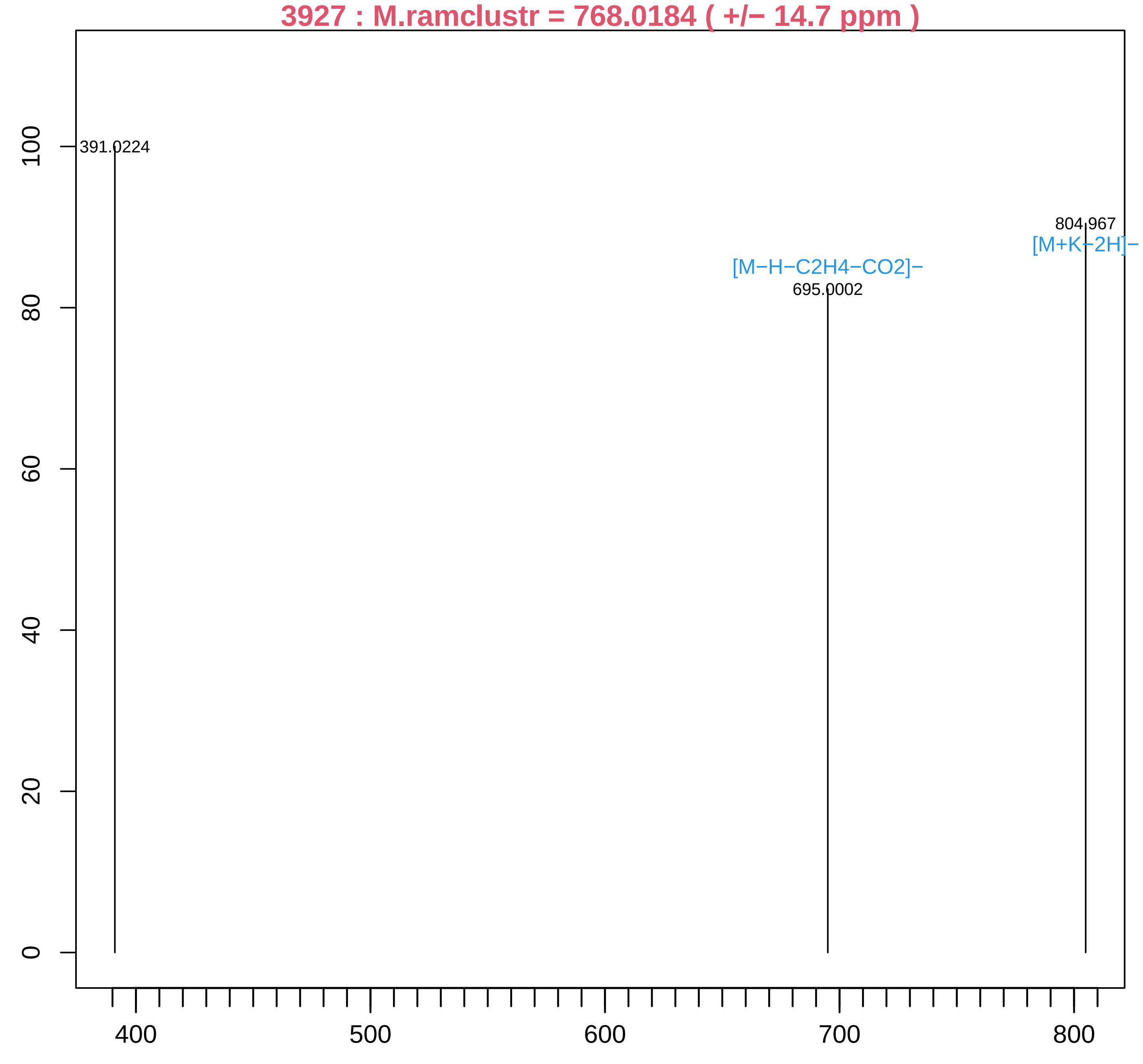
3927 : M.ramclustr = 768.0184 (+/− 14.7 ppm)
391.0224
[M−H−C2H4−CO2]−
695.0002
804.967
[M+K−2H]−
0
20
40
60
80
100
400
500
600
700
800

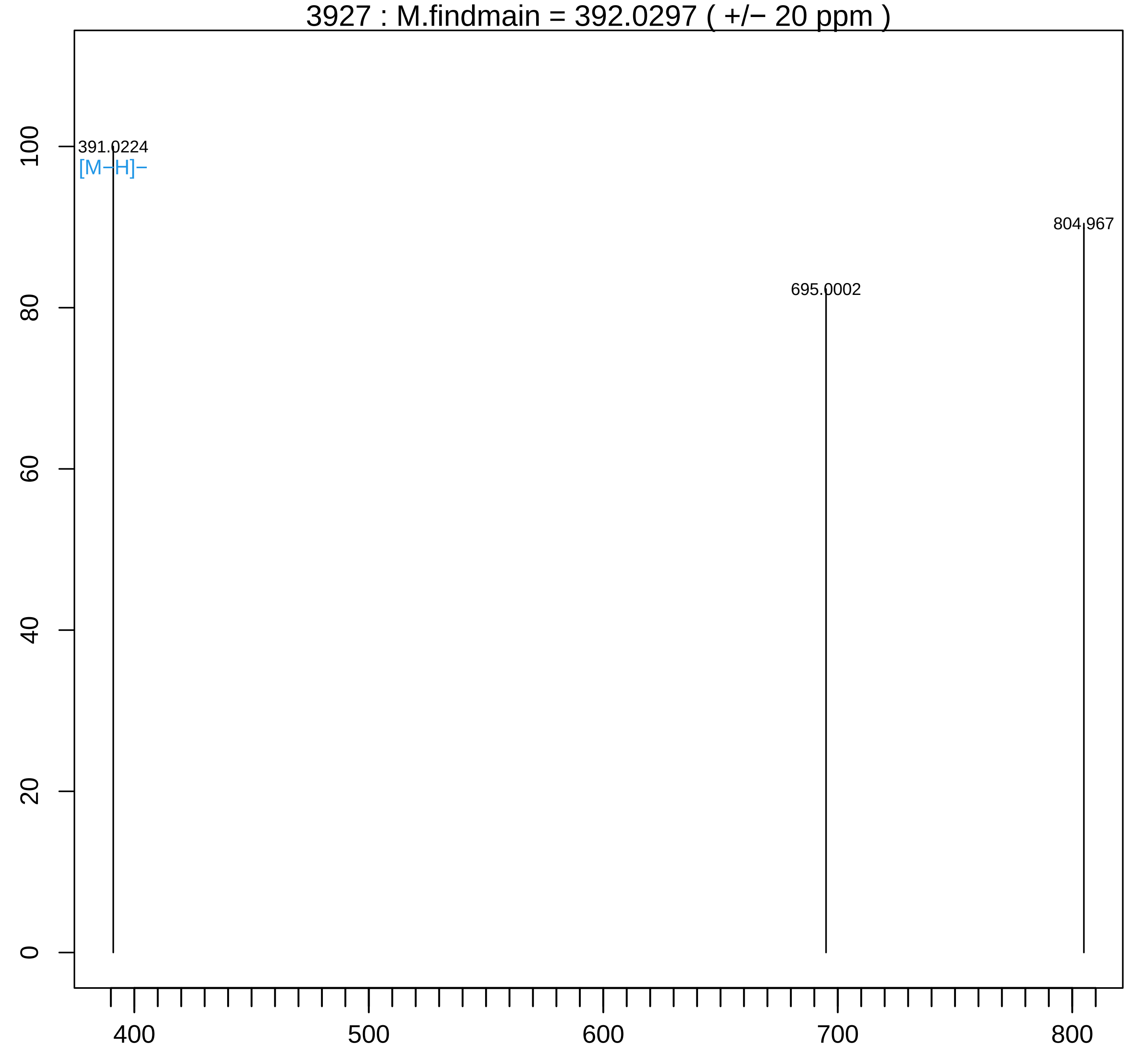
3927 : M.findmain = 392.0297 (+/− 20 ppm)
391.0224
[M−H]−
695.0002
804.967
0
20
40
60
80
100
400
500
600
700
800

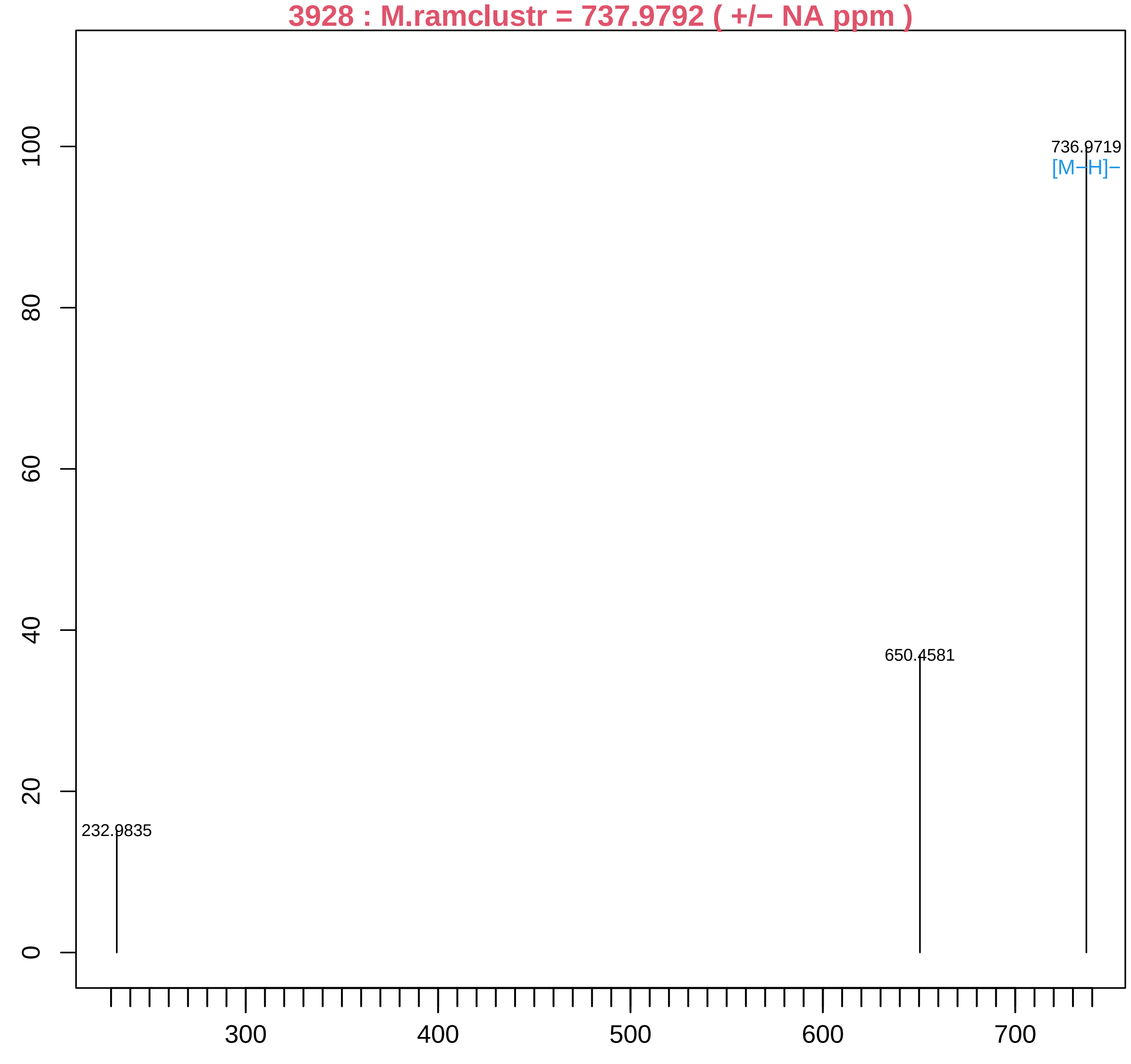
3928 : M.ramclustr = 737.9792 (+/− NA ppm)
736.9719
[M−H]−
650.4581
232.9835
0
20
40
60
80
100
300
400
500
600
700

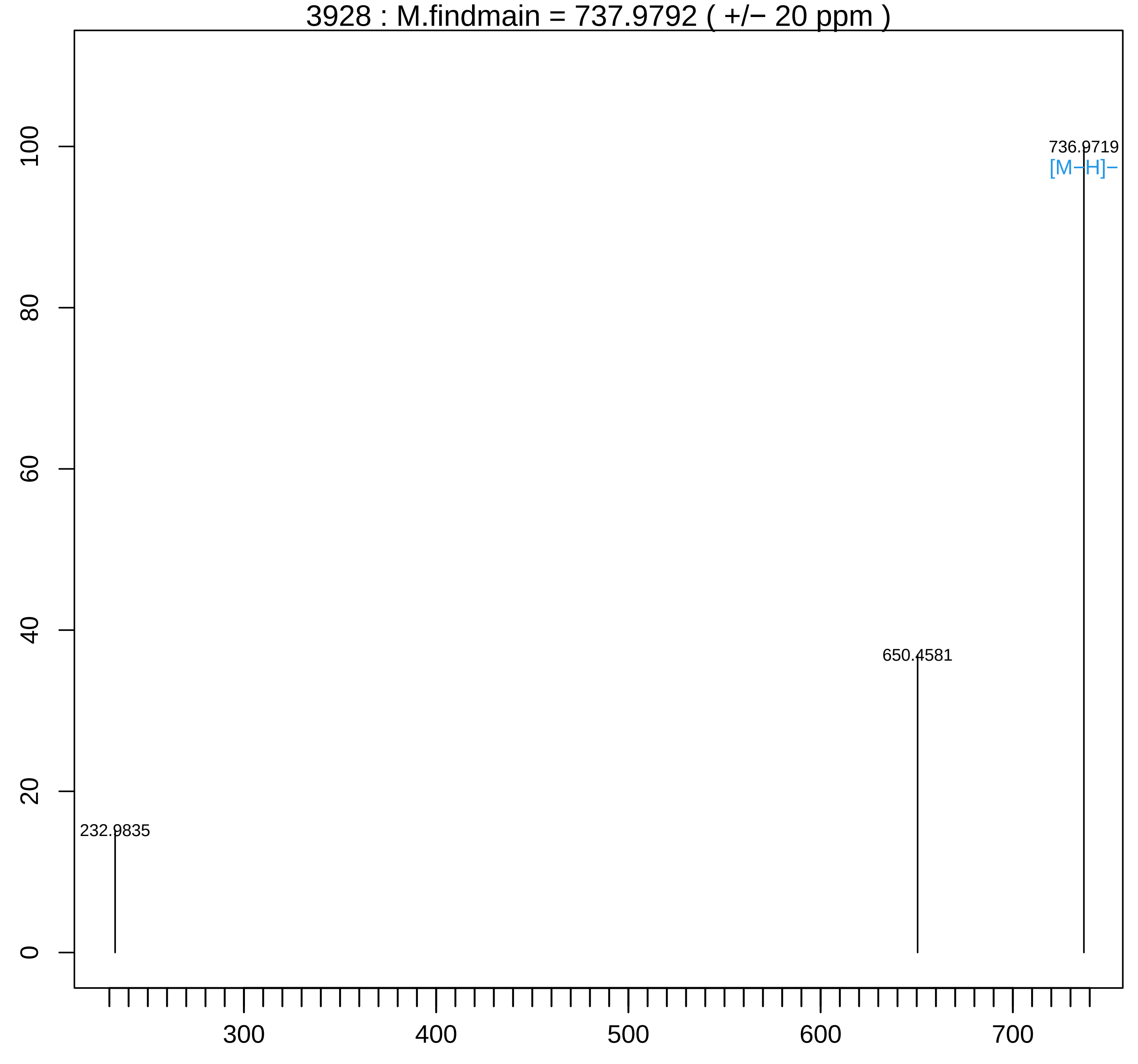
3928 : M.findmain = 737.9792 (+/− 20 ppm)
736.9719
[M−H]−
650.4581
232.9835
0
20
40
60
80
100
300
400
500
600
700

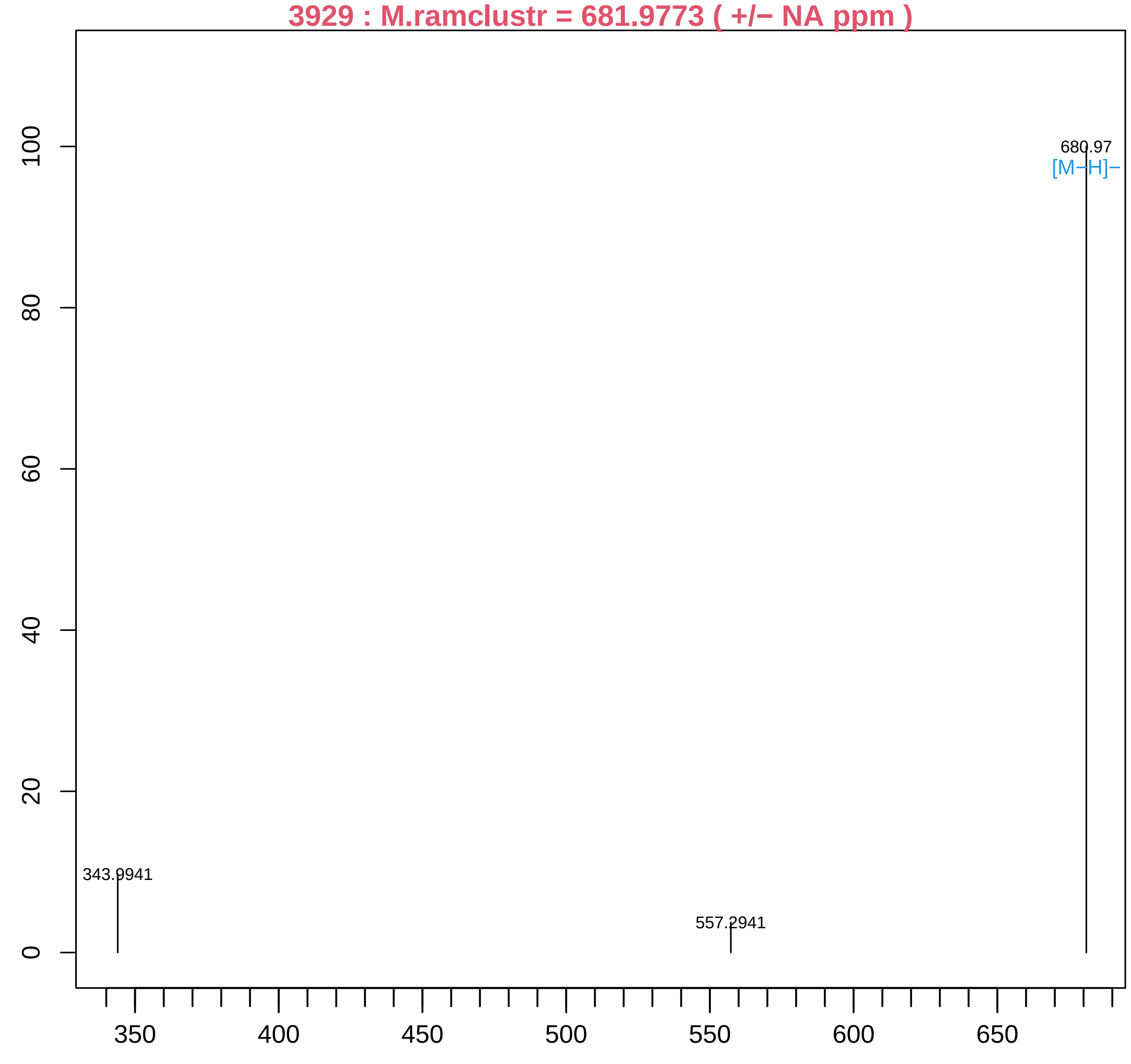
3929 : M.ramclustr = 681.9773 (+/− NA ppm)
680.97
[M−H]−
343.9941
557.2941
0
20
40
60
80
100
350
400
450
500
550
600
650

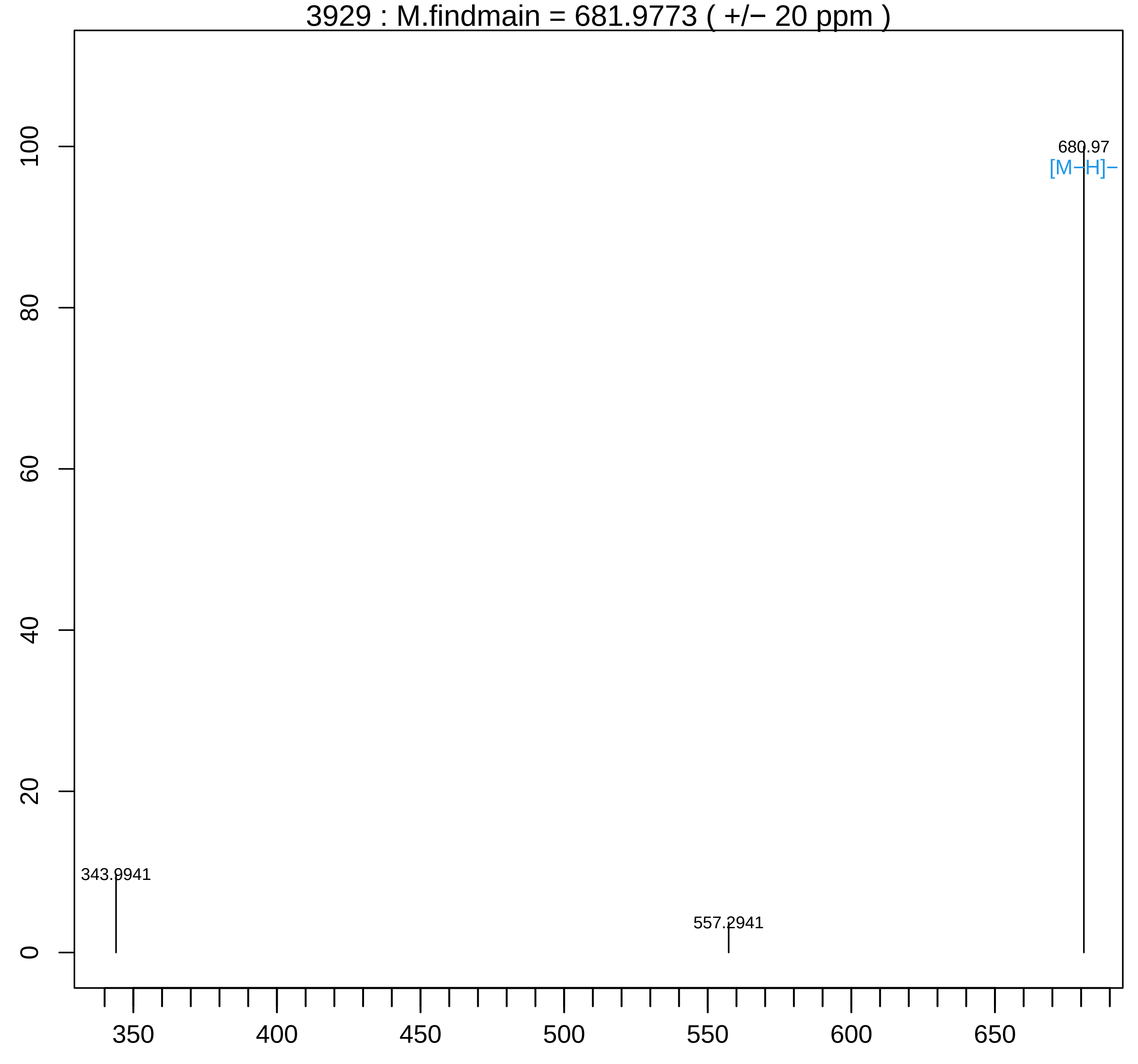
3929 : M.findmain = 681.9773 (+/− 20 ppm)
680.97
[M−H]−
343.9941
557.2941
0
20
40
60
80
100
350
400
450
500
550
600
650

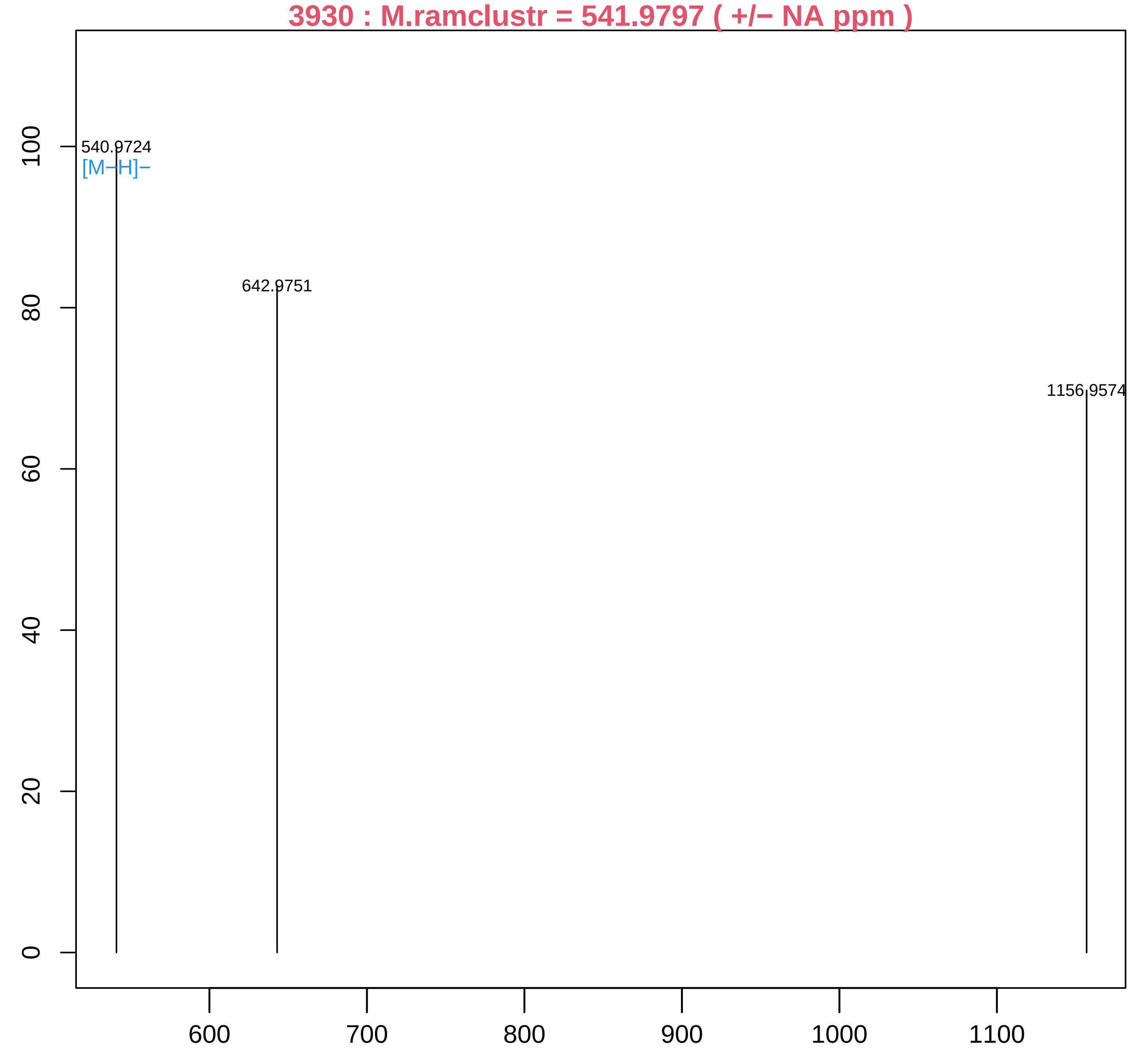
3930 : M.ramclustr = 541.9797 (+/− NA ppm)
540.9724
[M−H]−
642.9751
1156.9574
0
20
40
60
80
100
600
700
800
900
1000
1100

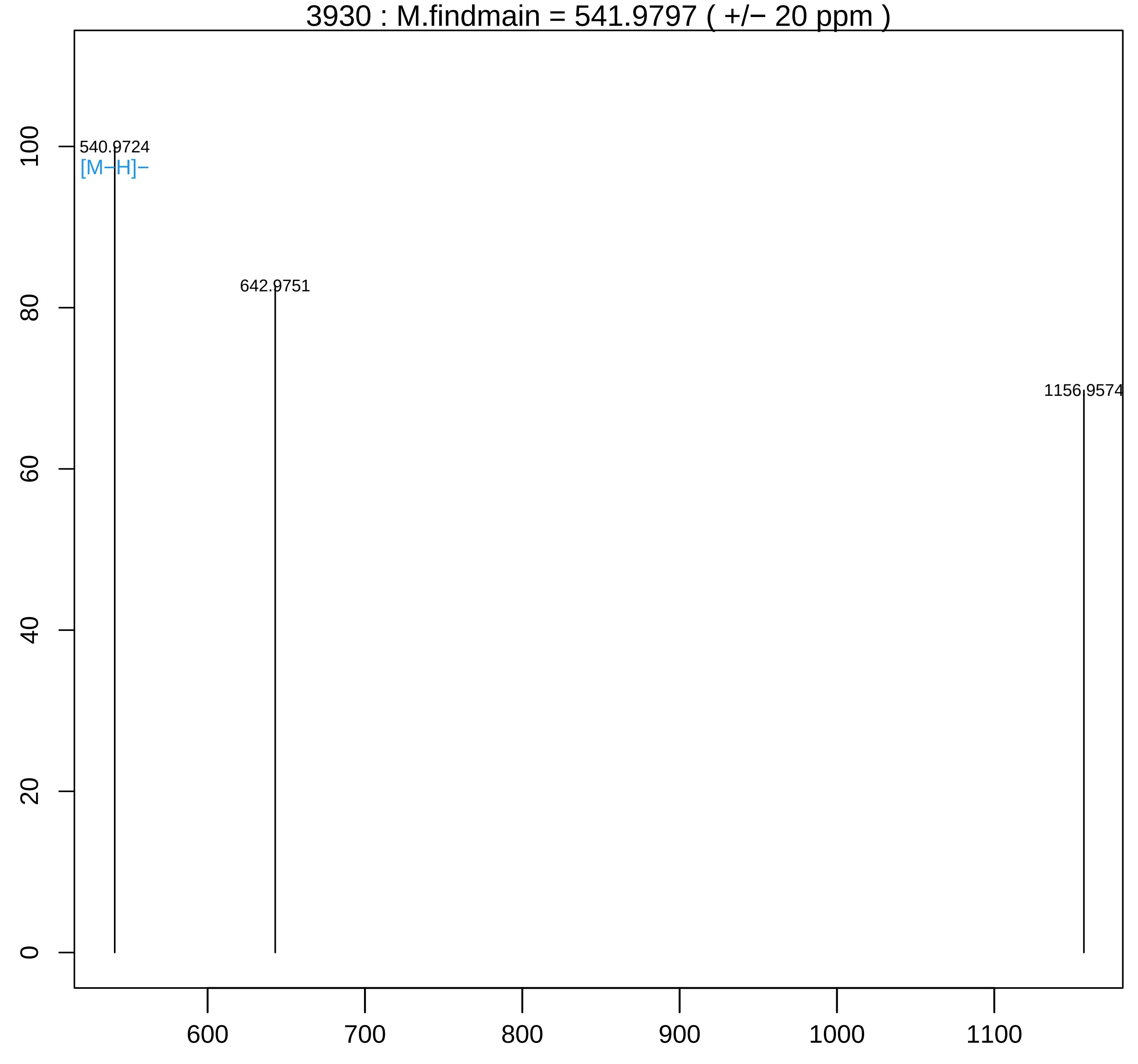
3930 : M.findmain = 541.9797 (+/− 20 ppm)
540.9724
[M−H]−
642.9751
1156.9574
0
20
40
60
80
100
600
700
800
900
1000
1100

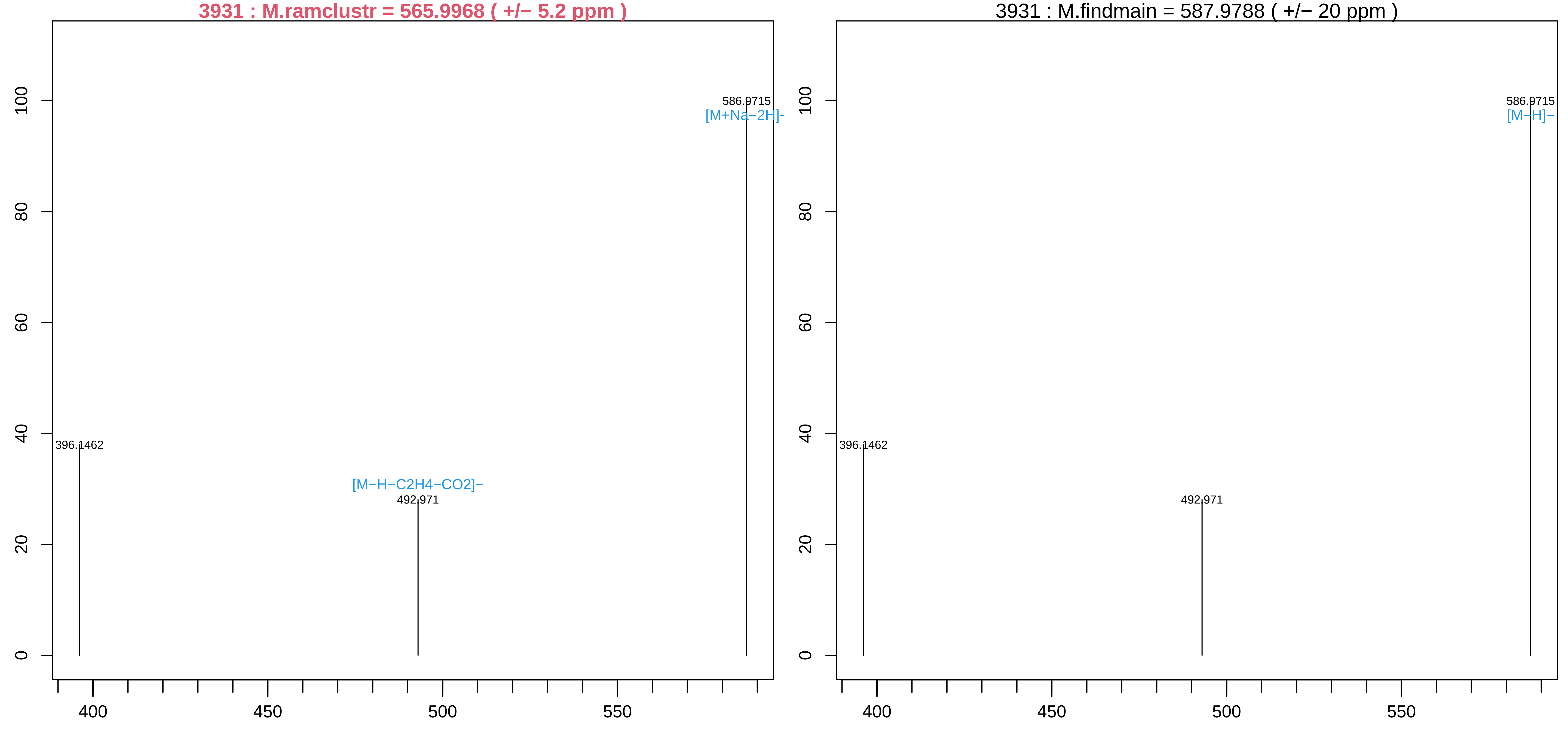
3931 : M.ramclustr = 565.9968 (+/− 5.2 ppm)
586.9715
[M+Na−2H]-
396.1462
[M−H−C2H4−CO2]−
492.971
3931 : M.findmain = 587.9788 (+/− 20 ppm)
586.9715
[M−H]−
396.1462
492.971
0
20
40
60
80
100
400
450
500
550

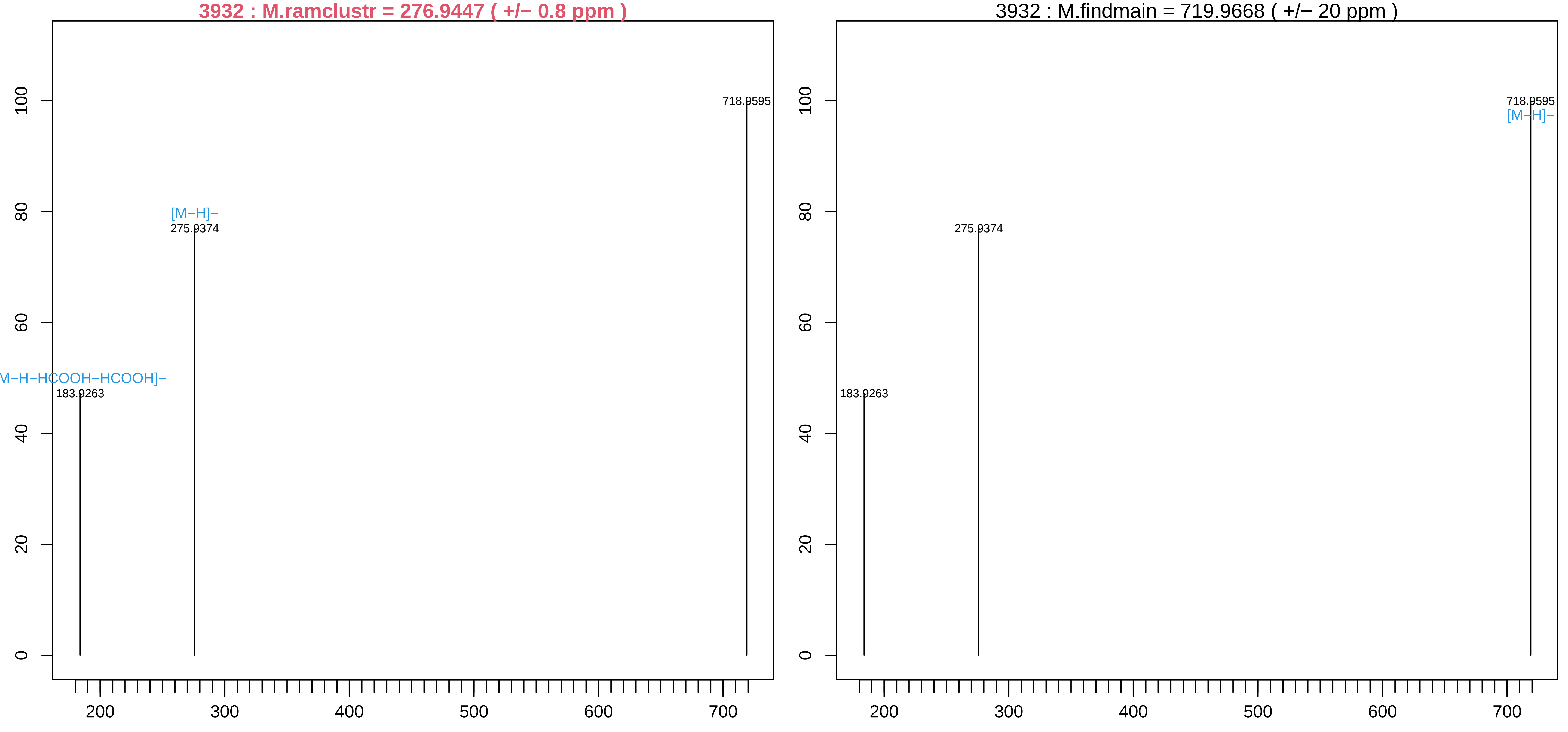
3932 : M.ramclustr = 276.9447 (+/− 0.8 ppm)
718.9595
[M−H]−
275.9374
M−H−HCOOH−HCOOH]−
183.9263
0
20
40
60
80
100
200
300
400
500
600
700
3932 : M.findmain = 719.9668 (+/− 20 ppm)
718.9595
[M−H]−
275.9374
183.9263
0
20
40
60
80
100
200
300
400
500
600
700

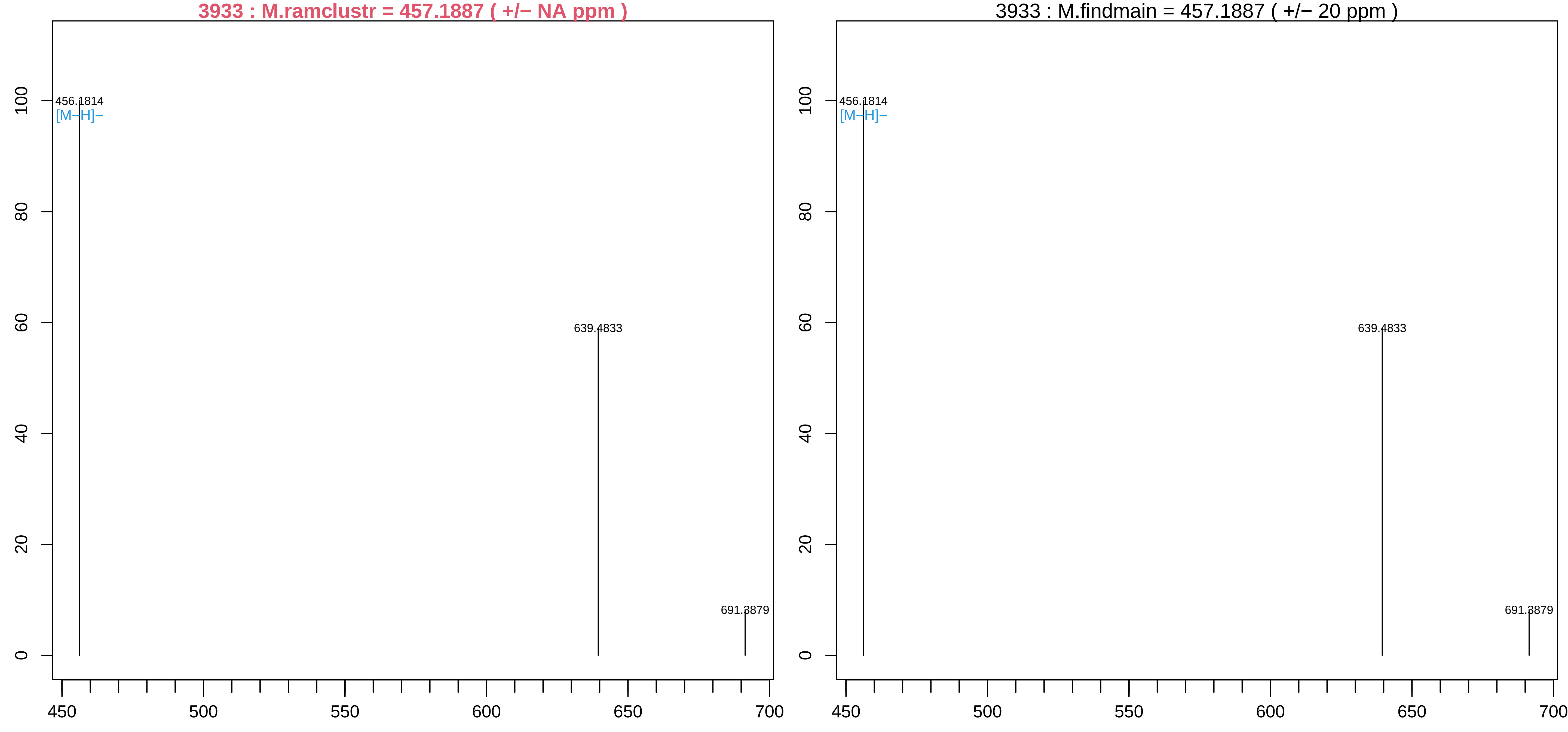
3933 : M.ramclustr = 457.1887 (+/− NA ppm)
456.1814
[M−H]−
639.4833
691.3879
3933 : M.findmain = 457.1887 (+/− 20 ppm)
456.1814
[M−H]−
639.4833
691.3879
0
20
40
60
80
100
450
500
550
600
650
700

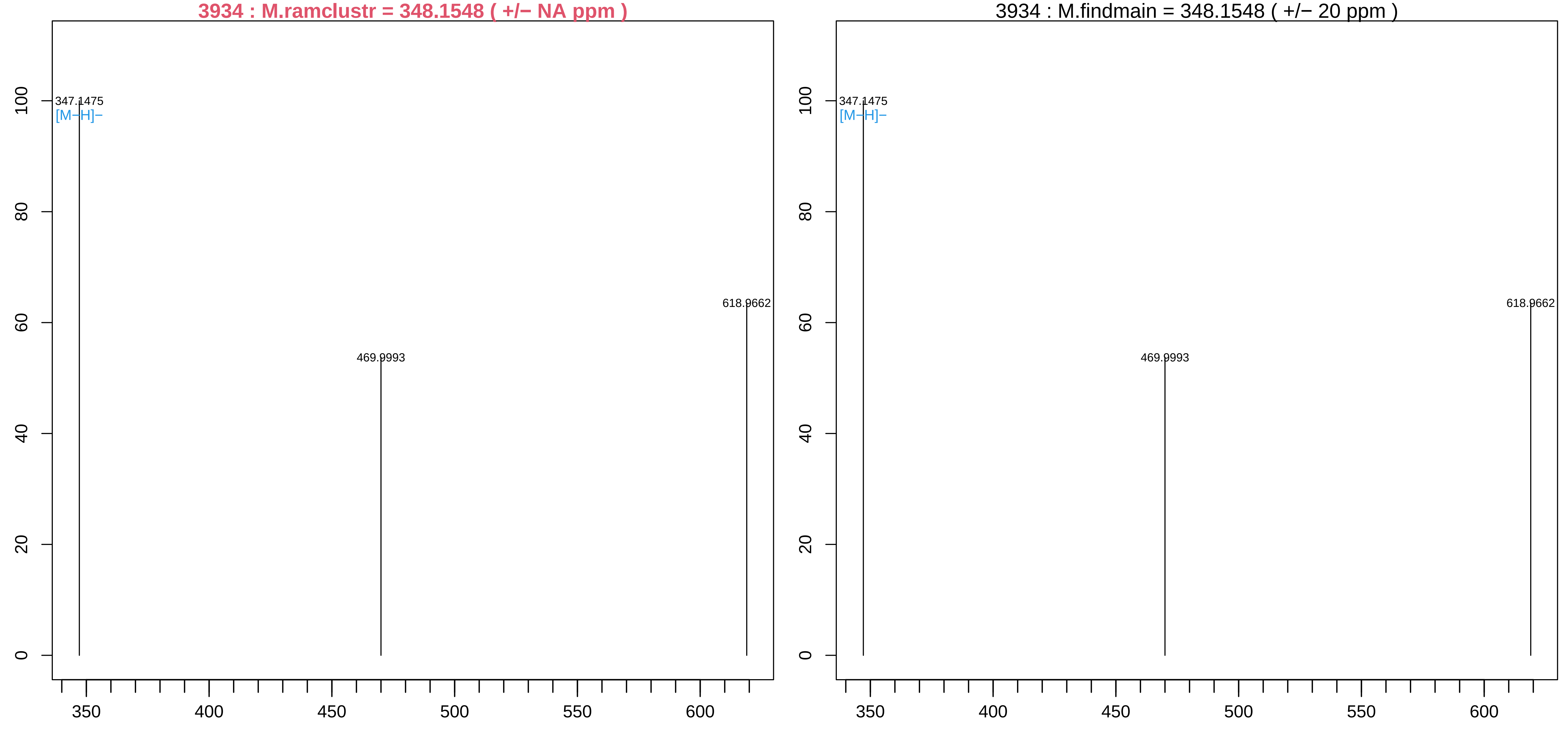
3934 : M.ramclustr = 348.1548 (+/− NA ppm)
347.1475
[M−H]−
469.9993
618.9662
3934 : M.findmain = 348.1548 (+/− 20 ppm)
347.1475
[M−H]−
469.9993
618.9662
0
20
40
60
80
100
350
400
450
500
550
600

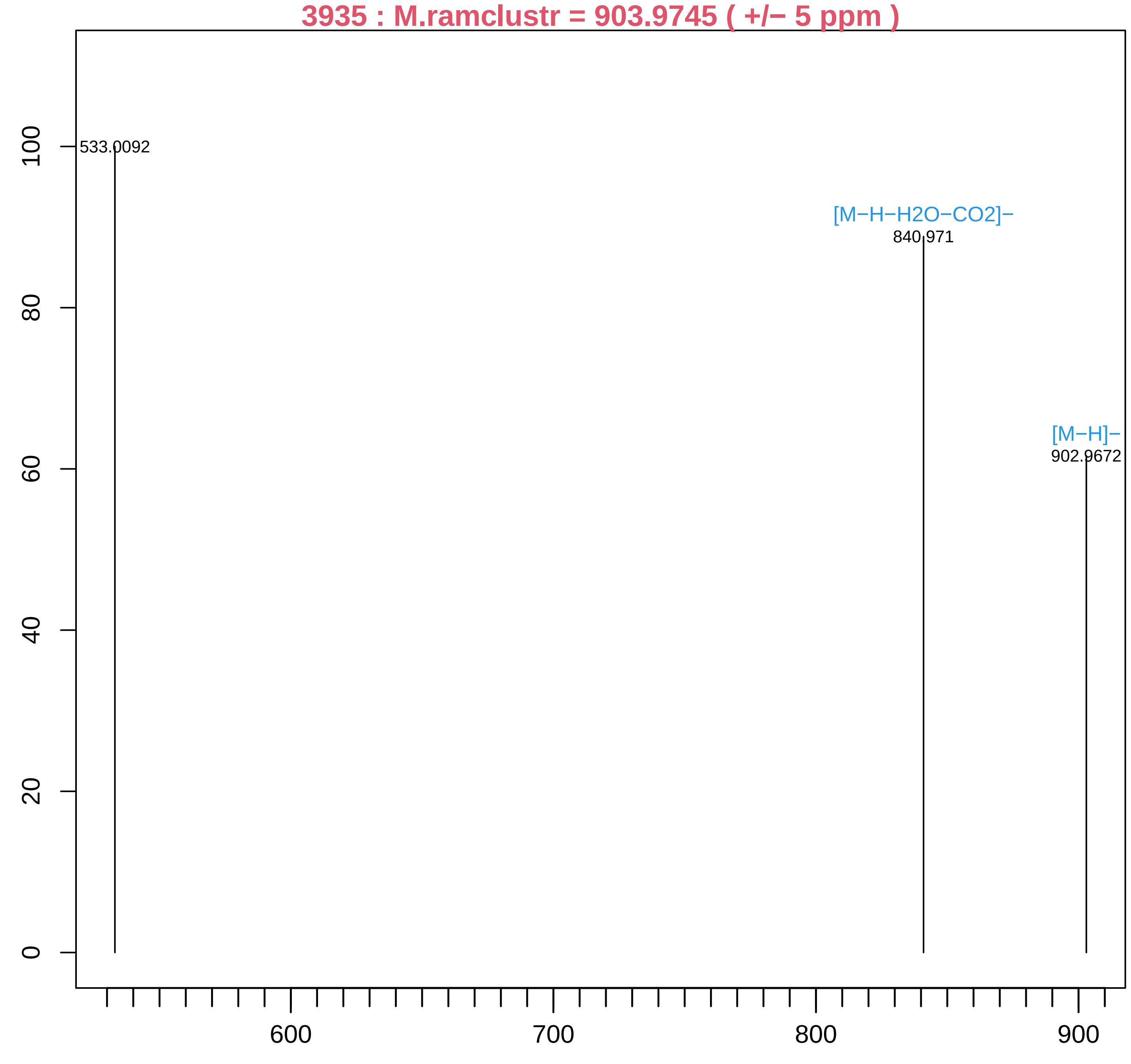
3935 : M.ramclustr = 903.9745 (+/− 5 ppm)
533.0092
[M−H−H2O−CO2]−
840.971
[M−H]−
902.9672
0
20
40
60
80
100
600
700
800
900

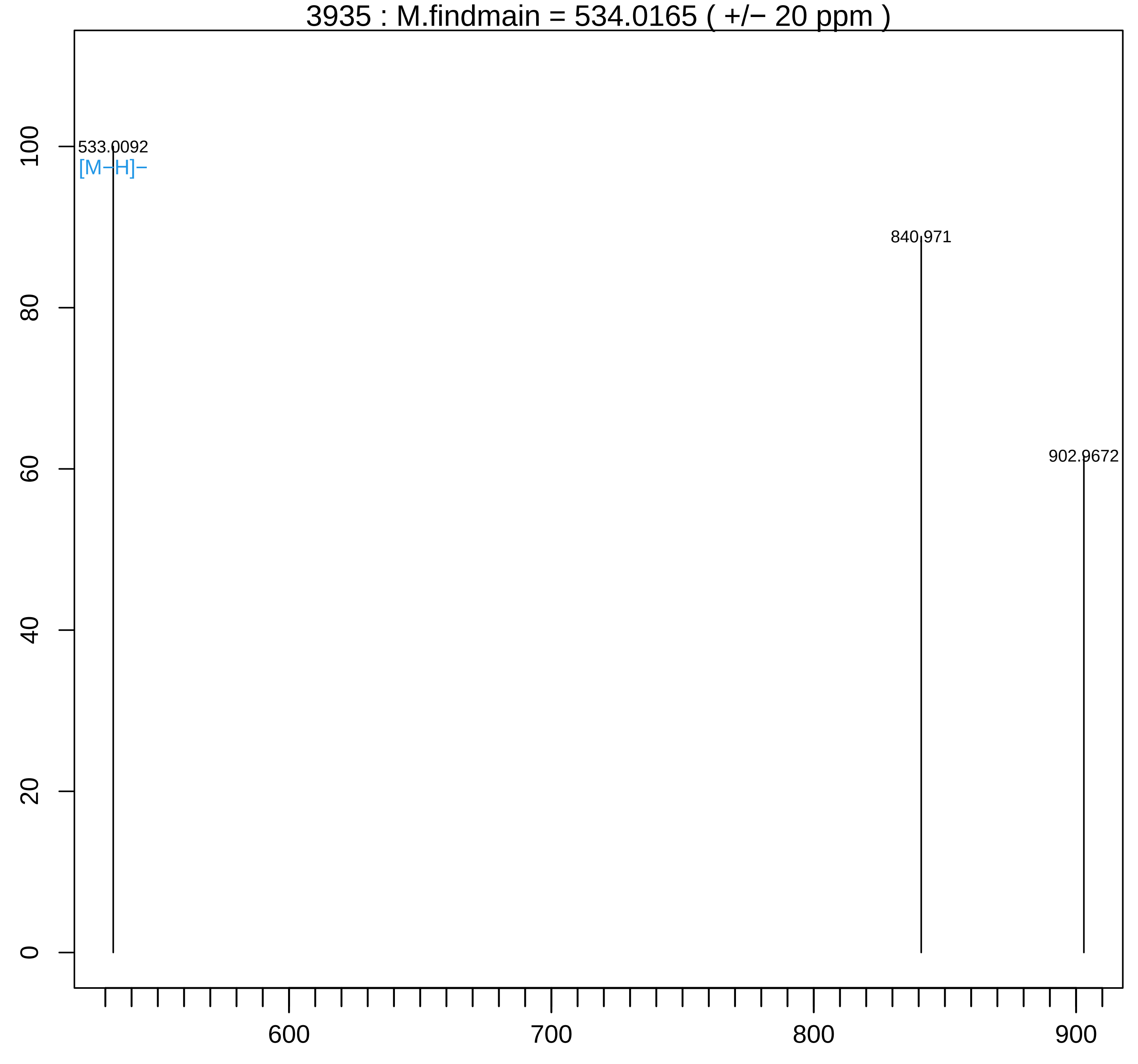
3935 : M.findmain = 534.0165 (+/− 20 ppm)
533.0092
[M−H]−
840.971
902.9672
0
20
40
60
80
100
600
700
800
900

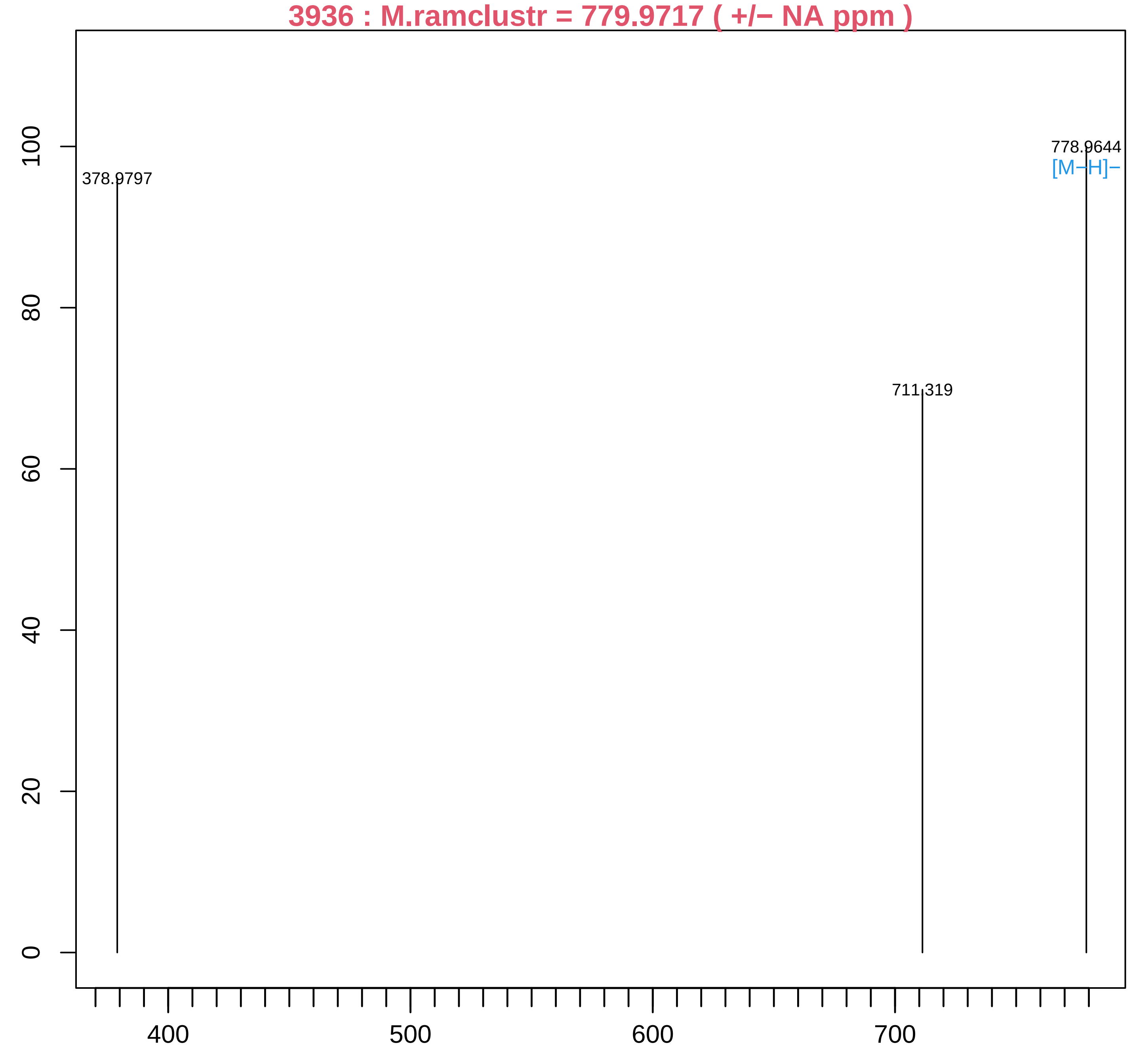
3936 : M.ramclustr = 779.9717 (+/− NA ppm)
378.9797
711.319
778.9644
[M−H]−
0
20
40
60
80
100
400
500
600
700

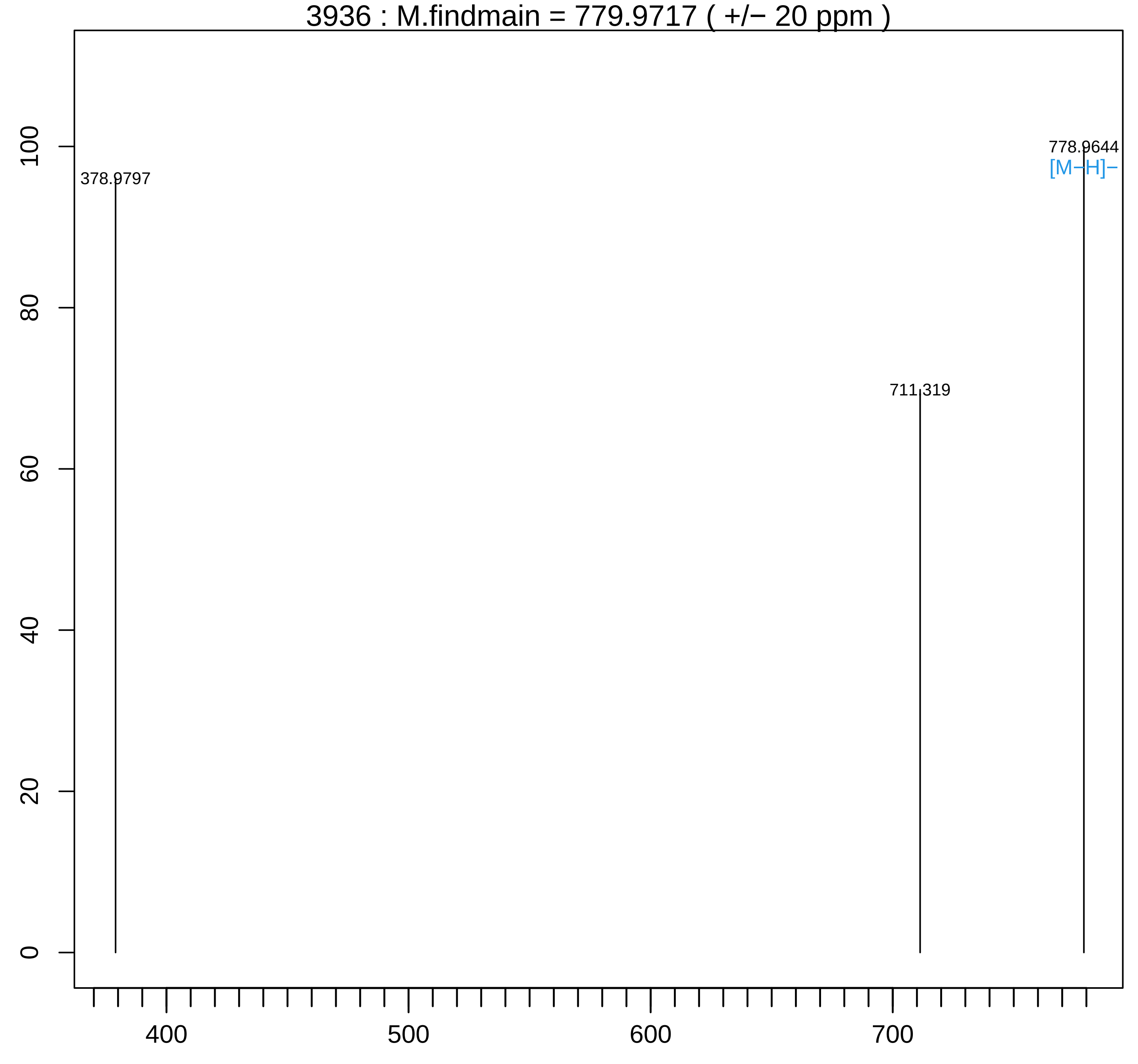
3936 : M.findmain = 779.9717 (+/− 20 ppm)
378.9797
711.319
778.9644
[M−H]−
0
20
40
60
80
100
400
500
600
700

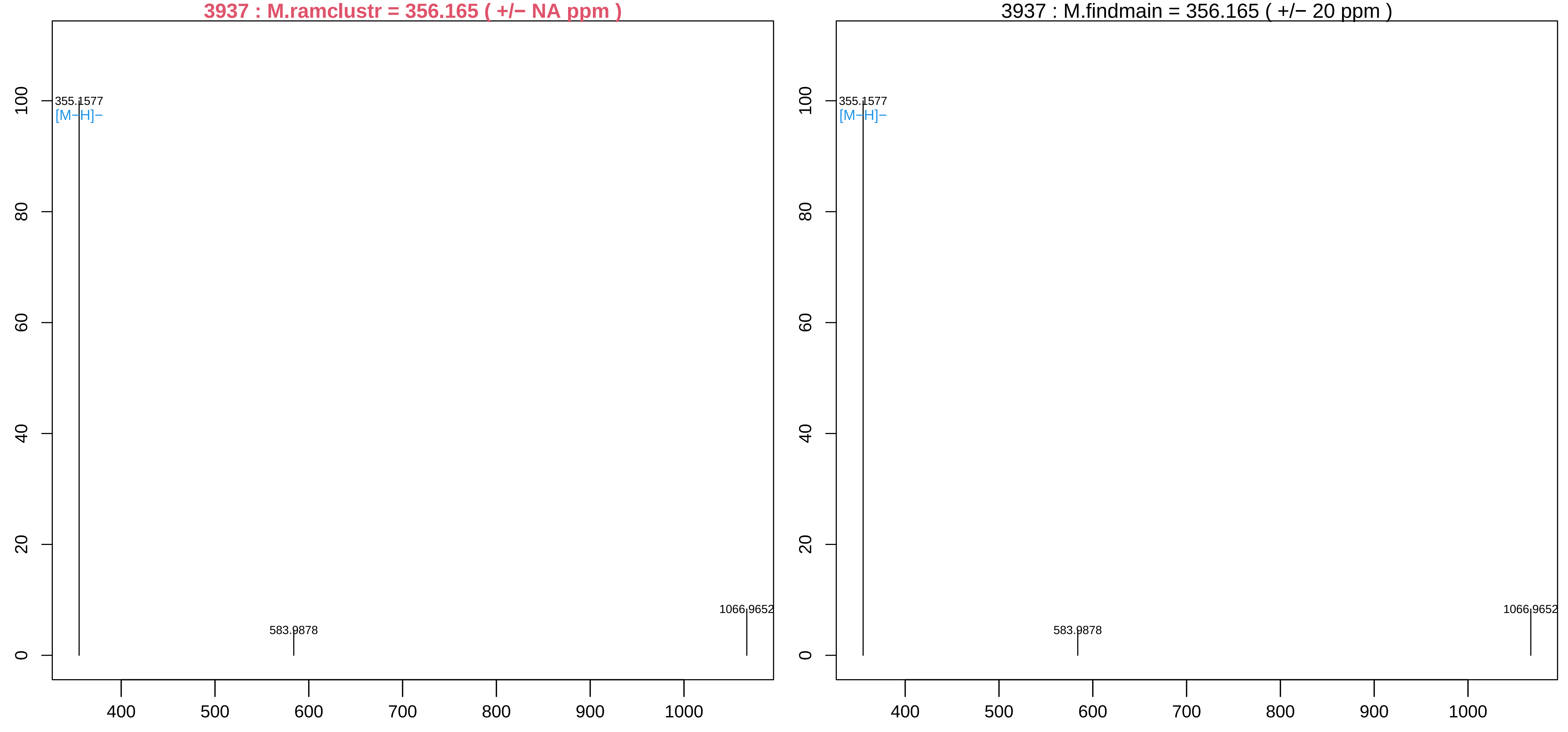
3937 : M.ramclustr = 356.165 (+/− NA ppm)
355.1577
[M−H]−
583.9878
1066.9652
0
20
40
60
80
100
400
500
600
700
800
900
1000
3937 : M.findmain = 356.165 (+/− 20 ppm)
355.1577
[M−H]−
583.9878
1066.9652
0
20
40
60
80
100
400
500
600
700
800
900
1000

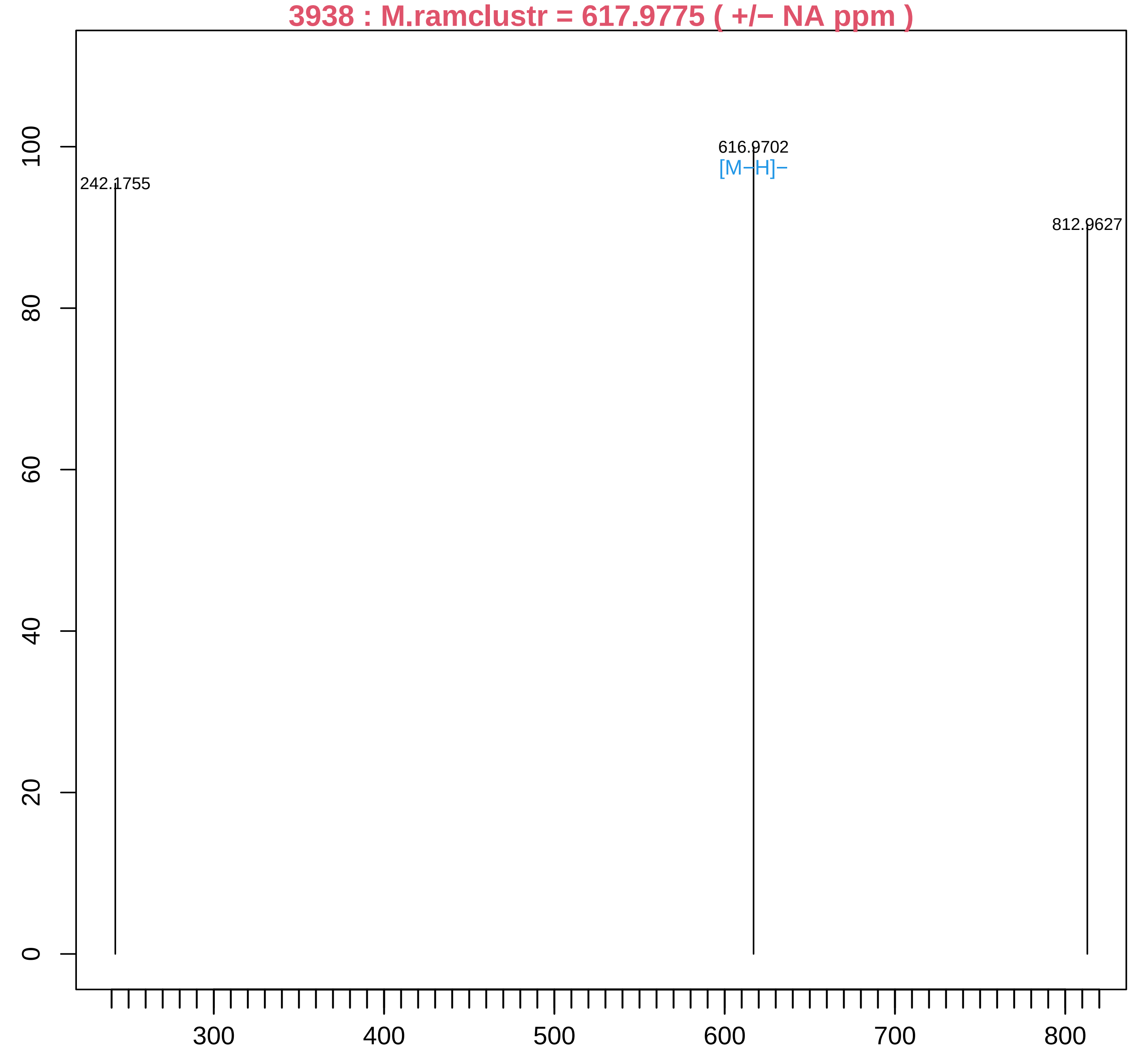
3938 : M.ramclustr = 617.9775 (+/− NA ppm)
242.1755
616.9702
[M−H]−
812.9627
0
20
40
60
80
100
300
400
500
600
700
800

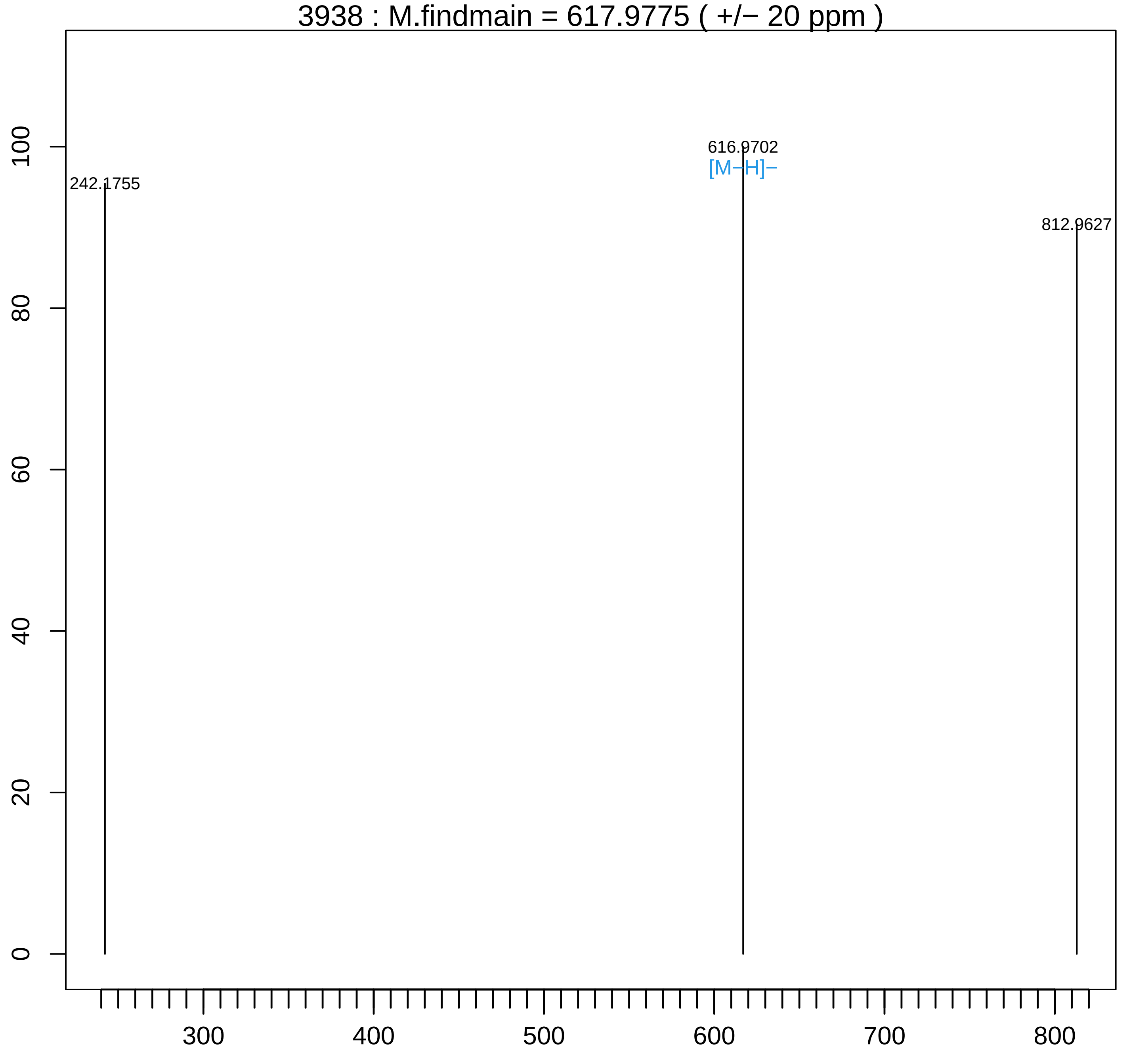
3938 : M.findmain = 617.9775 (+/− 20 ppm)
242.1755
616.9702
[M−H]−
812.9627
0
20
40
60
80
100
300
400
500
600
700
800

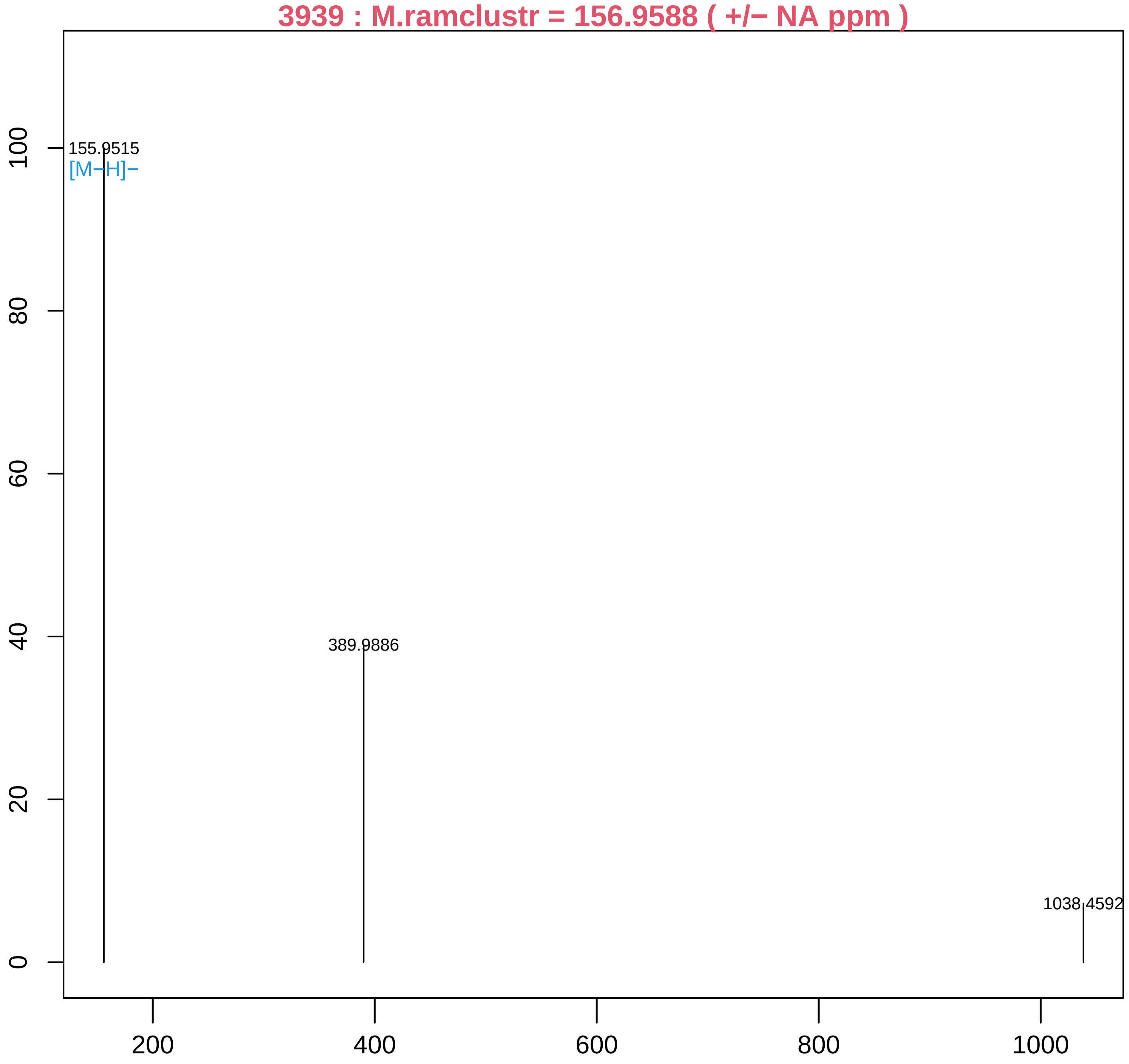
3939 : M.ramclustr = 156.9588 (+/− NA ppm)
155.9515
[M−H]−
389.9886
1038.4592
0
20
40
60
80
100
200
400
600
800
1000

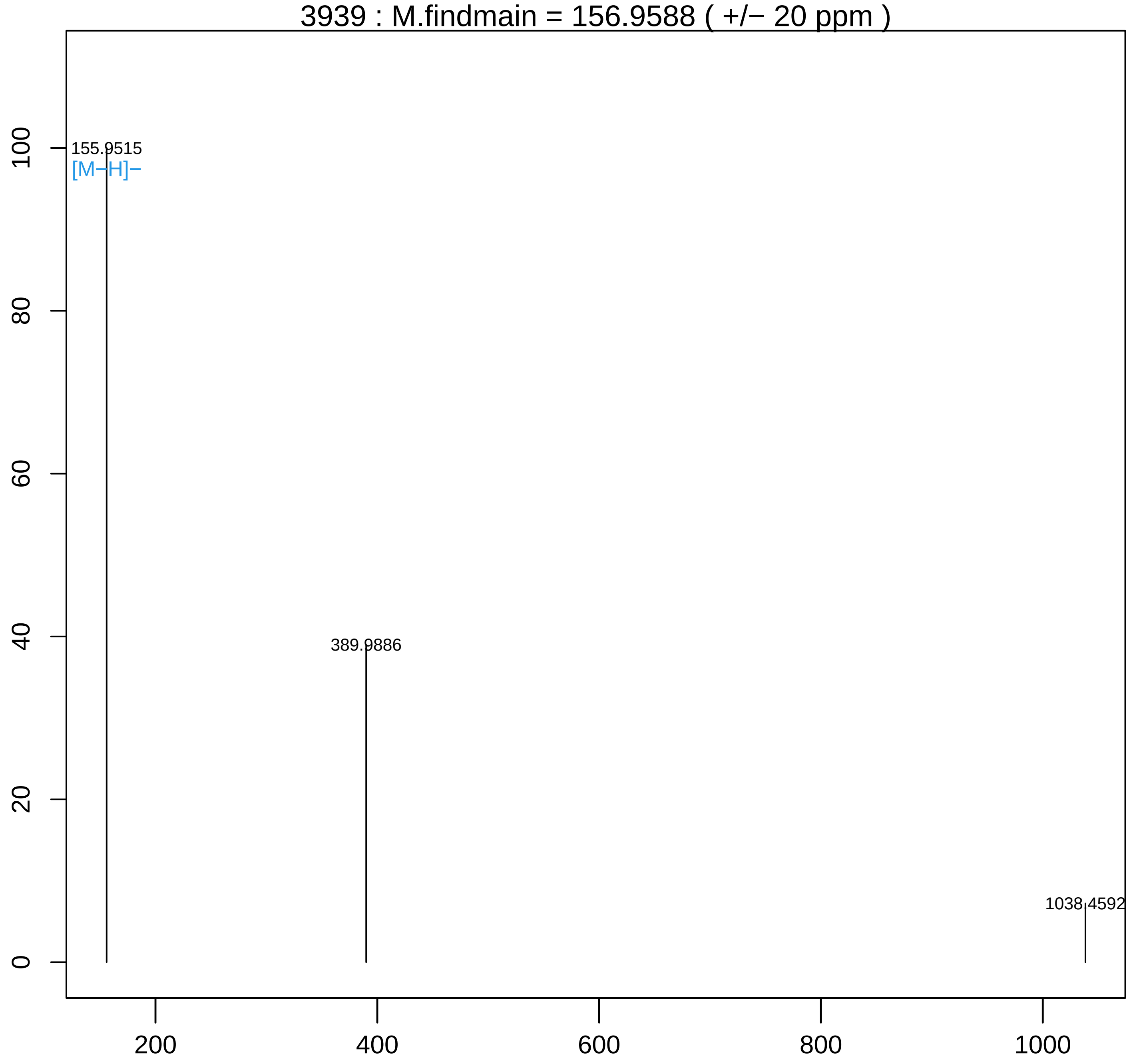
3939 : M.findmain = 156.9588 (+/− 20 ppm)
155.9515
[M−H]−
389.9886
1038.4592
0
20
40
60
80
100
200
400
600
800
1000

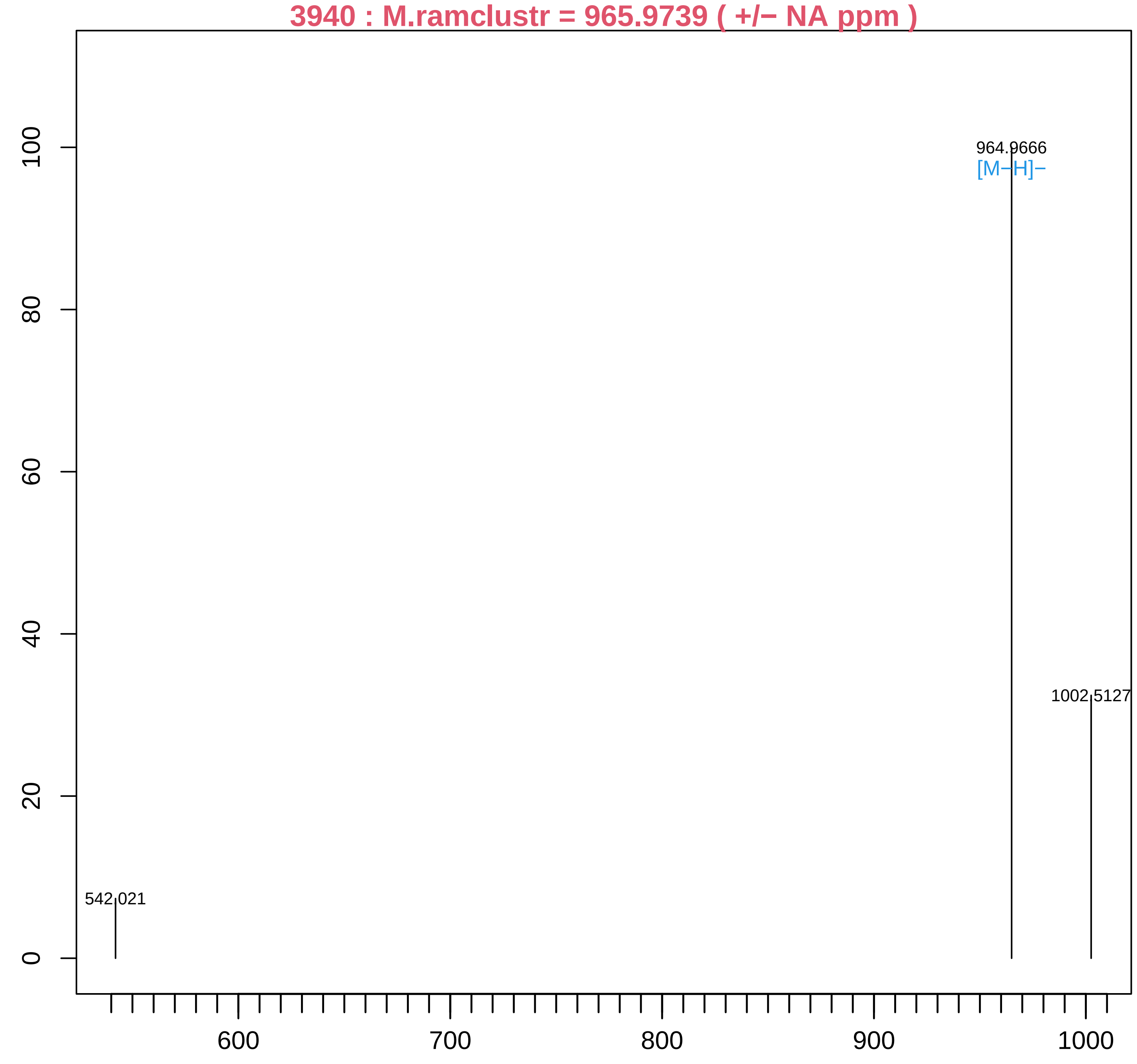
3940 : M.ramclustr = 965.9739 (+/− NA ppm)
964.9666
[M−H]−
1002.5127
542.021
0
20
40
60
80
100
600
700
800
900
1000

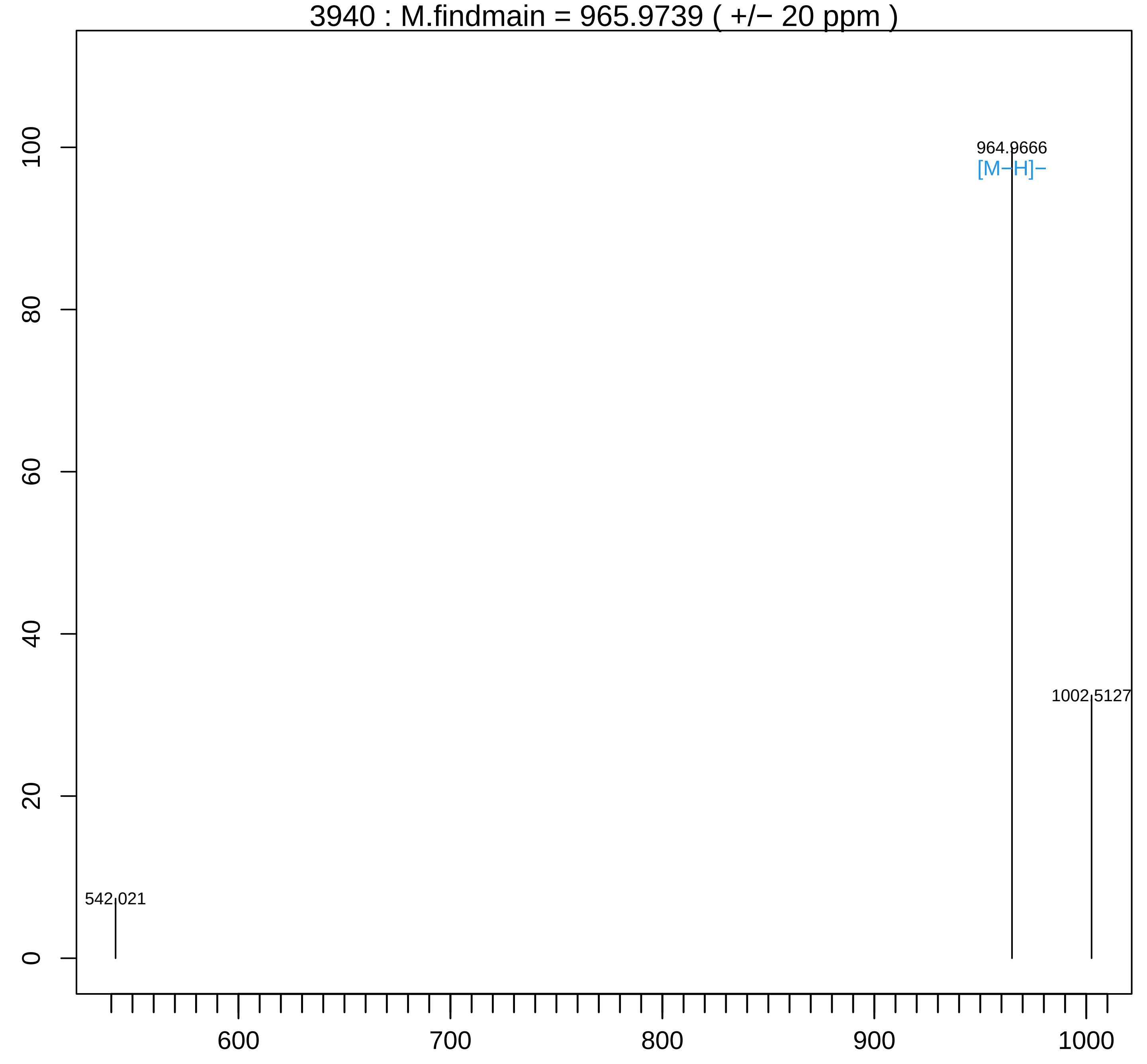
3940 : M.findmain = 965.9739 (+/− 20 ppm)
964.9666
[M−H]−
1002.5127
542.021
0
20
40
60
80
100
600
700
800
900
1000

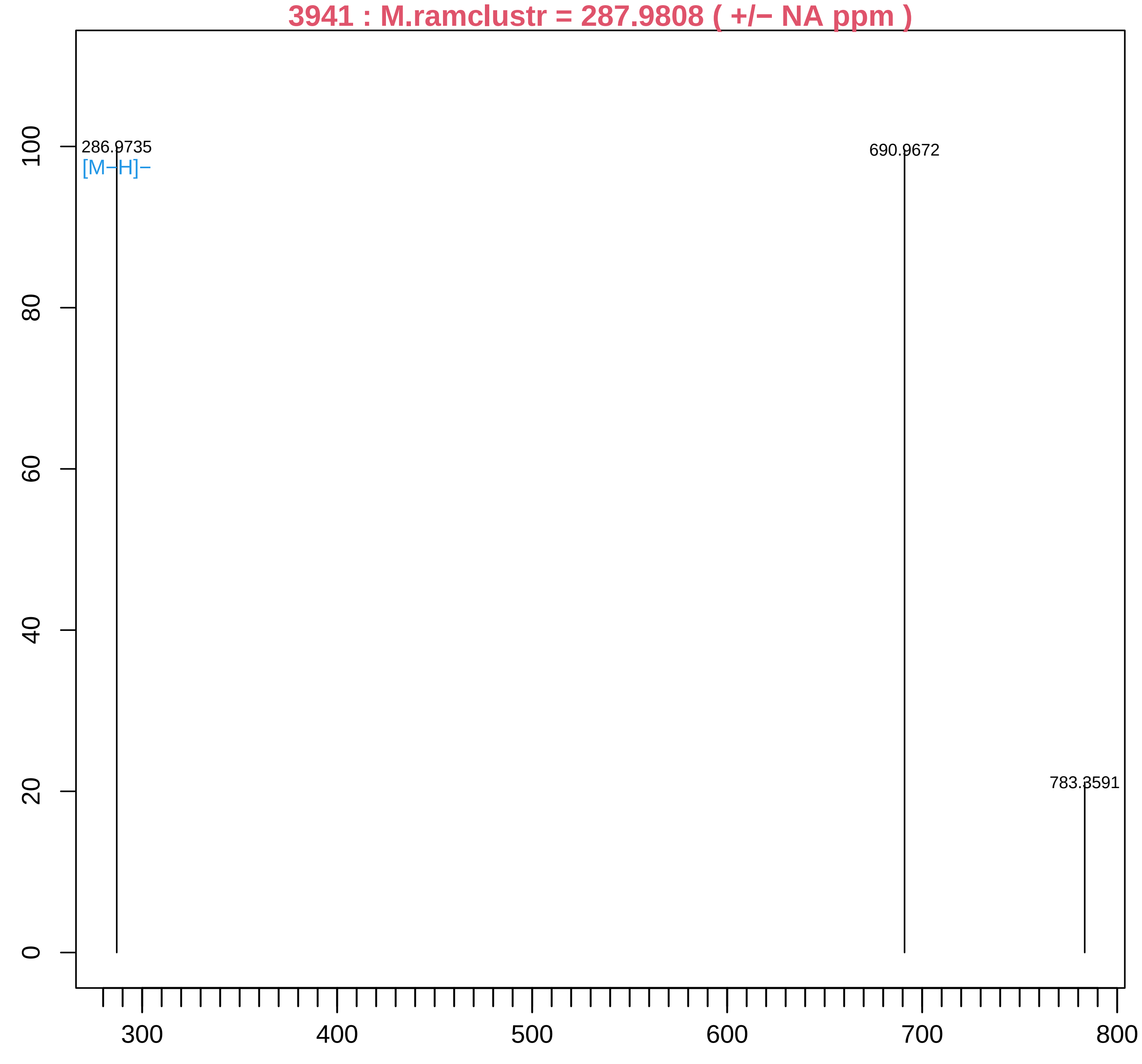
3941 : M.ramclustr = 287.9808 (+/− NA ppm)
286.9735
[M−H]−
690.9672
783.3591
0
20
40
60
80
100
300
400
500
600
700
800

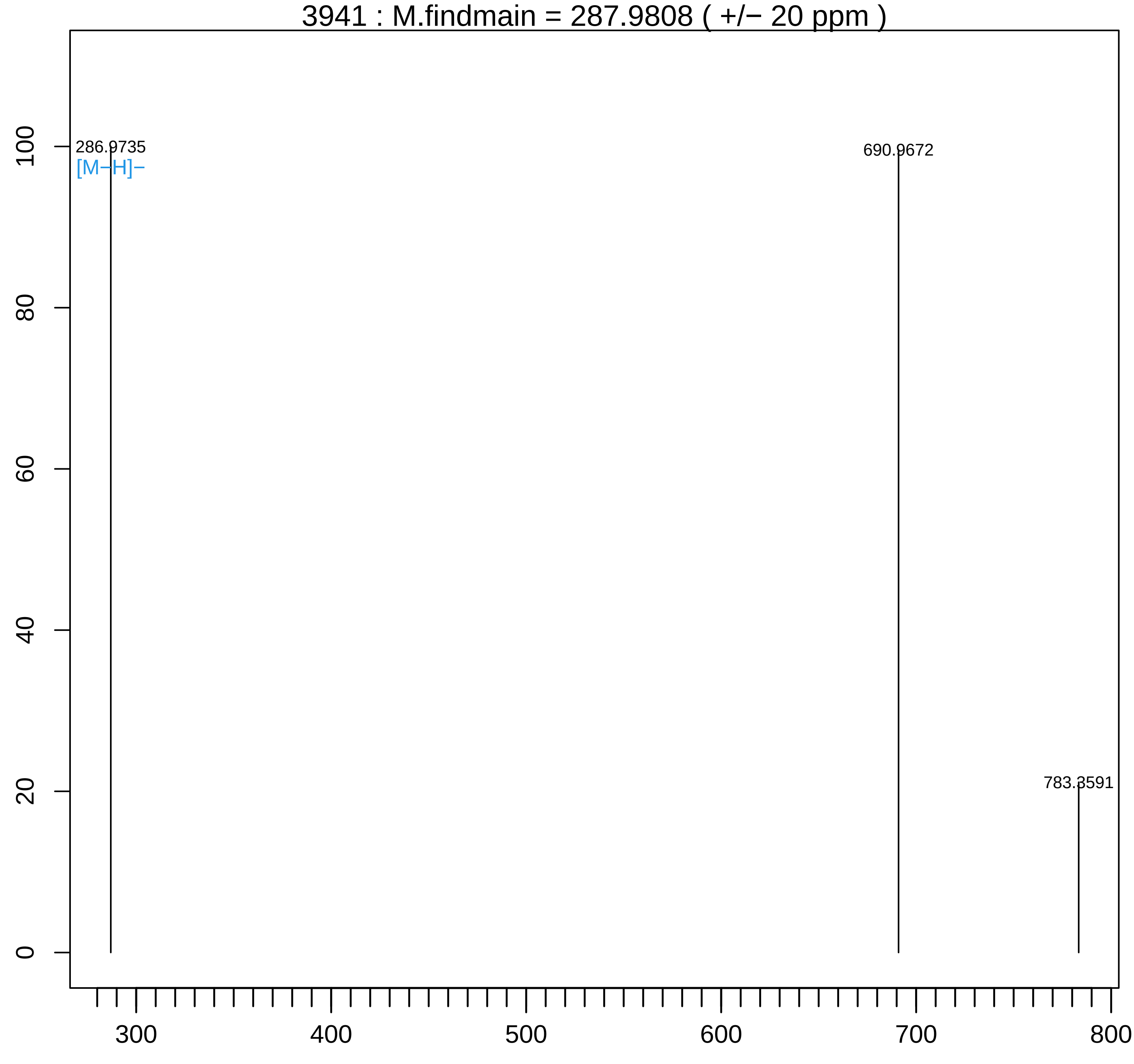
3941 : M.findmain = 287.9808 (+/− 20 ppm)
286.9735
[M−H]−
690.9672
783.3591
0
20
40
60
80
100
300
400
500
600
700
800

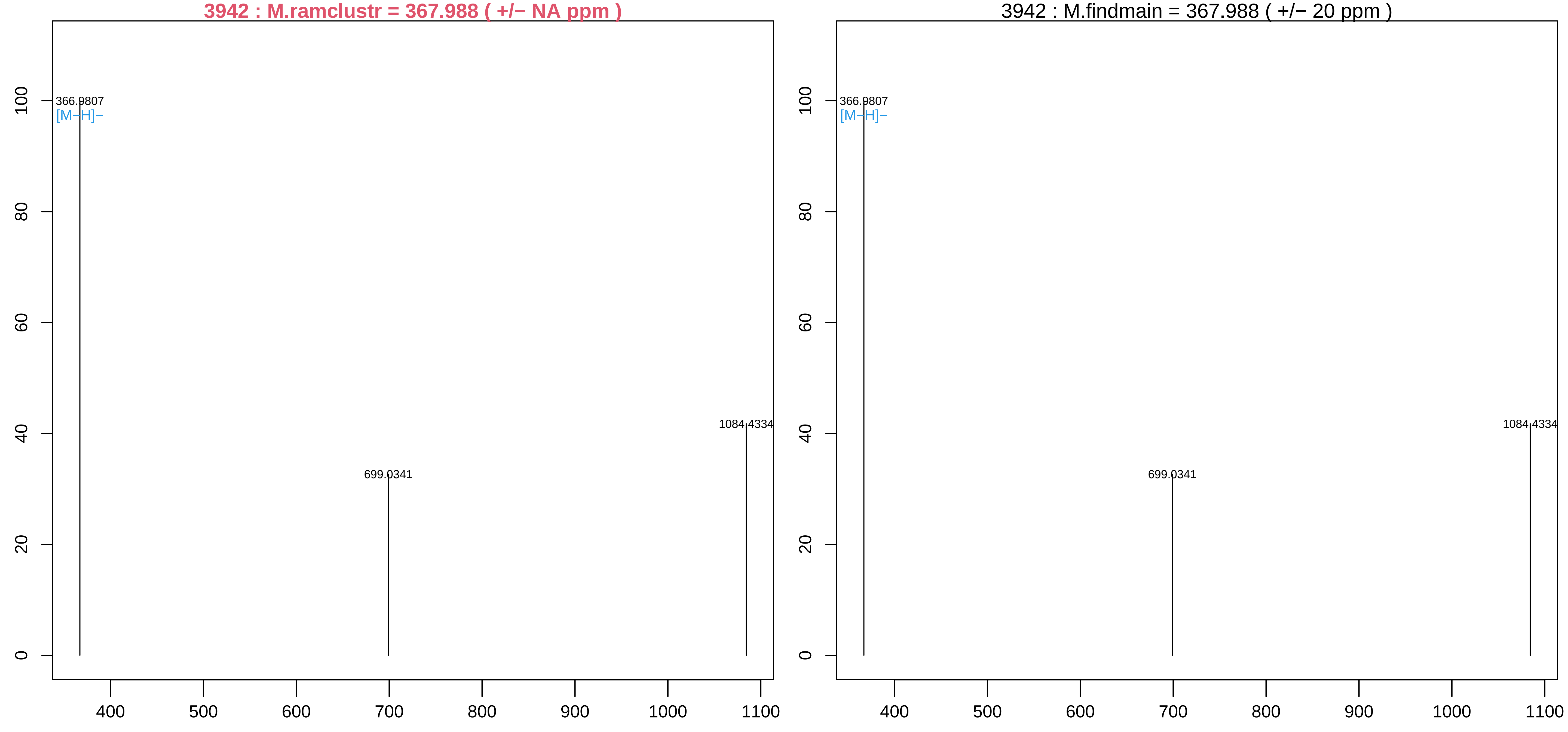
3942 : M.ramclustr = 367.988 (+/− NA ppm)
366.9807
[M−H]−
699.0341
1084.4334
0
20
40
60
80
100
400
500
600
700
800
900
1000
1100
3942 : M.findmain = 367.988 (+/− 20 ppm)
366.9807
[M−H]−
699.0341
1084.4334
0
20
40
60
80
100
400
500
600
700
800
900
1000
1100

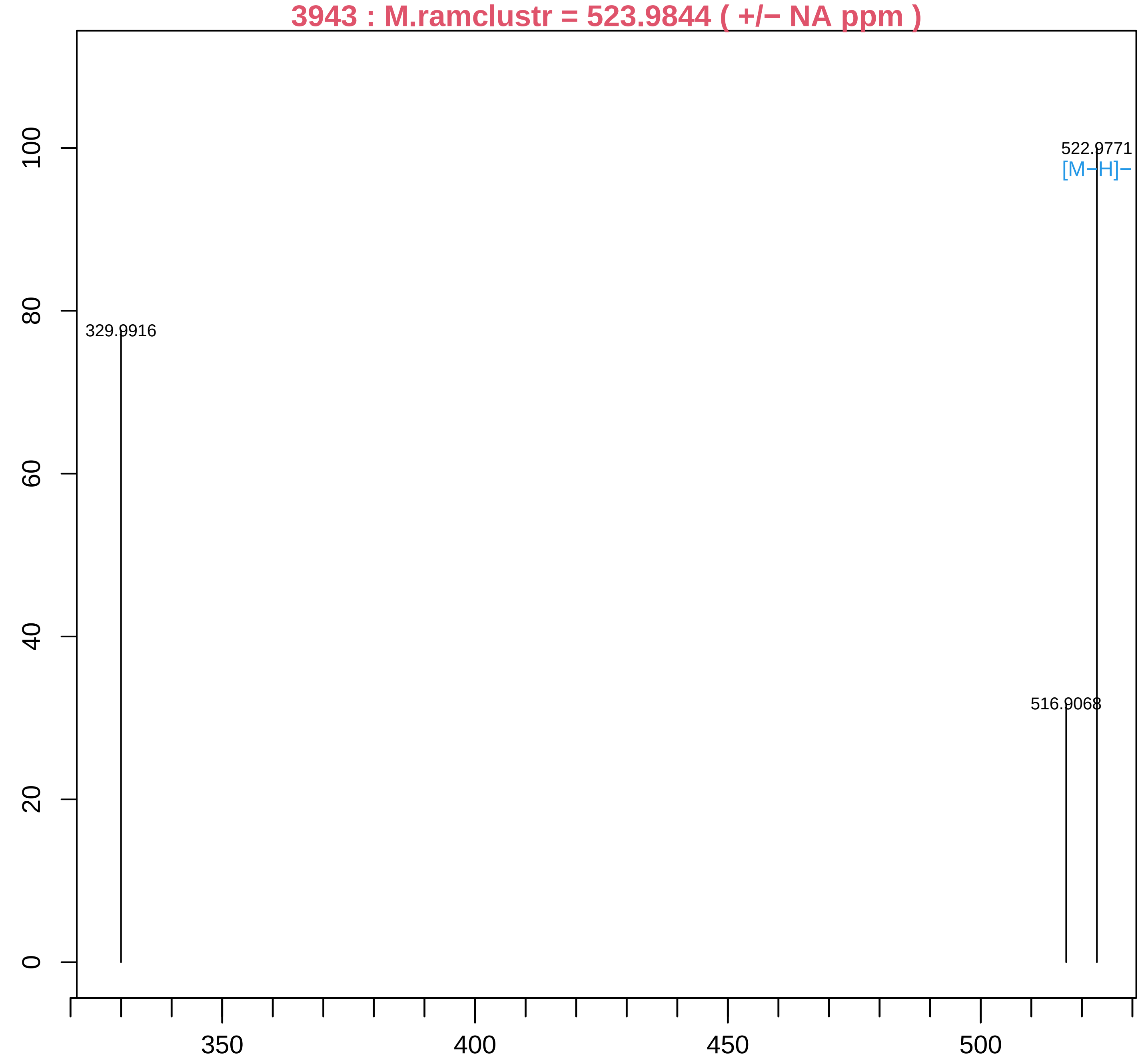
3943 : M.ramclustr = 523.9844 (+/− NA ppm)
522.9771
[M−H]−
329.9916
516.9068
0
20
40
60
80
100
350
400
450
500

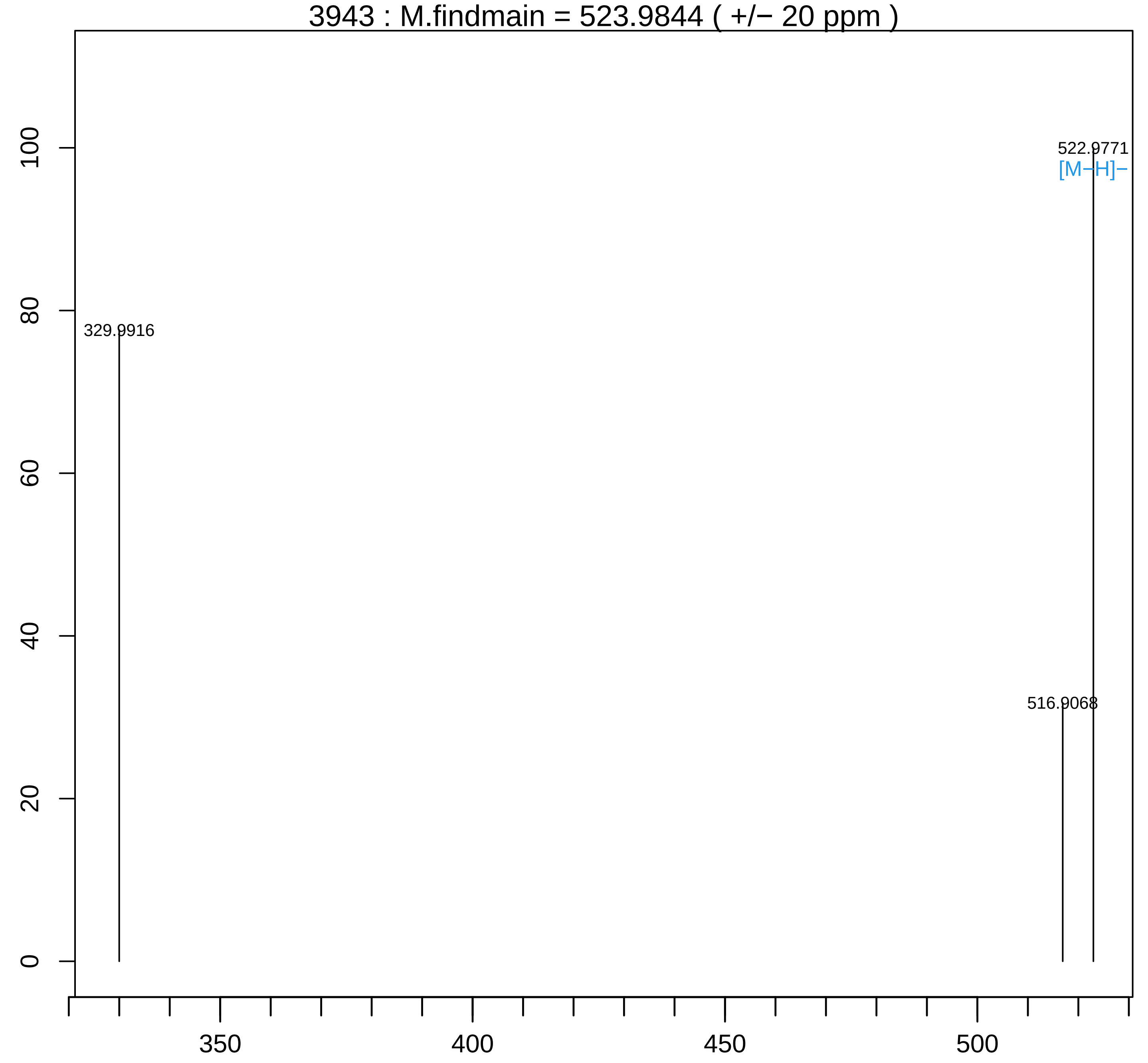
3943 : M.findmain = 523.9844 (+/− 20 ppm)
522.9771
[M−H]−
329.9916
516.9068
0
20
40
60
80
100
350
400
450
500

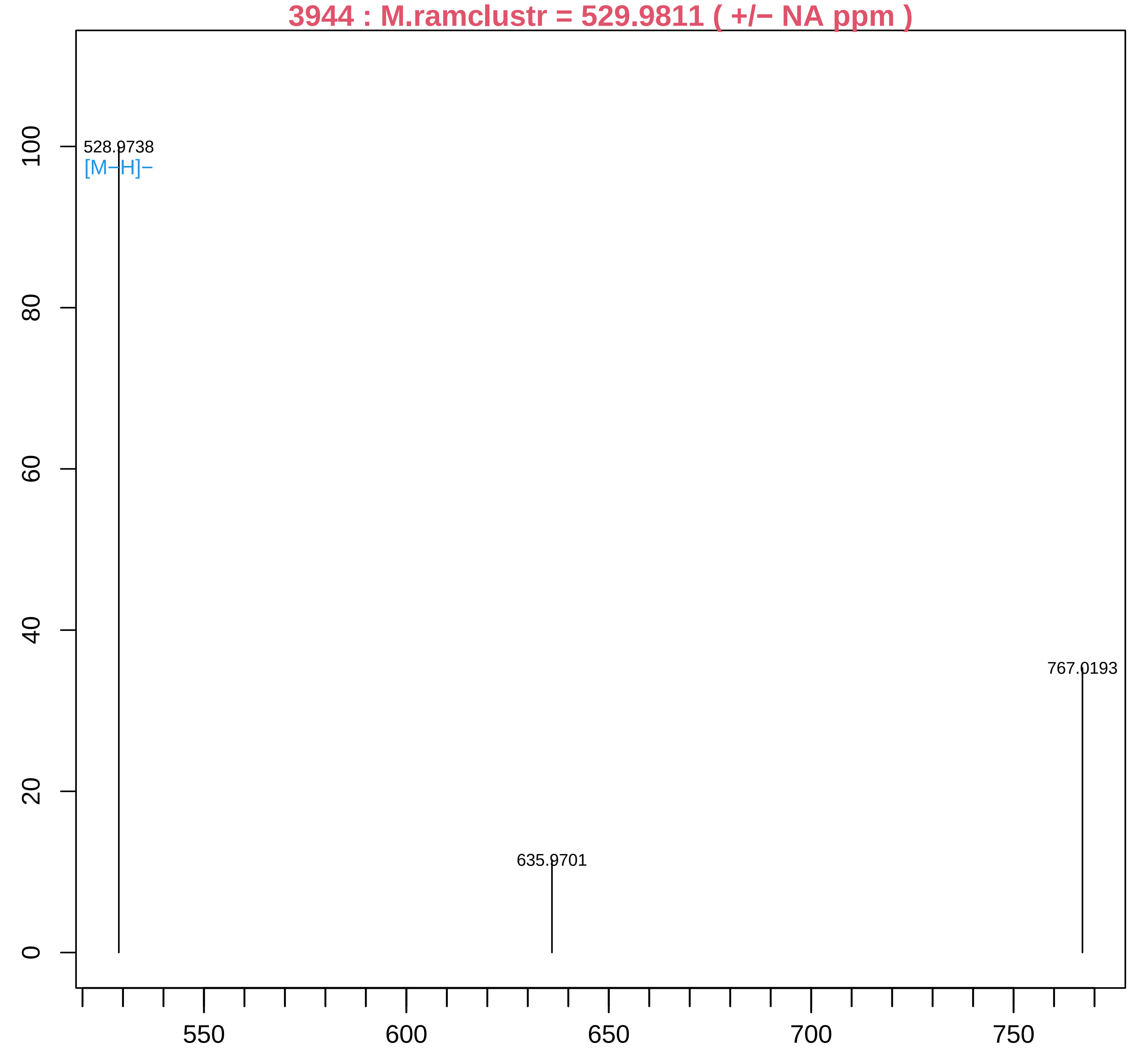

3944 : M.ramclustr = 529.9811 (+/− NA ppm)
528.9738
[M−H]−
635.9701
767.0193
0
20
40
60
80
100
550
600
650
700
750

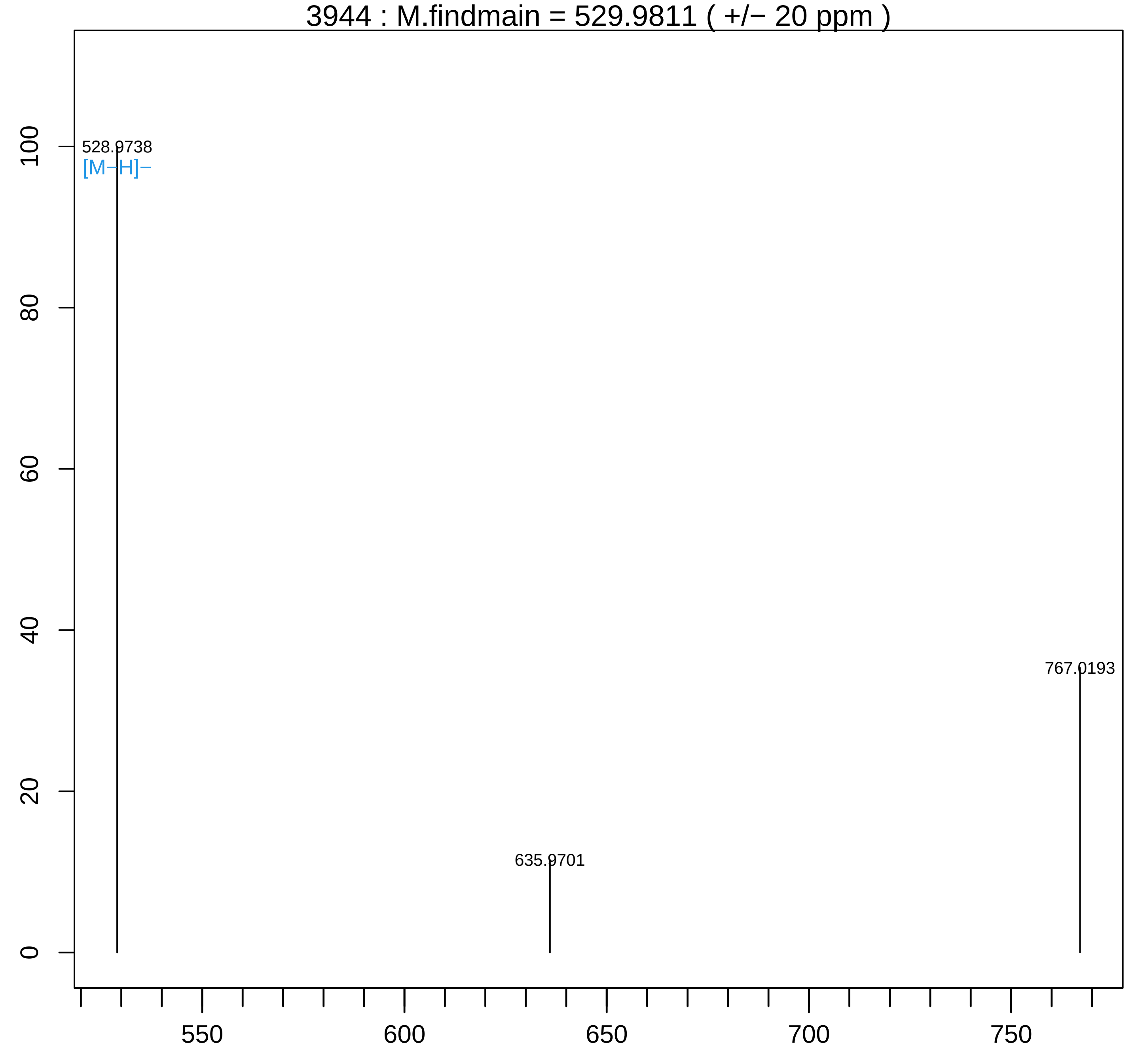

3944 : M.findmain = 529.9811 (+/− 20 ppm)
528.9738
[M−H]−
635.9701
767.0193
0
20
40
60
80
100
550
600
650
700
750

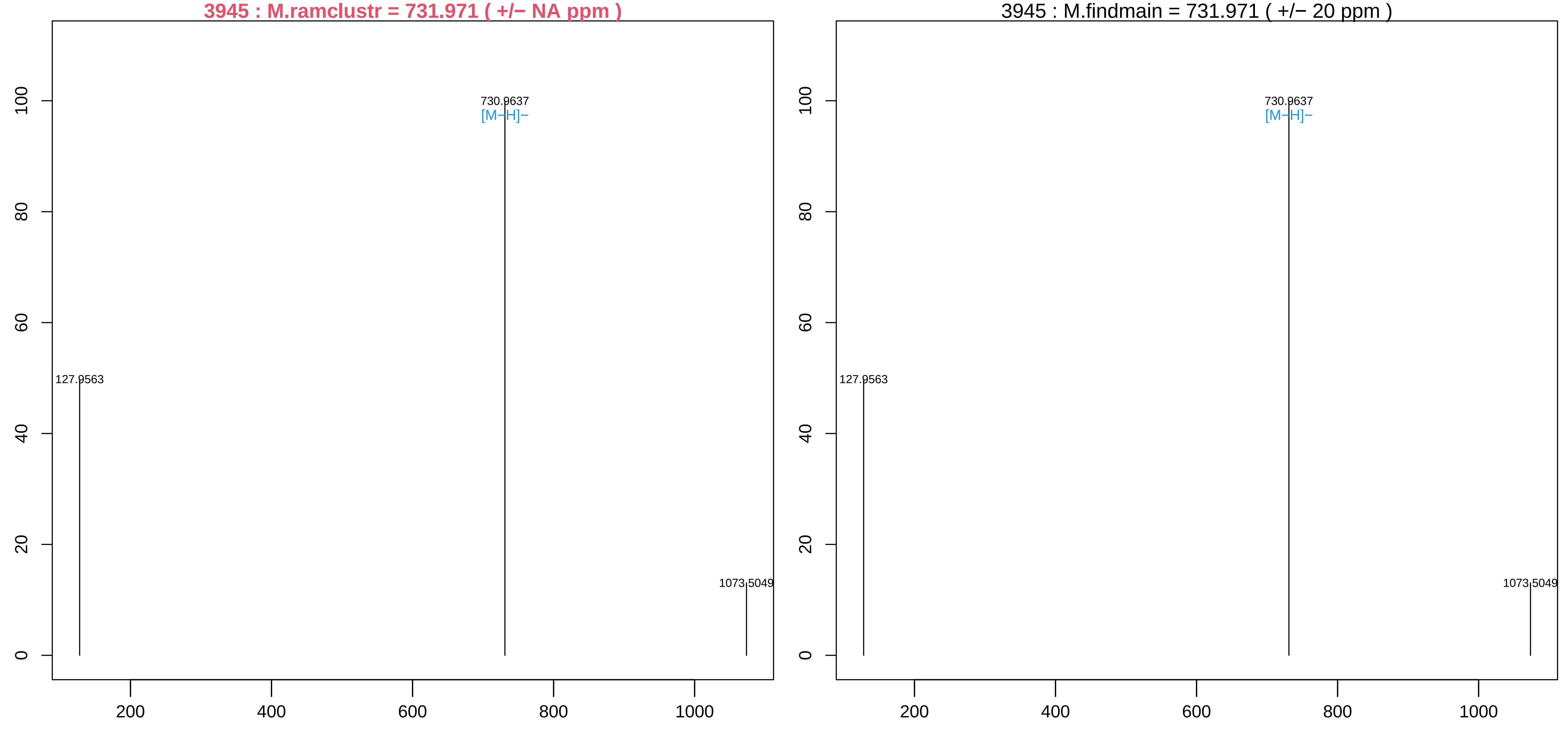
3945 : M.ramclustr = 731.971 (+/− NA ppm)
3945 : M.findmain = 731.971 (+/− 20 ppm)
730.9637
[M−H]−
127.9563
1073.5049
730.9637
[M−H]−
127.9563
1073.5049
0
20
40
60
80
100
200
400
600
800
1000

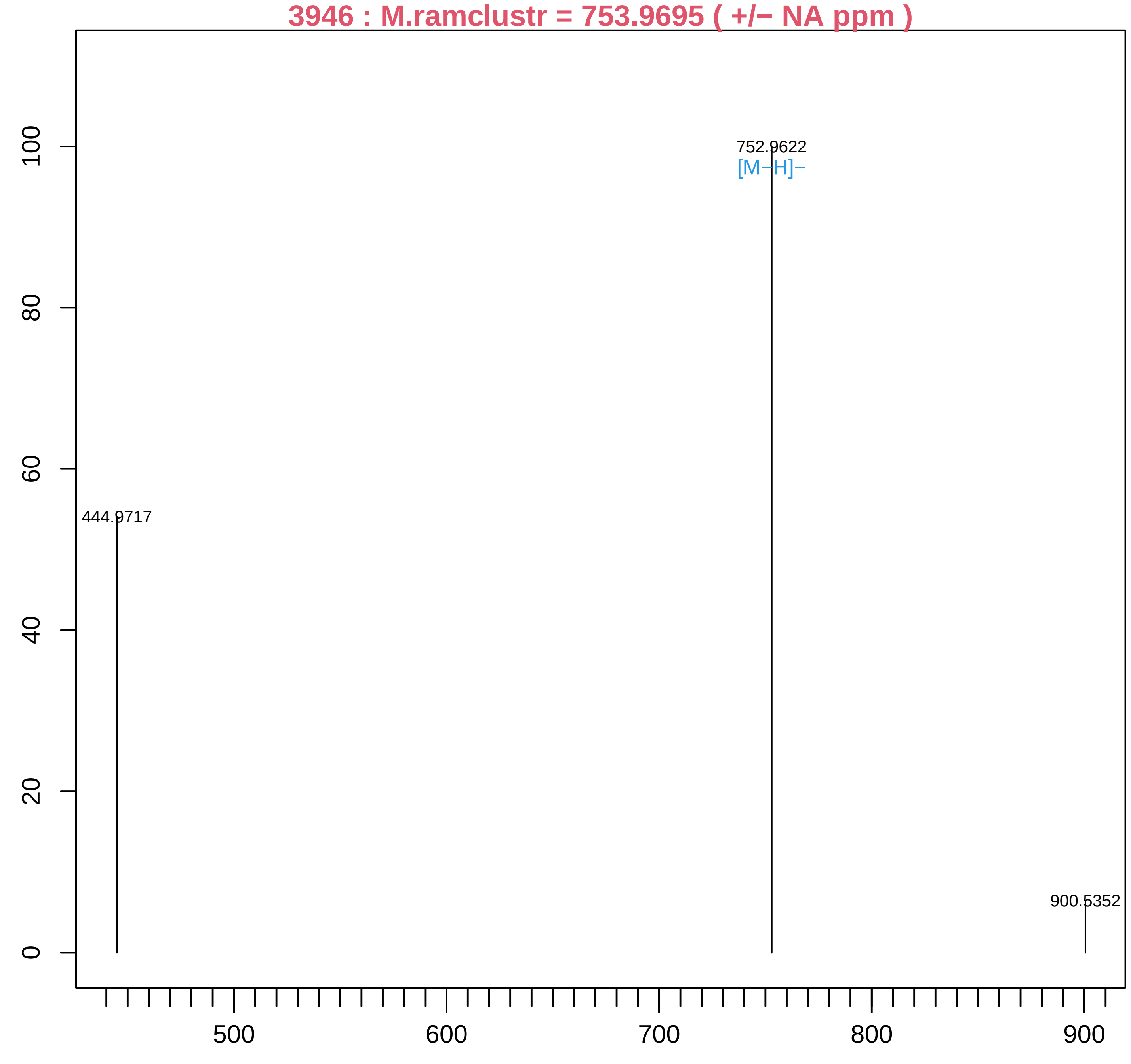
3946 : M.ramclustr = 753.9695 (+/− NA ppm)
752.9622
[M−H]−
444.9717
900.5352
0
20
40
60
80
100
500
600
700
800
900

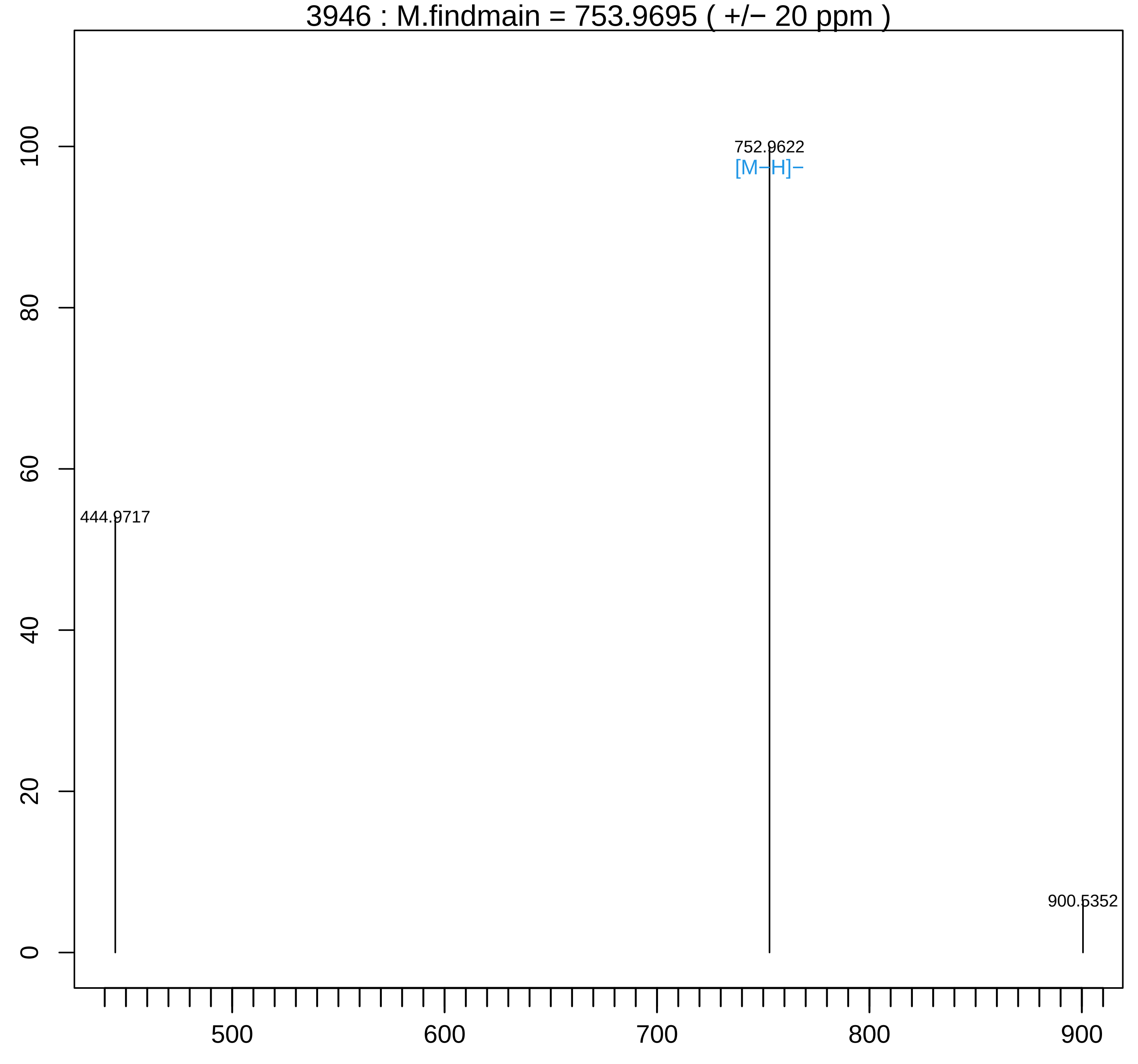
3946 : M.findmain = 753.9695 (+/− 20 ppm)
752.9622
[M−H]−
444.9717
900.5352
0
20
40
60
80
100
500
600
700
800
900

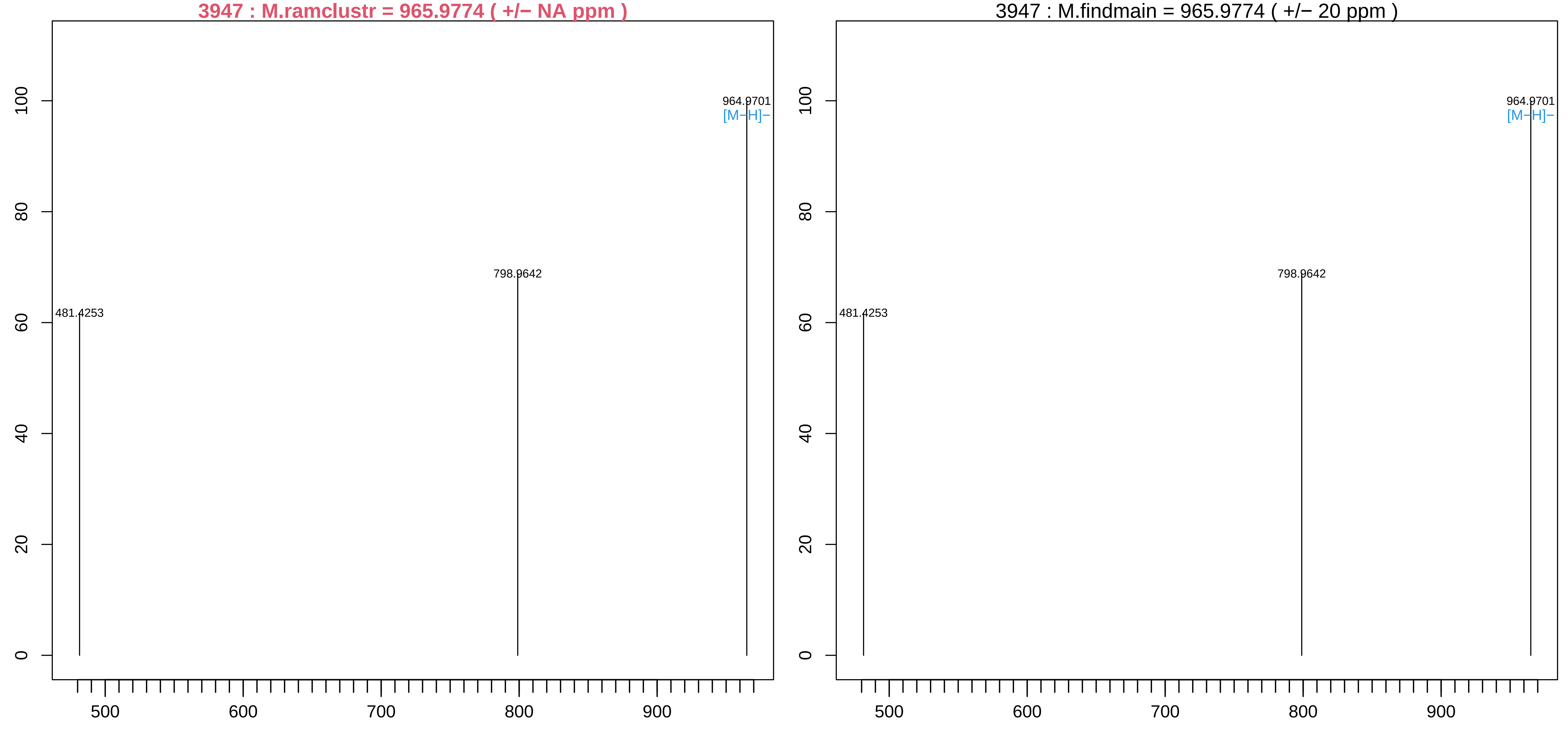
3947 : M.ramclustr = 965.9774 (+/− NA ppm)
964.9701
[M−H]−
798.9642
481.4253
0
20
40
60
80
100
500
600
700
800
900
3947 : M.findmain = 965.9774 (+/− 20 ppm)
964.9701
[M−H]−
798.9642
481.4253
0
20
40
60
80
100
500
600
700
800
900

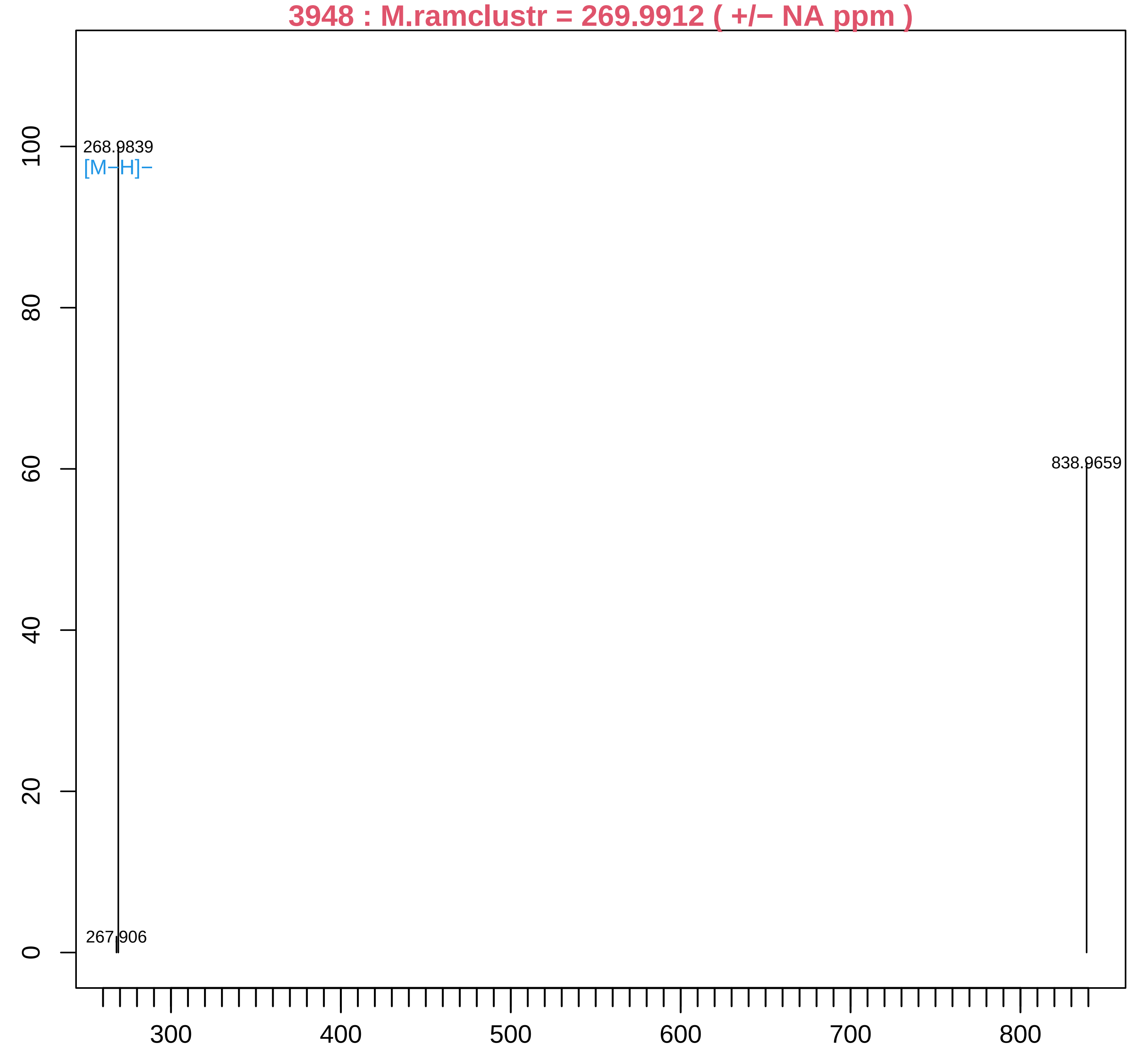
3948 : M.ramclustr = 269.9912 (+/− NA ppm)
268.9839
[M−H]−
838.9659
267.906
0
20
40
60
80
100
300
400
500
600
700
800

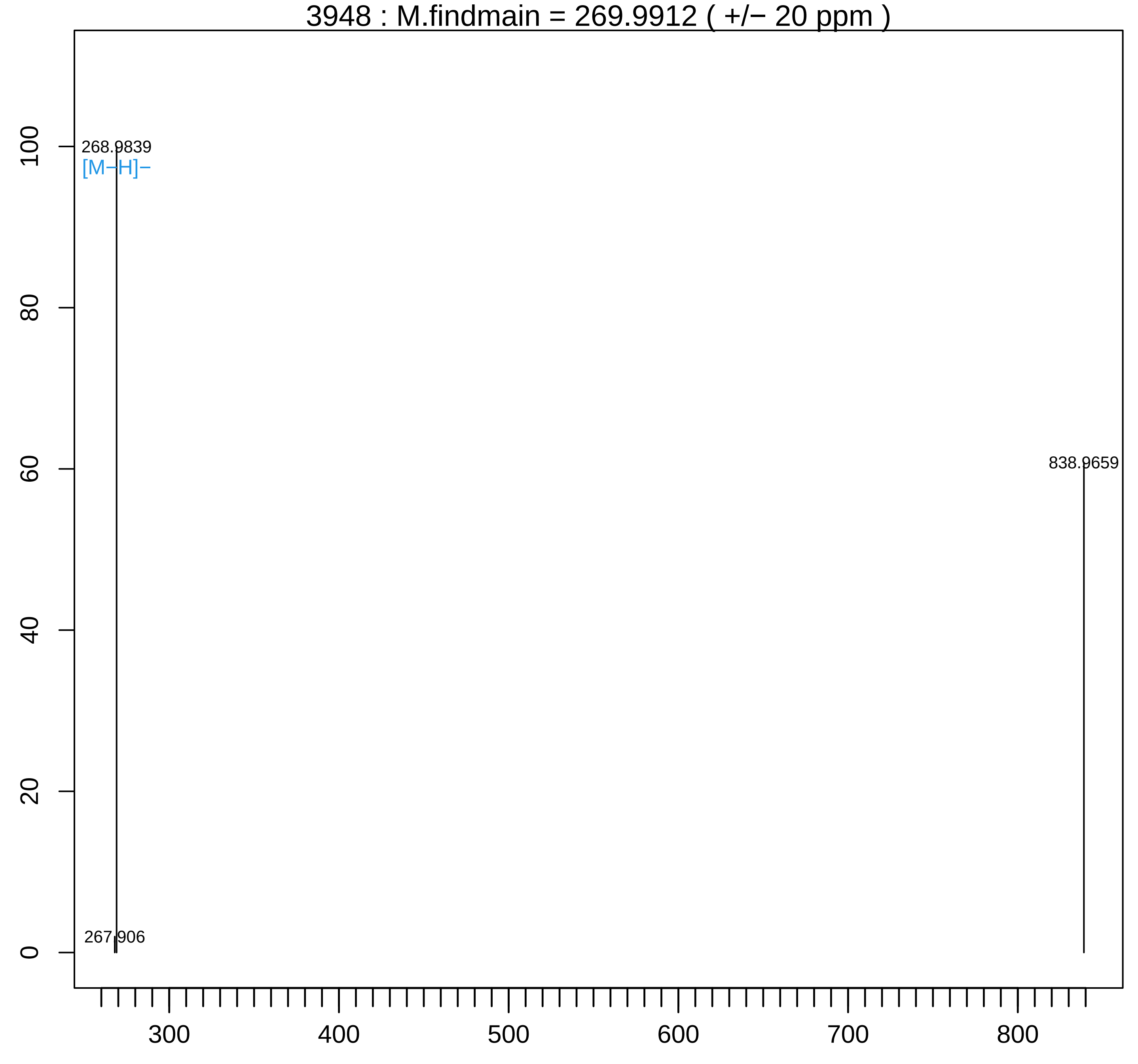
3948 : M.findmain = 269.9912 (+/− 20 ppm)
268.9839
[M−H]−
838.9659
267.906
0
20
40
60
80
100
300
400
500
600
700
800

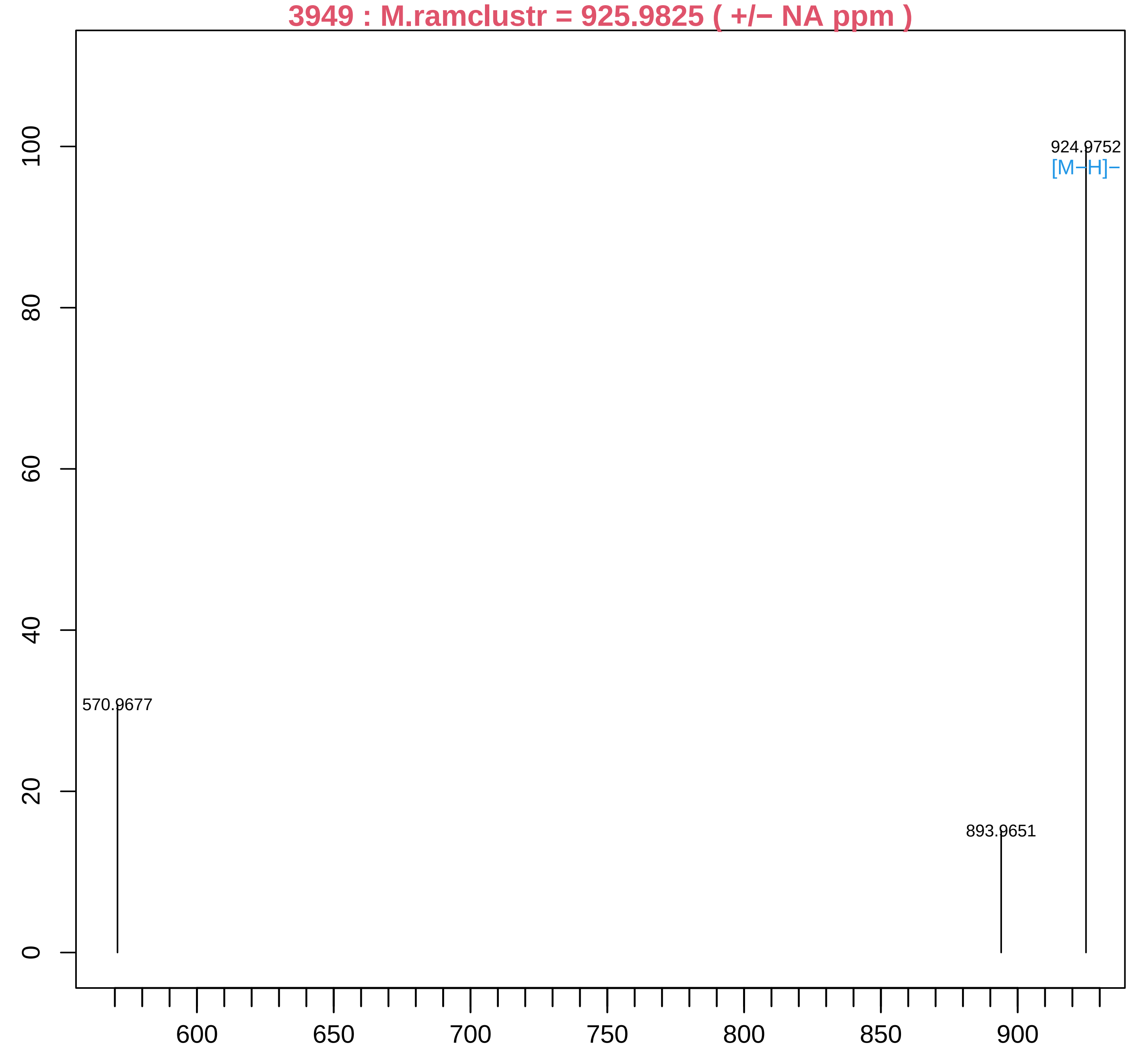
3949 : M.ramclustr = 925.9825 (+/− NA ppm)
924.9752
[M−H]−
570.9677
893.9651
0
20
40
60
80
100
600
650
700
750
800
850
900

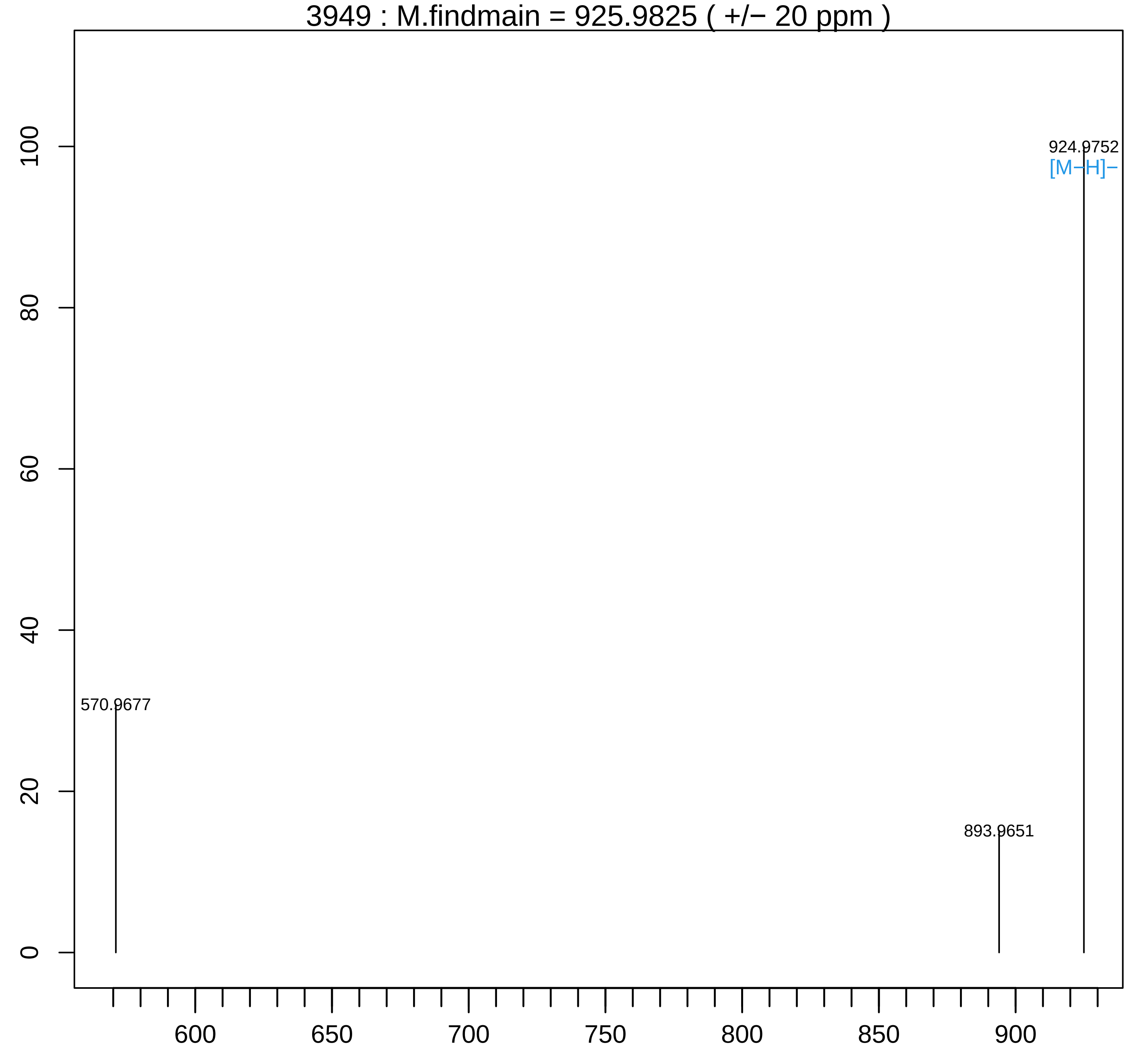
3949 : M.findmain = 925.9825 (+/− 20 ppm)
924.9752
[M−H]−
570.9677
893.9651
0
20
40
60
80
100
600
650
700
750
800
850
900

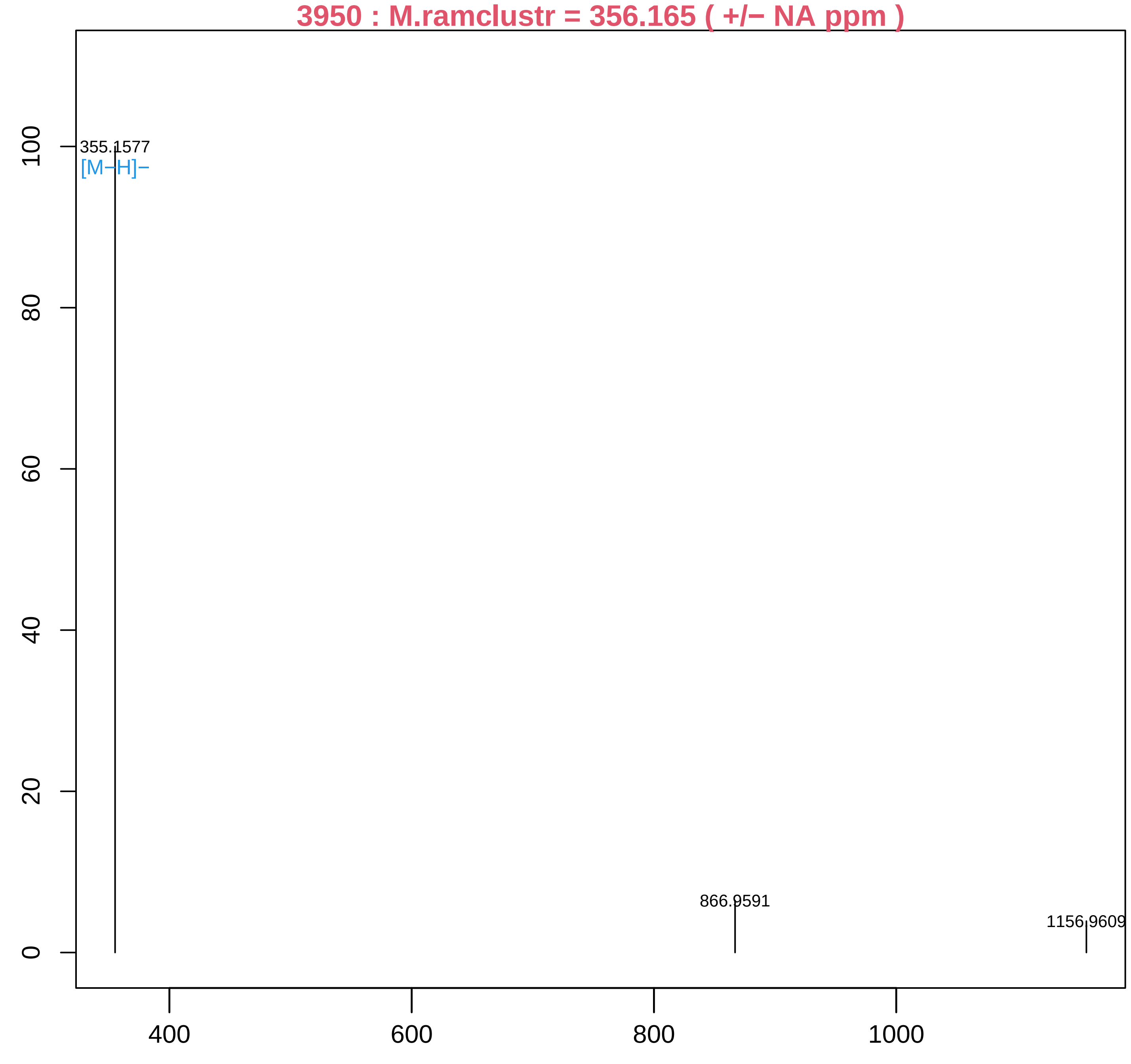
3950 : M.ramclustr = 356.165 (+/− NA ppm)
100
80
60
40
20
0
400
600
800
1000
355.1577
[M−H]−
866.9591
1156.9609

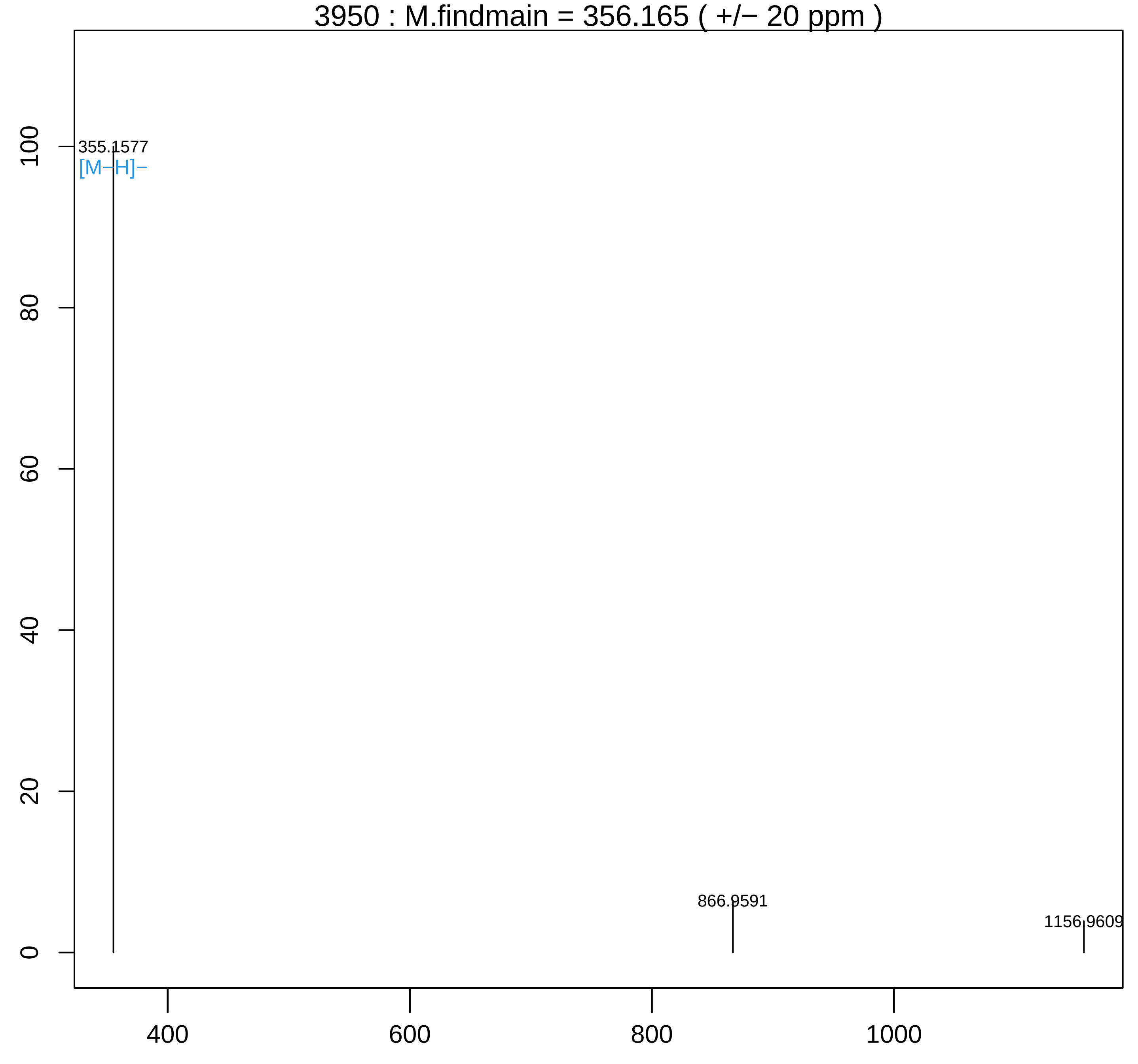
3950 : M.findmain = 356.165 (+/− 20 ppm)
100
80
60
40
20
0
400
600
800
1000
355.1577
[M−H]−
866.9591
1156.9609

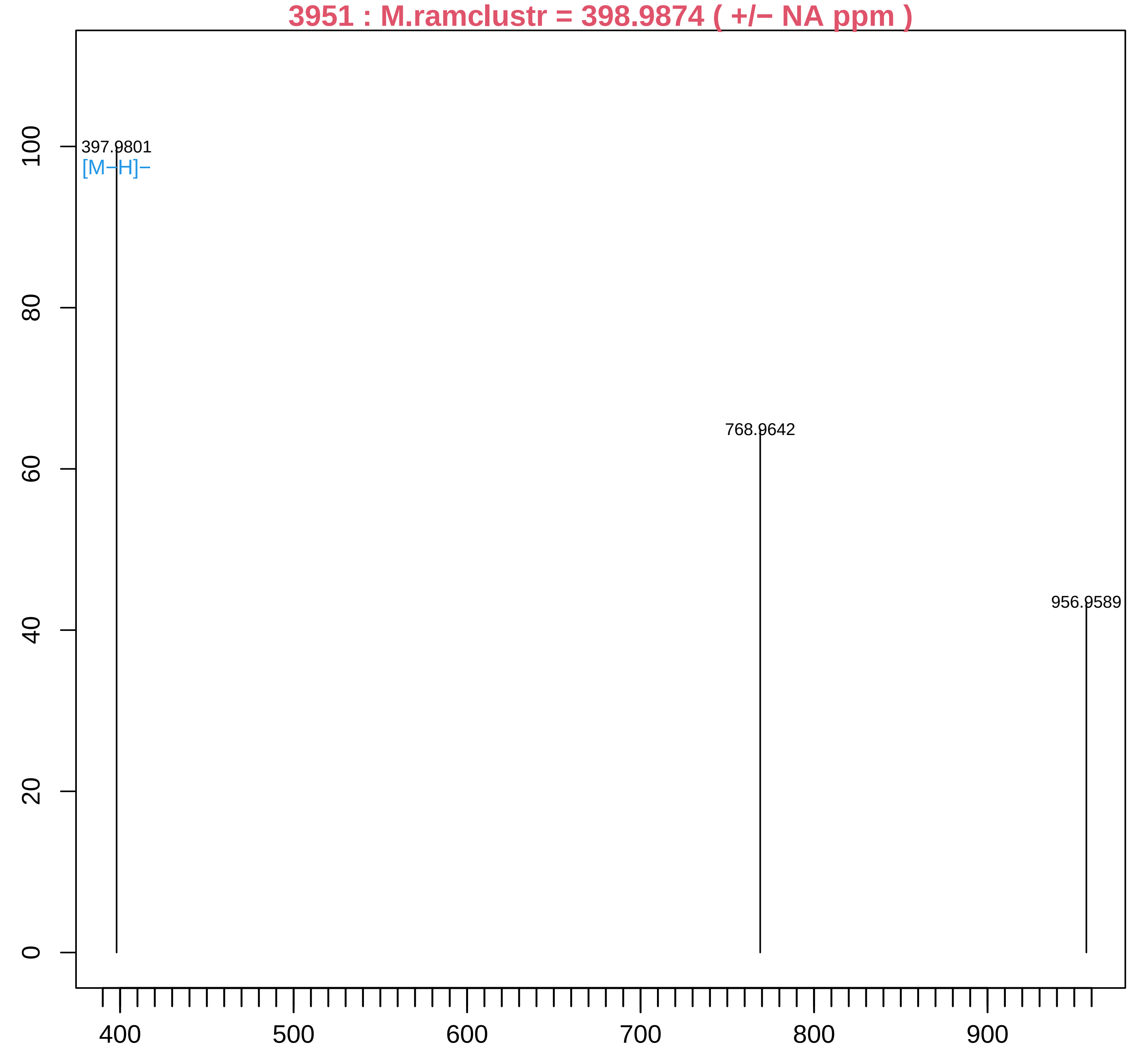

3951 : M.ramclustr = 398.9874 (+/− NA ppm)
397.9801
[M−H]−
768.9642
956.9589
0
20
40
60
80
100
400
500
600
700
800
900

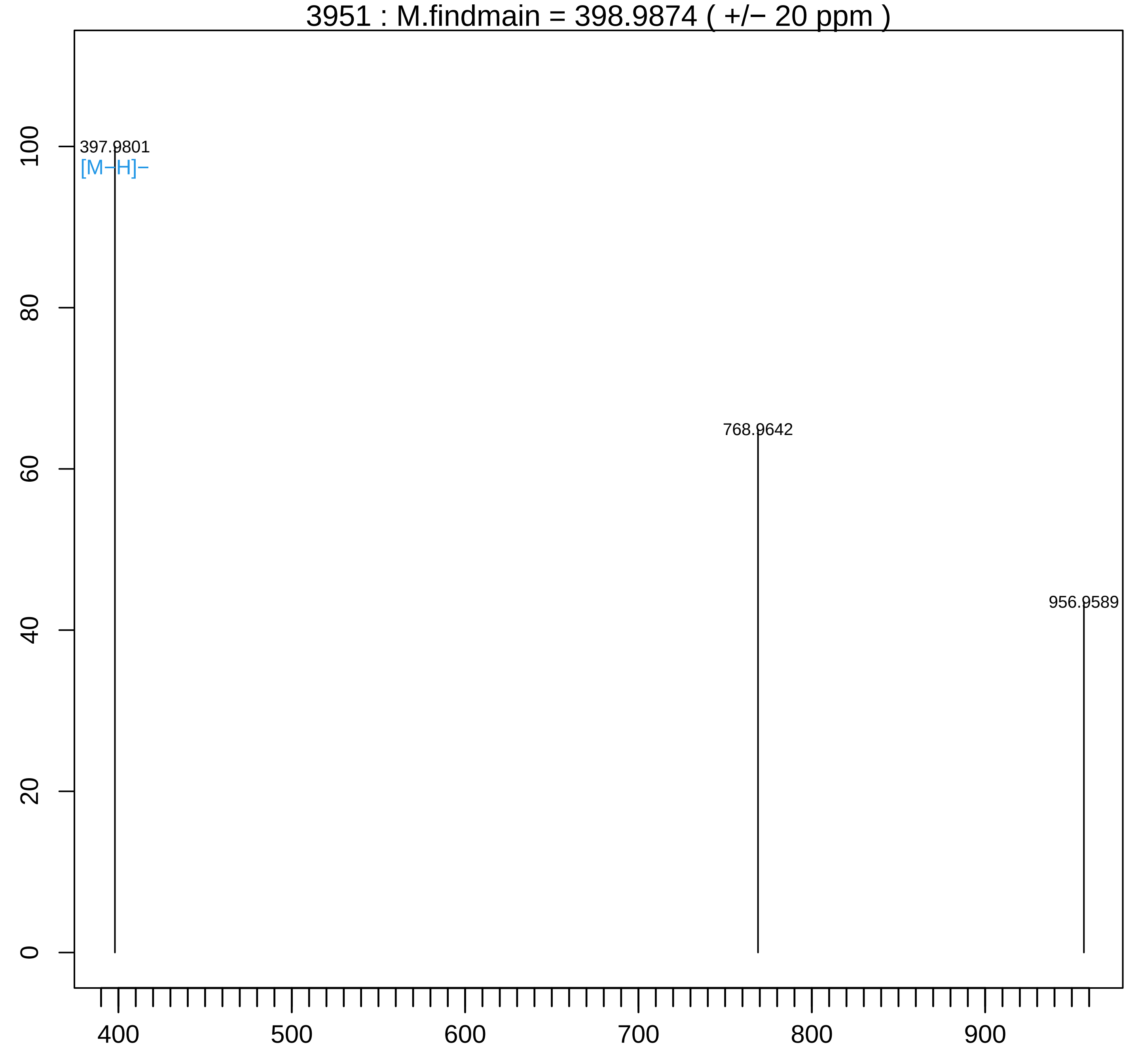

3951 : M.findmain = 398.9874 (+/− 20 ppm)
397.9801
[M−H]−
768.9642
956.9589
0
20
40
60
80
100
400
500
600
700
800
900

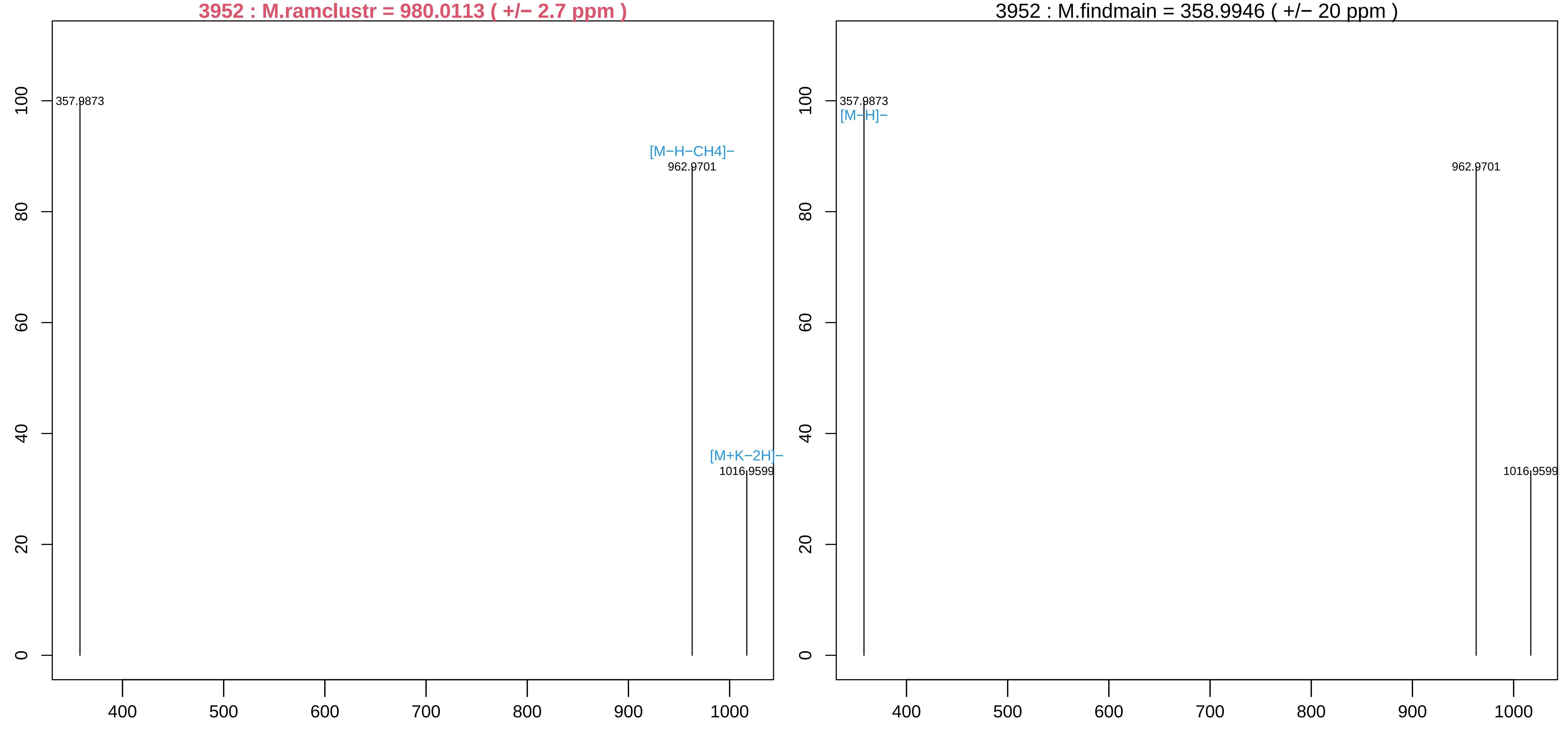
3952 : M.ramclustr = 980.0113 (+/− 2.7 ppm)
357.9873
[M−H−CH4]−
962.9701
[M+K−2H]−
1016.9599
3952 : M.findmain = 358.9946 (+/− 20 ppm)
357.9873
[M−H]−
962.9701
1016.9599
0
20
40
60
80
100
400
500
600
700
800
900
1000

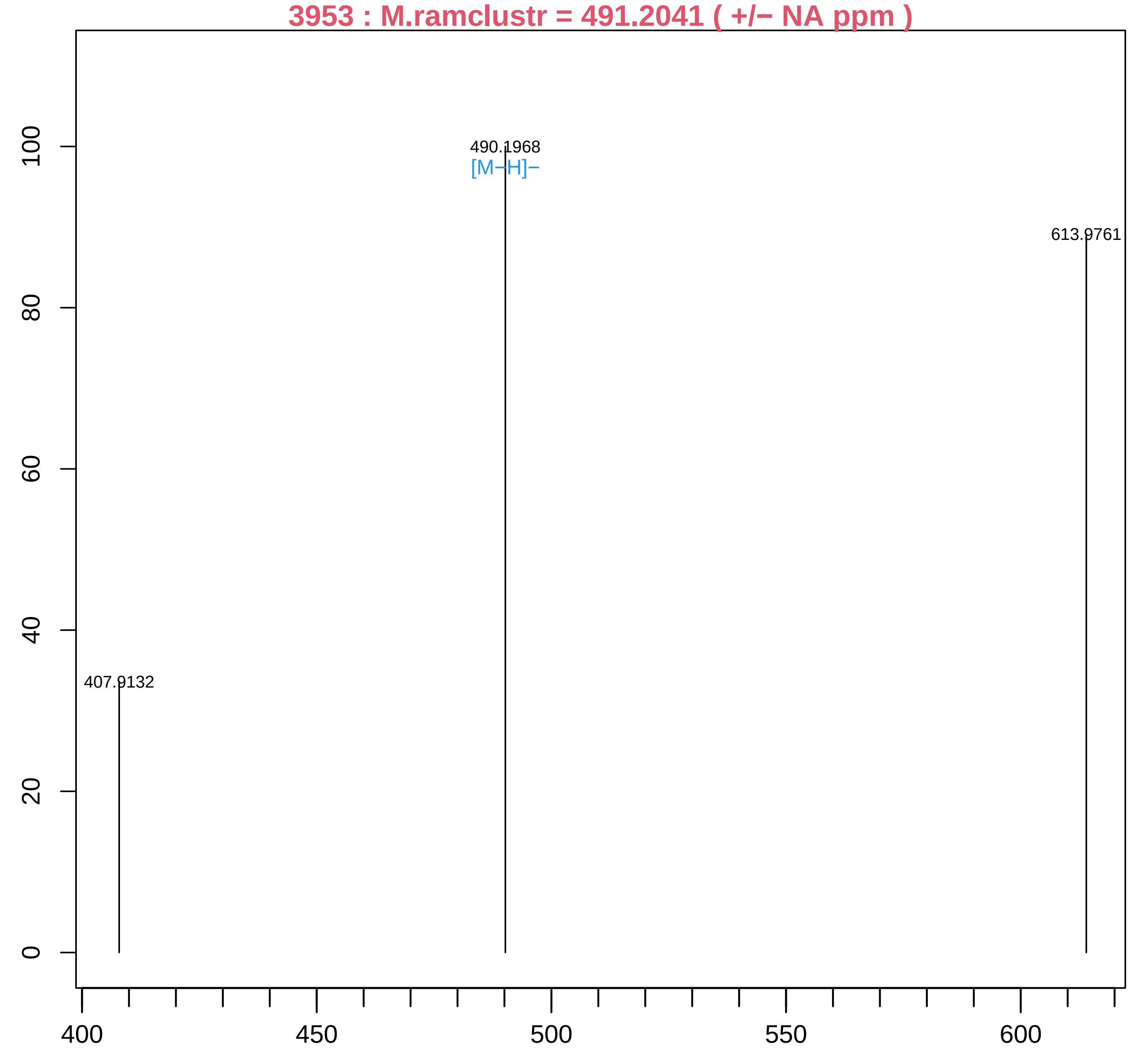
3953 : M.ramclustr = 491.2041 (+/− NA ppm)
490.1968
[M−H]−
613.9761
407.9132
0
20
40
60
80
100
400
450
500
550
600

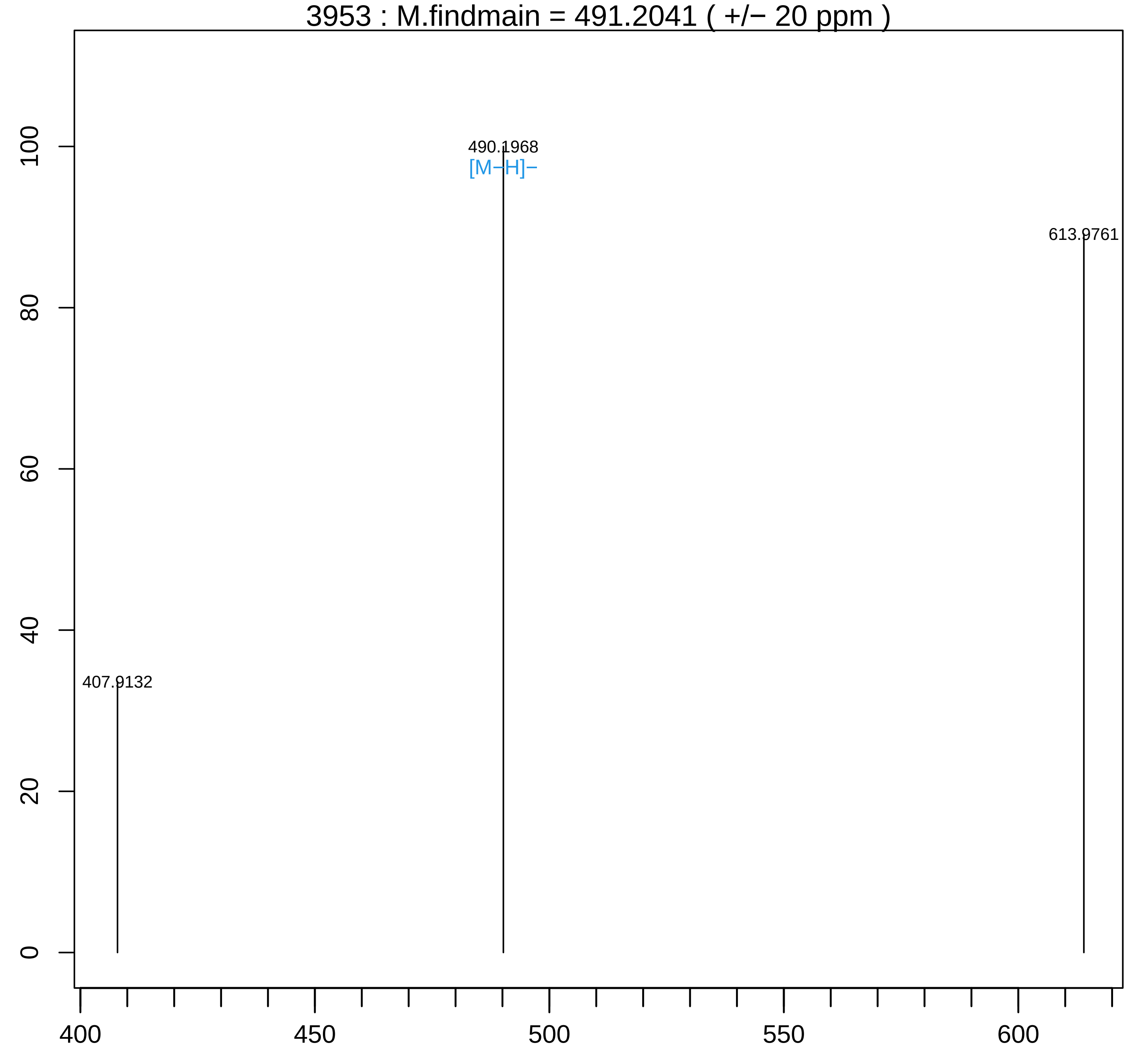
3953 : M.findmain = 491.2041 (+/− 20 ppm)
490.1968
[M−H]−
613.9761
407.9132
0
20
40
60
80
100
400
450
500
550
600

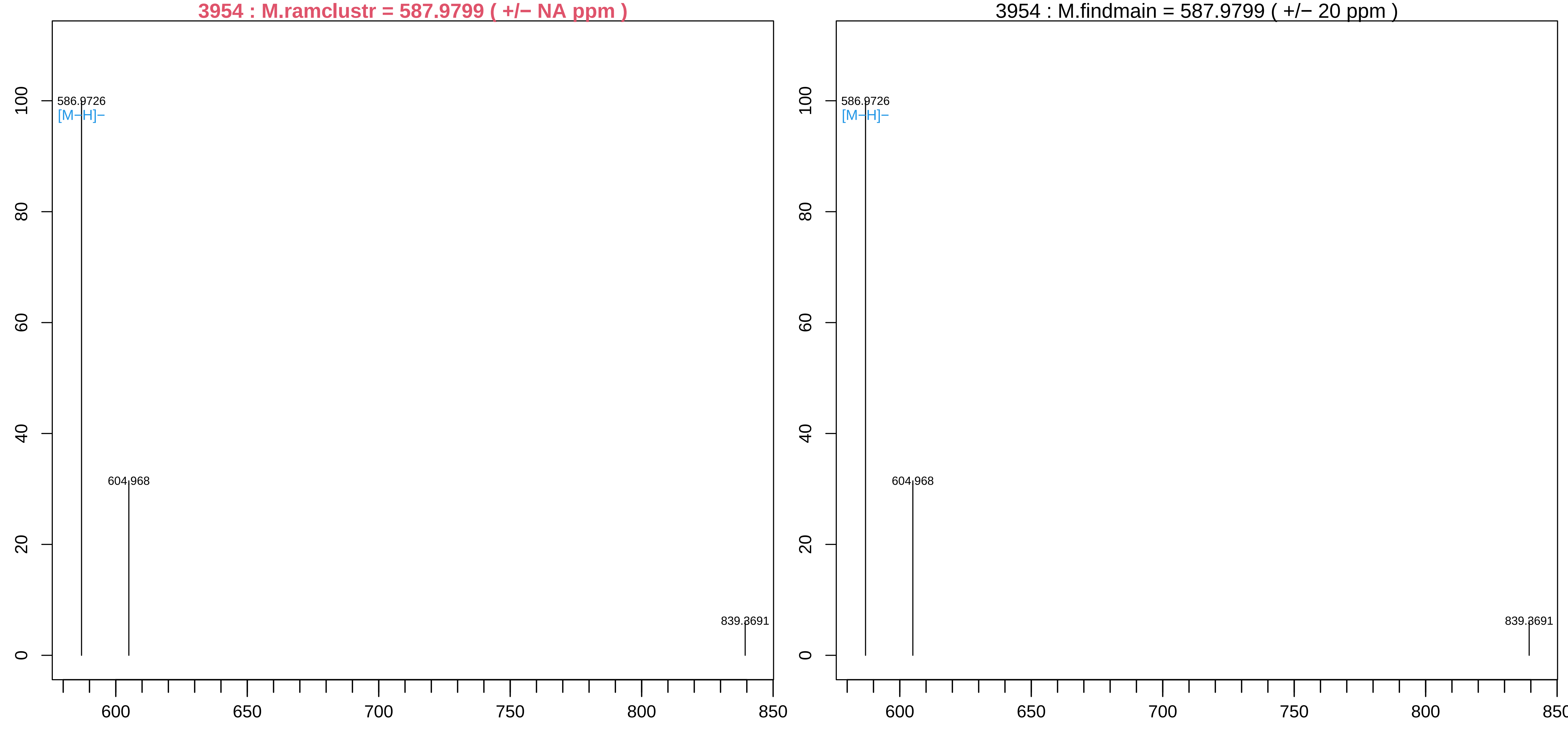

3954 : M.ramclustr = 587.9799 (+/− NA ppm)
586.9726
[M−H]−
604.968
839.3691
0
20
40
60
80
100
600
650
700
750
800
850
3954 : M.findmain = 587.9799 (+/− 20 ppm)
586.9726
[M−H]−
604.968
839.3691
0
20
40
60
80
100
600
650
700
750
800
850

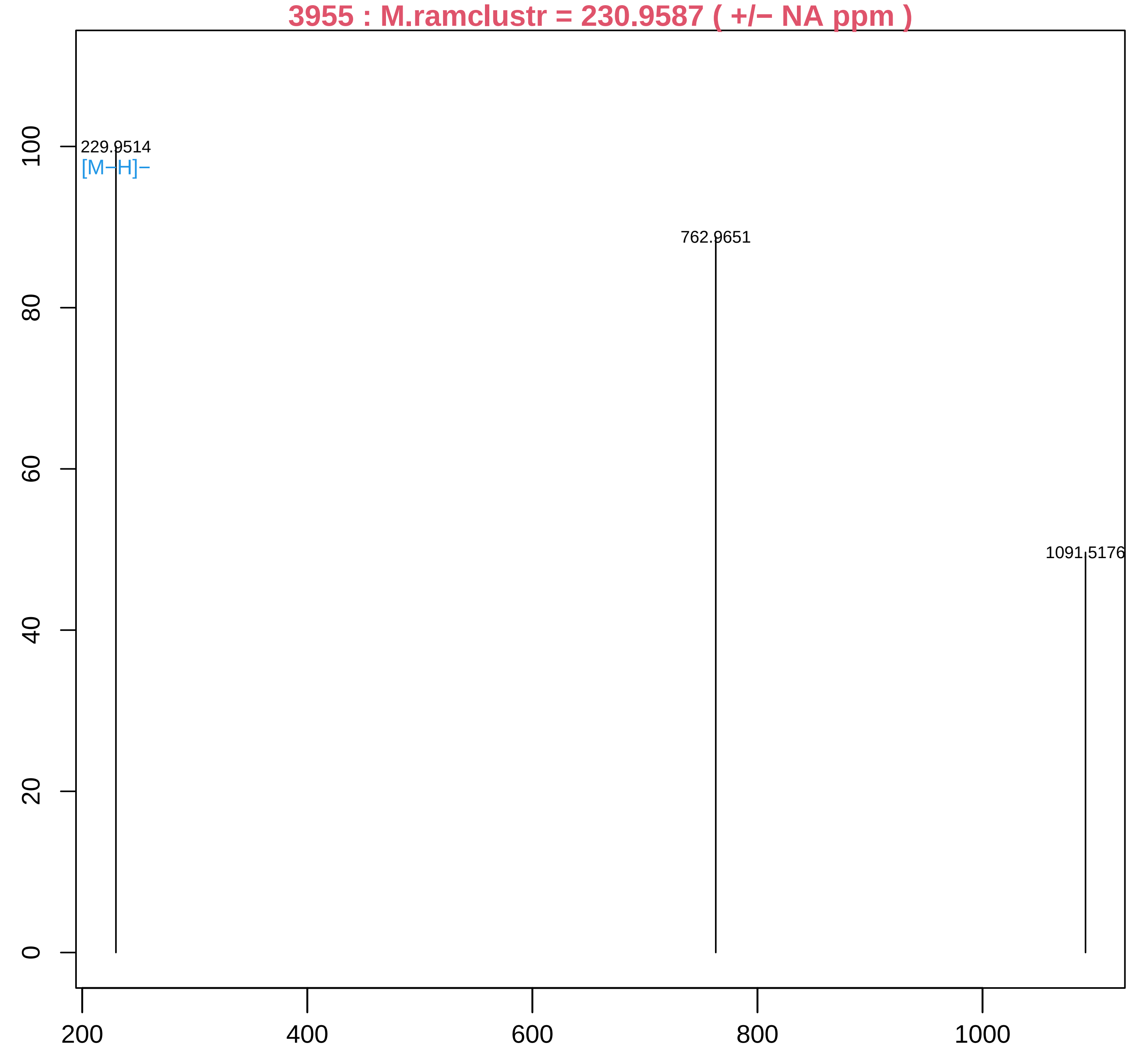
3955 : M.ramclustr = 230.9587 (+/− NA ppm)
229.9514
[M−H]−
762.9651
1091.5176
0
20
40
60
80
100
200
400
600
800
1000

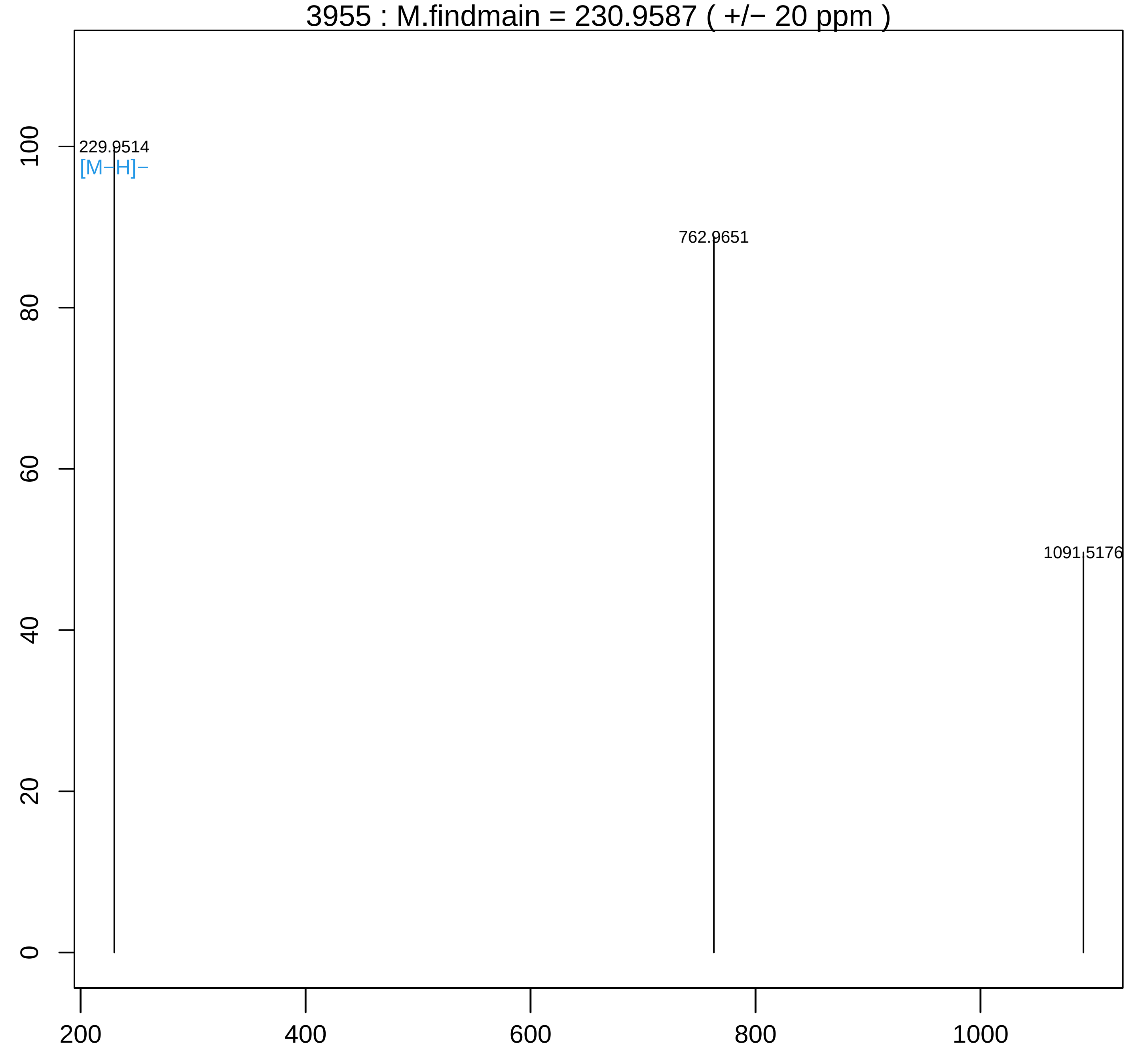
3955 : M.findmain = 230.9587 (+/− 20 ppm)
229.9514
[M−H]−
762.9651
1091.5176
0
20
40
60
80
100
200
400
600
800
1000

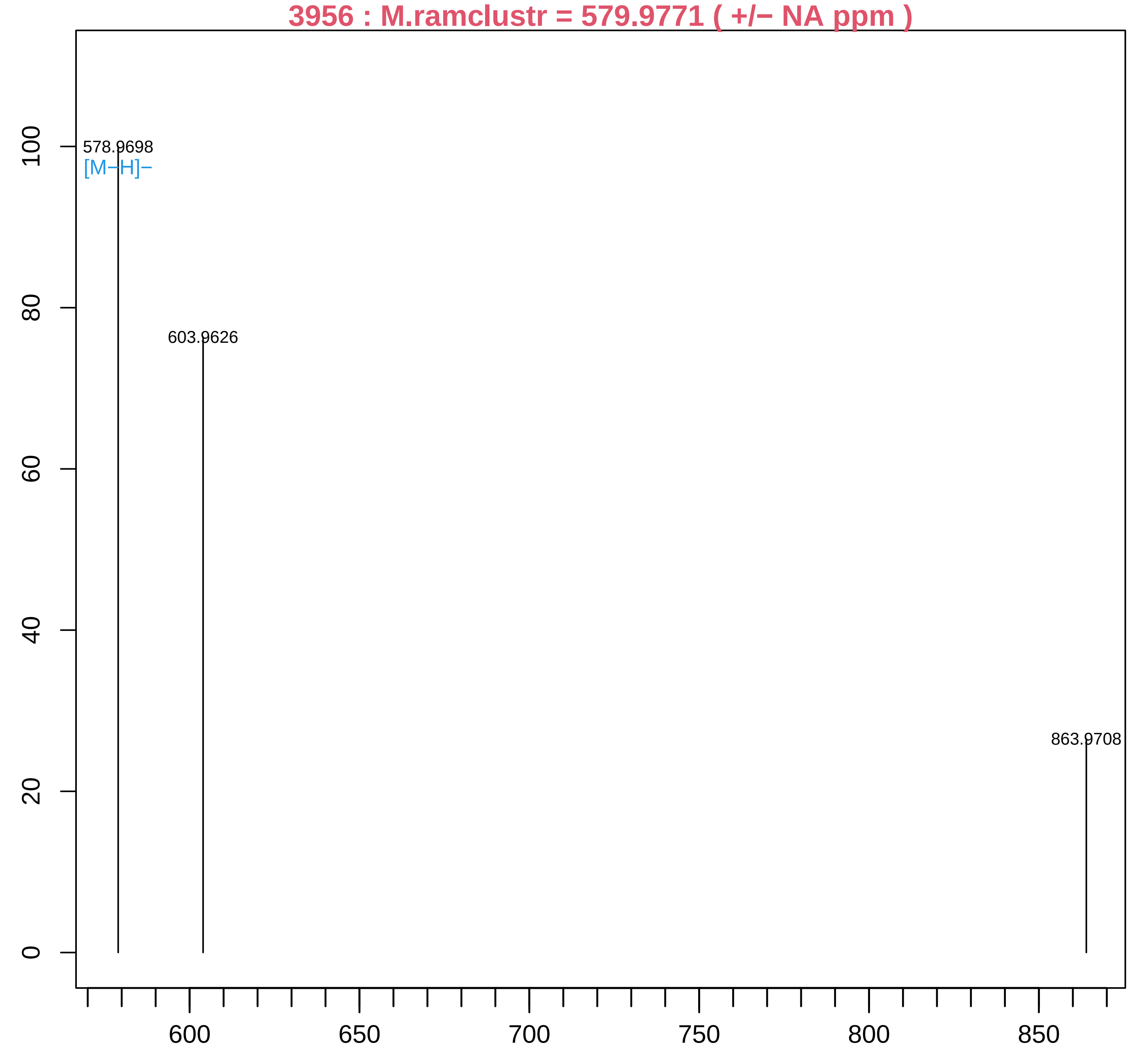
3956 : M.ramclustr = 579.9771 (+/− NA ppm)
578.9698
[M−H]−
603.9626
863.9708
0
20
40
60
80
100
600
650
700
750
800
850

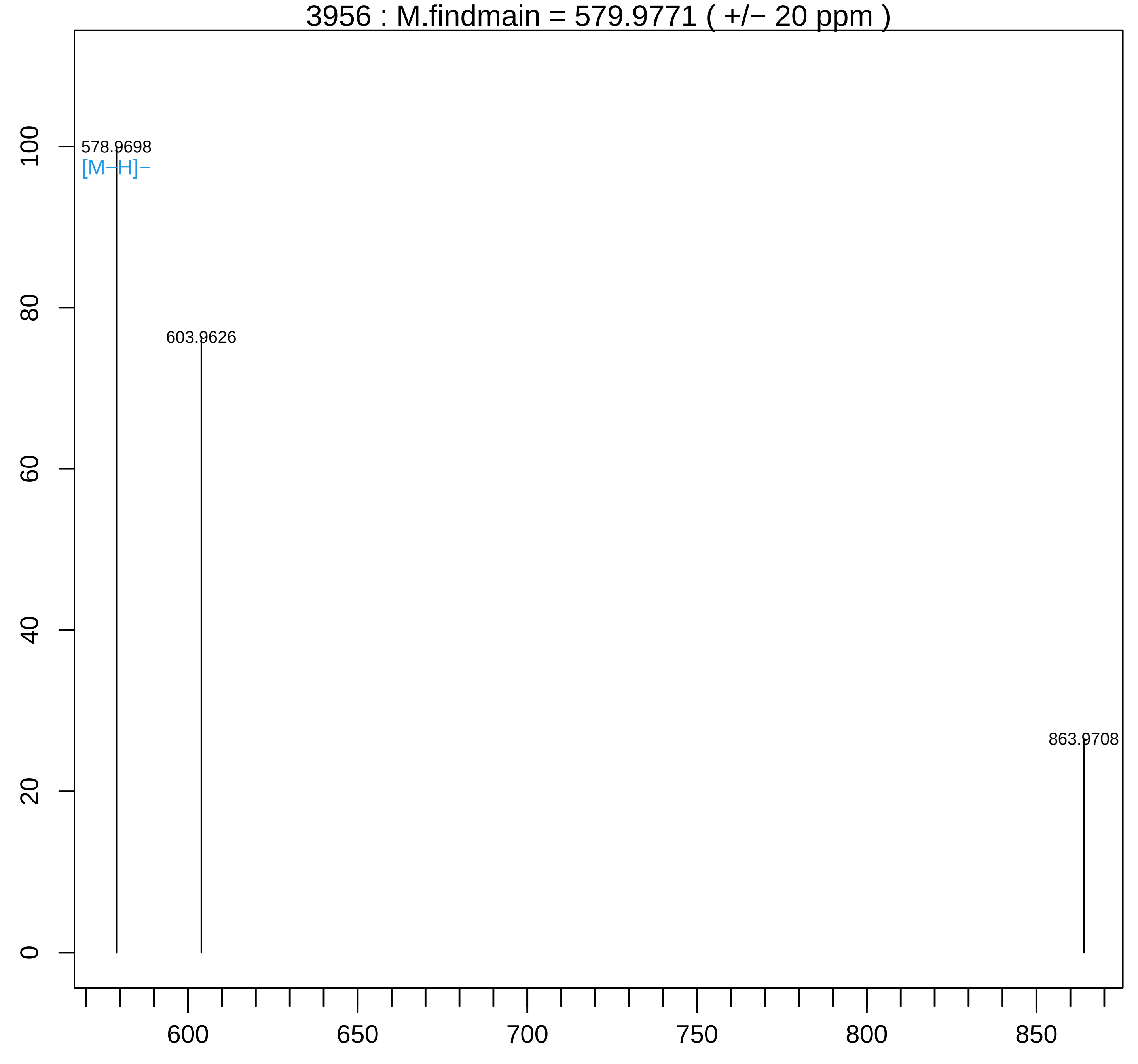
3956 : M.findmain = 579.9771 (+/− 20 ppm)
578.9698
[M−H]−
603.9626
863.9708
0
20
40
60
80
100
600
650
700
750
800
850

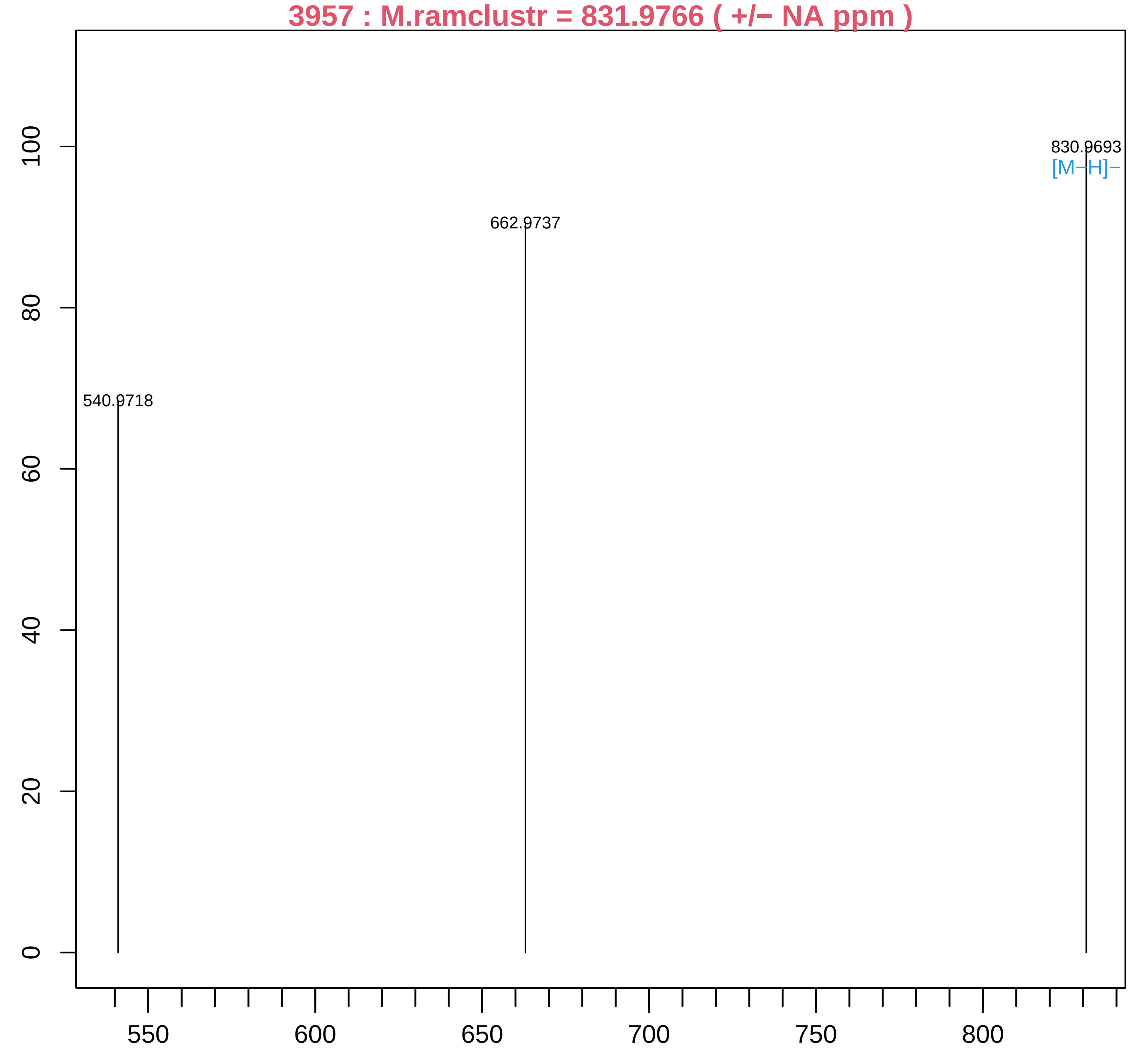
3957 : M.ramclustr = 831.9766 (+/− NA ppm)
540.9718
662.9737
830.9693
[M−H]−
0
20
40
60
80
100
550
600
650
700
750
800

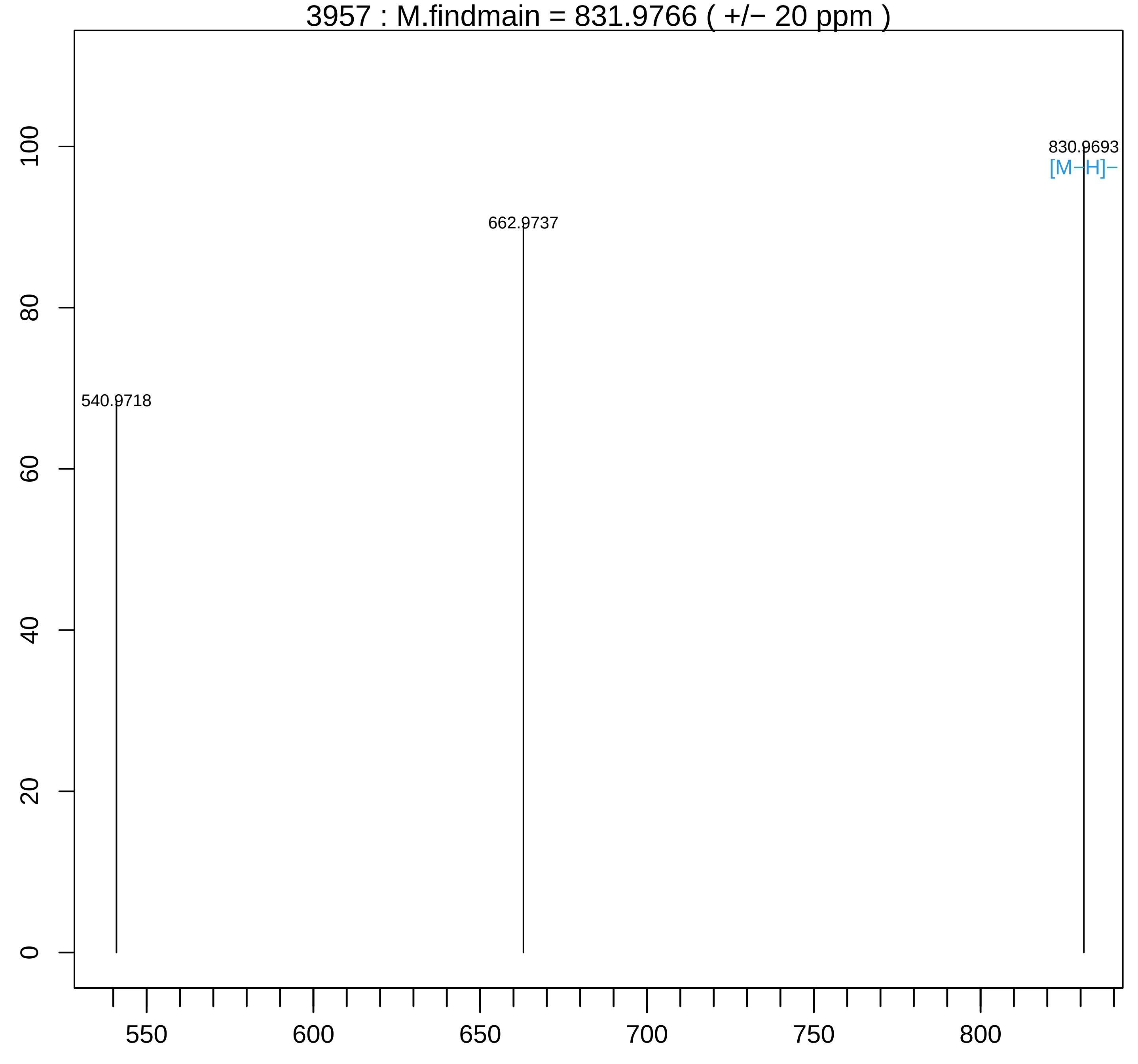
3957 : M.findmain = 831.9766 (+/− 20 ppm)
540.9718
662.9737
830.9693
[M−H]−
0
20
40
60
80
100
550
600
650
700
750
800

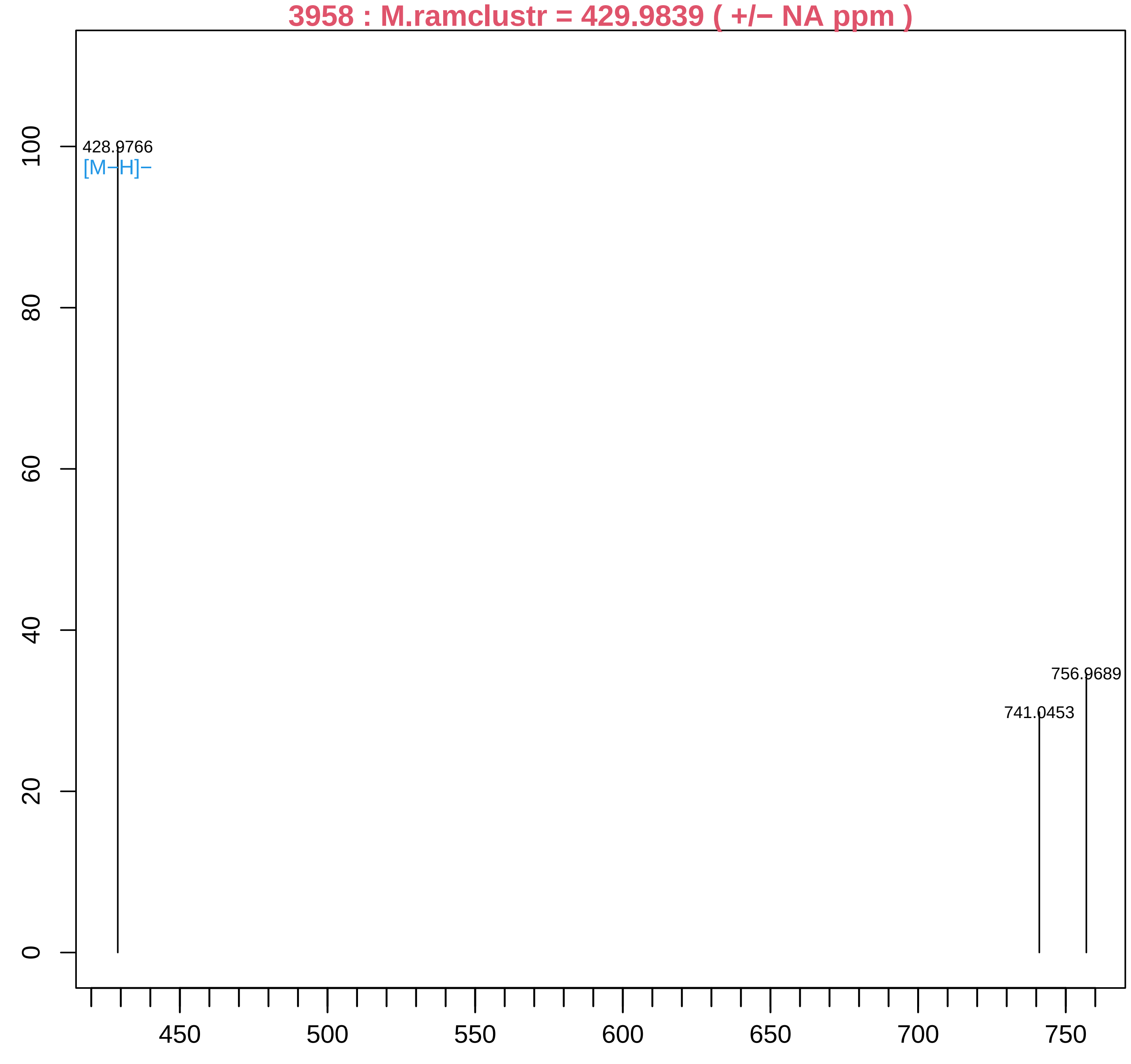
3958 : M.ramclustr = 429.9839 (+/− NA ppm)
428.9766
[M−H]−
741.0453
756.9689
0
20
40
60
80
100
450
500
550
600
650
700
750

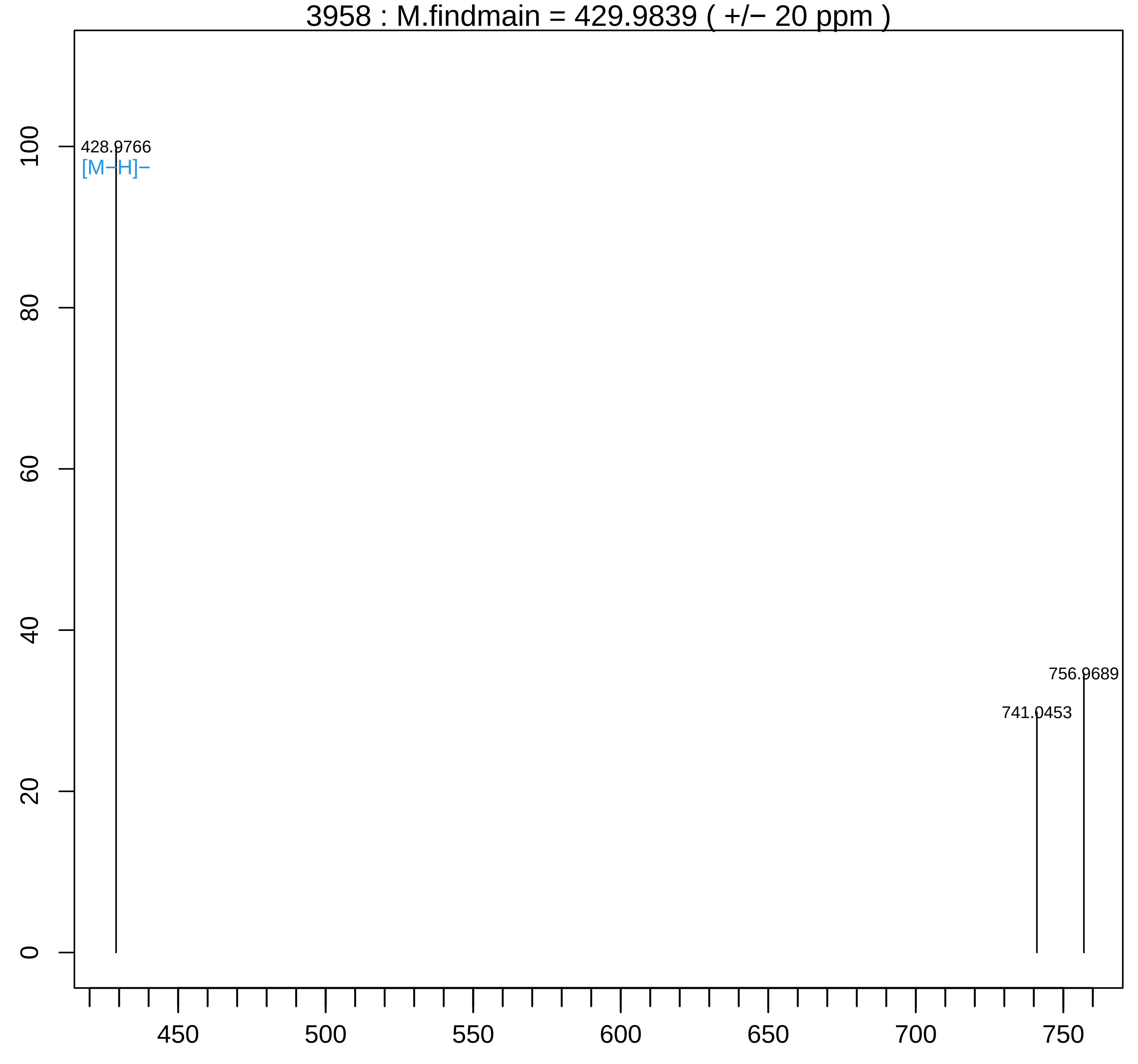
3958 : M.findmain = 429.9839 (+/− 20 ppm)
428.9766
[M−H]−
741.0453
756.9689
0
20
40
60
80
100
450
500
550
600
650
700
750

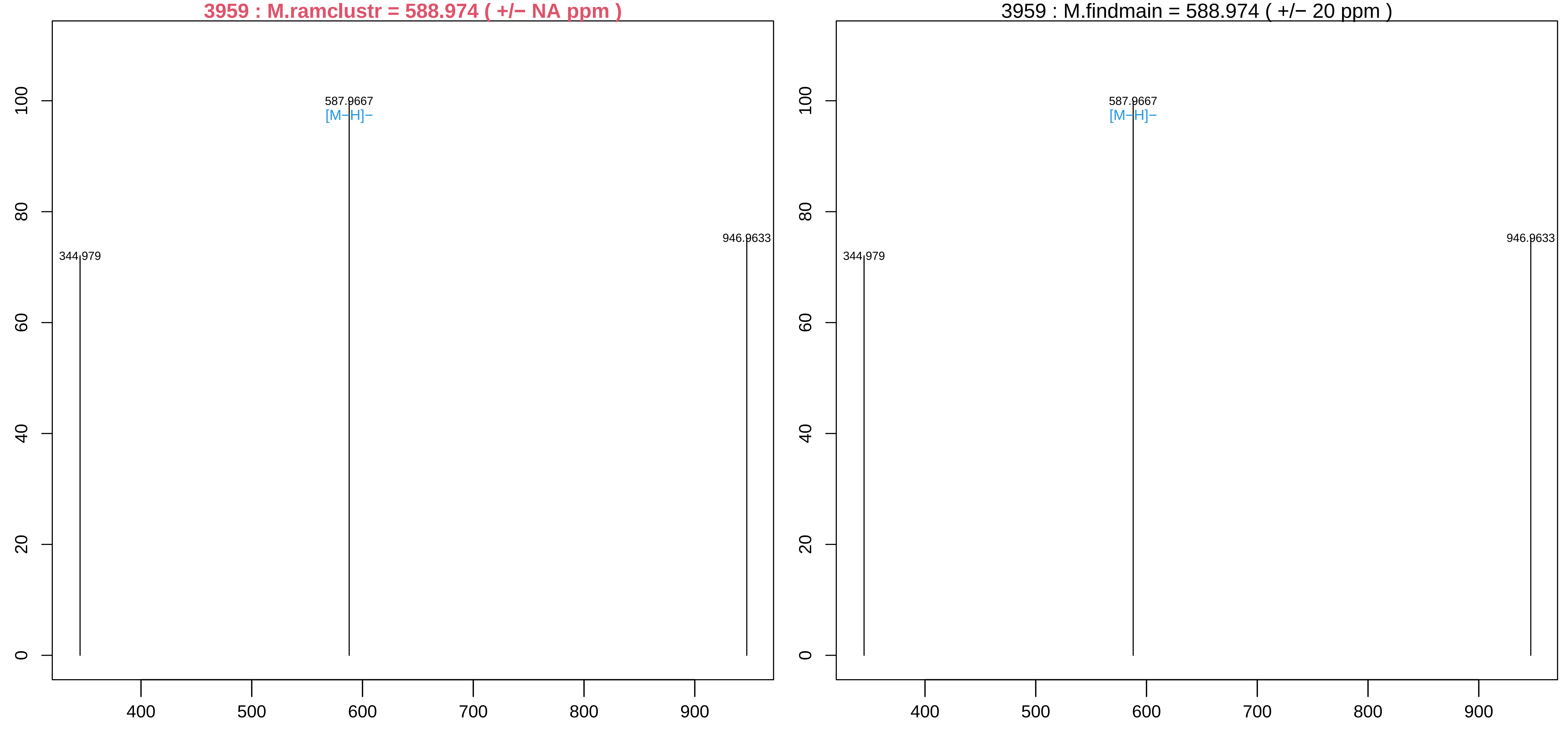
3959 : M.ramclustr = 588.974 (+/− NA ppm)
3959 : M.findmain = 588.974 (+/− 20 ppm)
587.9667
[M−H]−
344.979
946.9633
587.9667
[M−H]−
344.979
946.9633

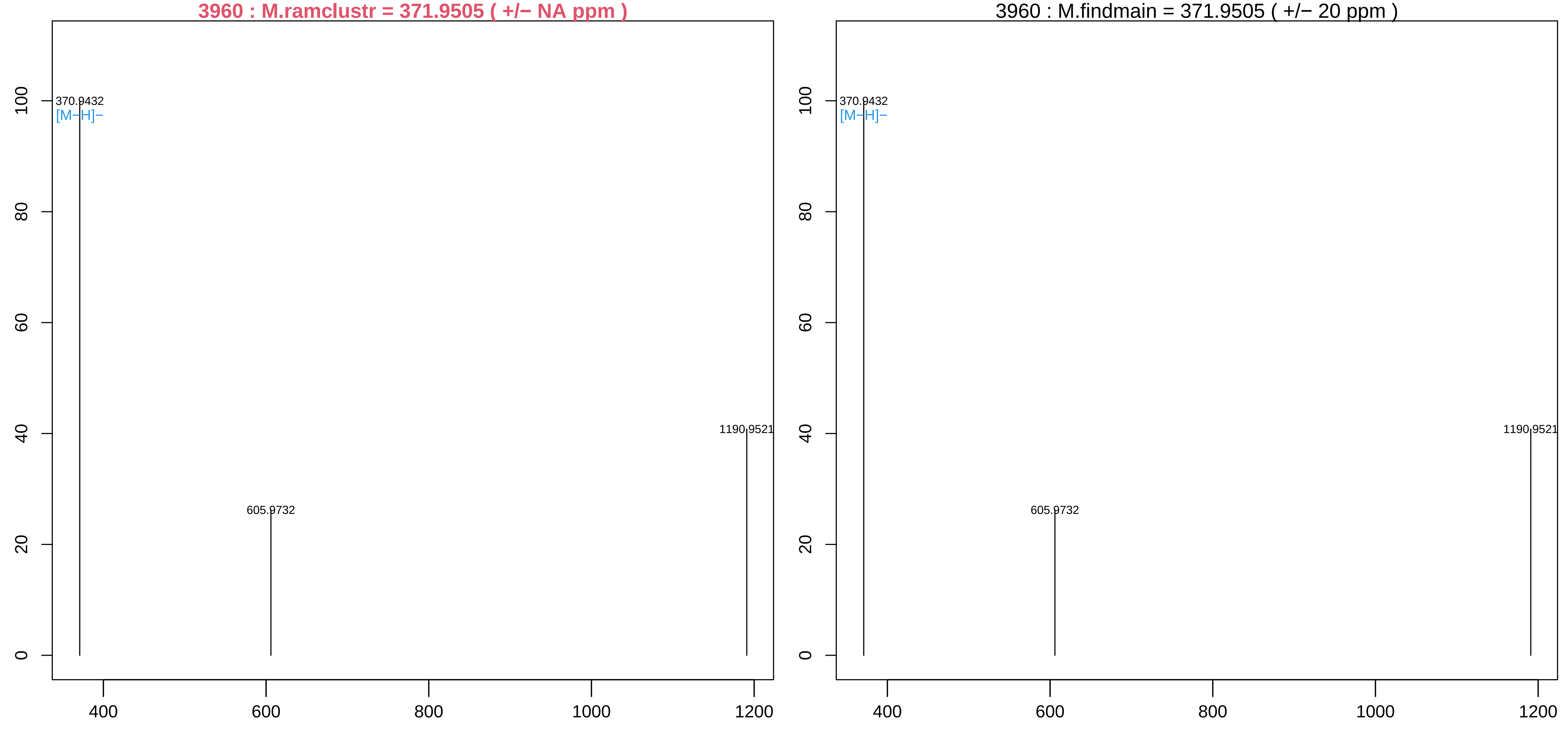
3960 : M.ramclustr = 371.9505 (+/− NA ppm)
370.9432
[M−H]−
605.9732
1190.9521
3960 : M.findmain = 371.9505 (+/− 20 ppm)
370.9432
[M−H]−
605.9732
1190.9521

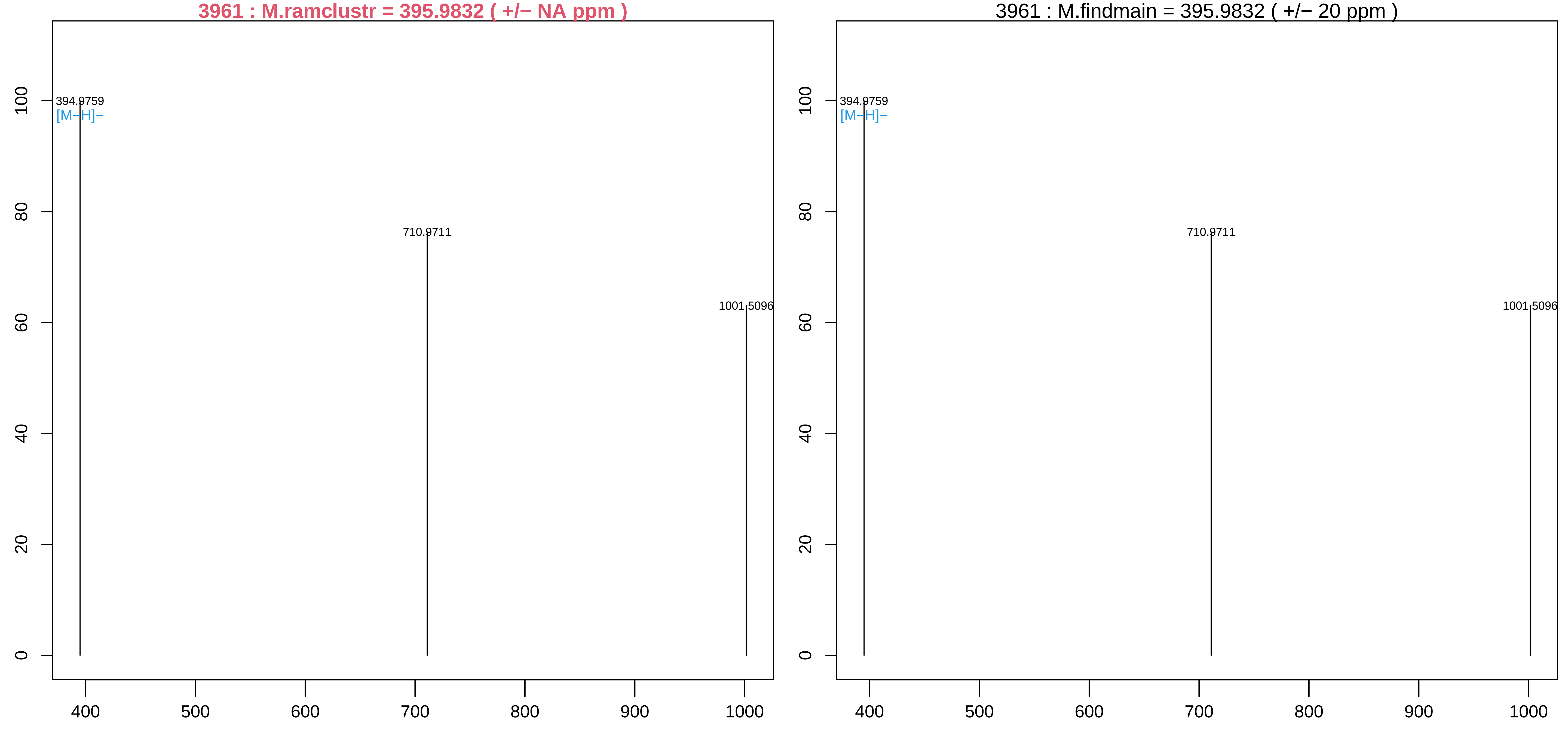

3961 : M.ramclustr = 395.9832 (+/− NA ppm)
394.9759
[M−H]−
710.9711
1001.5096
3961 : M.findmain = 395.9832 (+/− 20 ppm)
394.9759
[M−H]−
710.9711
1001.5096
0
20
40
60
80
100
400
500
600
700
800
900
1000

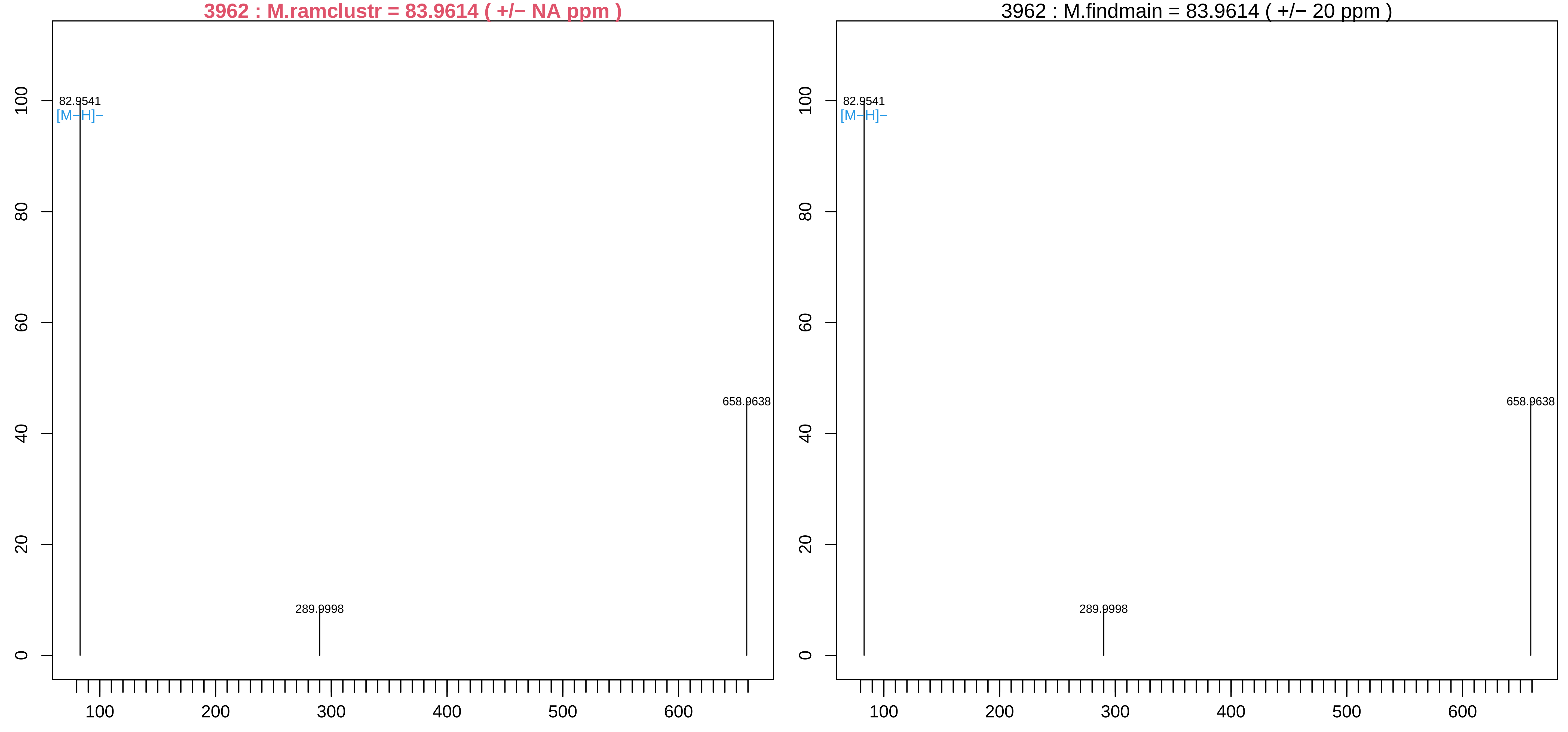
3962 : M.ramclustr = 83.9614 (+/− NA ppm)
82.9541
[M−H]−
289.9998
658.9638
0
20
40
60
80
100
200
300
400
500
600
3962 : M.findmain = 83.9614 (+/− 20 ppm)
82.9541
[M−H]−
289.9998
658.9638

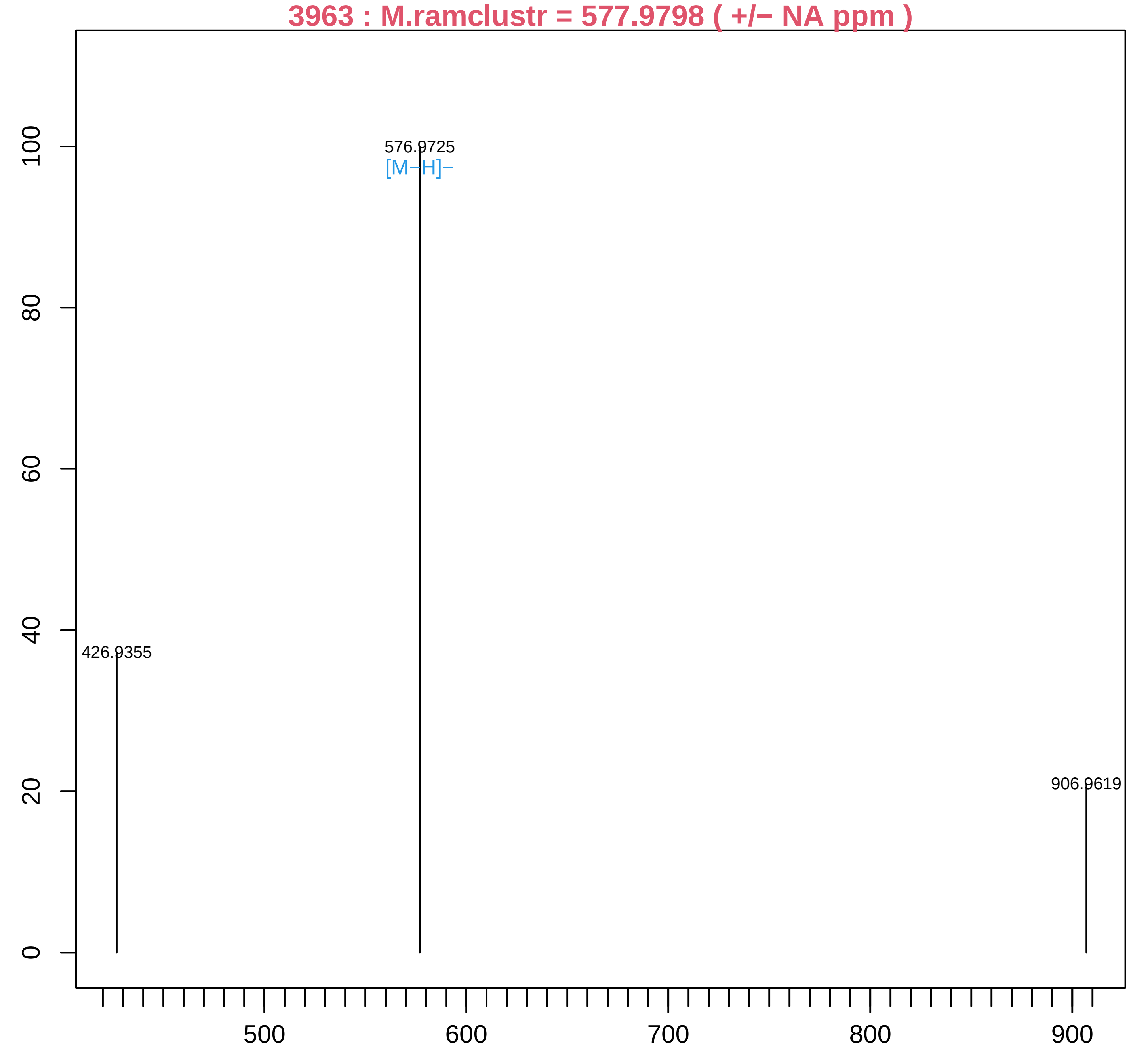
3963 : M.ramclustr = 577.9798 (+/− NA ppm)
576.9725
[M−H]−
426.9355
906.9619
0
20
40
60
80
100
500
600
700
800
900

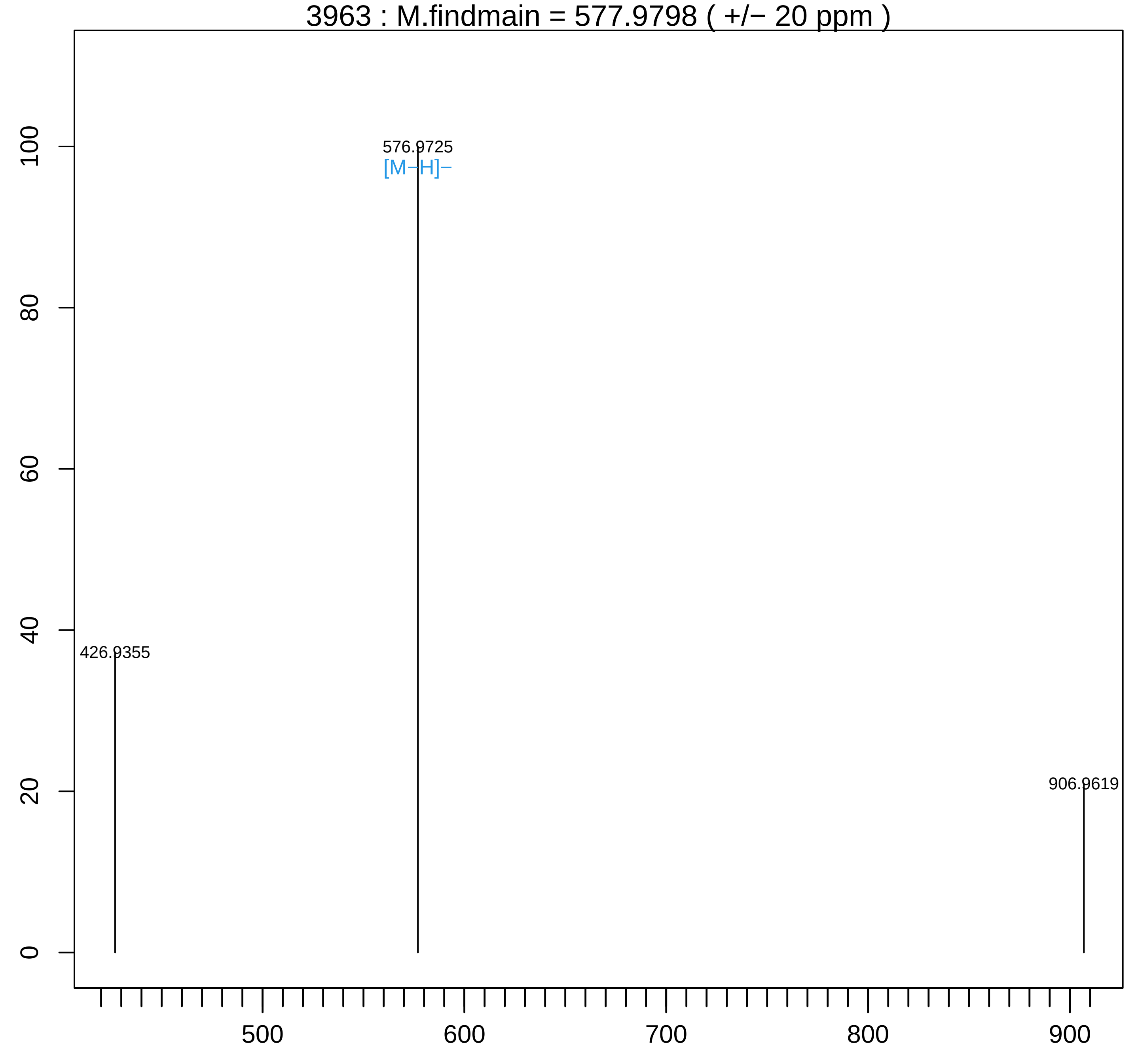
3963 : M.findmain = 577.9798 (+/− 20 ppm)
576.9725
[M−H]−
426.9355
906.9619
0
20
40
60
80
100
500
600
700
800
900

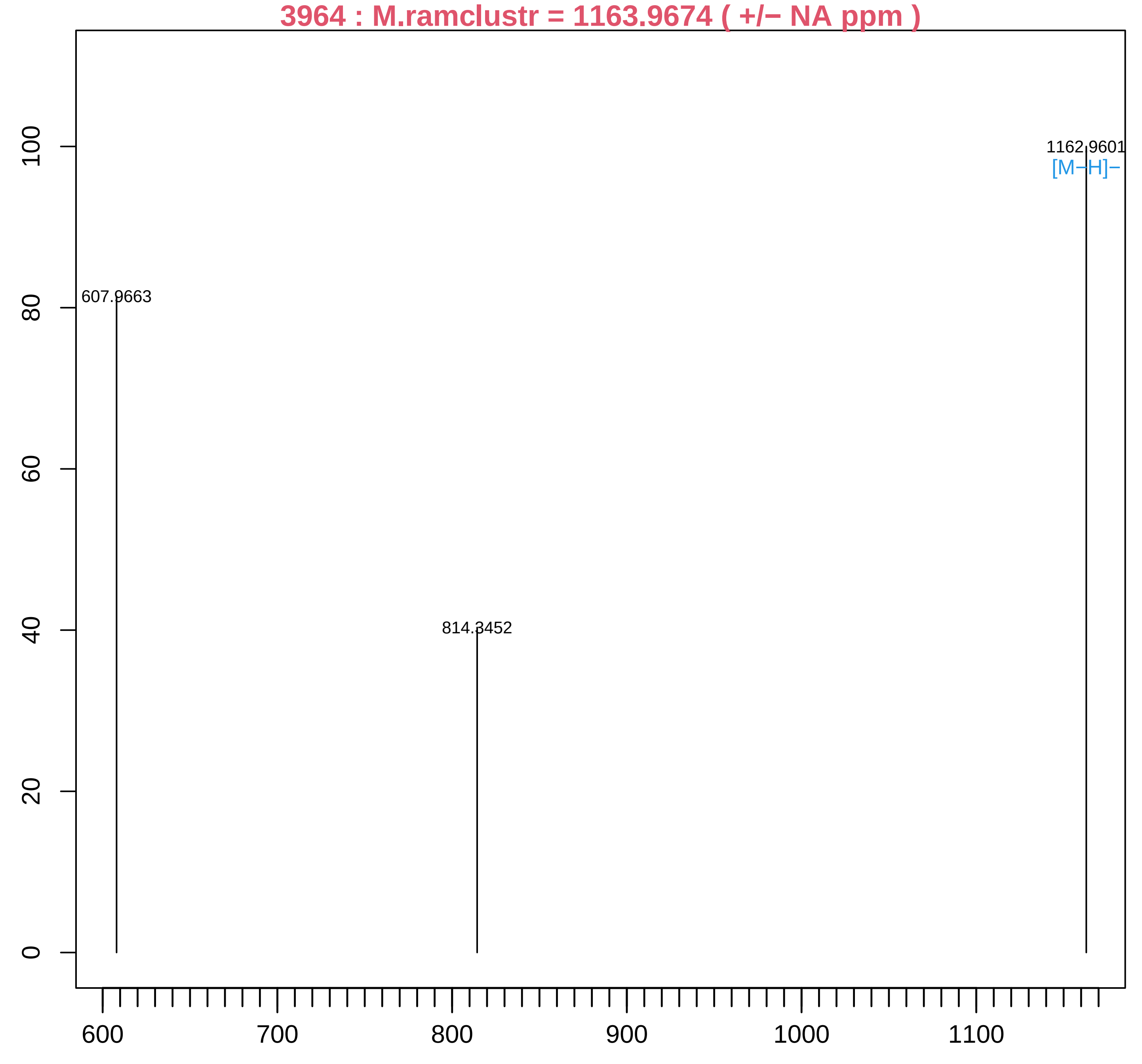
3964 : M.ramclustr = 1163.9674 (+/− NA ppm)
1162.9601
[M−H]−
607.9663
814.3452
0
20
40
60
80
100
600
700
800
900
1000
1100

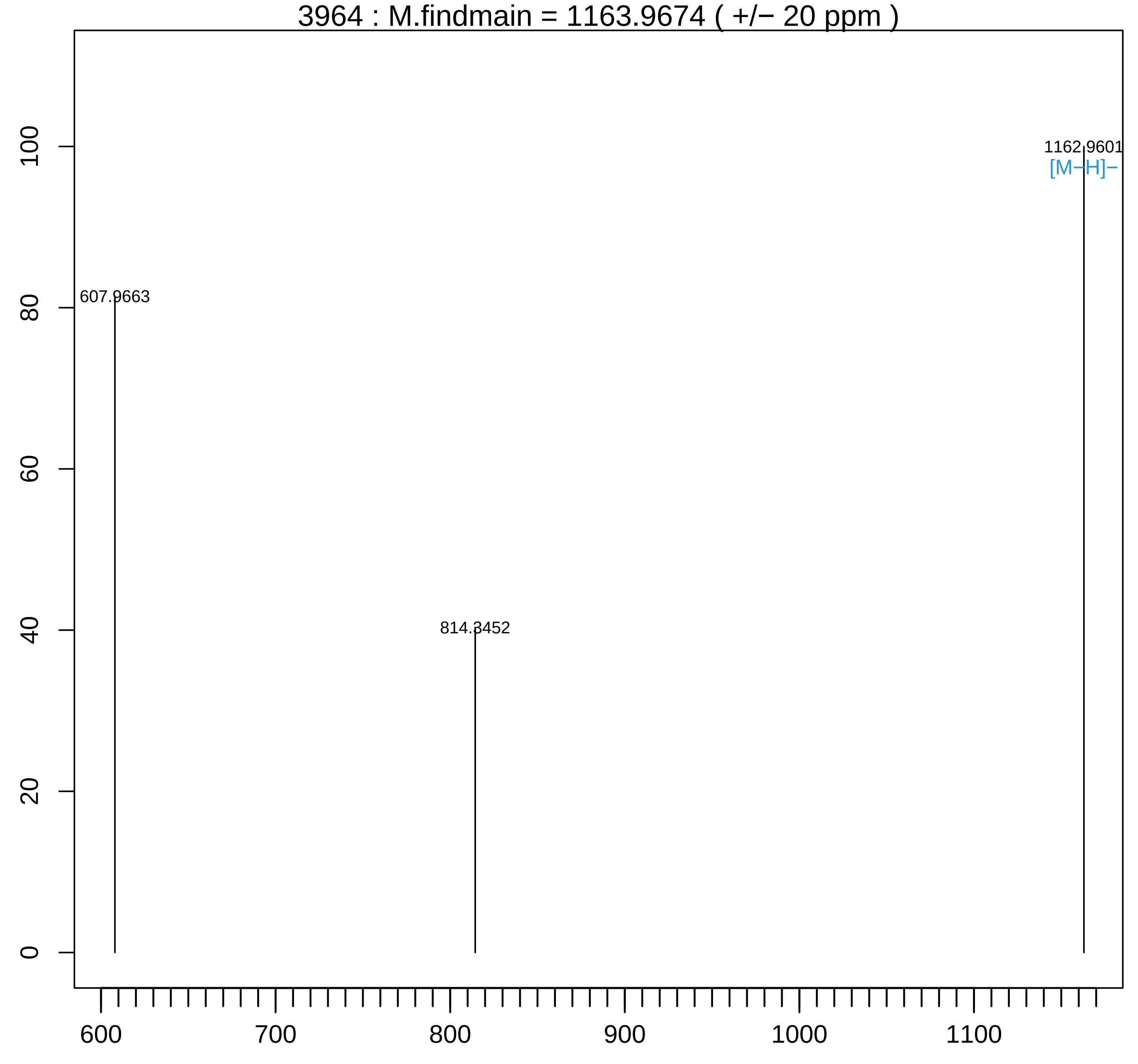
3964 : M.findmain = 1163.9674 (+/− 20 ppm)
1162.9601
[M−H]−
607.9663
814.3452
0
20
40
60
80
100
600
700
800
900
1000
1100

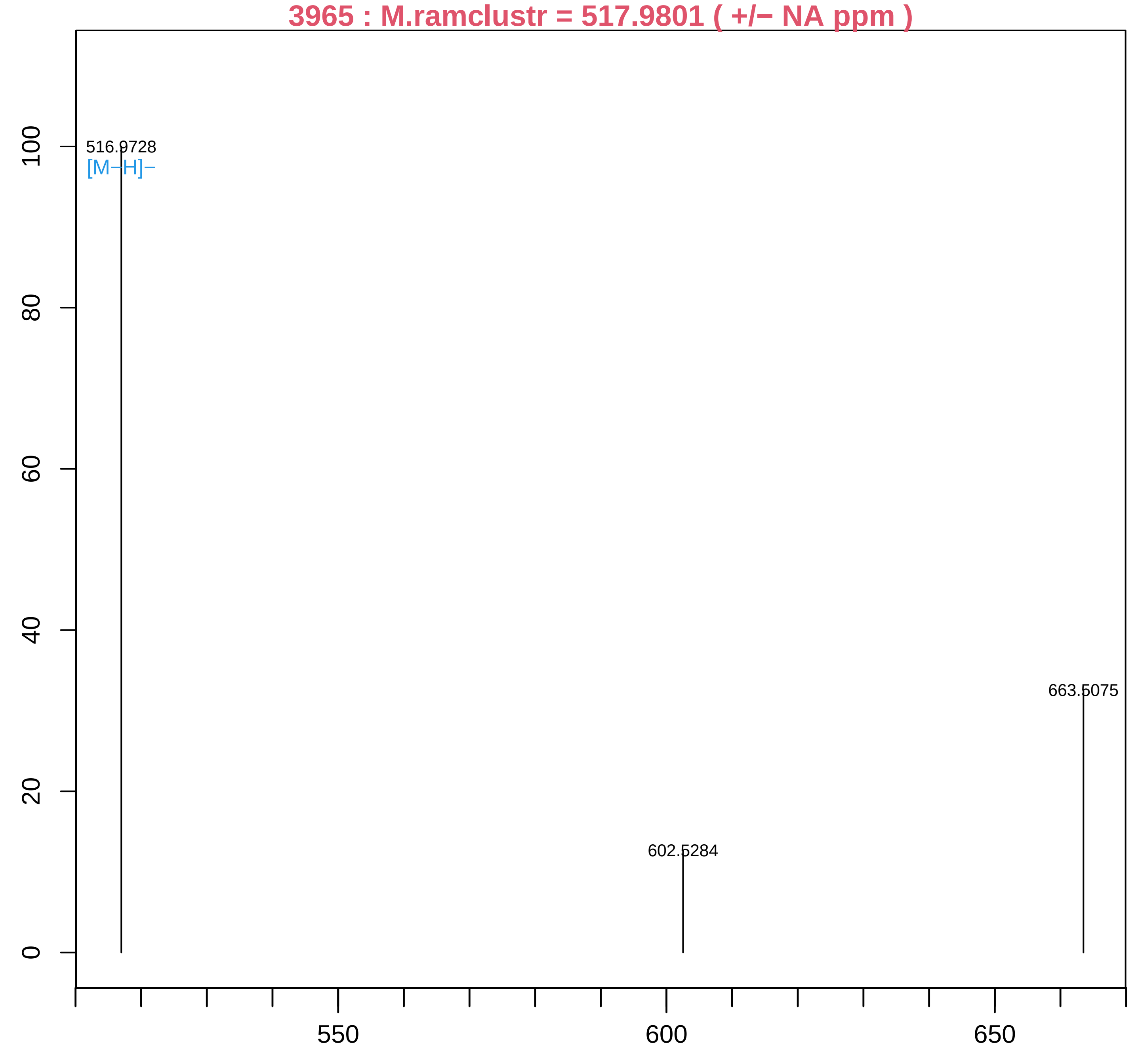
3965 : M.ramclustr = 517.9801 (+/− NA ppm)
516.9728
[M−H]−
602.5284
663.5075
0
20
40
60
80
100
550
600
650

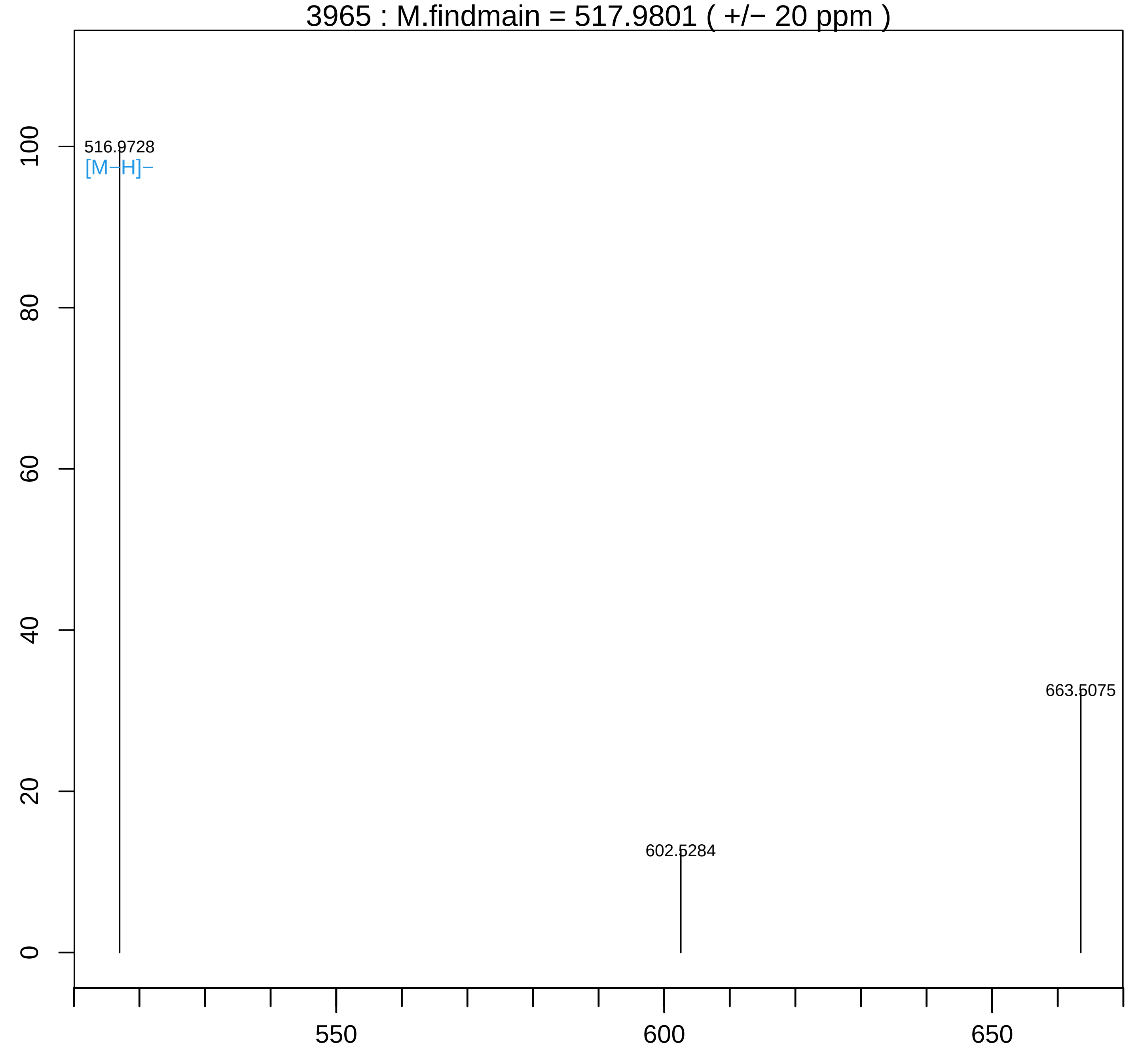
3965 : M.findmain = 517.9801 (+/− 20 ppm)
516.9728
[M−H]−
602.5284
663.5075
0
20
40
60
80
100
550
600
650

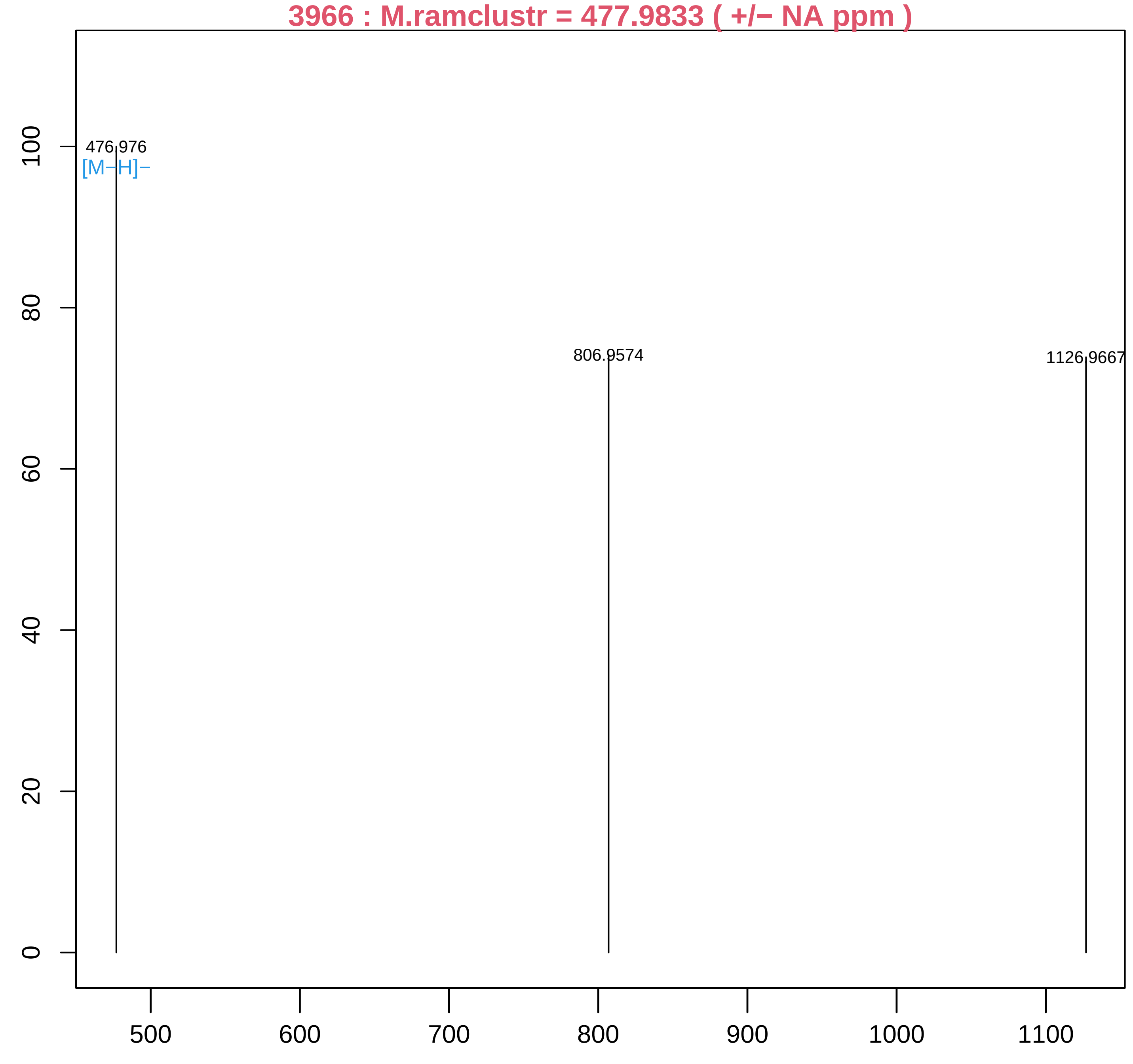
3966 : M.ramclustr = 477.9833 (+/− NA ppm)
476.976
[M−H]−
806.9574
1126.9667
0
20
40
60
80
100
500
600
700
800
900
1000
1100

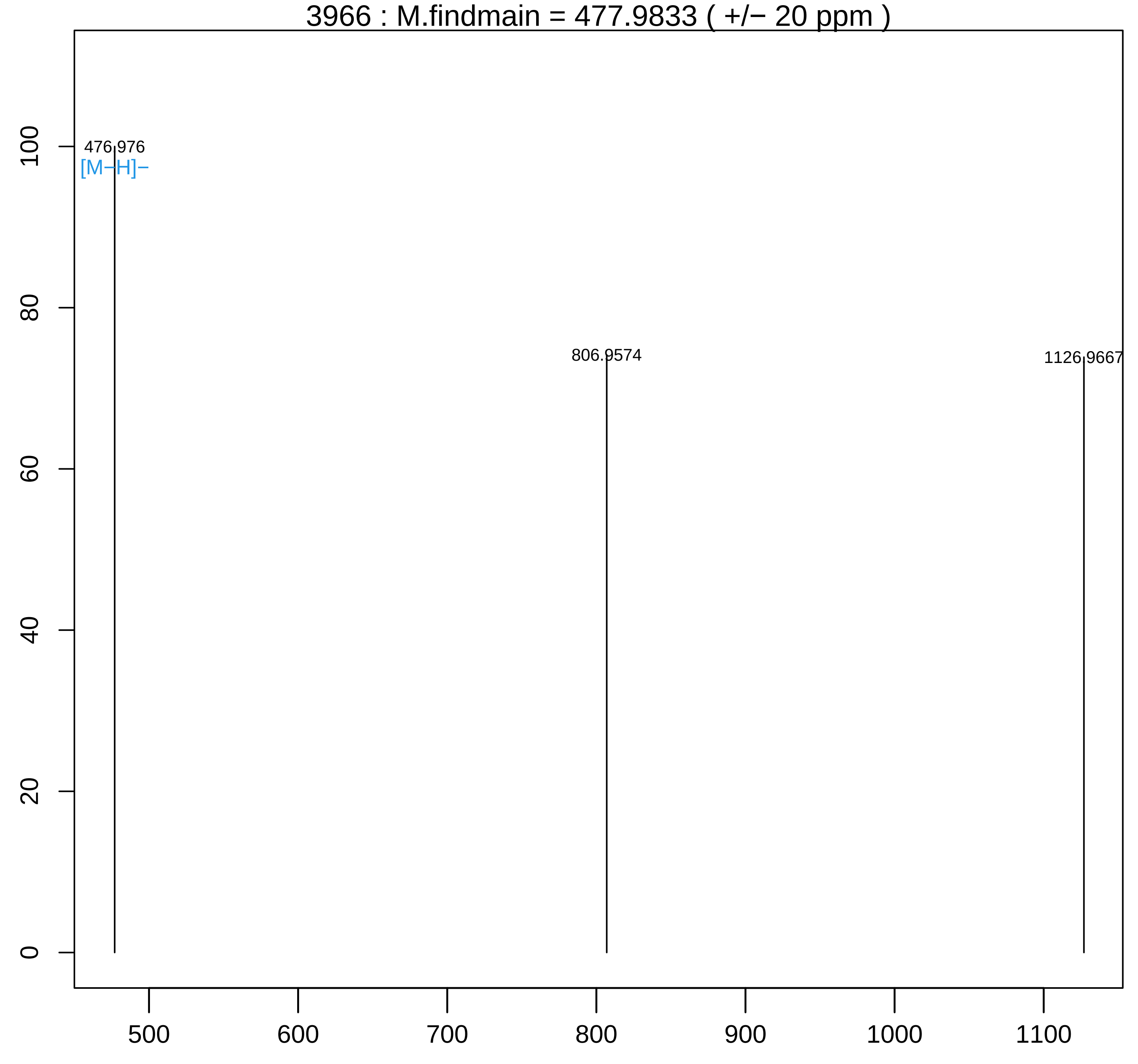
3966 : M.findmain = 477.9833 (+/− 20 ppm)
476.976
[M−H]−
806.9574
1126.9667
0
20
40
60
80
100
500
600
700
800
900
1000
1100

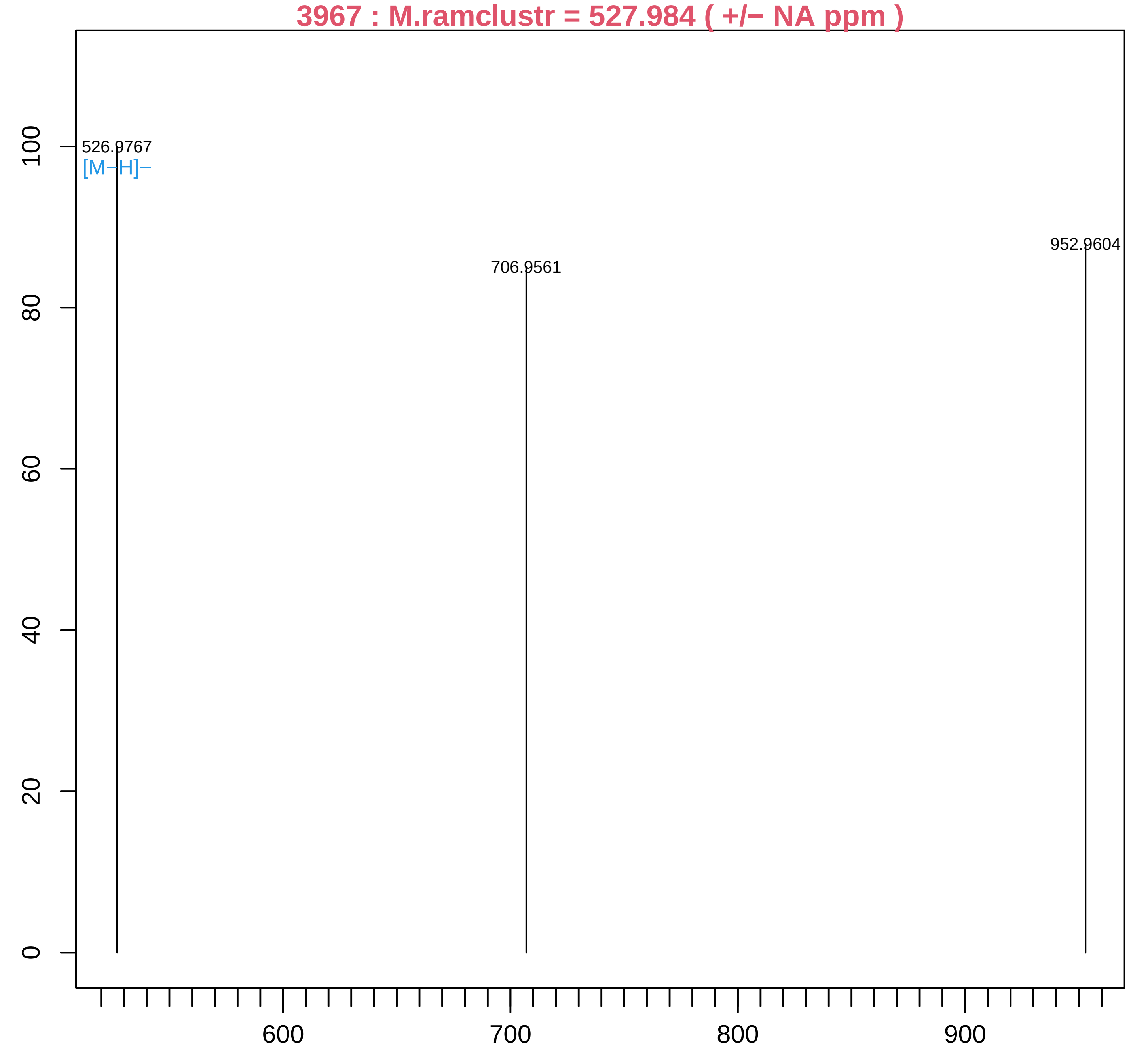
3967 : M.ramclustr = 527.984 (+/− NA ppm)
526.9767
[M−H]−
706.9561
952.9604
0
20
40
60
80
100
600
700
800
900

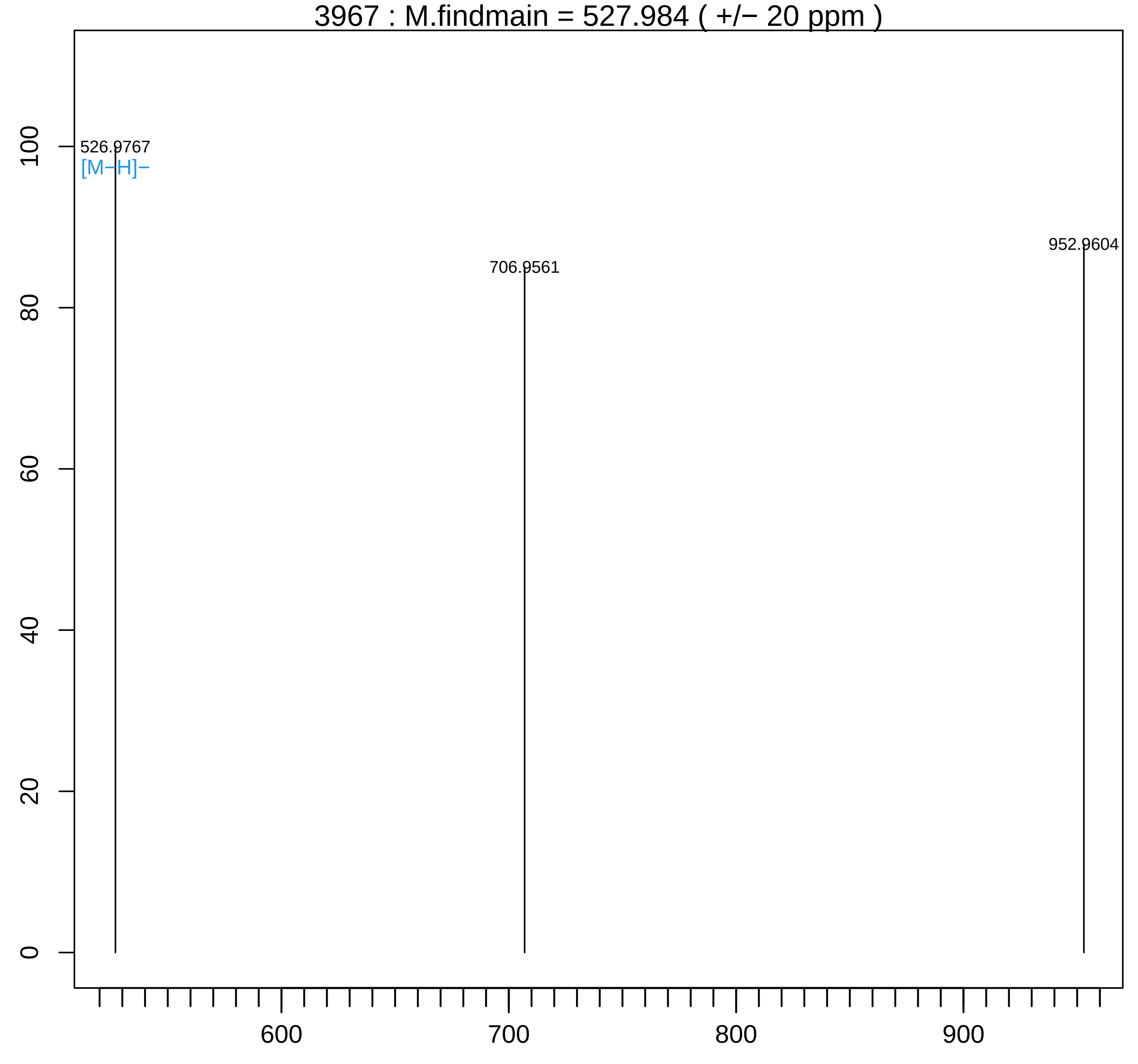
3967 : M.findmain = 527.984 (+/− 20 ppm)
526.9767
[M−H]−
706.9561
952.9604
0
20
40
60
80
100
600
700
800
900

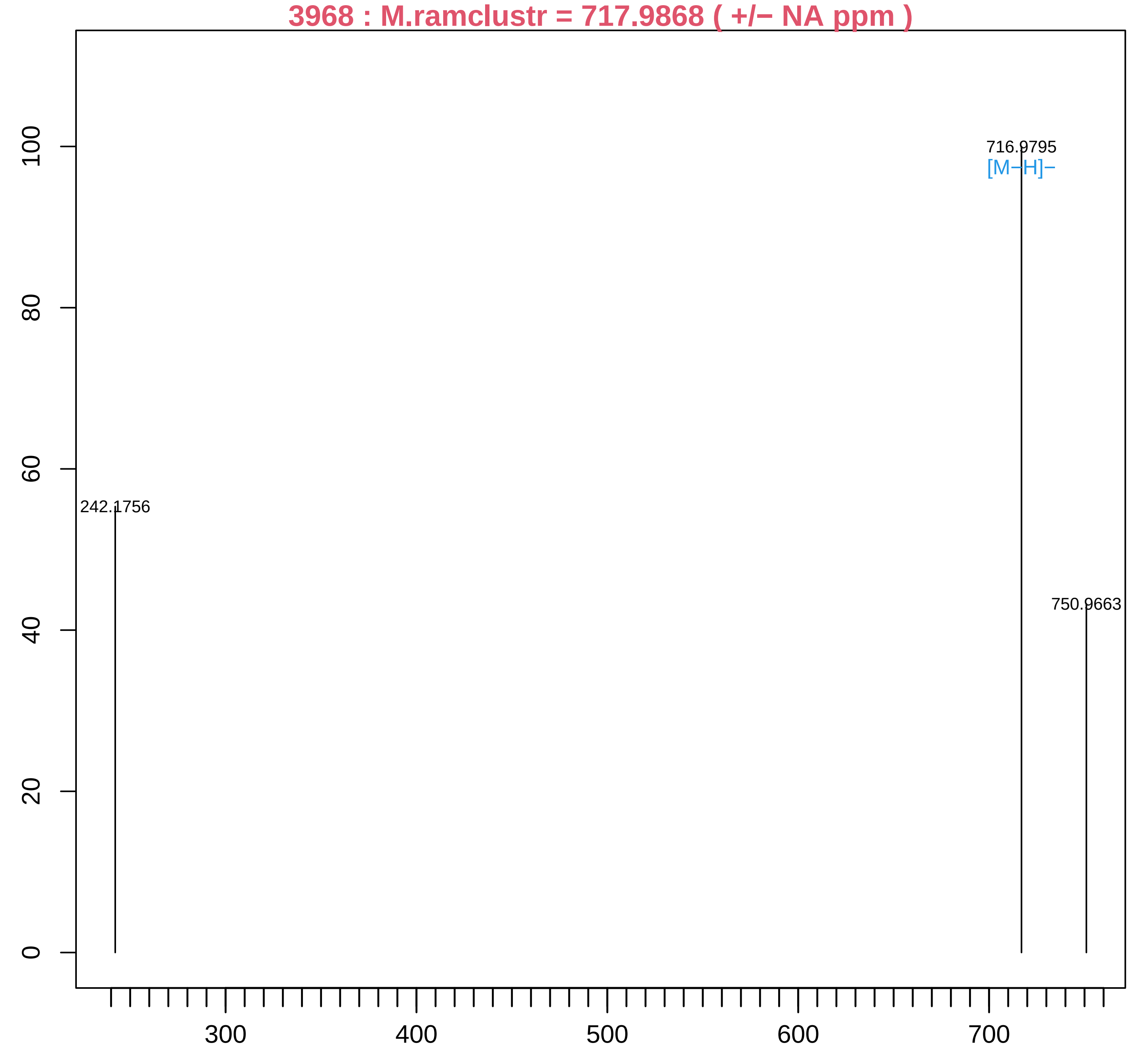

3968 : M.ramclustr = 717.9868 (+/− NA ppm)
716.9795
[M−H]−
242.1756
750.9663
0
20
40
60
80
100
300
400
500
600
700

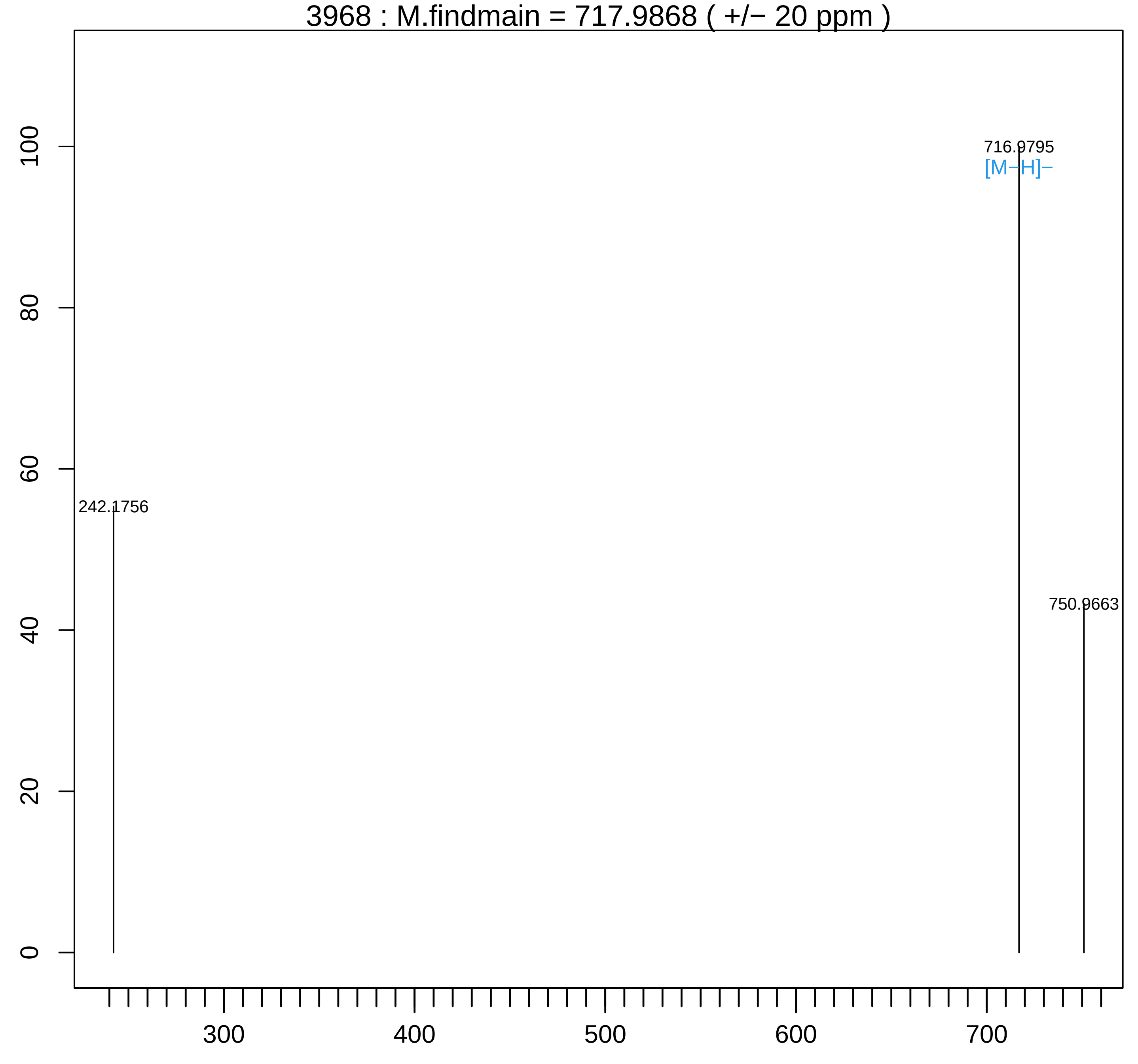

3968 : M.findmain = 717.9868 (+/− 20 ppm)
716.9795
[M−H]−
242.1756
750.9663
0
20
40
60
80
100
300
400
500
600
700

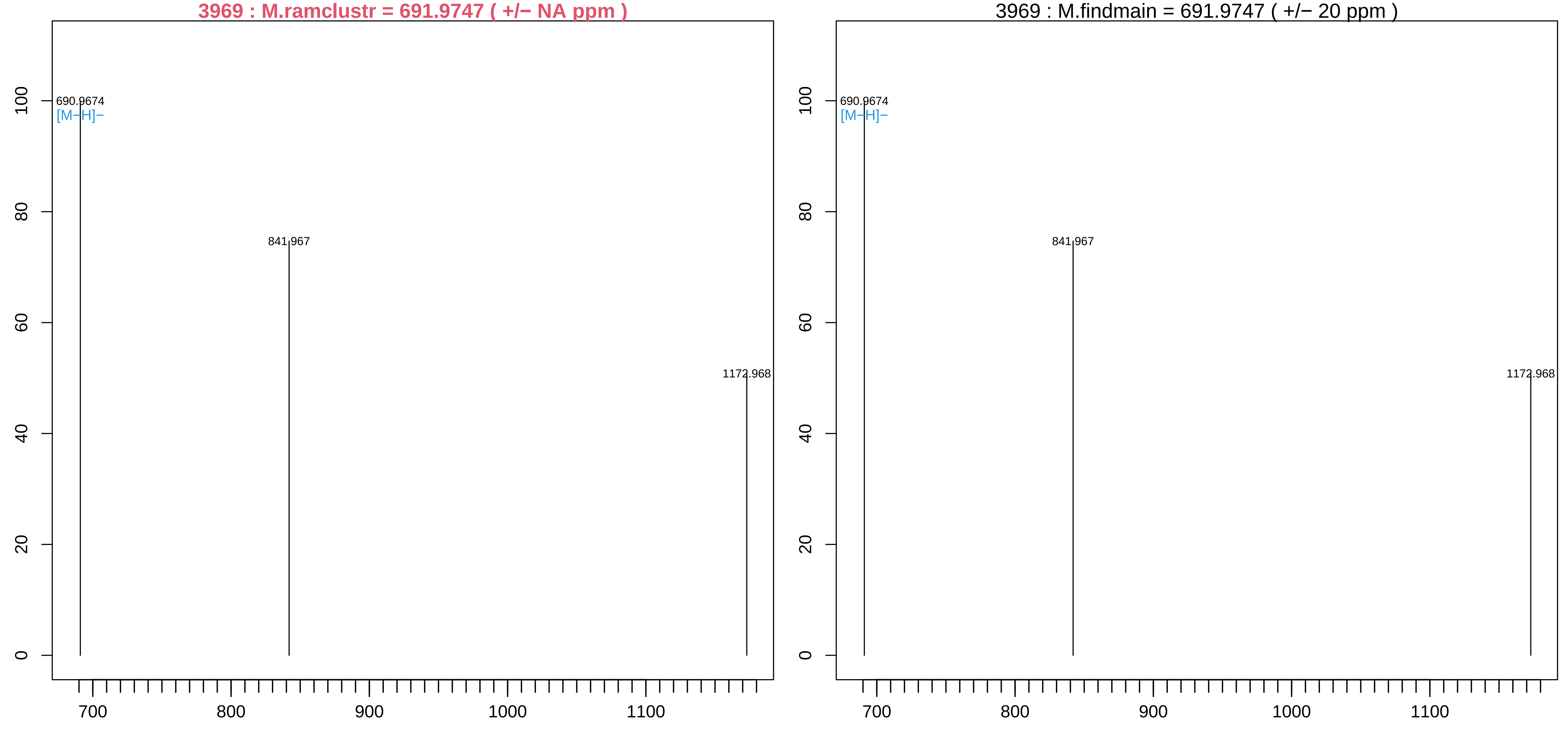
3969 : M.ramclustr = 691.9747 (+/− NA ppm)
690.9674
[M−H]−
841.967
1172.968
0
20
40
60
80
100
700
800
900
1000
1100
3969 : M.findmain = 691.9747 (+/− 20 ppm)
690.9674
[M−H]−
841.967
1172.968
0
20
40
60
80
100
700
800
900
1000
1100

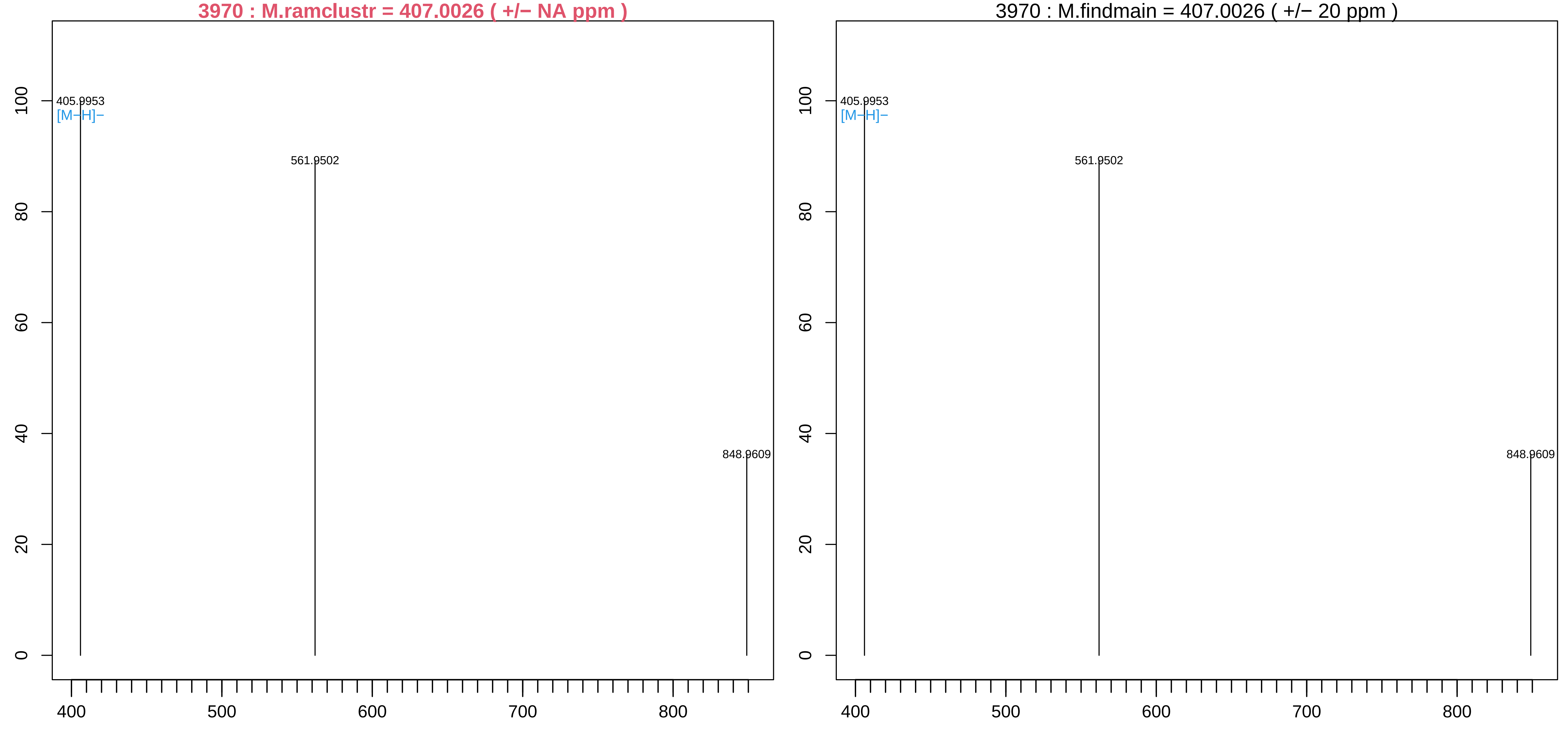

3970 : M.ramclustr = 407.0026 (+/− NA ppm)
405.9953
[M−H]−
561.9502
848.9609
3970 : M.findmain = 407.0026 (+/− 20 ppm)
405.9953
[M−H]−
561.9502
848.9609
0
20
40
60
80
100
400
500
600
700
800

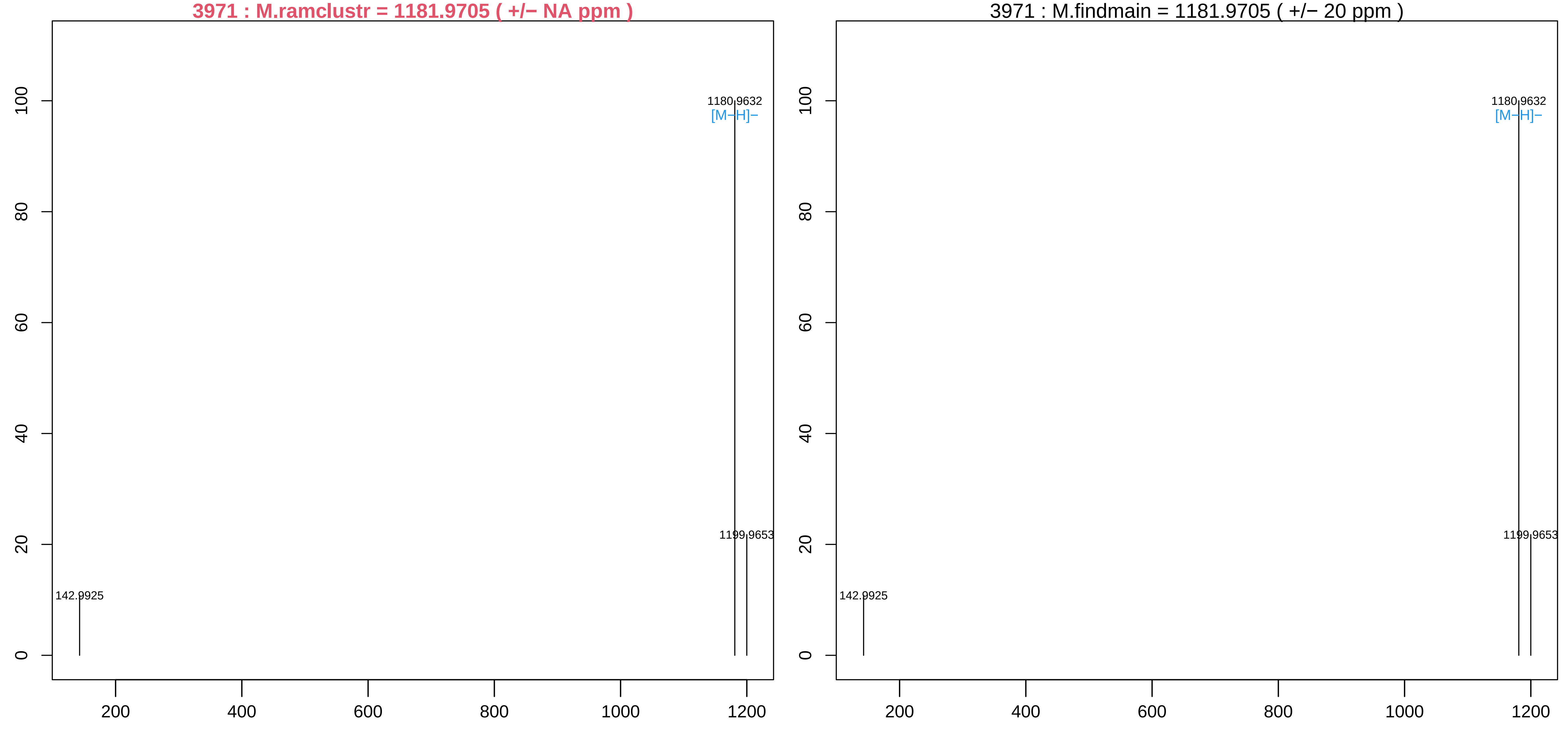
3971 : M.ramclustr = 1181.9705 (+/− NA ppm)
3971 : M.findmain = 1181.9705 (+/− 20 ppm)
1180.9632
[M−H]−
1199.9653
142.9925
0
20
40
60
80
100
200
400
600
800
1000
1200

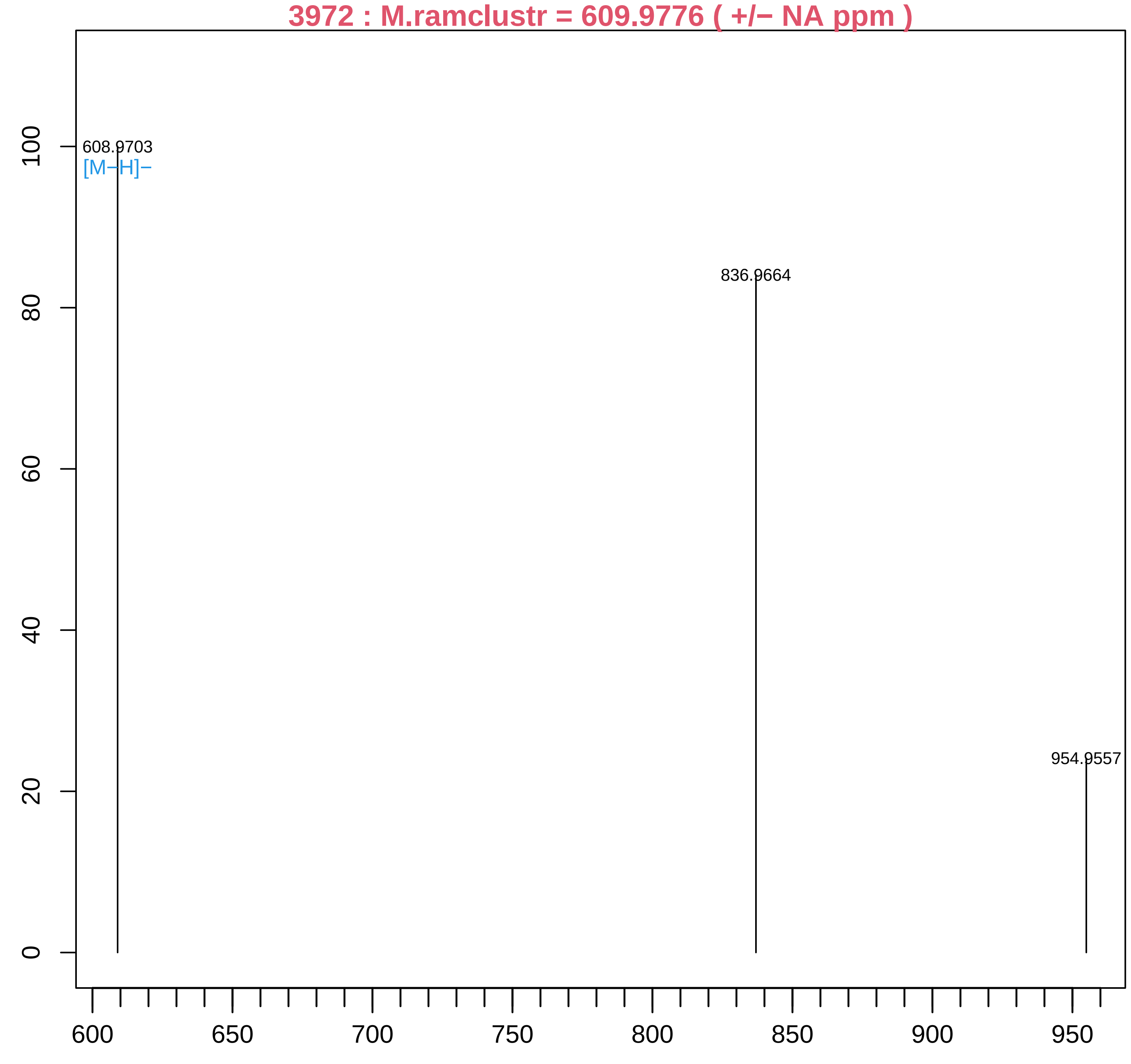
3972 : M.ramclustr = 609.9776 (+/− NA ppm)
608.9703
[M−H]−
836.9664
954.9557
0
20
40
60
80
100
600
650
700
750
800
850
900
950

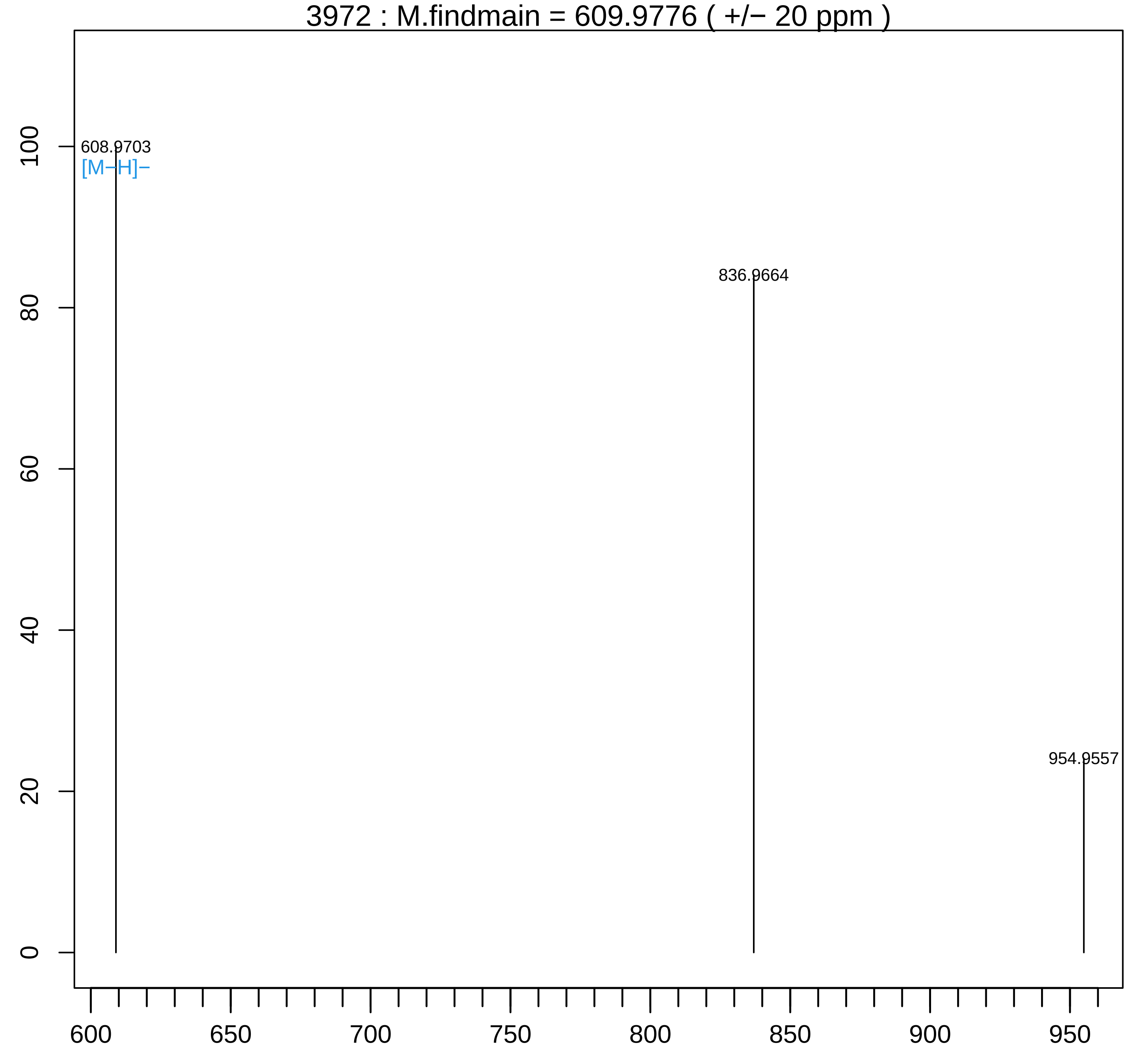
3972 : M.findmain = 609.9776 (+/− 20 ppm)
608.9703
[M−H]−
836.9664
954.9557
0
20
40
60
80
100
600
650
700
750
800
850
900
950

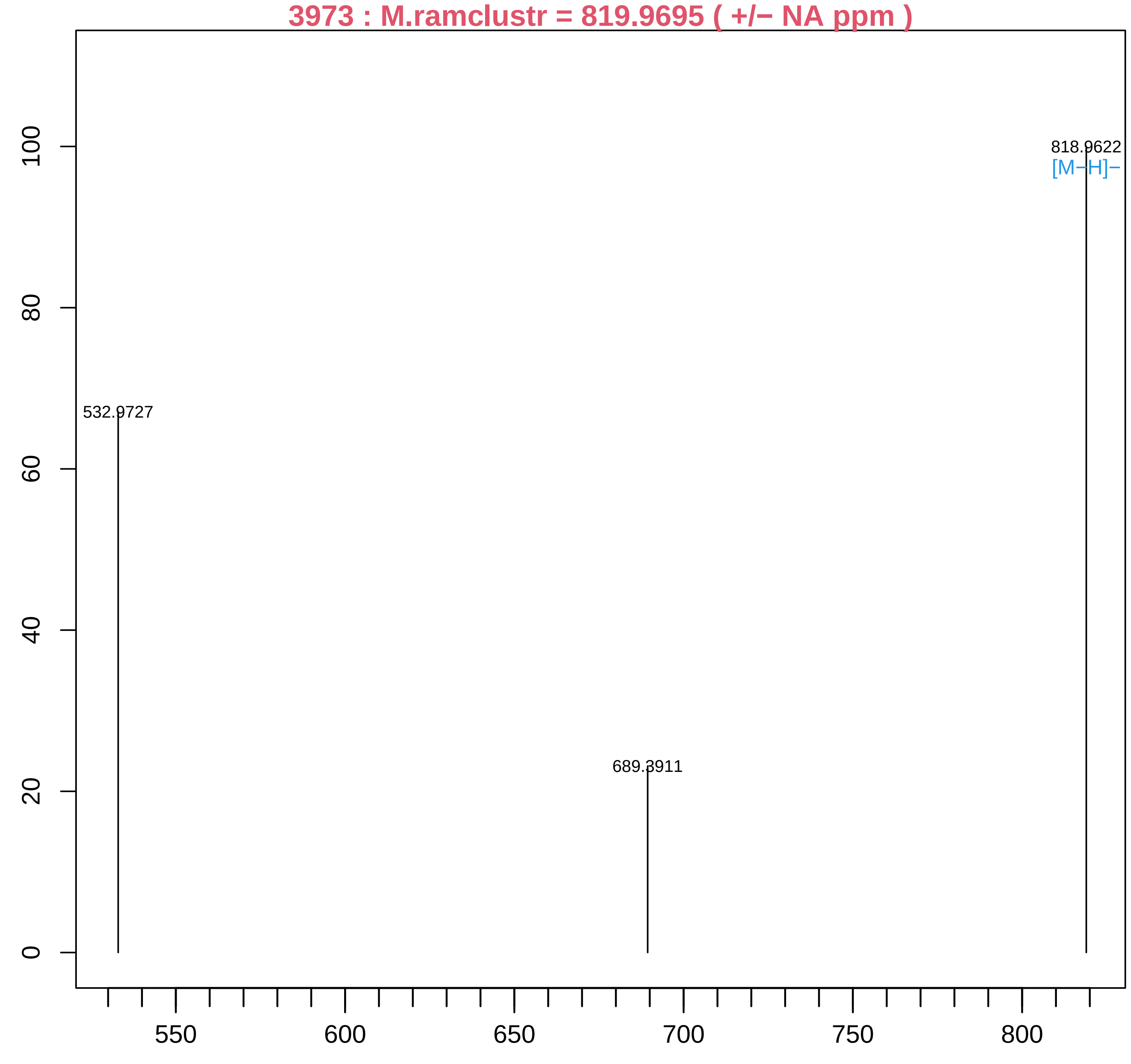

3973 : M.ramclustr = 819.9695 (+/− NA ppm)
818.9622
[M−H]−
532.9727
689.3911
0
20
40
60
80
100
550
600
650
700
750
800

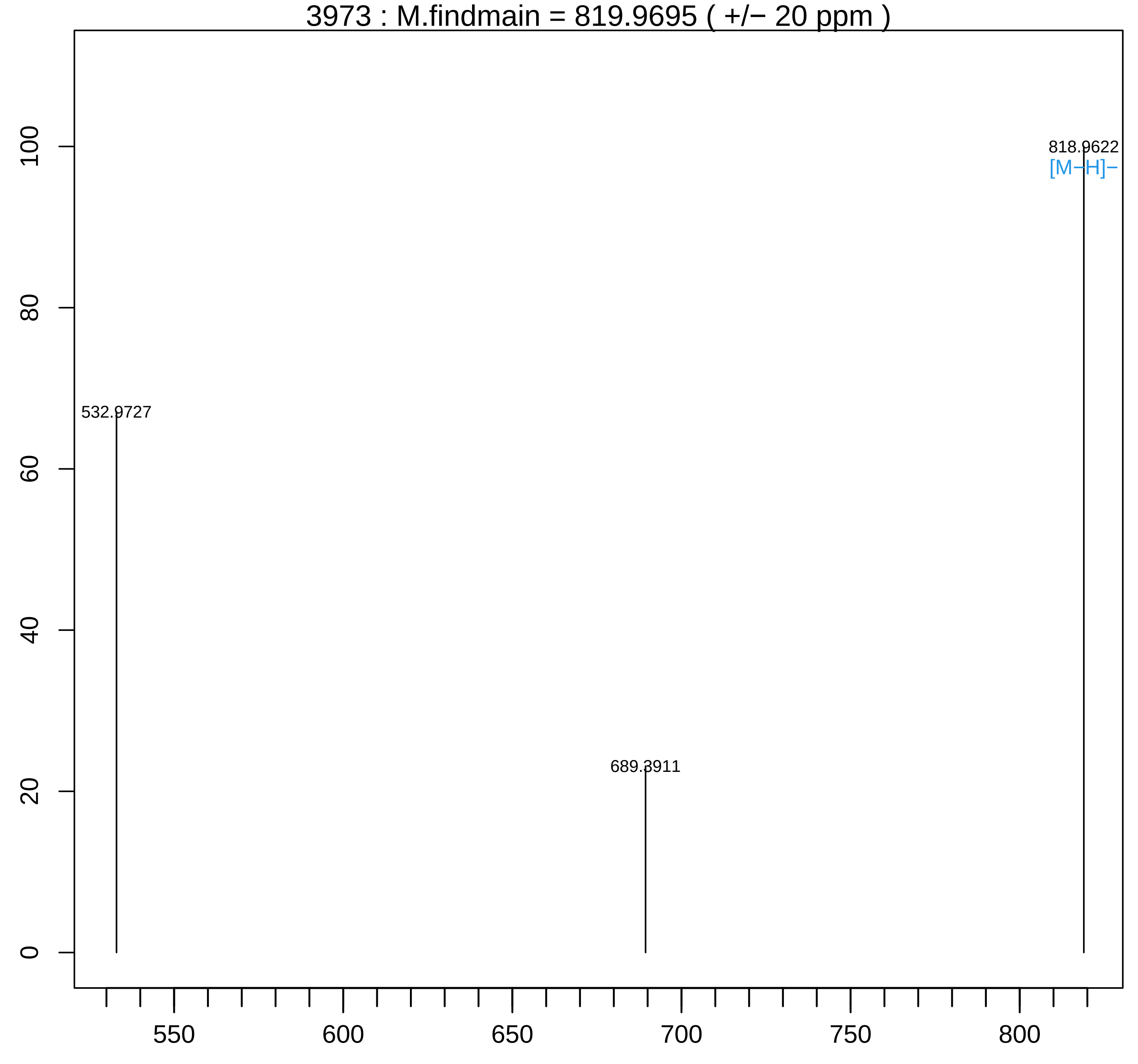

3973 : M.findmain = 819.9695 (+/− 20 ppm)
818.9622
[M−H]−
532.9727
689.3911
0
20
40
60
80
100
550
600
650
700
750
800

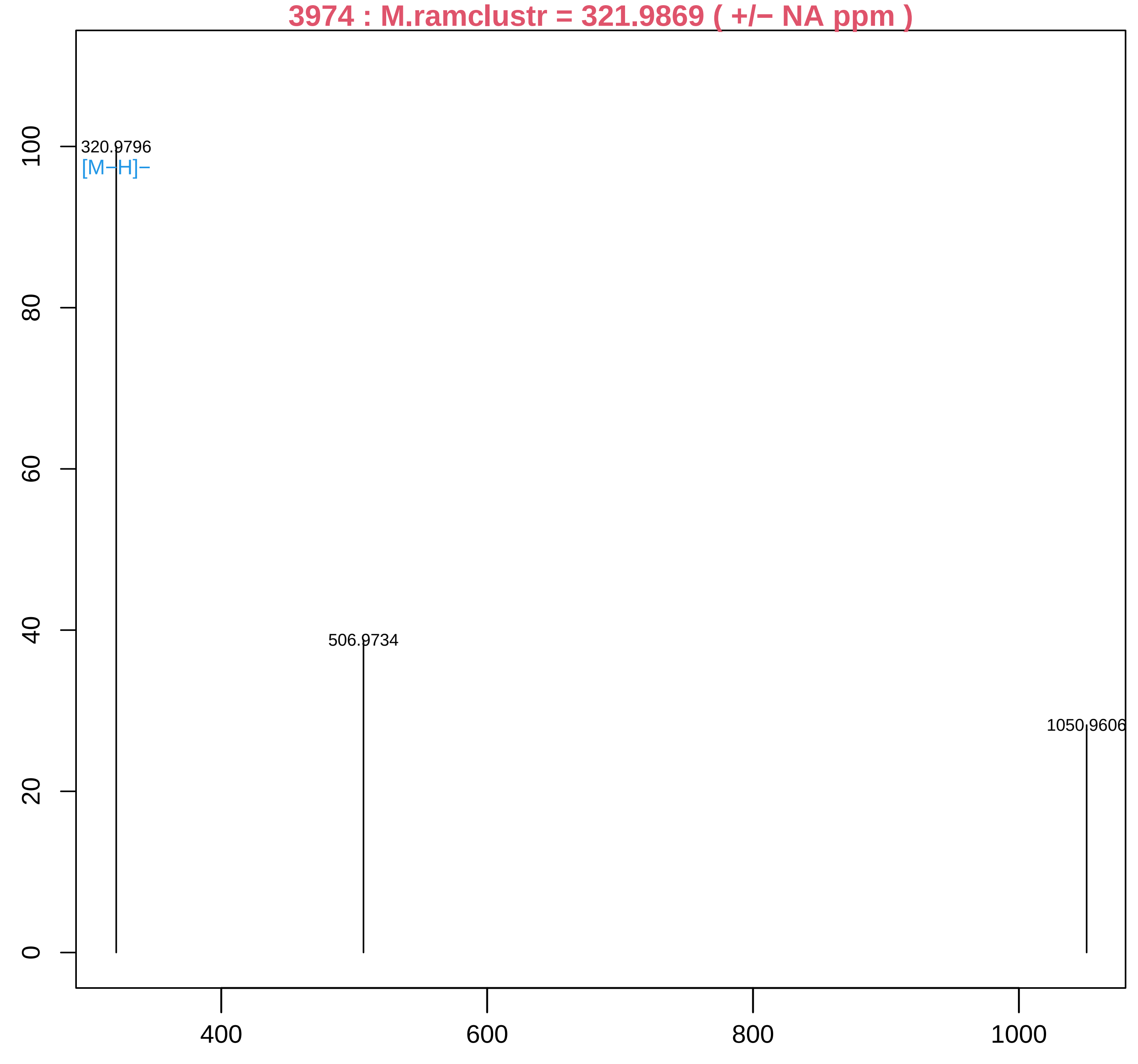
3974 : M.ramclustr = 321.9869 (+/− NA ppm)
320.9796
[M−H]−
506.9734
1050.9606
0
20
40
60
80
100
400
600
800
1000

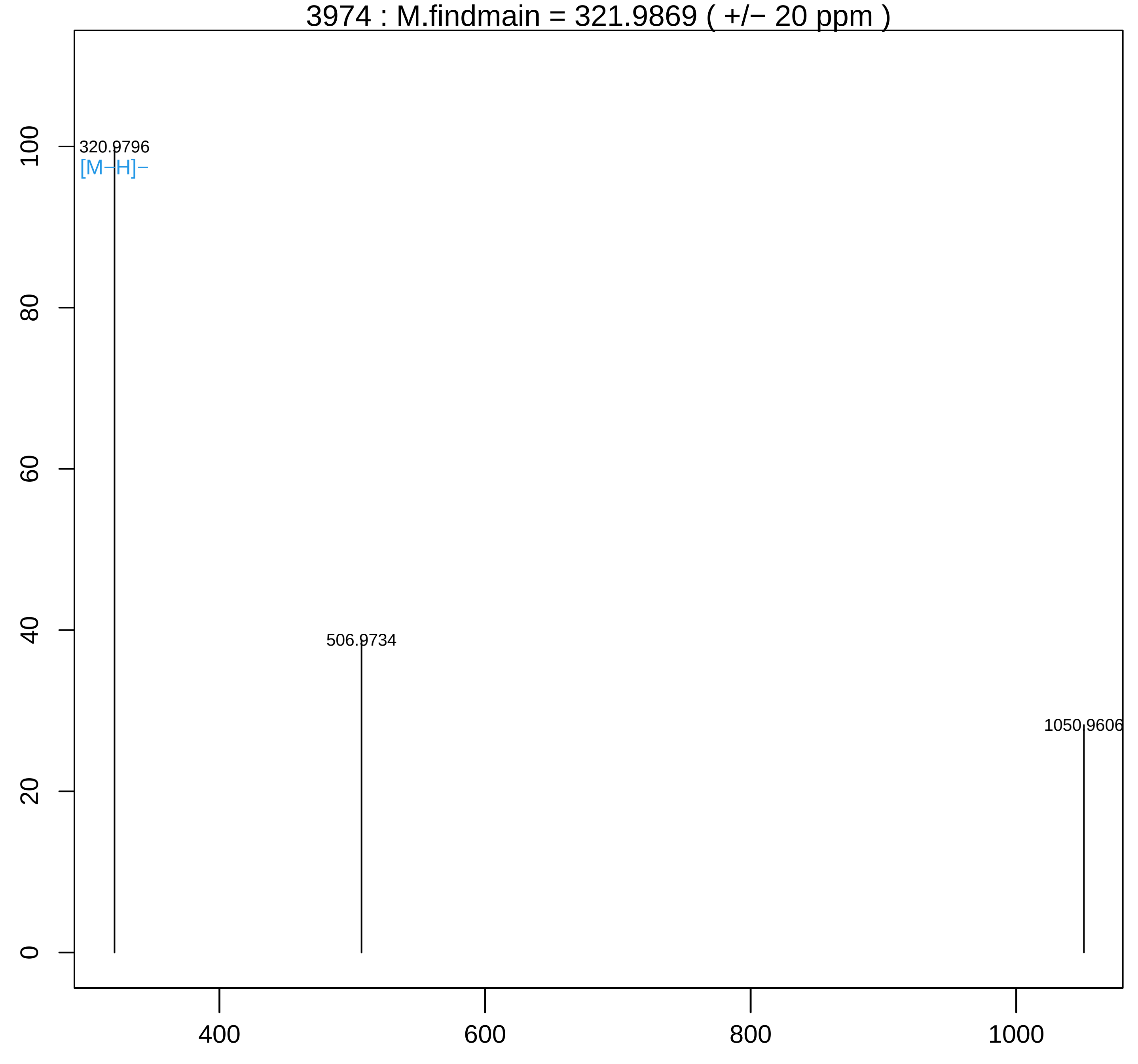
3974 : M.findmain = 321.9869 (+/− 20 ppm)
320.9796
[M−H]−
506.9734
1050.9606
0
20
40
60
80
100
400
600
800
1000

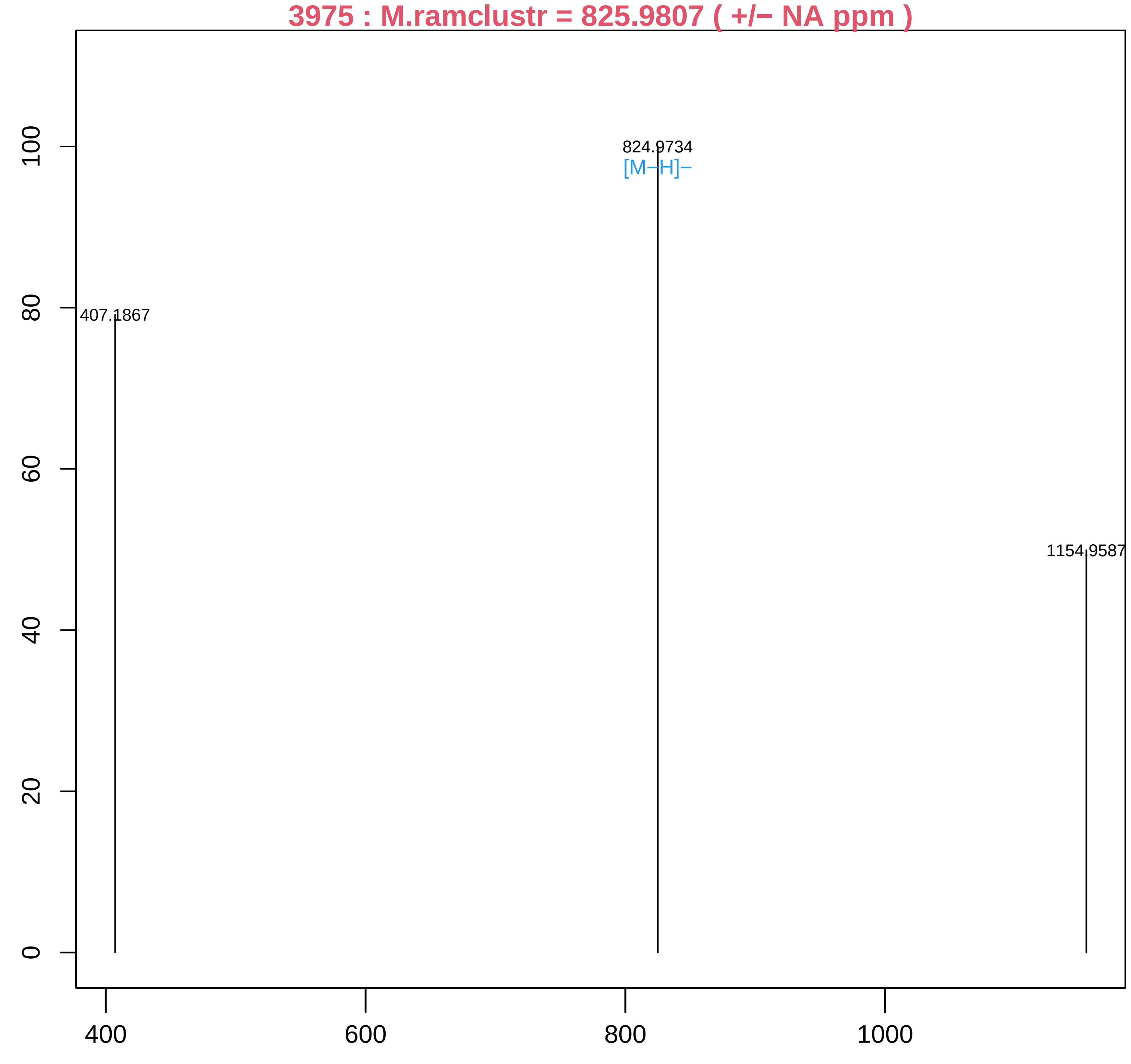
3975 : M.ramclustr = 825.9807 (+/− NA ppm)
407.1867
824.9734
[M−H]−
1154.9587
0
20
40
60
80
100
400
600
800
1000

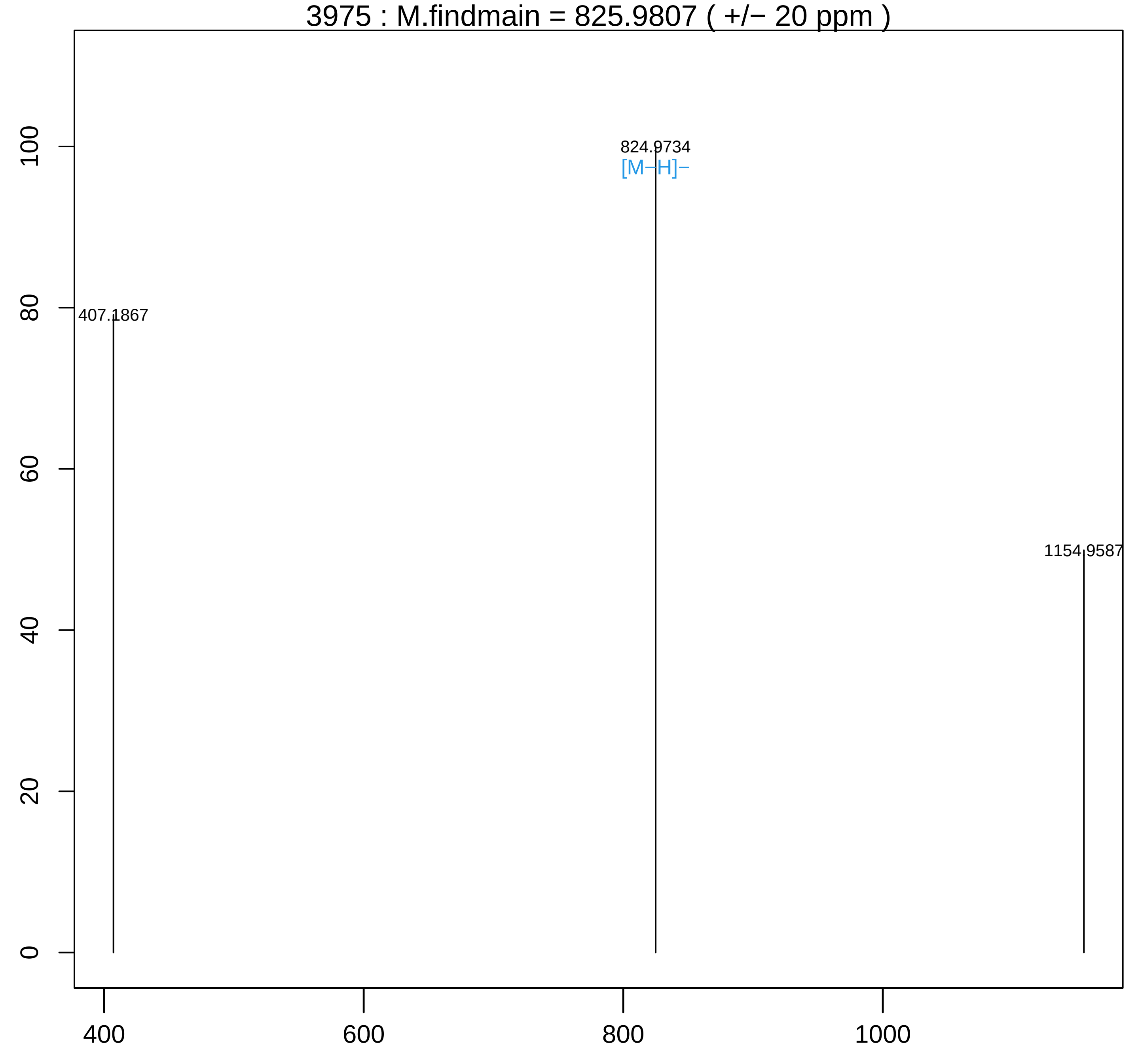
3975 : M.findmain = 825.9807 (+/− 20 ppm)
407.1867
824.9734
[M−H]−
1154.9587
0
20
40
60
80
100
400
600
800
1000

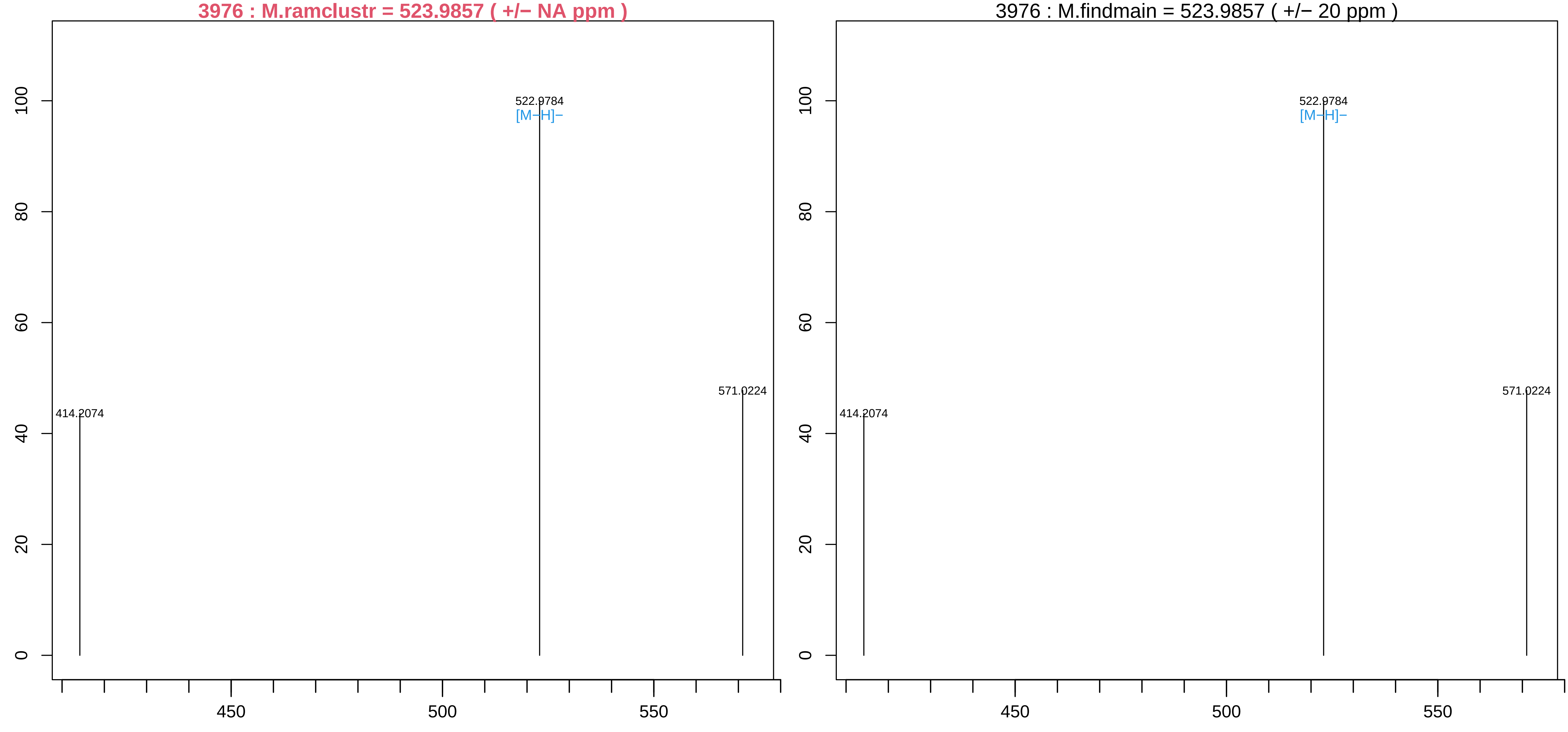

3976 : M.ramclustr = 523.9857 (+/− NA ppm)
3976 : M.findmain = 523.9857 (+/− 20 ppm)
522.9784
[M−H]−
414.2074
571.0224
0
20
40
60
80
100
450
500
550

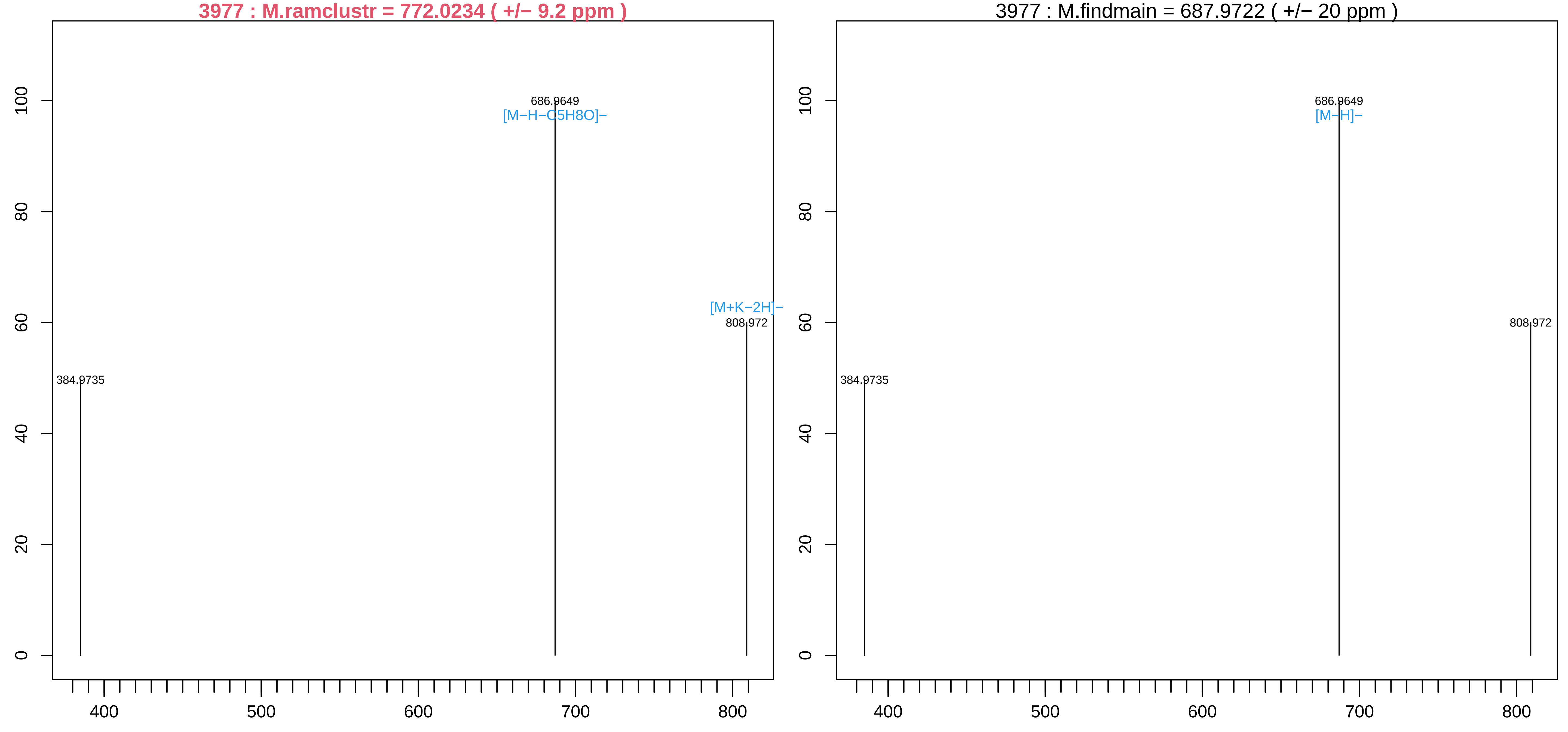
3977 : M.ramclustr = 772.0234 (+/− 9.2 ppm)
686.9649
[M−H−C5H8O]−
[M+K−2H]−
808.972
384.9735
3977 : M.findmain = 687.9722 (+/− 20 ppm)
686.9649
[M−H]−
808.972
384.9735
0
20
40
60
80
100
400
500
600
700
800

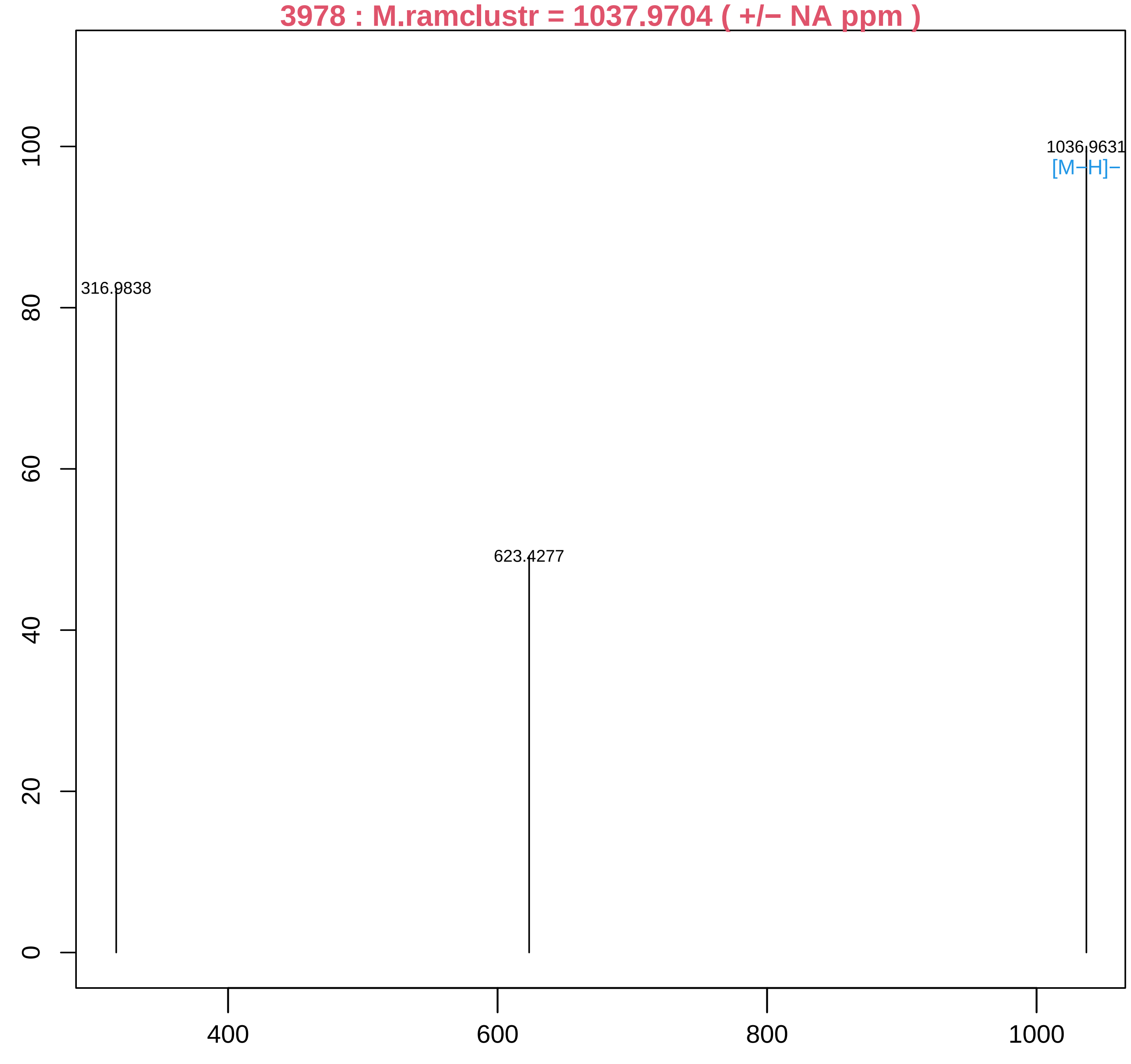

3978 : M.ramclustr = 1037.9704 (+/− NA ppm)
1036.9631
[M−H]−
316.9838
623.4277
0
20
40
60
80
100
400
600
800
1000

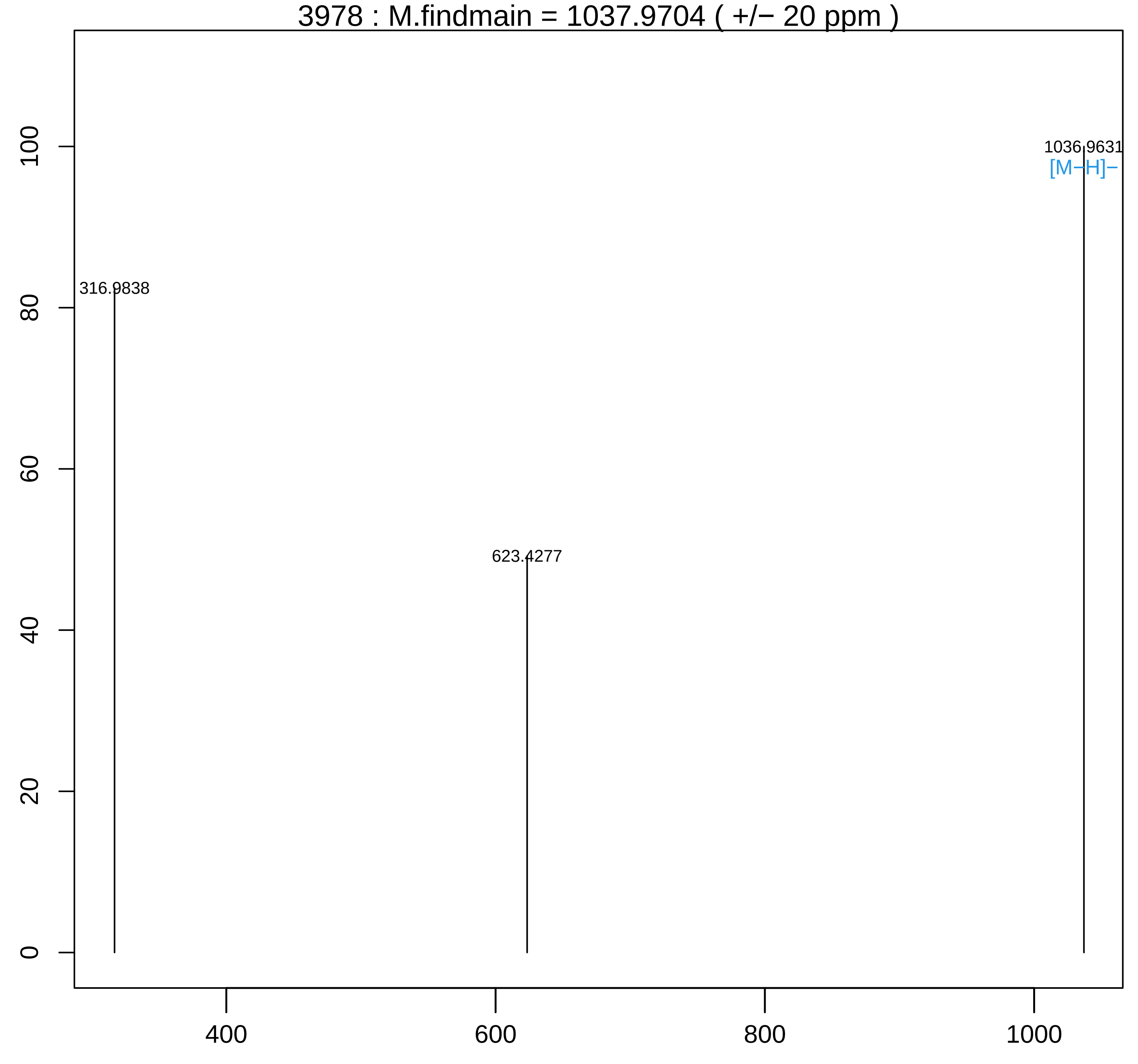

3978 : M.findmain = 1037.9704 (+/− 20 ppm)
1036.9631
[M−H]−
316.9838
623.4277
0
20
40
60
80
100
400
600
800
1000

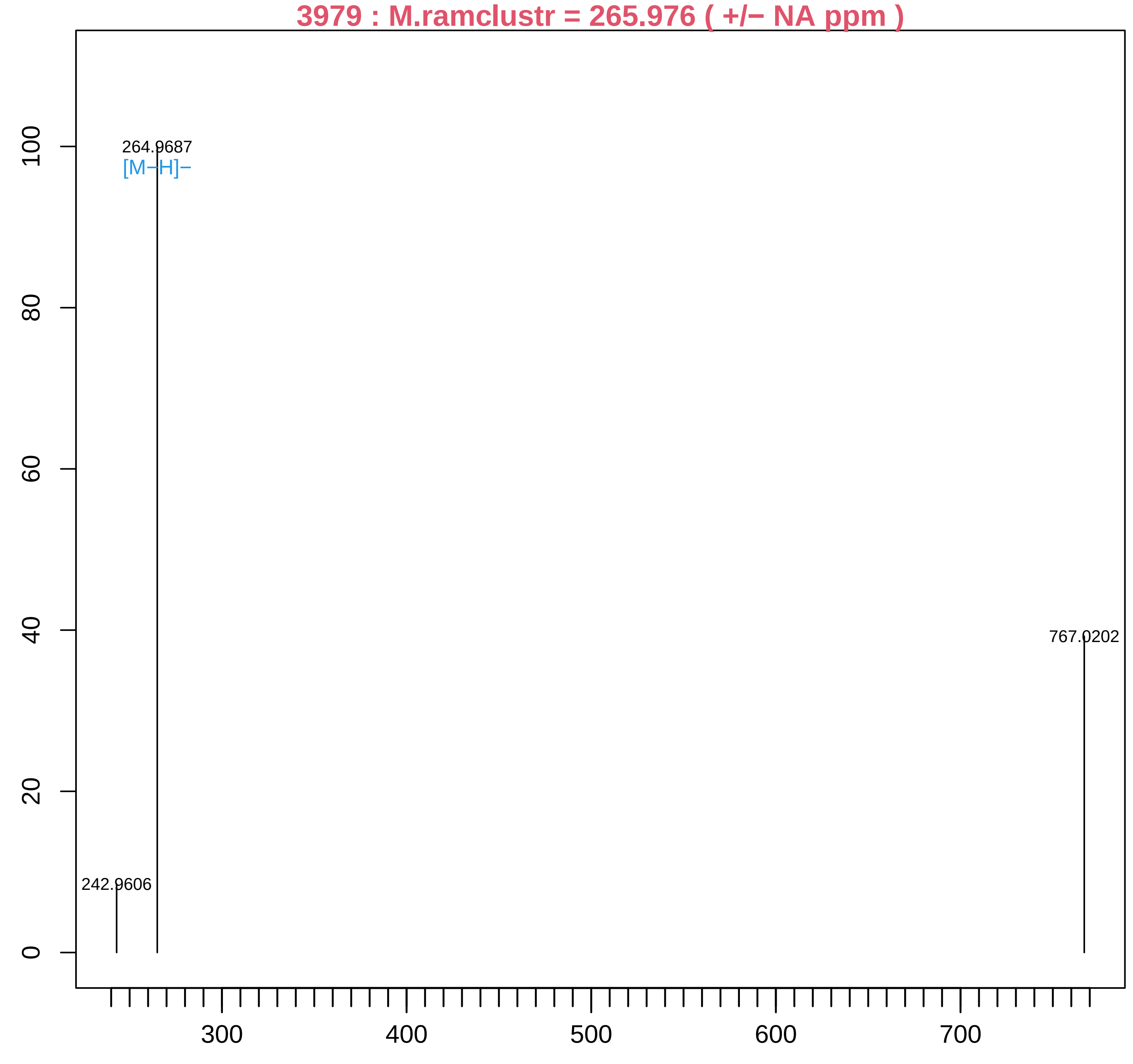
3979 : M.ramclustr = 265.976 (+/− NA ppm)
264.9687
[M−H]−
242.9606
767.0202
0
20
40
60
80
100
300
400
500
600
700

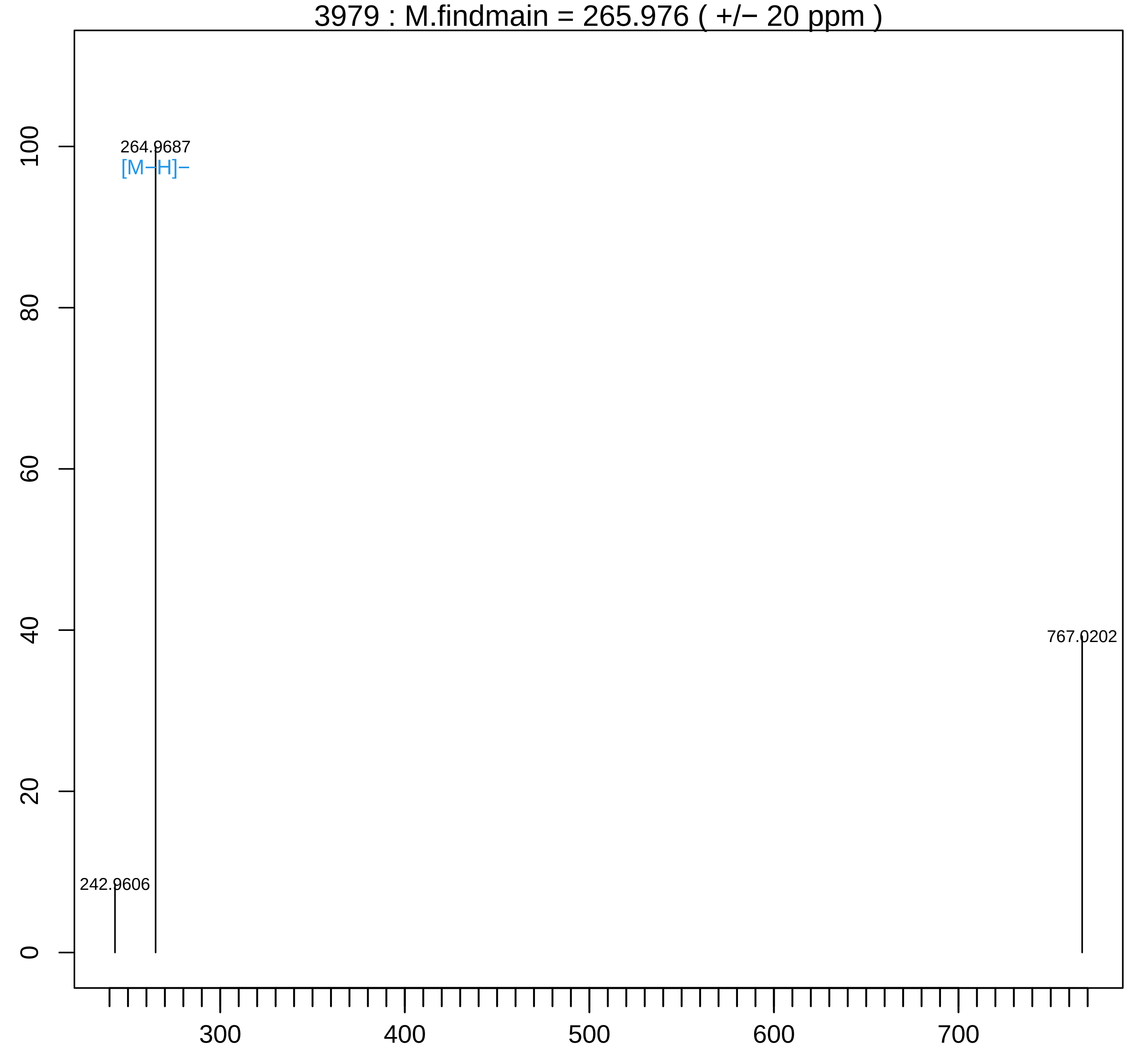
3979 : M.findmain = 265.976 (+/− 20 ppm)
264.9687
[M−H]−
242.9606
767.0202
0
20
40
60
80
100
300
400
500
600
700

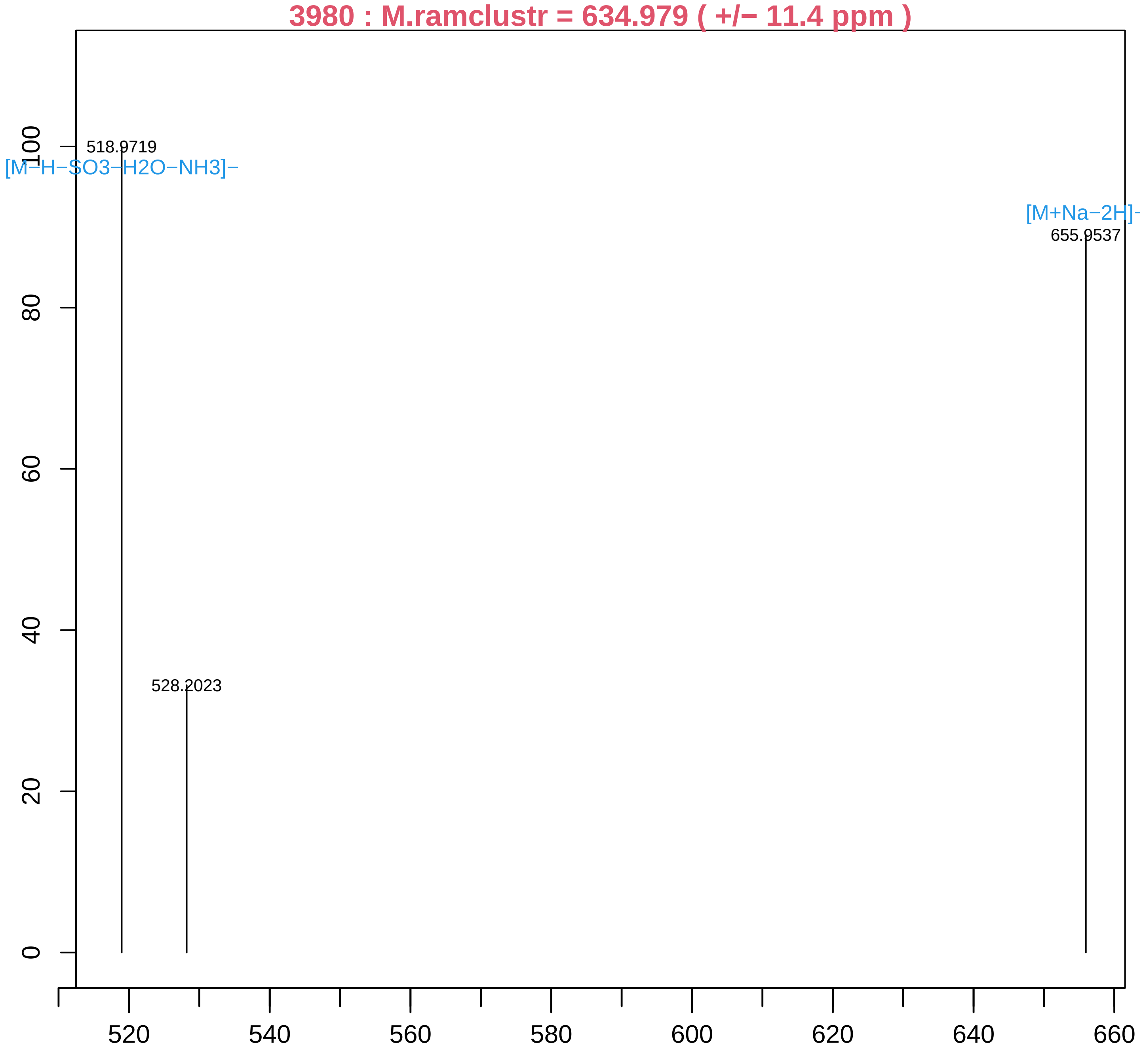
3980 : M.ramclustr = 634.979 (+/− 11.4 ppm)
518.9719
[M−H−SO3−H2O−NH3]−
528.2023
[M+Na−2H]-
655.9537
0
20
40
60
80
100
520
540
560
580
600
620
640
660

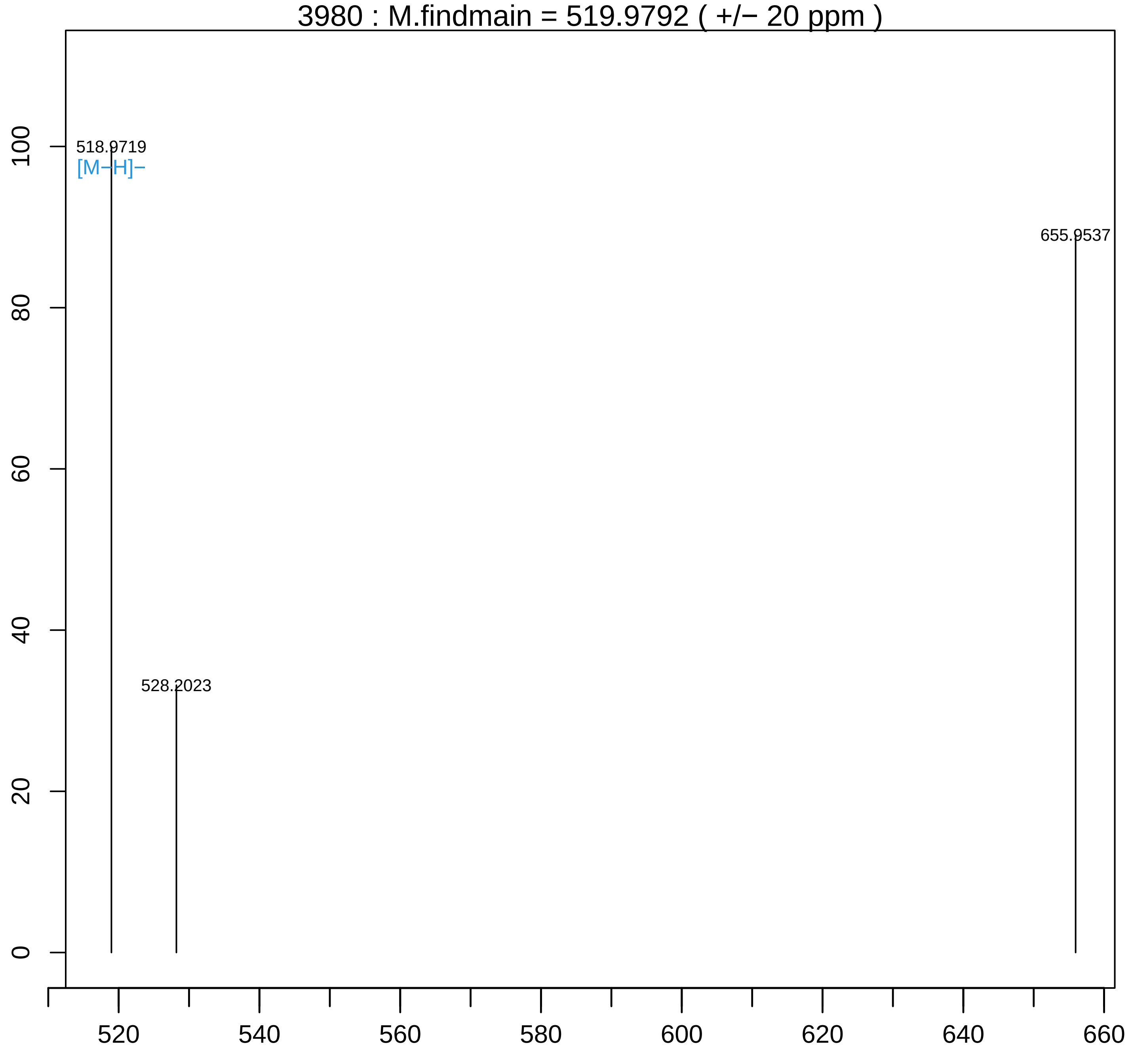
3980 : M.findmain = 519.9792 (+/− 20 ppm)
518.9719
[M−H]−
528.2023
655.9537
0
20
40
60
80
100
520
540
560
580
600
620
640
660

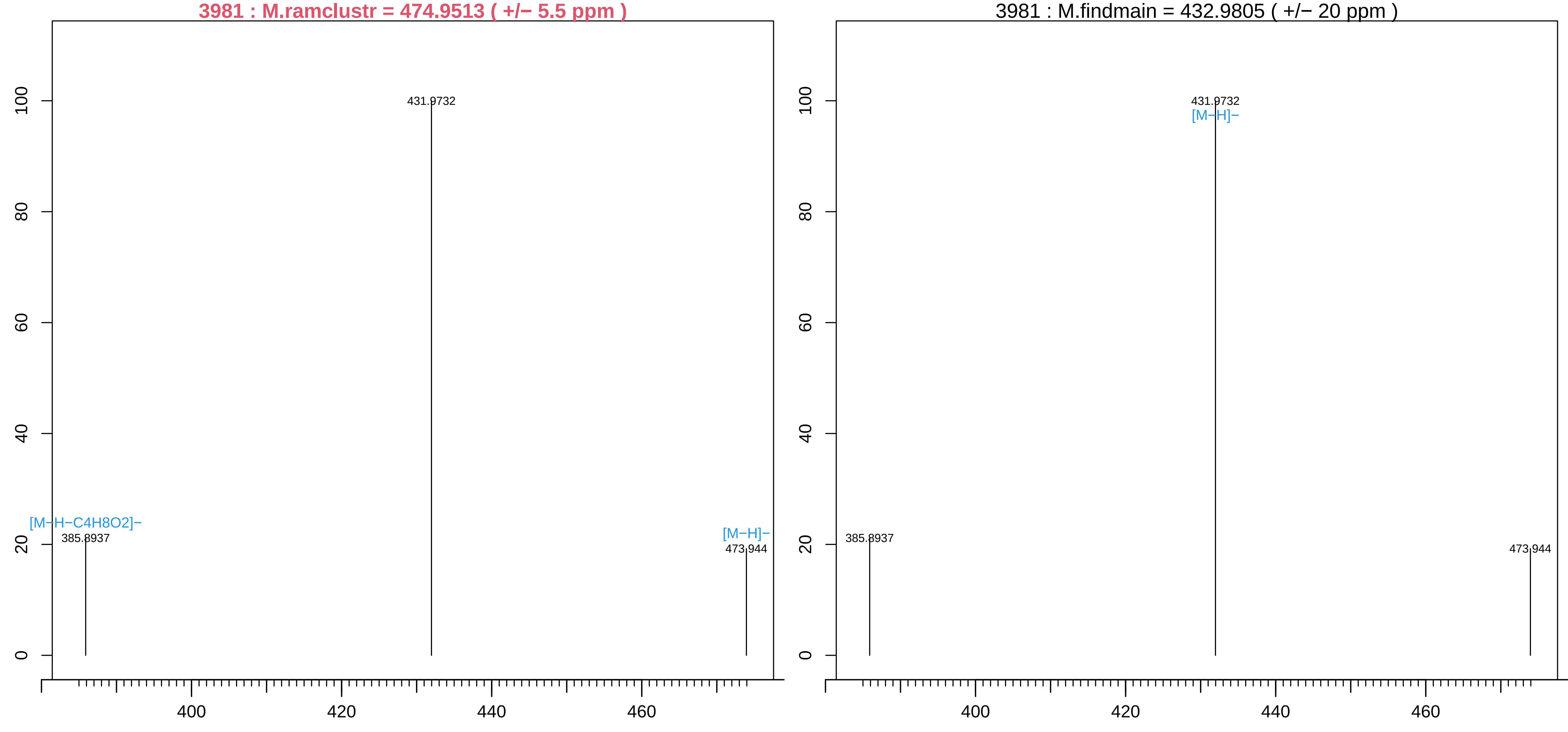

3981 : M.ramclustr = 474.9513 (+/− 5.5 ppm)
431.9732
[M−H−C4H8O2]−
385.8937
[M−H]−
473.944
0
20
40
60
80
100
400
420
440
460
3981 : M.findmain = 432.9805 (+/− 20 ppm)
431.9732
[M−H]−
385.8937
473.944

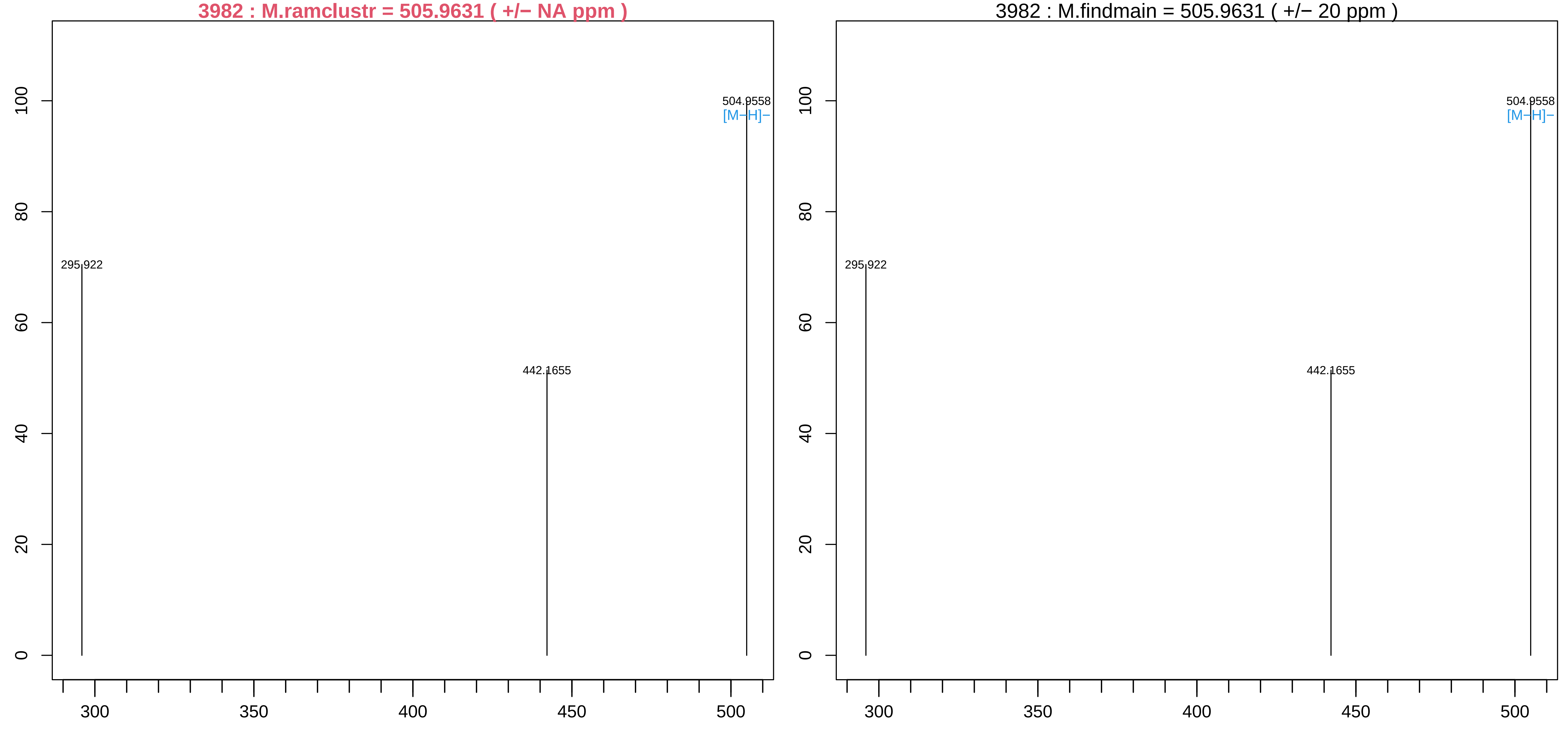
3982 : M.ramclustr = 505.9631 (+/− NA ppm)
3982 : M.findmain = 505.9631 (+/− 20 ppm)
504.9558
[M−H]−
295.922
442.1655
0
20
40
60
80
100
300
350
400
450
500

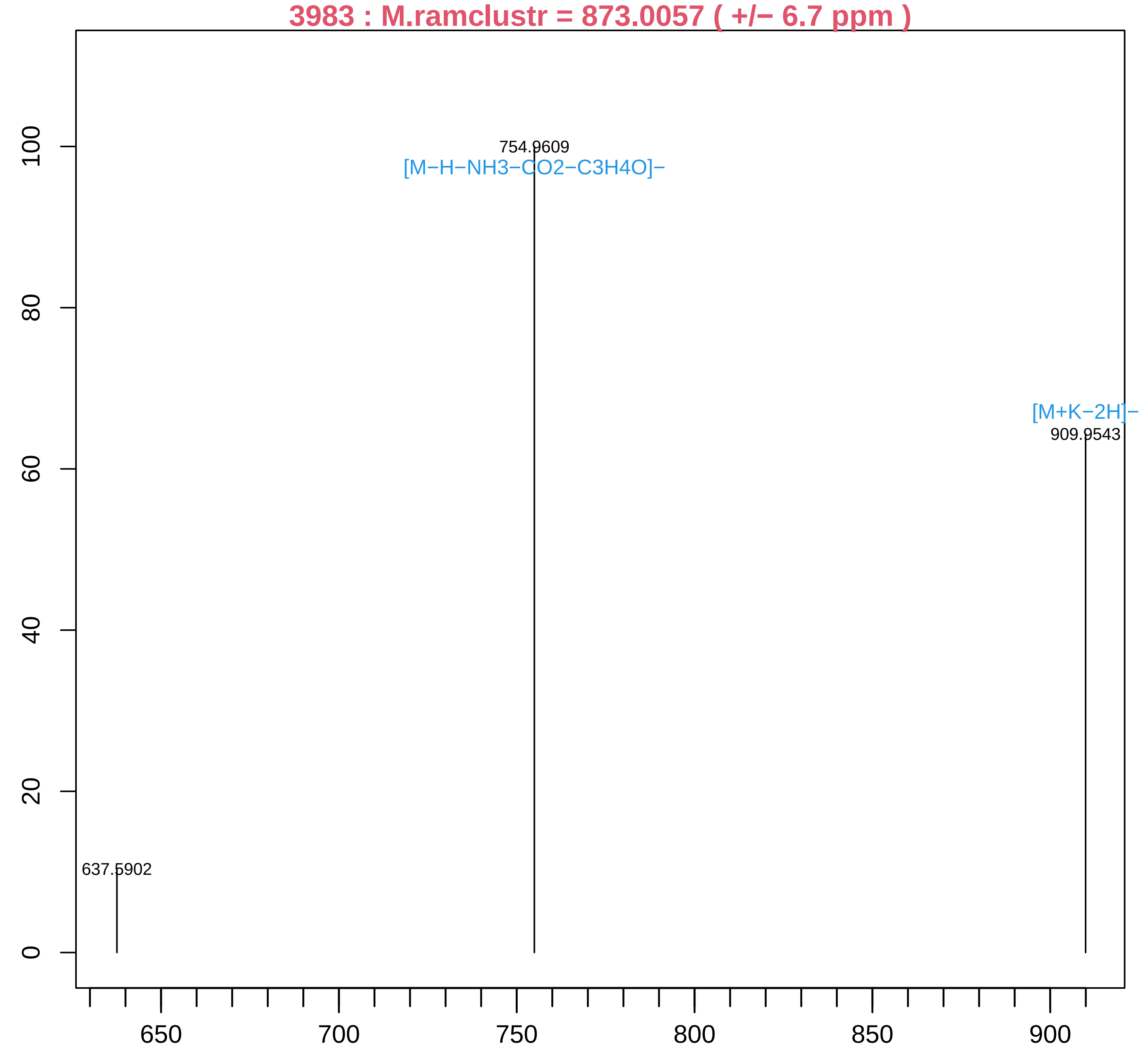
3983 : M.ramclustr = 873.0057 (+/− 6.7 ppm)
754.9609
[M−H−NH3−CO2−C3H4O]−
[M+K−2H]−
909.9543
637.5902
0
20
40
60
80
100
650
700
750
800
850
900
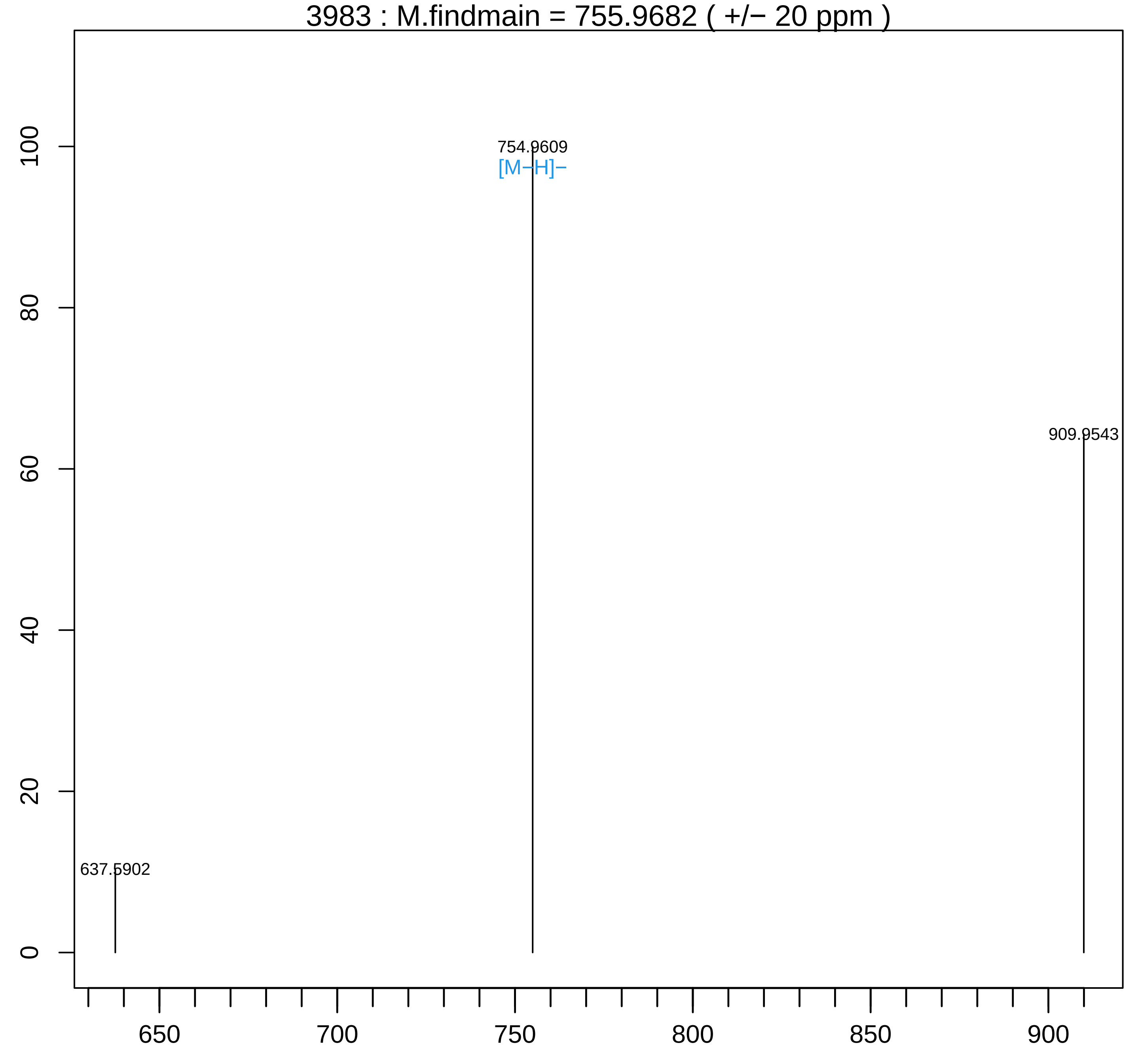
3983 : M.findmain = 755.9682 (+/− 20 ppm)
754.9609
[M−H]−
909.9543
637.5902
0
20
40
60
80
100
650
700
750
800
850
900

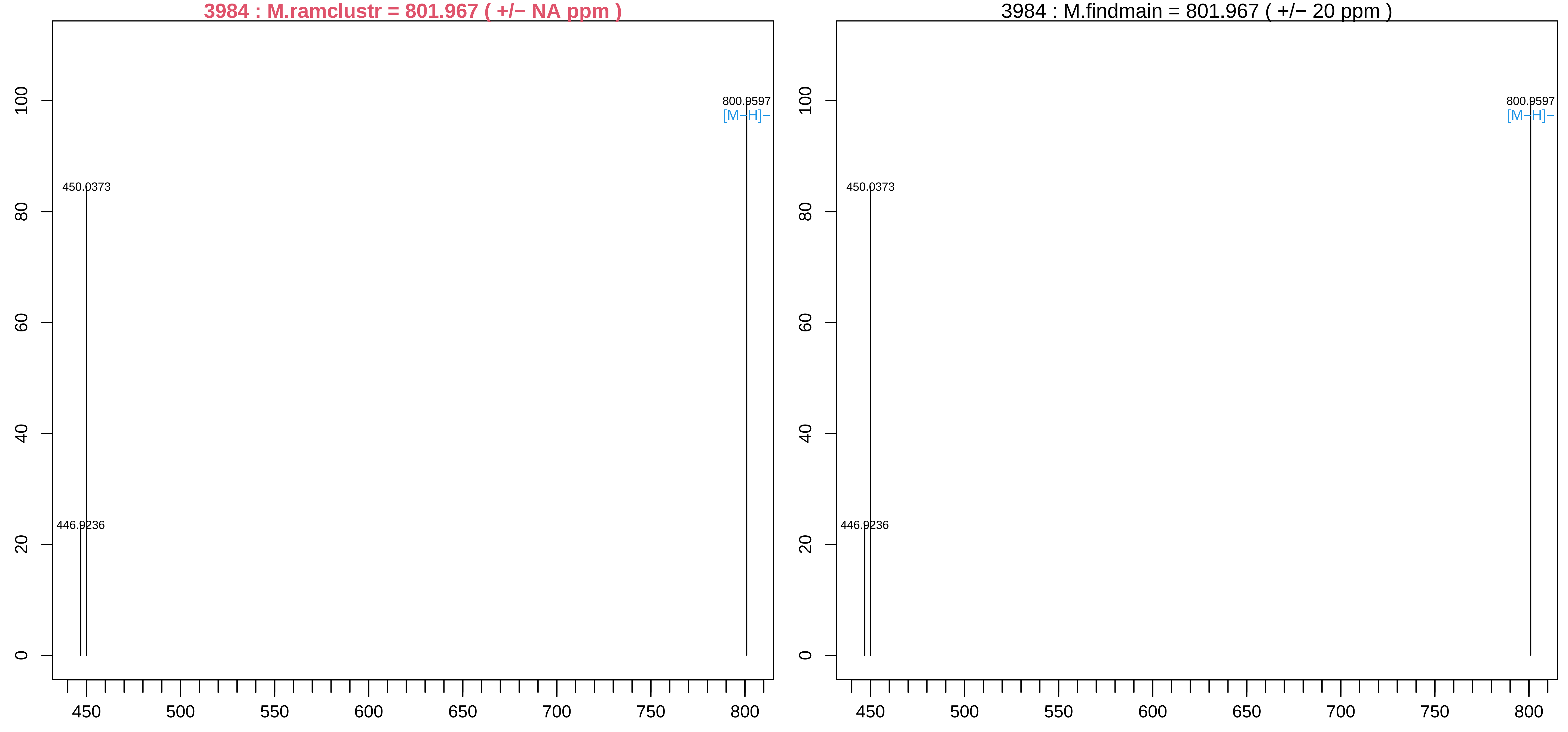
3984 : M.ramclustr = 801.967 (+/− NA ppm)
800.9597
[M−H]−
450.0373
446.9236
3984 : M.findmain = 801.967 (+/− 20 ppm)
800.9597
[M−H]−
450.0373
446.9236

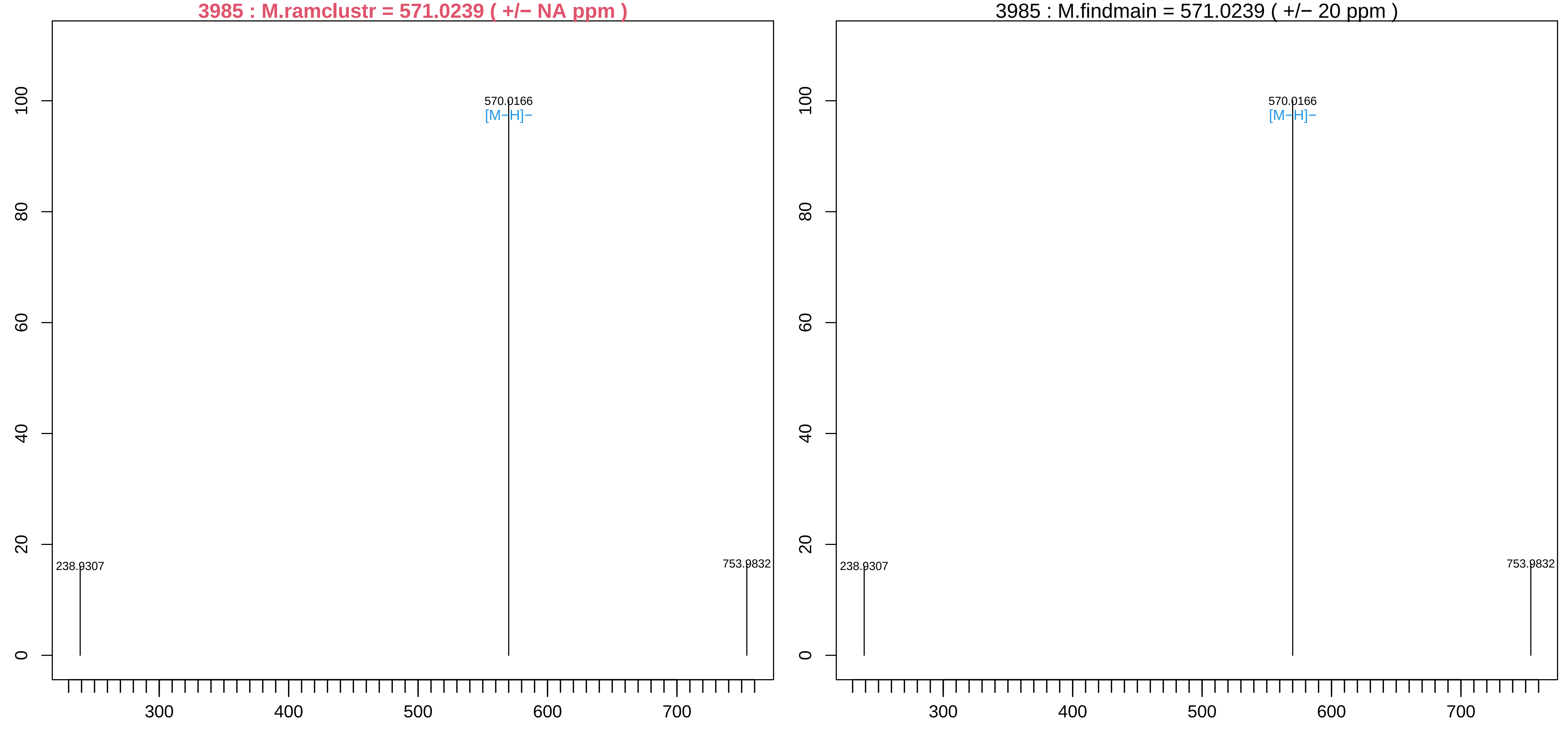
3985 : M.ramclustr = 571.0239 (+/− NA ppm)
570.0166
[M−H]−
238.9307
753.9832
3985 : M.findmain = 571.0239 (+/− 20 ppm)
570.0166
[M−H]−
238.9307
753.9832
0
20
40
60
80
100
300
400
500
600
700

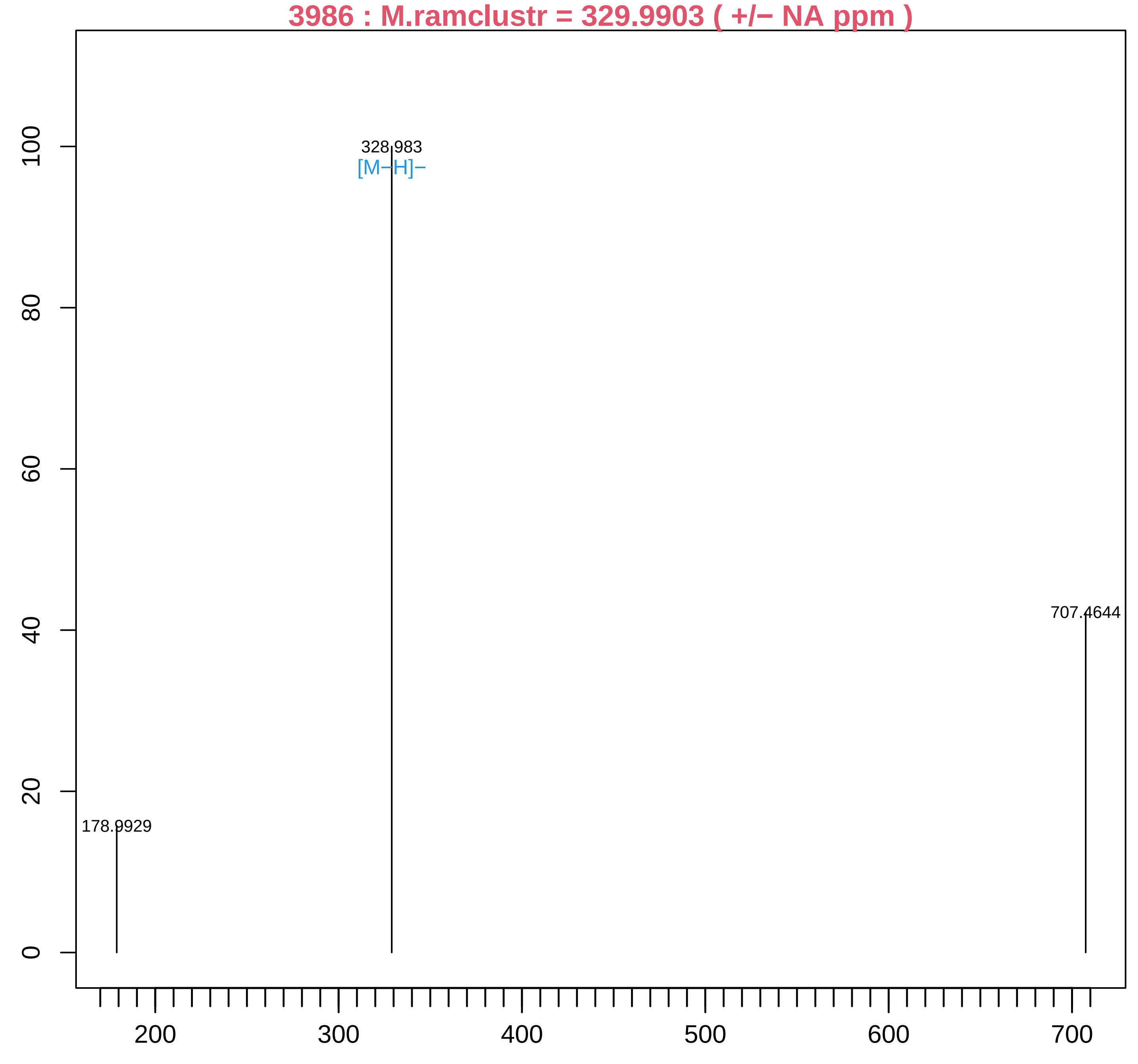
3986 : M.ramclustr = 329.9903 (+/− NA ppm)
328.983
[M−H]−
707.4644
178.9929
0
20
40
60
80
100
200
300
400
500
600
700

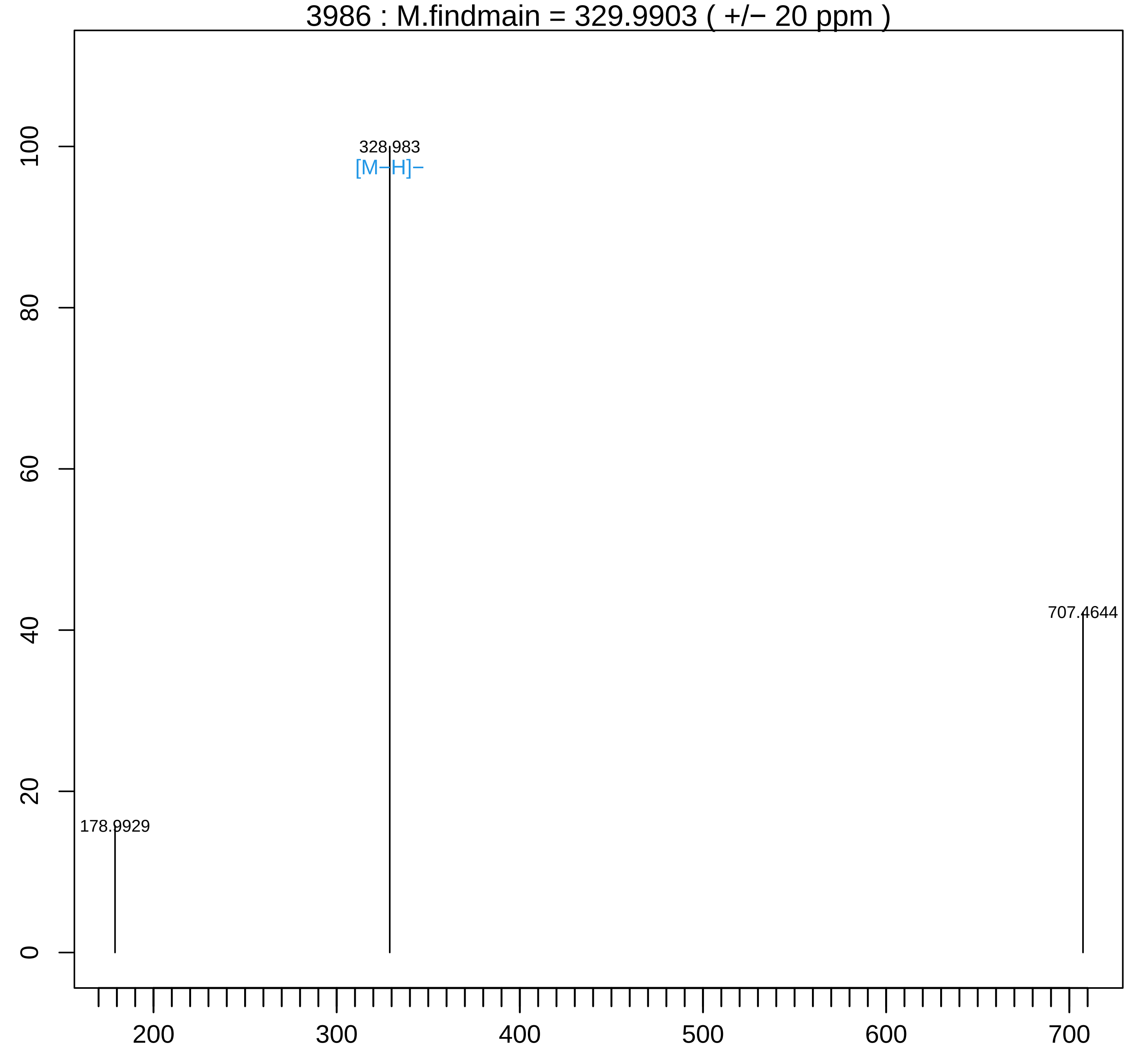
3986 : M.findmain = 329.9903 (+/− 20 ppm)
328.983
[M−H]−
707.4644
178.9929
0
20
40
60
80
100
200
300
400
500
600
700

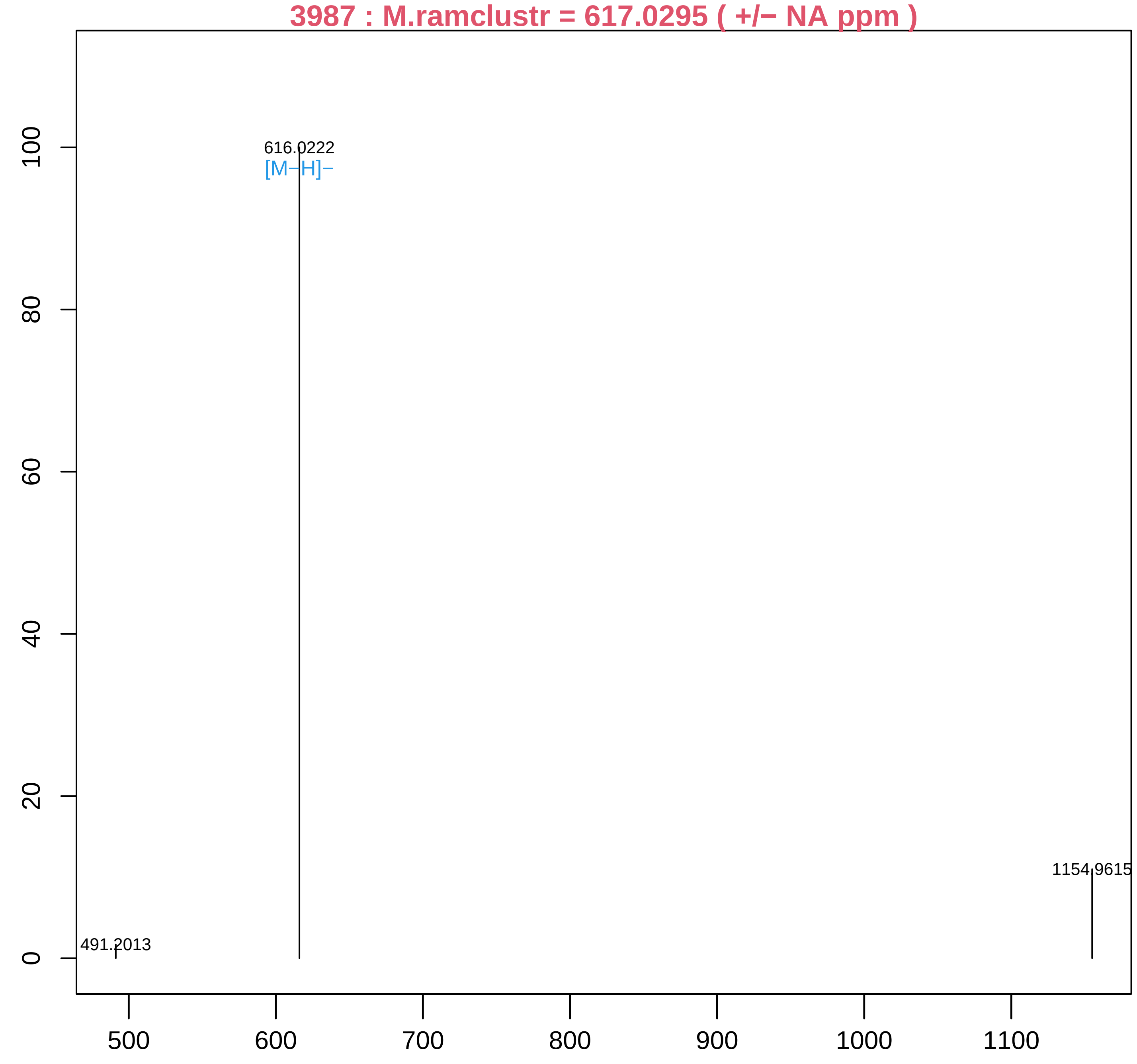
3987 : M.ramclustr = 617.0295 (+/− NA ppm)
616.0222
[M−H]−
491.2013
1154.9615
0
20
40
60
80
100
500
600
700
800
900
1000
1100

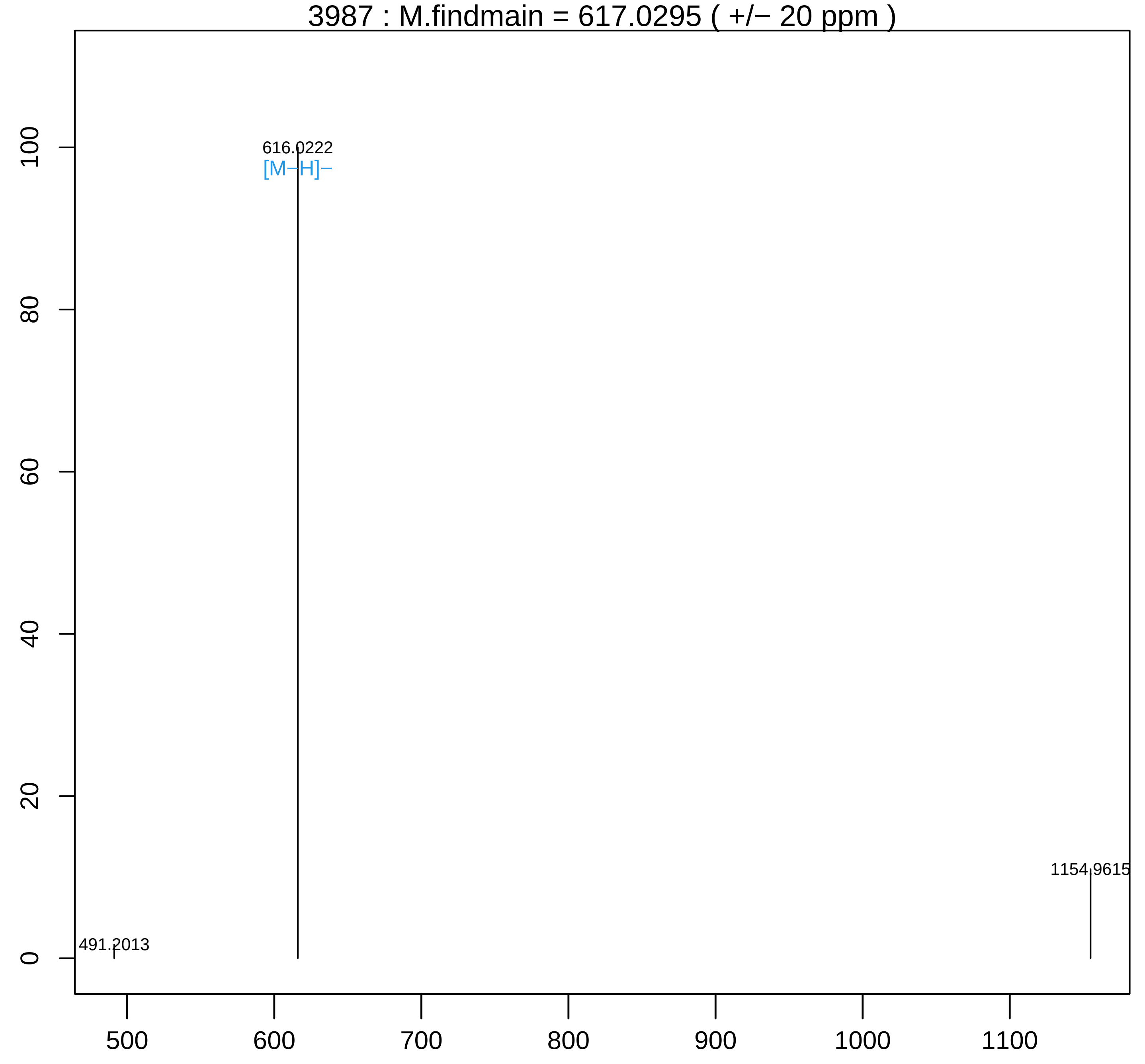
3987 : M.findmain = 617.0295 (+/− 20 ppm)
616.0222
[M−H]−
491.2013
1154.9615
0
20
40
60
80
100
500
600
700
800
900
1000
1100

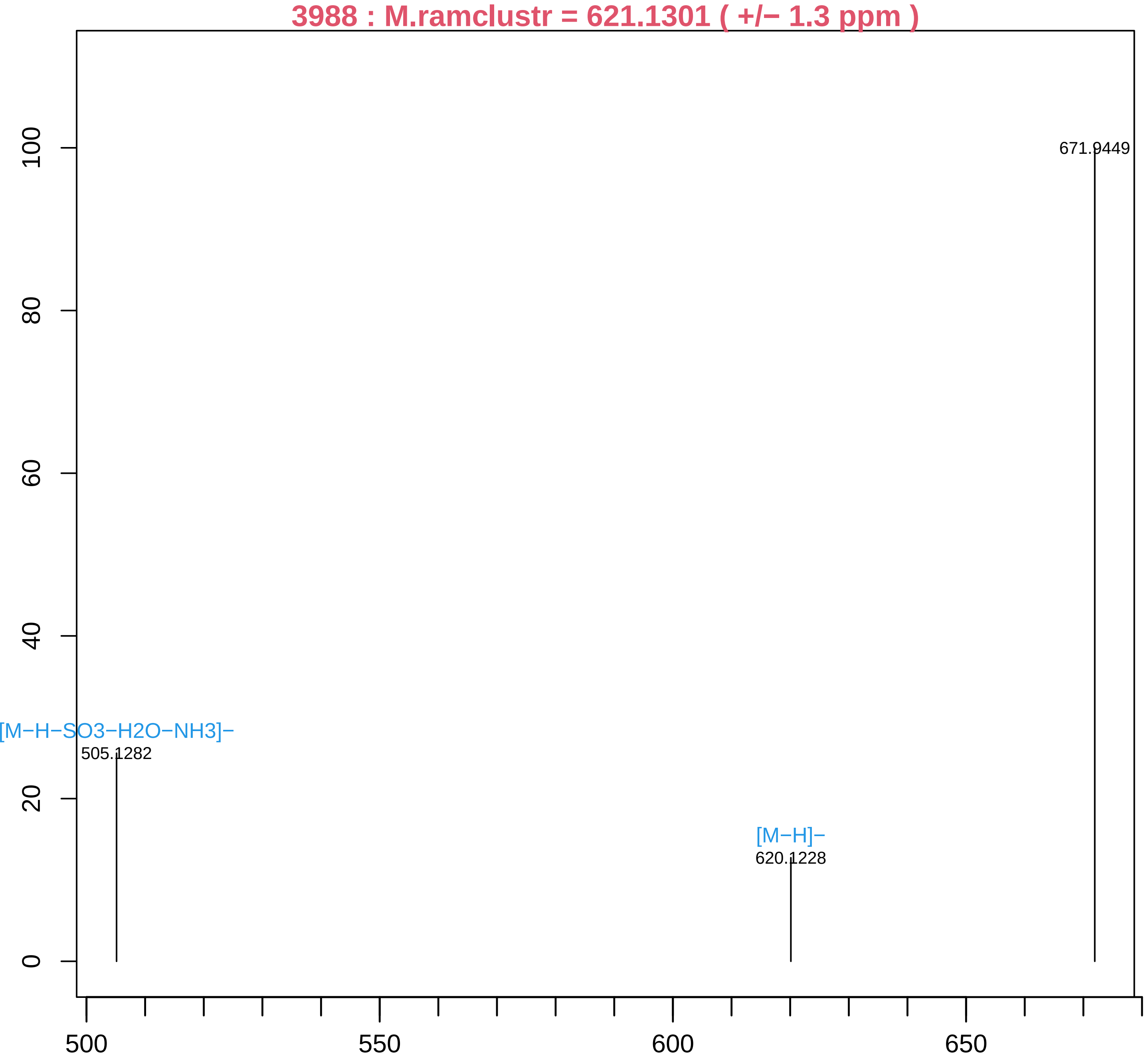
3988 : M.ramclustr = 621.1301 (+/− 1.3 ppm)
671.9449
[M−H−SO3−H2O−NH3]−
505.1282
[M−H]−
620.1228
0
20
40
60
80
100
500
550
600
650

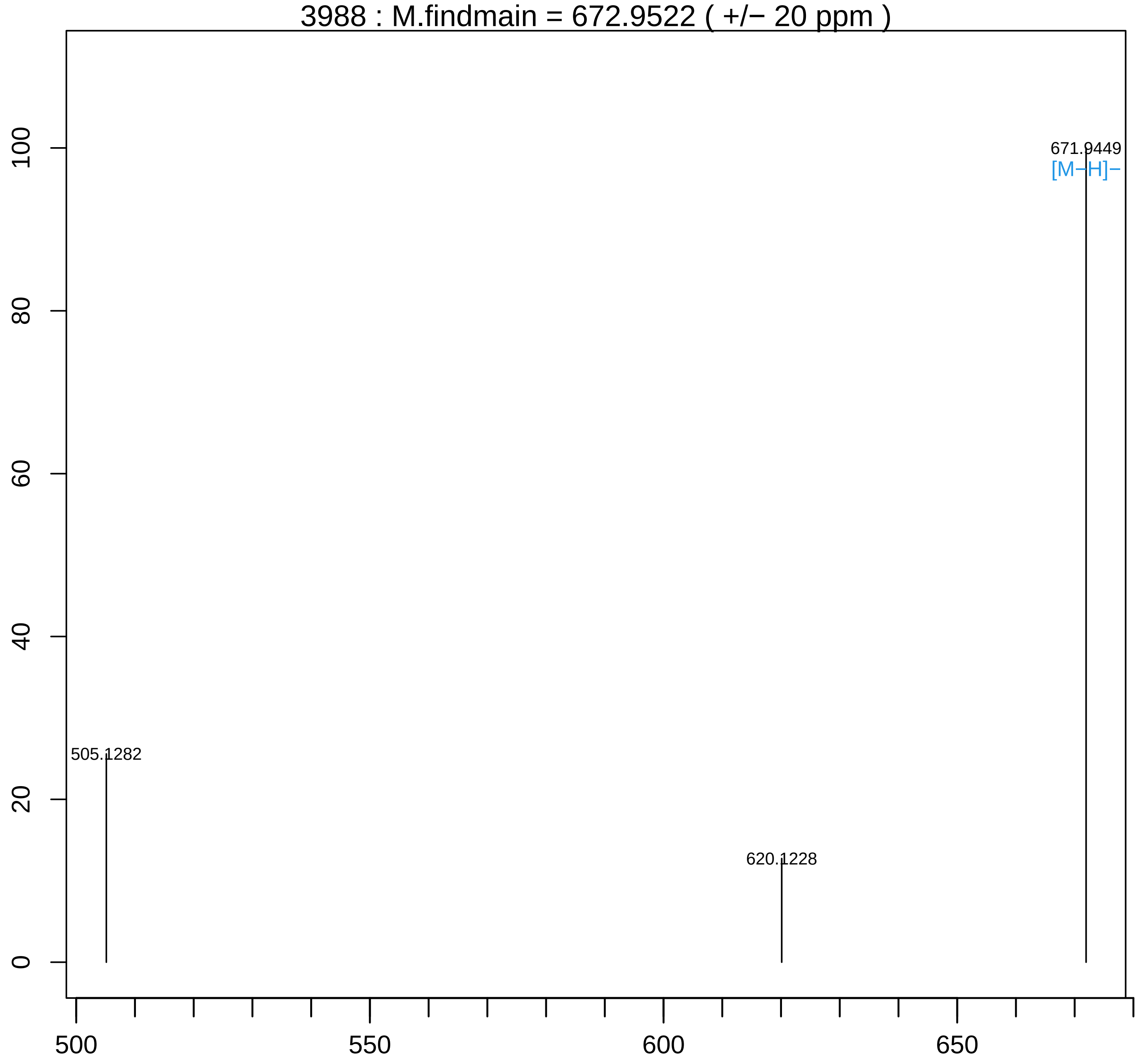
3988 : M.findmain = 672.9522 (+/− 20 ppm)
671.9449
[M−H]−
505.1282
620.1228
0
20
40
60
80
100
500
550
600
650

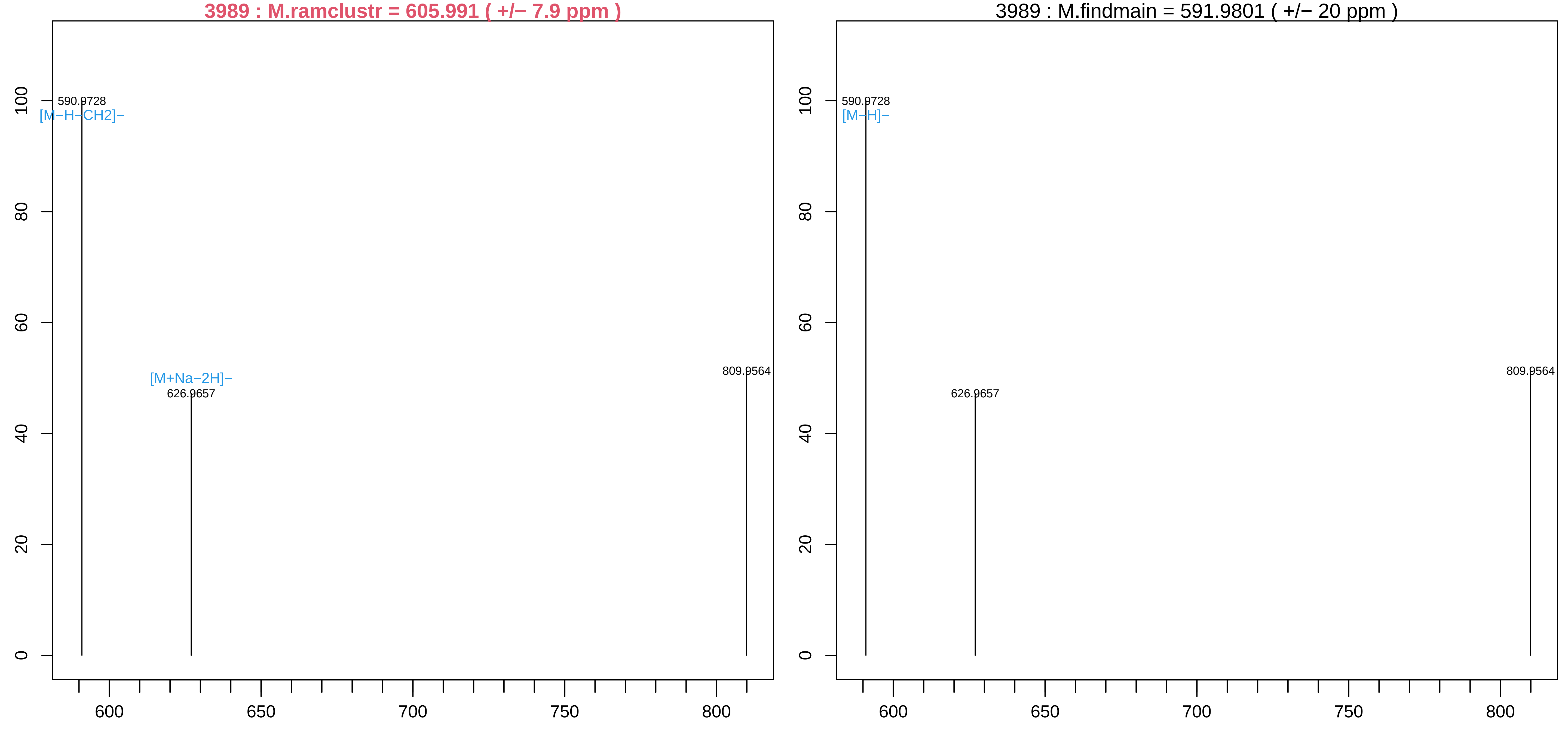
3989 : M.ramclustr = 605.991 (+/− 7.9 ppm)
590.9728
[M−H−CH2]−
[M+Na−2H]−
626.9657
809.9564
0
20
40
60
80
100
600
650
700
750
800
3989 : M.findmain = 591.9801 (+/− 20 ppm)
590.9728
[M−H]−
626.9657
809.9564
0
20
40
60
80
100
600
650
700
750
800

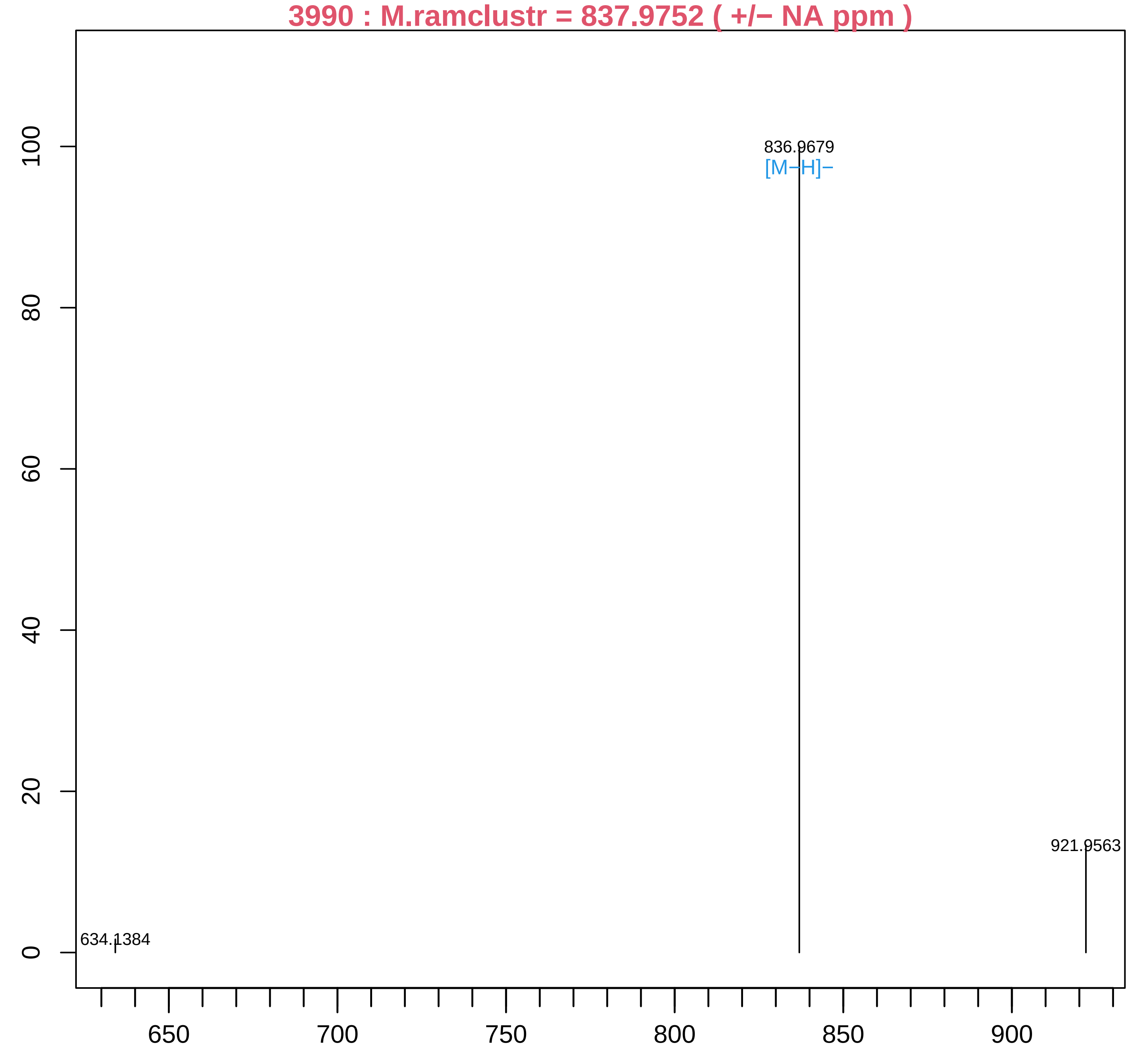
3990 : M.ramclustr = 837.9752 (+/− NA ppm)
836.9679
[M−H]−
921.9563
634.1384
0
20
40
60
80
100
650
700
750
800
850
900

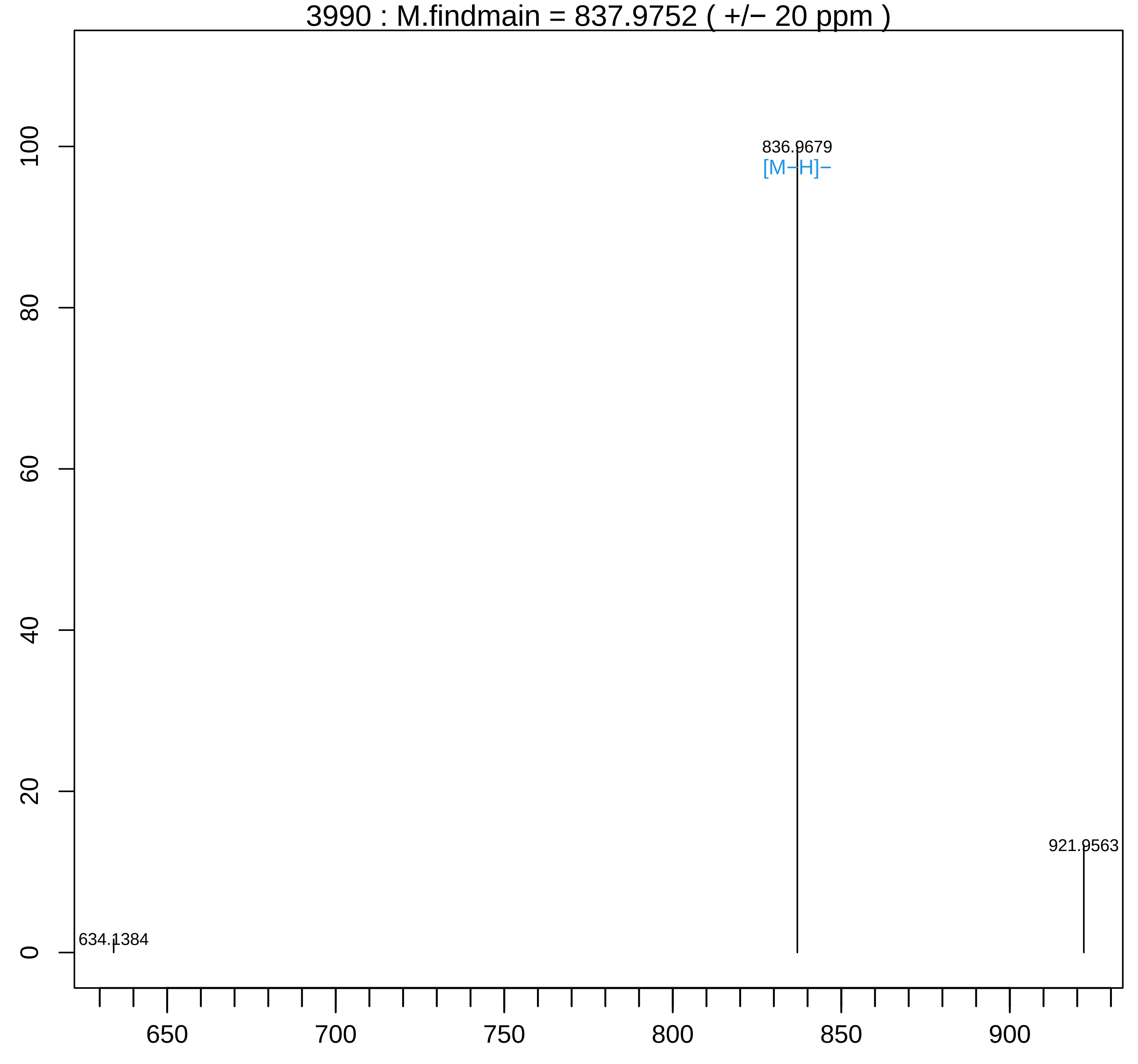
3990 : M.findmain = 837.9752 (+/− 20 ppm)
836.9679
[M−H]−
921.9563
634.1384
0
20
40
60
80
100
650
700
750
800
850
900

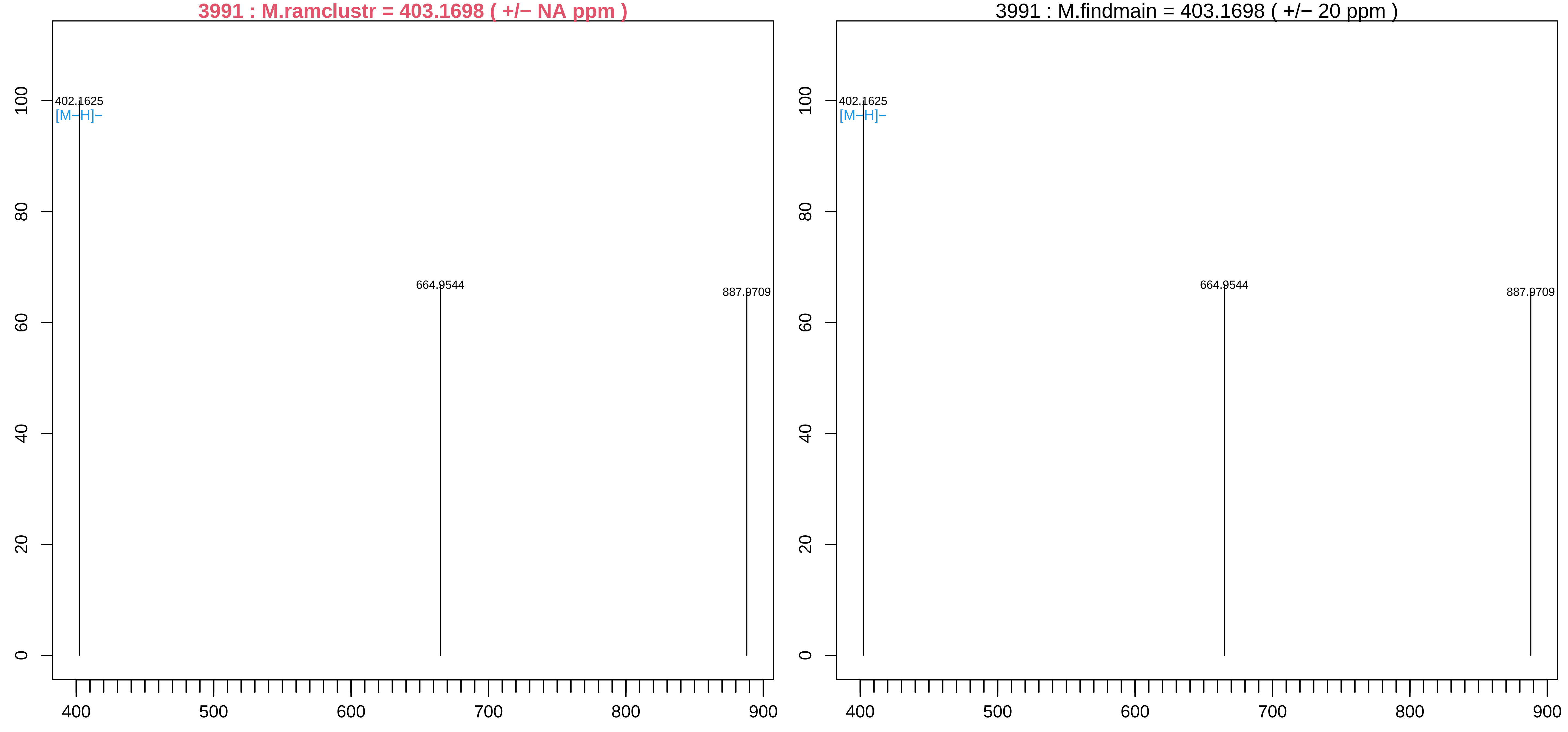
3991 : M.ramclustr = 403.1698 (+/− NA ppm)
402.1625
[M−H]−
664.9544
887.9709
0
20
40
60
80
100
400
500
600
700
800
900
3991 : M.findmain = 403.1698 (+/− 20 ppm)
402.1625
[M−H]−
664.9544
887.9709
0
20
40
60
80
100
400
500
600
700
800
900

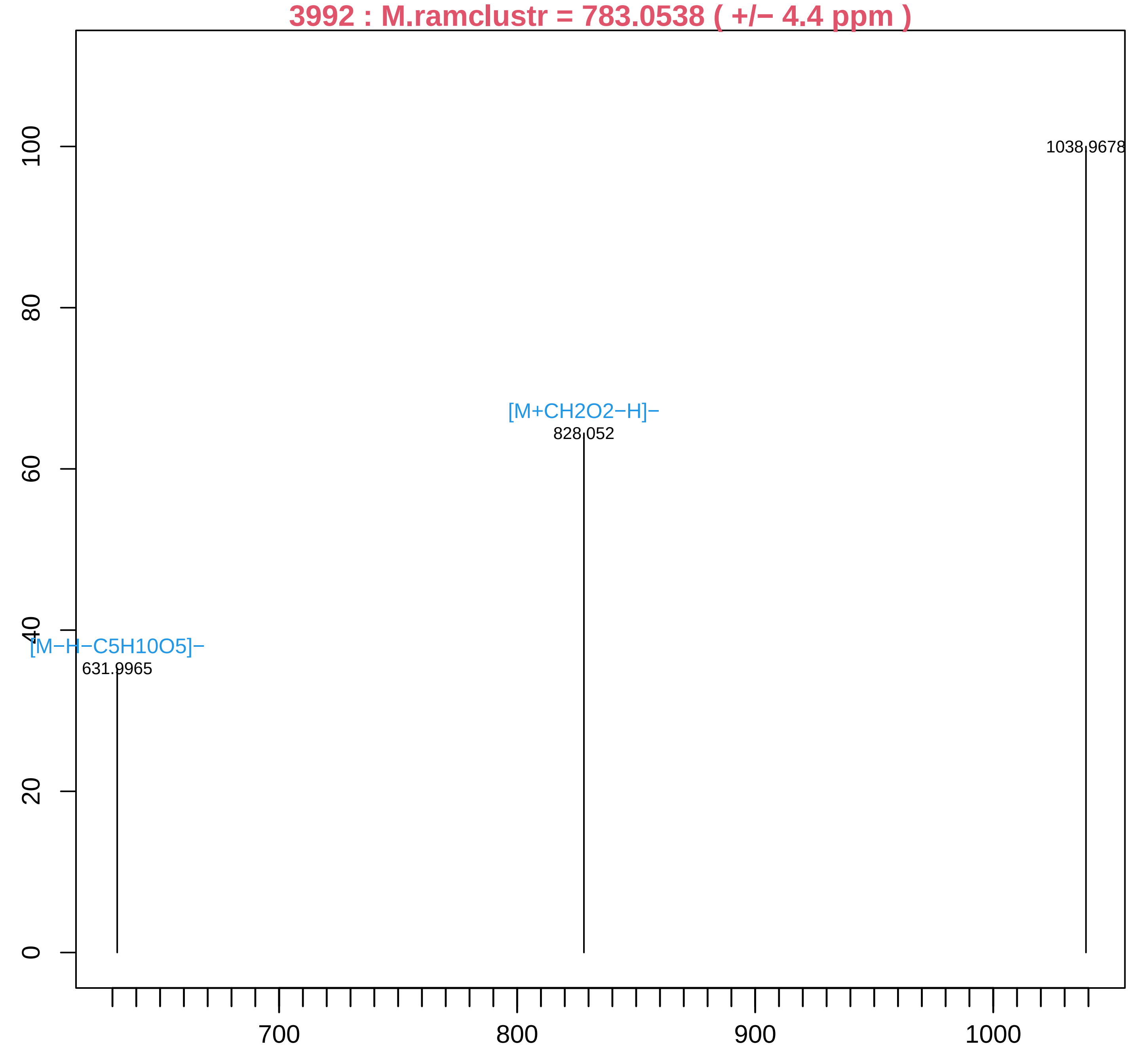

3992 : M.ramclustr = 783.0538 (+/− 4.4 ppm)
1038.9678
[M+CH2O2−H]−
828.052
[M−H−C5H10O5]−
631.9965
0
20
40
60
80
100
700
800
900
1000

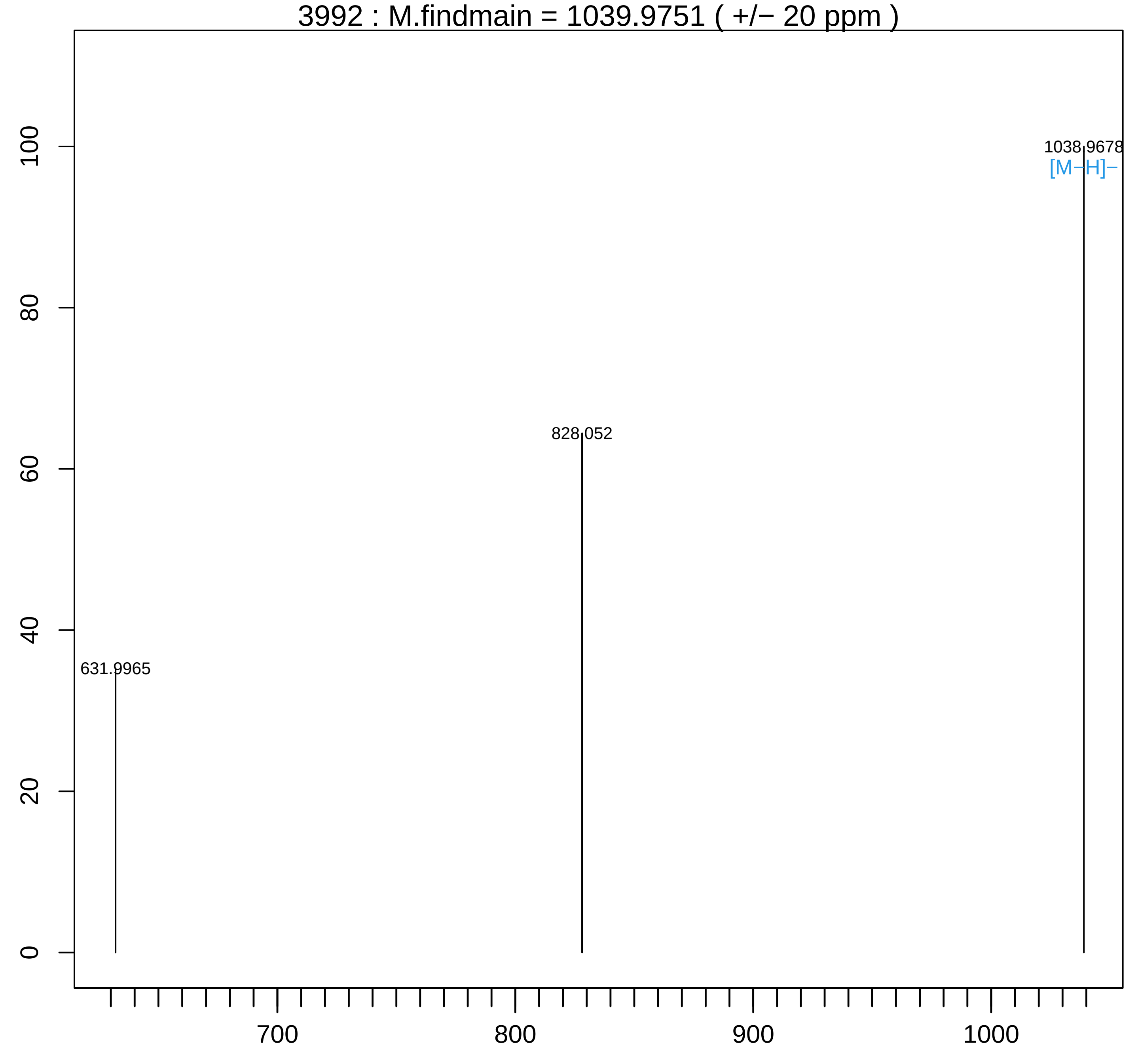

3992 : M.findmain = 1039.9751 (+/− 20 ppm)
1038.9678
[M−H]−
828.052
631.9965
0
20
40
60
80
100
700
800
900
1000

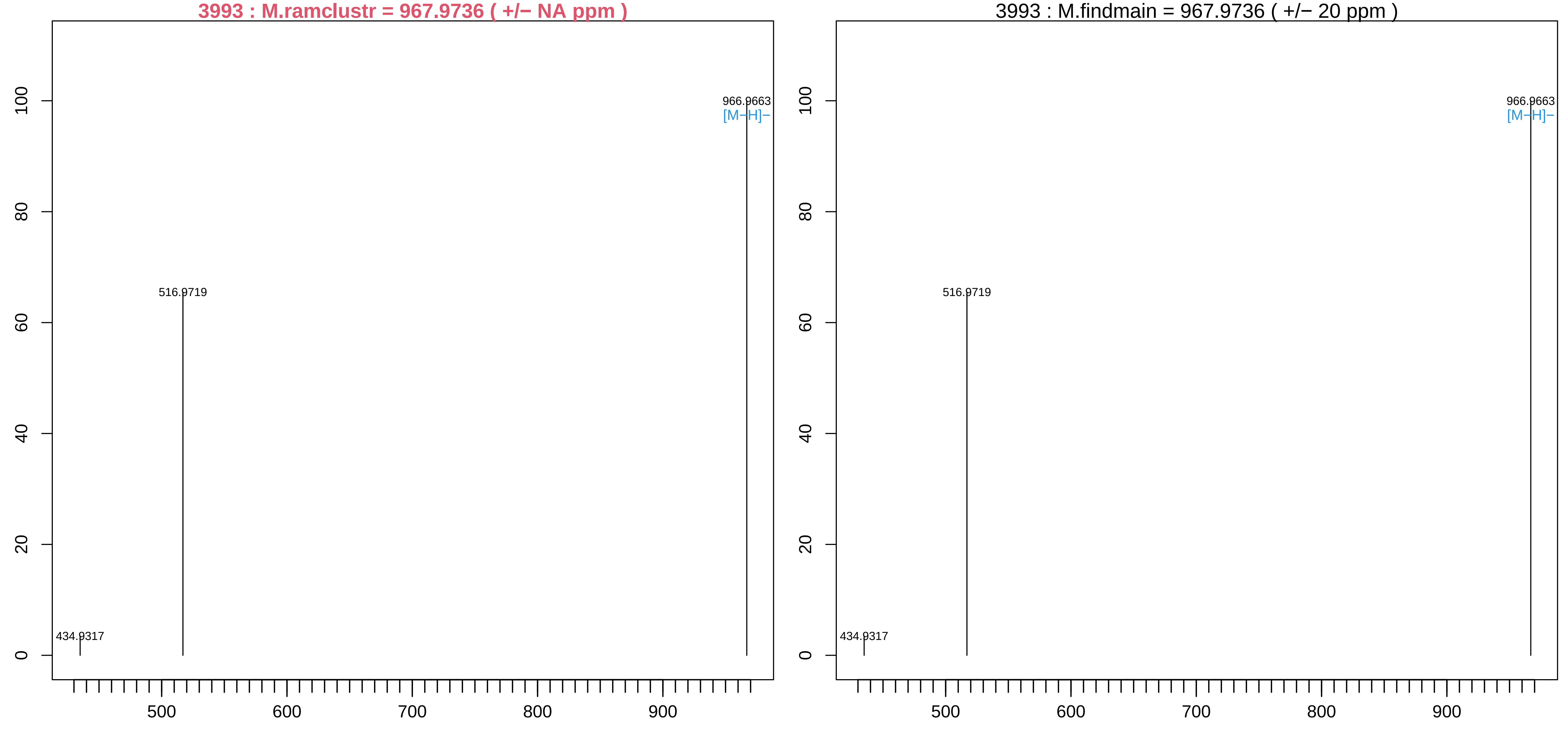
3993 : M.ramclustr = 967.9736 (+/− NA ppm)
966.9663
[M−H]−
516.9719
434.9317
3993 : M.findmain = 967.9736 (+/− 20 ppm)
966.9663
[M−H]−
516.9719
434.9317
0
20
40
60
80
100
500
600
700
800
900

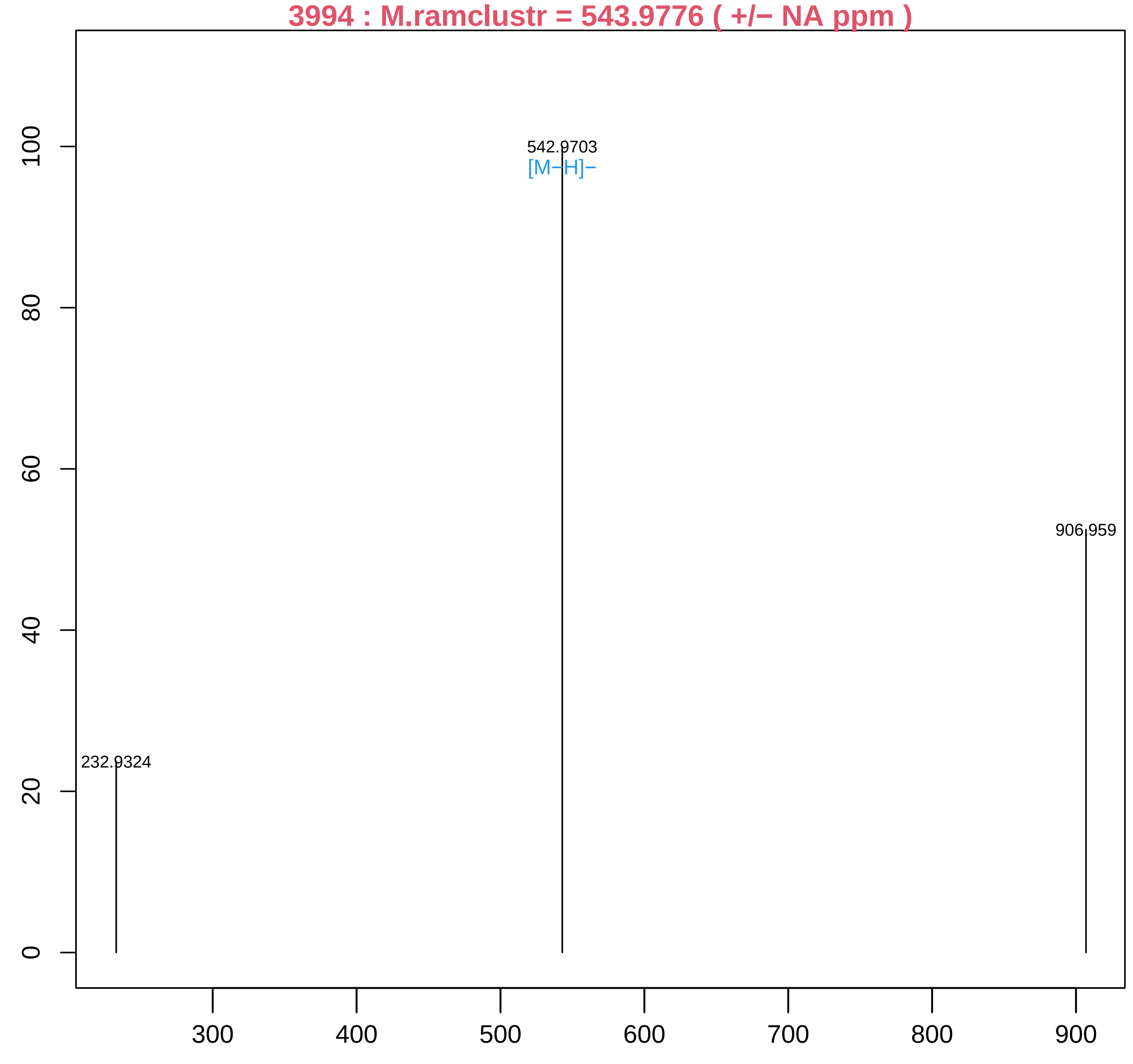
3994 : M.ramclustr = 543.9776 (+/− NA ppm)
542.9703
[M−H]−
906.959
232.9324
0
20
40
60
80
100
300
400
500
600
700
800
900

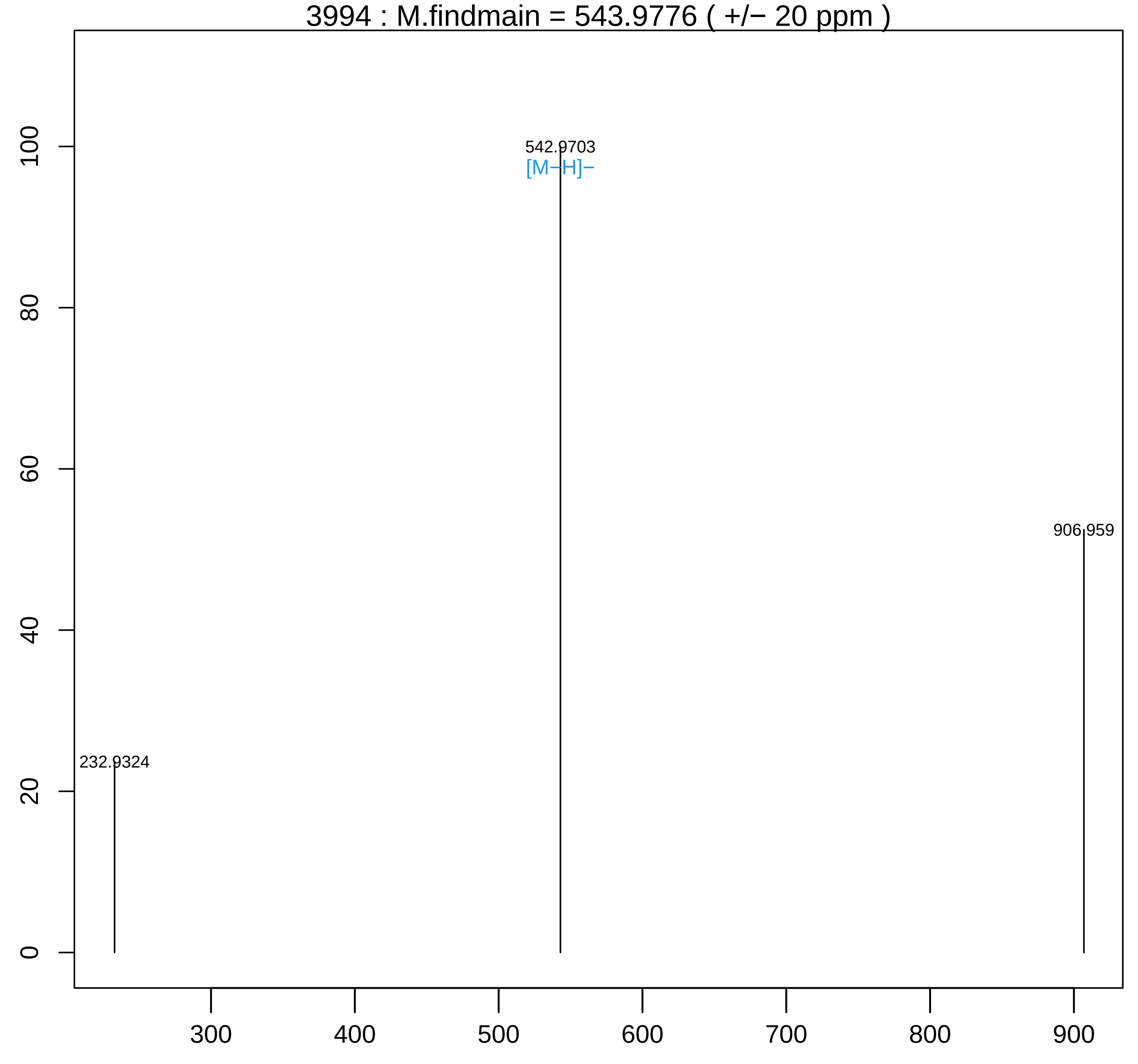
3994 : M.findmain = 543.9776 (+/− 20 ppm)
542.9703
[M−H]−
906.959
232.9324
0
20
40
60
80
100
300
400
500
600
700
800
900

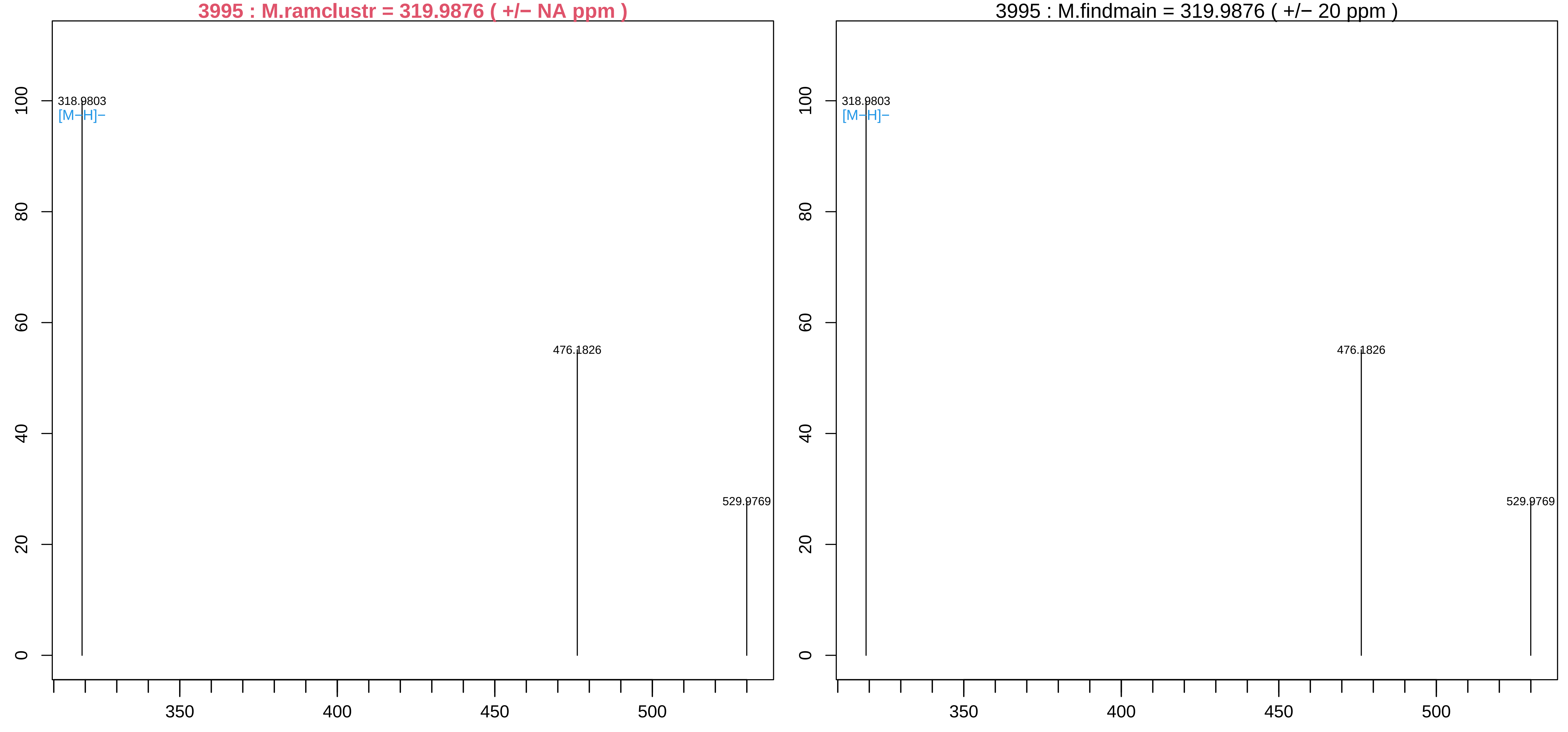
3995 : M.ramclustr = 319.9876 (+/− NA ppm)
318.9803
[M−H]−
476.1826
529.9769
3995 : M.findmain = 319.9876 (+/− 20 ppm)
318.9803
[M−H]−
476.1826
529.9769
0
20
40
60
80
100
350
400
450
500

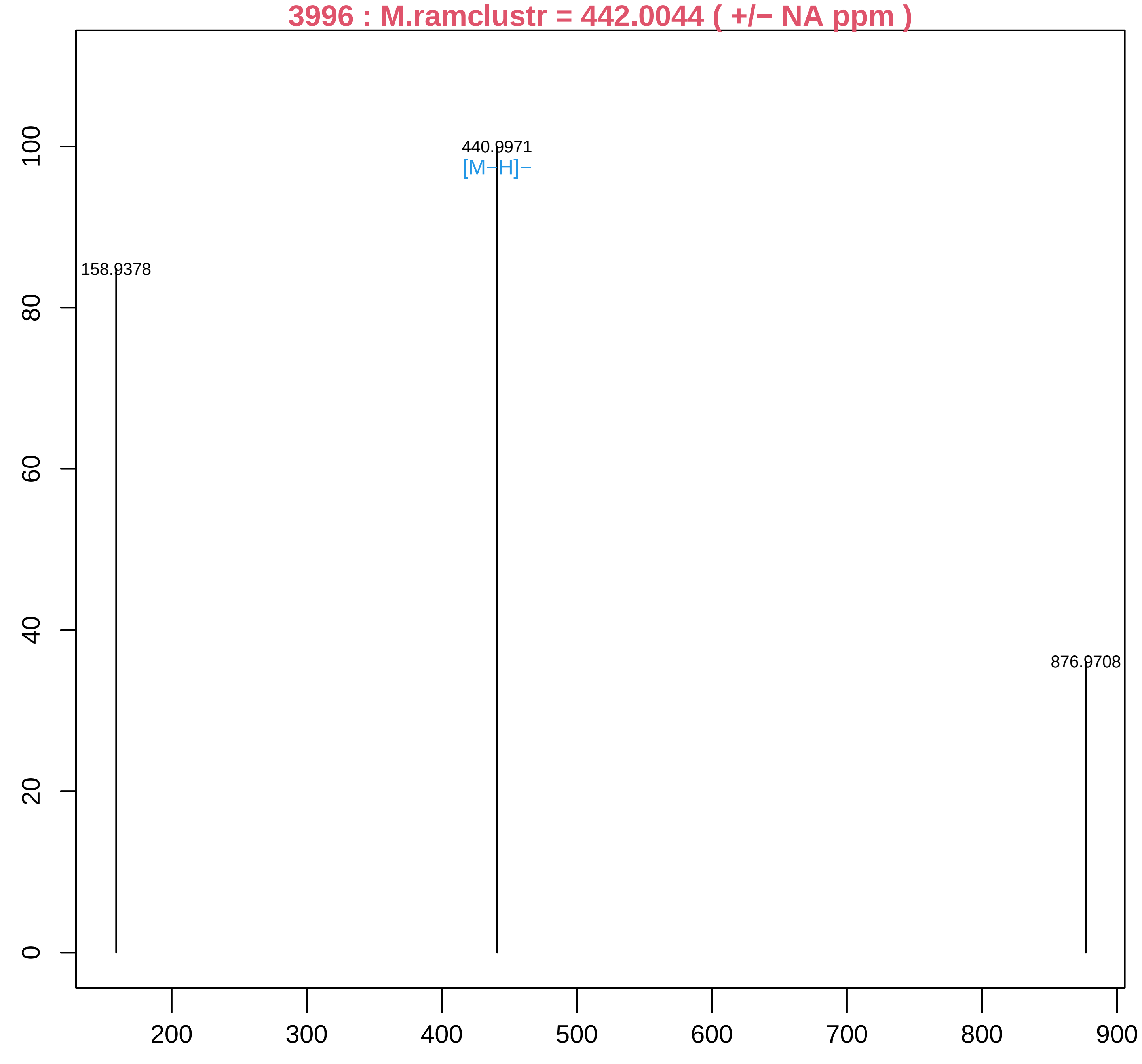
3996 : M.ramclustr = 442.0044 (+/− NA ppm)
440.9971
[M−H]−
158.9378
876.9708
0
20
40
60
80
100
200
300
400
500
600
700
800
900

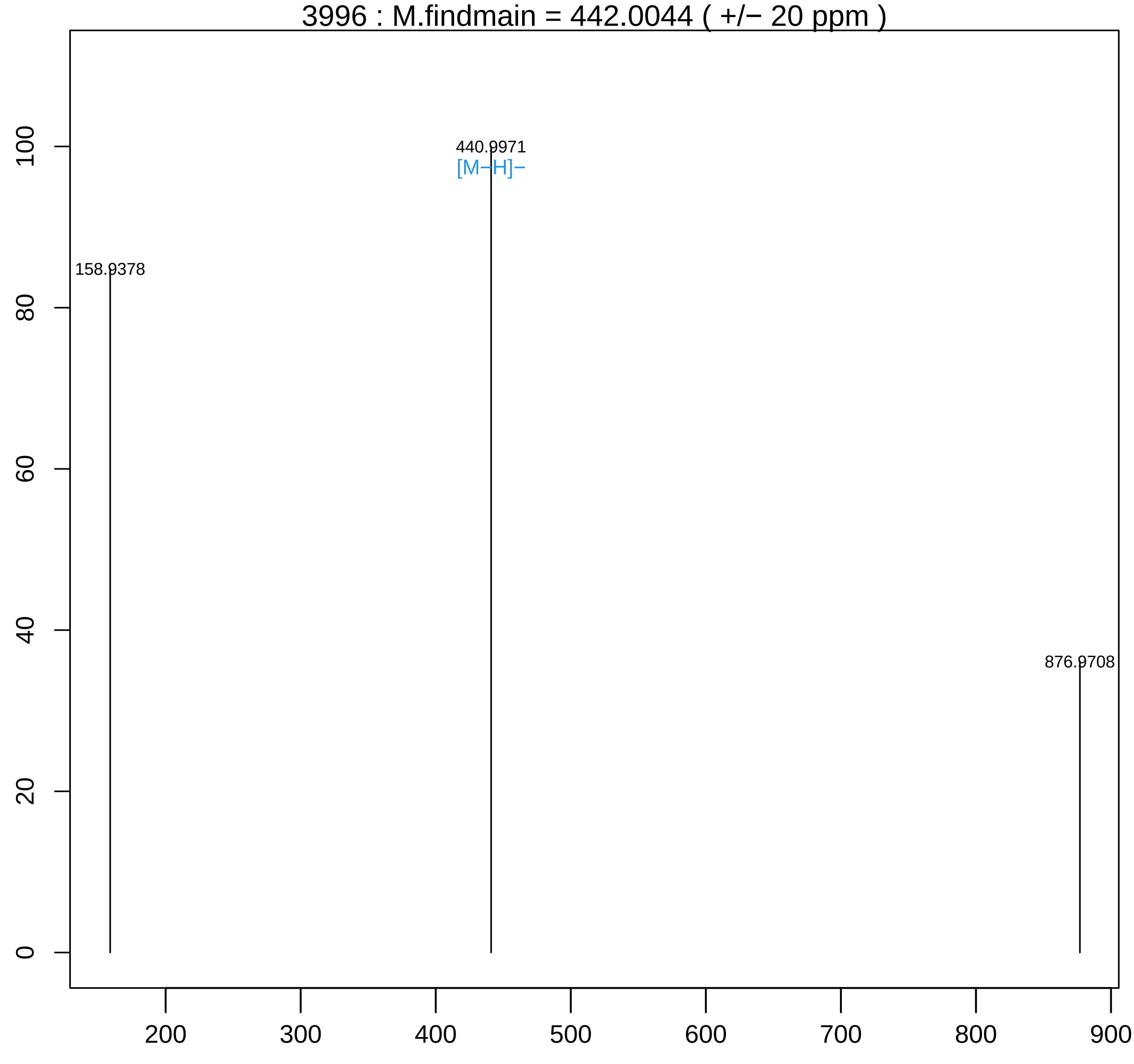
3996 : M.findmain = 442.0044 (+/− 20 ppm)
440.9971
[M−H]−
158.9378
876.9708
0
20
40
60
80
100
200
300
400
500
600
700
800
900

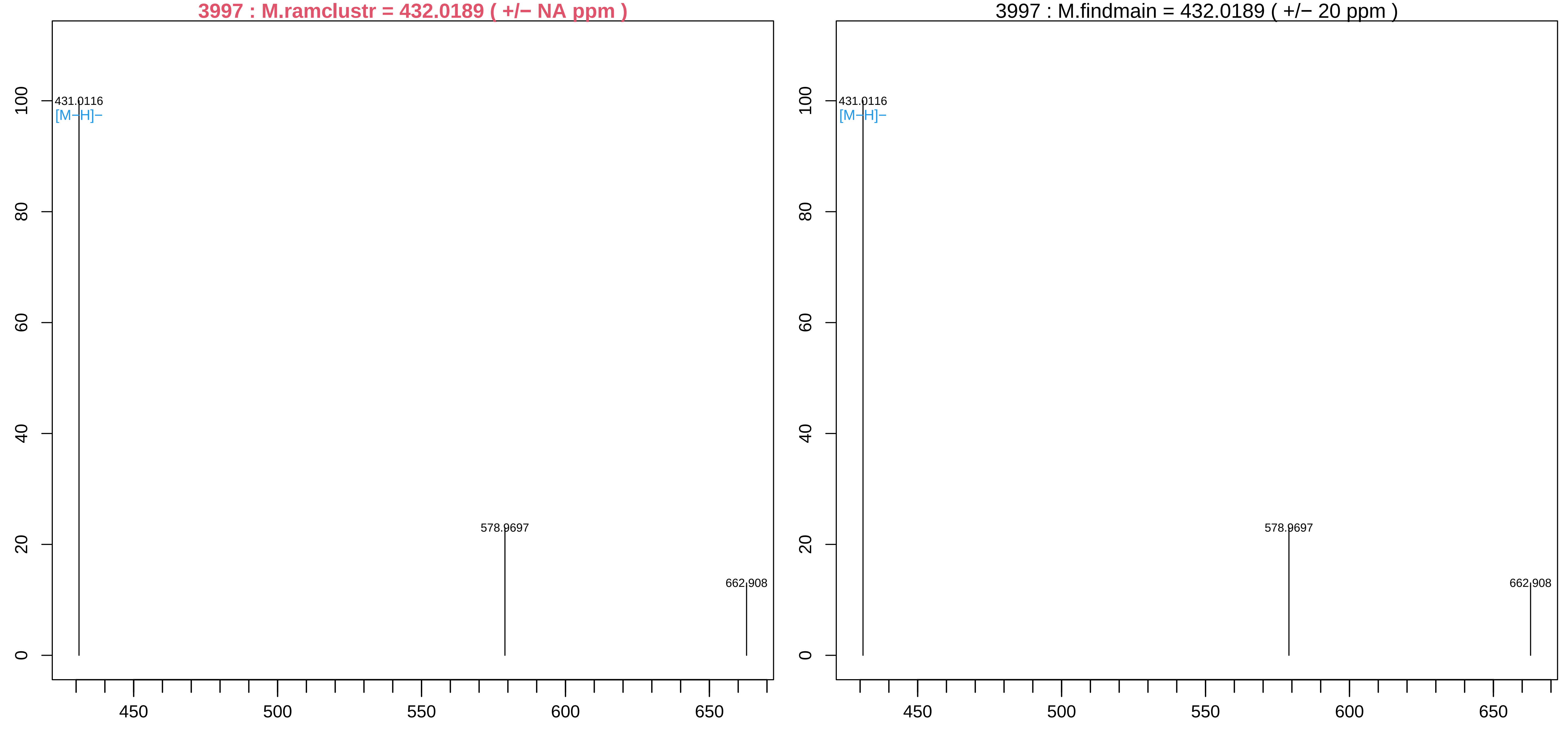
3997 : M.ramclustr = 432.0189 (+/− NA ppm)
431.0116
[M−H]−
578.9697
662.908
3997 : M.findmain = 432.0189 (+/− 20 ppm)
431.0116
[M−H]−
578.9697
662.908
0
20
40
60
80
100
450
500
550
600
650

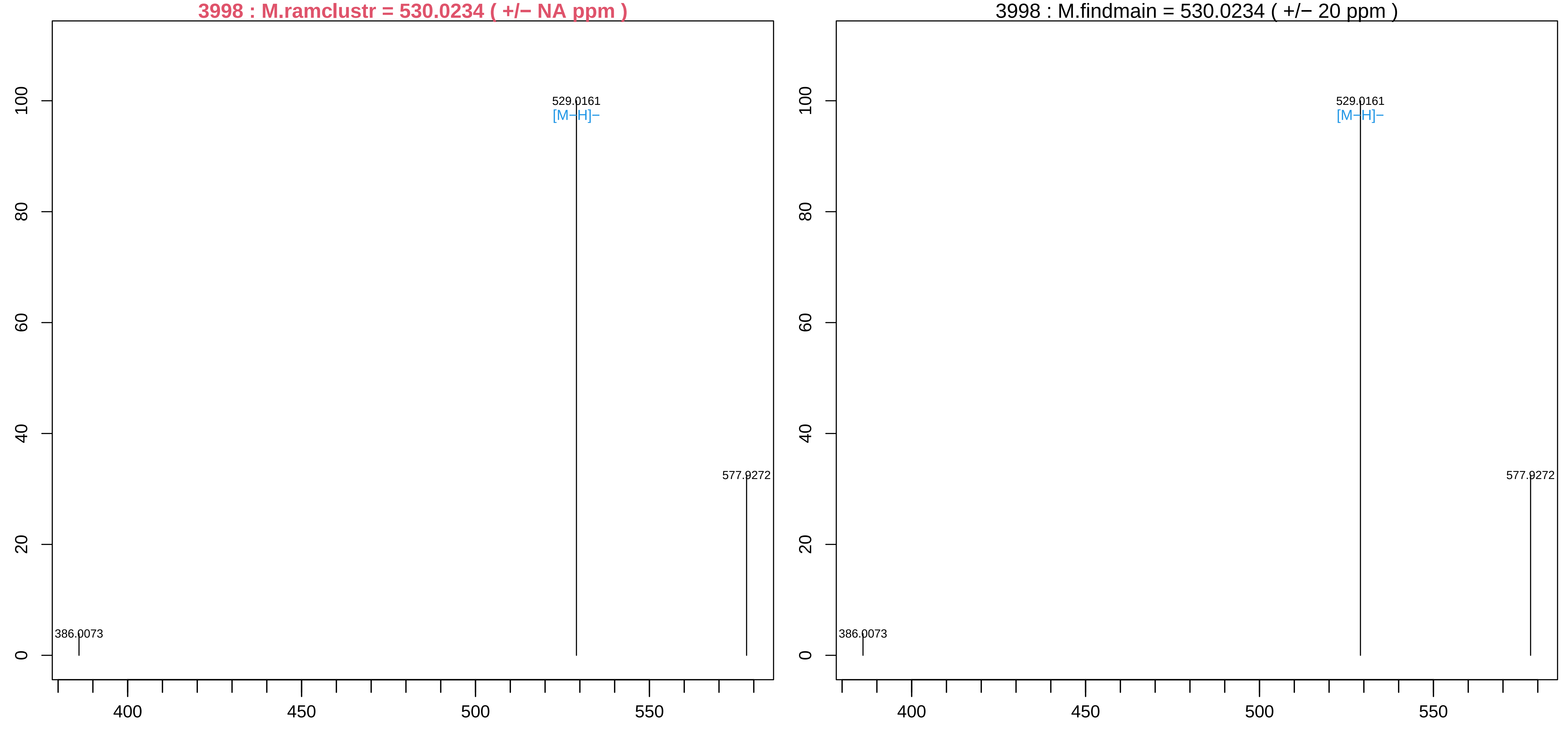
3998 : M.ramclustr = 530.0234 (+/− NA ppm)
529.0161
[M−H]−
577.9272
386.0073
3998 : M.findmain = 530.0234 (+/− 20 ppm)
529.0161
[M−H]−
577.9272
386.0073
0
20
40
60
80
100
400
450
500
550

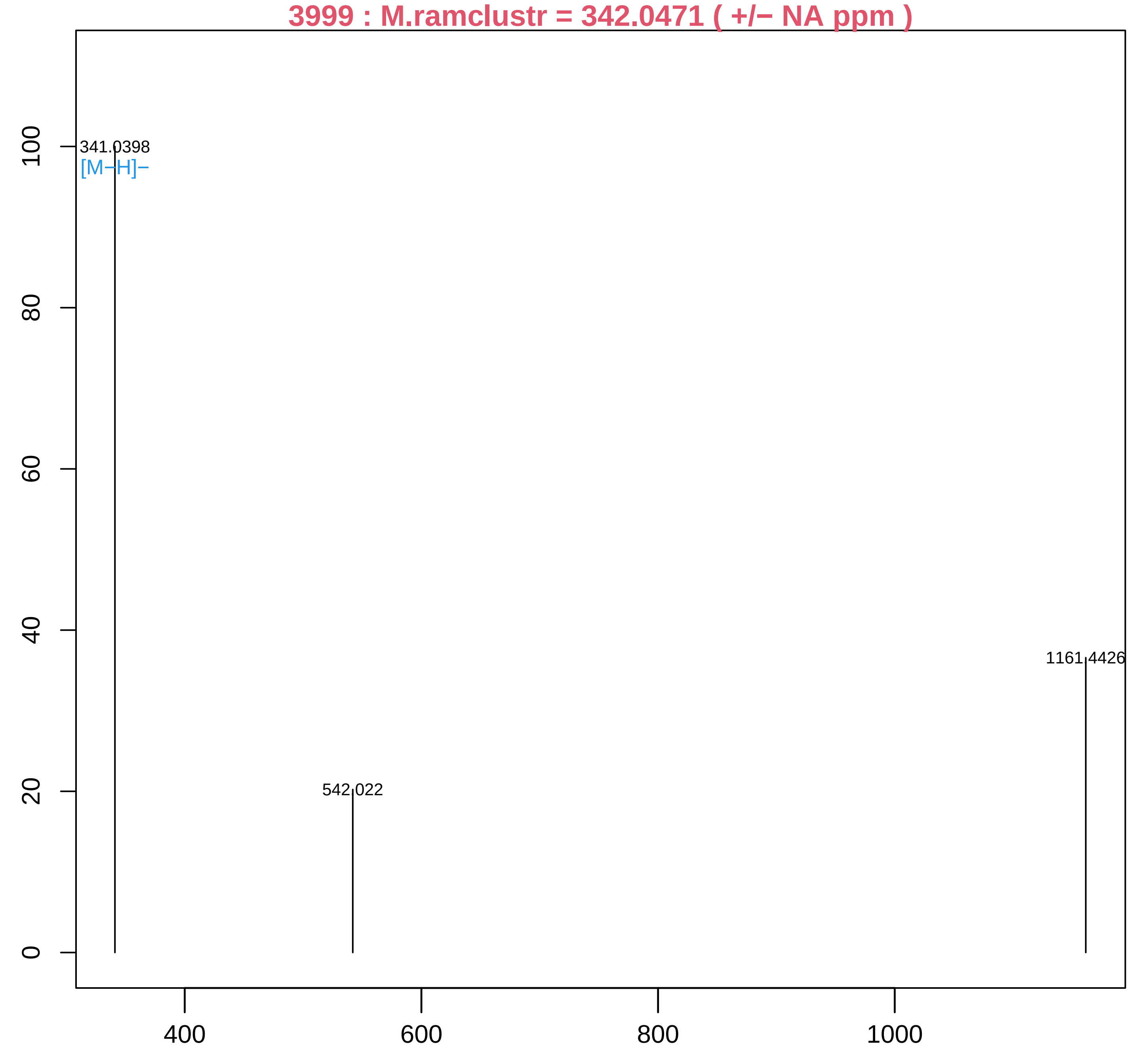

3999 : M.ramclustr = 342.0471 (+/− NA ppm)
341.0398
[M−H]−
542.022
1161.4426
0
20
40
60
80
100
400
600
800
1000

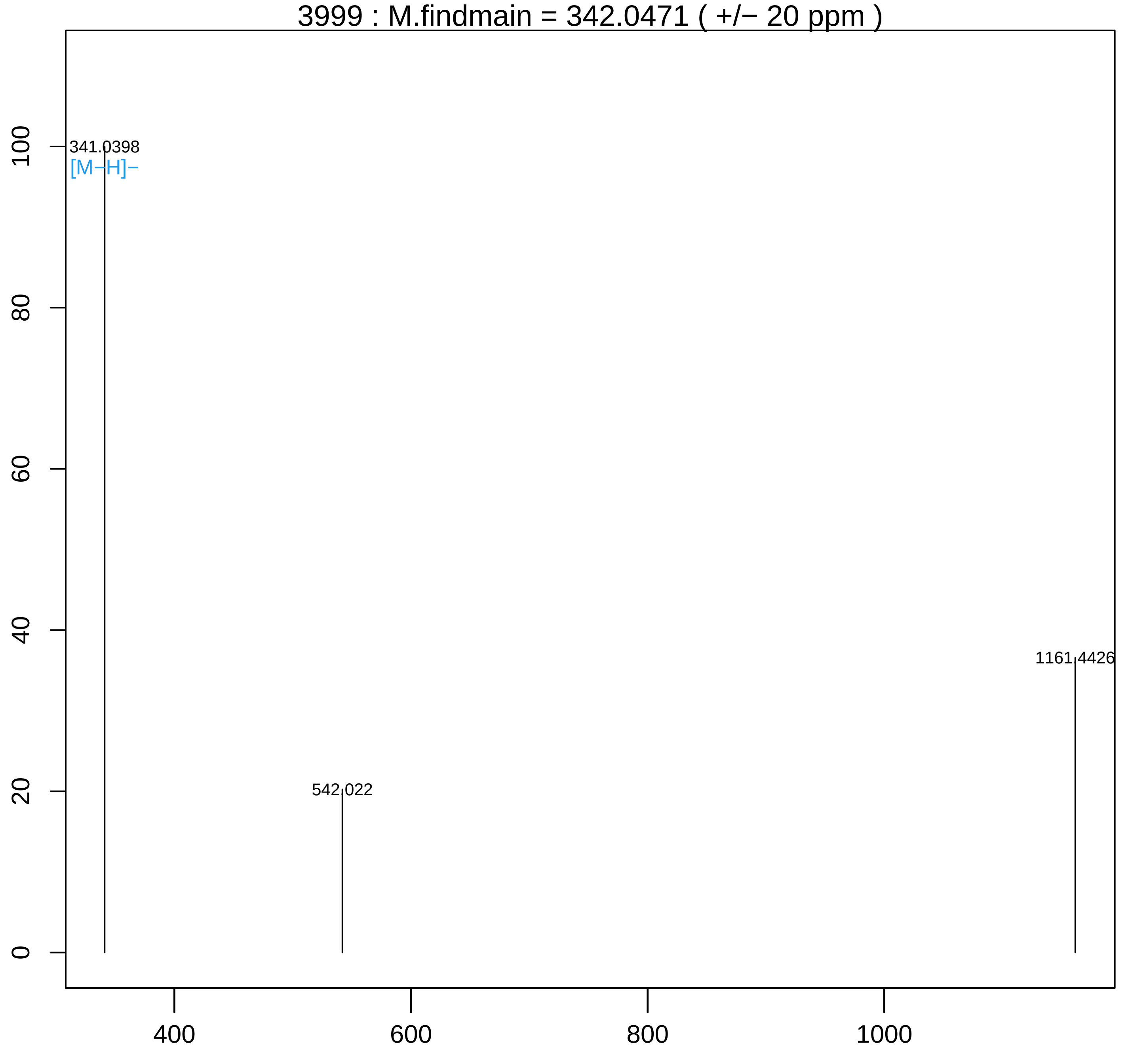

3999 : M.findmain = 342.0471 (+/− 20 ppm)
341.0398
[M−H]−
542.022
1161.4426
0
20
40
60
80
100
400
600
800
1000

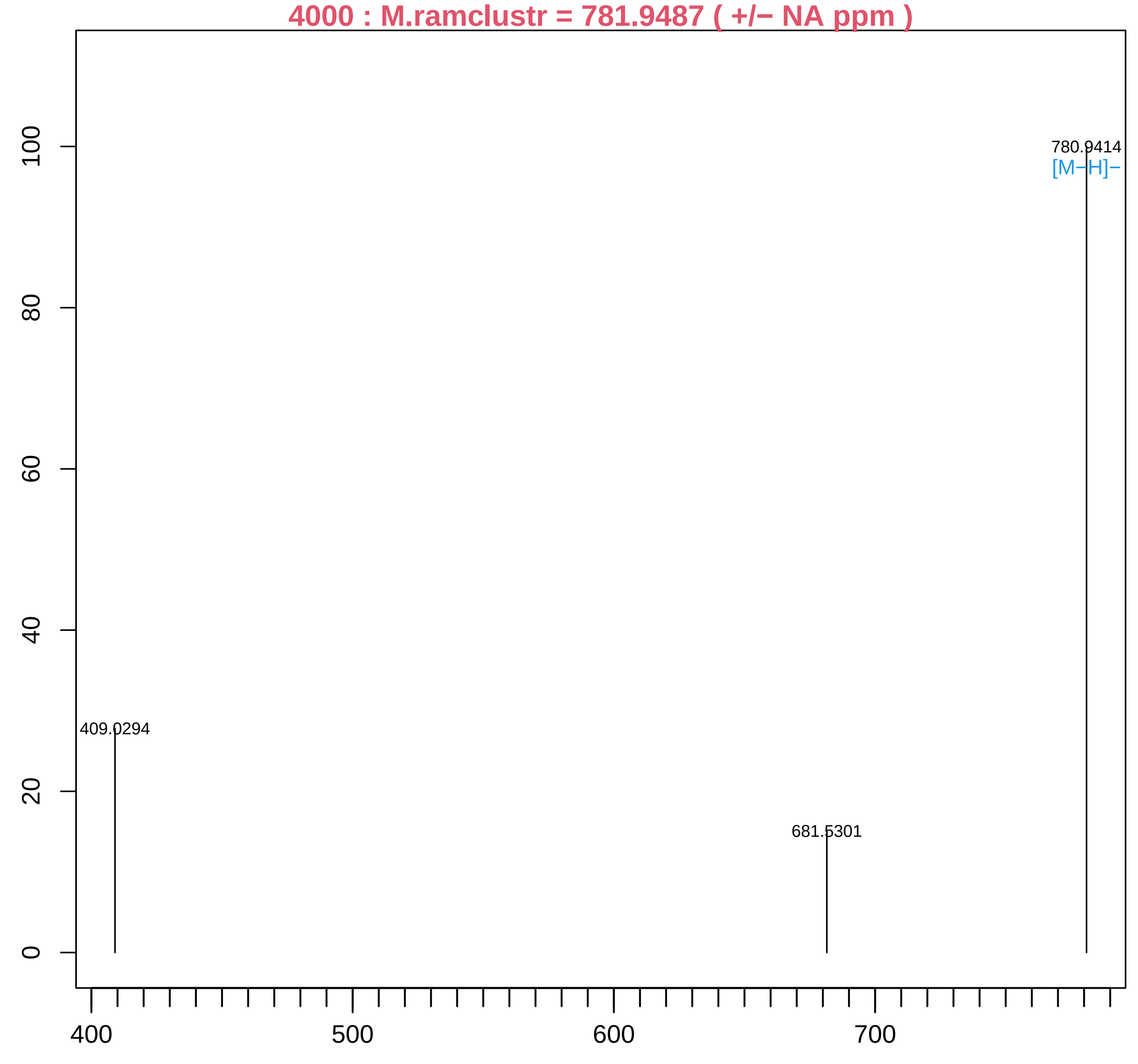
4000 : M.ramclustr = 781.9487 (+/− NA ppm)
780.9414
[M−H]−
409.0294
681.5301
0
20
40
60
80
100
400
500
600
700

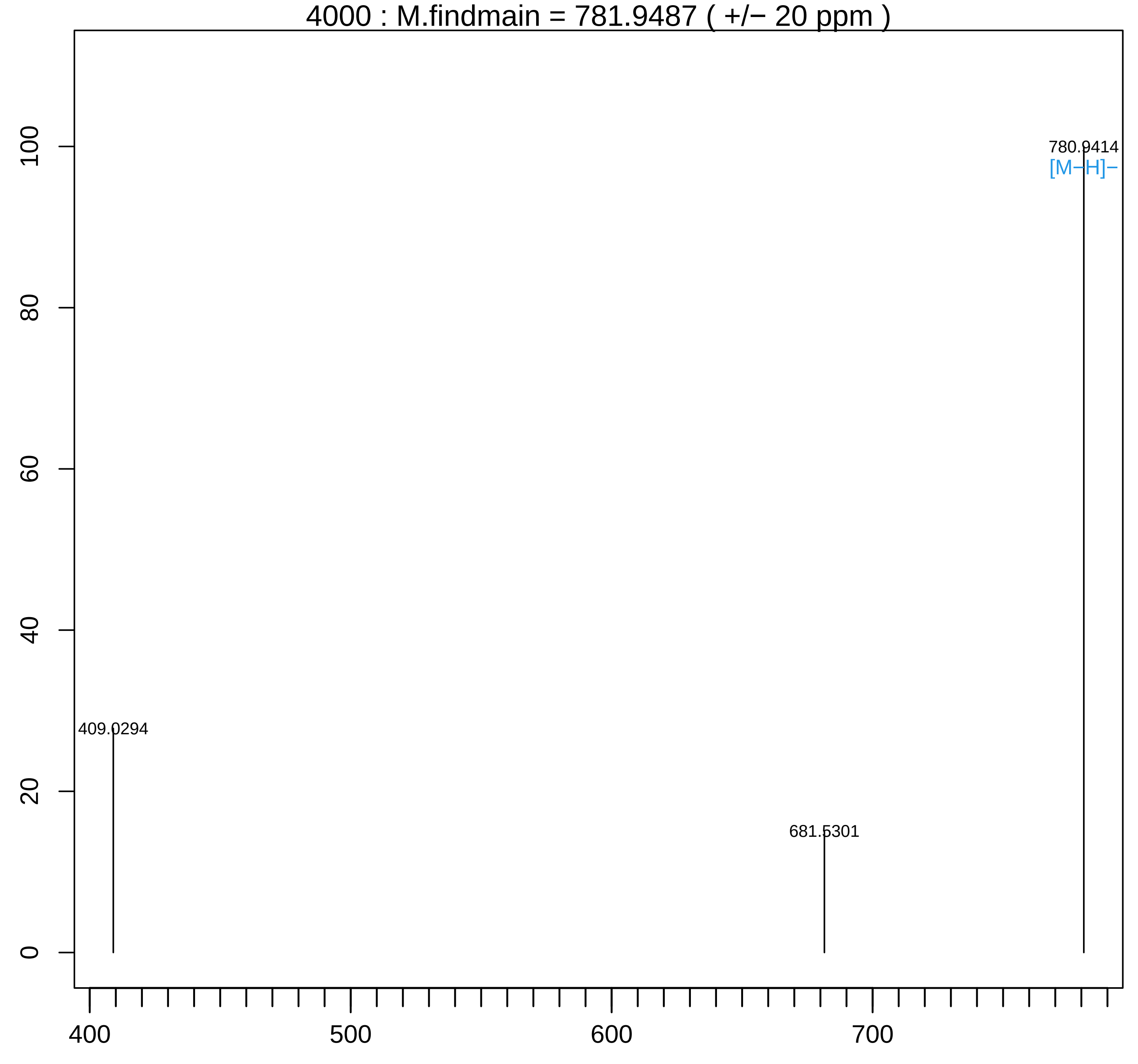
4000 : M.findmain = 781.9487 (+/− 20 ppm)
780.9414
[M−H]−
409.0294
681.5301
0
20
40
60
80
100
400
500
600
700

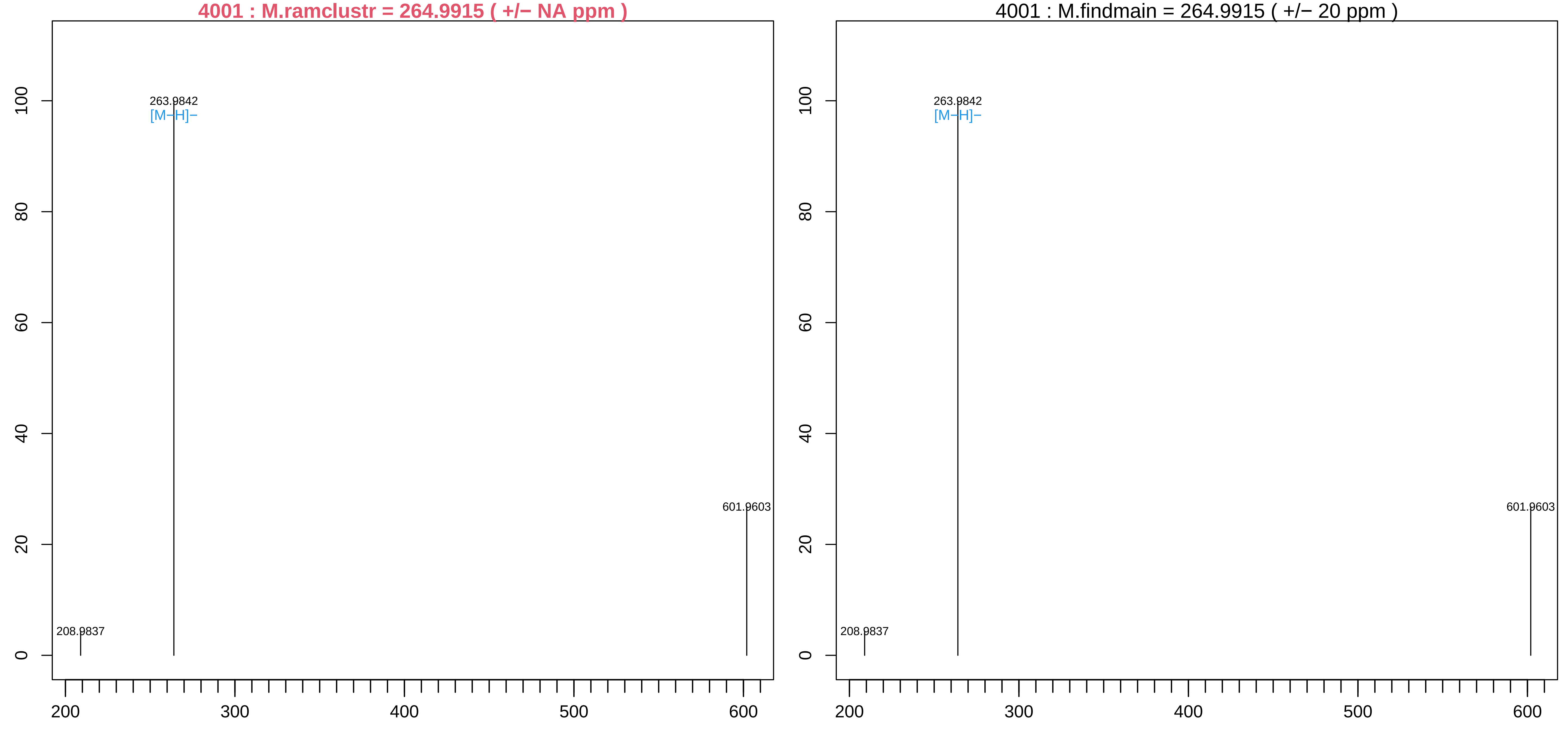
4001 : M.ramclustr = 264.9915 (+/− NA ppm)
263.9842
[M−H]−
601.9603
208.9837
0
20
40
60
80
100
200
300
400
500
600
4001 : M.findmain = 264.9915 (+/− 20 ppm)
263.9842
[M−H]−
601.9603
208.9837

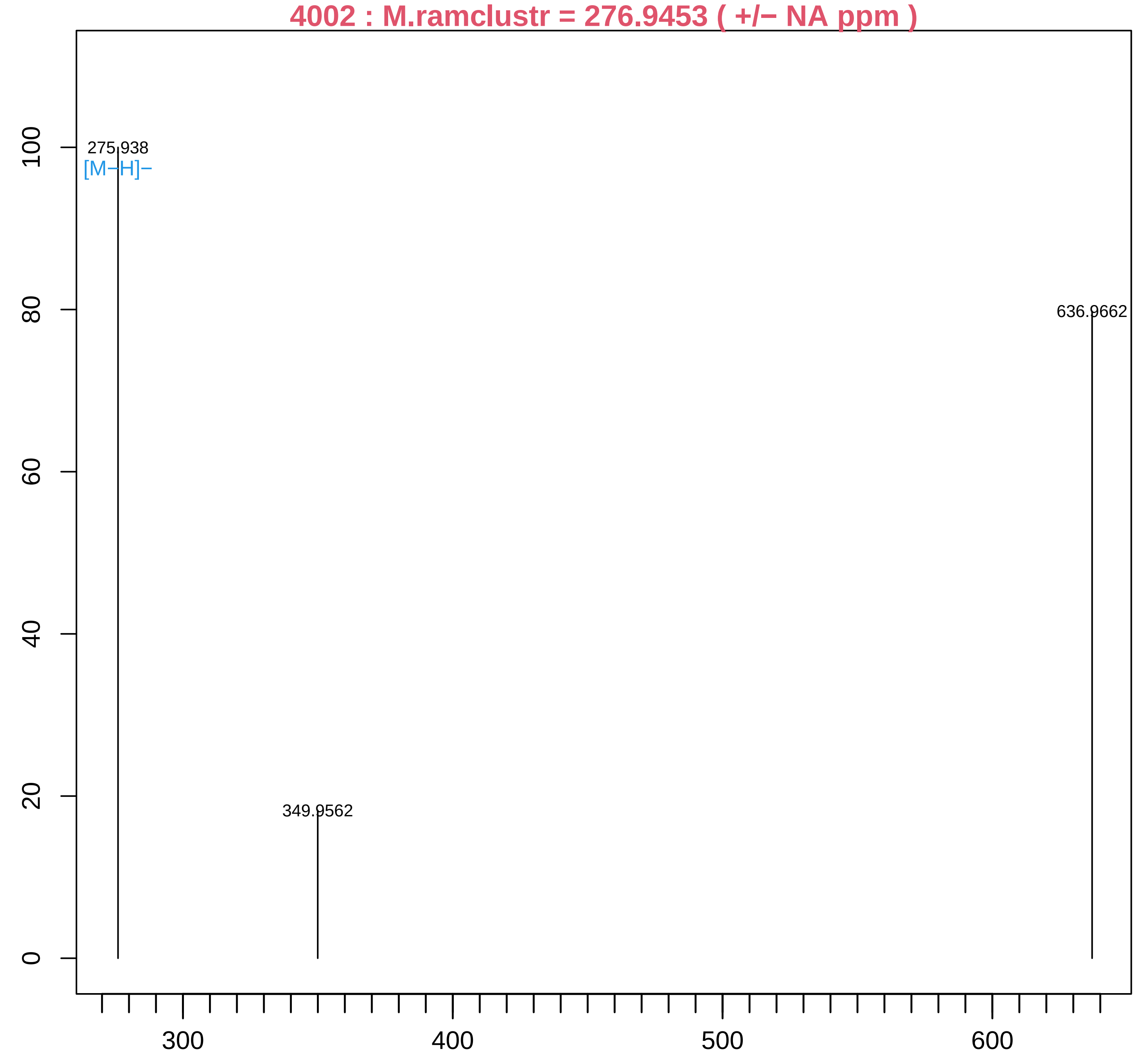
4002 : M.ramclustr = 276.9453 (+/− NA ppm)
275.938
[M−H]−
349.9562
636.9662
0
20
40
60
80
100
300
400
500
600

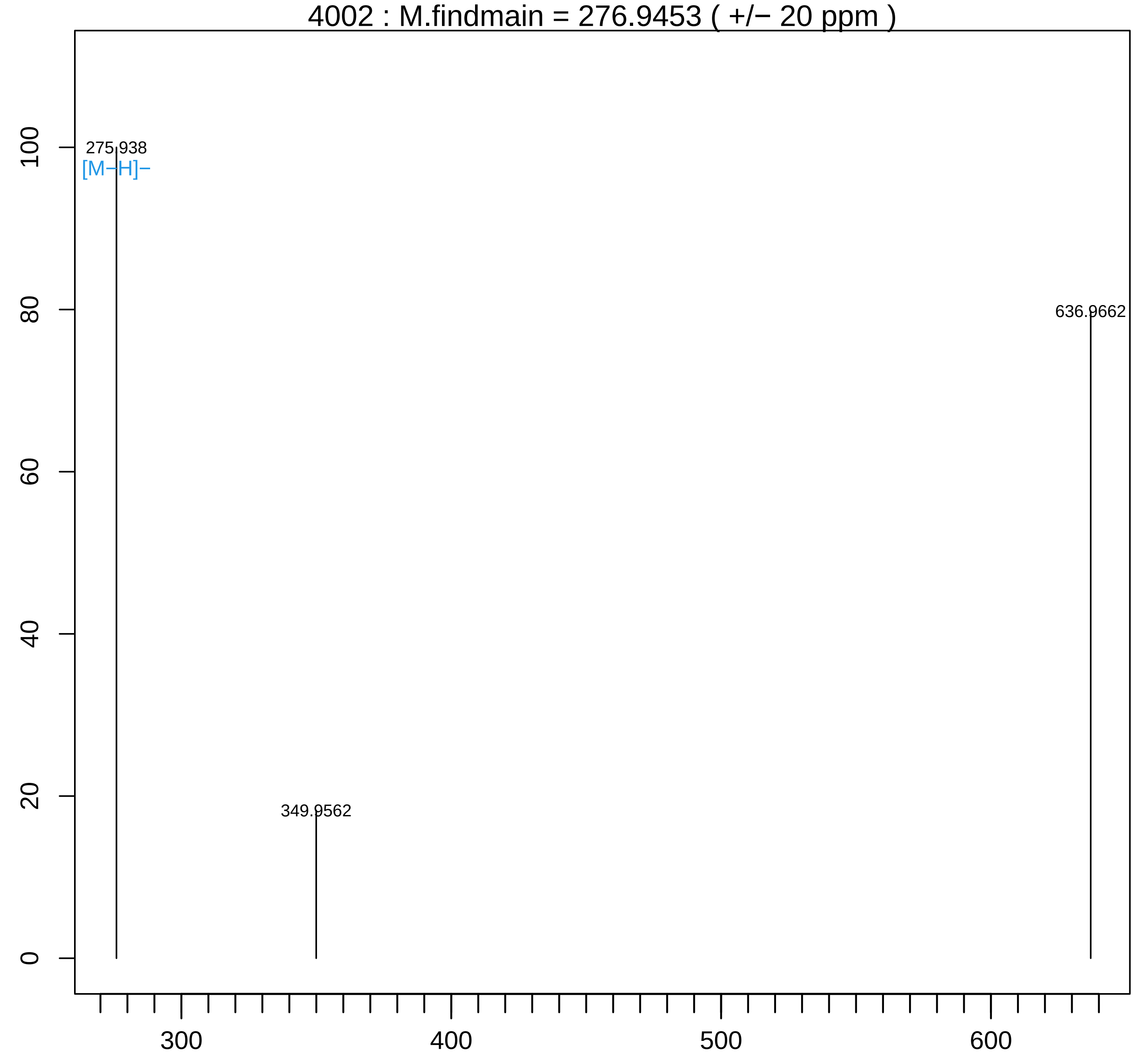
4002 : M.findmain = 276.9453 (+/− 20 ppm)
275.938
[M−H]−
349.9562
636.9662
0
20
40
60
80
100
300
400
500
600

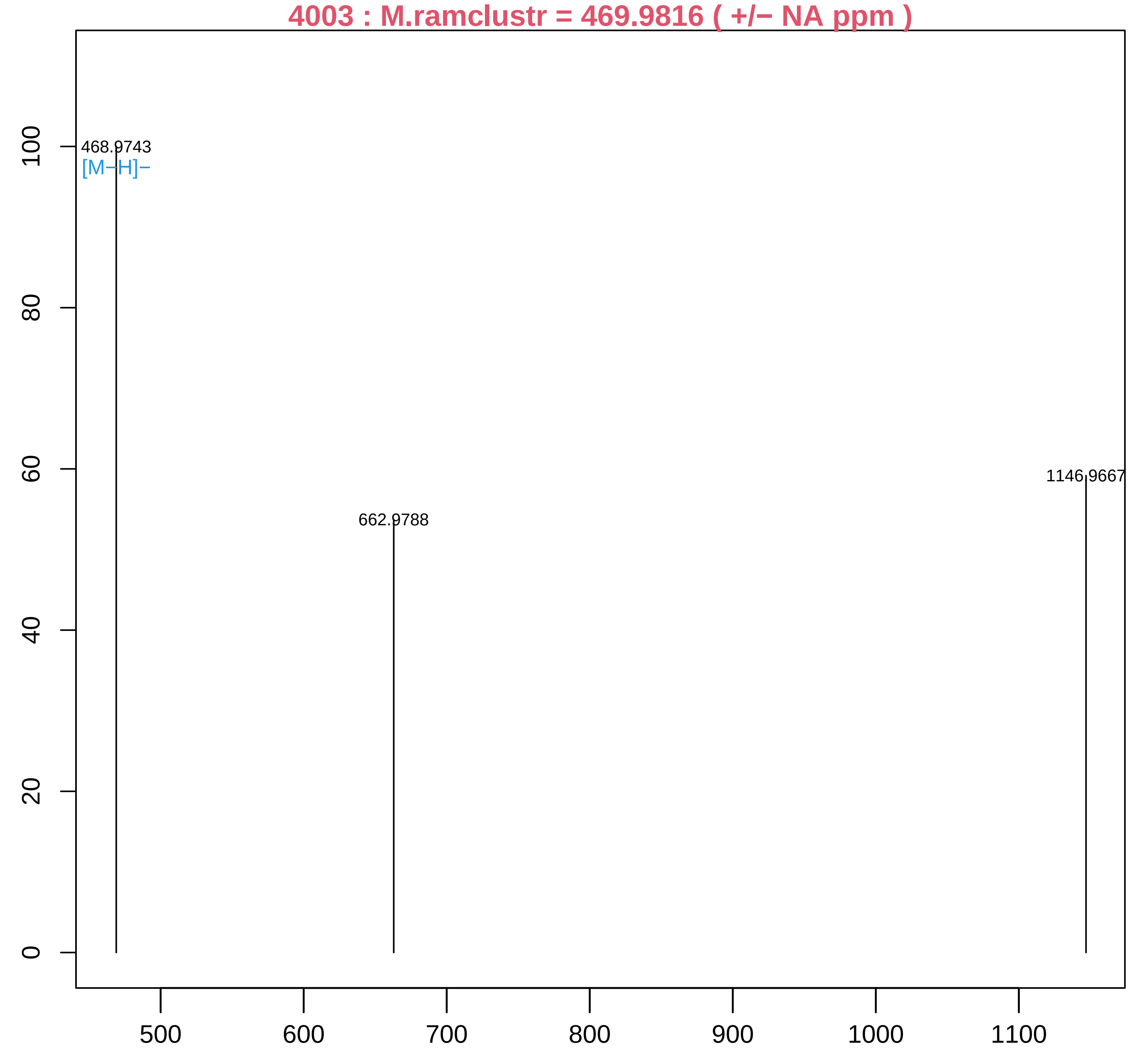
4003 : M.ramclustr = 469.9816 (+/− NA ppm)
468.9743
[M−H]−
662.9788
1146.9667
0
20
40
60
80
100
500
600
700
800
900
1000
1100

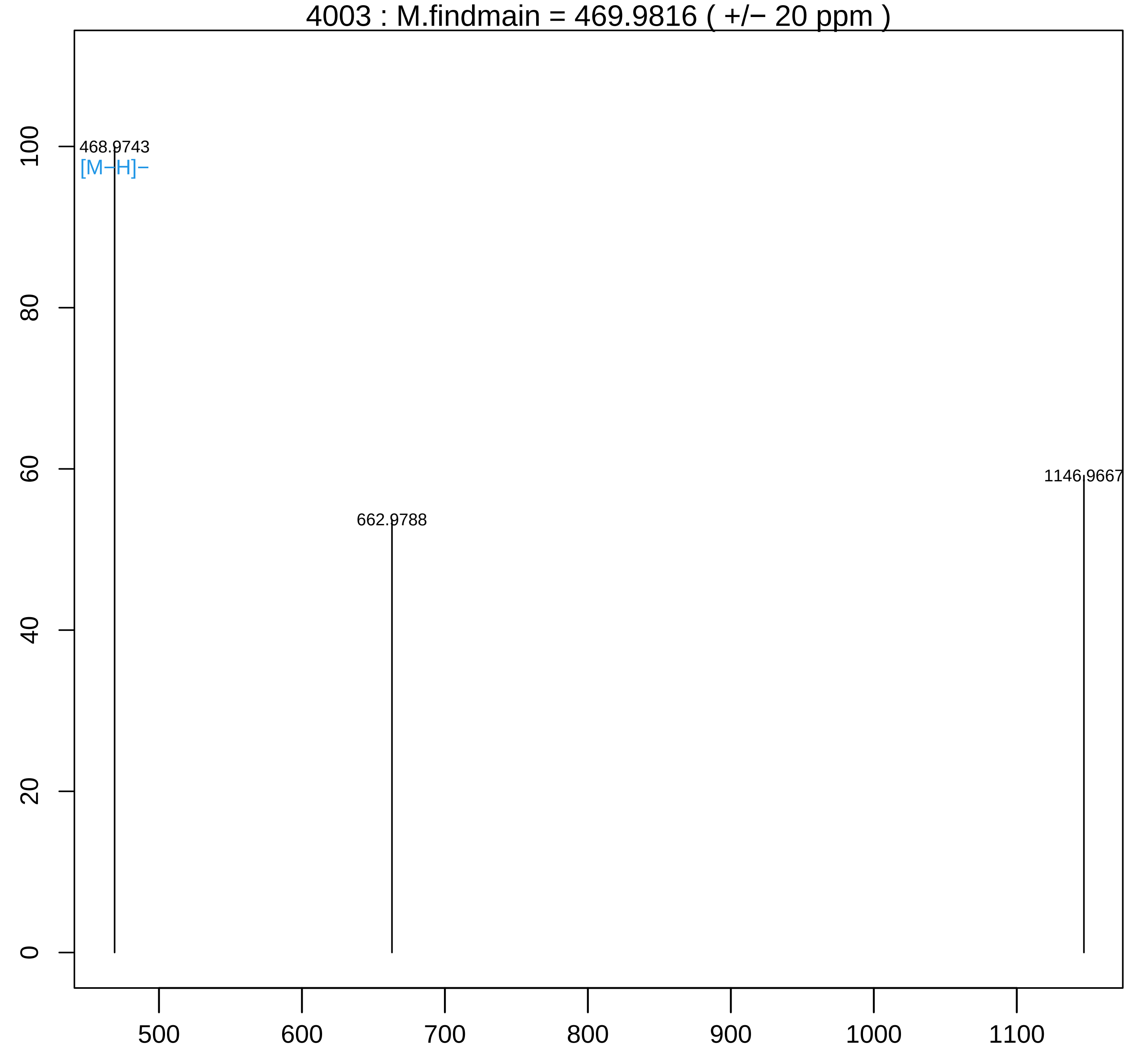
4003 : M.findmain = 469.9816 (+/− 20 ppm)
468.9743
[M−H]−
662.9788
1146.9667
0
20
40
60
80
100
500
600
700
800
900
1000
1100

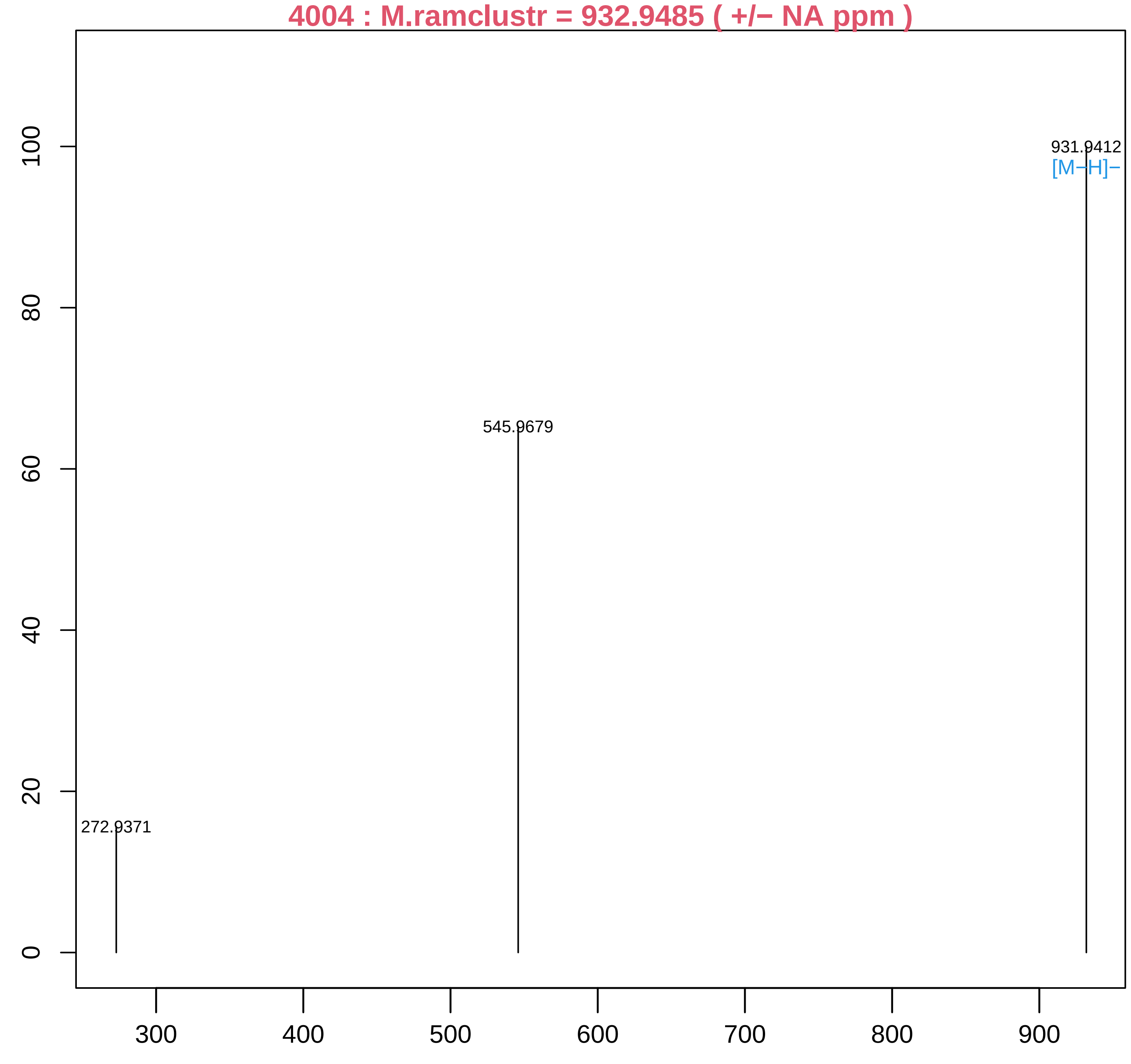
4004 : M.ramclustr = 932.9485 (+/− NA ppm)
931.9412
[M−H]−
545.9679
272.9371
0
20
40
60
80
100
300
400
500
600
700
800
900

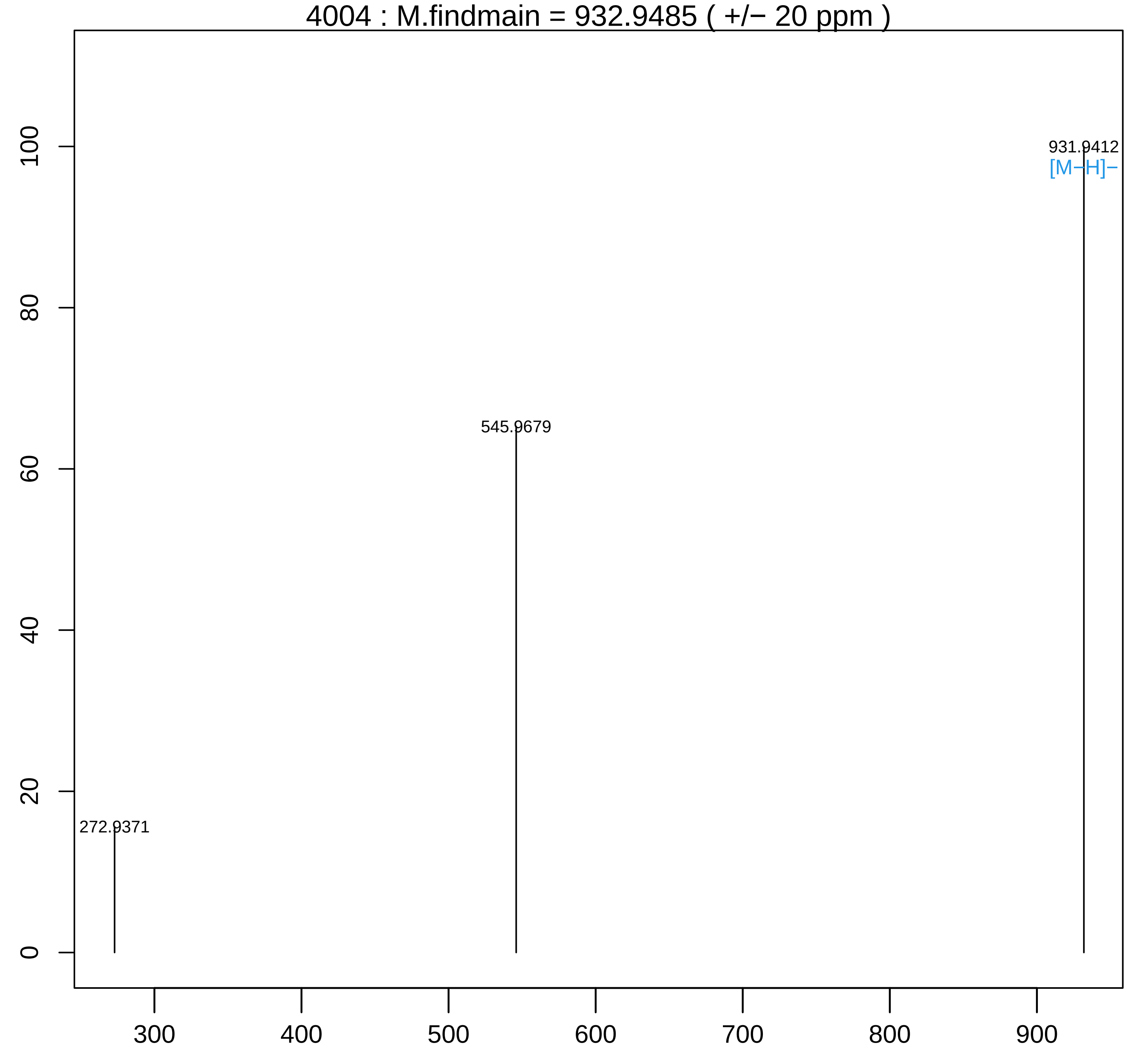
4004 : M.findmain = 932.9485 (+/− 20 ppm)
931.9412
[M−H]−
545.9679
272.9371
0
20
40
60
80
100
300
400
500
600
700
800
900

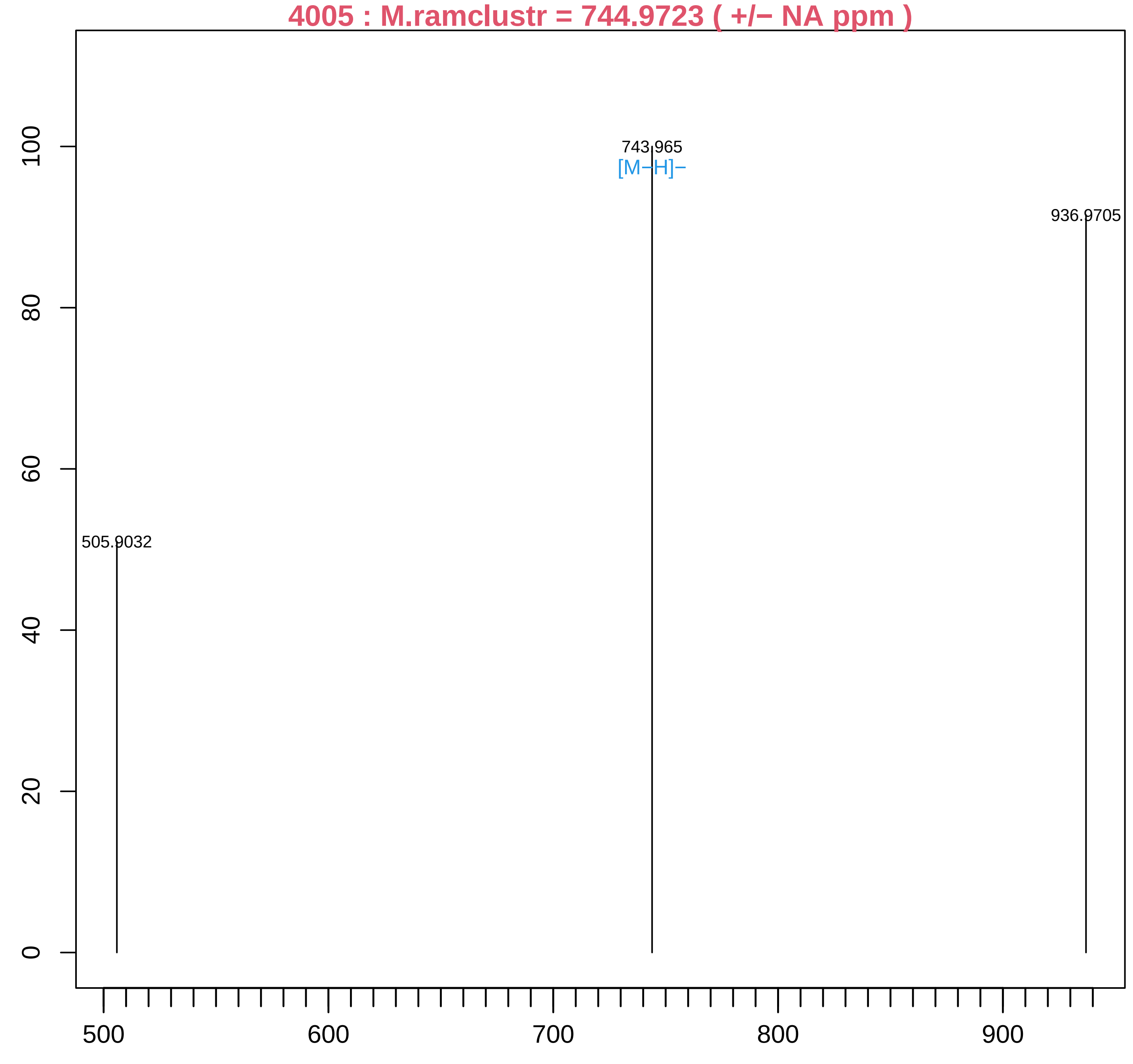
4005 : M.ramclustr = 744.9723 (+/− NA ppm)
743.965
[M−H]−
936.9705
505.9032
0
20
40
60
80
100
500
600
700
800
900

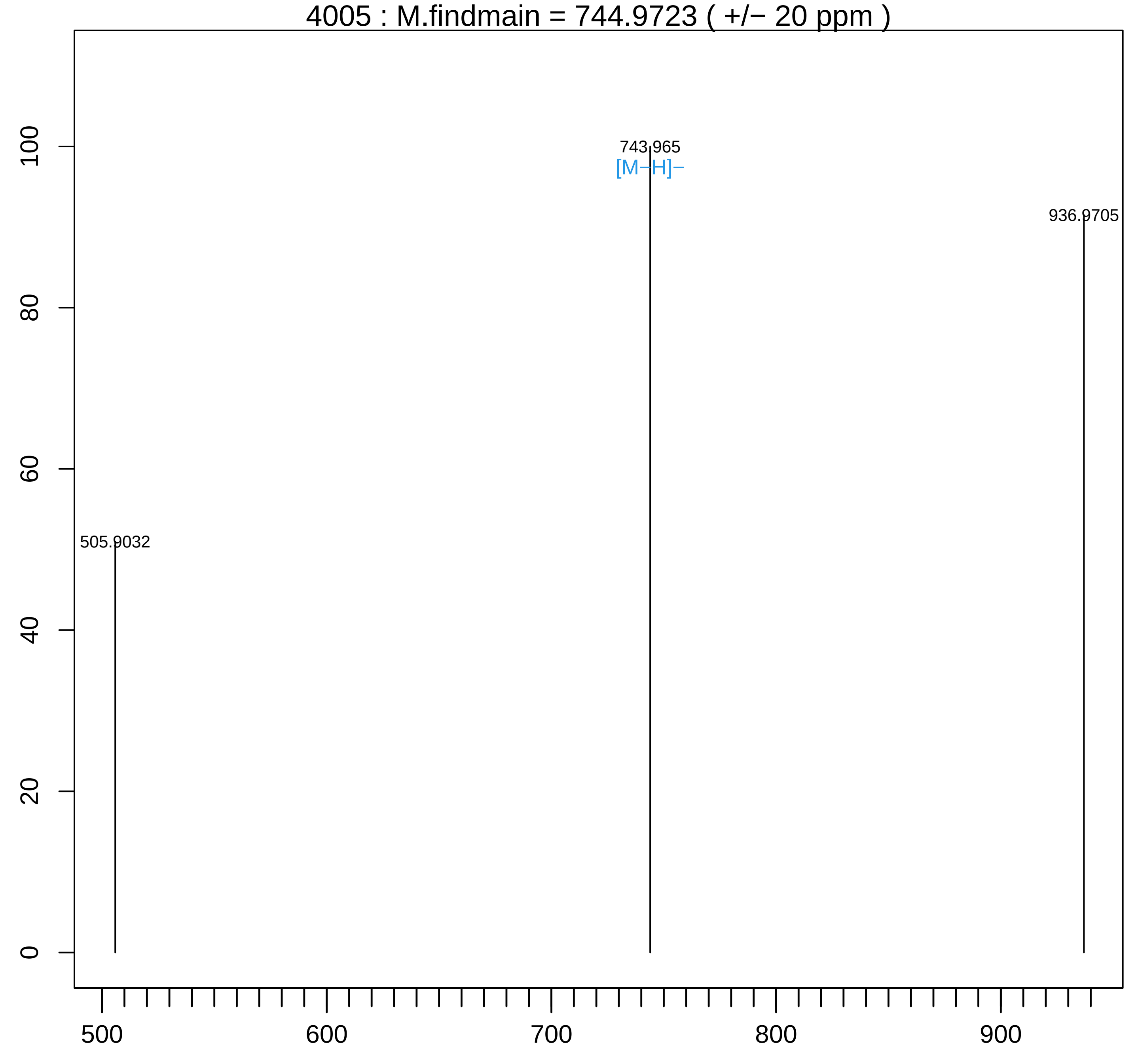
4005 : M.findmain = 744.9723 (+/− 20 ppm)
743.965
[M−H]−
936.9705
505.9032
0
20
40
60
80
100
500
600
700
800
900

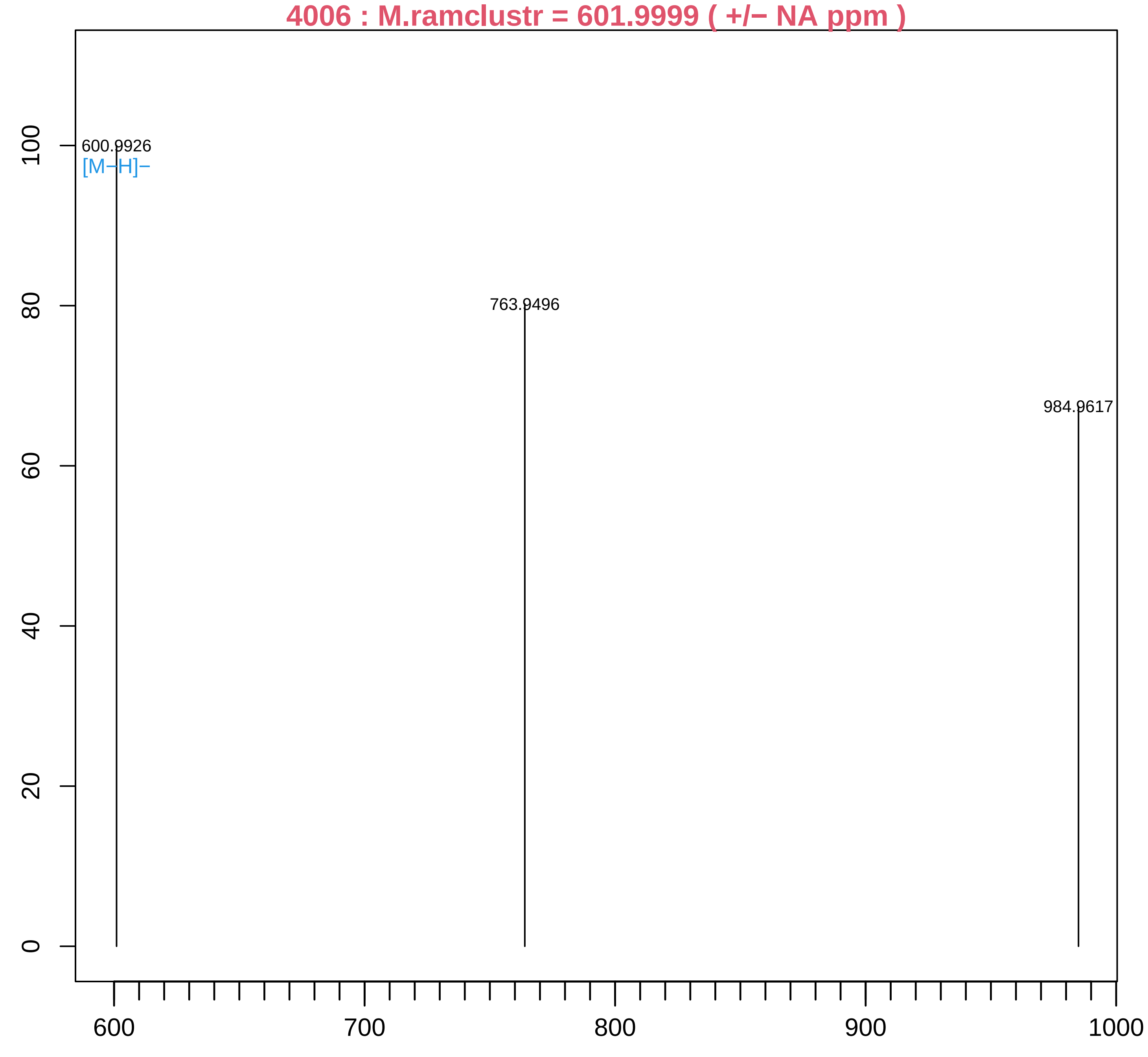
4006 : M.ramclustr = 601.9999 (+/− NA ppm)
600.9926
[M−H]−
763.9496
984.9617
0
20
40
60
80
100
600
700
800
900
1000

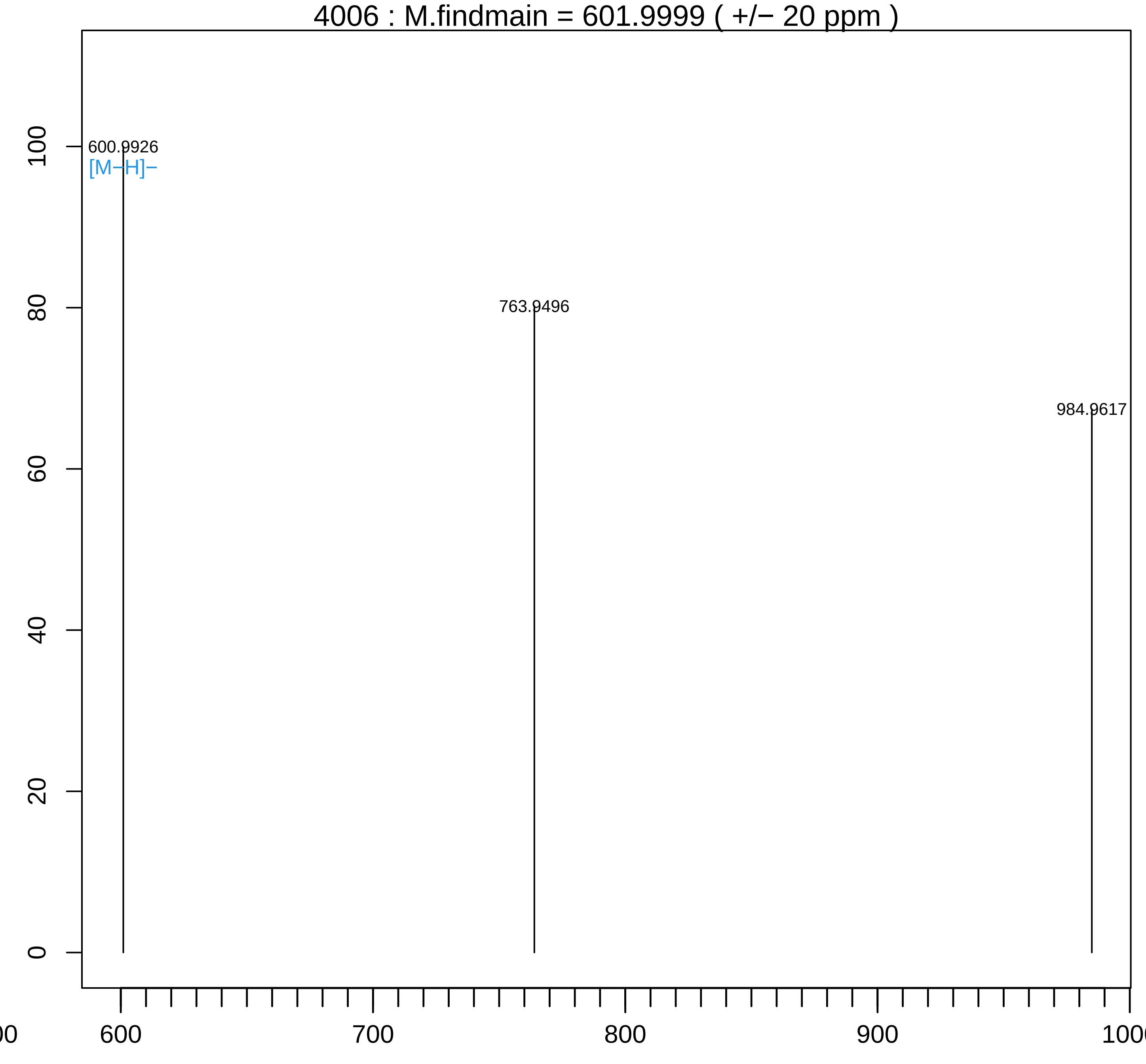
4006 : M.findmain = 601.9999 (+/− 20 ppm)
600.9926
[M−H]−
763.9496
984.9617
0
20
40
60
80
100
600
700
800
900
1000

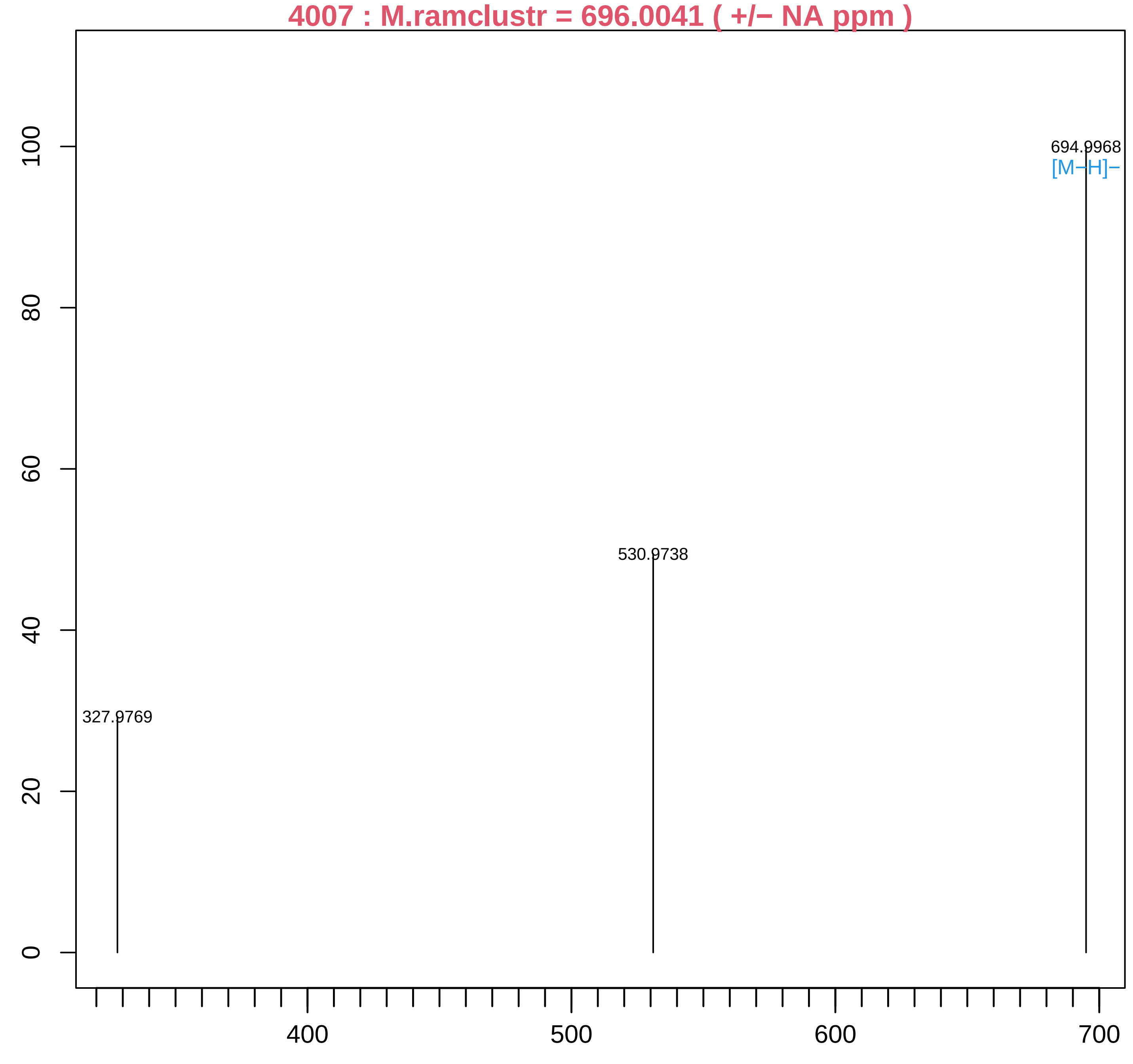
4007 : M.ramclustr = 696.0041 (+/− NA ppm)
694.9968
[M−H]−
530.9738
327.9769
0
20
40
60
80
100
400
500
600
700

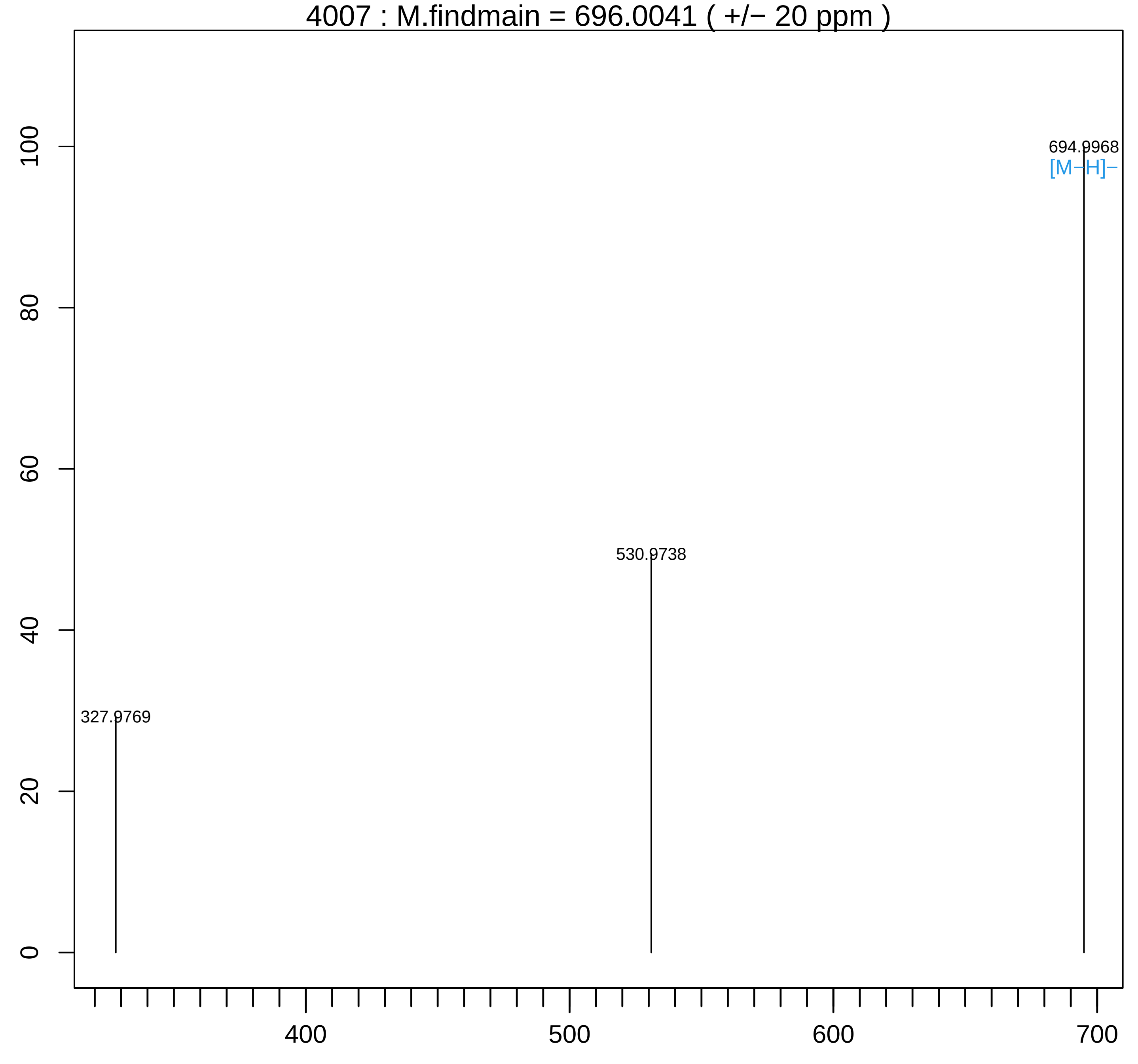
4007 : M.findmain = 696.0041 (+/− 20 ppm)
694.9968
[M−H]−
530.9738
327.9769
0
20
40
60
80
100
400
500
600
700

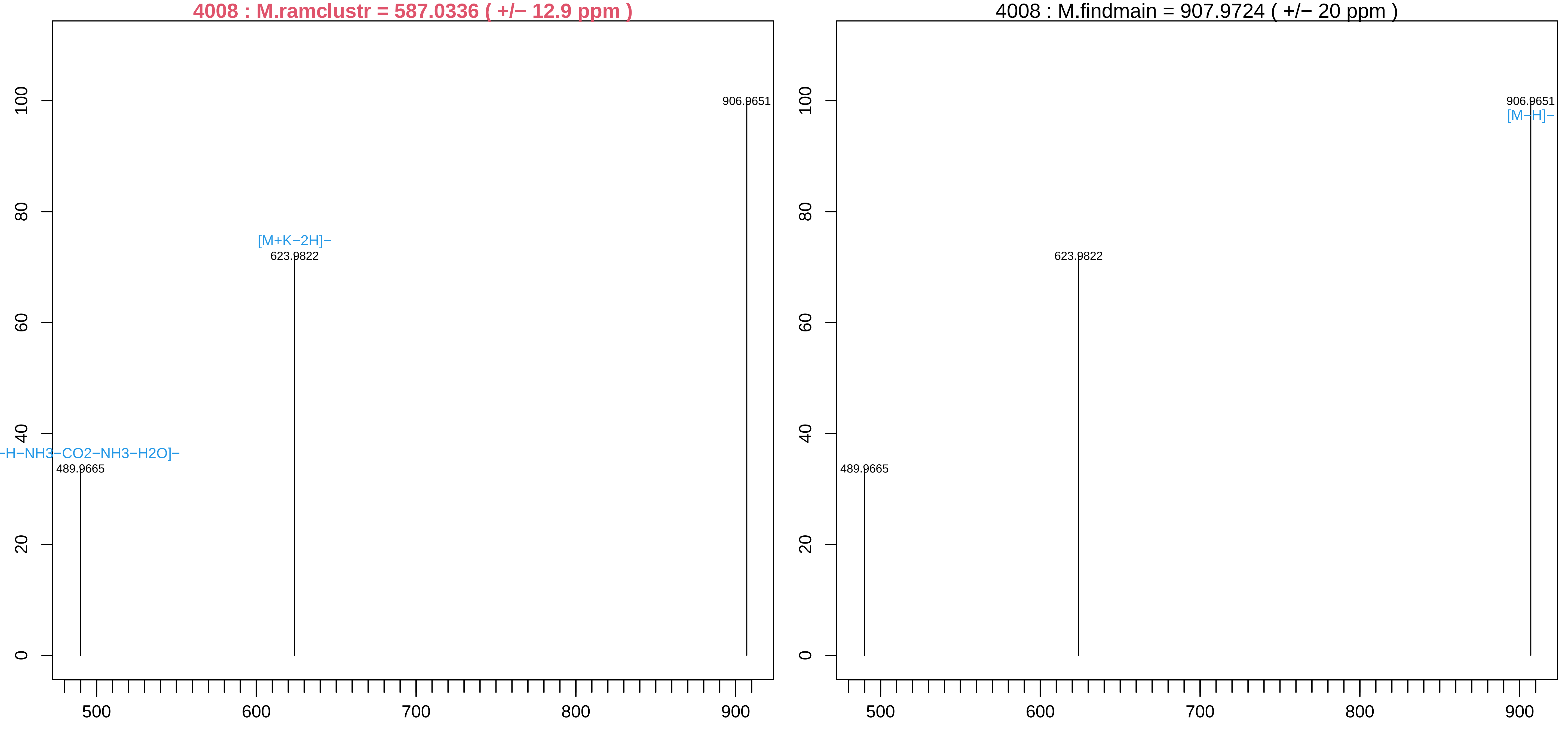

4008 : M.ramclustr = 587.0336 (+/− 12.9 ppm)
906.9651
[M+K−2H]−
623.9822
−H−NH3−CO2−NH3−H2O]−
489.9665
0
20
40
60
80
100
500
600
700
800
900
4008 : M.findmain = 907.9724 (+/− 20 ppm)
906.9651
[M−H]−
623.9822
489.9665
0
20
40
60
80
100
500
600
700
800
900

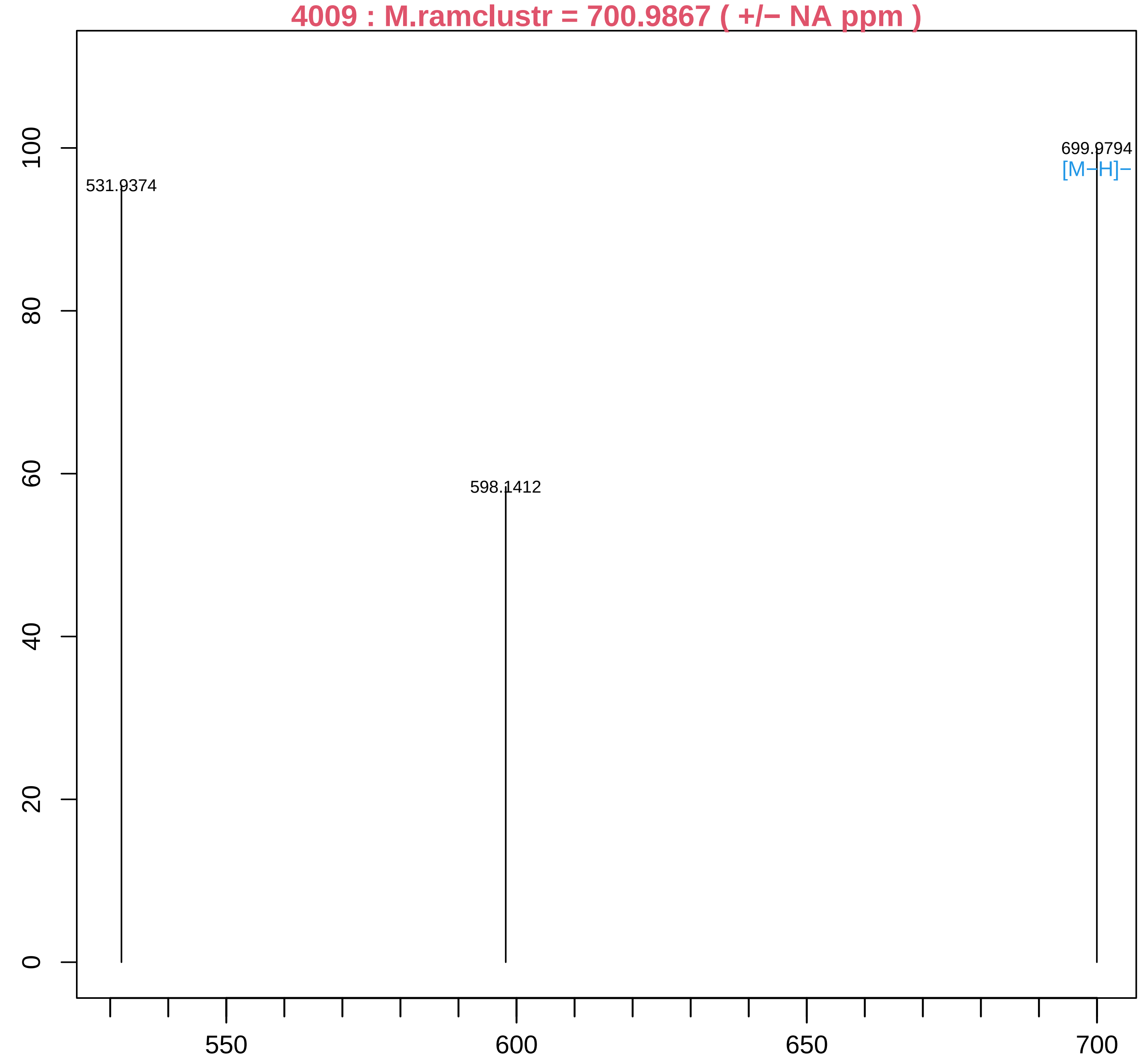
4009 : M.ramclustr = 700.9867 (+/− NA ppm)
531.9374
598.1412
699.9794
[M−H]−
0
20
40
60
80
100
550
600
650
700

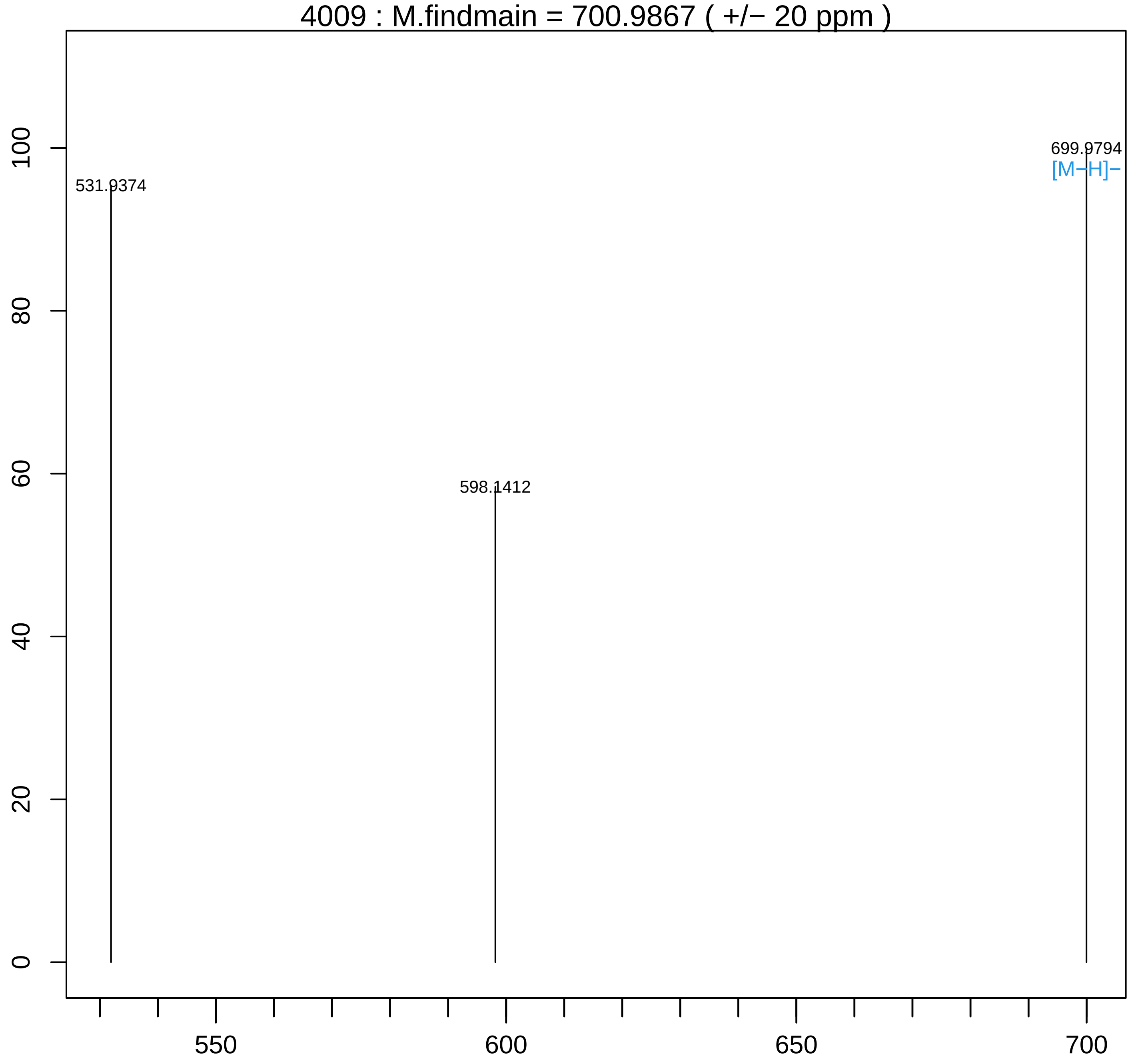
4009 : M.findmain = 700.9867 (+/− 20 ppm)
531.9374
598.1412
699.9794
[M−H]−
0
20
40
60
80
100
550
600
650
700

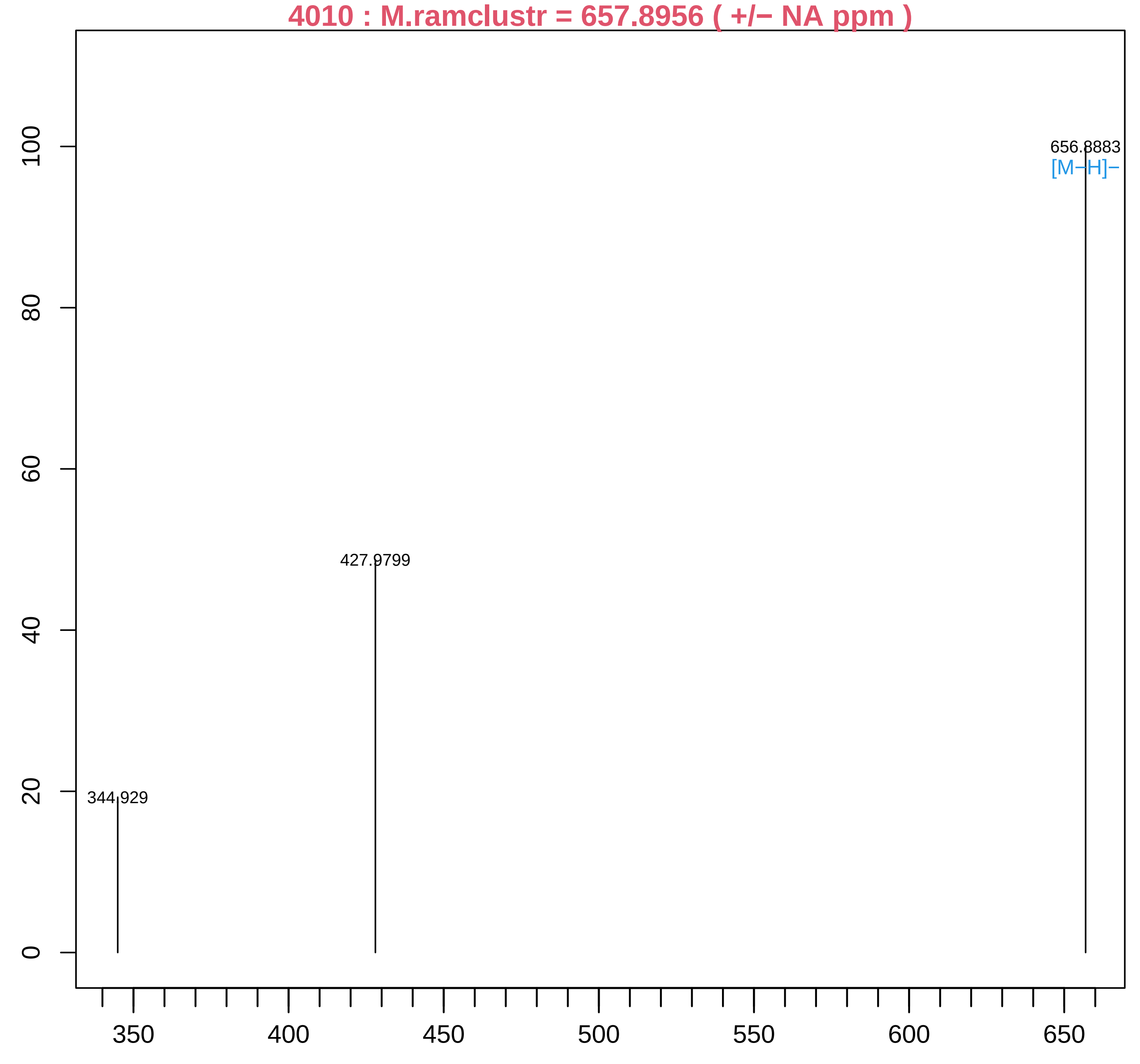
4010 : M.ramclustr = 657.8956 (+/− NA ppm)
656.8883
[M−H]−
427.9799
344.929
0
20
40
60
80
100
350
400
450
500
550
600
650
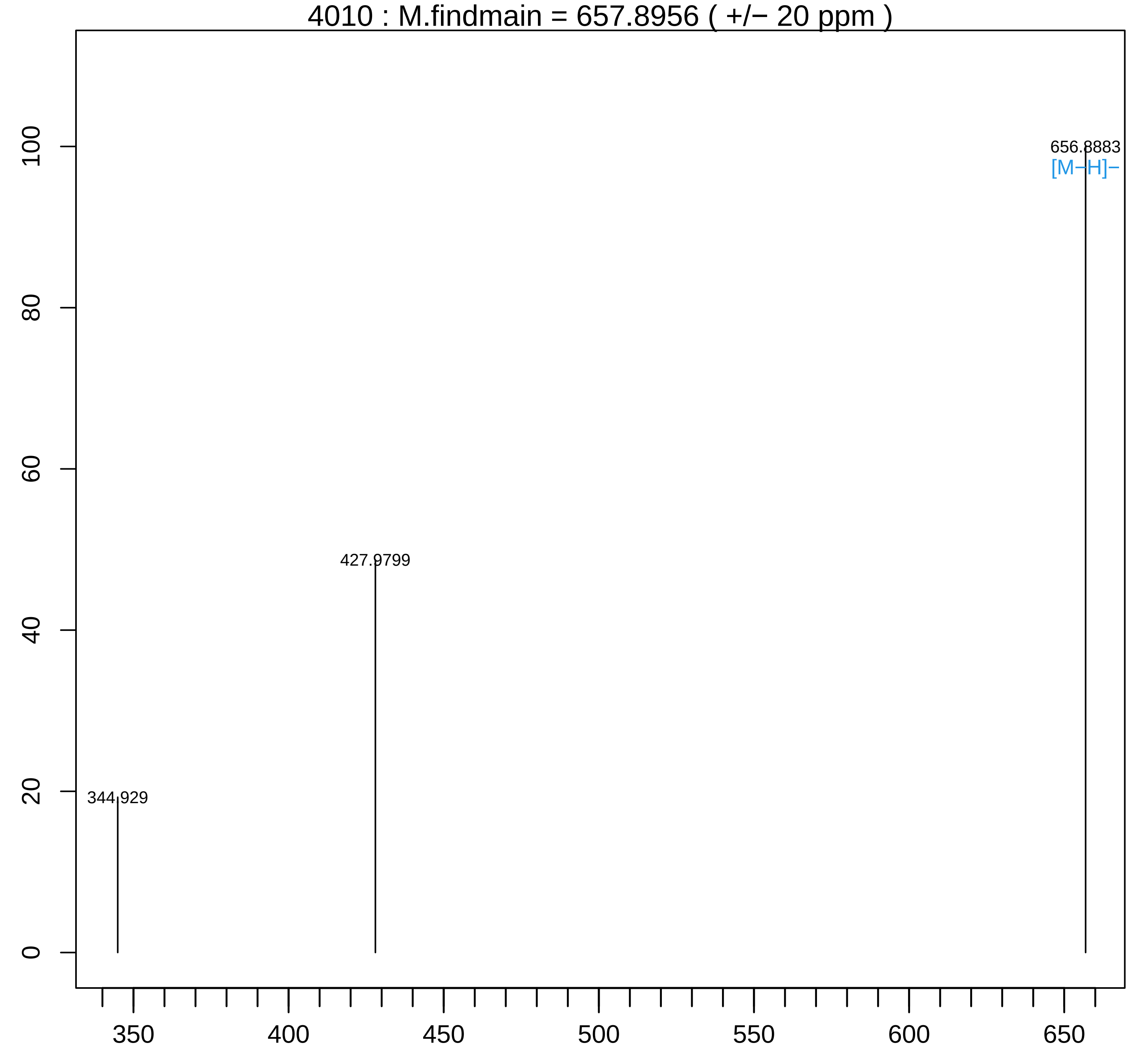
4010 : M.findmain = 657.8956 (+/− 20 ppm)
656.8883
[M−H]−
427.9799
344.929
0
20
40
60
80
100
350
400
450
500
550
600
650

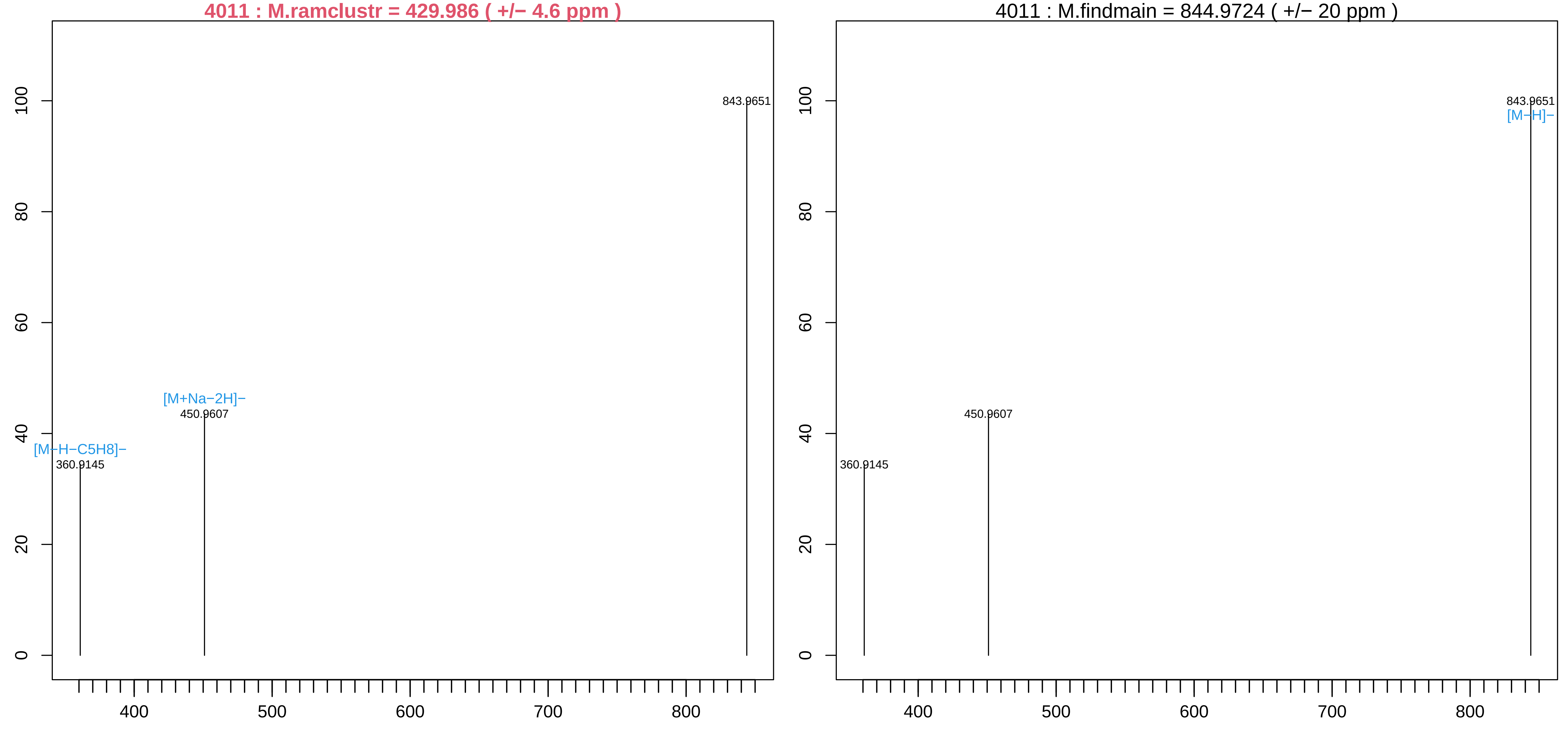
4011 : M.ramclustr = 429.986 (+/− 4.6 ppm)
843.9651
[M+Na−2H]−
450.9607
[M−H−C5H8]−
360.9145
0
20
40
60
80
100
400
500
600
700
800
4011 : M.findmain = 844.9724 (+/− 20 ppm)
843.9651
[M−H]−
450.9607
360.9145
0
20
40
60
80
100
400
500
600
700
800

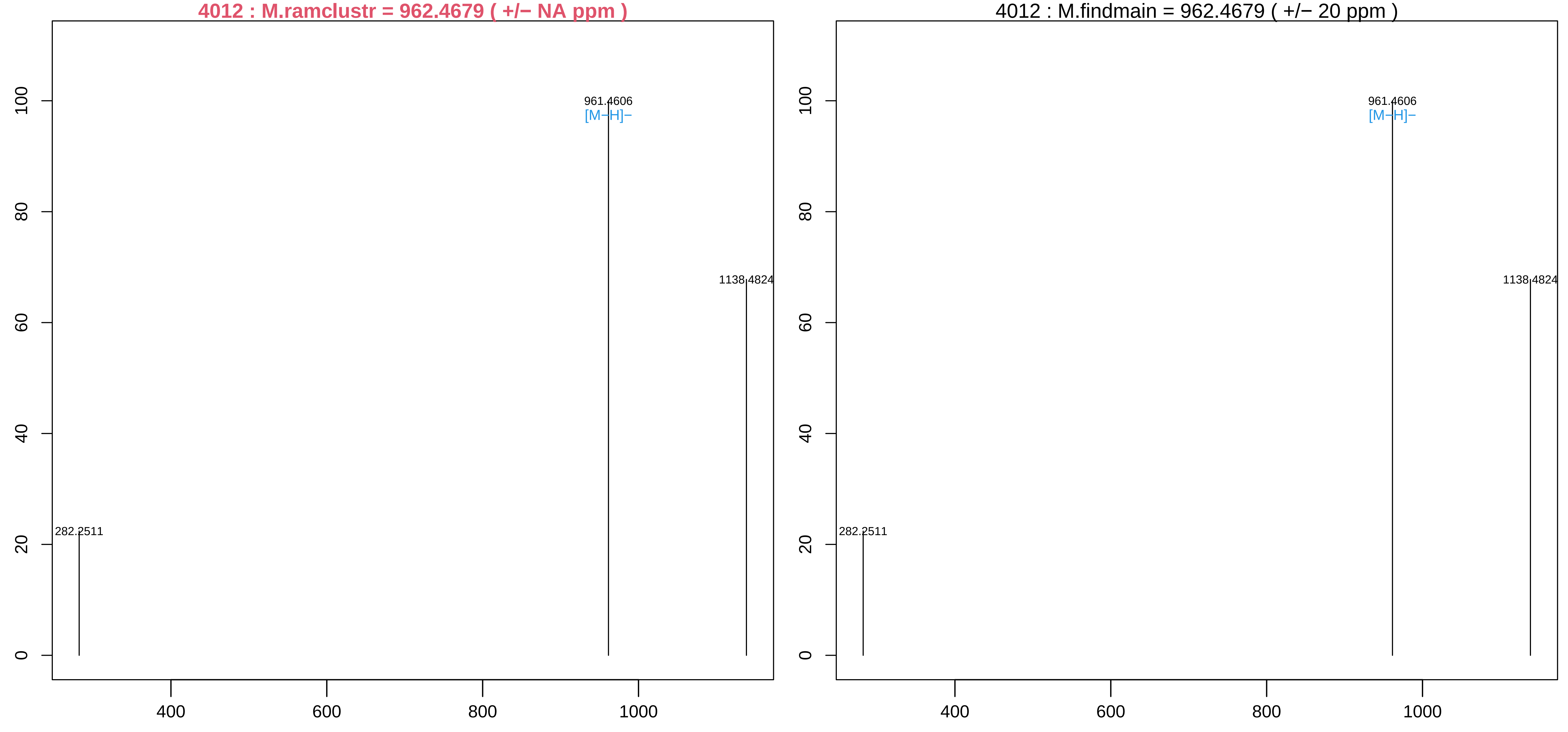
4012 : M.ramclustr = 962.4679 (+/− NA ppm)
961.4606
[M−H]−
1138.4824
282.2511
0
20
40
60
80
100
400
600
800
1000
4012 : M.findmain = 962.4679 (+/− 20 ppm)
961.4606
[M−H]−
1138.4824
282.2511
0
20
40
60
80
100
400
600
800
1000

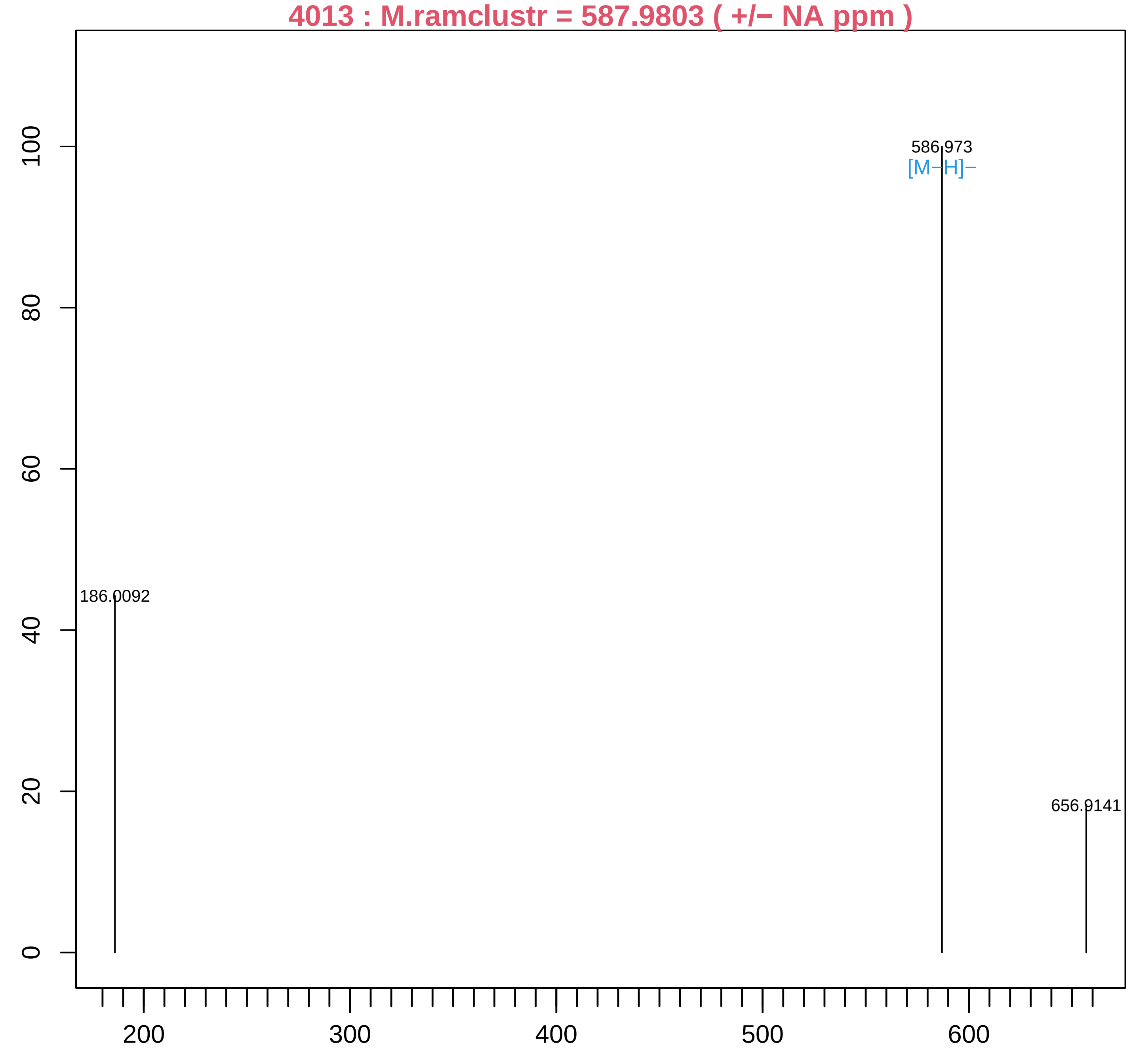
4013 : M.ramclustr = 587.9803 (+/− NA ppm)
186.0092
586.973
[M−H]−
656.9141
0
20
40
60
80
100
200
300
400
500
600
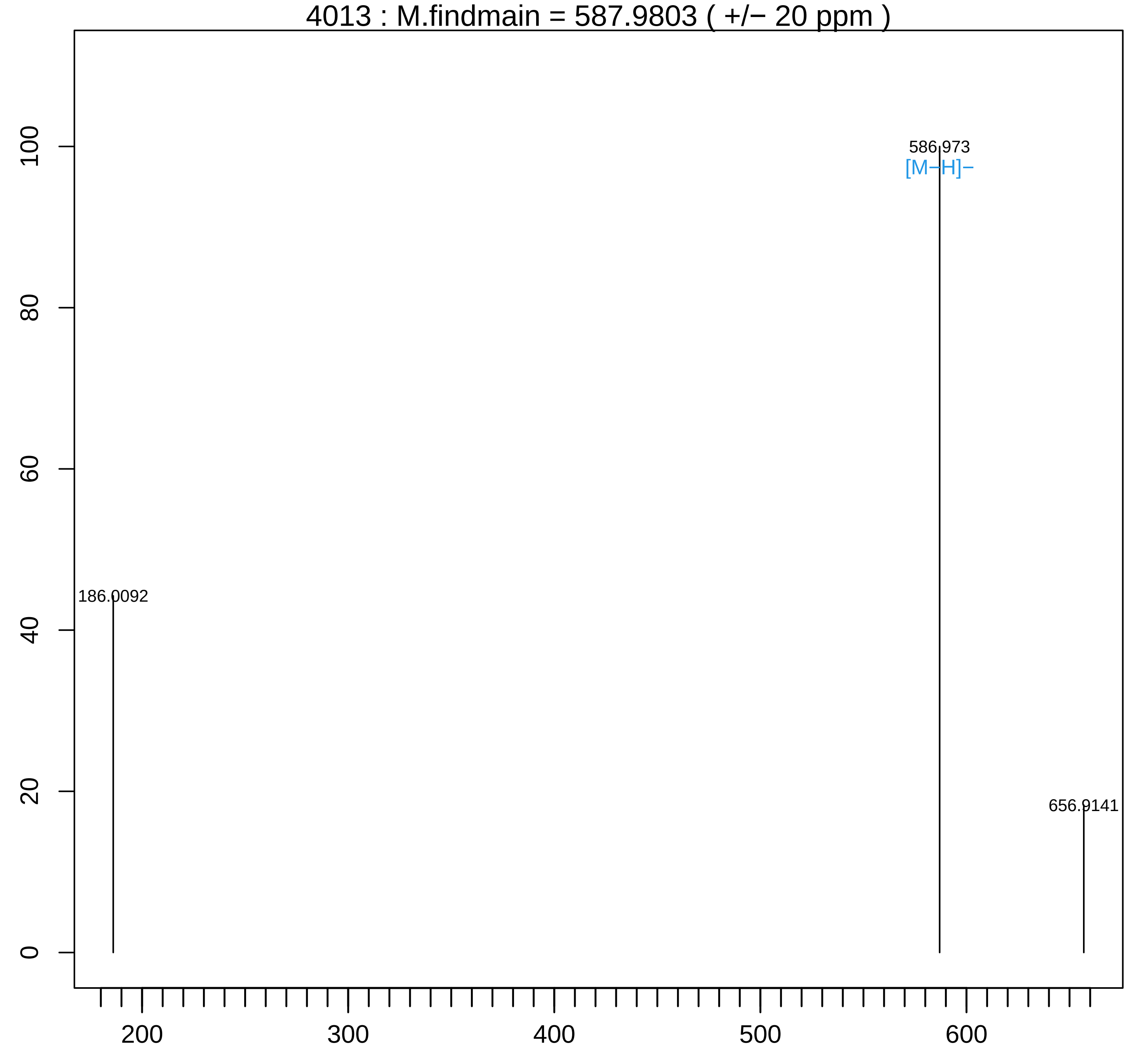
4013 : M.findmain = 587.9803 (+/− 20 ppm)
186.0092
586.973
[M−H]−
656.9141
0
20
40
60
80
100
200
300
400
500
600

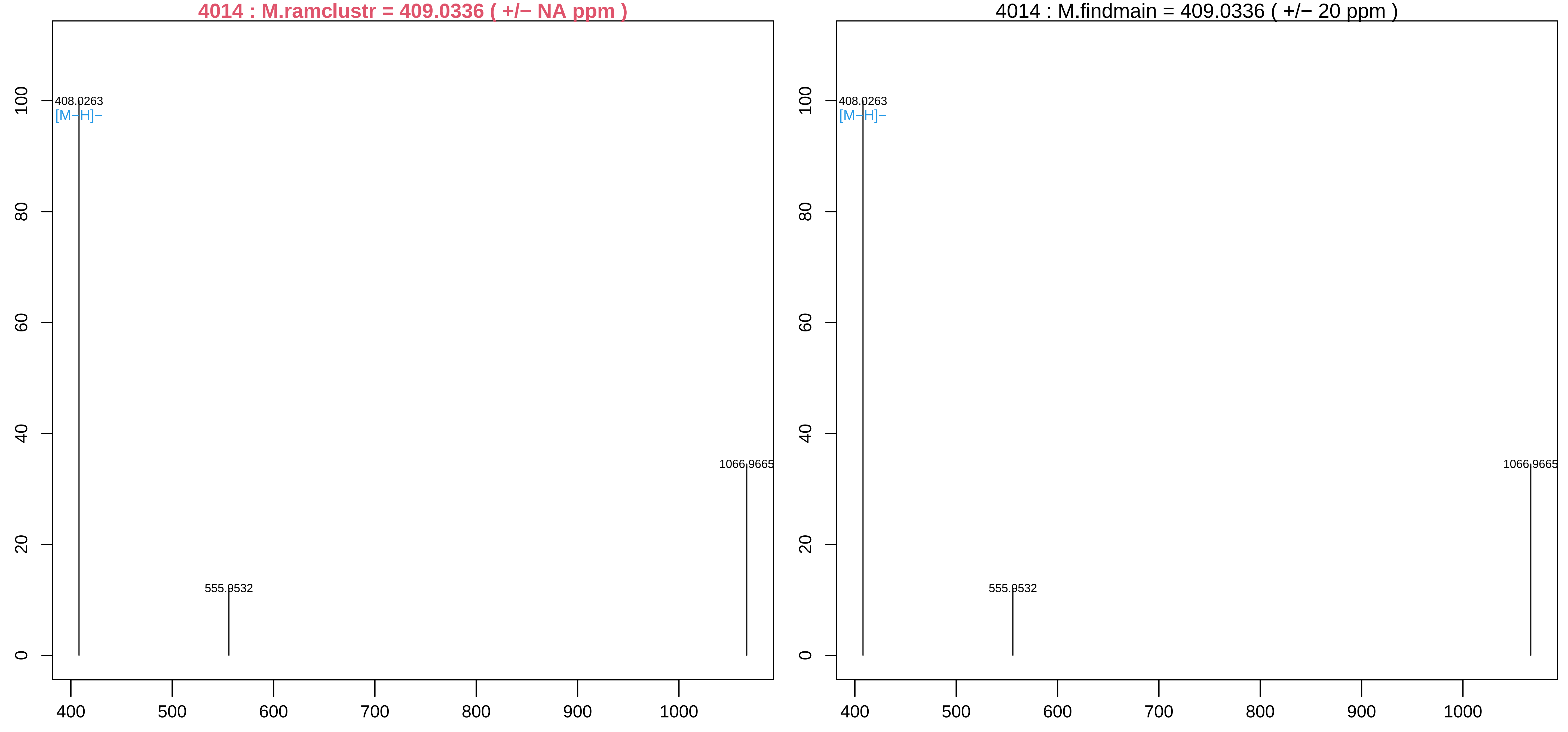
4014 : M.ramclustr = 409.0336 (+/− NA ppm)
408.0263
[M−H]−
555.9532
1066.9665
4014 : M.findmain = 409.0336 (+/− 20 ppm)
408.0263
[M−H]−
555.9532
1066.9665
0
20
40
60
80
100
400
500
600
700
800
900
1000

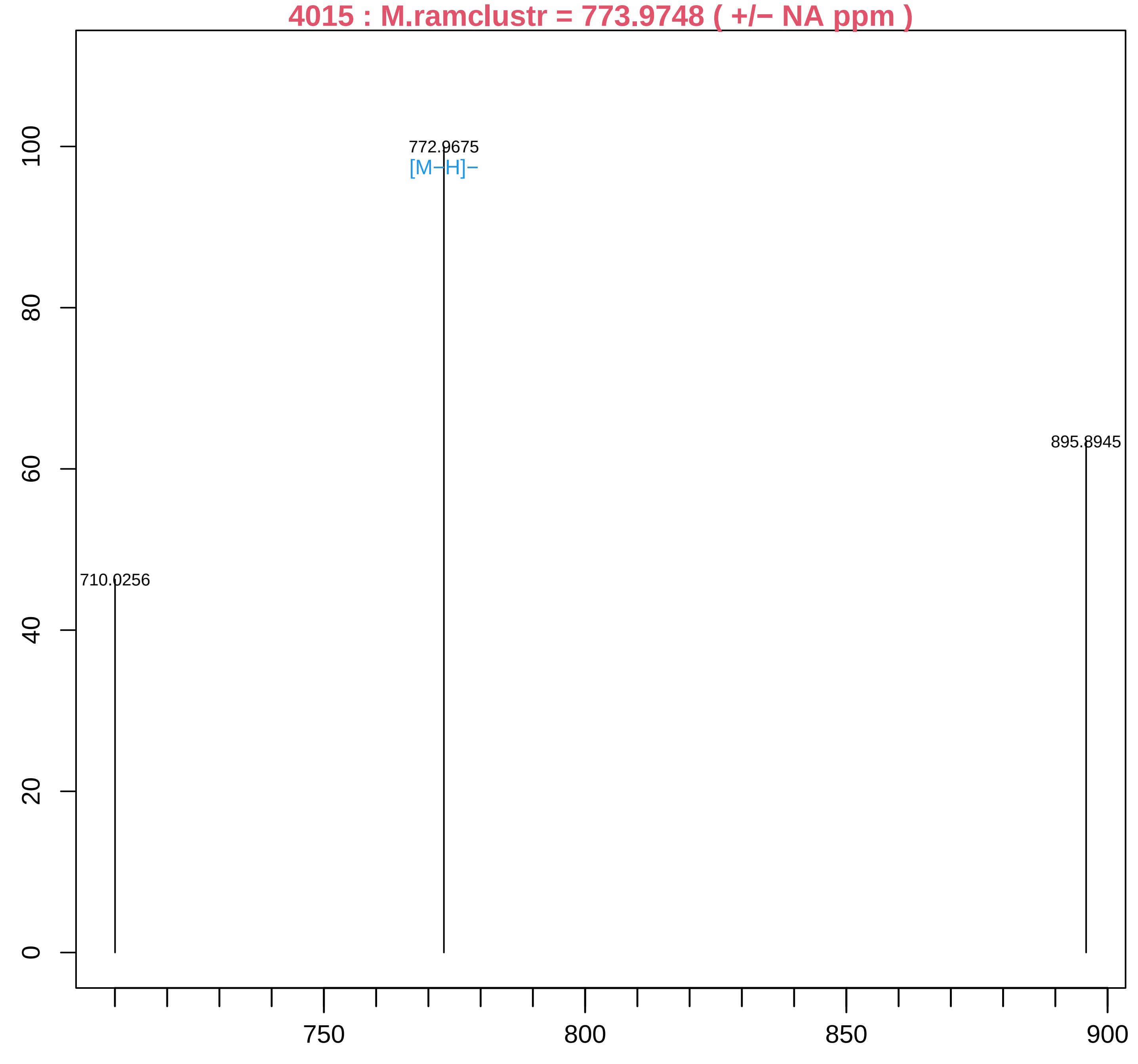
4015 : M.ramclustr = 773.9748 (+/− NA ppm)
772.9675
[M−H]−
895.8945
710.0256
0
20
40
60
80
100
750
800
850
900

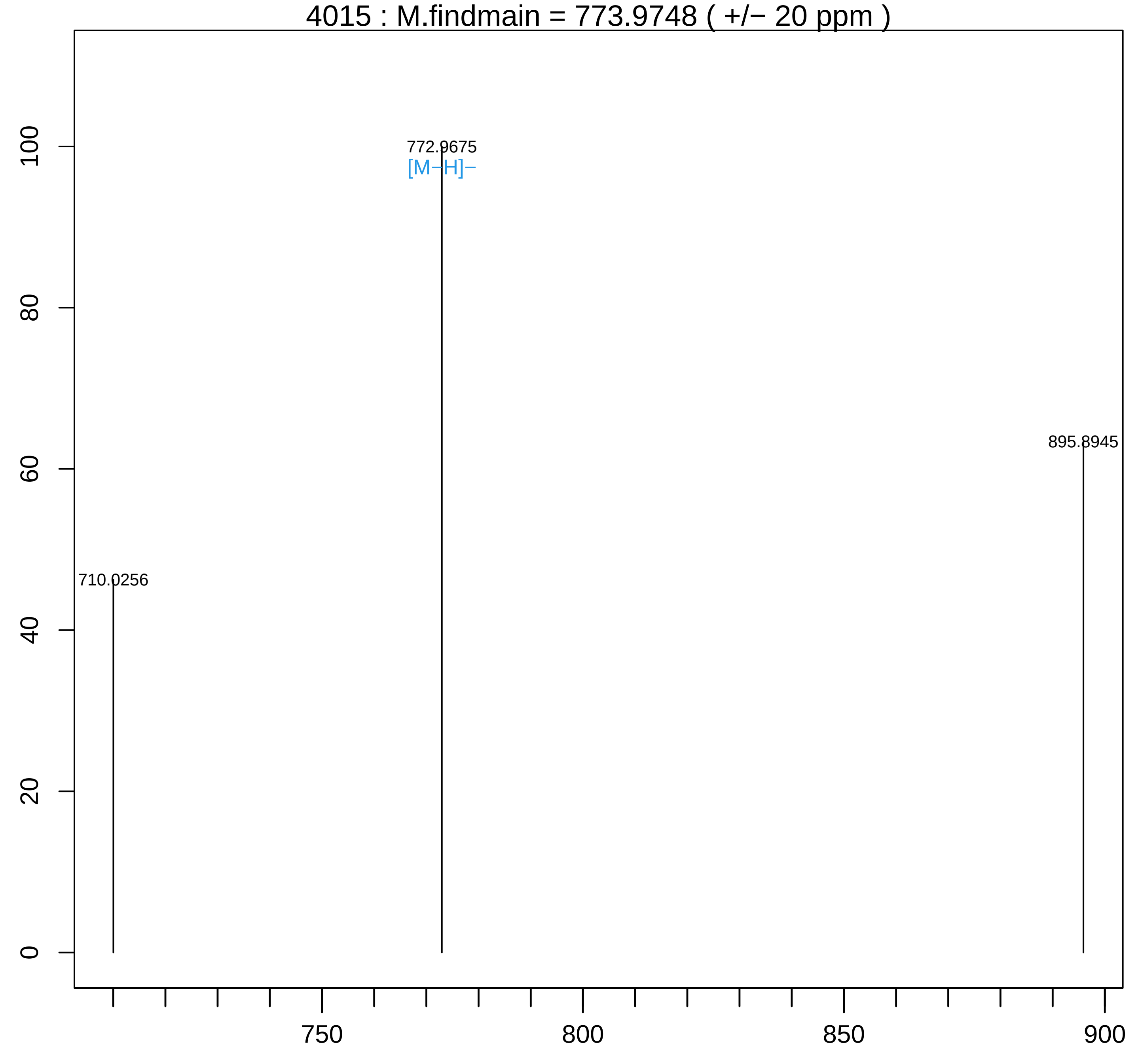
4015 : M.findmain = 773.9748 (+/− 20 ppm)
772.9675
[M−H]−
895.8945
710.0256
0
20
40
60
80
100
750
800
850
900

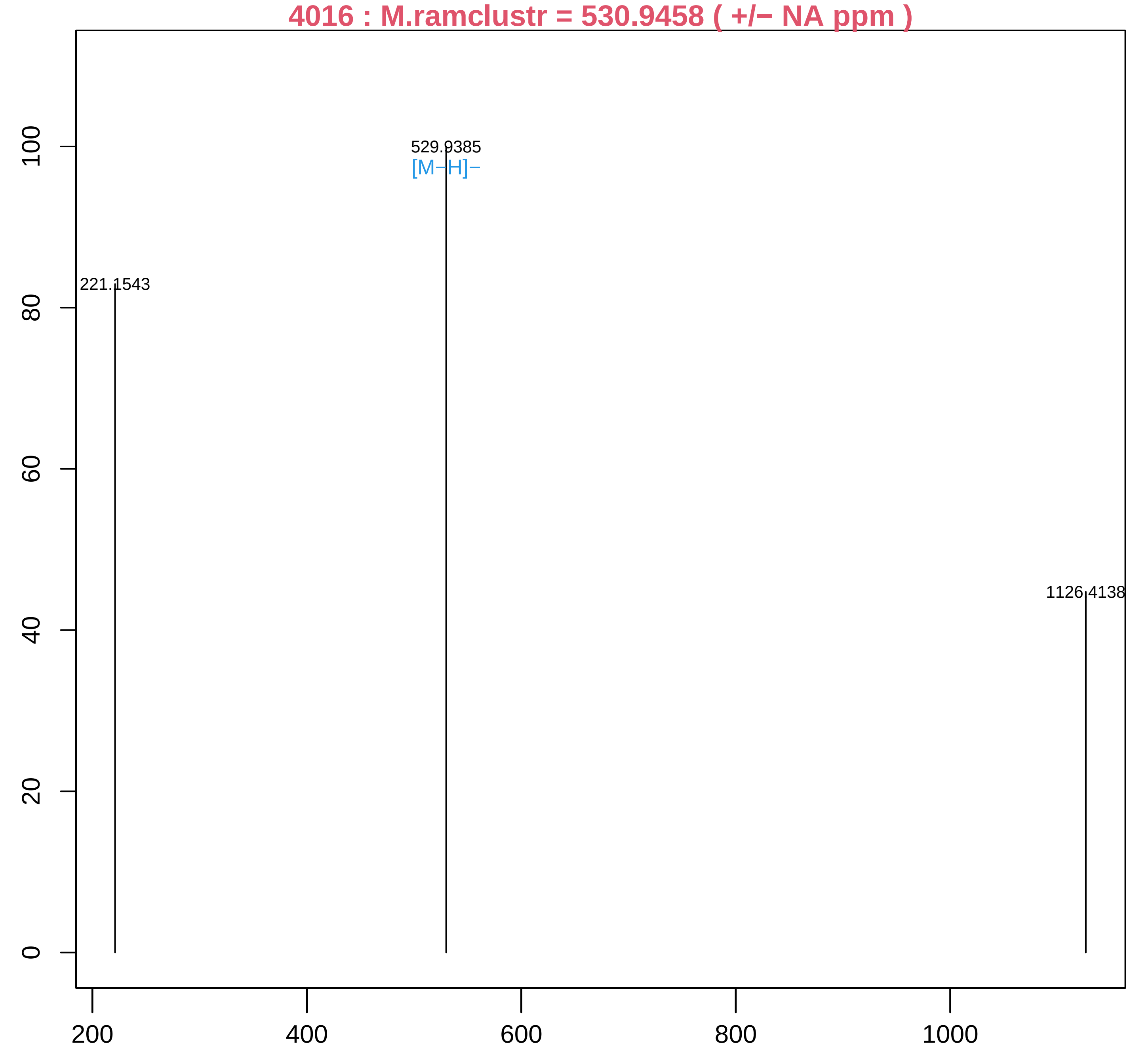

4016 : M.ramclustr = 530.9458 (+/− NA ppm)
529.9385
[M−H]−
221.1543
1126.4138
0
20
40
60
80
100
200
400
600
800
1000

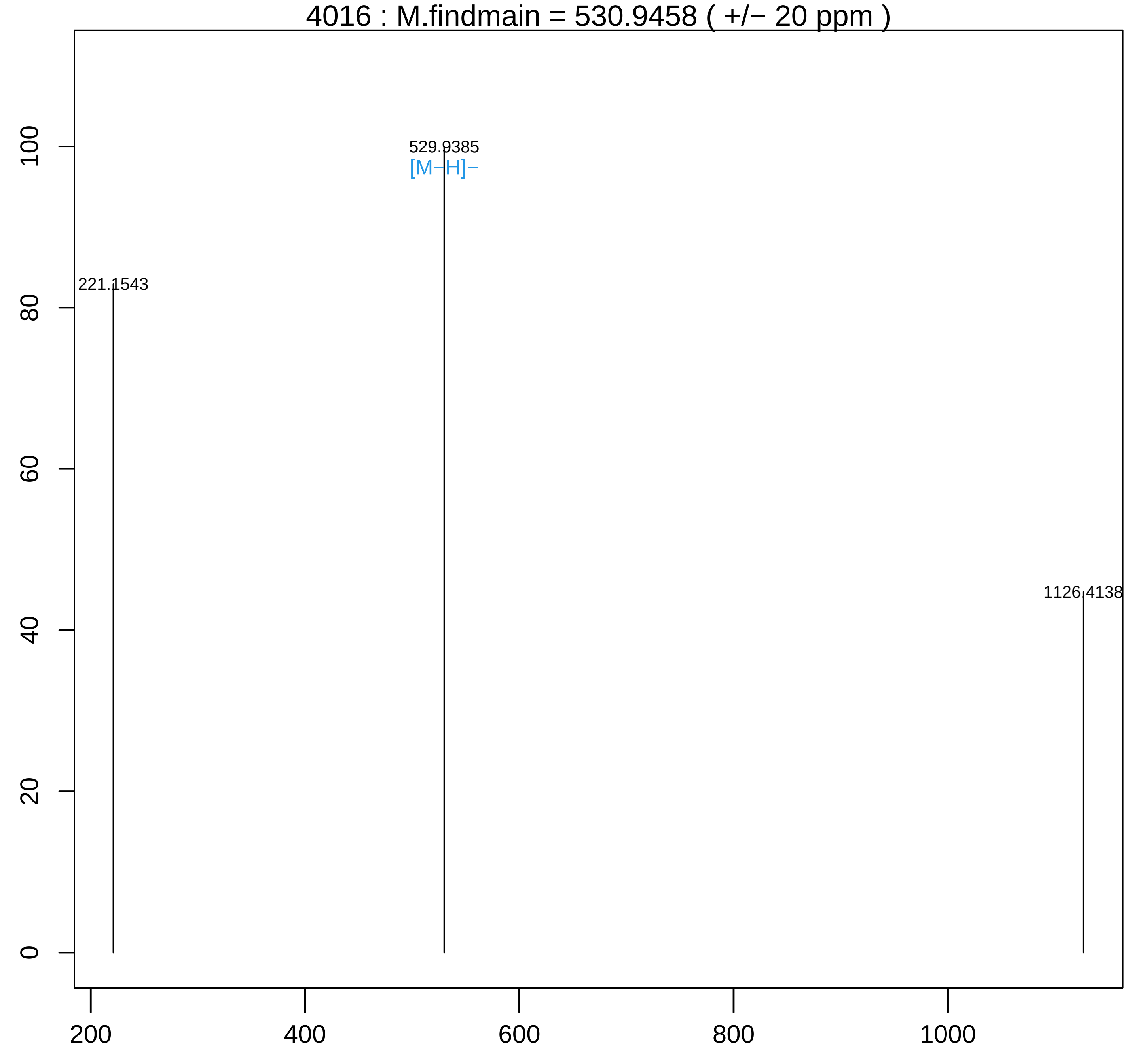

4016 : M.findmain = 530.9458 (+/− 20 ppm)
529.9385
[M−H]−
221.1543
1126.4138
0
20
40
60
80
100
200
400
600
800
1000

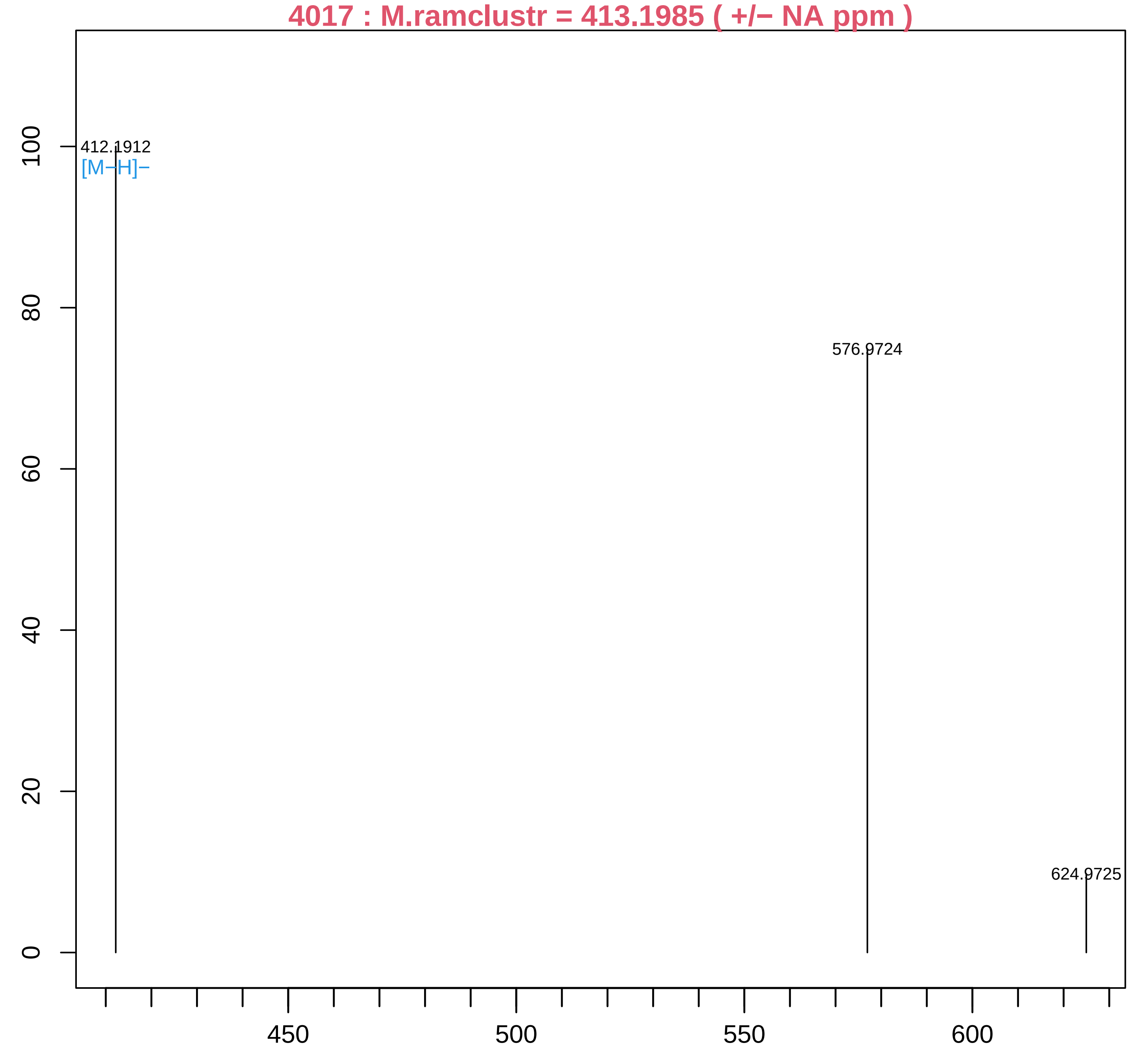
4017 : M.ramclustr = 413.1985 (+/− NA ppm)
412.1912
[M−H]−
576.9724
624.9725
0
20
40
60
80
100
450
500
550
600

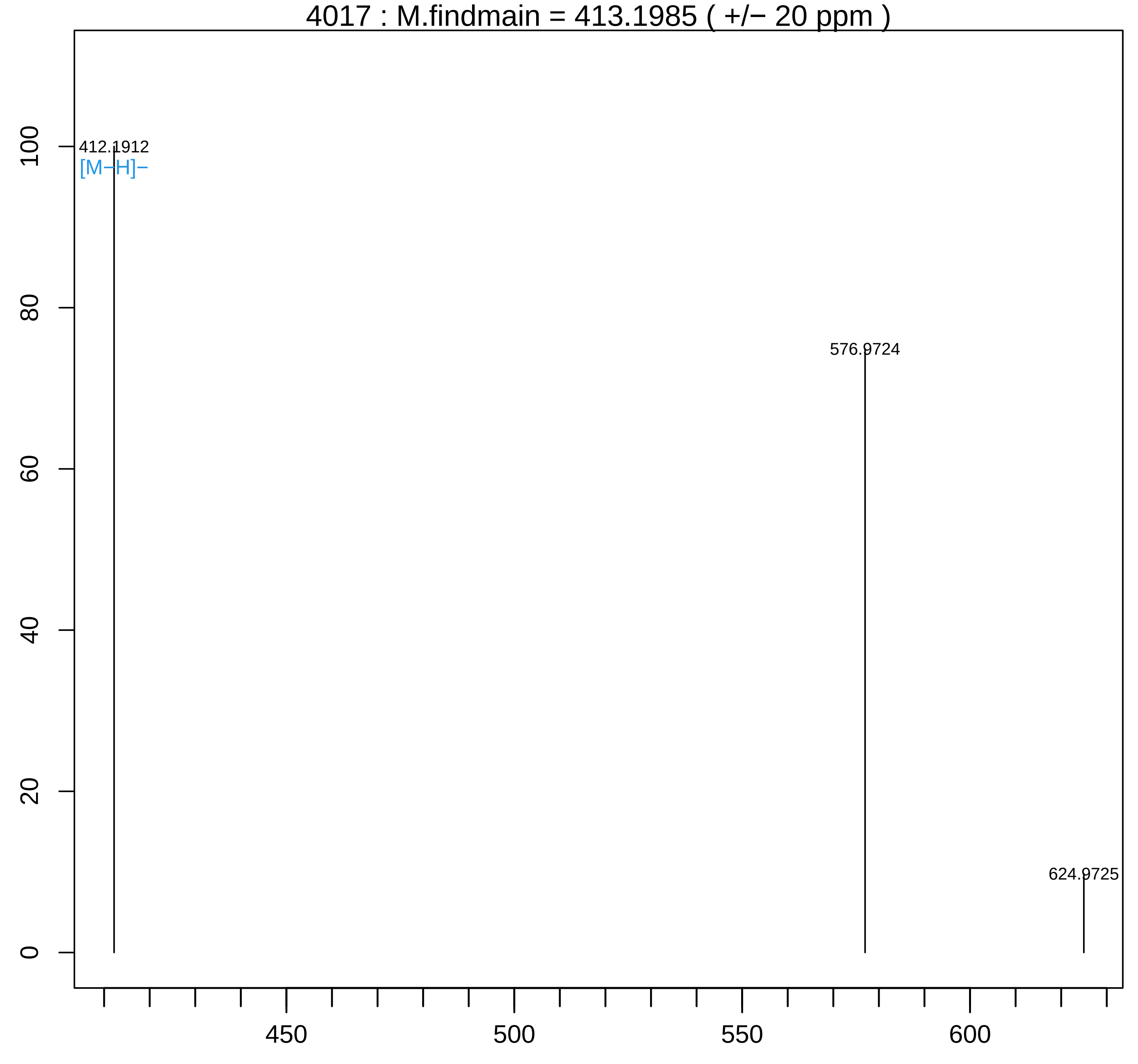
4017 : M.findmain = 413.1985 (+/− 20 ppm)
412.1912
[M−H]−
576.9724
624.9725
0
20
40
60
80
100
450
500
550
600

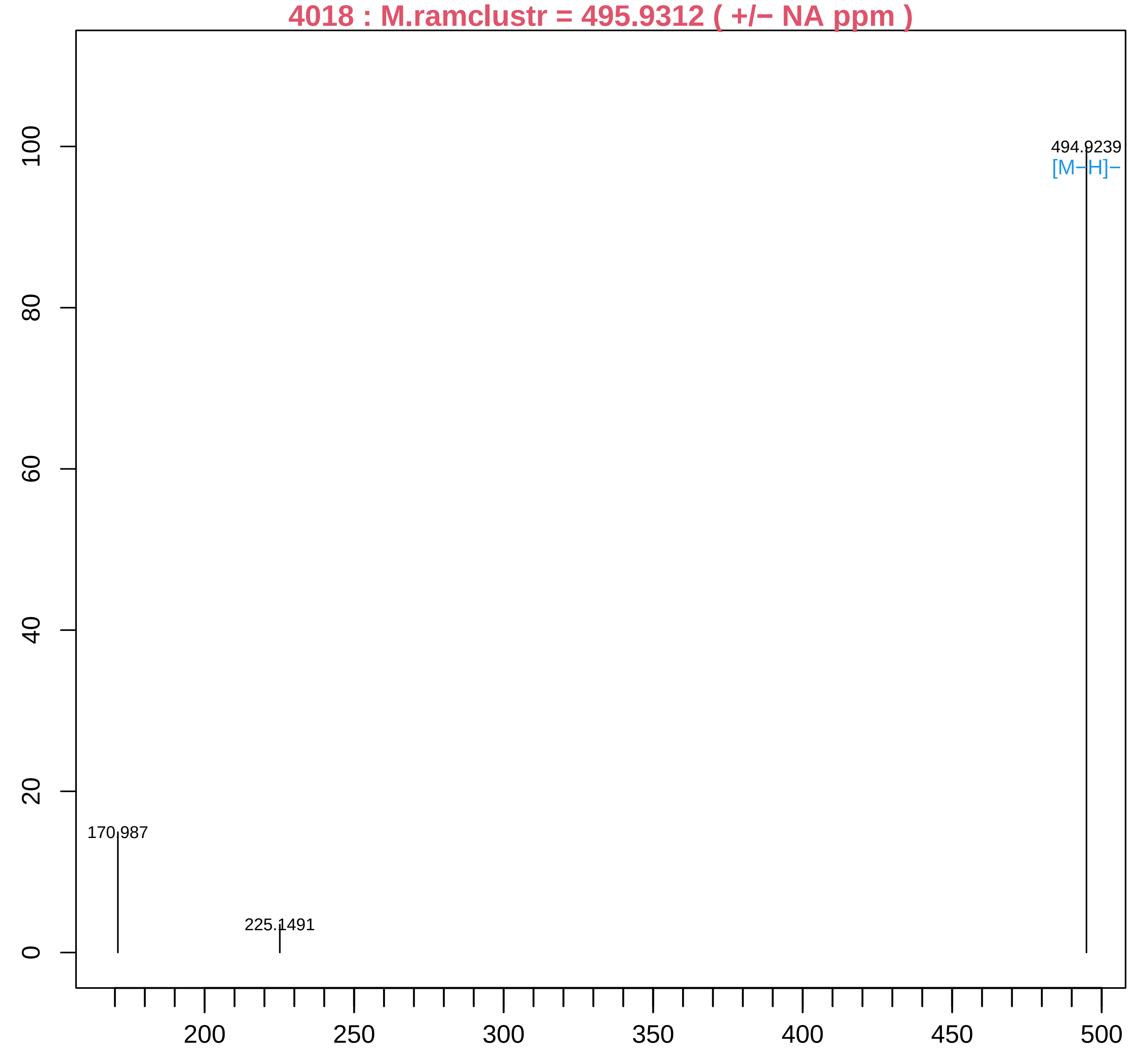
4018 : M.ramclustr = 495.9312 (+/− NA ppm)
494.9239
[M−H]−
170.987
225.1491
0
20
40
60
80
100
200
250
300
350
400
450
500

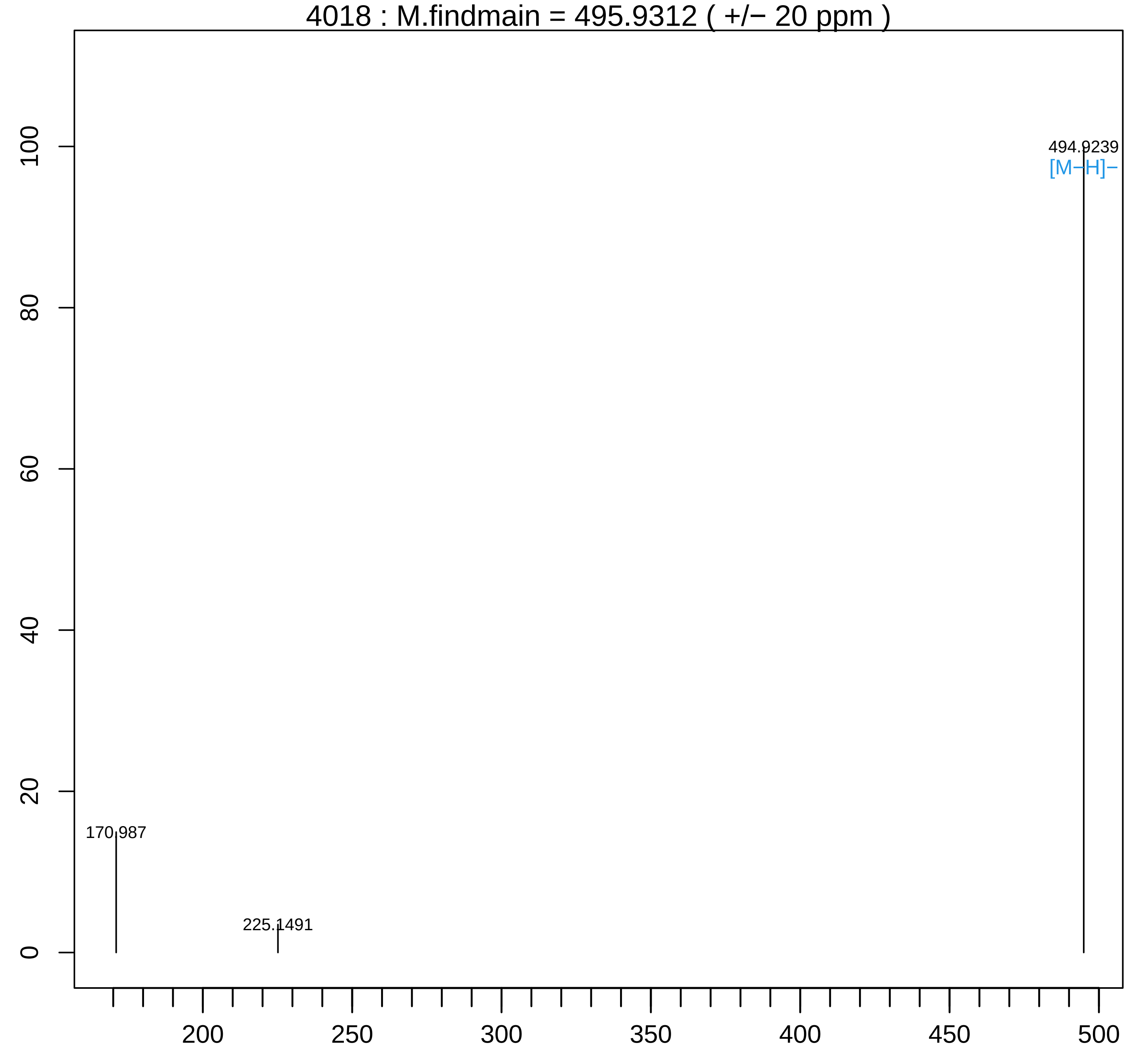
4018 : M.findmain = 495.9312 (+/− 20 ppm)
494.9239
[M−H]−
170.987
225.1491
0
20
40
60
80
100
200
250
300
350
400
450
500

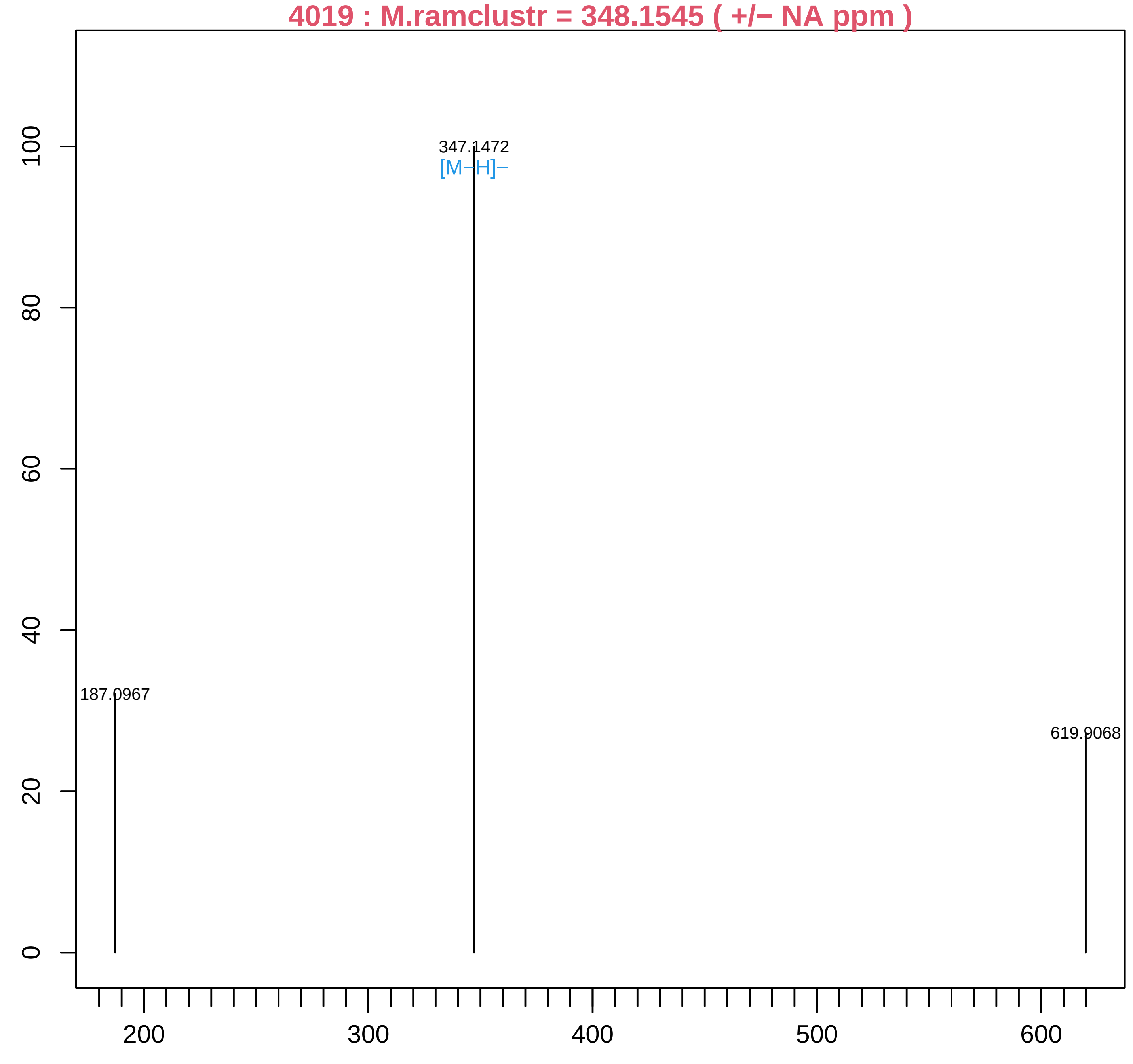

4019 : M.ramclustr = 348.1545 (+/− NA ppm)
347.1472
[M−H]−
187.0967
619.9068
0
20
40
60
80
100
200
300
400
500
600

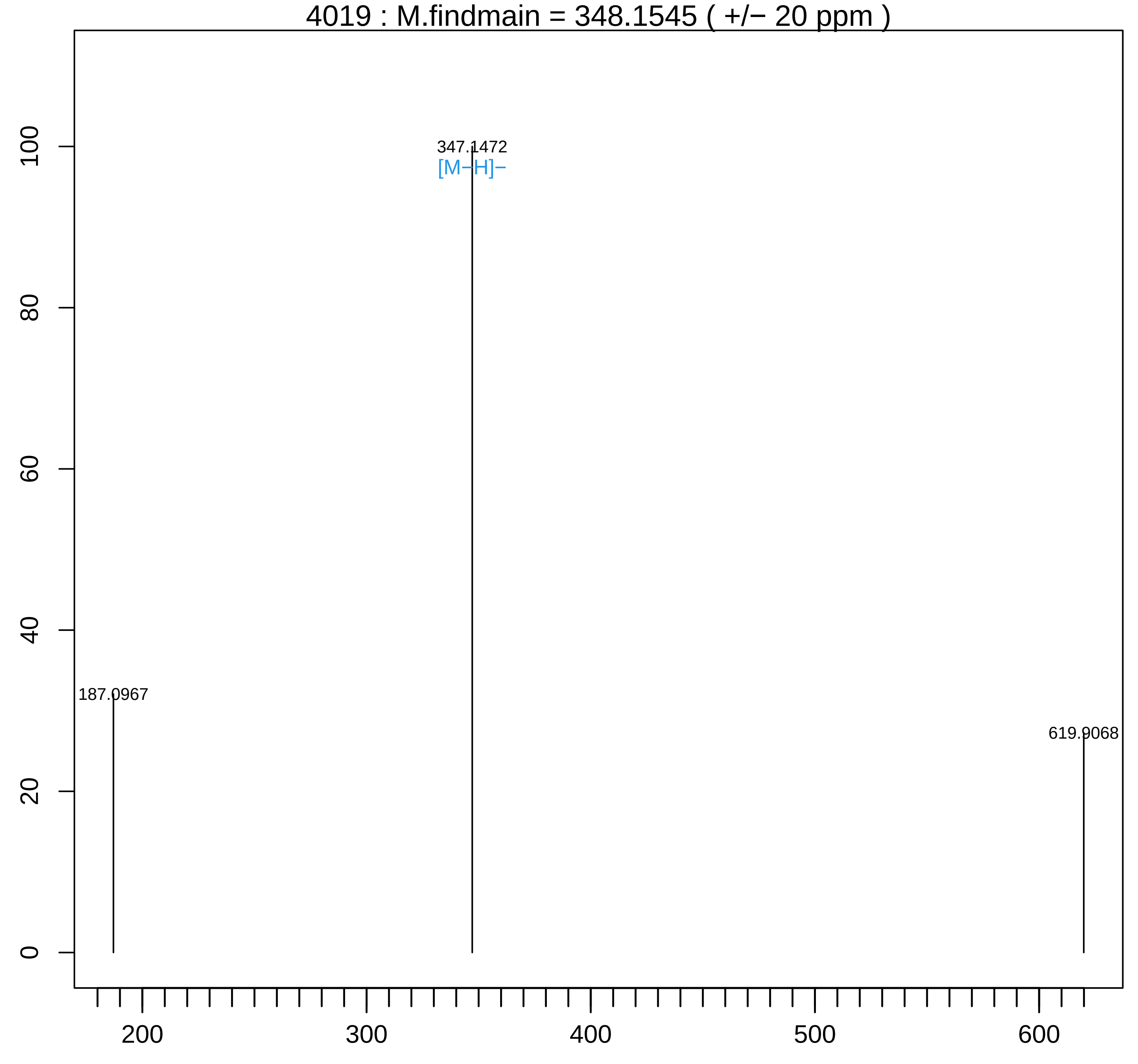

4019 : M.findmain = 348.1545 (+/− 20 ppm)
347.1472
[M−H]−
187.0967
619.9068
0
20
40
60
80
100
200
300
400
500
600

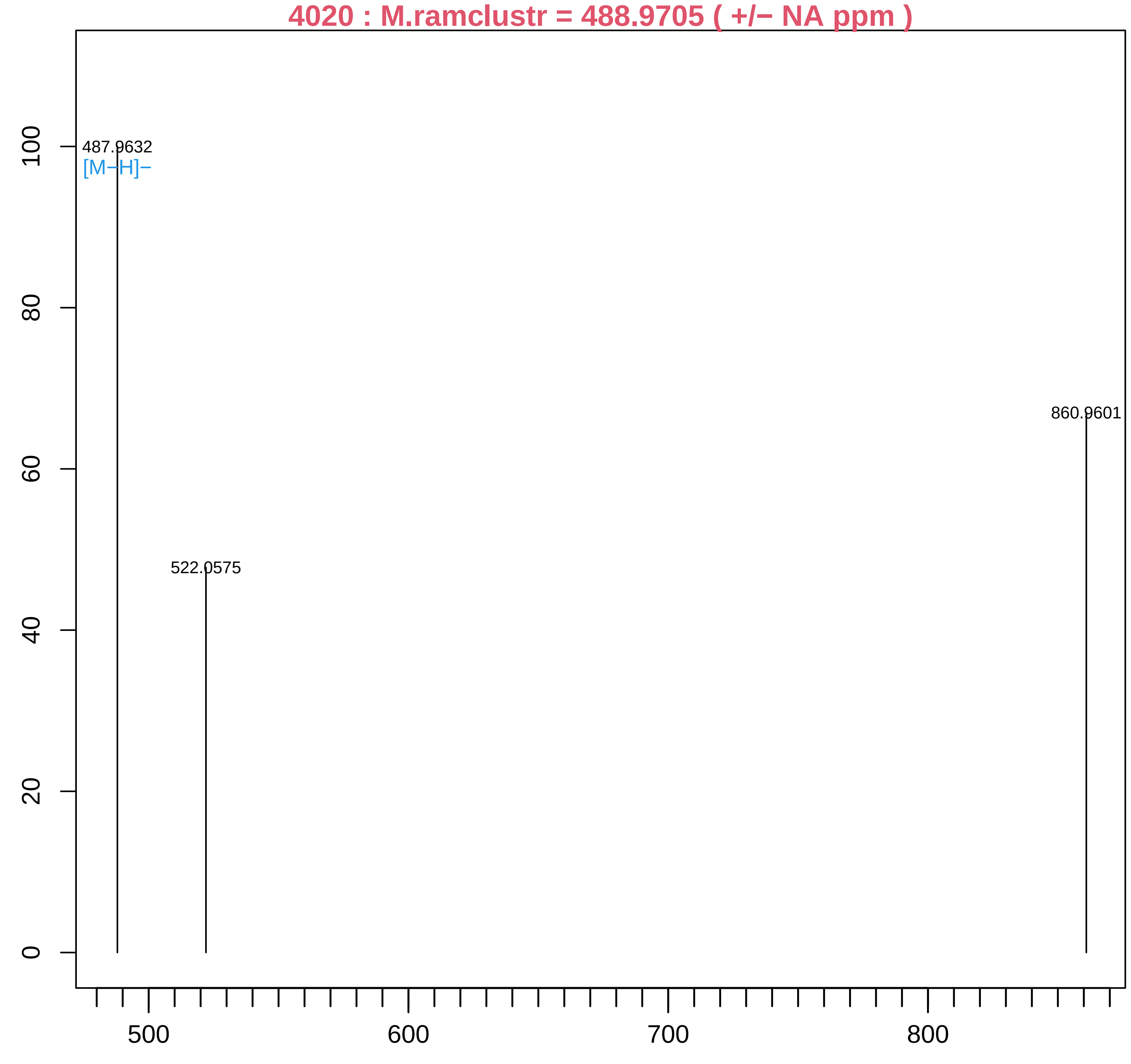
4020 : M.ramclustr = 488.9705 (+/− NA ppm)
487.9632
[M−H]−
522.0575
860.9601
0
20
40
60
80
100
500
600
700
800

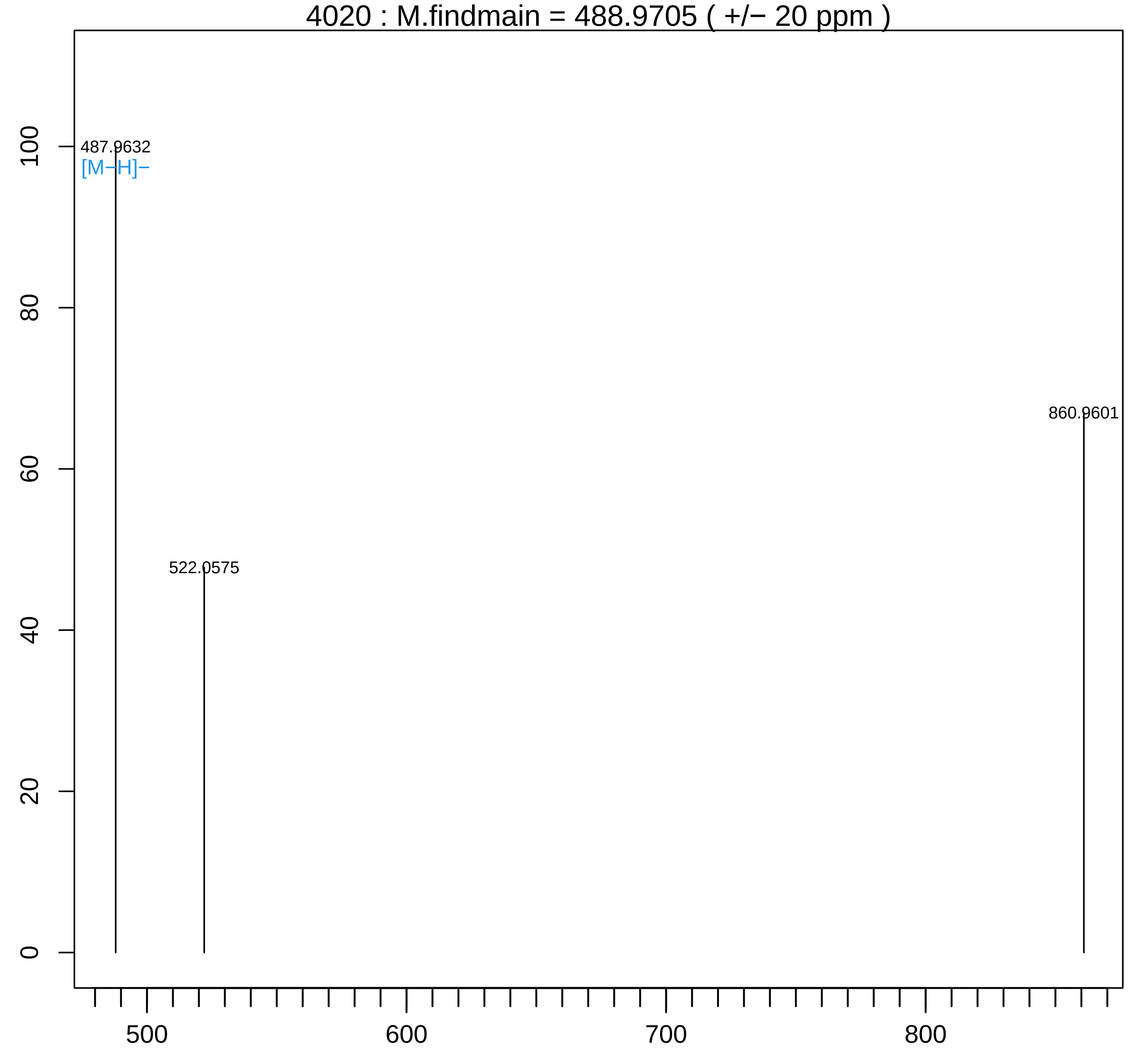
4020 : M.findmain = 488.9705 (+/− 20 ppm)
487.9632
[M−H]−
522.0575
860.9601
0
20
40
60
80
100
500
600
700
800

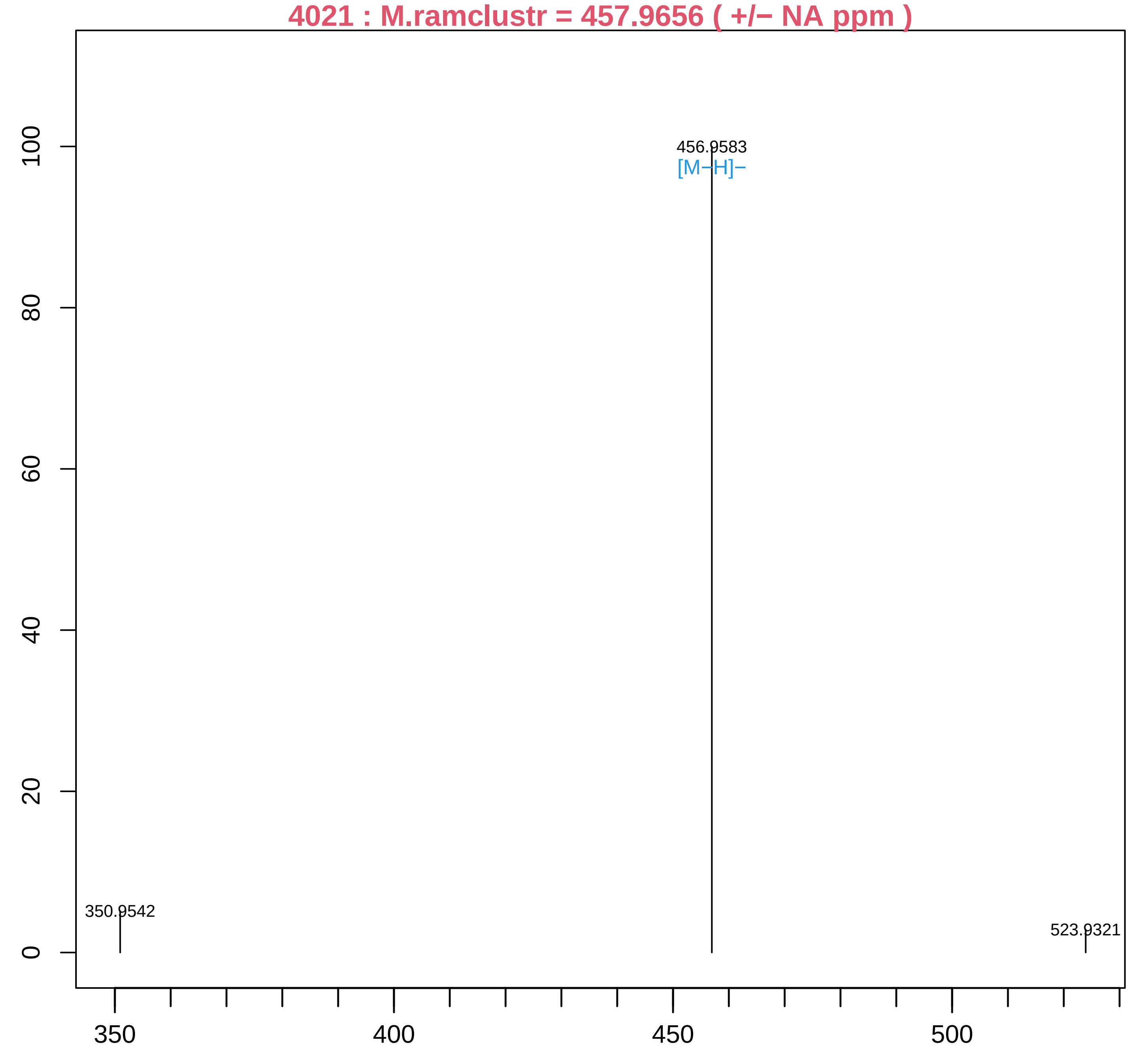

4021 : M.ramclustr = 457.9656 (+/− NA ppm)
456.9583
[M−H]−
350.9542
523.9321
0
20
40
60
80
100
350
400
450
500

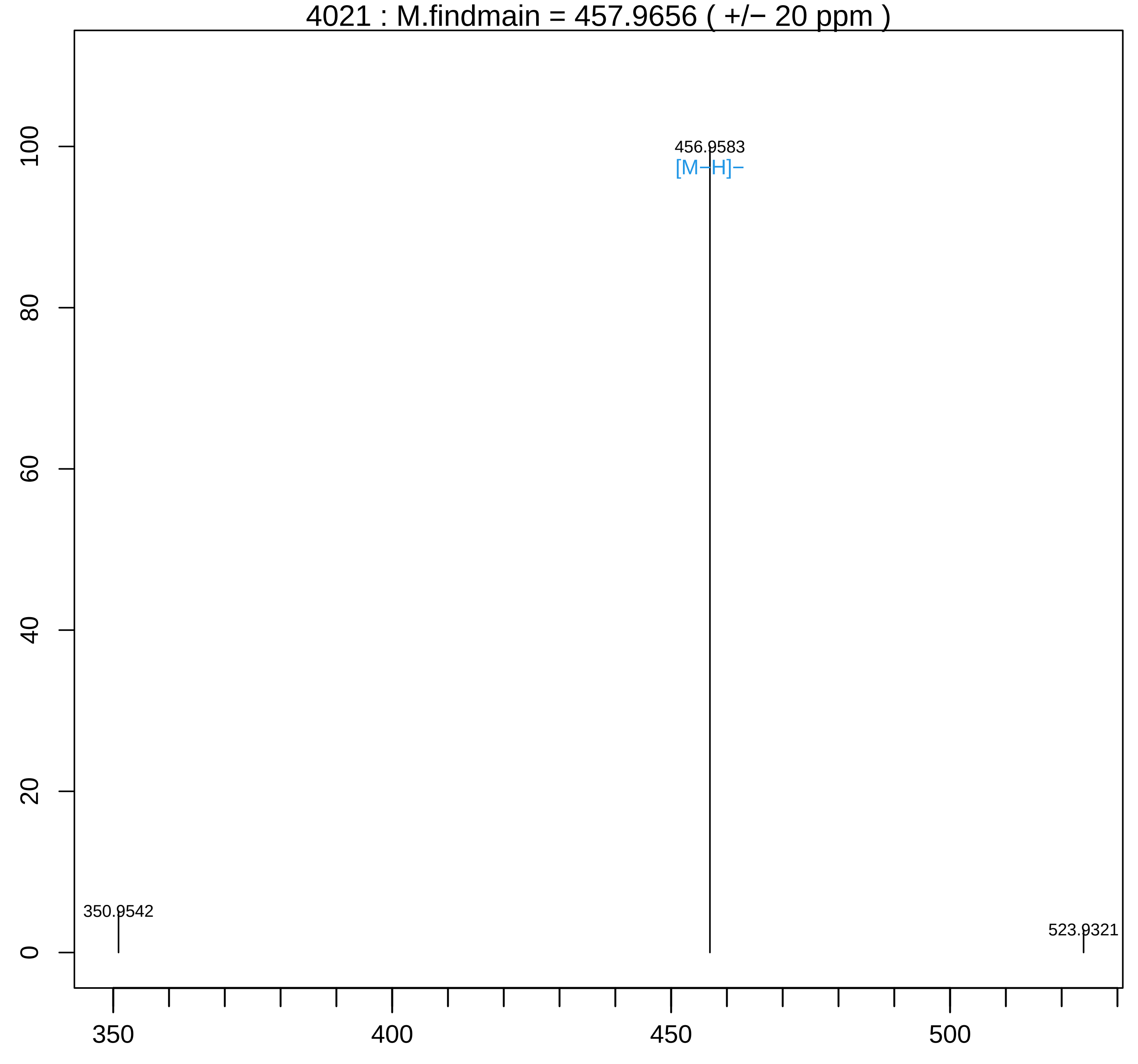

4021 : M.findmain = 457.9656 (+/− 20 ppm)
456.9583
[M−H]−
350.9542
523.9321
0
20
40
60
80
100
350
400
450
500

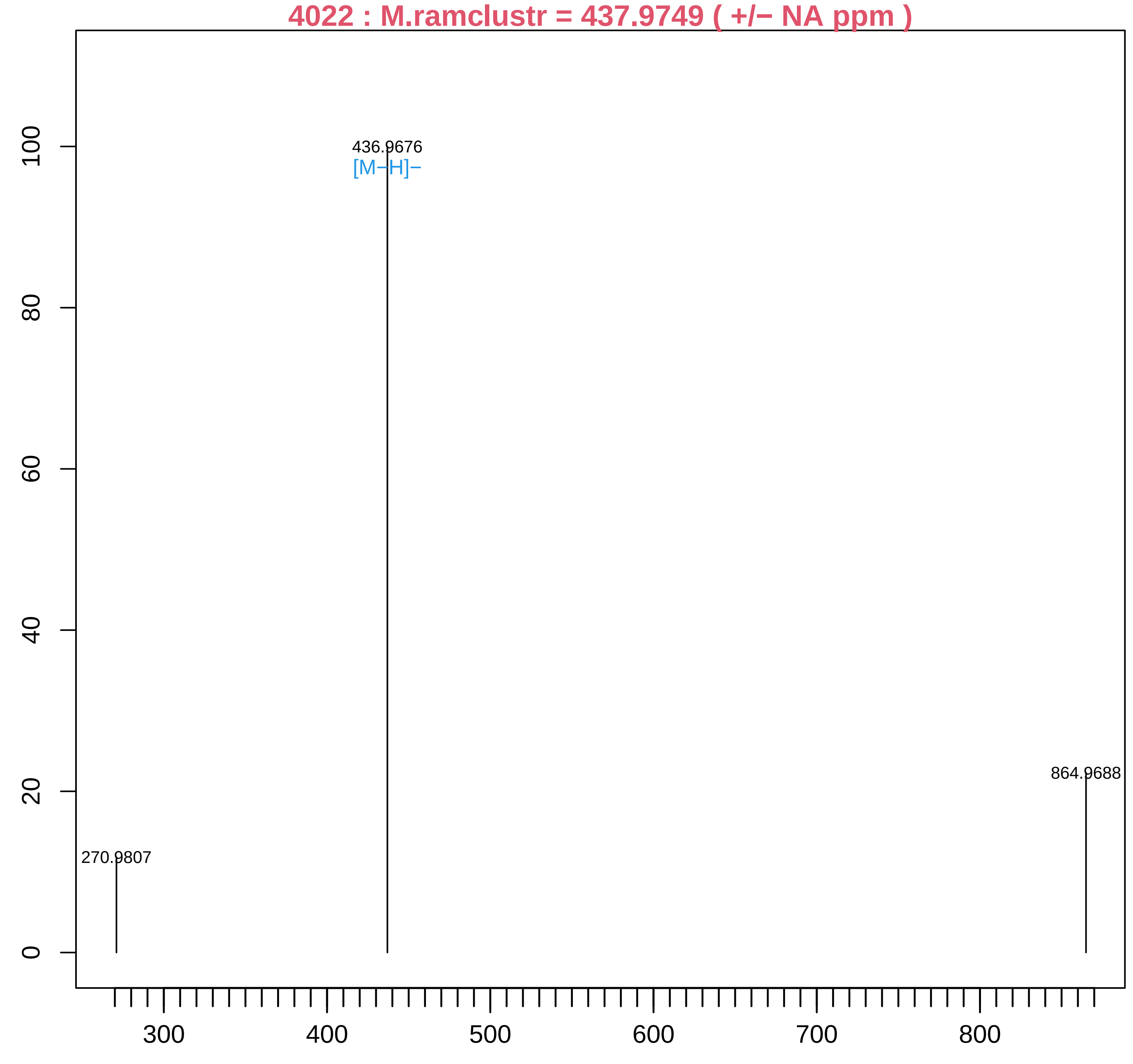
4022 : M.ramclustr = 437.9749 (+/− NA ppm)
436.9676
[M−H]−
864.9688
270.9807
0
20
40
60
80
100
300
400
500
600
700
800

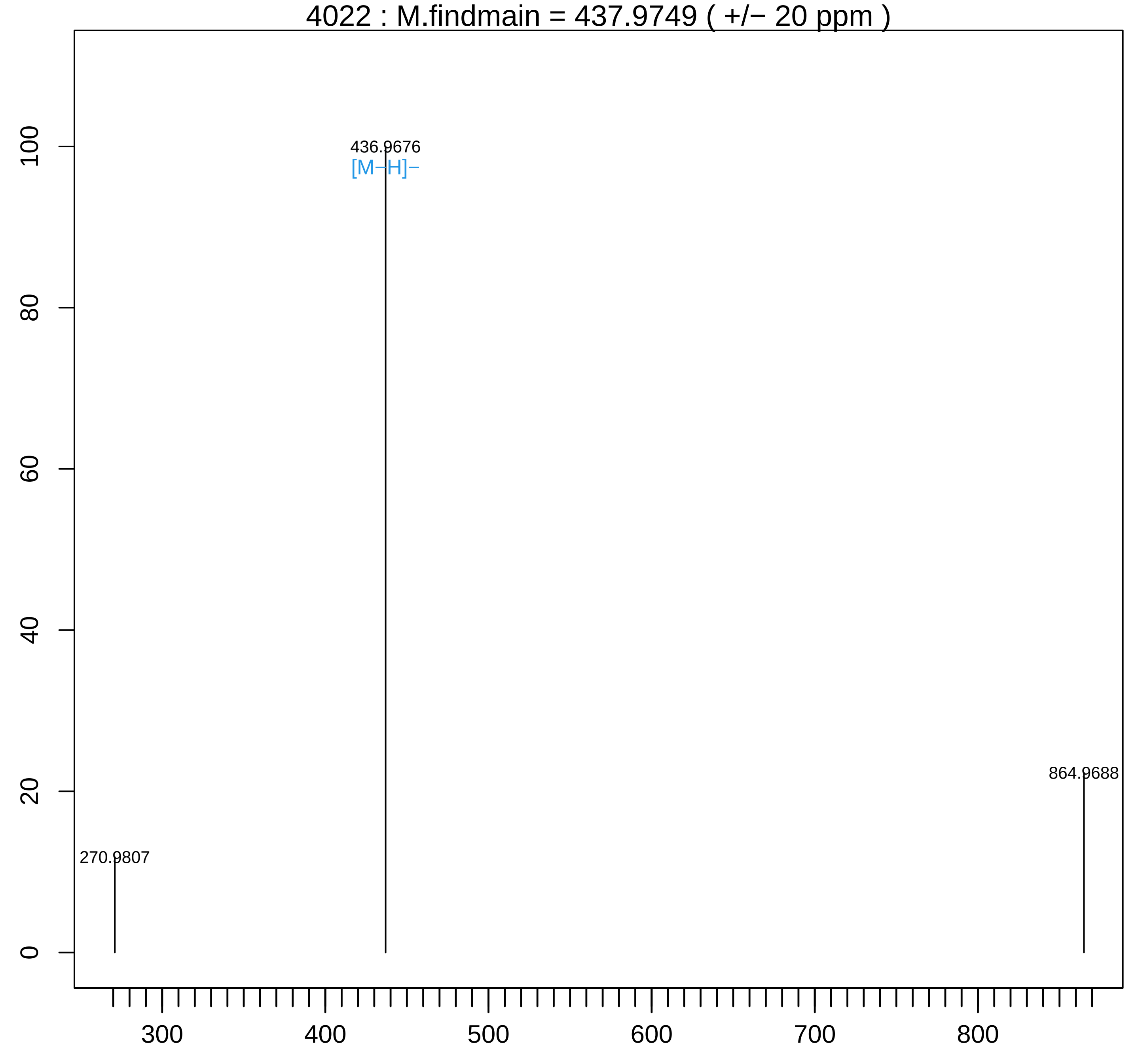
4022 : M.findmain = 437.9749 (+/− 20 ppm)
436.9676
[M−H]−
864.9688
270.9807
0
20
40
60
80
100
300
400
500
600
700
800

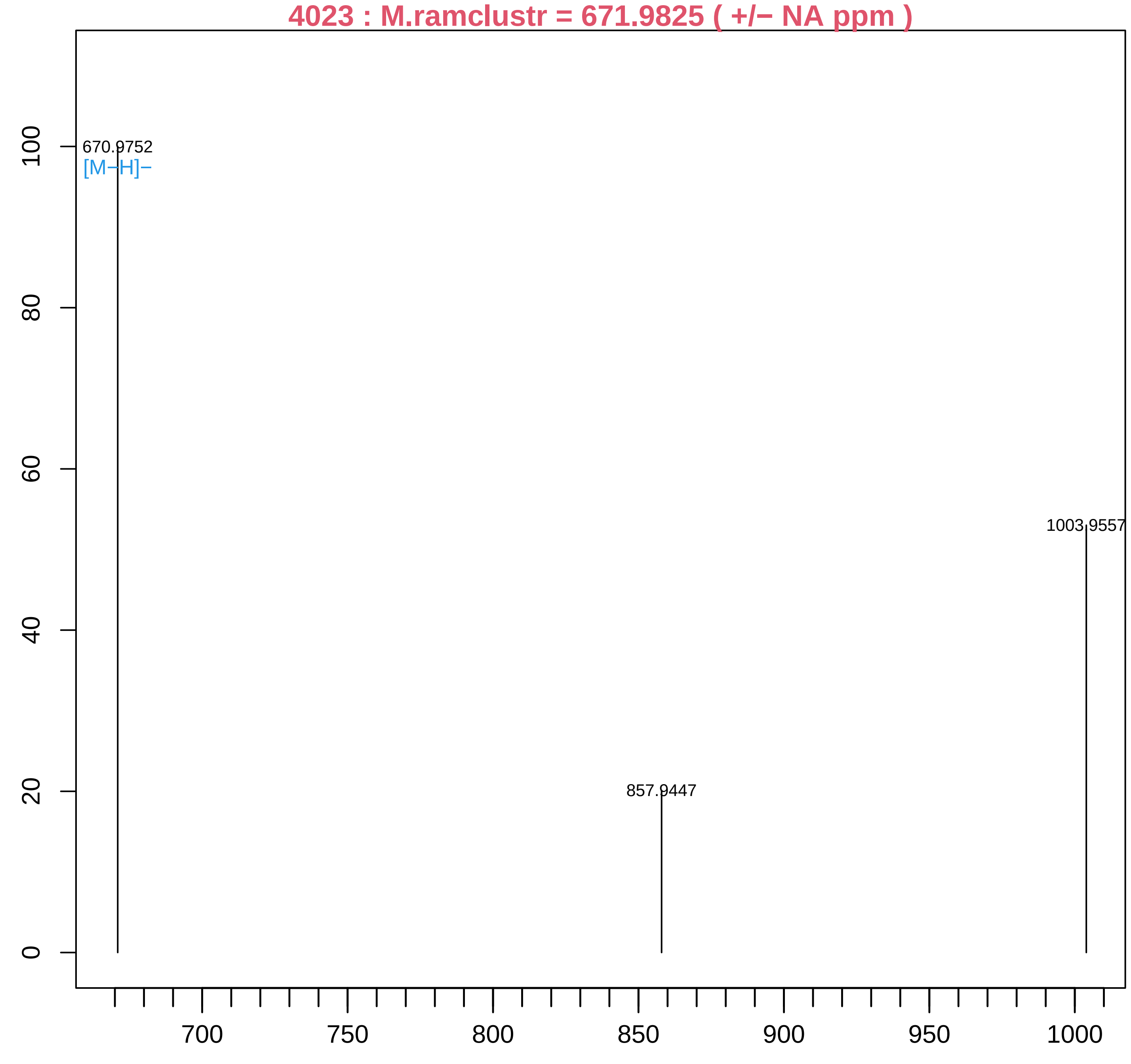
4023 : M.ramclustr = 671.9825 (+/− NA ppm)
670.9752
[M−H]−
857.9447
1003.9557
0
20
40
60
80
100
700
750
800
850
900
950
1000

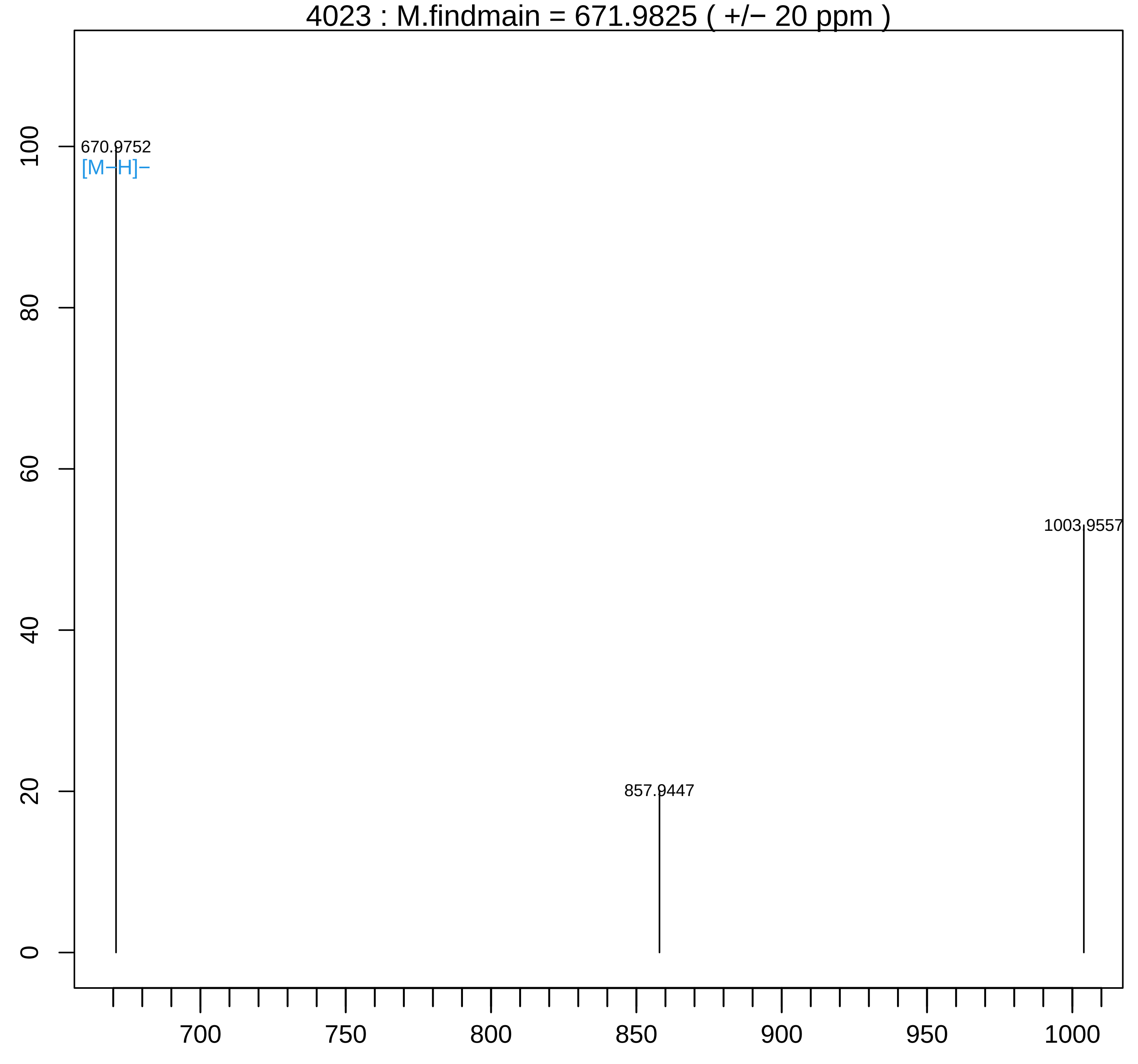
4023 : M.findmain = 671.9825 (+/− 20 ppm)
670.9752
[M−H]−
857.9447
1003.9557
0
20
40
60
80
100
700
750
800
850
900
950
1000

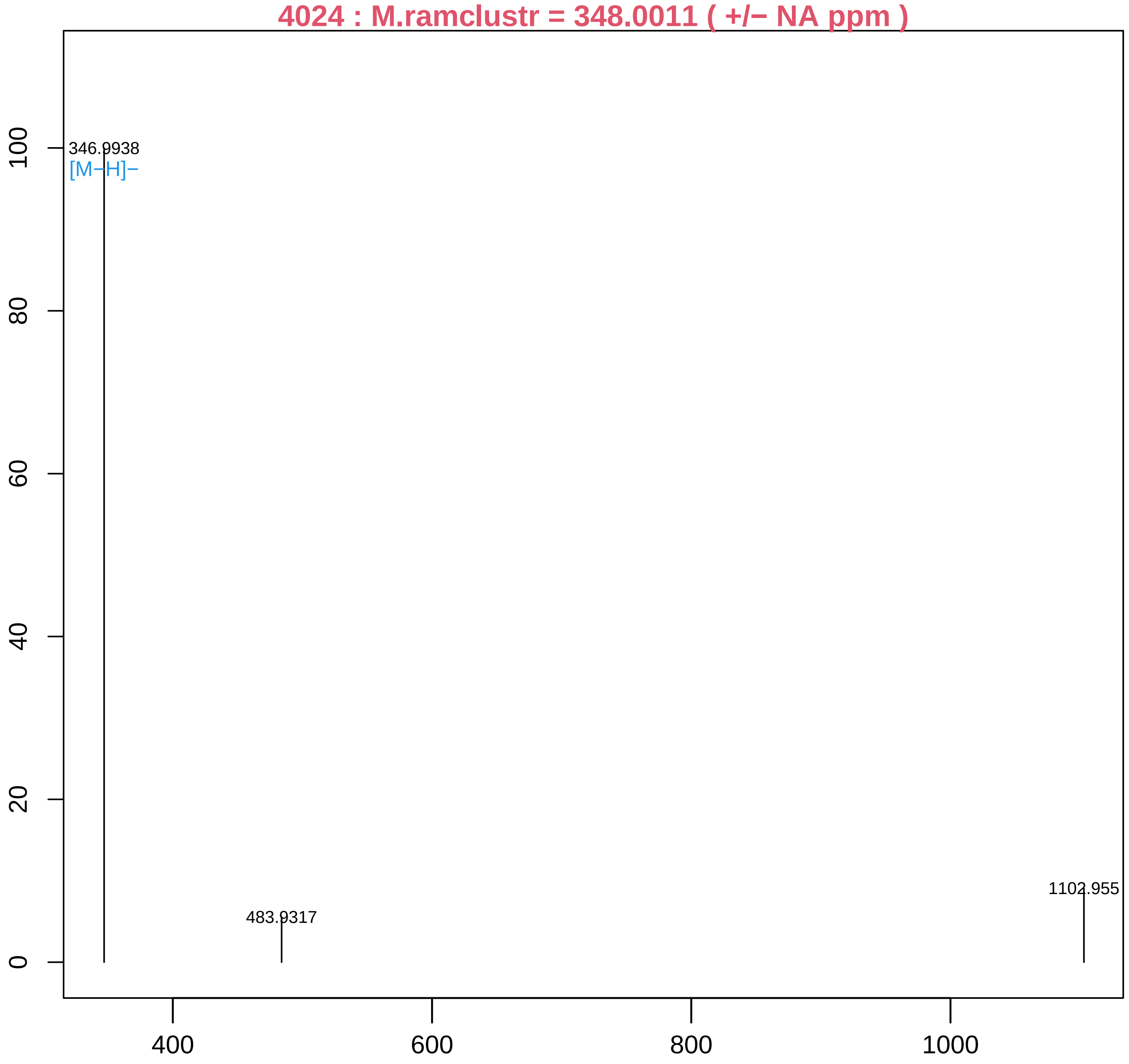
4024 : M.ramclustr = 348.0011 (+/− NA ppm)
346.9938
[M−H]−
483.9317
1102.955
0
20
40
60
80
100
400
600
800
1000
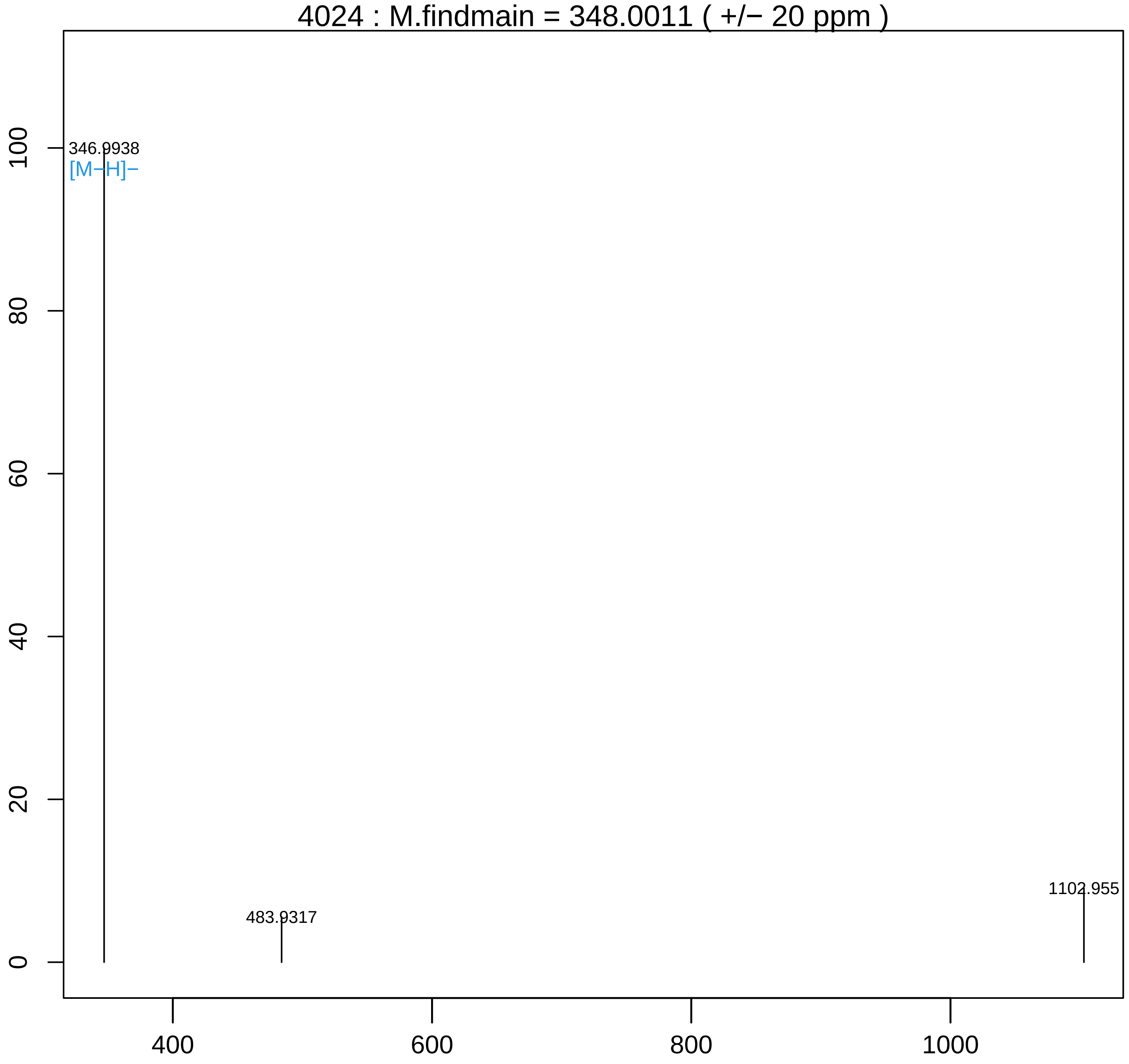
4024 : M.findmain = 348.0011 (+/− 20 ppm)
346.9938
[M−H]−
483.9317
1102.955
0
20
40
60
80
100
400
600
800
1000

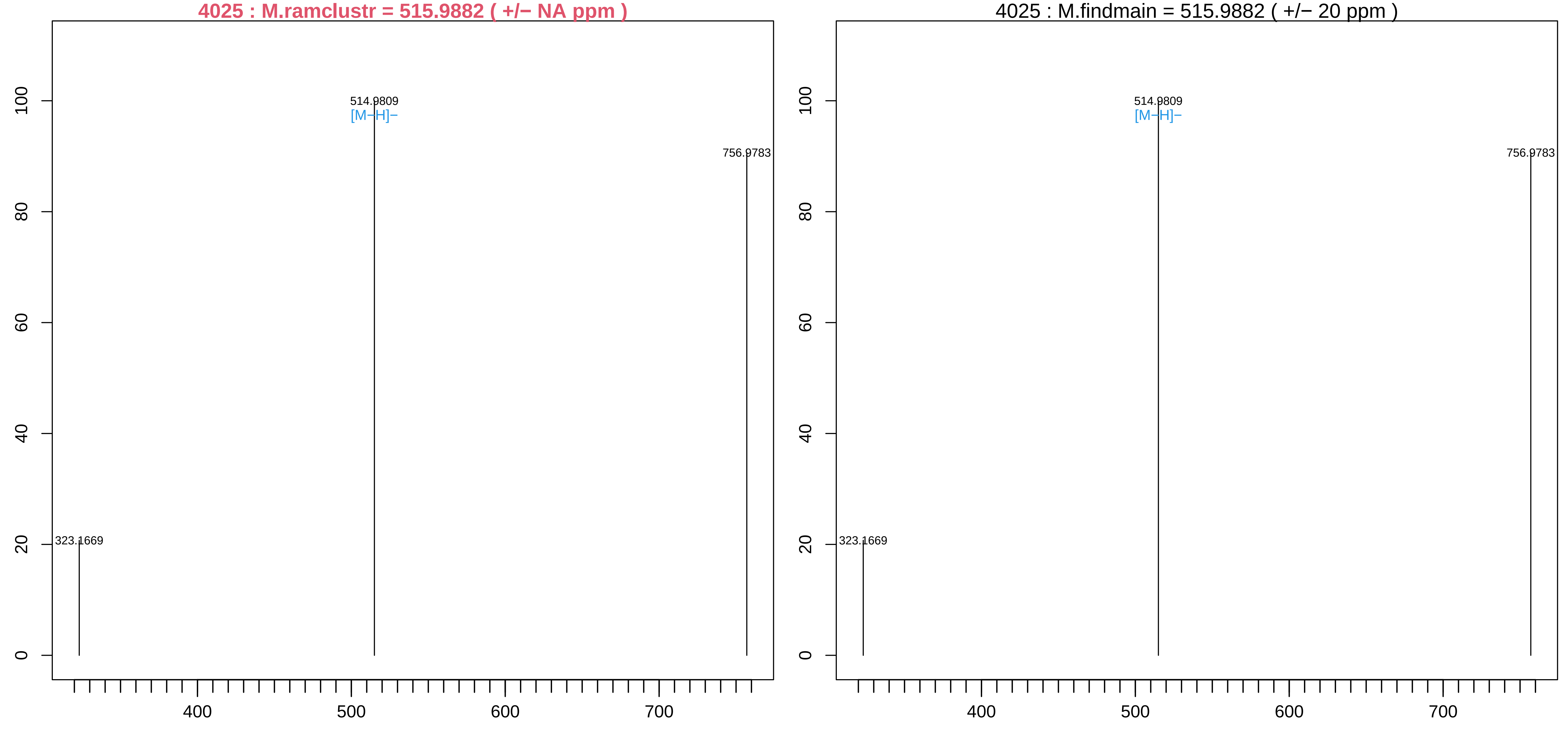
4025 : M.ramclustr = 515.9882 (+/− NA ppm)
323.1669
514.9809
[M−H]−
756.9783
4025 : M.findmain = 515.9882 (+/− 20 ppm)
323.1669
514.9809
[M−H]−
756.9783

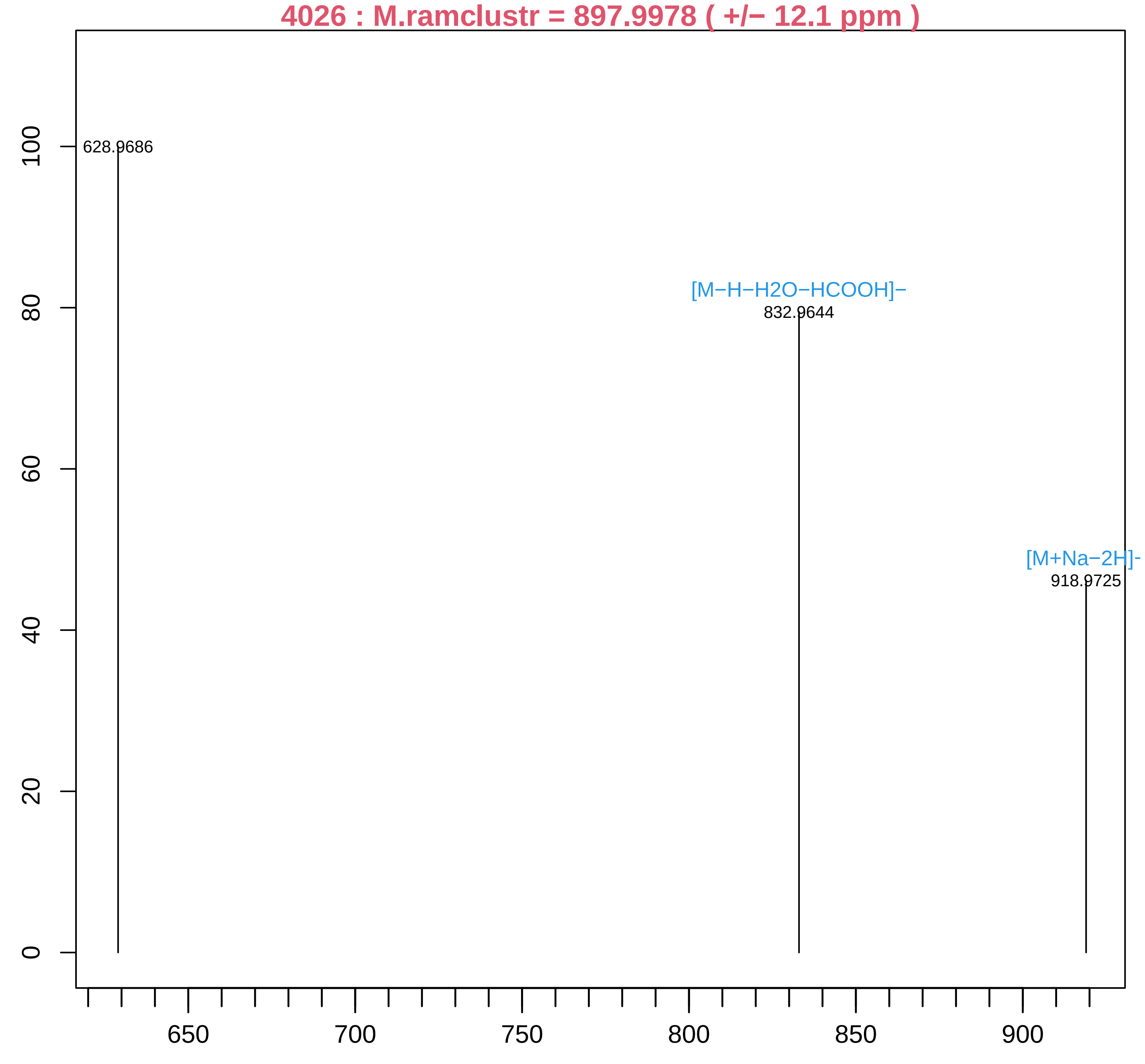
4026 : M.ramclustr = 897.9978 (+/− 12.1 ppm)
628.9686
[M−H−H2O−HCOOH]−
832.9644
[M+Na−2H]-
918.9725
0
20
40
60
80
100
650
700
750
800
850
900

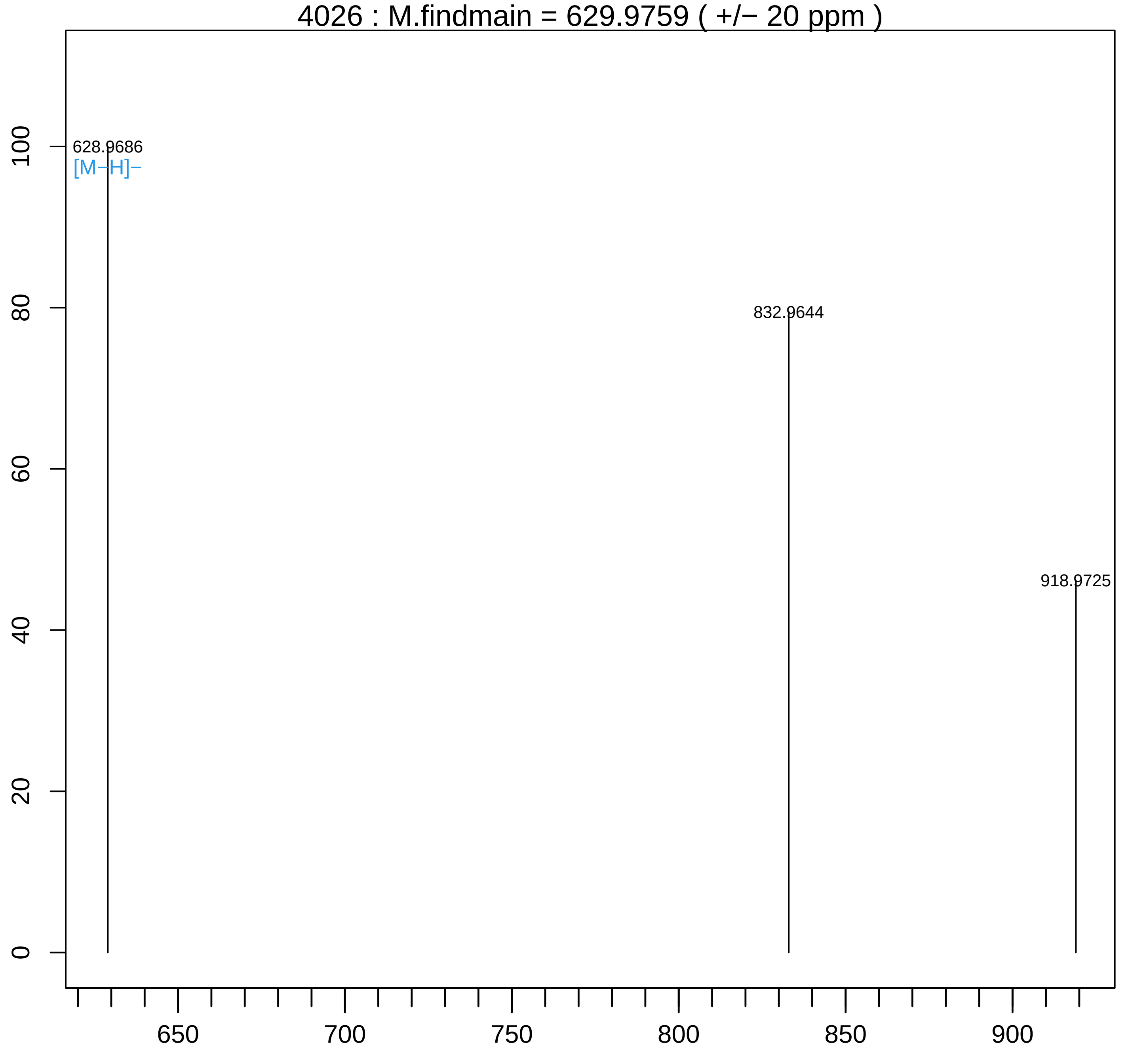
4026 : M.findmain = 629.9759 (+/− 20 ppm)
628.9686
[M−H]−
832.9644
918.9725
0
20
40
60
80
100
650
700
750
800
850
900

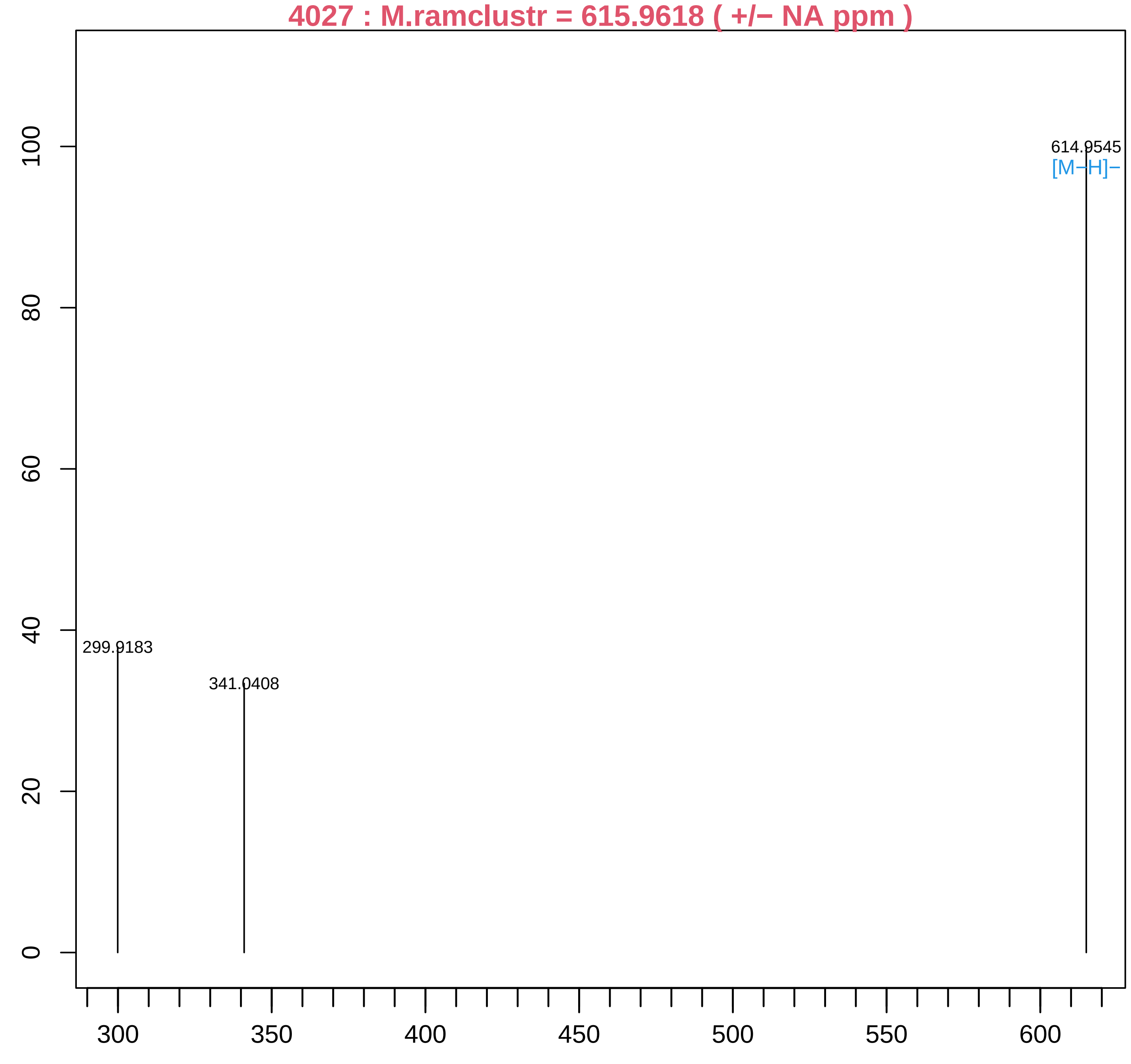
4027 : M.ramclustr = 615.9618 (+/− NA ppm)
614.9545
[M−H]−
299.9183
341.0408
0
20
40
60
80
100
300
350
400
450
500
550
600

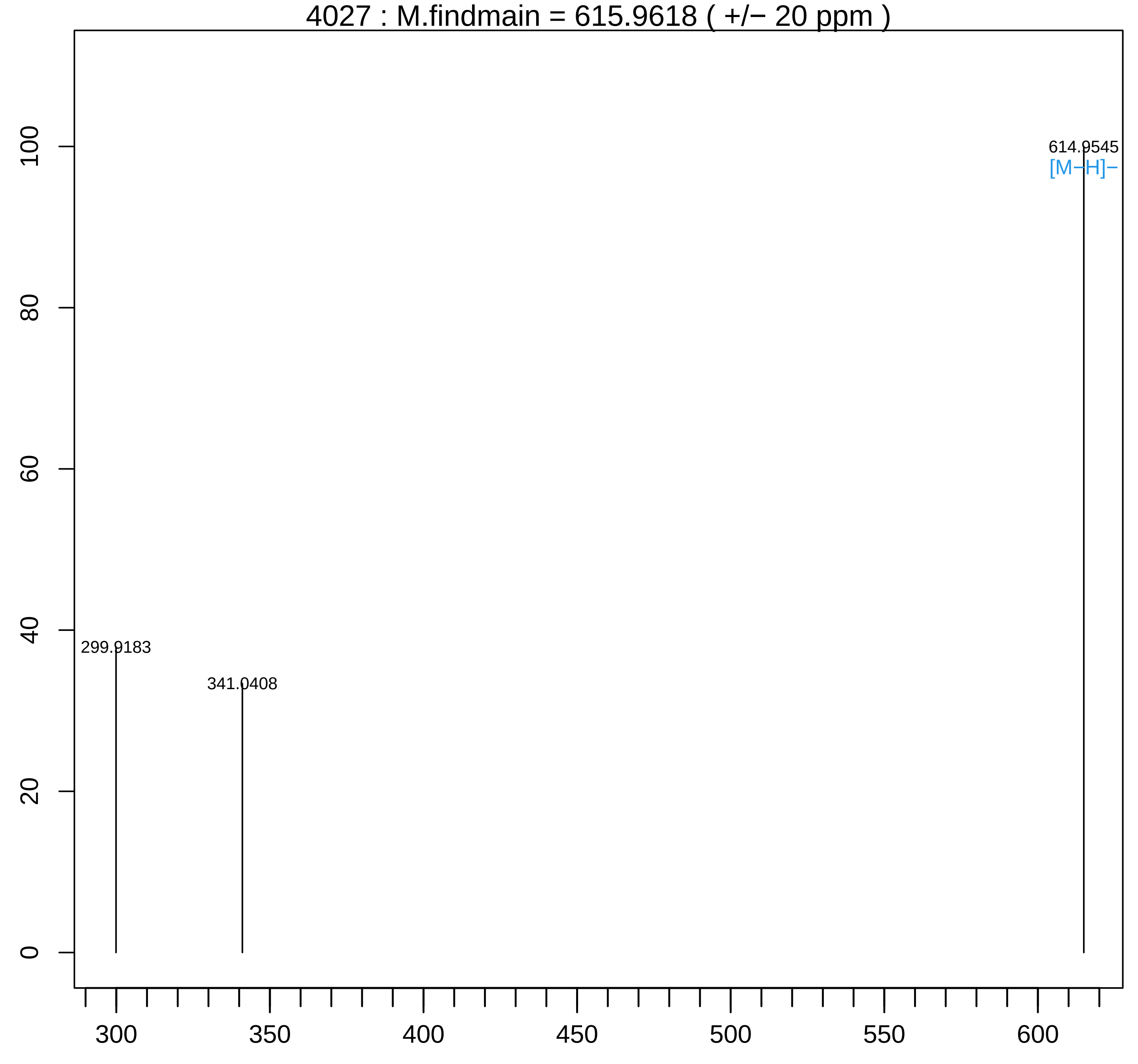
4027 : M.findmain = 615.9618 (+/− 20 ppm)
614.9545
[M−H]−
299.9183
341.0408
0
20
40
60
80
100
300
350
400
450
500
550
600

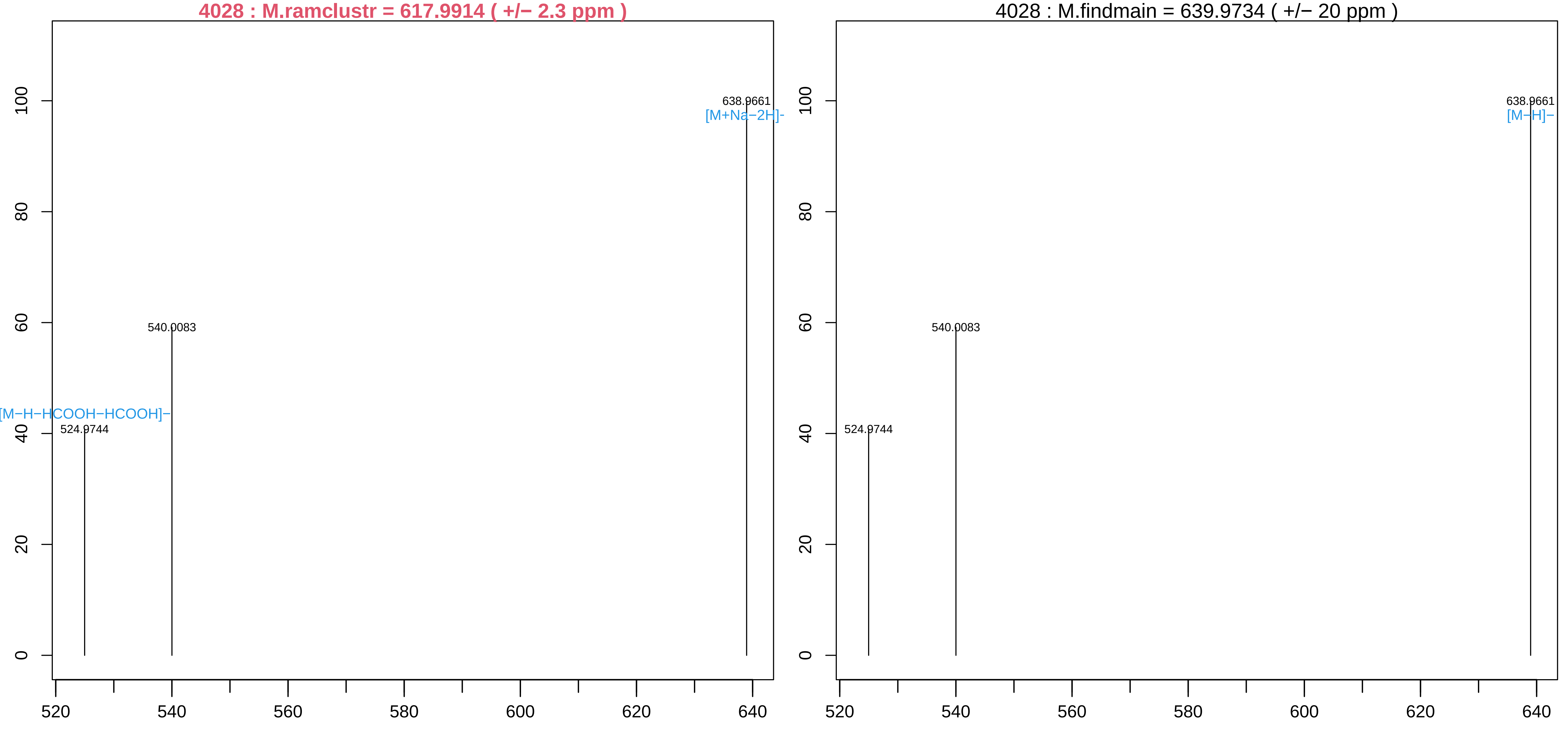

4028 : M.ramclustr = 617.9914 (+/− 2.3 ppm)
638.9661
[M+Na−2H]-
540.0083
[M−H−HCOOH−HCOOH]−
524.9744
4028 : M.findmain = 639.9734 (+/− 20 ppm)
638.9661
[M−H]−
540.0083
524.9744

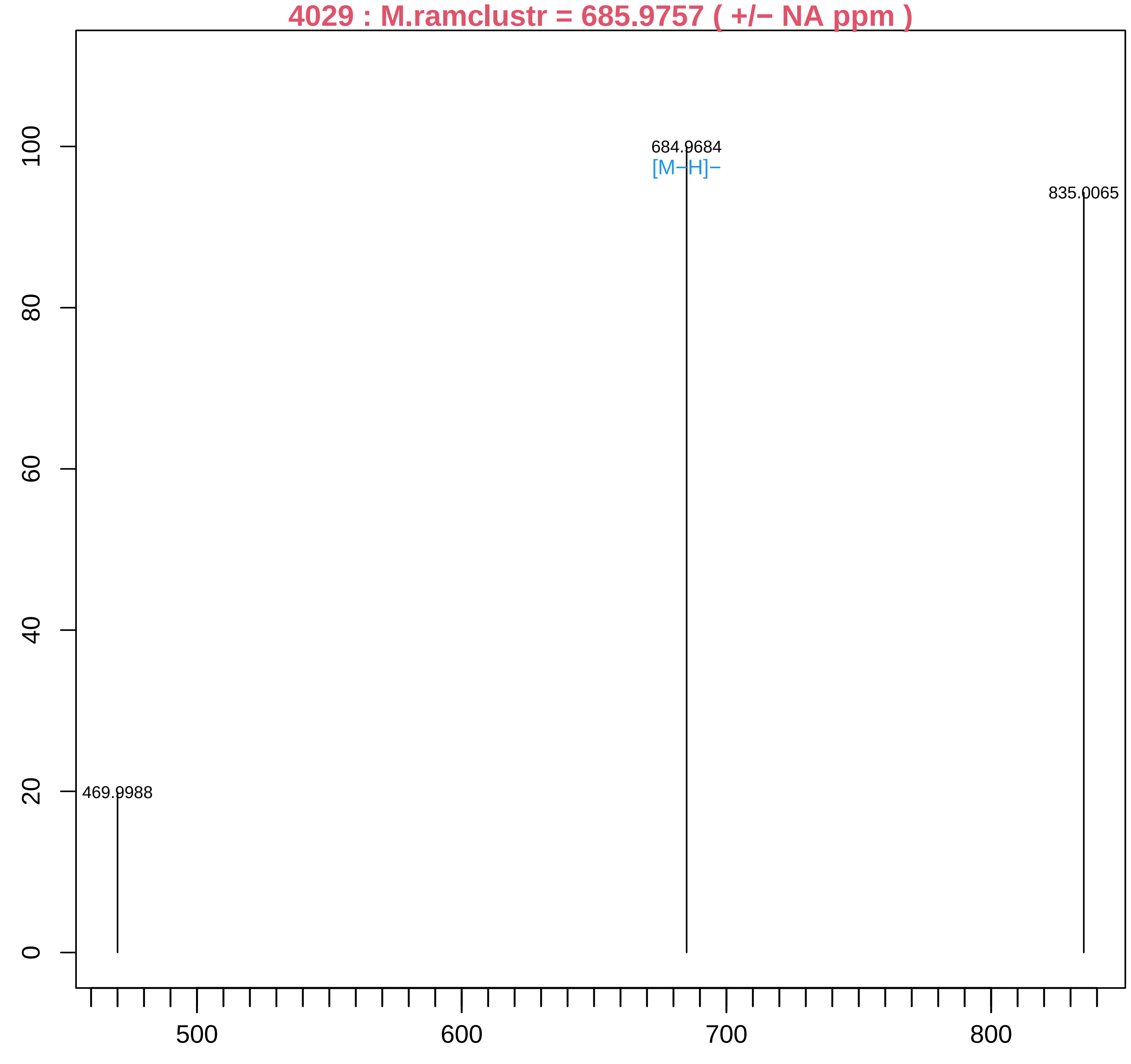
4029 : M.ramclustr = 685.9757 (+/− NA ppm)
684.9684
[M−H]−
835.0065
469.9988
0
20
40
60
80
100
500
600
700
800

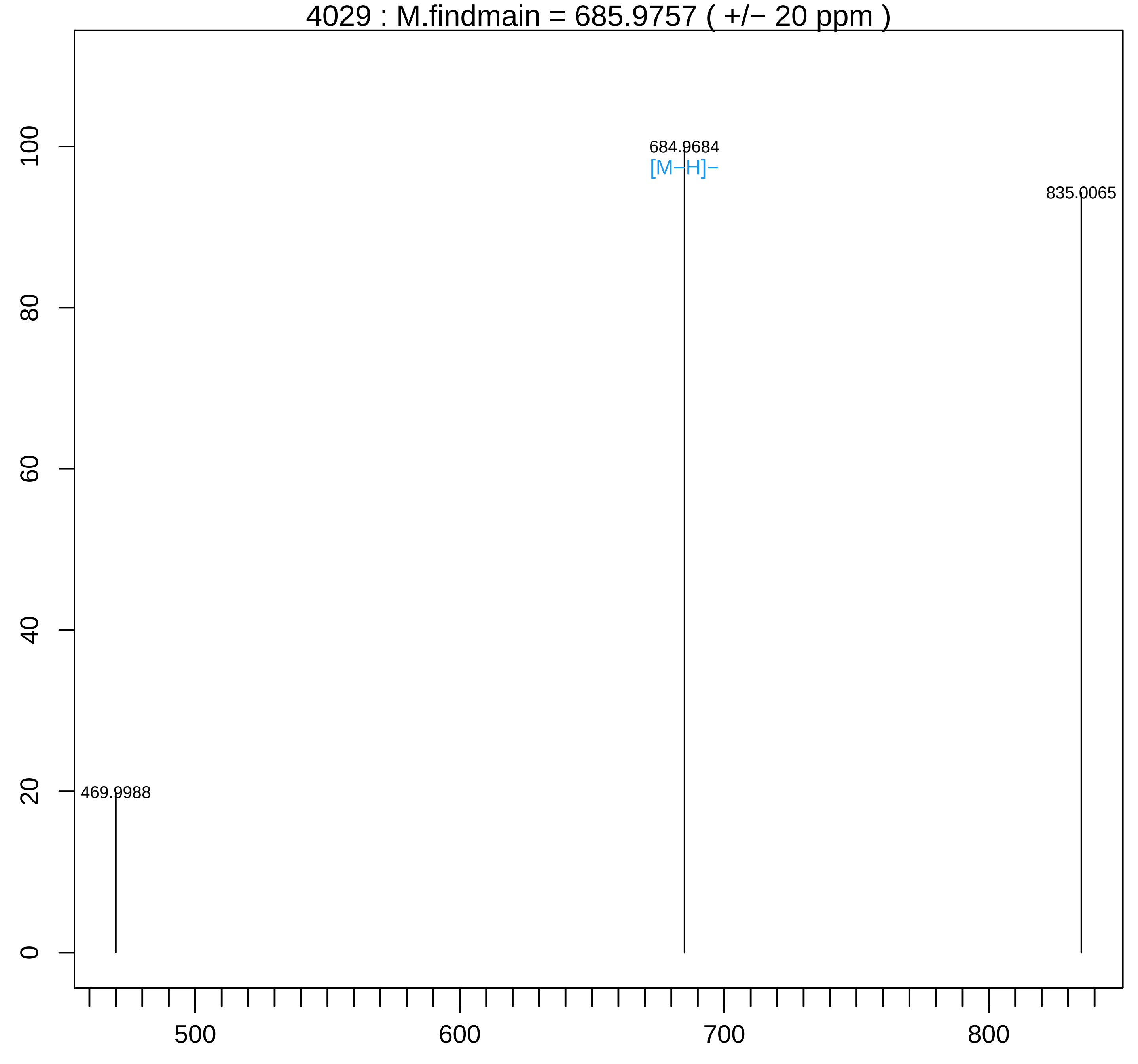
4029 : M.findmain = 685.9757 (+/− 20 ppm)
684.9684
[M−H]−
835.0065
469.9988
0
20
40
60
80
100
500
600
700
800

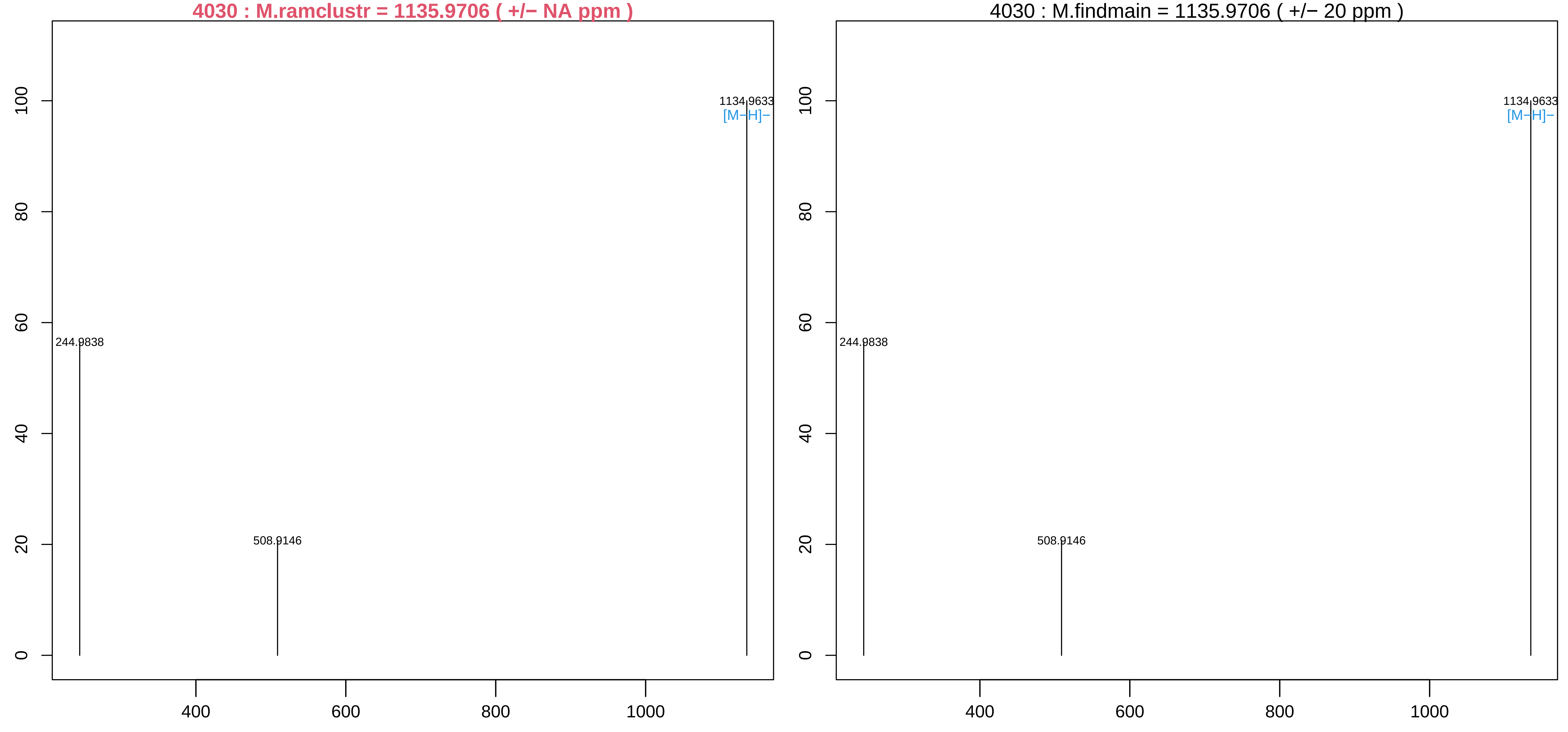
4030 : M.ramclustr = 1135.9706 (+/− NA ppm)
4030 : M.findmain = 1135.9706 (+/− 20 ppm)
1134.9633
[M−H]−
244.9838
508.9146
0
20
40
60
80
100
400
600
800
1000

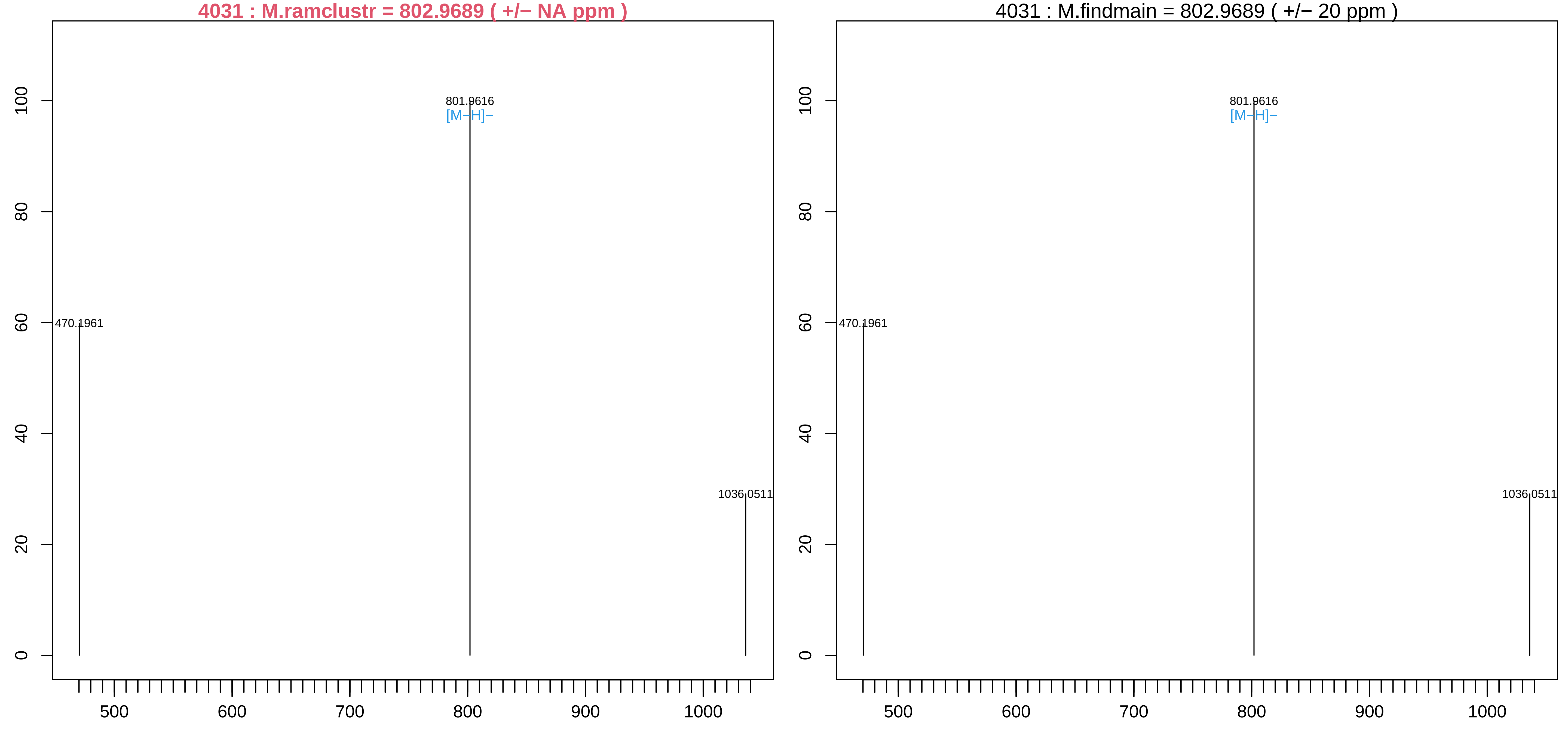
4031 : M.ramclustr = 802.9689 (+/− NA ppm)
470.1961
801.9616
[M−H]−
1036.0511
0
20
40
60
80
100
500
600
700
800
900
1000
4031 : M.findmain = 802.9689 (+/− 20 ppm)
470.1961
801.9616
[M−H]−
1036.0511
0
20
40
60
80
100
500
600
700
800
900
1000

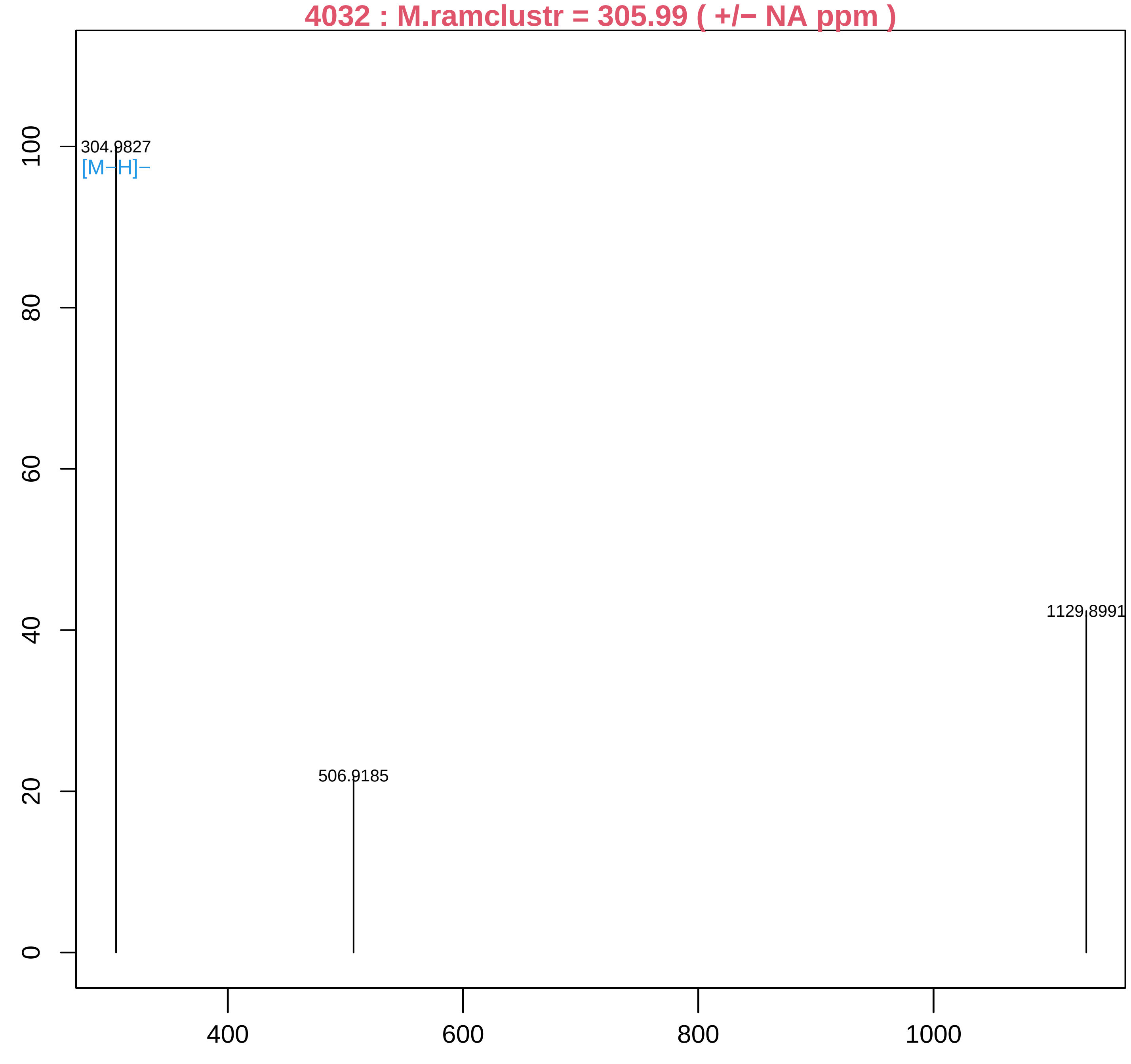

4032 : M.ramclustr = 305.99 (+/− NA ppm)
304.9827
[M−H]−
506.9185
1129.8991
0
20
40
60
80
100
400
600
800
1000

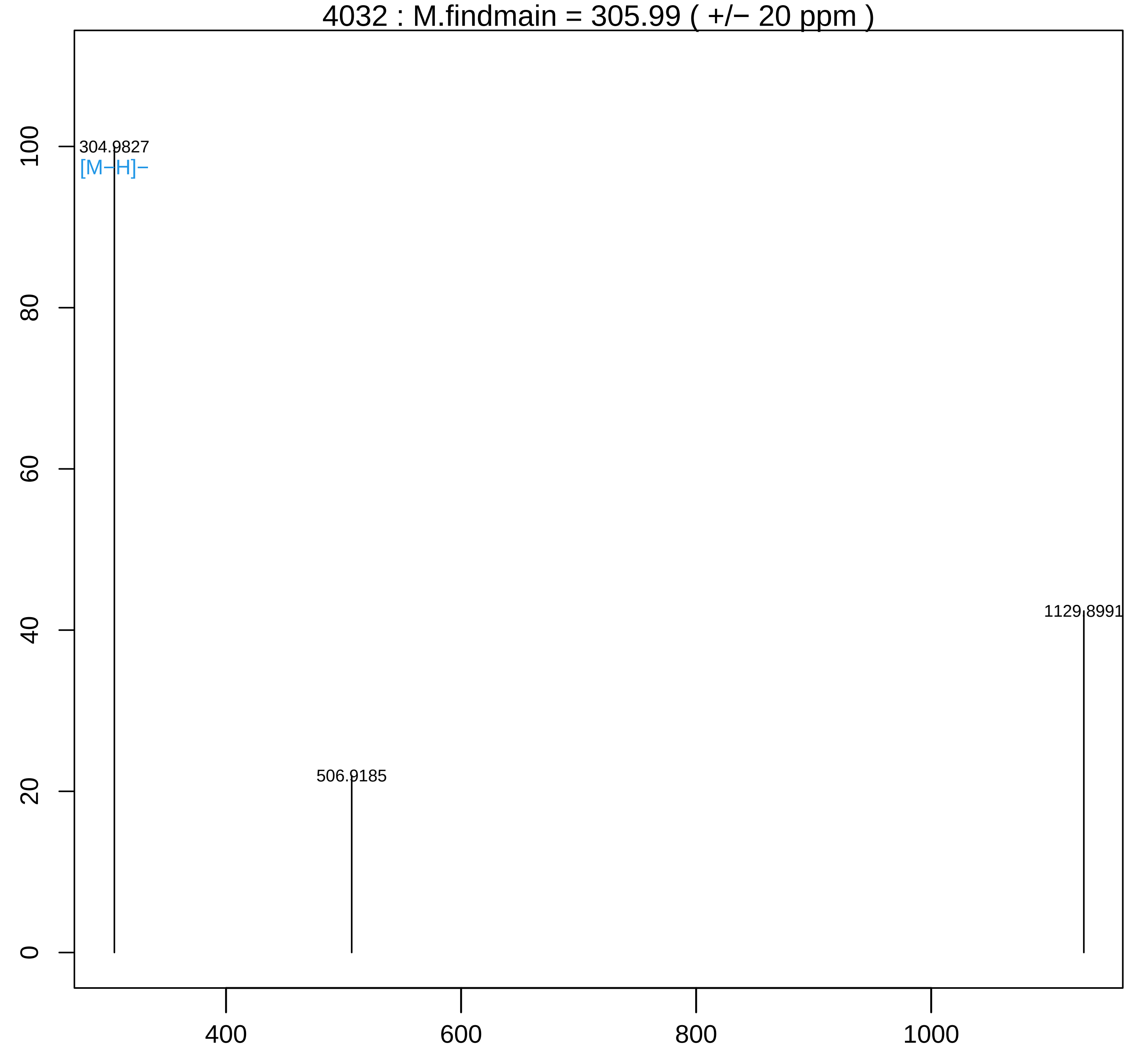

4032 : M.findmain = 305.99 (+/− 20 ppm)
304.9827
[M−H]−
506.9185
1129.8991
0
20
40
60
80
100
400
600
800
1000

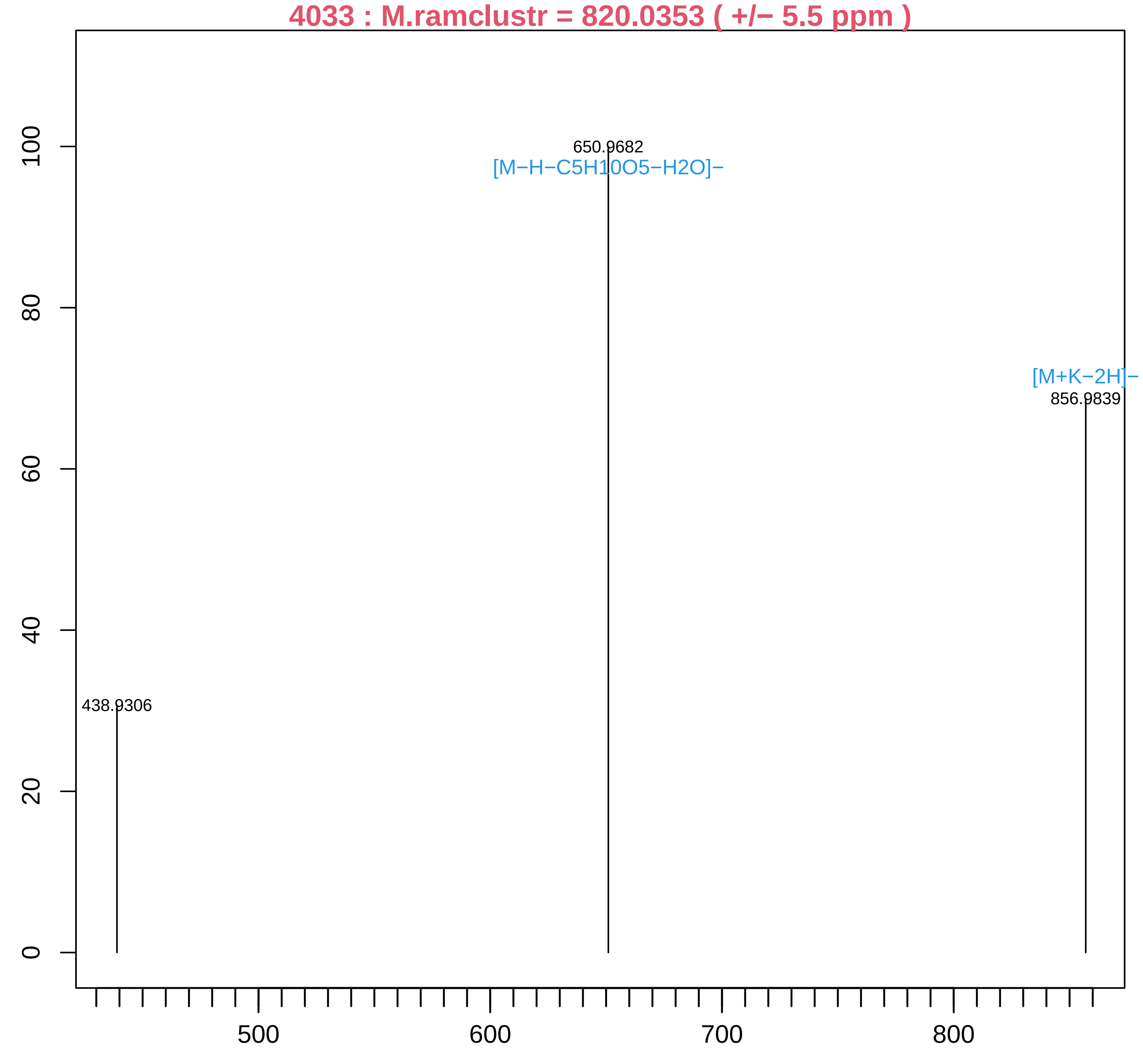
4033 : M.ramclustr = 820.0353 (+/− 5.5 ppm)
650.9682
[M−H−C5H10O5−H2O]−
[M+K−2H]−
856.9839
438.9306
0
20
40
60
80
100
500
600
700
800

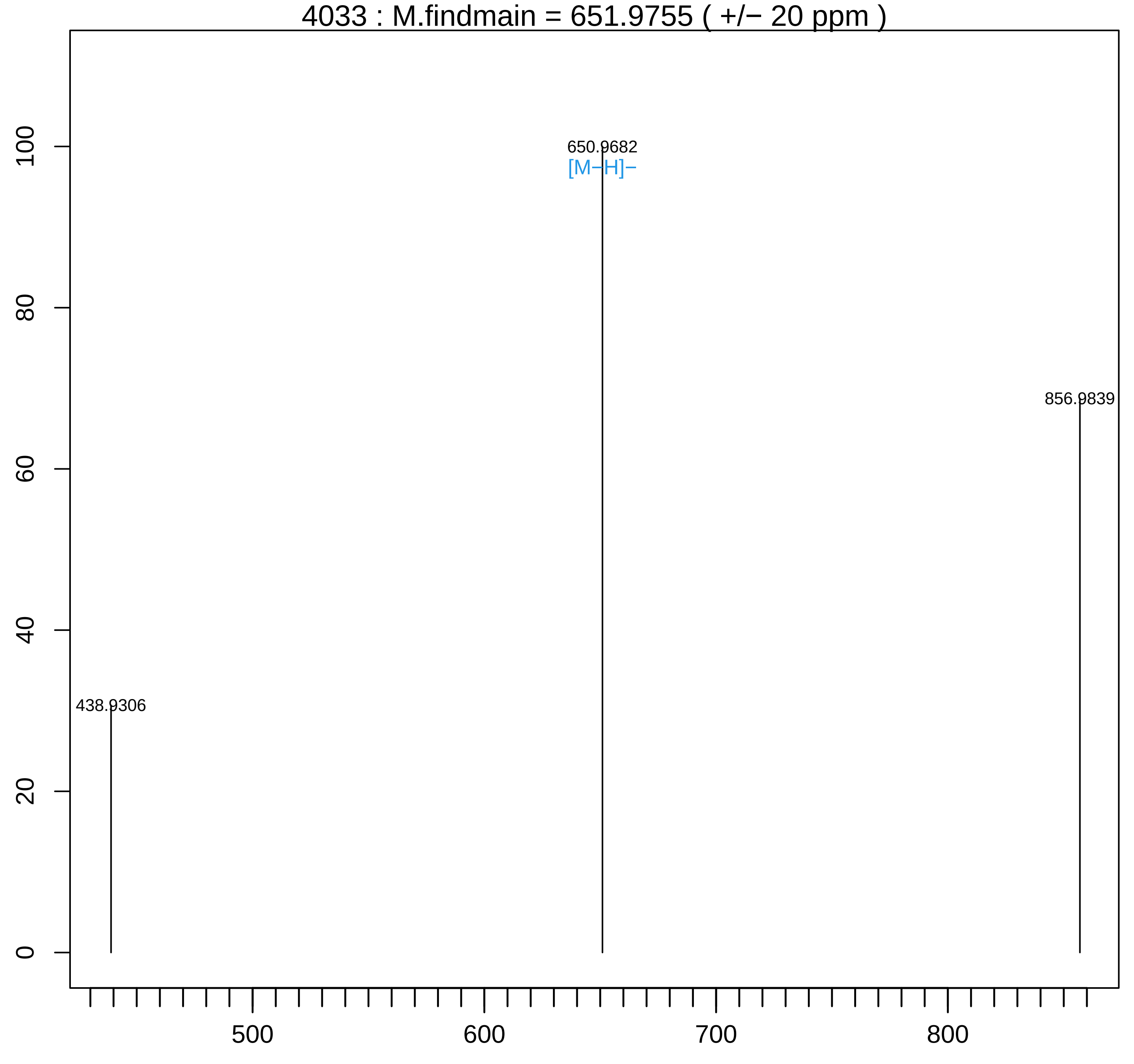
4033 : M.findmain = 651.9755 (+/− 20 ppm)
650.9682
[M−H]−
856.9839
438.9306
0
20
40
60
80
100
500
600
700
800

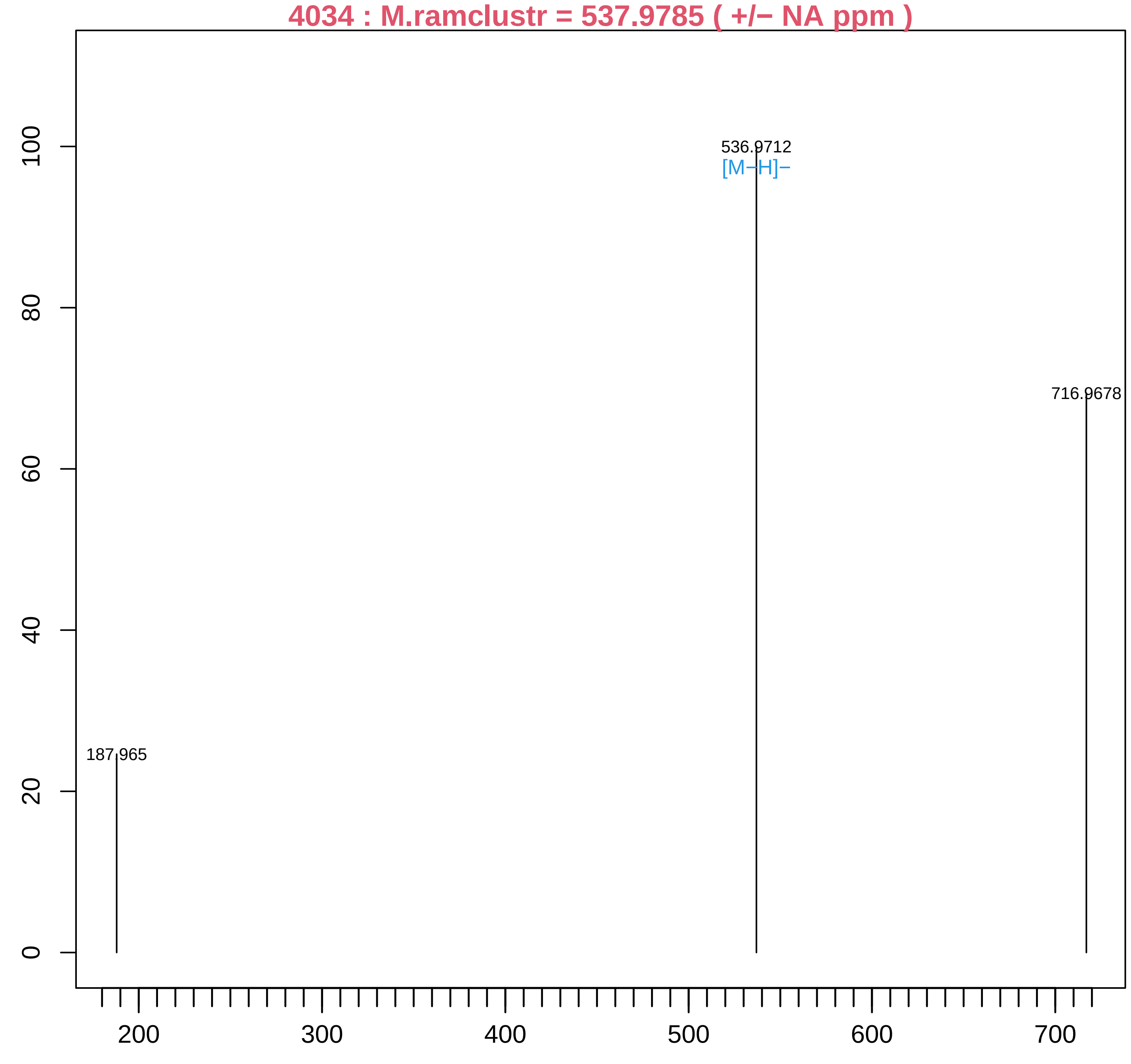
4034 : M.ramclustr = 537.9785 (+/− NA ppm)
536.9712
[M−H]−
716.9678
187.965
0
20
40
60
80
100
200
300
400
500
600
700

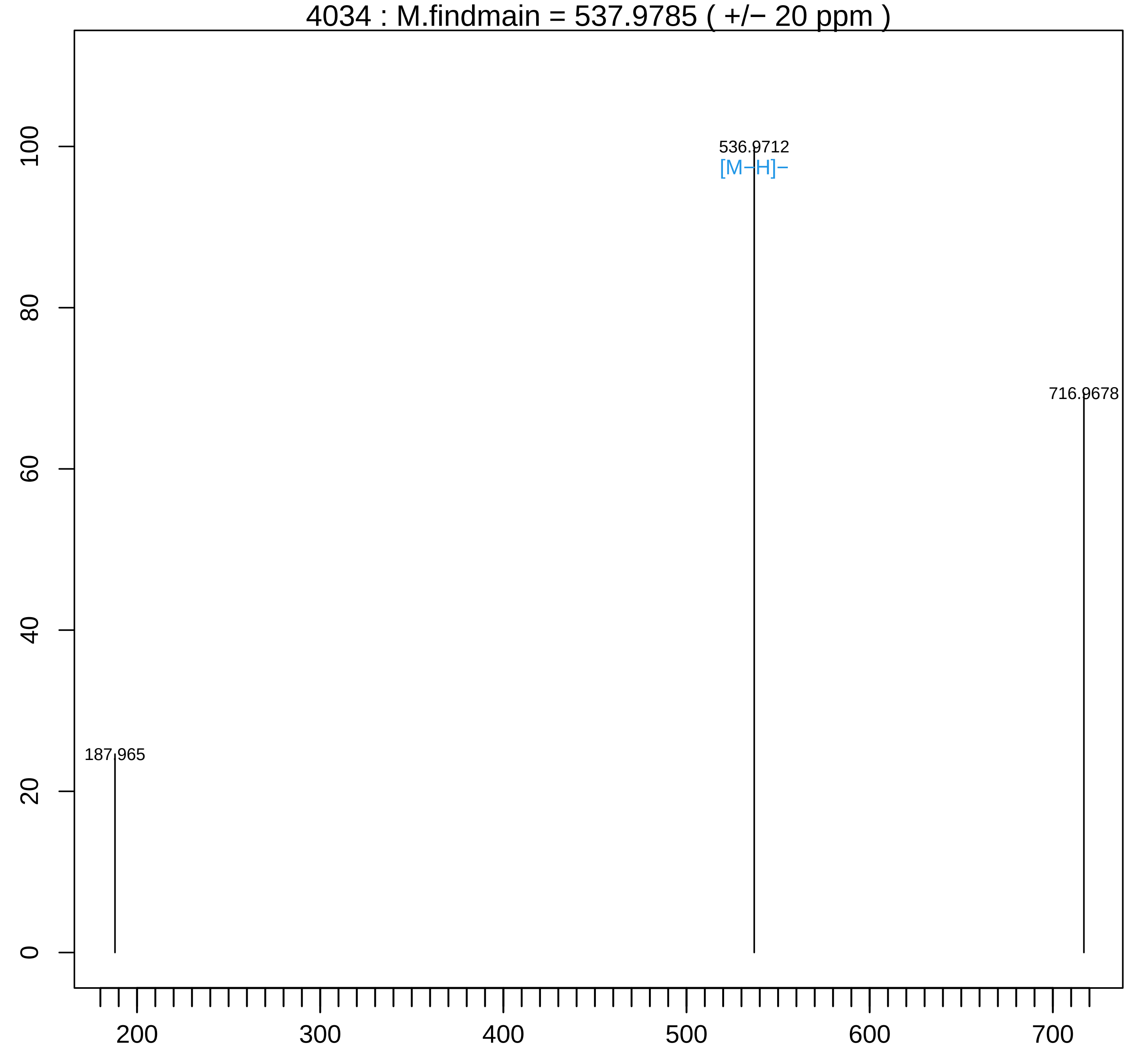
4034 : M.findmain = 537.9785 (+/− 20 ppm)
536.9712
[M−H]−
716.9678
187.965
0
20
40
60
80
100
200
300
400
500
600
700

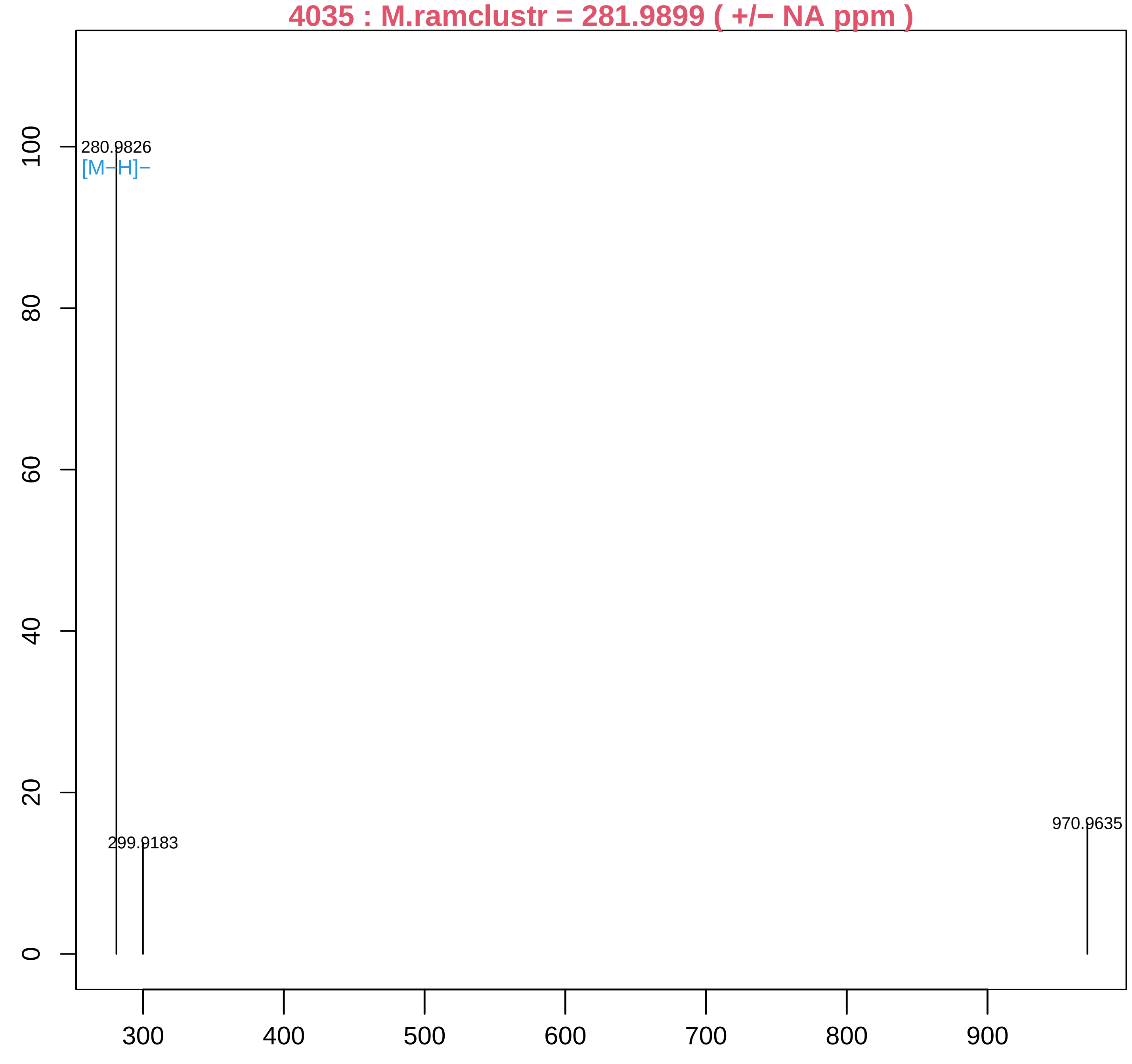
4035 : M.ramclustr = 281.9899 (+/− NA ppm)
280.9826
[M−H]−
299.9183
970.9635
0
20
40
60
80
100
300
400
500
600
700
800
900

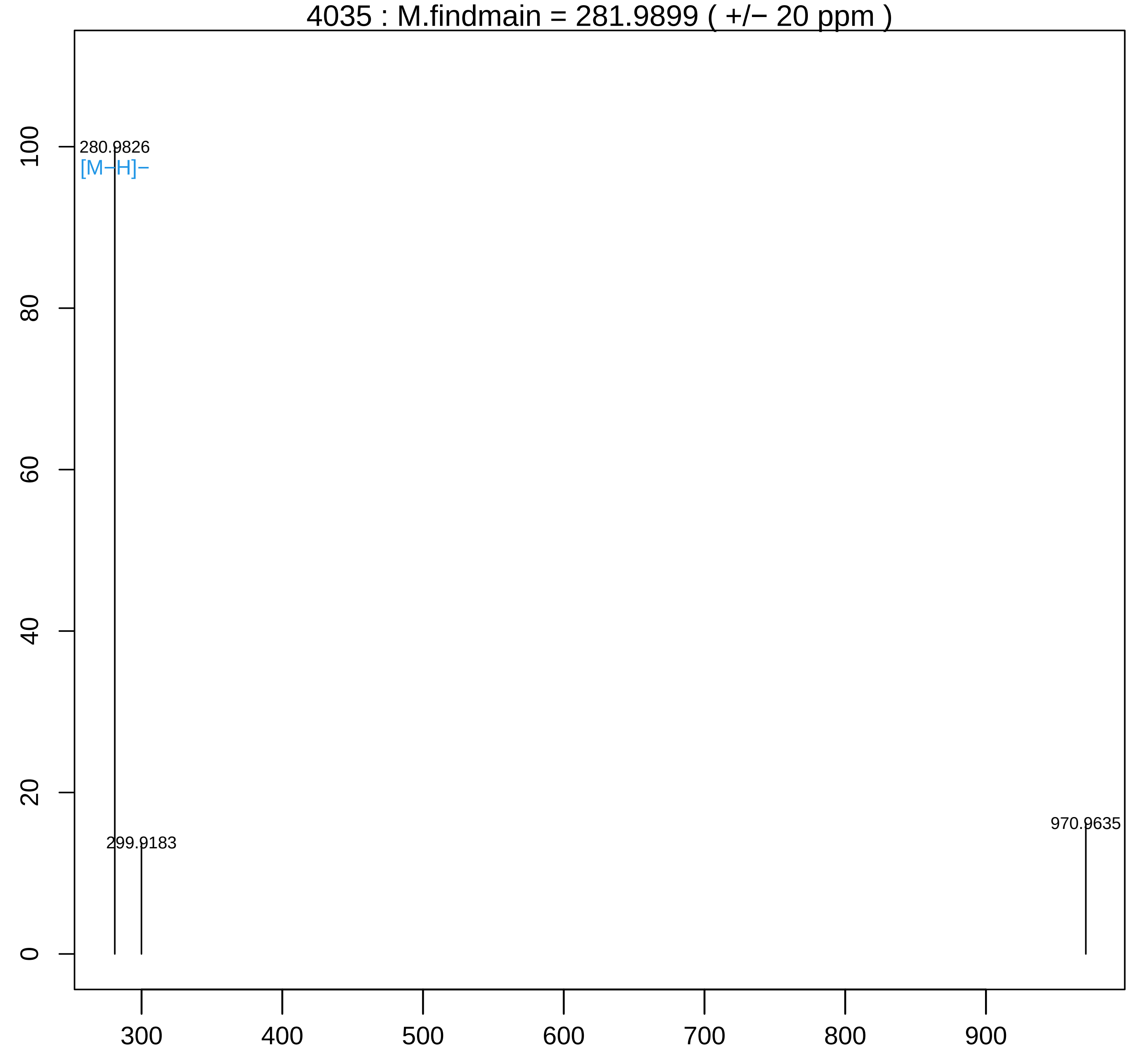
4035 : M.findmain = 281.9899 (+/− 20 ppm)
280.9826
[M−H]−
299.9183
970.9635
0
20
40
60
80
100
300
400
500
600
700
800
900

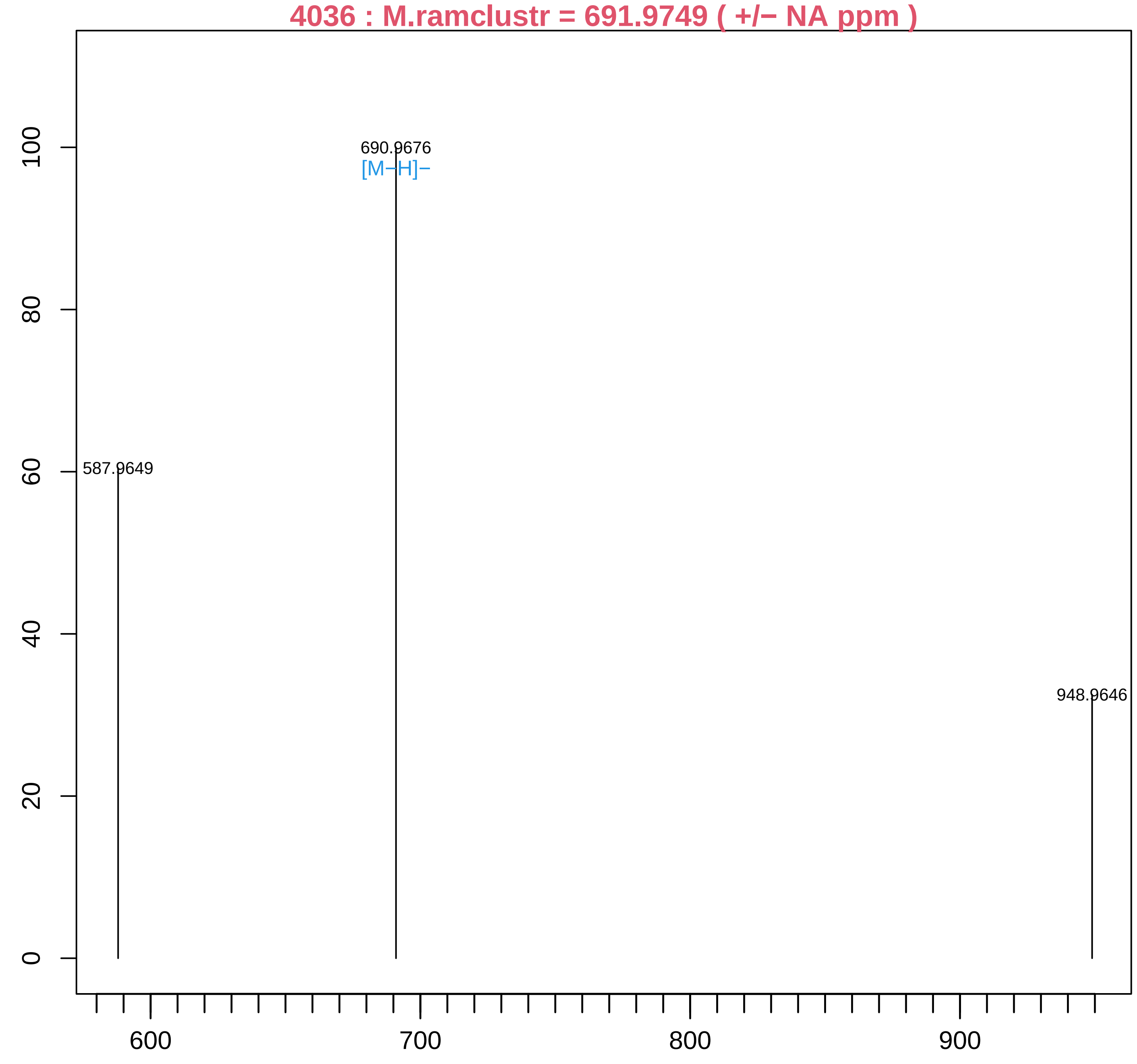

4036 : M.ramclustr = 691.9749 (+/− NA ppm)
690.9676
[M−H]−
587.9649
948.9646
0
20
40
60
80
100
600
700
800
900

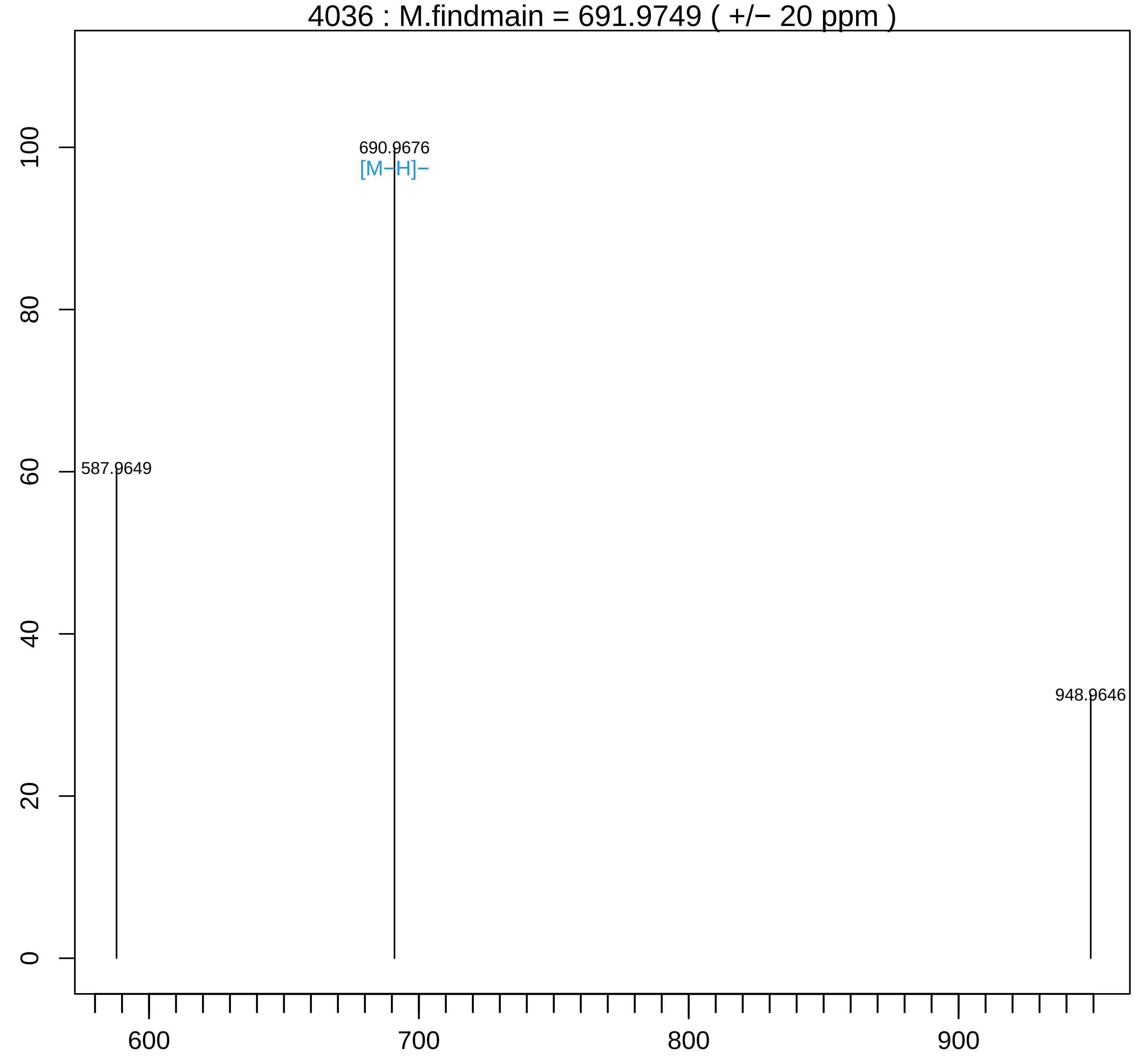

4036 : M.findmain = 691.9749 (+/− 20 ppm)
690.9676
[M−H]−
587.9649
948.9646
0
20
40
60
80
100
600
700
800
900

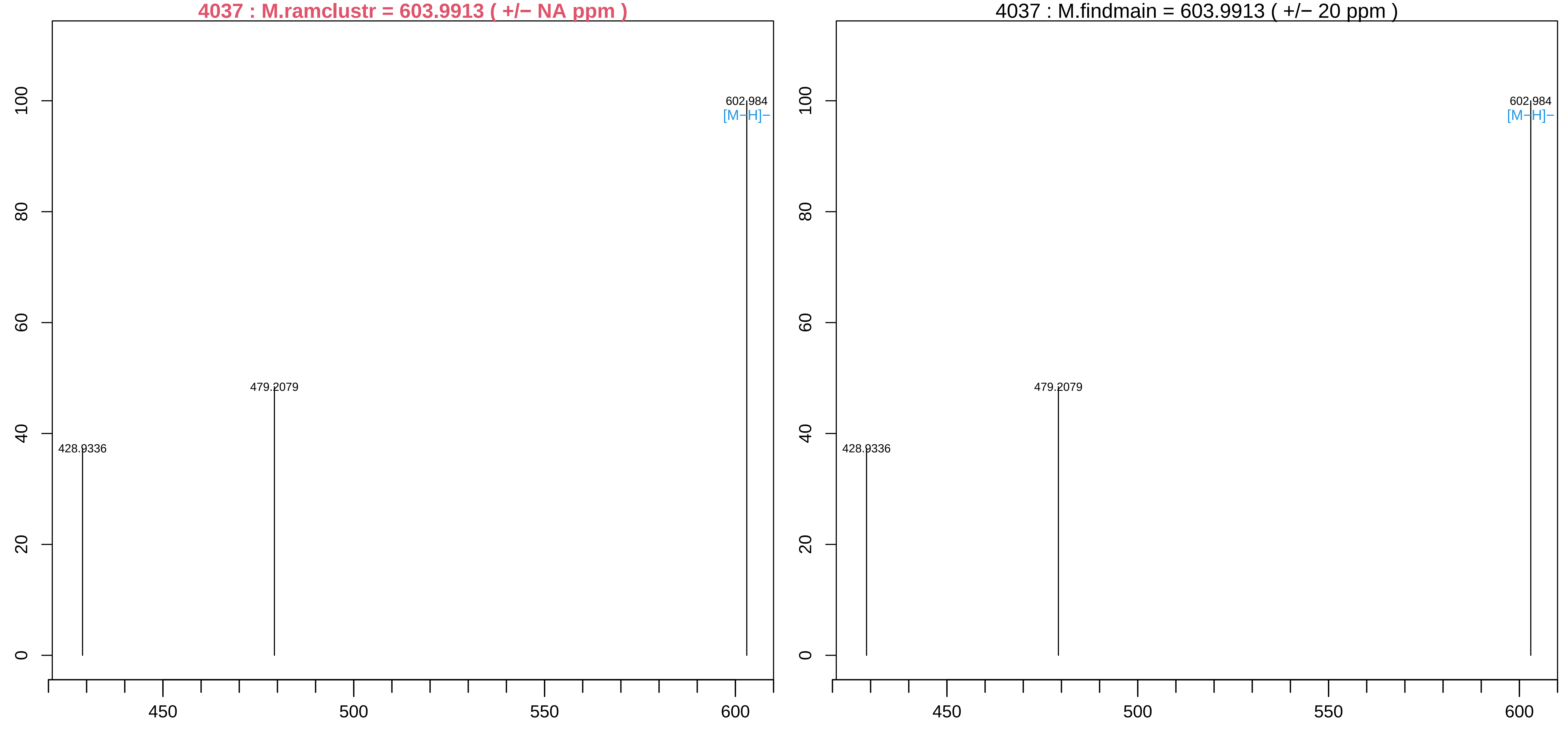

4037 : M.ramclustr = 603.9913 (+/− NA ppm)
4037 : M.findmain = 603.9913 (+/− 20 ppm)
602.984
[M−H]−
479.2079
428.9336
0
20
40
60
80
100
450
500
550
600

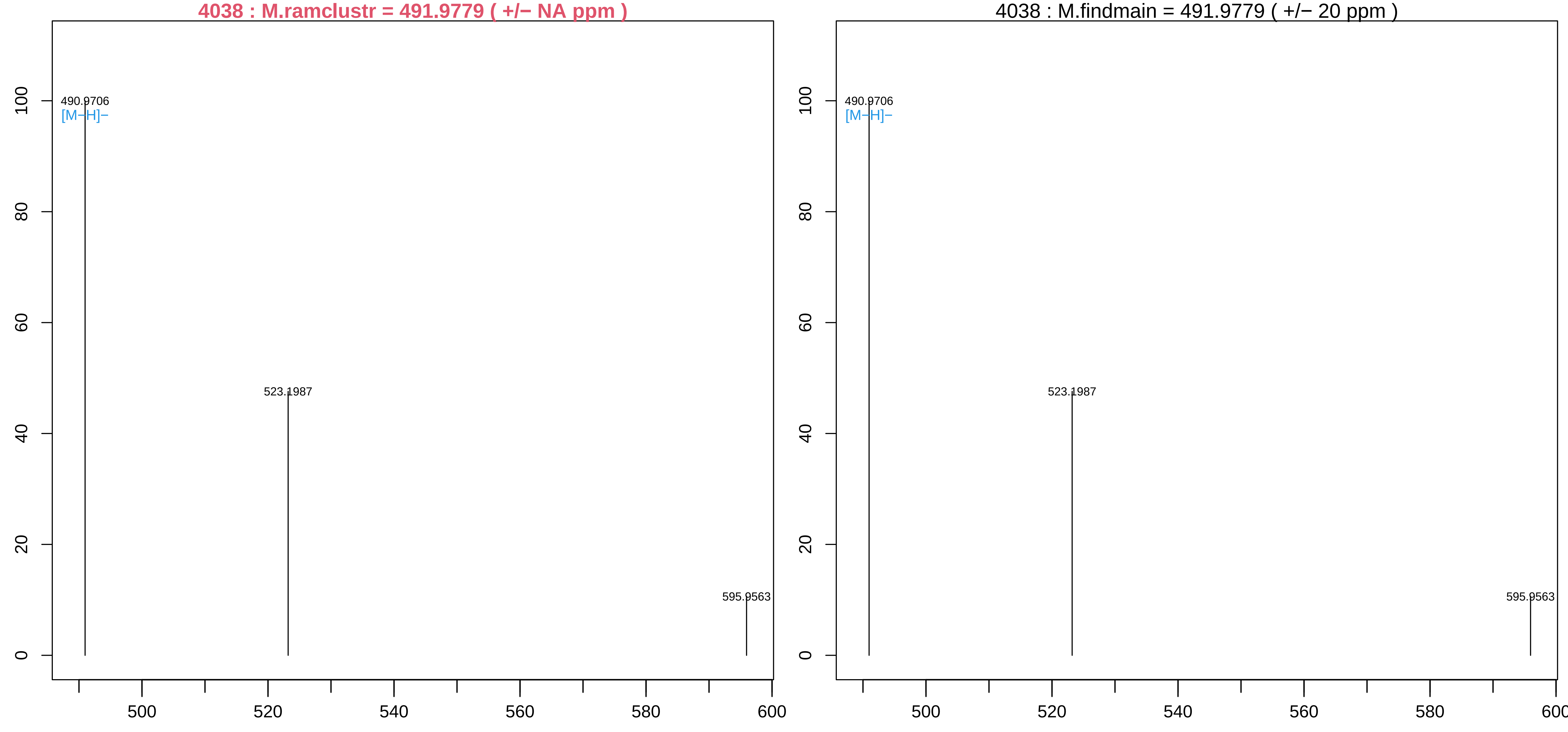

4038 : M.ramclustr = 491.9779 (+/− NA ppm)
490.9706
[M−H]−
523.1987
595.9563
4038 : M.findmain = 491.9779 (+/− 20 ppm)
490.9706
[M−H]−
523.1987
595.9563

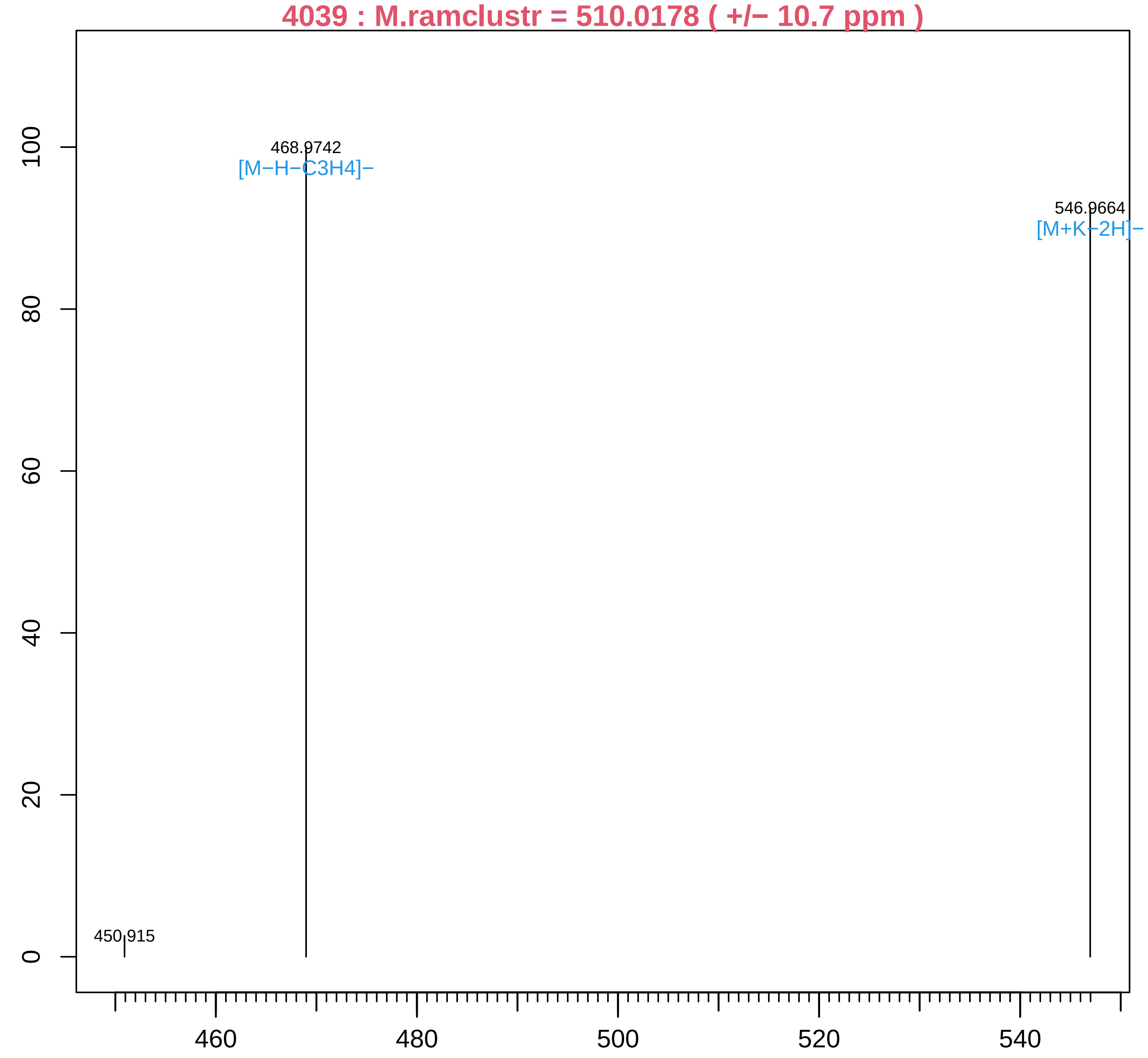
4039 : M.ramclustr = 510.0178 (+/− 10.7 ppm)
468.9742
[M−H−C3H4]−
546.9664
[M+K−2H]−
450.915
0
20
40
60
80
100
460
480
500
520
540

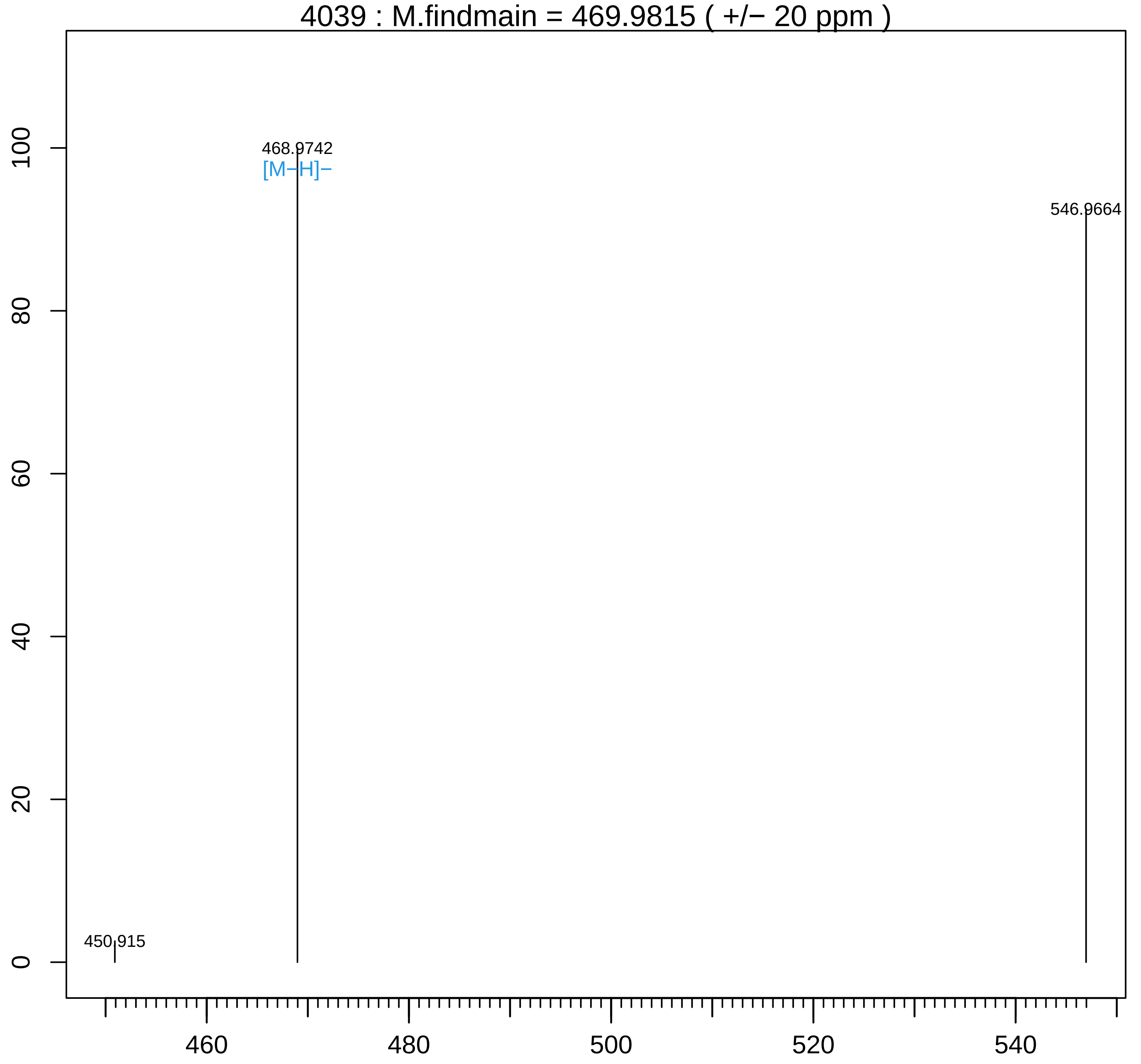
4039 : M.findmain = 469.9815 (+/− 20 ppm)
468.9742
[M−H]−
546.9664
450.915
0
20
40
60
80
100
460
480
500
520
540

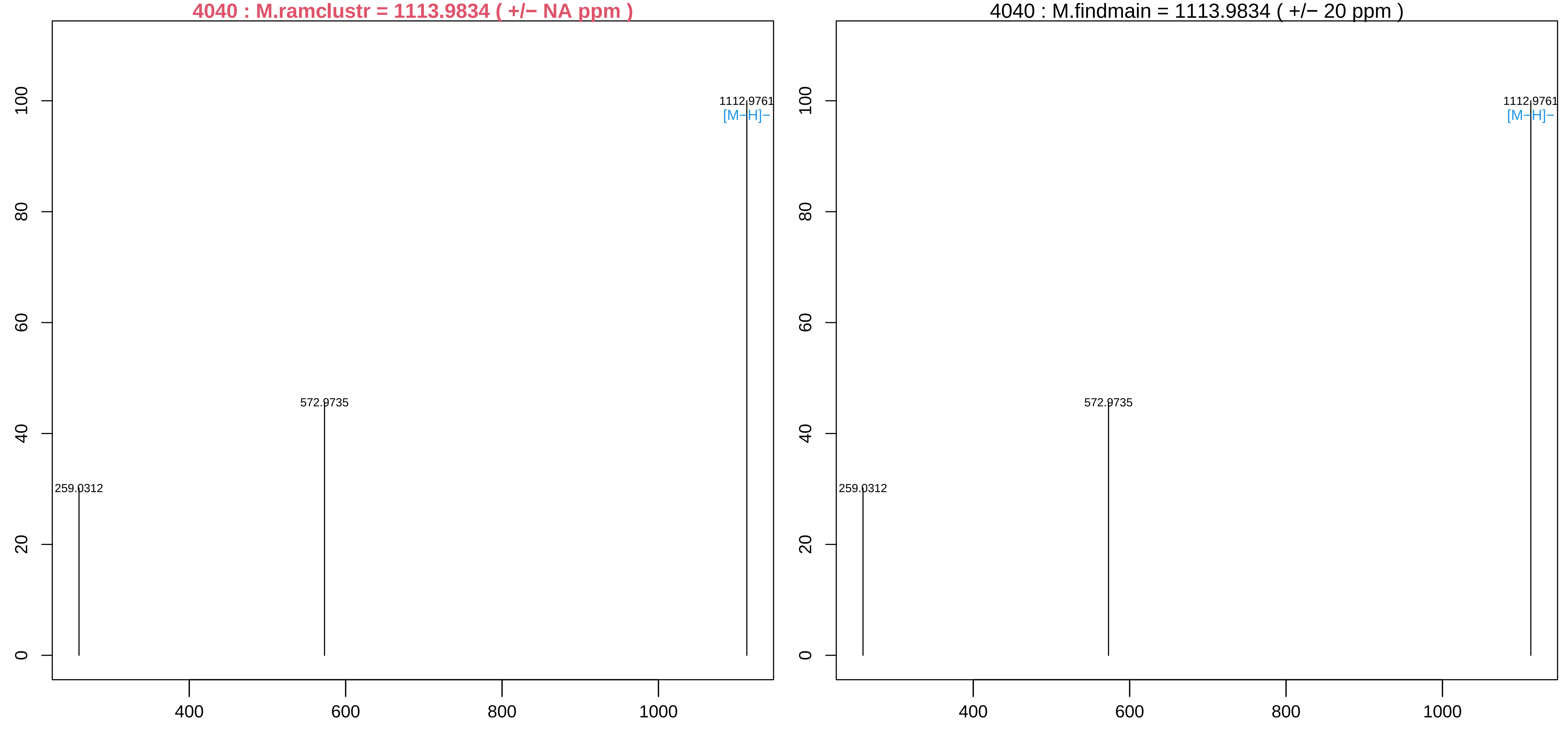
4040 : M.ramclustr = 1113.9834 (+/− NA ppm)
1112.9761
[M−H]−
572.9735
259.0312
4040 : M.findmain = 1113.9834 (+/− 20 ppm)
1112.9761
[M−H]−
572.9735
259.0312
0
20
40
60
80
100
400
600
800
1000

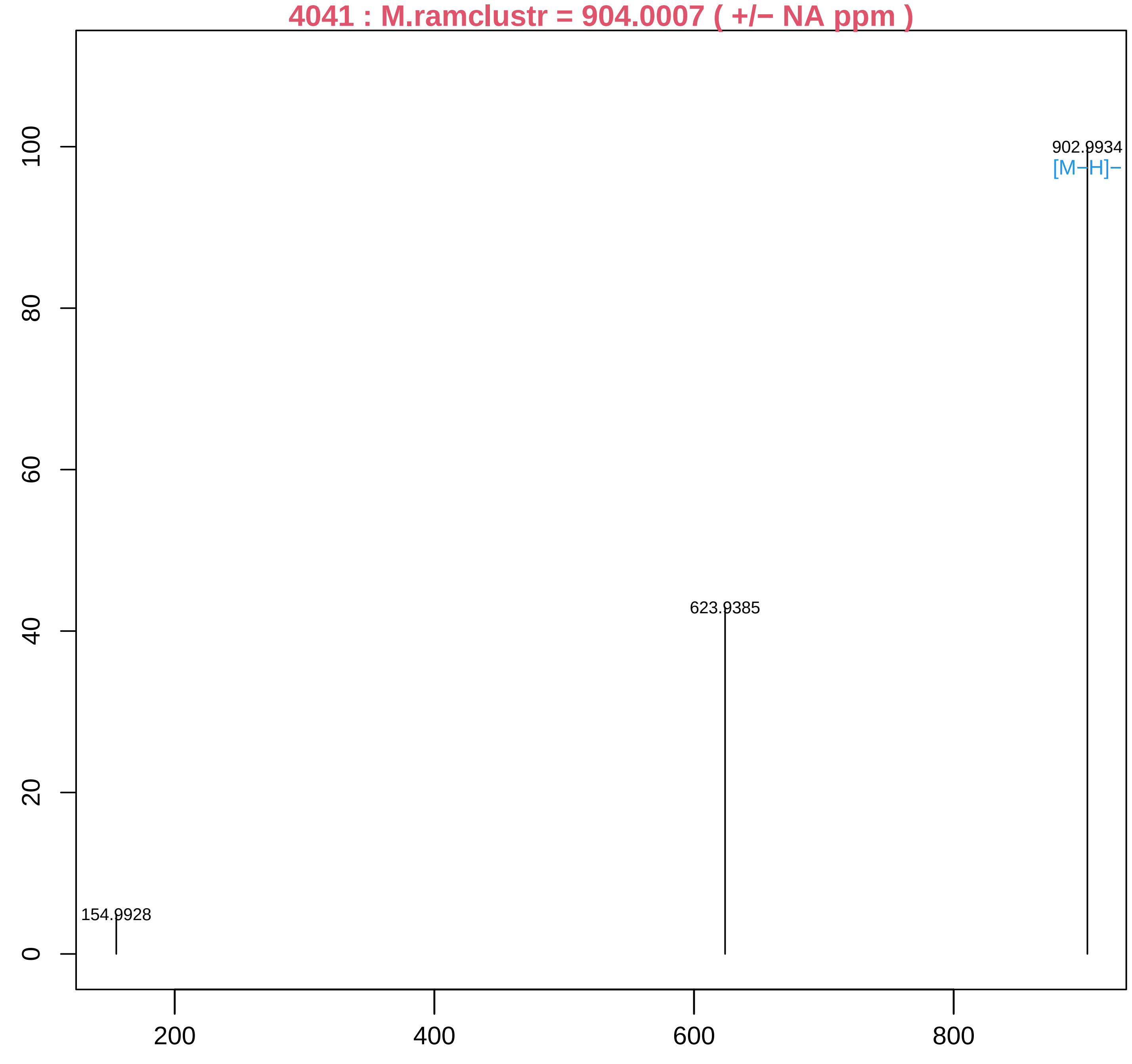
4041 : M.ramclustr = 904.0007 (+/− NA ppm)
902.9934
[M−H]−
623.9385
154.9928
0
20
40
60
80
100
200
400
600
800

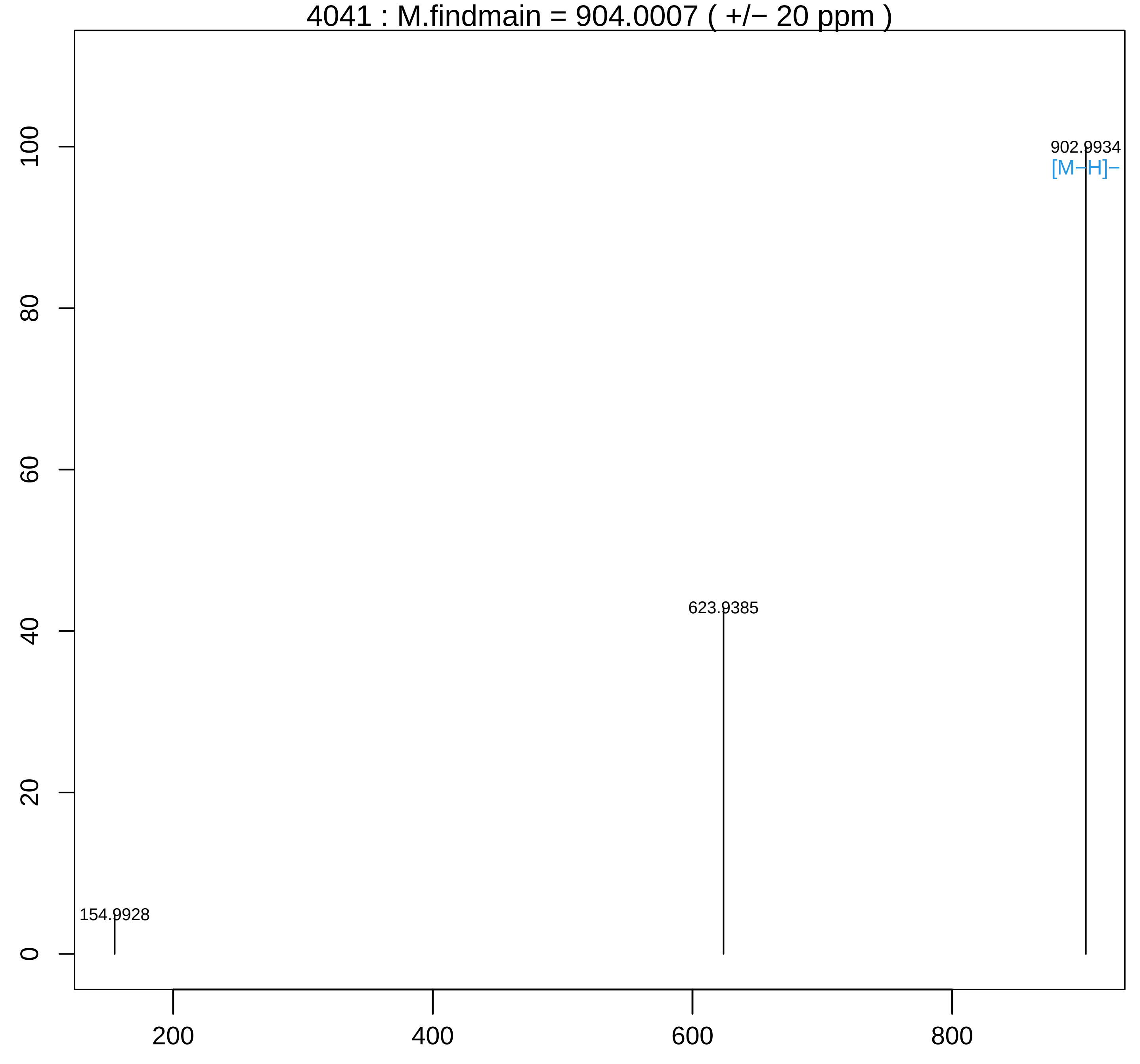
4041 : M.findmain = 904.0007 (+/− 20 ppm)
902.9934
[M−H]−
623.9385
154.9928
0
20
40
60
80
100
200
400
600
800

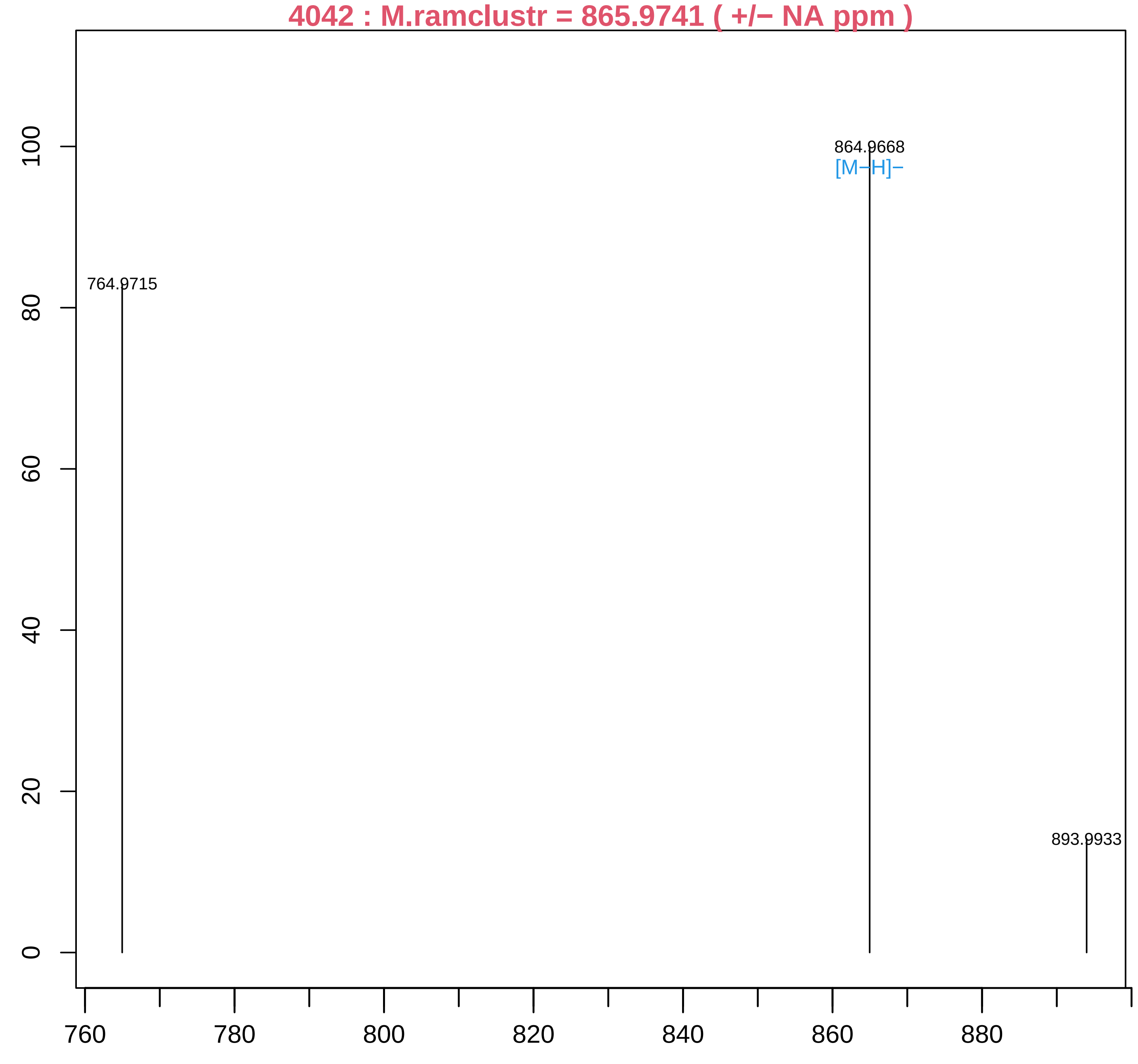
4042 : M.ramclustr = 865.9741 (+/− NA ppm)
764.9715
864.9668
[M−H]−
893.9933
0
20
40
60
80
100
760
780
800
820
840
860
880
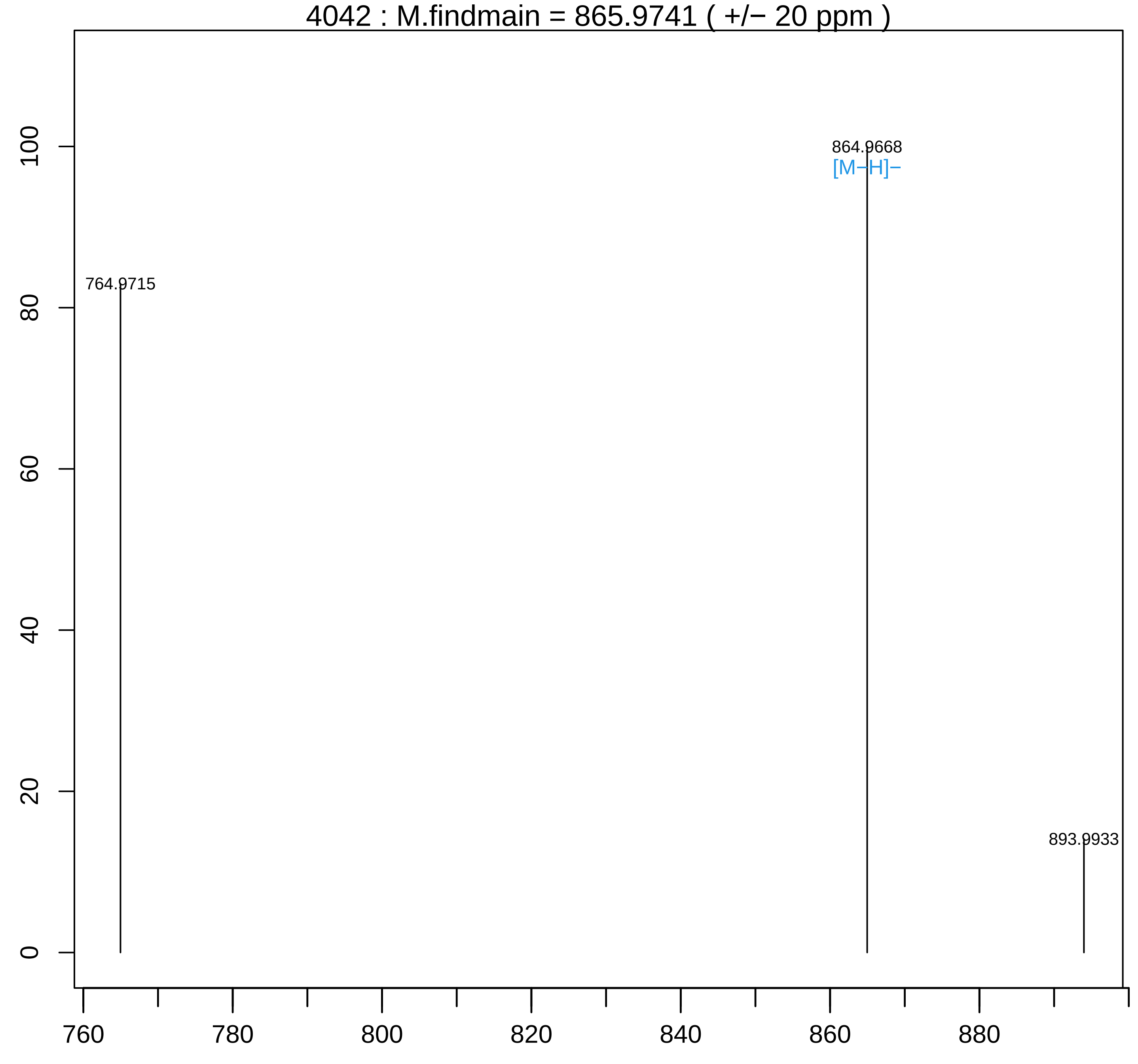
4042 : M.findmain = 865.9741 (+/− 20 ppm)
764.9715
864.9668
[M−H]−
893.9933
0
20
40
60
80
100
760
780
800
820
840
860
880

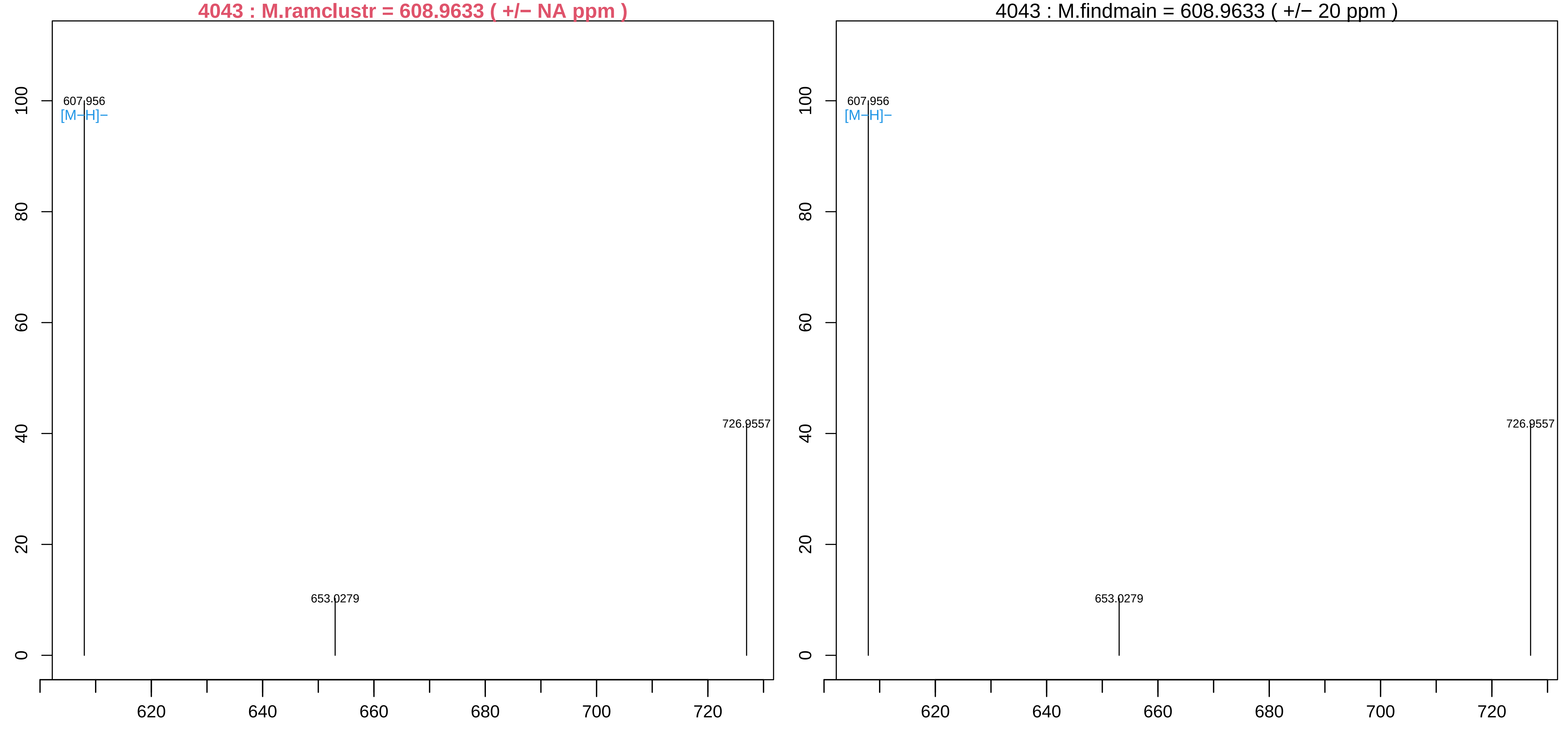
4043 : M.ramclustr = 608.9633 (+/− NA ppm)
607.956
[M−H]−
653.0279
726.9557
0
20
40
60
80
100
620
640
660
680
700
720
4043 : M.findmain = 608.9633 (+/− 20 ppm)
607.956
[M−H]−
653.0279
726.9557
0
20
40
60
80
100
620
640
660
680
700
720

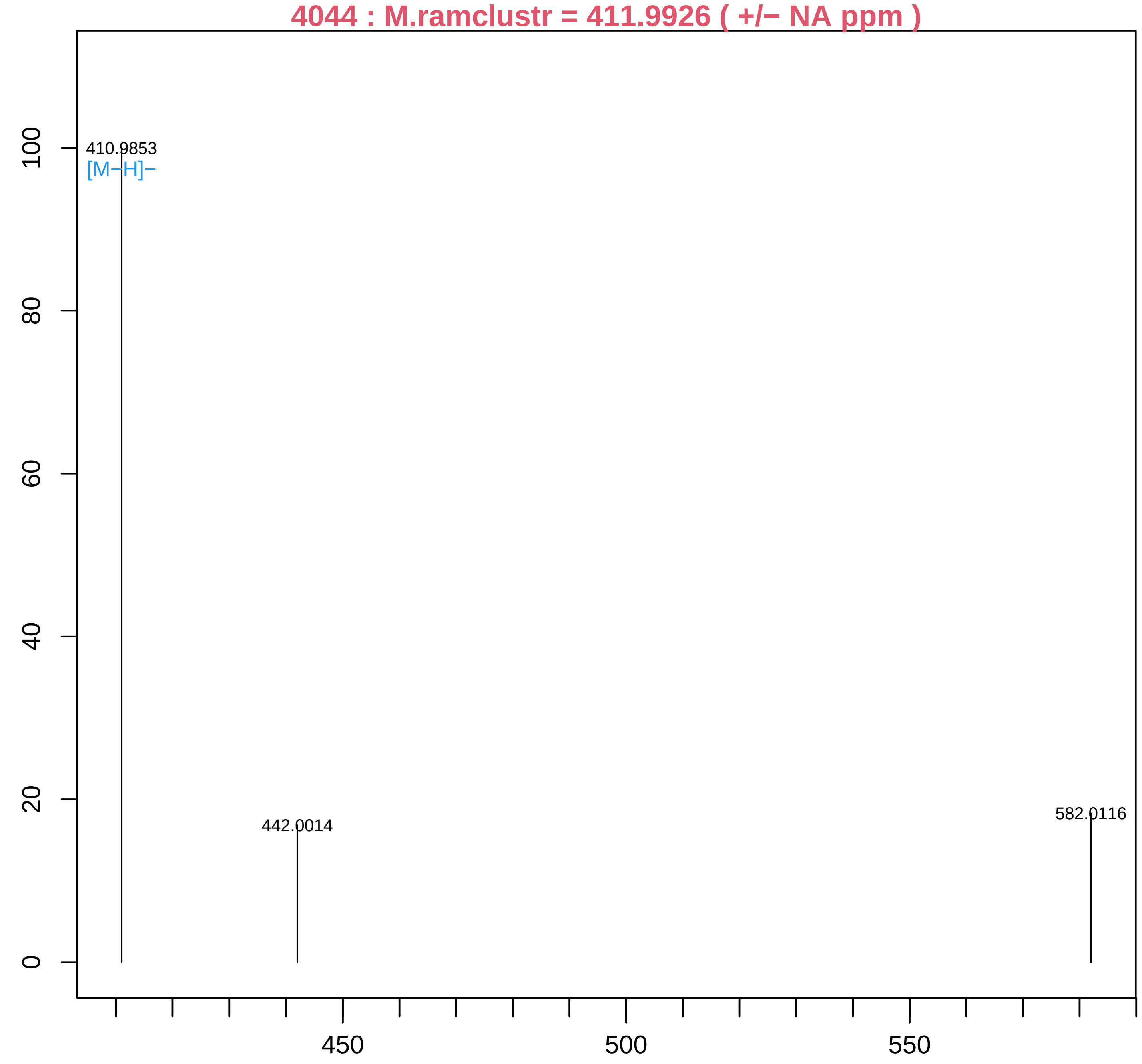
4044 : M.ramclustr = 411.9926 (+/− NA ppm)
410.9853
[M−H]−
442.0014
582.0116
0
20
40
60
80
100
450
500
550

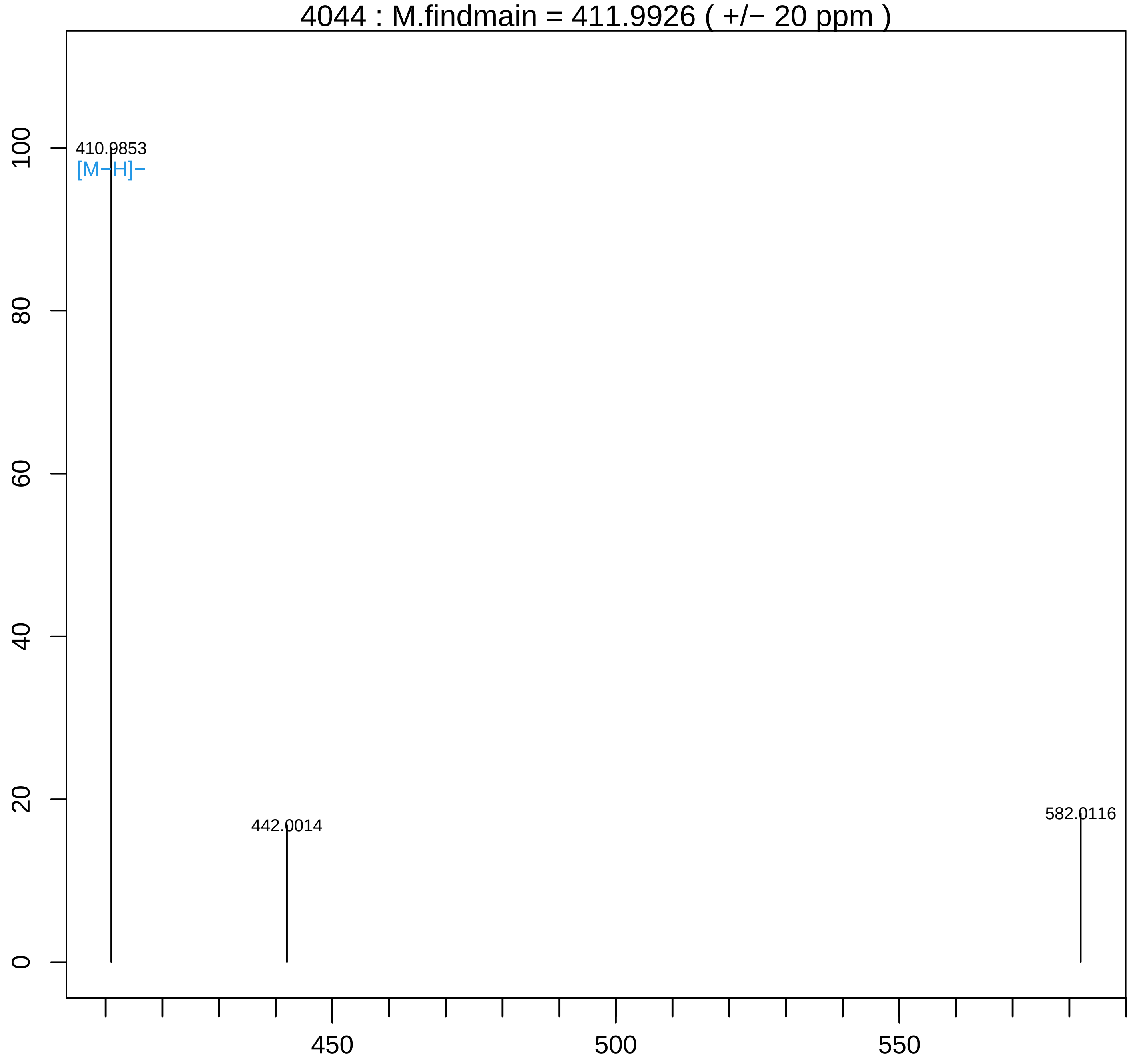
4044 : M.findmain = 411.9926 (+/− 20 ppm)
410.9853
[M−H]−
442.0014
582.0116
0
20
40
60
80
100
450
500
550

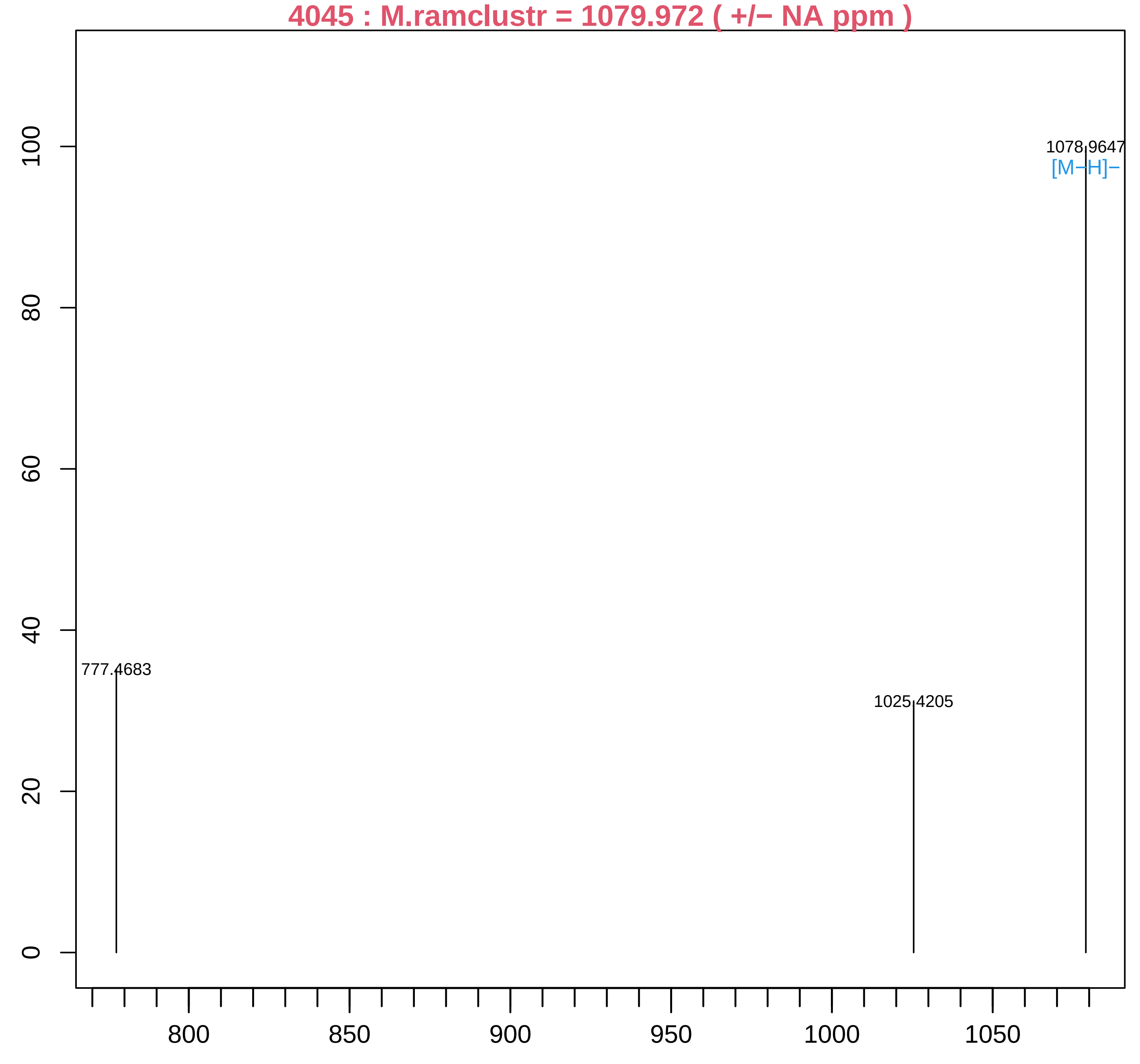
4045 : M.ramclustr = 1079.972 (+/− NA ppm)
1078.9647
[M−H]−
777.4683
1025.4205
0
20
40
60
80
100
800
850
900
950
1000
1050

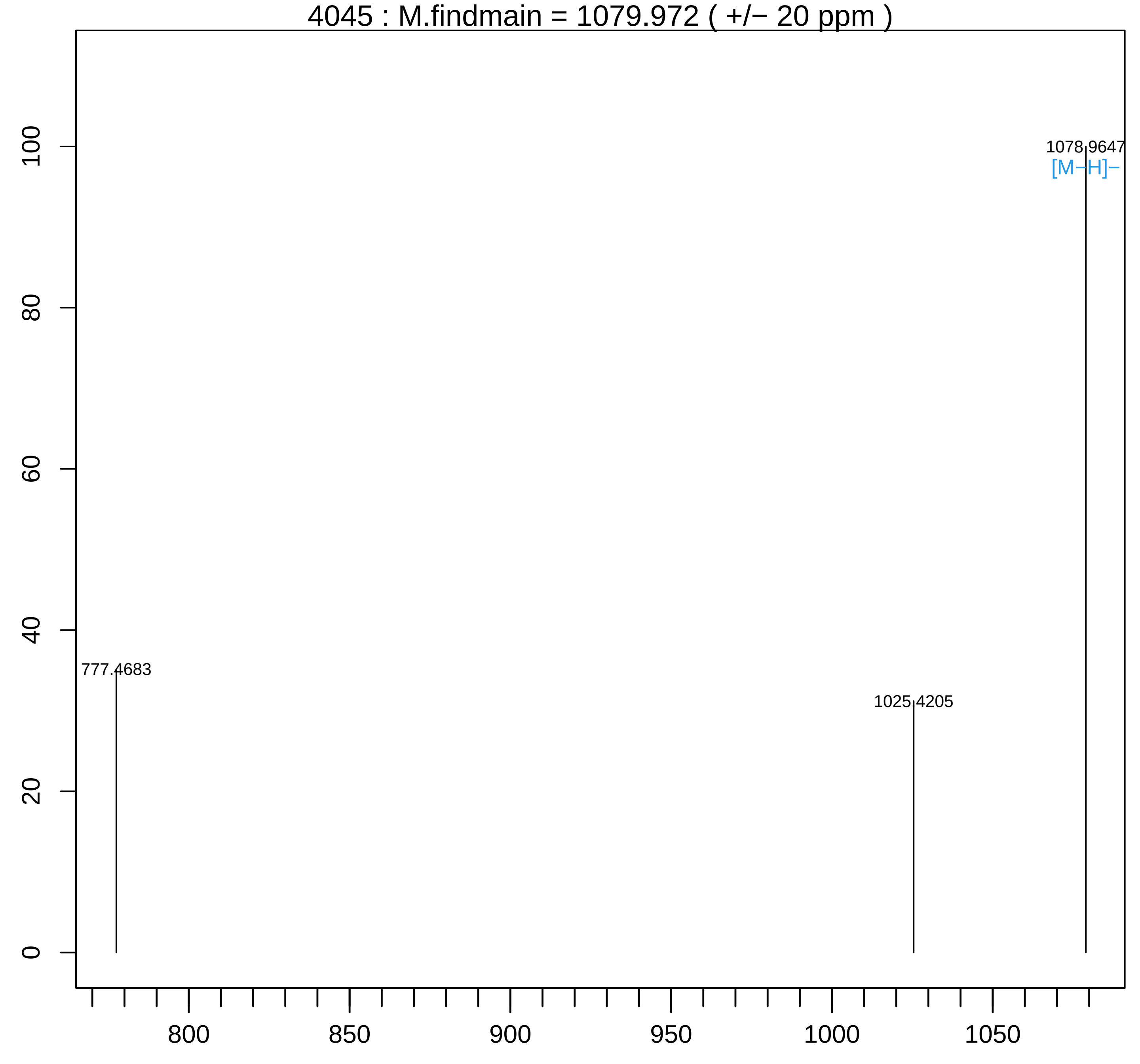
4045 : M.findmain = 1079.972 (+/− 20 ppm)
1078.9647
[M−H]−
777.4683
1025.4205
0
20
40
60
80
100
800
850
900
950
1000
1050

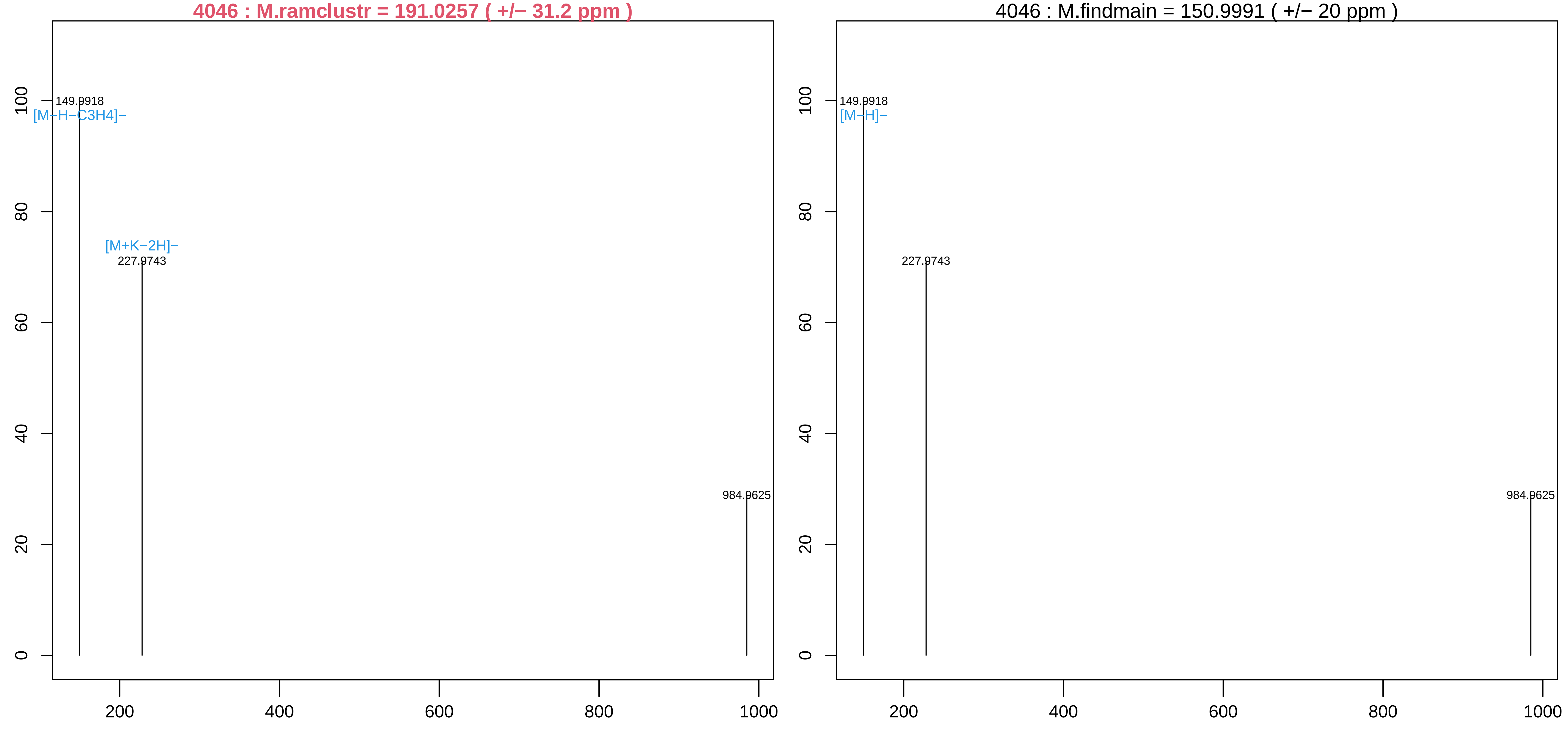
4046 : M.ramclustr = 191.0257 (+/− 31.2 ppm)
149.9918
[M−H−C3H4]−
[M+K−2H]−
227.9743
984.9625
0
20
40
60
80
100
200
400
600
800
1000
4046 : M.findmain = 150.9991 (+/− 20 ppm)
149.9918
[M−H]−
227.9743
984.9625

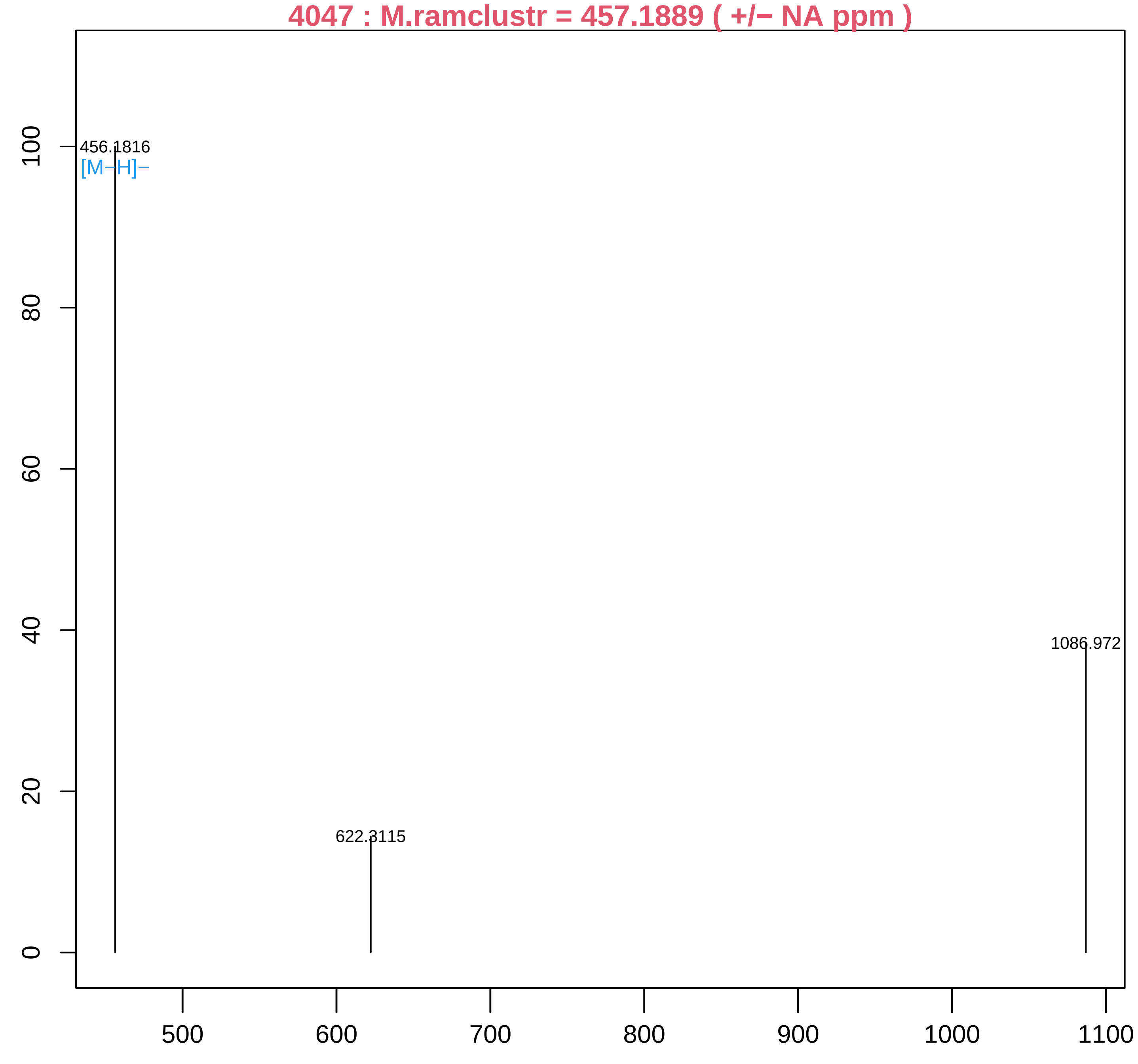

4047 : M.ramclustr = 457.1889 (+/− NA ppm)
456.1816
[M−H]−
622.3115
1086.972
0
20
40
60
80
100
500
600
700
800
900
1000
1100

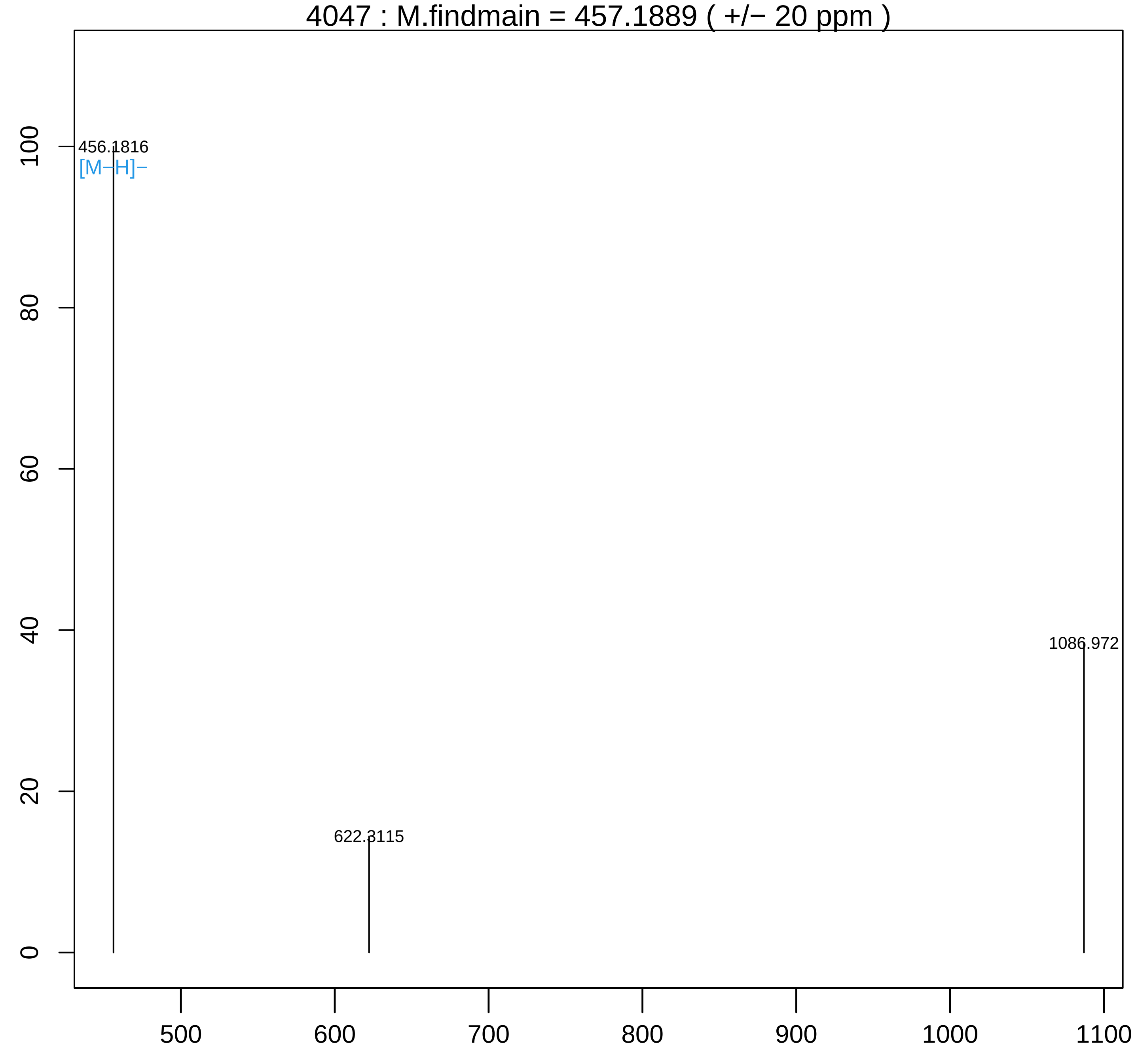

4047 : M.findmain = 457.1889 (+/− 20 ppm)
456.1816
[M−H]−
622.3115
1086.972
0
20
40
60
80
100
500
600
700
800
900
1000
1100

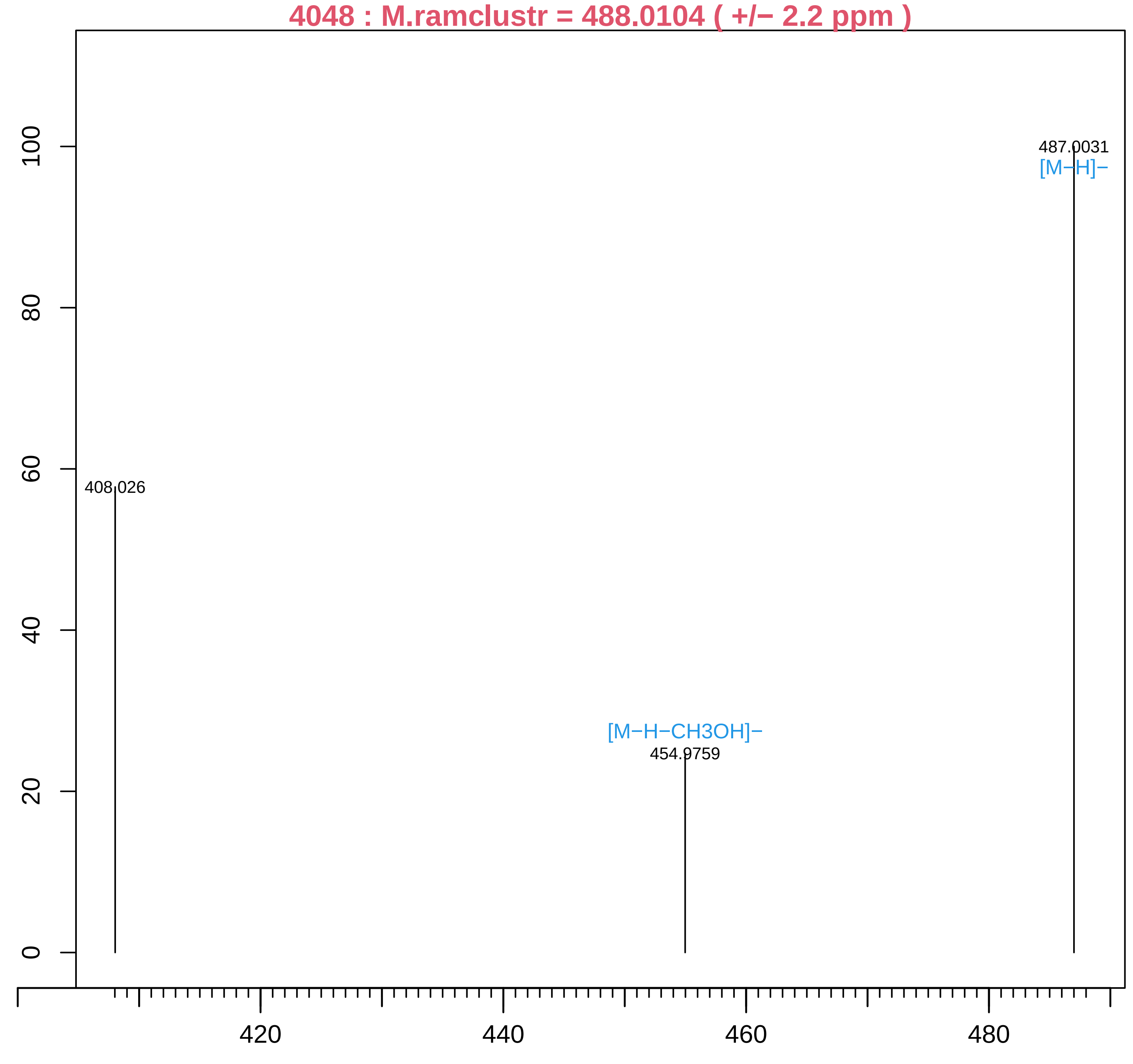
4048 : M.ramclustr = 488.0104 (+/− 2.2 ppm)
487.0031
[M−H]−
408.026
[M−H−CH3OH]−
454.9759
0
20
40
60
80
100
420
440
460
480

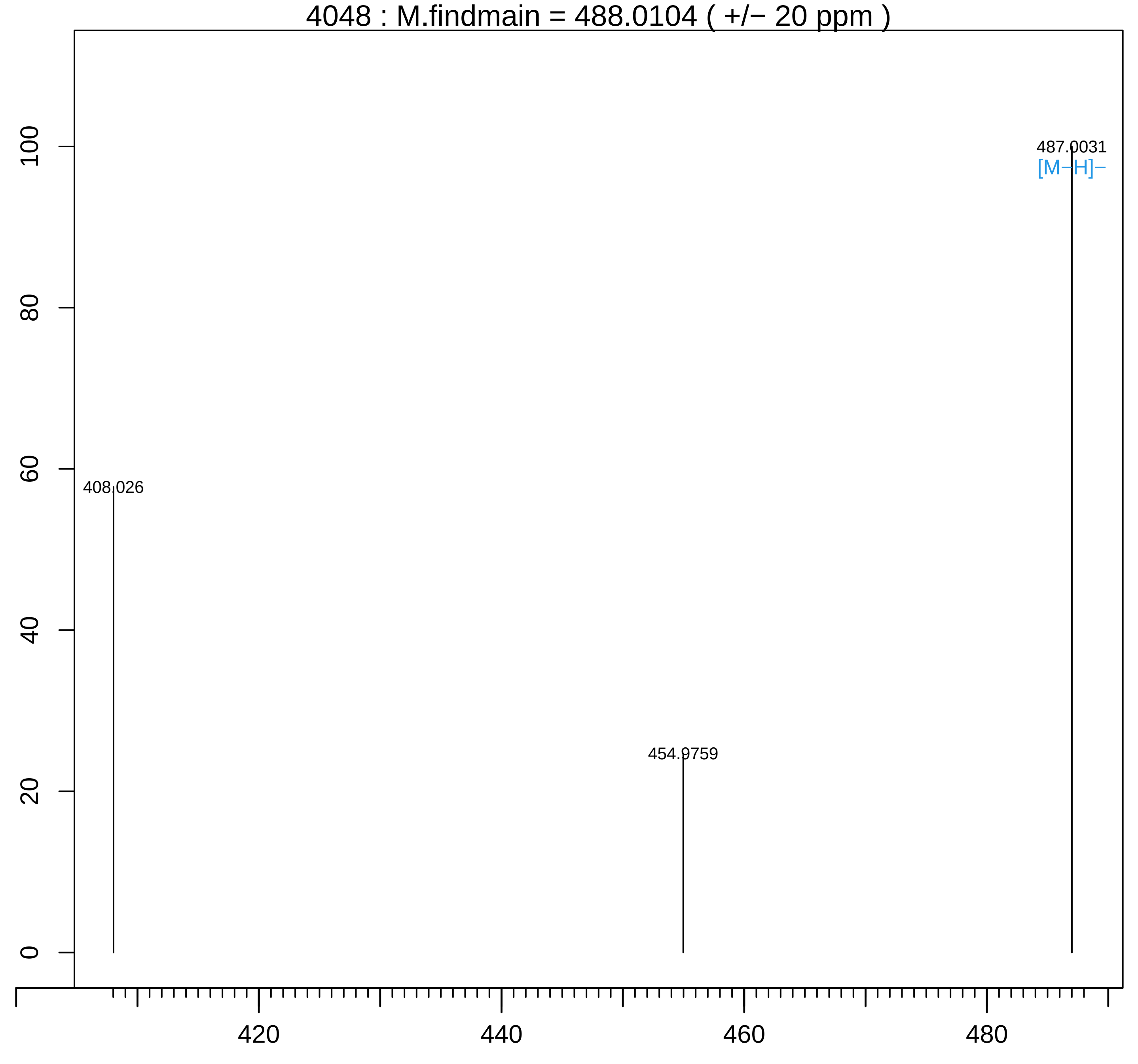
4048 : M.findmain = 488.0104 (+/− 20 ppm)
487.0031
[M−H]−
408.026
454.9759
0
20
40
60
80
100
420
440
460
480

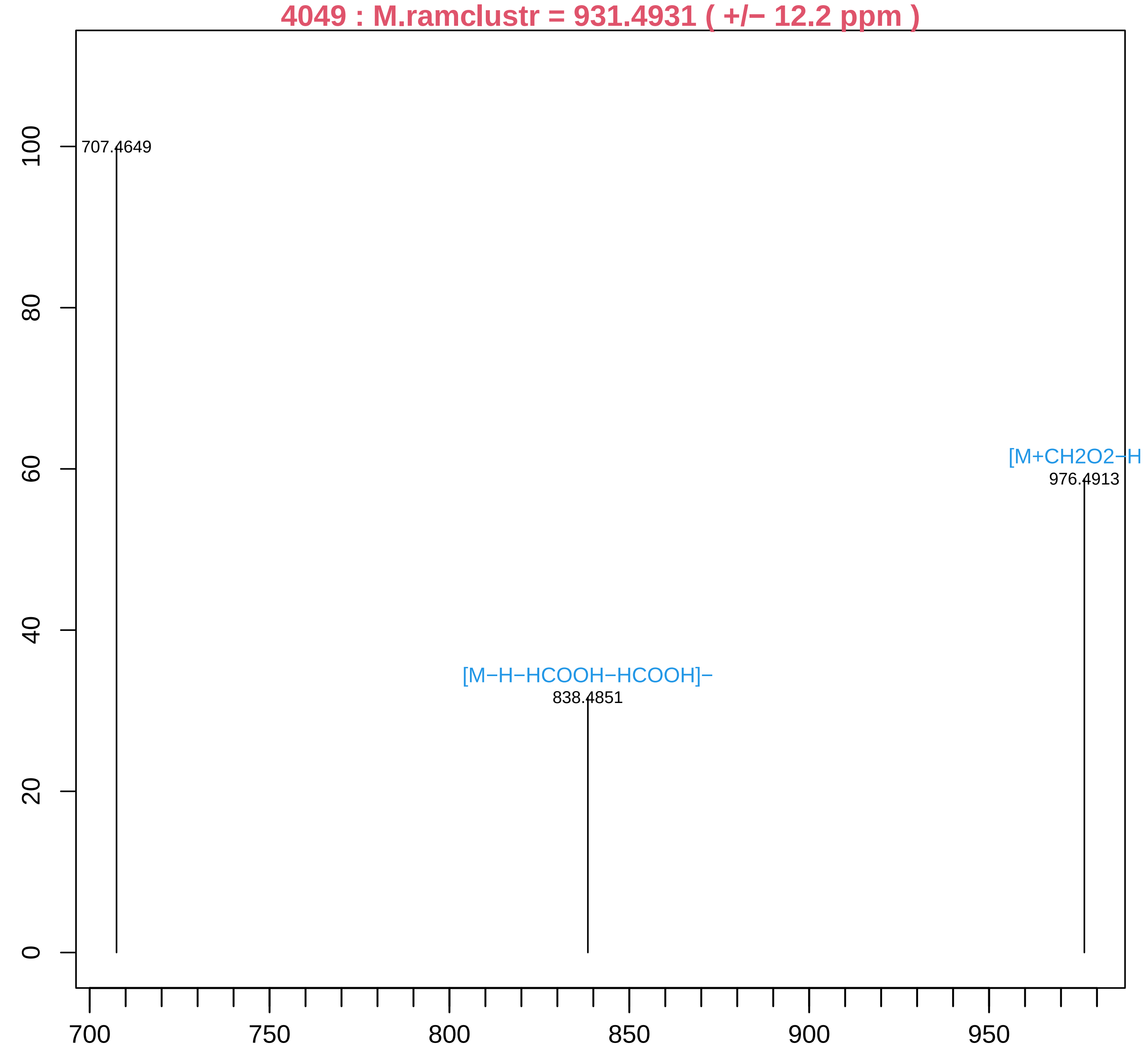
4049 : M.ramclustr = 931.4931 (+/− 12.2 ppm)
707.4649
[M−H−HCOOH−HCOOH]−
838.4851
[M+CH2O2−H
976.4913
0
20
40
60
80
100
700
750
800
850
900
950

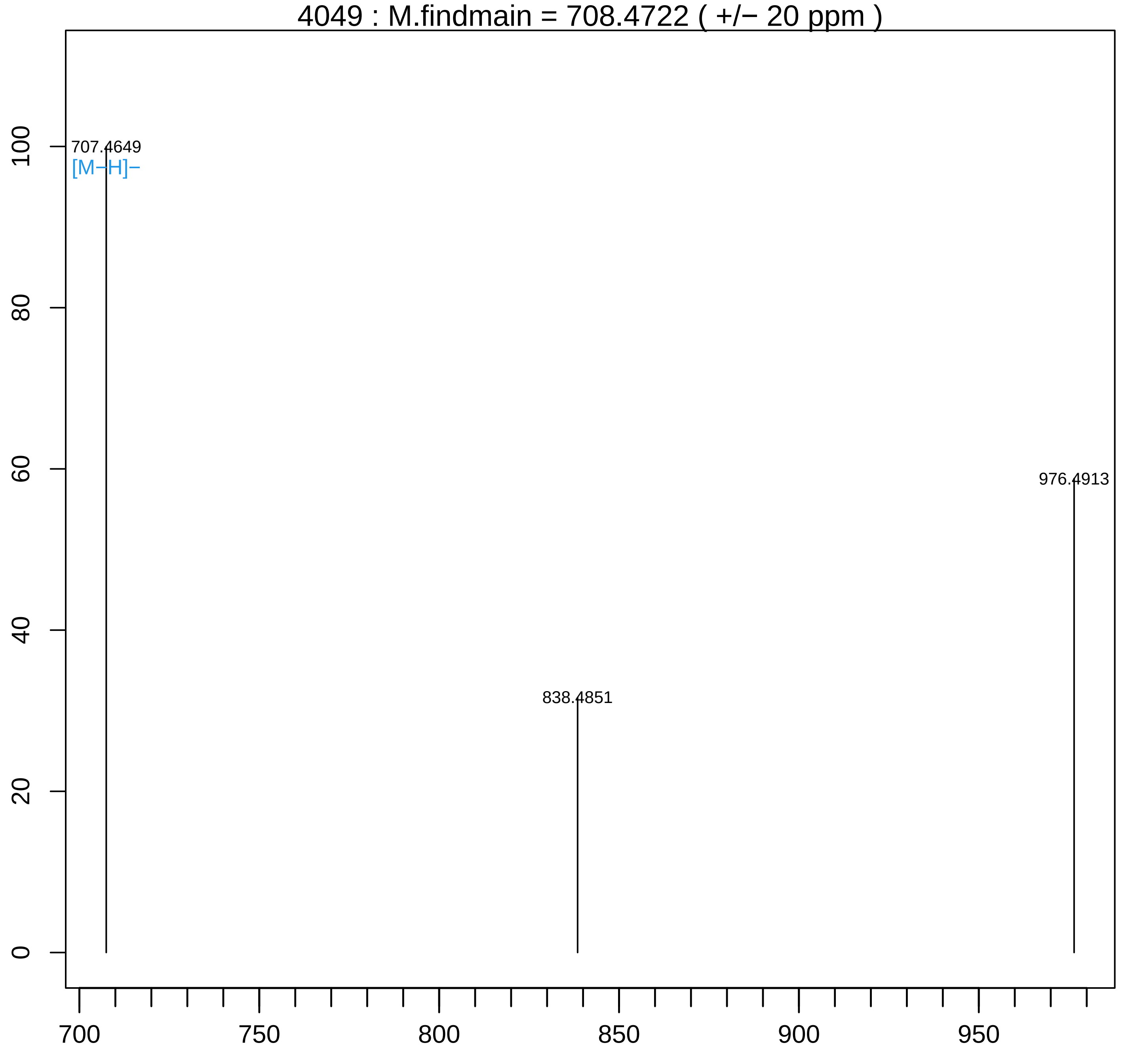
4049 : M.findmain = 708.4722 (+/− 20 ppm)
707.4649
[M−H]−
838.4851
976.4913
0
20
40
60
80
100
700
750
800
850
900
950

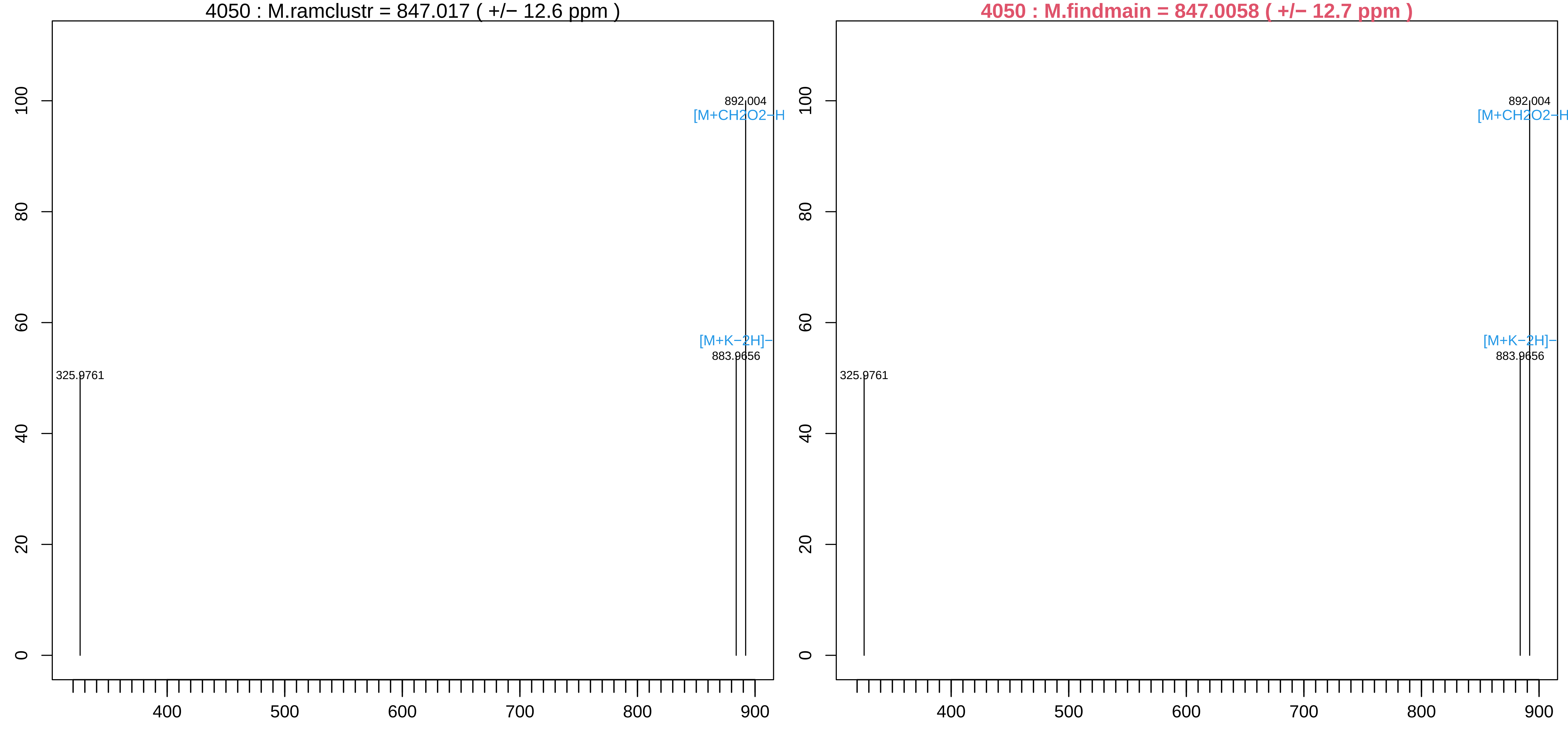
4050 : M.ramclustr = 847.017 (+/− 12.6 ppm)
892.004
[M+CH2O2−H
[M+K−2H]−
883.9656
325.9761
4050 : M.findmain = 847.0058 (+/− 12.7 ppm)
892.004
[M+CH2O2−H
[M+K−2H]−
883.9656
325.9761
0
20
40
60
80
100
400
500
600
700
800
900

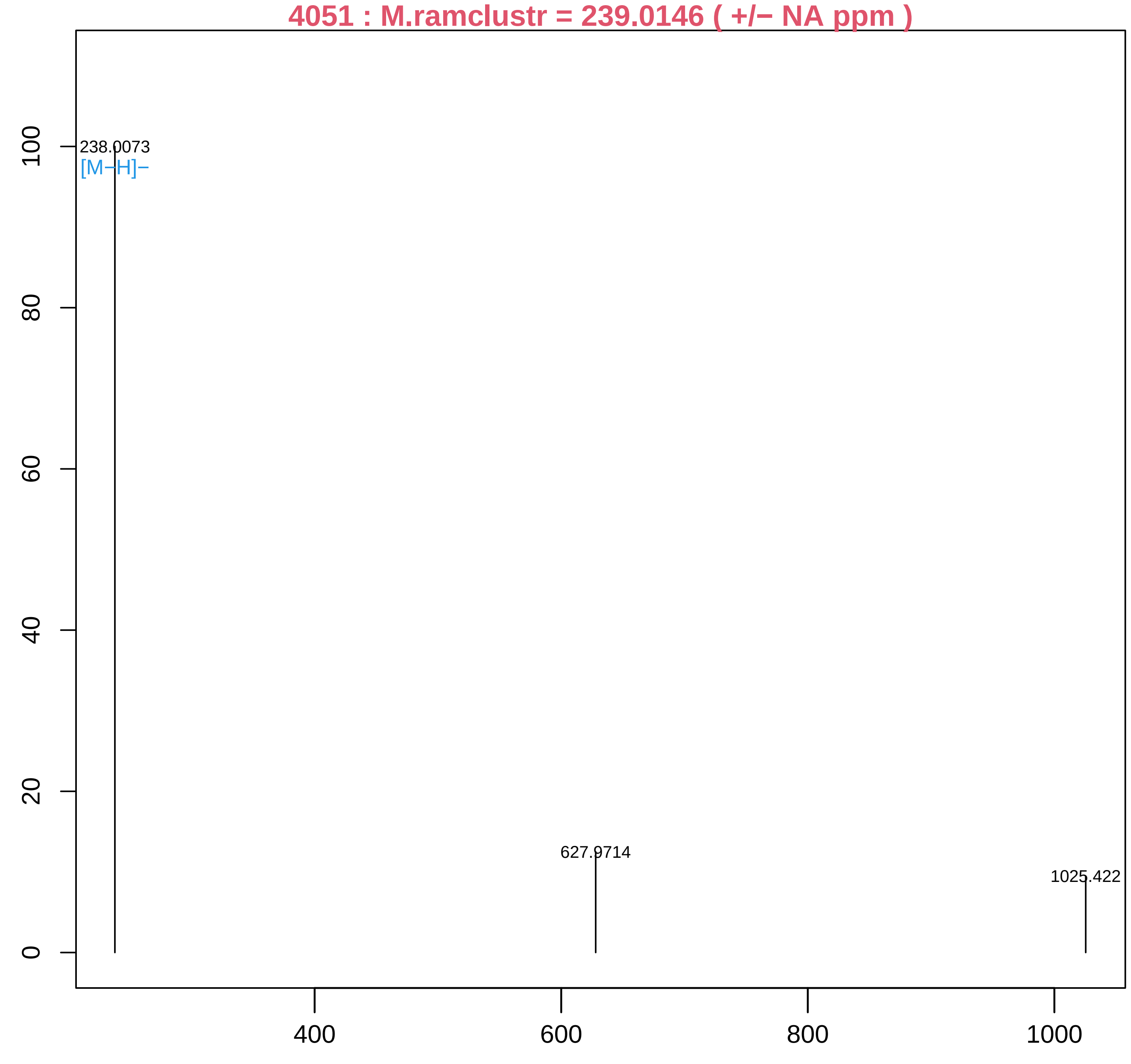
4051 : M.ramclustr = 239.0146 (+/− NA ppm)
238.0073
[M−H]−
627.9714
1025.422
0
20
40
60
80
100
400
600
800
1000

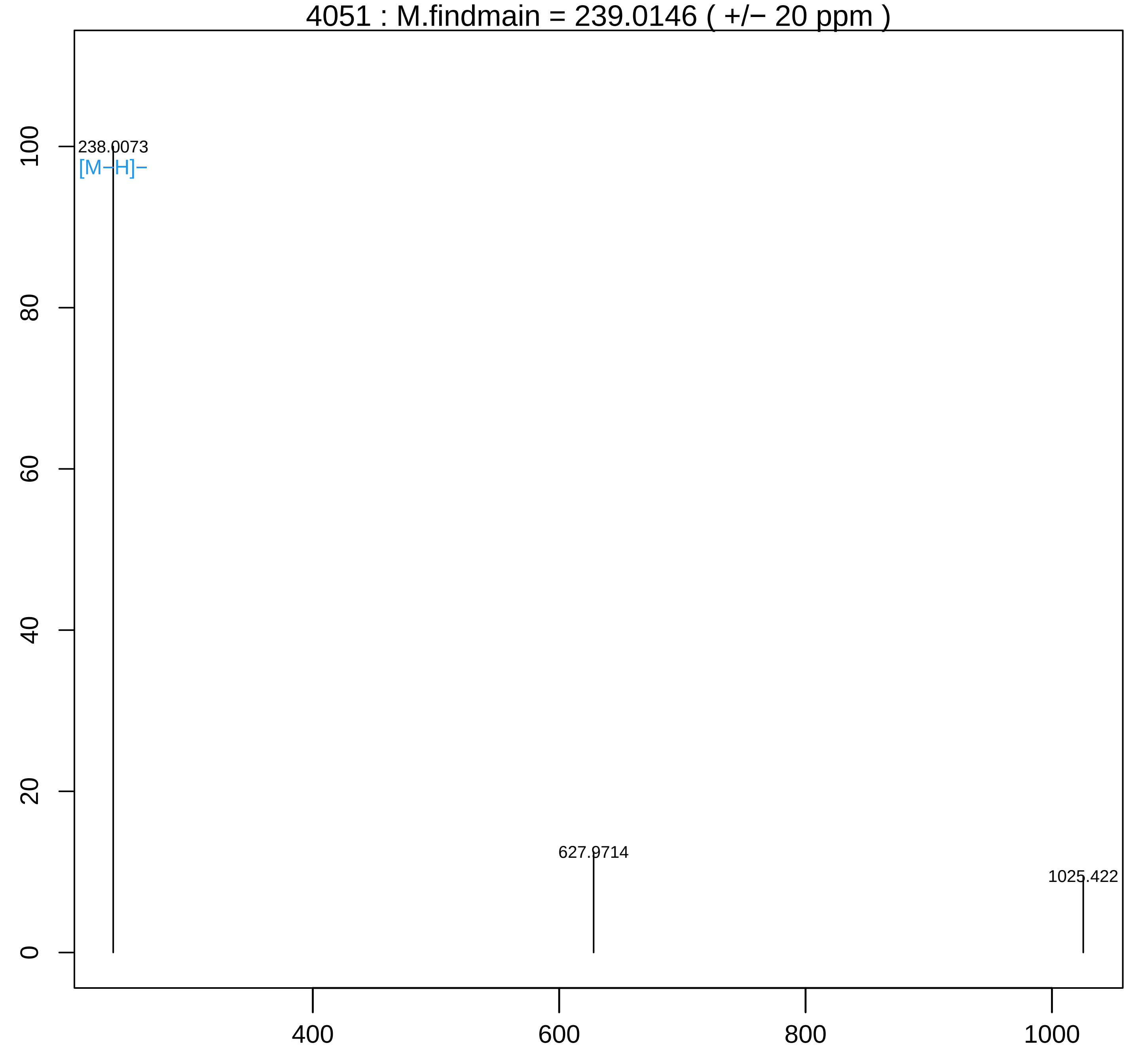
4051 : M.findmain = 239.0146 (+/− 20 ppm)
238.0073
[M−H]−
627.9714
1025.422
0
20
40
60
80
100
400
600
800
1000

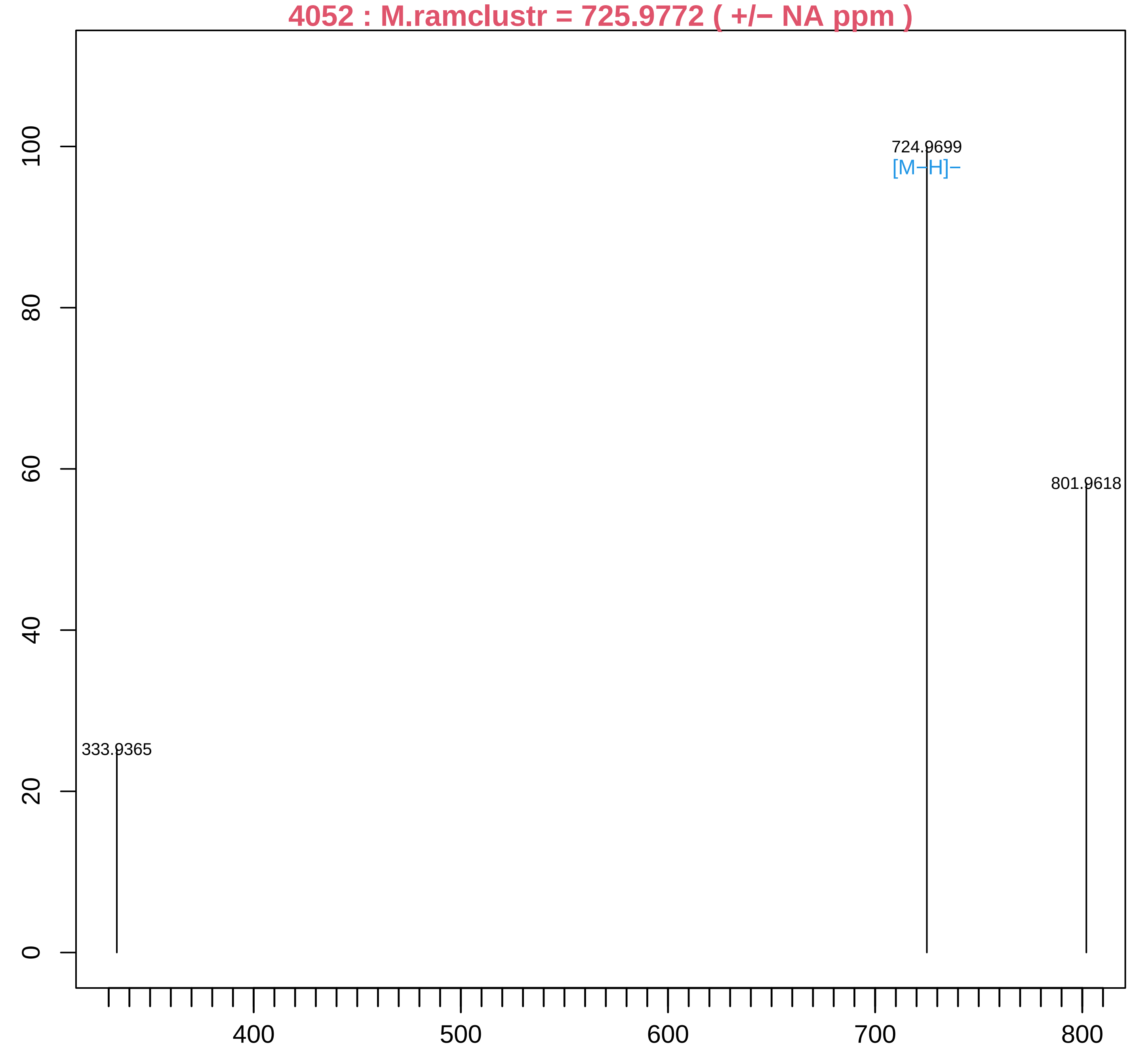
4052 : M.ramclustr = 725.9772 (+/− NA ppm)
724.9699
[M−H]−
801.9618
333.9365
0
20
40
60
80
100
400
500
600
700
800

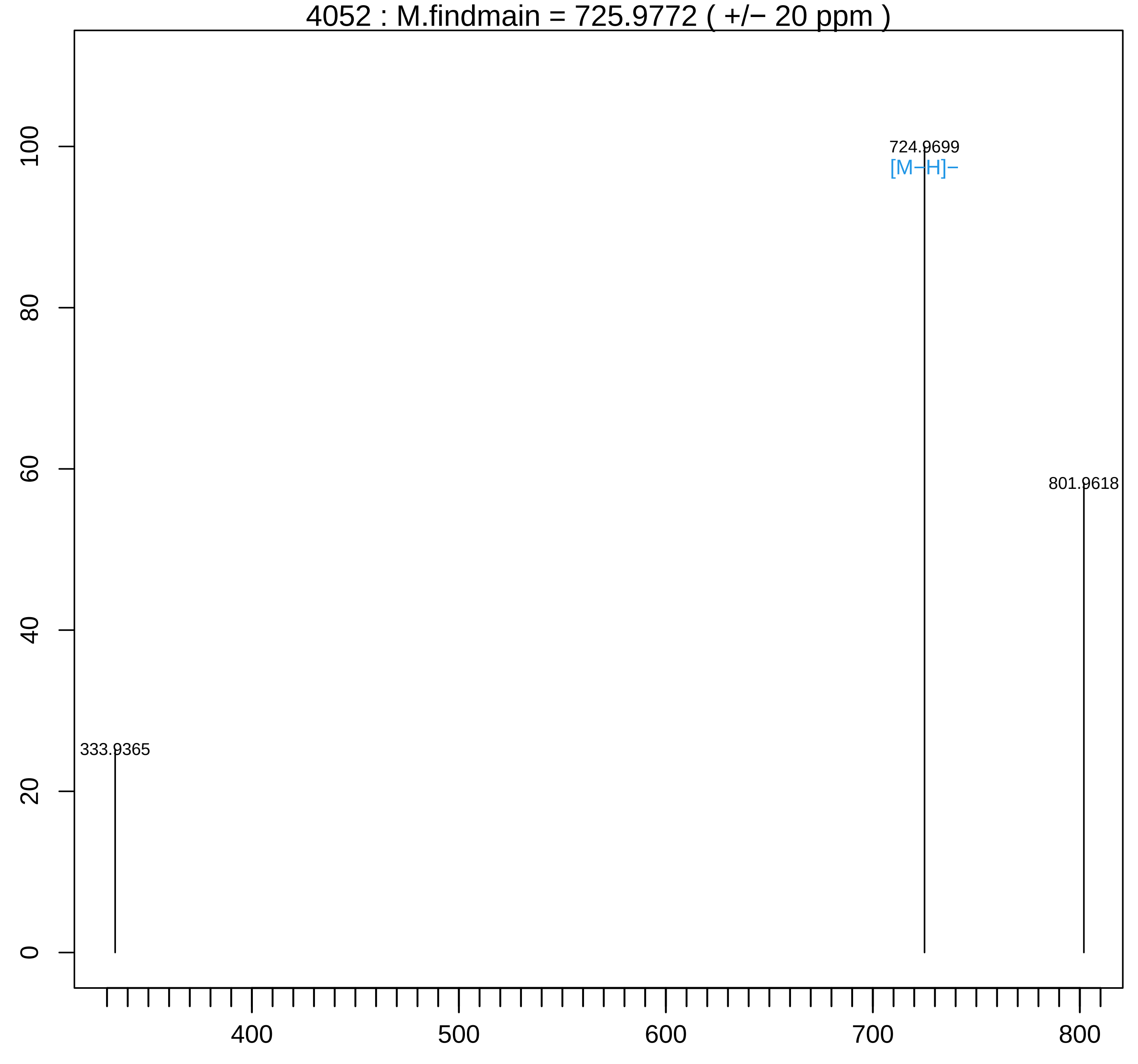
4052 : M.findmain = 725.9772 (+/− 20 ppm)
724.9699
[M−H]−
801.9618
333.9365
0
20
40
60
80
100
400
500
600
700
800

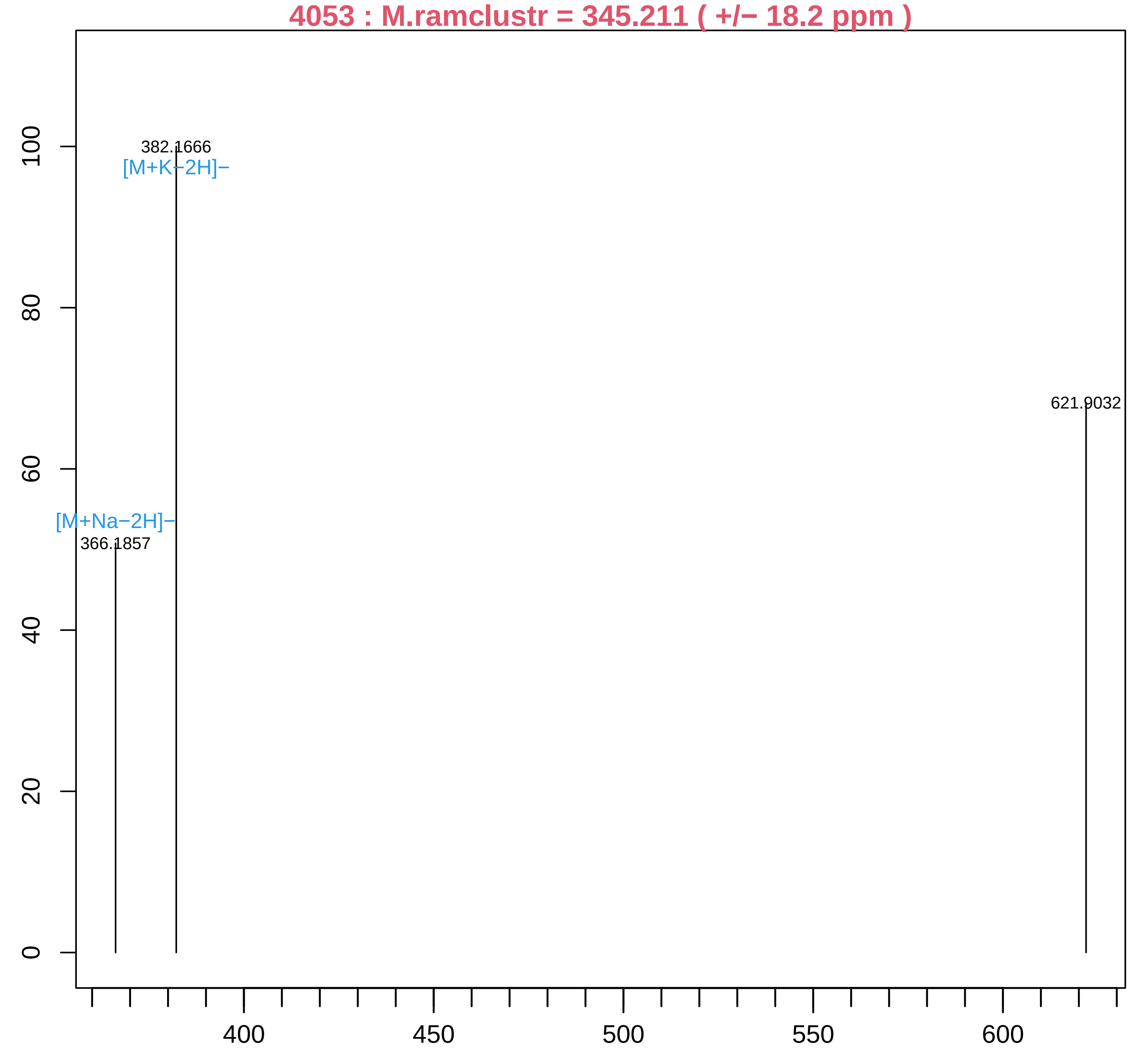
4053 : M.ramclustr = 345.211 (+/− 18.2 ppm)
382.1666
[M+K−2H]−
621.9032
[M+Na−2H]−
366.1857
0
20
40
60
80
100
400
450
500
550
600

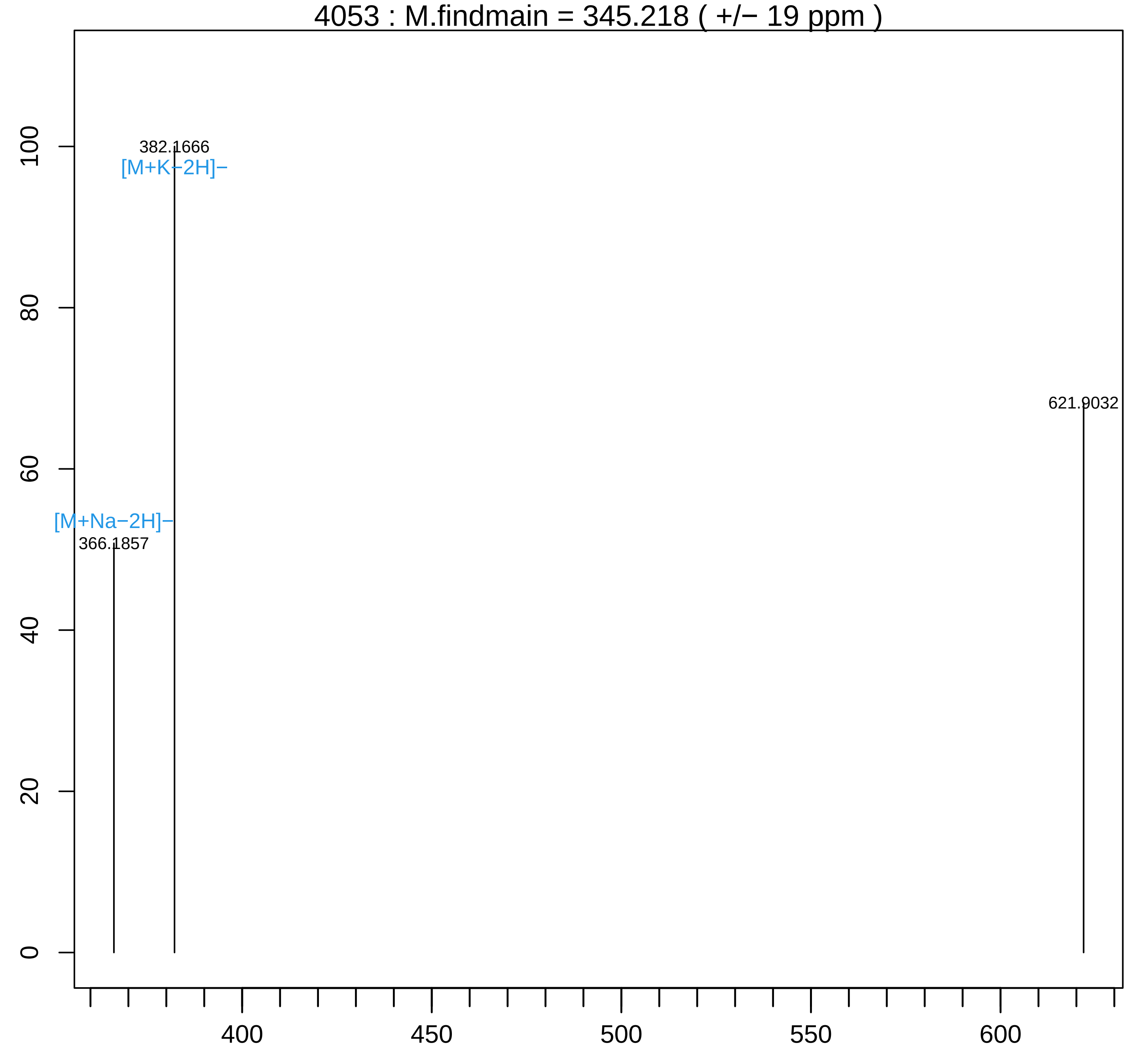
4053 : M.findmain = 345.218 (+/− 19 ppm)
382.1666
[M+K−2H]−
621.9032
[M+Na−2H]−
366.1857
0
20
40
60
80
100
400
450
500
550
600

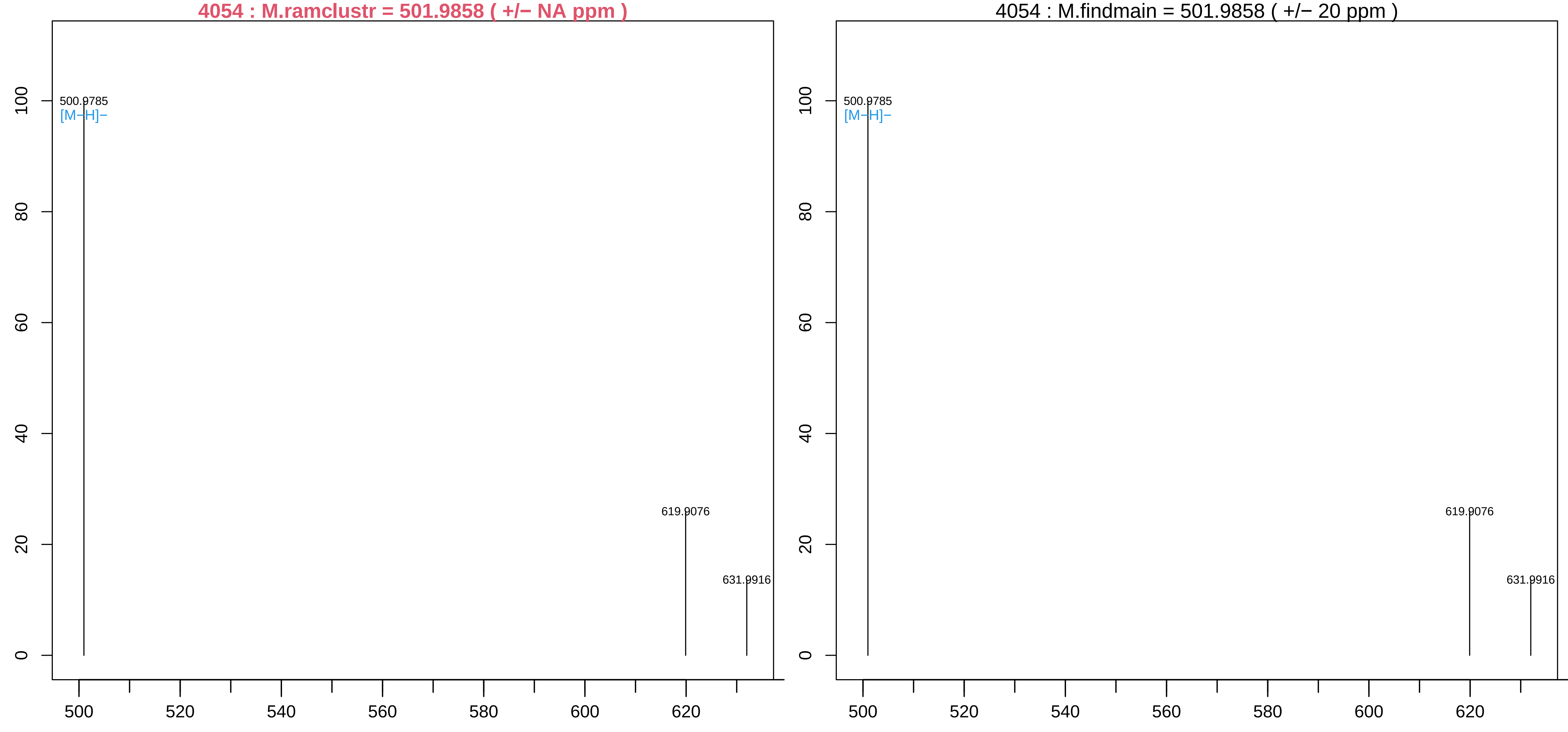

4054 : M.ramclustr = 501.9858 (+/− NA ppm)
500.9785
[M−H]−
619.9076
631.9916
0
20
40
60
80
100
500
520
540
560
580
600
620
4054 : M.findmain = 501.9858 (+/− 20 ppm)
500.9785
[M−H]−
619.9076
631.9916
0
20
40
60
80
100
500
520
540
560
580
600
620

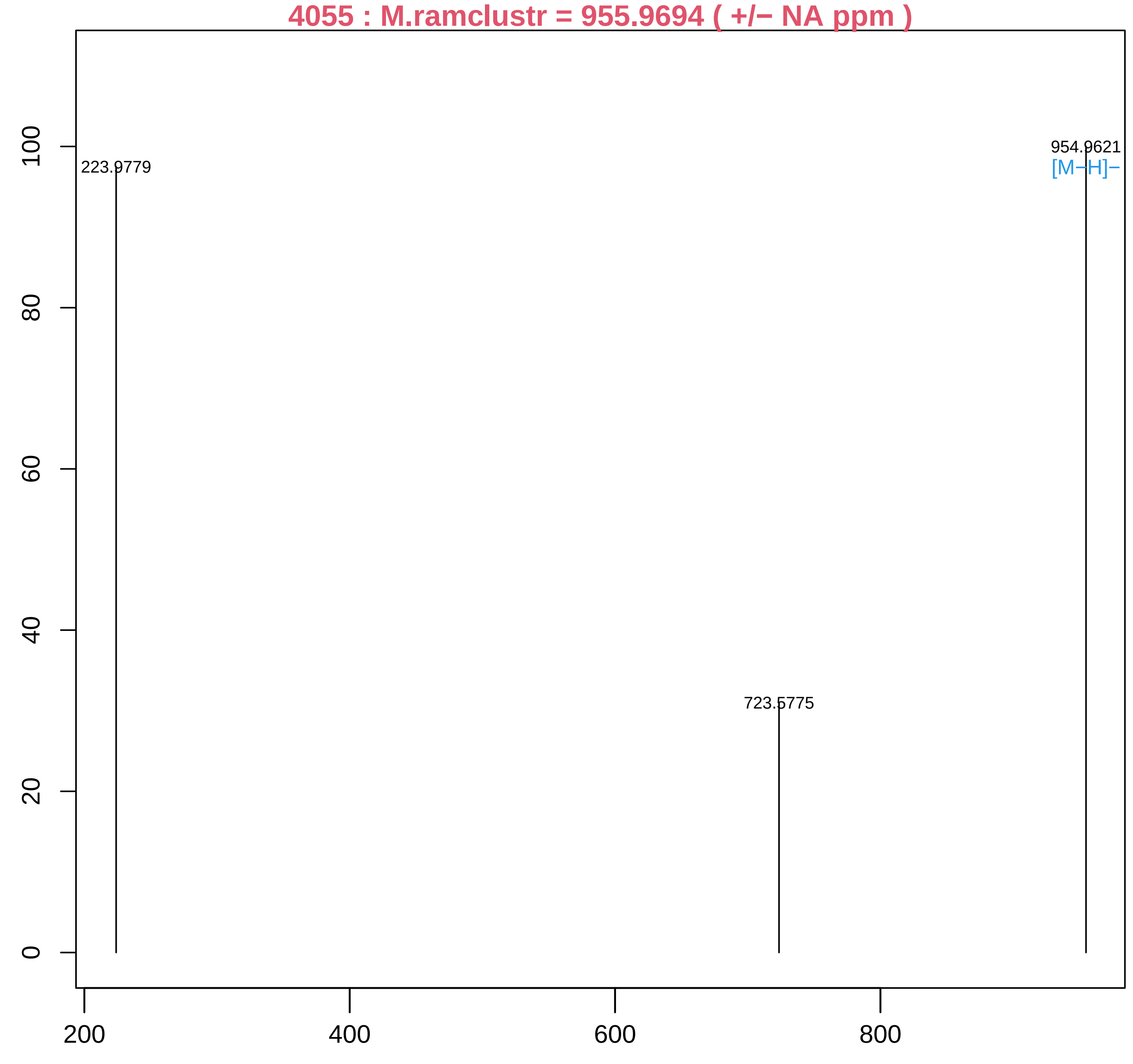
4055 : M.ramclustr = 955.9694 (+/− NA ppm)
223.9779
723.5775
954.9621
[M−H]−
0
20
40
60
80
100
200
400
600
800

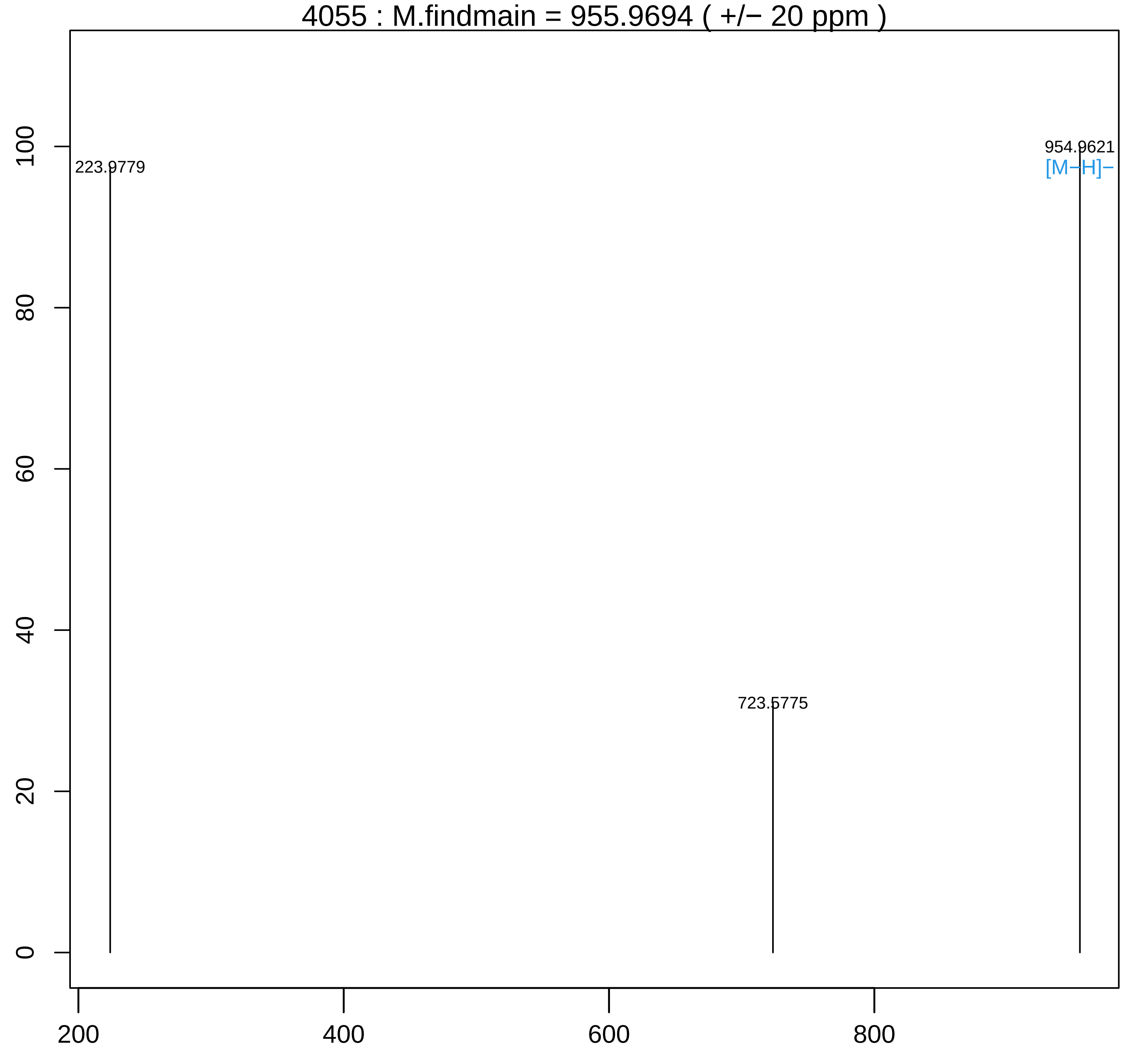
4055 : M.findmain = 955.9694 (+/− 20 ppm)
223.9779
723.5775
954.9621
[M−H]−
0
20
40
60
80
100
200
400
600
800

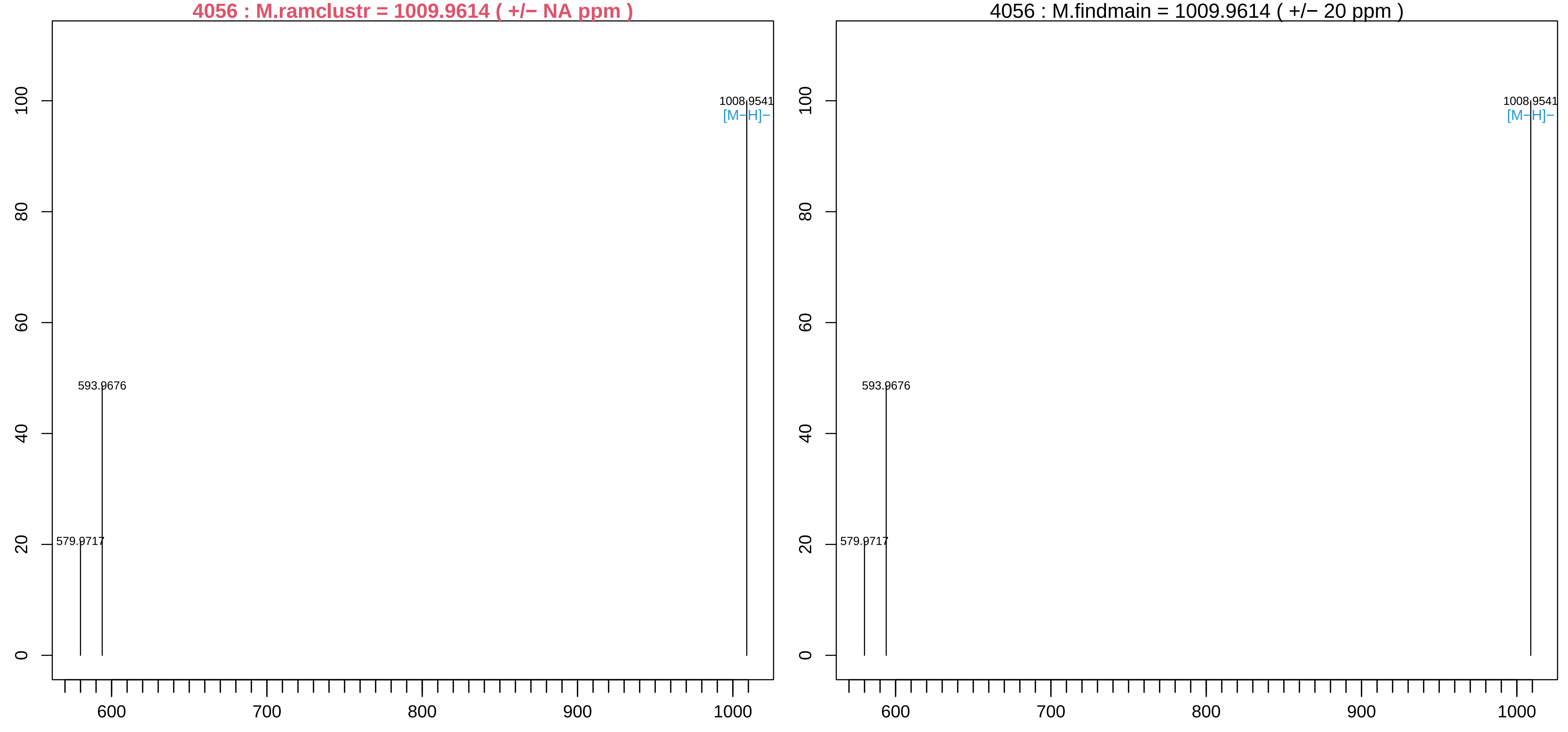
4056 : M.ramclustr = 1009.9614 (+/− NA ppm)
1008.9541
[M−H]−
593.9676
579.9717
4056 : M.findmain = 1009.9614 (+/− 20 ppm)
1008.9541
[M−H]−
593.9676
579.9717
0
20
40
60
80
100
600
700
800
900
1000

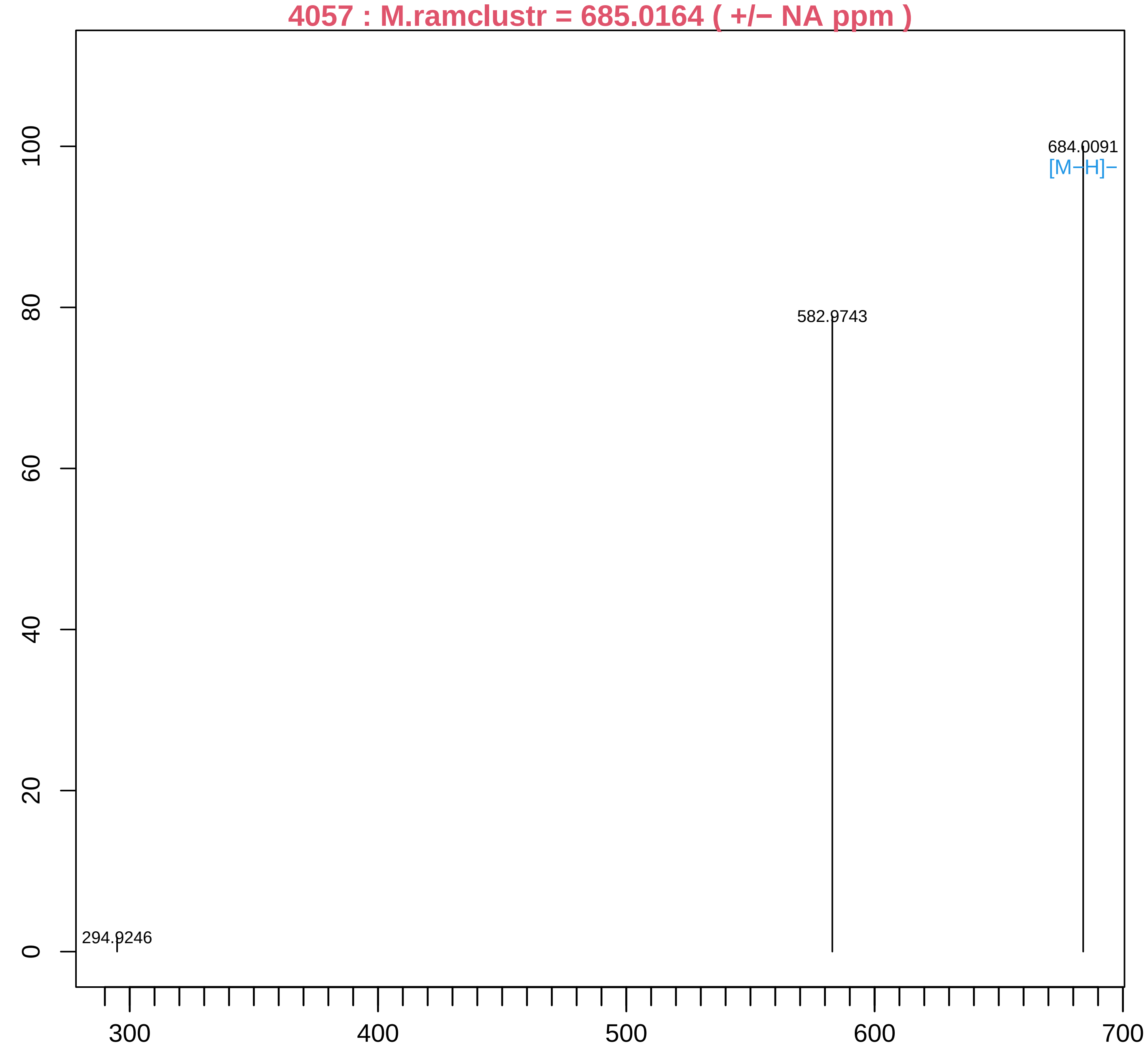
4057 : M.ramclustr = 685.0164 (+/− NA ppm)
684.0091
[M−H]−
582.9743
294.9246
0
20
40
60
80
100
300
400
500
600
700

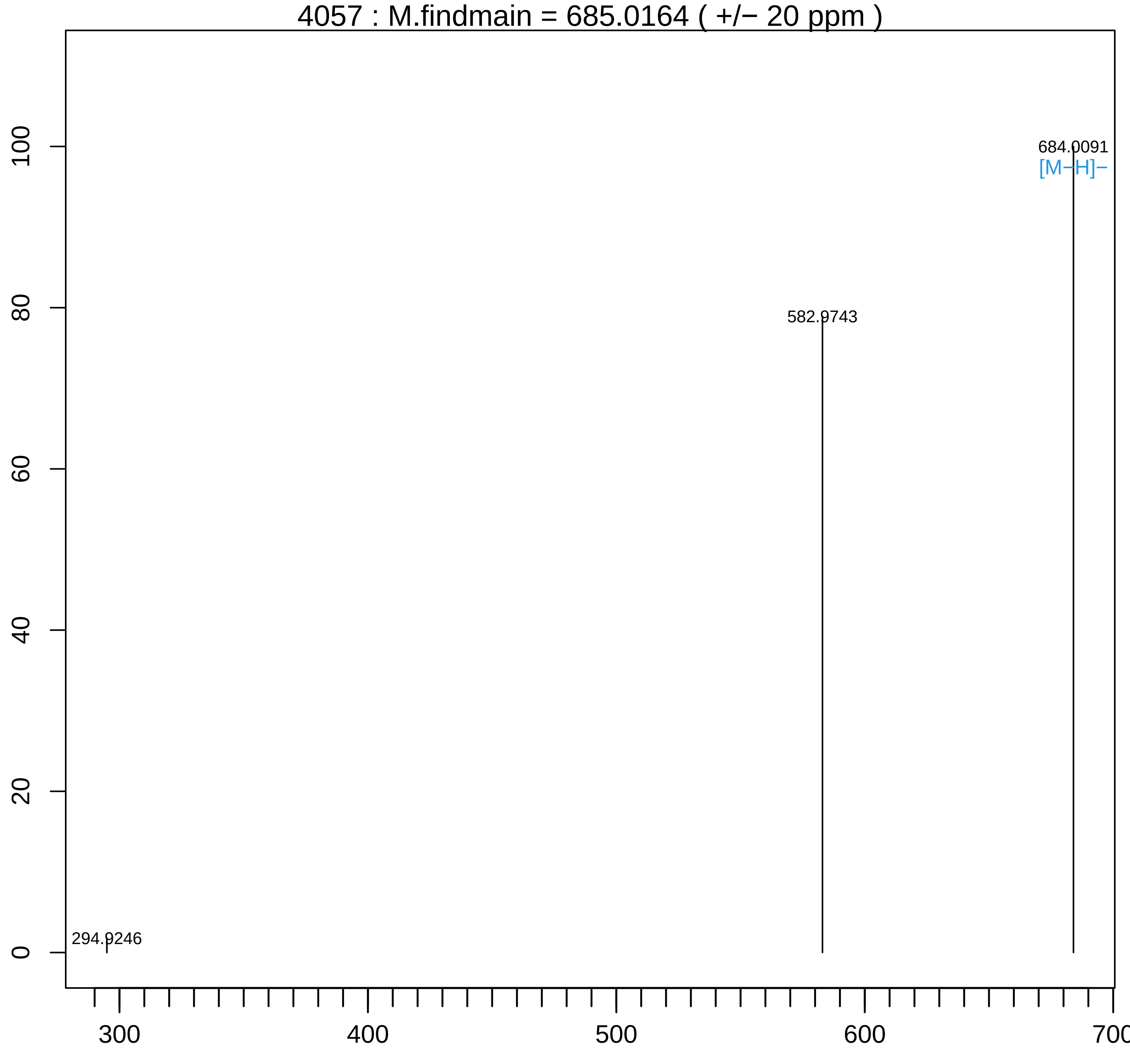
4057 : M.findmain = 685.0164 (+/− 20 ppm)
684.0091
[M−H]−
582.9743
294.9246
0
20
40
60
80
100
300
400
500
600
700

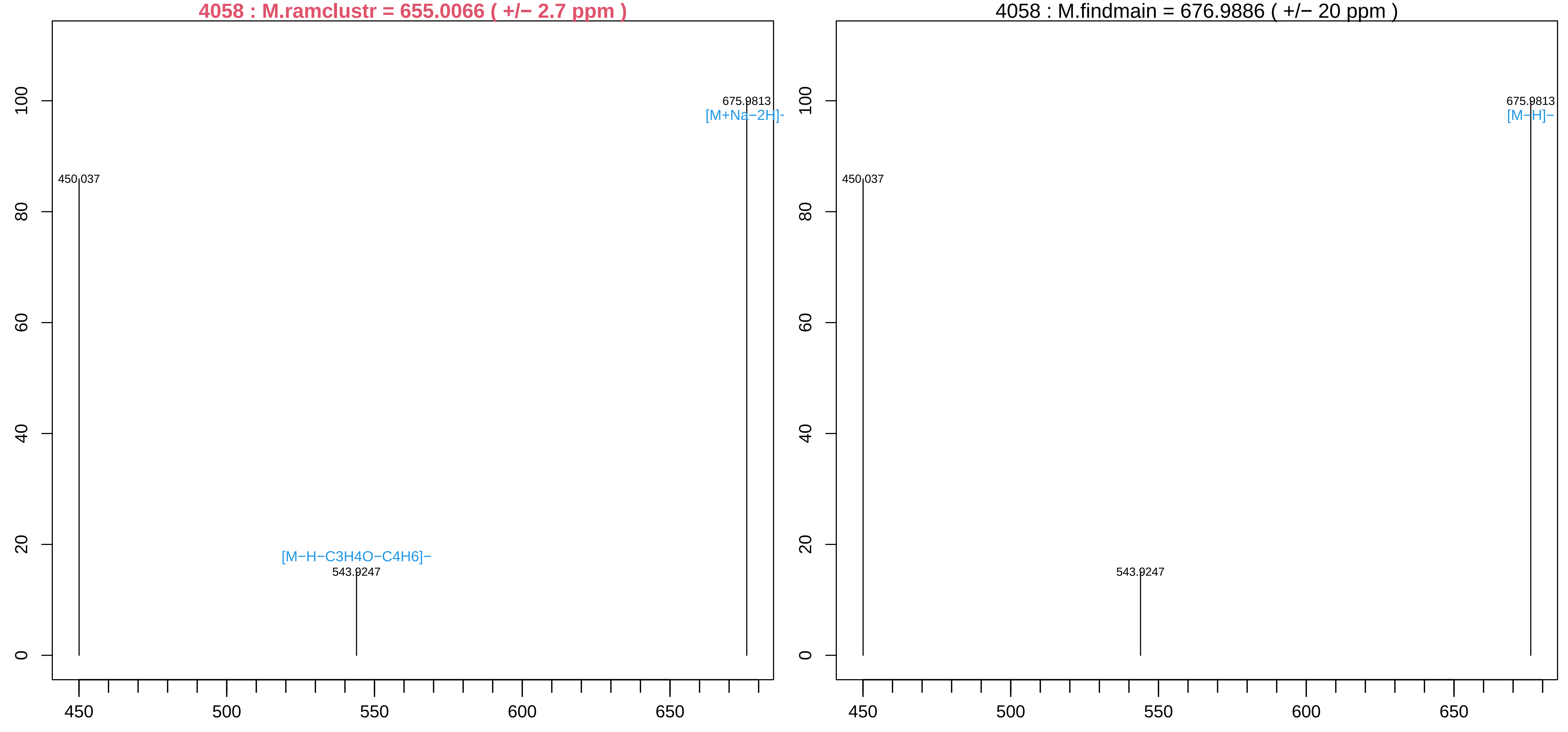

4058 : M.ramclustr = 655.0066 (+/− 2.7 ppm)
675.9813
[M+Na−2H]-
450.037
[M−H−C3H4O−C4H6]−
543.9247
4058 : M.findmain = 676.9886 (+/− 20 ppm)
675.9813
[M−H]−
450.037
543.9247

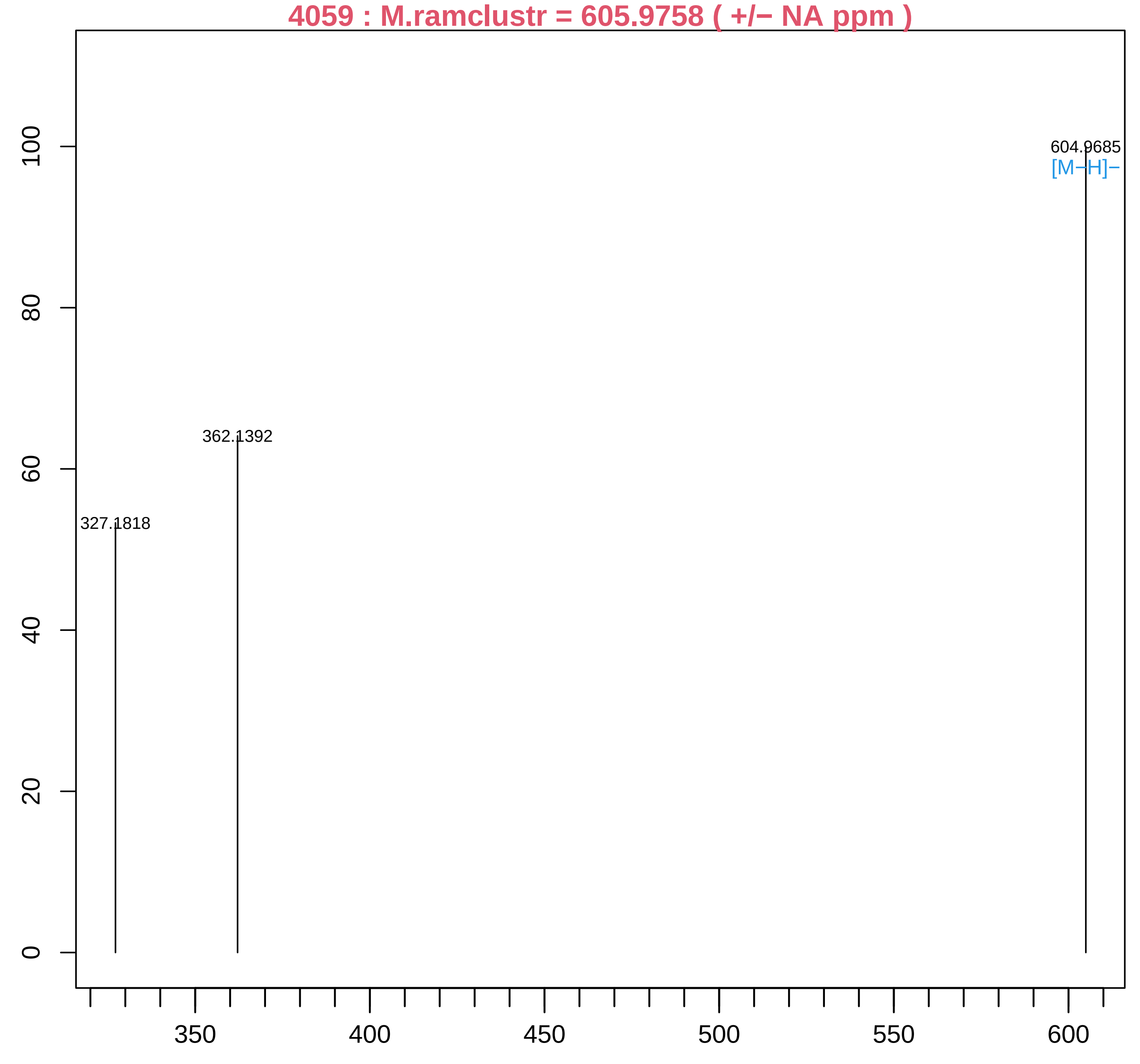
4059 : M.ramclustr = 605.9758 (+/− NA ppm)
604.9685
[M−H]−
362.1392
327.1818
0
20
40
60
80
100
350
400
450
500
550
600

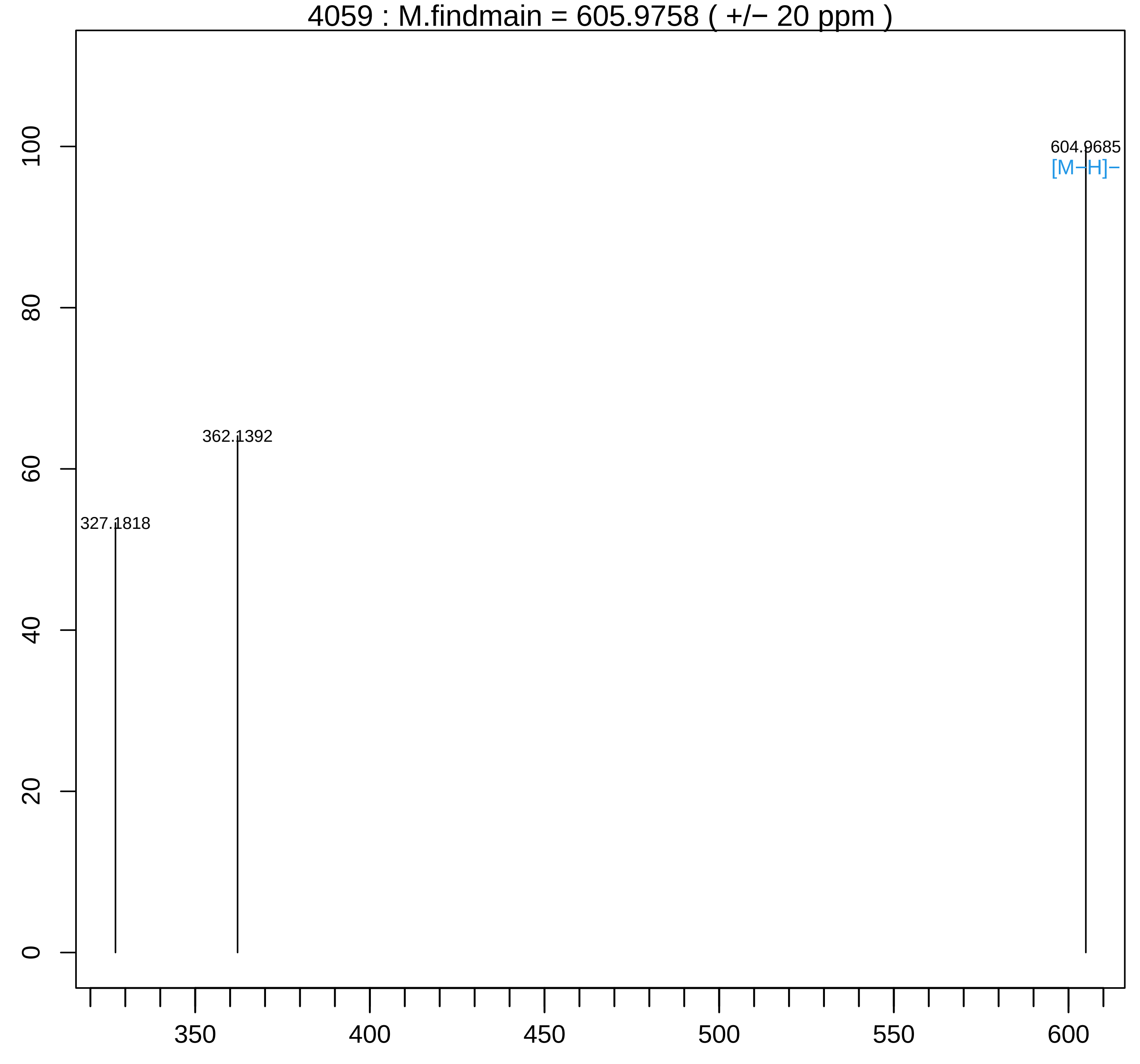
4059 : M.findmain = 605.9758 (+/− 20 ppm)
604.9685
[M−H]−
362.1392
327.1818
0
20
40
60
80
100
350
400
450
500
550
600

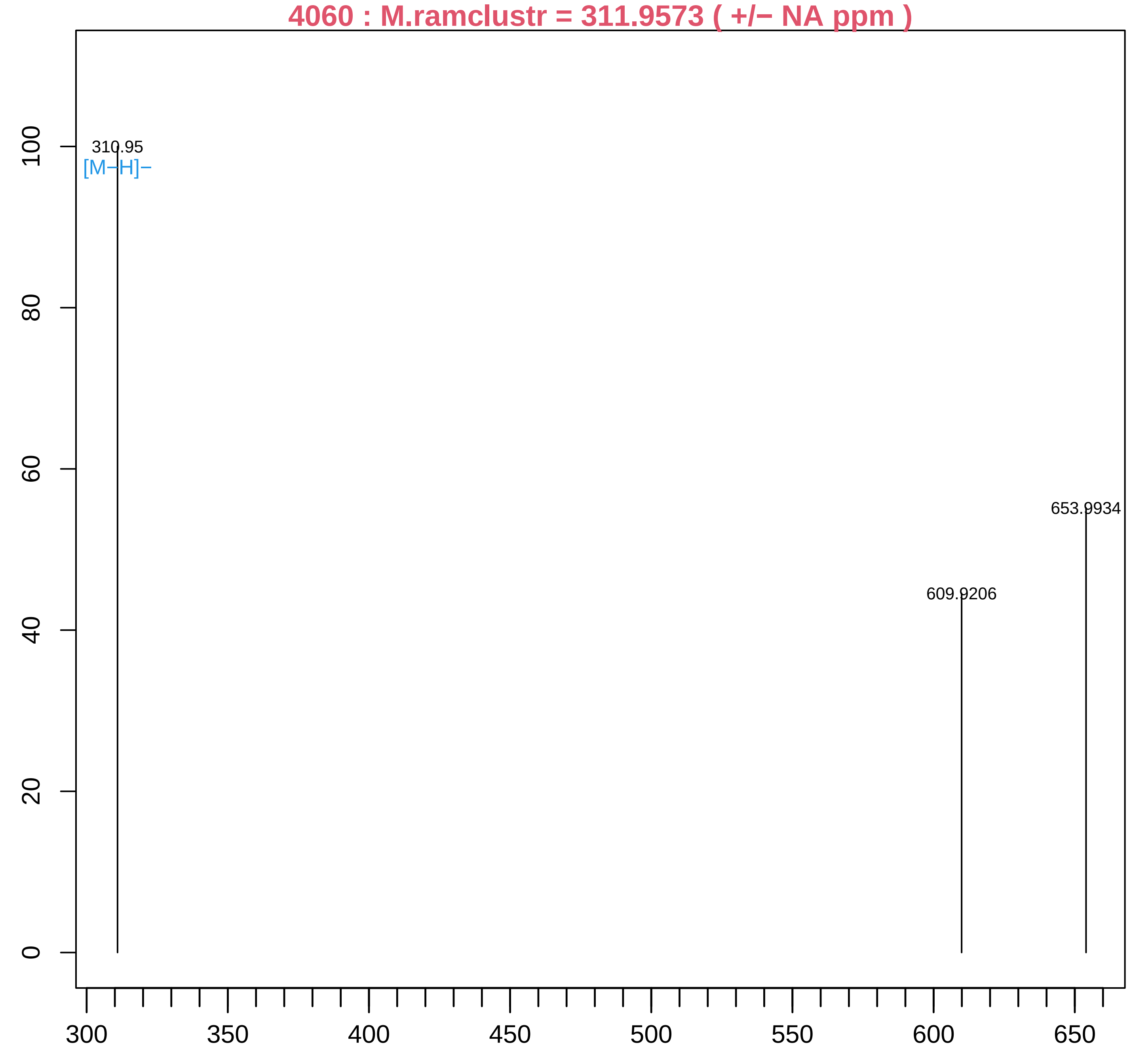
4060 : M.ramclustr = 311.9573 (+/− NA ppm)
310.95
[M−H]−
609.9206
653.9934
0
20
40
60
80
100
300
350
400
450
500
550
600
650

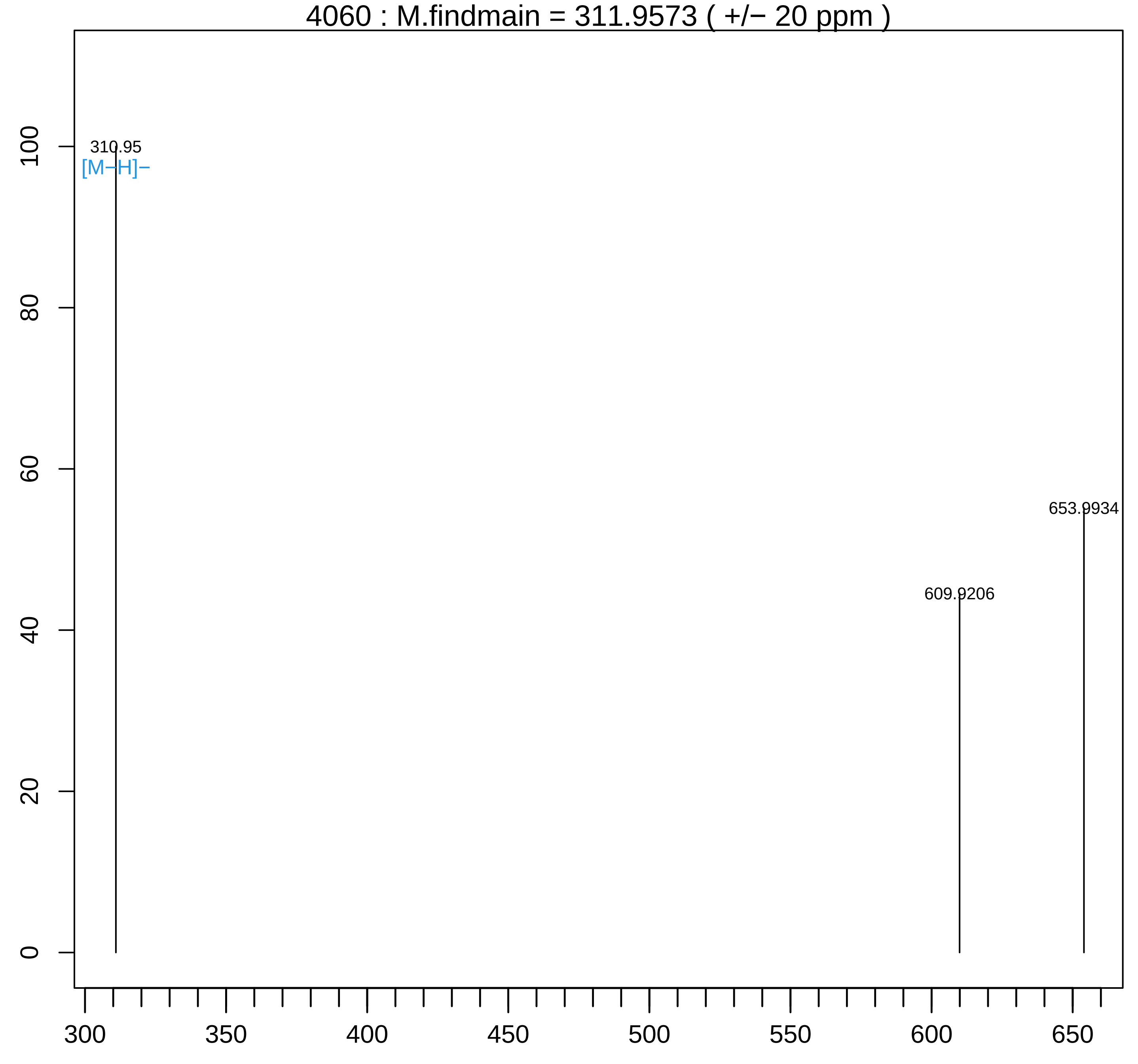
4060 : M.findmain = 311.9573 (+/− 20 ppm)
310.95
[M−H]−
609.9206
653.9934
0
20
40
60
80
100
300
350
400
450
500
550
600
650

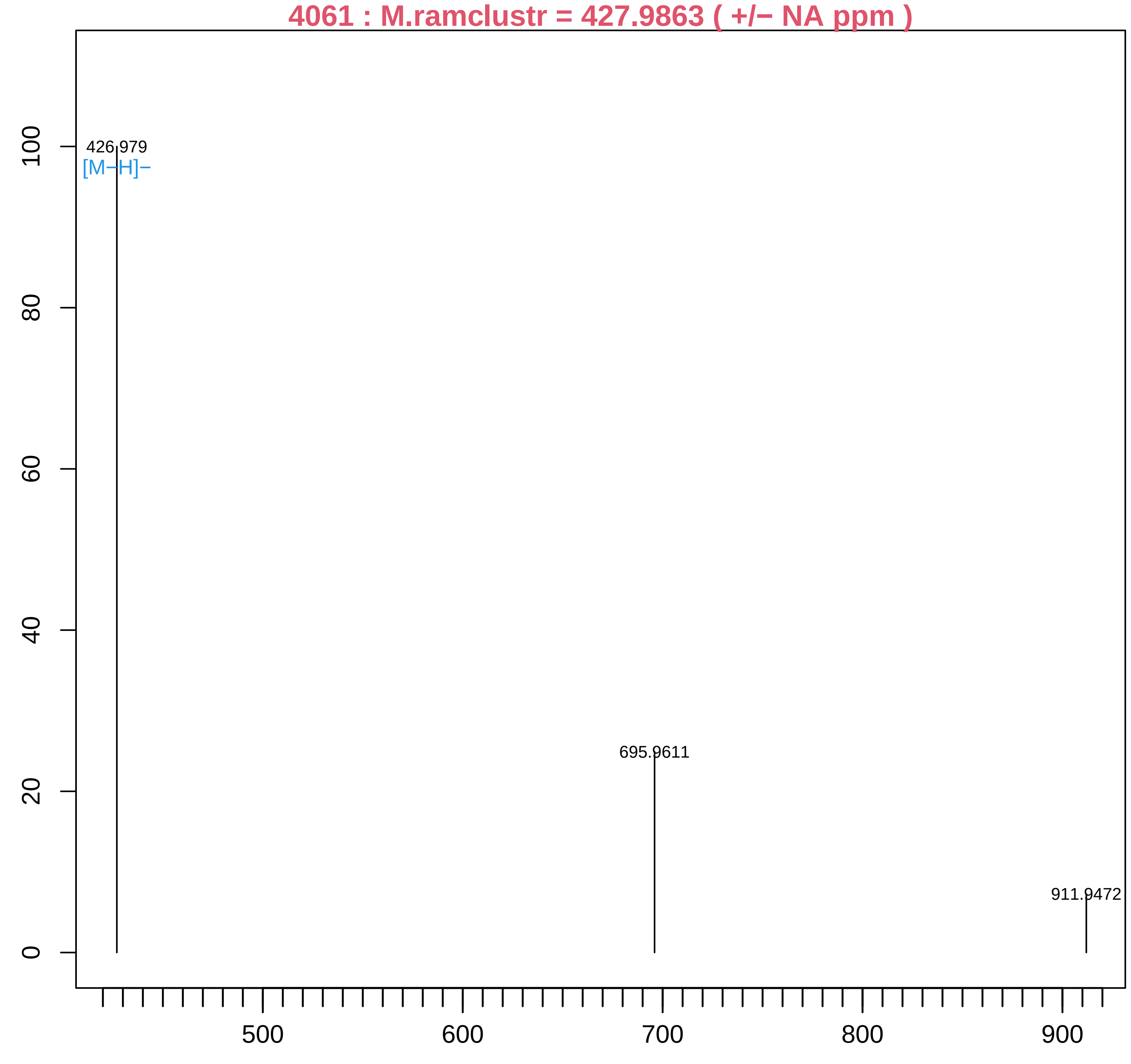
4061 : M.ramclustr = 427.9863 (+/− NA ppm)
426.979
[M−H]−
695.9611
911.9472
0
20
40
60
80
100
500
600
700
800
900

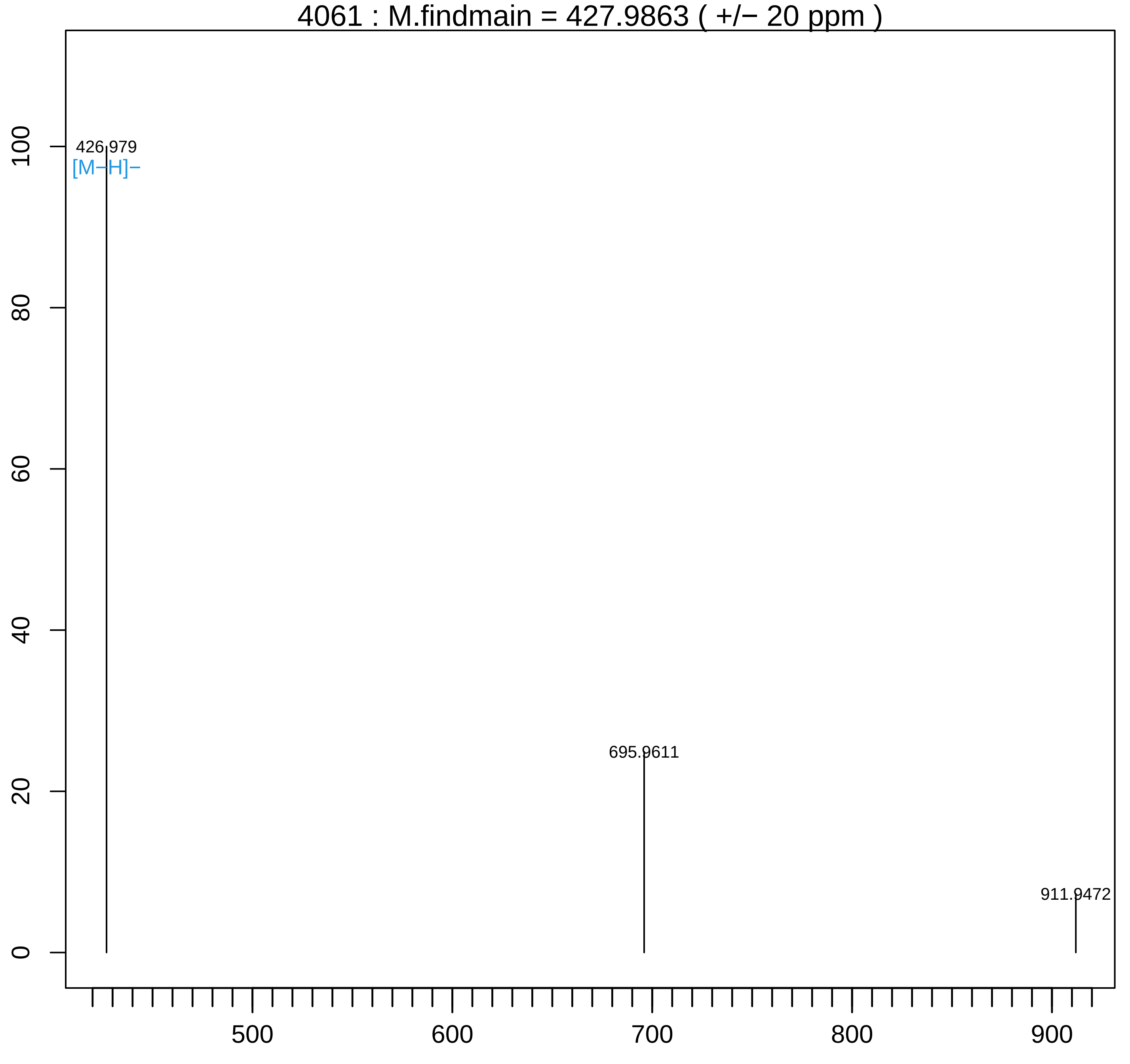
4061 : M.findmain = 427.9863 (+/− 20 ppm)
426.979
[M−H]−
695.9611
911.9472
0
20
40
60
80
100
500
600
700
800
900

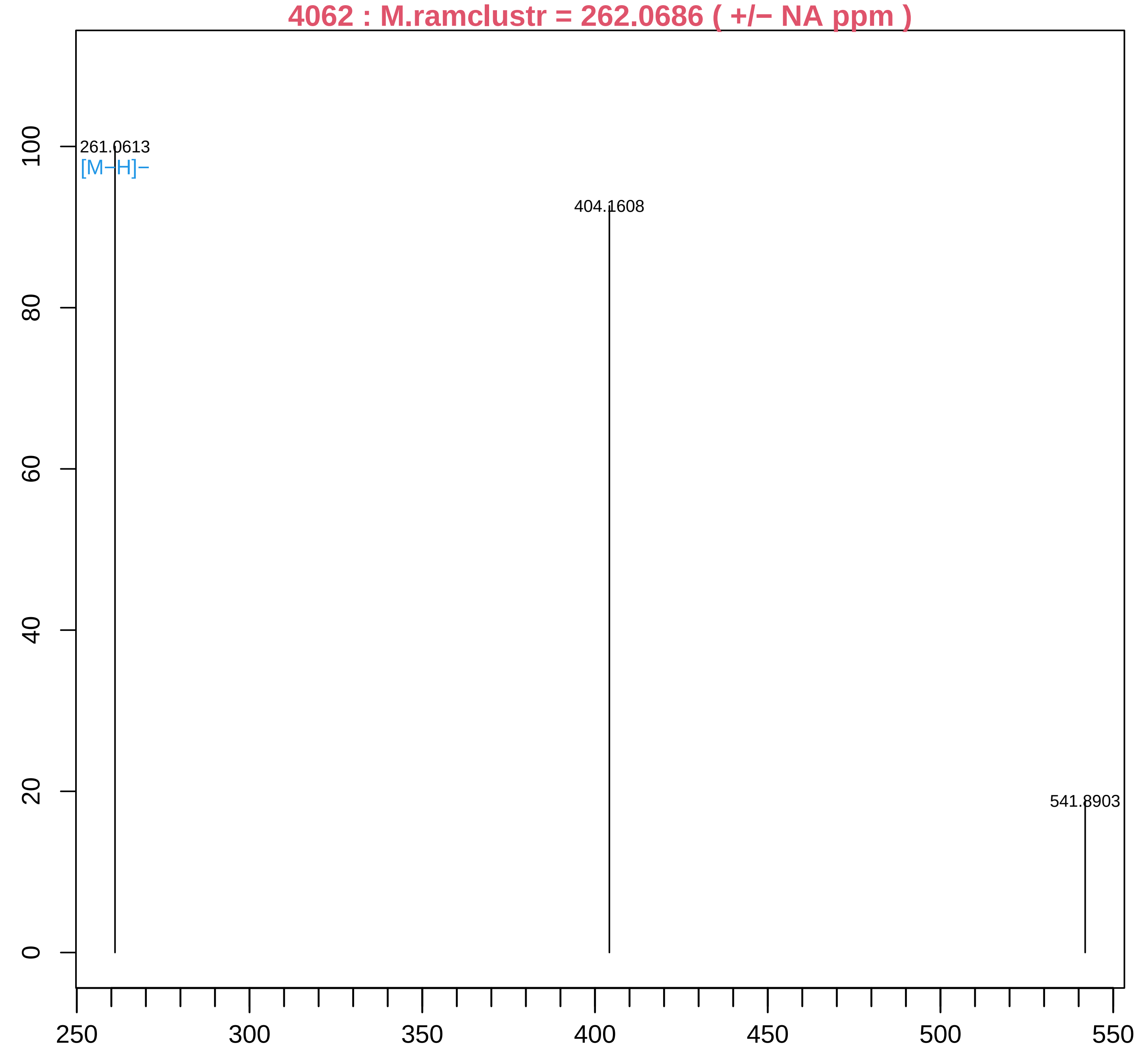
4062 : M.ramclustr = 262.0686 (+/− NA ppm)
261.0613
[M−H]−
404.1608
541.8903
0
20
40
60
80
100
250
300
350
400
450
500
550

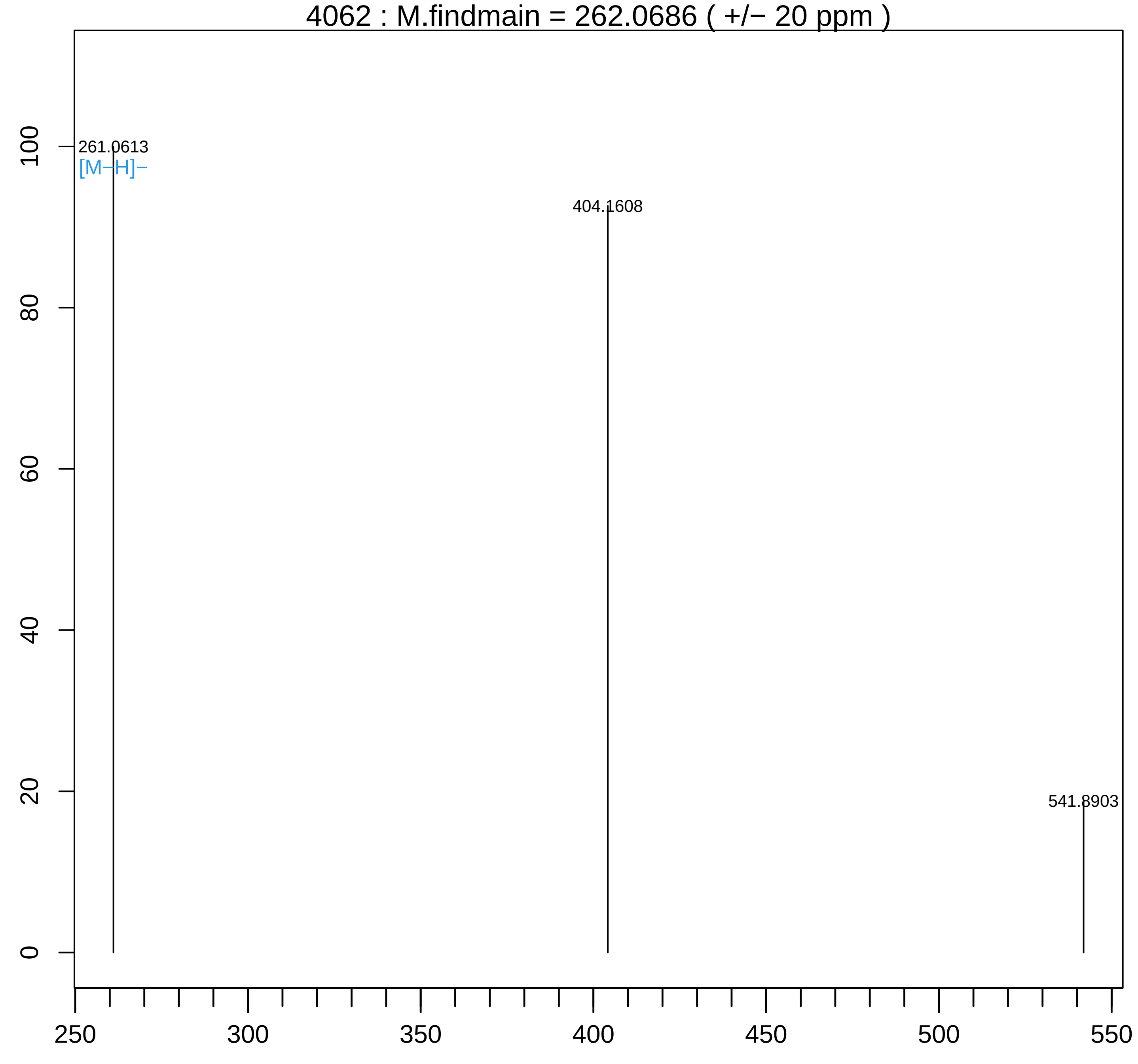
4062 : M.findmain = 262.0686 (+/− 20 ppm)
261.0613
[M−H]−
404.1608
541.8903
0
20
40
60
80
100
250
300
350
400
450
500
550

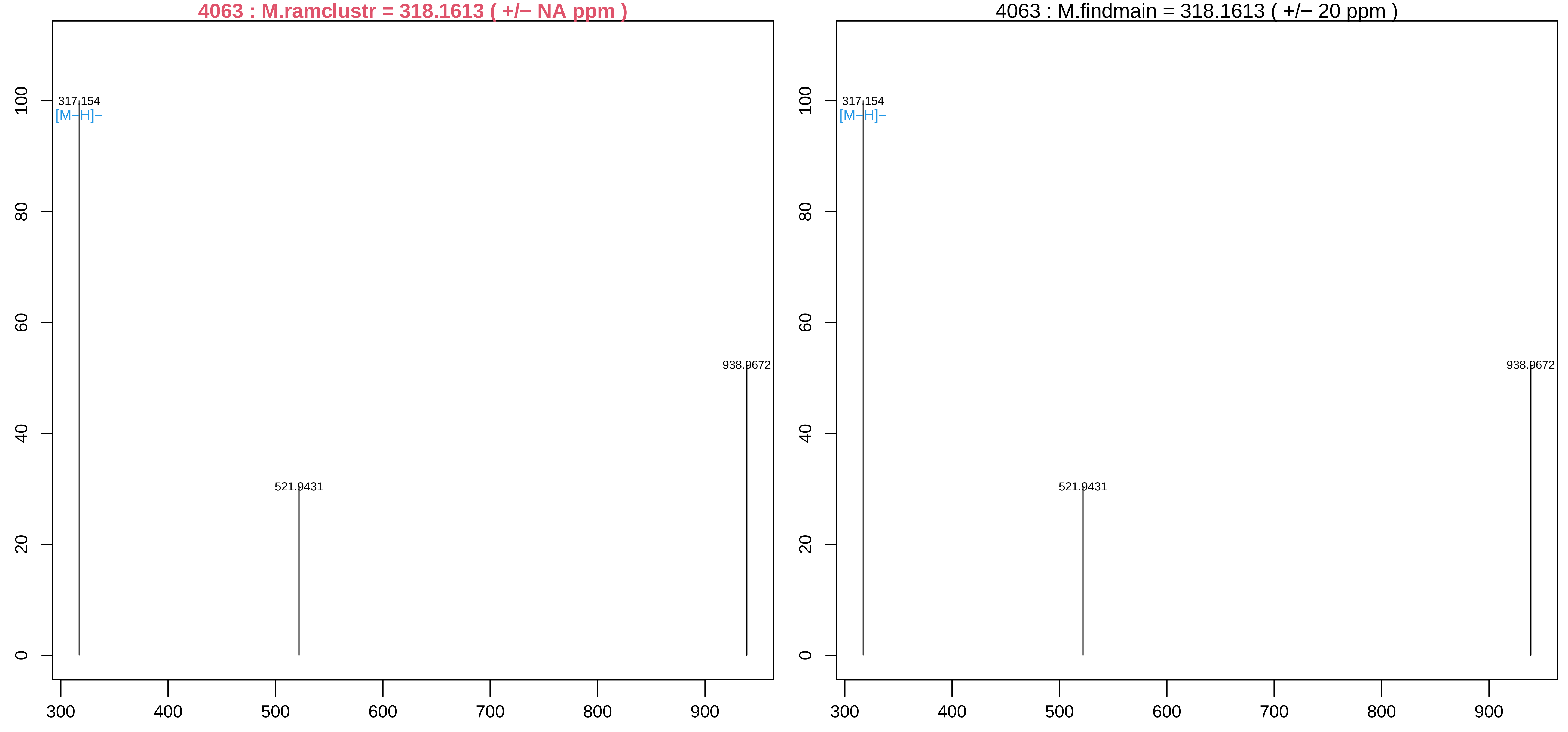

4063 : M.ramclustr = 318.1613 (+/− NA ppm)
317.154
[M−H]−
521.9431
938.9672
4063 : M.findmain = 318.1613 (+/− 20 ppm)
317.154
[M−H]−
521.9431
938.9672

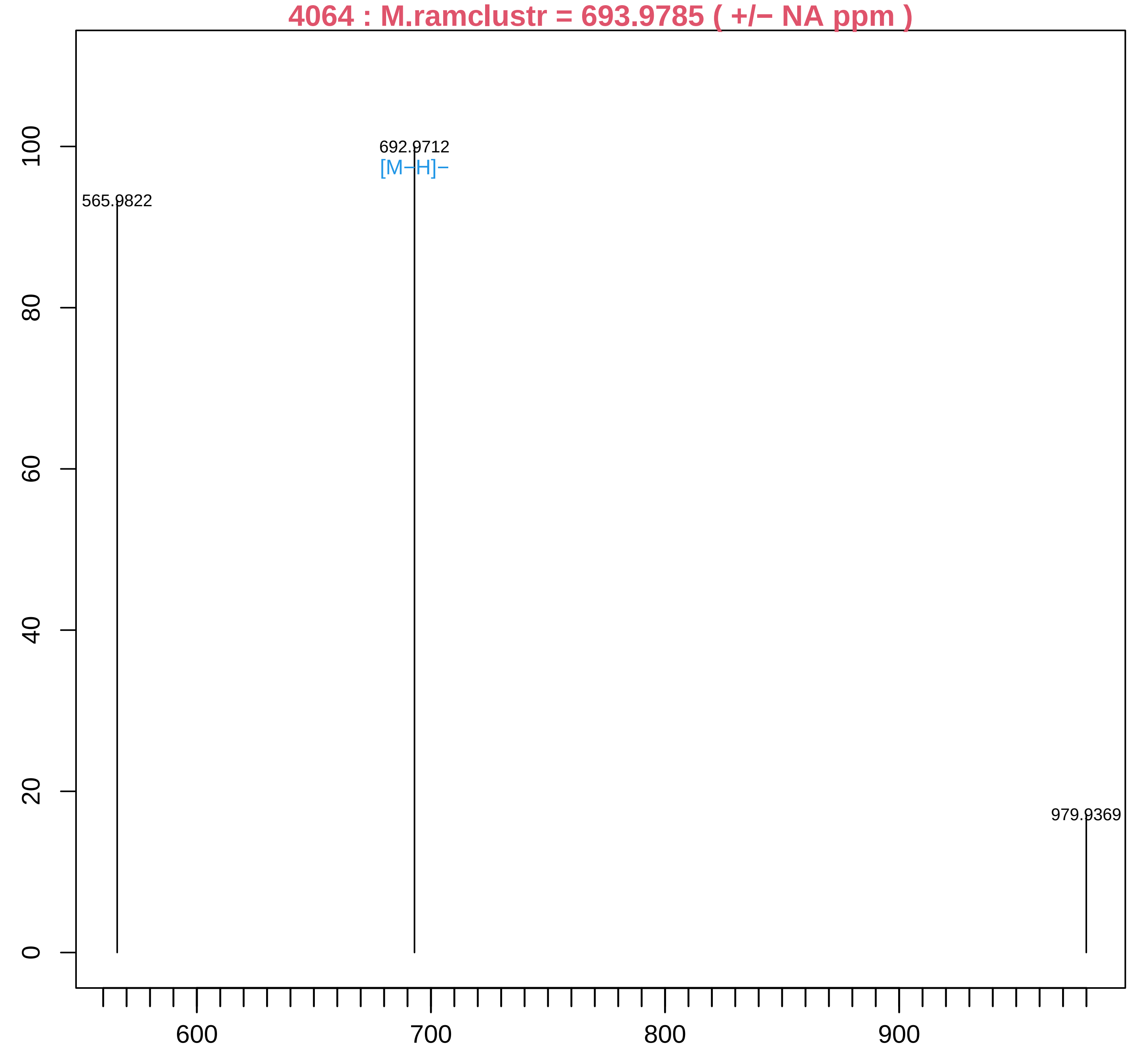
4064 : M.ramclustr = 693.9785 (+/− NA ppm)
565.9822
692.9712
[M−H]−
979.9369
0
20
40
60
80
100
600
700
800
900

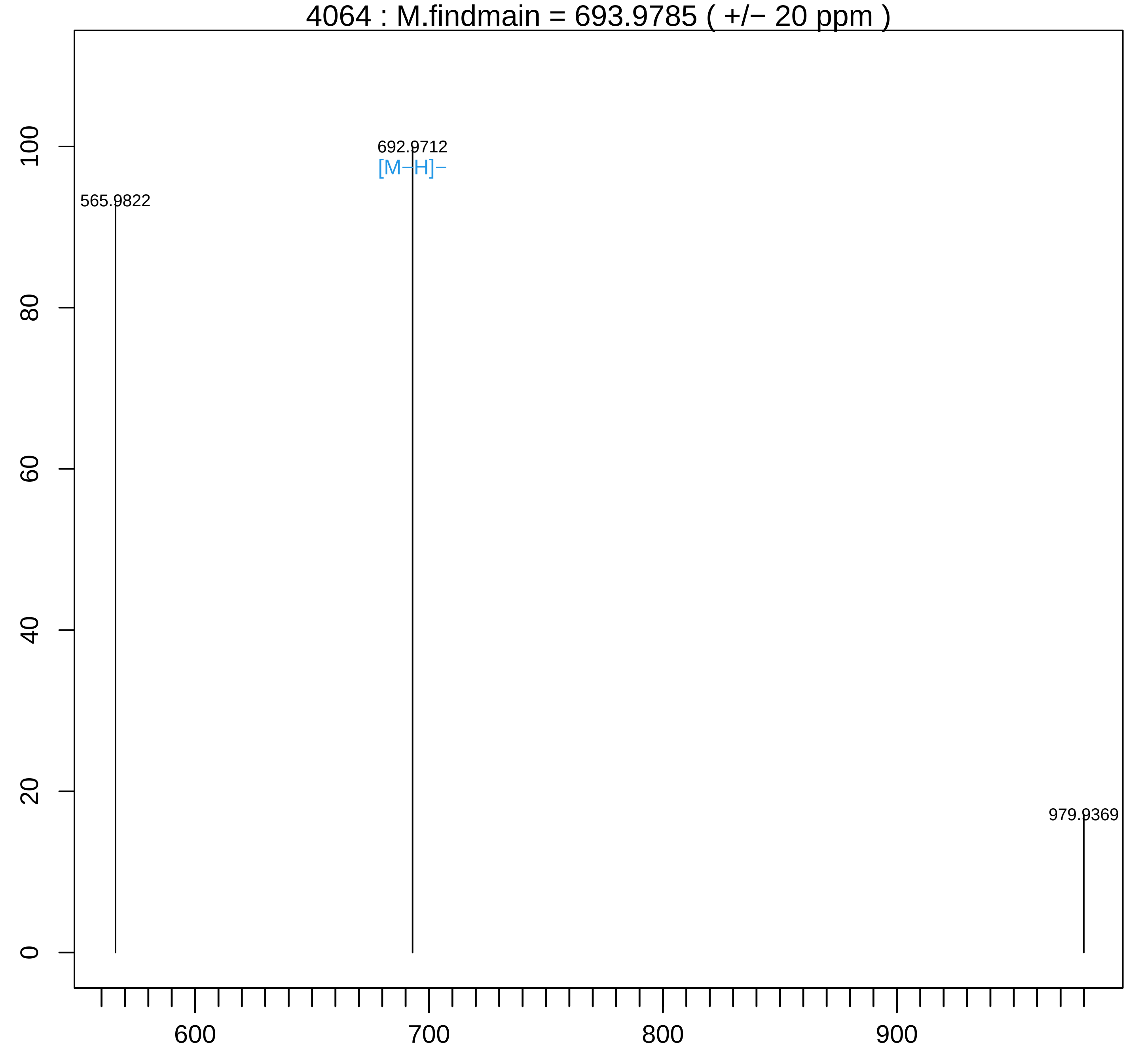
4064 : M.findmain = 693.9785 (+/− 20 ppm)
565.9822
692.9712
[M−H]−
979.9369
0
20
40
60
80
100
600
700
800
900

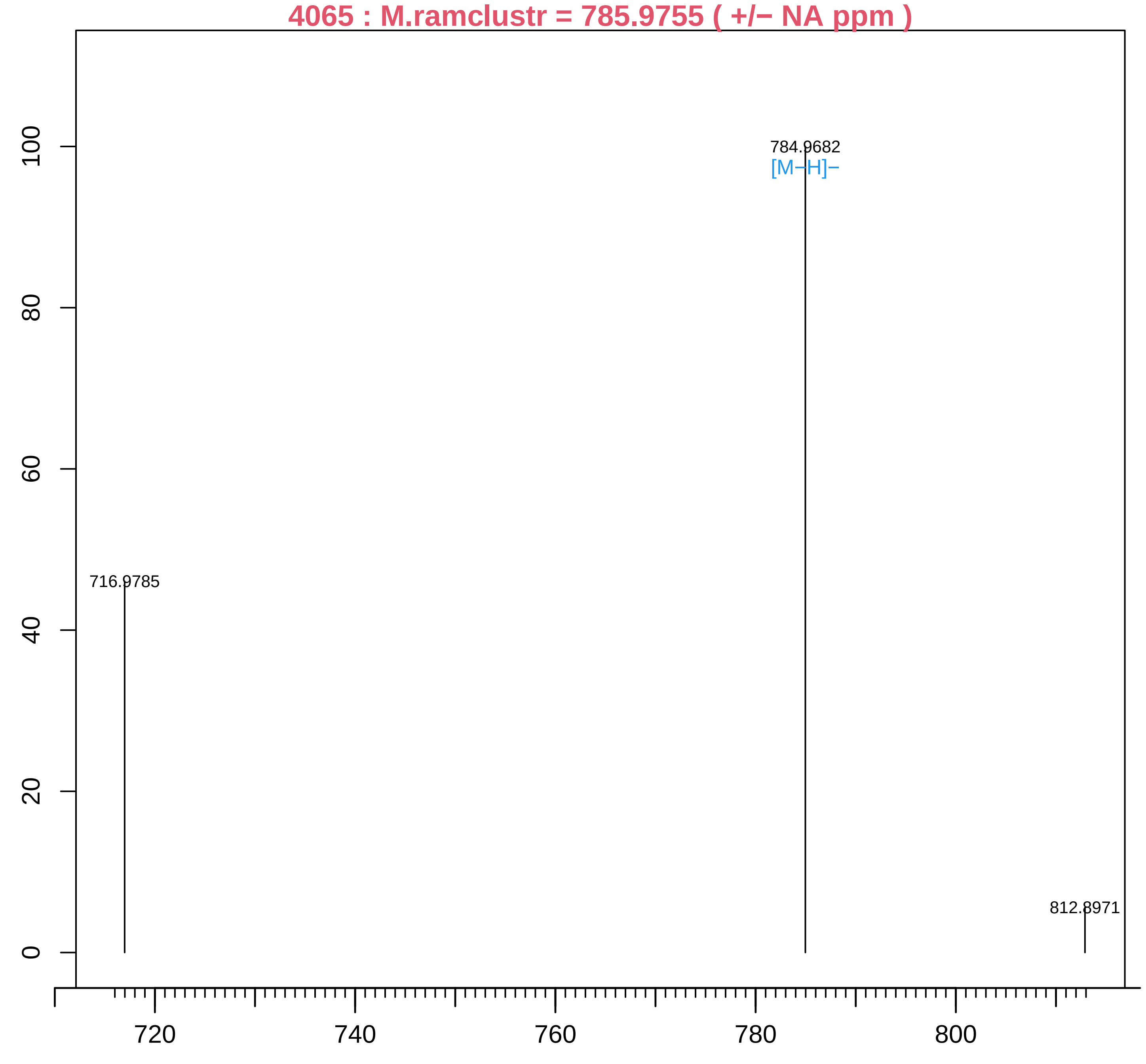
4065 : M.ramclustr = 785.9755 (+/− NA ppm)
784.9682
[M−H]−
716.9785
812.8971
0
20
40
60
80
100
720
740
760
780
800

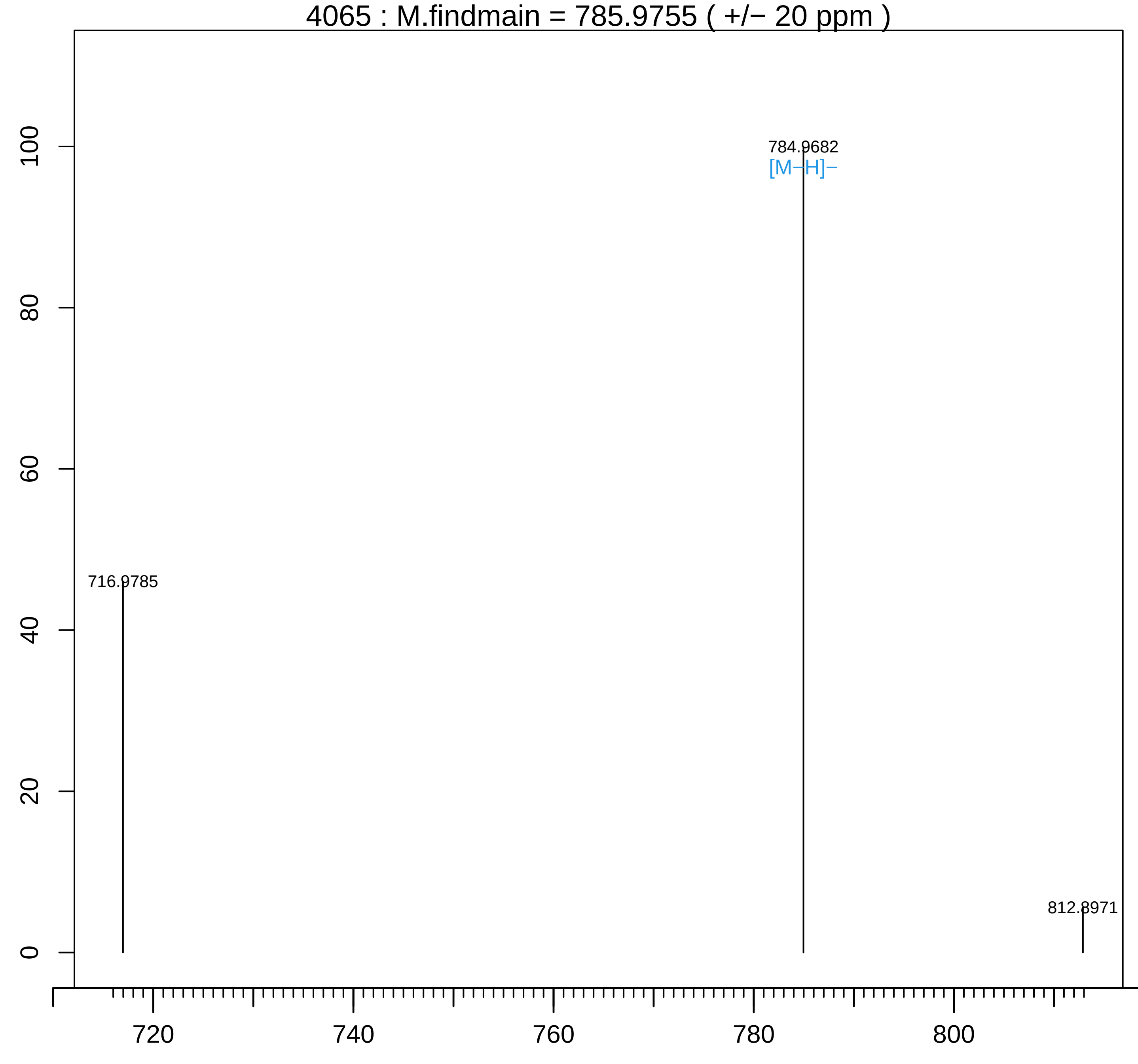
4065 : M.findmain = 785.9755 (+/− 20 ppm)
784.9682
[M−H]−
716.9785
812.8971
0
20
40
60
80
100
720
740
760
780
800

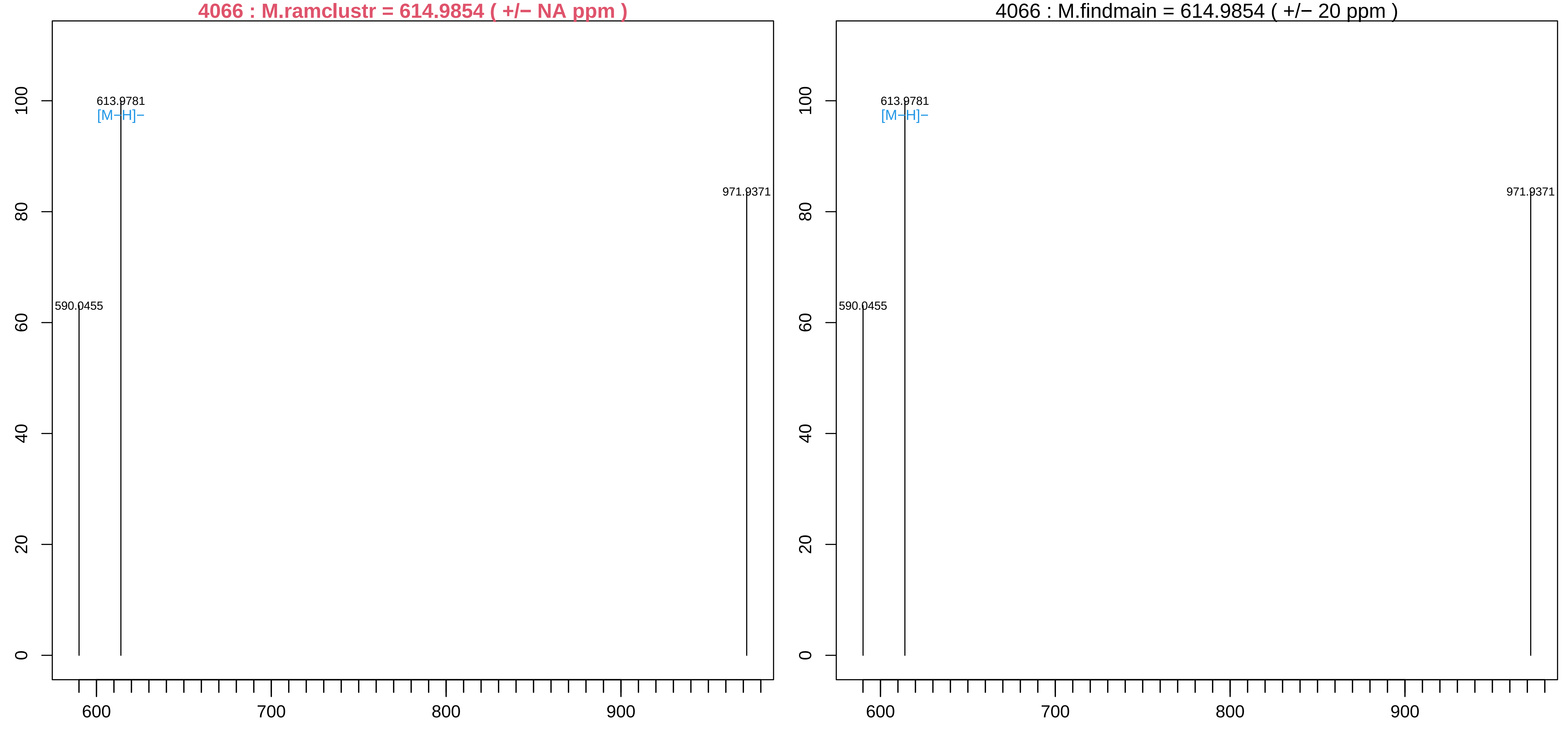

4066 : M.ramclustr = 614.9854 (+/− NA ppm)
613.9781
[M−H]−
590.0455
971.9371
0
20
40
60
80
100
600
700
800
900
4066 : M.findmain = 614.9854 (+/− 20 ppm)
613.9781
[M−H]−
590.0455
971.9371
0
20
40
60
80
100
600
700
800
900

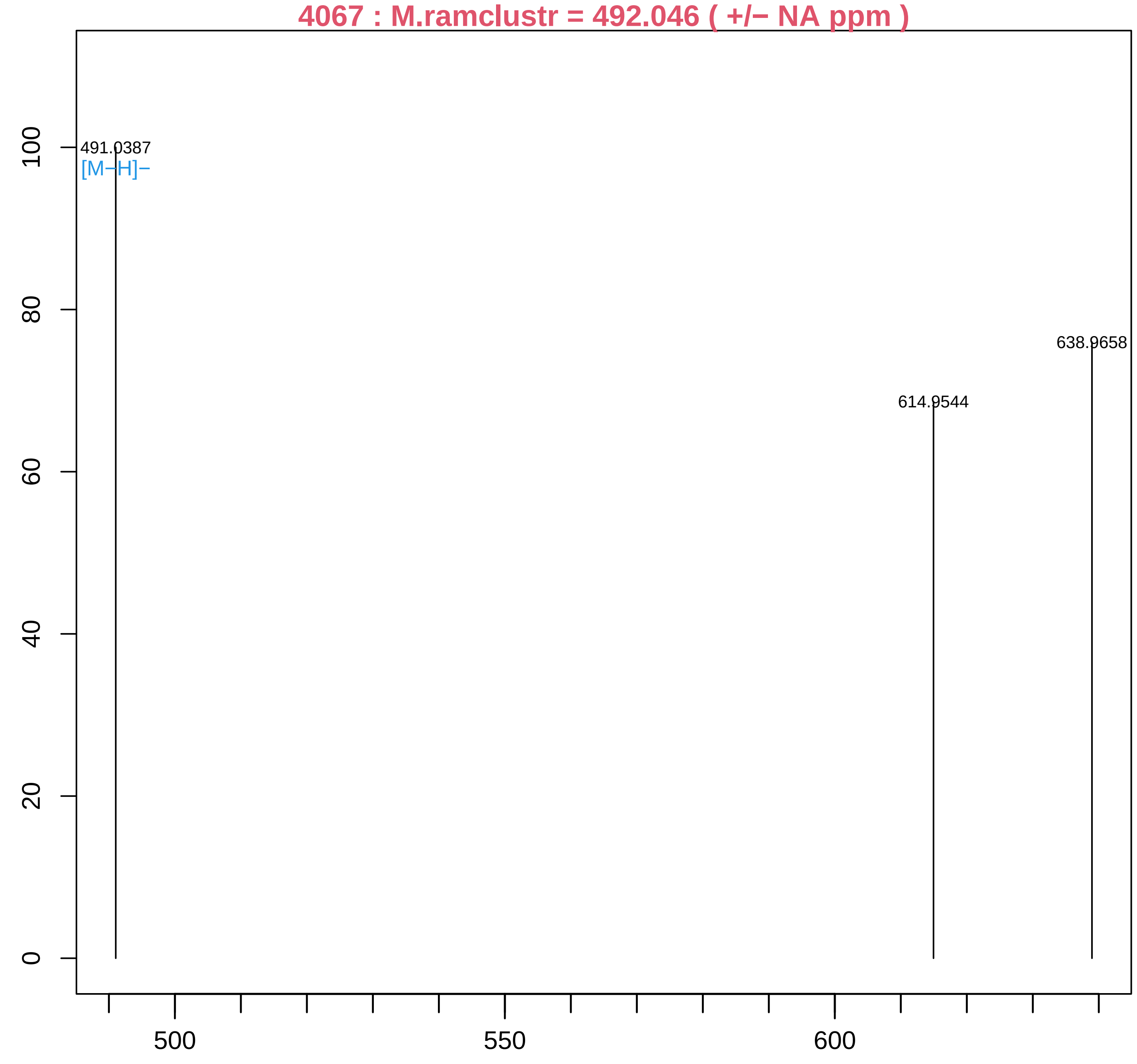
4067 : M.ramclustr = 492.046 (+/− NA ppm)
491.0387
[M−H]−
614.9544
638.9658
0
20
40
60
80
100
500
550
600

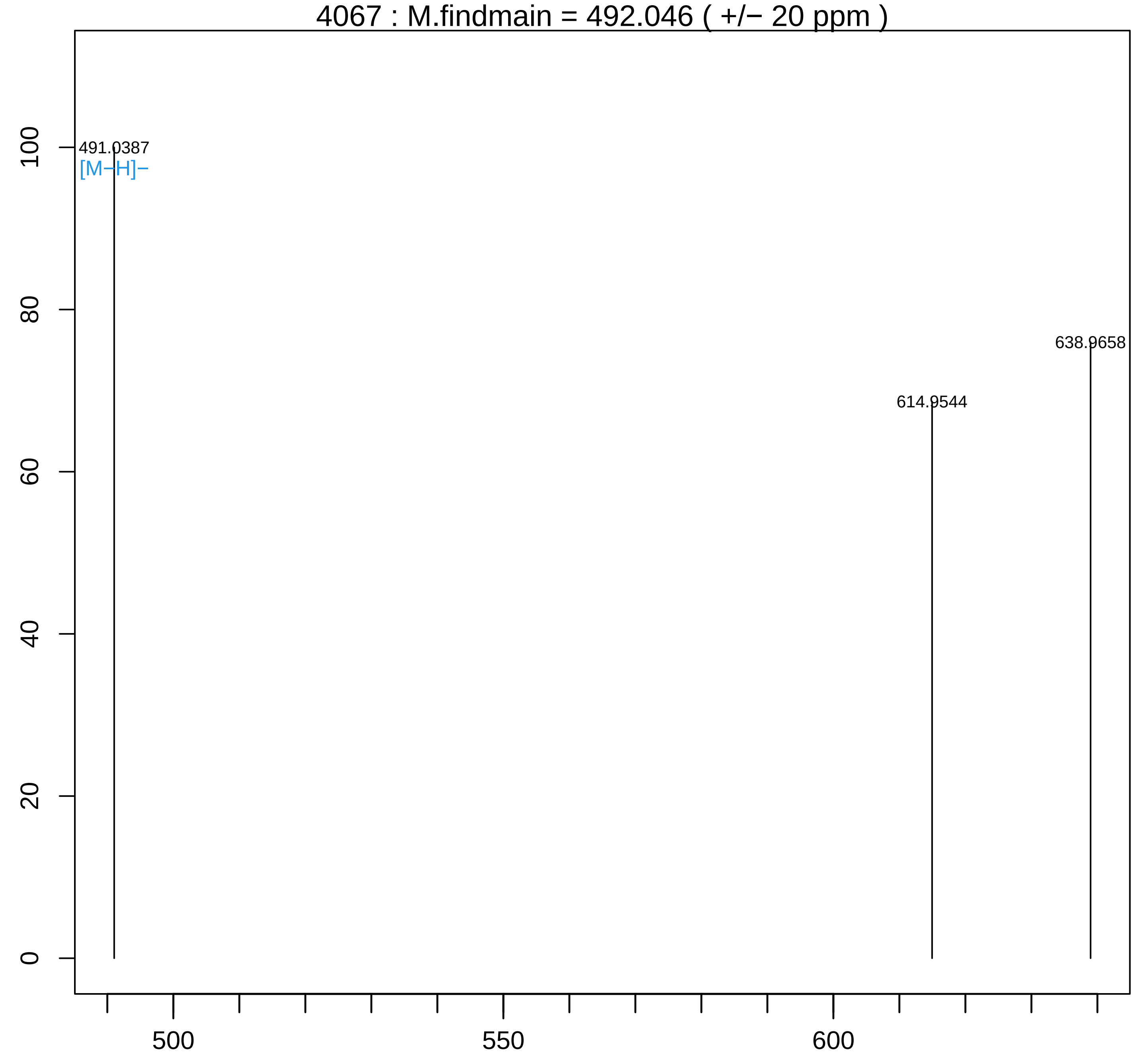
4067 : M.findmain = 492.046 (+/− 20 ppm)
491.0387
[M−H]−
614.9544
638.9658
0
20
40
60
80
100
500
550
600

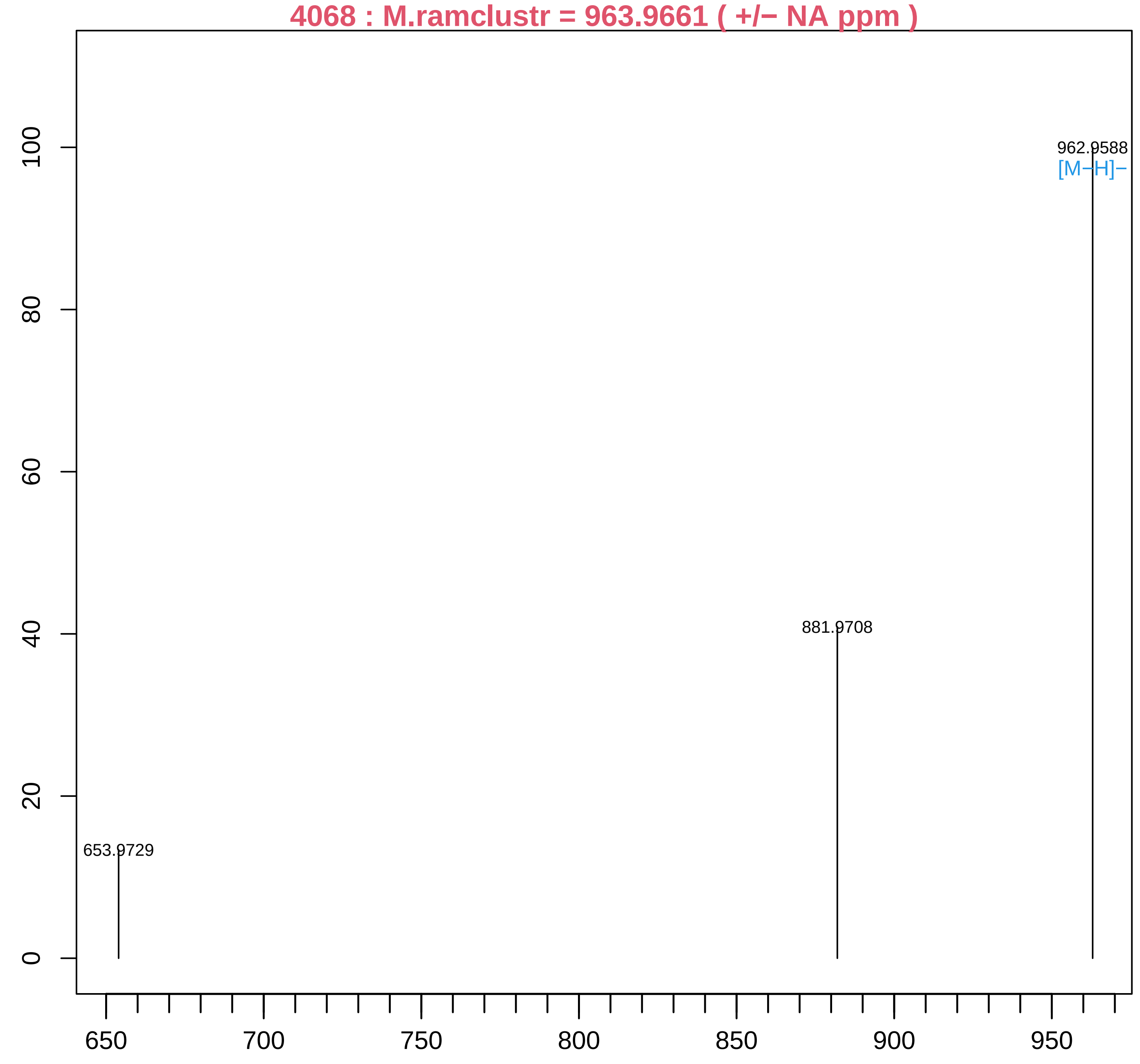
4068 : M.ramclustr = 963.9661 (+/− NA ppm)
962.9588
[M−H]−
881.9708
653.9729
0
20
40
60
80
100
650
700
750
800
850
900
950

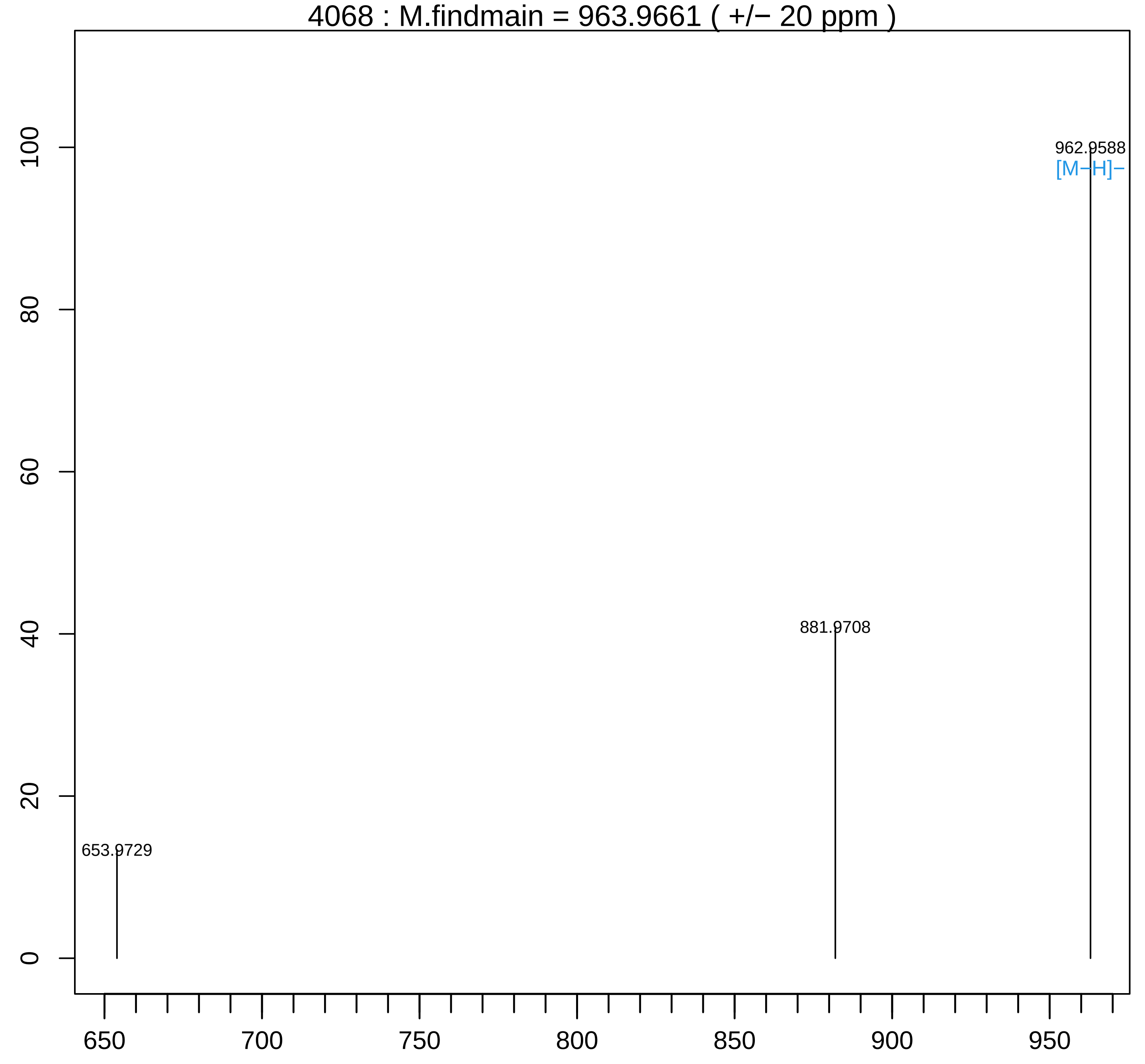
4068 : M.findmain = 963.9661 (+/− 20 ppm)
962.9588
[M−H]−
881.9708
653.9729
0
20
40
60
80
100
650
700
750
800
850
900
950

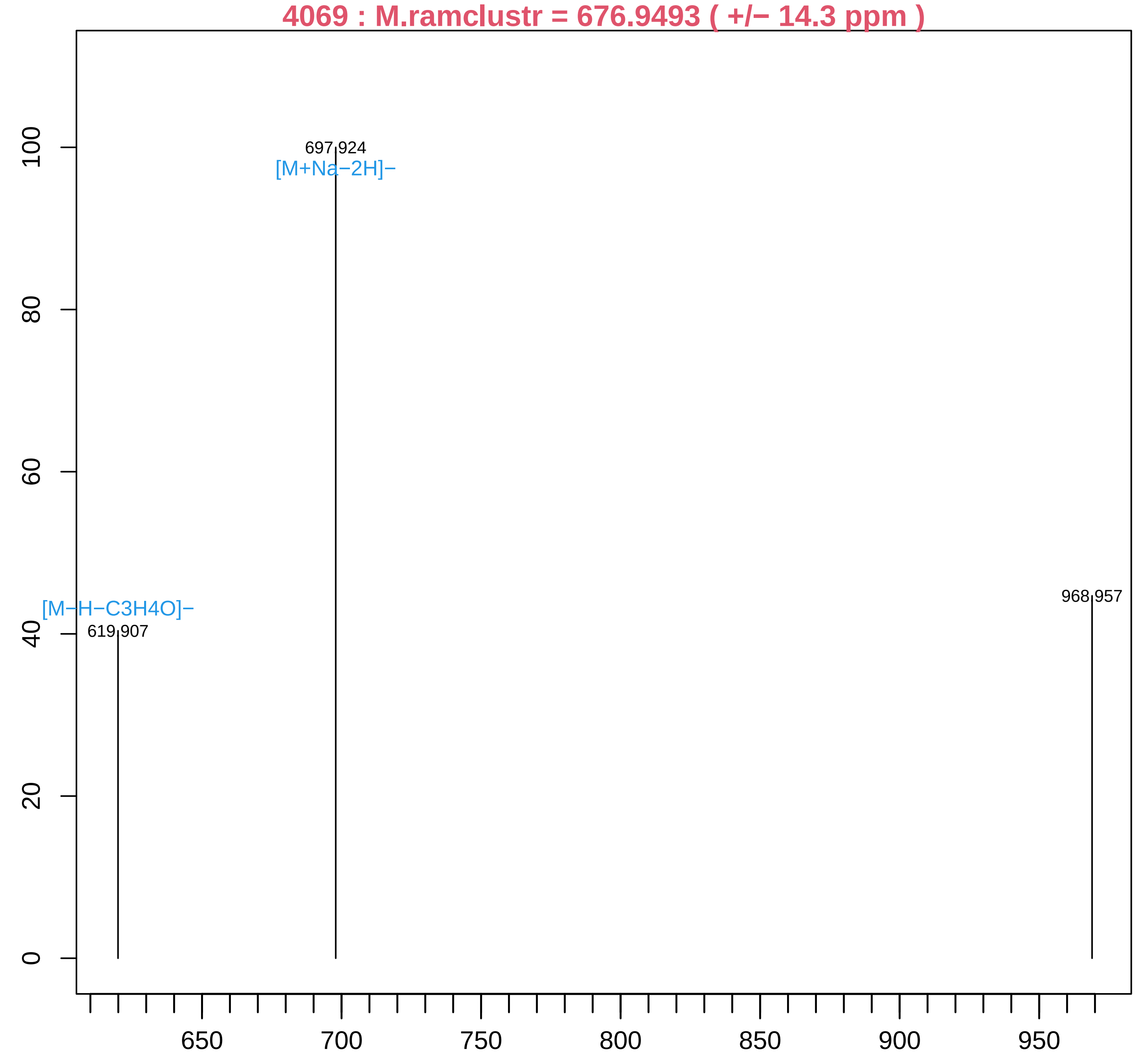
4069 : M.ramclustr = 676.9493 (+/− 14.3 ppm)
697.924
[M+Na−2H]−
[M−H−C3H4O]−
619.907
968.957
0
20
40
60
80
100
650
700
750
800
850
900
950

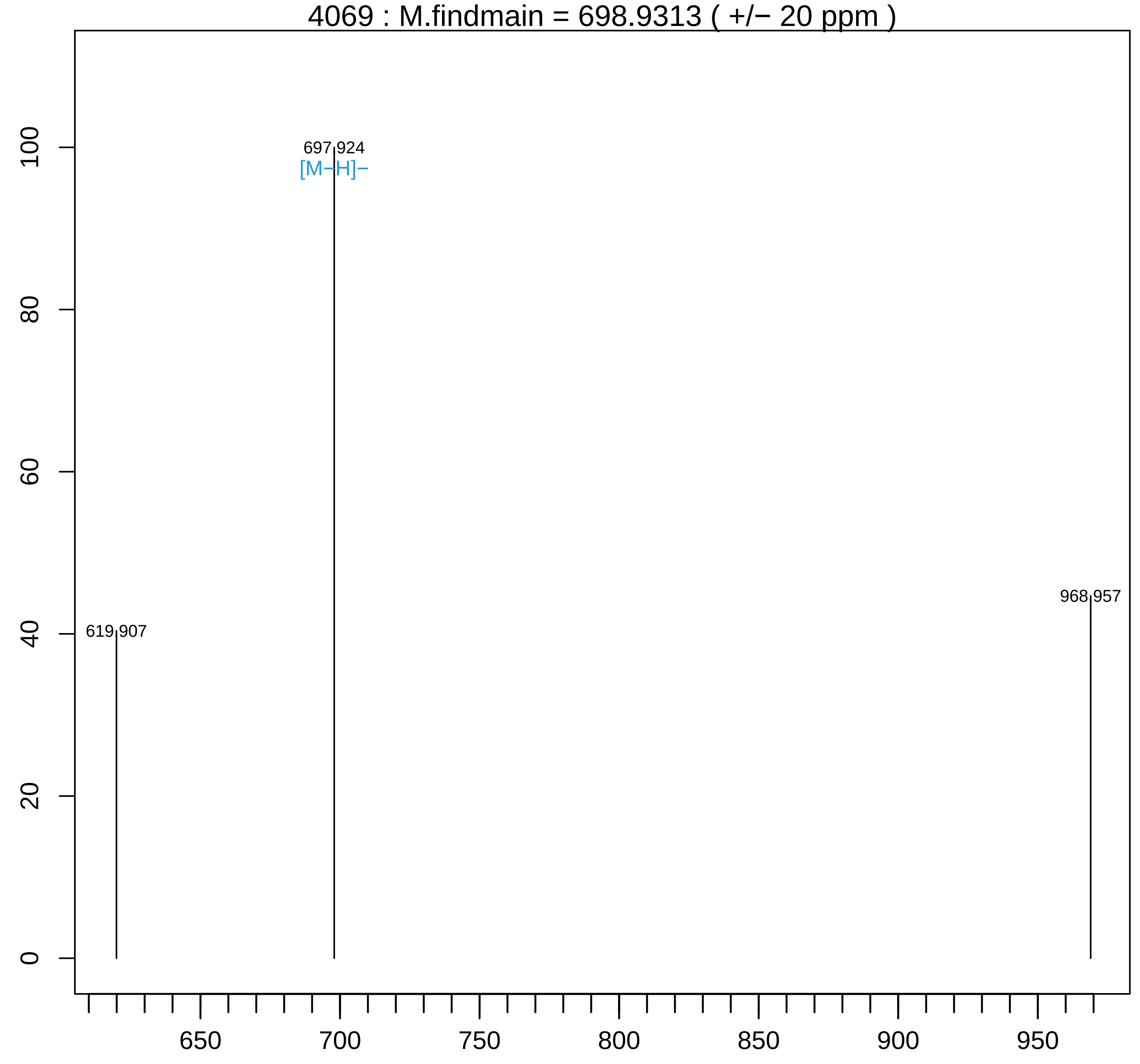
4069 : M.findmain = 698.9313 (+/− 20 ppm)
697.924
[M−H]−
619.907
968.957
0
20
40
60
80
100
650
700
750
800
850
900
950

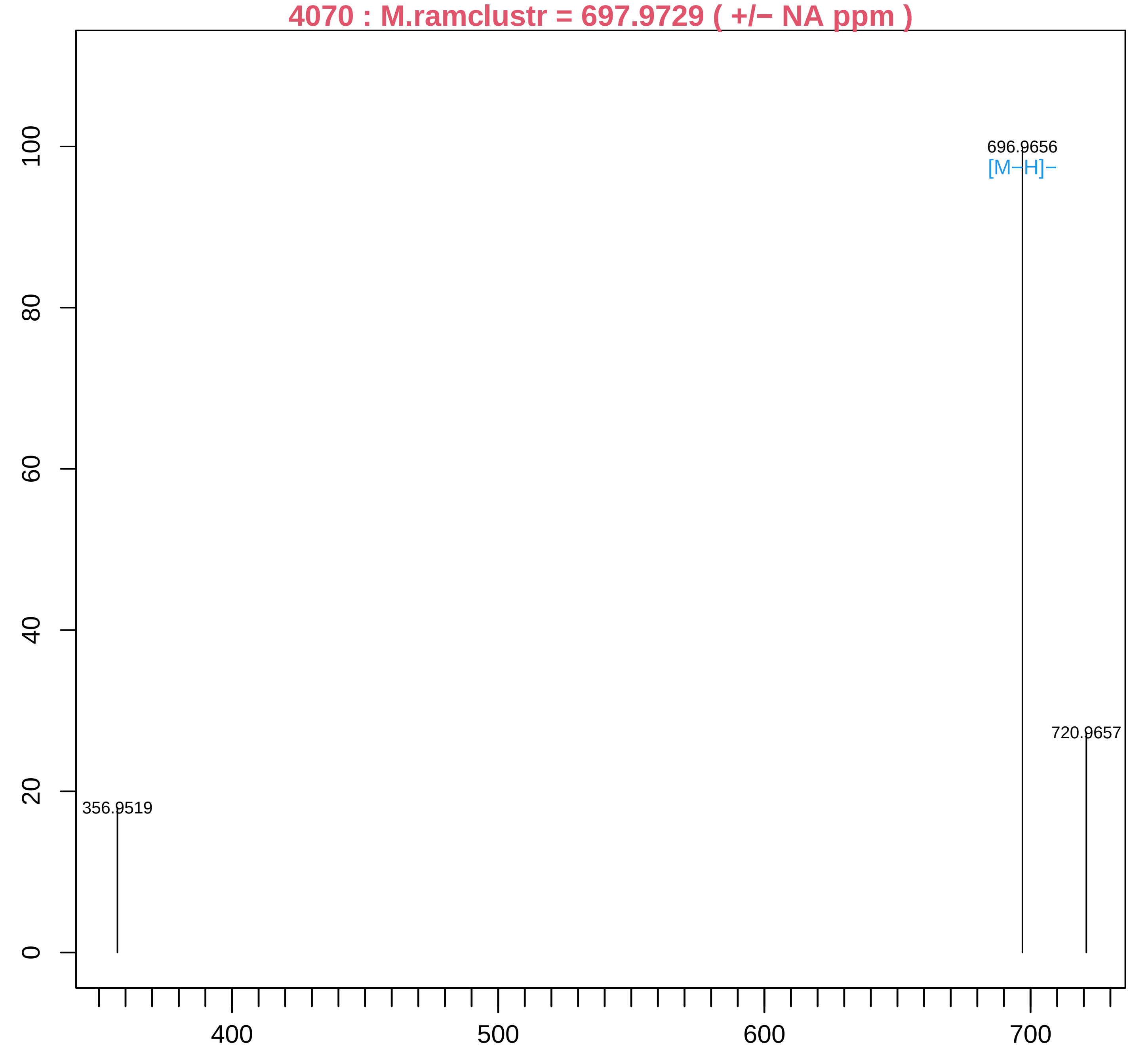
4070 : M.ramclustr = 697.9729 (+/− NA ppm)
696.9656
[M−H]−
720.9657
356.9519
0
20
40
60
80
100
400
500
600
700

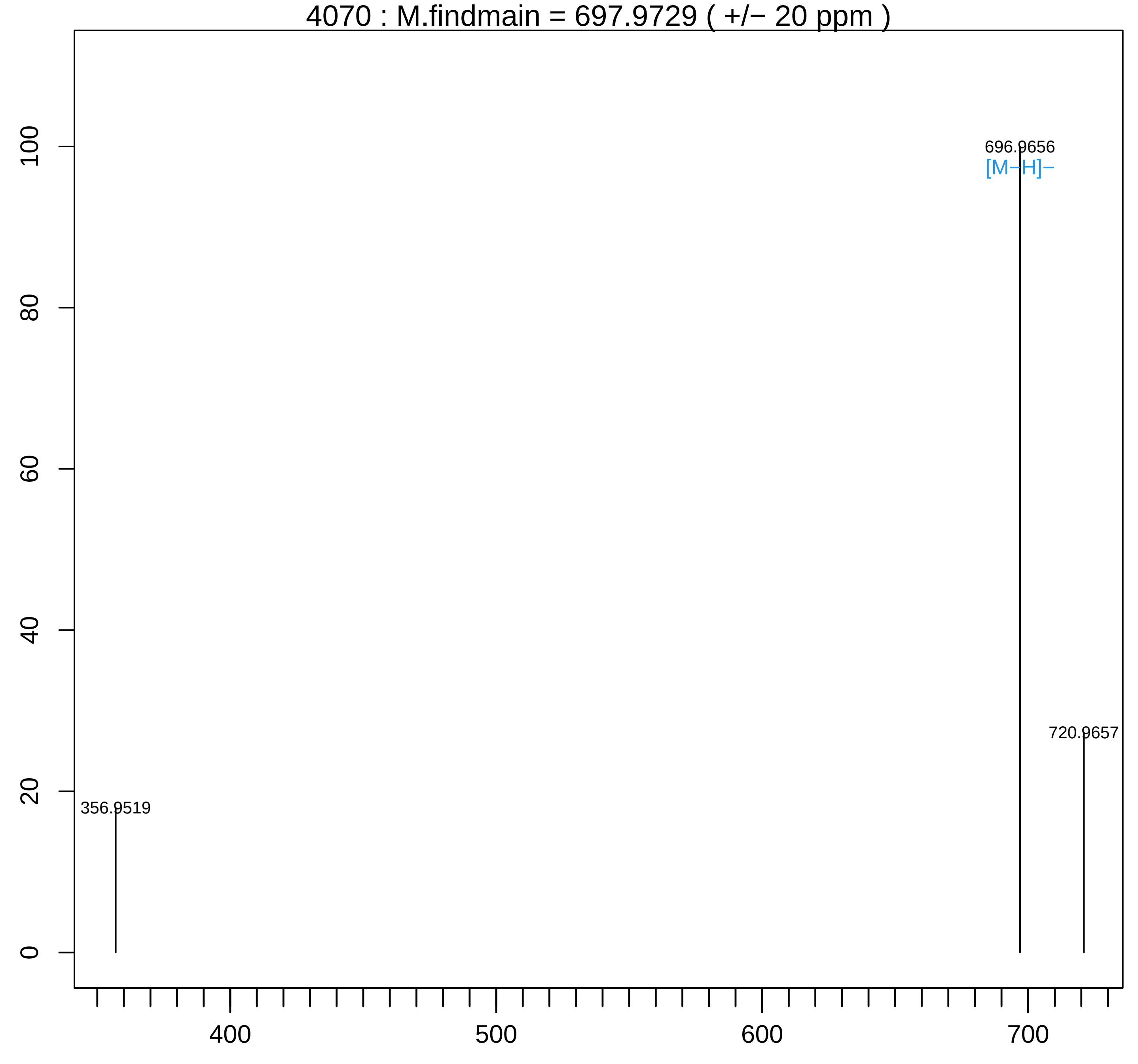
4070 : M.findmain = 697.9729 (+/− 20 ppm)
696.9656
[M−H]−
720.9657
356.9519
0
20
40
60
80
100
400
500
600
700

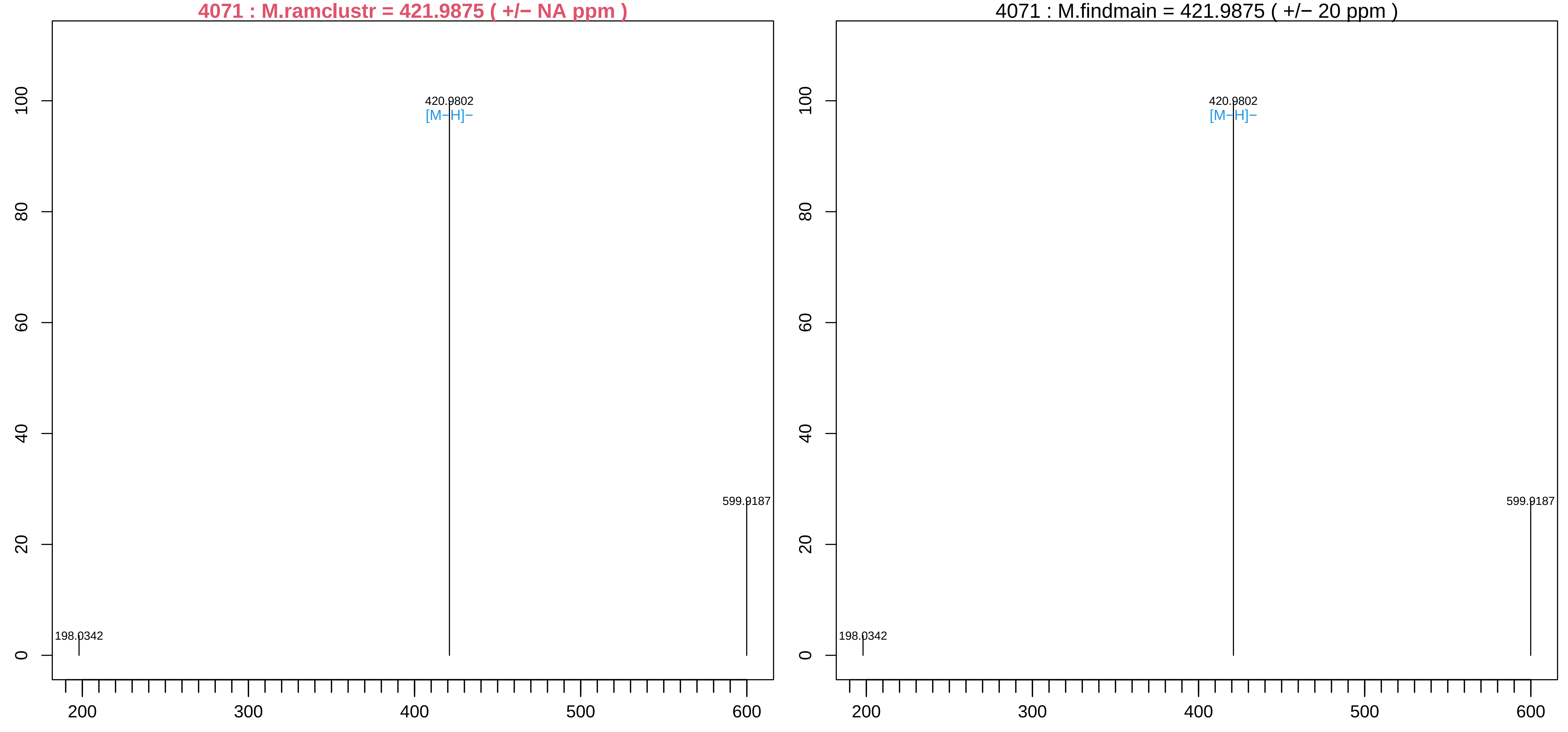
4071 : M.ramclustr = 421.9875 (+/− NA ppm)
420.9802
[M−H]−
599.9187
198.0342
0
20
40
60
80
100
200
300
400
500
600
4071 : M.findmain = 421.9875 (+/− 20 ppm)
420.9802
[M−H]−
599.9187
198.0342
0
20
40
60
80
100
200
300
400
500
600

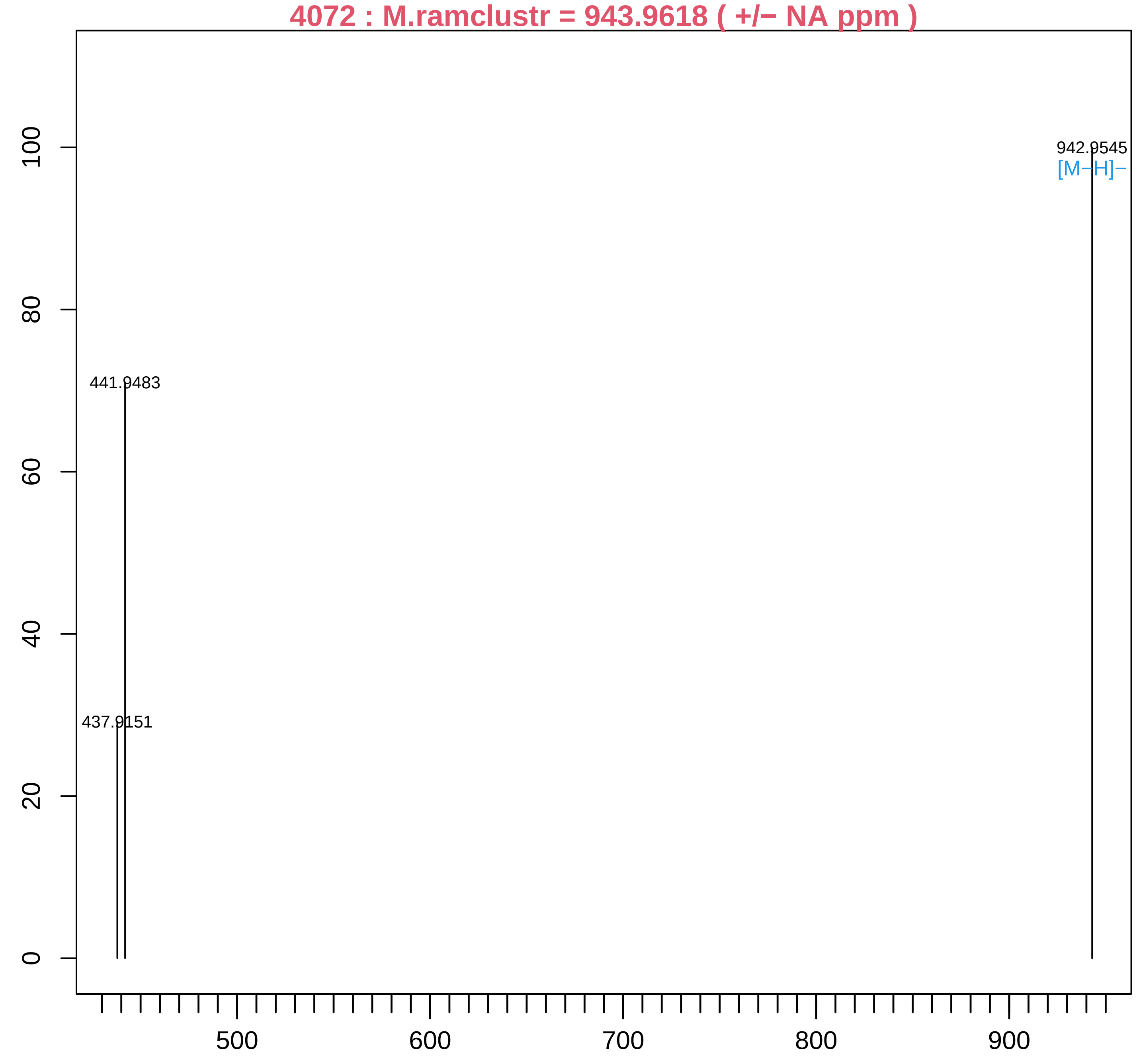
4072 : M.ramclustr = 943.9618 (+/− NA ppm)
942.9545
[M−H]−
441.9483
437.9151
0
20
40
60
80
100
500
600
700
800
900

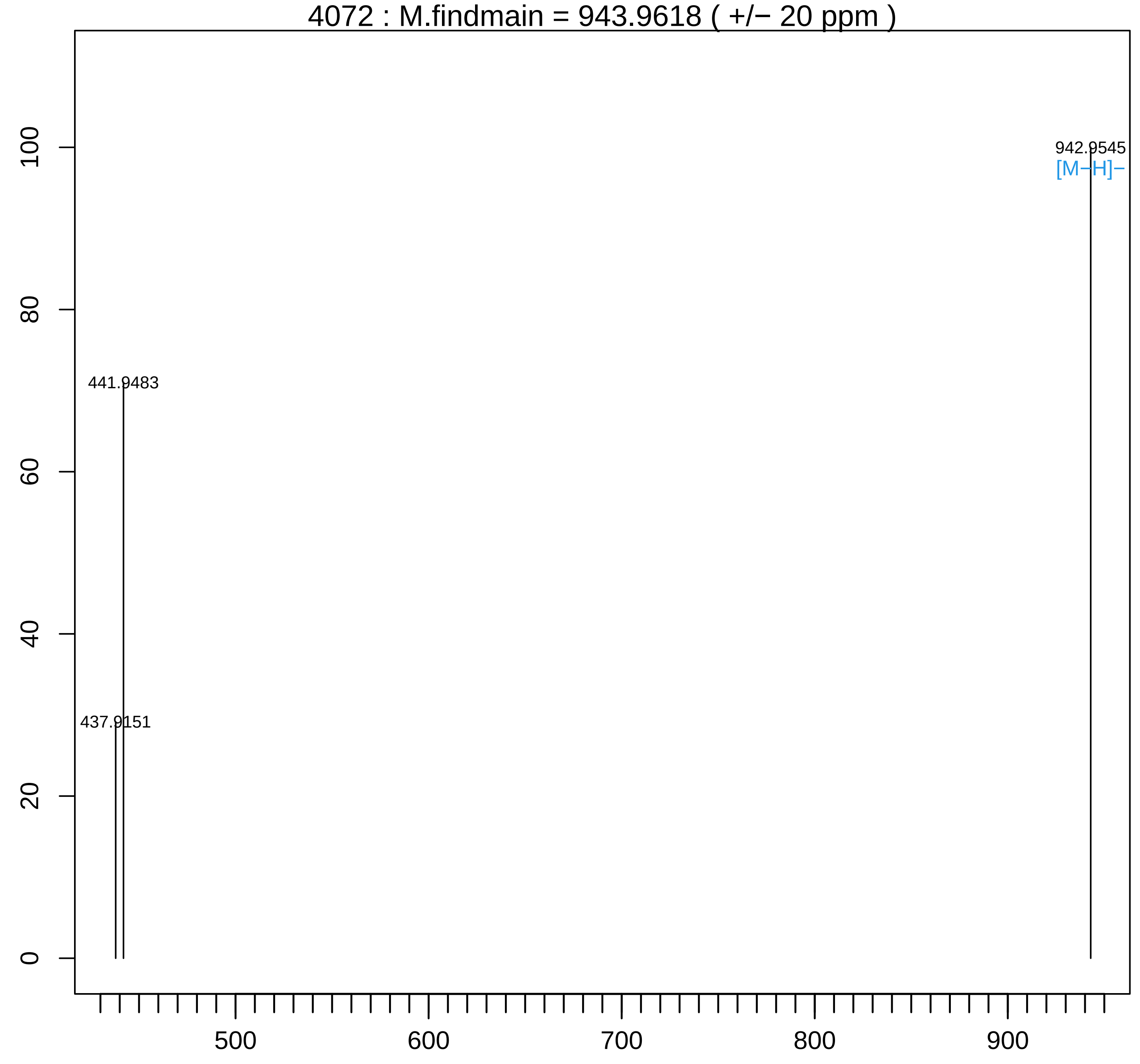
4072 : M.findmain = 943.9618 (+/− 20 ppm)
942.9545
[M−H]−
441.9483
437.9151
0
20
40
60
80
100
500
600
700
800
900

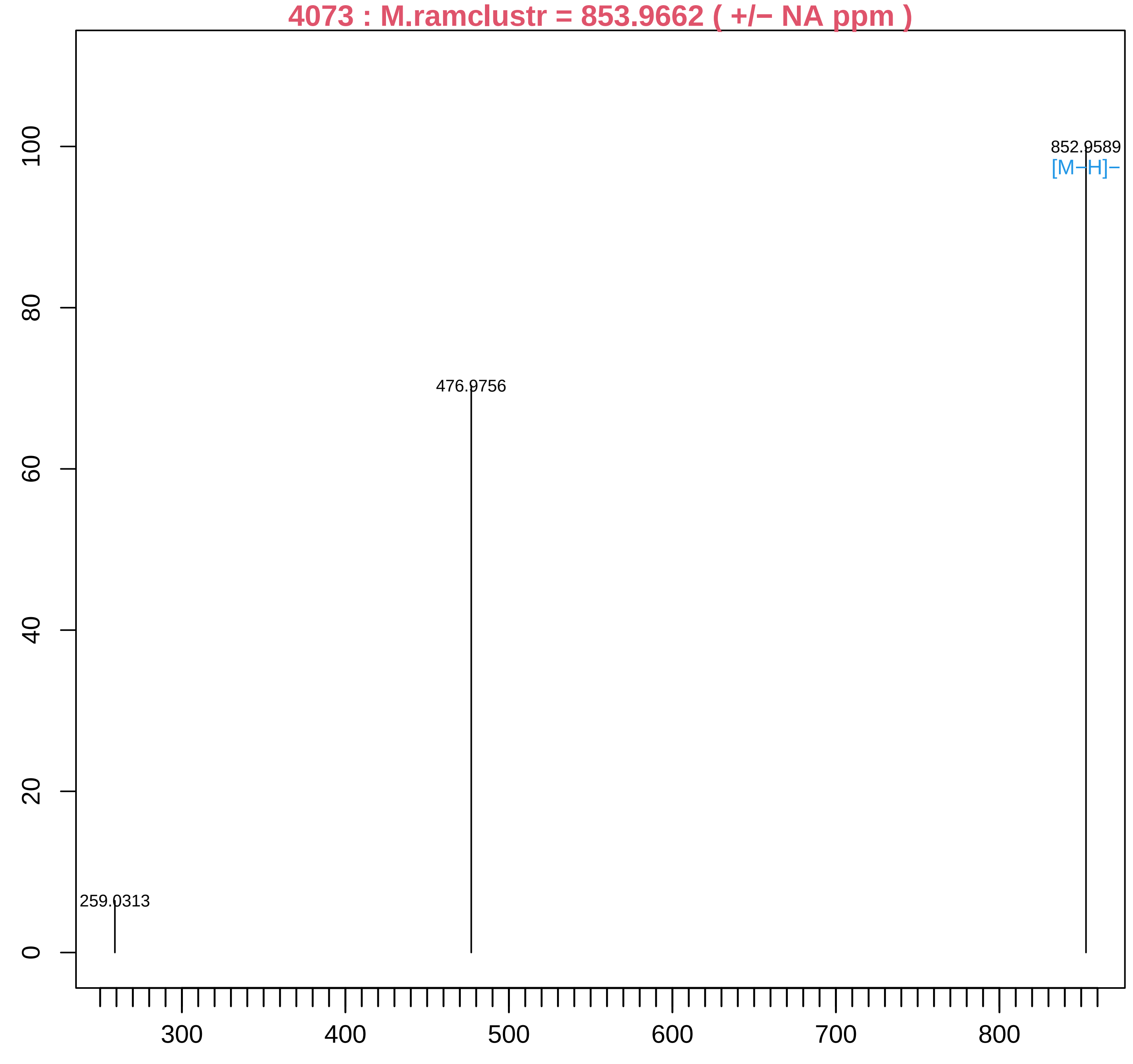
4073 : M.ramclustr = 853.9662 (+/− NA ppm)
852.9589
[M−H]−
476.9756
259.0313
0
20
40
60
80
100
300
400
500
600
700
800

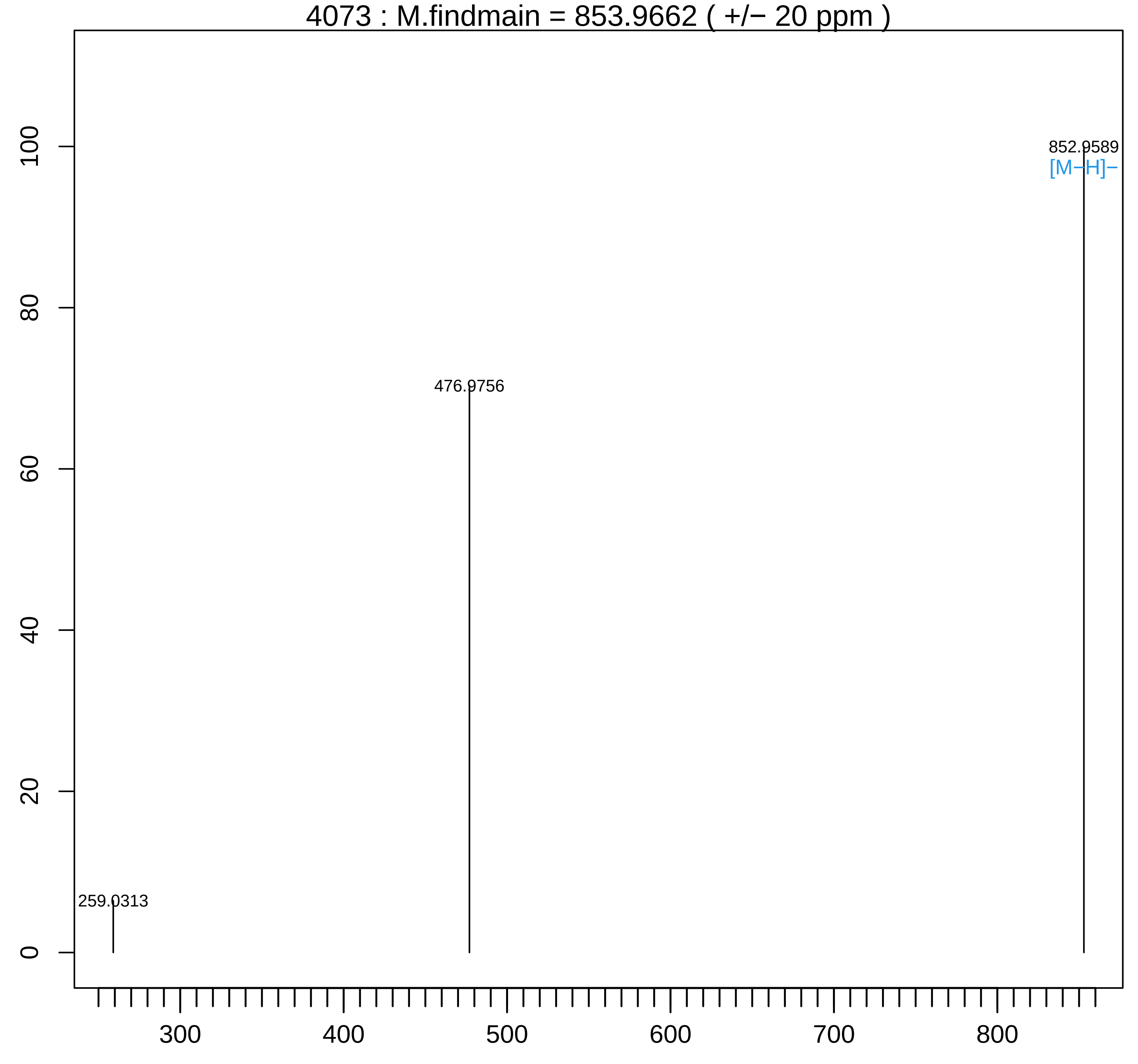
4073 : M.findmain = 853.9662 (+/− 20 ppm)
852.9589
[M−H]−
476.9756
259.0313
0
20
40
60
80
100
300
400
500
600
700
800

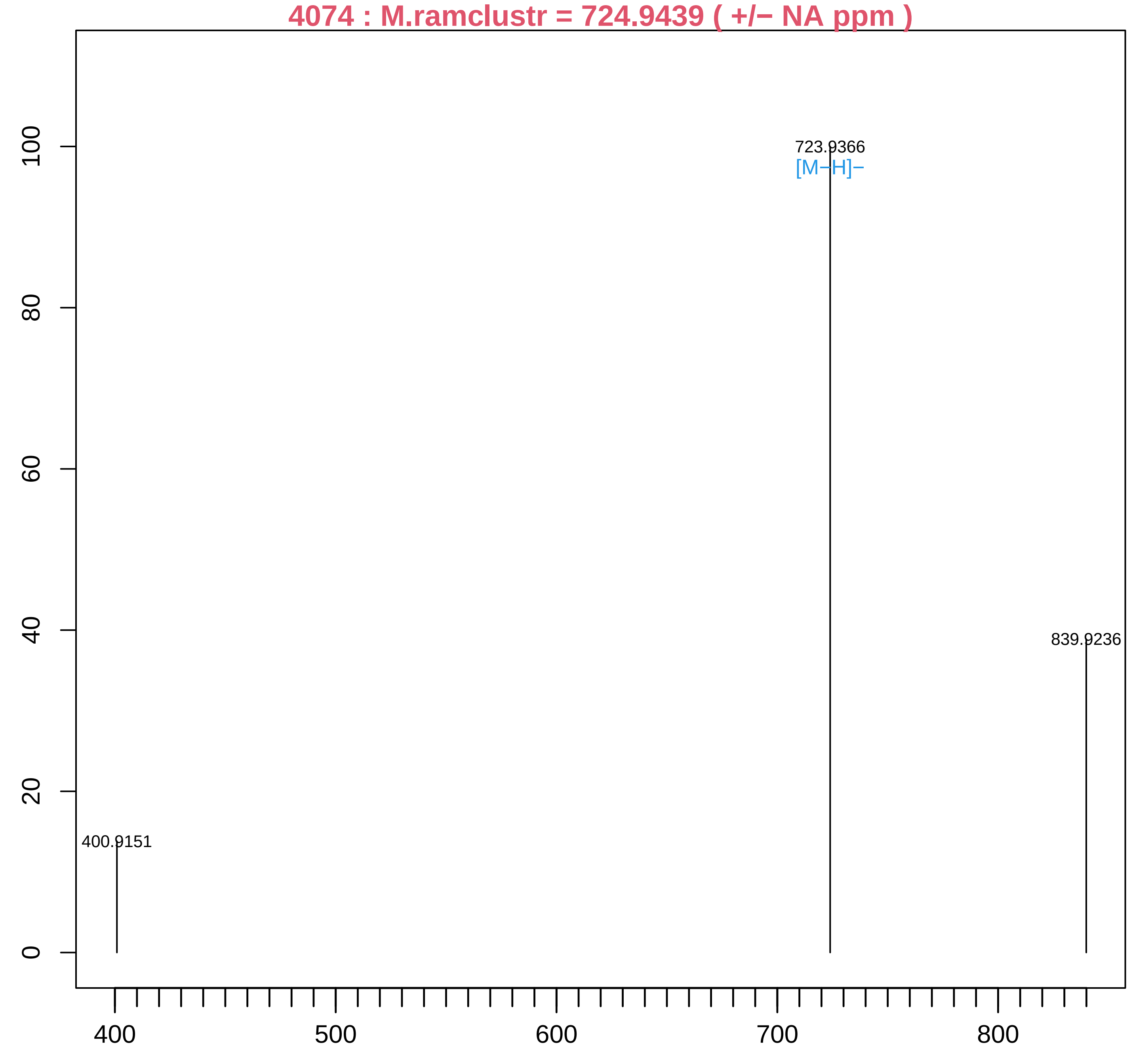
4074 : M.ramclustr = 724.9439 (+/− NA ppm)
723.9366
[M−H]−
839.9236
400.9151
0
20
40
60
80
100
400
500
600
700
800

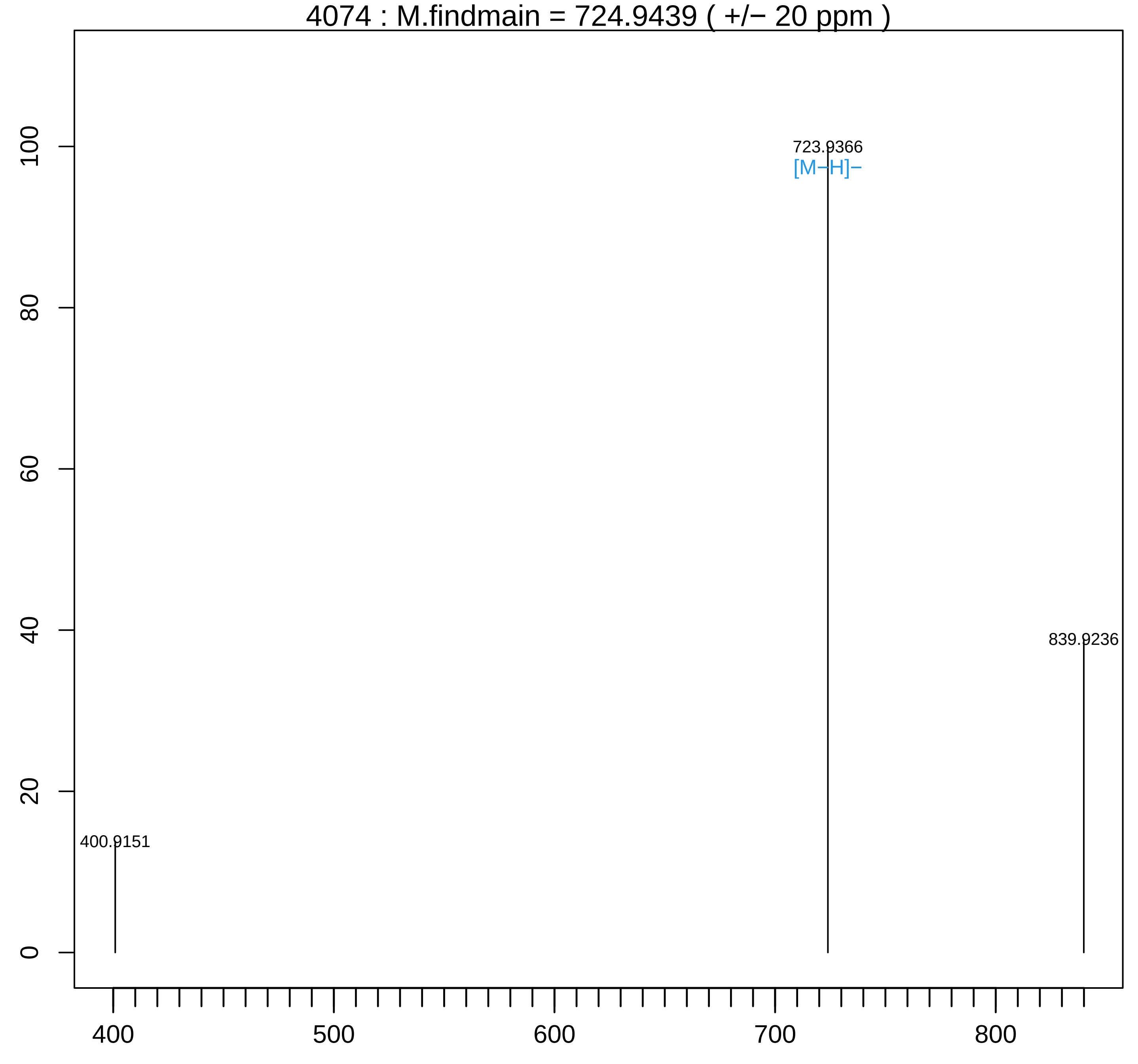
4074 : M.findmain = 724.9439 (+/− 20 ppm)
723.9366
[M−H]−
839.9236
400.9151
0
20
40
60
80
100
400
500
600
700
800

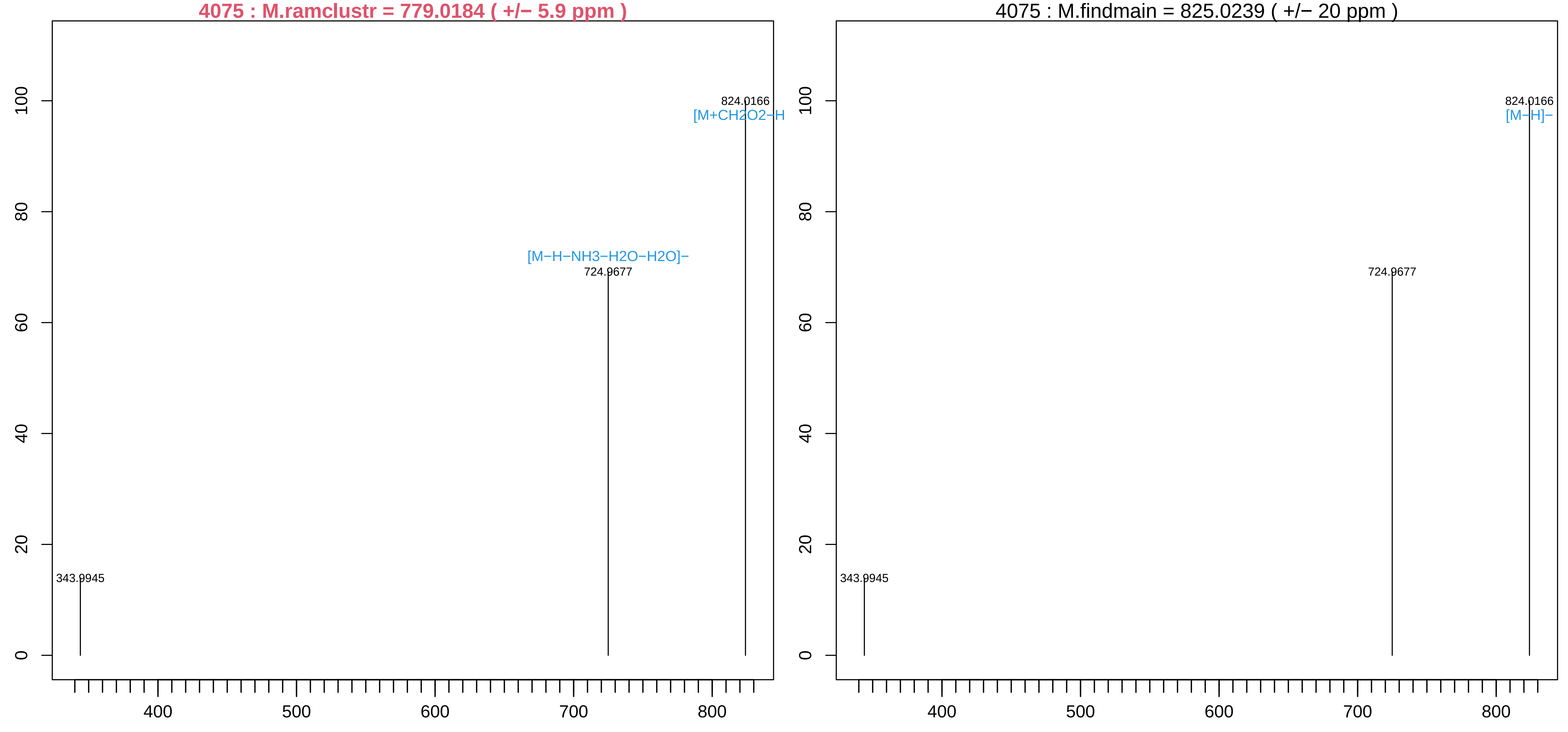
4075 : M.ramclustr = 779.0184 (+/− 5.9 ppm)
824.0166
[M+CH2O2−H
[M−H−NH3−H2O−H2O]−
724.9677
343.9945
4075 : M.findmain = 825.0239 (+/− 20 ppm)
824.0166
[M−H]−
724.9677
343.9945
0
20
40
60
80
100
400
500
600
700
800

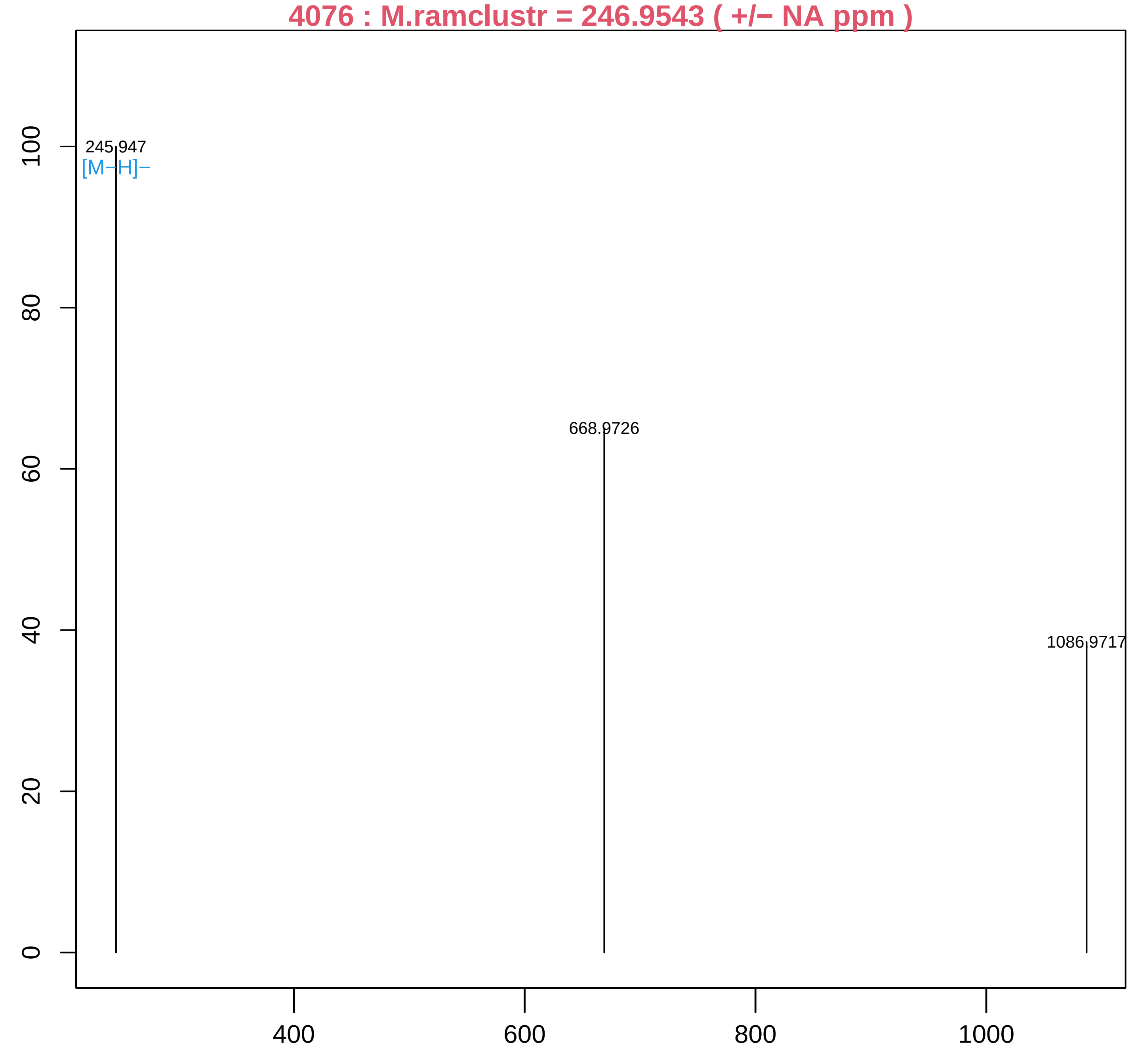
4076 : M.ramclustr = 246.9543 (+/− NA ppm)
245.947
[M−H]−
668.9726
1086.9717
0
20
40
60
80
100
400
600
800
1000

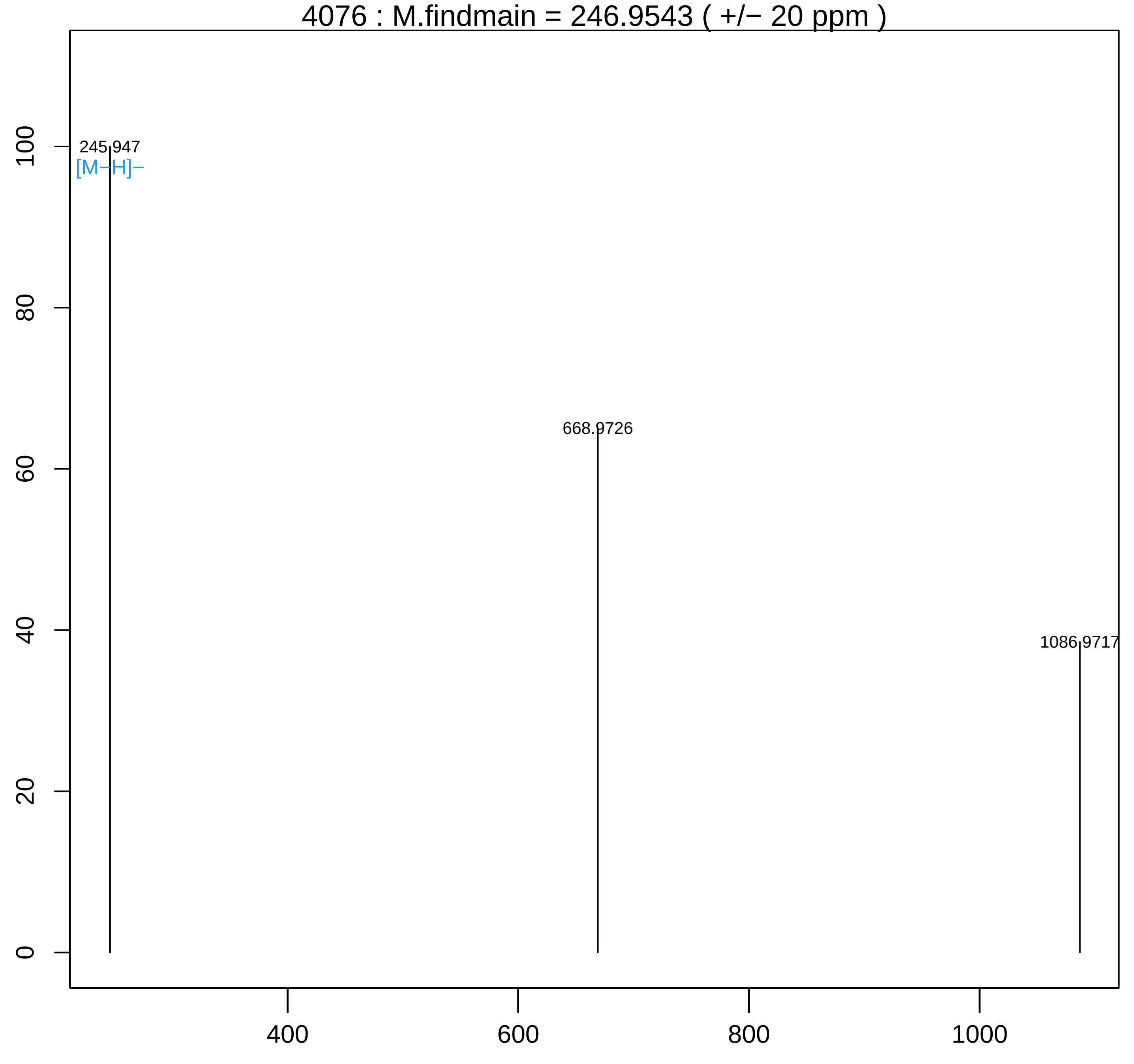
4076 : M.findmain = 246.9543 (+/− 20 ppm)
245.947
[M−H]−
668.9726
1086.9717
0
20
40
60
80
100
400
600
800
1000

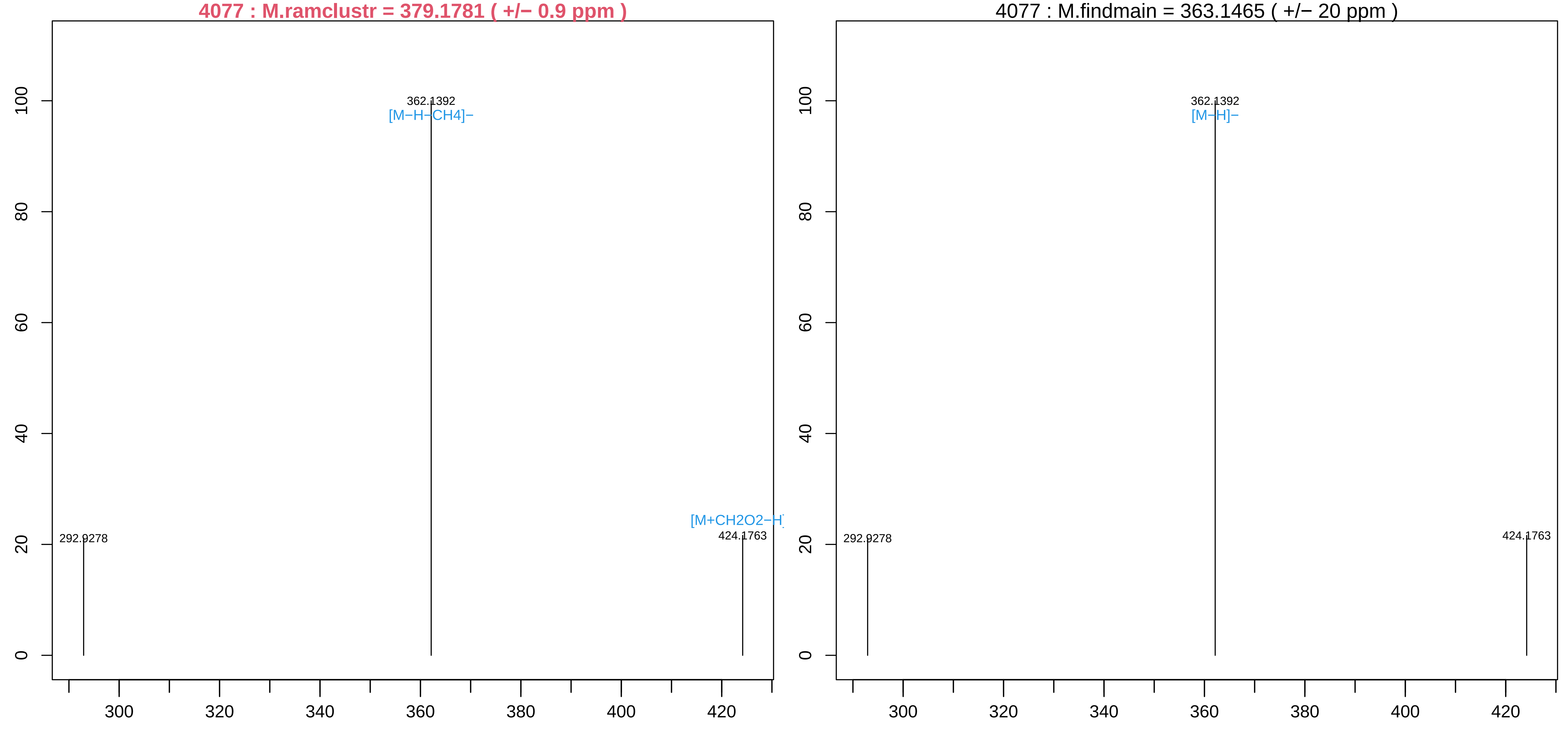

4077 : M.ramclustr = 379.1781 (+/− 0.9 ppm)
362.1392
[M−H−CH4]−
[M+CH2O2−H]
424.1763
292.9278
4077 : M.findmain = 363.1465 (+/− 20 ppm)
362.1392
[M−H]−
292.9278
424.1763
0
20
40
60
80
100
300
320
340
360
380
400
420

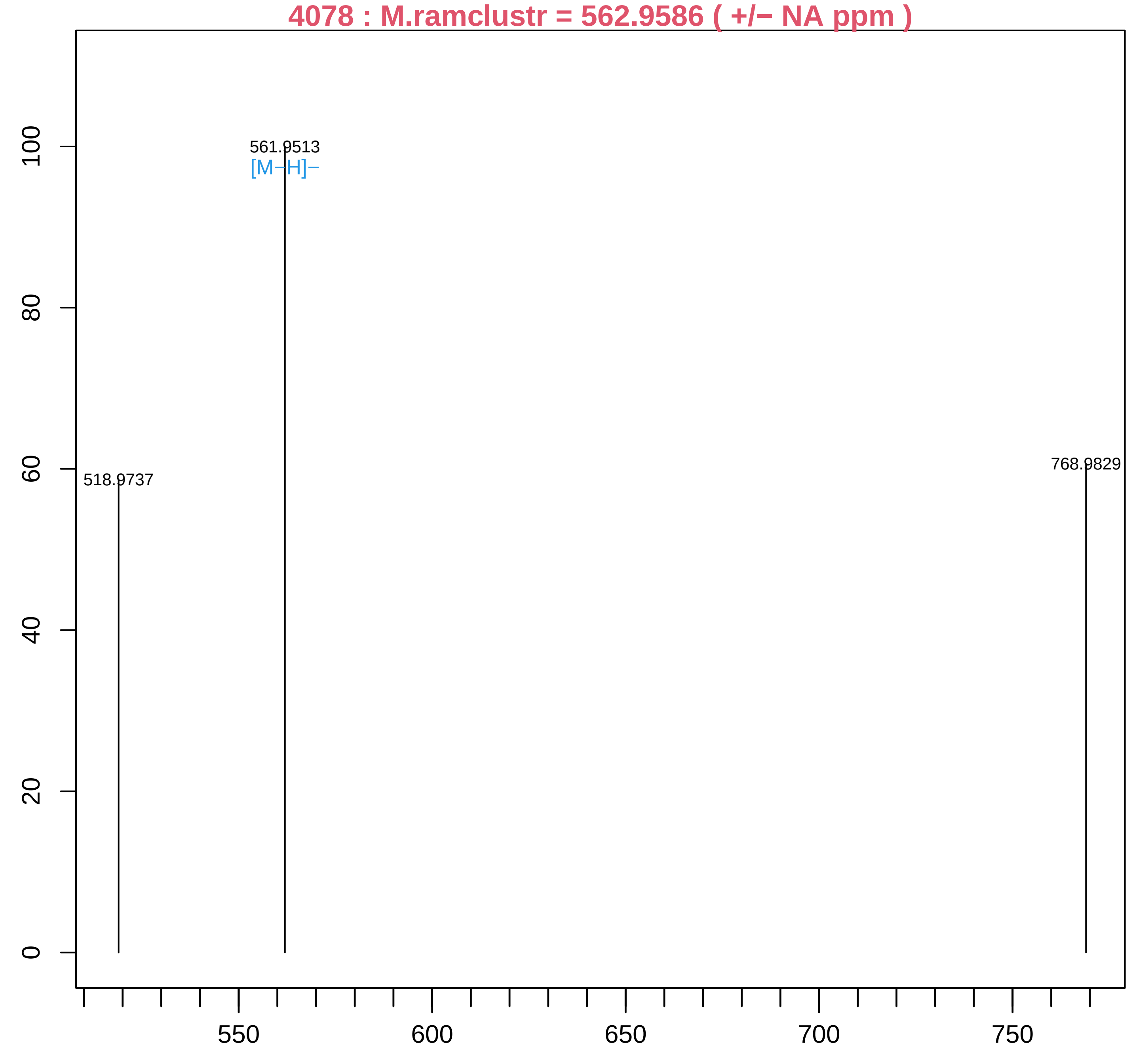

4078 : M.ramclustr = 562.9586 (+/− NA ppm)
561.9513
[M−H]−
518.9737
768.9829
0
20
40
60
80
100
550
600
650
700
750

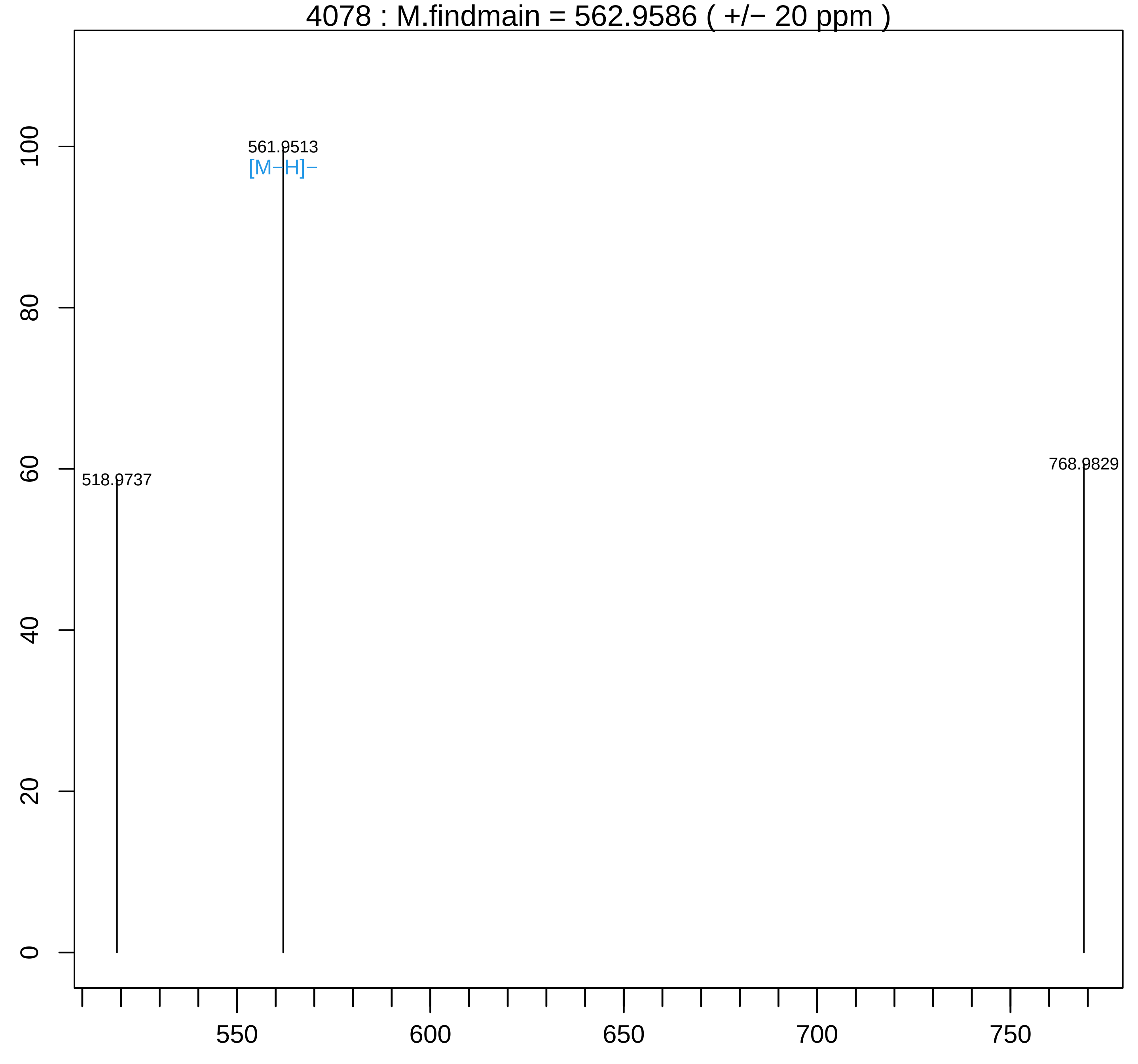

4078 : M.findmain = 562.9586 (+/− 20 ppm)
561.9513
[M−H]−
518.9737
768.9829
0
20
40
60
80
100
550
600
650
700
750

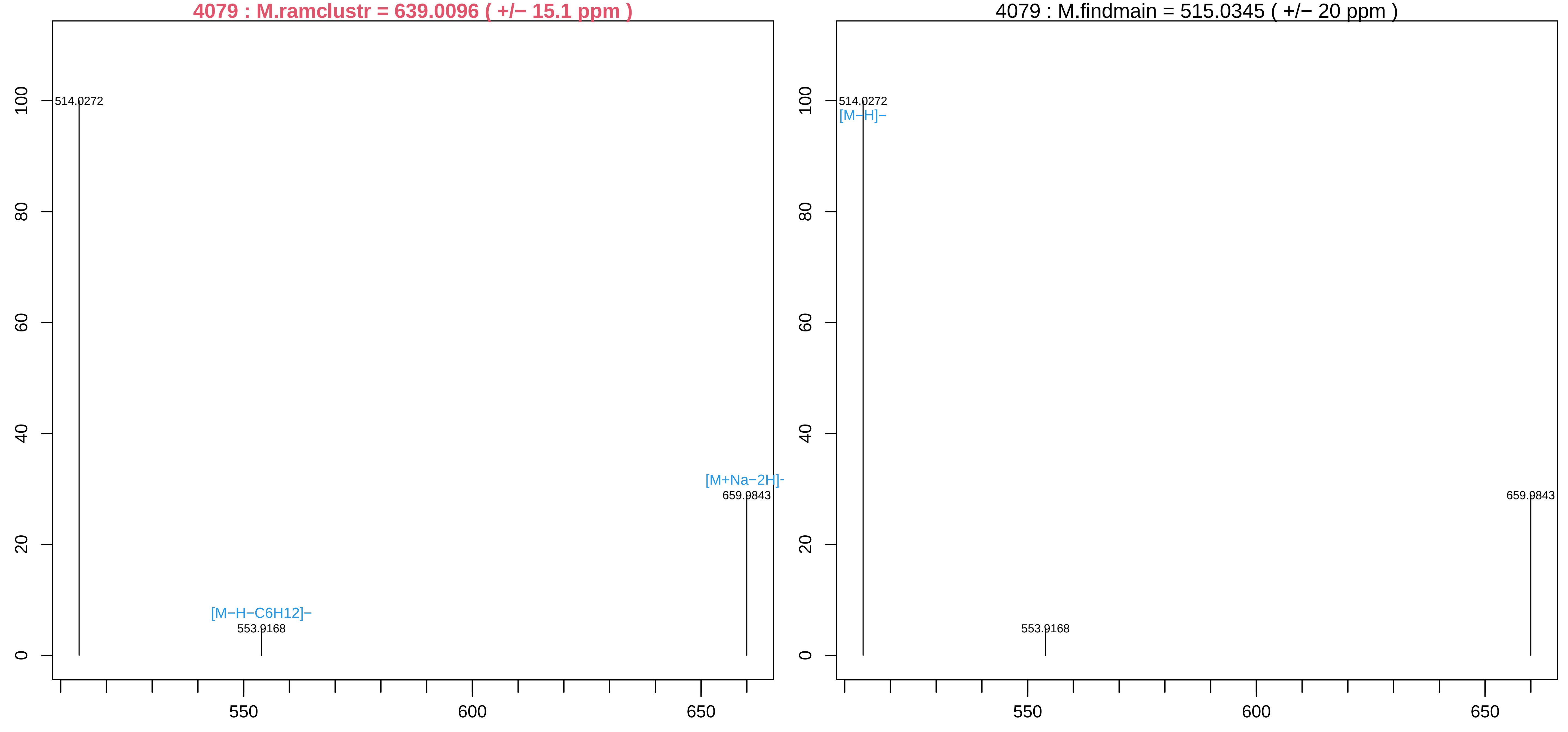
4079 : M.ramclustr = 639.0096 (+/− 15.1 ppm)
514.0272
[M−H−C6H12]−
553.9168
[M+Na−2H]-
659.9843
100
80
60
40
20
0
550
600
650
4079 : M.findmain = 515.0345 (+/− 20 ppm)
514.0272
[M−H]−
553.9168
659.9843
100
80
60
40
20
0
550
600
650

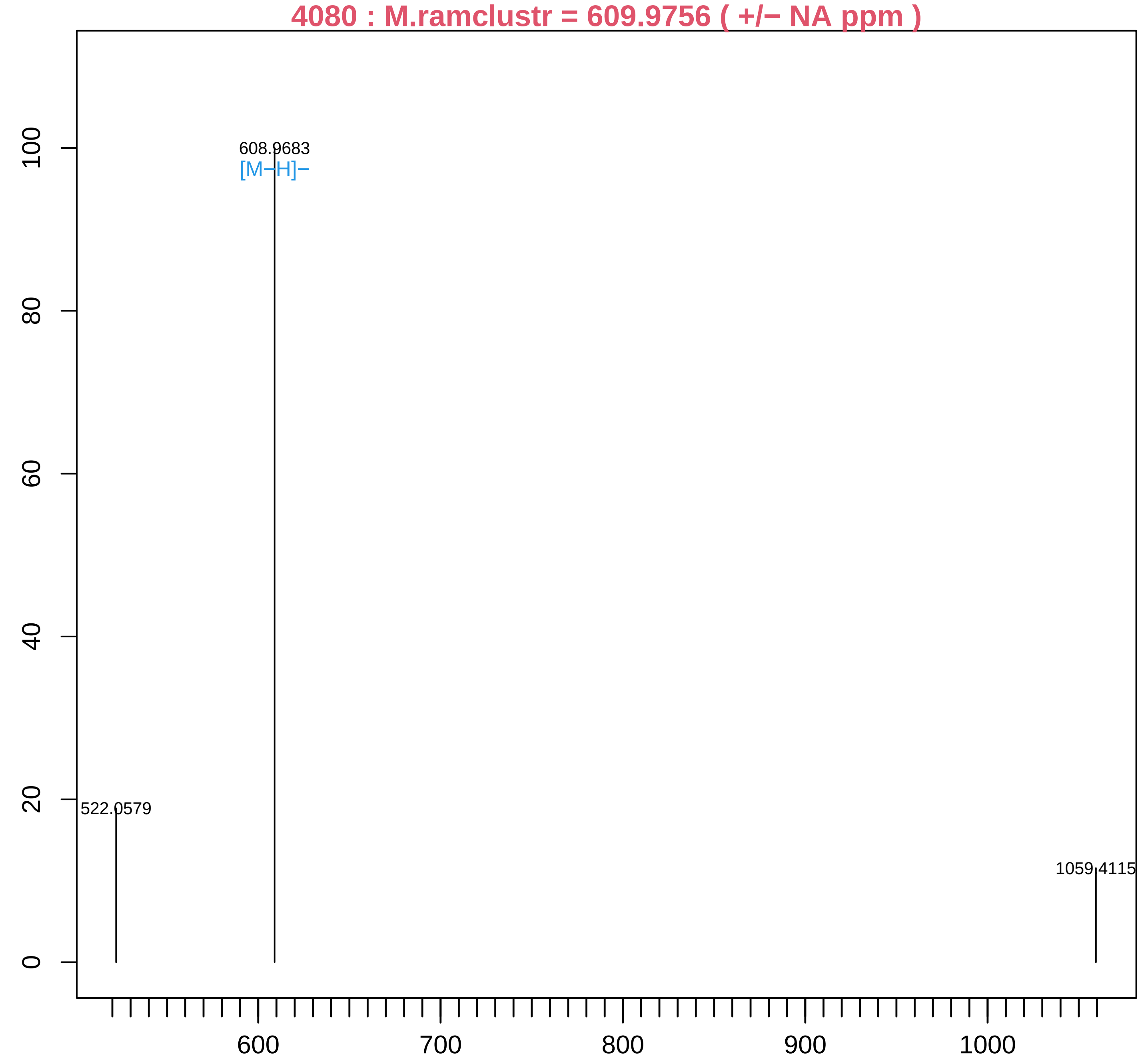
4080 : M.ramclustr = 609.9756 (+/− NA ppm)
608.9683
[M−H]−
522.0579
1059.4115
0
20
40
60
80
100
600
700
800
900
1000

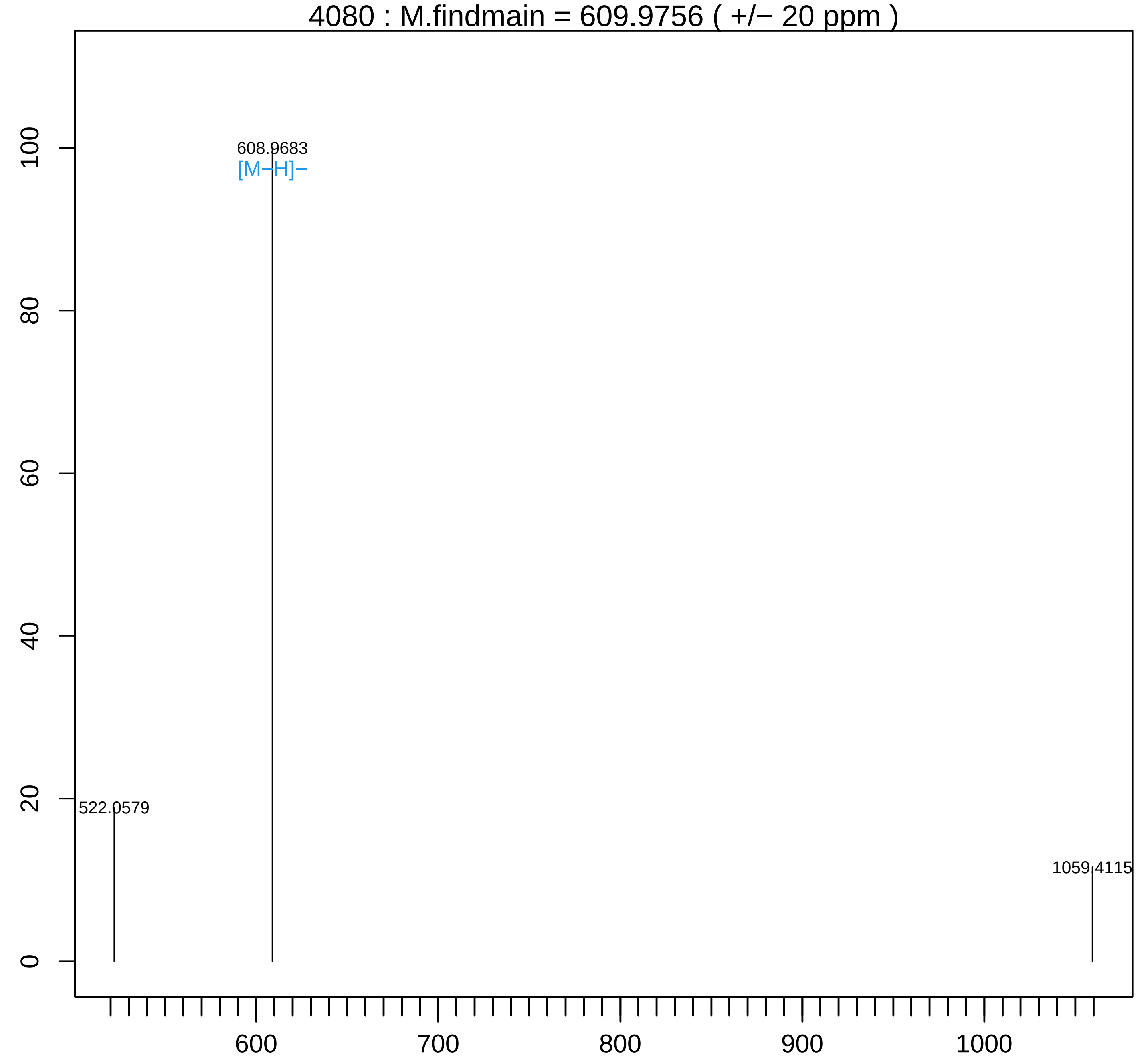
4080 : M.findmain = 609.9756 (+/− 20 ppm)
608.9683
[M−H]−
522.0579
1059.4115
0
20
40
60
80
100
600
700
800
900
1000

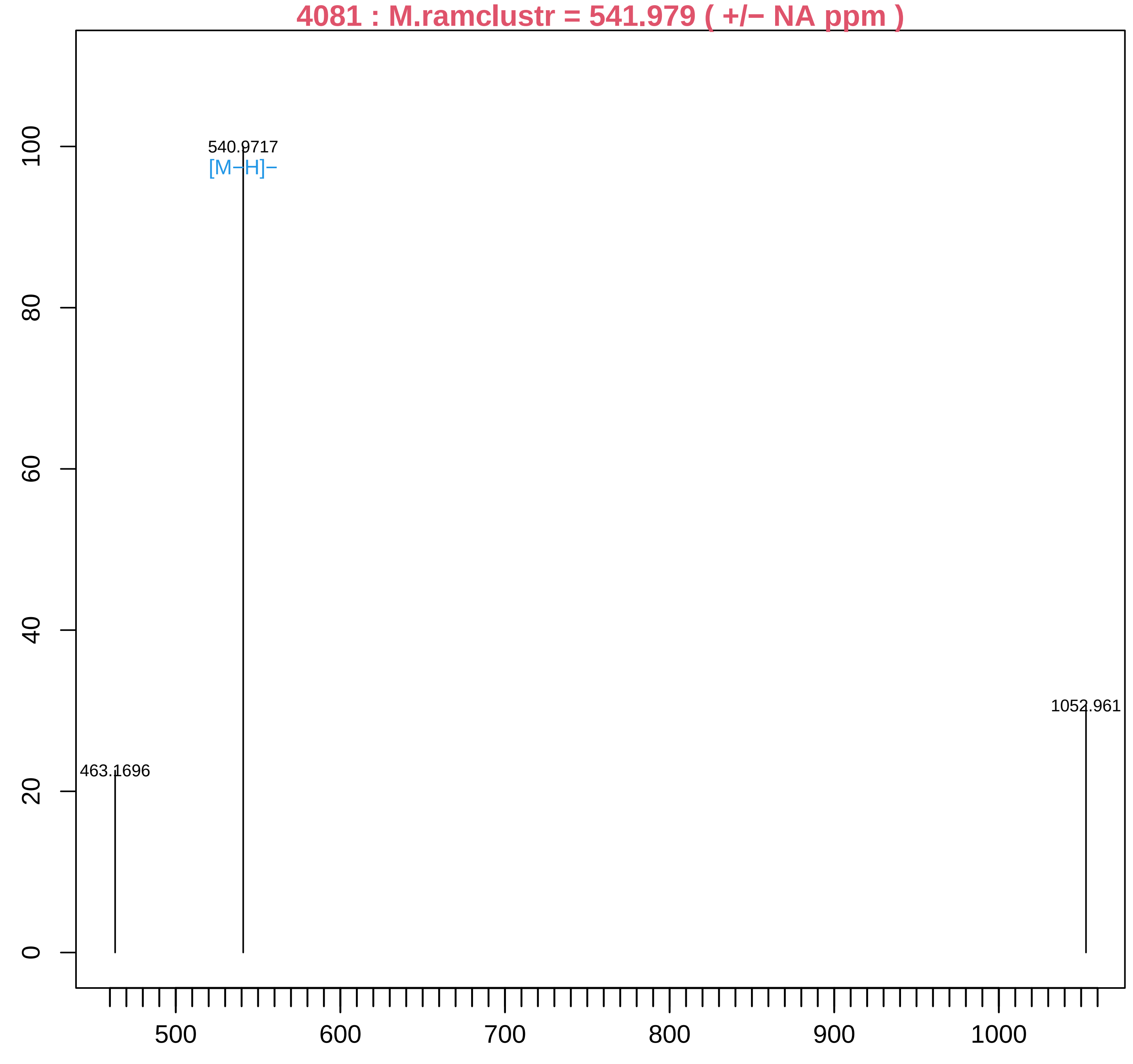
4081 : M.ramclustr = 541.979 (+/− NA ppm)
540.9717
[M−H]−
463.1696
1052.961
0
20
40
60
80
100
500
600
700
800
900
1000
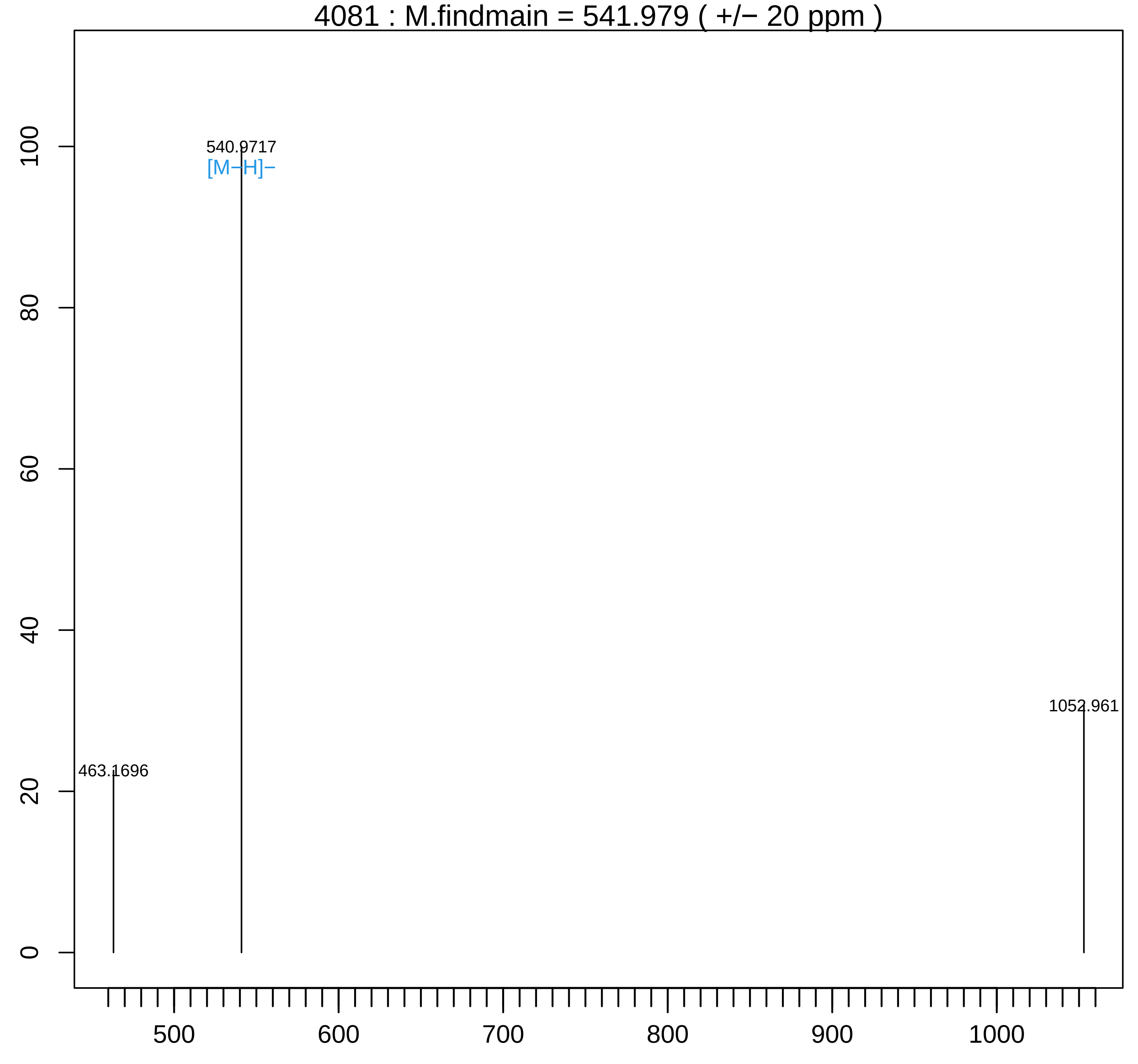
4081 : M.findmain = 541.979 (+/− 20 ppm)
540.9717
[M−H]−
463.1696
1052.961
0
20
40
60
80
100
500
600
700
800
900
1000

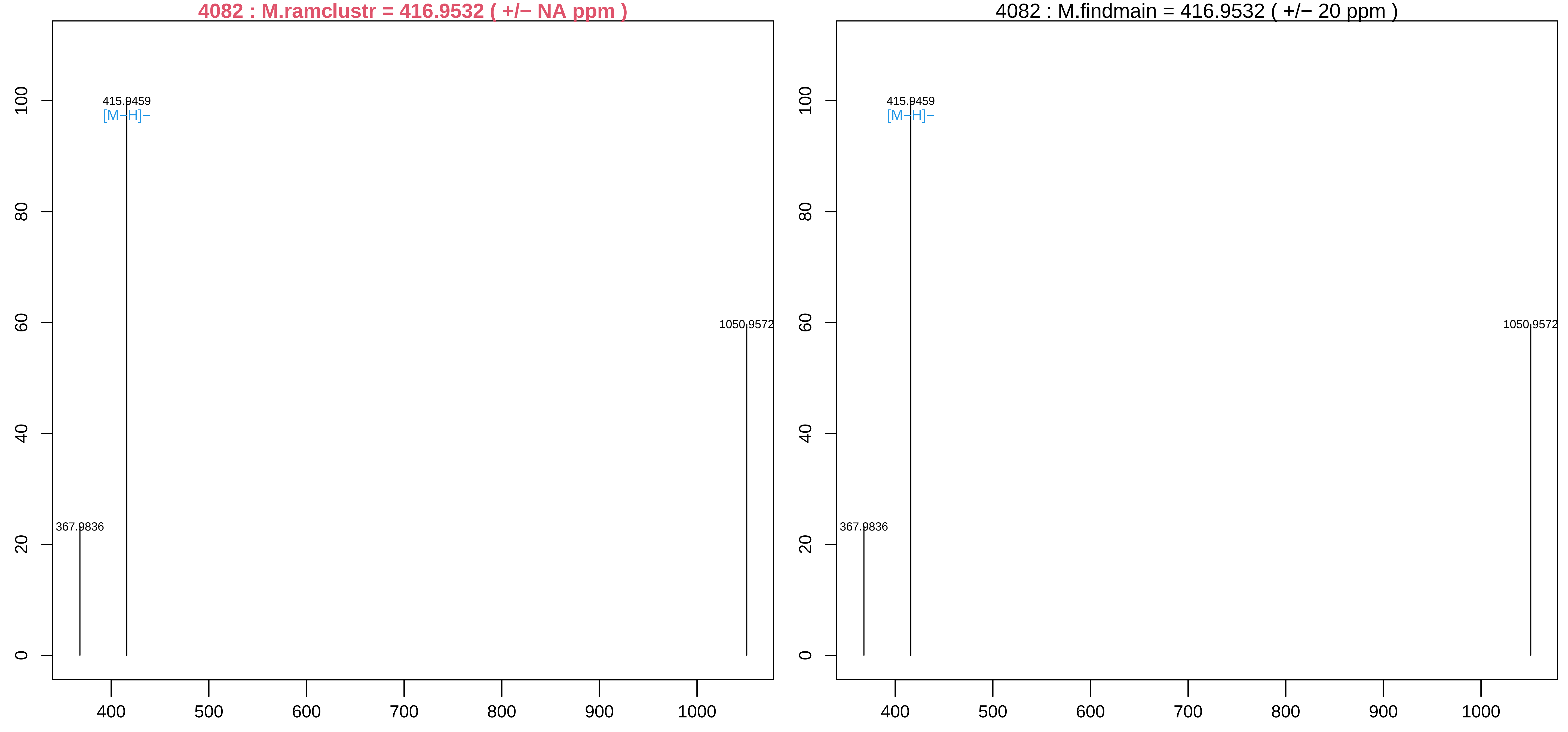

4082 : M.ramclustr = 416.9532 (+/− NA ppm)
4082 : M.findmain = 416.9532 (+/− 20 ppm)
415.9459
[M−H]−
1050.9572
367.9836
0
20
40
60
80
100
400
500
600
700
800
900
1000

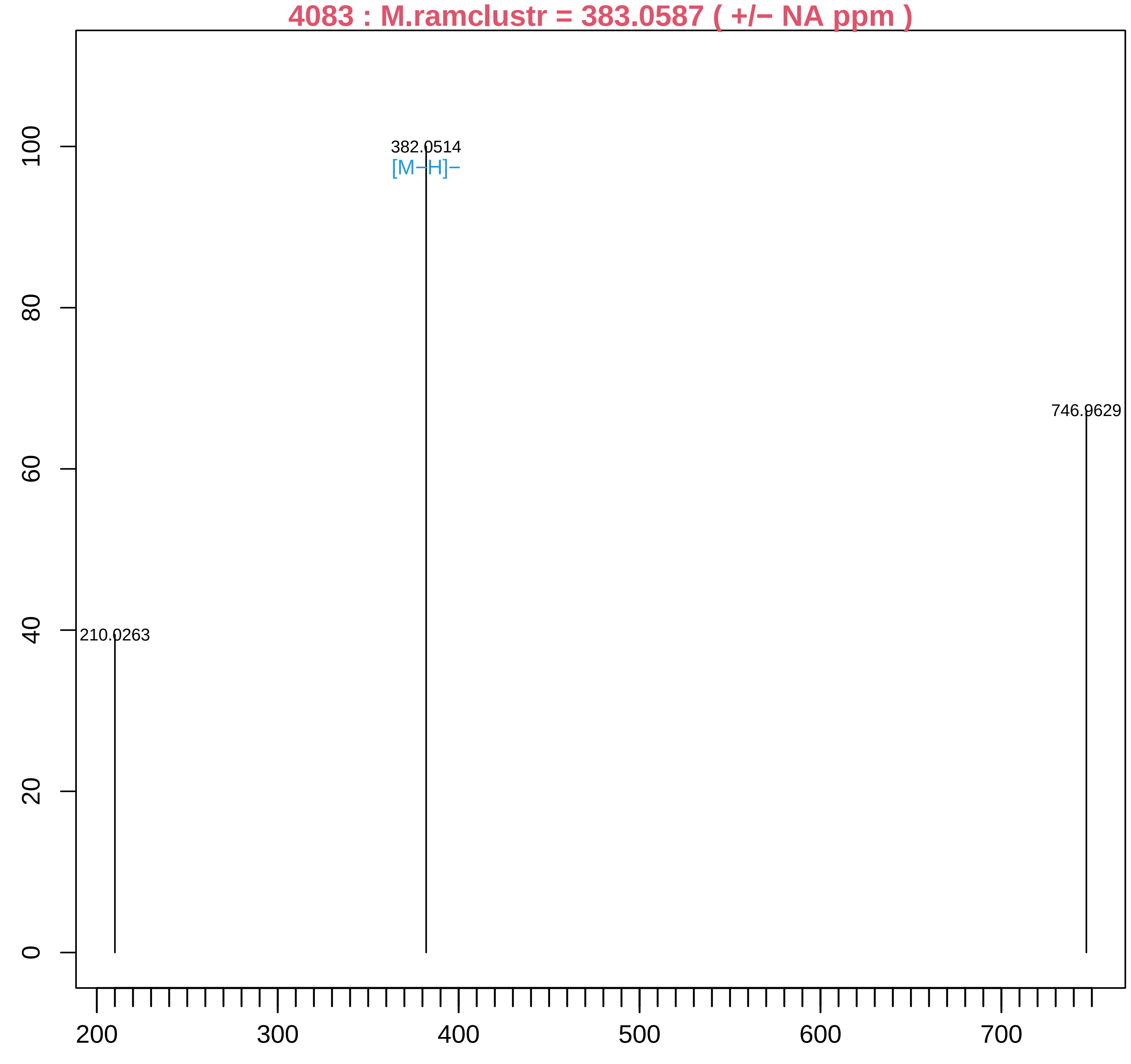
4083 : M.ramclustr = 383.0587 (+/− NA ppm)
382.0514
[M−H]−
746.9629
210.0263
0
20
40
60
80
100
200
300
400
500
600
700
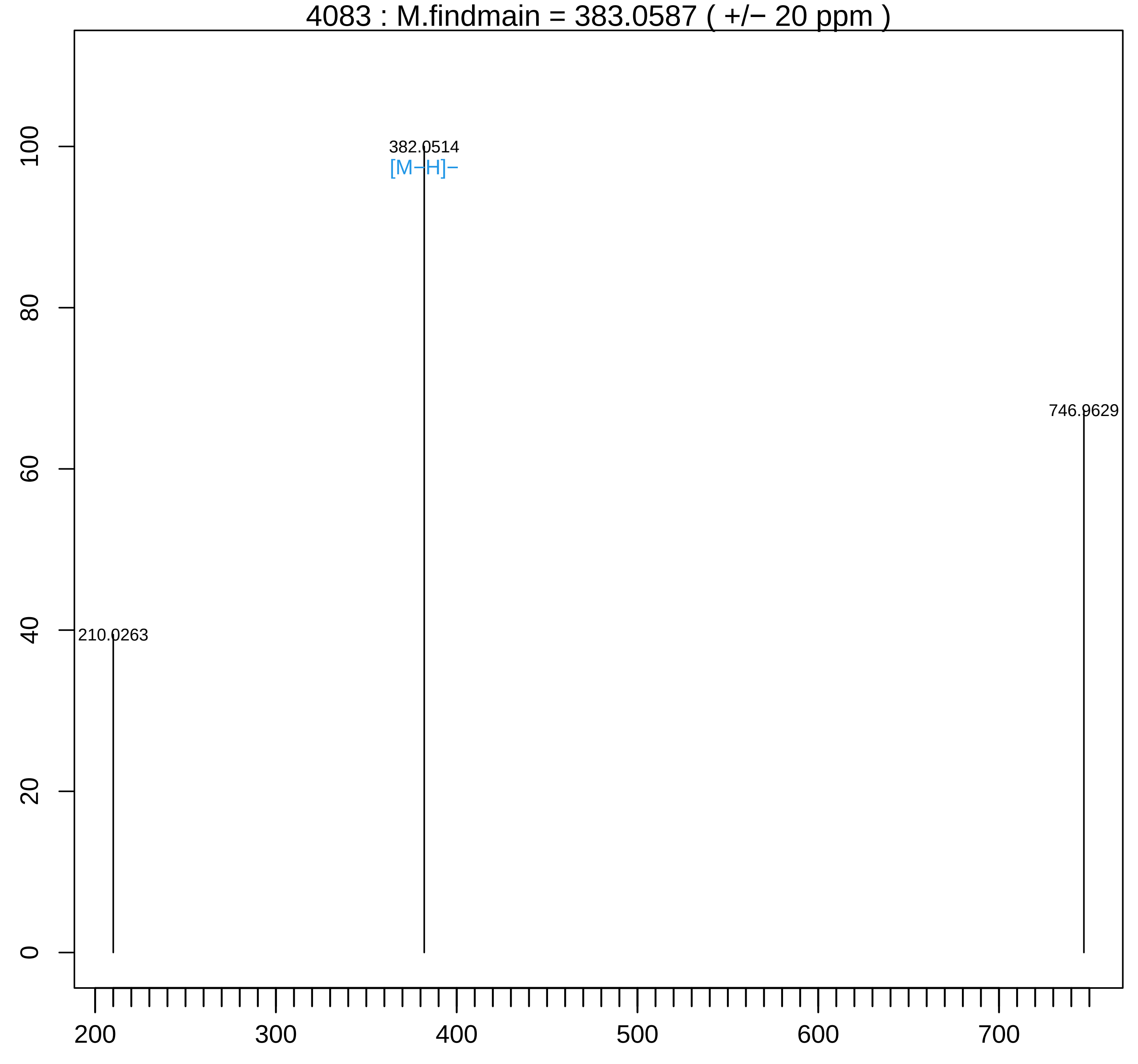
4083 : M.findmain = 383.0587 (+/− 20 ppm)
382.0514
[M−H]−
746.9629
210.0263
0
20
40
60
80
100
200
300
400
500
600
700

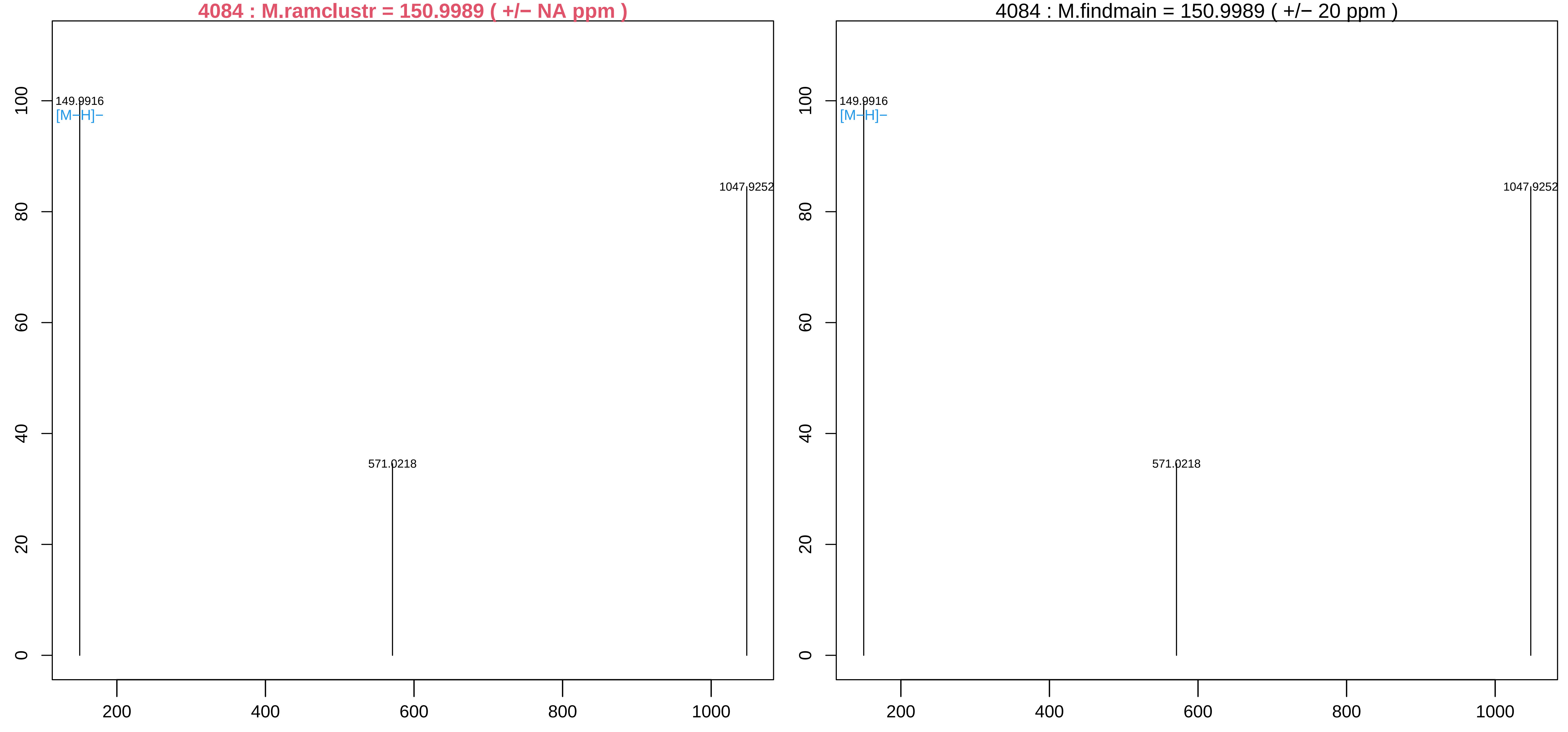
4084 : M.ramclustr = 150.9989 (+/− NA ppm)
149.9916
[M−H]−
1047.9252
571.0218
4084 : M.findmain = 150.9989 (+/− 20 ppm)
149.9916
[M−H]−
1047.9252
571.0218

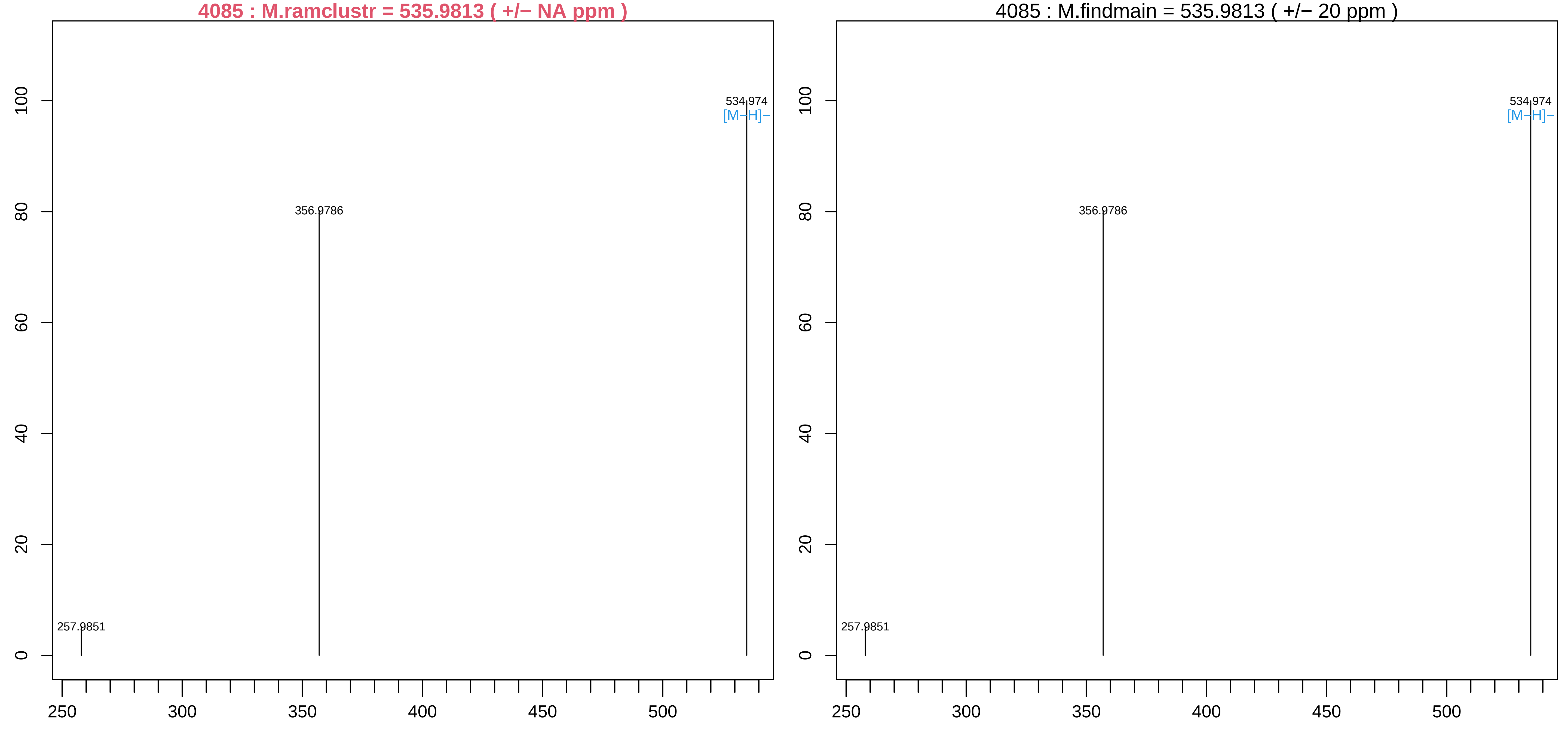
4085 : M.ramclustr = 535.9813 (+/− NA ppm)
534.974
[M−H]−
356.9786
257.9851
4085 : M.findmain = 535.9813 (+/− 20 ppm)
534.974
[M−H]−
356.9786
257.9851
0
20
40
60
80
100
250
300
350
400
450
500

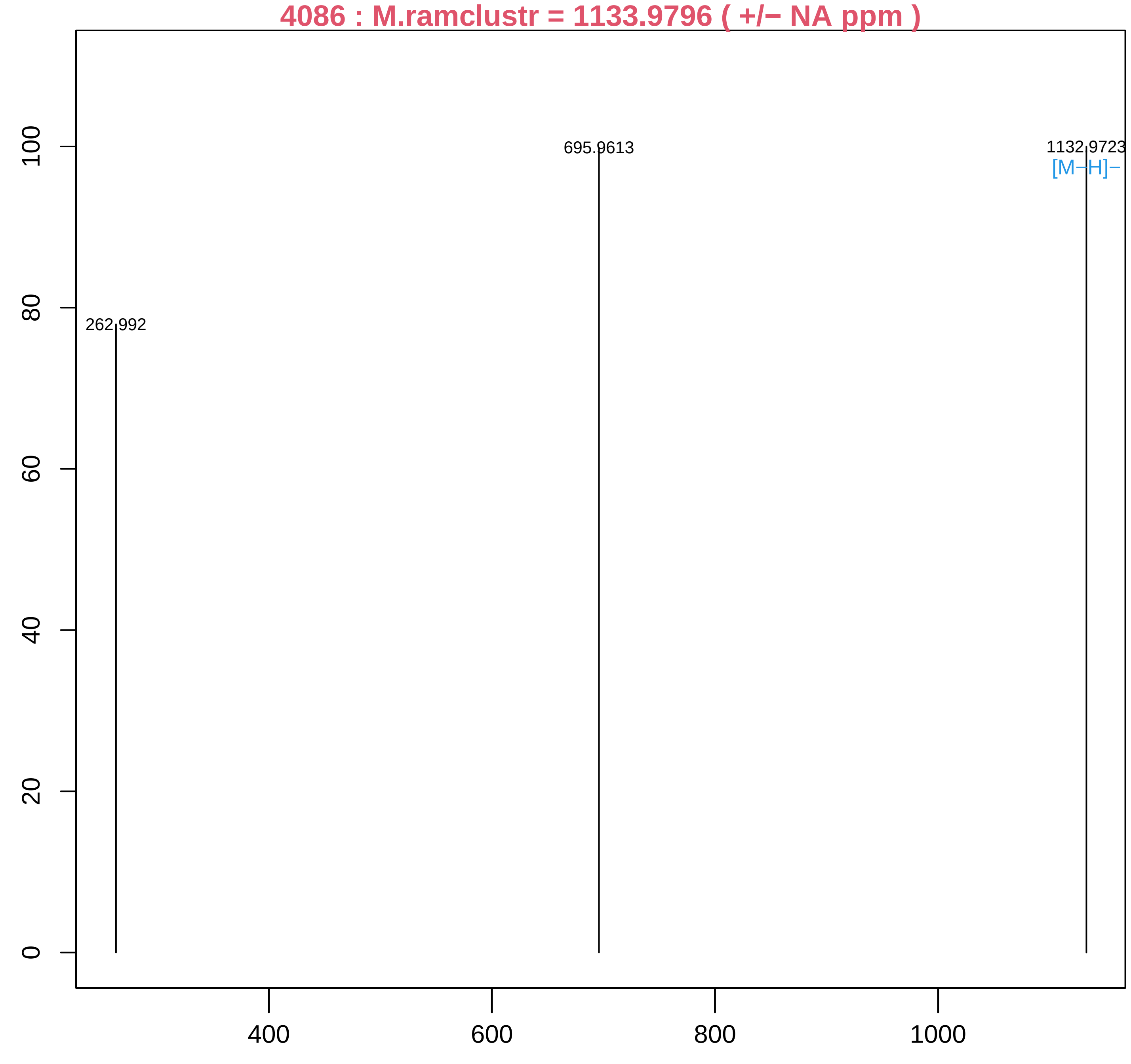
4086 : M.ramclustr = 1133.9796 (+/− NA ppm)
262.992
695.9613
1132.9723
[M−H]−
0
20
40
60
80
100
400
600
800
1000

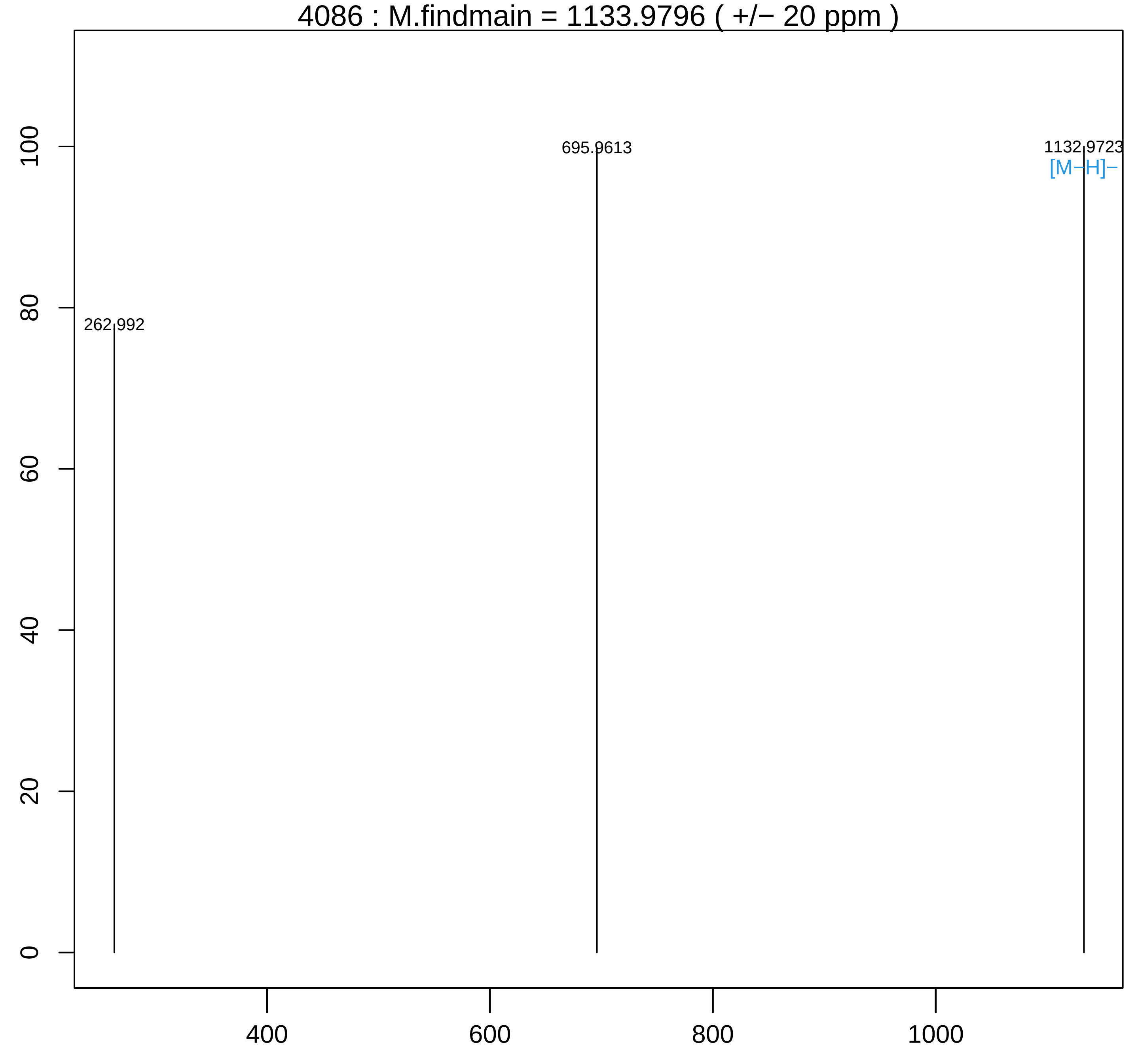
4086 : M.findmain = 1133.9796 (+/− 20 ppm)
262.992
695.9613
1132.9723
[M−H]−
0
20
40
60
80
100
400
600
800
1000

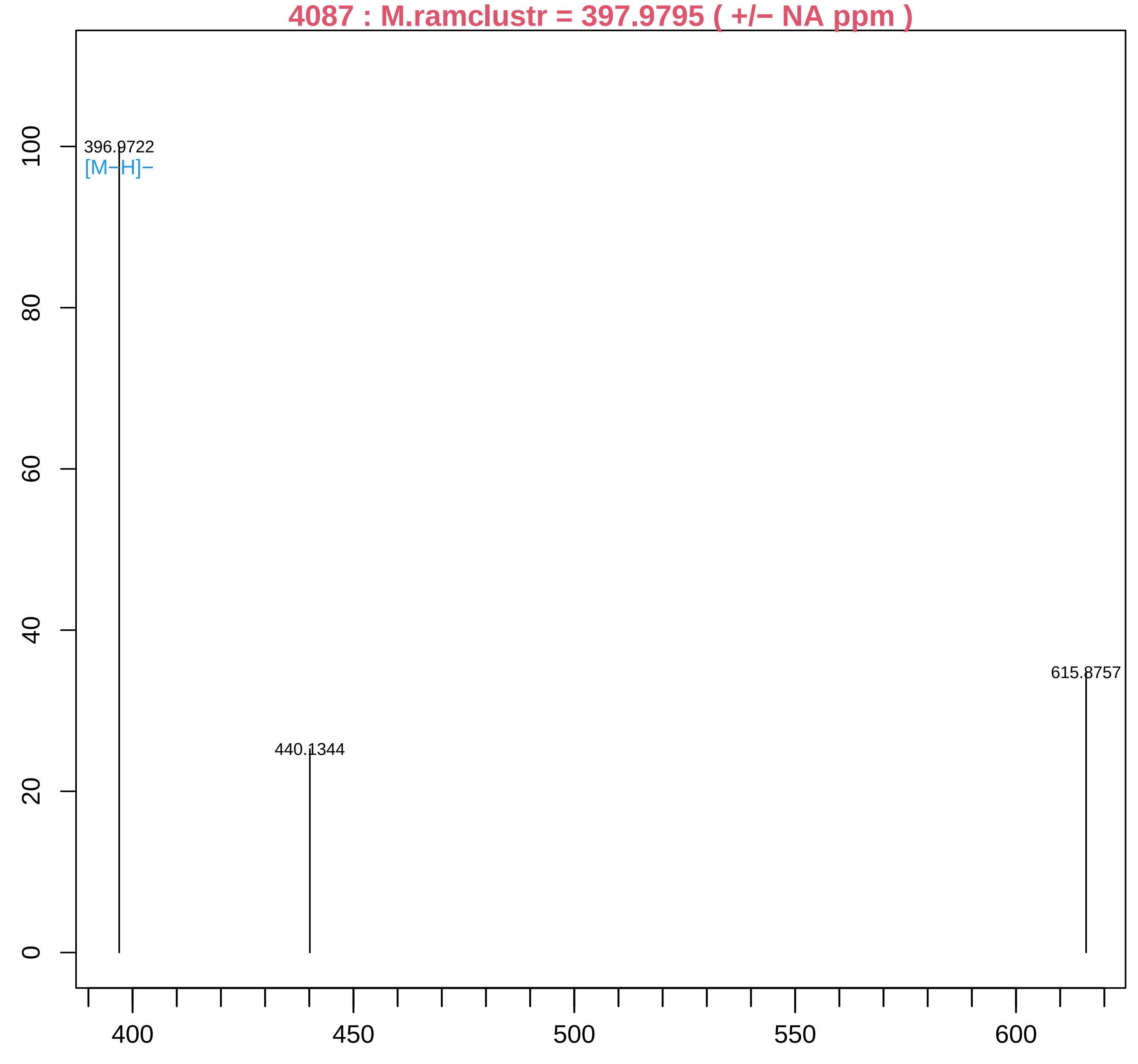
4087 : M.ramclustr = 397.9795 (+/− NA ppm)
396.9722
[M−H]−
440.1344
615.8757
0
20
40
60
80
100
400
450
500
550
600

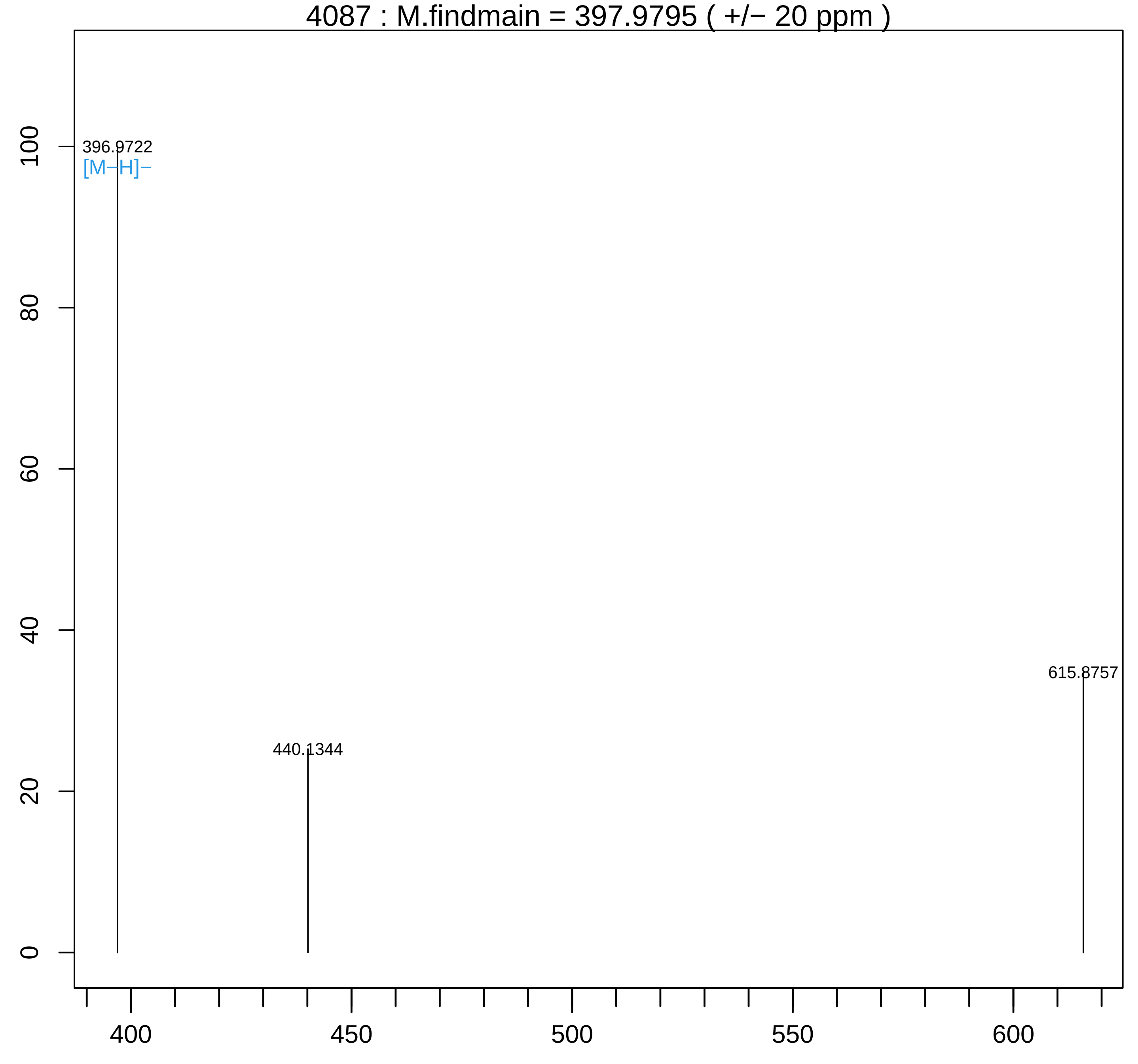
4087 : M.findmain = 397.9795 (+/− 20 ppm)
396.9722
[M−H]−
440.1344
615.8757
0
20
40
60
80
100
400
450
500
550
600

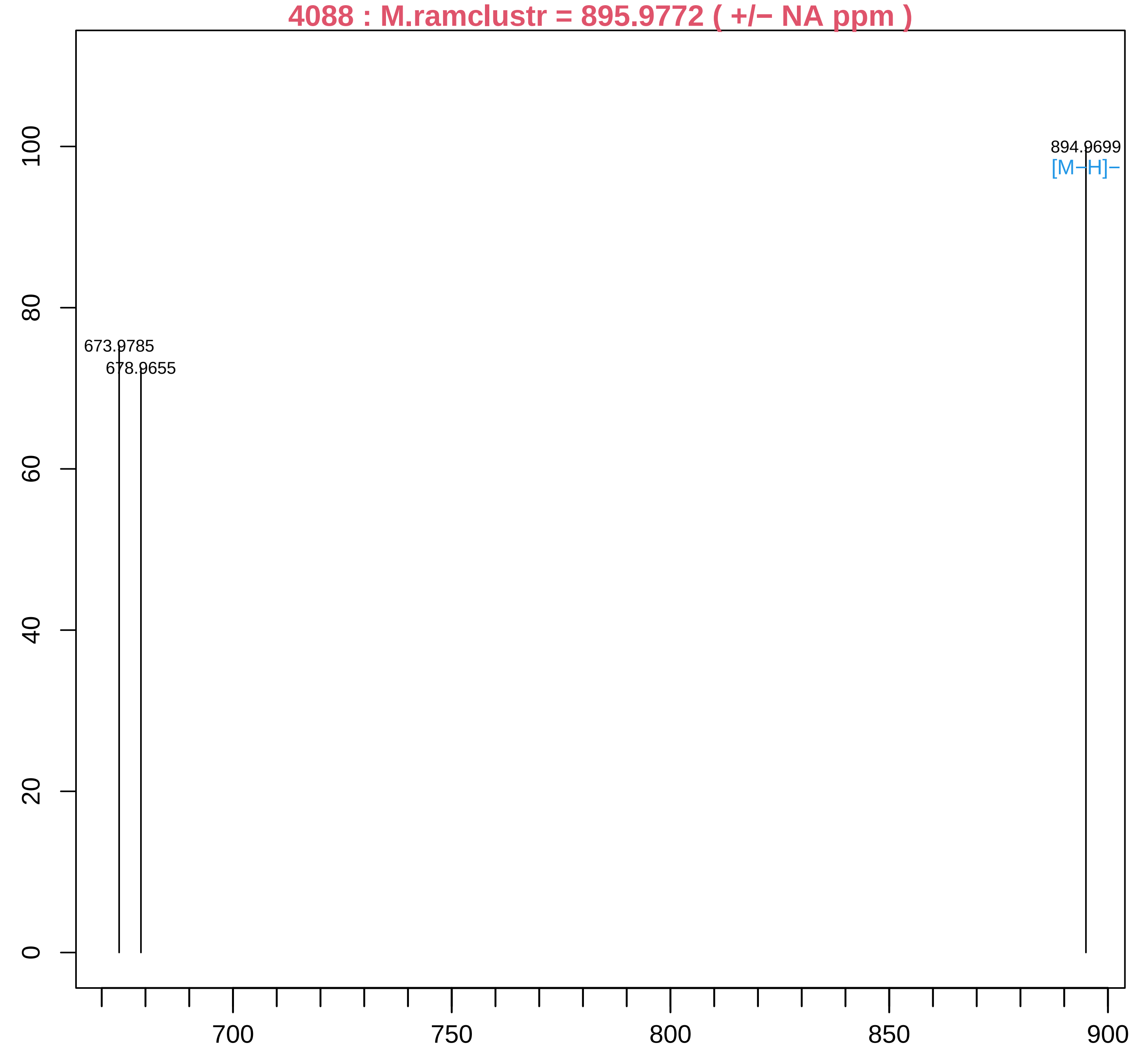
4088 : M.ramclustr = 895.9772 (+/− NA ppm)
894.9699
[M−H]−
673.9785
678.9655
0
20
40
60
80
100
700
750
800
850
900

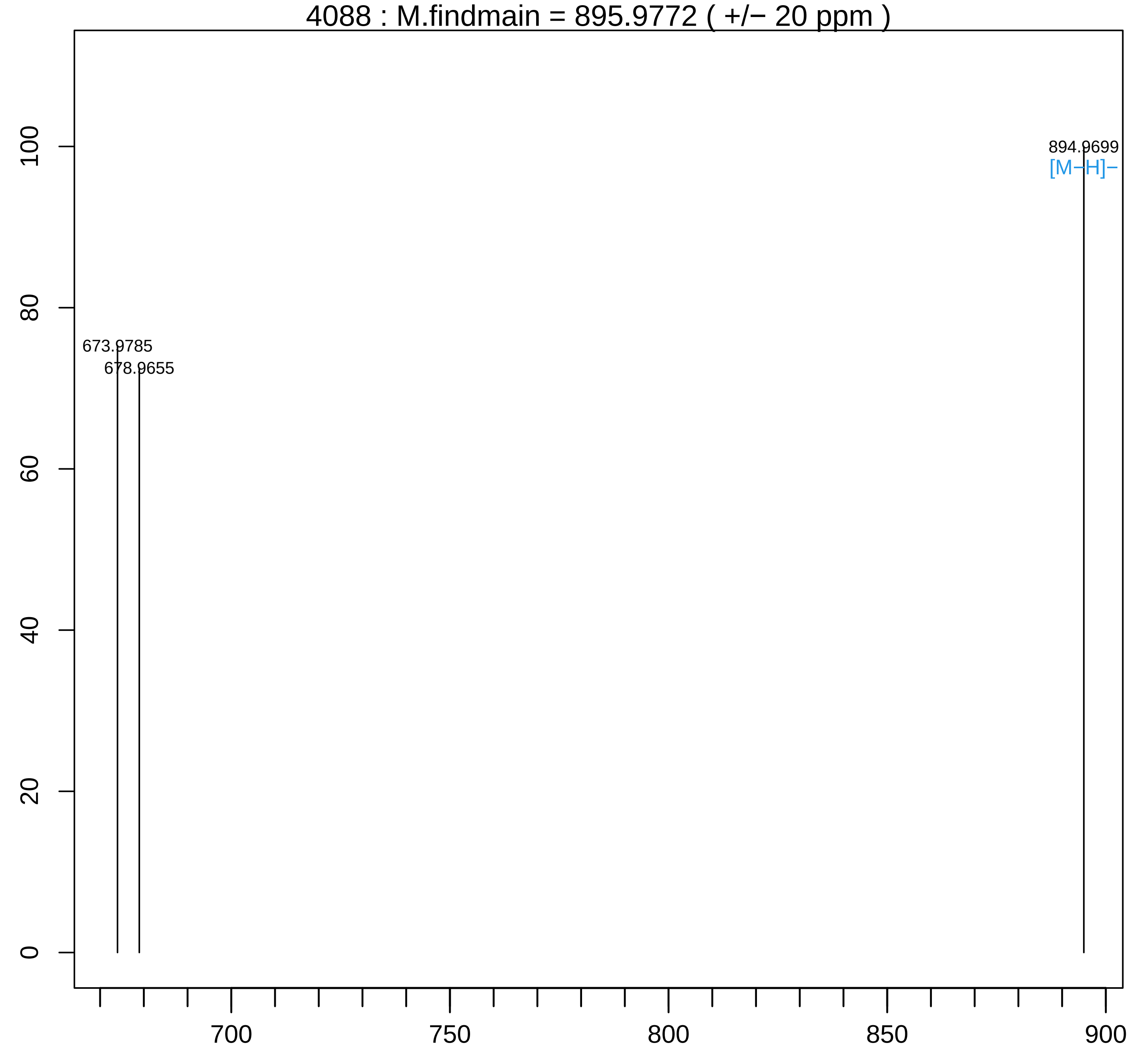
4088 : M.findmain = 895.9772 (+/− 20 ppm)
894.9699
[M−H]−
673.9785
678.9655
0
20
40
60
80
100
700
750
800
850
900

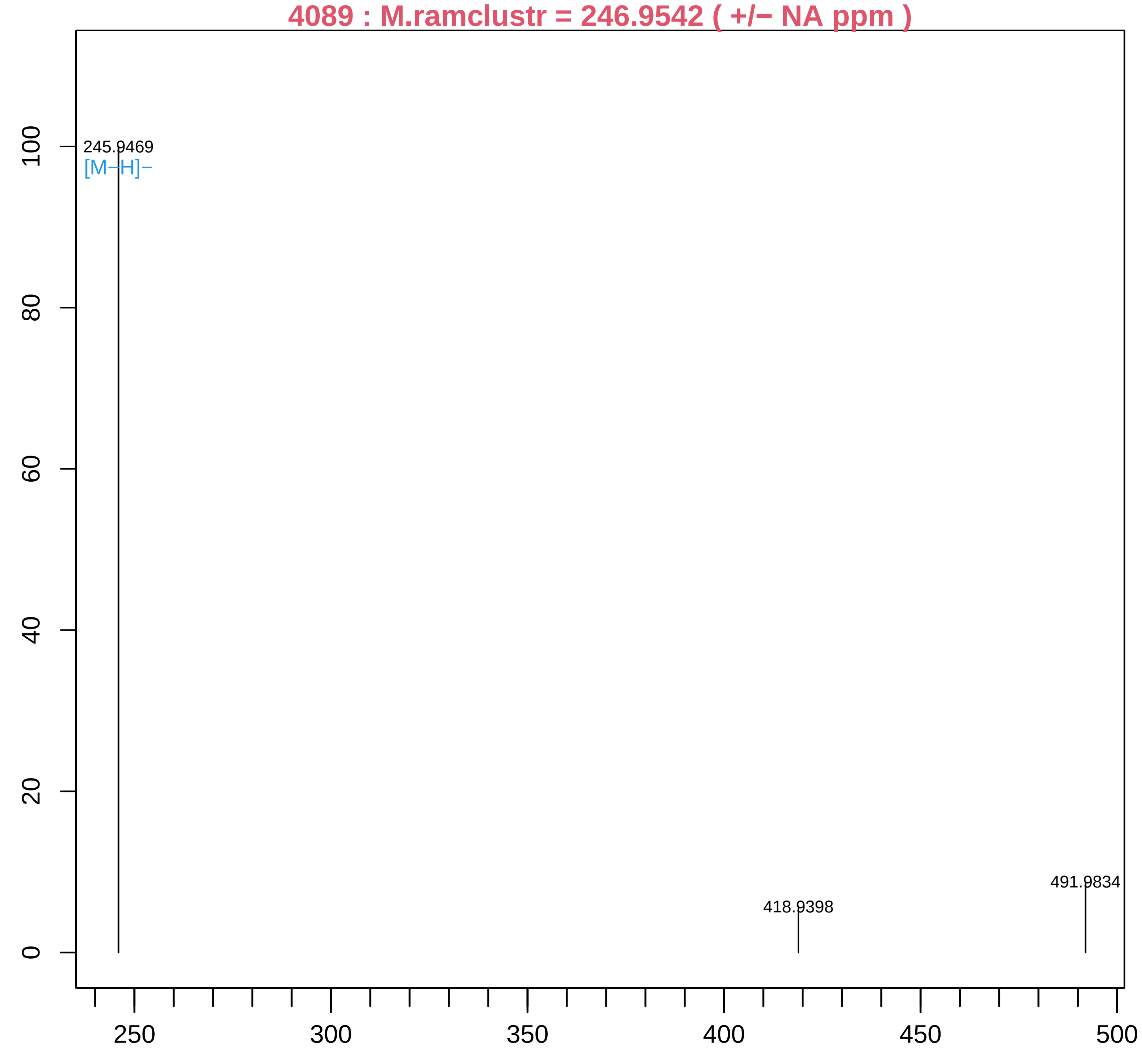
4089 : M.ramclustr = 246.9542 (+/− NA ppm)
245.9469
[M−H]−
418.9398
491.9834
0
20
40
60
80
100
250
300
350
400
450
500

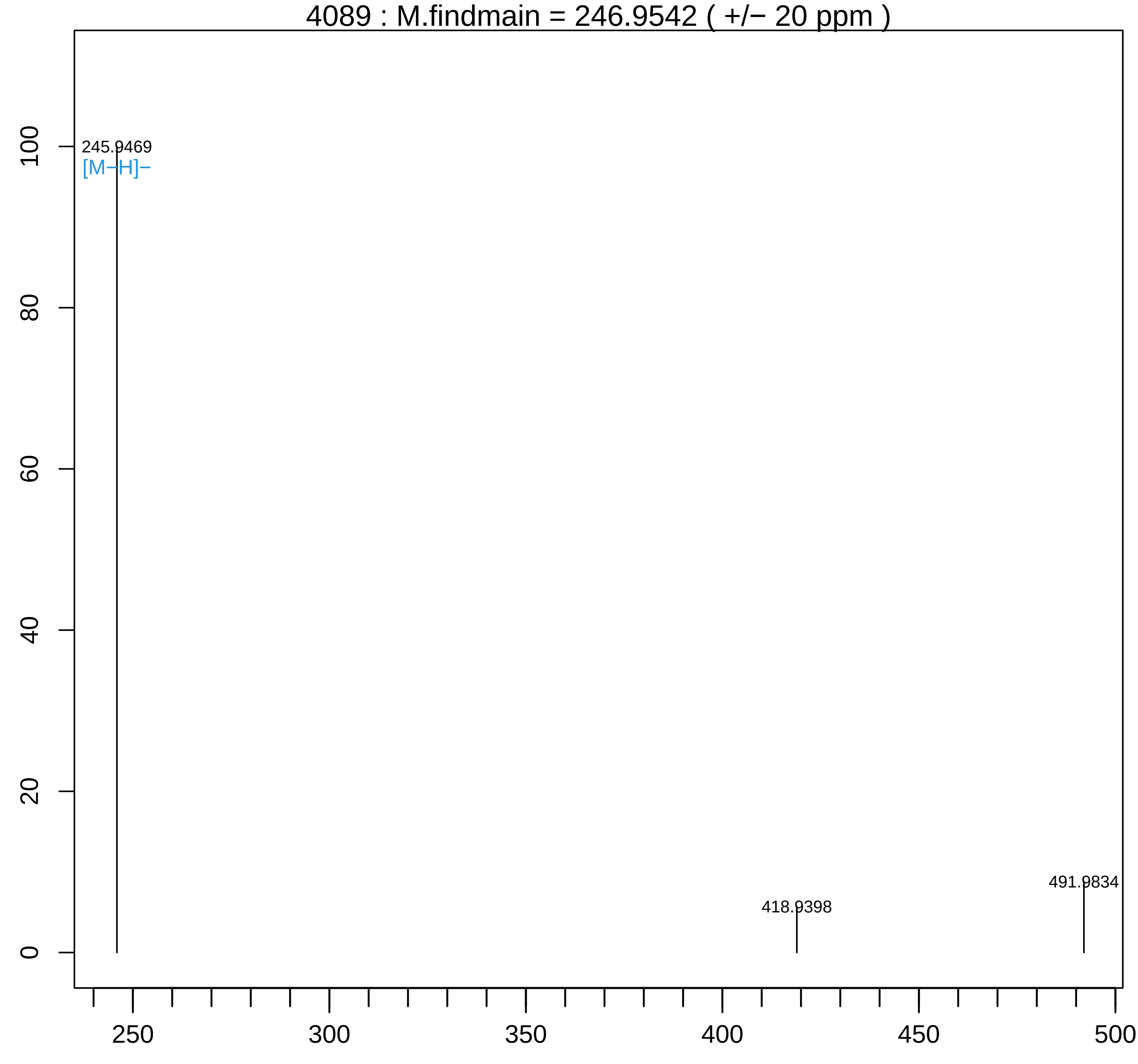
4089 : M.findmain = 246.9542 (+/− 20 ppm)
245.9469
[M−H]−
418.9398
491.9834
0
20
40
60
80
100
250
300
350
400
450
500

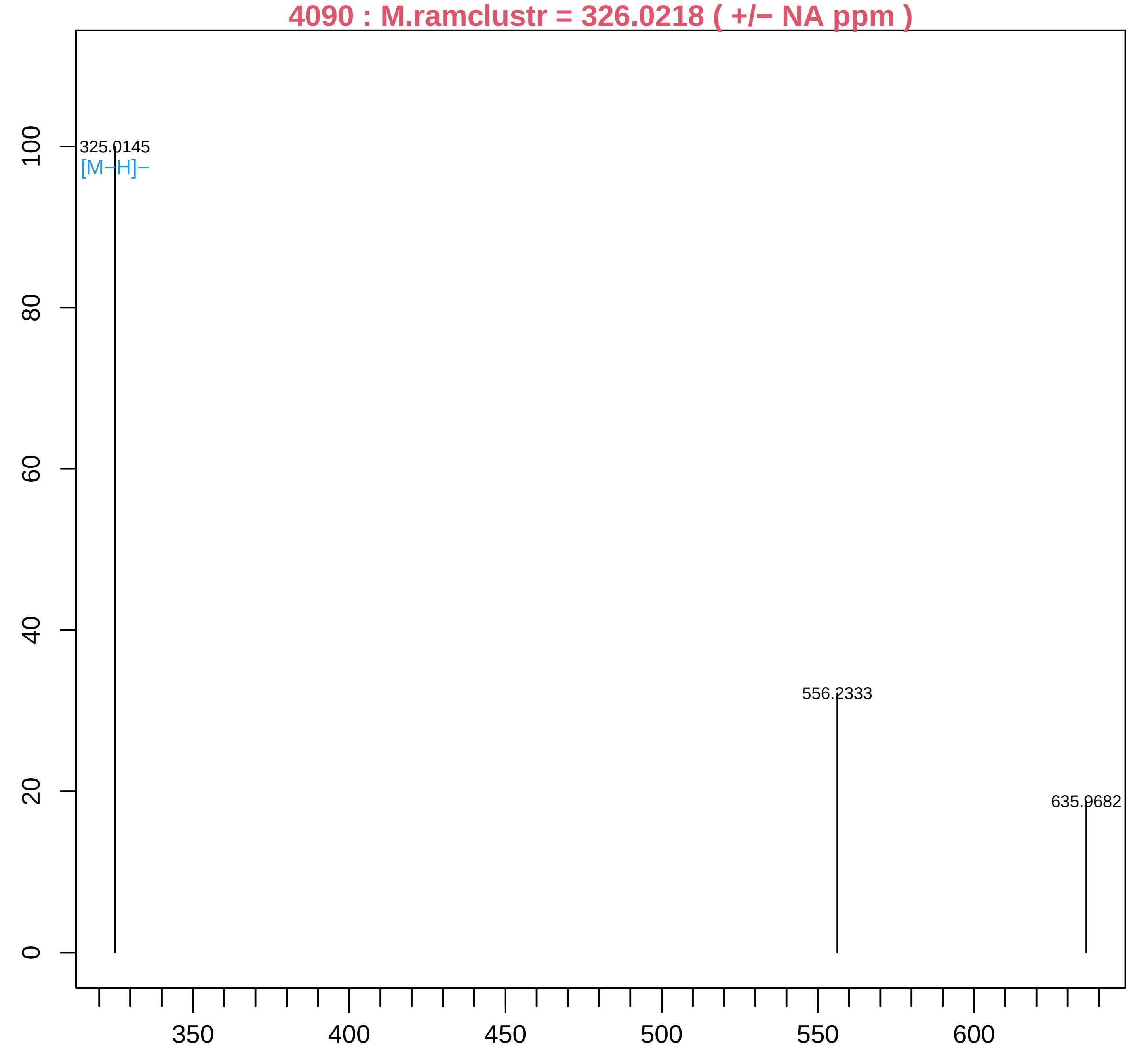
4090 : M.ramclustr = 326.0218 (+/− NA ppm)
325.0145
[M−H]−
556.2333
635.9682
0
20
40
60
80
100
350
400
450
500
550
600

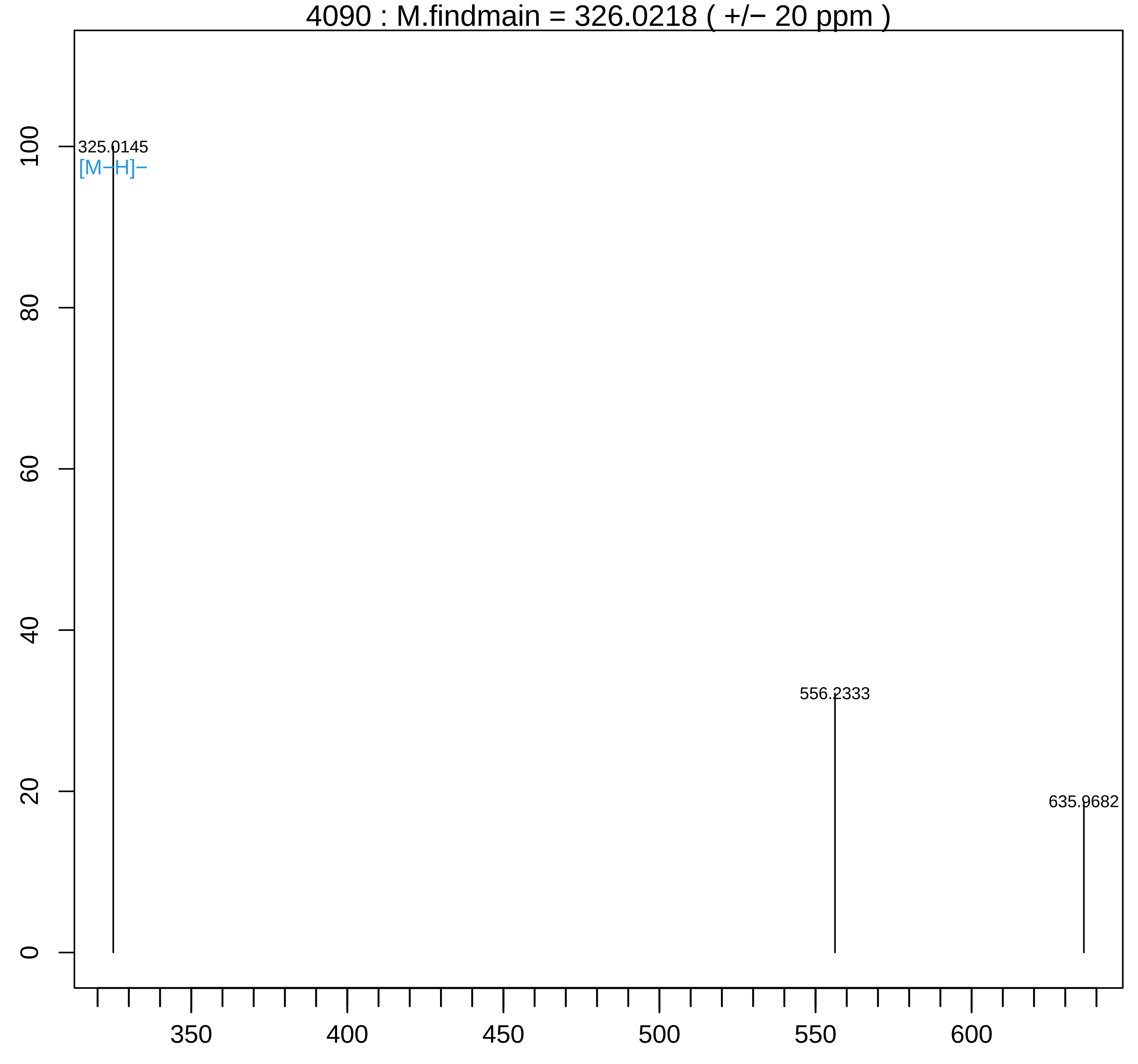
4090 : M.findmain = 326.0218 (+/− 20 ppm)
325.0145
[M−H]−
556.2333
635.9682
0
20
40
60
80
100
350
400
450
500
550
600

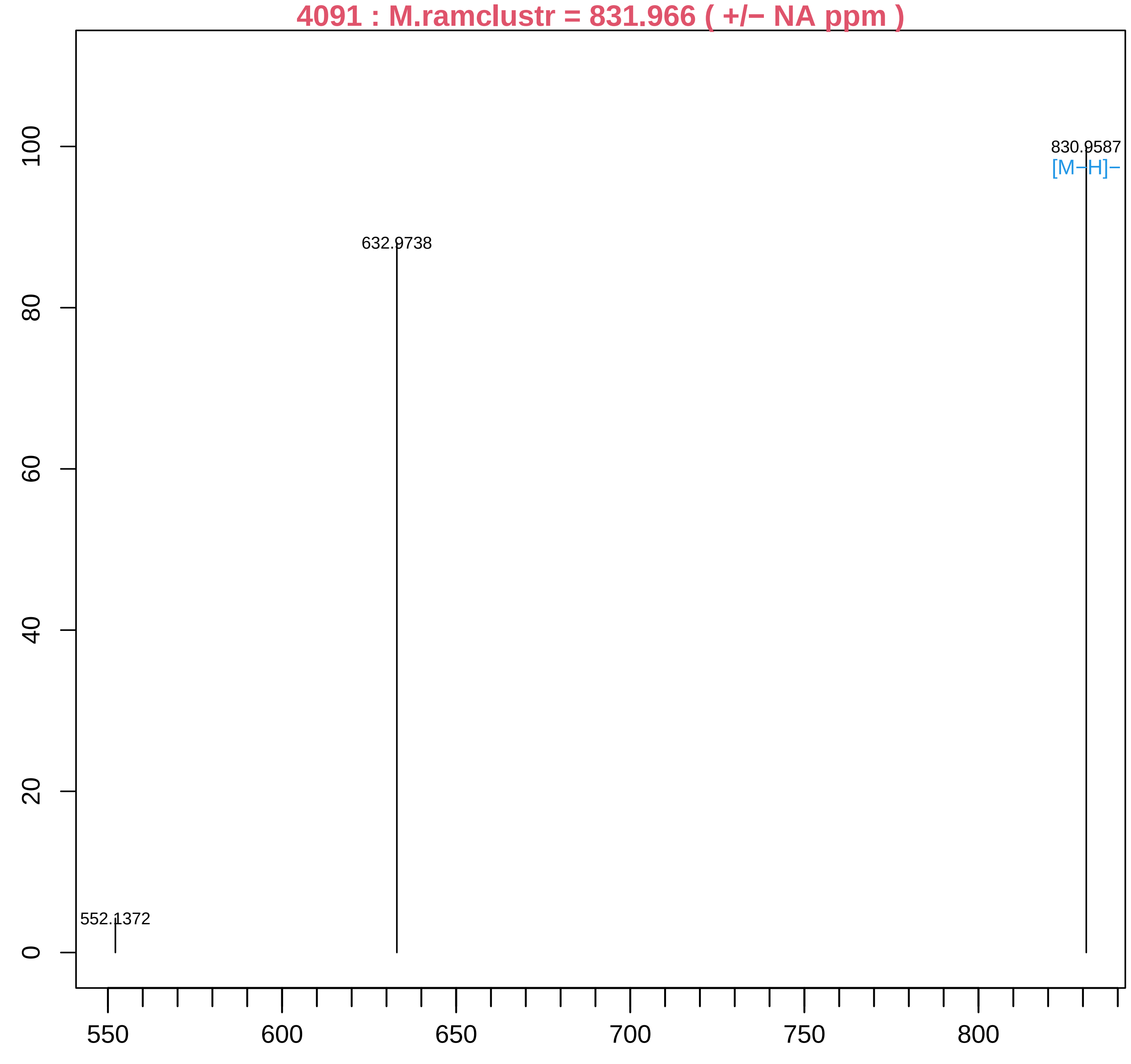
4091 : M.ramclustr = 831.966 (+/− NA ppm)
0
20
40
60
80
100
550
600
650
700
750
800
552.1372
632.9738
830.9587
[M−H]−

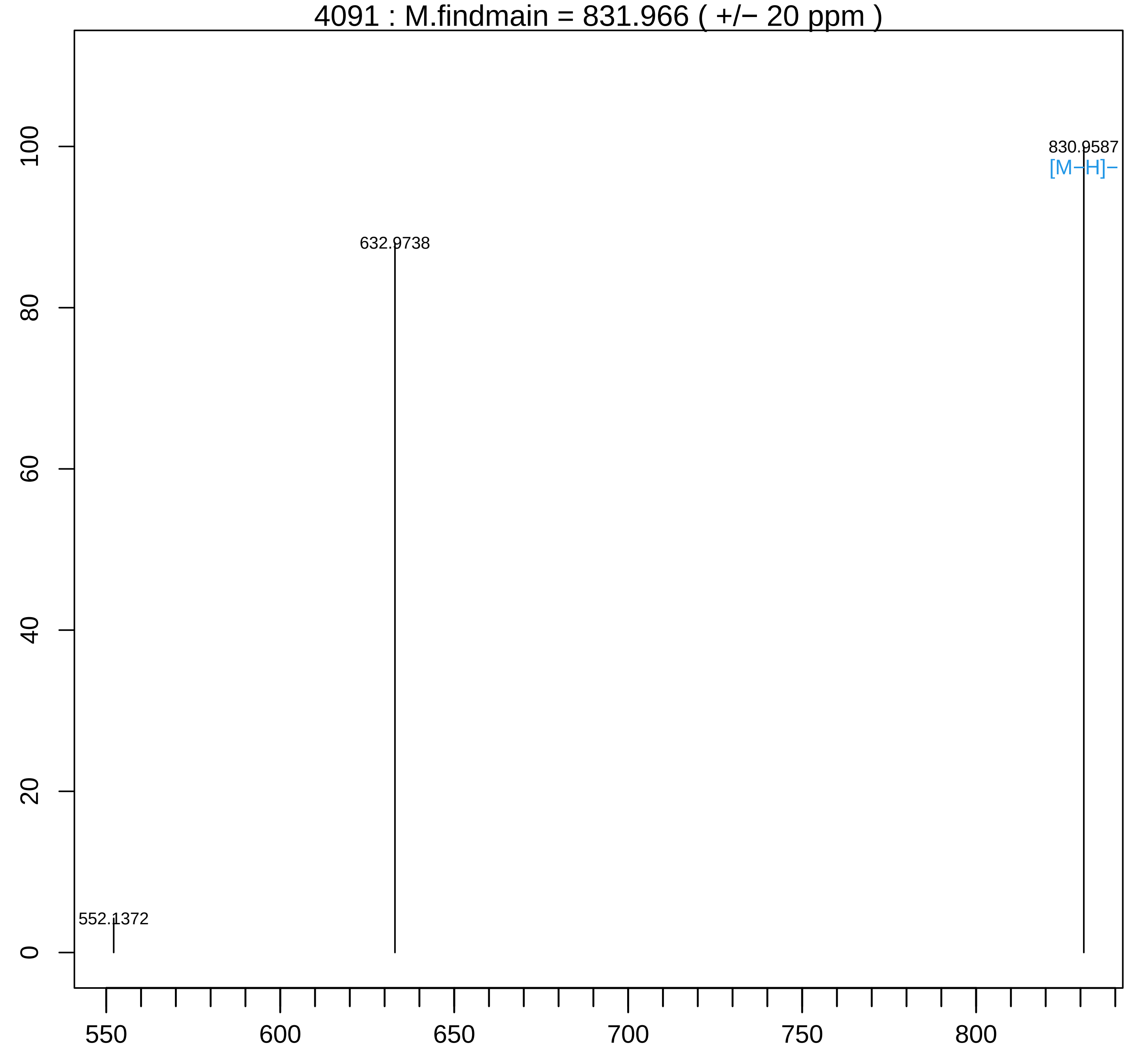
4091 : M.findmain = 831.966 (+/− 20 ppm)
0
20
40
60
80
100
550
600
650
700
750
800
552.1372
632.9738
830.9587
[M−H]−

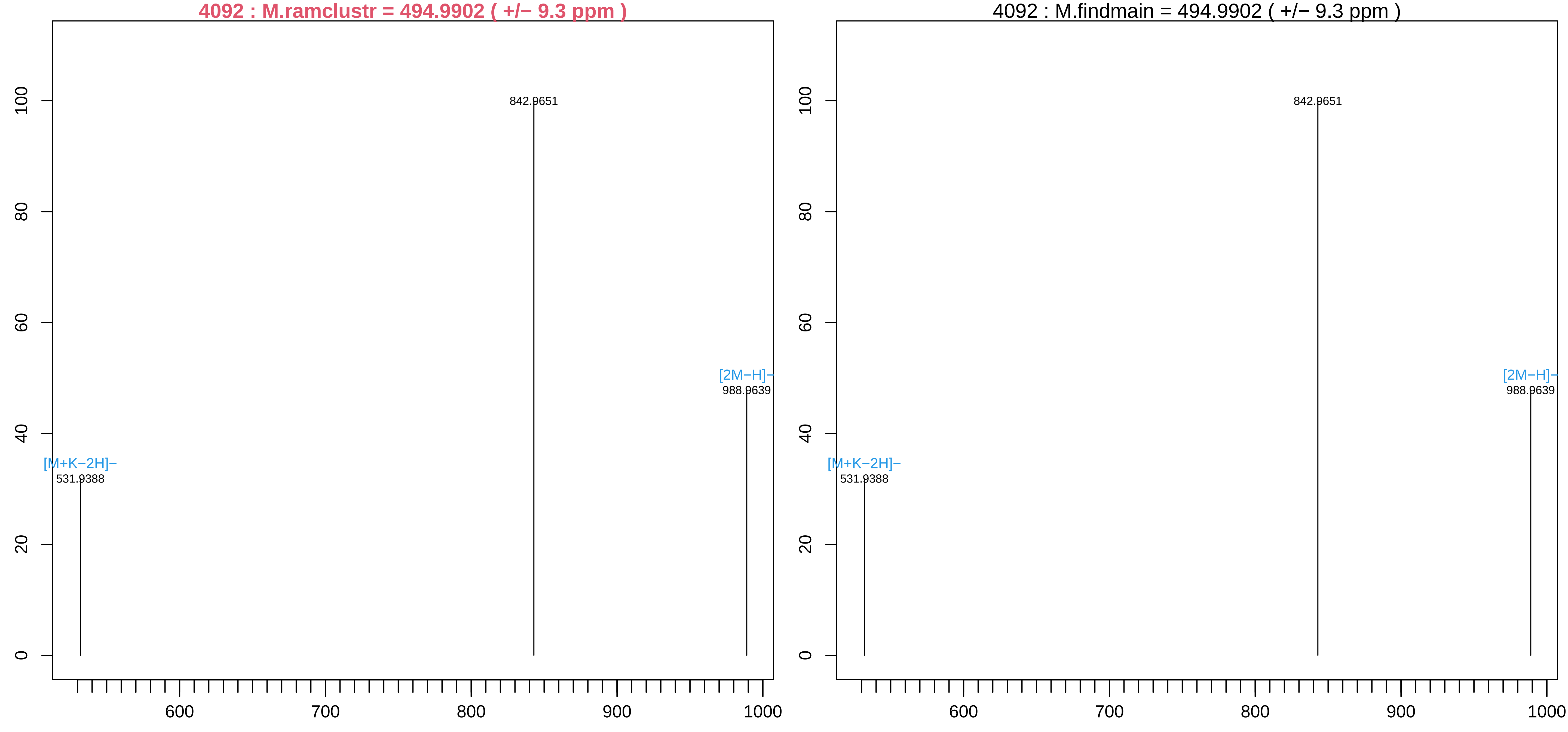
4092 : M.ramclustr = 494.9902 (+/− 9.3 ppm)
842.9651
[2M−H]−
988.9639
[M+K−2H]−
531.9388
0
20
40
60
80
100
600
700
800
900
1000
4092 : M.findmain = 494.9902 (+/− 9.3 ppm)
842.9651
[2M−H]−
988.9639
[M+K−2H]−
531.9388
0
20
40
60
80
100
600
700
800
900
1000

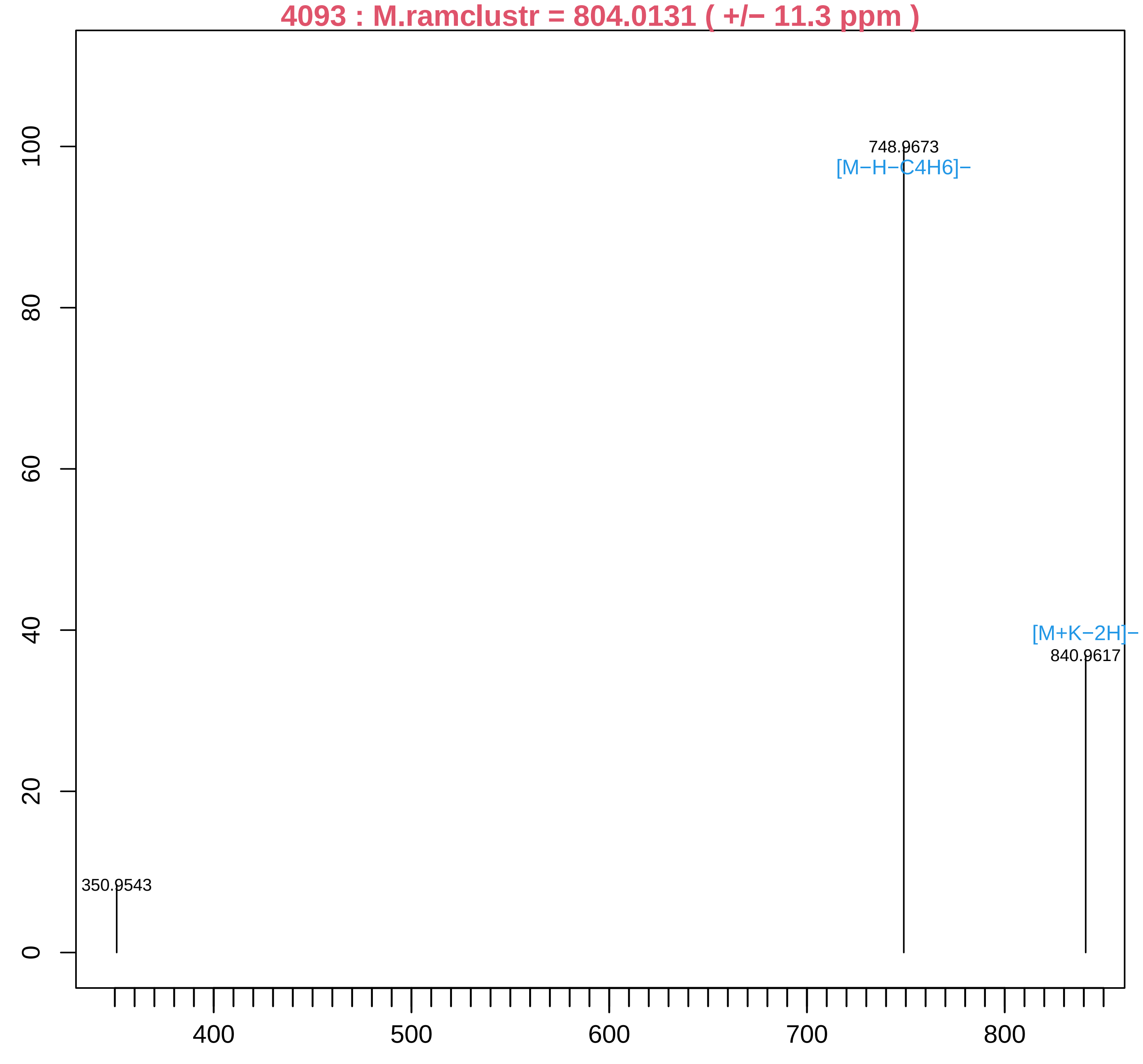
4093 : M.ramclustr = 804.0131 (+/− 11.3 ppm)
748.9673
[M−H−C4H6]−
[M+K−2H]−
840.9617
350.9543
0
20
40
60
80
100
400
500
600
700
800

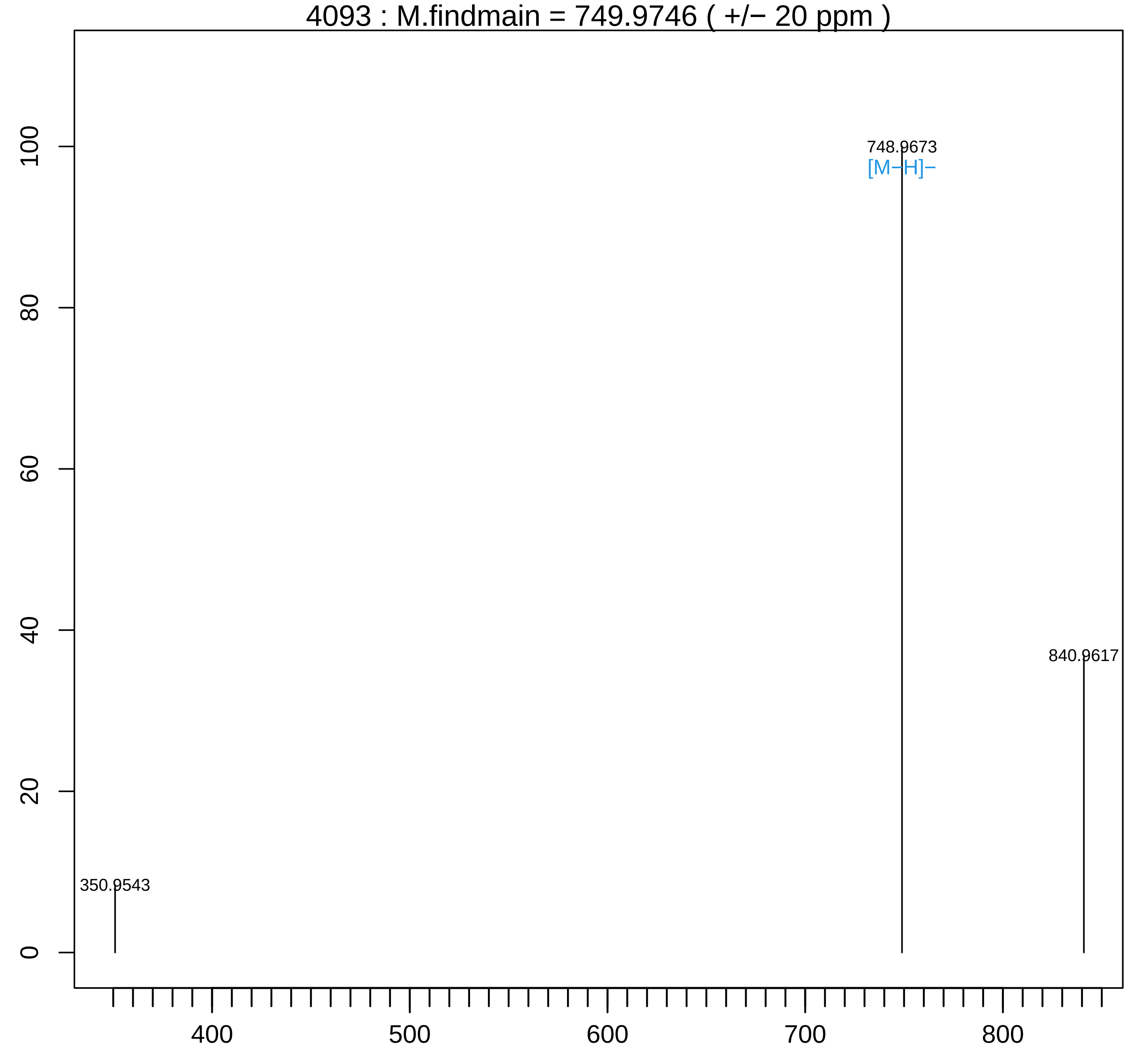
4093 : M.findmain = 749.9746 (+/− 20 ppm)
748.9673
[M−H]−
840.9617
350.9543
0
20
40
60
80
100
400
500
600
700
800

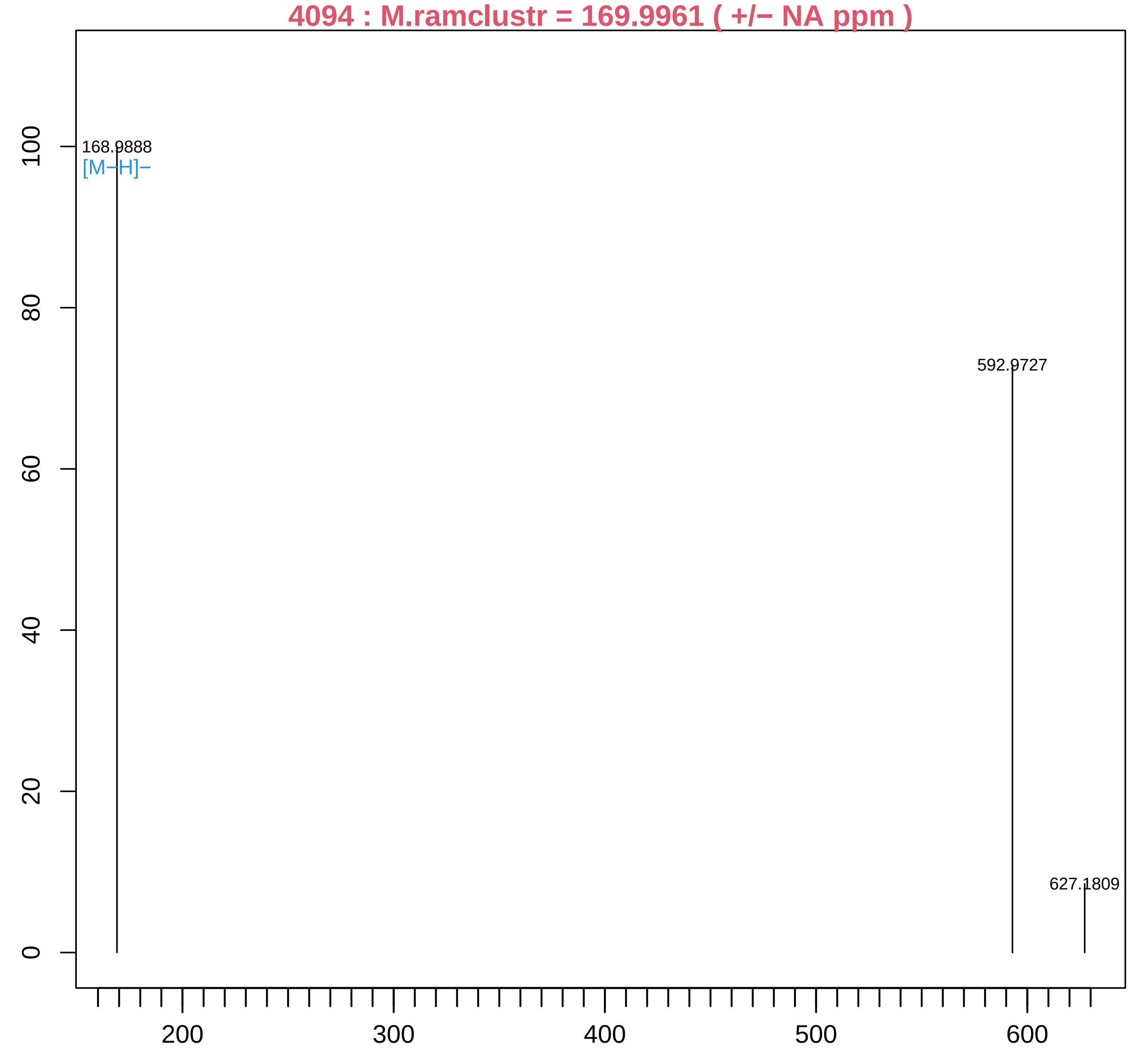

4094 : M.ramclustr = 169.9961 (+/− NA ppm)
168.9888
[M−H]−
592.9727
627.1809
0
20
40
60
80
100
200
300
400
500
600

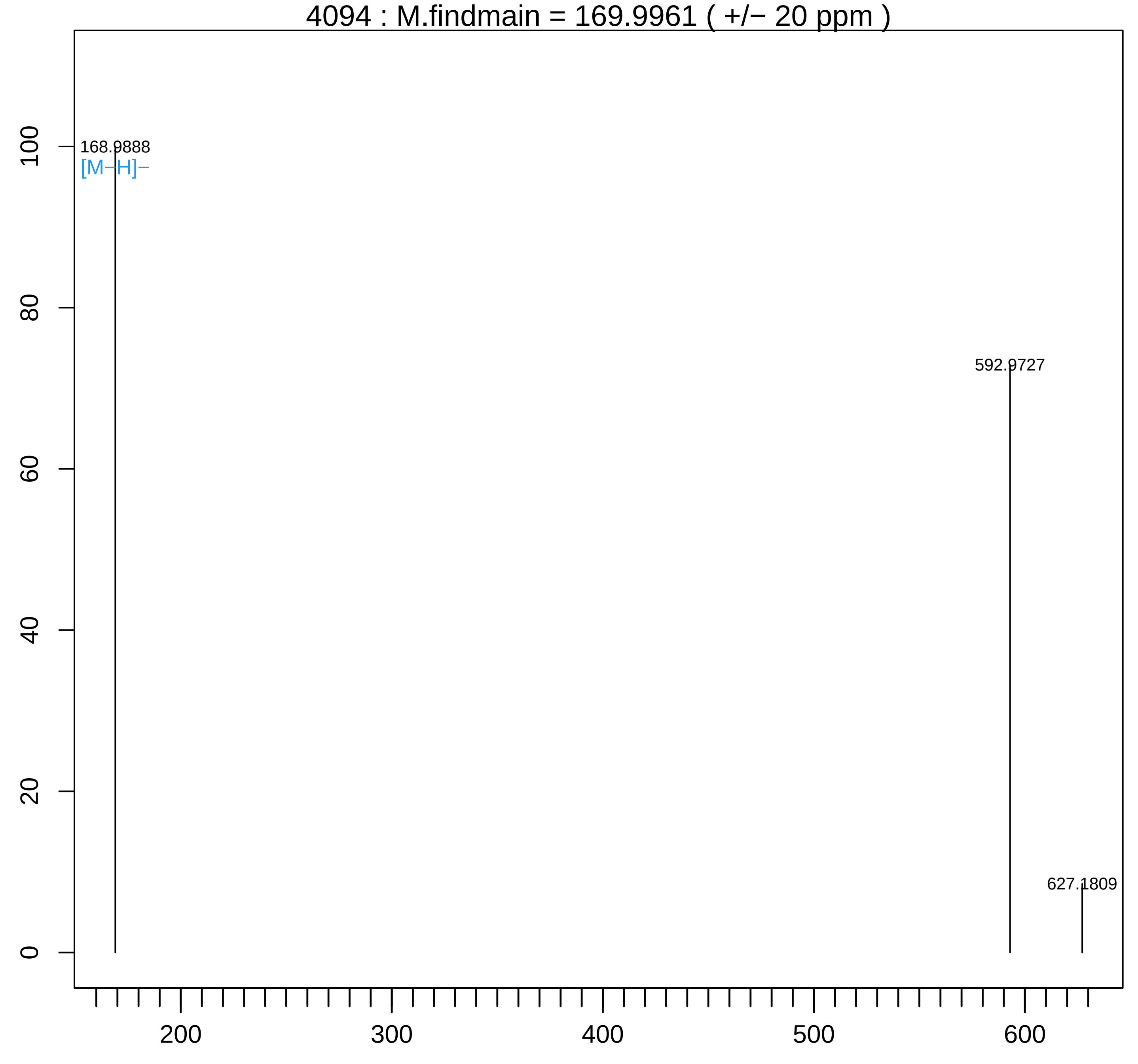

4094 : M.findmain = 169.9961 (+/− 20 ppm)
168.9888
[M−H]−
592.9727
627.1809
0
20
40
60
80
100
200
300
400
500
600

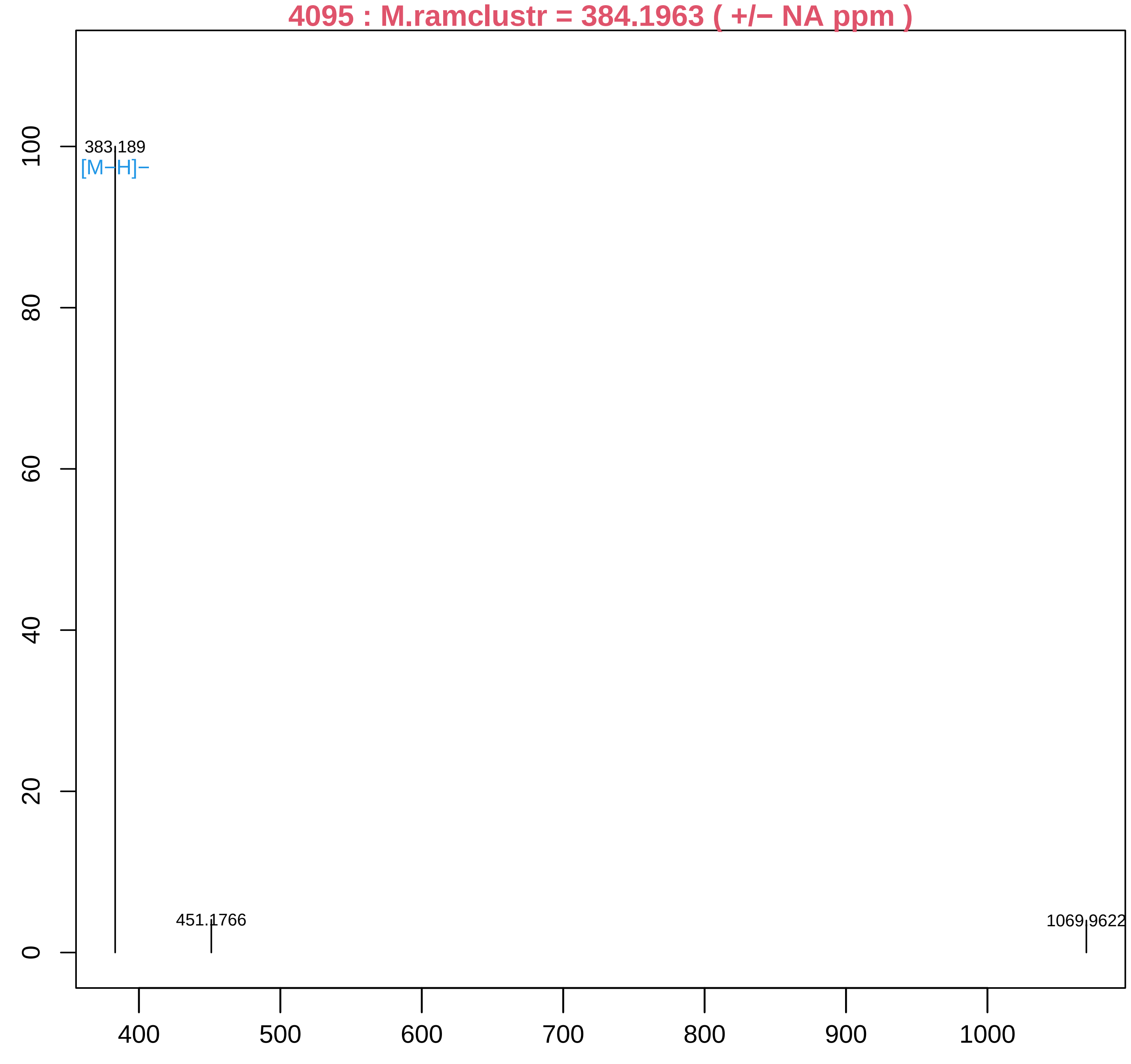

4095 : M.ramclustr = 384.1963 (+/− NA ppm)
383.189
[M−H]−
451.1766
1069.9622
0
20
40
60
80
100
400
500
600
700
800
900
1000

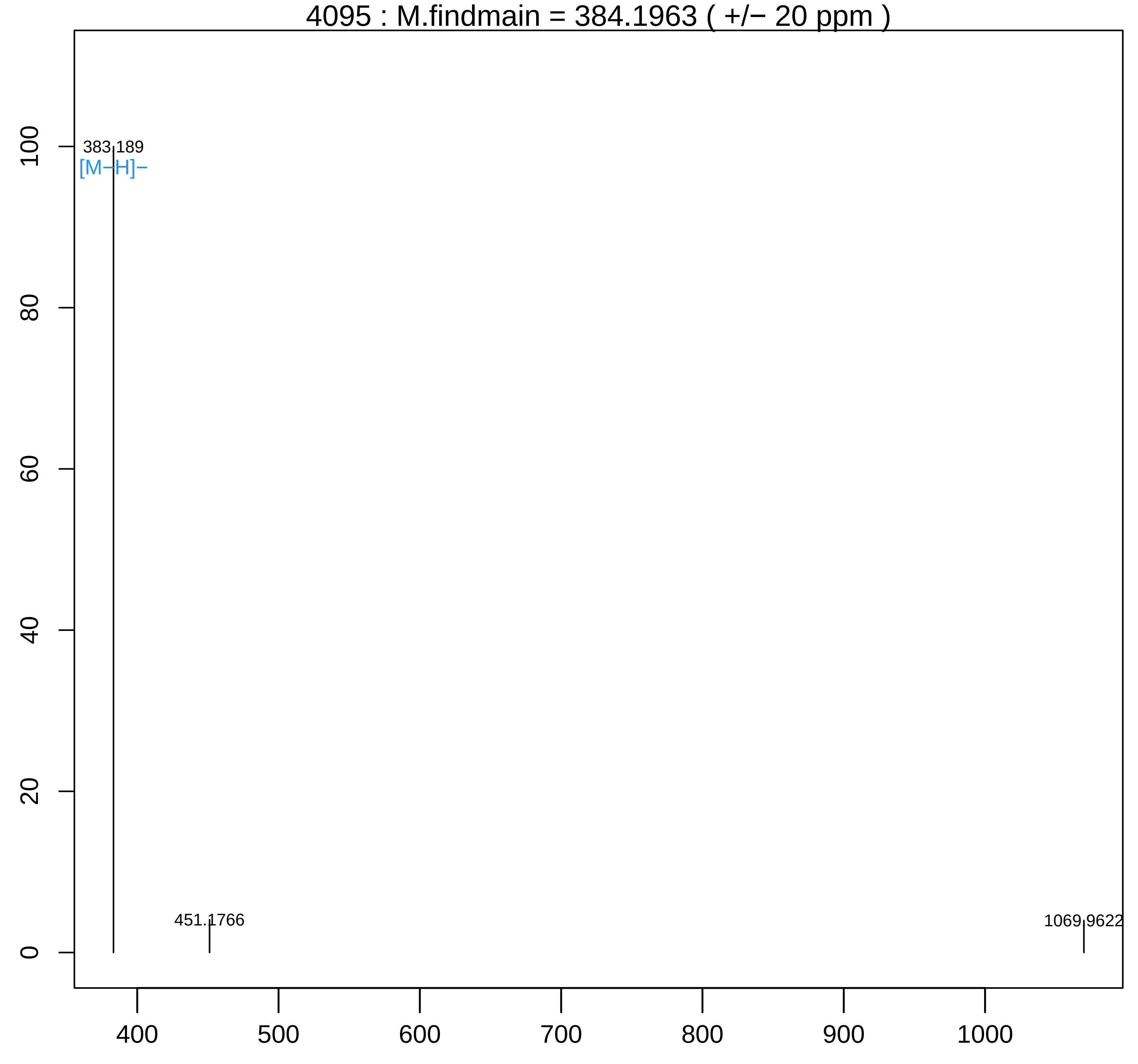

4095 : M.findmain = 384.1963 (+/− 20 ppm)
383.189
[M−H]−
451.1766
1069.9622
0
20
40
60
80
100
400
500
600
700
800
900
1000

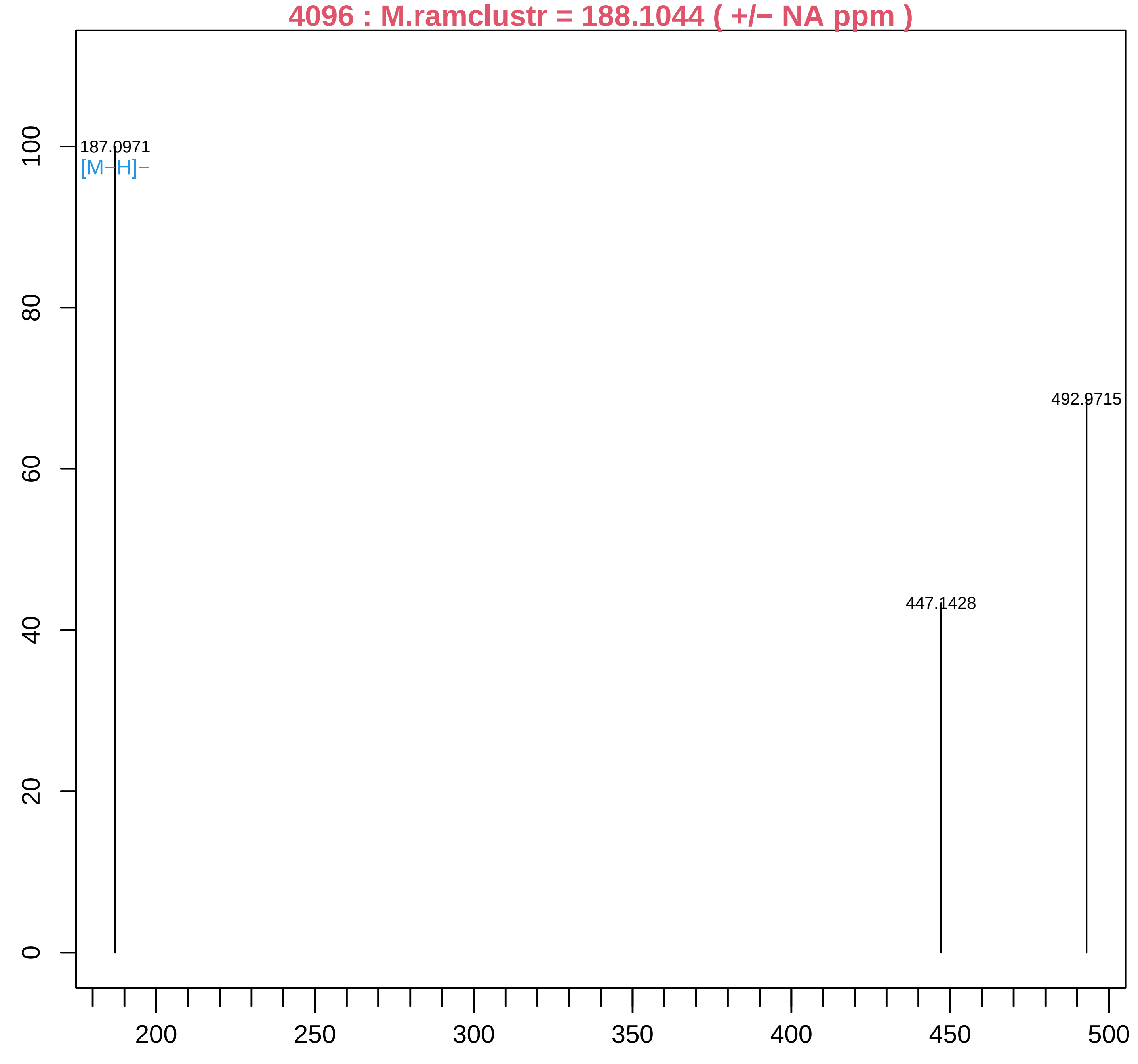
4096 : M.ramclustr = 188.1044 (+/− NA ppm)
187.0971
[M−H]−
492.9715
447.1428
0
20
40
60
80
100
200
250
300
350
400
450
500

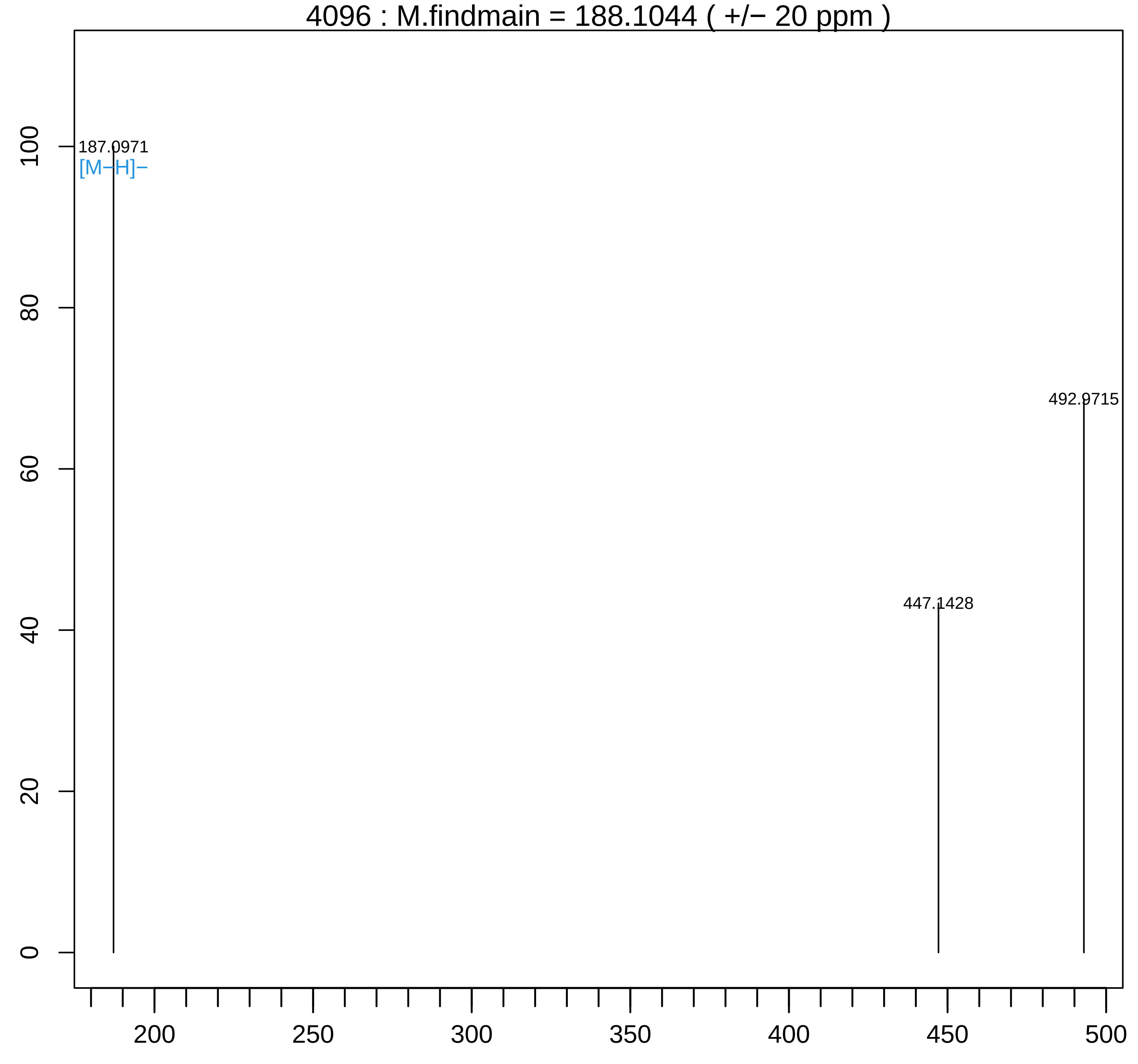
4096 : M.findmain = 188.1044 (+/− 20 ppm)
187.0971
[M−H]−
492.9715
447.1428
0
20
40
60
80
100
200
250
300
350
400
450
500

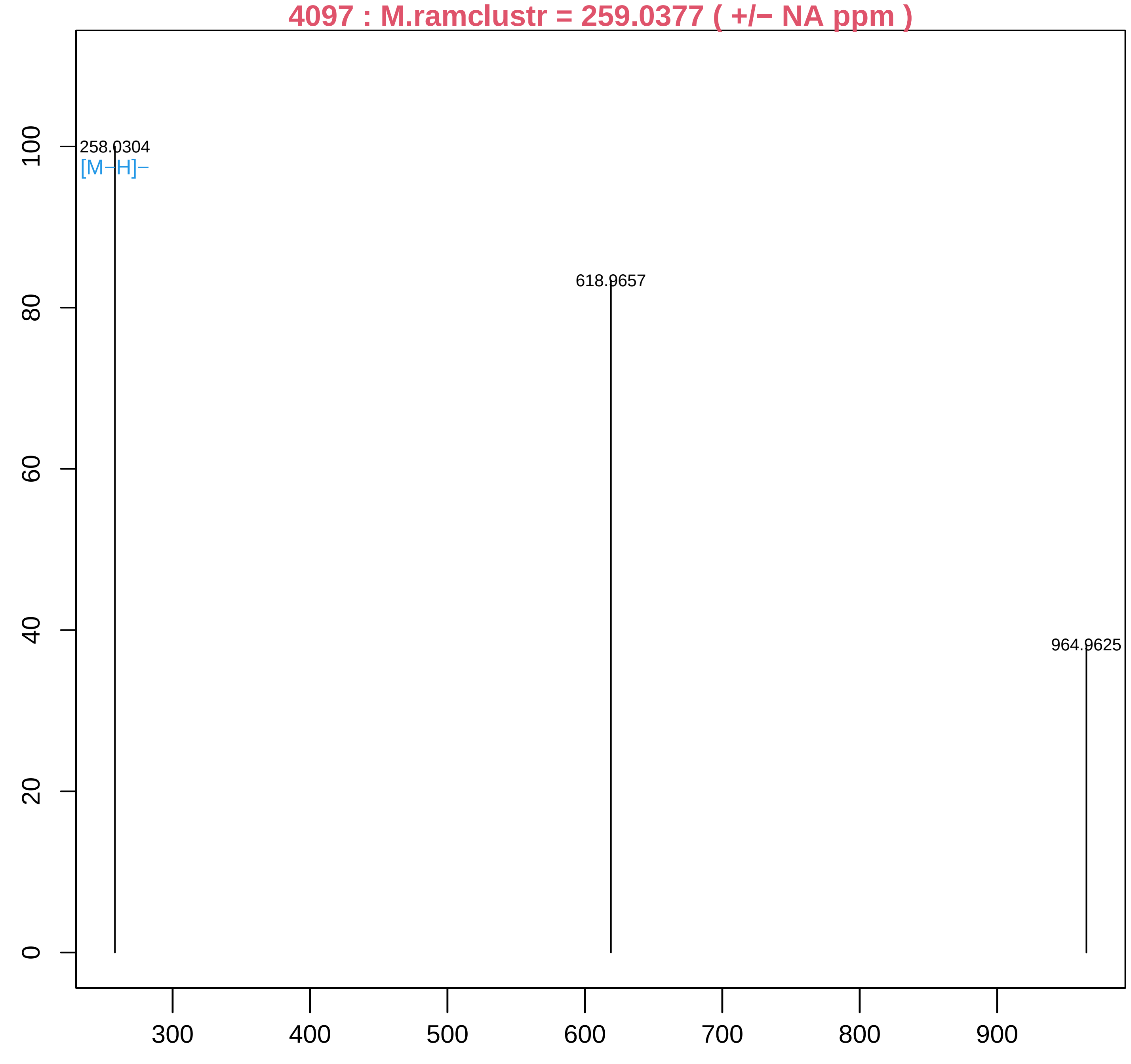
4097 : M.ramclustr = 259.0377 (+/− NA ppm)
258.0304
[M−H]−
618.9657
964.9625
0
20
40
60
80
100
300
400
500
600
700
800
900
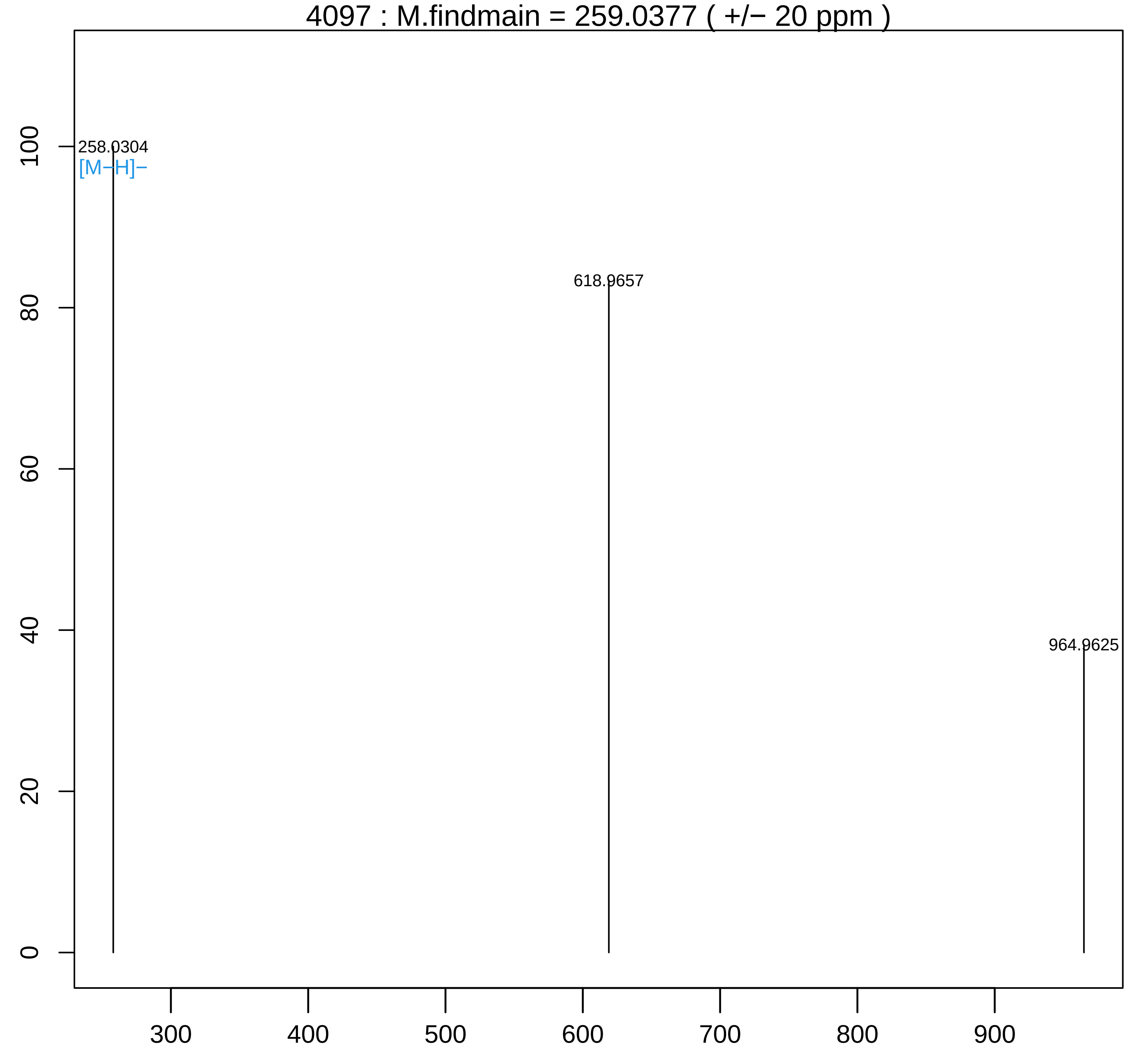
4097 : M.findmain = 259.0377 (+/− 20 ppm)
258.0304
[M−H]−
618.9657
964.9625
0
20
40
60
80
100
300
400
500
600
700
800
900

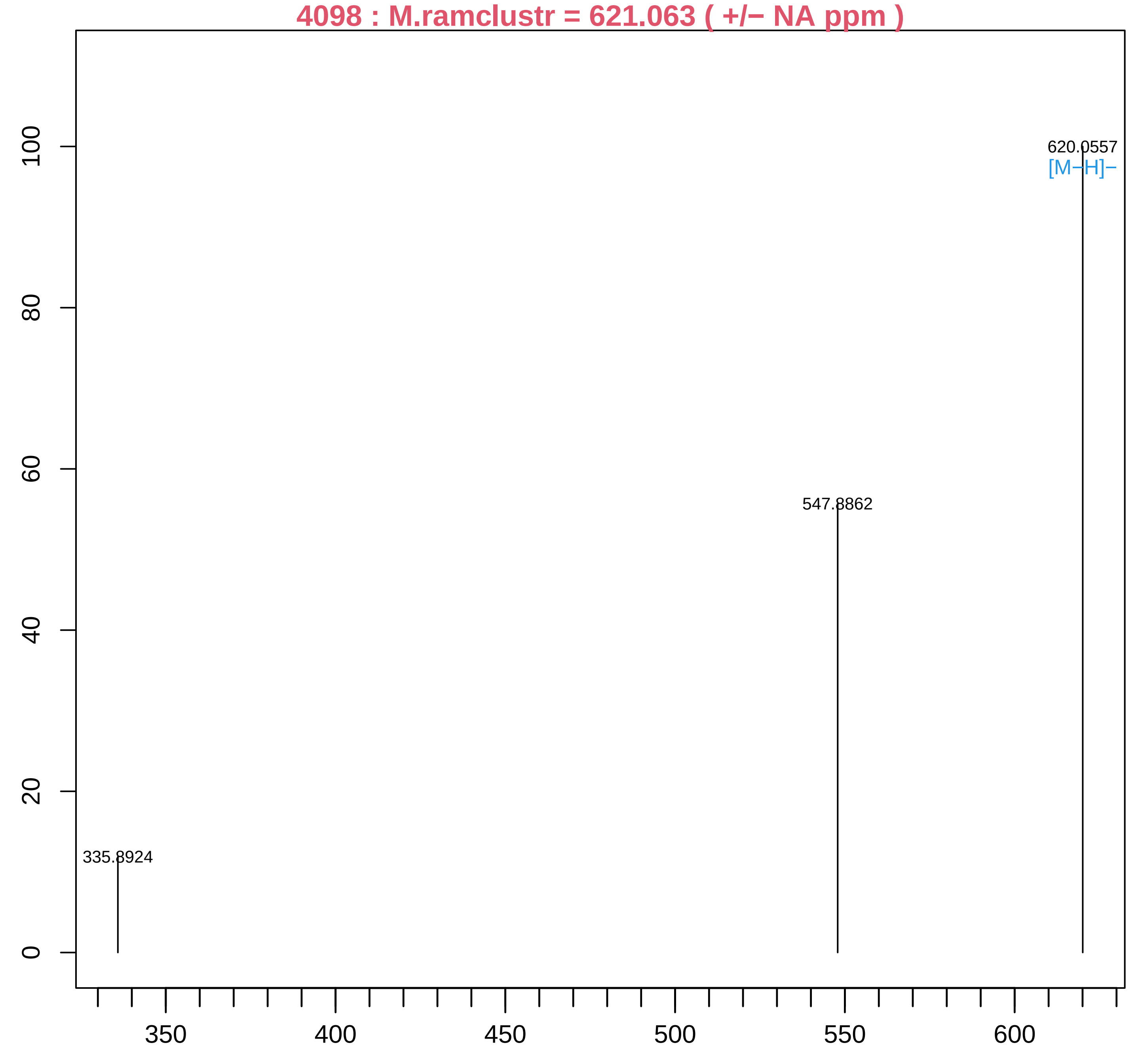
4098 : M.ramclustr = 621.063 (+/− NA ppm)
620.0557
[M−H]−
547.8862
335.8924
0
20
40
60
80
100
350
400
450
500
550
600

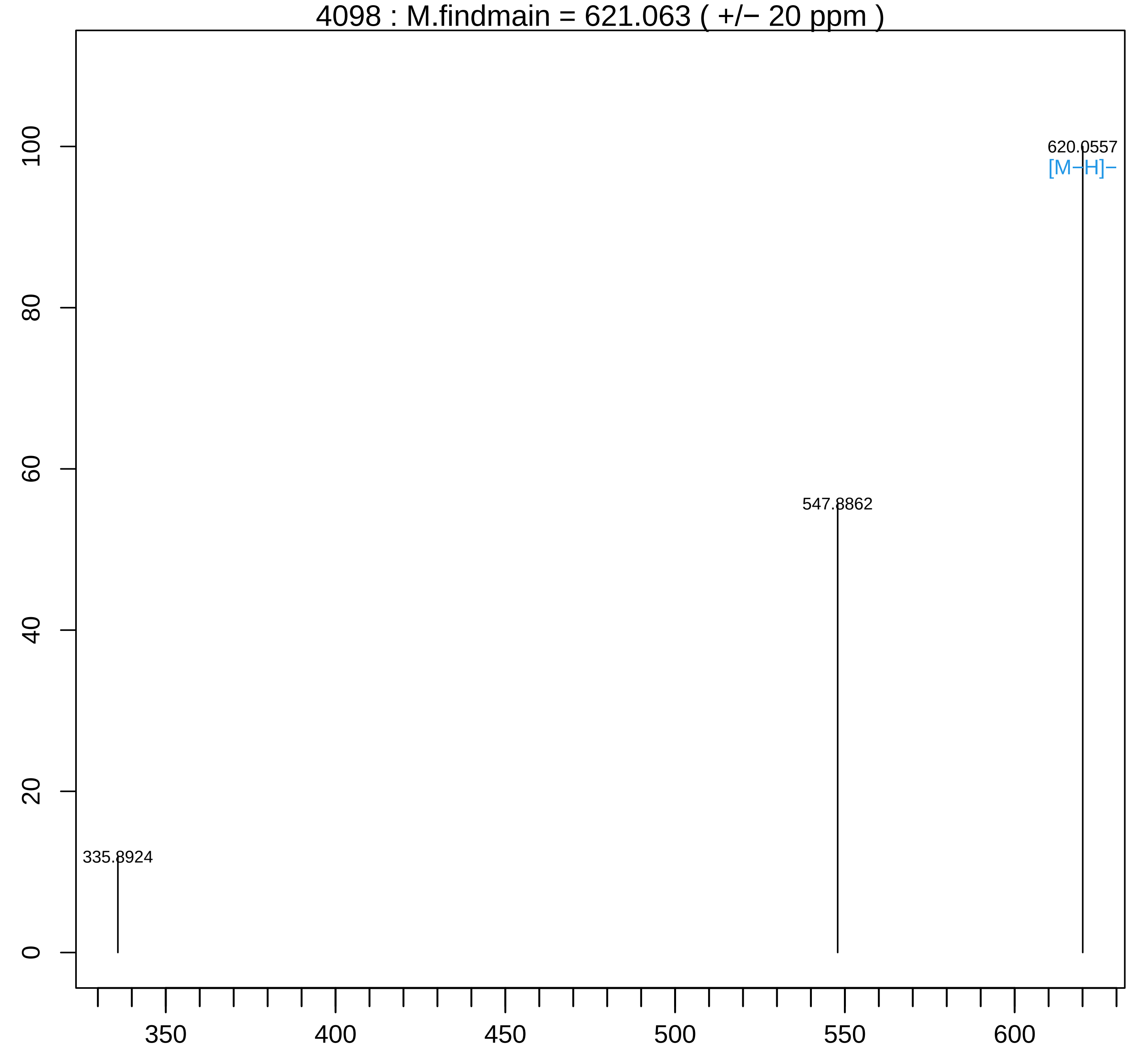
4098 : M.findmain = 621.063 (+/− 20 ppm)
620.0557
[M−H]−
547.8862
335.8924
0
20
40
60
80
100
350
400
450
500
550
600

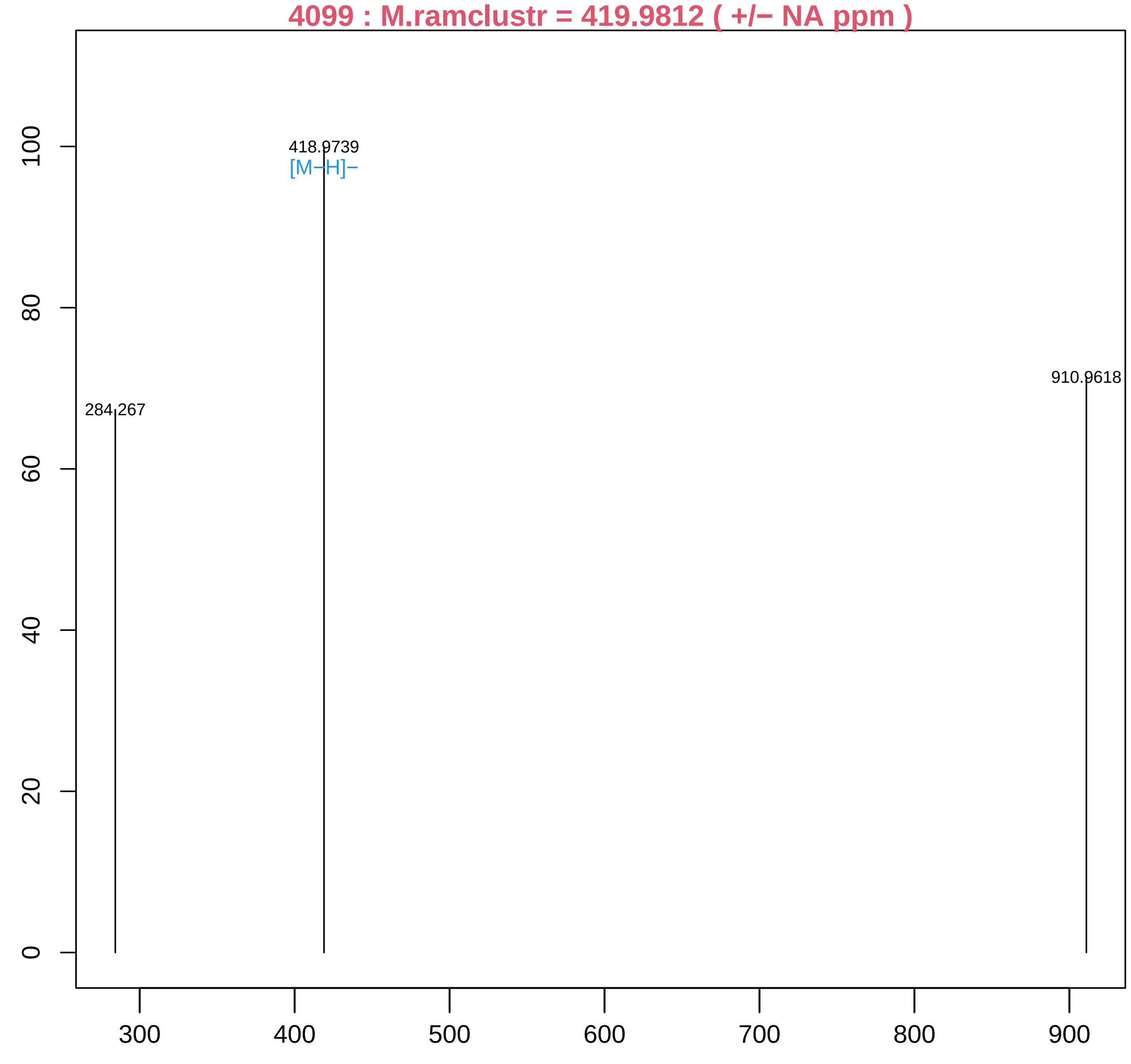

4099 : M.ramclustr = 419.9812 (+/− NA ppm)
418.9739
[M−H]−
910.9618
284.267
0
20
40
60
80
100
300
400
500
600
700
800
900

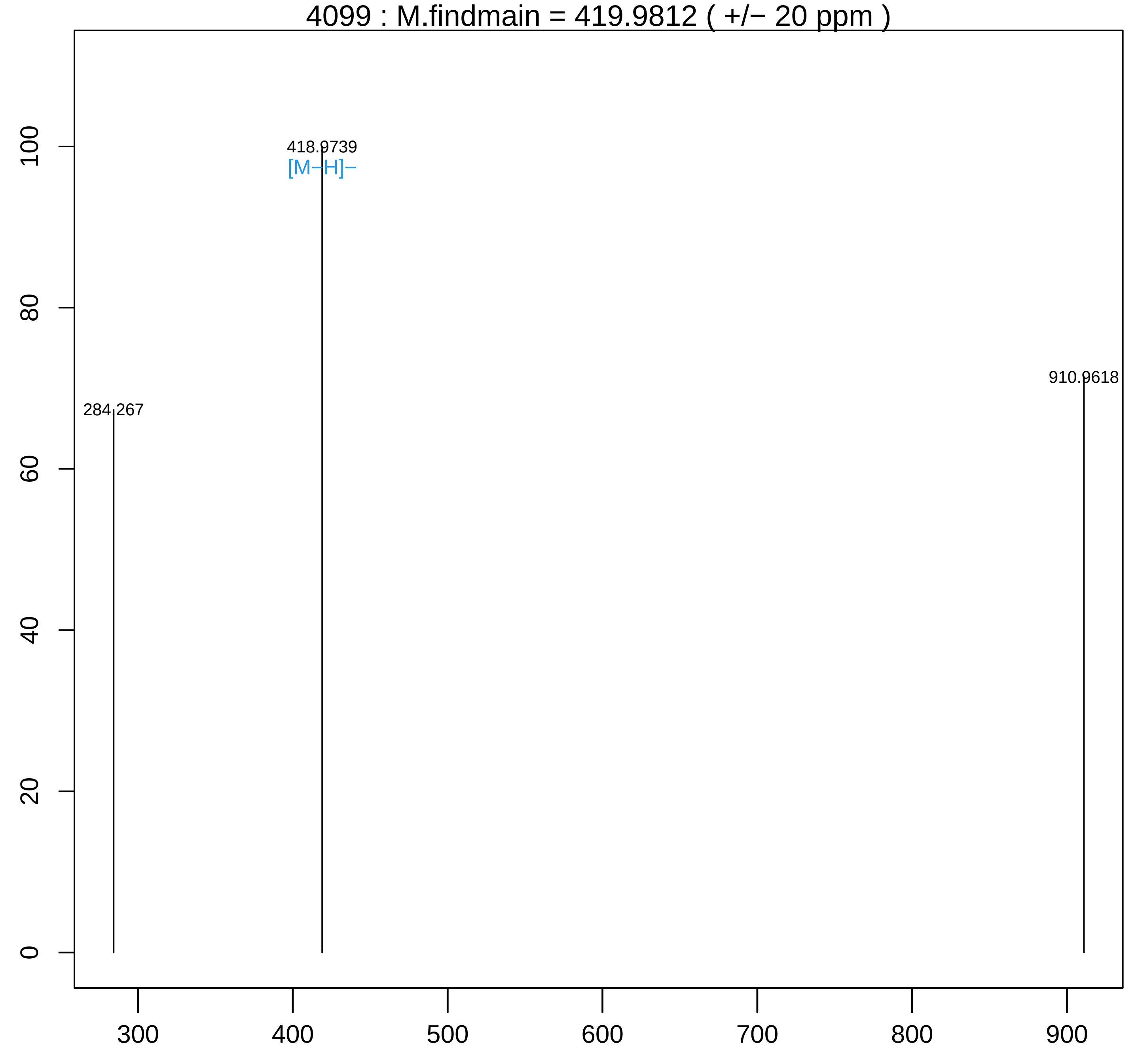

4099 : M.findmain = 419.9812 (+/− 20 ppm)
418.9739
[M−H]−
910.9618
284.267
0
20
40
60
80
100
300
400
500
600
700
800
900

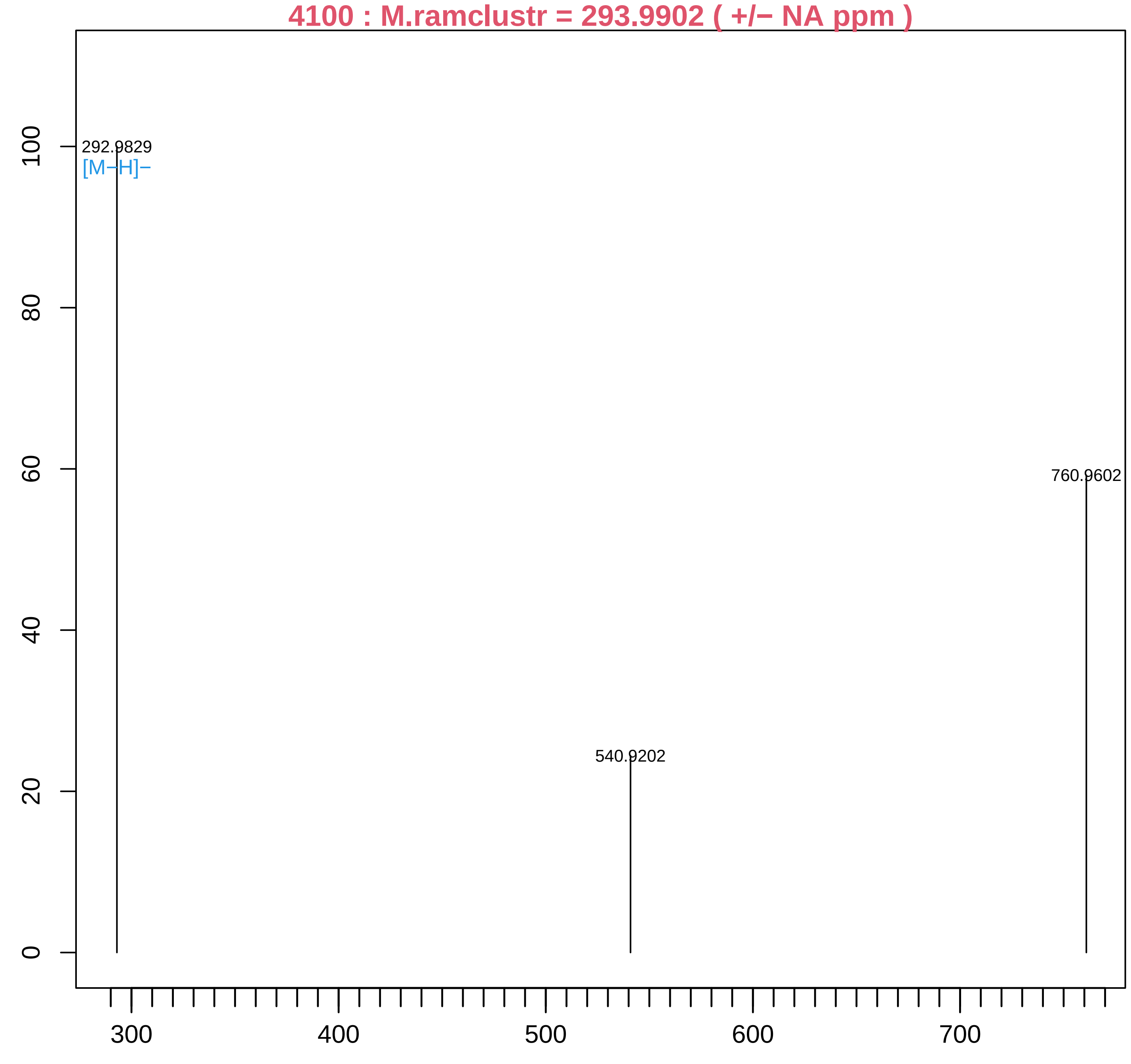

4100 : M.ramclustr = 293.9902 (+/− NA ppm)
292.9829
[M−H]−
540.9202
760.9602
0
20
40
60
80
100
300
400
500
600
700

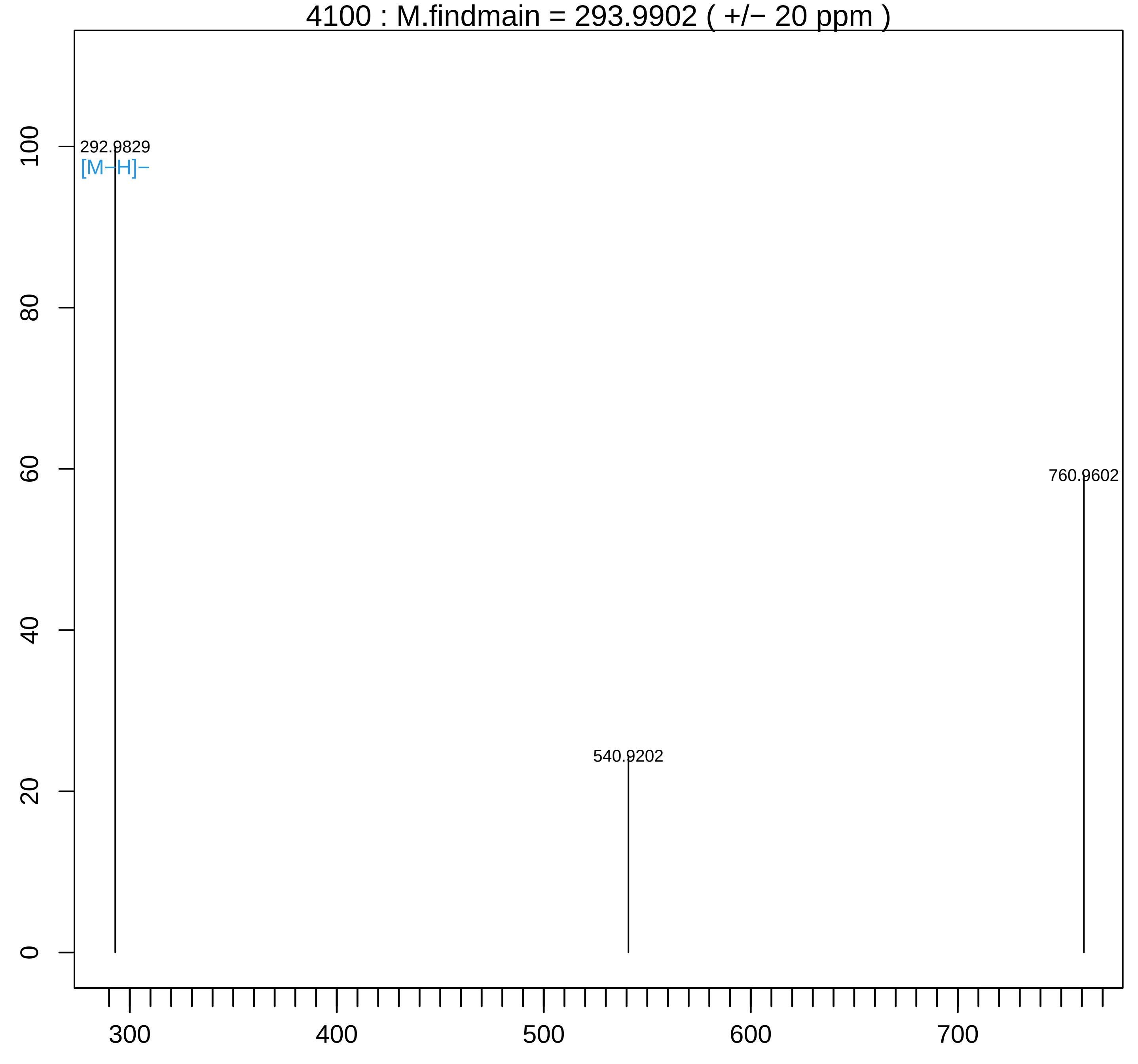

4100 : M.findmain = 293.9902 (+/− 20 ppm)
292.9829
[M−H]−
540.9202
760.9602
0
20
40
60
80
100
300
400
500
600
700

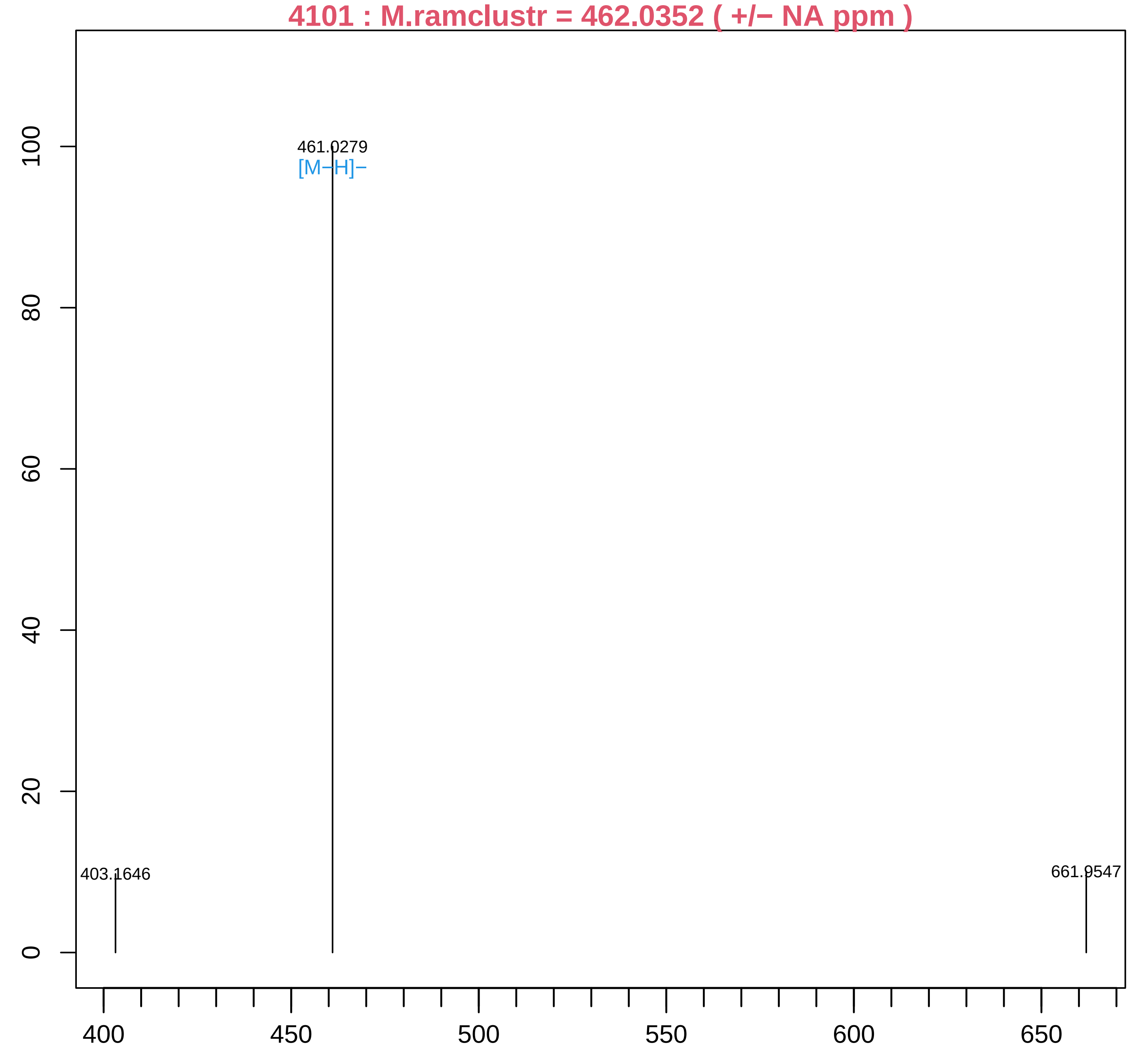
4101 : M.ramclustr = 462.0352 (+/− NA ppm)
461.0279
[M−H]−
403.1646
661.9547
0
20
40
60
80
100
400
450
500
550
600
650

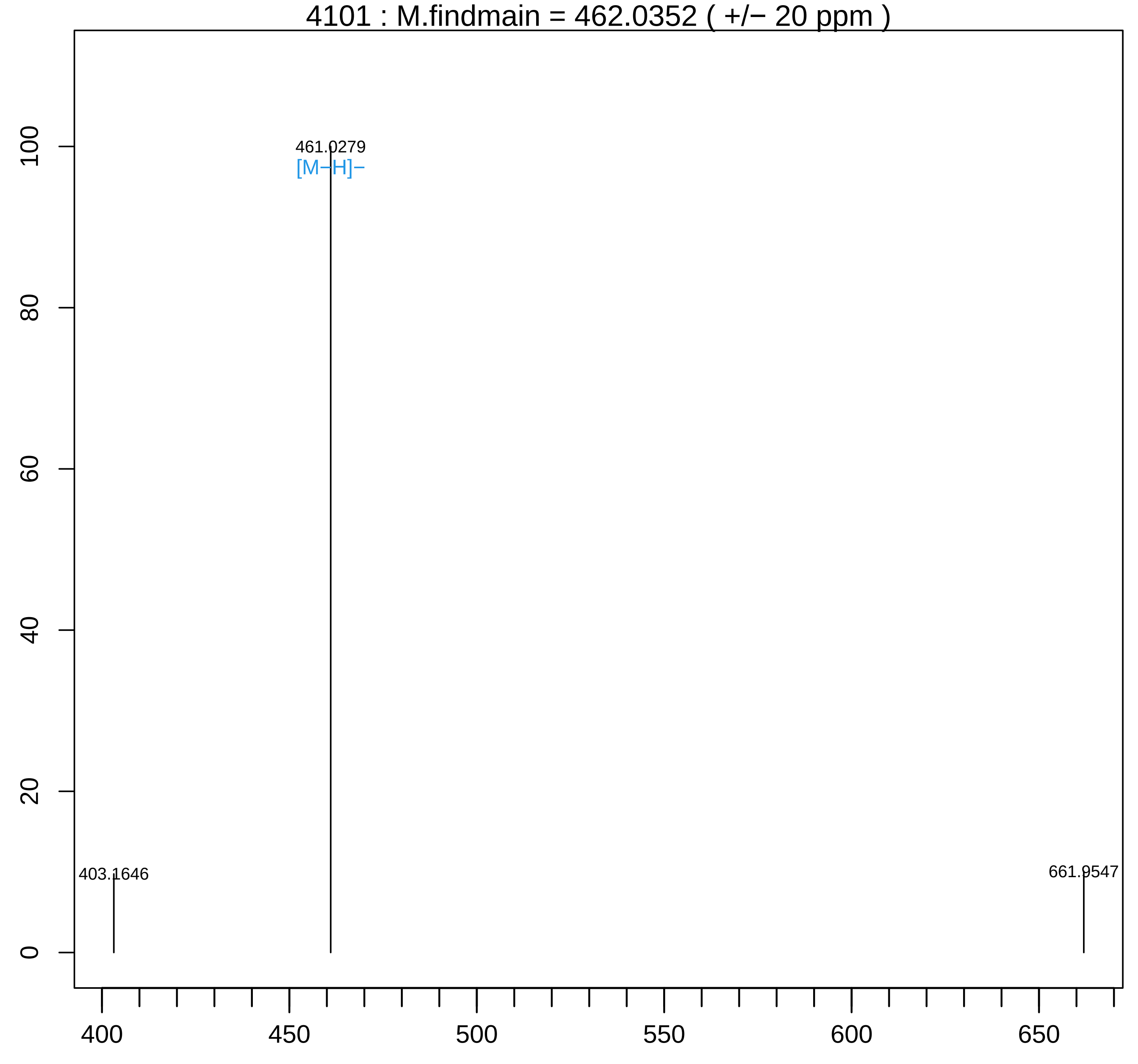
4101 : M.findmain = 462.0352 (+/− 20 ppm)
461.0279
[M−H]−
403.1646
661.9547
0
20
40
60
80
100
400
450
500
550
600
650

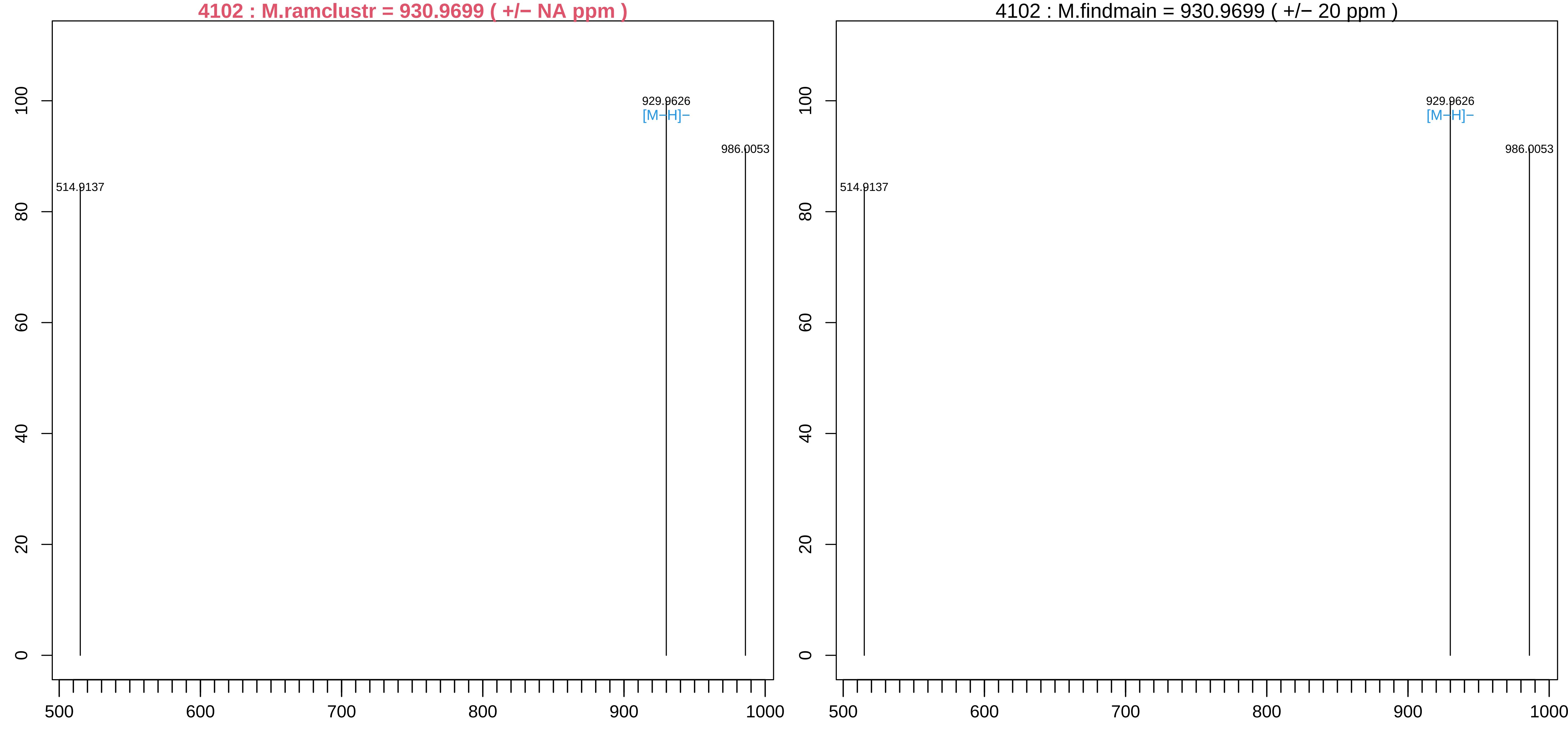
4102 : M.ramclustr = 930.9699 (+/− NA ppm)
514.9137
929.9626
[M−H]−
986.0053
4102 : M.findmain = 930.9699 (+/− 20 ppm)
514.9137
929.9626
[M−H]−
986.0053
0
20
40
60
80
100
500
600
700
800
900
1000

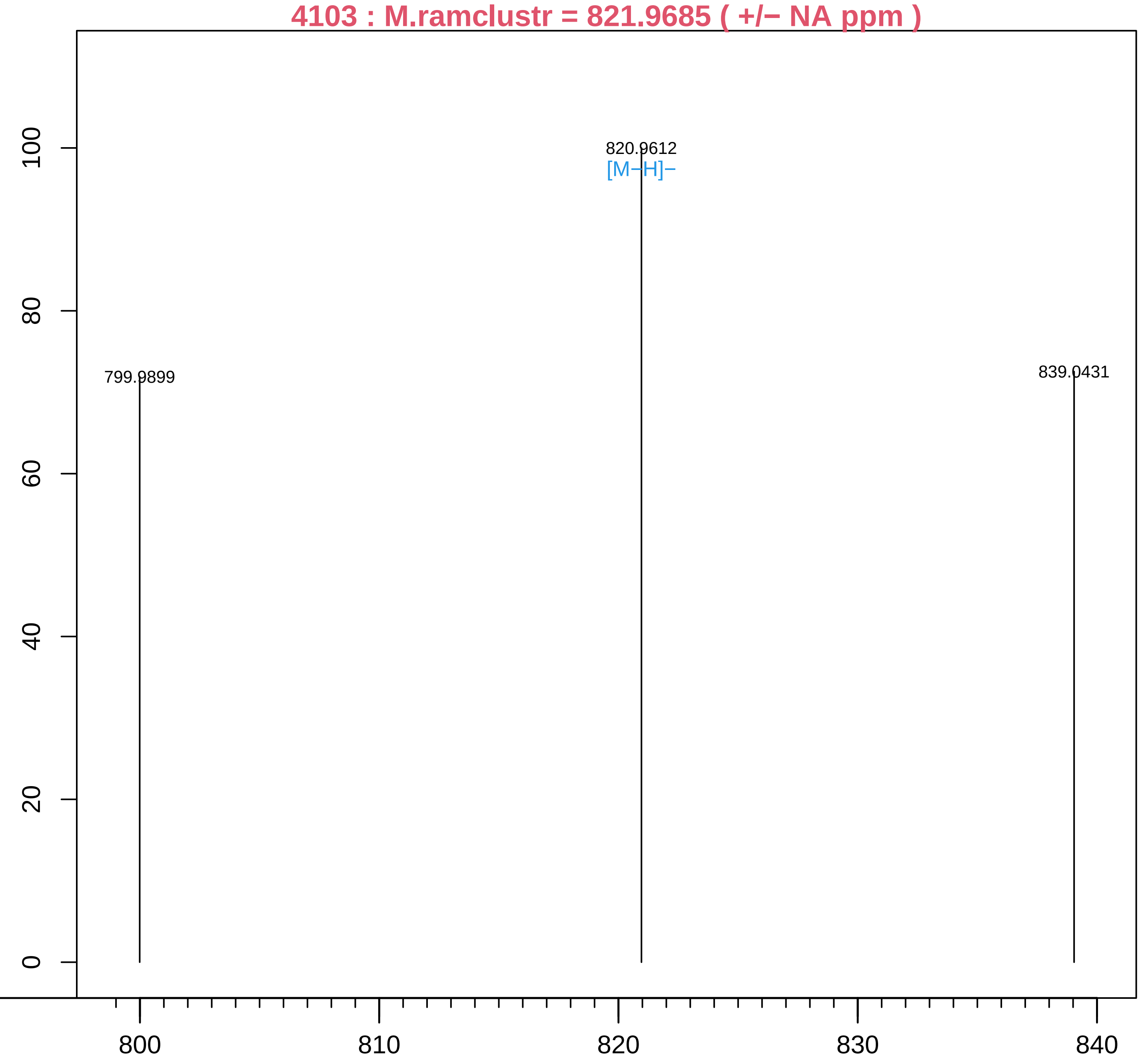

4103 : M.ramclustr = 821.9685 (+/− NA ppm)
799.9899
820.9612
[M−H]−
839.0431
0
20
40
60
80
100
800
810
820
830
840

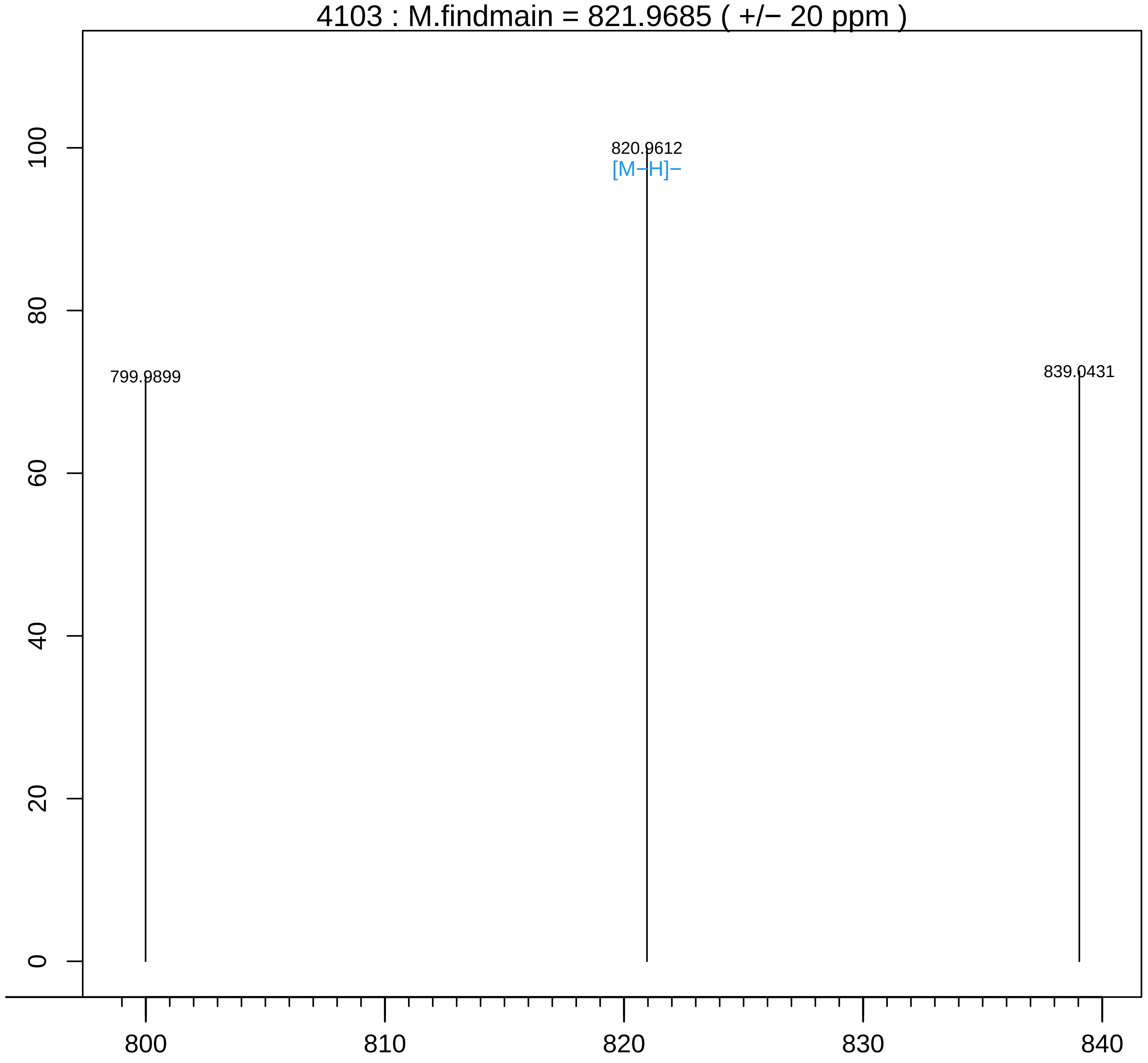

4103 : M.findmain = 821.9685 (+/− 20 ppm)
799.9899
820.9612
[M−H]−
839.0431
0
20
40
60
80
100
800
810
820
830
840

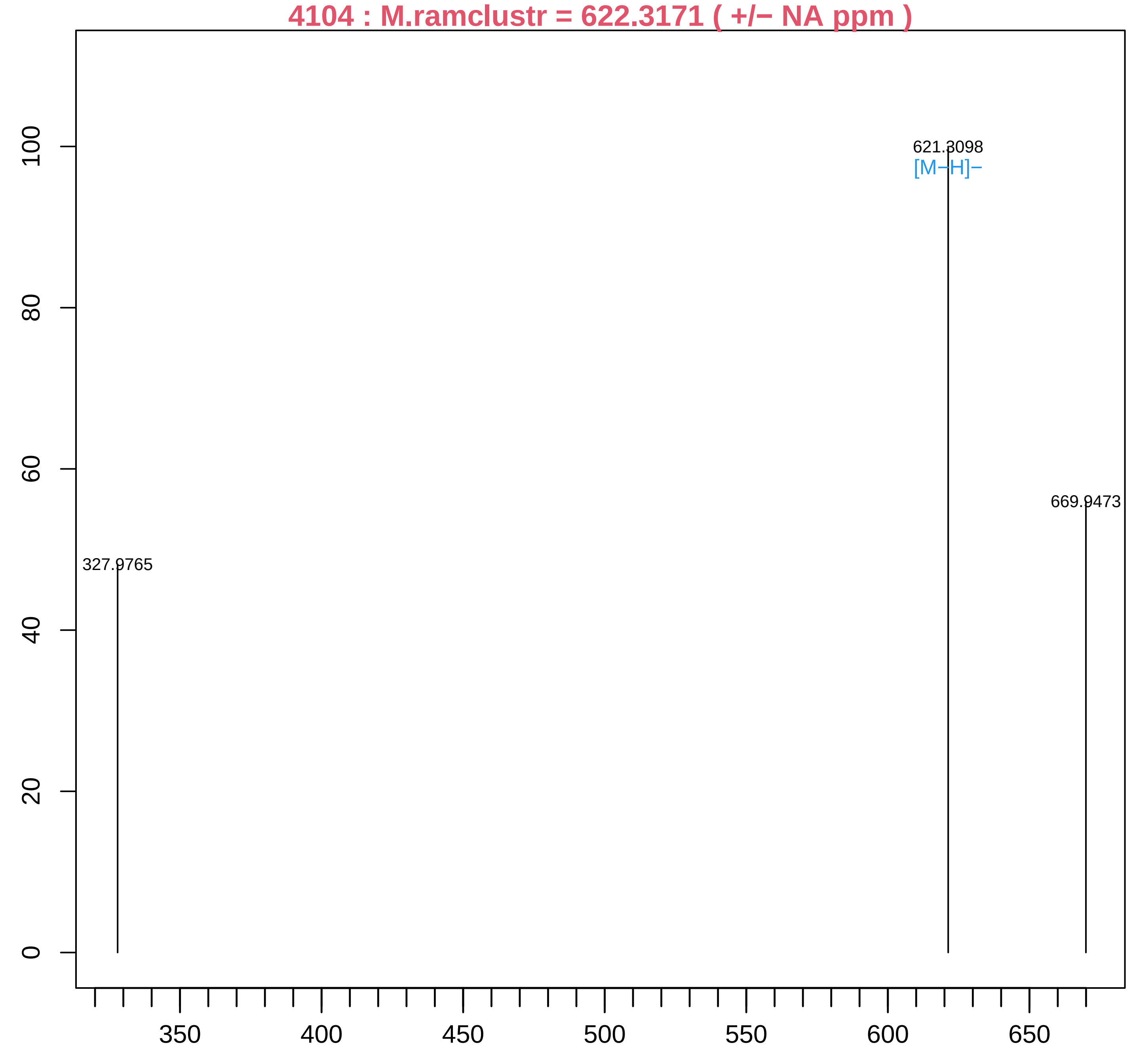
4104 : M.ramclustr = 622.3171 (+/− NA ppm)
621.3098
[M−H]−
669.9473
327.9765
0
20
40
60
80
100
350
400
450
500
550
600
650

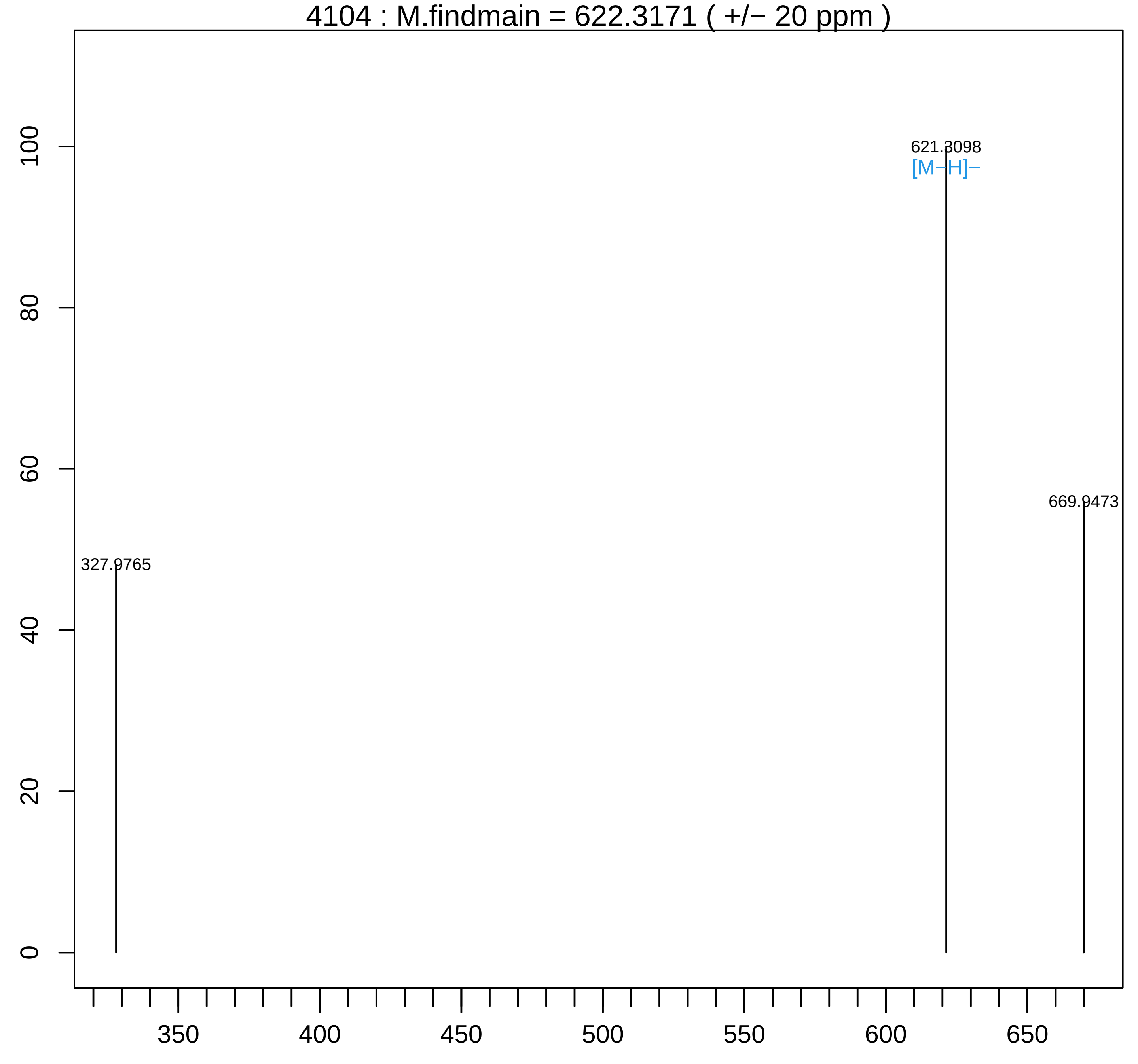
4104 : M.findmain = 622.3171 (+/− 20 ppm)
621.3098
[M−H]−
669.9473
327.9765
0
20
40
60
80
100
350
400
450
500
550
600
650

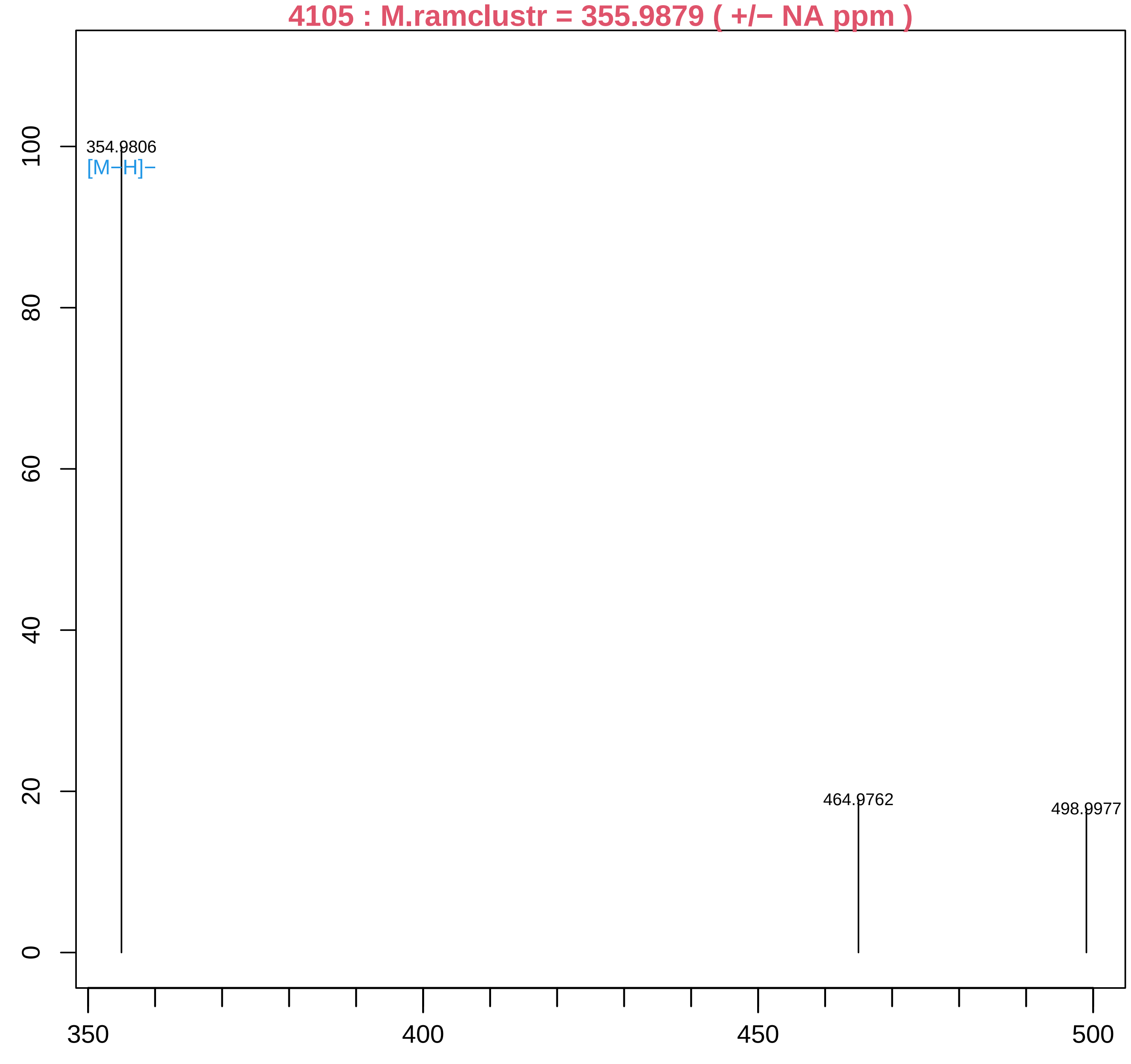
4105 : M.ramclustr = 355.9879 (+/− NA ppm)
354.9806
[M−H]−
464.9762
498.9977
0
20
40
60
80
100
350
400
450
500

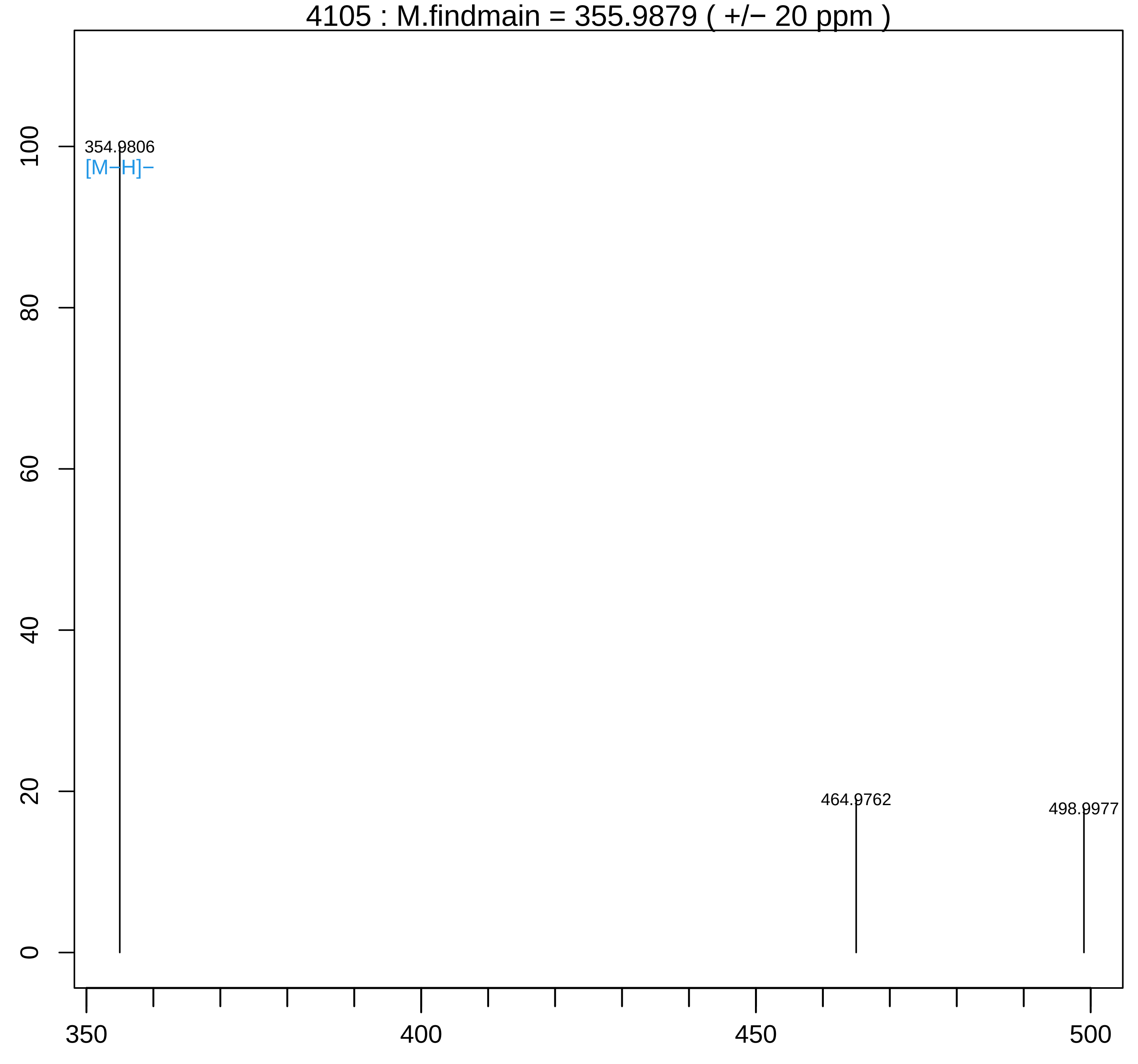
4105 : M.findmain = 355.9879 (+/− 20 ppm)
354.9806
[M−H]−
464.9762
498.9977
0
20
40
60
80
100
350
400
450
500

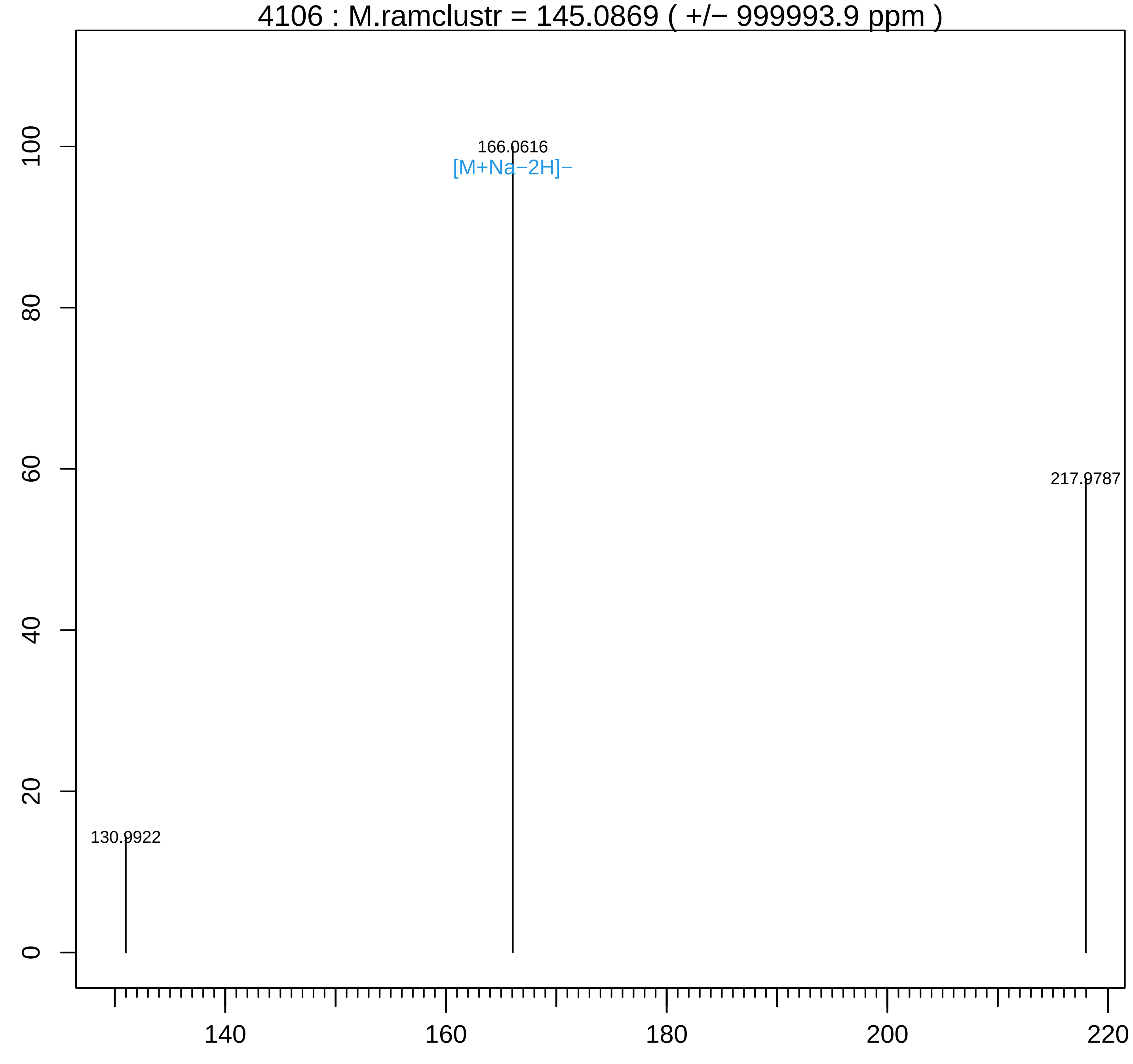
4106 : M.ramclustr = 145.0869 (+/− 999993.9 ppm)
166.0616
[M+Na−2H]−
217.9787
130.9922
0
20
40
60
80
100
140
160
180
200
220

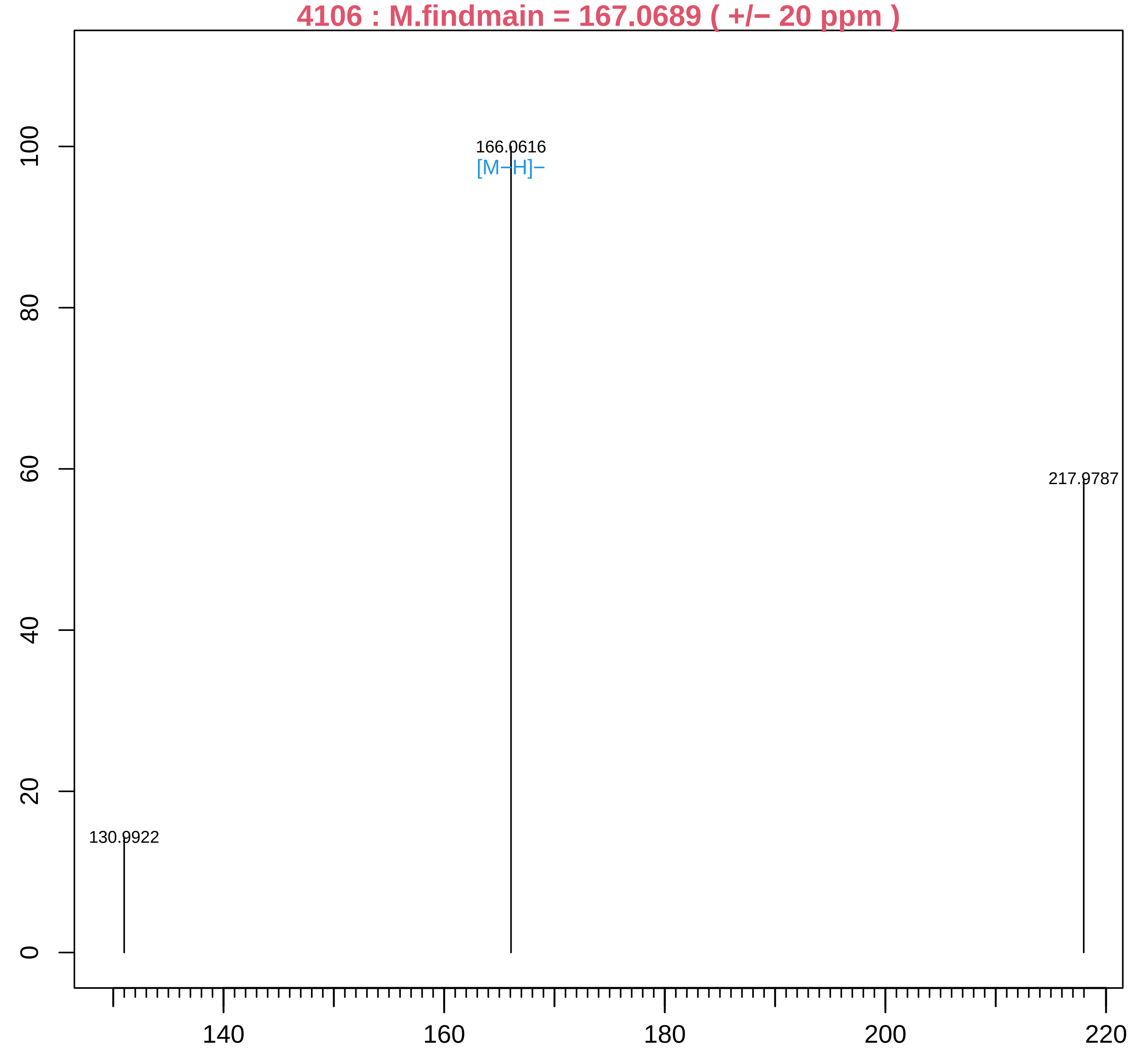
4106 : M.findmain = 167.0689 (+/− 20 ppm)
166.0616
[M−H]−
217.9787
130.9922
0
20
40
60
80
100
140
160
180
200
220

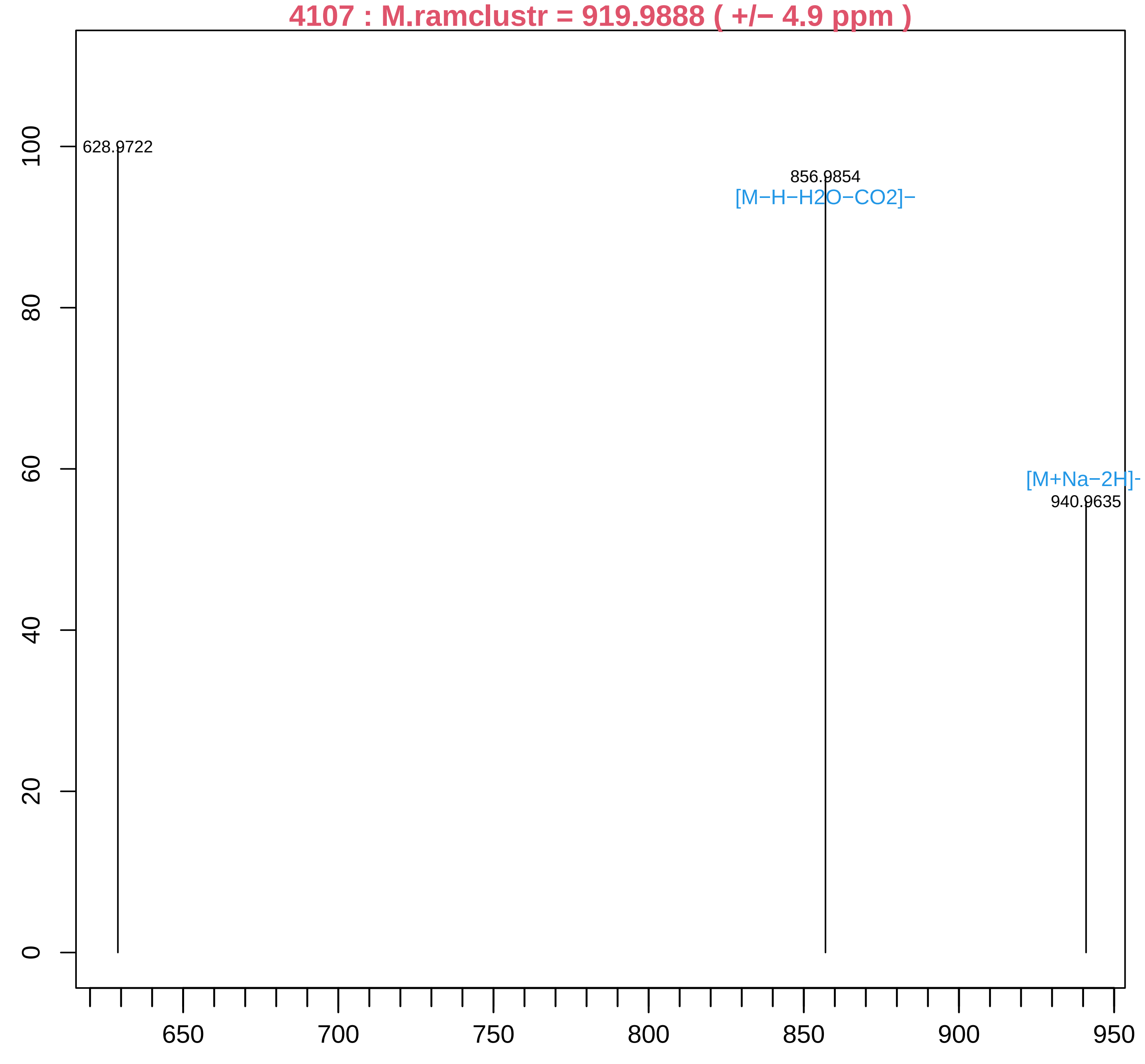

4107 : M.ramclustr = 919.9888 (+/− 4.9 ppm)
628.9722
856.9854
[M−H−H2O−CO2]−
[M+Na−2H]-
940.9635
0
20
40
60
80
100
650
700
750
800
850
900
950

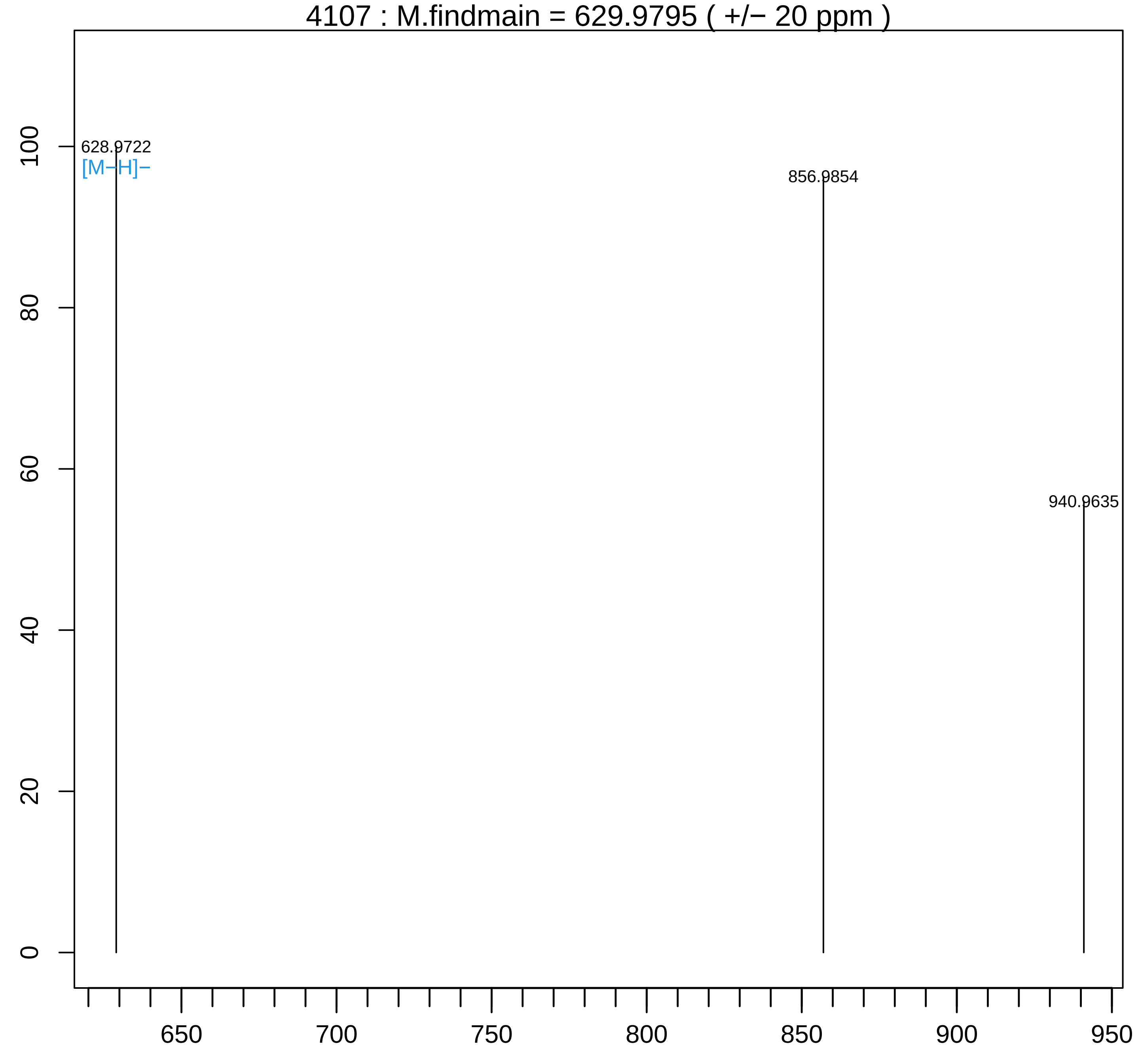

4107 : M.findmain = 629.9795 (+/− 20 ppm)
628.9722
[M−H]−
856.9854
940.9635
0
20
40
60
80
100
650
700
750
800
850
900
950

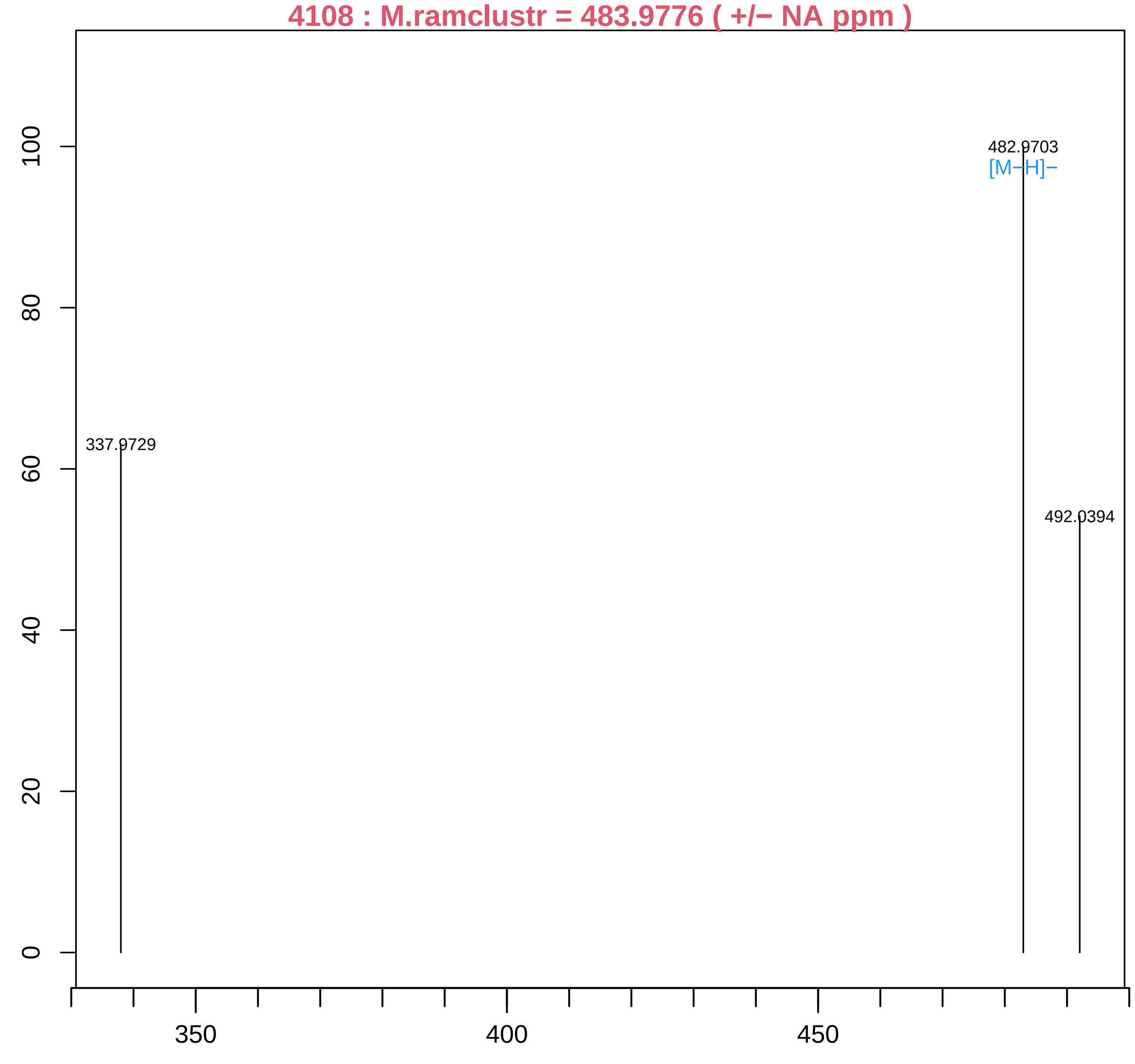
4108 : M.ramclustr = 483.9776 (+/− NA ppm)
482.9703
[M−H]−
337.9729
492.0394
0
20
40
60
80
100
350
400
450

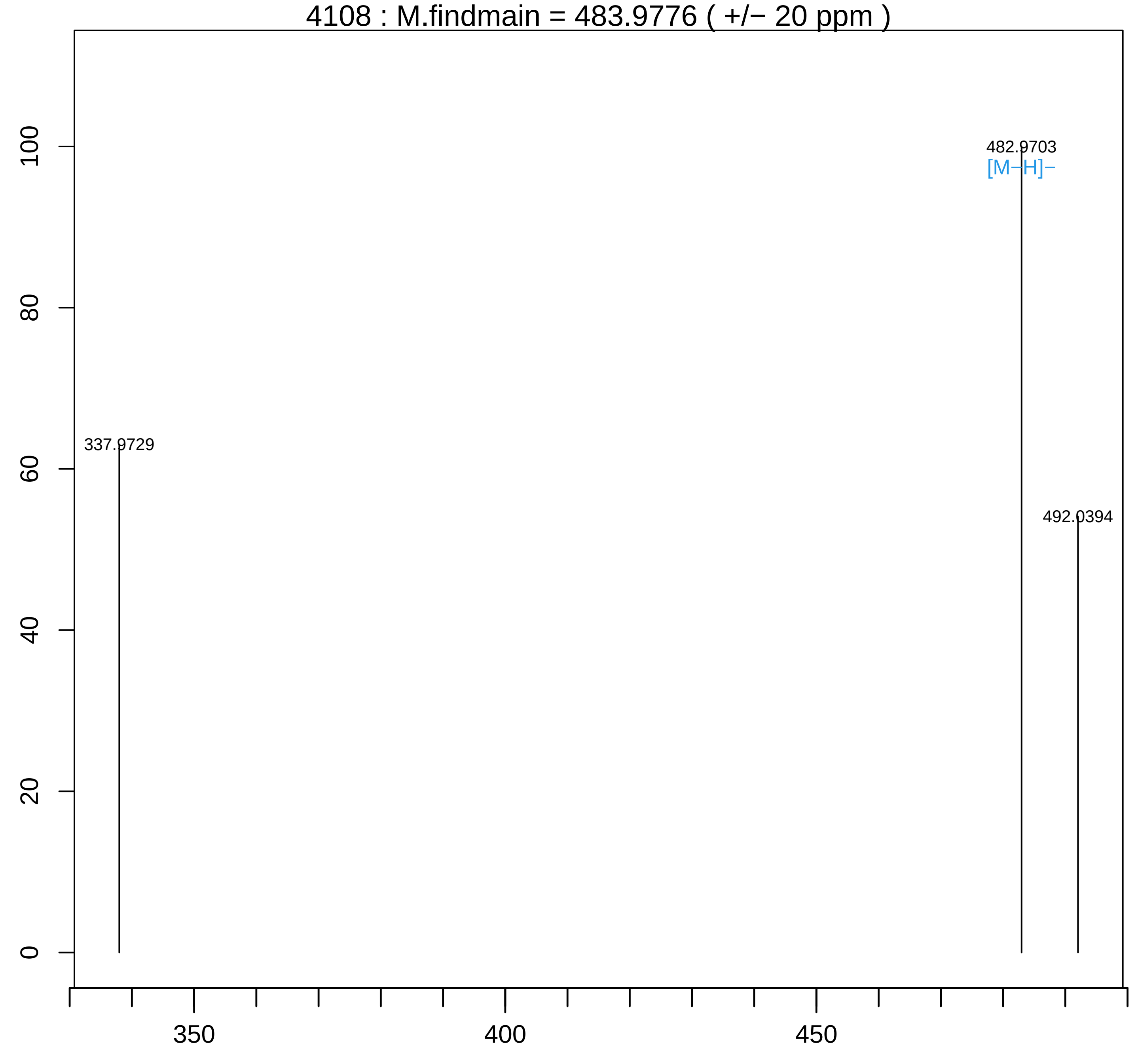
4108 : M.findmain = 483.9776 (+/− 20 ppm)
482.9703
[M−H]−
337.9729
492.0394
0
20
40
60
80
100
350
400
450

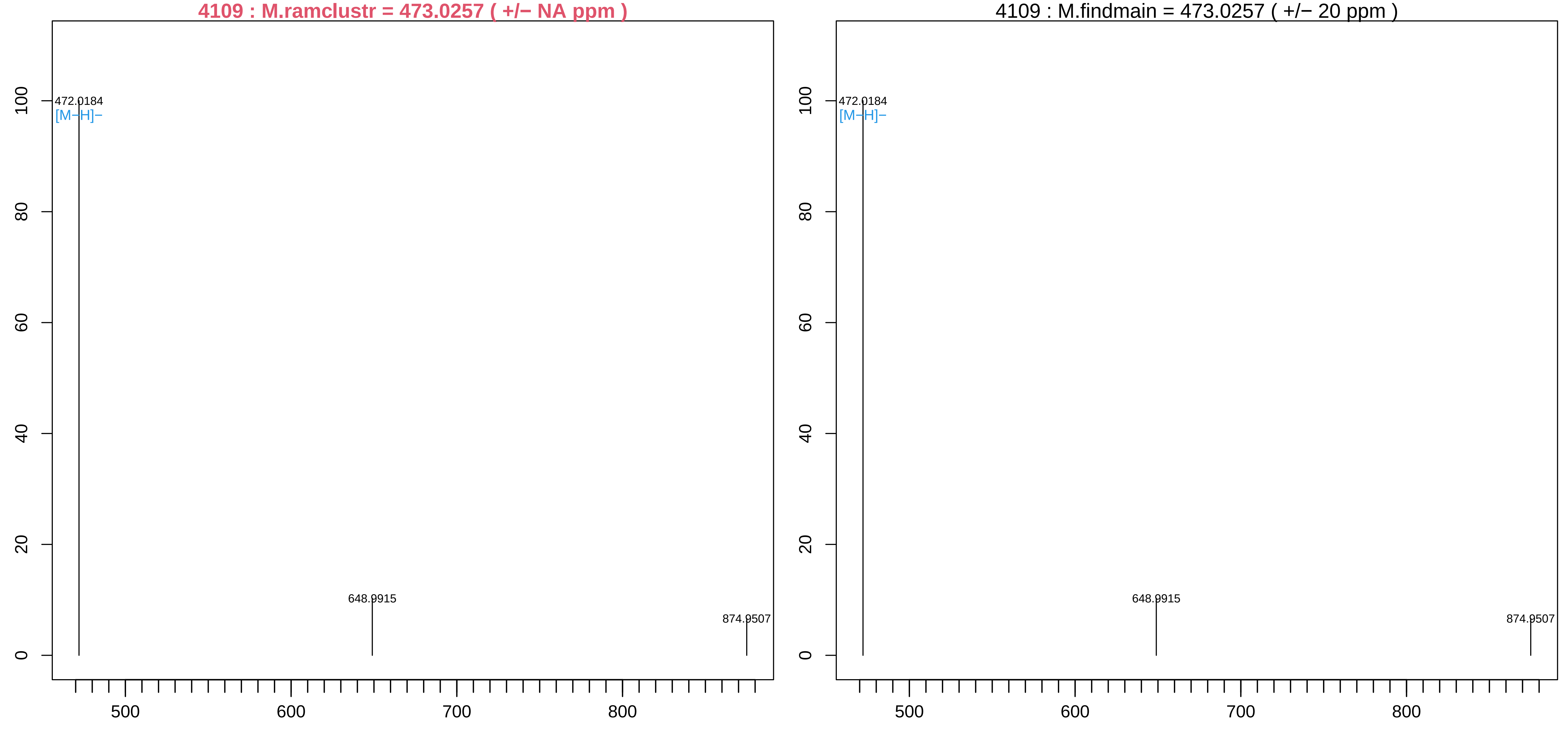

4109 : M.ramclustr = 473.0257 (+/− NA ppm)
472.0184
[M−H]−
648.9915
874.9507
4109 : M.findmain = 473.0257 (+/− 20 ppm)
472.0184
[M−H]−
648.9915
874.9507
0
20
40
60
80
100
500
600
700
800

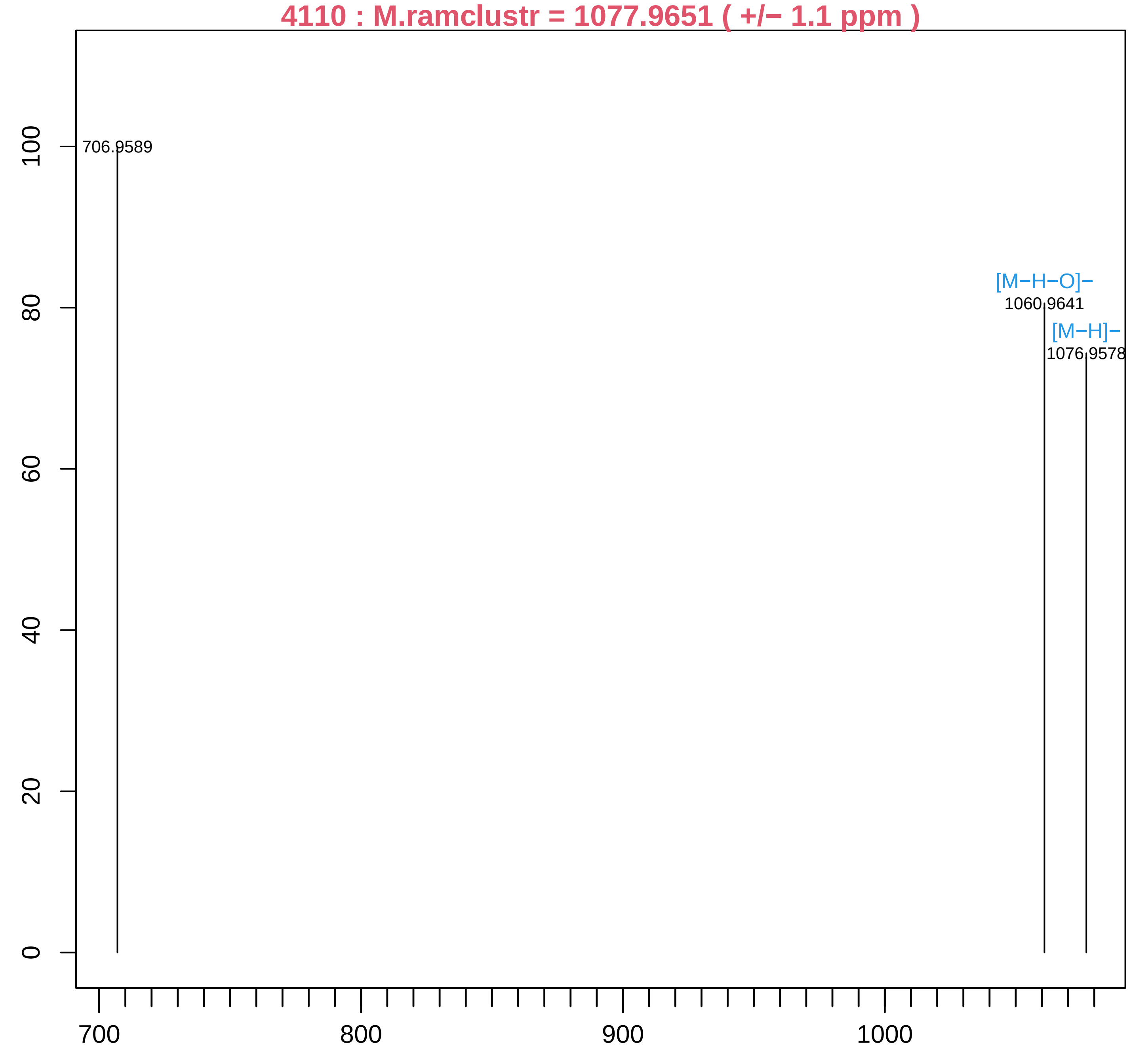
4110 : M.ramclustr = 1077.9651 (+/− 1.1 ppm)
706.9589
[M−H−O]−
1060.9641
[M−H]−
1076.9578
0
20
40
60
80
100
700
800
900
1000

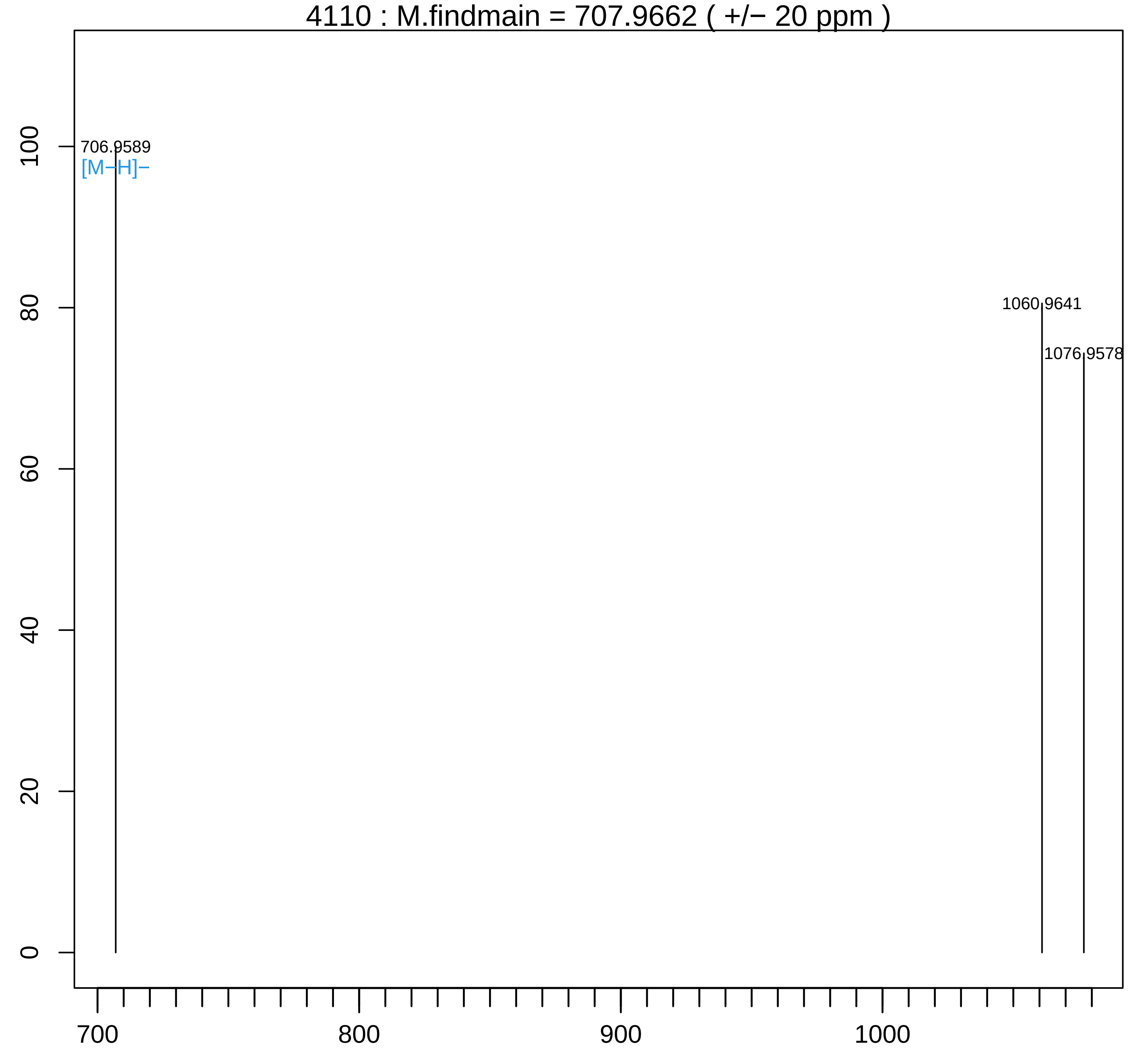
4110 : M.findmain = 707.9662 (+/− 20 ppm)
706.9589
[M−H]−
1060.9641
1076.9578
0
20
40
60
80
100
700
800
900
1000

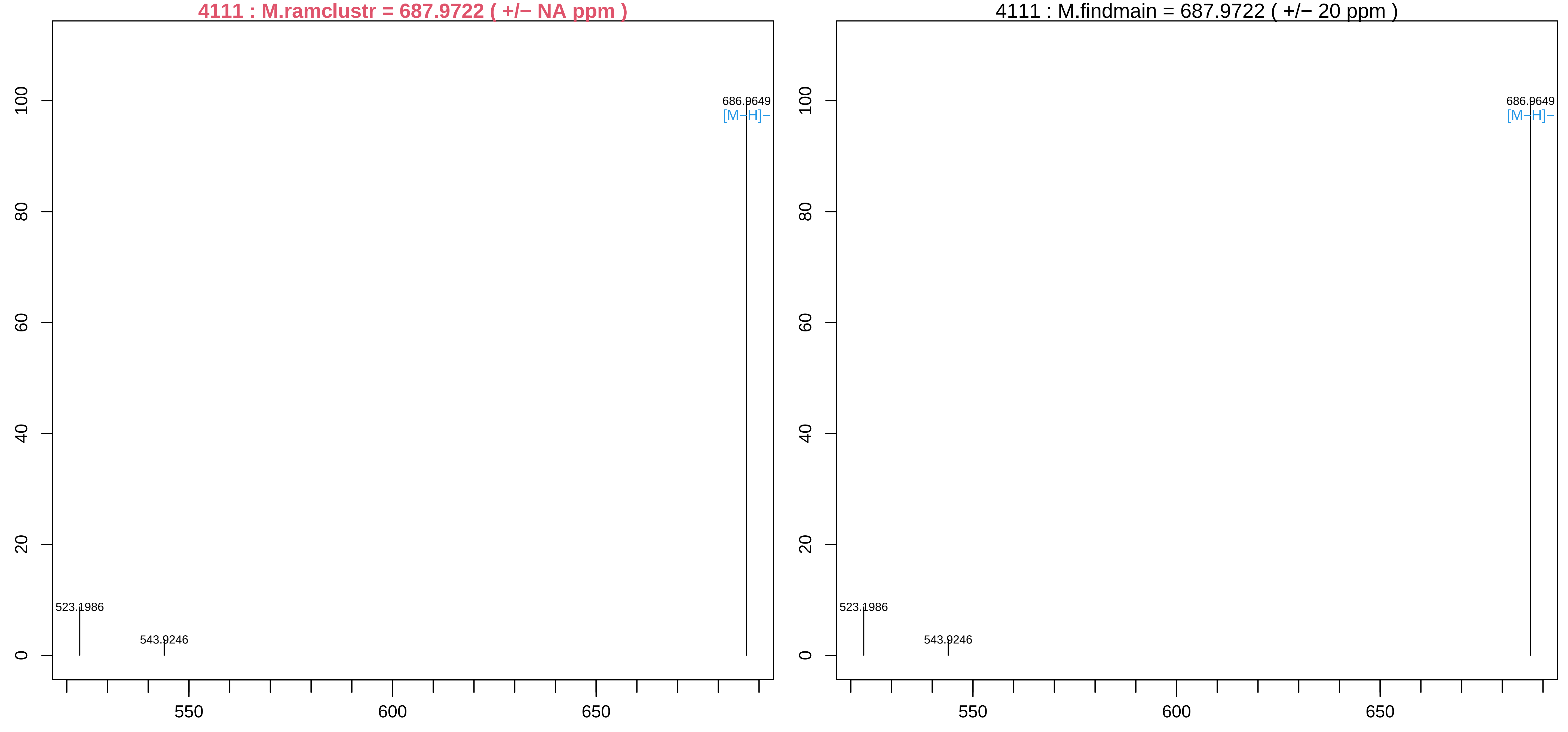
4111 : M.ramclustr = 687.9722 (+/− NA ppm)
4111 : M.findmain = 687.9722 (+/− 20 ppm)
686.9649
[M−H]−
523.1986
543.9246
0
20
40
60
80
100
550
600
650

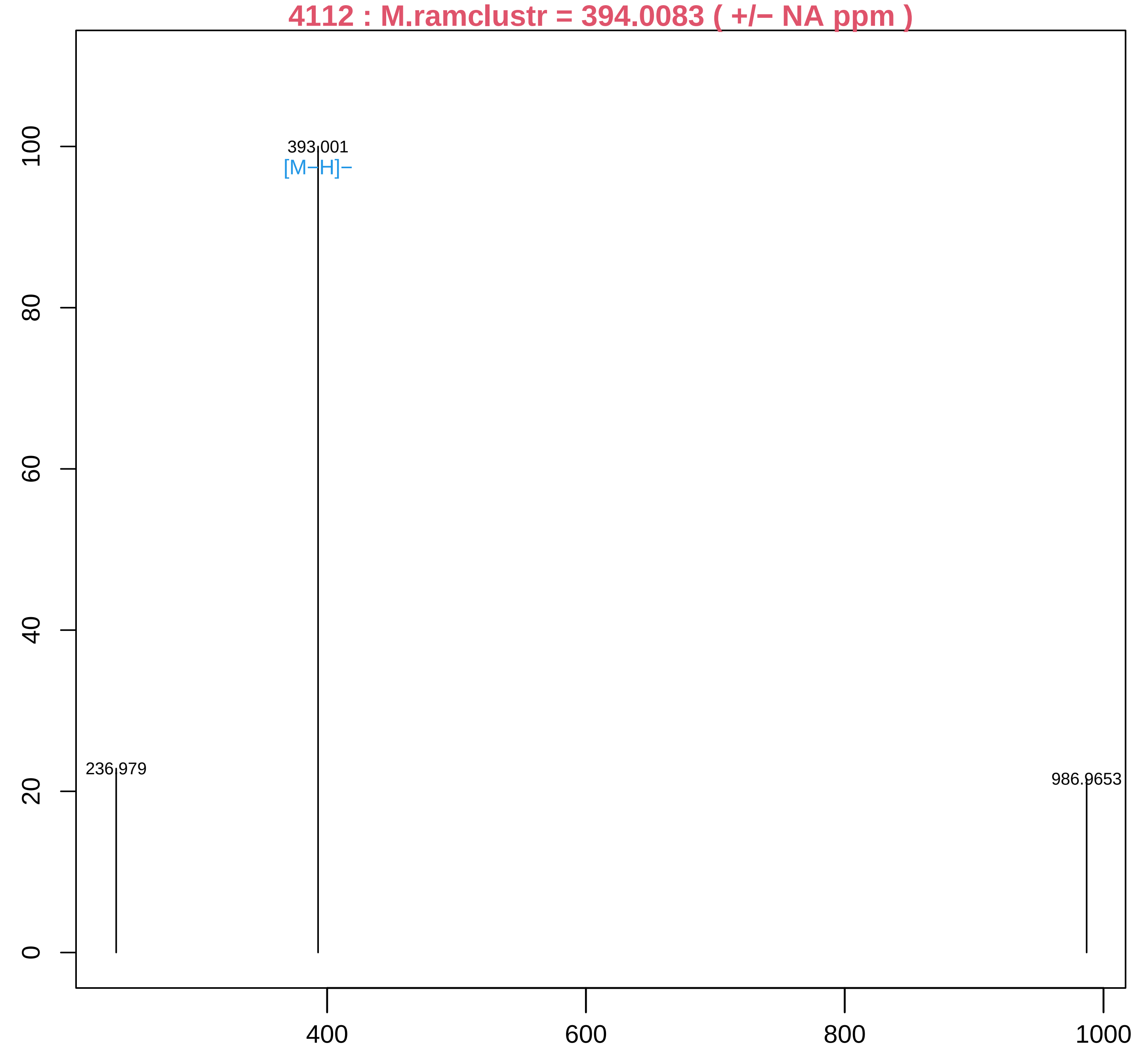
4112 : M.ramclustr = 394.0083 (+/− NA ppm)
393.001
[M−H]−
236.979
986.9653
0
20
40
60
80
100
400
600
800
1000

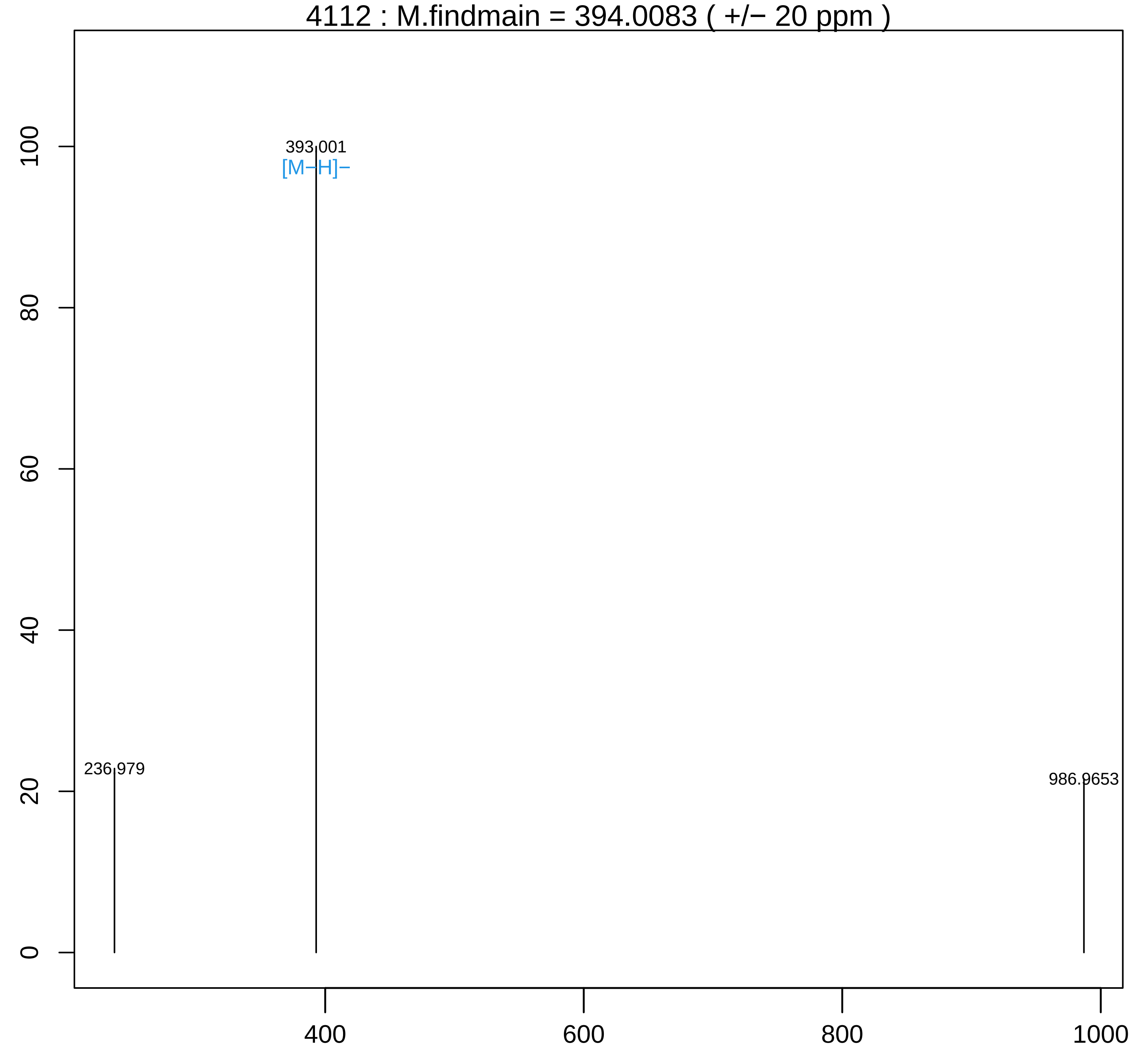
4112 : M.findmain = 394.0083 (+/− 20 ppm)
393.001
[M−H]−
236.979
986.9653
0
20
40
60
80
100
400
600
800
1000

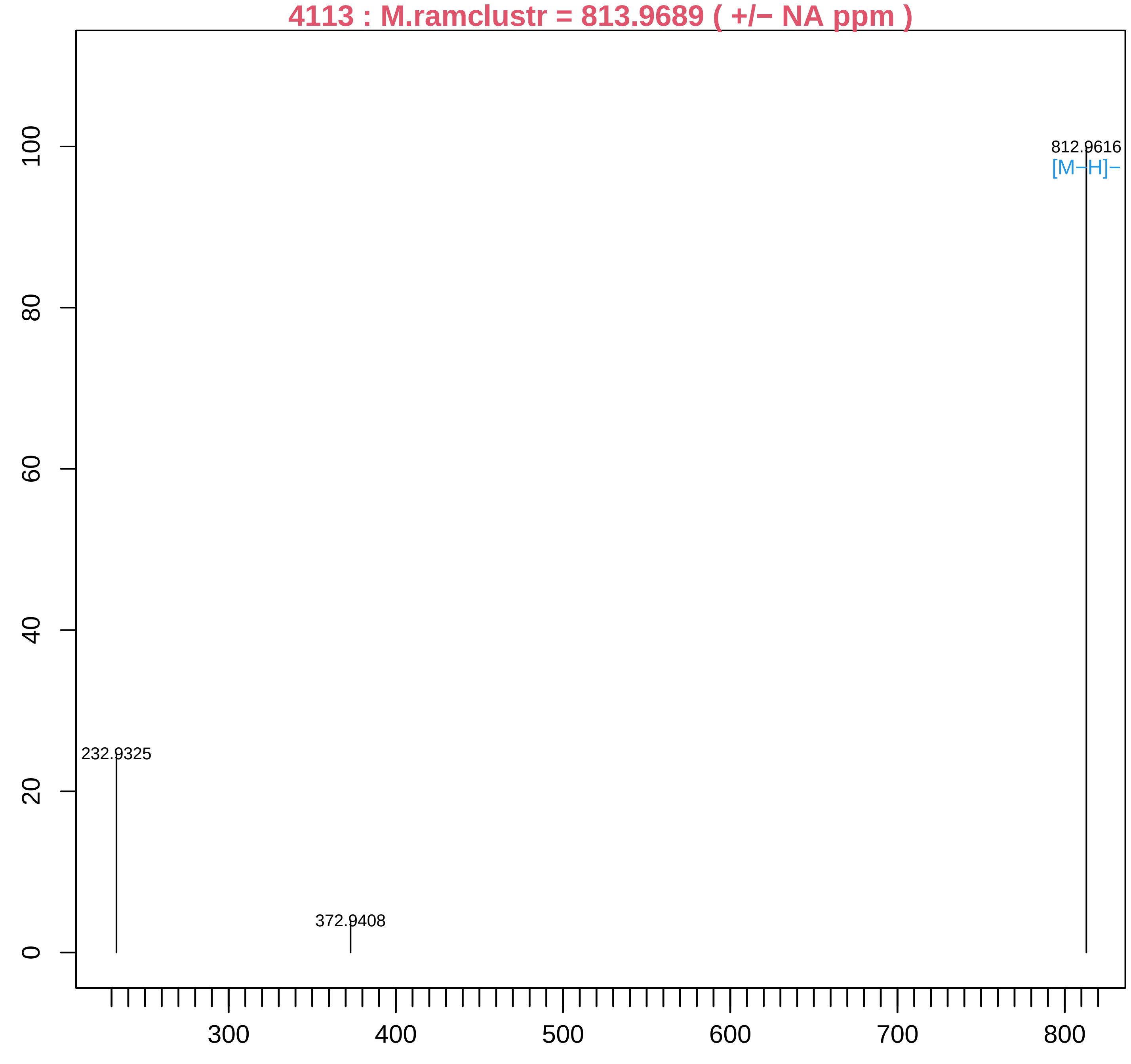

4113 : M.ramclustr = 813.9689 (+/− NA ppm)
812.9616
[M−H]−
232.9325
372.9408
0
20
40
60
80
100
300
400
500
600
700
800

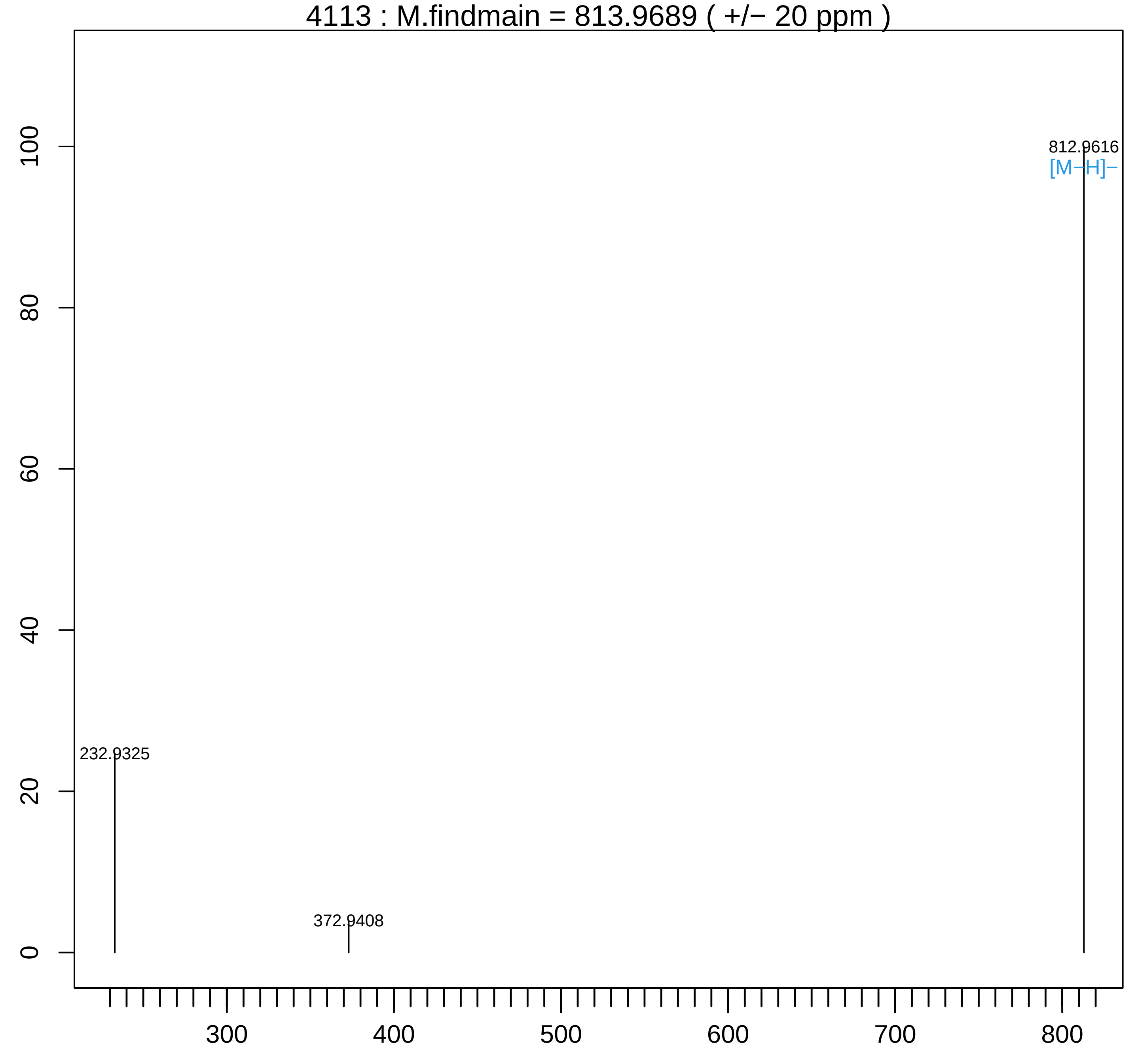

4113 : M.findmain = 813.9689 (+/− 20 ppm)
812.9616
[M−H]−
232.9325
372.9408
0
20
40
60
80
100
300
400
500
600
700
800

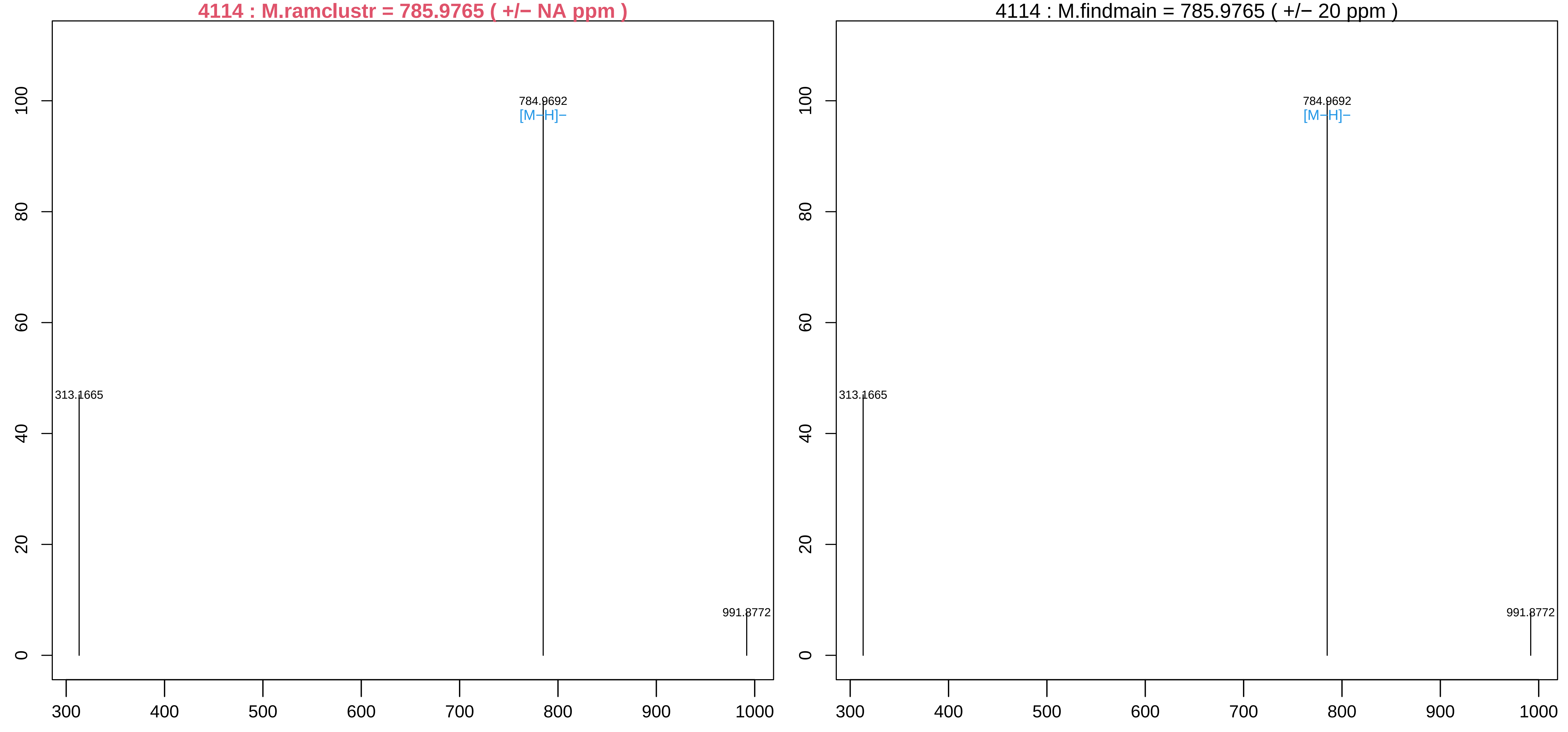
4114 : M.ramclustr = 785.9765 (+/− NA ppm)
784.9692
[M−H]−
313.1665
991.8772
4114 : M.findmain = 785.9765 (+/− 20 ppm)
784.9692
[M−H]−
313.1665
991.8772
0
20
40
60
80
100
300
400
500
600
700
800
900
1000

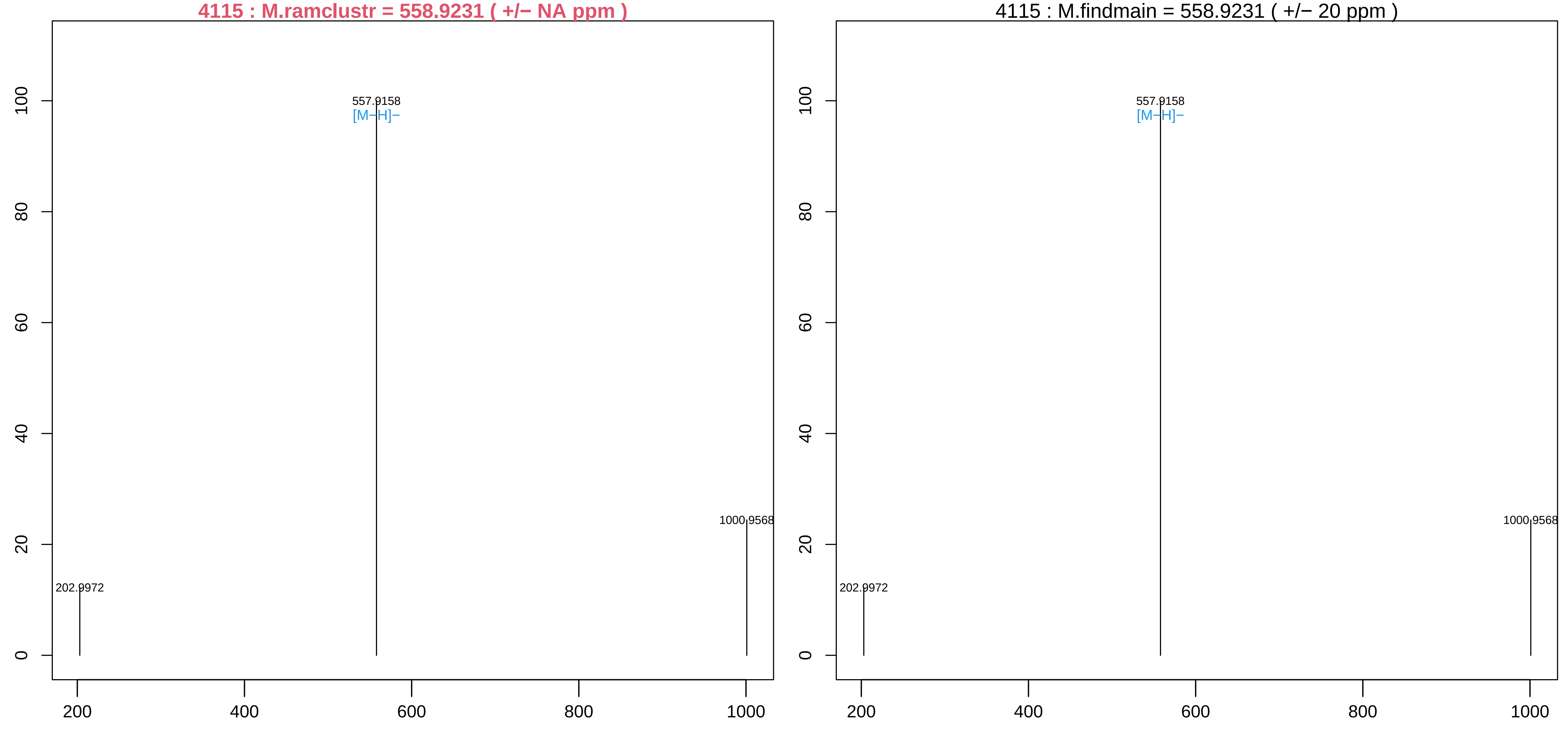

4115 : M.ramclustr = 558.9231 (+/− NA ppm)
557.9158
[M−H]−
1000.9568
202.9972
4115 : M.findmain = 558.9231 (+/− 20 ppm)
557.9158
[M−H]−
1000.9568
202.9972

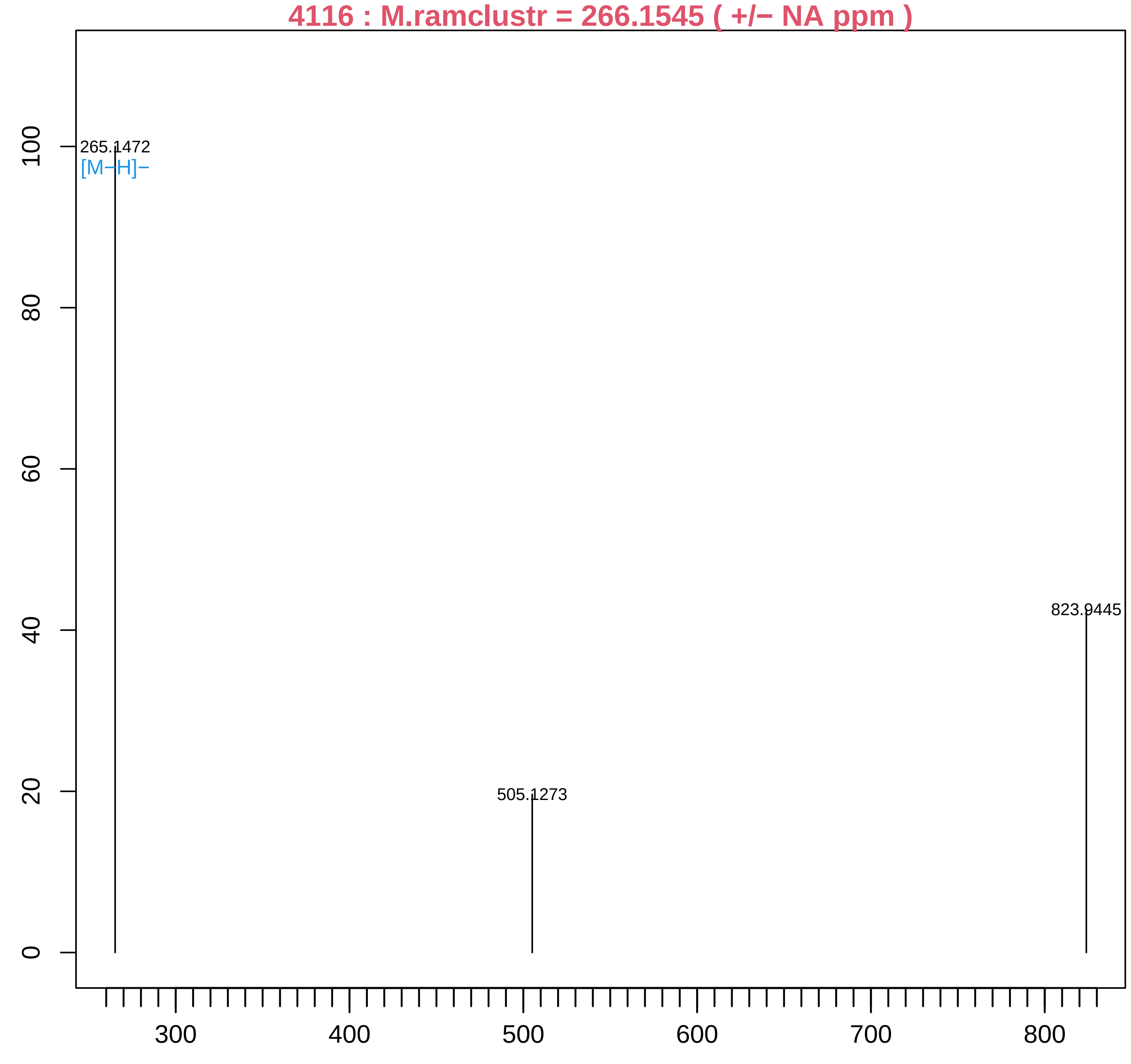
4116 : M.ramclustr = 266.1545 (+/− NA ppm)
265.1472
[M−H]−
505.1273
823.9445
0
20
40
60
80
100
300
400
500
600
700
800

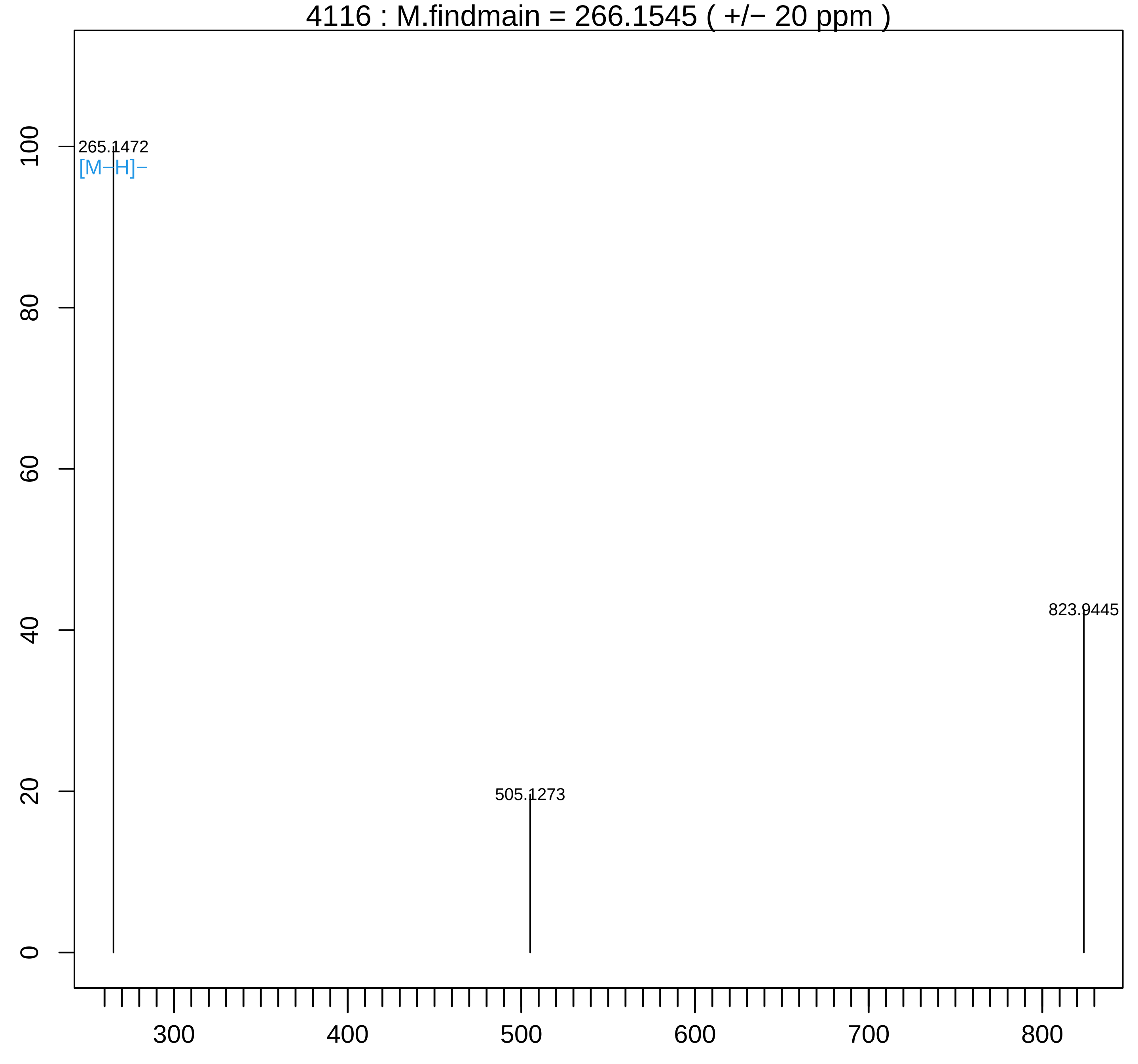
4116 : M.findmain = 266.1545 (+/− 20 ppm)
265.1472
[M−H]−
505.1273
823.9445
0
20
40
60
80
100
300
400
500
600
700
800

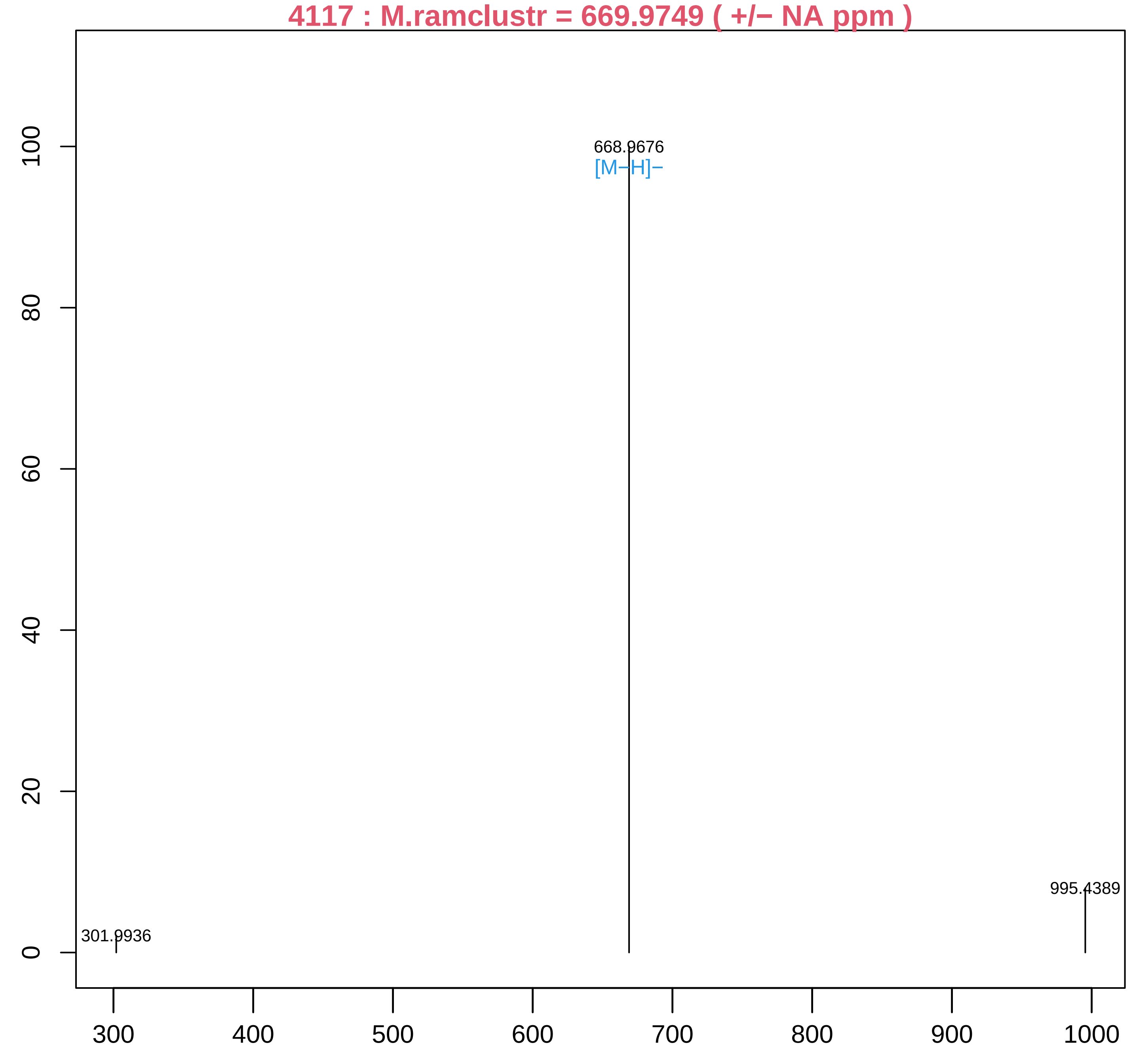
4117 : M.ramclustr = 669.9749 (+/− NA ppm)
668.9676
[M−H]−
995.4389
301.9936
0
20
40
60
80
100
300
400
500
600
700
800
900
1000

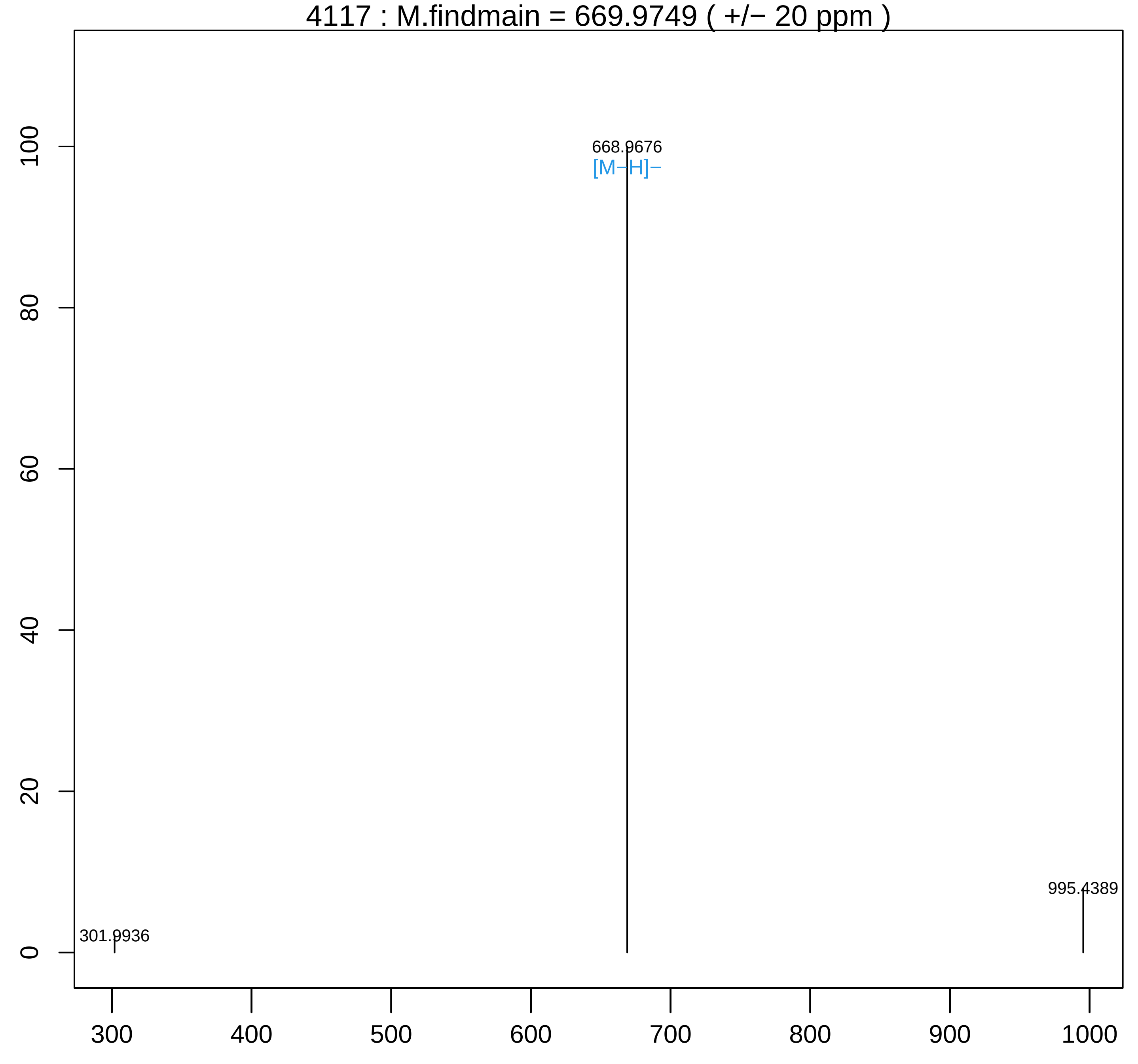
4117 : M.findmain = 669.9749 (+/− 20 ppm)
668.9676
[M−H]−
995.4389
301.9936
0
20
40
60
80
100
300
400
500
600
700
800
900
1000

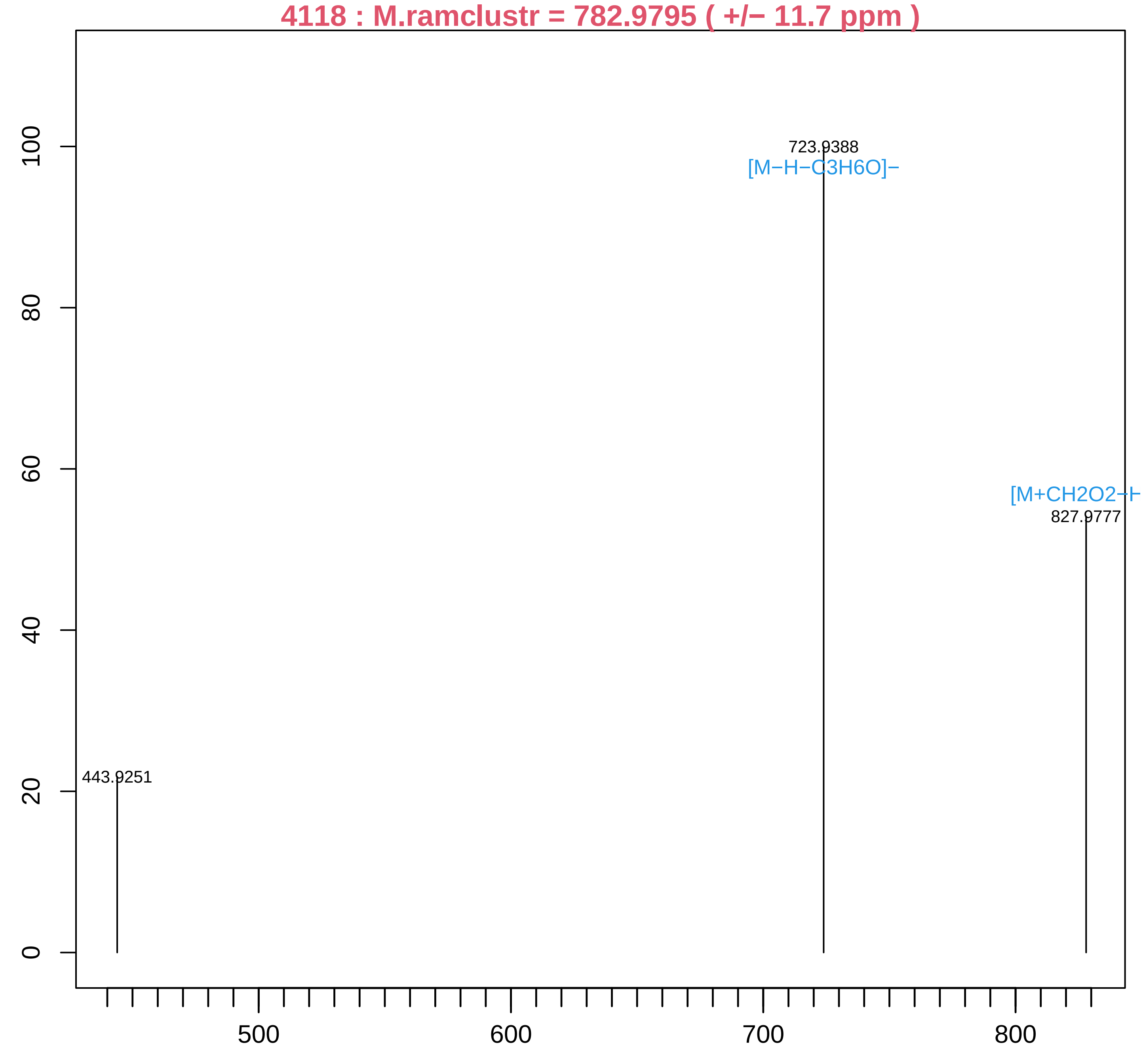
4118 : M.ramclustr = 782.9795 (+/− 11.7 ppm)
723.9388
[M−H−C3H6O]−
[M+CH2O2−H
827.9777
443.9251
0
20
40
60
80
100
500
600
700
800

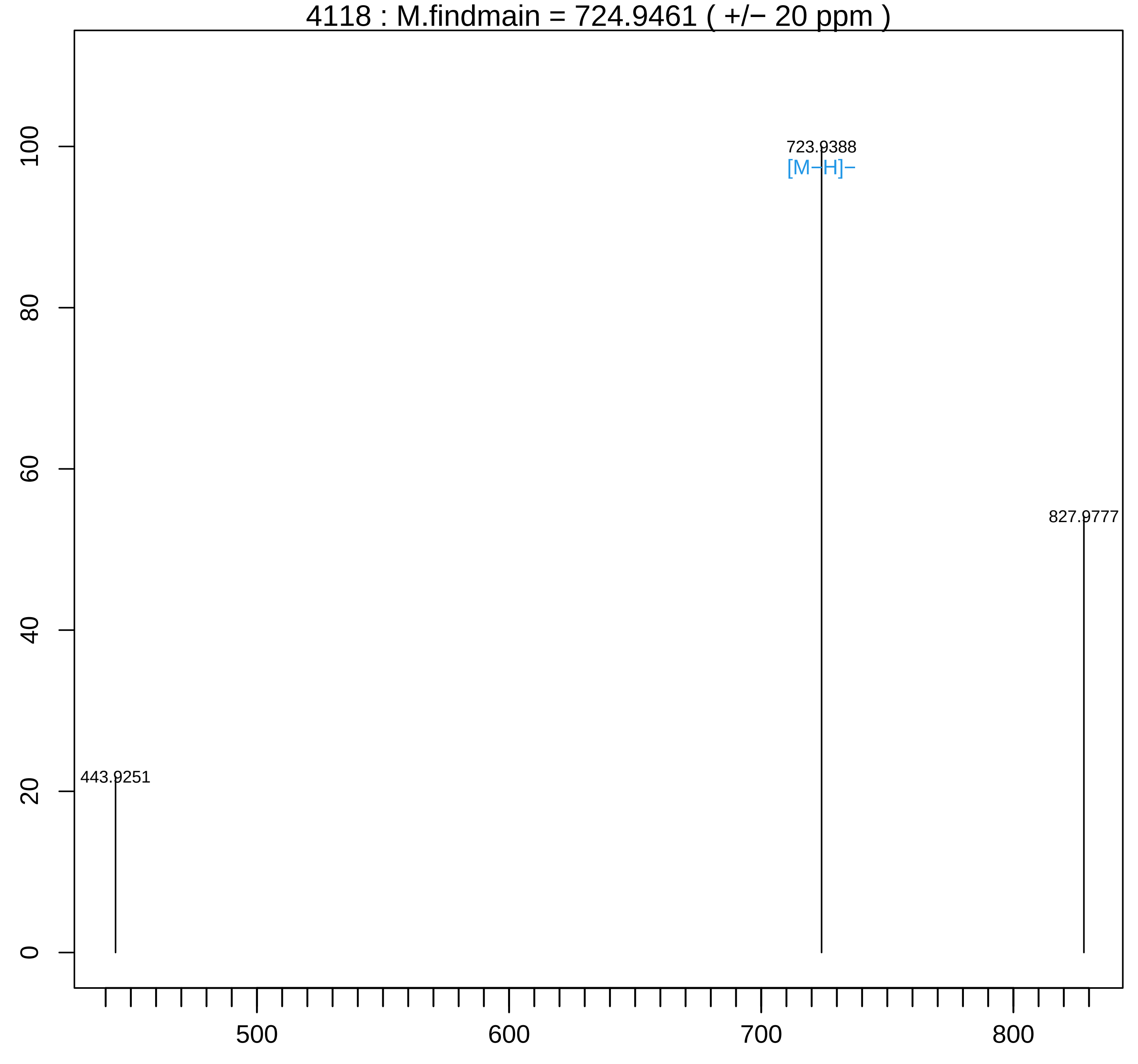
4118 : M.findmain = 724.9461 (+/− 20 ppm)
723.9388
[M−H]−
827.9777
443.9251
0
20
40
60
80
100
500
600
700
800

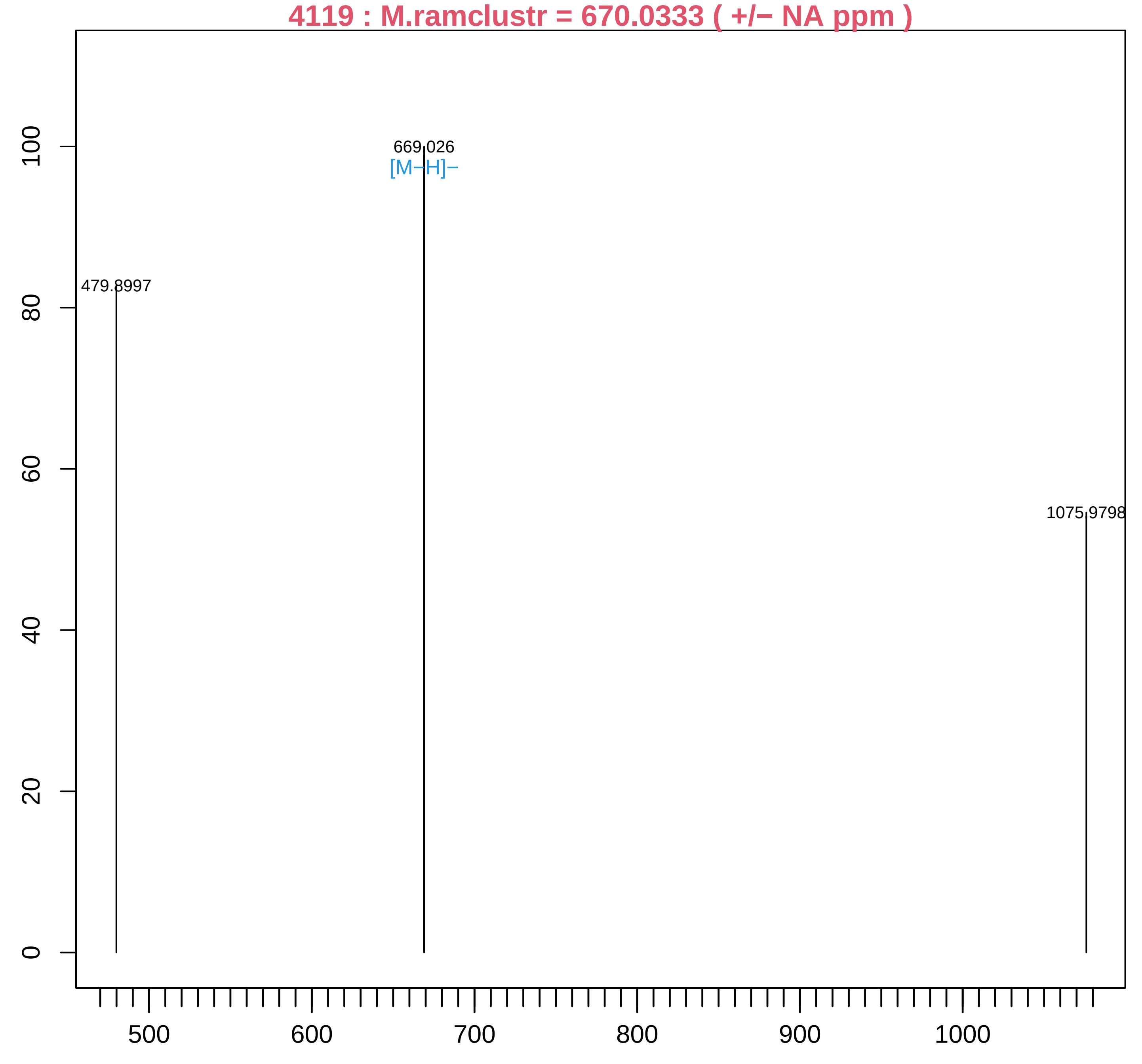
4119 : M.ramclustr = 670.0333 (+/− NA ppm)
669.026
[M−H]−
479.8997
1075.9798
0
20
40
60
80
100
500
600
700
800
900
1000

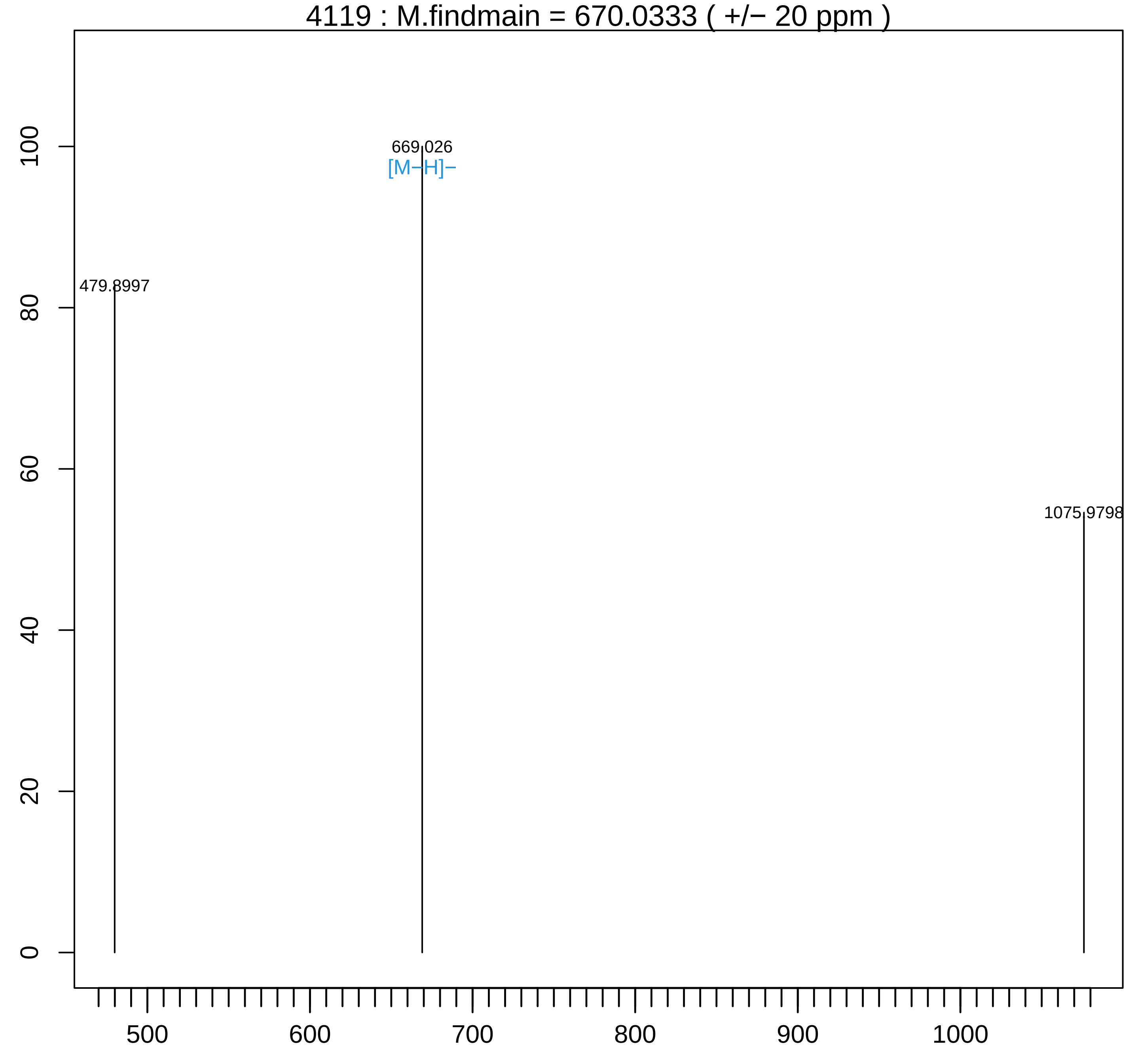
4119 : M.findmain = 670.0333 (+/− 20 ppm)
669.026
[M−H]−
479.8997
1075.9798
0
20
40
60
80
100
500
600
700
800
900
1000

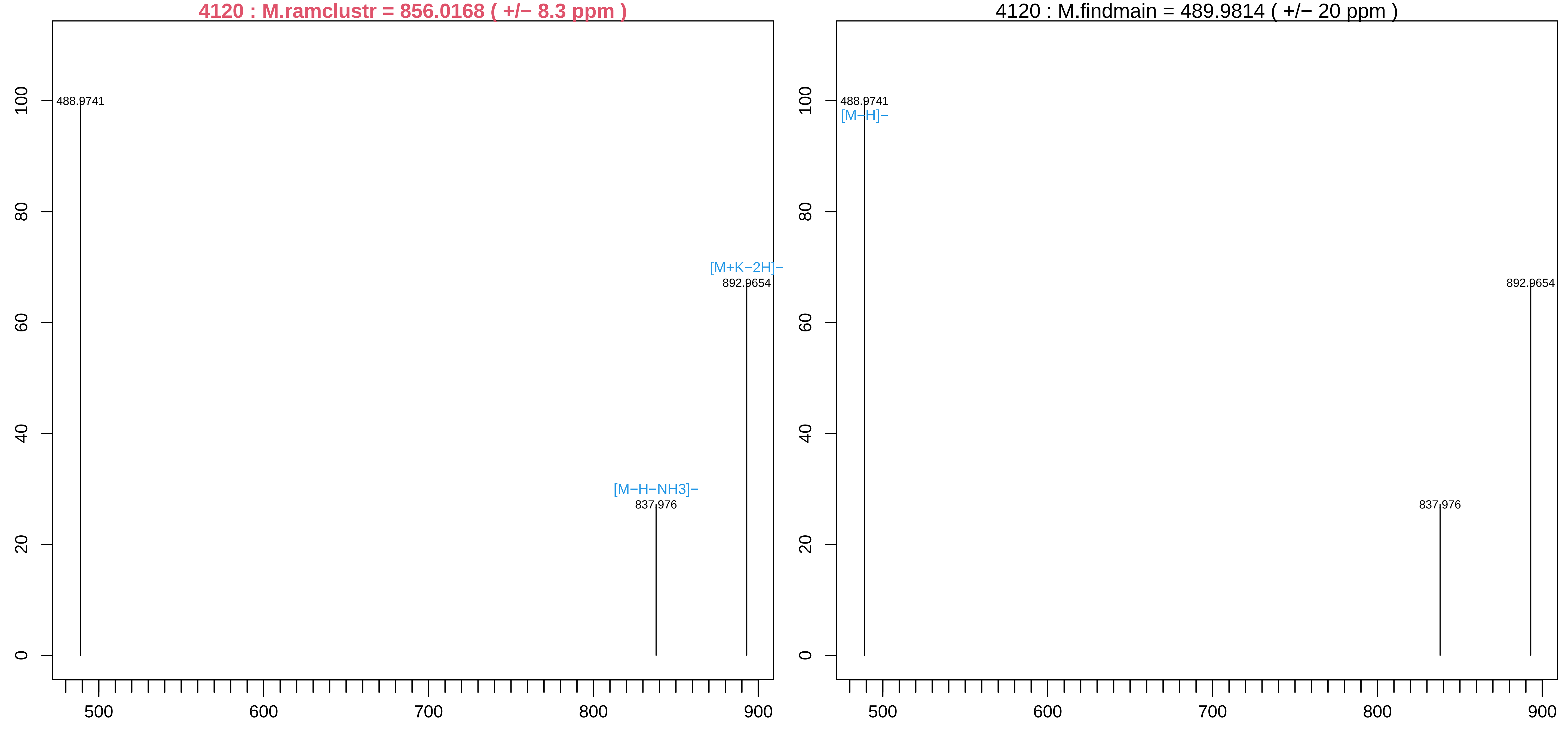
4120 : M.ramclustr = 856.0168 (+/− 8.3 ppm)
488.9741
[M+K−2H]−
892.9654
[M−H−NH3]−
837.976
0
20
40
60
80
100
500
600
700
800
900
4120 : M.findmain = 489.9814 (+/− 20 ppm)
488.9741
[M−H]−
892.9654
837.976

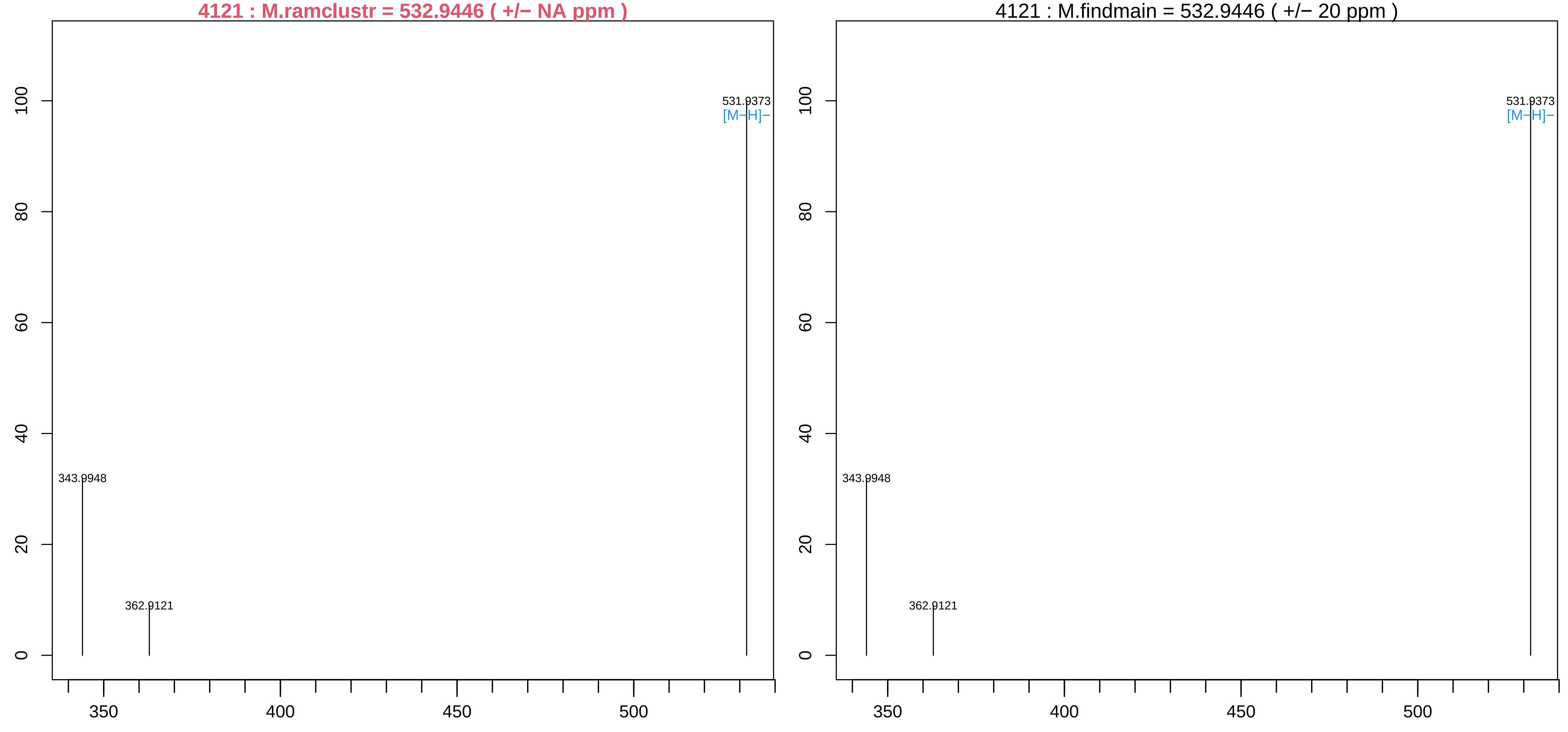
4121 : M.ramclustr = 532.9446 (+/− NA ppm)
531.9373
[M−H]−
343.9948
362.9121
4121 : M.findmain = 532.9446 (+/− 20 ppm)
531.9373
[M−H]−
343.9948
362.9121

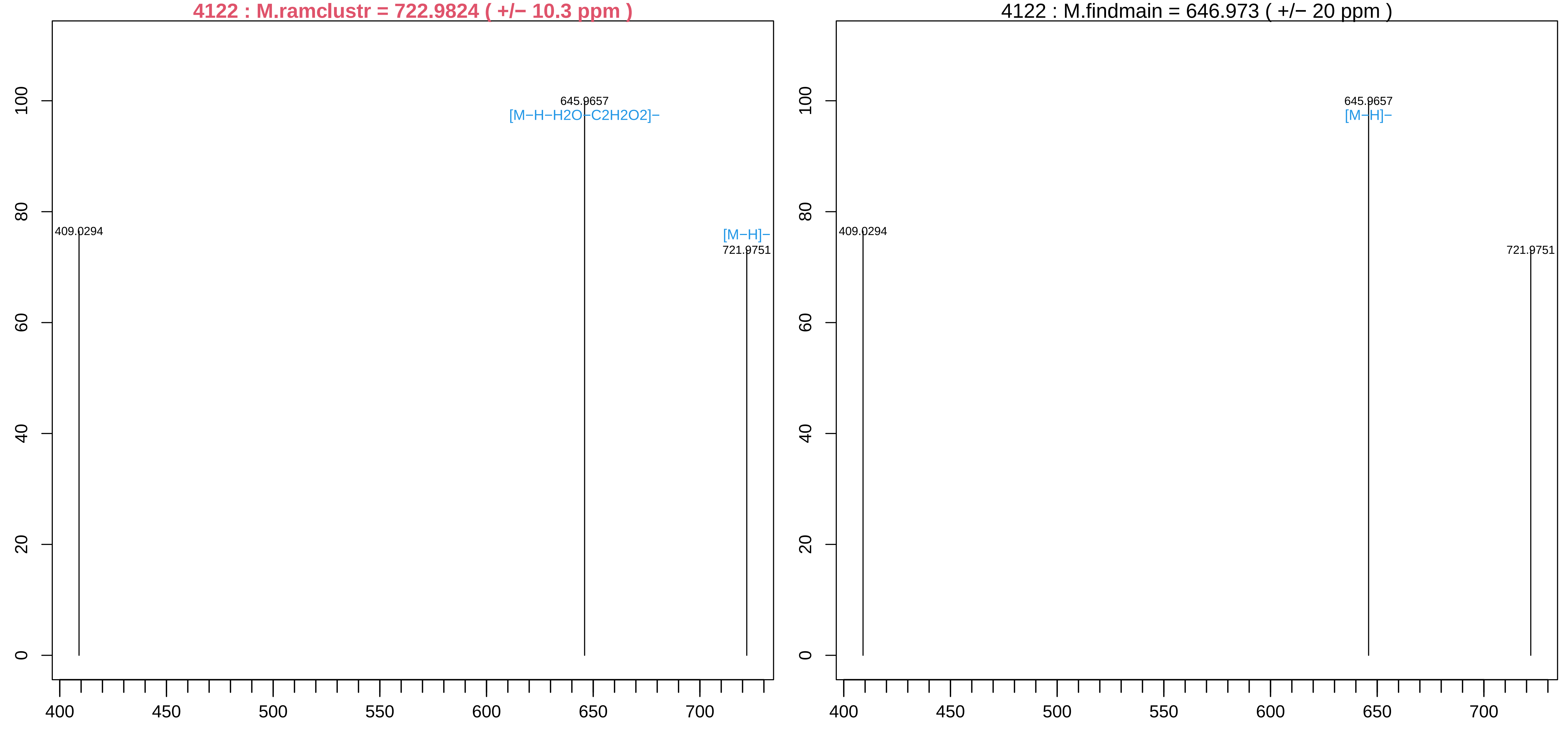
4122 : M.ramclustr = 722.9824 (+/− 10.3 ppm)
645.9657
[M−H−H2O−C2H2O2]−
409.0294
[M−H]−
721.9751
4122 : M.findmain = 646.973 (+/− 20 ppm)
645.9657
[M−H]−
409.0294
721.9751

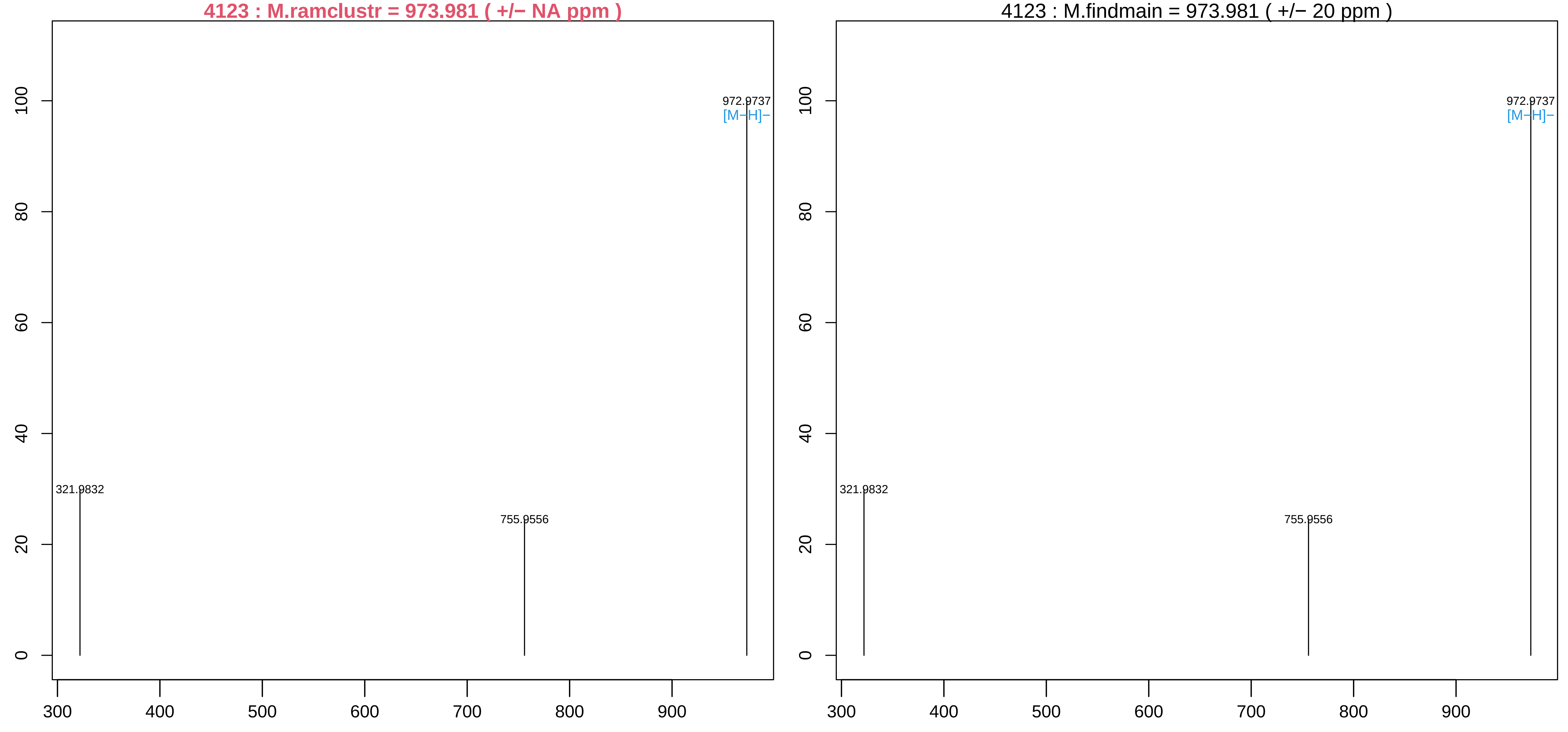
4123 : M.ramclustr = 973.981 (+/− NA ppm)
4123 : M.findmain = 973.981 (+/− 20 ppm)
972.9737
[M−H]−
321.9832
755.9556
0
20
40
60
80
100
300
400
500
600
700
800
900

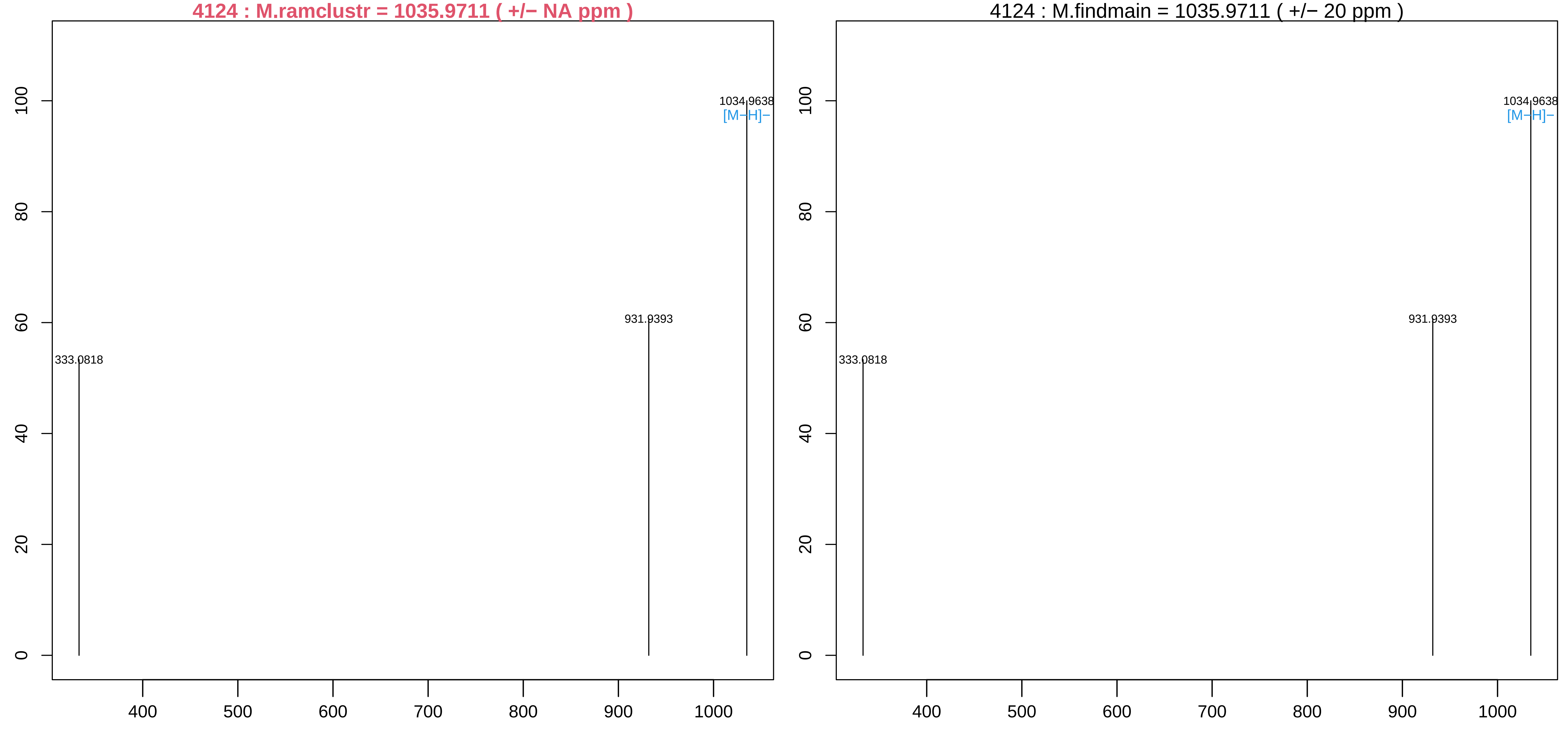
4124 : M.ramclustr = 1035.9711 (+/− NA ppm)
1034.9638
[M−H]−
931.9393
333.0818
0
20
40
60
80
100
400
500
600
700
800
900
1000
4124 : M.findmain = 1035.9711 (+/− 20 ppm)
1034.9638
[M−H]−
931.9393
333.0818
0
20
40
60
80
100
400
500
600
700
800
900
1000

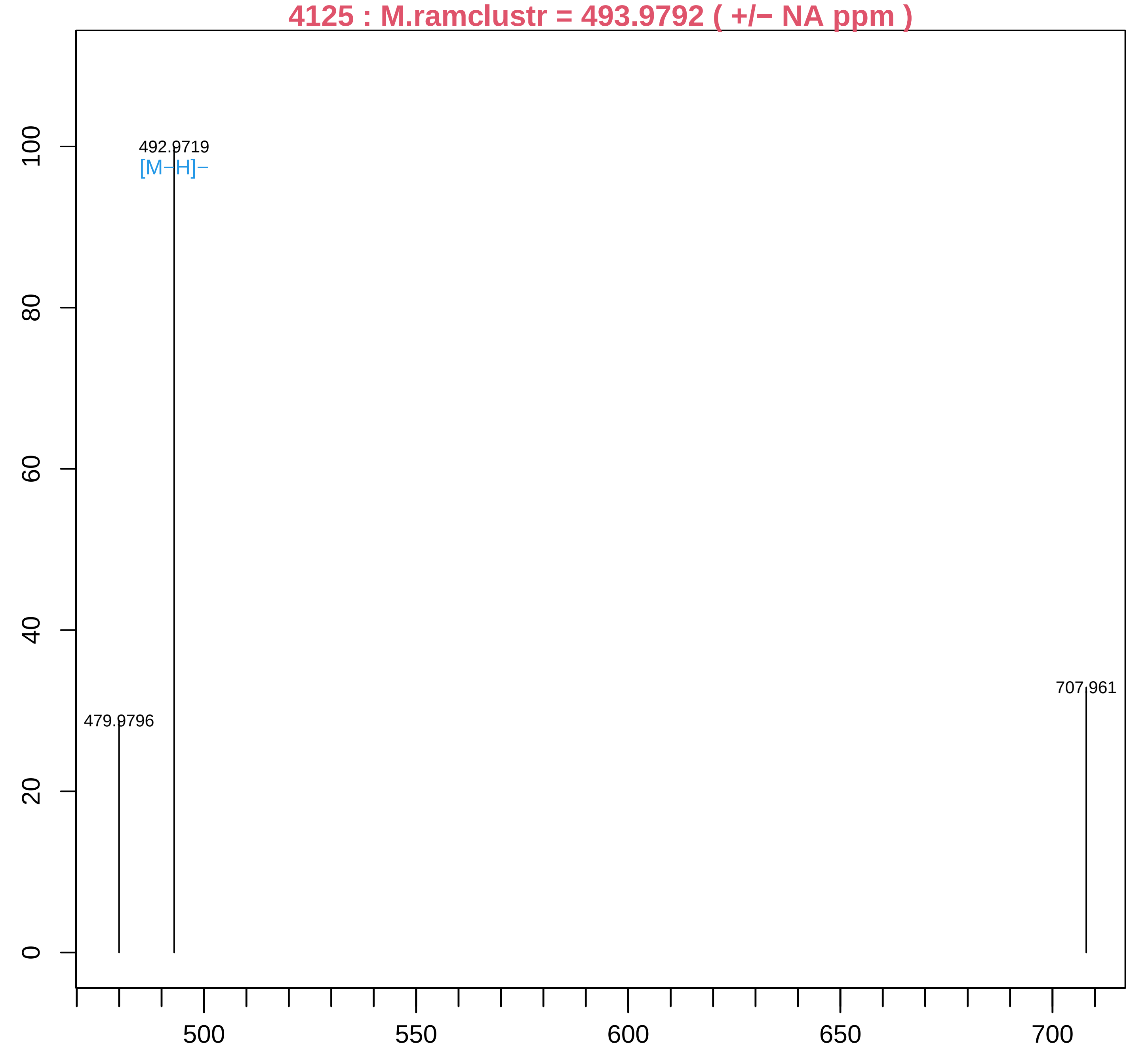
4125 : M.ramclustr = 493.9792 (+/− NA ppm)
492.9719
[M−H]−
479.9796
707.961
0
20
40
60
80
100
500
550
600
650
700

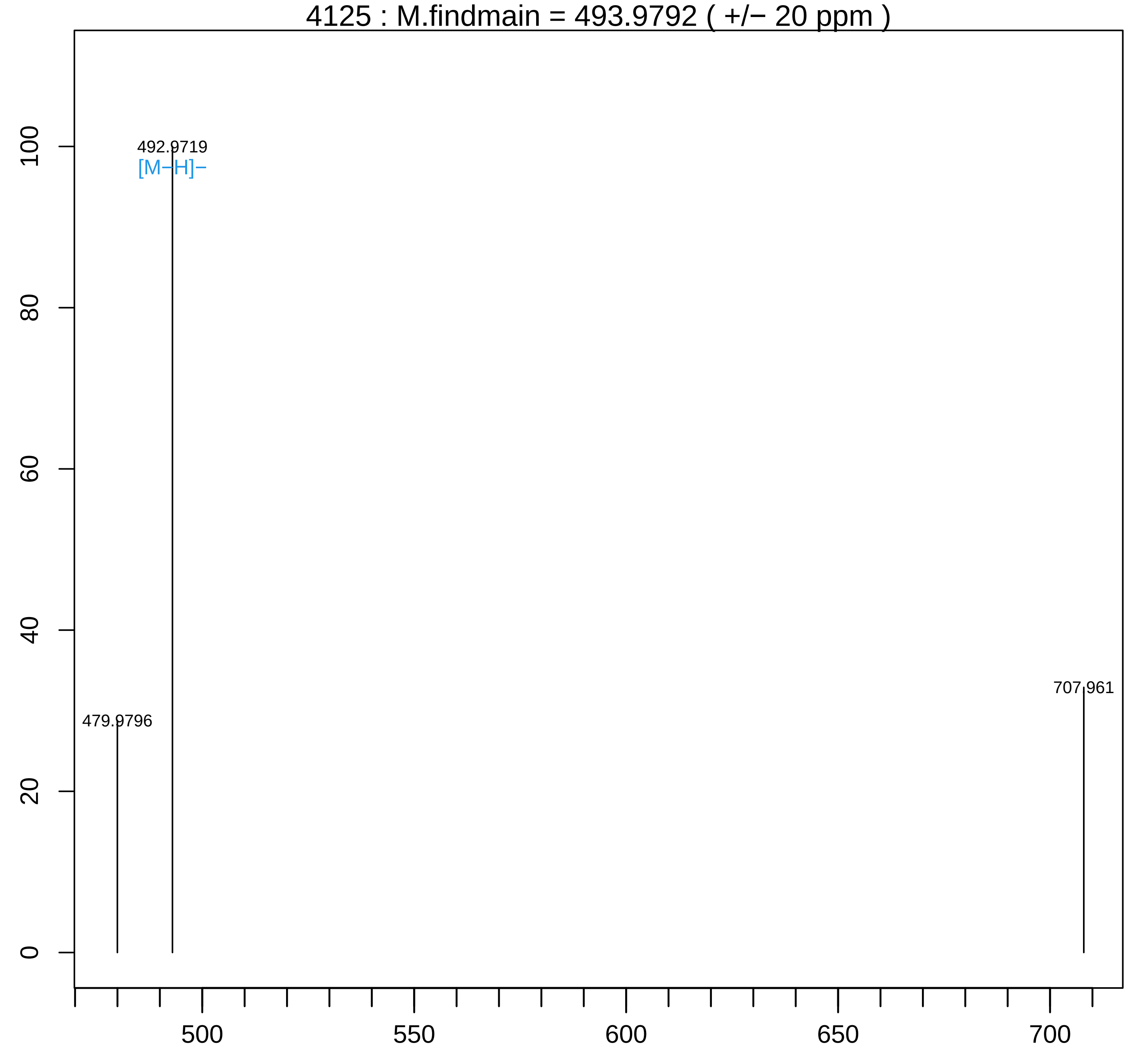
4125 : M.findmain = 493.9792 (+/− 20 ppm)
492.9719
[M−H]−
479.9796
707.961
0
20
40
60
80
100
500
550
600
650
700

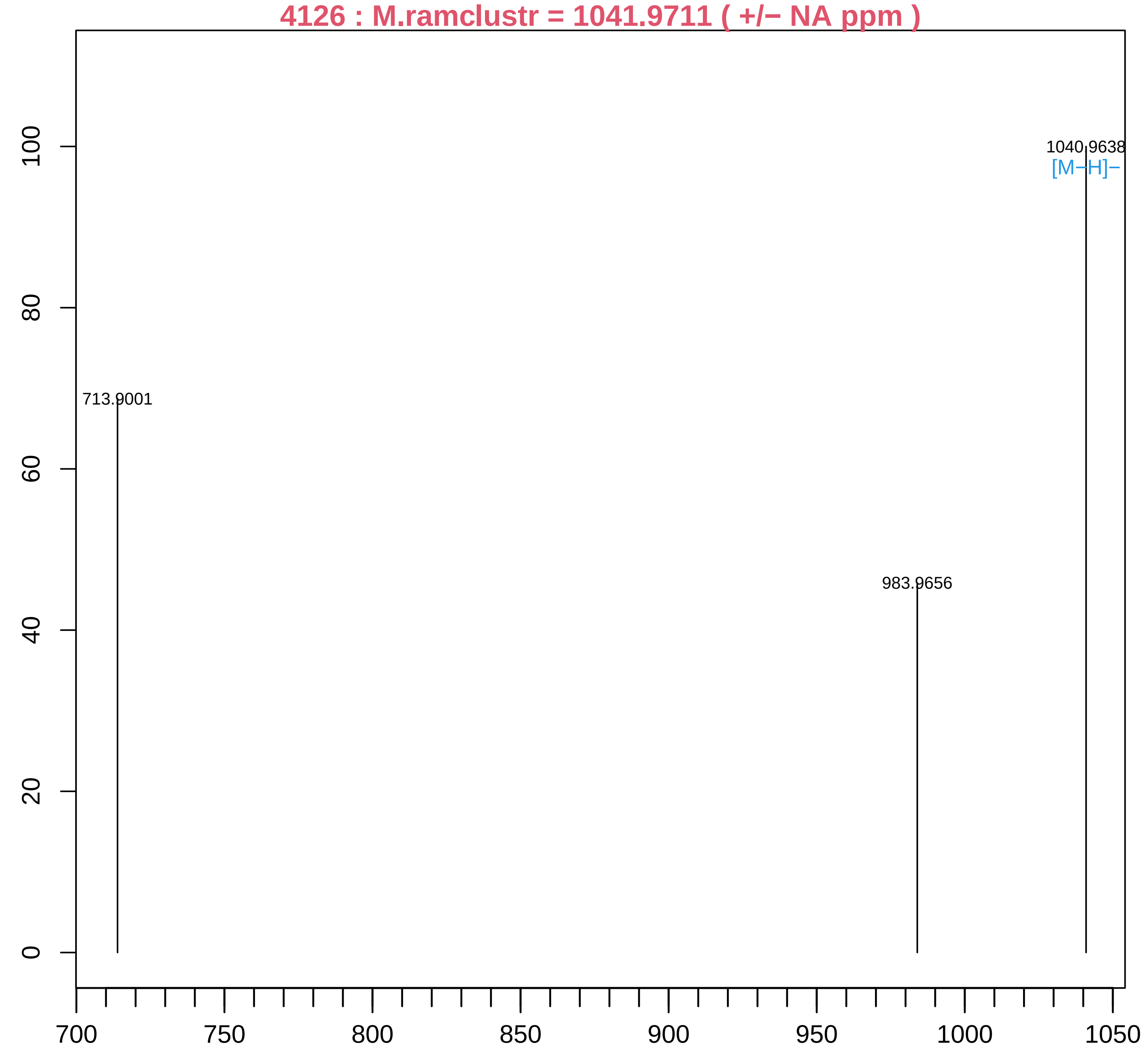
4126 : M.ramclustr = 1041.9711 (+/− NA ppm)
1040.9638
[M−H]−
713.9001
983.9656
0
20
40
60
80
100
700
750
800
850
900
950
1000
1050

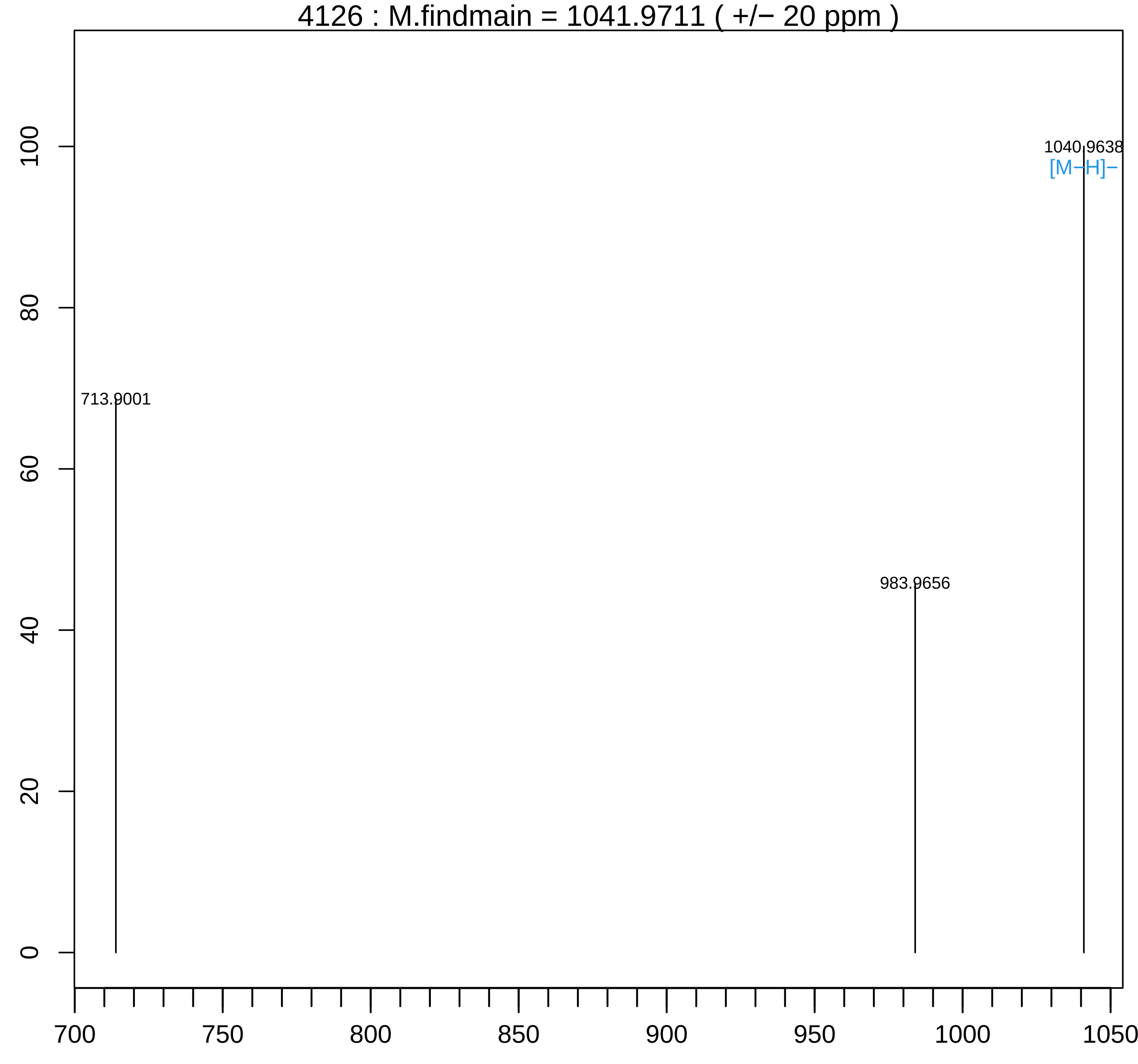
4126 : M.findmain = 1041.9711 (+/− 20 ppm)
1040.9638
[M−H]−
713.9001
983.9656
0
20
40
60
80
100
700
750
800
850
900
950
1000
1050

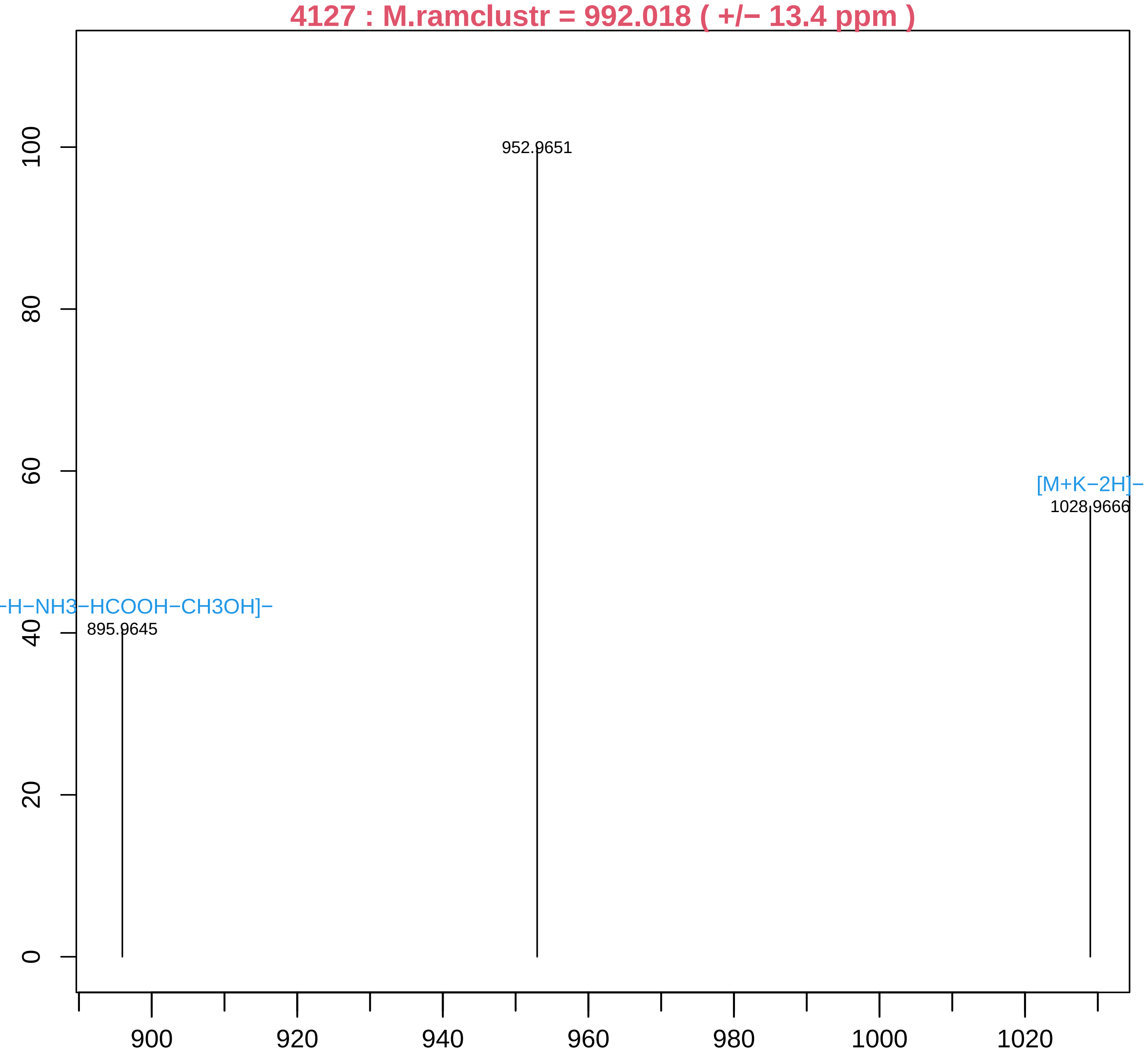
4127 : M.ramclustr = 992.018 (+/− 13.4 ppm)
952.9651
[M+K−2H]−
1028.9666
−H−NH3−HCOOH−CH3OH]−
895.9645
0
20
40
60
80
100
900
920
940
960
980
1000
1020

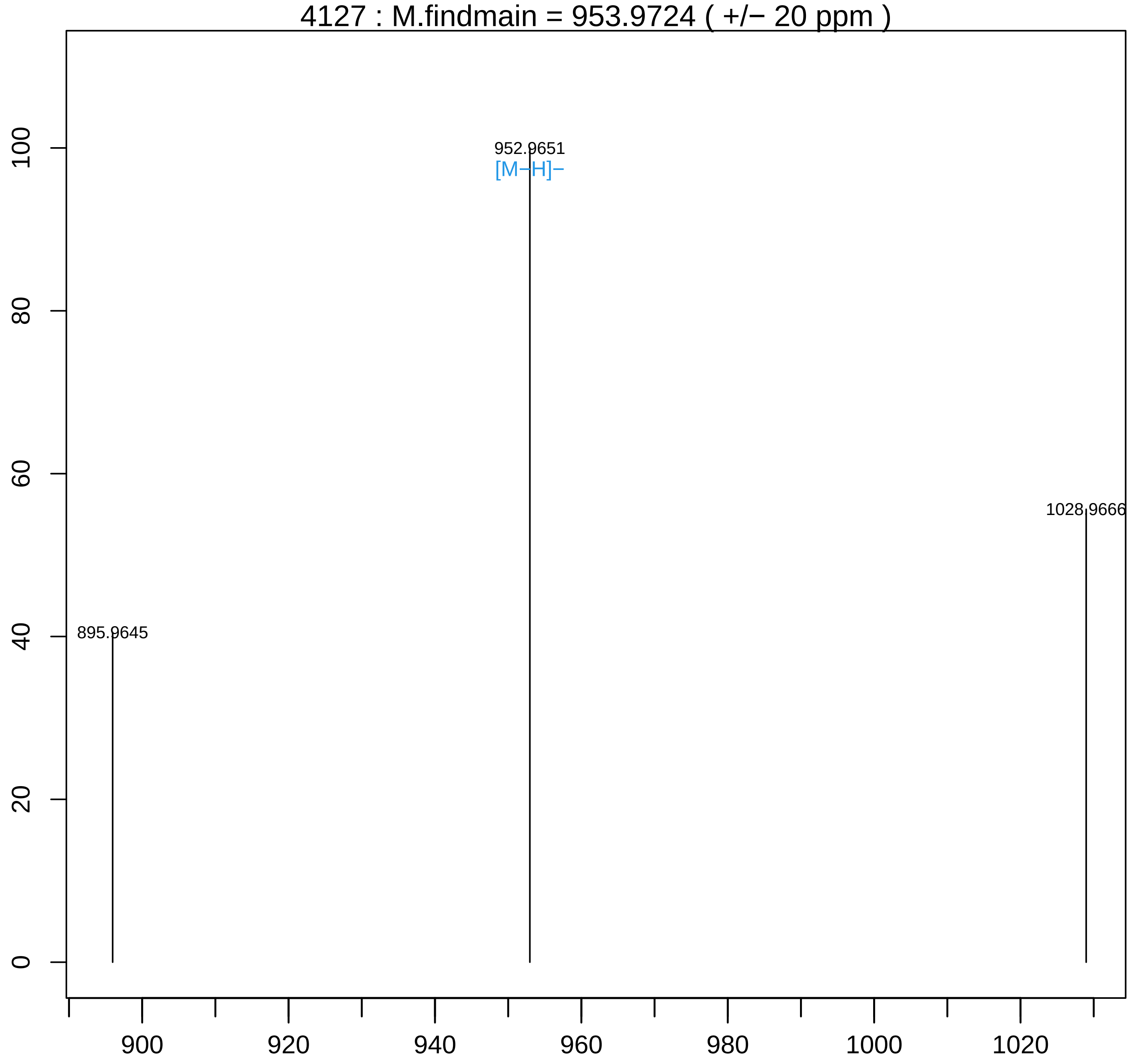
4127 : M.findmain = 953.9724 (+/− 20 ppm)
952.9651
[M−H]−
1028.9666
895.9645
0
20
40
60
80
100
900
920
940
960
980
1000
1020

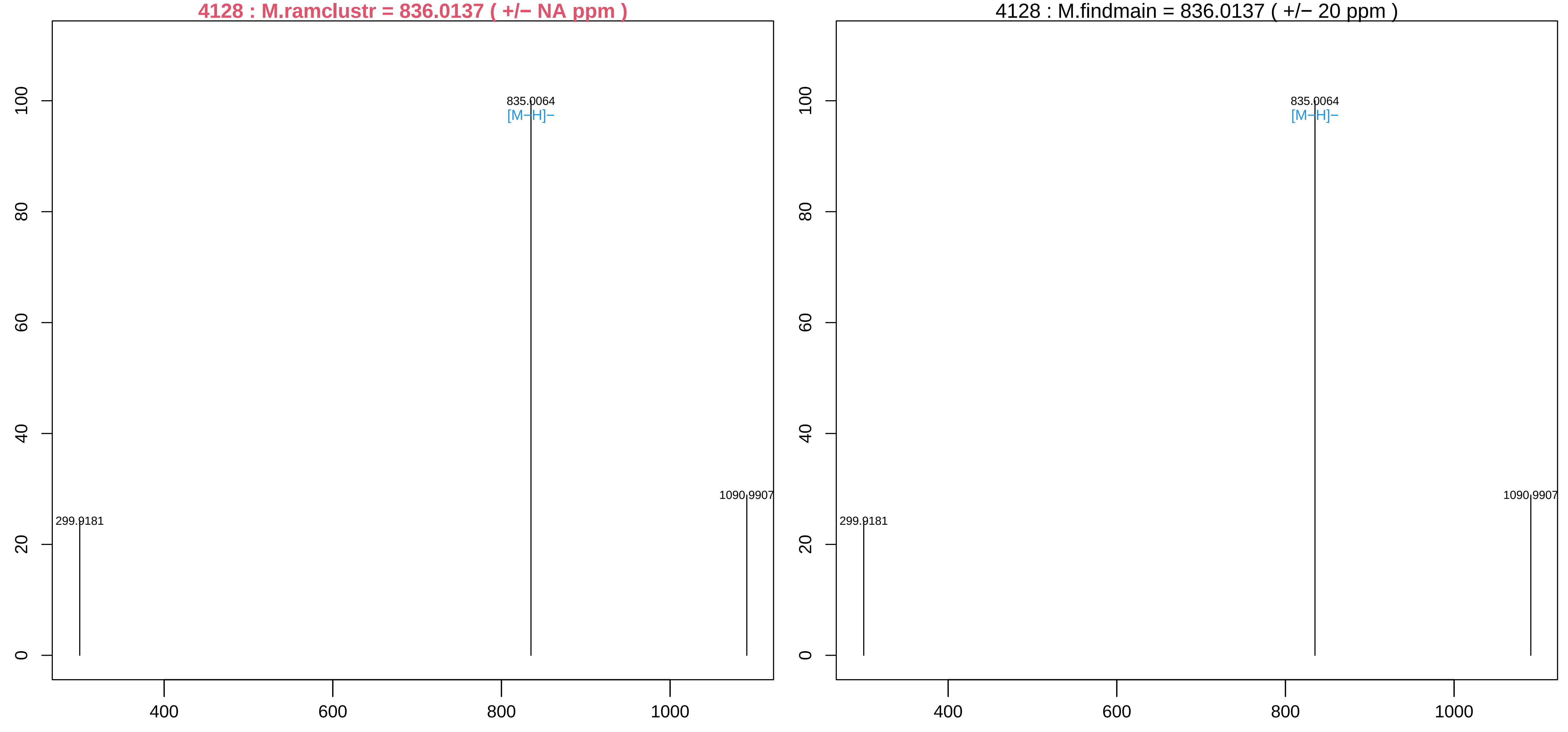

4128 : M.ramclustr = 836.0137 (+/− NA ppm)
4128 : M.findmain = 836.0137 (+/− 20 ppm)
835.0064
[M−H]−
299.9181
1090.9907
0
20
40
60
80
100
400
600
800
1000

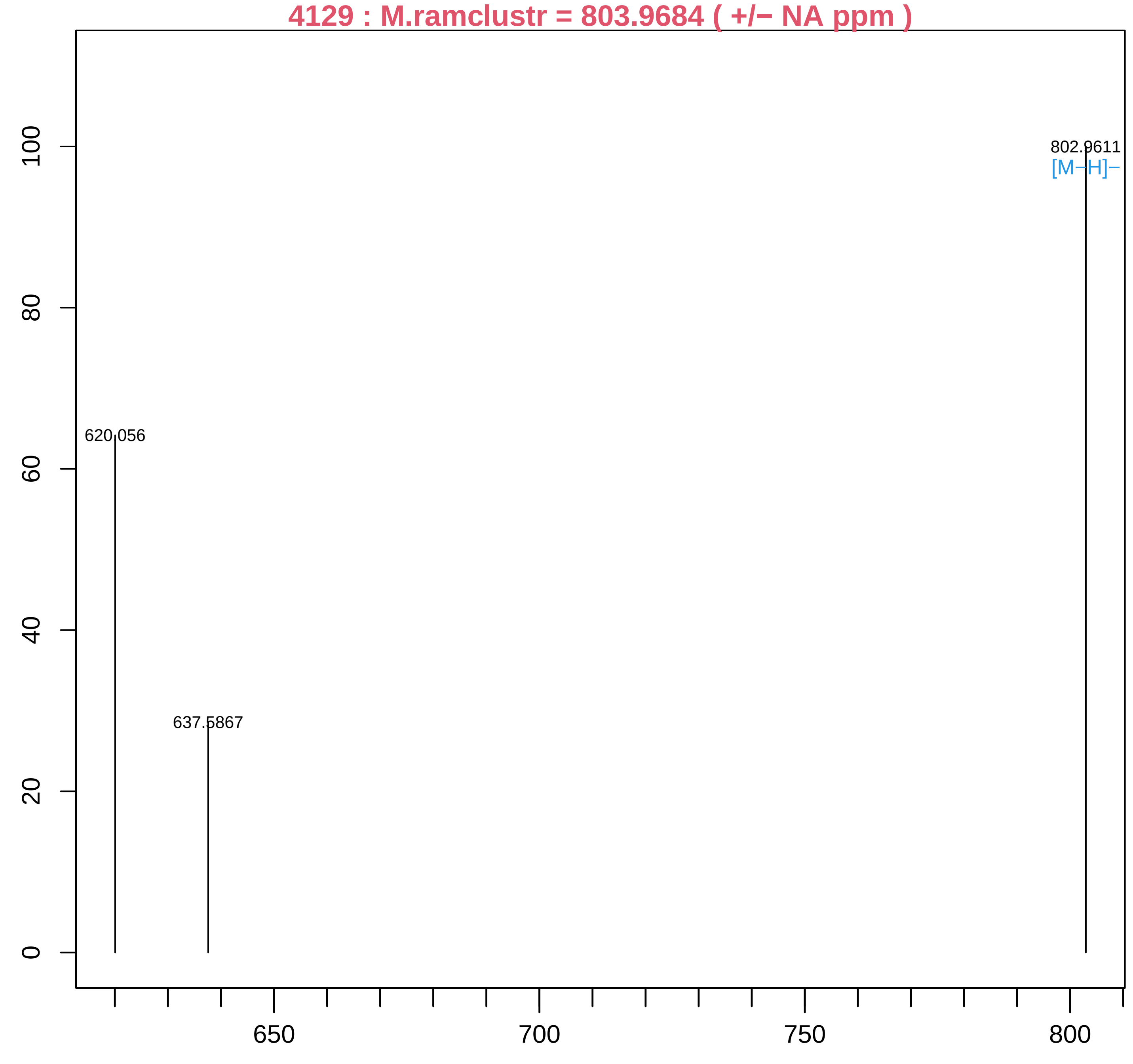

4129 : M.ramclustr = 803.9684 (+/− NA ppm)
802.9611
[M−H]−
620.056
637.5867
0
20
40
60
80
100
650
700
750
800

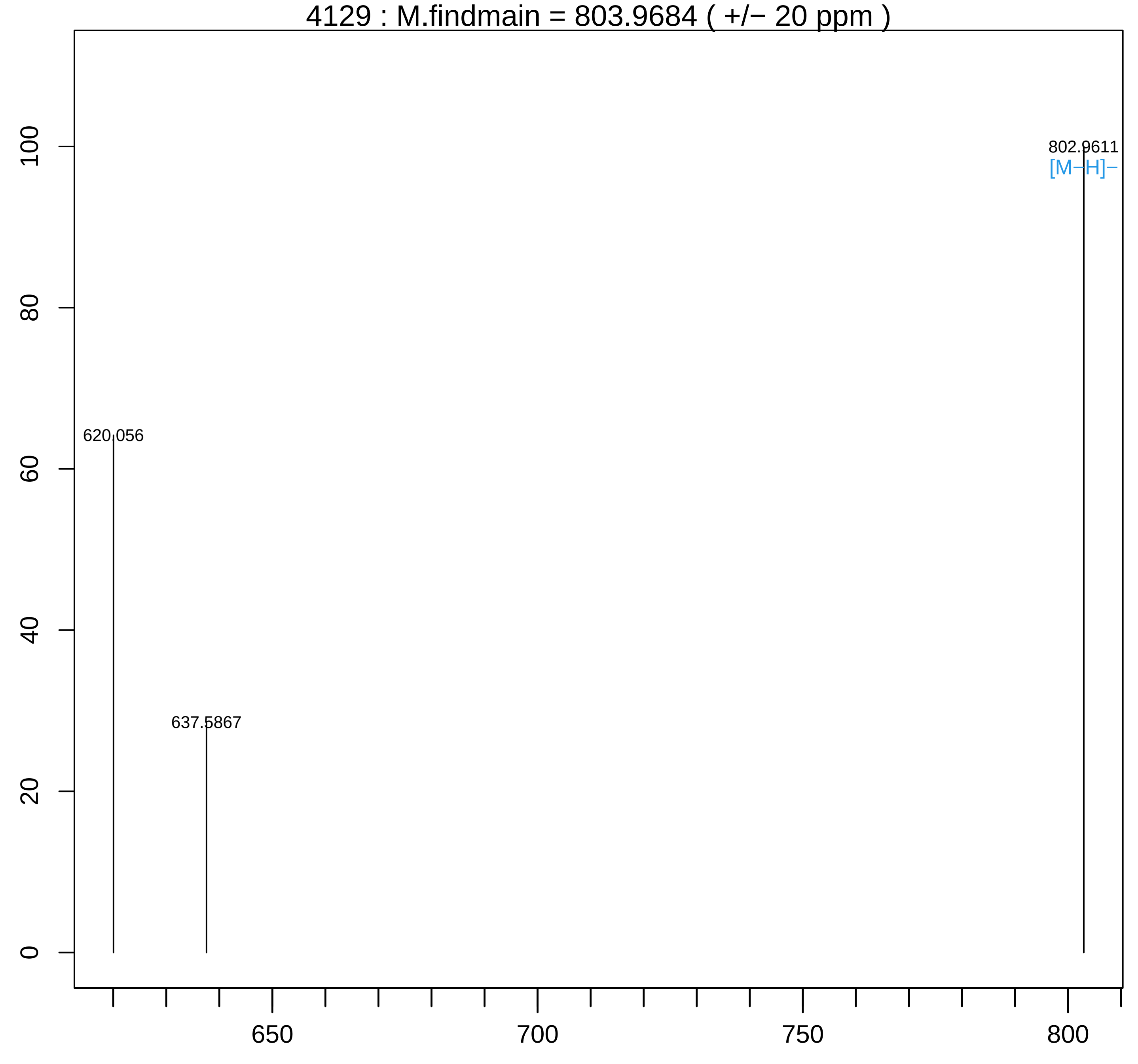

4129 : M.findmain = 803.9684 (+/− 20 ppm)
802.9611
[M−H]−
620.056
637.5867
0
20
40
60
80
100
650
700
750
800

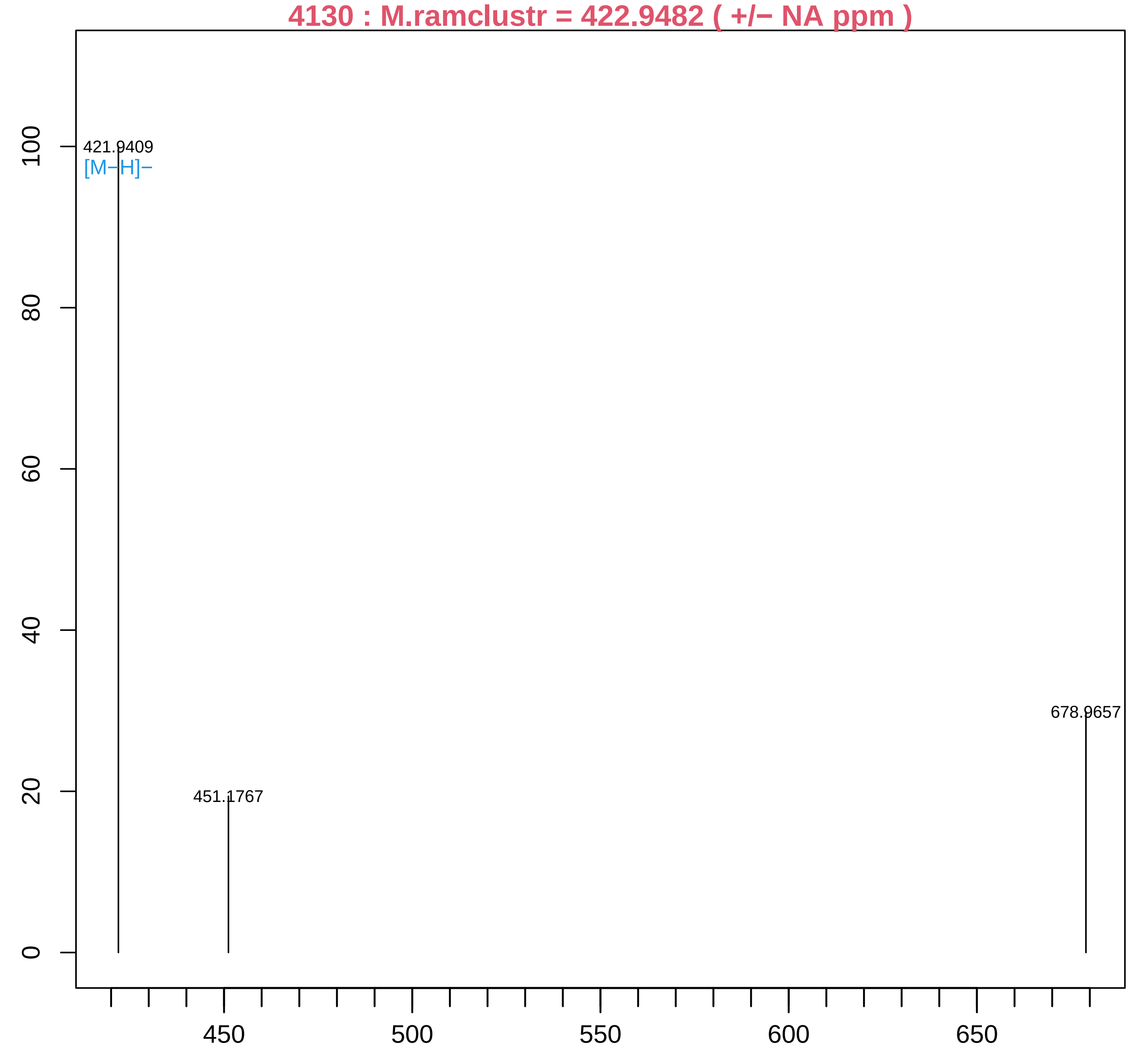

4130 : M.ramclustr = 422.9482 (+/− NA ppm)
421.9409
[M−H]−
451.1767
678.9657
0
20
40
60
80
100
450
500
550
600
650

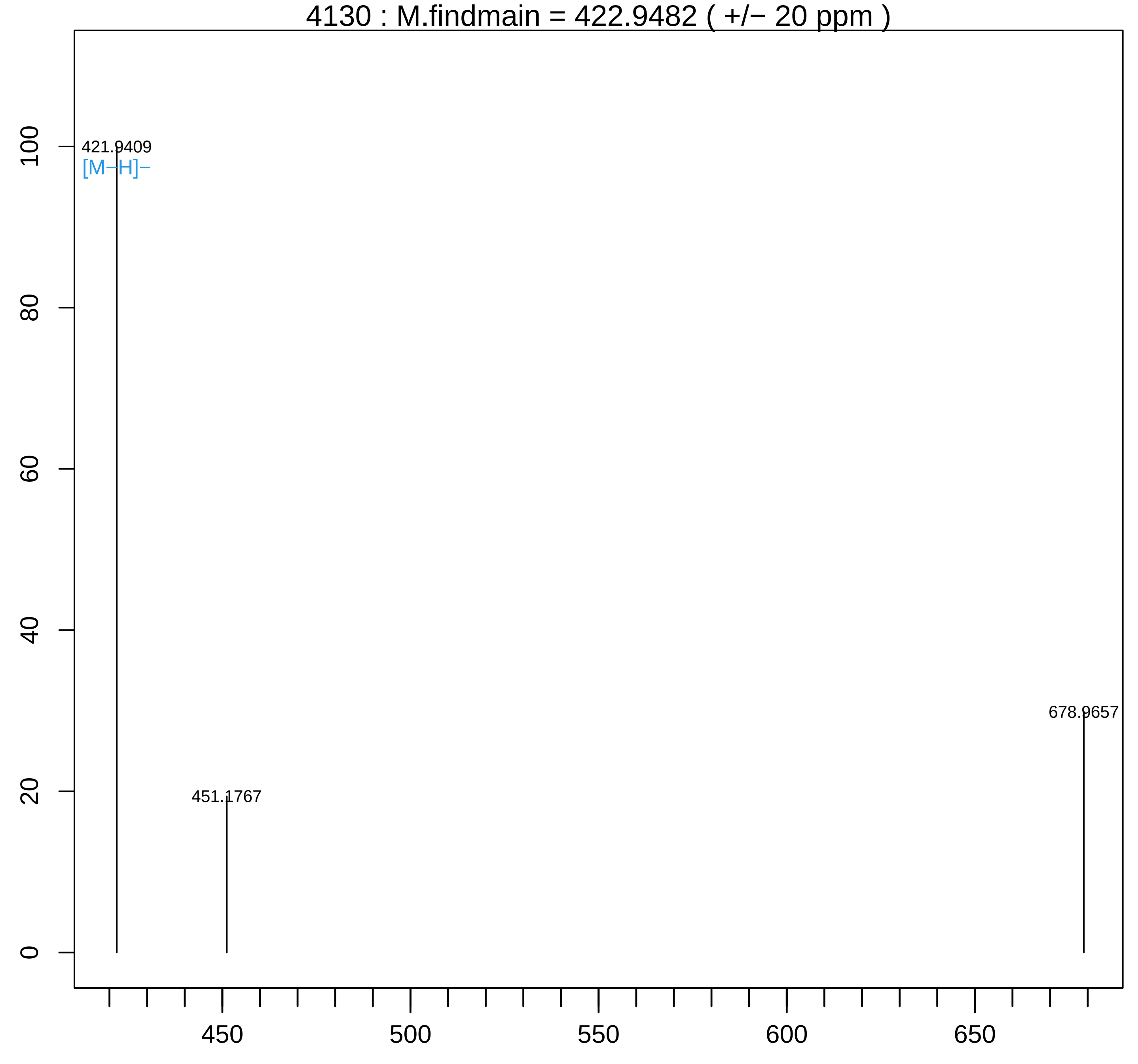

4130 : M.findmain = 422.9482 (+/− 20 ppm)
421.9409
[M−H]−
451.1767
678.9657
0
20
40
60
80
100
450
500
550
600
650

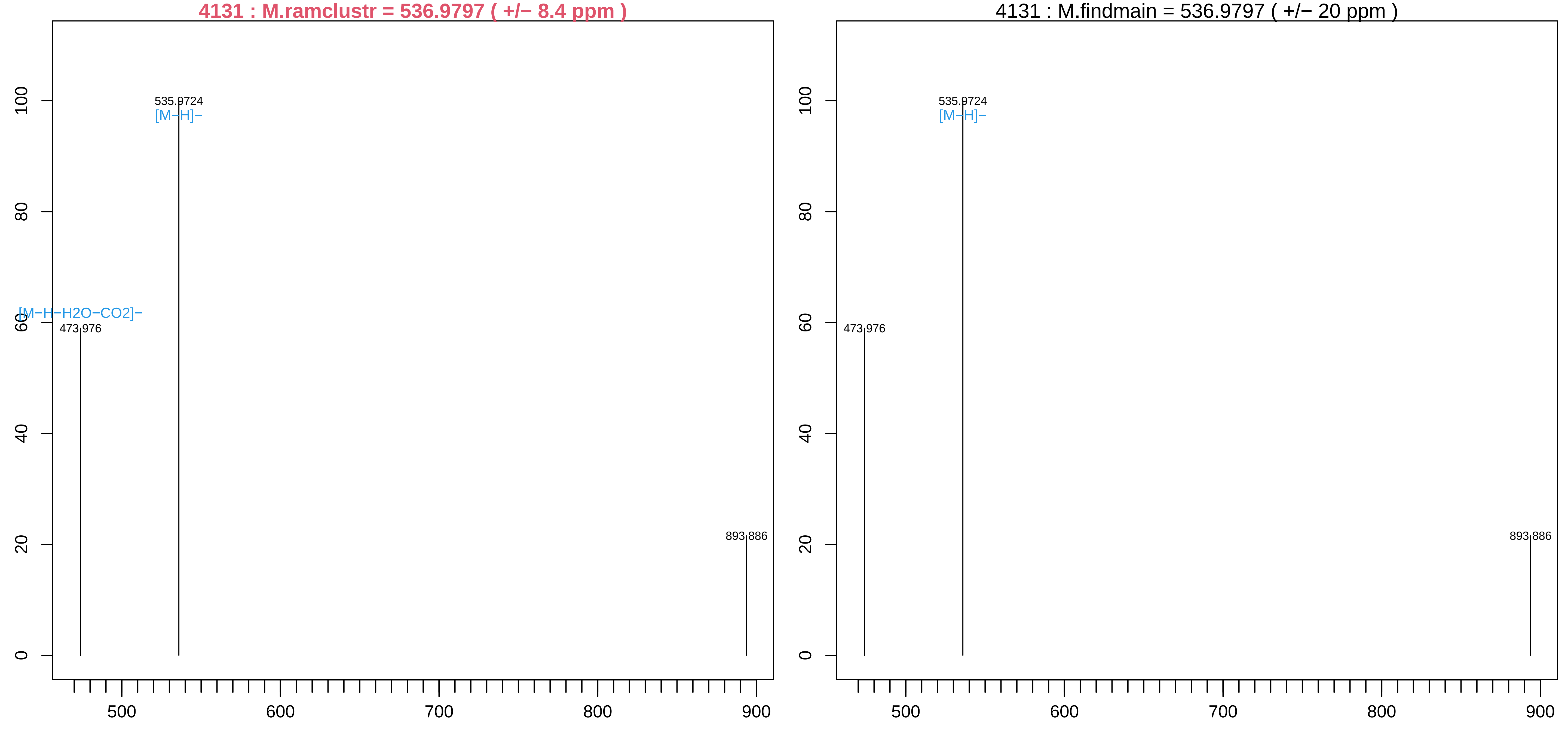
4131 : M.ramclustr = 536.9797 (+/− 8.4 ppm)
535.9724
[M−H]−
[M−H−H2O−CO2]−
473.976
893.886
0
20
40
60
80
100
500
600
700
800
900
4131 : M.findmain = 536.9797 (+/− 20 ppm)
535.9724
[M−H]−
473.976
893.886
0
20
40
60
80
100
500
600
700
800
900

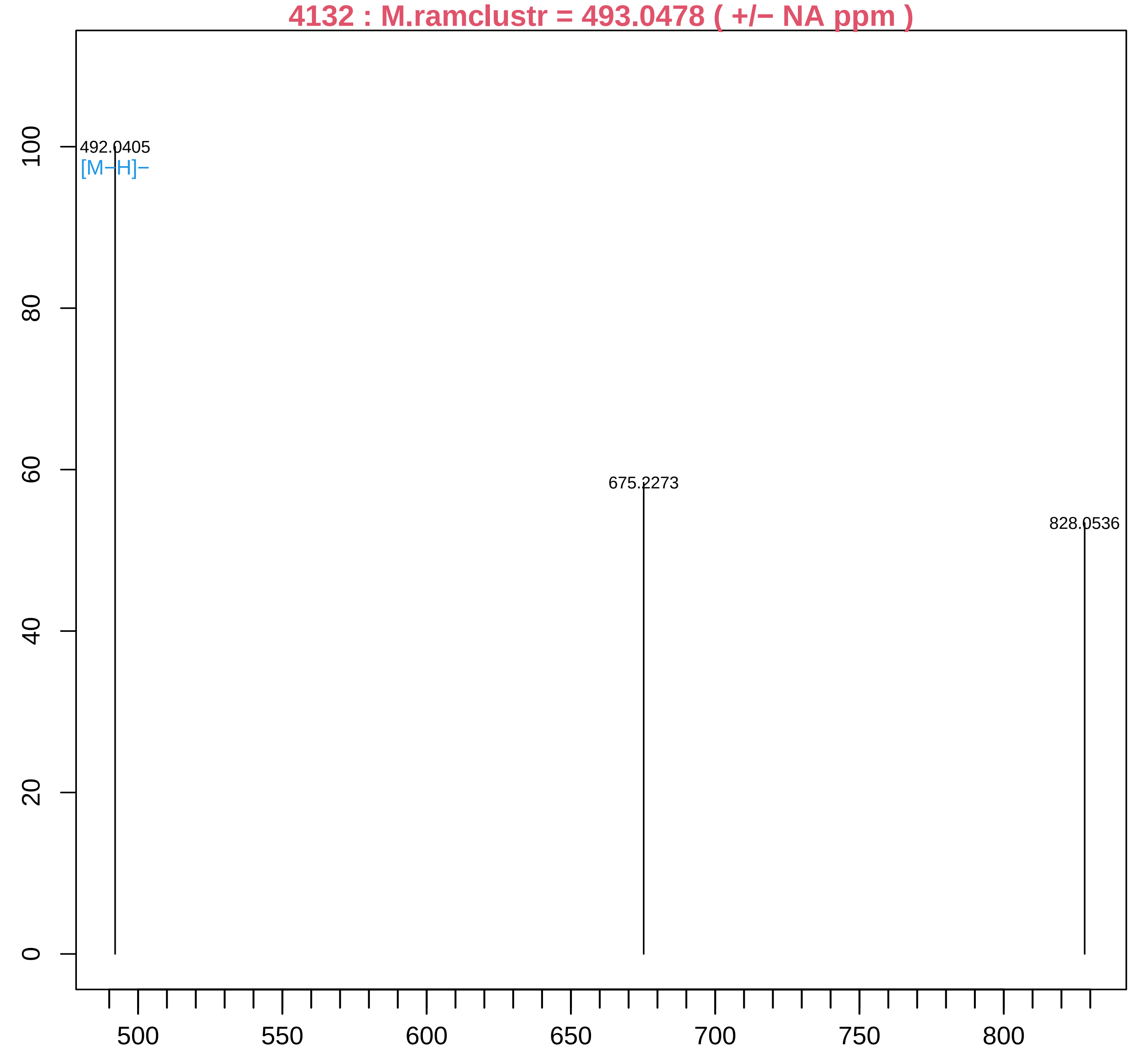
4132 : M.ramclustr = 493.0478 (+/− NA ppm)
492.0405
[M−H]−
675.2273
828.0536
0
20
40
60
80
100
500
550
600
650
700
750
800
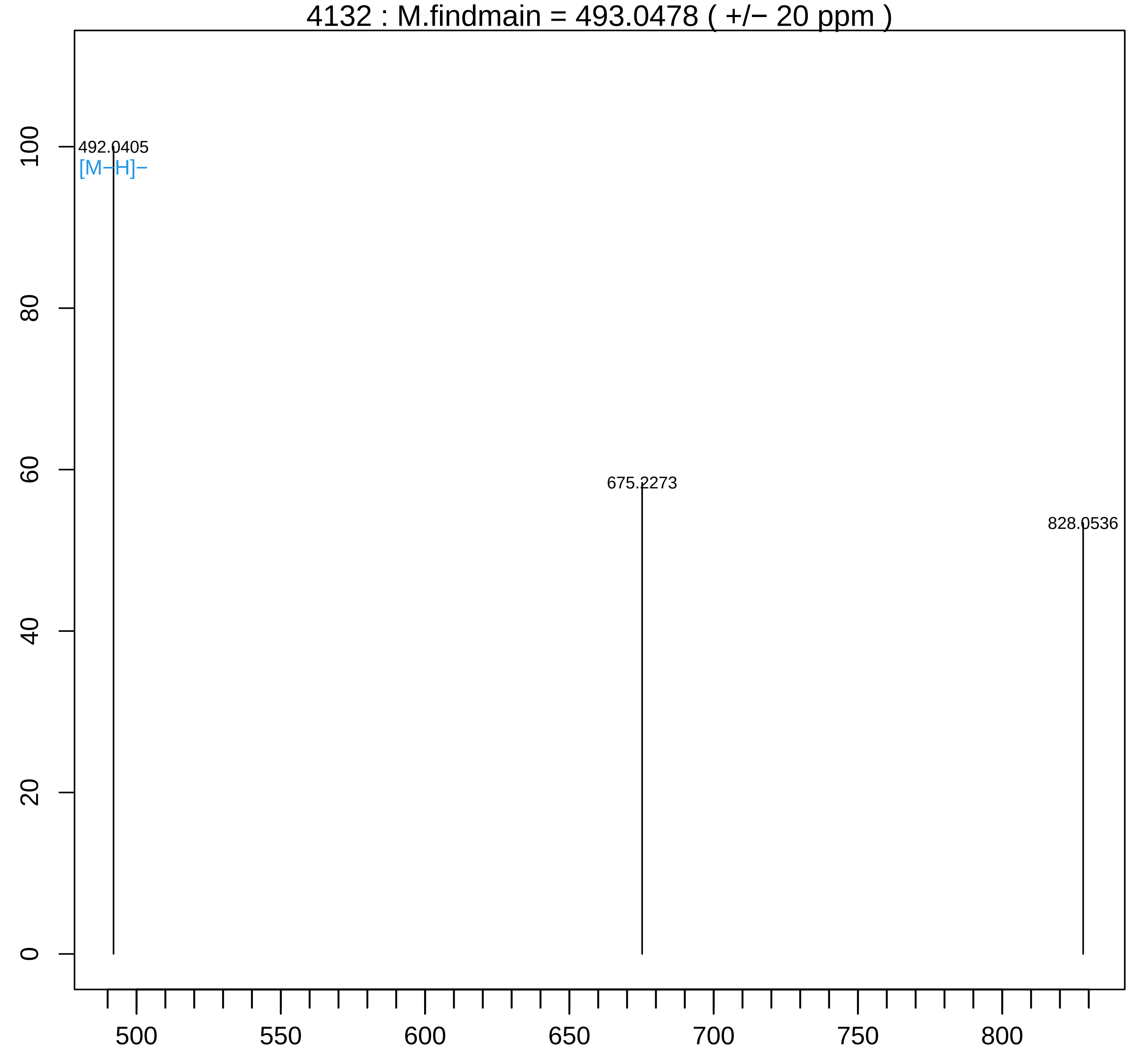
4132 : M.findmain = 493.0478 (+/− 20 ppm)
492.0405
[M−H]−
675.2273
828.0536
0
20
40
60
80
100
500
550
600
650
700
750
800

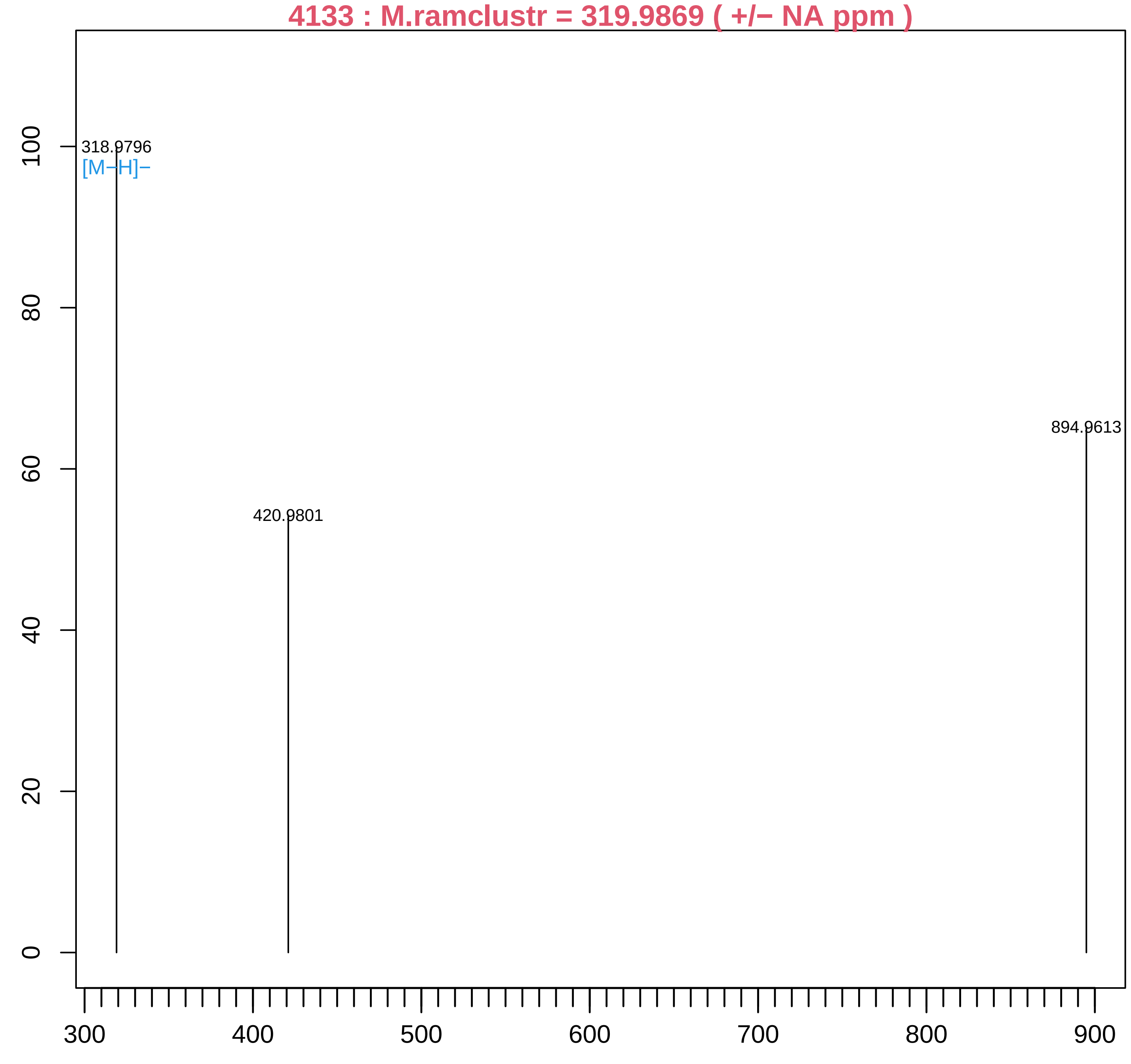
4133 : M.ramclustr = 319.9869 (+/− NA ppm)
318.9796
[M−H]−
420.9801
894.9613
0
20
40
60
80
100
300
400
500
600
700
800
900

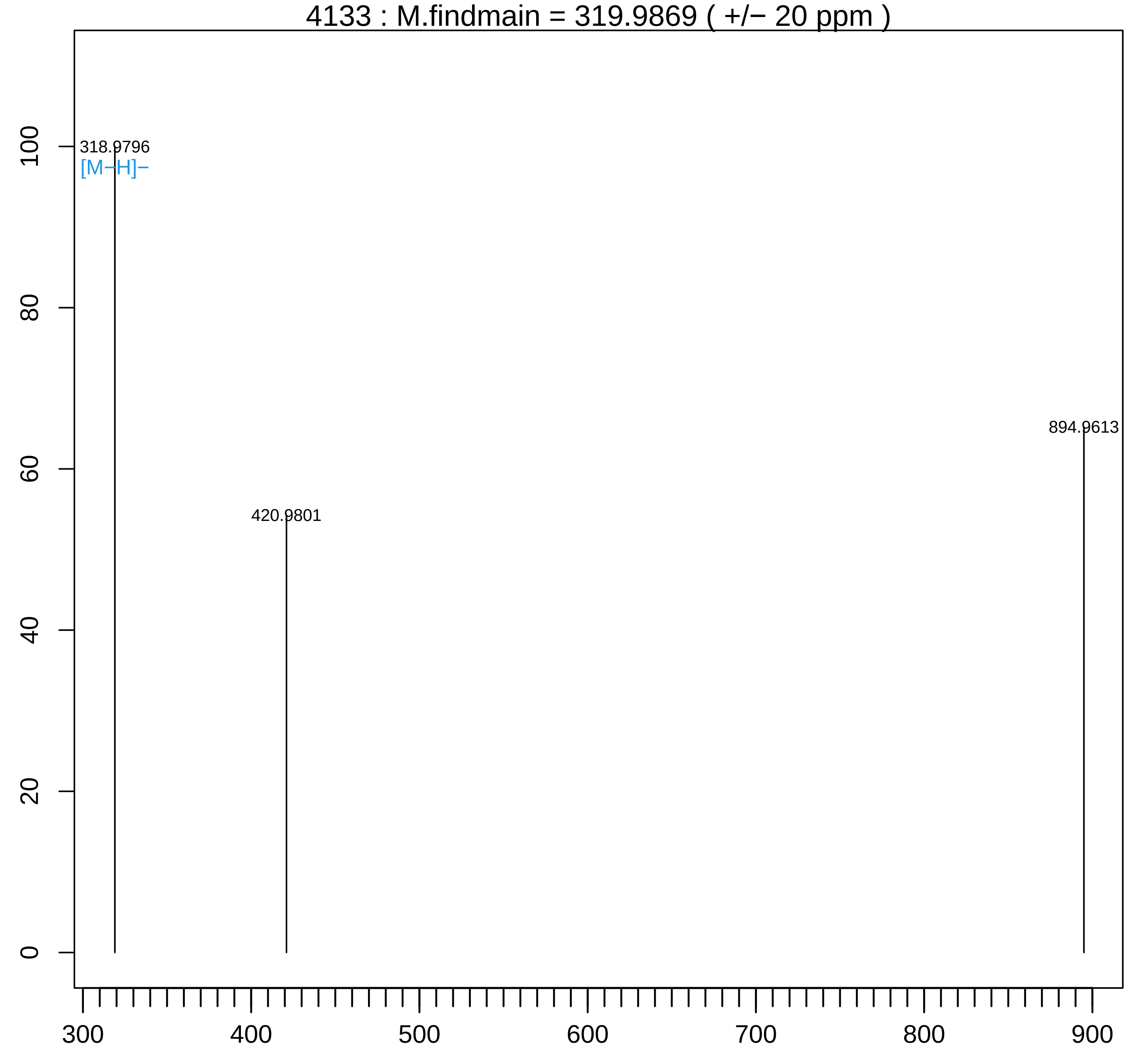
4133 : M.findmain = 319.9869 (+/− 20 ppm)
318.9796
[M−H]−
420.9801
894.9613
0
20
40
60
80
100
300
400
500
600
700
800
900

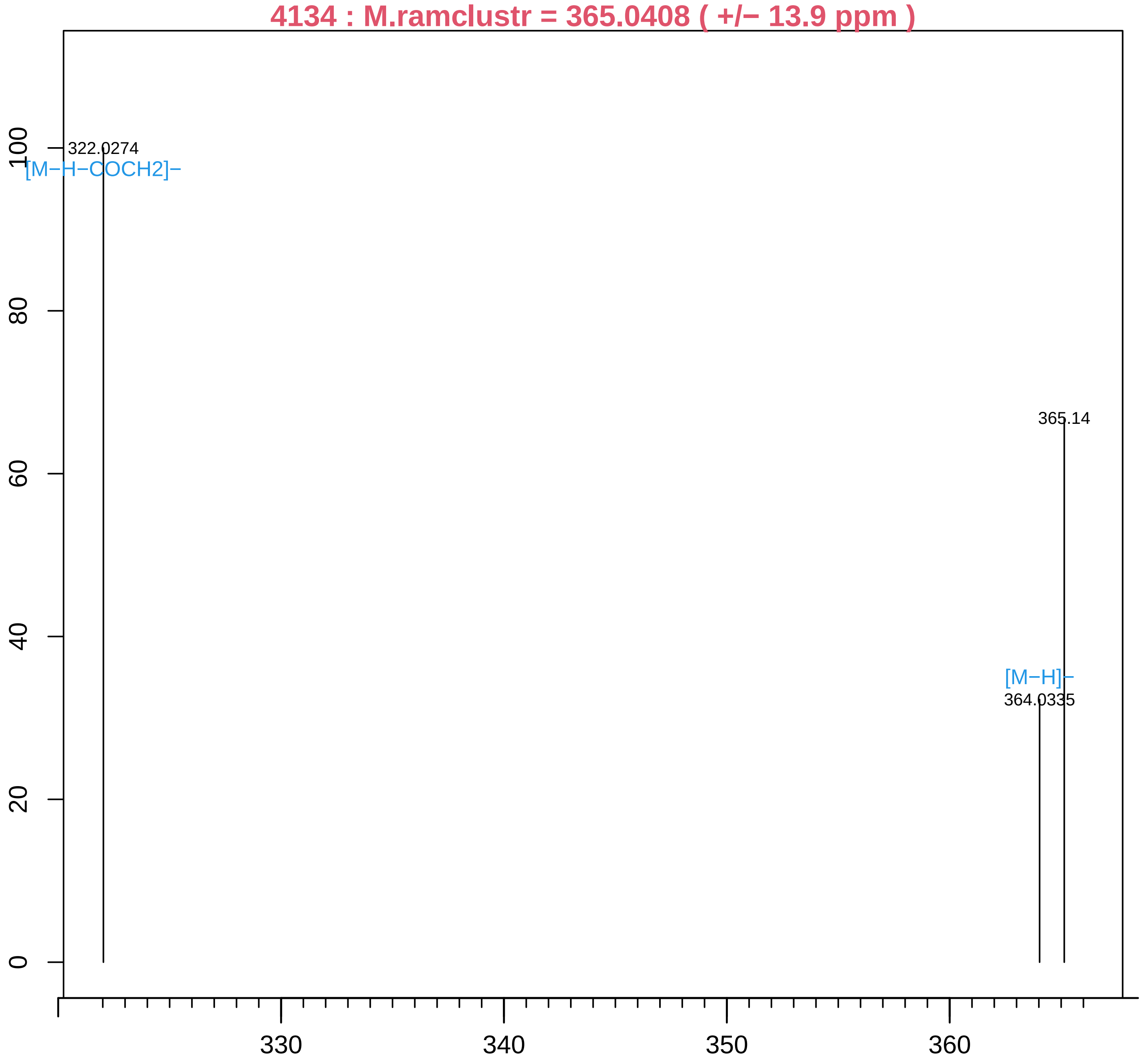
4134 : M.ramclustr = 365.0408 (+/− 13.9 ppm)
322.0274
[M−H−COCH2]−
365.14
[M−H]−
364.0335
0
20
40
60
80
100
330
340
350
360

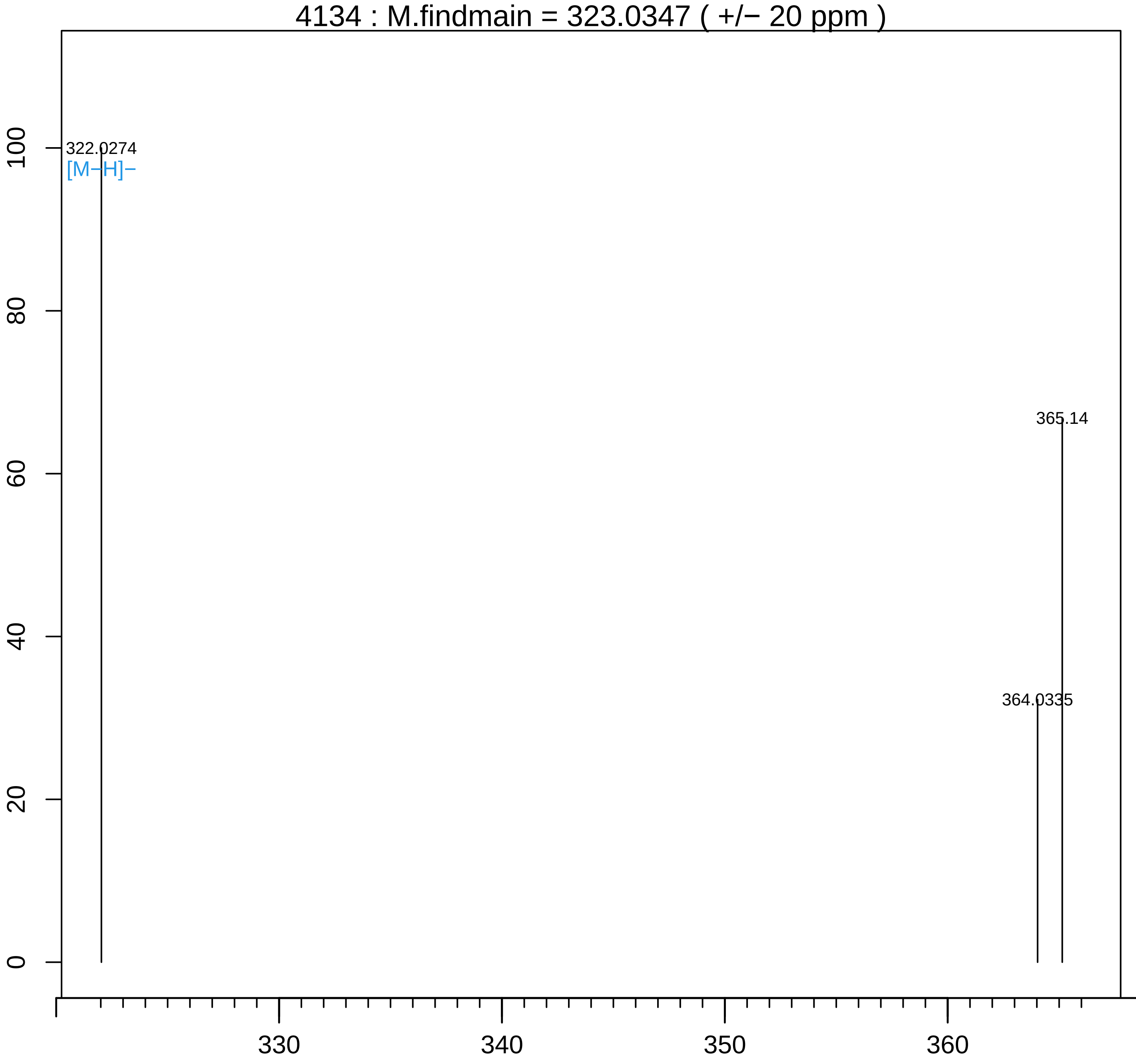
4134 : M.findmain = 323.0347 (+/− 20 ppm)
322.0274
[M−H]−
365.14
364.0335
0
20
40
60
80
100
330
340
350
360

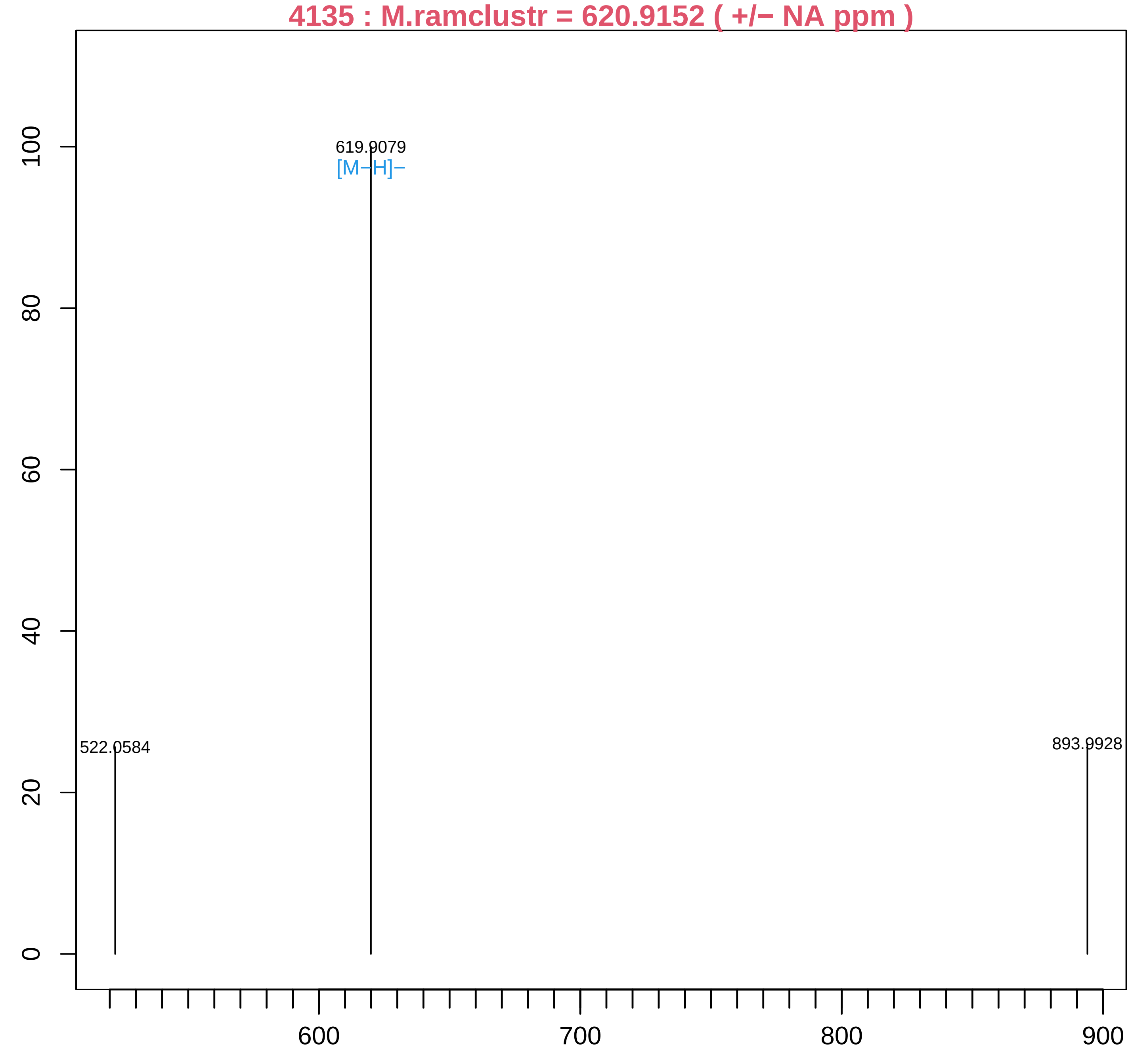
4135 : M.ramclustr = 620.9152 (+/− NA ppm)
619.9079
[M−H]−
522.0584
893.9928
0
20
40
60
80
100
600
700
800
900

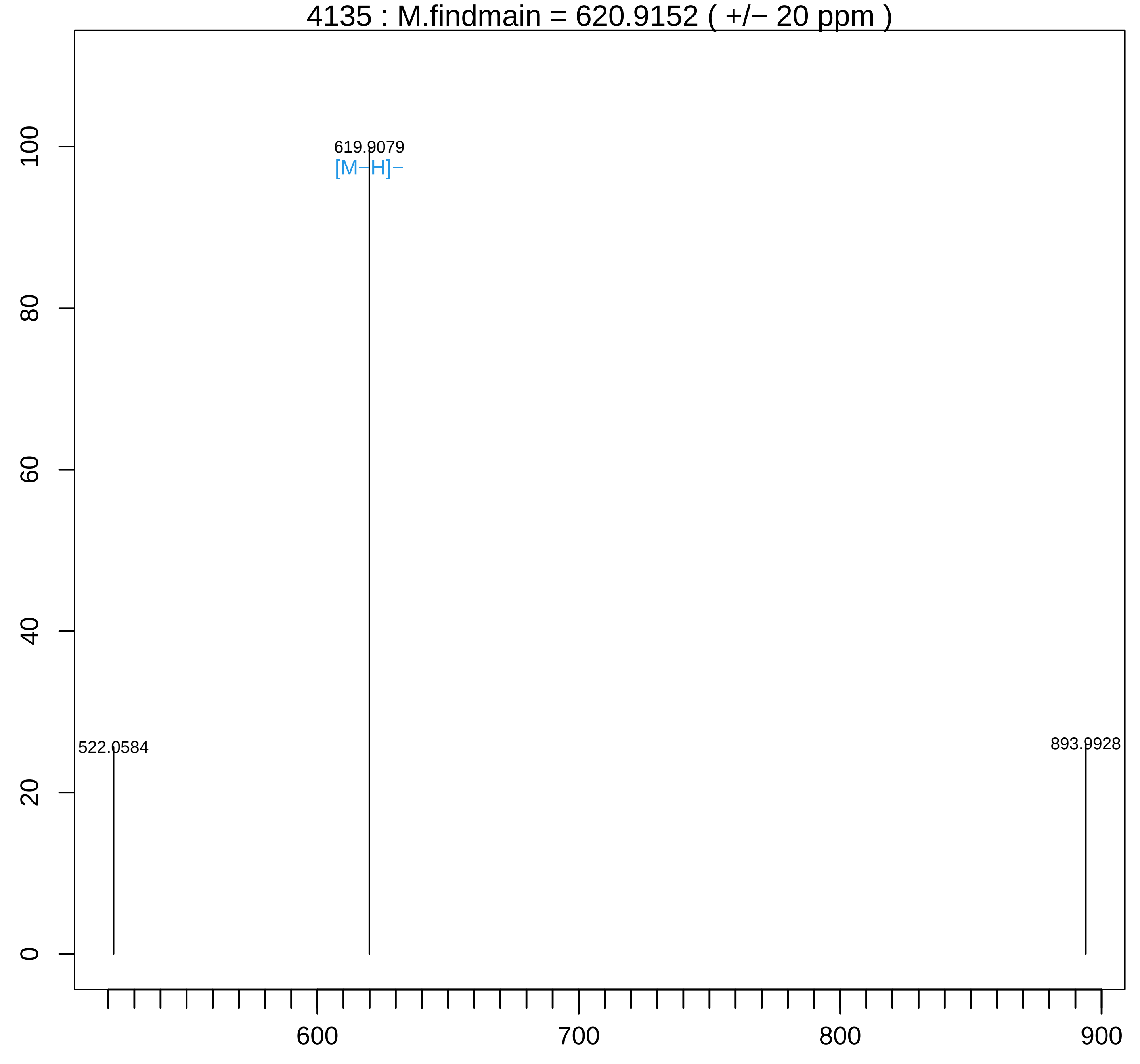
4135 : M.findmain = 620.9152 (+/− 20 ppm)
619.9079
[M−H]−
522.0584
893.9928
0
20
40
60
80
100
600
700
800
900

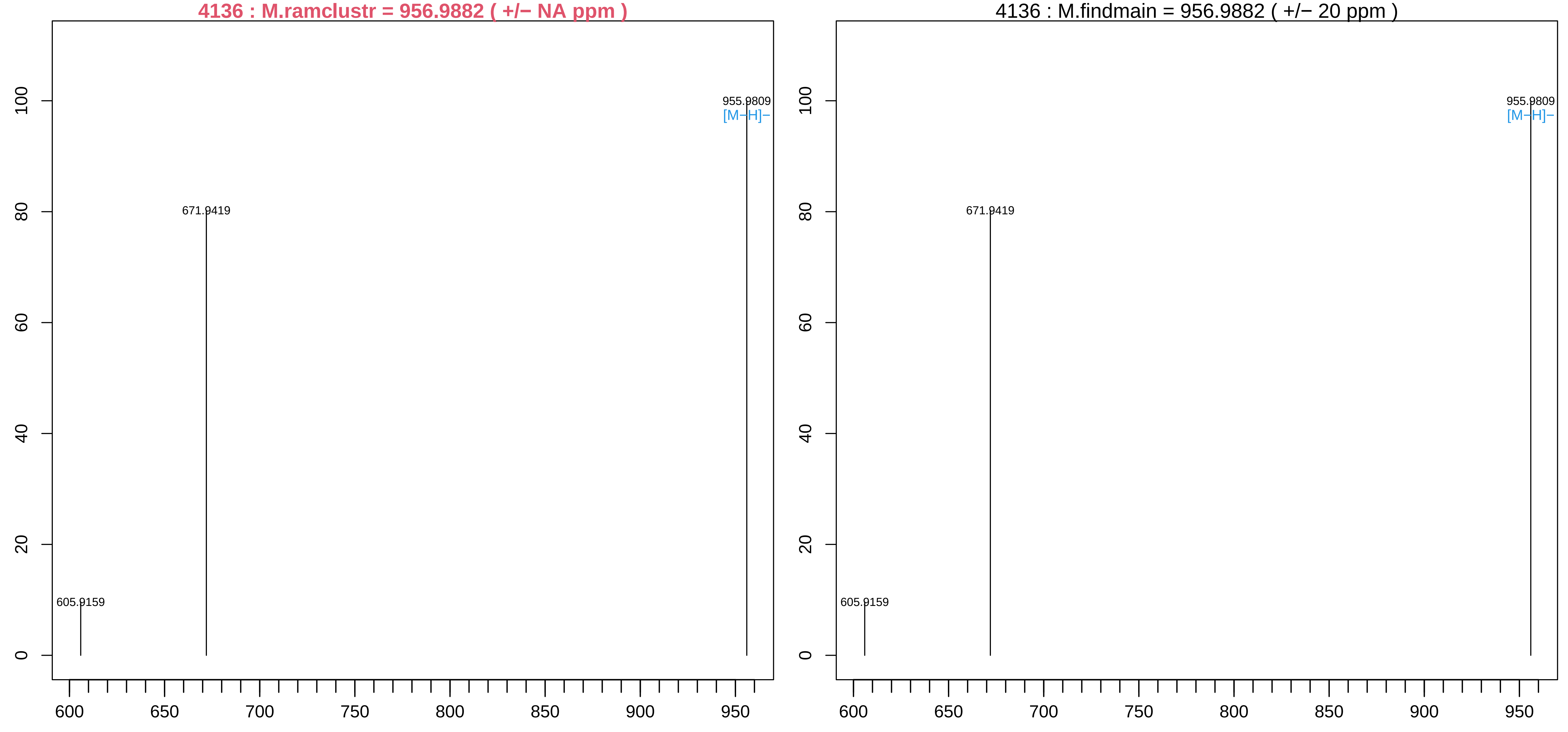
4136 : M.ramclustr = 956.9882 (+/− NA ppm)
955.9809
[M−H]−
671.9419
605.9159
0
20
40
60
80
100
600
650
700
750
800
850
900
950
4136 : M.findmain = 956.9882 (+/− 20 ppm)
955.9809
[M−H]−
671.9419
605.9159
0
20
40
60
80
100
600
650
700
750
800
850
900
950

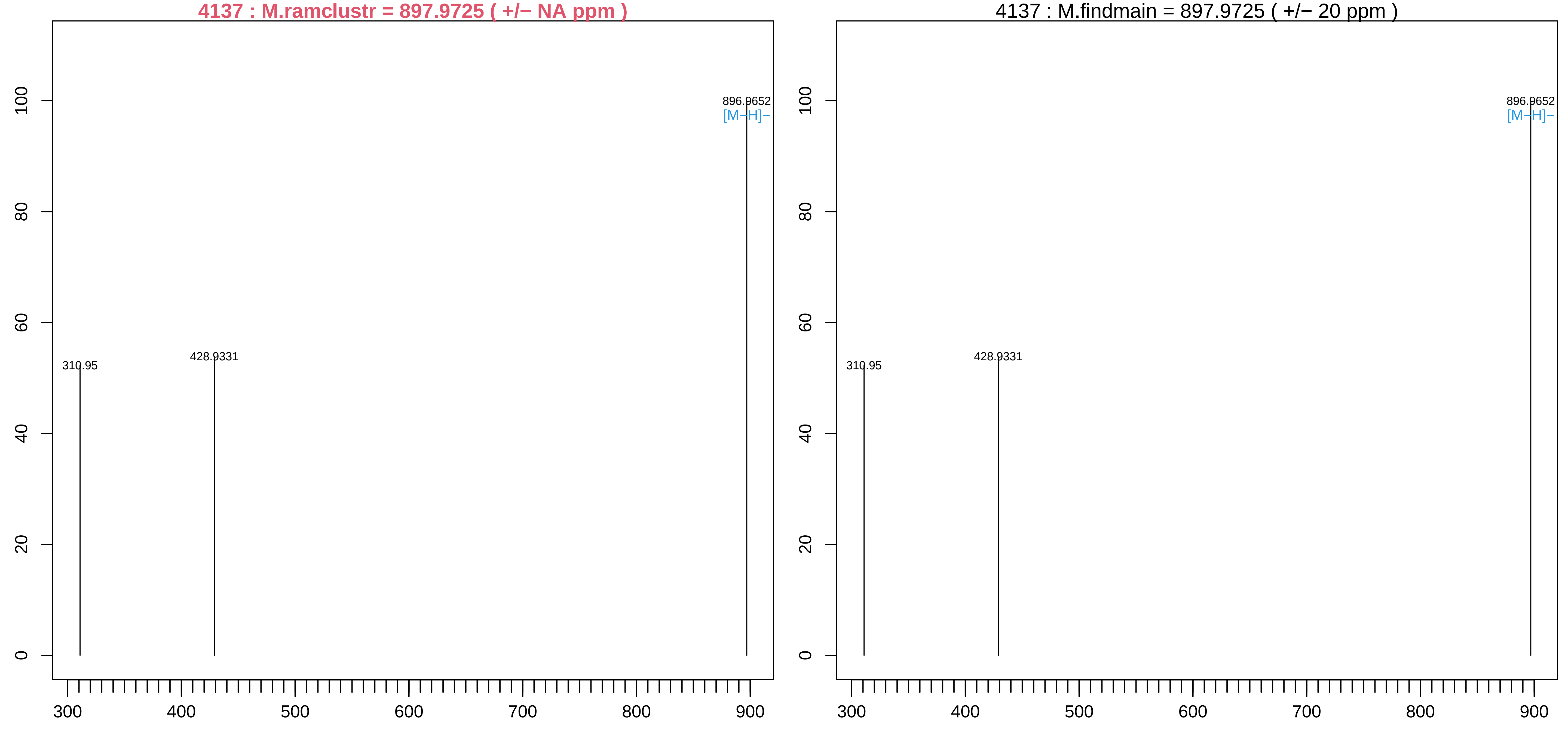

4137 : M.ramclustr = 897.9725 (+/− NA ppm)
896.9652
[M−H]−
310.95
428.9331
0
20
40
60
80
100
300
400
500
600
700
800
900
4137 : M.findmain = 897.9725 (+/− 20 ppm)
896.9652
[M−H]−
310.95
428.9331
0
20
40
60
80
100
300
400
500
600
700
800
900

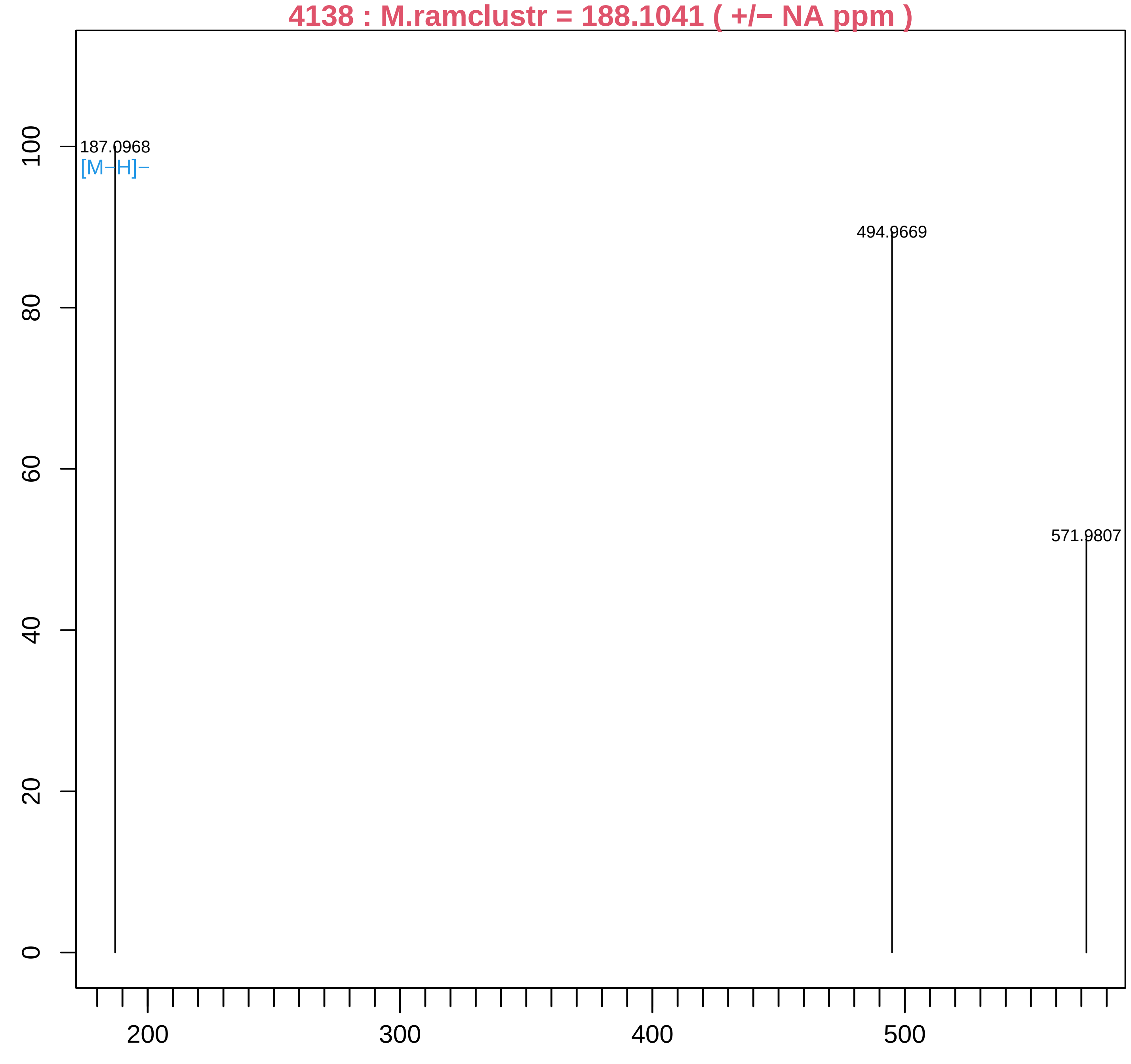
4138 : M.ramclustr = 188.1041 (+/− NA ppm)
187.0968
[M−H]−
494.9669
571.9807
0
20
40
60
80
100
200
300
400
500

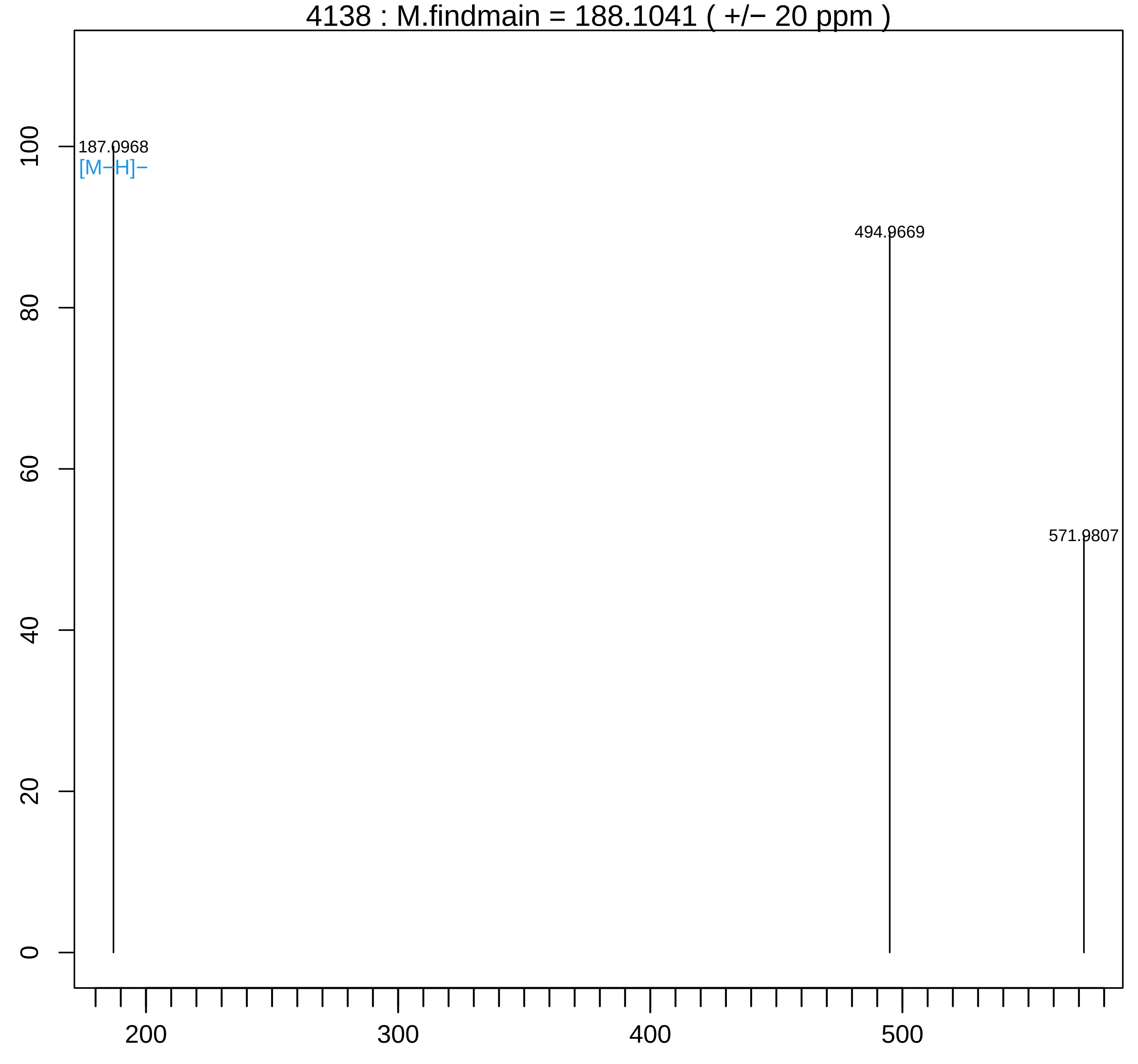
4138 : M.findmain = 188.1041 (+/− 20 ppm)
187.0968
[M−H]−
494.9669
571.9807
0
20
40
60
80
100
200
300
400
500

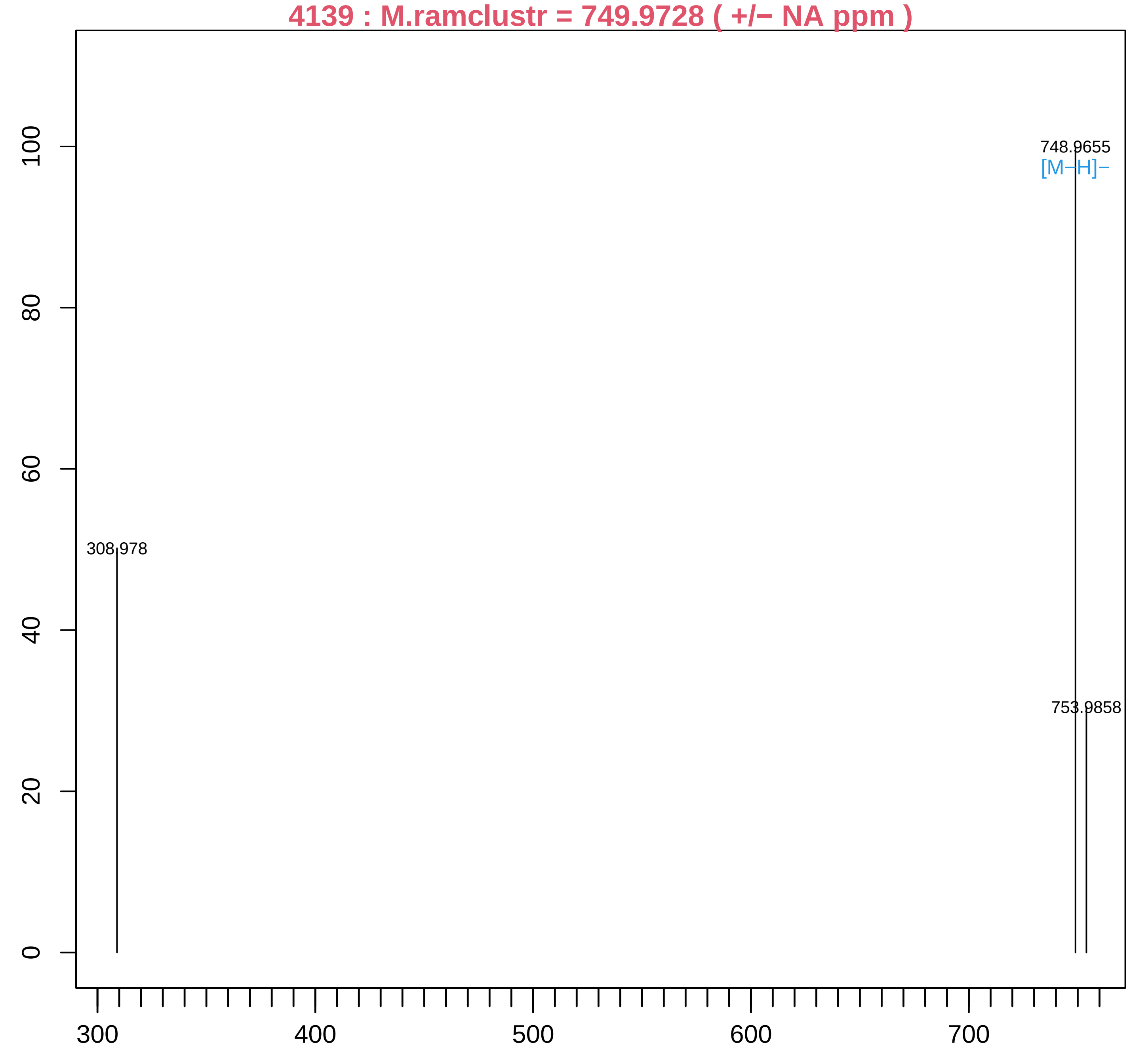
4139 : M.ramclustr = 749.9728 (+/− NA ppm)
748.9655
[M−H]−
308.978
753.9858
0
20
40
60
80
100
300
400
500
600
700

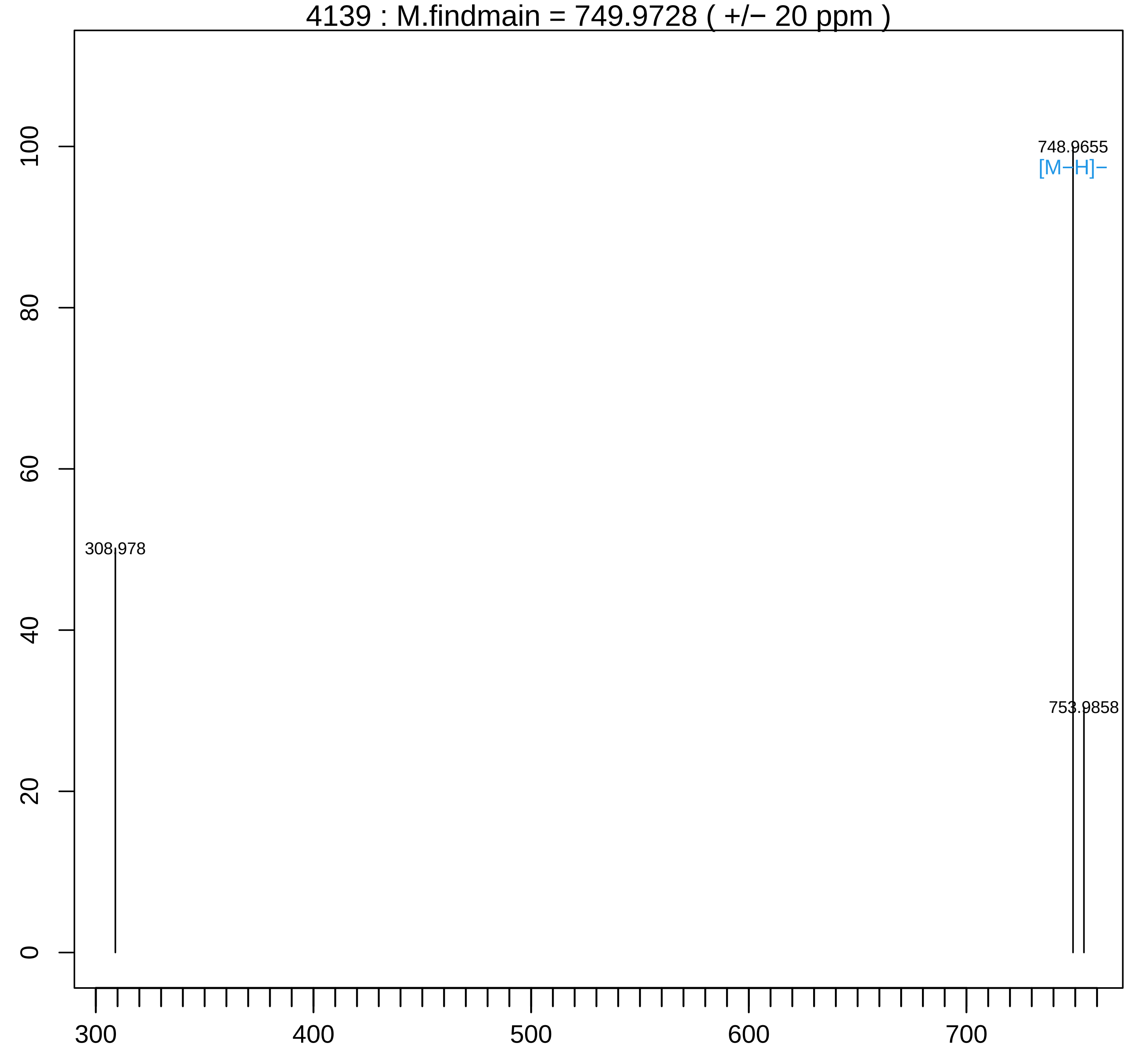
4139 : M.findmain = 749.9728 (+/− 20 ppm)
748.9655
[M−H]−
308.978
753.9858
0
20
40
60
80
100
300
400
500
600
700

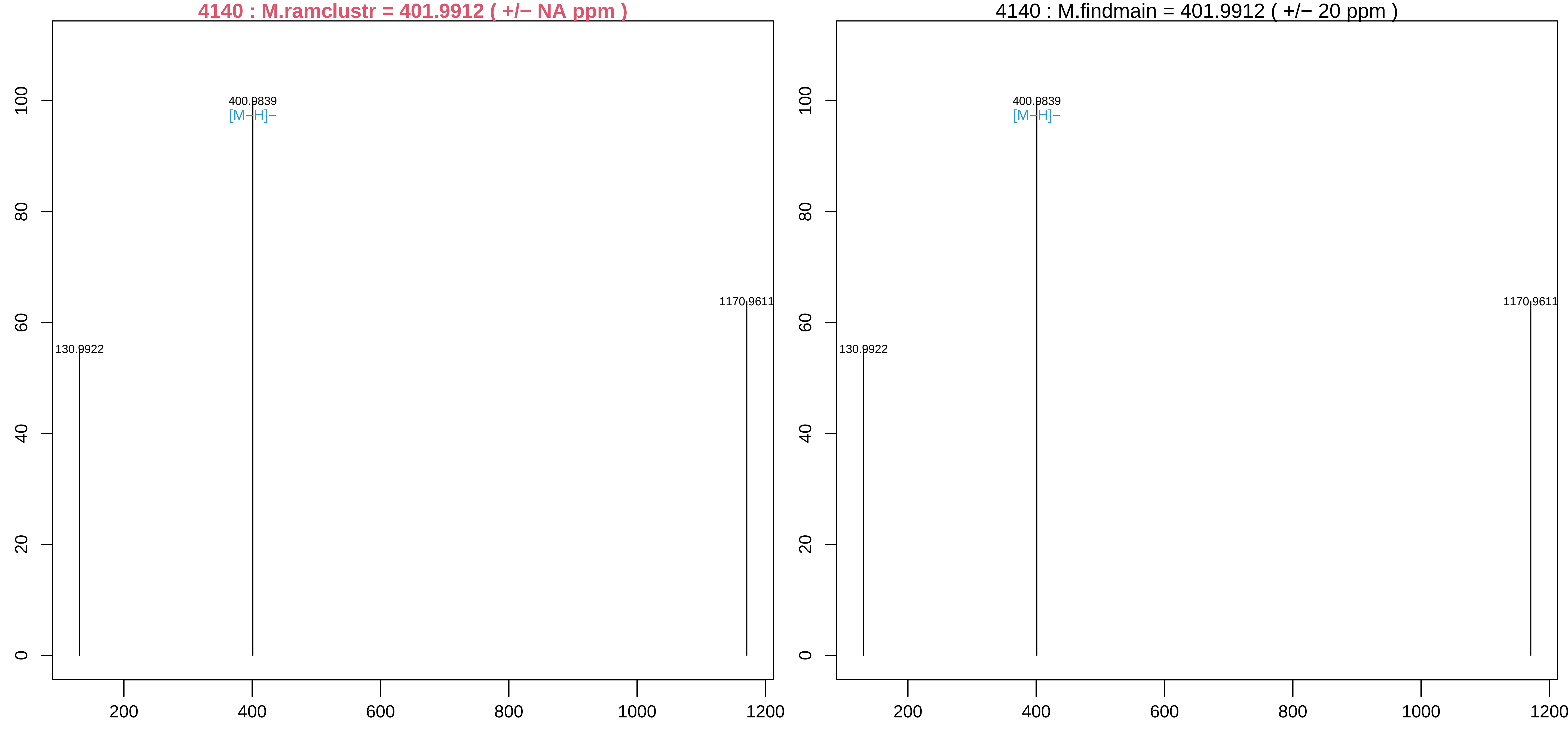
4140 : M.ramclustr = 401.9912 (+/− NA ppm)
400.9839
[M−H]−
1170.9611
130.9922
0
20
40
60
80
100
200
400
600
800
1000
1200
4140 : M.findmain = 401.9912 (+/− 20 ppm)
400.9839
[M−H]−
1170.9611
130.9922
0
20
40
60
80
100
200
400
600
800
1000
1200

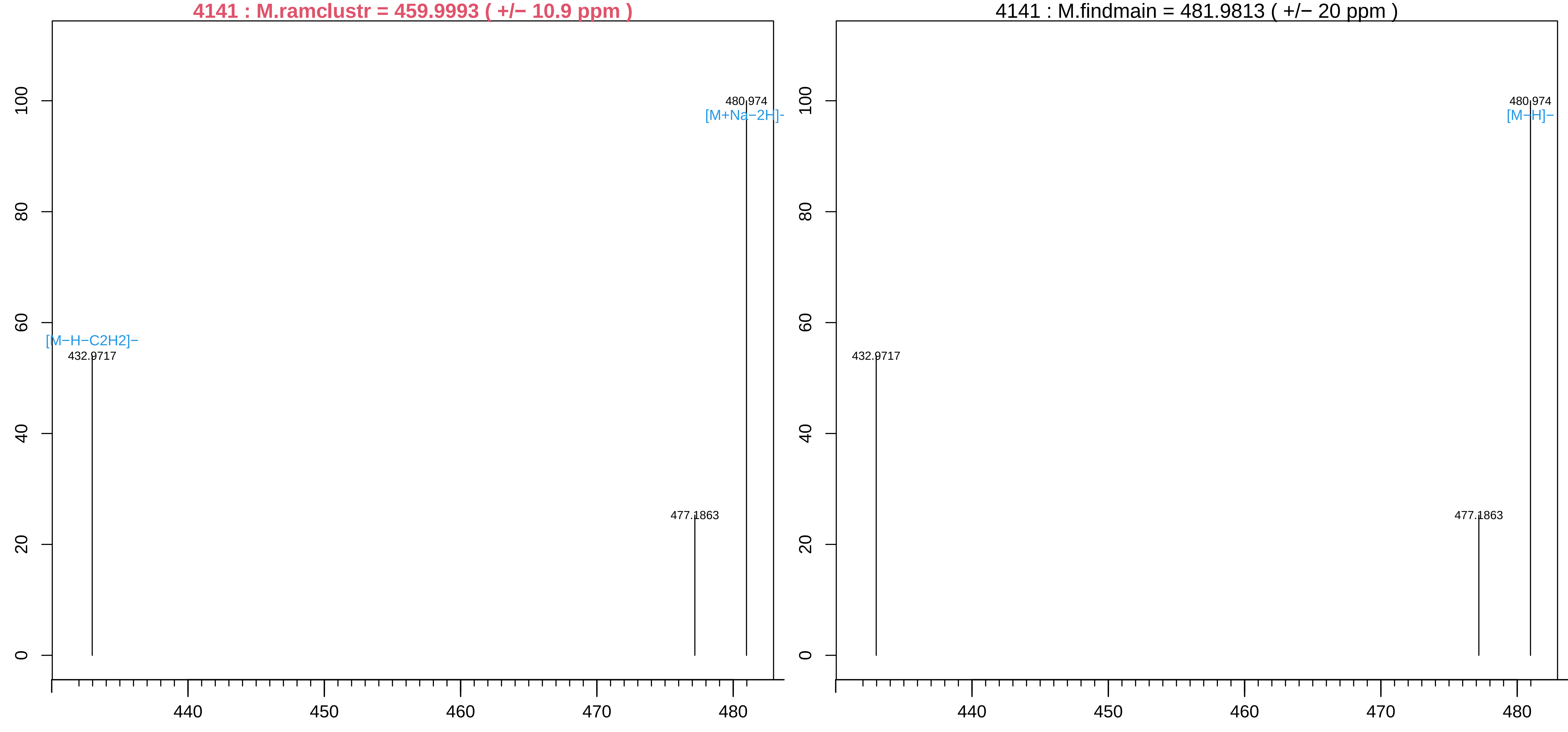
4141 : M.ramclustr = 459.9993 (+/− 10.9 ppm)
4141 : M.findmain = 481.9813 (+/− 20 ppm)
480.974
[M+Na−2H]−
[M−H−C2H2]−
432.9717
477.1863
480.974
[M−H]−
432.9717
477.1863
0
20
40
60
80
100
440
450
460
470
480

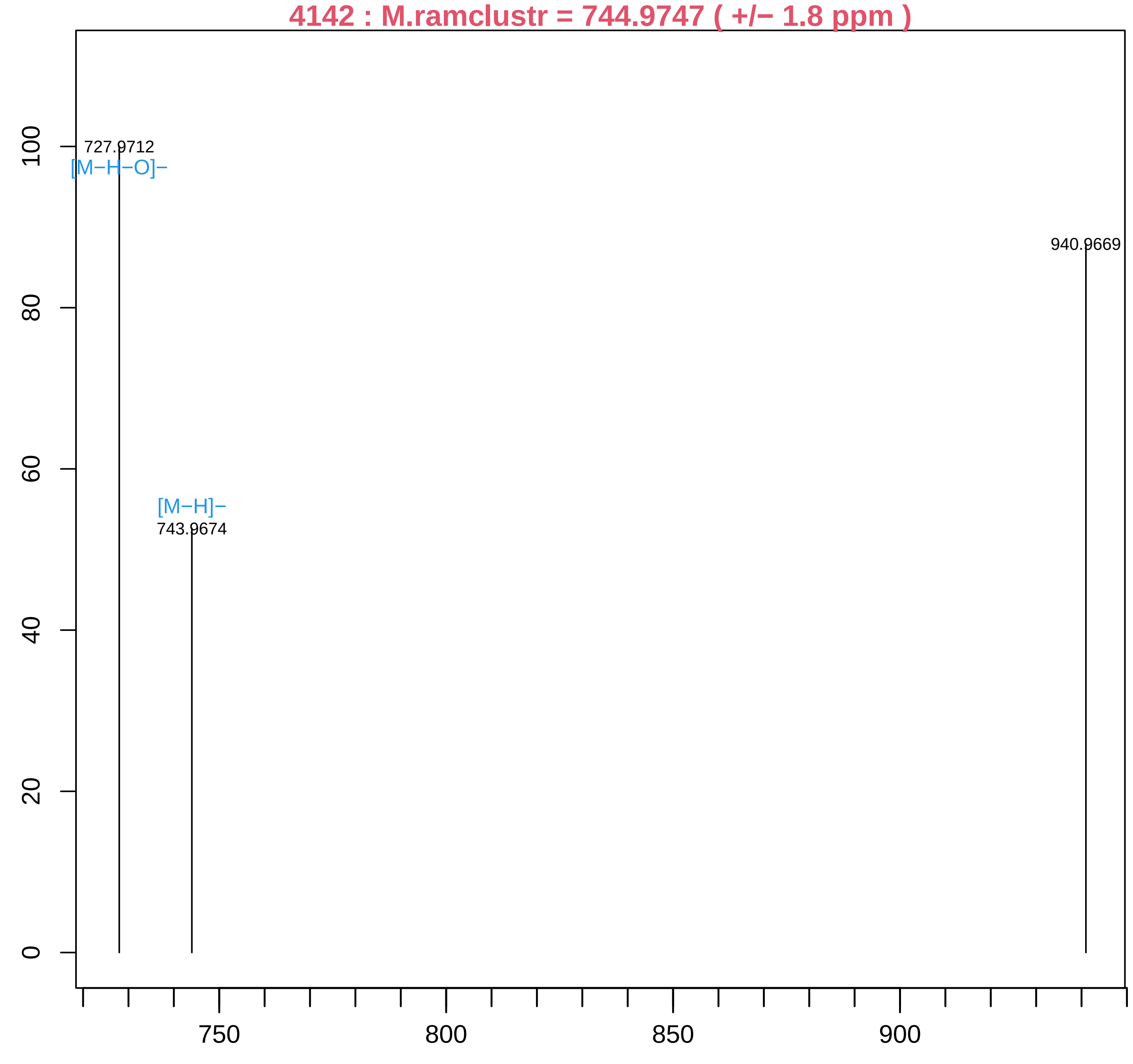
4142 : M.ramclustr = 744.9747 (+/− 1.8 ppm)
727.9712
[M−H−O]−
[M−H]−
743.9674
940.9669
0
20
40
60
80
100
750
800
850
900

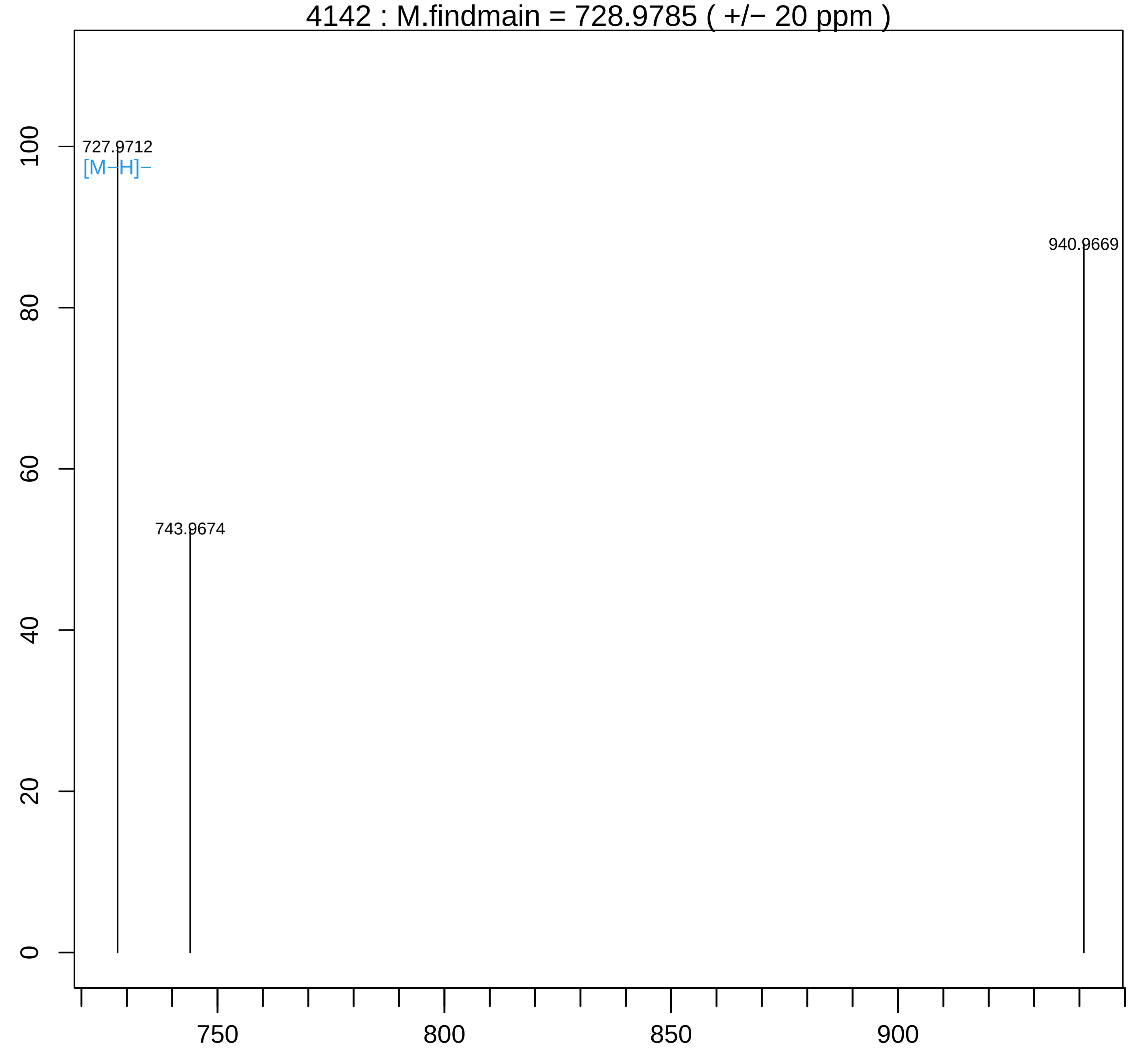
4142 : M.findmain = 728.9785 (+/− 20 ppm)
727.9712
[M−H]−
743.9674
940.9669
0
20
40
60
80
100
750
800
850
900

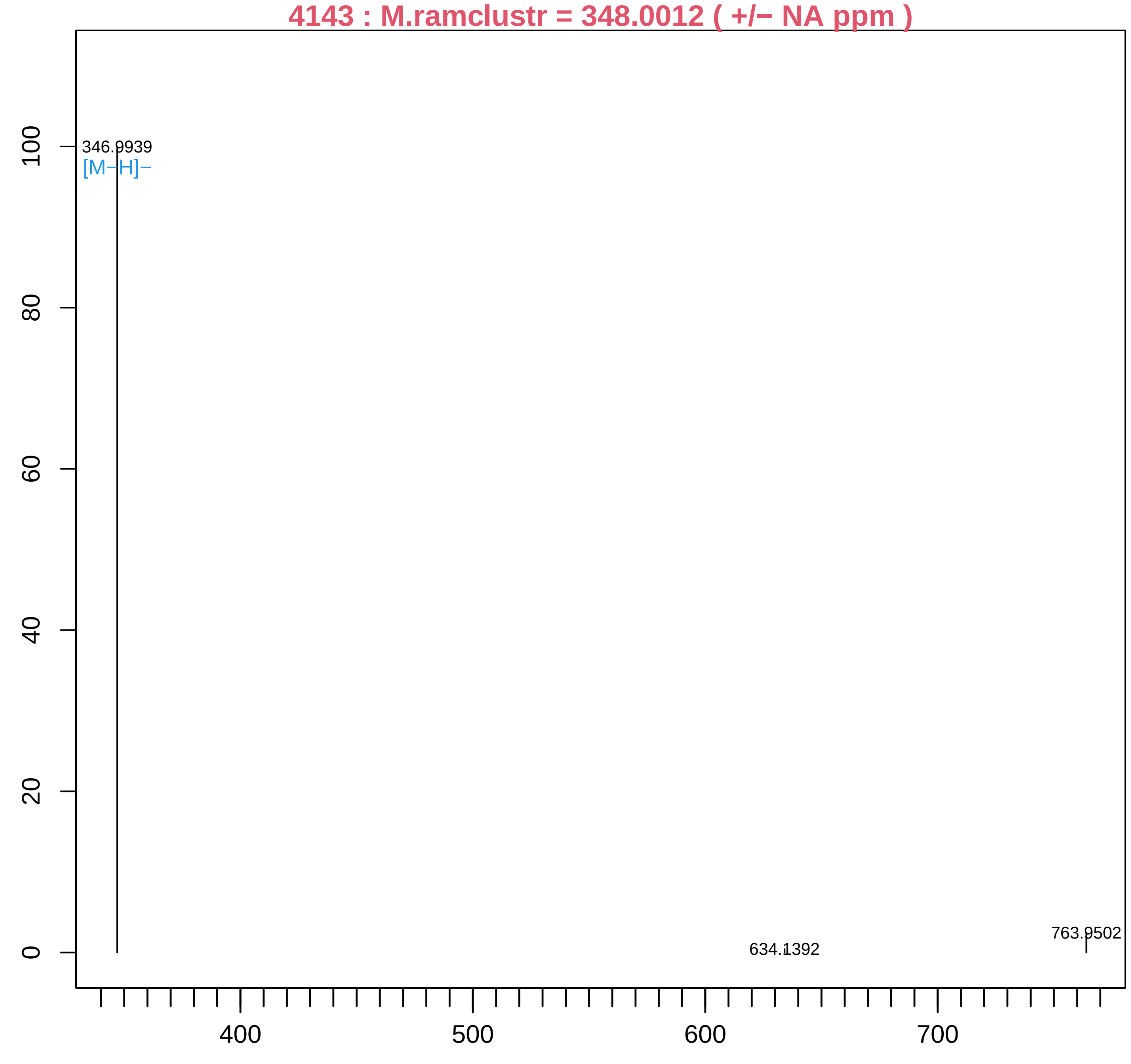

4143 : M.ramclustr = 348.0012 (+/− NA ppm)
346.9939
[M−H]−
634.1392
763.9502
0
20
40
60
80
100
400
500
600
700

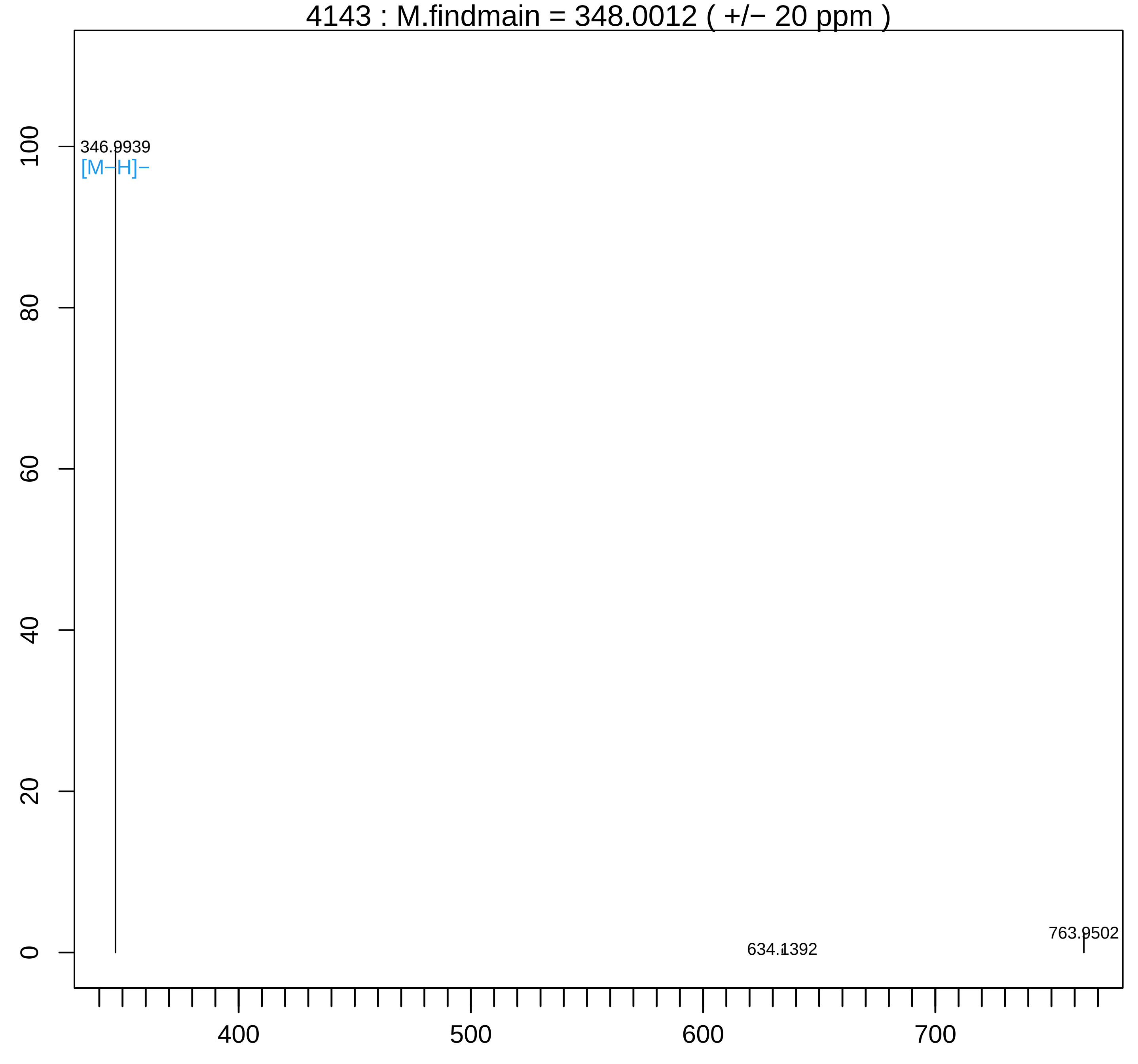

4143 : M.findmain = 348.0012 (+/− 20 ppm)
346.9939
[M−H]−
634.1392
763.9502
0
20
40
60
80
100
400
500
600
700

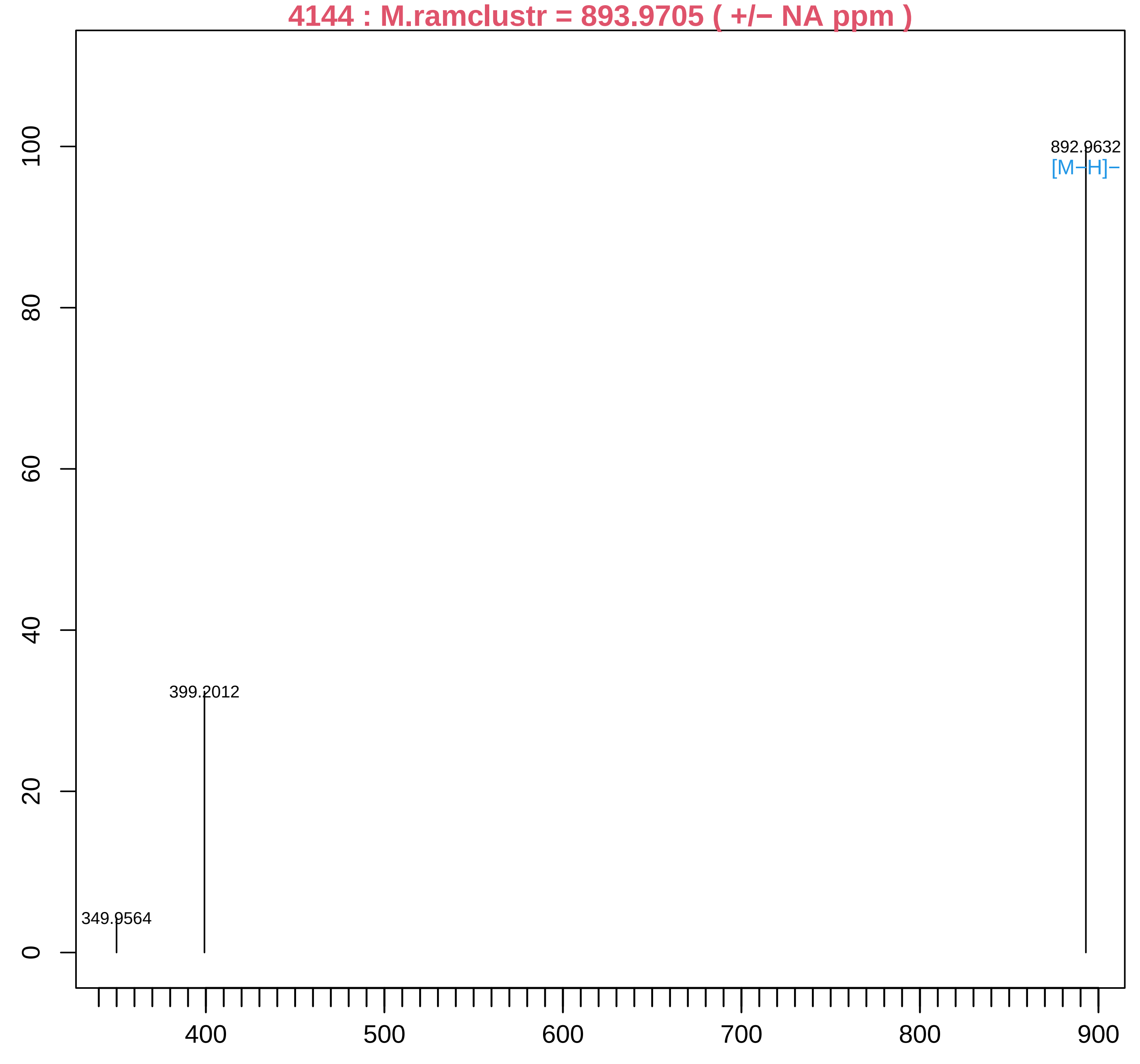
4144 : M.ramclustr = 893.9705 (+/− NA ppm)
892.9632
[M−H]−
399.2012
349.9564
0
20
40
60
80
100
400
500
600
700
800
900
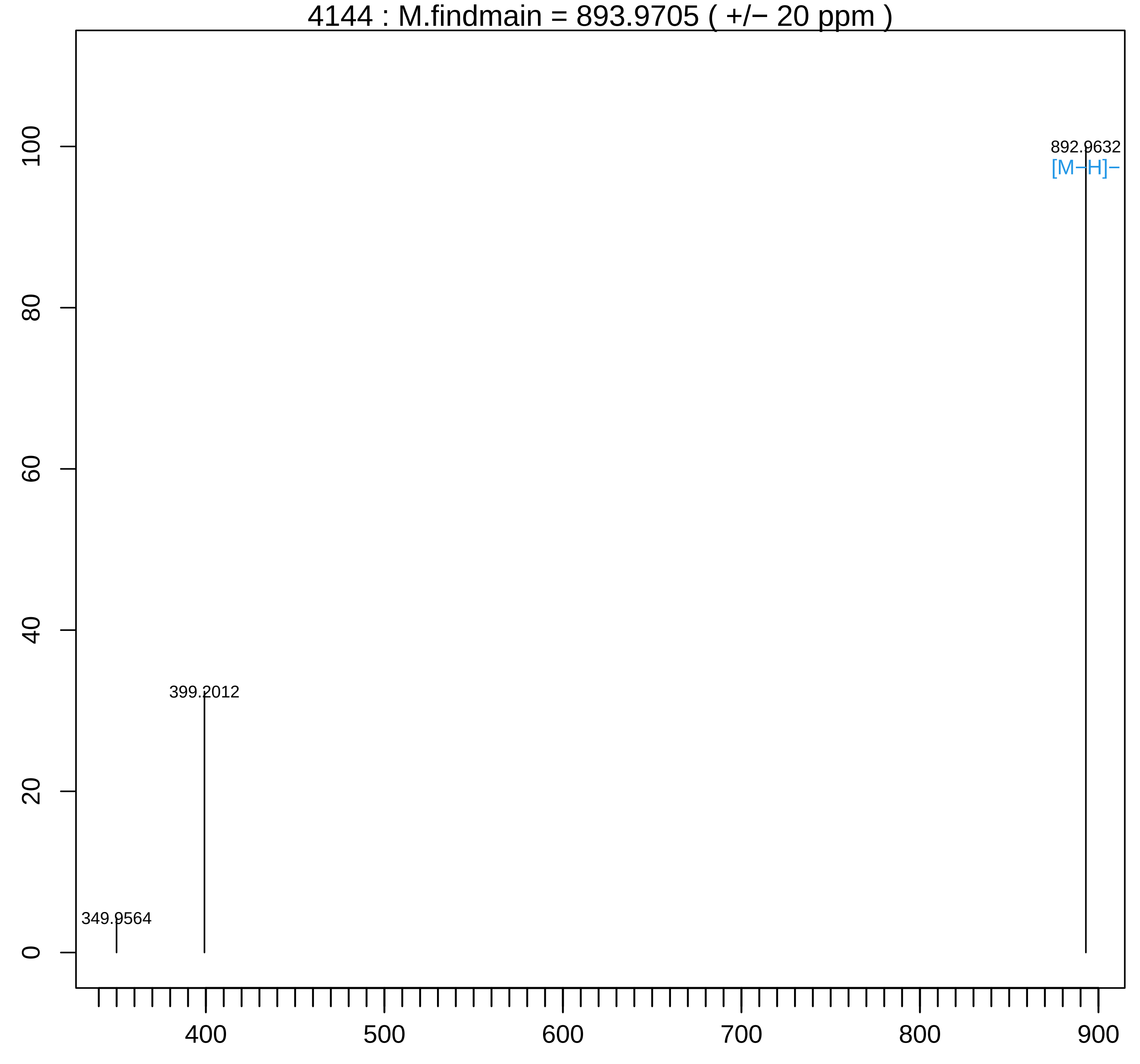
4144 : M.findmain = 893.9705 (+/− 20 ppm)
892.9632
[M−H]−
399.2012
349.9564
0
20
40
60
80
100
400
500
600
700
800
900

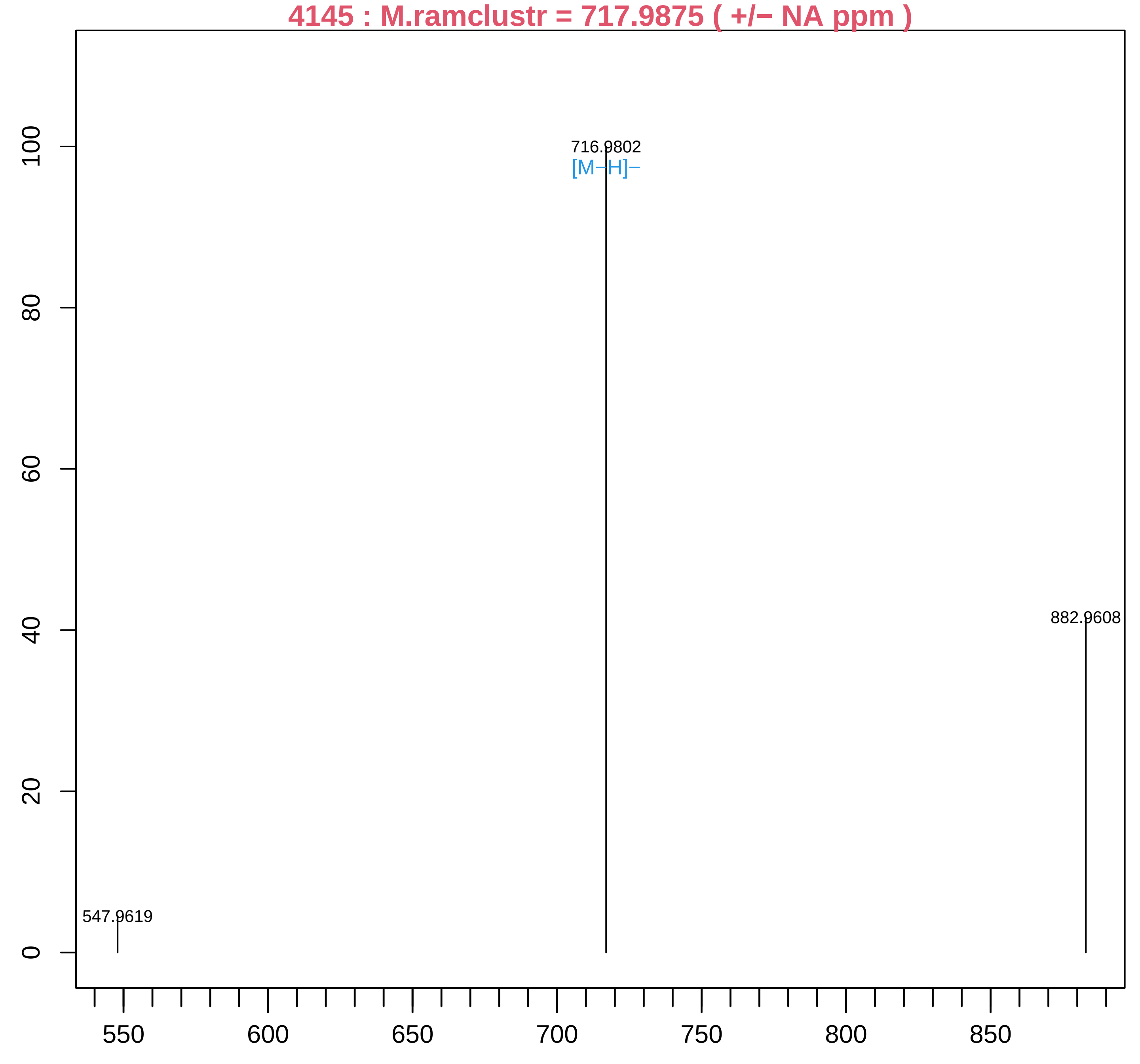

4145 : M.ramclustr = 717.9875 (+/− NA ppm)
716.9802
[M−H]−
882.9608
547.9619
0
20
40
60
80
100
550
600
650
700
750
800
850

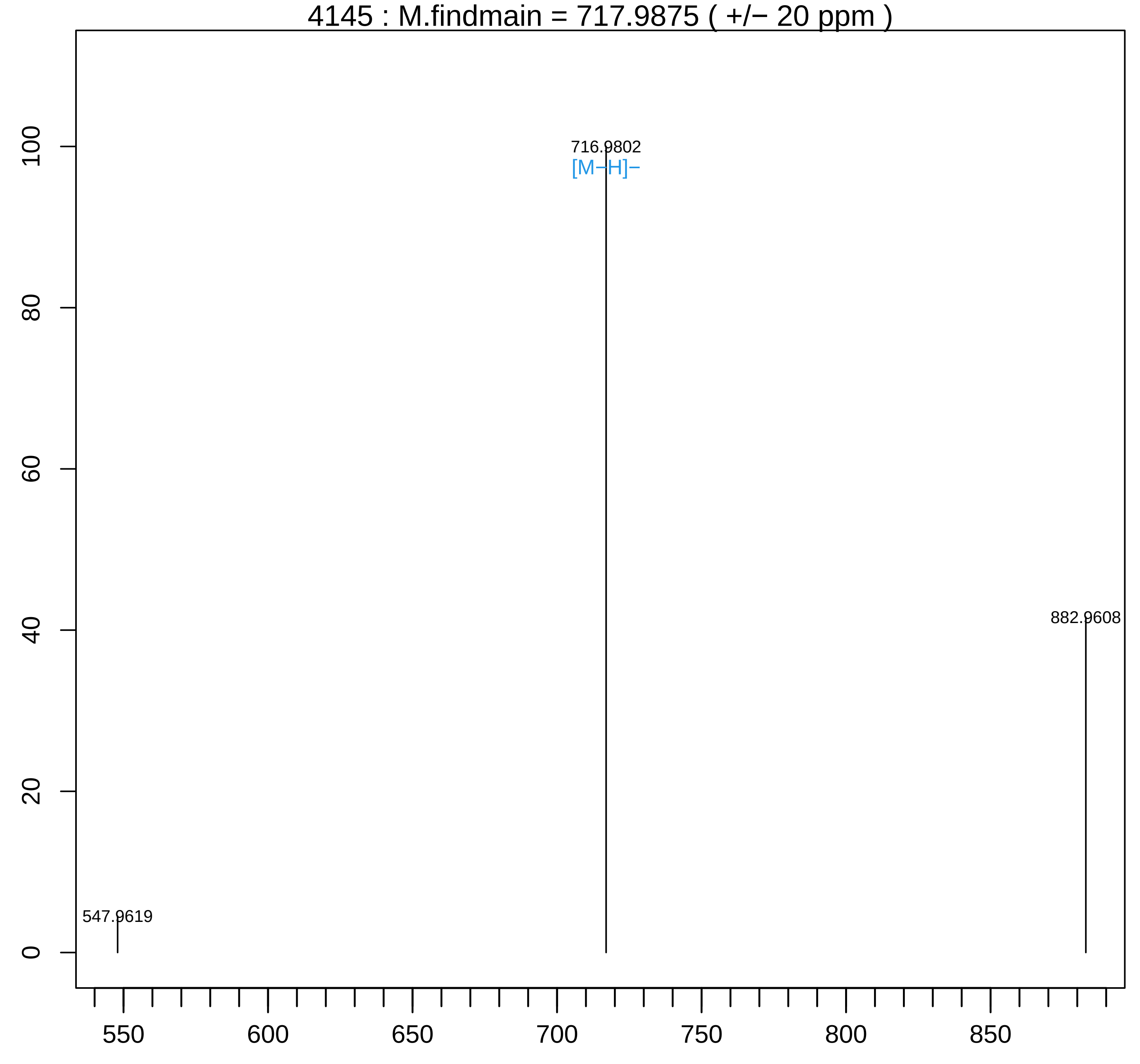

4145 : M.findmain = 717.9875 (+/− 20 ppm)
716.9802
[M−H]−
882.9608
547.9619
0
20
40
60
80
100
550
600
650
700
750
800
850

4146 : M.ramclustr = 1015.9667 (+/− NA ppm)

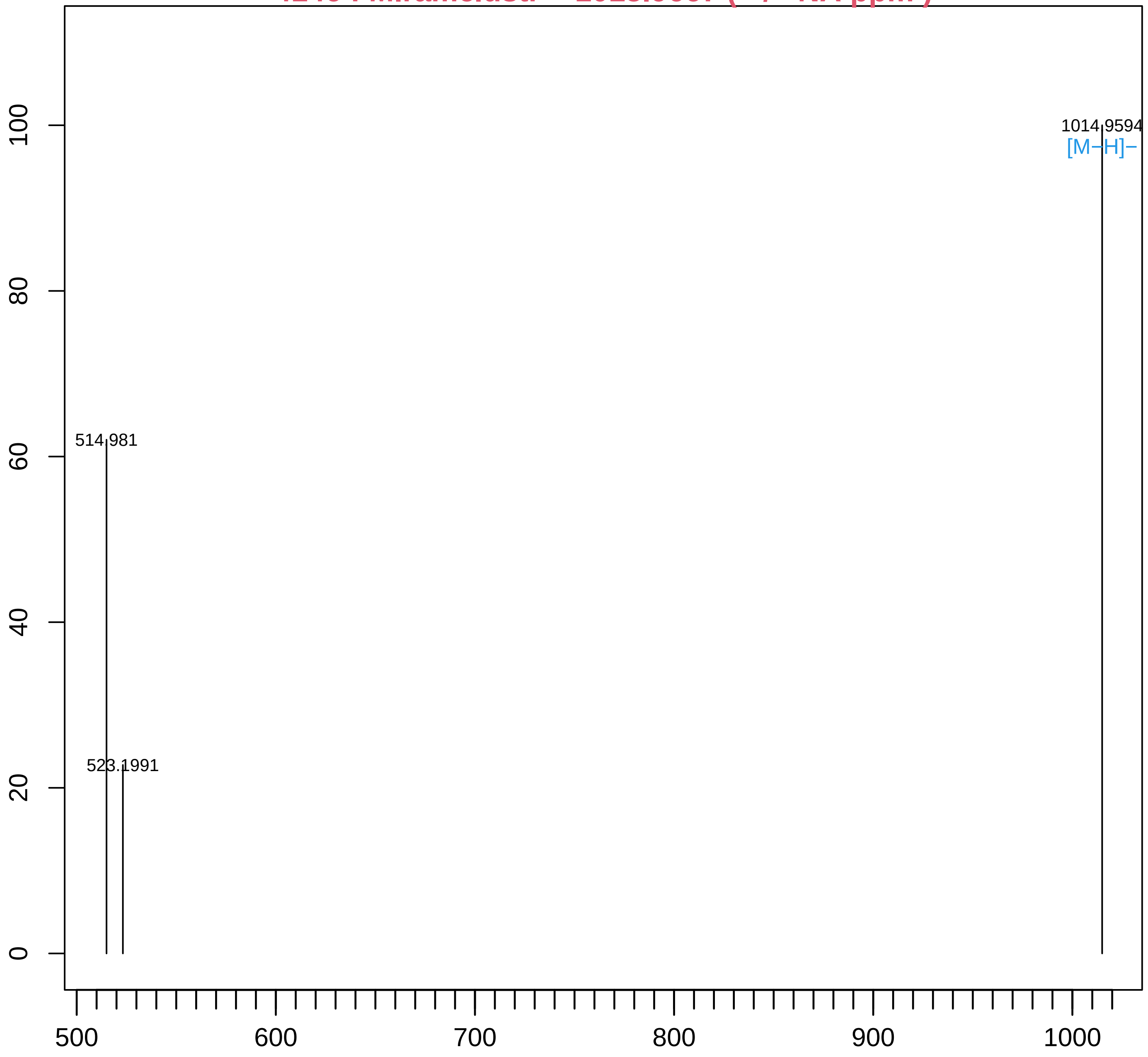

4146 : M.findmain = 1015.9667 (+/− 20 ppm)

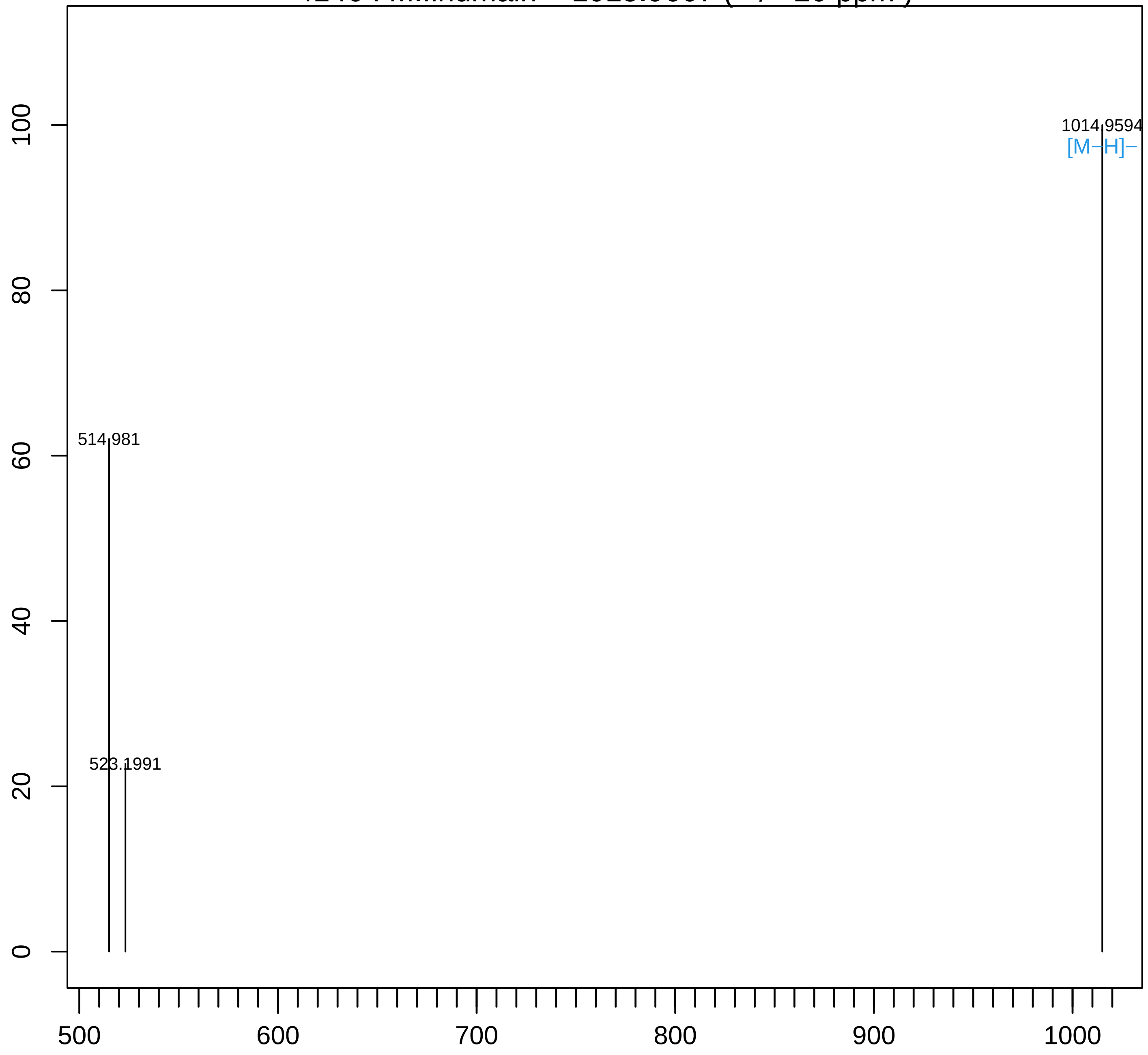

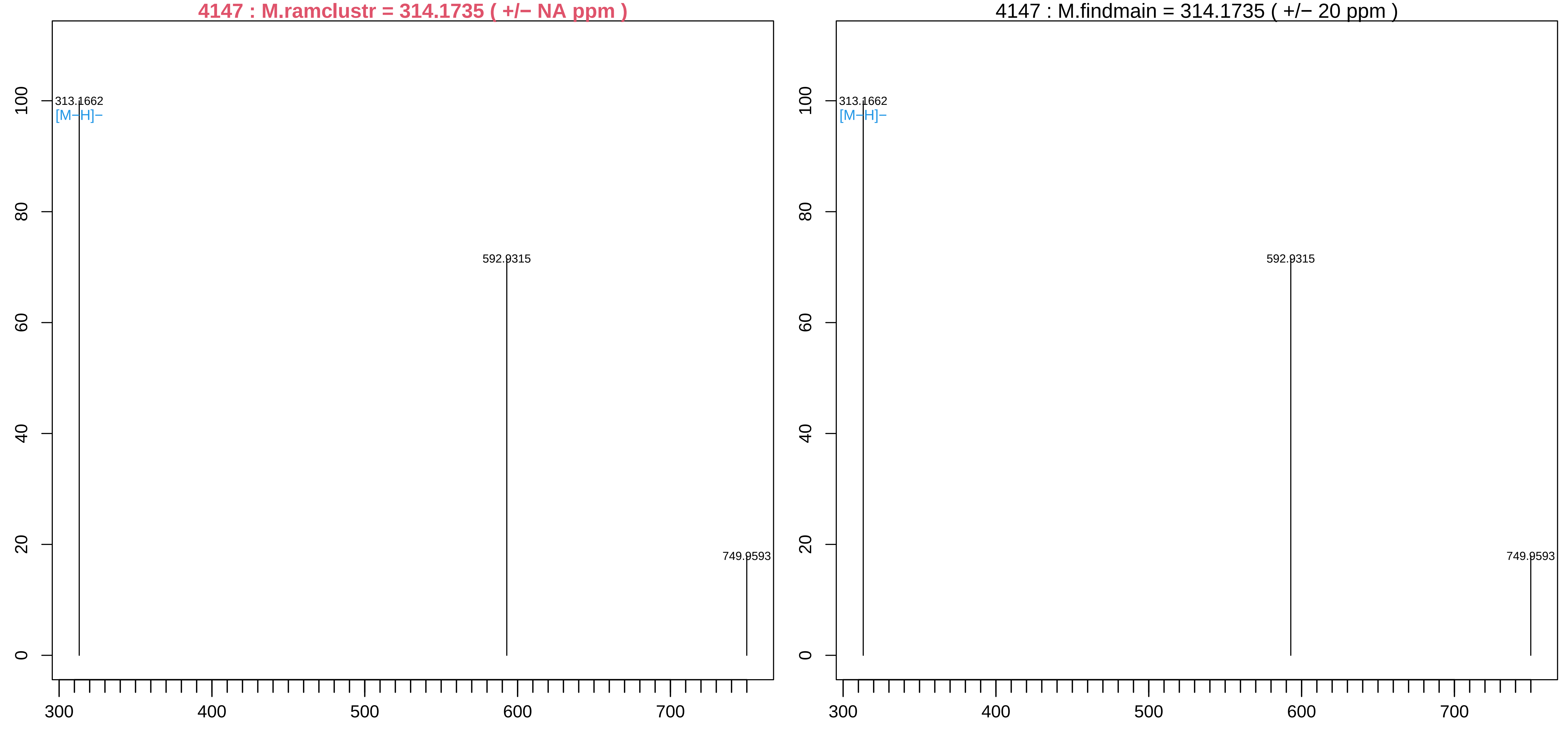

4147 : M.ramclustr = 314.1735 (+/− NA ppm)
313.1662
[M−H]−
592.9315
749.9593
0
20
40
60
80
100
300
400
500
600
700
4147 : M.findmain = 314.1735 (+/− 20 ppm)
313.1662
[M−H]−
592.9315
749.9593
0
20
40
60
80
100
300
400
500
600
700

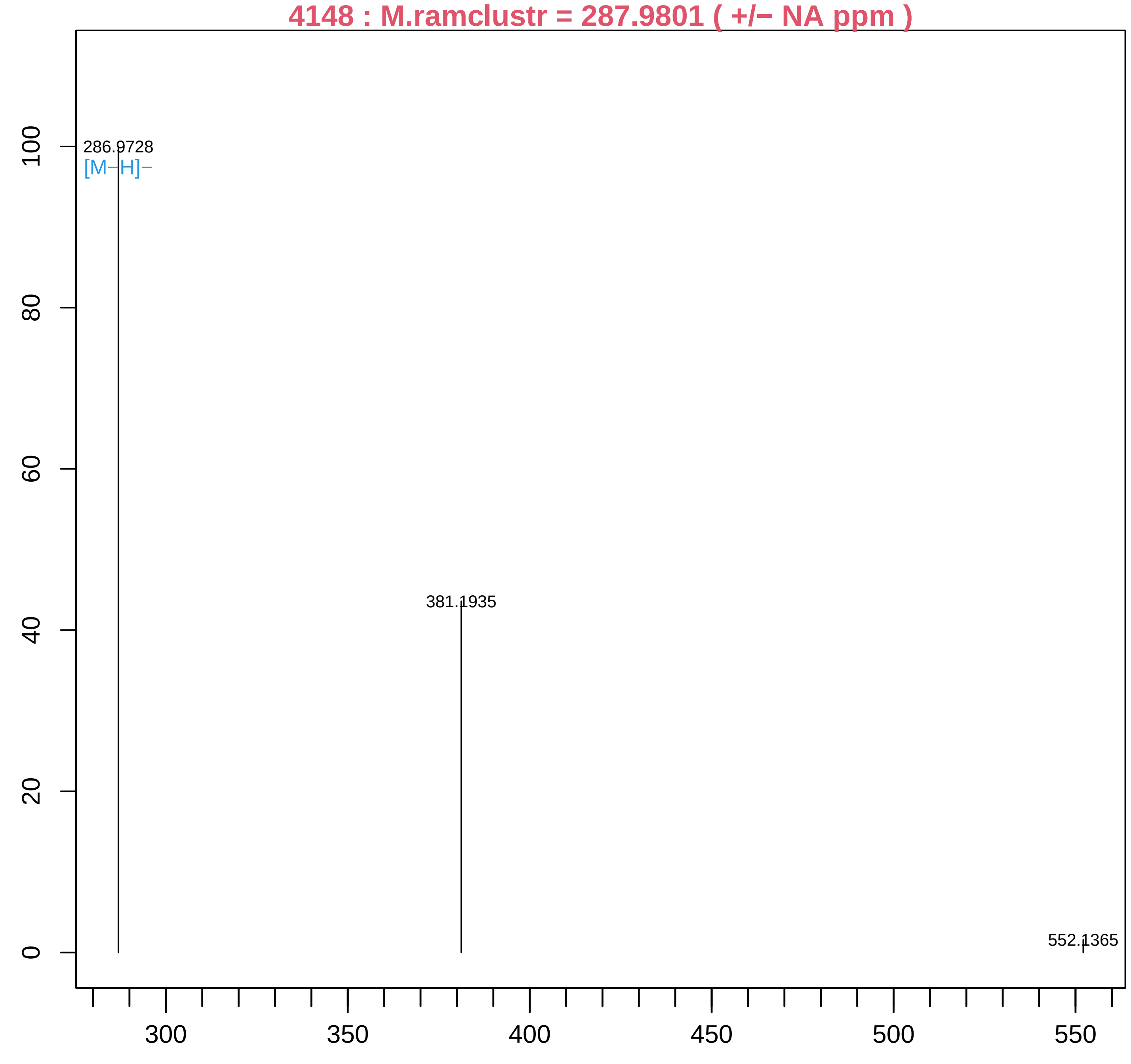
4148 : M.ramclustr = 287.9801 (+/− NA ppm)
286.9728
[M−H]−
381.1935
552.1365
0
20
40
60
80
100
300
350
400
450
500
550

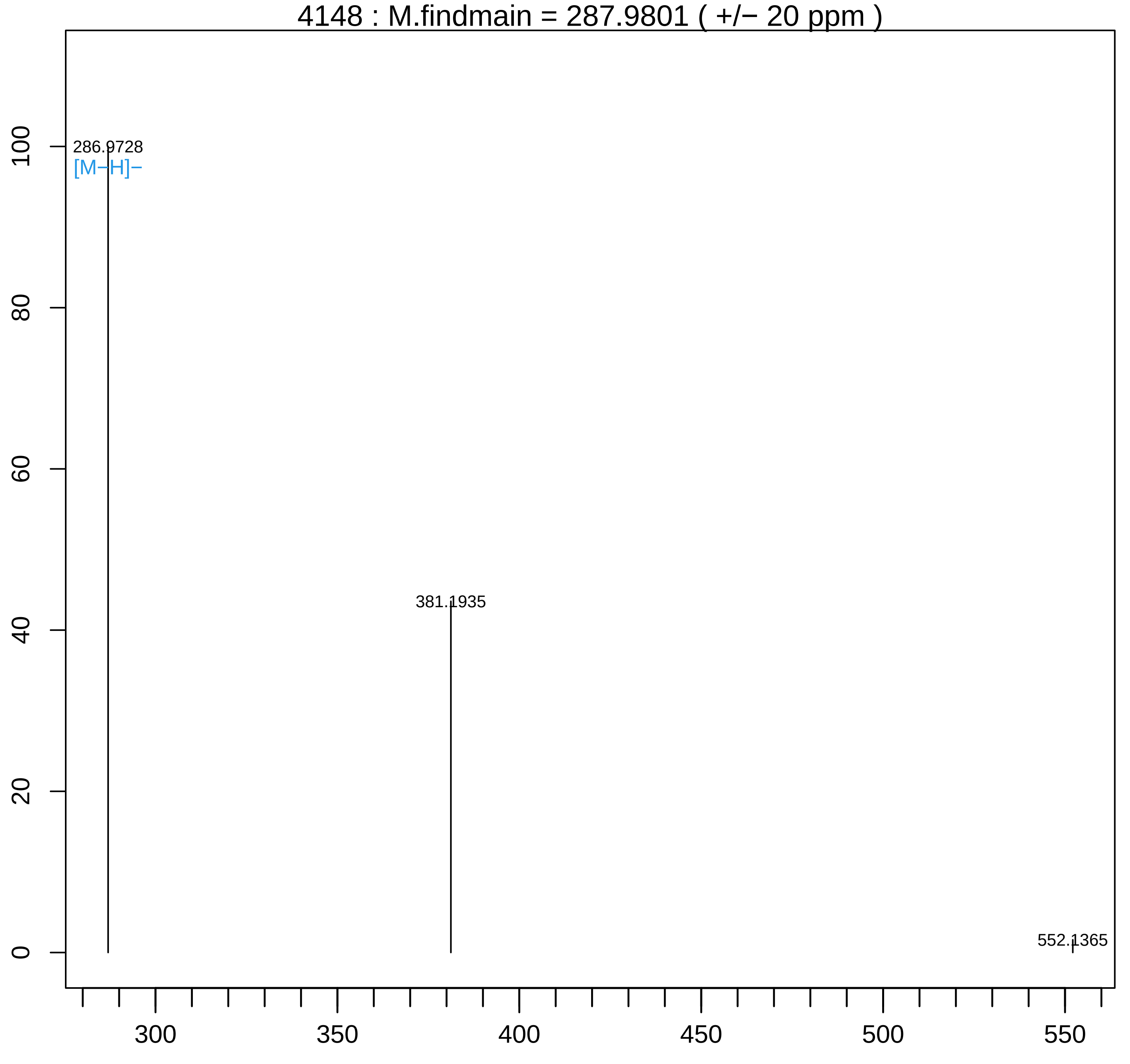
4148 : M.findmain = 287.9801 (+/− 20 ppm)
286.9728
[M−H]−
381.1935
552.1365
0
20
40
60
80
100
300
350
400
450
500
550

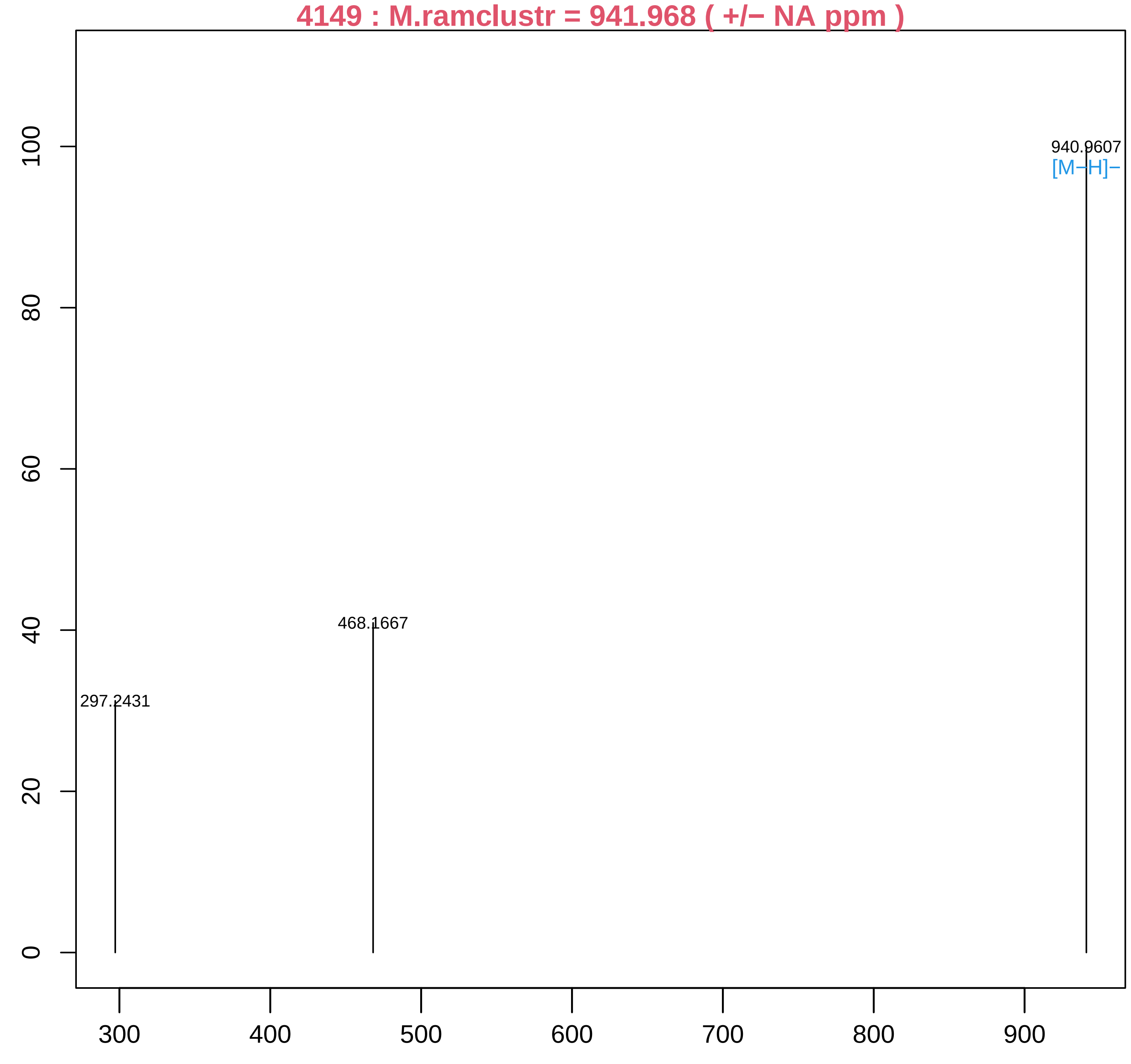

4149 : M.ramclustr = 941.968 (+/− NA ppm)
940.9607
[M−H]−
468.1667
297.2431
0
20
40
60
80
100
300
400
500
600
700
800
900

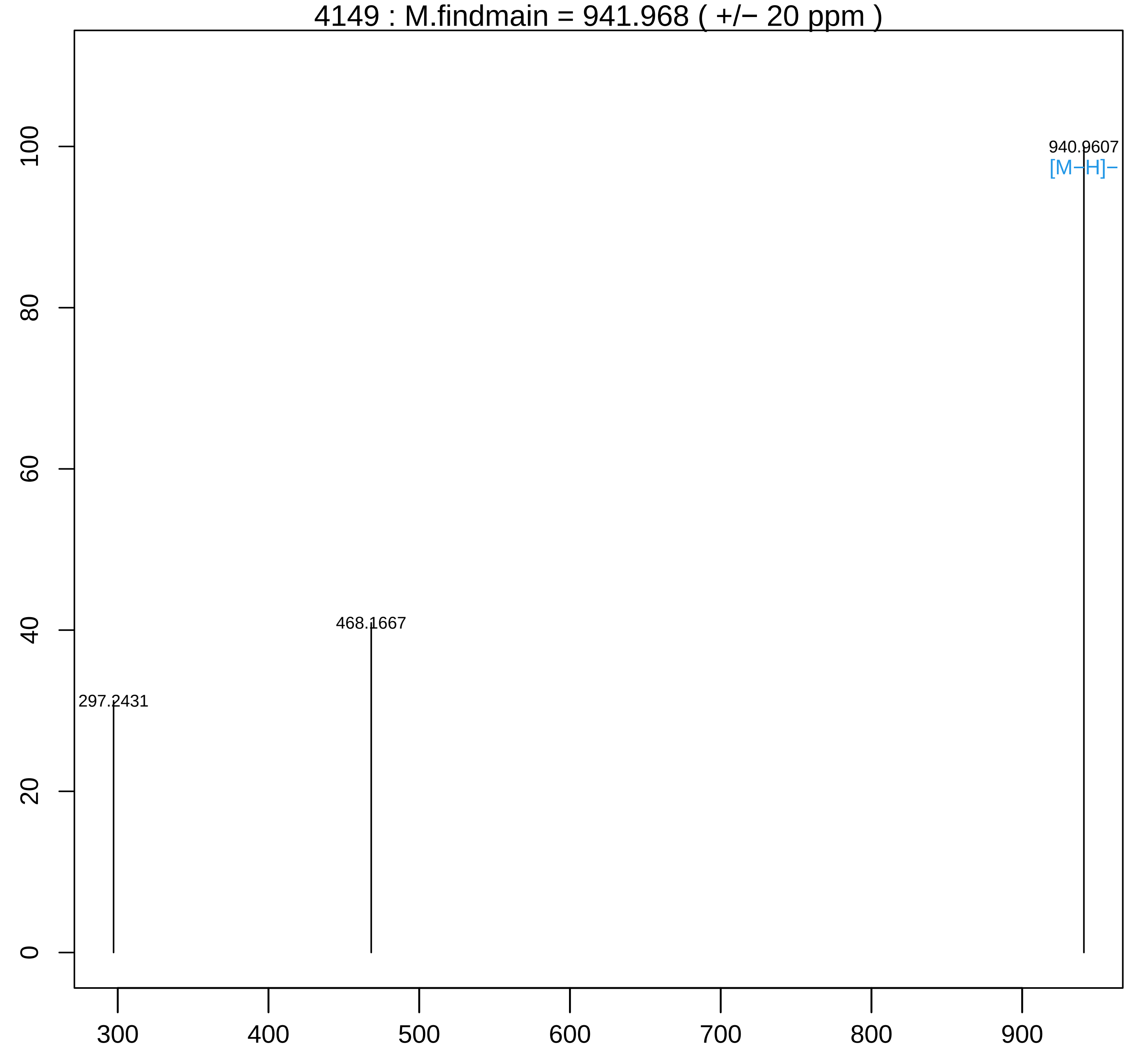

4149 : M.findmain = 941.968 (+/− 20 ppm)
940.9607
[M−H]−
468.1667
297.2431
0
20
40
60
80
100
300
400
500
600
700
800
900

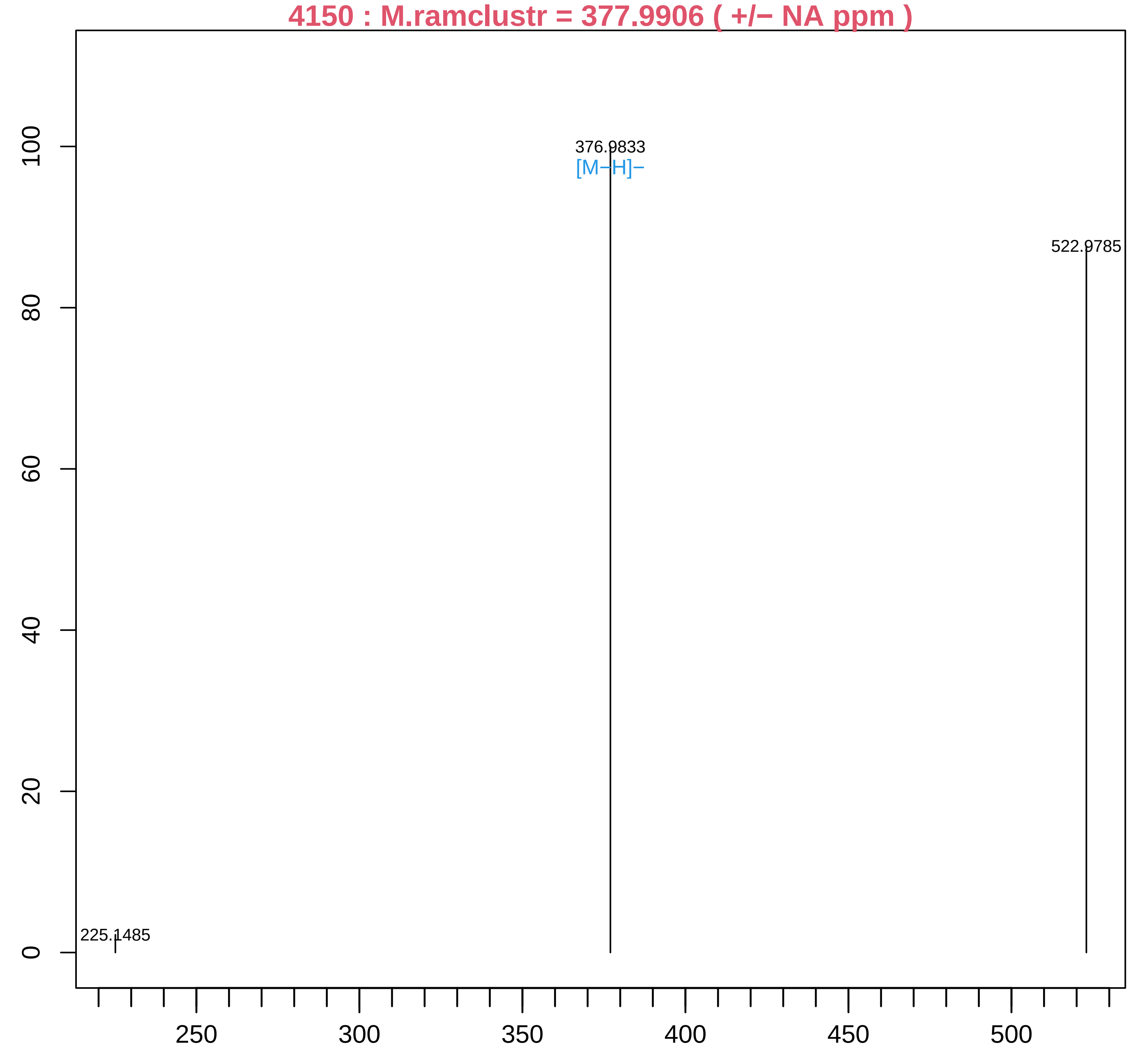
4150 : M.ramclustr = 377.9906 (+/− NA ppm)
376.9833
[M−H]−
522.9785
225.1485
0
20
40
60
80
100
250
300
350
400
450
500

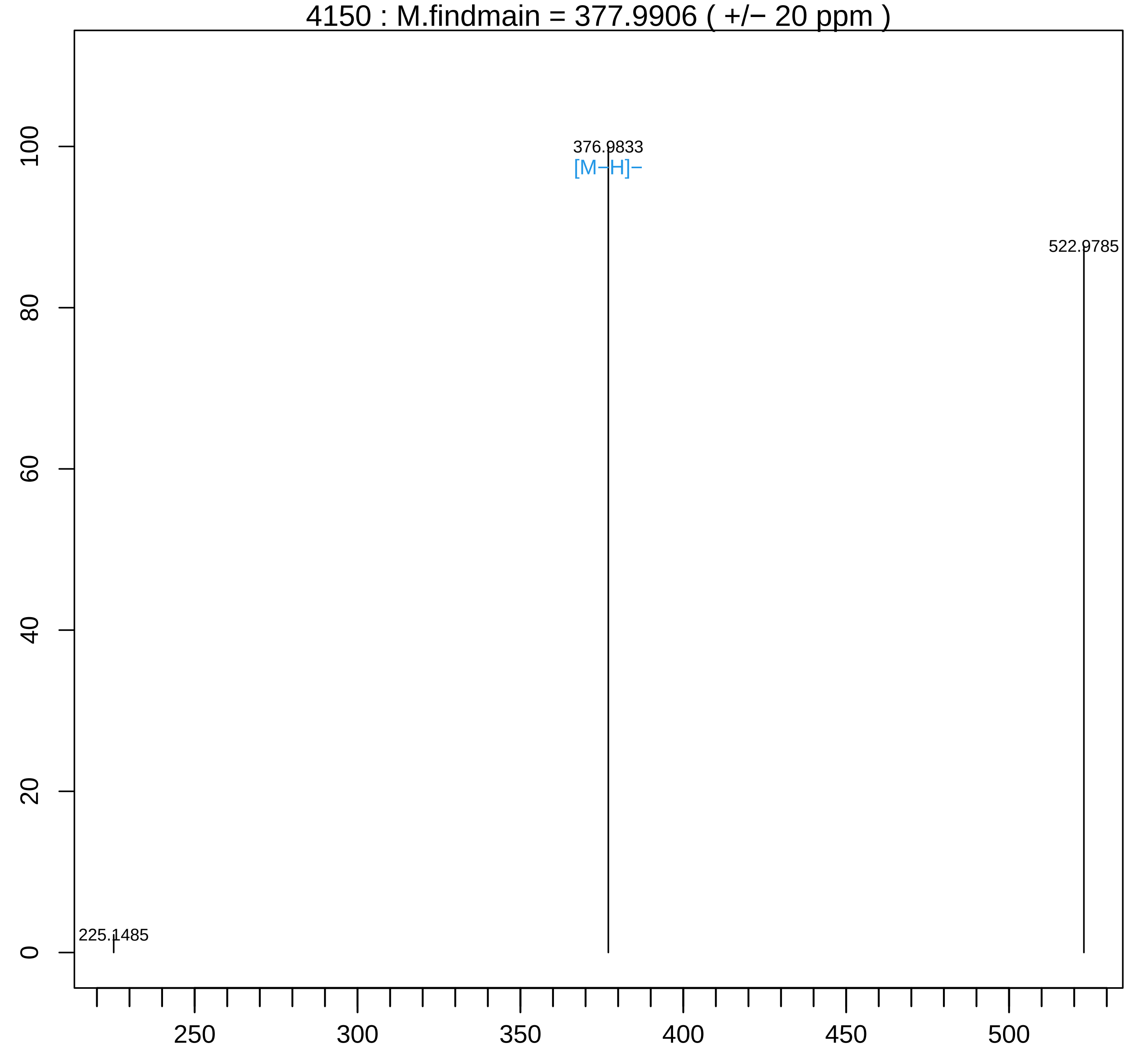
4150 : M.findmain = 377.9906 (+/− 20 ppm)
376.9833
[M−H]−
522.9785
225.1485
0
20
40
60
80
100
250
300
350
400
450
500

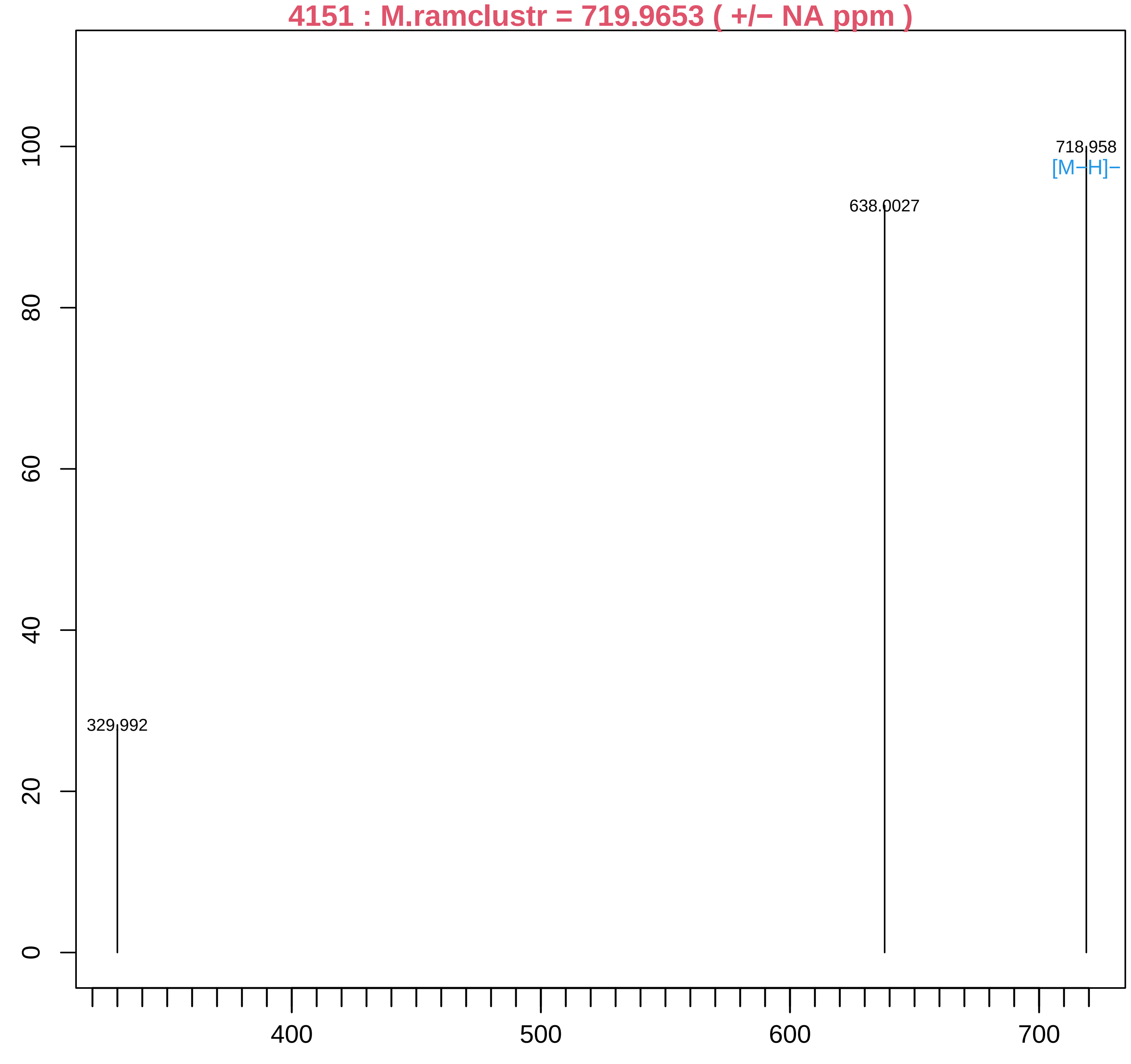
4151 : M.ramclustr = 719.9653 (+/− NA ppm)
718.958
[M−H]−
638.0027
329.992
0
20
40
60
80
100
400
500
600
700
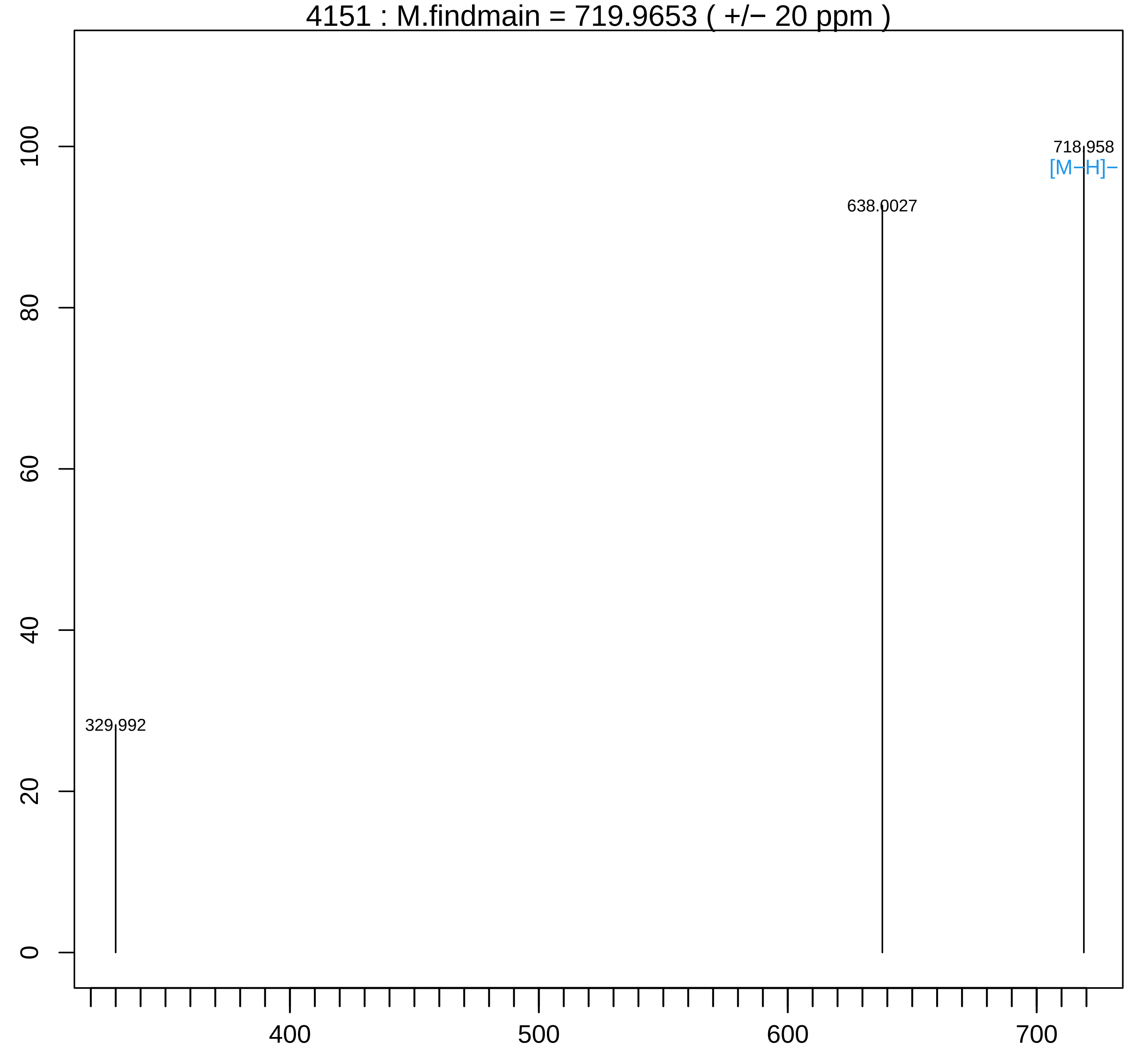
4151 : M.findmain = 719.9653 (+/− 20 ppm)
718.958
[M−H]−
638.0027
329.992
0
20
40
60
80
100
400
500
600
700

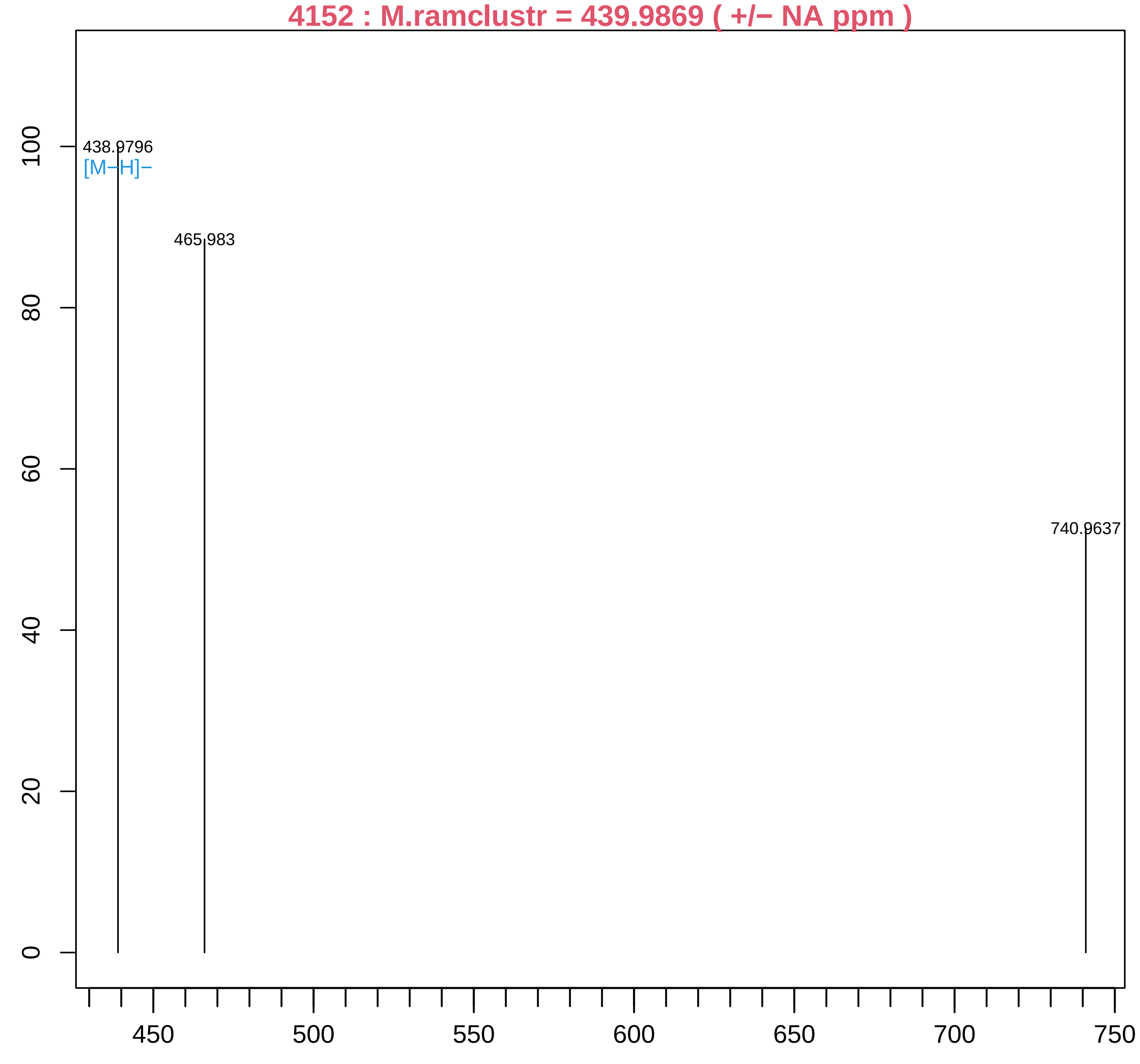
4152 : M.ramclustr = 439.9869 (+/− NA ppm)
438.9796
[M−H]−
465.983
740.9637
0
20
40
60
80
100
450
500
550
600
650
700
750

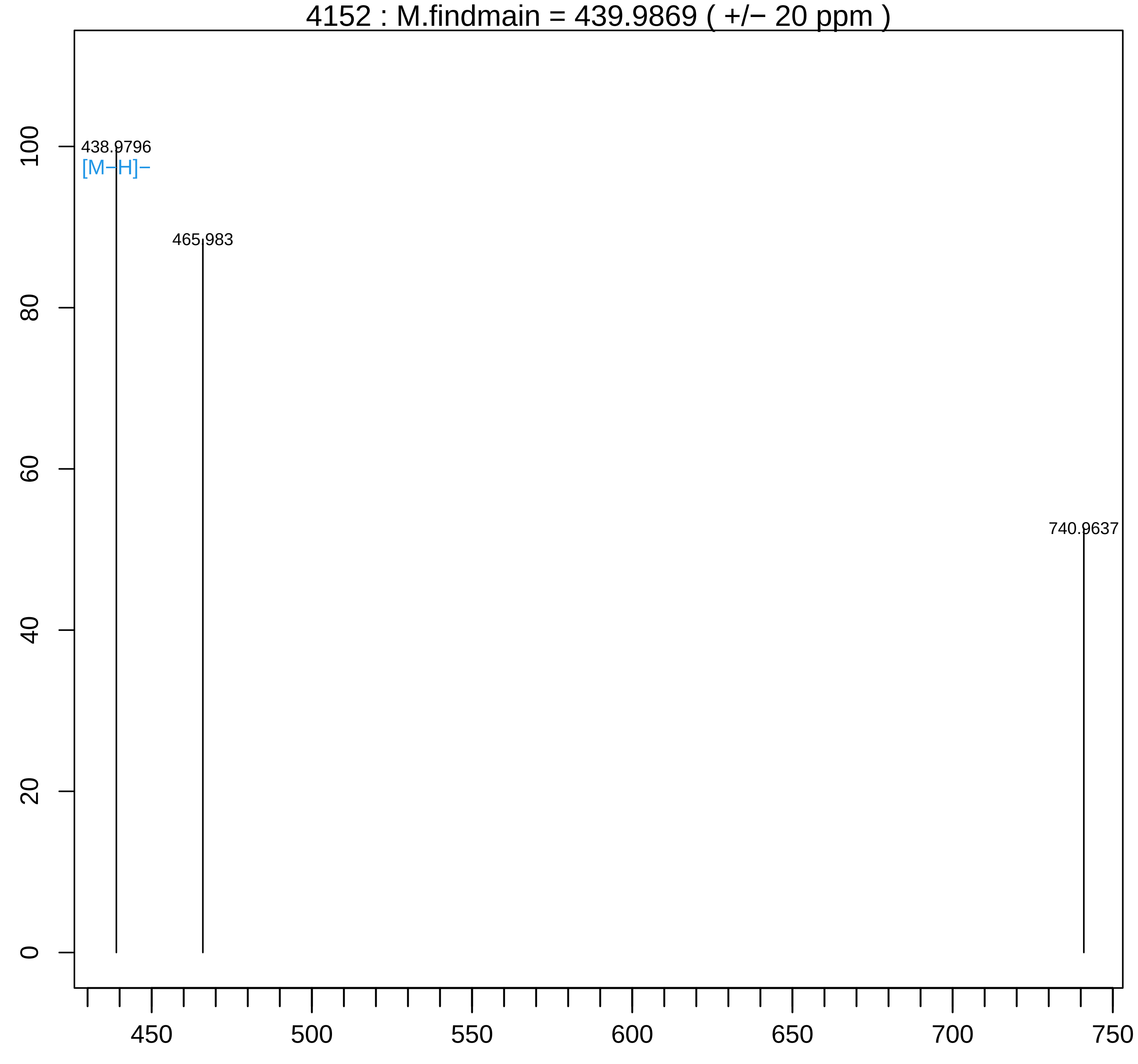
4152 : M.findmain = 439.9869 (+/− 20 ppm)
438.9796
[M−H]−
465.983
740.9637
0
20
40
60
80
100
450
500
550
600
650
700
750

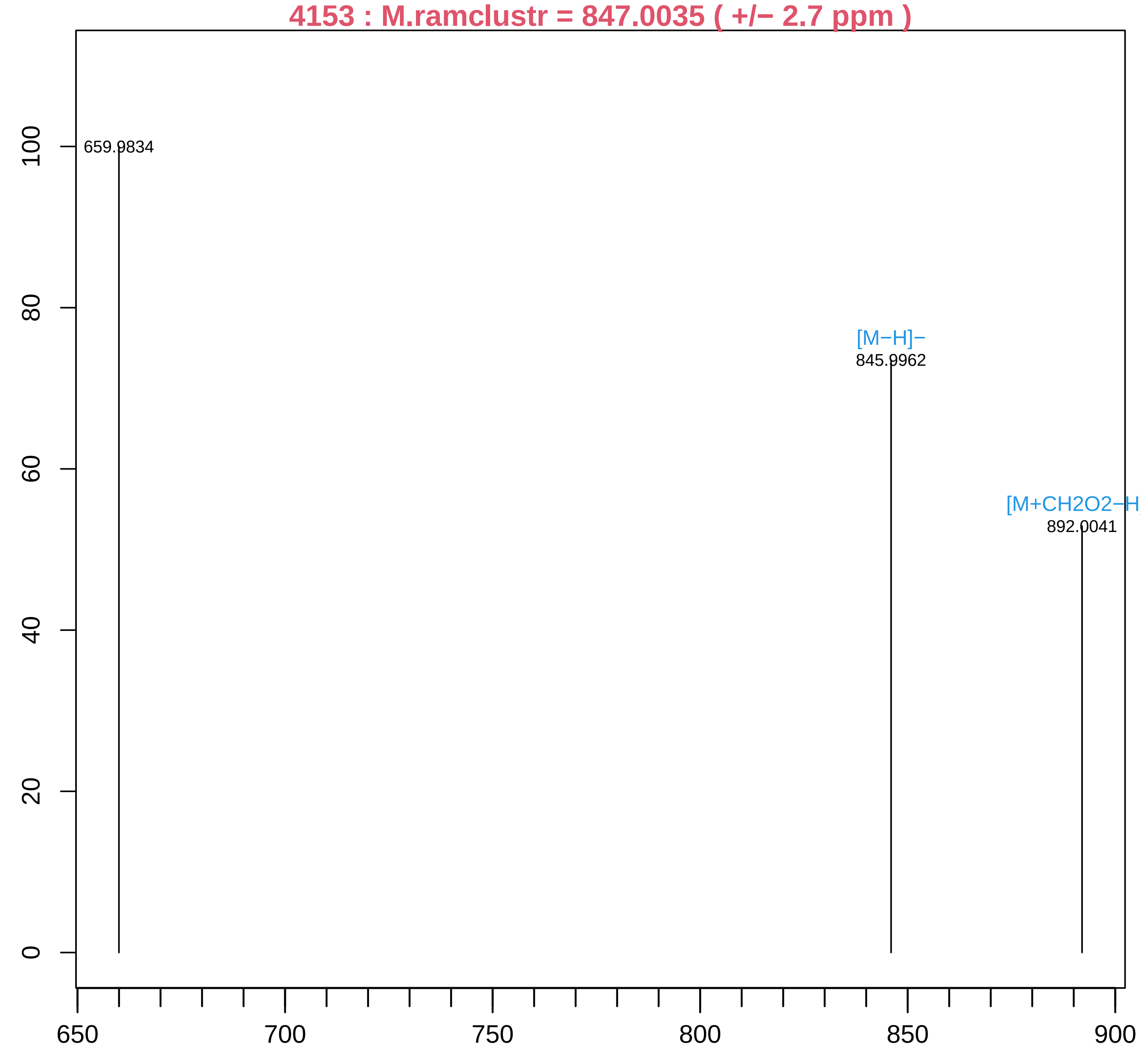

4153 : M.ramclustr = 847.0035 (+/− 2.7 ppm)
659.9834
[M−H]−
845.9962
[M+CH2O2−H
892.0041
0
20
40
60
80
100
650
700
750
800
850
900

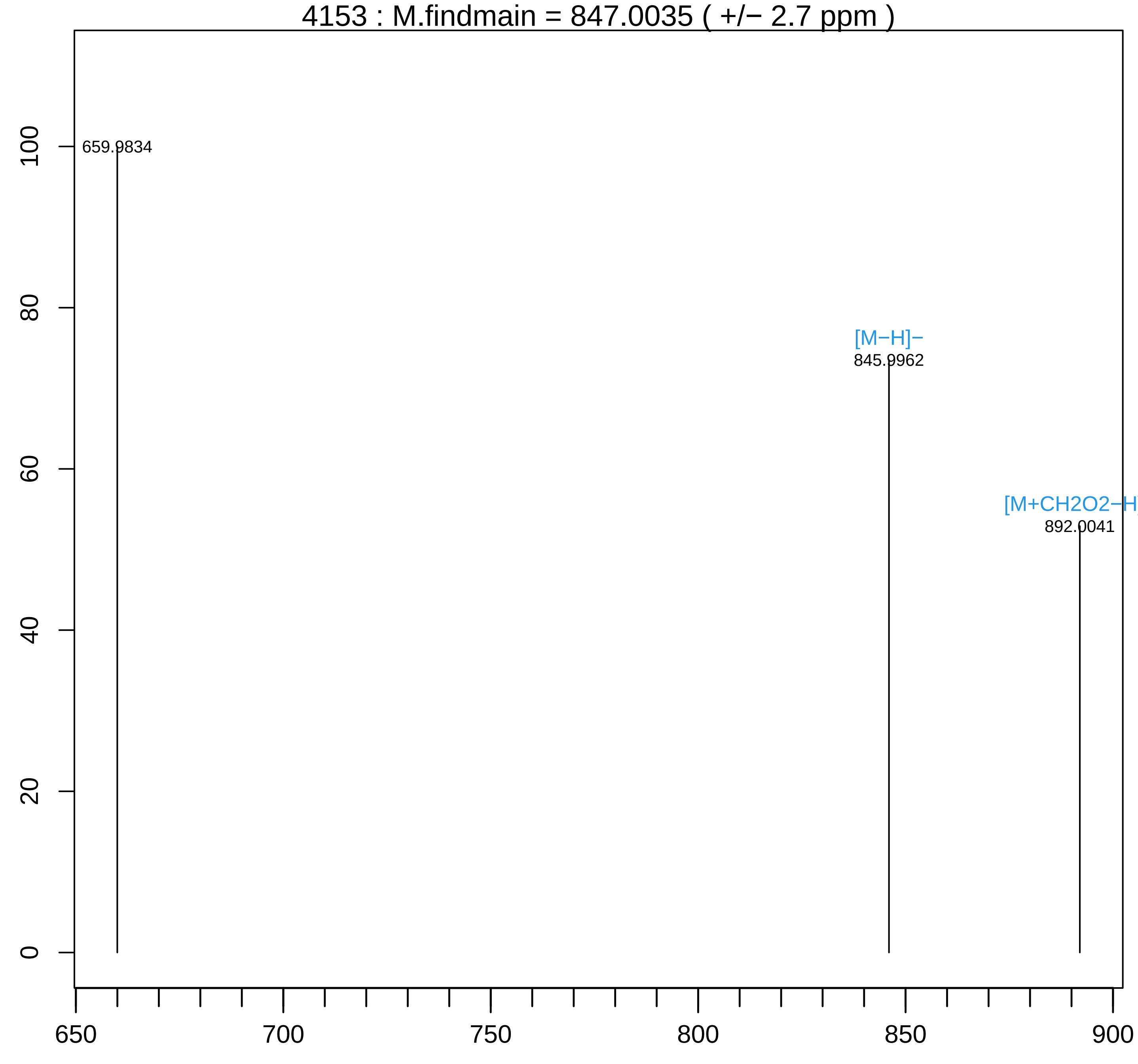

4153 : M.findmain = 847.0035 (+/− 2.7 ppm)
659.9834
[M−H]−
845.9962
[M+CH2O2−H
892.0041
0
20
40
60
80
100
650
700
750
800
850
900

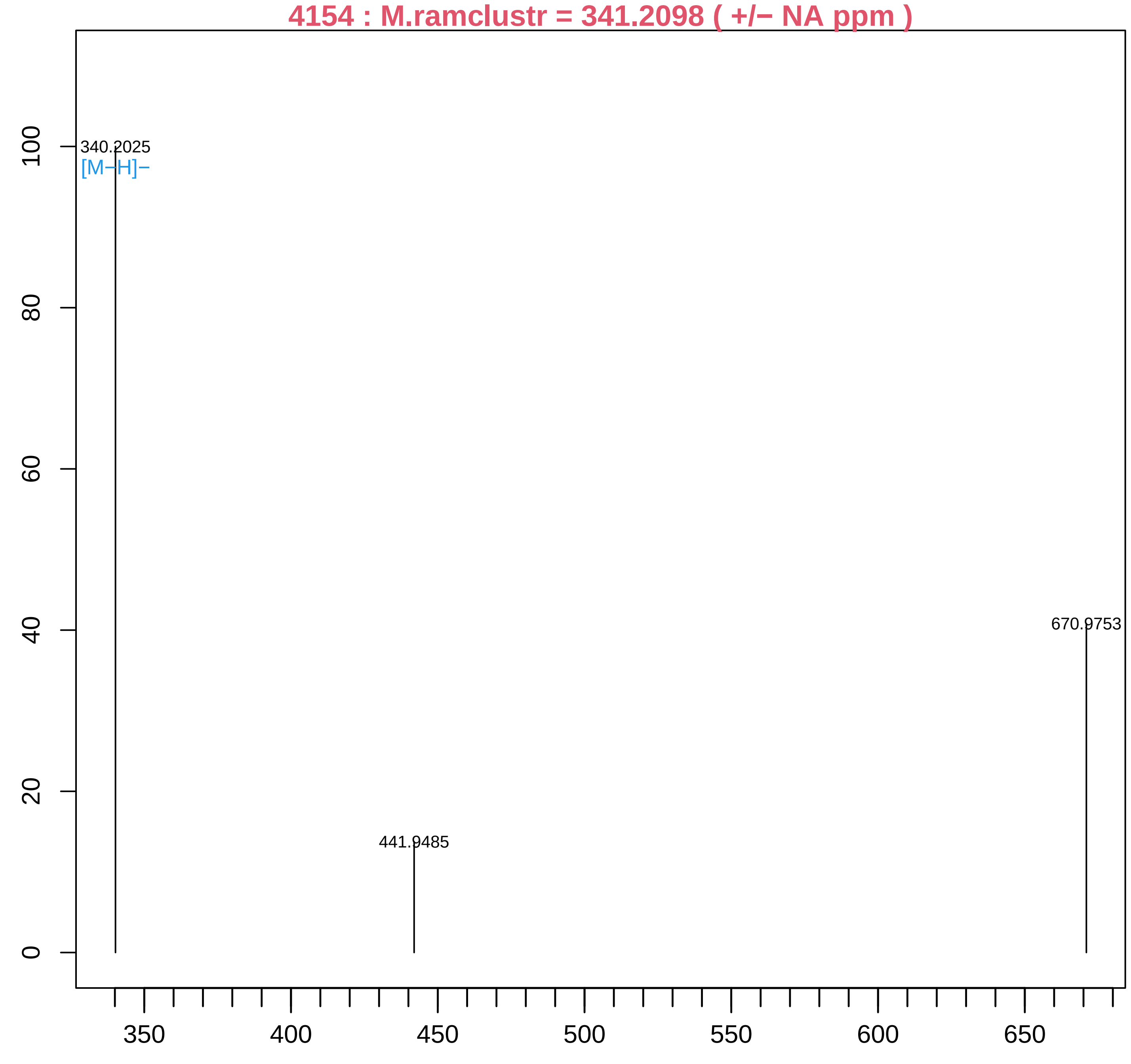
4154 : M.ramclustr = 341.2098 (+/− NA ppm)
340.2025
[M−H]−
441.9485
670.9753
0
20
40
60
80
100
350
400
450
500
550
600
650

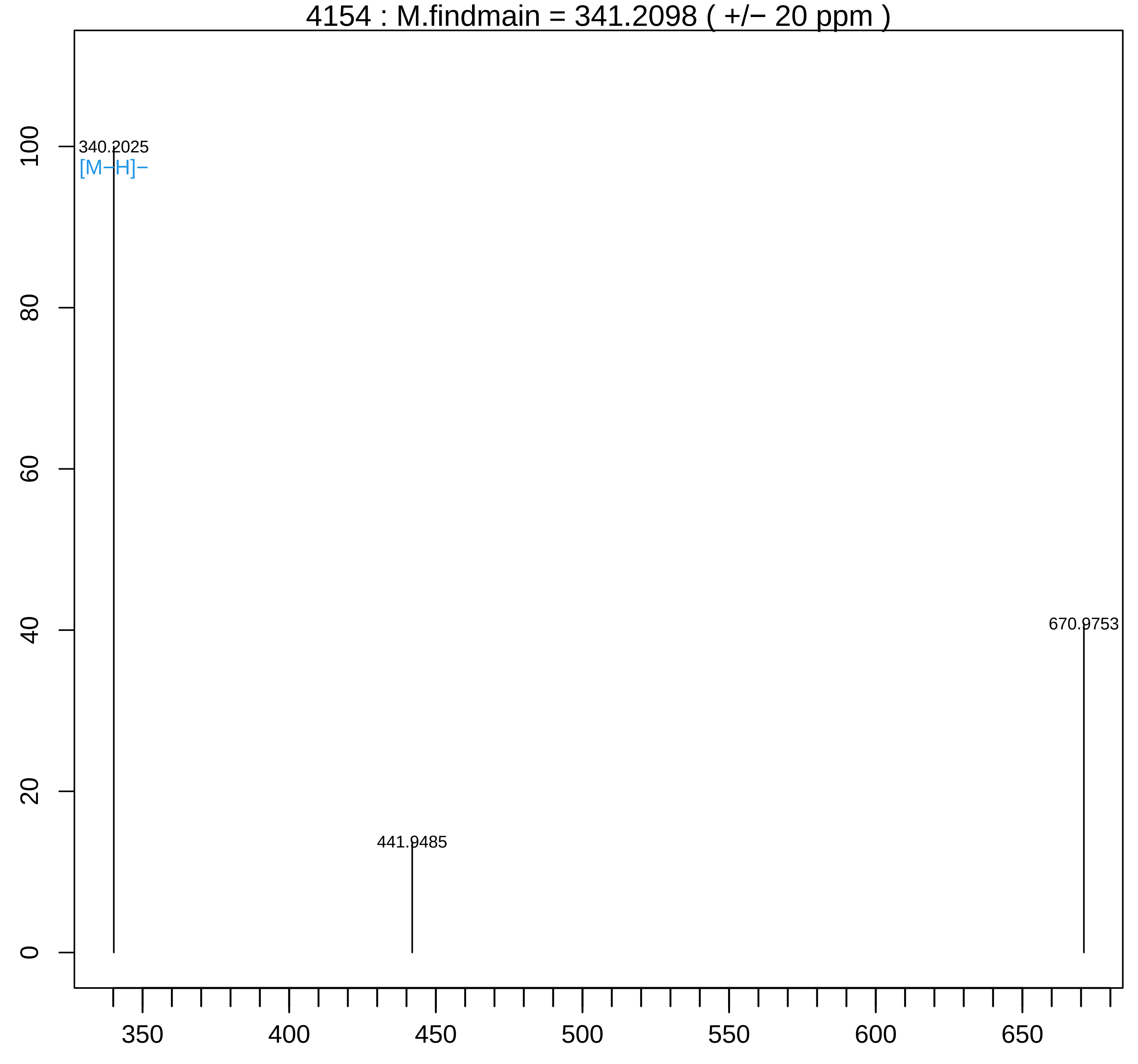
4154 : M.findmain = 341.2098 (+/− 20 ppm)
340.2025
[M−H]−
441.9485
670.9753
0
20
40
60
80
100
350
400
450
500
550
600
650

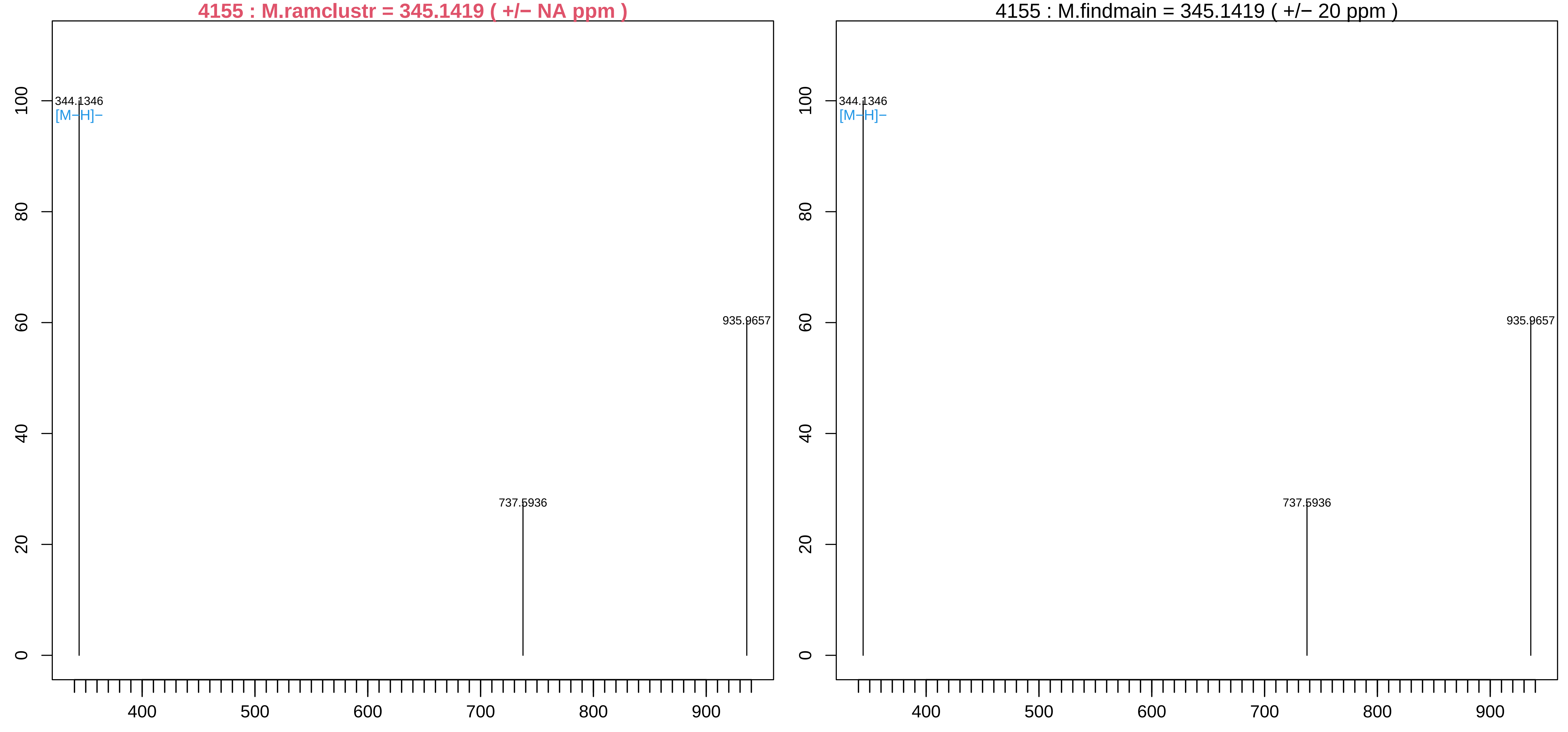
4155 : M.ramclustr = 345.1419 (+/− NA ppm)
344.1346
[M−H]−
935.9657
737.5936
4155 : M.findmain = 345.1419 (+/− 20 ppm)
344.1346
[M−H]−
935.9657
737.5936
0
20
40
60
80
100
400
500
600
700
800
900

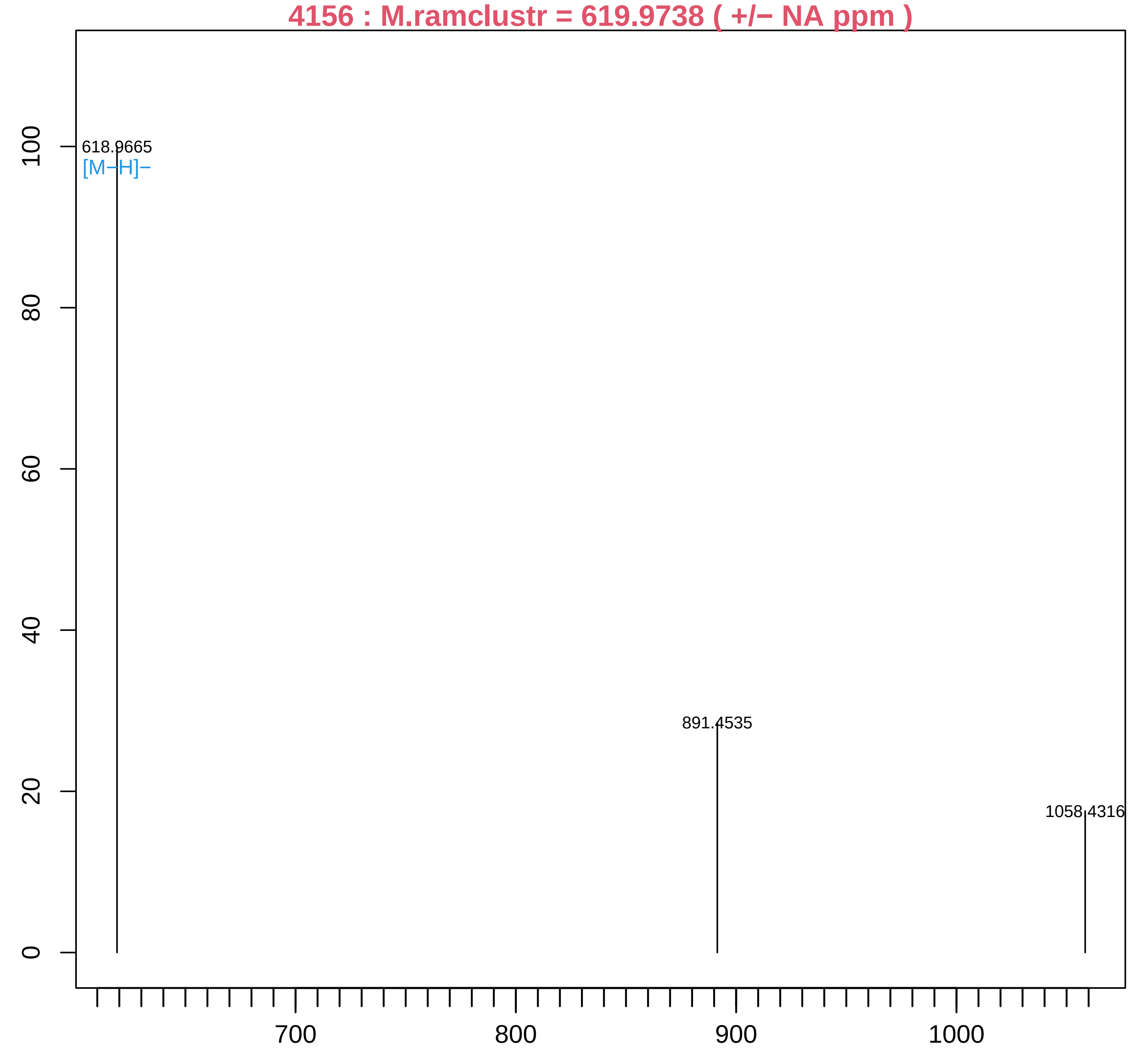

4156 : M.ramclustr = 619.9738 (+/− NA ppm)
618.9665
[M−H]−
891.4535
1058.4316
0
20
40
60
80
100
700
800
900
1000

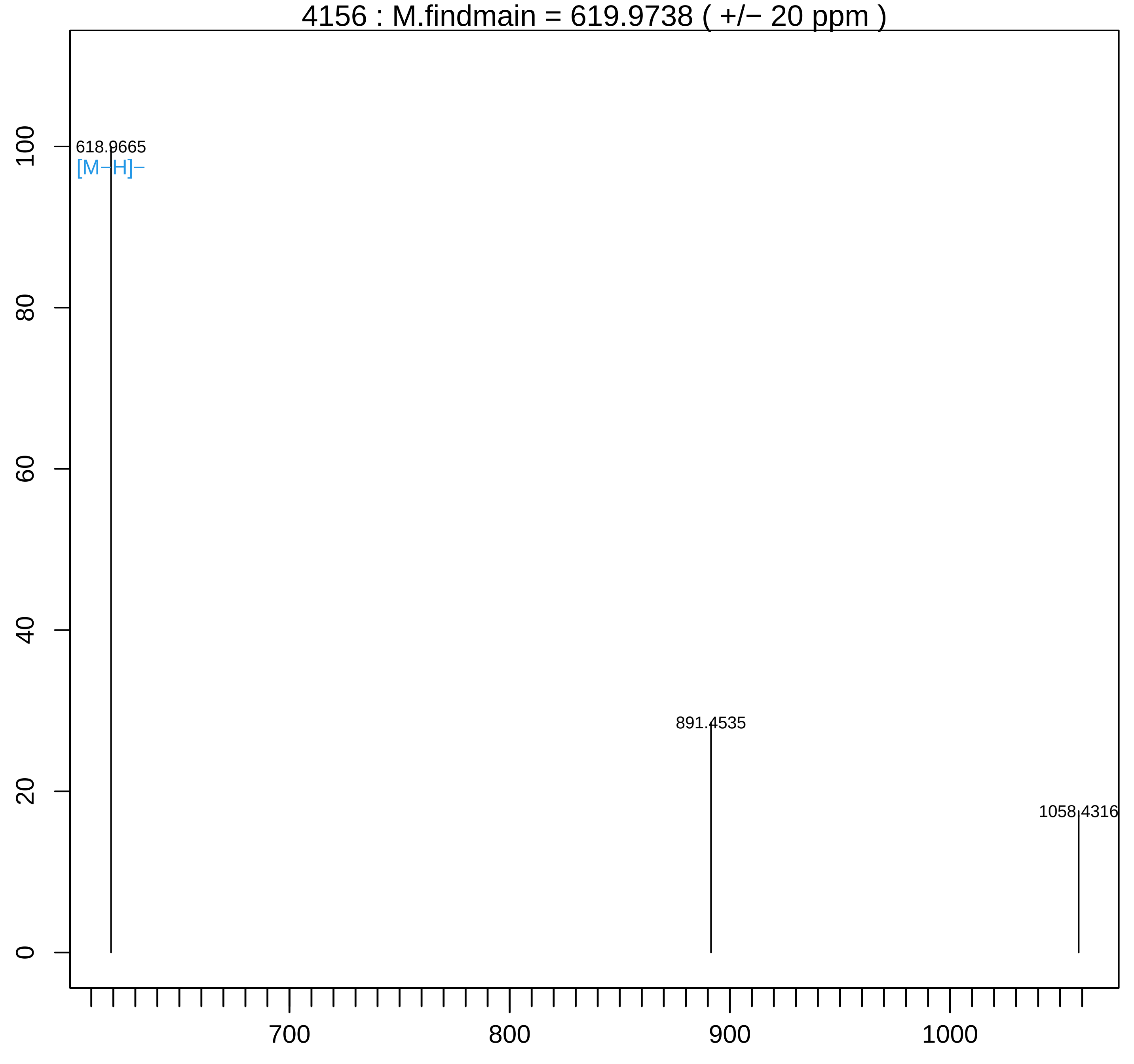

4156 : M.findmain = 619.9738 (+/− 20 ppm)
618.9665
[M−H]−
891.4535
1058.4316
0
20
40
60
80
100
700
800
900
1000

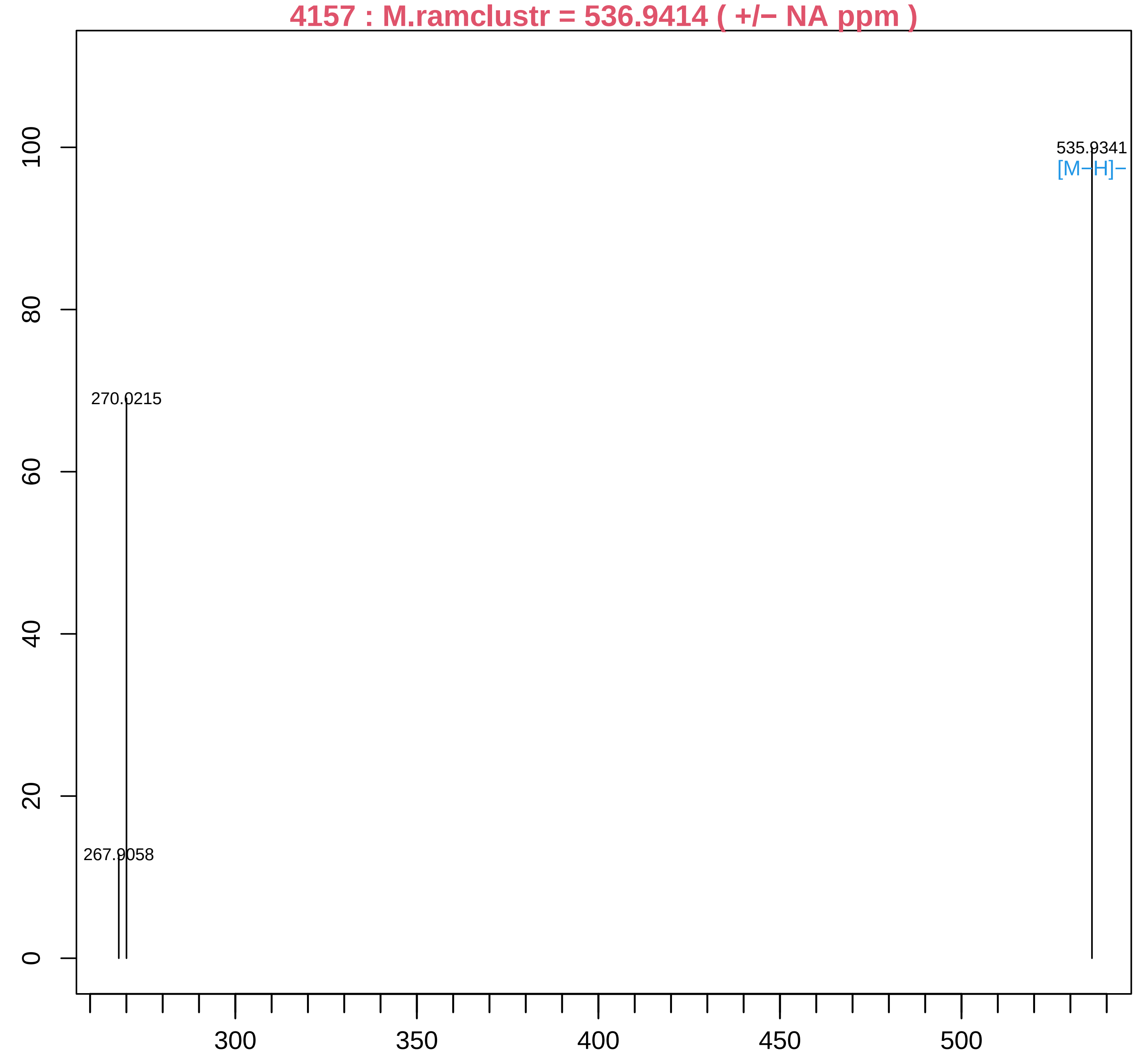
4157 : M.ramclustr = 536.9414 (+/− NA ppm)
535.9341
[M−H]−
270.0215
267.9058
0
20
40
60
80
100
300
350
400
450
500

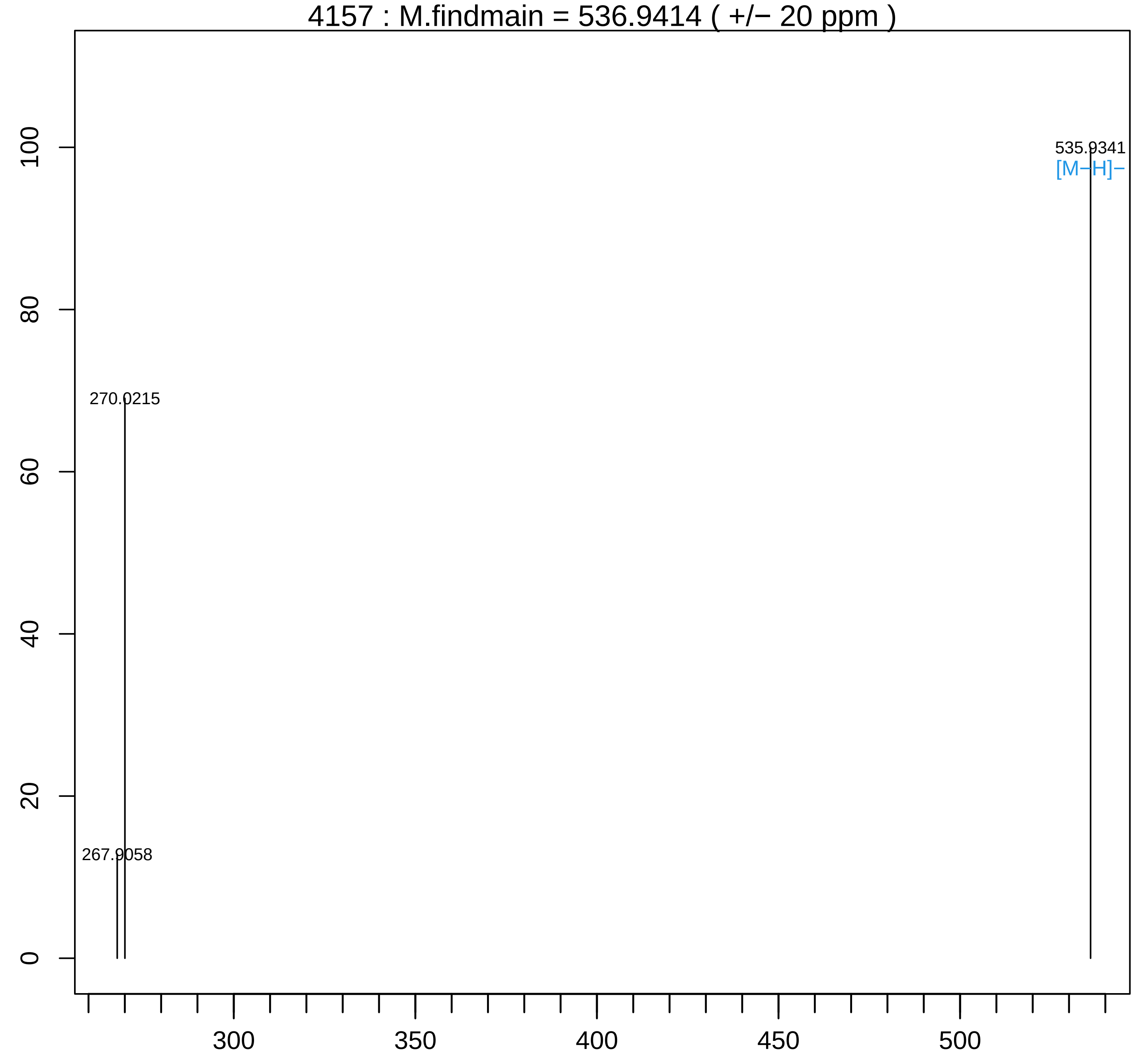
4157 : M.findmain = 536.9414 (+/− 20 ppm)
535.9341
[M−H]−
270.0215
267.9058
0
20
40
60
80
100
300
350
400
450
500

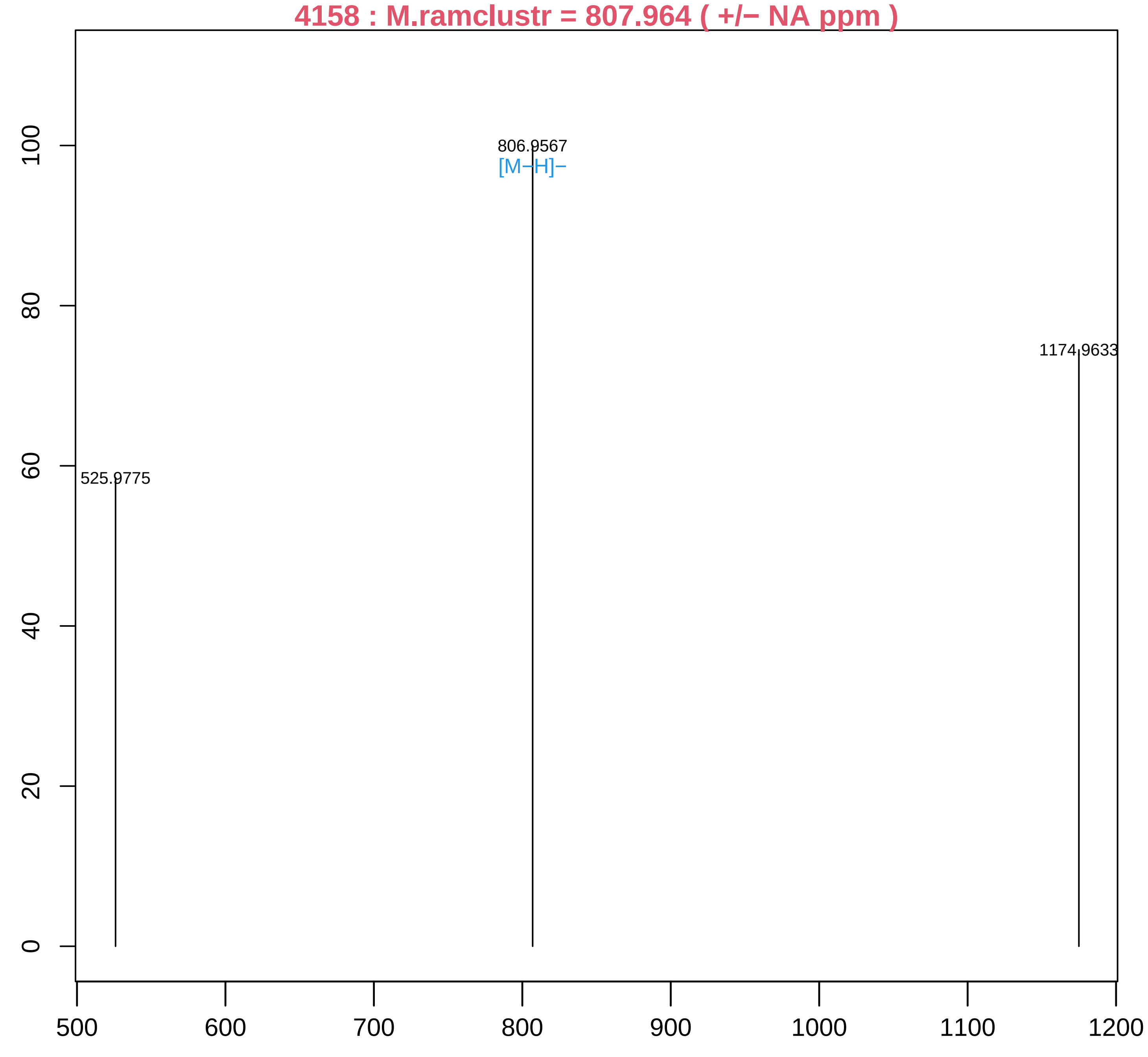
4158 : M.ramclustr = 807.964 (+/− NA ppm)
806.9567
[M−H]−
1174.9633
525.9775
0
20
40
60
80
100
500
600
700
800
900
1000
1100
1200

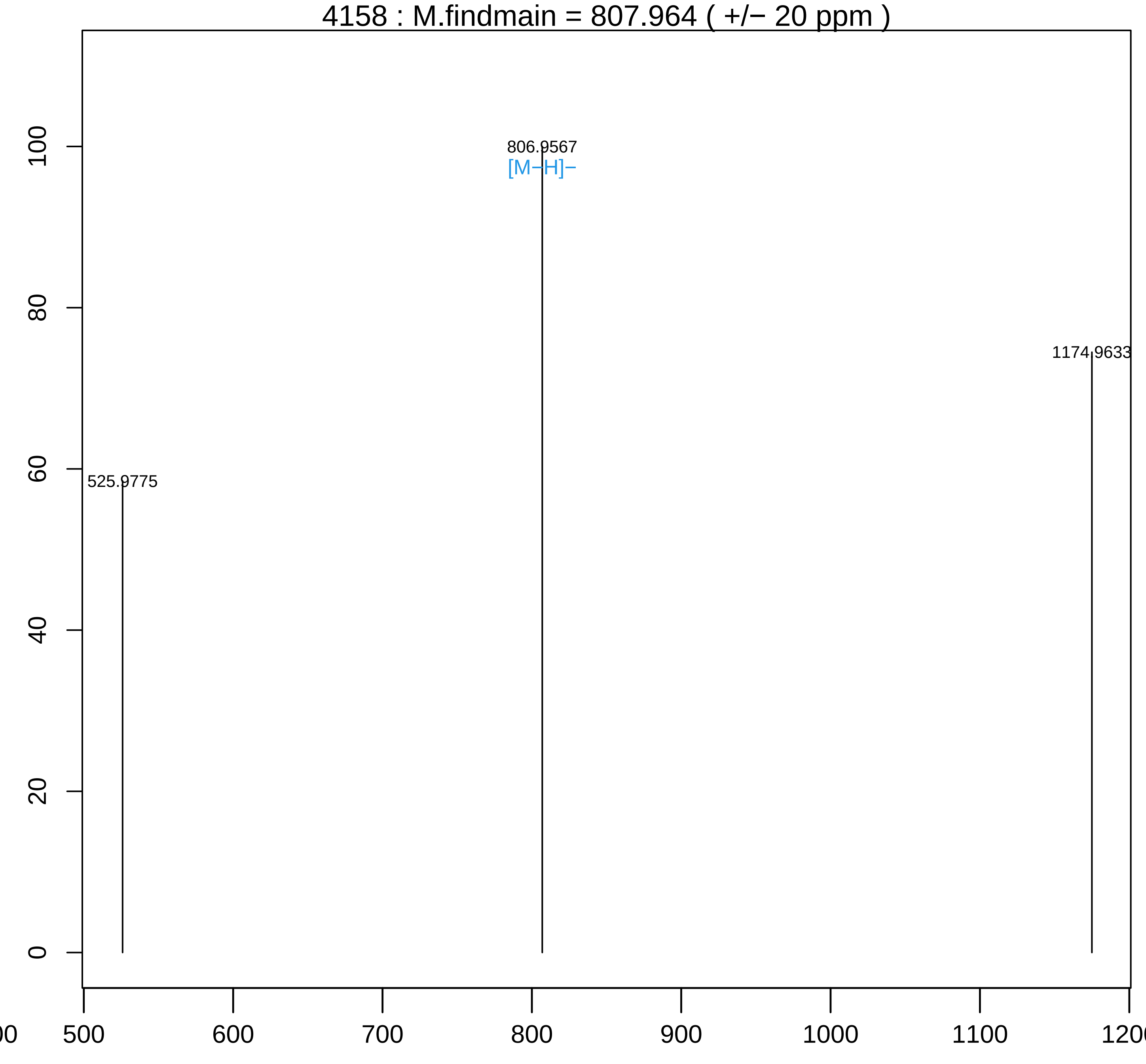
4158 : M.findmain = 807.964 (+/− 20 ppm)
806.9567
[M−H]−
1174.9633
525.9775
0
20
40
60
80
100
500
600
700
800
900
1000
1100

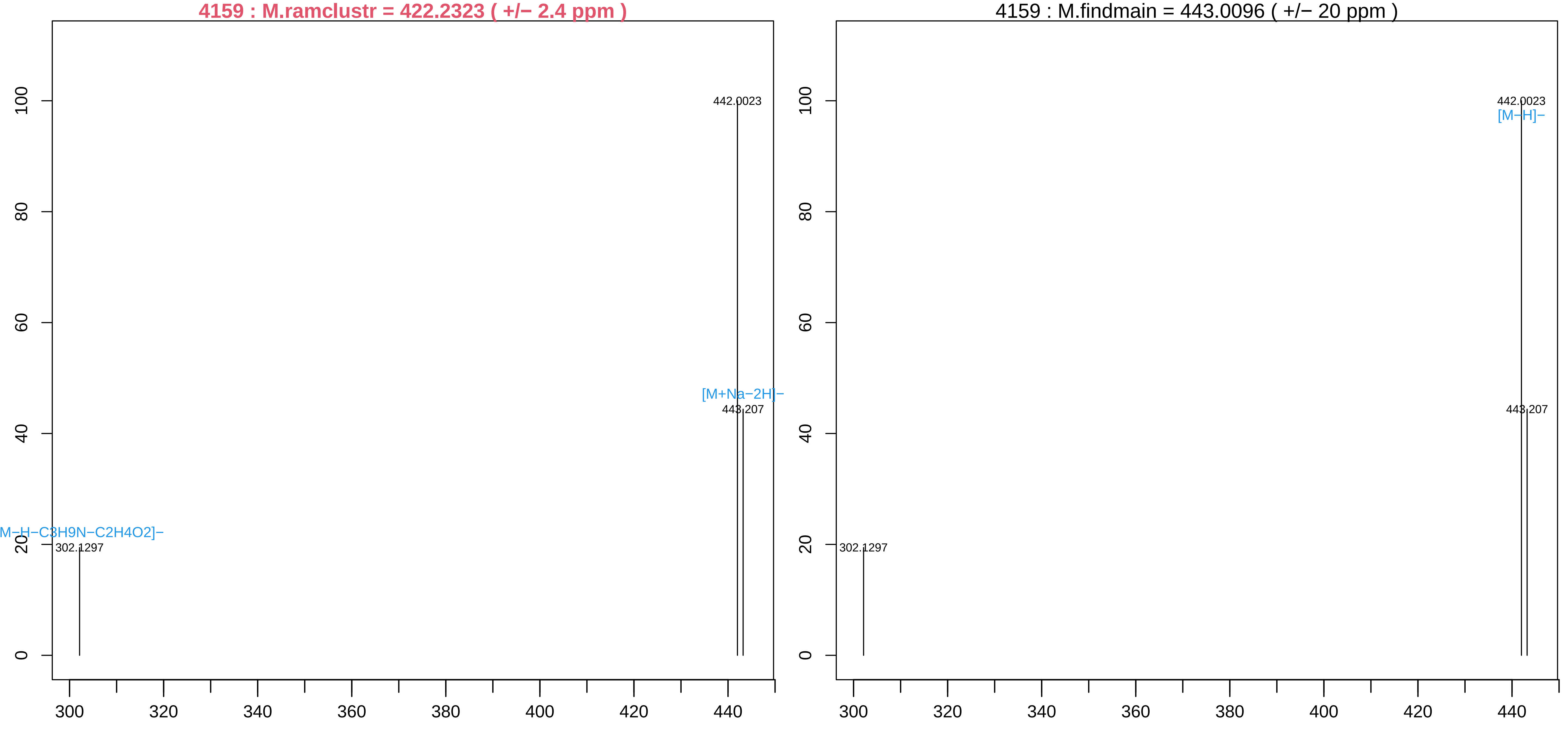

4159 : M.ramclustr = 422.2323 (+/− 2.4 ppm)
442.0023
[M+Na−2H]−
443.207
M−H−C3H9N−C2H4O2]−
302.1297
4159 : M.findmain = 443.0096 (+/− 20 ppm)
442.0023
[M−H]−
443.207
302.1297
0
20
40
60
80
100
300
320
340
360
380
400
420
440

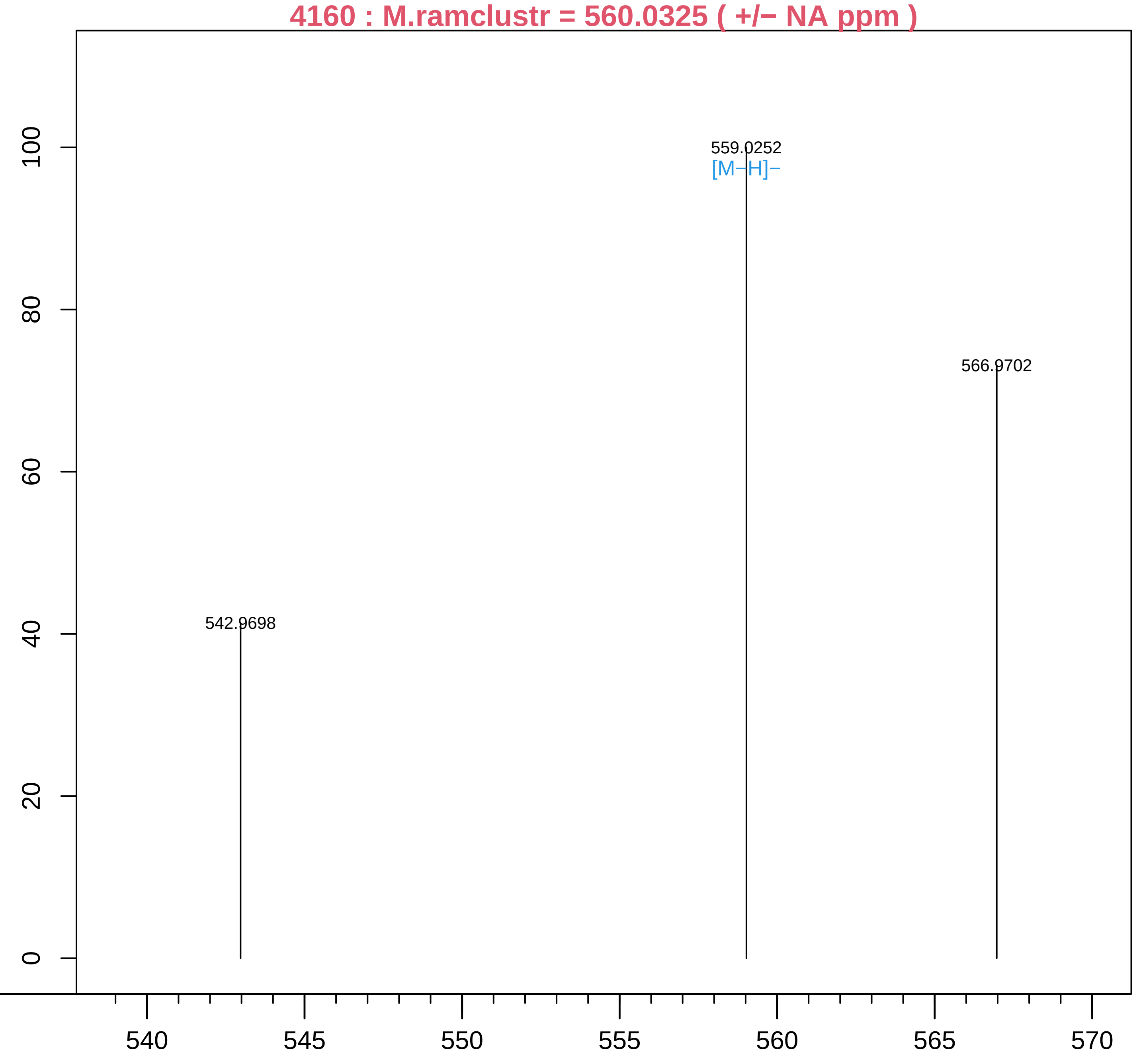
4160 : M.ramclustr = 560.0325 (+/− NA ppm)
559.0252
[M−H]−
566.9702
542.9698
0
20
40
60
80
100
540
545
550
555
560
565
570

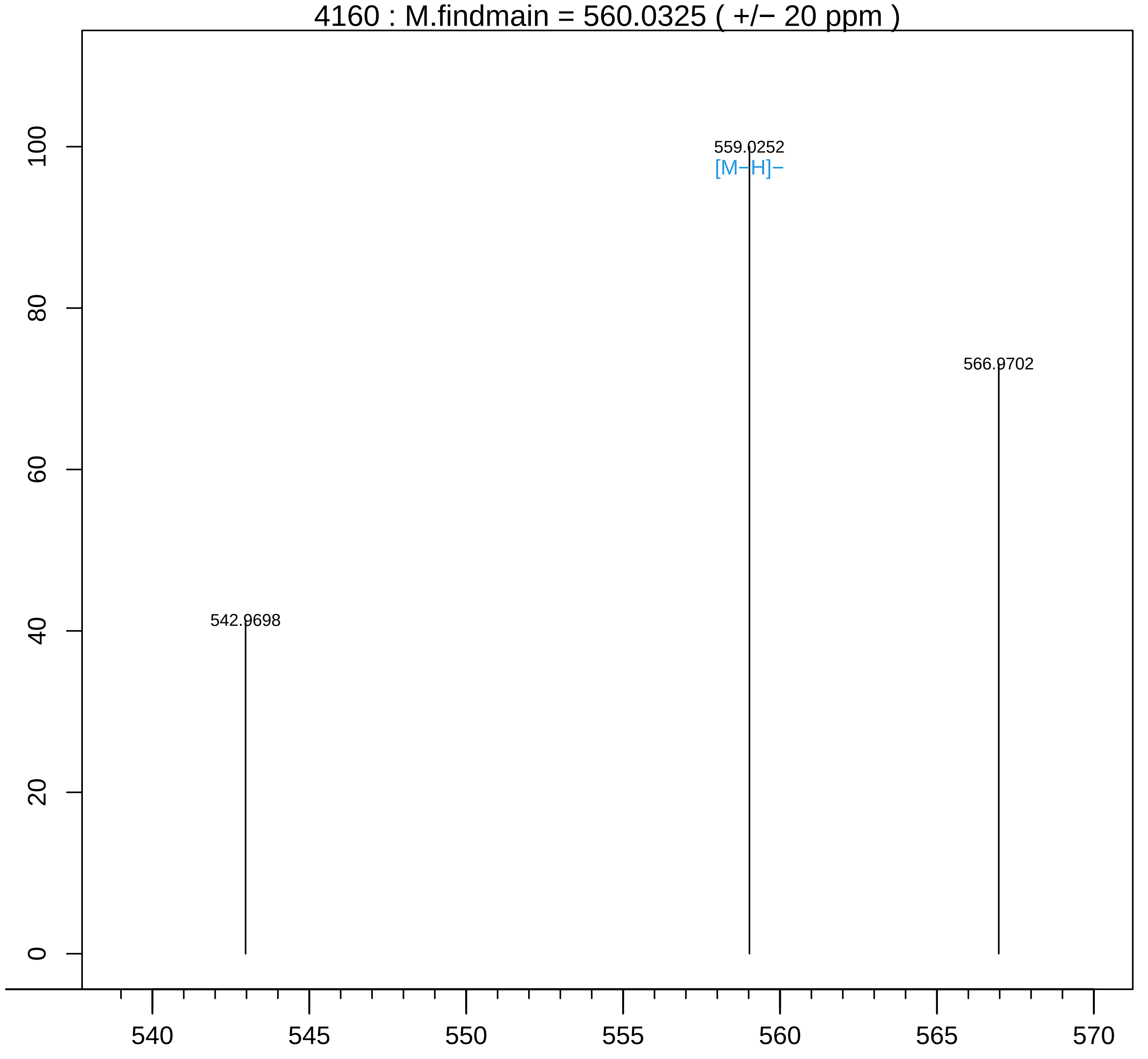
4160 : M.findmain = 560.0325 (+/− 20 ppm)
559.0252
[M−H]−
566.9702
542.9698
0
20
40
60
80
100
540
545
550
555
560
565
570

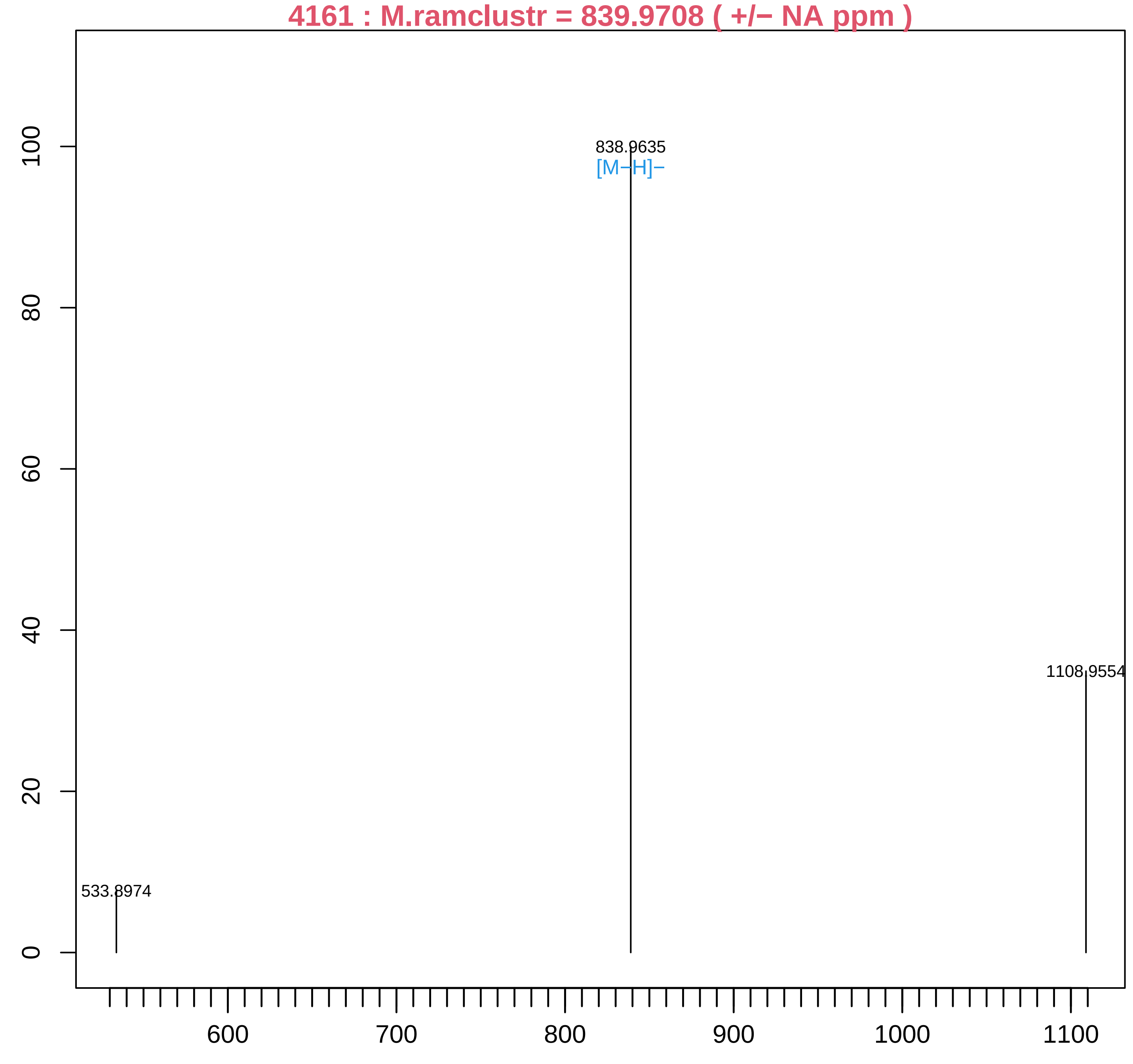
4161 : M.ramclustr = 839.9708 (+/− NA ppm)
838.9635
[M−H]−
1108.9554
533.8974
0
20
40
60
80
100
600
700
800
900
1000
1100

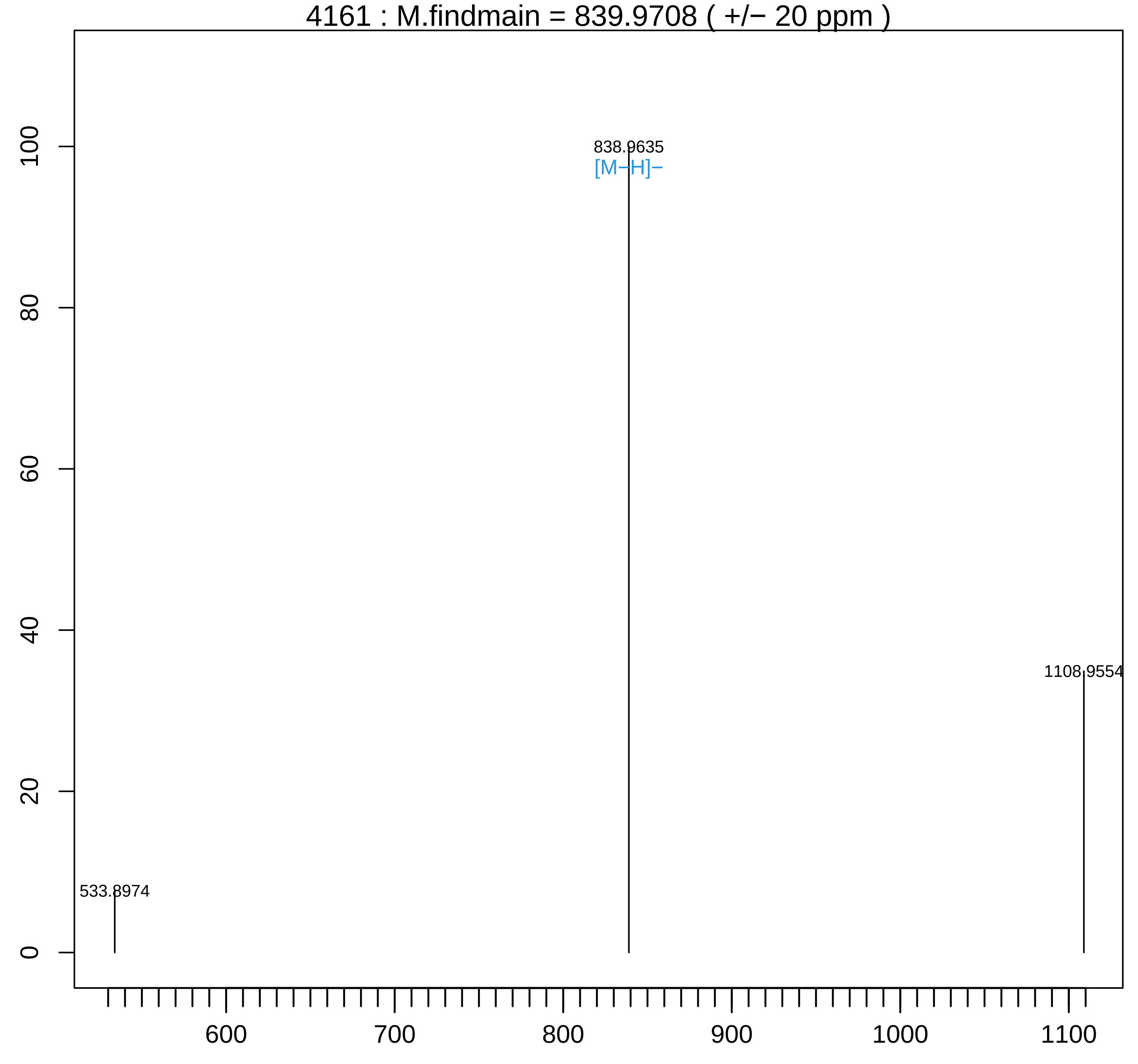
4161 : M.findmain = 839.9708 (+/− 20 ppm)
838.9635
[M−H]−
1108.9554
533.8974
0
20
40
60
80
100
600
700
800
900
1000
1100

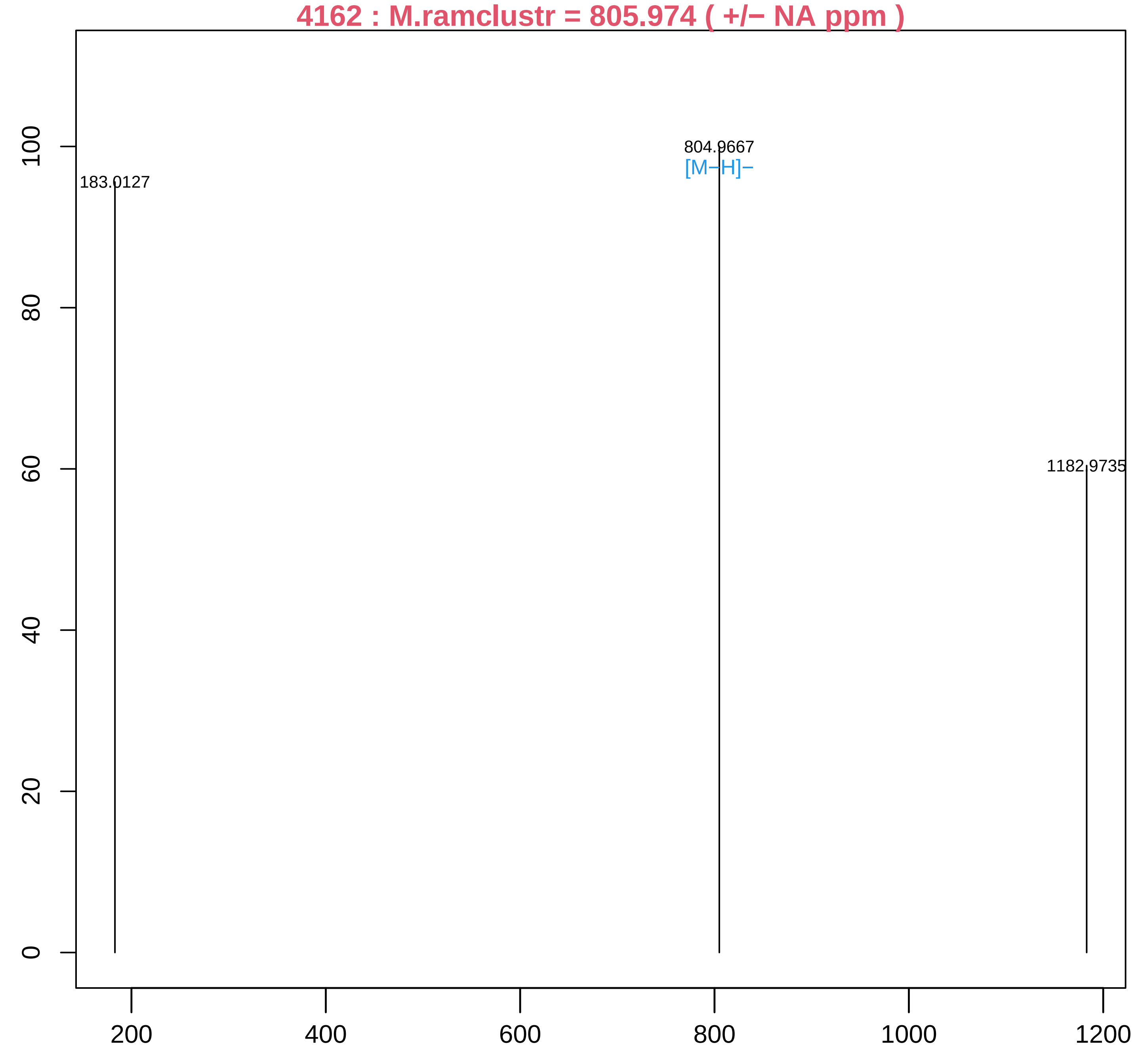
4162 : M.ramclustr = 805.974 (+/− NA ppm)
183.0127
804.9667
[M−H]−
1182.9735
0
20
40
60
80
100
200
400
600
800
1000
1200

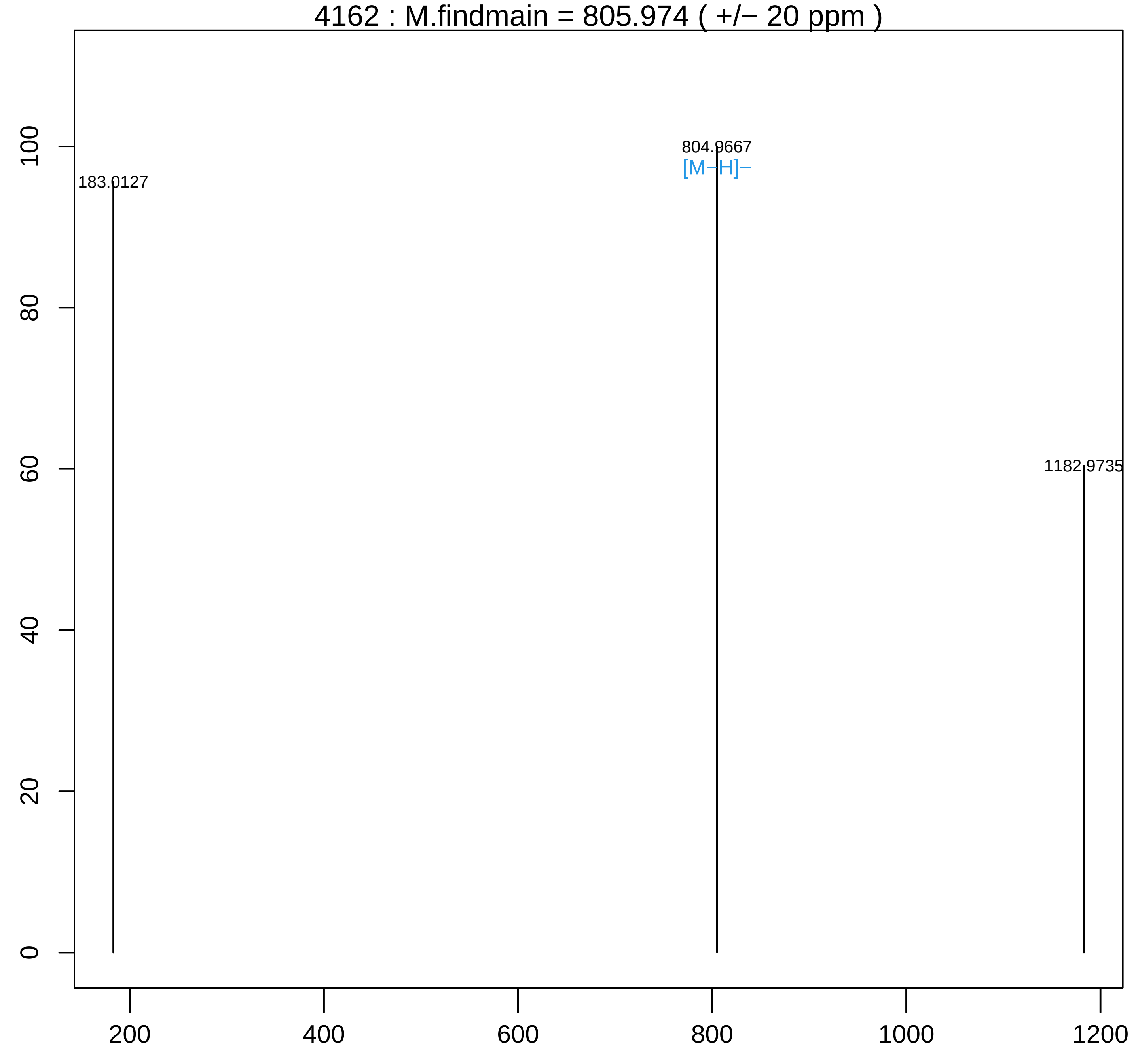
4162 : M.findmain = 805.974 (+/− 20 ppm)
183.0127
804.9667
[M−H]−
1182.9735
0
20
40
60
80
100
200
400
600
800
1000
1200

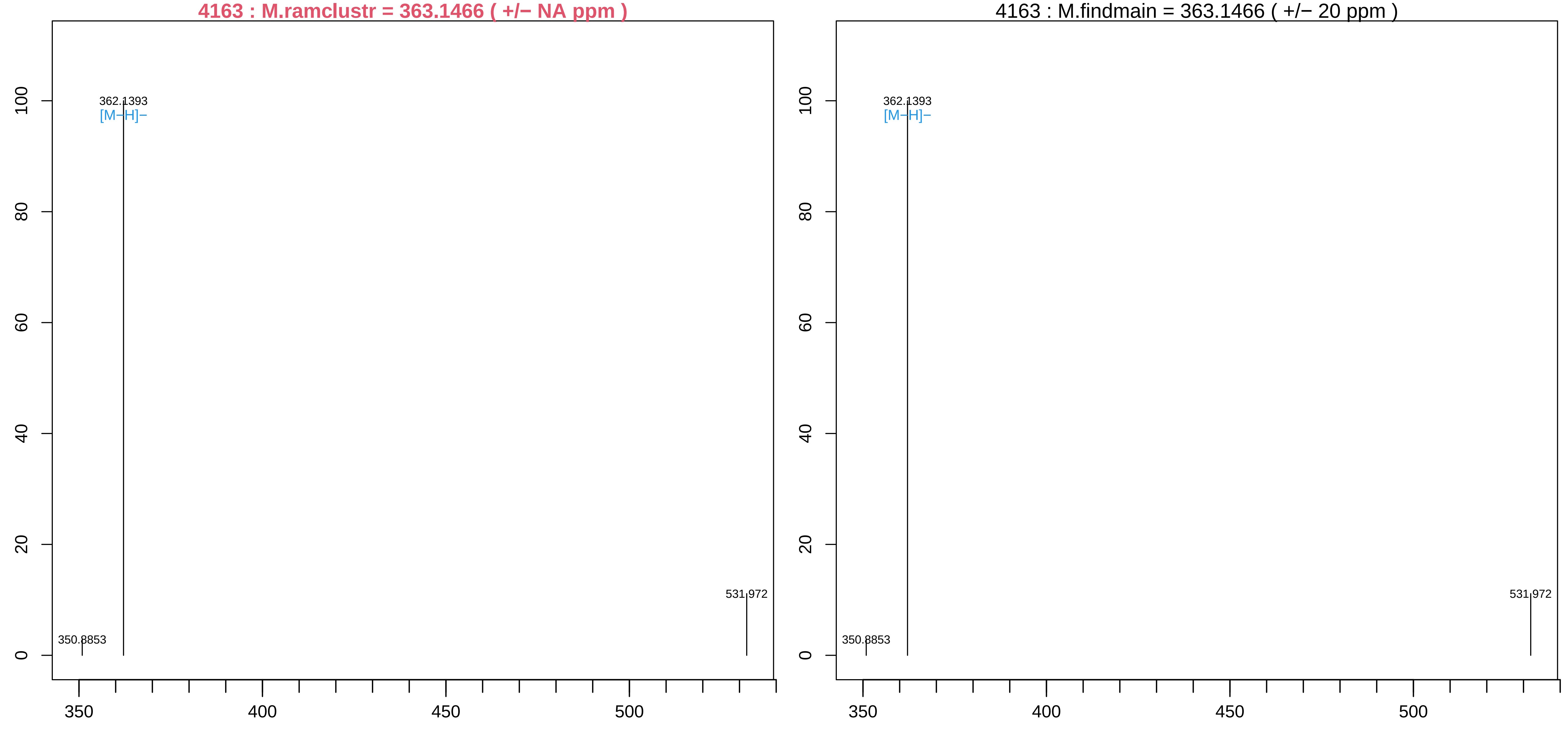
4163 : M.ramclustr = 363.1466 (+/− NA ppm)
362.1393
[M−H]−
350.8853
531.972
0
20
40
60
80
100
350
400
450
500
4163 : M.findmain = 363.1466 (+/− 20 ppm)
362.1393
[M−H]−
350.8853
531.972
0
20
40
60
80
100
350
400
450
500

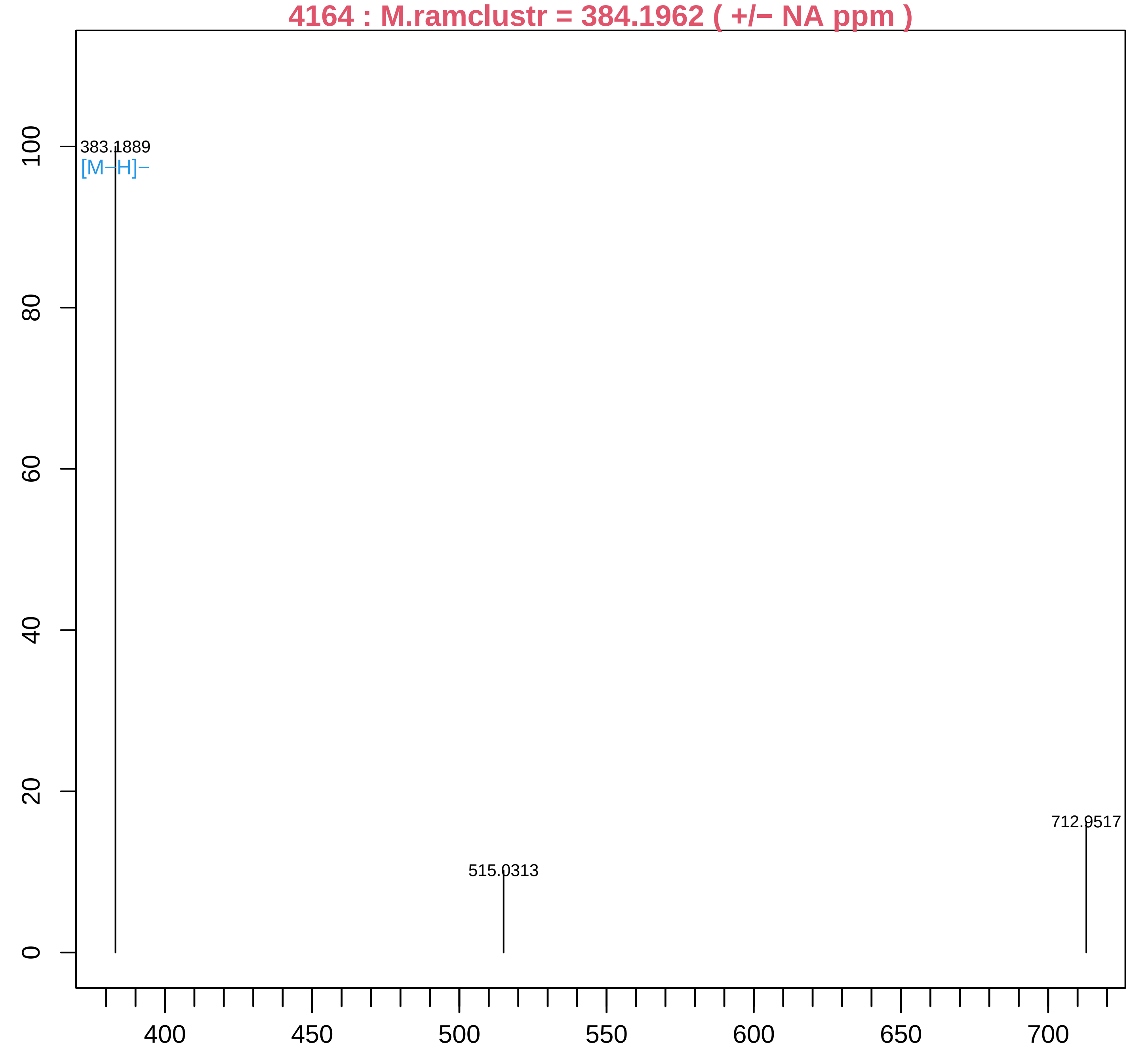
4164 : M.ramclustr = 384.1962 (+/− NA ppm)
383.1889
[M−H]−
515.0313
712.9517
0
20
40
60
80
100
400
450
500
550
600
650
700

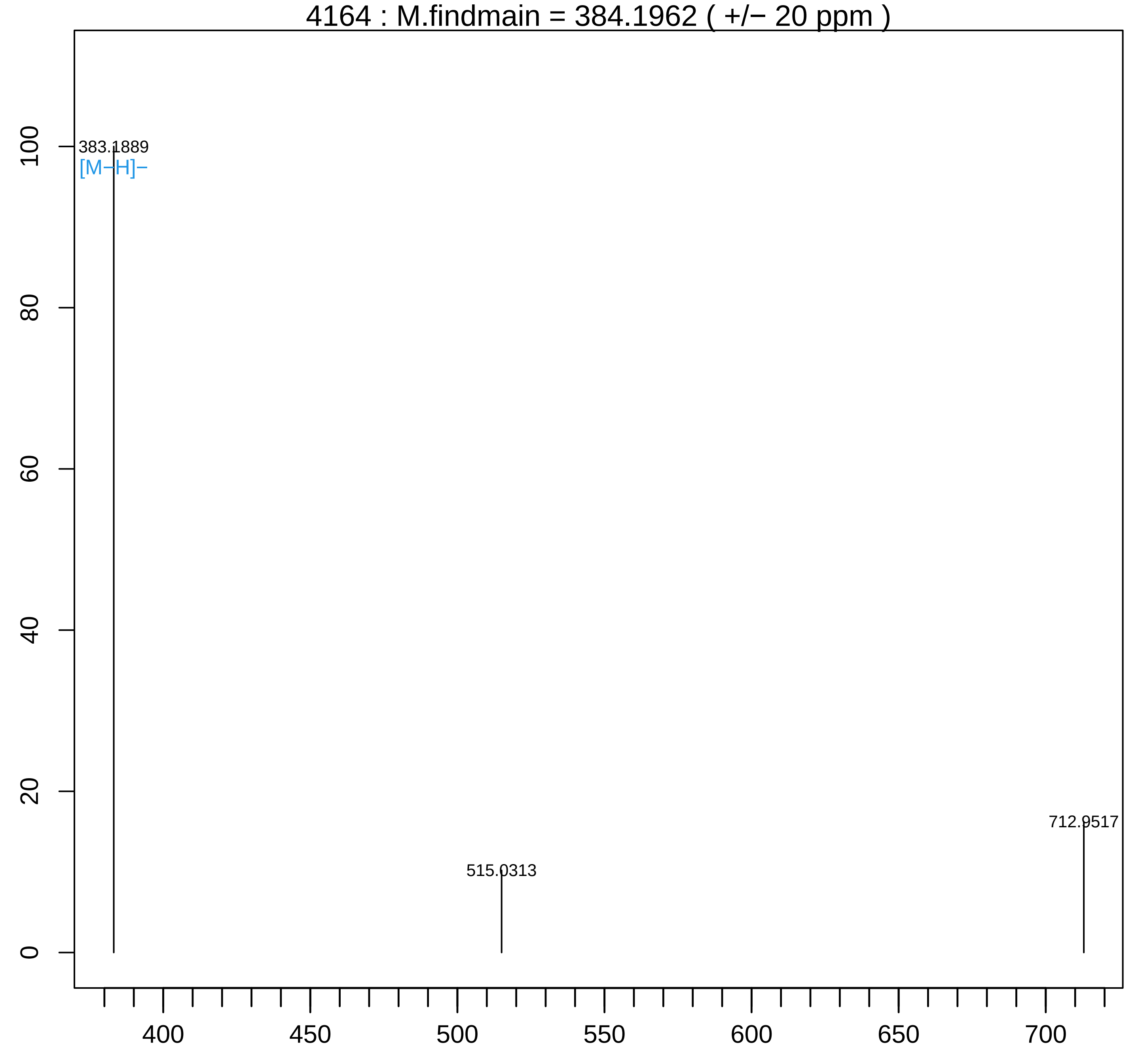
4164 : M.findmain = 384.1962 (+/− 20 ppm)
383.1889
[M−H]−
515.0313
712.9517
0
20
40
60
80
100
400
450
500
550
600
650
700

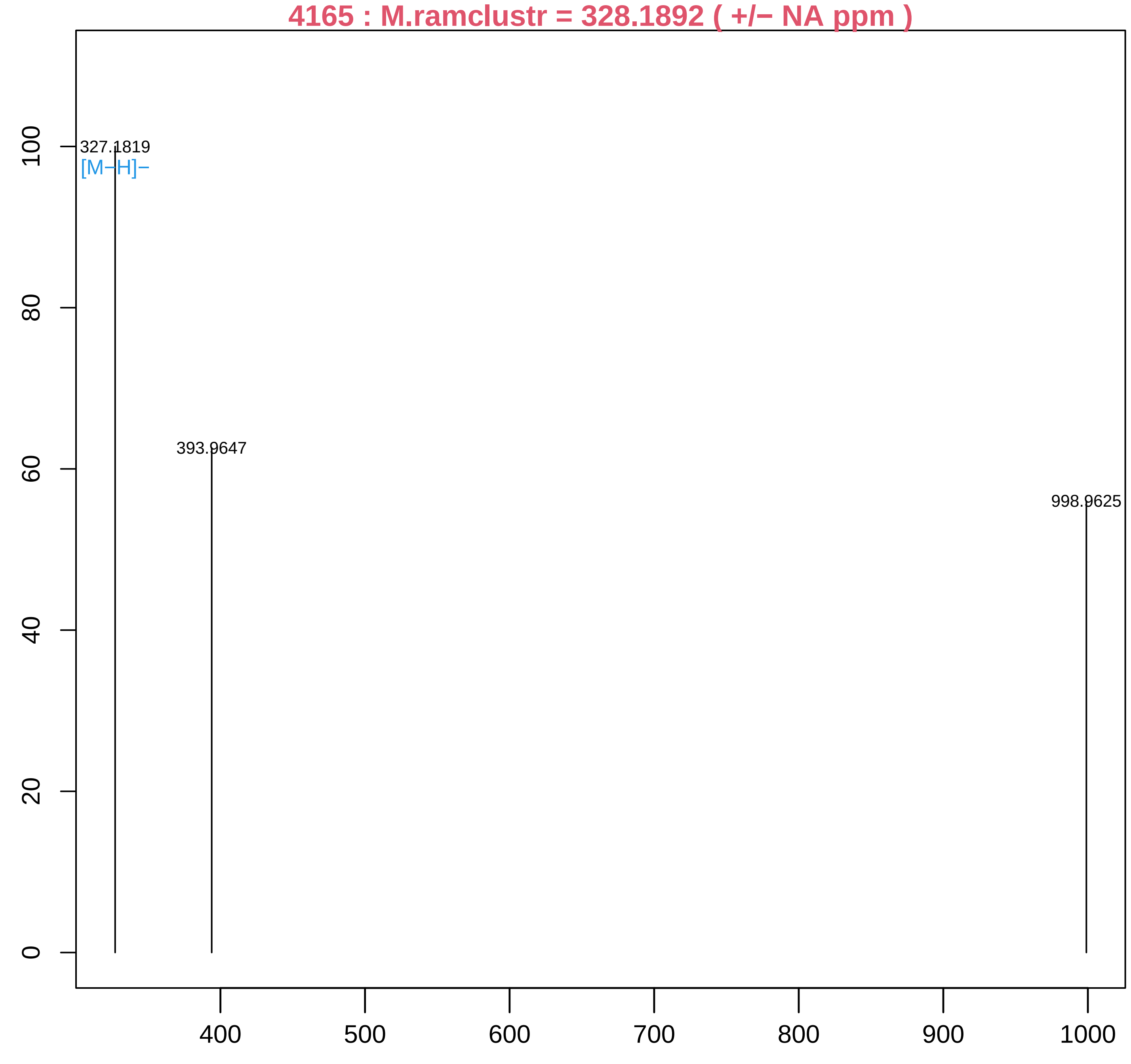
4165 : M.ramclustr = 328.1892 (+/− NA ppm)
327.1819
[M−H]−
393.9647
998.9625
0
20
40
60
80
100
400
500
600
700
800
900
1000

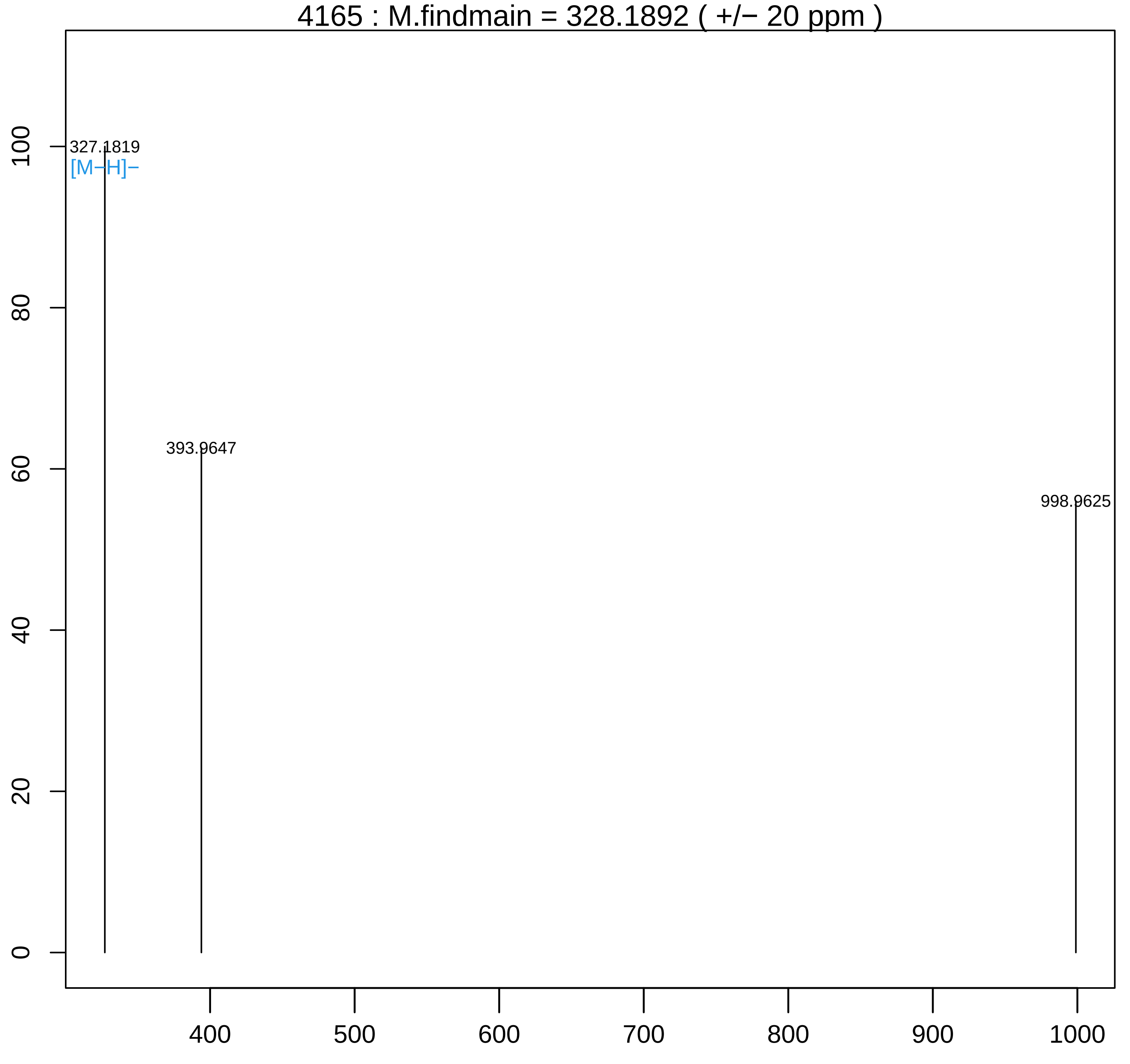
4165 : M.findmain = 328.1892 (+/− 20 ppm)
327.1819
[M−H]−
393.9647
998.9625
0
20
40
60
80
100
400
500
600
700
800
900
1000

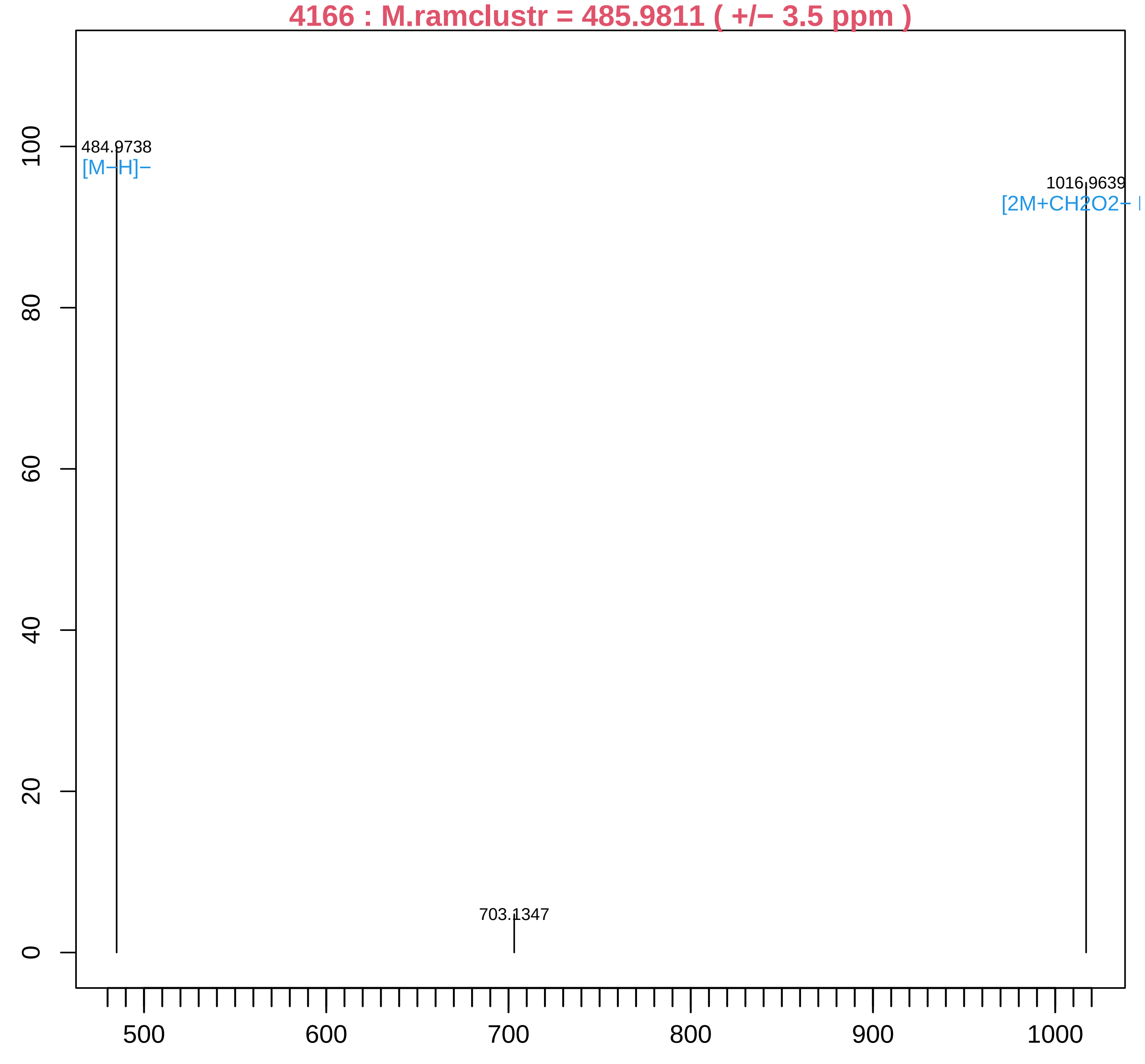
4166 : M.ramclustr = 485.9811 (+/− 3.5 ppm)
484.9738
[M−H]−
1016.9639
[2M+CH2O2−
703.1347
0
20
40
60
80
100
500
600
700
800
900
1000

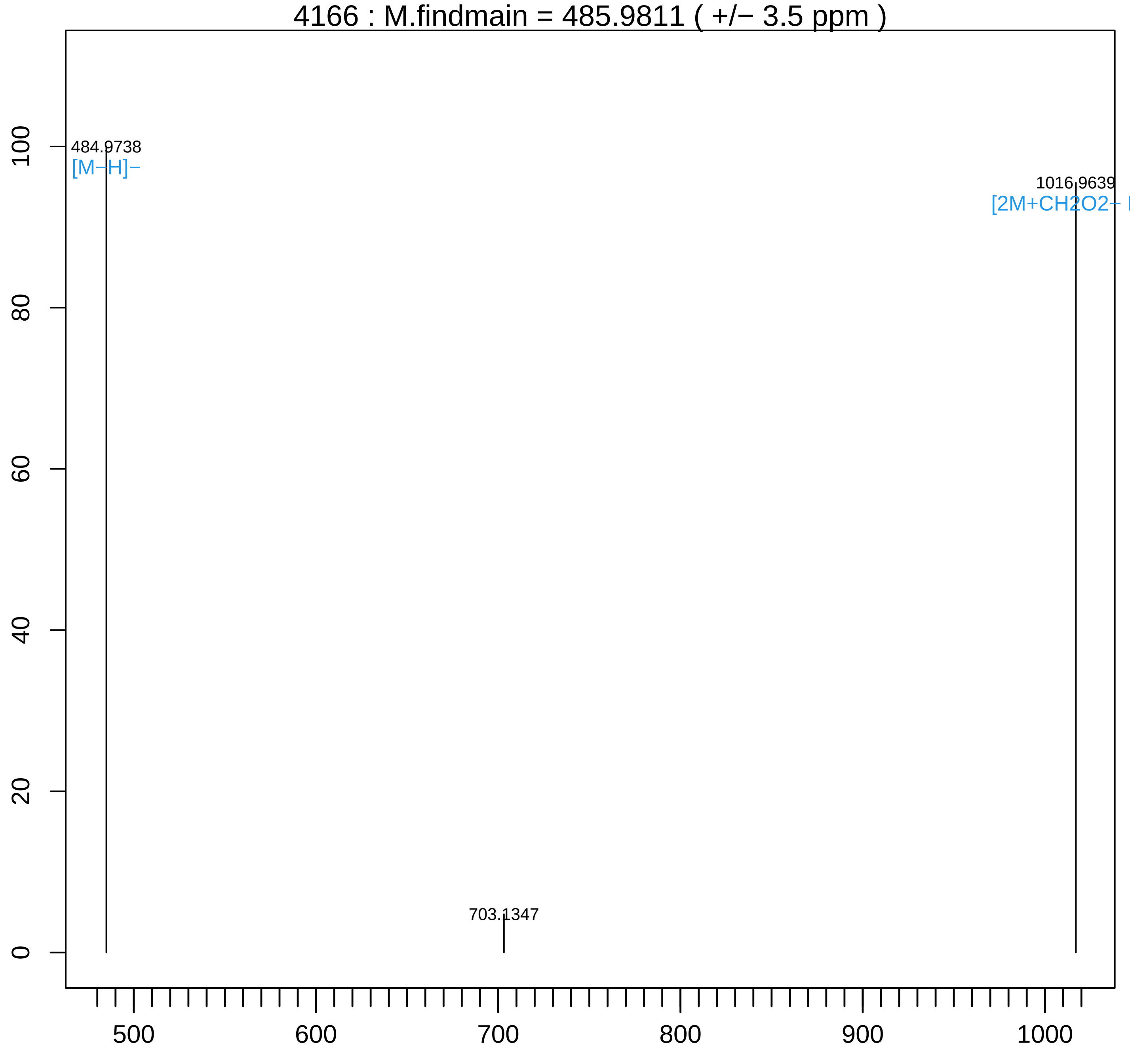
4166 : M.findmain = 485.9811 (+/− 3.5 ppm)
484.9738
[M−H]−
1016.9639
[2M+CH2O2−
703.1347
0
20
40
60
80
100
500
600
700
800
900
1000

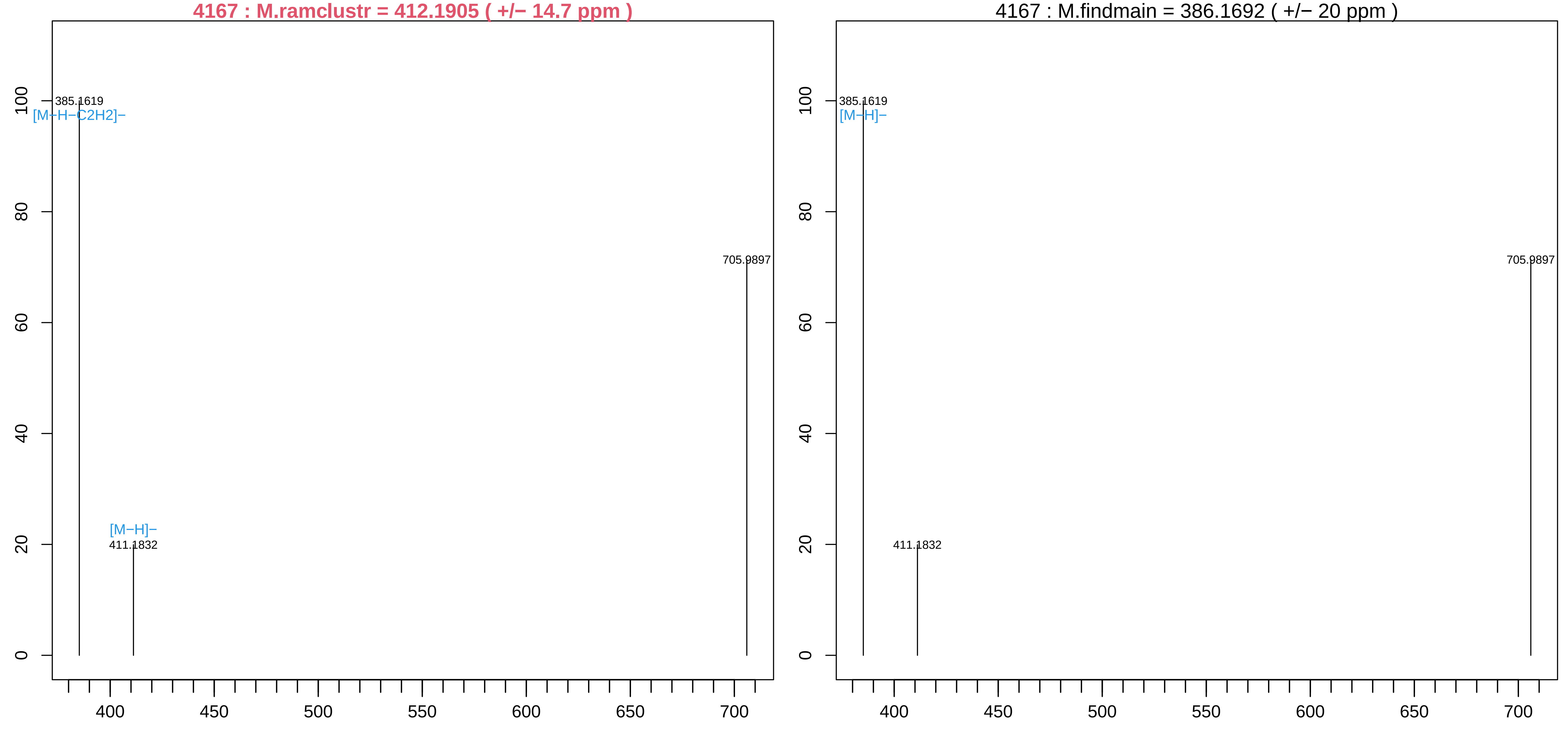
4167 : M.ramclustr = 412.1905 (+/− 14.7 ppm)
385.1619
[M−H−C2H2]−
[M−H]−
411.1832
705.9897
0
20
40
60
80
100
400
450
500
550
600
650
700
4167 : M.findmain = 386.1692 (+/− 20 ppm)
385.1619
[M−H]−
411.1832
705.9897
0
20
40
60
80
100
400
450
500
550
600
650
700

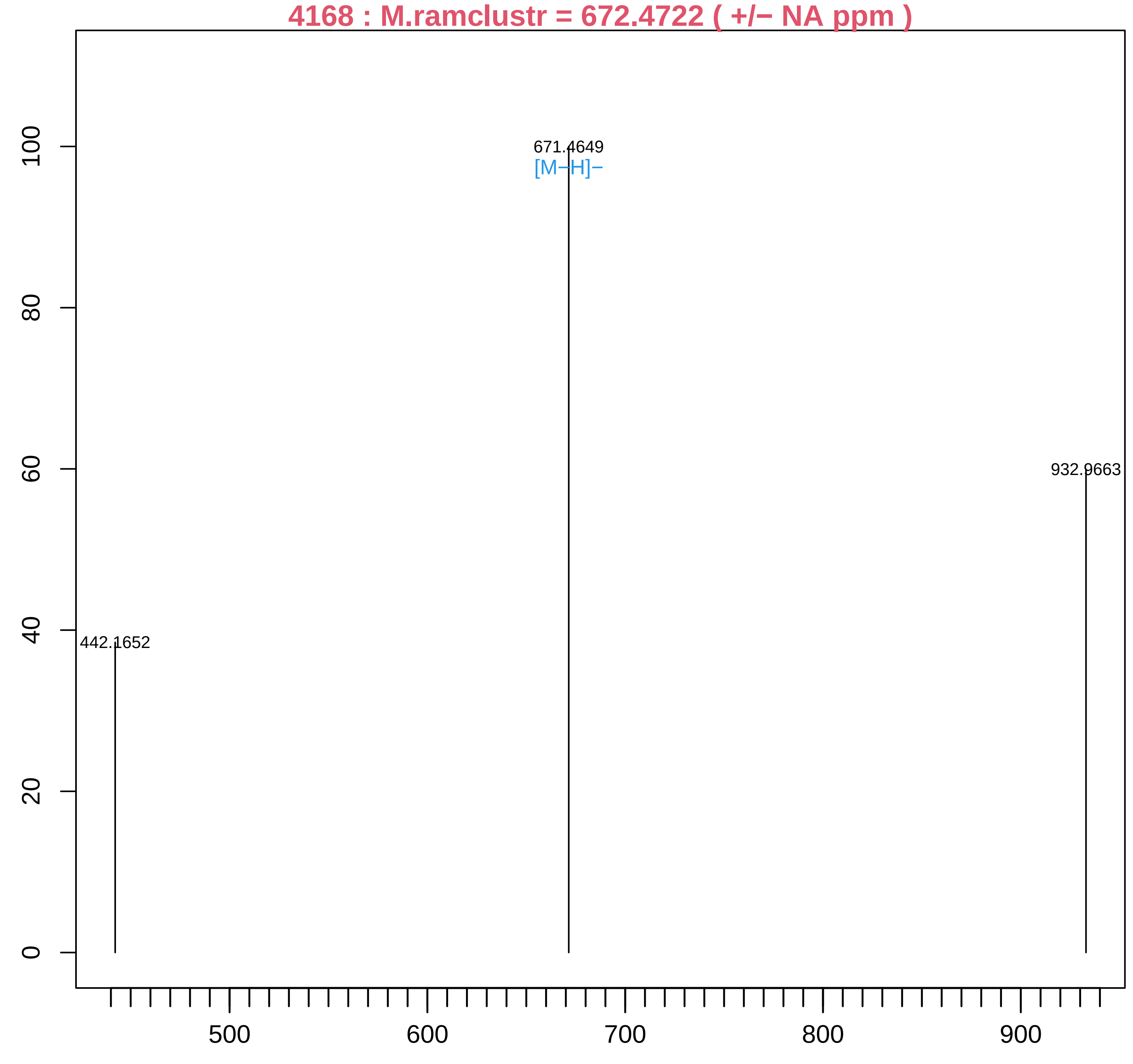

4168 : M.ramclustr = 672.4722 (+/− NA ppm)
671.4649
[M−H]−
932.9663
442.1652

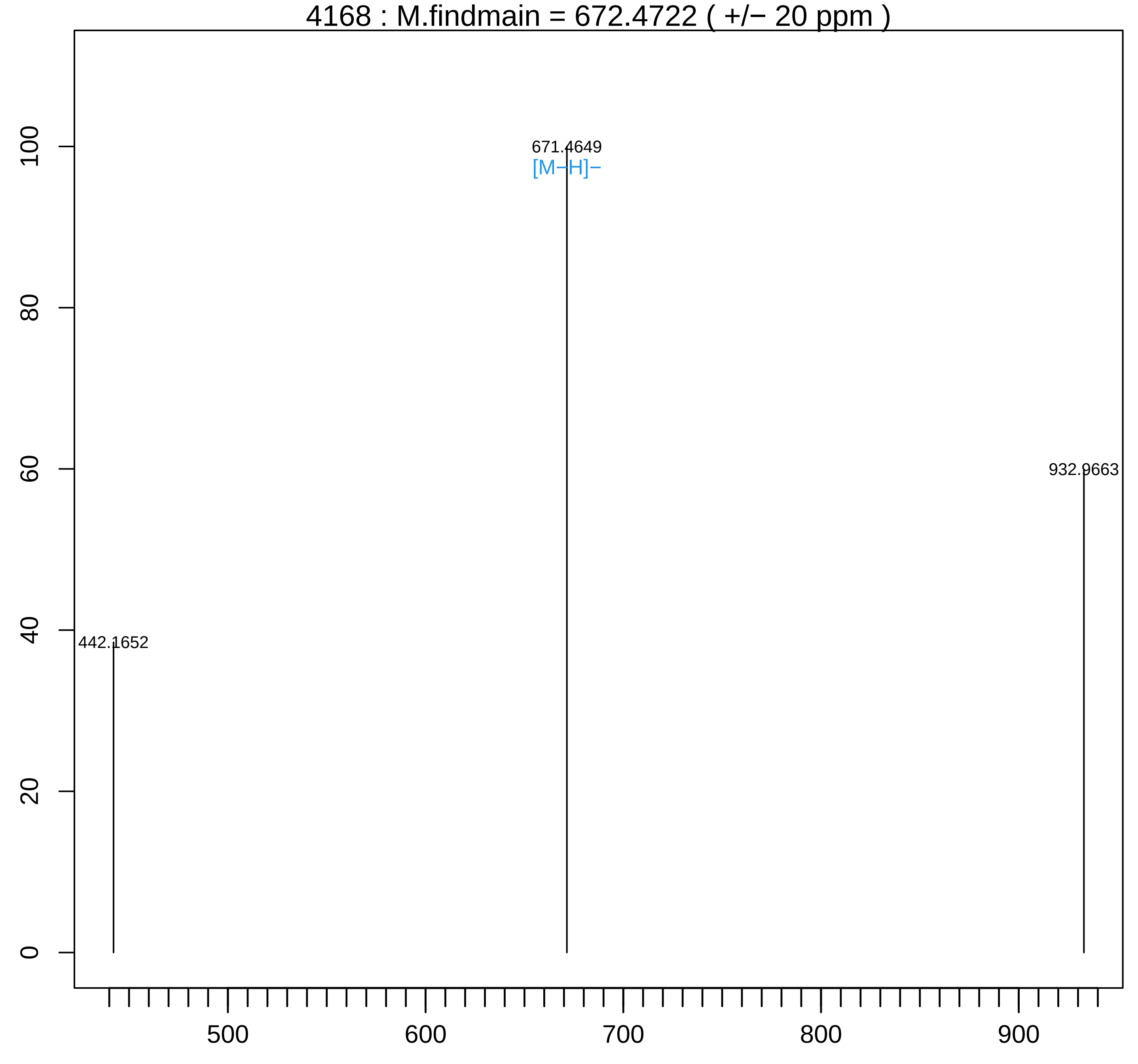

4168 : M.findmain = 672.4722 (+/− 20 ppm)
671.4649
[M−H]−
932.9663
442.1652

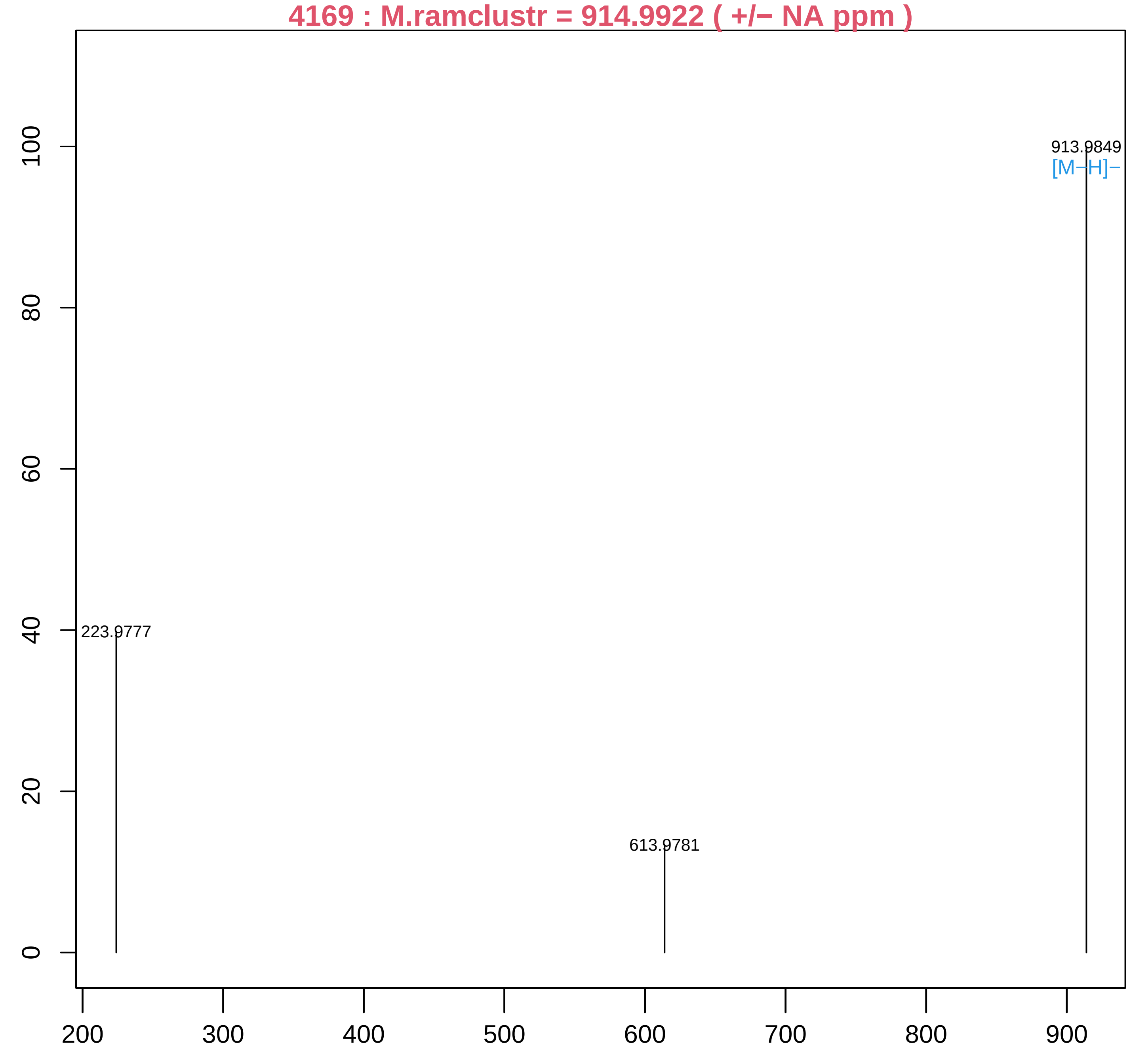
4169 : M.ramclustr = 914.9922 (+/− NA ppm)
913.9849
[M−H]−
223.9777
613.9781
0
20
40
60
80
100
200
300
400
500
600
700
800
900

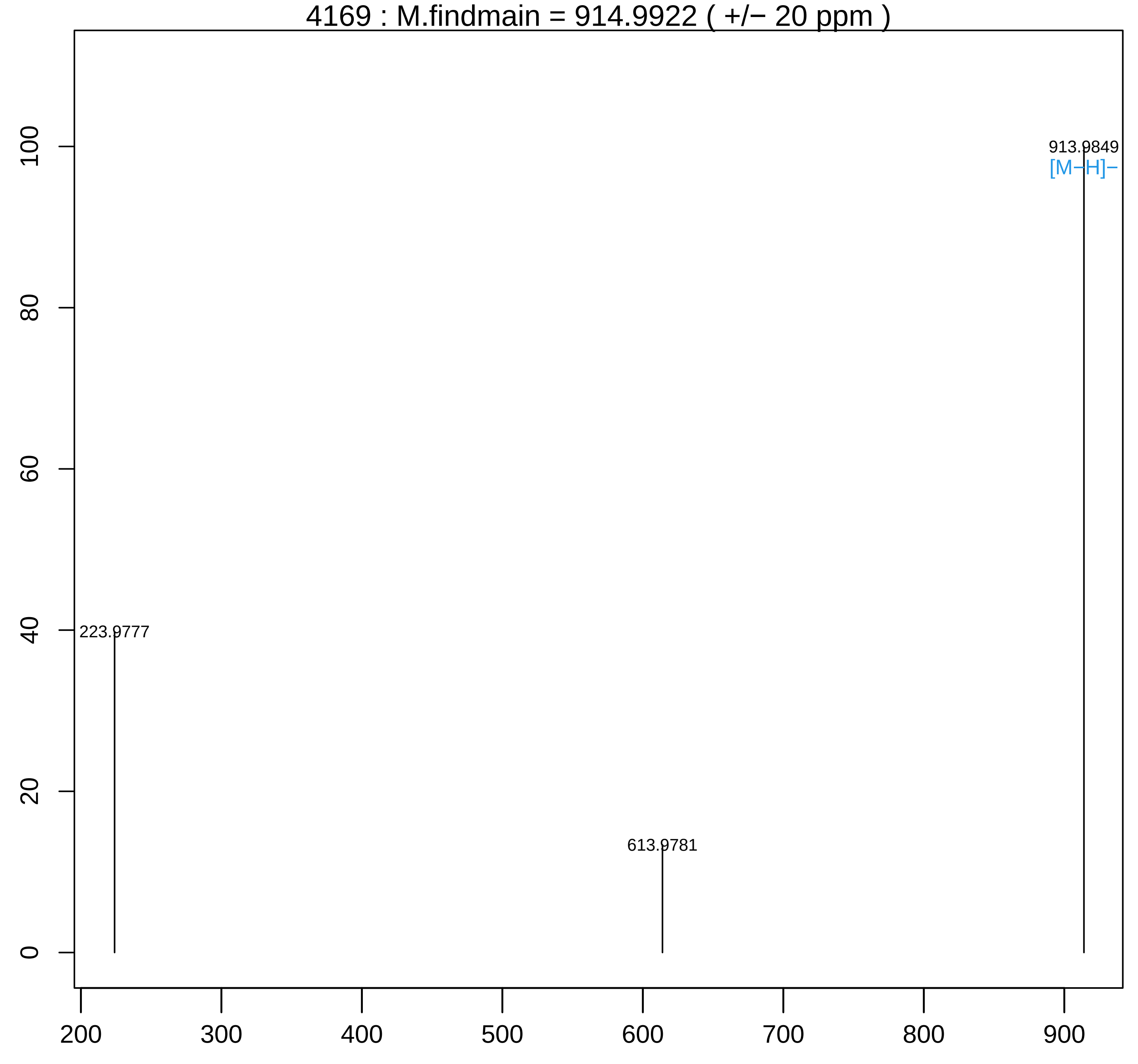
4169 : M.findmain = 914.9922 (+/− 20 ppm)
913.9849
[M−H]−
223.9777
613.9781
0
20
40
60
80
100
200
300
400
500
600
700
800
900

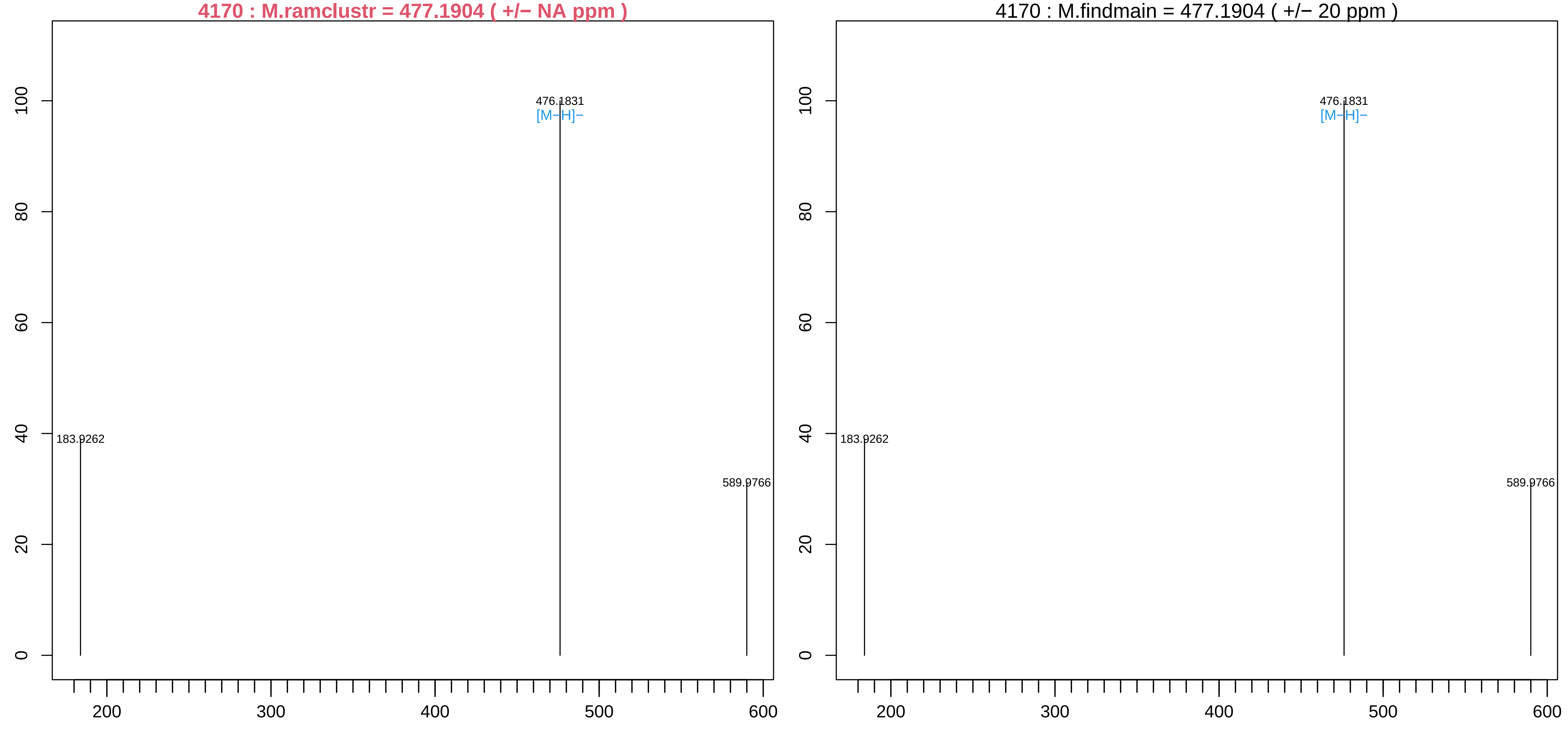
4170 : M.ramclustr = 477.1904 (+/− NA ppm)
476.1831
[M−H]−
183.9262
589.9766
0
20
40
60
80
100
200
300
400
500
600
4170 : M.findmain = 477.1904 (+/− 20 ppm)
476.1831
[M−H]−
183.9262
589.9766
0
20
40
60
80
100
200
300
400
500
600

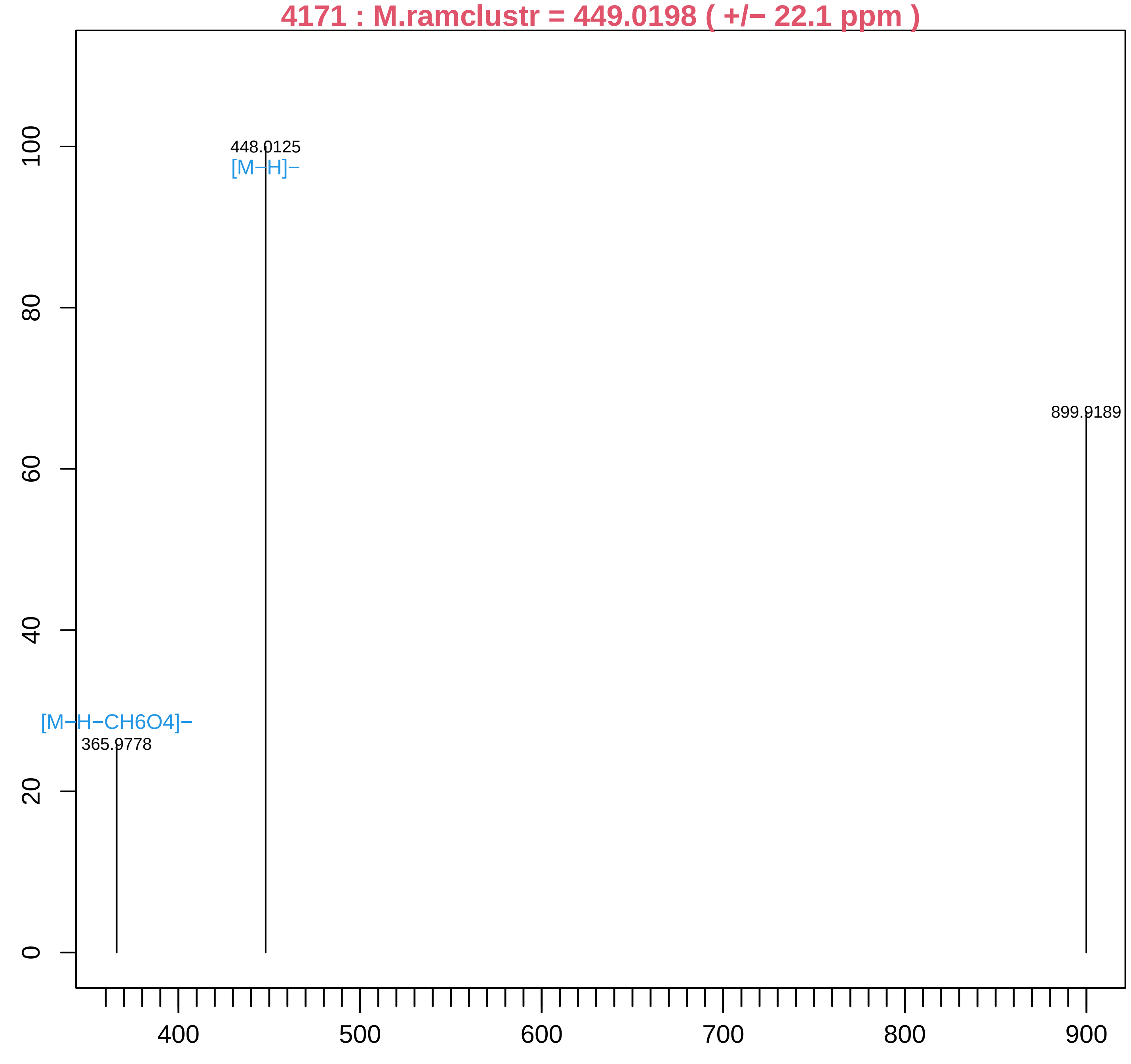

4171 : M.ramclustr = 449.0198 (+/− 22.1 ppm)
448.0125
[M−H]−
899.9189
[M−H−CH6O4]−
365.9778
0
20
40
60
80
100
400
500
600
700
800
900

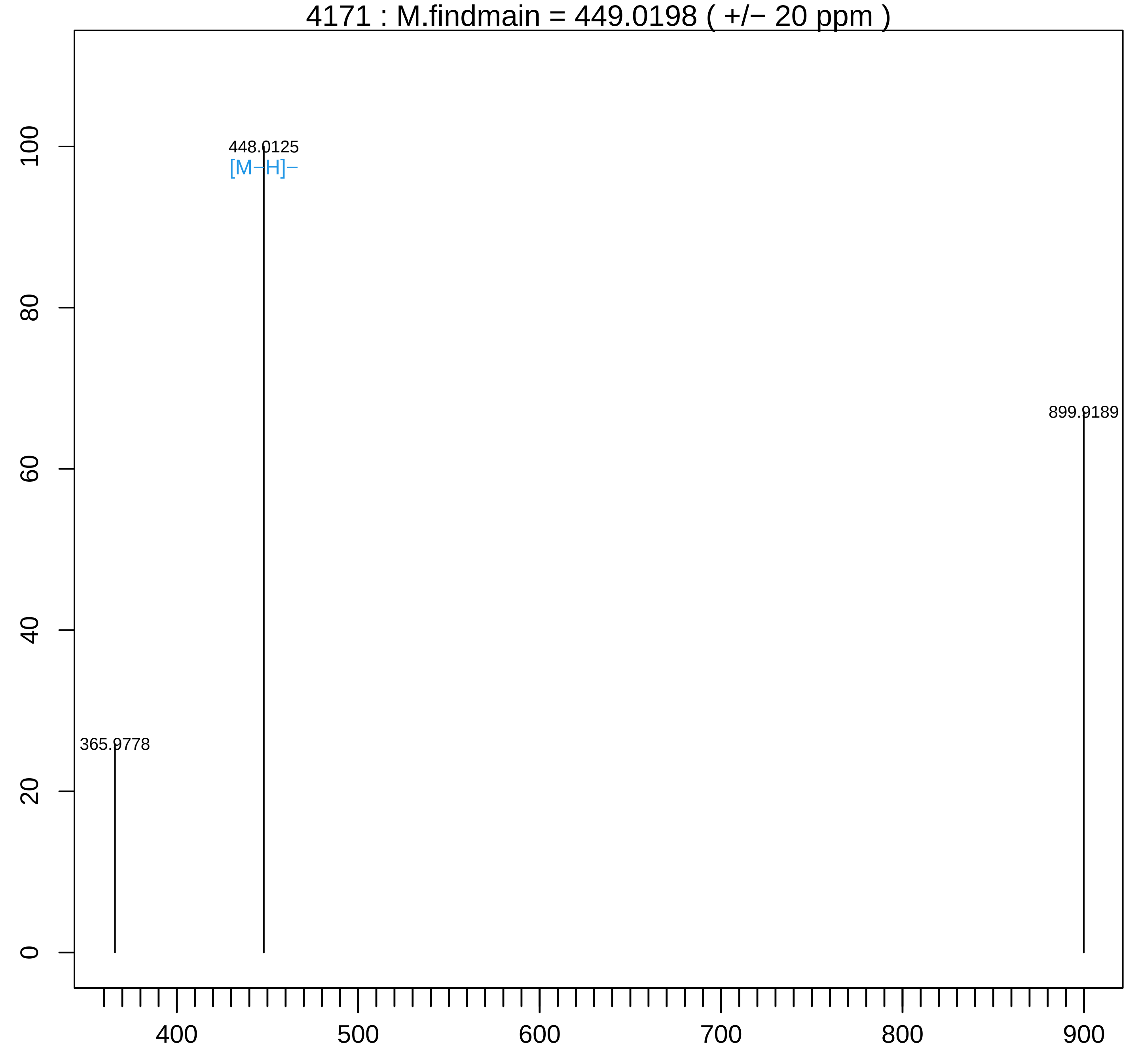

4171 : M.findmain = 449.0198 (+/− 20 ppm)
448.0125
[M−H]−
899.9189
365.9778
0
20
40
60
80
100
400
500
600
700
800
900

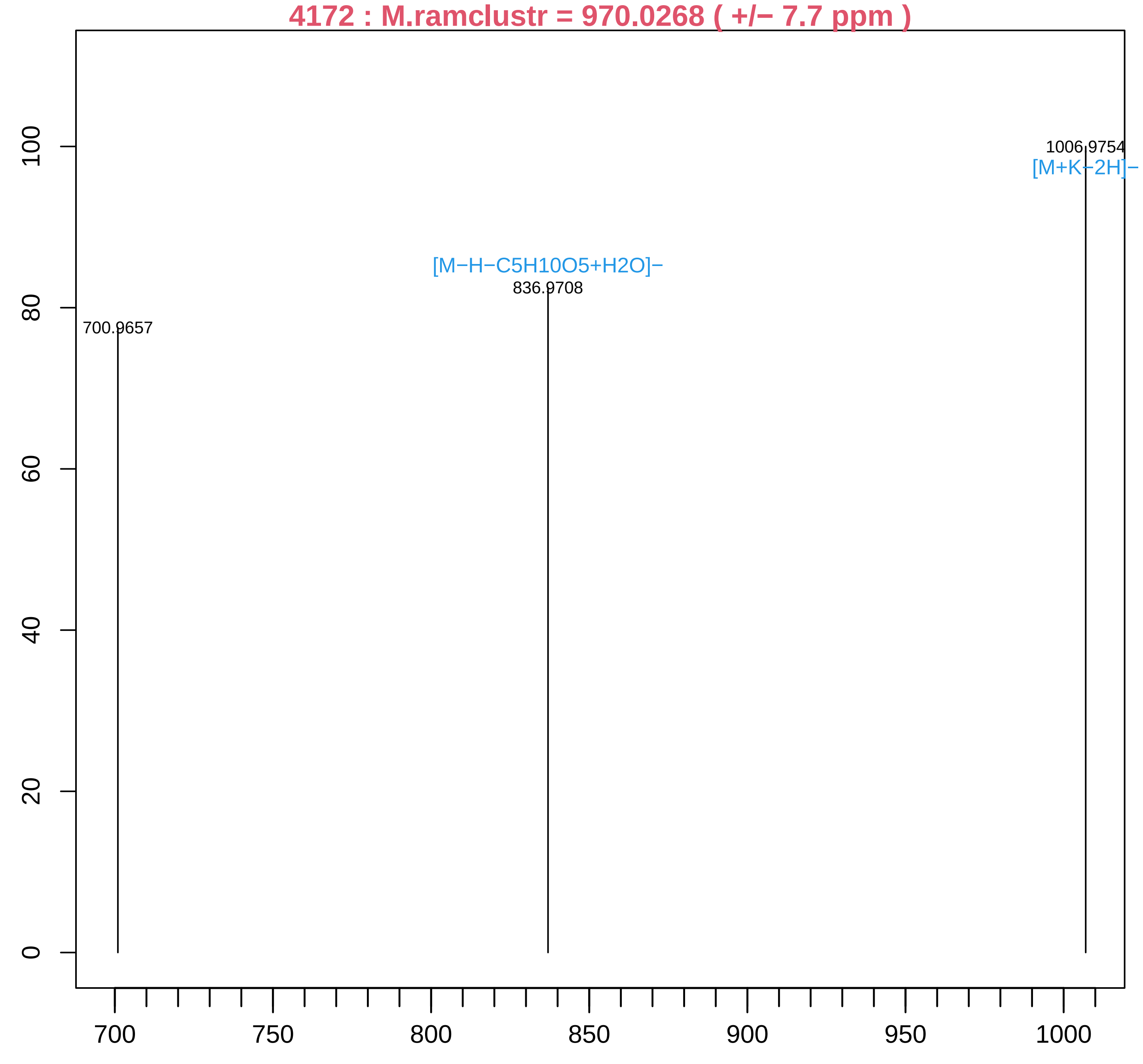
4172 : M.ramclustr = 970.0268 (+/− 7.7 ppm)
1006.9754
[M+K−2H]−
[M−H−C5H10O5+H2O]−
836.9708
700.9657
0
20
40
60
80
100
700
750
800
850
900
950
1000

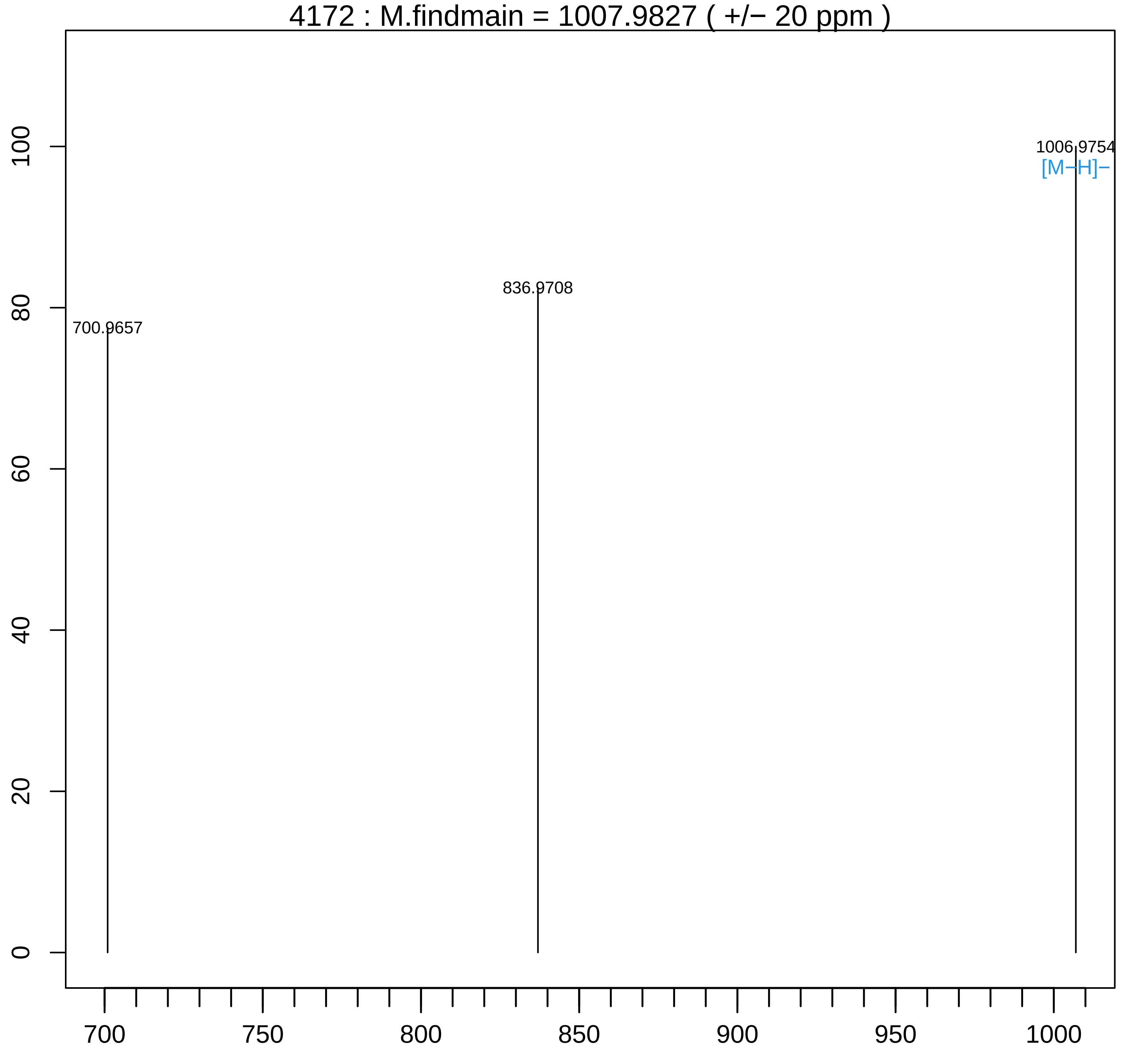
4172 : M.findmain = 1007.9827 (+/− 20 ppm)
1006.9754
[M−H]−
836.9708
700.9657
0
20
40
60
80
100
700
750
800
850
900
950
1000

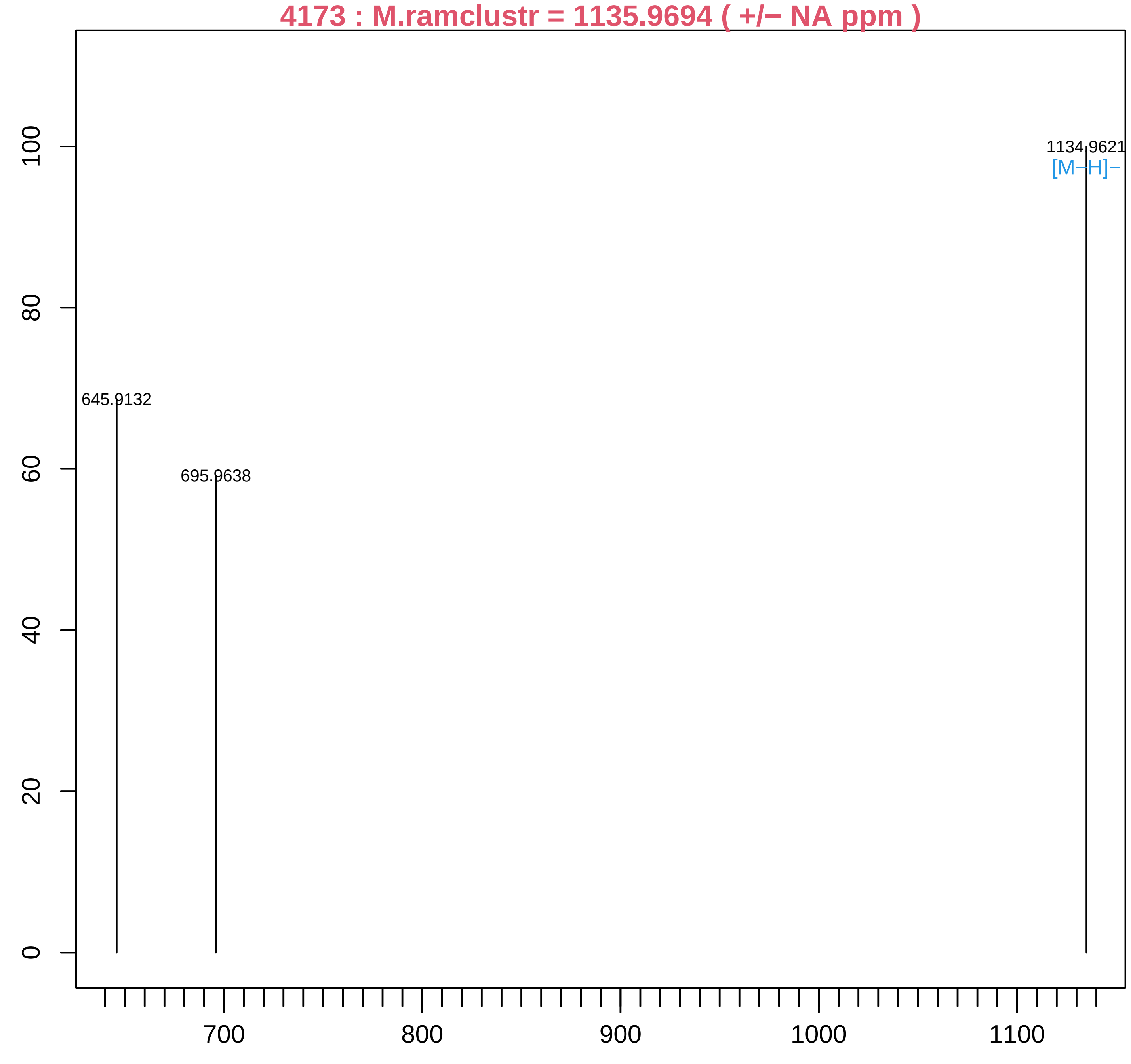

4173 : M.ramclustr = 1135.9694 (+/− NA ppm)
645.9132
695.9638
1134.9621
[M−H]−
0
20
40
60
80
100
700
800
900
1000
1100

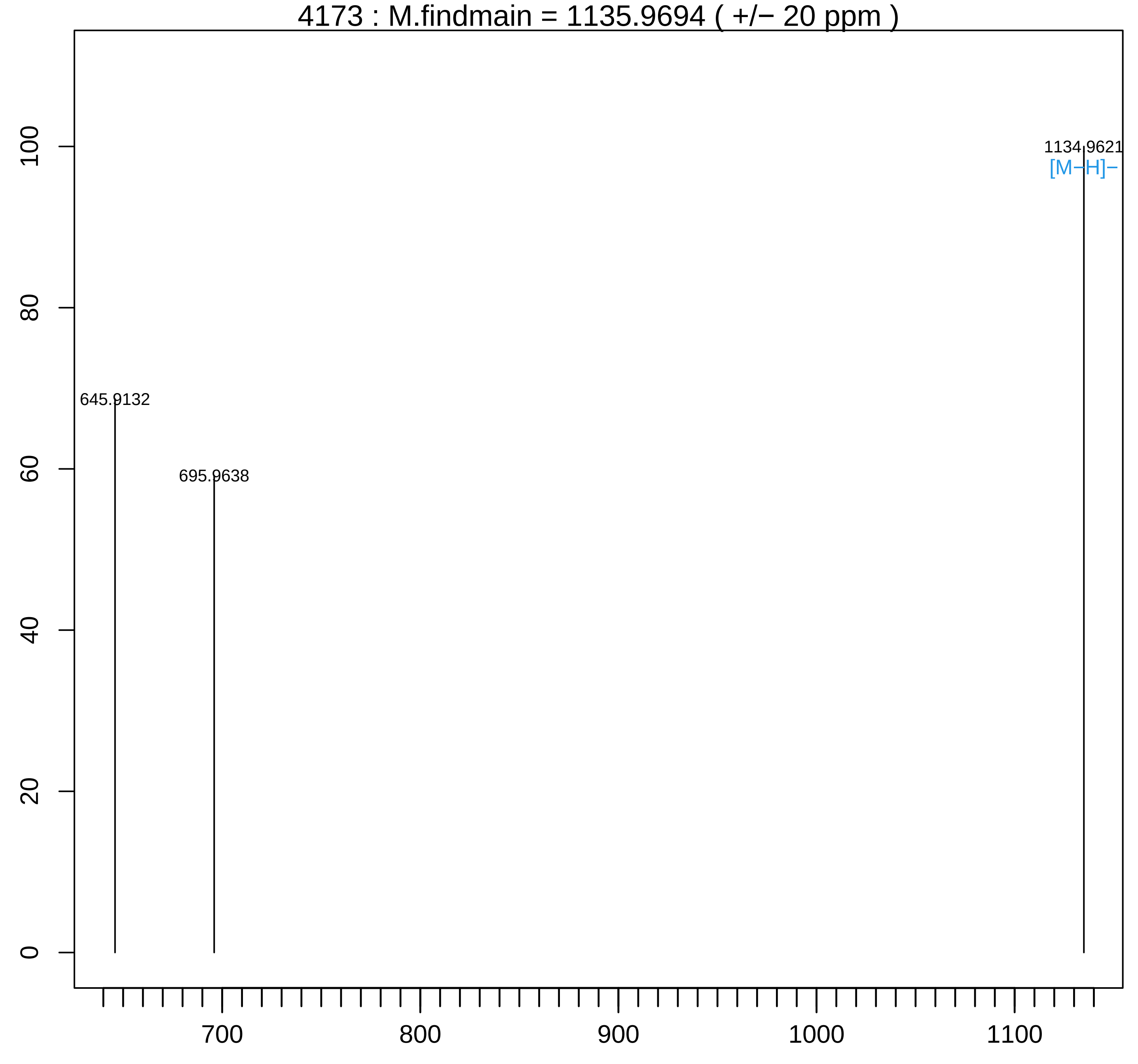

4173 : M.findmain = 1135.9694 (+/− 20 ppm)
645.9132
695.9638
1134.9621
[M−H]−
0
20
40
60
80
100
700
800
900
1000
1100

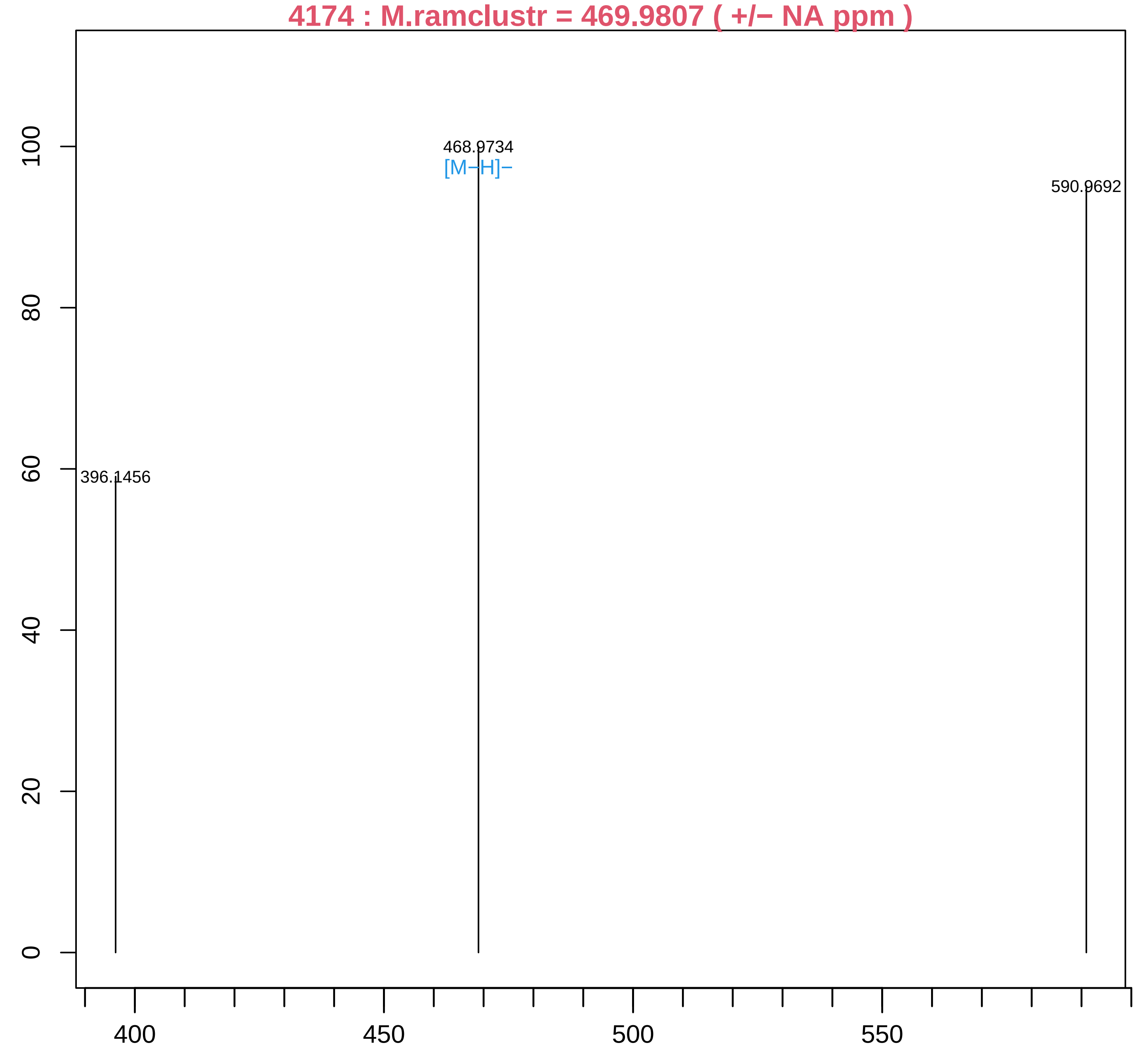
4174 : M.ramclustr = 469.9807 (+/− NA ppm)
468.9734
[M−H]−
590.9692
396.1456
0
20
40
60
80
100
400
450
500
550

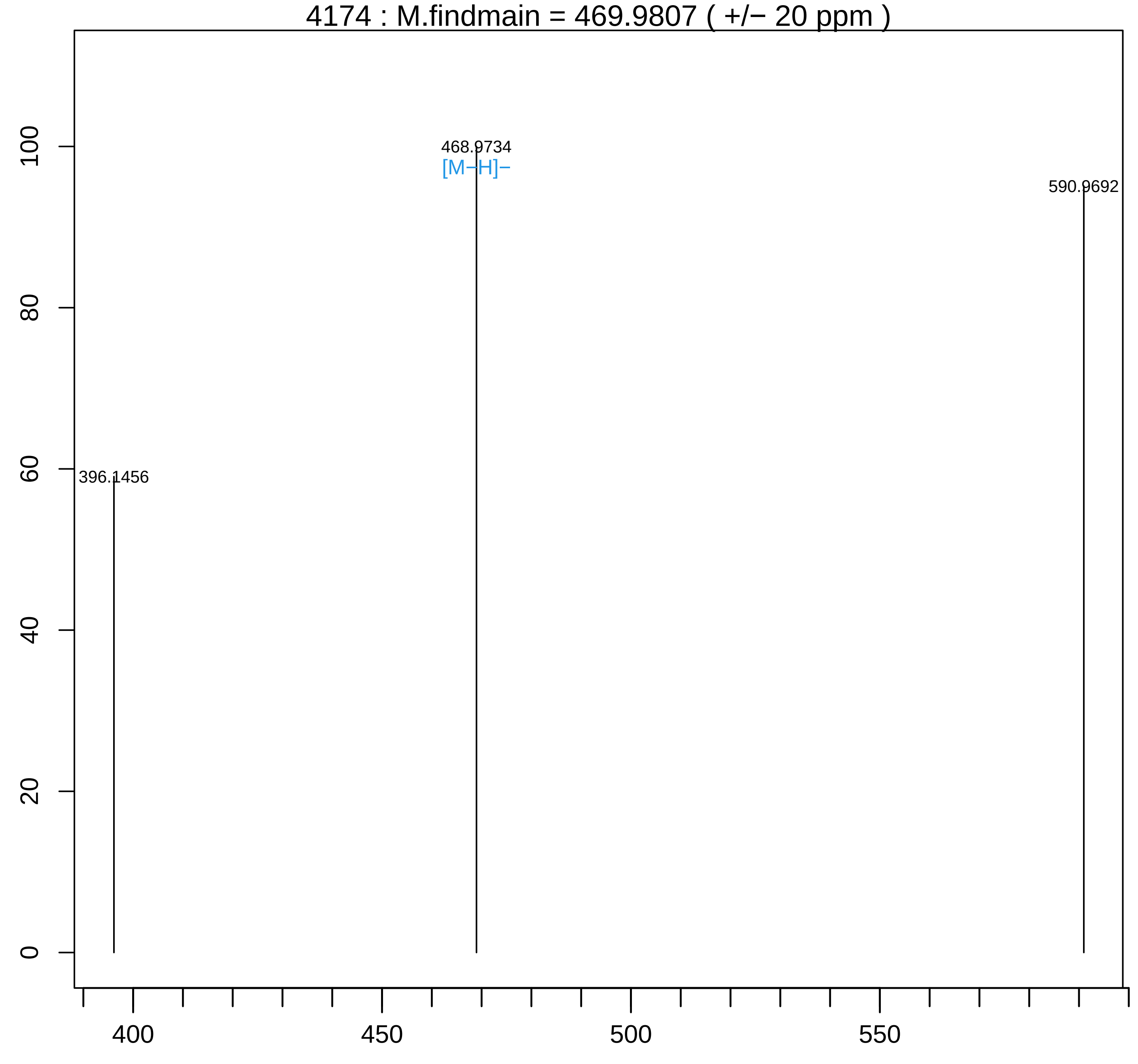
4174 : M.findmain = 469.9807 (+/− 20 ppm)
468.9734
[M−H]−
590.9692
396.1456
0
20
40
60
80
100
400
450
500
550

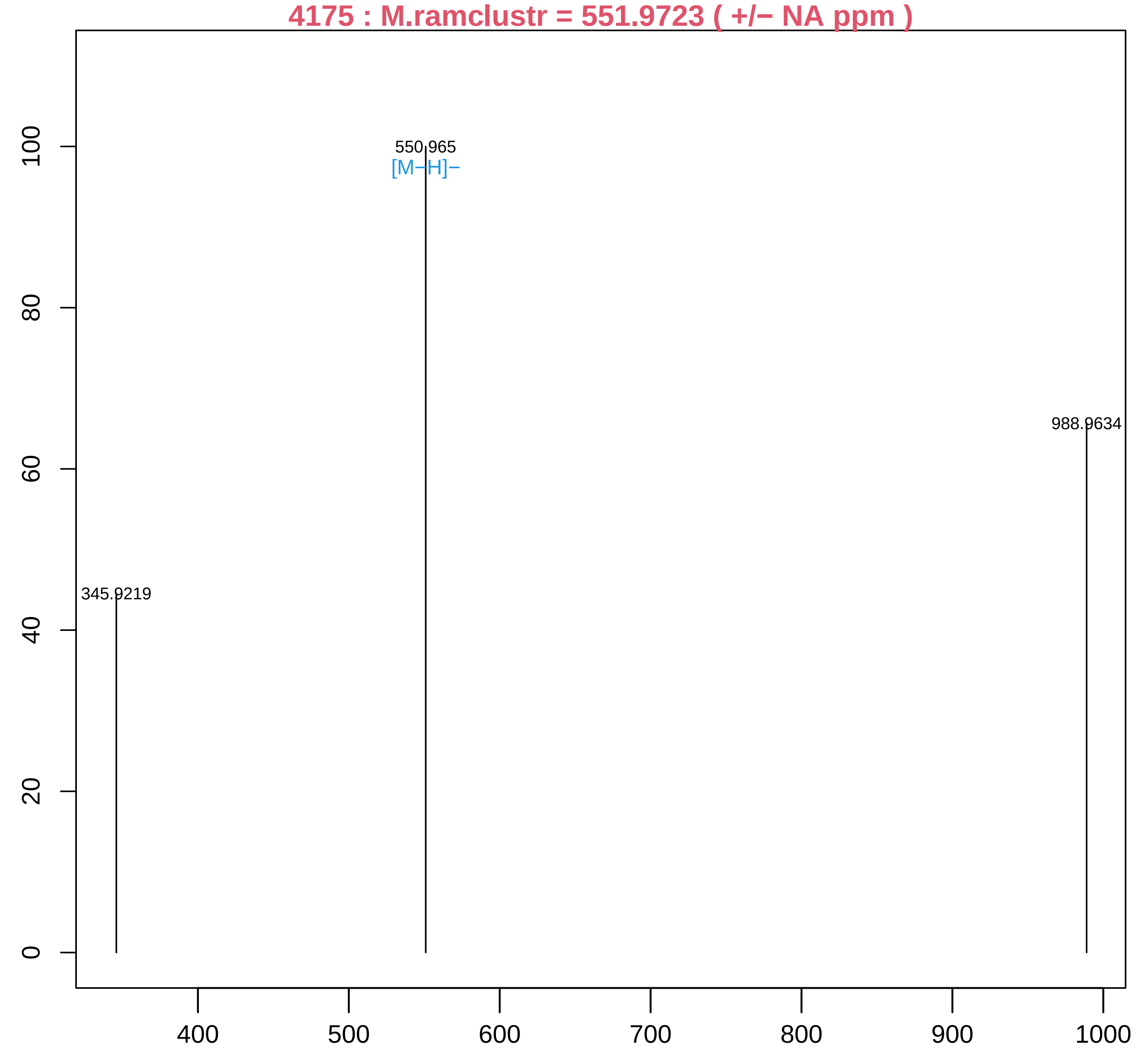
4175 : M.ramclustr = 551.9723 (+/− NA ppm)
550.965
[M−H]−
988.9634
345.9219
0
20
40
60
80
100
400
500
600
700
800
900
1000
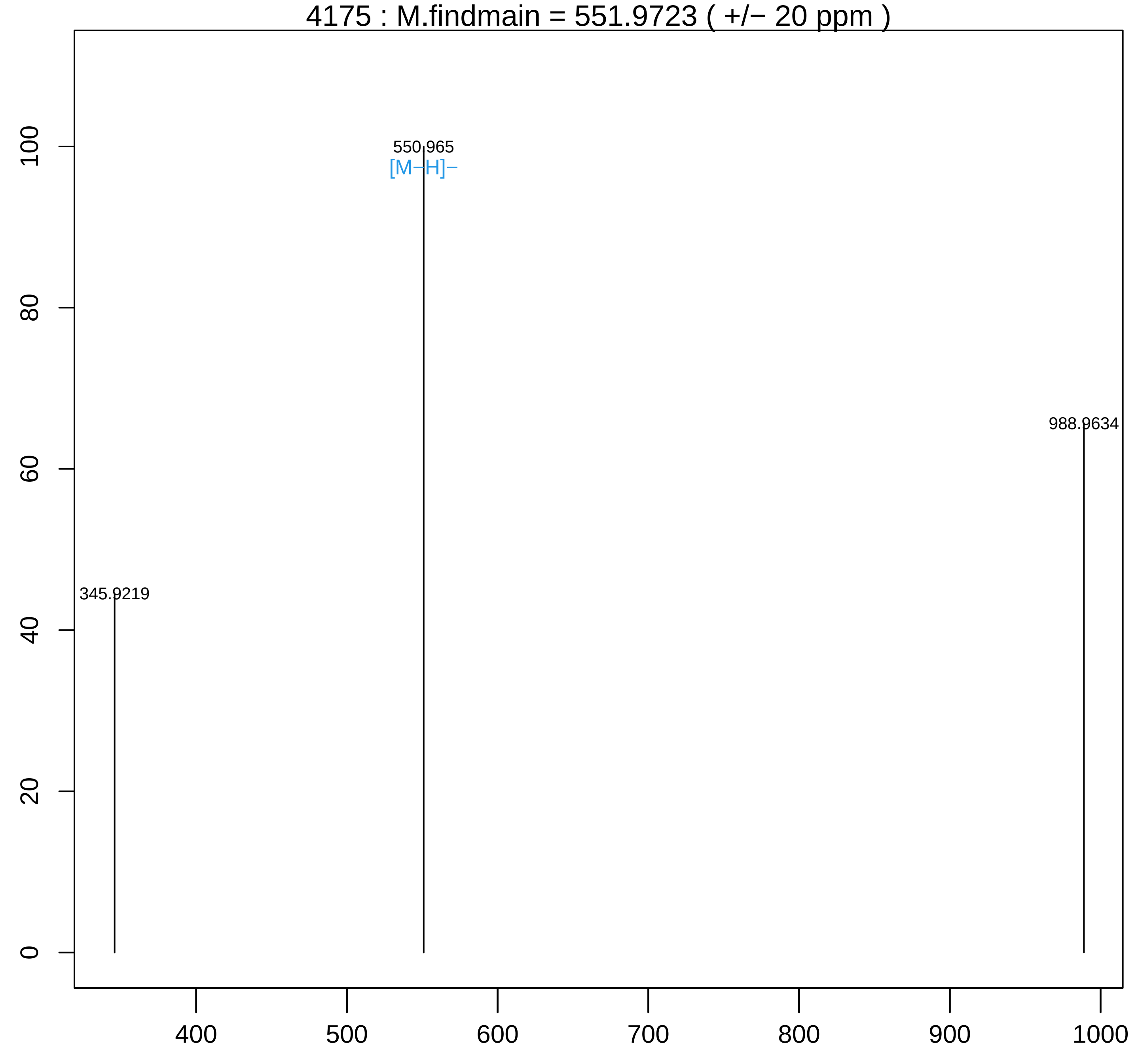
4175 : M.findmain = 551.9723 (+/− 20 ppm)
550.965
[M−H]−
988.9634
345.9219
0
20
40
60
80
100
400
500
600
700
800
900
1000

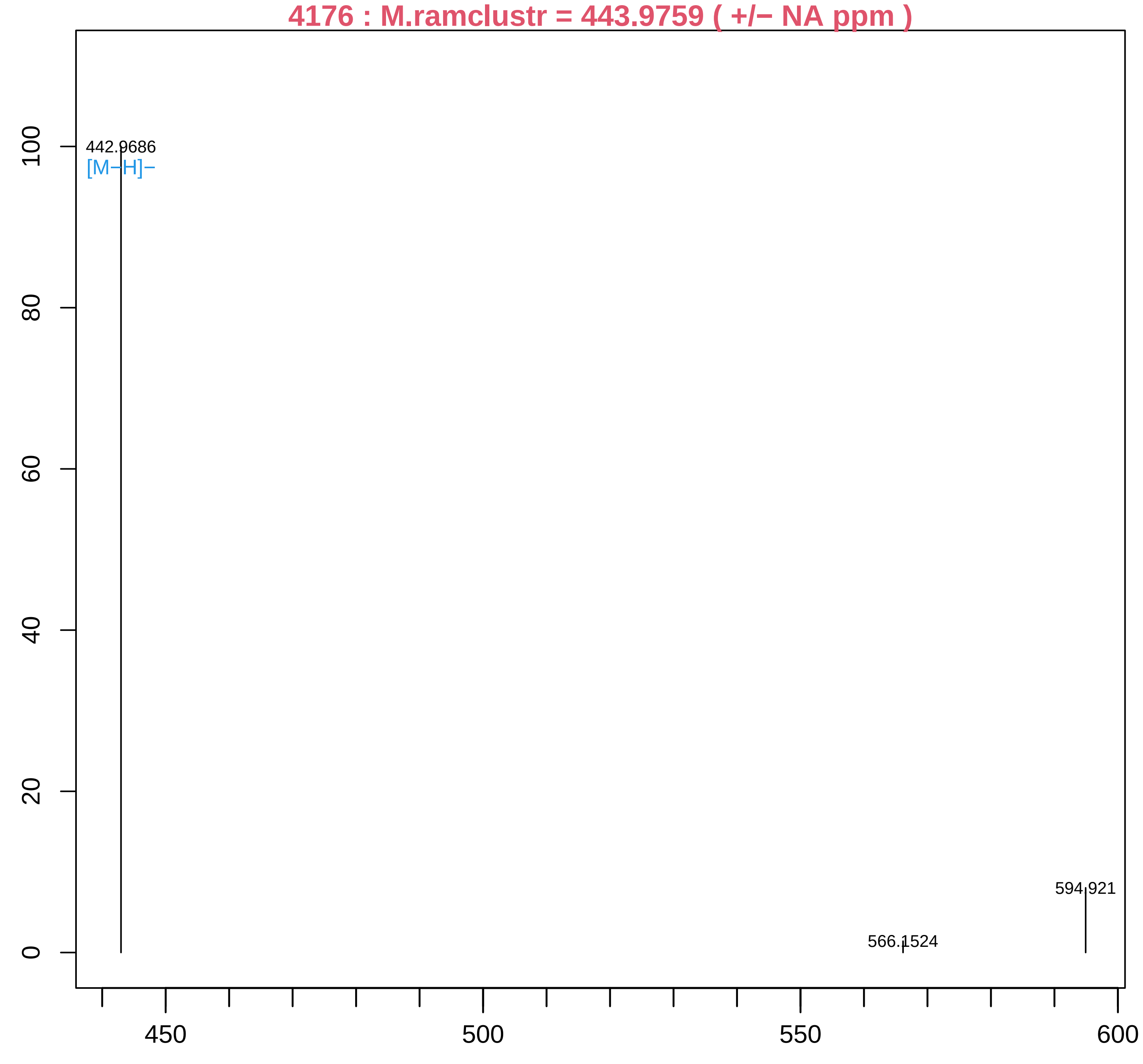
4176 : M.ramclustr = 443.9759 (+/− NA ppm)
442.9686
[M−H]−
566.1524
594.921
0
20
40
60
80
100
450
500
550
600

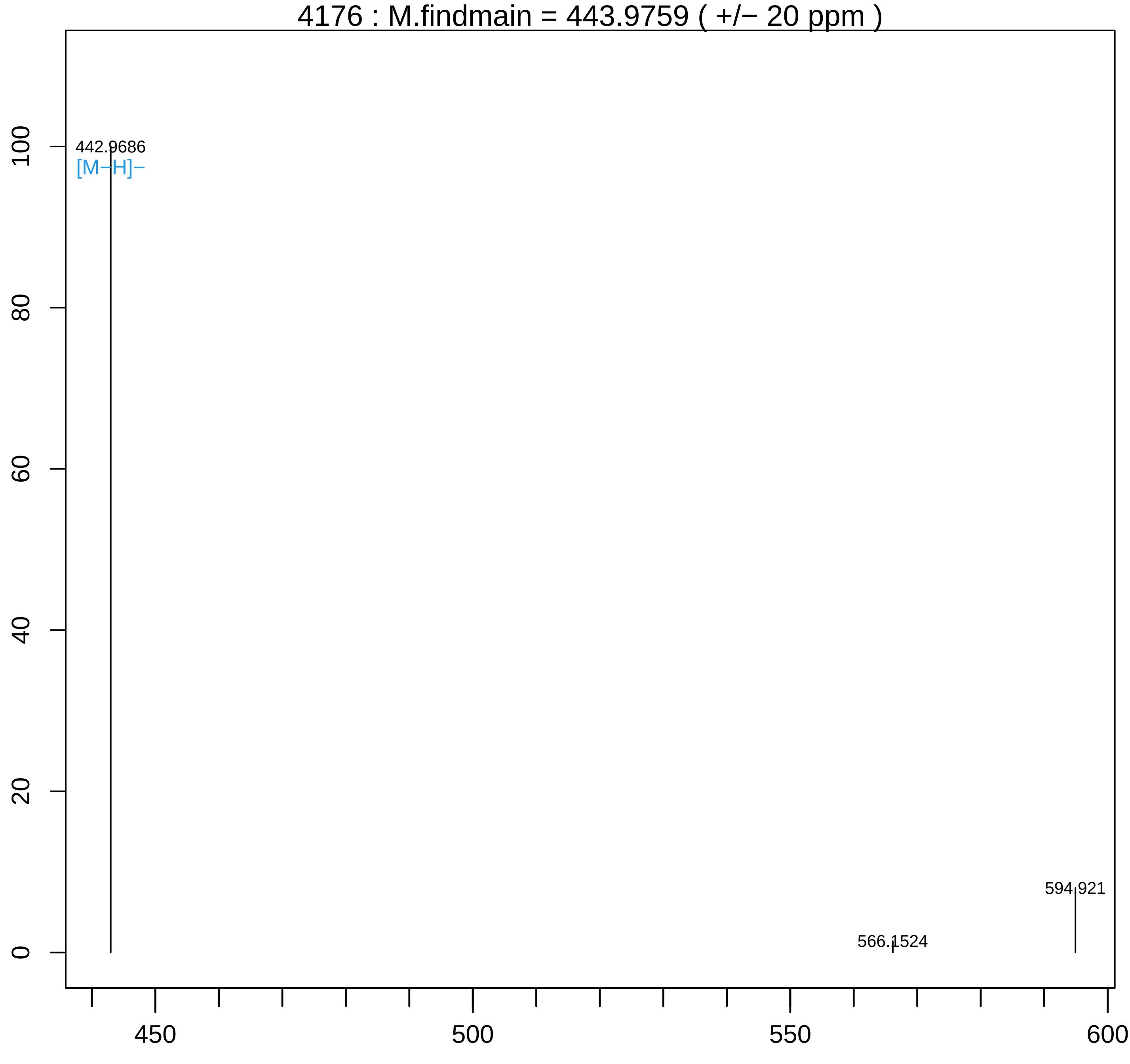
4176 : M.findmain = 443.9759 (+/− 20 ppm)
442.9686
[M−H]−
566.1524
594.921
0
20
40
60
80
100
450
500
550
600

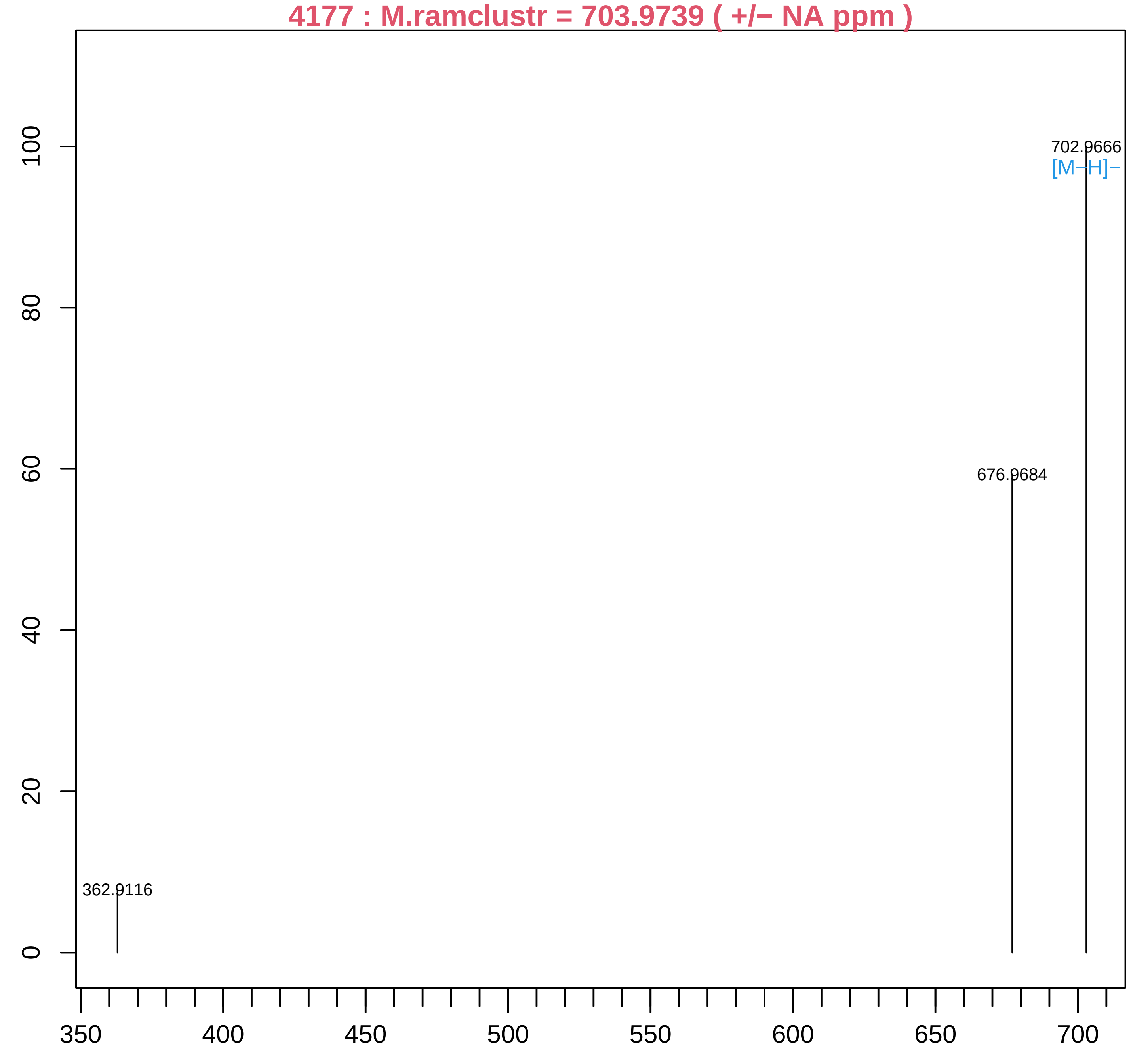

4177 : M.ramclustr = 703.9739 (+/− NA ppm)
702.9666
[M−H]−
676.9684
362.9116
0
20
40
60
80
100
350
400
450
500
550
600
650
700

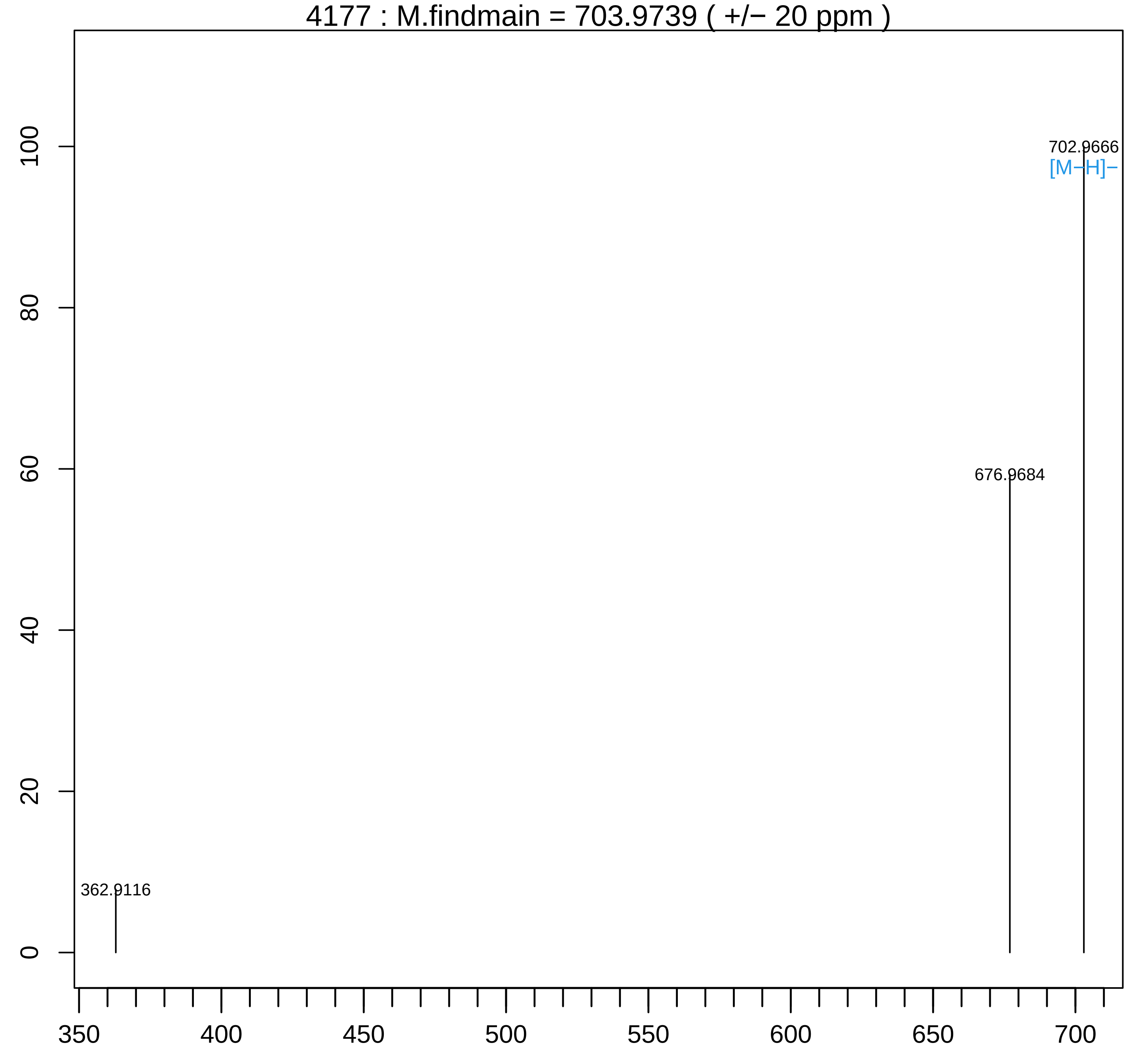

4177 : M.findmain = 703.9739 (+/− 20 ppm)
702.9666
[M−H]−
676.9684
362.9116
0
20
40
60
80
100
350
400
450
500
550
600
650
700

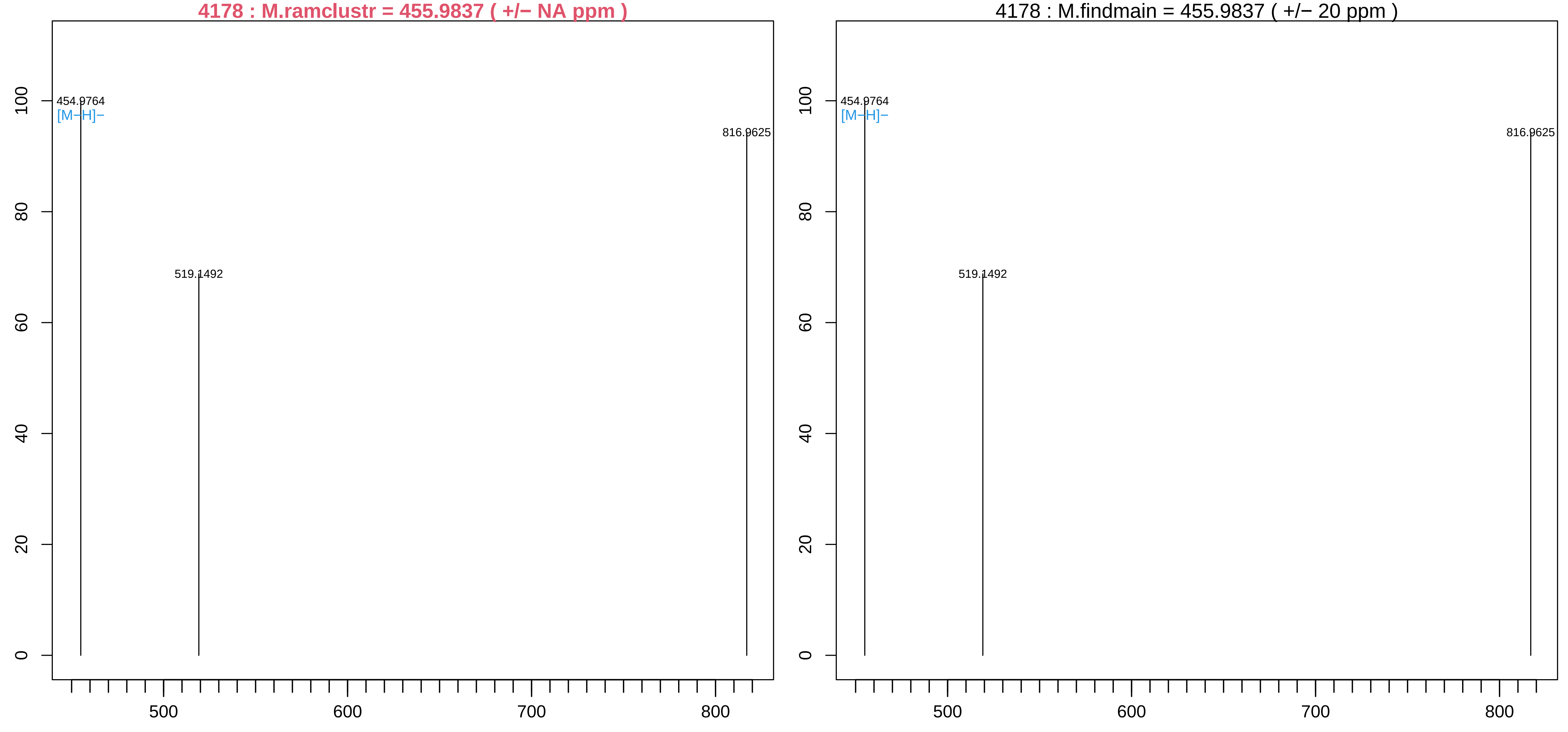
4178 : M.ramclustr = 455.9837 (+/− NA ppm)
454.9764
[M−H]−
519.1492
816.9625
4178 : M.findmain = 455.9837 (+/− 20 ppm)
454.9764
[M−H]−
519.1492
816.9625
0
20
40
60
80
100
500
600
700
800

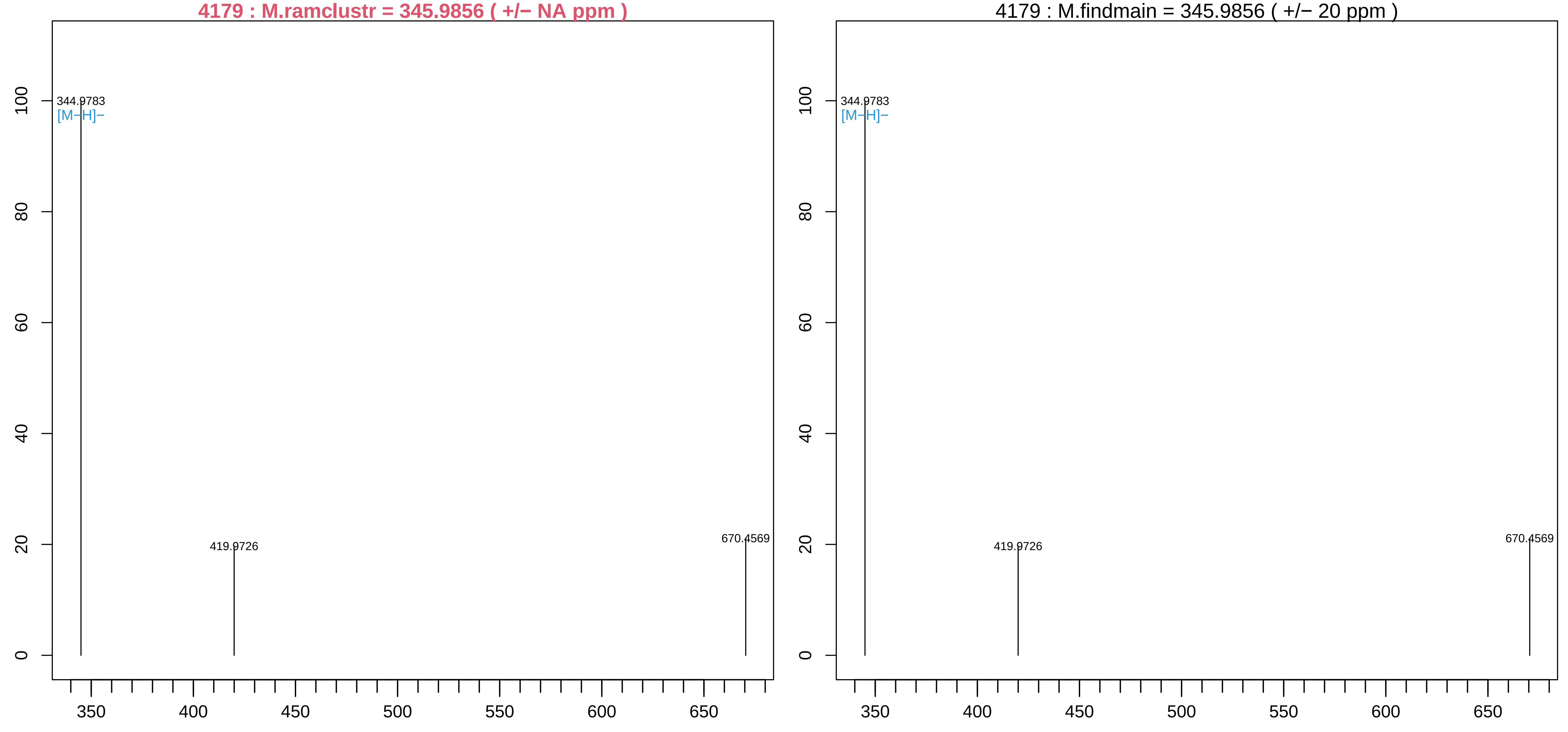

4179 : M.ramclustr = 345.9856 (+/− NA ppm)
344.9783
[M−H]−
419.9726
670.4569
4179 : M.findmain = 345.9856 (+/− 20 ppm)
344.9783
[M−H]−
419.9726
670.4569

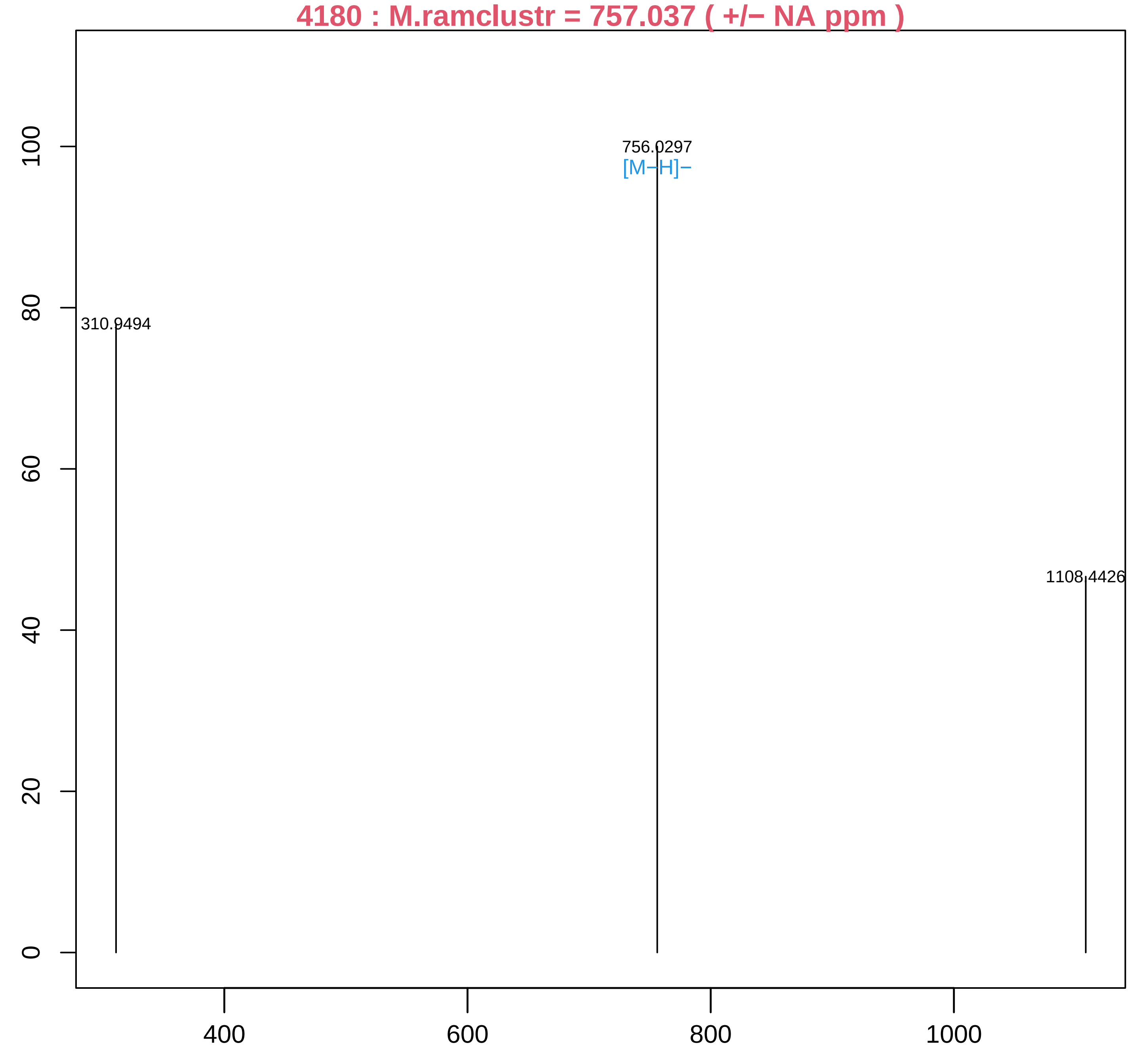

4180 : M.ramclustr = 757.037 (+/− NA ppm)
310.9494
756.0297
[M−H]−
1108.4426
0
20
40
60
80
100
400
600
800
1000

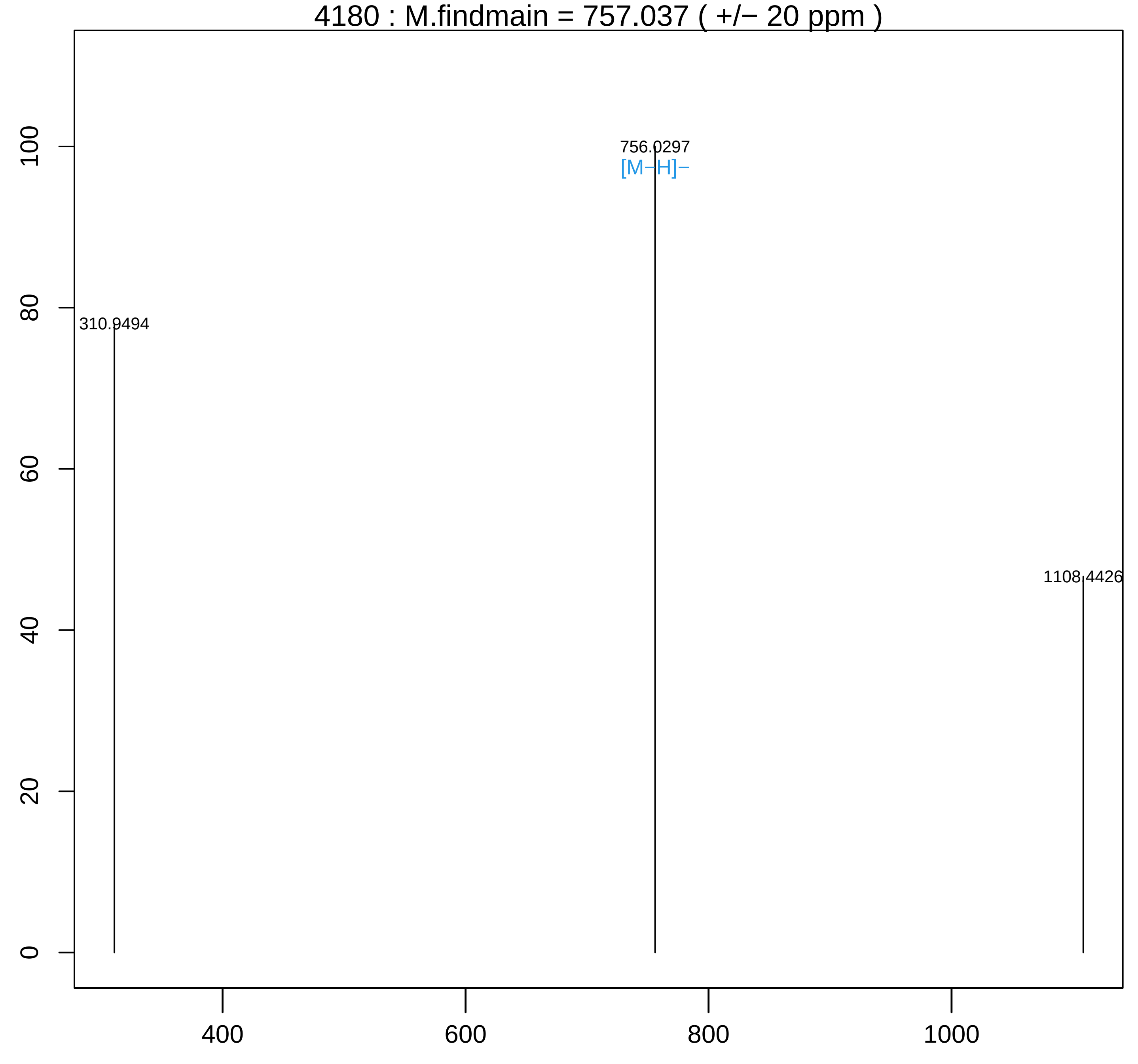

4180 : M.findmain = 757.037 (+/− 20 ppm)
310.9494
756.0297
[M−H]−
1108.4426
0
20
40
60
80
100
400
600
800
1000

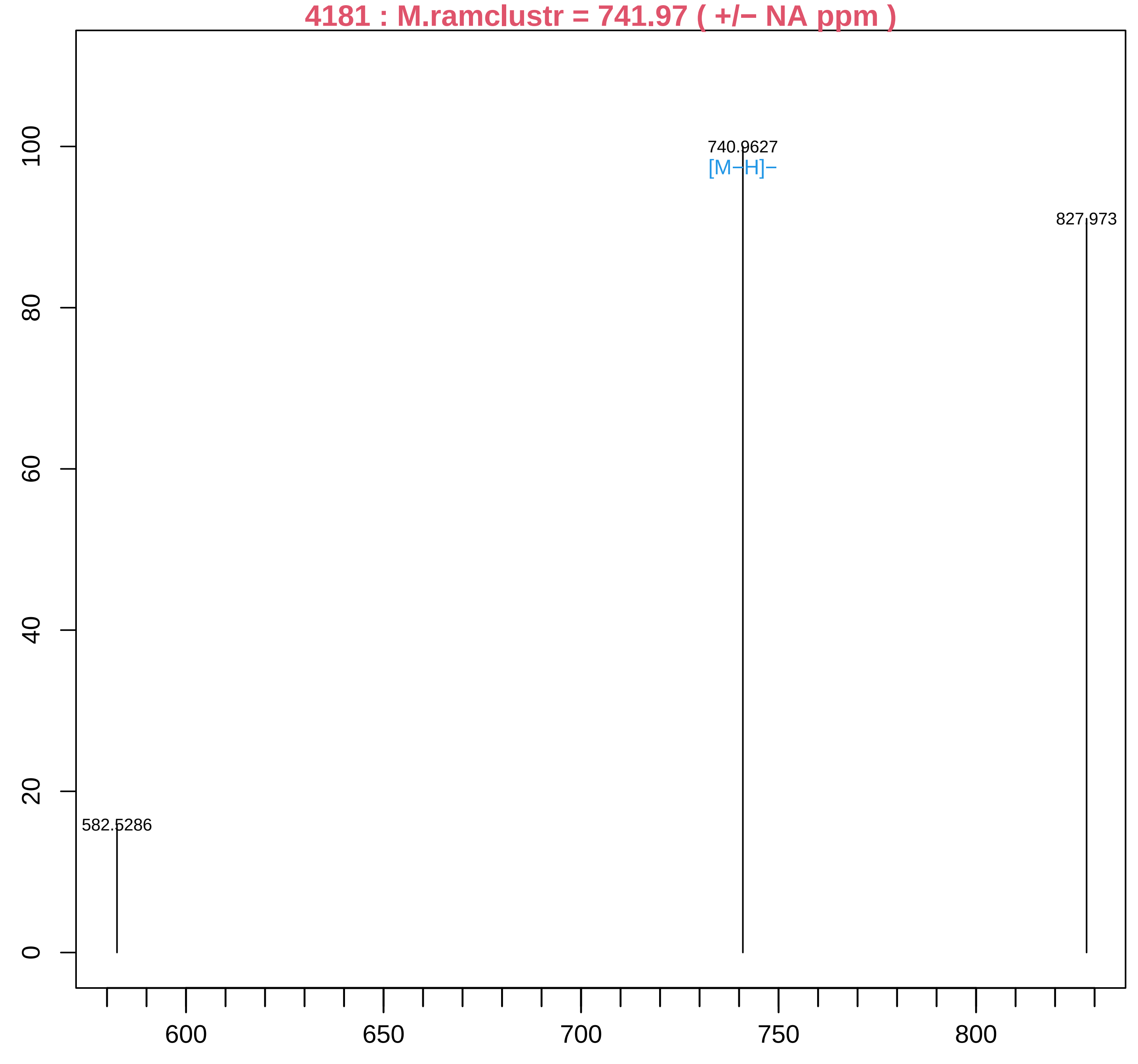
4181 : M.ramclustr = 741.97 (+/− NA ppm)
740.9627
[M−H]−
827.973
582.5286
0
20
40
60
80
100
600
650
700
750
800

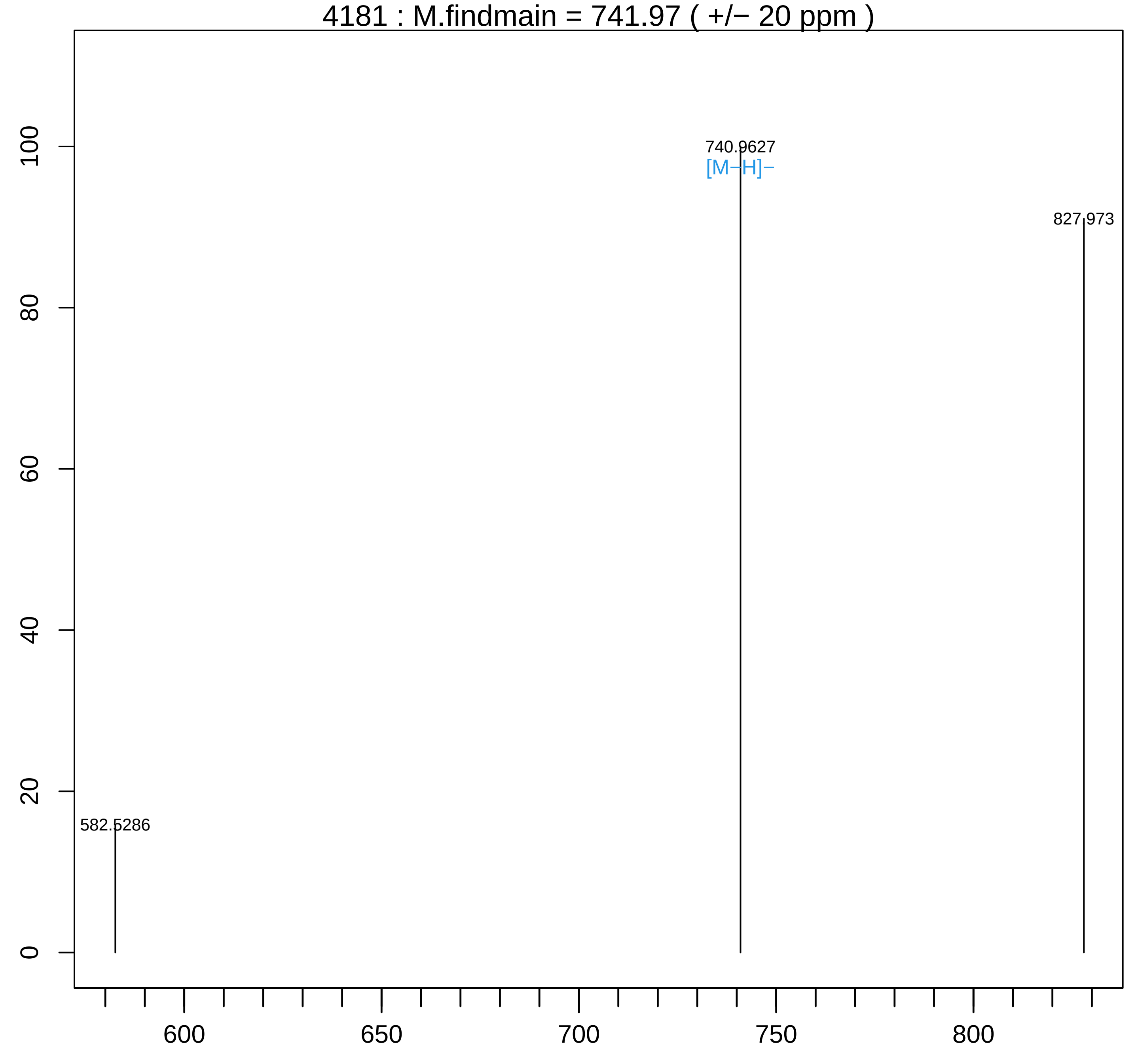
4181 : M.findmain = 741.97 (+/− 20 ppm)
740.9627
[M−H]−
827.973
582.5286
0
20
40
60
80
100
600
650
700
750
800

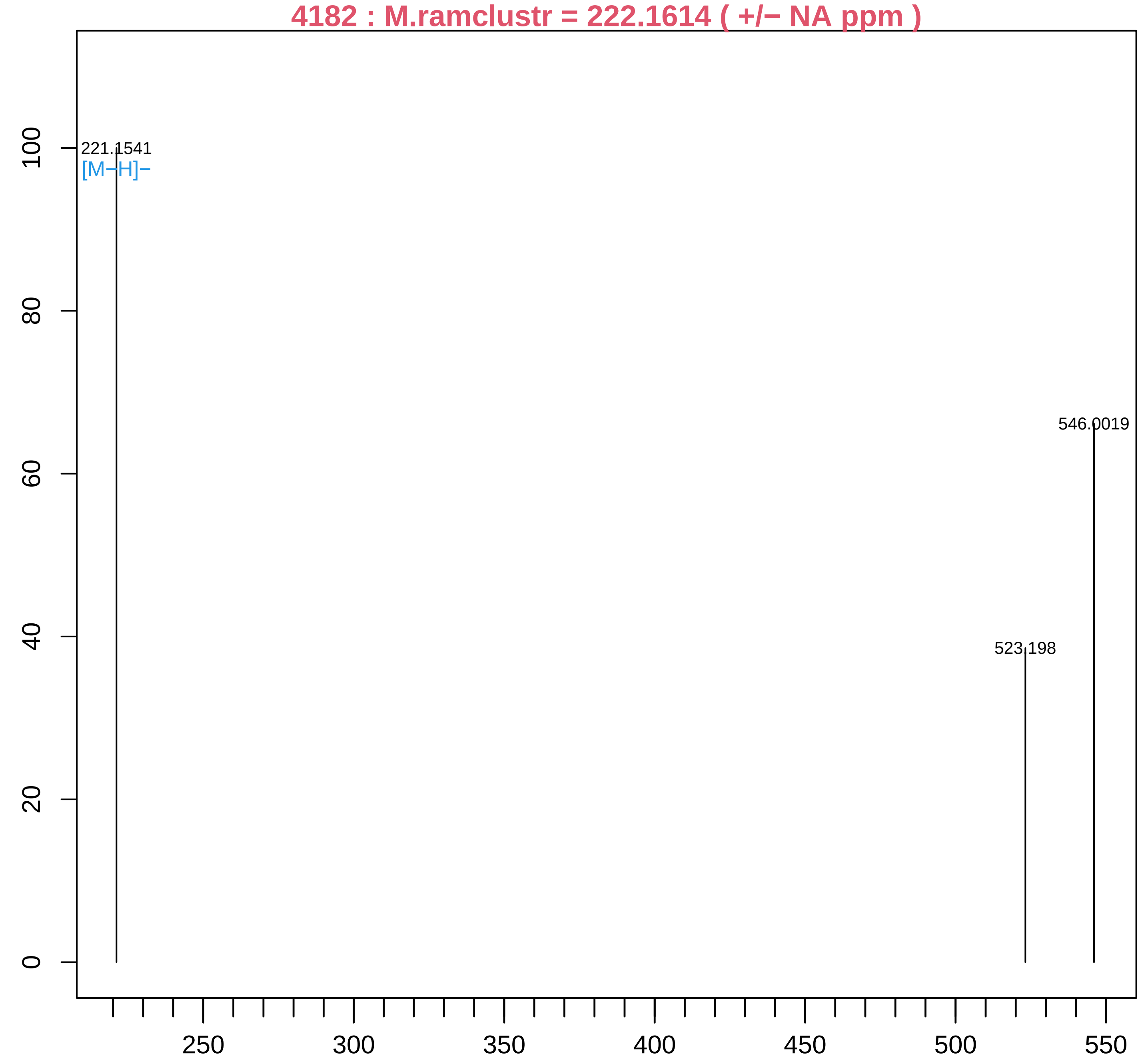
4182 : M.ramclustr = 222.1614 (+/− NA ppm)
221.1541
[M−H]−
546.0019
523.198
0
20
40
60
80
100
250
300
350
400
450
500
550

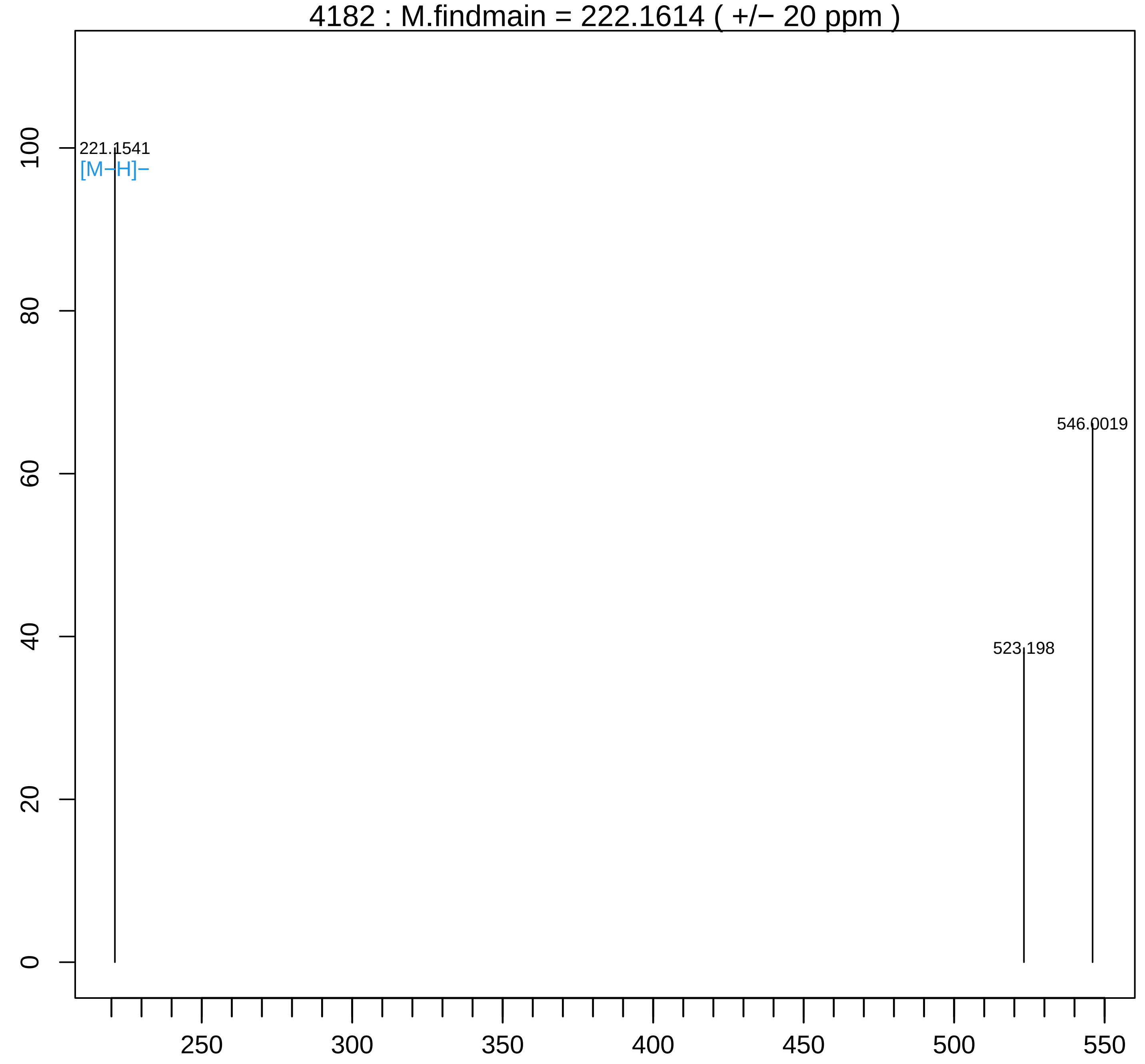
4182 : M.findmain = 222.1614 (+/− 20 ppm)
221.1541
[M−H]−
546.0019
523.198
0
20
40
60
80
100
250
300
350
400
450
500
550

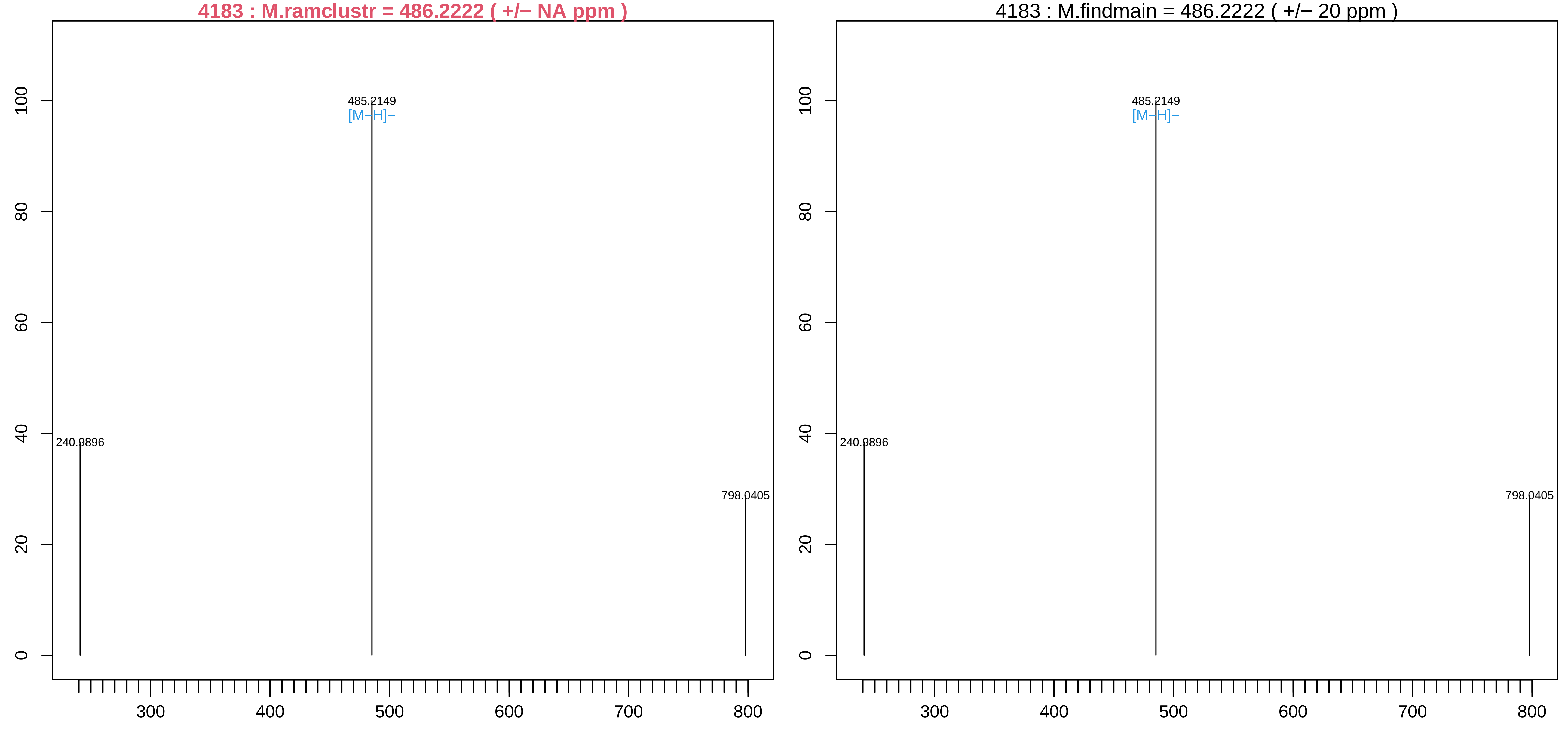
4183 : M.ramclustr = 486.2222 (+/− NA ppm)
485.2149
[M−H]−
240.9896
798.0405
4183 : M.findmain = 486.2222 (+/− 20 ppm)
485.2149
[M−H]−
240.9896
798.0405
0
20
40
60
80
100
300
400
500
600
700
800

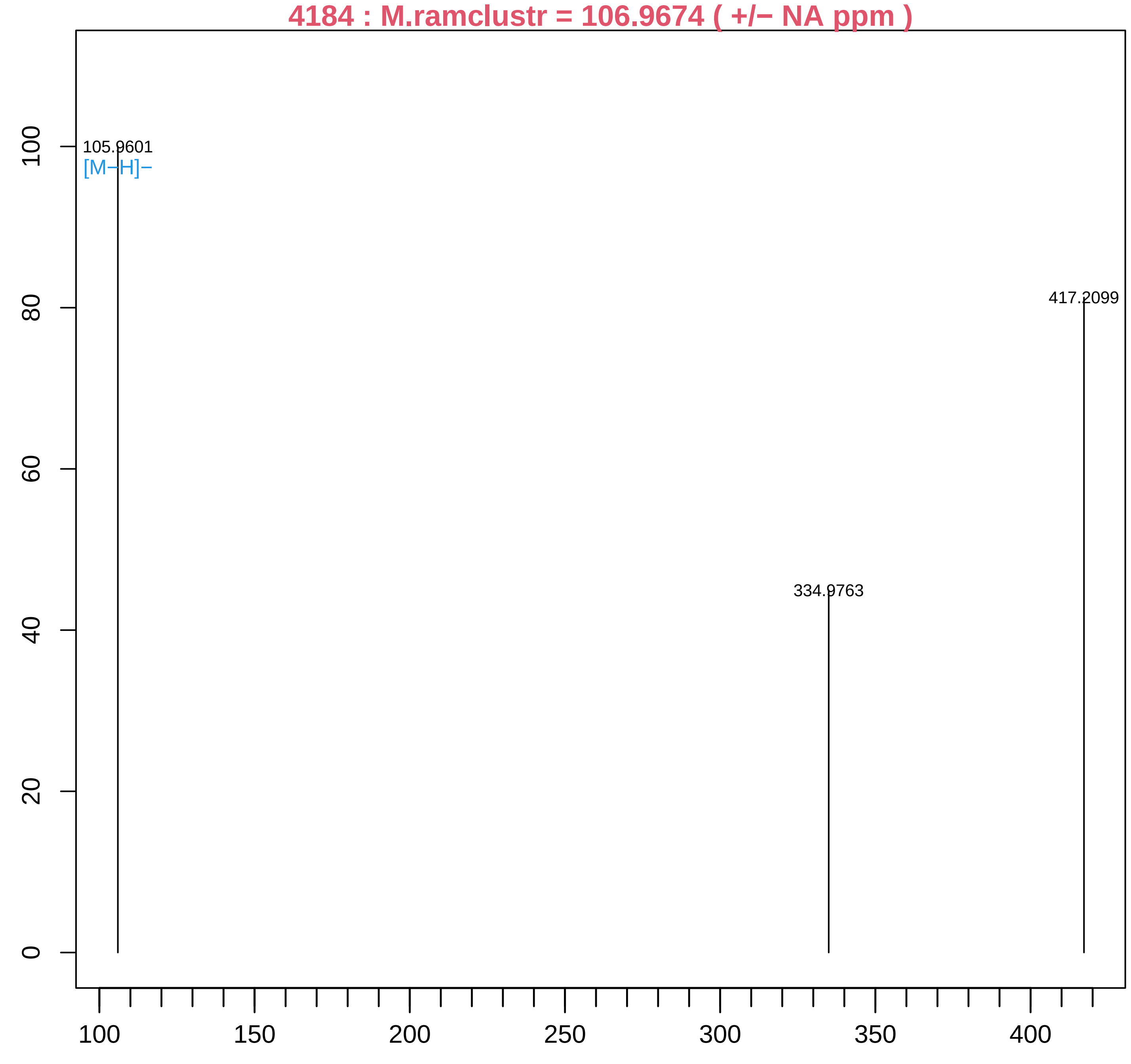

4184 : M.ramclustr = 106.9674 (+/− NA ppm)
105.9601
[M−H]−
417.2099
334.9763
0
20
40
60
80
100
100
150
200
250
300
350
400

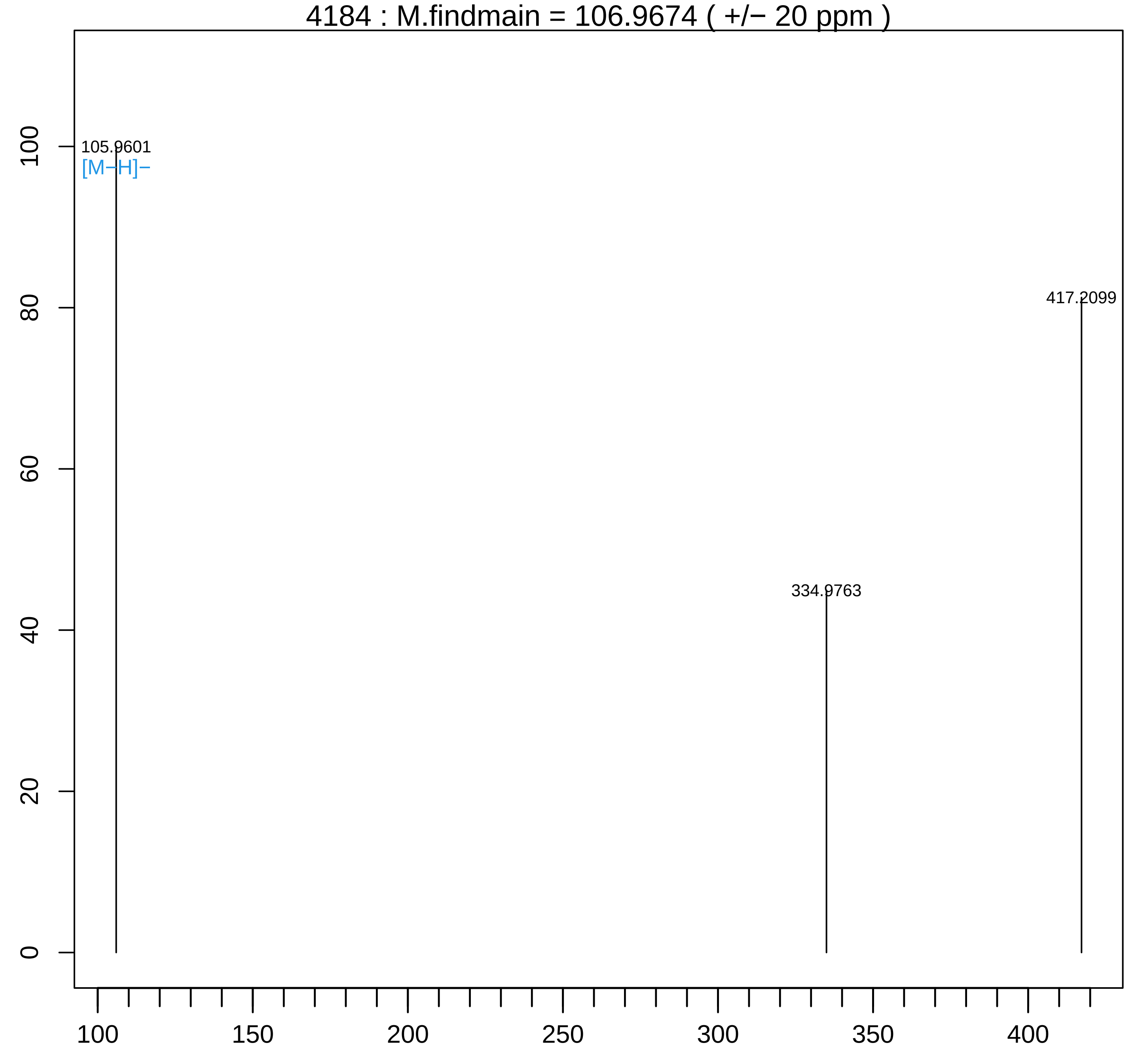

4184 : M.findmain = 106.9674 (+/− 20 ppm)
105.9601
[M−H]−
417.2099
334.9763
0
20
40
60
80
100
100
150
200
250
300
350
400

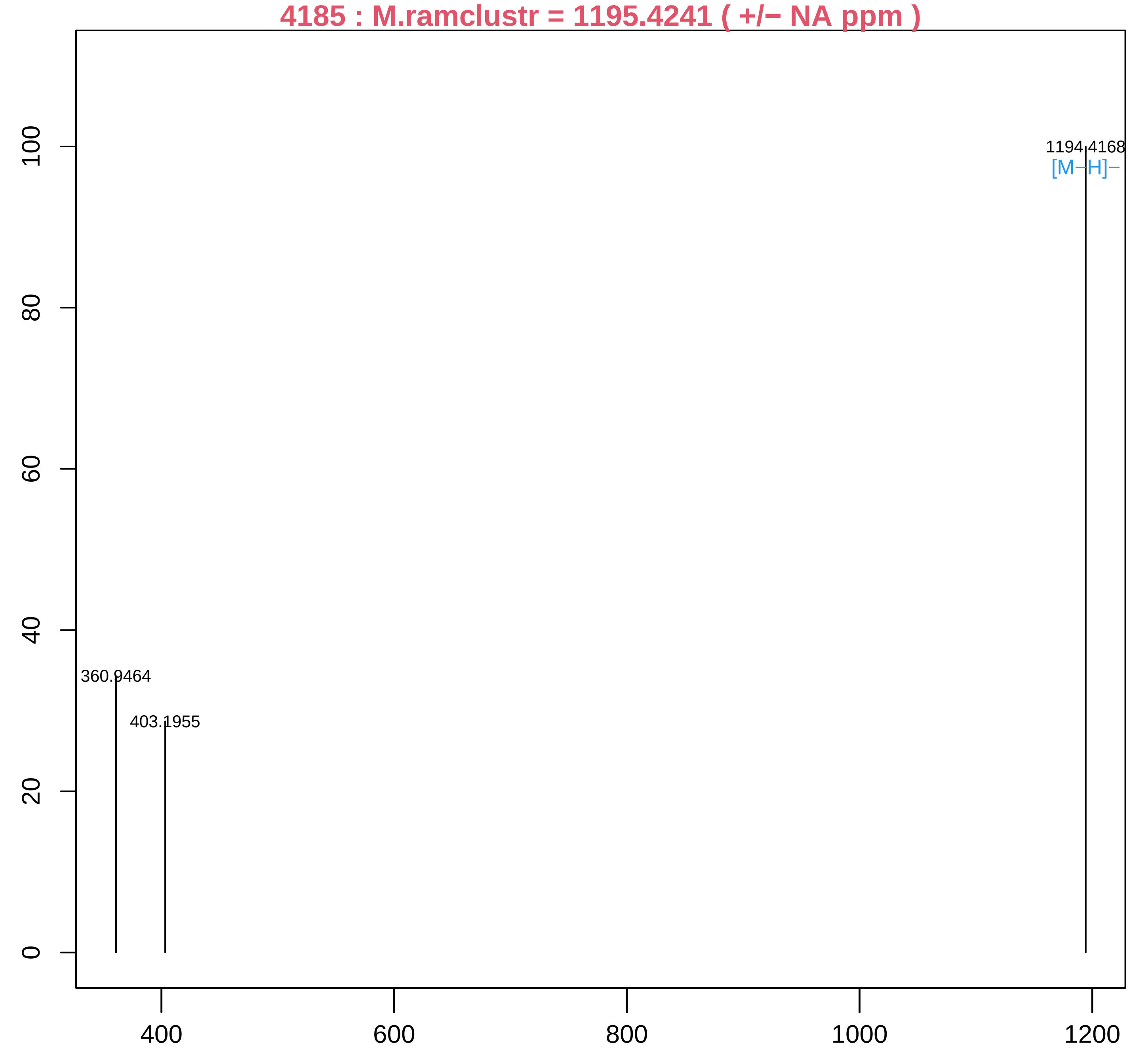
4185 : M.ramclustr = 1195.4241 (+/− NA ppm)
1194.4168
[M−H]−
360.9464
403.1955
0
20
40
60
80
100
400
600
800
1000
1200

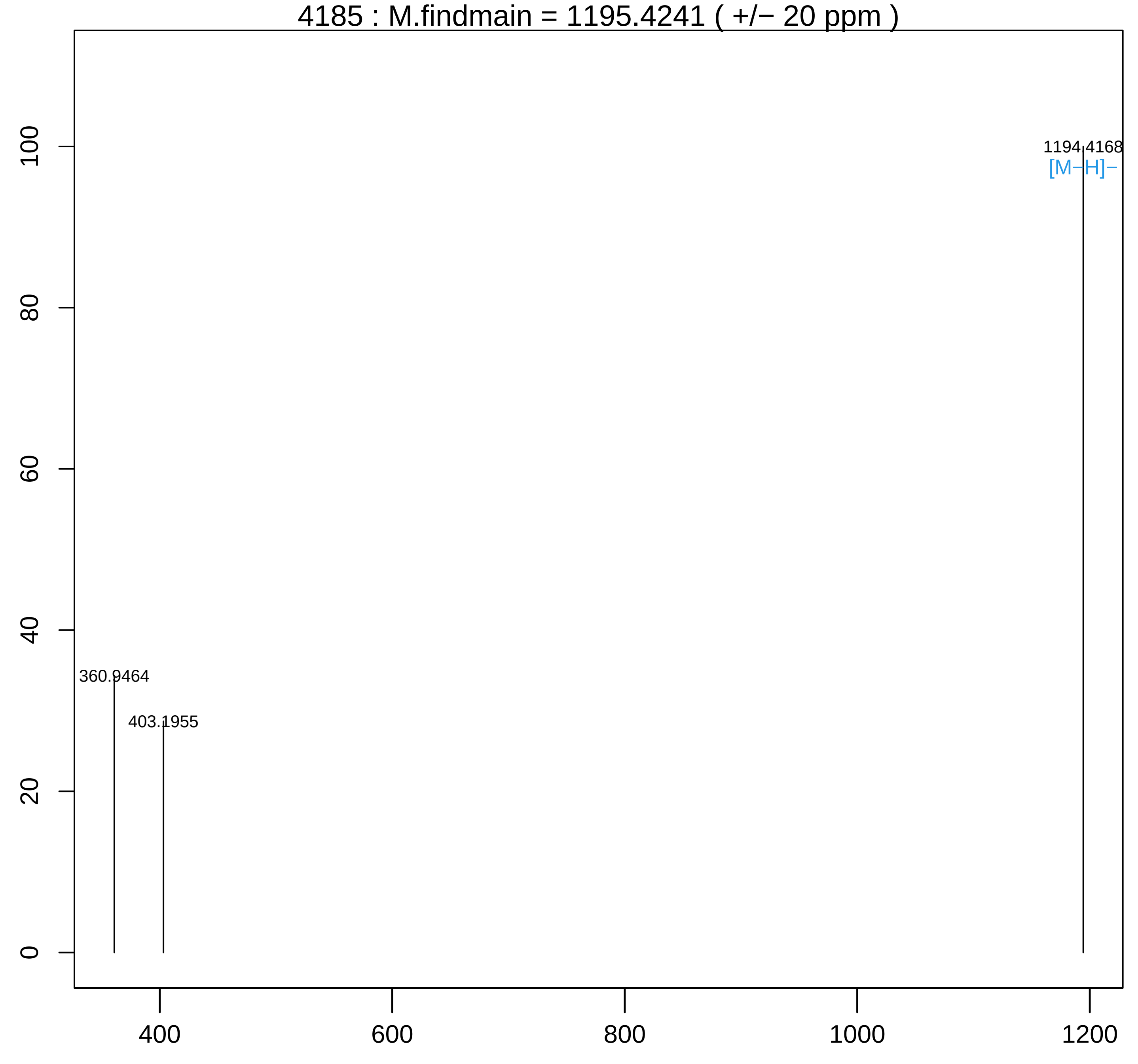
4185 : M.findmain = 1195.4241 (+/− 20 ppm)
1194.4168
[M−H]−
360.9464
403.1955
0
20
40
60
80
100
400
600
800
1000
1200

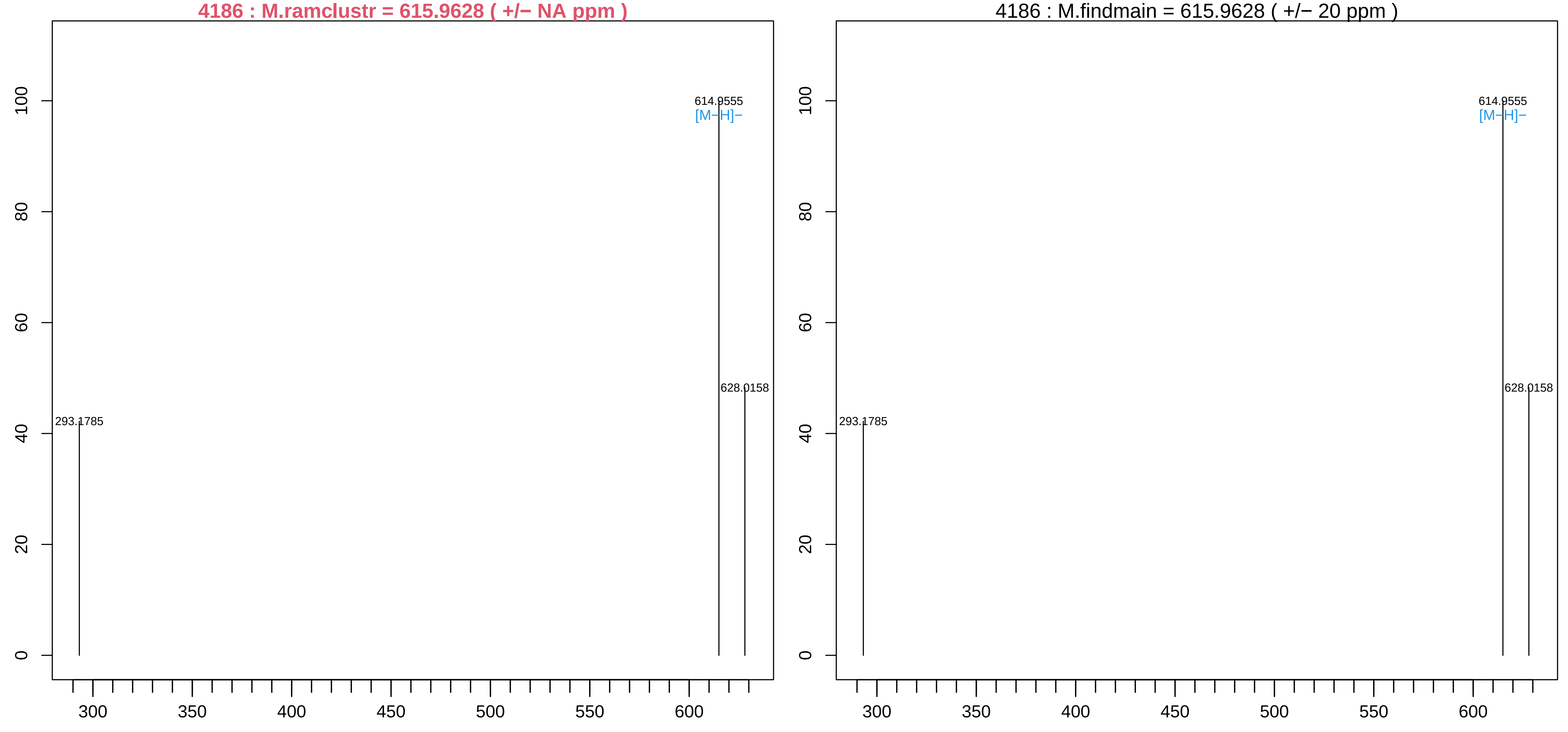
4186 : M.ramclustr = 615.9628 (+/− NA ppm)
614.9555
[M−H]−
628.0158
293.1785
0
20
40
60
80
100
300
350
400
450
500
550
600
4186 : M.findmain = 615.9628 (+/− 20 ppm)
614.9555
[M−H]−
628.0158
293.1785
0
20
40
60
80
100
300
350
400
450
500
550
600

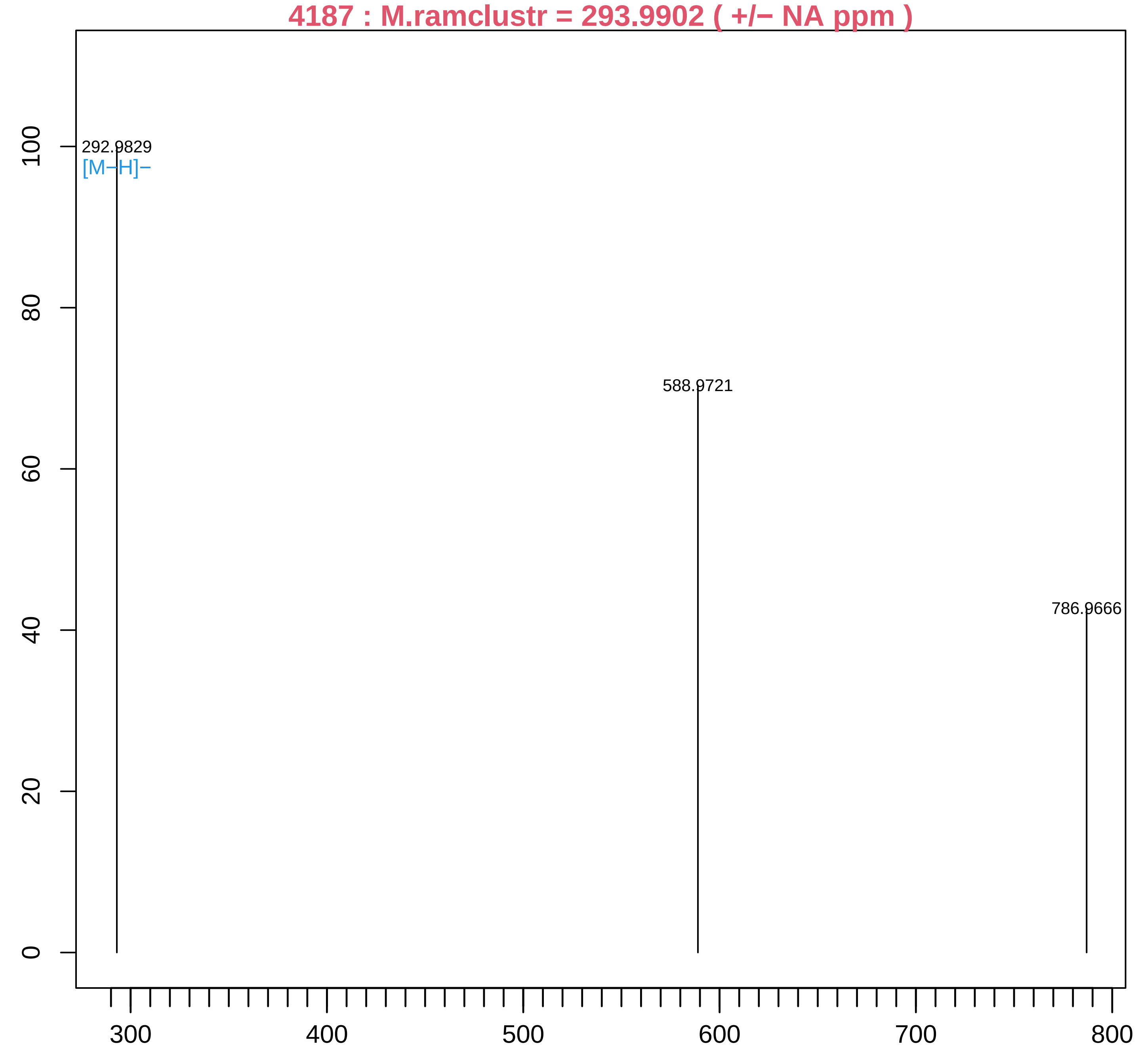
4187 : M.ramclustr = 293.9902 (+/− NA ppm)
292.9829
[M−H]−
588.9721
786.9666
0
20
40
60
80
100
300
400
500
600
700
800

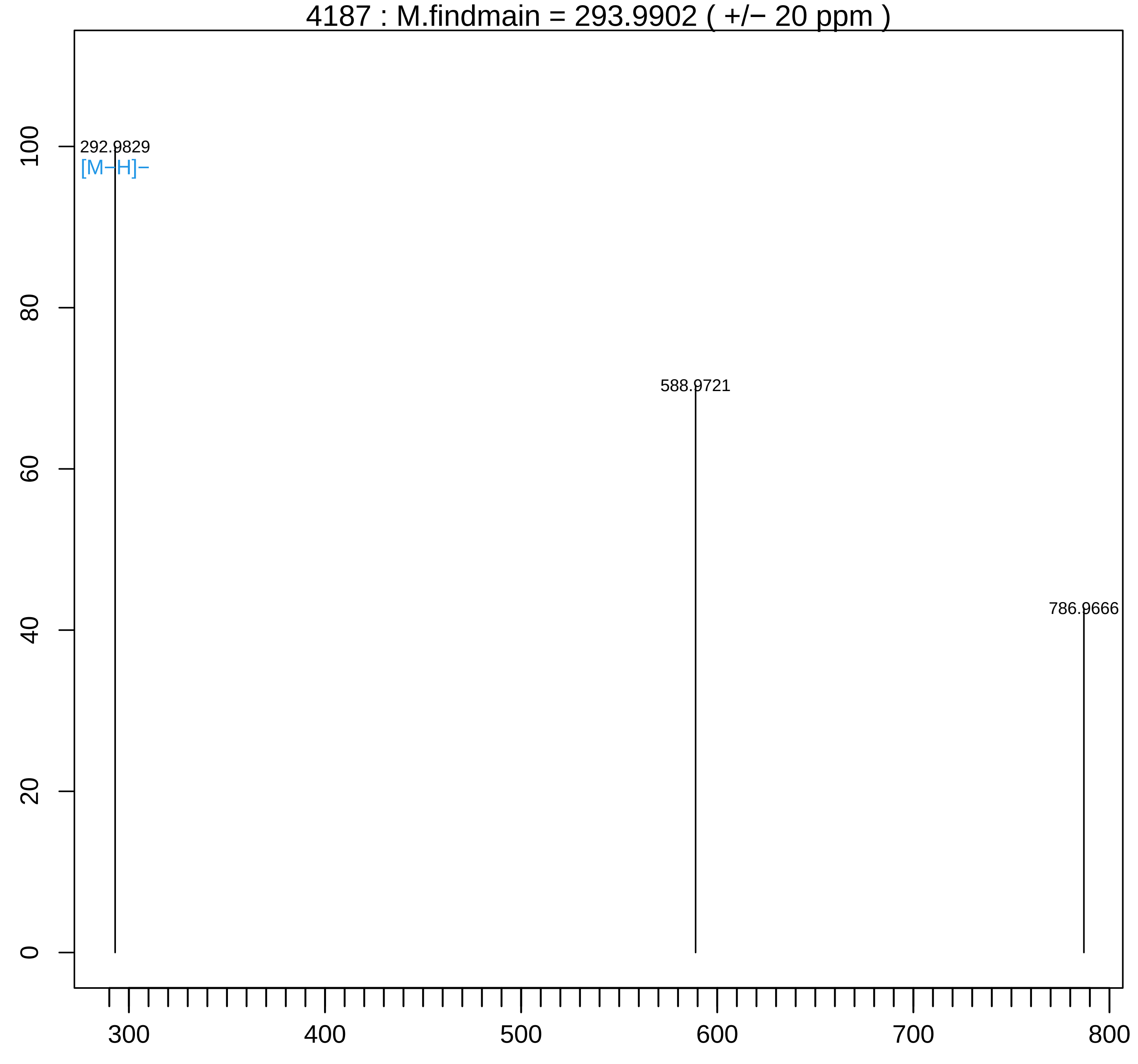
4187 : M.findmain = 293.9902 (+/− 20 ppm)
292.9829
[M−H]−
588.9721
786.9666
0
20
40
60
80
100
300
400
500
600
700
800

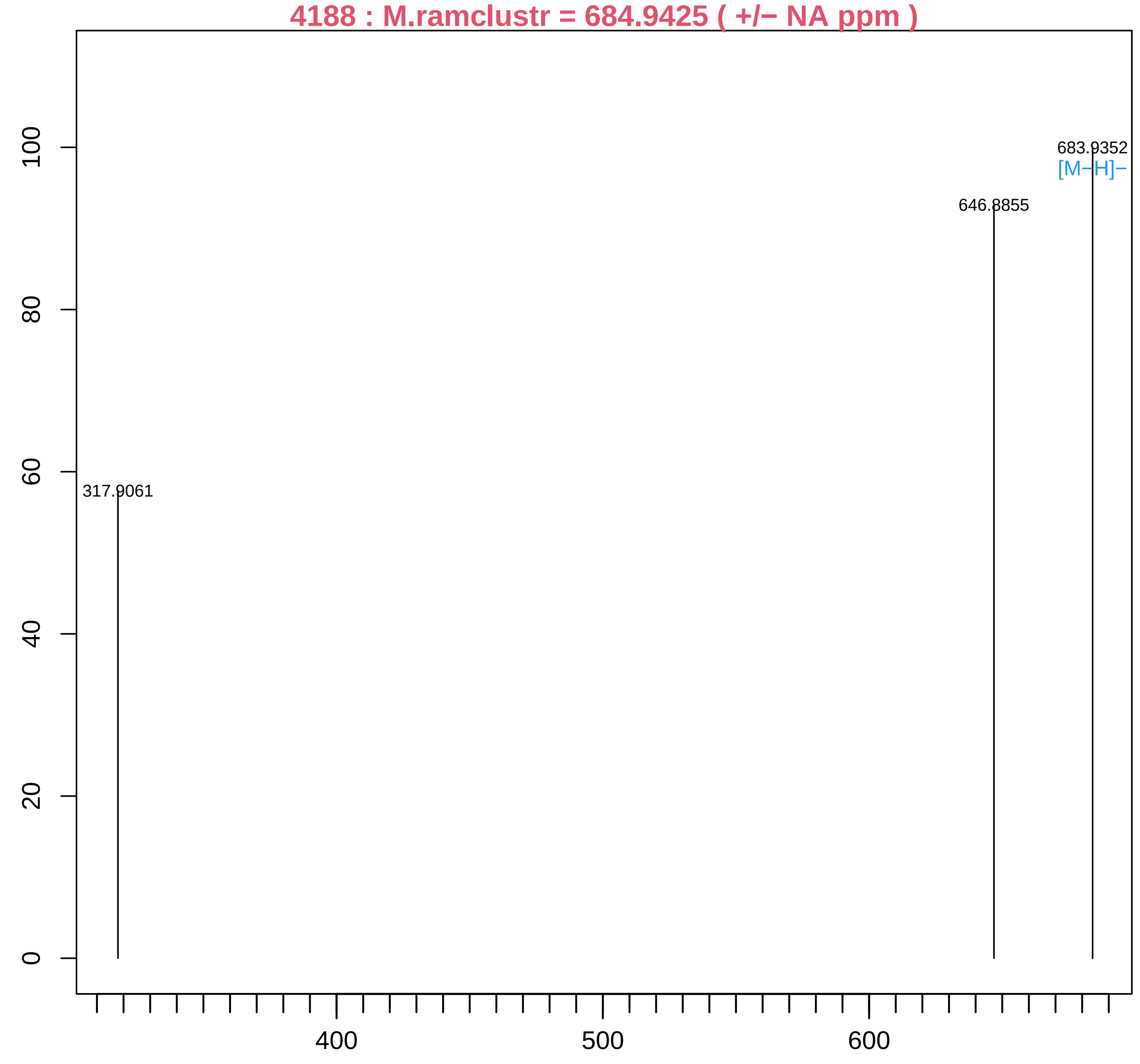
4188 : M.ramclustr = 684.9425 (+/− NA ppm)
683.9352
[M−H]−
646.8855
317.9061
0
20
40
60
80
100
400
500
600

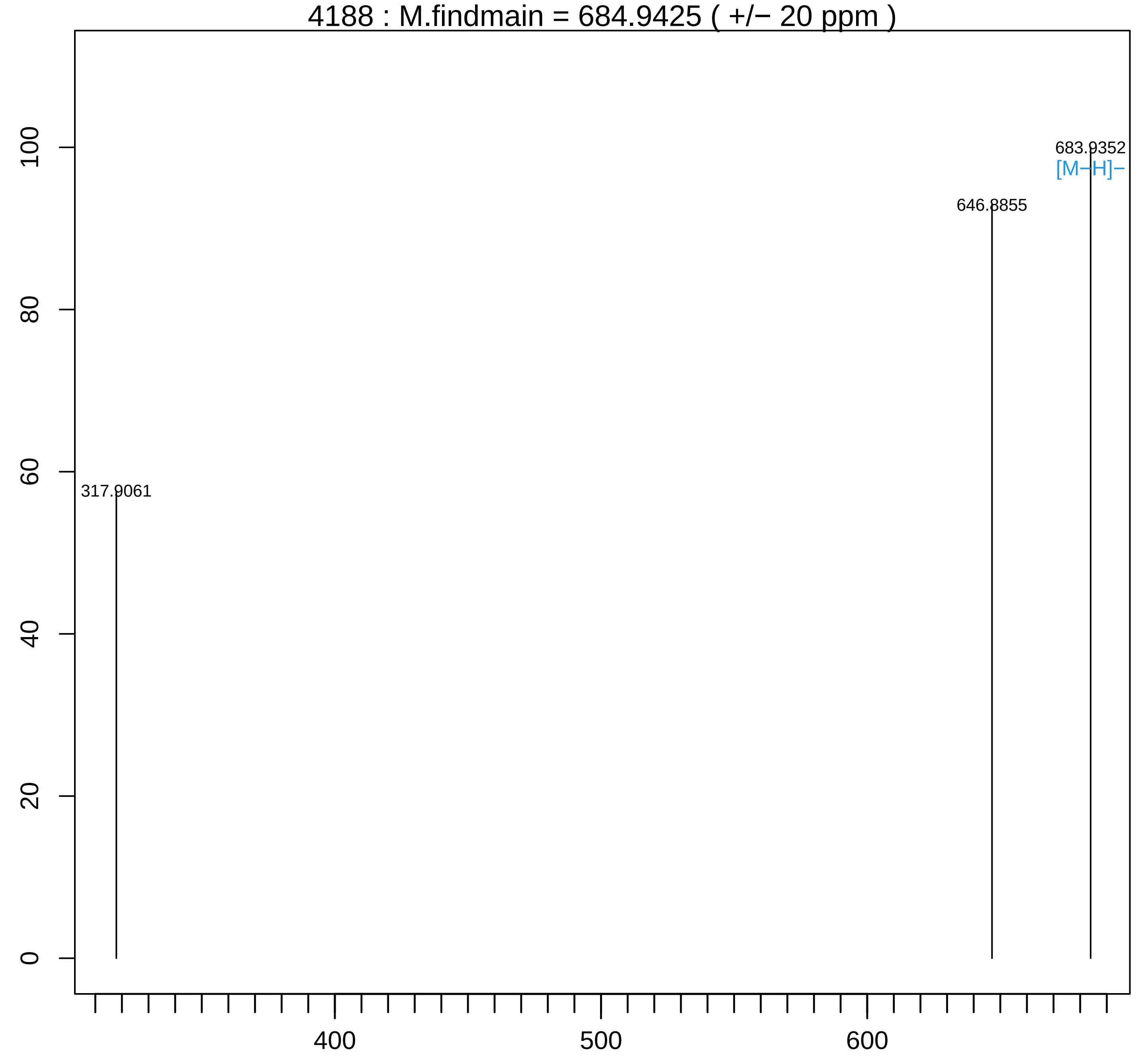
4188 : M.findmain = 684.9425 (+/− 20 ppm)
683.9352
[M−H]−
646.8855
317.9061
0
20
40
60
80
100
400
500
600

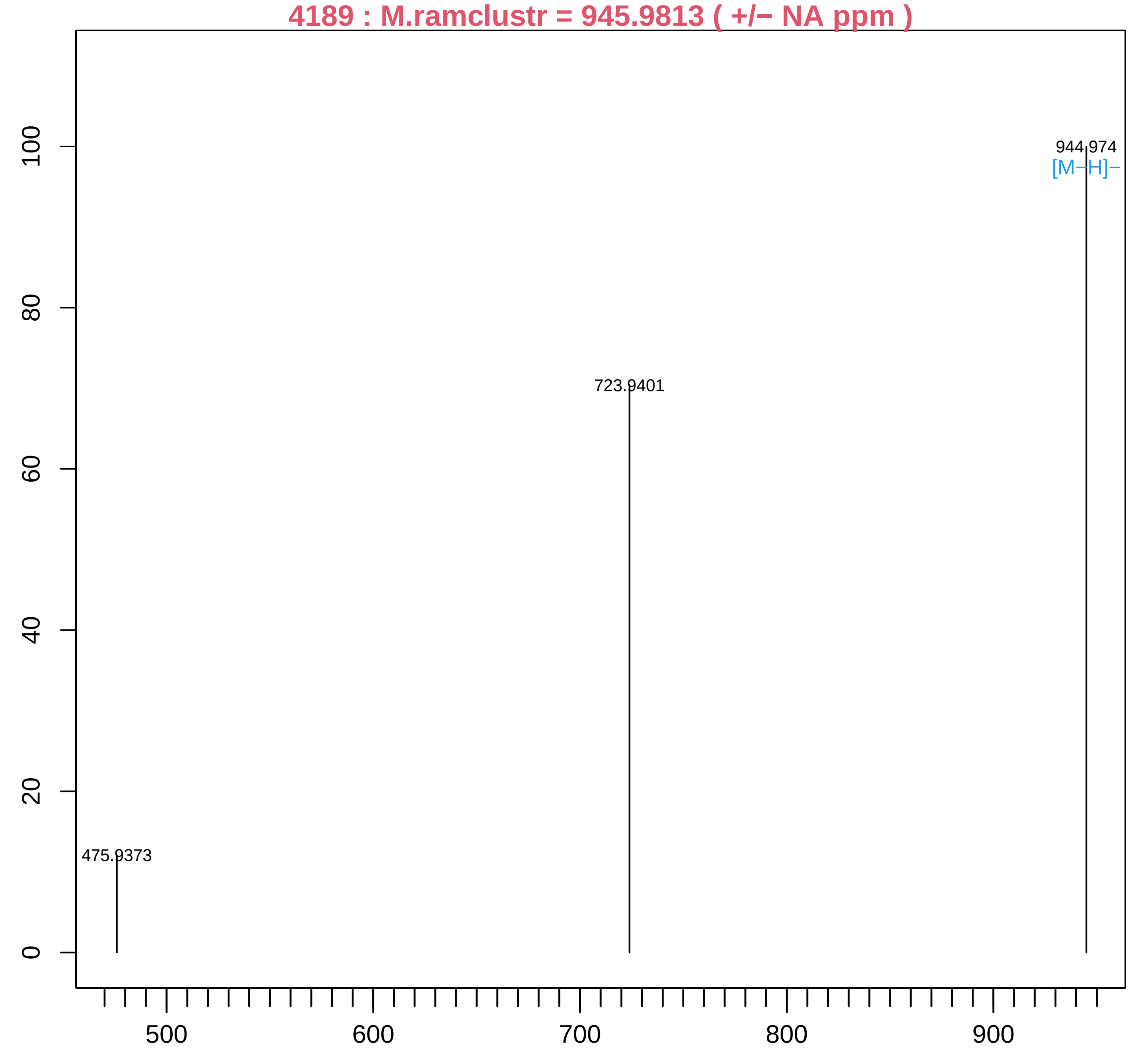

4189 : M.ramclustr = 945.9813 (+/− NA ppm)
944.974
[M−H]−
723.9401
475.9373
0
20
40
60
80
100
500
600
700
800
900

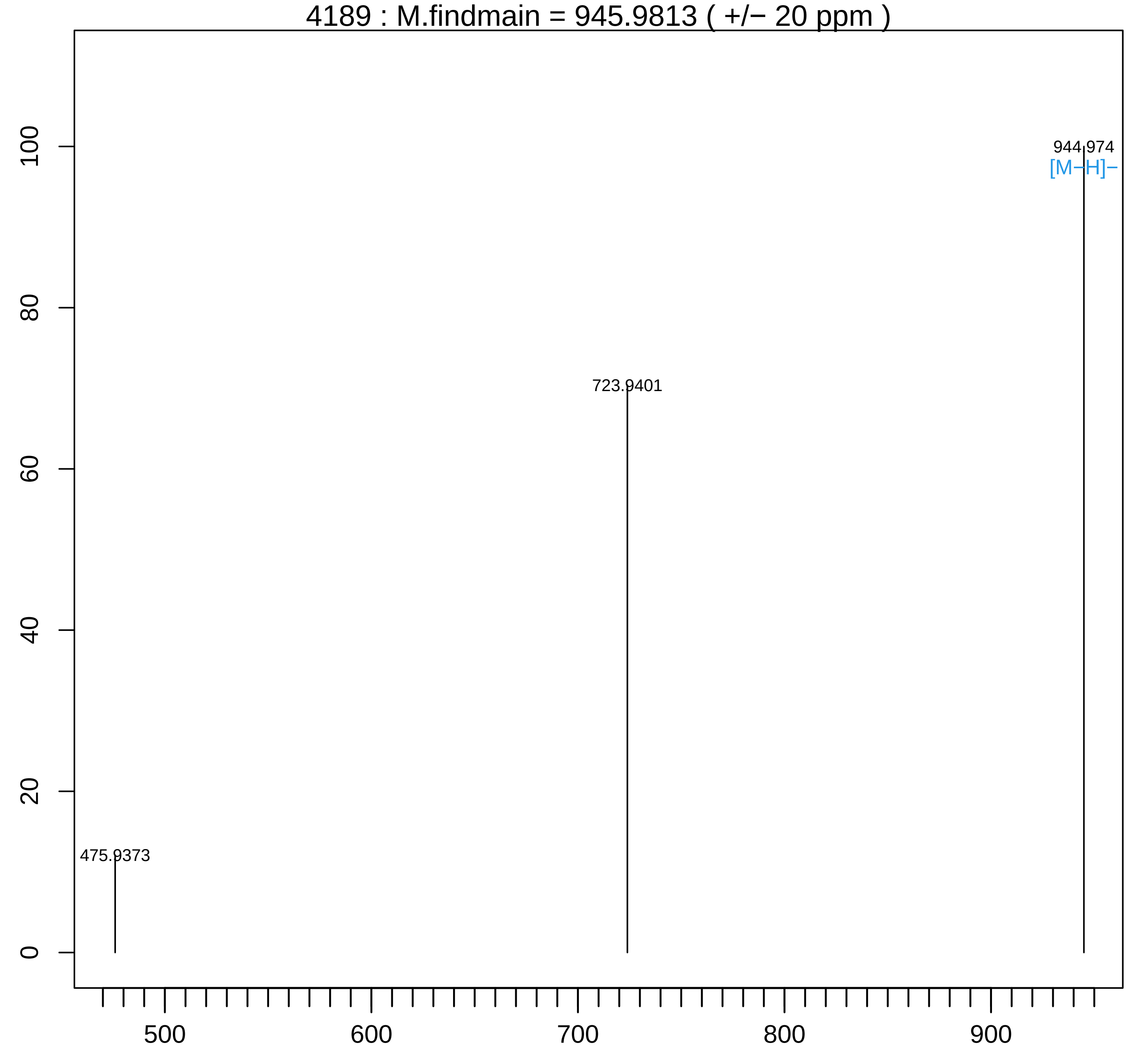

4189 : M.findmain = 945.9813 (+/− 20 ppm)
944.974
[M−H]−
723.9401
475.9373
0
20
40
60
80
100
500
600
700
800
900

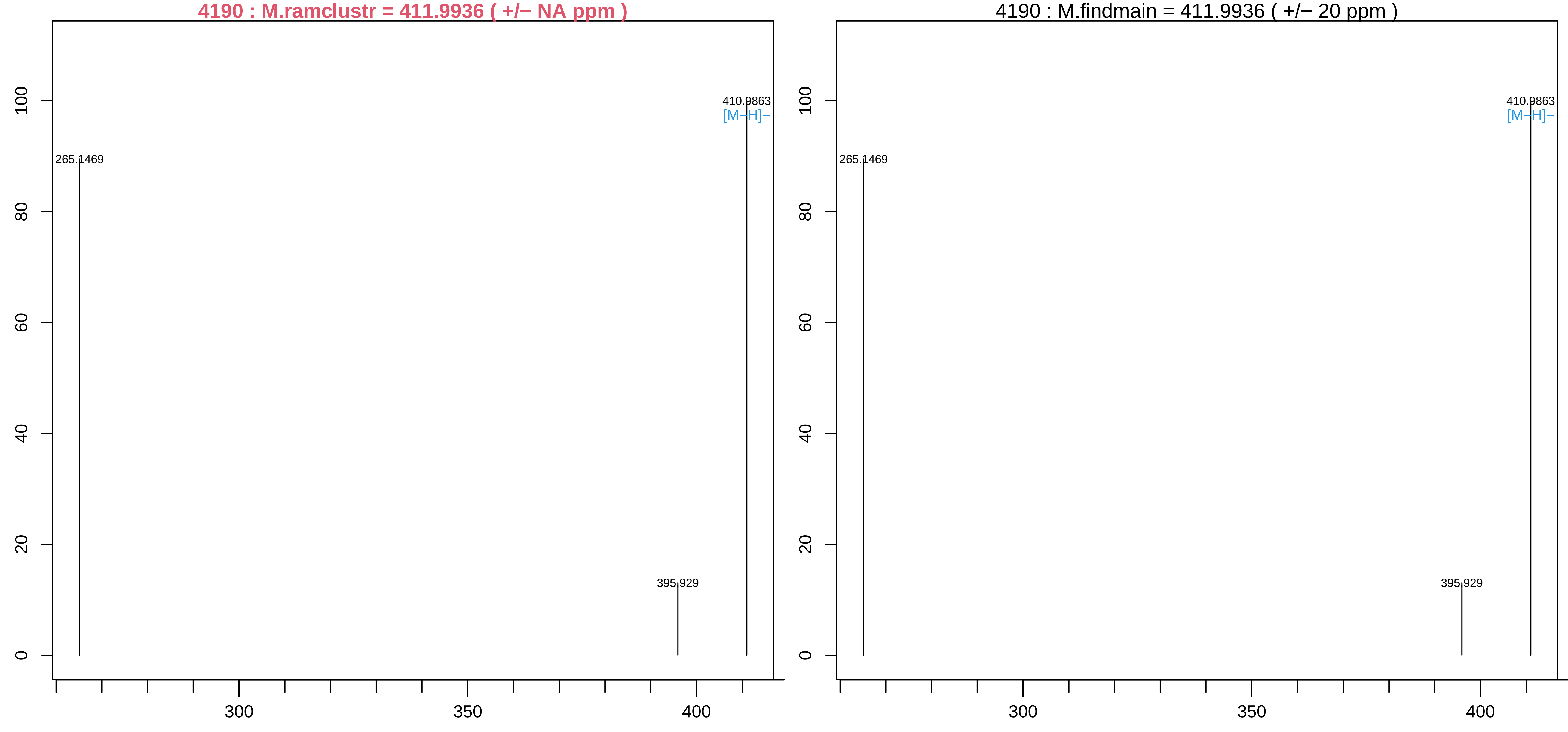

4190 : M.ramclustr = 411.9936 (+/− NA ppm)
265.1469
395.929
410.9863
[M−H]−
0
20
40
60
80
100
300
350
400
4190 : M.findmain = 411.9936 (+/− 20 ppm)
265.1469
395.929
410.9863
[M−H]−
0
20
40
60
80
100
300
350
400

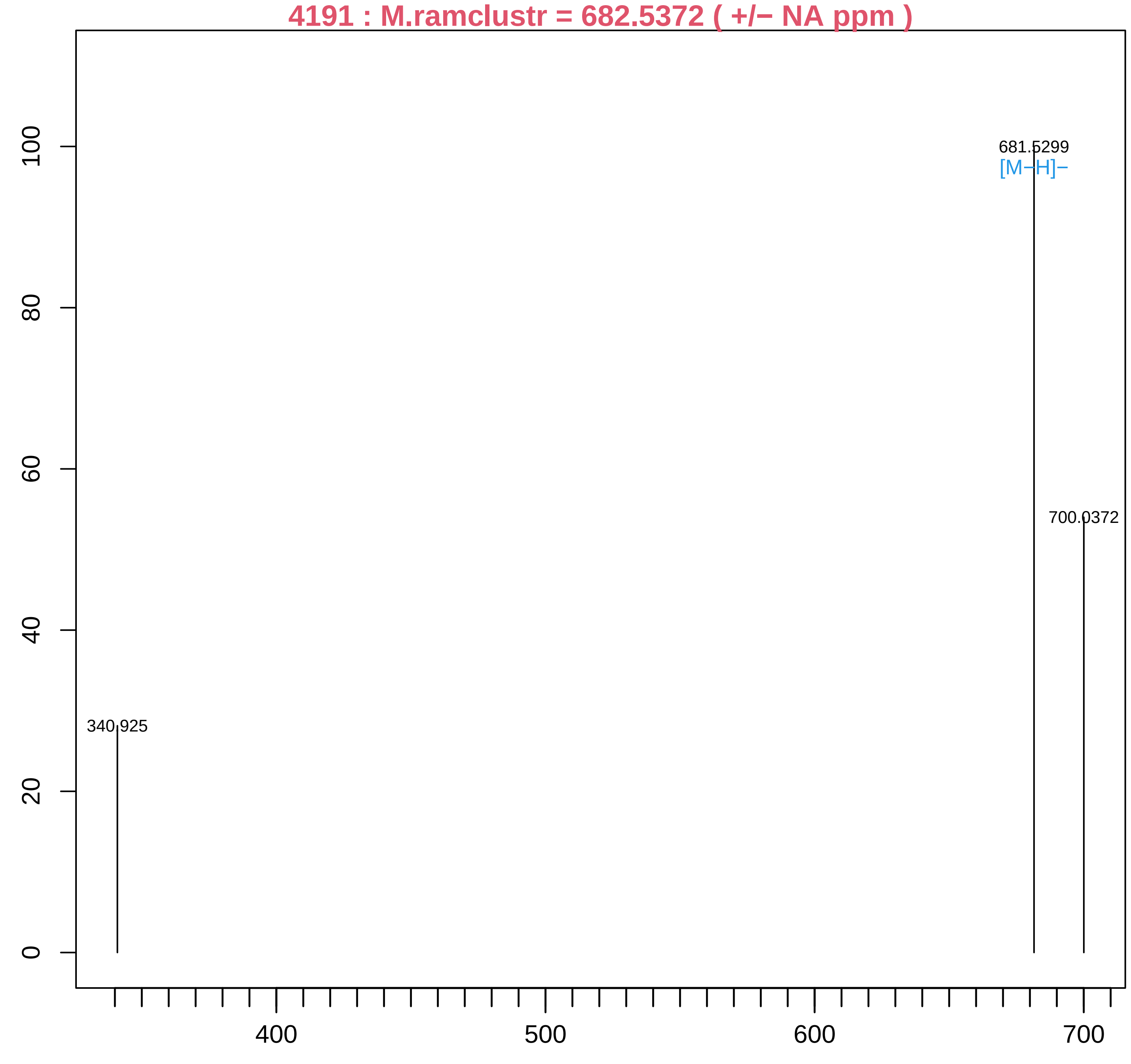

4191 : M.ramclustr = 682.5372 (+/− NA ppm)
681.5299
[M−H]−
700.0372
340.925
0
20
40
60
80
100
400
500
600
700

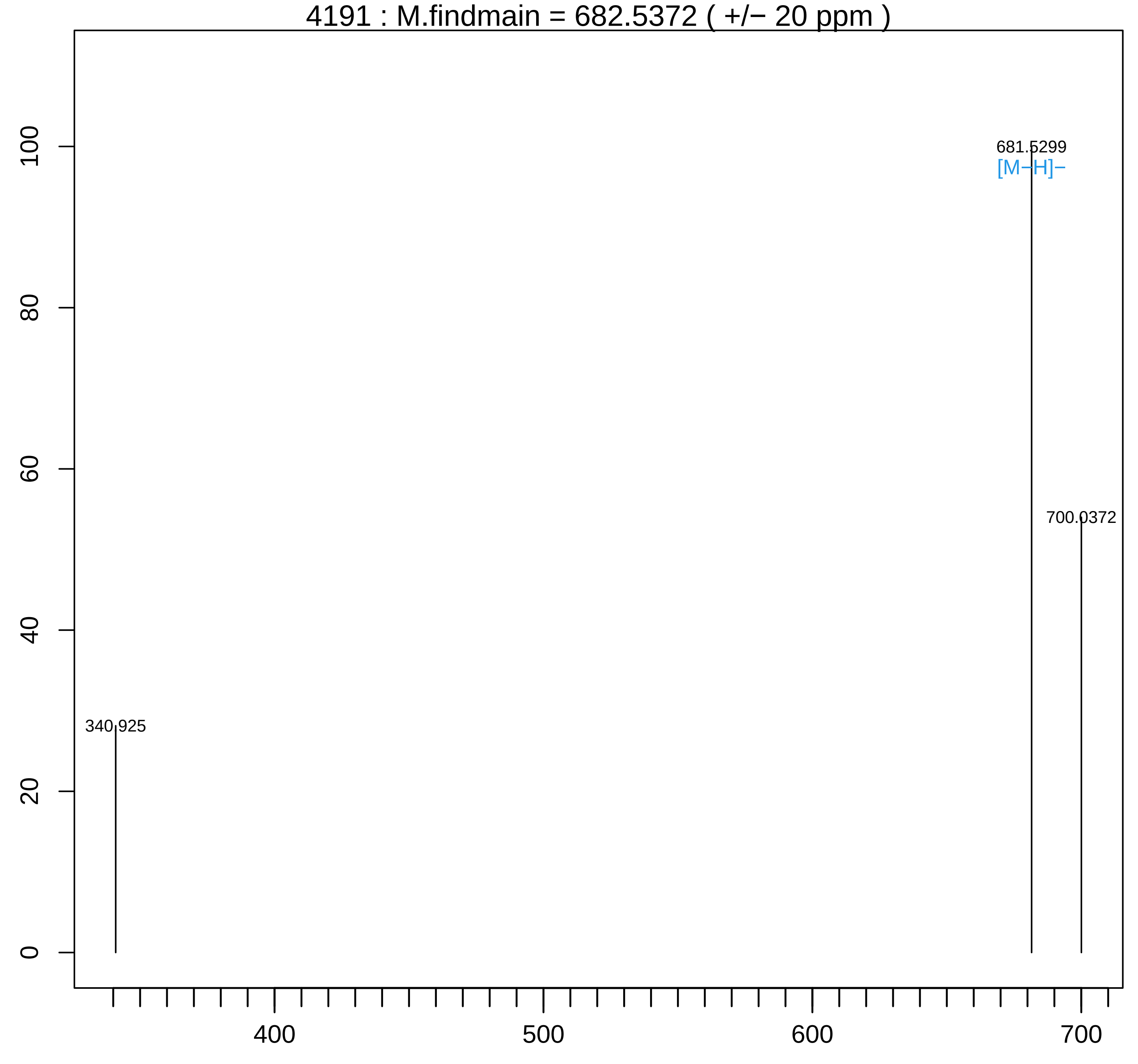

4191 : M.findmain = 682.5372 (+/− 20 ppm)
681.5299
[M−H]−
700.0372
340.925
0
20
40
60
80
100
400
500
600
700

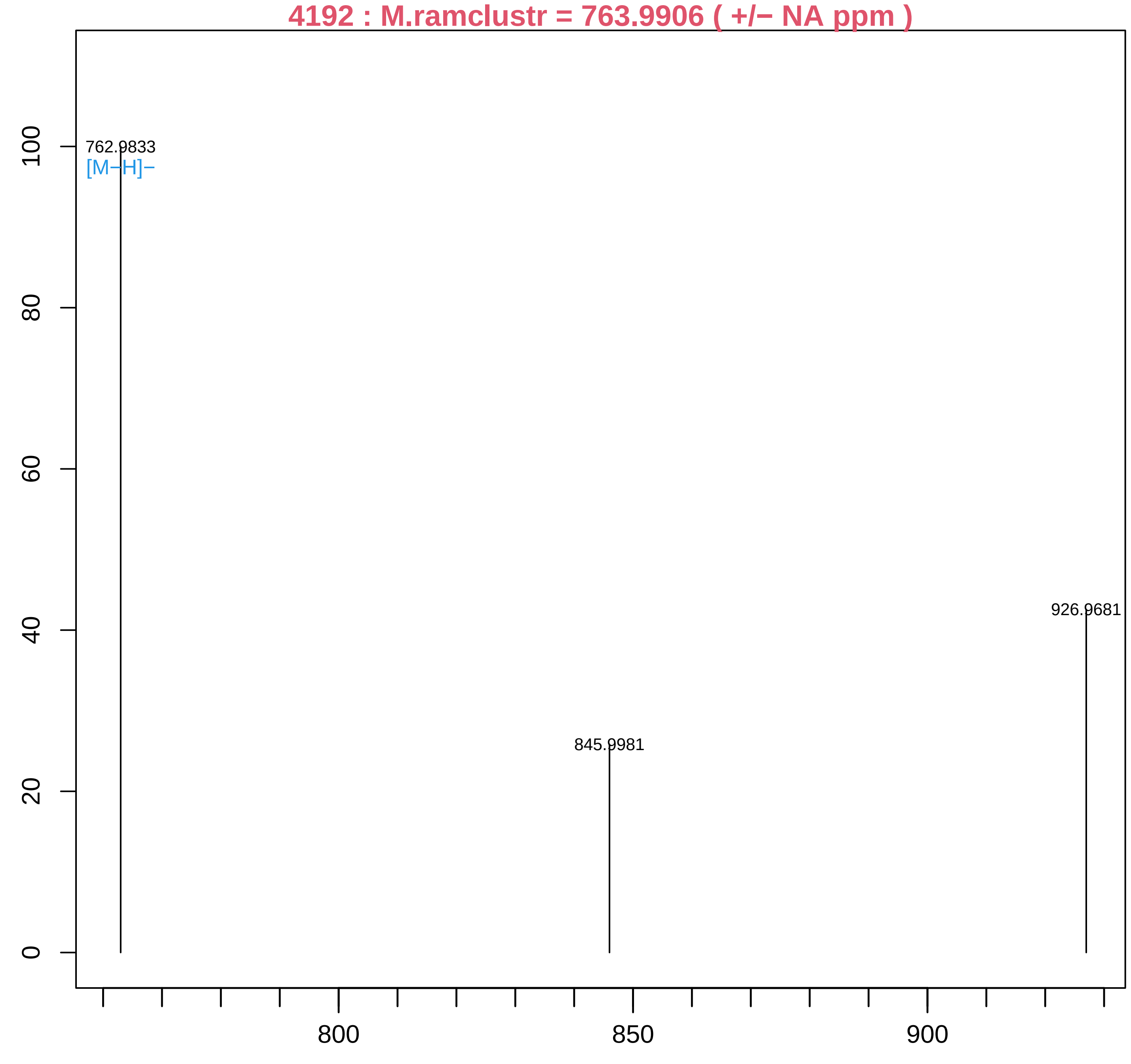

4192 : M.ramclustr = 763.9906 (+/− NA ppm)
762.9833
[M−H]−
845.9981
926.9681
0
20
40
60
80
100
800
850
900

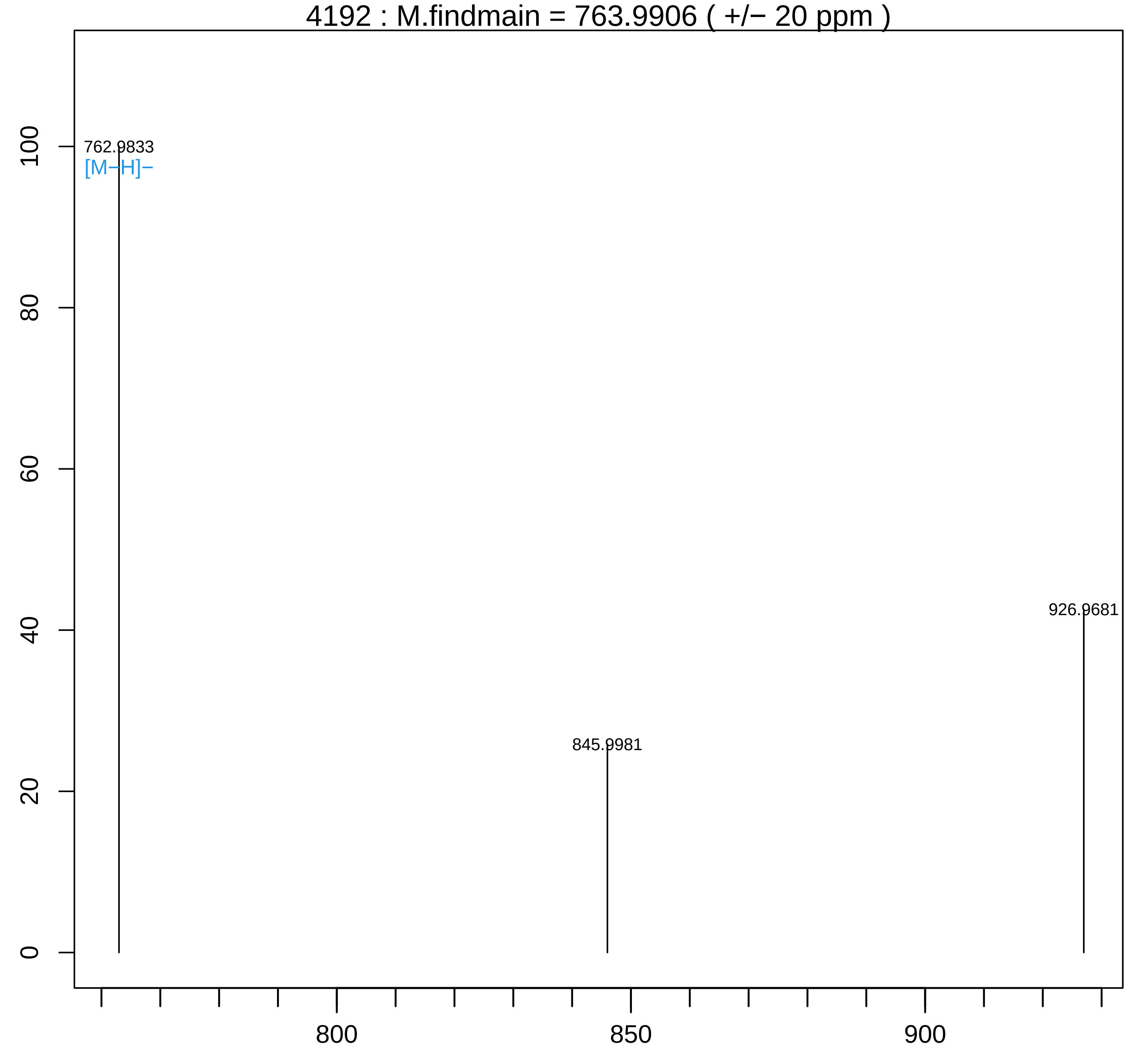

4192 : M.findmain = 763.9906 (+/− 20 ppm)
762.9833
[M−H]−
845.9981
926.9681
0
20
40
60
80
100
800
850
900

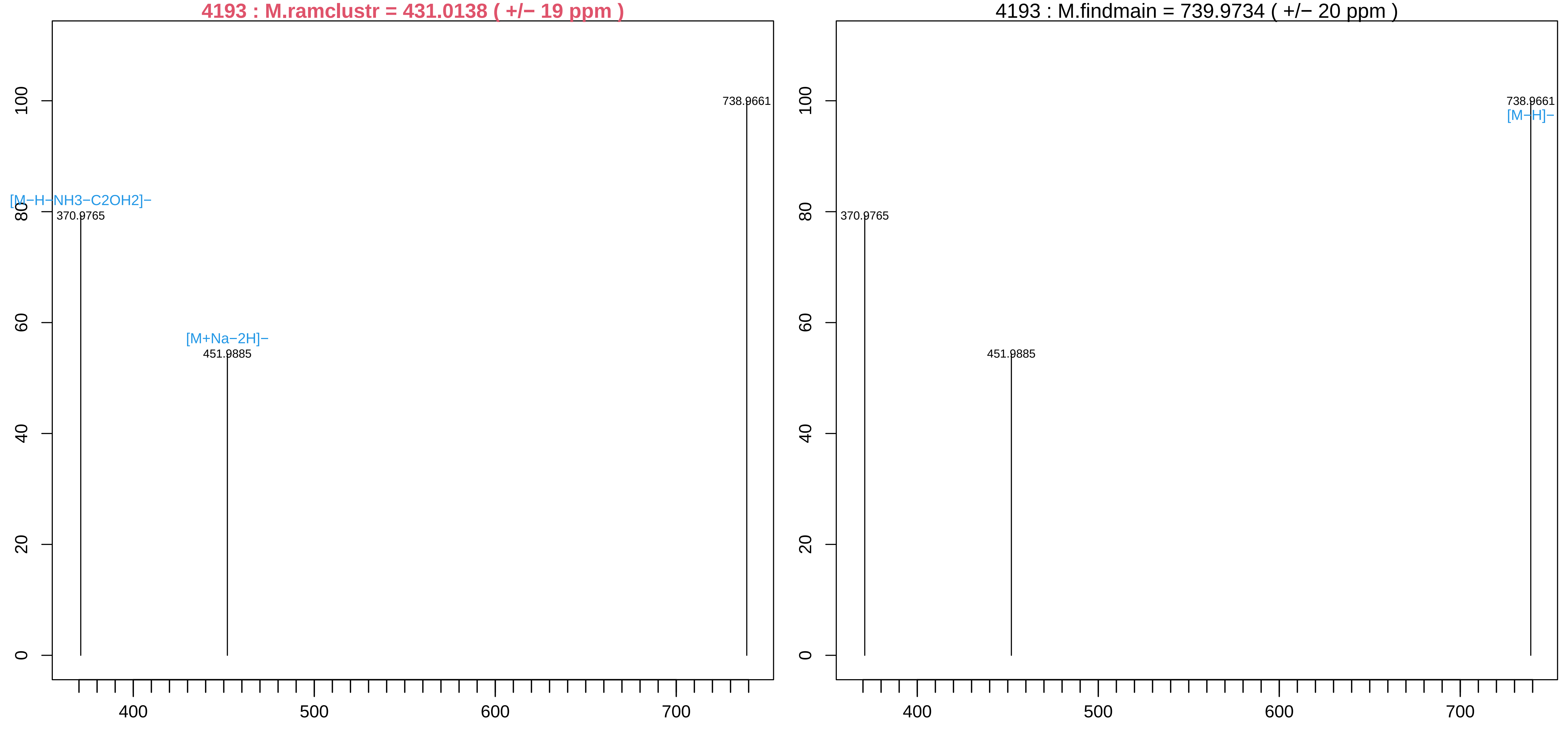
4193 : M.ramclustr = 431.0138 (+/− 19 ppm)
[M−H−NH3−C2OH2]−
370.9765
[M+Na−2H]−
451.9885
738.9661
4193 : M.findmain = 739.9734 (+/− 20 ppm)
370.9765
451.9885
738.9661
[M−H]−
0
20
40
60
80
100
400
500
600
700

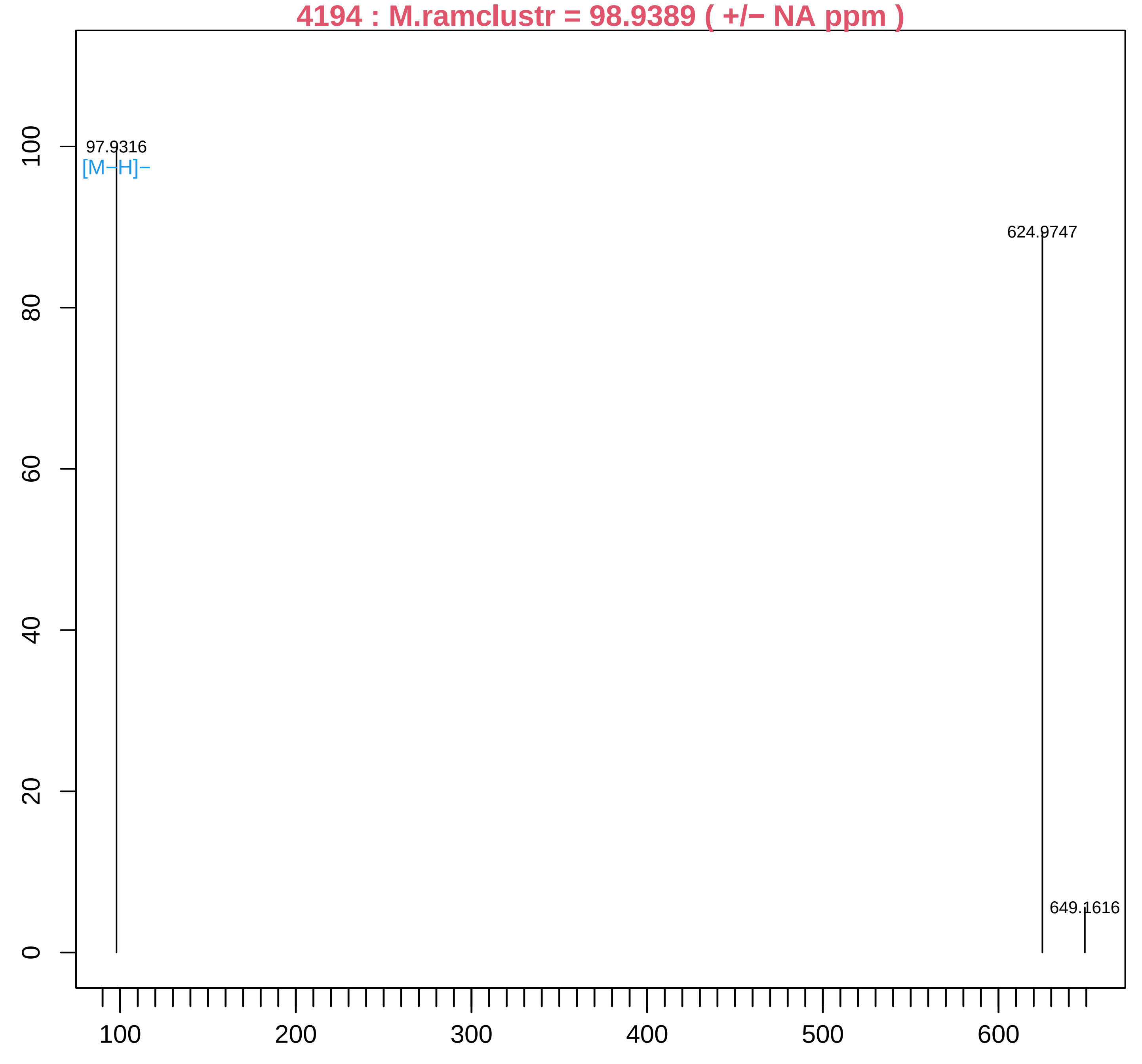
4194 : M.ramclustr = 98.9389 (+/− NA ppm)
97.9316
[M−H]−
624.9747
649.1616
0
20
40
60
80
100
100
200
300
400
500
600

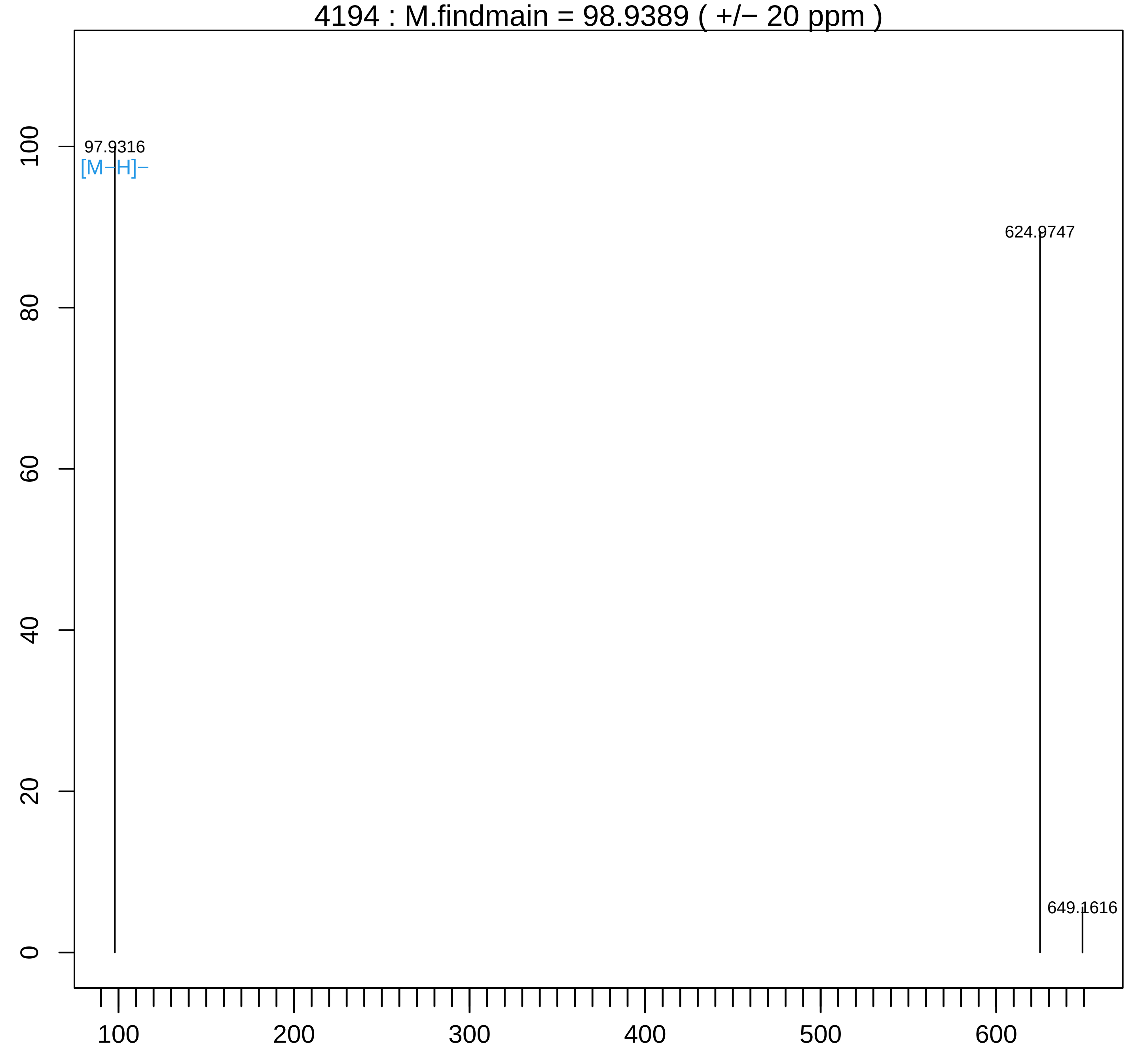
4194 : M.findmain = 98.9389 (+/− 20 ppm)
97.9316
[M−H]−
624.9747
649.1616
0
20
40
60
80
100
100
200
300
400
500
600

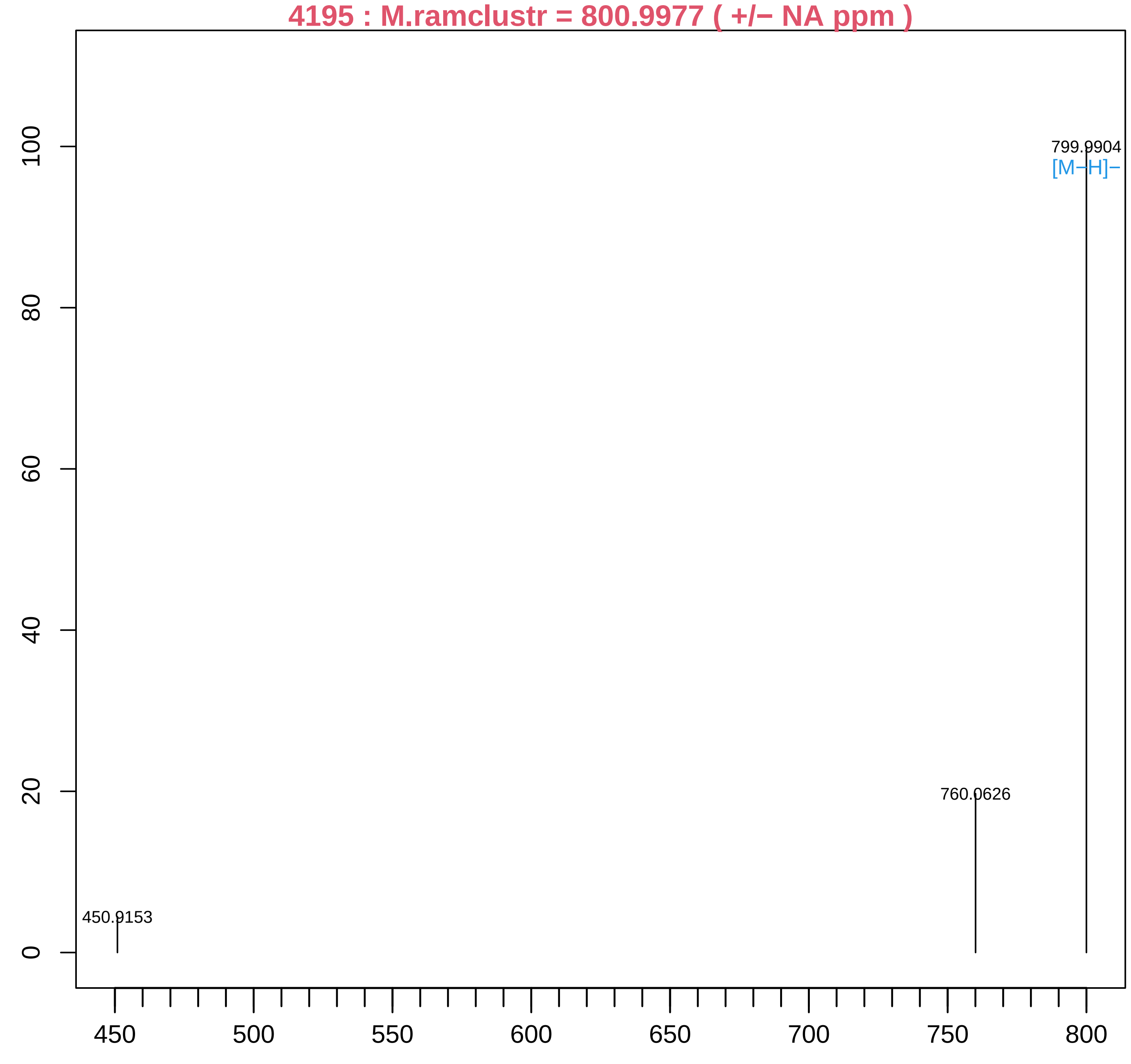
4195 : M.ramclustr = 800.9977 (+/− NA ppm)
799.9904
[M−H]−
760.0626
450.9153
0
20
40
60
80
100
450
500
550
600
650
700
750
800

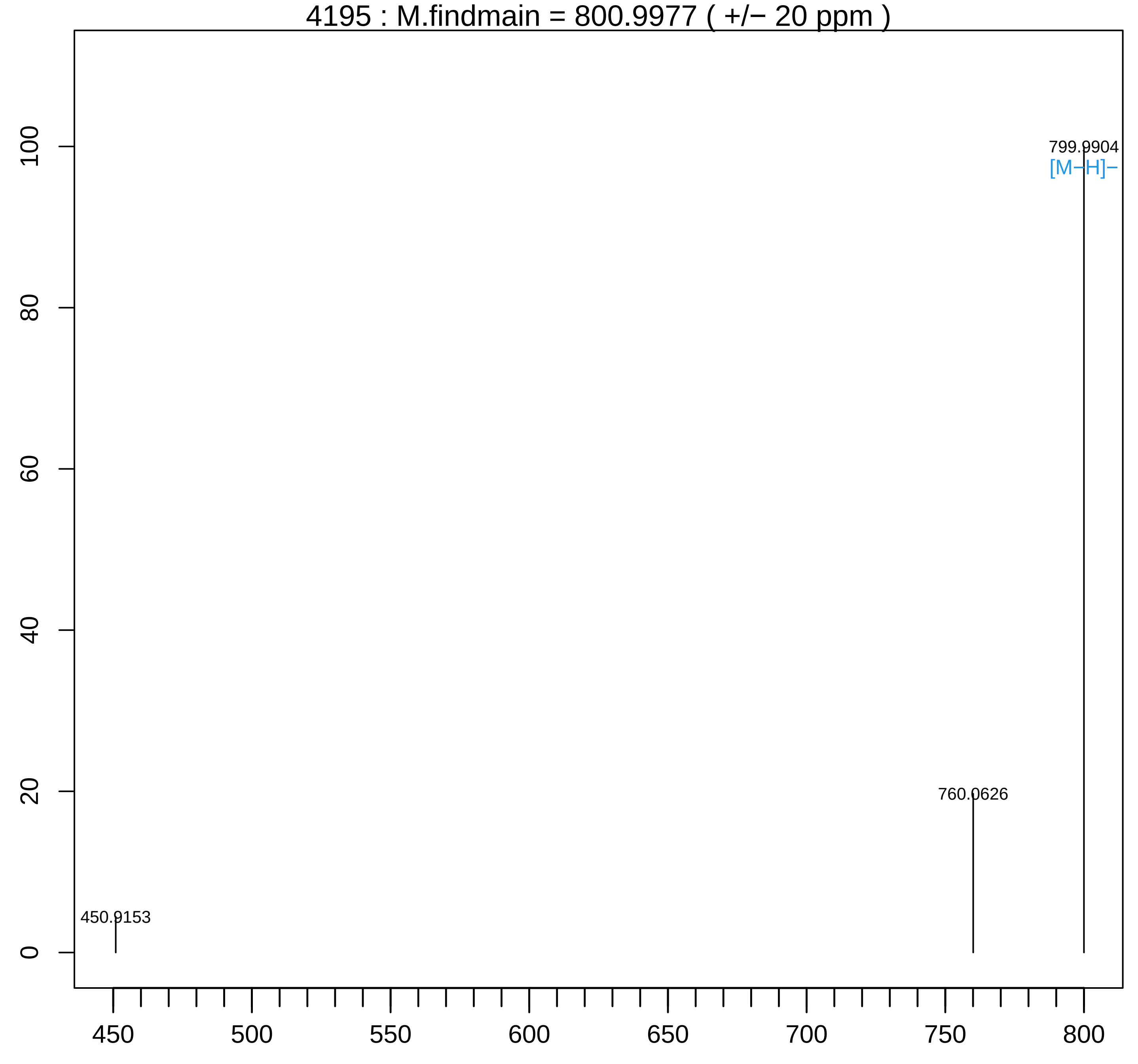
4195 : M.findmain = 800.9977 (+/− 20 ppm)
799.9904
[M−H]−
760.0626
450.9153
0
20
40
60
80
100
450
500
550
600
650
700
750
800

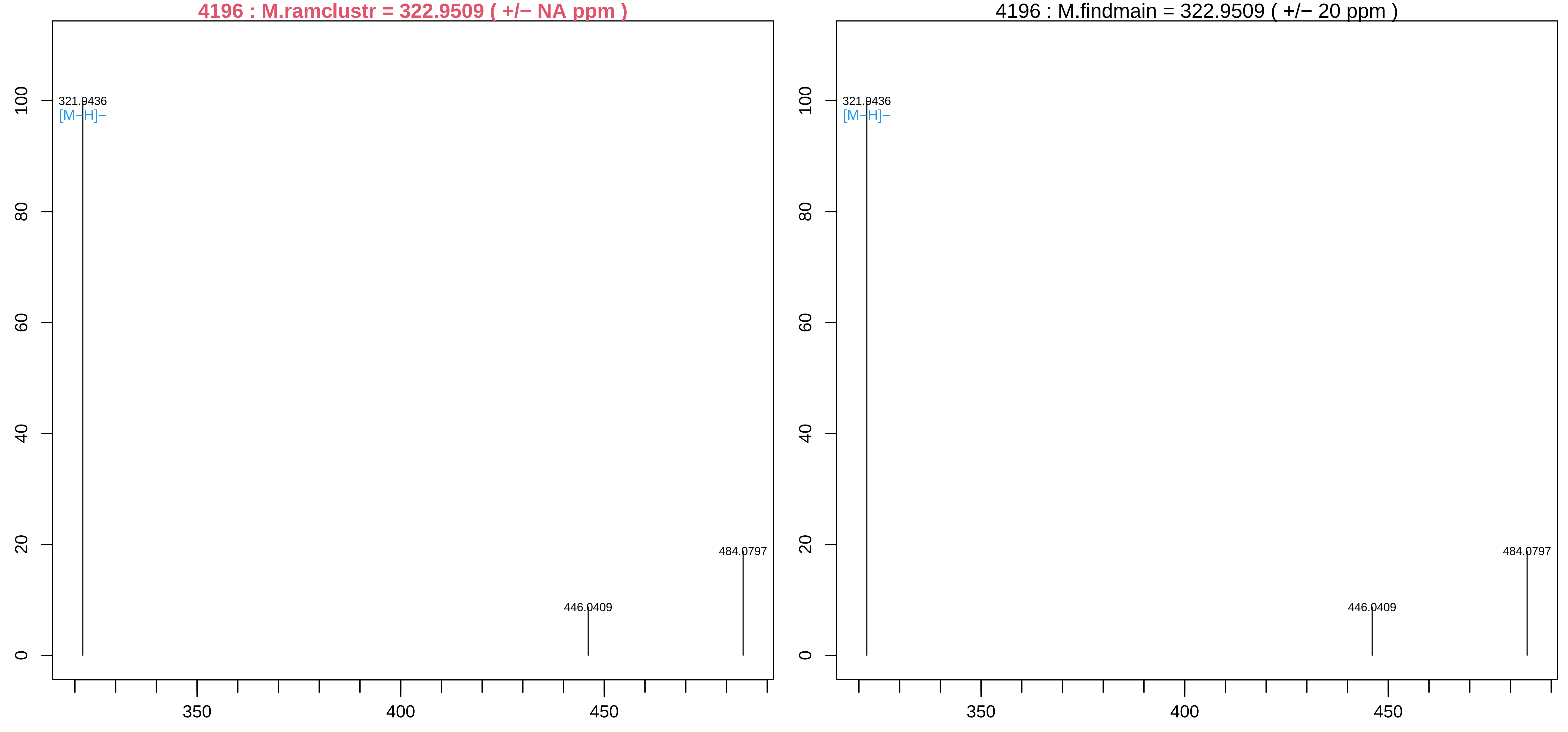
4196 : M.ramclustr = 322.9509 (+/− NA ppm)
321.9436
[M−H]−
446.0409
484.0797
4196 : M.findmain = 322.9509 (+/− 20 ppm)
321.9436
[M−H]−
446.0409
484.0797
0
20
40
60
80
100
350
400
450

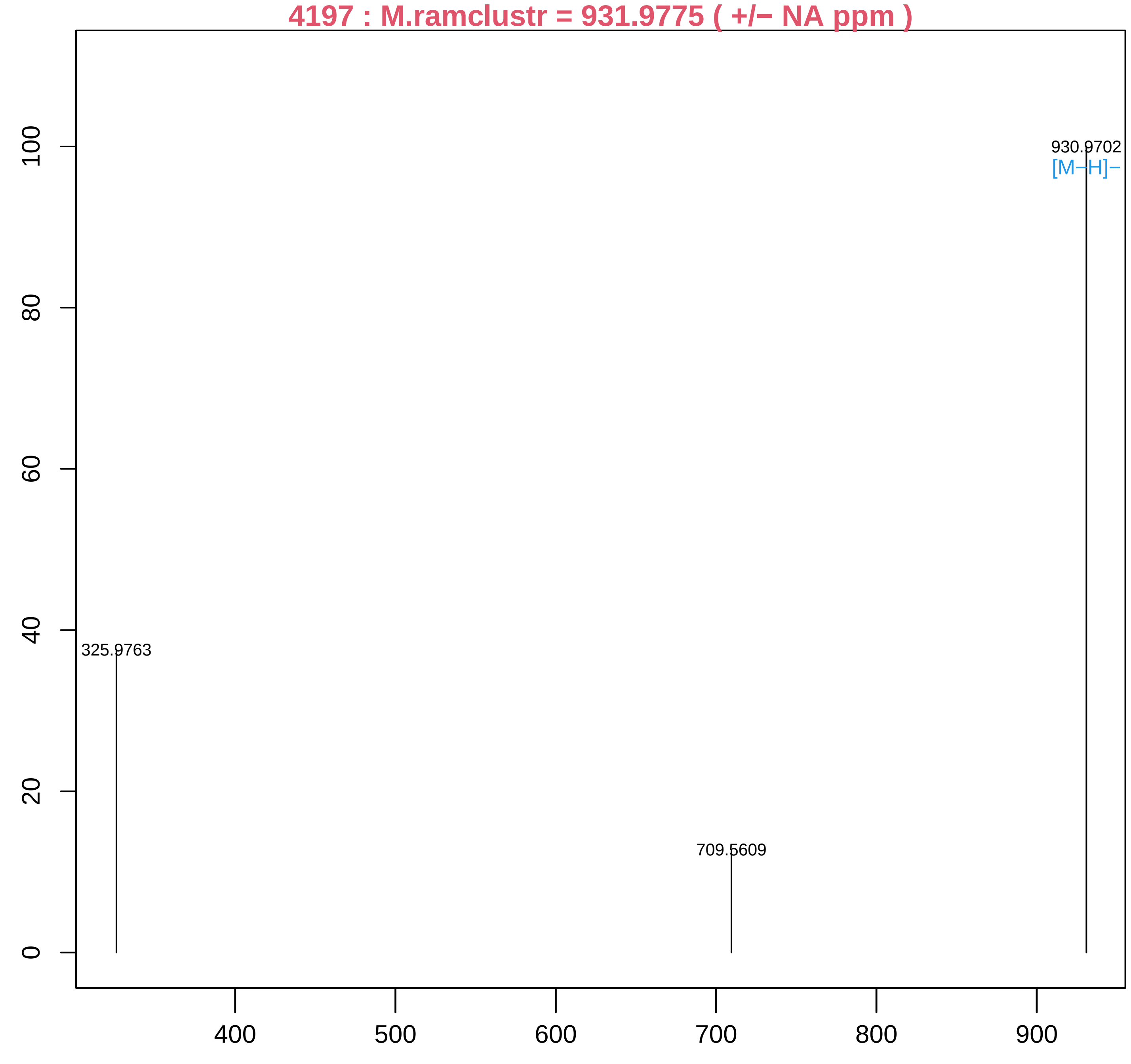
4197 : M.ramclustr = 931.9775 (+/− NA ppm)
930.9702
[M−H]−
325.9763
709.5609
0
20
40
60
80
100
400
500
600
700
800
900

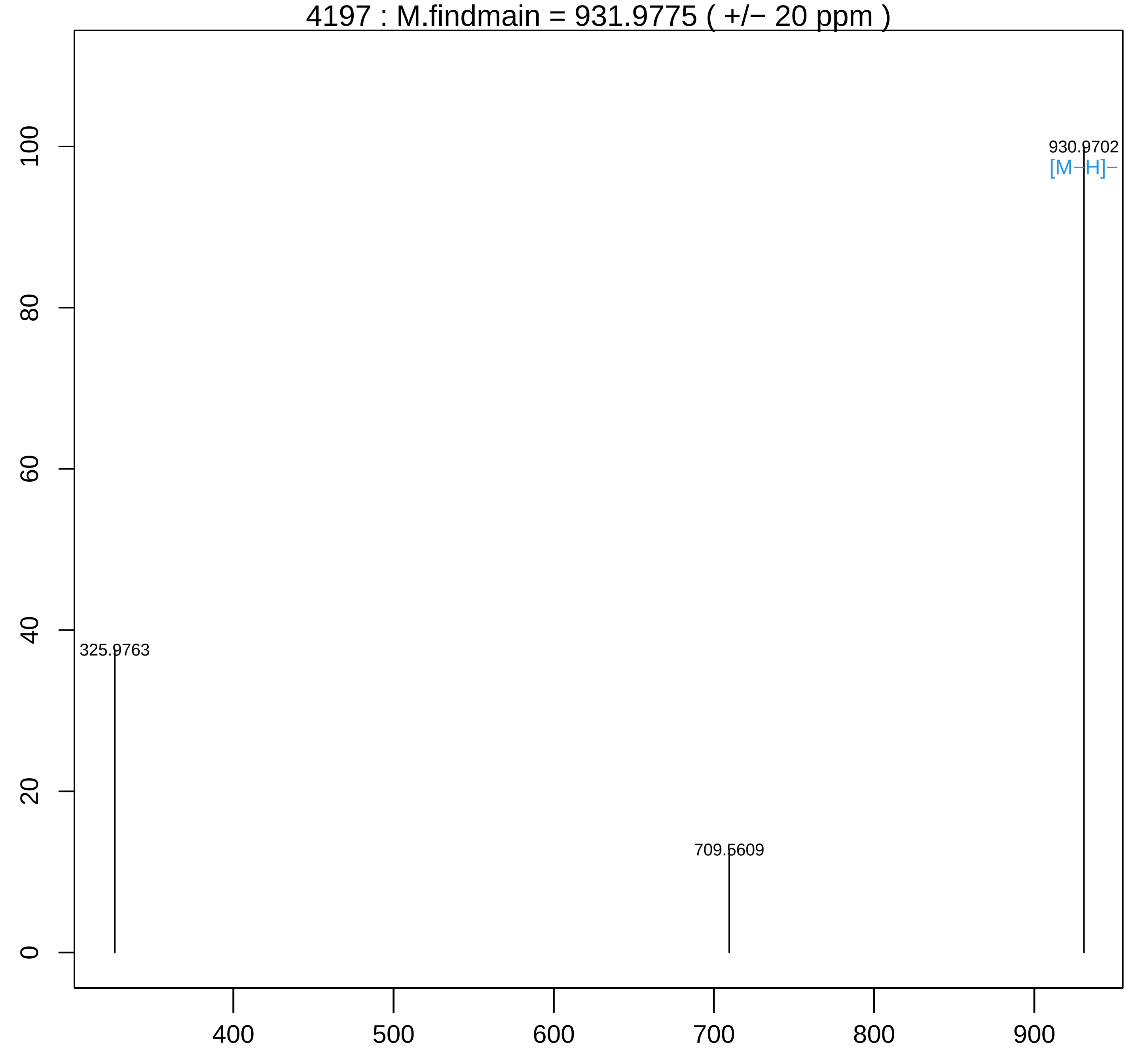
4197 : M.findmain = 931.9775 (+/− 20 ppm)
930.9702
[M−H]−
325.9763
709.5609
0
20
40
60
80
100
400
500
600
700
800
900

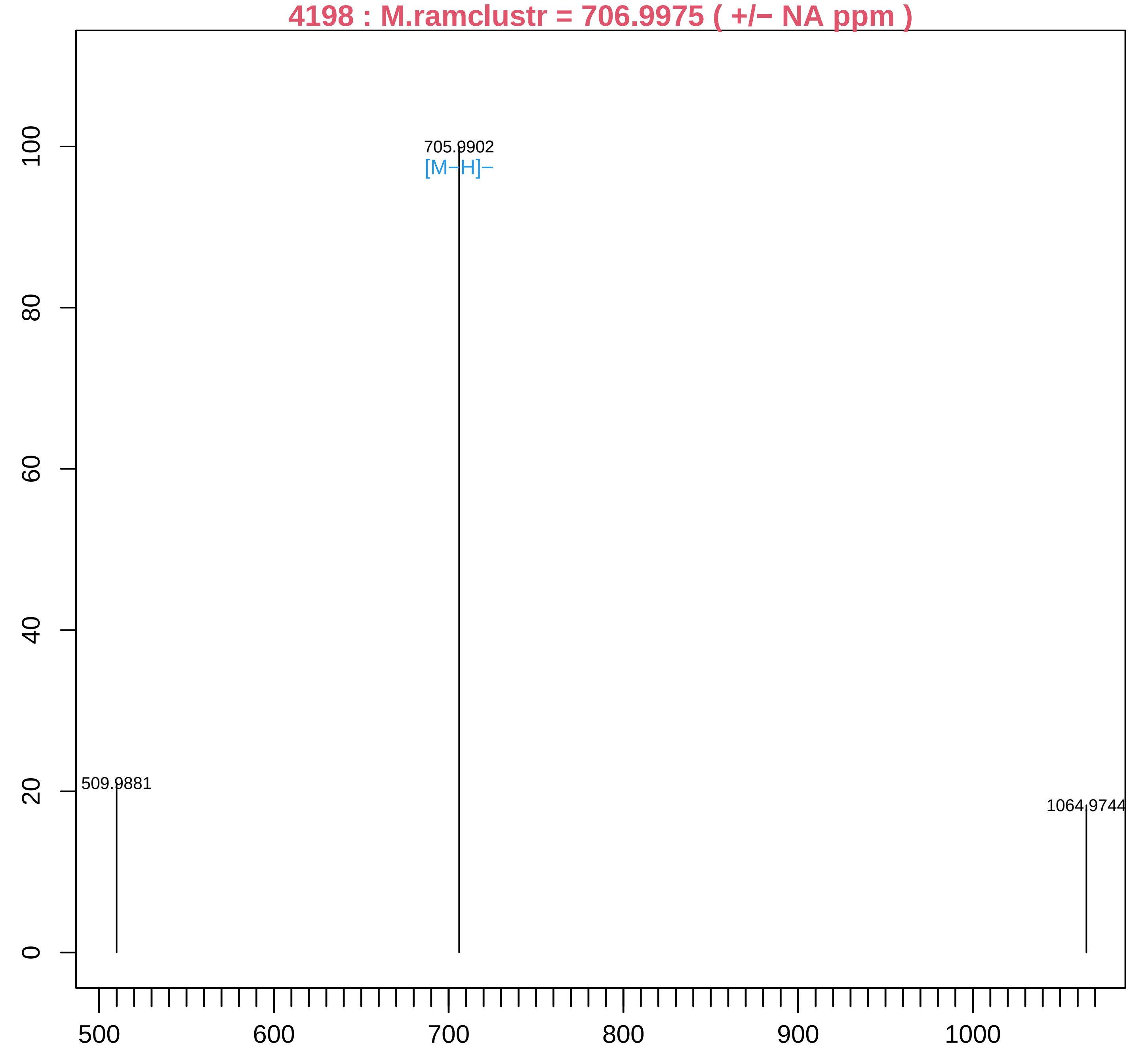
4198 : M.ramclustr = 706.9975 (+/− NA ppm)
705.9902
[M−H]−
509.9881
1064.9744
0
20
40
60
80
100
500
600
700
800
900
1000

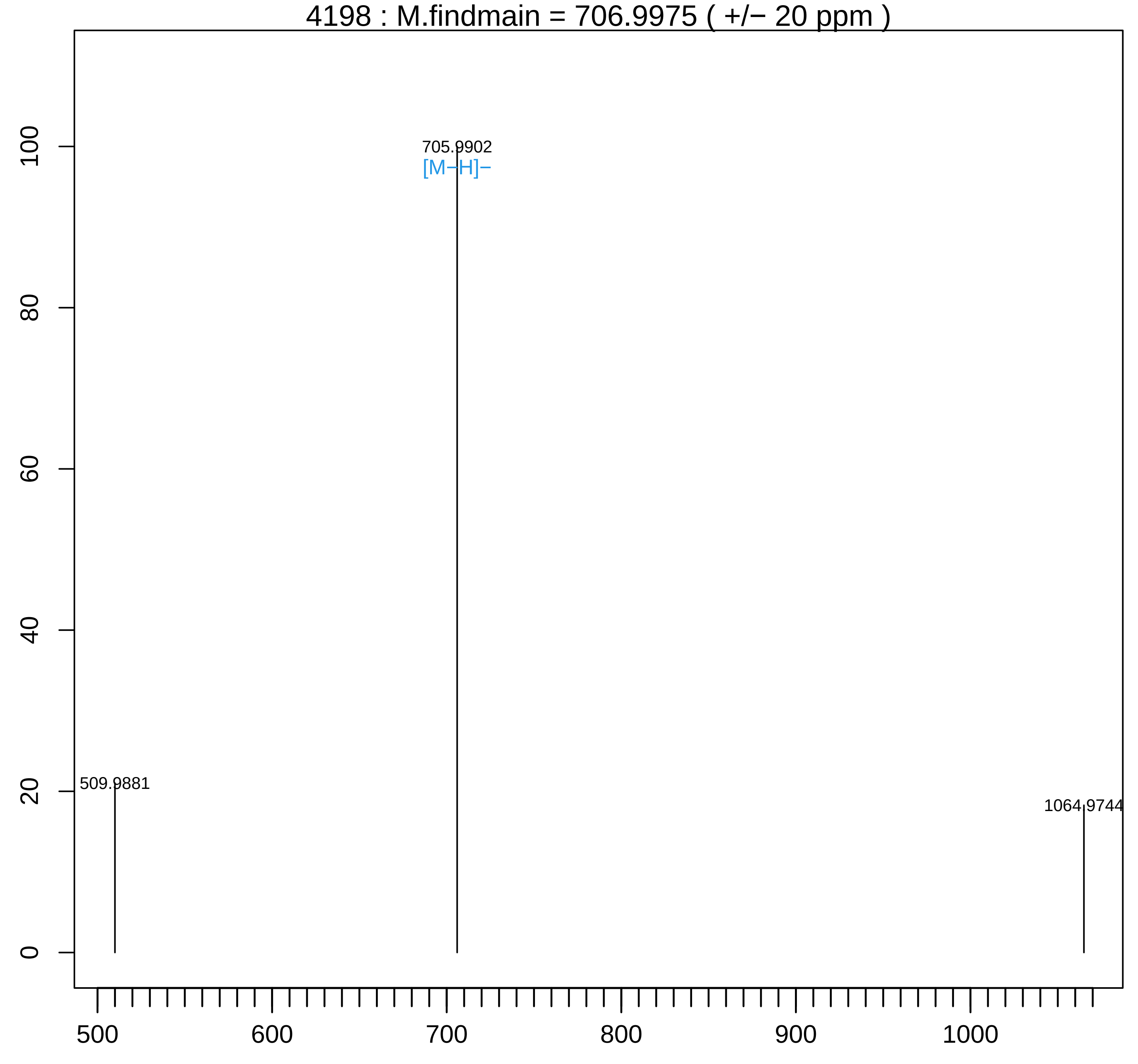
4198 : M.findmain = 706.9975 (+/− 20 ppm)
705.9902
[M−H]−
509.9881
1064.9744
0
20
40
60
80
100
500
600
700
800
900
1000

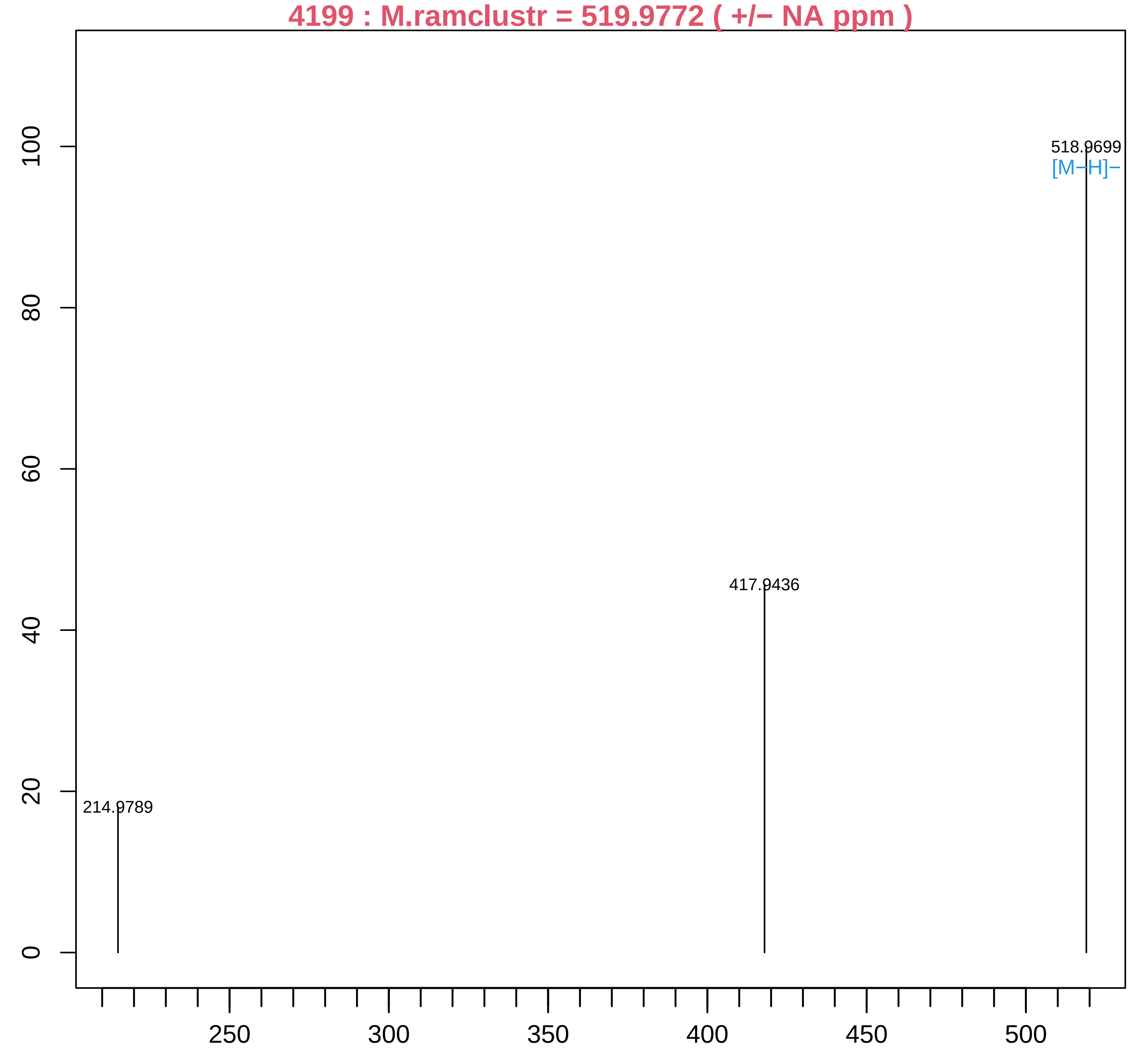
4199 : M.ramclustr = 519.9772 (+/− NA ppm)
518.9699
[M−H]−
417.9436
214.9789
0
20
40
60
80
100
250
300
350
400
450
500

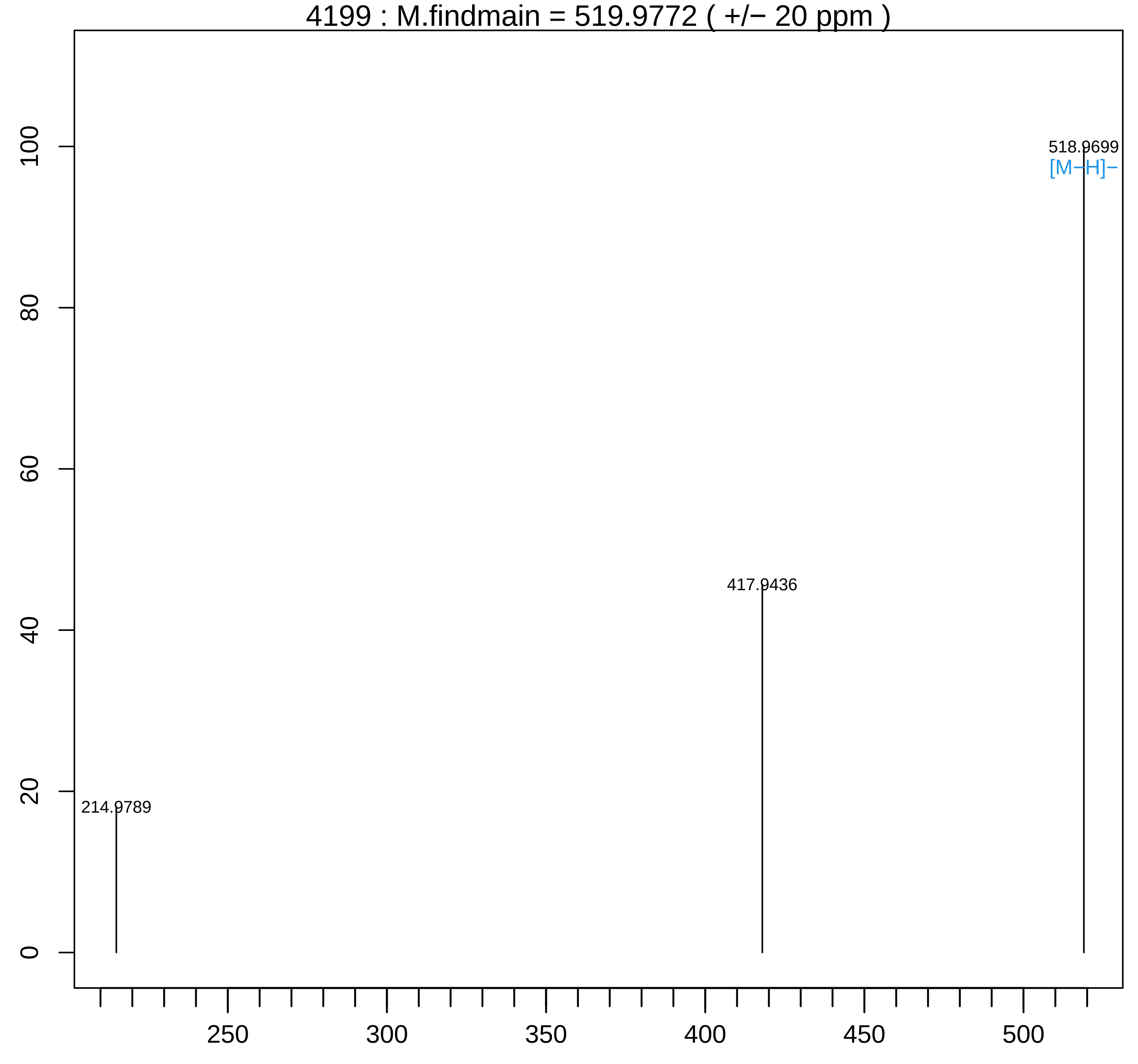
4199 : M.findmain = 519.9772 (+/− 20 ppm)
518.9699
[M−H]−
417.9436
214.9789
0
20
40
60
80
100
250
300
350
400
450
500

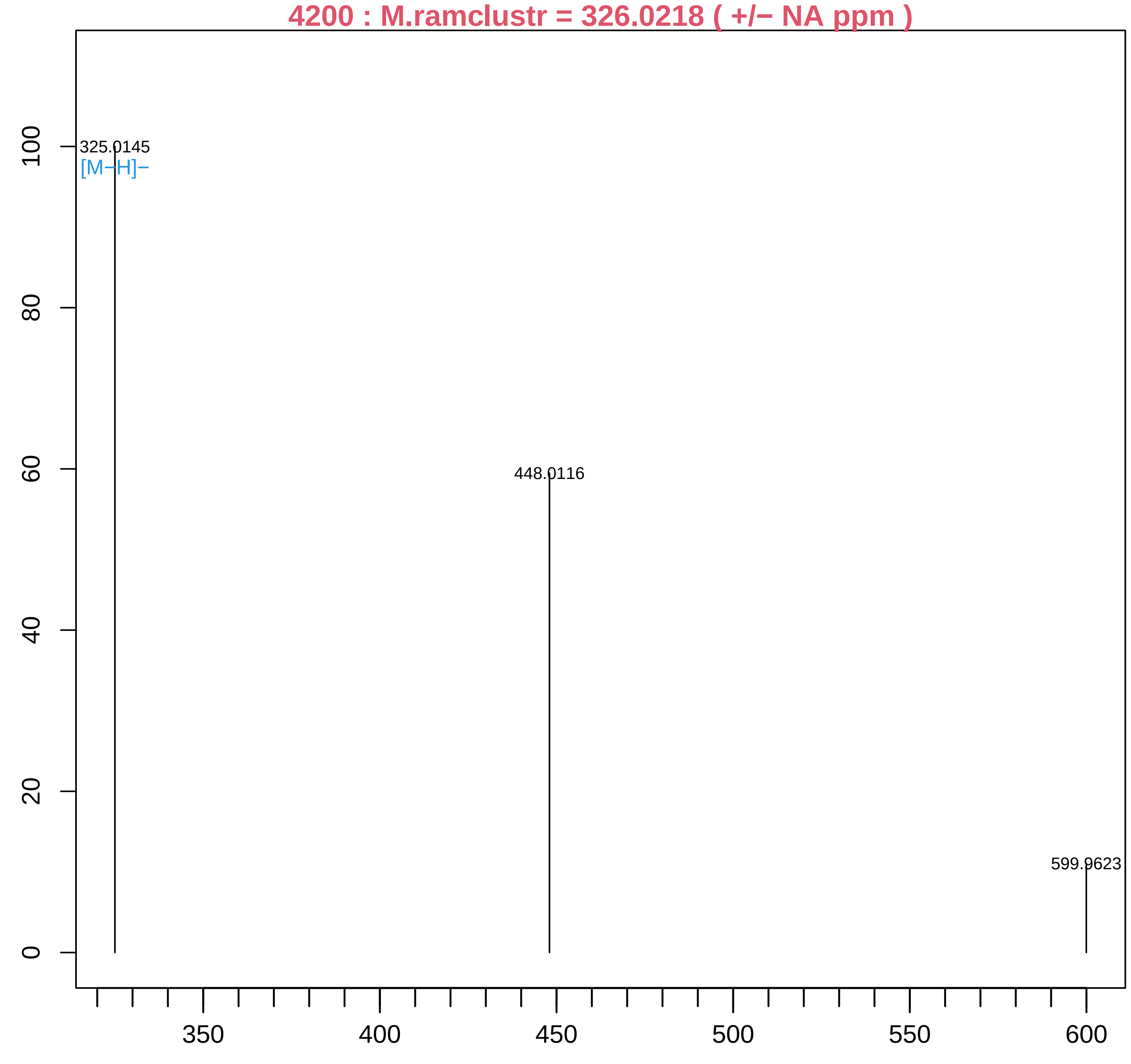
4200 : M.ramclustr = 326.0218 (+/− NA ppm)
325.0145
[M−H]−
448.0116
599.9623
0
20
40
60
80
100
350
400
450
500
550
600

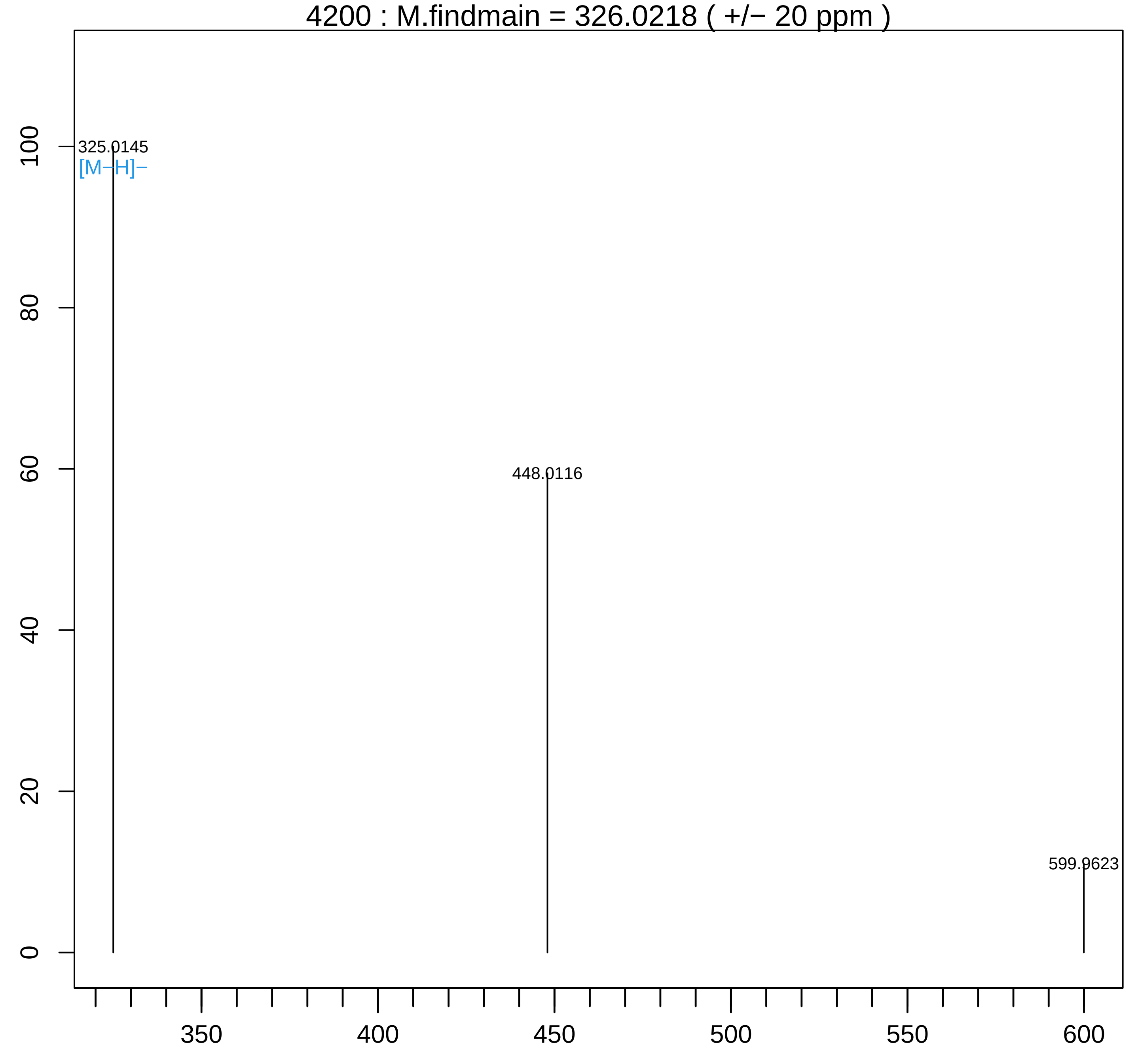
4200 : M.findmain = 326.0218 (+/− 20 ppm)
325.0145
[M−H]−
448.0116
599.9623
0
20
40
60
80
100
350
400
450
500
550
600

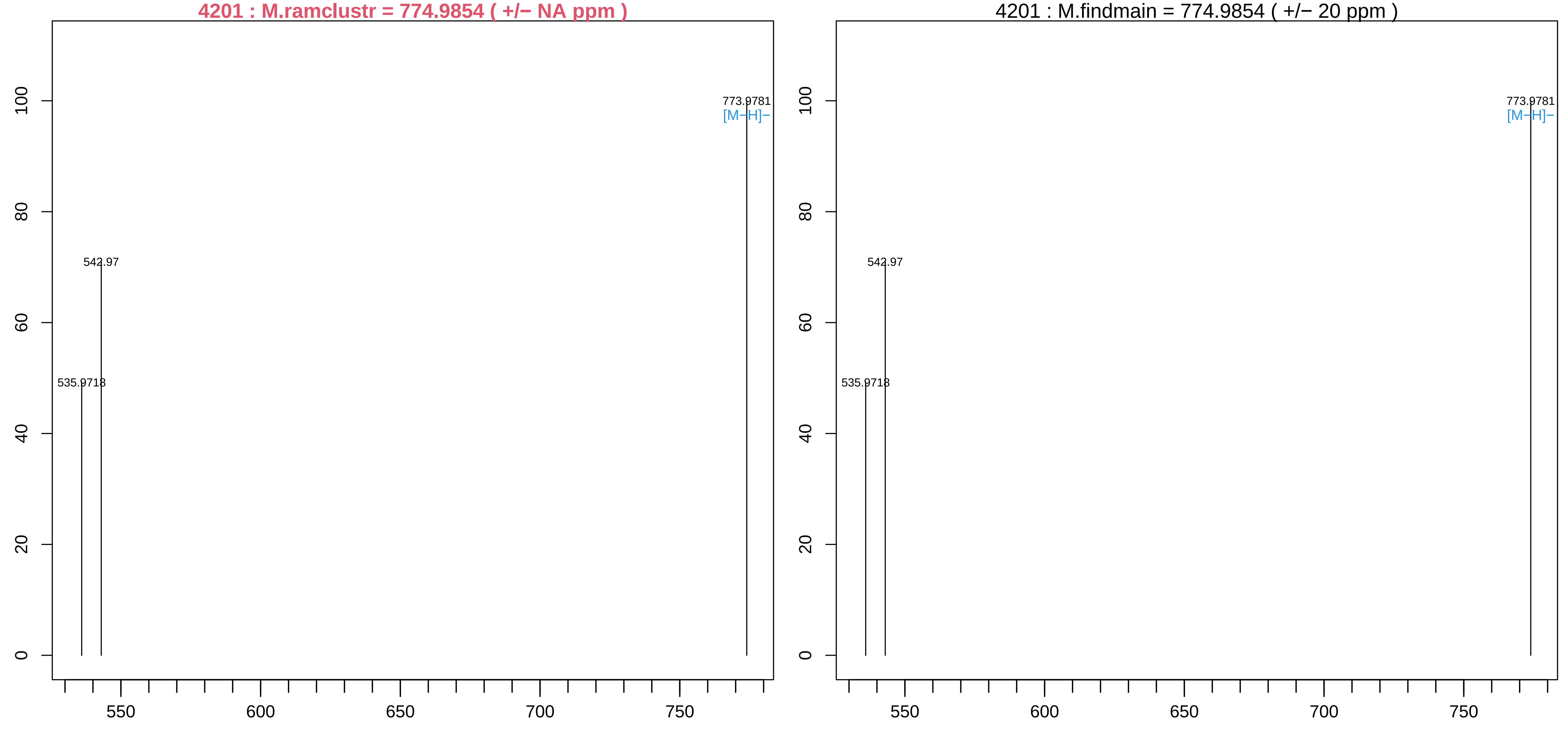
4201 : M.ramclustr = 774.9854 (+/− NA ppm)
773.9781
[M−H]−
542.97
535.9718
4201 : M.findmain = 774.9854 (+/− 20 ppm)
773.9781
[M−H]−
542.97
535.9718

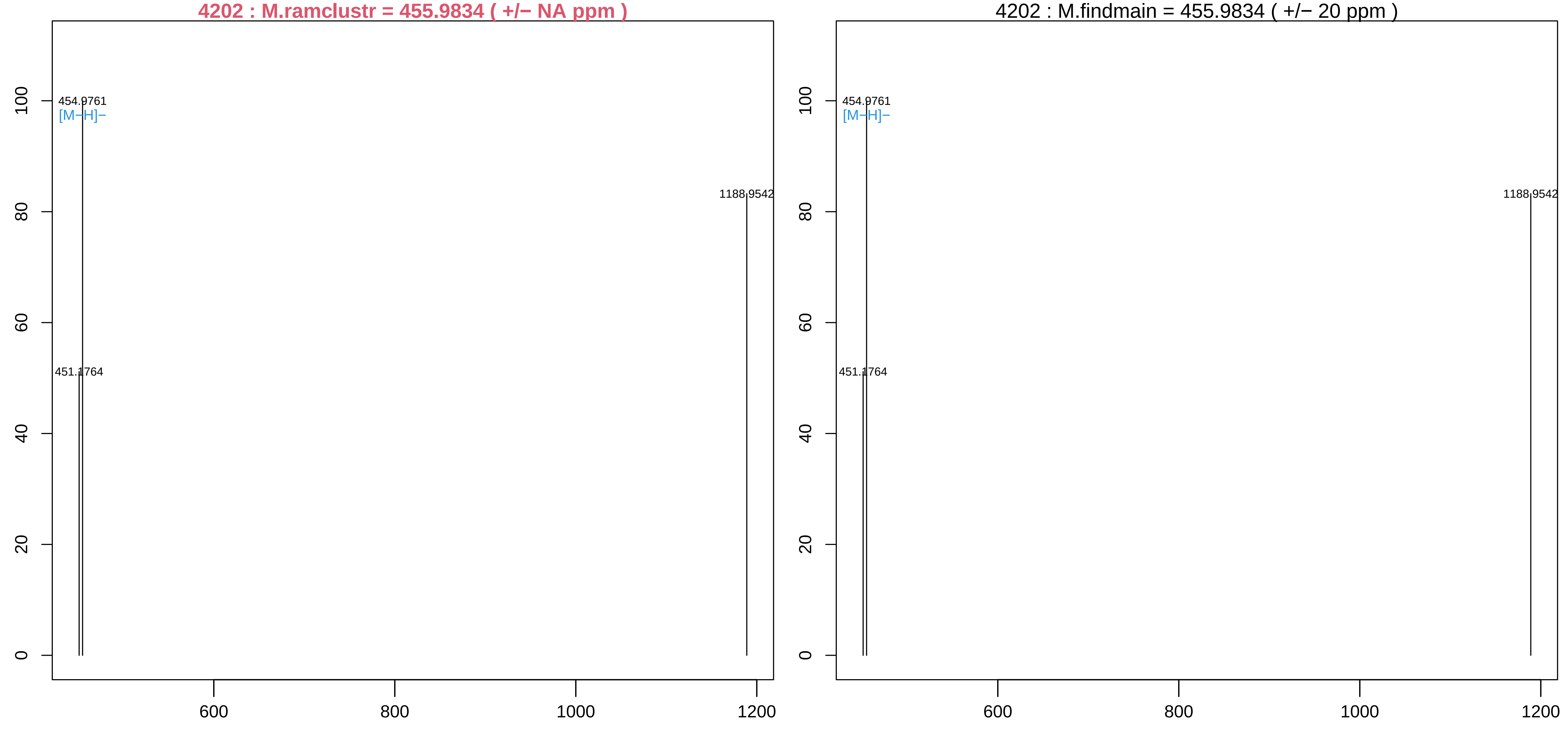
4202 : M.ramclustr = 455.9834 (+/− NA ppm)
454.9761
[M−H]−
451.1764
1188.9542
0
20
40
60
80
100
600
800
1000
1200
4202 : M.findmain = 455.9834 (+/− 20 ppm)
454.9761
[M−H]−
451.1764
1188.9542
0
20
40
60
80
100
600
800
1000
1200

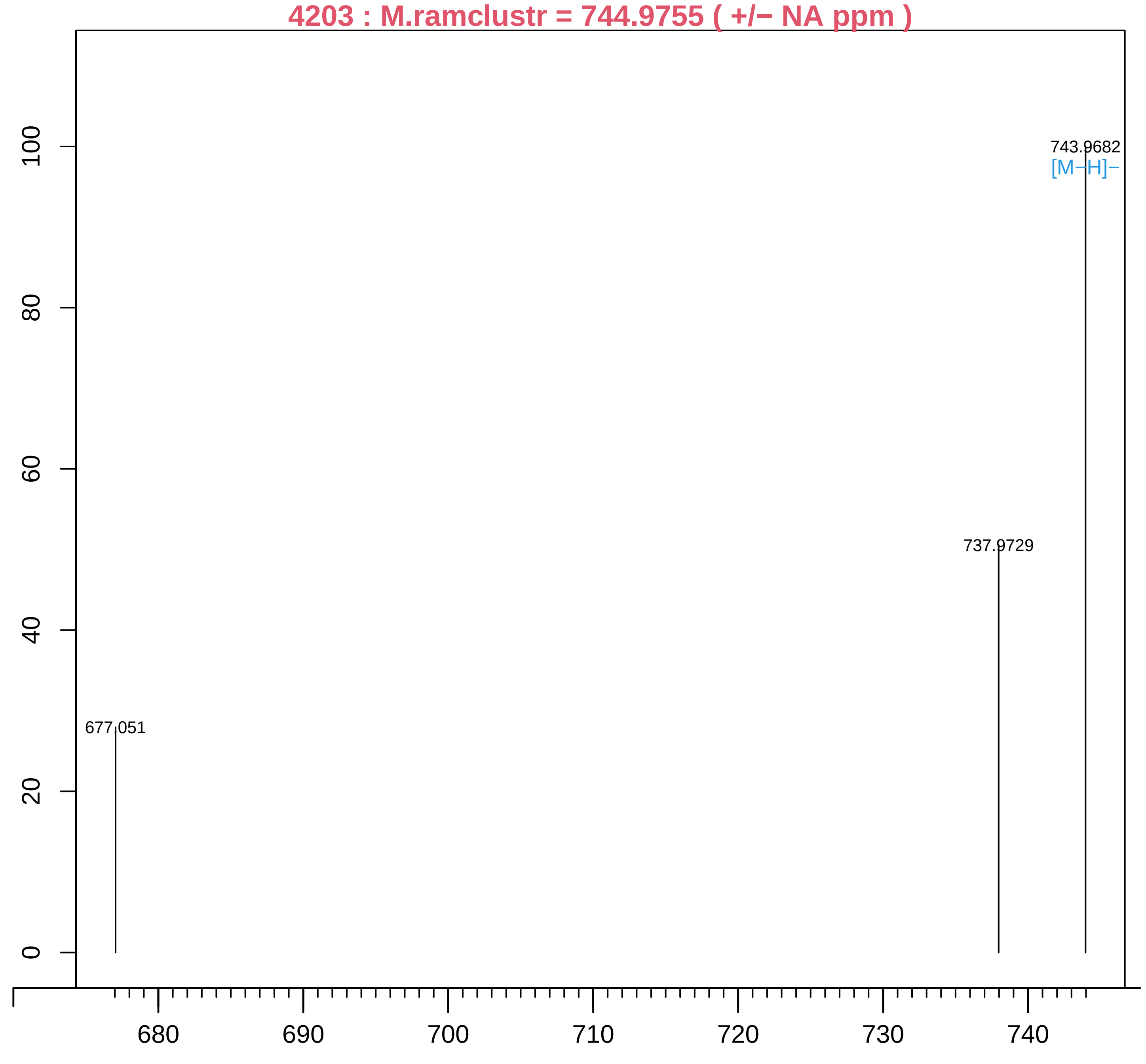

4203 : M.ramclustr = 744.9755 (+/− NA ppm)
743.9682
[M−H]−
737.9729
677.051
0
20
40
60
80
100
680
690
700
710
720
730
740

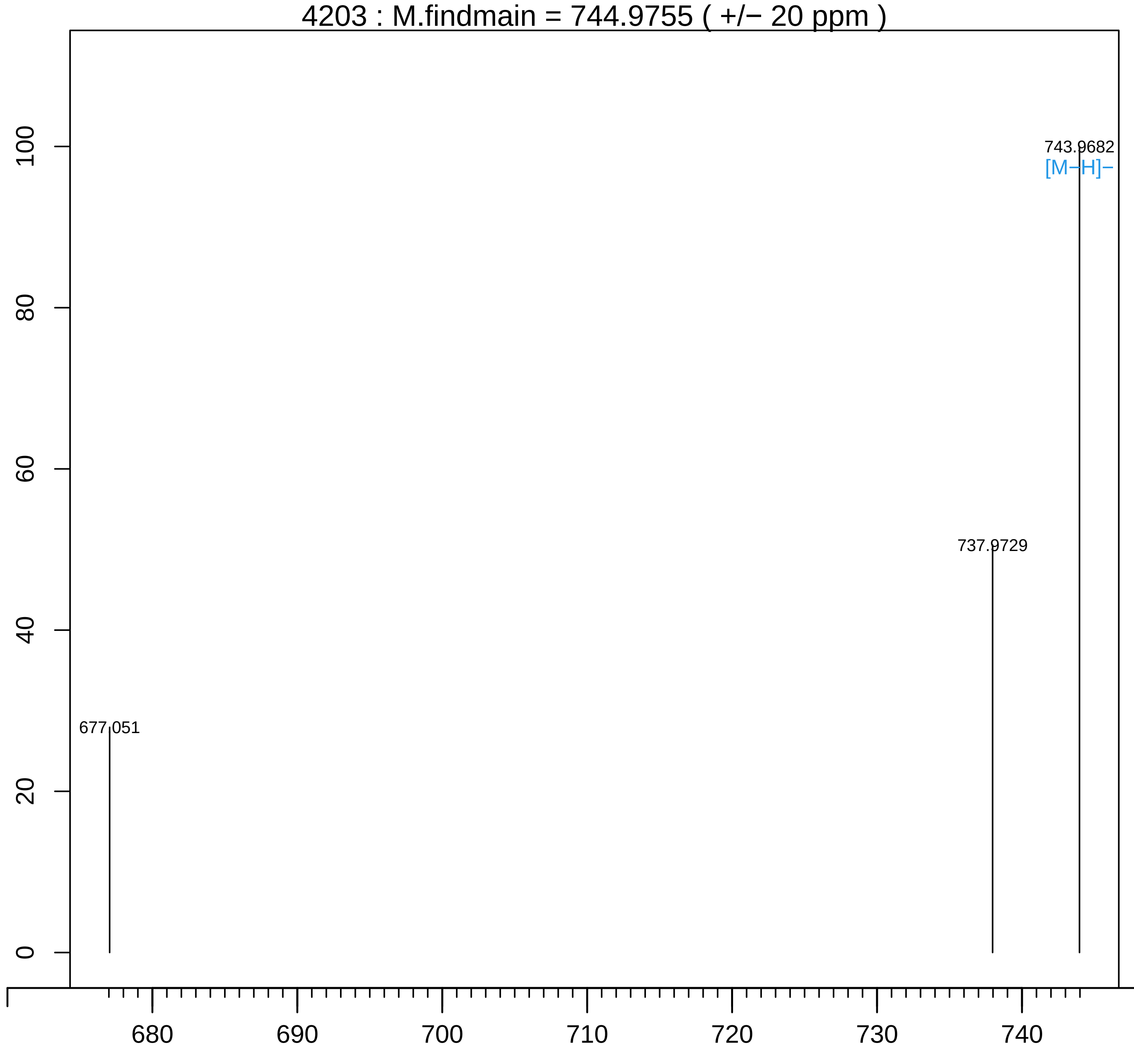

4203 : M.findmain = 744.9755 (+/− 20 ppm)
743.9682
[M−H]−
737.9729
677.051
0
20
40
60
80
100
680
690
700
710
720
730
740

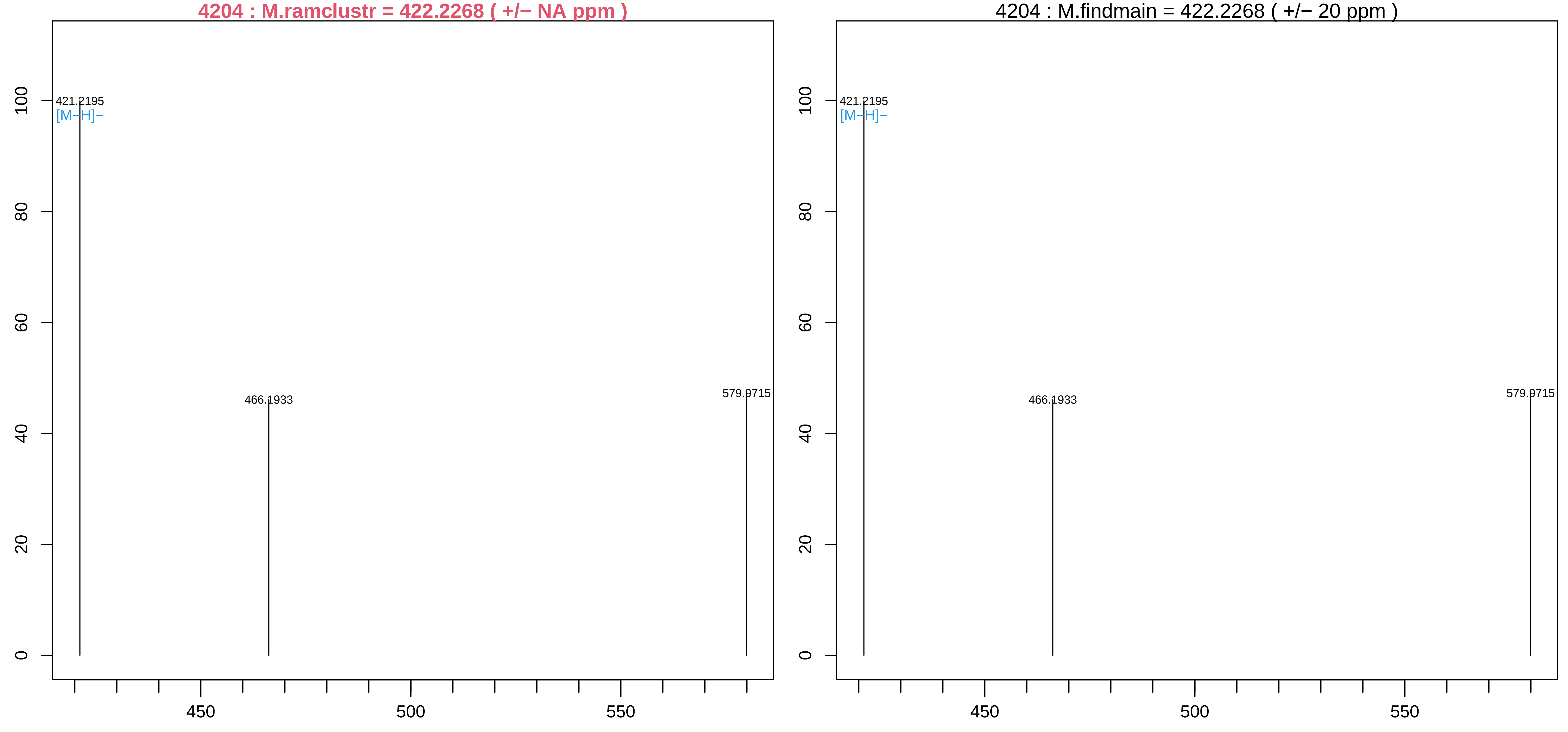
4204 : M.ramclustr = 422.2268 (+/− NA ppm)
421.2195
[M−H]−
466.1933
579.9715
0
20
40
60
80
100
450
500
550
4204 : M.findmain = 422.2268 (+/− 20 ppm)
421.2195
[M−H]−
466.1933
579.9715
0
20
40
60
80
100
450
500
550

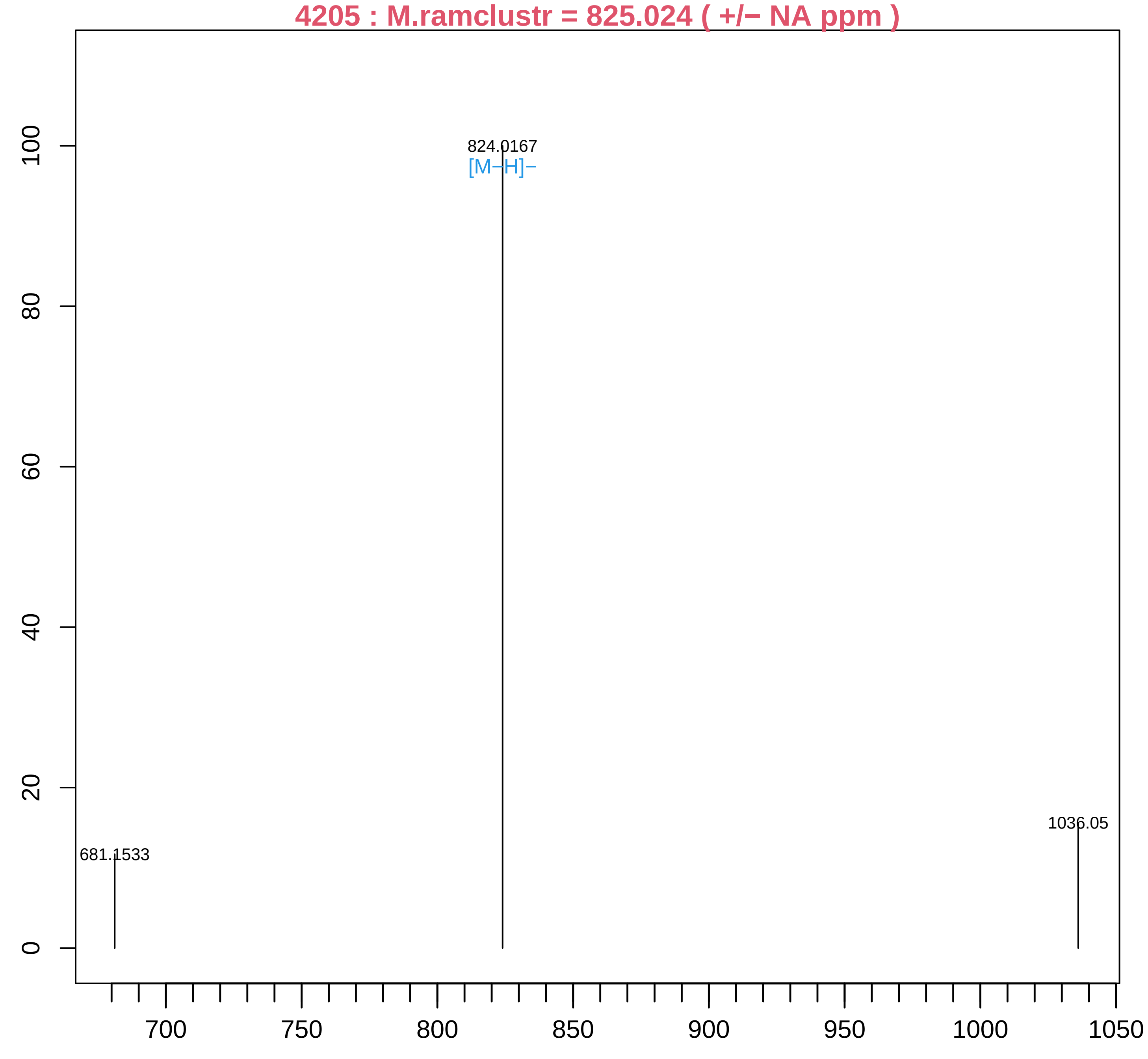
4205 : M.ramclustr = 825.024 (+/− NA ppm)
824.0167
[M−H]−
681.1533
1036.05
0
20
40
60
80
100
700
750
800
850
900
950
1000
1050

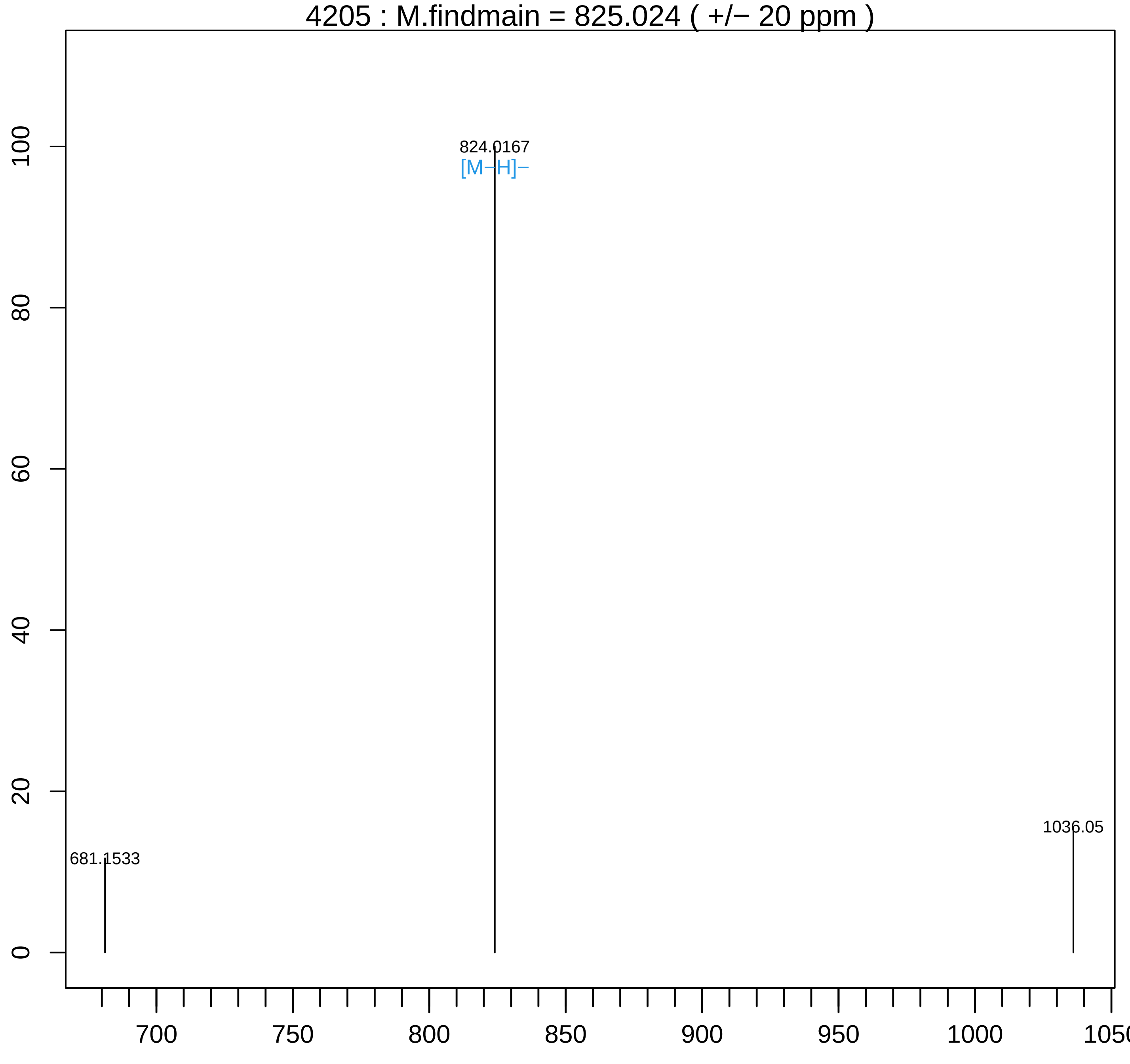
4205 : M.findmain = 825.024 (+/− 20 ppm)
824.0167
[M−H]−
681.1533
1036.05
0
20
40
60
80
100
700
750
800
850
900
950
1000

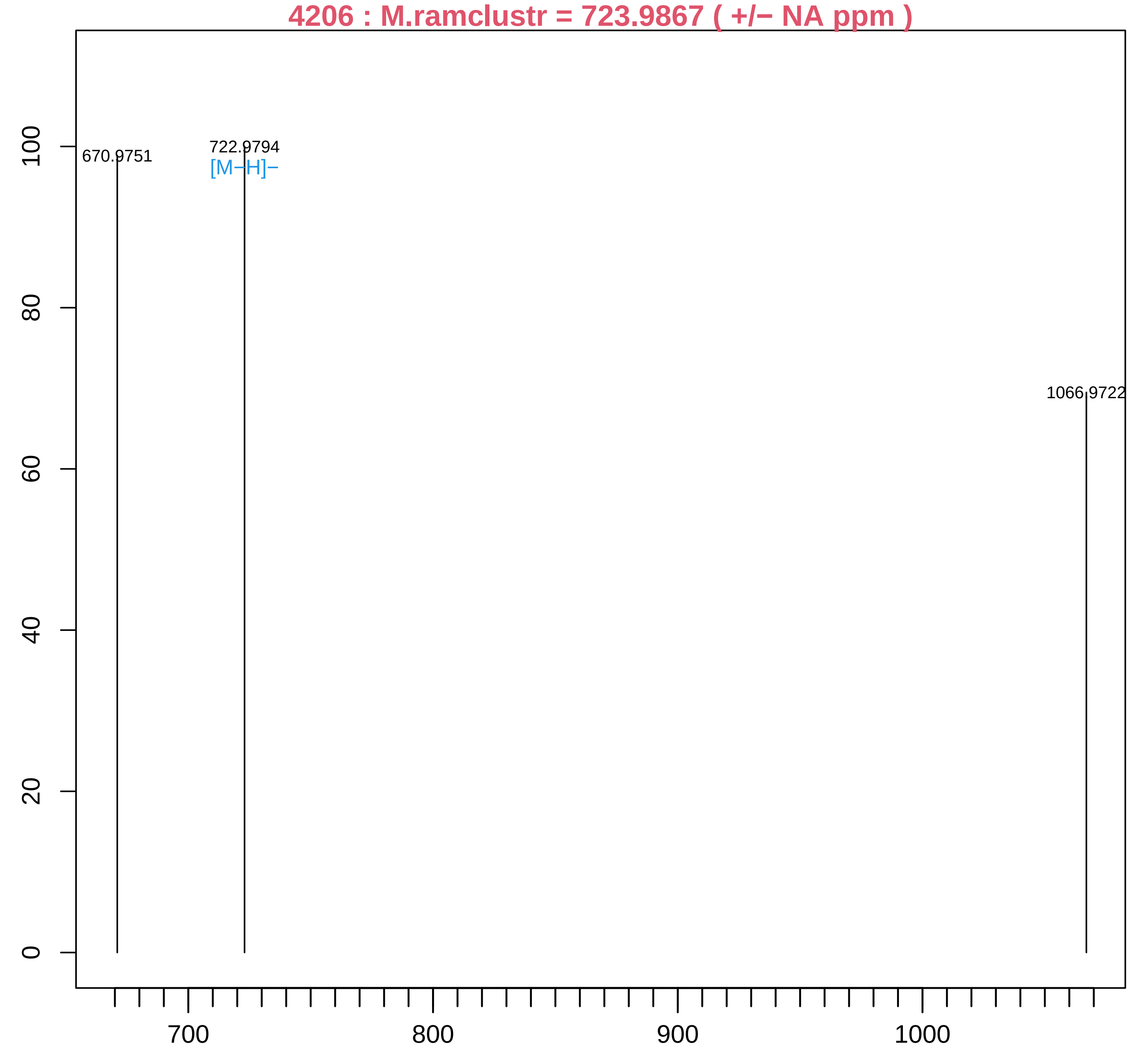
4206 : M.ramclustr = 723.9867 (+/− NA ppm)
670.9751
722.9794
[M−H]−
1066.9722
0
20
40
60
80
100
700
800
900
1000

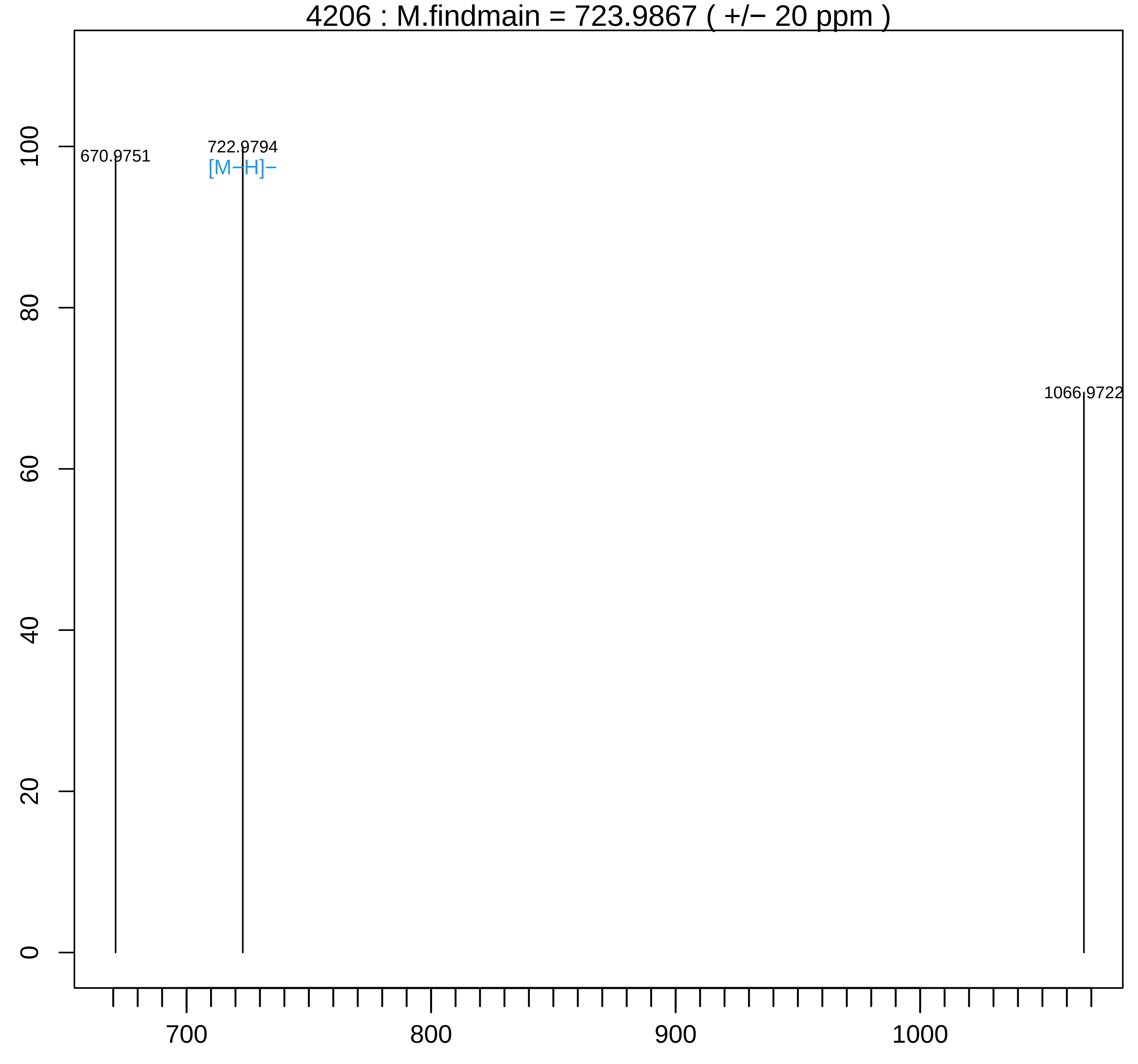
4206 : M.findmain = 723.9867 (+/− 20 ppm)
670.9751
722.9794
[M−H]−
1066.9722
0
20
40
60
80
100
700
800
900
1000

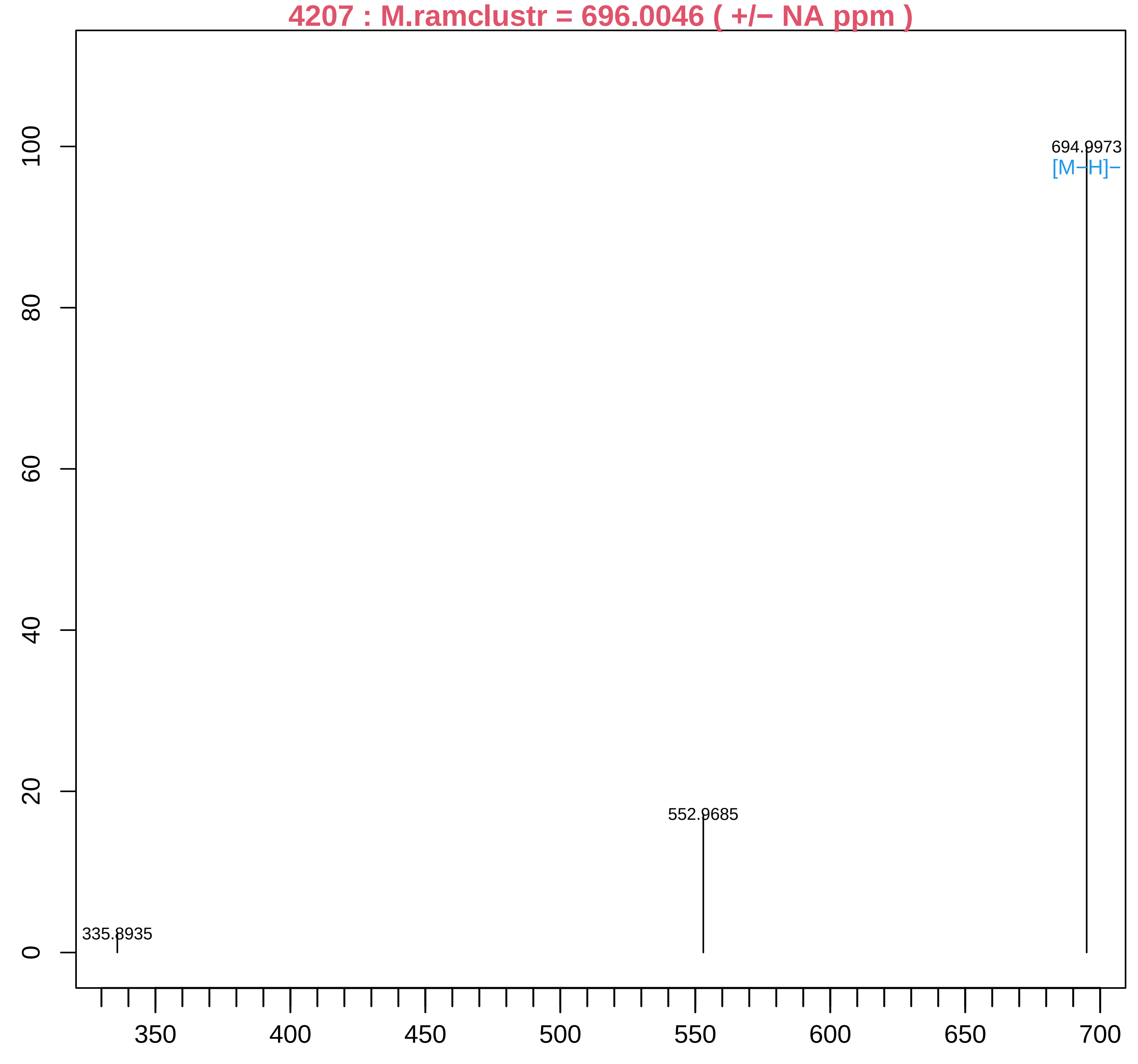

4207 : M.ramclustr = 696.0046 (+/− NA ppm)
694.9973
[M−H]−
552.9685
335.8935
0
20
40
60
80
100
350
400
450
500
550
600
650
700

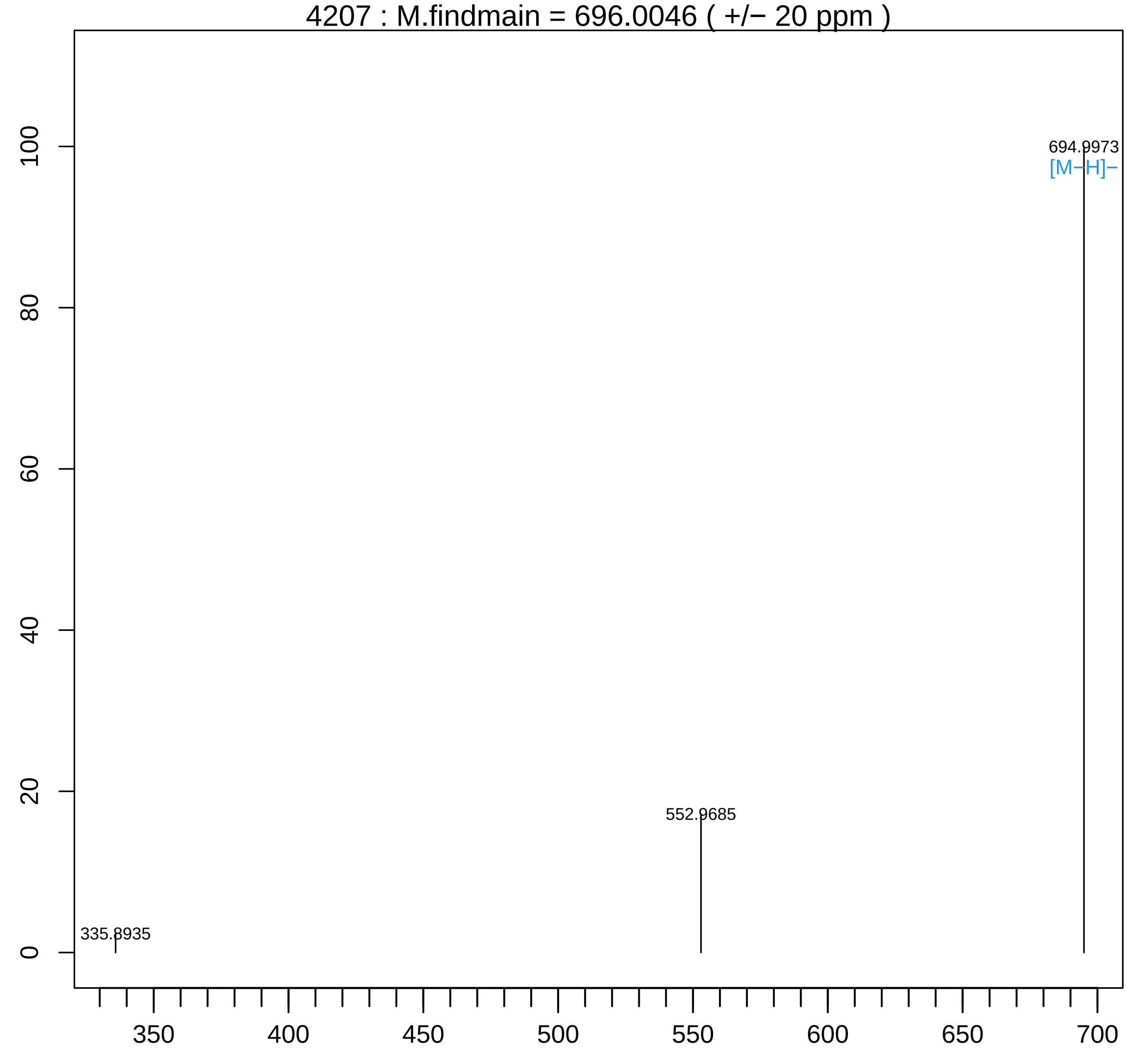

4207 : M.findmain = 696.0046 (+/− 20 ppm)
694.9973
[M−H]−
552.9685
335.8935
0
20
40
60
80
100
350
400
450
500
550
600
650
700

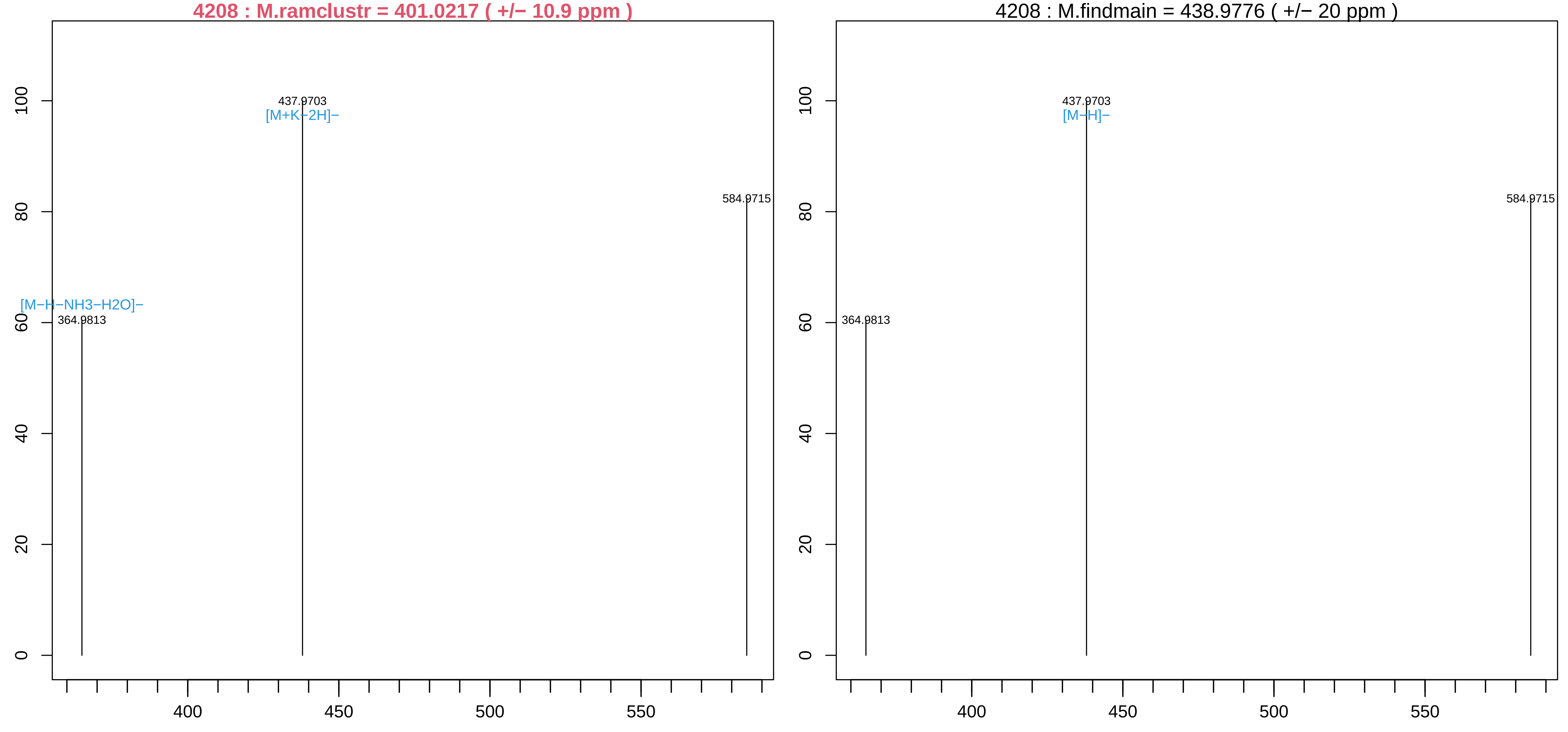
4208 : M.ramclustr = 401.0217 (+/− 10.9 ppm)
437.9703
[M+K−2H]−
584.9715
[M−H−NH3−H2O]−
364.9813
4208 : M.findmain = 438.9776 (+/− 20 ppm)
437.9703
[M−H]−
584.9715
364.9813
0
20
40
60
80
100
400
450
500
550

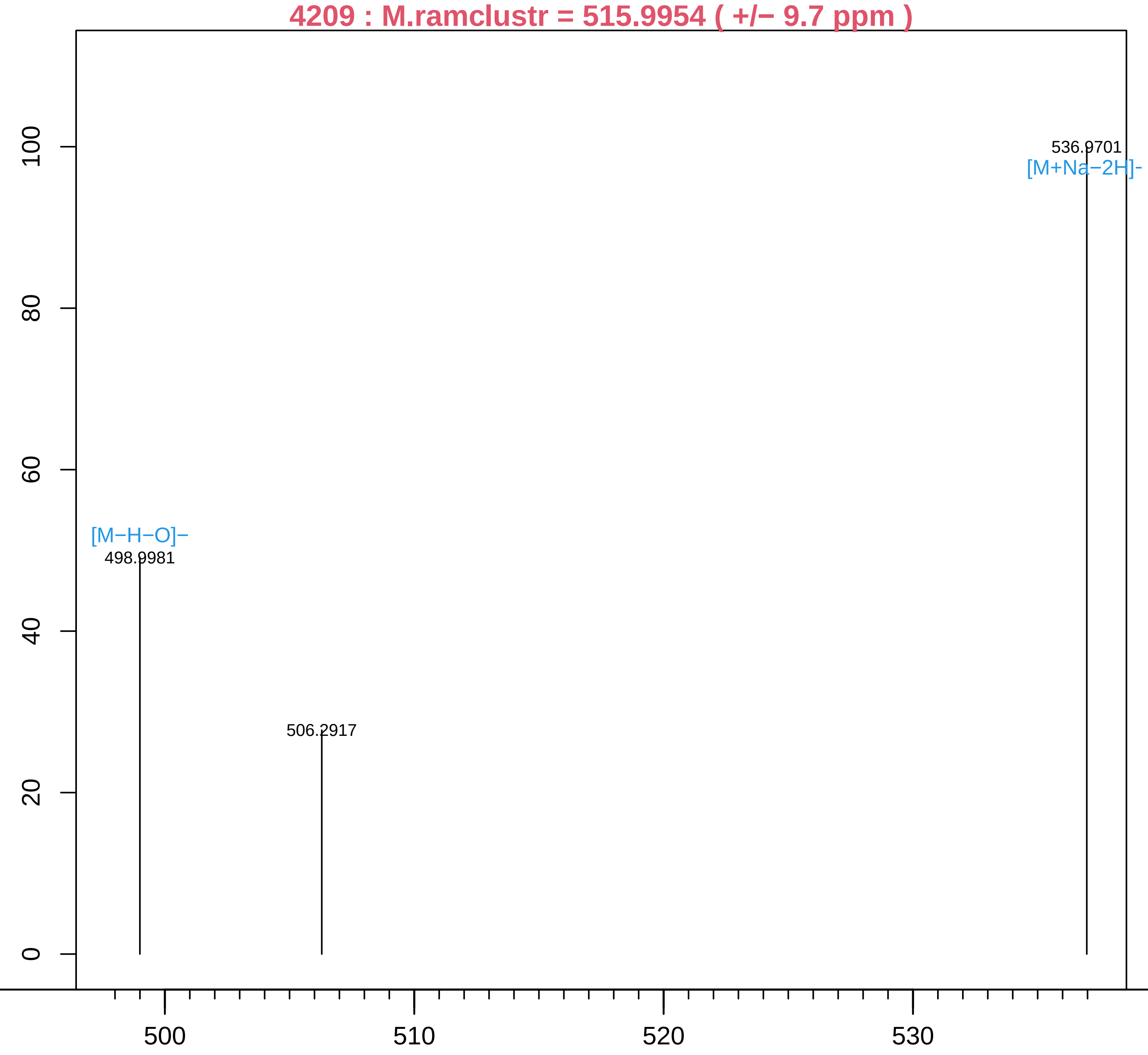
4209 : M.ramclustr = 515.9954 (+/− 9.7 ppm)
536.9701
[M+Na−2H]-
[M−H−O]−
498.9981
506.2917
0
20
40
60
80
100
500
510
520
530

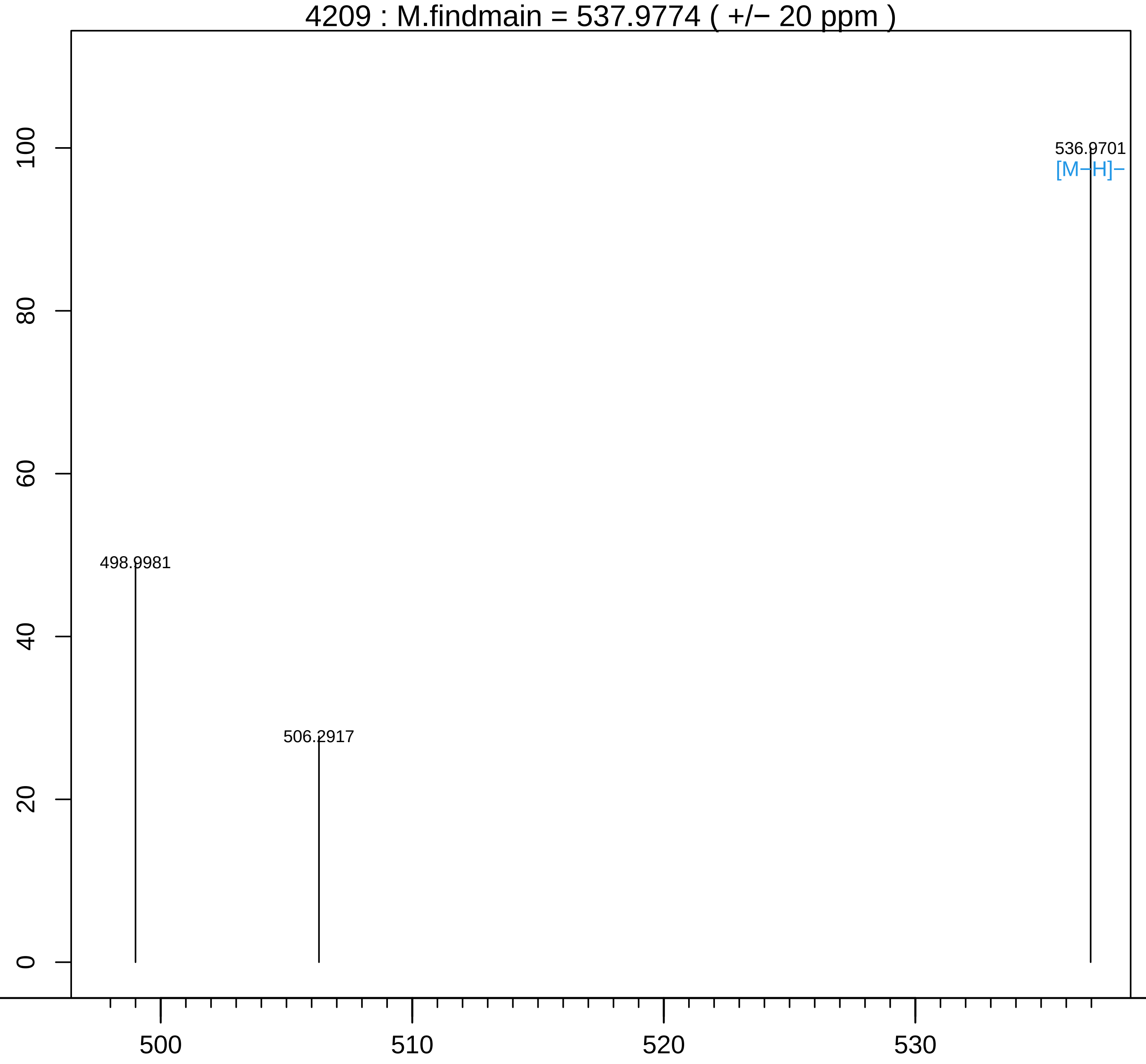
4209 : M.findmain = 537.9774 (+/− 20 ppm)
536.9701
[M−H]−
498.9981
506.2917
0
20
40
60
80
100
500
510
520
530

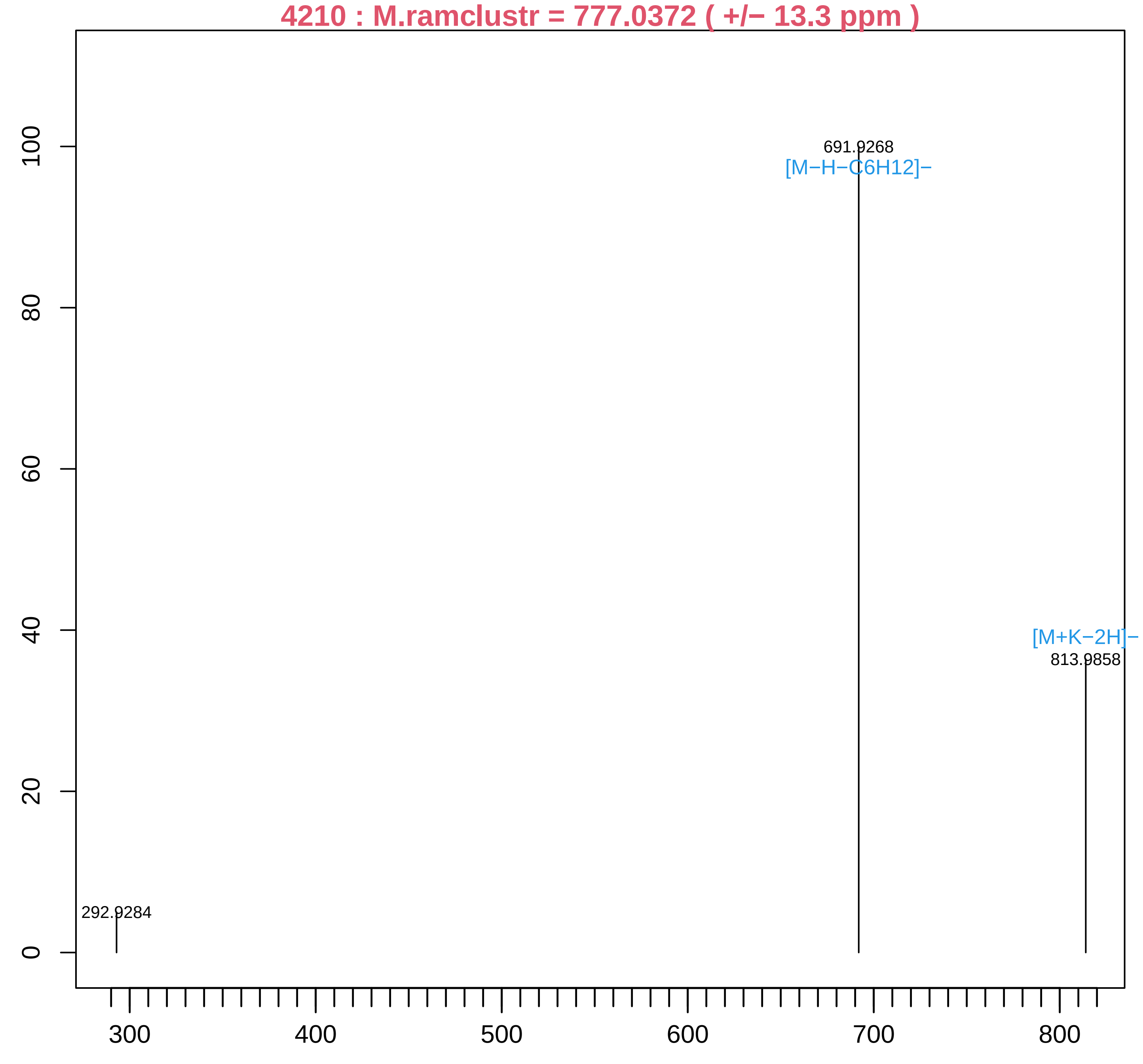
4210 : M.ramclustr = 777.0372 (+/− 13.3 ppm)
691.9268
[M−H−C6H12]−
[M+K−2H]−
813.9858
292.9284
0
20
40
60
80
100
300
400
500
600
700
800

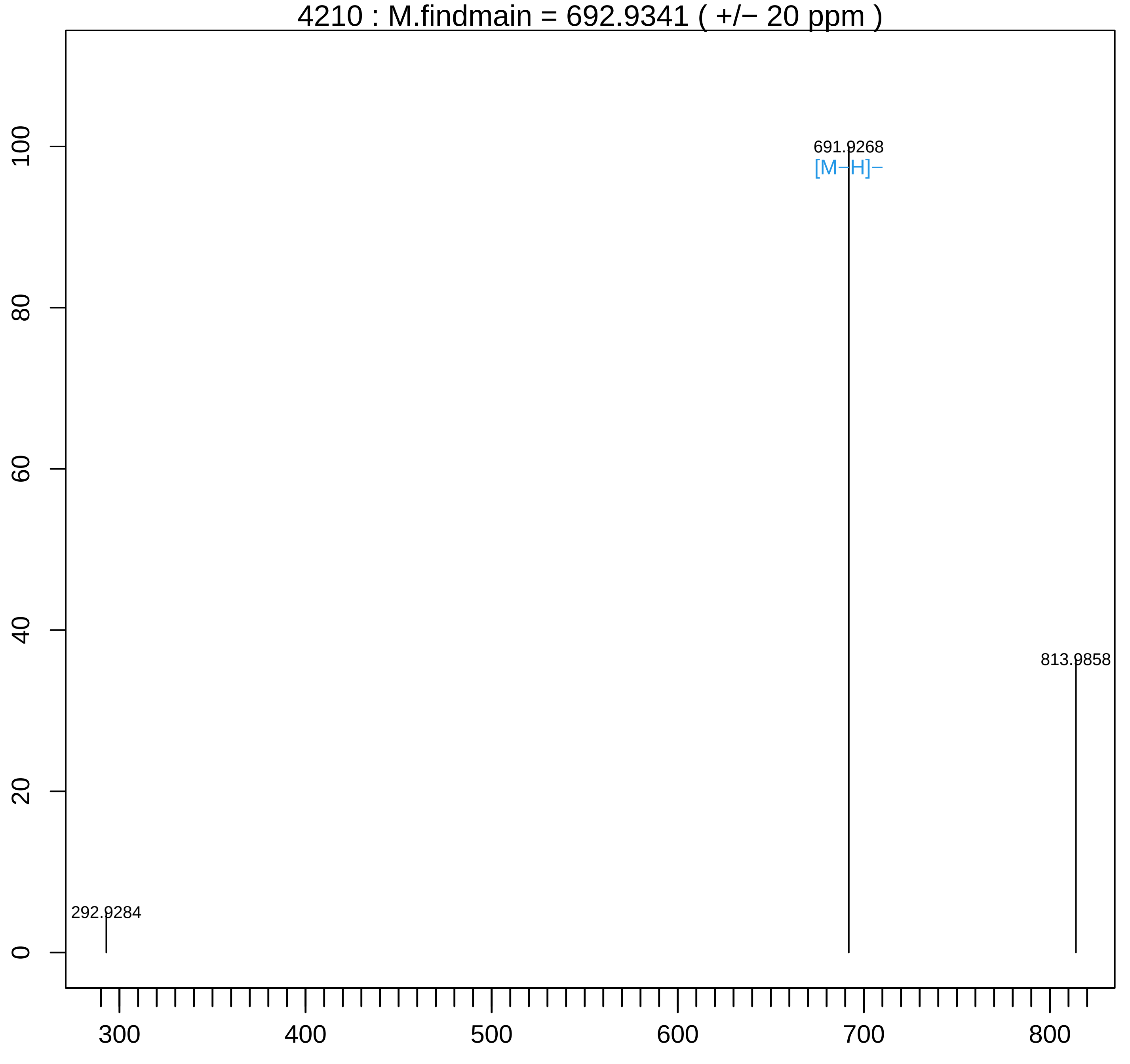
4210 : M.findmain = 692.9341 (+/− 20 ppm)
691.9268
[M−H]−
813.9858
292.9284
0
20
40
60
80
100
300
400
500
600
700
800

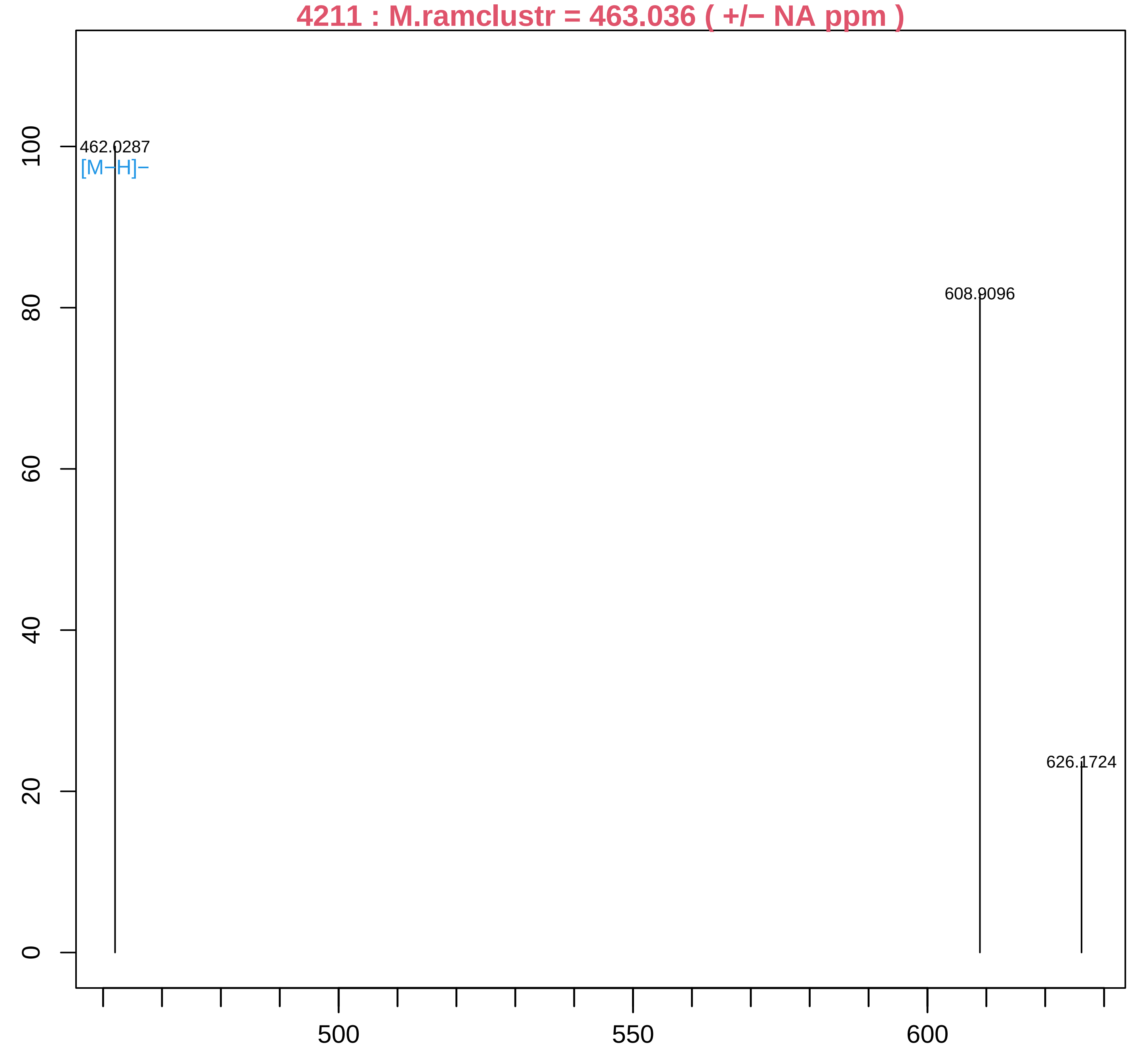
4211 : M.ramclustr = 463.036 (+/− NA ppm)
462.0287
[M−H]−
608.9096
626.1724
0
20
40
60
80
100
500
550
600

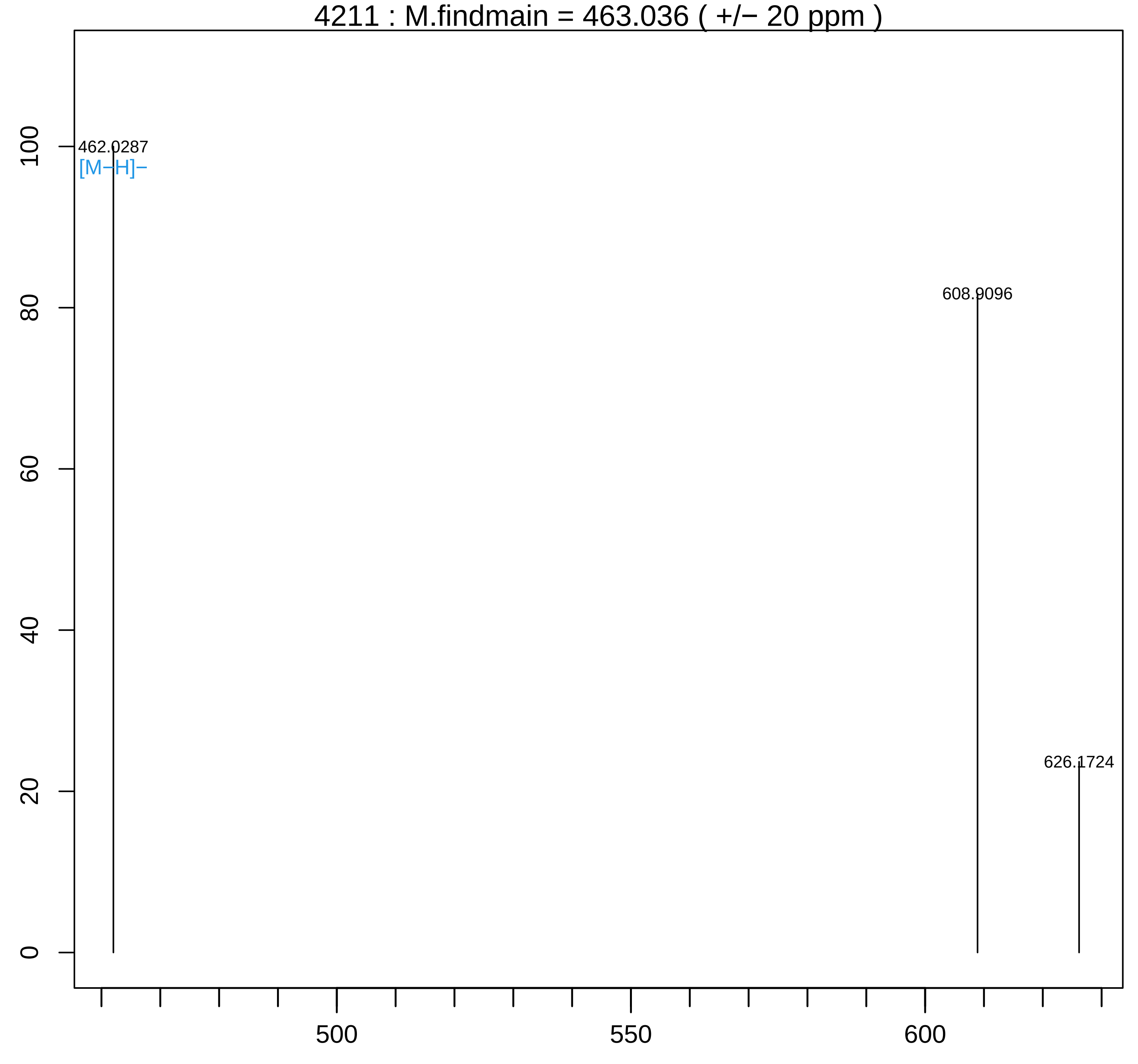
4211 : M.findmain = 463.036 (+/− 20 ppm)
462.0287
[M−H]−
608.9096
626.1724
0
20
40
60
80
100
500
550
600

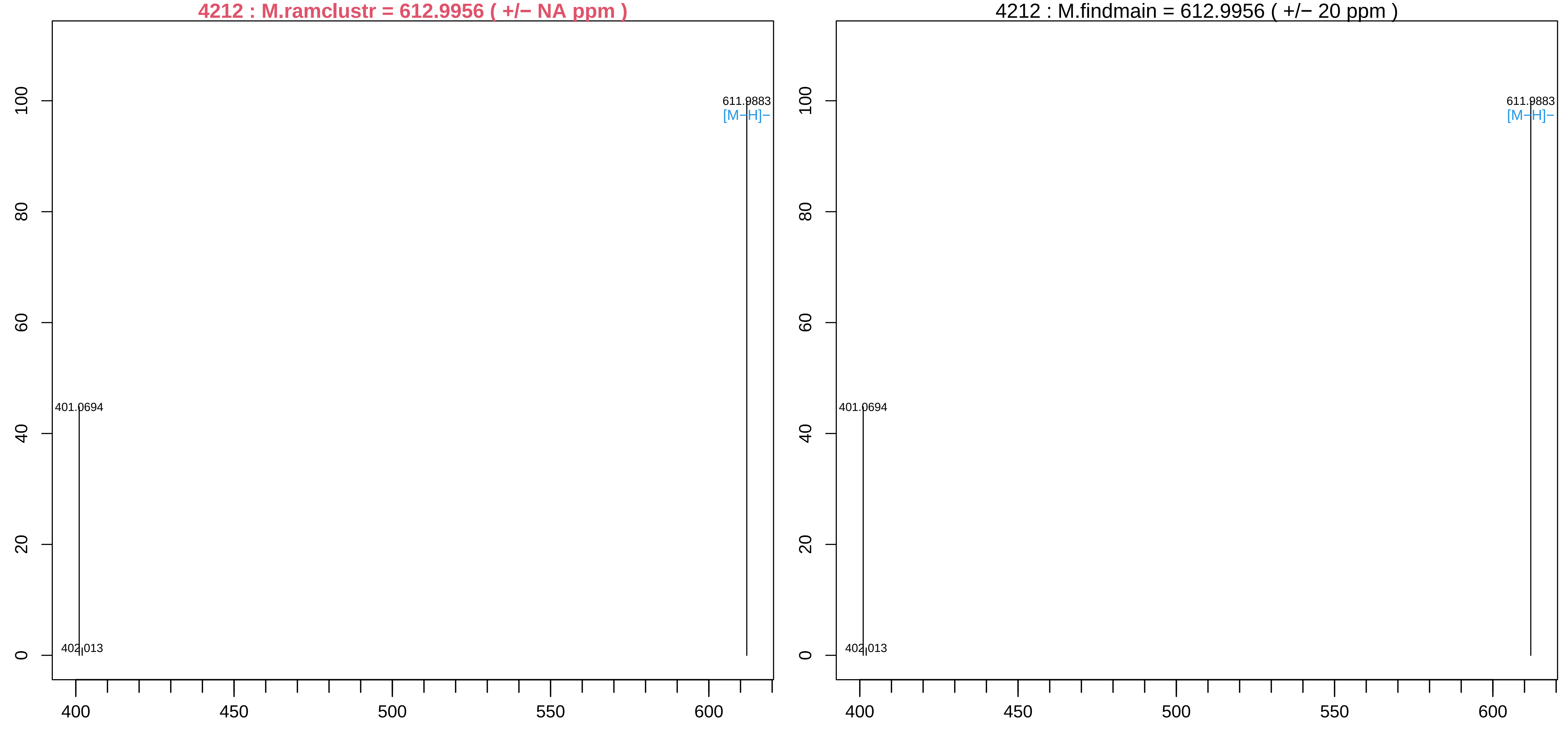
4212 : M.ramclustr = 612.9956 (+/− NA ppm)
4212 : M.findmain = 612.9956 (+/− 20 ppm)
611.9883
[M−H]−
401.0694
402.013
0
20
40
60
80
100
400
450
500
550
600

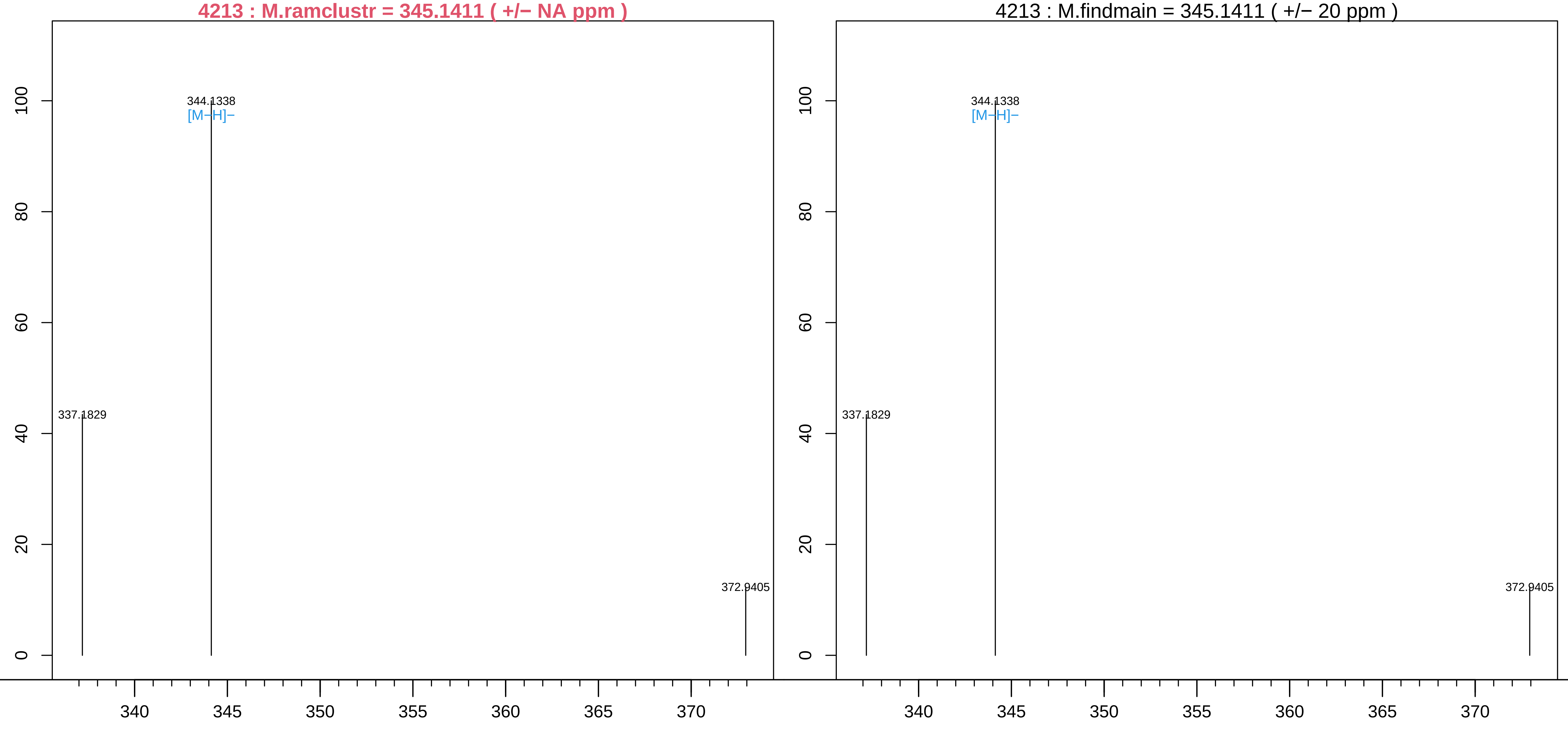

4213 : M.ramclustr = 345.1411 (+/− NA ppm)
4213 : M.findmain = 345.1411 (+/− 20 ppm)
344.1338
[M−H]−
337.1829
372.9405
0
20
40
60
80
100
340
345
350
355
360
365
370

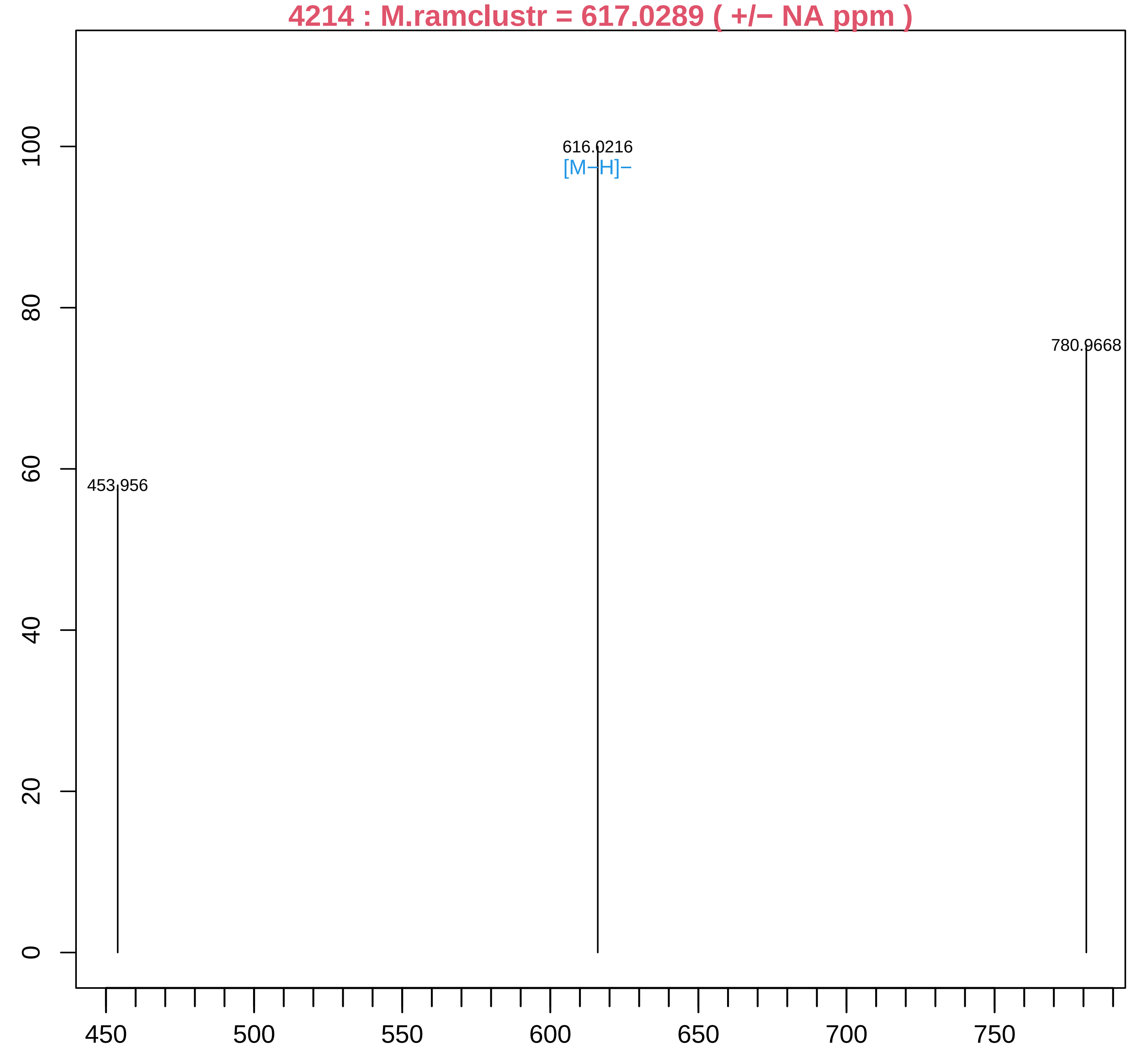
4214 : M.ramclustr = 617.0289 (+/− NA ppm)
616.0216
[M−H]−
780.9668
453.956
0
20
40
60
80
100
450
500
550
600
650
700
750

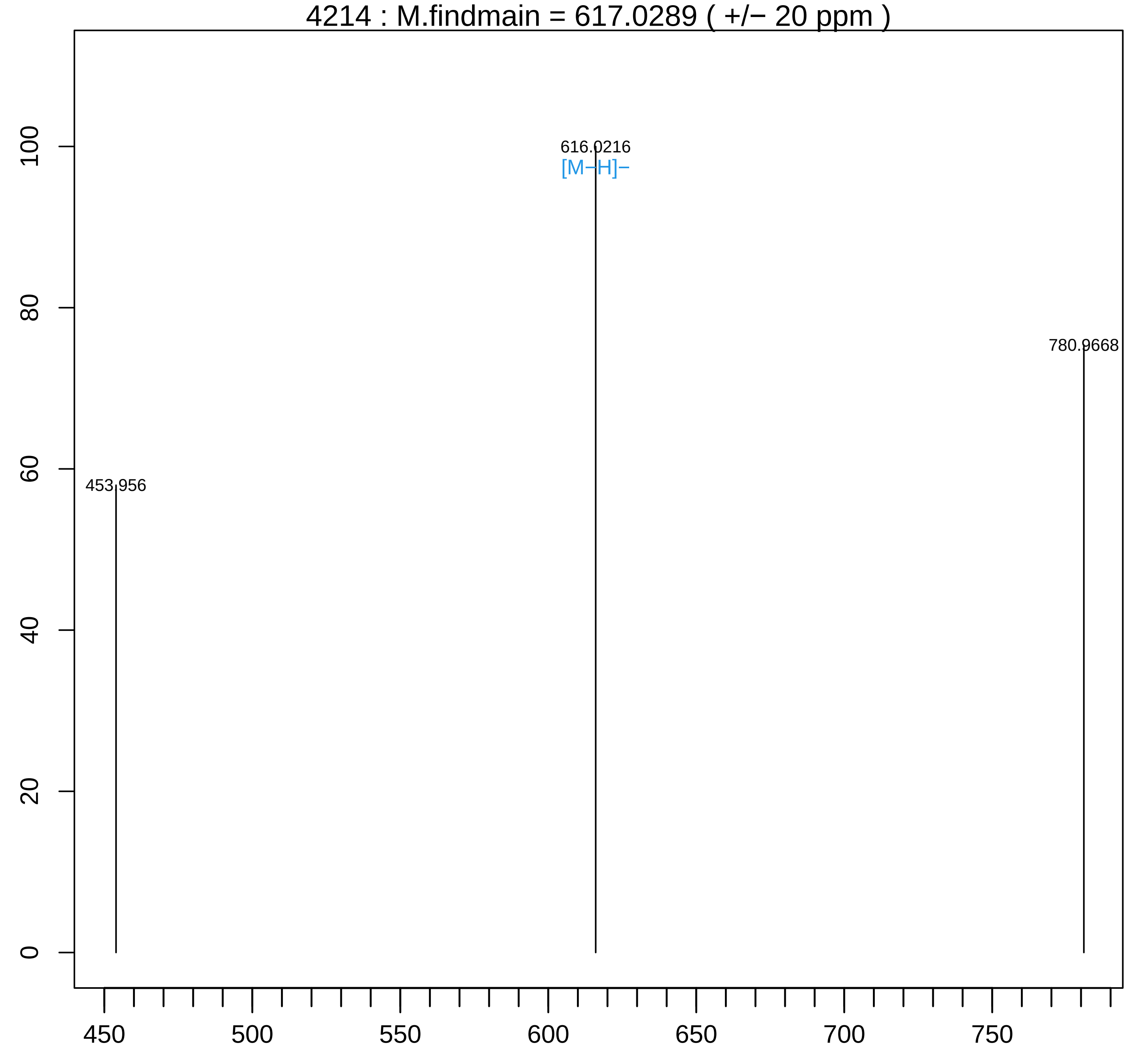
4214 : M.findmain = 617.0289 (+/− 20 ppm)
616.0216
[M−H]−
780.9668
453.956
0
20
40
60
80
100
450
500
550
600
650
700
750

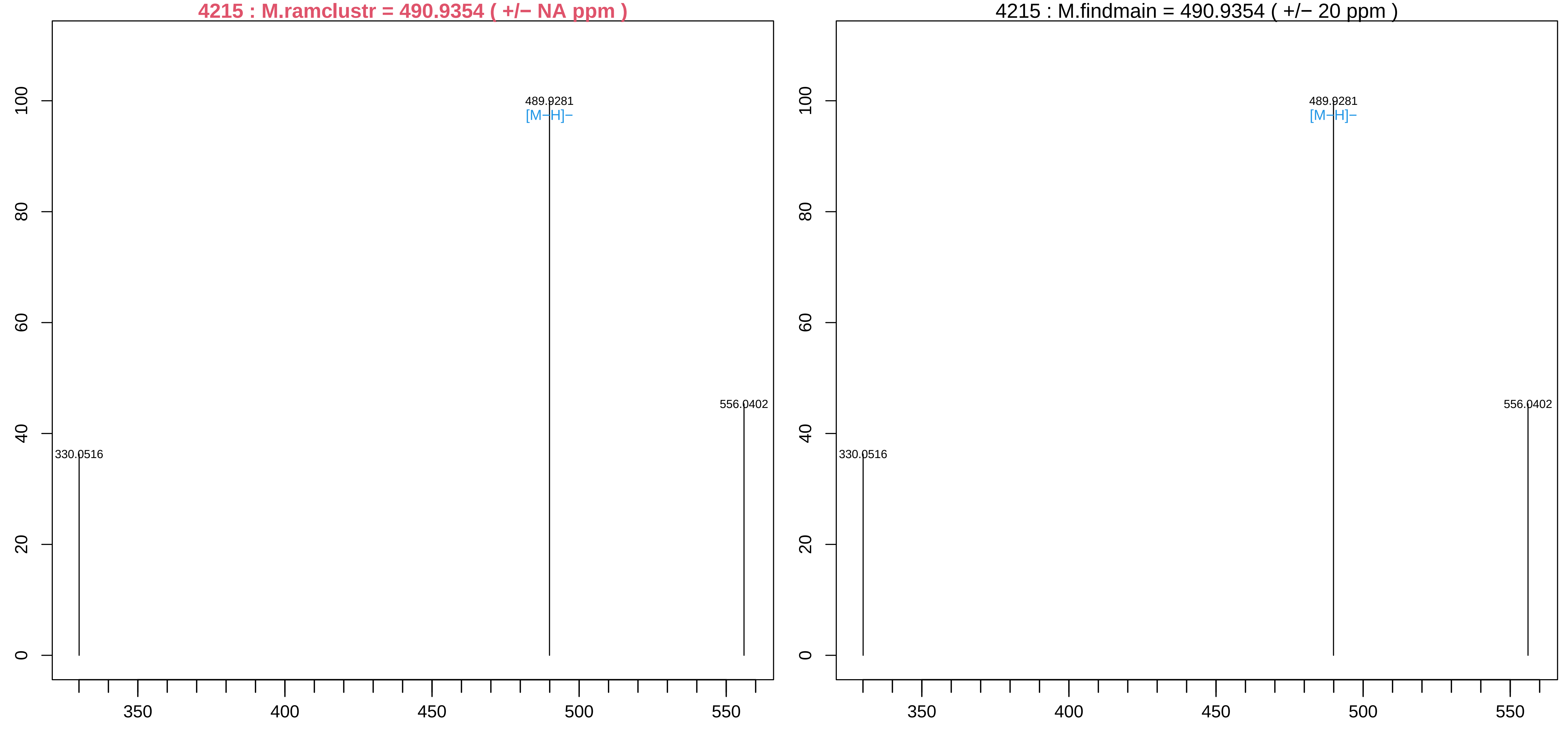
4215 : M.ramclustr = 490.9354 (+/− NA ppm)
4215 : M.findmain = 490.9354 (+/− 20 ppm)
489.9281
[M−H]−
556.0402
330.0516
0
20
40
60
80
100
350
400
450
500
550

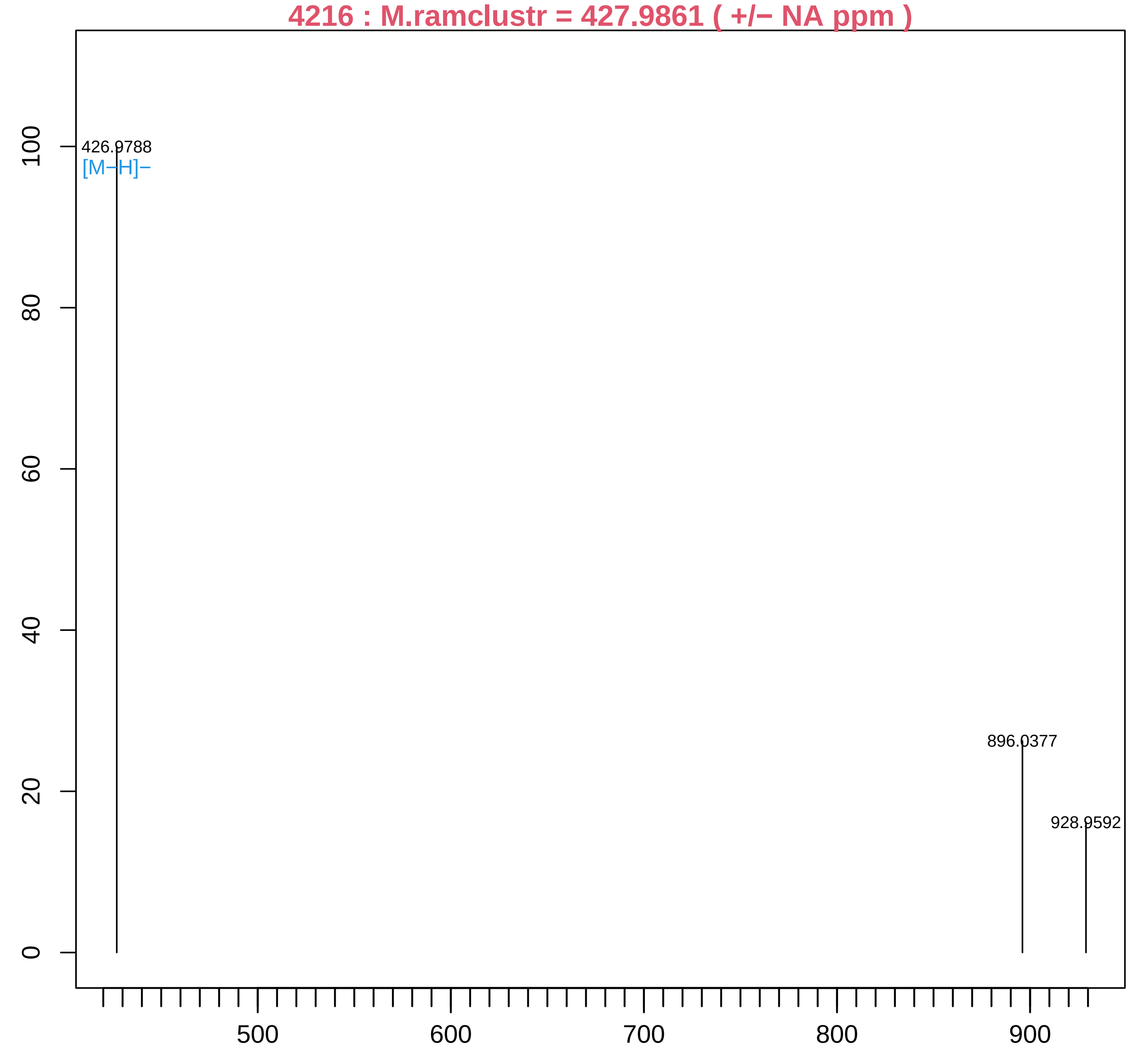
4216 : M.ramclustr = 427.9861 (+/− NA ppm)
426.9788
[M−H]−
896.0377
928.9592
0
20
40
60
80
100
500
600
700
800
900

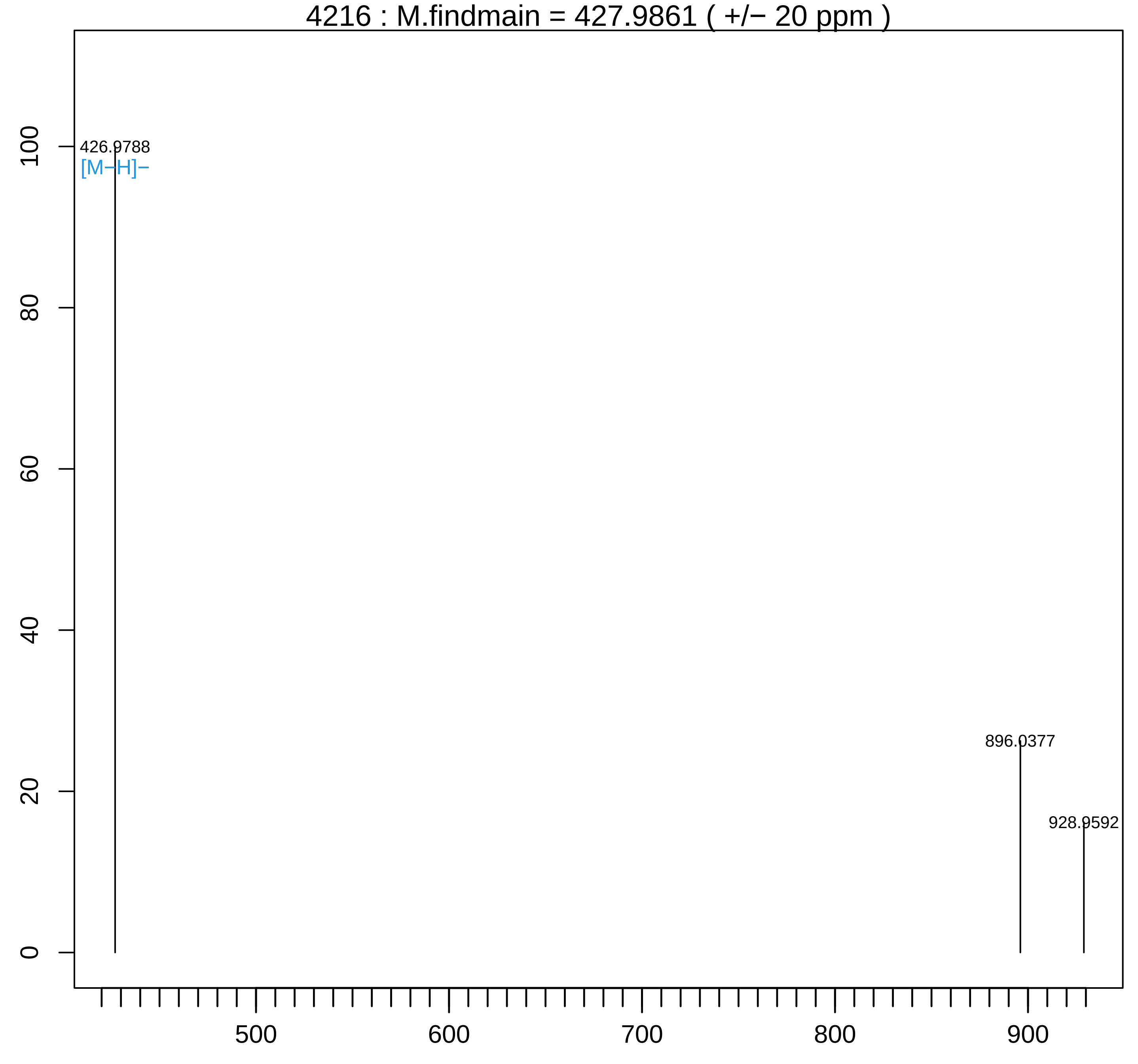
4216 : M.findmain = 427.9861 (+/− 20 ppm)
426.9788
[M−H]−
896.0377
928.9592
0
20
40
60
80
100
500
600
700
800
900

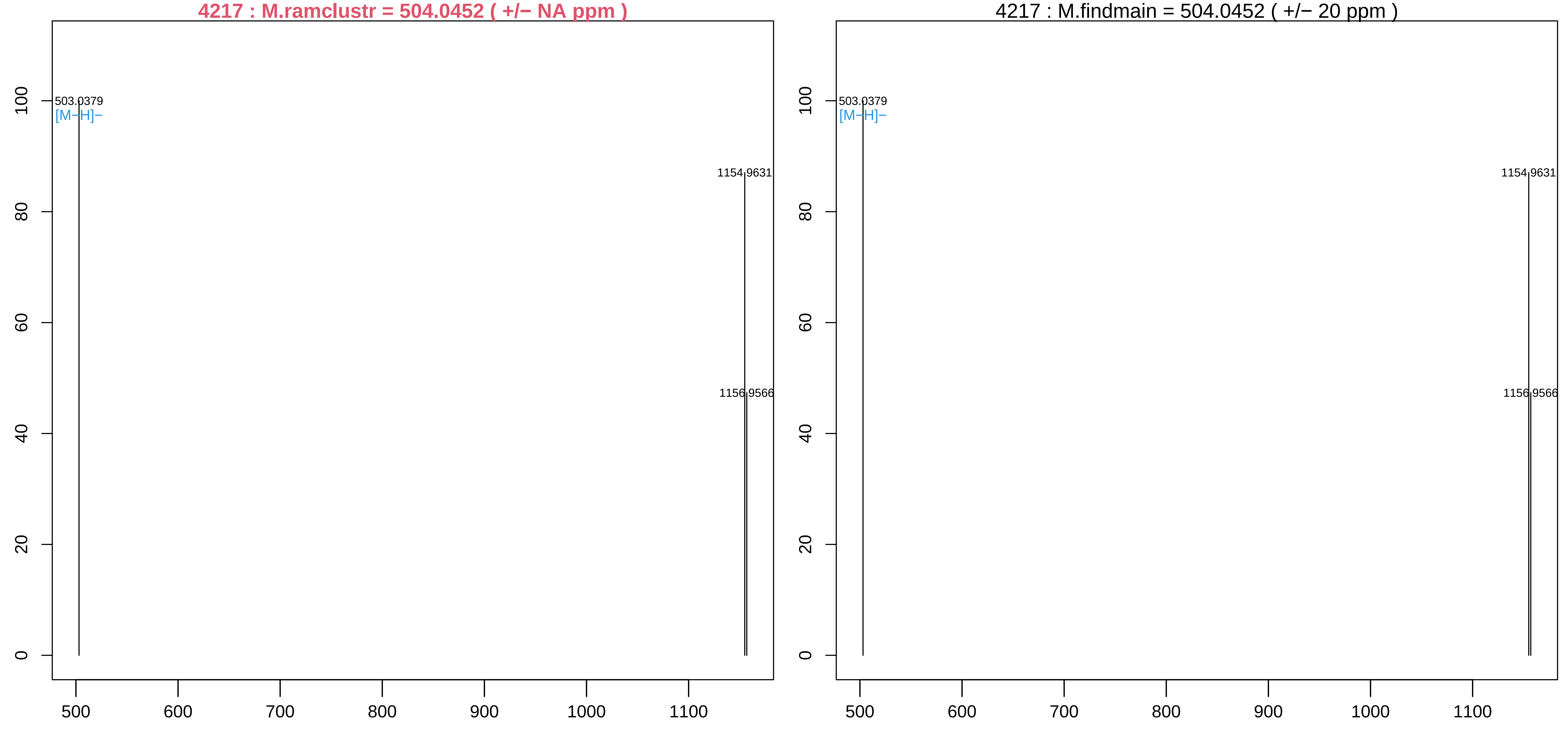

4217 : M.ramclustr = 504.0452 (+/− NA ppm)
503.0379
[M−H]−
1154.9631
1156.9566
4217 : M.findmain = 504.0452 (+/− 20 ppm)
503.0379
[M−H]−
1154.9631
1156.9566
0
20
40
60
80
100
500
600
700
800
900
1000
1100

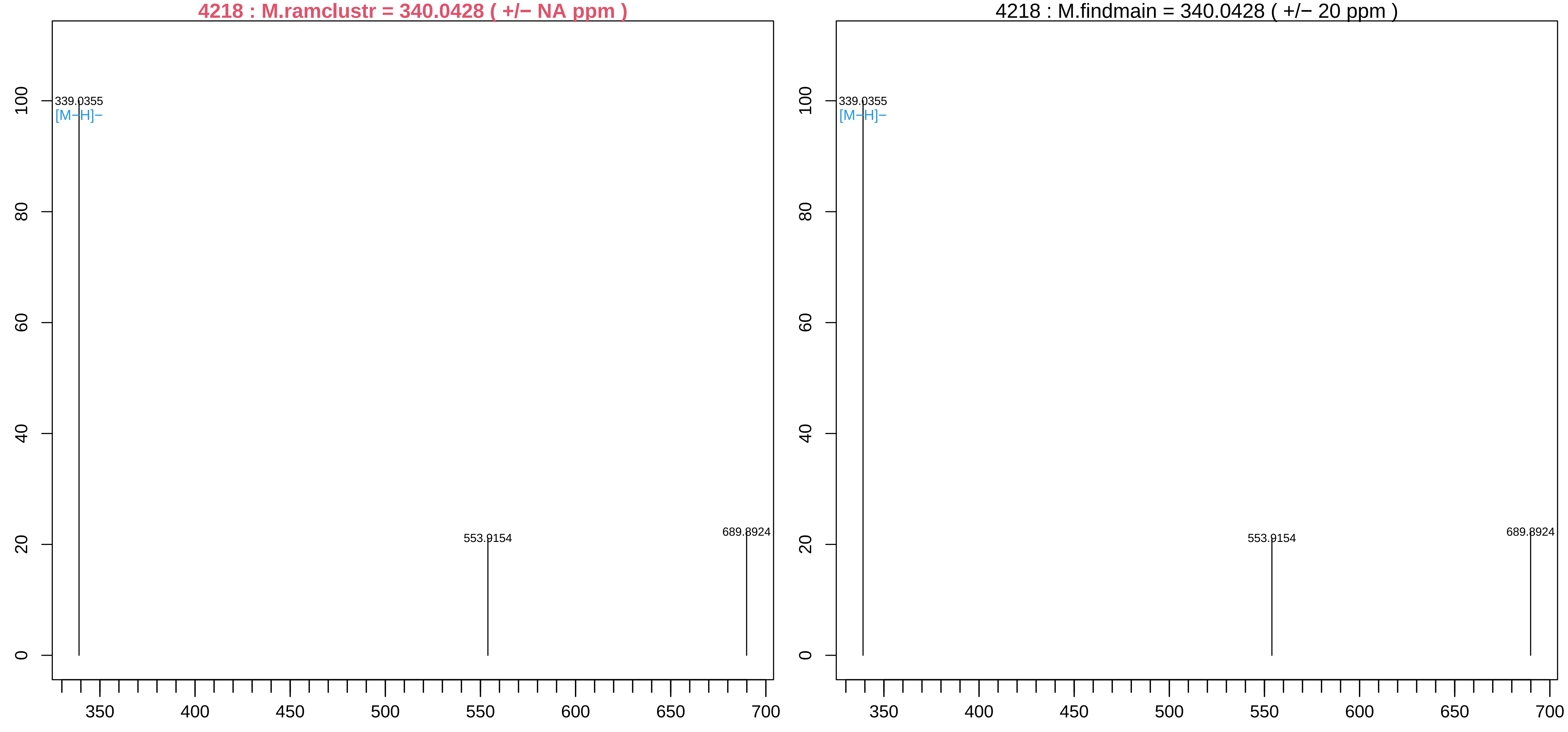
4218 : M.ramclustr = 340.0428 (+/− NA ppm)
339.0355
[M−H]−
553.9154
689.8924
4218 : M.findmain = 340.0428 (+/− 20 ppm)
339.0355
[M−H]−
553.9154
689.8924
0
20
40
60
80
100
350
400
450
500
550
600
650
700

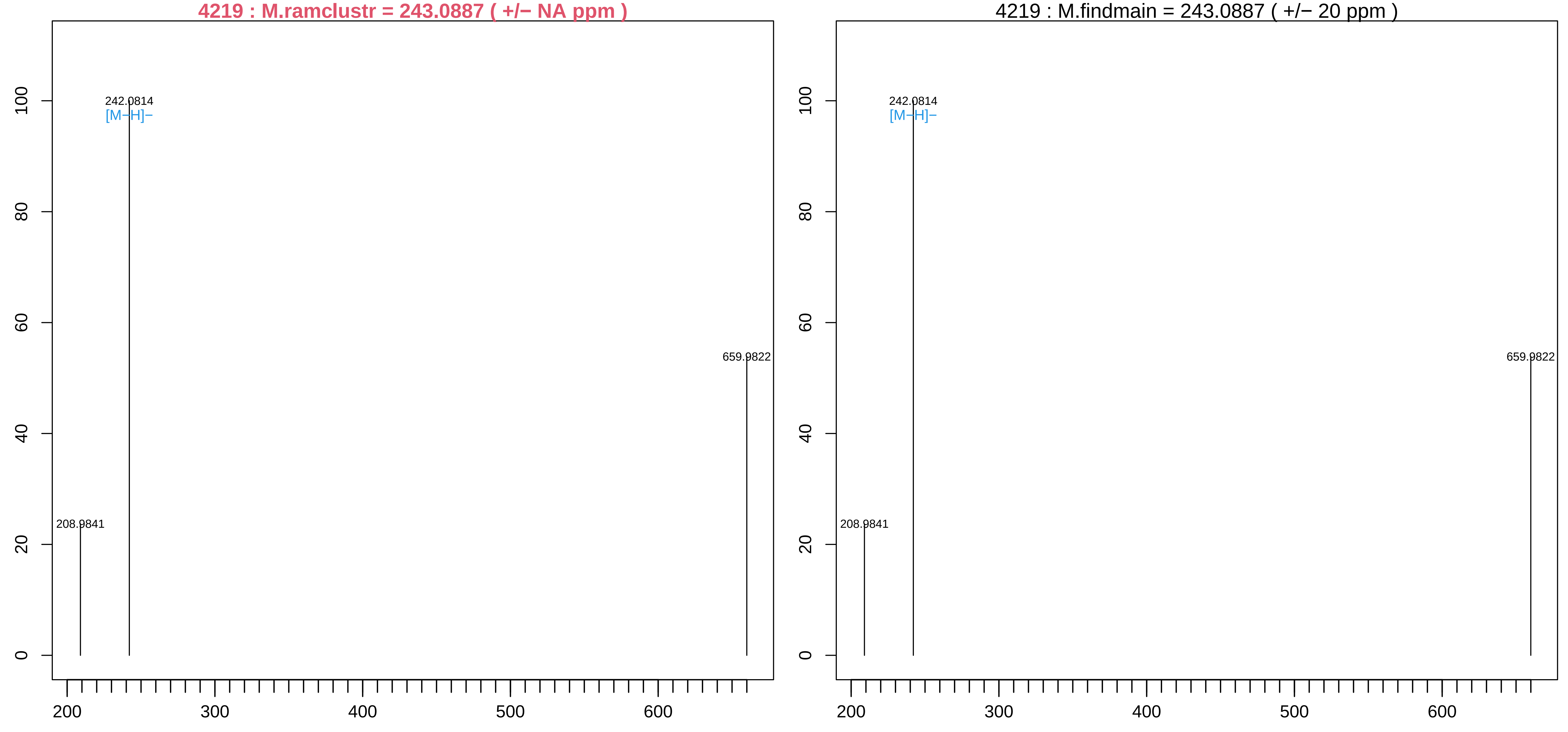
4219 : M.ramclustr = 243.0887 (+/− NA ppm)
242.0814
[M−H]−
659.9822
208.9841
4219 : M.findmain = 243.0887 (+/− 20 ppm)
242.0814
[M−H]−
659.9822
208.9841
0
20
40
60
80
100
200
300
400
500
600

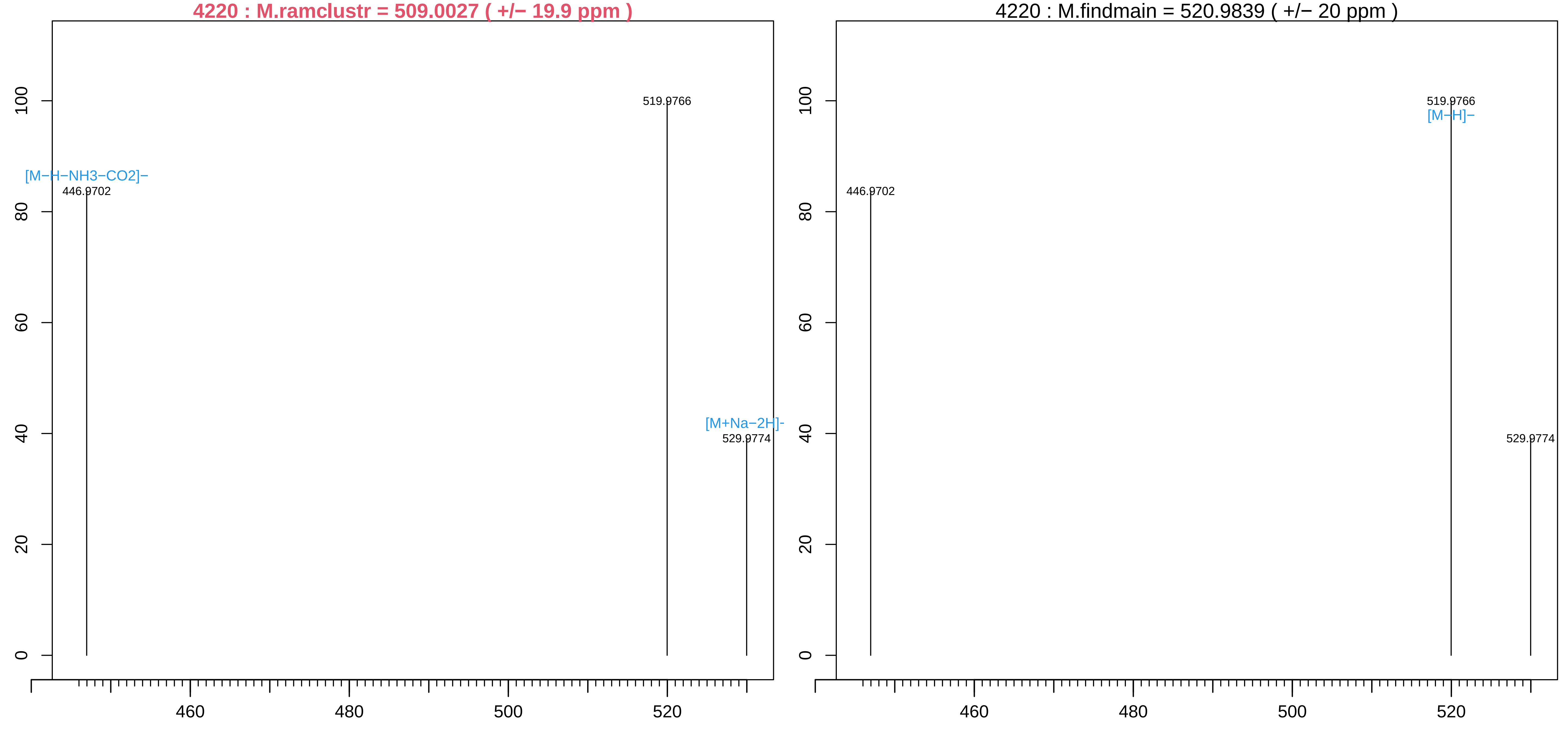

4220 : M.ramclustr = 509.0027 (+/− 19.9 ppm)
[M−H−NH3−CO2]−
446.9702
519.9766
[M+Na−2H]-
529.9774
4220 : M.findmain = 520.9839 (+/− 20 ppm)
446.9702
519.9766
[M−H]−
529.9774
0
20
40
60
80
100
460
480
500
520

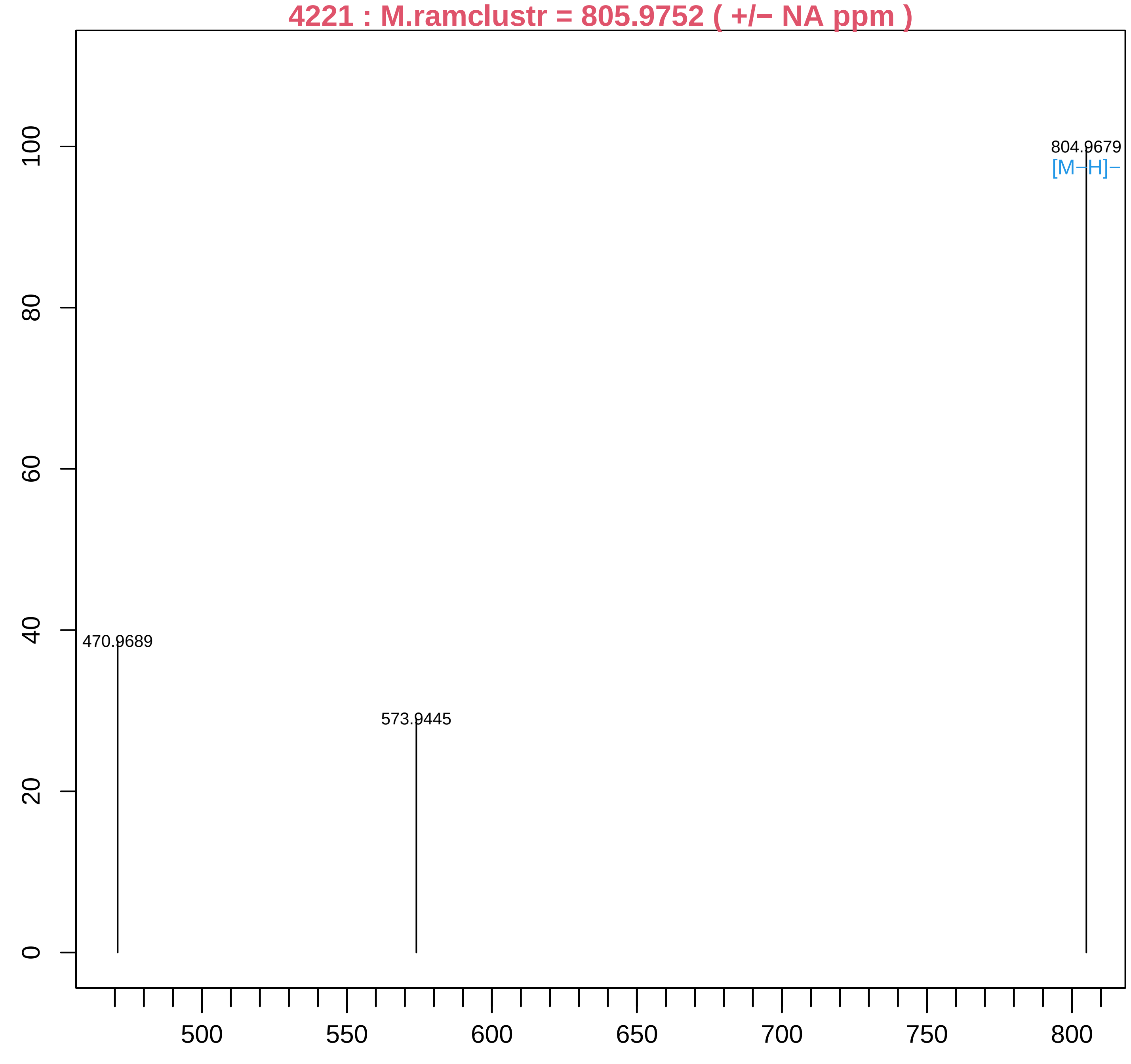
4221 : M.ramclustr = 805.9752 (+/− NA ppm)
804.9679
[M−H]−
470.9689
573.9445
0
20
40
60
80
100
500
550
600
650
700
750
800

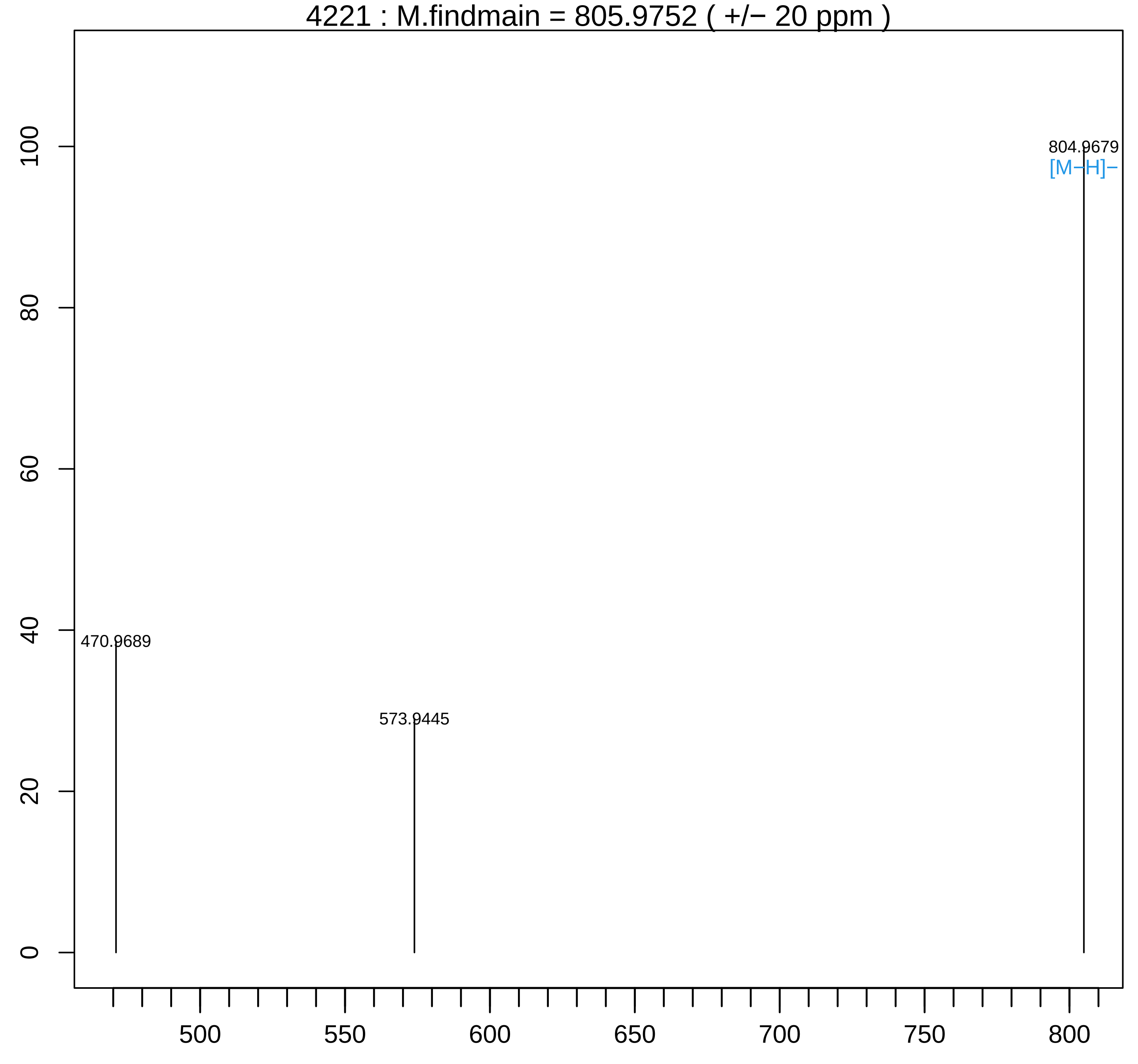
4221 : M.findmain = 805.9752 (+/− 20 ppm)
804.9679
[M−H]−
470.9689
573.9445
0
20
40
60
80
100
500
550
600
650
700
750
800

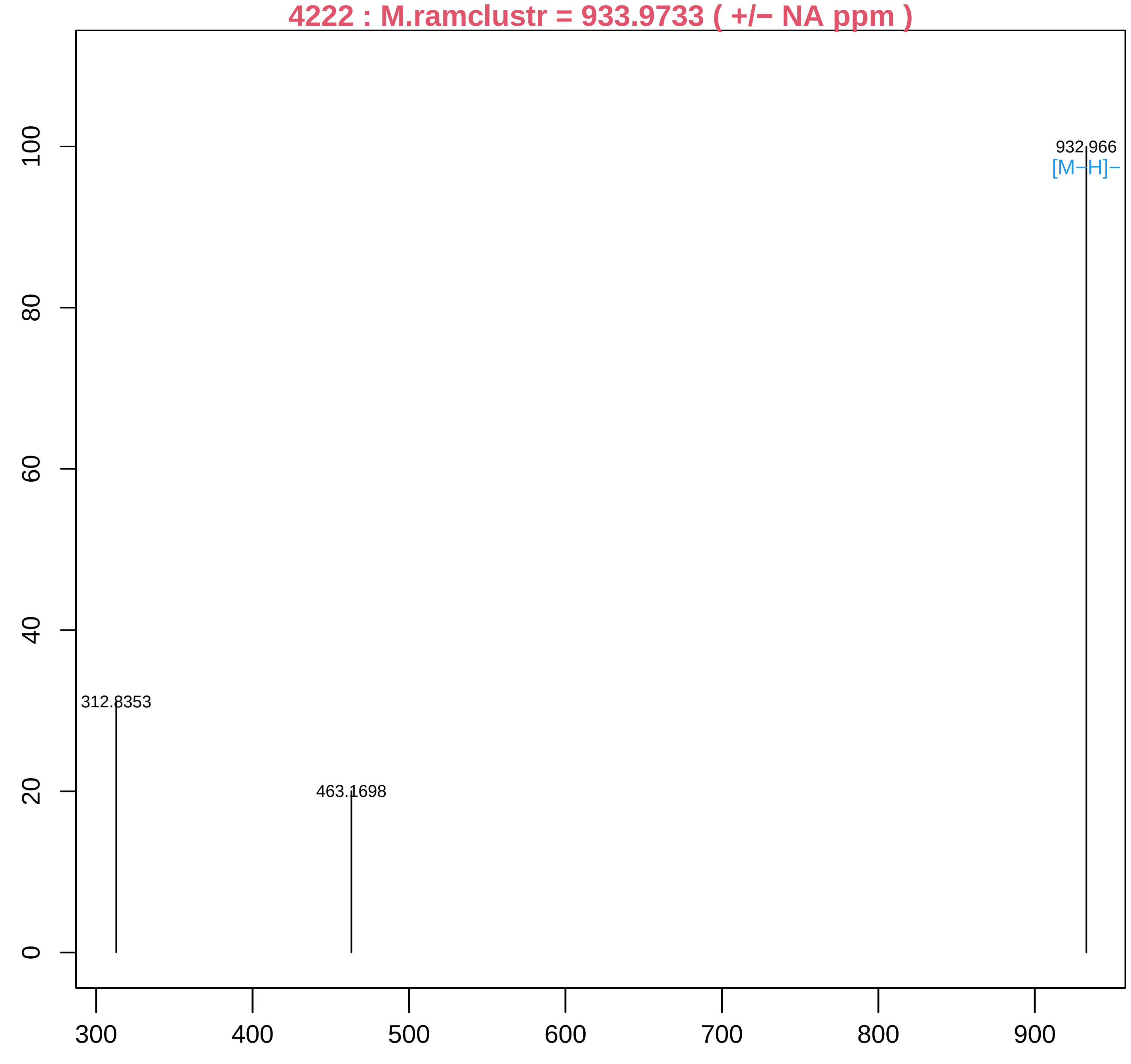
4222 : M.ramclustr = 933.9733 (+/− NA ppm)
932.966
[M−H]−
312.8353
463.1698
0
20
40
60
80
100
300
400
500
600
700
800
900

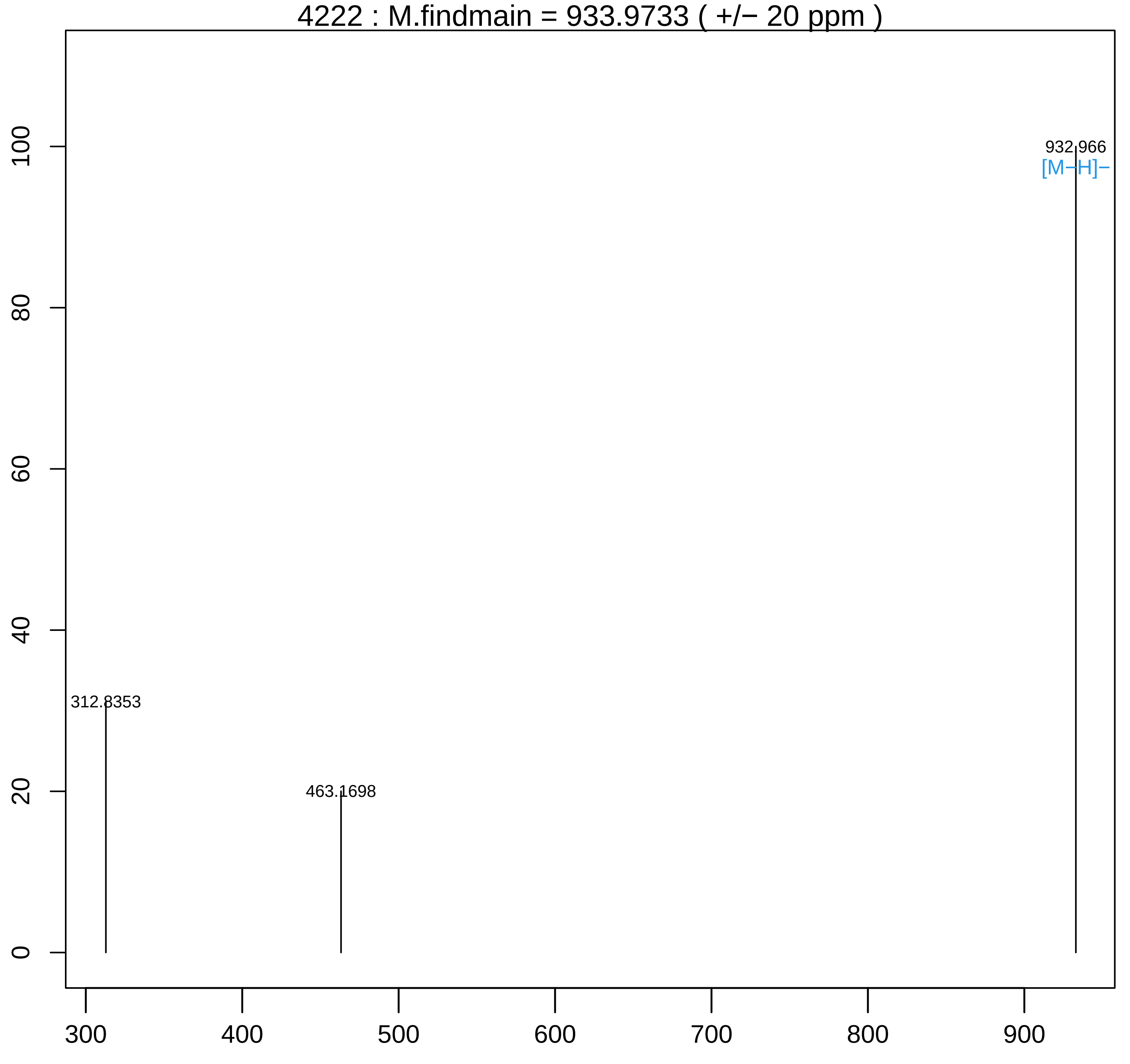
4222 : M.findmain = 933.9733 (+/− 20 ppm)
932.966
[M−H]−
312.8353
463.1698
0
20
40
60
80
100
300
400
500
600
700
800
900

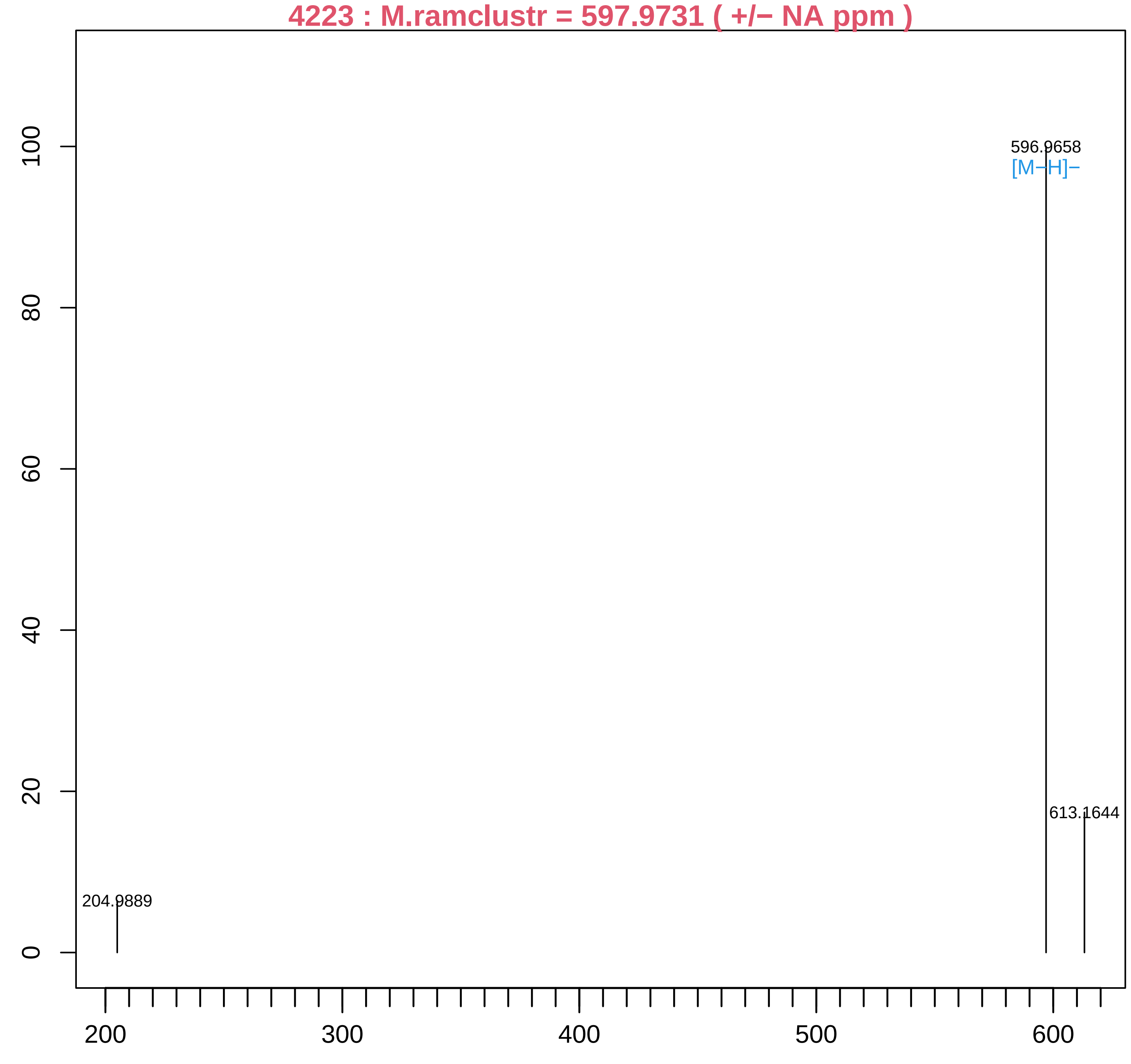
4223 : M.ramclustr = 597.9731 (+/− NA ppm)
596.9658
[M−H]−
613.1644
204.9889
0
20
40
60
80
100
200
300
400
500
600

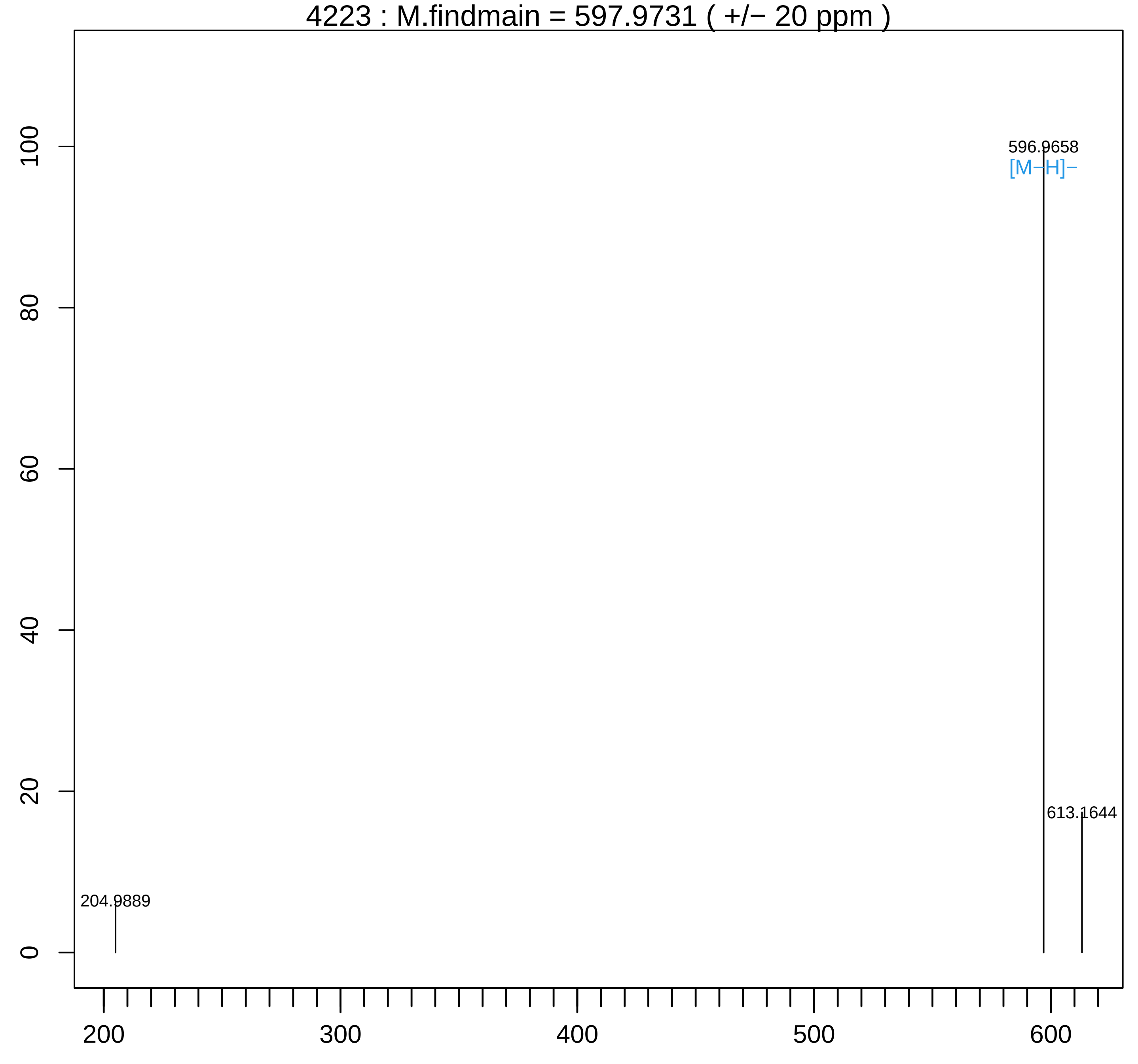
4223 : M.findmain = 597.9731 (+/− 20 ppm)
596.9658
[M−H]−
613.1644
204.9889
0
20
40
60
80
100
200
300
400
500
600

4224 : M.ramclustr = 696.9665 (+/− NA ppm)

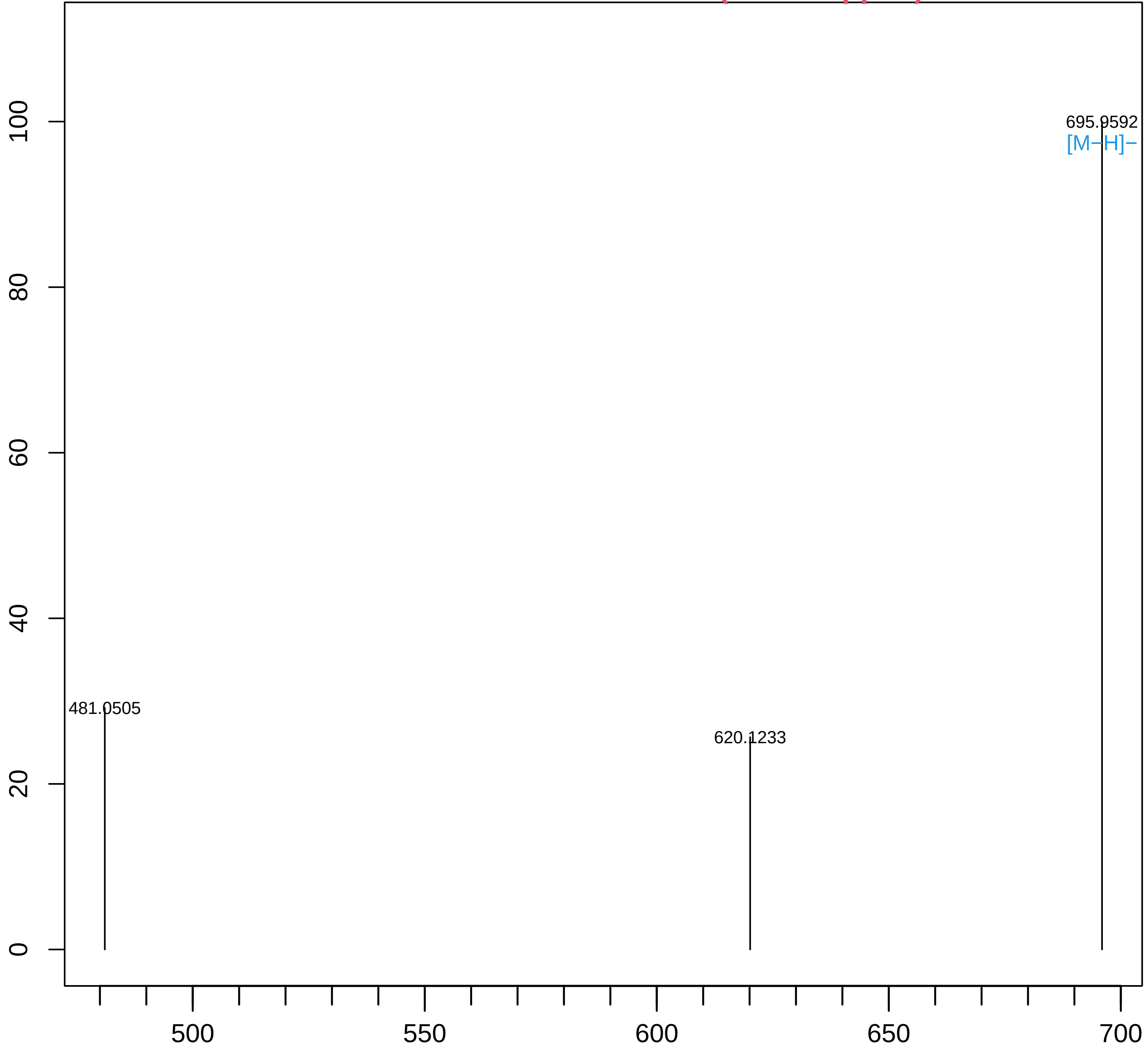

4224 : M.findmain = 696.9665 (+/− 20 ppm)

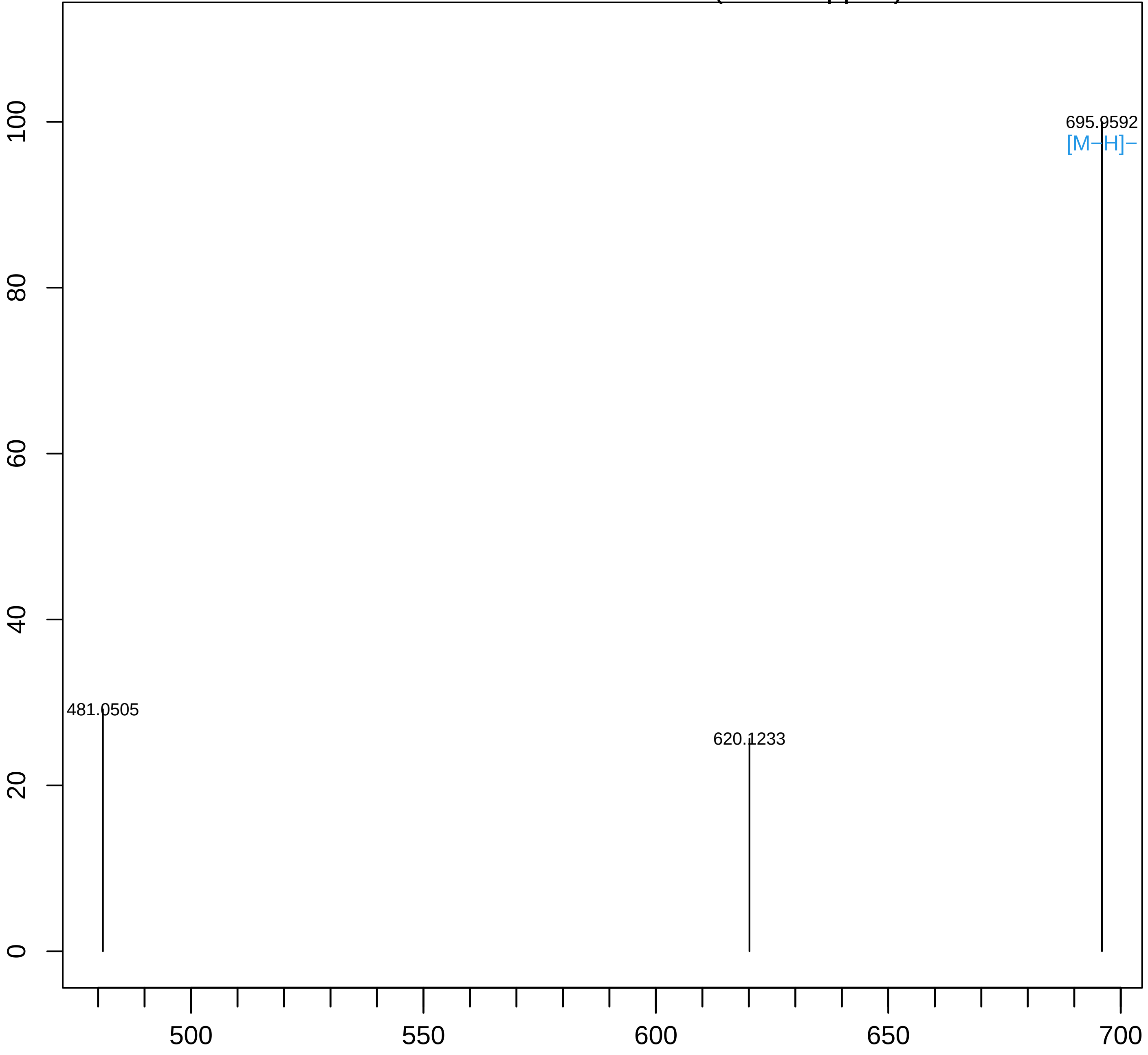

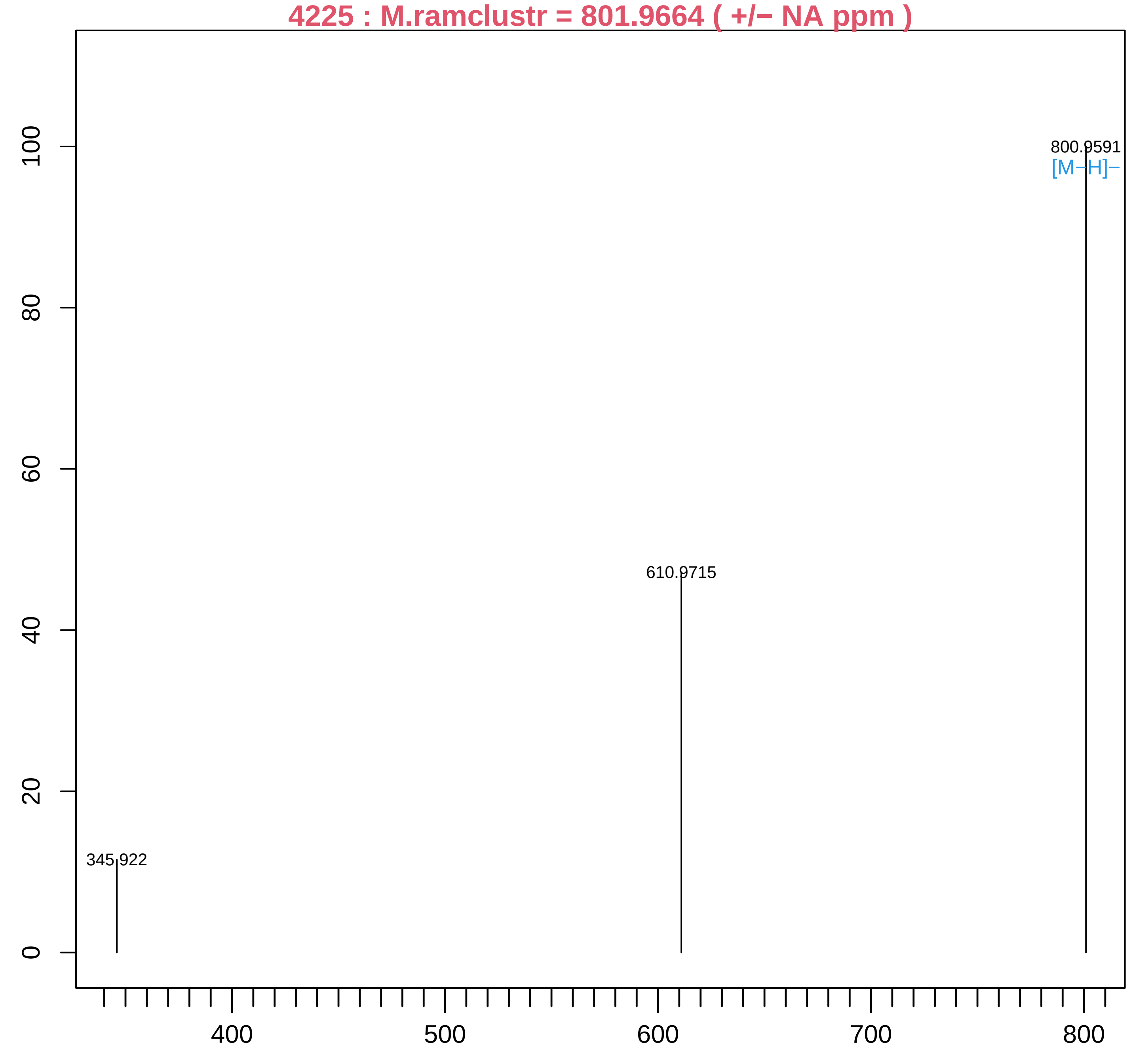
4225 : M.ramclustr = 801.9664 (+/− NA ppm)
800.9591
[M−H]−
610.9715
345.922
0
20
40
60
80
100
400
500
600
700
800

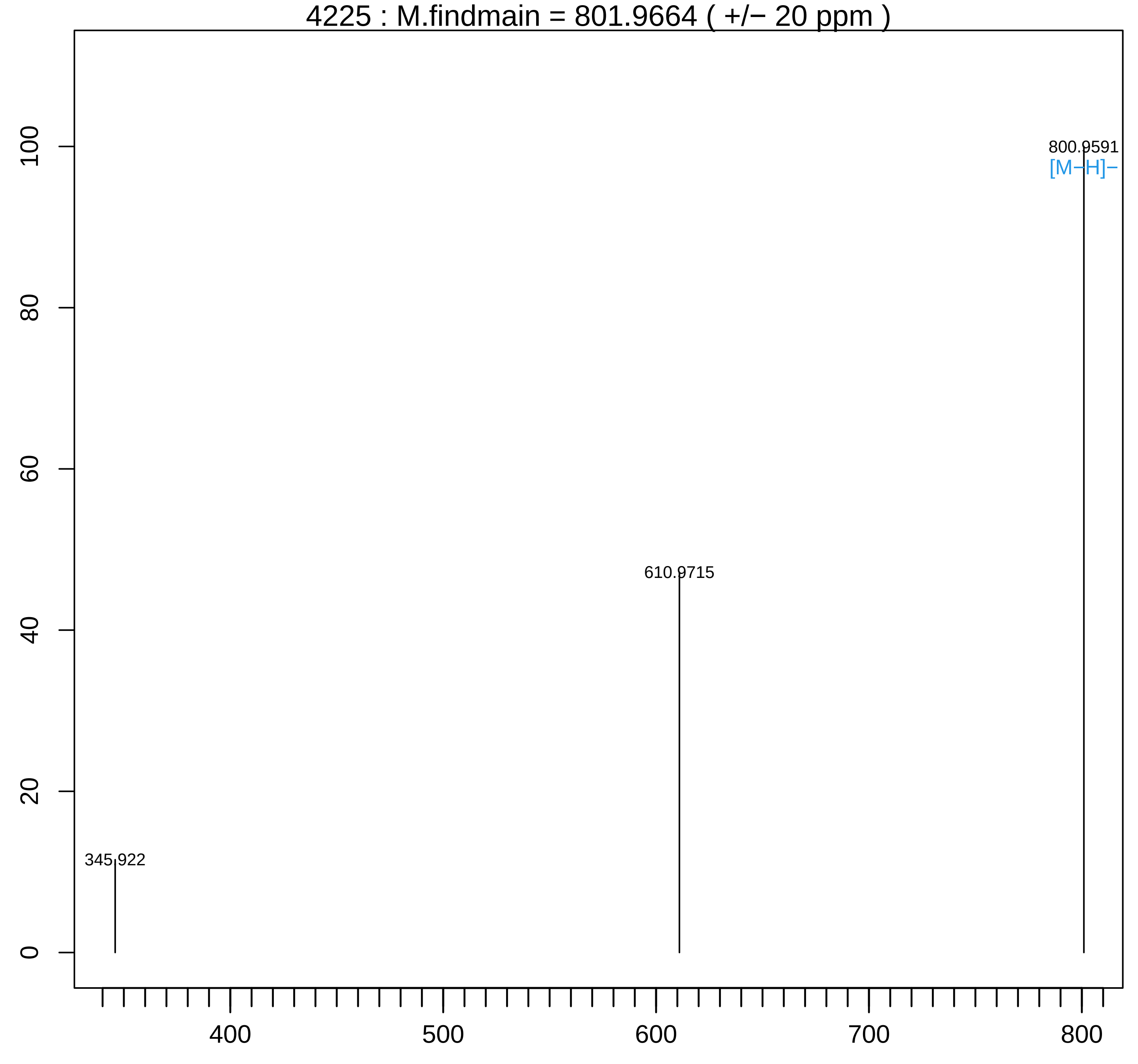
4225 : M.findmain = 801.9664 (+/− 20 ppm)
800.9591
[M−H]−
610.9715
345.922
0
20
40
60
80
100
400
500
600
700
800

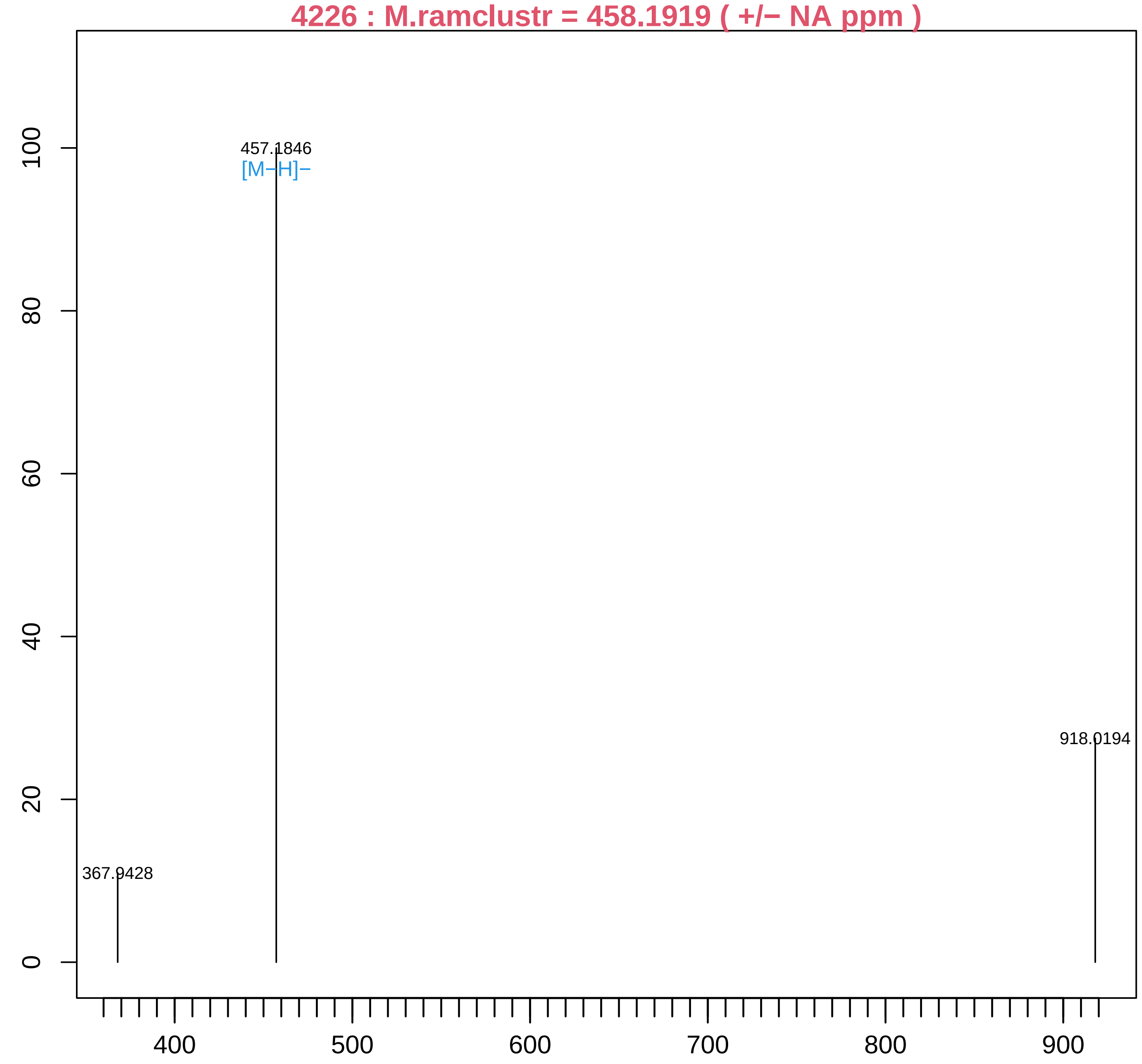
4226 : M.ramclustr = 458.1919 (+/− NA ppm)
457.1846
[M−H]−
918.0194
367.9428
0
20
40
60
80
100
400
500
600
700
800
900

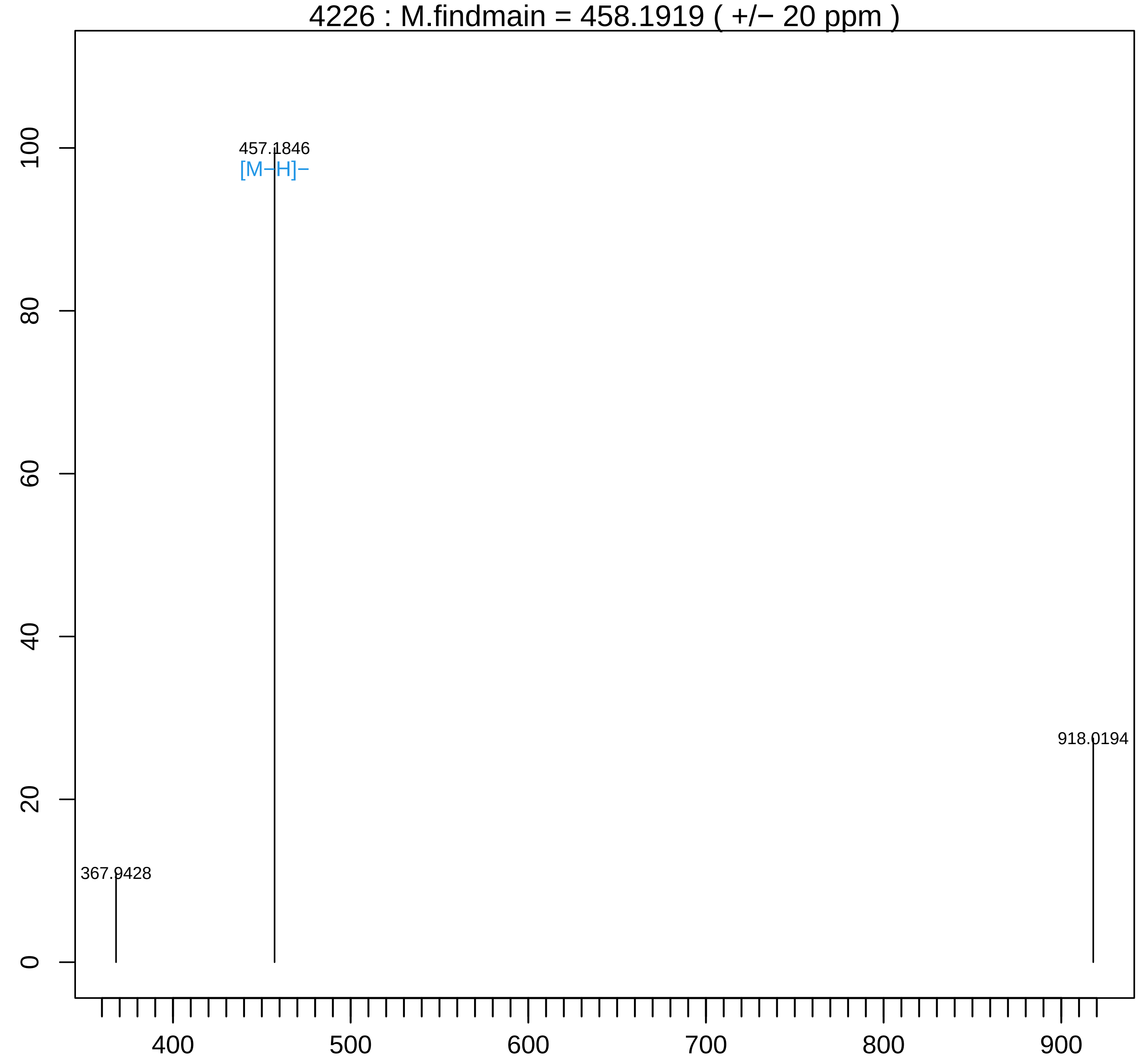
4226 : M.findmain = 458.1919 (+/− 20 ppm)
457.1846
[M−H]−
918.0194
367.9428
0
20
40
60
80
100
400
500
600
700
800
900

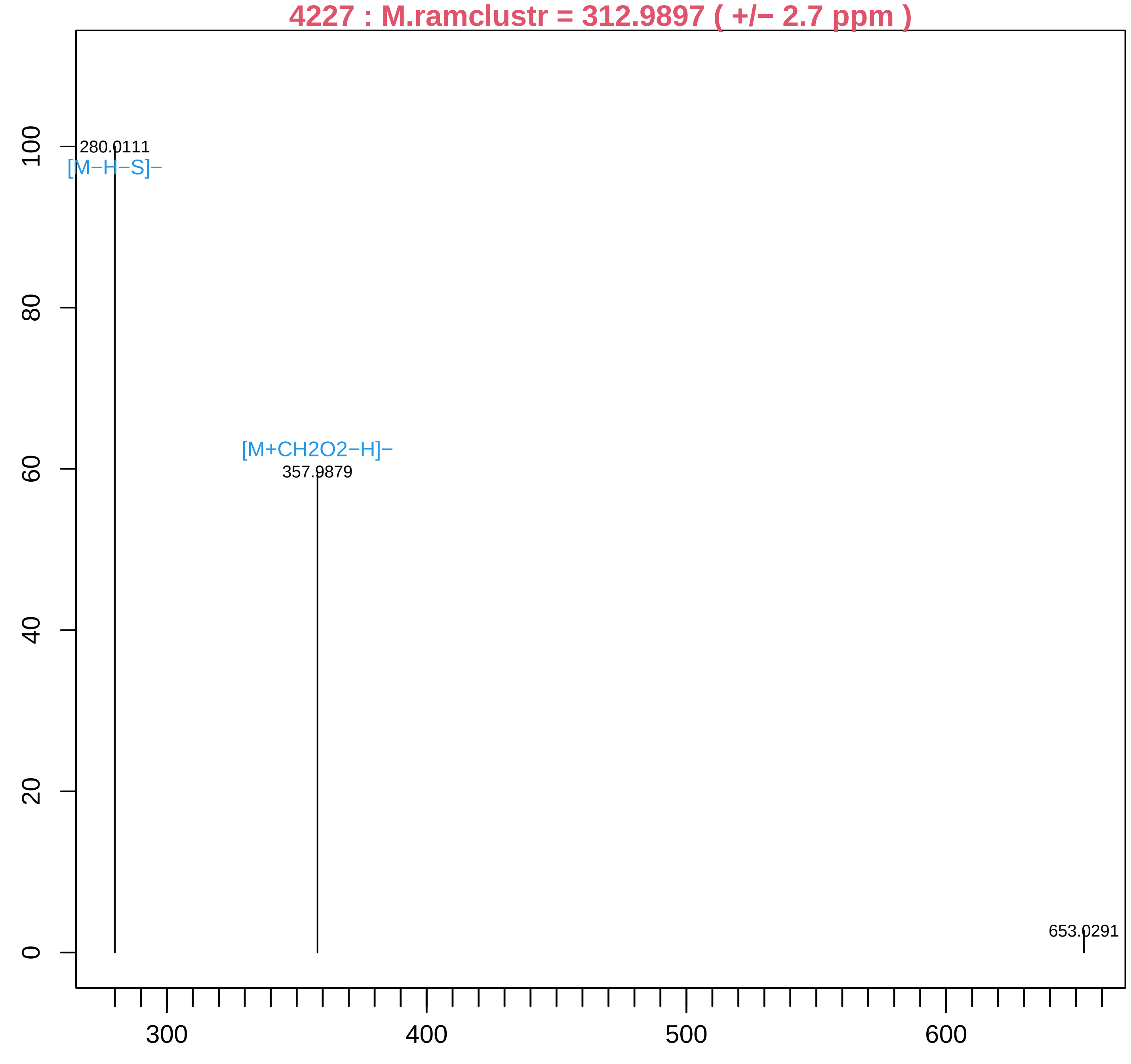

4227 : M.ramclustr = 312.9897 (+/− 2.7 ppm)
100
80
60
40
20
0
280.0111
[M−H−S]−
[M+CH2O2−H]−
357.9879
653.0291
300
400
500
600

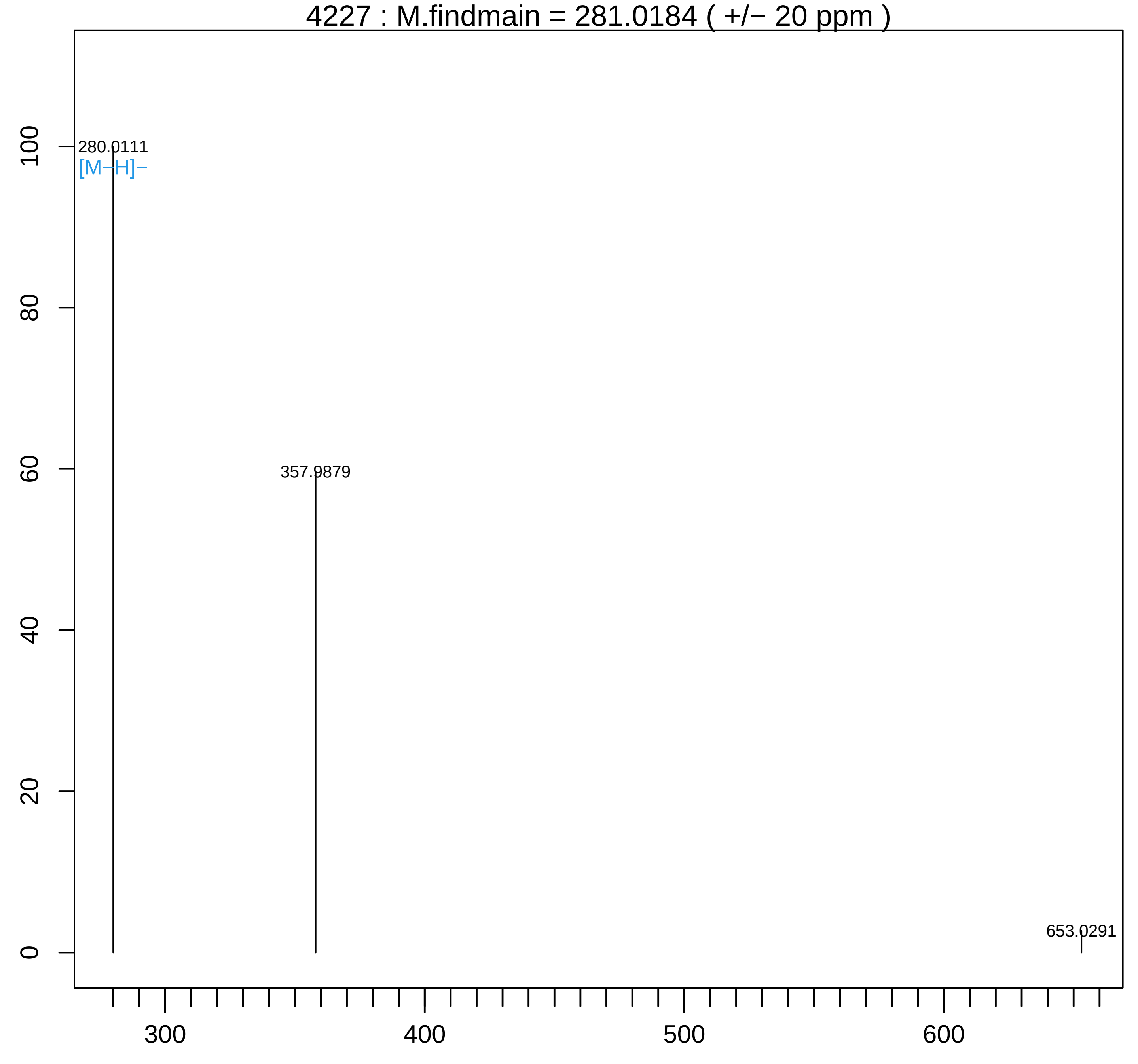

4227 : M.findmain = 281.0184 (+/− 20 ppm)
100
80
60
40
20
0
280.0111
[M−H]−
357.9879
653.0291
300
400
500
600

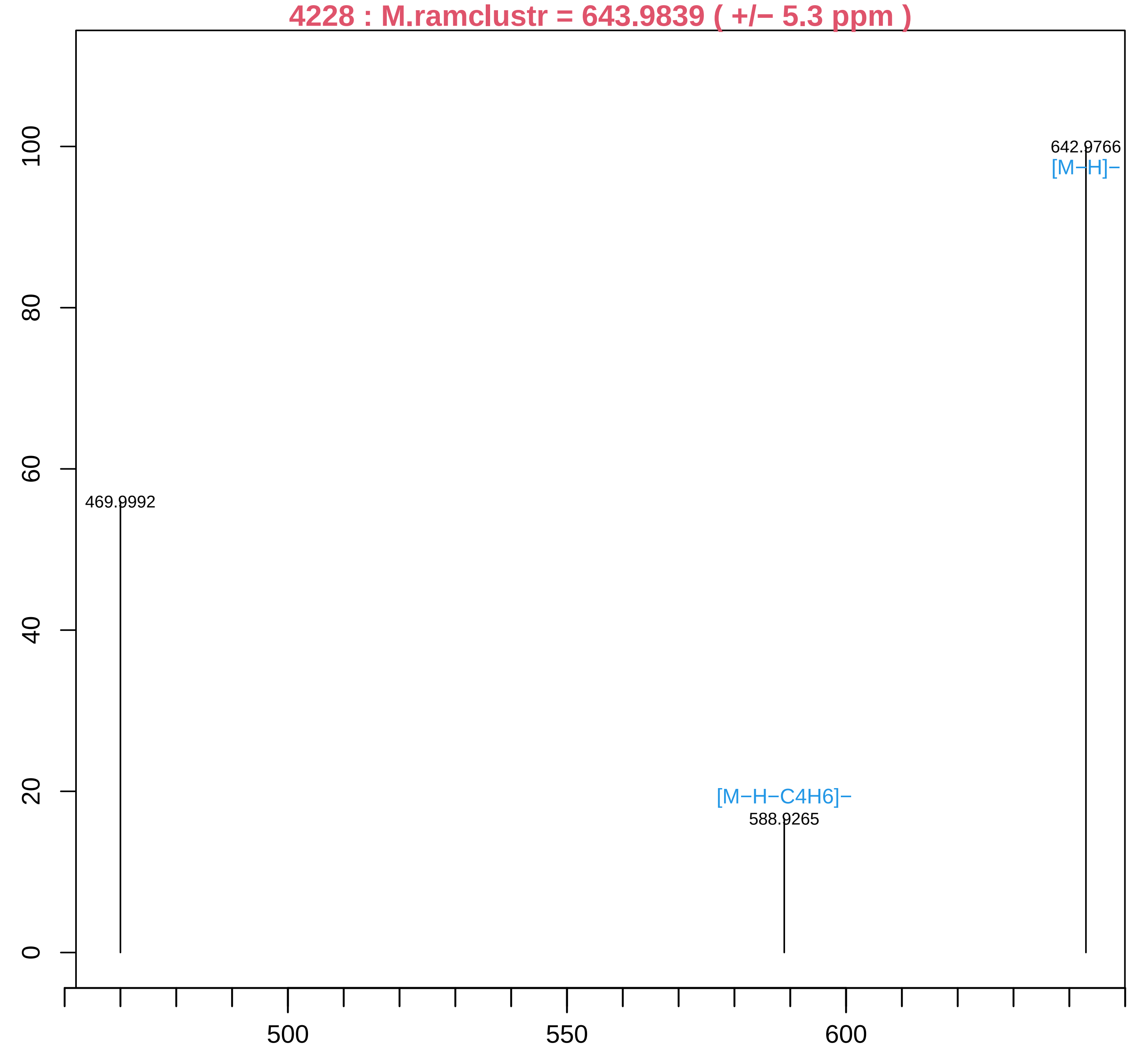

4228 : M.ramclustr = 643.9839 (+/− 5.3 ppm)
642.9766
[M−H]−
469.9992
[M−H−C4H6]−
588.9265
0
20
40
60
80
100
500
550
600

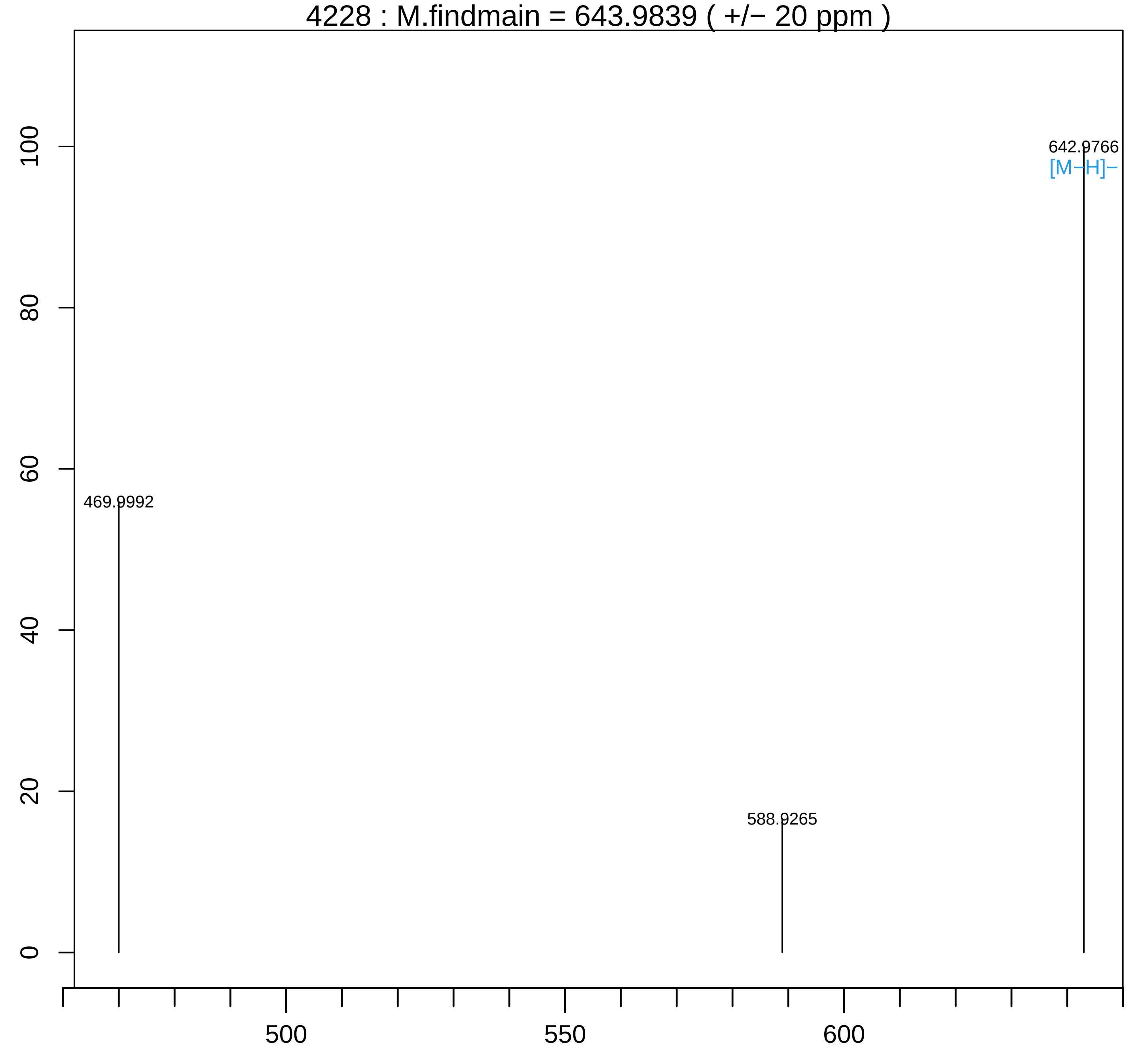

4228 : M.findmain = 643.9839 (+/− 20 ppm)
642.9766
[M−H]−
469.9992
588.9265
0
20
40
60
80
100
500
550
600

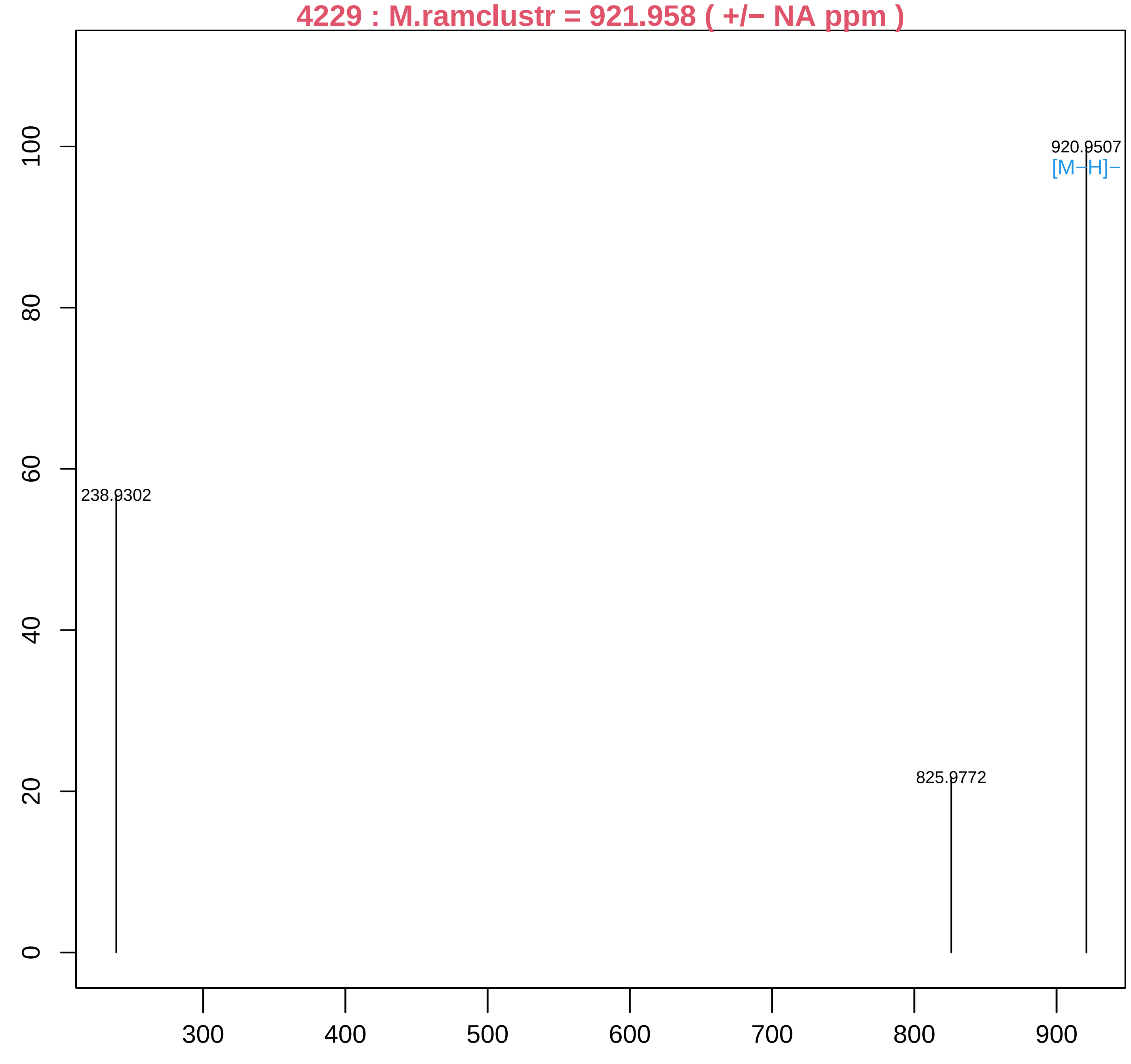
4229 : M.ramclustr = 921.958 (+/− NA ppm)
920.9507
[M−H]−
238.9302
825.9772
0
20
40
60
80
100
300
400
500
600
700
800
900

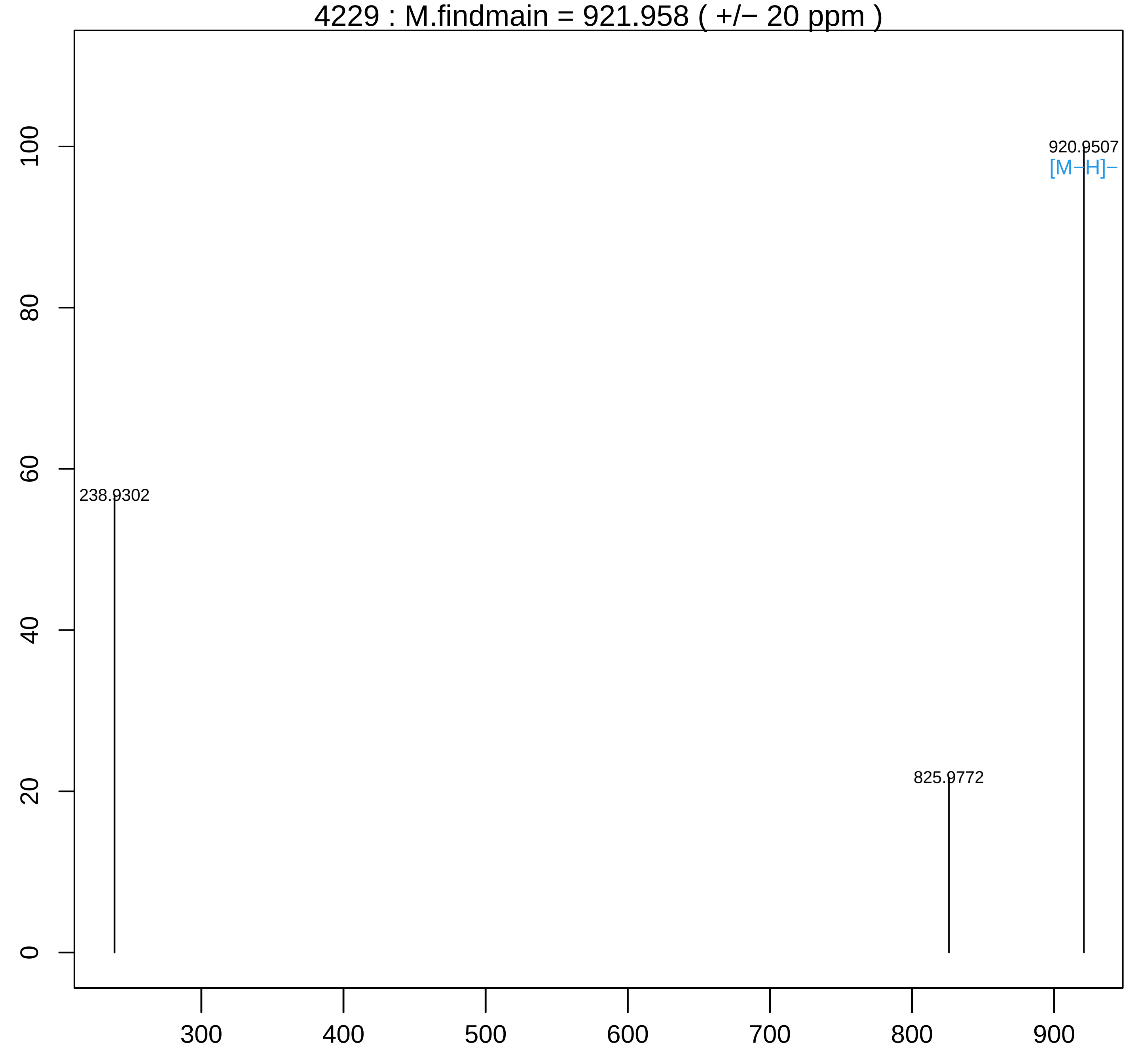
4229 : M.findmain = 921.958 (+/− 20 ppm)
920.9507
[M−H]−
238.9302
825.9772
0
20
40
60
80
100
300
400
500
600
700
800
900

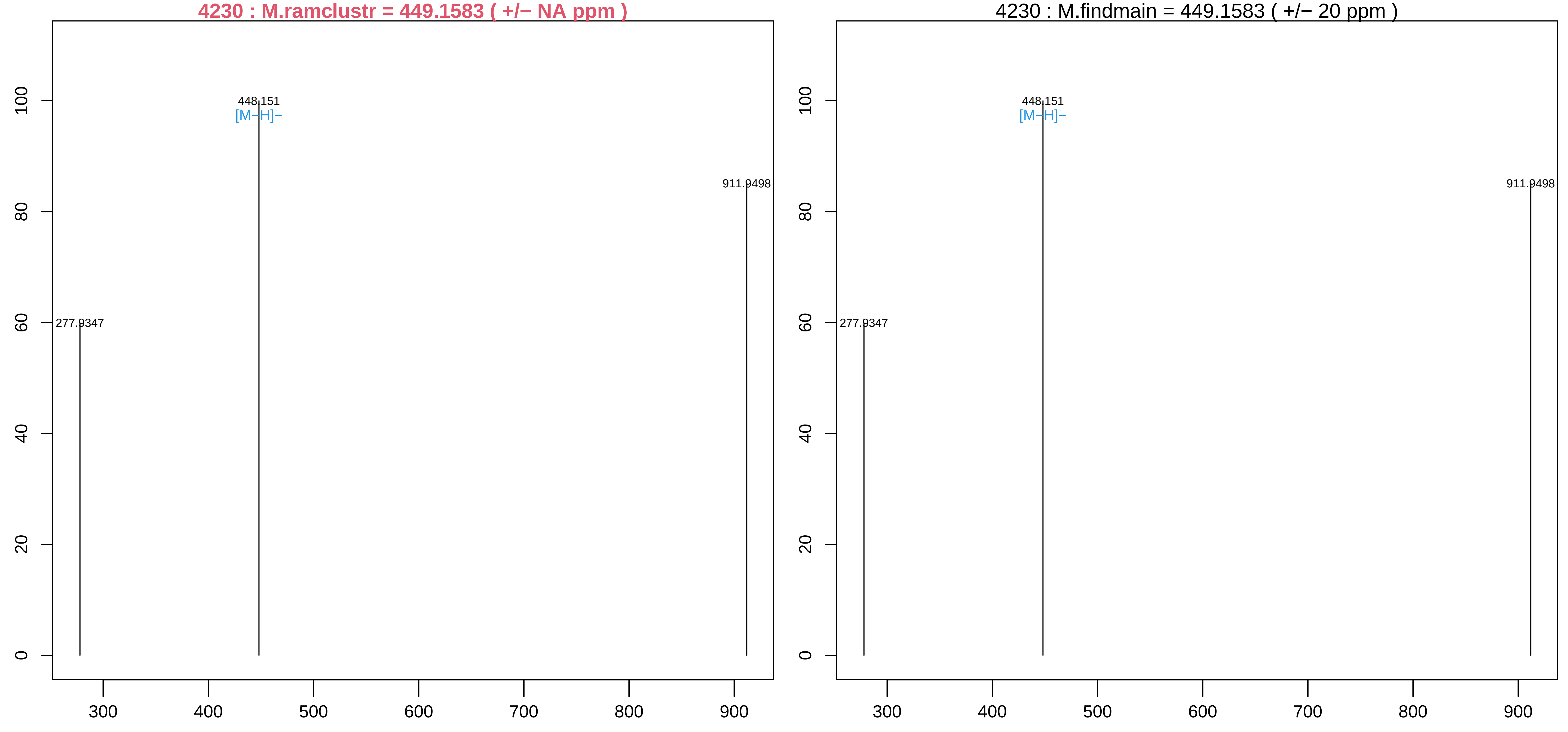

4230 : M.ramclustr = 449.1583 (+/− NA ppm)
448.151
[M−H]−
911.9498
277.9347
0
20
40
60
80
100
300
400
500
600
700
800
900
4230 : M.findmain = 449.1583 (+/− 20 ppm)
448.151
[M−H]−
911.9498
277.9347
0
20
40
60
80
100
300
400
500
600
700
800
900

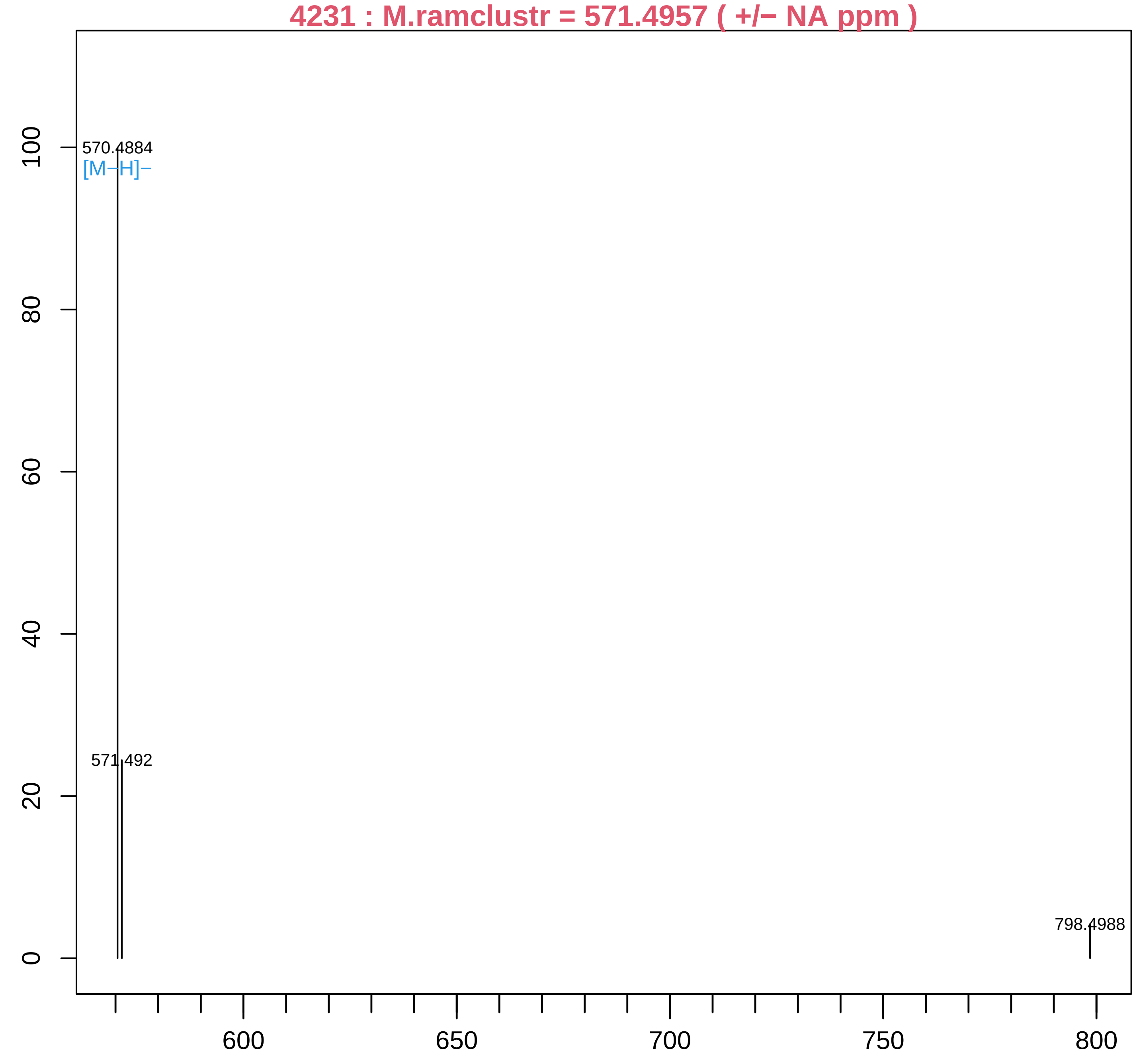

4231 : M.ramclustr = 571.4957 (+/− NA ppm)
570.4884
[M−H]−
571.492
798.4988
0
20
40
60
80
100
600
650
700
750
800

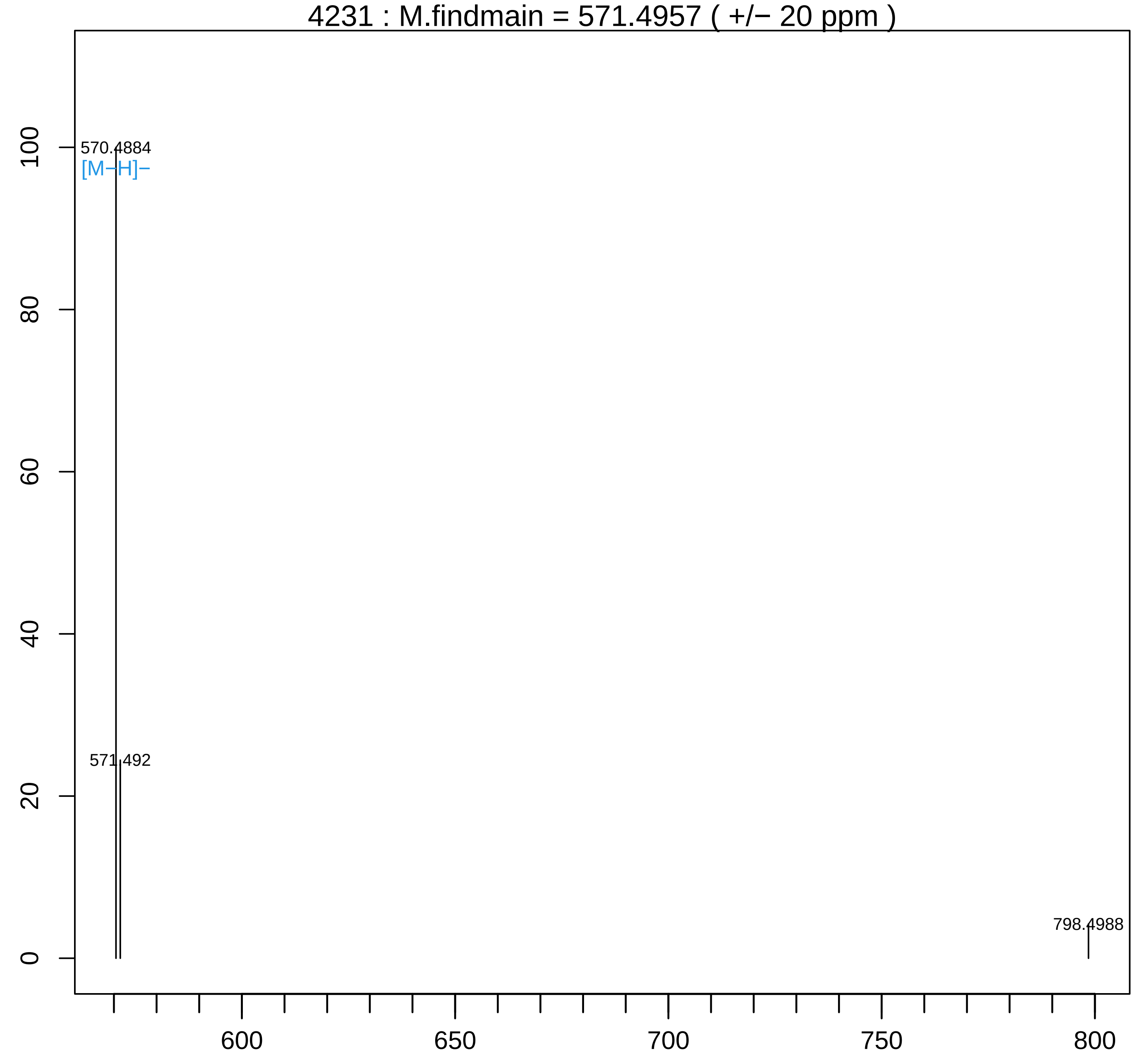

4231 : M.findmain = 571.4957 (+/− 20 ppm)
570.4884
[M−H]−
571.492
798.4988
0
20
40
60
80
100
600
650
700
750
800

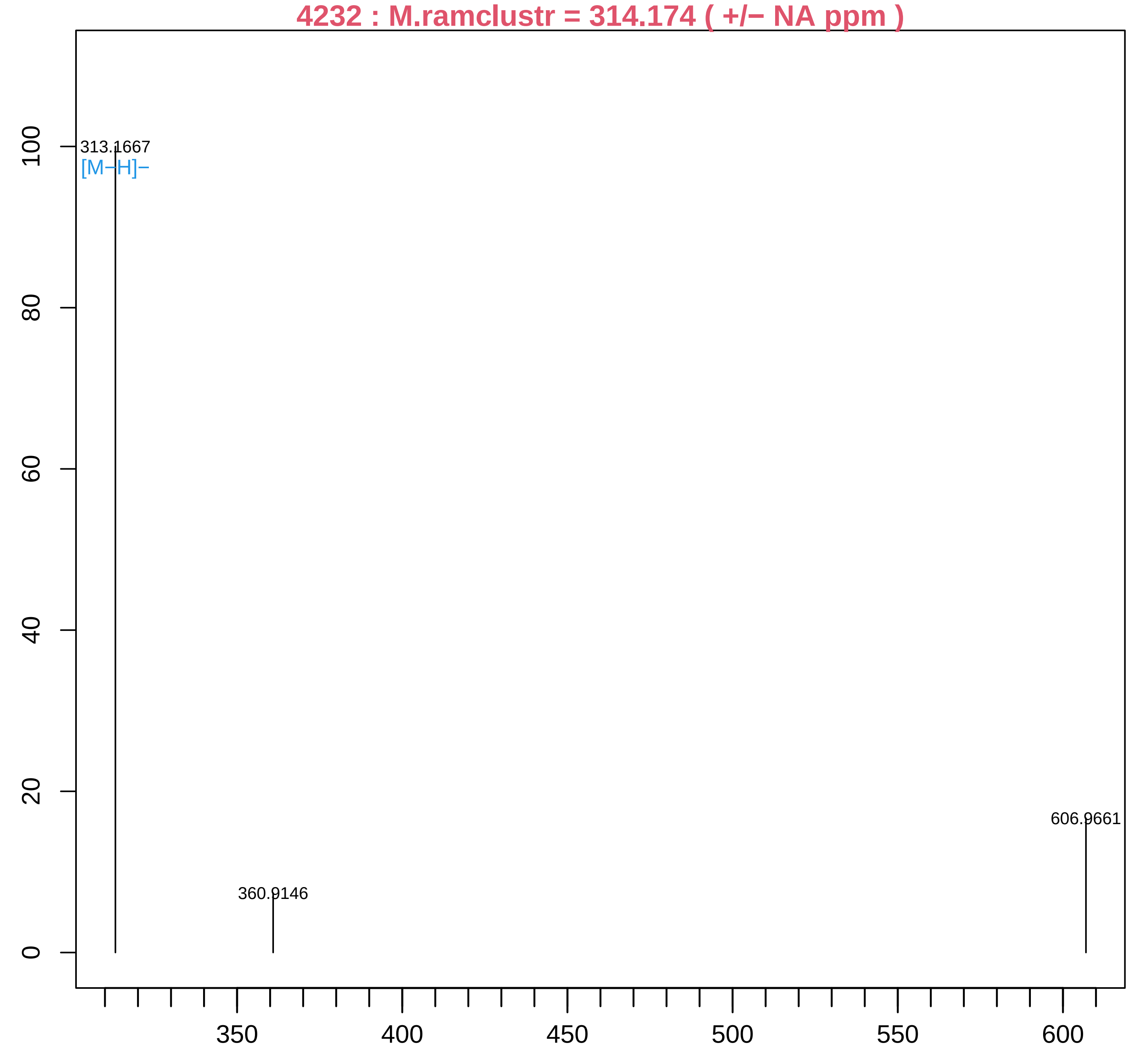
4232 : M.ramclustr = 314.174 (+/− NA ppm)
313.1667
[M−H]−
360.9146
606.9661
0
20
40
60
80
100
350
400
450
500
550
600

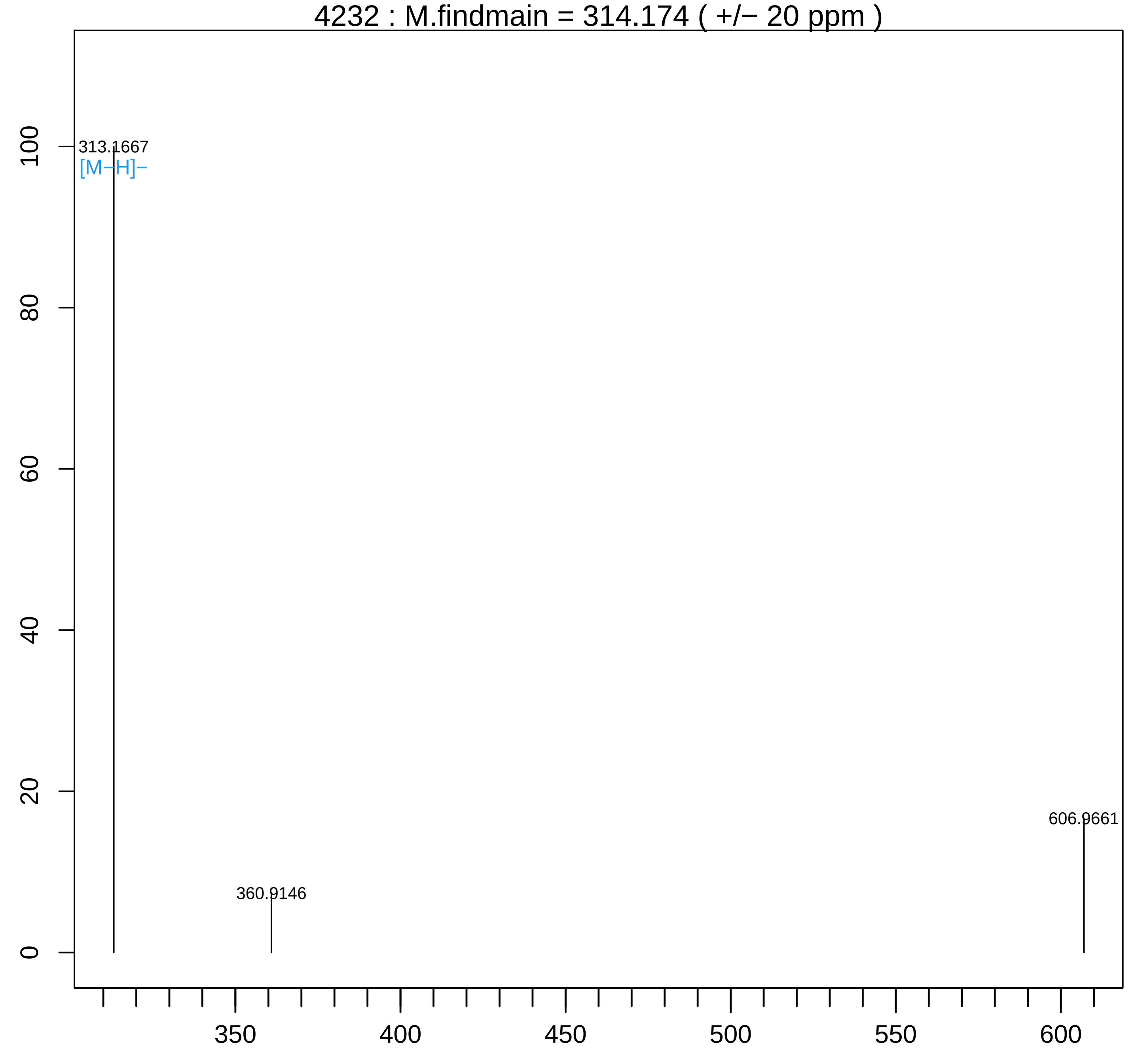
4232 : M.findmain = 314.174 (+/− 20 ppm)
313.1667
[M−H]−
360.9146
606.9661
0
20
40
60
80
100
350
400
450
500
550
600

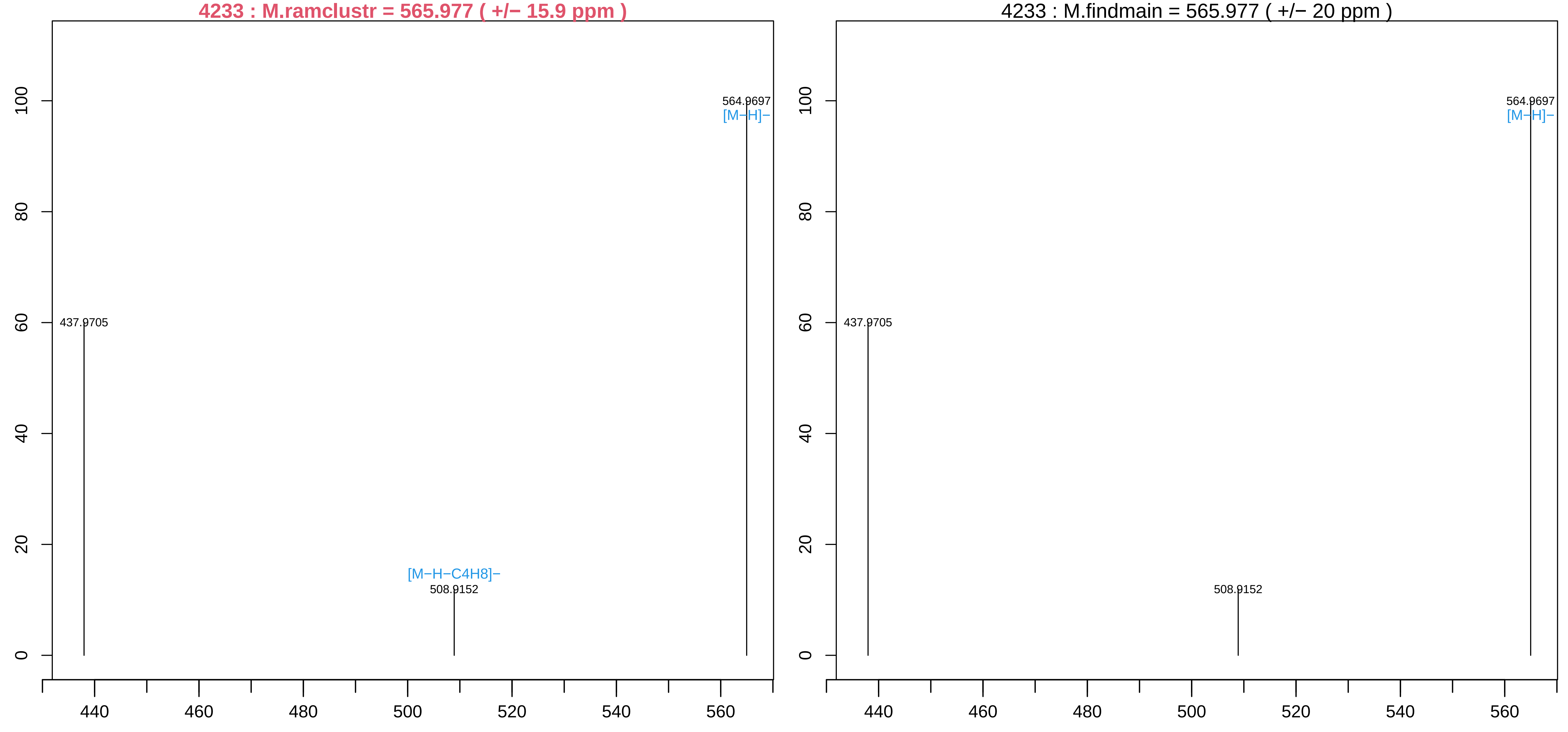

4233 : M.ramclustr = 565.977 (+/− 15.9 ppm)
564.9697
[M−H]−
437.9705
[M−H−C4H8]−
508.9152
4233 : M.findmain = 565.977 (+/− 20 ppm)
564.9697
[M−H]−
437.9705
508.9152

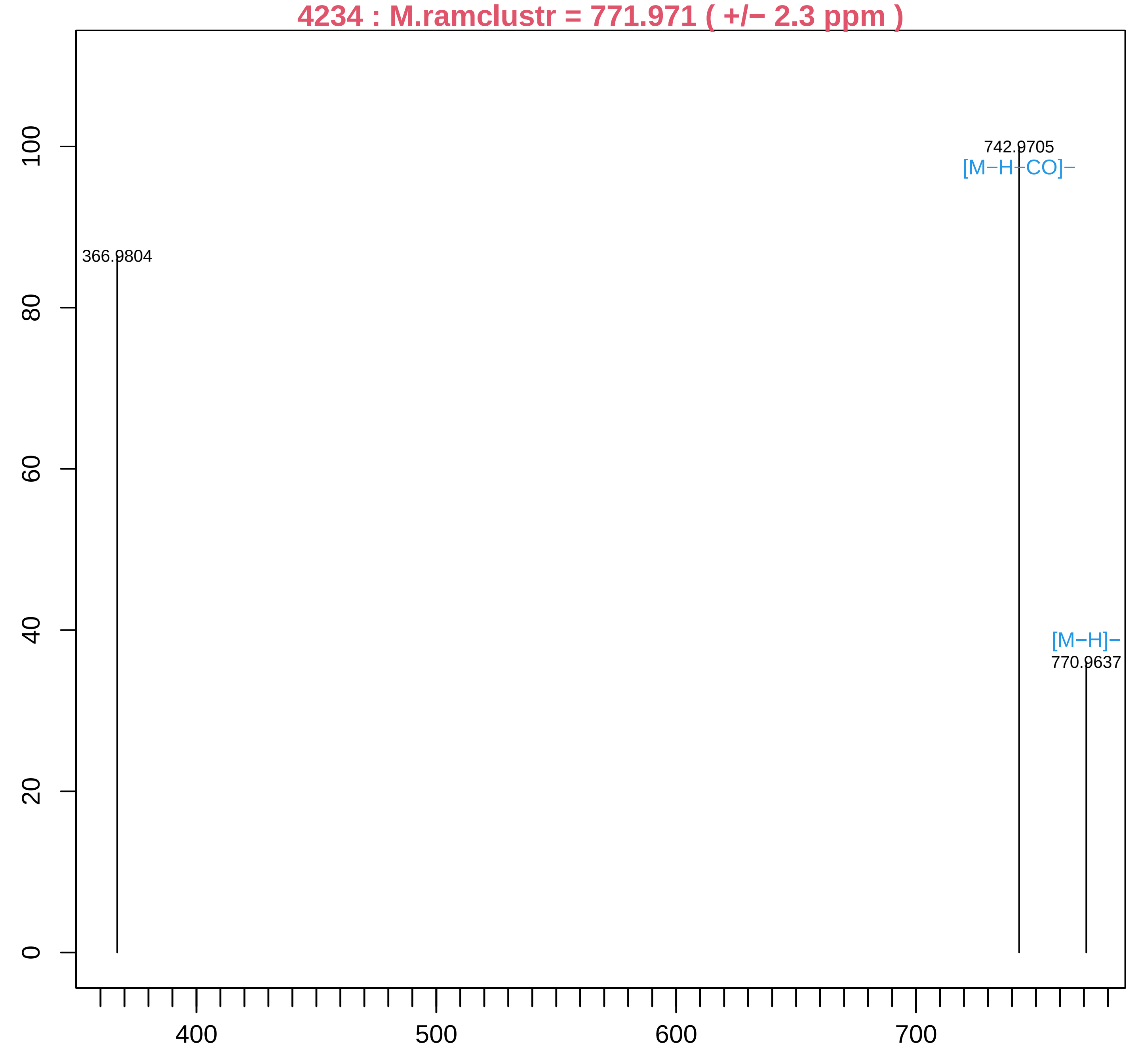
4234 : M.ramclustr = 771.971 (+/− 2.3 ppm)
366.9804
742.9705
[M−H−CO]−
[M−H]−
770.9637
0
20
40
60
80
100
400
500
600
700

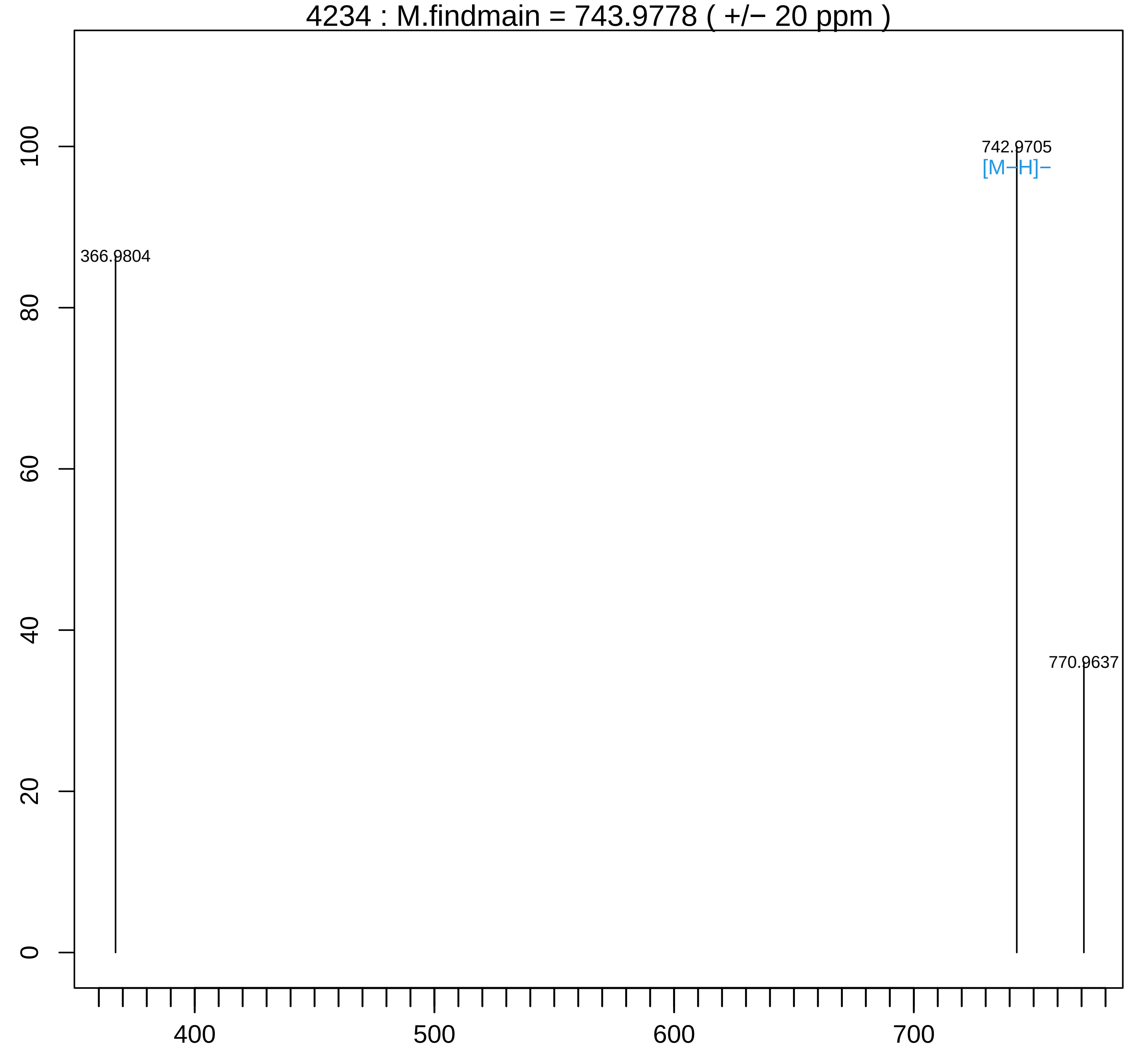
4234 : M.findmain = 743.9778 (+/− 20 ppm)
366.9804
742.9705
[M−H]−
770.9637
0
20
40
60
80
100
400
500
600
700

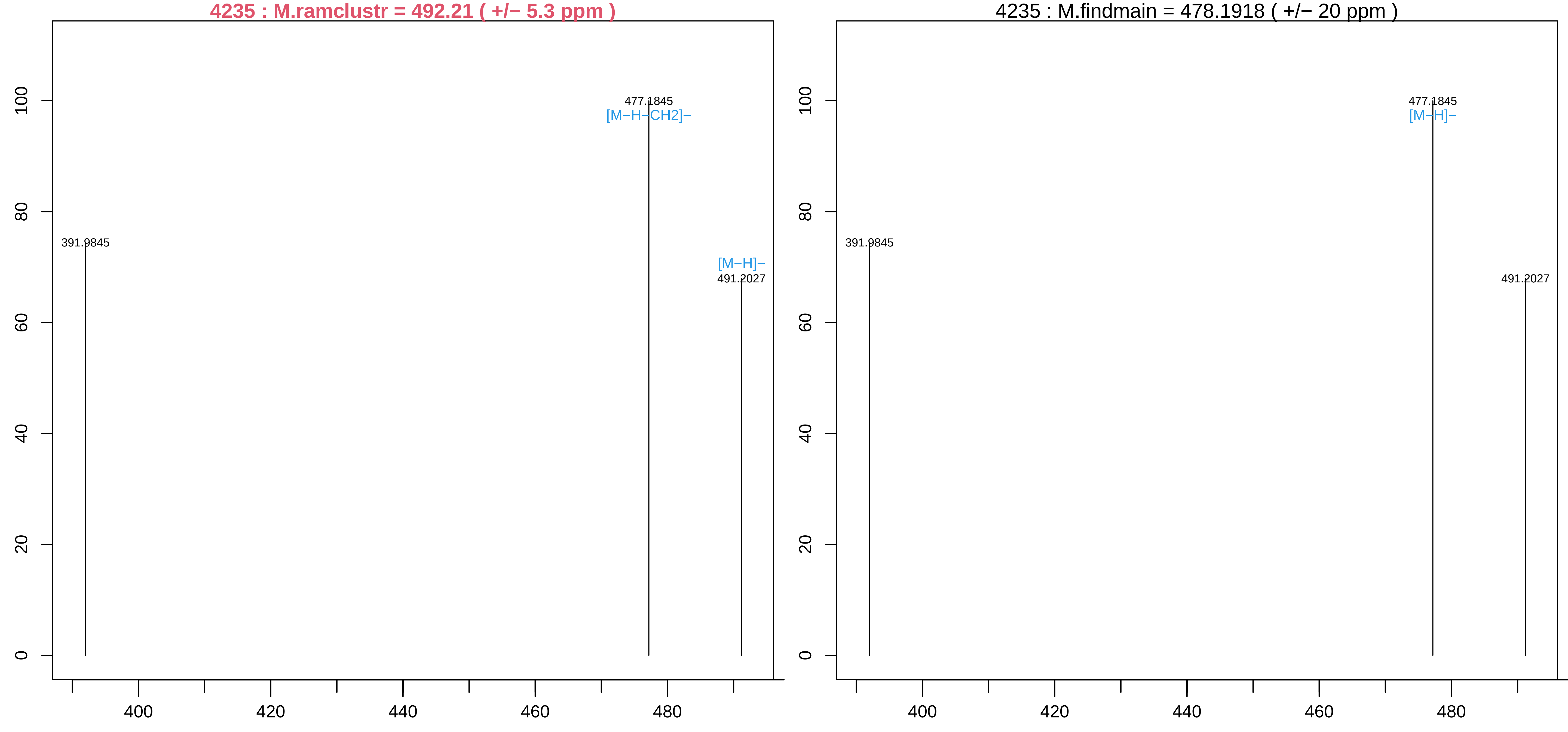

4235 : M.ramclustr = 492.21 (+/− 5.3 ppm)
477.1845
[M−H−CH2]−
391.9845
[M−H]−
491.2027
0
20
40
60
80
100
400
420
440
460
480
4235 : M.findmain = 478.1918 (+/− 20 ppm)
477.1845
[M−H]−
391.9845
491.2027
0
20
40
60
80
100
400
420
440
460
480

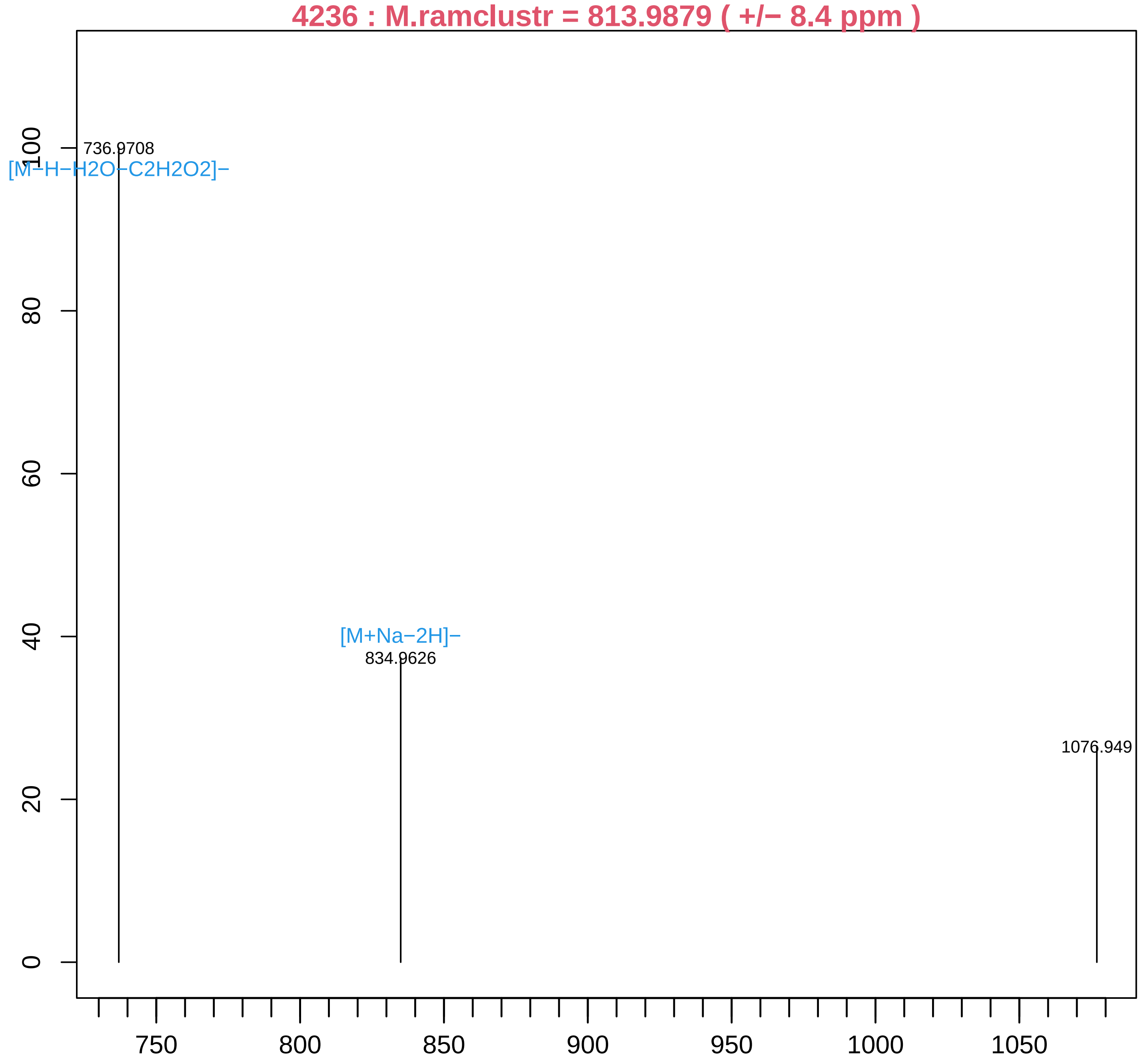
4236 : M.ramclustr = 813.9879 (+/− 8.4 ppm)
736.9708
[M−H−H2O−C2H2O2]−
[M+Na−2H]−
834.9626
1076.949
0
20
40
60
80
100
750
800
850
900
950
1000
1050

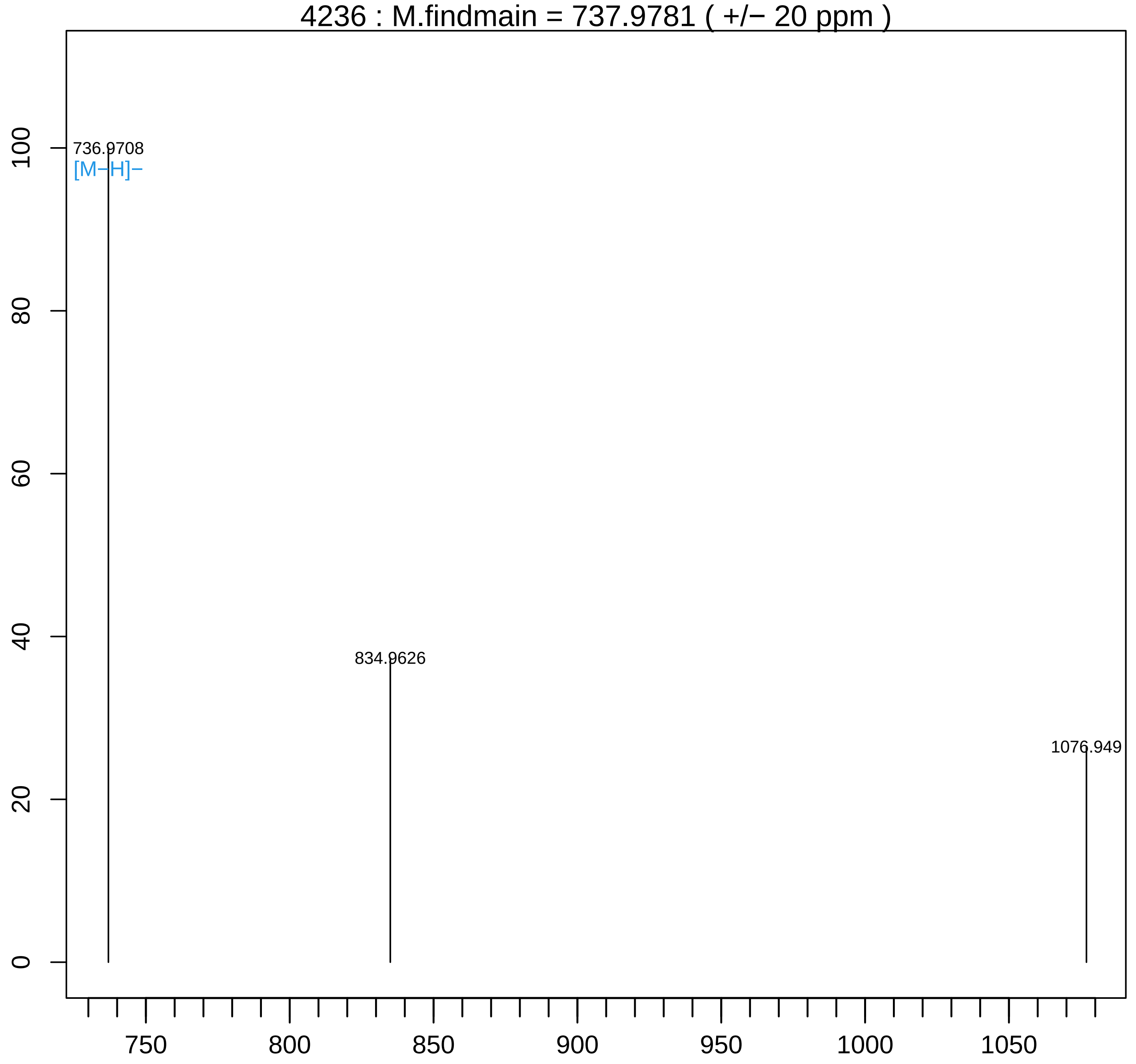
4236 : M.findmain = 737.9781 (+/− 20 ppm)
736.9708
[M−H]−
834.9626
1076.949
0
20
40
60
80
100
750
800
850
900
950
1000
1050

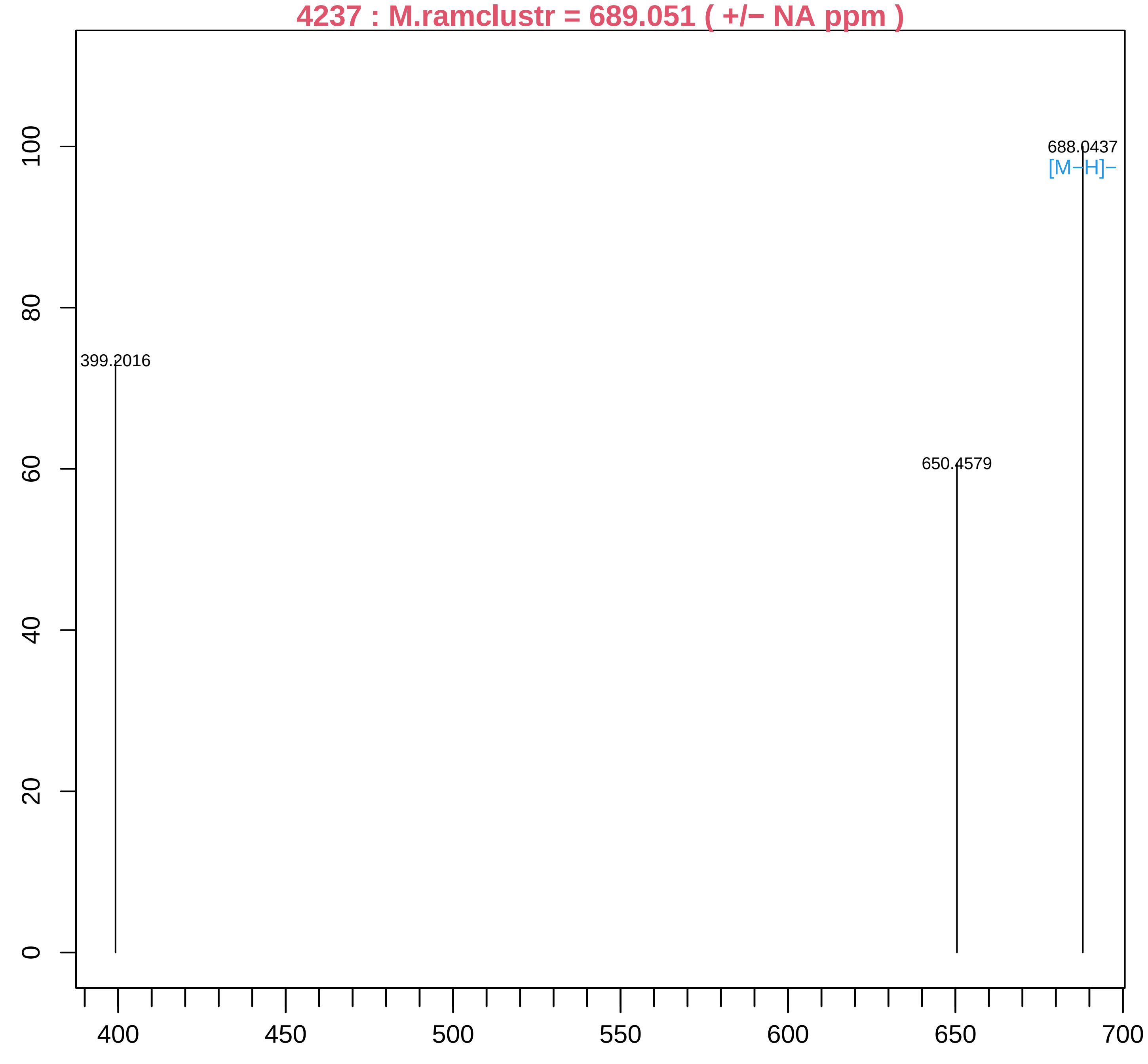

4237 : M.ramclustr = 689.051 (+/− NA ppm)
688.0437
[M−H]−
399.2016
650.4579
0
20
40
60
80
100
400
450
500
550
600
650
700

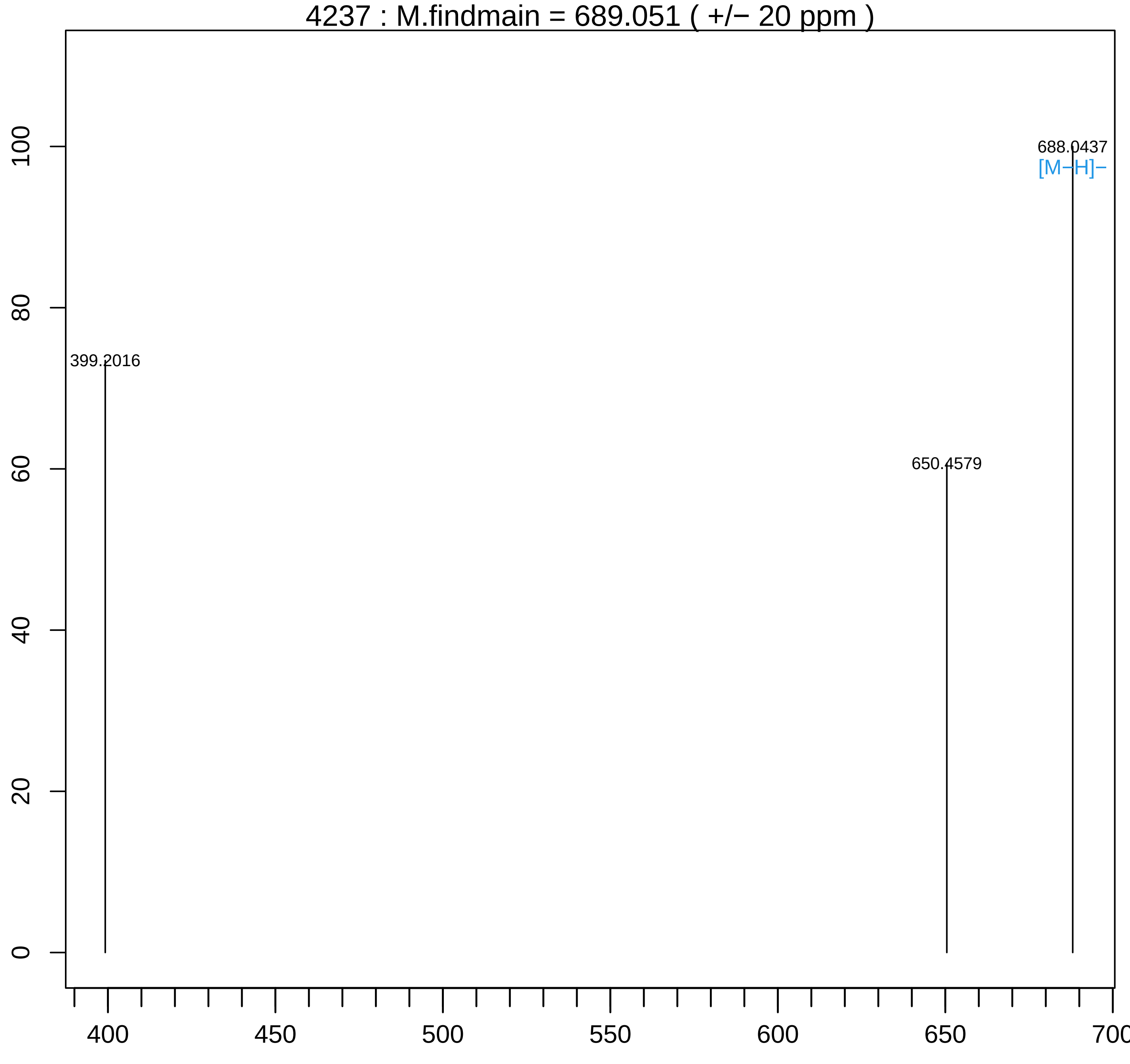

4237 : M.findmain = 689.051 (+/− 20 ppm)
688.0437
[M−H]−
399.2016
650.4579
0
20
40
60
80
100
400
450
500
550
600
650
700

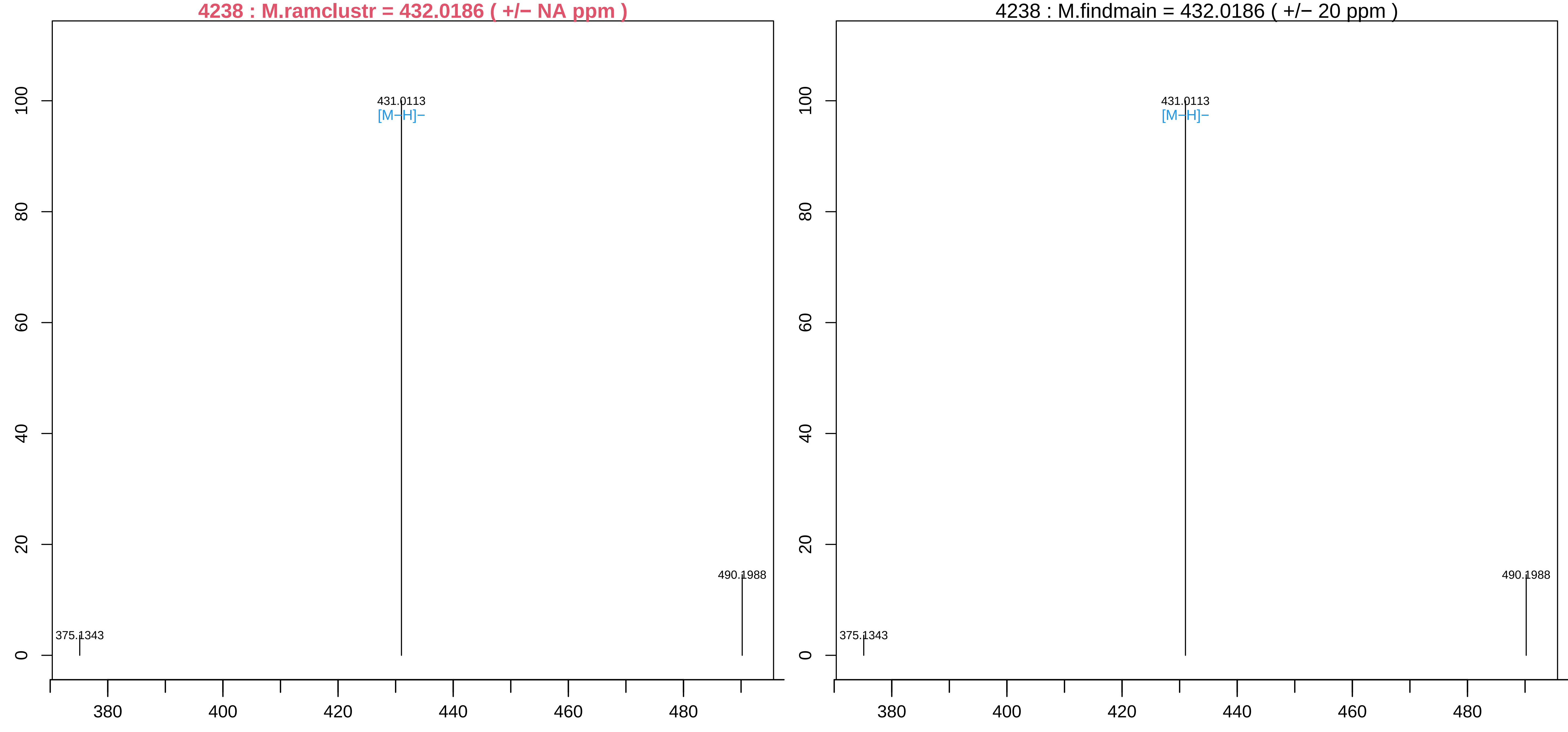
4238 : M.ramclustr = 432.0186 (+/− NA ppm)
431.0113
[M−H]−
490.1988
375.1343
4238 : M.findmain = 432.0186 (+/− 20 ppm)
431.0113
[M−H]−
490.1988
375.1343
0
20
40
60
80
100
380
400
420
440
460
480

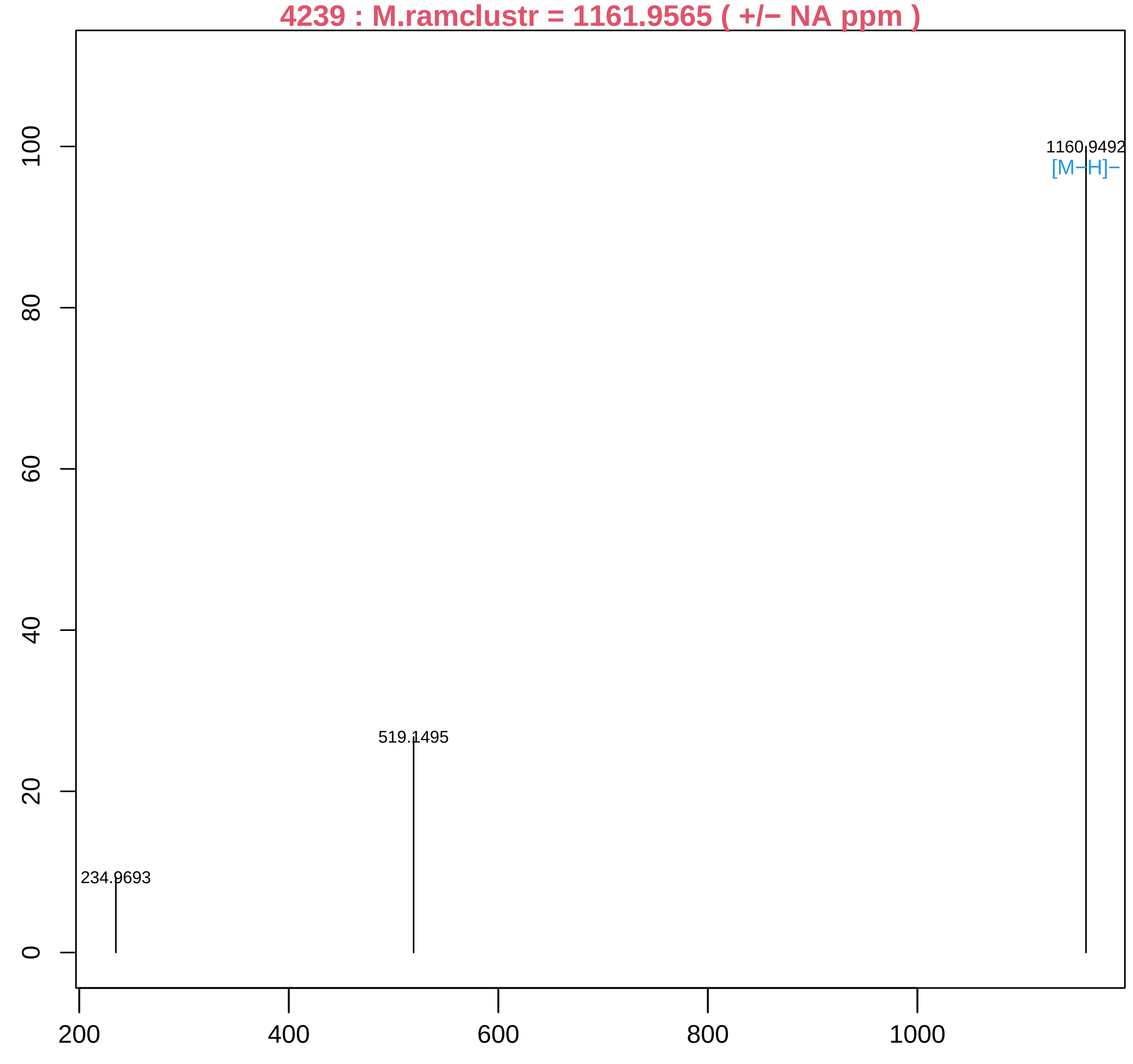
4239 : M.ramclustr = 1161.9565 (+/− NA ppm)
1160.9492
[M−H]−
519.1495
234.9693
0
20
40
60
80
100
200
400
600
800
1000
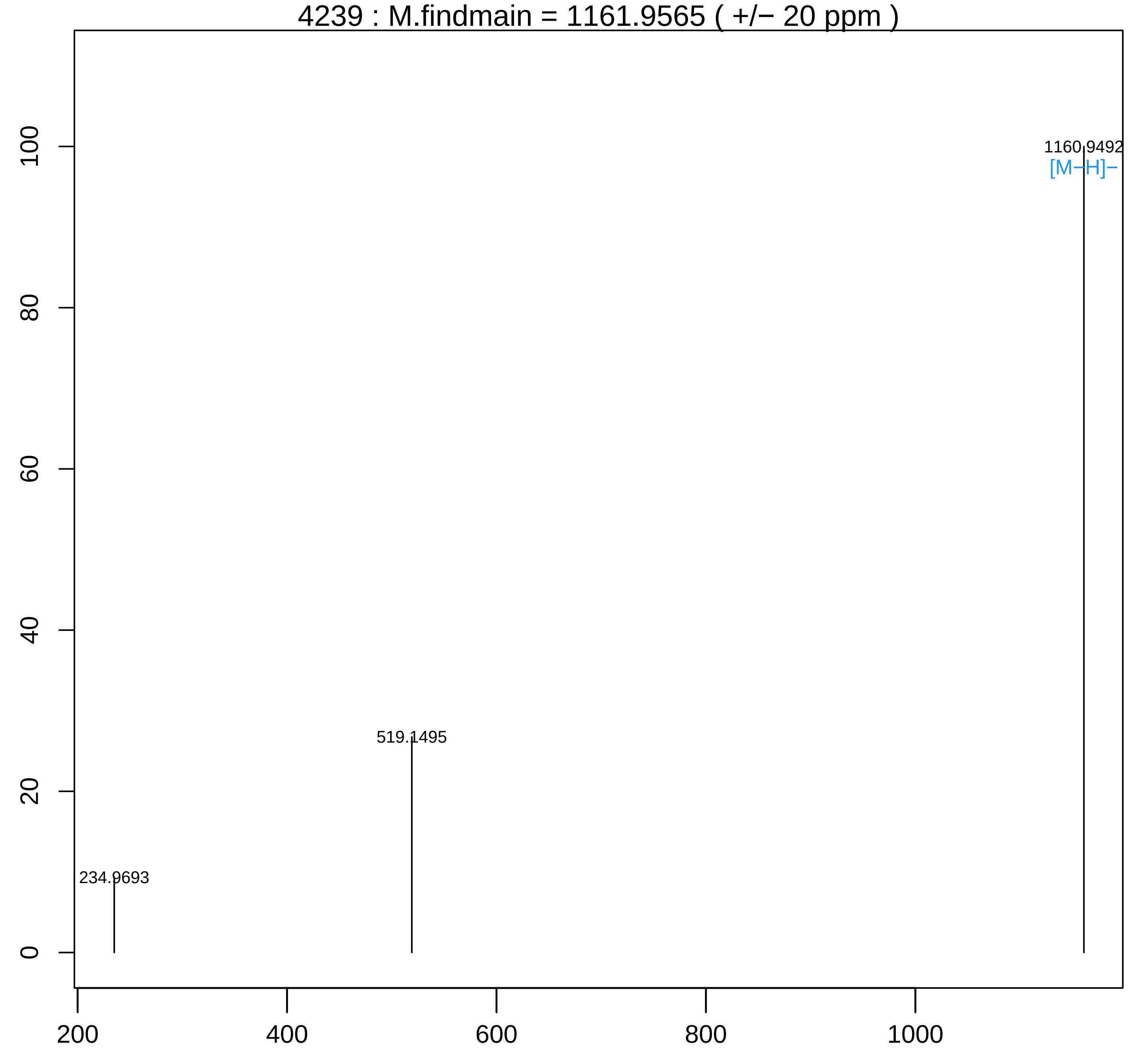
4239 : M.findmain = 1161.9565 (+/− 20 ppm)
1160.9492
[M−H]−
519.1495
234.9693
0
20
40
60
80
100
200
400
600
800
1000

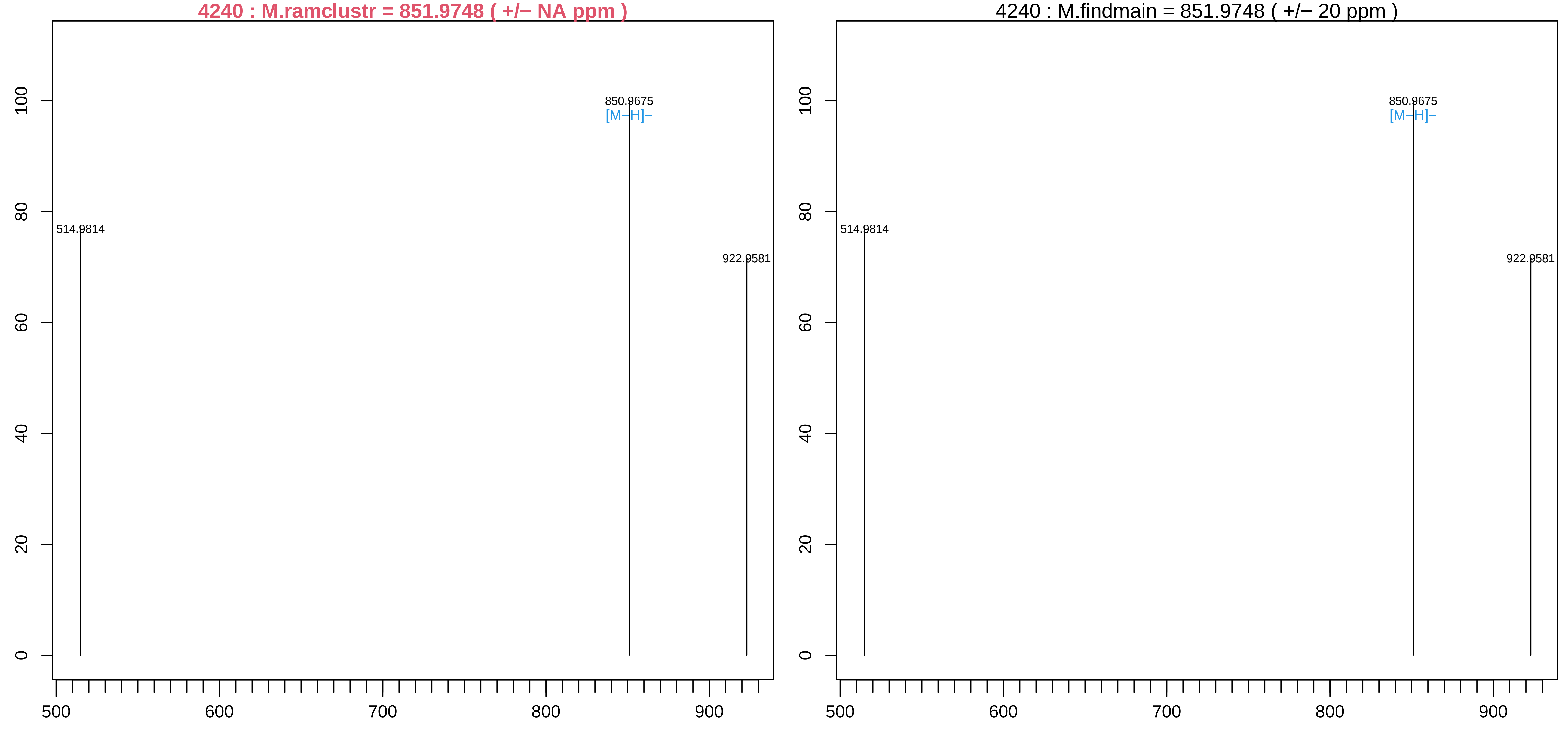
4240 : M.ramclustr = 851.9748 (+/− NA ppm)
850.9675
[M−H]−
514.9814
922.9581
4240 : M.findmain = 851.9748 (+/− 20 ppm)
850.9675
[M−H]−
514.9814
922.9581
0
20
40
60
80
100
500
600
700
800
900

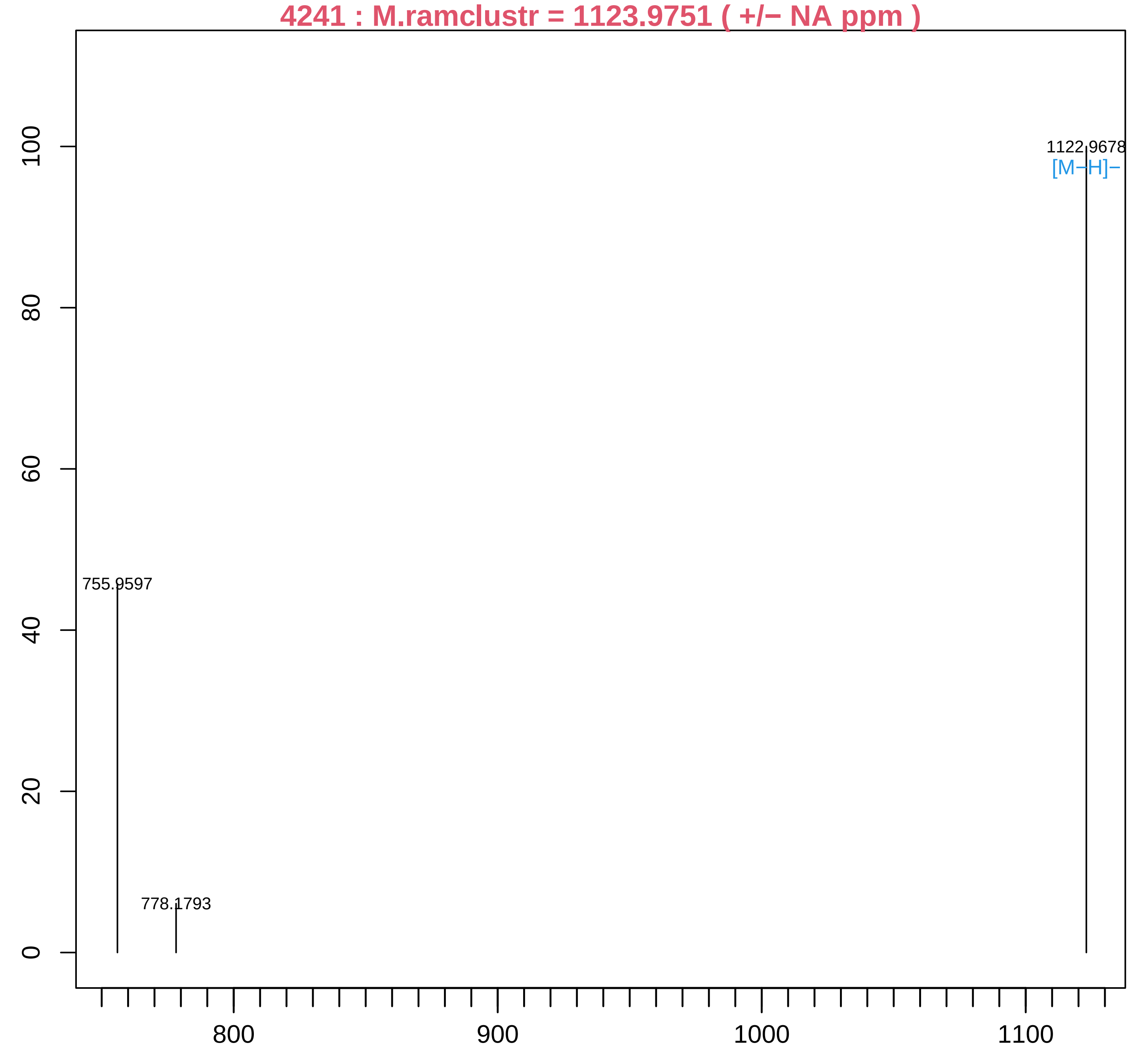
4241 : M.ramclustr = 1123.9751 (+/− NA ppm)
1122.9678
[M−H]−
755.9597
778.1793
0
20
40
60
80
100
800
900
1000
1100

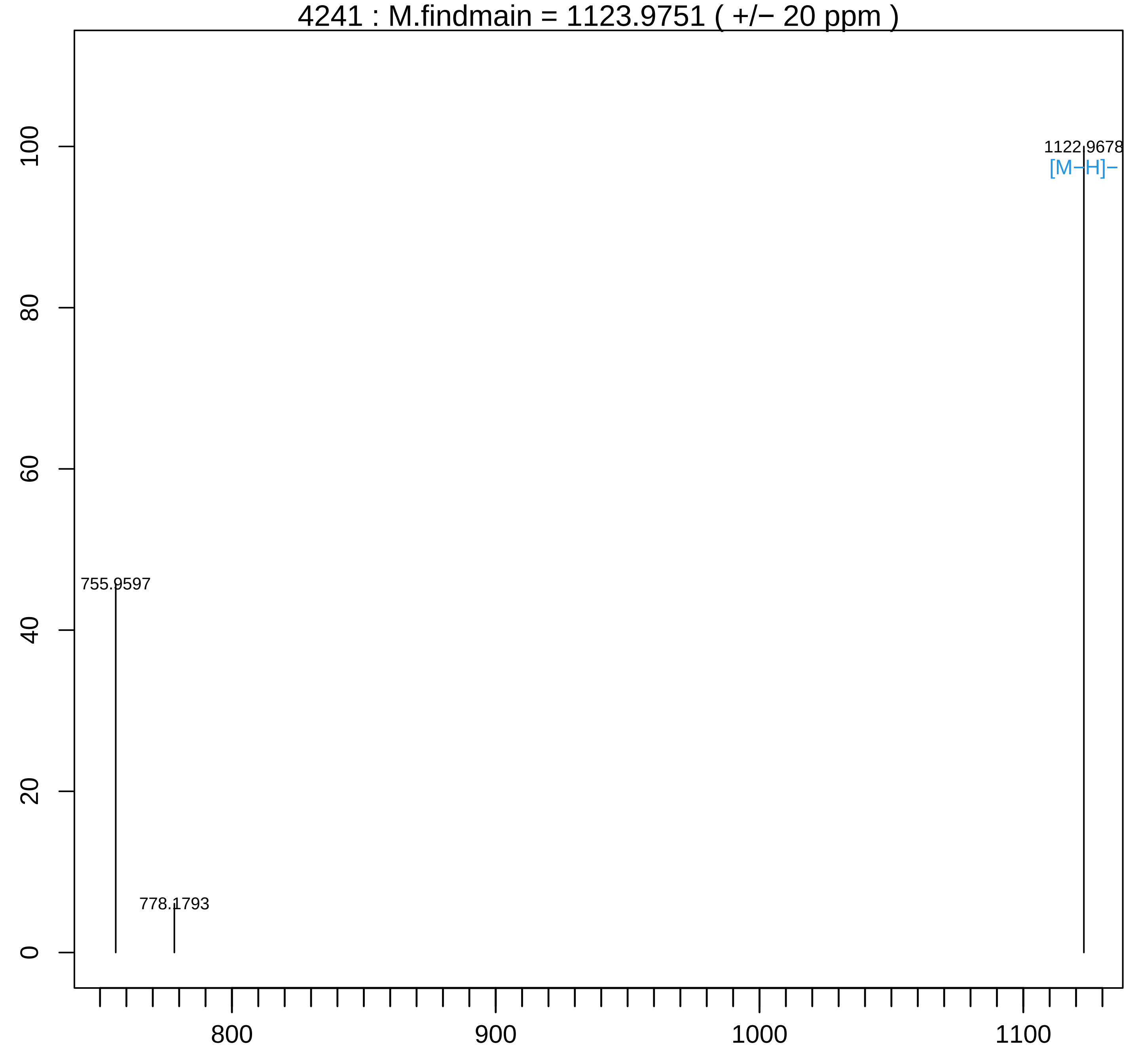
4241 : M.findmain = 1123.9751 (+/− 20 ppm)
1122.9678
[M−H]−
755.9597
778.1793
0
20
40
60
80
100
800
900
1000
1100

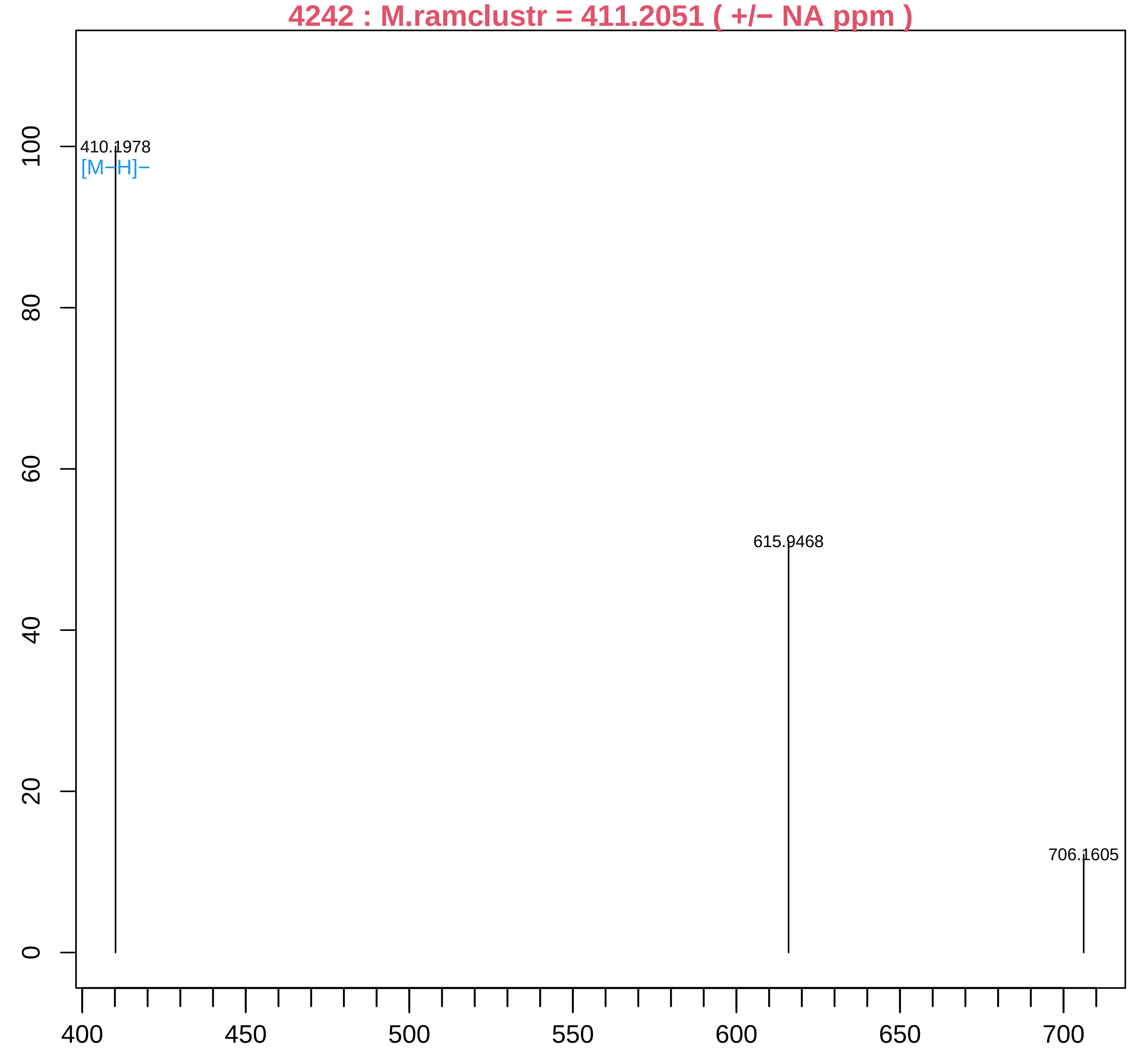
4242 : M.ramclustr = 411.2051 (+/− NA ppm)
410.1978
[M−H]−
615.9468
706.1605
0
20
40
60
80
100
400
450
500
550
600
650
700

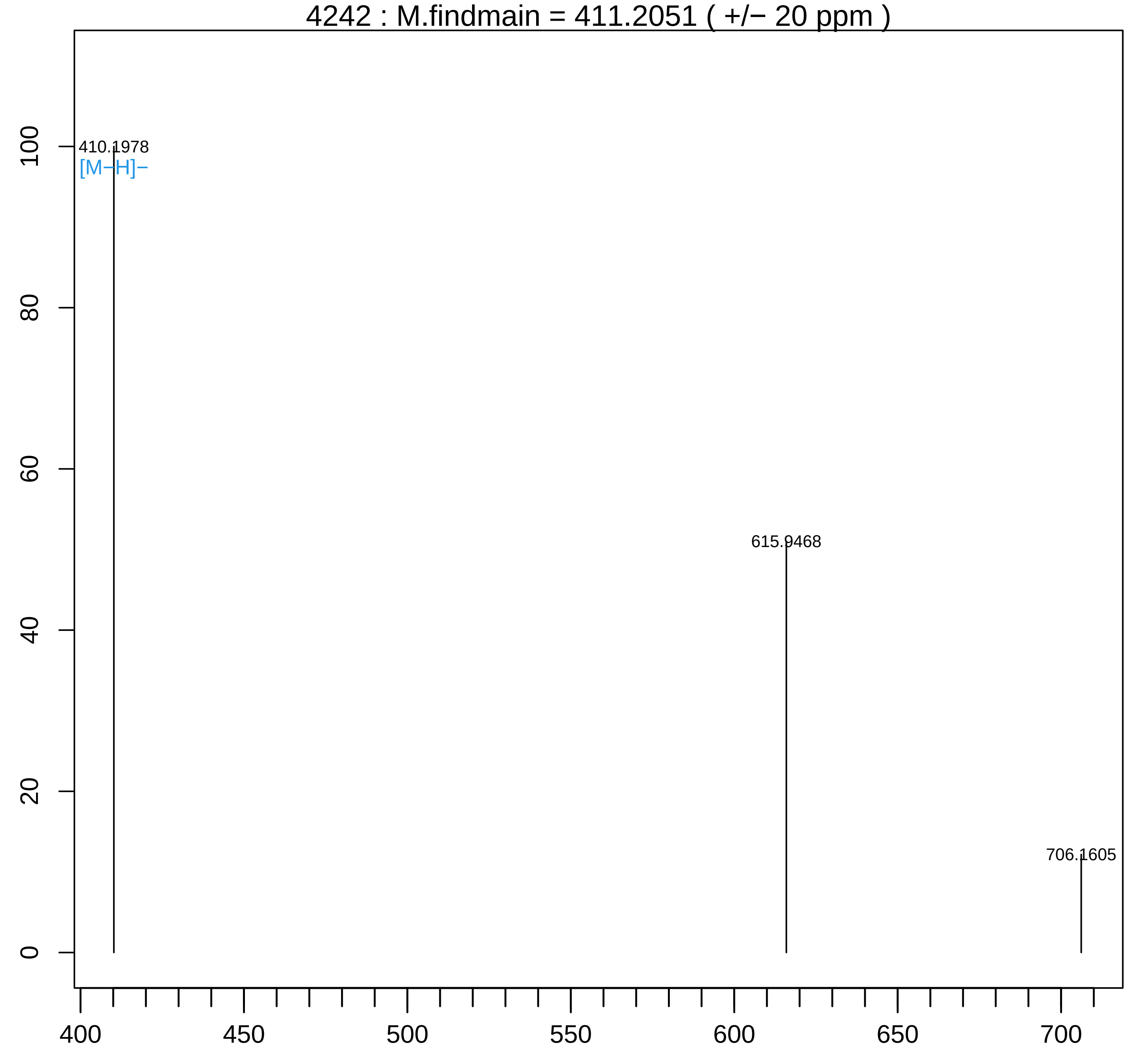
4242 : M.findmain = 411.2051 (+/− 20 ppm)
410.1978
[M−H]−
615.9468
706.1605
0
20
40
60
80
100
400
450
500
550
600
650
700

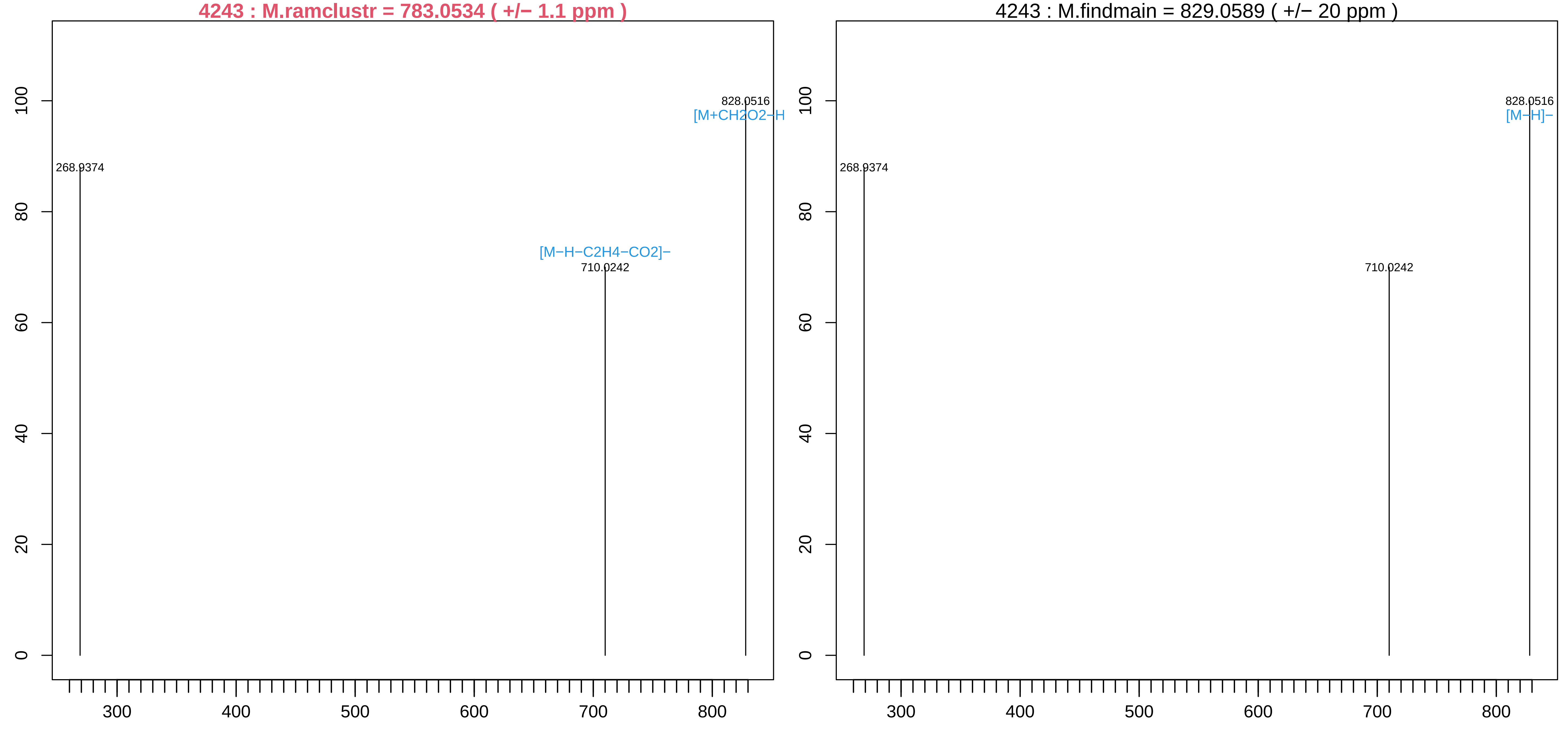

4243 : M.ramclustr = 783.0534 (+/− 1.1 ppm)
828.0516
[M+CH2O2−H
268.9374
[M−H−C2H4−CO2]−
710.0242
0
20
40
60
80
100
300
400
500
600
700
800
4243 : M.findmain = 829.0589 (+/− 20 ppm)
828.0516
[M−H]−
268.9374
710.0242

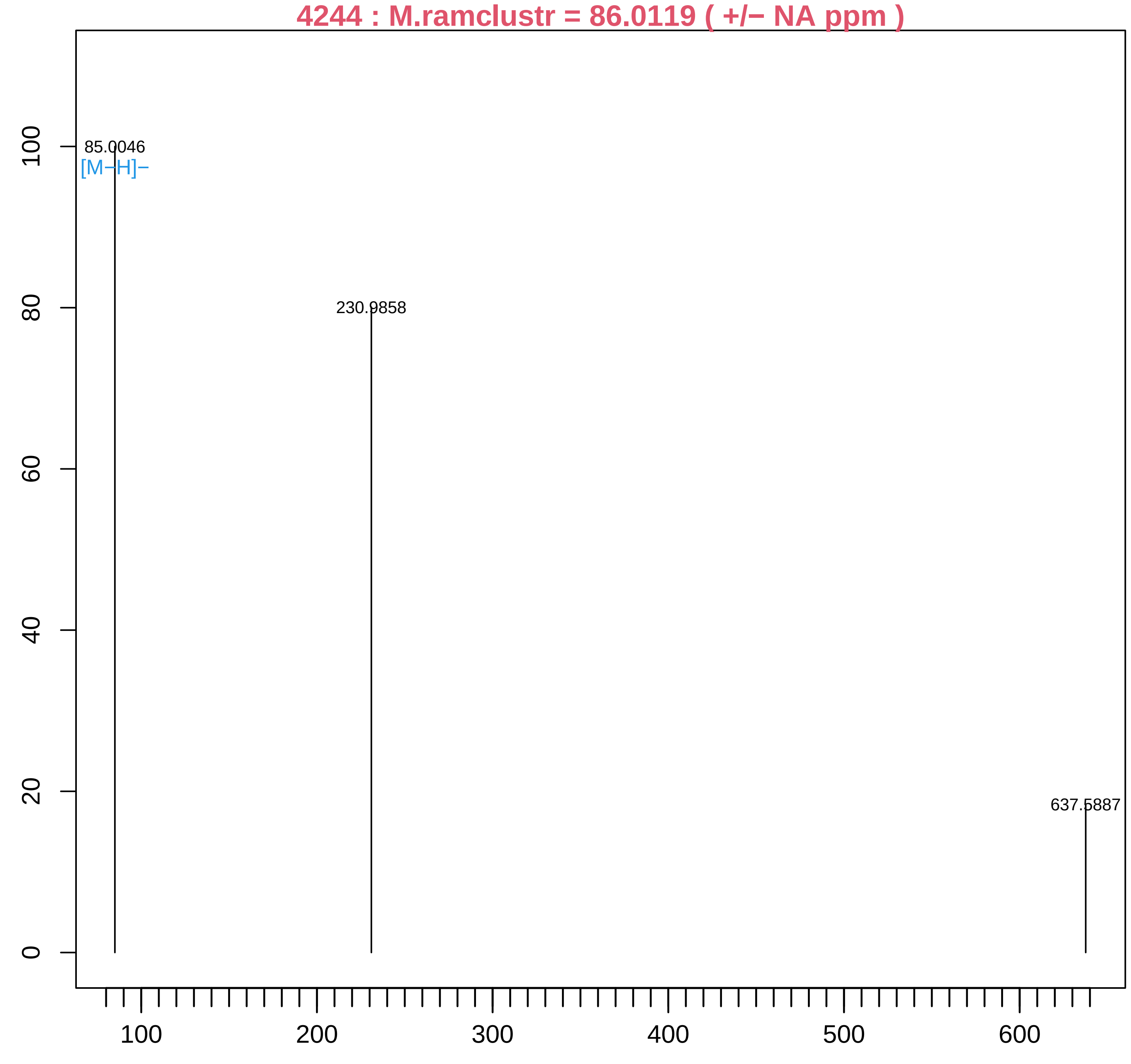
4244 : M.ramclustr = 86.0119 (+/− NA ppm)
85.0046
[M−H]−
230.9858
637.5887

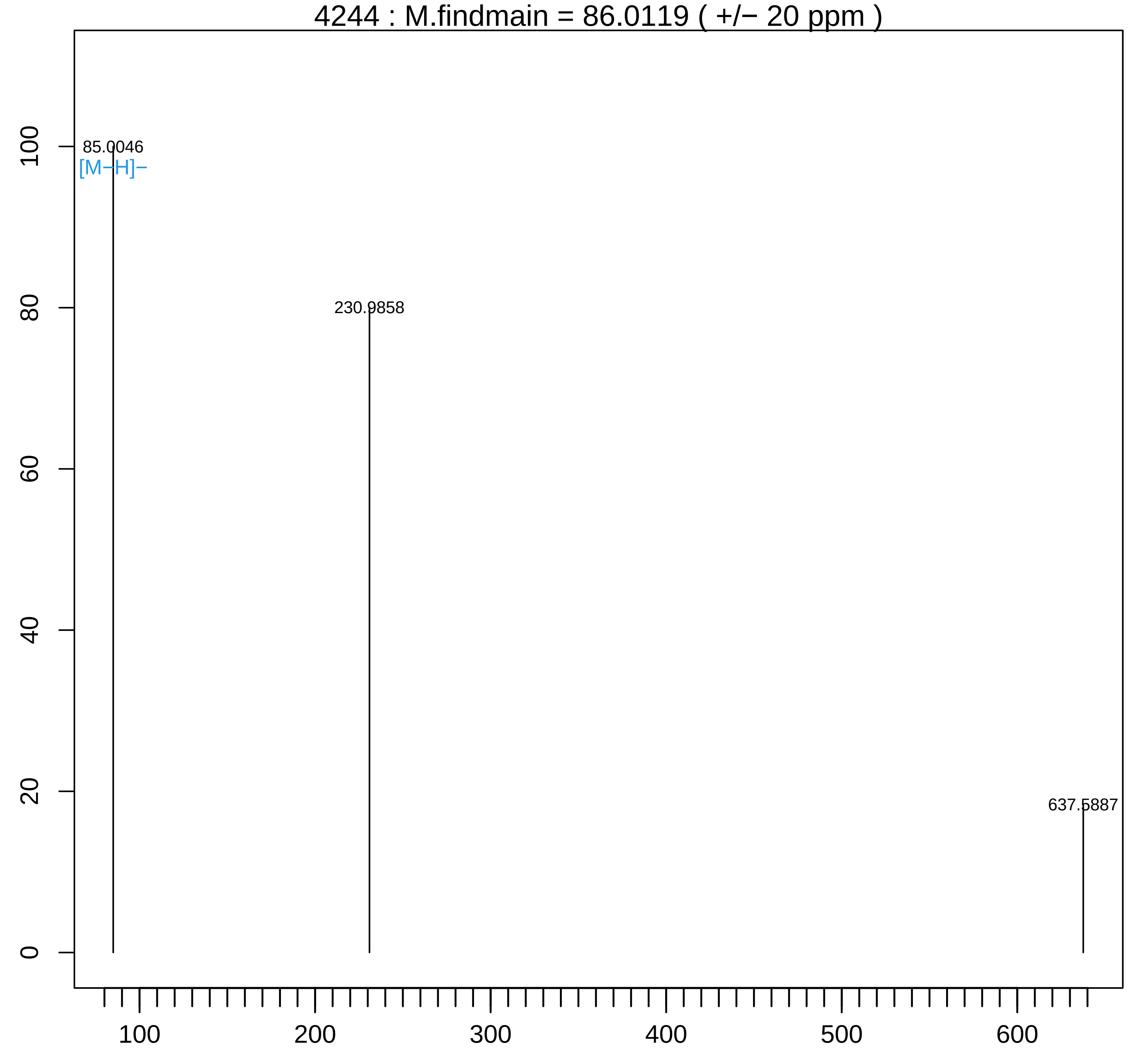
4244 : M.findmain = 86.0119 (+/− 20 ppm)
85.0046
[M−H]−
230.9858
637.5887

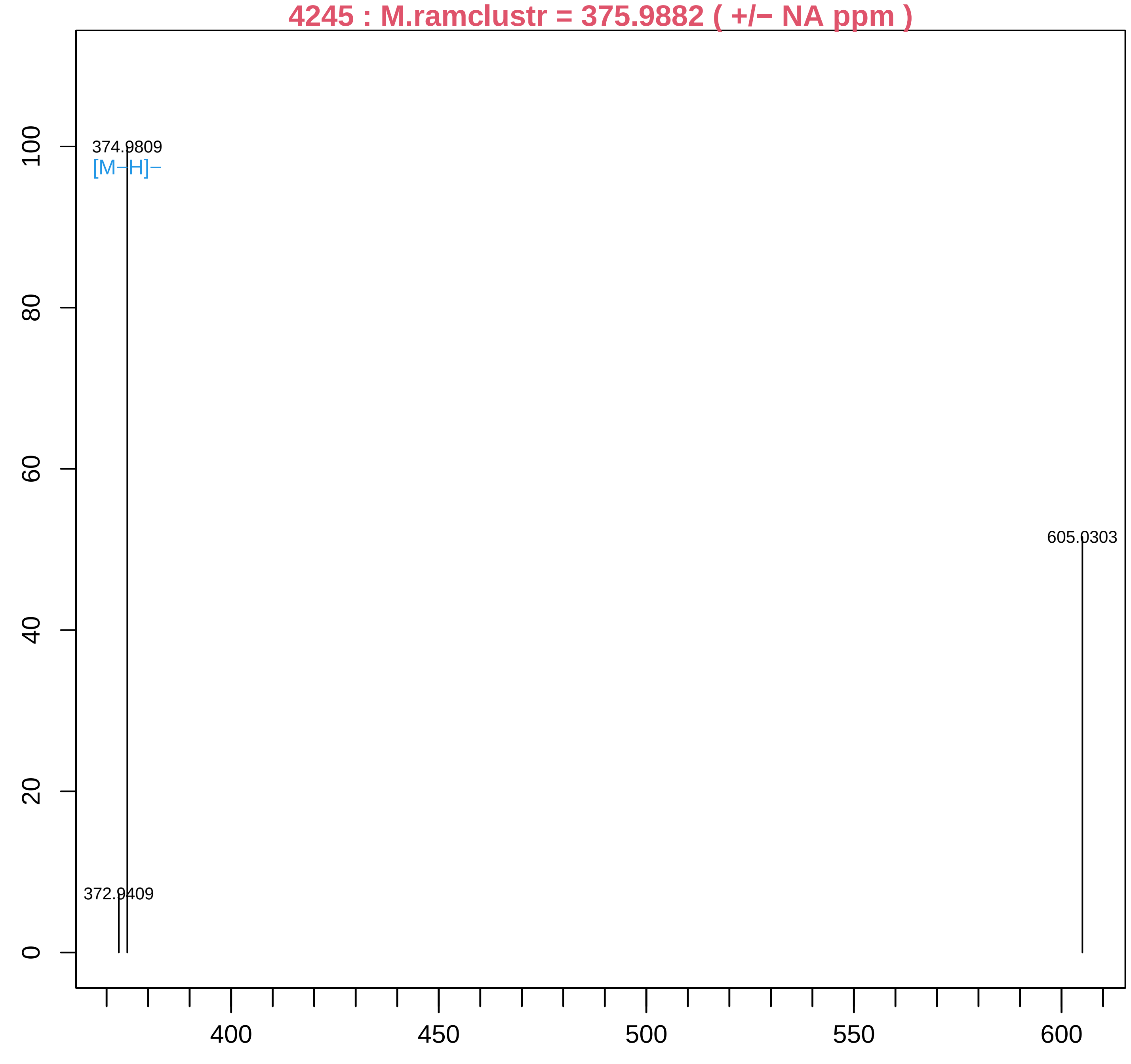
4245 : M.ramclustr = 375.9882 (+/− NA ppm)
374.9809
[M−H]−
605.0303
372.9409
0
20
40
60
80
100
400
450
500
550
600

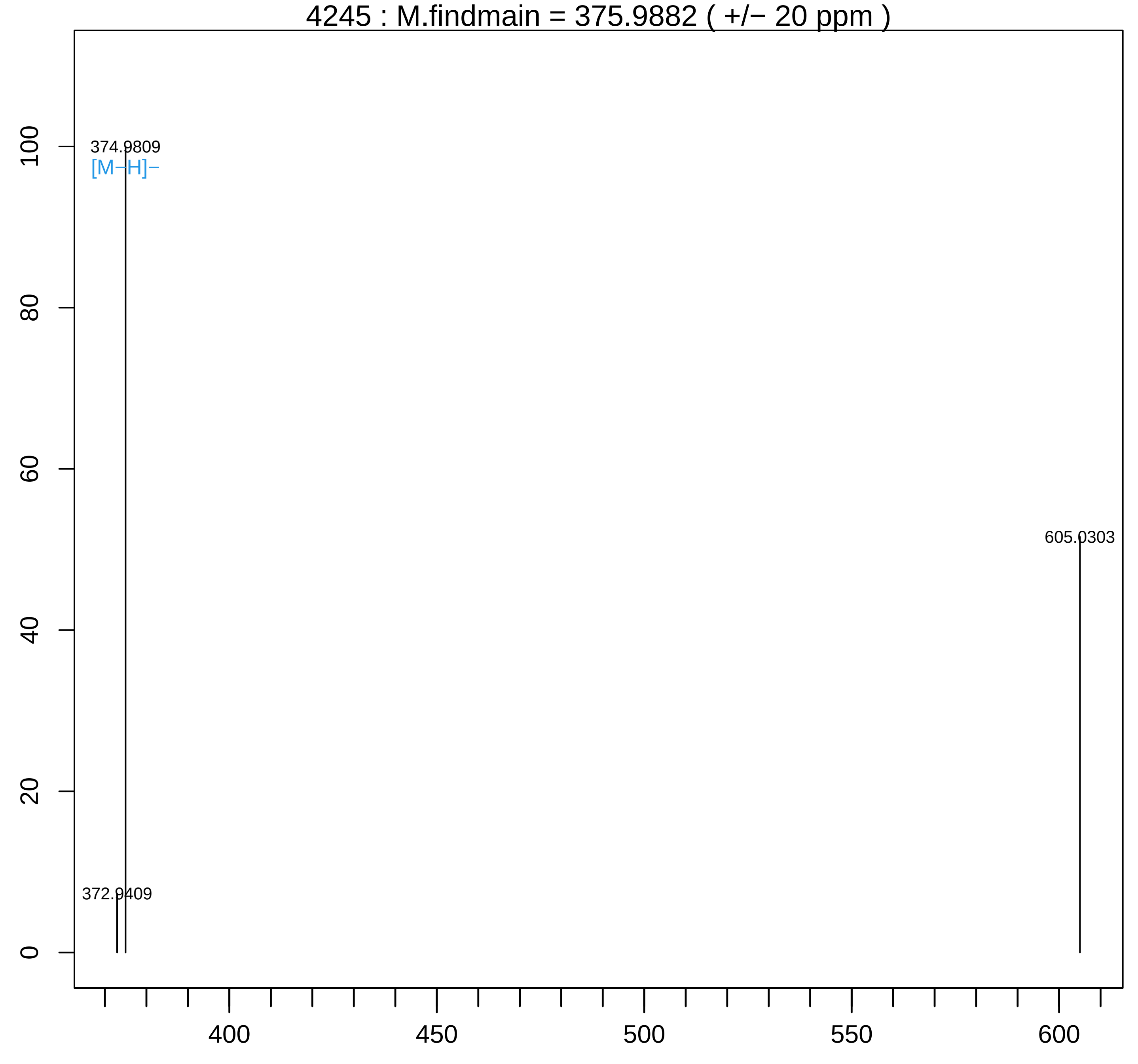
4245 : M.findmain = 375.9882 (+/− 20 ppm)
374.9809
[M−H]−
605.0303
372.9409
0
20
40
60
80
100
400
450
500
550
600

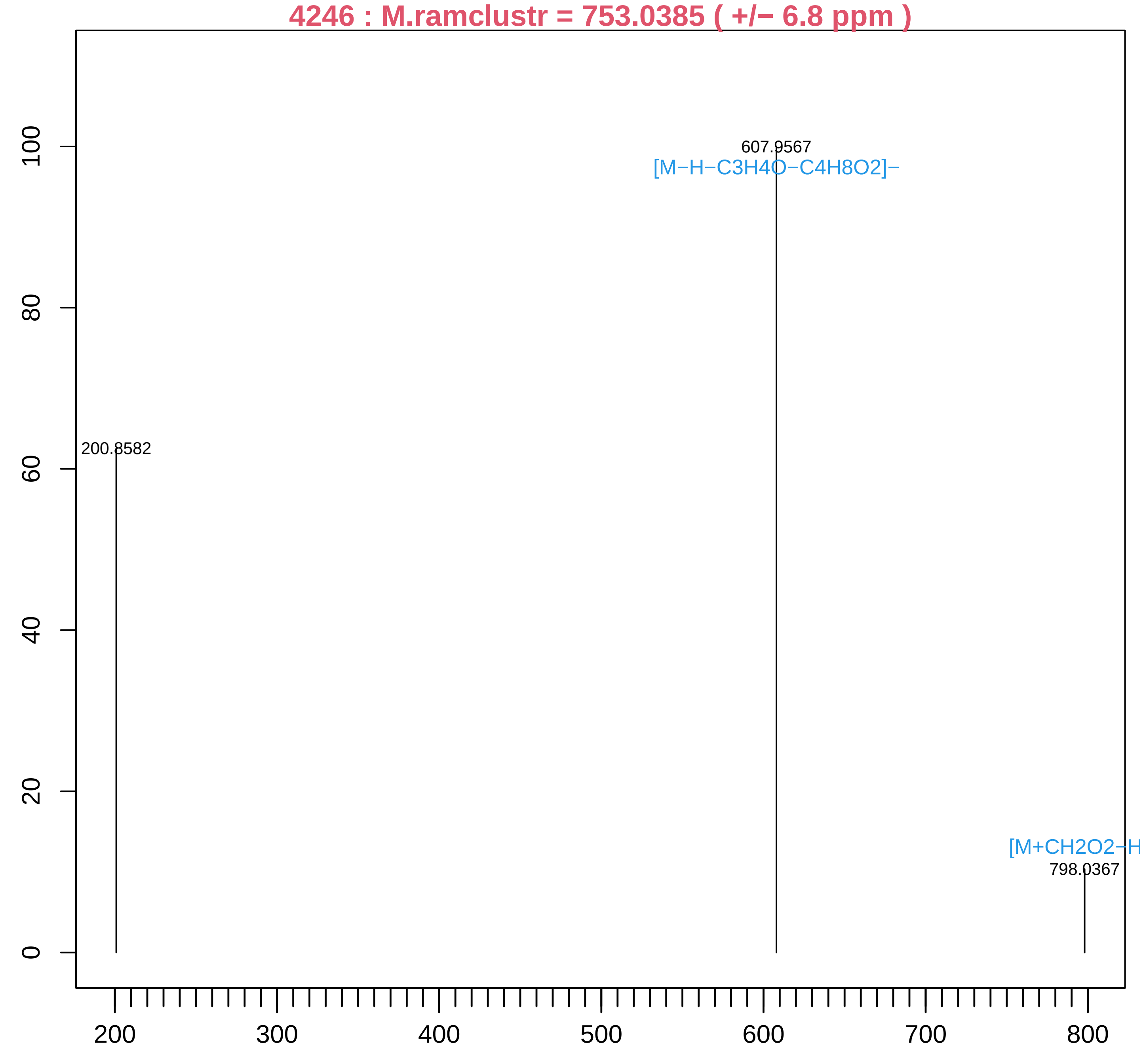
4246 : M.ramclustr = 753.0385 (+/− 6.8 ppm)
607.9567
[M−H−C3H4O−C4H8O2]−
200.8582
[M+CH2O2−H
798.0367
0
20
40
60
80
100
200
300
400
500
600
700
800

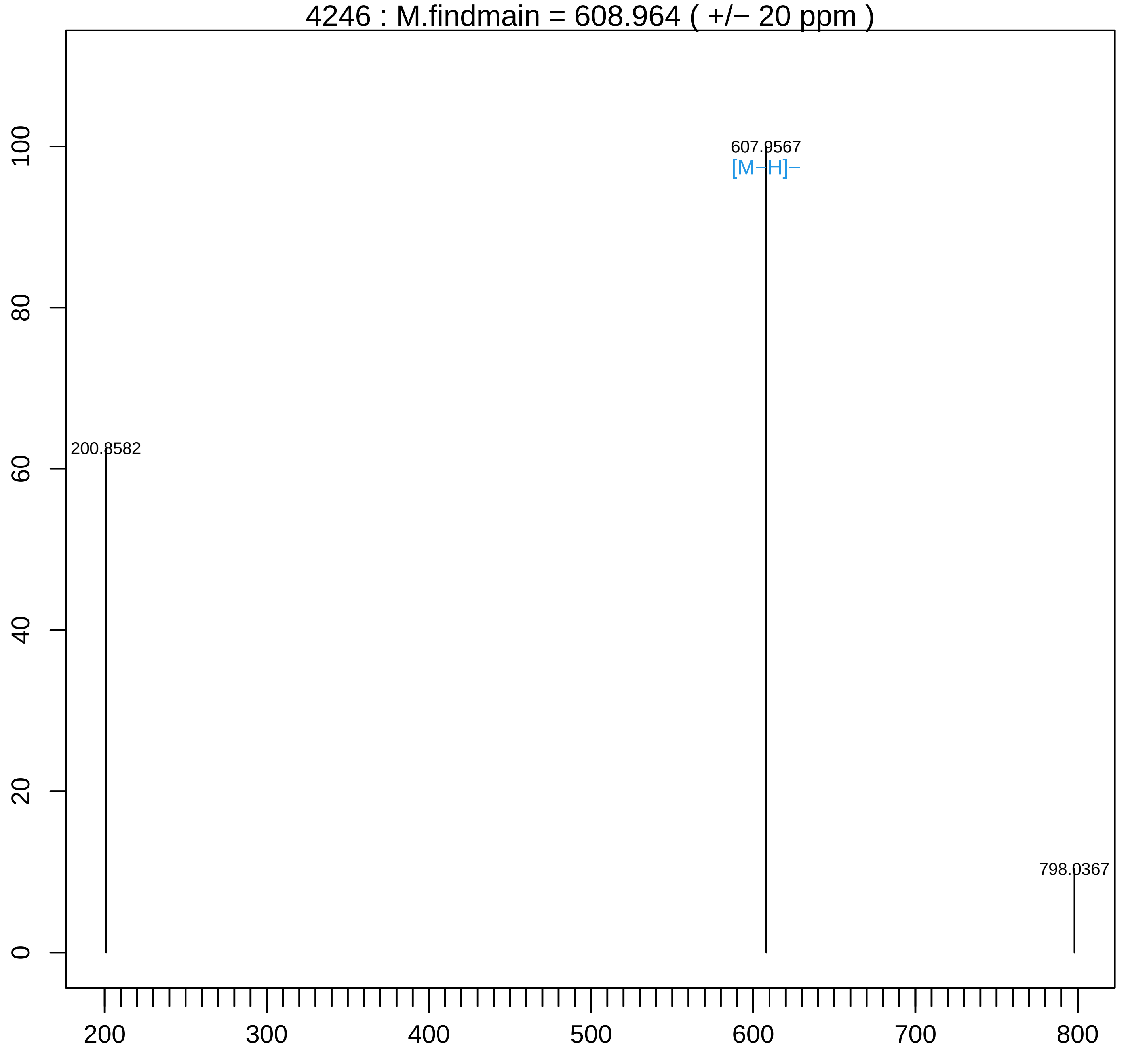
4246 : M.findmain = 608.964 (+/− 20 ppm)
607.9567
[M−H]−
200.8582
798.0367
0
20
40
60
80
100
200
300
400
500
600
700
800

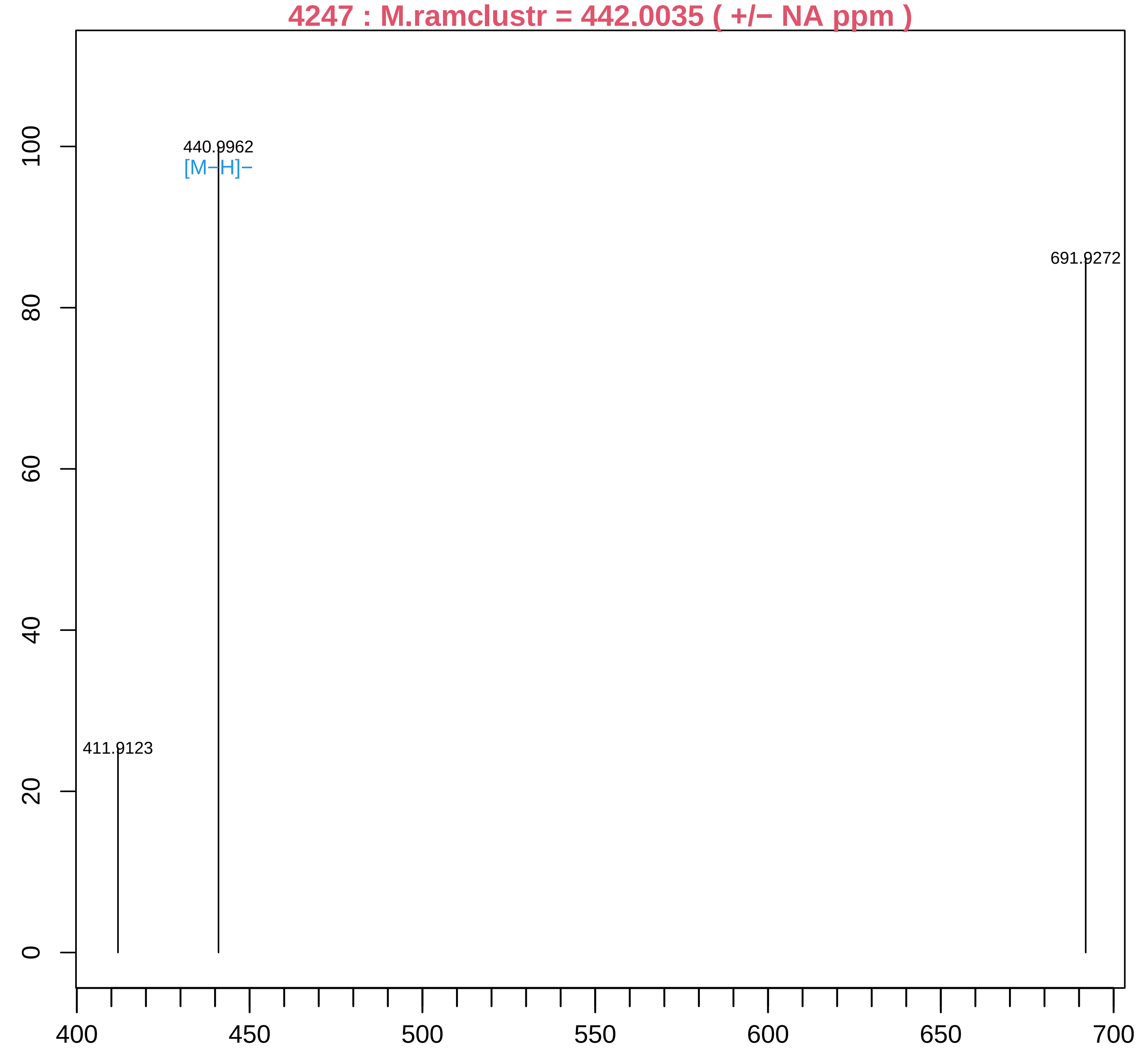
4247 : M.ramclustr = 442.0035 (+/− NA ppm)
440.9962
[M−H]−
691.9272
411.9123
0
20
40
60
80
100
400
450
500
550
600
650
700

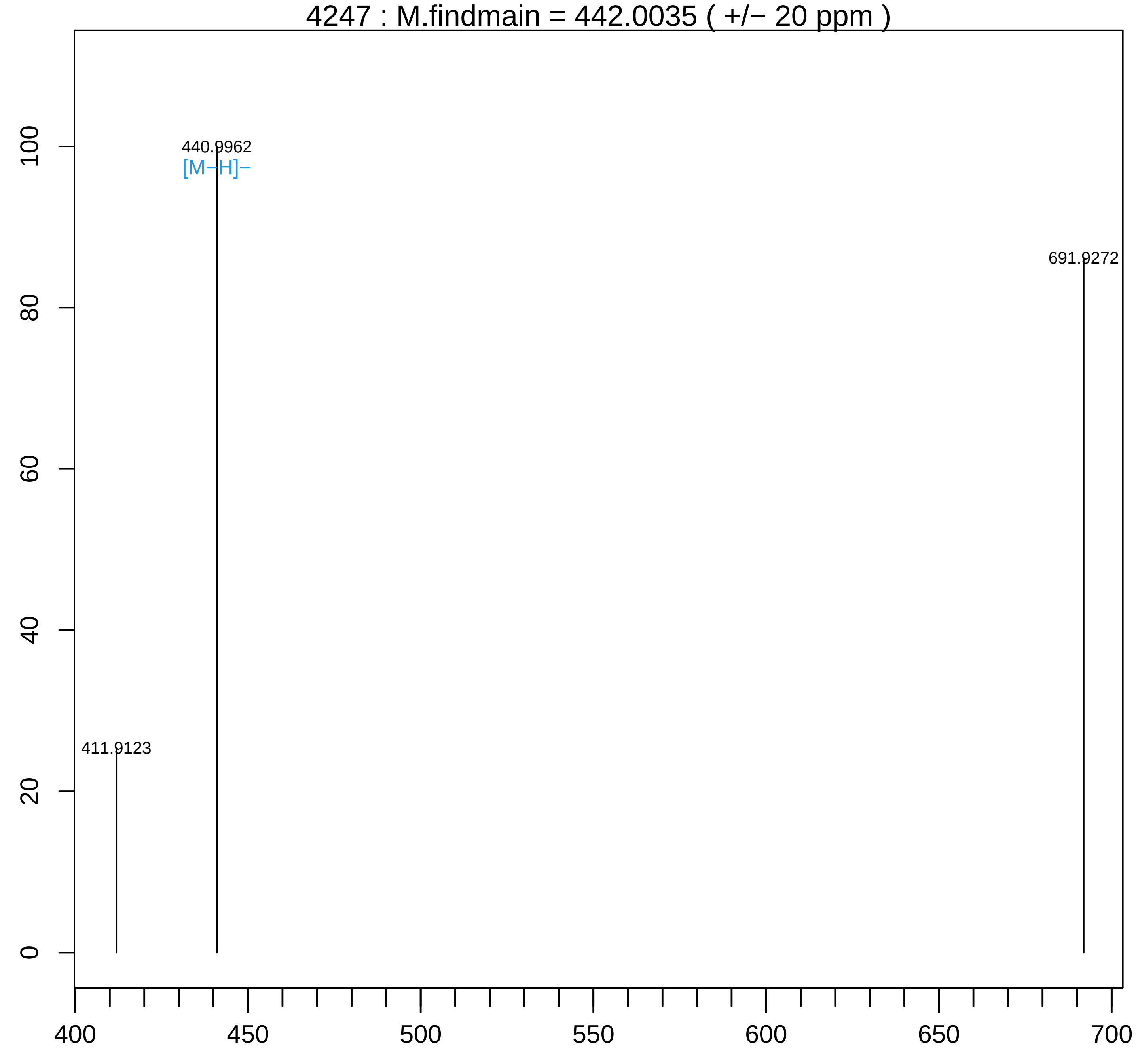
4247 : M.findmain = 442.0035 (+/− 20 ppm)
440.9962
[M−H]−
691.9272
411.9123
0
20
40
60
80
100
400
450
500
550
600
650
700

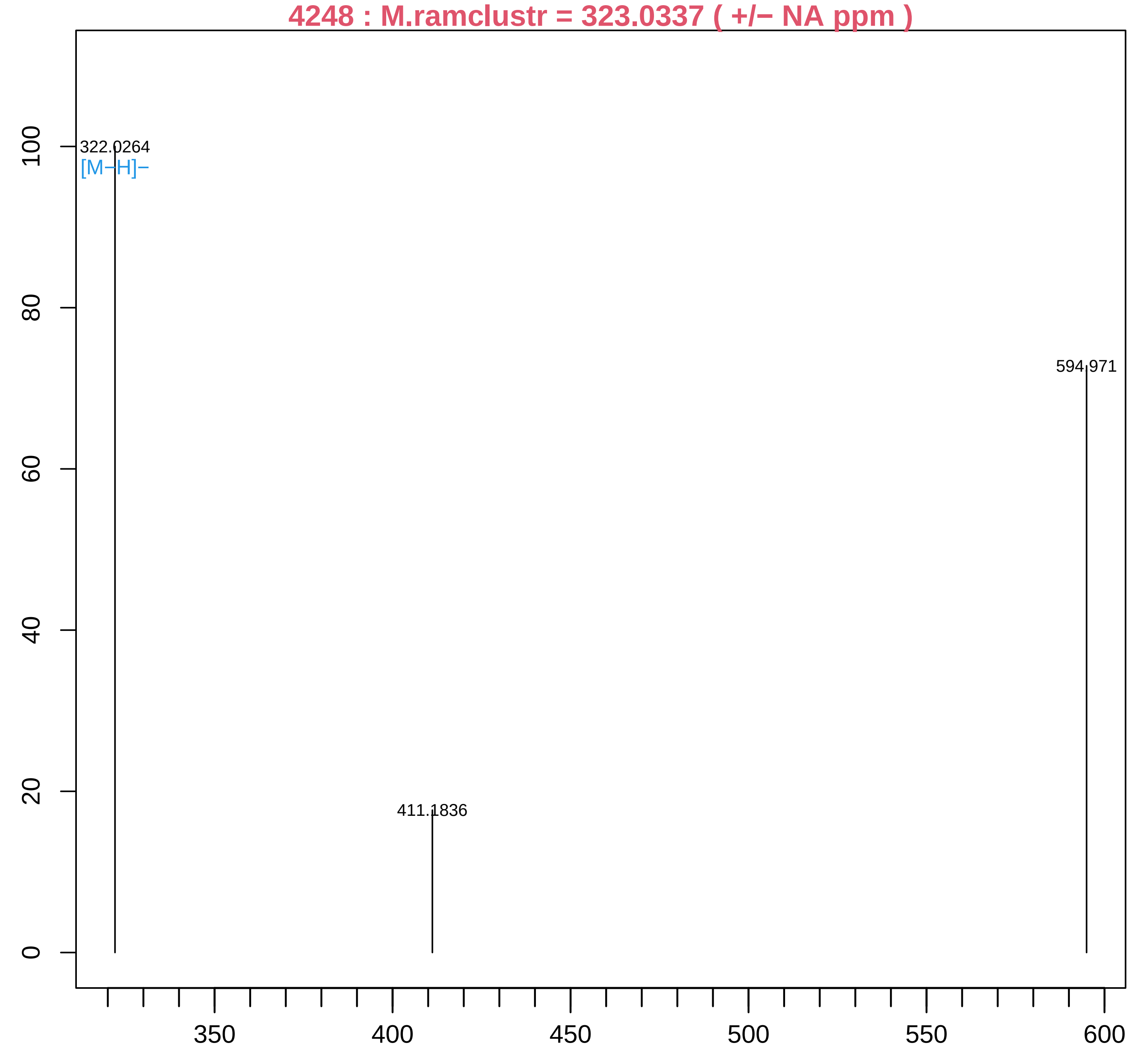

4248 : M.ramclustr = 323.0337 (+/− NA ppm)
322.0264
[M−H]−
594.971
411.1836
0
20
40
60
80
100
350
400
450
500
550
600

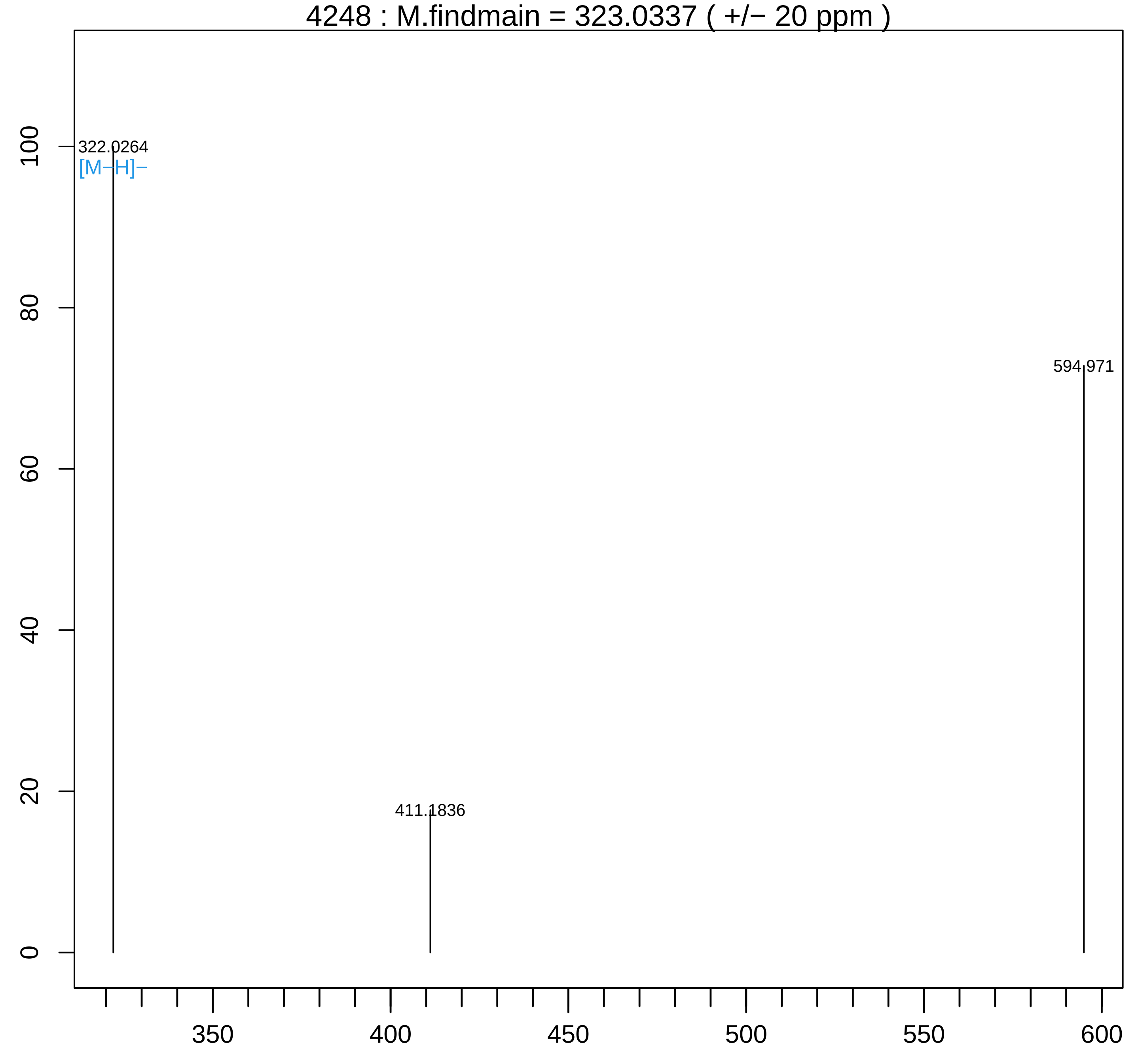

4248 : M.findmain = 323.0337 (+/− 20 ppm)
322.0264
[M−H]−
594.971
411.1836
0
20
40
60
80
100
350
400
450
500
550
600

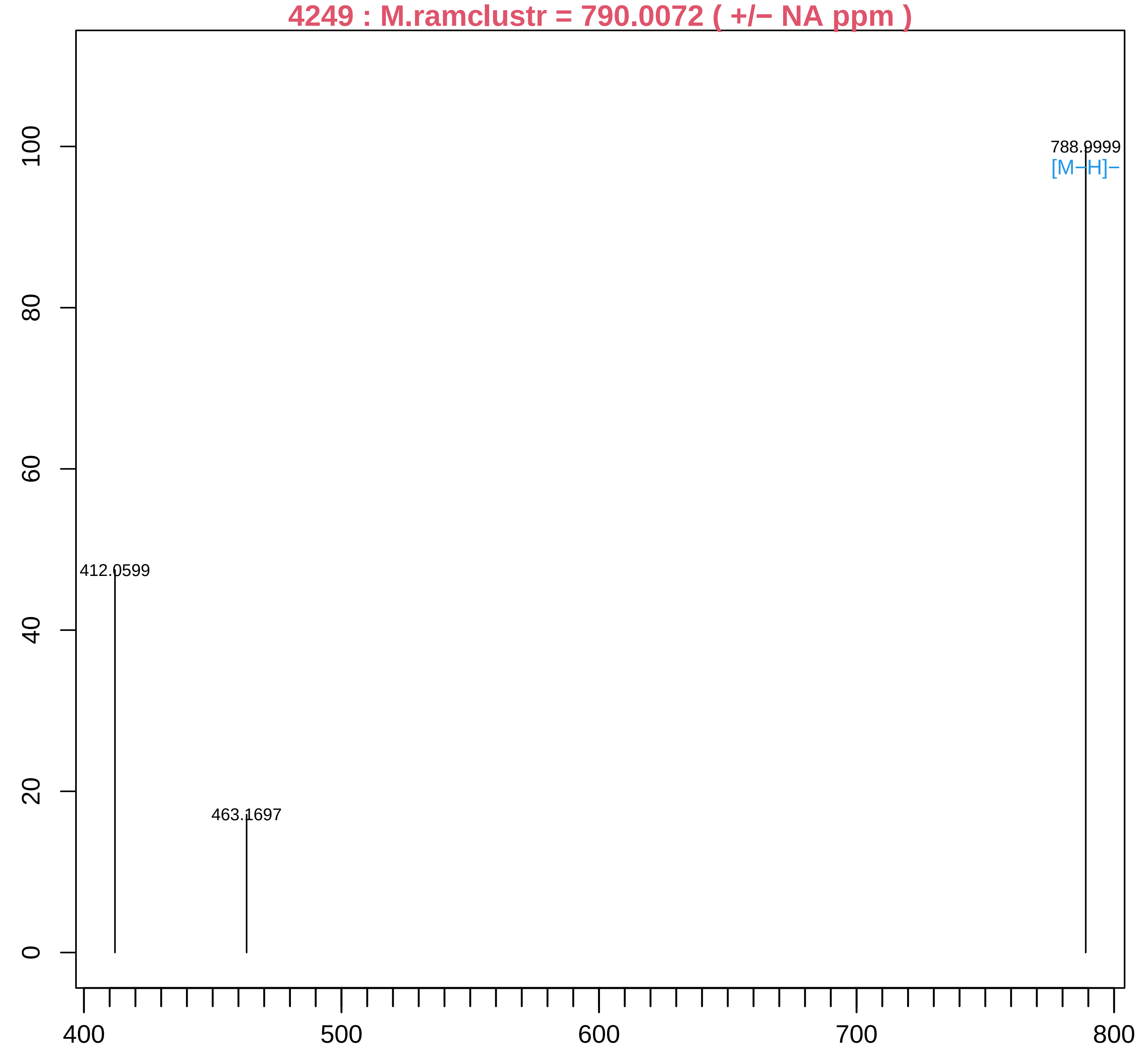
4249 : M.ramclustr = 790.0072 (+/− NA ppm)
788.9999
[M−H]−
412.0599
463.1697
0
20
40
60
80
100
400
500
600
700
800

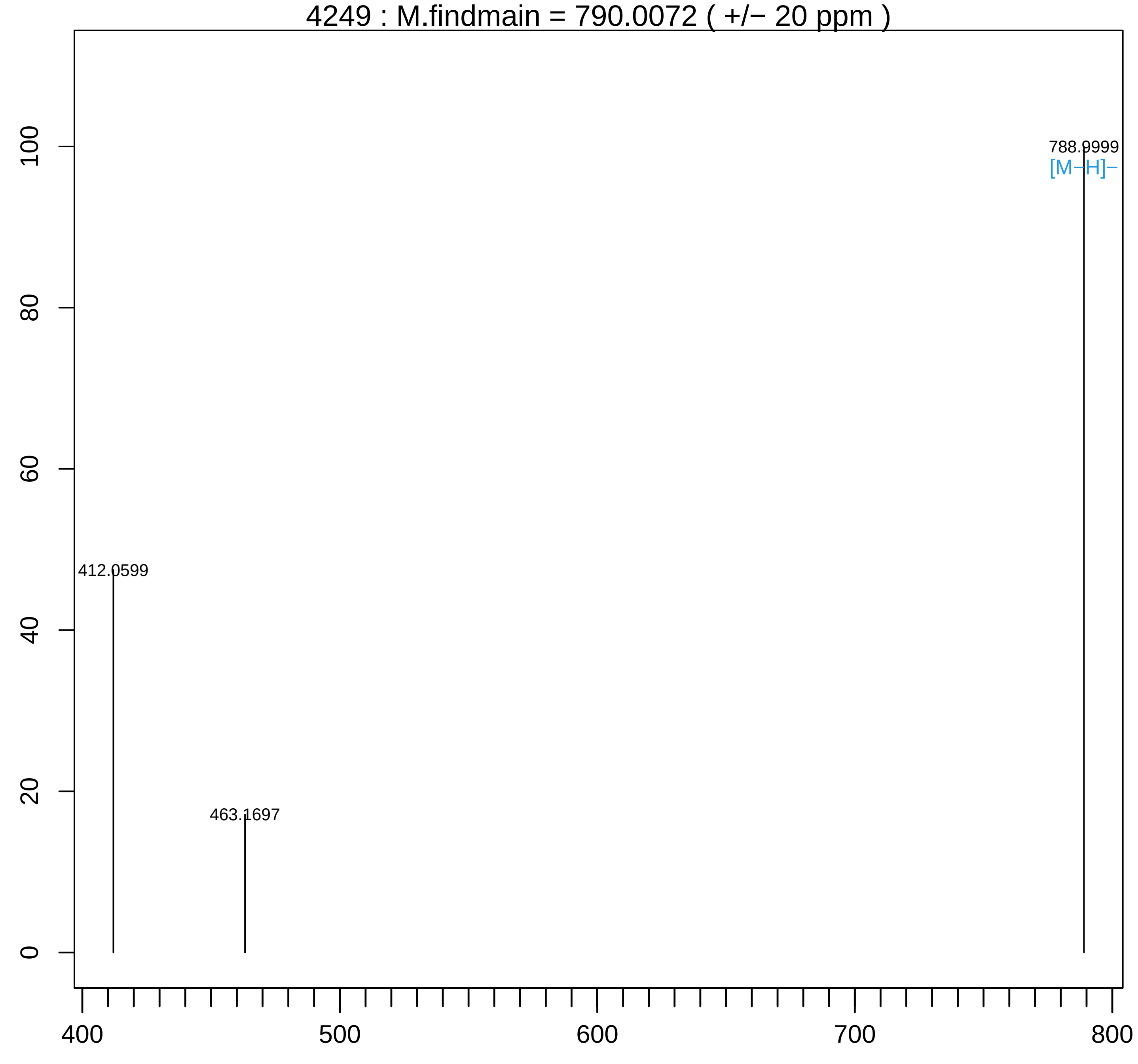
4249 : M.findmain = 790.0072 (+/− 20 ppm)
788.9999
[M−H]−
412.0599
463.1697
0
20
40
60
80
100
400
500
600
700
800

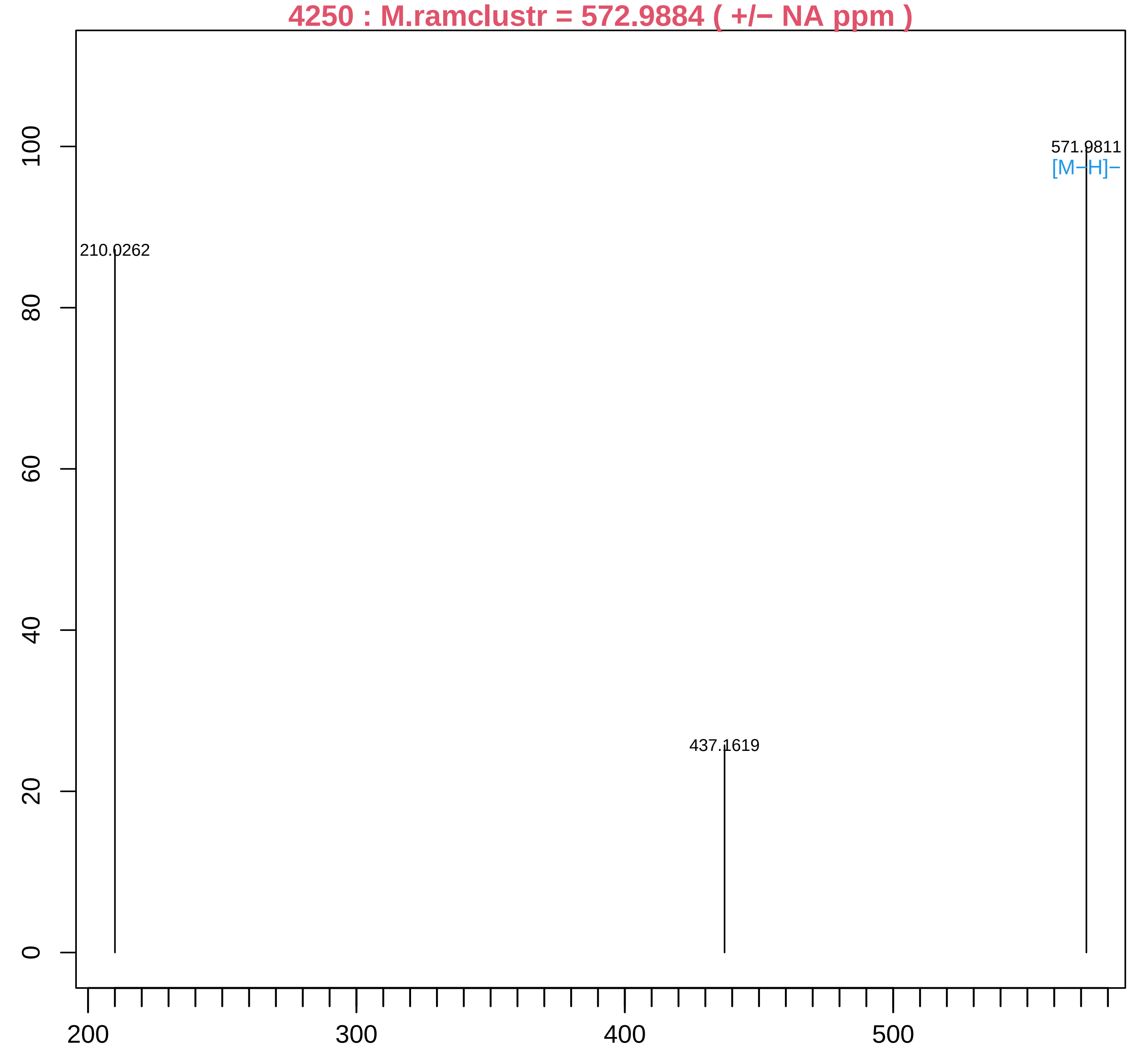

4250 : M.ramclustr = 572.9884 (+/− NA ppm)
571.9811
[M−H]−
210.0262
437.1619
0
20
40
60
80
100
200
300
400
500

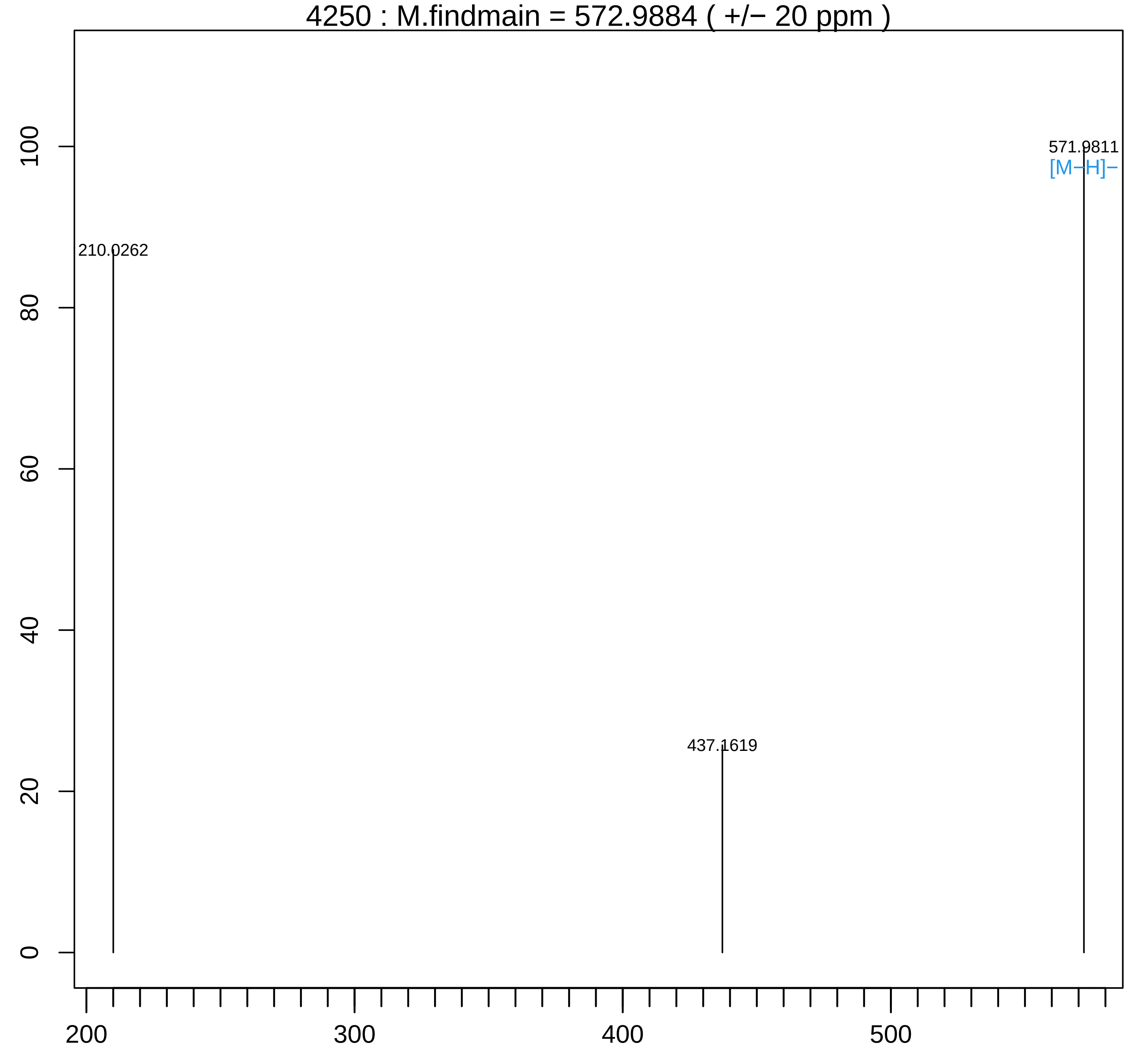

4250 : M.findmain = 572.9884 (+/− 20 ppm)
571.9811
[M−H]−
210.0262
437.1619
0
20
40
60
80
100
200
300
400
500

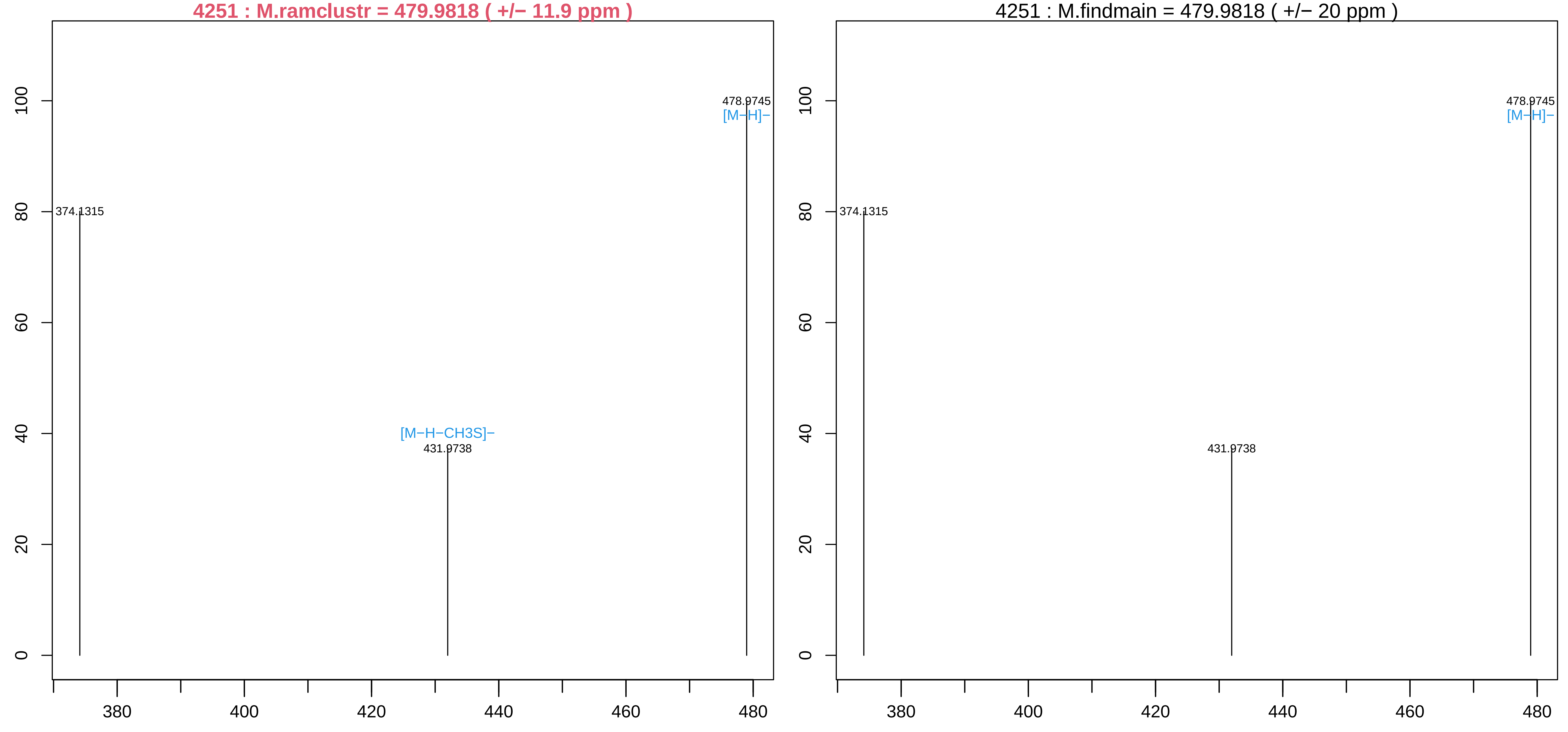
4251 : M.ramclustr = 479.9818 (+/− 11.9 ppm)
478.9745
[M−H]−
374.1315
[M−H−CH3S]−
431.9738
4251 : M.findmain = 479.9818 (+/− 20 ppm)
478.9745
[M−H]−
374.1315
431.9738

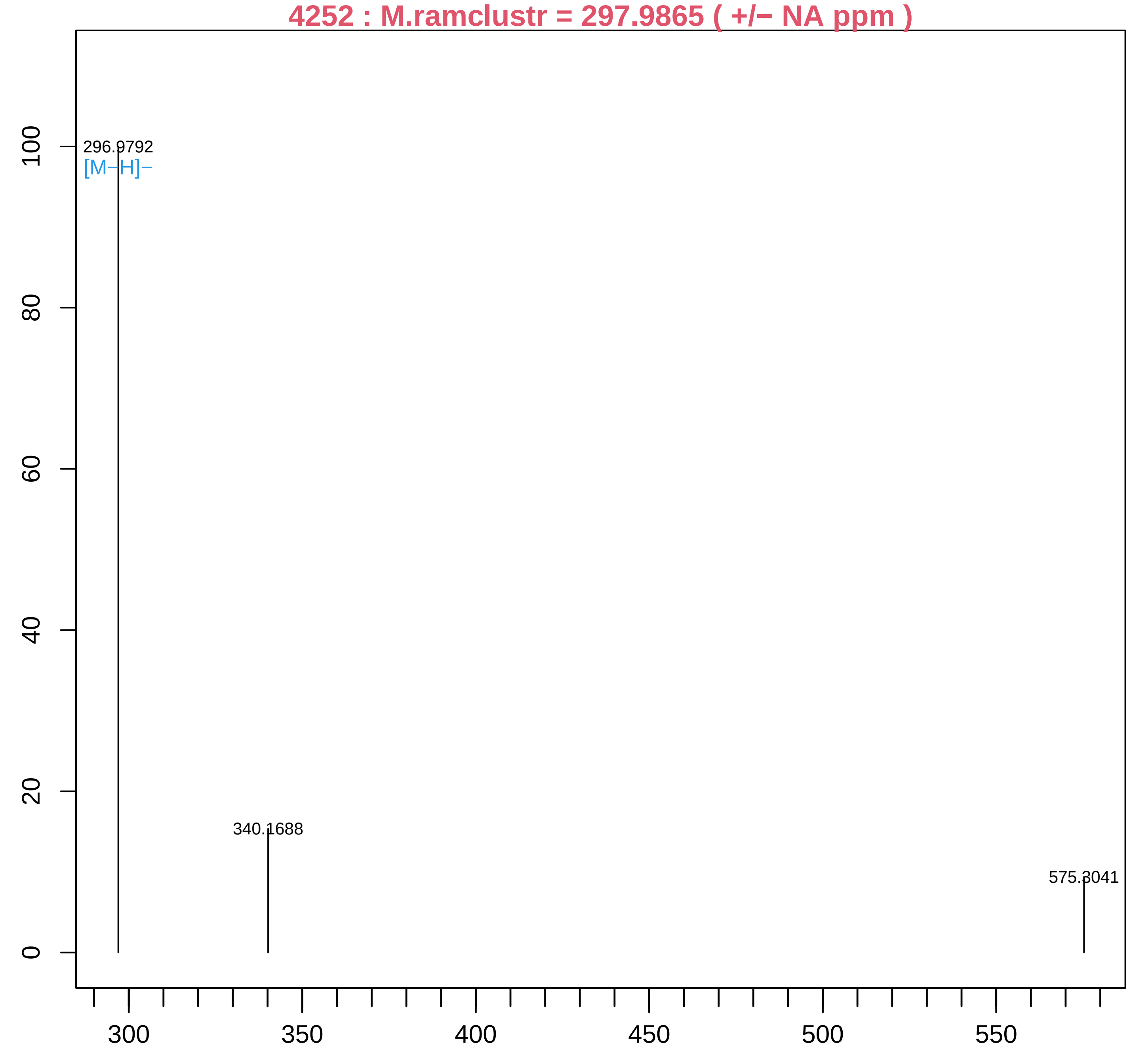
4252 : M.ramclustr = 297.9865 (+/− NA ppm)
296.9792
[M−H]−
340.1688
575.3041
0
20
40
60
80
100
300
350
400
450
500
550

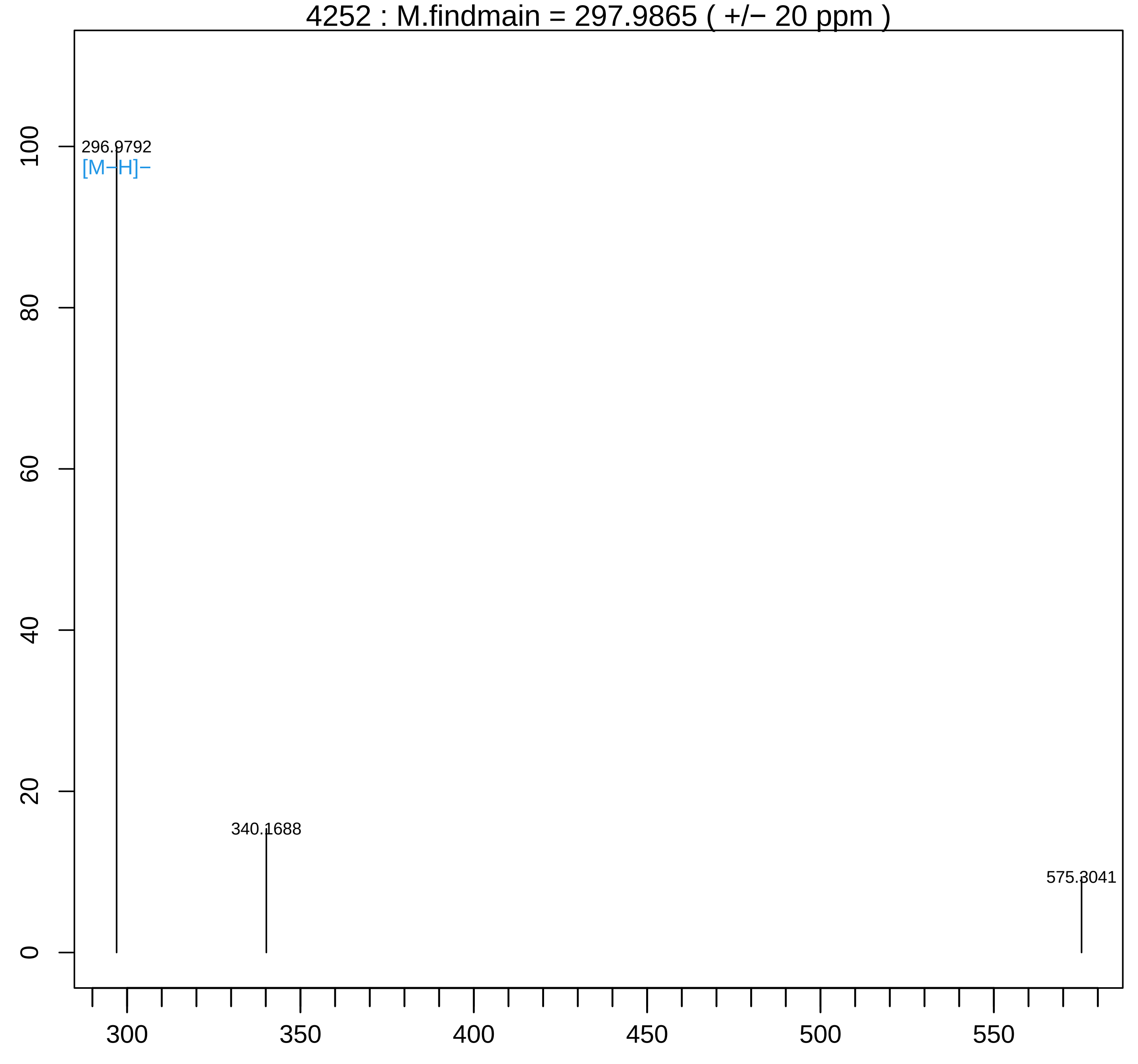
4252 : M.findmain = 297.9865 (+/− 20 ppm)
296.9792
[M−H]−
340.1688
575.3041
0
20
40
60
80
100
300
350
400
450
500
550

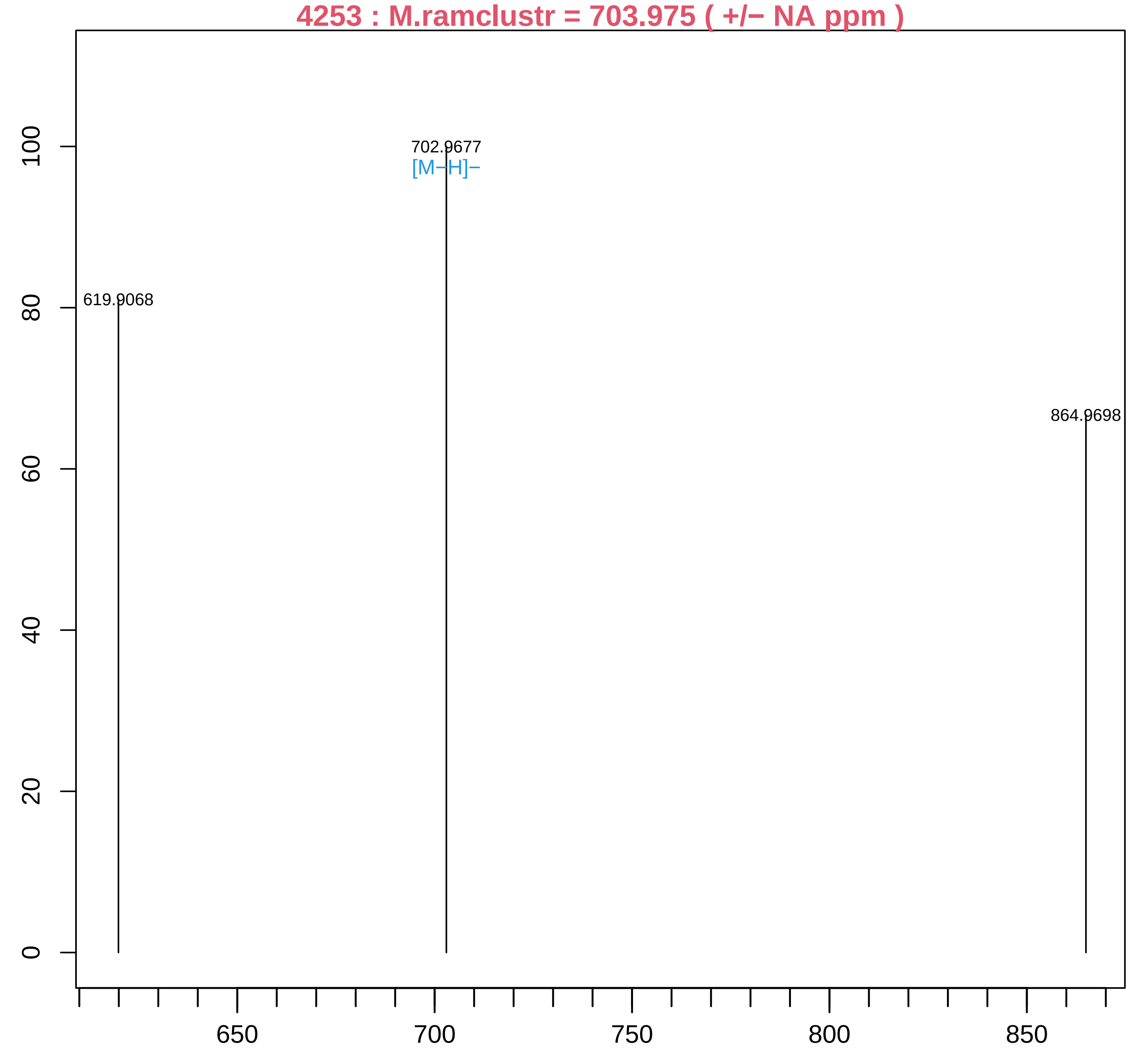
4253 : M.ramclustr = 703.975 (+/− NA ppm)
702.9677
[M−H]−
619.9068
864.9698
0
20
40
60
80
100
650
700
750
800
850

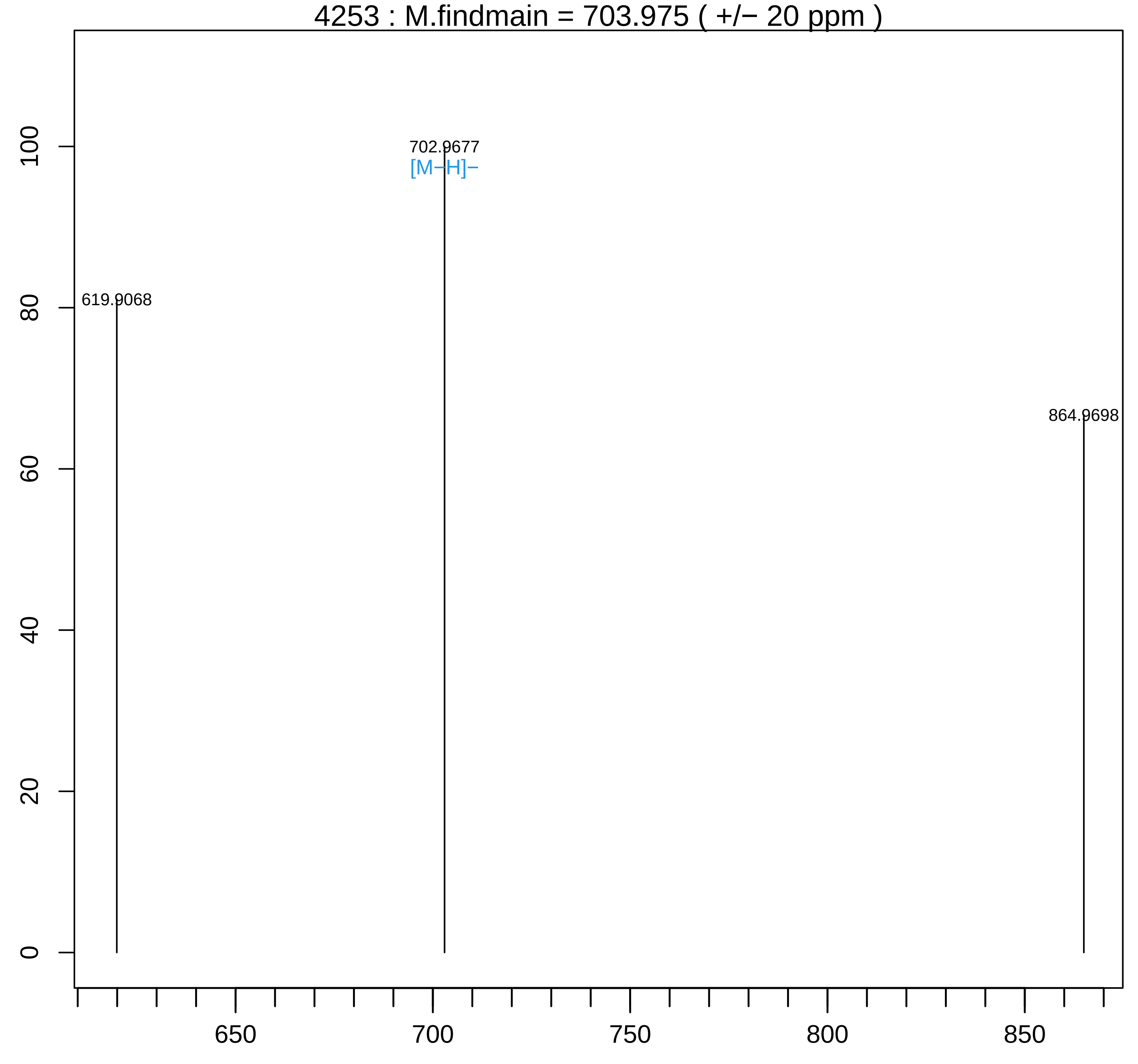
4253 : M.findmain = 703.975 (+/− 20 ppm)
702.9677
[M−H]−
619.9068
864.9698
0
20
40
60
80
100
650
700
750
800
850

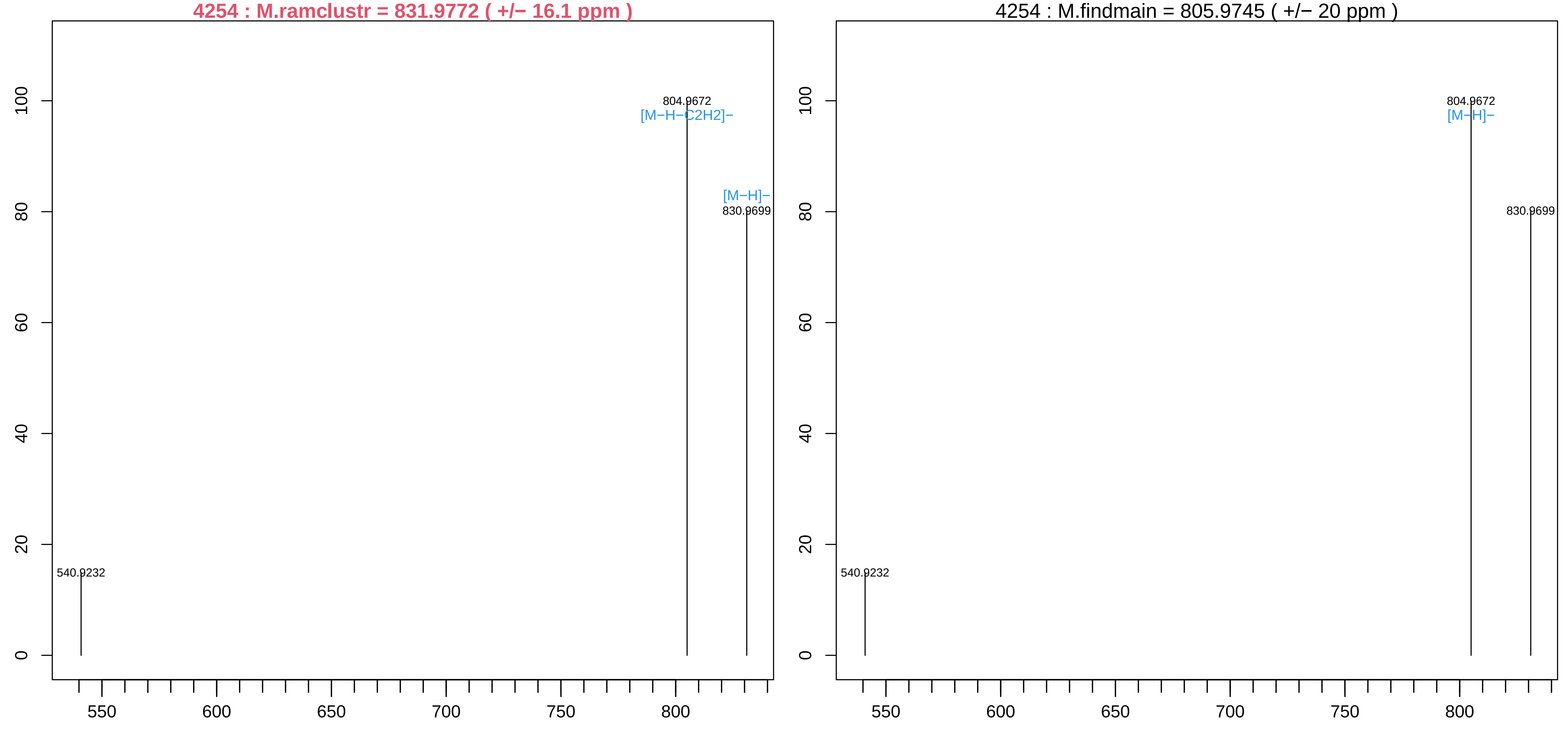

4254 : M.ramclustr = 831.9772 (+/− 16.1 ppm)
804.9672
[M−H−C2H2]−
[M−H]−
830.9699
540.9232
0
20
40
60
80
100
550
600
650
700
750
800
4254 : M.findmain = 805.9745 (+/− 20 ppm)
804.9672
[M−H]−
830.9699
540.9232
0
20
40
60
80
100
550
600
650
700
750
800

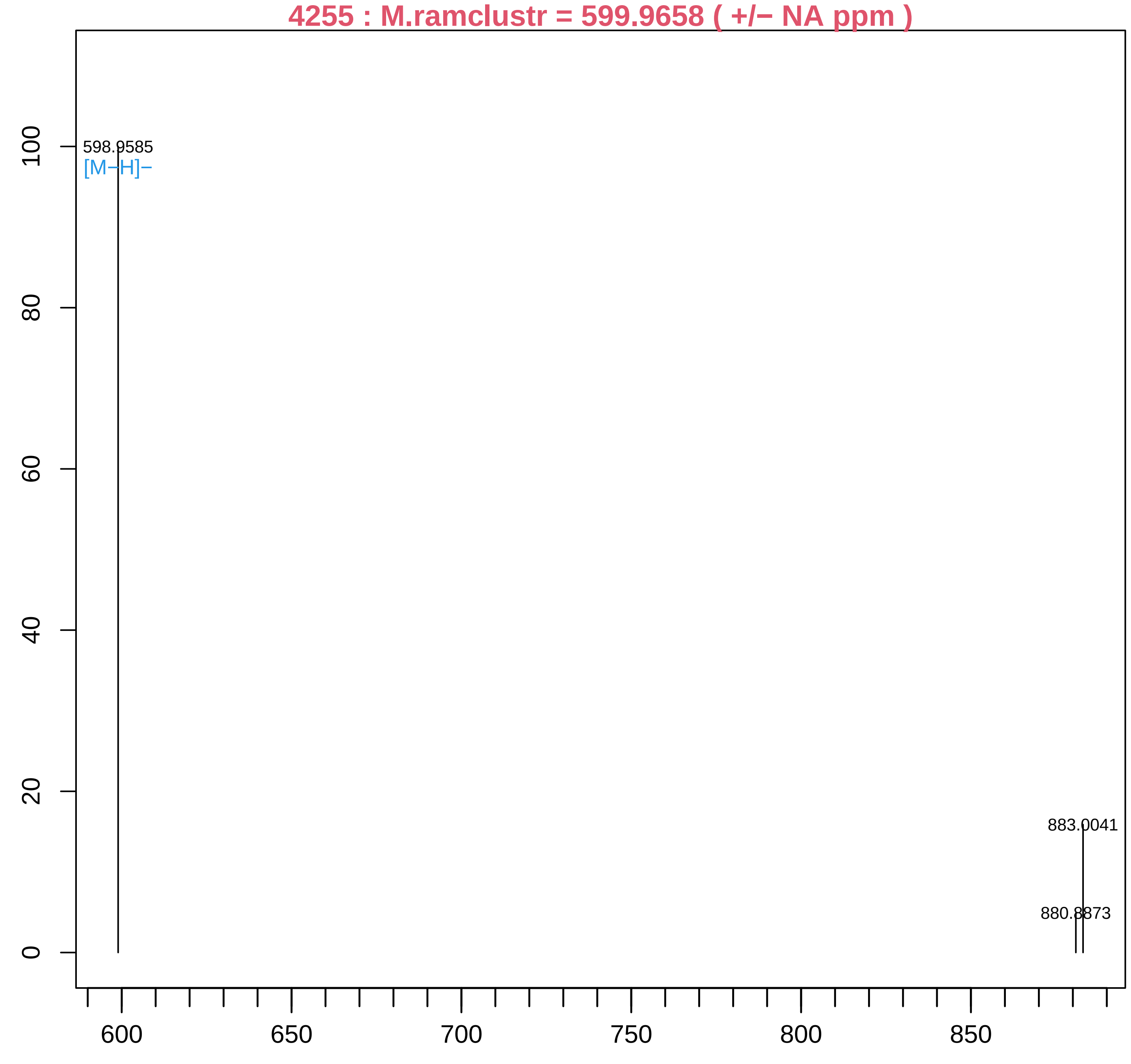
4255 : M.ramclustr = 599.9658 (+/− NA ppm)
598.9585
[M−H]−
883.0041
880.8873
0
20
40
60
80
100
600
650
700
750
800
850

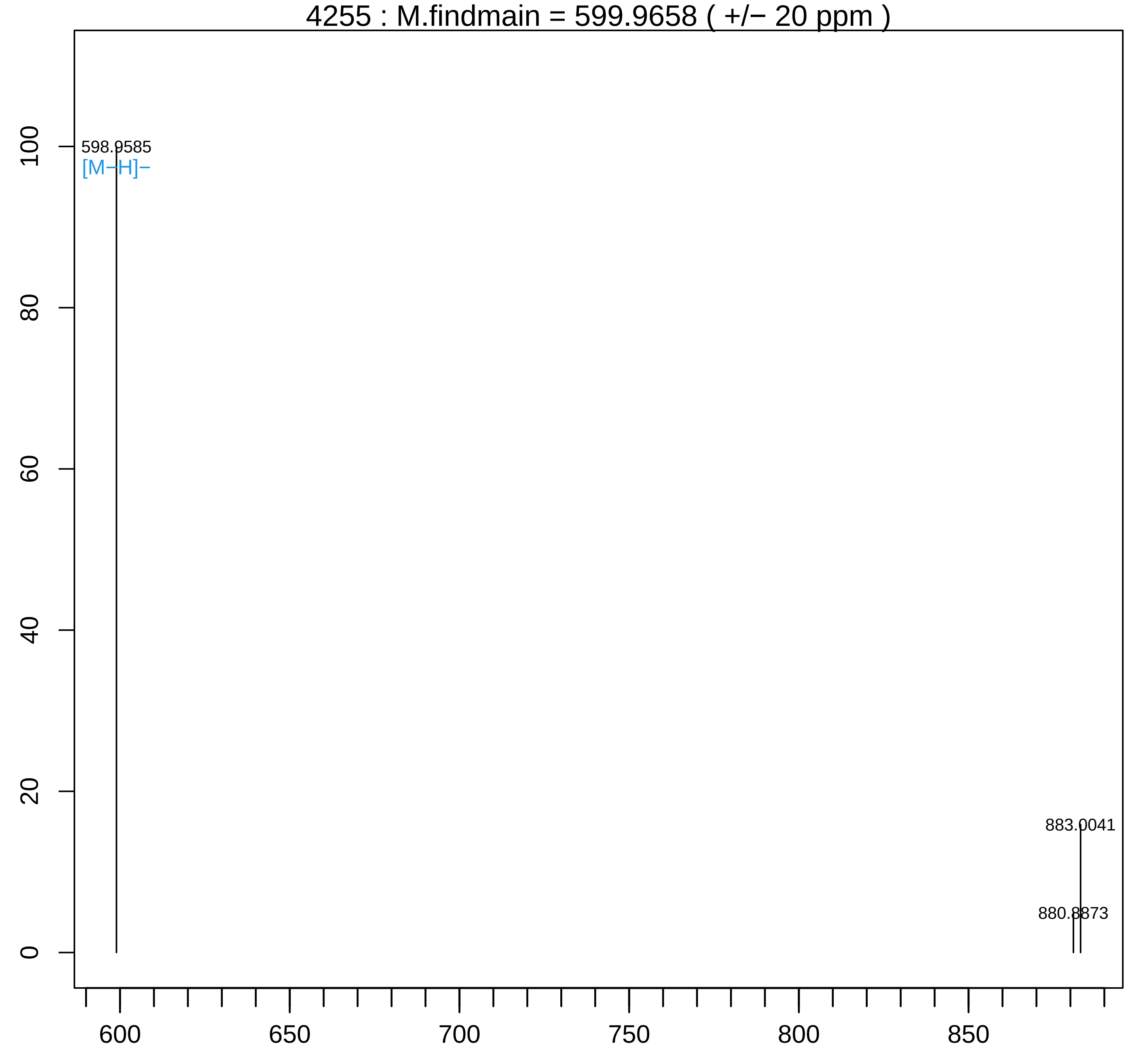
4255 : M.findmain = 599.9658 (+/− 20 ppm)
598.9585
[M−H]−
883.0041
880.8873
0
20
40
60
80
100
600
650
700
750
800
850

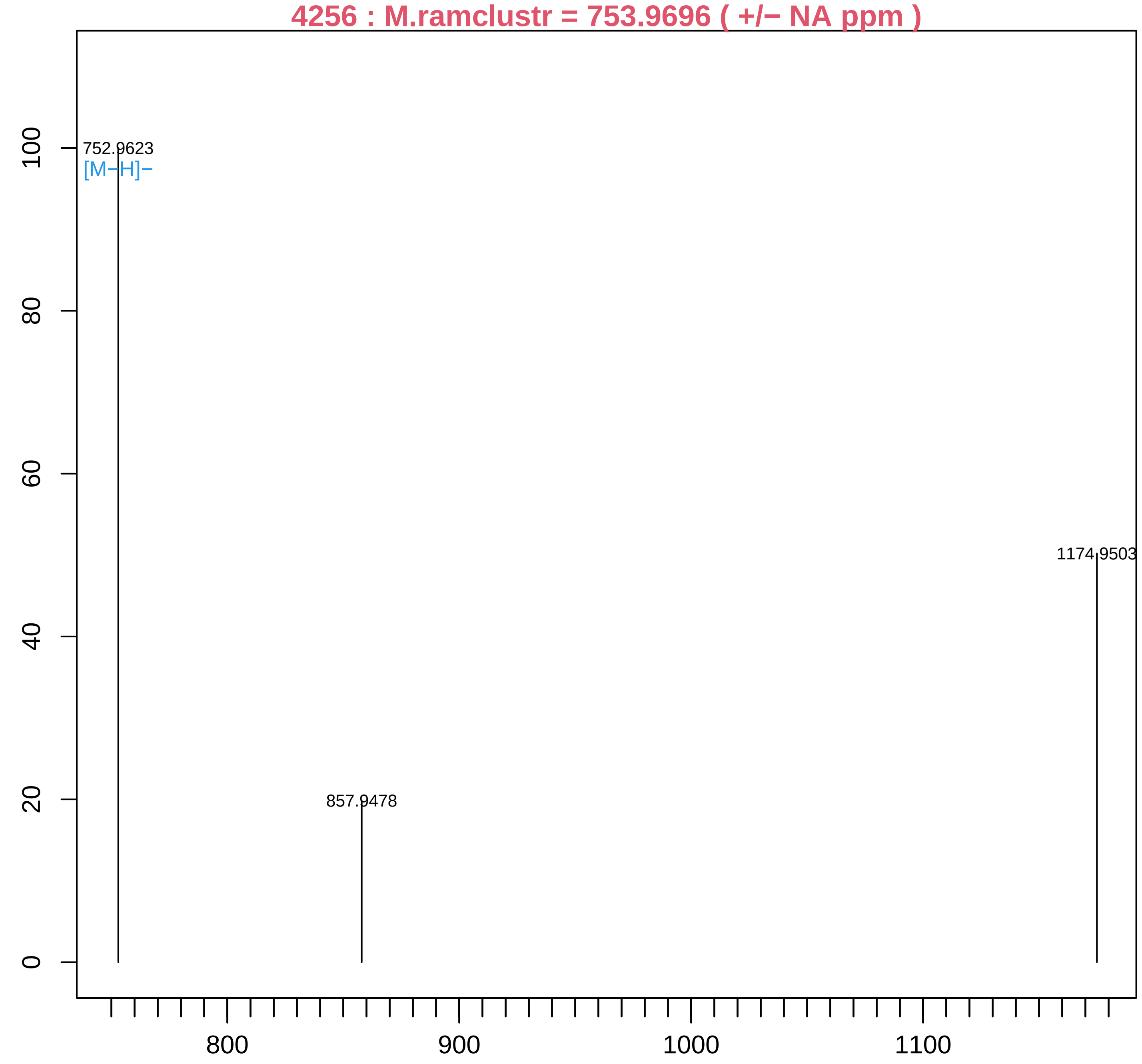
4256 : M.ramclustr = 753.9696 (+/− NA ppm)
752.9623
[M−H]−
857.9478
1174.9503
0
20
40
60
80
100
800
900
1000
1100

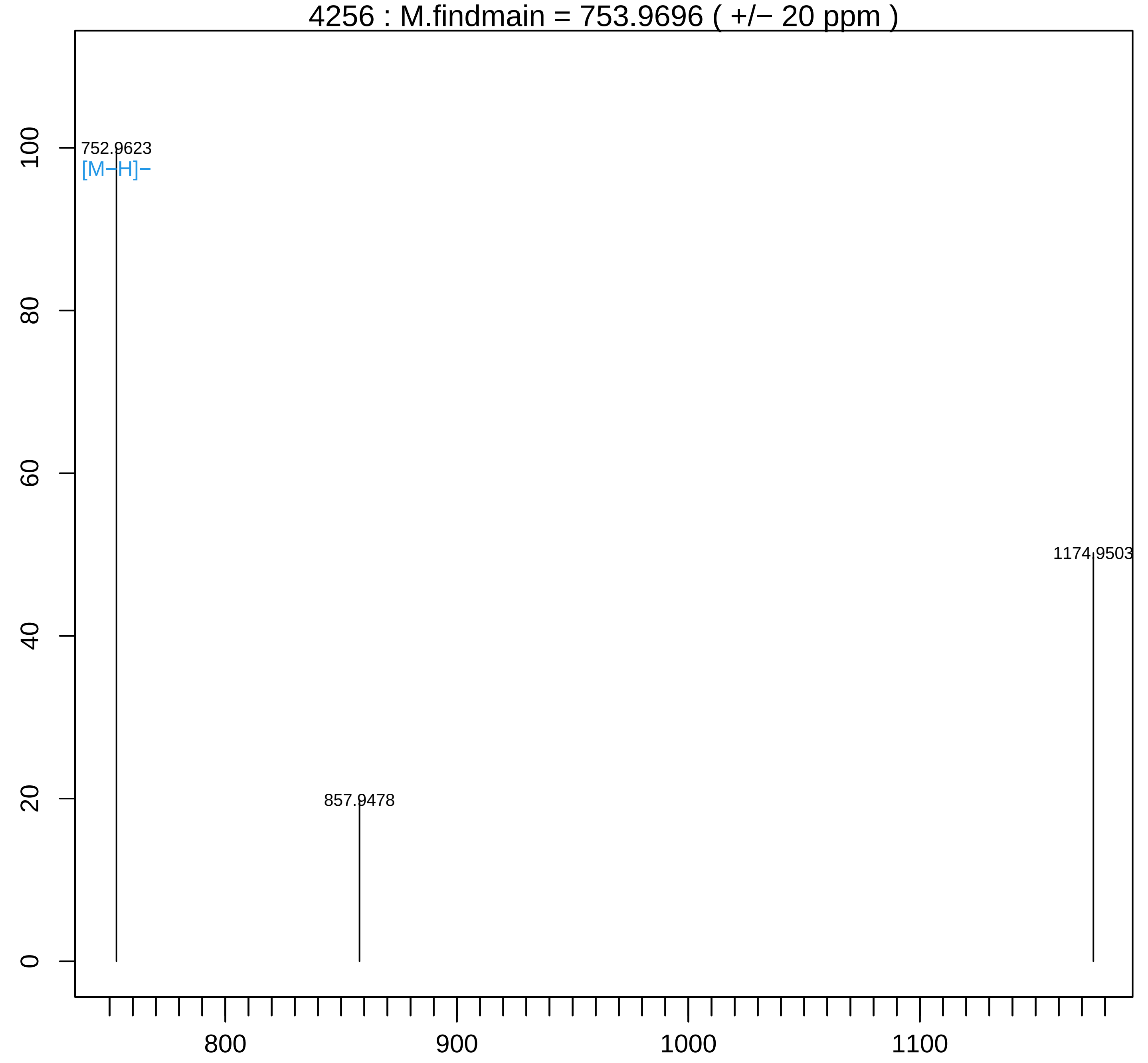
4256 : M.findmain = 753.9696 (+/− 20 ppm)
752.9623
[M−H]−
857.9478
1174.9503
0
20
40
60
80
100
800
900
1000
1100

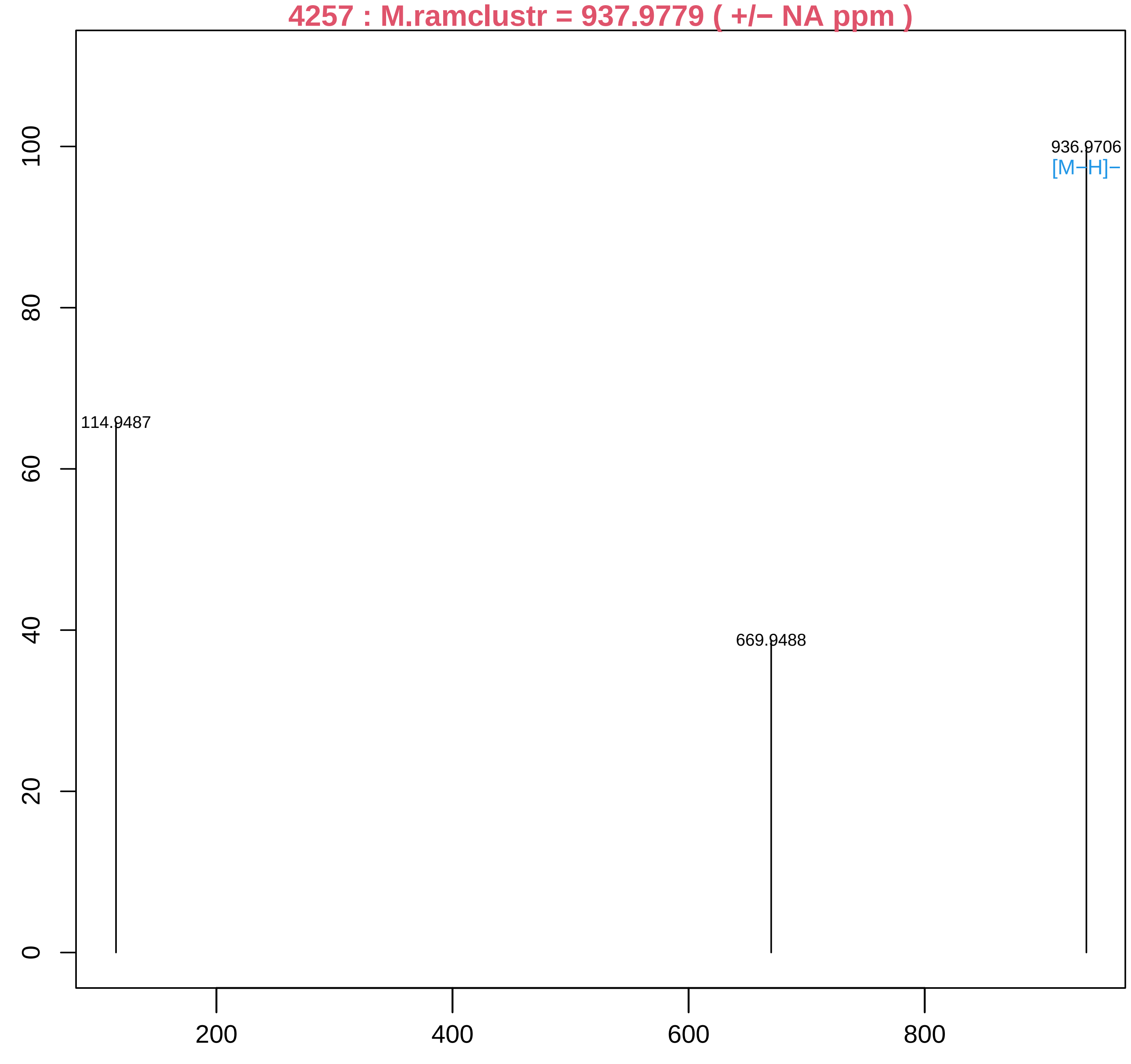

4257 : M.ramclustr = 937.9779 (+/− NA ppm)
936.9706
[M−H]−
114.9487
669.9488
0
20
40
60
80
100
200
400
600
800

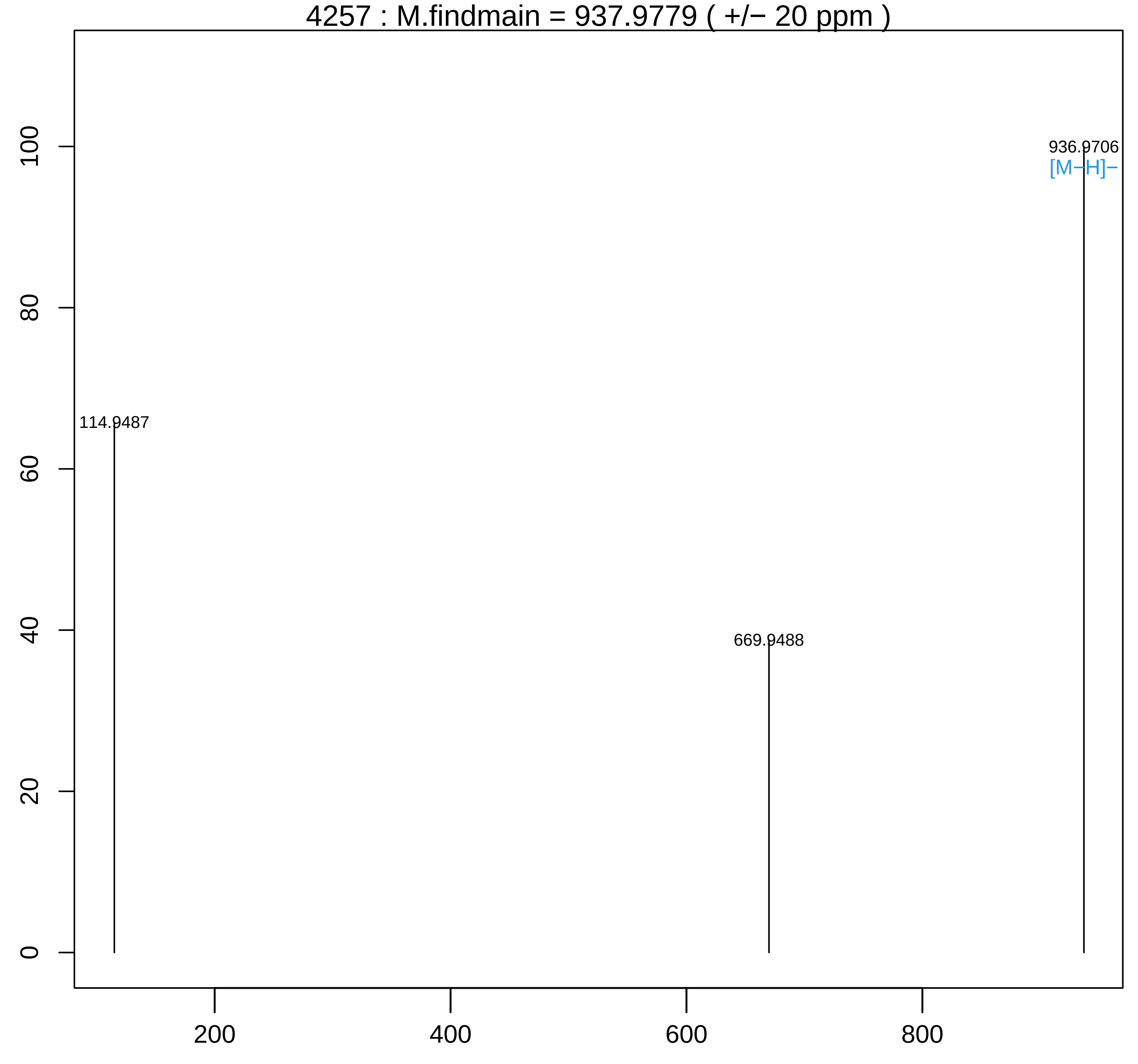

4257 : M.findmain = 937.9779 (+/− 20 ppm)
936.9706
[M−H]−
114.9487
669.9488
0
20
40
60
80
100
200
400
600
800

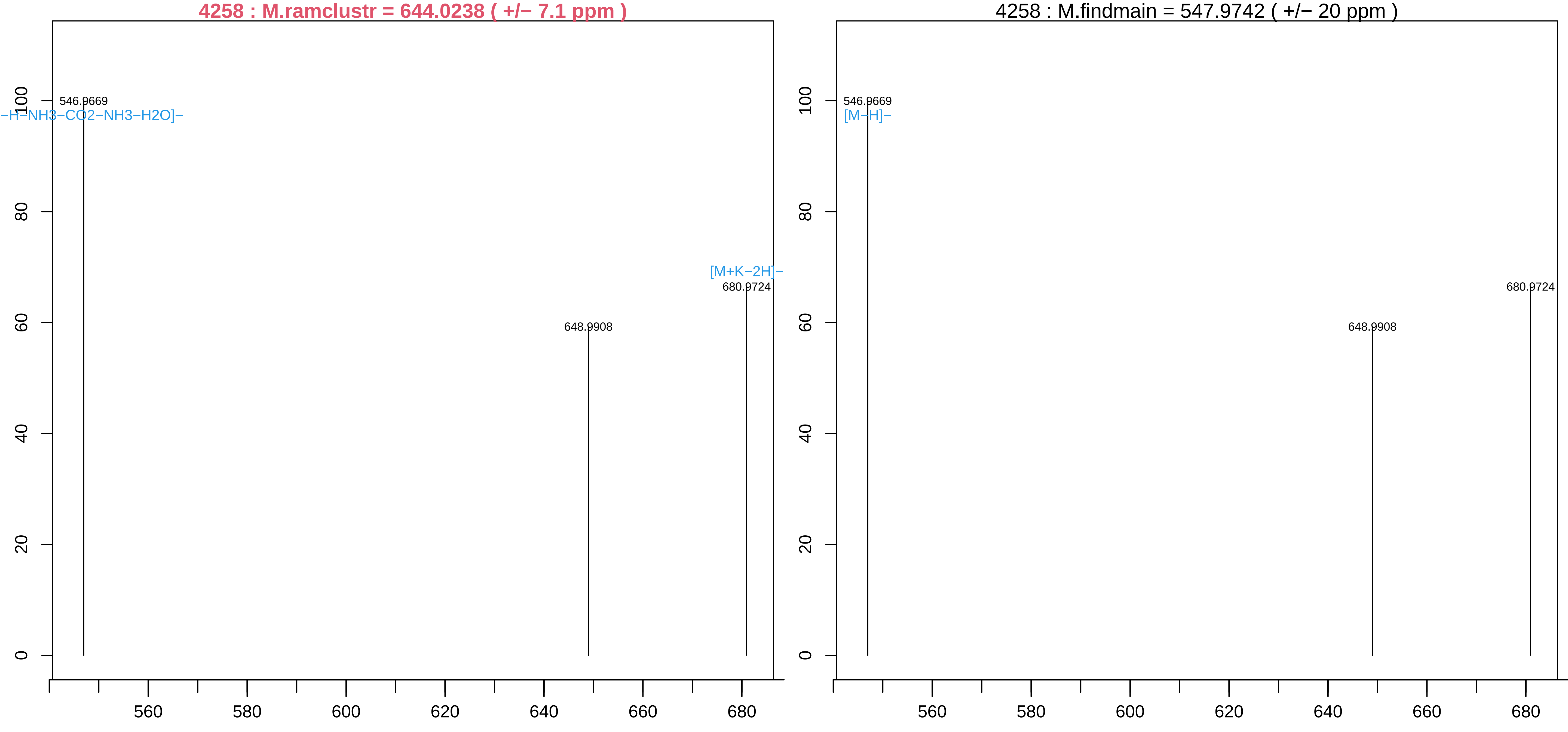

4258 : M.ramclustr = 644.0238 (+/− 7.1 ppm)
546.9669
−H−NH3−CO2−NH3−H2O]−
648.9908
[M+K−2H]−
680.9724
4258 : M.findmain = 547.9742 (+/− 20 ppm)
546.9669
[M−H]−
648.9908
680.9724
0
20
40
60
80
100
560
580
600
620
640
660
680

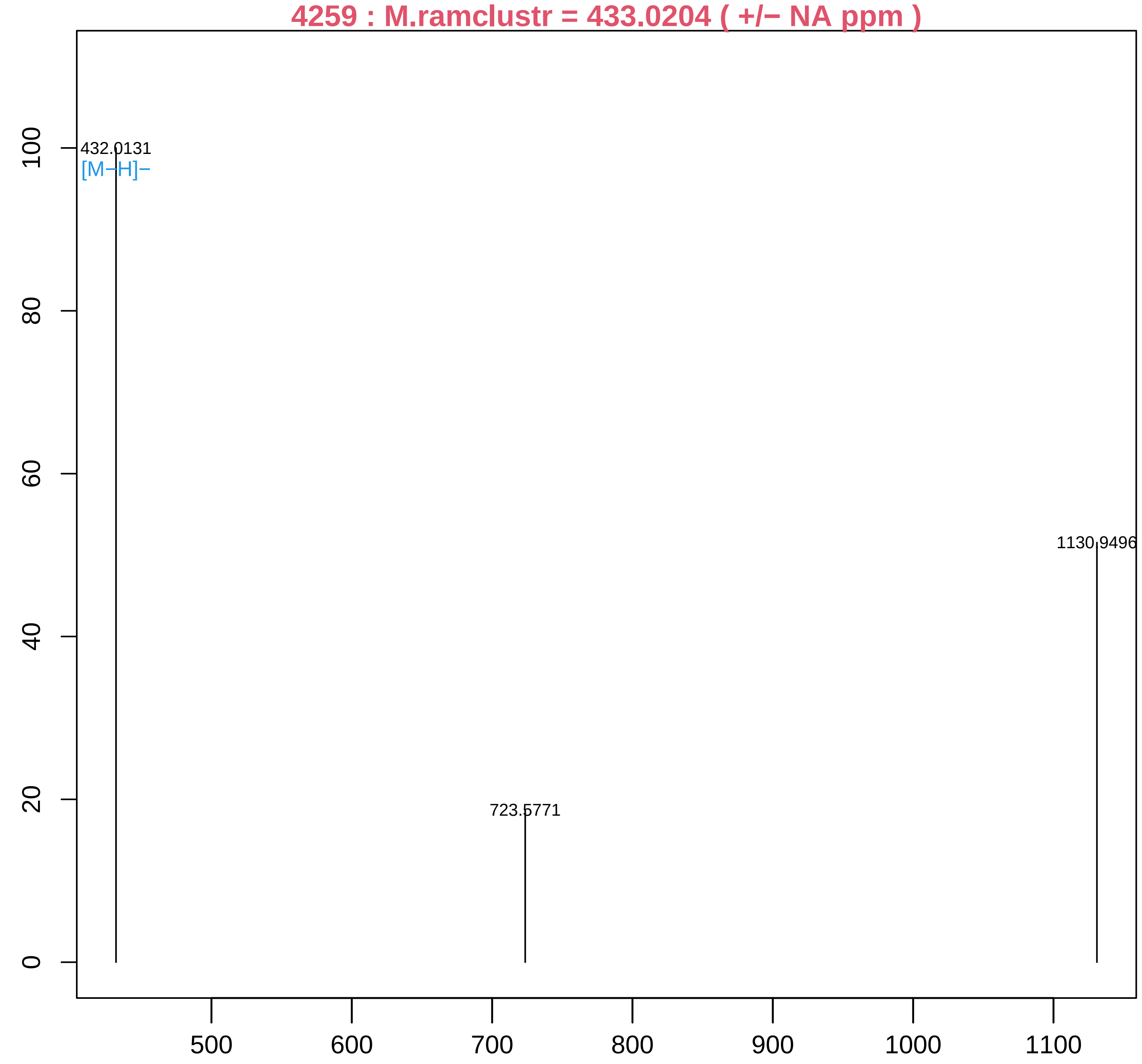
4259 : M.ramclustr = 433.0204 (+/− NA ppm)
432.0131
[M−H]−
723.5771
1130.9496
0
20
40
60
80
100
500
600
700
800
900
1000
1100
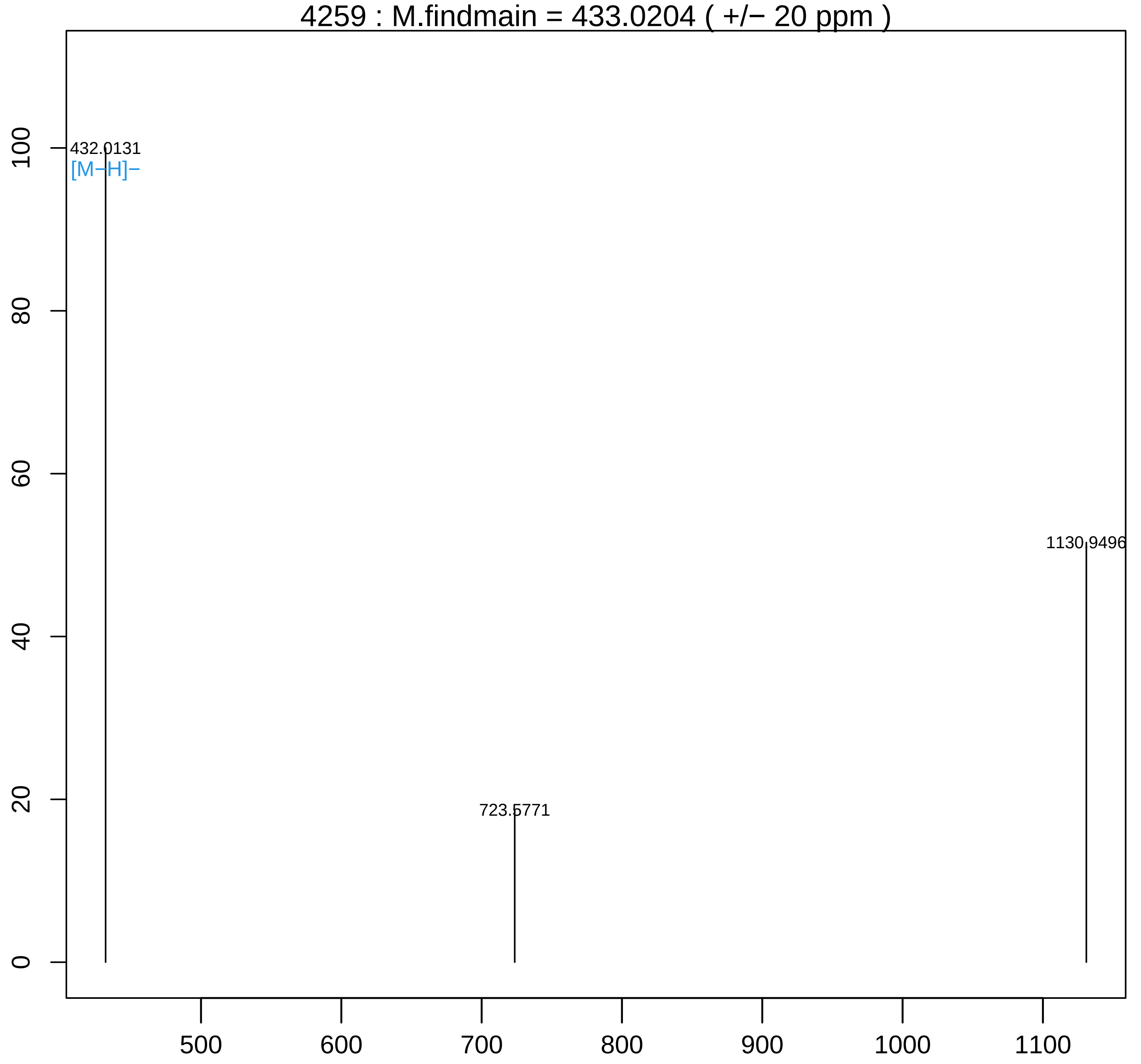
4259 : M.findmain = 433.0204 (+/− 20 ppm)
432.0131
[M−H]−
723.5771
1130.9496
0
20
40
60
80
100
500
600
700
800
900
1000
1100

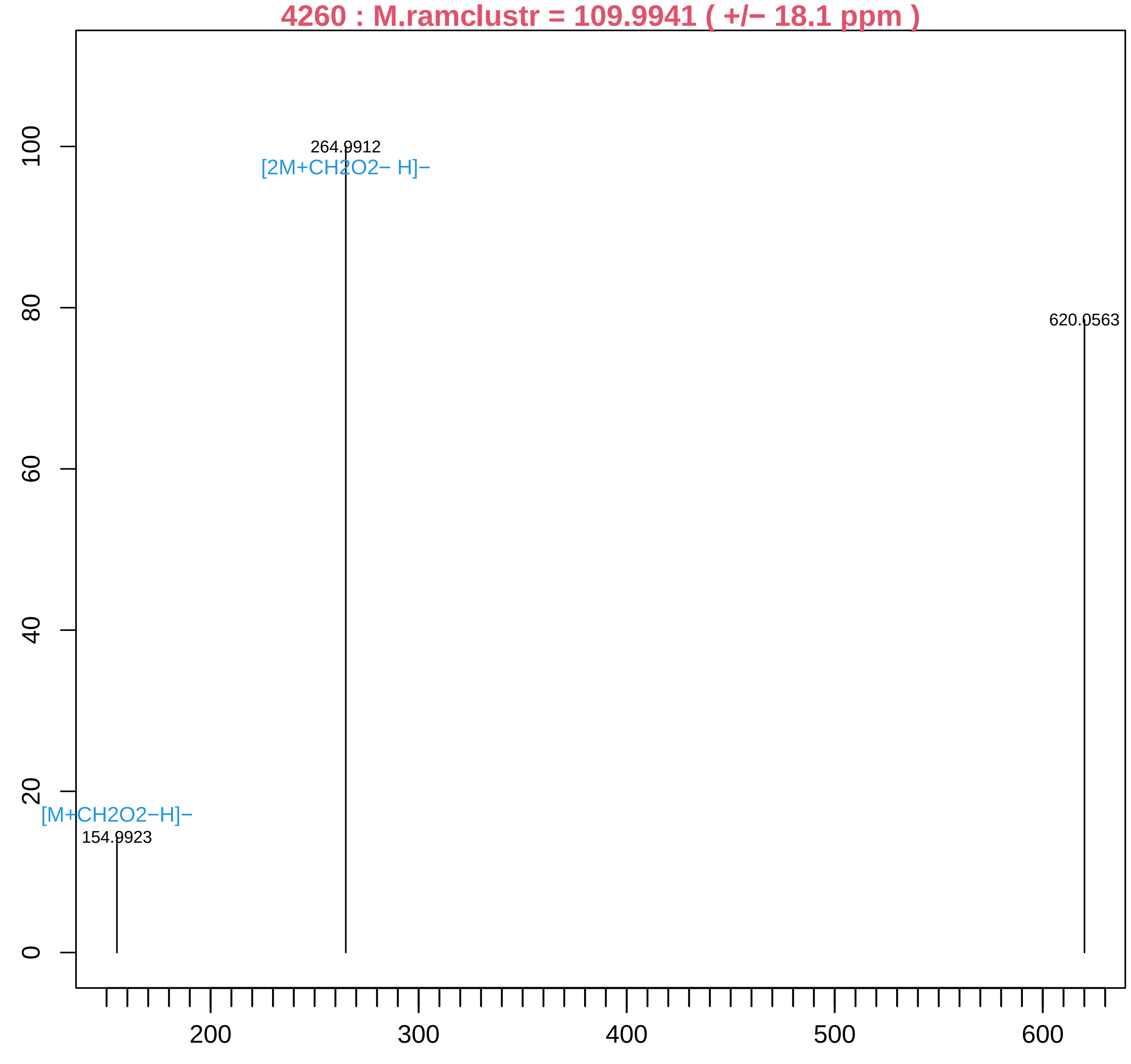

4260 : M.ramclustr = 109.9941 (+/− 18.1 ppm)
264.9912
[2M+CH2O2− H]−
620.0563
[M+CH2O2−H]−
154.9923
0
20
40
60
80
100
200
300
400
500
600

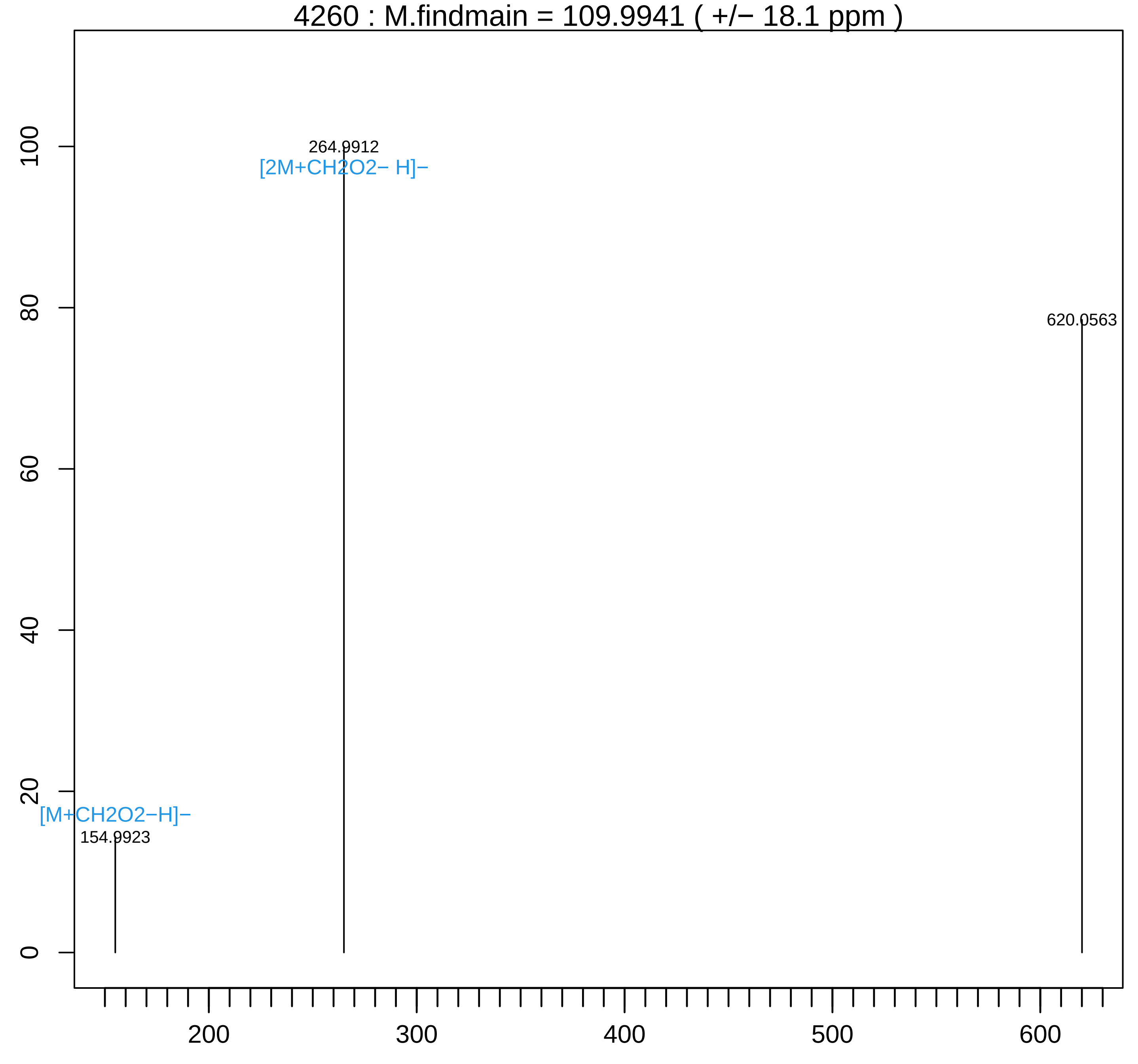

4260 : M.findmain = 109.9941 (+/− 18.1 ppm)
264.9912
[2M+CH2O2− H]−
620.0563
[M+CH2O2−H]−
154.9923
0
20
40
60
80
100
200
300
400
500
600

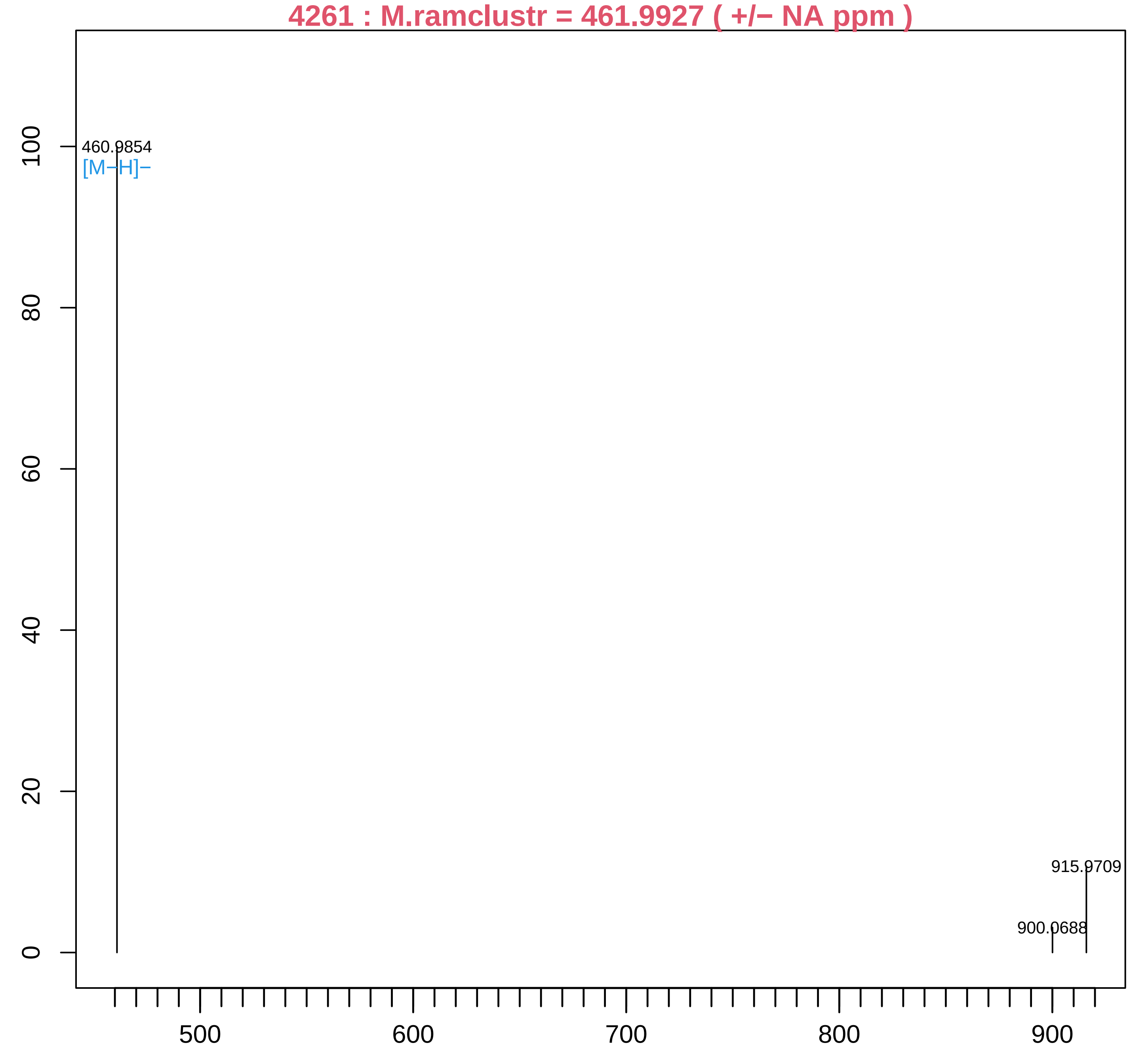
4261 : M.ramclustr = 461.9927 (+/− NA ppm)
460.9854
[M−H]−
915.9709
900.0688
0
20
40
60
80
100
500
600
700
800
900

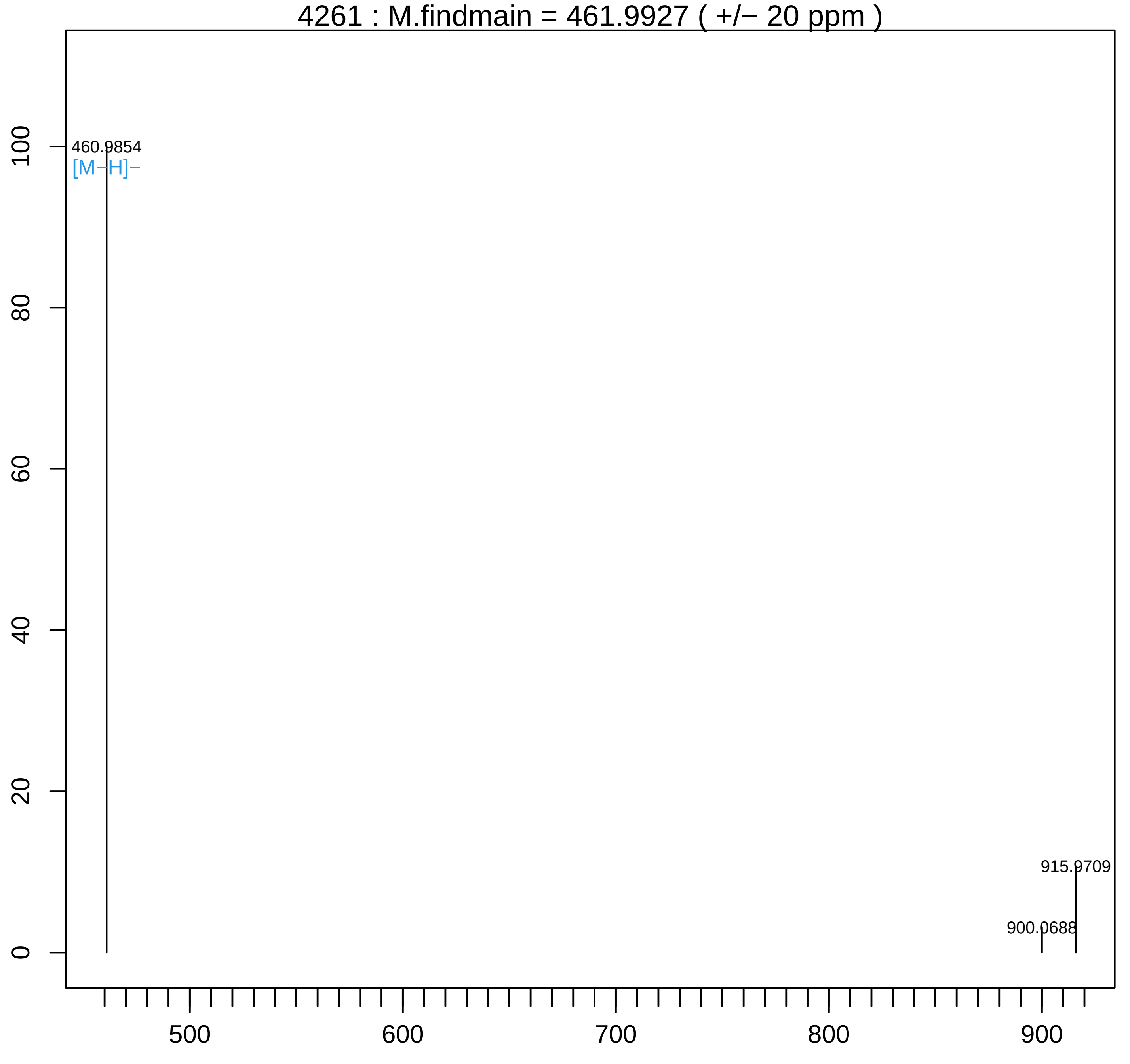
4261 : M.findmain = 461.9927 (+/− 20 ppm)
460.9854
[M−H]−
915.9709
900.0688
0
20
40
60
80
100
500
600
700
800
900

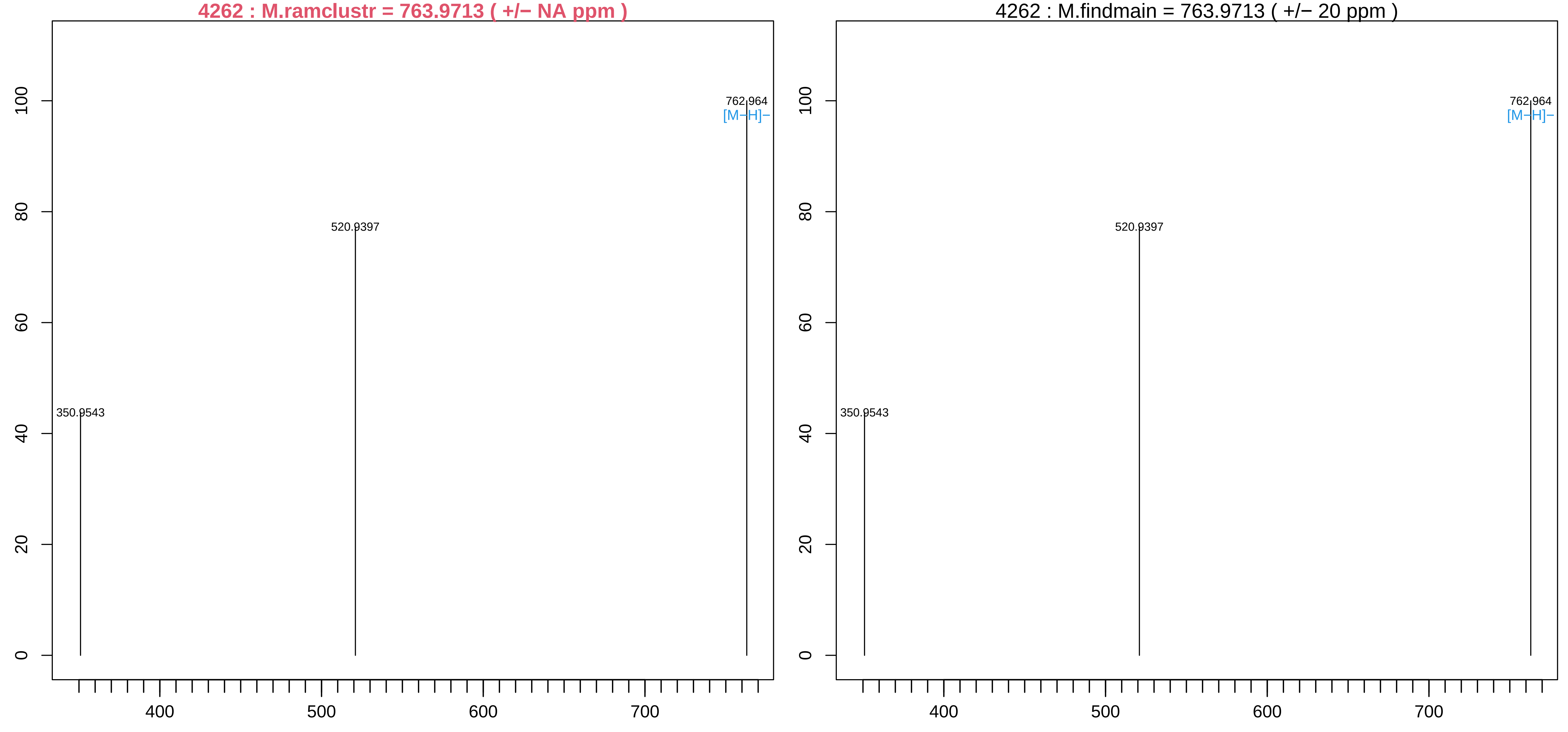

4262 : M.ramclustr = 763.9713 (+/− NA ppm)
762.964
[M−H]−
520.9397
350.9543
0
20
40
60
80
100
400
500
600
700
4262 : M.findmain = 763.9713 (+/− 20 ppm)
762.964
[M−H]−
520.9397
350.9543
0
20
40
60
80
100
400
500
600
700

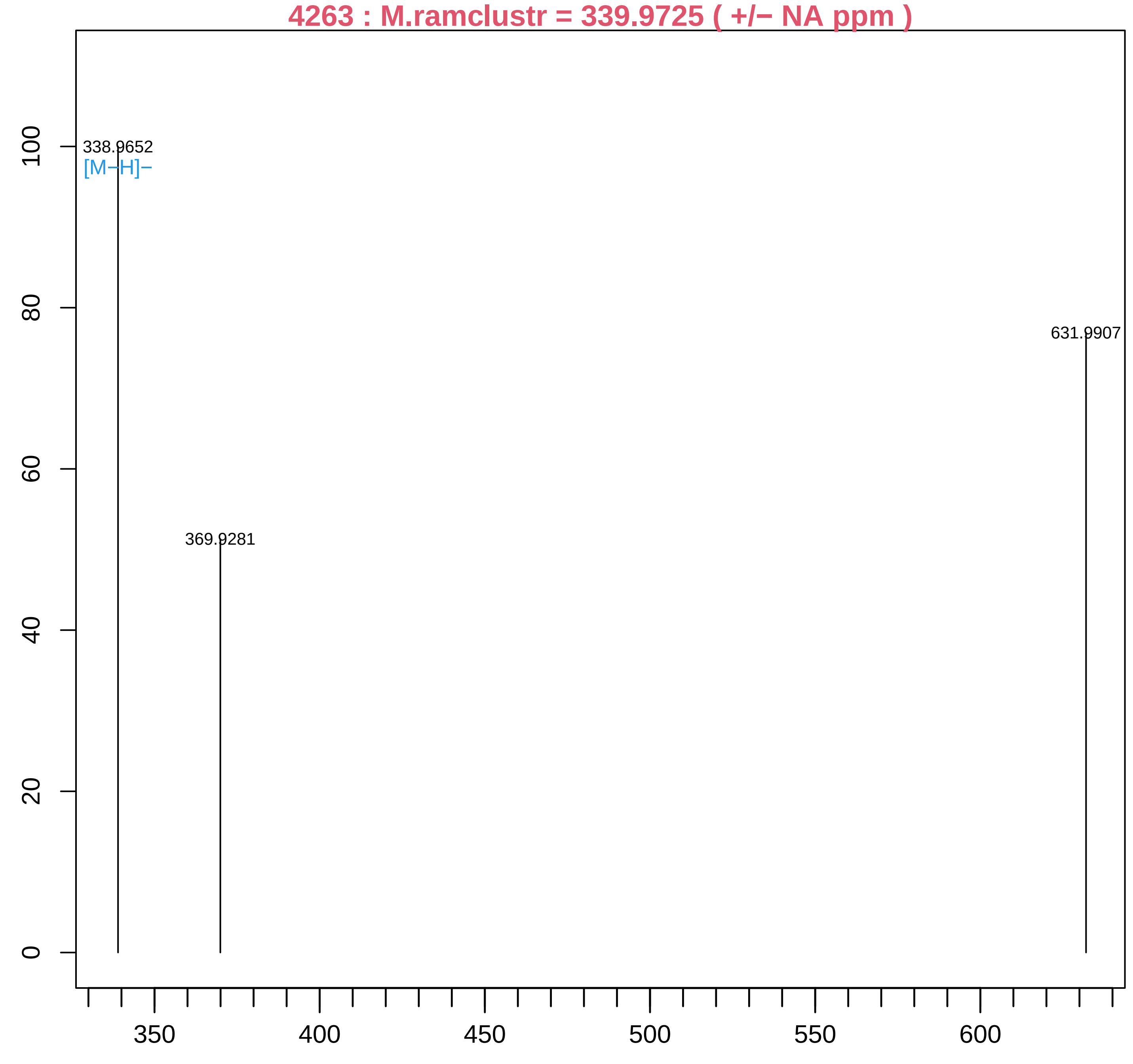

4263 : M.ramclustr = 339.9725 (+/− NA ppm)
338.9652
[M−H]−
369.9281
631.9907
0
20
40
60
80
100
350
400
450
500
550
600

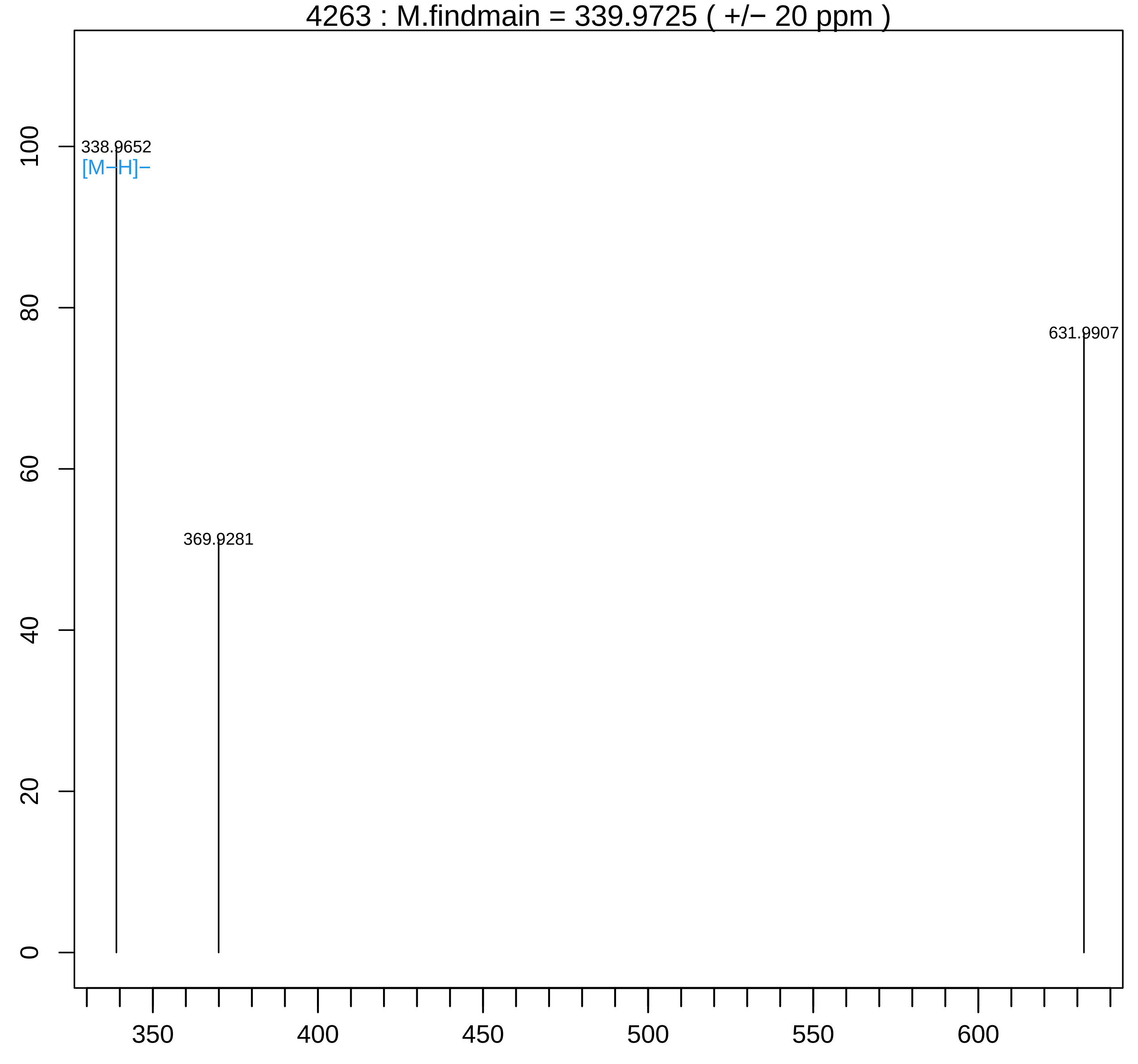

4263 : M.findmain = 339.9725 (+/− 20 ppm)
338.9652
[M−H]−
369.9281
631.9907
0
20
40
60
80
100
350
400
450
500
550
600

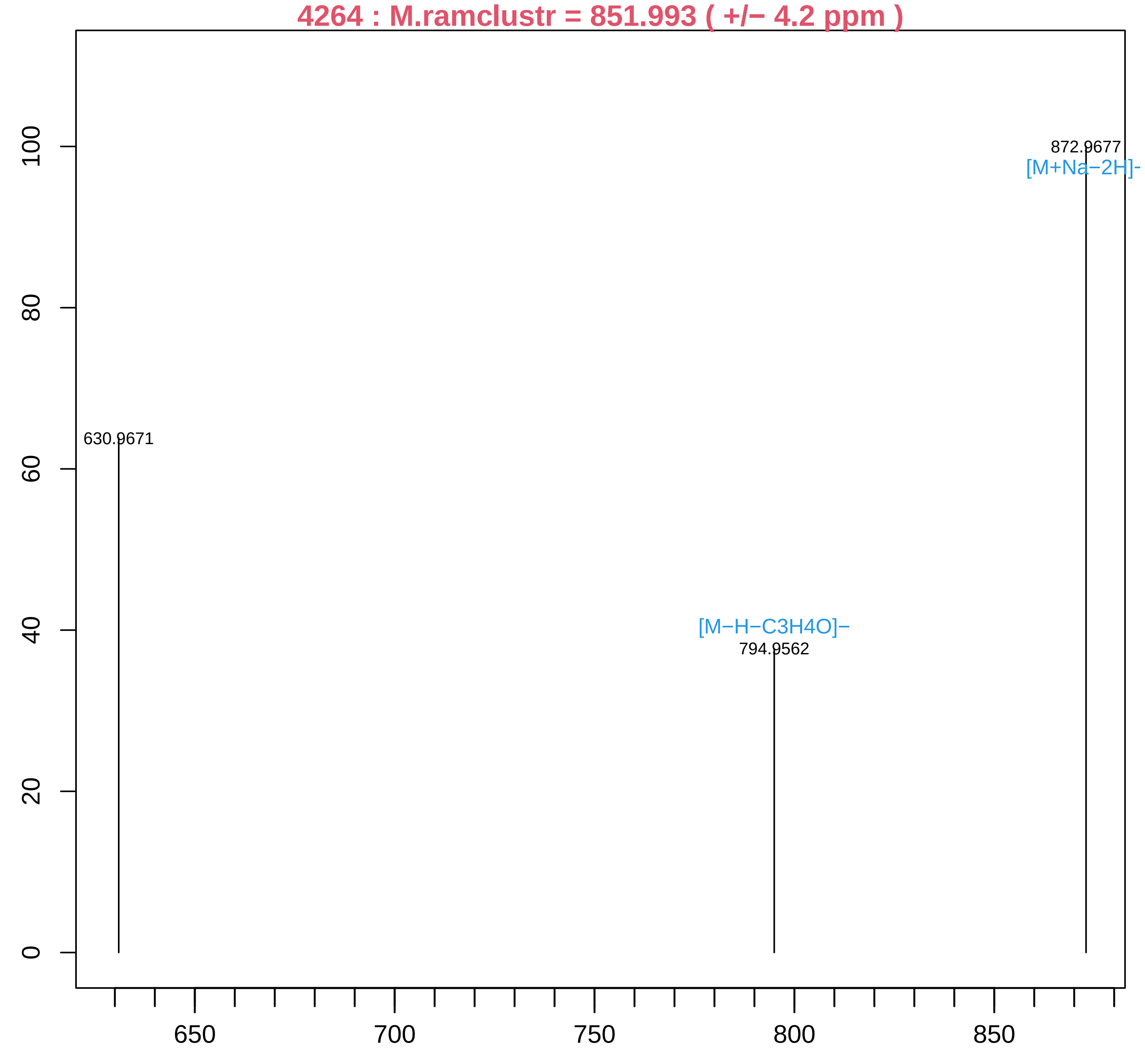
4264 : M.ramclustr = 851.993 (+/− 4.2 ppm)
872.9677
[M+Na−2H]-
630.9671
[M−H−C3H4O]−
794.9562
0
20
40
60
80
100
650
700
750
800
850

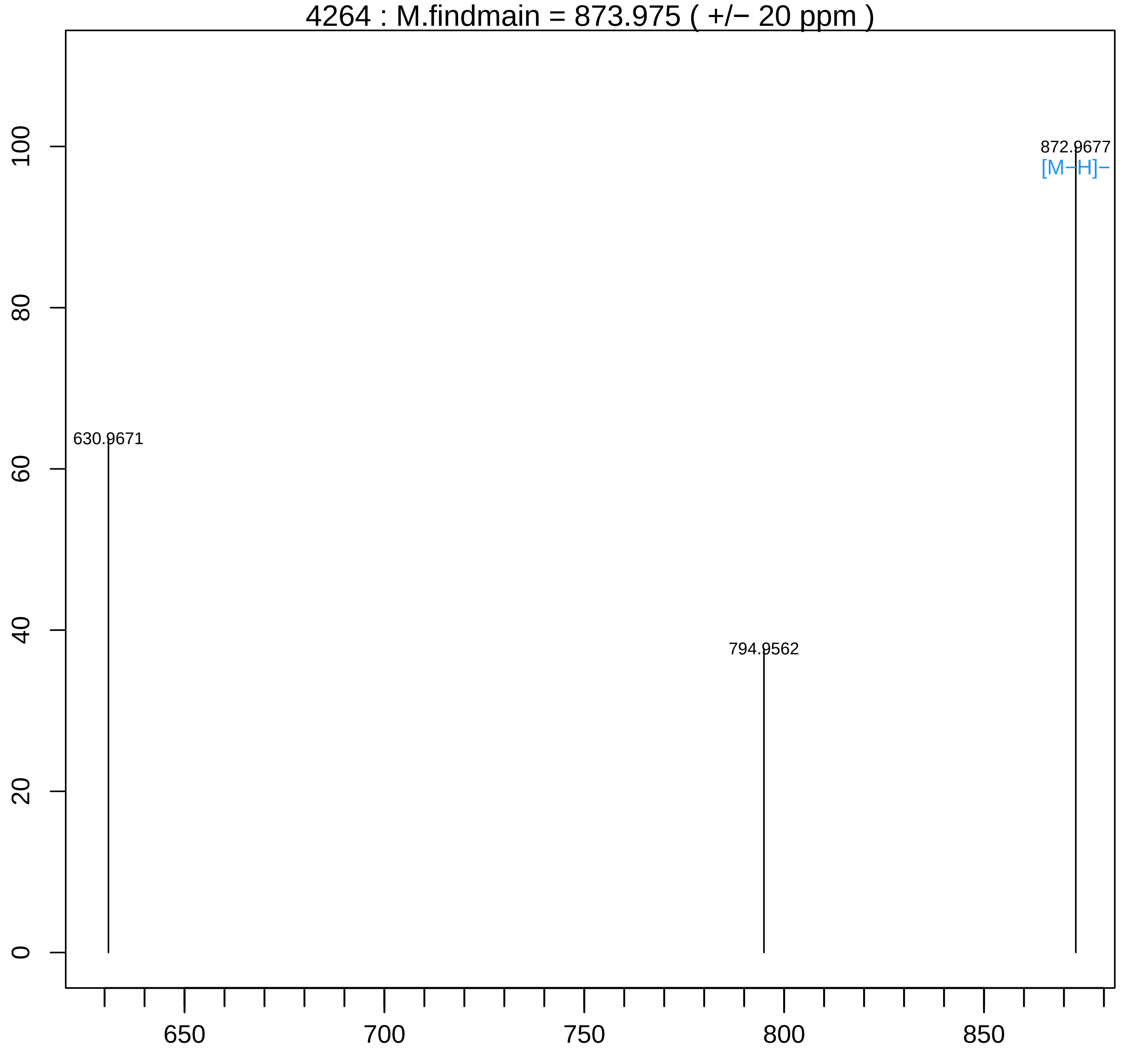
4264 : M.findmain = 873.975 (+/− 20 ppm)
872.9677
[M−H]−
630.9671
794.9562
0
20
40
60
80
100
650
700
750
800
850

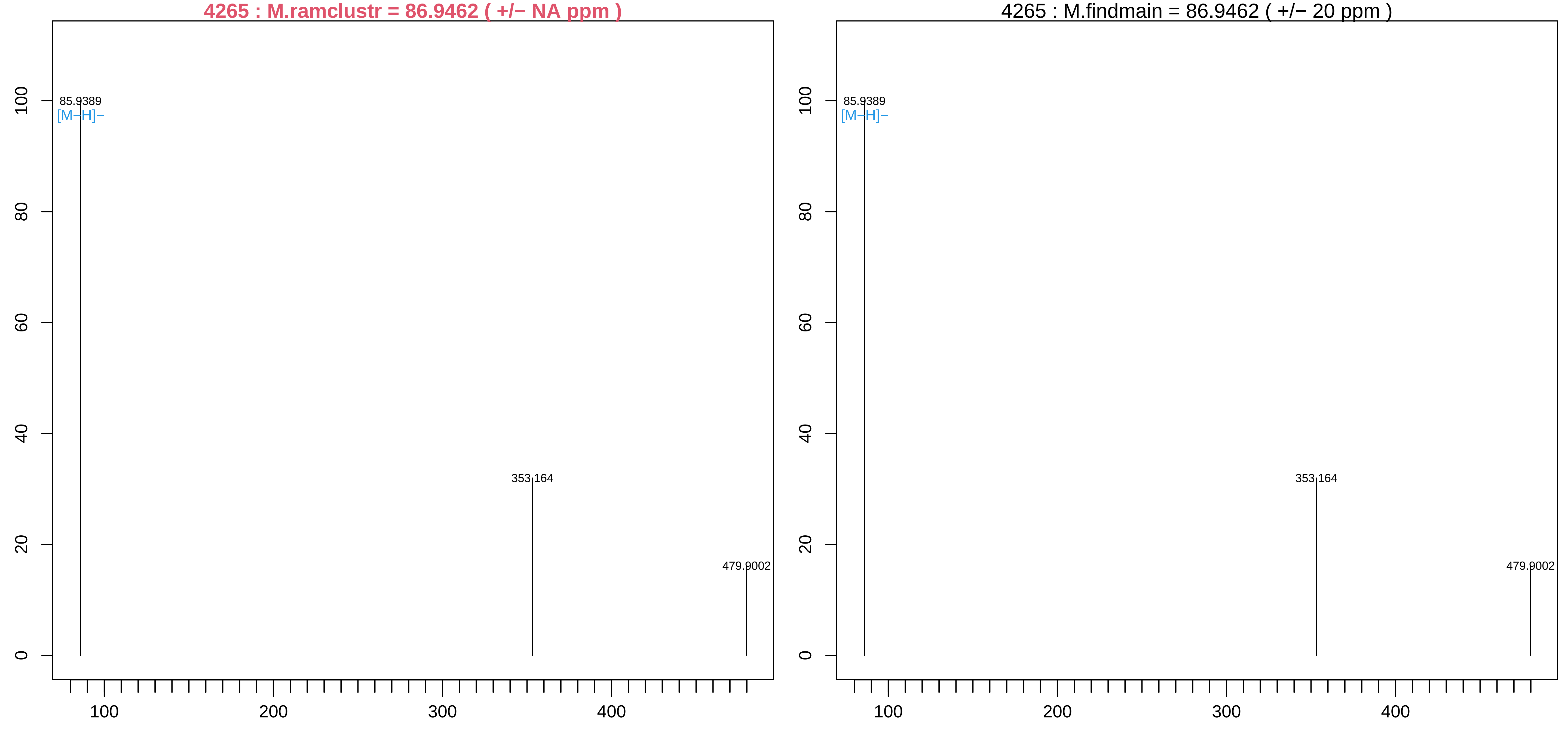
4265 : M.ramclustr = 86.9462 (+/− NA ppm)
4265 : M.findmain = 86.9462 (+/− 20 ppm)
85.9389
[M−H]−
353.164
479.9002
0
20
40
60
80
100
100
200
300
400

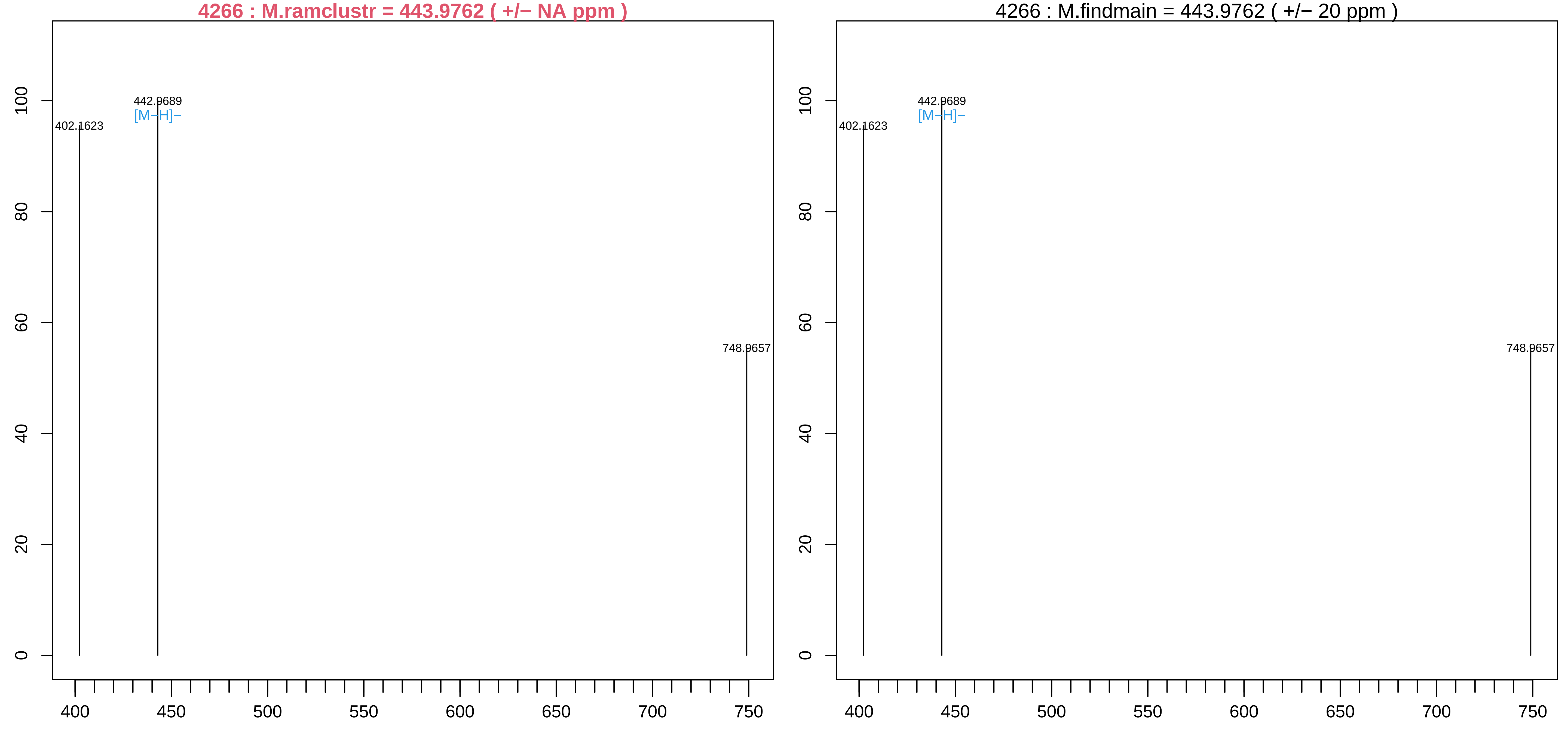
4266 : M.ramclustr = 443.9762 (+/− NA ppm)
442.9689
[M−H]−
402.1623
748.9657
4266 : M.findmain = 443.9762 (+/− 20 ppm)
442.9689
[M−H]−
402.1623
748.9657

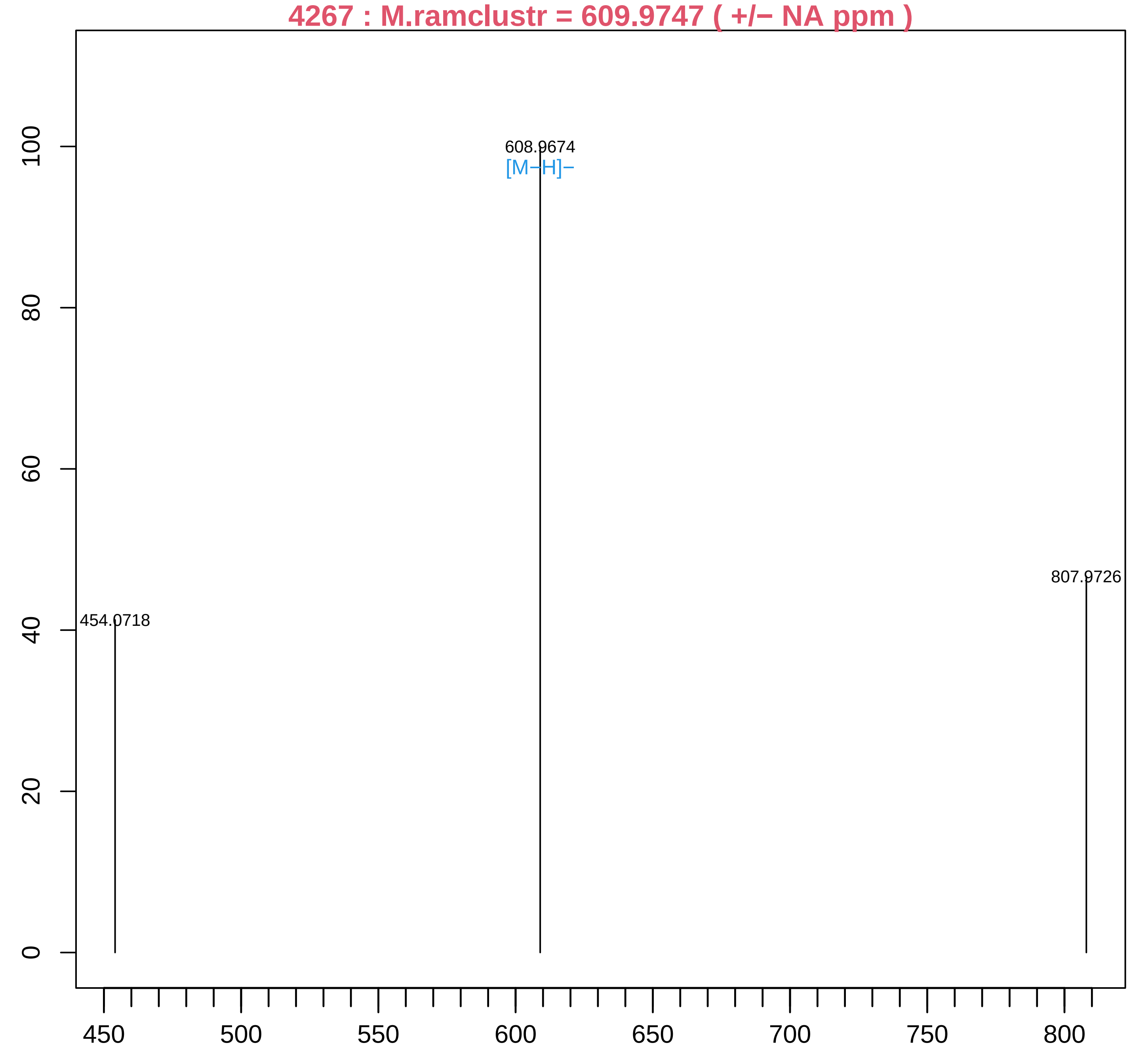
4267 : M.ramclustr = 609.9747 (+/− NA ppm)
608.9674
[M−H]−
454.0718
807.9726
0
20
40
60
80
100
450
500
550
600
650
700
750
800

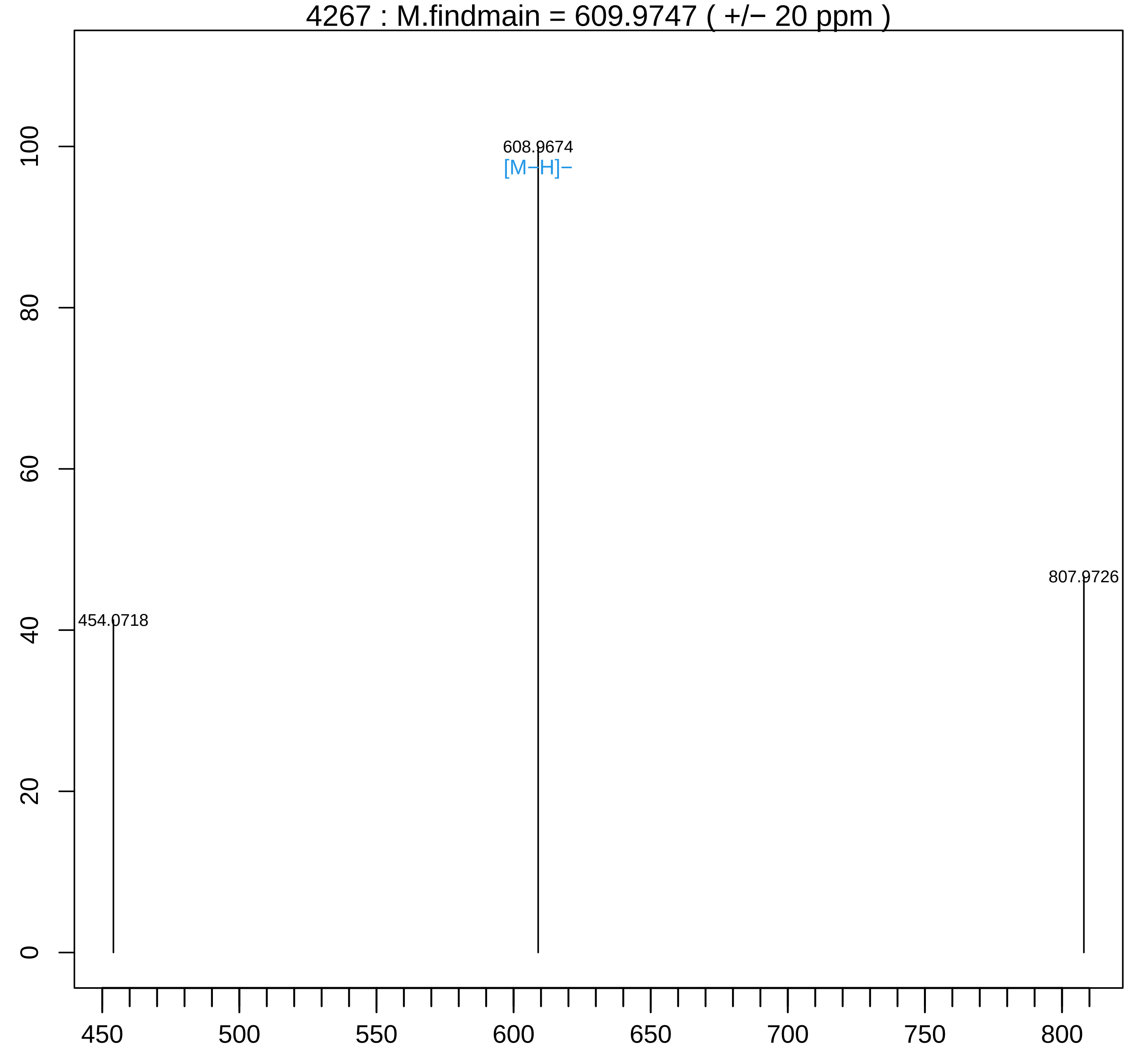
4267 : M.findmain = 609.9747 (+/− 20 ppm)
608.9674
[M−H]−
454.0718
807.9726
0
20
40
60
80
100
450
500
550
600
650
700
750
800

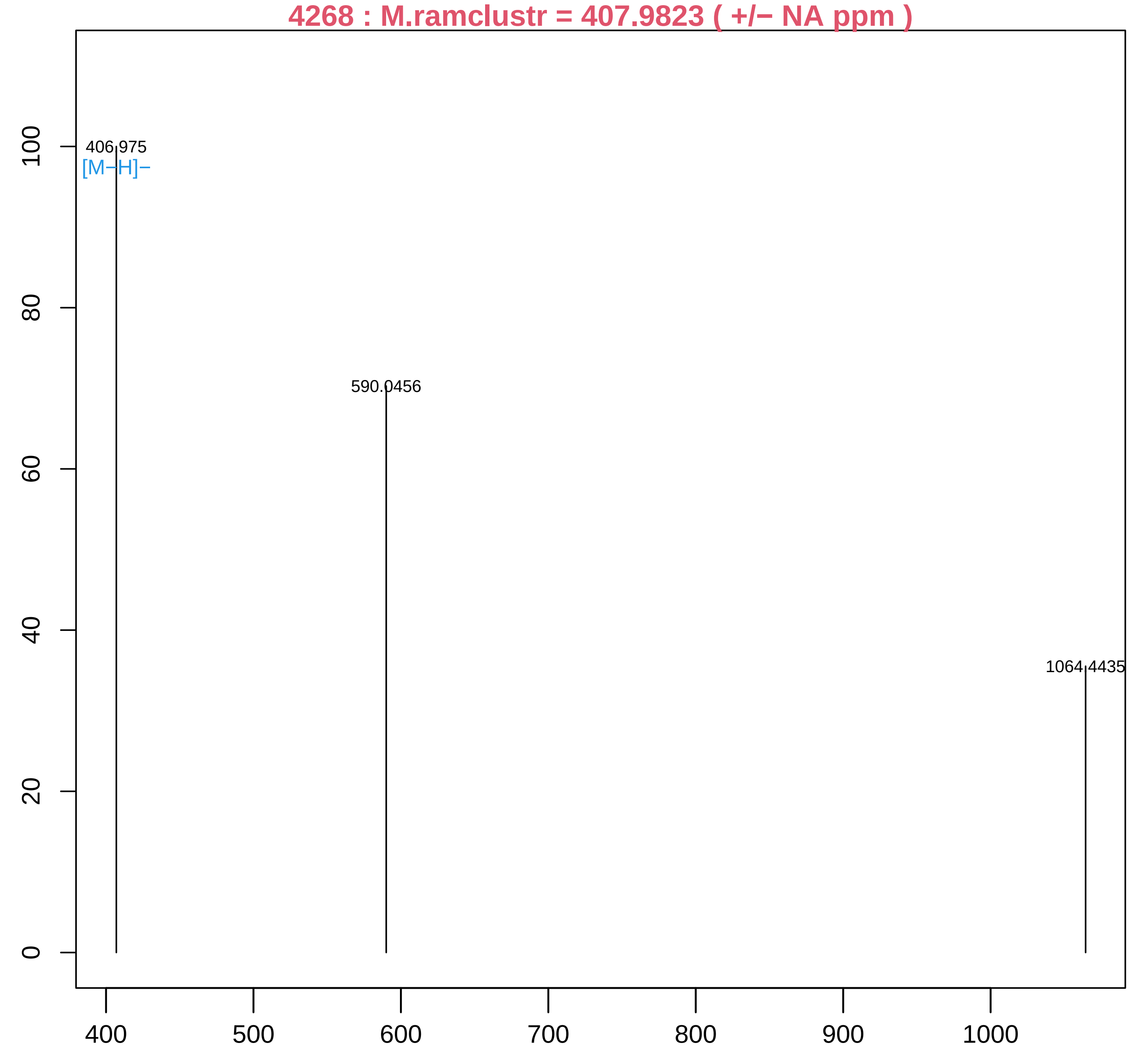

4268 : M.ramclustr = 407.9823 (+/− NA ppm)
406.975
[M−H]−
590.0456
1064.4435

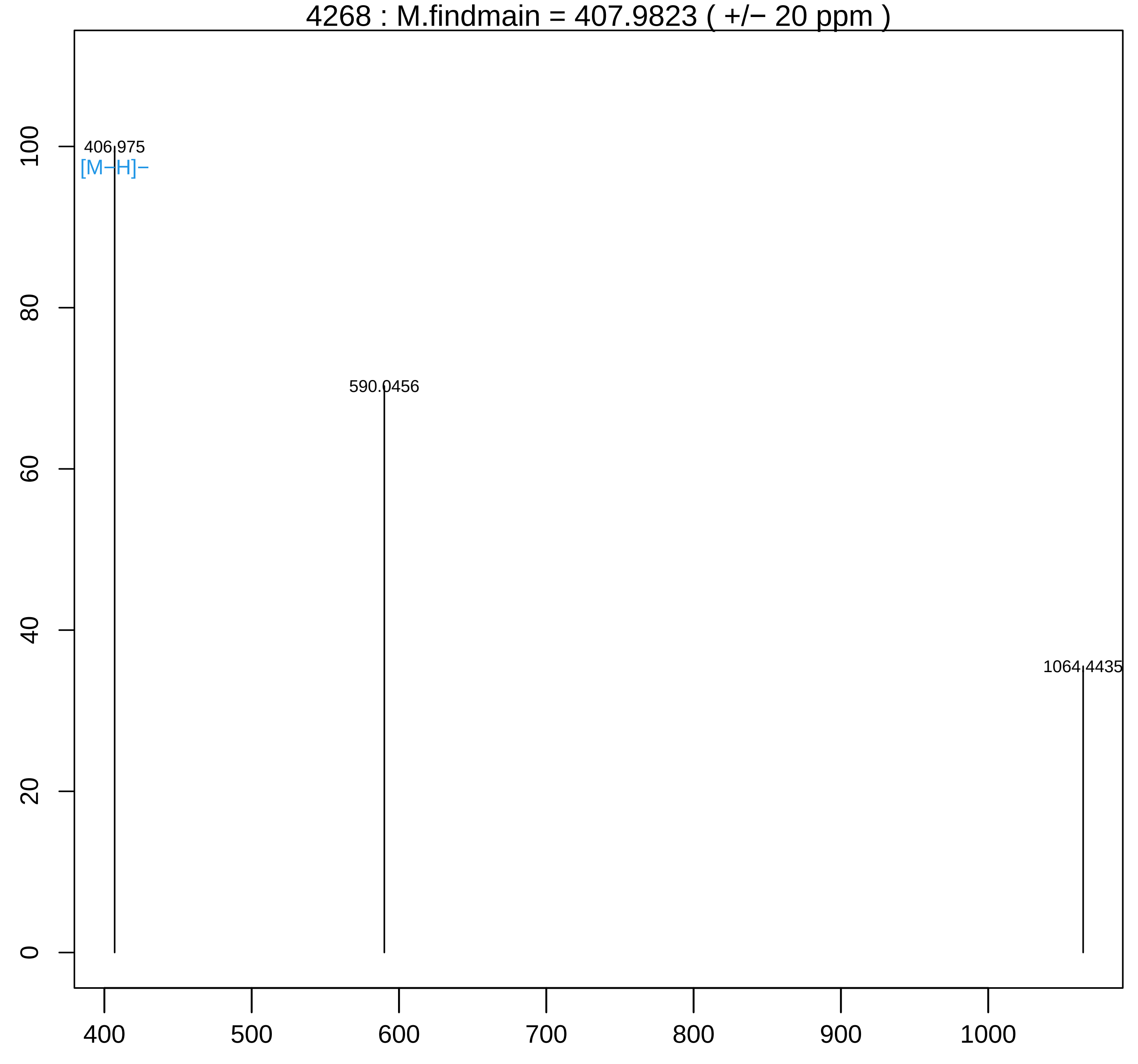

4268 : M.findmain = 407.9823 (+/− 20 ppm)
406.975
[M−H]−
590.0456
1064.4435

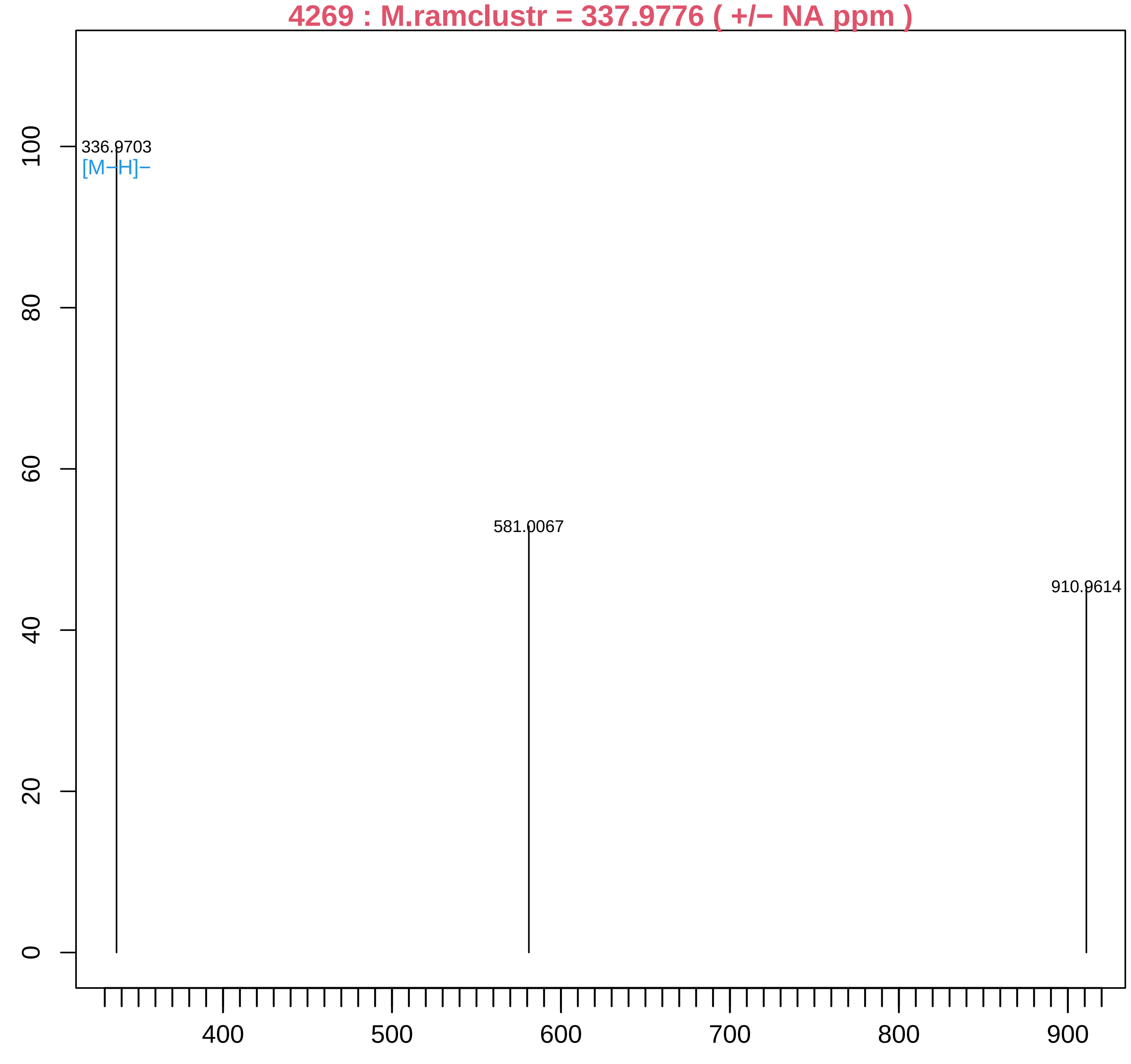
4269 : M.ramclustr = 337.9776 (+/− NA ppm)
336.9703
[M−H]−
581.0067
910.9614
0
20
40
60
80
100
400
500
600
700
800
900

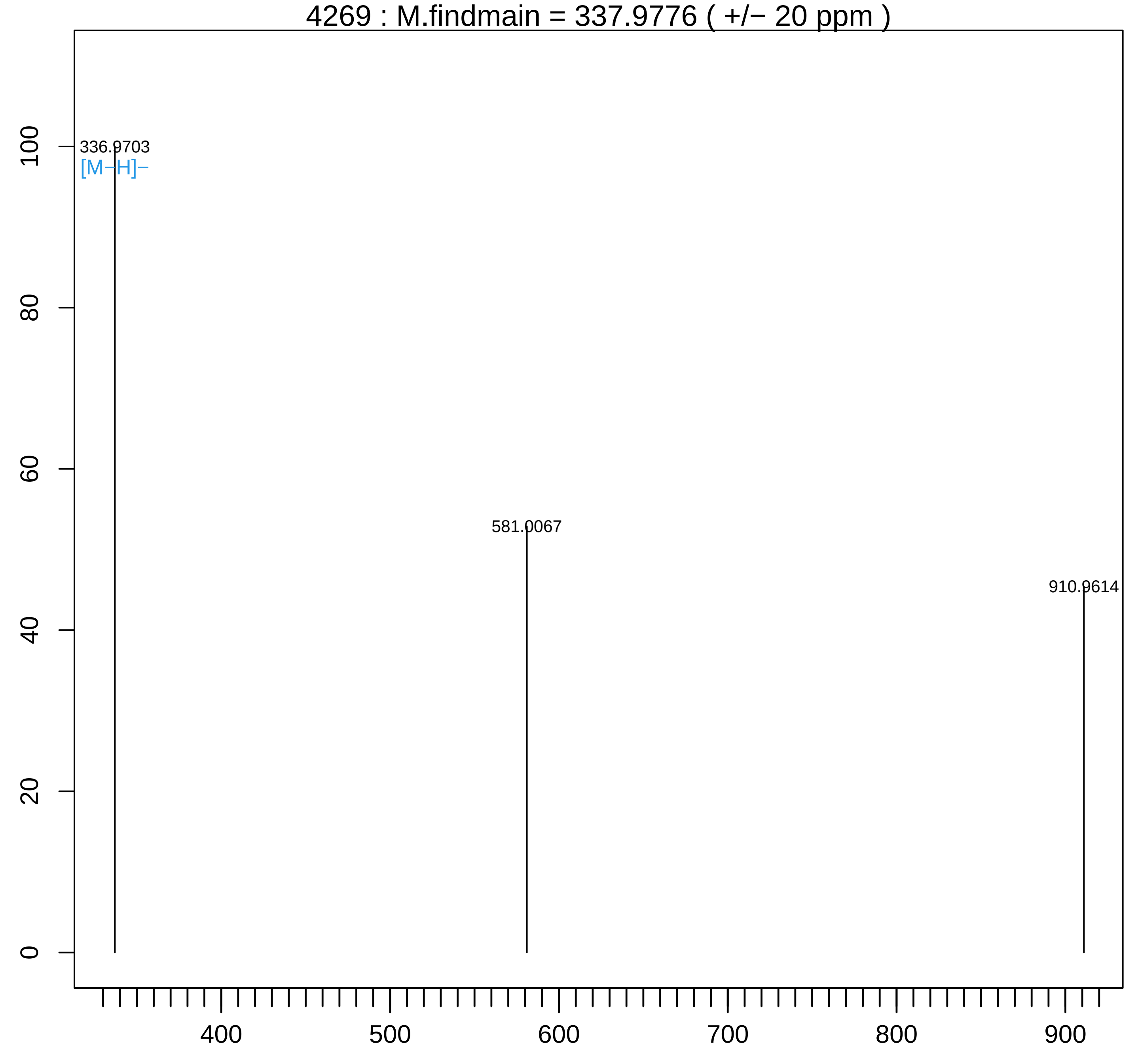
4269 : M.findmain = 337.9776 (+/− 20 ppm)
336.9703
[M−H]−
581.0067
910.9614
0
20
40
60
80
100
400
500
600
700
800
900

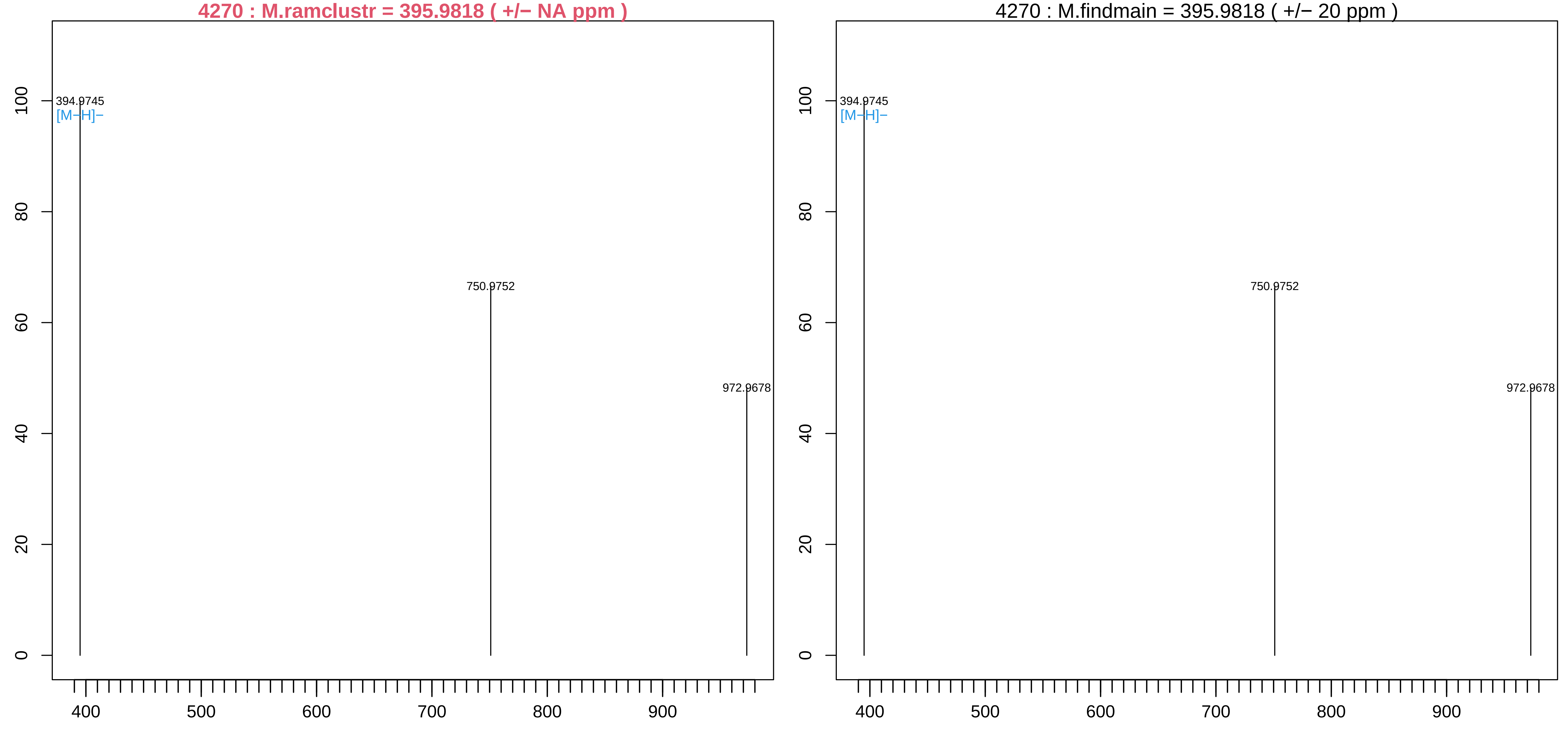
4270 : M.ramclustr = 395.9818 (+/− NA ppm)
394.9745
[M−H]−
750.9752
972.9678
4270 : M.findmain = 395.9818 (+/− 20 ppm)
394.9745
[M−H]−
750.9752
972.9678
0
20
40
60
80
100
400
500
600
700
800
900

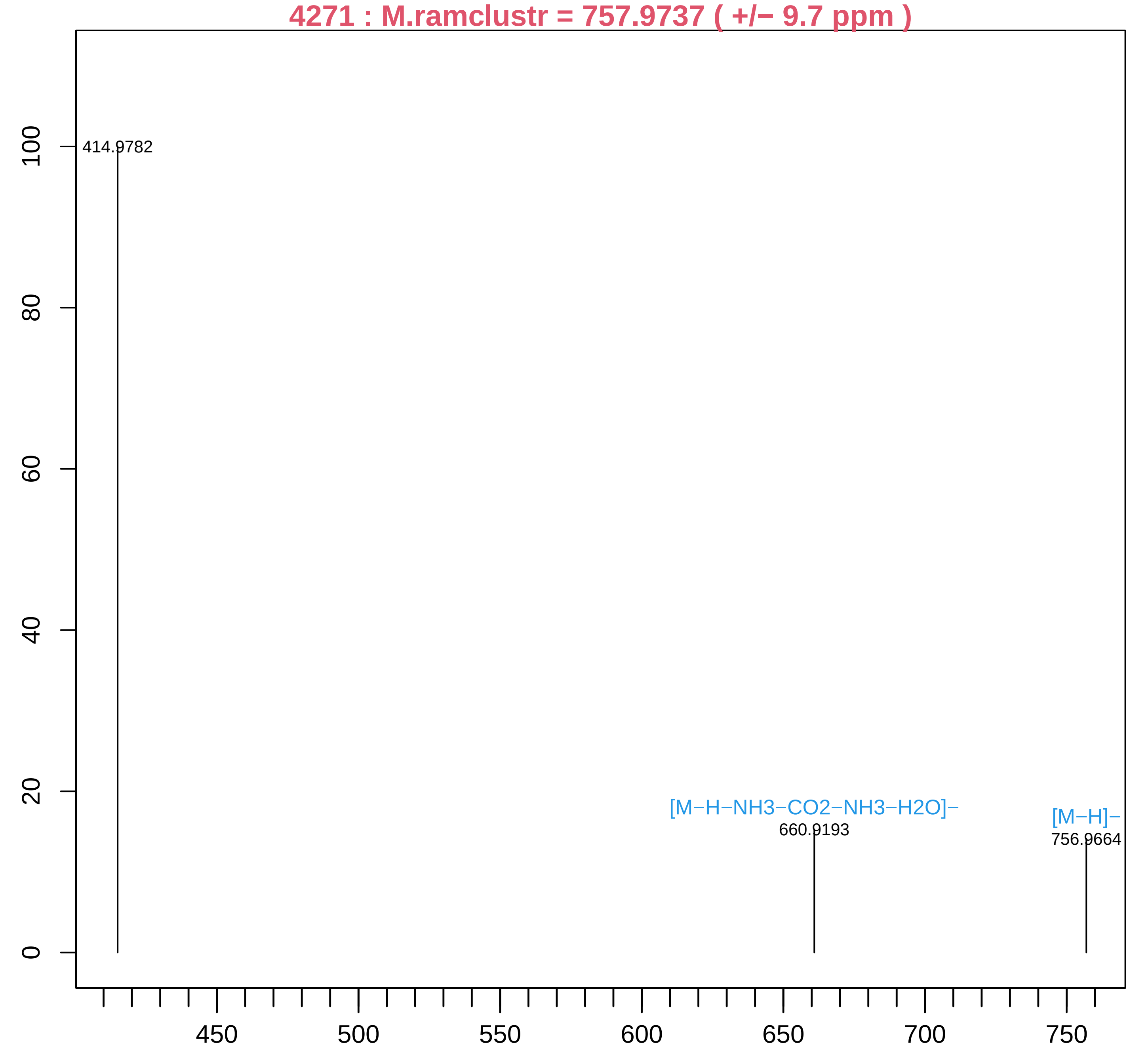

4271 : M.ramclustr = 757.9737 (+/− 9.7 ppm)
414.9782
[M−H−NH3−CO2−NH3−H2O]−
660.9193
[M−H]−
756.9664
0
20
40
60
80
100
450
500
550
600
650
700
750

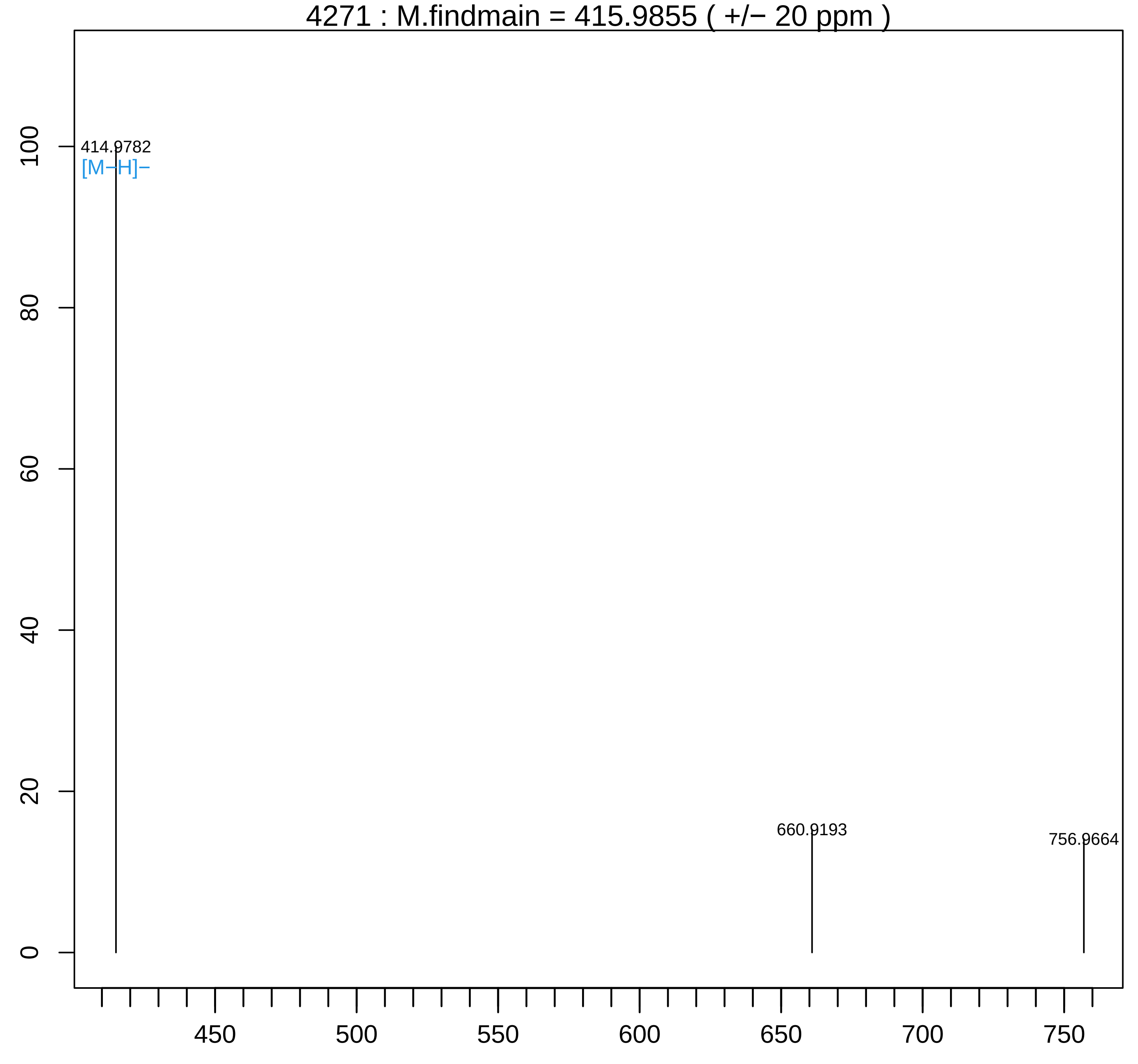

4271 : M.findmain = 415.9855 (+/− 20 ppm)
414.9782
[M−H]−
660.9193
756.9664
0
20
40
60
80
100
450
500
550
600
650
700
750

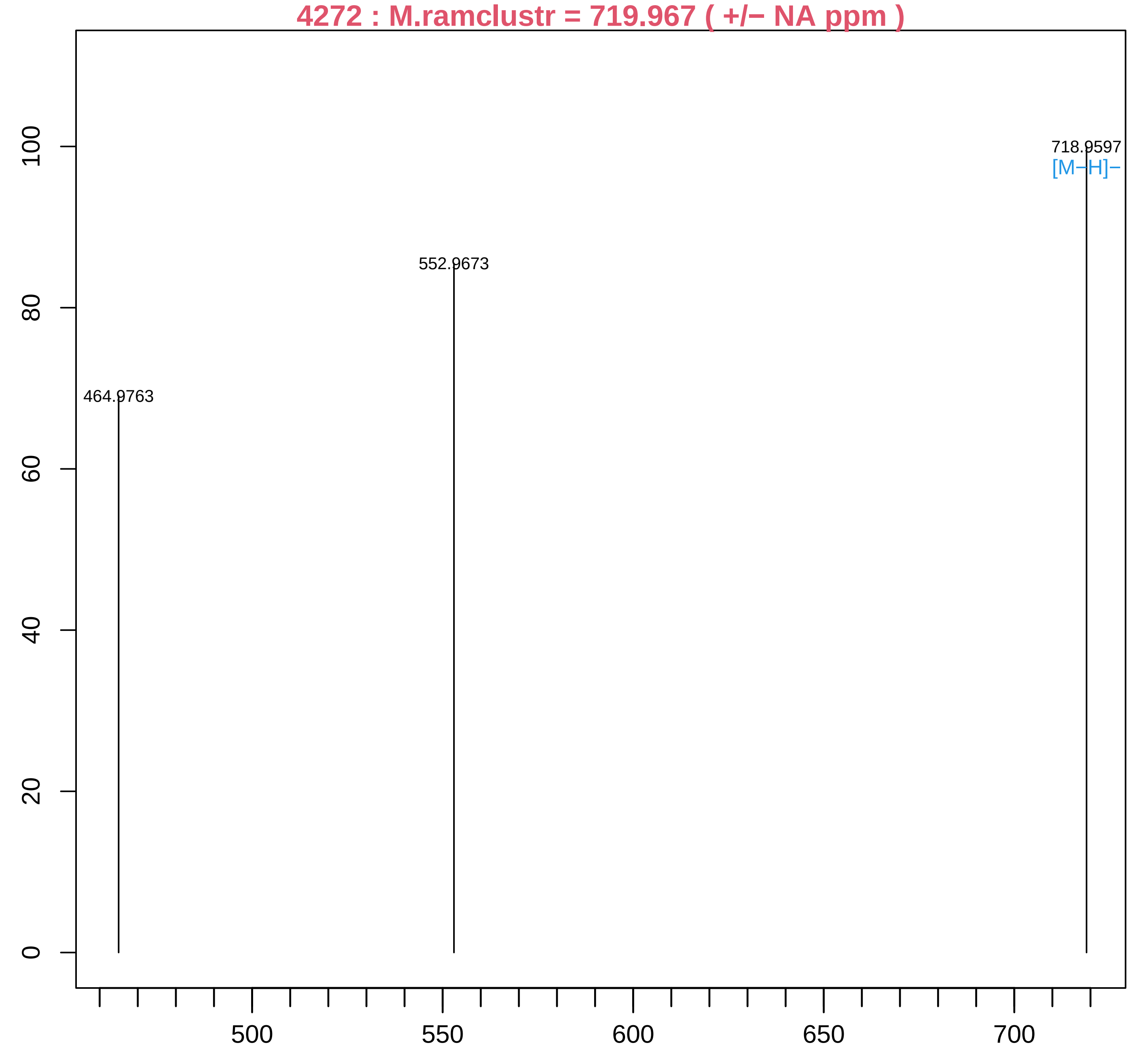
4272 : M.ramclustr = 719.967 (+/− NA ppm)
718.9597
[M−H]−
552.9673
464.9763
0
20
40
60
80
100
500
550
600
650
700

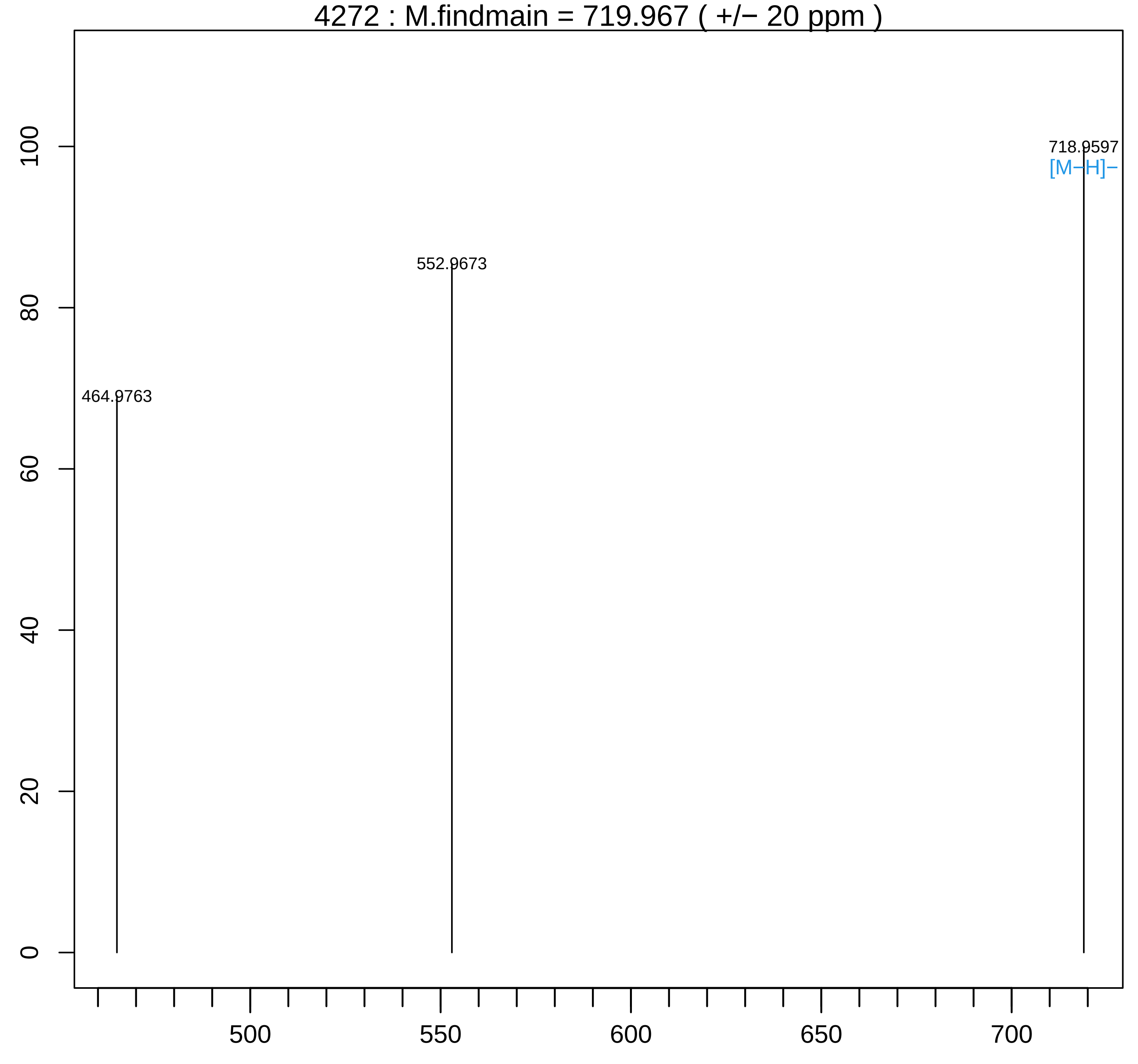
4272 : M.findmain = 719.967 (+/− 20 ppm)
718.9597
[M−H]−
552.9673
464.9763
0
20
40
60
80
100
500
550
600
650
700

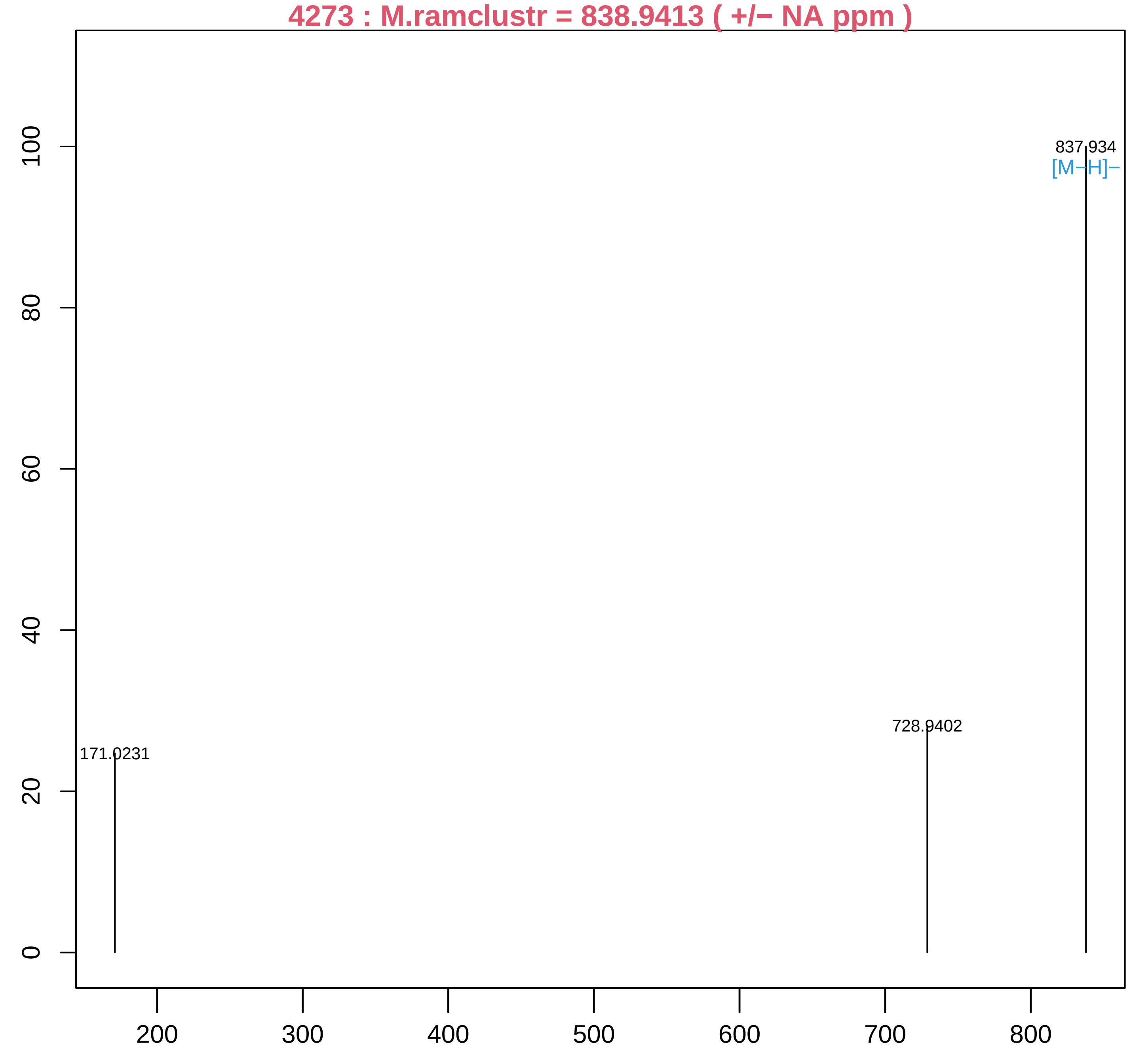
4273 : M.ramclustr = 838.9413 (+/− NA ppm)
837.934
[M−H]−
728.9402
171.0231
0
20
40
60
80
100
200
300
400
500
600
700
800

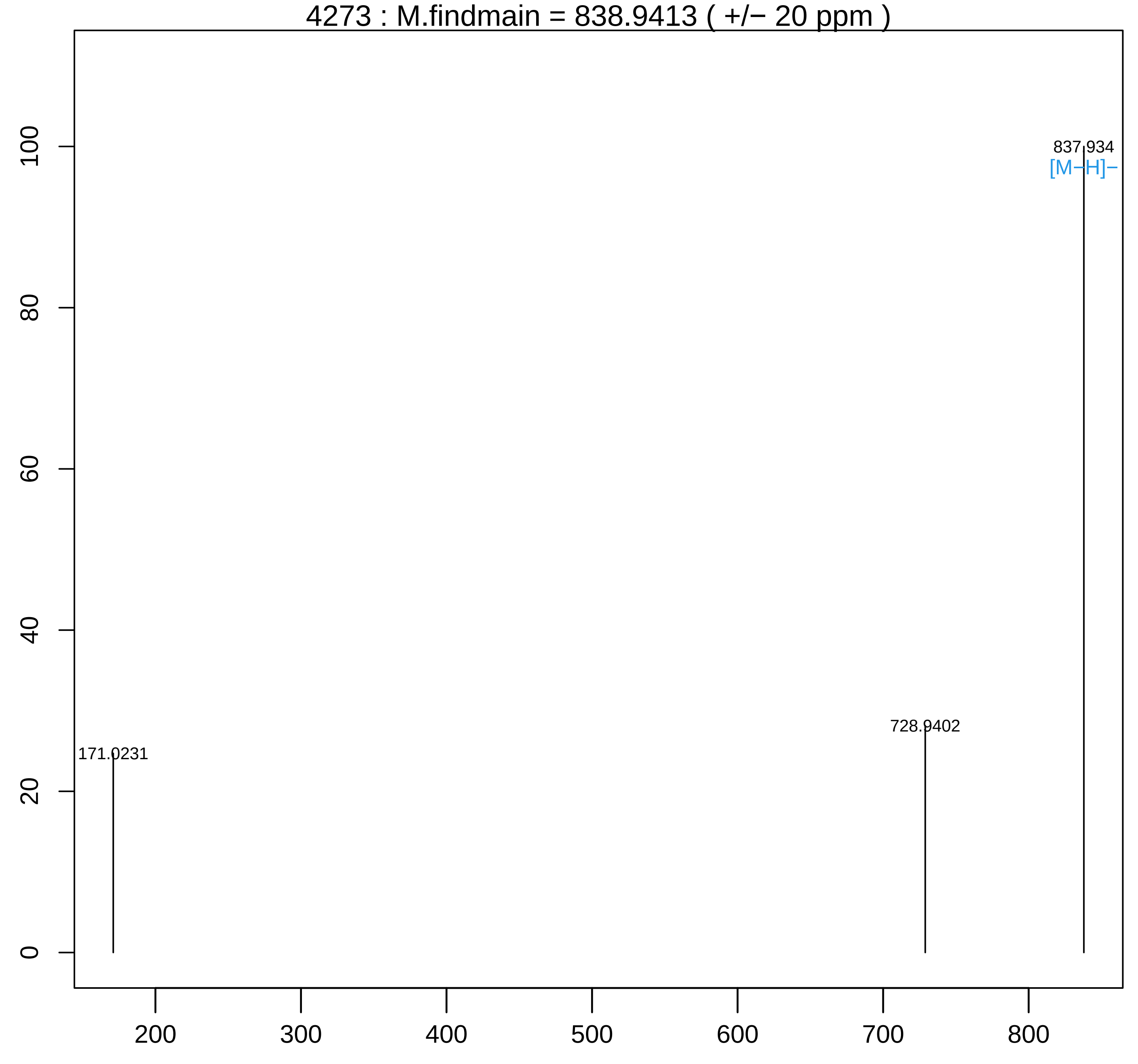
4273 : M.findmain = 838.9413 (+/− 20 ppm)
837.934
[M−H]−
728.9402
171.0231
0
20
40
60
80
100
200
300
400
500
600
700
800

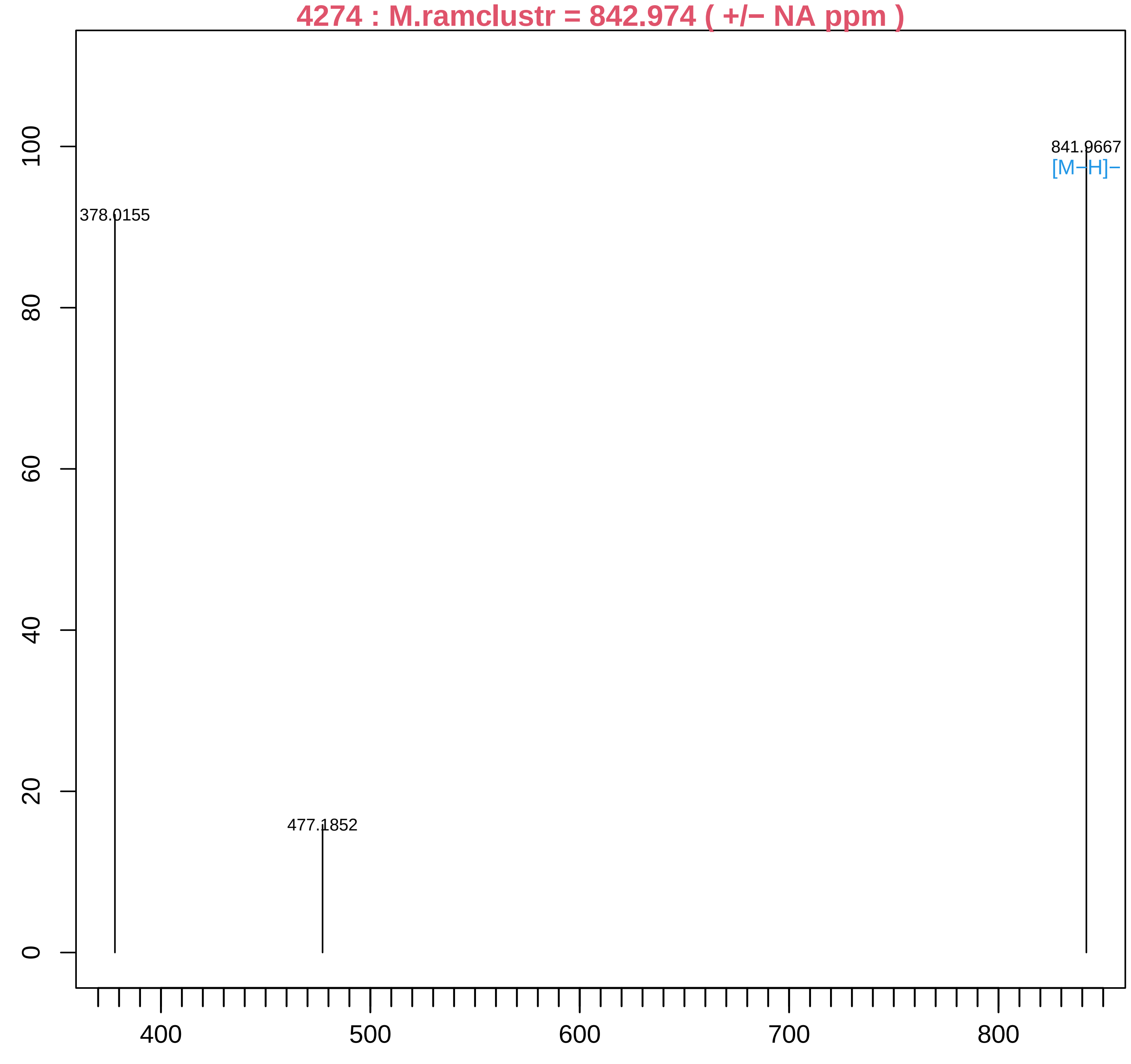
4274 : M.ramclustr = 842.974 (+/− NA ppm)
378.0155
477.1852
841.9667
[M−H]−
0
20
40
60
80
100
400
500
600
700
800

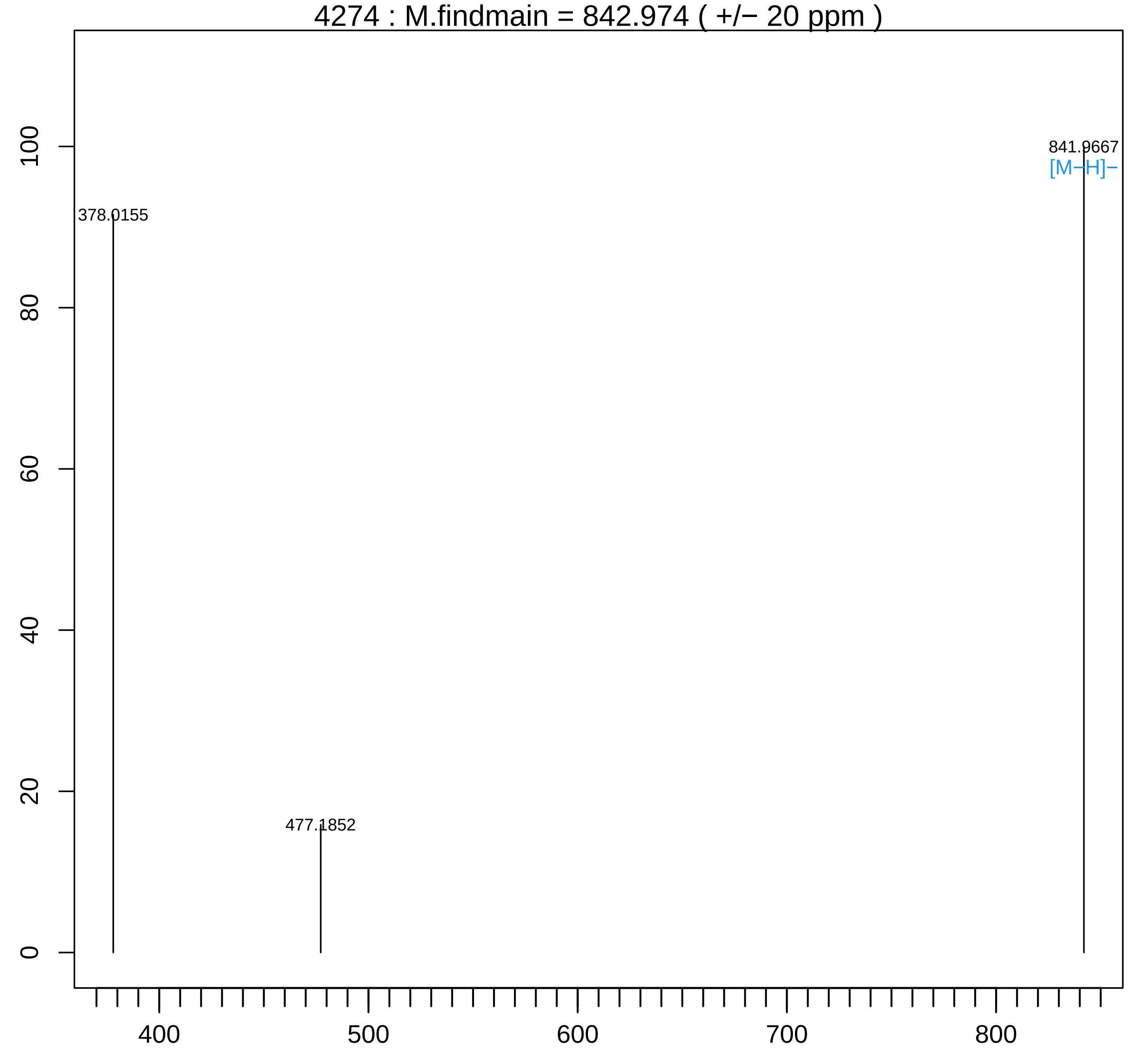
4274 : M.findmain = 842.974 (+/− 20 ppm)
378.0155
477.1852
841.9667
[M−H]−
0
20
40
60
80
100
400
500
600
700
800

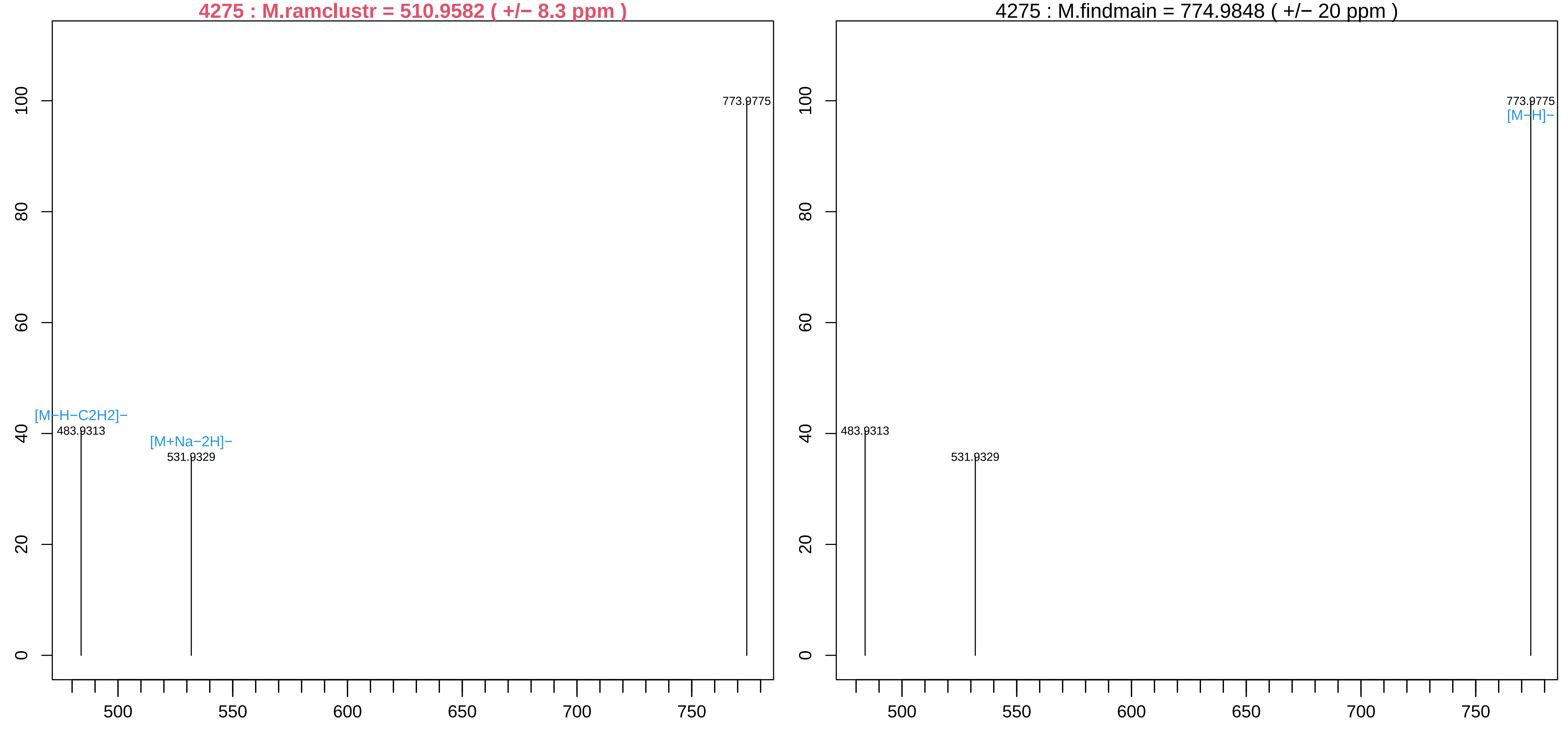
4275 : M.ramclustr = 510.9582 (+/− 8.3 ppm)
773.9775
[M−H−C2H2]−
483.9313
[M+Na−2H]−
531.9329
4275 : M.findmain = 774.9848 (+/− 20 ppm)
773.9775
[M−H]−
483.9313
531.9329
0
20
40
60
80
100
500
550
600
650
700
750

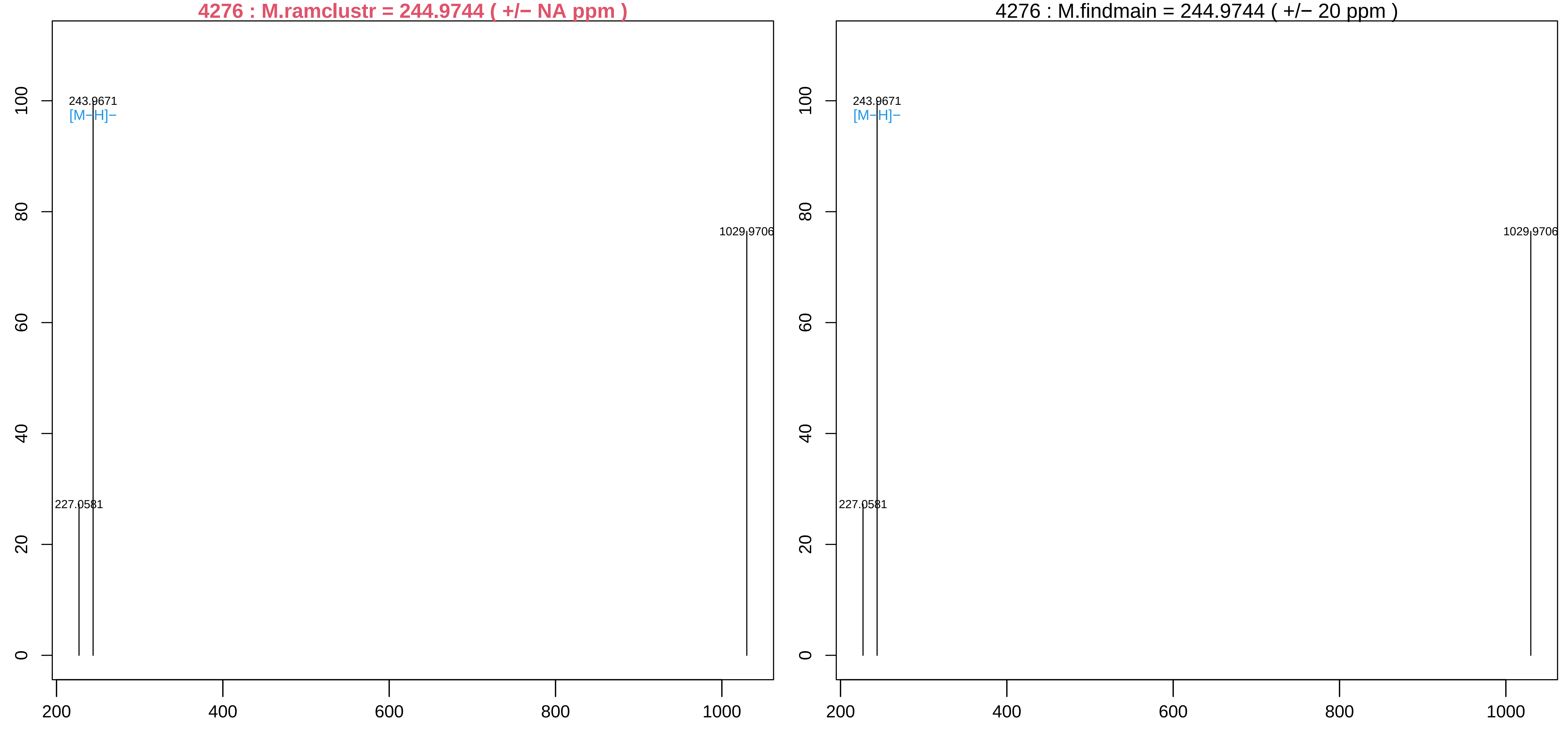

4276 : M.ramclustr = 244.9744 (+/− NA ppm)
243.9671
[M−H]−
1029.9706
227.0581
0
20
40
60
80
100
200
400
600
800
1000
4276 : M.findmain = 244.9744 (+/− 20 ppm)
243.9671
[M−H]−
1029.9706
227.0581
0
20
40
60
80
100
200
400
600
800
1000

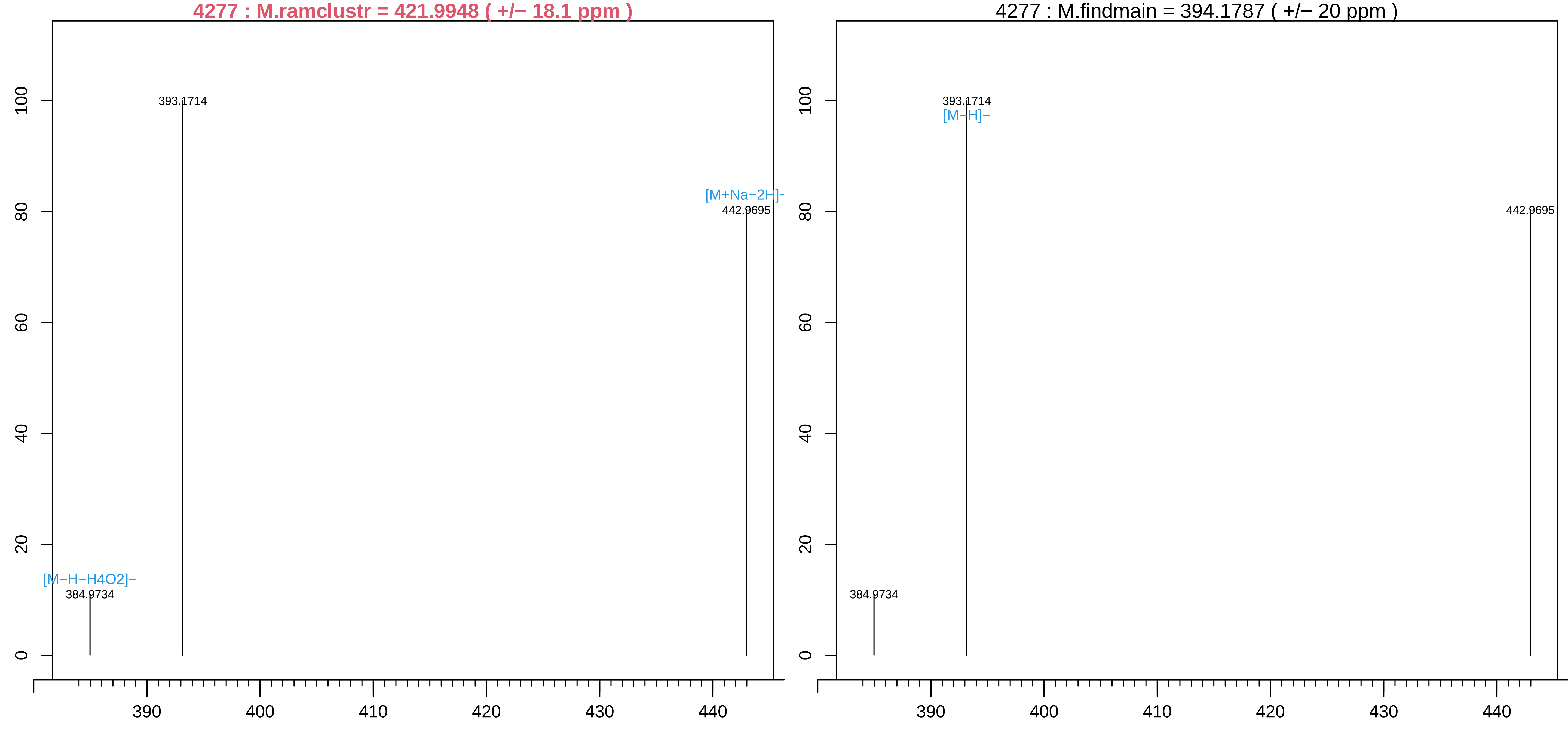

4277 : M.ramclustr = 421.9948 (+/− 18.1 ppm)
393.1714
[M+Na−2H]-
442.9695
[M−H−H4O2]−
384.9734
0
20
40
60
80
100
390
400
410
420
430
440
4277 : M.findmain = 394.1787 (+/− 20 ppm)
393.1714
[M−H]−
442.9695
384.9734
0
20
40
60
80
100
390
400
410
420
430
440

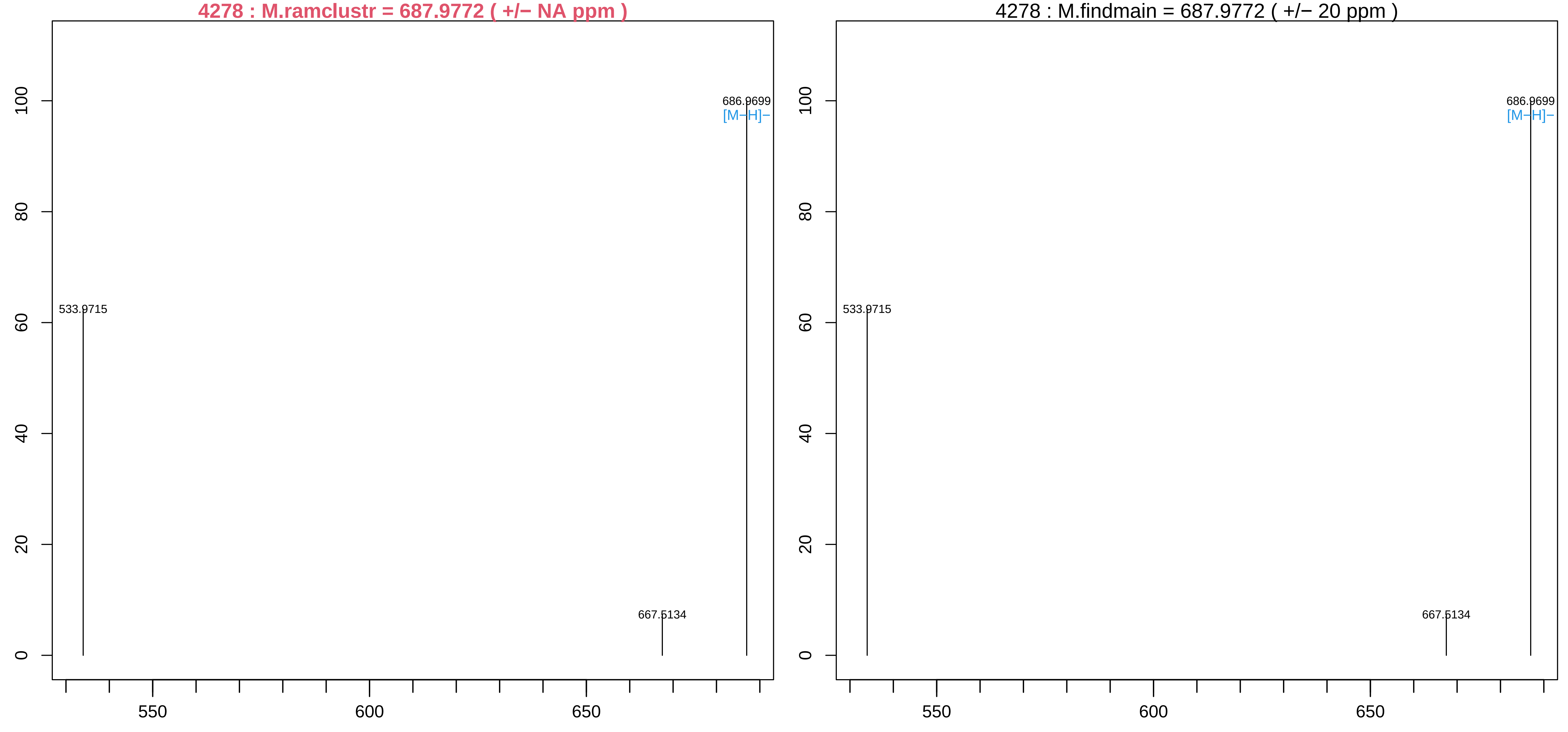
4278 : M.ramclustr = 687.9772 (+/− NA ppm)
533.9715
667.5134
686.9699
[M−H]−
0
20
40
60
80
100
550
600
650
4278 : M.findmain = 687.9772 (+/− 20 ppm)
533.9715
667.5134
686.9699
[M−H]−
0
20
40
60
80
100
550
600
650

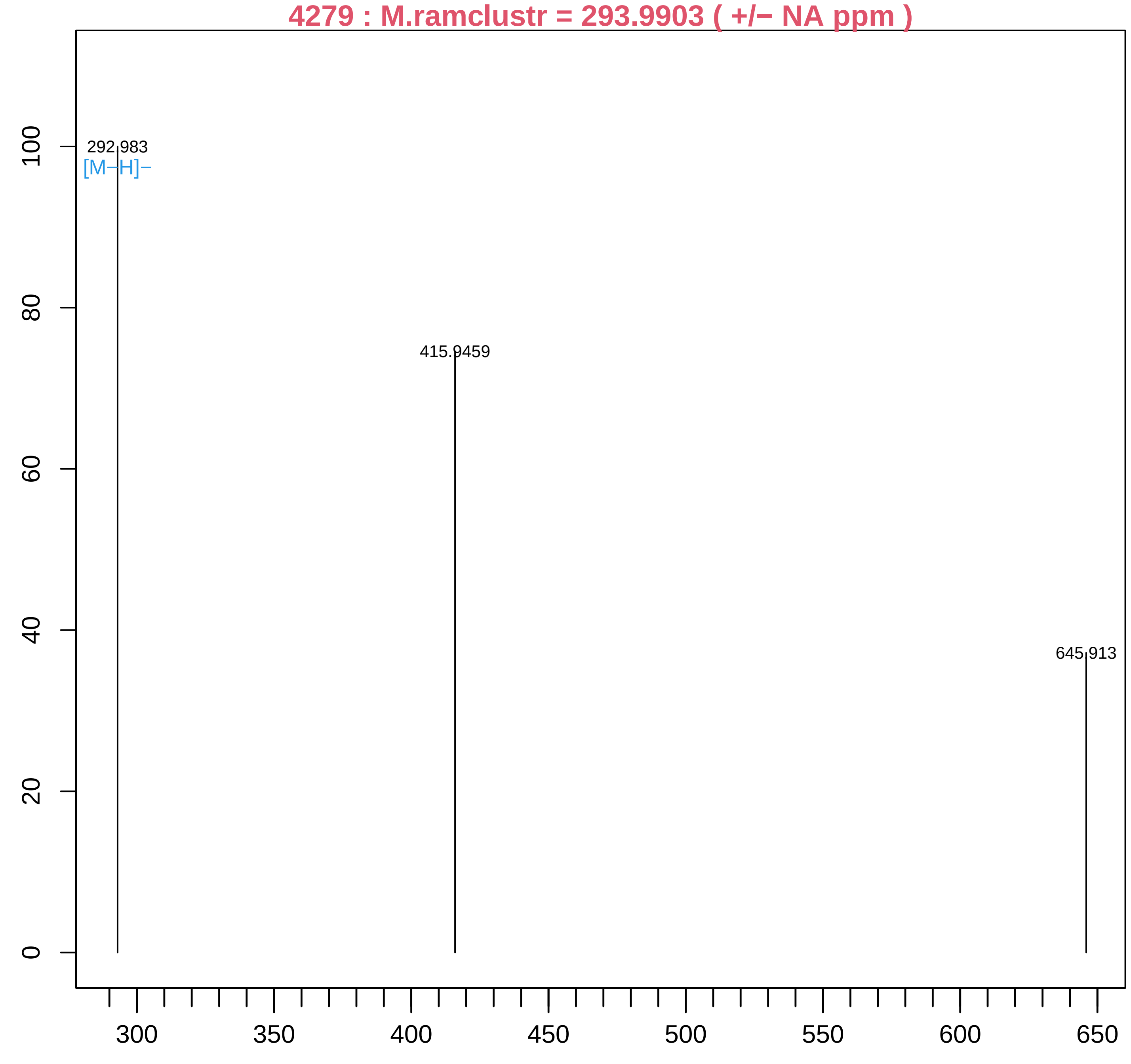

4279 : M.ramclustr = 293.9903 (+/− NA ppm)
292.983
[M−H]−
415.9459
645.913
0
20
40
60
80
100
300
350
400
450
500
550
600
650

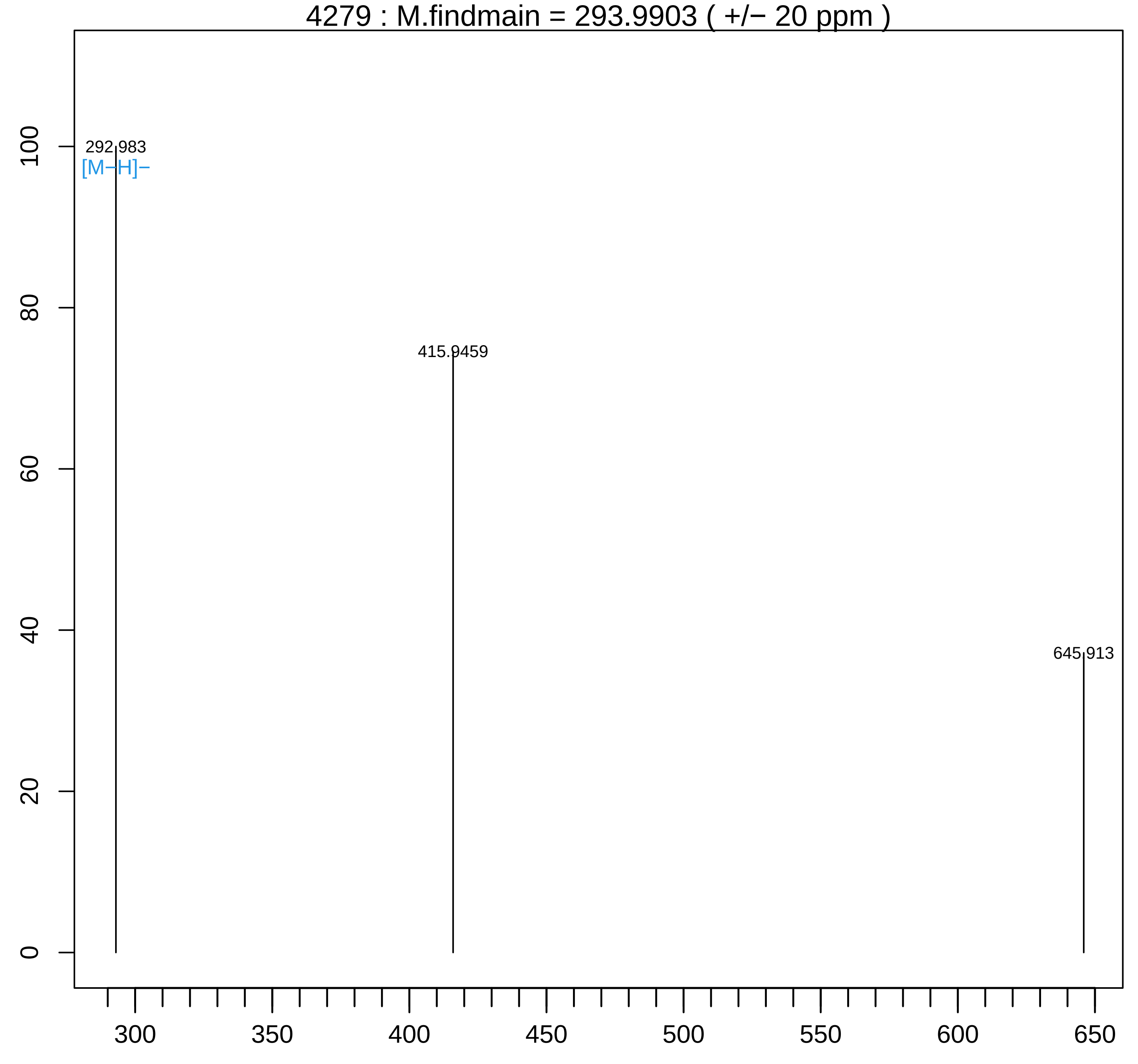

4279 : M.findmain = 293.9903 (+/− 20 ppm)
292.983
[M−H]−
415.9459
645.913
0
20
40
60
80
100
300
350
400
450
500
550
600
650

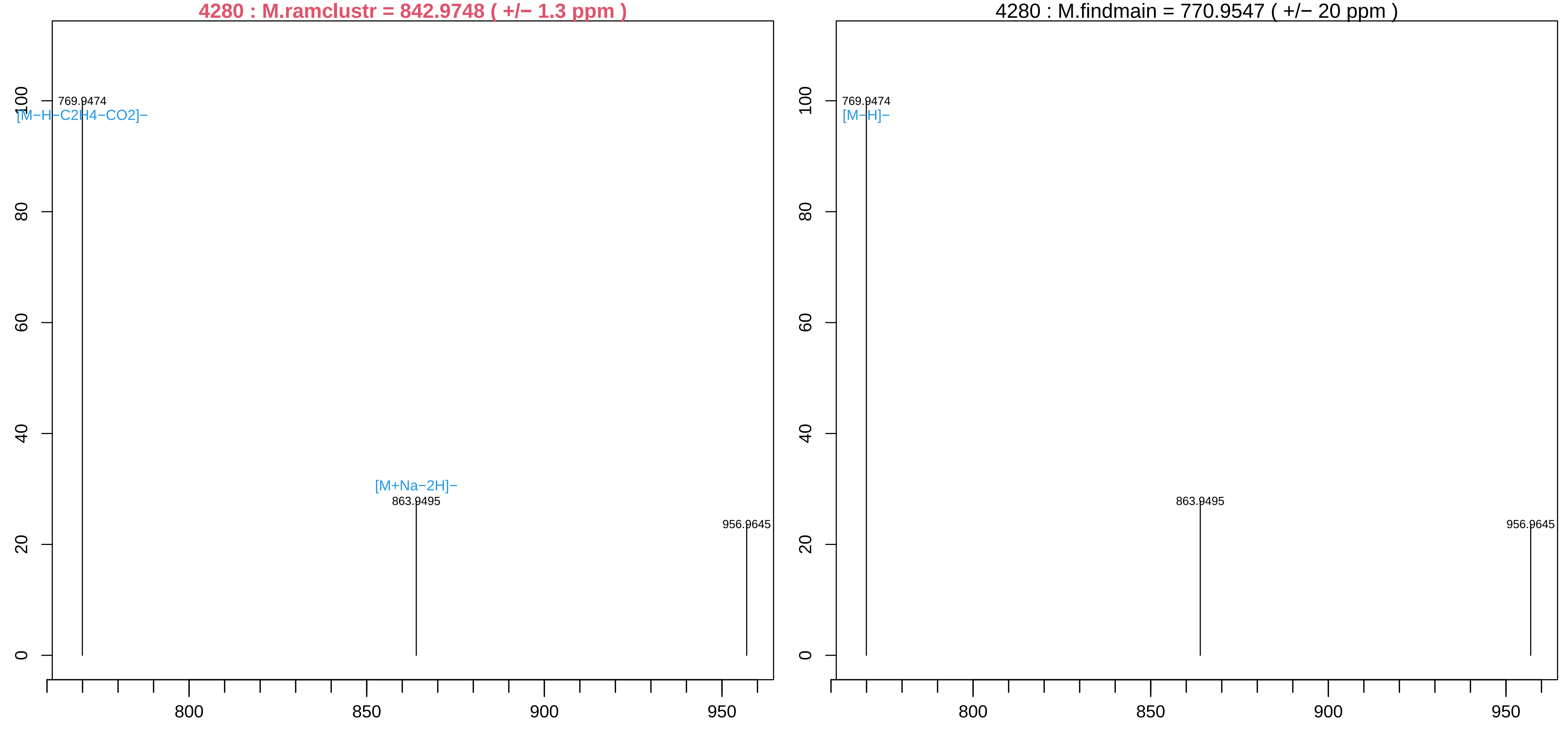
4280 : M.ramclustr = 842.9748 (+/− 1.3 ppm)
769.9474
[M−H−C2H4−CO2]−
[M+Na−2H]−
863.9495
956.9645
4280 : M.findmain = 770.9547 (+/− 20 ppm)
769.9474
[M−H]−
863.9495
956.9645
0
20
40
60
80
100
800
850
900
950

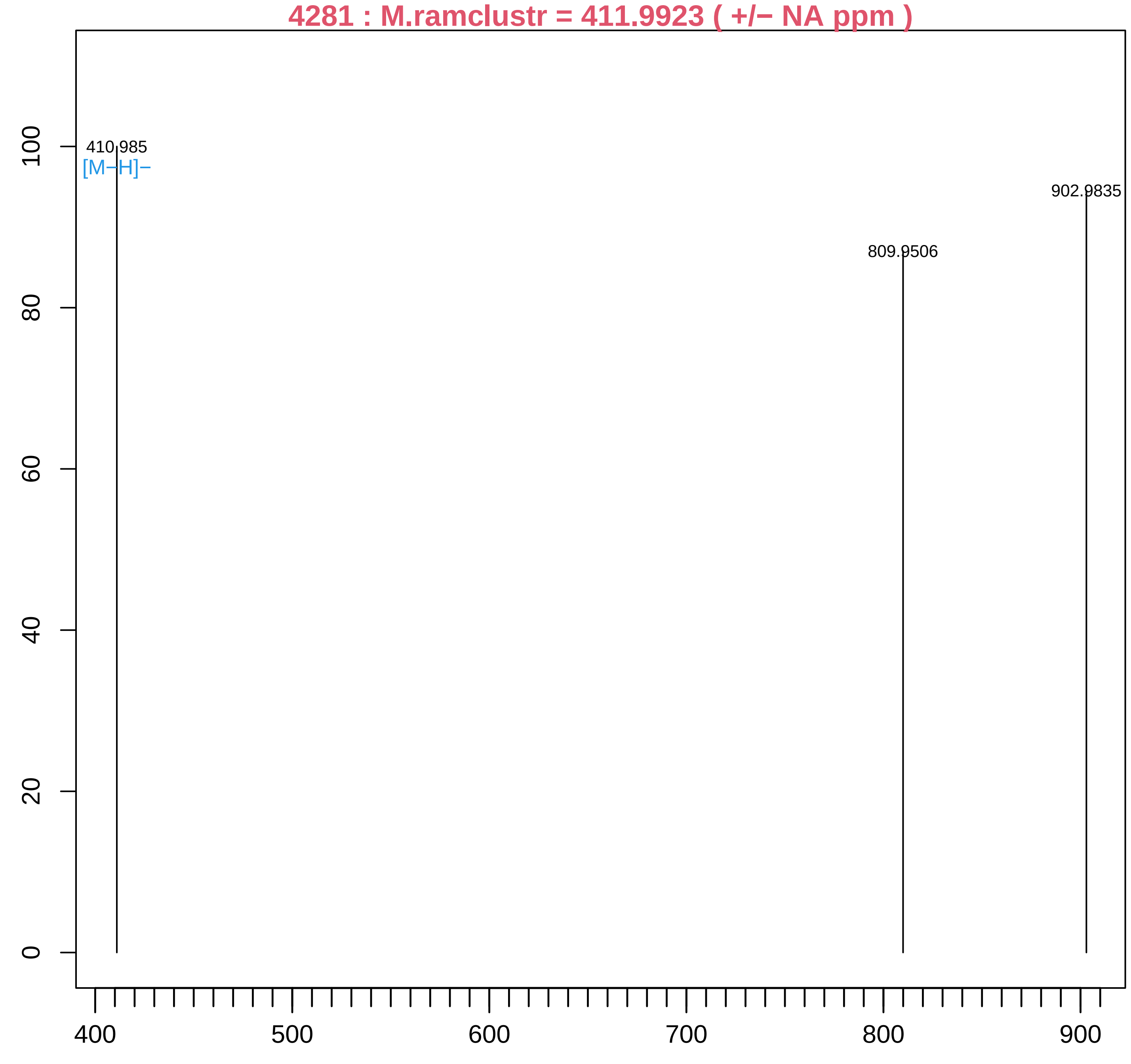
4281 : M.ramclustr = 411.9923 (+/− NA ppm)
410.985
[M−H]−
809.9506
902.9835
0
20
40
60
80
100
400
500
600
700
800
900

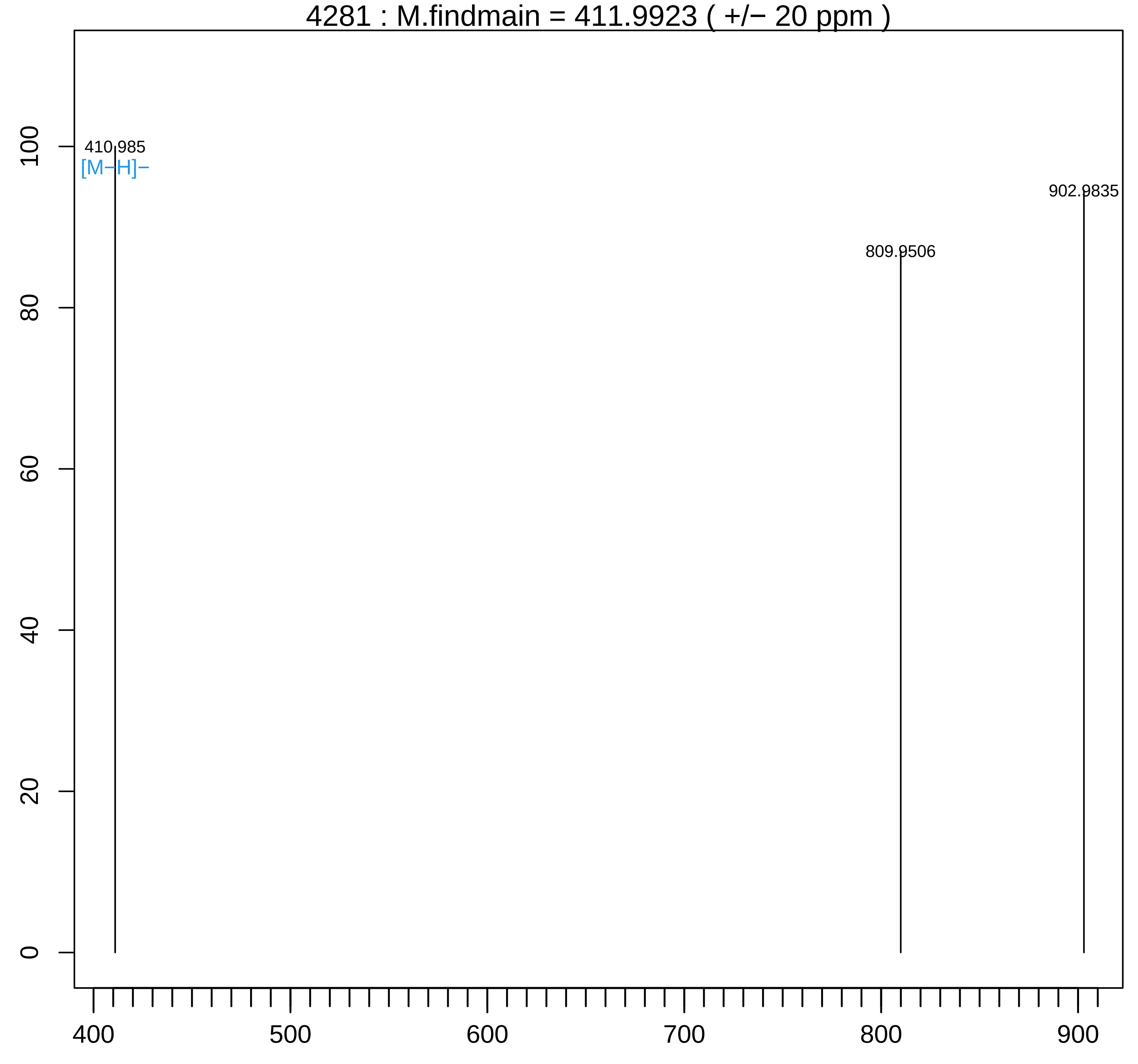
4281 : M.findmain = 411.9923 (+/− 20 ppm)
410.985
[M−H]−
809.9506
902.9835
0
20
40
60
80
100
400
500
600
700
800
900

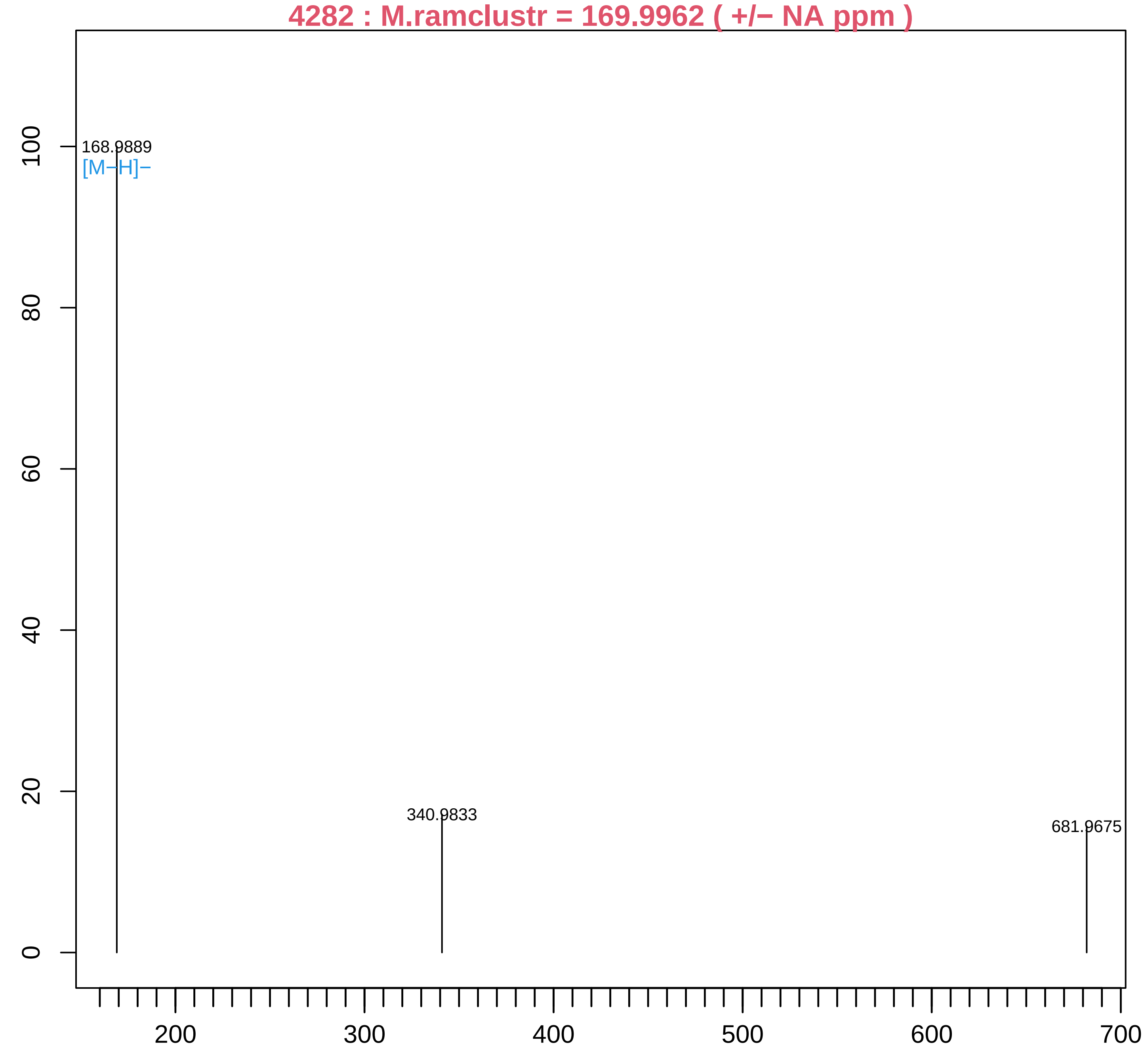
4282 : M.ramclustr = 169.9962 (+/− NA ppm)
168.9889
[M−H]−
340.9833
681.9675
0
20
40
60
80
100
200
300
400
500
600
700

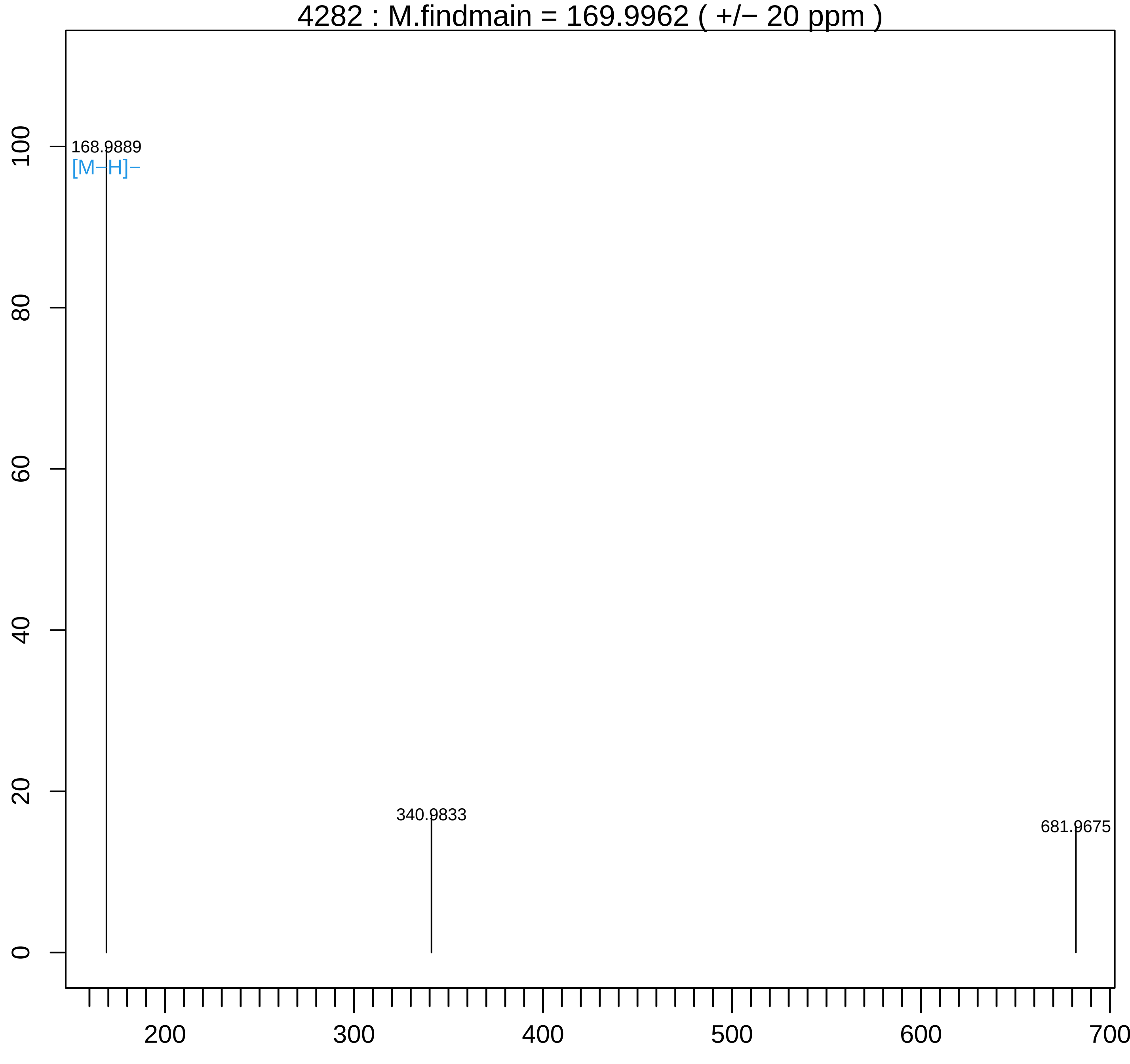
4282 : M.findmain = 169.9962 (+/− 20 ppm)
168.9889
[M−H]−
340.9833
681.9675
0
20
40
60
80
100
200
300
400
500
600
700

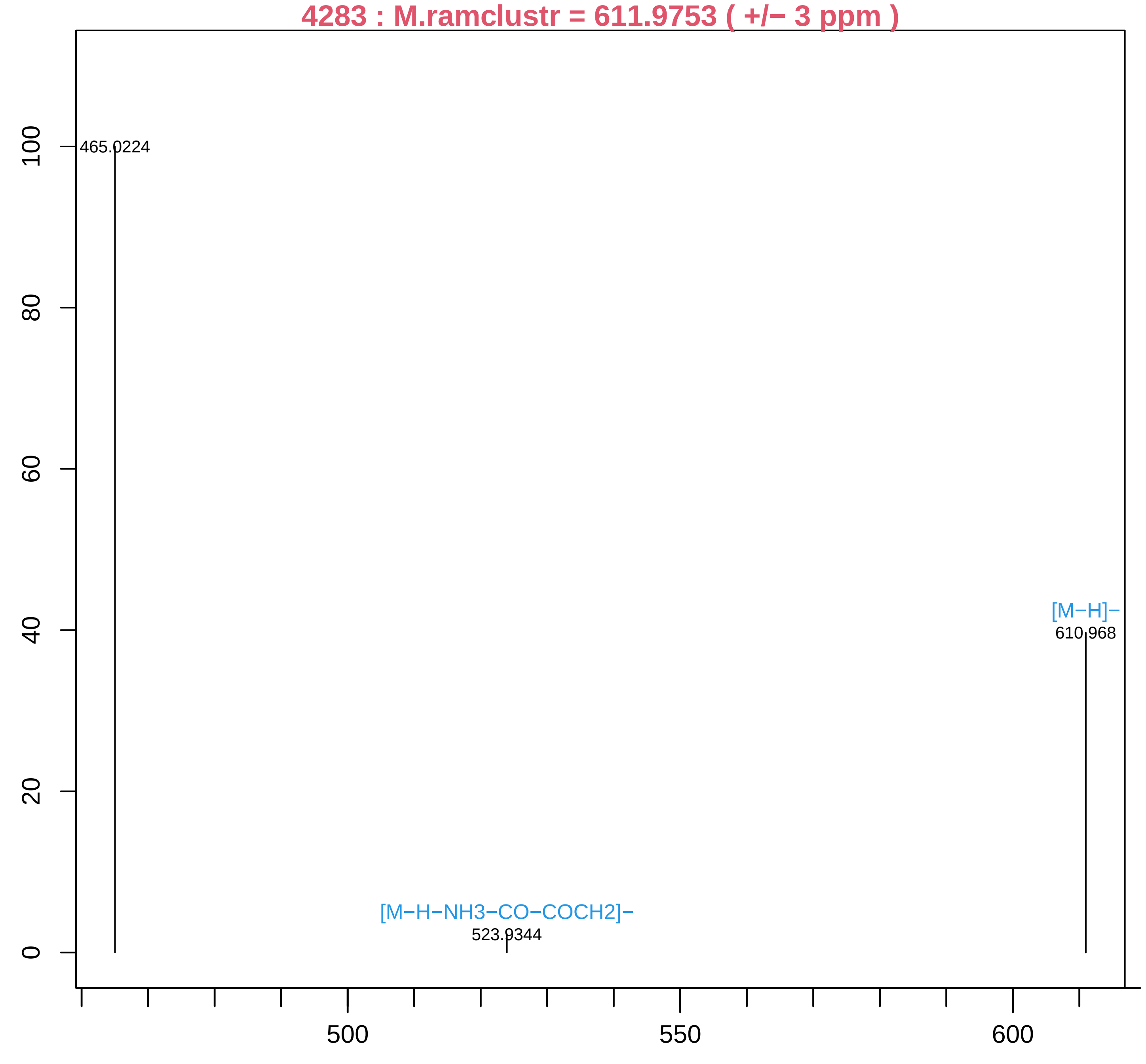
4283 : M.ramclustr = 611.9753 (+/− 3 ppm)
465.0224
[M−H]−
610.968
[M−H−NH3−CO−COCH2]−
523.9344
0
20
40
60
80
100
500
550
600

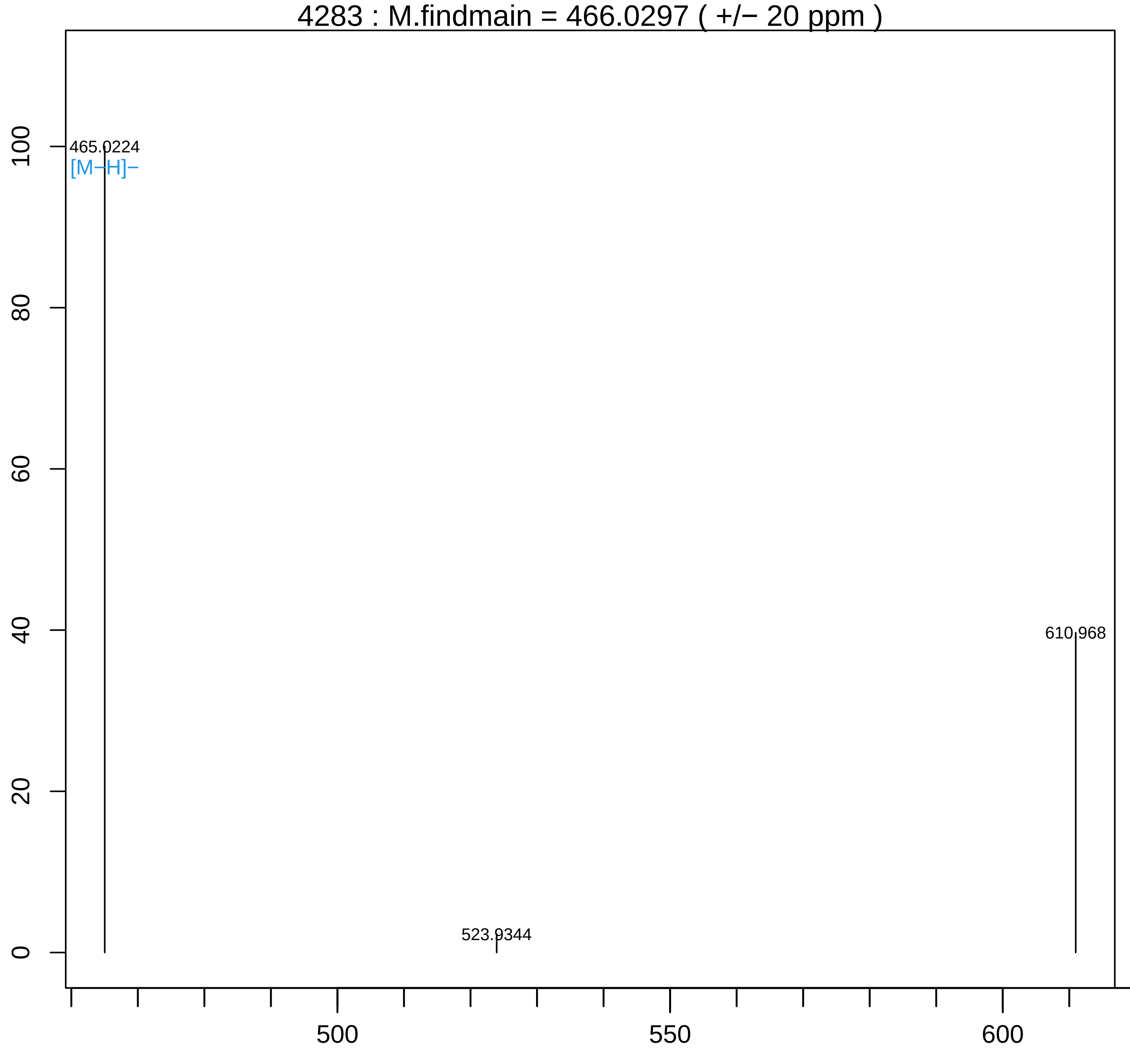
4283 : M.findmain = 466.0297 (+/− 20 ppm)
465.0224
[M−H]−
610.968
523.9344
0
20
40
60
80
100
500
550
600

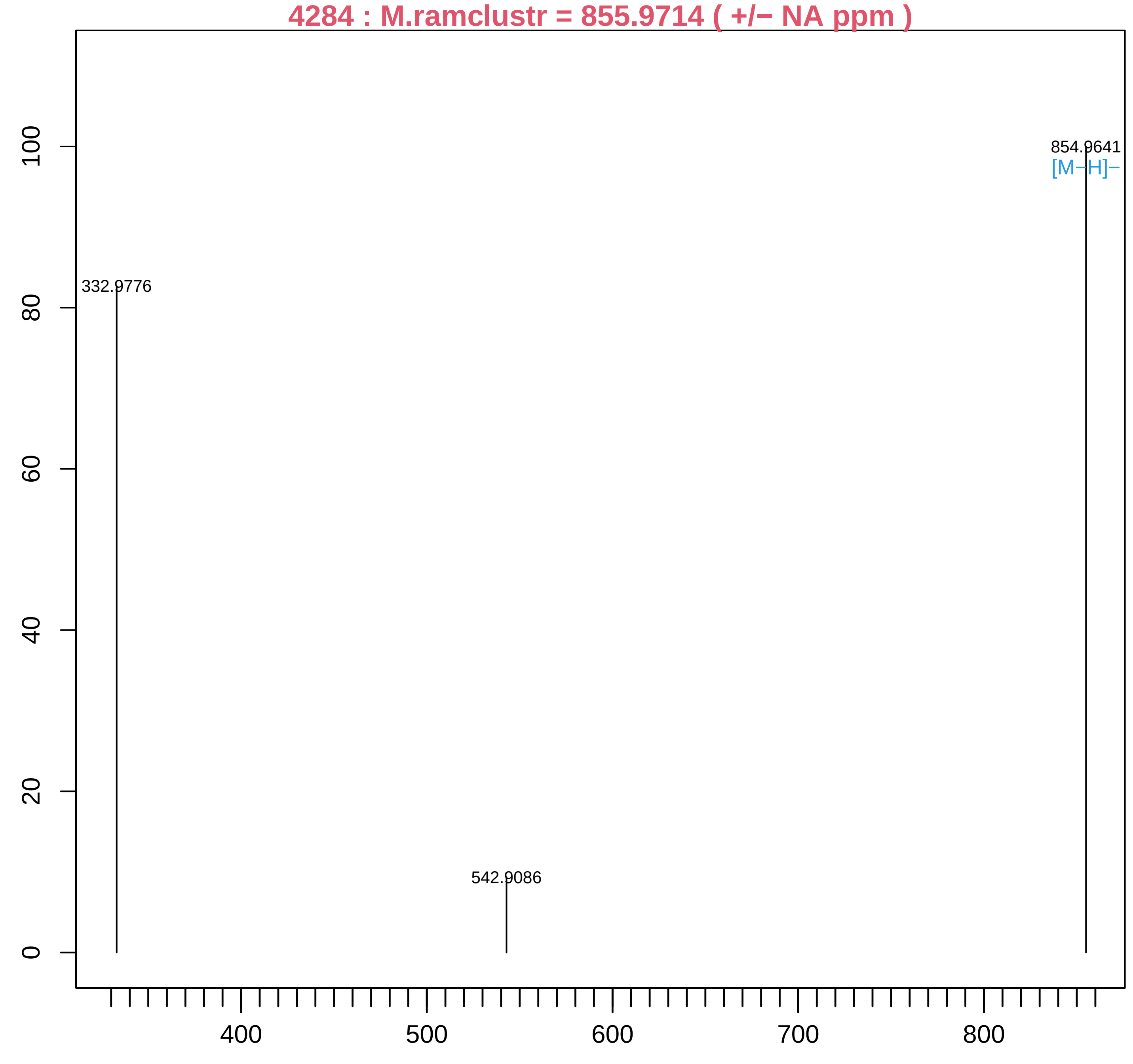
4284 : M.ramclustr = 855.9714 (+/− NA ppm)
854.9641
[M−H]−
332.9776
542.9086
0
20
40
60
80
100
400
500
600
700
800

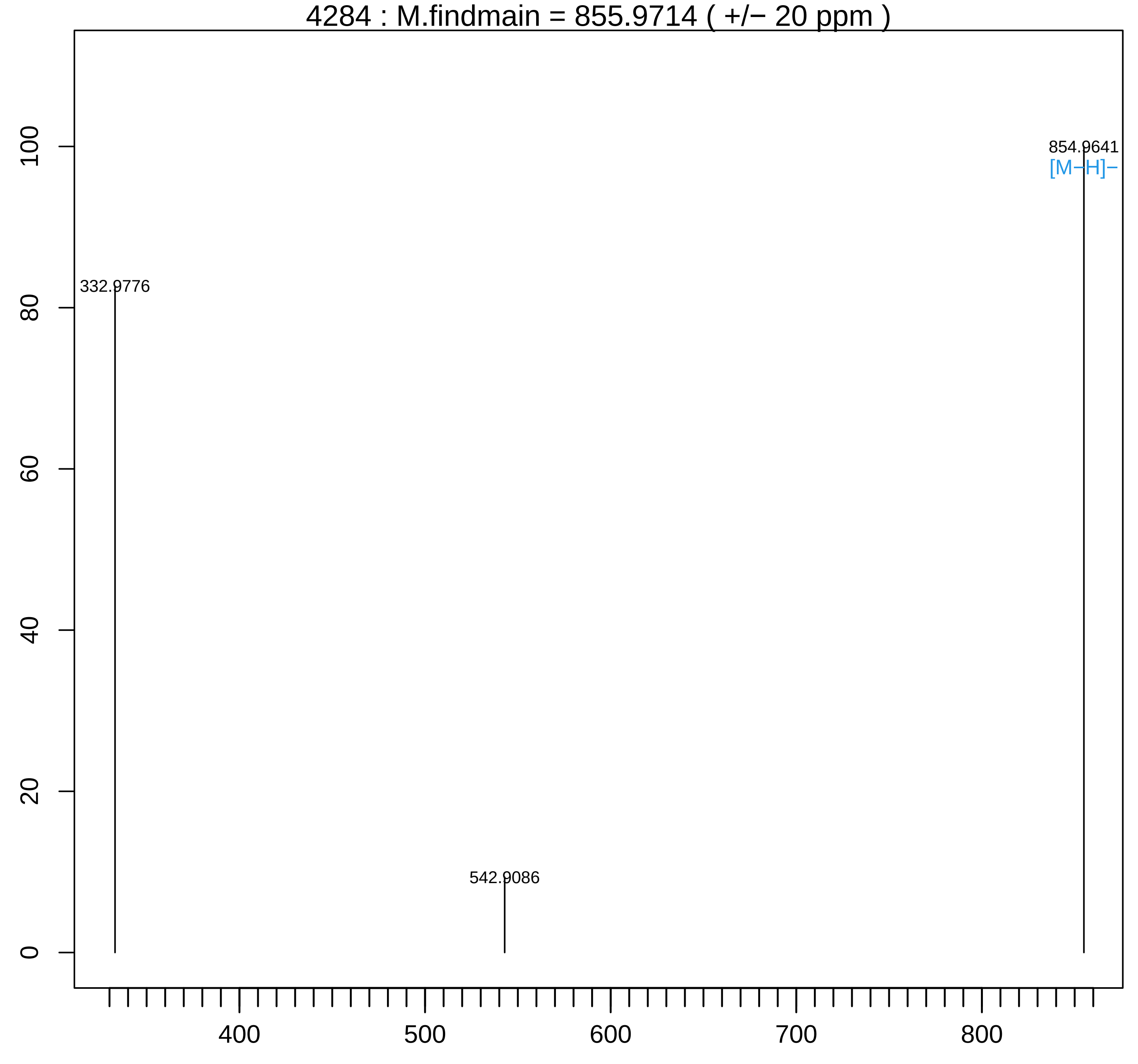
4284 : M.findmain = 855.9714 (+/− 20 ppm)
854.9641
[M−H]−
332.9776
542.9086
0
20
40
60
80
100
400
500
600
700
800

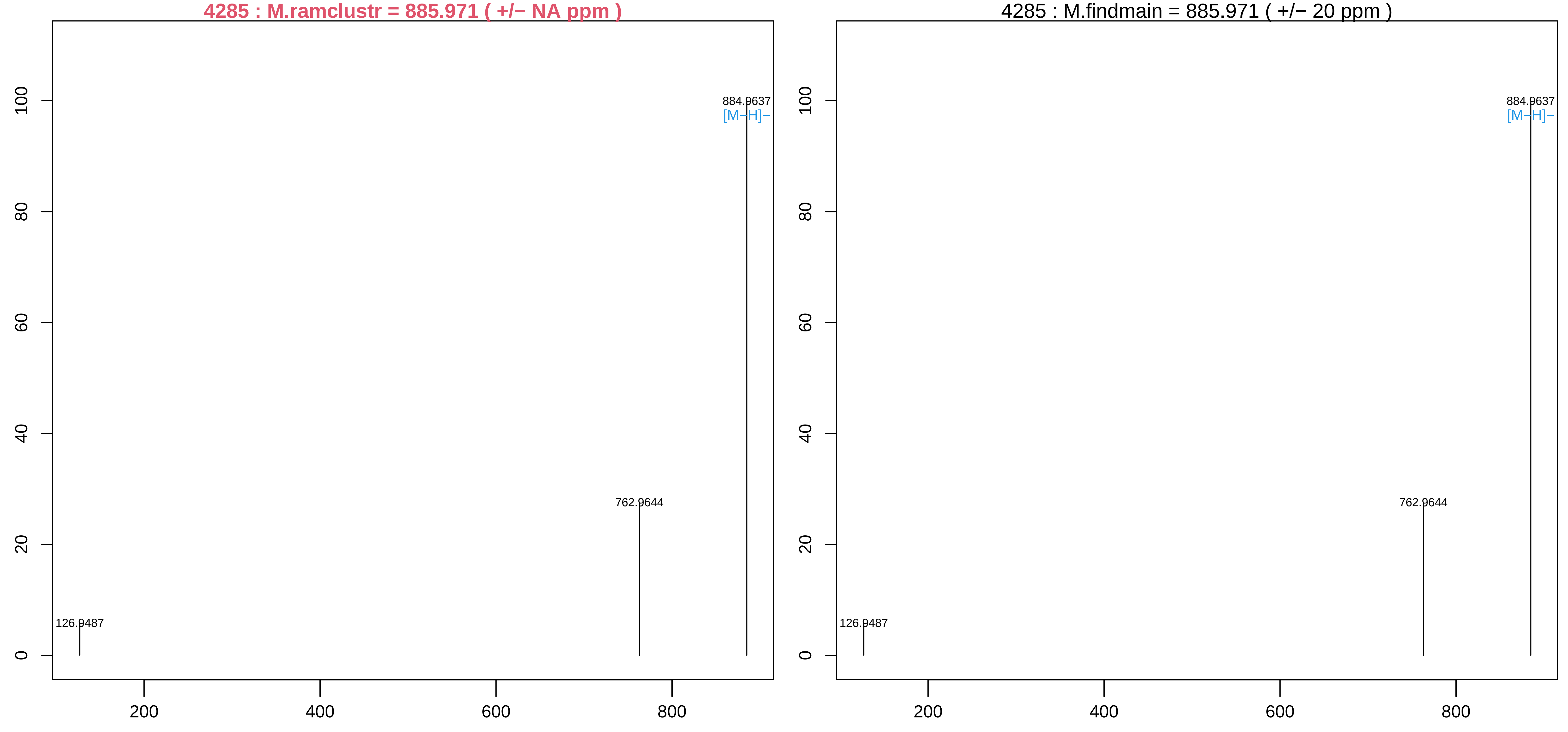

4285 : M.ramclustr = 885.971 (+/− NA ppm)
884.9637
[M−H]−
762.9644
126.9487
4285 : M.findmain = 885.971 (+/− 20 ppm)
884.9637
[M−H]−
762.9644
126.9487
0
20
40
60
80
100
200
400
600
800

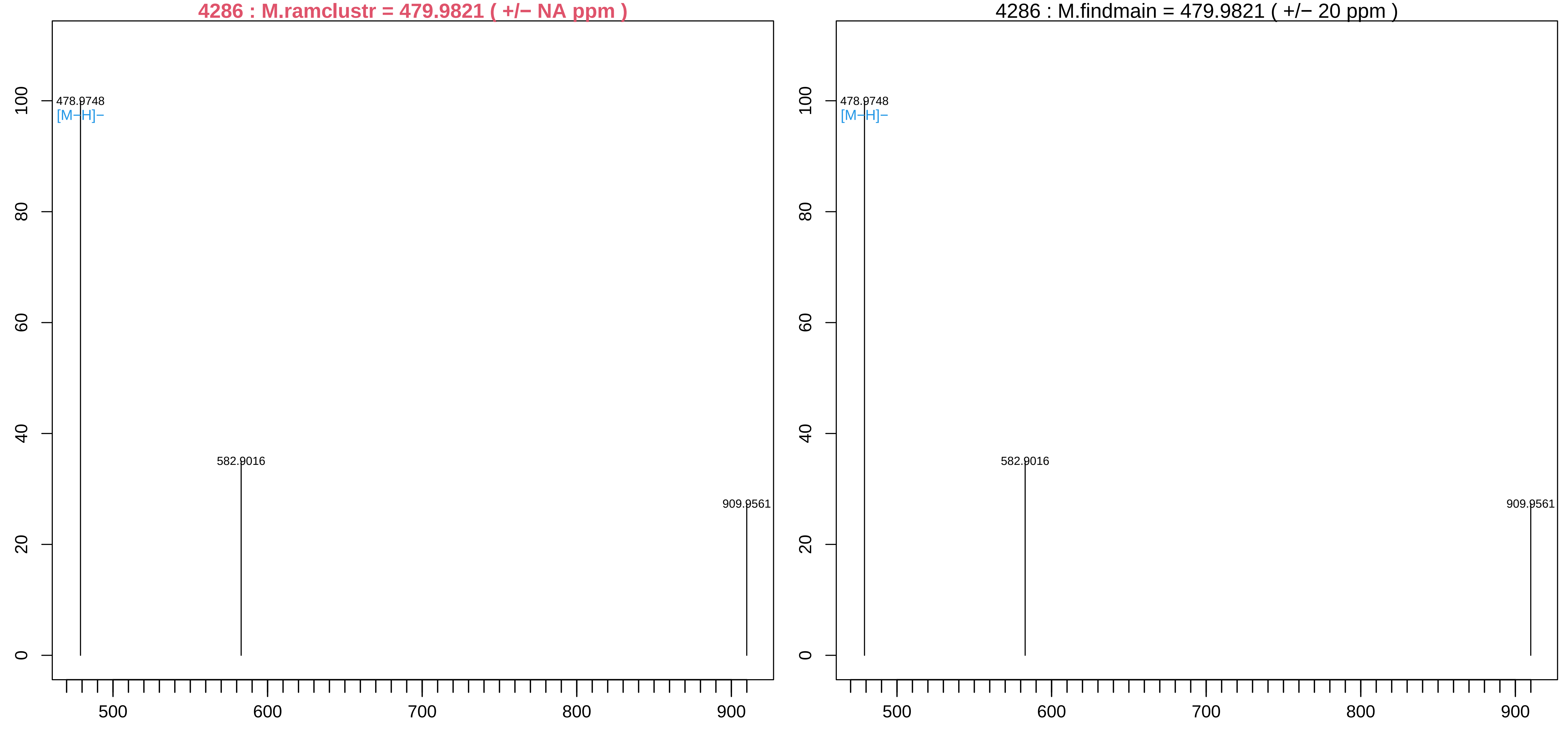

4286 : M.ramclustr = 479.9821 (+/− NA ppm)
4286 : M.findmain = 479.9821 (+/− 20 ppm)
478.9748
[M−H]−
582.9016
909.9561
0
20
40
60
80
100
500
600
700
800
900

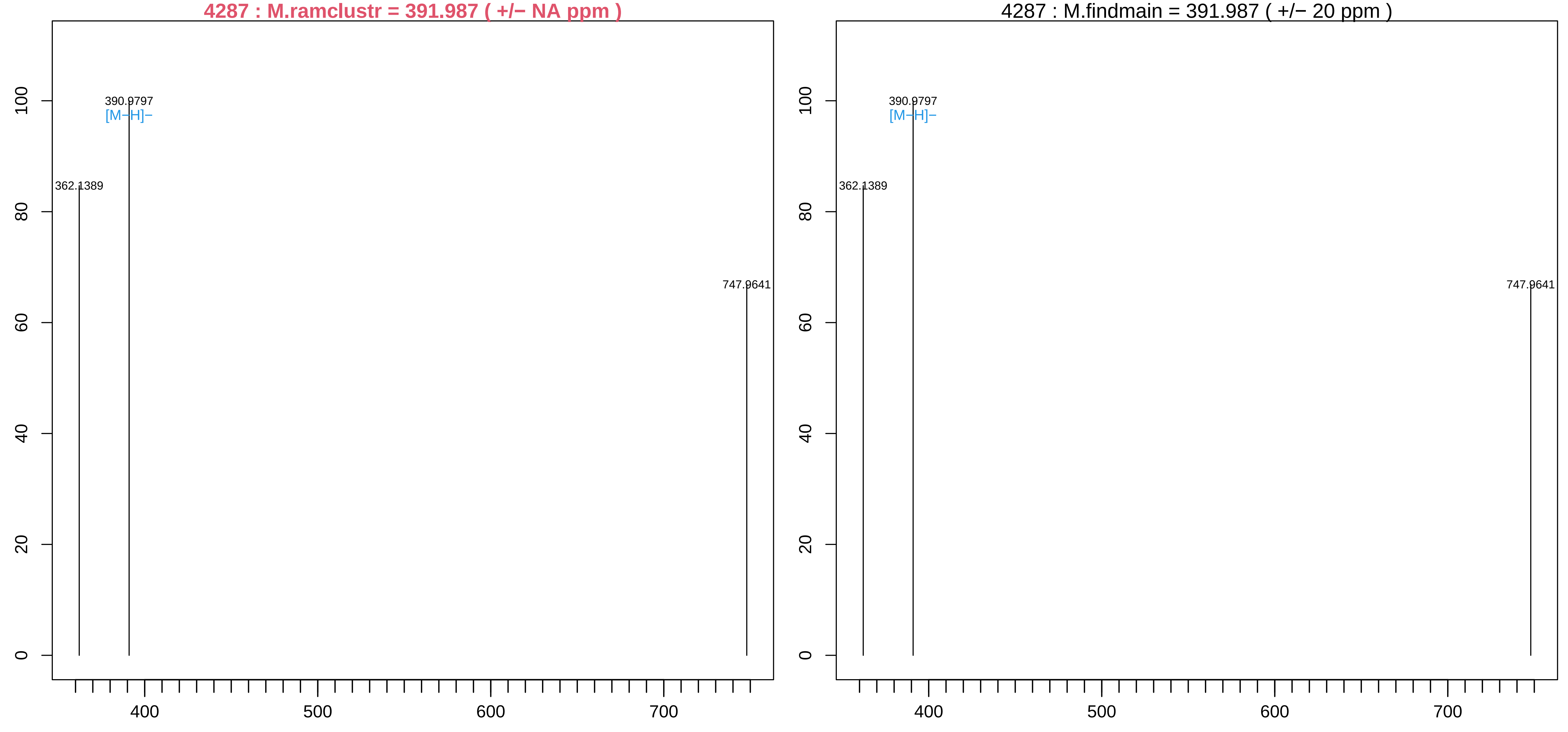

4287 : M.ramclustr = 391.987 (+/− NA ppm)
390.9797
[M−H]−
362.1389
747.9641
0
20
40
60
80
100
400
500
600
700
4287 : M.findmain = 391.987 (+/− 20 ppm)
390.9797
[M−H]−
362.1389
747.9641
0
20
40
60
80
100
400
500
600
700

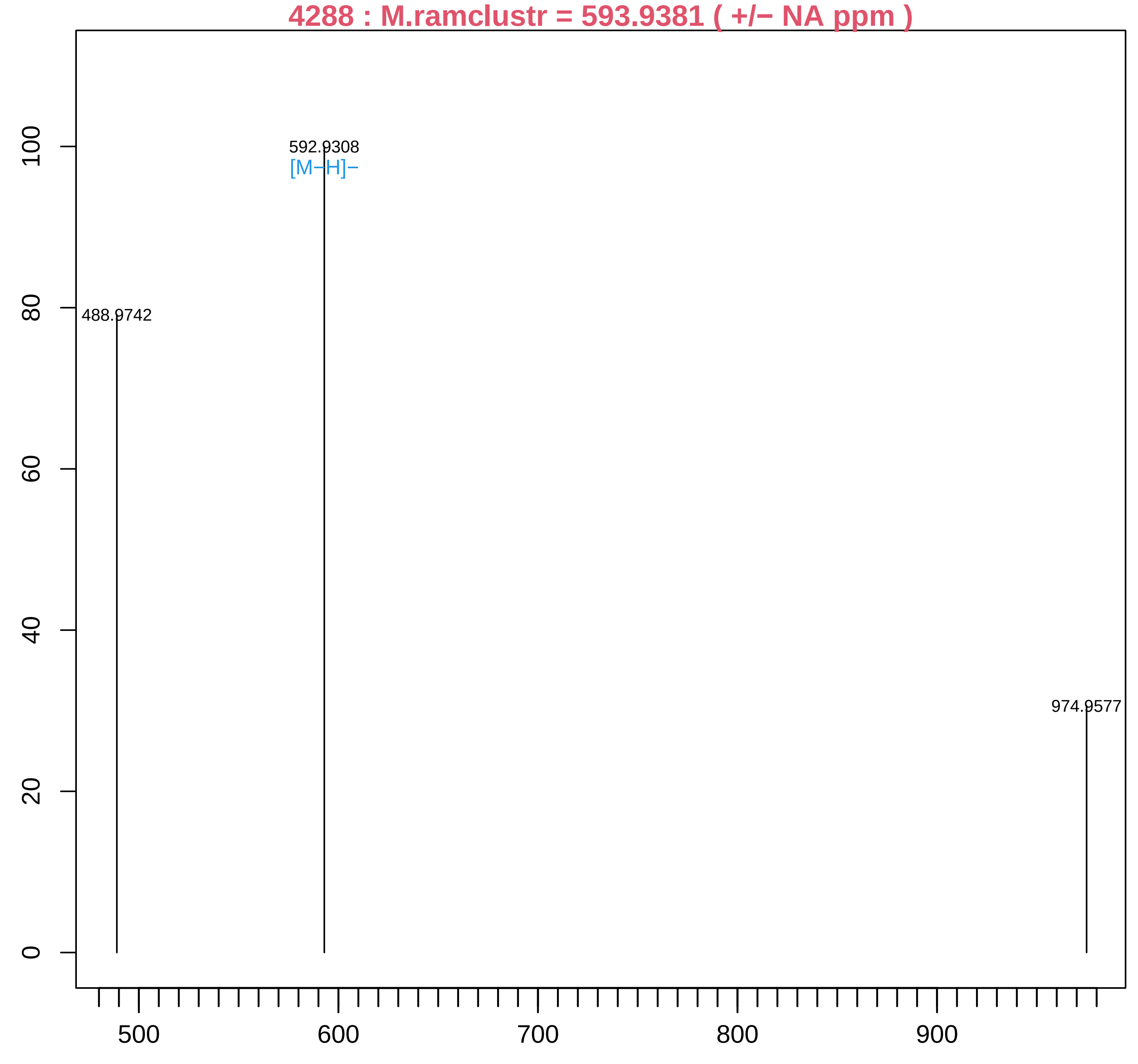

4288 : M.ramclustr = 593.9381 (+/− NA ppm)
592.9308
[M−H]−
488.9742
974.9577
0
20
40
60
80
100
500
600
700
800
900

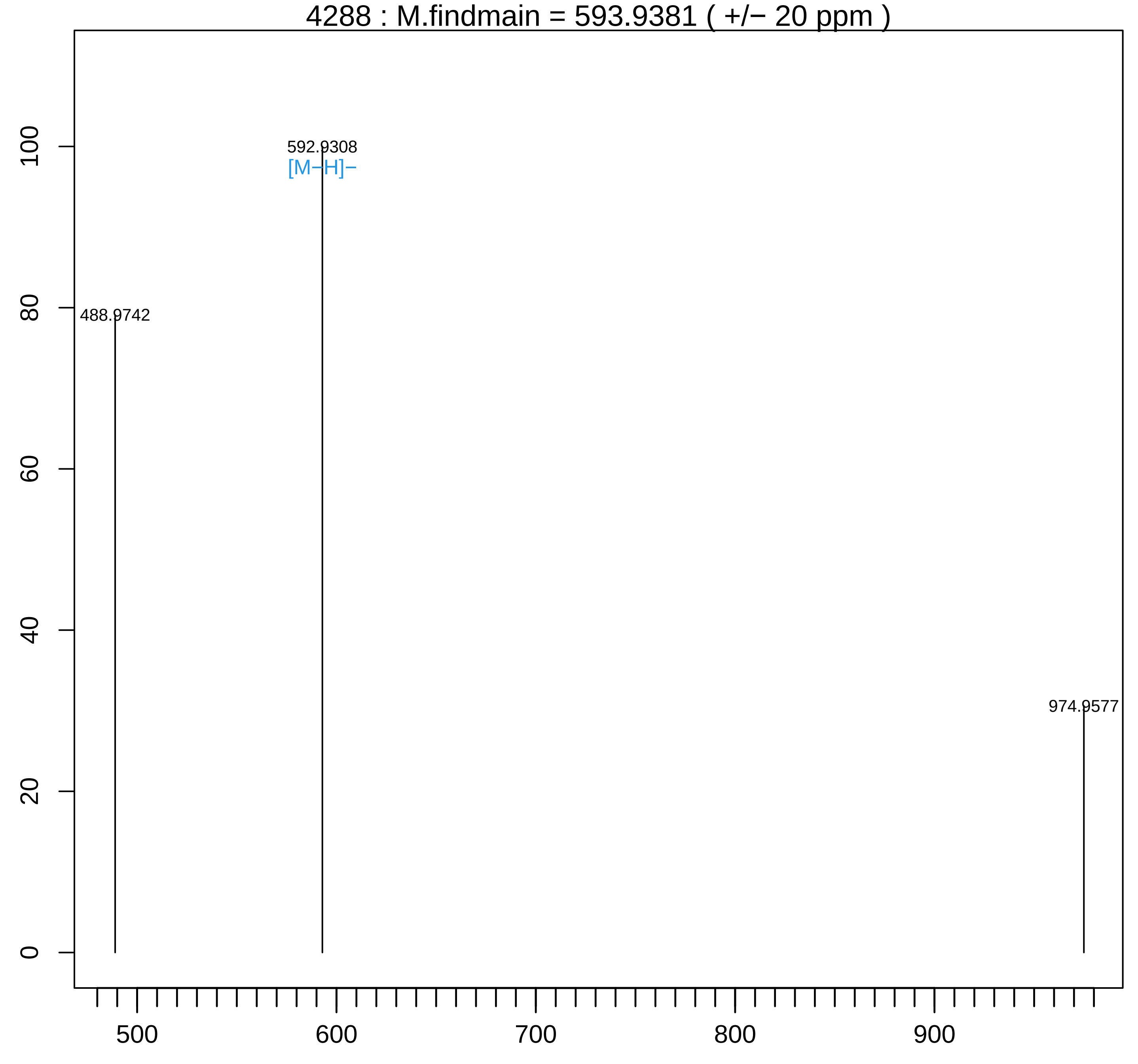

4288 : M.findmain = 593.9381 (+/− 20 ppm)
592.9308
[M−H]−
488.9742
974.9577
0
20
40
60
80
100
500
600
700
800
900

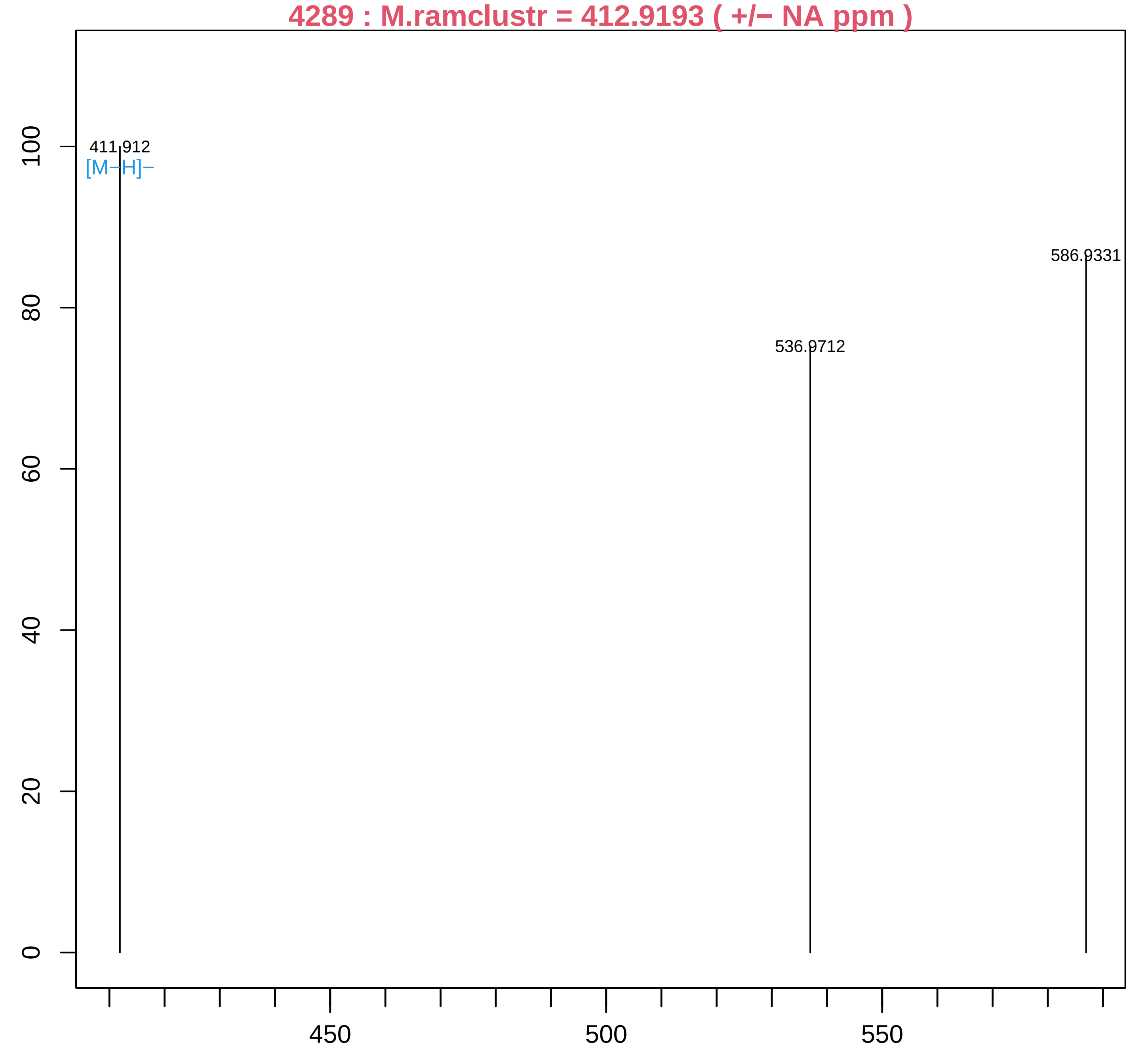
4289 : M.ramclustr = 412.9193 (+/− NA ppm)
411.912
[M−H]−
536.9712
586.9331
0
20
40
60
80
100
450
500
550

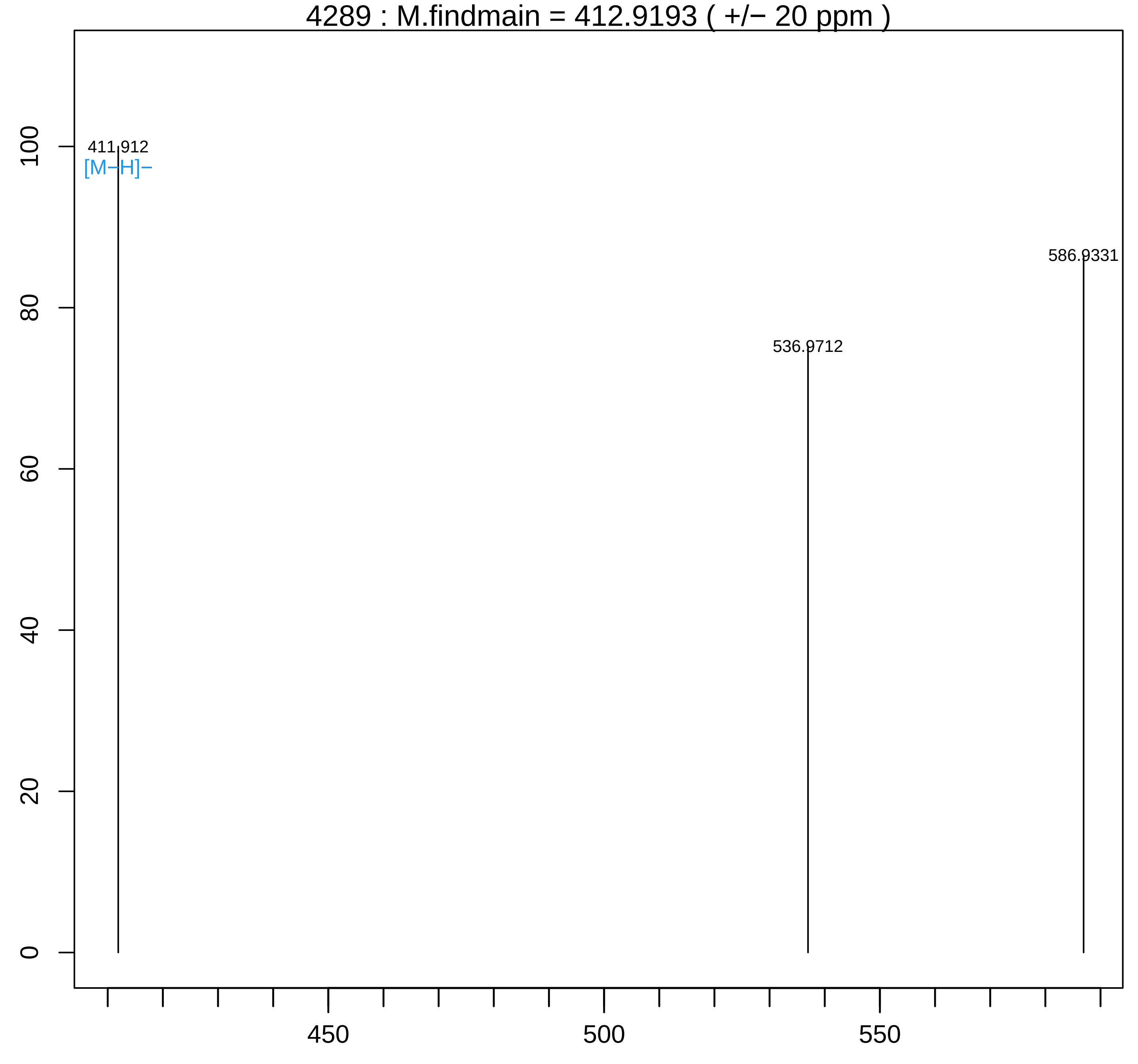
4289 : M.findmain = 412.9193 (+/− 20 ppm)
411.912
[M−H]−
536.9712
586.9331
0
20
40
60
80
100
450
500
550

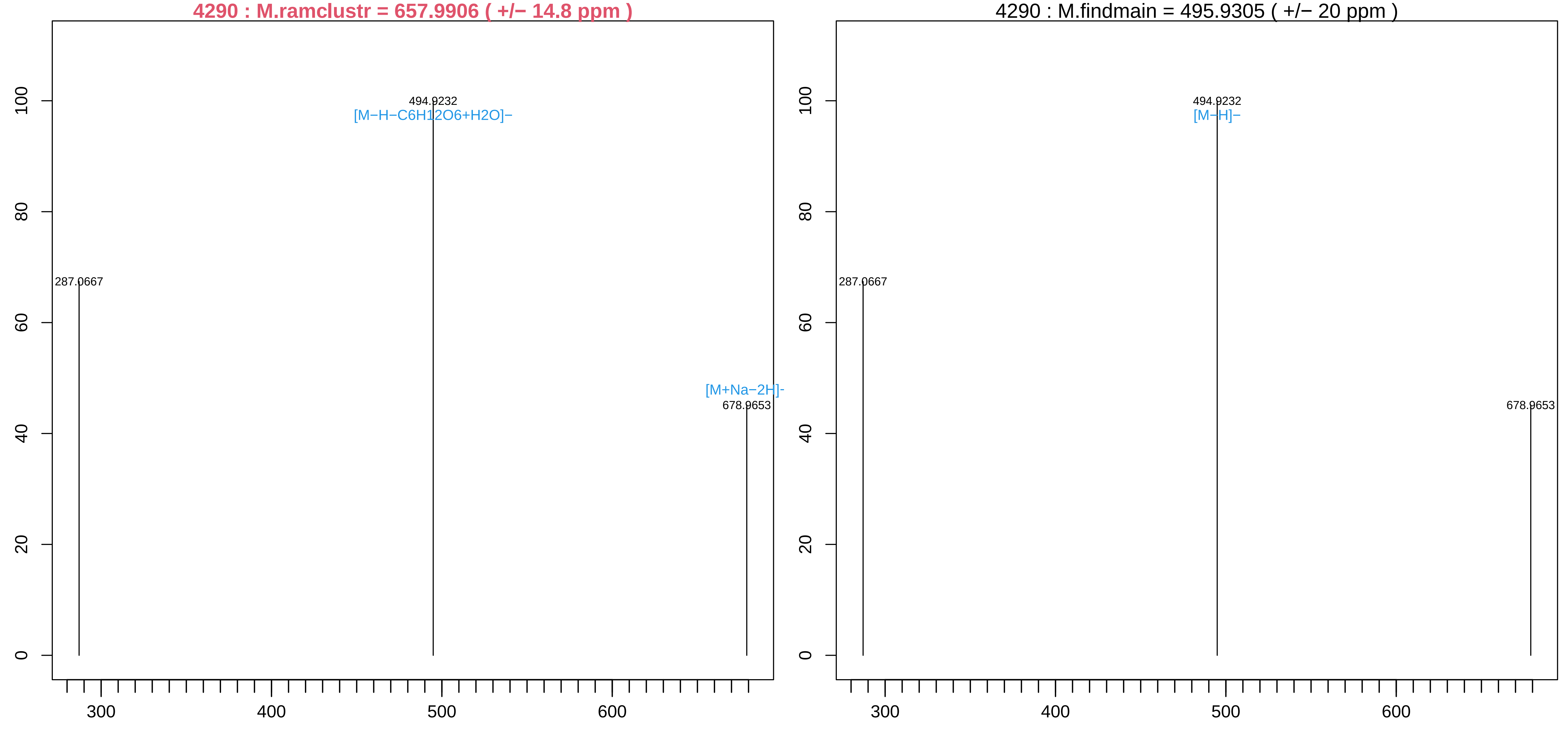
4290 : M.ramclustr = 657.9906 (+/− 14.8 ppm)
494.9232
[M−H−C6H12O6+H2O]−
287.0667
[M+Na−2H]-
678.9653
4290 : M.findmain = 495.9305 (+/− 20 ppm)
494.9232
[M−H]−
287.0667
678.9653
0
20
40
60
80
100
300
400
500
600

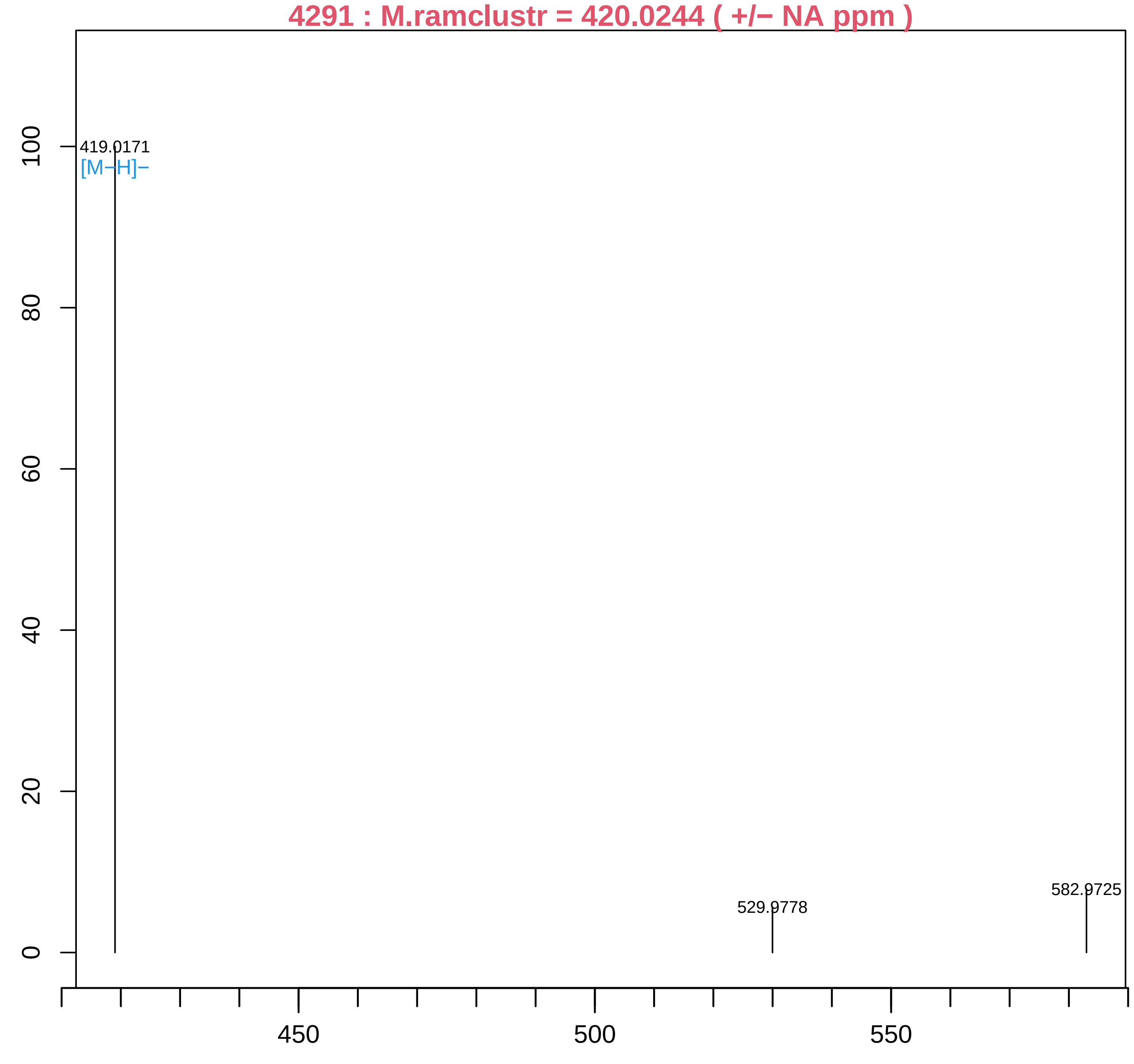
4291 : M.ramclustr = 420.0244 (+/− NA ppm)
419.0171
[M−H]−
529.9778
582.9725
0
20
40
60
80
100
450
500
550

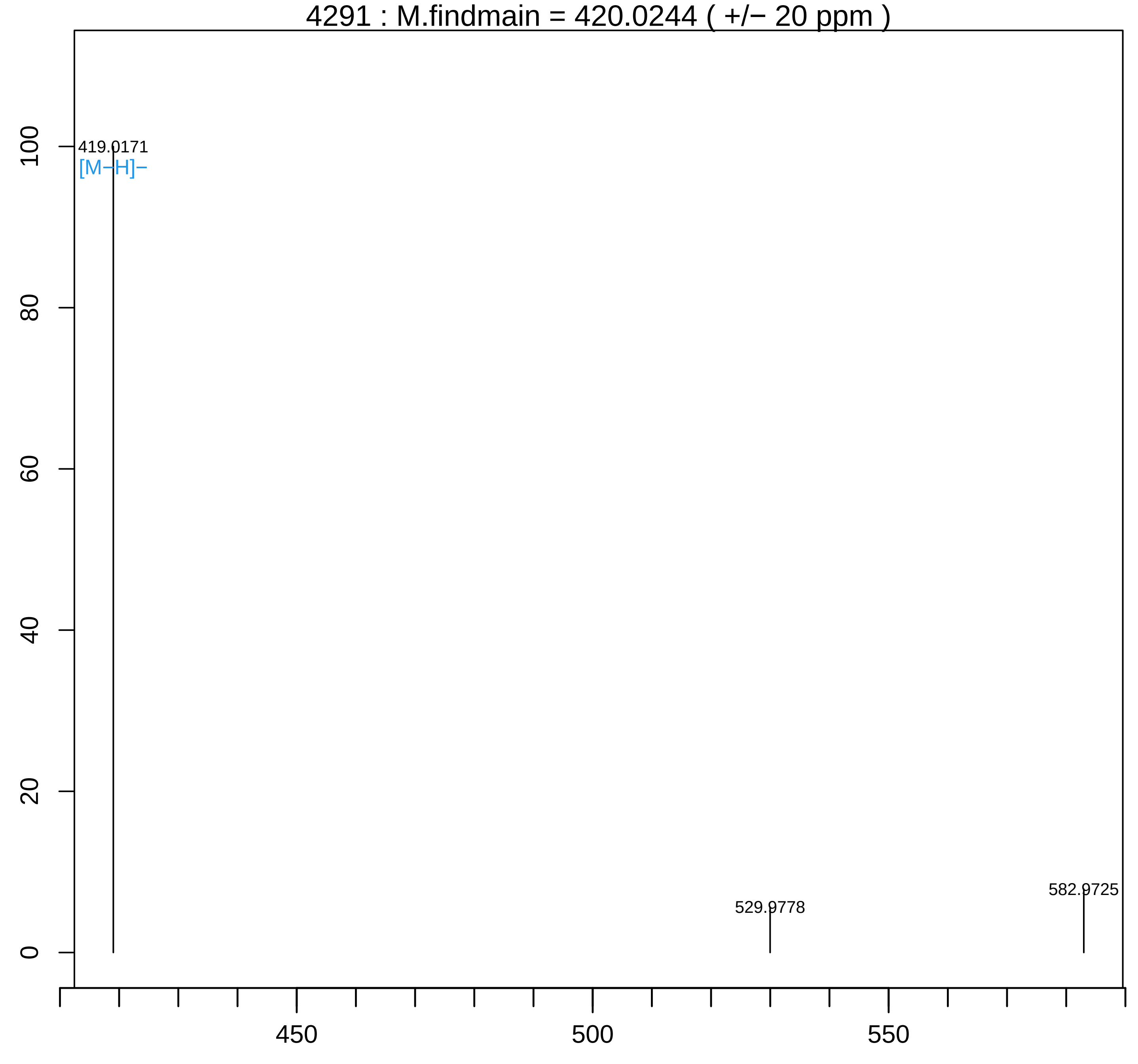
4291 : M.findmain = 420.0244 (+/− 20 ppm)
419.0171
[M−H]−
529.9778
582.9725
0
20
40
60
80
100
450
500
550

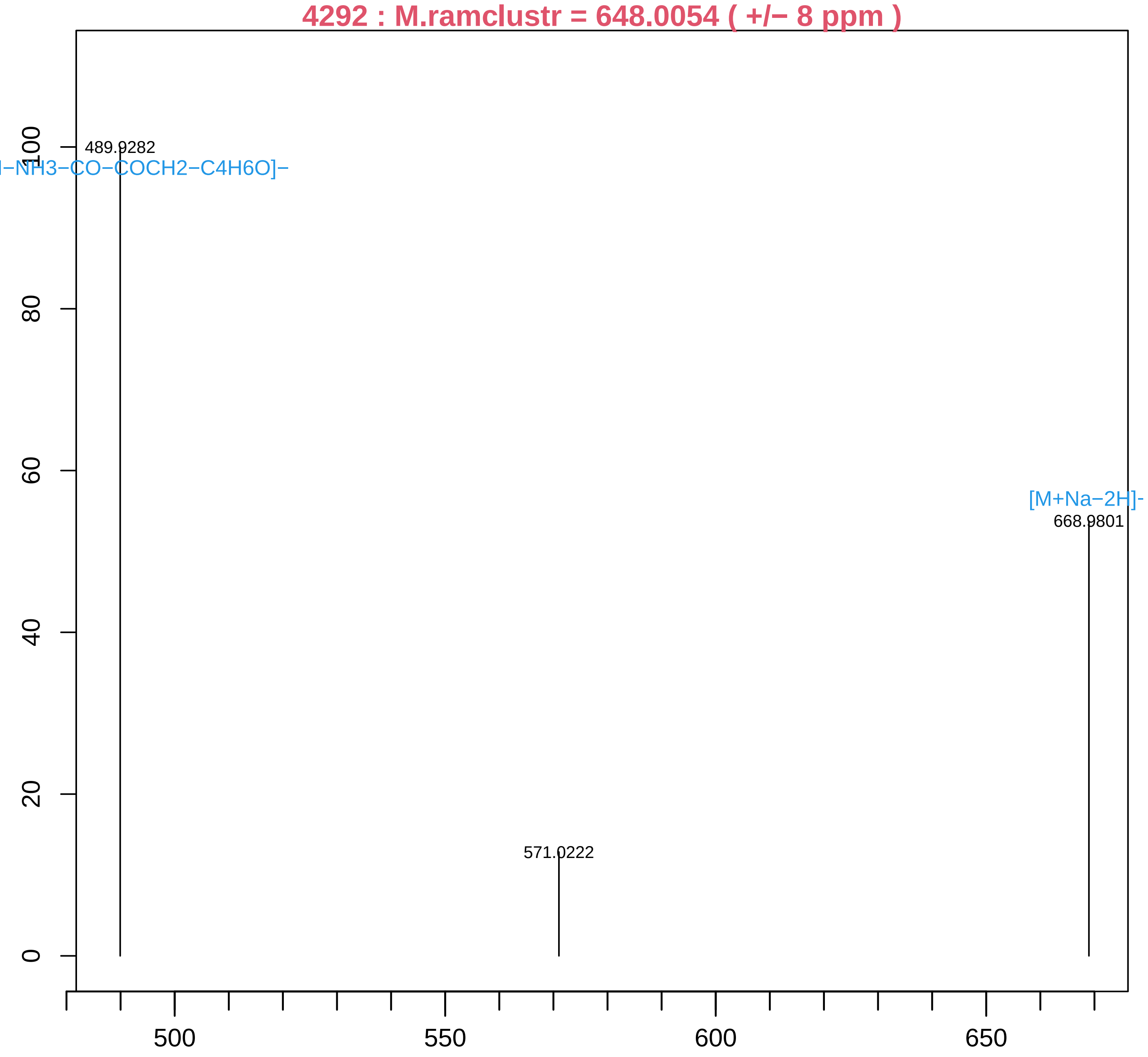

4292 : M.ramclustr = 648.0054 (+/− 8 ppm)
489.9282
[M−NH3−CO−COCH2−C4H6O]−
[M+Na−2H]-
668.9801
571.0222
0
20
40
60
80
100
500
550
600
650

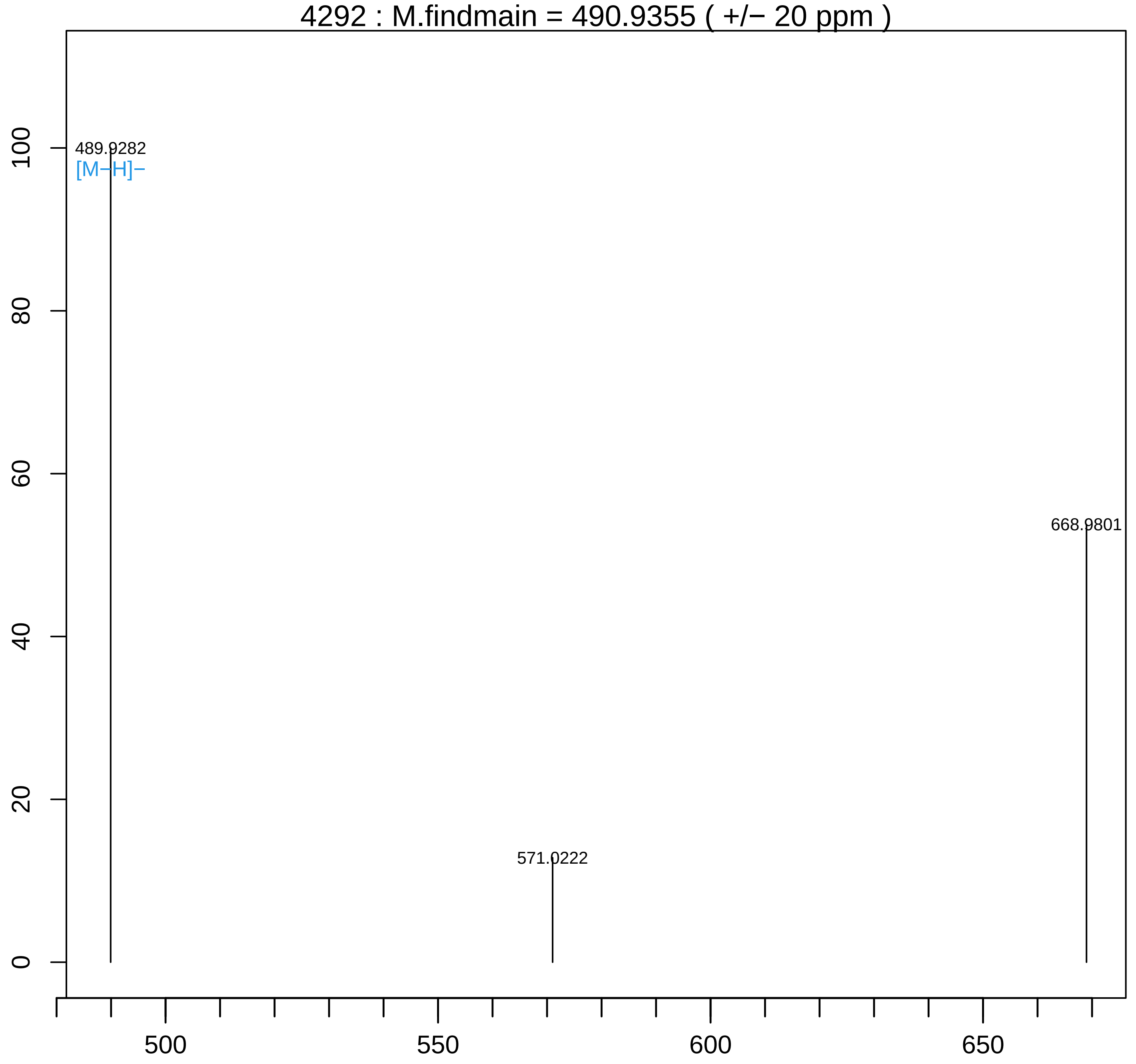

4292 : M.findmain = 490.9355 (+/− 20 ppm)
489.9282
[M−H]−
668.9801
571.0222
0
20
40
60
80
100
500
550
600
650

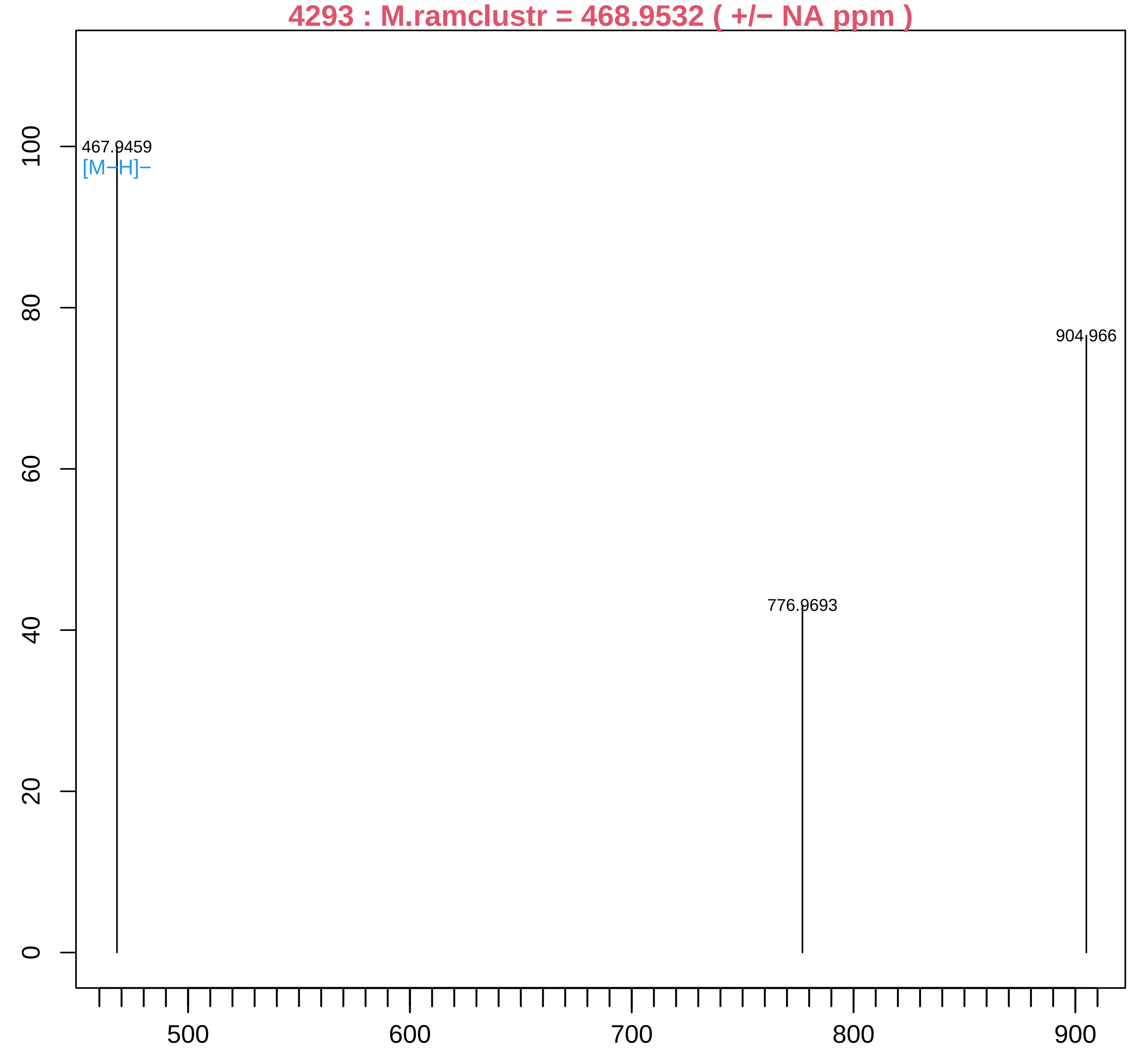
4293 : M.ramclustr = 468.9532 (+/− NA ppm)
467.9459
[M−H]−
776.9693
904.966
0
20
40
60
80
100
500
600
700
800
900

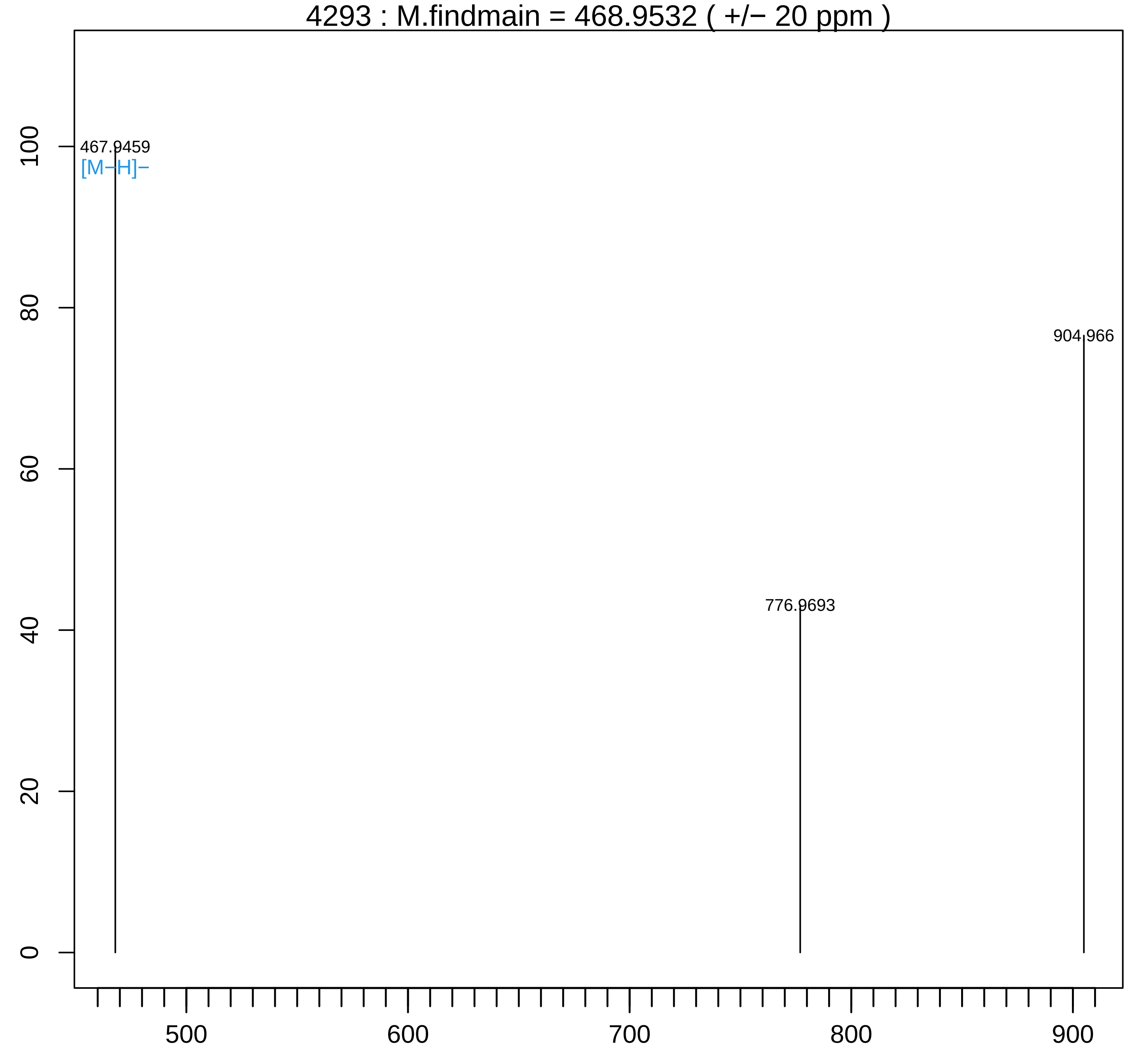
4293 : M.findmain = 468.9532 (+/− 20 ppm)
467.9459
[M−H]−
776.9693
904.966
0
20
40
60
80
100
500
600
700
800
900

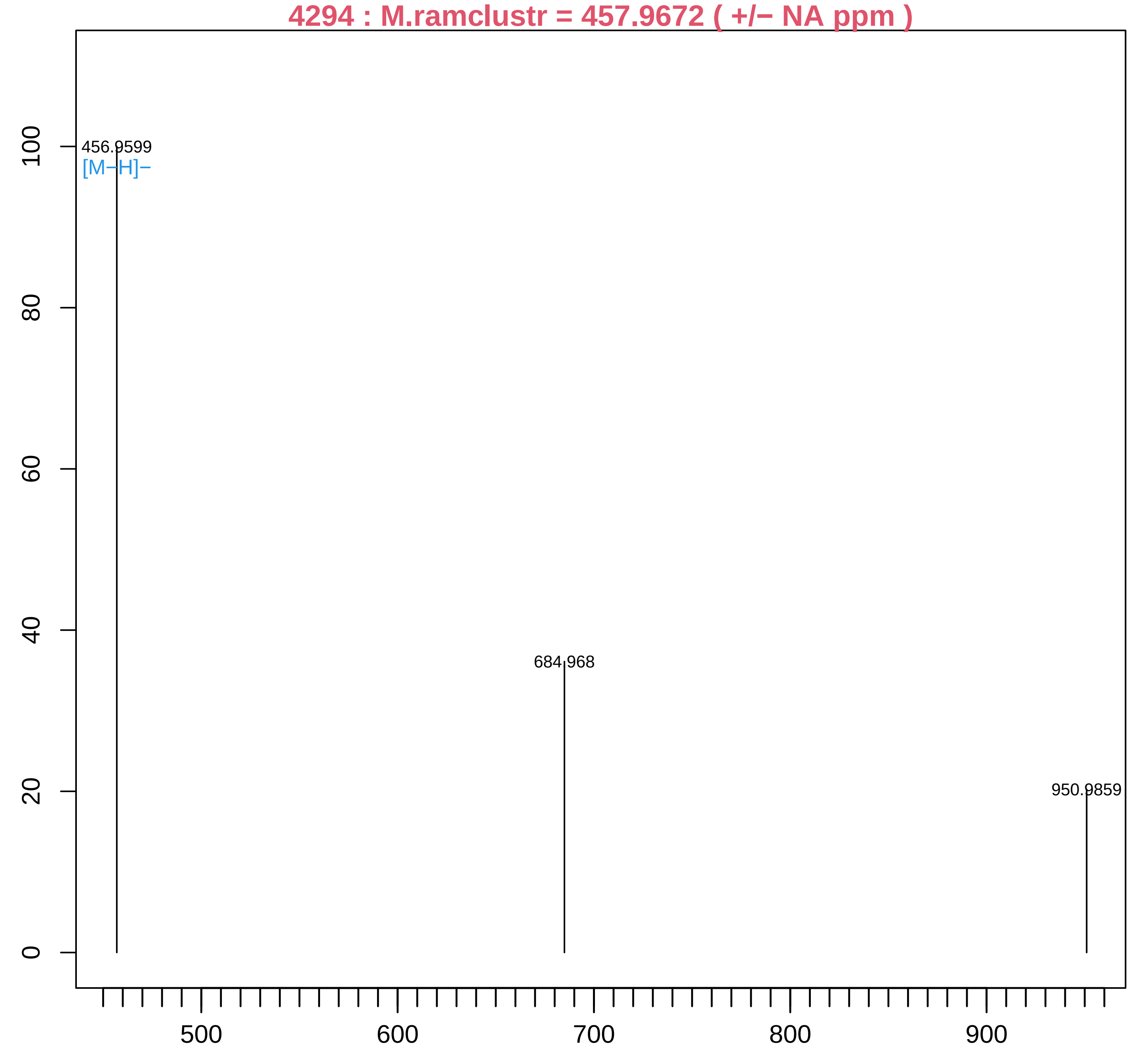

4294 : M.ramclustr = 457.9672 (+/− NA ppm)
456.9599
[M−H]−
684.968
950.9859
0
20
40
60
80
100
500
600
700
800
900

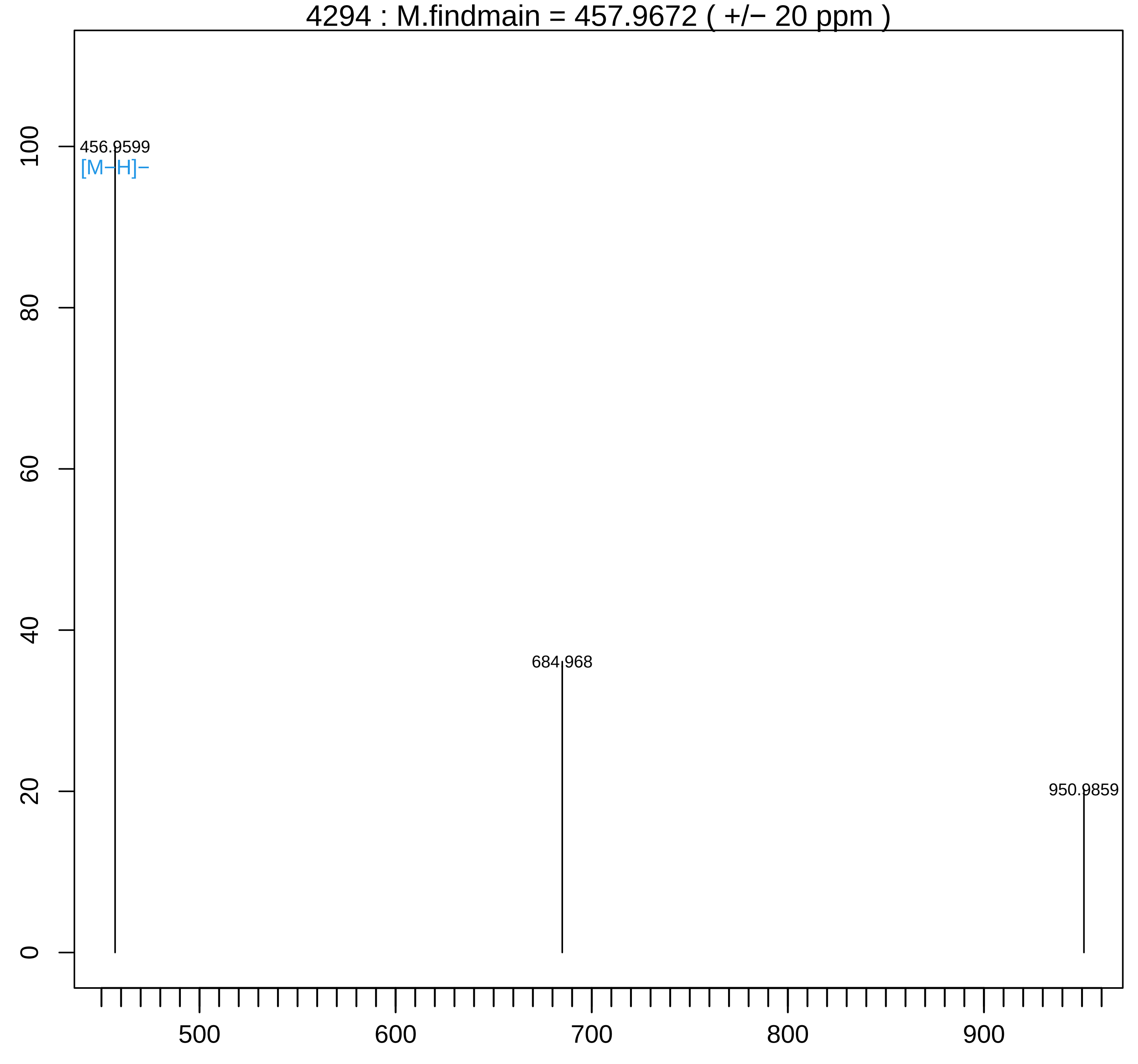

4294 : M.findmain = 457.9672 (+/− 20 ppm)
456.9599
[M−H]−
684.968
950.9859
0
20
40
60
80
100
500
600
700
800
900

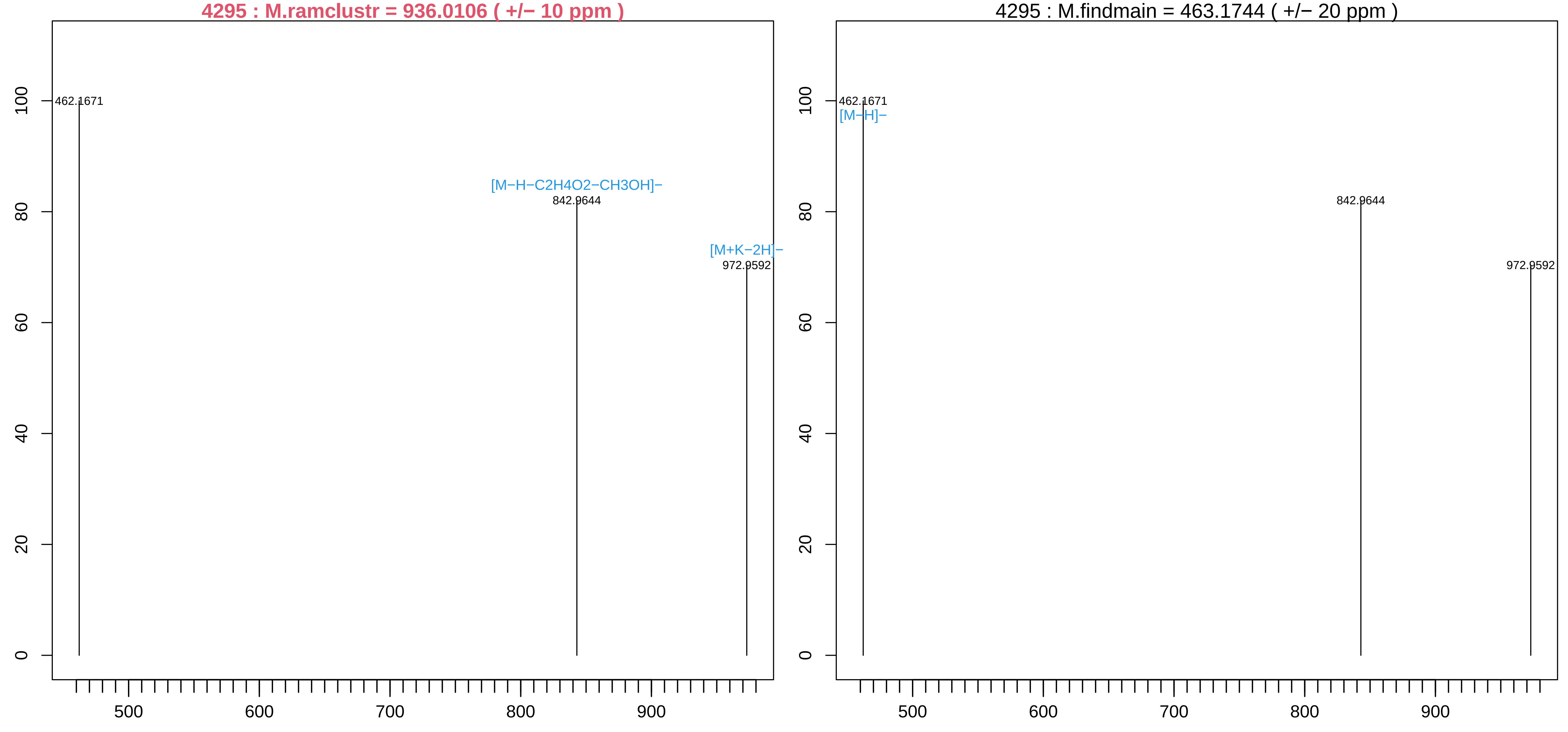

4295 : M.ramclustr = 936.0106 (+/− 10 ppm)
462.1671
[M−H−C2H4O2−CH3OH]−
842.9644
[M+K−2H]−
972.9592
4295 : M.findmain = 463.1744 (+/− 20 ppm)
462.1671
[M−H]−
842.9644
972.9592
0
20
40
60
80
100
500
600
700
800
900

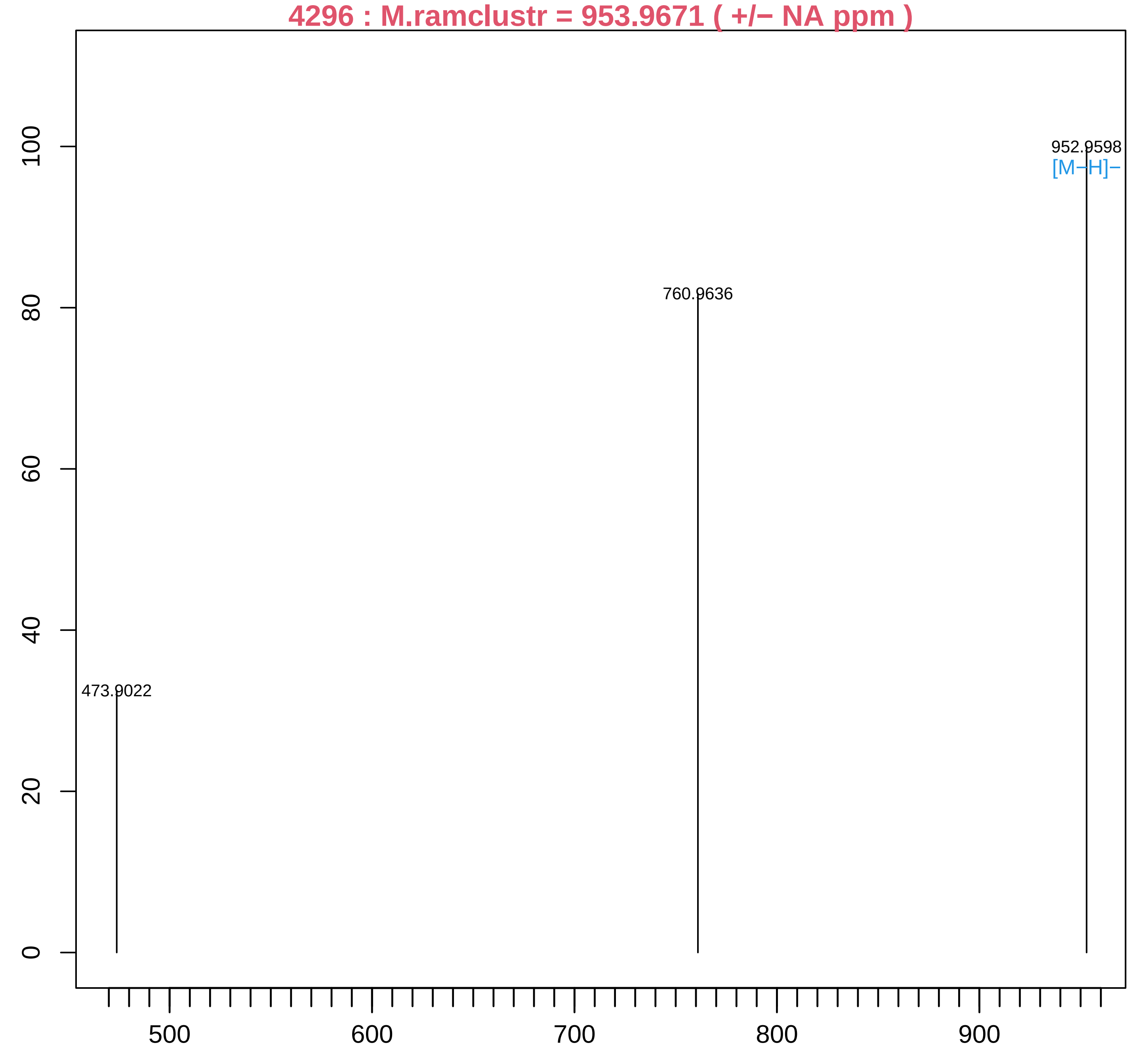

4296 : M.ramclustr = 953.9671 (+/− NA ppm)
952.9598
[M−H]−
760.9636
473.9022
0
20
40
60
80
100
500
600
700
800
900

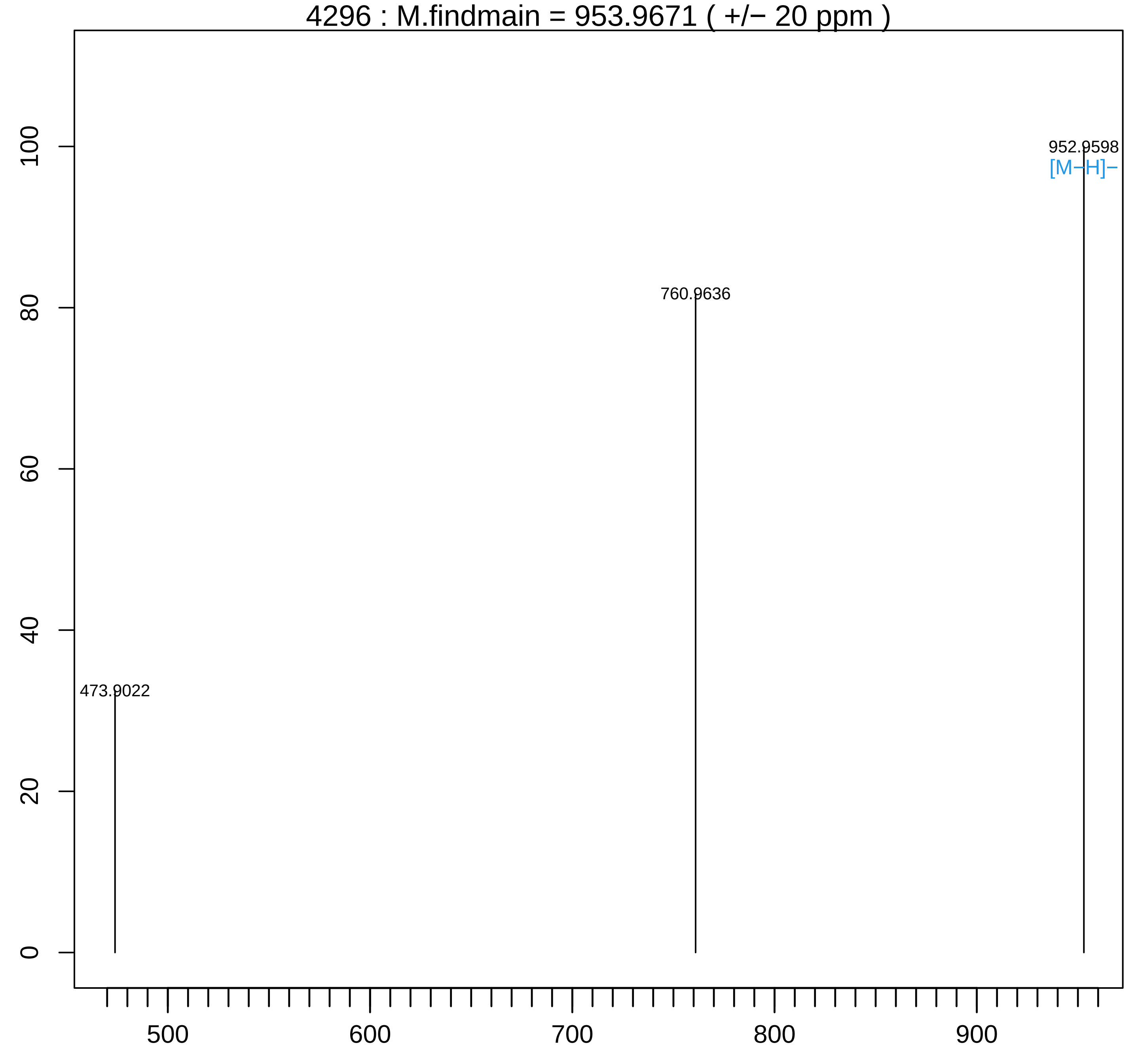

4296 : M.findmain = 953.9671 (+/− 20 ppm)
952.9598
[M−H]−
760.9636
473.9022
0
20
40
60
80
100
500
600
700
800
900

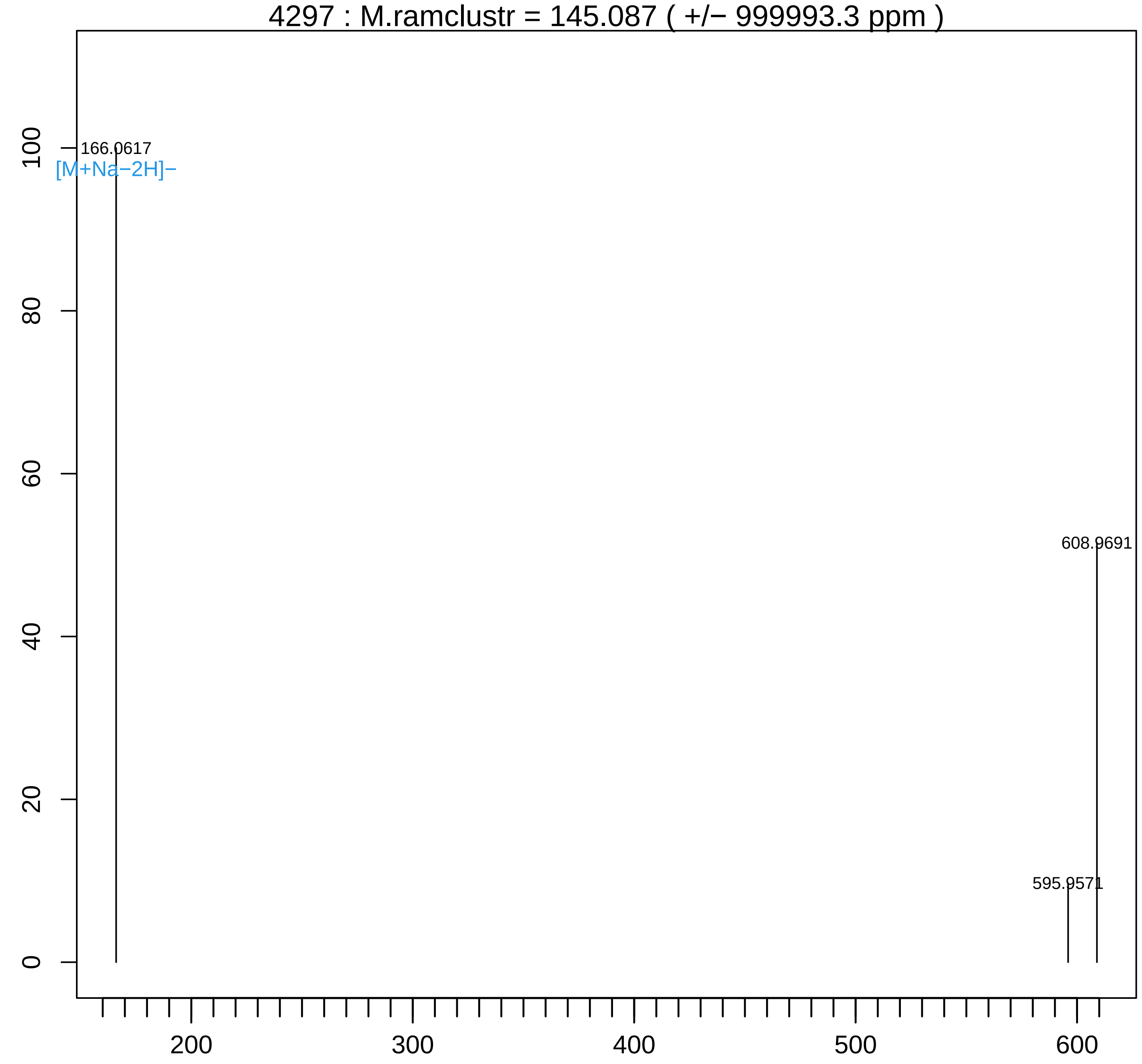
4297 : M.ramclustr = 145.087 (+/− 999993.3 ppm)
166.0617
[M+Na−2H]−
608.9691
595.9571
0
20
40
60
80
100
200
300
400
500
600

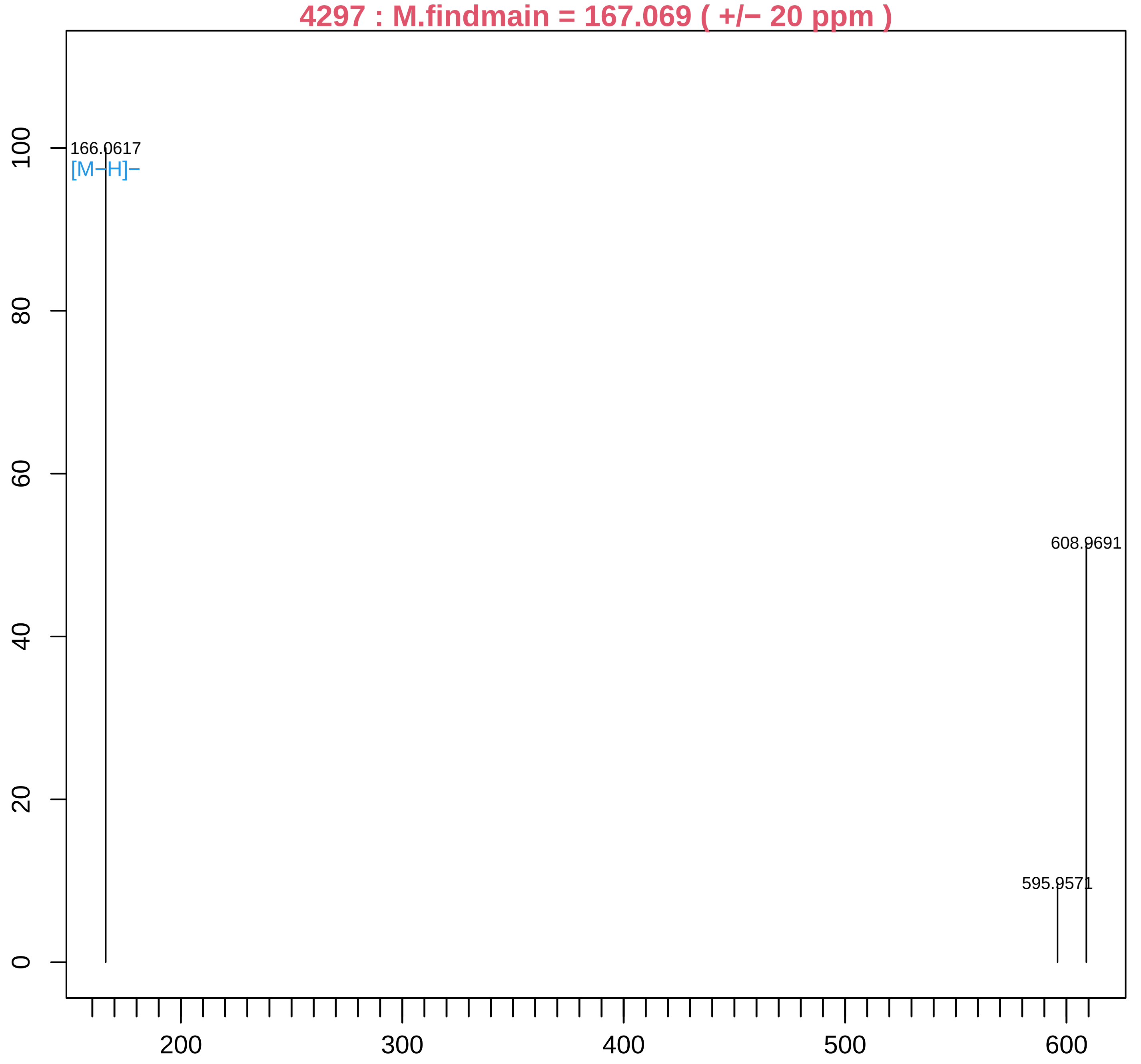
4297 : M.findmain = 167.069 (+/− 20 ppm)
166.0617
[M−H]−
608.9691
595.9571
0
20
40
60
80
100
200
300
400
500
600

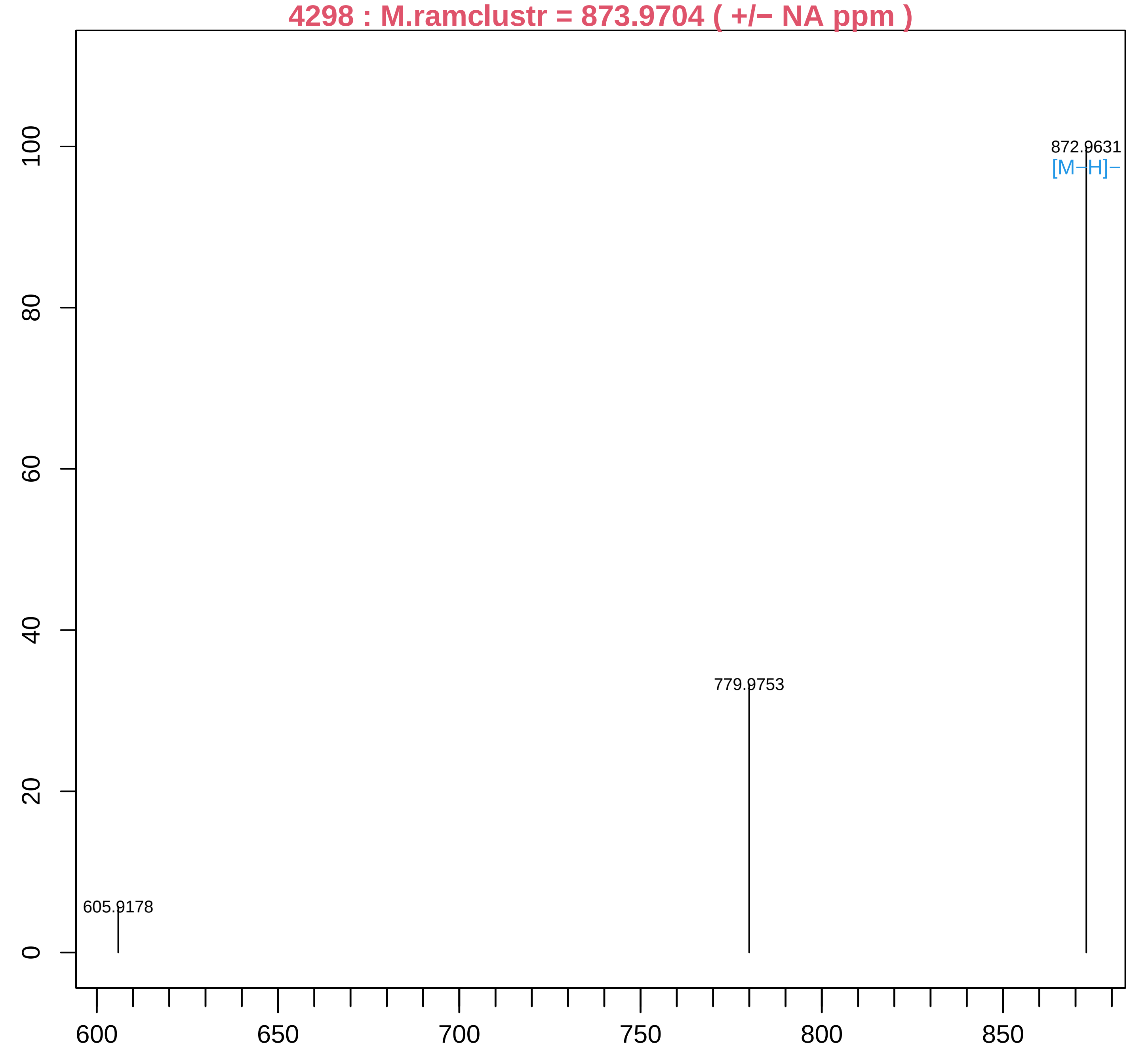

4298 : M.ramclustr = 873.9704 (+/− NA ppm)
872.9631
[M−H]−
779.9753
605.9178
0
20
40
60
80
100
600
650
700
750
800
850

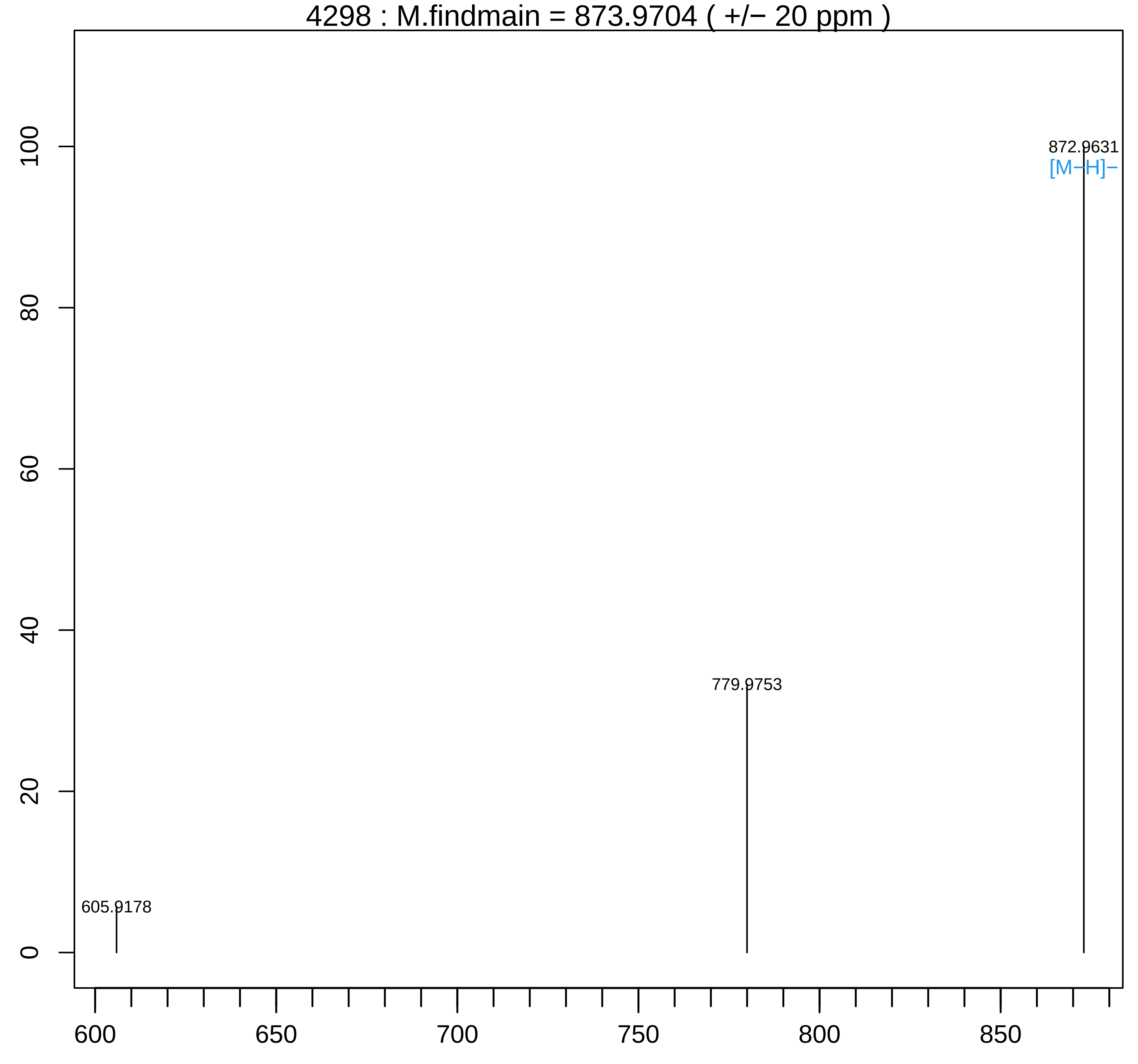

4298 : M.findmain = 873.9704 (+/− 20 ppm)
872.9631
[M−H]−
779.9753
605.9178
0
20
40
60
80
100
600
650
700
750
800
850

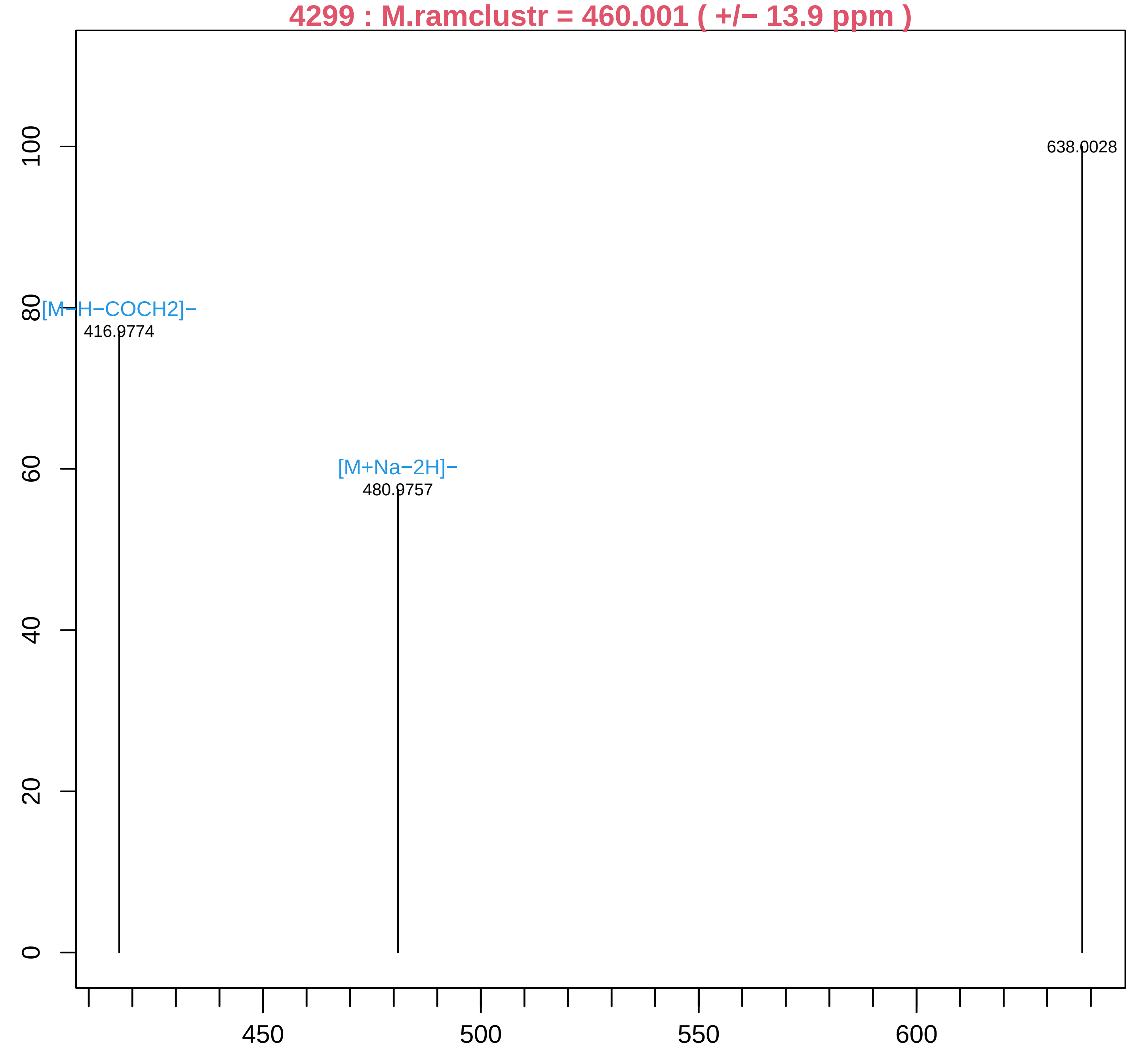
4299 : M.ramclustr = 460.001 (+/− 13.9 ppm)
[M−H−COCH2]−
416.9774
[M+Na−2H]−
480.9757
638.0028
0
20
40
60
80
100
450
500
550
600

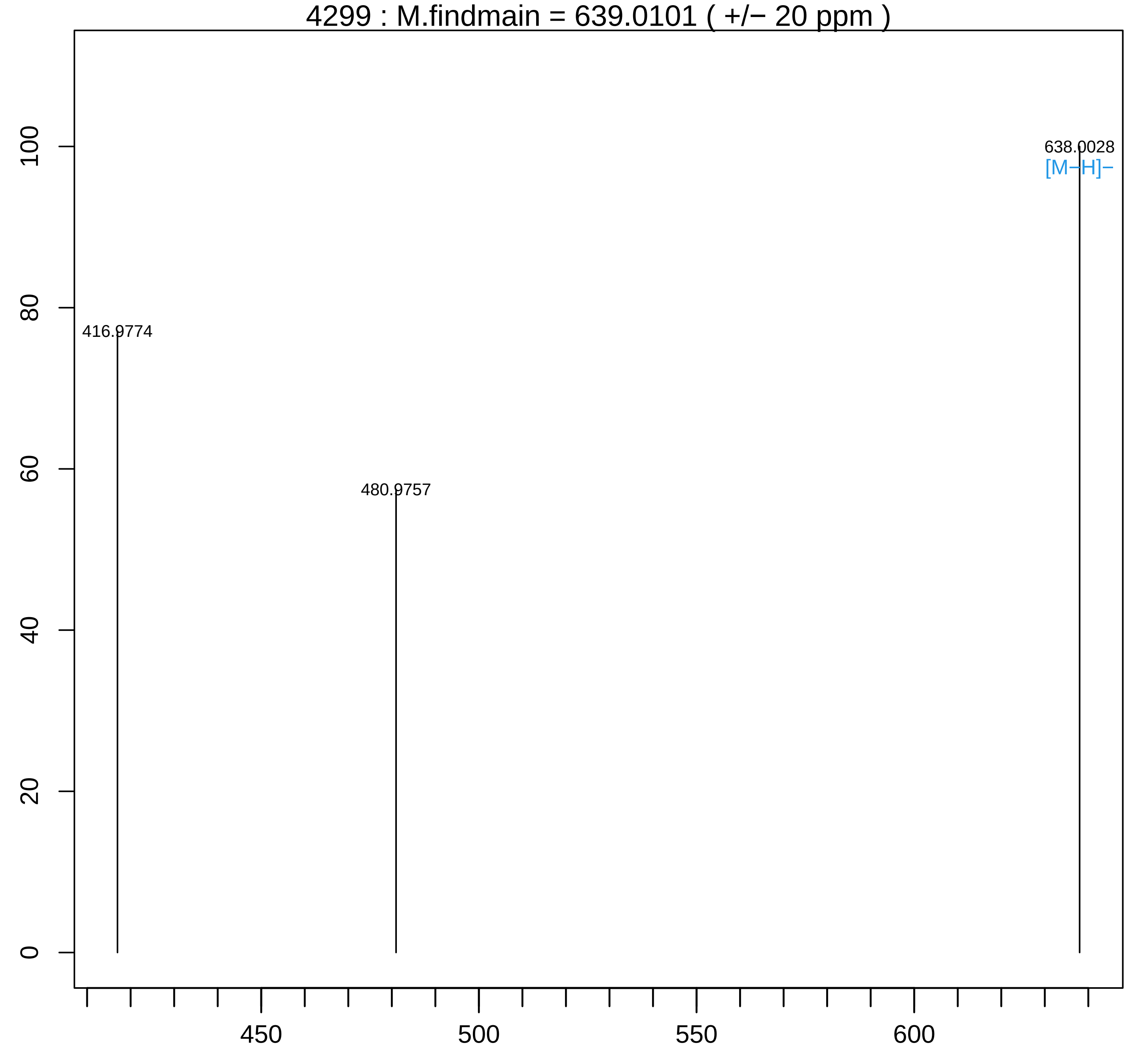
4299 : M.findmain = 639.0101 (+/− 20 ppm)
638.0028
[M−H]−
416.9774
480.9757
0
20
40
60
80
100
450
500
550
600

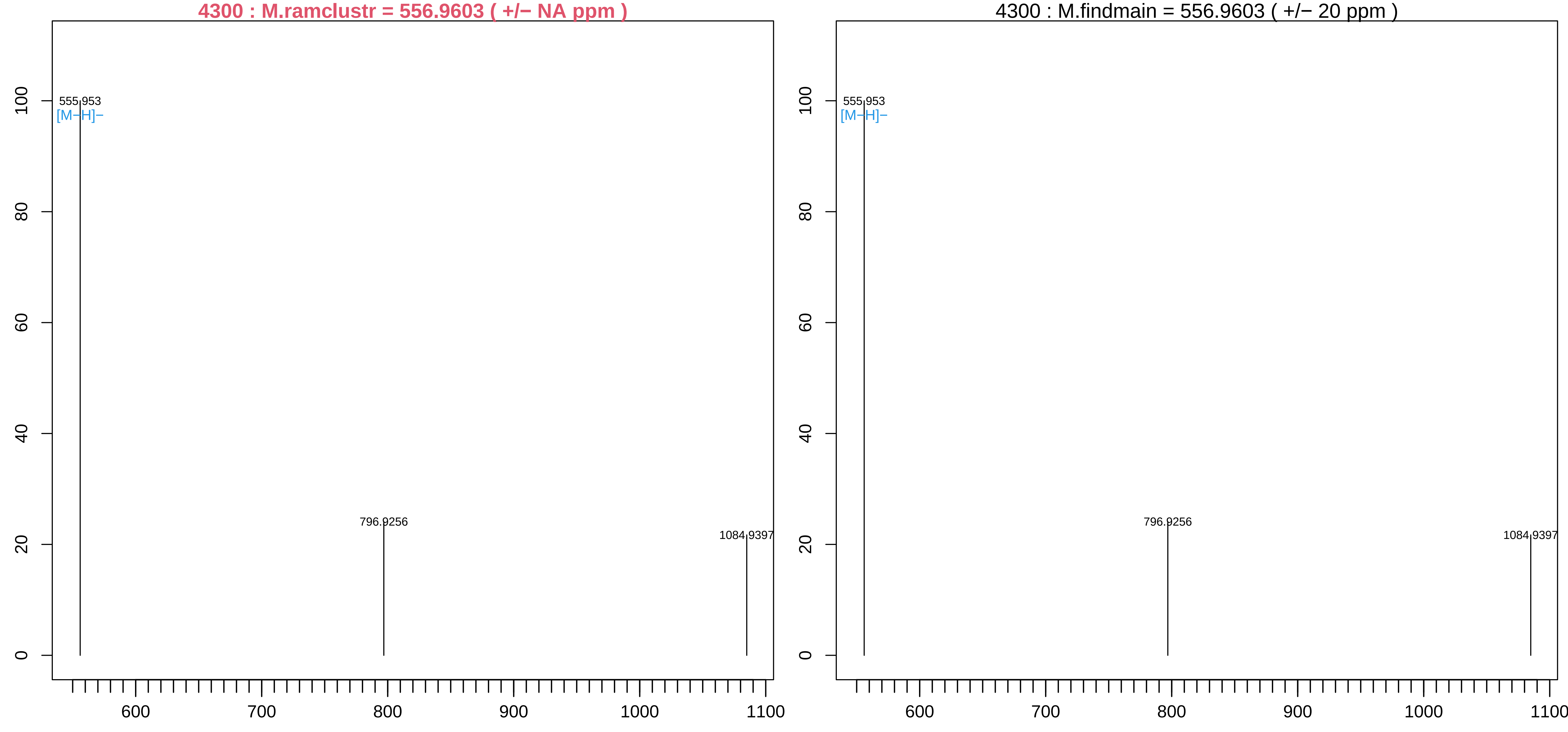

4300 : M.ramclustr = 556.9603 (+/− NA ppm)
555.953
[M−H]−
796.9256
1084.9397
0
20
40
60
80
100
600
700
800
900
1000
1100
4300 : M.findmain = 556.9603 (+/− 20 ppm)
555.953
[M−H]−
796.9256
1084.9397
0
20
40
60
80
100
600
700
800
900
1000
1100

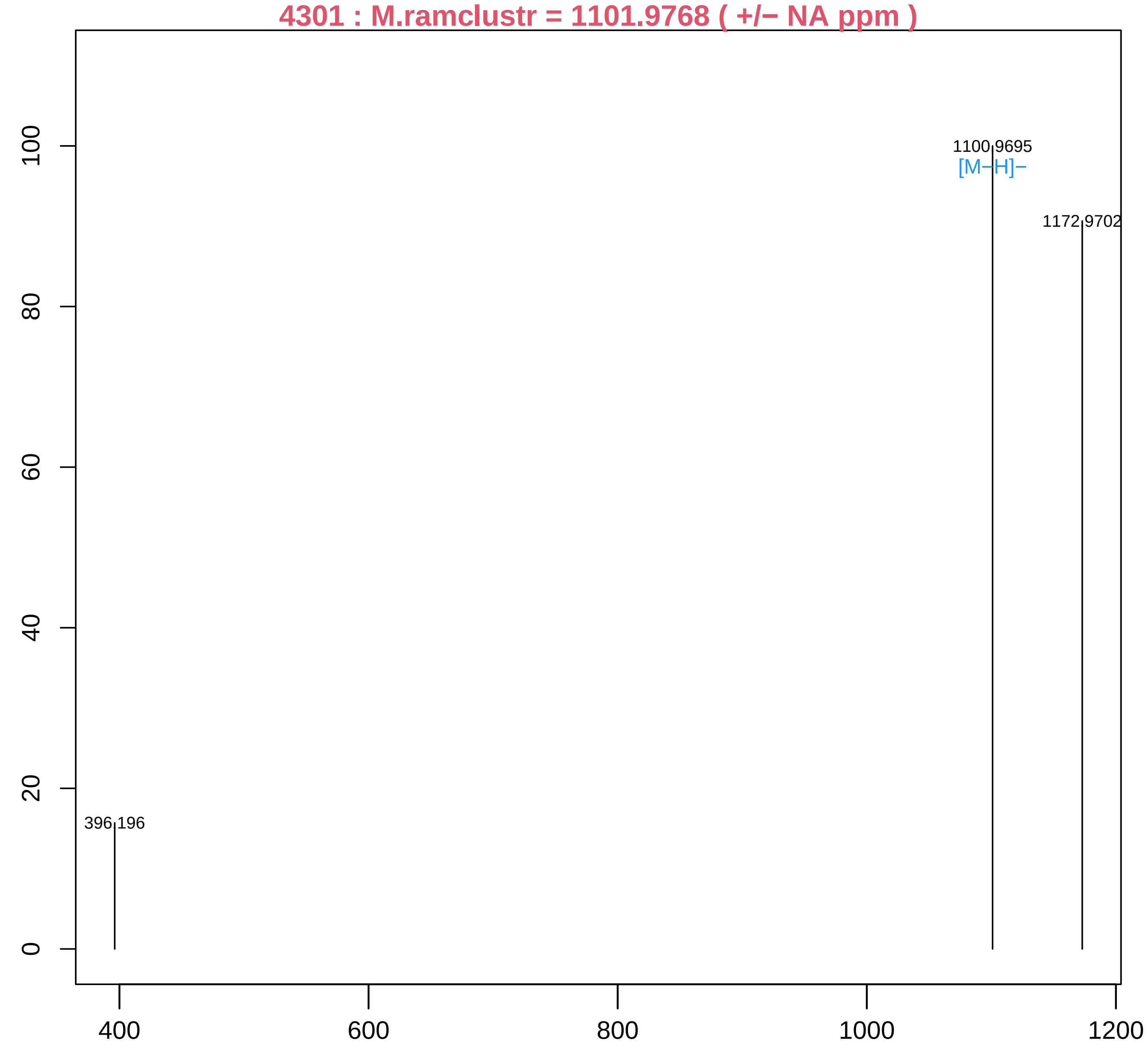
4301 : M.ramclustr = 1101.9768 (+/− NA ppm)
1100.9695
[M−H]−
1172.9702
396.196
0
20
40
60
80
100
400
600
800
1000
1200

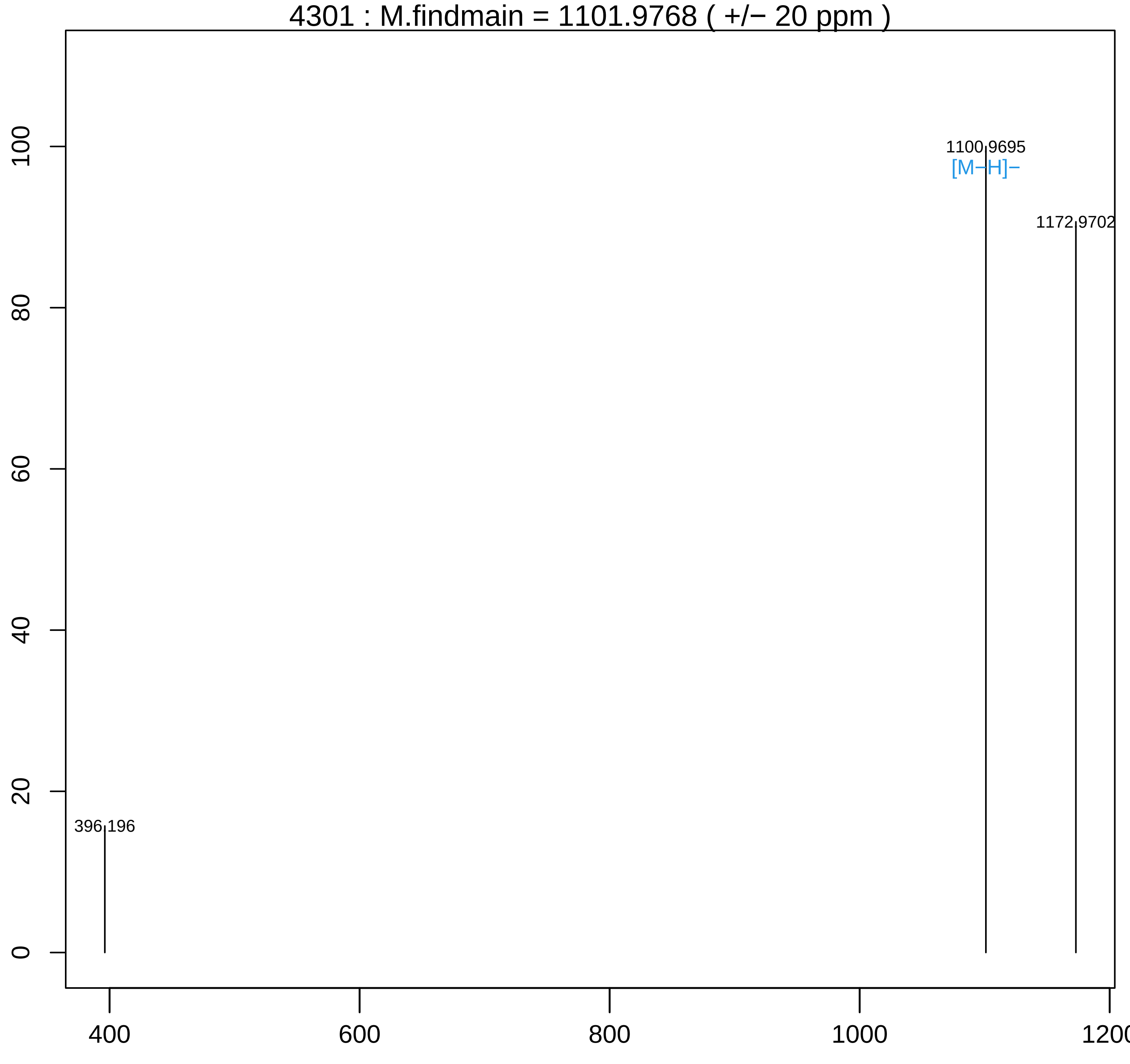
4301 : M.findmain = 1101.9768 (+/− 20 ppm)
1100.9695
[M−H]−
1172.9702
396.196
0
20
40
60
80
100
400
600
800
1000
1200

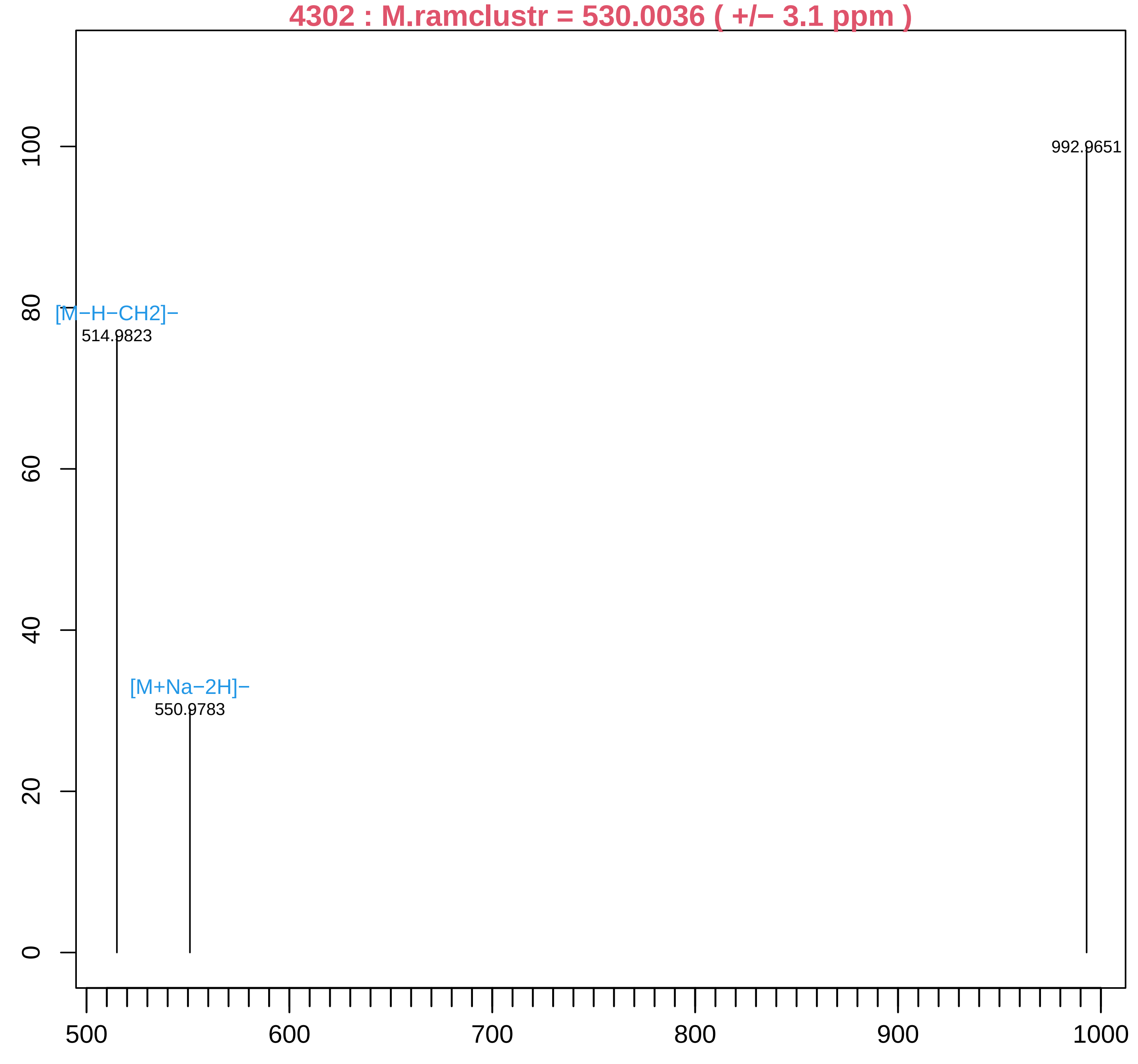
4302 : M.ramclustr = 530.0036 (+/− 3.1 ppm)
992.9651
[M−H−CH2]−
514.9823
[M+Na−2H]−
550.9783
0
20
40
60
80
100
500
600
700
800
900
1000

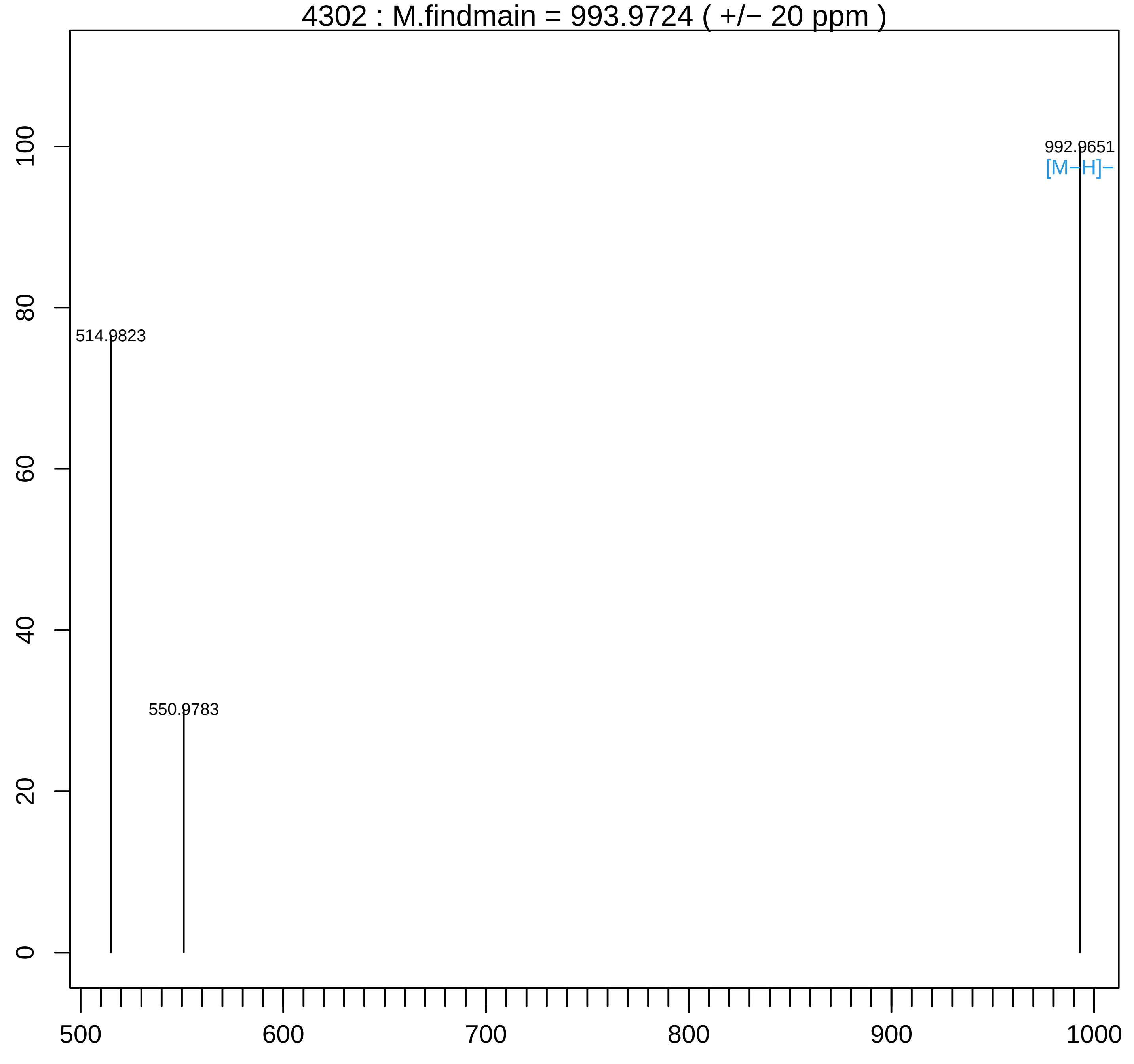
4302 : M.findmain = 993.9724 (+/− 20 ppm)
992.9651
[M−H]−
514.9823
550.9783
0
20
40
60
80
100
500
600
700
800
900
1000

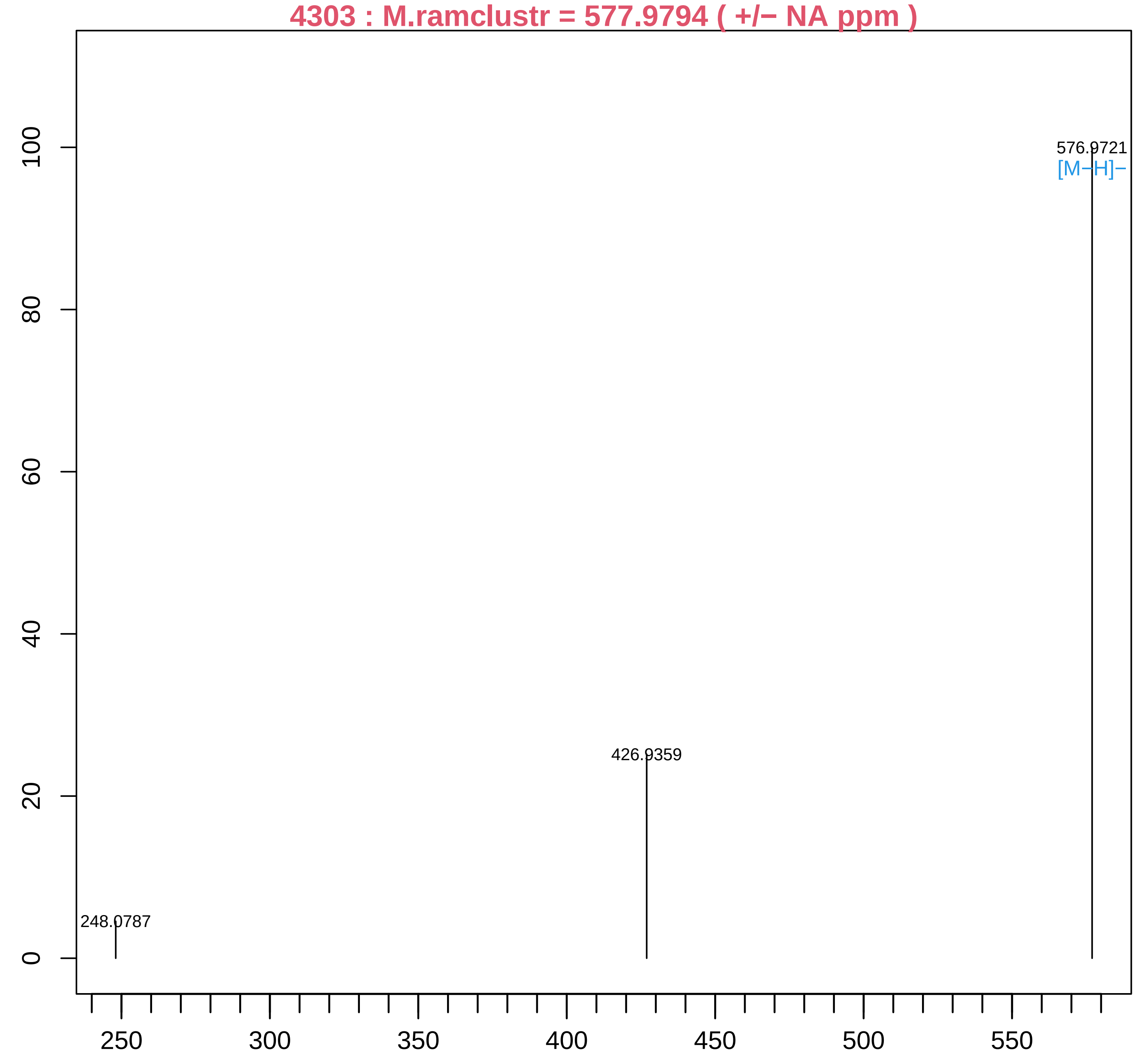
4303 : M.ramclustr = 577.9794 (+/− NA ppm)
576.9721
[M−H]−
426.9359
248.0787
0
20
40
60
80
100
250
300
350
400
450
500
550

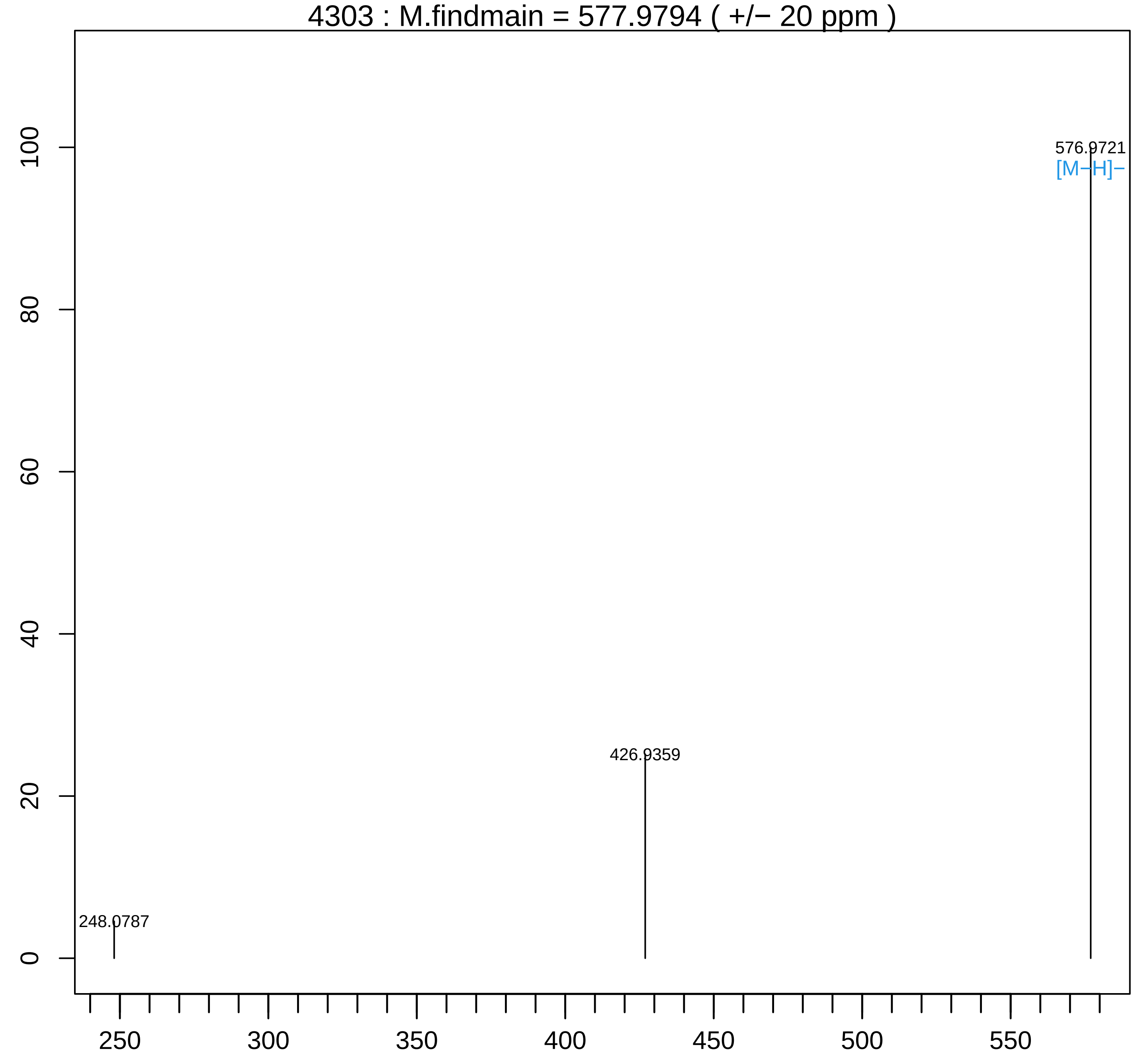
4303 : M.findmain = 577.9794 (+/− 20 ppm)
576.9721
[M−H]−
426.9359
248.0787
0
20
40
60
80
100
250
300
350
400
450
500
550

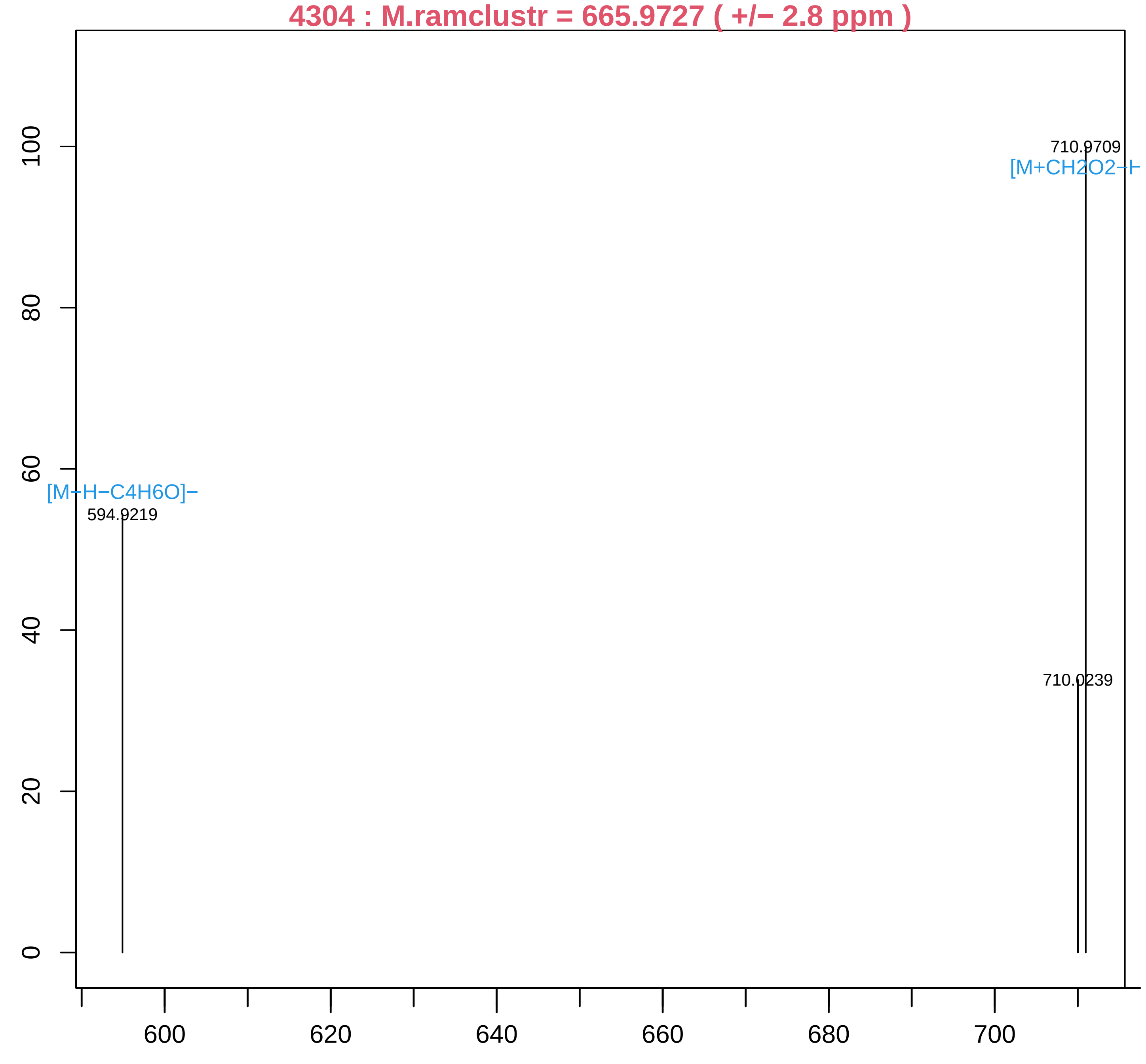
4304 : M.ramclustr = 665.9727 (+/− 2.8 ppm)
710.9709
[M+CH2O2−H
[M−H−C4H6O]−
594.9219
710.0239
0
20
40
60
80
100
600
620
640
660
680
700

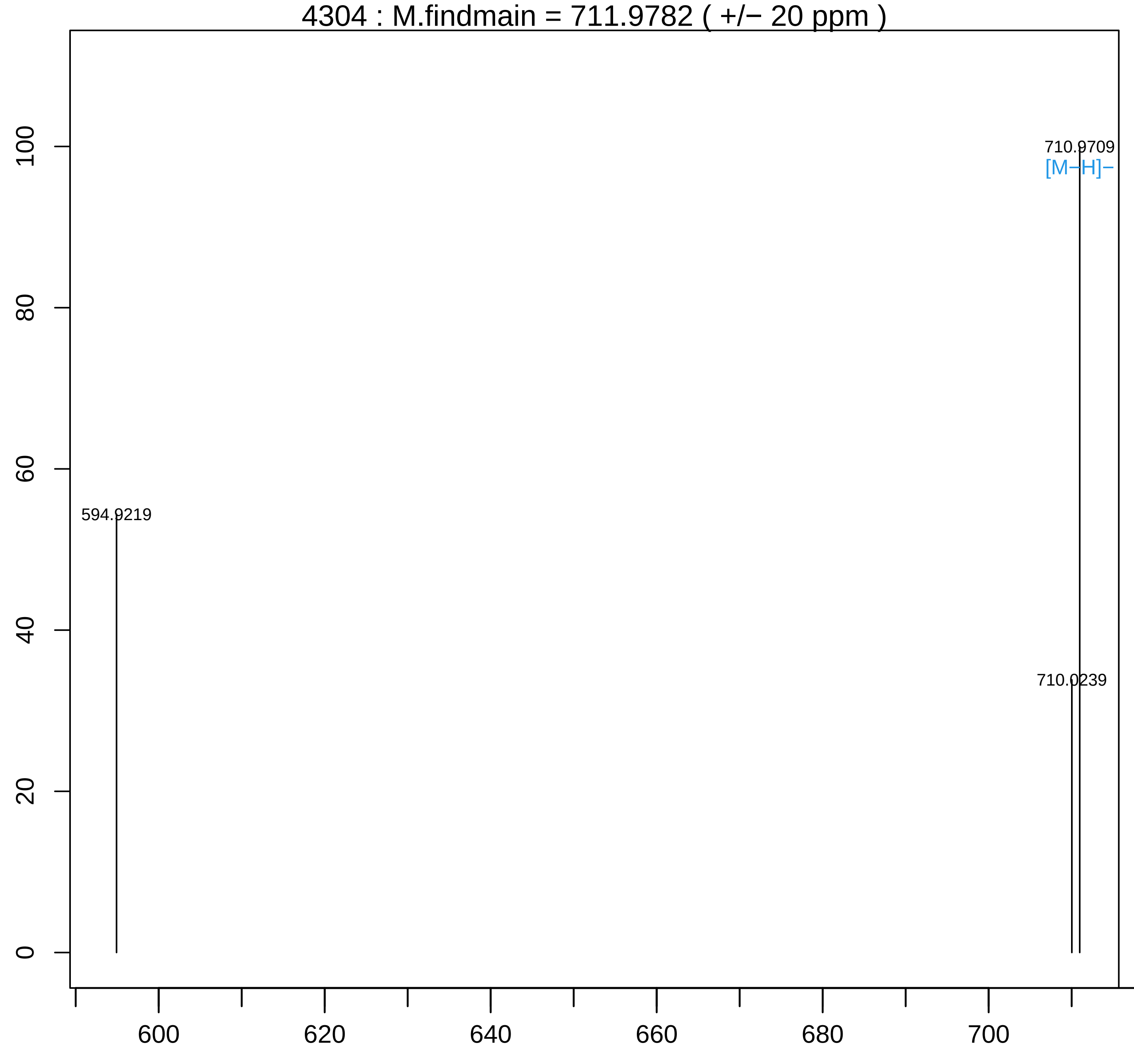
4304 : M.findmain = 711.9782 (+/− 20 ppm)
710.9709
[M−H]−
594.9219
710.0239
0
20
40
60
80
100
600
620
640
660
680
700

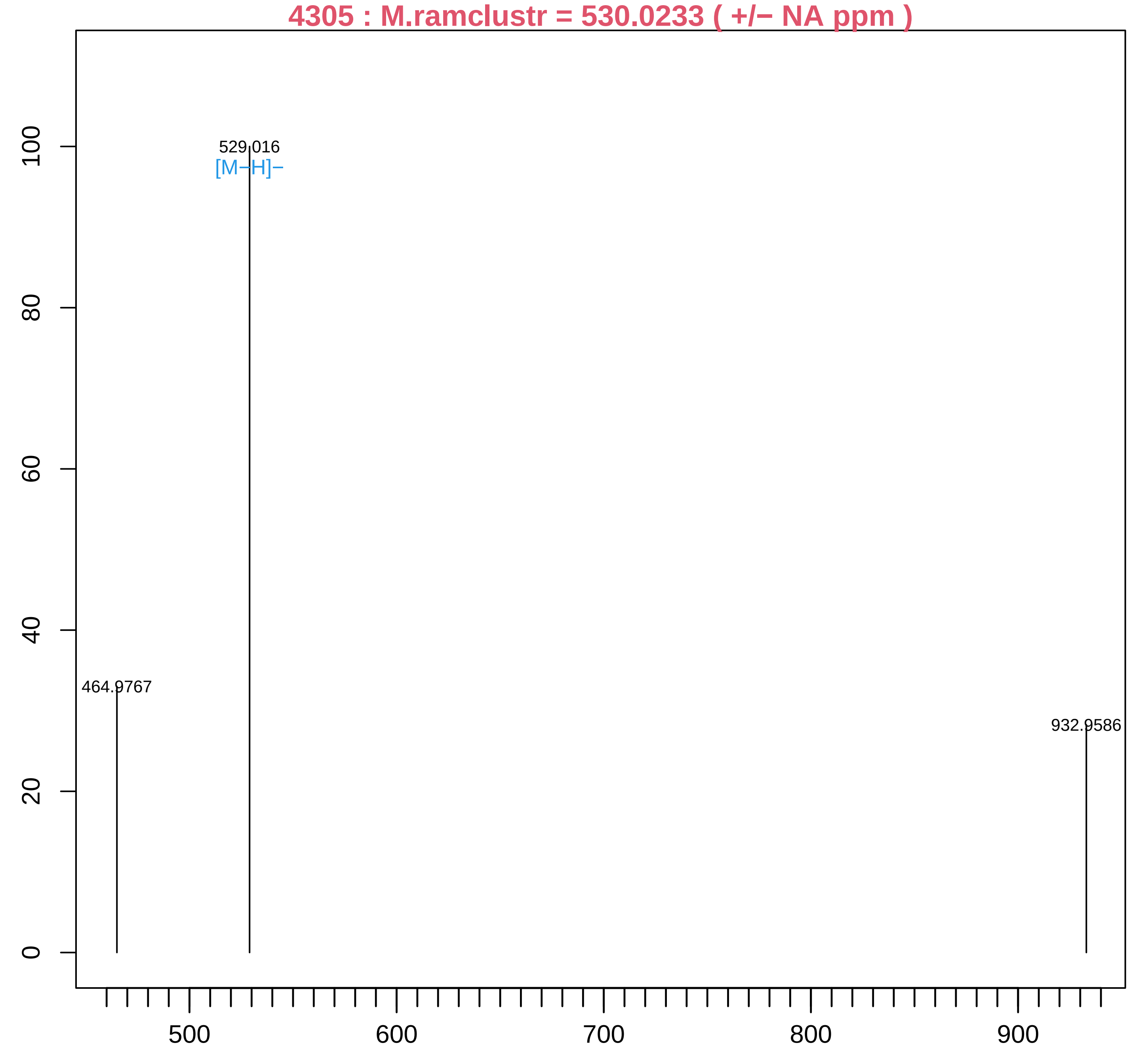
4305 : M.ramclustr = 530.0233 (+/− NA ppm)
529.016
[M−H]−
464.9767
932.9586
0
20
40
60
80
100
500
600
700
800
900

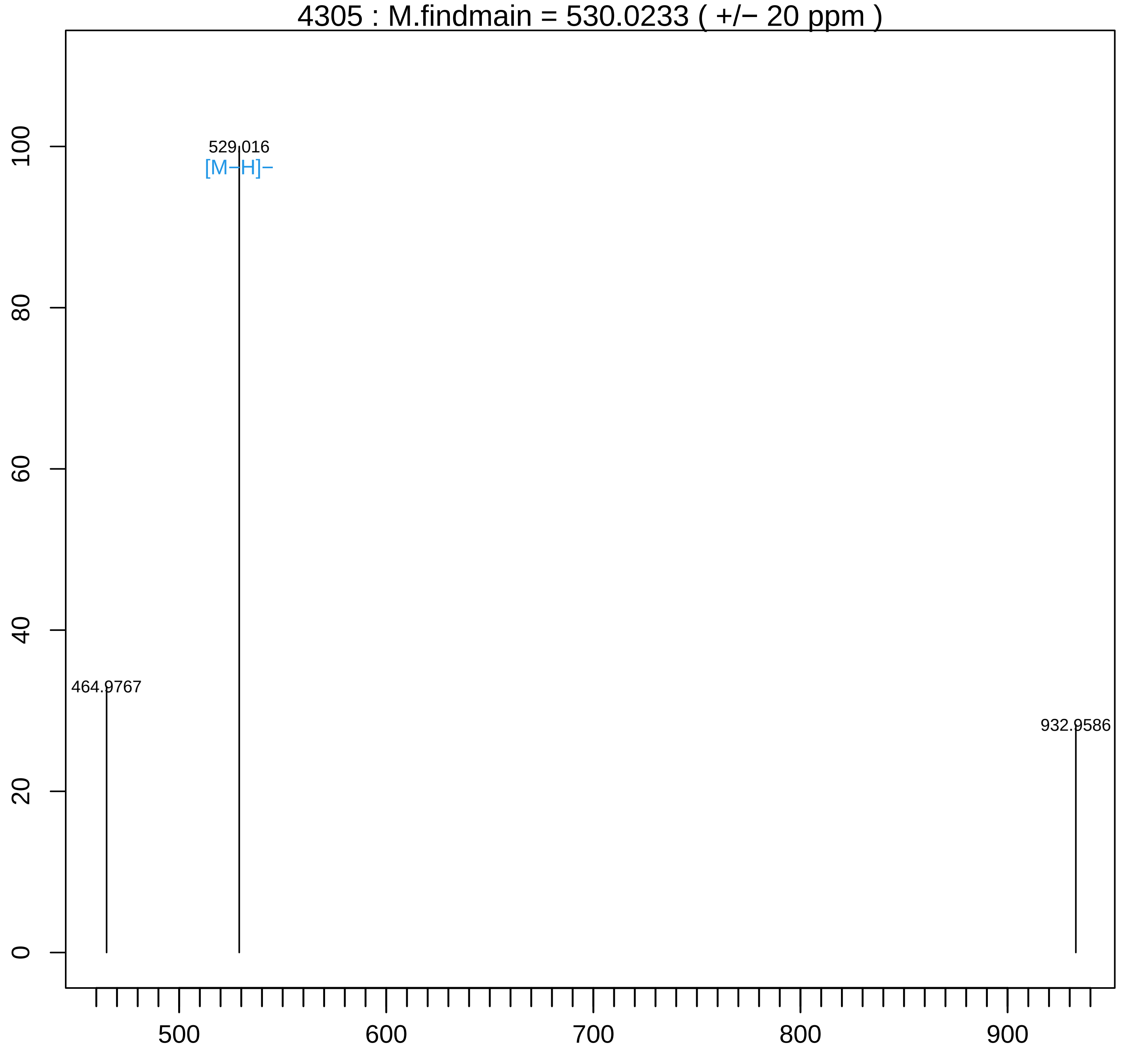
4305 : M.findmain = 530.0233 (+/− 20 ppm)
529.016
[M−H]−
464.9767
932.9586
0
20
40
60
80
100
500
600
700
800
900

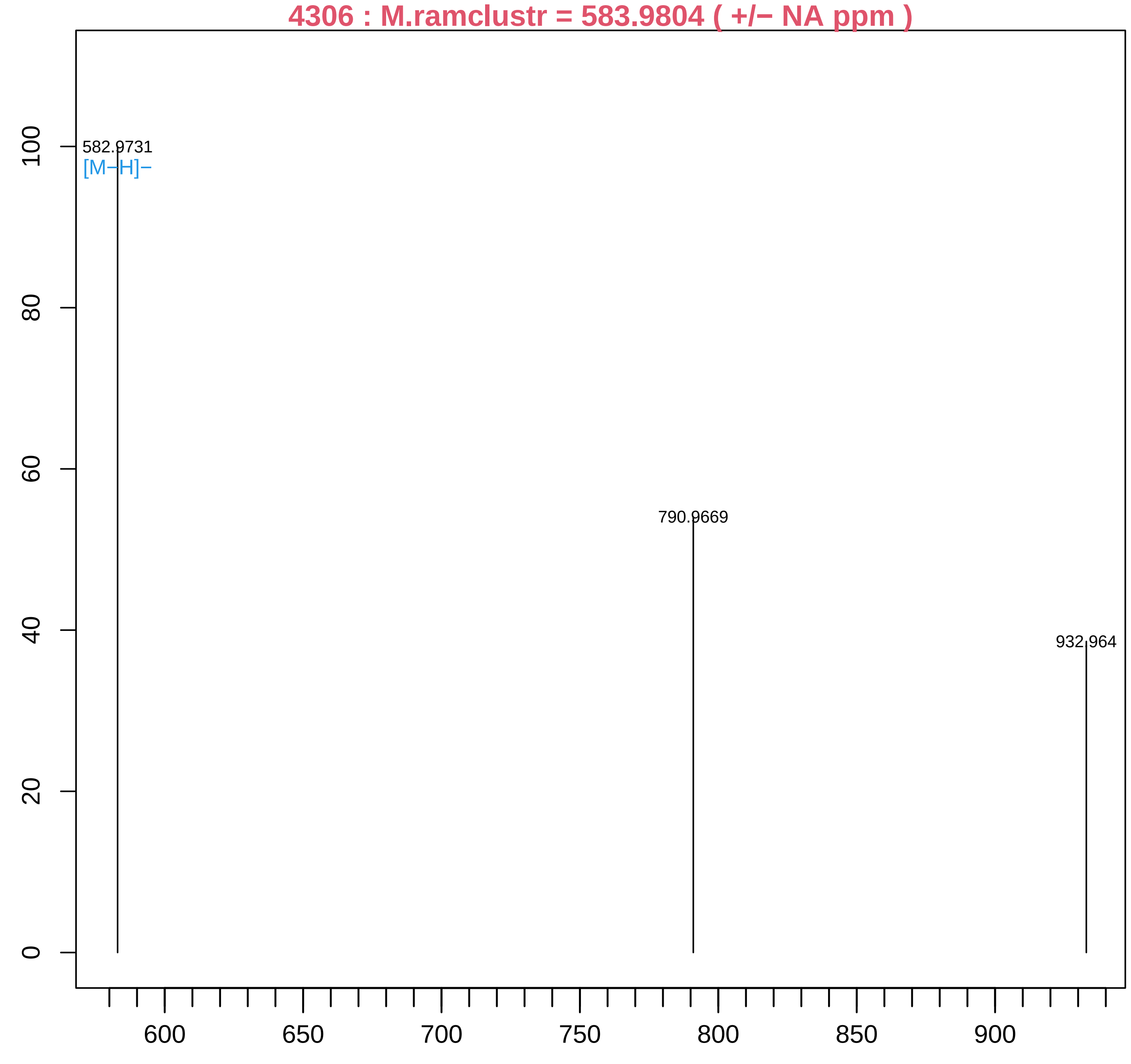
4306 : M.ramclustr = 583.9804 (+/− NA ppm)
582.9731
[M−H]−
790.9669
932.964
0
20
40
60
80
100
600
650
700
750
800
850
900

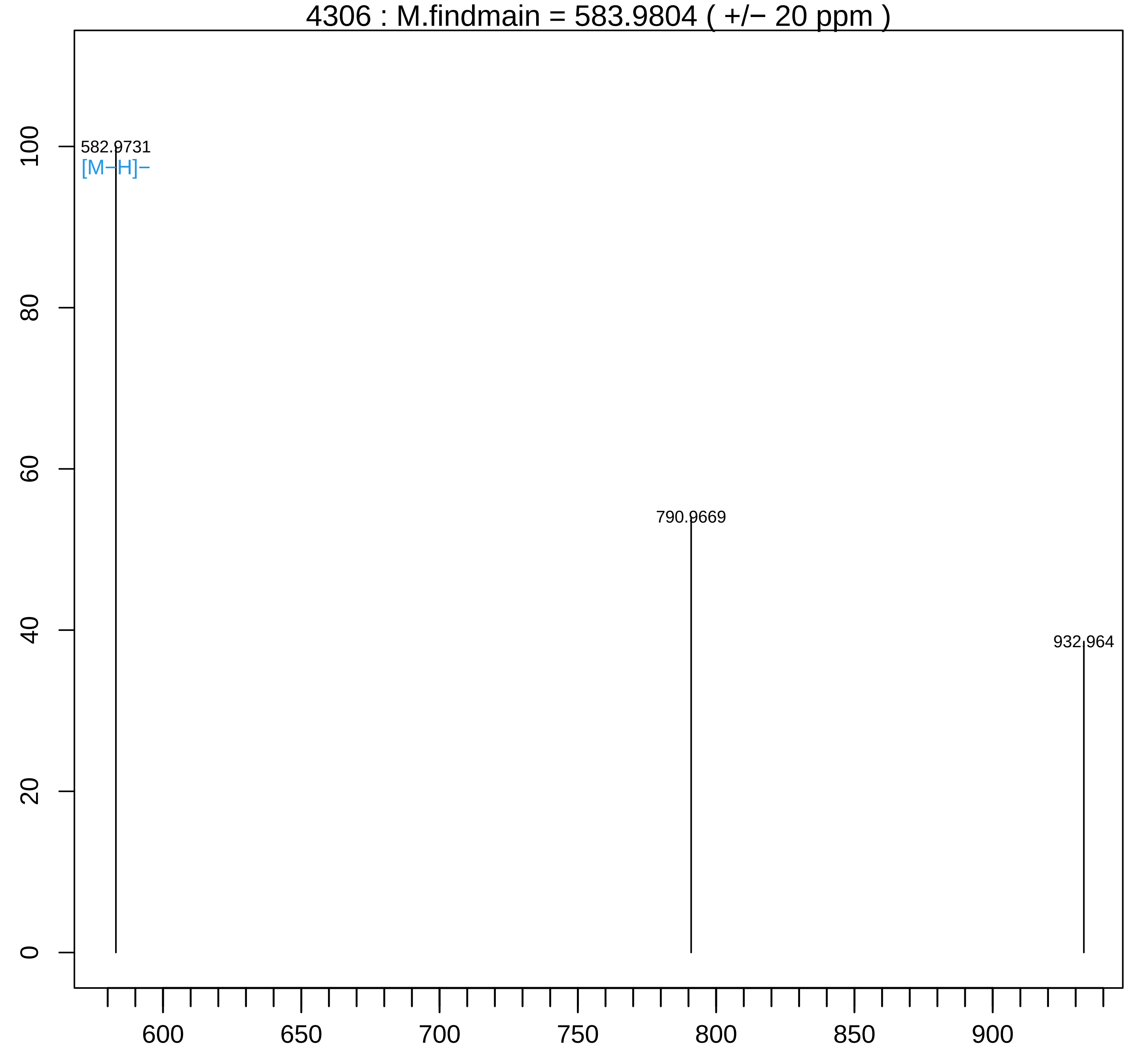
4306 : M.findmain = 583.9804 (+/− 20 ppm)
582.9731
[M−H]−
790.9669
932.964
0
20
40
60
80
100
600
650
700
750
800
850
900

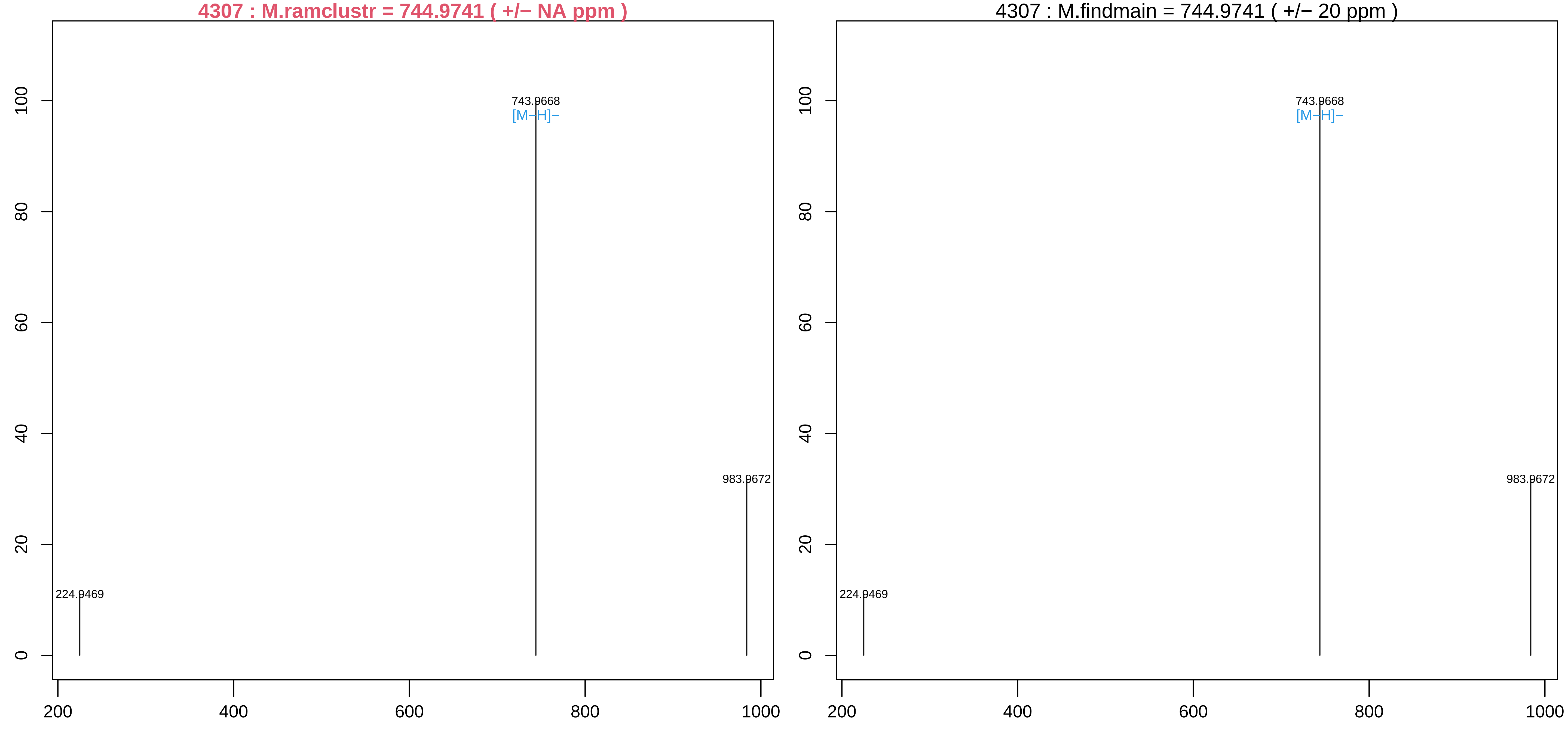
4307 : M.ramclustr = 744.9741 (+/− NA ppm)
743.9668
[M−H]−
983.9672
224.9469
0
20
40
60
80
100
200
400
600
800
1000
4307 : M.findmain = 744.9741 (+/− 20 ppm)
743.9668
[M−H]−
983.9672
224.9469
0
20
40
60
80
100
200
400
600
800
1000

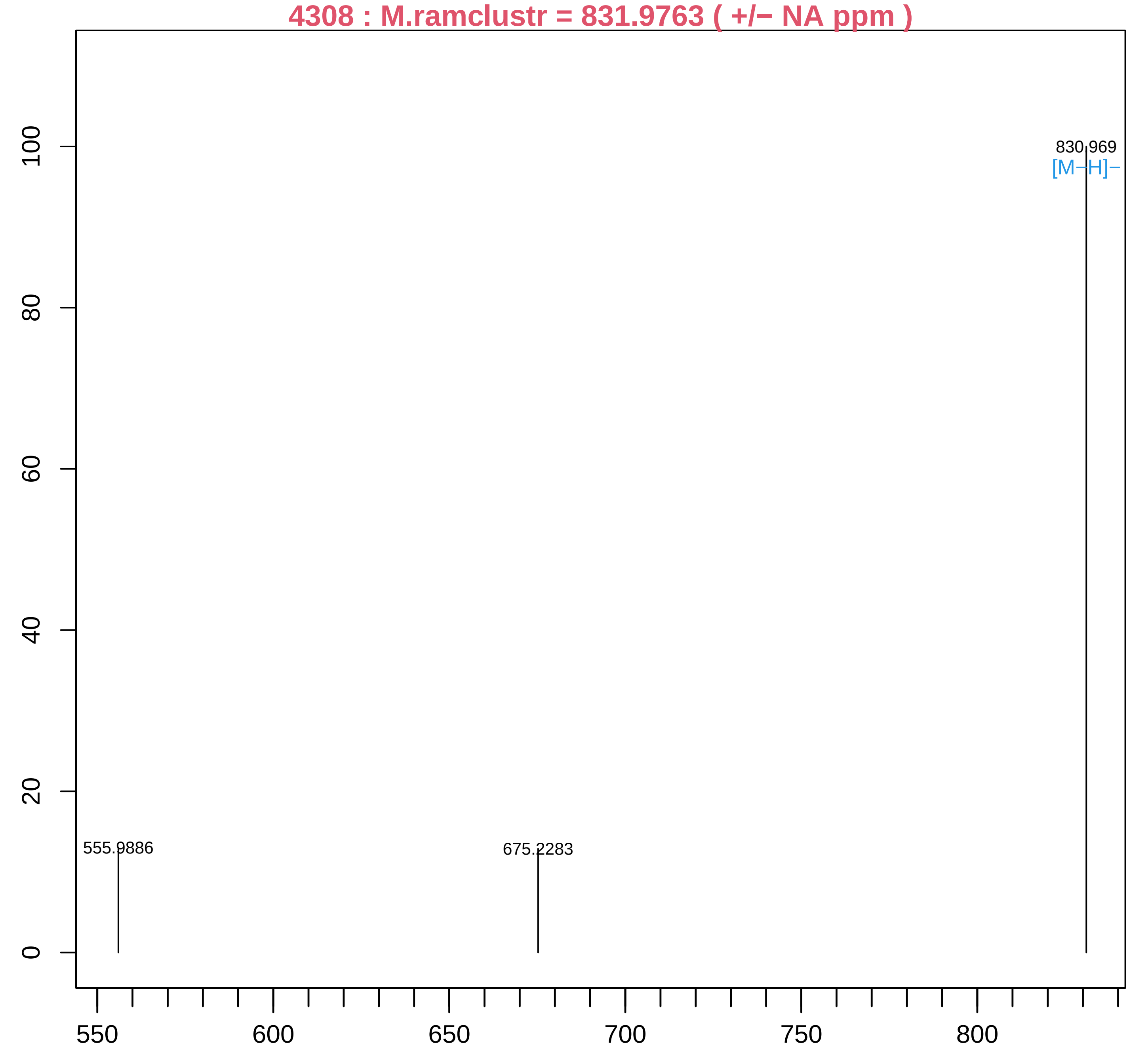
4308 : M.ramclustr = 831.9763 (+/− NA ppm)
830.969
[M−H]−
555.9886
675.2283
0
20
40
60
80
100
550
600
650
700
750
800

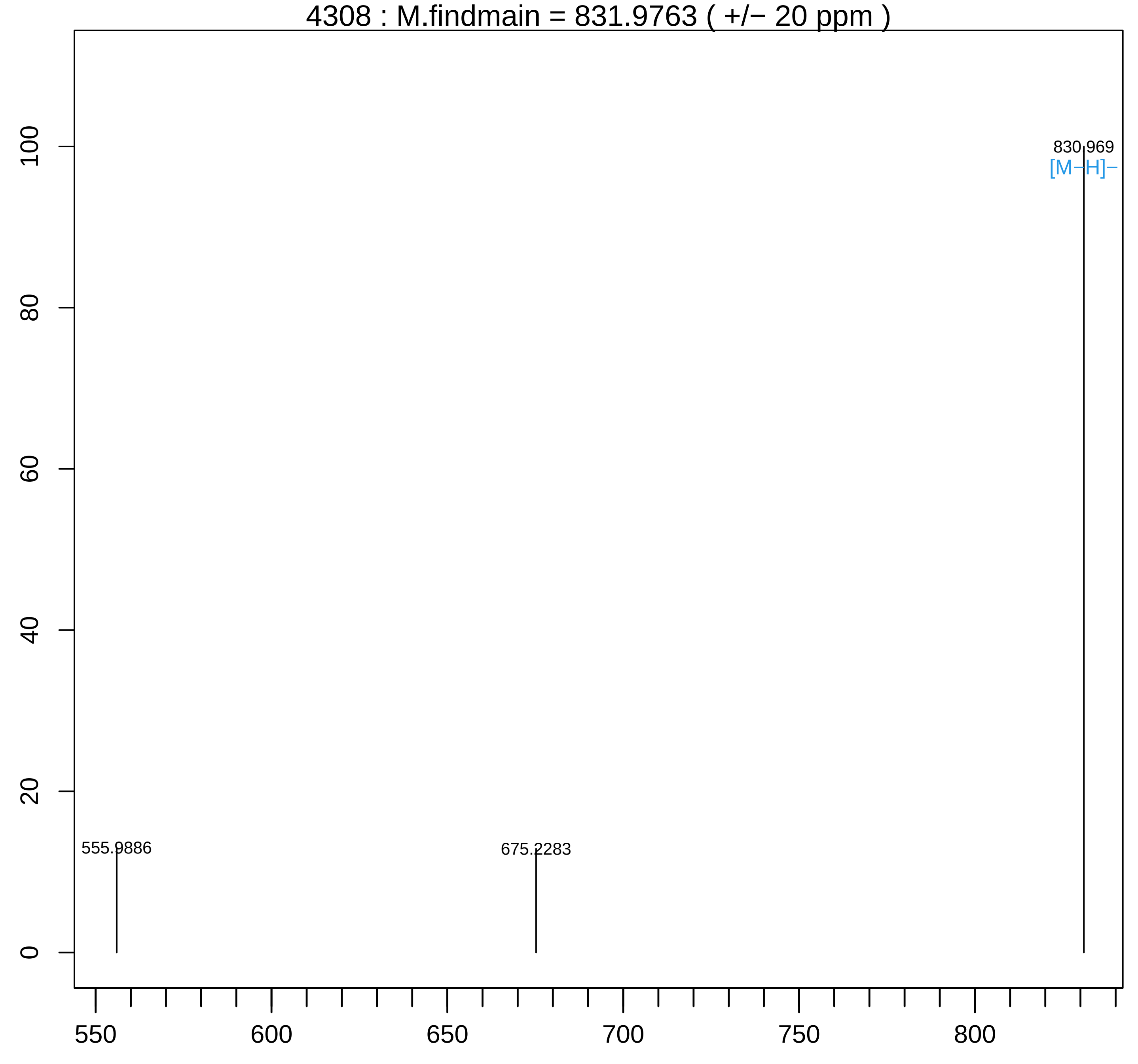
4308 : M.findmain = 831.9763 (+/− 20 ppm)
830.969
[M−H]−
555.9886
675.2283
0
20
40
60
80
100
550
600
650
700
750
800

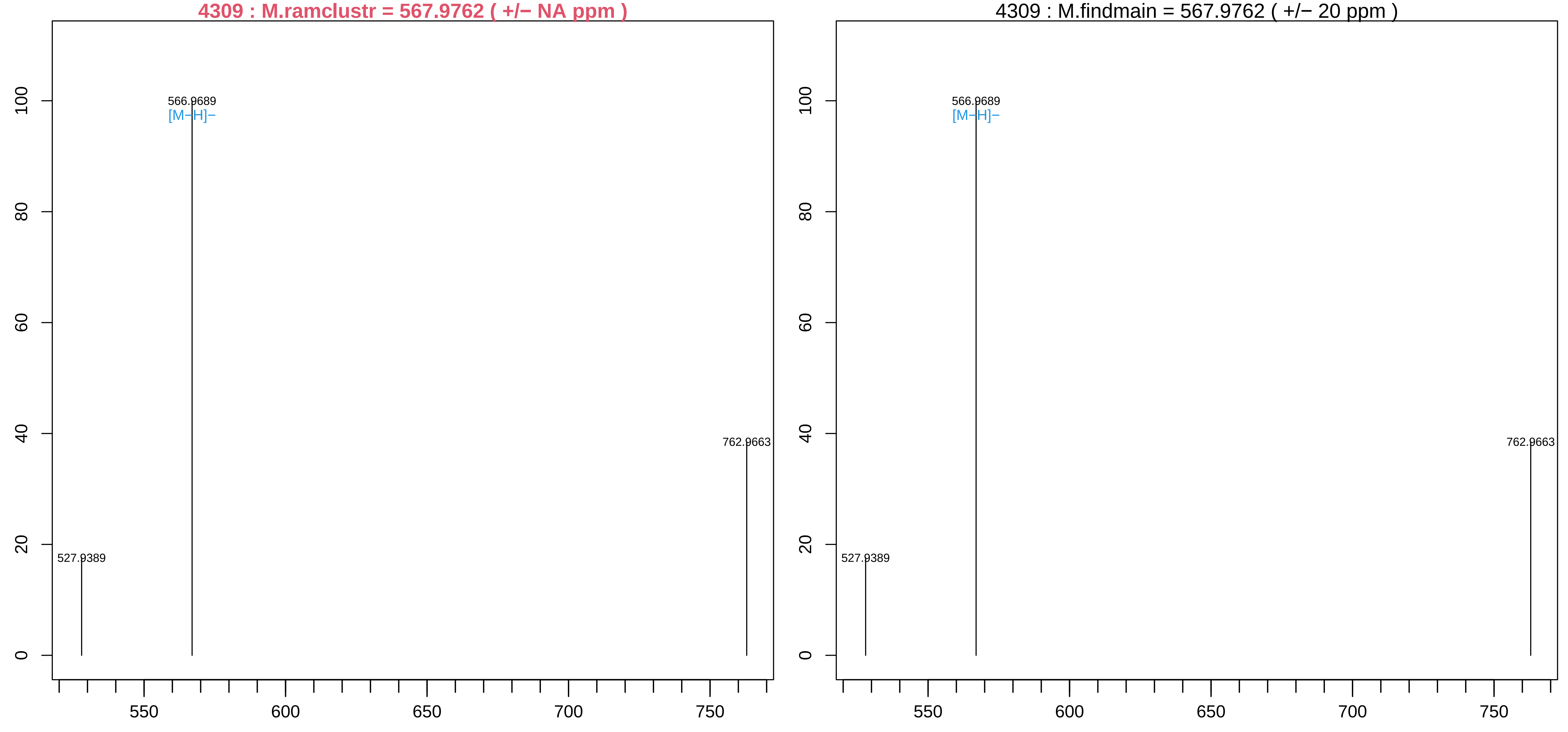

4309 : M.ramclustr = 567.9762 (+/− NA ppm)
566.9689
[M−H]−
762.9663
527.9389
0
20
40
60
80
100
550
600
650
700
750
4309 : M.findmain = 567.9762 (+/− 20 ppm)
566.9689
[M−H]−
762.9663
527.9389
0
20
40
60
80
100
550
600
650
700
750

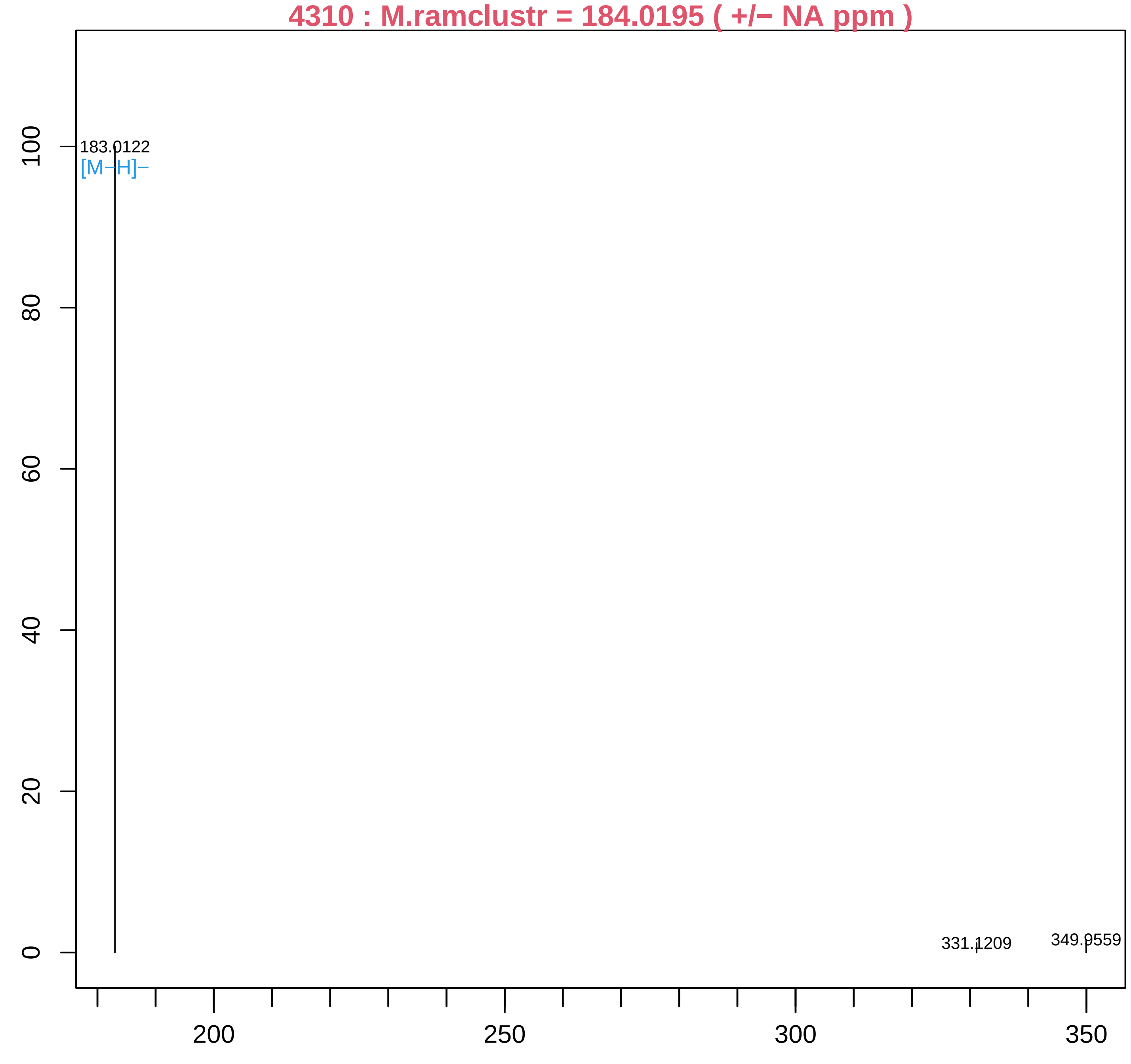

4310 : M.ramclustr = 184.0195 (+/− NA ppm)
183.0122
[M−H]−
331.1209
349.9559
0
20
40
60
80
100
200
250
300
350

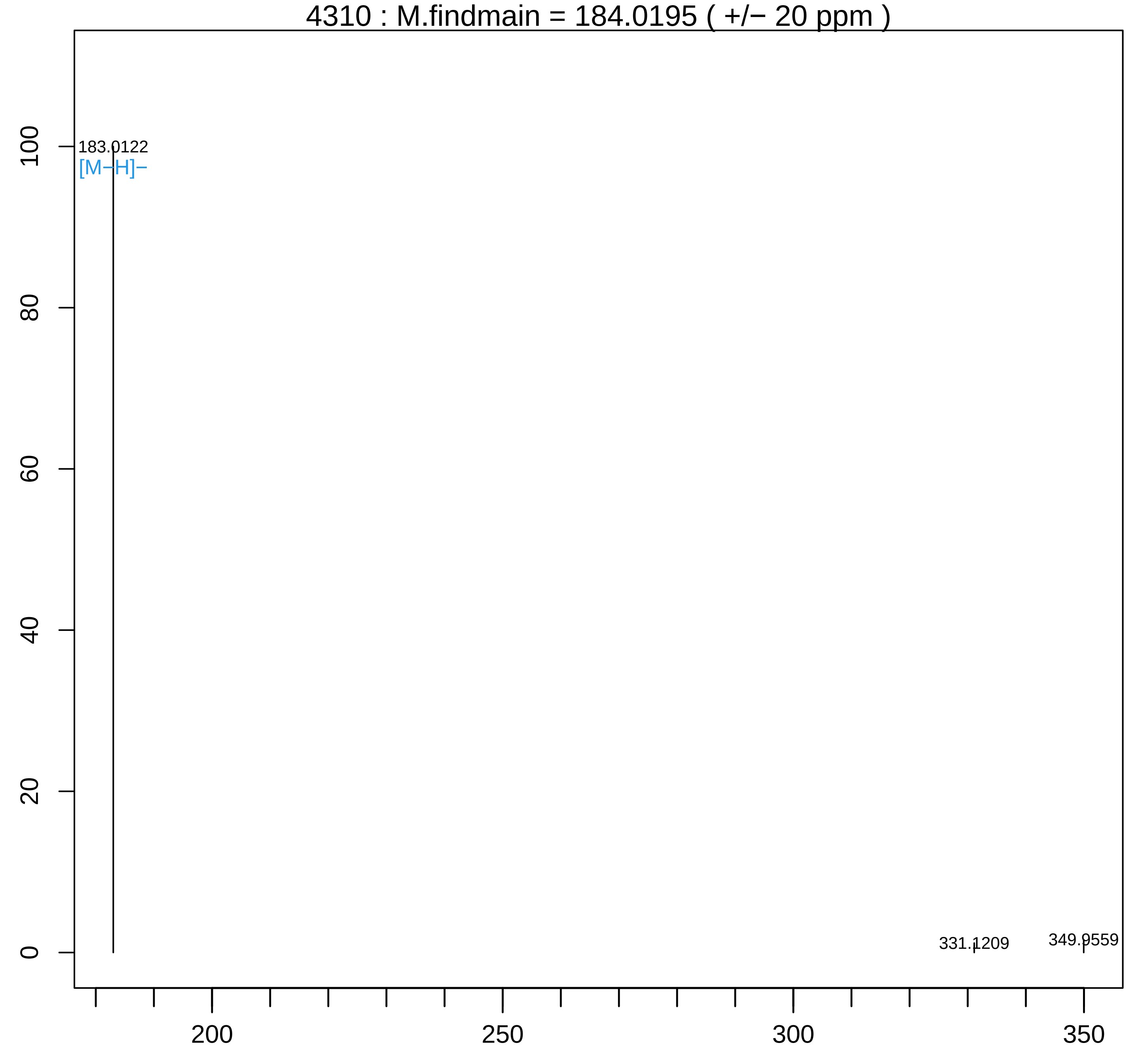

4310 : M.findmain = 184.0195 (+/− 20 ppm)
183.0122
[M−H]−
331.1209
349.9559
0
20
40
60
80
100
200
250
300
350

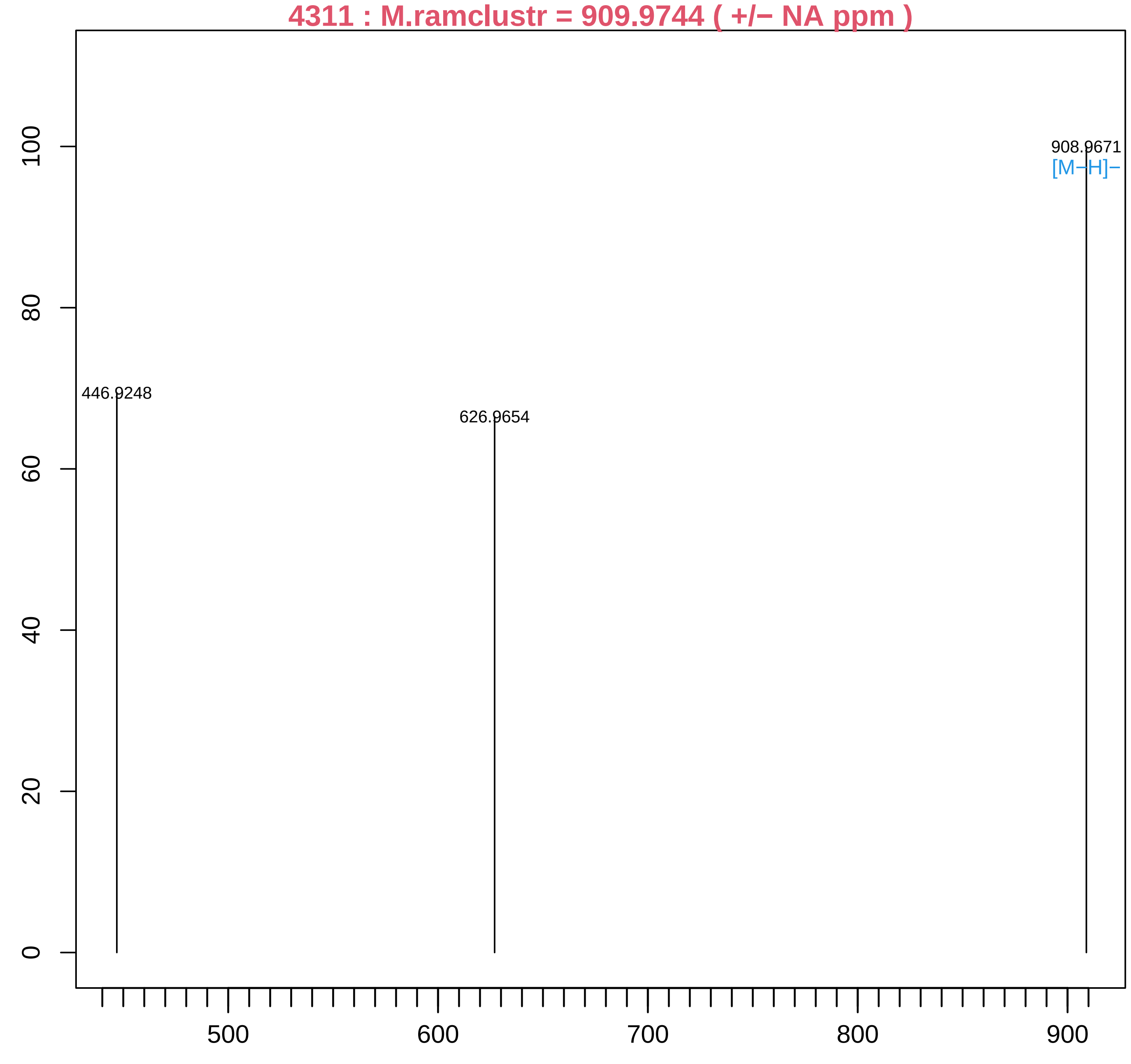

4311 : M.ramclustr = 909.9744 (+/− NA ppm)
908.9671
[M−H]−
446.9248
626.9654
0
20
40
60
80
100
500
600
700
800
900

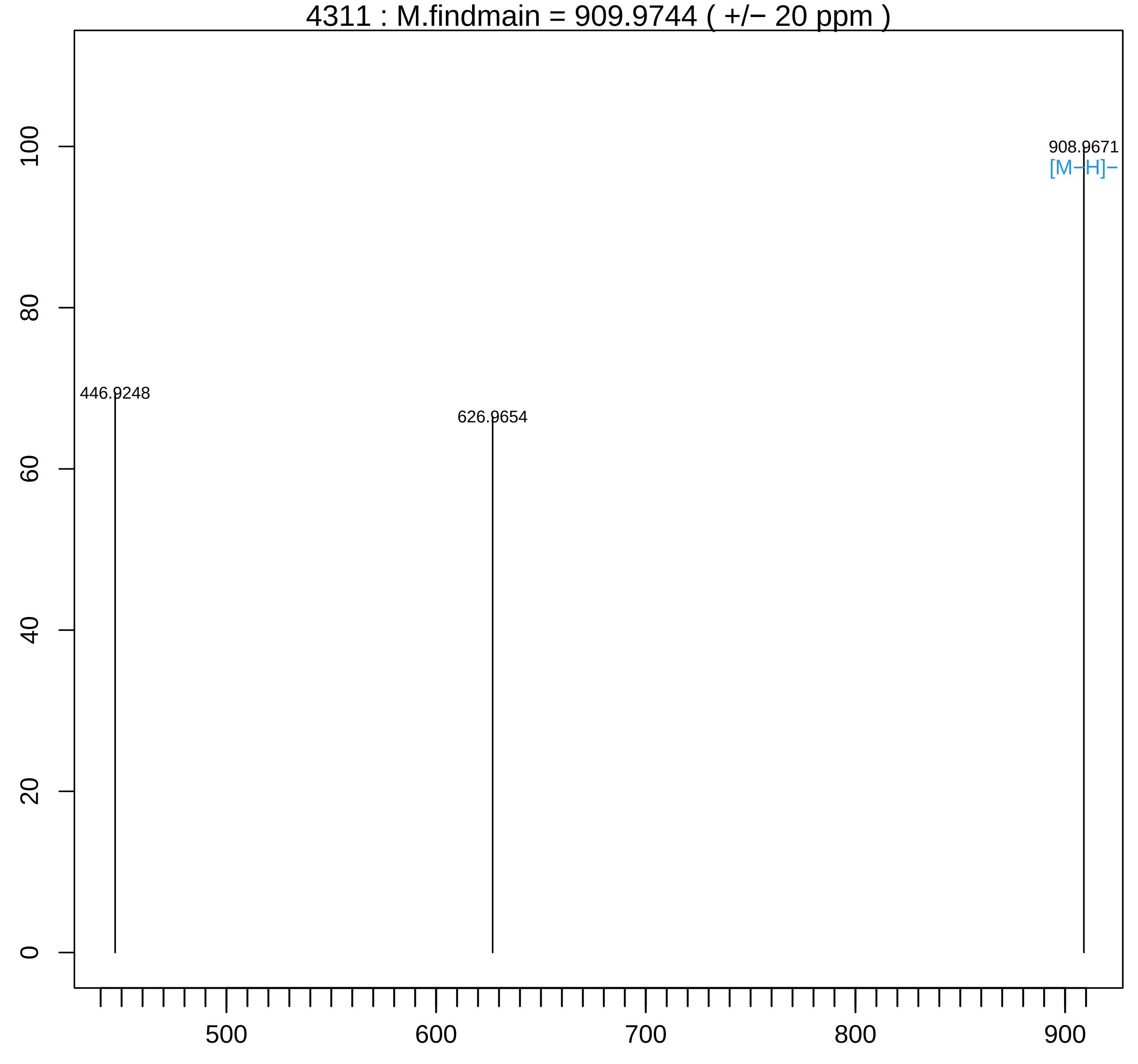

4311 : M.findmain = 909.9744 (+/− 20 ppm)
908.9671
[M−H]−
446.9248
626.9654
0
20
40
60
80
100
500
600
700
800
900

4312 : M.ramclustr = 214.0224 (+/− NA ppm)

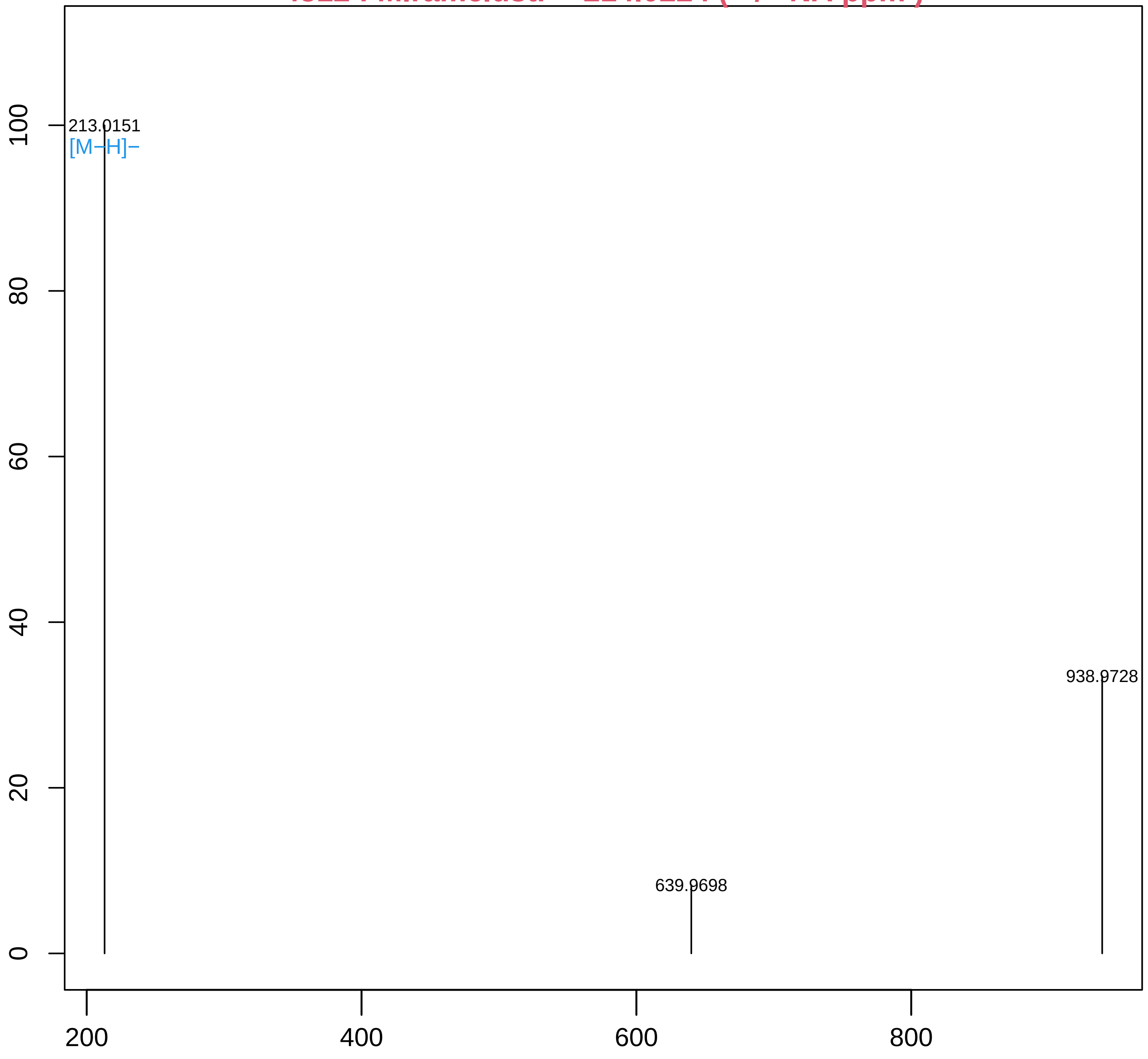

4312 : M.findmain = 214.0224 (+/− 20 ppm)

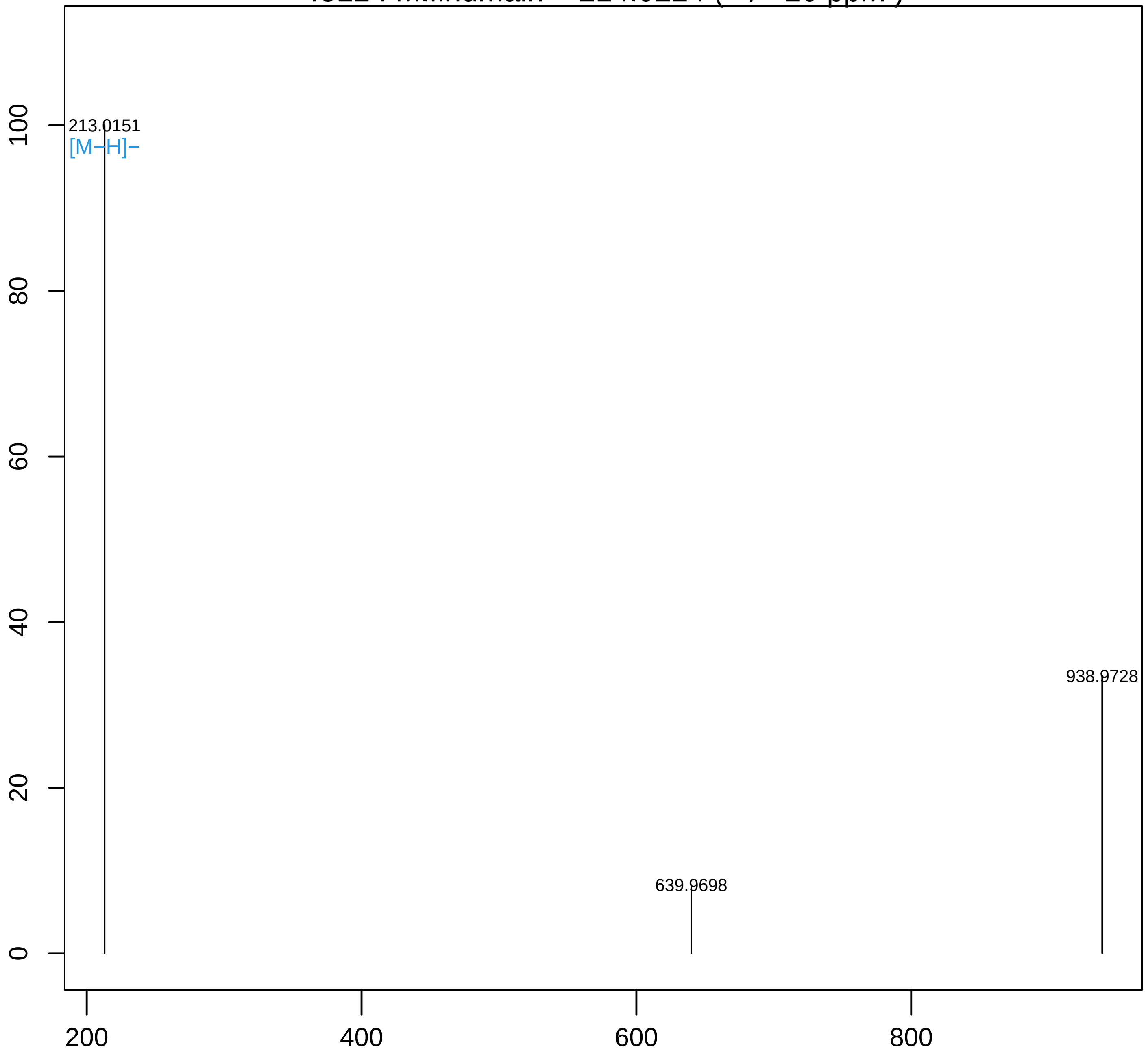

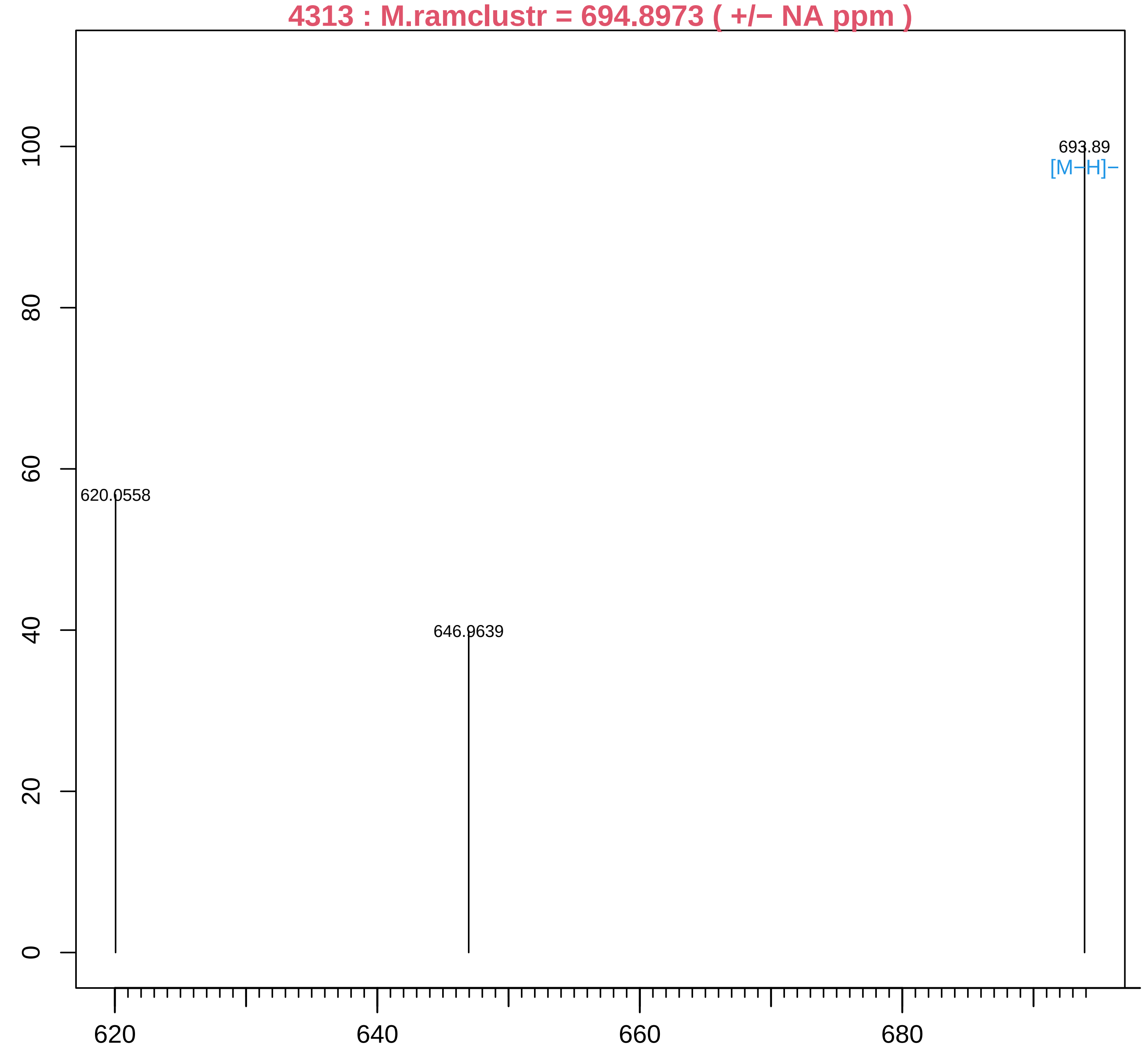
4313 : M.ramclustr = 694.8973 (+/− NA ppm)
693.89
[M−H]−
620.0558
646.9639
0
20
40
60
80
100
620
640
660
680

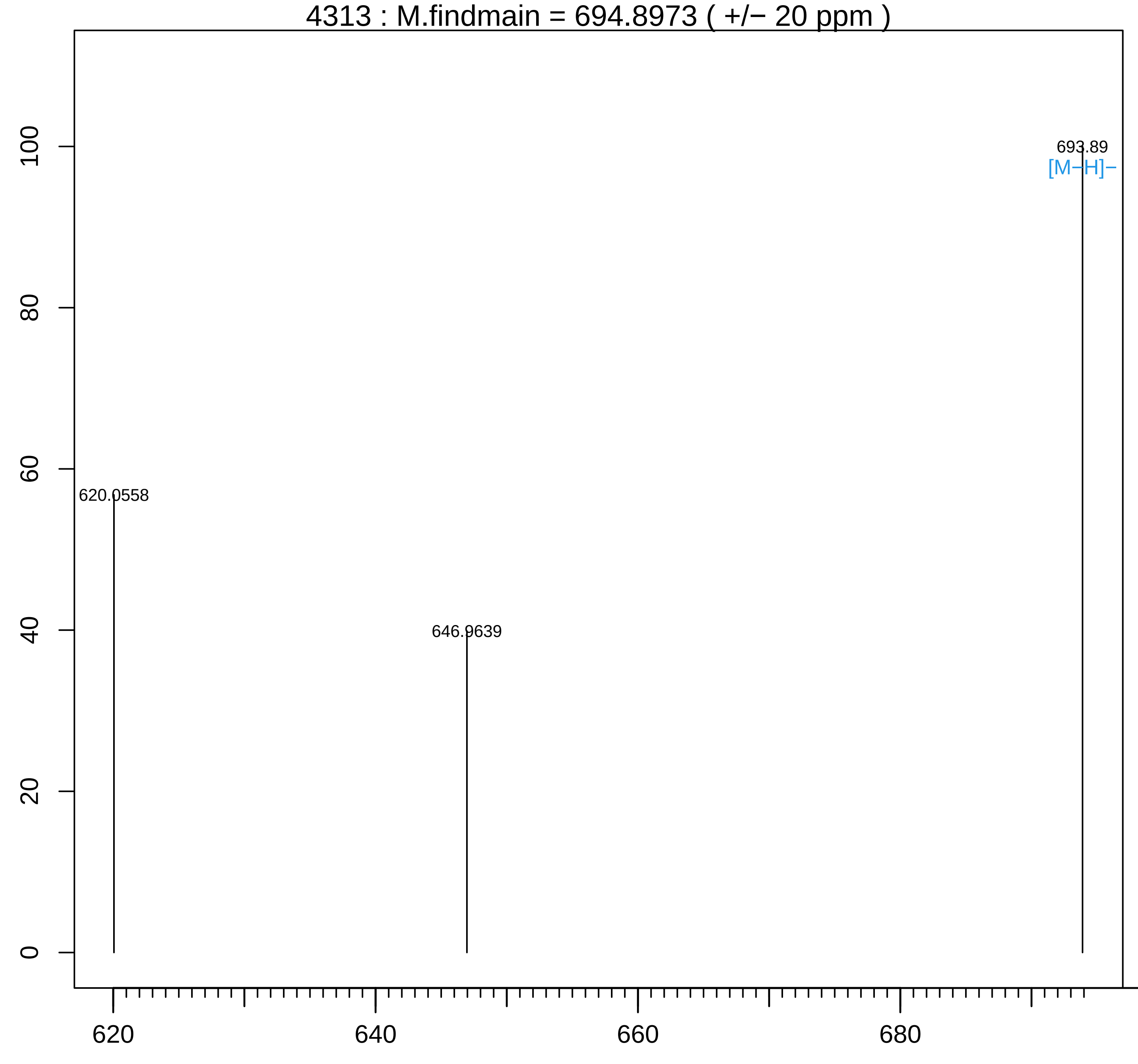
4313 : M.findmain = 694.8973 (+/− 20 ppm)
693.89
[M−H]−
620.0558
646.9639
0
20
40
60
80
100
620
640
660
680

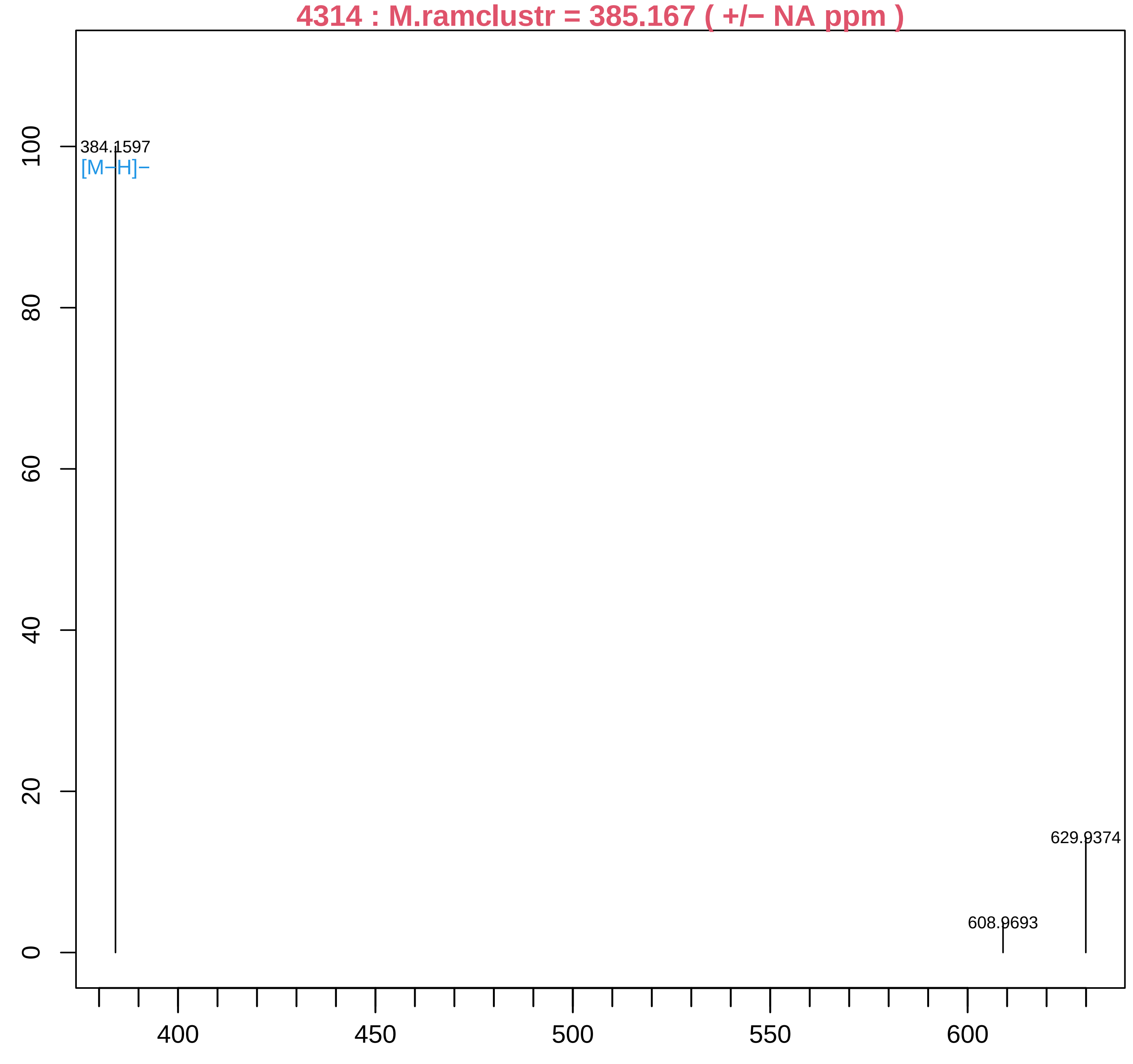

4314 : M.ramclustr = 385.167 (+/− NA ppm)
384.1597
[M−H]−
608.9693
629.9374
0
20
40
60
80
100
400
450
500
550
600

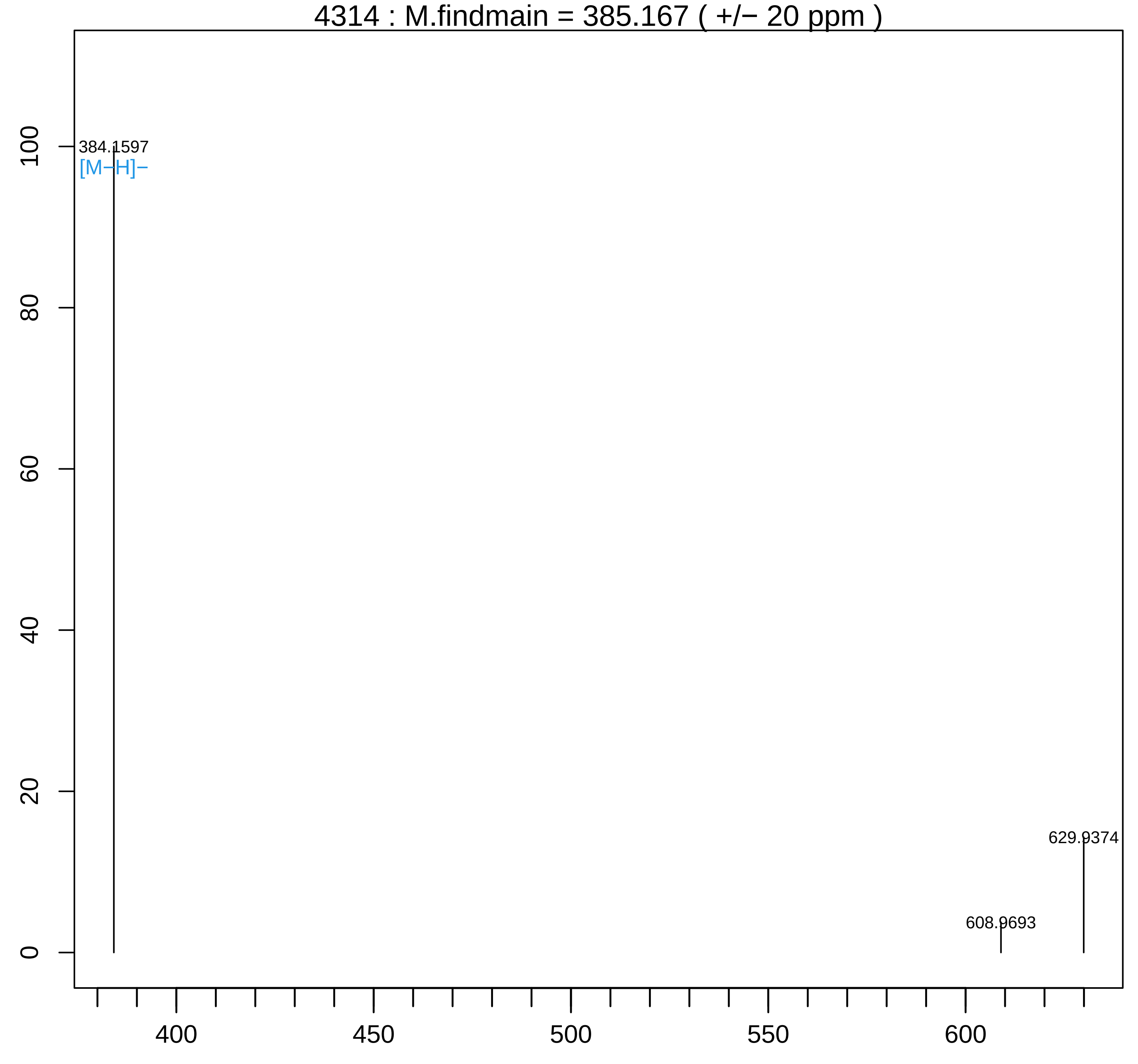

4314 : M.findmain = 385.167 (+/− 20 ppm)
384.1597
[M−H]−
608.9693
629.9374
0
20
40
60
80
100
400
450
500
550
600

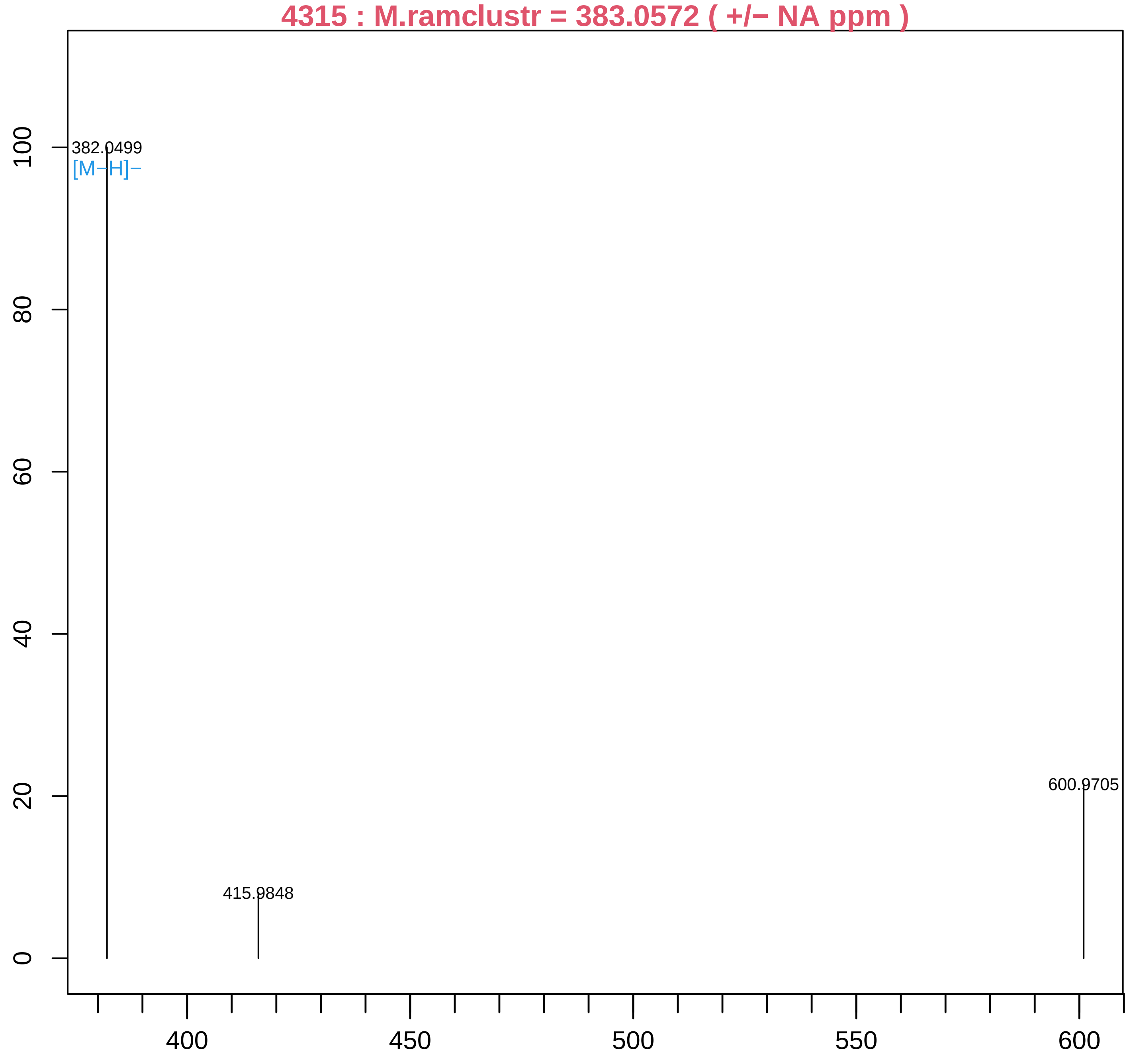
4315 : M.ramclustr = 383.0572 (+/− NA ppm)
382.0499
[M−H]−
415.9848
600.9705
0
20
40
60
80
100
400
450
500
550
600

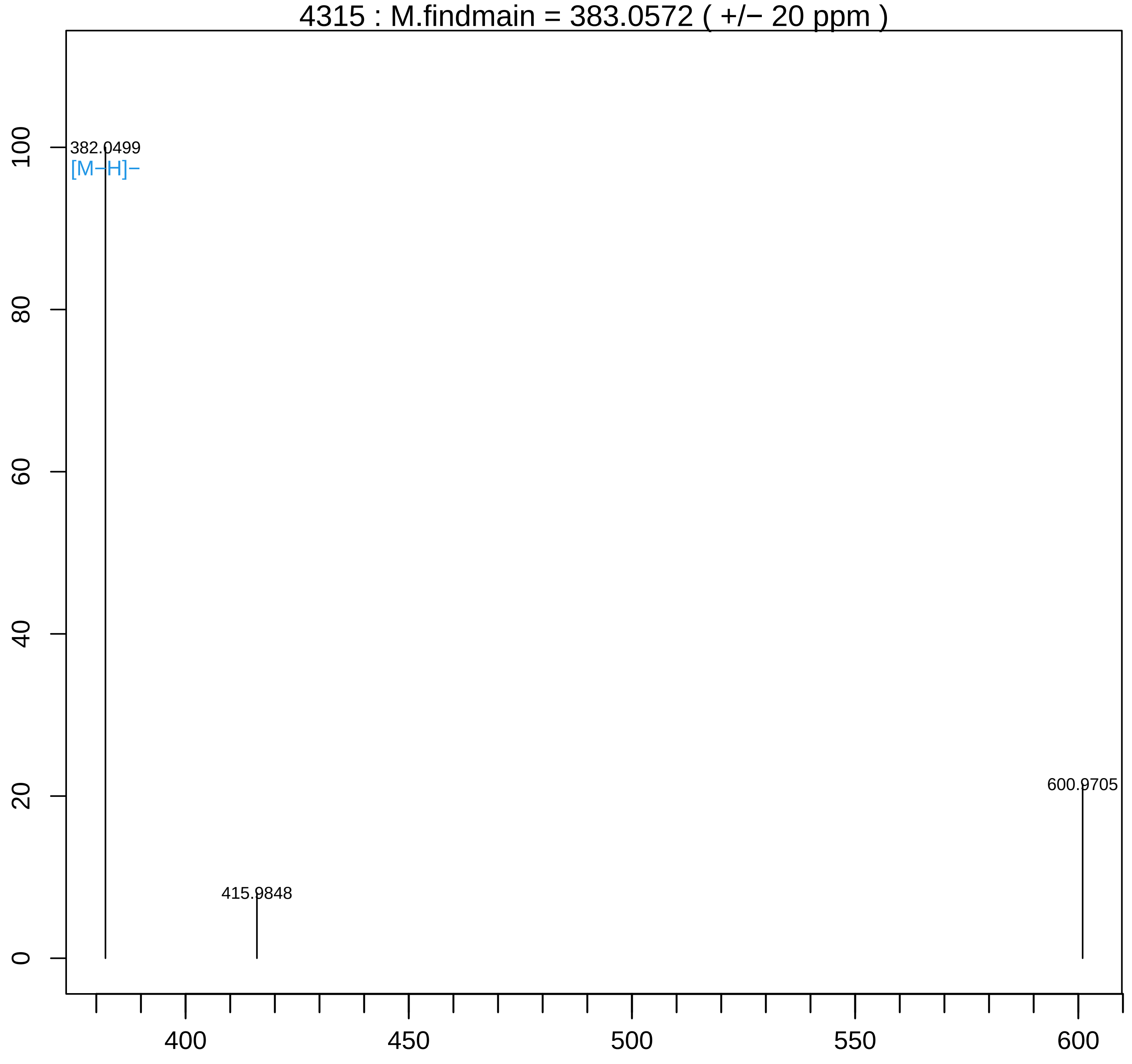
4315 : M.findmain = 383.0572 (+/− 20 ppm)
382.0499
[M−H]−
415.9848
600.9705
0
20
40
60
80
100
400
450
500
550
600

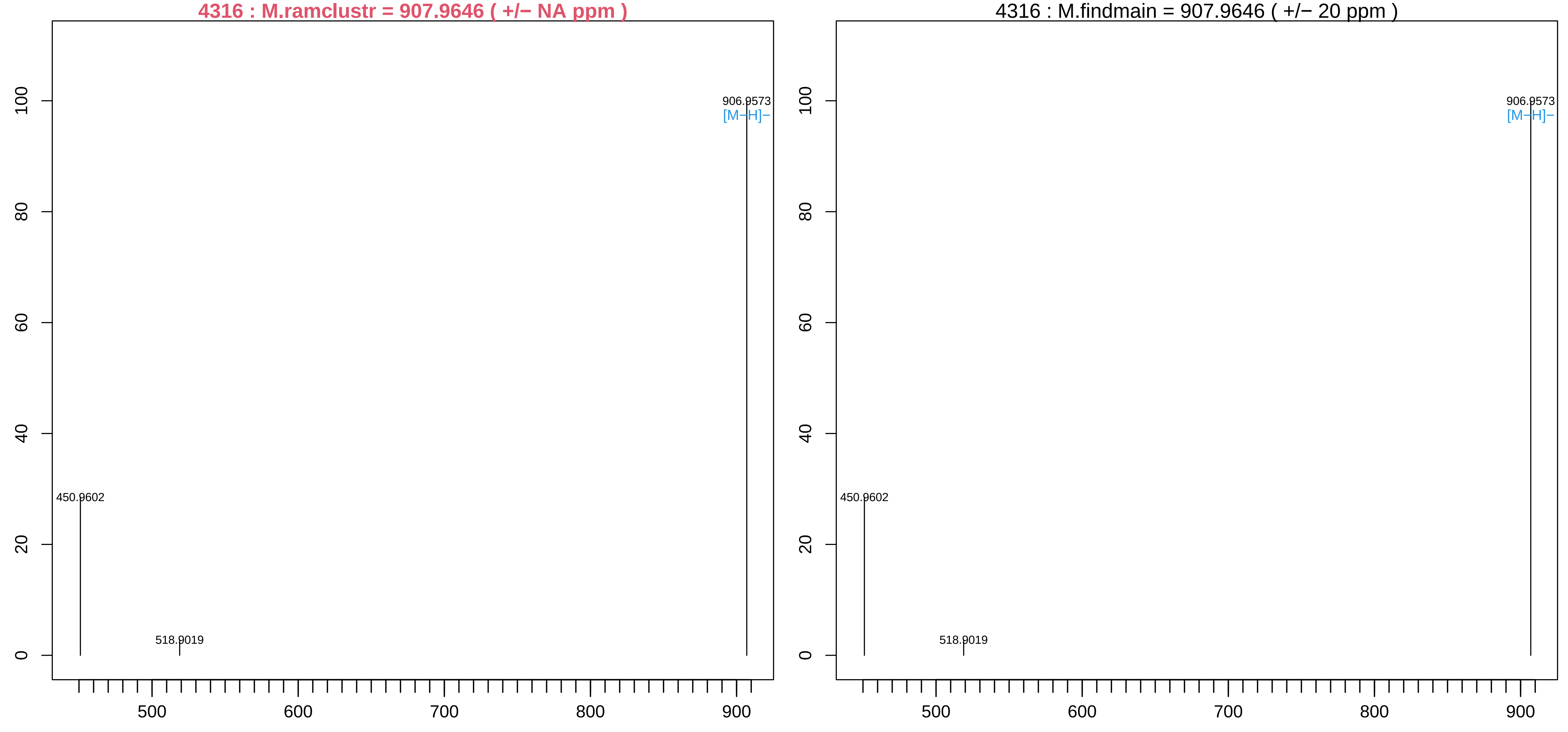
4316 : M.ramclustr = 907.9646 (+/− NA ppm)
4316 : M.findmain = 907.9646 (+/− 20 ppm)
906.9573
[M−H]−
450.9602
518.9019
906.9573
[M−H]−
450.9602
518.9019
0
20
40
60
80
100
500
600
700
800
900

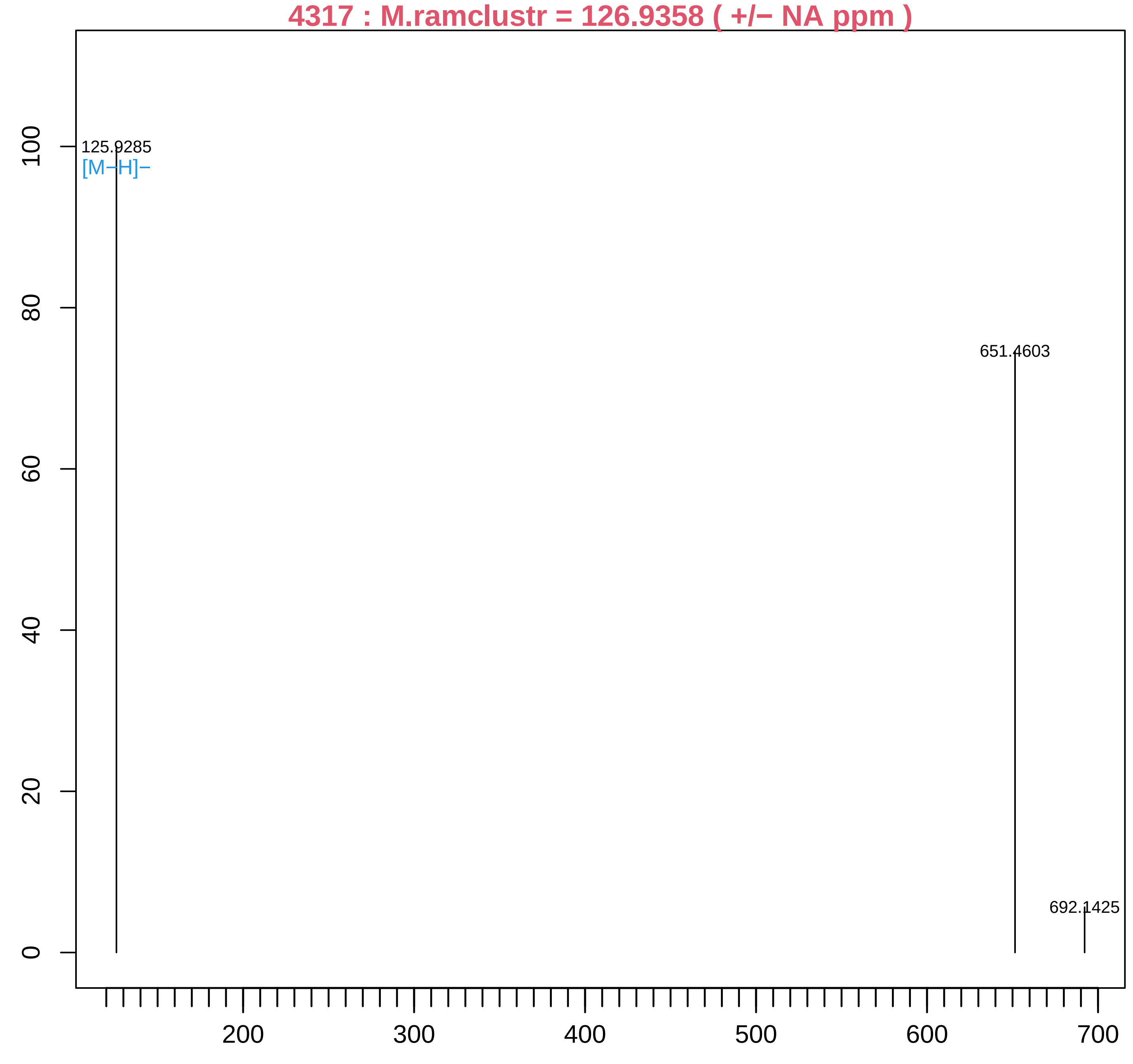
4317 : M.ramclustr = 126.9358 (+/− NA ppm)
125.9285
[M−H]−
651.4603
692.1425
0
20
40
60
80
100
200
300
400
500
600
700

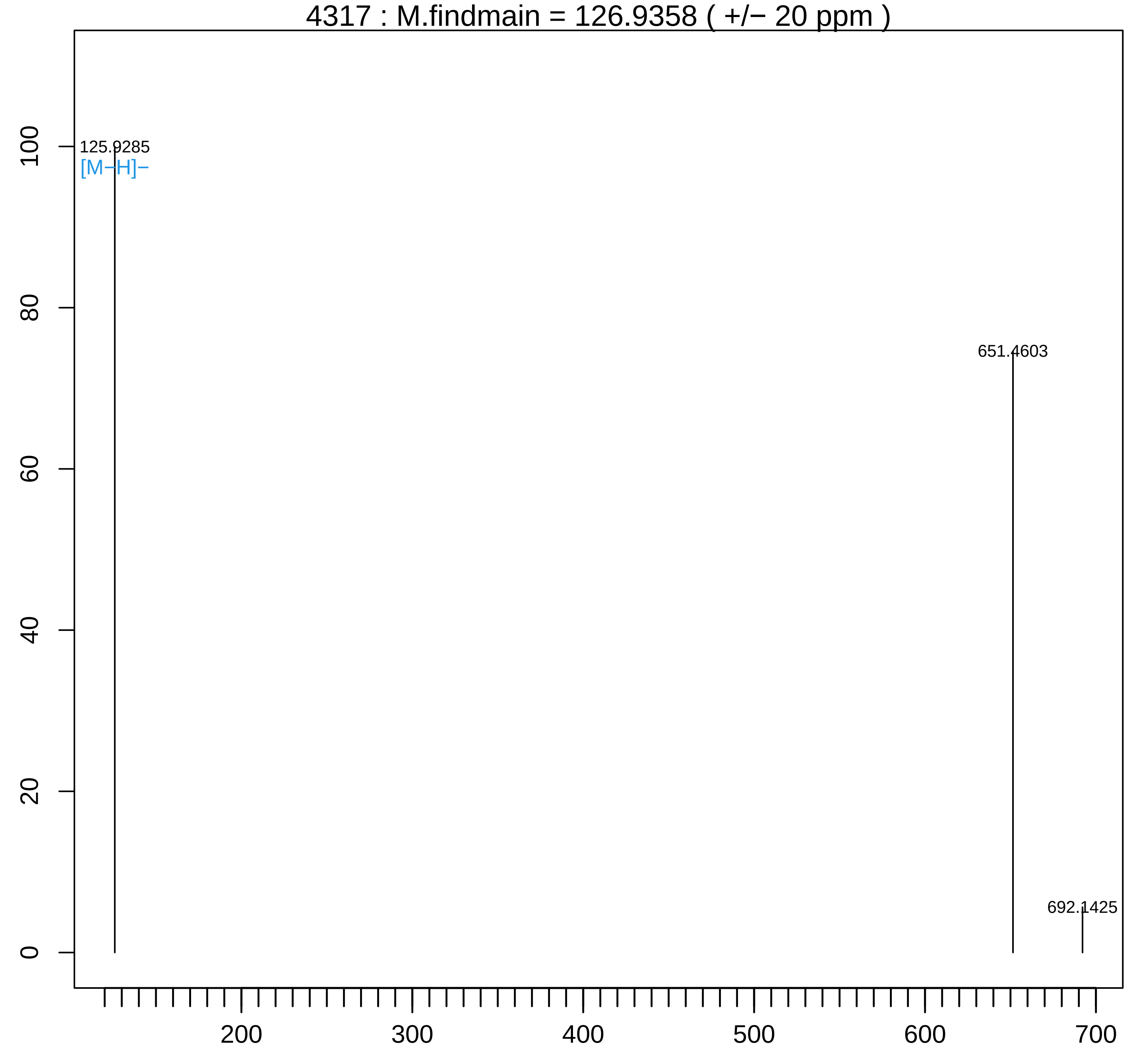
4317 : M.findmain = 126.9358 (+/− 20 ppm)
125.9285
[M−H]−
651.4603
692.1425
0
20
40
60
80
100
200
300
400
500
600
700

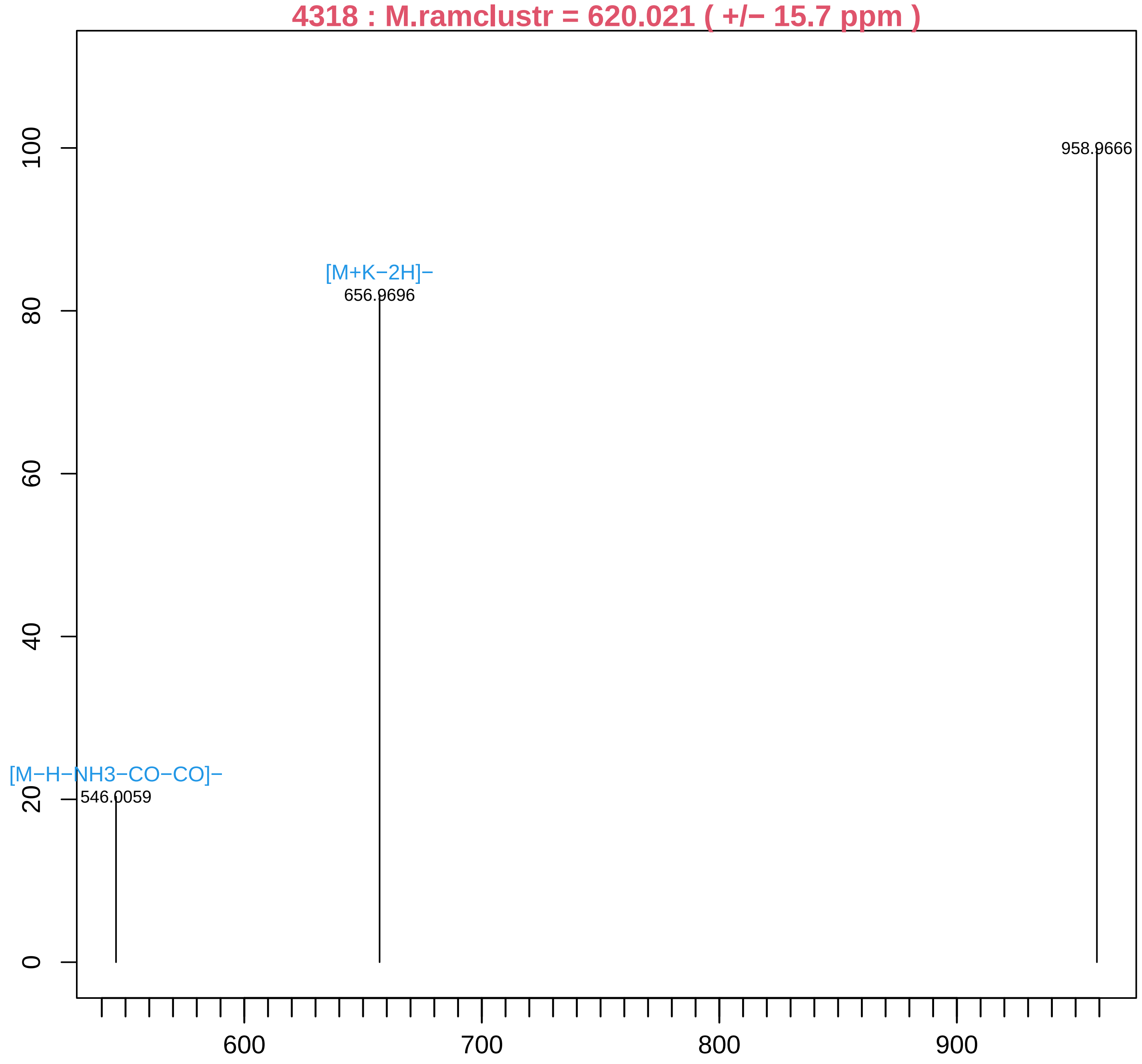
4318 : M.ramclustr = 620.021 (+/− 15.7 ppm)
958.9666
[M+K−2H]−
656.9696
[M−H−NH3−CO−CO]−
546.0059
0
20
40
60
80
100
600
700
800
900

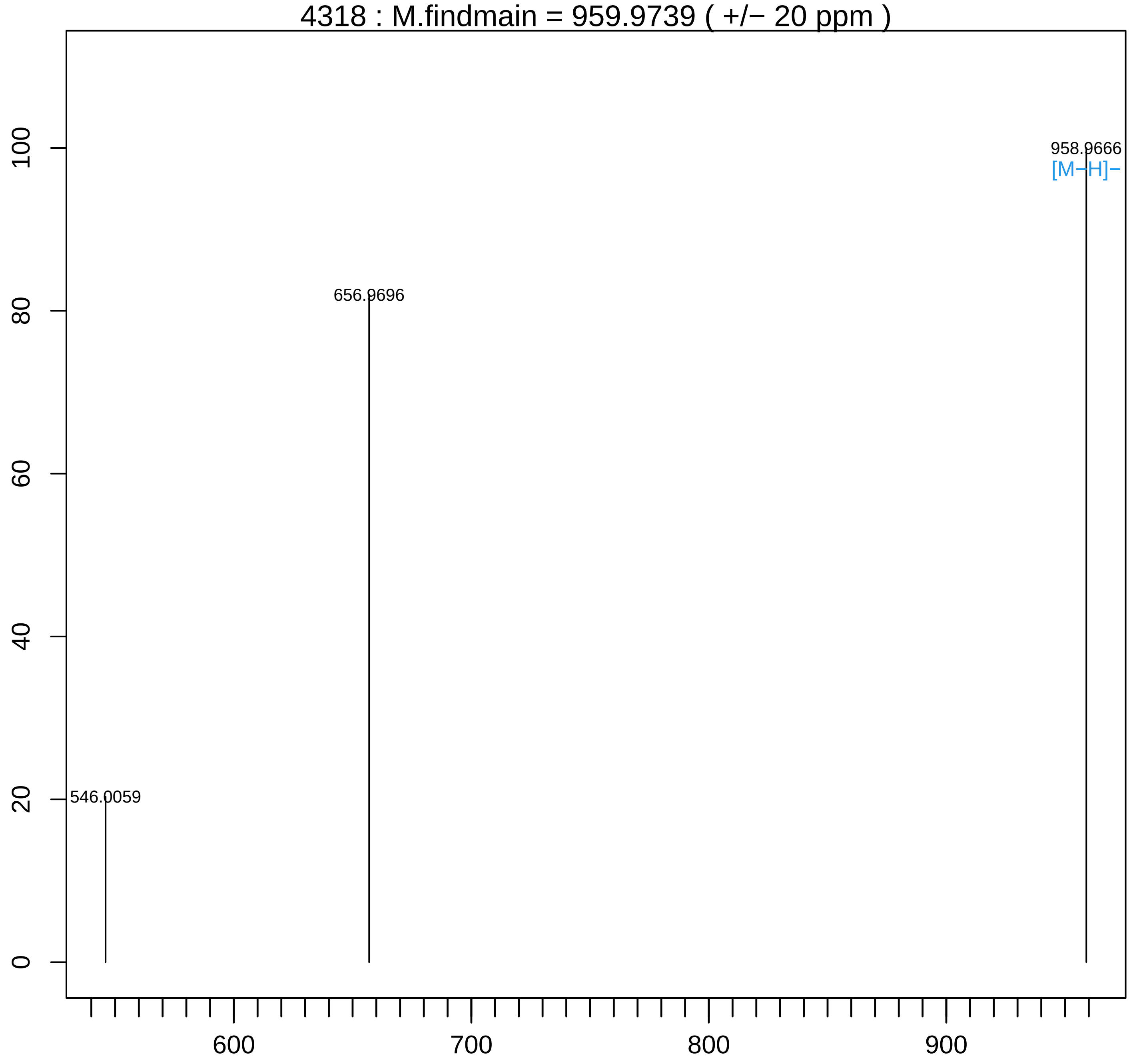
4318 : M.findmain = 959.9739 (+/− 20 ppm)
958.9666
[M−H]−
656.9696
546.0059
0
20
40
60
80
100
600
700
800
900

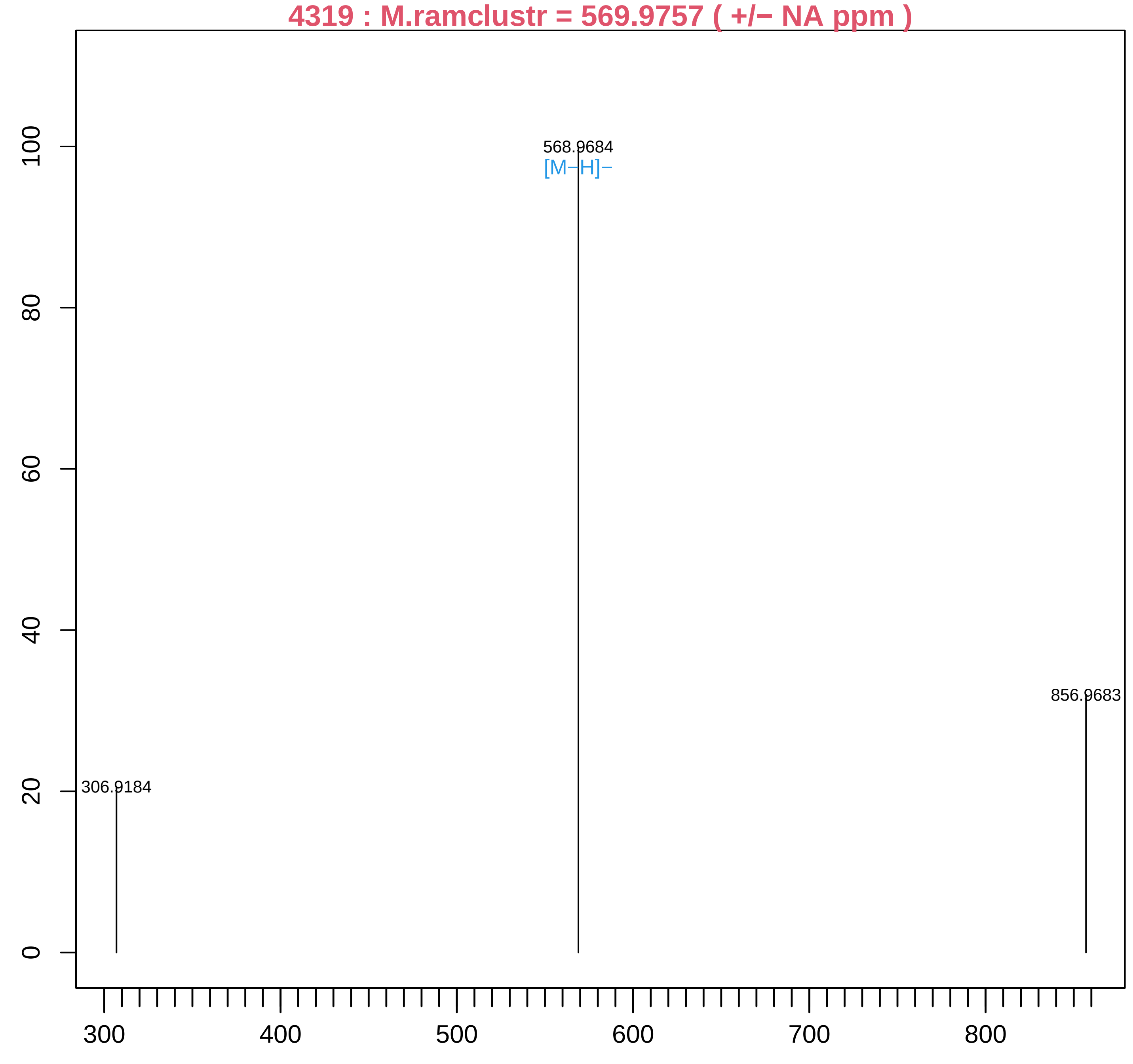
4319 : M.ramclustr = 569.9757 (+/− NA ppm)
568.9684
[M−H]−
856.9683
306.9184
0
20
40
60
80
100
300
400
500
600
700
800

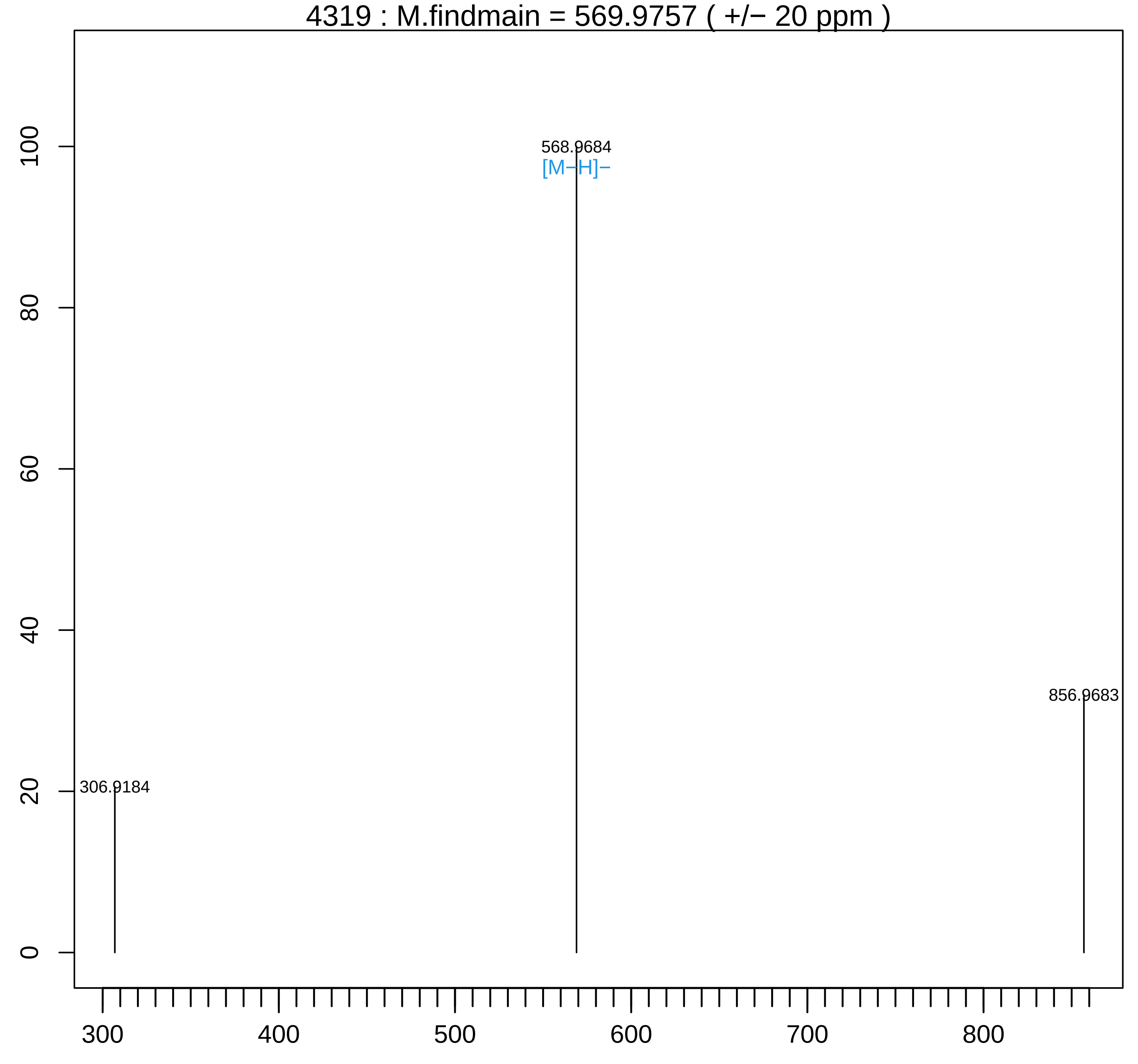
4319 : M.findmain = 569.9757 (+/− 20 ppm)
568.9684
[M−H]−
856.9683
306.9184
0
20
40
60
80
100
300
400
500
600
700
800

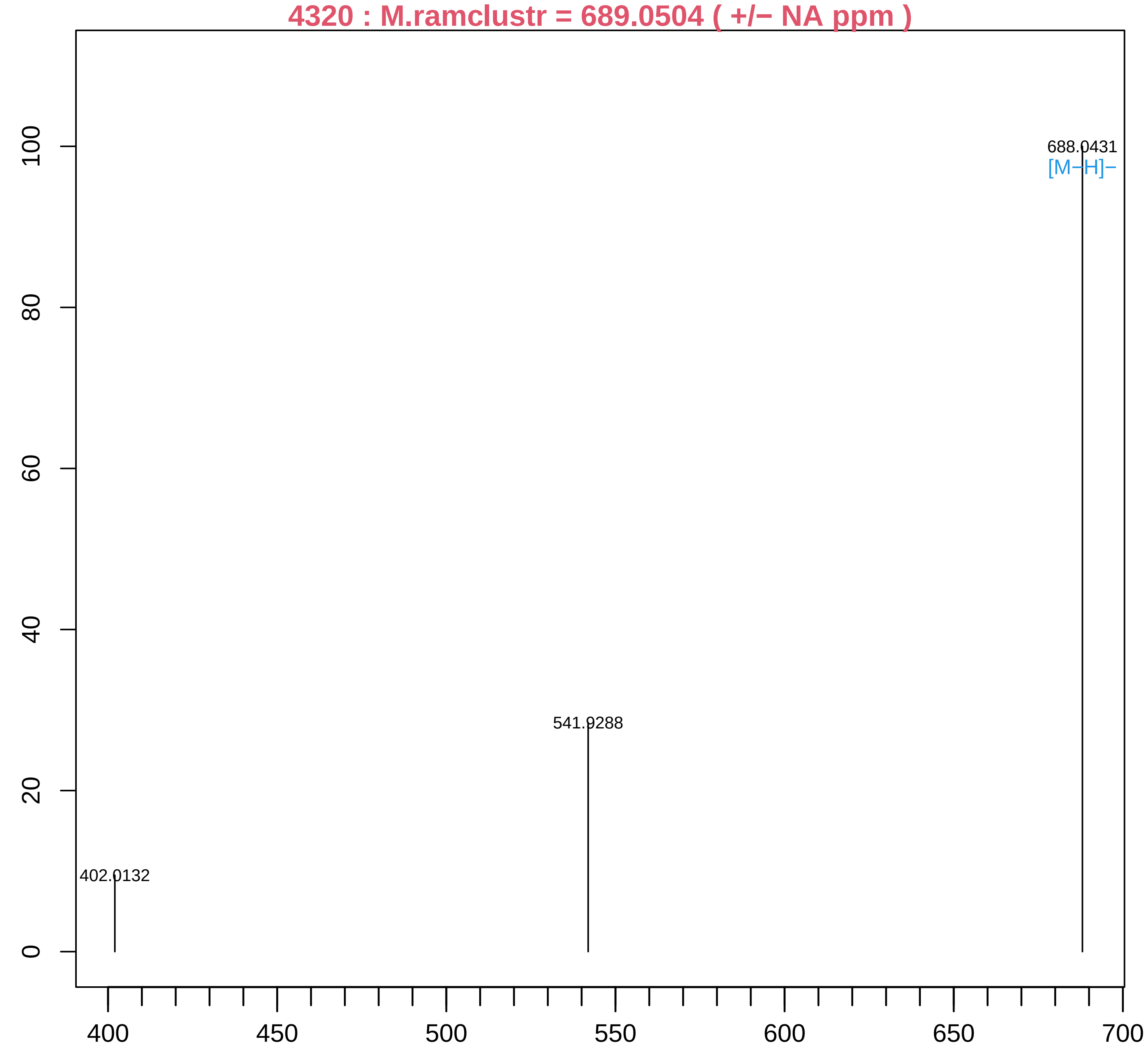
4320 : M.ramclustr = 689.0504 (+/− NA ppm)
688.0431
[M−H]−
541.9288
402.0132
0
20
40
60
80
100
400
450
500
550
600
650
700

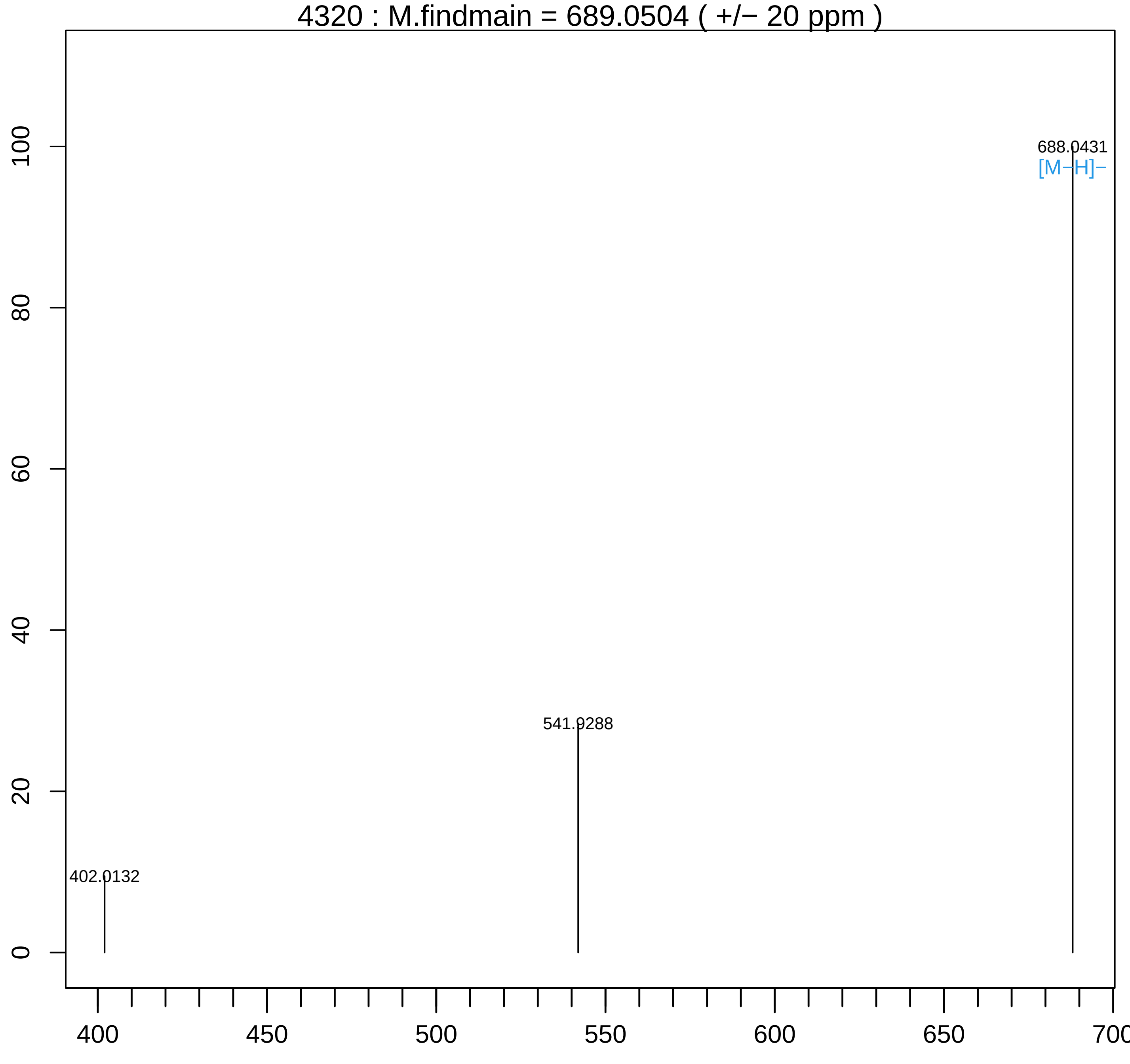
4320 : M.findmain = 689.0504 (+/− 20 ppm)
688.0431
[M−H]−
541.9288
402.0132
0
20
40
60
80
100
400
450
500
550
600
650
700

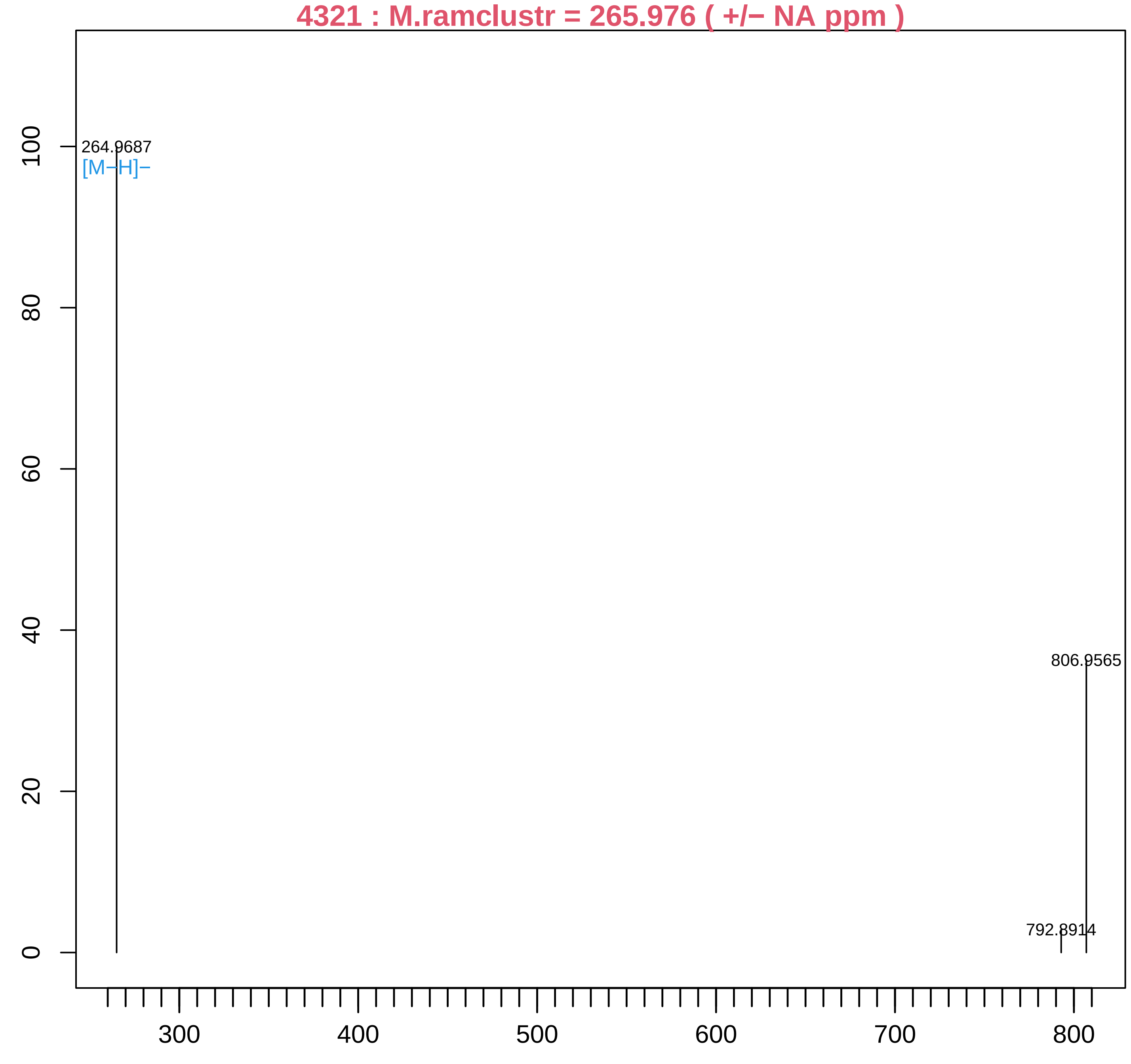
4321 : M.ramclustr = 265.976 (+/− NA ppm)
264.9687
[M−H]−
806.9565
792.8914
0
20
40
60
80
100
300
400
500
600
700
800

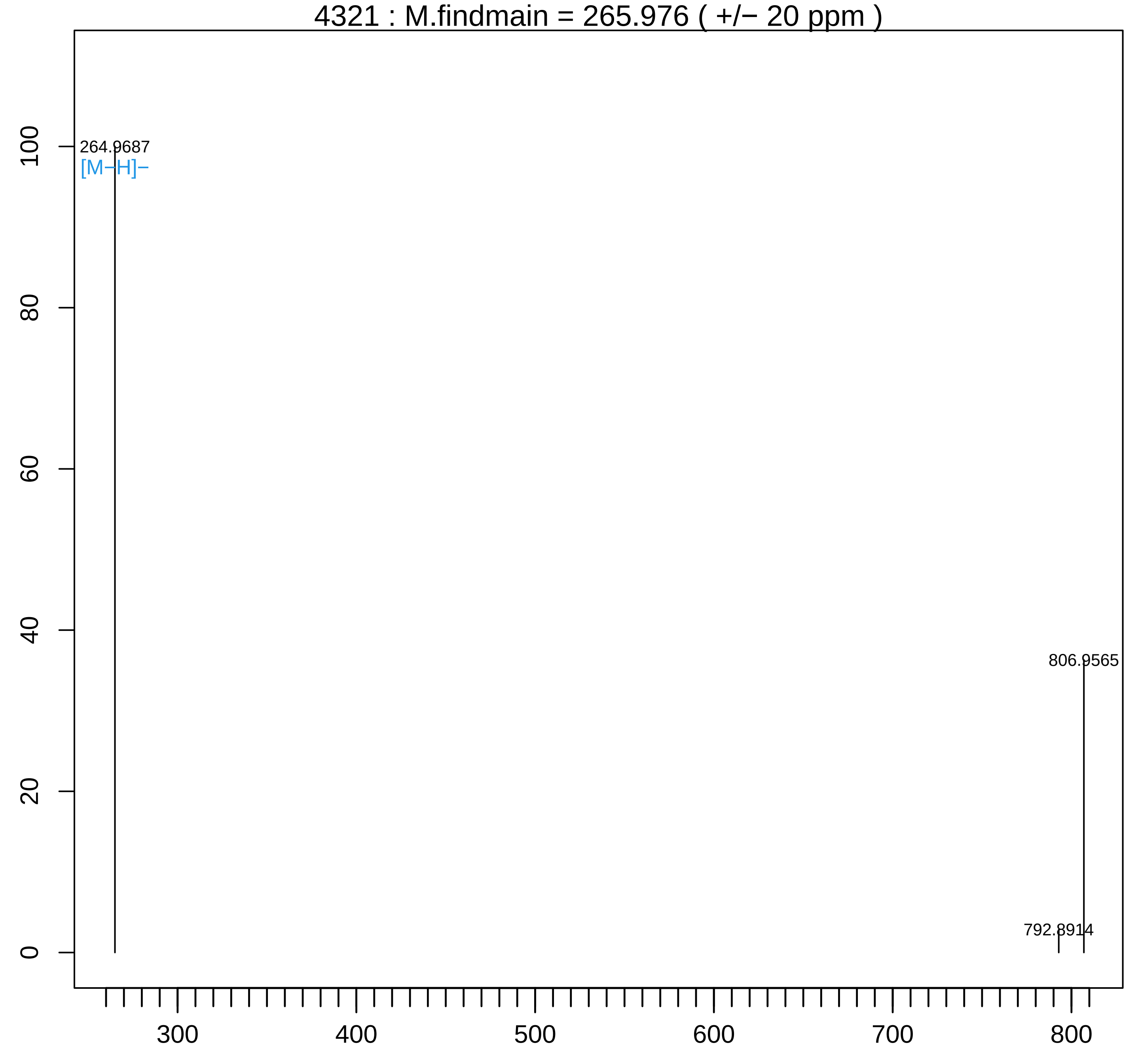
4321 : M.findmain = 265.976 (+/− 20 ppm)
264.9687
[M−H]−
806.9565
792.8914
0
20
40
60
80
100
300
400
500
600
700
800

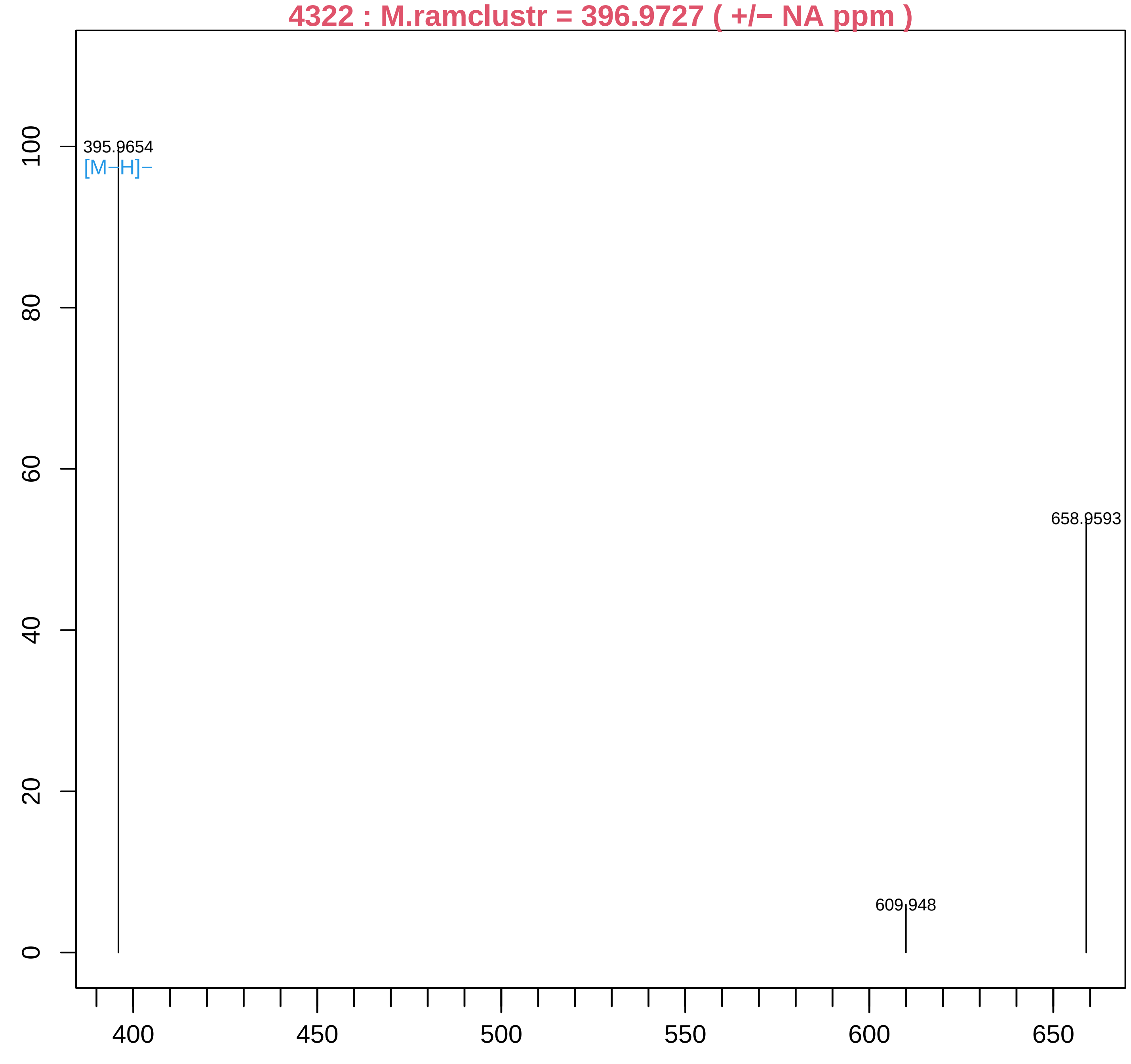

4322 : M.ramclustr = 396.9727 (+/− NA ppm)
395.9654
[M−H]−
658.9593
609.948
0
20
40
60
80
100
400
450
500
550
600
650

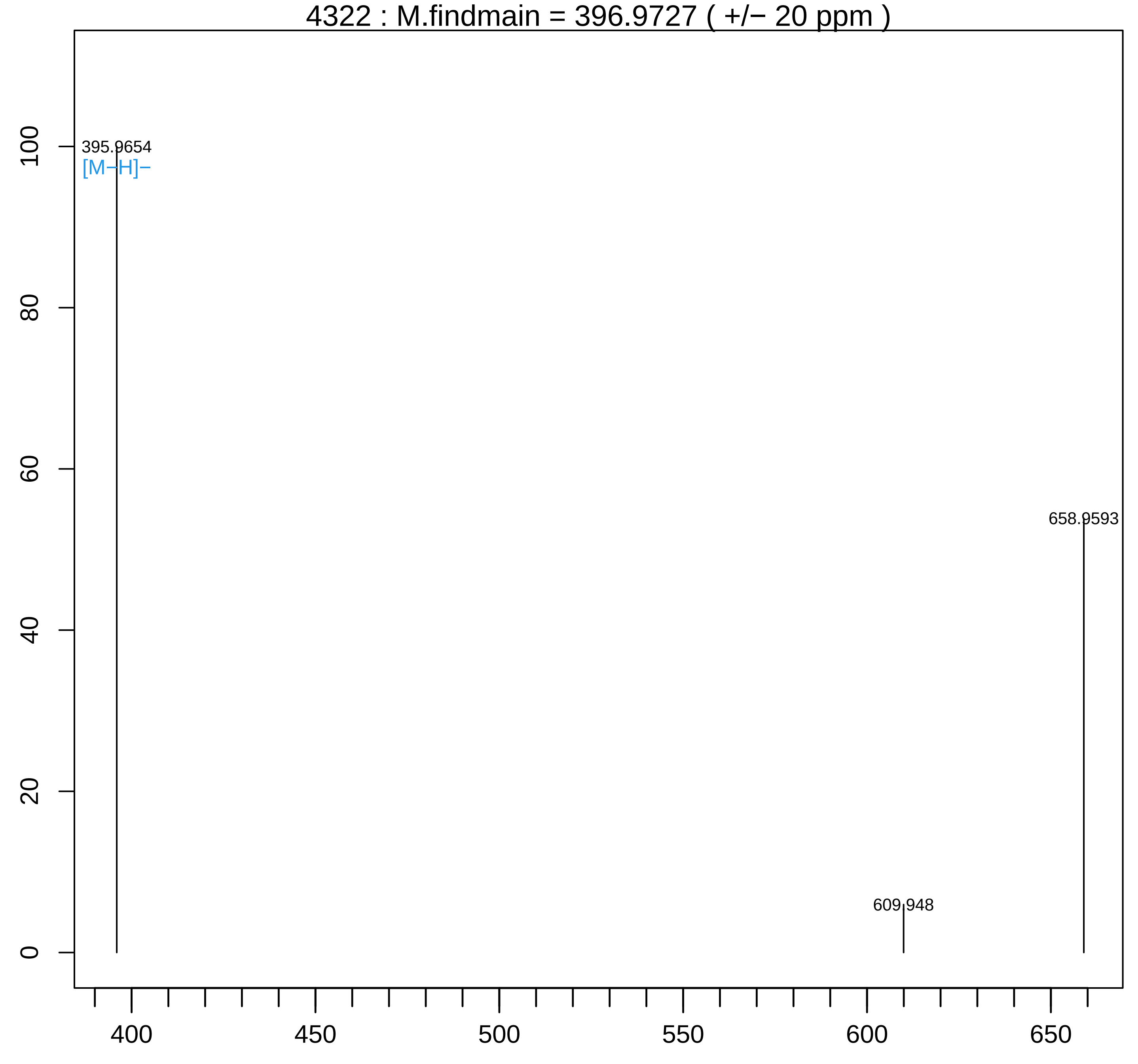

4322 : M.findmain = 396.9727 (+/− 20 ppm)
395.9654
[M−H]−
658.9593
609.948
0
20
40
60
80
100
400
450
500
550
600
650

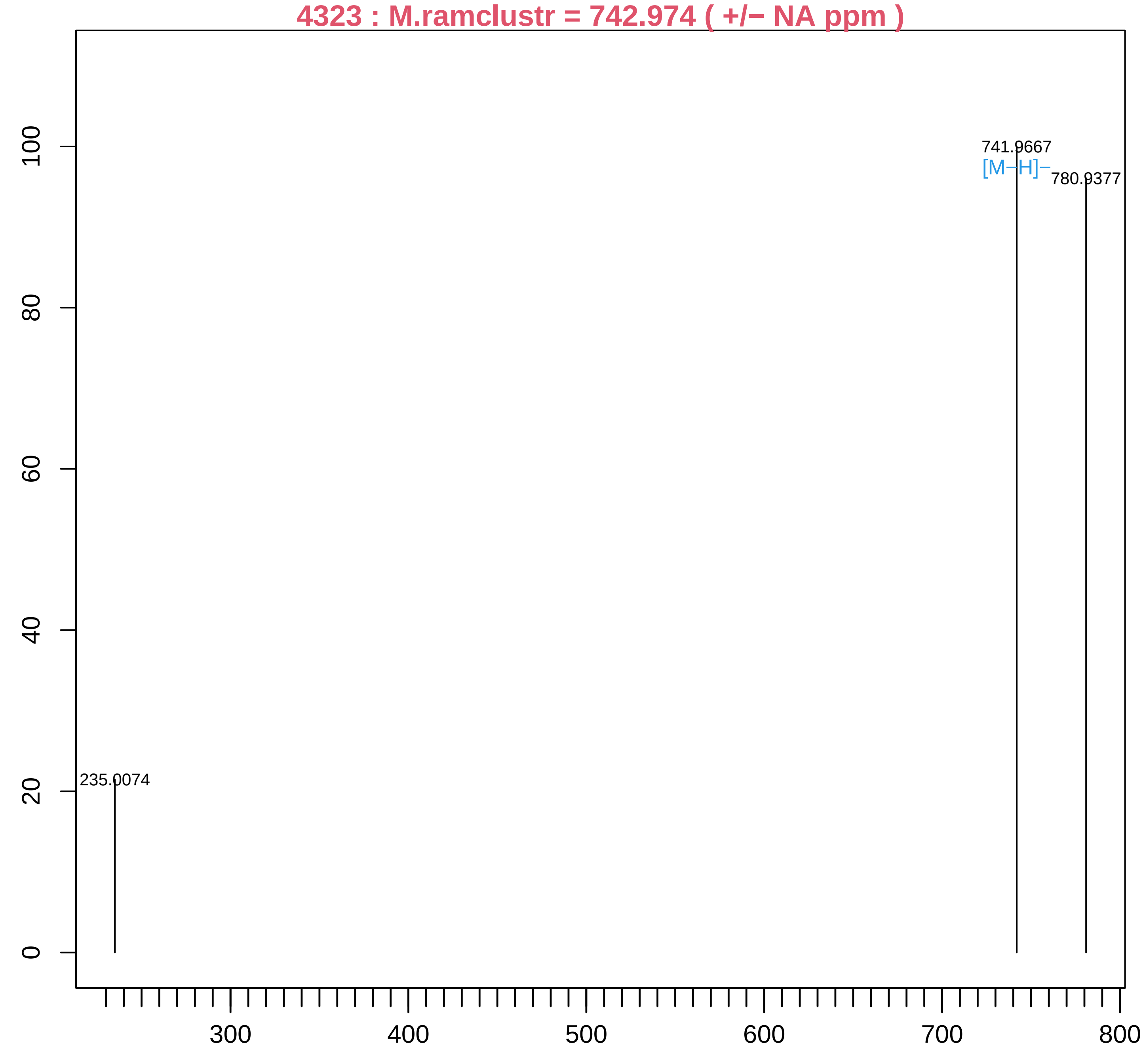
4323 : M.ramclustr = 742.974 (+/− NA ppm)
741.9667
[M−H]−
780.9377
235.0074
0
20
40
60
80
100
300
400
500
600
700
800

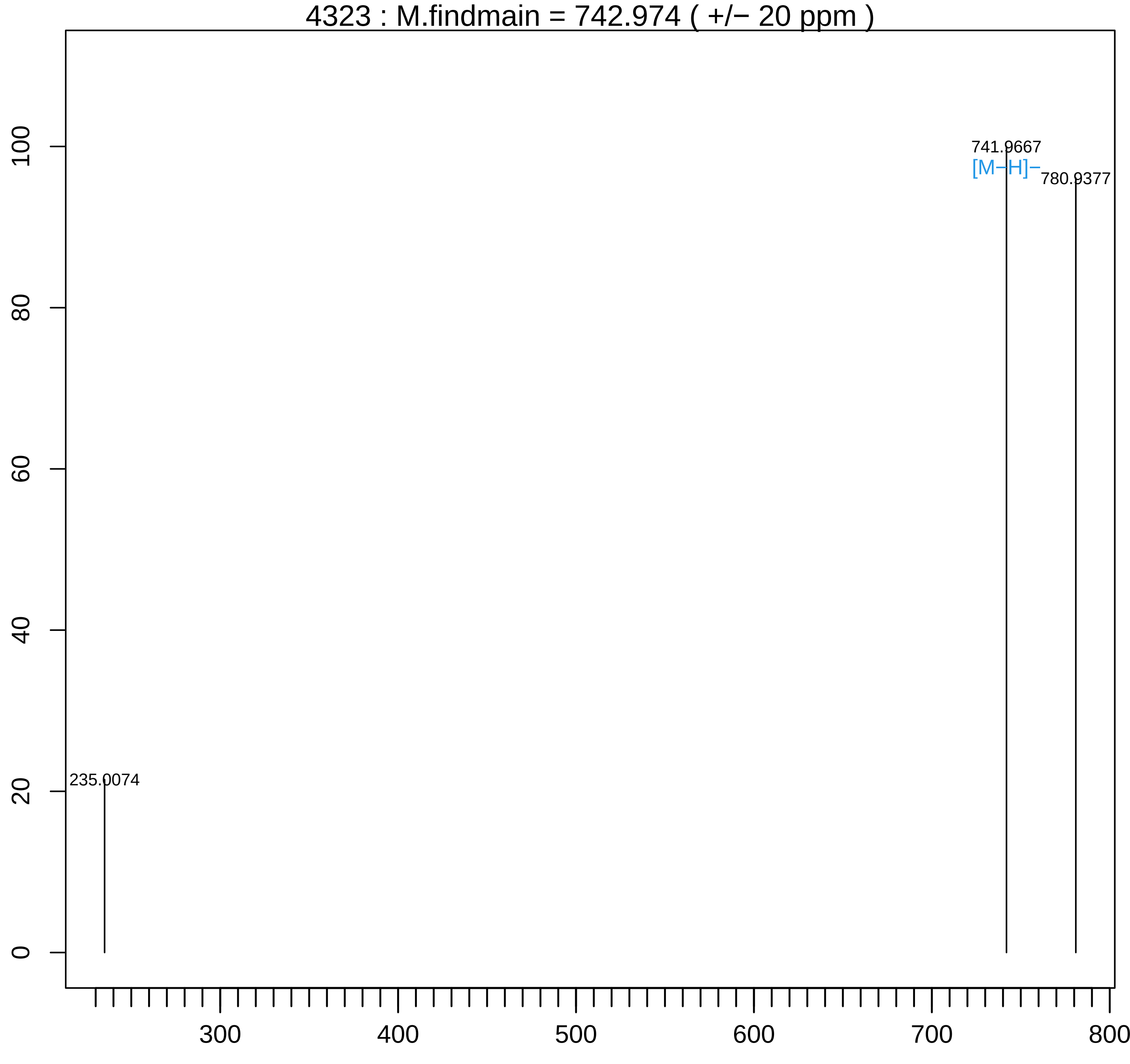
4323 : M.findmain = 742.974 (+/− 20 ppm)
741.9667
[M−H]−
780.9377
235.0074
0
20
40
60
80
100
300
400
500
600
700
800

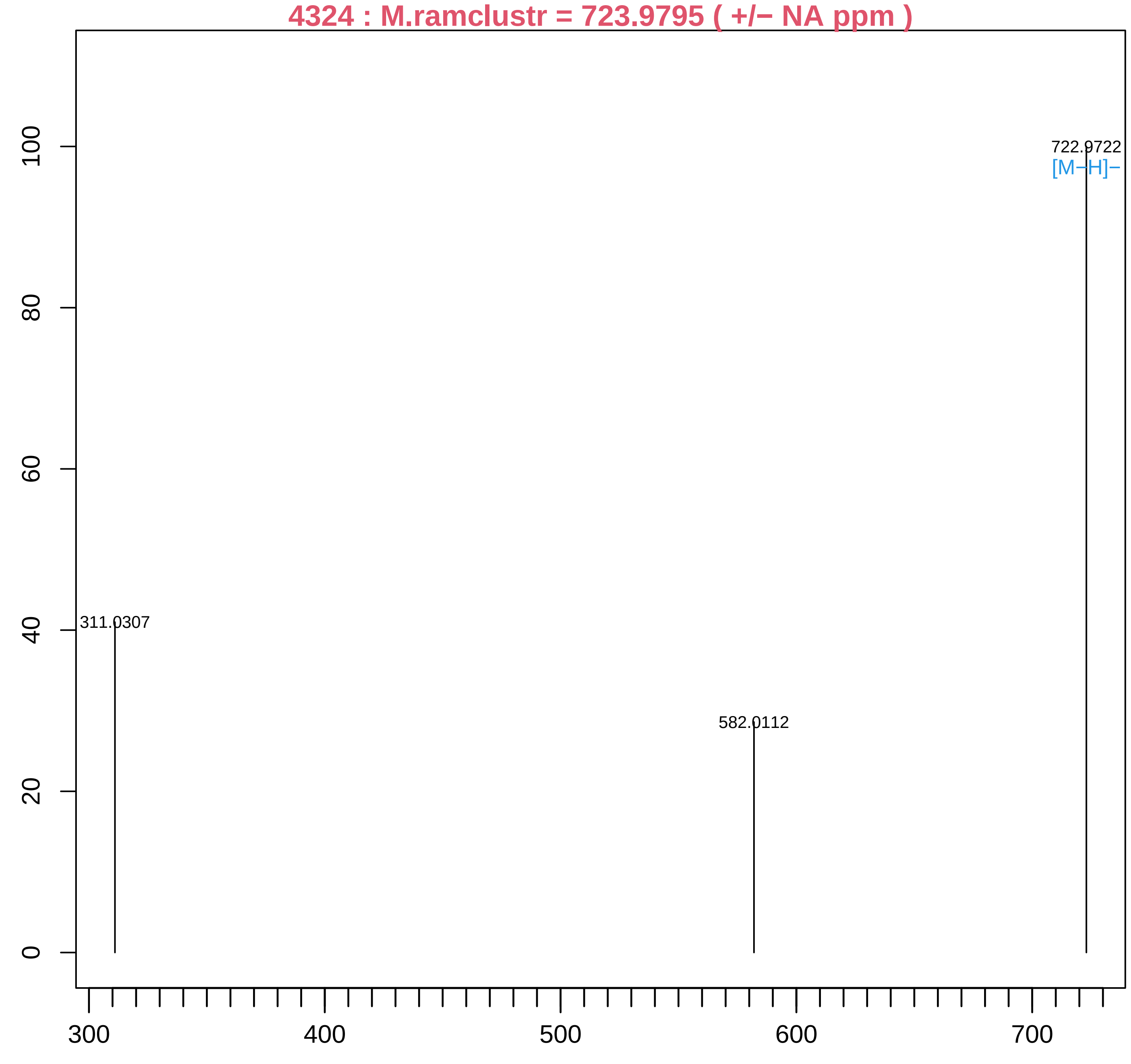

4324 : M.ramclustr = 723.9795 (+/− NA ppm)
722.9722
[M−H]−
311.0307
582.0112
0
20
40
60
80
100
300
400
500
600
700

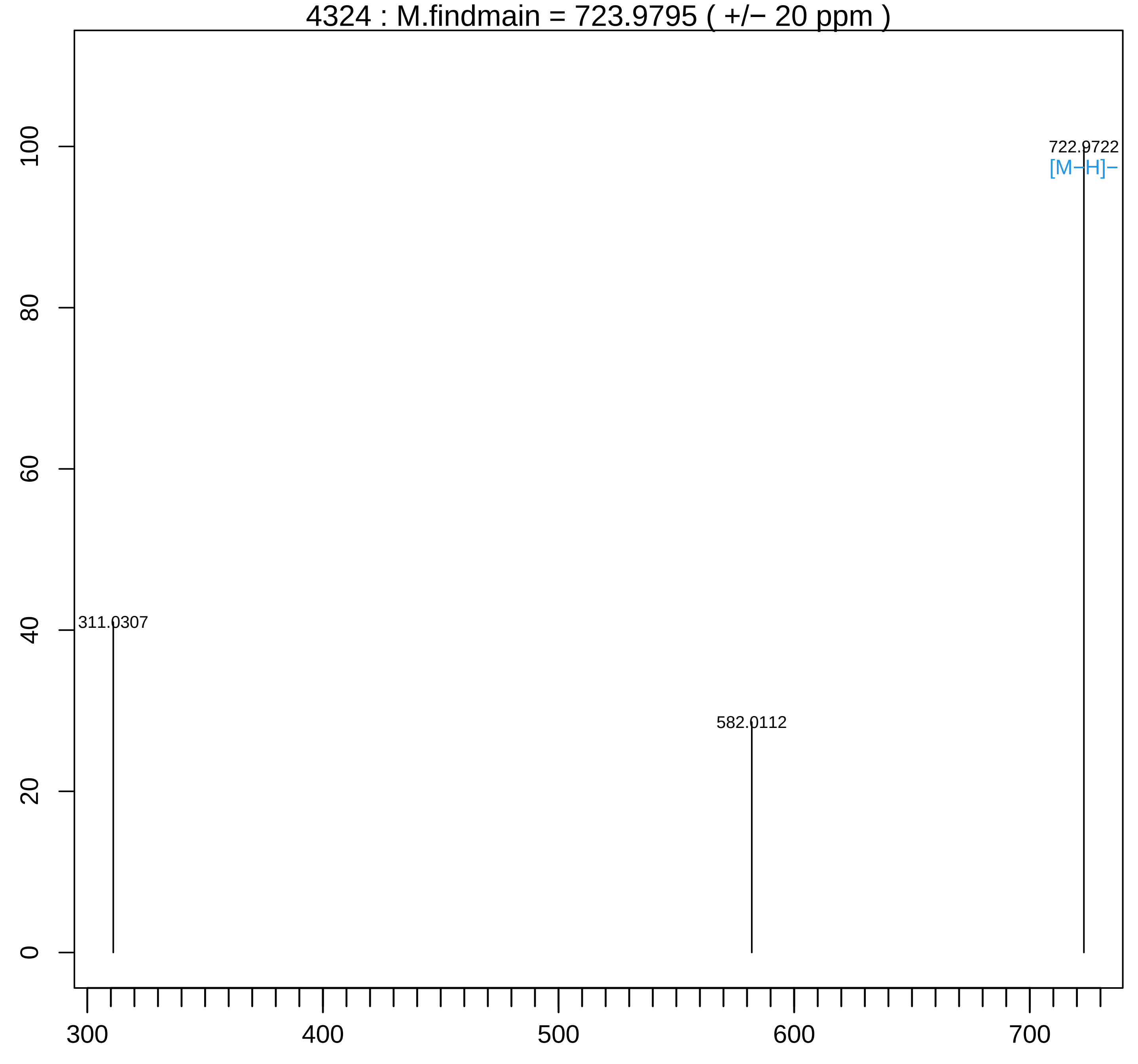

4324 : M.findmain = 723.9795 (+/− 20 ppm)
722.9722
[M−H]−
311.0307
582.0112
0
20
40
60
80
100
300
400
500
600
700

4325 : M.ramclustr = 853.9685 (+/− NA ppm)

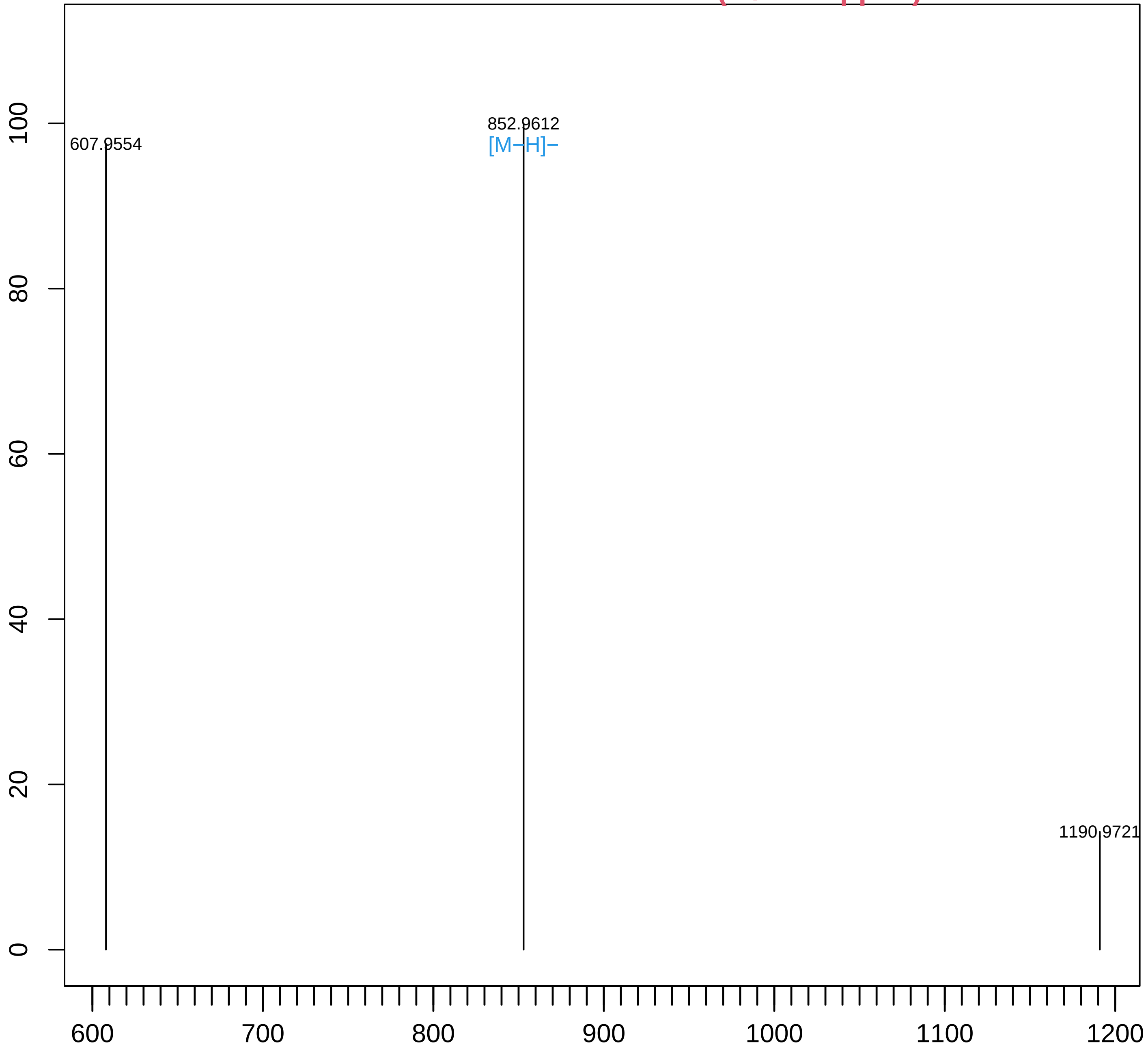

4325 : M.findmain = 853.9685 (+/− 20 ppm)

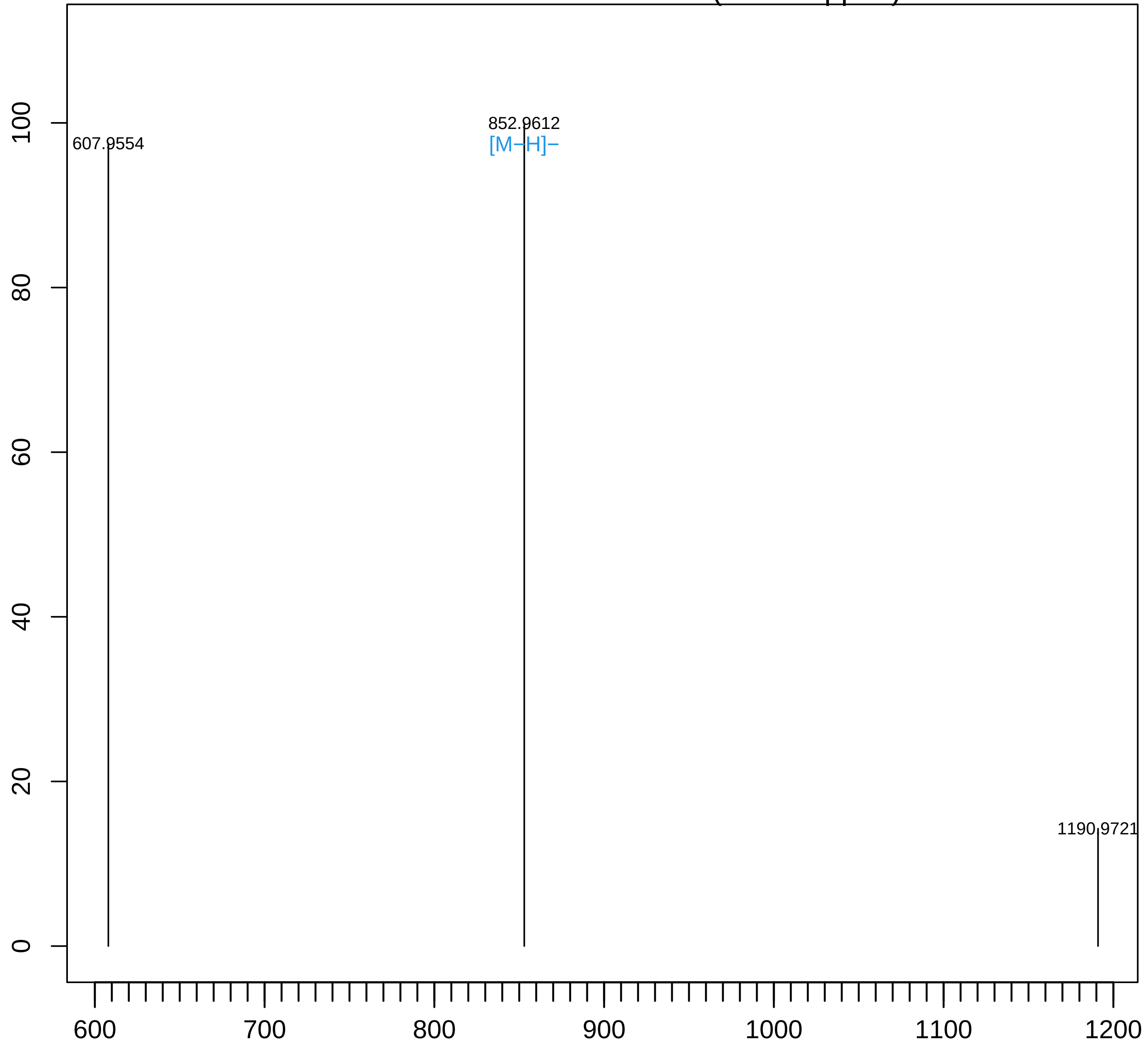

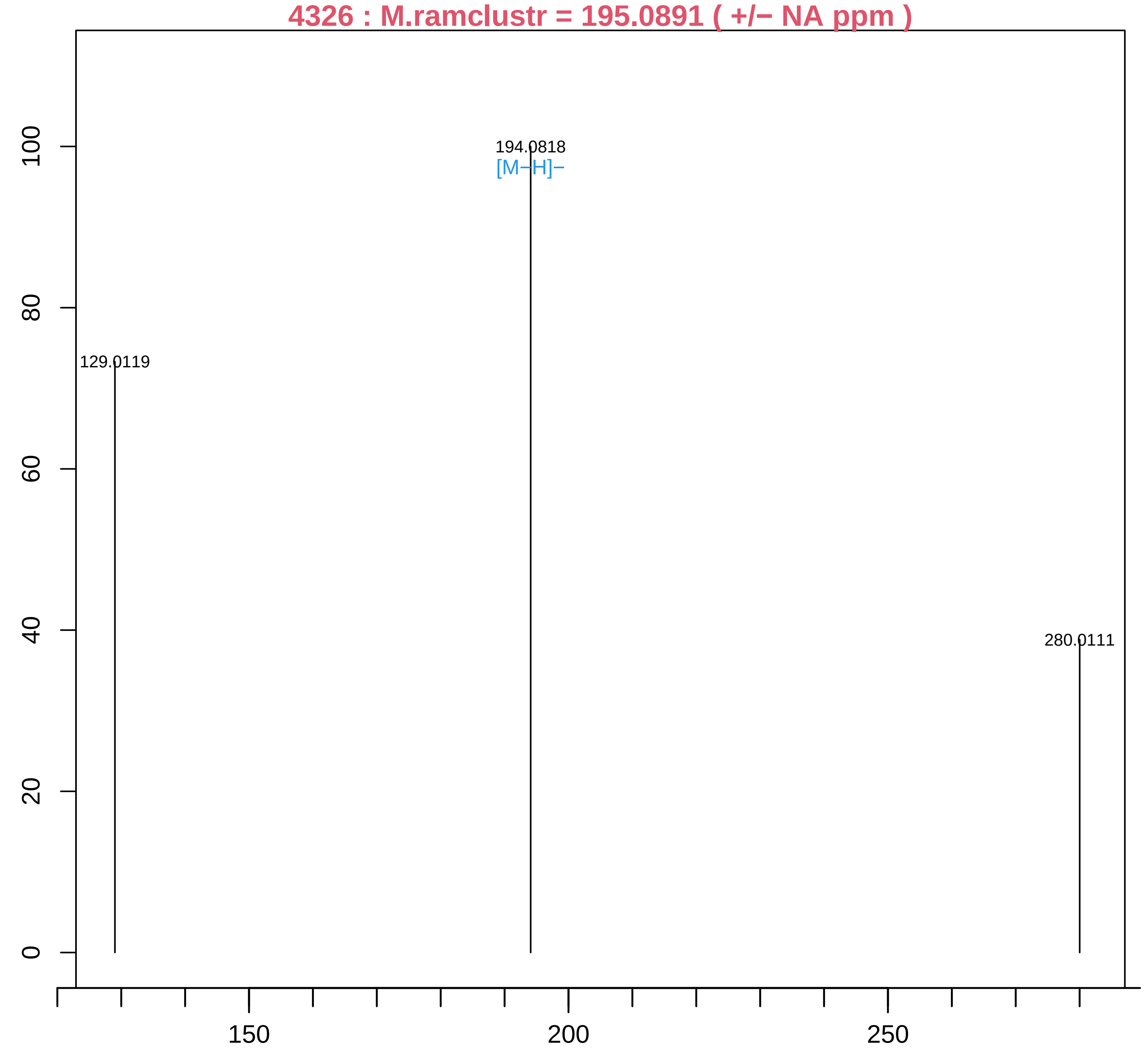
4326 : M.ramclustr = 195.0891 (+/− NA ppm)
194.0818
[M−H]−
129.0119
280.0111
0
20
40
60
80
100
150
200
250

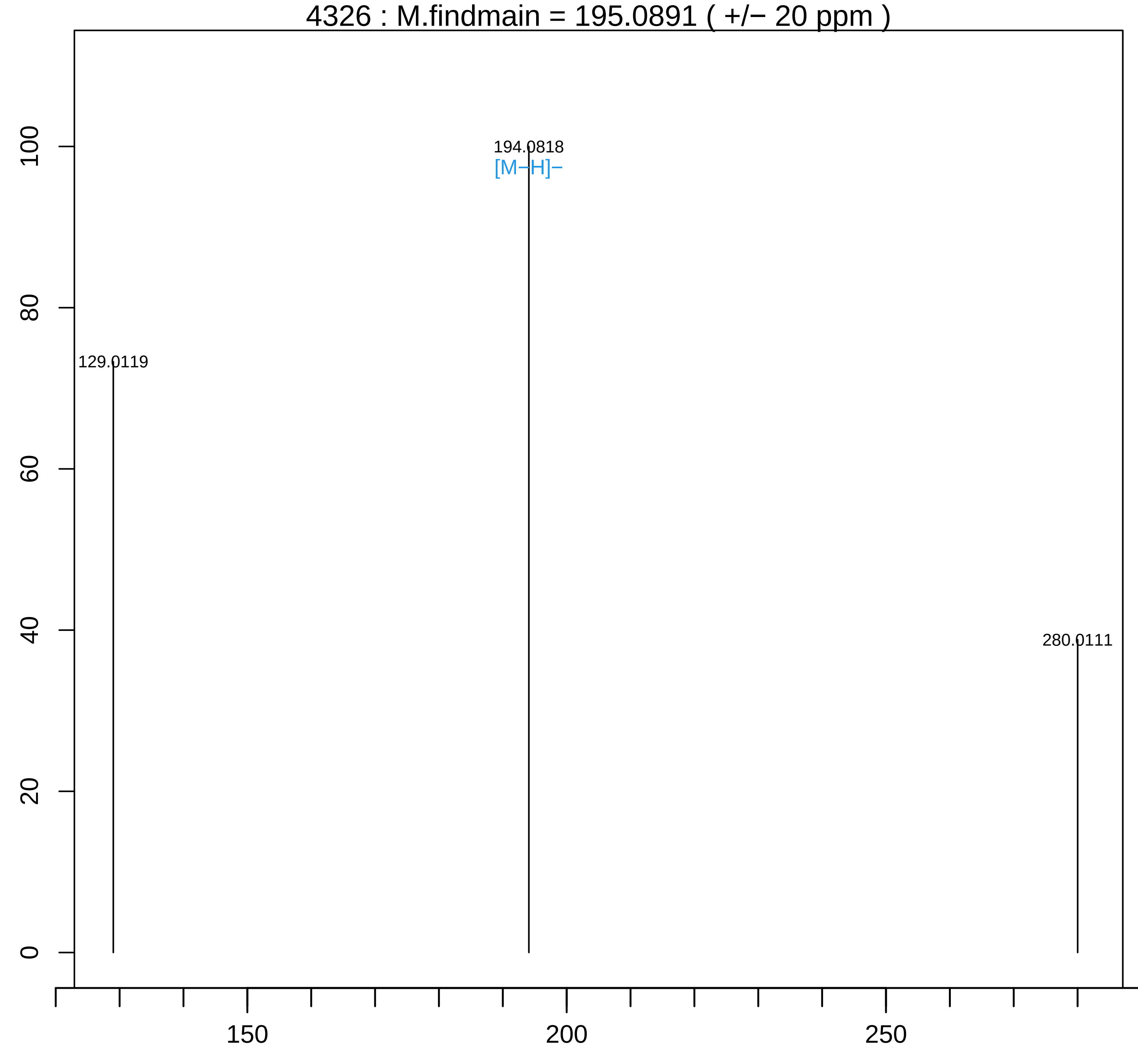
4326 : M.findmain = 195.0891 (+/− 20 ppm)
194.0818
[M−H]−
129.0119
280.0111
0
20
40
60
80
100
150
200
250

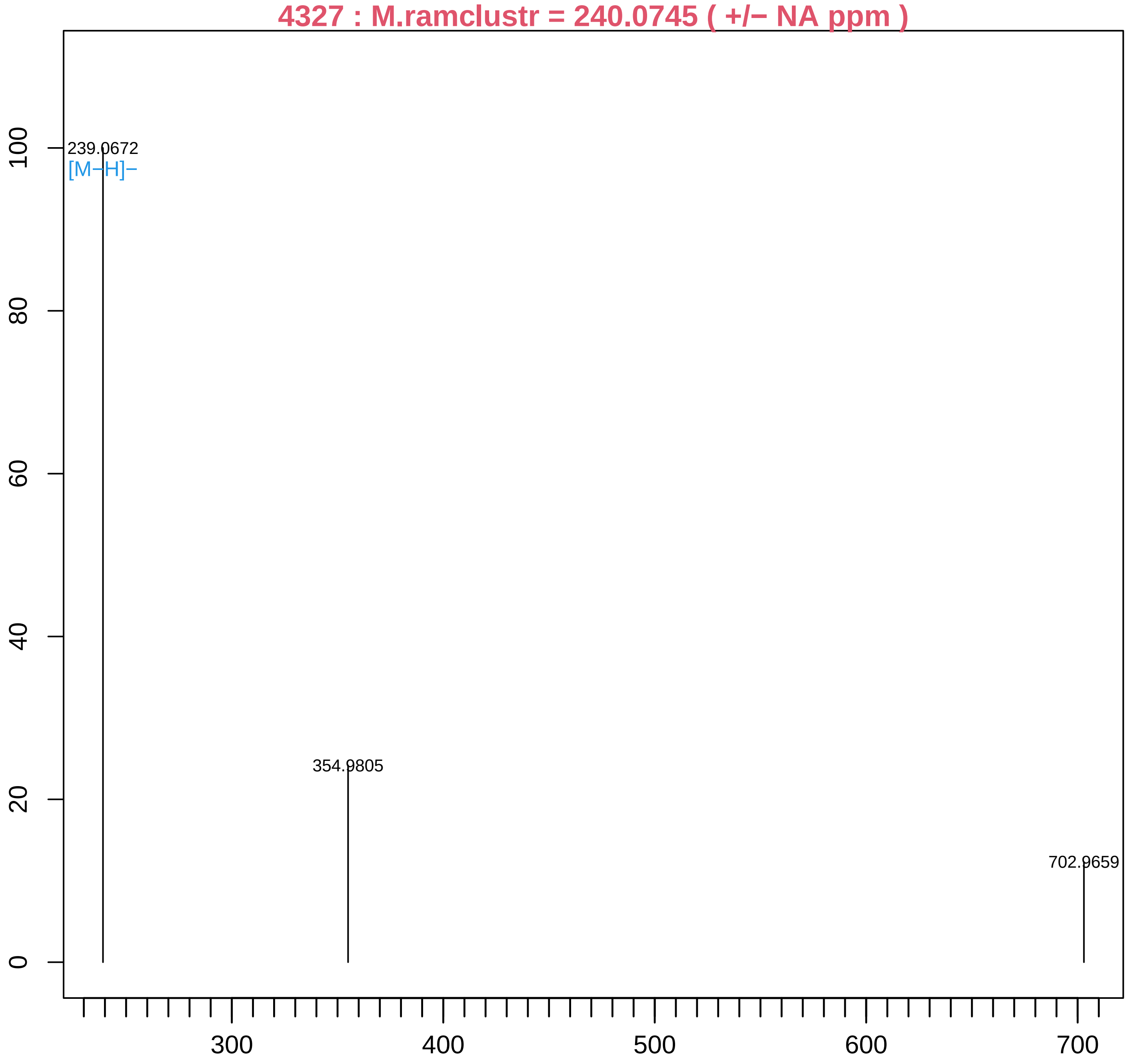
4327 : M.ramclustr = 240.0745 (+/− NA ppm)
239.0672
[M−H]−
354.9805
702.9659
0
20
40
60
80
100
300
400
500
600
700

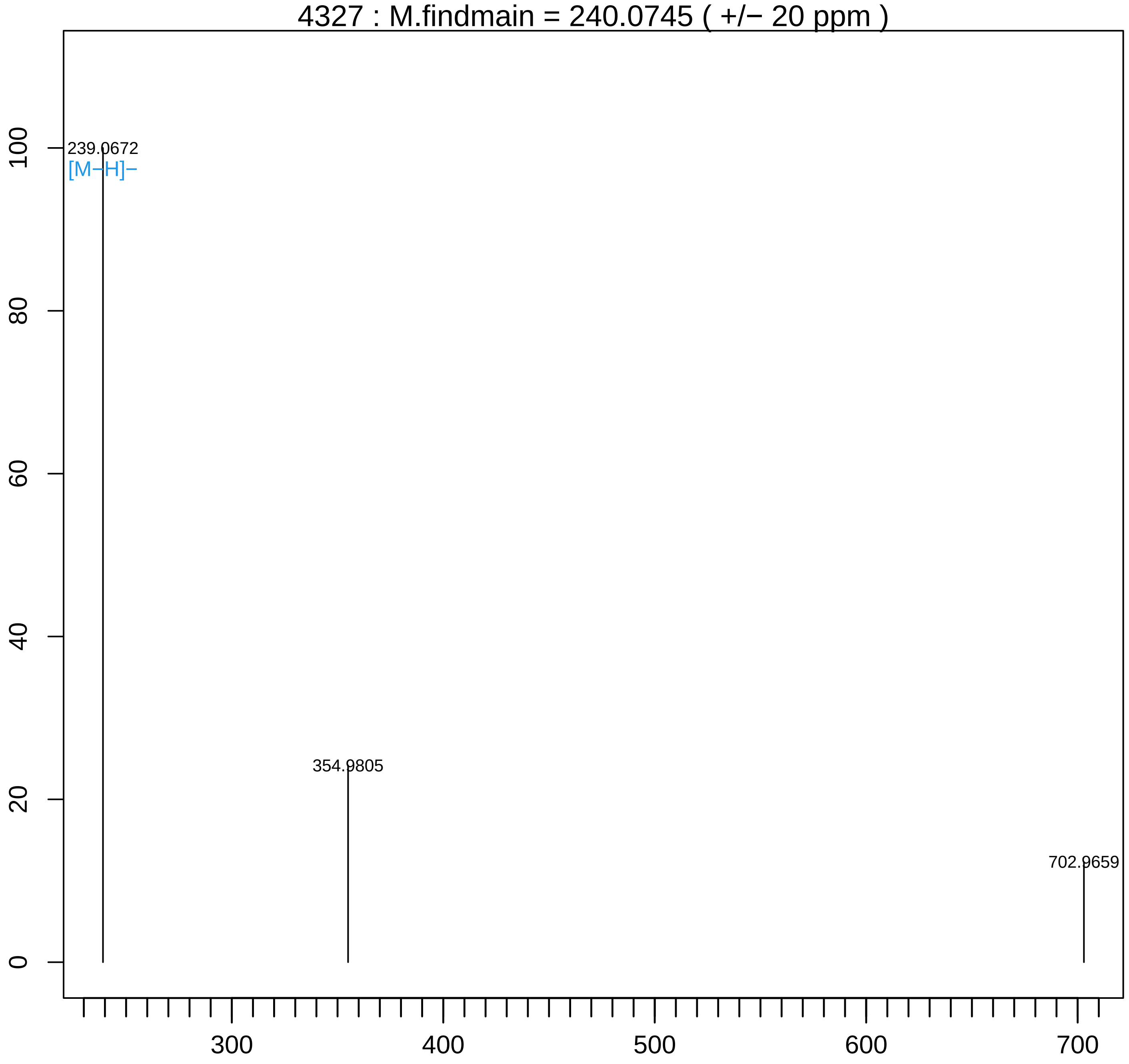
4327 : M.findmain = 240.0745 (+/− 20 ppm)
239.0672
[M−H]−
354.9805
702.9659
0
20
40
60
80
100
300
400
500
600
700

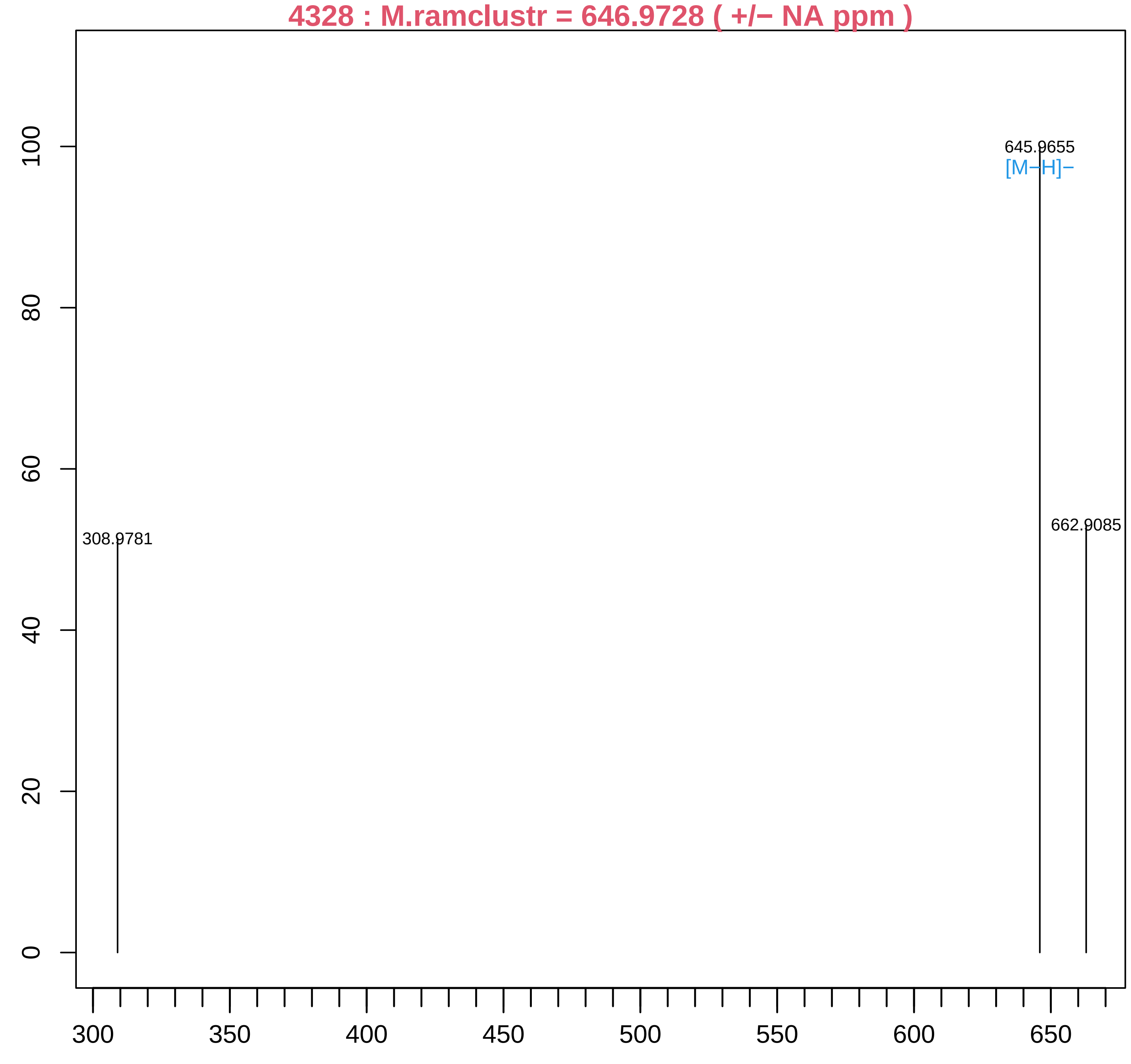
4328 : M.ramclustr = 646.9728 (+/− NA ppm)
645.9655
[M−H]−
662.9085
308.9781
0
20
40
60
80
100
300
350
400
450
500
550
600
650

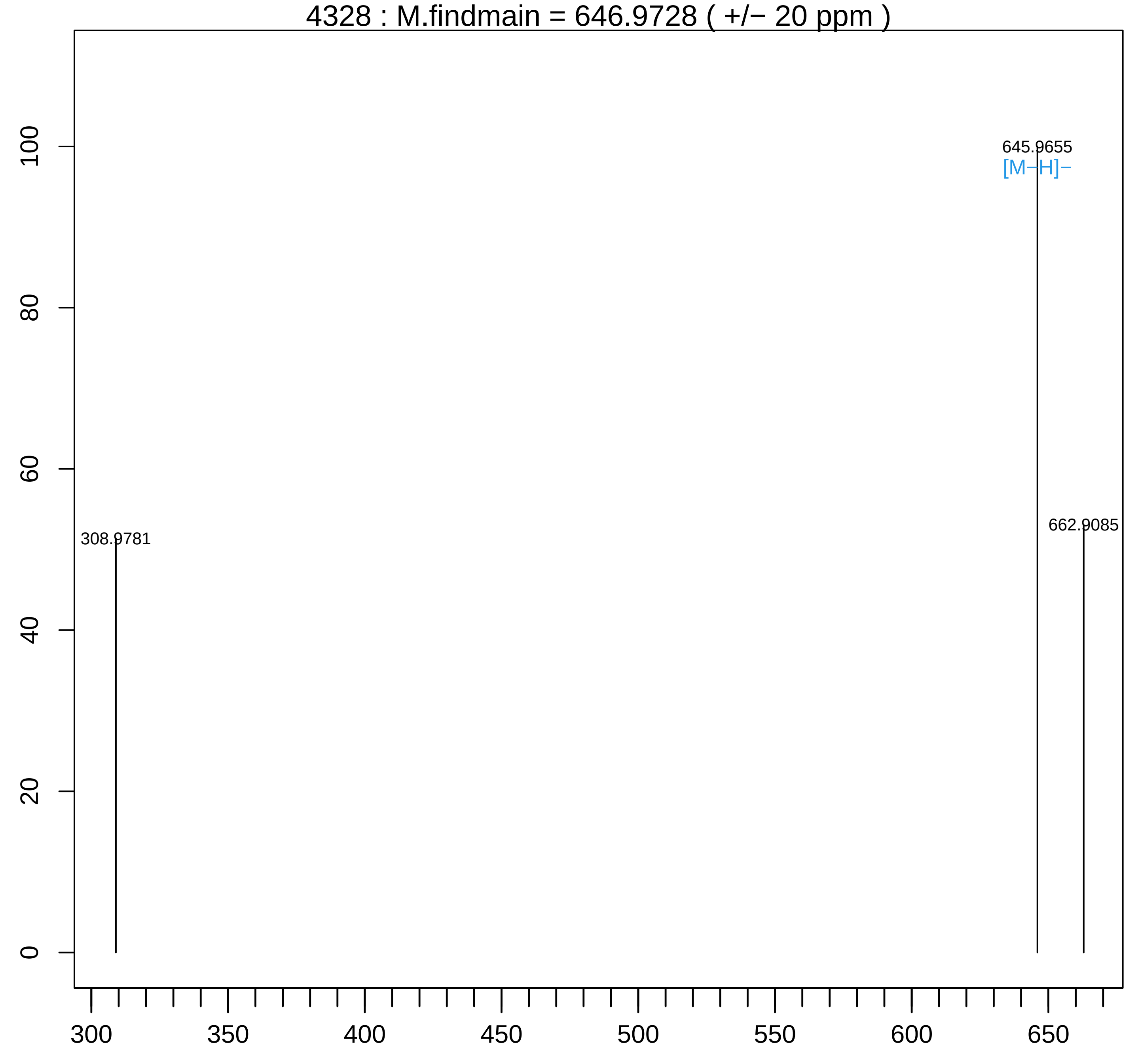
4328 : M.findmain = 646.9728 (+/− 20 ppm)
645.9655
[M−H]−
662.9085
308.9781
0
20
40
60
80
100
300
350
400
450
500
550
600
650

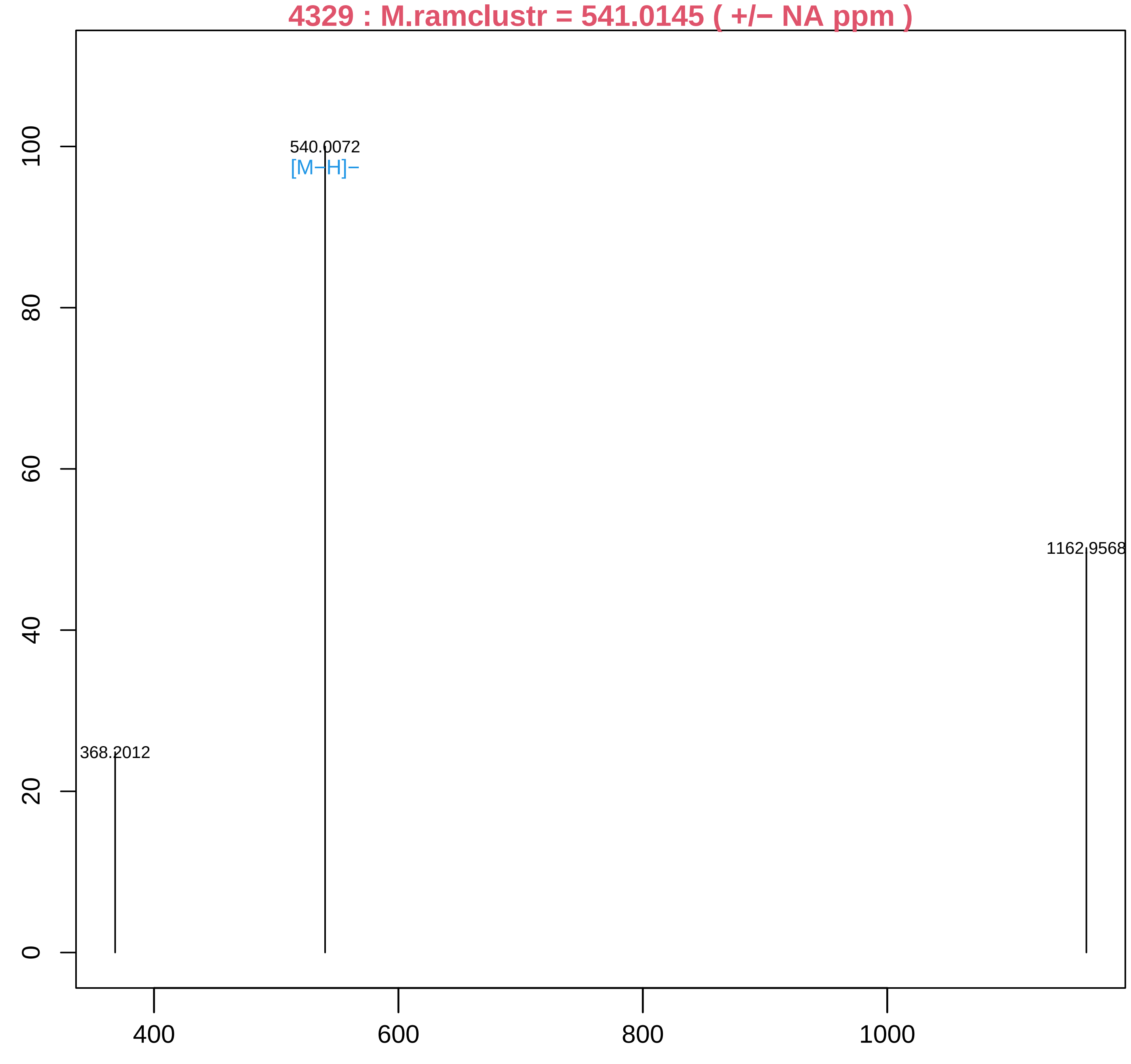
4329 : M.ramclustr = 541.0145 (+/− NA ppm)
540.0072
[M−H]−
1162.9568
368.2012
0
20
40
60
80
100
400
600
800
1000

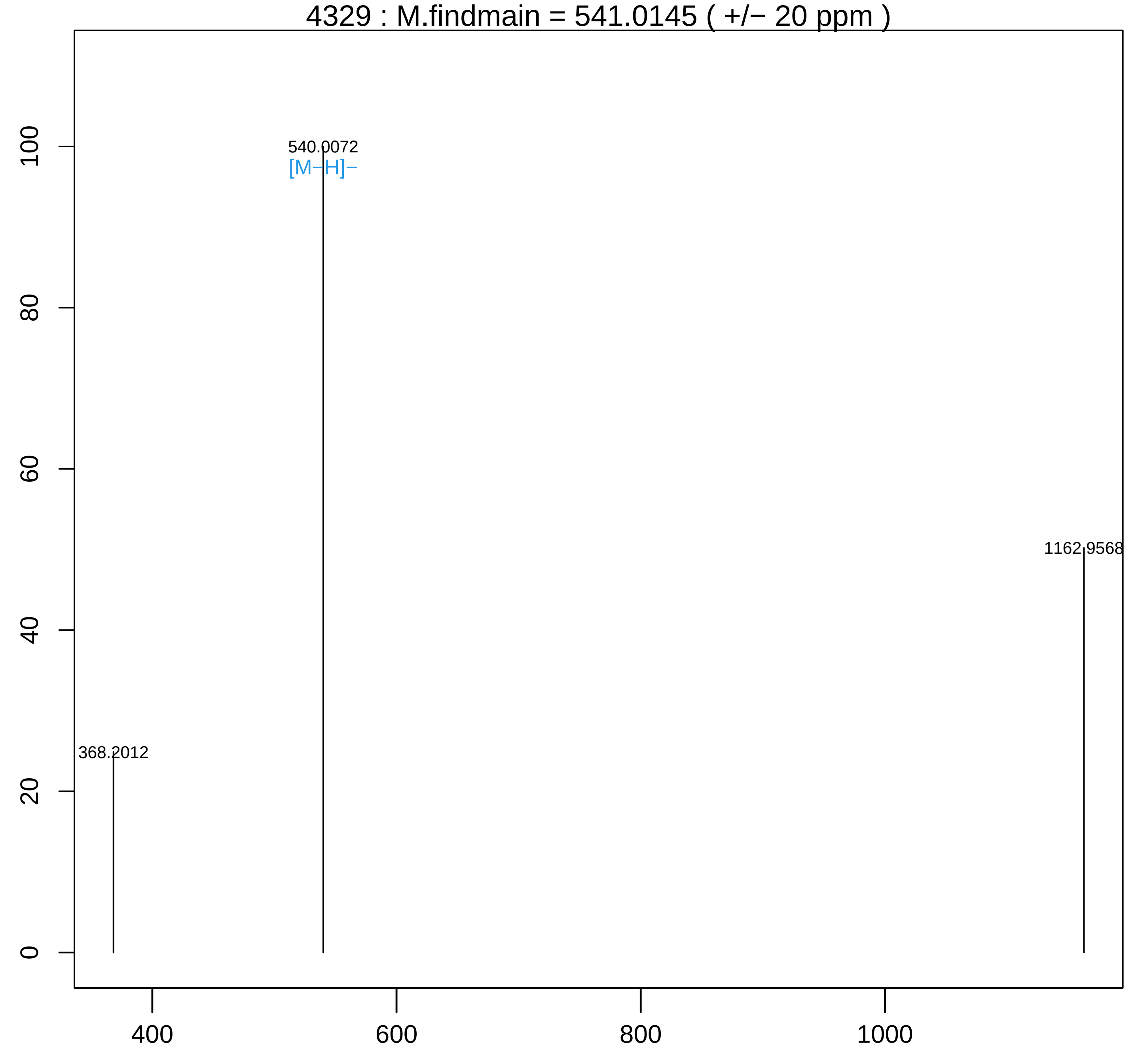
4329 : M.findmain = 541.0145 (+/− 20 ppm)
540.0072
[M−H]−
1162.9568
368.2012
0
20
40
60
80
100
400
600
800
1000

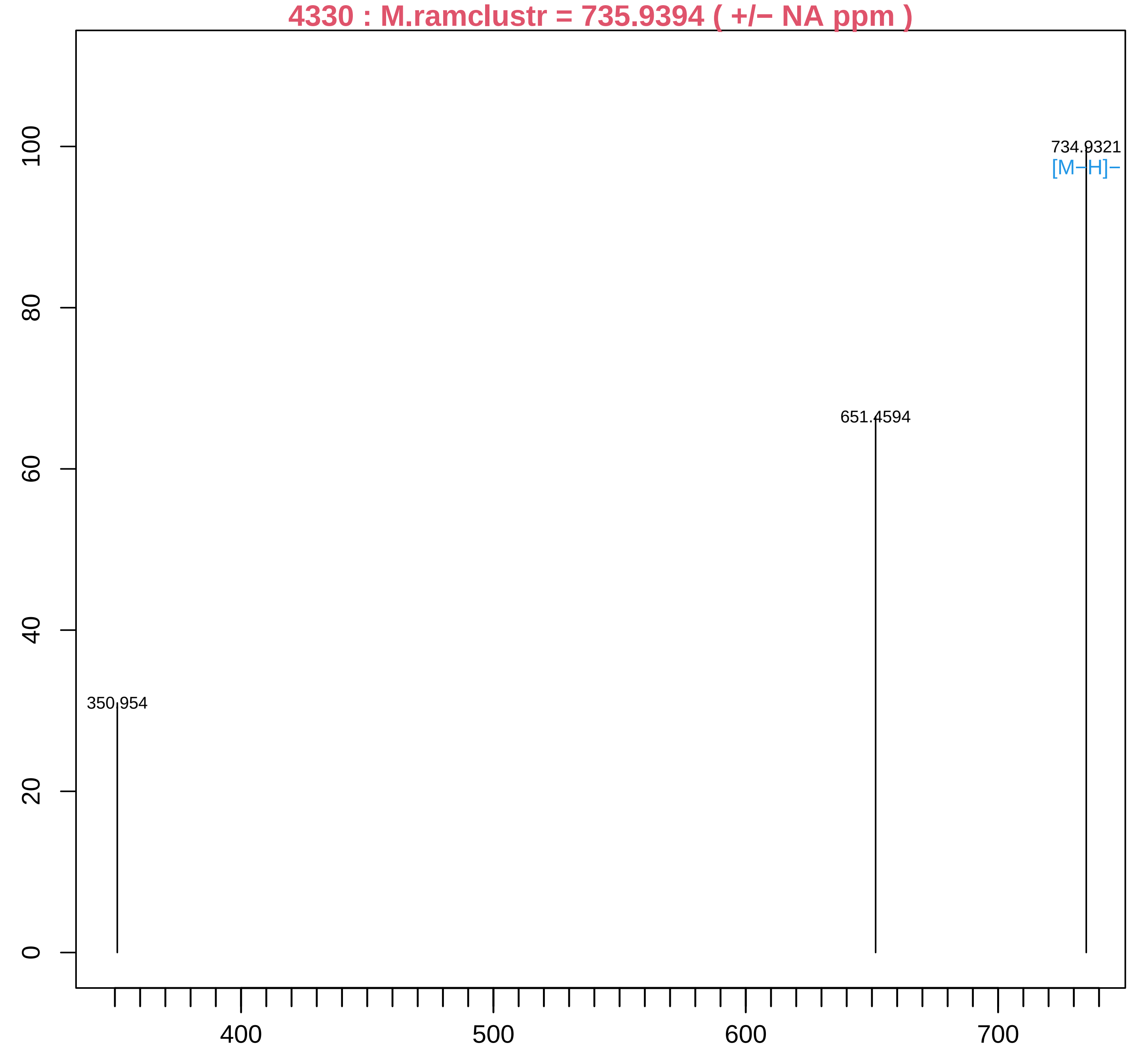
4330 : M.ramclustr = 735.9394 (+/− NA ppm)
734.9321
[M−H]−
651.4594
350.954
0
20
40
60
80
100
400
500
600
700

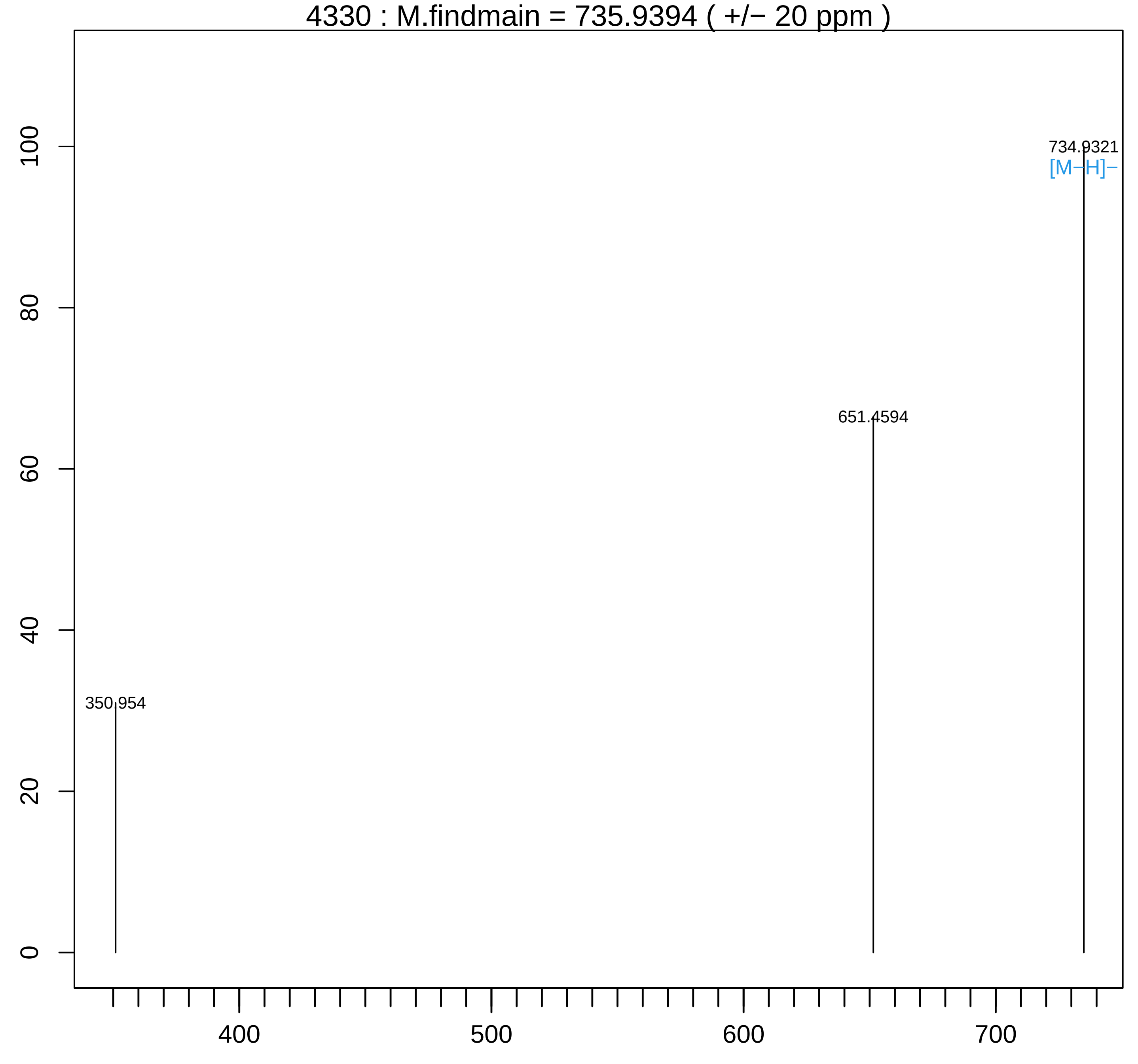
4330 : M.findmain = 735.9394 (+/− 20 ppm)
734.9321
[M−H]−
651.4594
350.954
0
20
40
60
80
100
400
500
600
700

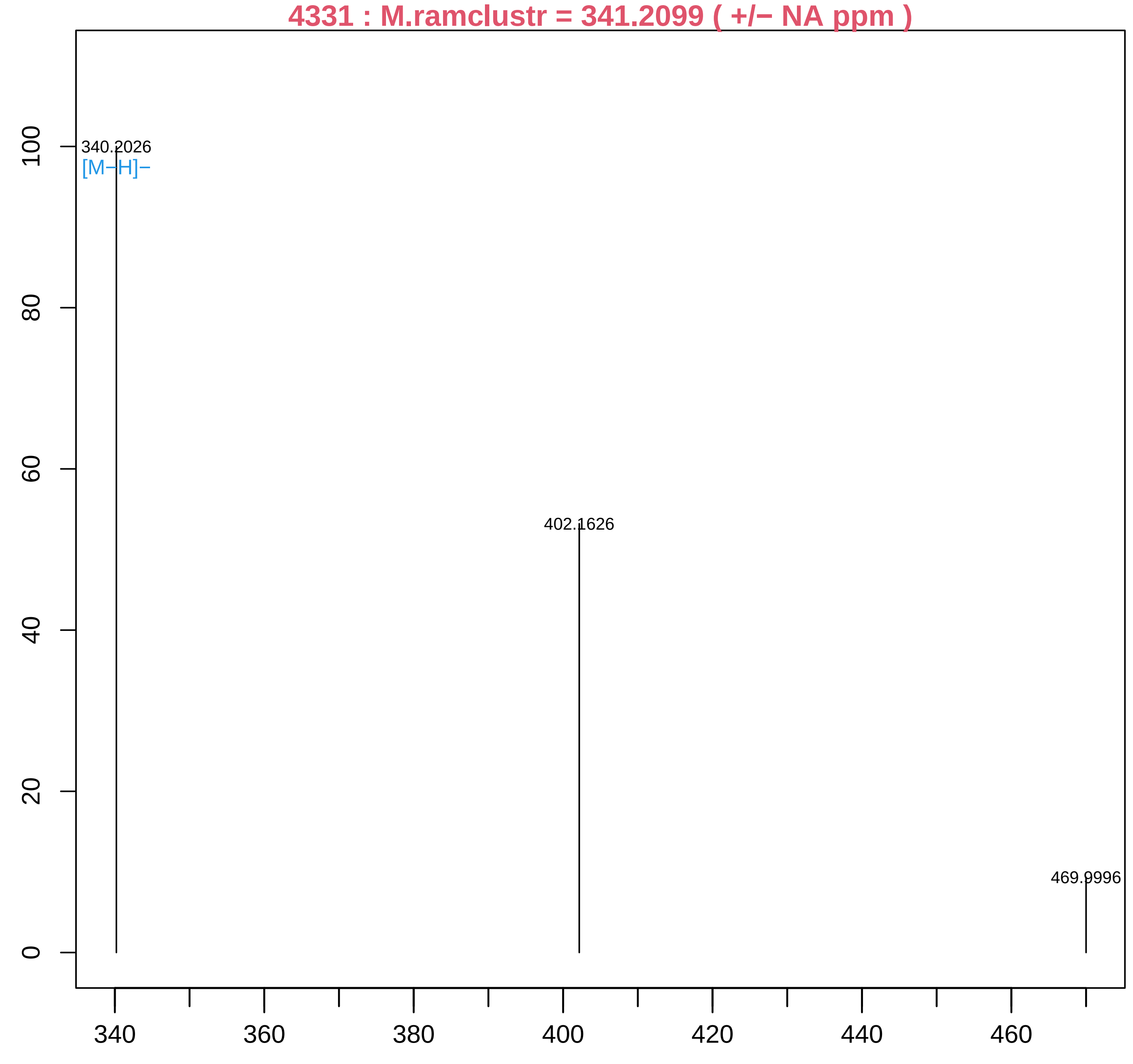
4331 : M.ramclustr = 341.2099 (+/− NA ppm)
340.2026
[M−H]−
402.1626
469.9996
0
20
40
60
80
100
340
360
380
400
420
440
460

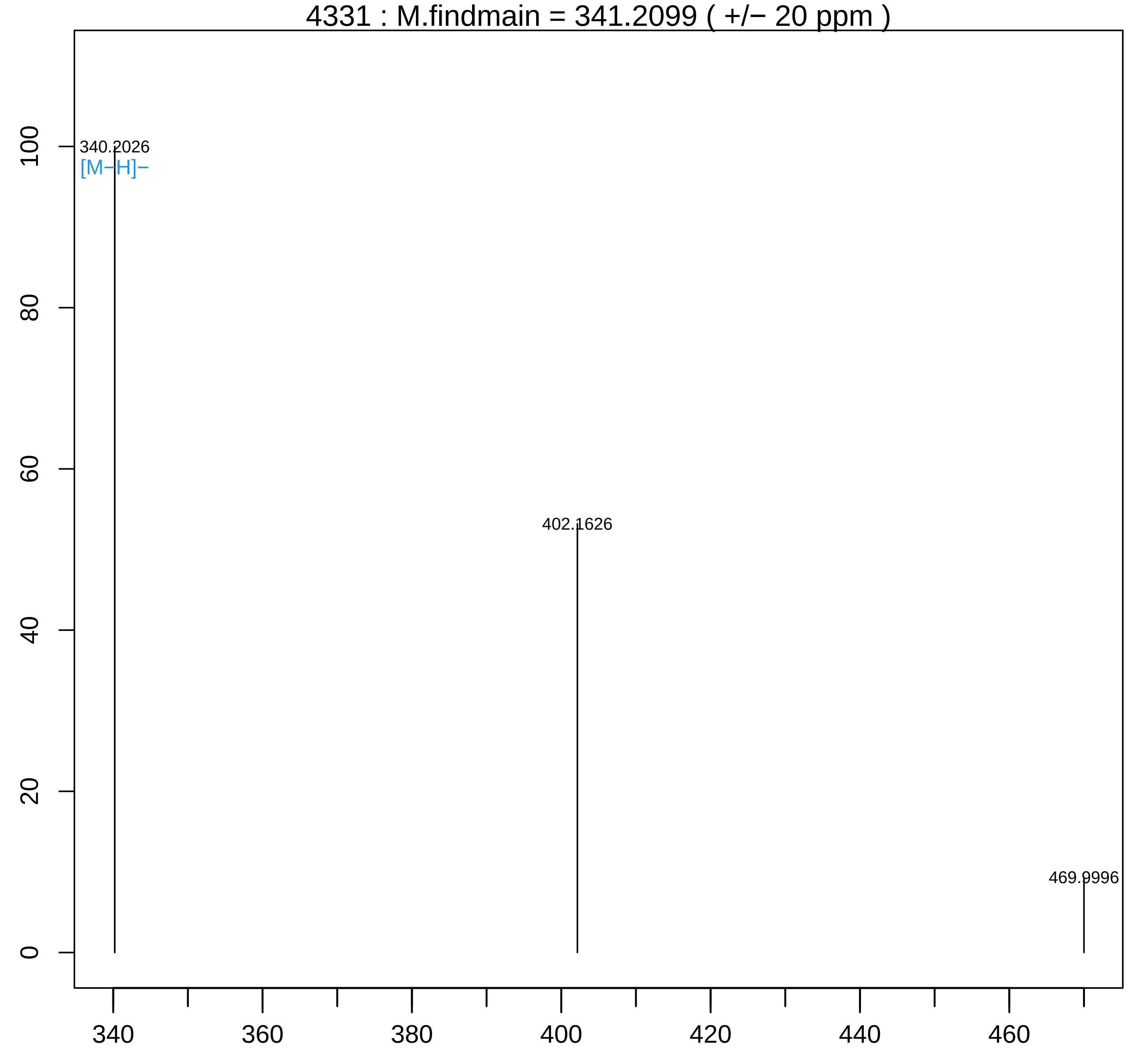
4331 : M.findmain = 341.2099 (+/− 20 ppm)
340.2026
[M−H]−
402.1626
469.9996
0
20
40
60
80
100
340
360
380
400
420
440
460

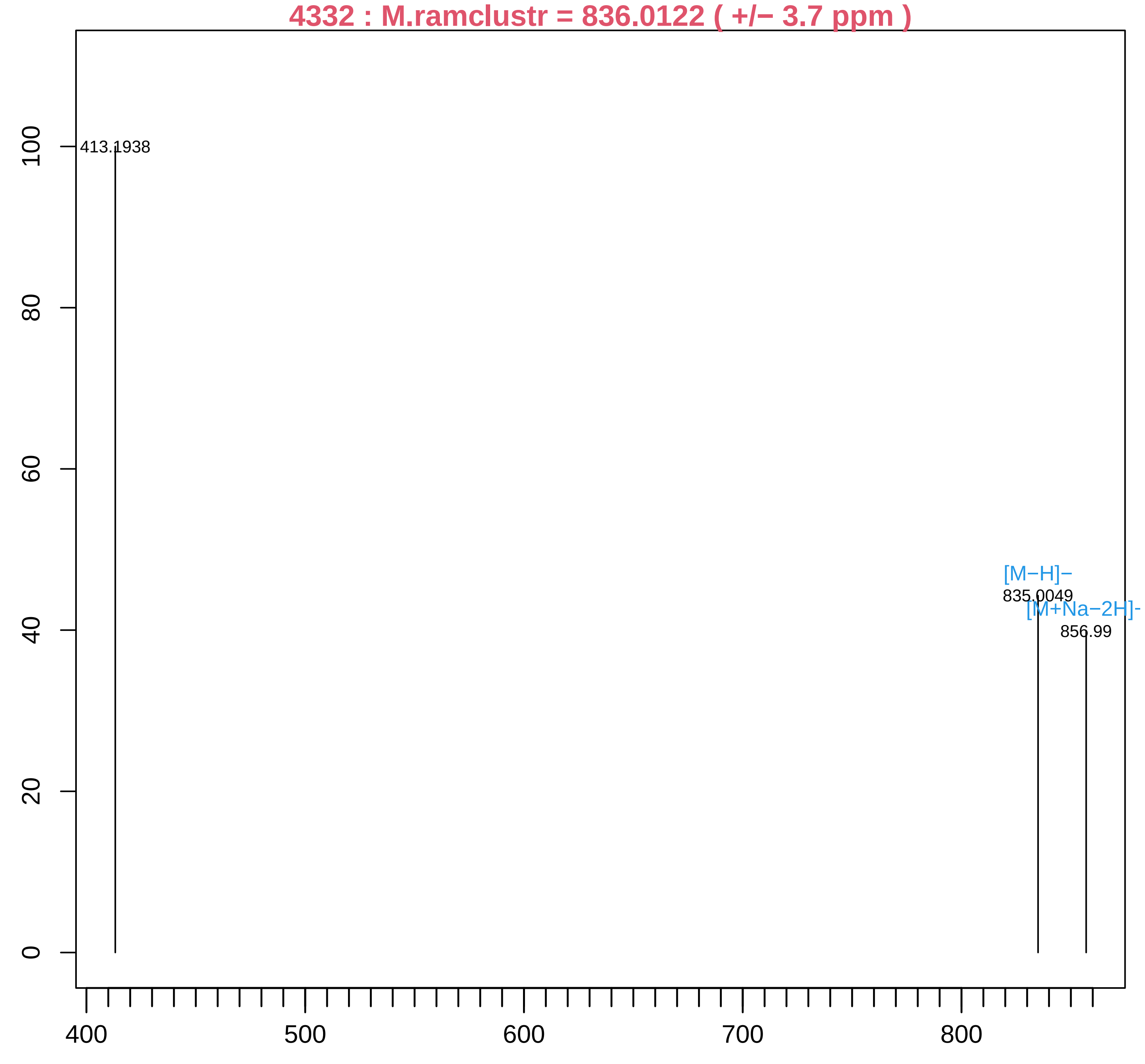
4332 : M.ramclustr = 836.0122 (+/− 3.7 ppm)
413.1938
[M−H]−
835.0049
[M+Na−2H]-
856.99
0
20
40
60
80
100
400
500
600
700
800

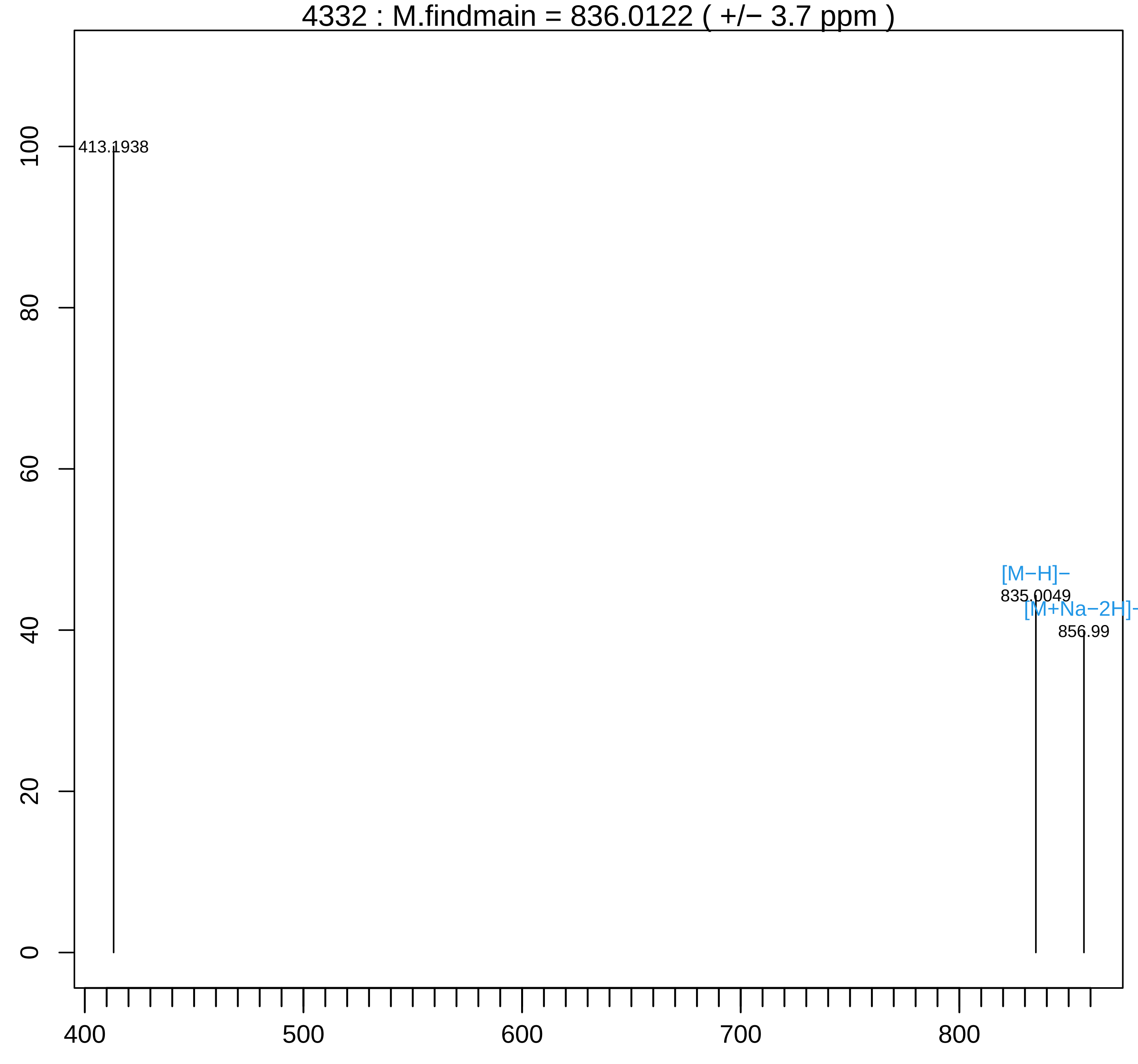
4332 : M.findmain = 836.0122 (+/− 3.7 ppm)
413.1938
[M−H]−
835.0049
[M+Na−2H]-
856.99
0
20
40
60
80
100
400
500
600
700
800

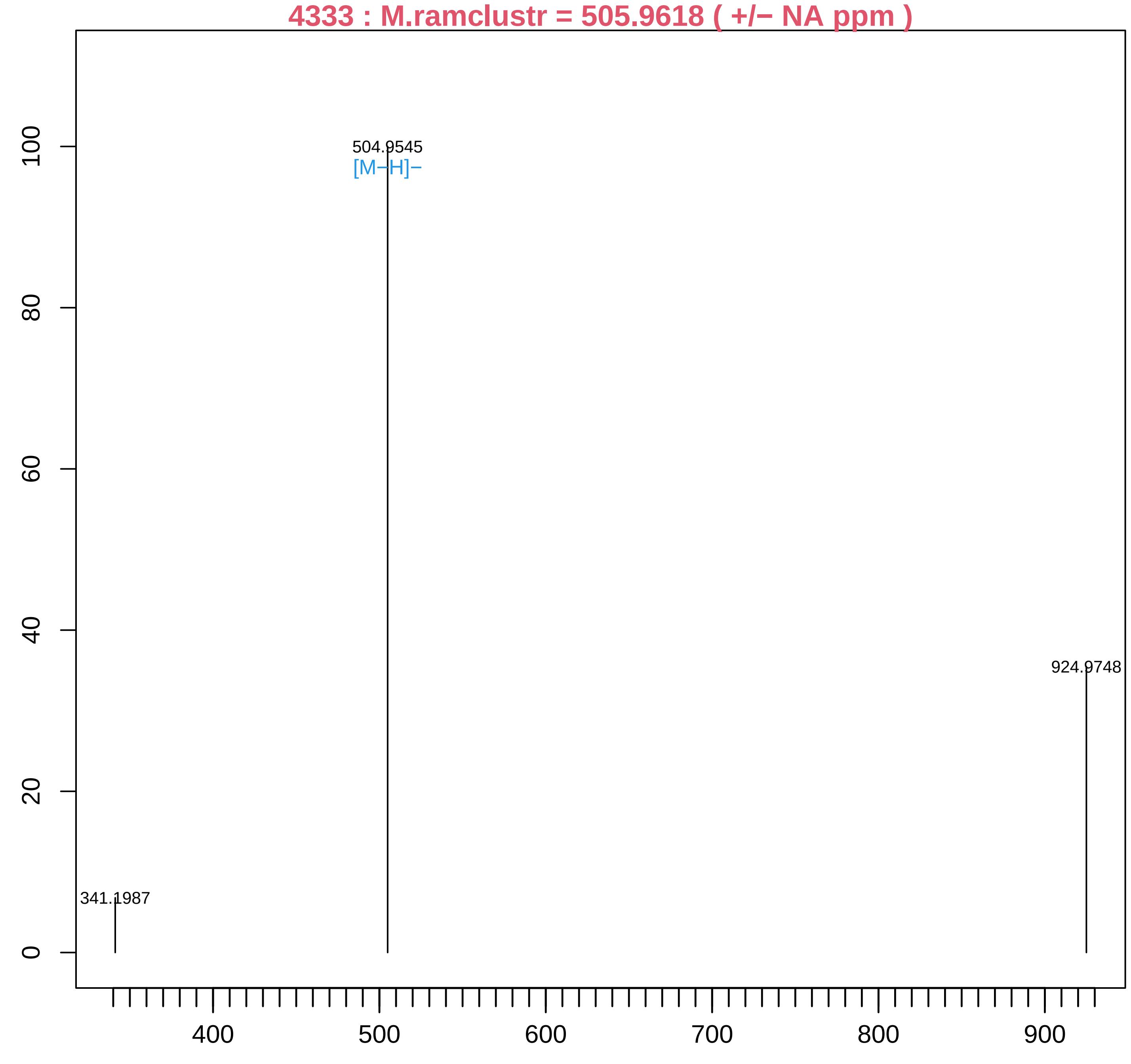
4333 : M.ramclustr = 505.9618 (+/− NA ppm)
504.9545
[M−H]−
924.9748
341.1987
0
20
40
60
80
100
400
500
600
700
800
900

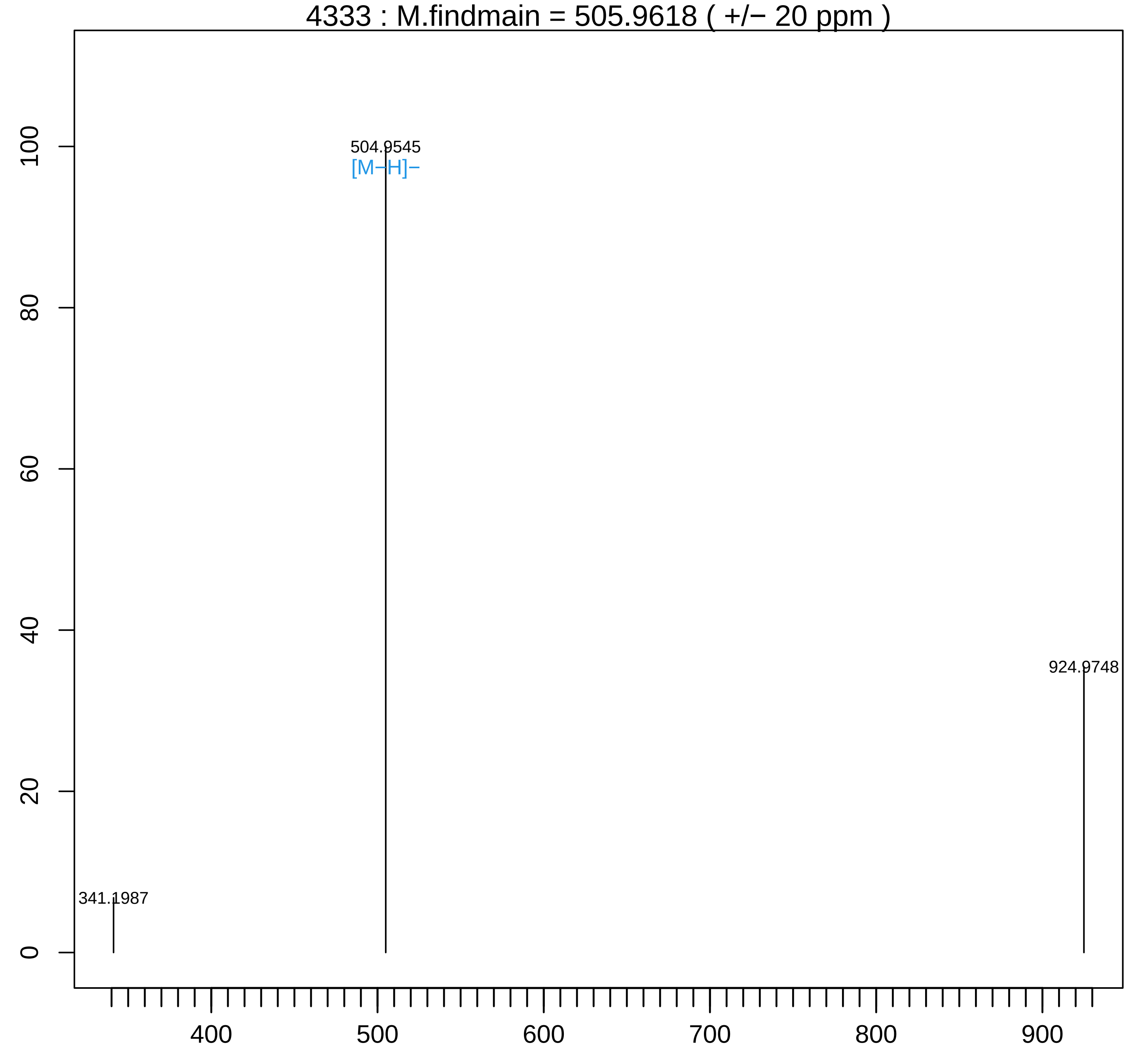
4333 : M.findmain = 505.9618 (+/− 20 ppm)
504.9545
[M−H]−
924.9748
341.1987
0
20
40
60
80
100
400
500
600
700
800
900

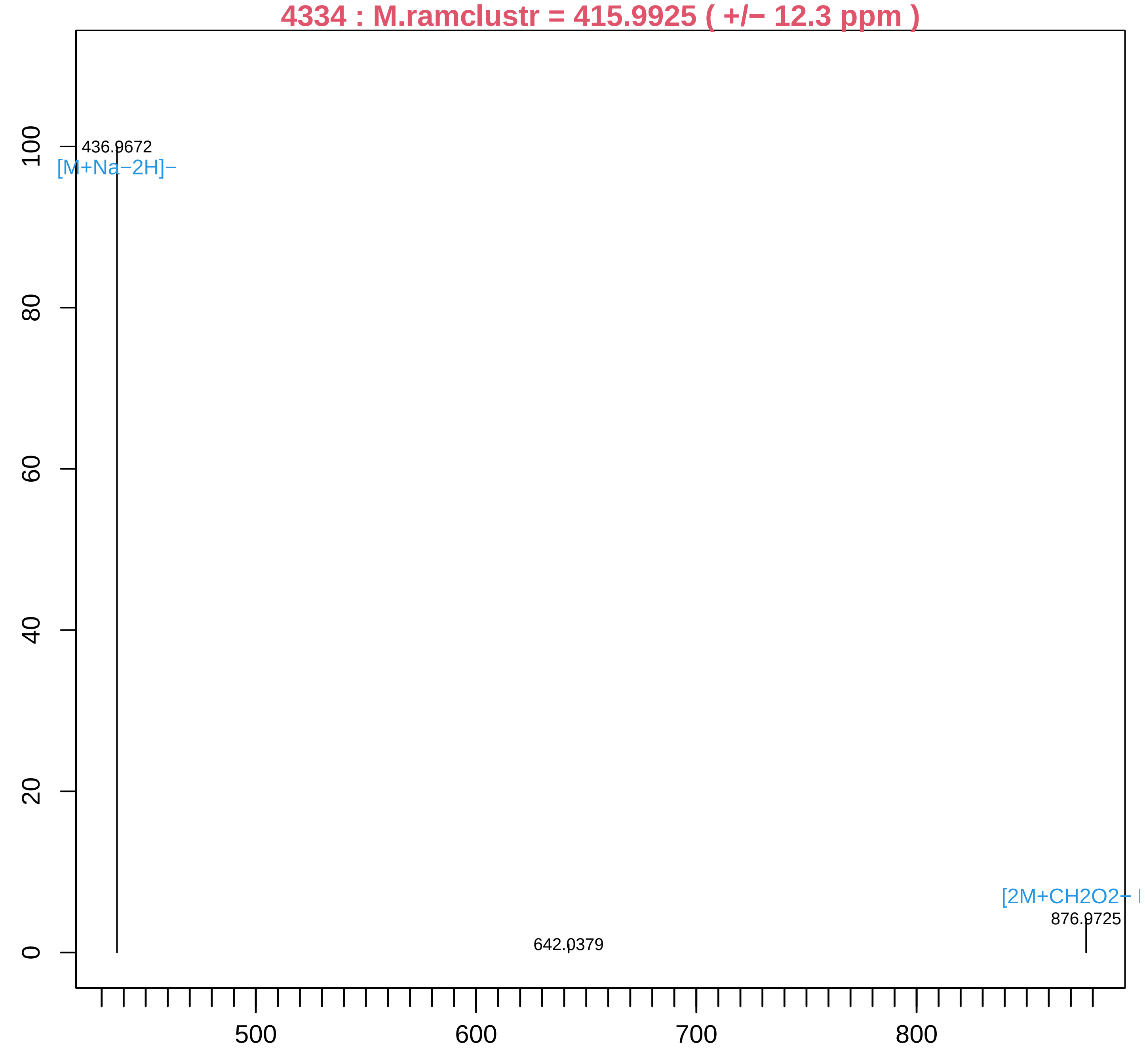

4334 : M.ramclustr = 415.9925 (+/− 12.3 ppm)
436.9672
[M+Na−2H]−
642.0379
876.9725
[2M+CH2O2−
0
20
40
60
80
100
500
600
700
800

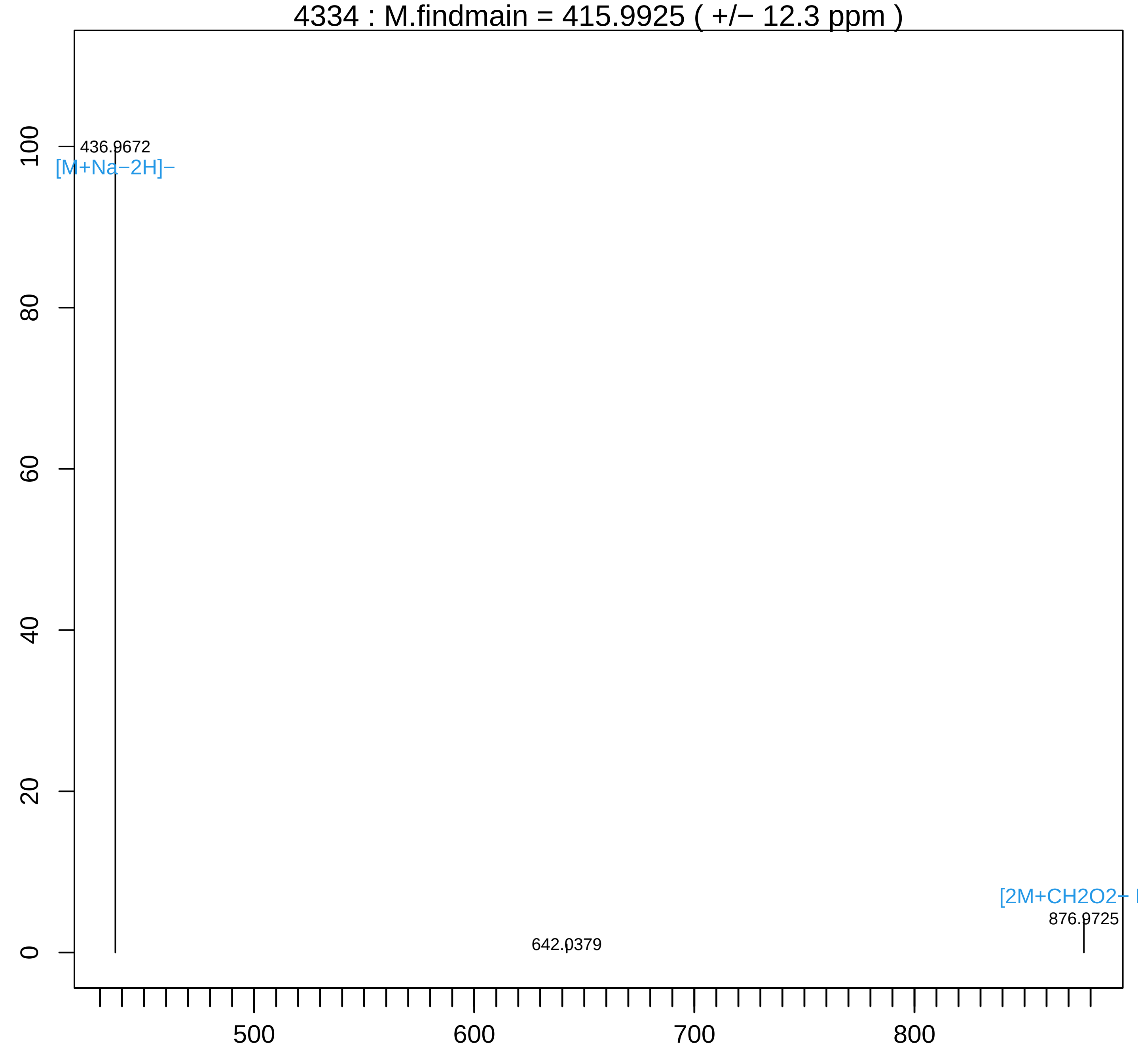

4334 : M.findmain = 415.9925 (+/− 12.3 ppm)
436.9672
[M+Na−2H]−
642.0379
876.9725
[2M+CH2O2−
0
20
40
60
80
100
500
600
700
800

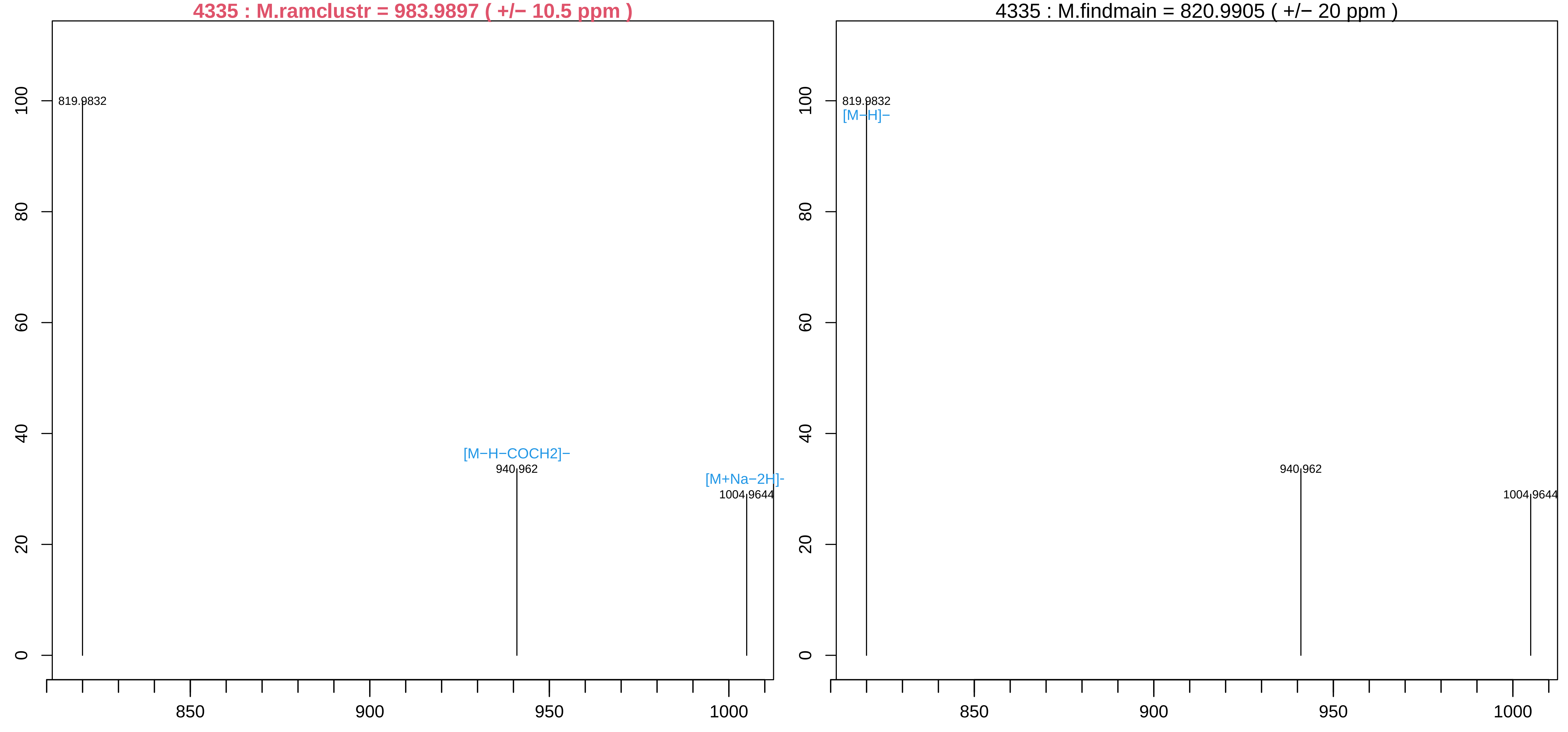
4335 : M.ramclustr = 983.9897 (+/− 10.5 ppm)
819.9832
[M−H−COCH2]−
940.962
[M+Na−2H]-
1004.9644
4335 : M.findmain = 820.9905 (+/− 20 ppm)
819.9832
[M−H]−
940.962
1004.9644
0
20
40
60
80
100
850
900
950
1000

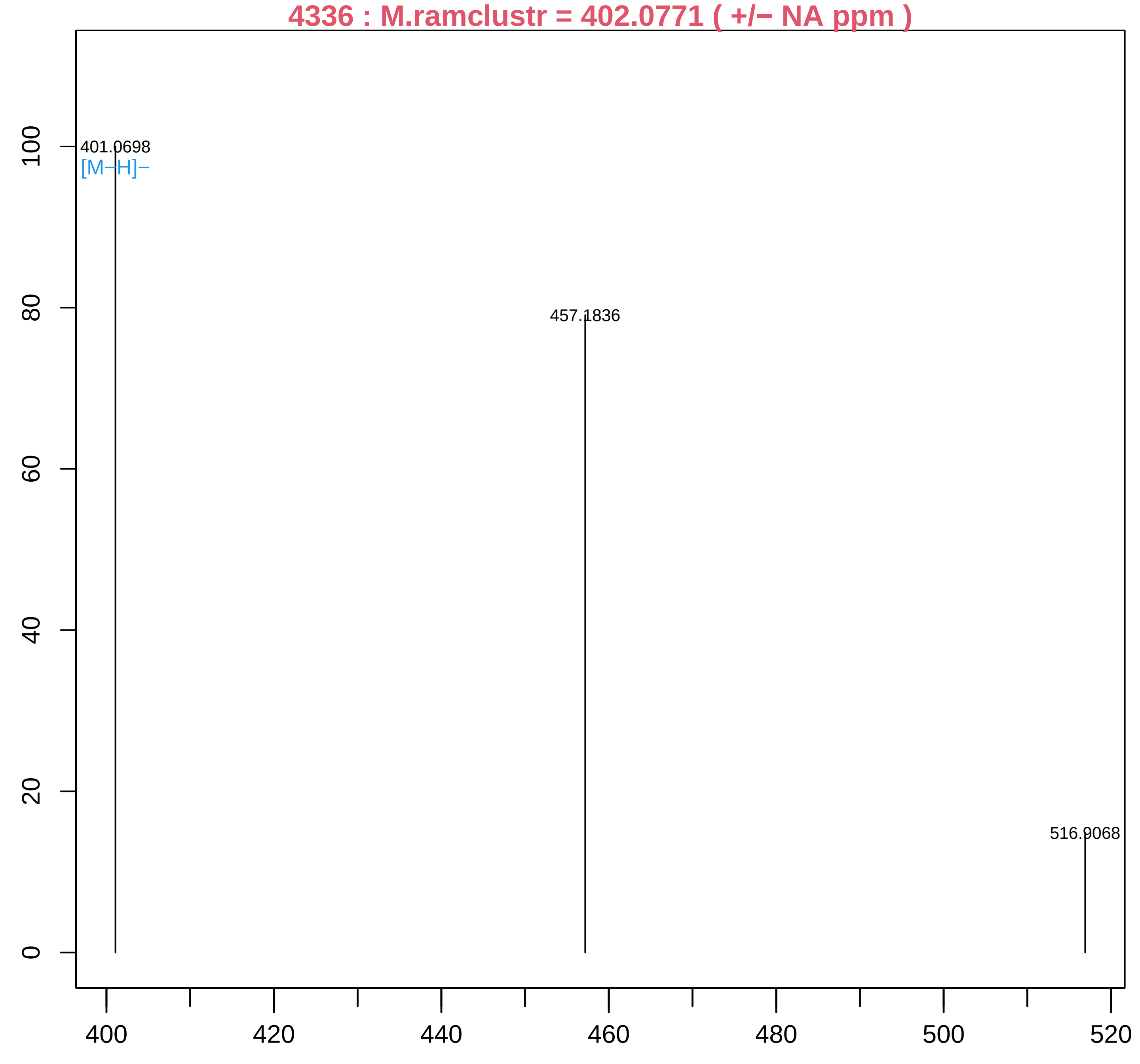
4336 : M.ramclustr = 402.0771 (+/− NA ppm)
401.0698
[M−H]−
457.1836
516.9068
0
20
40
60
80
100
400
420
440
460
480
500
520

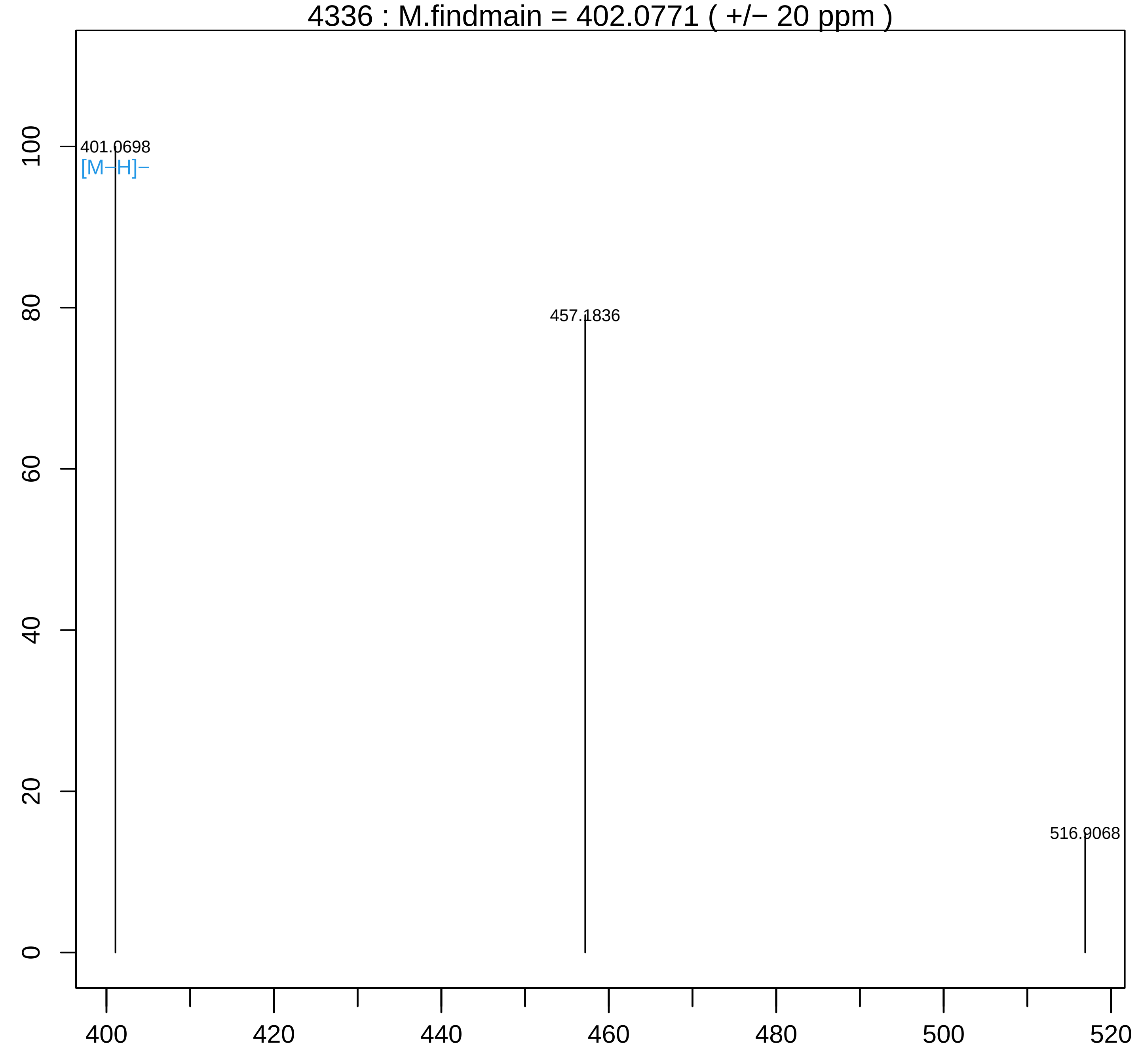
4336 : M.findmain = 402.0771 (+/− 20 ppm)
401.0698
[M−H]−
457.1836
516.9068
0
20
40
60
80
100
400
420
440
460
480
500
520

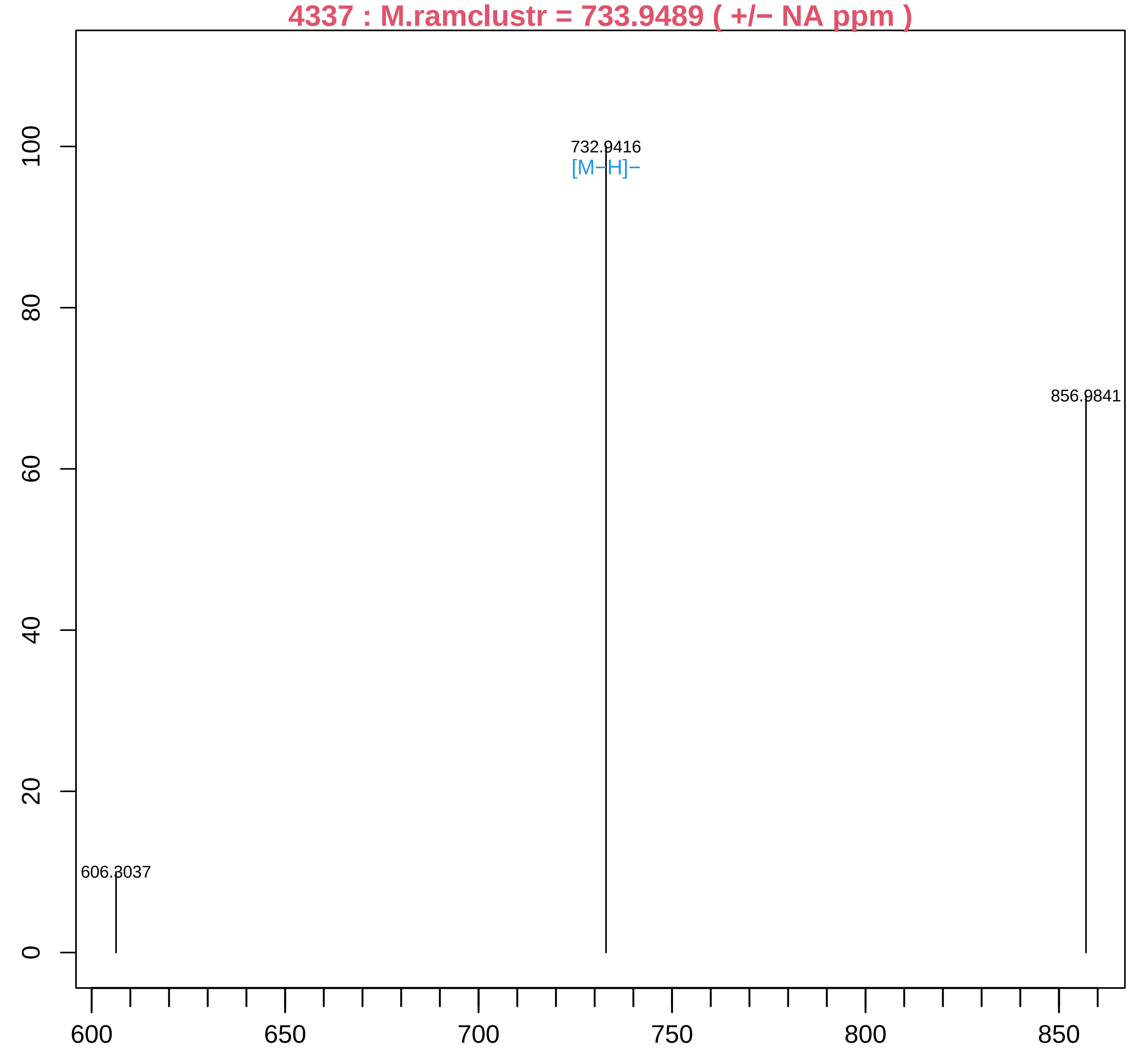

4337 : M.ramclustr = 733.9489 (+/− NA ppm)
732.9416
[M−H]−
856.9841
606.3037
0
20
40
60
80
100
600
650
700
750
800
850

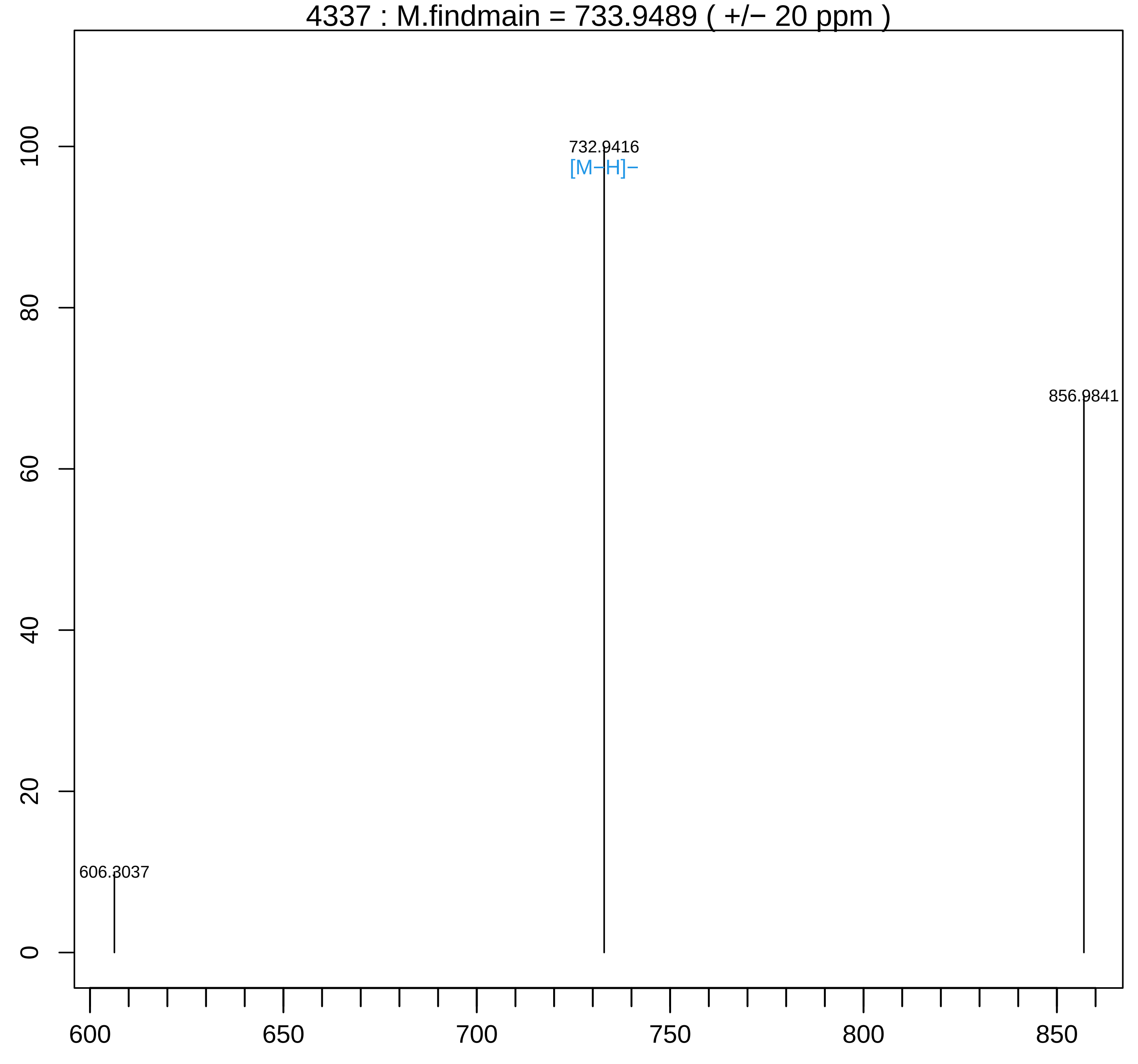

4337 : M.findmain = 733.9489 (+/− 20 ppm)
732.9416
[M−H]−
856.9841
606.3037
0
20
40
60
80
100
600
650
700
750
800
850

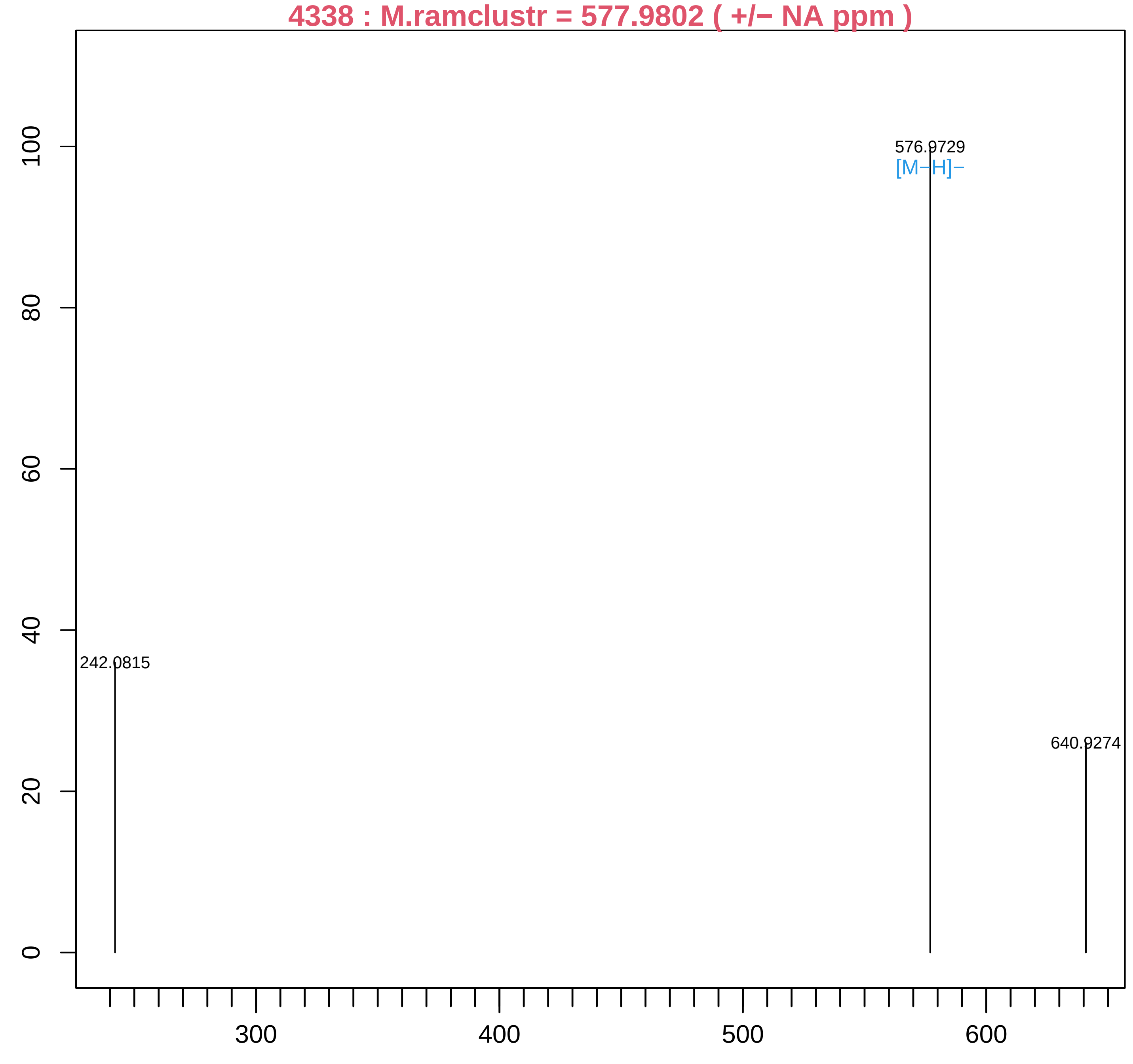
4338 : M.ramclustr = 577.9802 (+/− NA ppm)
576.9729
[M−H]−
242.0815
640.9274
0
20
40
60
80
100
300
400
500
600

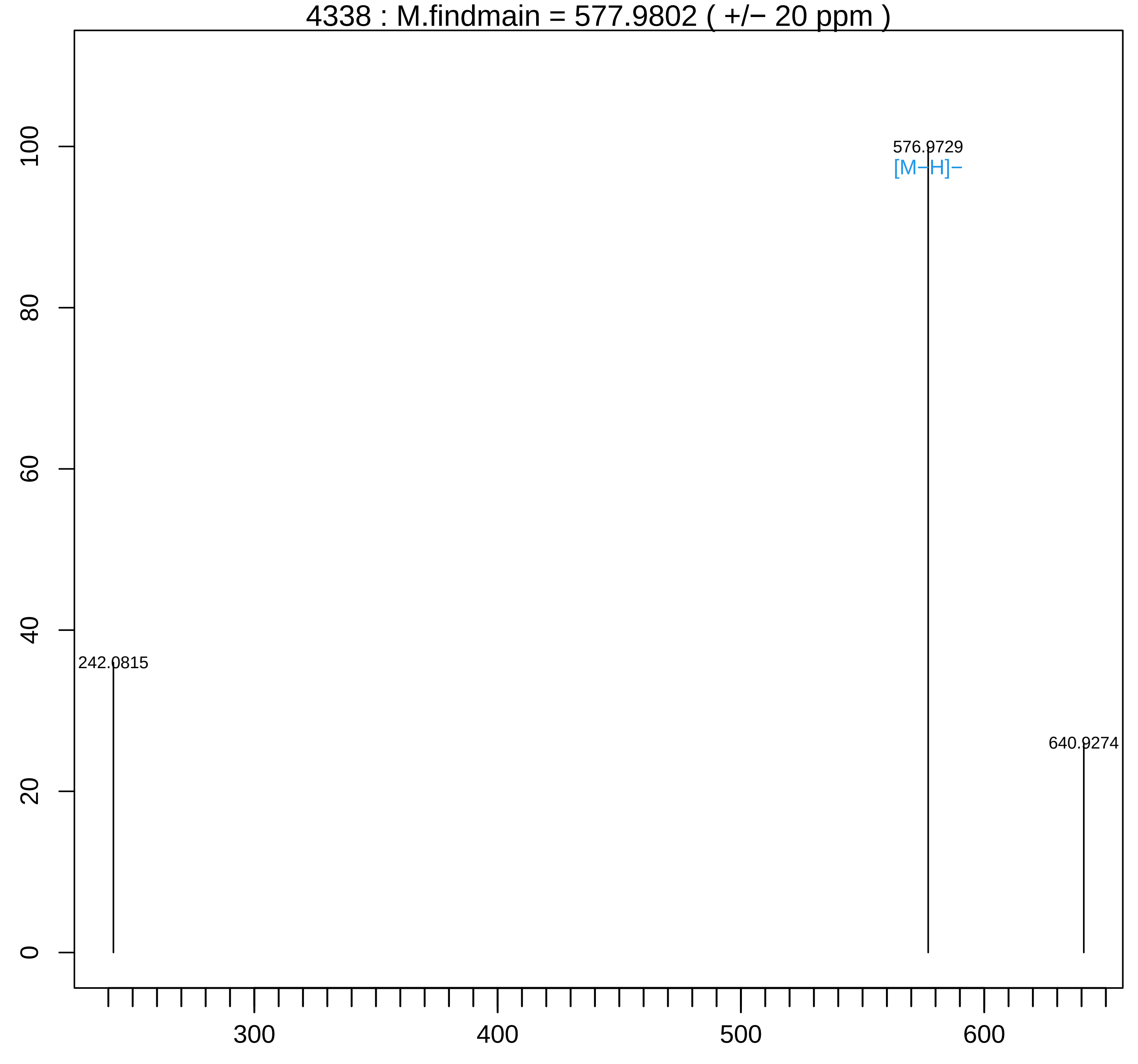
4338 : M.findmain = 577.9802 (+/− 20 ppm)
576.9729
[M−H]−
242.0815
640.9274
0
20
40
60
80
100
300
400
500
600

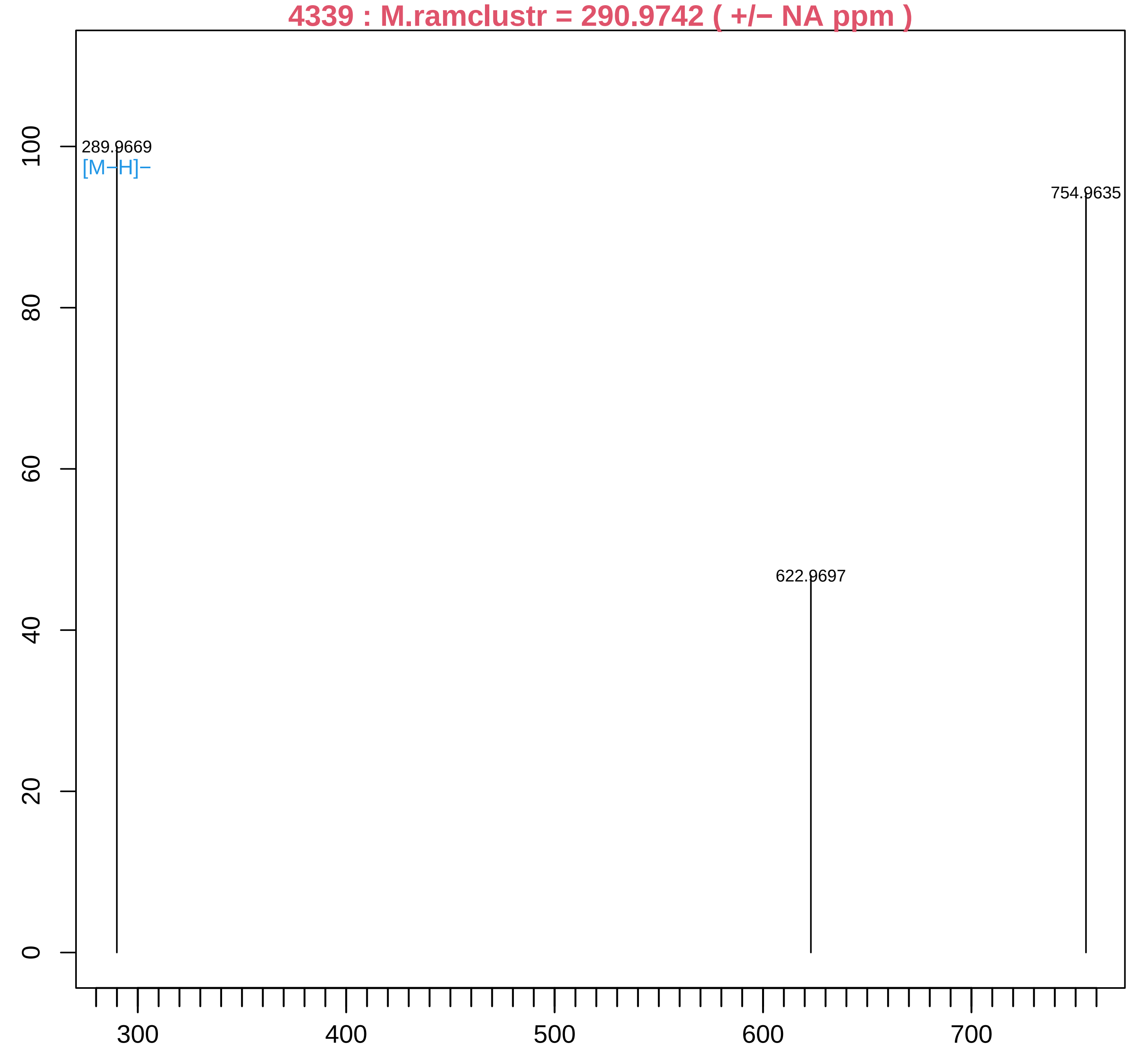
4339 : M.ramclustr = 290.9742 (+/− NA ppm)
289.9669
[M−H]−
622.9697
754.9635
0
20
40
60
80
100
300
400
500
600
700

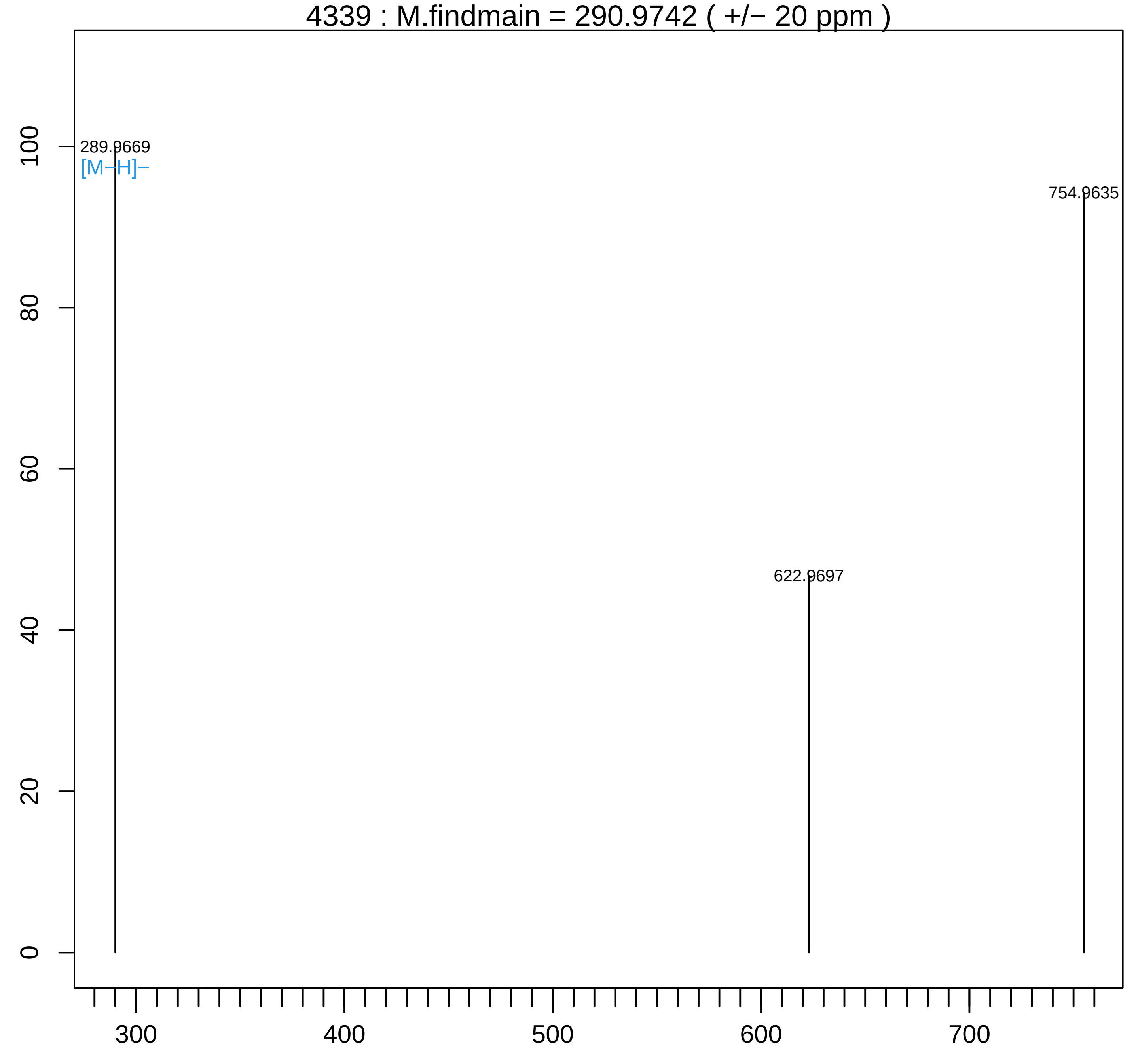
4339 : M.findmain = 290.9742 (+/− 20 ppm)
289.9669
[M−H]−
622.9697
754.9635
0
20
40
60
80
100
300
400
500
600
700

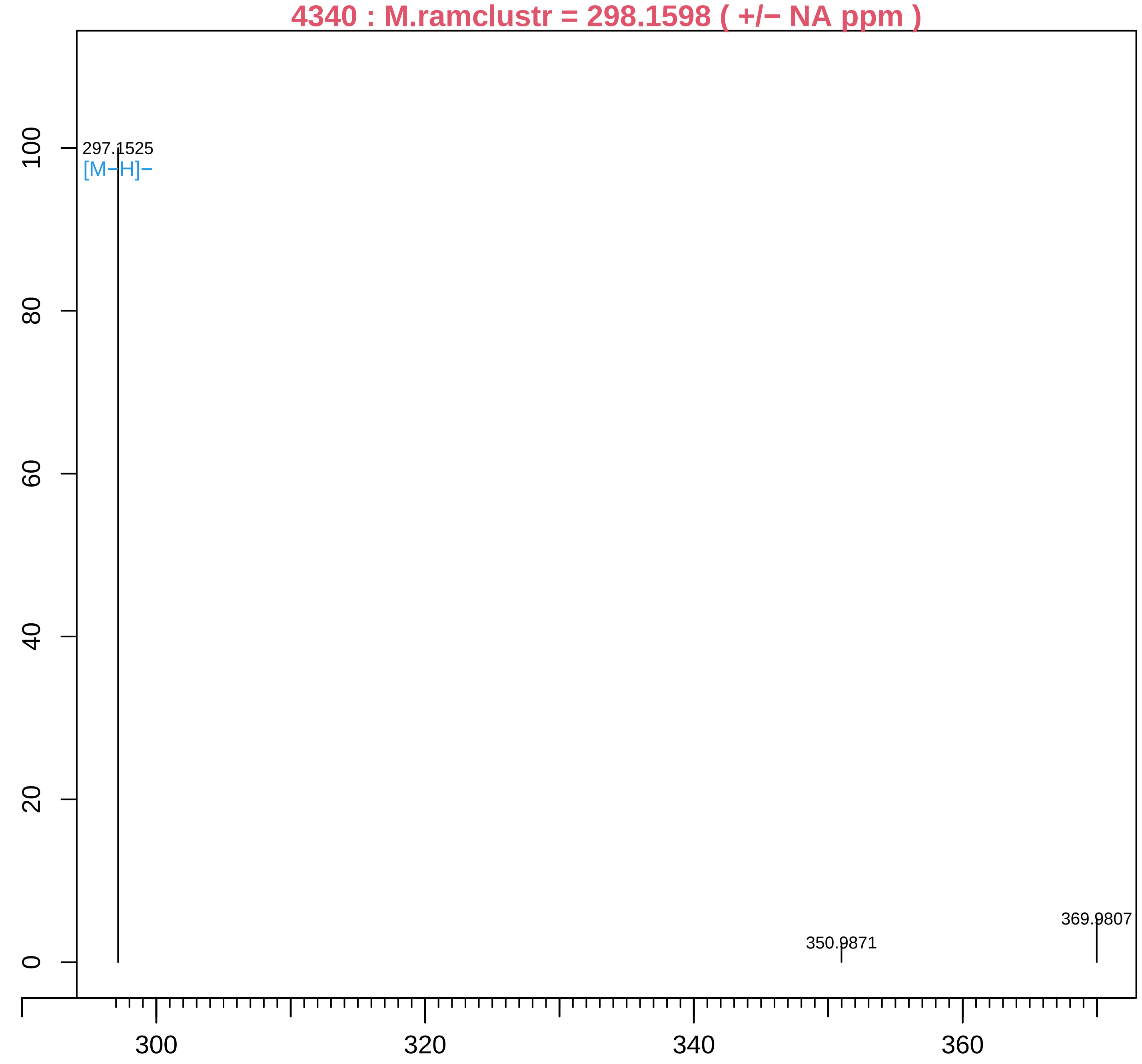
4340 : M.ramclustr = 298.1598 (+/− NA ppm)
297.1525
[M−H]−
350.9871
369.9807
0
20
40
60
80
100
300
320
340
360

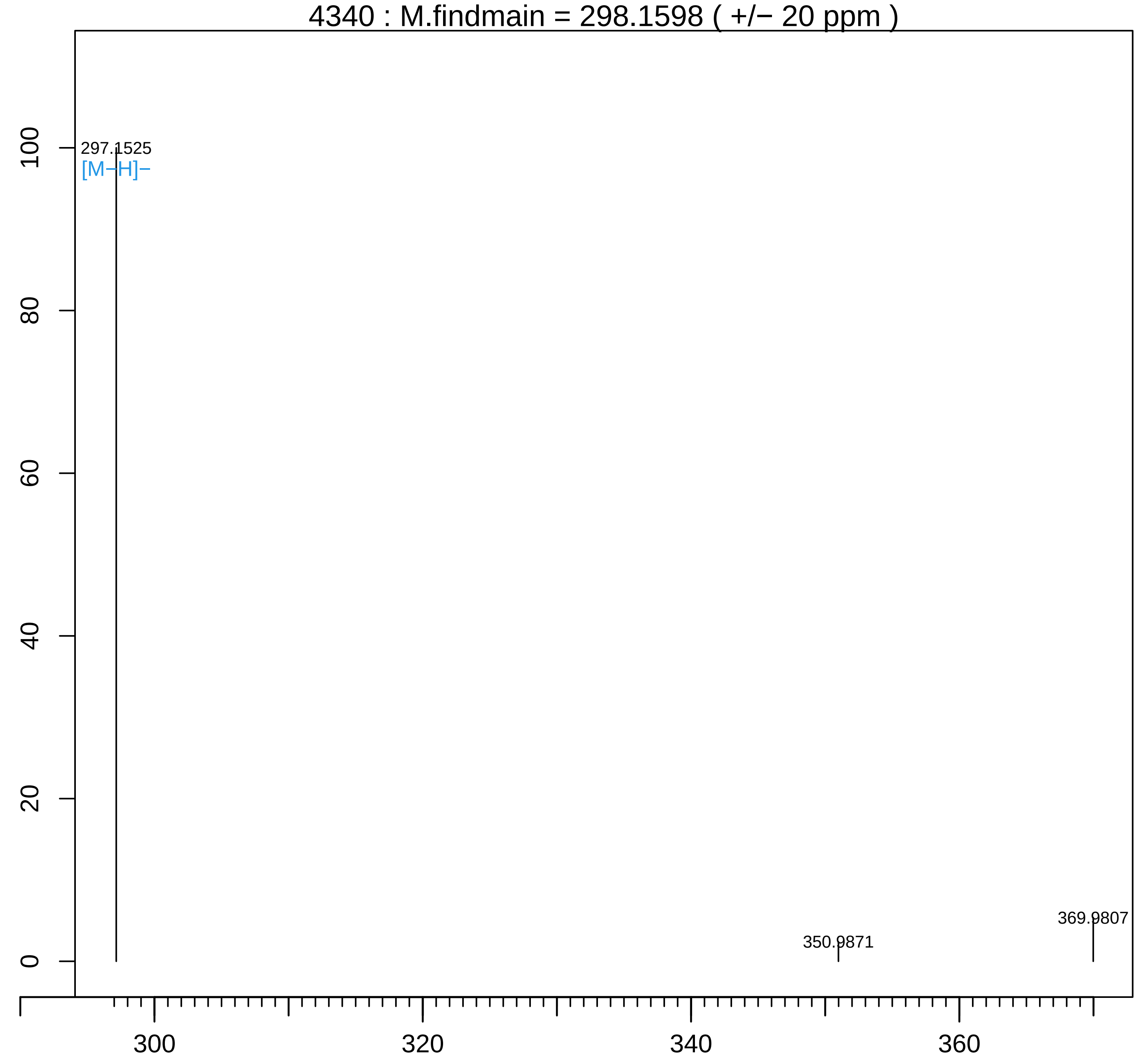
4340 : M.findmain = 298.1598 (+/− 20 ppm)
297.1525
[M−H]−
350.9871
369.9807
0
20
40
60
80
100
300
320
340
360

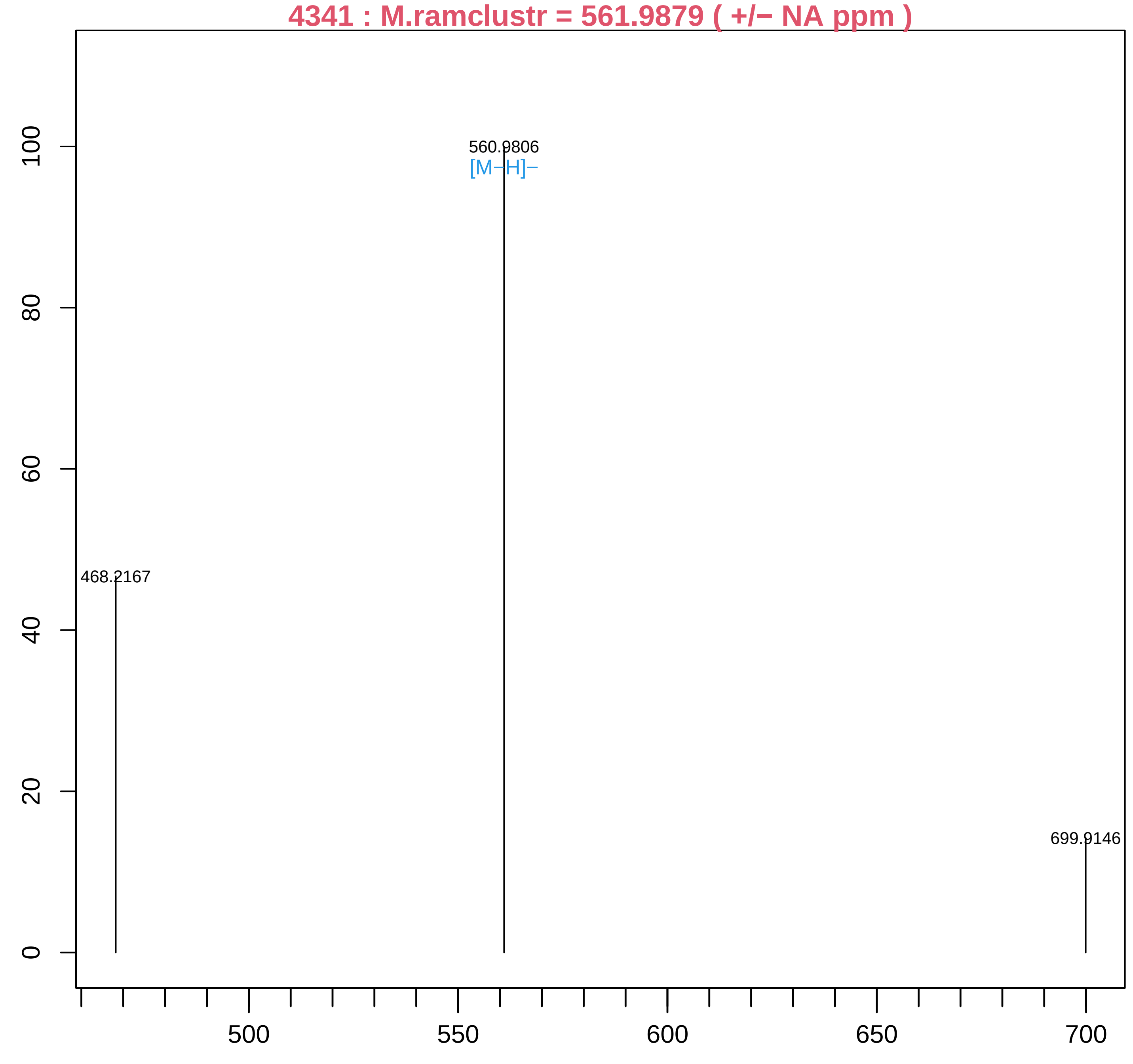
4341 : M.ramclustr = 561.9879 (+/− NA ppm)
560.9806
[M−H]−
468.2167
699.9146
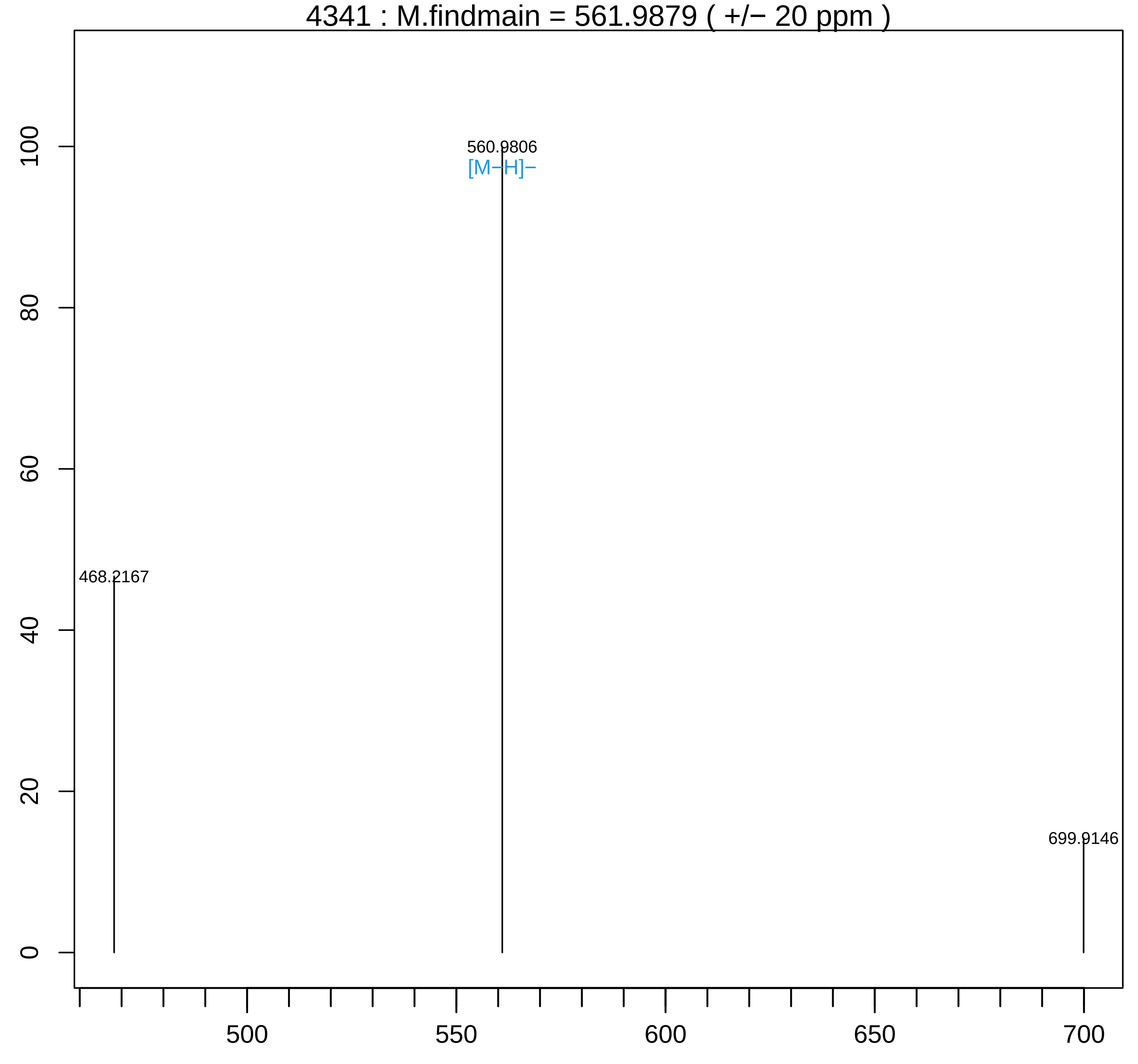
4341 : M.findmain = 561.9879 (+/− 20 ppm)
560.9806
[M−H]−
468.2167
699.9146

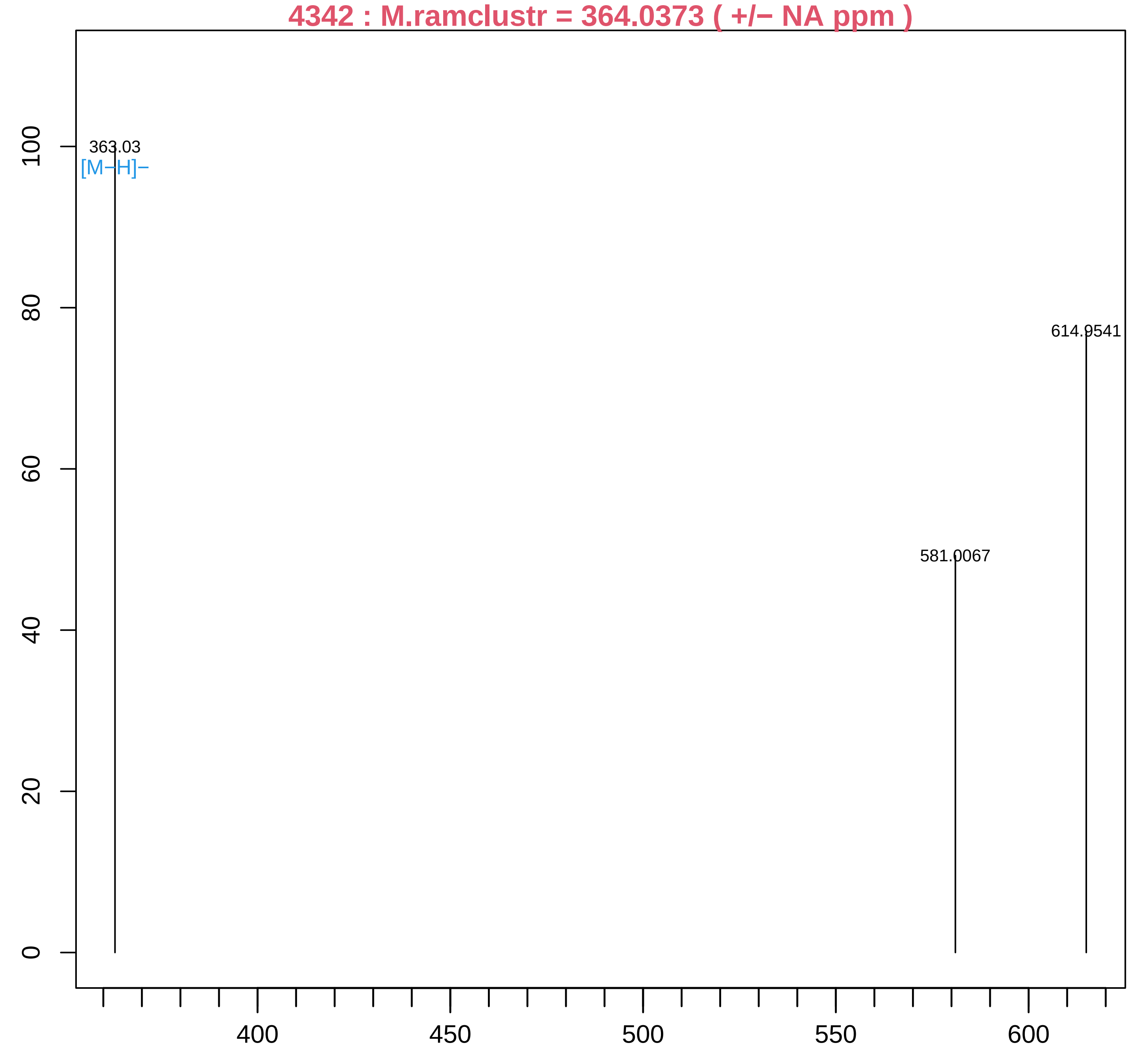

4342 : M.ramclustr = 364.0373 (+/− NA ppm)
363.03
[M−H]−
614.9541
581.0067
0
20
40
60
80
100
400
450
500
550
600

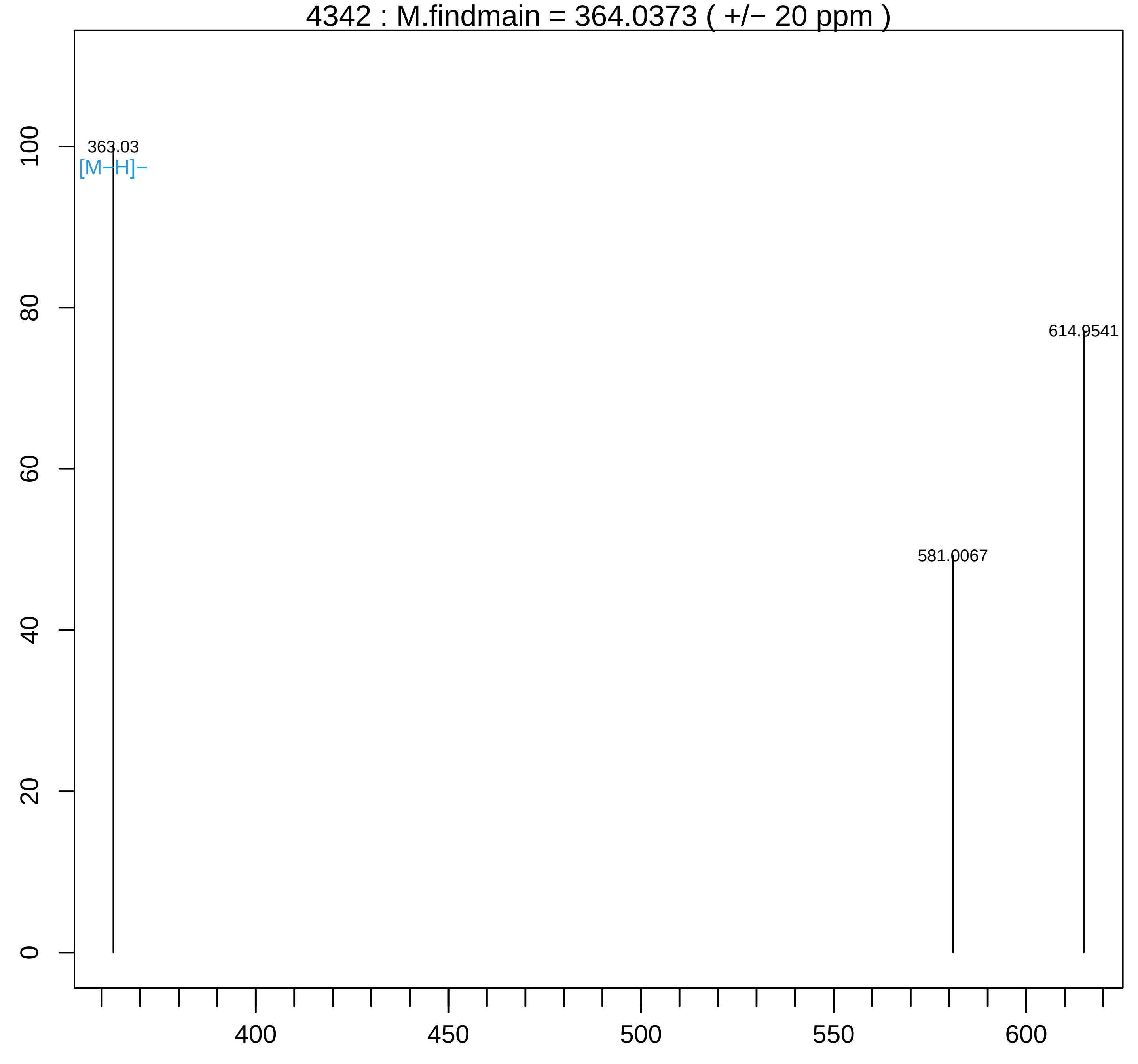

4342 : M.findmain = 364.0373 (+/− 20 ppm)
363.03
[M−H]−
614.9541
581.0067
0
20
40
60
80
100
400
450
500
550
600

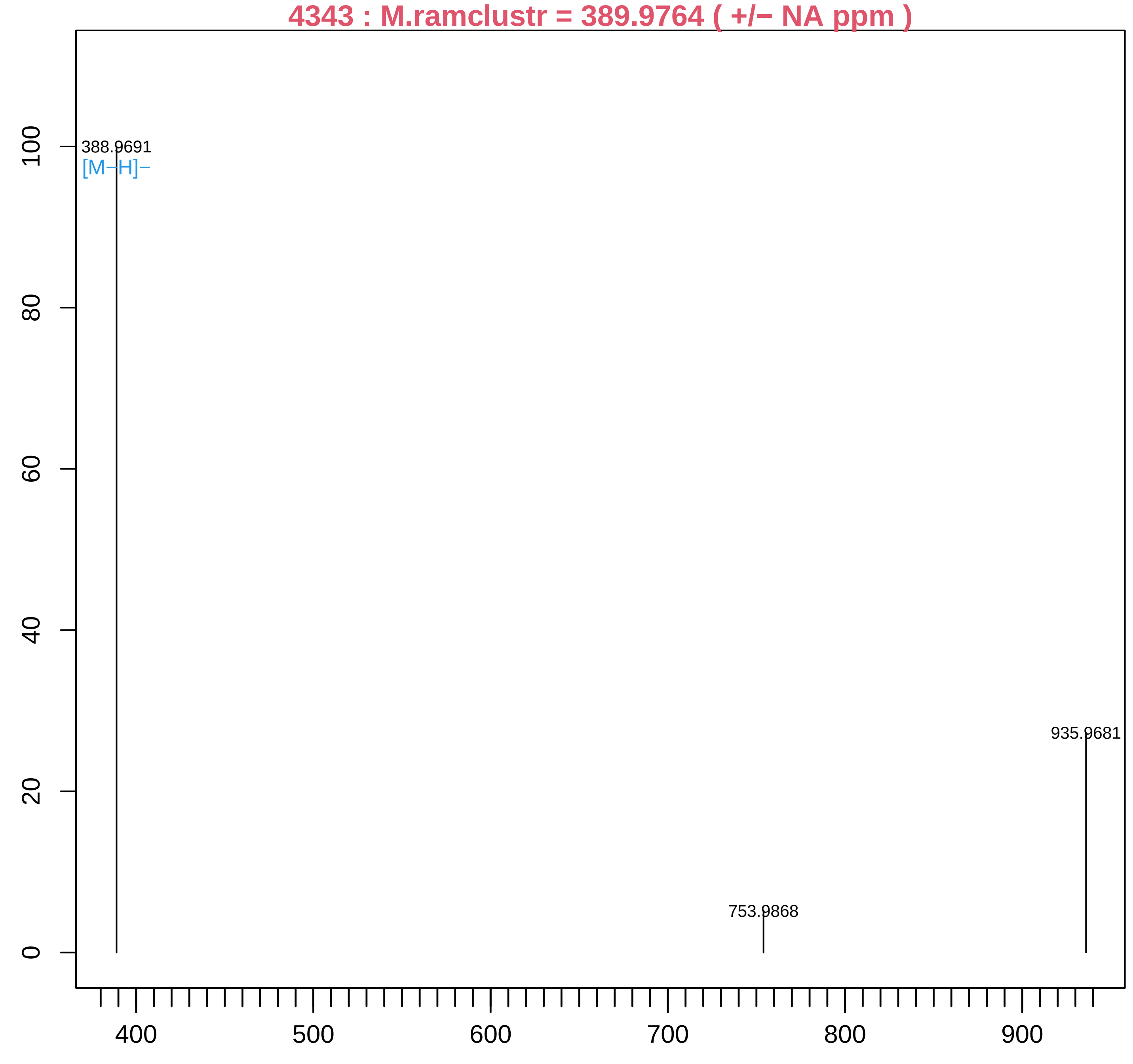
4343 : M.ramclustr = 389.9764 (+/− NA ppm)
388.9691
[M−H]−
753.9868
935.9681
0
20
40
60
80
100
400
500
600
700
800
900

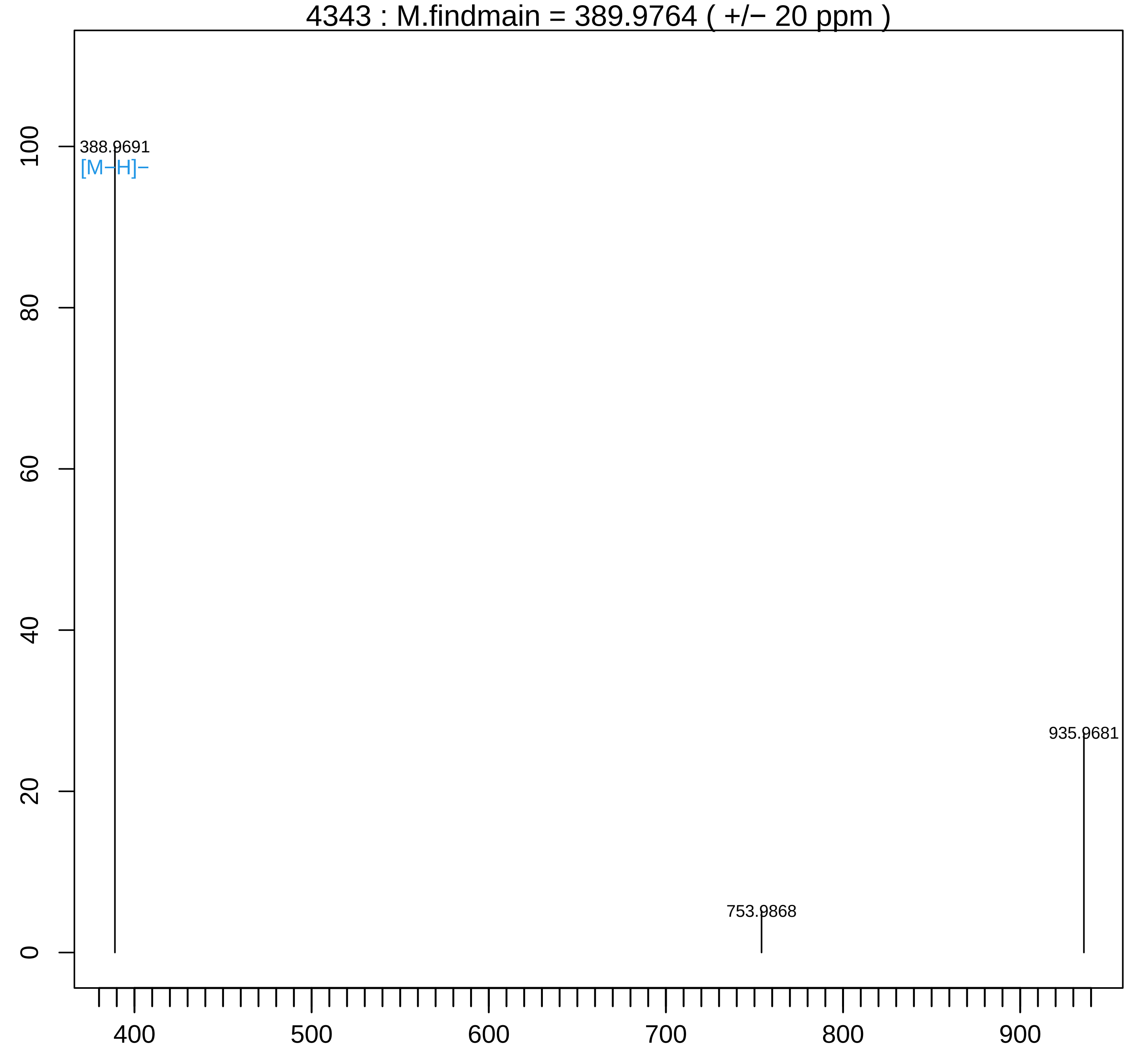
4343 : M.findmain = 389.9764 (+/− 20 ppm)
388.9691
[M−H]−
753.9868
935.9681
0
20
40
60
80
100
400
500
600
700
800
900

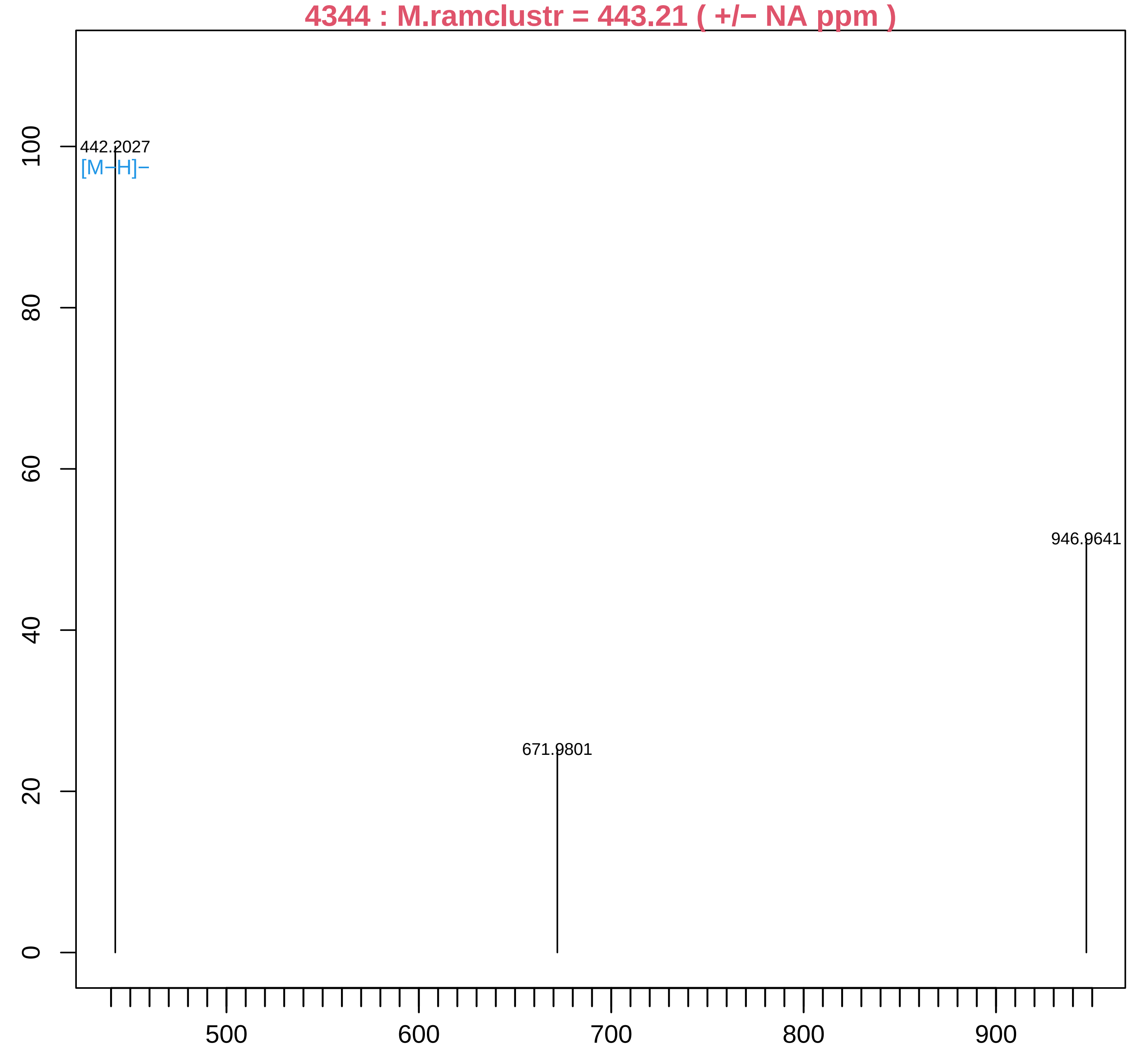

4344 : M.ramclustr = 443.21 (+/− NA ppm)
442.2027
[M−H]−
671.9801
946.9641
0
20
40
60
80
100
500
600
700
800
900

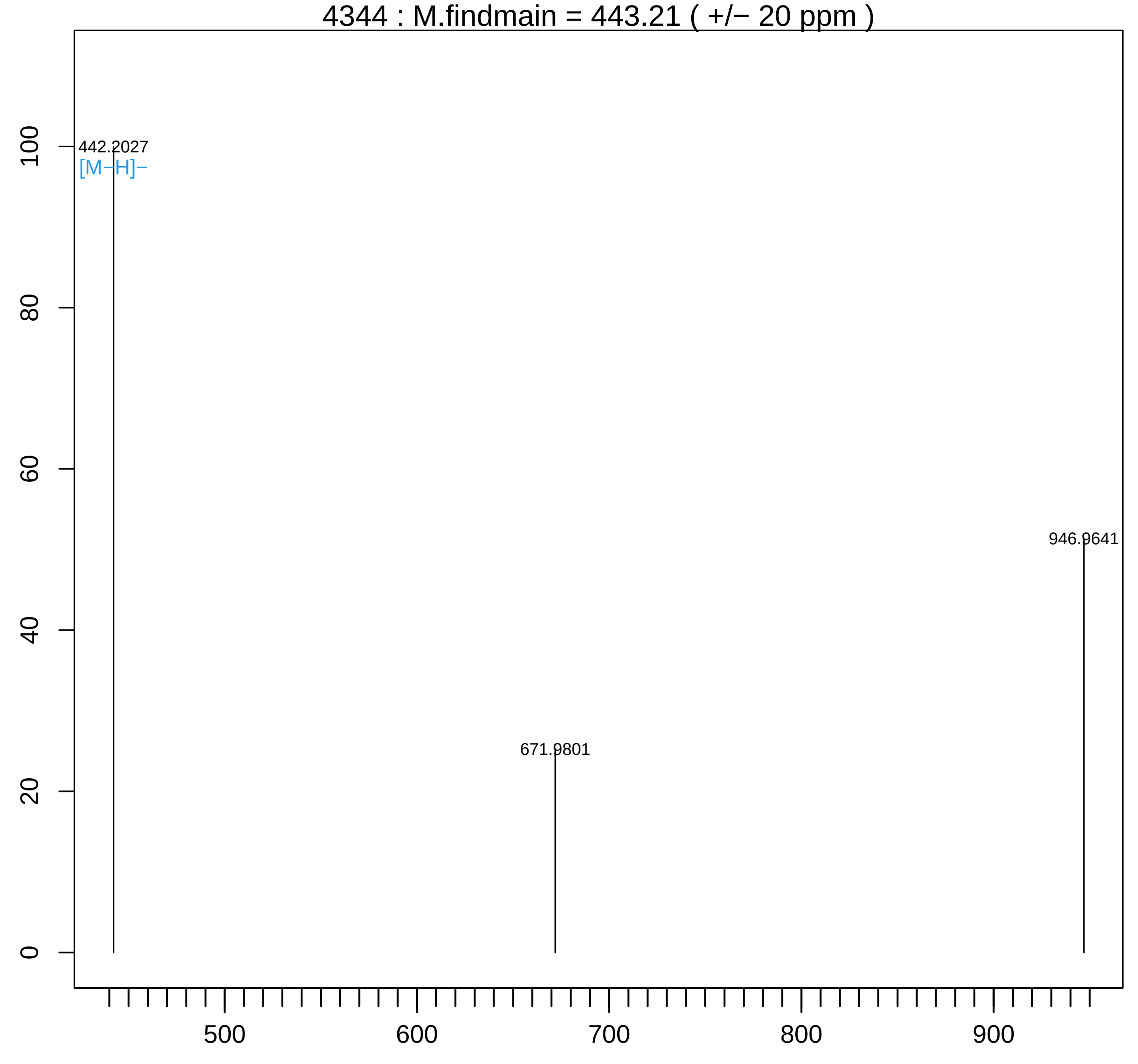

4344 : M.findmain = 443.21 (+/− 20 ppm)
442.2027
[M−H]−
671.9801
946.9641
0
20
40
60
80
100
500
600
700
800
900

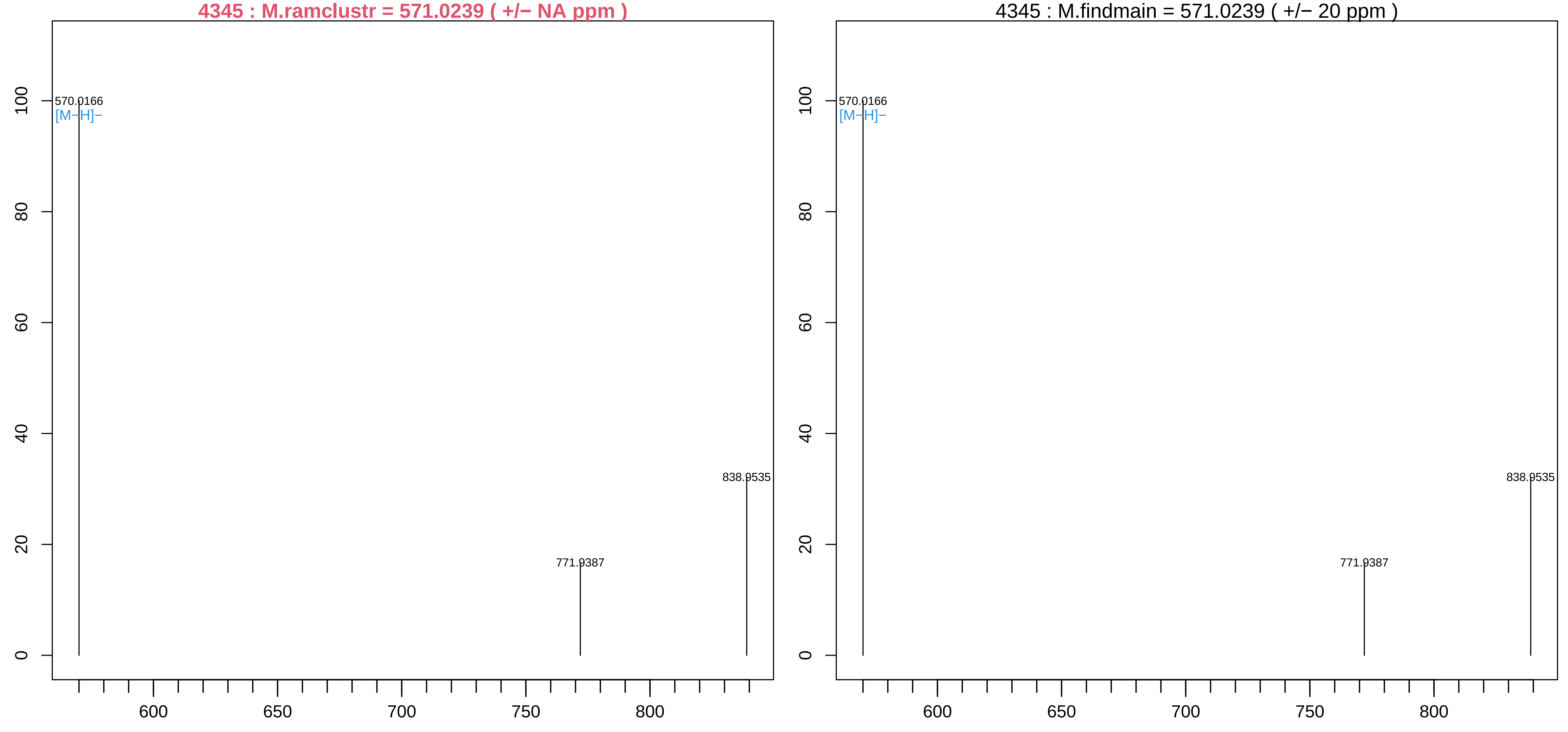
4345 : M.ramclustr = 571.0239 (+/− NA ppm)
570.0166
[M−H]−
771.9387
838.9535
0
20
40
60
80
100
600
650
700
750
800
4345 : M.findmain = 571.0239 (+/− 20 ppm)
570.0166
[M−H]−
771.9387
838.9535
0
20
40
60
80
100
600
650
700
750
800

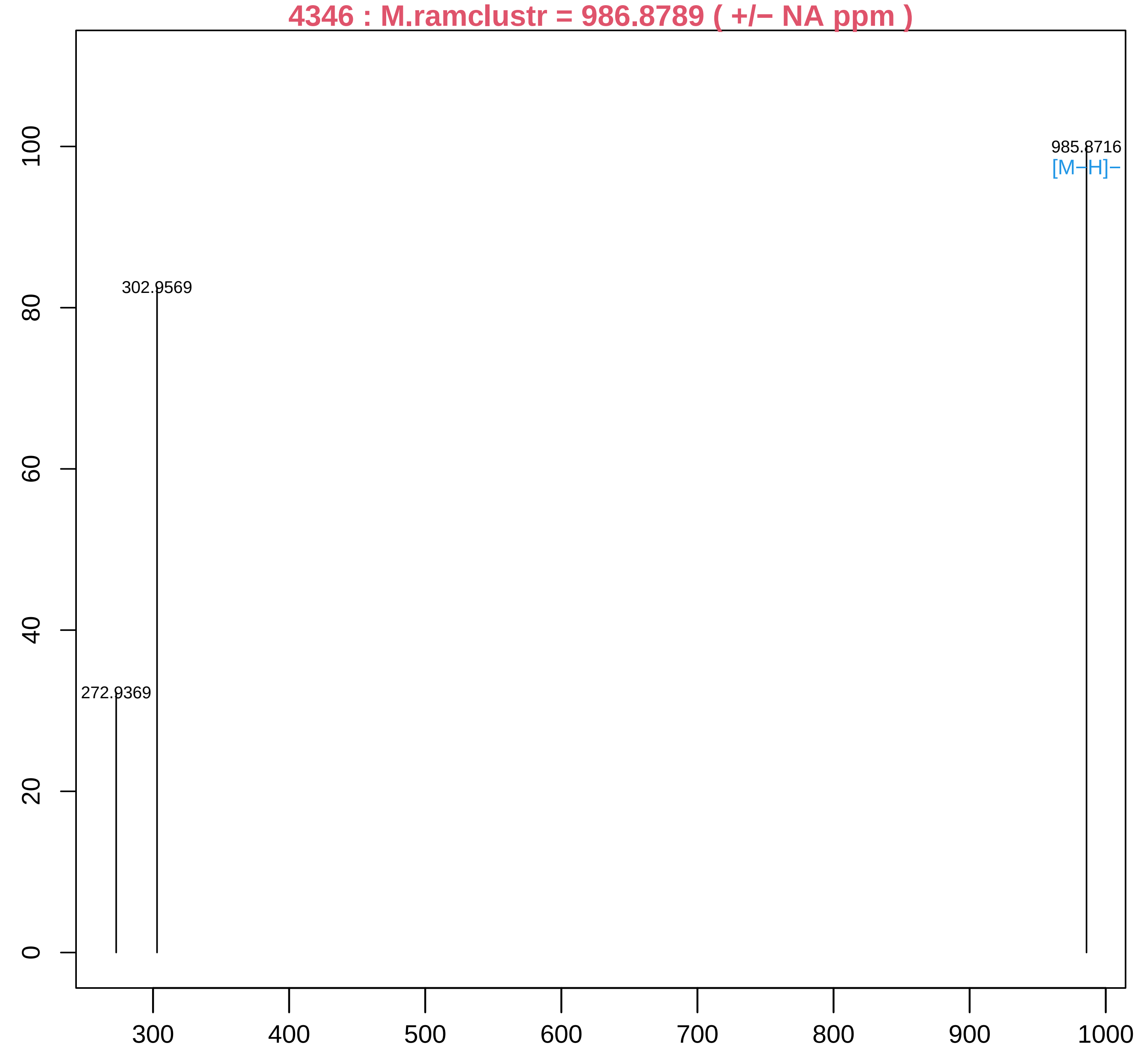
4346 : M.ramclustr = 986.8789 (+/− NA ppm)
985.8716
[M−H]−
302.9569
272.9369
0
20
40
60
80
100
300
400
500
600
700
800
900
1000

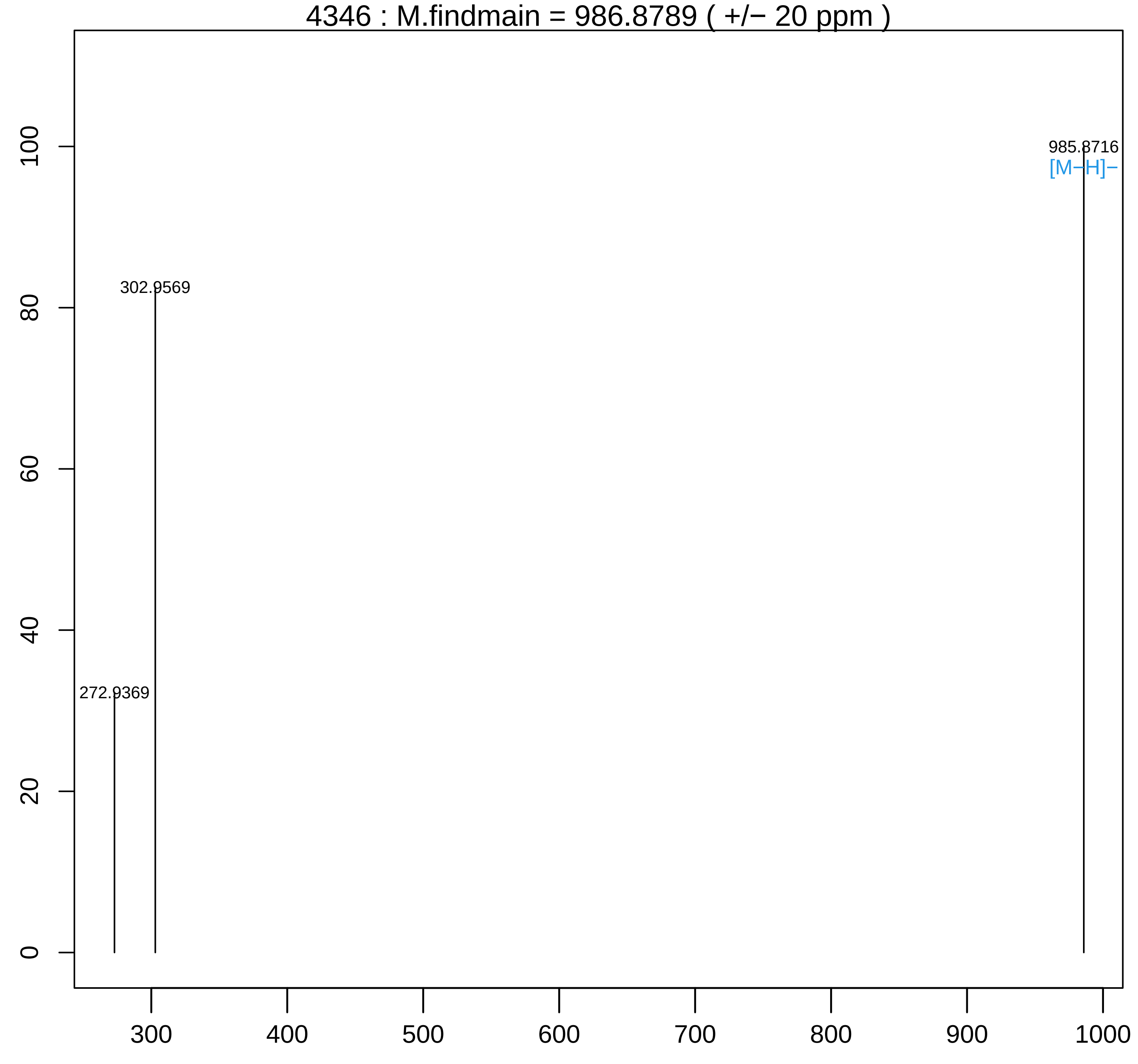
4346 : M.findmain = 986.8789 (+/− 20 ppm)
985.8716
[M−H]−
302.9569
272.9369
0
20
40
60
80
100
300
400
500
600
700
800
900
1000

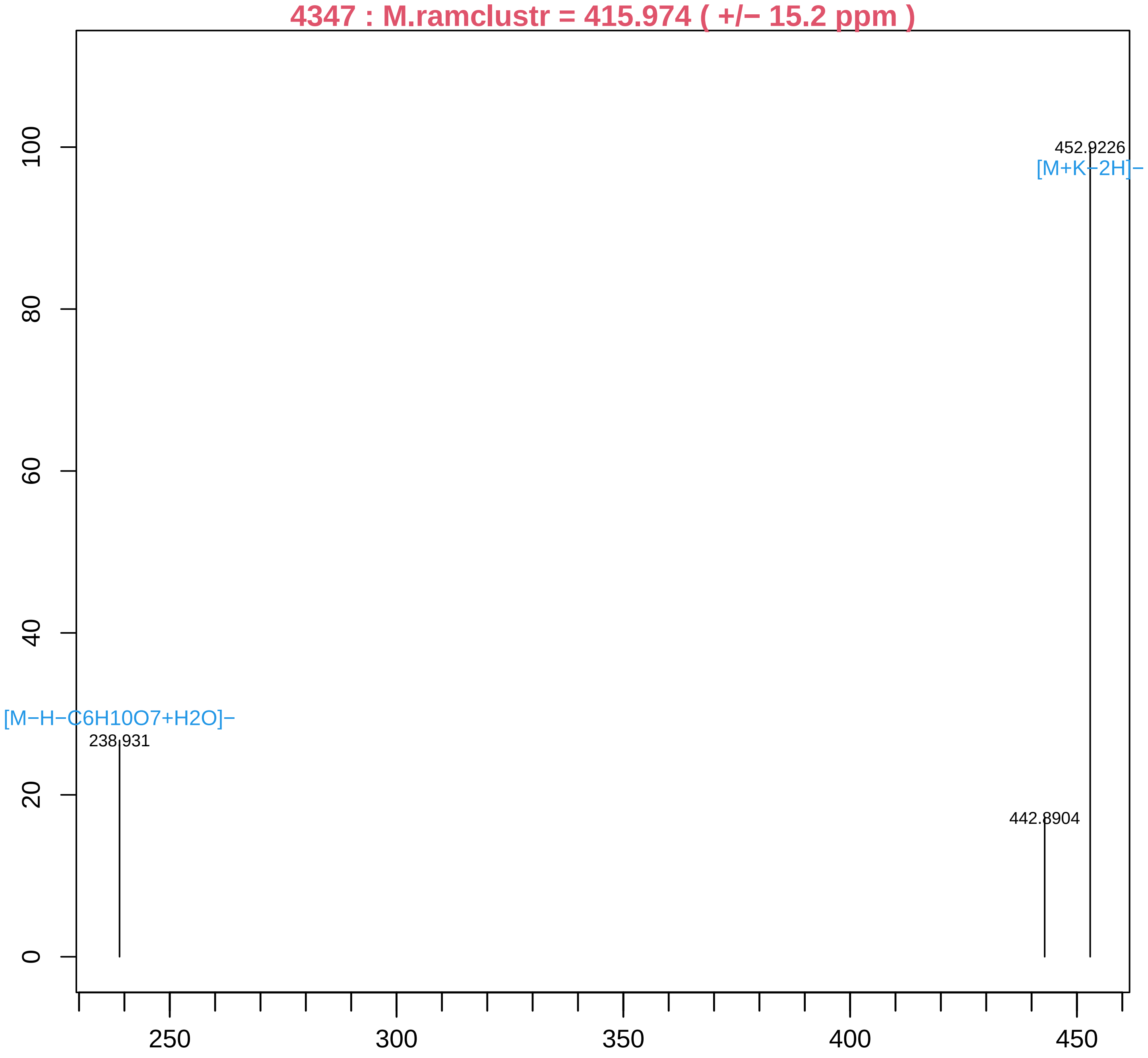

4347 : M.ramclustr = 415.974 (+/− 15.2 ppm)
452.9226
[M+K−2H]−
[M−H−C6H10O7+H2O]−
238.931
442.8904
0
20
40
60
80
100
250
300
350
400
450

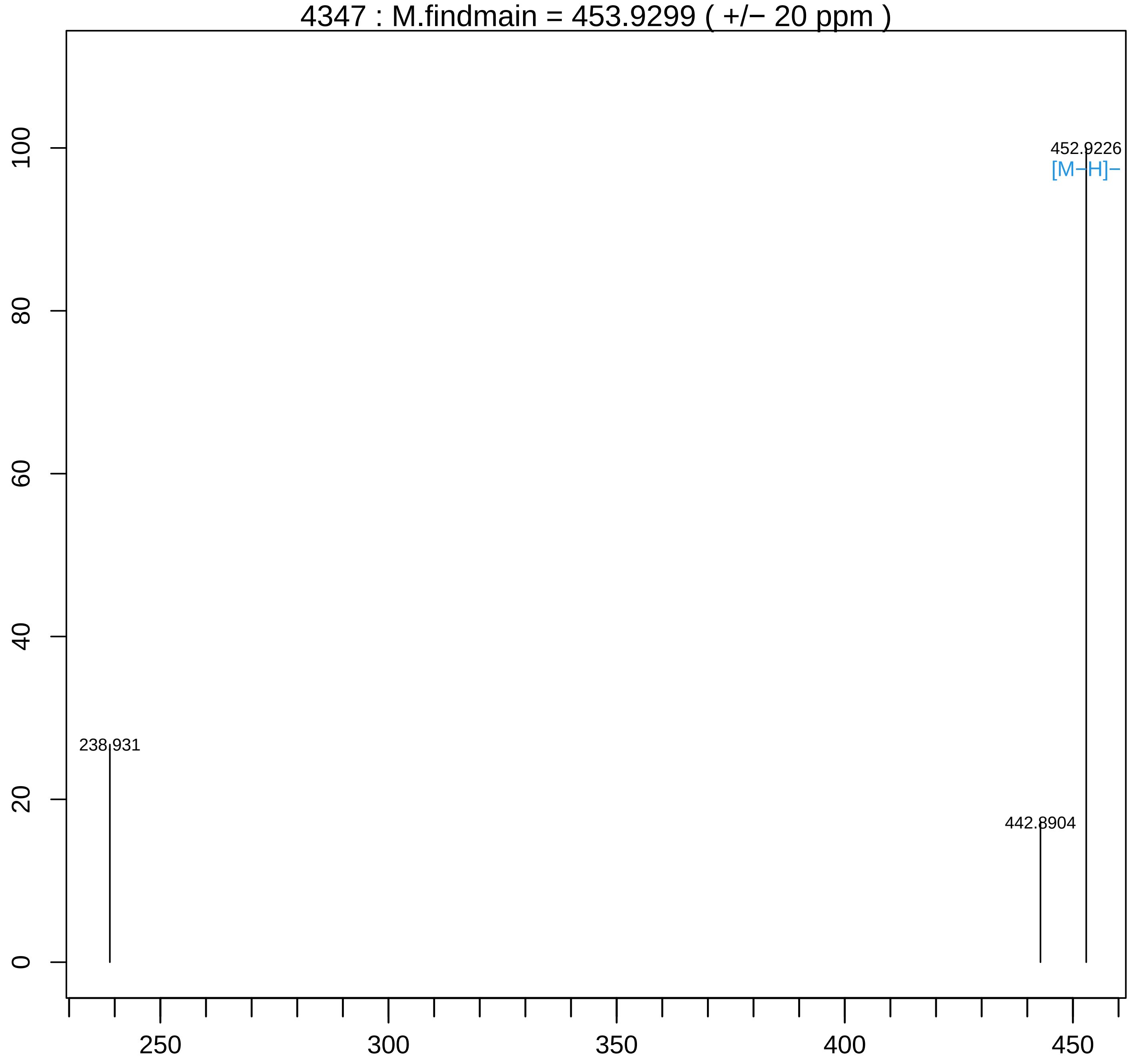

4347 : M.findmain = 453.9299 (+/− 20 ppm)
452.9226
[M−H]−
238.931
442.8904
0
20
40
60
80
100
250
300
350
400
450

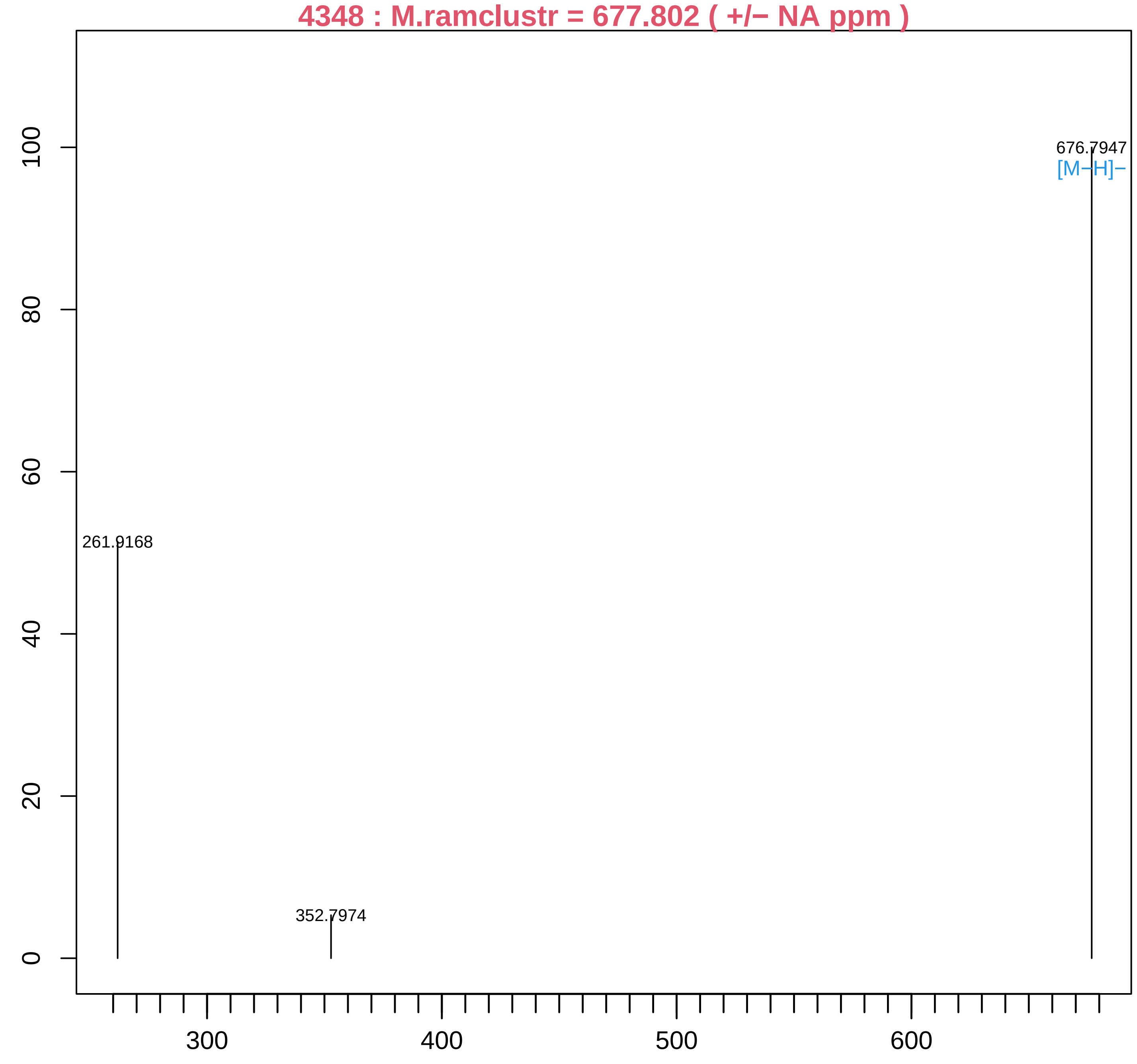
4348 : M.ramclustr = 677.802 (+/− NA ppm)
676.7947
[M−H]−
261.9168
352.7974
0
20
40
60
80
100
300
400
500
600

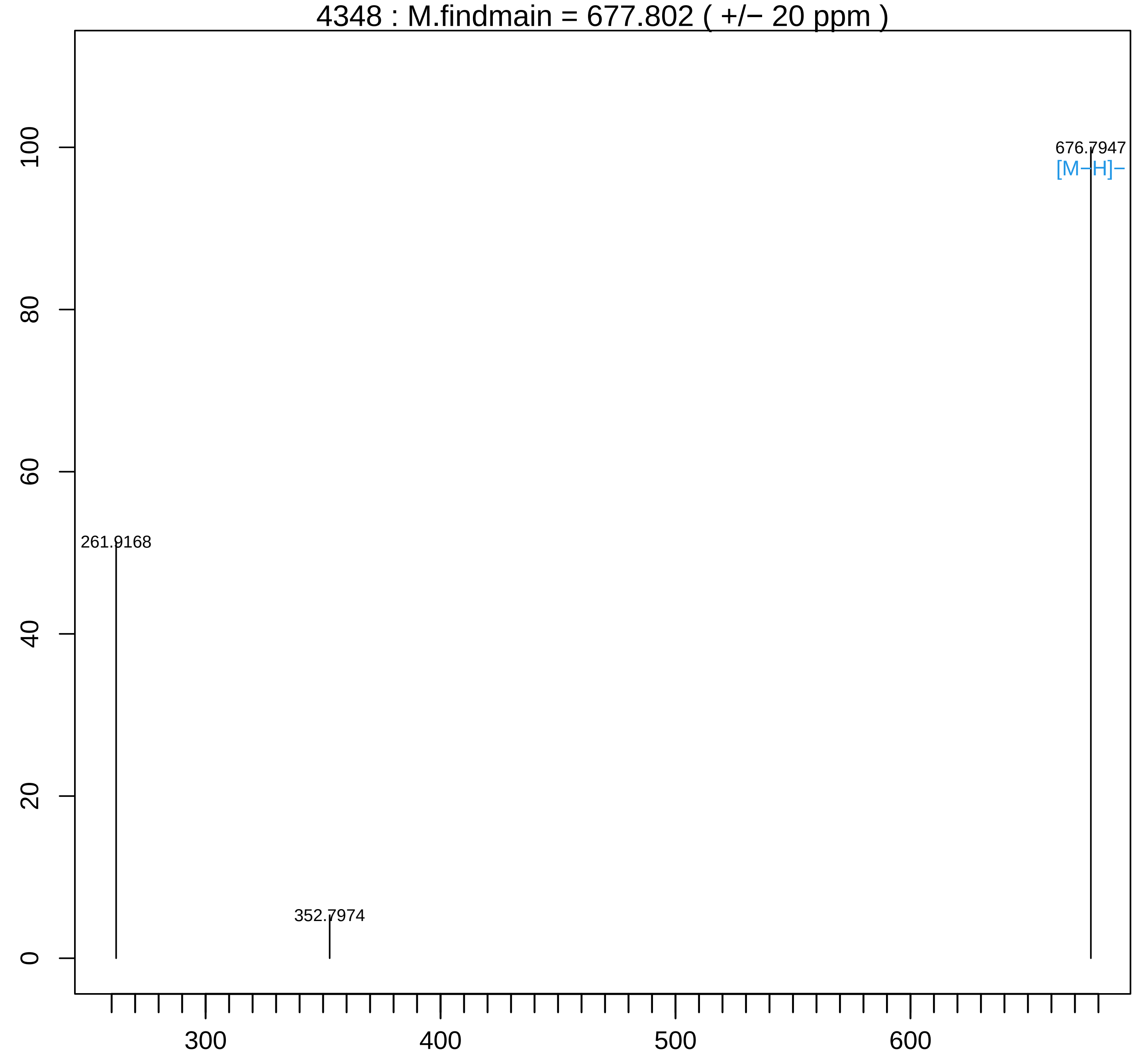
4348 : M.findmain = 677.802 (+/− 20 ppm)
676.7947
[M−H]−
261.9168
352.7974
0
20
40
60
80
100
300
400
500
600

4349 : M.ramclustr = 576.1307 (+/− NA ppm)

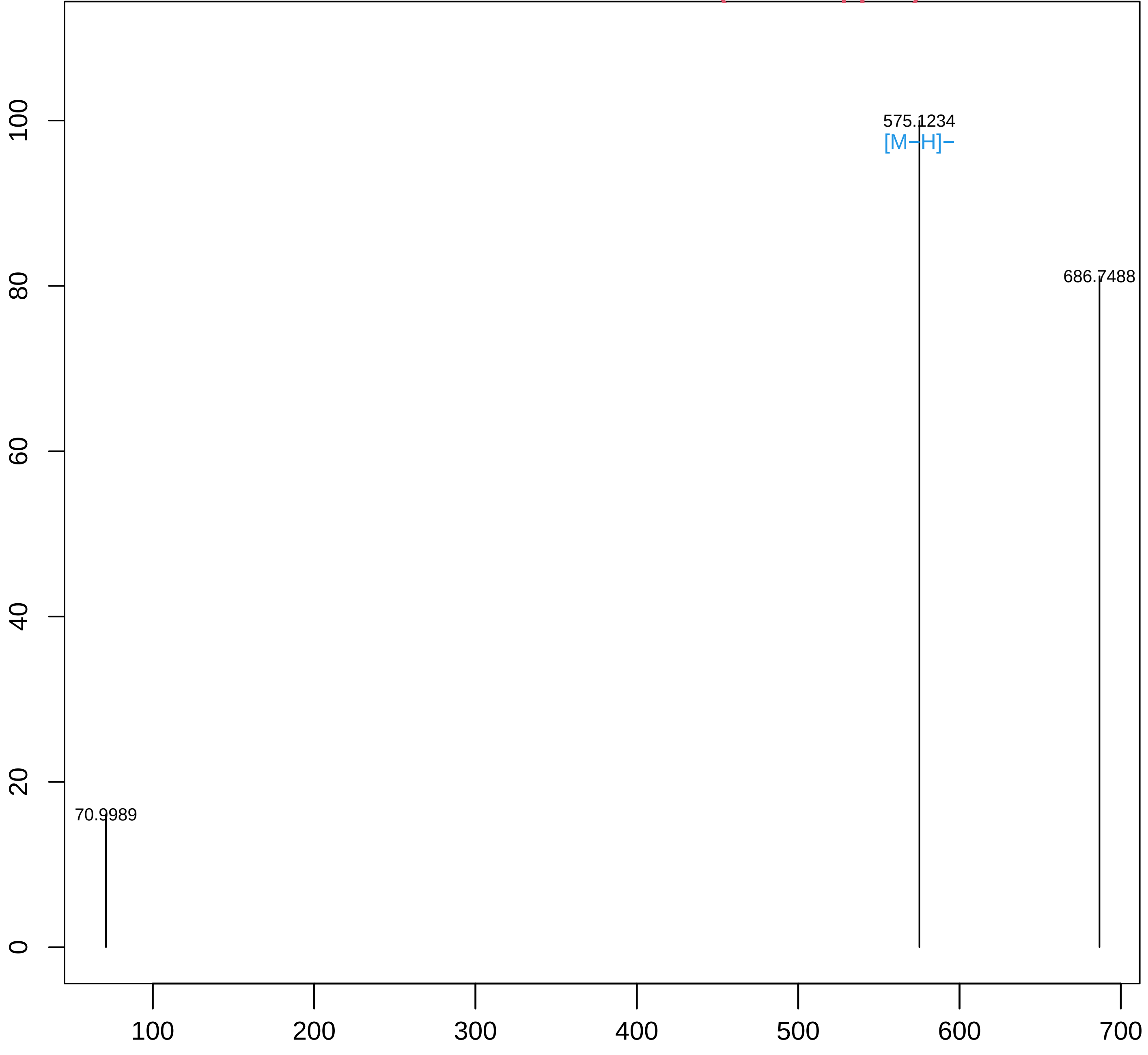

4349 : M.findmain = 576.1307 (+/− 20 ppm)

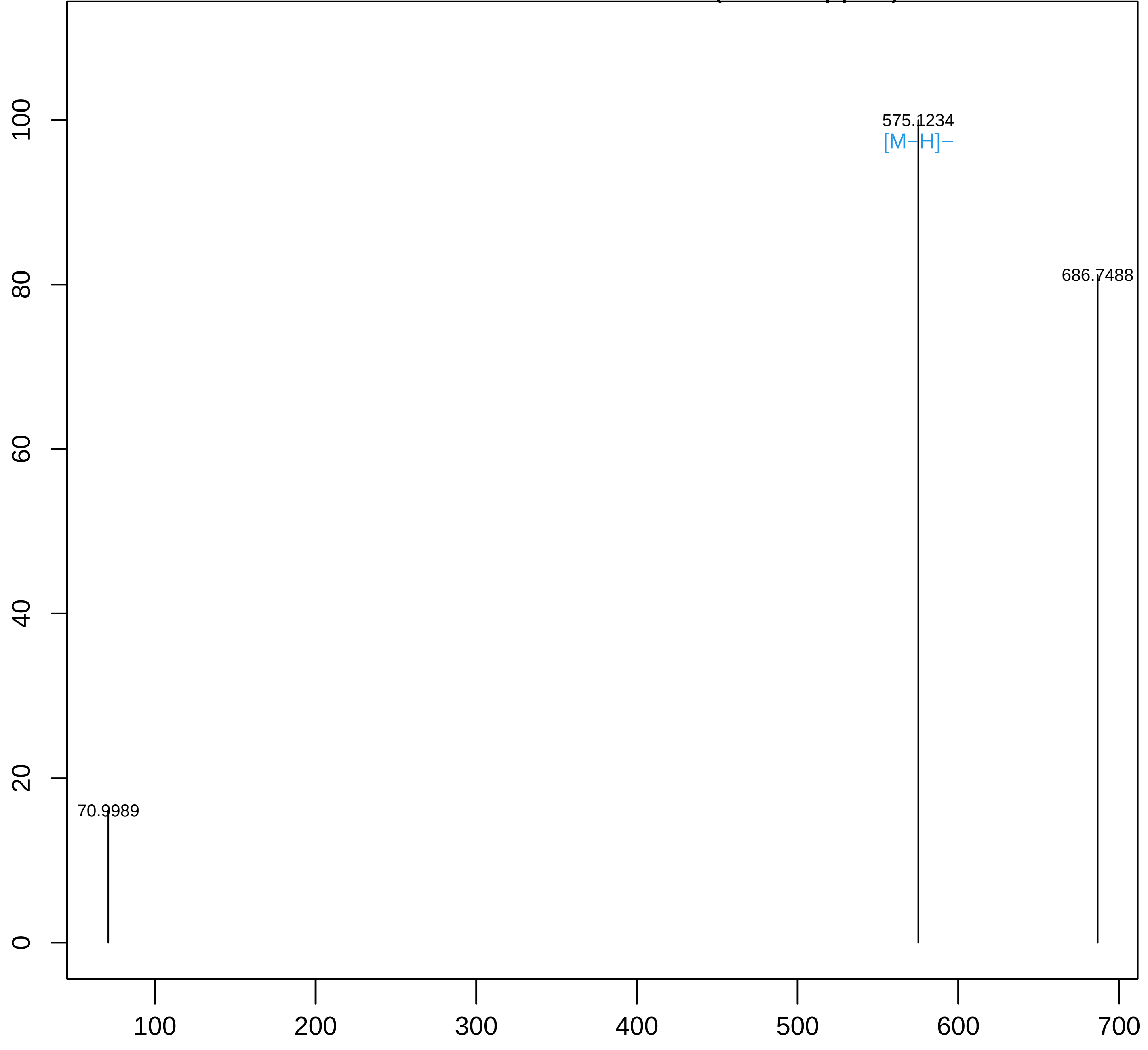

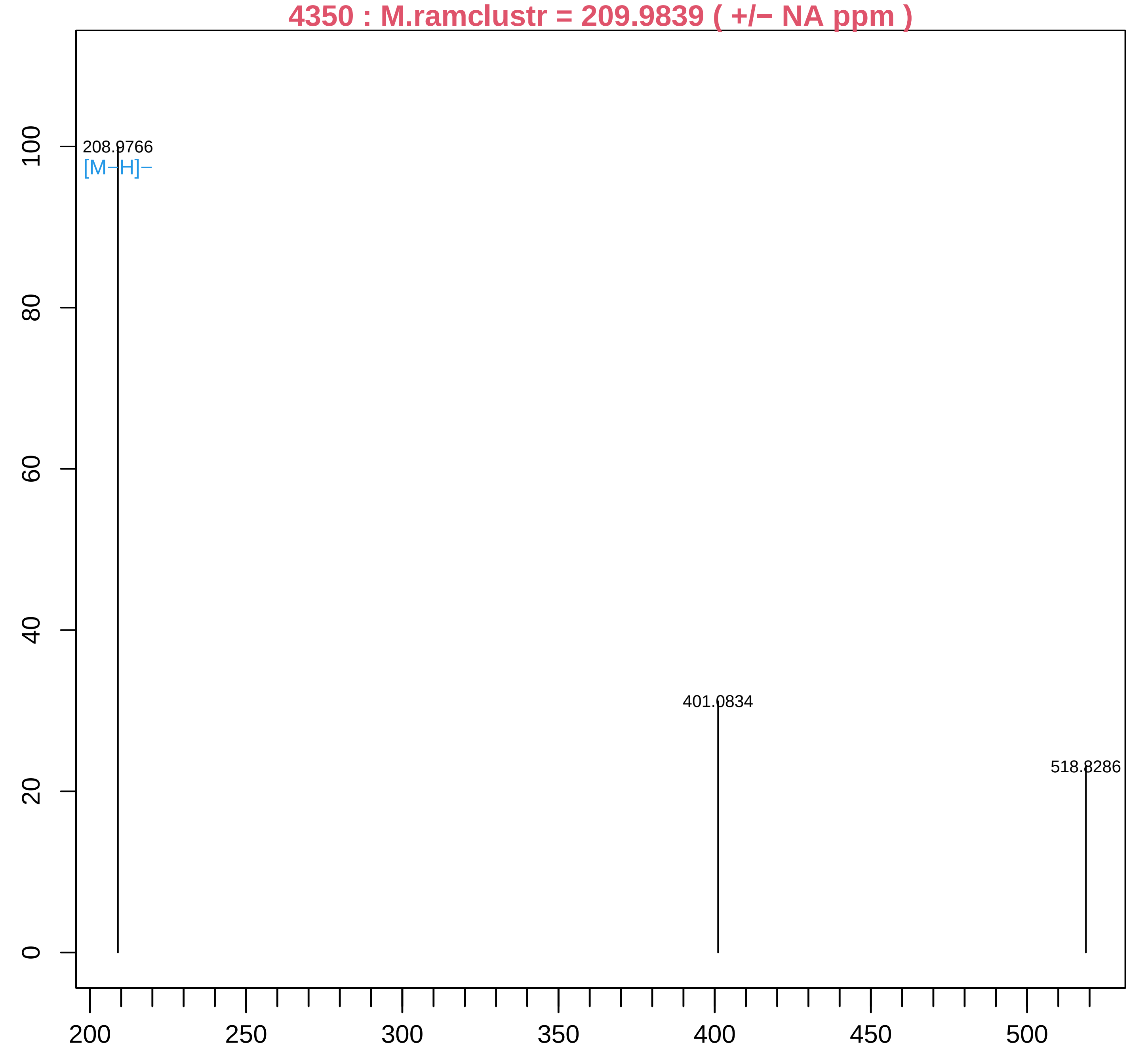
4350 : M.ramclustr = 209.9839 (+/− NA ppm)
208.9766
[M−H]−
401.0834
518.8286
0
20
40
60
80
100
200
250
300
350
400
450
500

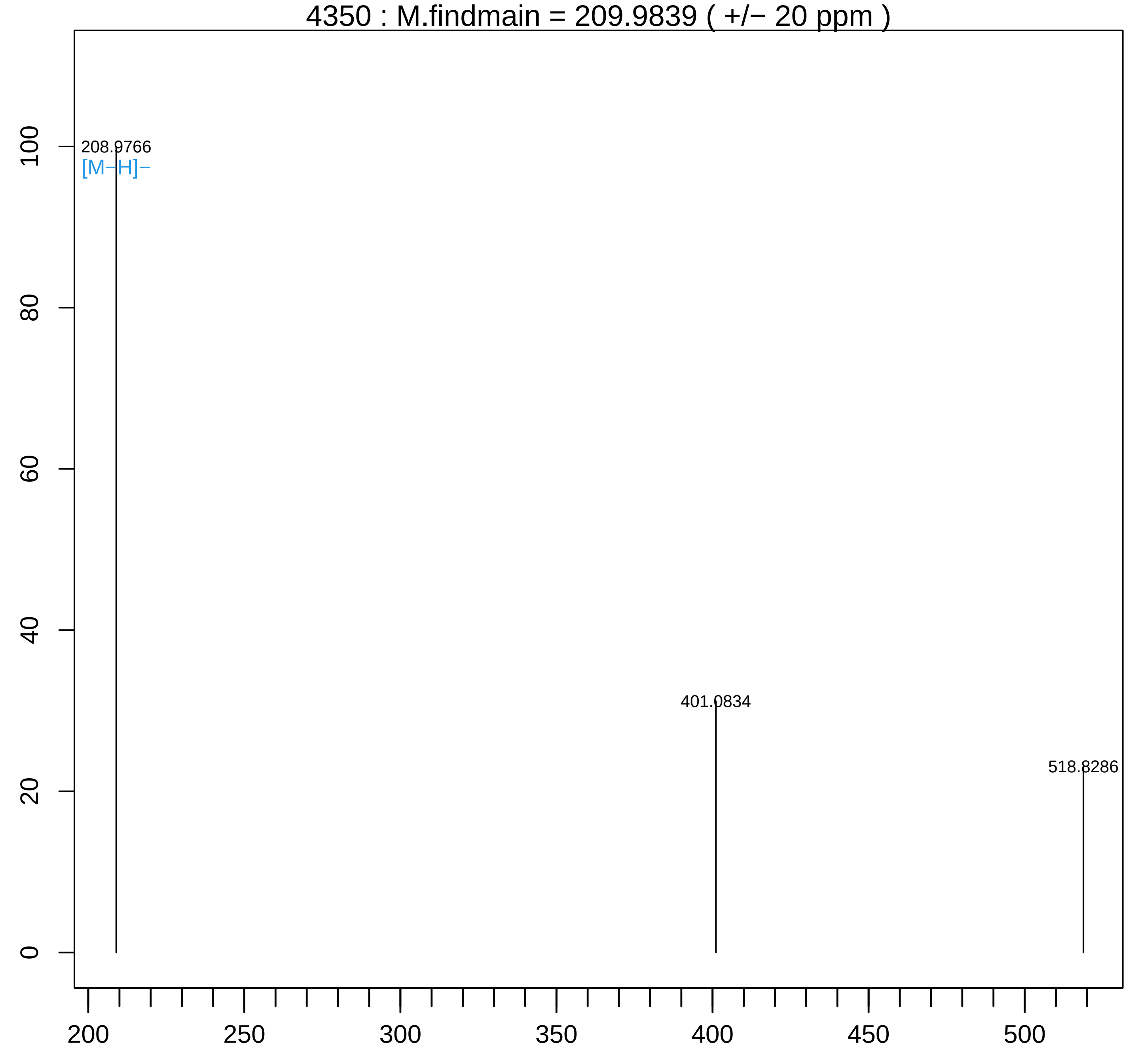
4350 : M.findmain = 209.9839 (+/− 20 ppm)
208.9766
[M−H]−
401.0834
518.8286
0
20
40
60
80
100
200
250
300
350
400
450
500

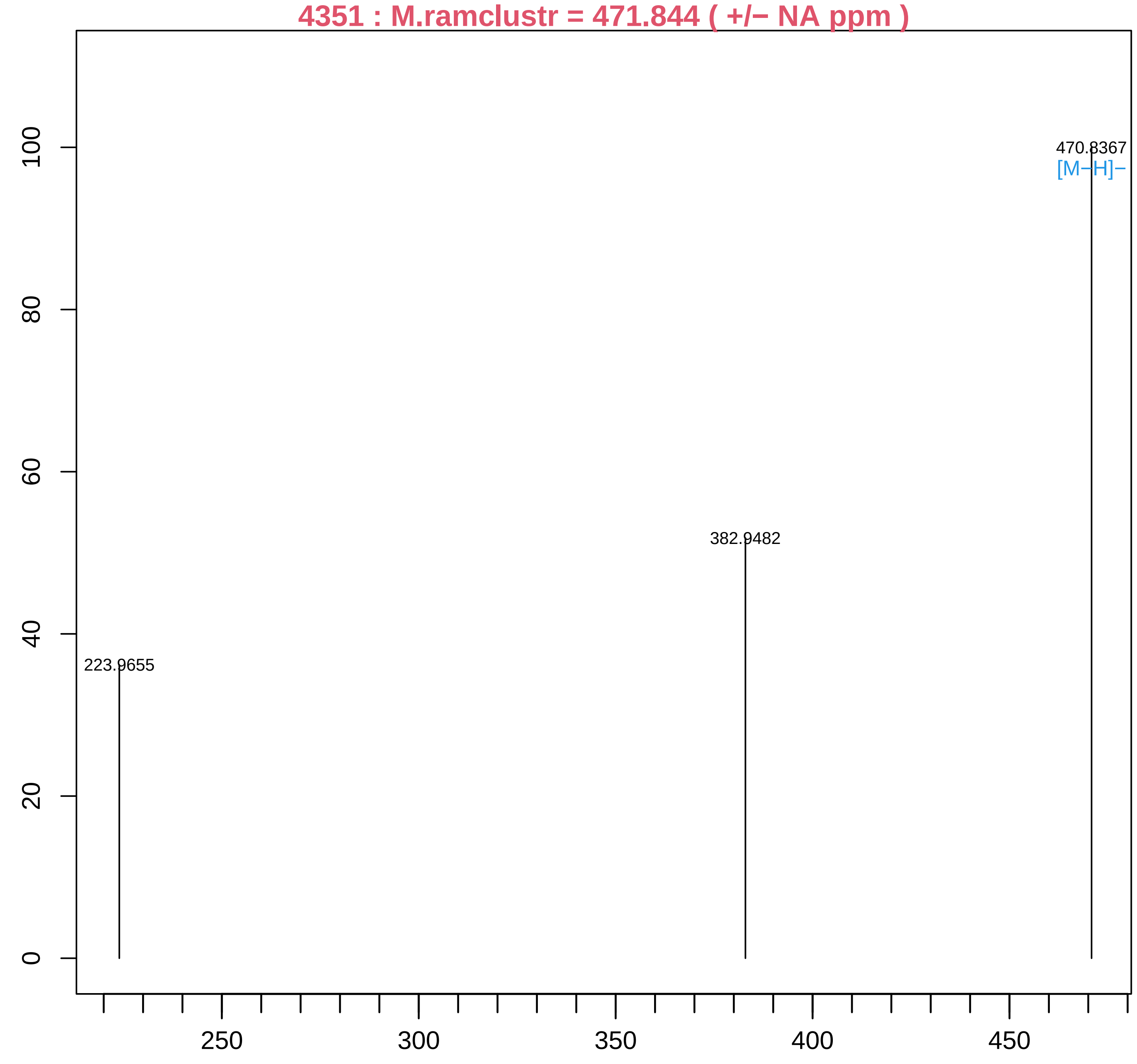
4351 : M.ramclustr = 471.844 (+/− NA ppm)
470.8367
[M−H]−
382.9482
223.9655
0
20
40
60
80
100
250
300
350
400
450

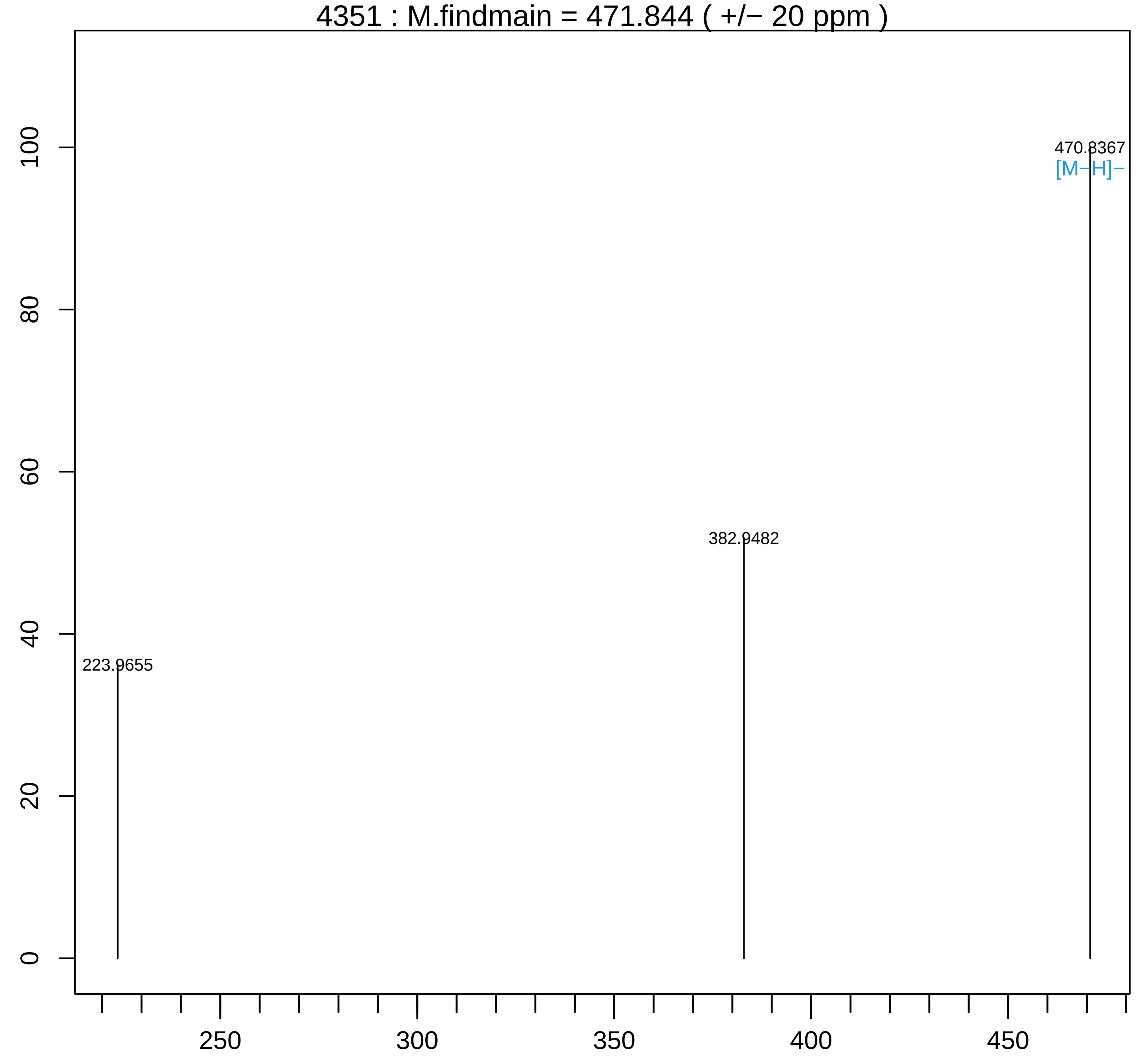
4351 : M.findmain = 471.844 (+/− 20 ppm)
470.8367
[M−H]−
382.9482
223.9655
0
20
40
60
80
100
250
300
350
400
450

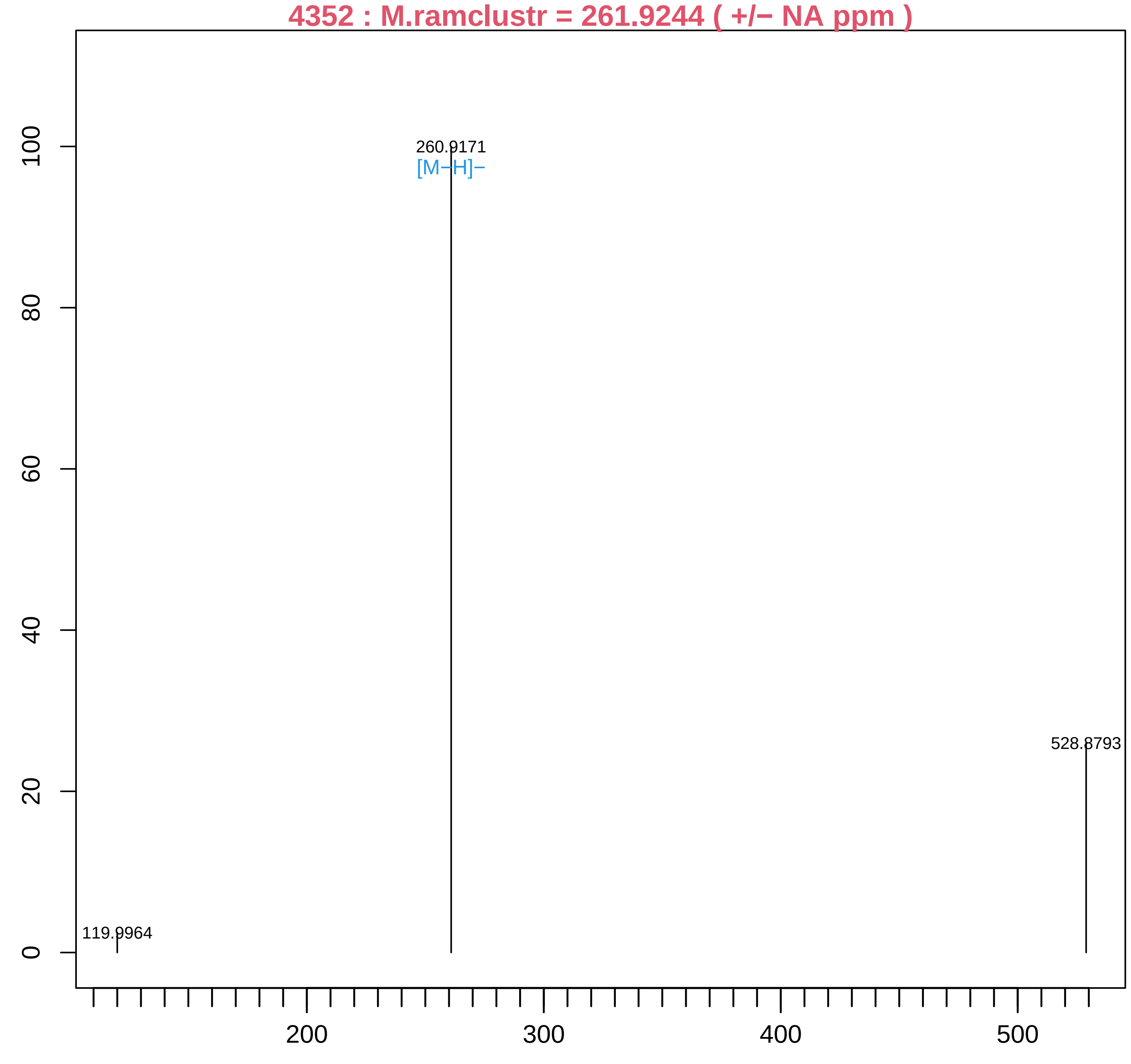
4352 : M.ramclustr = 261.9244 (+/− NA ppm)
260.9171
[M−H]−
528.8793
119.9964
0
20
40
60
80
100
200
300
400
500

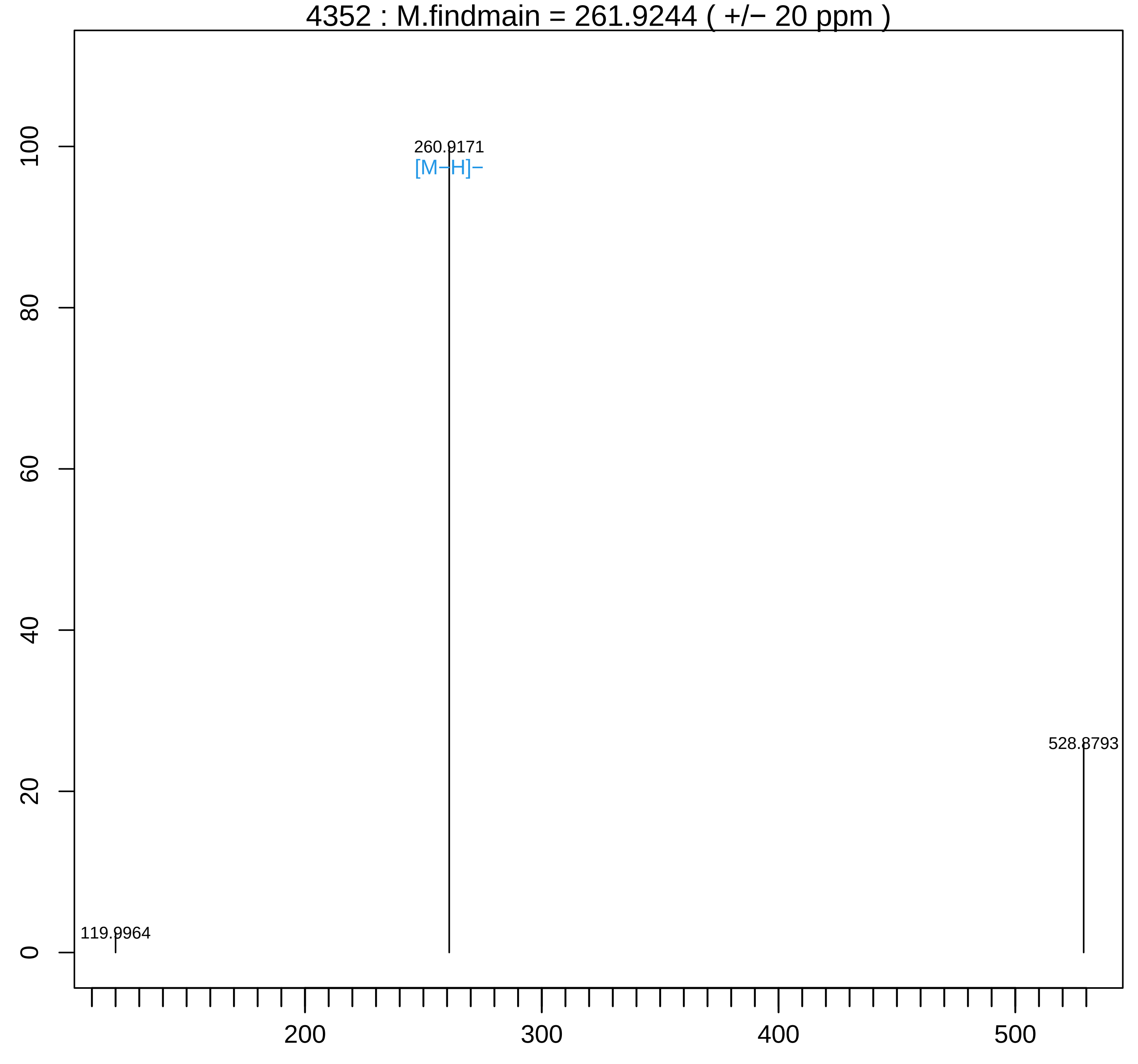
4352 : M.findmain = 261.9244 (+/− 20 ppm)
260.9171
[M−H]−
528.8793
119.9964
0
20
40
60
80
100
200
300
400
500

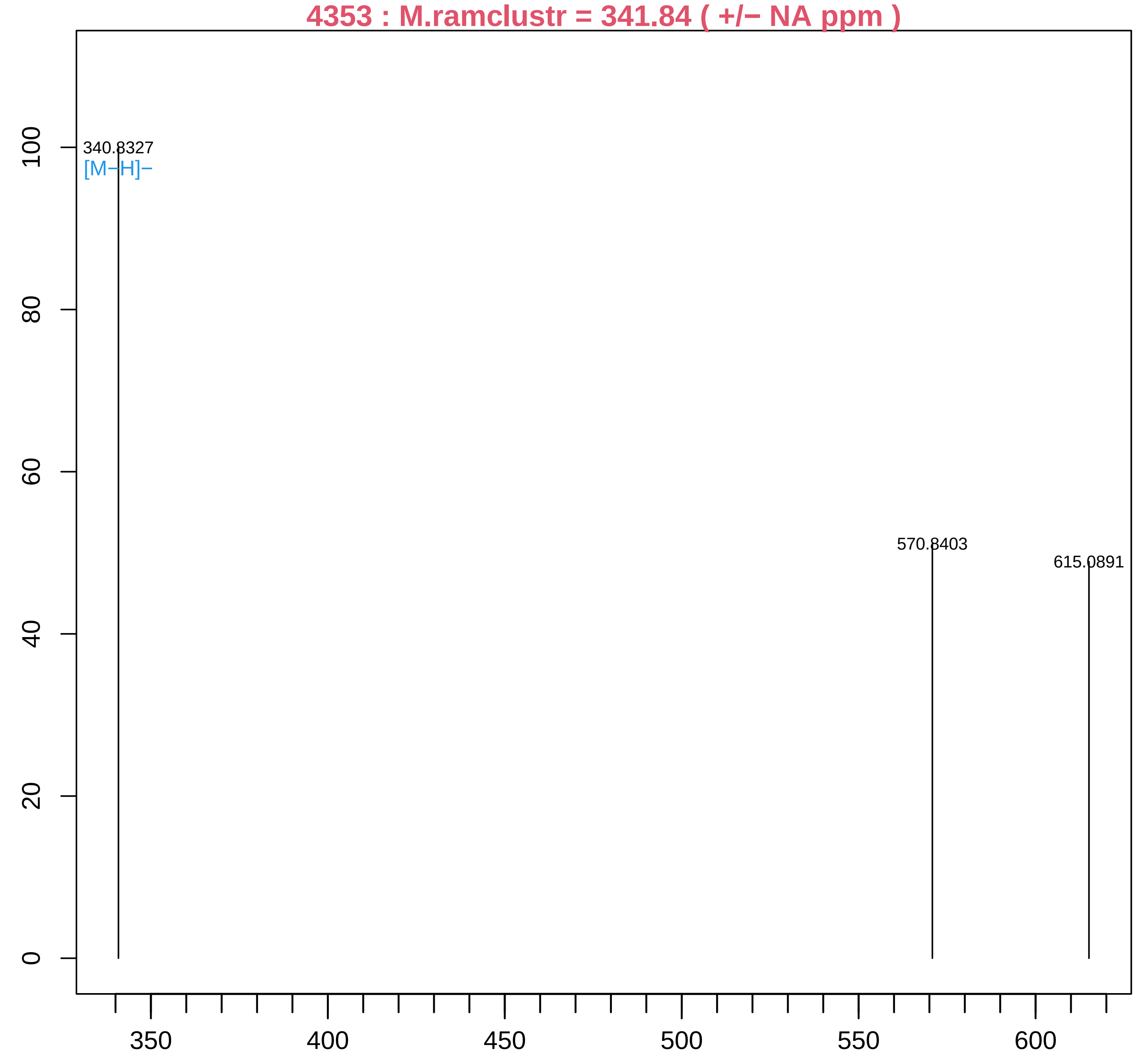

4353 : M.ramclustr = 341.84 (+/− NA ppm)
340.8327
[M−H]−
570.8403
615.0891
0
20
40
60
80
100
350
400
450
500
550
600

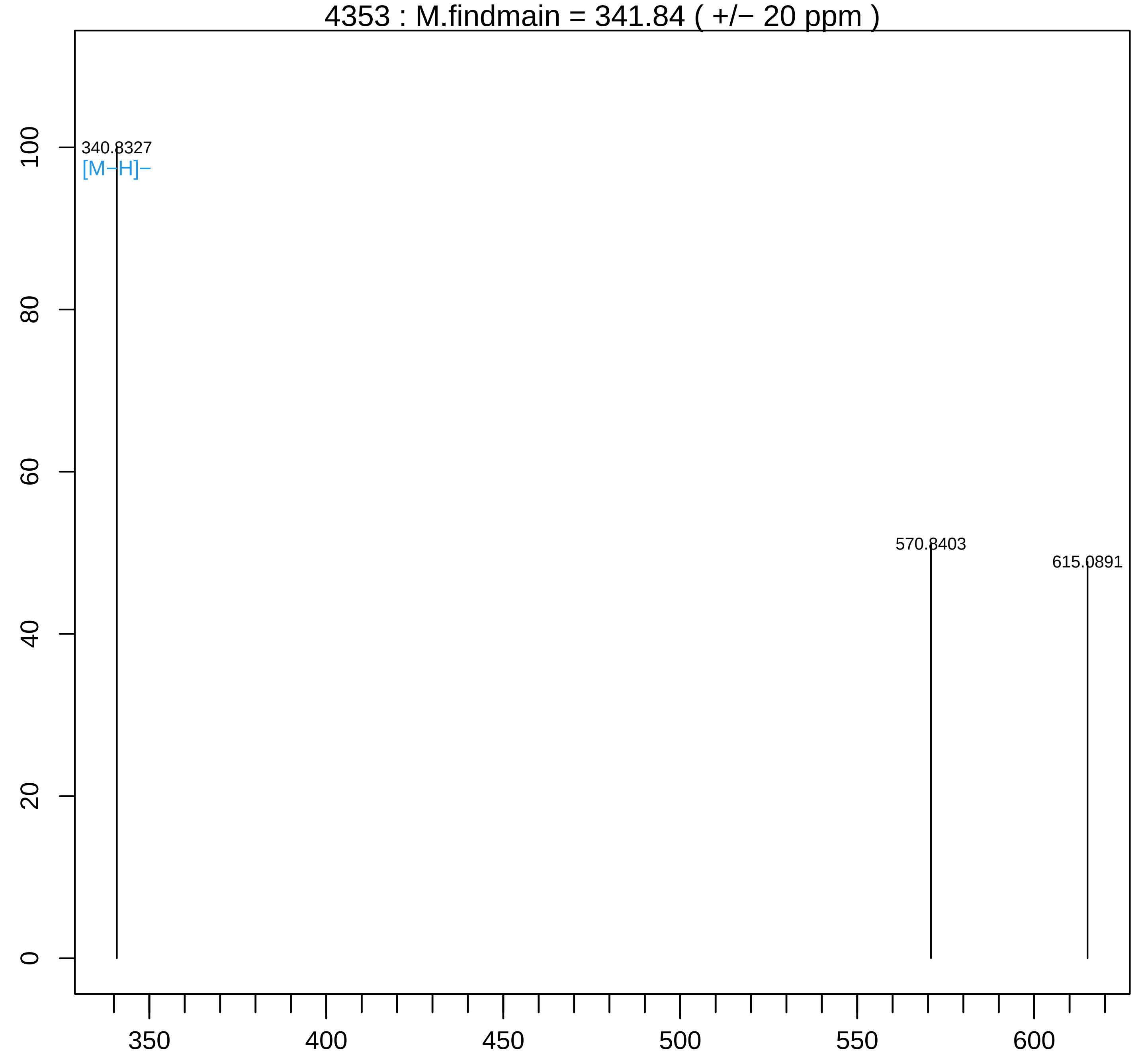

4353 : M.findmain = 341.84 (+/− 20 ppm)
340.8327
[M−H]−
570.8403
615.0891
0
20
40
60
80
100
350
400
450
500
550
600

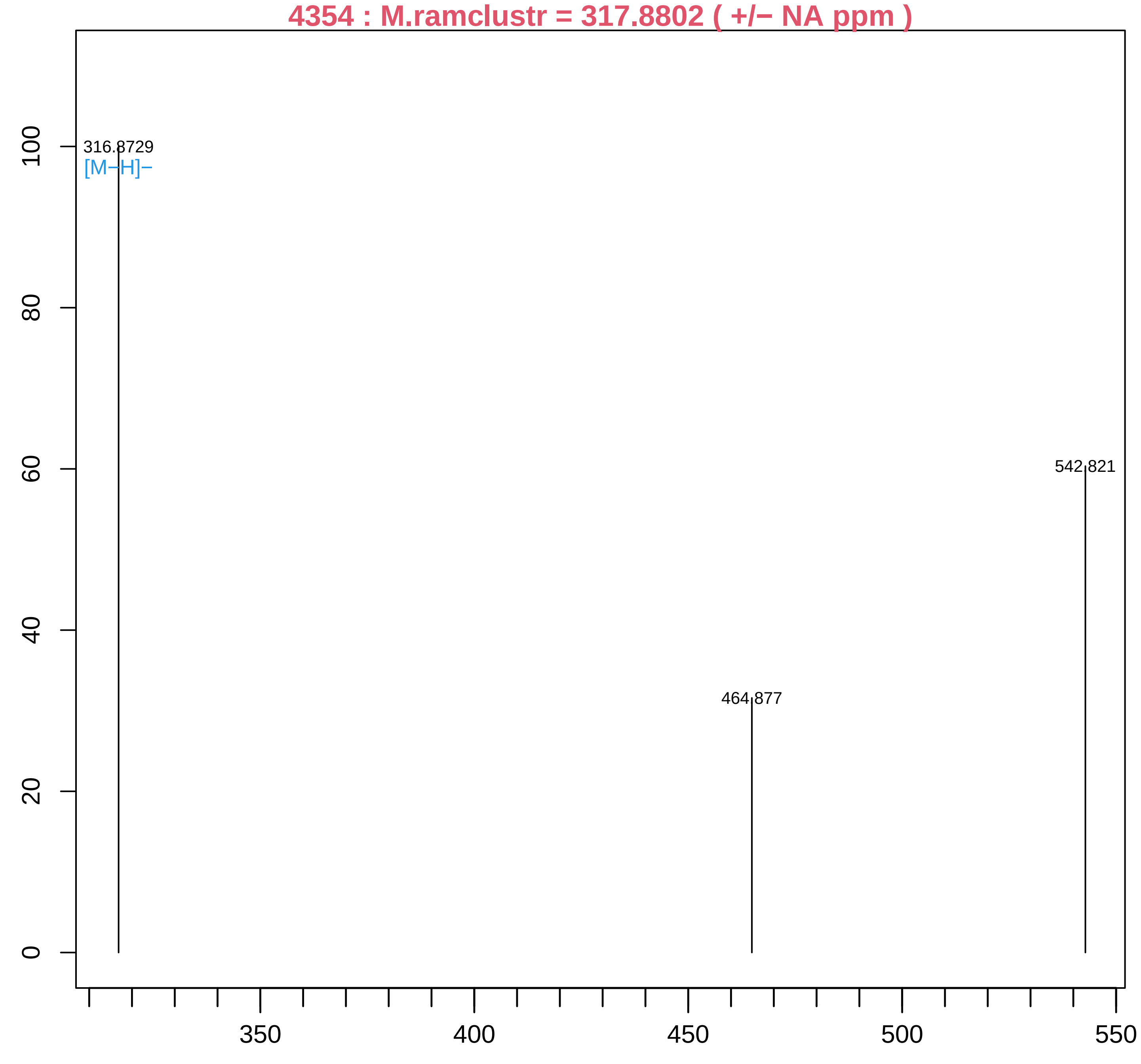
4354 : M.ramclustr = 317.8802 (+/− NA ppm)
316.8729
[M−H]−
464.877
542.821
0
20
40
60
80
100
350
400
450
500
550

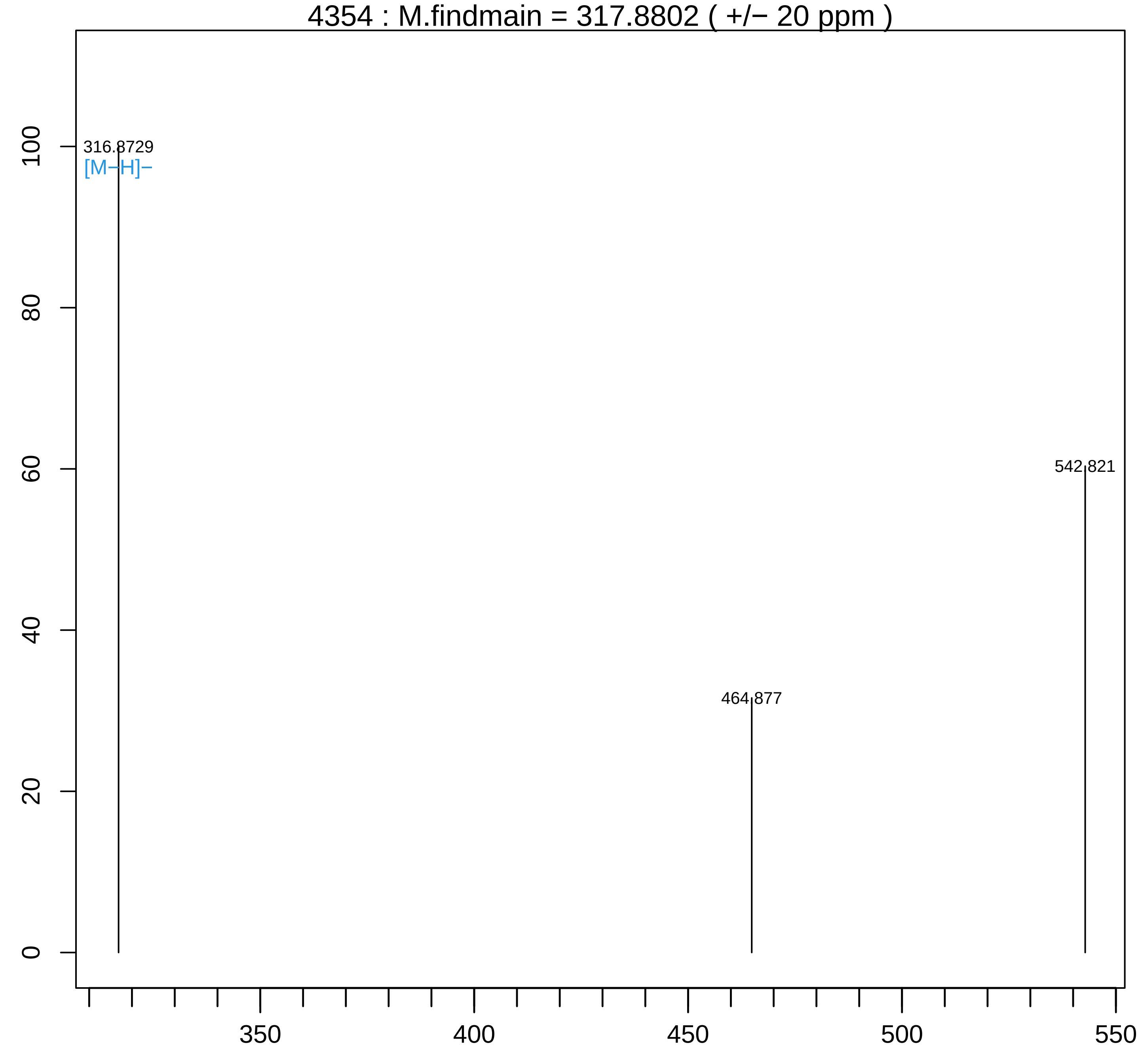
4354 : M.findmain = 317.8802 (+/− 20 ppm)
316.8729
[M−H]−
464.877
542.821
0
20
40
60
80
100
350
400
450
500
550

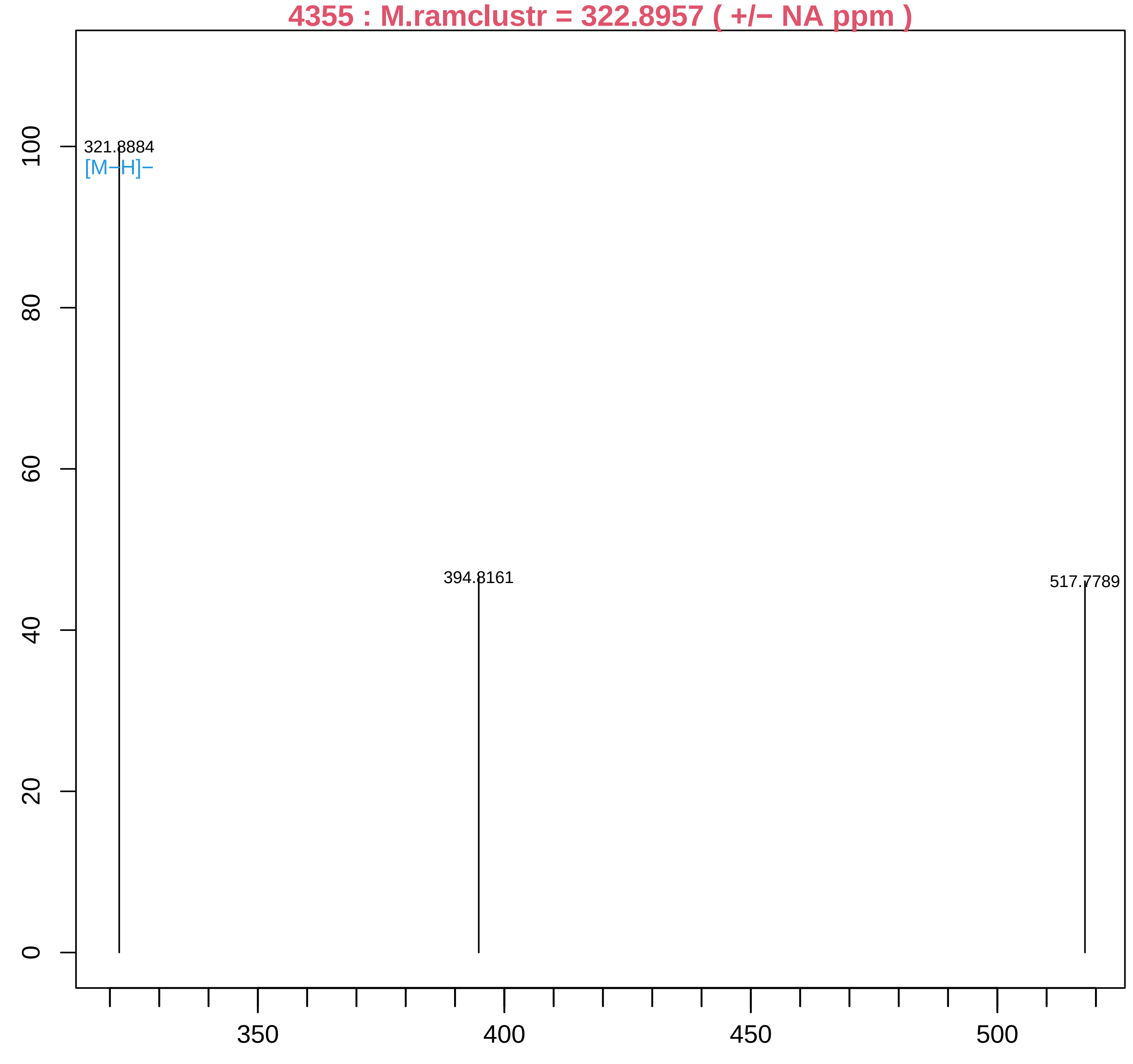
4355 : M.ramclustr = 322.8957 (+/− NA ppm)
321.8884
[M−H]−
394.8161
517.7789
0
20
40
60
80
100
350
400
450
500

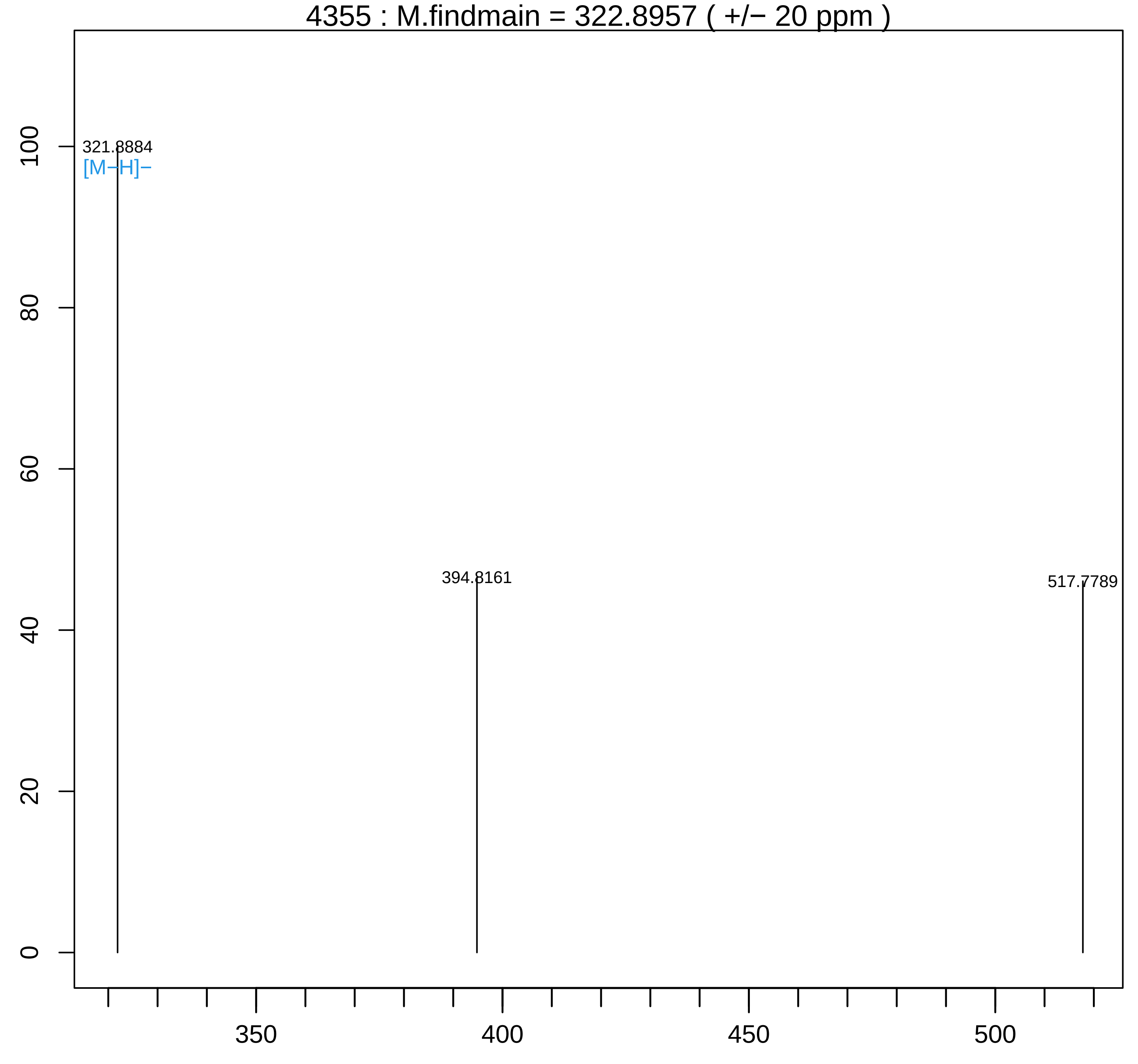
4355 : M.findmain = 322.8957 (+/− 20 ppm)
321.8884
[M−H]−
394.8161
517.7789
0
20
40
60
80
100
350
400
450
500

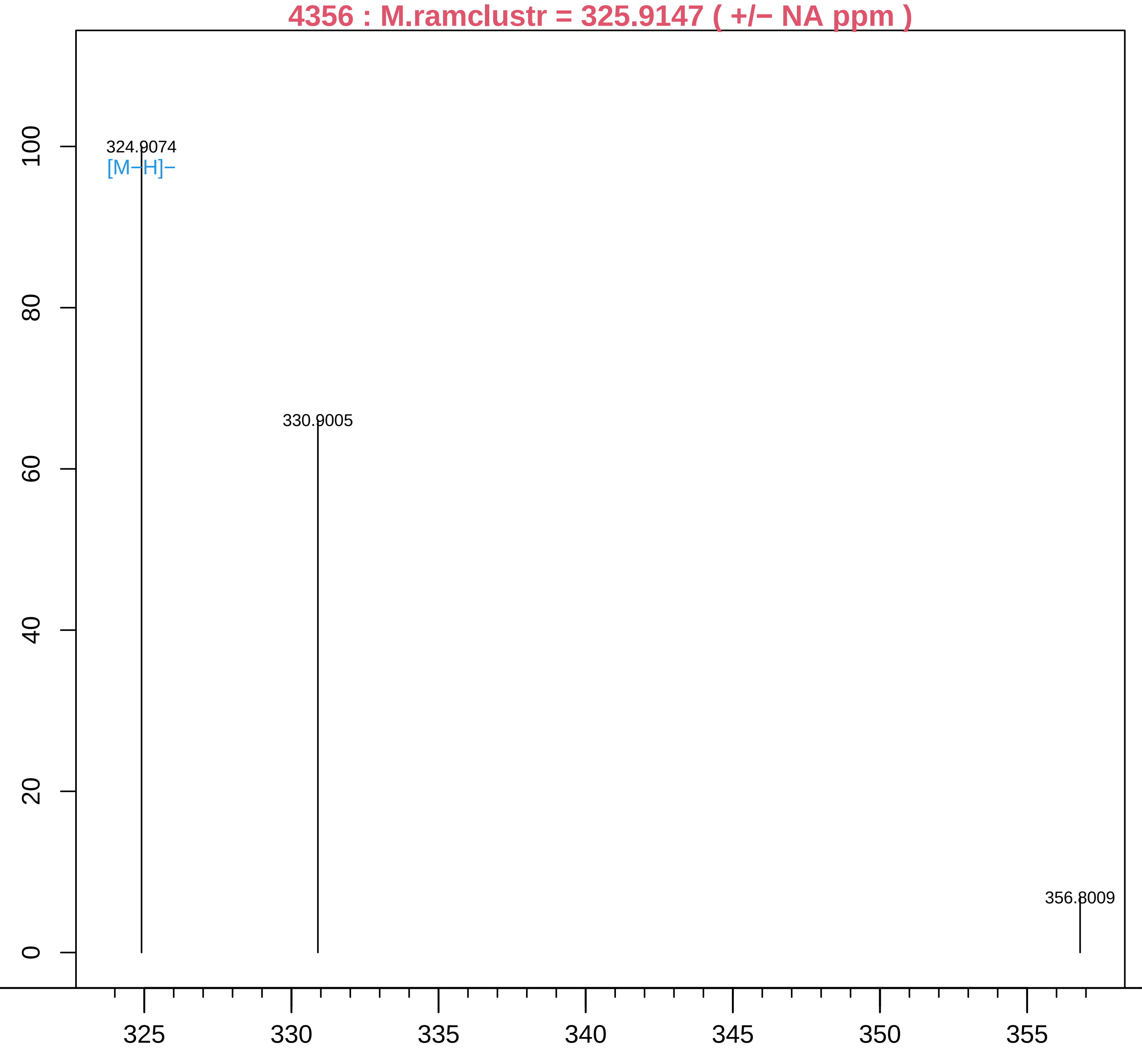
4356 : M.ramclustr = 325.9147 (+/− NA ppm)
324.9074
[M−H]−
330.9005
356.8009

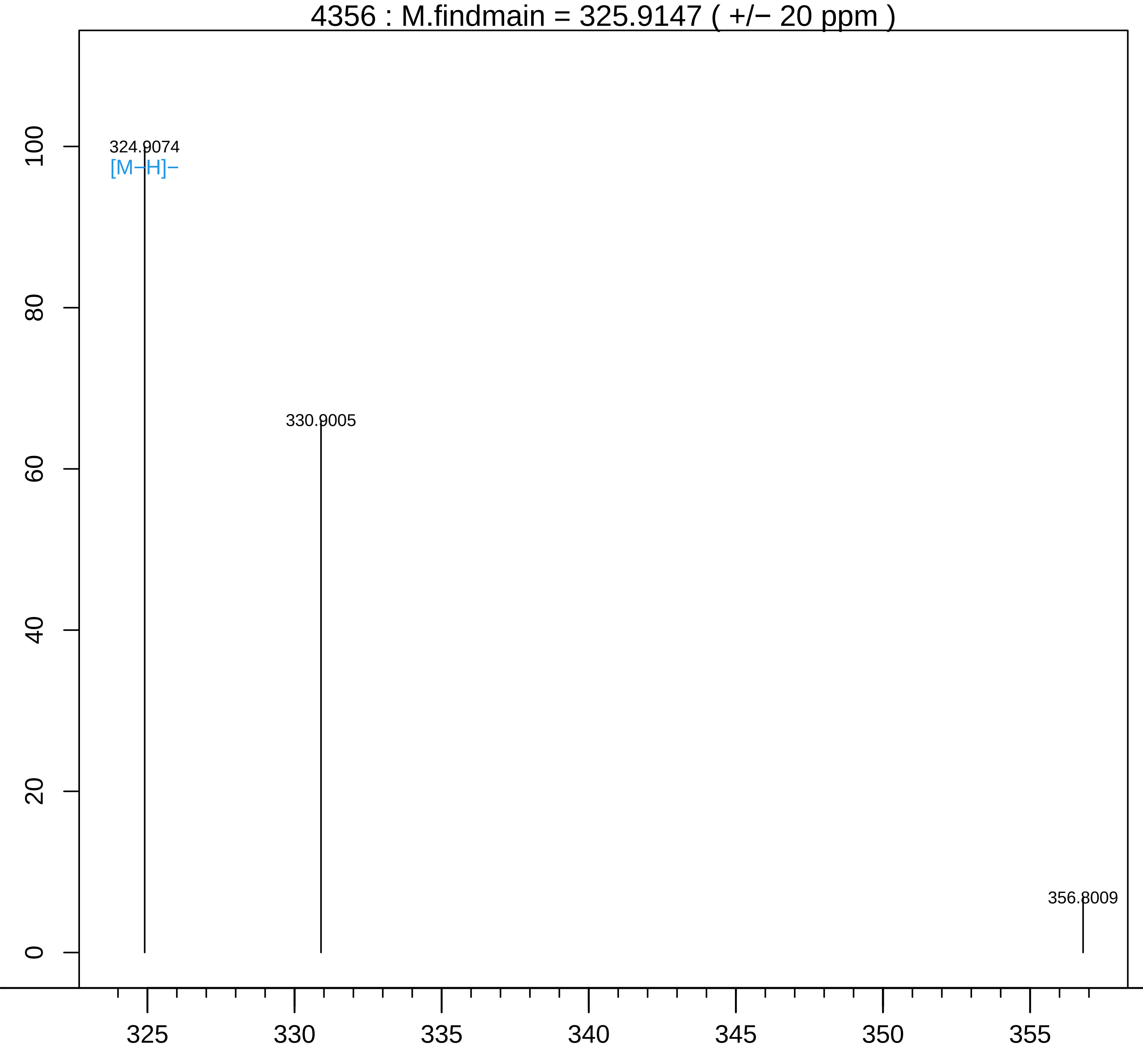
4356 : M.findmain = 325.9147 (+/− 20 ppm)
324.9074
[M−H]−
330.9005
356.8009

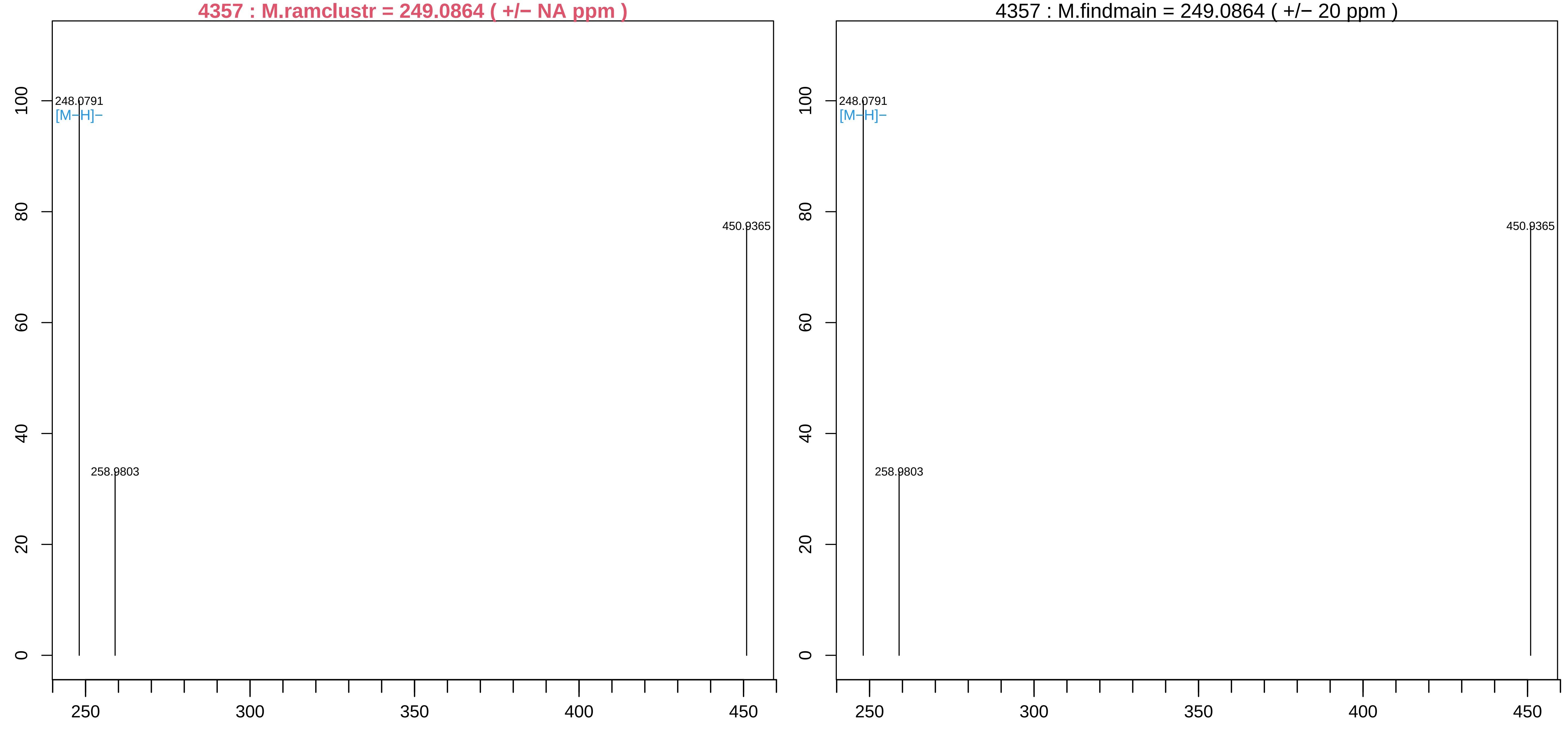
4357 : M.ramclustr = 249.0864 (+/− NA ppm)
248.0791
[M−H]−
258.9803
450.9365
4357 : M.findmain = 249.0864 (+/− 20 ppm)
248.0791
[M−H]−
258.9803
450.9365
0
20
40
60
80
100
250
300
350
400
450

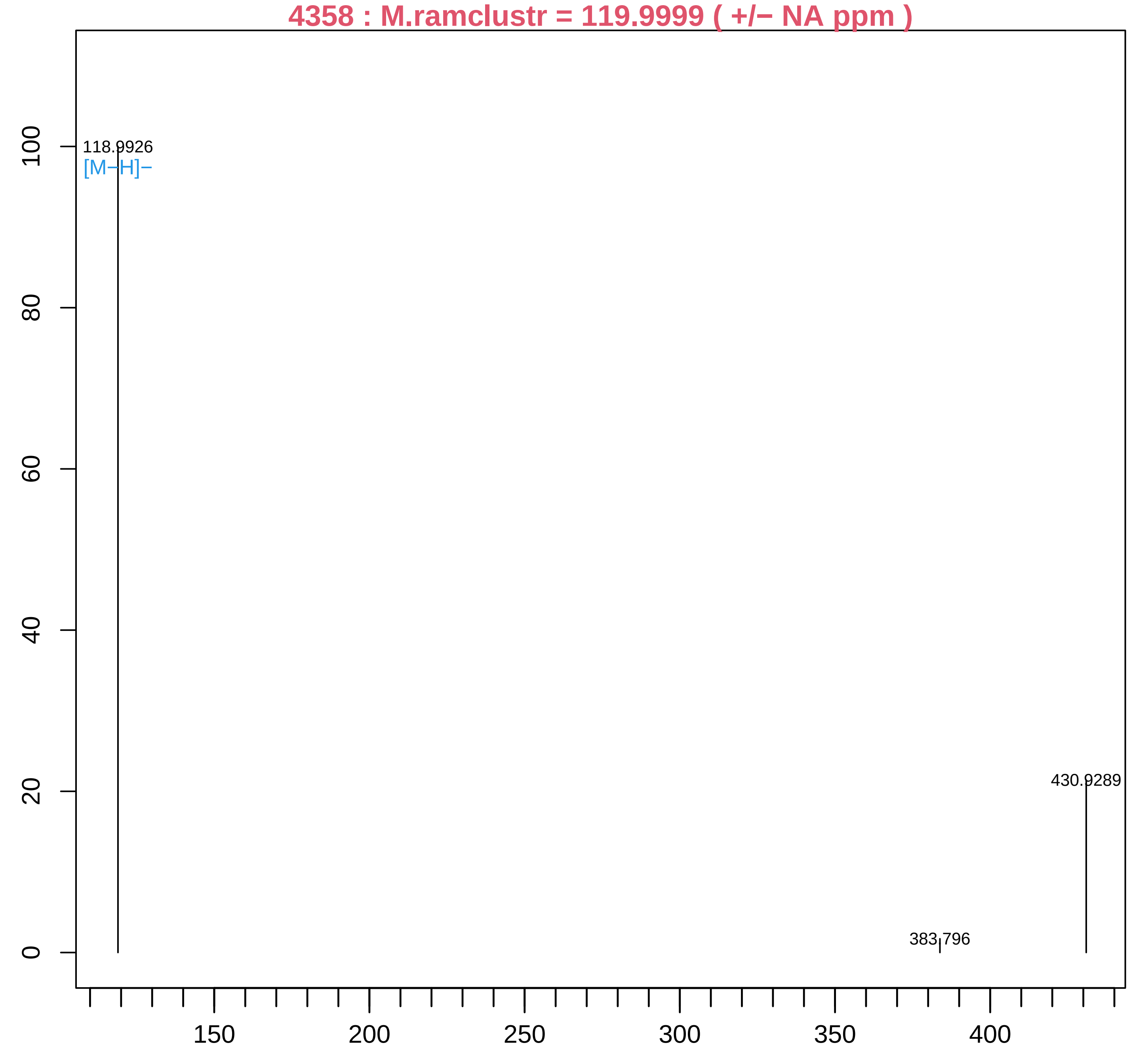
4358 : M.ramclustr = 119.9999 (+/− NA ppm)
118.9926
[M−H]−
430.9289
383.796
0
20
40
60
80
100
150
200
250
300
350
400

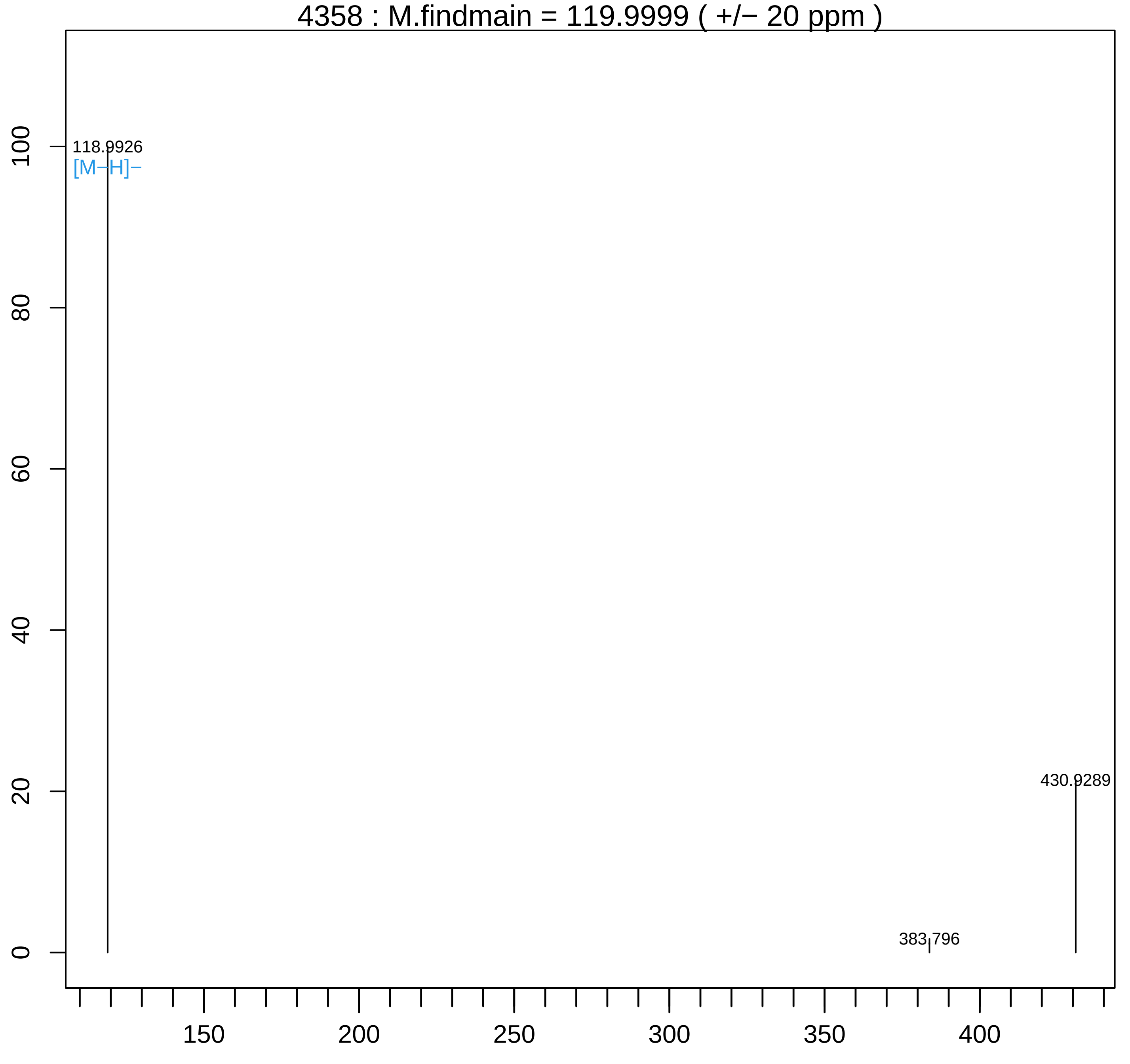
4358 : M.findmain = 119.9999 (+/− 20 ppm)
118.9926
[M−H]−
430.9289
383.796
0
20
40
60
80
100
150
200
250
300
350
400

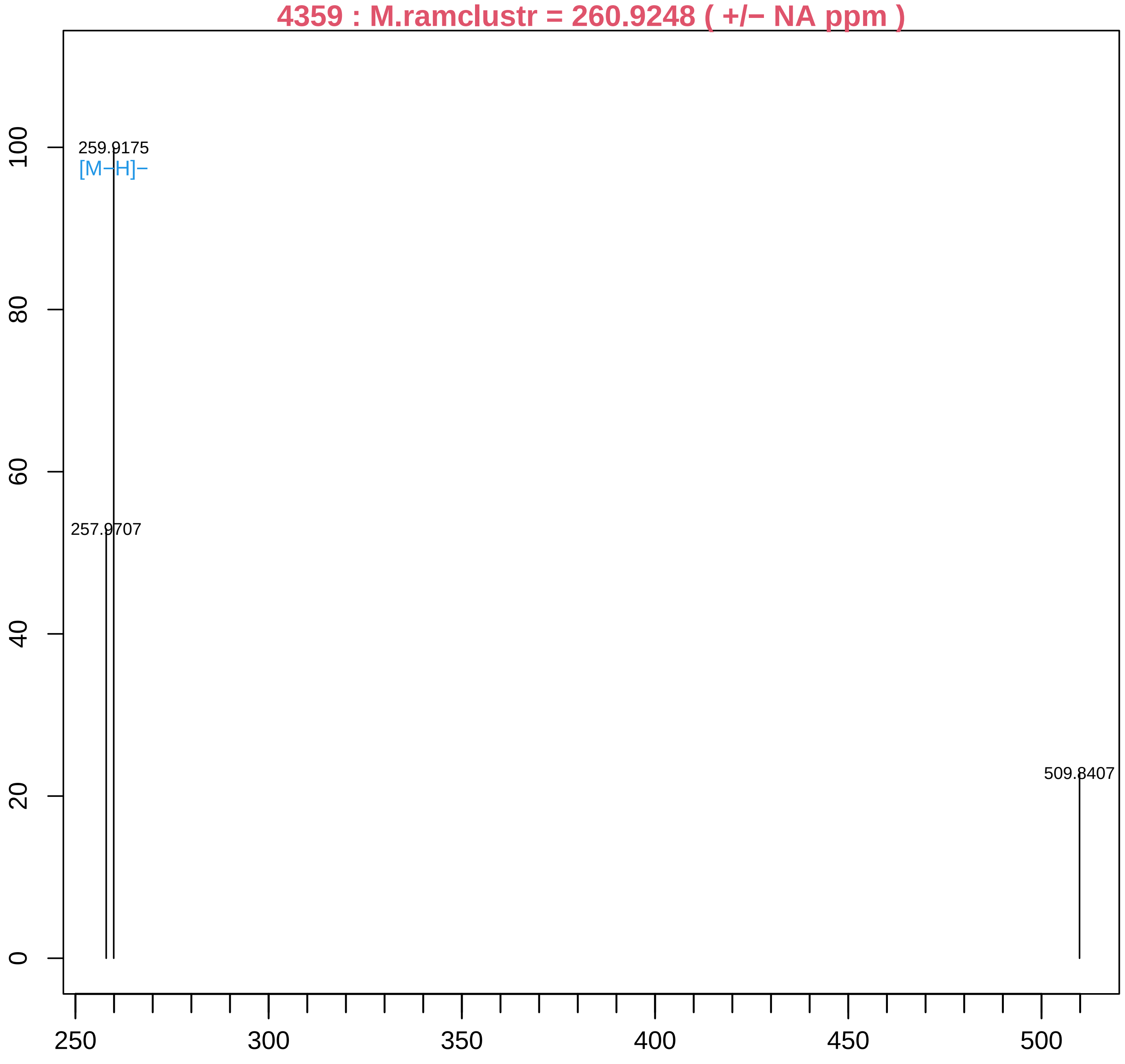
4359 : M.ramclustr = 260.9248 (+/− NA ppm)
259.9175
[M−H]−
257.9707
509.8407
0
20
40
60
80
100
250
300
350
400
450
500

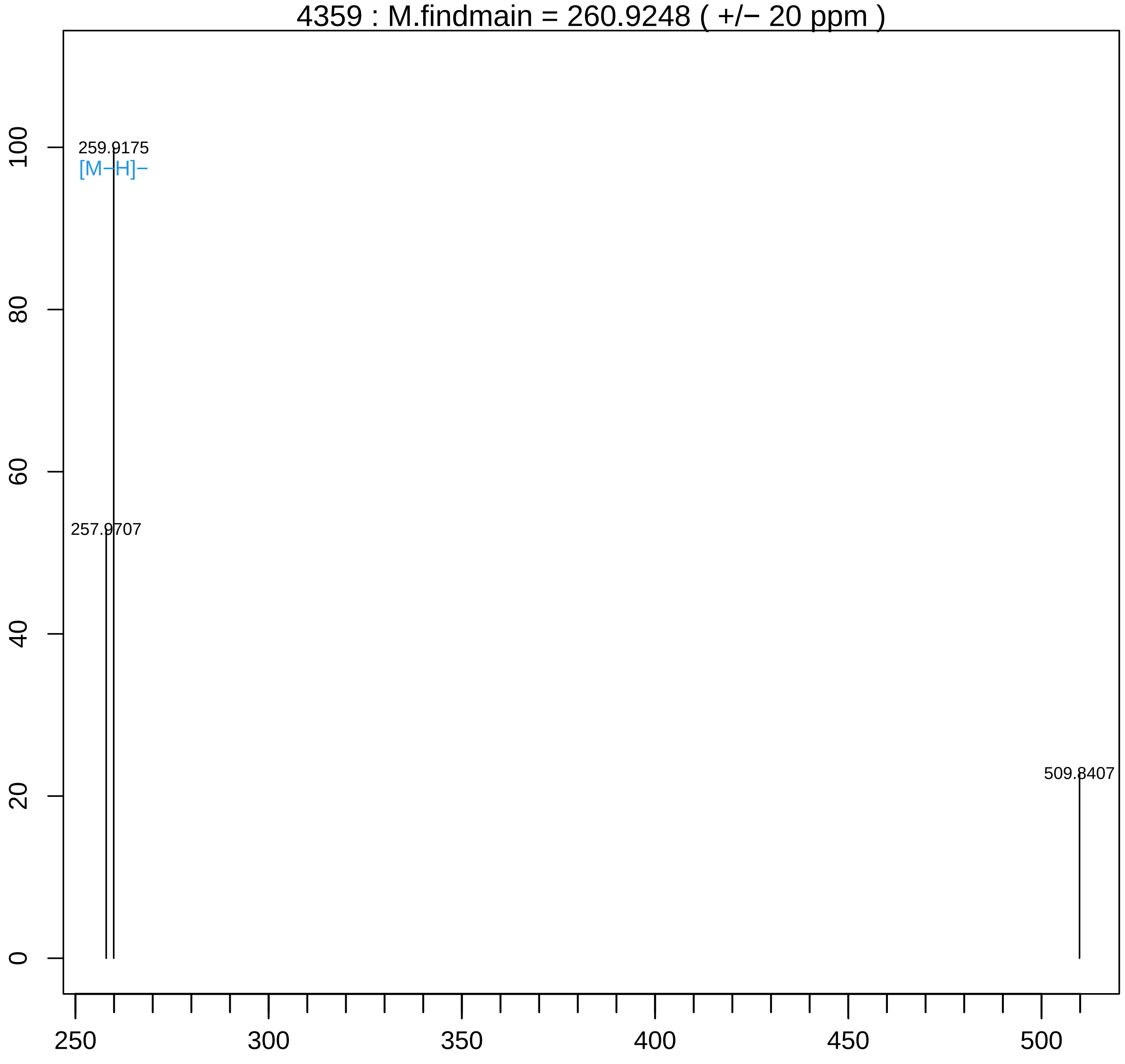
4359 : M.findmain = 260.9248 (+/− 20 ppm)
259.9175
[M−H]−
257.9707
509.8407
0
20
40
60
80
100
250
300
350
400
450
500

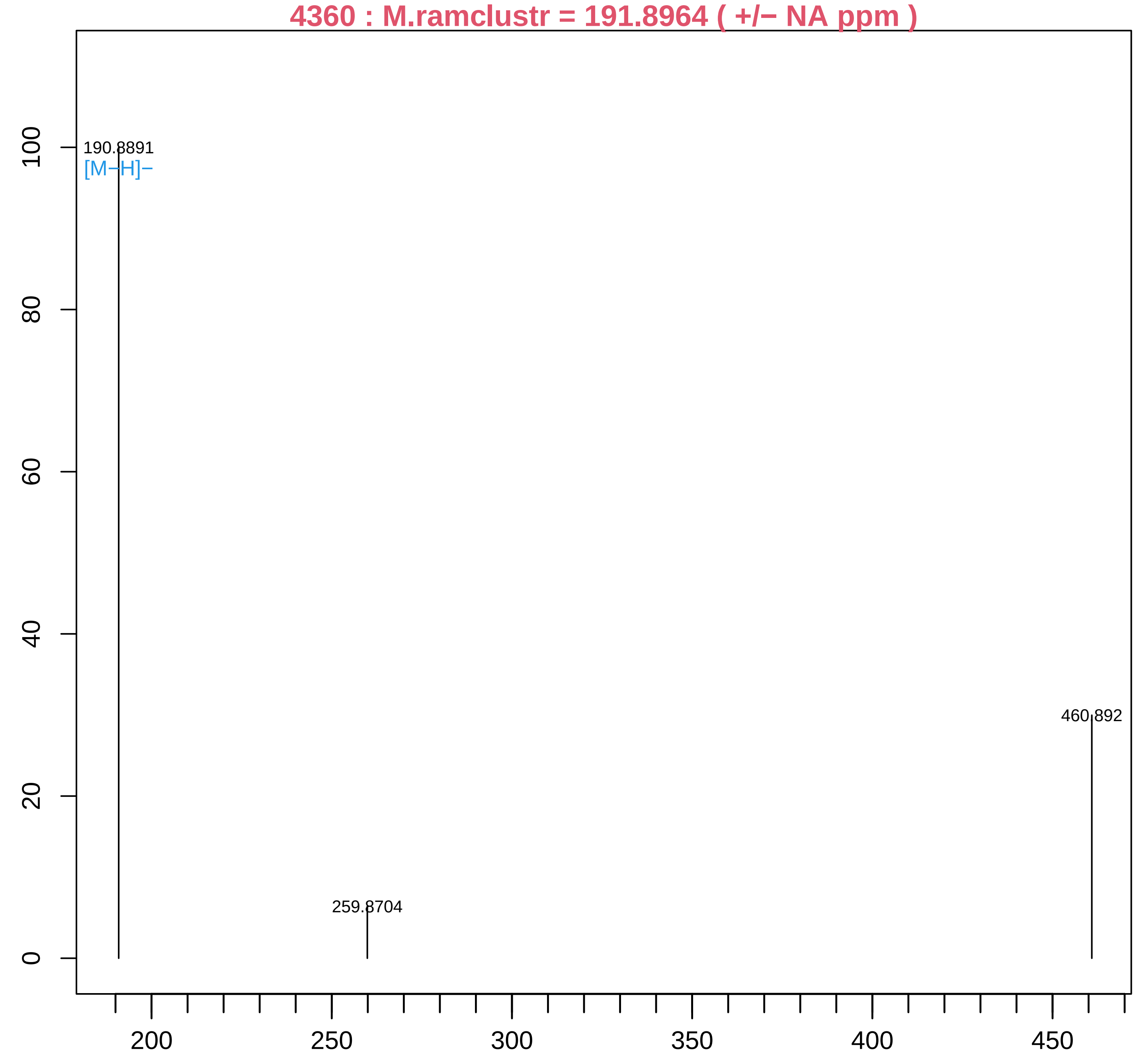

4360 : M.ramclustr = 191.8964 (+/− NA ppm)
190.8891
[M−H]−
259.8704
460.892
0
20
40
60
80
100
200
250
300
350
400
450

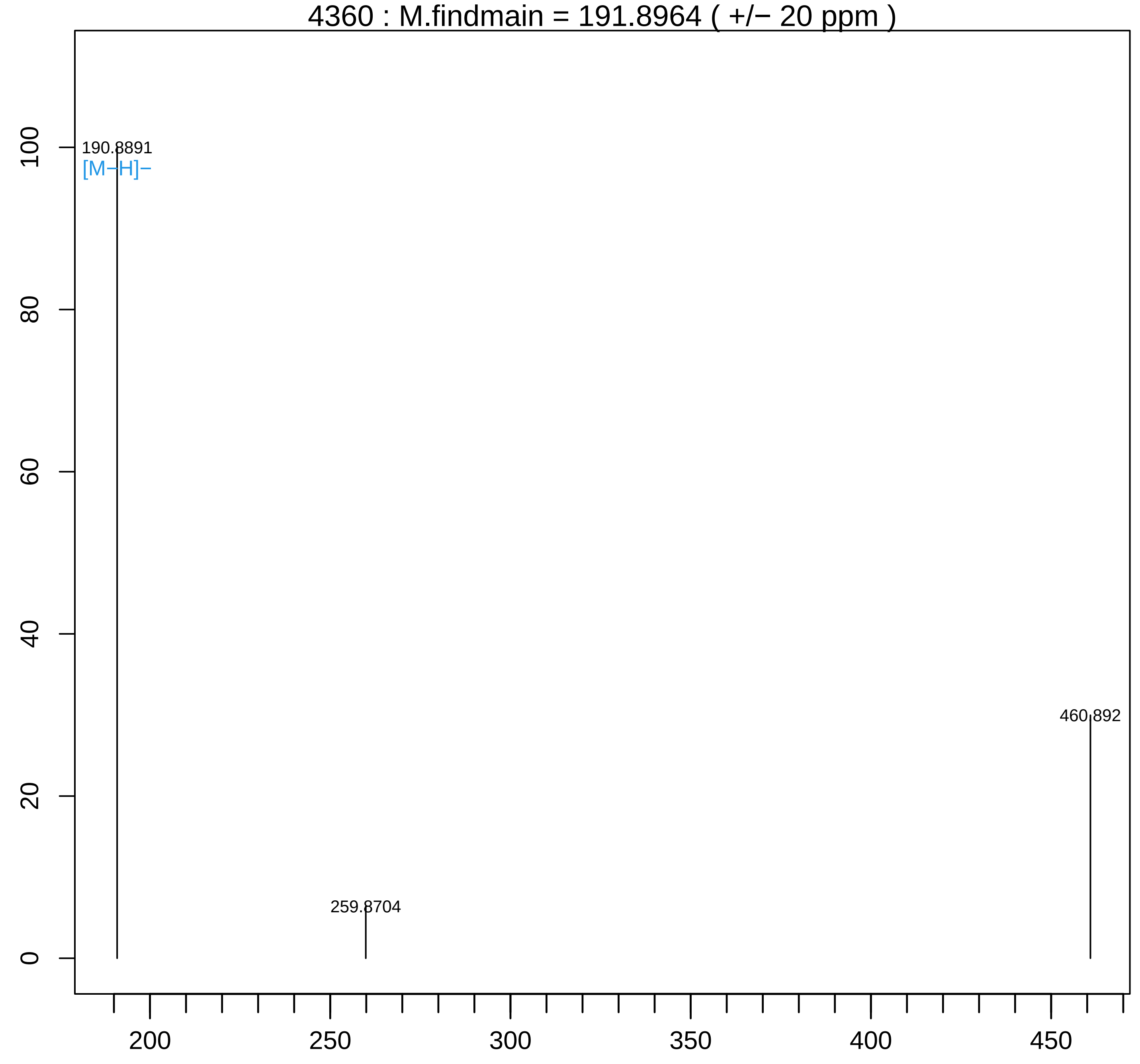

4360 : M.findmain = 191.8964 (+/− 20 ppm)
190.8891
[M−H]−
259.8704
460.892
0
20
40
60
80
100
200
250
300
350
400
450

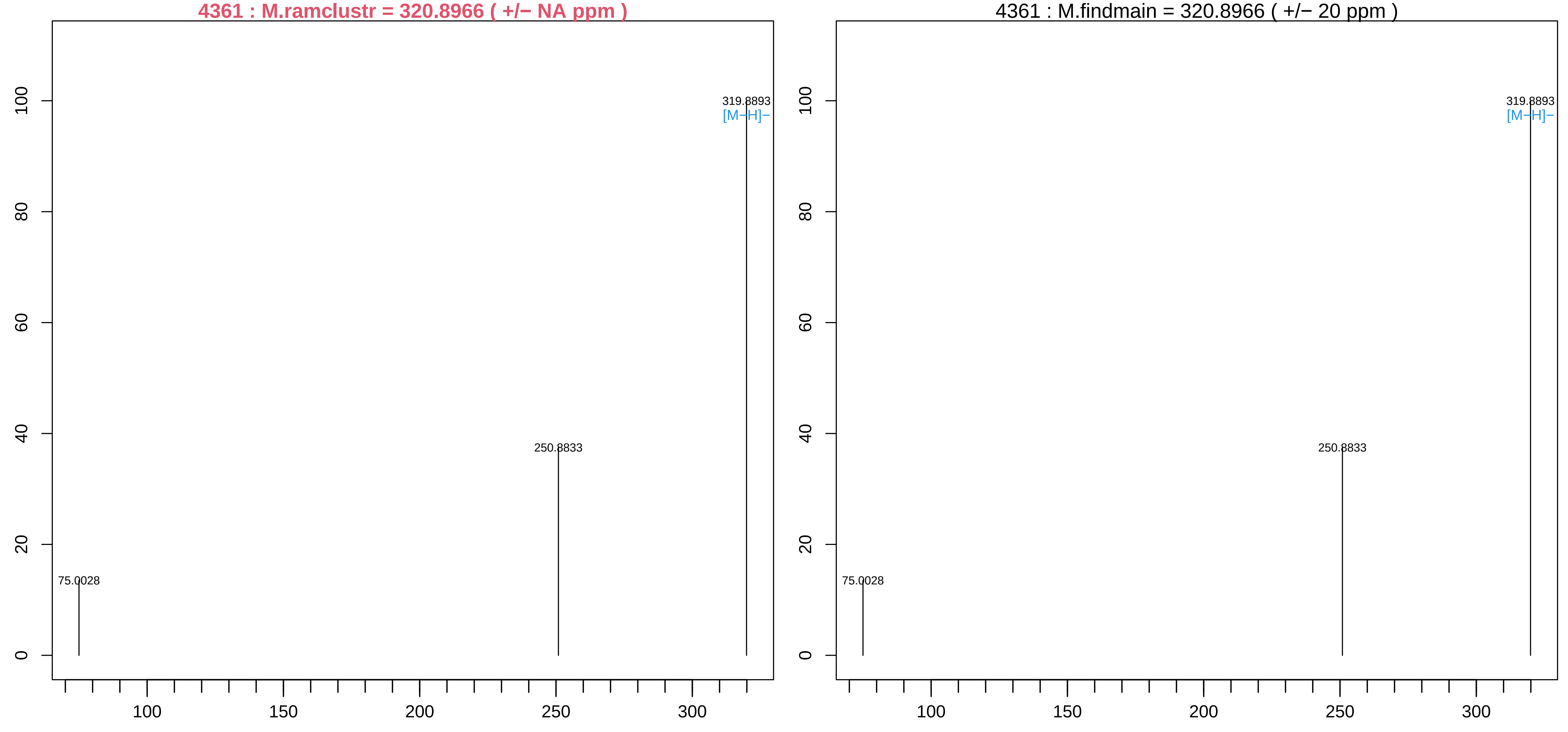

4361 : M.ramclustr = 320.8966 (+/− NA ppm)
4361 : M.findmain = 320.8966 (+/− 20 ppm)
319.8893
[M−H]−
250.8833
75.0028
0
20
40
60
80
100
100
150
200
250
300

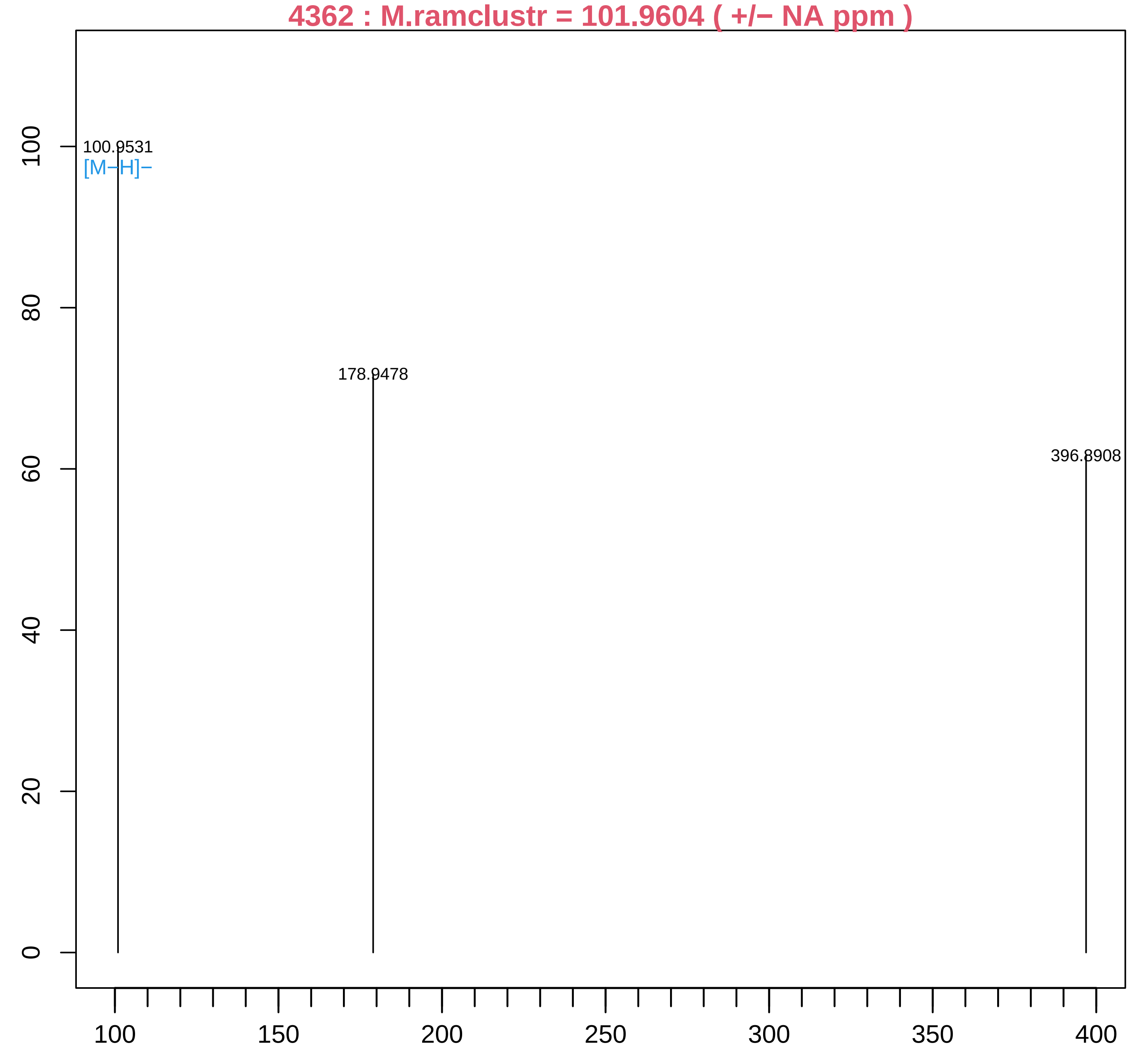
4362 : M.ramclustr = 101.9604 (+/− NA ppm)
100.9531
[M−H]−
178.9478
396.8908
0
20
40
60
80
100
100
150
200
250
300
350
400

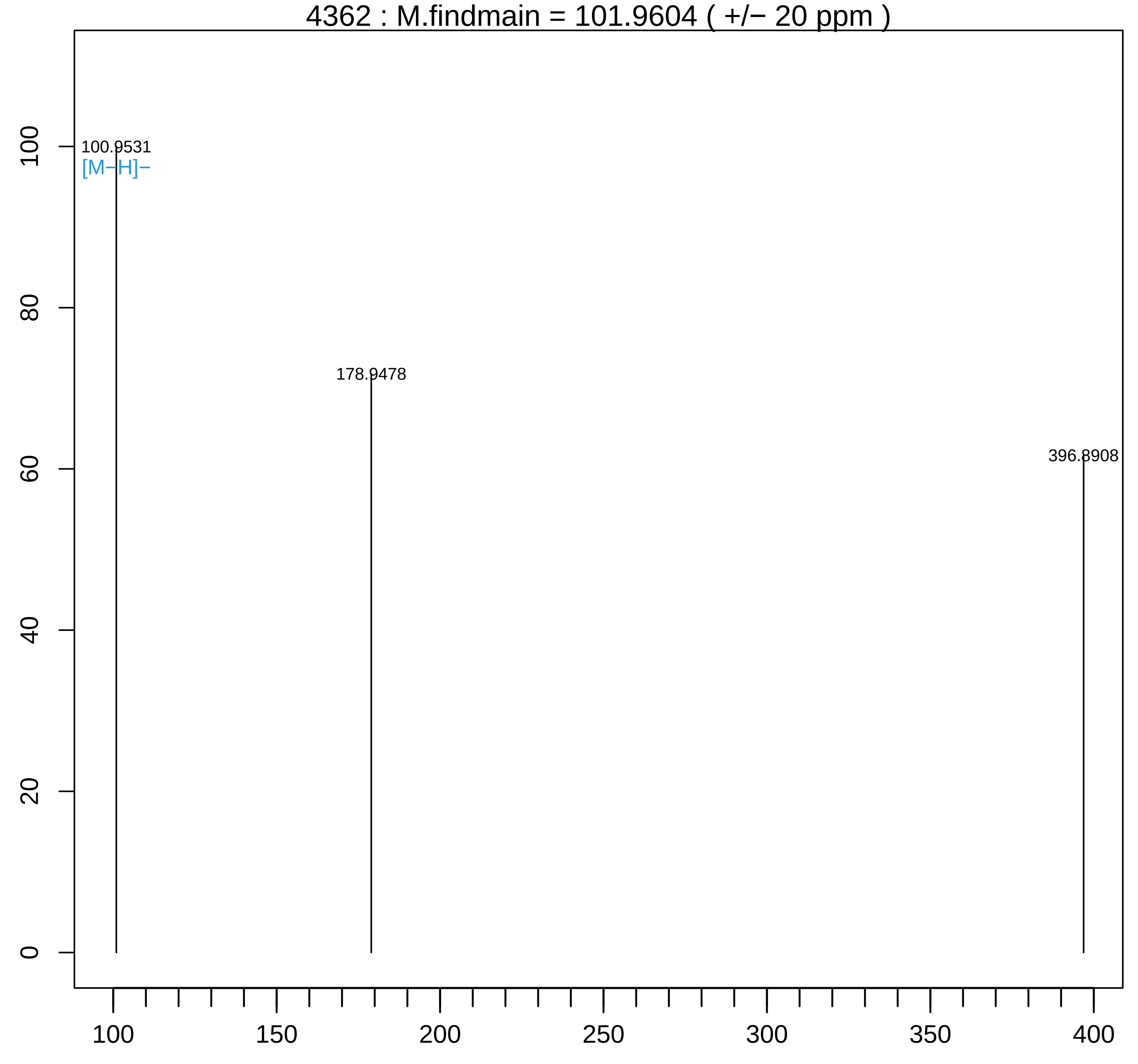
4362 : M.findmain = 101.9604 (+/− 20 ppm)
100.9531
[M−H]−
178.9478
396.8908
0
20
40
60
80
100
100
150
200
250
300
350
400

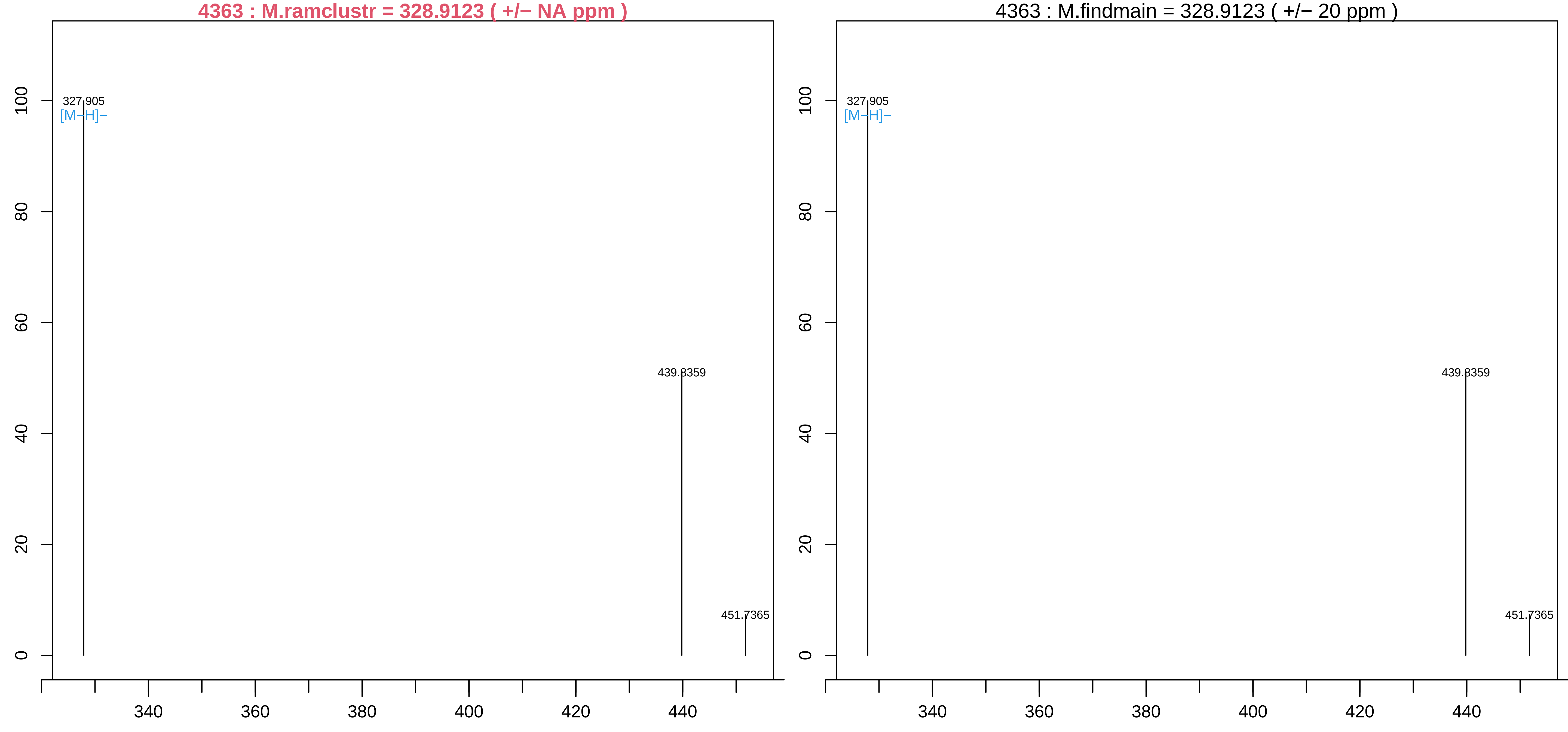

4363 : M.ramclustr = 328.9123 (+/− NA ppm)
327.905
[M−H]−
439.8359
451.7365
4363 : M.findmain = 328.9123 (+/− 20 ppm)
327.905
[M−H]−
439.8359
451.7365
0
20
40
60
80
100
340
360
380
400
420
440

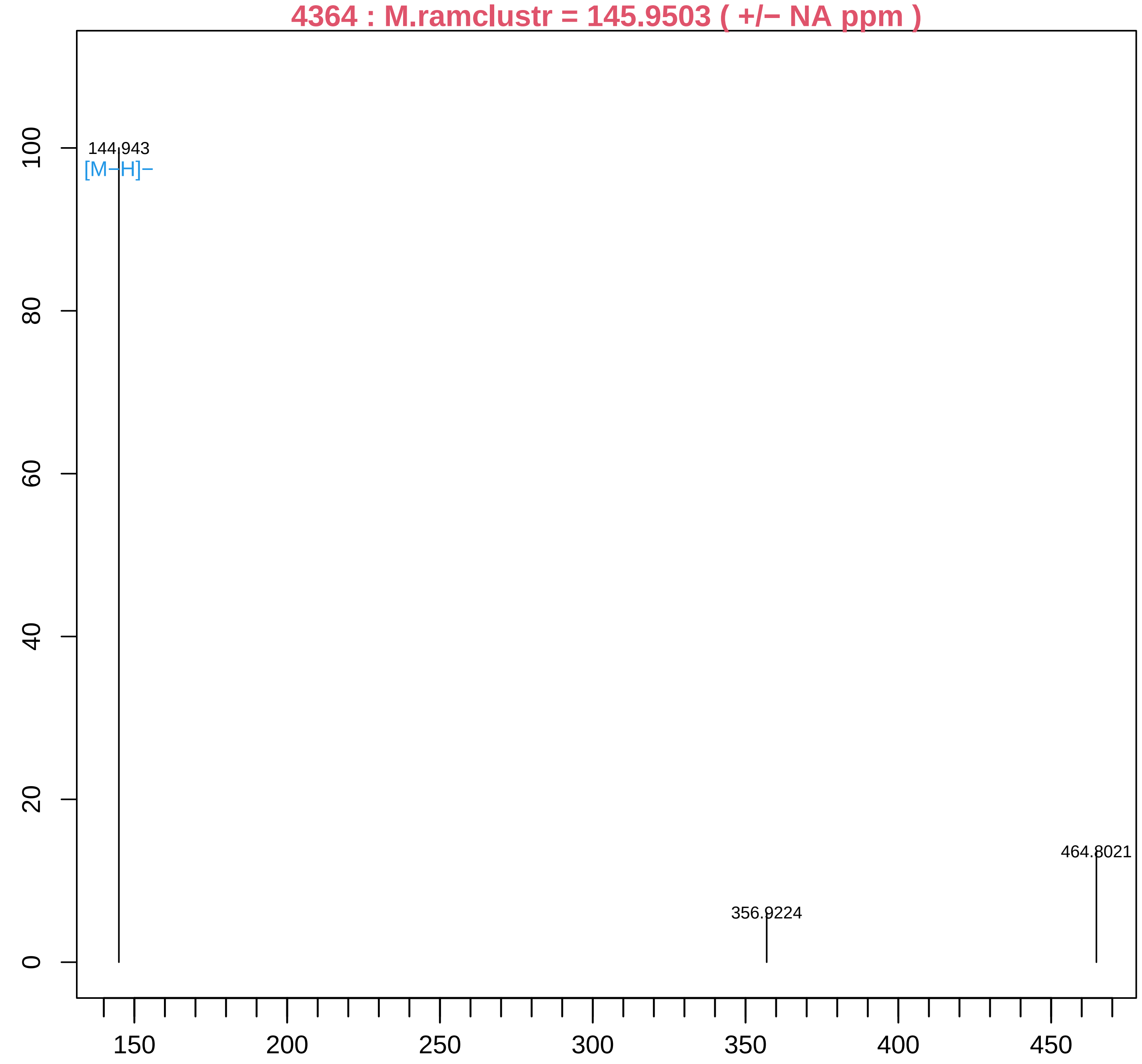
4364 : M.ramclustr = 145.9503 (+/− NA ppm)
144.943
[M−H]−
356.9224
464.8021
0
20
40
60
80
100
150
200
250
300
350
400
450

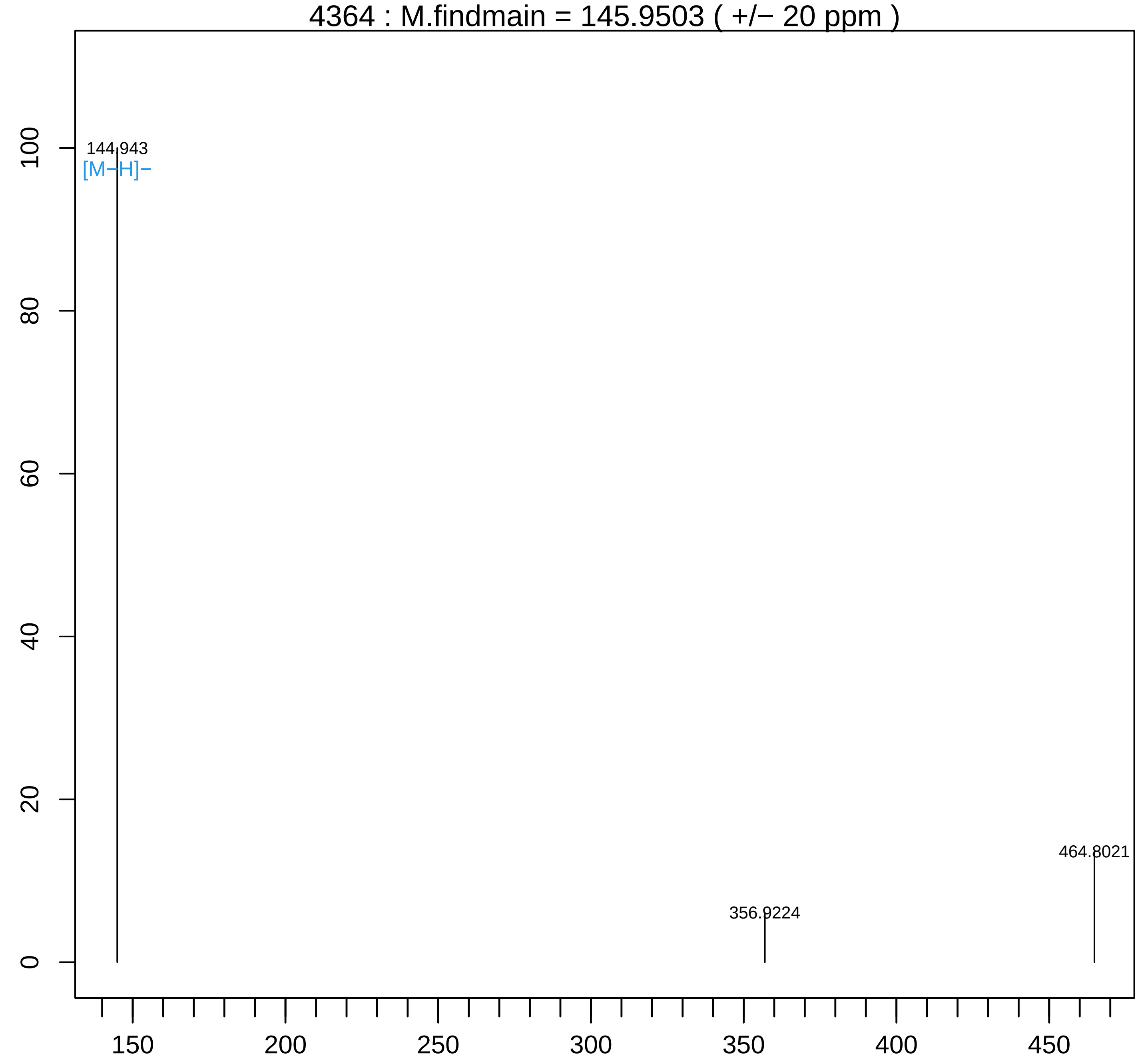
4364 : M.findmain = 145.9503 (+/− 20 ppm)
144.943
[M−H]−
356.9224
464.8021
0
20
40
60
80
100
150
200
250
300
350
400
450

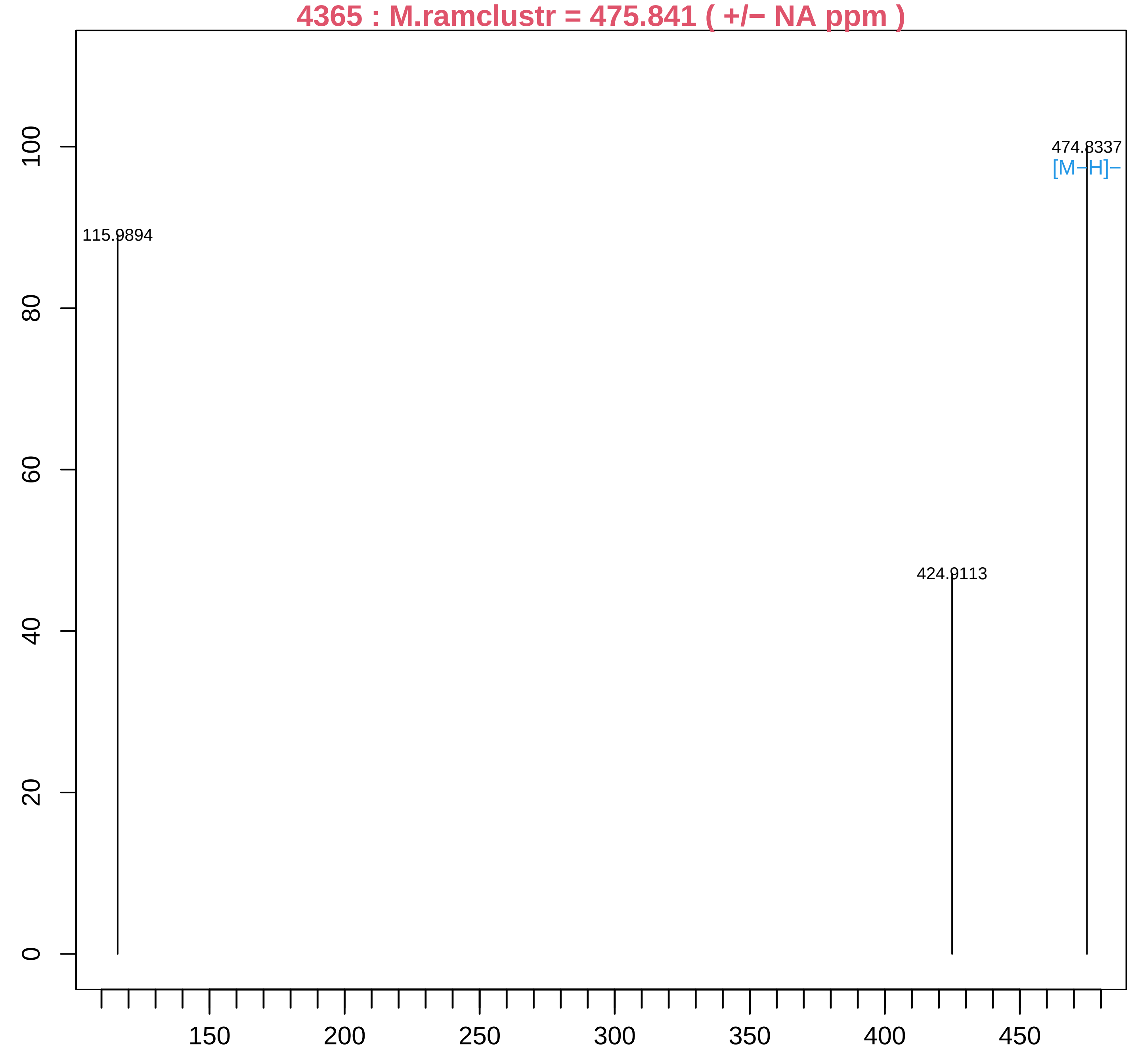

4365 : M.ramclustr = 475.841 (+/− NA ppm)
474.8337
[M−H]−
115.9894
424.9113
0
20
40
60
80
100
150
200
250
300
350
400
450

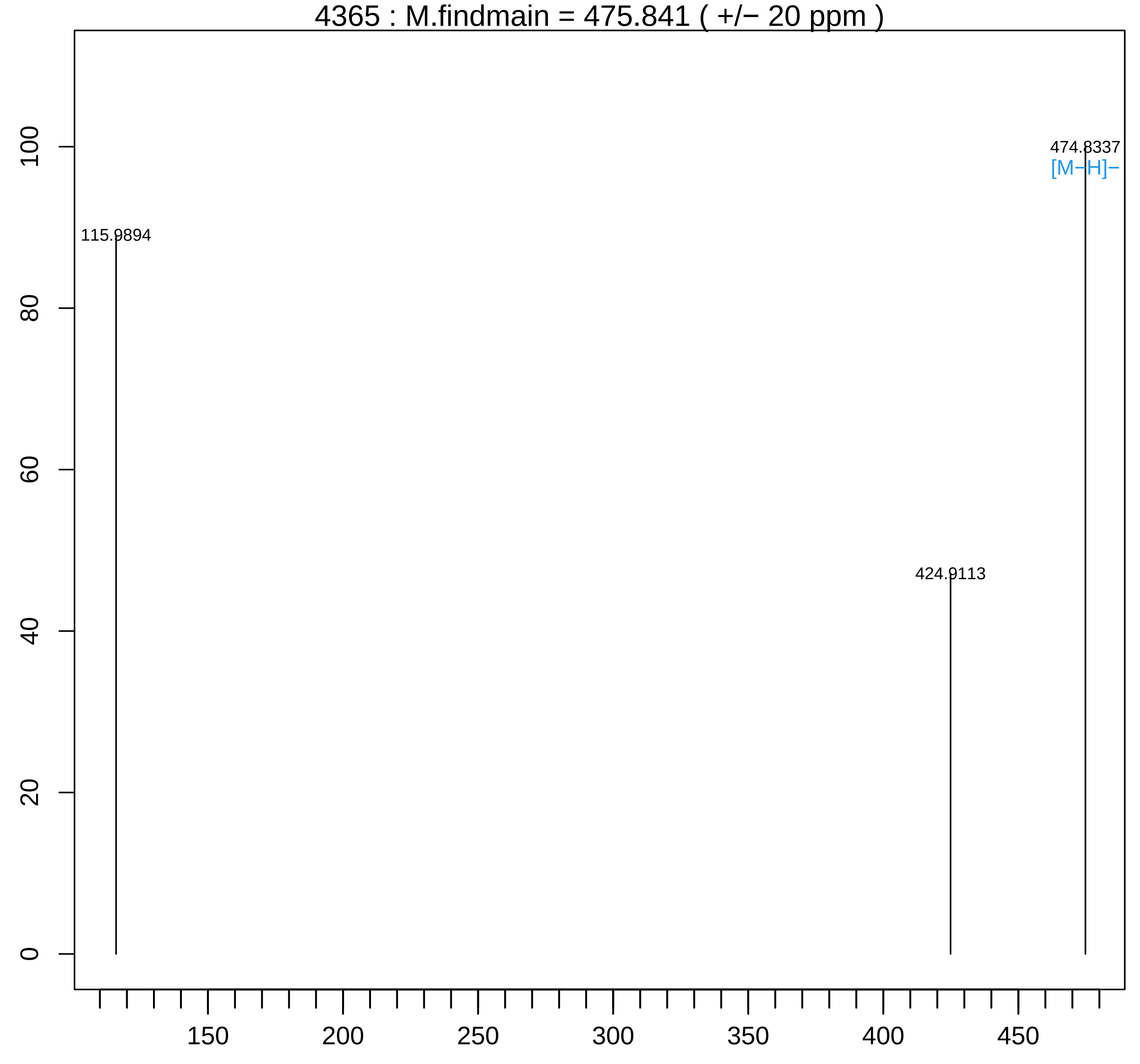

4365 : M.findmain = 475.841 (+/− 20 ppm)
474.8337
[M−H]−
115.9894
424.9113
0
20
40
60
80
100
150
200
250
300
350
400
450

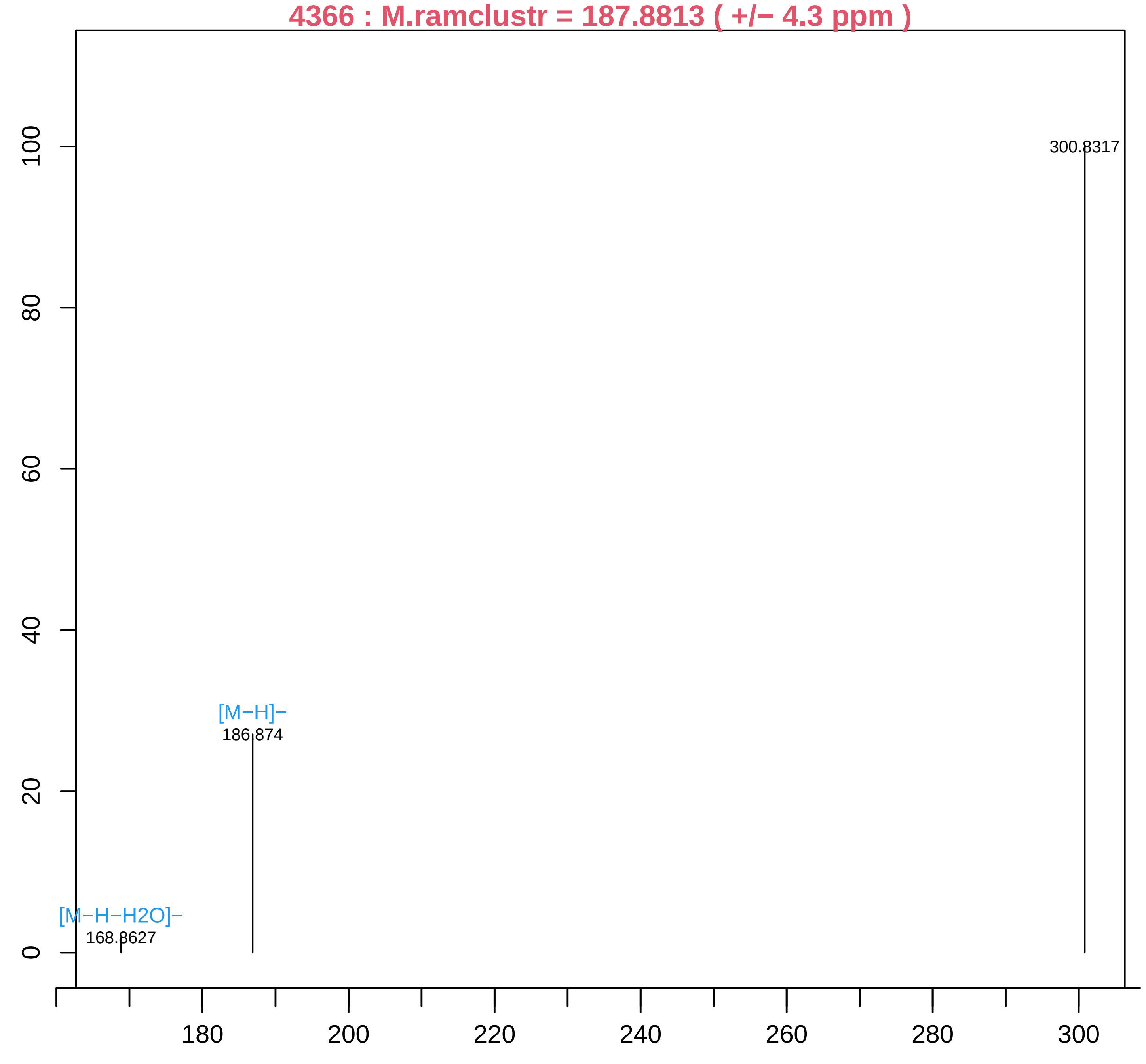

4366 : M.ramclustr = 187.8813 (+/− 4.3 ppm)
300.8317
[M−H]−
186.874
[M−H−H2O]−
168.8627
0
20
40
60
80
100
180
200
220
240
260
280
300

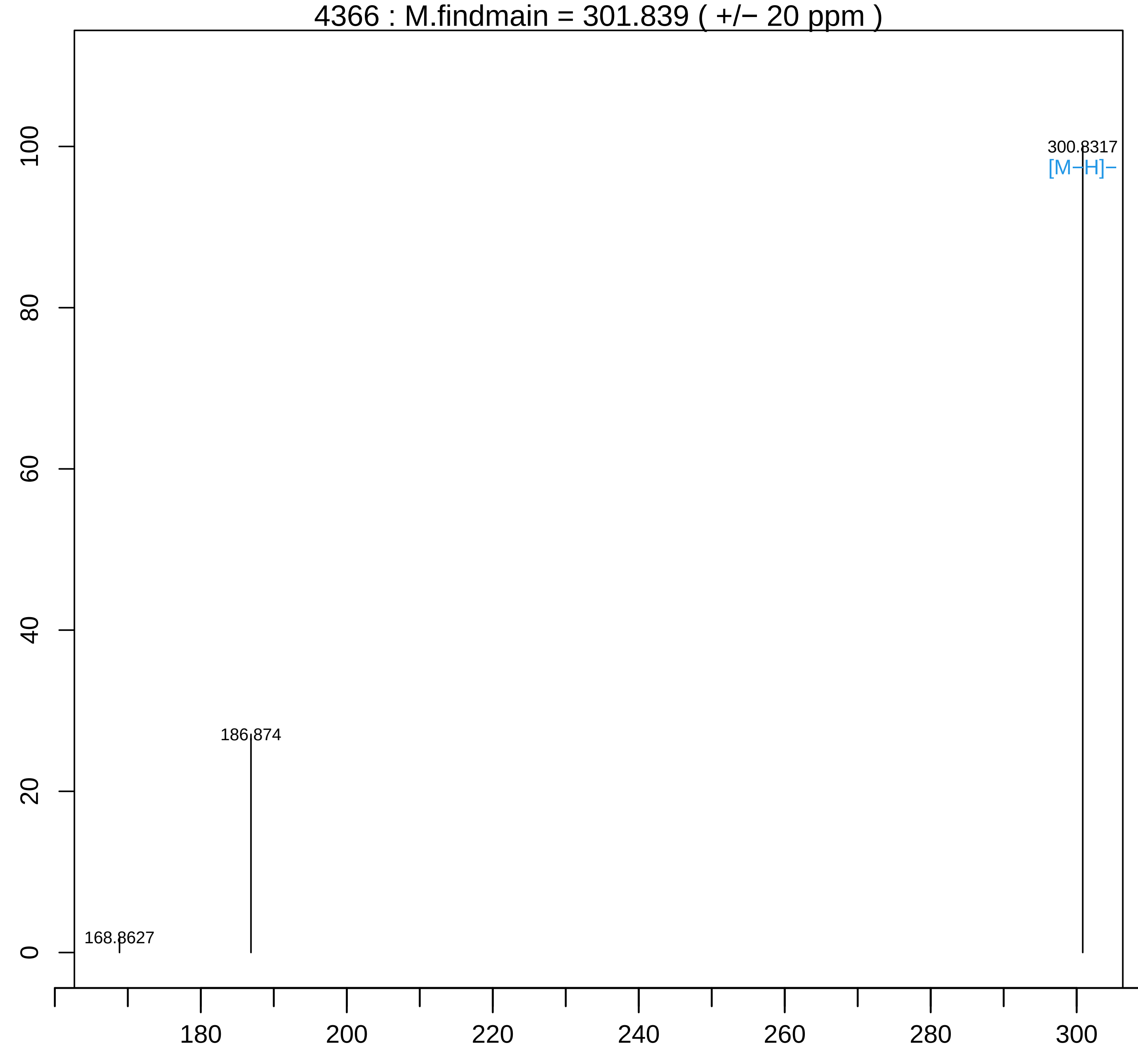

4366 : M.findmain = 301.839 (+/− 20 ppm)
300.8317
[M−H]−
186.874
168.8627
0
20
40
60
80
100
180
200
220
240
260
280
300

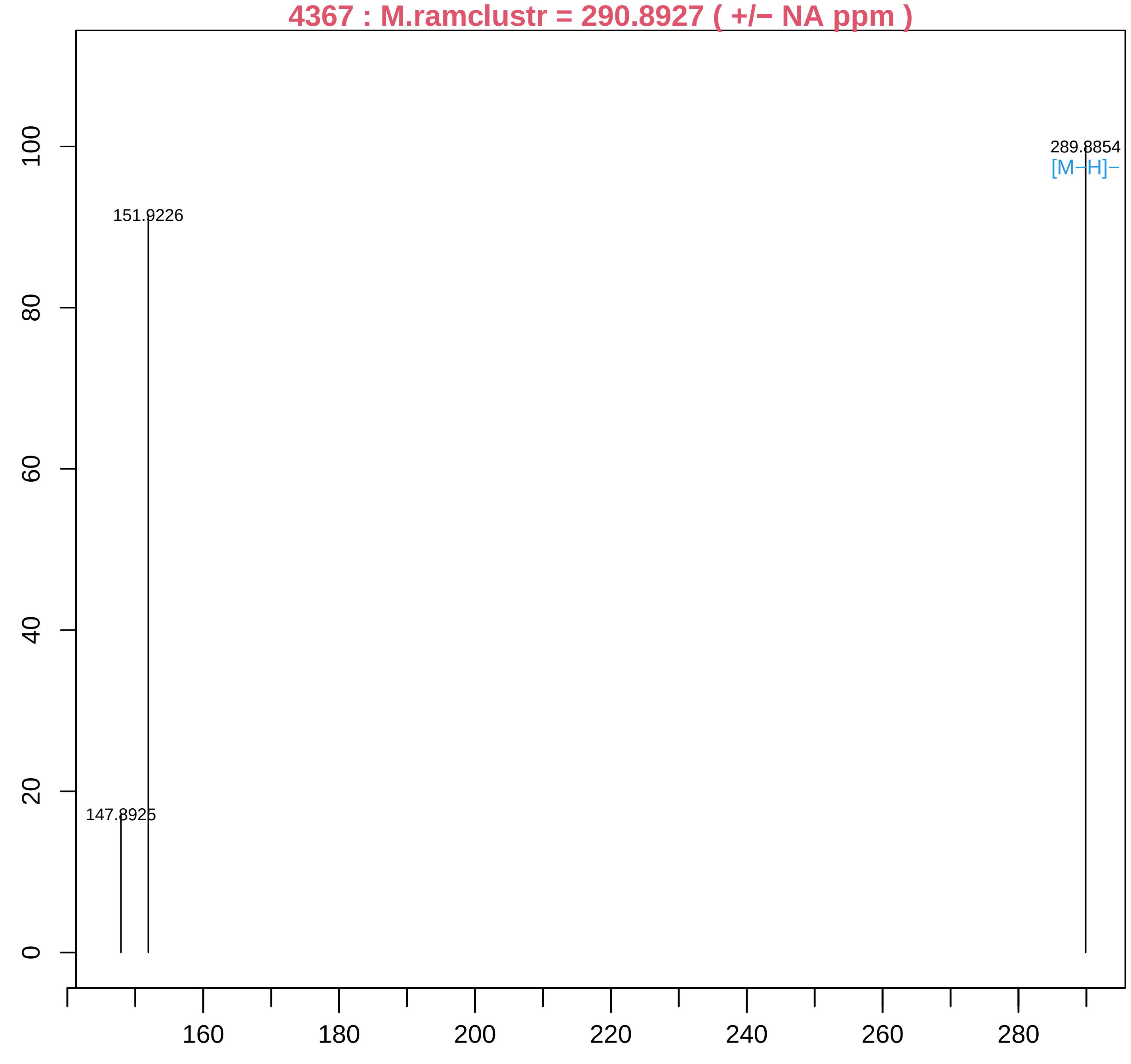

4367 : M.ramclustr = 290.8927 (+/− NA ppm)
289.8854
[M−H]−
151.9226
147.8925
0
20
40
60
80
100
160
180
200
220
240
260
280

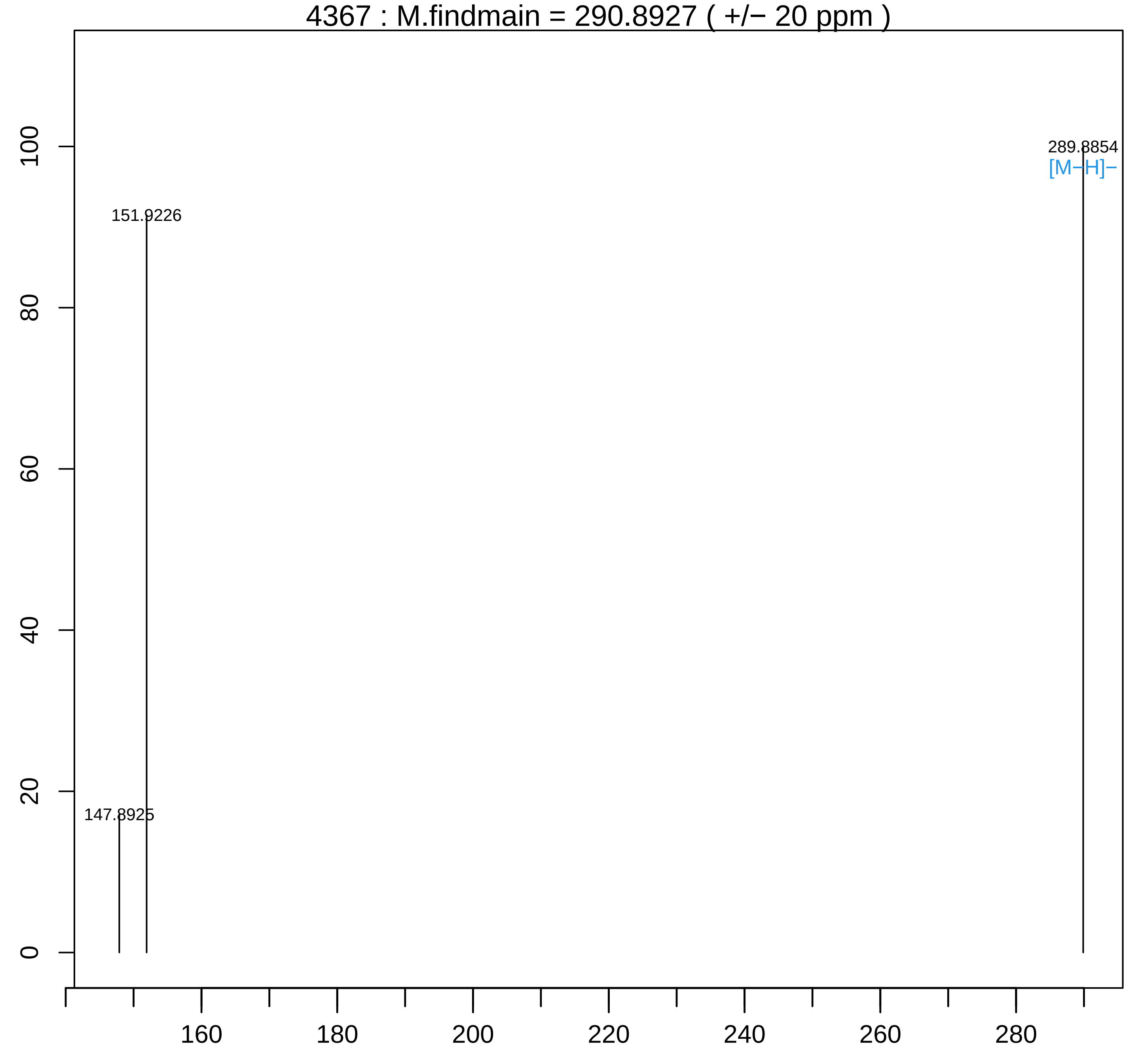

4367 : M.findmain = 290.8927 (+/− 20 ppm)
289.8854
[M−H]−
151.9226
147.8925
0
20
40
60
80
100
160
180
200
220
240
260
280

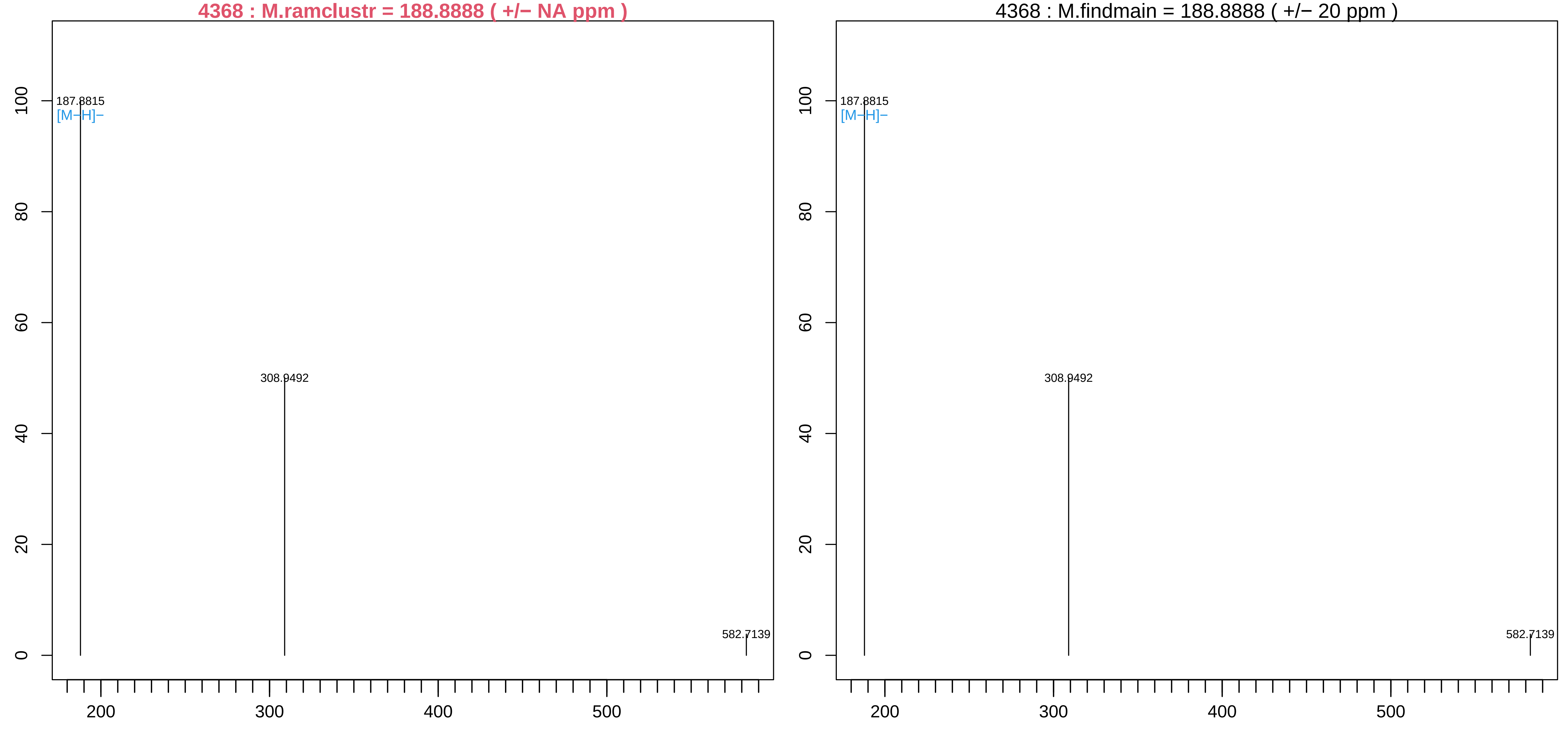
4368 : M.ramclustr = 188.8888 (+/− NA ppm)
187.8815
[M−H]−
308.9492
582.7139
4368 : M.findmain = 188.8888 (+/− 20 ppm)
187.8815
[M−H]−
308.9492
582.7139
0
20
40
60
80
100
200
300
400
500

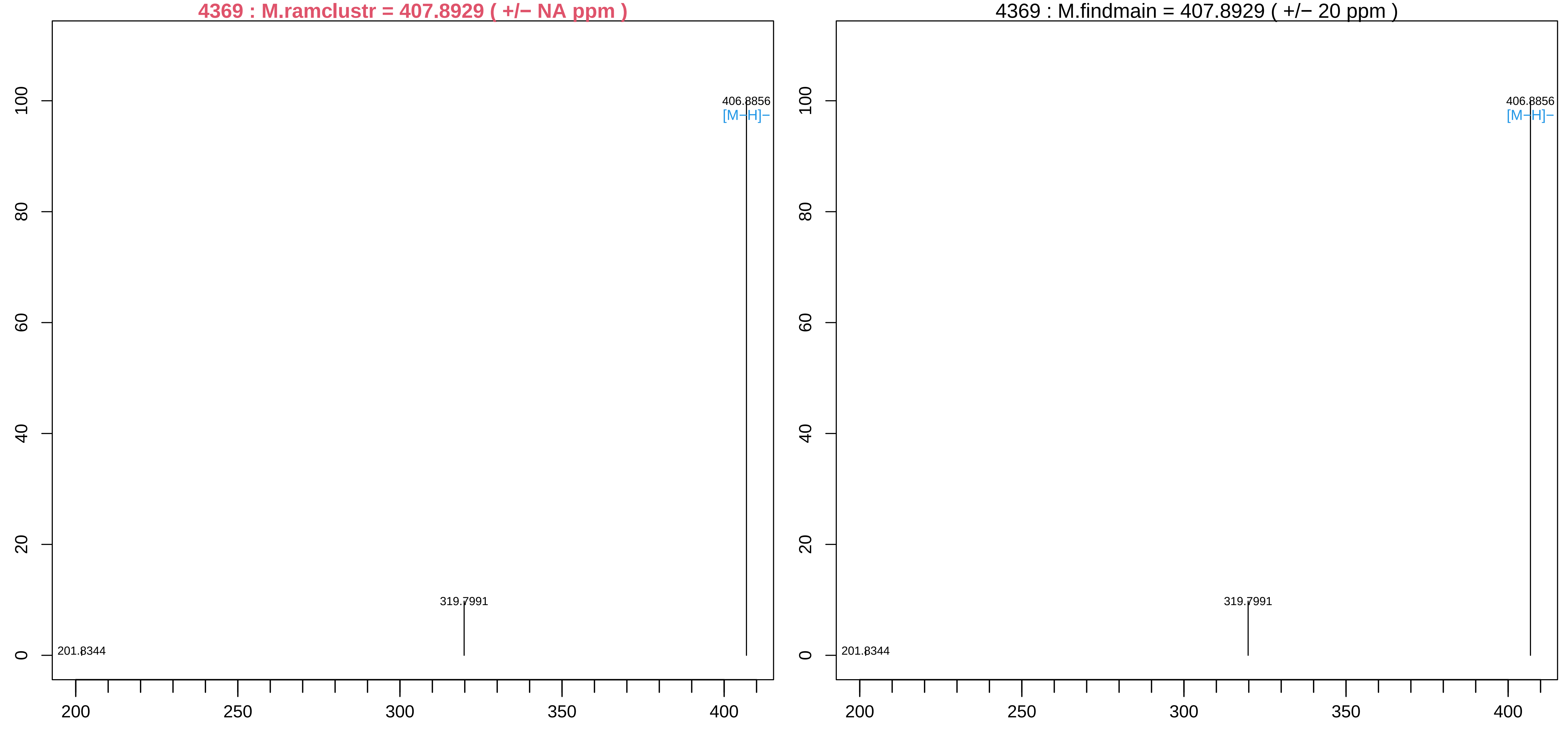

4369 : M.ramclustr = 407.8929 (+/− NA ppm)
406.8856
[M−H]−
319.7991
201.8344
0
20
40
60
80
100
200
250
300
350
400
4369 : M.findmain = 407.8929 (+/− 20 ppm)
406.8856
[M−H]−
319.7991
201.8344
0
20
40
60
80
100
200
250
300
350
400

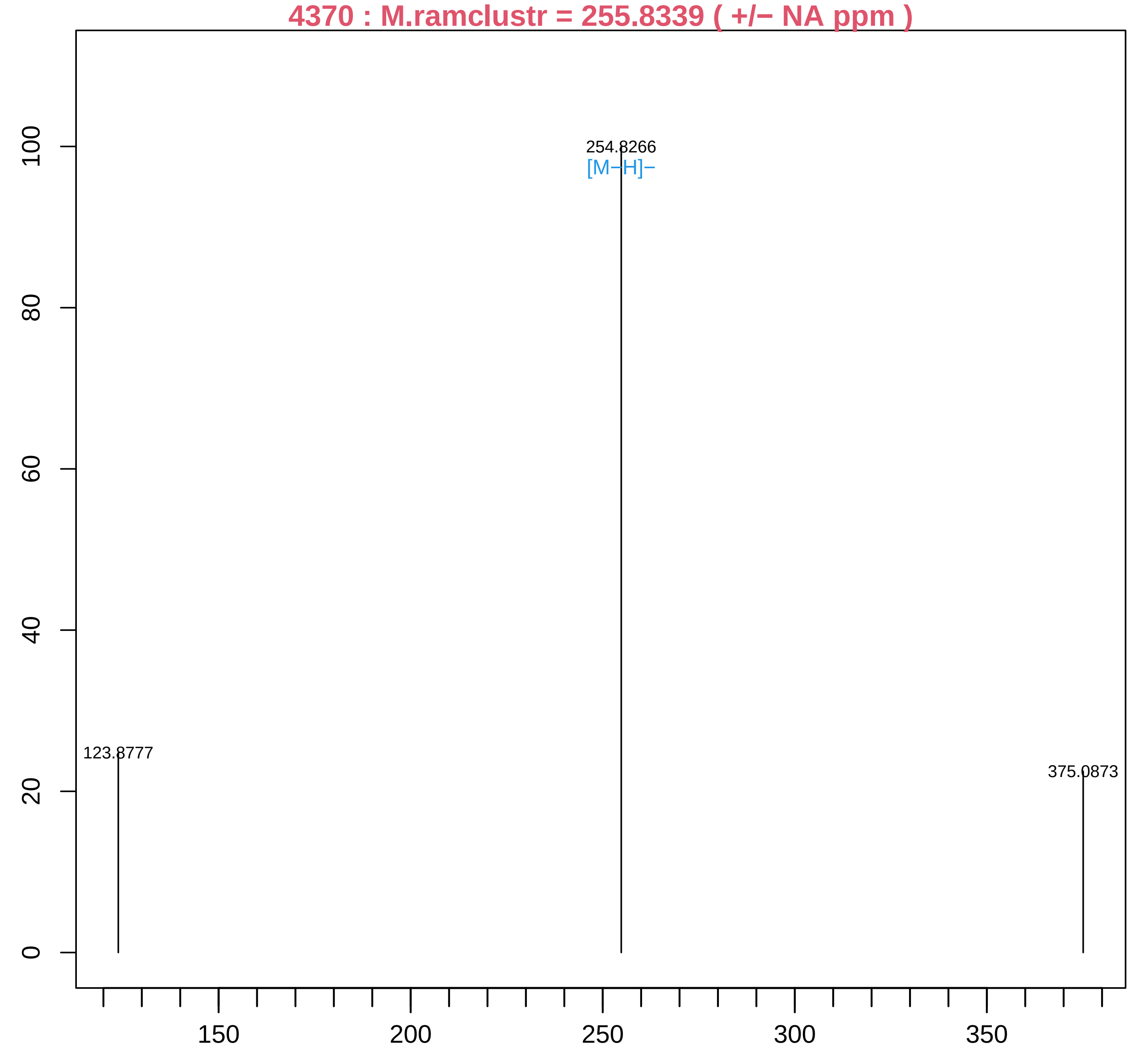

4370 : M.ramclustr = 255.8339 (+/− NA ppm)
254.8266
[M−H]−
123.8777
375.0873
0
20
40
60
80
100
150
200
250
300
350

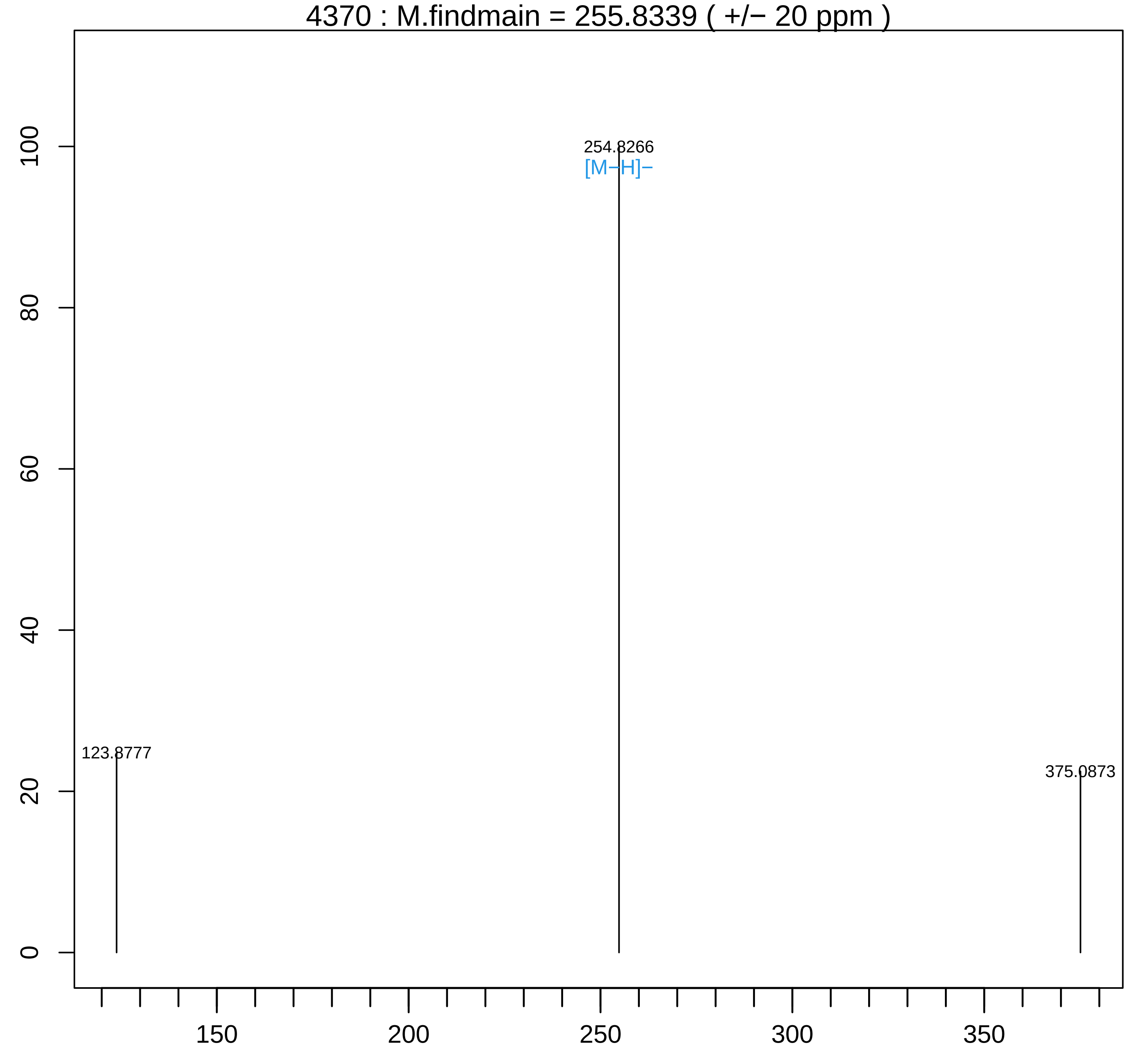

4370 : M.findmain = 255.8339 (+/− 20 ppm)
254.8266
[M−H]−
123.8777
375.0873
0
20
40
60
80
100
150
200
250
300
350

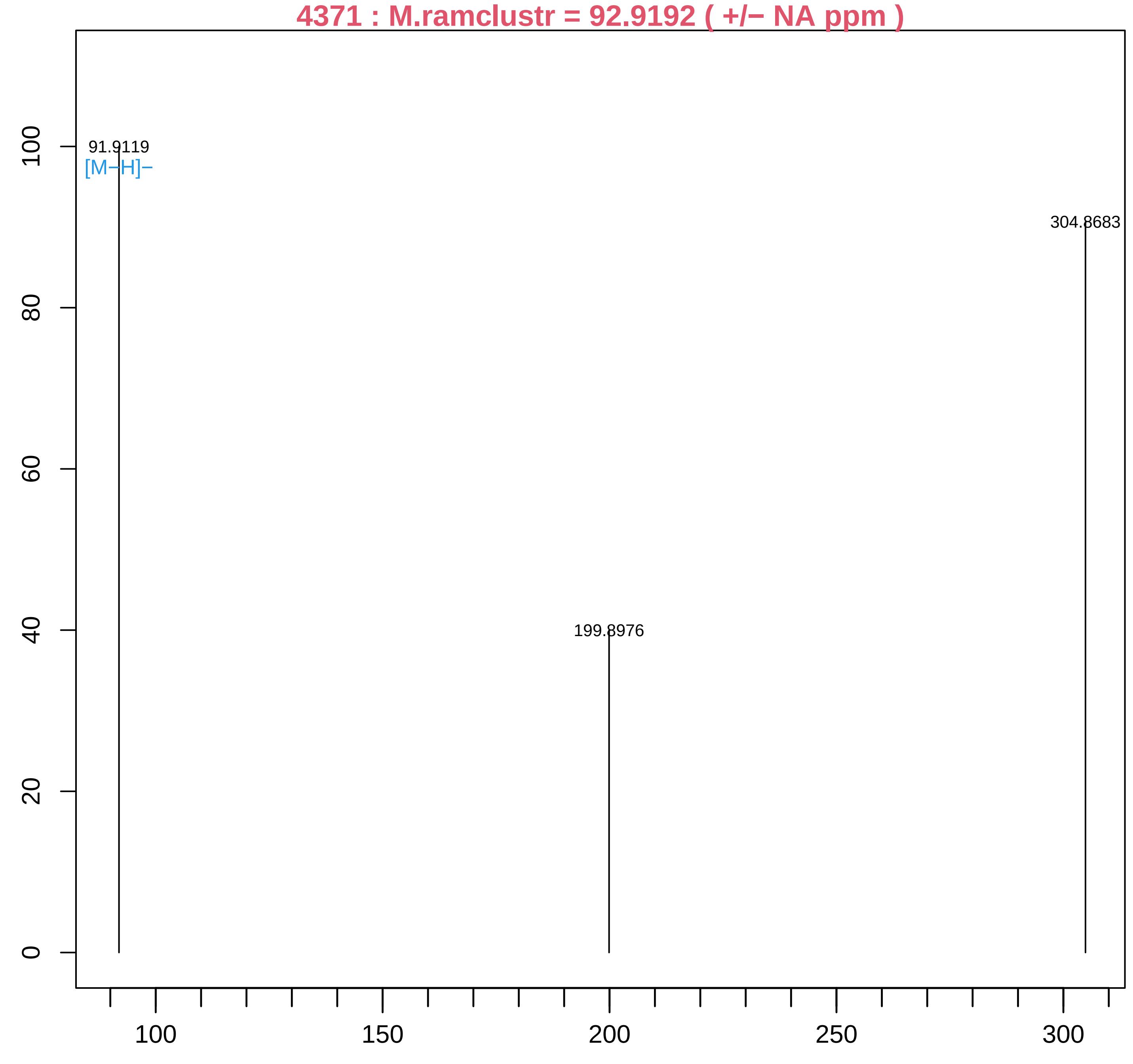

4371 : M.ramclustr = 92.9192 (+/− NA ppm)
91.9119
[M−H]−
199.8976
304.8683
0
20
40
60
80
100
100
150
200
250
300

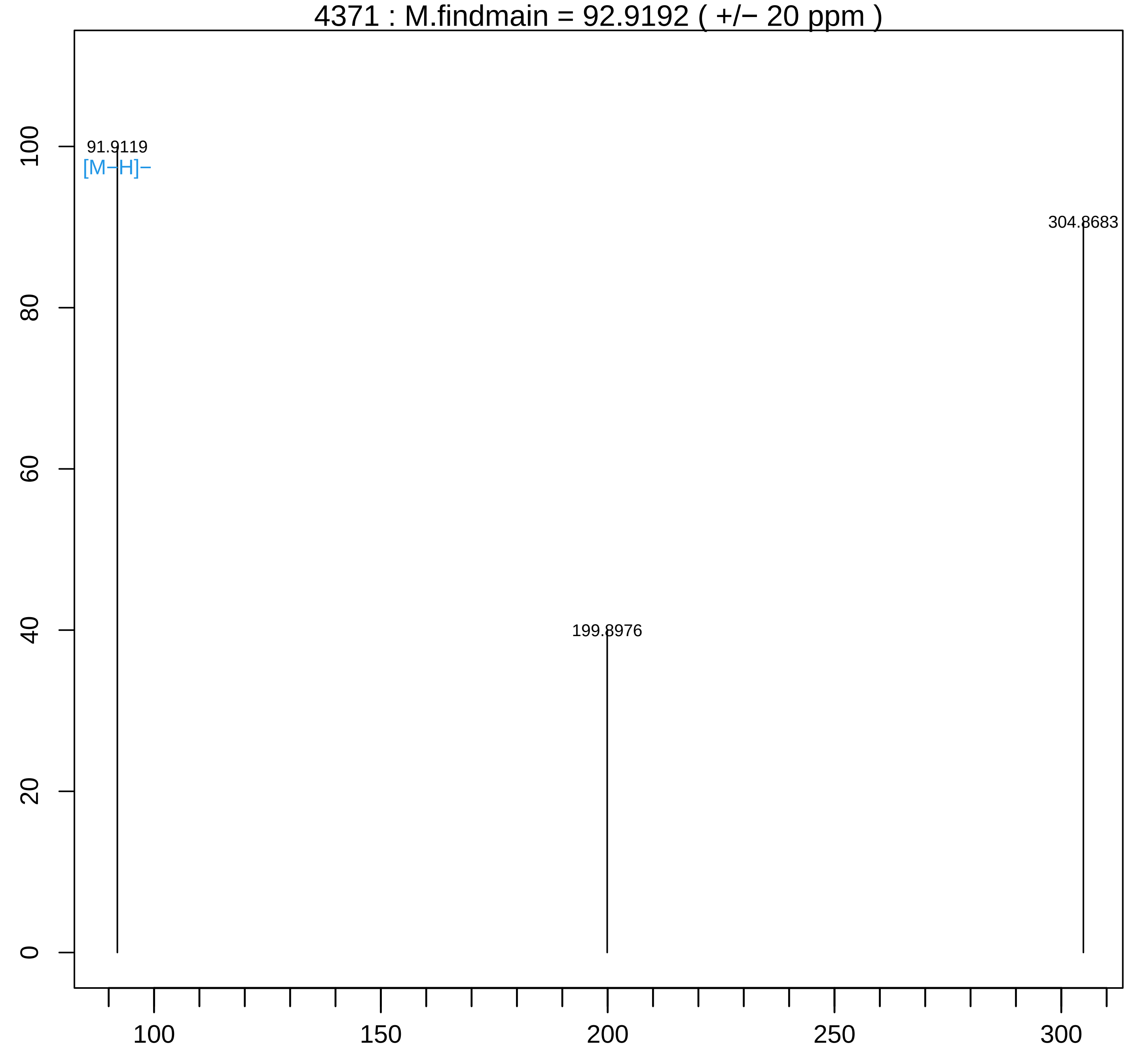

4371 : M.findmain = 92.9192 (+/− 20 ppm)
91.9119
[M−H]−
199.8976
304.8683
0
20
40
60
80
100
100
150
200
250
300

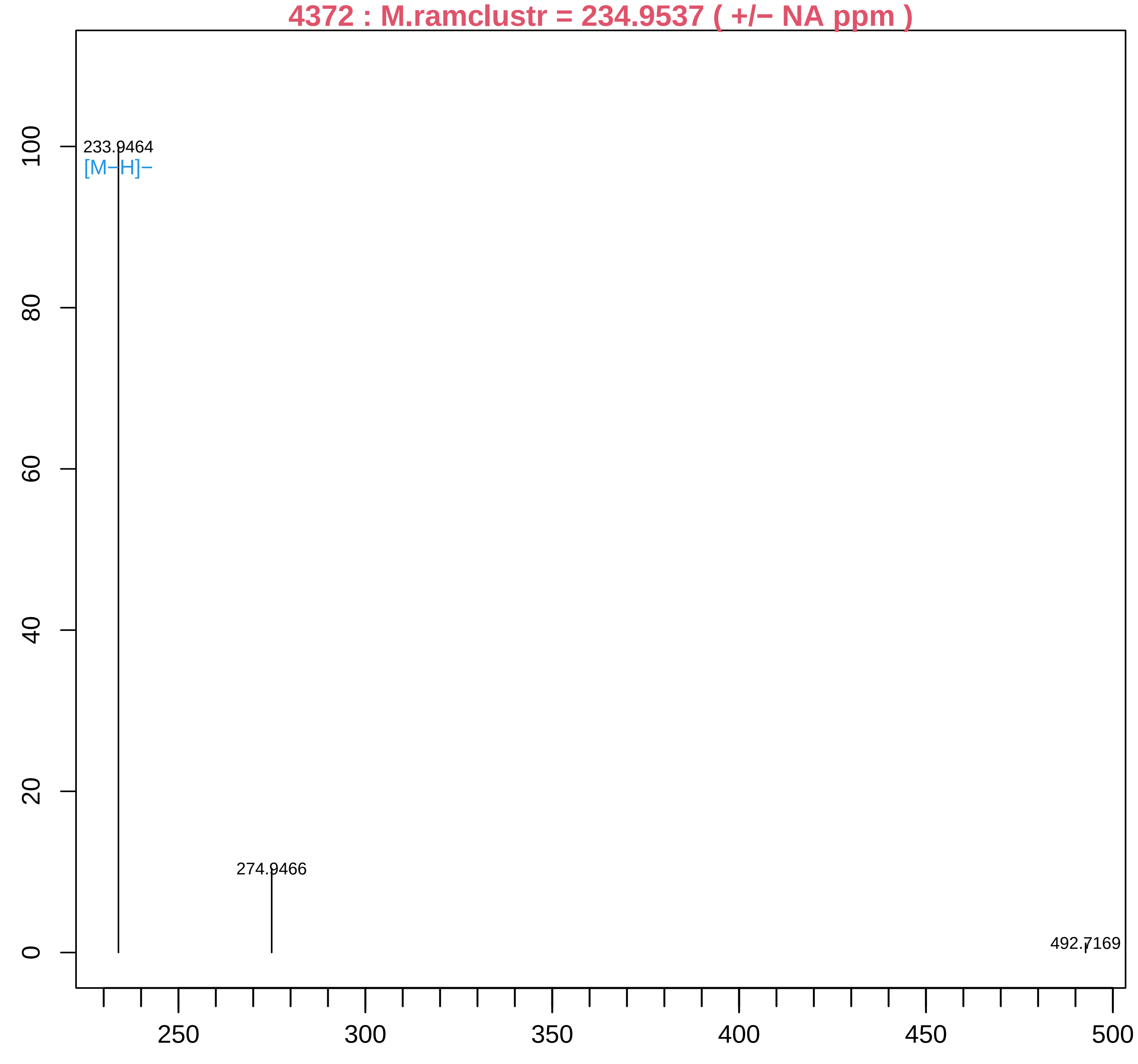
4372 : M.ramclustr = 234.9537 (+/− NA ppm)
233.9464
[M−H]−
274.9466
492.7169
0
20
40
60
80
100
250
300
350
400
450
500

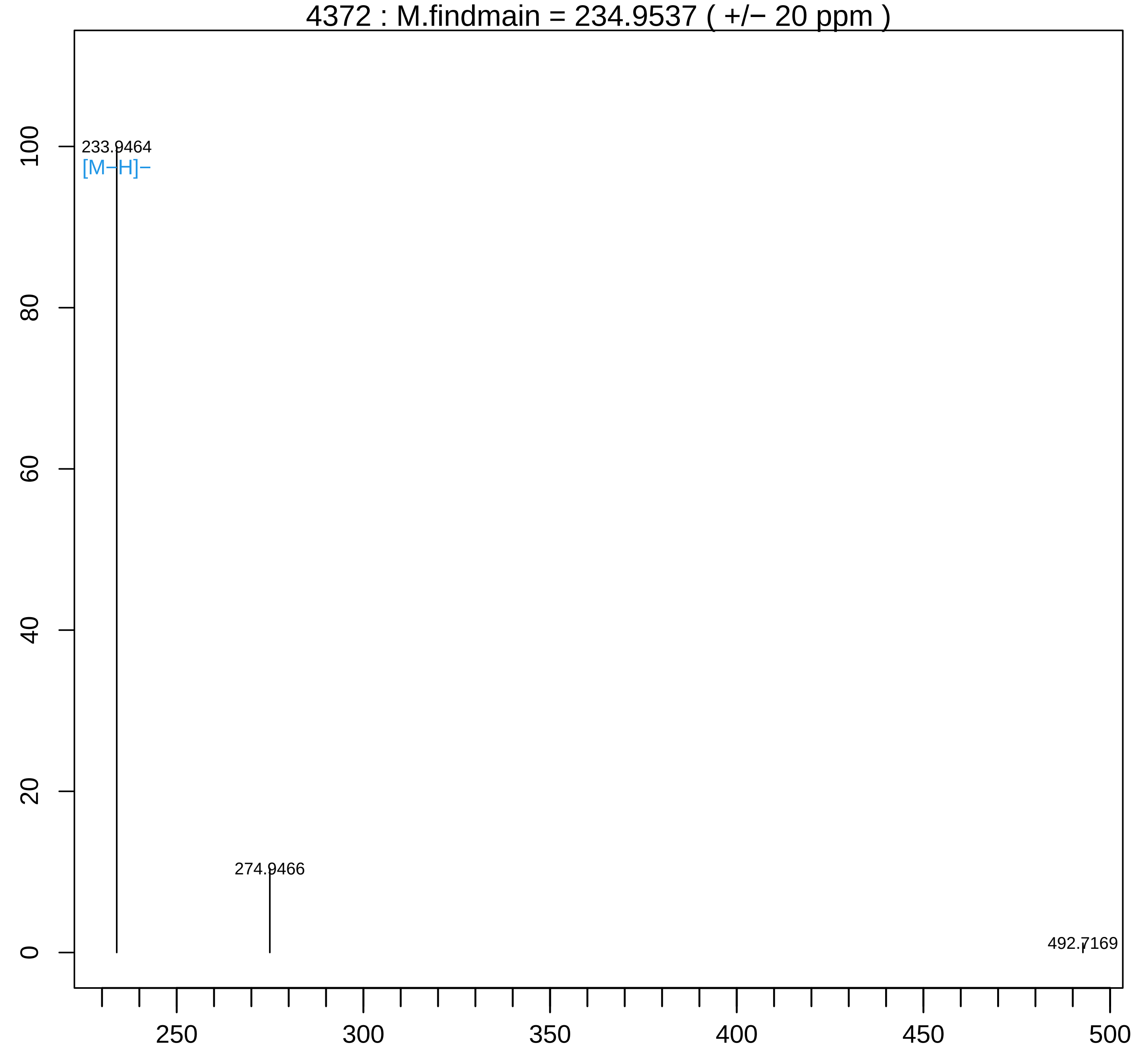
4372 : M.findmain = 234.9537 (+/− 20 ppm)
233.9464
[M−H]−
274.9466
492.7169
0
20
40
60
80
100
250
300
350
400
450
500

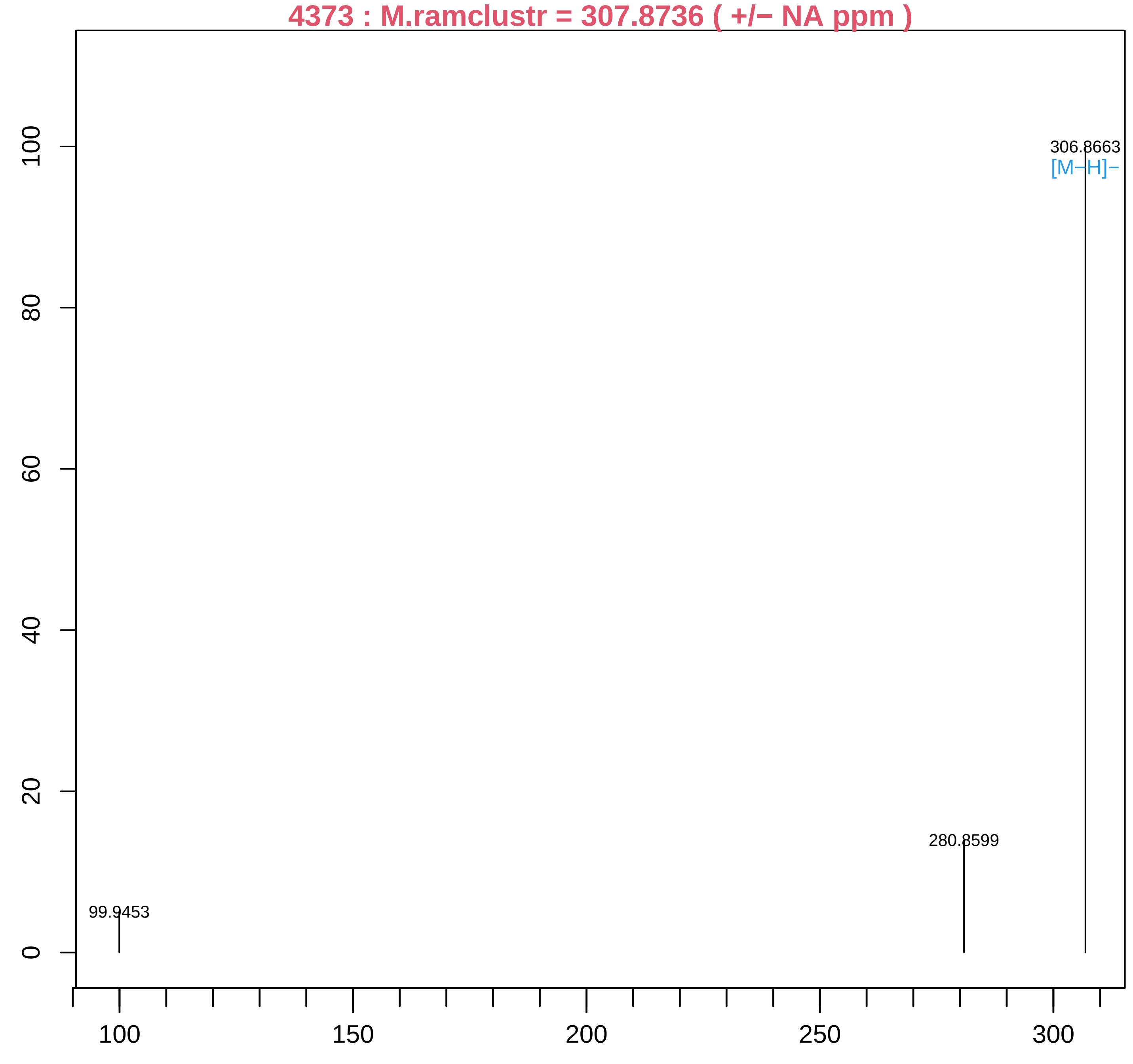
4373 : M.ramclustr = 307.8736 (+/− NA ppm)
306.8663
[M−H]−
280.8599
99.9453
0
20
40
60
80
100
100
150
200
250
300

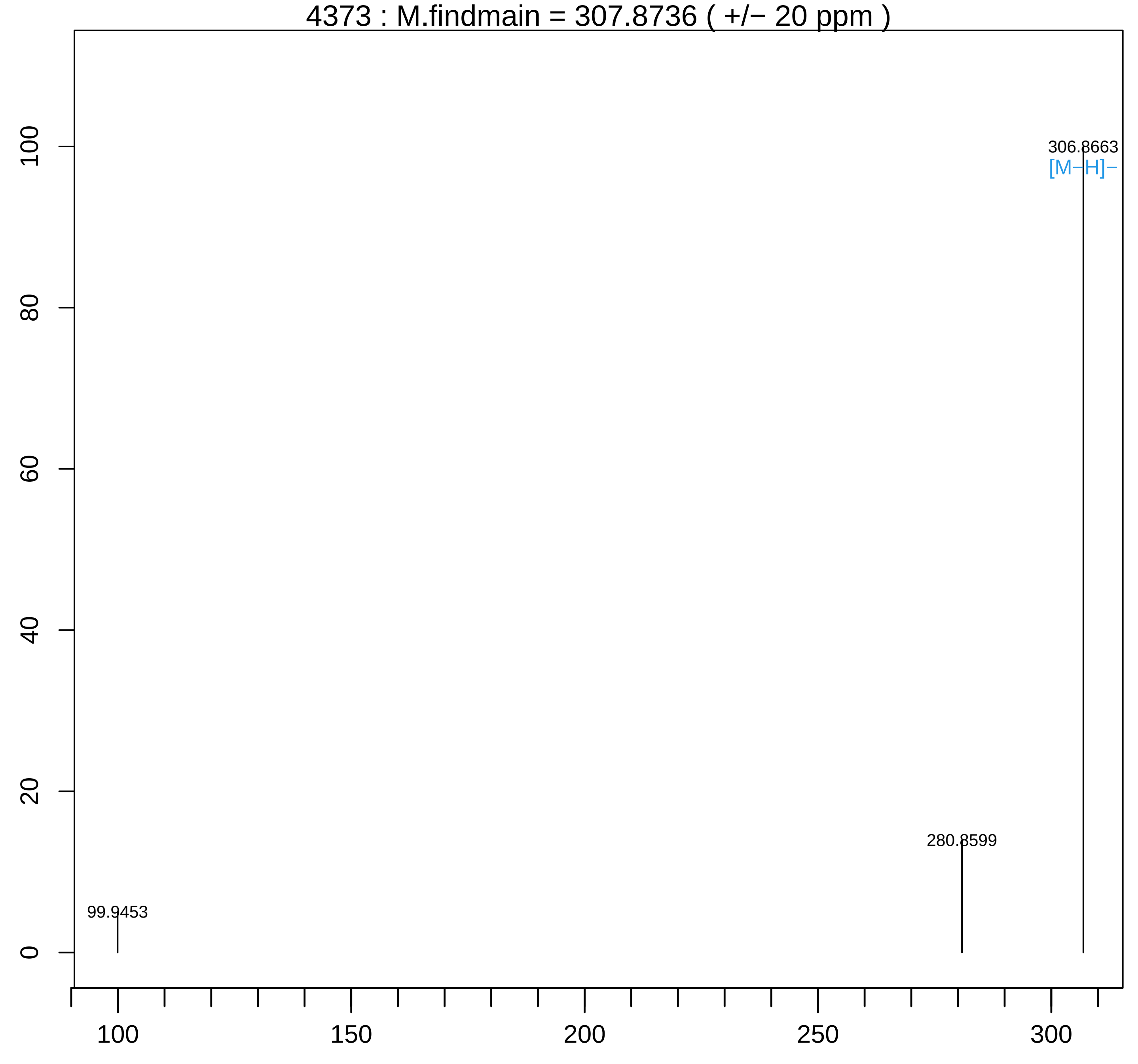
4373 : M.findmain = 307.8736 (+/− 20 ppm)
306.8663
[M−H]−
280.8599
99.9453
0
20
40
60
80
100
100
150
200
250
300

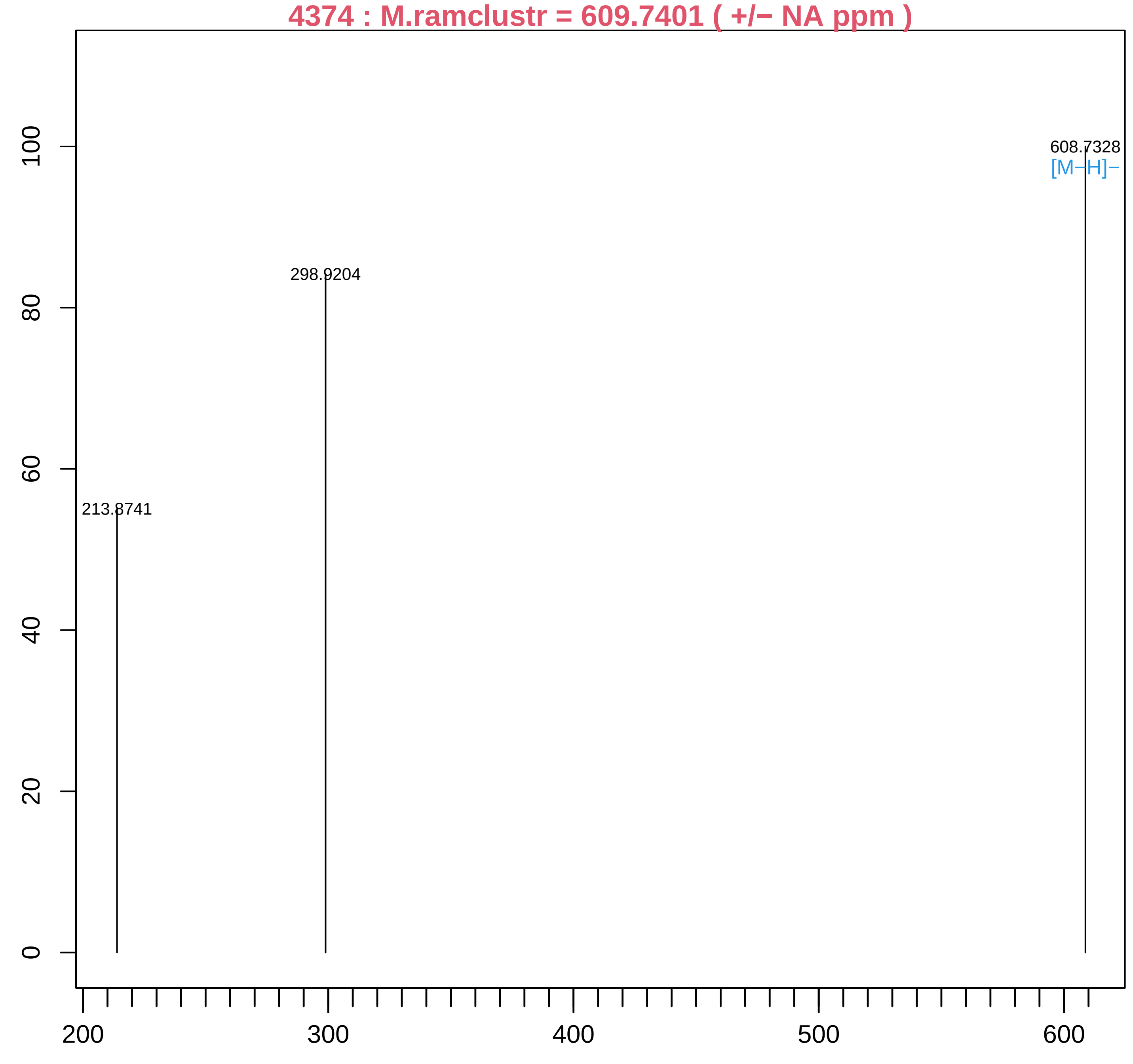
4374 : M.ramclustr = 609.7401 (+/− NA ppm)
608.7328
[M−H]−
298.9204
213.8741
0
20
40
60
80
100
200
300
400
500
600

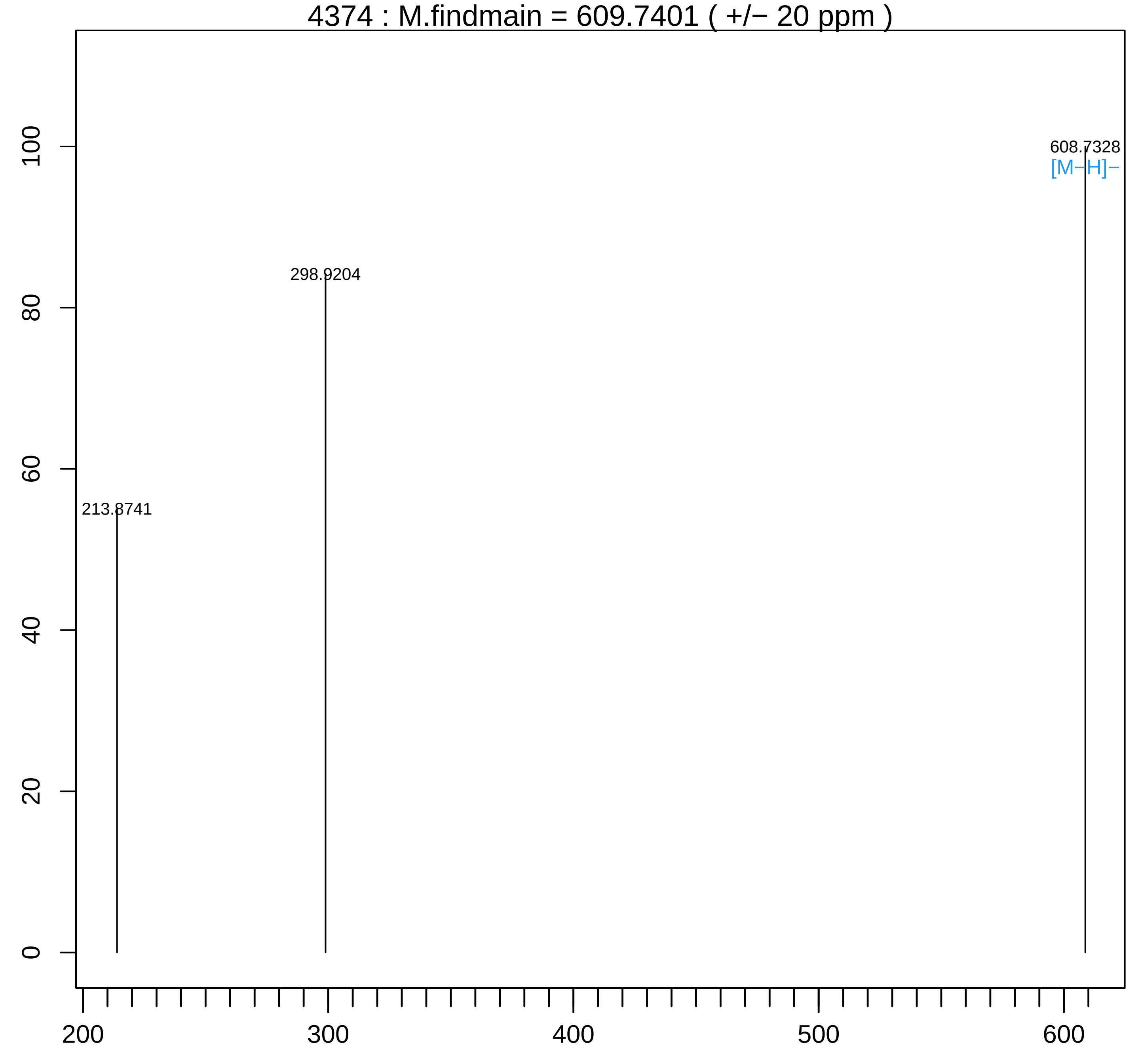
4374 : M.findmain = 609.7401 (+/− 20 ppm)
608.7328
[M−H]−
298.9204
213.8741
0
20
40
60
80
100
200
300
400
500
600

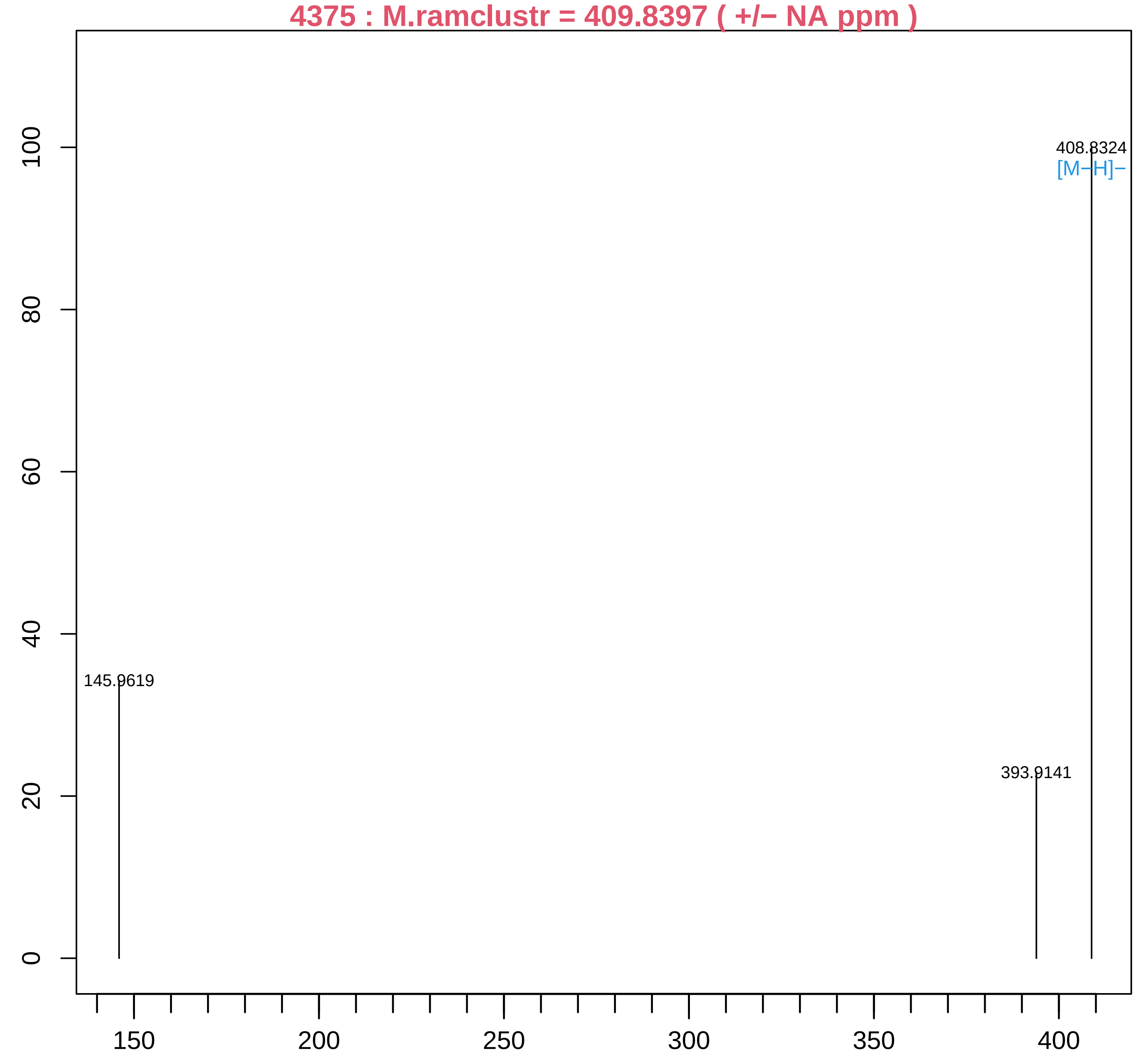
4375 : M.ramclustr = 409.8397 (+/− NA ppm)
408.8324
[M−H]−
145.9619
393.9141
0
20
40
60
80
100
150
200
250
300
350
400

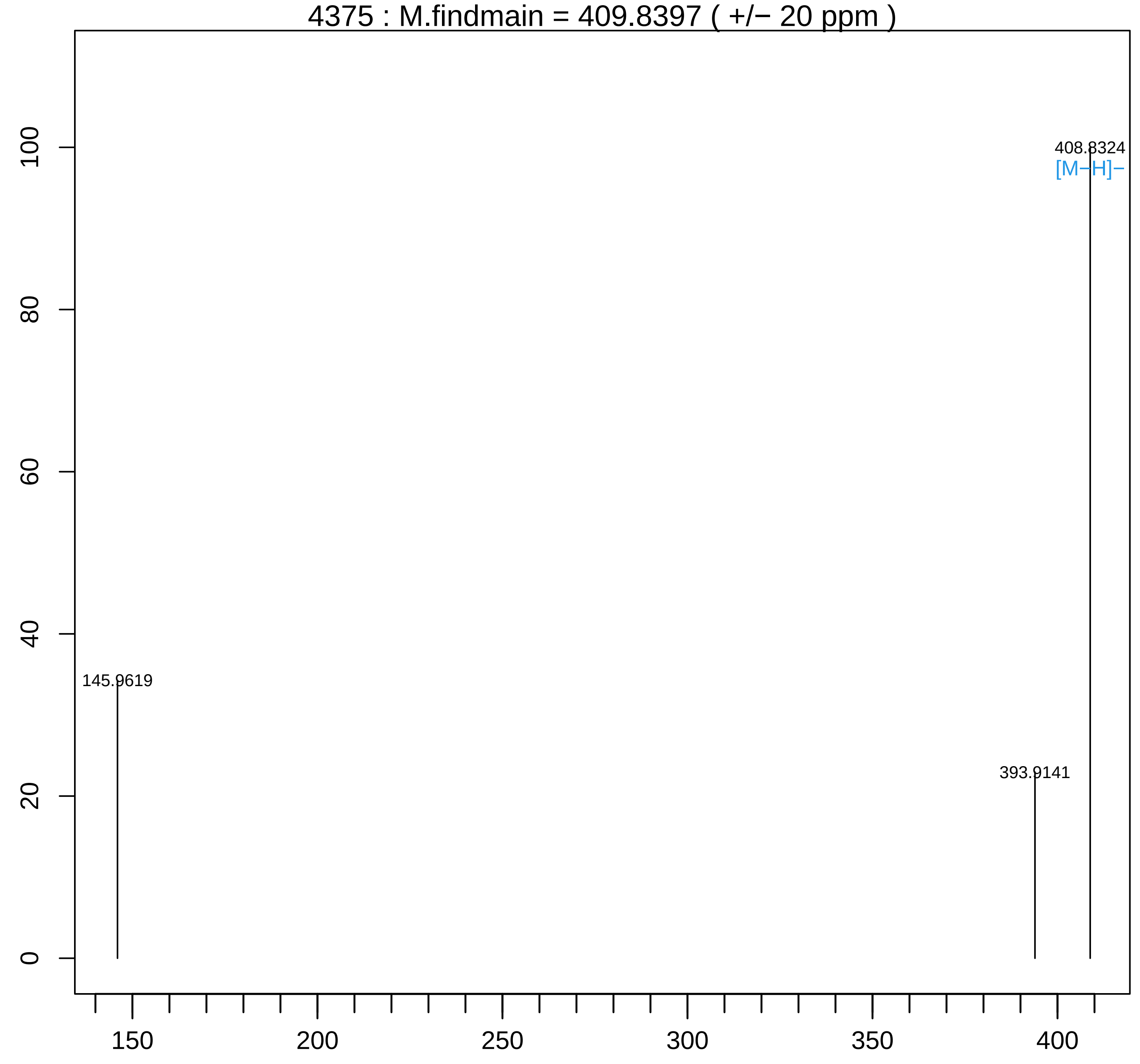
4375 : M.findmain = 409.8397 (+/− 20 ppm)
408.8324
[M−H]−
145.9619
393.9141
0
20
40
60
80
100
150
200
250
300
350
400

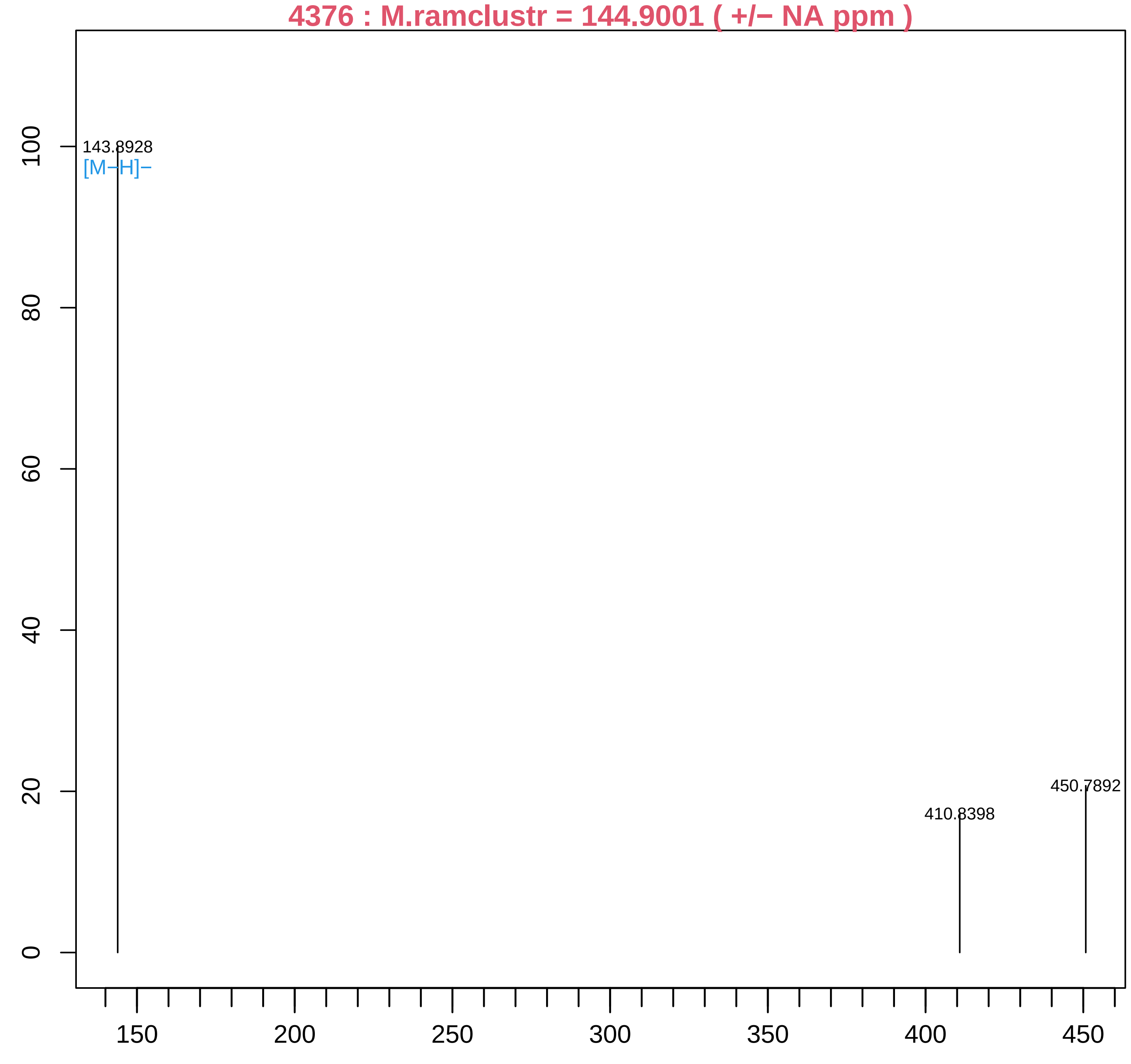
4376 : M.ramclustr = 144.9001 (+/− NA ppm)
143.8928
[M−H]−
410.8398
450.7892
0
20
40
60
80
100
150
200
250
300
350
400
450

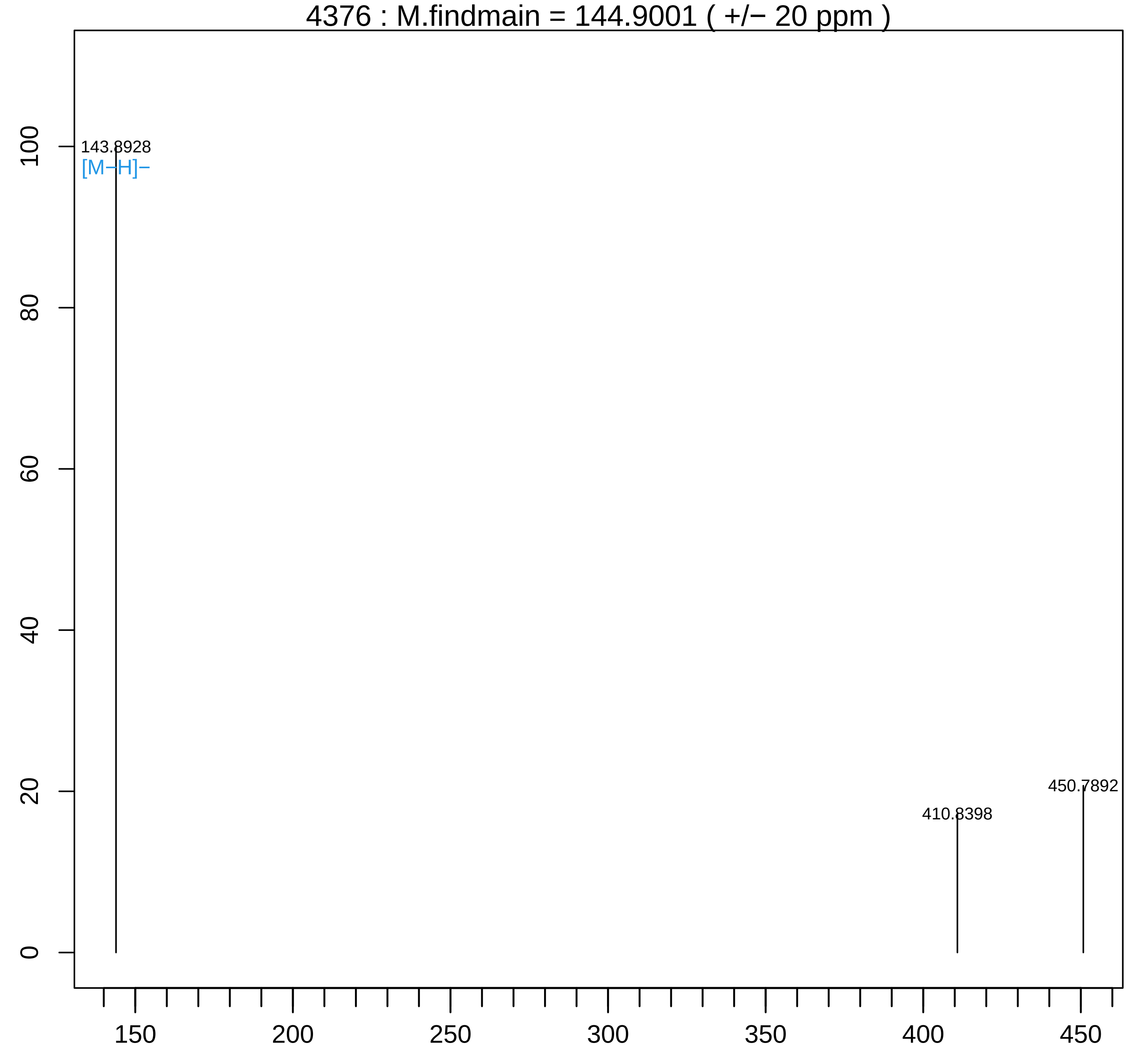
4376 : M.findmain = 144.9001 (+/− 20 ppm)
143.8928
[M−H]−
410.8398
450.7892
0
20
40
60
80
100
150
200
250
300
350
400
450

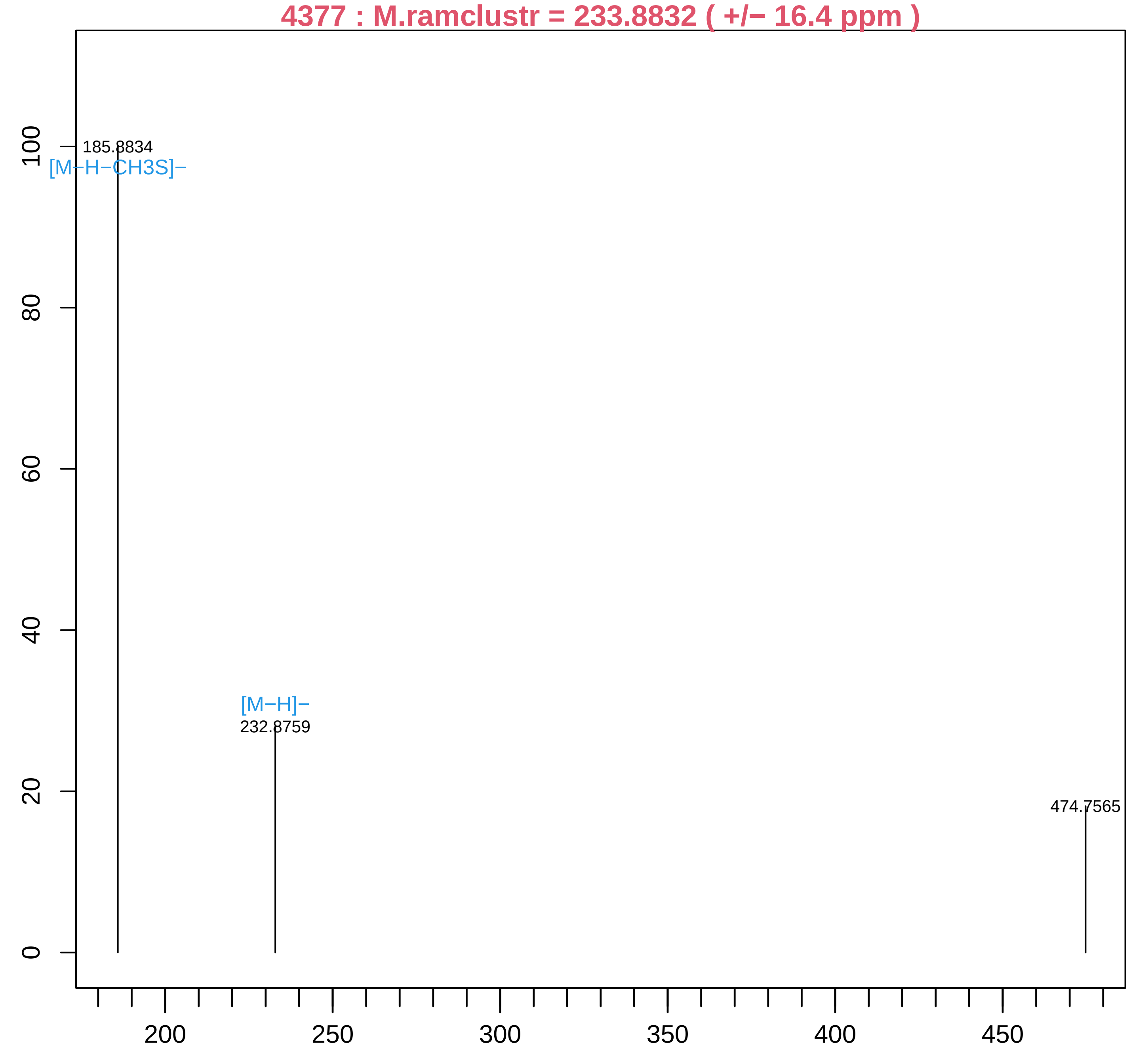
4377 : M.ramclustr = 233.8832 (+/− 16.4 ppm)
185.8834
[M−H−CH3S]−
[M−H]−
232.8759
474.7565
0
20
40
60
80
100
200
250
300
350
400
450

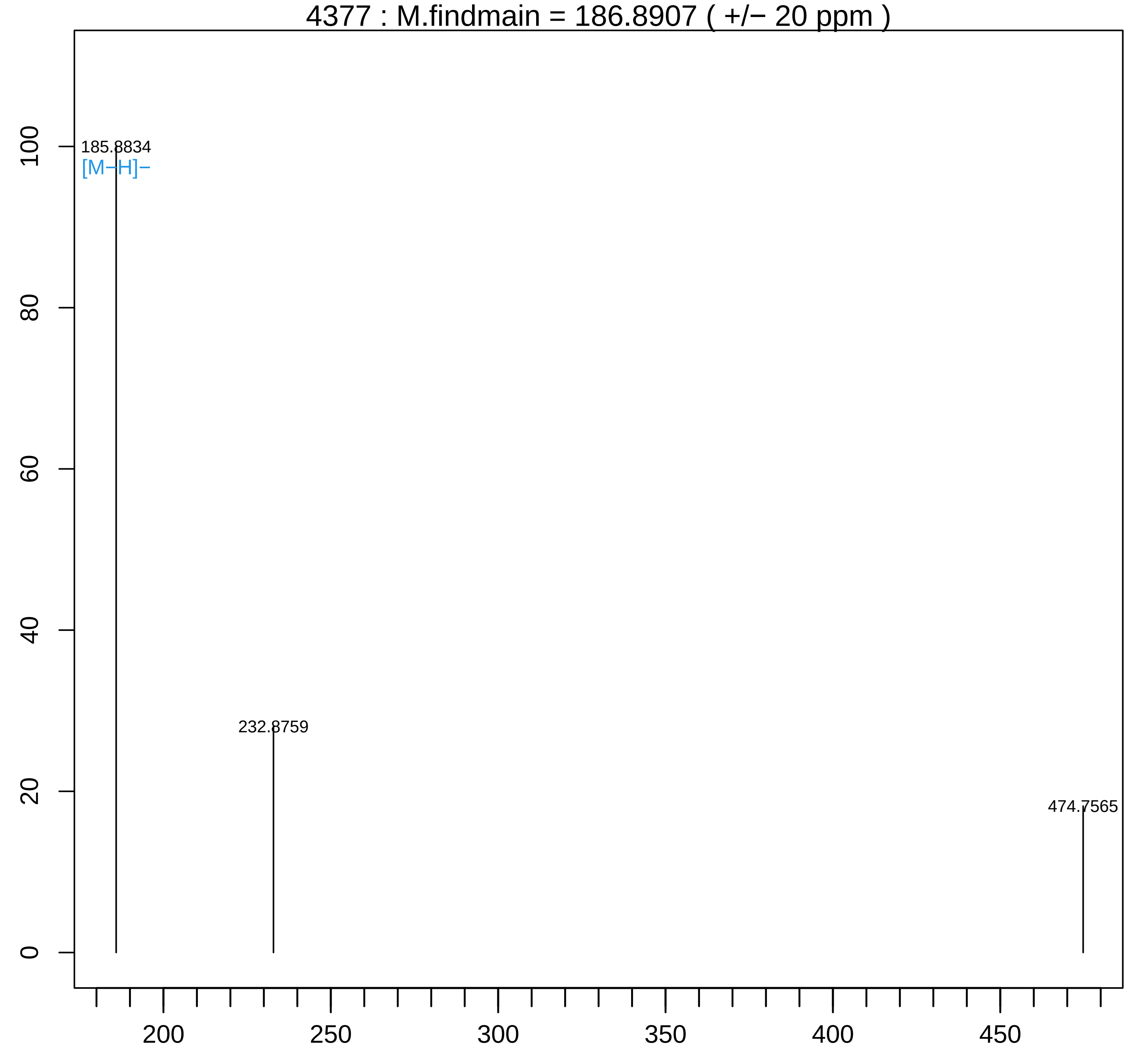
4377 : M.findmain = 186.8907 (+/− 20 ppm)
185.8834
[M−H]−
232.8759
474.7565
0
20
40
60
80
100
200
250
300
350
400
450

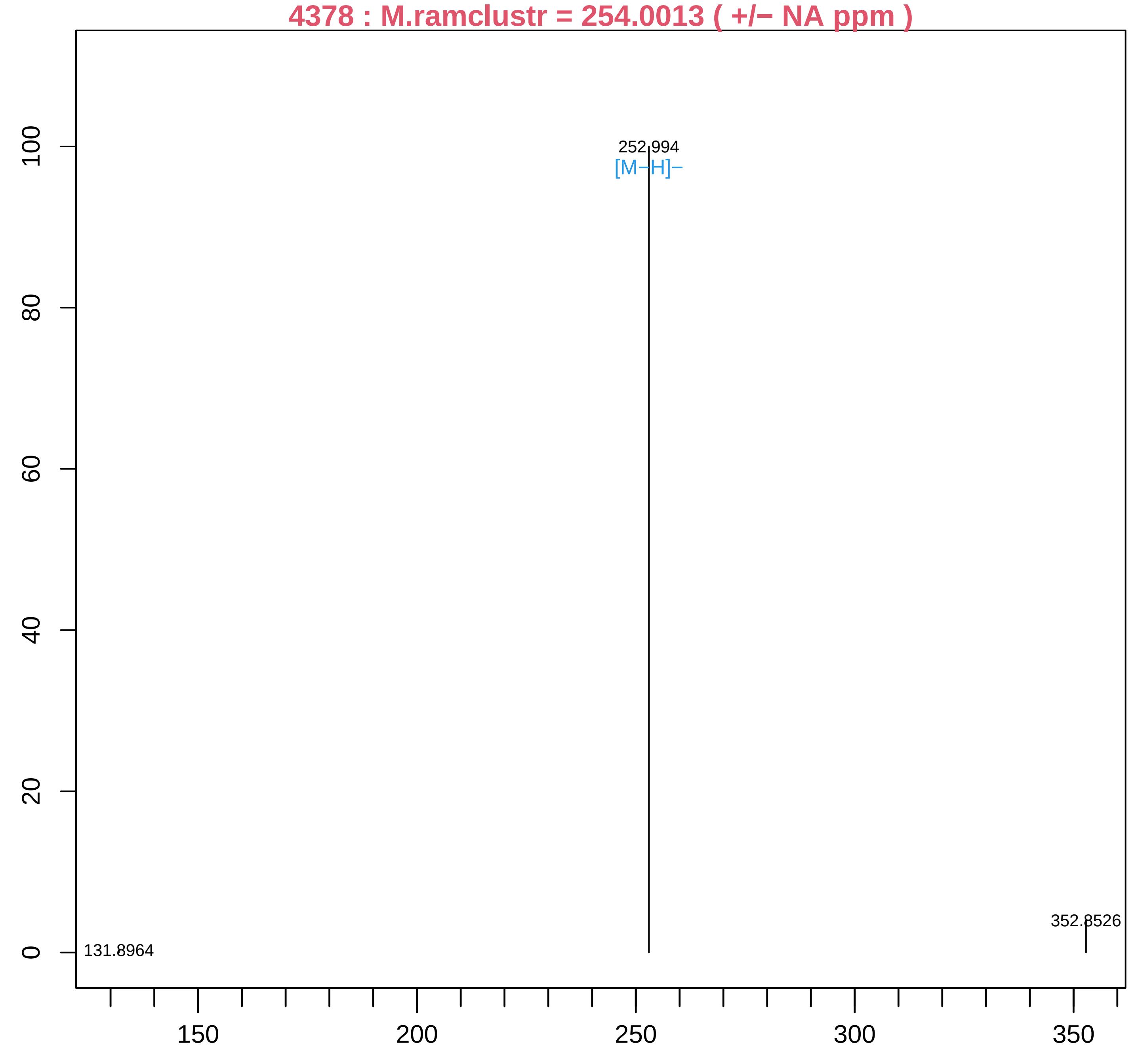
4378 : M.ramclustr = 254.0013 (+/− NA ppm)
252.994
[M−H]−
352.8526
131.8964
0
20
40
60
80
100
150
200
250
300
350

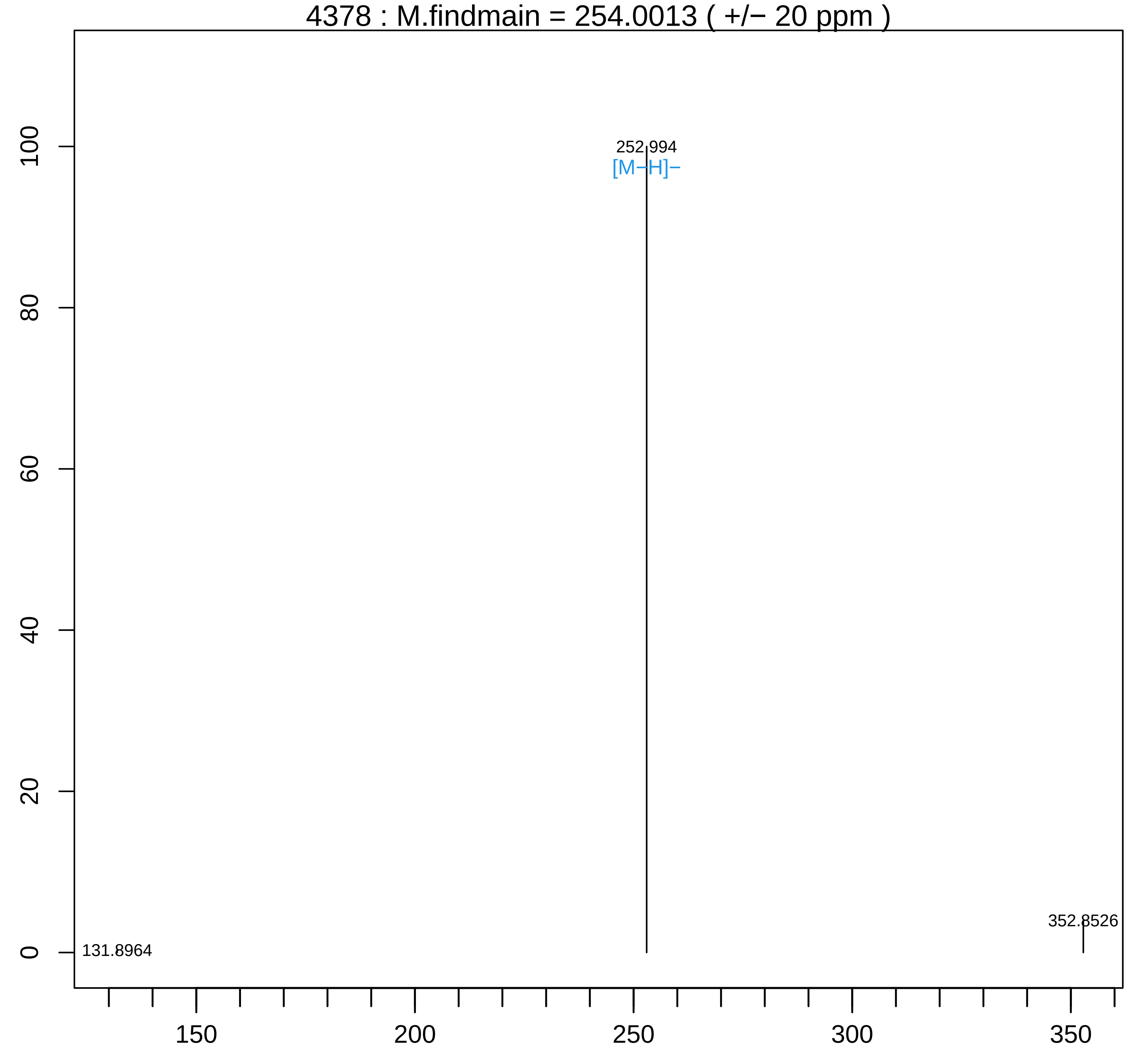
4378 : M.findmain = 254.0013 (+/− 20 ppm)
252.994
[M−H]−
352.8526
131.8964
0
20
40
60
80
100
150
200
250
300
350

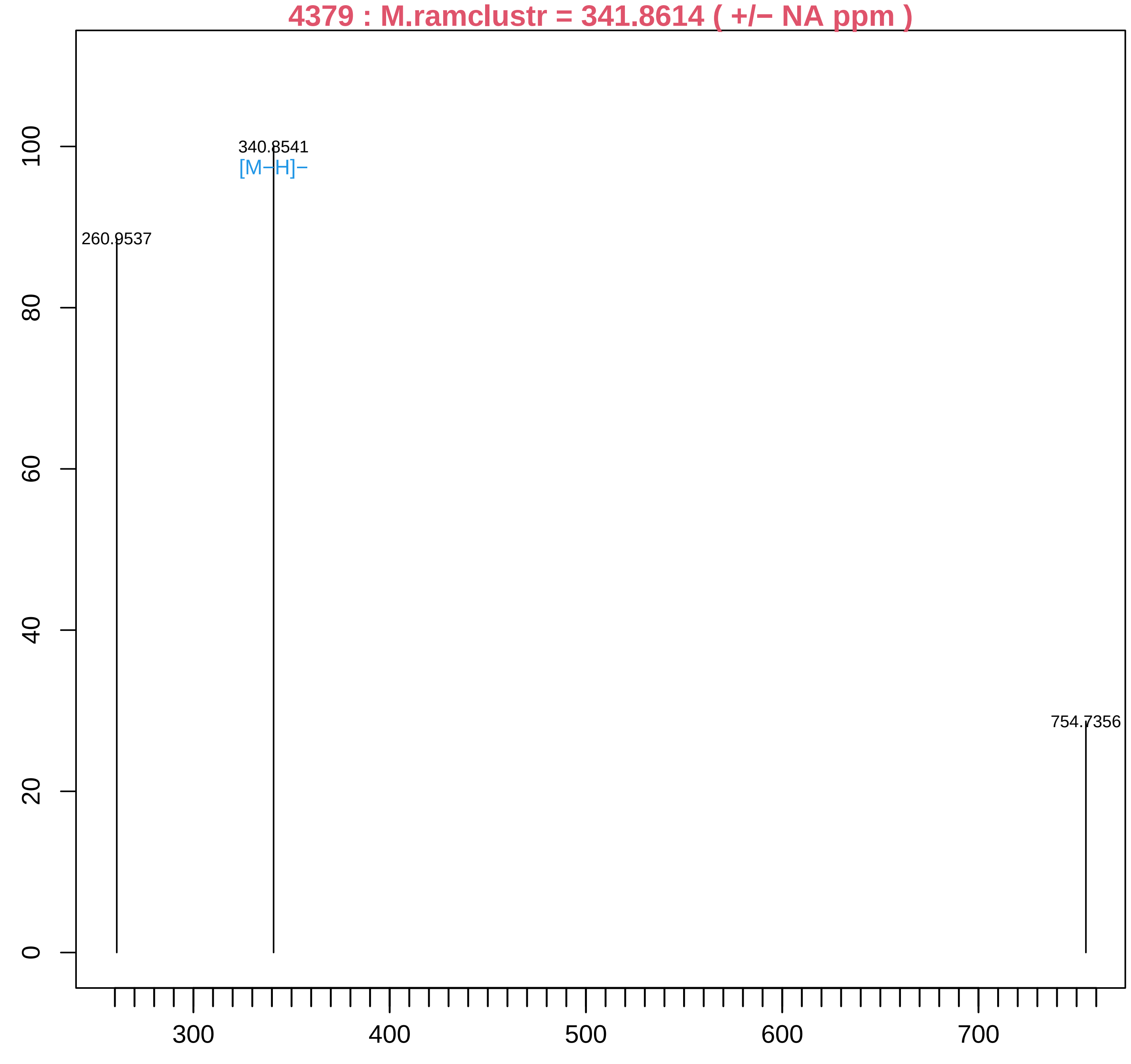
4379 : M.ramclustr = 341.8614 (+/− NA ppm)
340.8541
[M−H]−
260.9537
754.7356
0
20
40
60
80
100
300
400
500
600
700

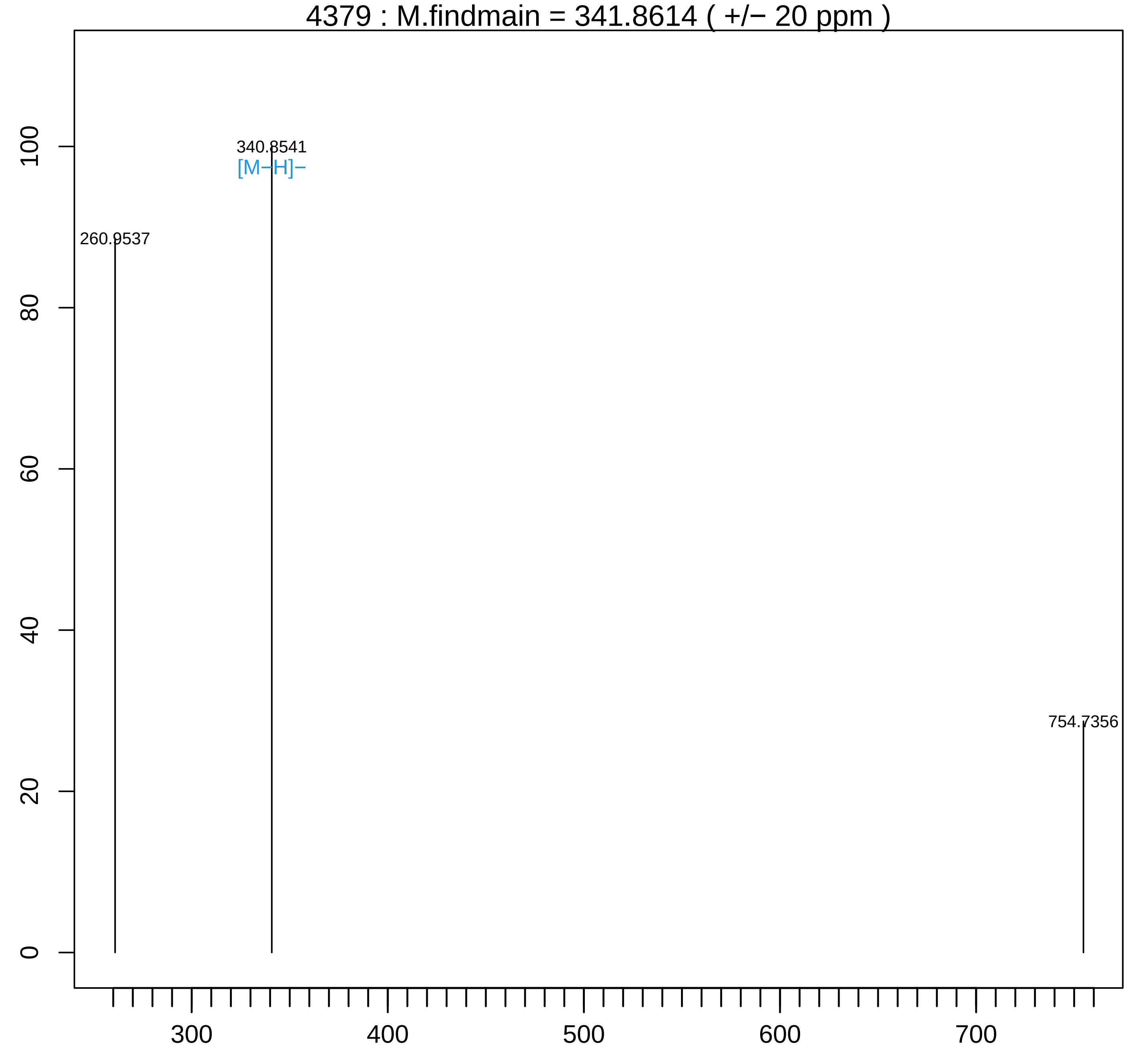
4379 : M.findmain = 341.8614 (+/− 20 ppm)
340.8541
[M−H]−
260.9537
754.7356
0
20
40
60
80
100
300
400
500
600
700

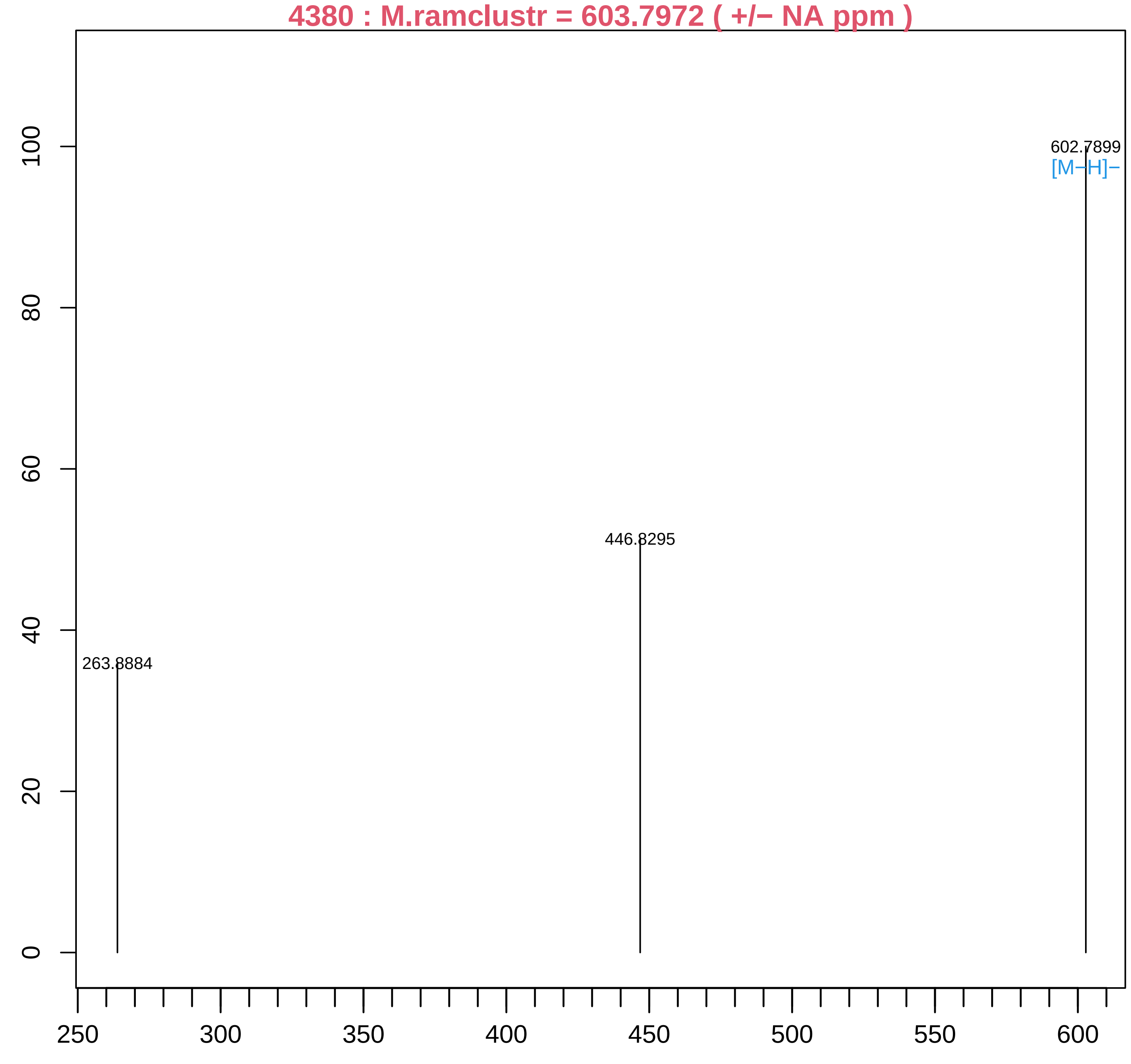
4380 : M.ramclustr = 603.7972 (+/− NA ppm)
602.7899
[M−H]−
446.8295
263.8884
0
20
40
60
80
100
250
300
350
400
450
500
550
600

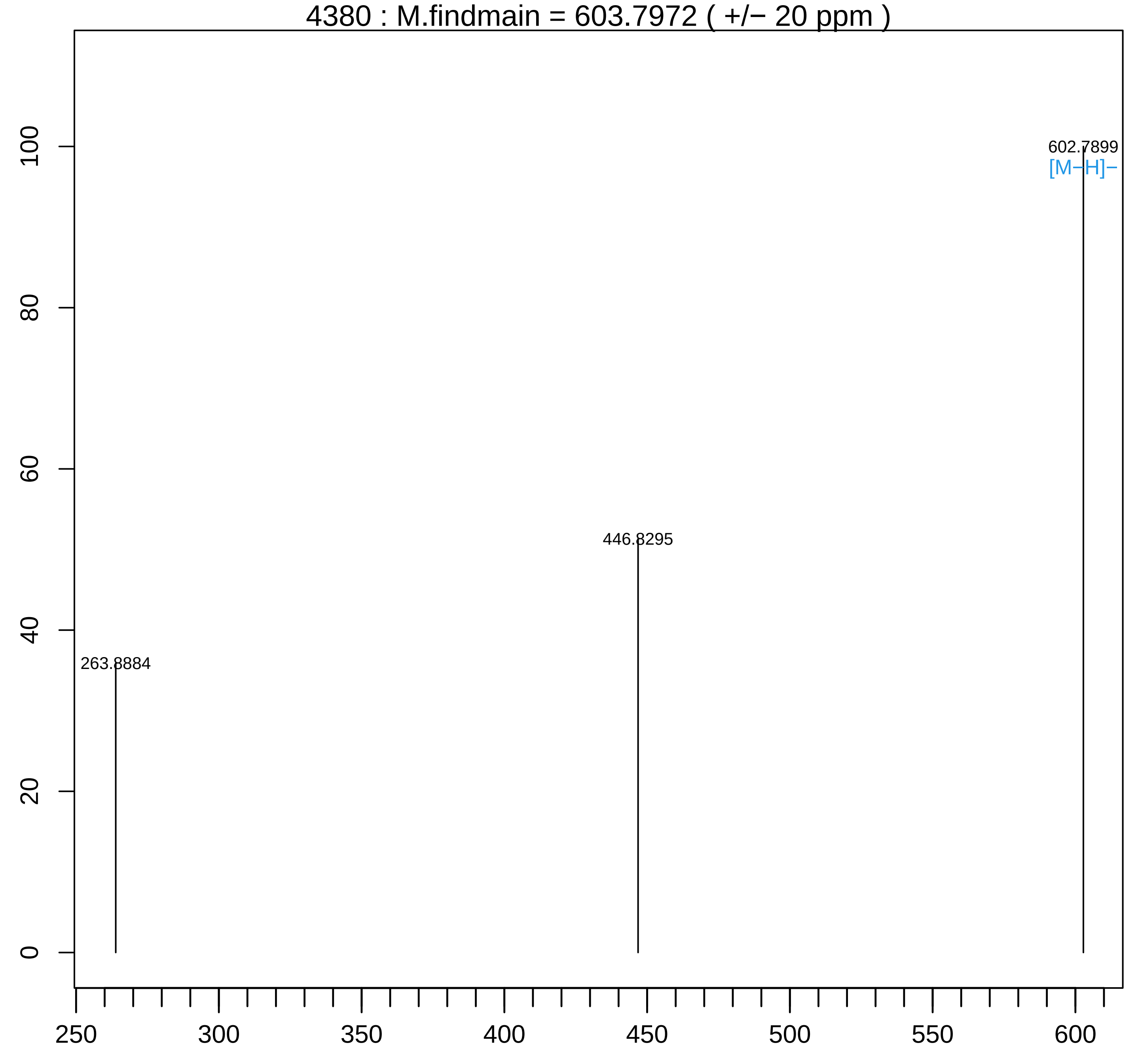
4380 : M.findmain = 603.7972 (+/− 20 ppm)
602.7899
[M−H]−
446.8295
263.8884
0
20
40
60
80
100
250
300
350
400
450
500
550
600

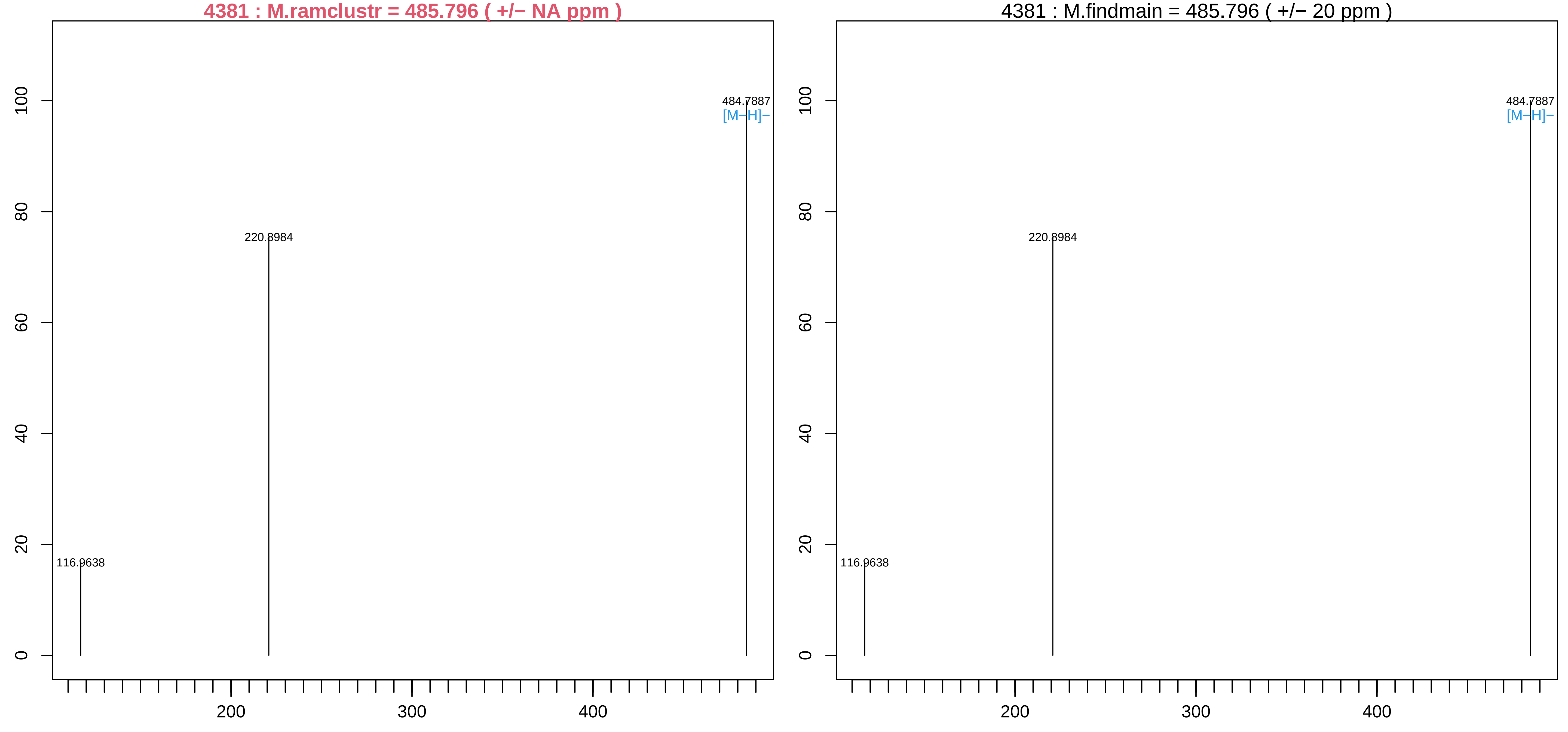

4381 : M.ramclustr = 485.796 (+/− NA ppm)
116.9638
220.8984
484.7887
[M−H]−
4381 : M.findmain = 485.796 (+/− 20 ppm)
116.9638
220.8984
484.7887
[M−H]−

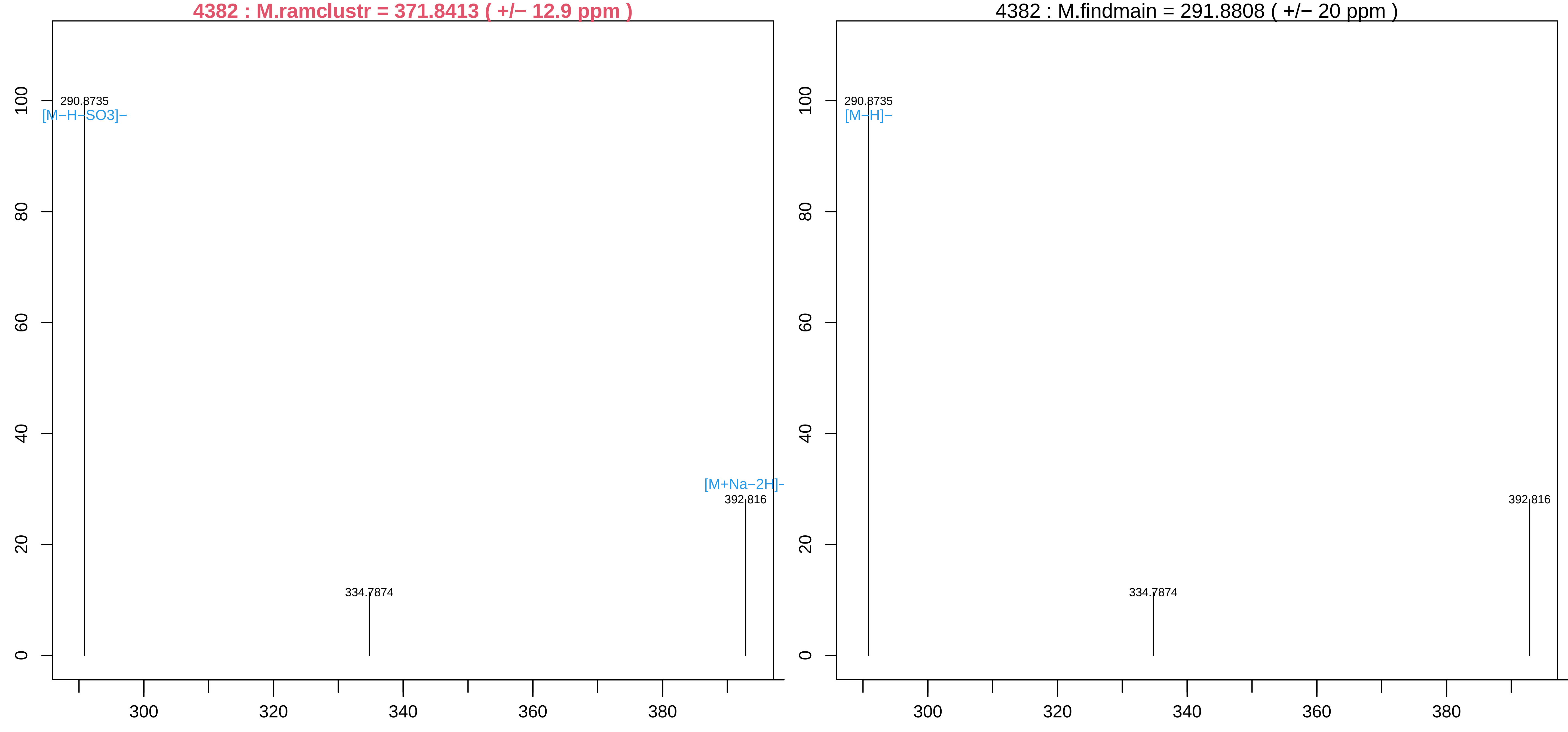
4382 : M.ramclustr = 371.8413 (+/− 12.9 ppm)
290.8735
[M−H−SO3]−
334.7874
[M+Na−2H]−
392.816
4382 : M.findmain = 291.8808 (+/− 20 ppm)
290.8735
[M−H]−
334.7874
392.816
0
20
40
60
80
100
300
320
340
360
380

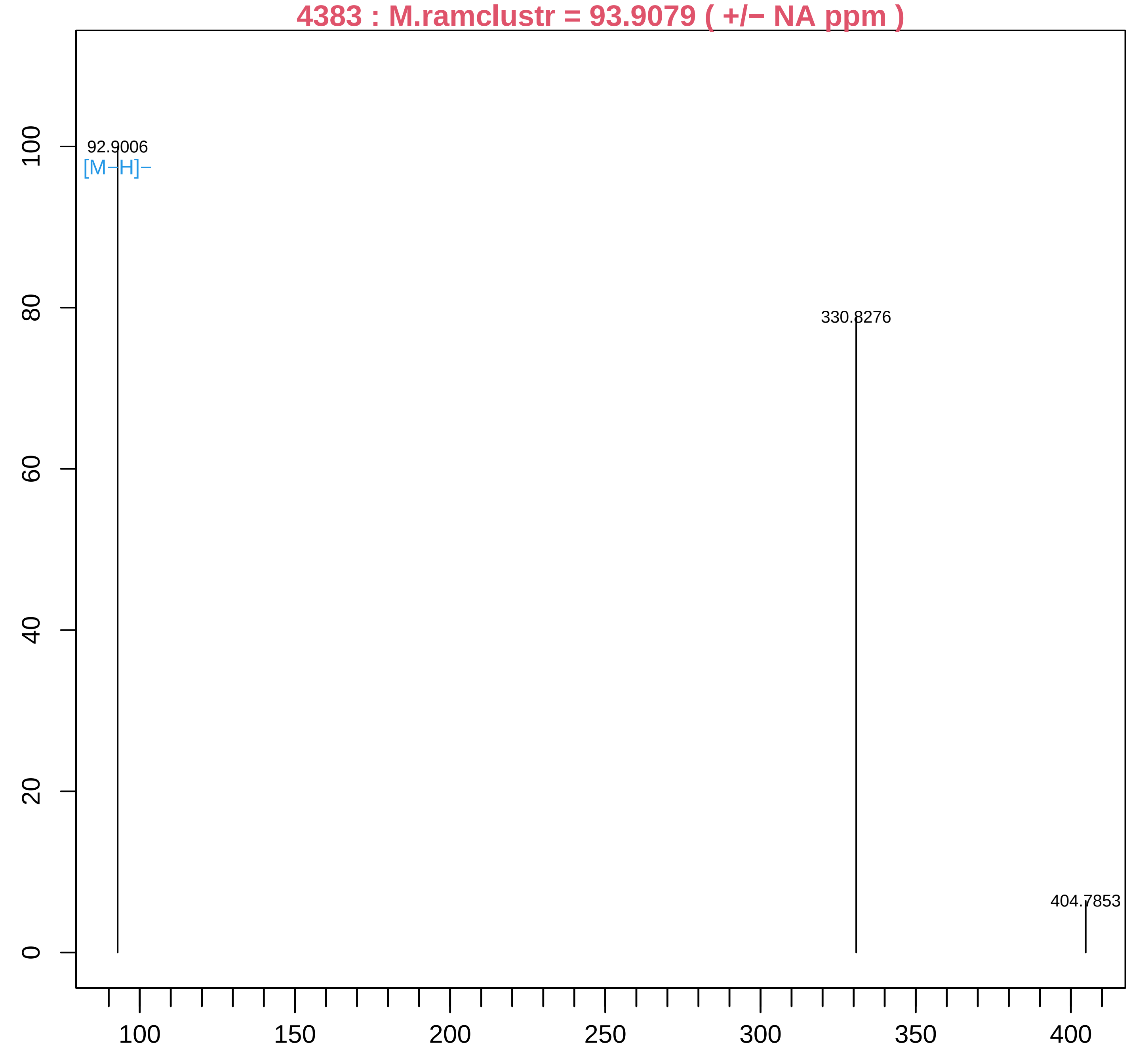

4383 : M.ramclustr = 93.9079 (+/− NA ppm)
92.9006
[M−H]−
330.8276
404.7853
0
20
40
60
80
100
100
150
200
250
300
350
400

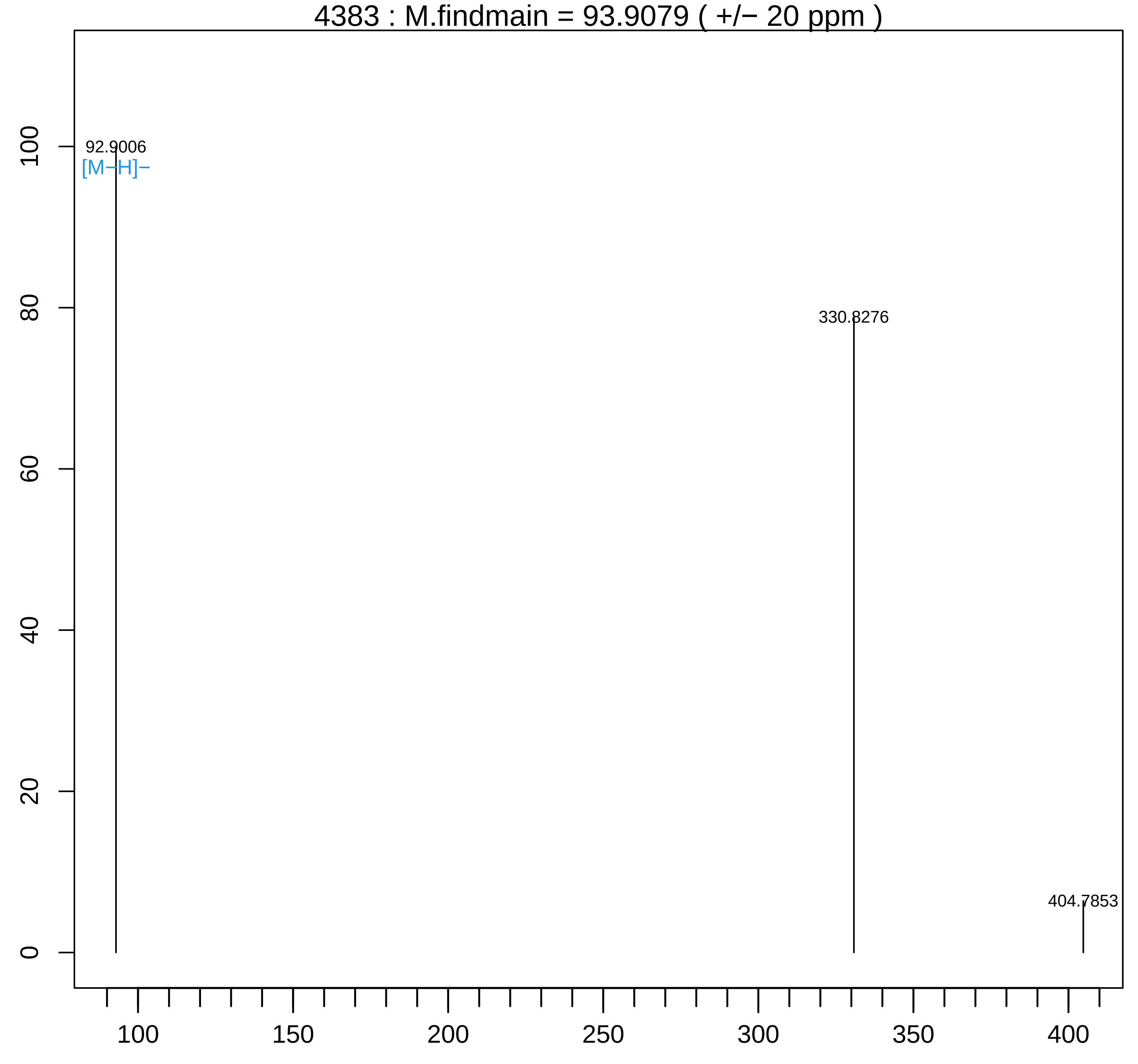

4383 : M.findmain = 93.9079 (+/− 20 ppm)
92.9006
[M−H]−
330.8276
404.7853
0
20
40
60
80
100
100
150
200
250
300
350
400

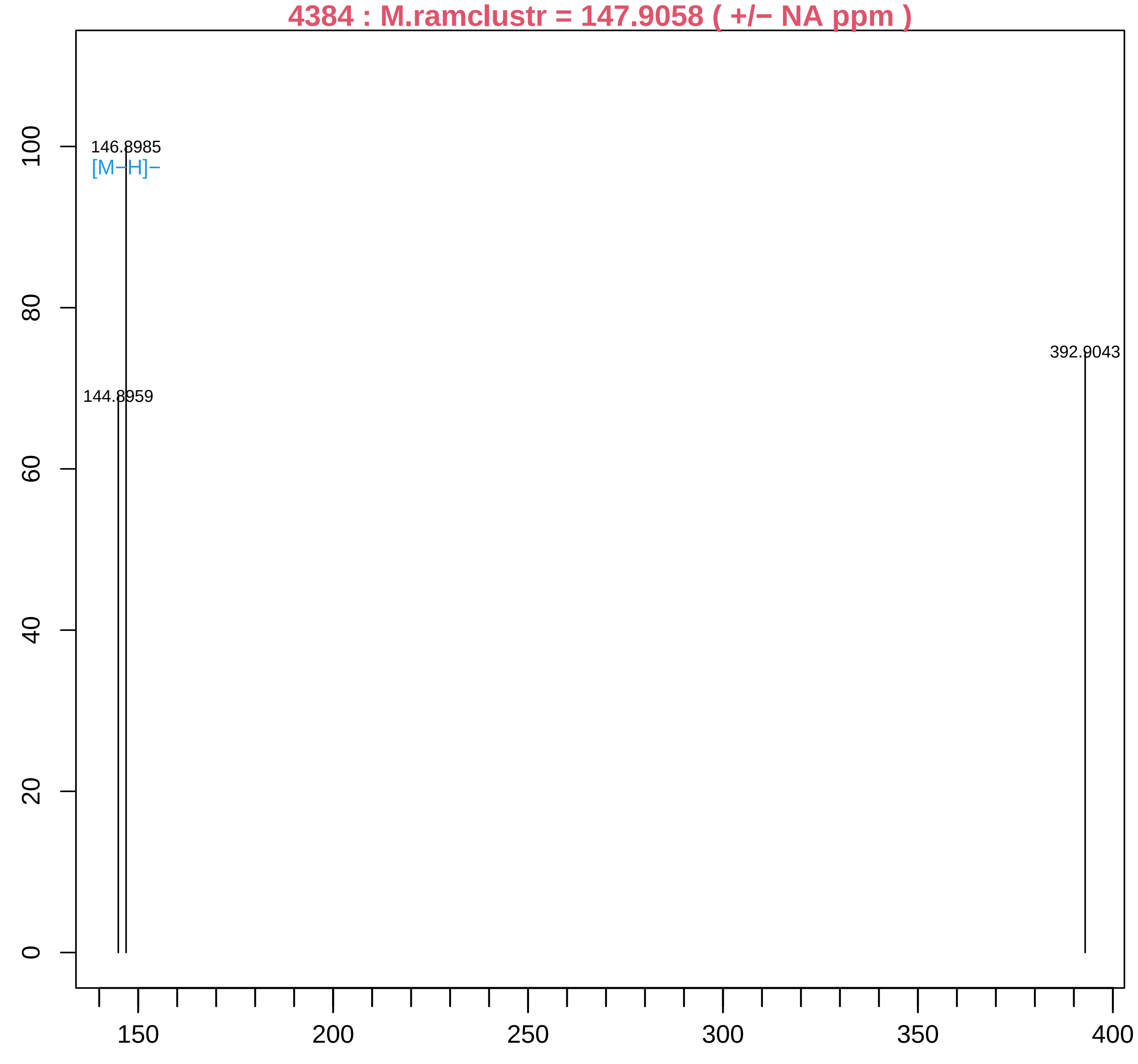
4384 : M.ramclustr = 147.9058 (+/− NA ppm)
146.8985
[M−H]−
144.8959
392.9043
0
20
40
60
80
100
150
200
250
300
350
400
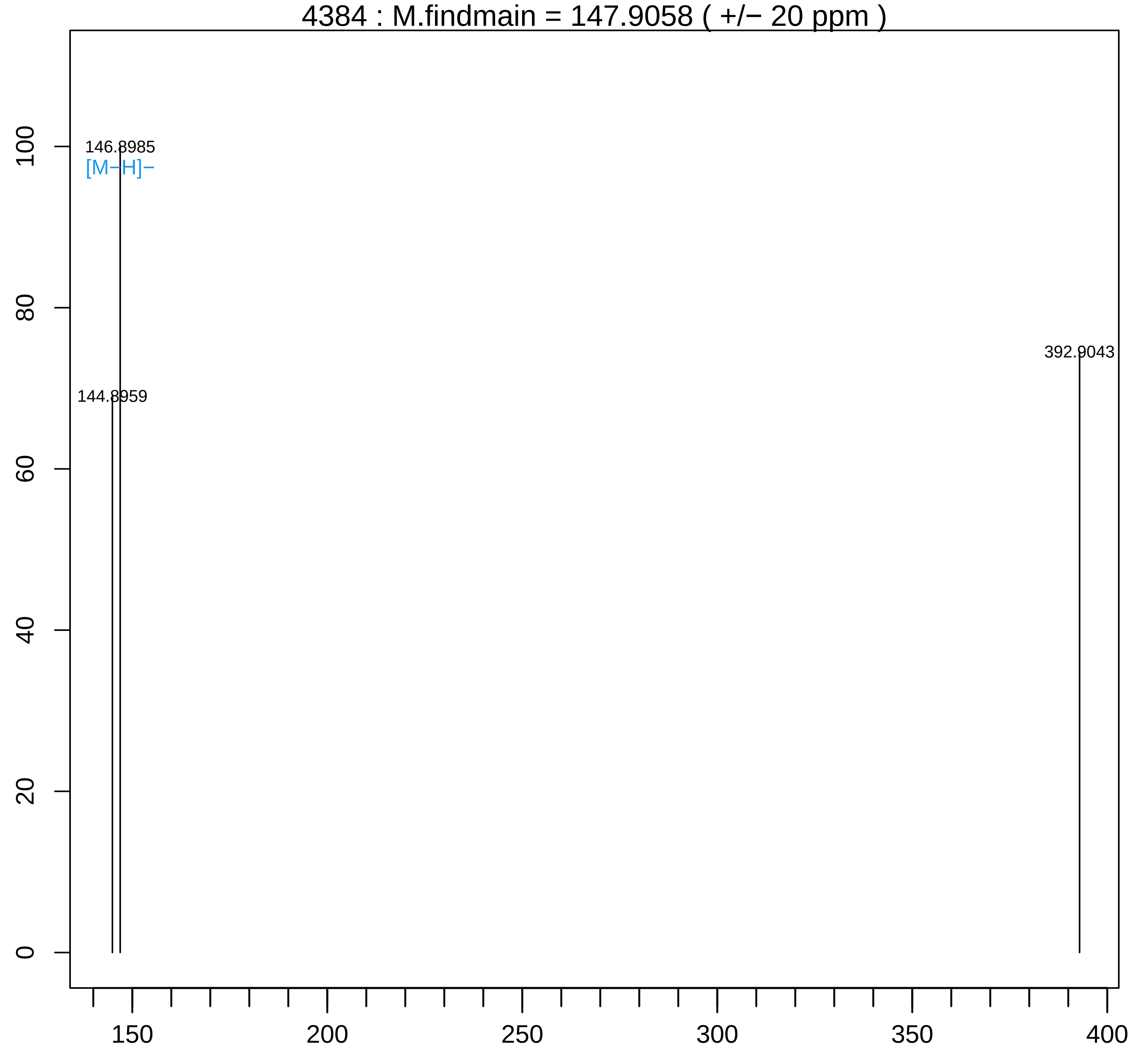
4384 : M.findmain = 147.9058 (+/− 20 ppm)
146.8985
[M−H]−
144.8959
392.9043
0
20
40
60
80
100
150
200
250
300
350
400

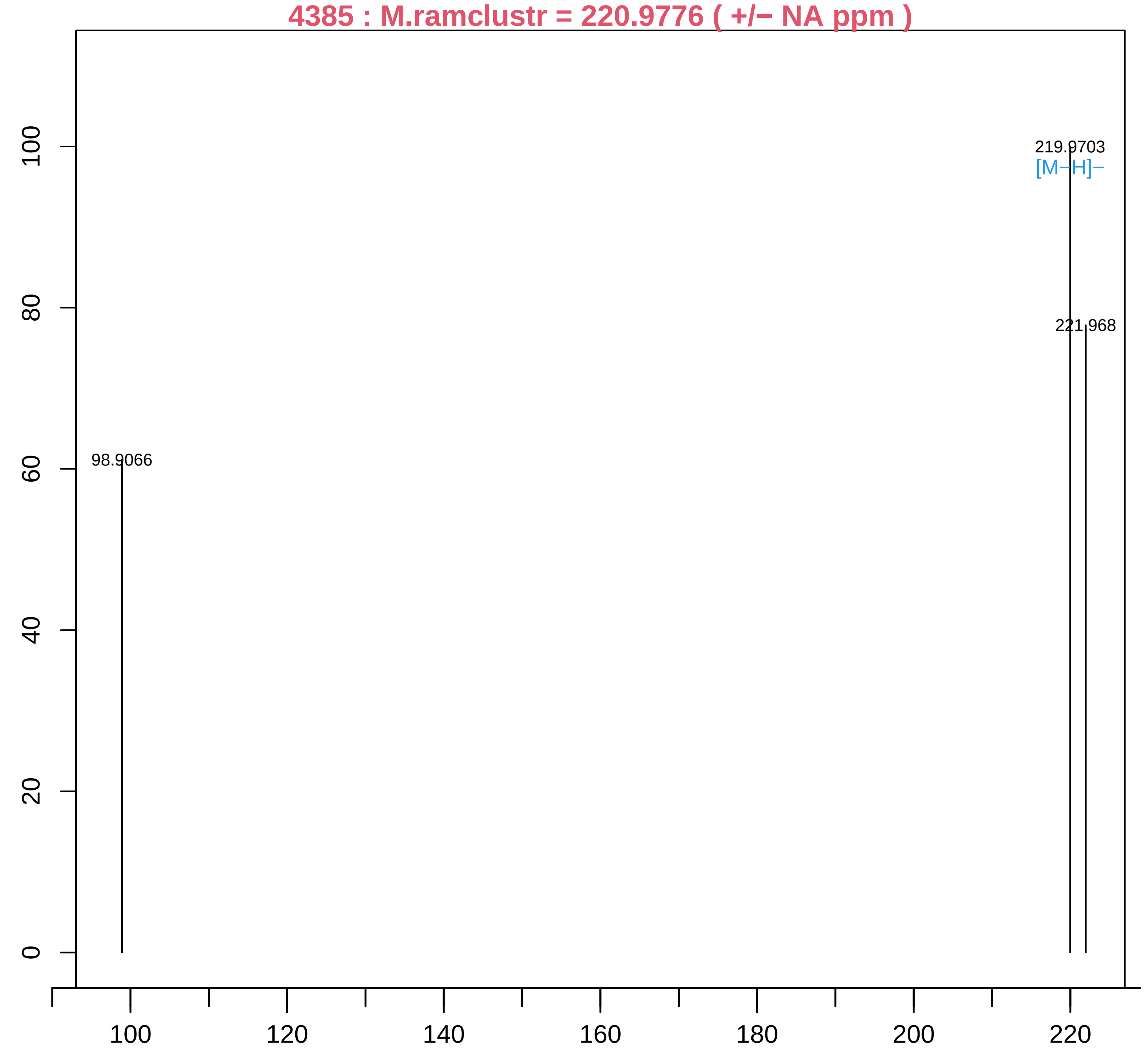
4385 : M.ramclustr = 220.9776 (+/− NA ppm)
219.9703
[M−H]−
221.968
98.9066
0
20
40
60
80
100
120
140
160
180
200
220

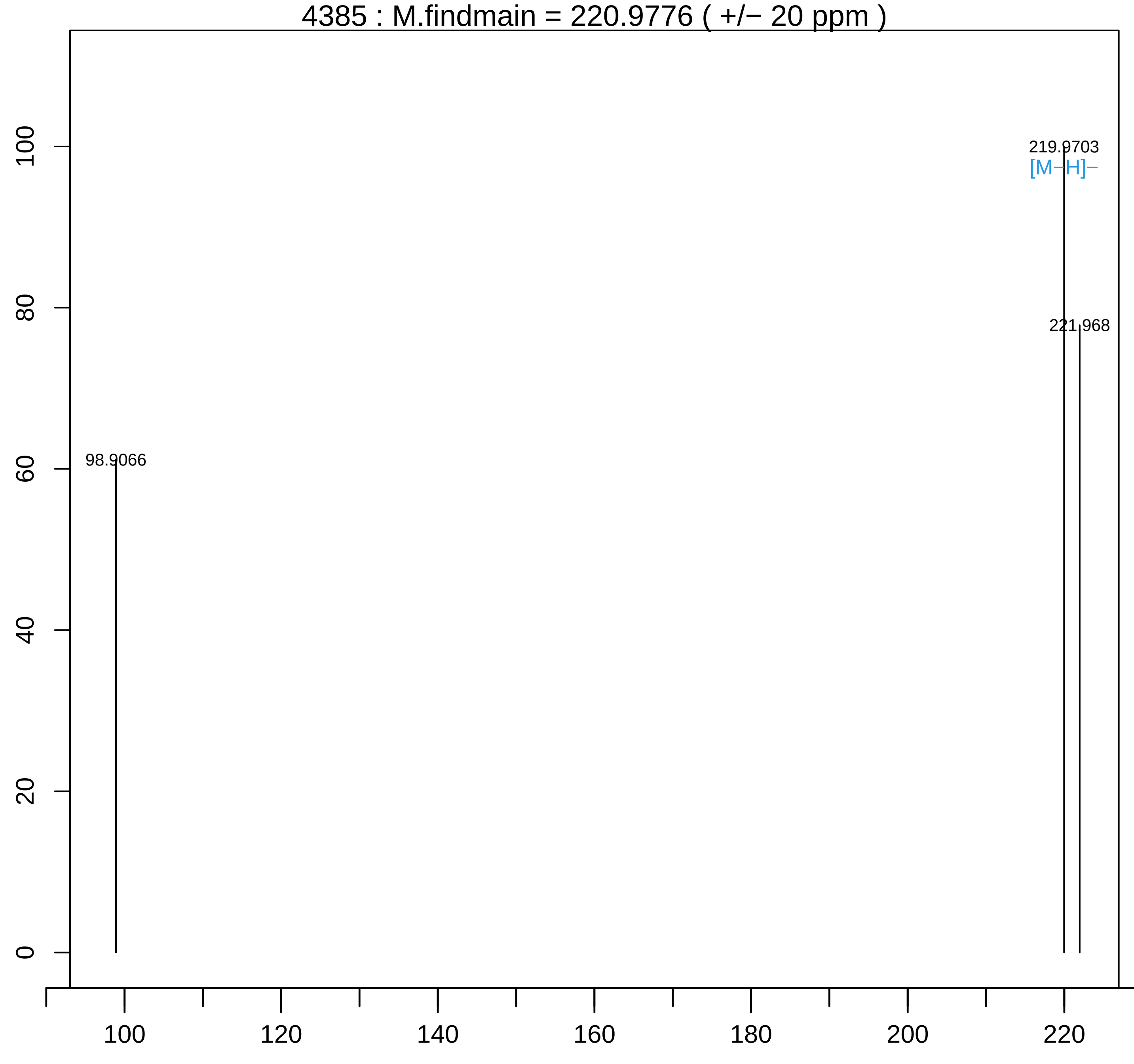
4385 : M.findmain = 220.9776 (+/− 20 ppm)
219.9703
[M−H]−
221.968
98.9066
0
20
40
60
80
100
120
140
160
180
200
220

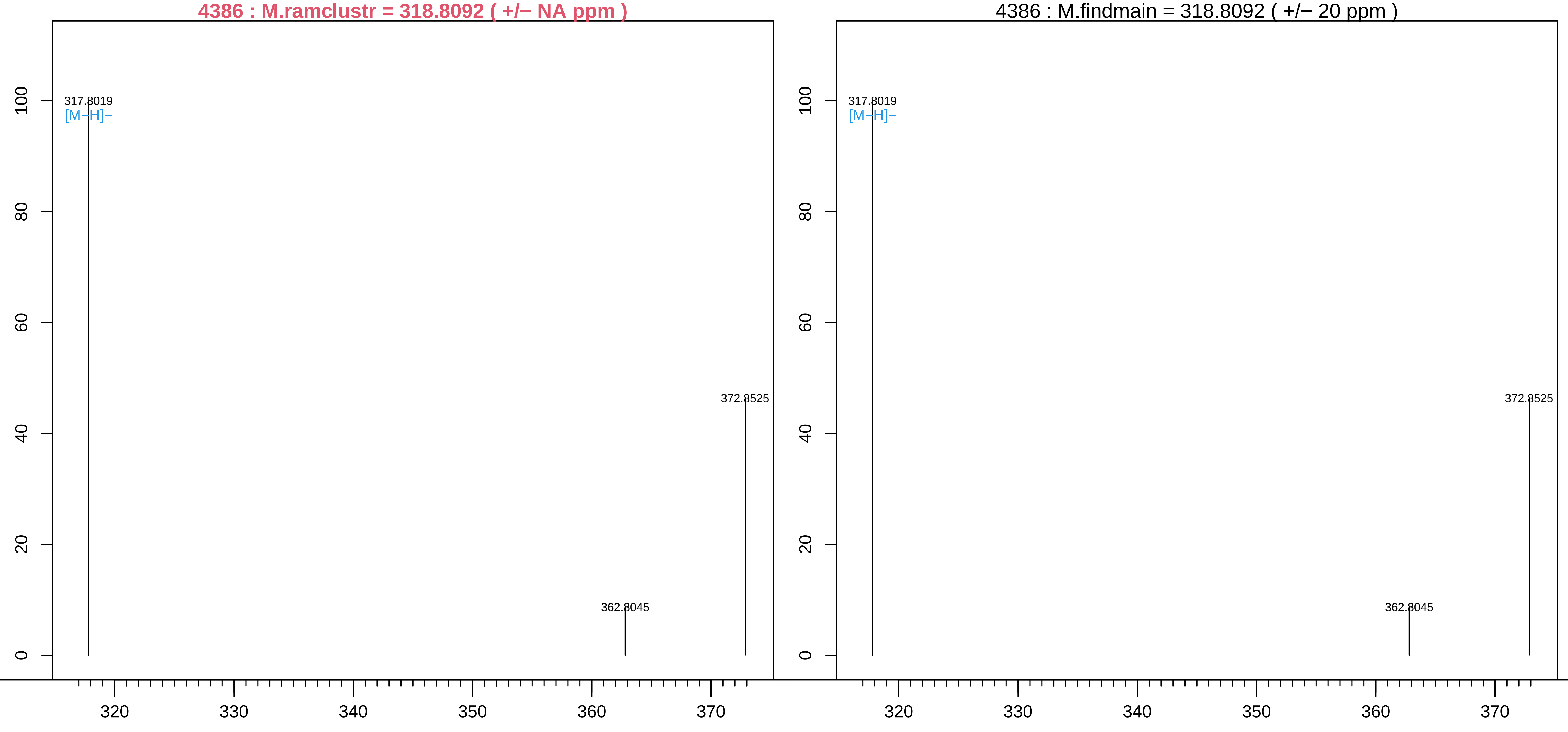
4386 : M.ramclustr = 318.8092 (+/− NA ppm)
317.8019
[M−H]−
372.8525
362.8045
4386 : M.findmain = 318.8092 (+/− 20 ppm)
317.8019
[M−H]−
372.8525
362.8045
0
20
40
60
80
100
320
330
340
350
360
370

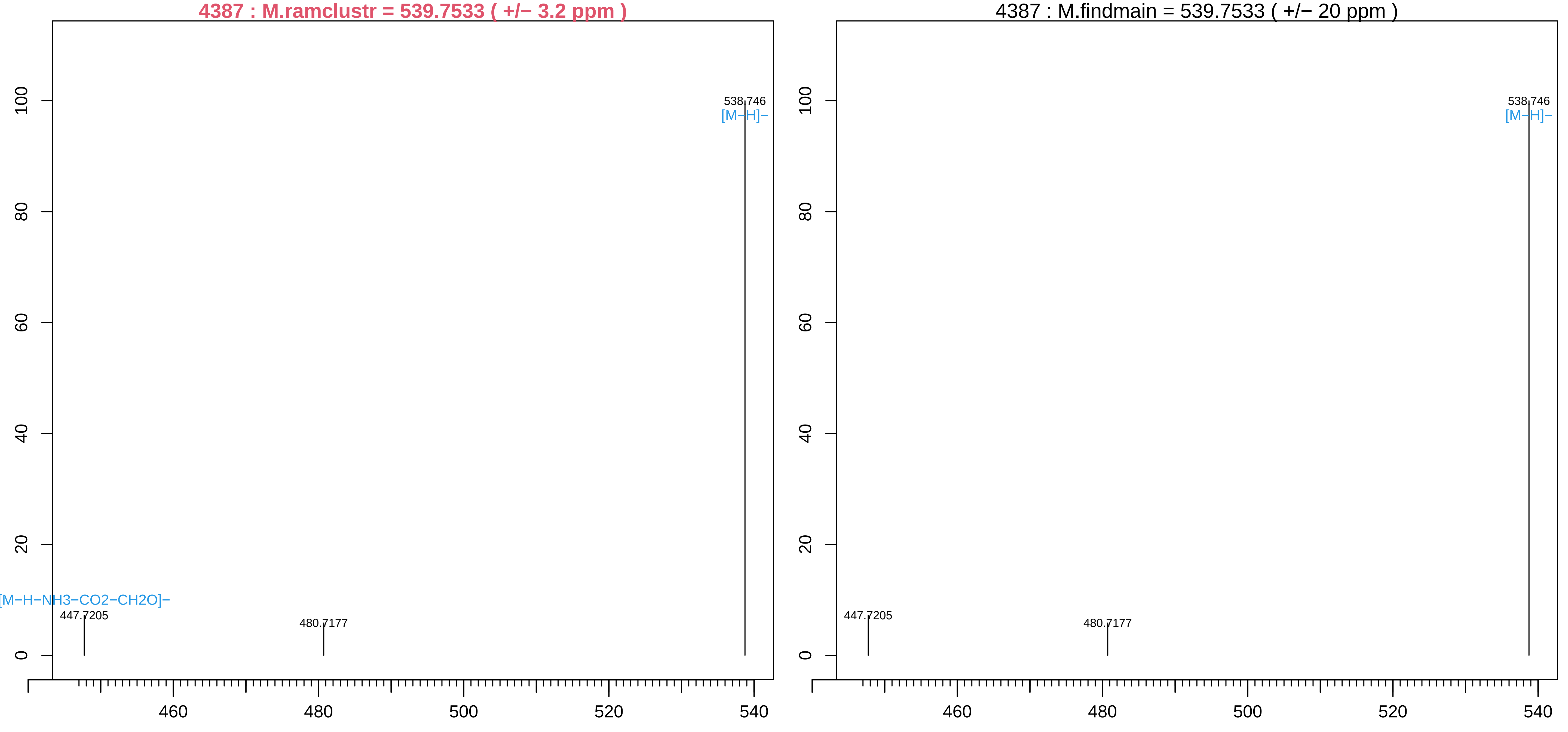
4387 : M.ramclustr = 539.7533 (+/− 3.2 ppm)
538.746
[M−H]−
[M−H−NH3−CO2−CH2O]−
447.7205
480.7177
0
20
40
60
80
100
460
480
500
520
540
4387 : M.findmain = 539.7533 (+/− 20 ppm)
538.746
[M−H]−
447.7205
480.7177

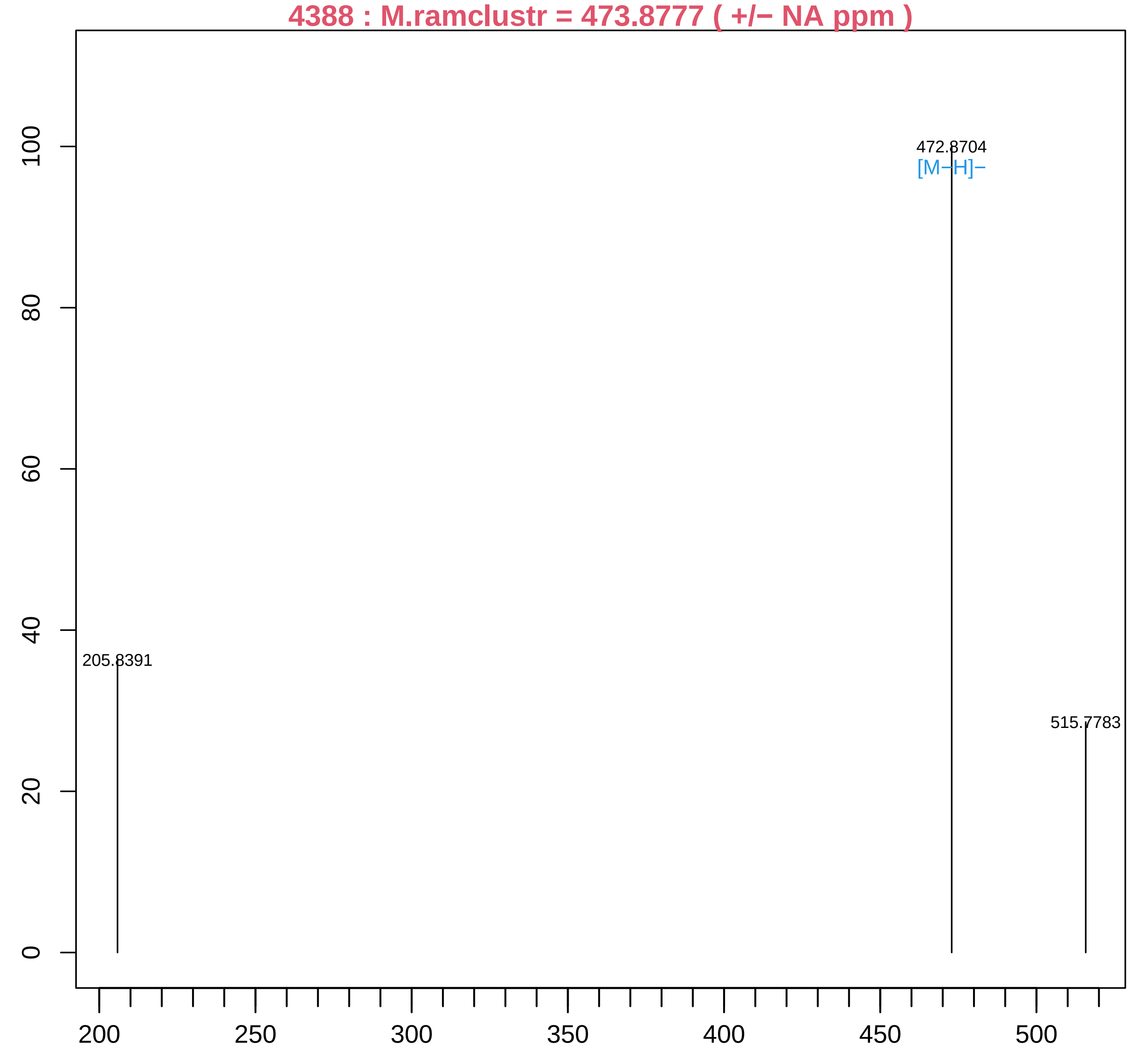
4388 : M.ramclustr = 473.8777 (+/− NA ppm)
472.8704
[M−H]−
205.8391
515.7783
0
20
40
60
80
100
200
250
300
350
400
450
500

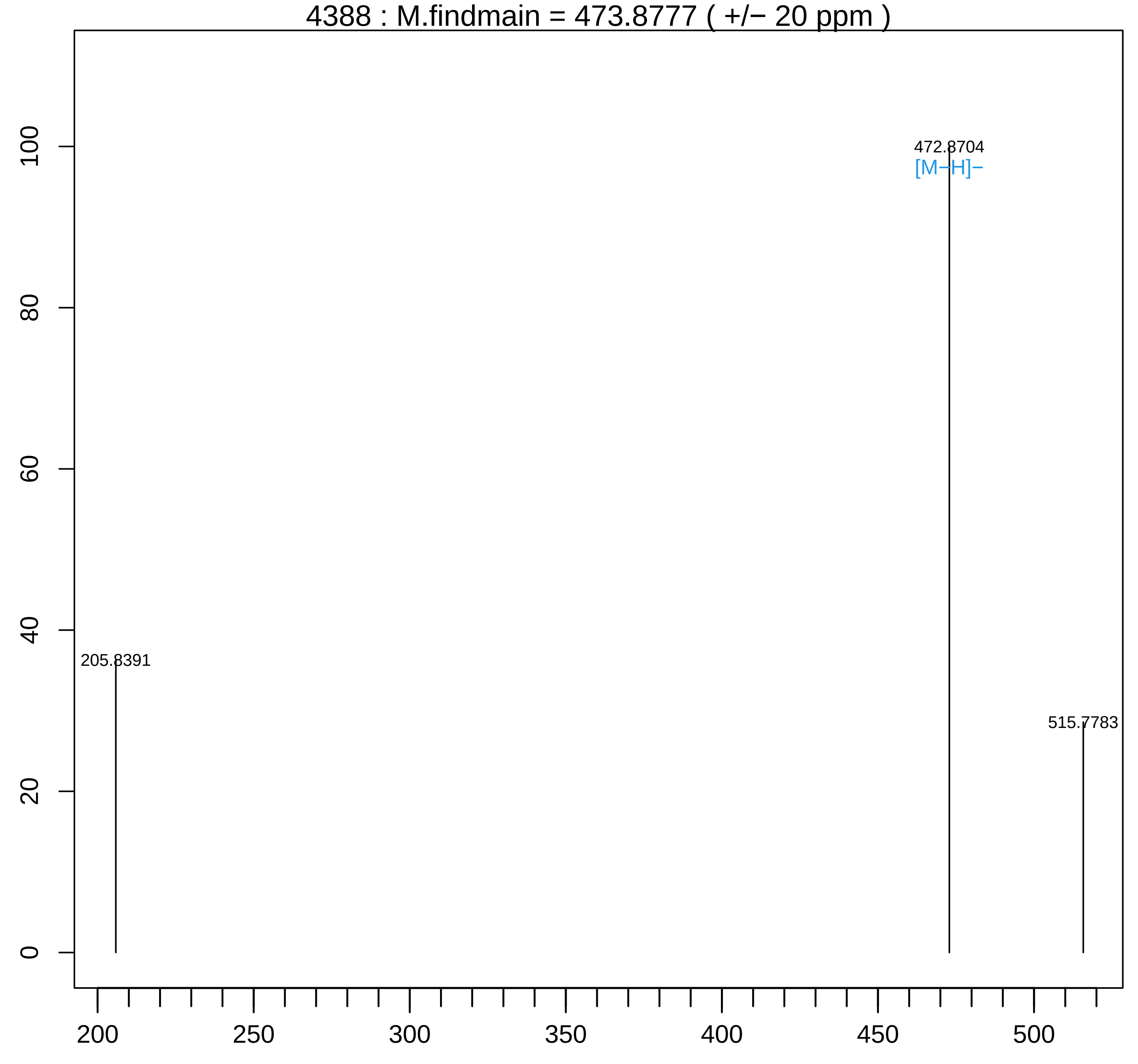
4388 : M.findmain = 473.8777 (+/− 20 ppm)
472.8704
[M−H]−
205.8391
515.7783
0
20
40
60
80
100
200
250
300
350
400
450
500

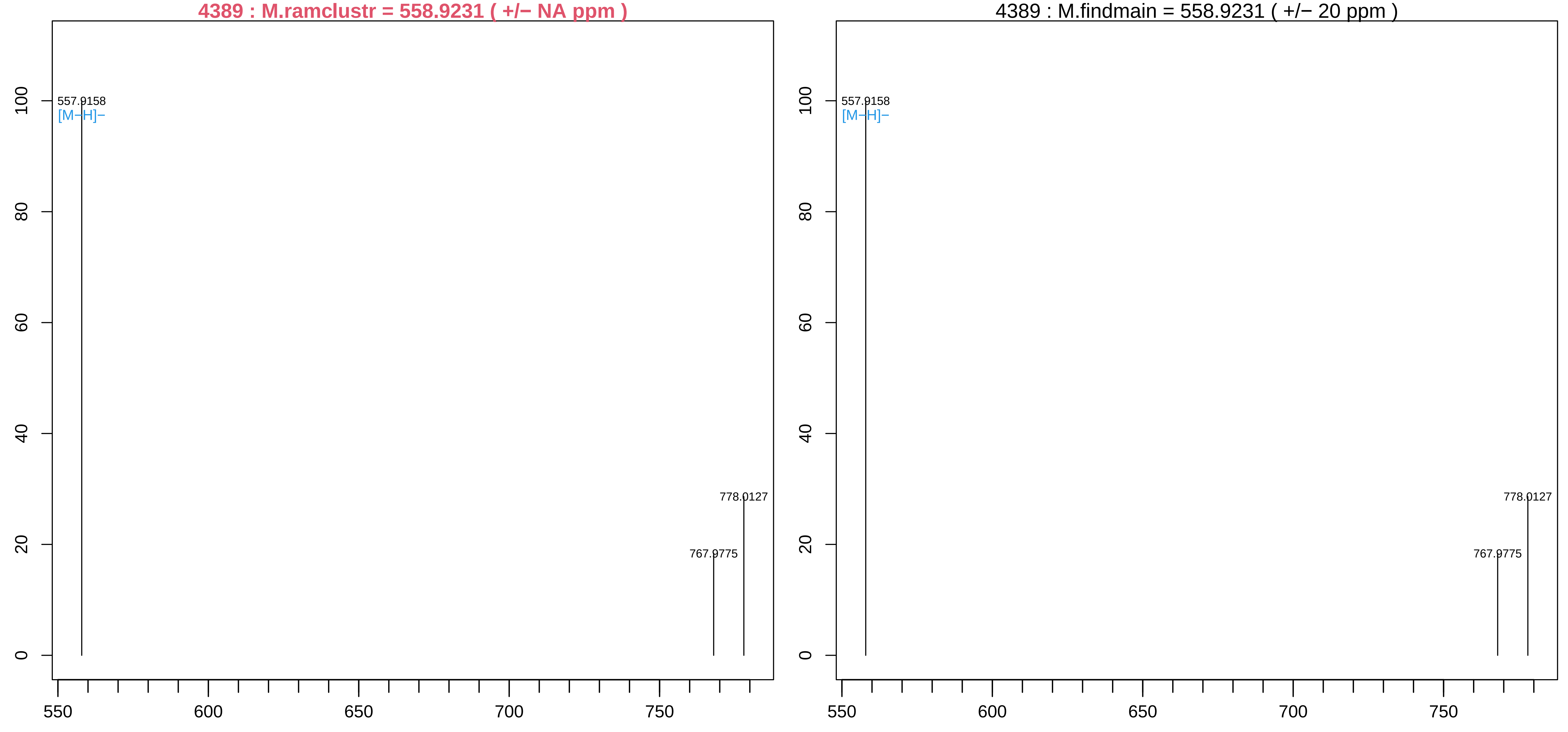
4389 : M.ramclustr = 558.9231 (+/− NA ppm)
4389 : M.findmain = 558.9231 (+/− 20 ppm)
557.9158
[M−H]−
767.9775
778.0127
0
20
40
60
80
100
550
600
650
700
750

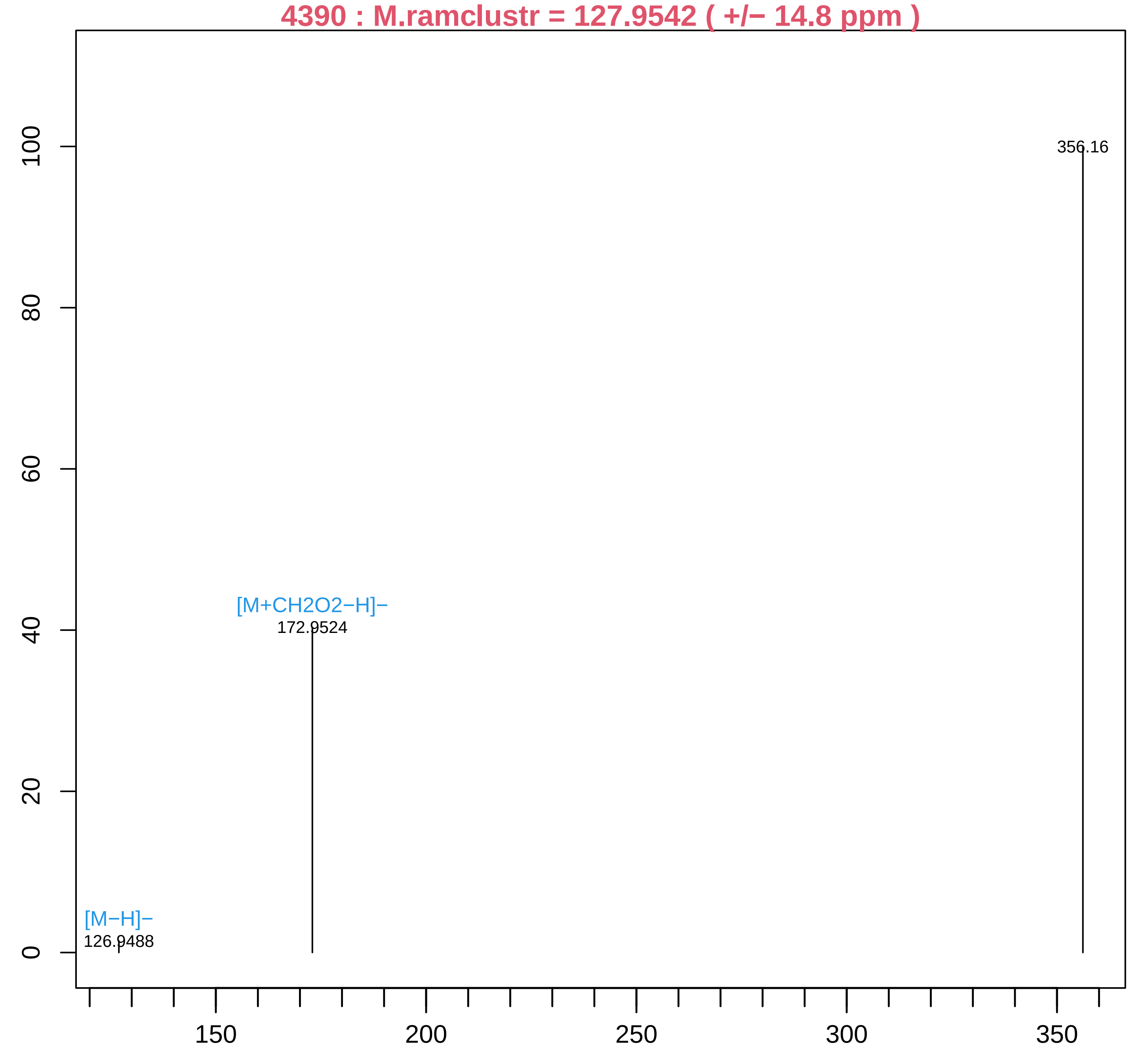
4390 : M.ramclustr = 127.9542 (+/− 14.8 ppm)
356.16
[M+CH2O2−H]−
172.9524
[M−H]−
126.9488
0
20
40
60
80
100
150
200
250
300
350

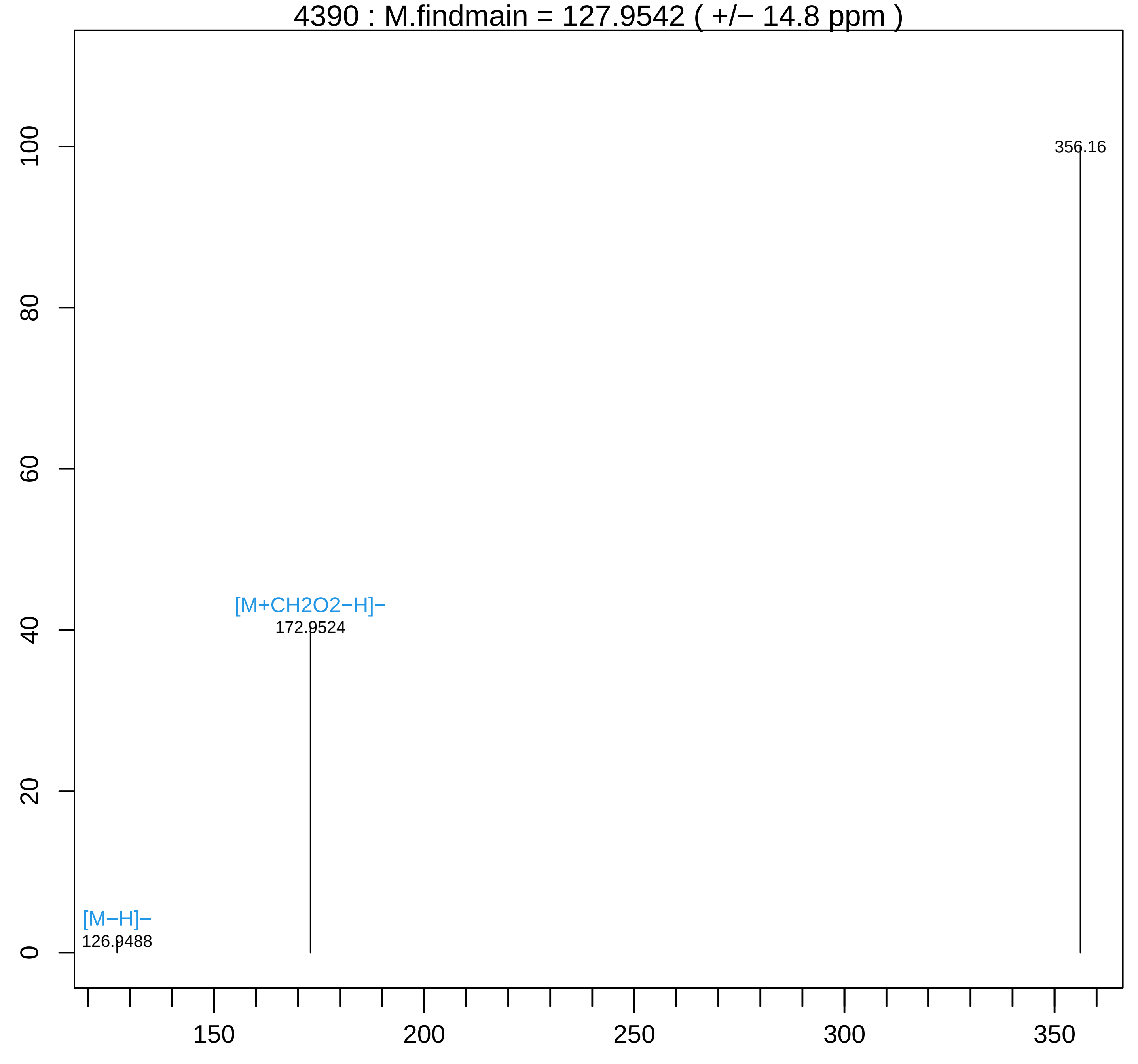
4390 : M.findmain = 127.9542 (+/− 14.8 ppm)
356.16
[M+CH2O2−H]−
172.9524
[M−H]−
126.9488
0
20
40
60
80
100
150
200
250
300
350

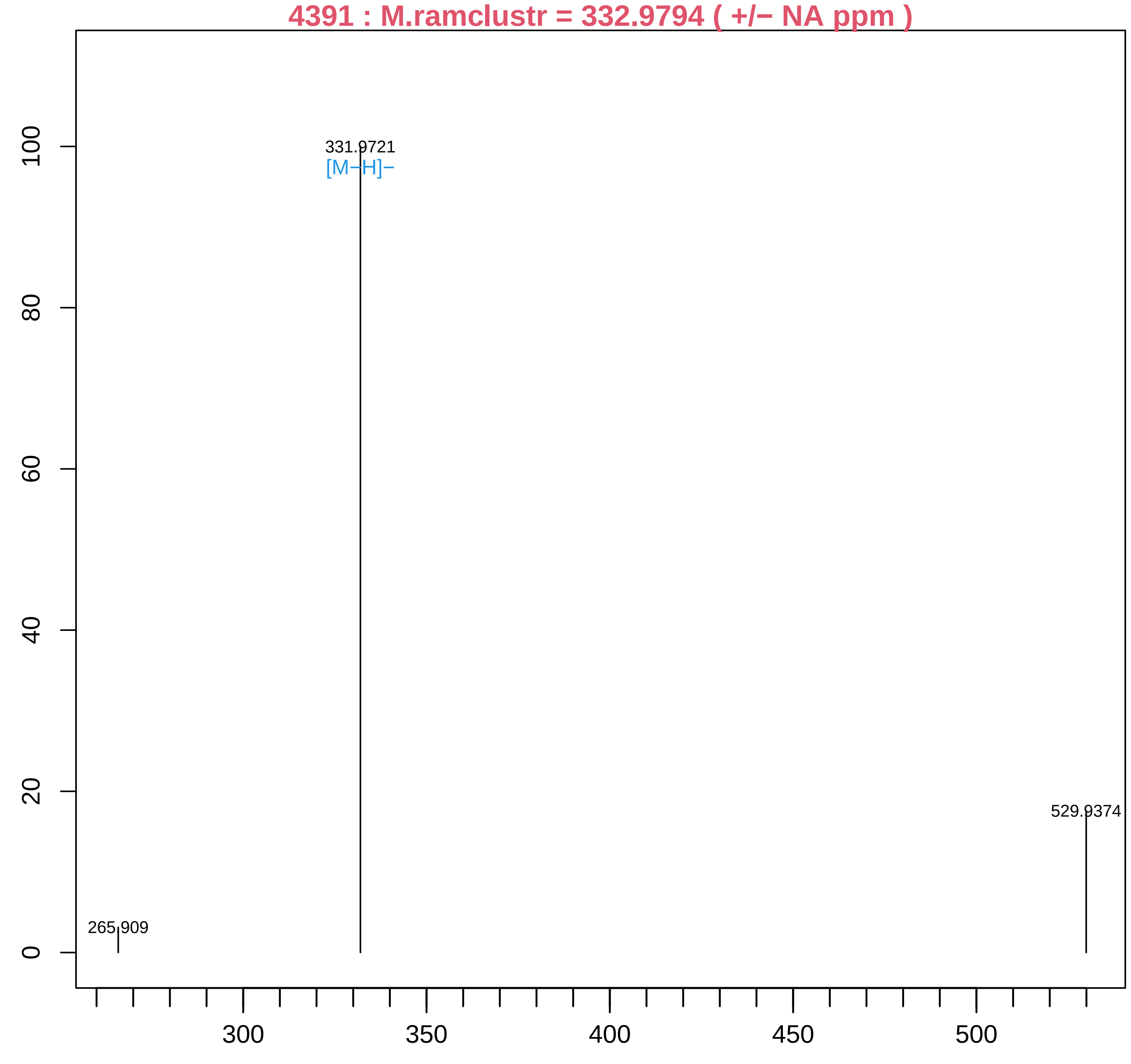
4391 : M.ramclustr = 332.9794 (+/− NA ppm)
331.9721
[M−H]−
529.9374
265.909
0
20
40
60
80
100
300
350
400
450
500

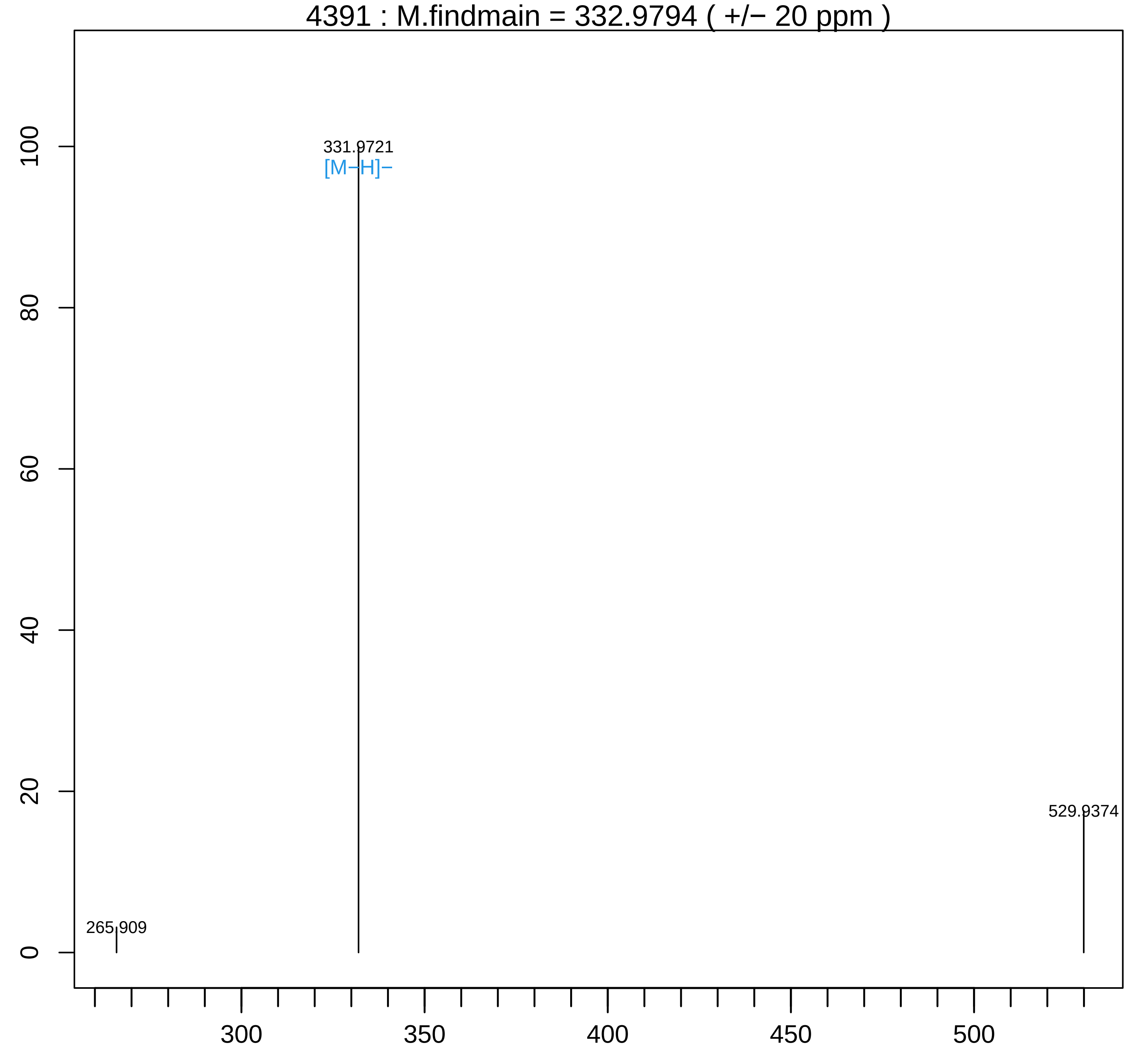
4391 : M.findmain = 332.9794 (+/− 20 ppm)
331.9721
[M−H]−
529.9374
265.909
0
20
40
60
80
100
300
350
400
450
500

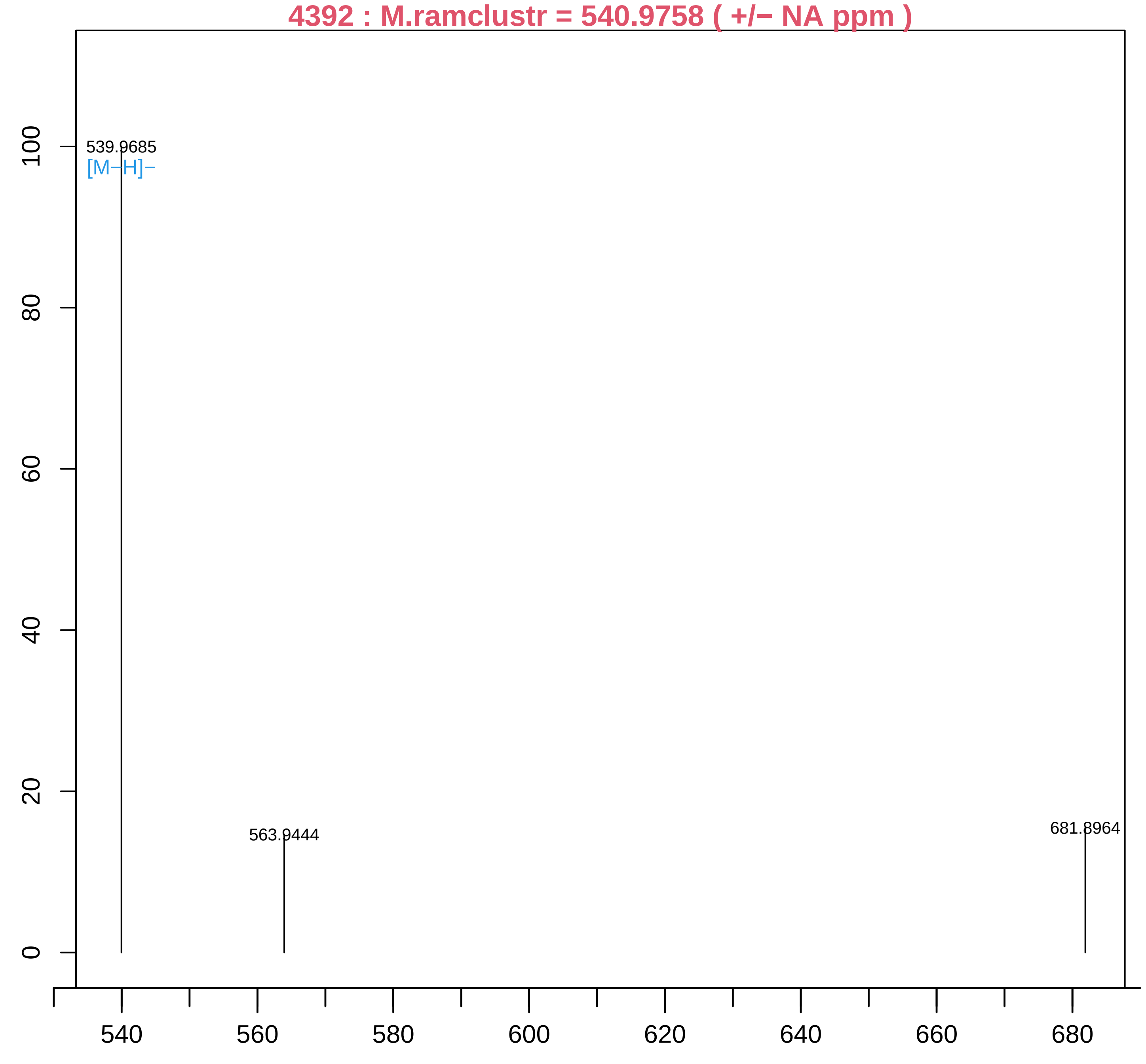

4392 : M.ramclustr = 540.9758 (+/− NA ppm)
539.9685
[M−H]−
563.9444
681.8964
0
20
40
60
80
100
540
560
580
600
620
640
660
680

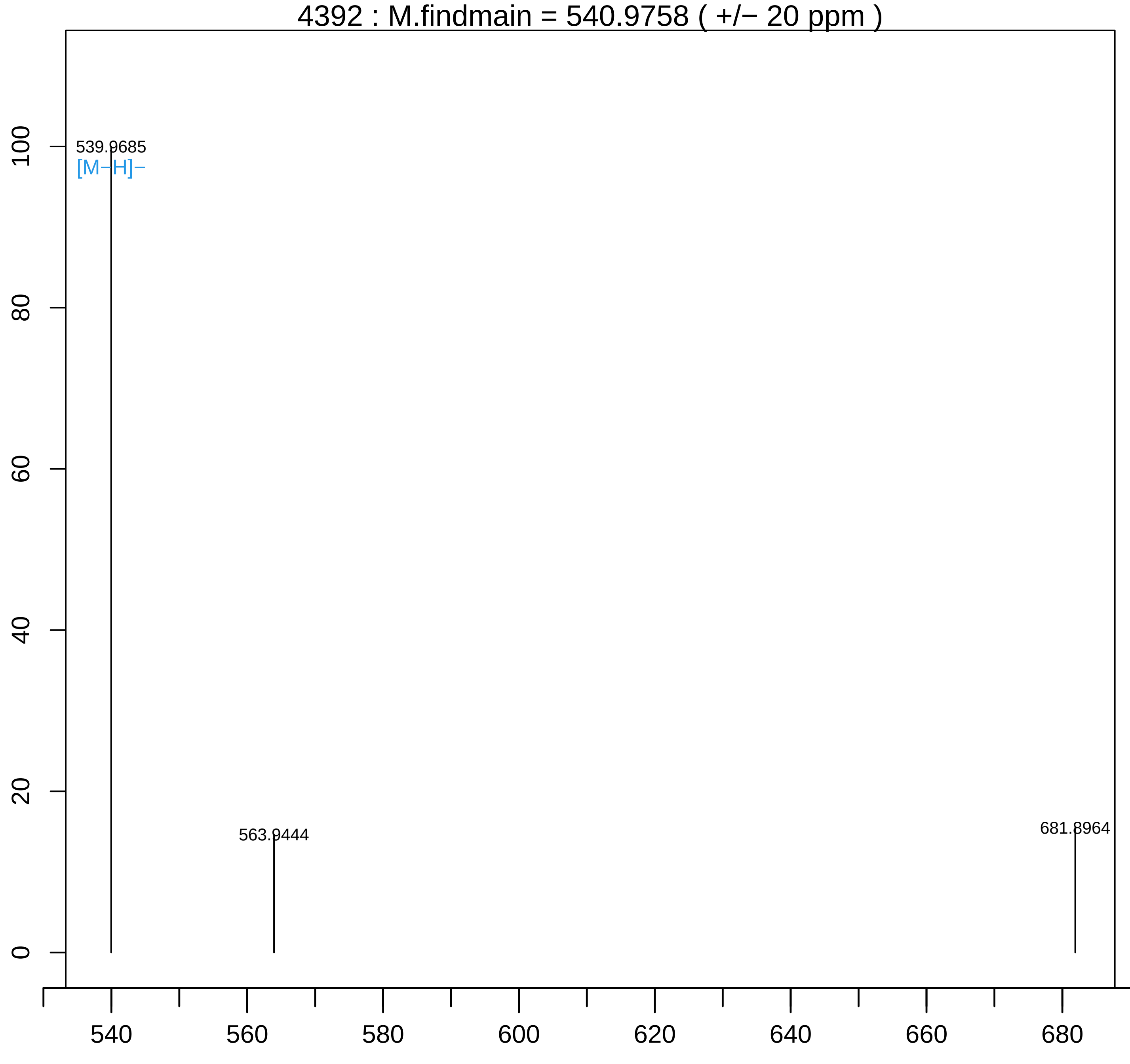

4392 : M.findmain = 540.9758 (+/− 20 ppm)
539.9685
[M−H]−
563.9444
681.8964
0
20
40
60
80
100
540
560
580
600
620
640
660
680

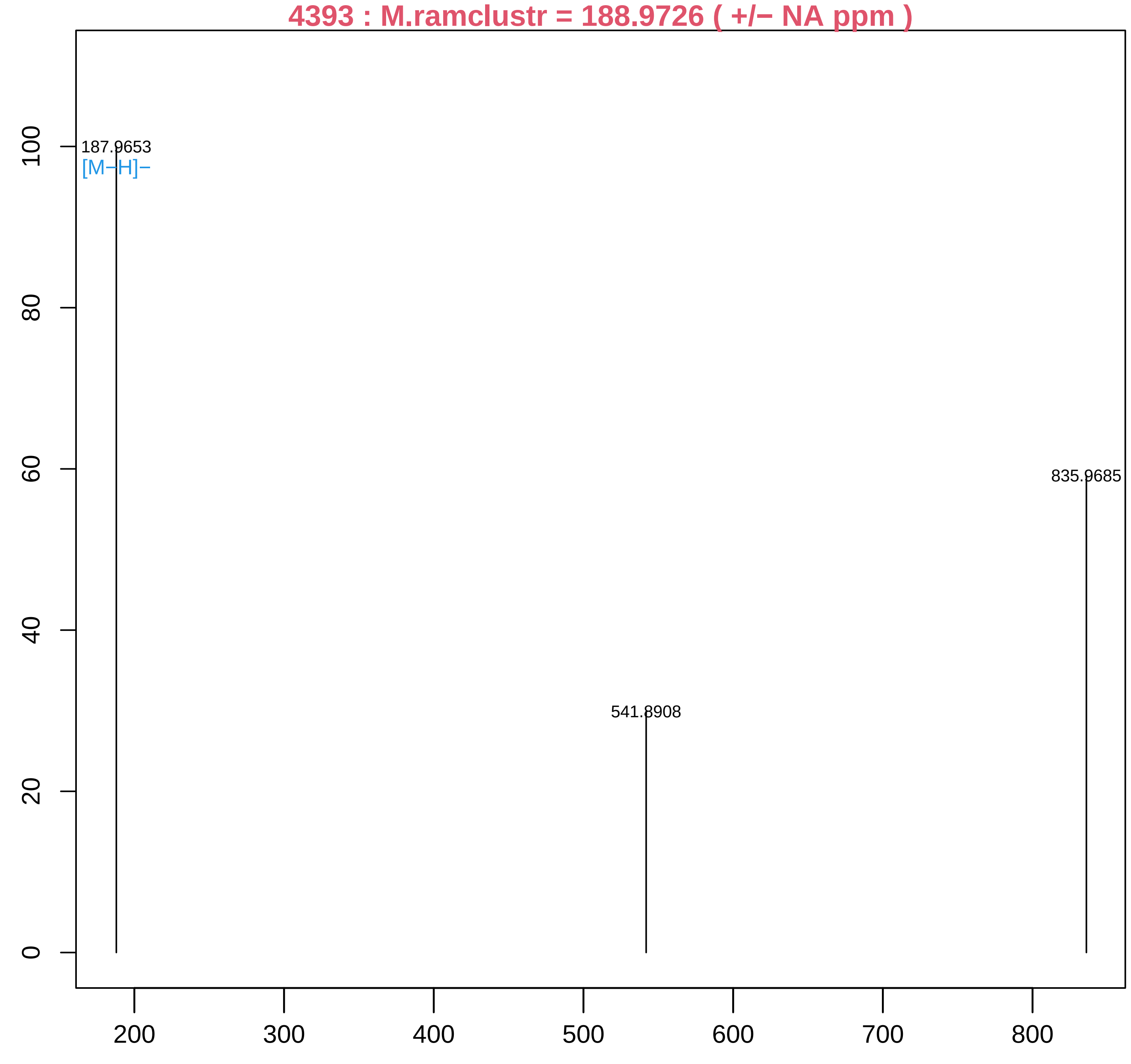
4393 : M.ramclustr = 188.9726 (+/− NA ppm)
187.9653
[M−H]−
541.8908
835.9685
0
20
40
60
80
100
200
300
400
500
600
700
800

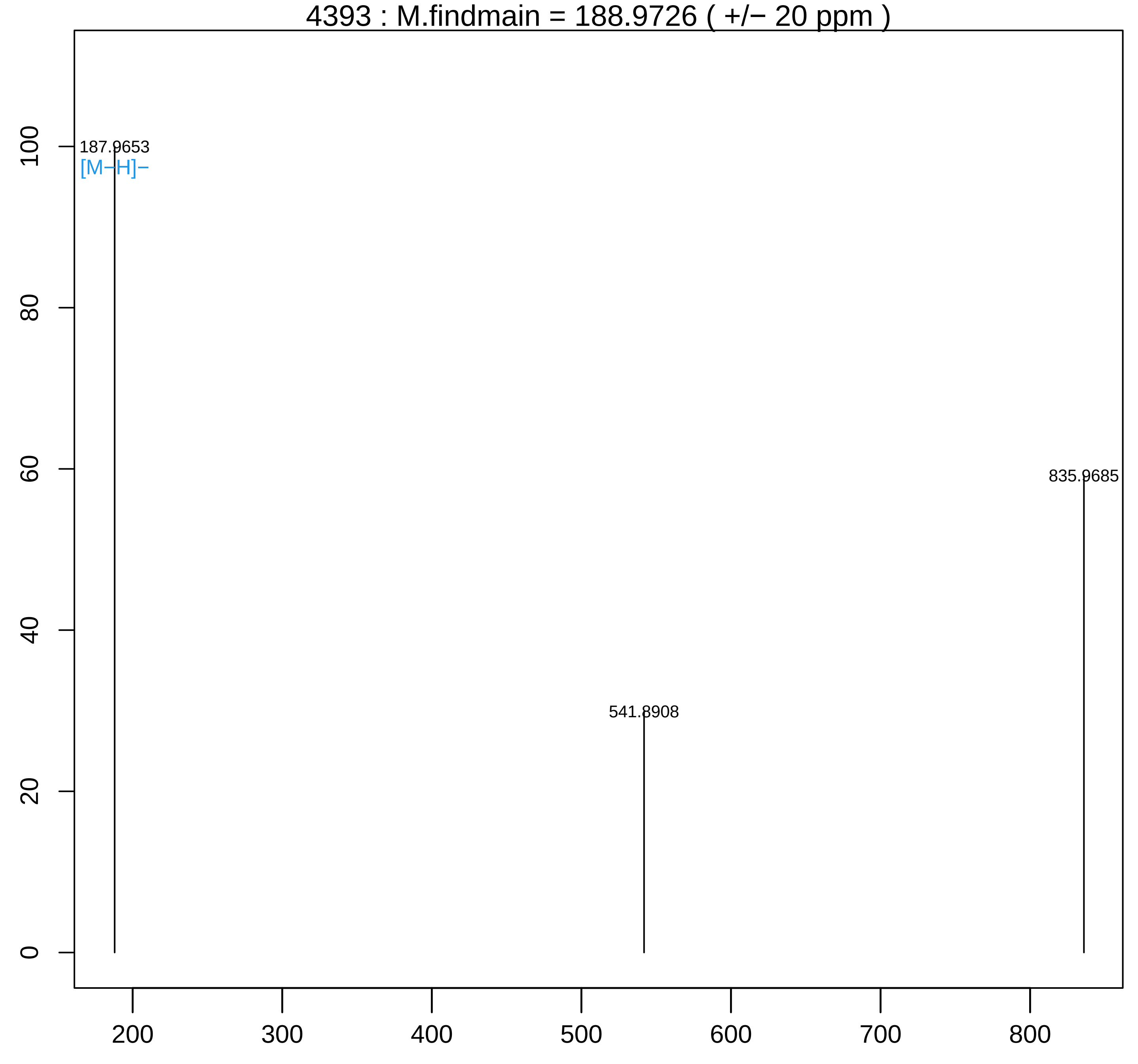
4393 : M.findmain = 188.9726 (+/− 20 ppm)
187.9653
[M−H]−
541.8908
835.9685
0
20
40
60
80
100
200
300
400
500
600
700
800

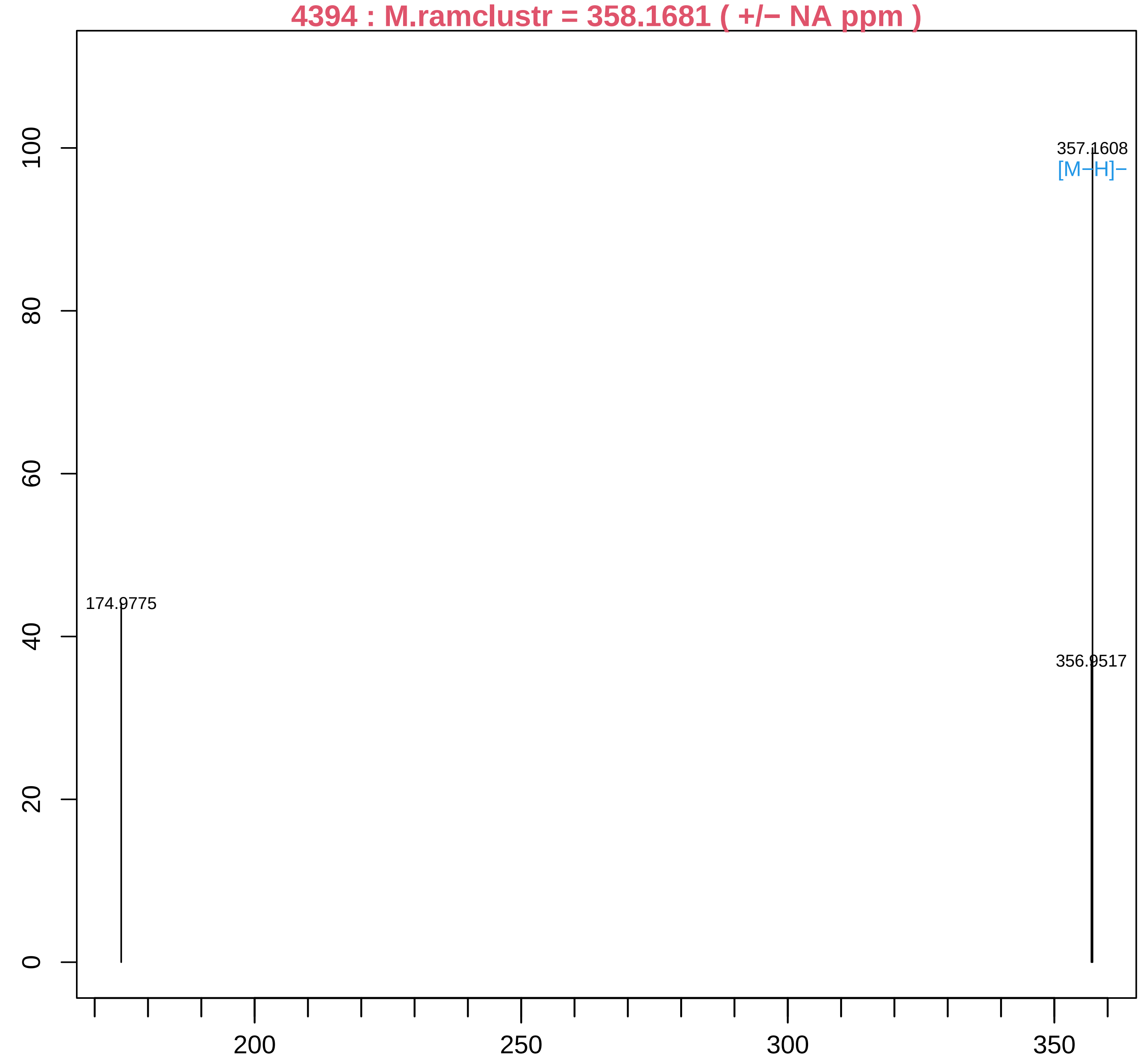
4394 : M.ramclustr = 358.1681 (+/− NA ppm)
357.1608
[M−H]−
174.9775
356.9517
0
20
40
60
80
100
200
250
300
350

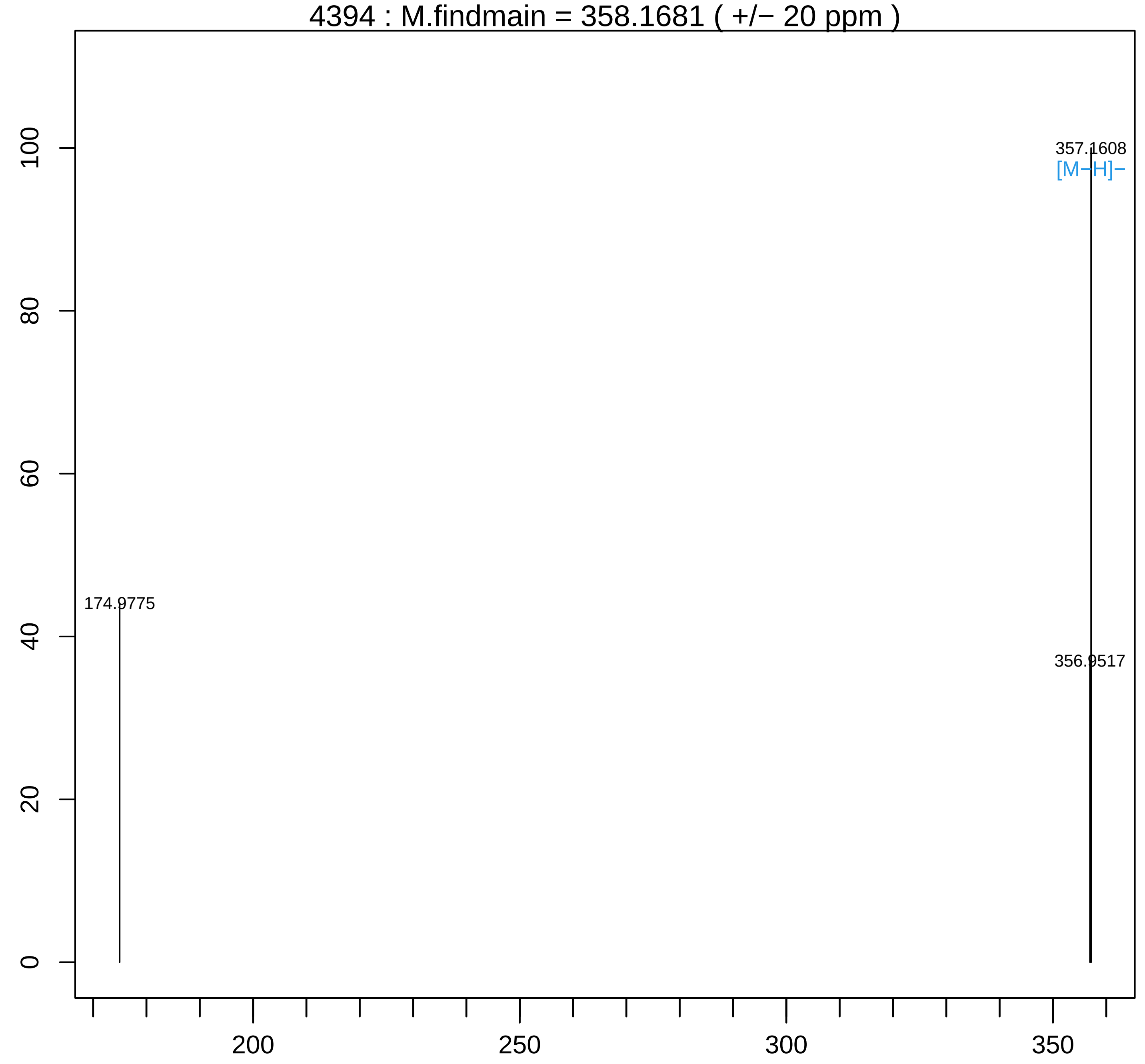
4394 : M.findmain = 358.1681 (+/− 20 ppm)
357.1608
[M−H]−
174.9775
356.9517
0
20
40
60
80
100
200
250
300
350

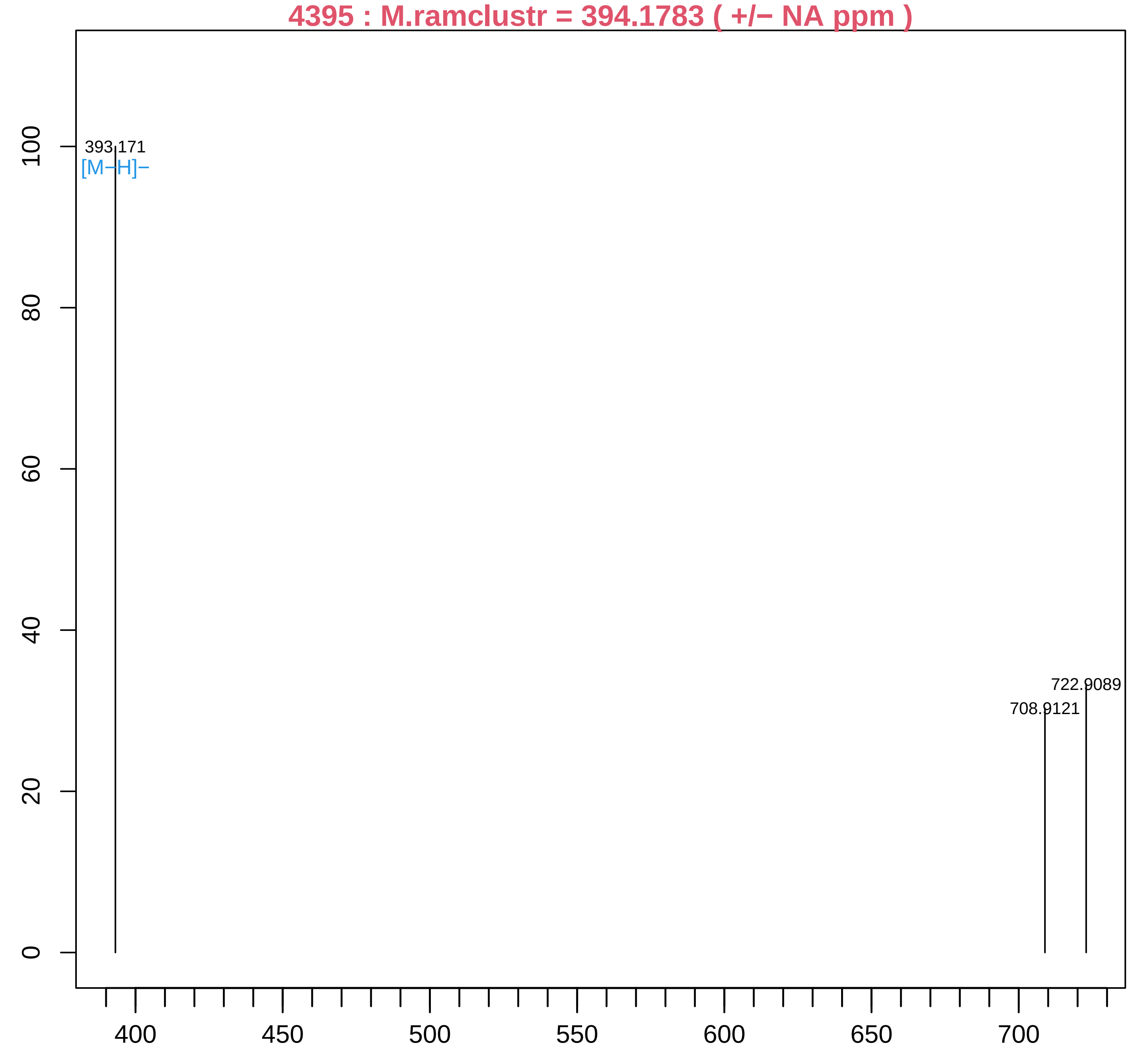

4395 : M.ramclustr = 394.1783 (+/− NA ppm)
393.171
[M−H]−
708.9121
722.9089
0
20
40
60
80
100
400
450
500
550
600
650
700

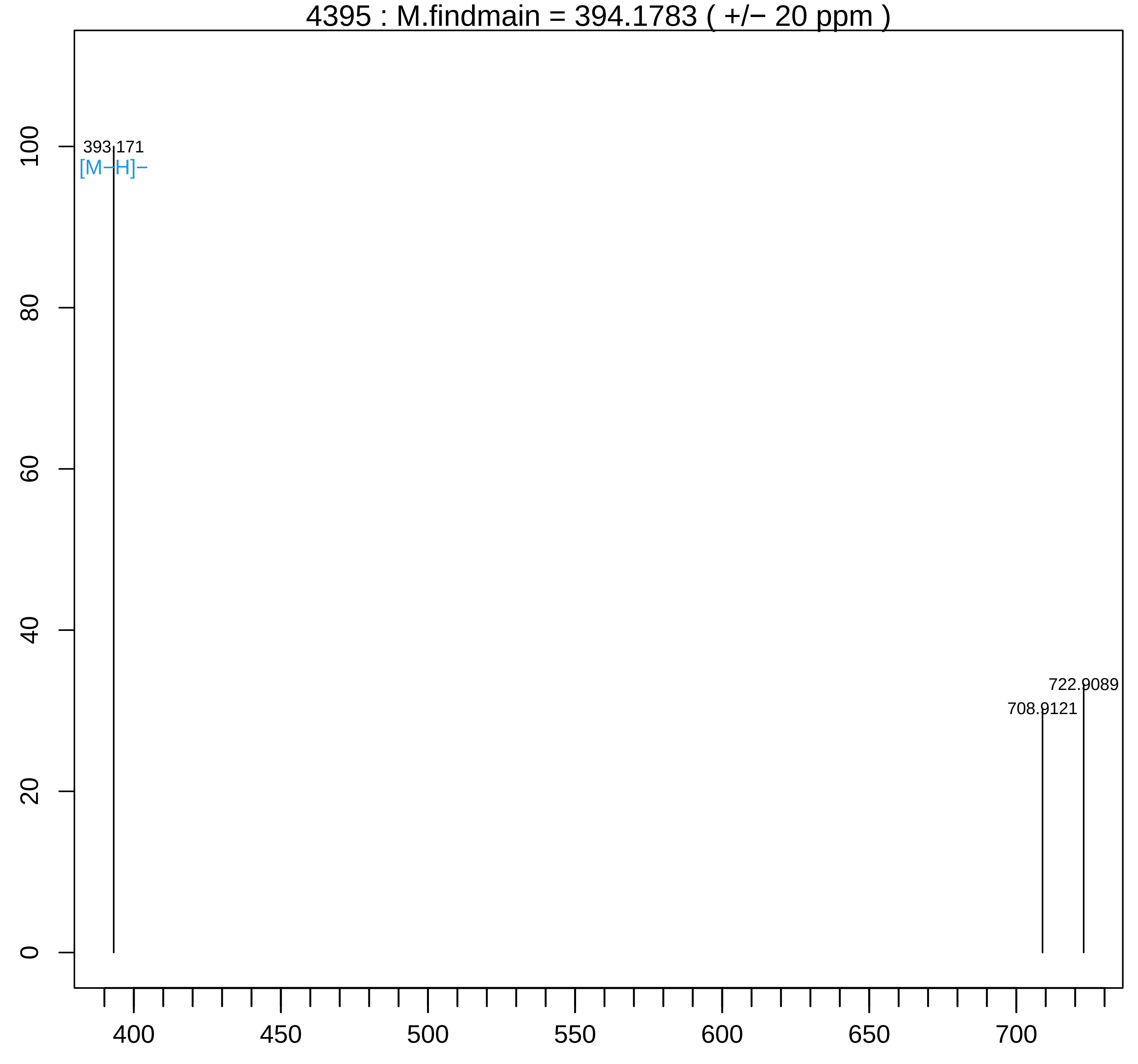

4395 : M.findmain = 394.1783 (+/− 20 ppm)
393.171
[M−H]−
708.9121
722.9089
0
20
40
60
80
100
400
450
500
550
600
650
700

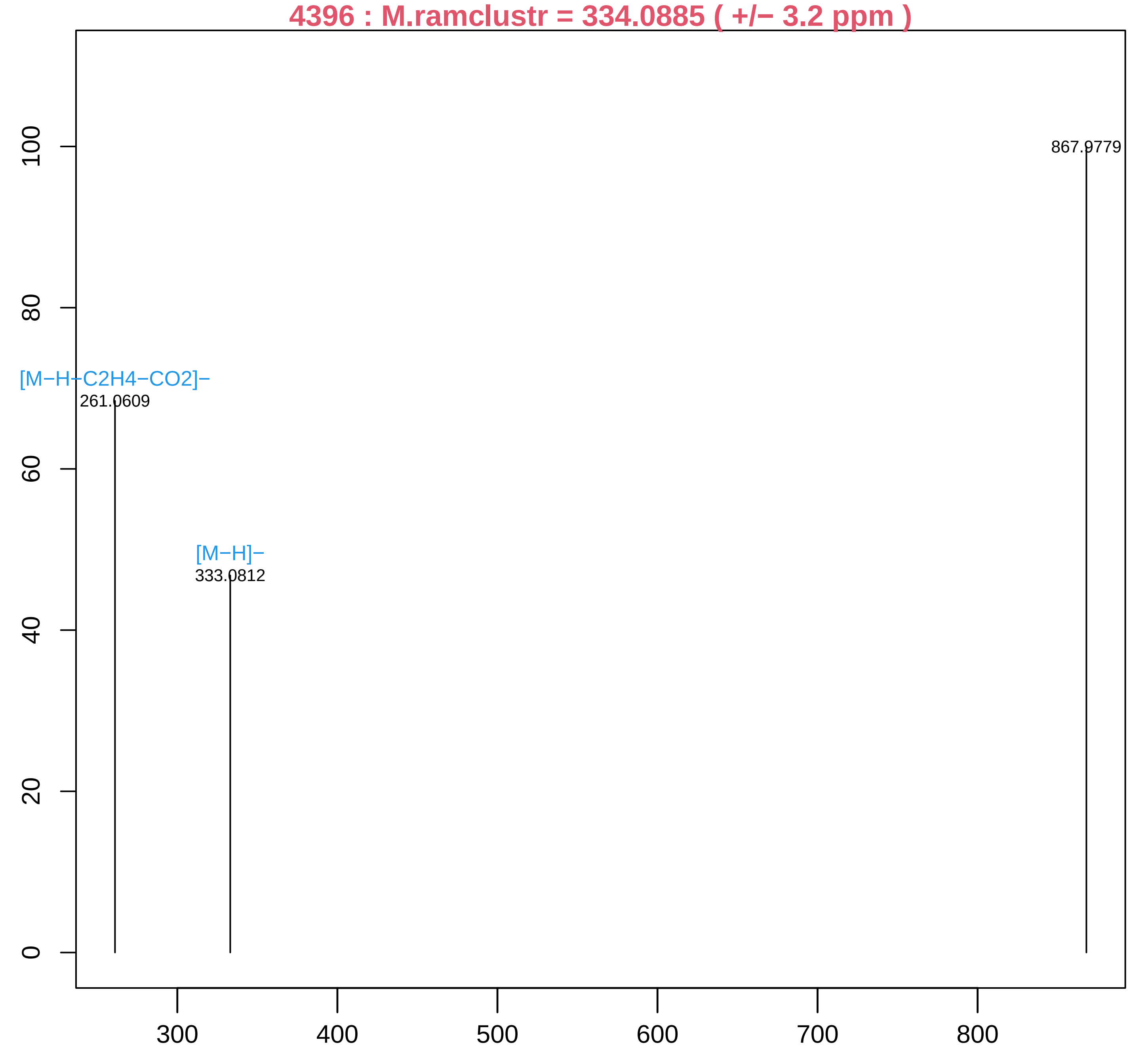
4396 : M.ramclustr = 334.0885 (+/− 3.2 ppm)
[M−H−C2H4−CO2]−
261.0609
[M−H]−
333.0812
867.9779
0
20
40
60
80
100
300
400
500
600
700
800

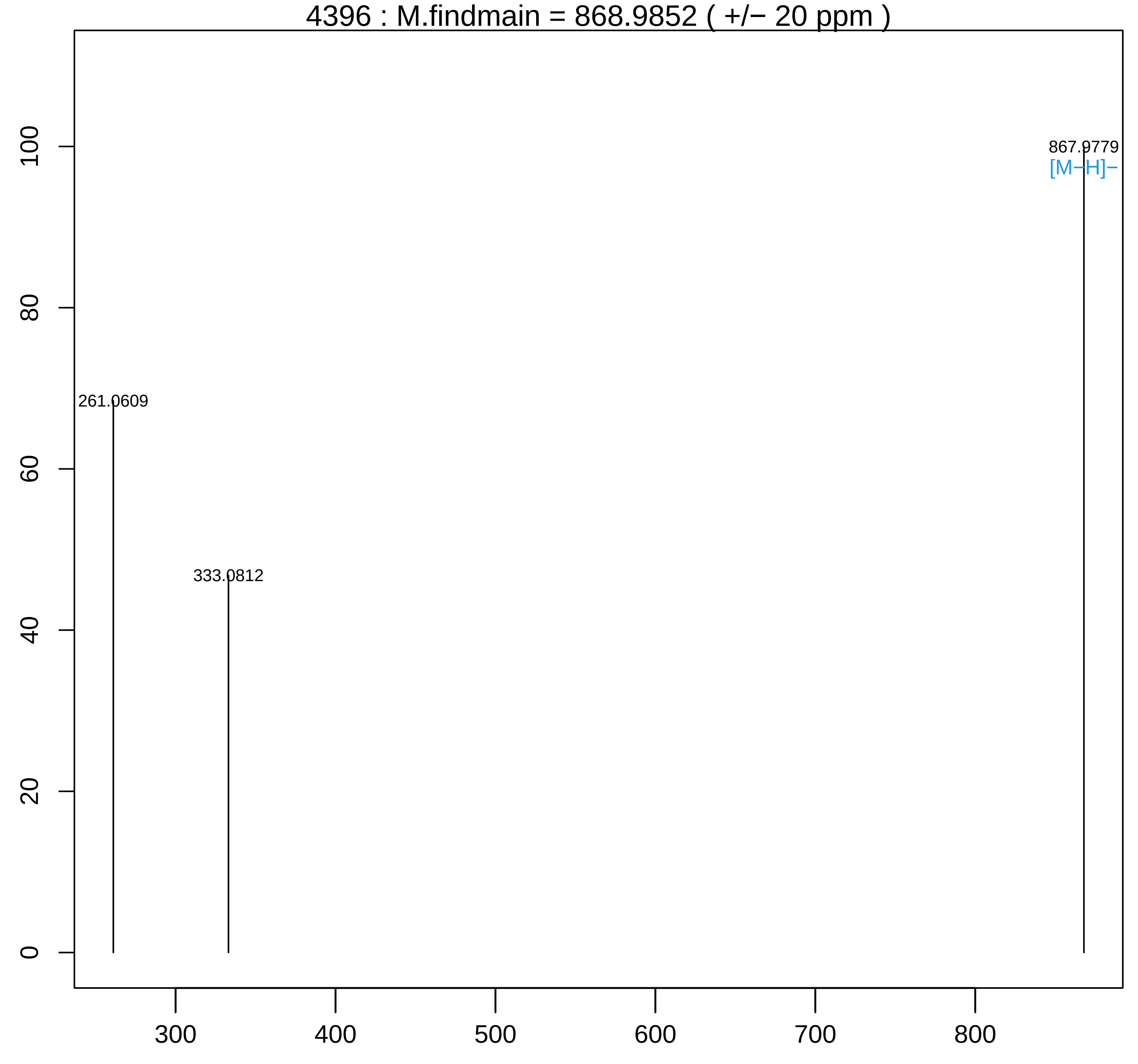
4396 : M.findmain = 868.9852 (+/− 20 ppm)
261.0609
333.0812
867.9779
[M−H]−
0
20
40
60
80
100
300
400
500
600
700
800

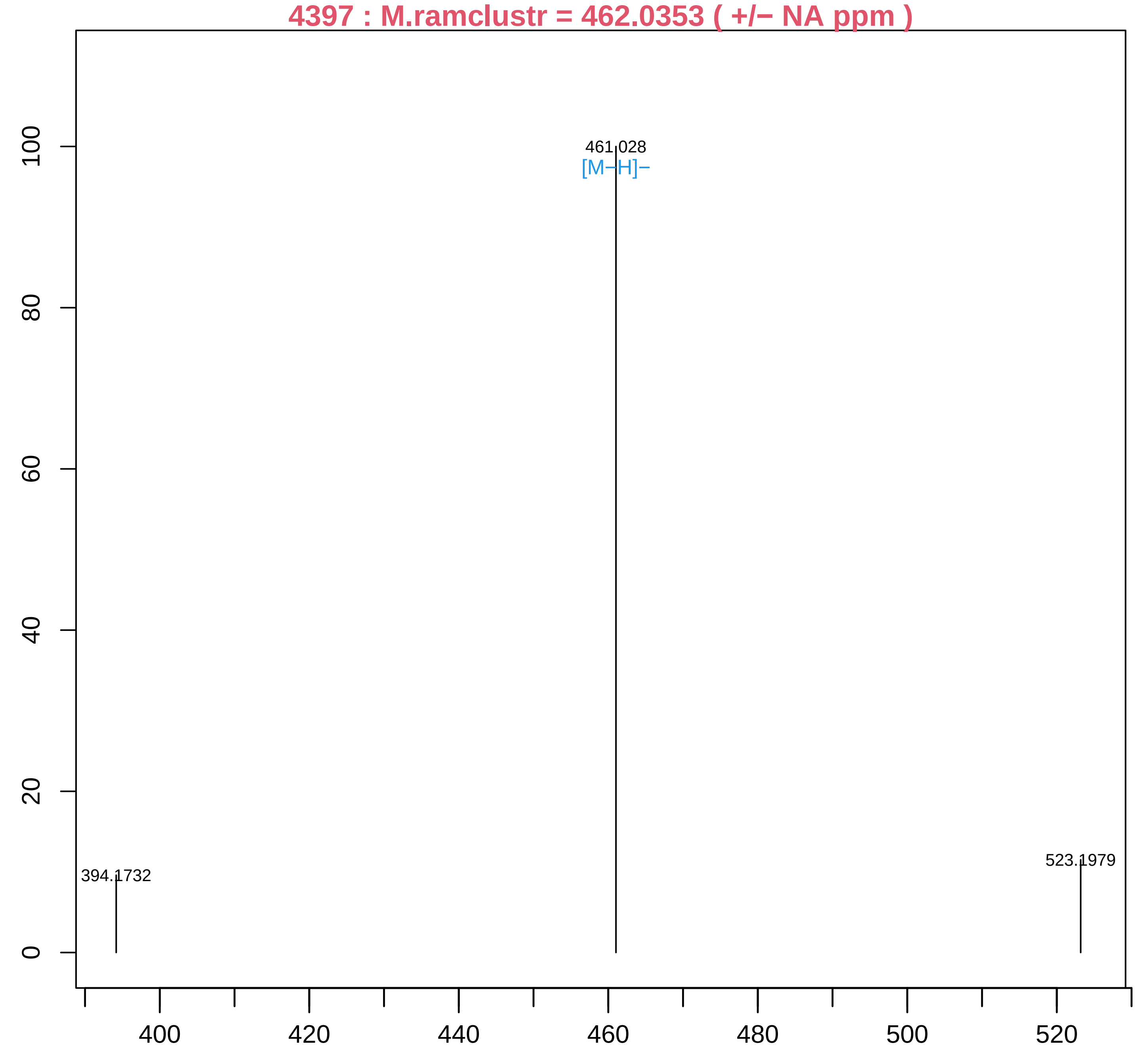
4397 : M.ramclustr = 462.0353 (+/− NA ppm)
461.028
[M−H]−
394.1732
523.1979
0
20
40
60
80
100
400
420
440
460
480
500
520

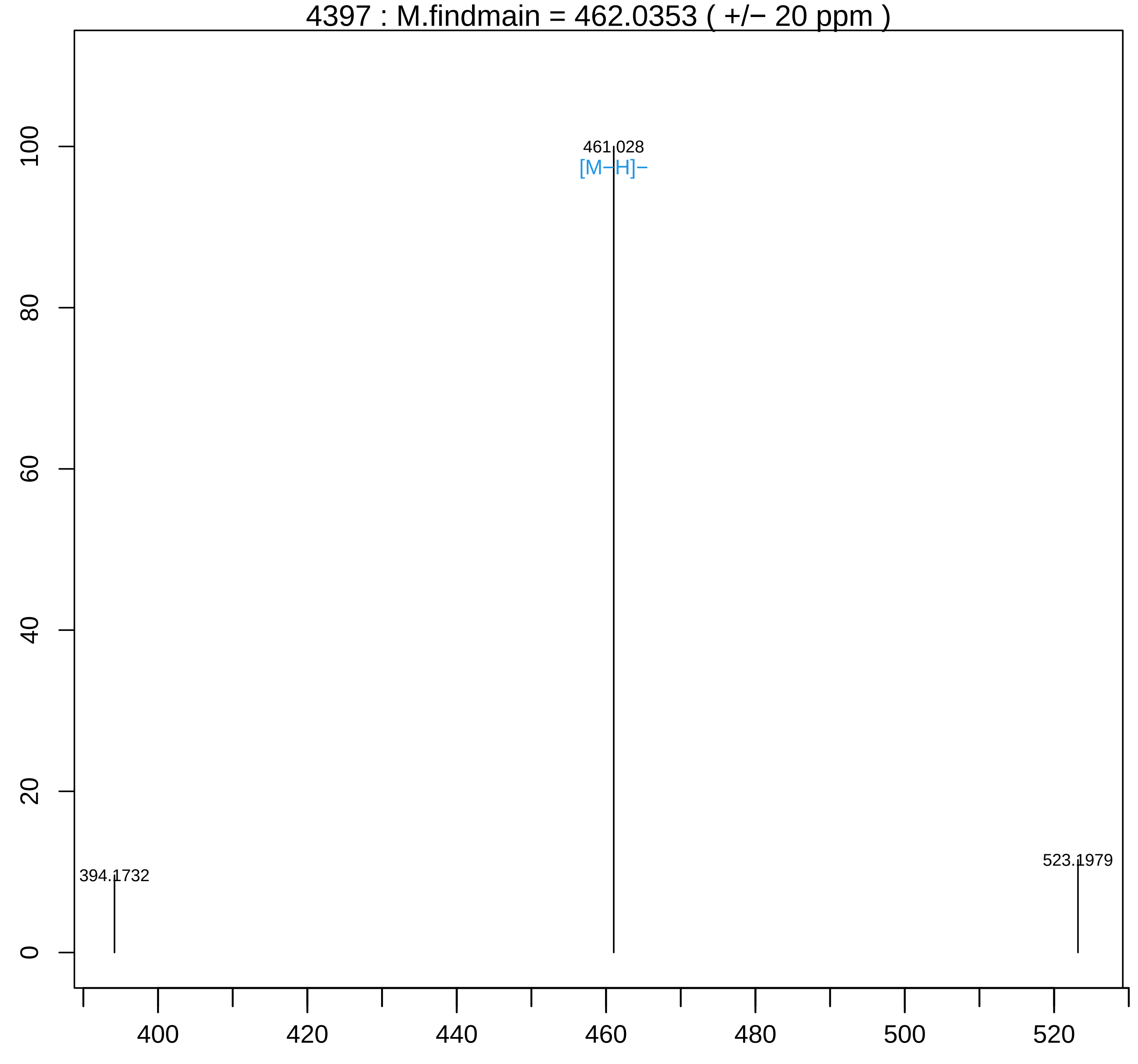
4397 : M.findmain = 462.0353 (+/− 20 ppm)
461.028
[M−H]−
394.1732
523.1979
0
20
40
60
80
100
400
420
440
460
480
500
520

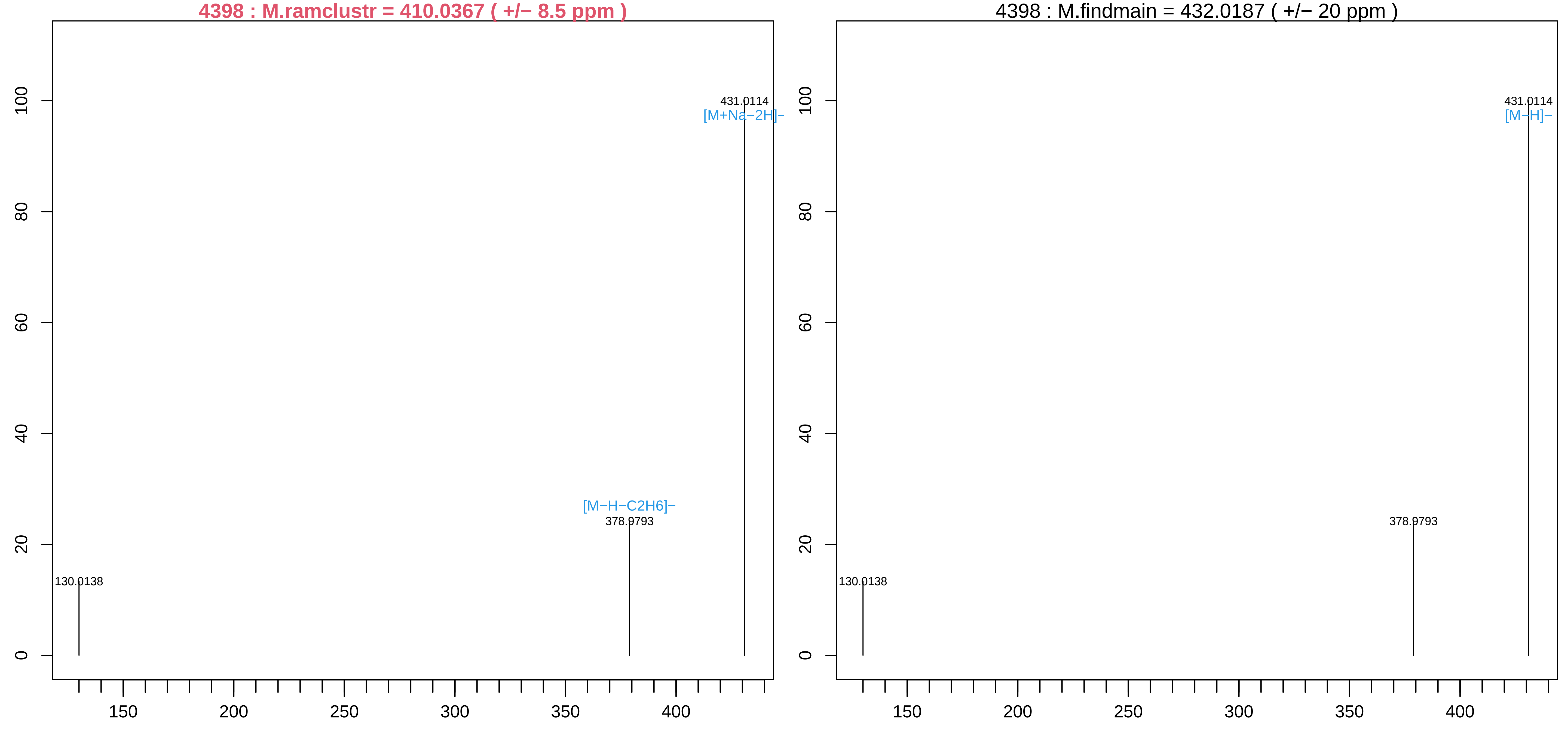
4398 : M.ramclustr = 410.0367 (+/− 8.5 ppm)
431.0114
[M+Na−2H]−
[M−H−C2H6]−
378.9793
130.0138
4398 : M.findmain = 432.0187 (+/− 20 ppm)
431.0114
[M−H]−
378.9793
130.0138
0
20
40
60
80
100
150
200
250
300
350
400

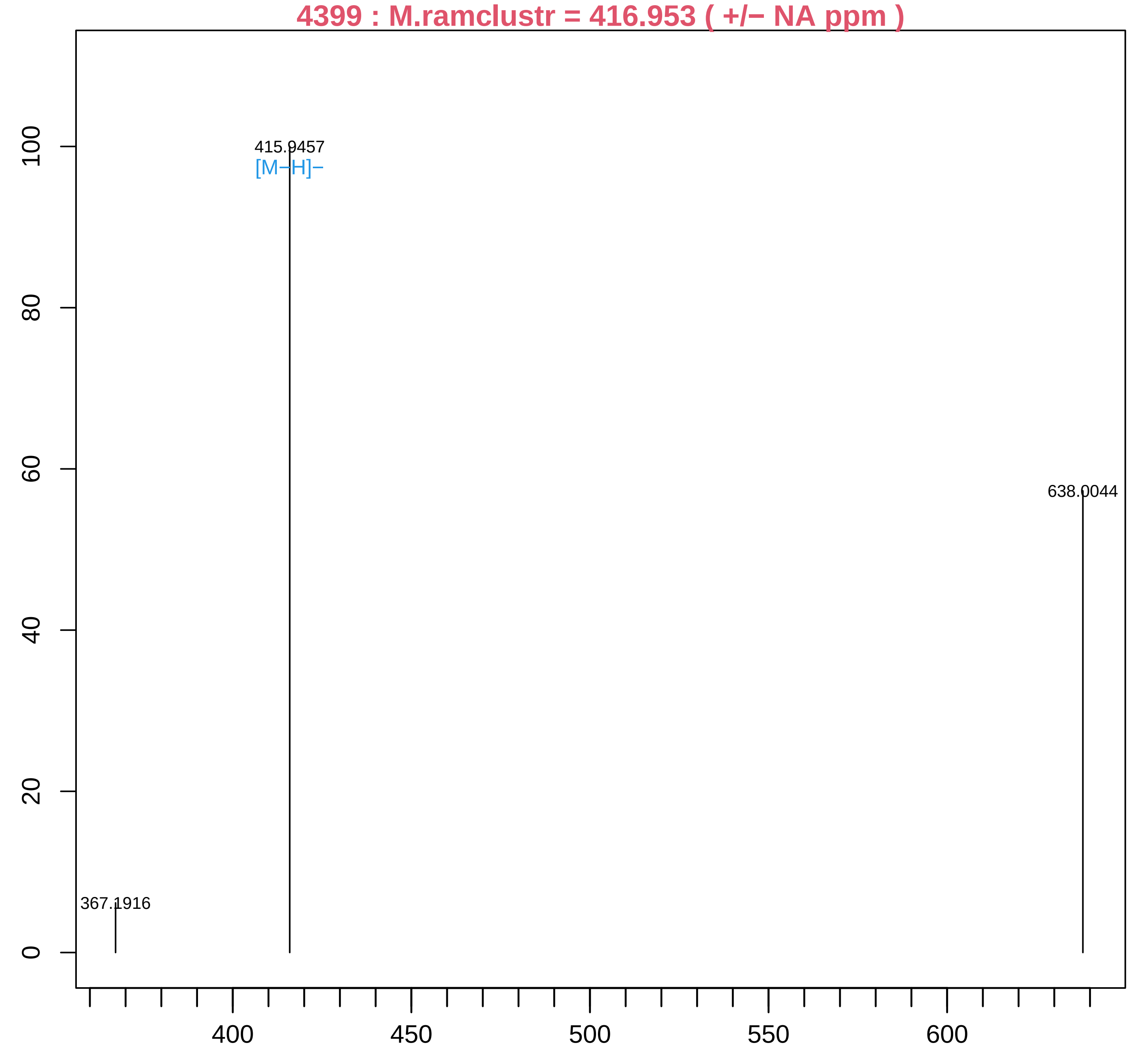
4399 : M.ramclustr = 416.953 (+/− NA ppm)
415.9457
[M−H]−
638.0044
367.1916
0
20
40
60
80
100
400
450
500
550
600

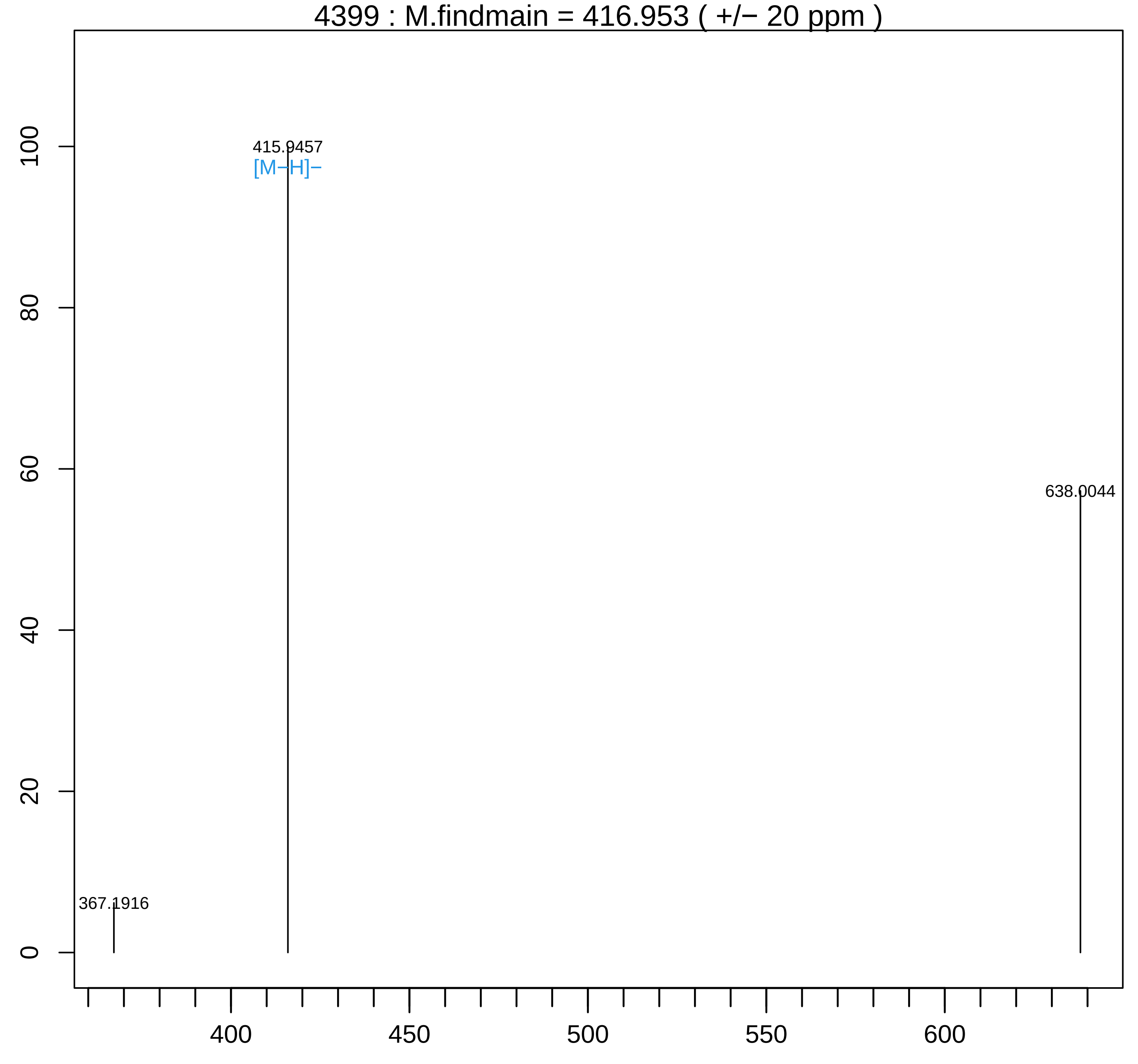
4399 : M.findmain = 416.953 (+/− 20 ppm)
415.9457
[M−H]−
638.0044
367.1916
0
20
40
60
80
100
400
450
500
550
600

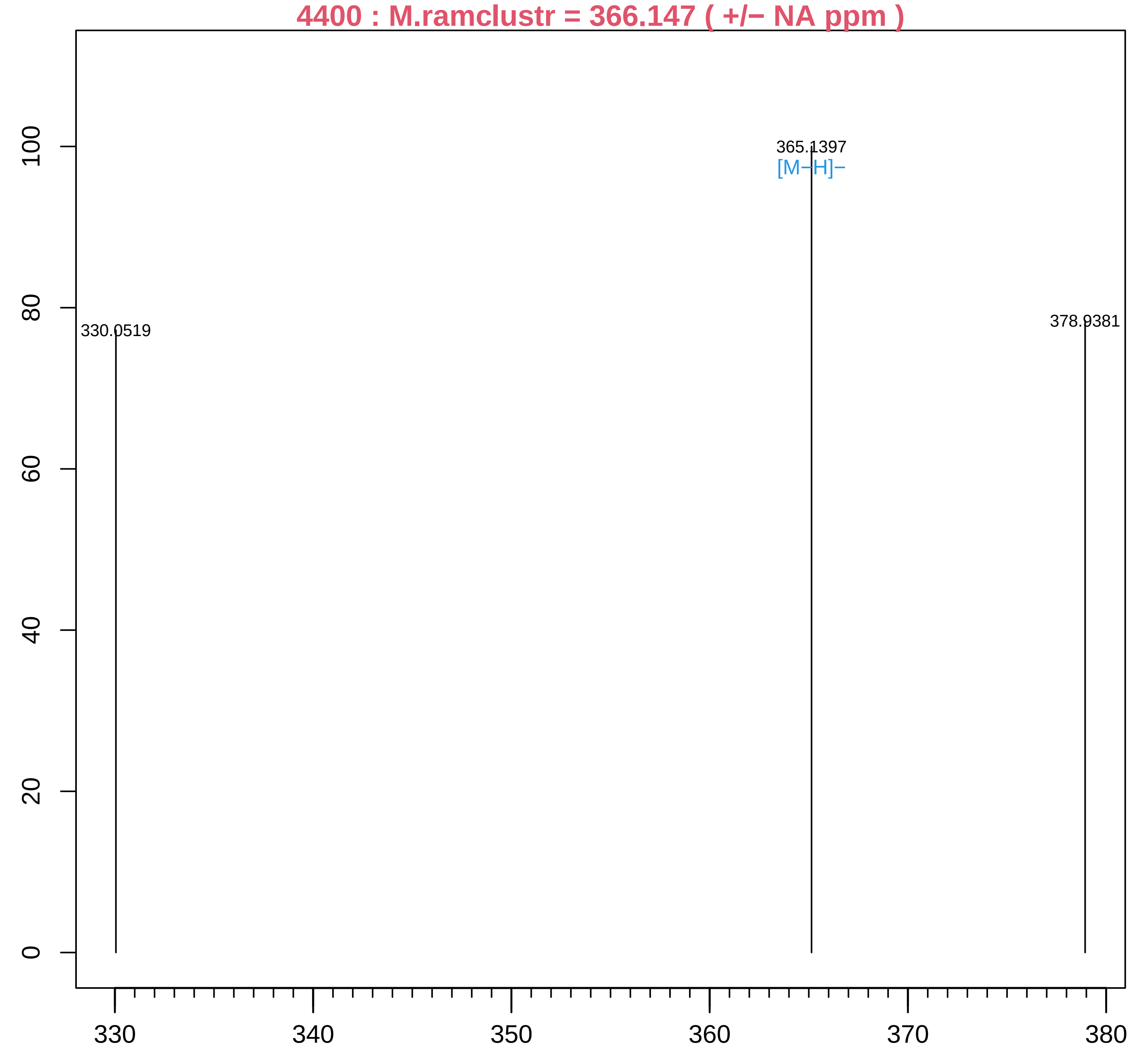
4400 : M.ramclustr = 366.147 (+/− NA ppm)
365.1397
[M−H]−
330.0519
378.9381
0
20
40
60
80
100
330
340
350
360
370
380

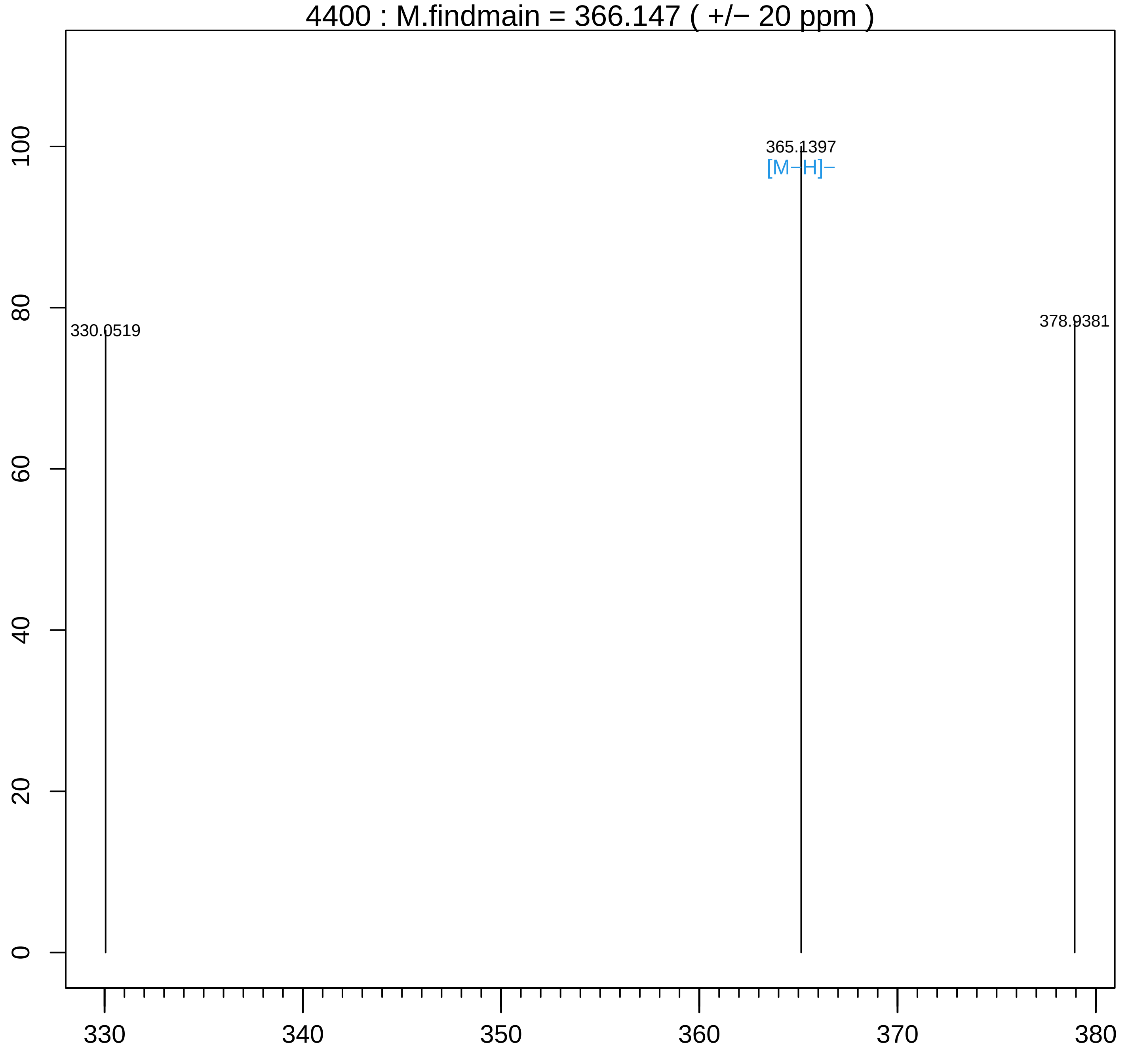
4400 : M.findmain = 366.147 (+/− 20 ppm)
365.1397
[M−H]−
330.0519
378.9381
0
20
40
60
80
100
330
340
350
360
370
380

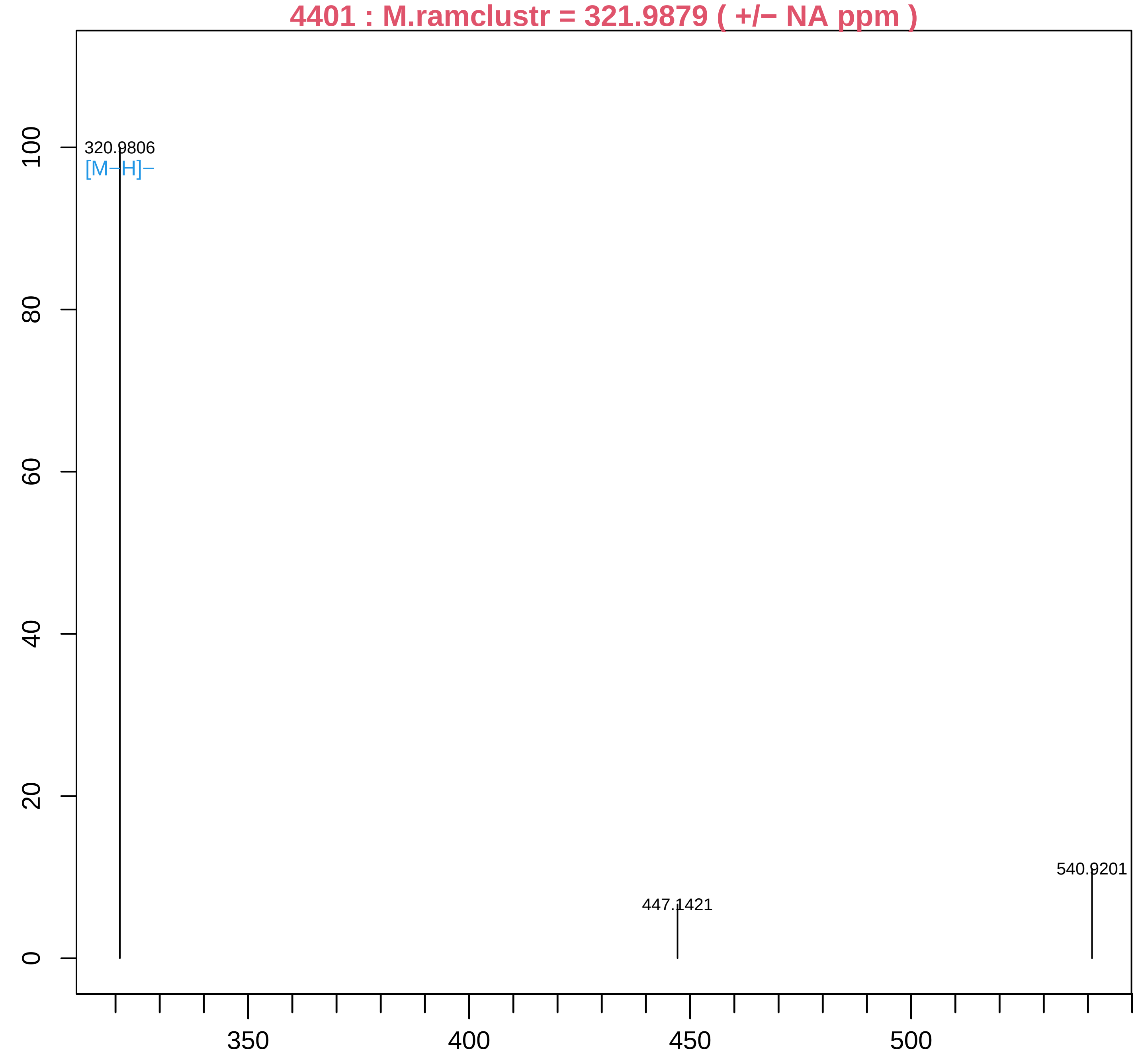
4401 : M.ramclustr = 321.9879 (+/− NA ppm)
320.9806
[M−H]−
447.1421
540.9201
0
20
40
60
80
100
350
400
450
500

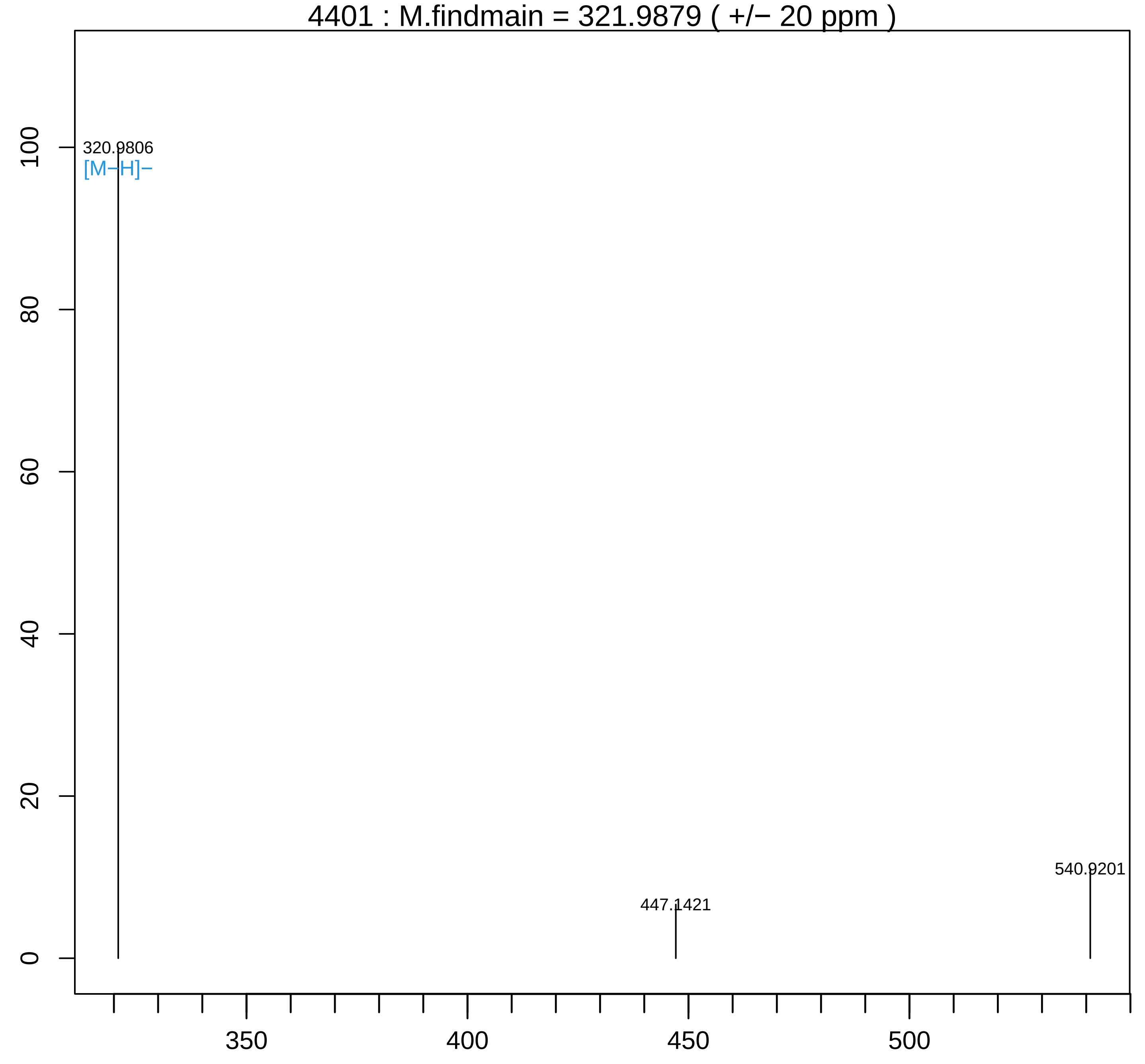
4401 : M.findmain = 321.9879 (+/− 20 ppm)
320.9806
[M−H]−
447.1421
540.9201
0
20
40
60
80
100
350
400
450
500

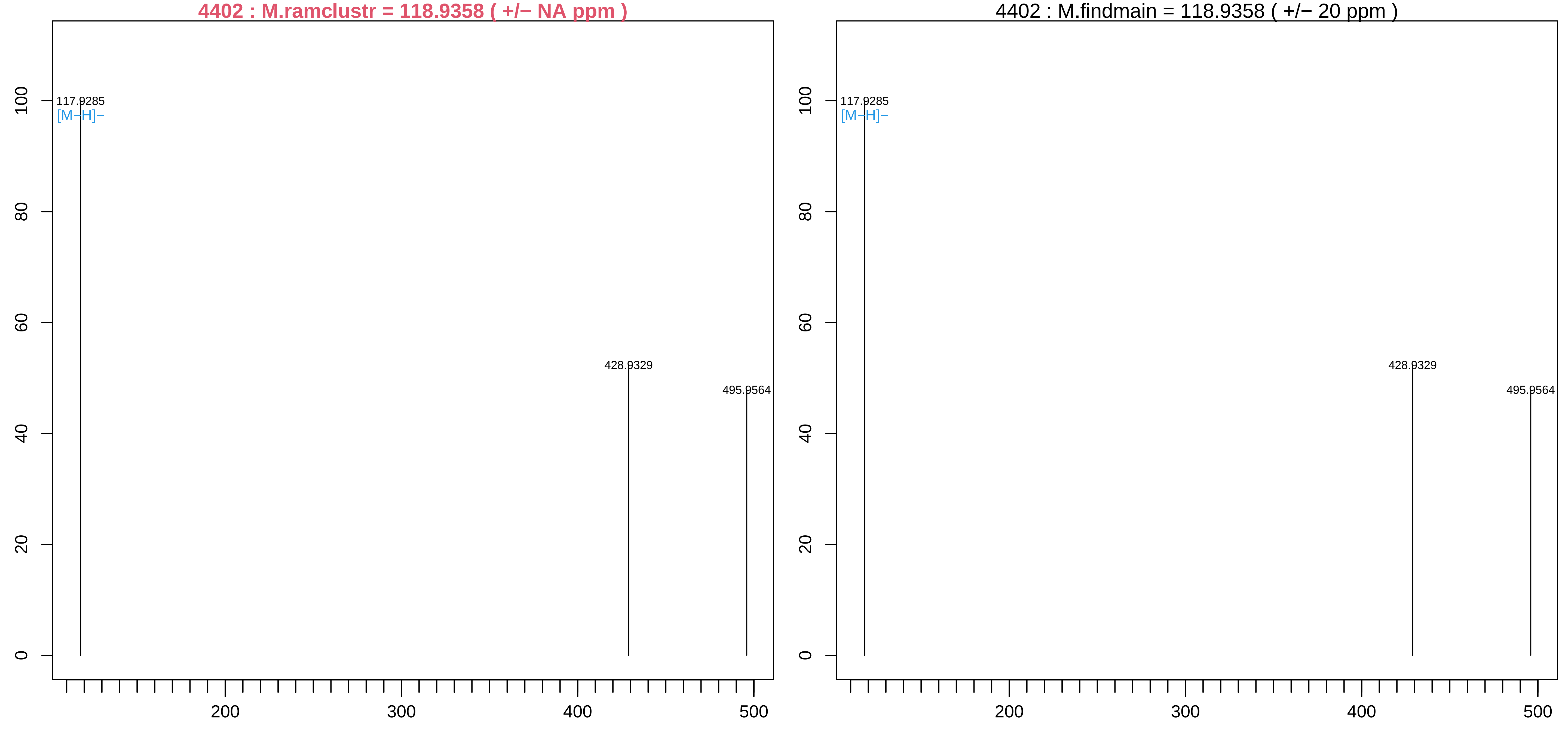
4402 : M.ramclustr = 118.9358 (+/− NA ppm)
117.9285
[M−H]−
428.9329
495.9564
4402 : M.findmain = 118.9358 (+/− 20 ppm)
117.9285
[M−H]−
428.9329
495.9564

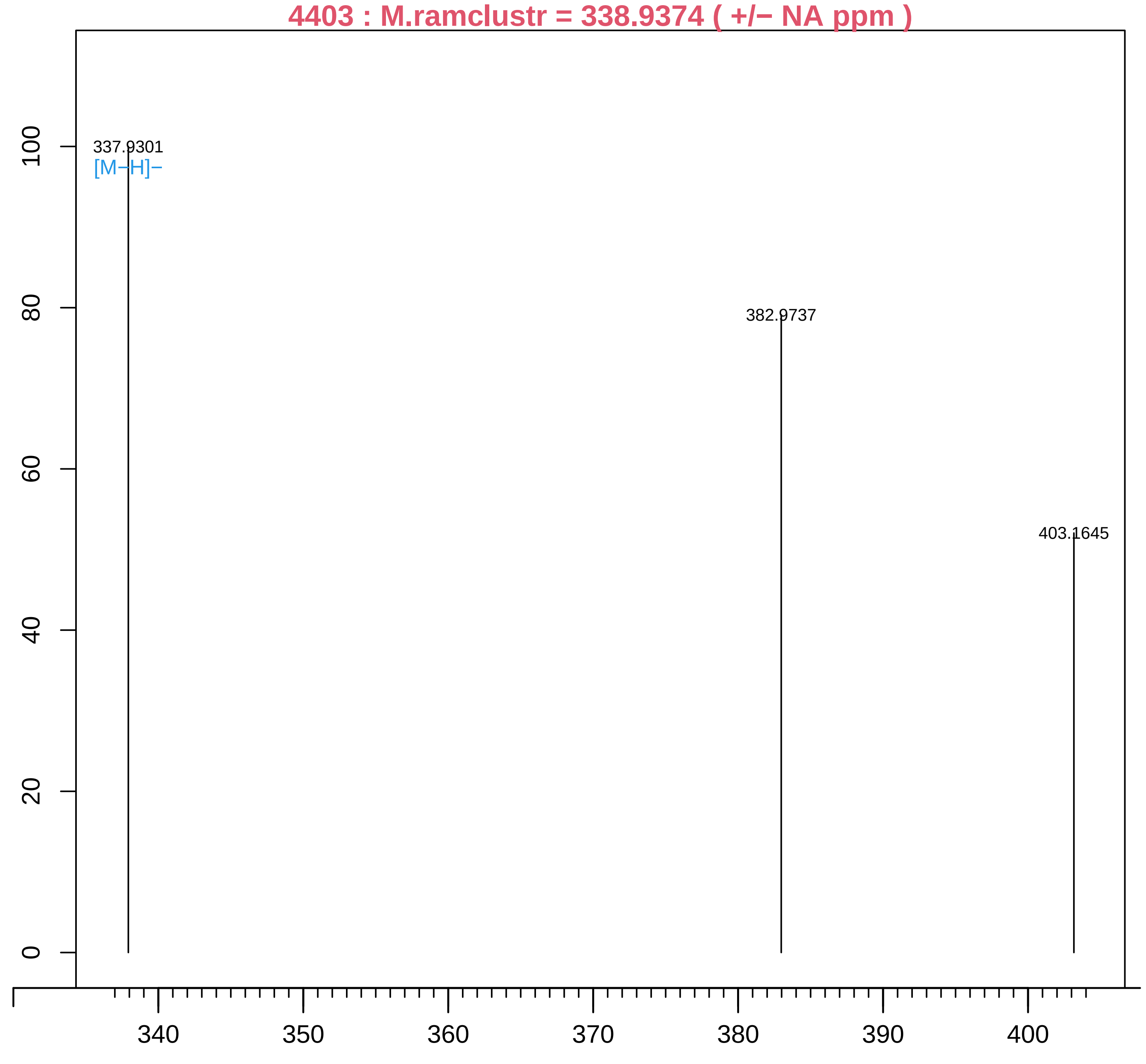
4403 : M.ramclustr = 338.9374 (+/− NA ppm)
337.9301
[M−H]−
382.9737
403.1645
0
20
40
60
80
100
340
350
360
370
380
390
400

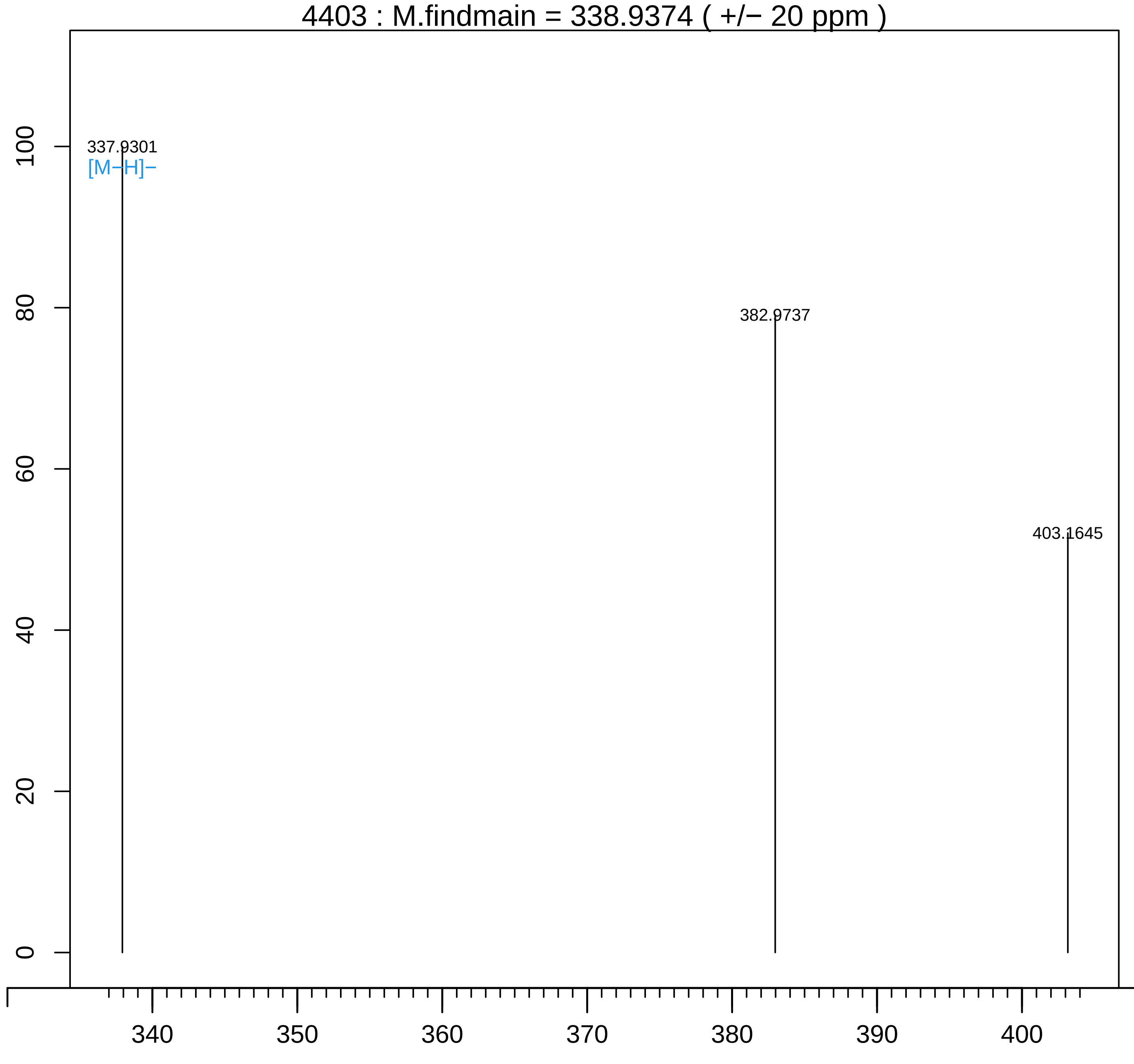
4403 : M.findmain = 338.9374 (+/− 20 ppm)
337.9301
[M−H]−
382.9737
403.1645
0
20
40
60
80
100
340
350
360
370
380
390
400

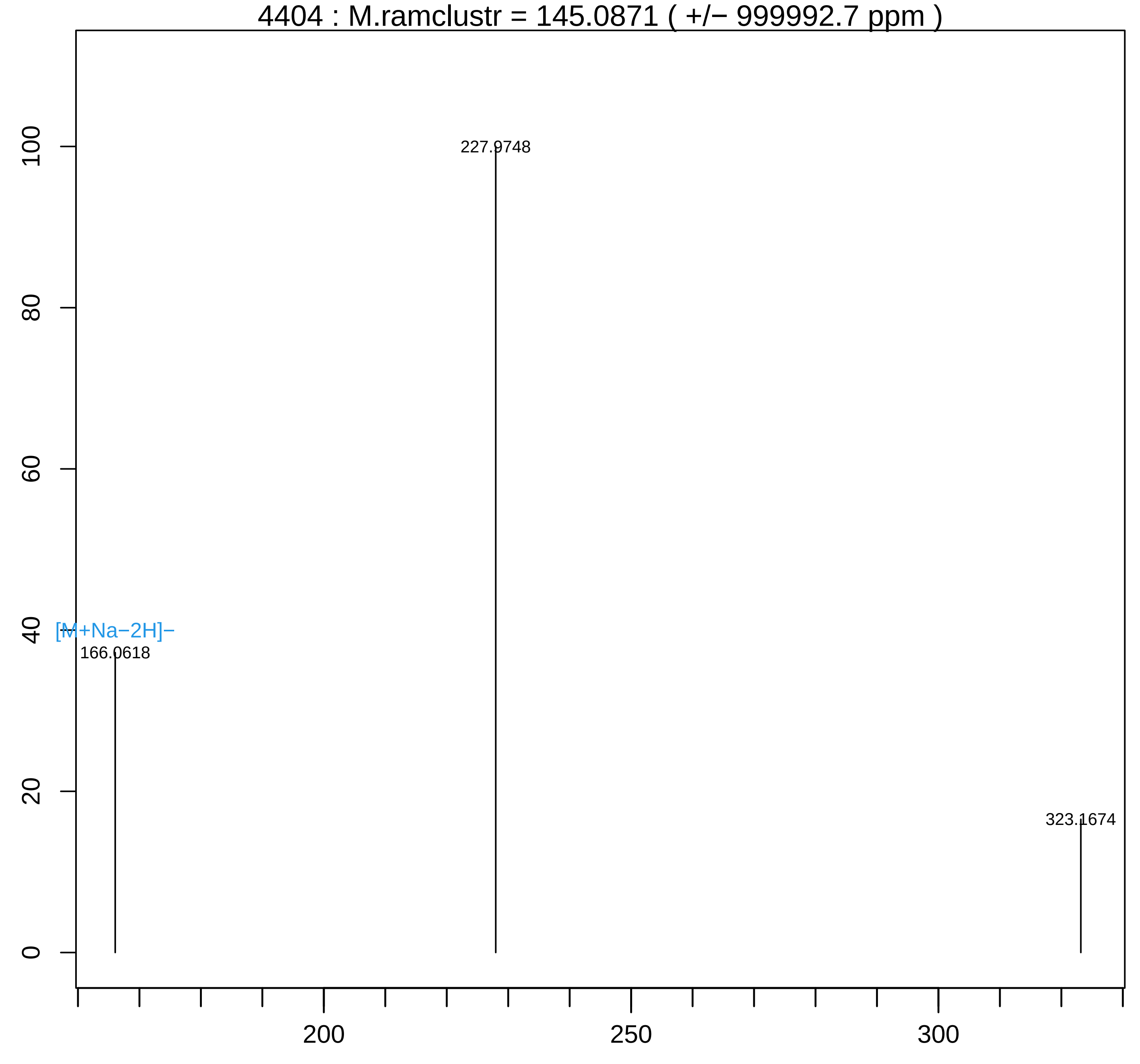
4404 : M.ramclustr = 145.0871 (+/− 999992.7 ppm)
227.9748
[M+Na−2H]−
166.0618
323.1674
0
20
40
60
80
100
200
250
300

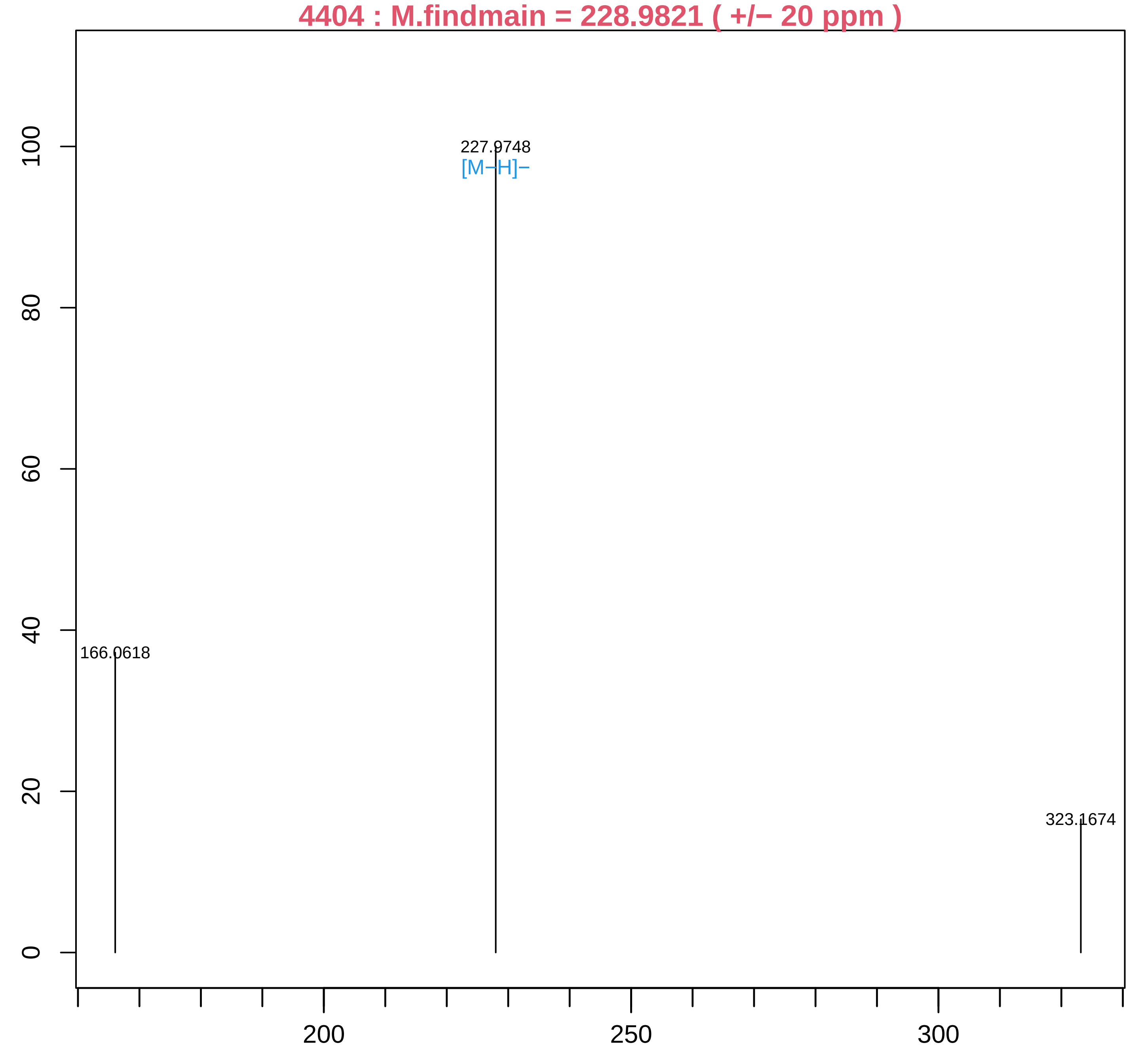
4404 : M.findmain = 228.9821 (+/− 20 ppm)
227.9748
[M−H]−
166.0618
323.1674
0
20
40
60
80
100
200
250
300

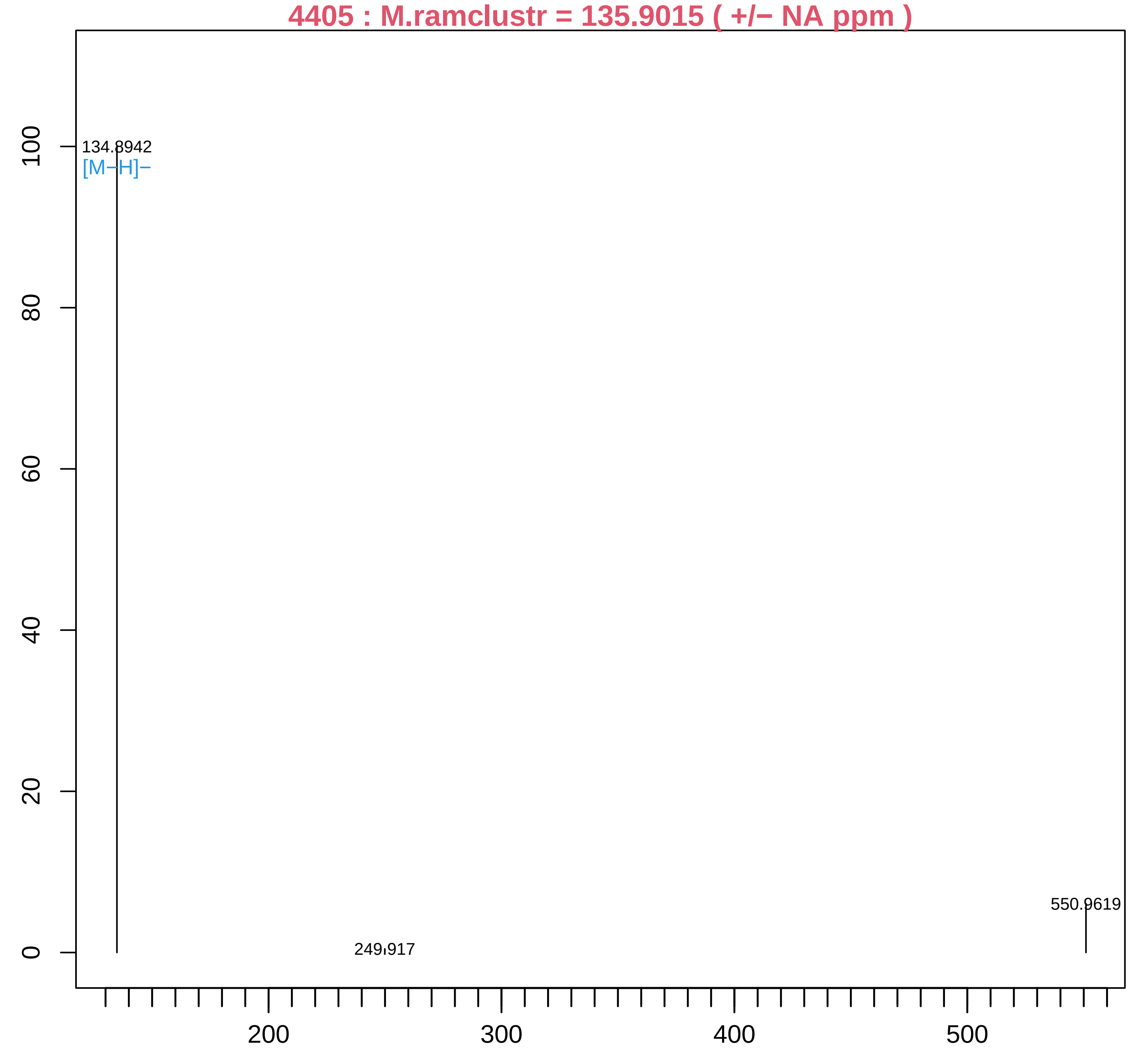
4405 : M.ramclustr = 135.9015 (+/− NA ppm)
134.8942
[M−H]−
249.917
550.9619
0
20
40
60
80
100
200
300
400
500

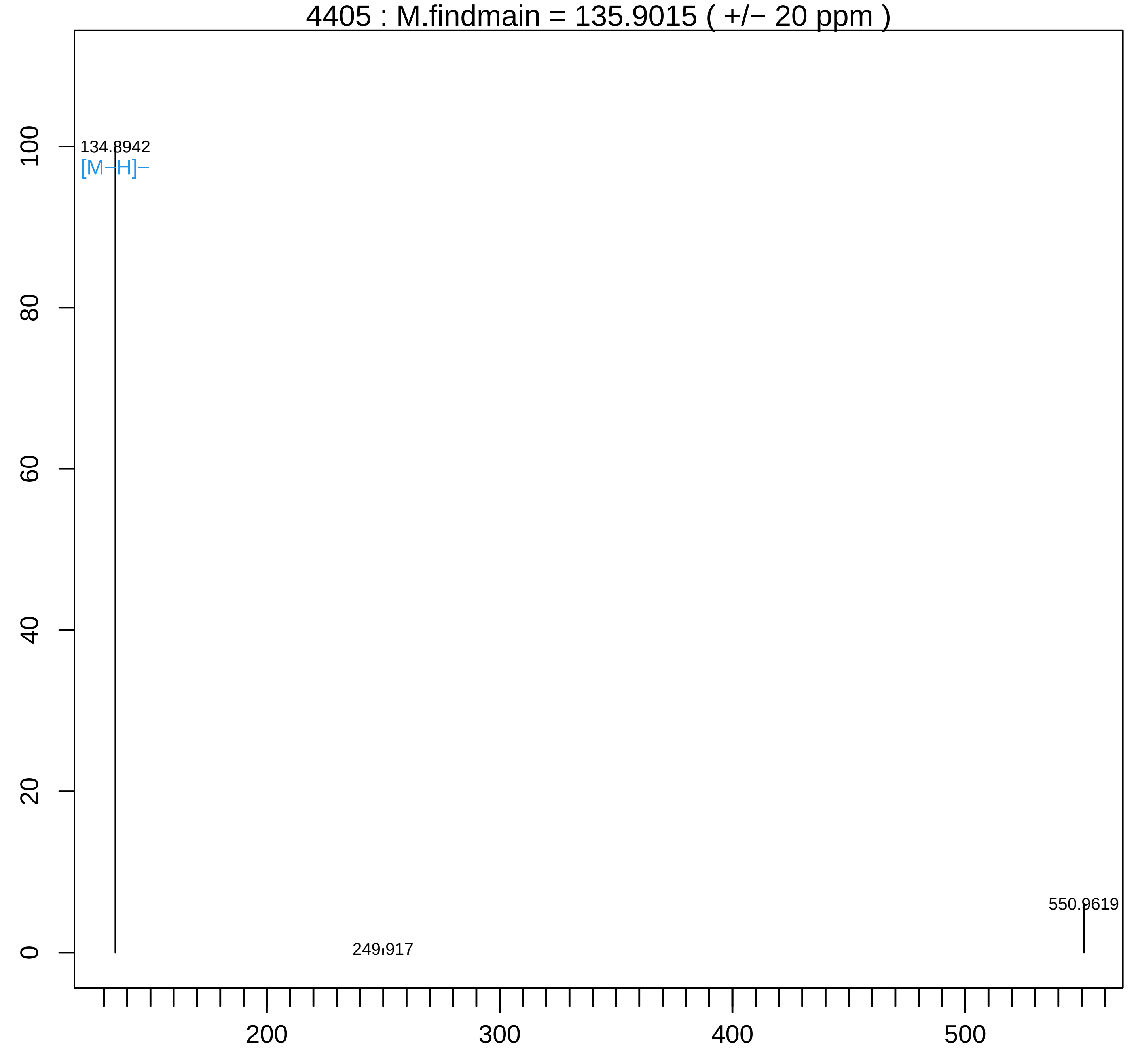
4405 : M.findmain = 135.9015 (+/− 20 ppm)
134.8942
[M−H]−
249.917
550.9619
0
20
40
60
80
100
200
300
400
500

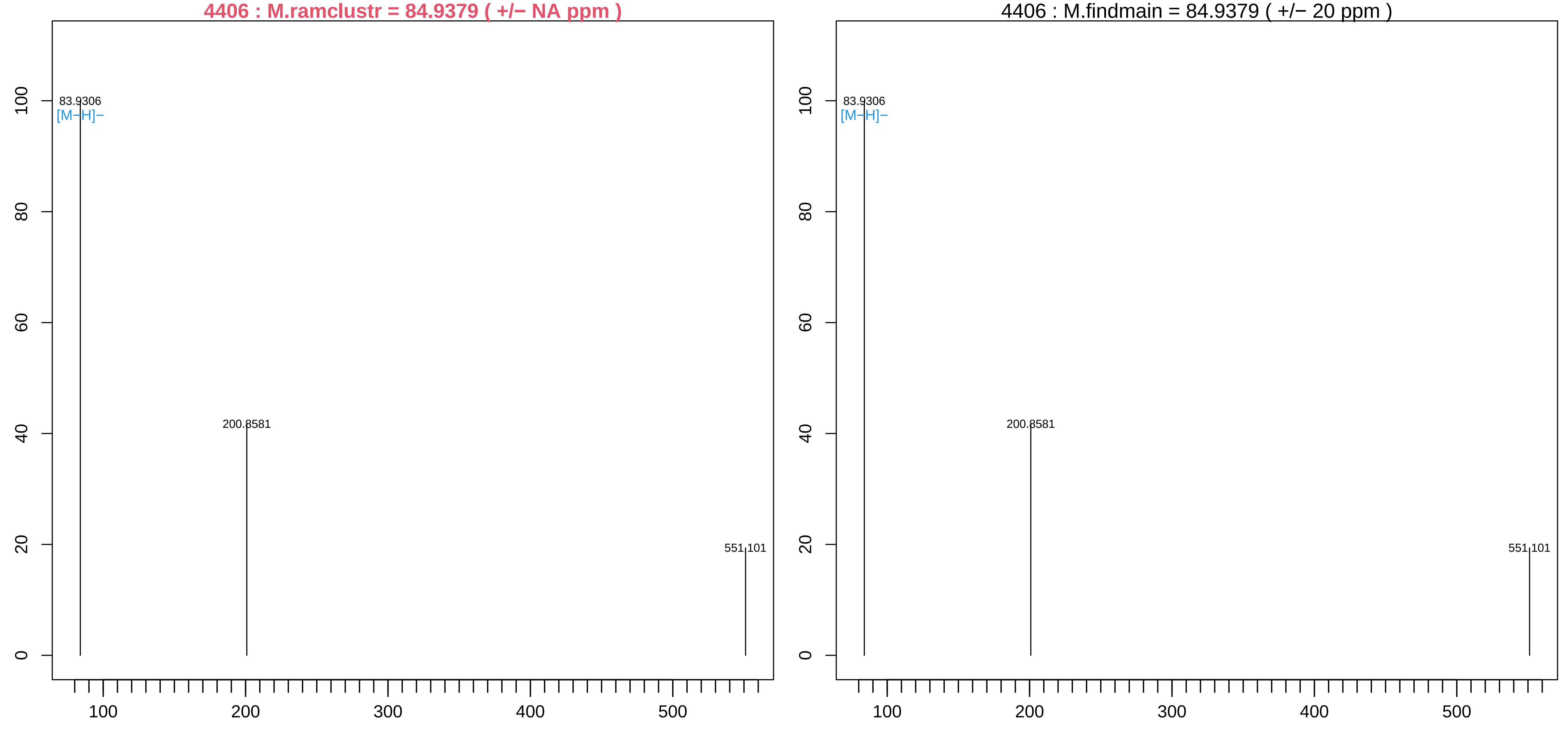
4406 : M.ramclustr = 84.9379 (+/− NA ppm)
83.9306
[M−H]−
200.8581
551.101
4406 : M.findmain = 84.9379 (+/− 20 ppm)
83.9306
[M−H]−
200.8581
551.101
0
20
40
60
80
100
100
200
300
400
500

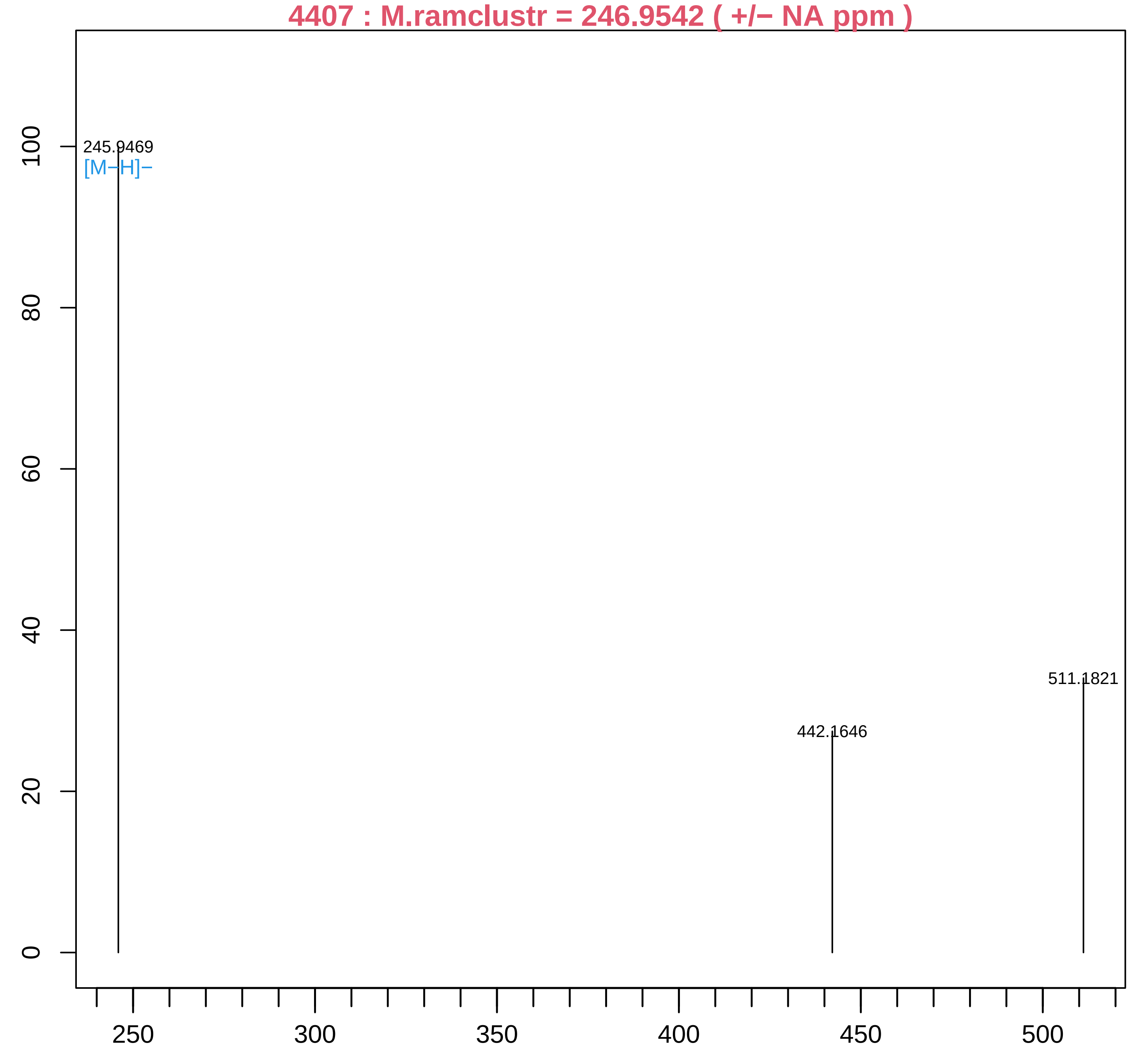
4407 : M.ramclustr = 246.9542 (+/− NA ppm)
245.9469
[M−H]−
442.1646
511.1821
0
20
40
60
80
100
250
300
350
400
450
500

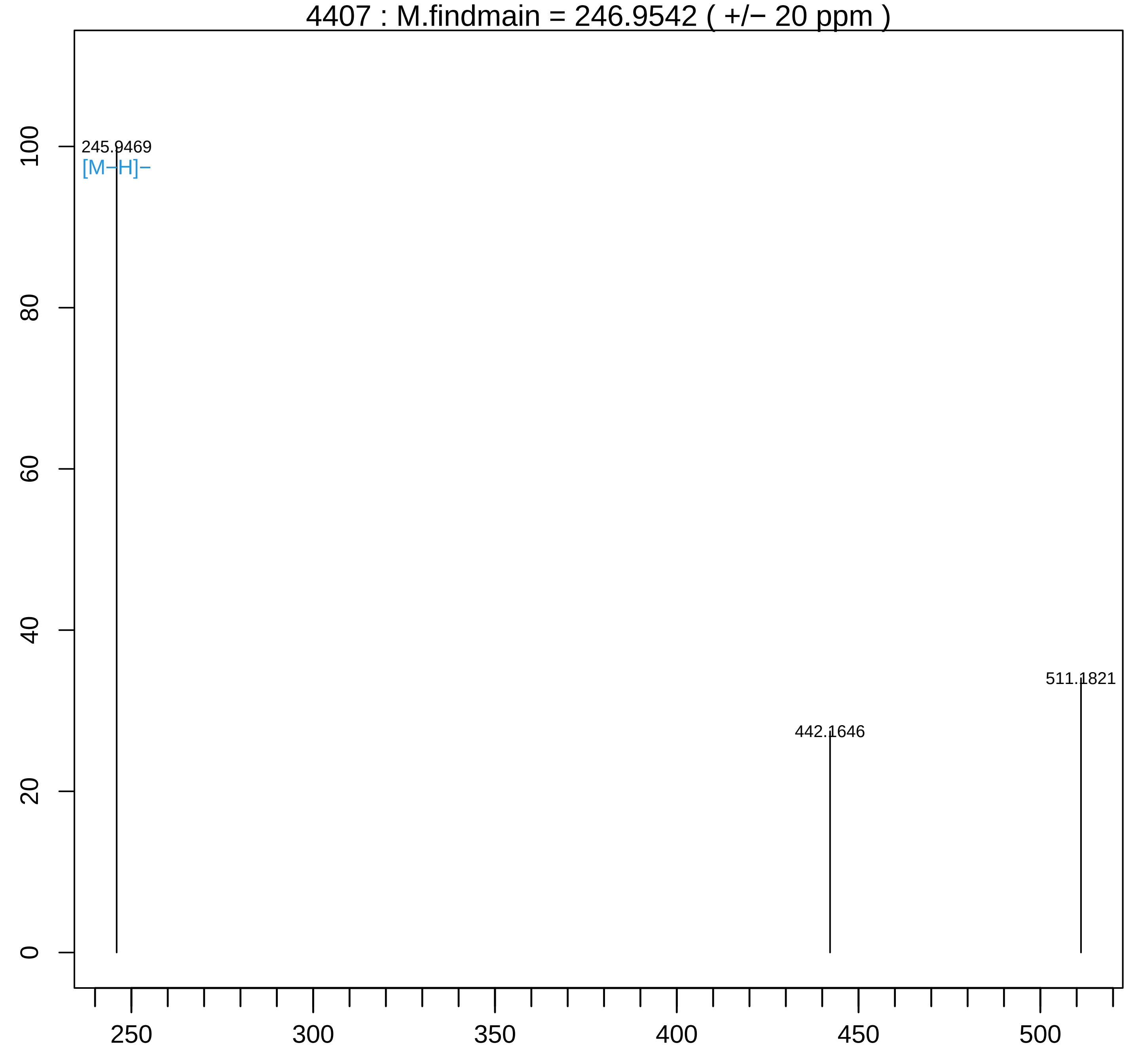
4407 : M.findmain = 246.9542 (+/− 20 ppm)
245.9469
[M−H]−
442.1646
511.1821
0
20
40
60
80
100
250
300
350
400
450
500

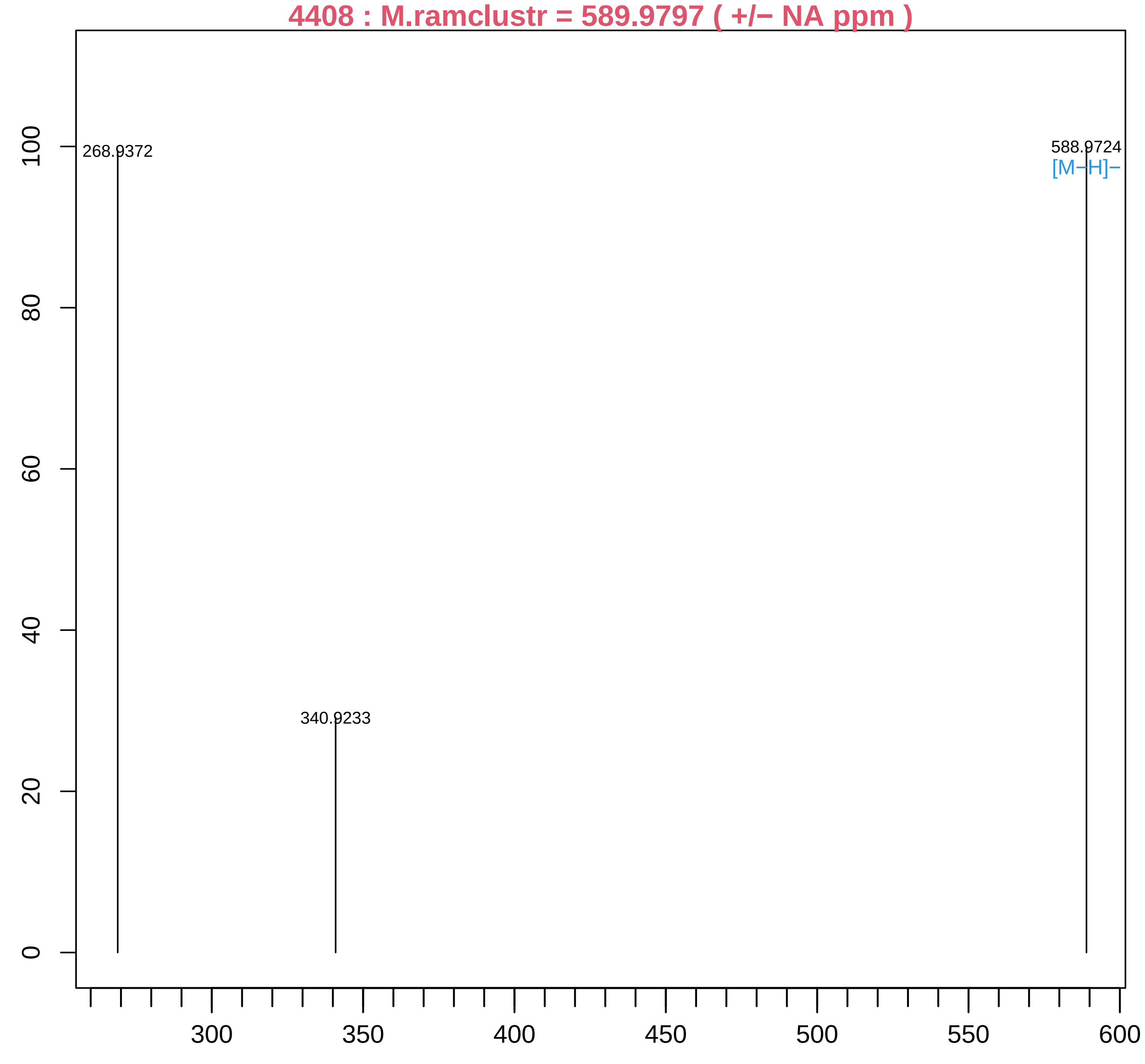

4408 : M.ramclustr = 589.9797 (+/− NA ppm)
268.9372
340.9233
588.9724
[M−H]−
0
20
40
60
80
100
300
350
400
450
500
550
600

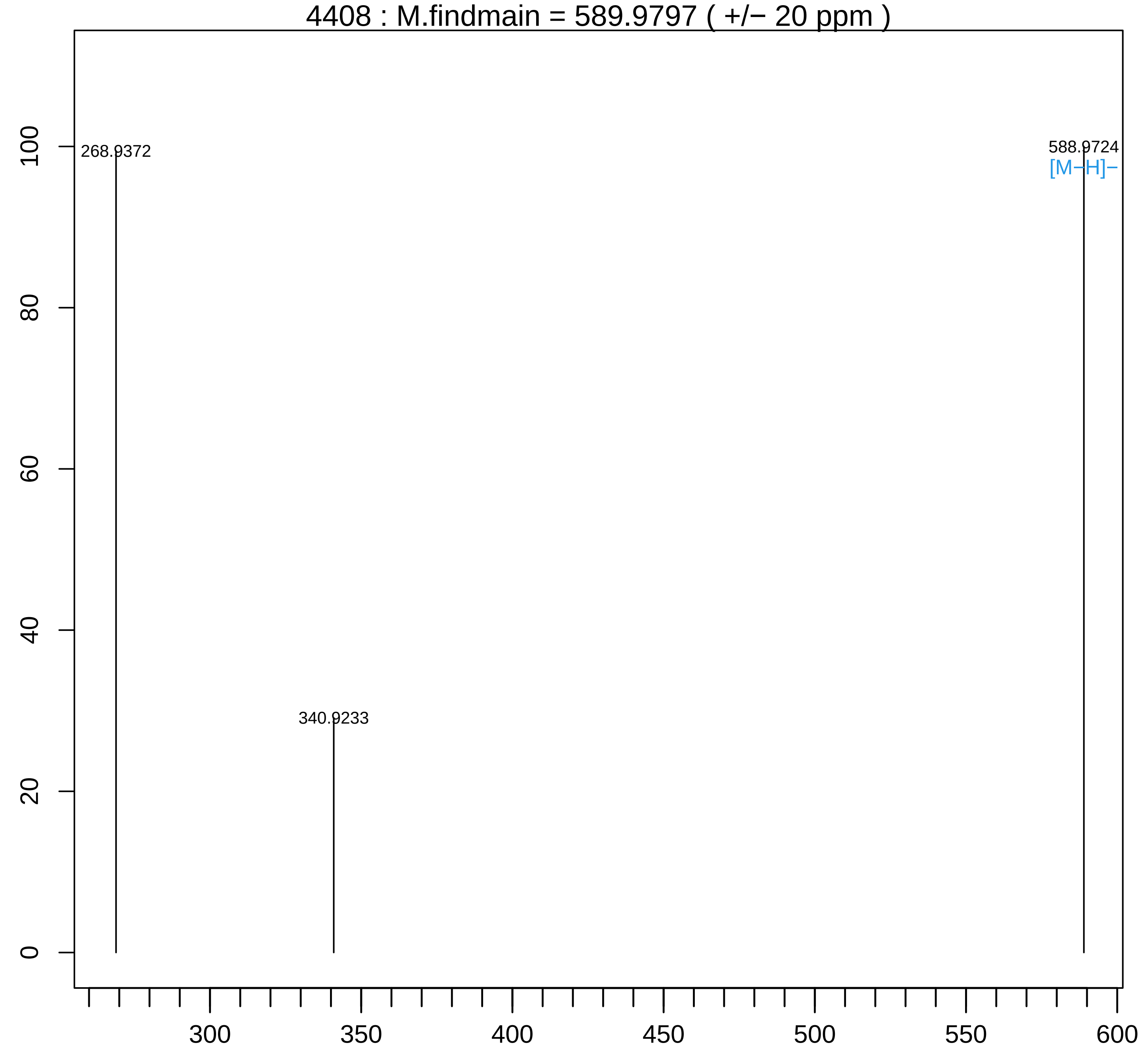

4408 : M.findmain = 589.9797 (+/− 20 ppm)
268.9372
340.9233
588.9724
[M−H]−
0
20
40
60
80
100
300
350
400
450
500
550
600

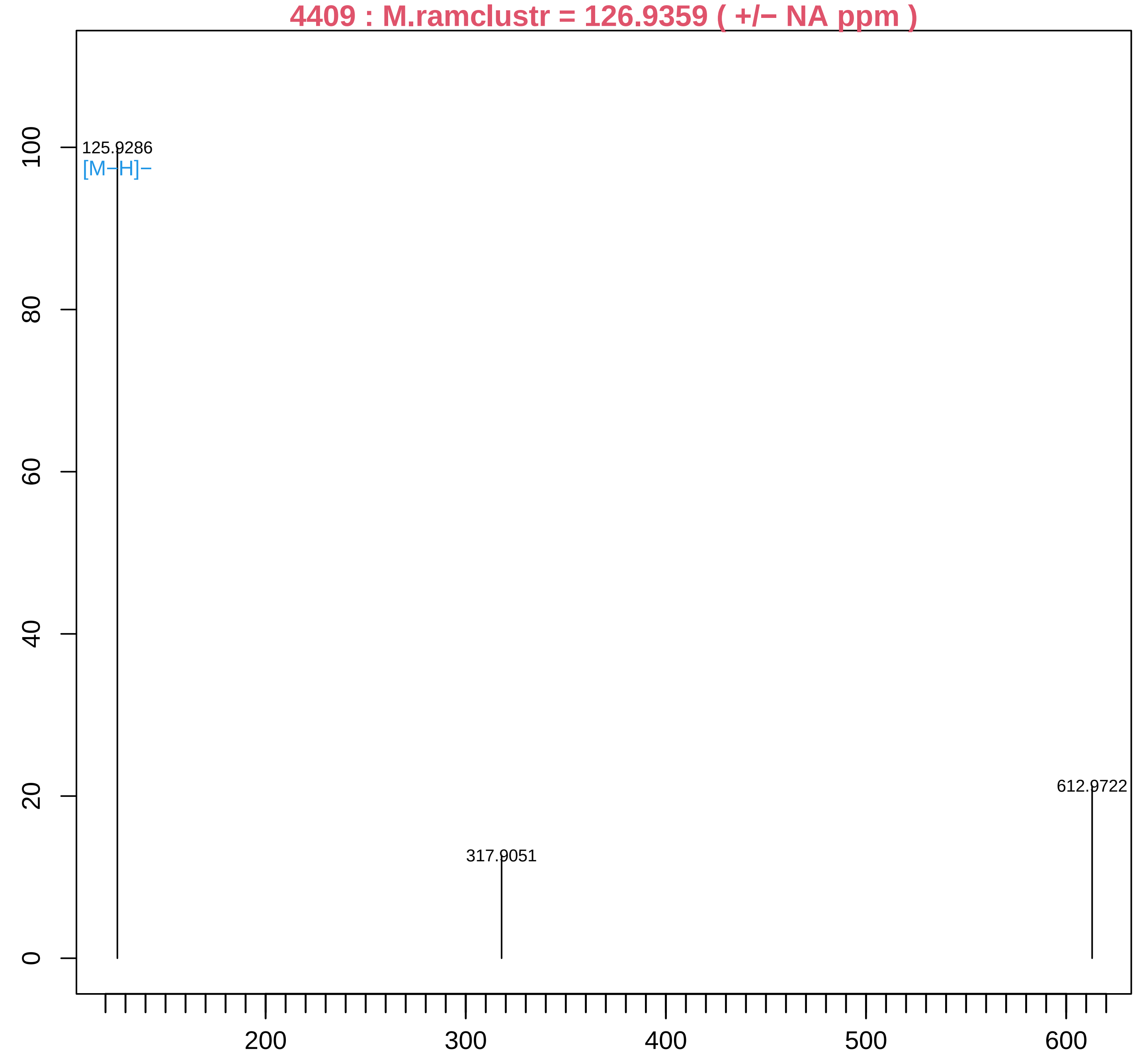
4409 : M.ramclustr = 126.9359 (+/− NA ppm)
125.9286
[M−H]−
317.9051
612.9722
0
20
40
60
80
100
200
300
400
500
600

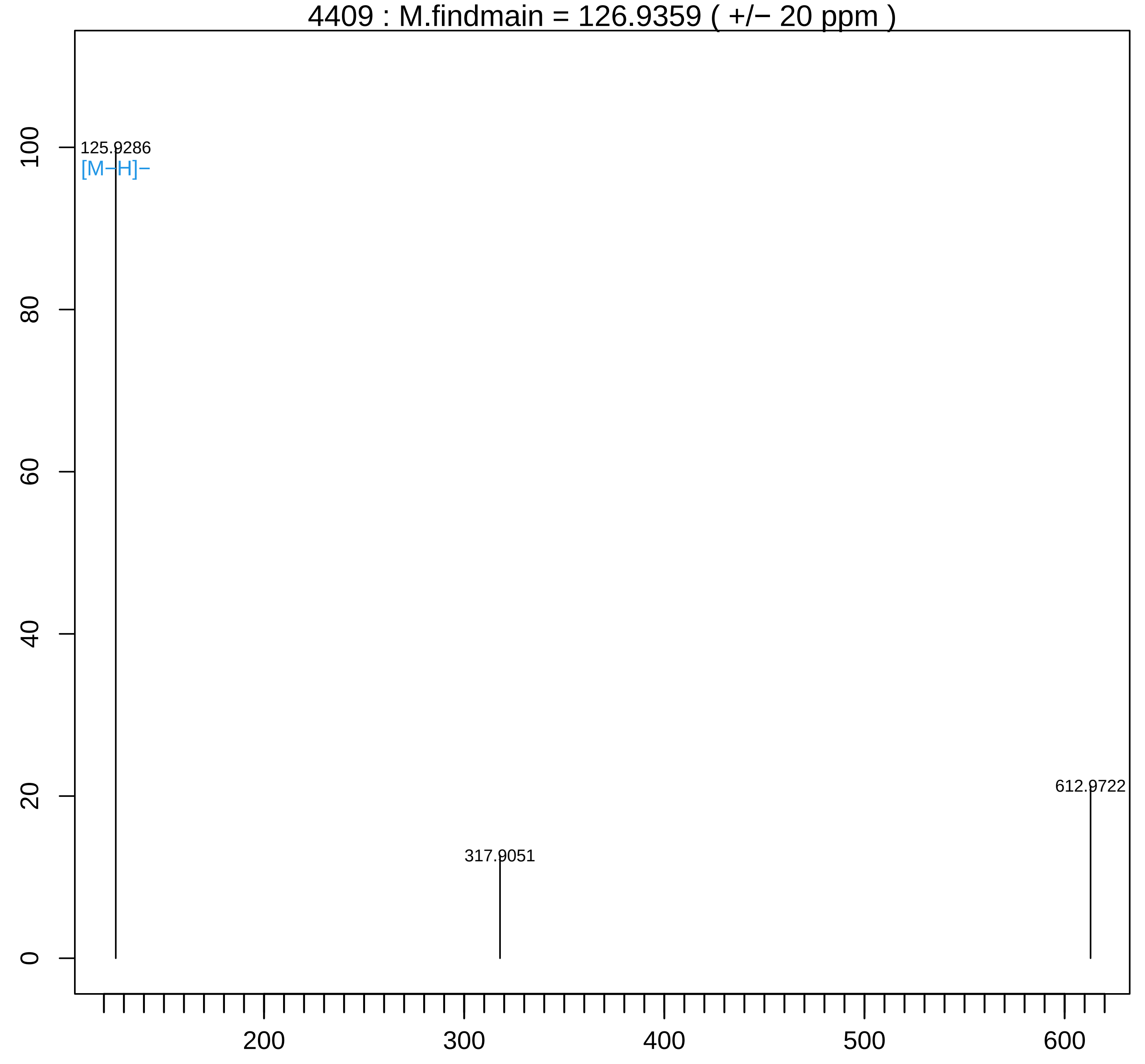
4409 : M.findmain = 126.9359 (+/− 20 ppm)
125.9286
[M−H]−
317.9051
612.9722
0
20
40
60
80
100
200
300
400
500
600

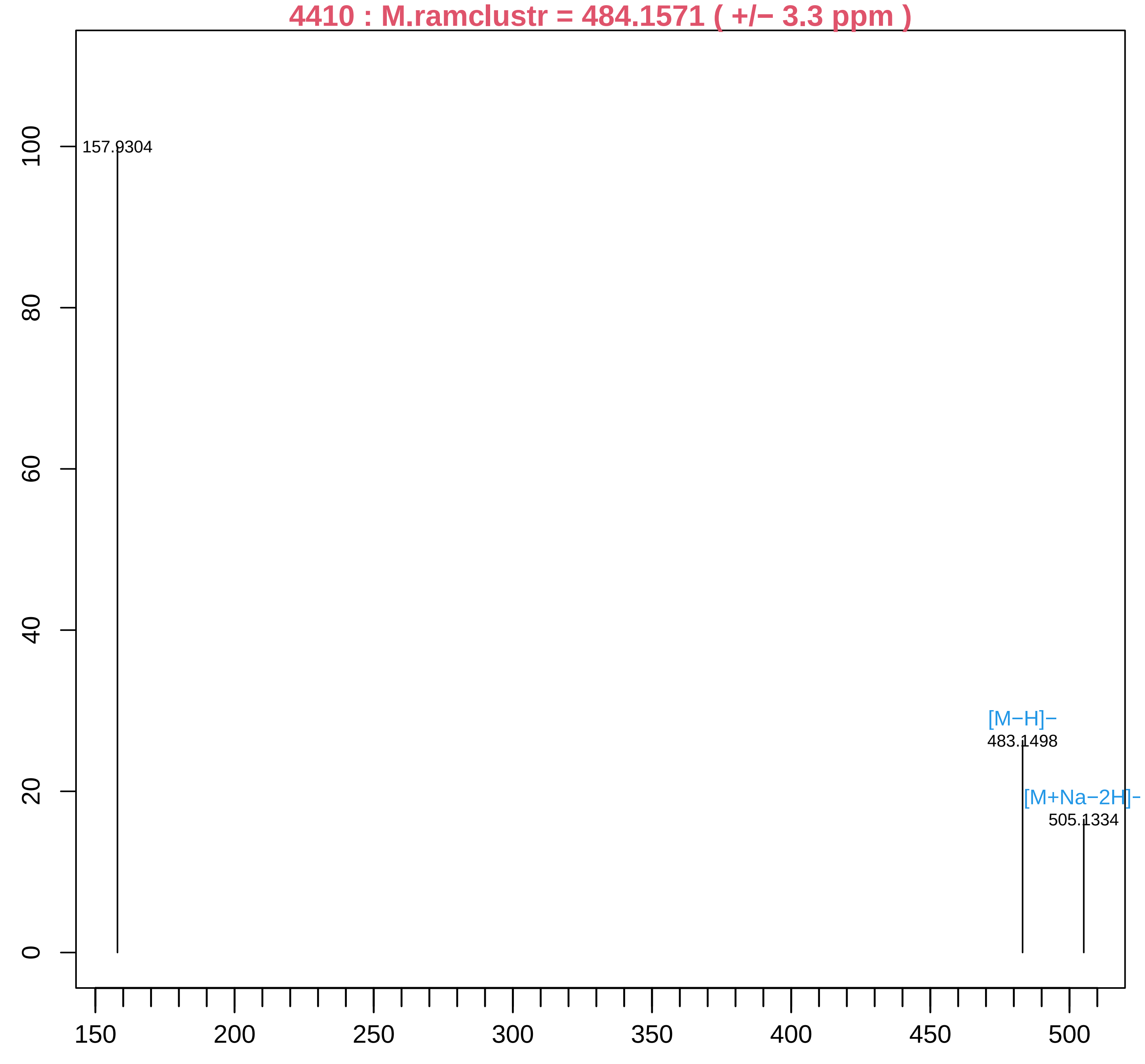

4410 : M.ramclustr = 484.1571 (+/− 3.3 ppm)
157.9304
[M−H]−
483.1498
[M+Na−2H]−
505.1334
0
20
40
60
80
100
150
200
250
300
350
400
450
500

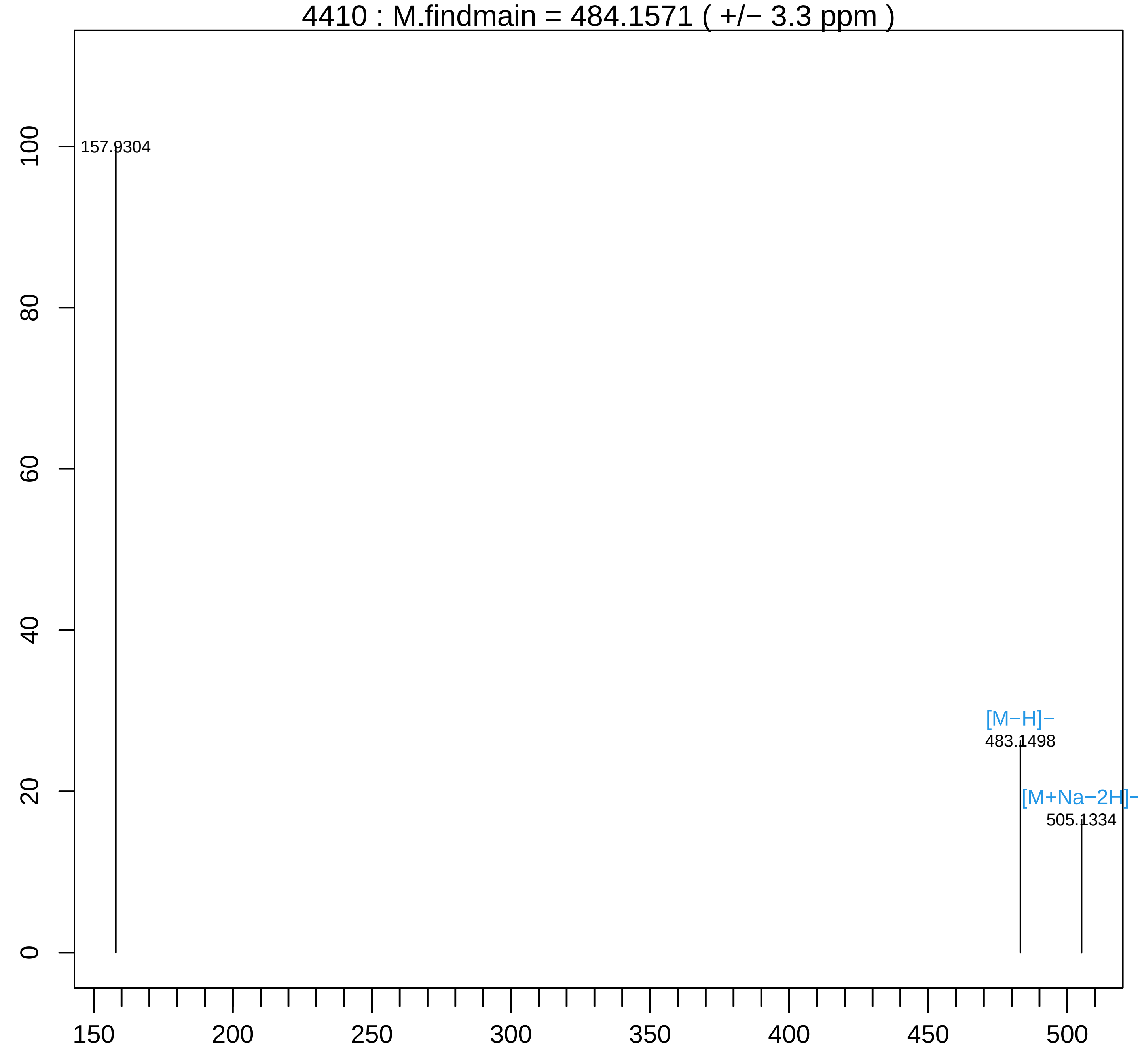

4410 : M.findmain = 484.1571 (+/− 3.3 ppm)
157.9304
[M−H]−
483.1498
[M+Na−2H]−
505.1334
0
20
40
60
80
100
150
200
250
300
350
400
450
500

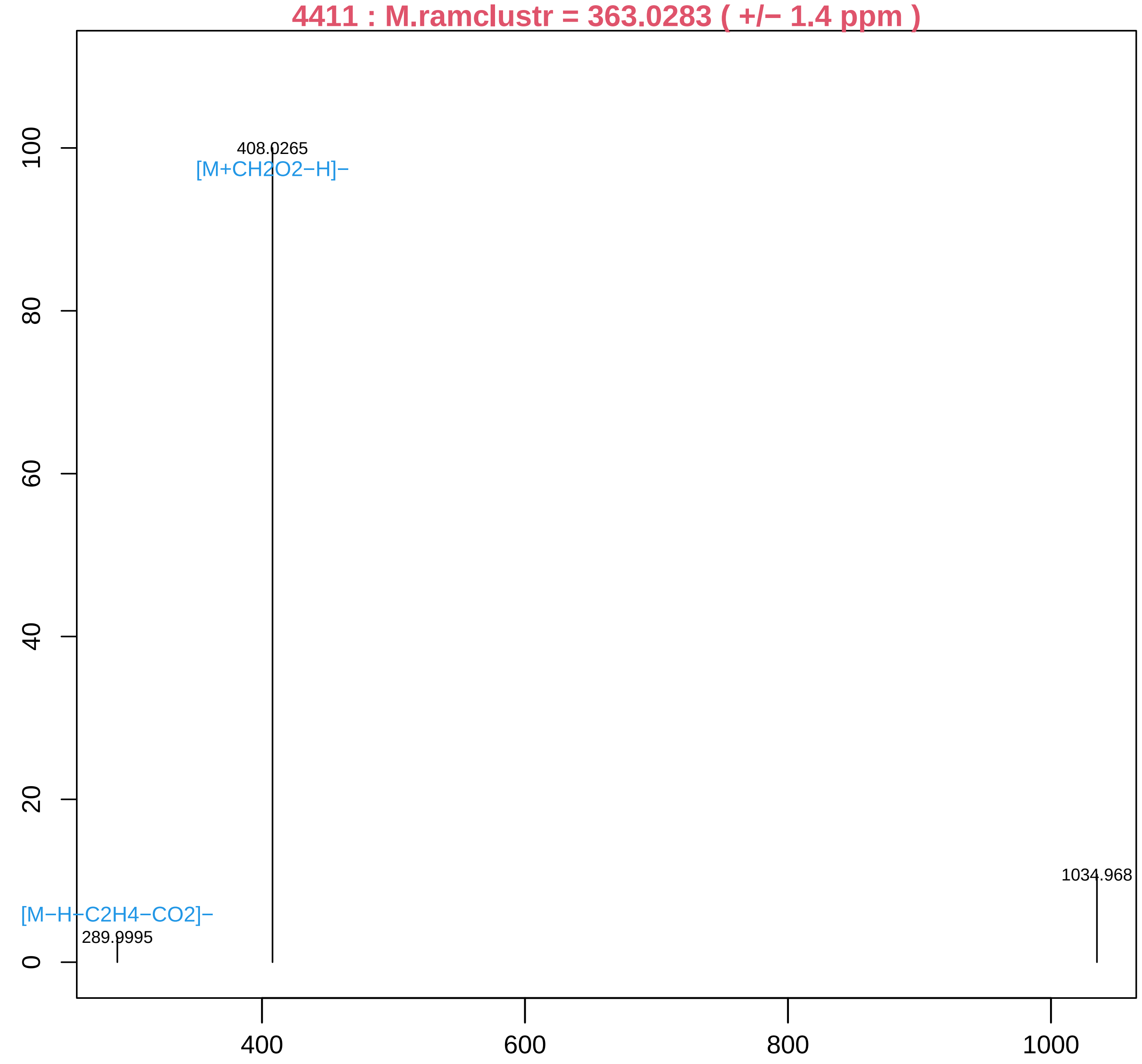

4411 : M.ramclustr = 363.0283 (+/− 1.4 ppm)
408.0265
[M+CH2O2−H]−
1034.968
[M−H−C2H4−CO2]−
289.9995
0
20
40
60
80
100
400
600
800
1000

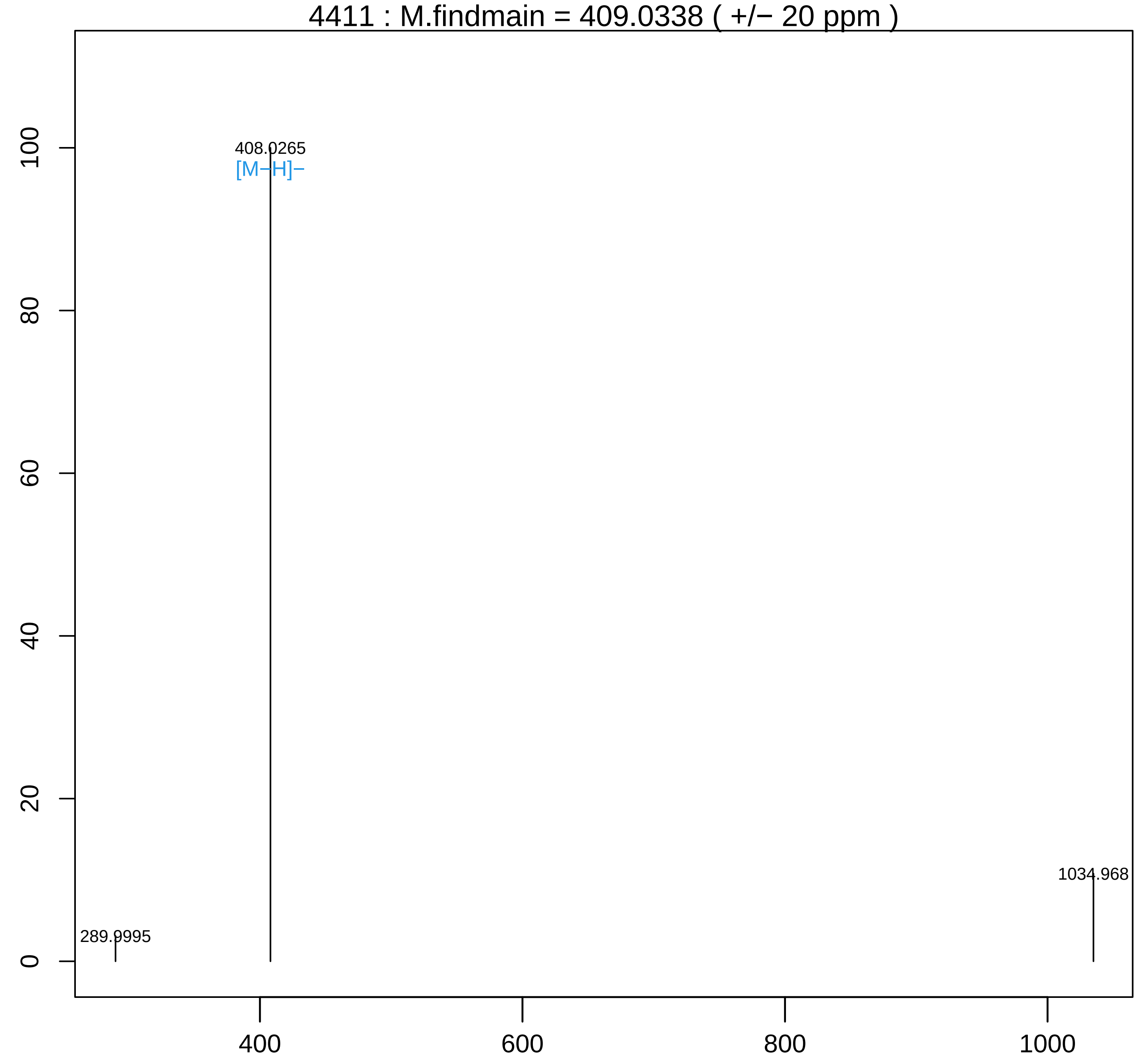

4411 : M.findmain = 409.0338 (+/− 20 ppm)
408.0265
[M−H]−
1034.968
289.9995
0
20
40
60
80
100
400
600
800
1000

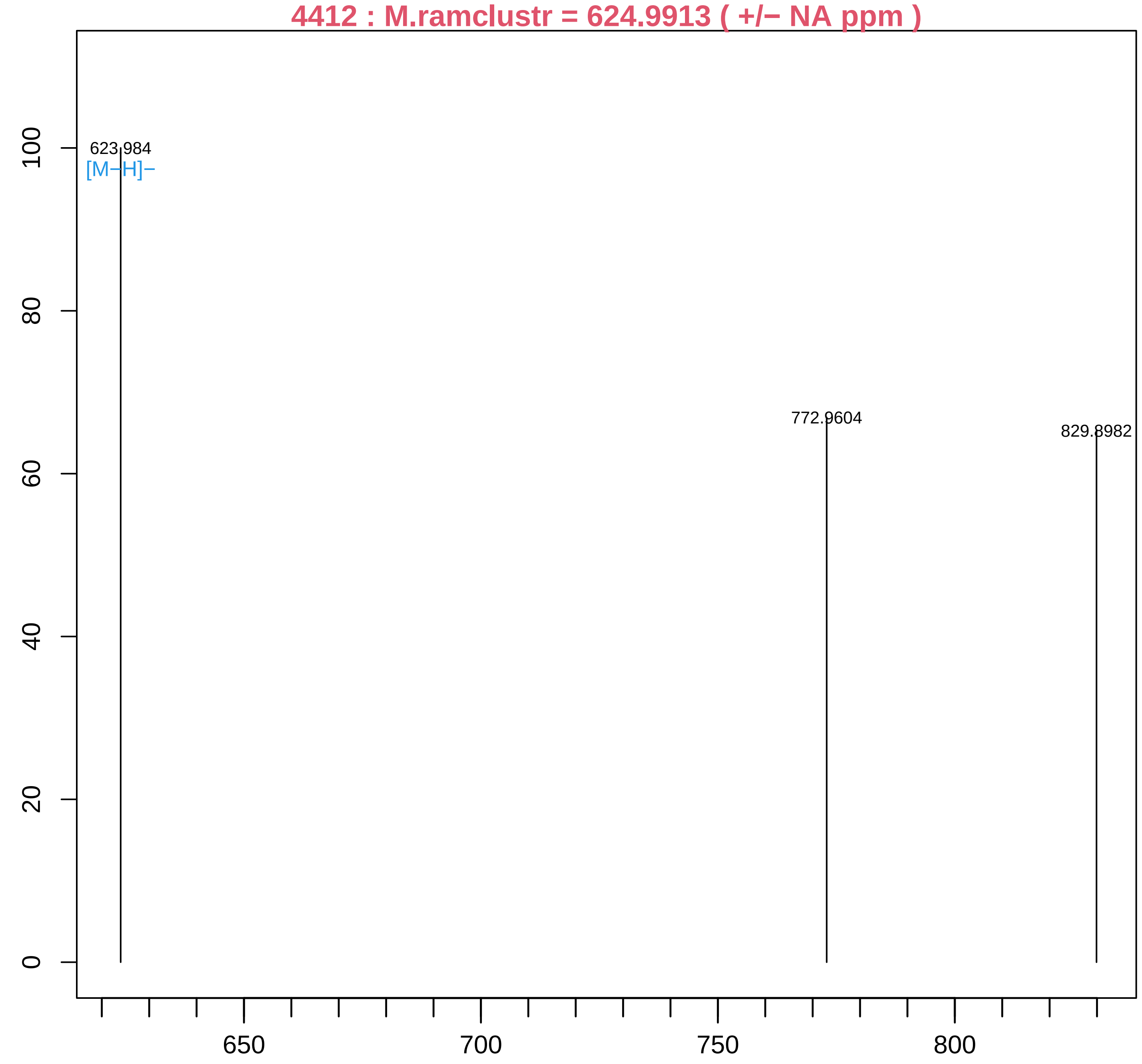
4412 : M.ramclustr = 624.9913 (+/− NA ppm)
623.984
[M−H]−
772.9604
829.8982
0
20
40
60
80
100
650
700
750
800

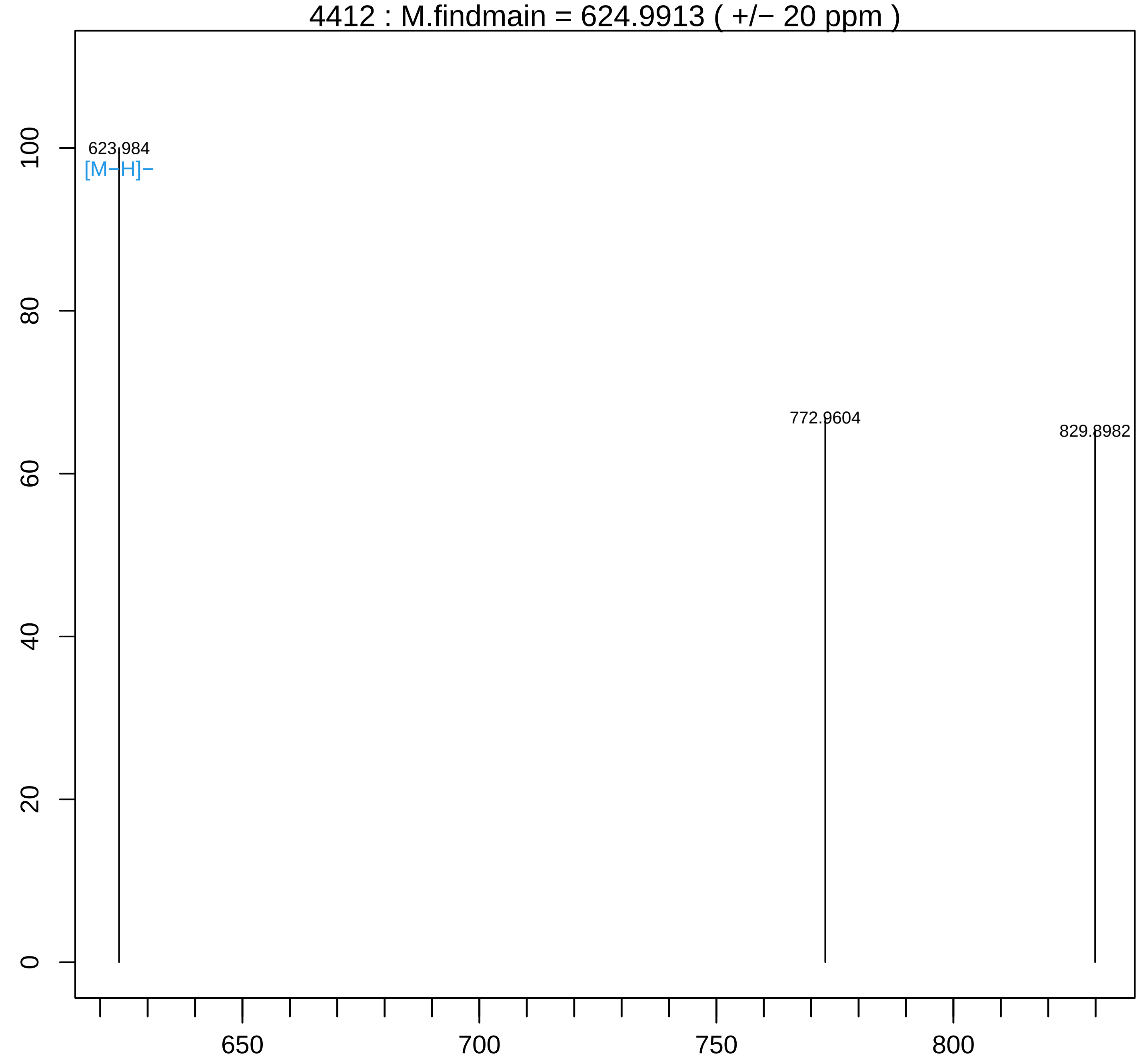
4412 : M.findmain = 624.9913 (+/− 20 ppm)
623.984
[M−H]−
772.9604
829.8982
0
20
40
60
80
100
650
700
750
800

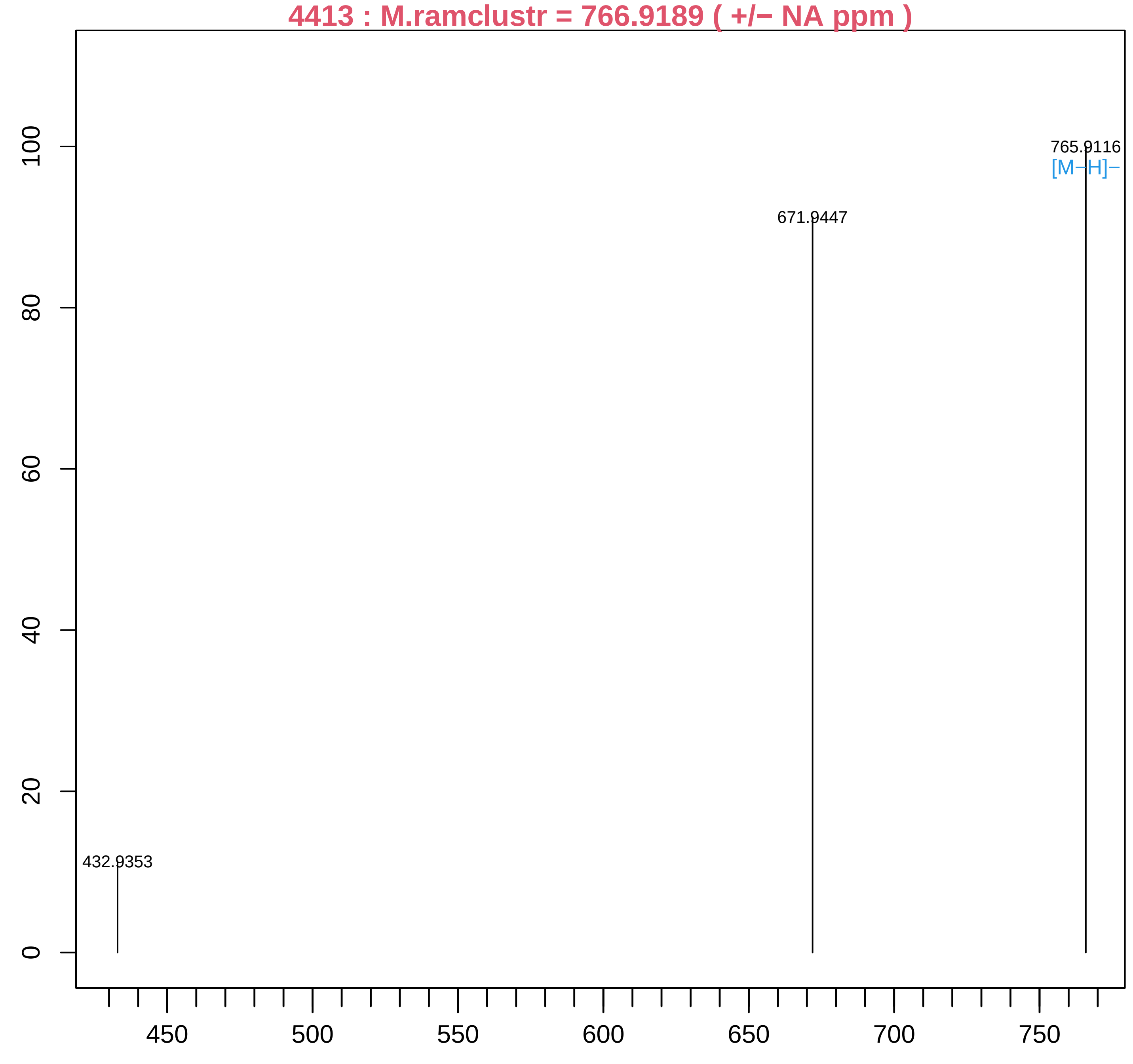
4413 : M.ramclustr = 766.9189 (+/− NA ppm)
765.9116
[M−H]−
671.9447
432.9353
0
20
40
60
80
100
450
500
550
600
650
700
750

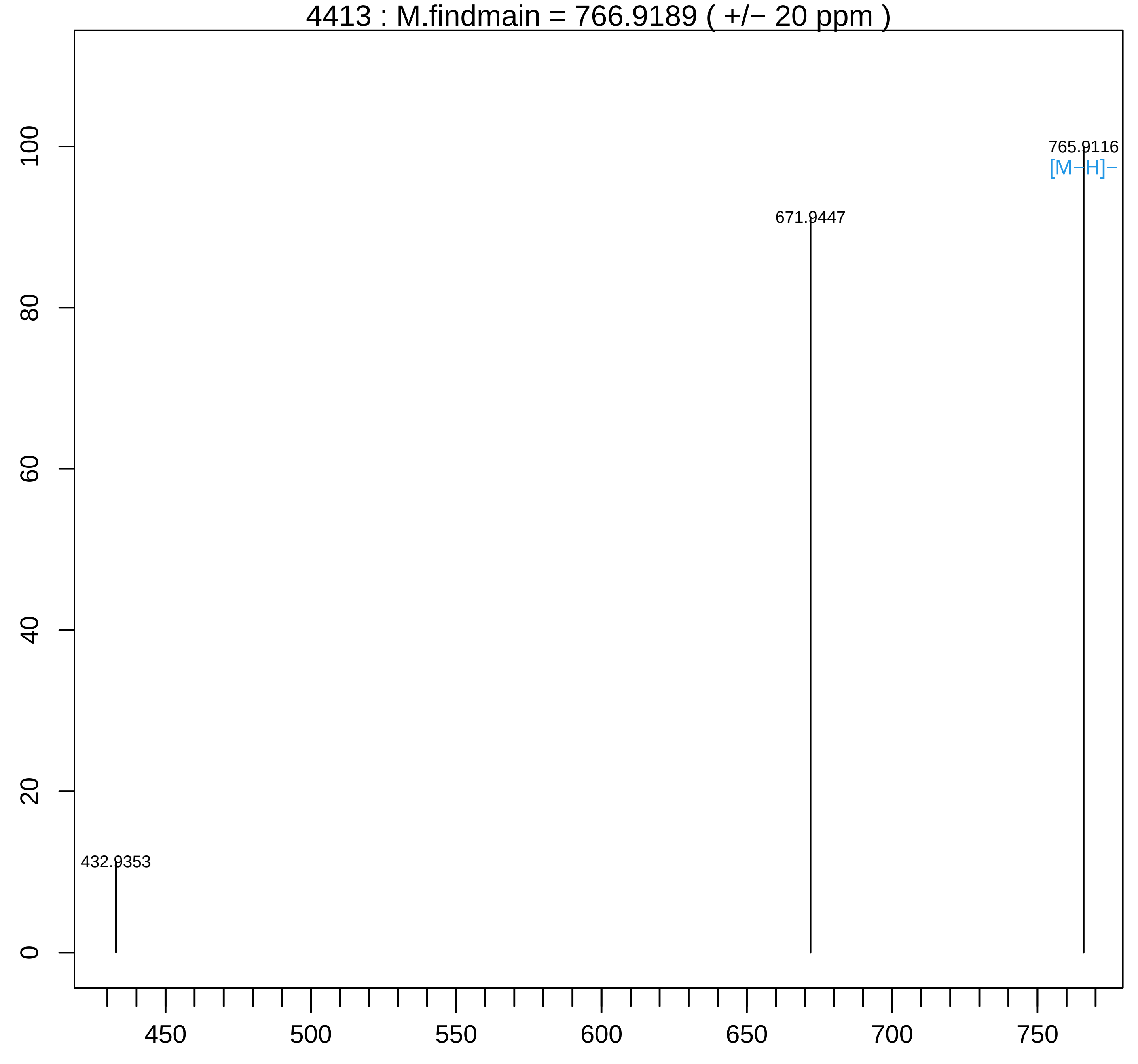
4413 : M.findmain = 766.9189 (+/− 20 ppm)
765.9116
[M−H]−
671.9447
432.9353
0
20
40
60
80
100
450
500
550
600
650
700
750

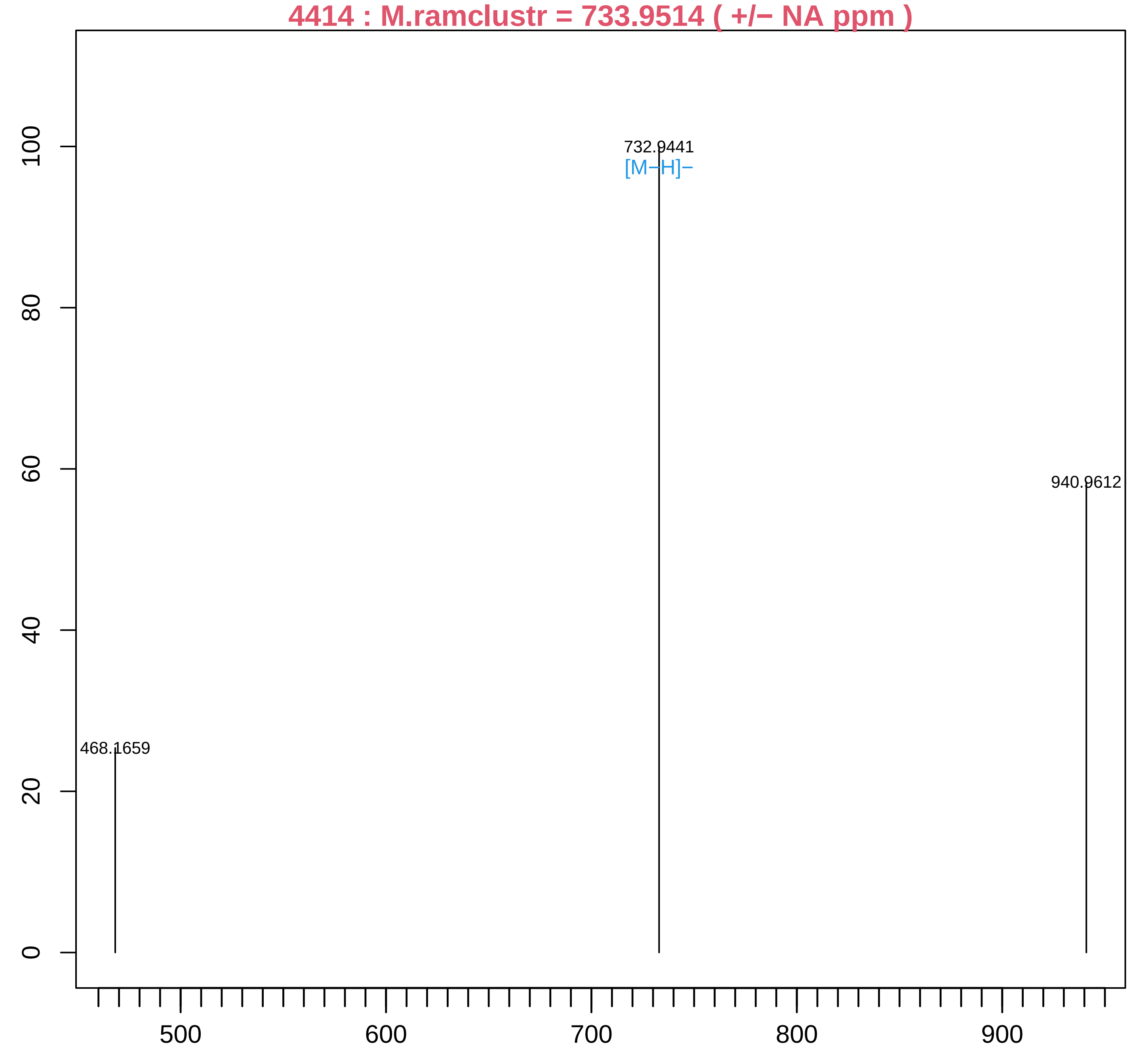
4414 : M.ramclustr = 733.9514 (+/− NA ppm)
732.9441
[M−H]−
940.9612
468.1659
0
20
40
60
80
100
500
600
700
800
900

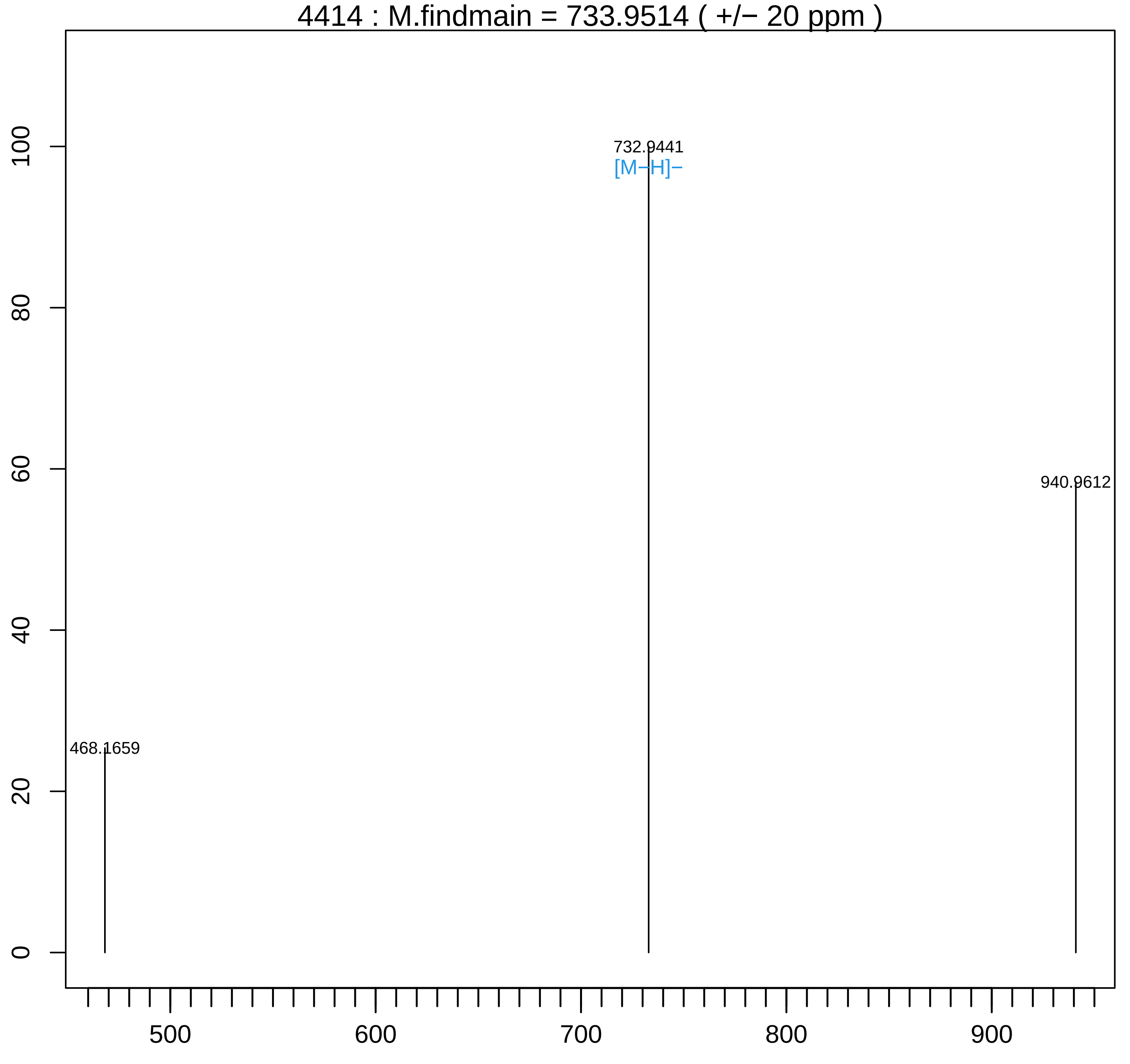
4414 : M.findmain = 733.9514 (+/− 20 ppm)
732.9441
[M−H]−
940.9612
468.1659
0
20
40
60
80
100
500
600
700
800
900

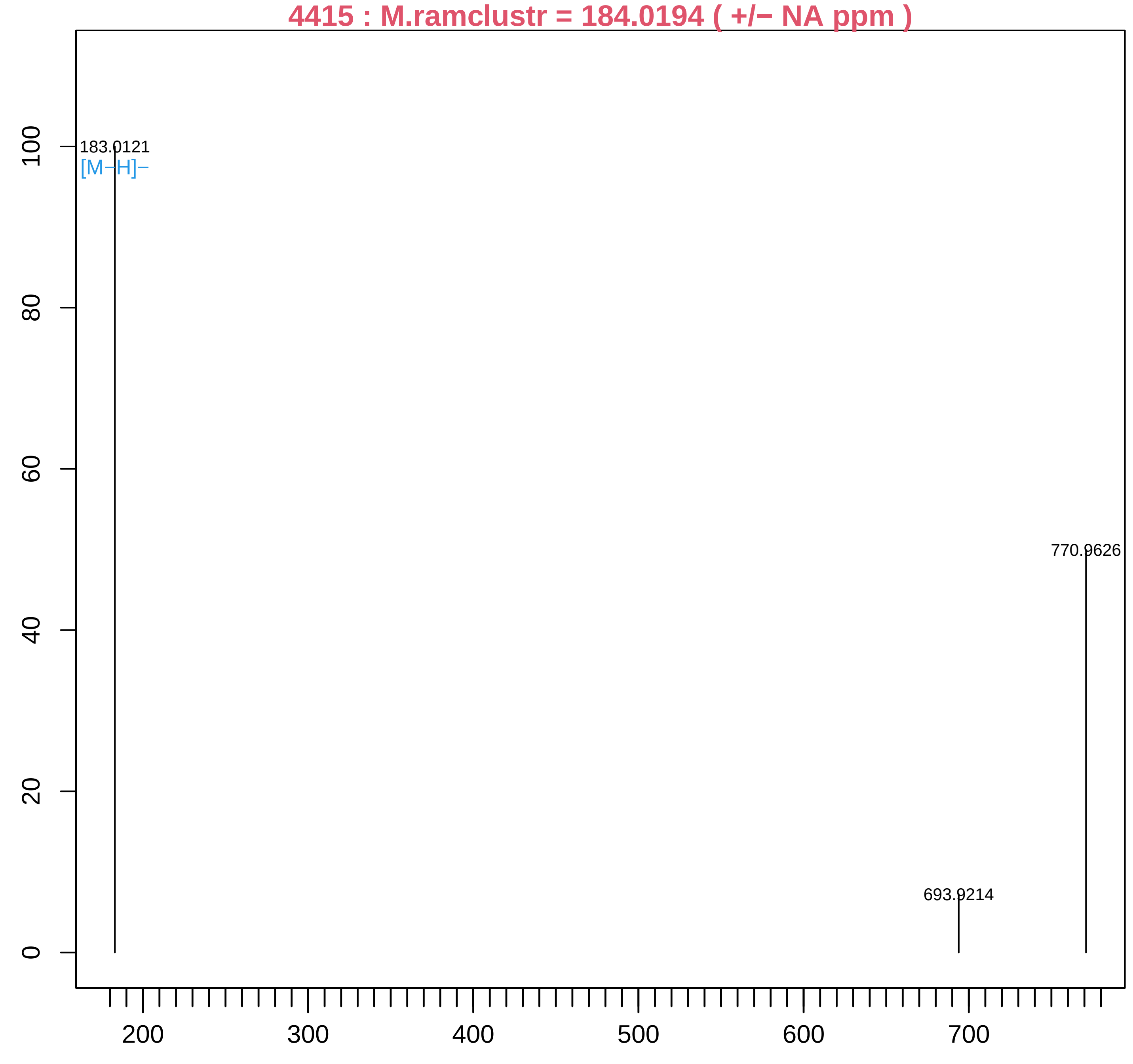
4415 : M.ramclustr = 184.0194 (+/− NA ppm)
183.0121
[M−H]−
693.9214
770.9626
0
20
40
60
80
100
200
300
400
500
600
700

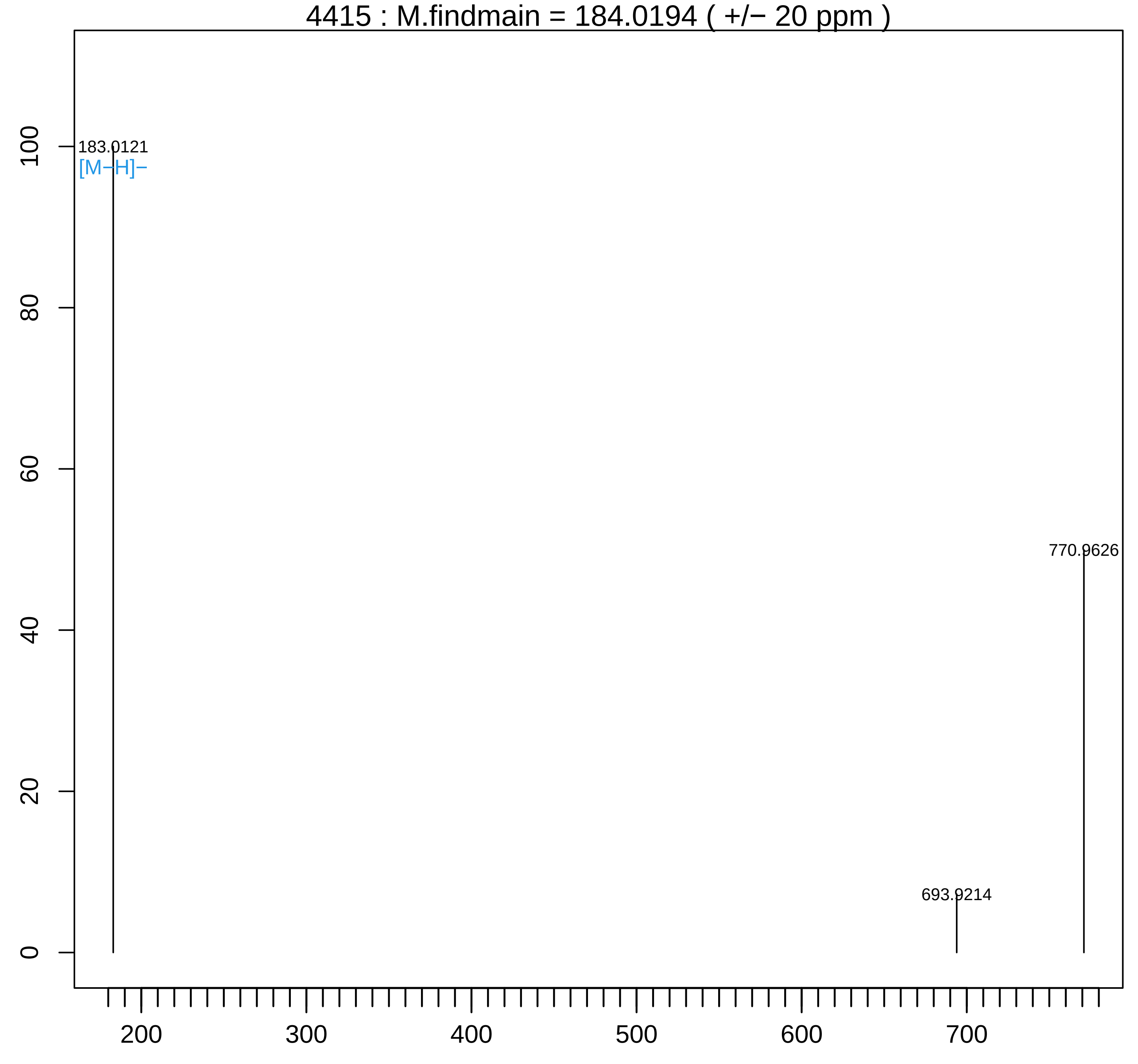
4415 : M.findmain = 184.0194 (+/− 20 ppm)
183.0121
[M−H]−
693.9214
770.9626
0
20
40
60
80
100
200
300
400
500
600
700

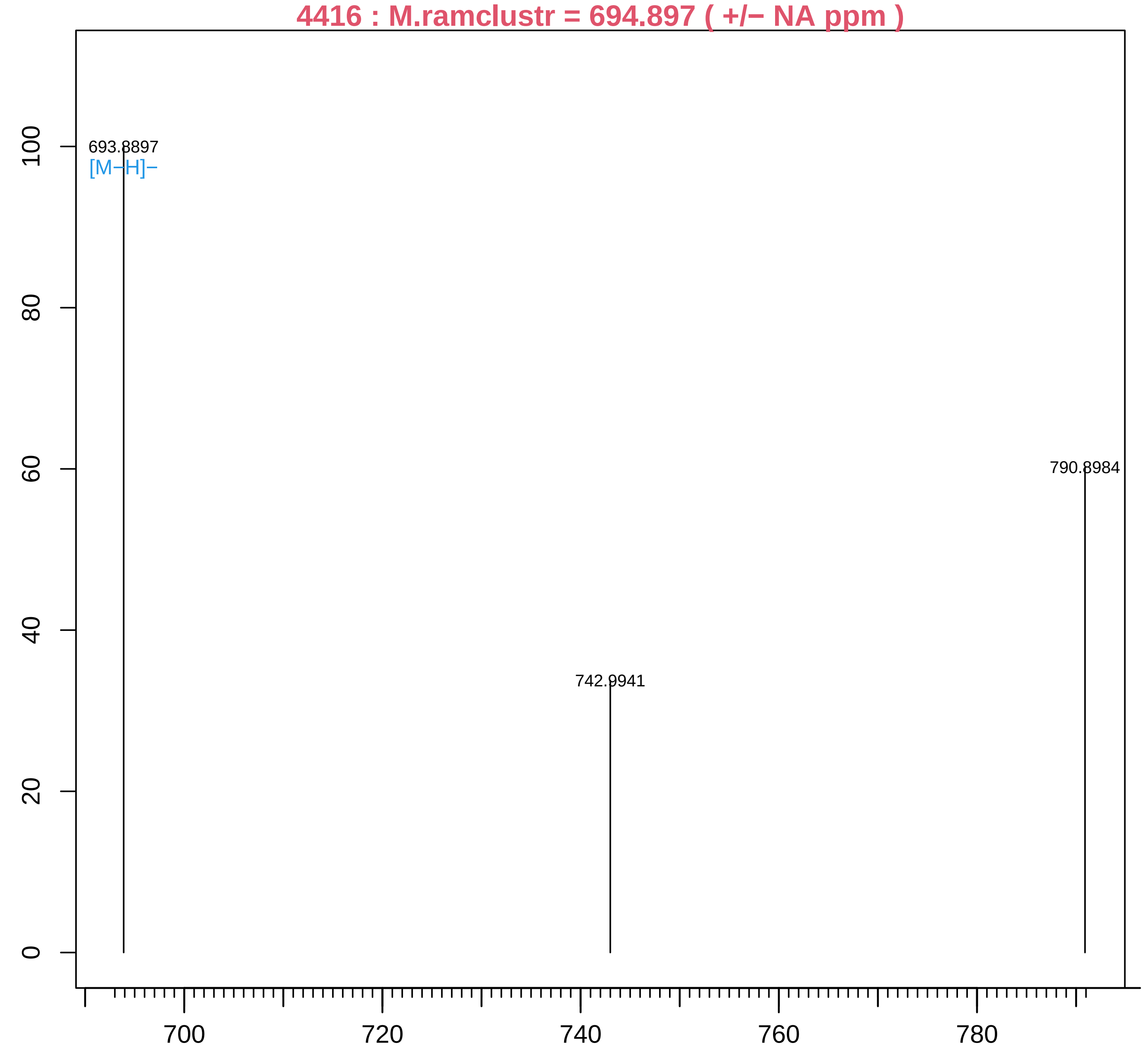
4416 : M.ramclustr = 694.897 (+/− NA ppm)
693.8897
[M−H]−
742.9941
790.8984
0
20
40
60
80
100
700
720
740
760
780

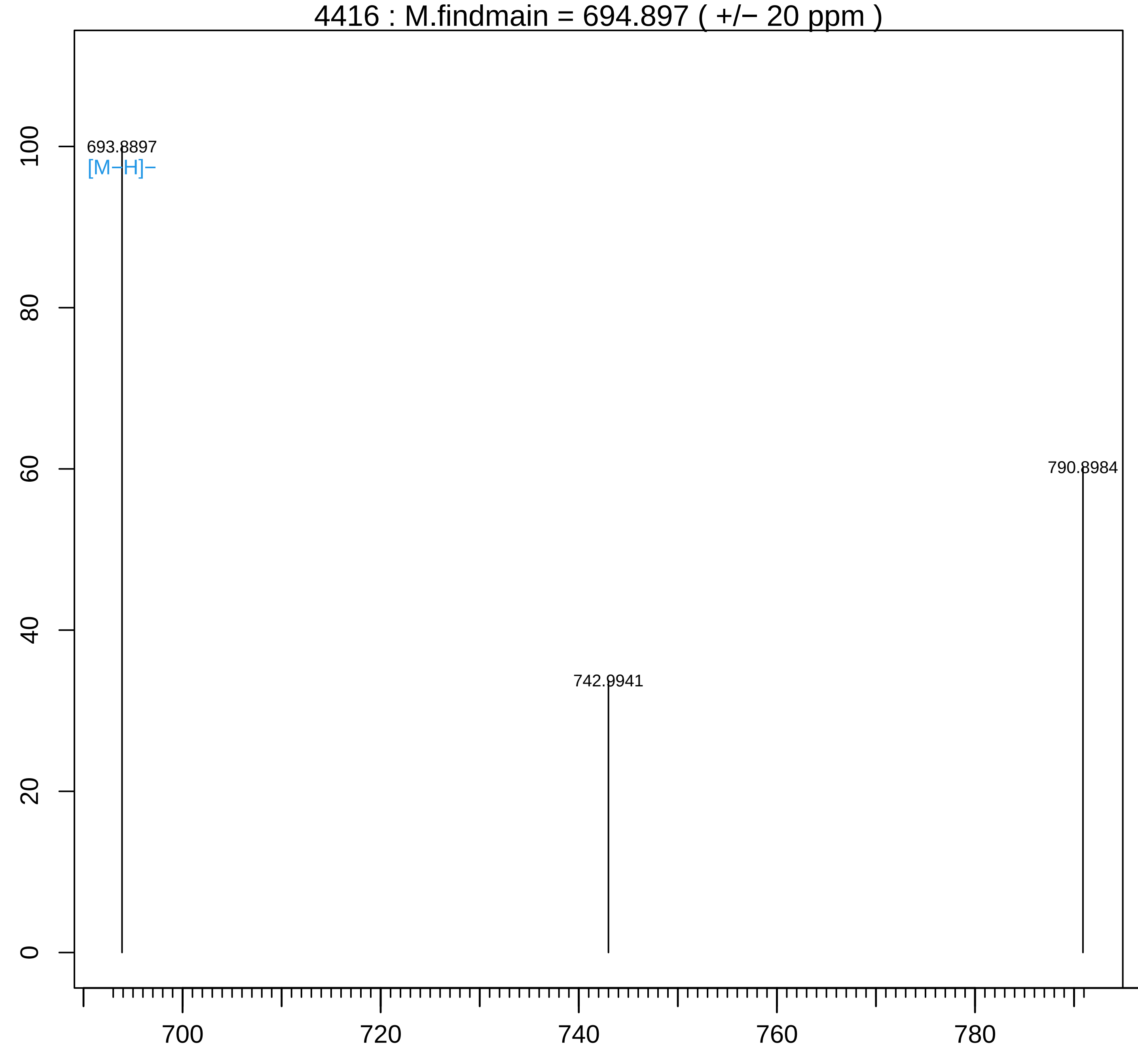
4416 : M.findmain = 694.897 (+/− 20 ppm)
693.8897
[M−H]−
742.9941
790.8984
0
20
40
60
80
100
700
720
740
760
780

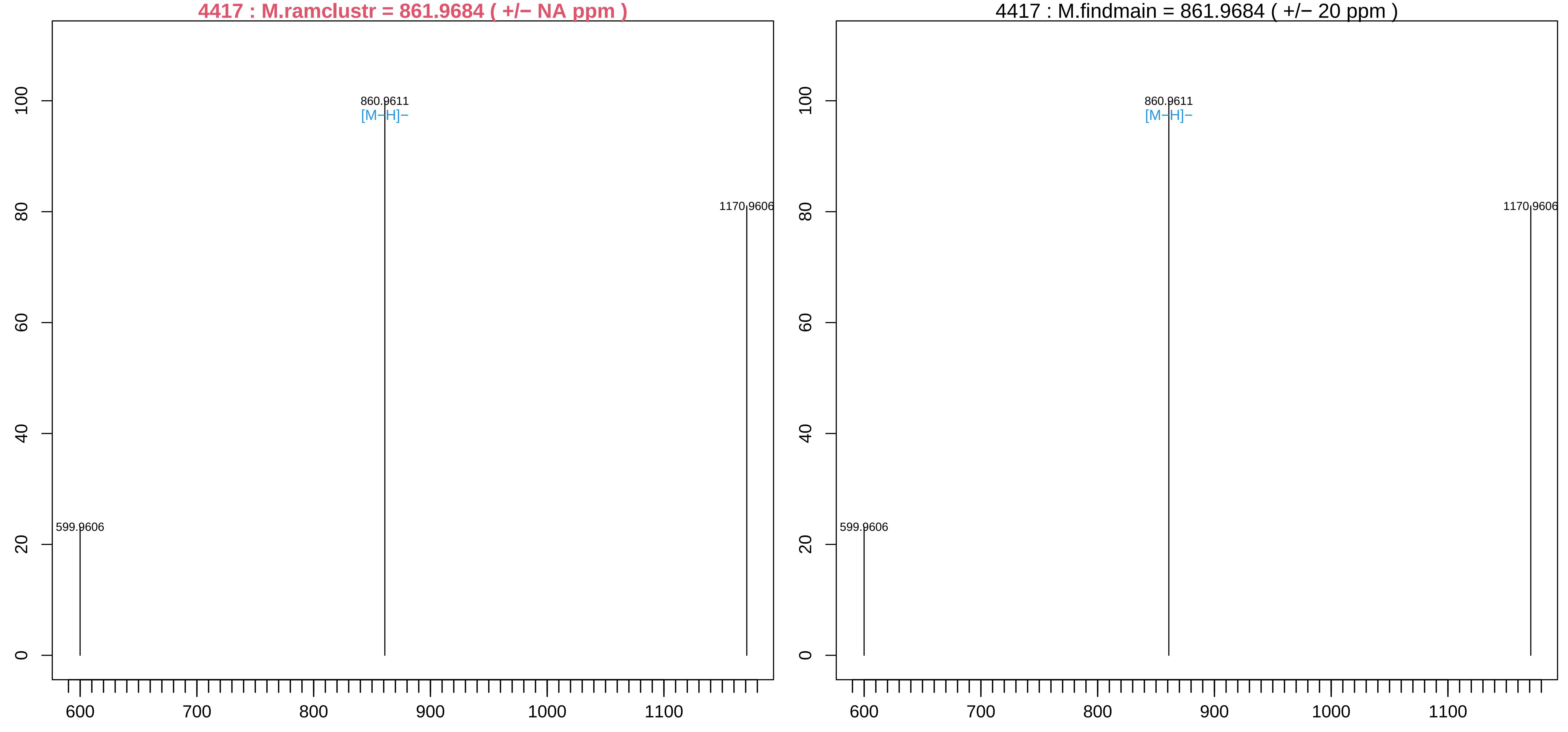
4417 : M.ramclustr = 861.9684 (+/− NA ppm)
4417 : M.findmain = 861.9684 (+/− 20 ppm)
599.9606
860.9611
[M−H]−
1170.9606
0
20
40
60
80
100
600
700
800
900
1000
1100

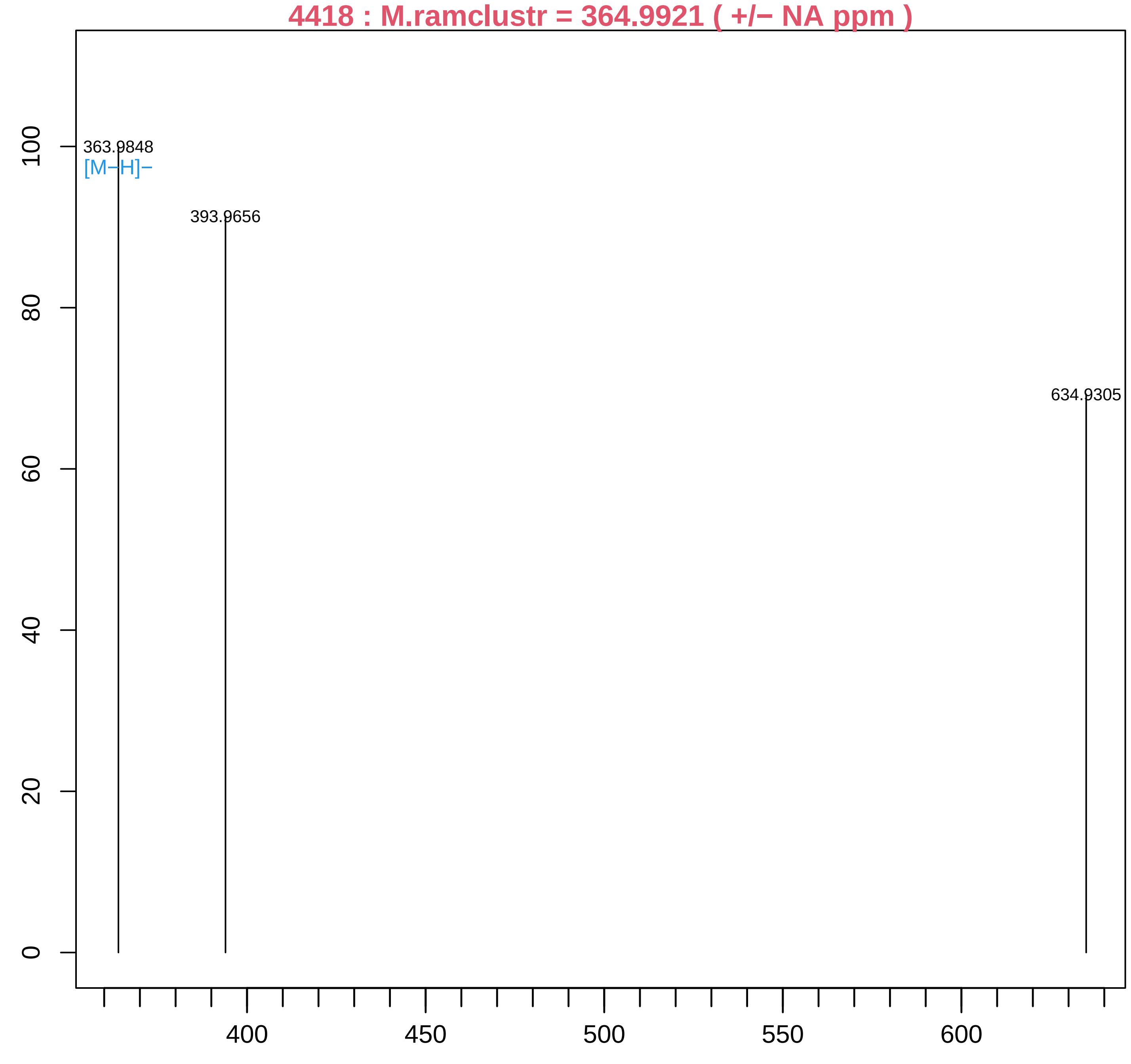
4418 : M.ramclustr = 364.9921 (+/− NA ppm)
363.9848
[M−H]−
393.9656
634.9305
0
20
40
60
80
100
400
450
500
550
600

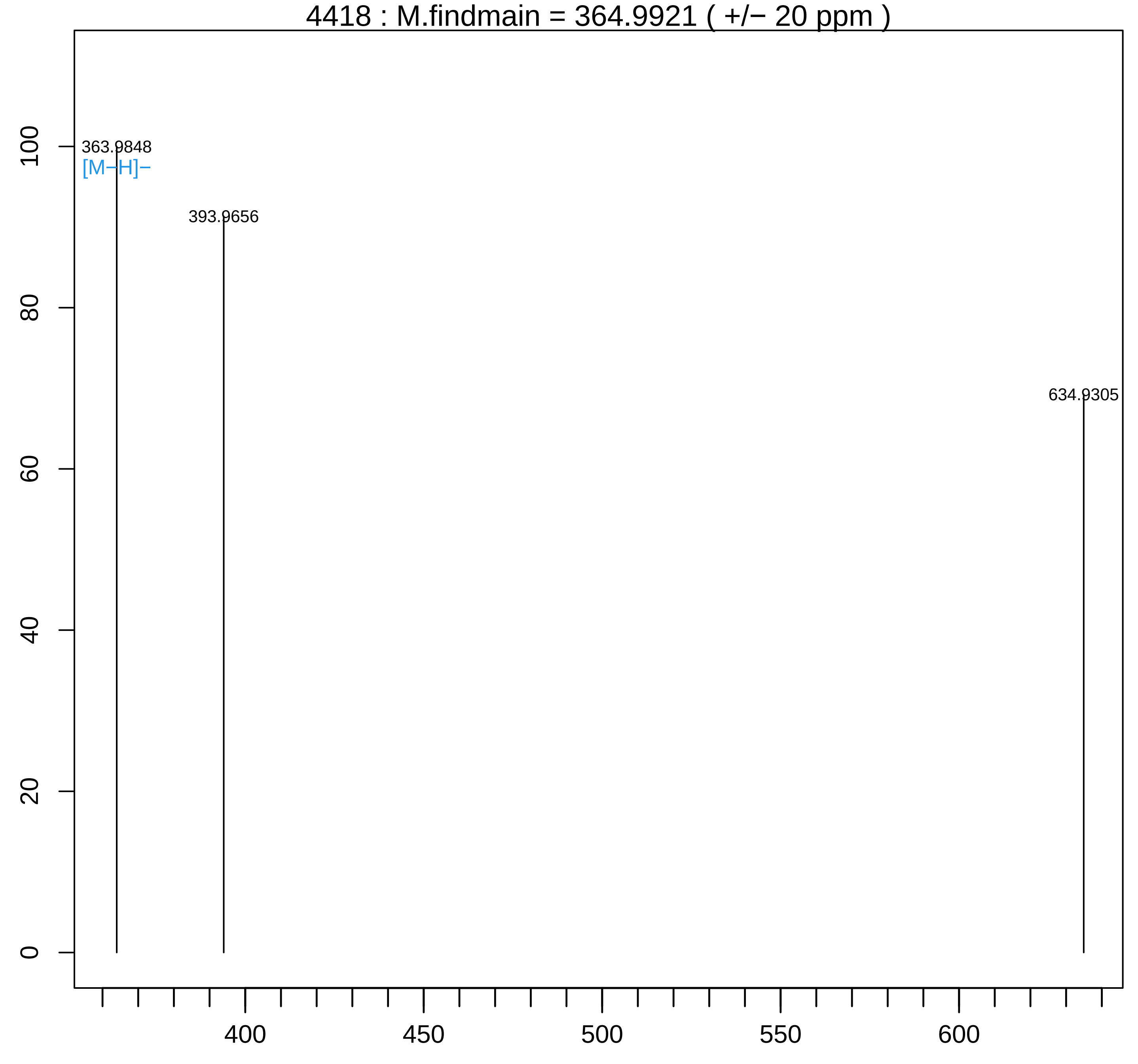
4418 : M.findmain = 364.9921 (+/− 20 ppm)
363.9848
[M−H]−
393.9656
634.9305
0
20
40
60
80
100
400
450
500
550
600

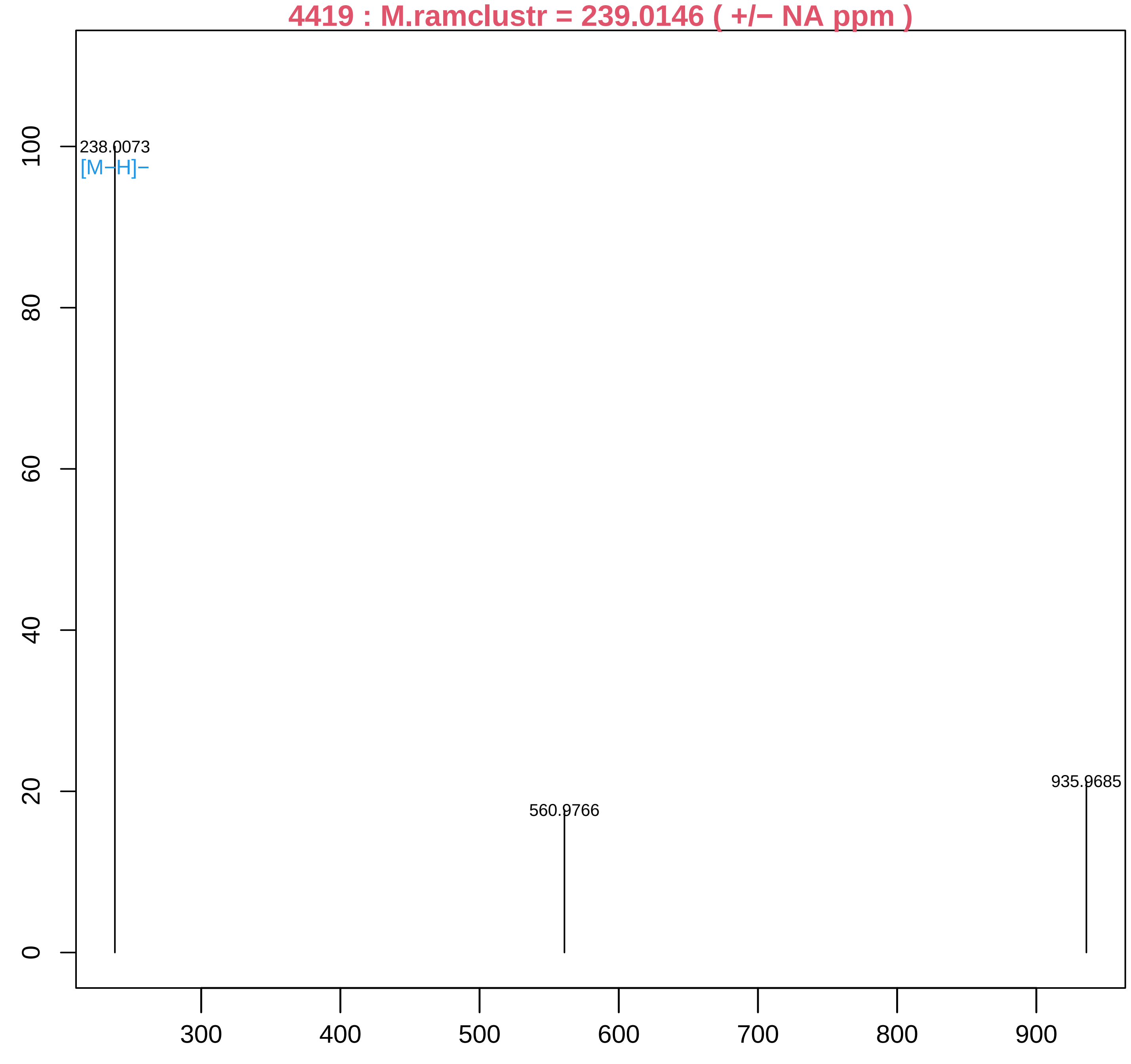
4419 : M.ramclustr = 239.0146 (+/− NA ppm)
238.0073
[M−H]−
560.9766
935.9685
0
20
40
60
80
100
300
400
500
600
700
800
900

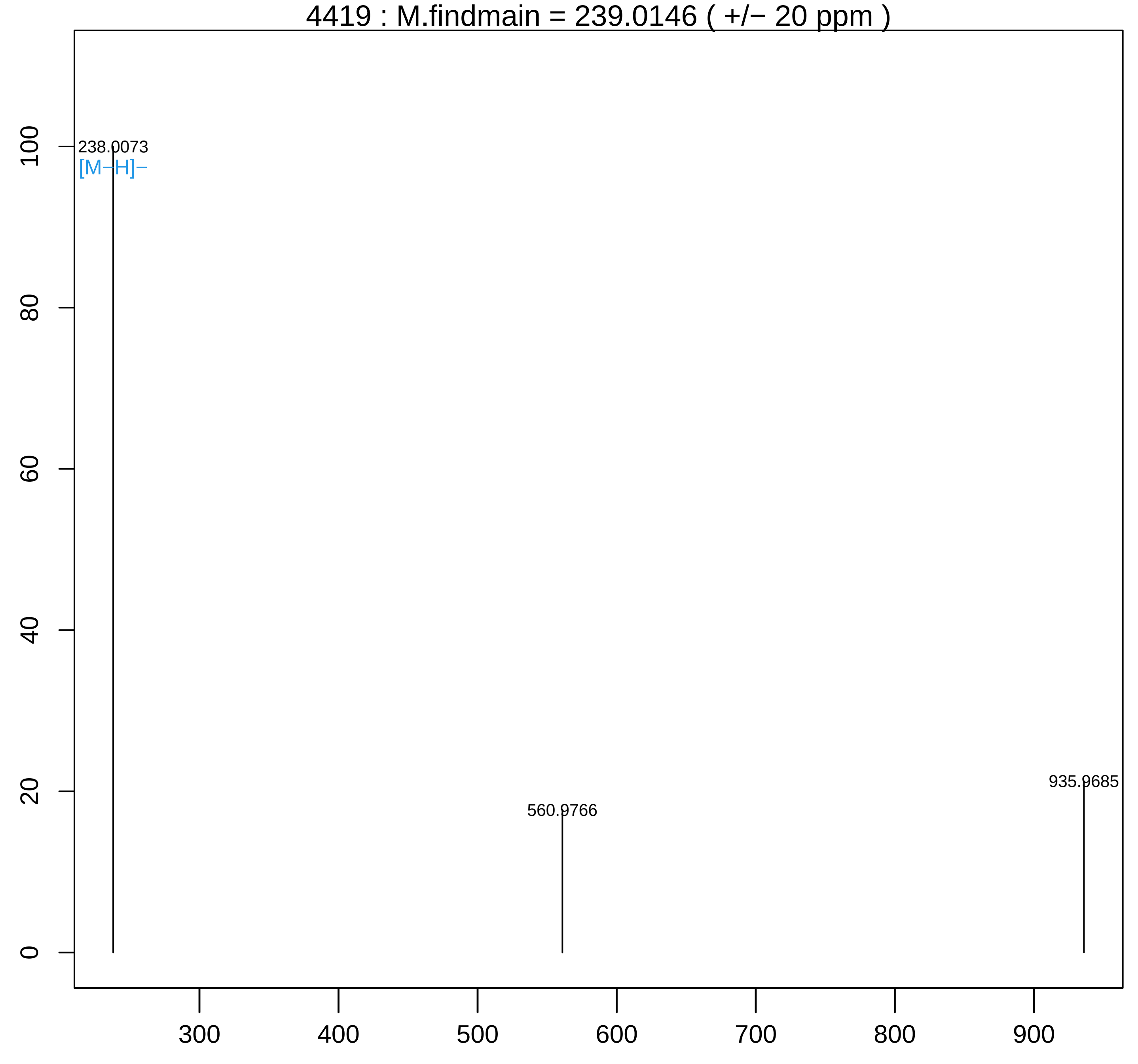
4419 : M.findmain = 239.0146 (+/− 20 ppm)
238.0073
[M−H]−
560.9766
935.9685
0
20
40
60
80
100
300
400
500
600
700
800
900

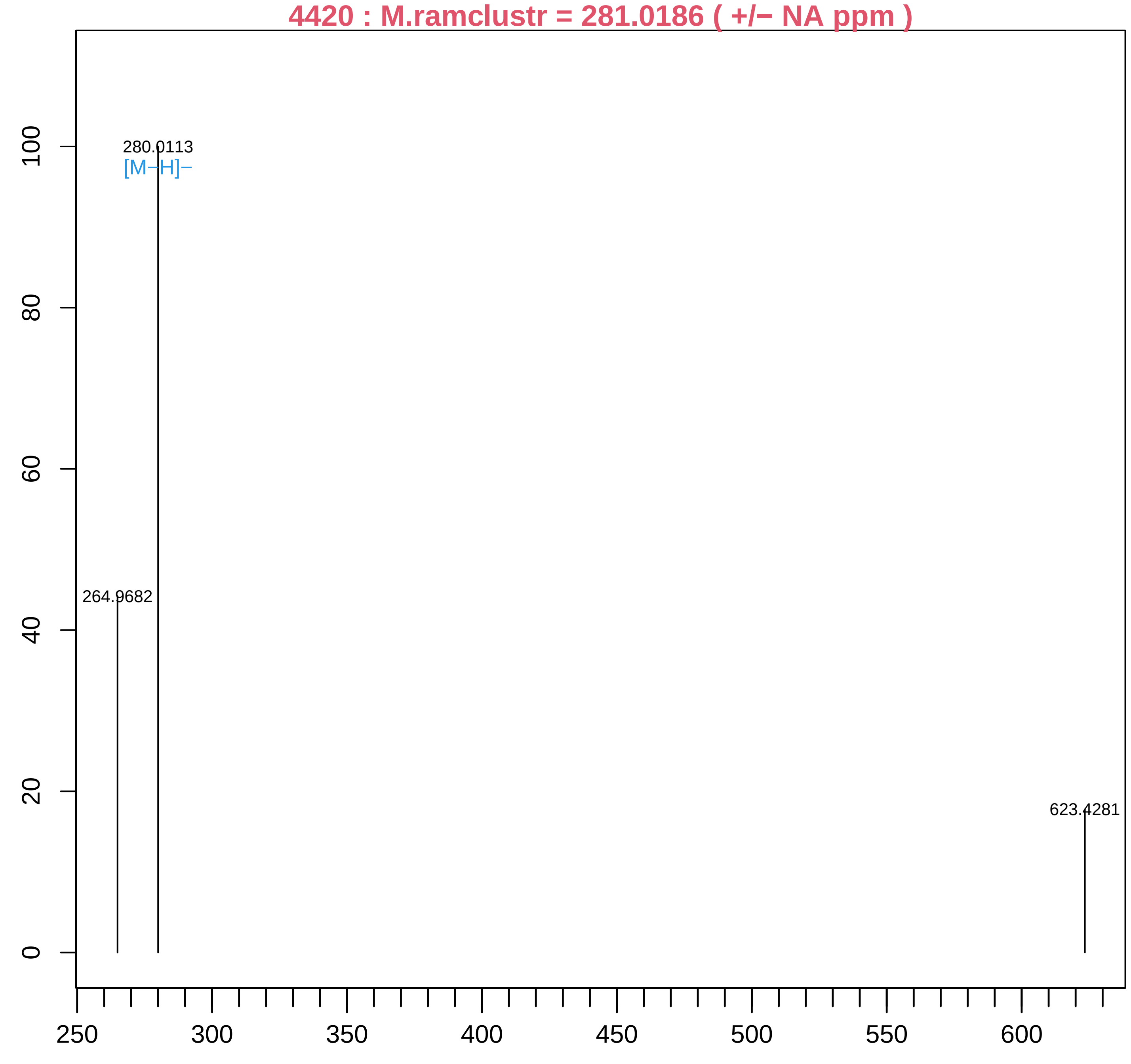

4420 : M.ramclustr = 281.0186 (+/− NA ppm)
280.0113
[M−H]−
264.9682
623.4281
0
20
40
60
80
100
250
300
350
400
450
500
550
600

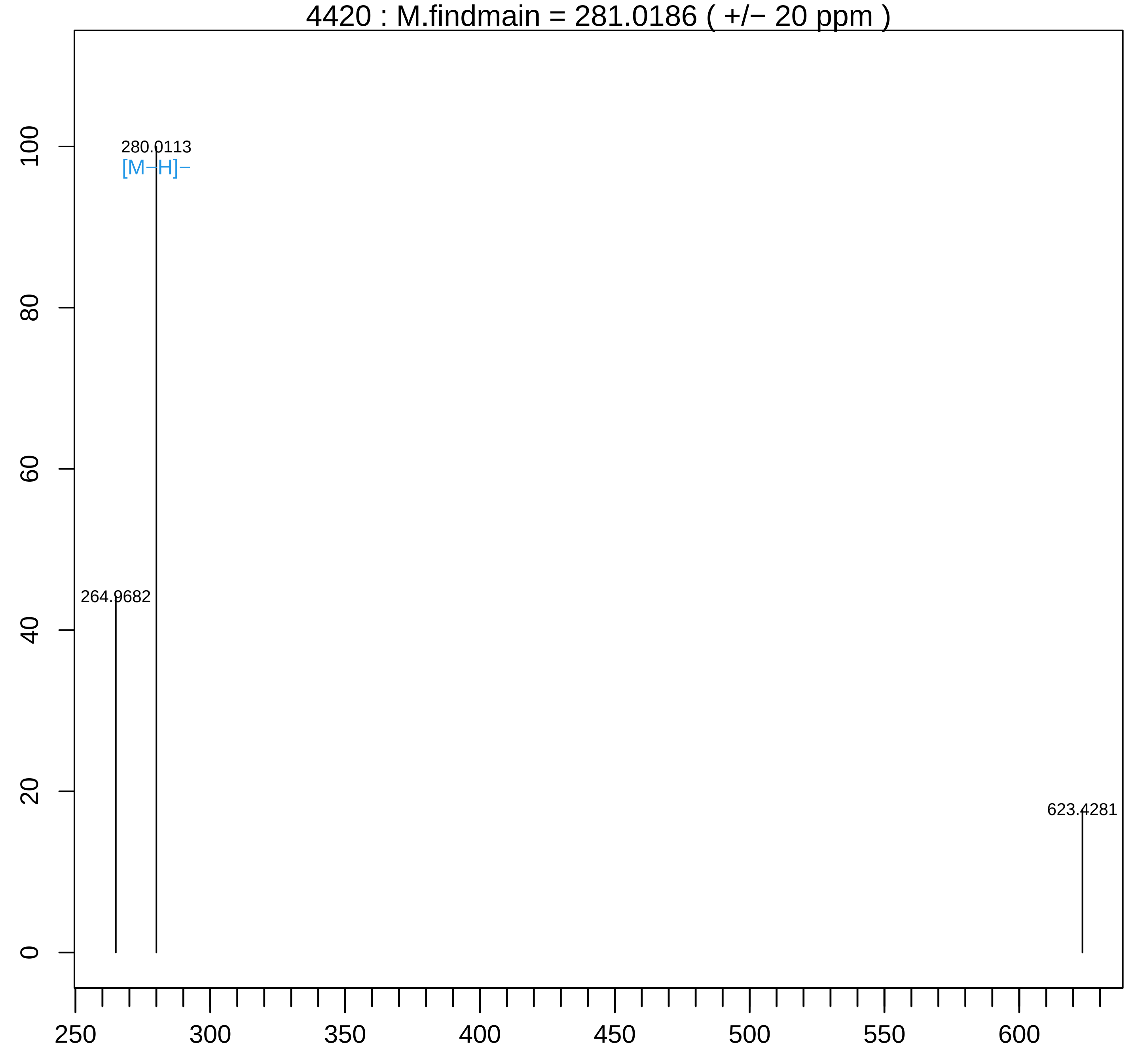

4420 : M.findmain = 281.0186 (+/− 20 ppm)
280.0113
[M−H]−
264.9682
623.4281
0
20
40
60
80
100
250
300
350
400
450
500
550
600

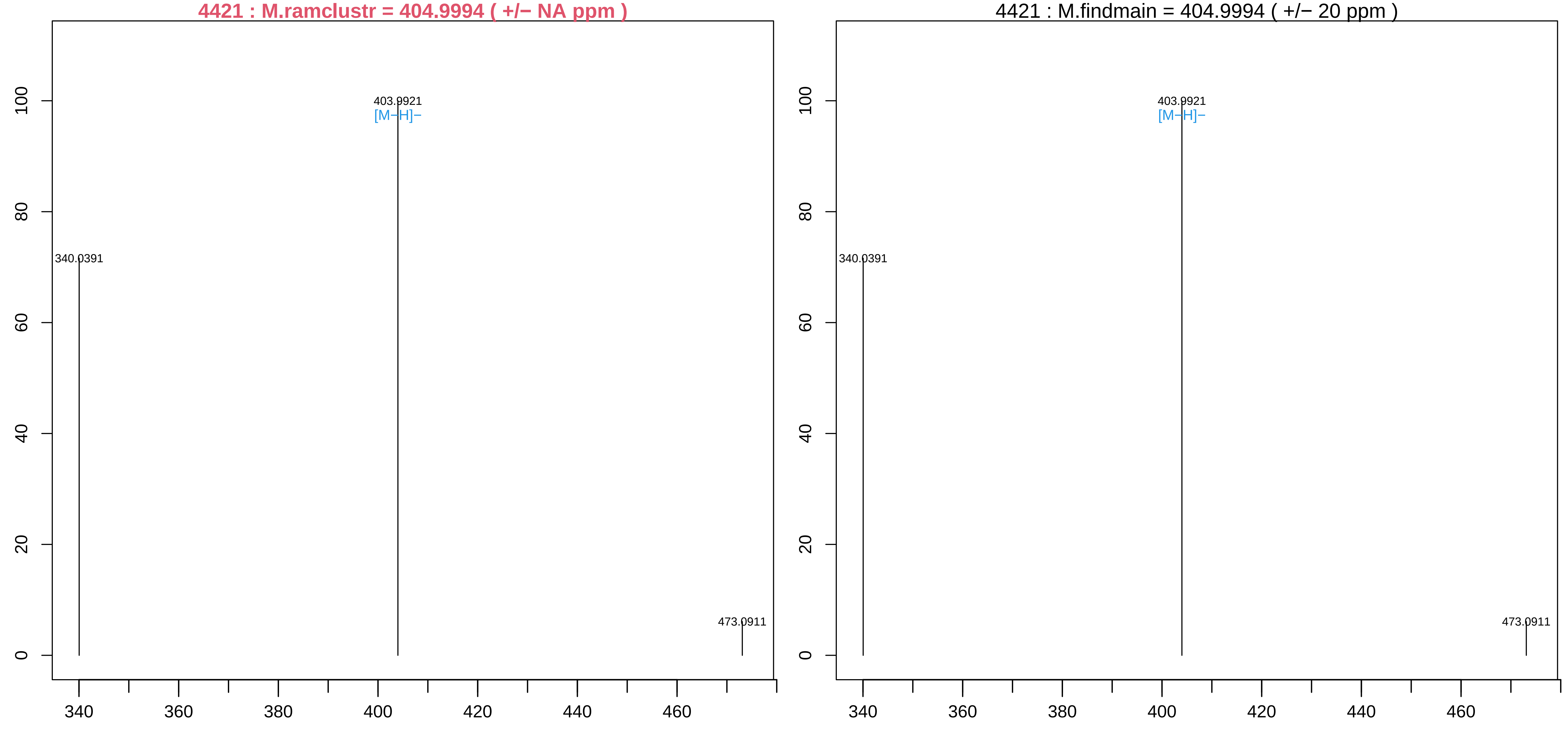
4421 : M.ramclustr = 404.9994 (+/− NA ppm)
4421 : M.findmain = 404.9994 (+/− 20 ppm)
340.0391
403.9921
[M−H]−
473.0911
0
20
40
60
80
100
340
360
380
400
420
440
460

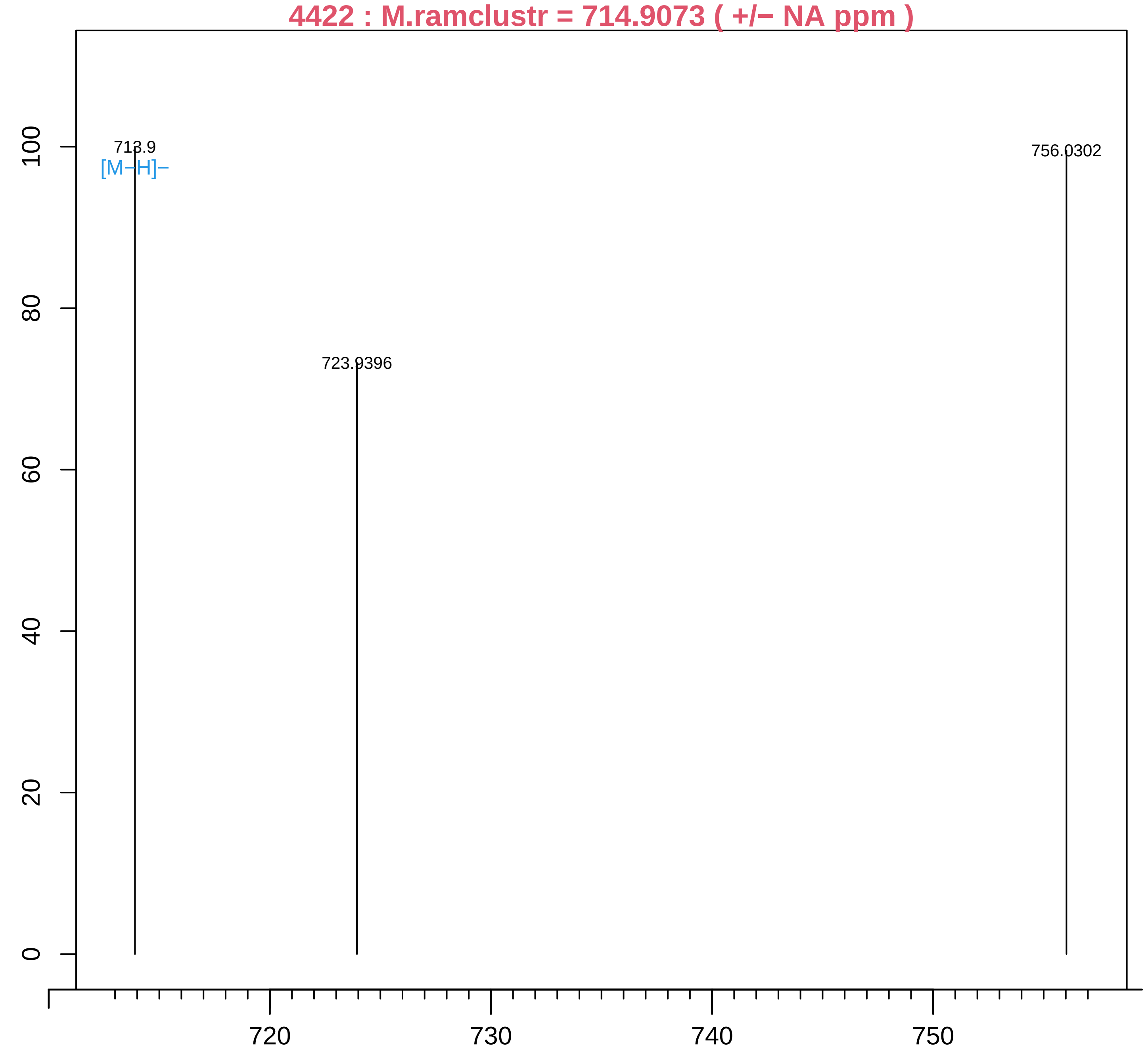
4422 : M.ramclustr = 714.9073 (+/− NA ppm)
713.9
[M−H]−
723.9396
756.0302
0
20
40
60
80
100
720
730
740
750

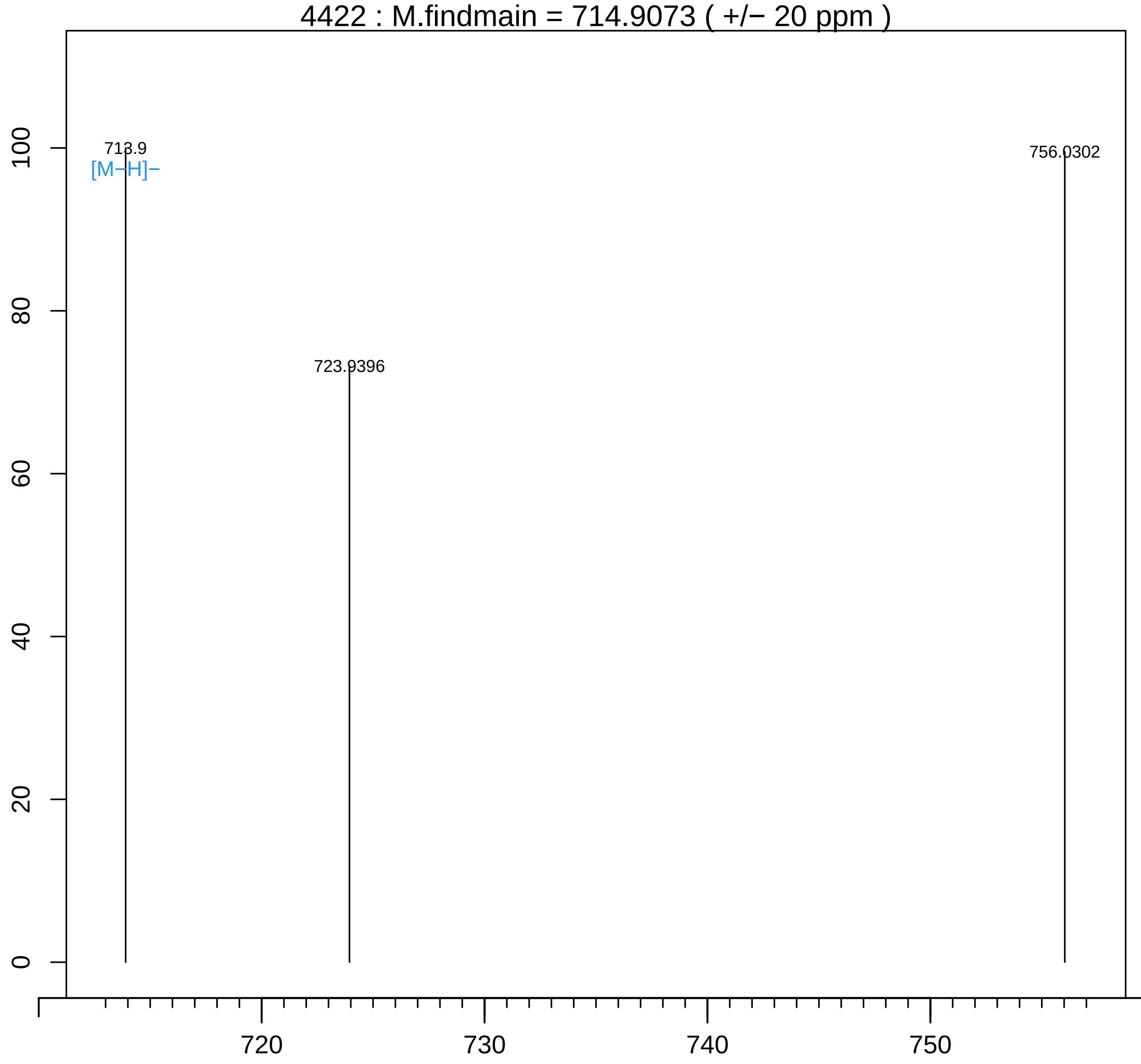
4422 : M.findmain = 714.9073 (+/− 20 ppm)
713.9
[M−H]−
723.9396
756.0302
0
20
40
60
80
100
720
730
740
750

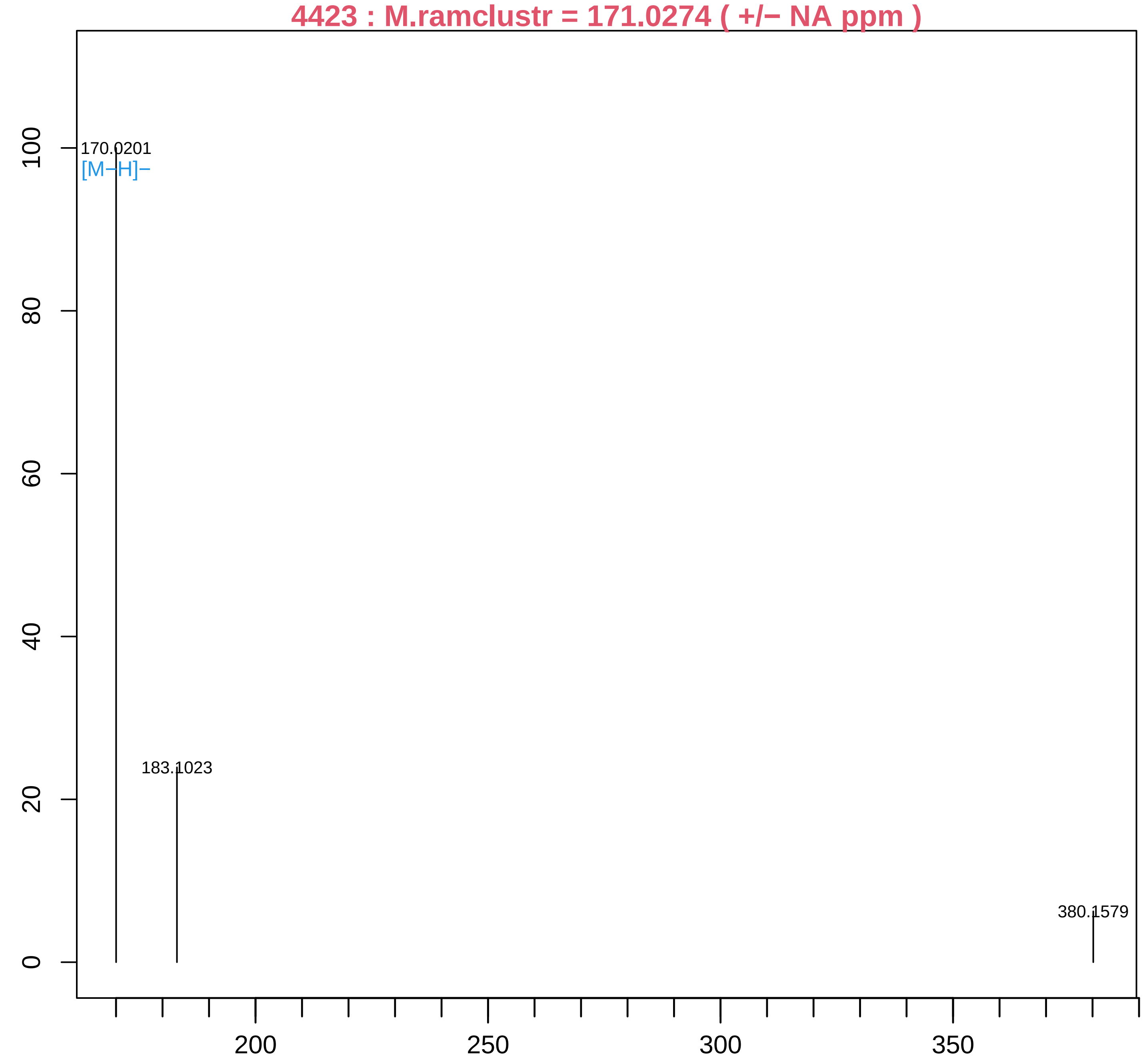
4423 : M.ramclustr = 171.0274 (+/− NA ppm)
170.0201
[M−H]−
183.1023
380.1579
0
20
40
60
80
100
200
250
300
350

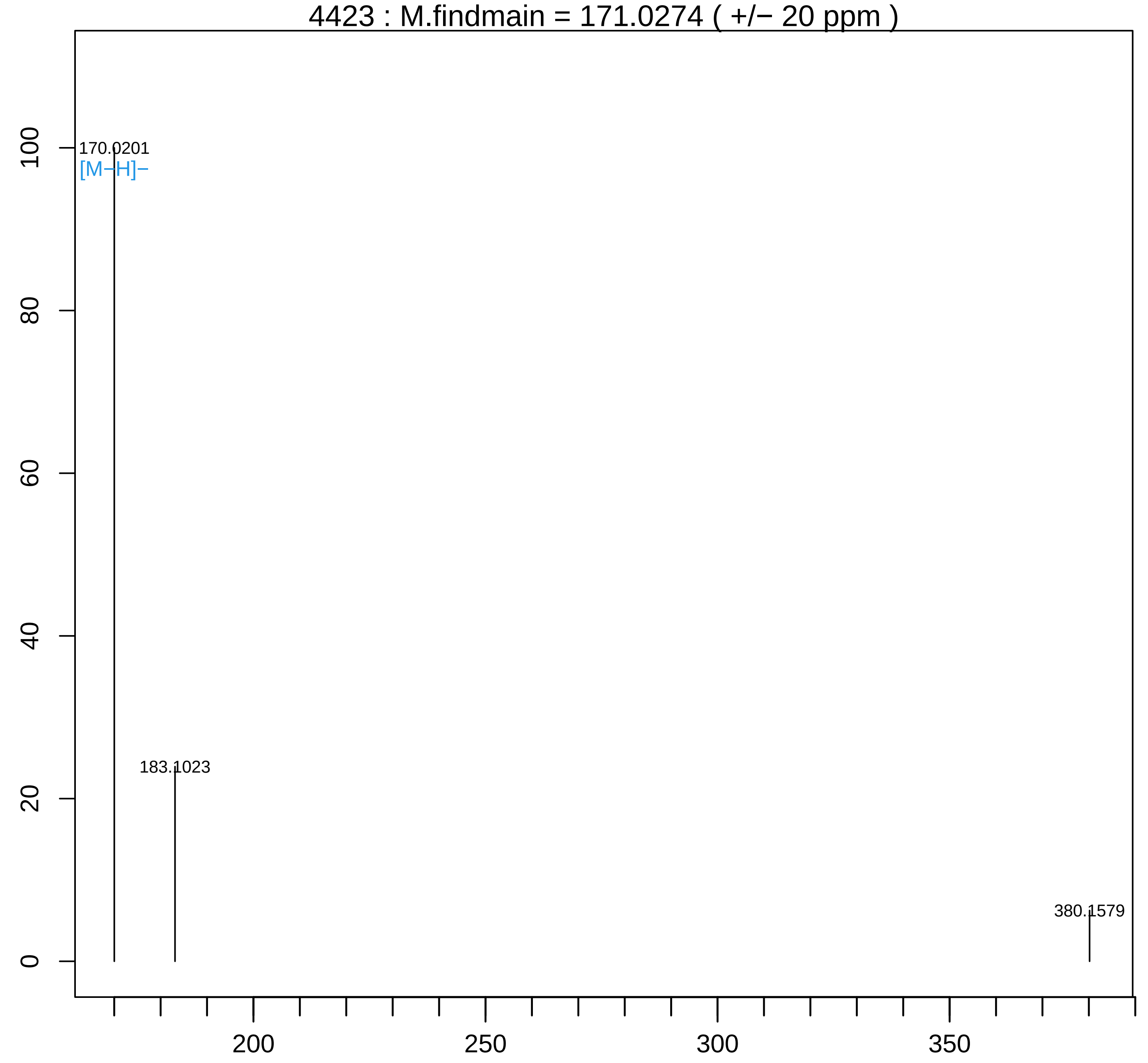
4423 : M.findmain = 171.0274 (+/− 20 ppm)
170.0201
[M−H]−
183.1023
380.1579
0
20
40
60
80
100
200
250
300
350

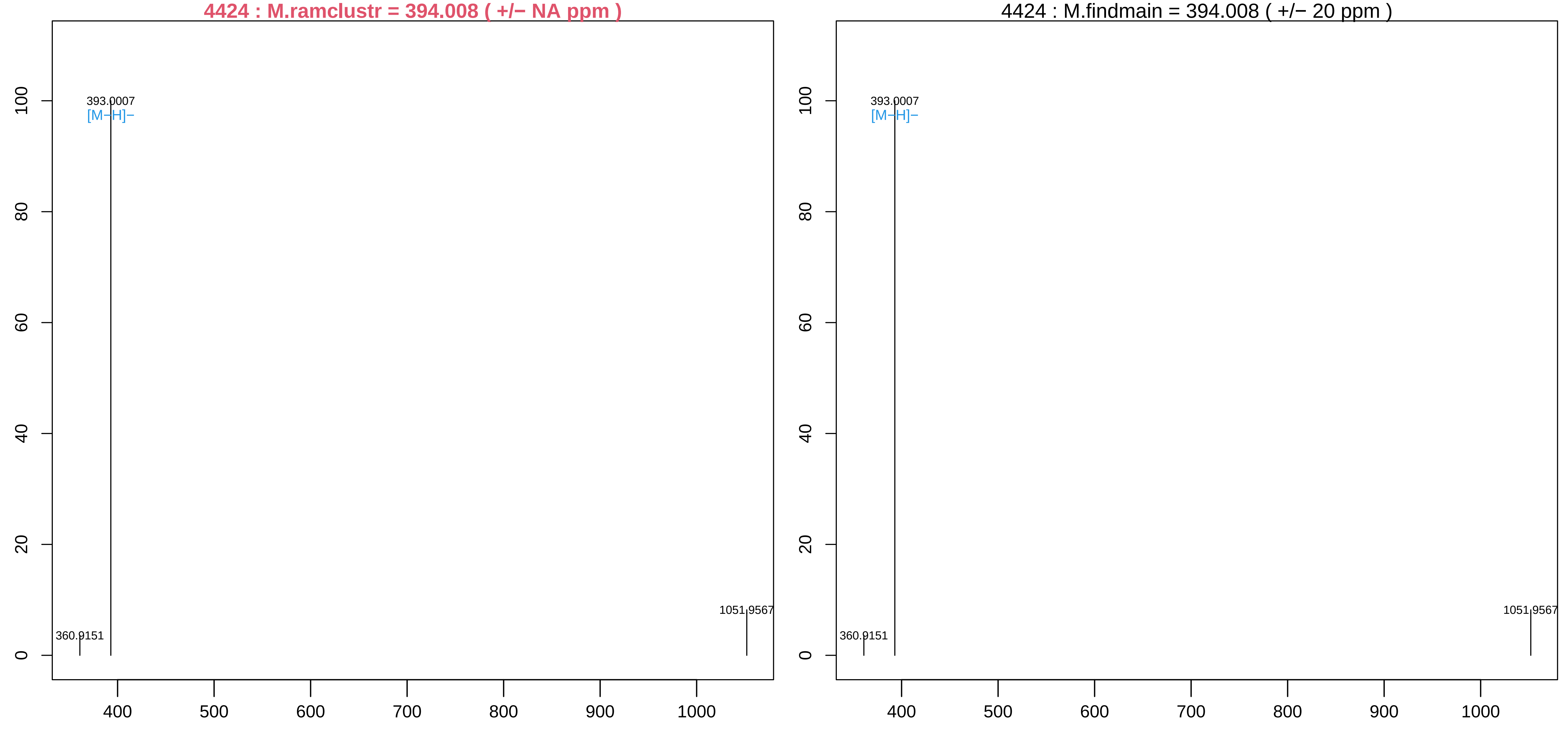
4424 : M.ramclustr = 394.008 (+/− NA ppm)
393.0007
[M−H]−
360.9151
1051.9567
0
20
40
60
80
100
400
500
600
700
800
900
1000
4424 : M.findmain = 394.008 (+/− 20 ppm)
393.0007
[M−H]−
360.9151
1051.9567

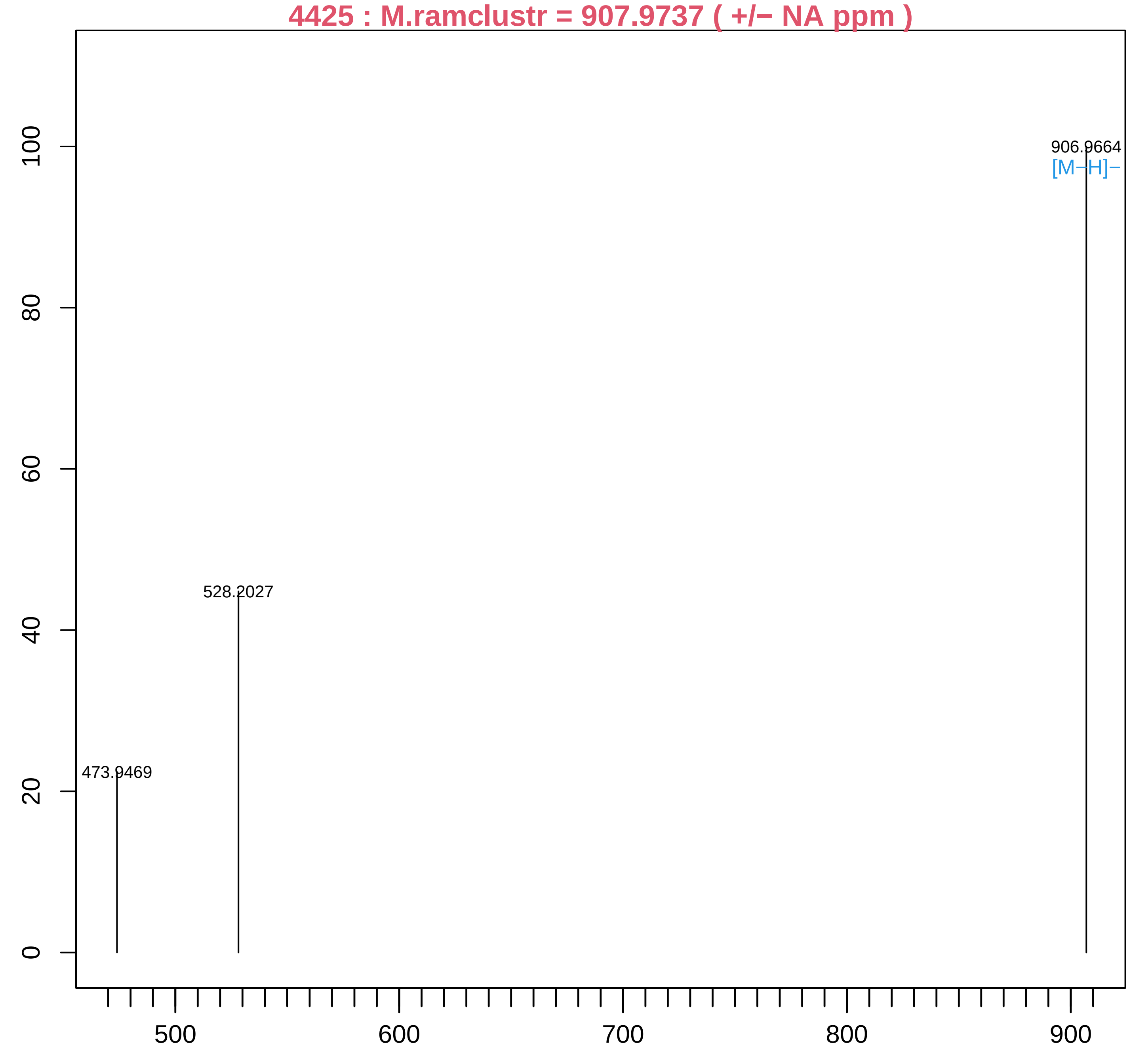
4425 : M.ramclustr = 907.9737 (+/− NA ppm)
906.9664
[M−H]−
528.2027
473.9469
0
20
40
60
80
100
500
600
700
800
900

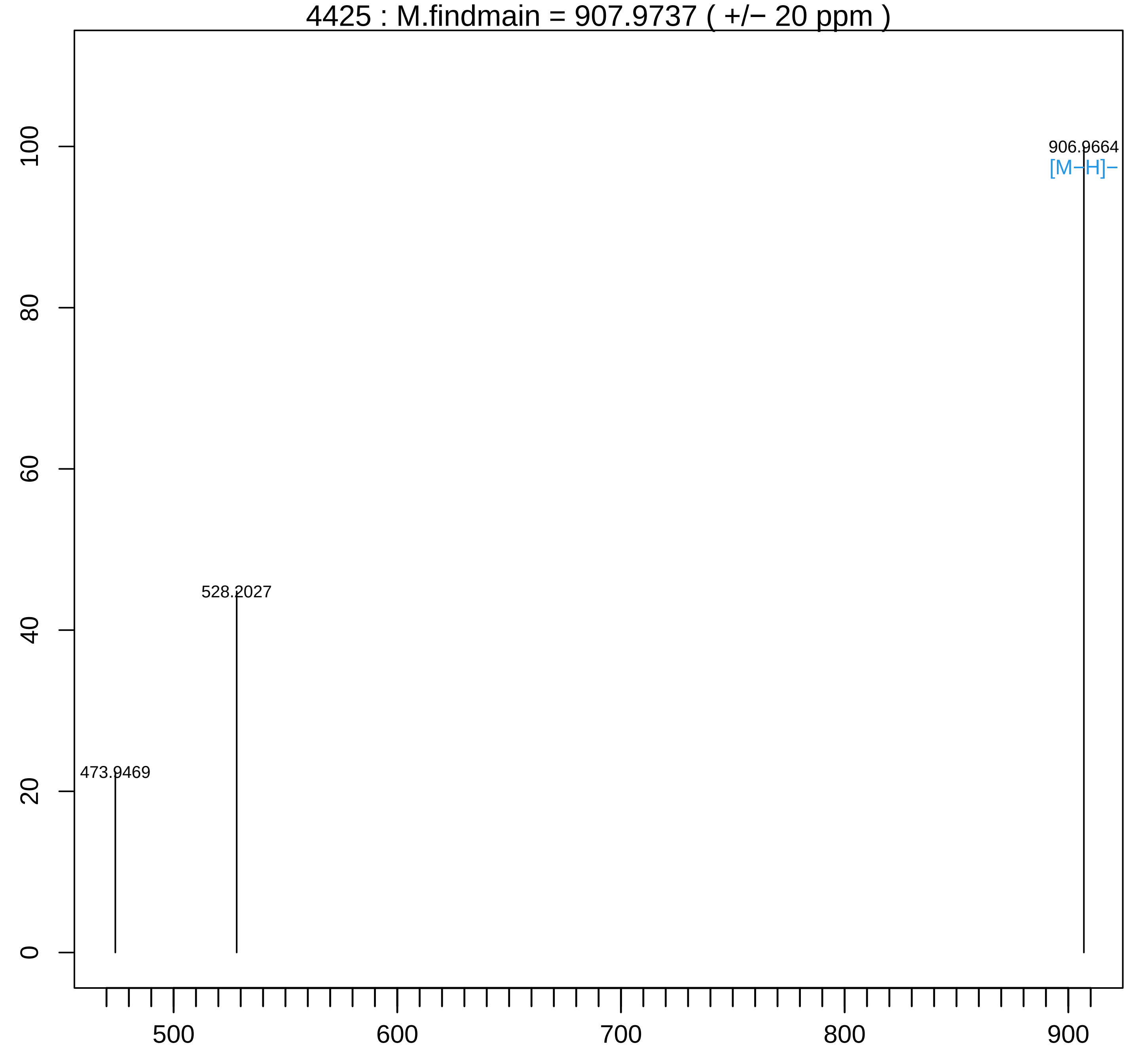
4425 : M.findmain = 907.9737 (+/− 20 ppm)
906.9664
[M−H]−
528.2027
473.9469
0
20
40
60
80
100
500
600
700
800
900

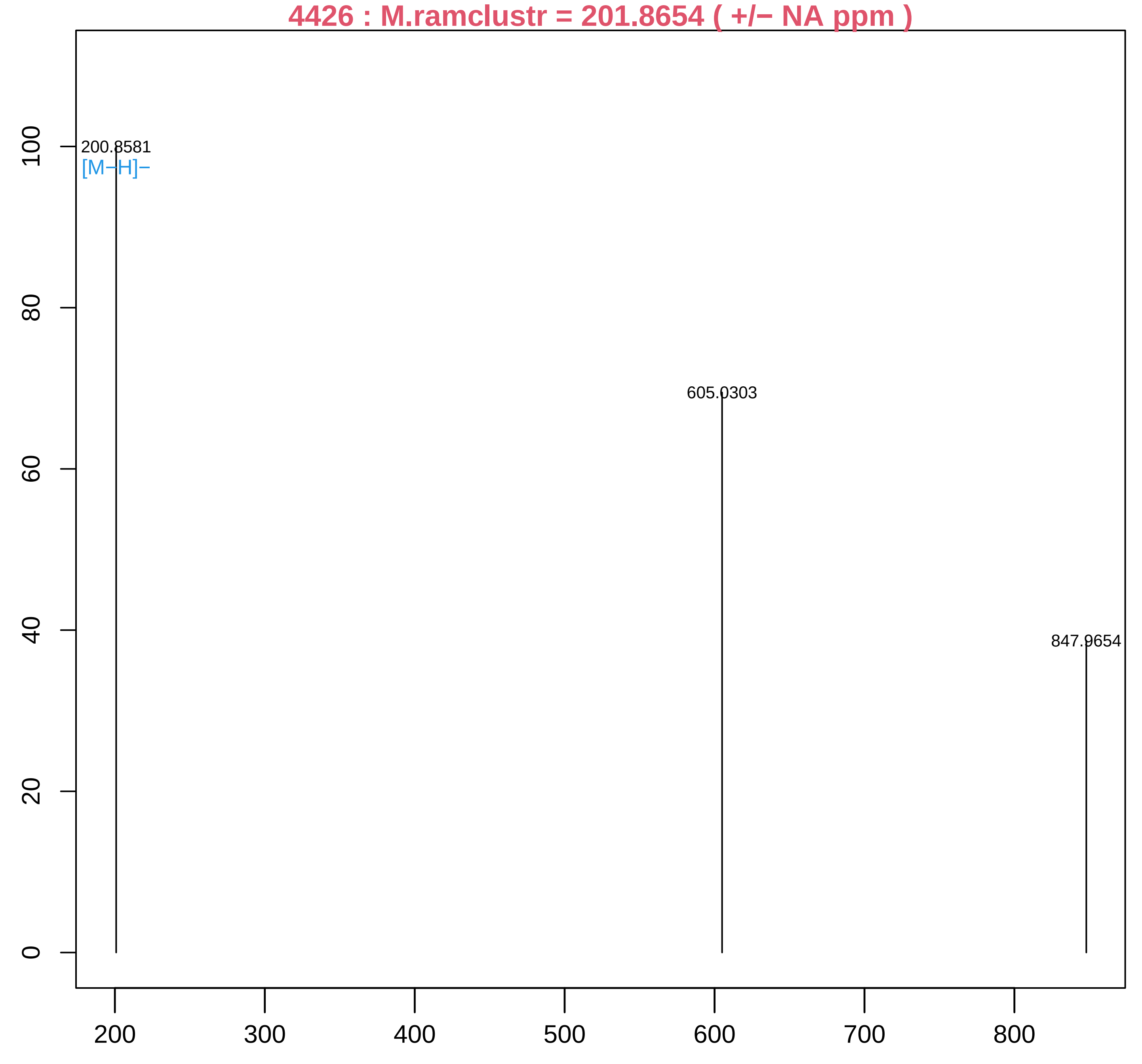
4426 : M.ramclustr = 201.8654 (+/− NA ppm)
200.8581
[M−H]−
605.0303
847.9654
0
20
40
60
80
100
200
300
400
500
600
700
800

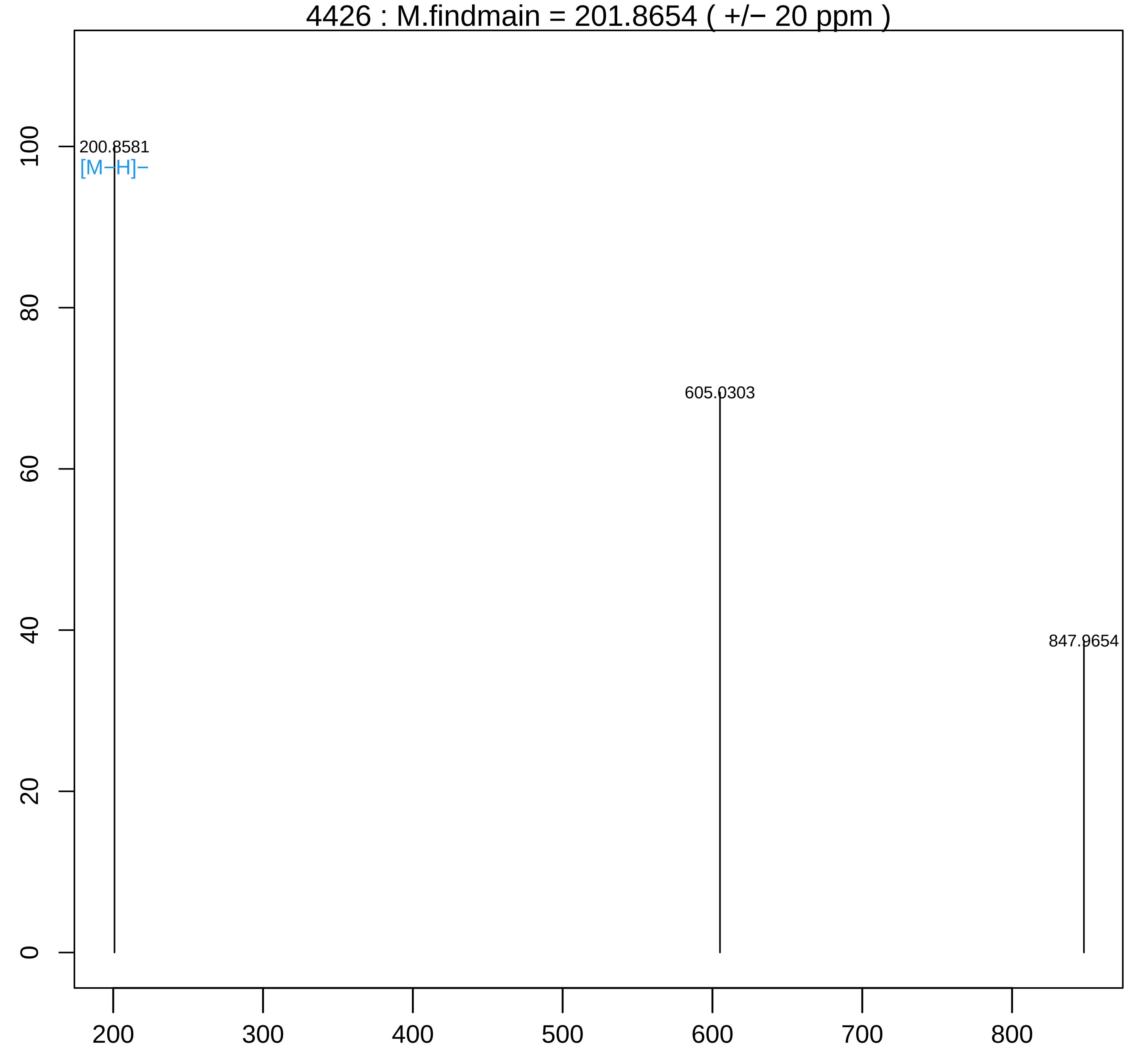
4426 : M.findmain = 201.8654 (+/− 20 ppm)
200.8581
[M−H]−
605.0303
847.9654
0
20
40
60
80
100
200
300
400
500
600
700
800

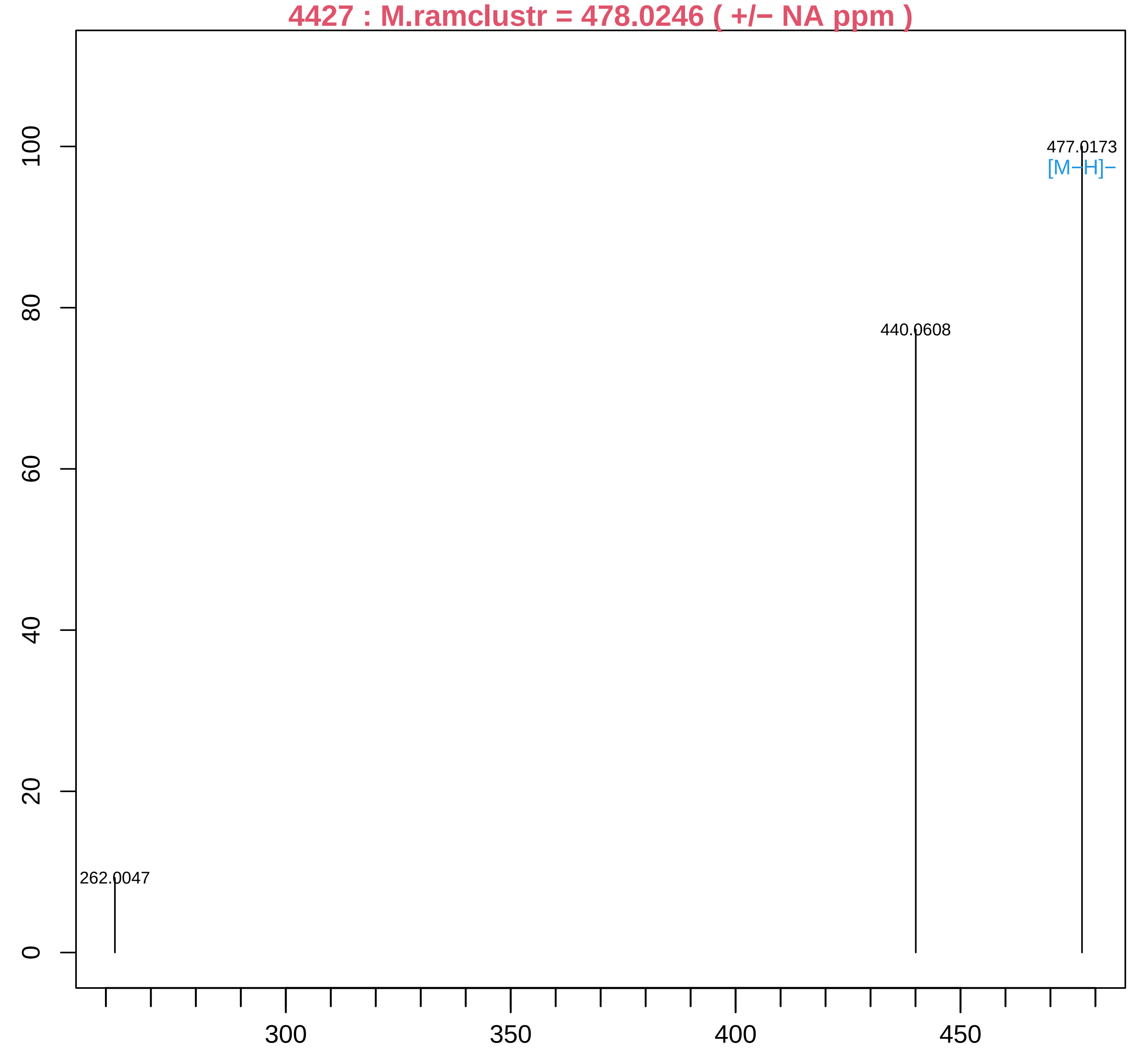
4427 : M.ramclustr = 478.0246 (+/− NA ppm)
477.0173
[M−H]−
440.0608
262.0047
0
20
40
60
80
100
300
350
400
450

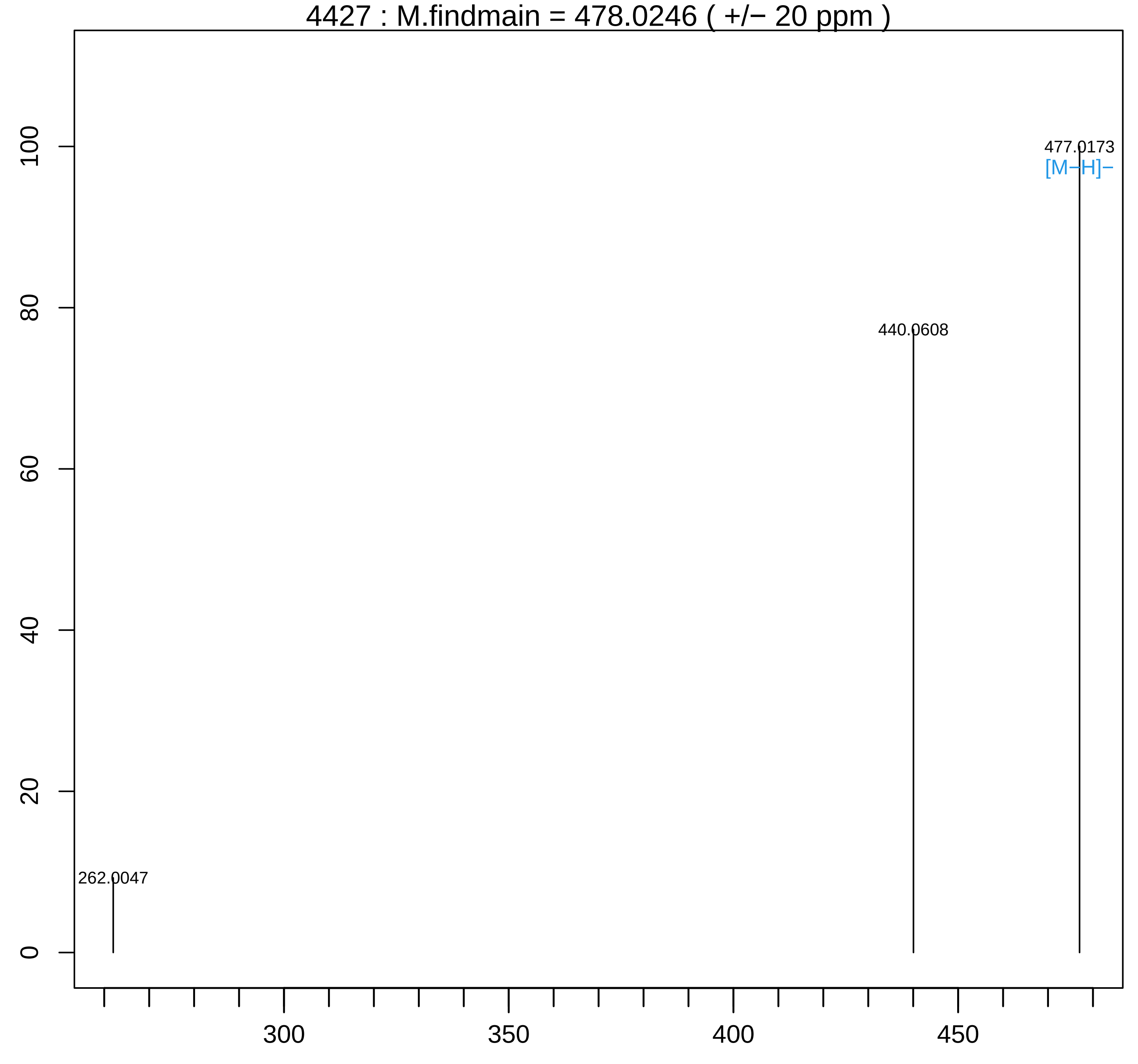
4427 : M.findmain = 478.0246 (+/− 20 ppm)
477.0173
[M−H]−
440.0608
262.0047
0
20
40
60
80
100
300
350
400
450

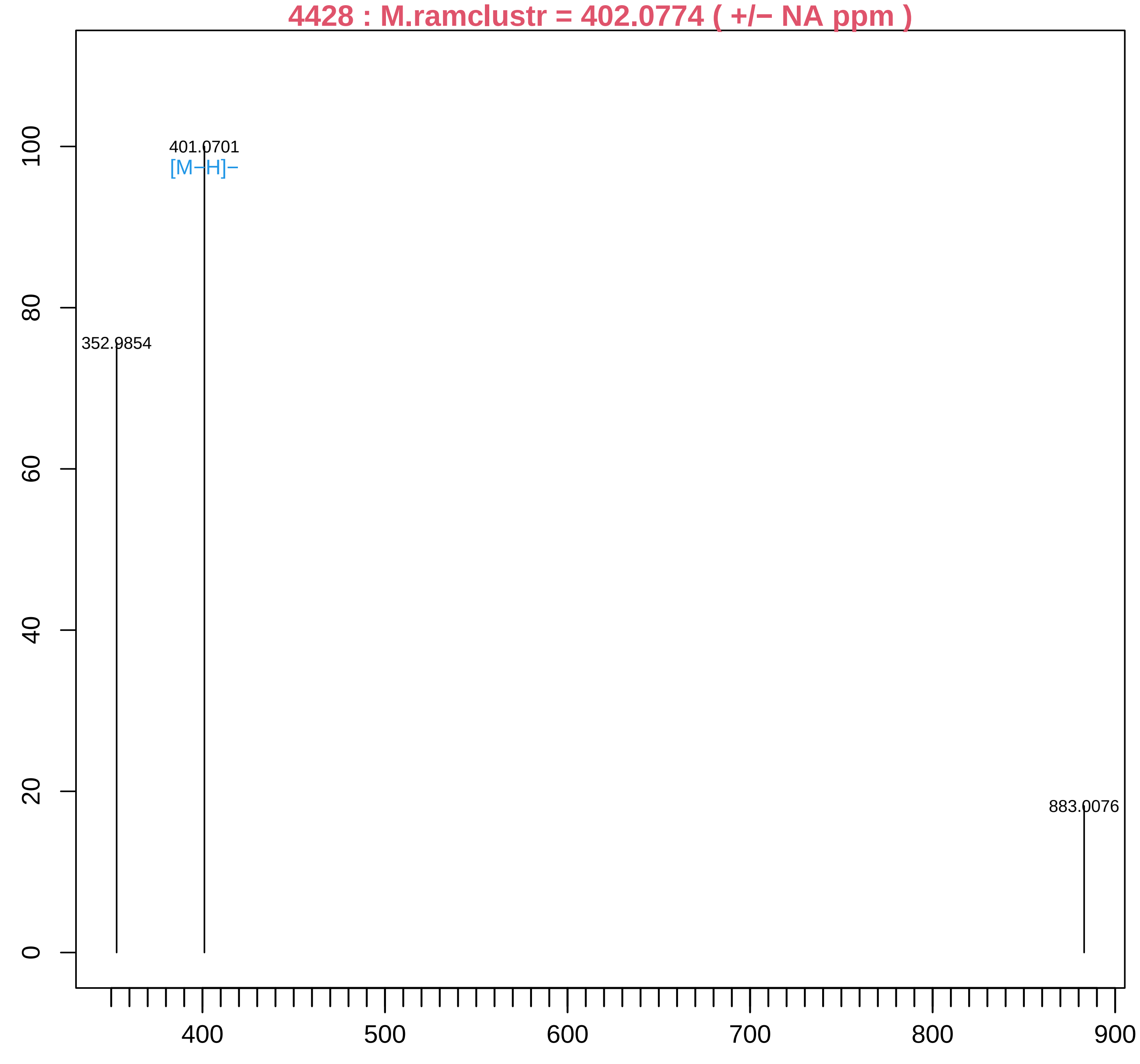
4428 : M.ramclustr = 402.0774 (+/− NA ppm)
401.0701
[M−H]−
352.9854
883.0076
0
20
40
60
80
100
400
500
600
700
800
900

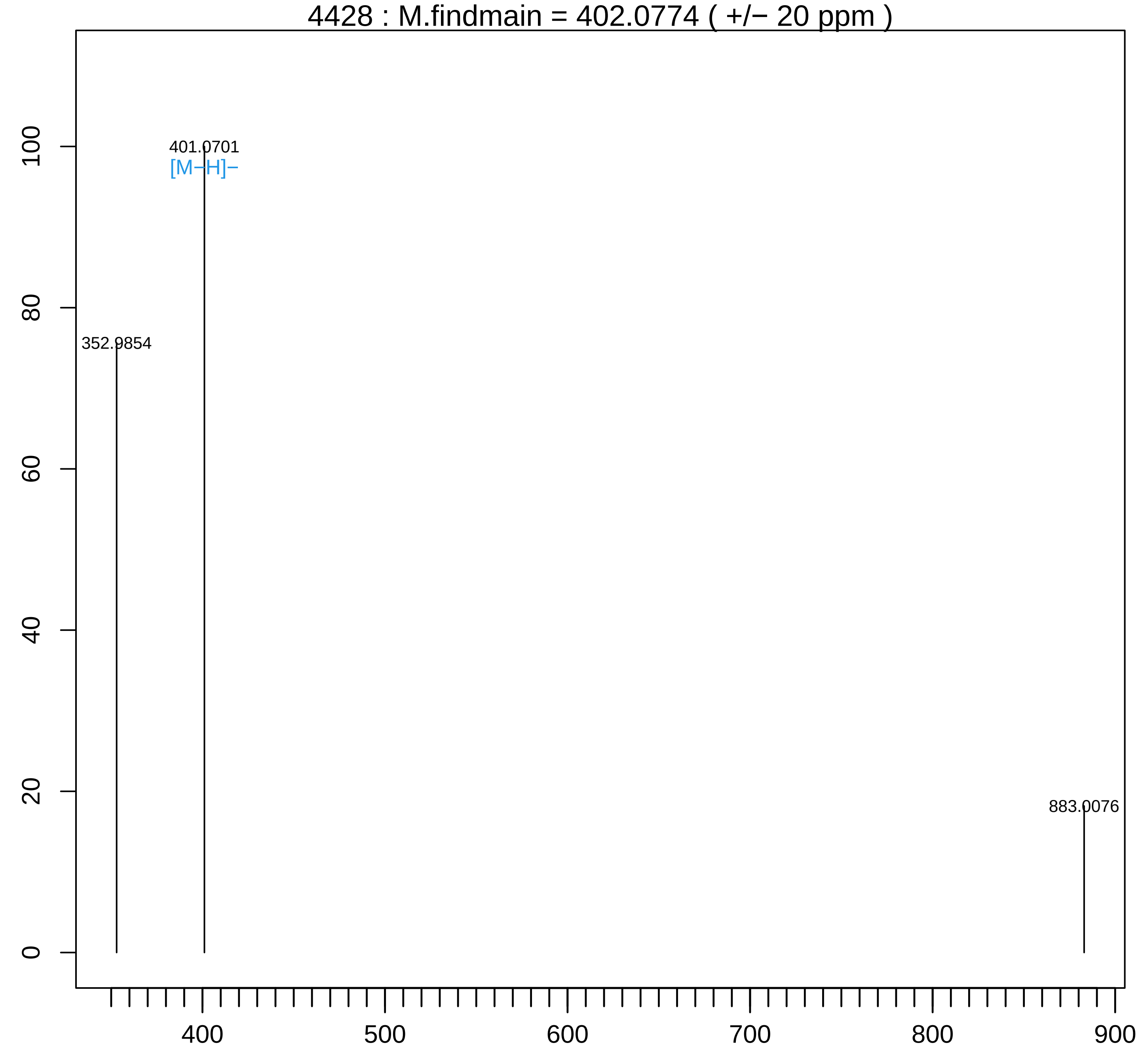
4428 : M.findmain = 402.0774 (+/− 20 ppm)
401.0701
[M−H]−
352.9854
883.0076
0
20
40
60
80
100
400
500
600
700
800
900

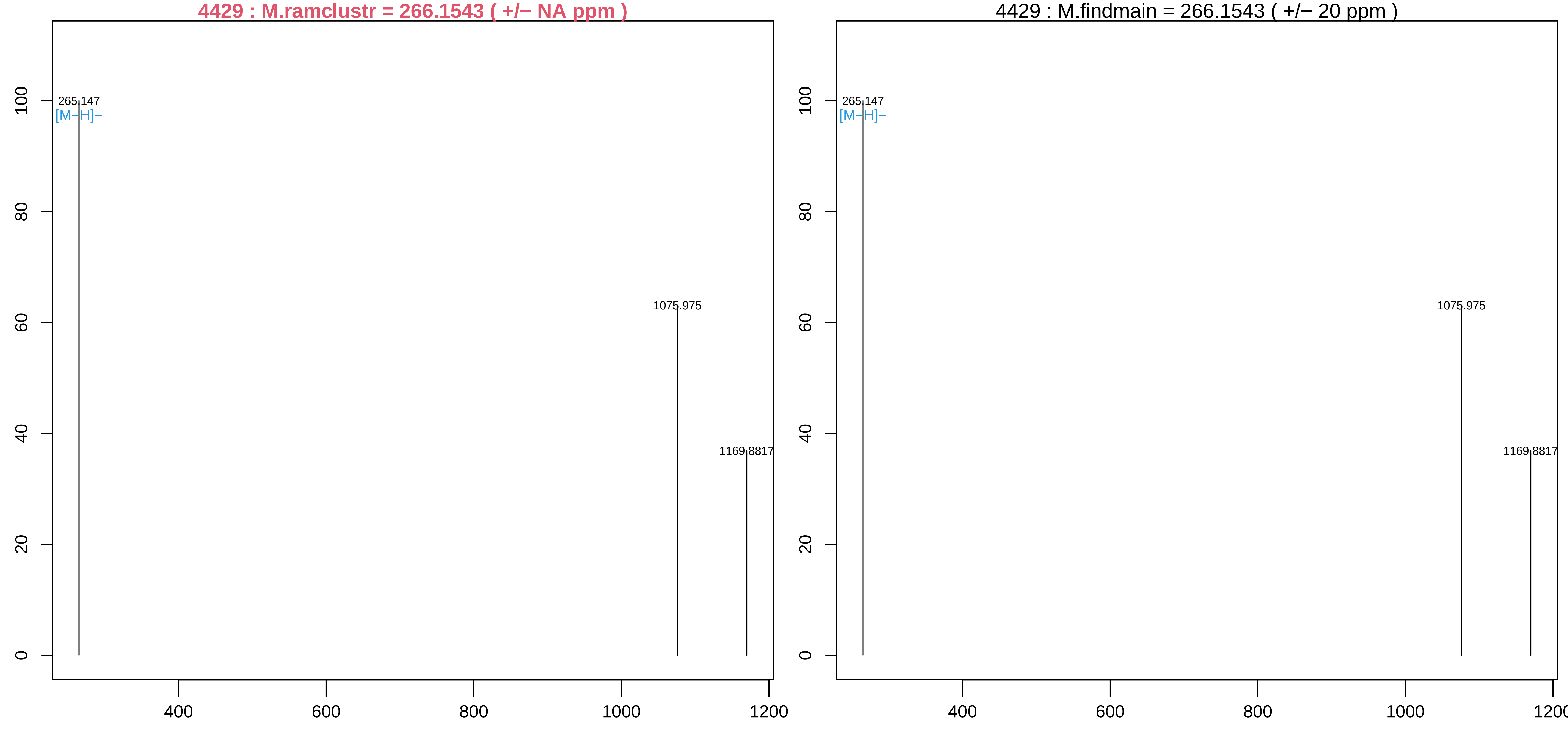
4429 : M.ramclustr = 266.1543 (+/− NA ppm)
265.147
[M−H]−
1075.975
1169.8817
0
20
40
60
80
100
400
600
800
1000
1200
4429 : M.findmain = 266.1543 (+/− 20 ppm)
265.147
[M−H]−
1075.975
1169.8817
0
20
40
60
80
100
400
600
800
1000
1200

4430 : M.ramclustr = 469.9819 (+/− NA ppm)

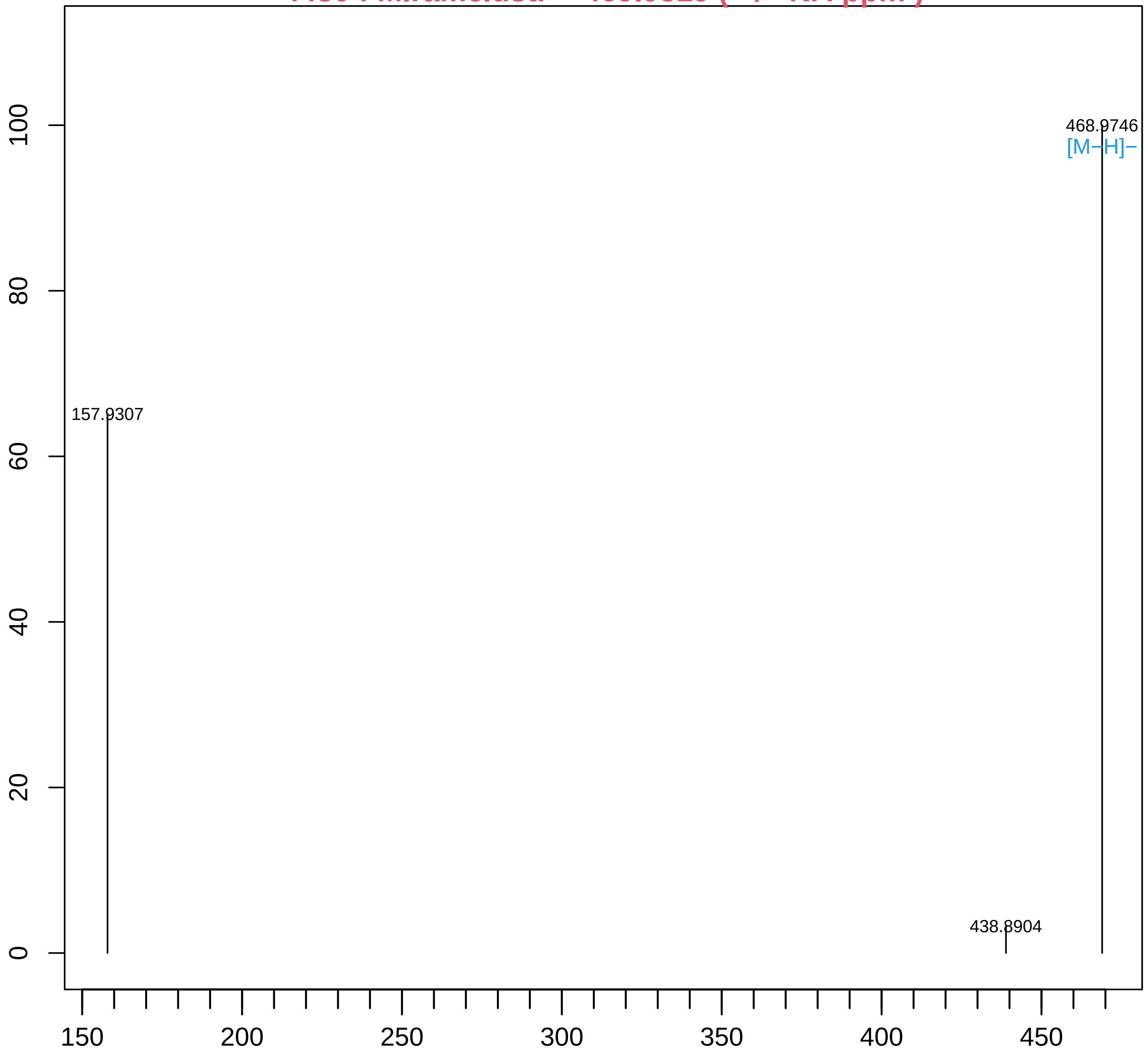

4430 : M.findmain = 469.9819 (+/− 20 ppm)

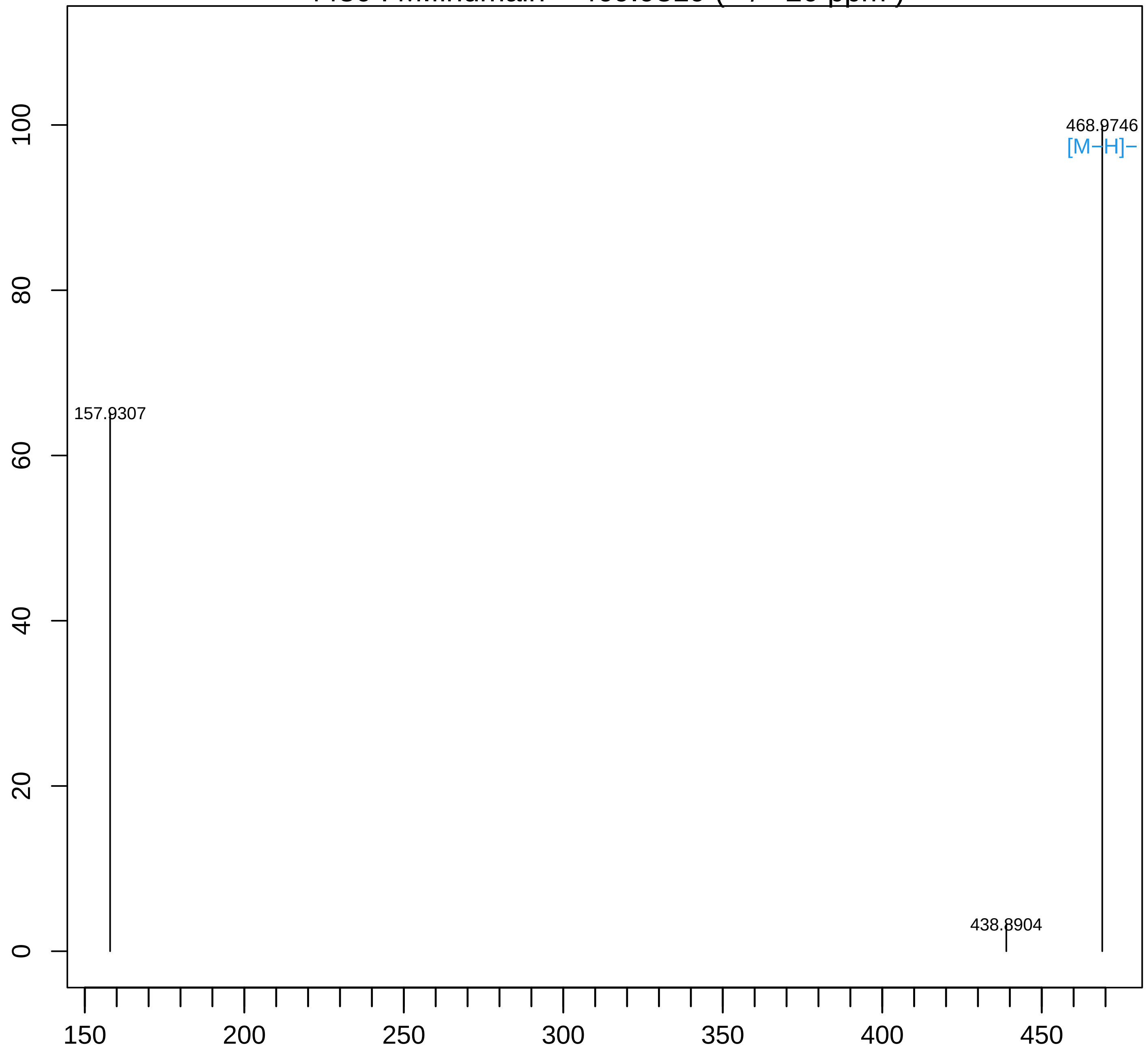

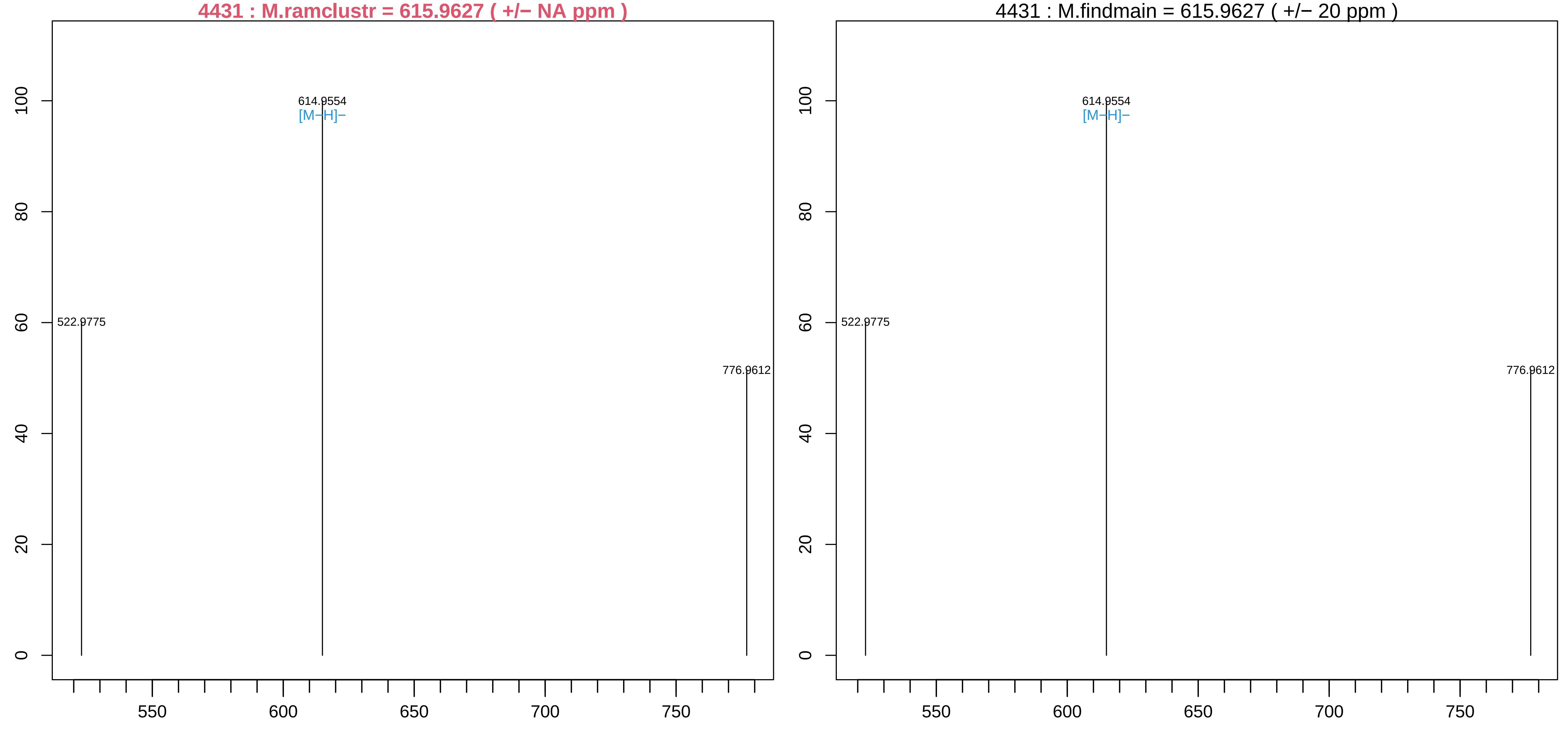
4431 : M.ramclustr = 615.9627 (+/− NA ppm)
4431 : M.findmain = 615.9627 (+/− 20 ppm)
614.9554
[M−H]−
522.9775
776.9612
614.9554
[M−H]−
522.9775
776.9612
0
20
40
60
80
100
550
600
650
700
750

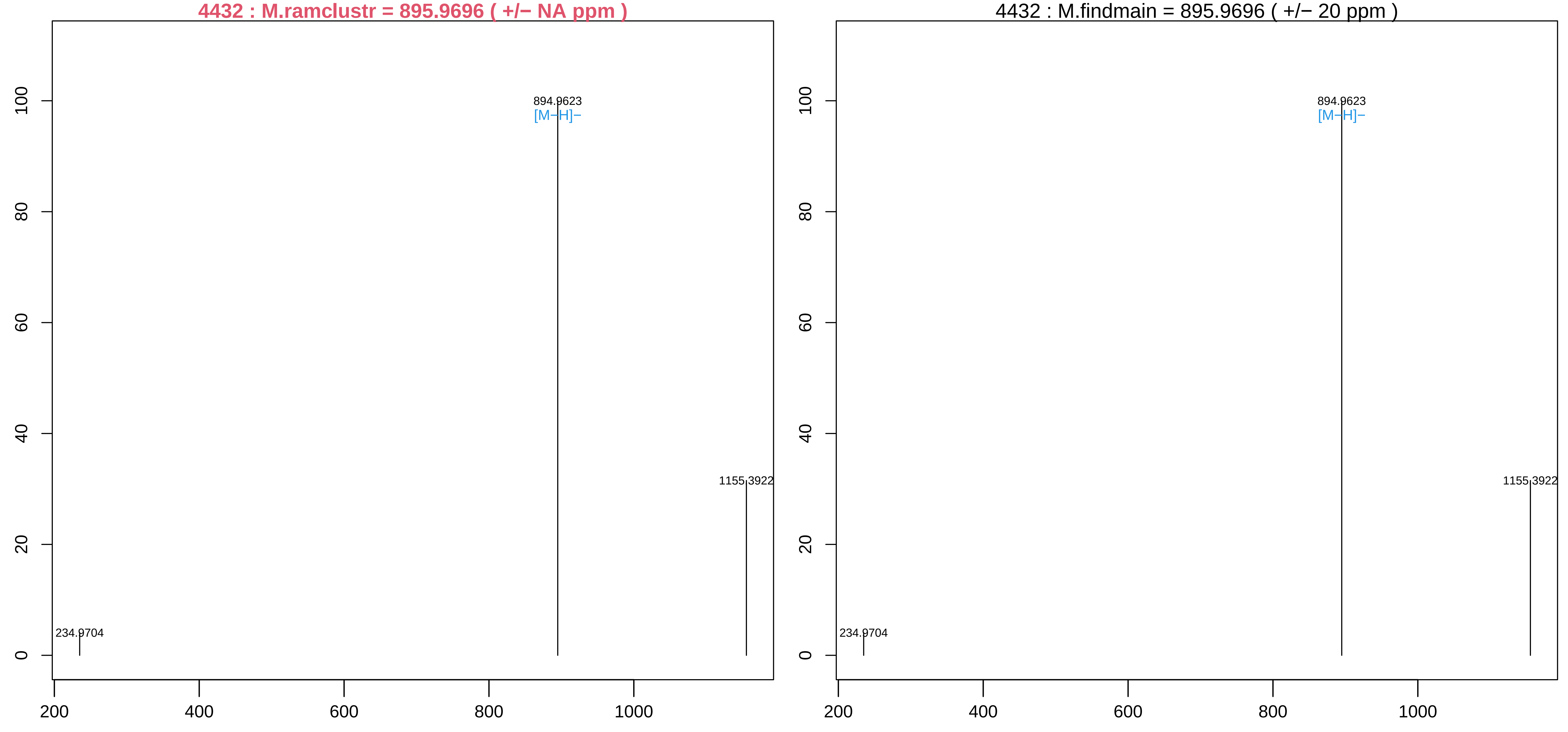
4432 : M.ramclustr = 895.9696 (+/− NA ppm)
894.9623
[M−H]−
1155.3922
234.9704
4432 : M.findmain = 895.9696 (+/− 20 ppm)
894.9623
[M−H]−
1155.3922
234.9704
0
20
40
60
80
100
200
400
600
800
1000

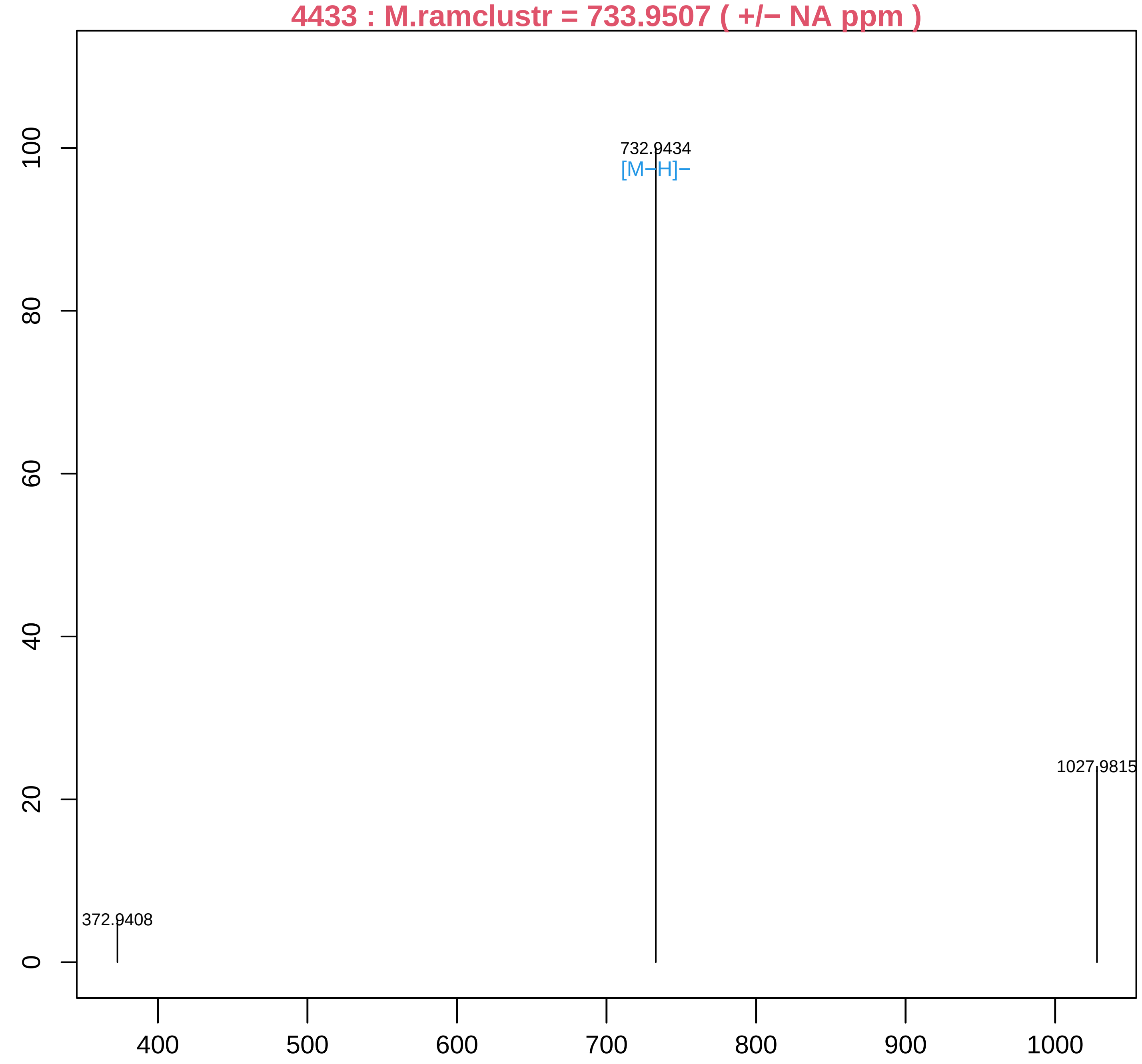
4433 : M.ramclustr = 733.9507 (+/− NA ppm)
732.9434
[M−H]−
1027.9815
372.9408
0
20
40
60
80
100
400
500
600
700
800
900
1000

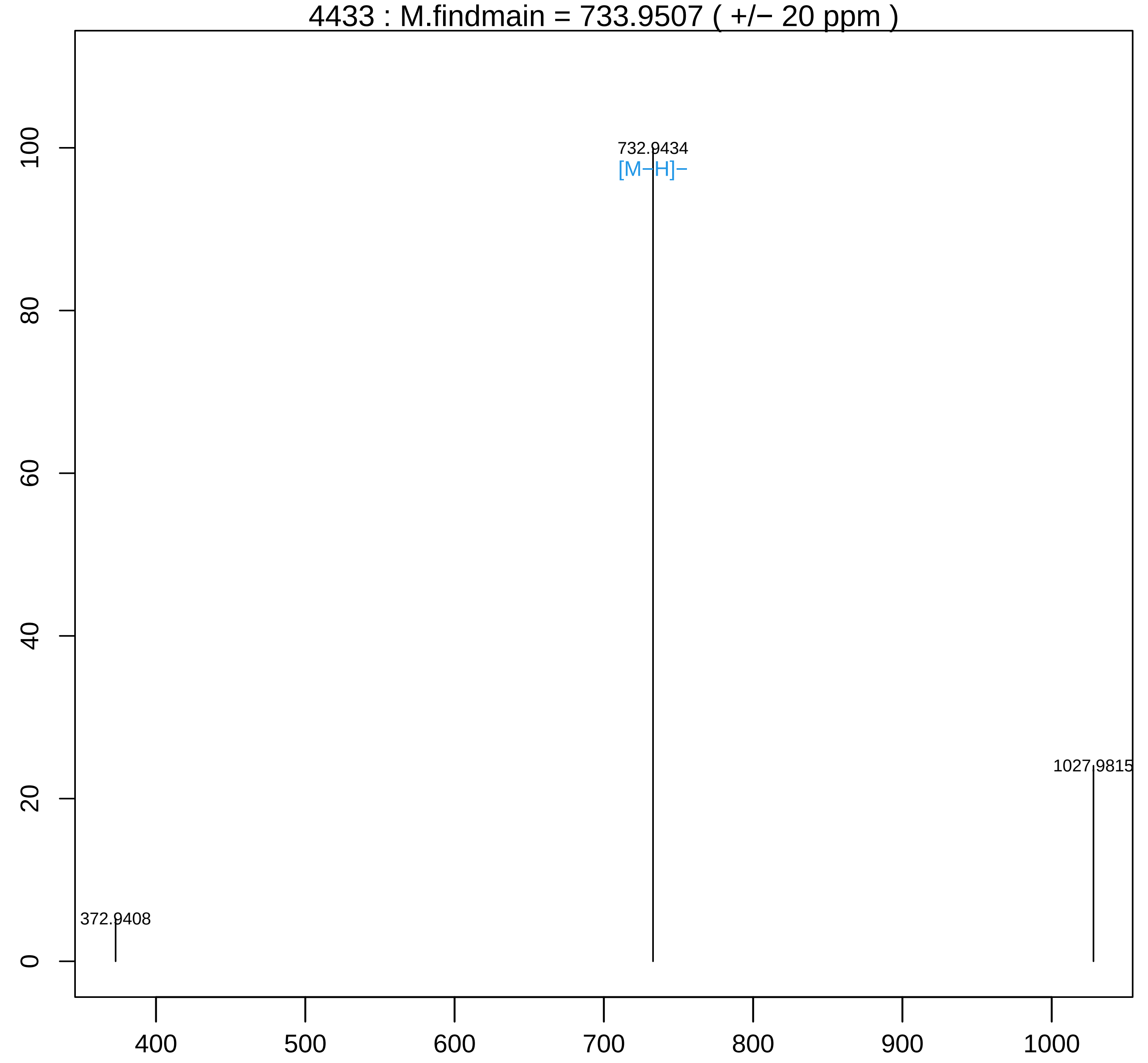
4433 : M.findmain = 733.9507 (+/− 20 ppm)
732.9434
[M−H]−
1027.9815
372.9408
0
20
40
60
80
100
400
500
600
700
800
900
1000

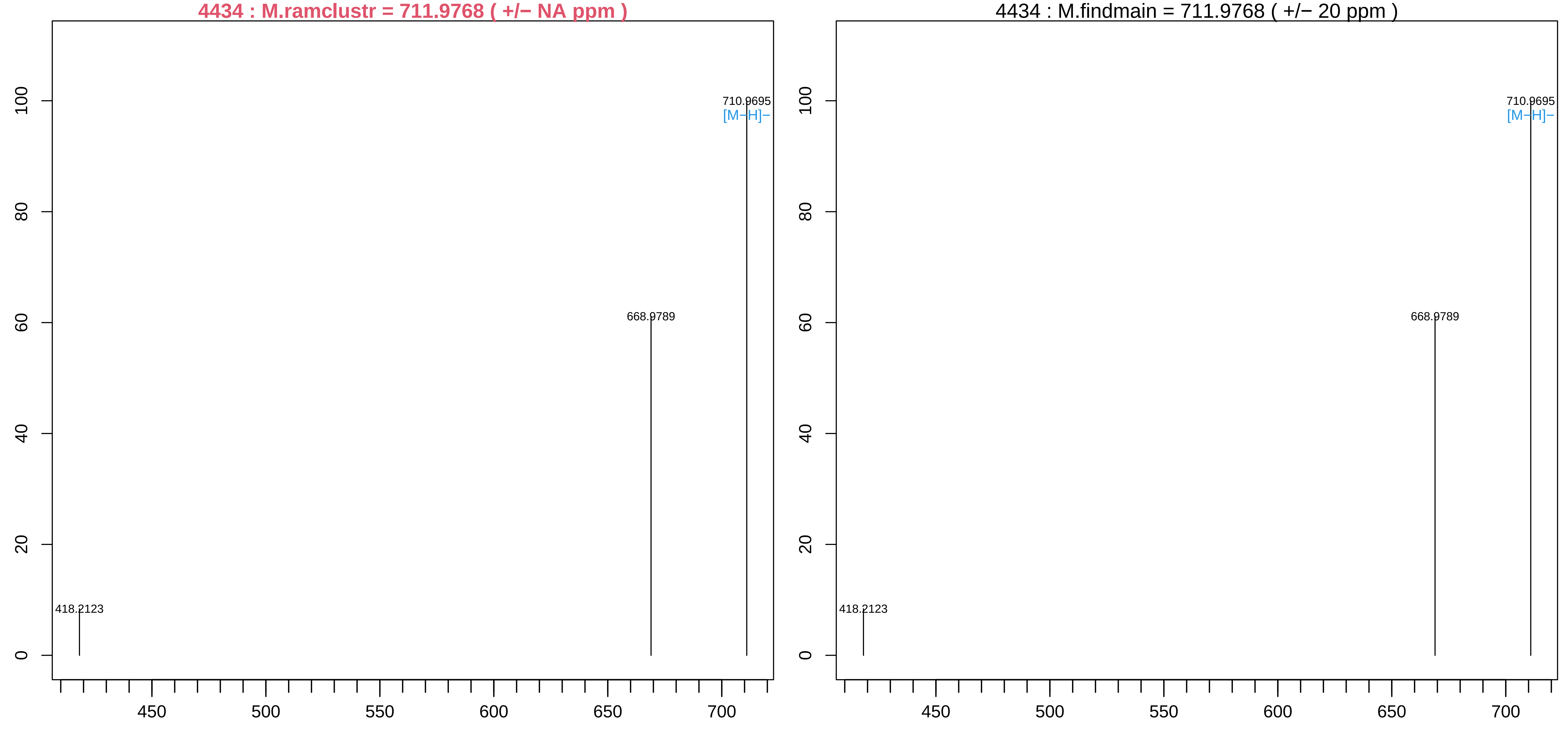

4434 : M.ramclustr = 711.9768 (+/− NA ppm)
710.9695
[M−H]−
668.9789
418.2123
0
20
40
60
80
100
450
500
550
600
650
700
4434 : M.findmain = 711.9768 (+/− 20 ppm)
710.9695
[M−H]−
668.9789
418.2123
0
20
40
60
80
100
450
500
550
600
650
700

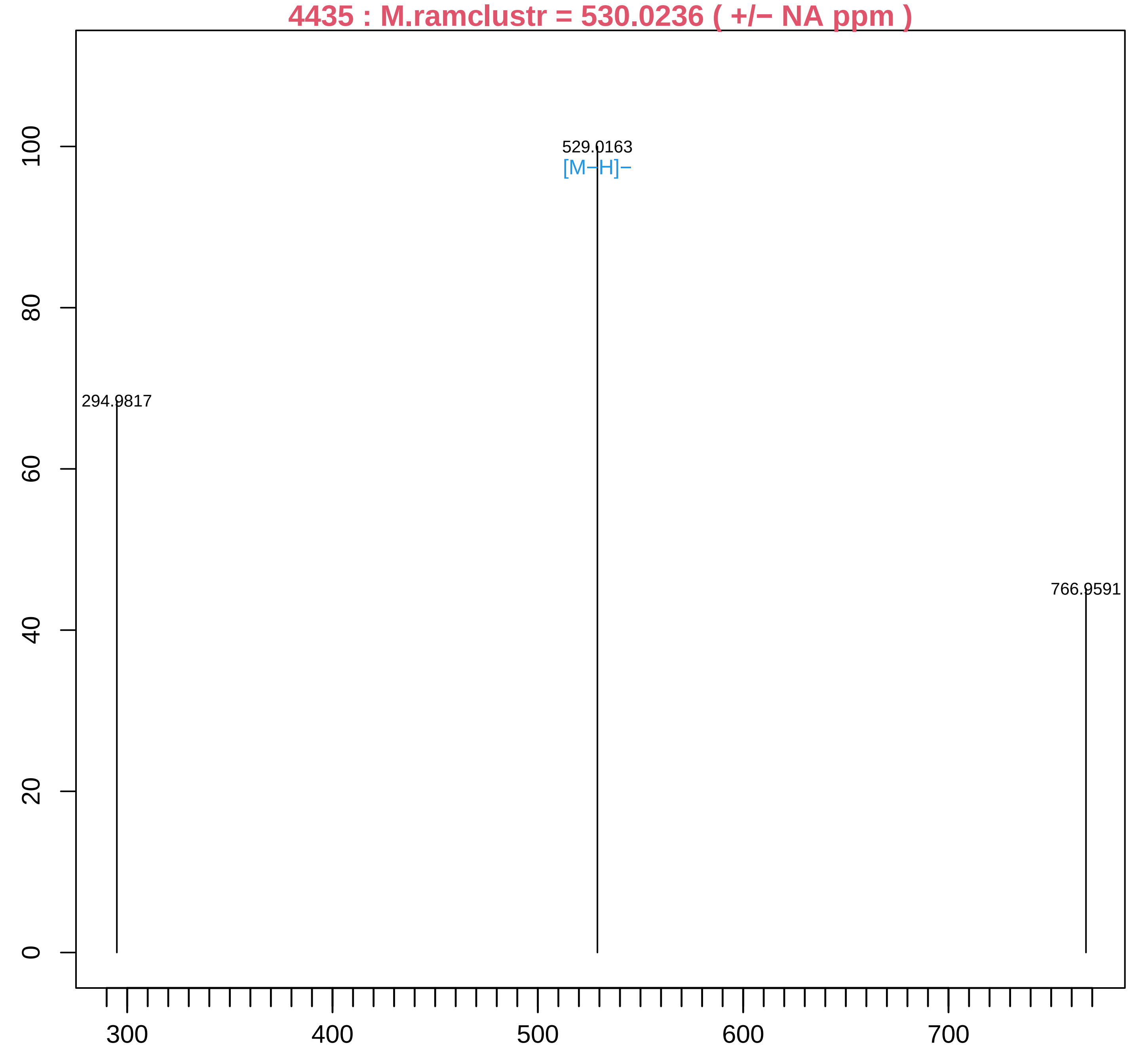

4435 : M.ramclustr = 530.0236 (+/− NA ppm)
529.0163
[M−H]−
294.9817
766.9591
0
20
40
60
80
100
300
400
500
600
700

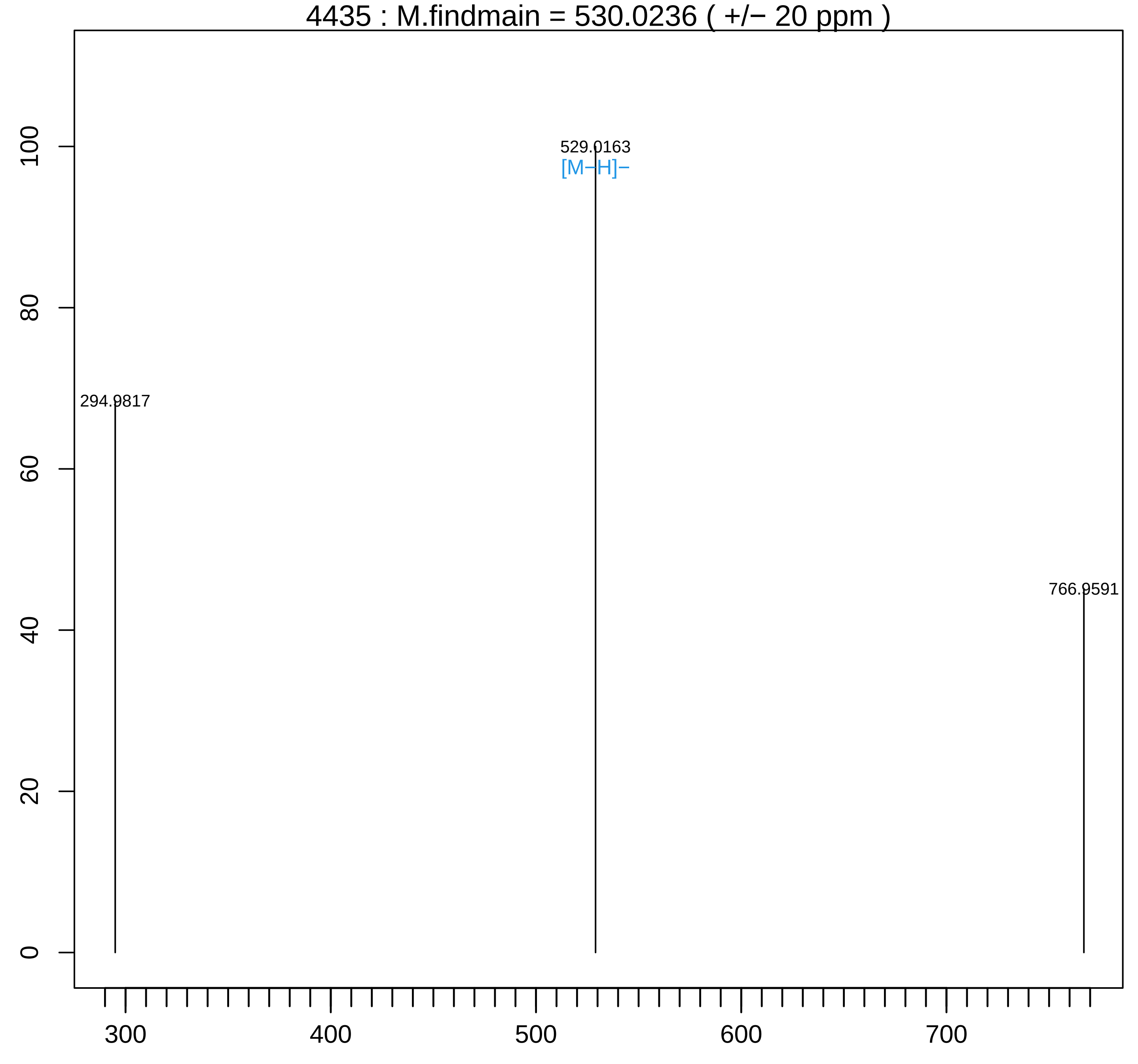

4435 : M.findmain = 530.0236 (+/− 20 ppm)
529.0163
[M−H]−
294.9817
766.9591
0
20
40
60
80
100
300
400
500
600
700

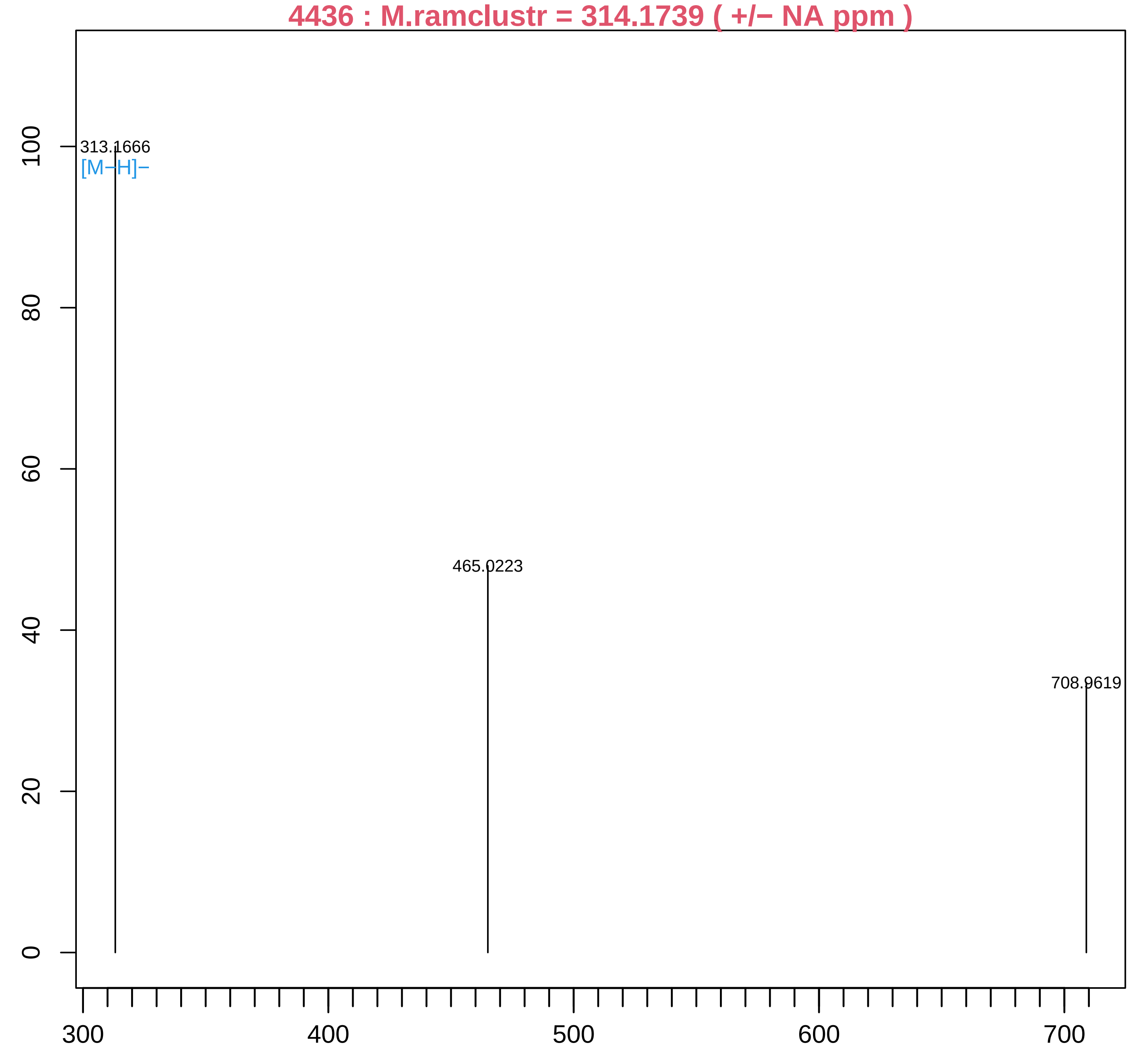
4436 : M.ramclustr = 314.1739 (+/− NA ppm)
313.1666
[M−H]−
465.0223
708.9619

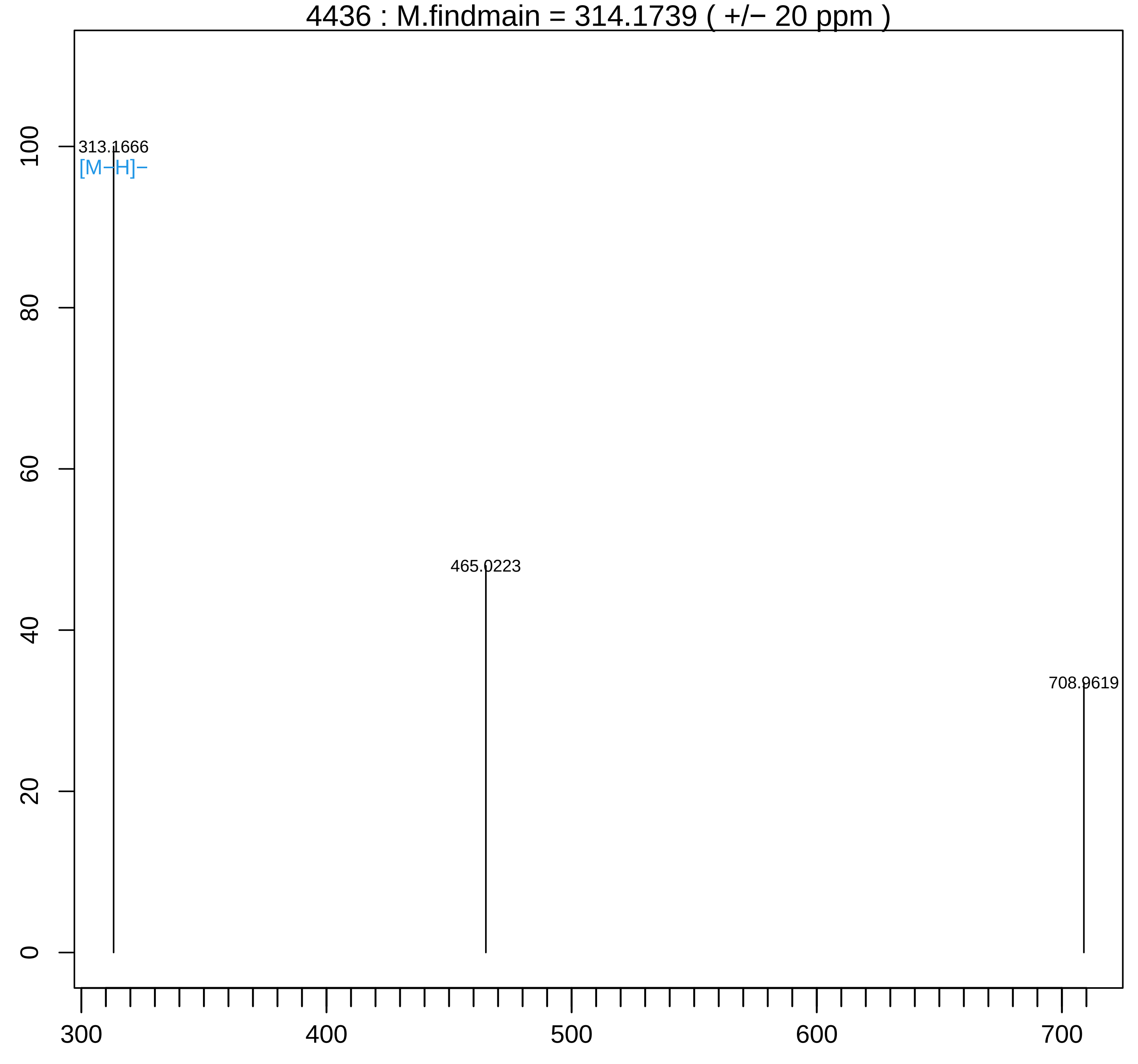
4436 : M.findmain = 314.1739 (+/− 20 ppm)
313.1666
[M−H]−
465.0223
708.9619

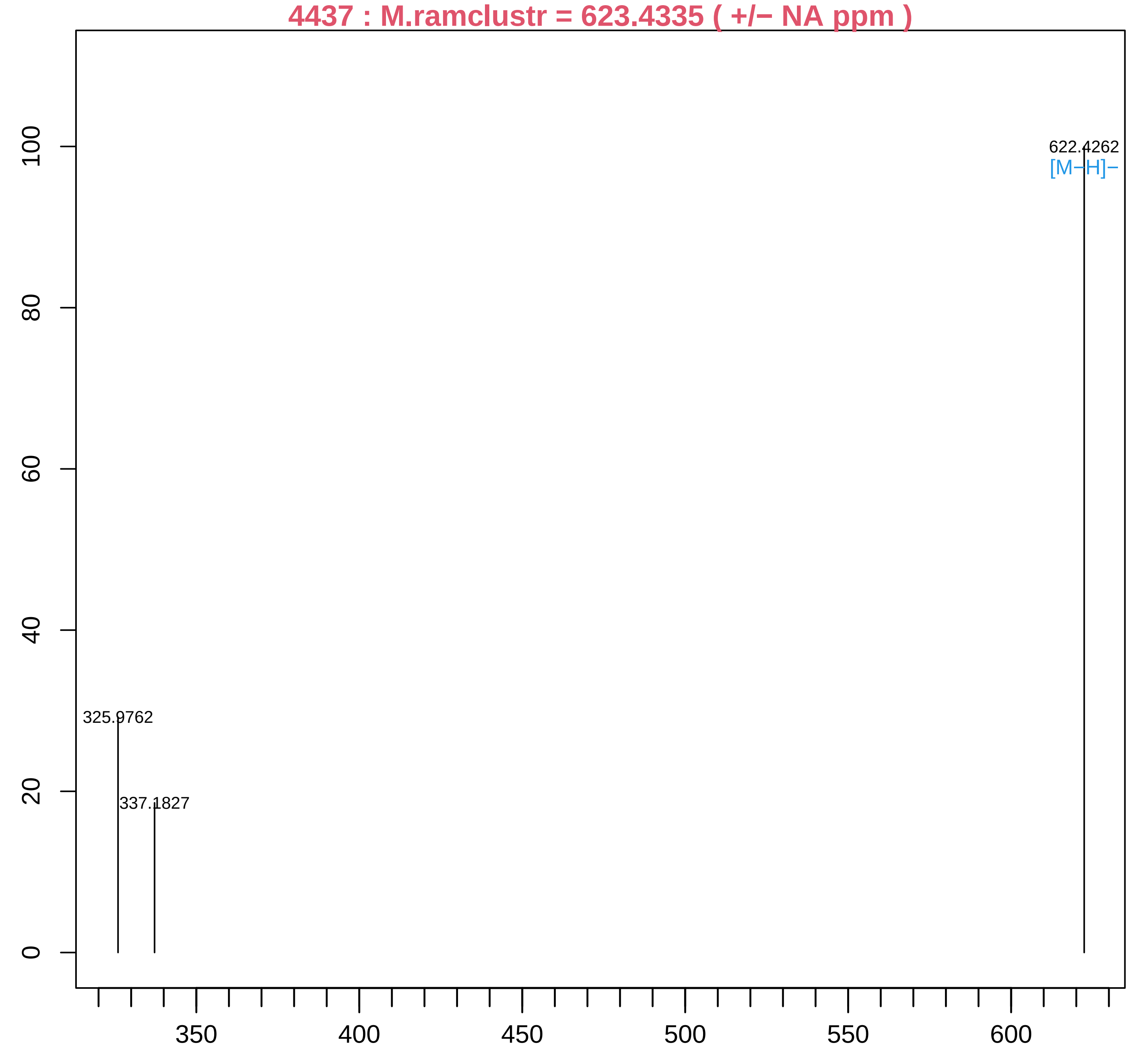
4437 : M.ramclustr = 623.4335 (+/− NA ppm)
622.4262
[M−H]−
325.9762
337.1827
0
20
40
60
80
100
350
400
450
500
550
600

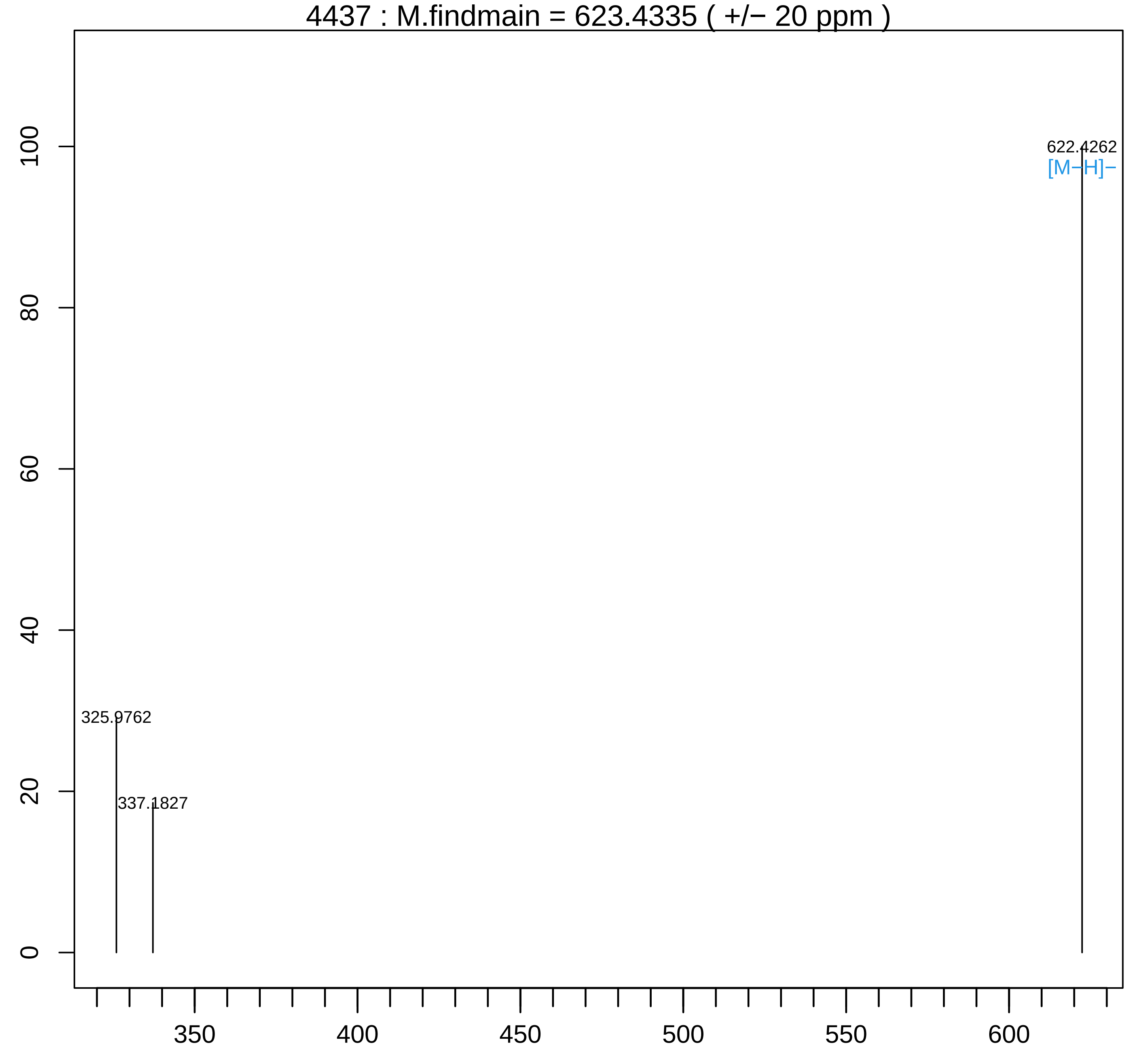
4437 : M.findmain = 623.4335 (+/− 20 ppm)
622.4262
[M−H]−
325.9762
337.1827
0
20
40
60
80
100
350
400
450
500
550
600

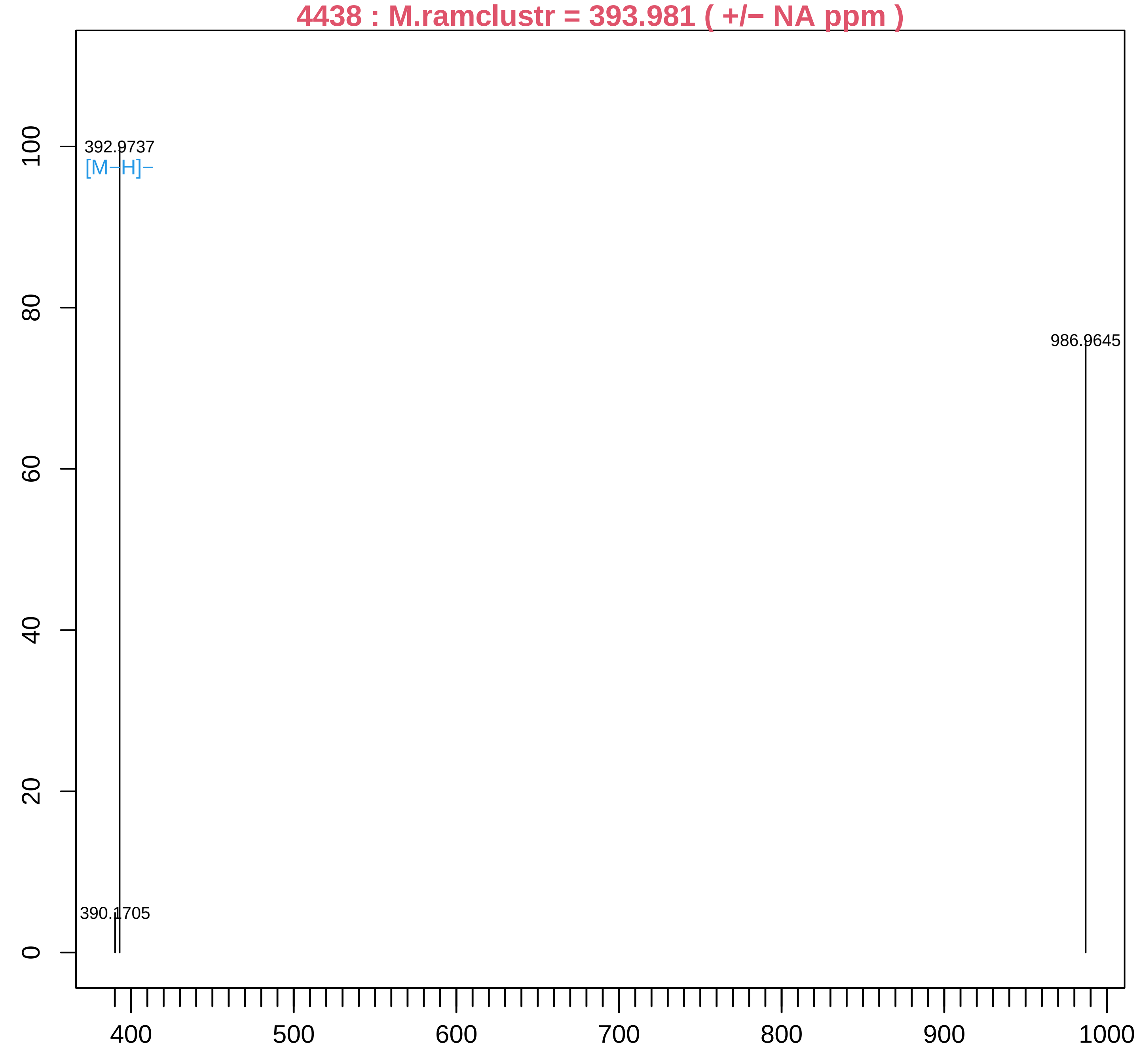
4438 : M.ramclustr = 393.981 (+/− NA ppm)
392.9737
[M−H]−
986.9645
390.1705
0
20
40
60
80
100
400
500
600
700
800
900
1000

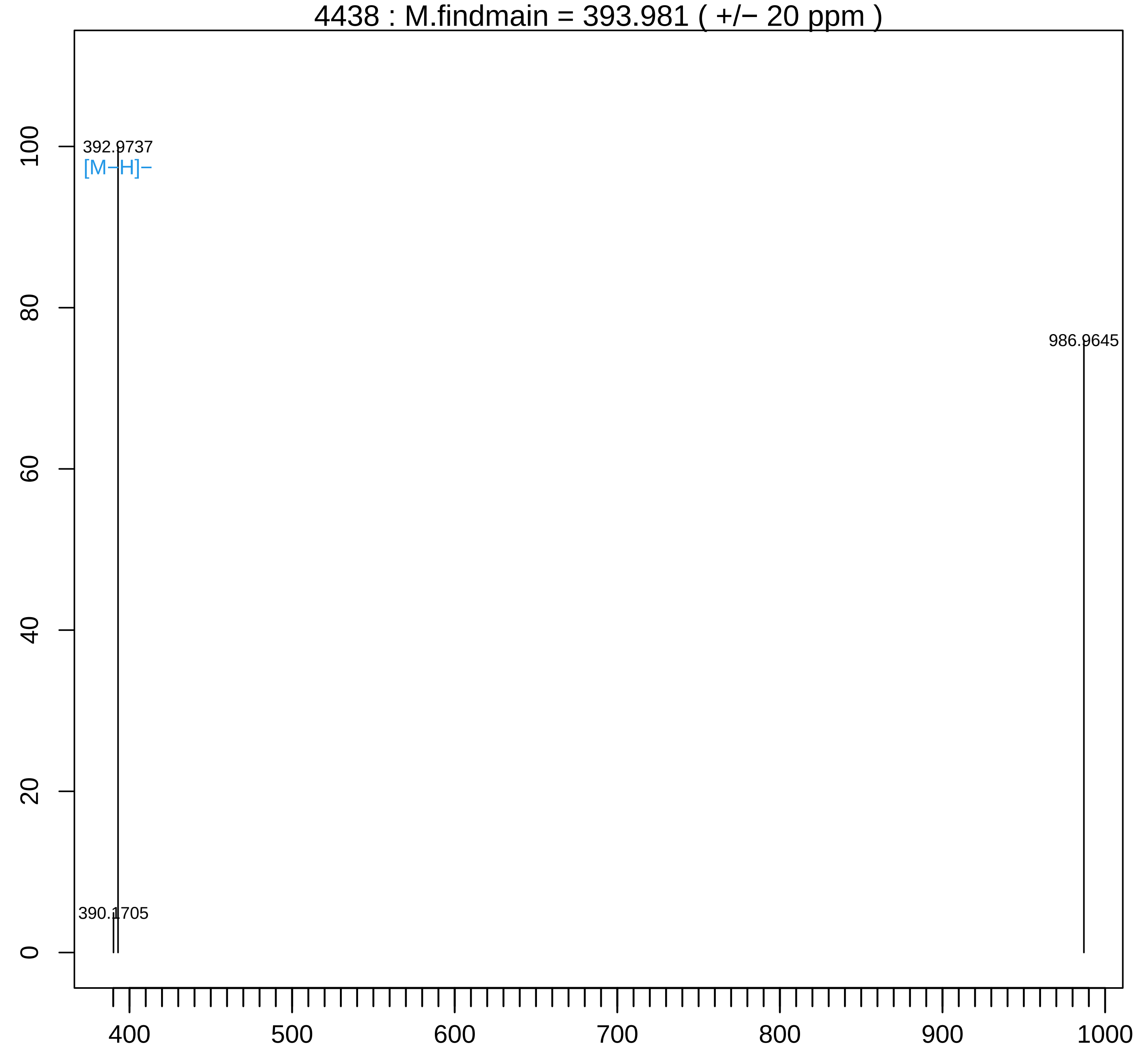
4438 : M.findmain = 393.981 (+/− 20 ppm)
392.9737
[M−H]−
986.9645
390.1705
0
20
40
60
80
100
400
500
600
700
800
900
1000

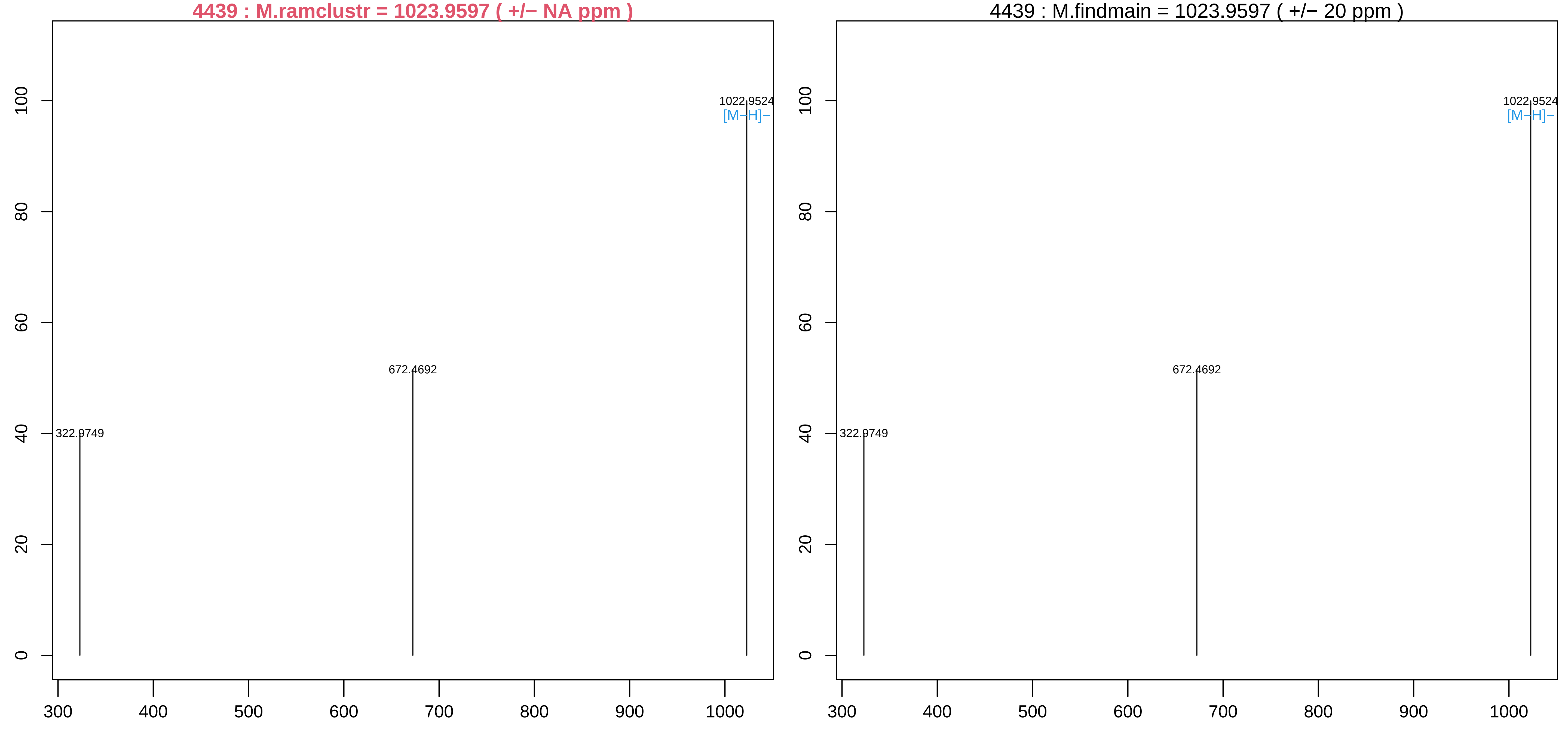
4439 : M.ramclustr = 1023.9597 (+/− NA ppm)
1022.9524
[M−H]−
672.4692
322.9749
4439 : M.findmain = 1023.9597 (+/− 20 ppm)
1022.9524
[M−H]−
672.4692
322.9749
0
20
40
60
80
100
300
400
500
600
700
800
900
1000

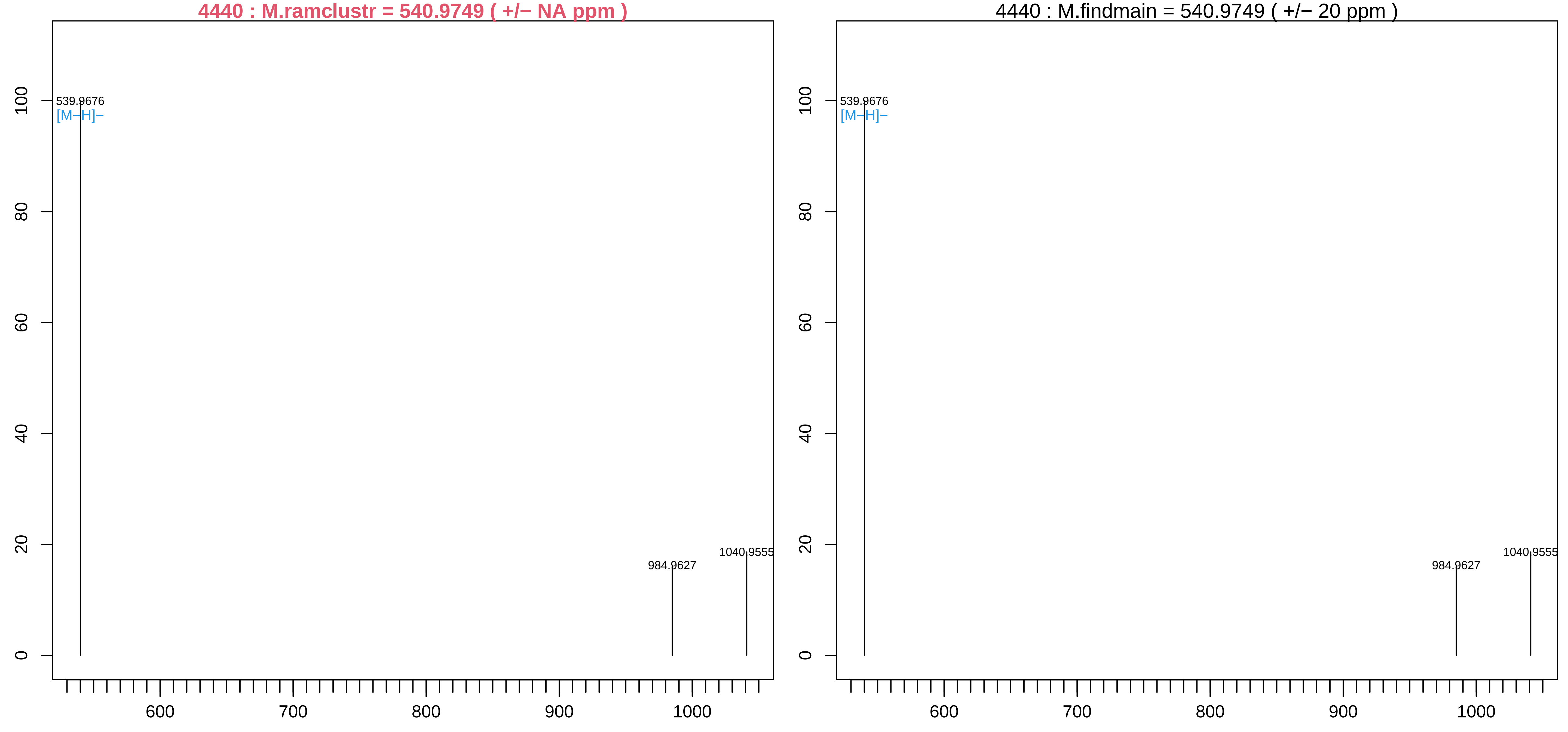
4440 : M.ramclustr = 540.9749 (+/− NA ppm)
539.9676
[M−H]−
984.9627
1040.9555
4440 : M.findmain = 540.9749 (+/− 20 ppm)
539.9676
[M−H]−
984.9627
1040.9555
0
20
40
60
80
100
600
700
800
900
1000

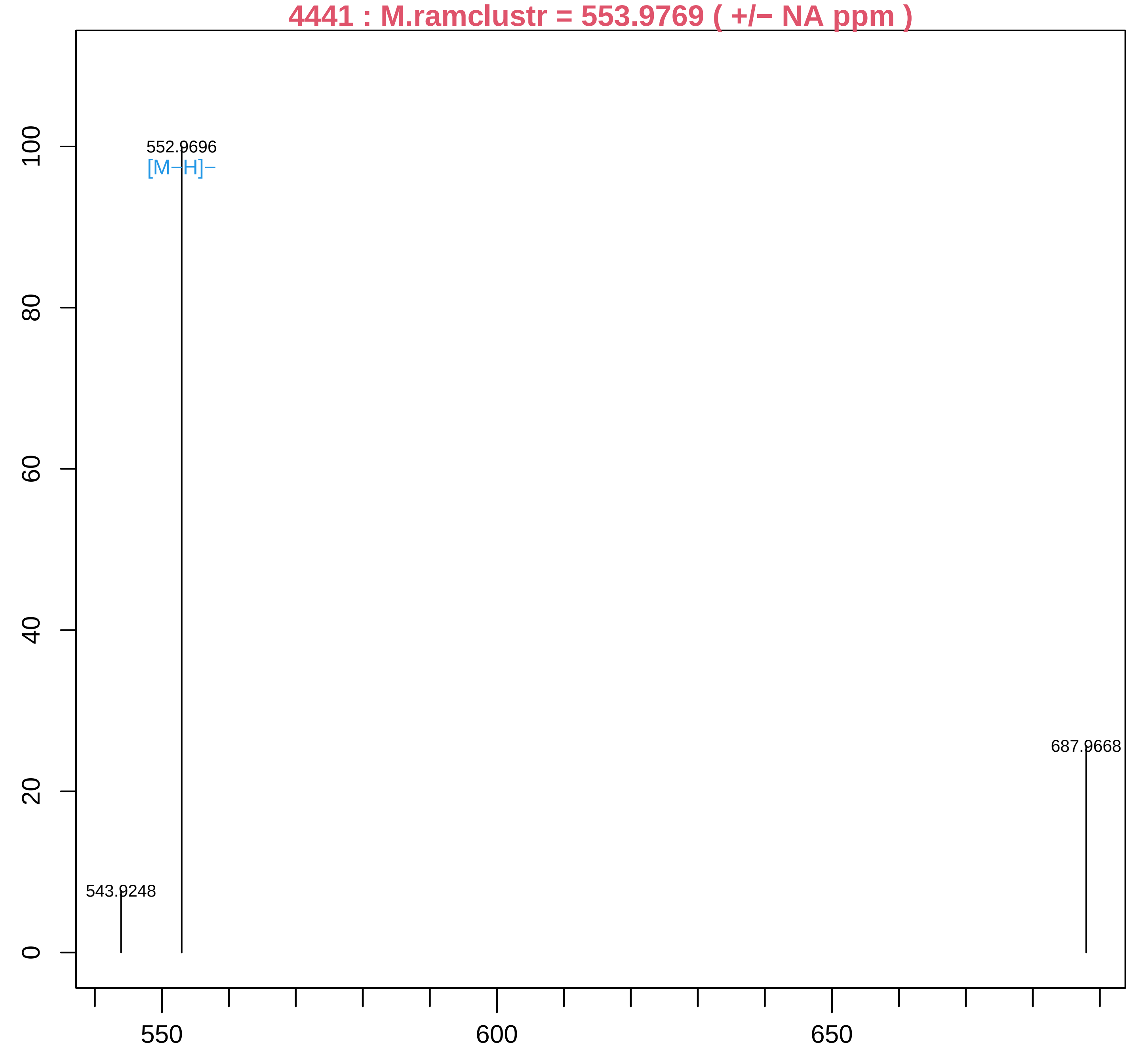
4441 : M.ramclustr = 553.9769 (+/− NA ppm)
552.9696
[M−H]−
687.9668
543.9248
0
20
40
60
80
100
550
600
650

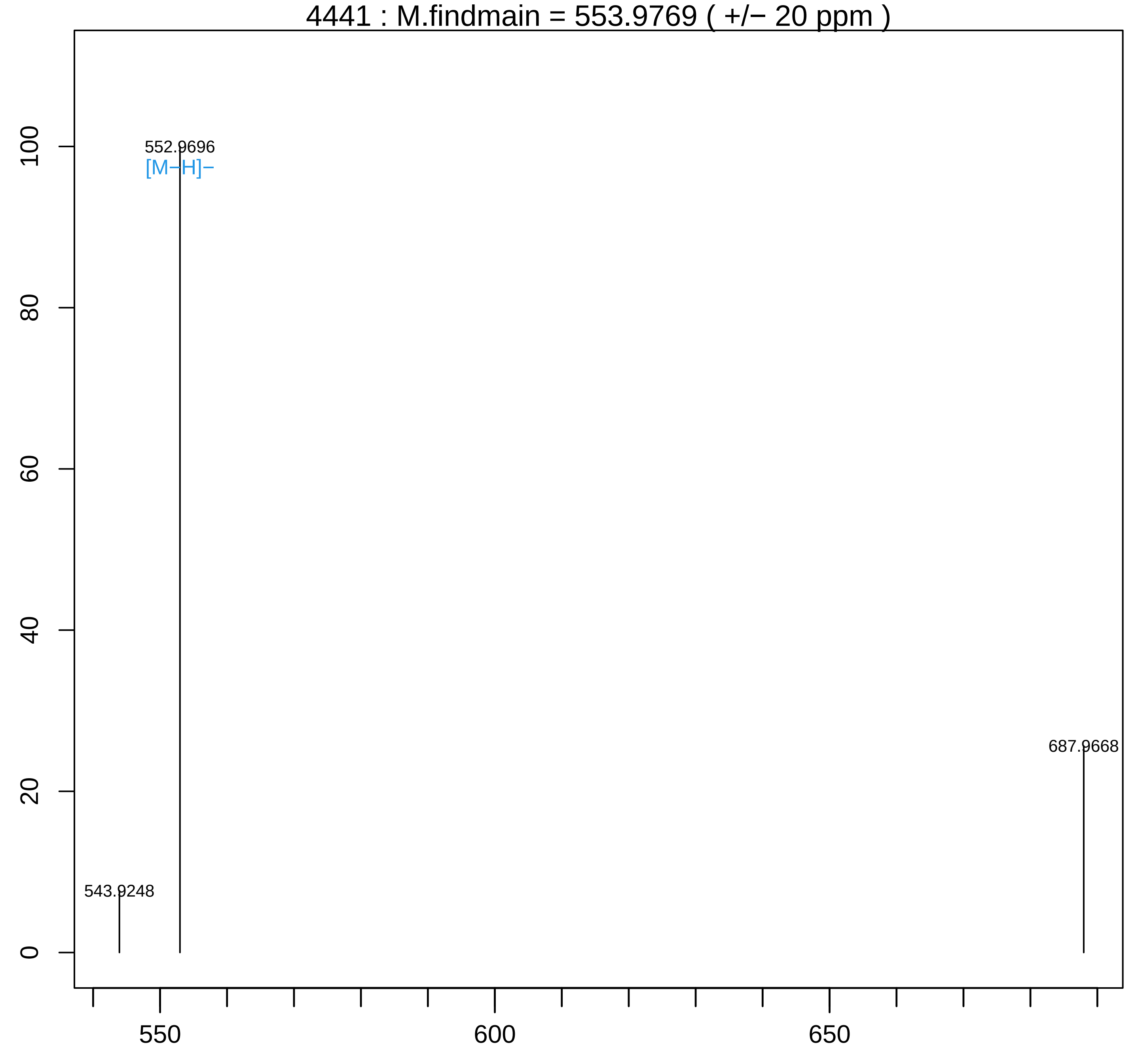
4441 : M.findmain = 553.9769 (+/− 20 ppm)
552.9696
[M−H]−
687.9668
543.9248
0
20
40
60
80
100
550
600
650

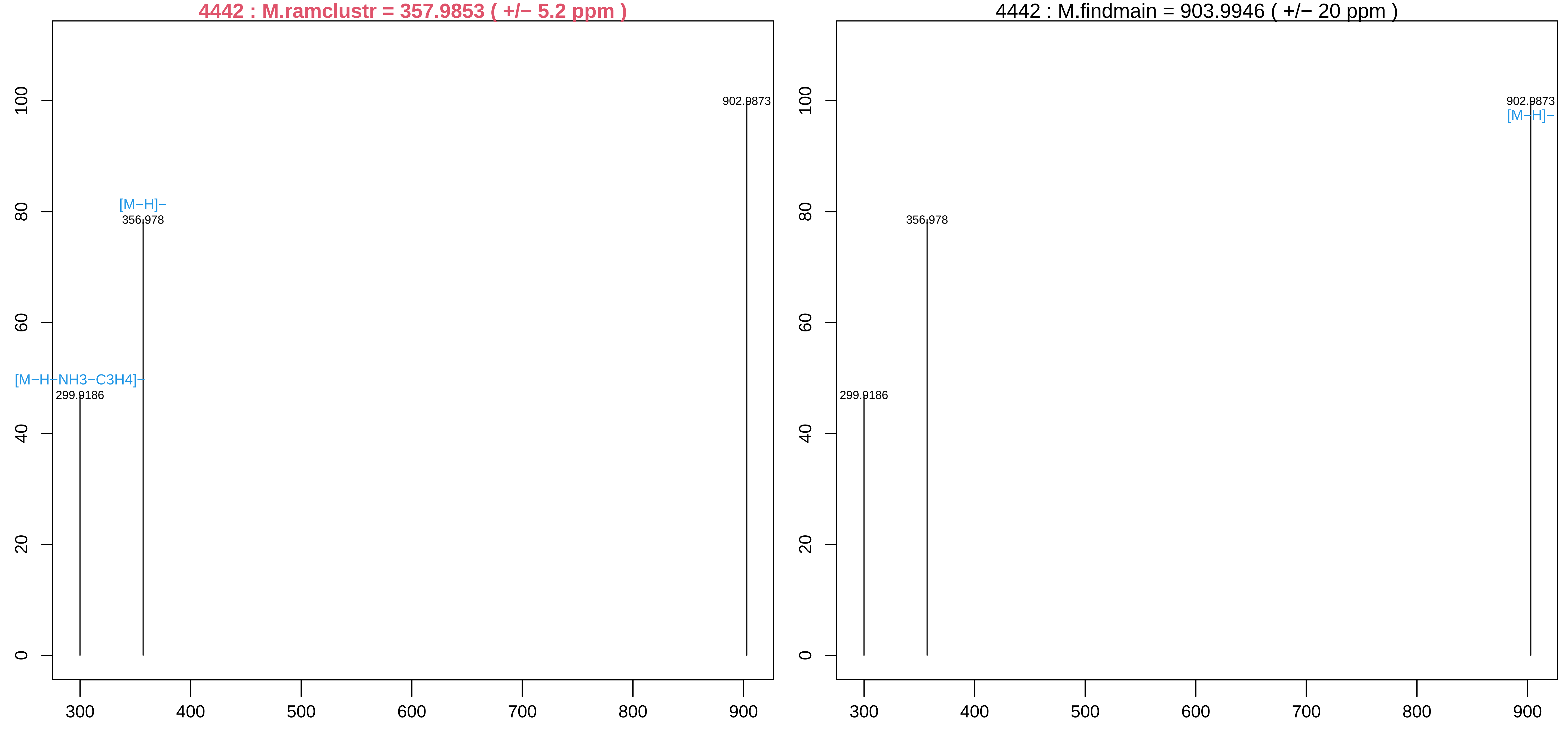
4442 : M.ramclustr = 357.9853 (+/− 5.2 ppm)
[M−H]−
356.978
[M−H−NH3−C3H4]−
299.9186
902.9873
0
20
40
60
80
100
300
400
500
600
700
800
900
4442 : M.findmain = 903.9946 (+/− 20 ppm)
902.9873
[M−H]−
356.978
299.9186
0
20
40
60
80
100
300
400
500
600
700
800
900

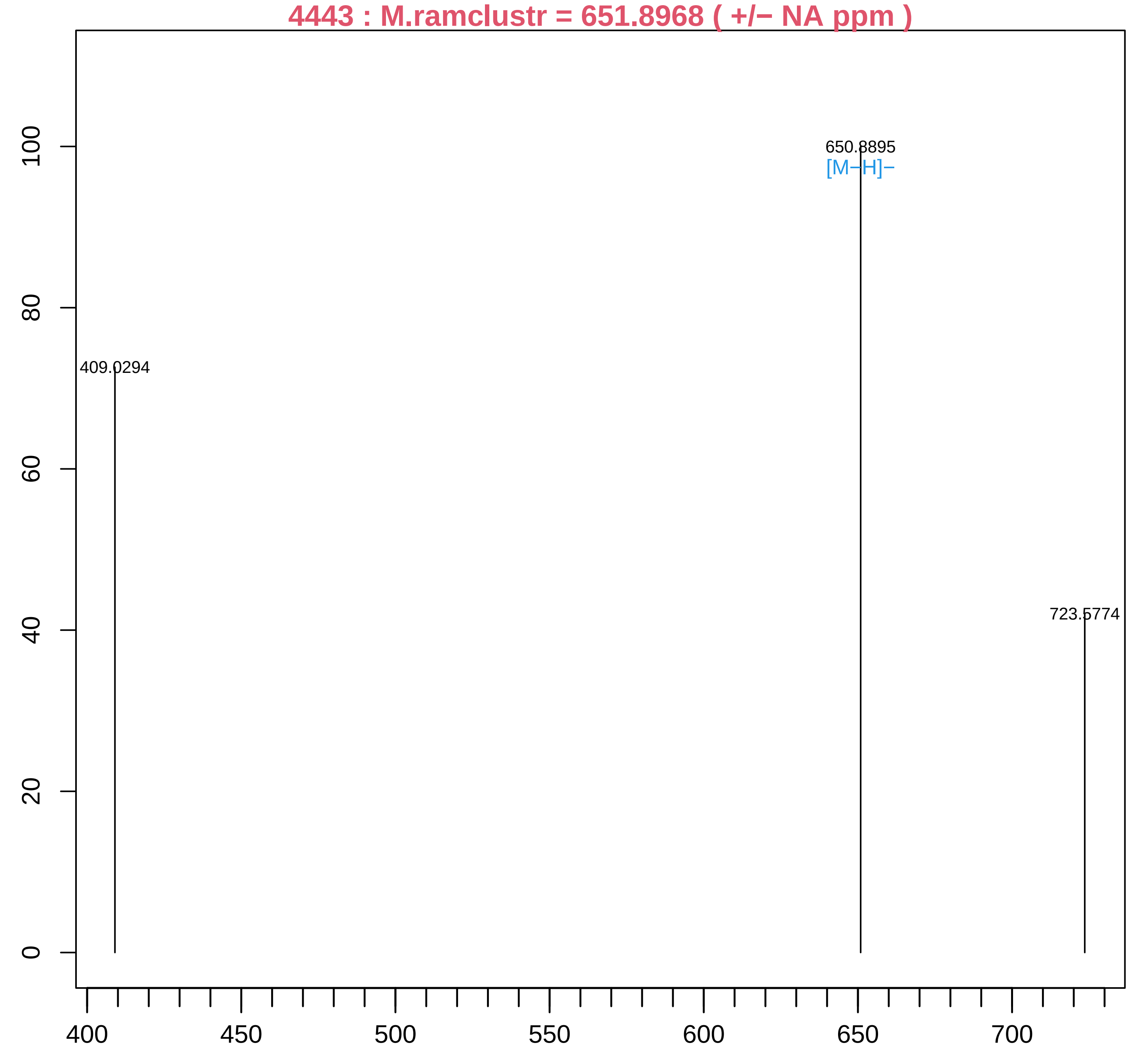
4443 : M.ramclustr = 651.8968 (+/− NA ppm)
409.0294
650.8895
[M−H]−
723.5774
0
20
40
60
80
100
400
450
500
550
600
650
700

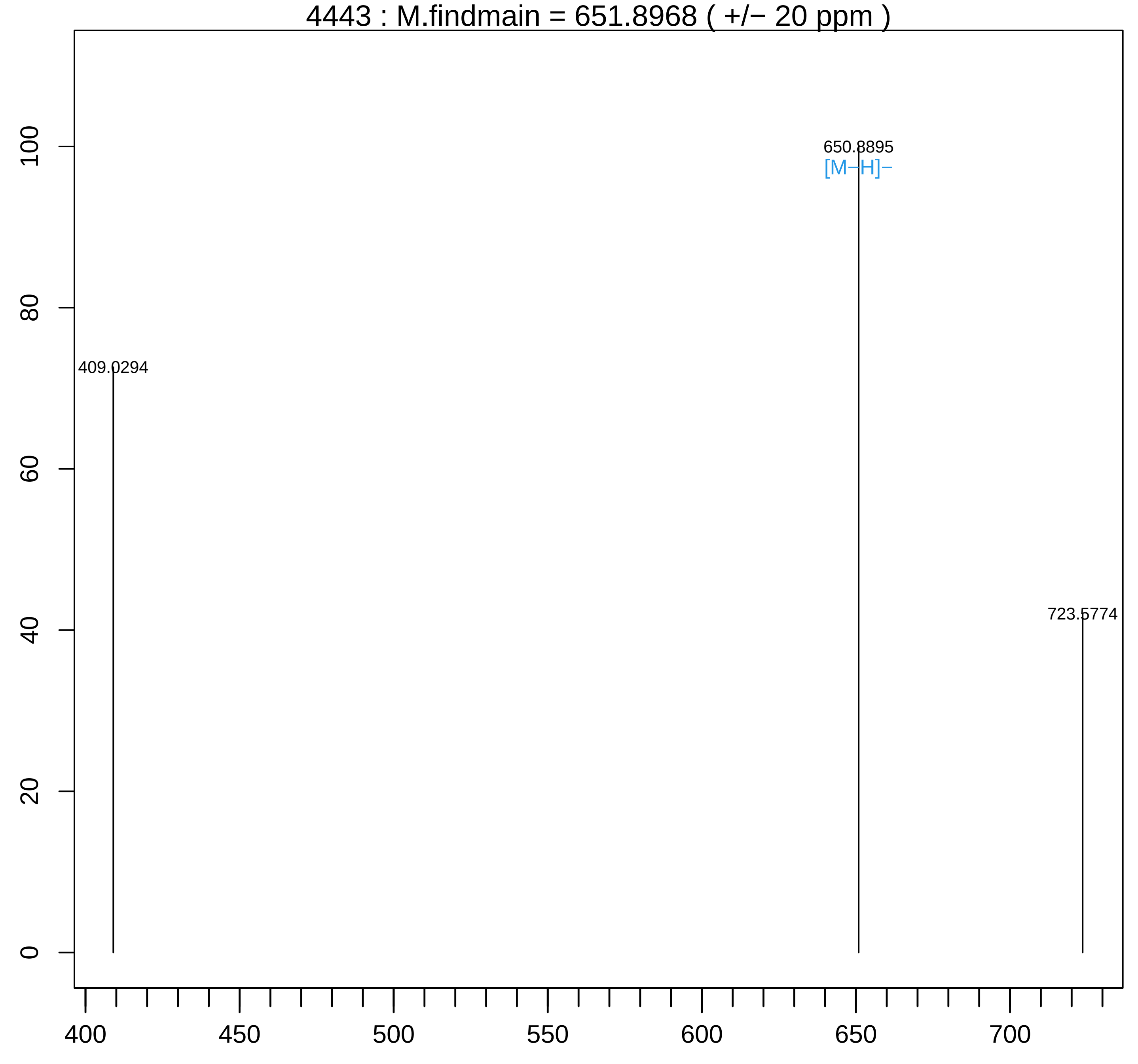
4443 : M.findmain = 651.8968 (+/− 20 ppm)
409.0294
650.8895
[M−H]−
723.5774
0
20
40
60
80
100
400
450
500
550
600
650
700

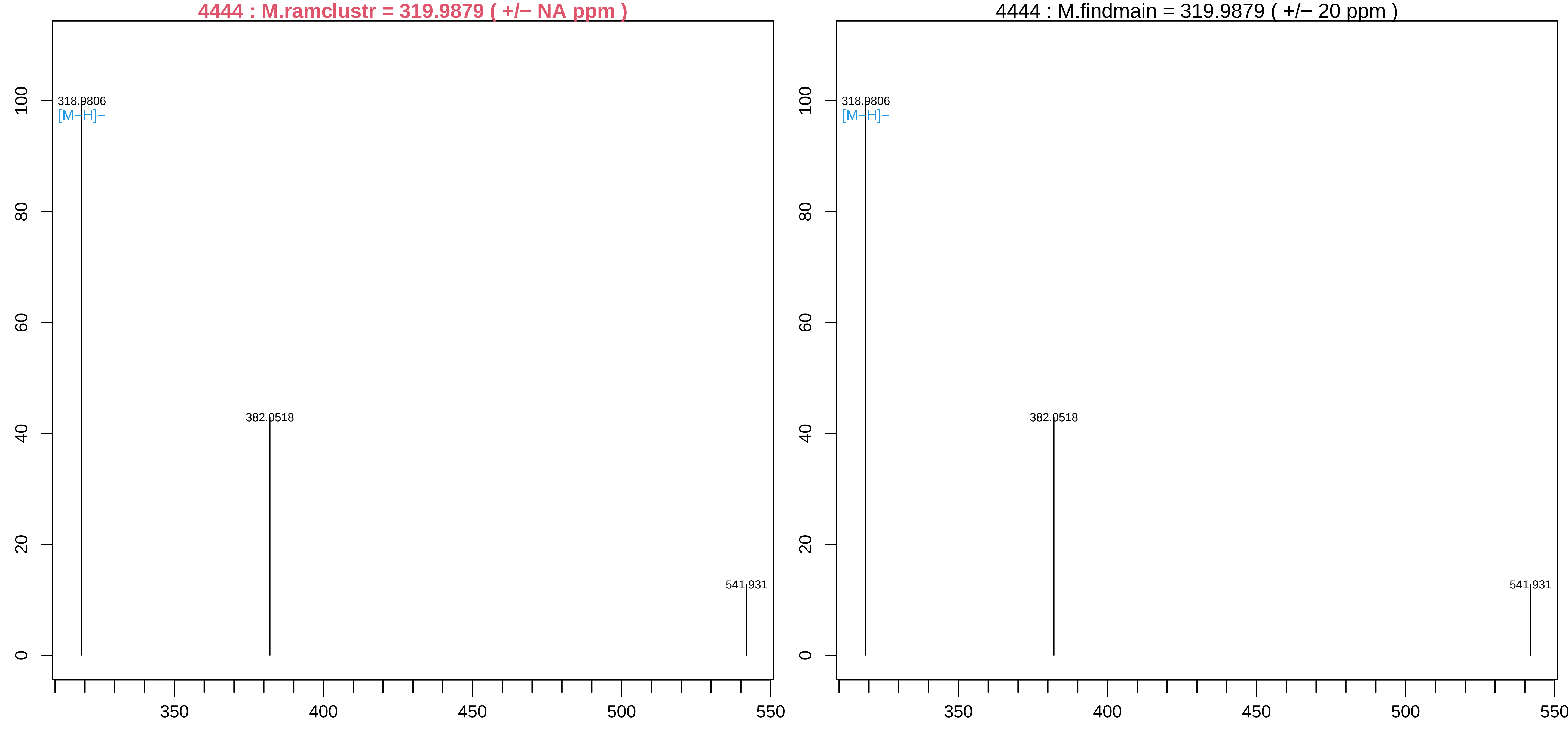
4444 : M.ramclustr = 319.9879 (+/− NA ppm)
318.9806
[M−H]−
382.0518
541.931
0
20
40
60
80
100
350
400
450
500
550
4444 : M.findmain = 319.9879 (+/− 20 ppm)
318.9806
[M−H]−
382.0518
541.931
0
20
40
60
80
100
350
400
450
500
550

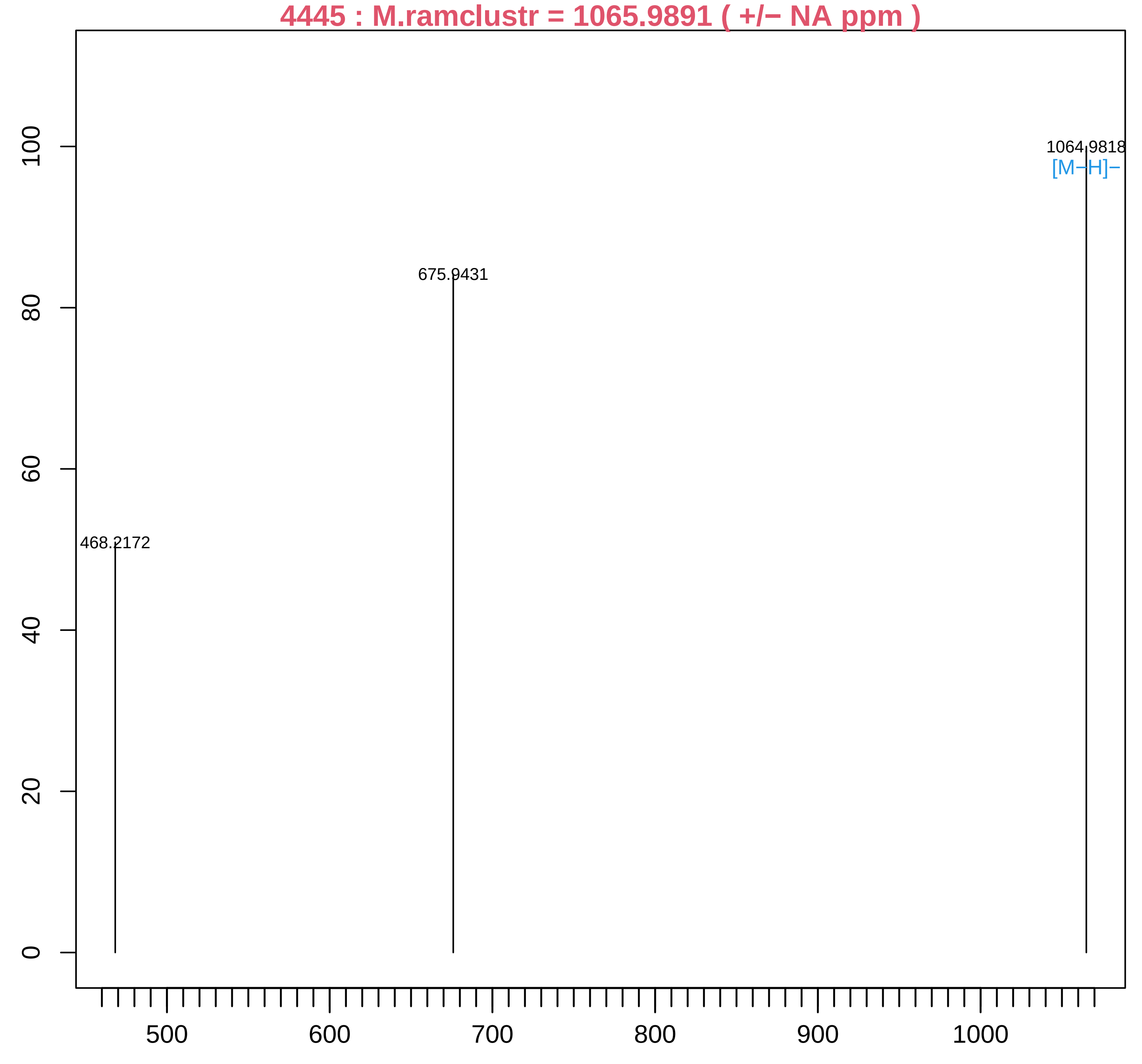
4445 : M.ramclustr = 1065.9891 (+/− NA ppm)
1064.9818
[M−H]−
675.9431
468.2172
0
20
40
60
80
100
500
600
700
800
900
1000

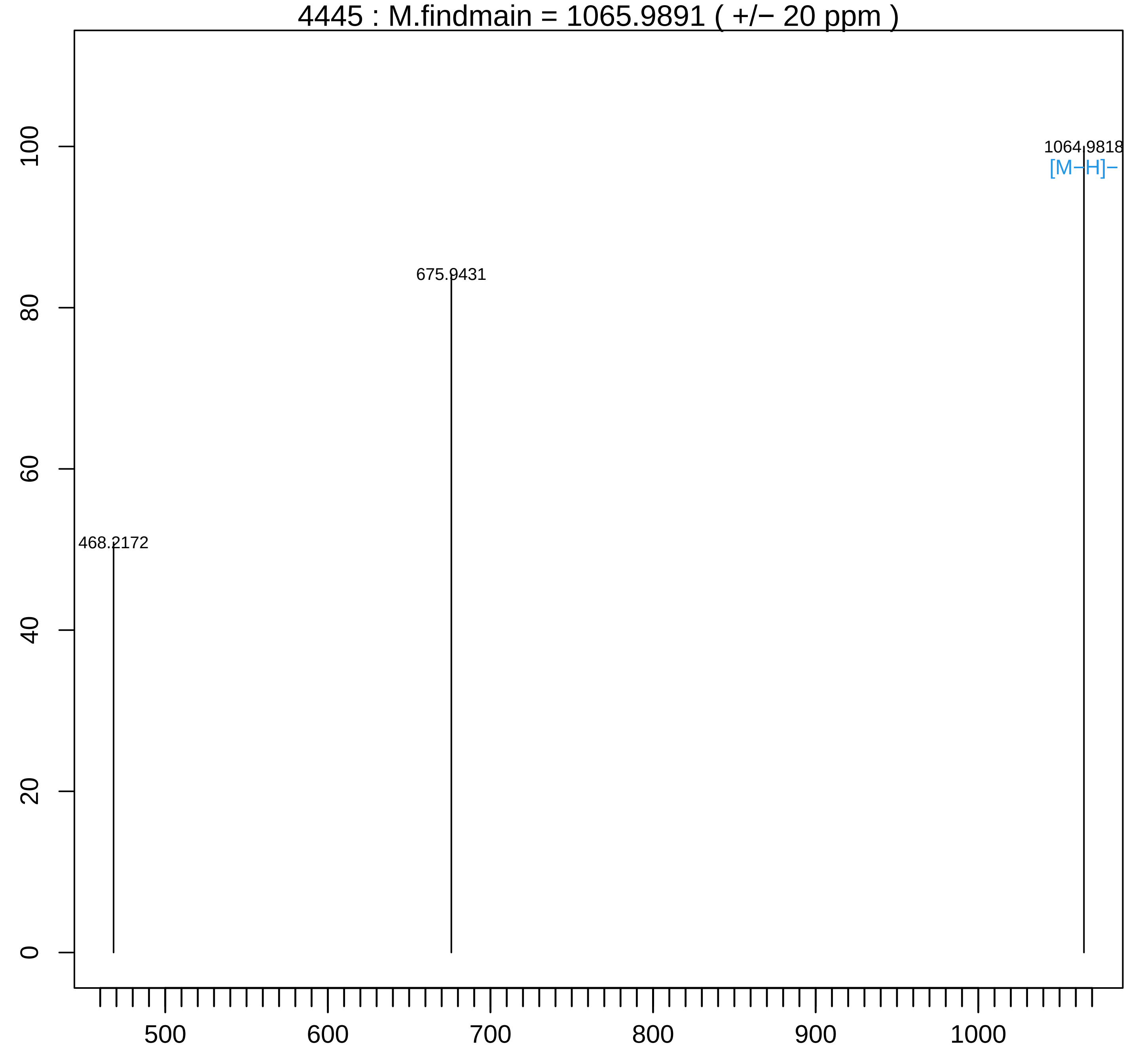
4445 : M.findmain = 1065.9891 (+/− 20 ppm)
1064.9818
[M−H]−
675.9431
468.2172
0
20
40
60
80
100
500
600
700
800
900
1000

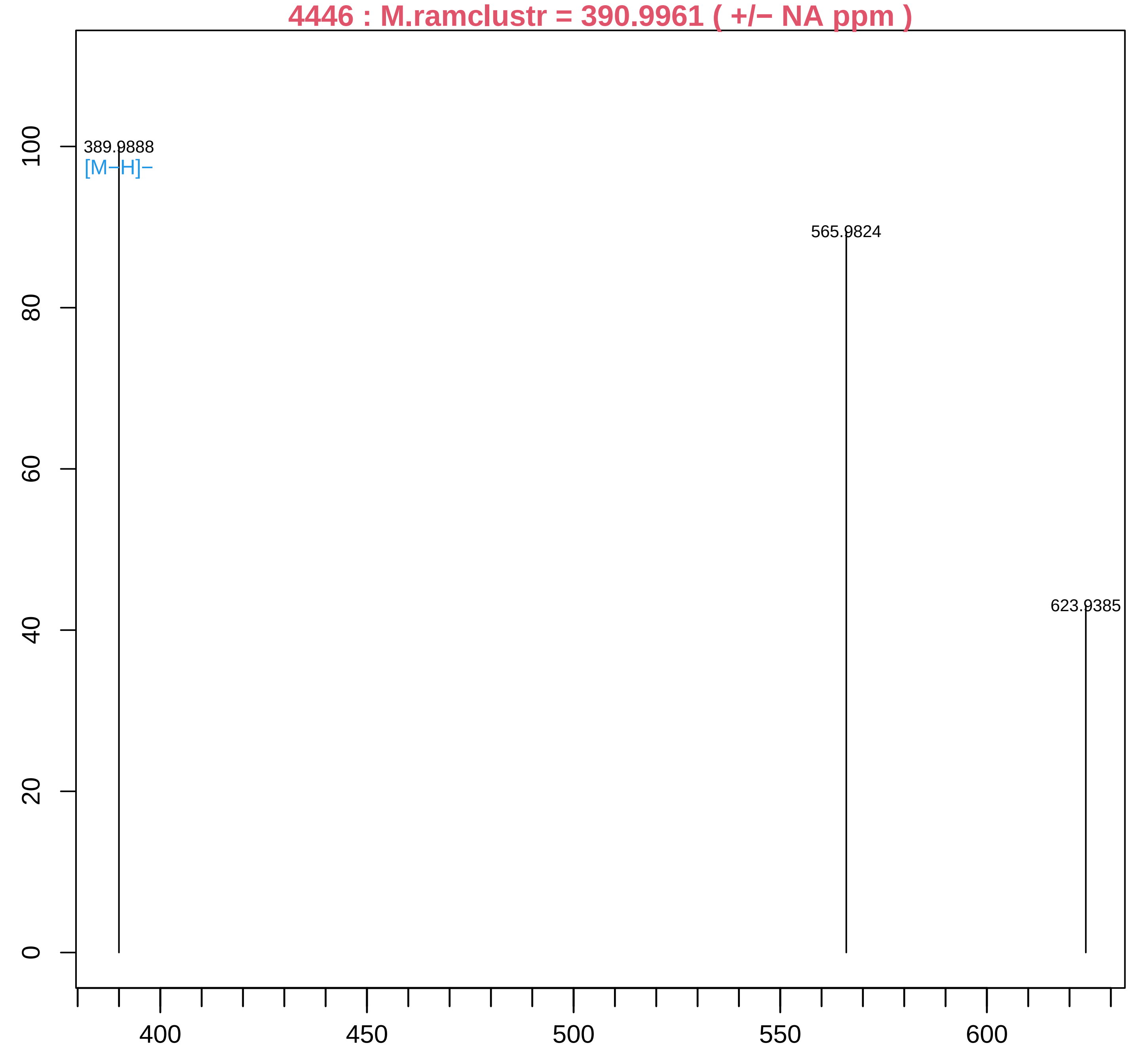
4446 : M.ramclustr = 390.9961 (+/− NA ppm)
389.9888
[M−H]−
565.9824
623.9385
0
20
40
60
80
100
400
450
500
550
600

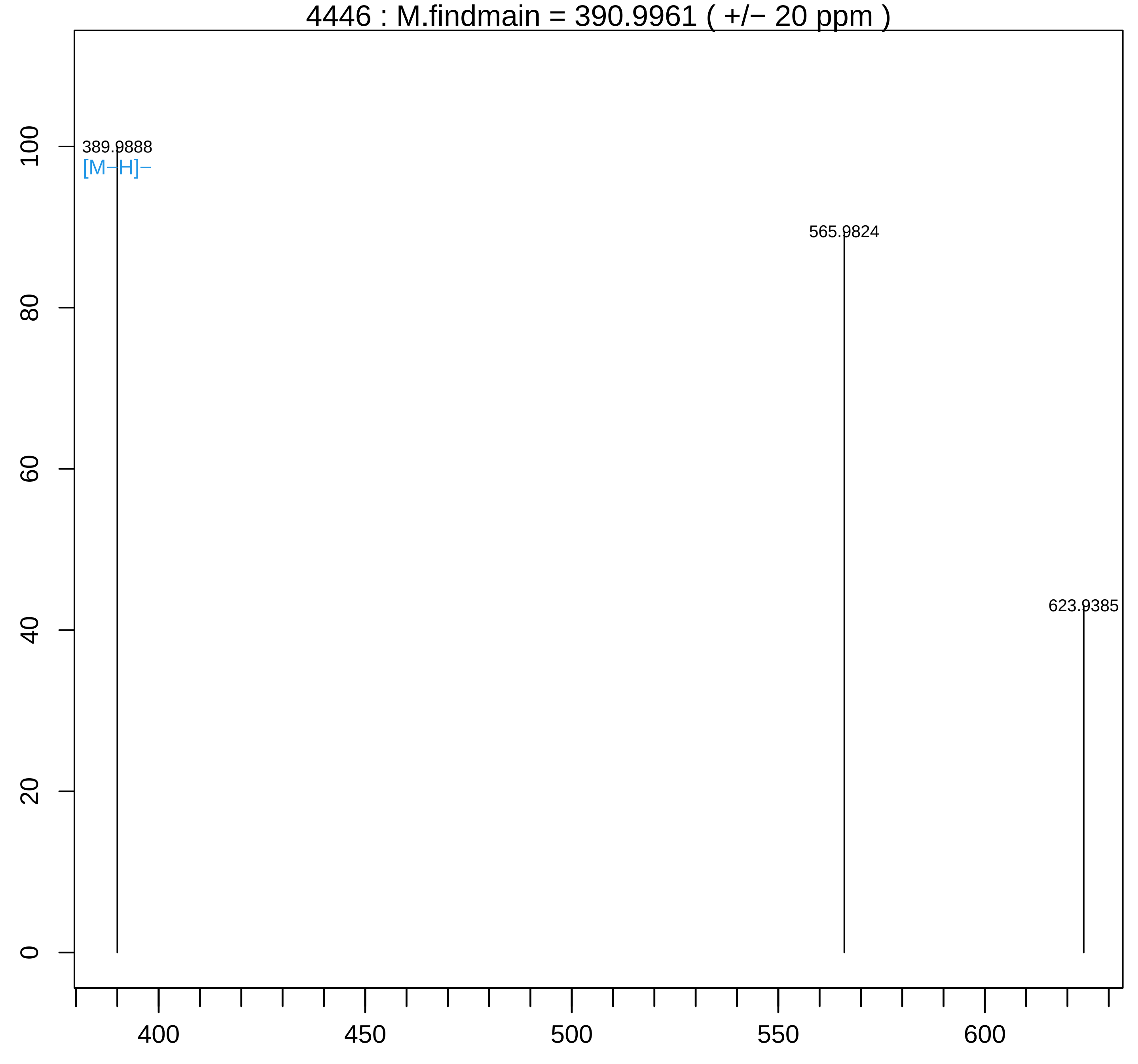
4446 : M.findmain = 390.9961 (+/− 20 ppm)
389.9888
[M−H]−
565.9824
623.9385
0
20
40
60
80
100
400
450
500
550
600

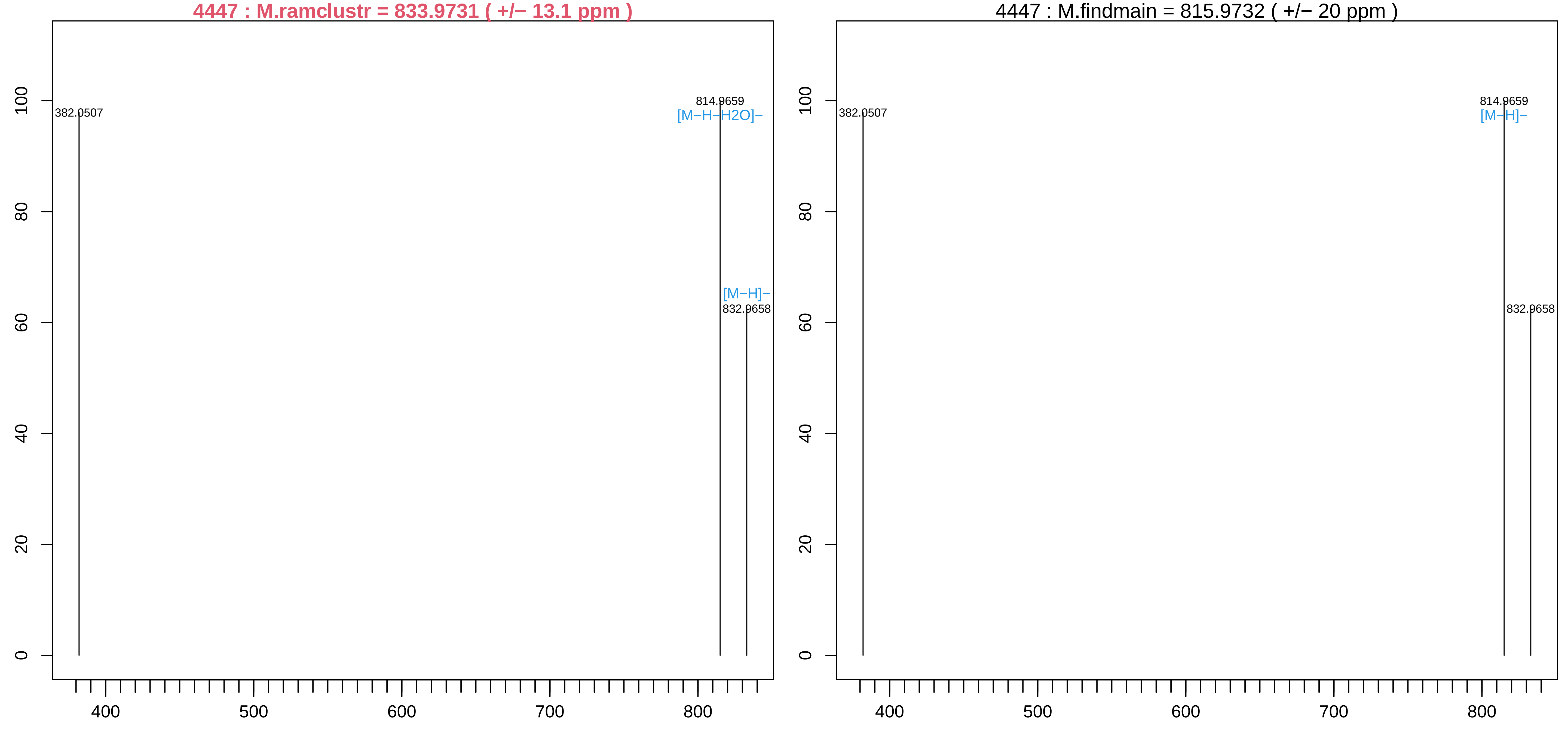

4447 : M.ramclustr = 833.9731 (+/− 13.1 ppm)
382.0507
814.9659
[M−H−H2O]−
[M−H]−
832.9658
4447 : M.findmain = 815.9732 (+/− 20 ppm)
382.0507
814.9659
[M−H]−
832.9658

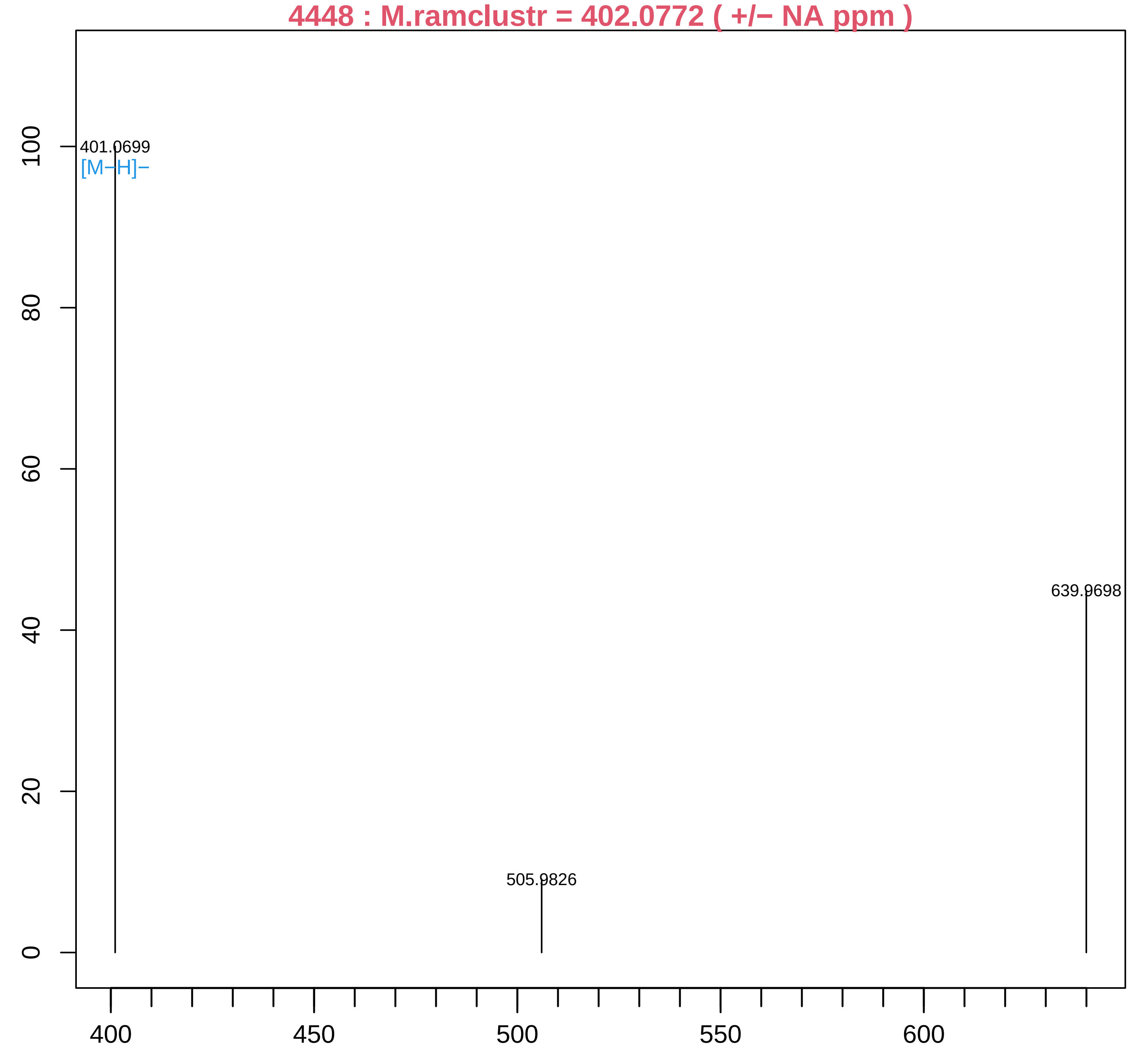
4448 : M.ramclustr = 402.0772 (+/− NA ppm)
401.0699
[M−H]−
505.9826
639.9698
0
20
40
60
80
100
400
450
500
550
600

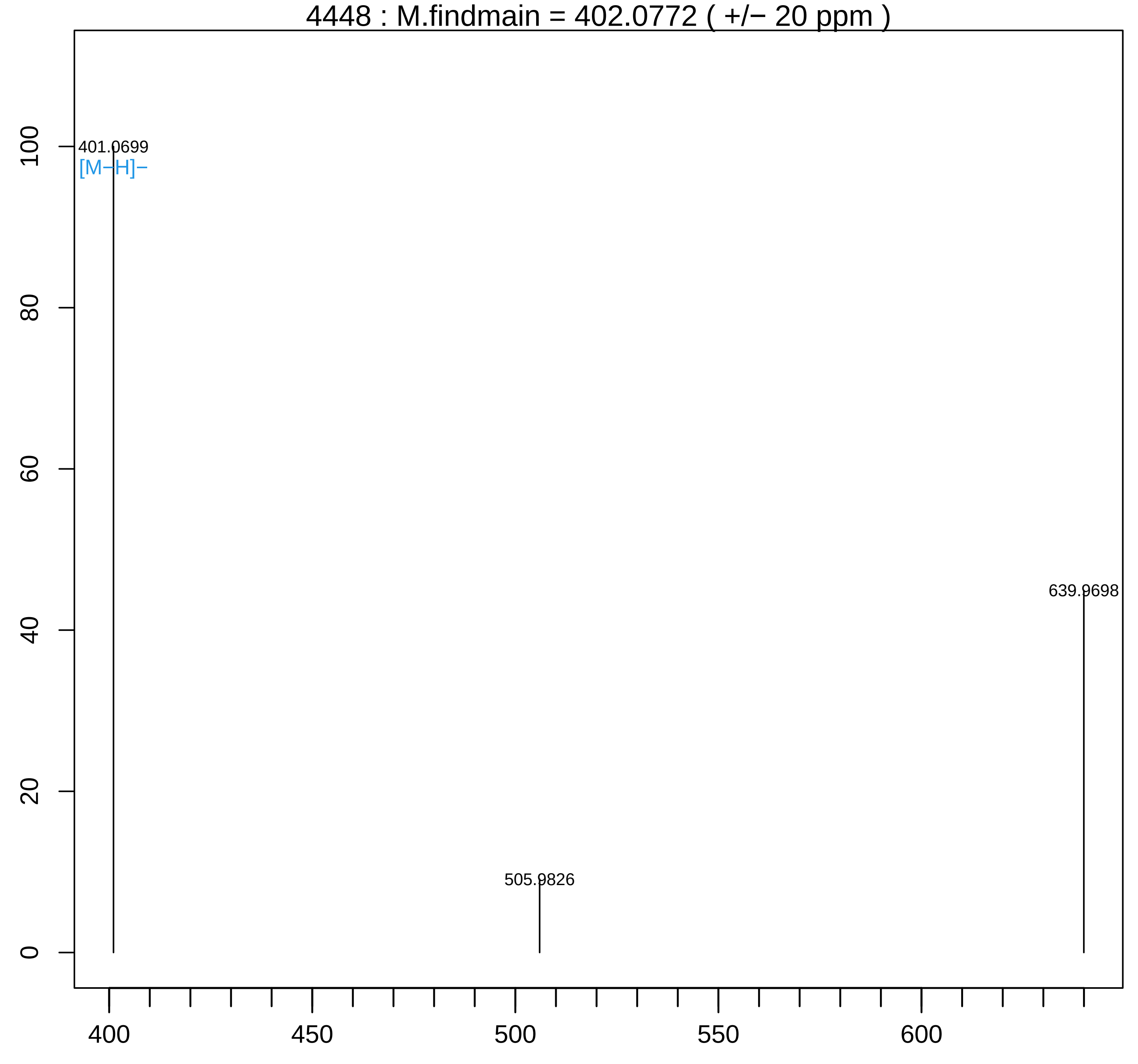
4448 : M.findmain = 402.0772 (+/− 20 ppm)
401.0699
[M−H]−
505.9826
639.9698
0
20
40
60
80
100
400
450
500
550
600

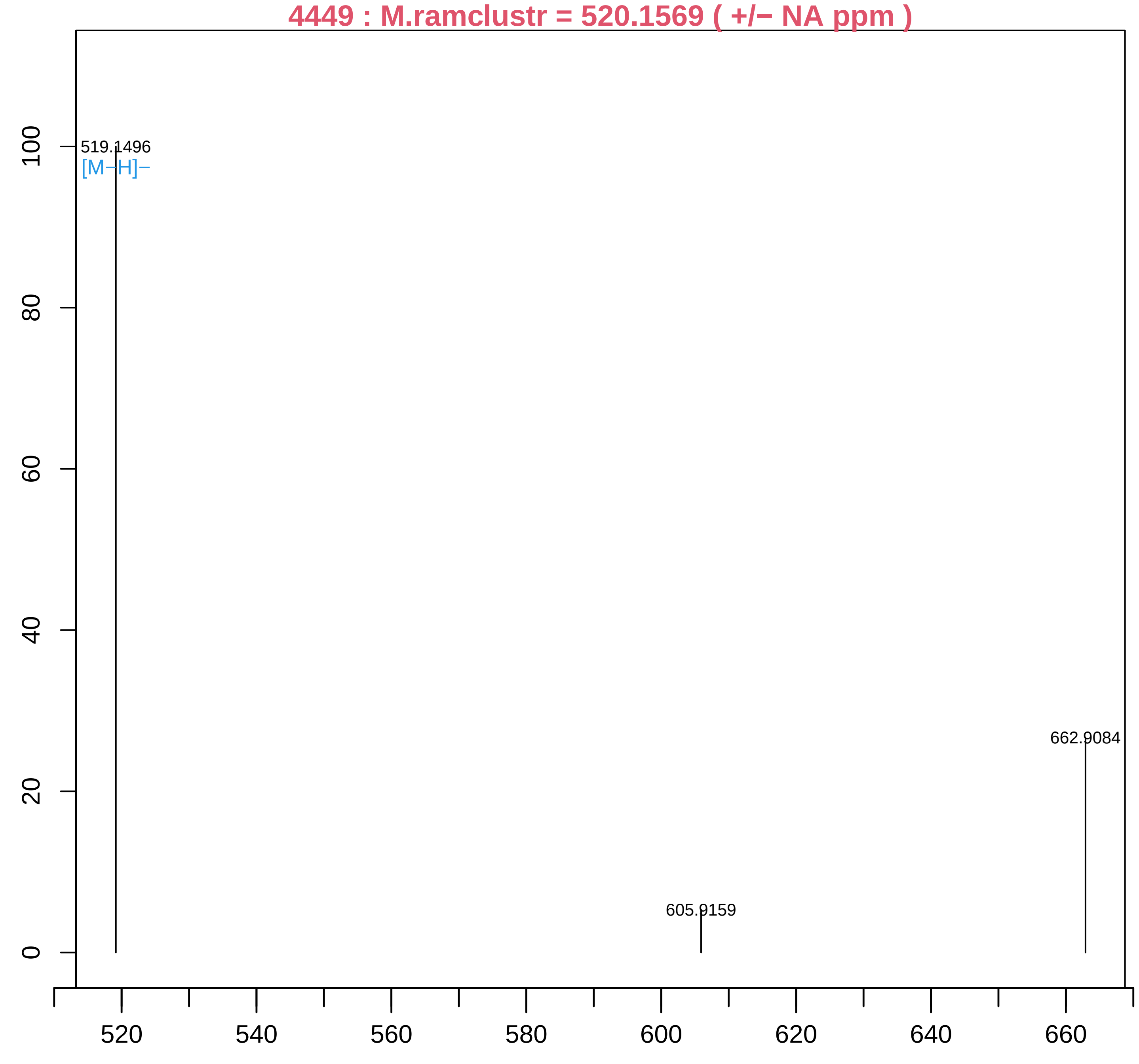
4449 : M.ramclustr = 520.1569 (+/− NA ppm)
519.1496
[M−H]−
605.9159
662.9084
0
20
40
60
80
100
520
540
560
580
600
620
640
660

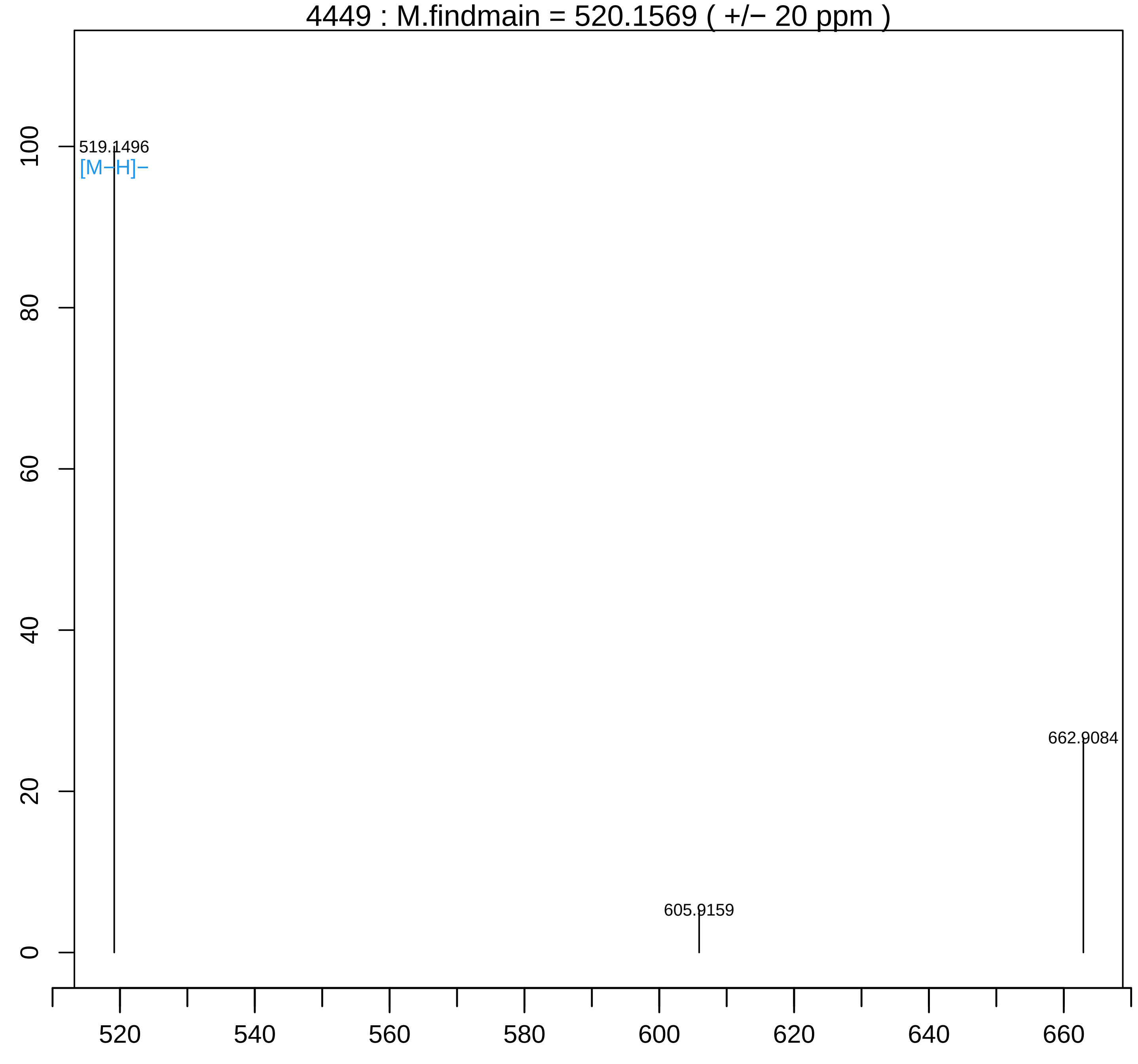
4449 : M.findmain = 520.1569 (+/− 20 ppm)
519.1496
[M−H]−
605.9159
662.9084
0
20
40
60
80
100
520
540
560
580
600
620
640
660

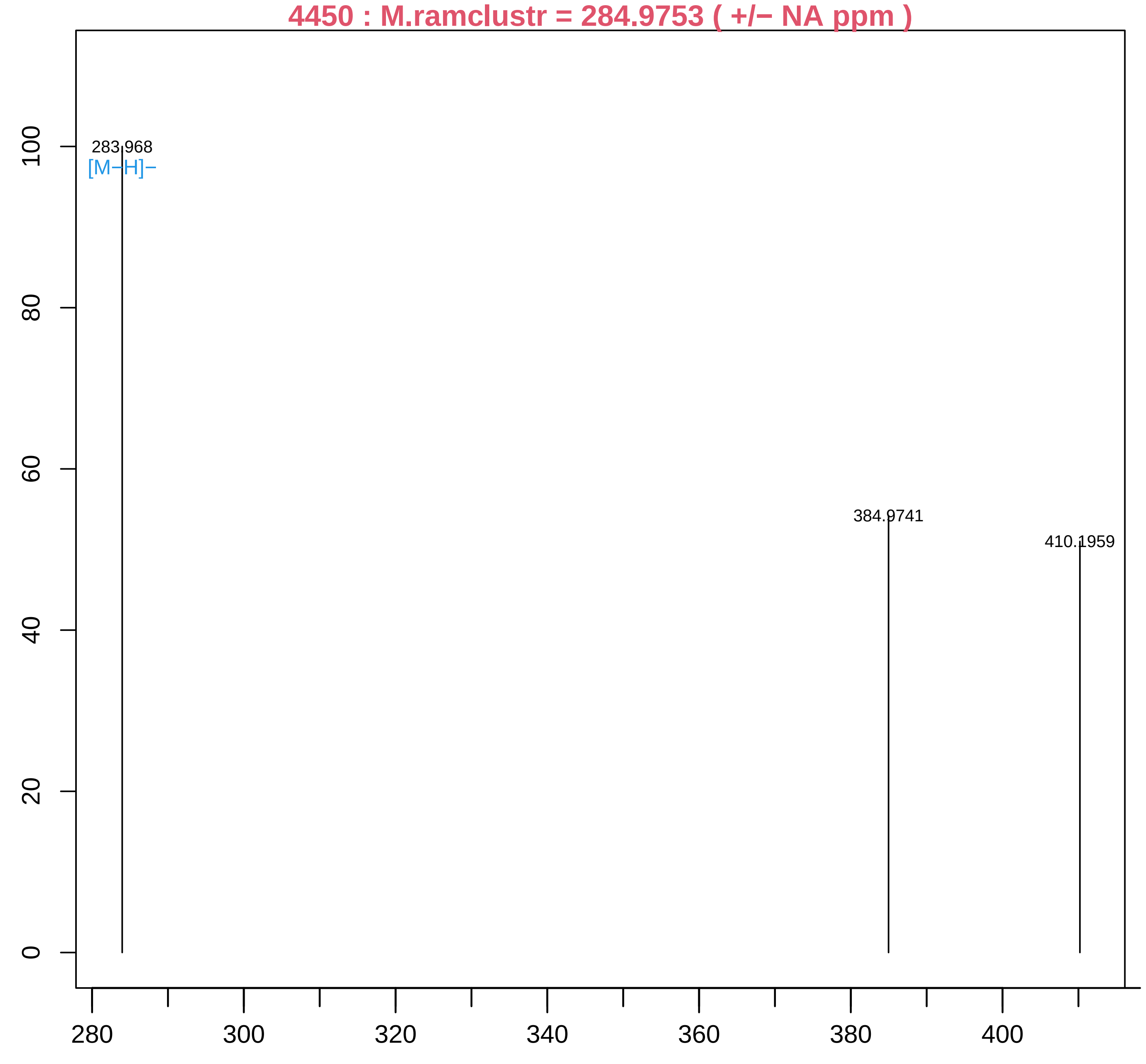
4450 : M.ramclustr = 284.9753 (+/− NA ppm)
283.968
[M−H]−
384.9741
410.1959
0
20
40
60
80
100
280
300
320
340
360
380
400

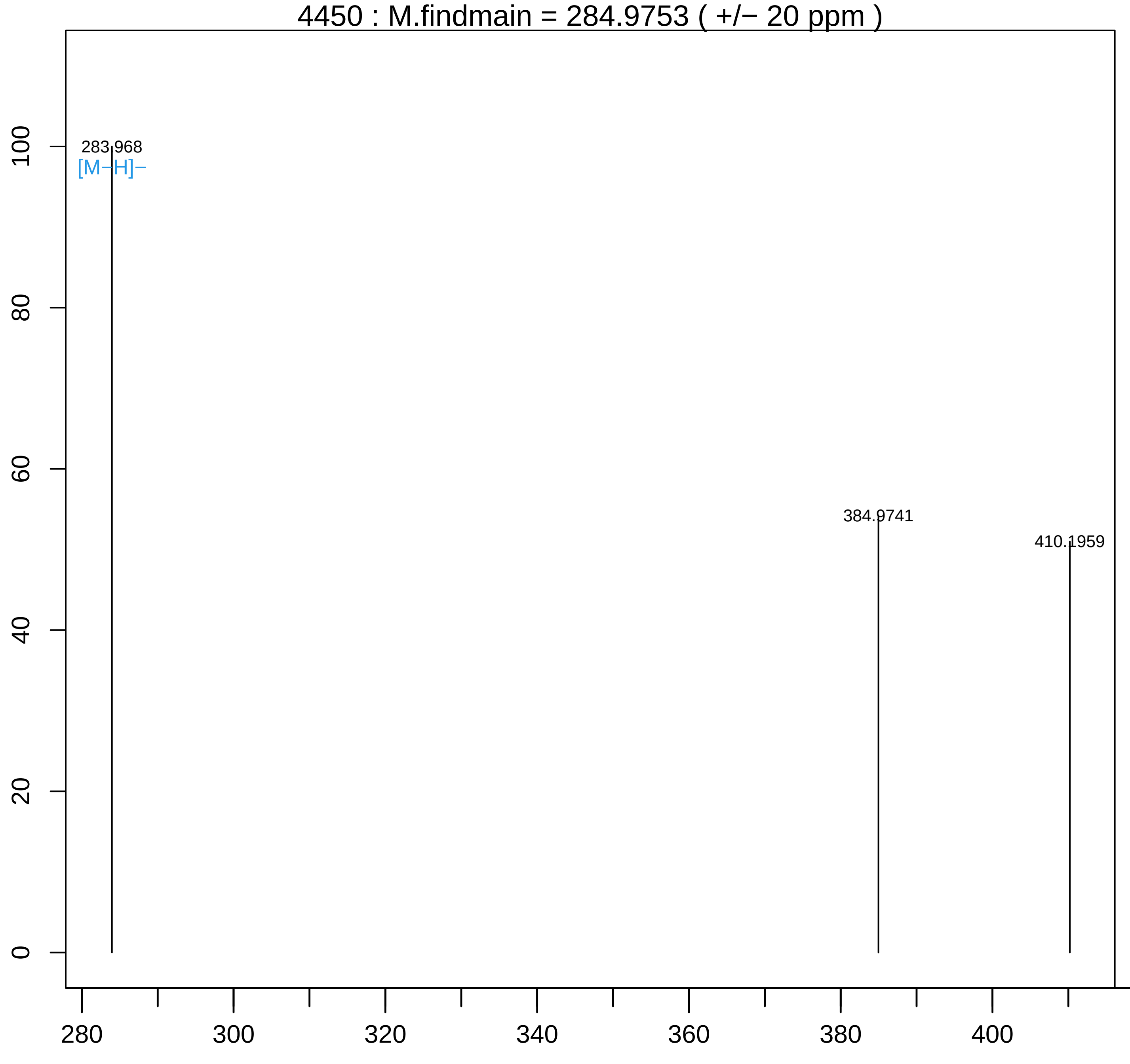
4450 : M.findmain = 284.9753 (+/− 20 ppm)
283.968
[M−H]−
384.9741
410.1959
0
20
40
60
80
100
280
300
320
340
360
380
400

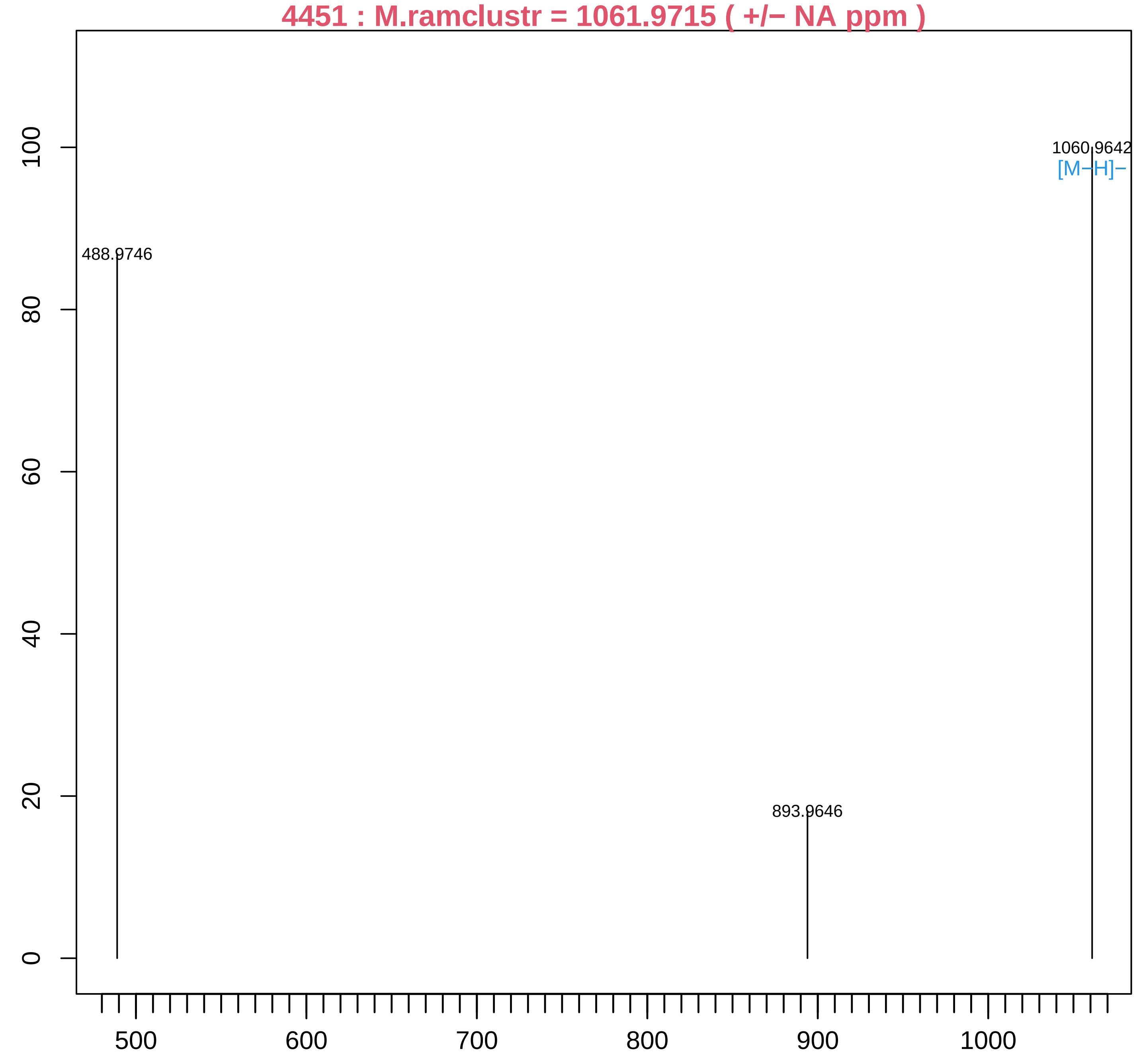
4451 : M.ramclustr = 1061.9715 (+/− NA ppm)
1060.9642
[M−H]−
488.9746
893.9646
0
20
40
60
80
100
500
600
700
800
900
1000

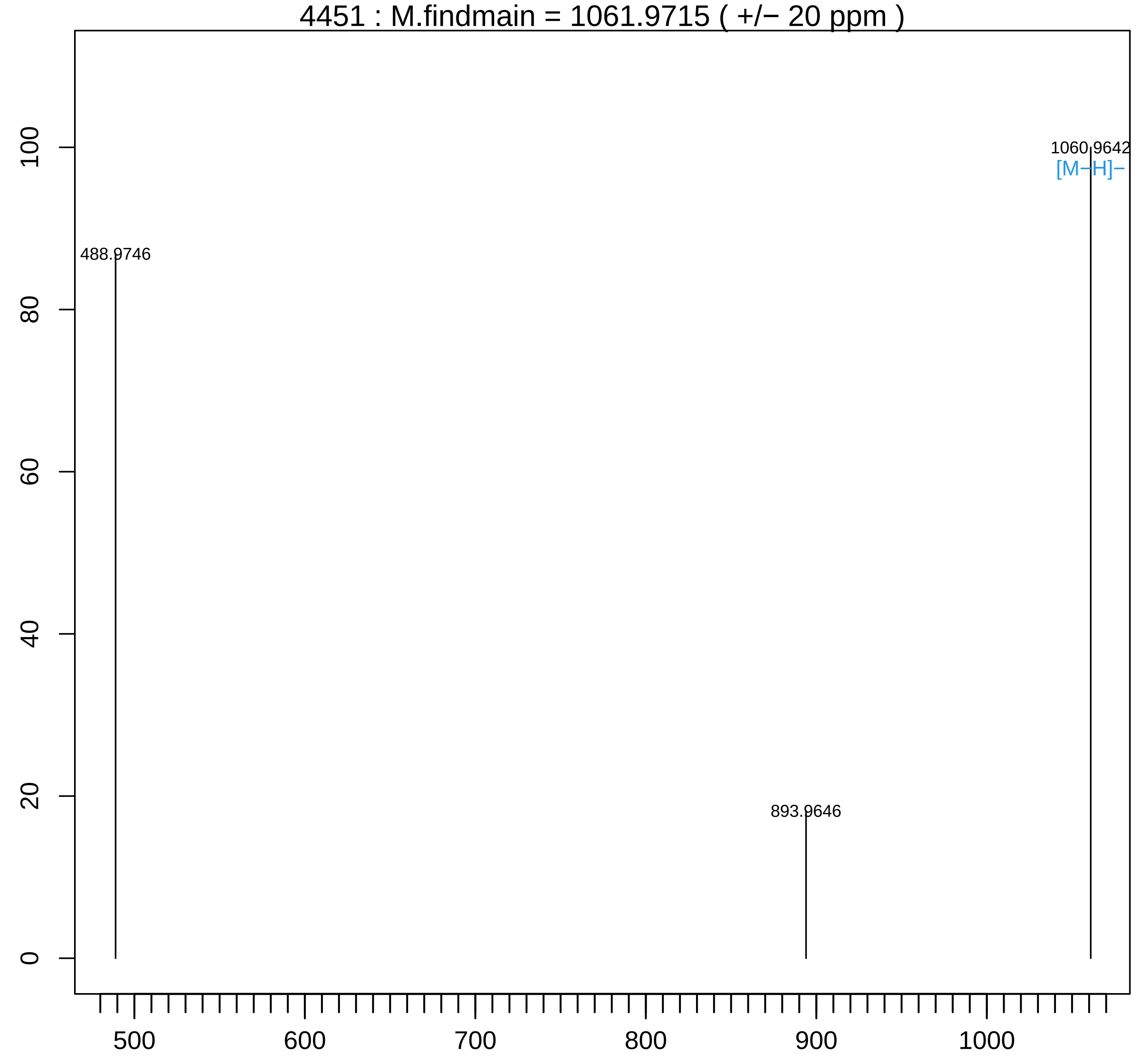
4451 : M.findmain = 1061.9715 (+/− 20 ppm)
1060.9642
[M−H]−
488.9746
893.9646
0
20
40
60
80
100
500
600
700
800
900
1000

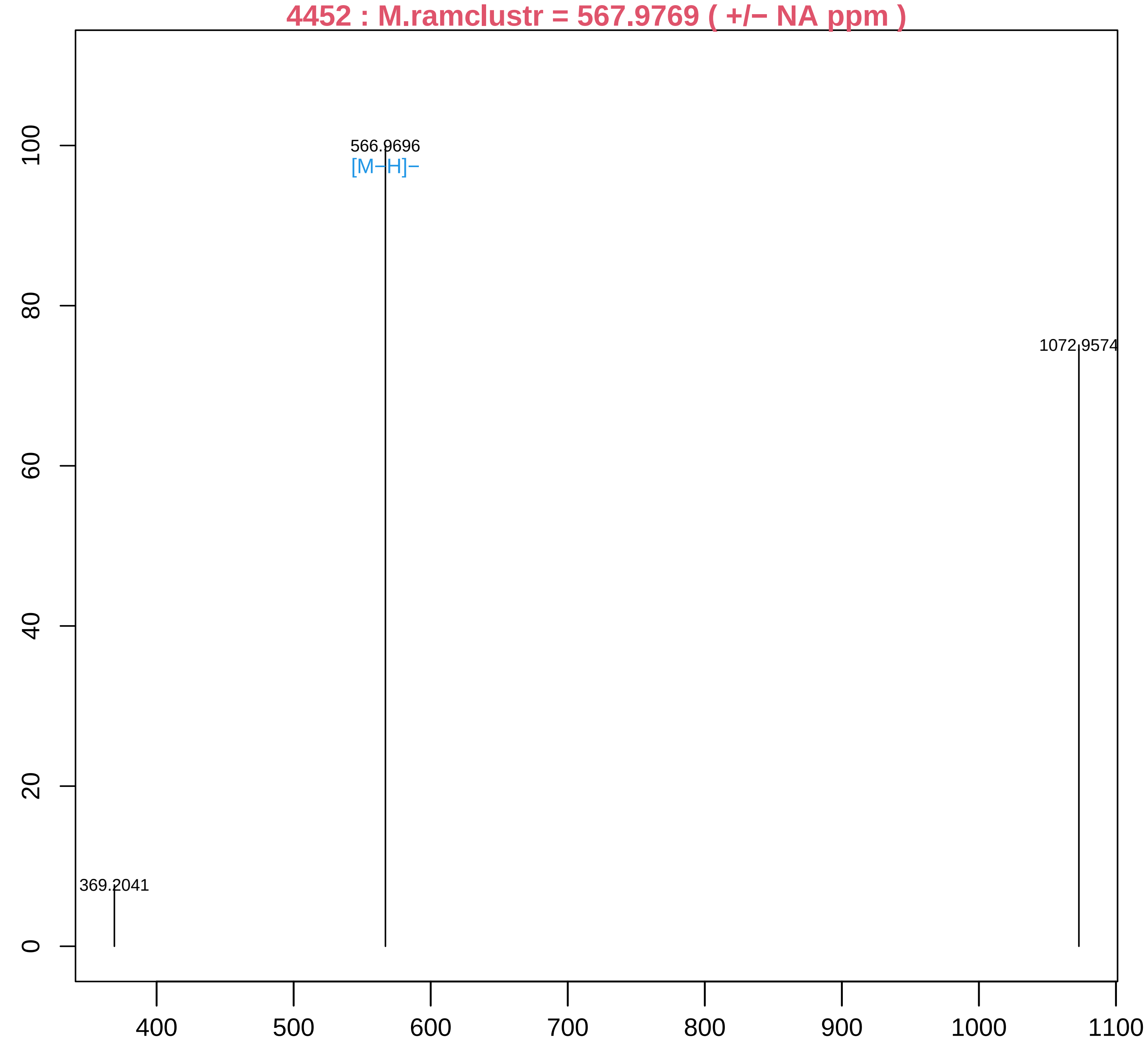

4452 : M.ramclustr = 567.9769 (+/− NA ppm)
566.9696
[M−H]−
1072.9574
369.2041
0
20
40
60
80
100
400
500
600
700
800
900
1000
1100

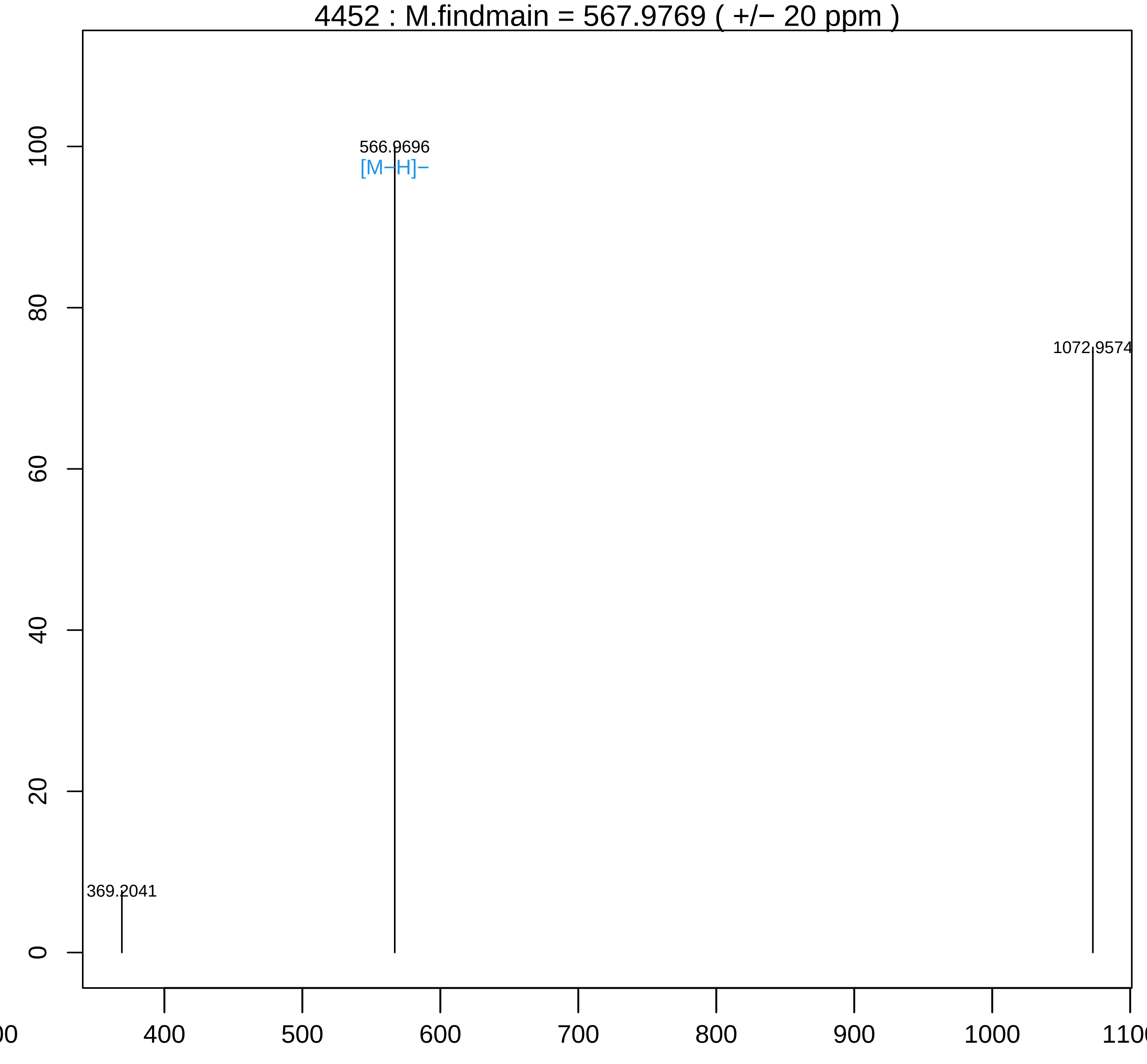

4452 : M.findmain = 567.9769 (+/− 20 ppm)
566.9696
[M−H]−
1072.9574
369.2041
0
20
40
60
80
100
400
500
600
700
800
900
1000
1100

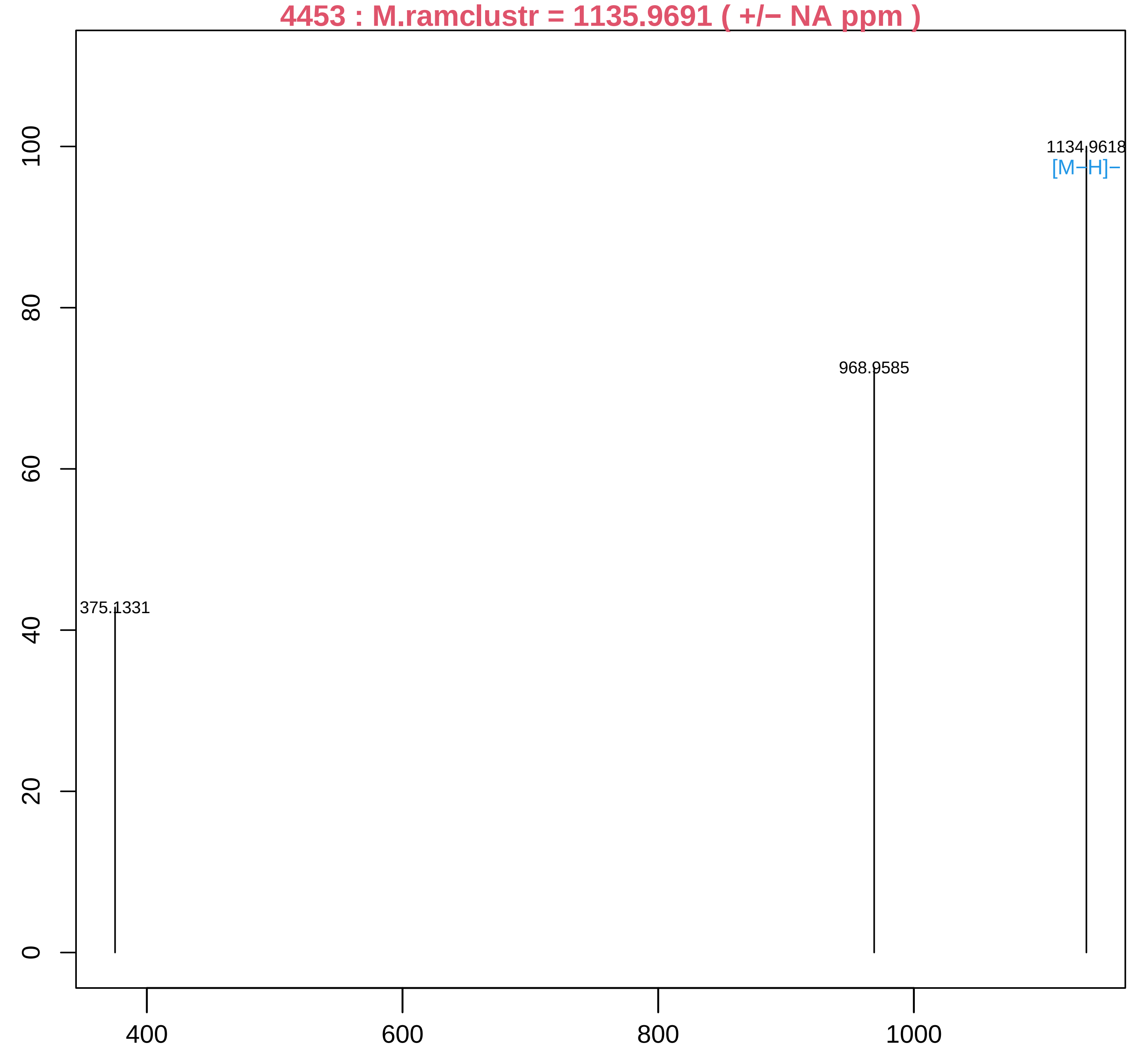
4453 : M.ramclustr = 1135.9691 (+/− NA ppm)
1134.9618
[M−H]−
968.9585
375.1331
0
20
40
60
80
100
400
600
800
1000

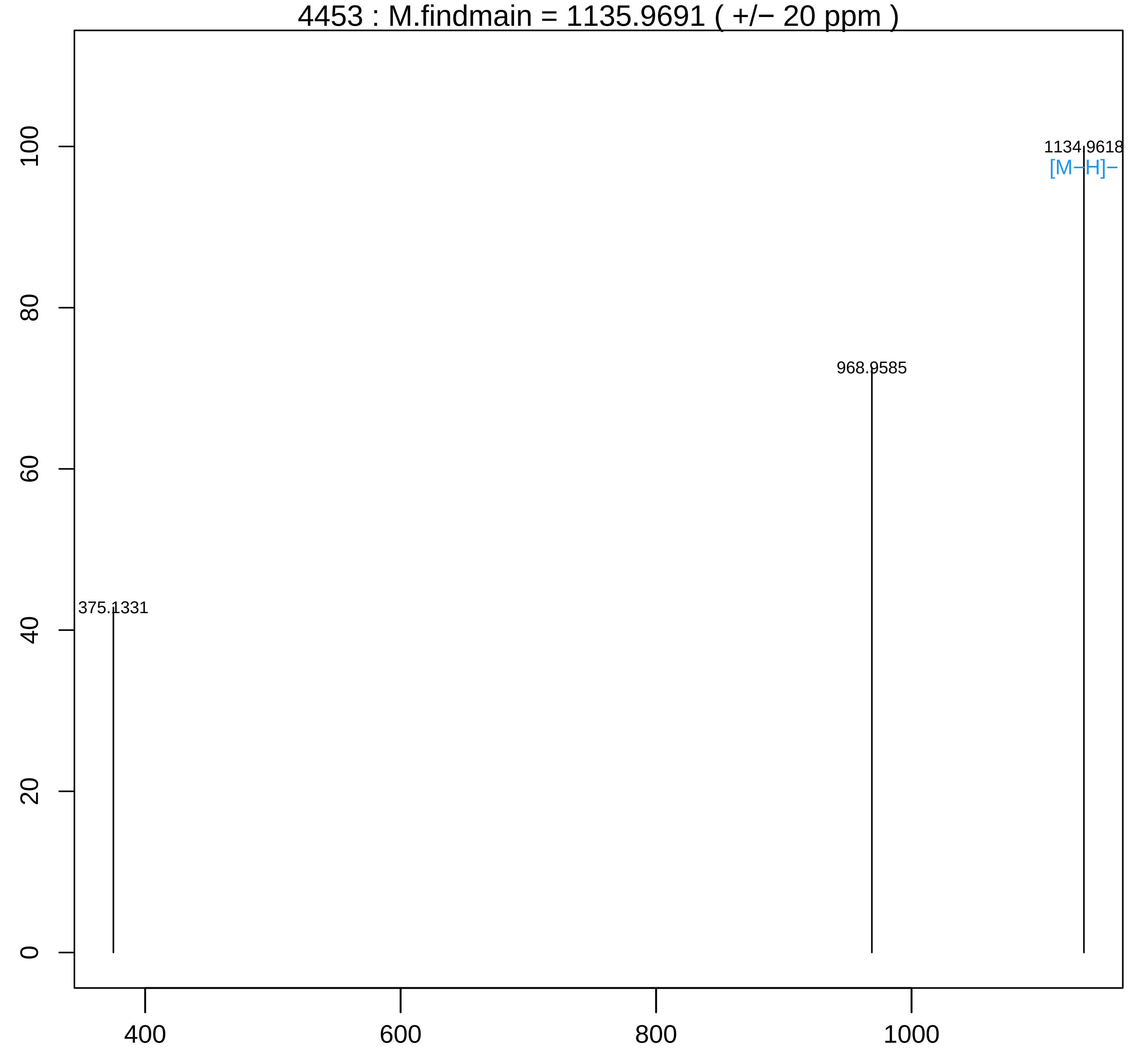
4453 : M.findmain = 1135.9691 (+/− 20 ppm)
1134.9618
[M−H]−
968.9585
375.1331
0
20
40
60
80
100
400
600
800
1000

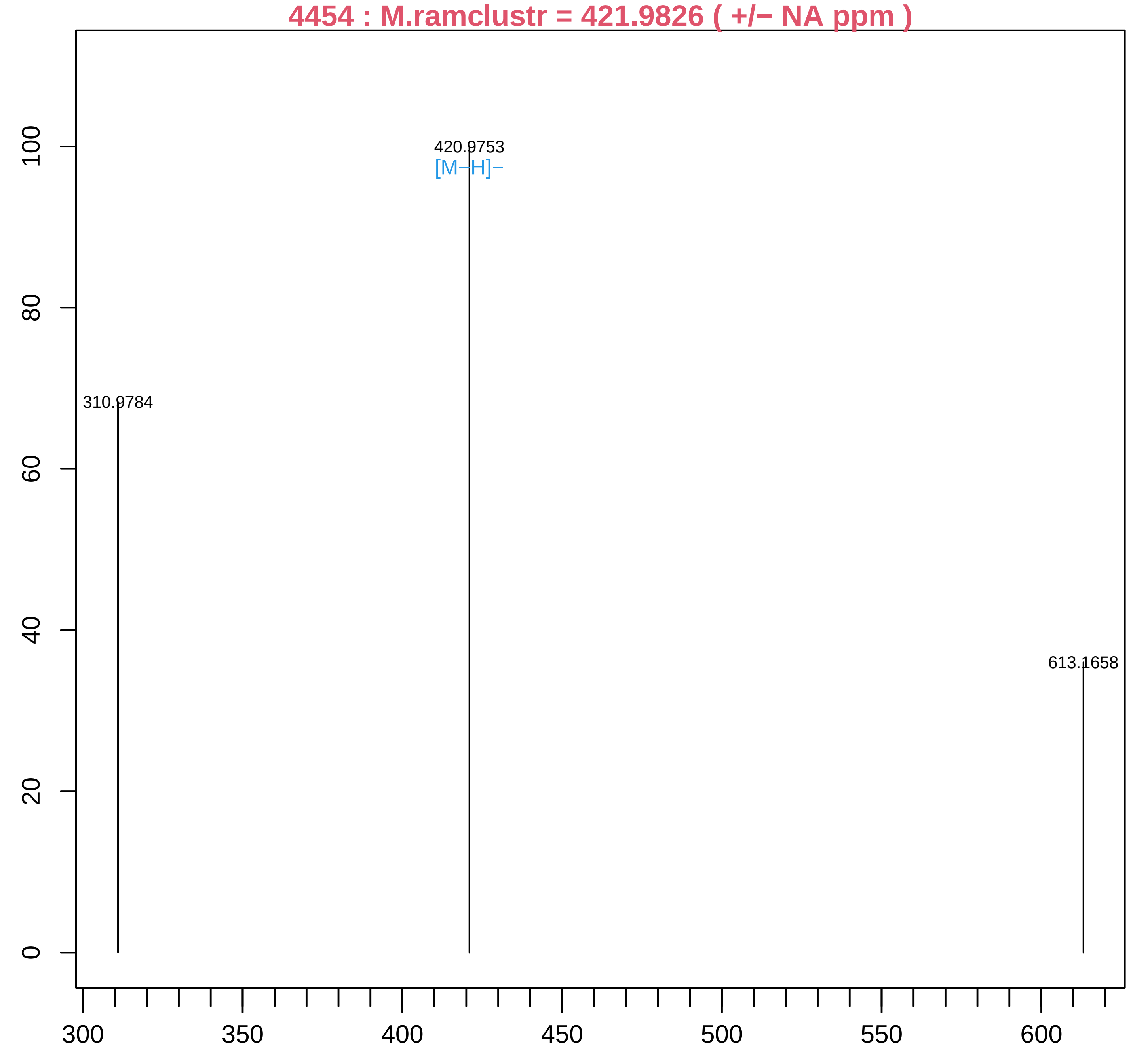
4454 : M.ramclustr = 421.9826 (+/− NA ppm)
420.9753
[M−H]−
310.9784
613.1658
0
20
40
60
80
100
300
350
400
450
500
550
600

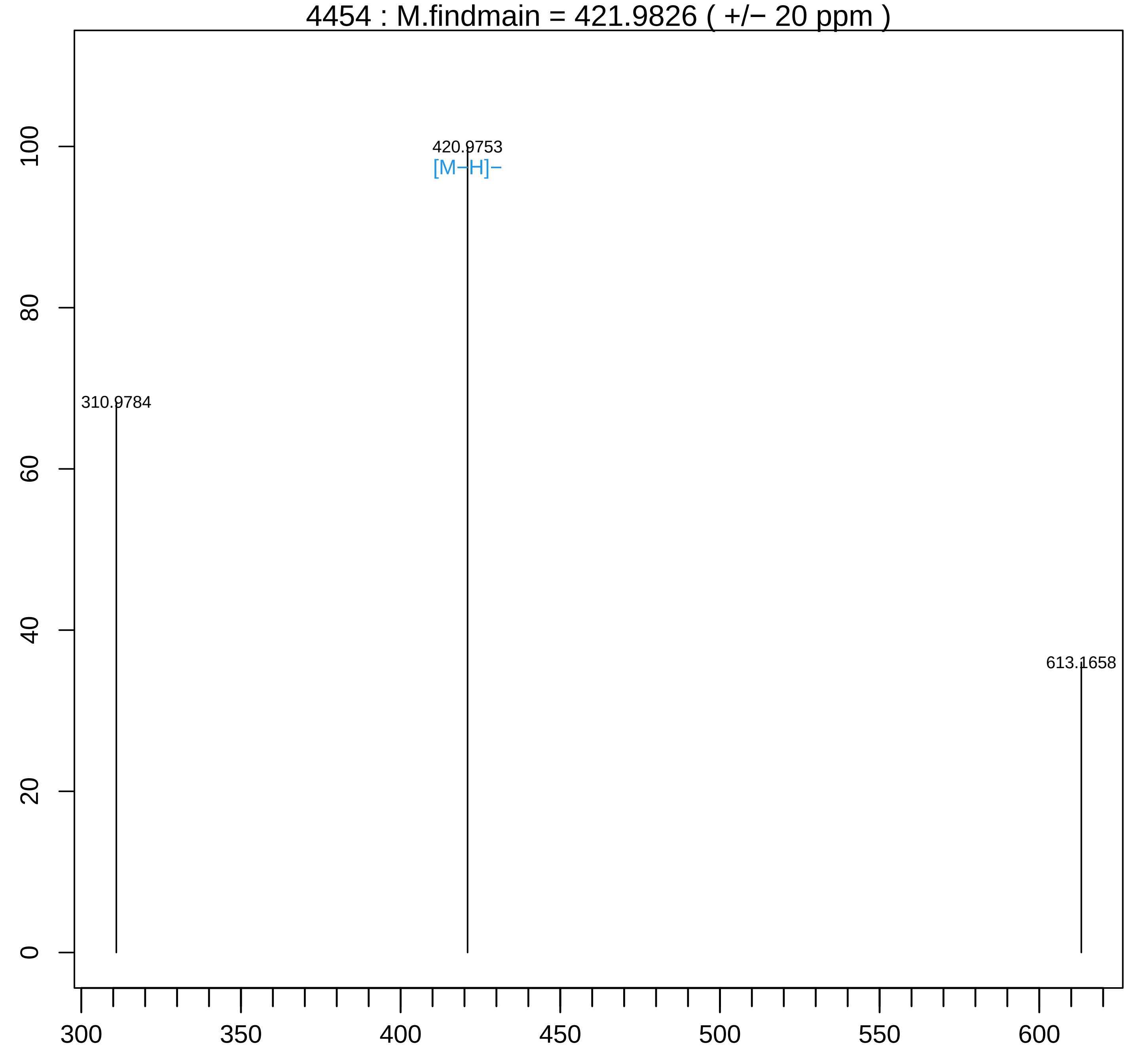
4454 : M.findmain = 421.9826 (+/− 20 ppm)
420.9753
[M−H]−
310.9784
613.1658
0
20
40
60
80
100
300
350
400
450
500
550
600

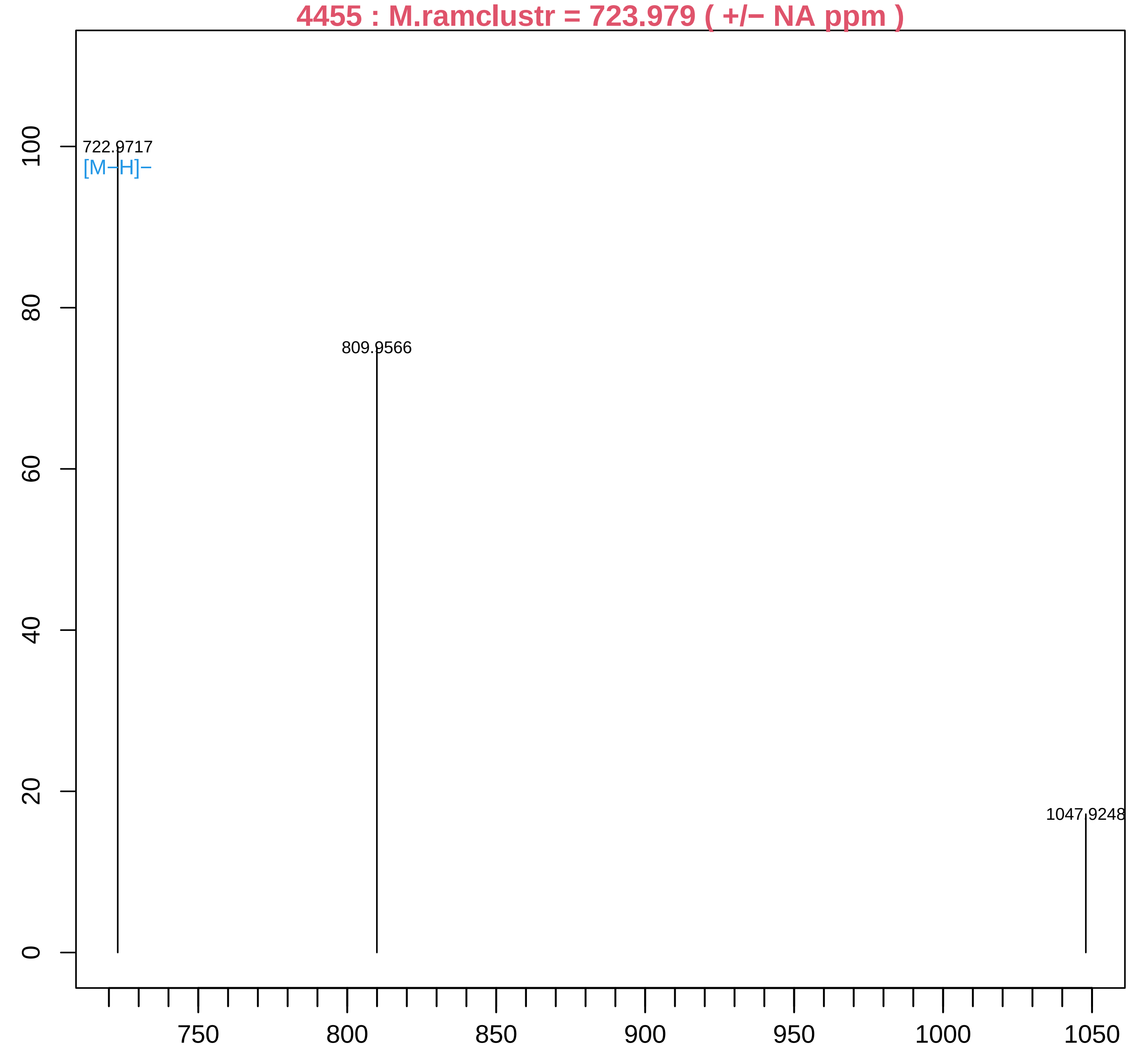
4455 : M.ramclustr = 723.979 (+/− NA ppm)
722.9717
[M−H]−
809.9566
1047.9248
0
20
40
60
80
100
750
800
850
900
950
1000
1050

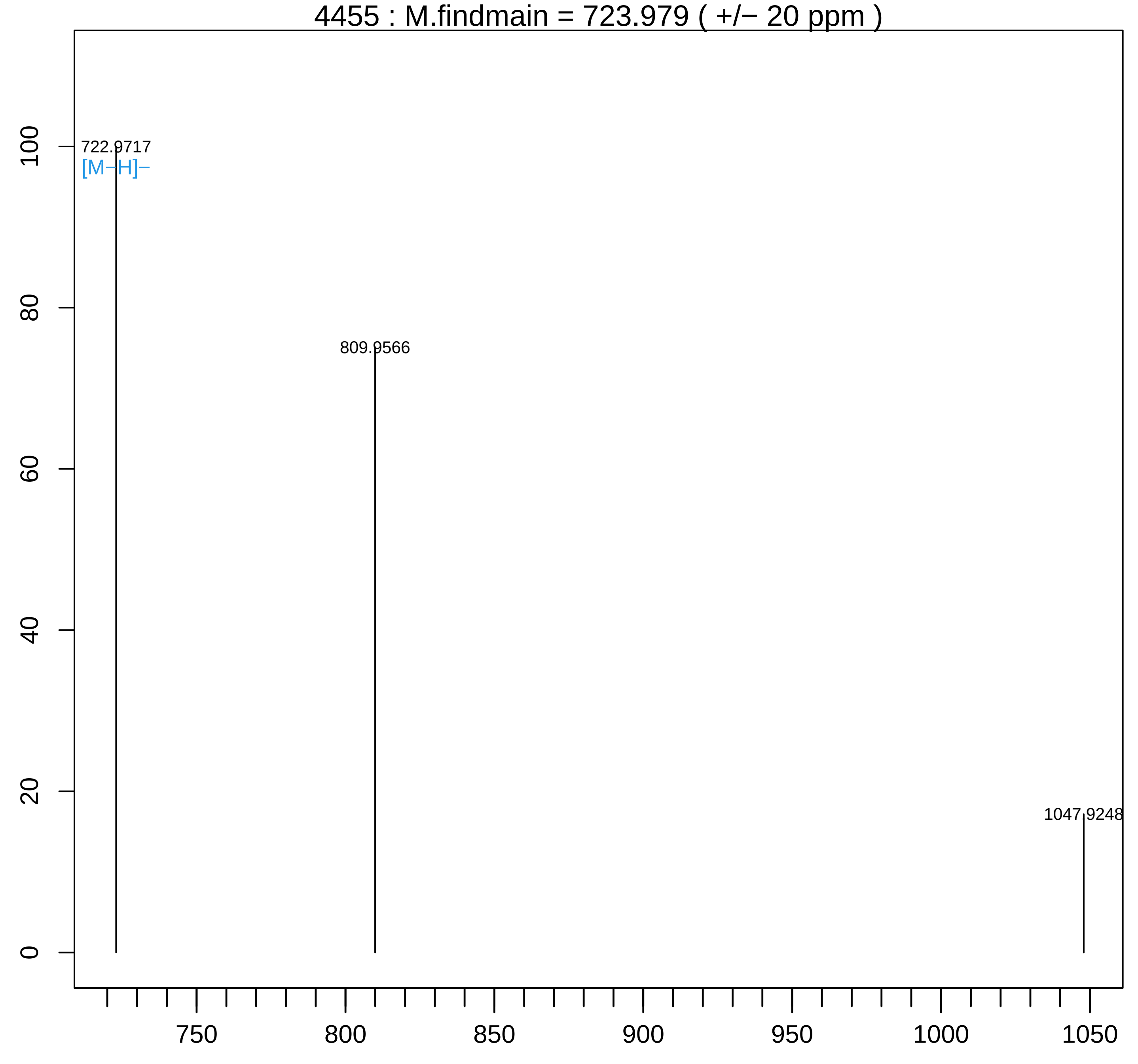
4455 : M.findmain = 723.979 (+/− 20 ppm)
722.9717
[M−H]−
809.9566
1047.9248
0
20
40
60
80
100
750
800
850
900
950
1000
1050

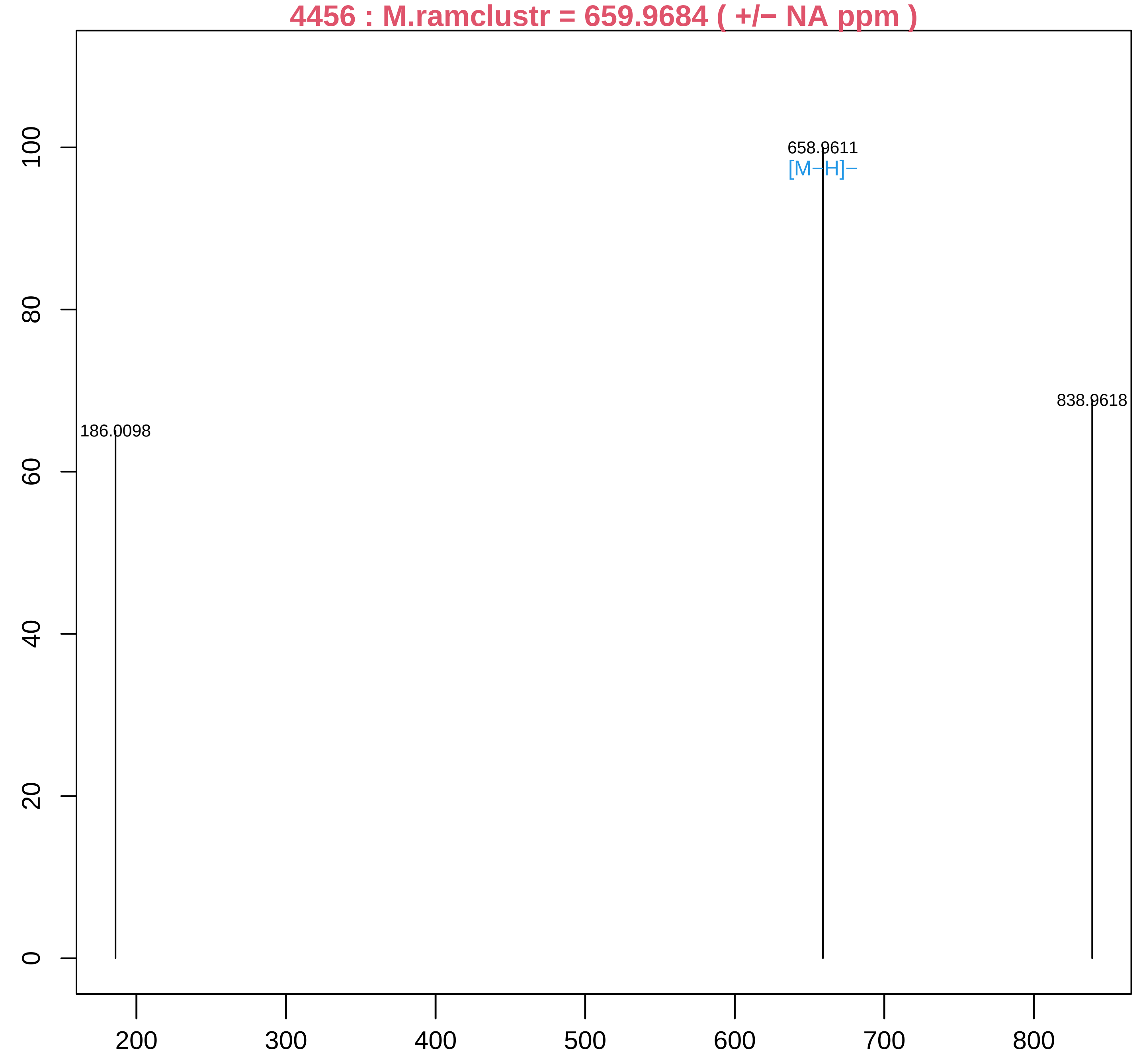
4456 : M.ramclustr = 659.9684 (+/− NA ppm)
186.0098
658.9611
[M−H]−
838.9618
0
20
40
60
80
100
200
300
400
500
600
700
800

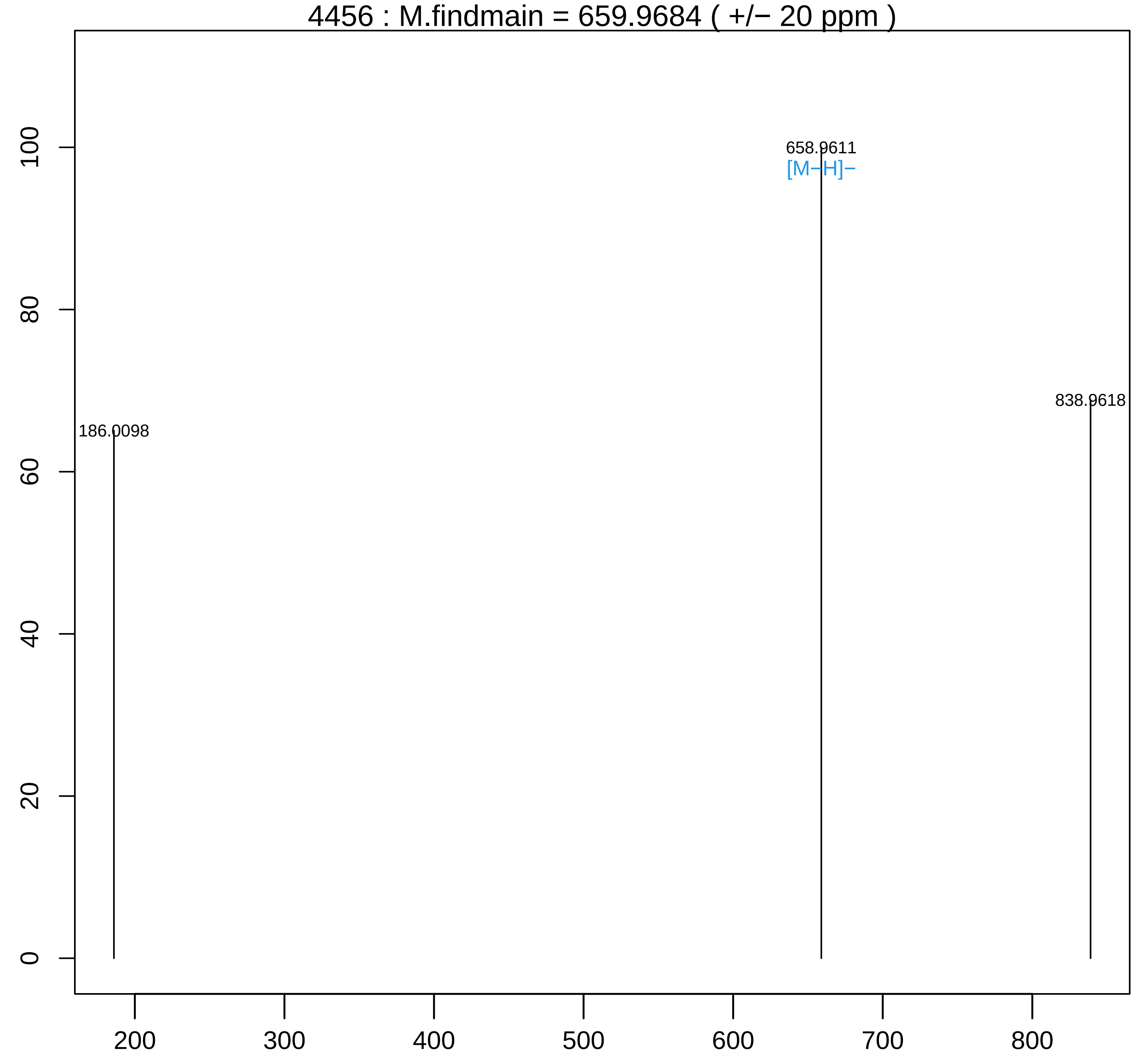
4456 : M.findmain = 659.9684 (+/− 20 ppm)
186.0098
658.9611
[M−H]−
838.9618
0
20
40
60
80
100
200
300
400
500
600
700
800

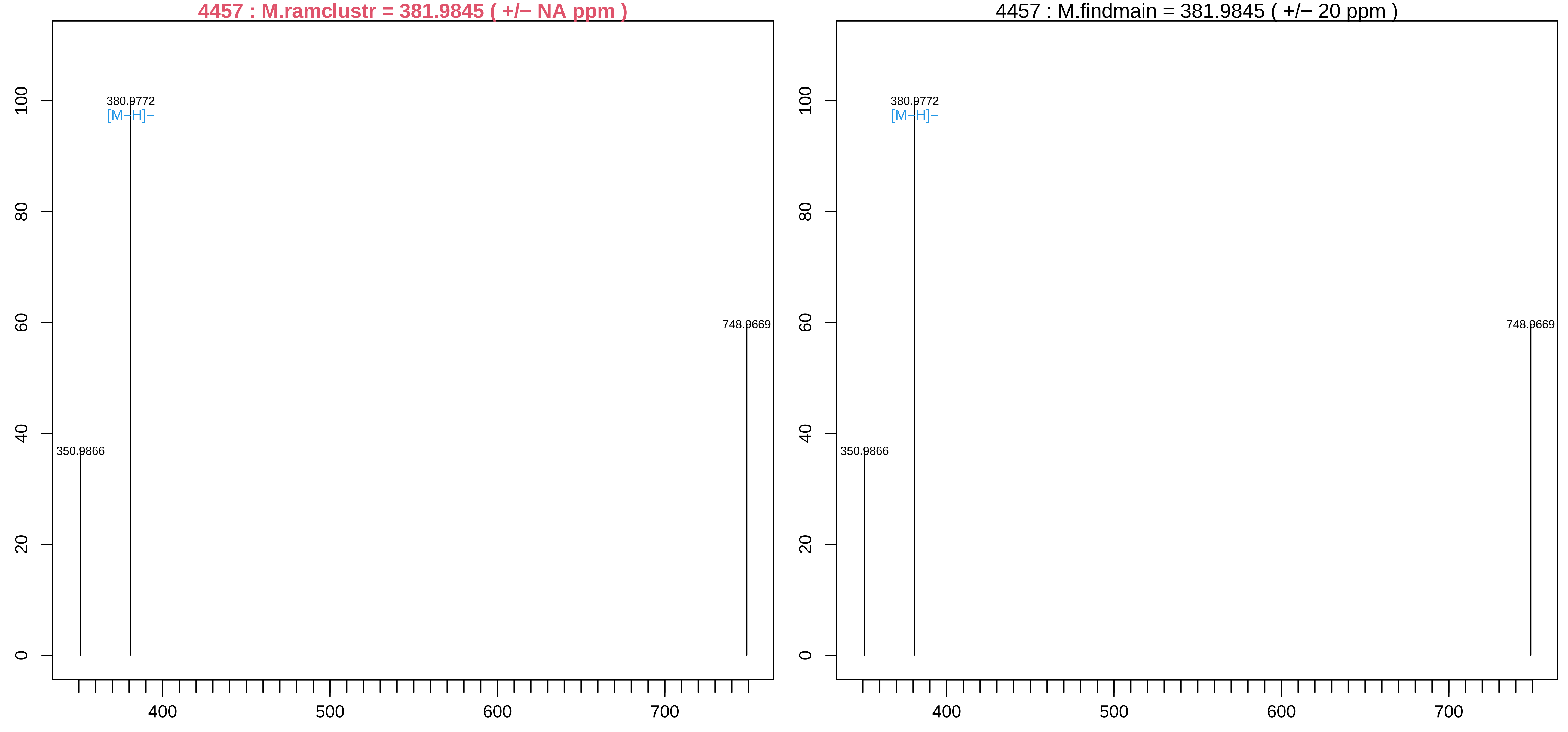

4457 : M.ramclustr = 381.9845 (+/− NA ppm)
380.9772
[M−H]−
350.9866
748.9669
0
20
40
60
80
100
400
500
600
700
4457 : M.findmain = 381.9845 (+/− 20 ppm)
380.9772
[M−H]−
350.9866
748.9669
0
20
40
60
80
100
400
500
600
700

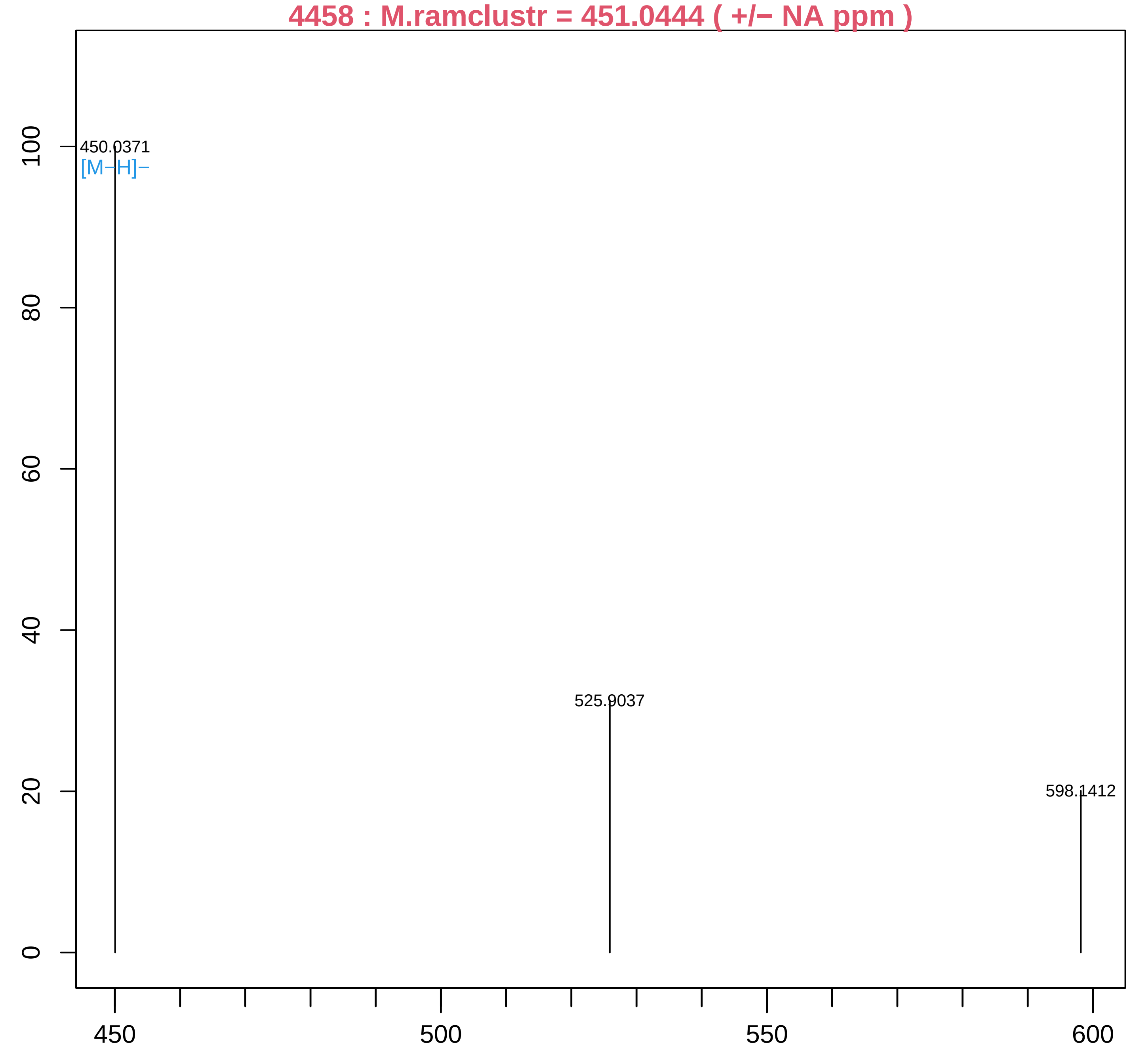

4458 : M.ramclustr = 451.0444 (+/− NA ppm)
450.0371
[M−H]−
525.9037
598.1412
0
20
40
60
80
100
450
500
550
600

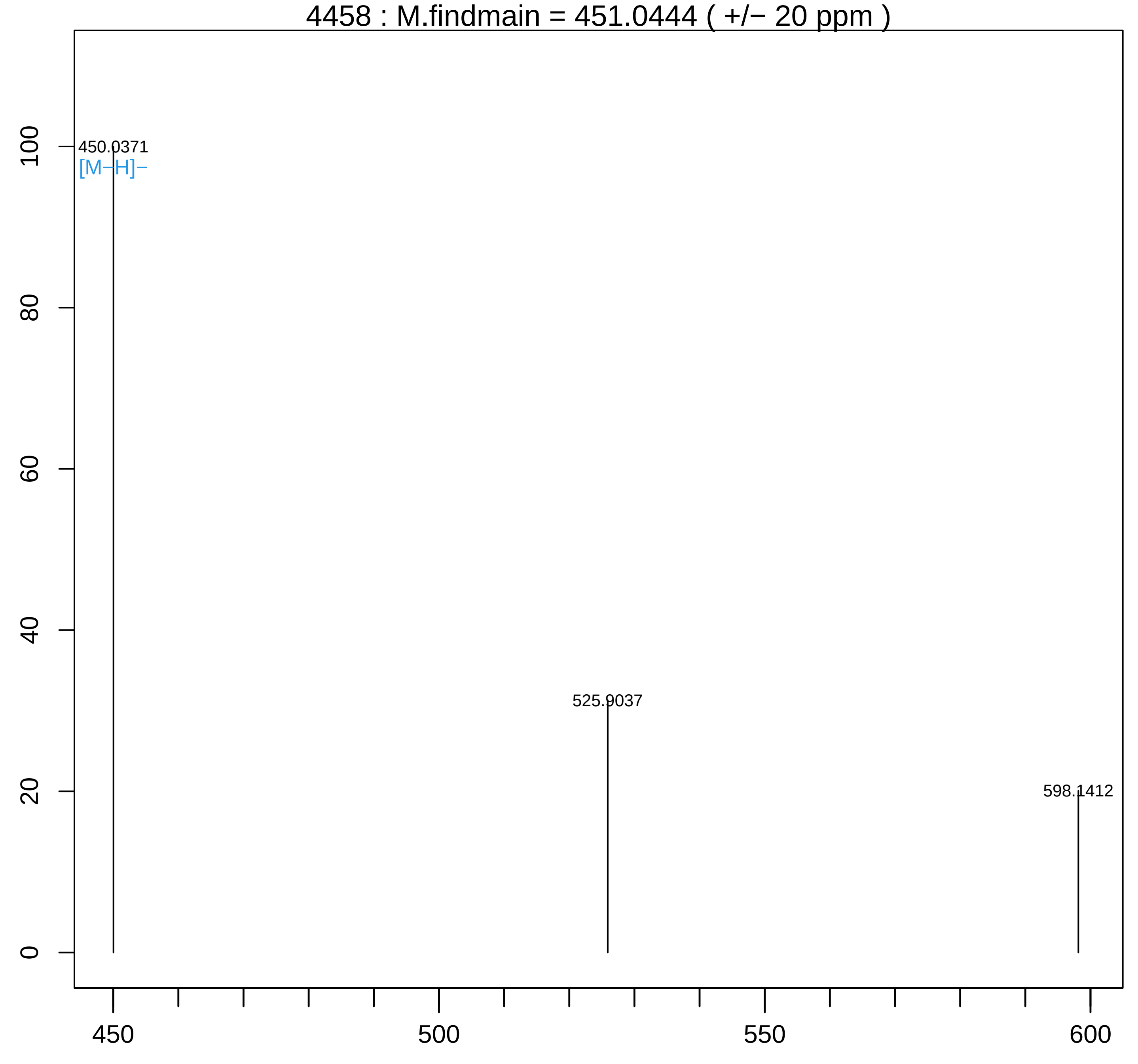

4458 : M.findmain = 451.0444 (+/− 20 ppm)
450.0371
[M−H]−
525.9037
598.1412
0
20
40
60
80
100
450
500
550
600

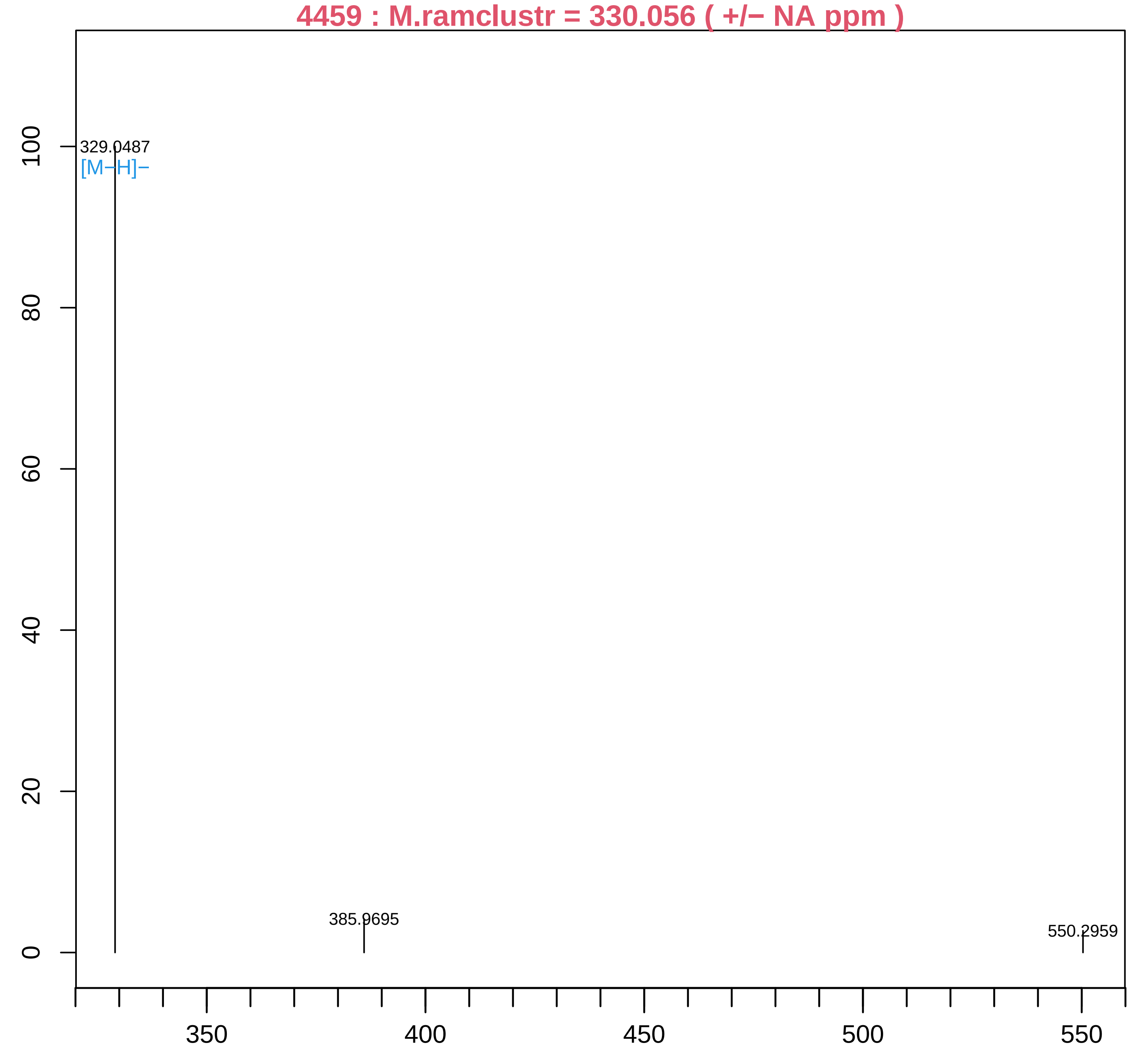
4459 : M.ramclustr = 330.056 (+/− NA ppm)
329.0487
[M−H]−
385.9695
550.2959
0
20
40
60
80
100
350
400
450
500
550

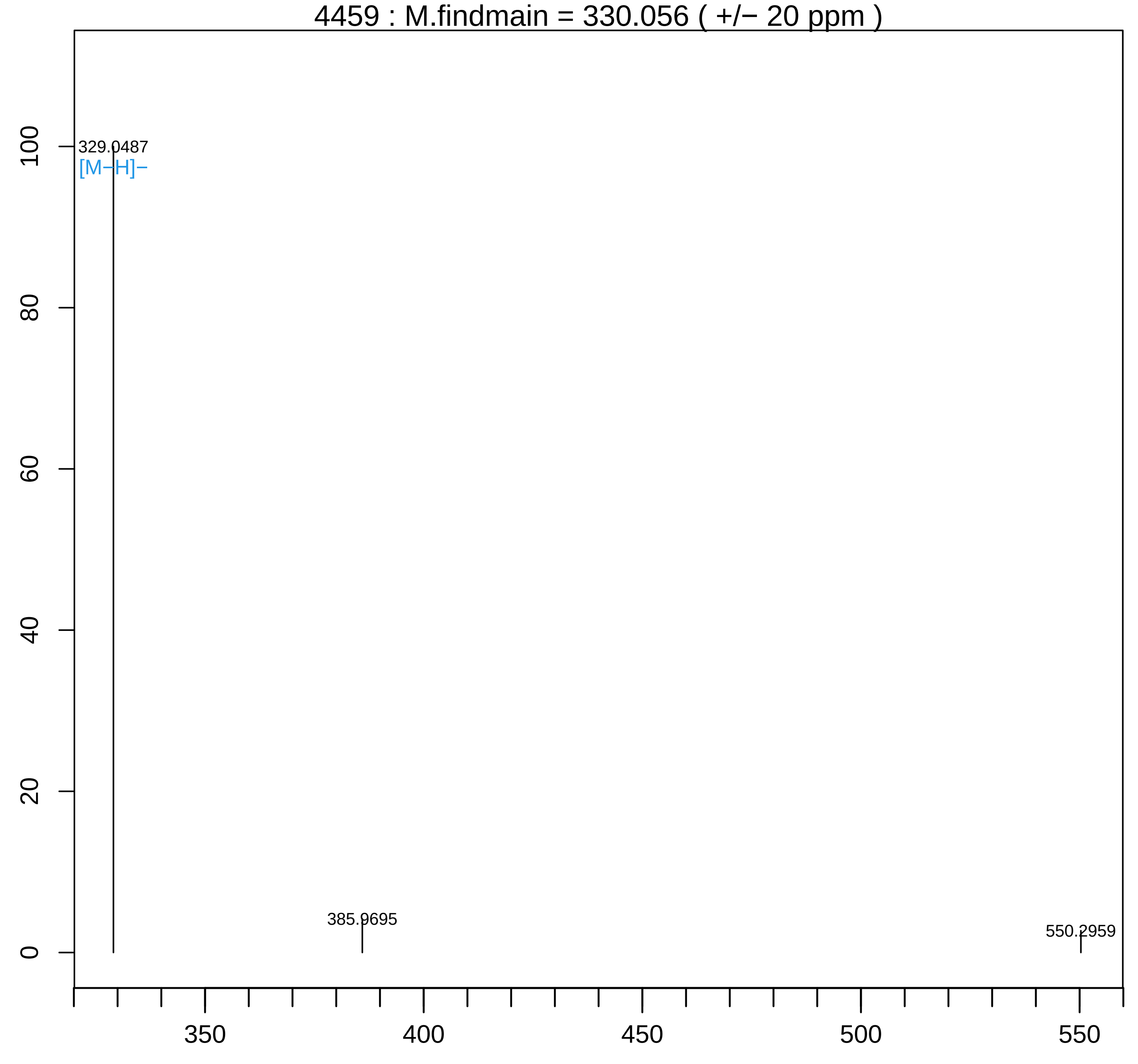
4459 : M.findmain = 330.056 (+/− 20 ppm)
329.0487
[M−H]−
385.9695
550.2959
0
20
40
60
80
100
350
400
450
500
550

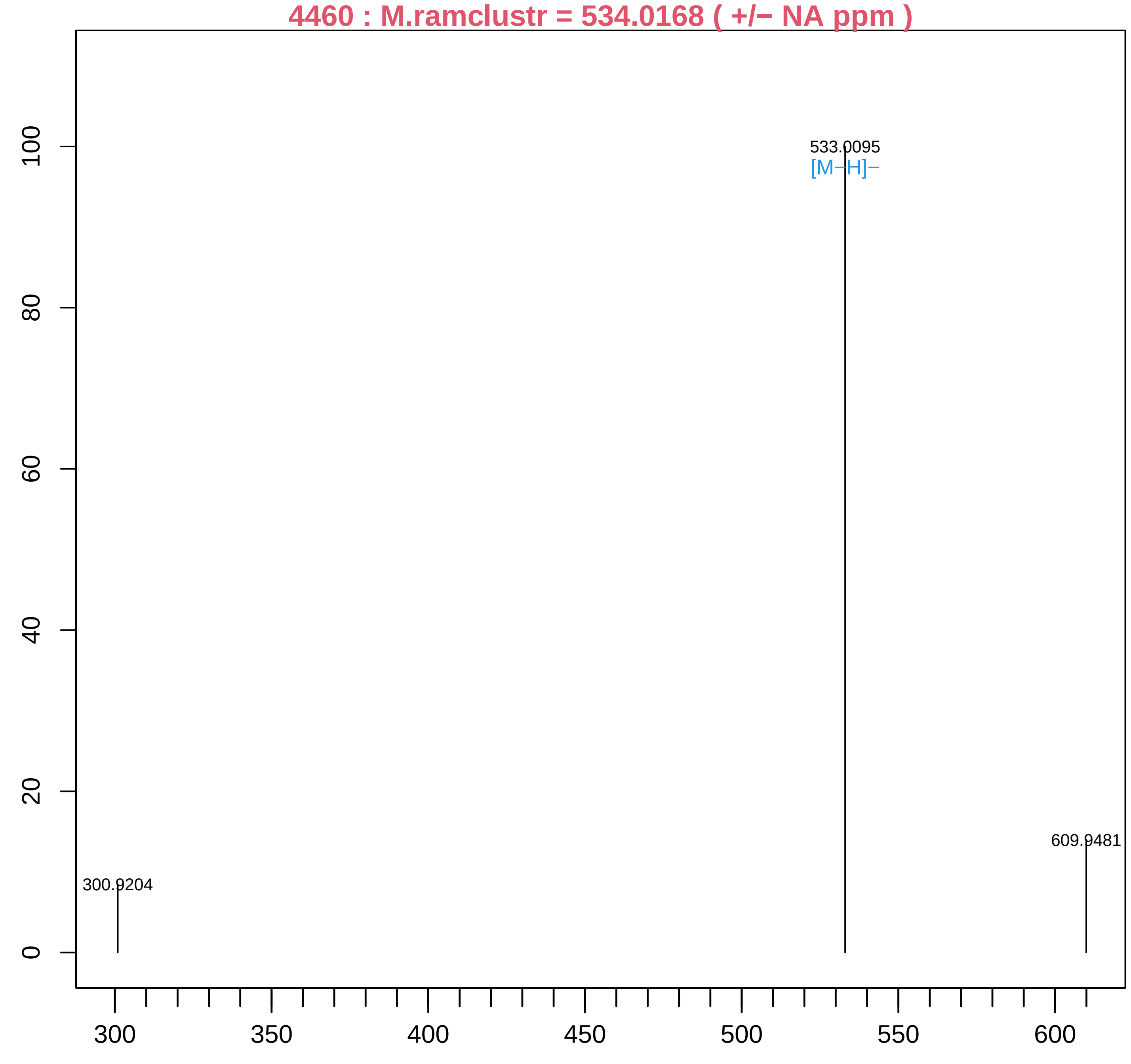
4460 : M.ramclustr = 534.0168 (+/− NA ppm)
533.0095
[M−H]−
300.9204
609.9481
0
20
40
60
80
100
300
350
400
450
500
550
600

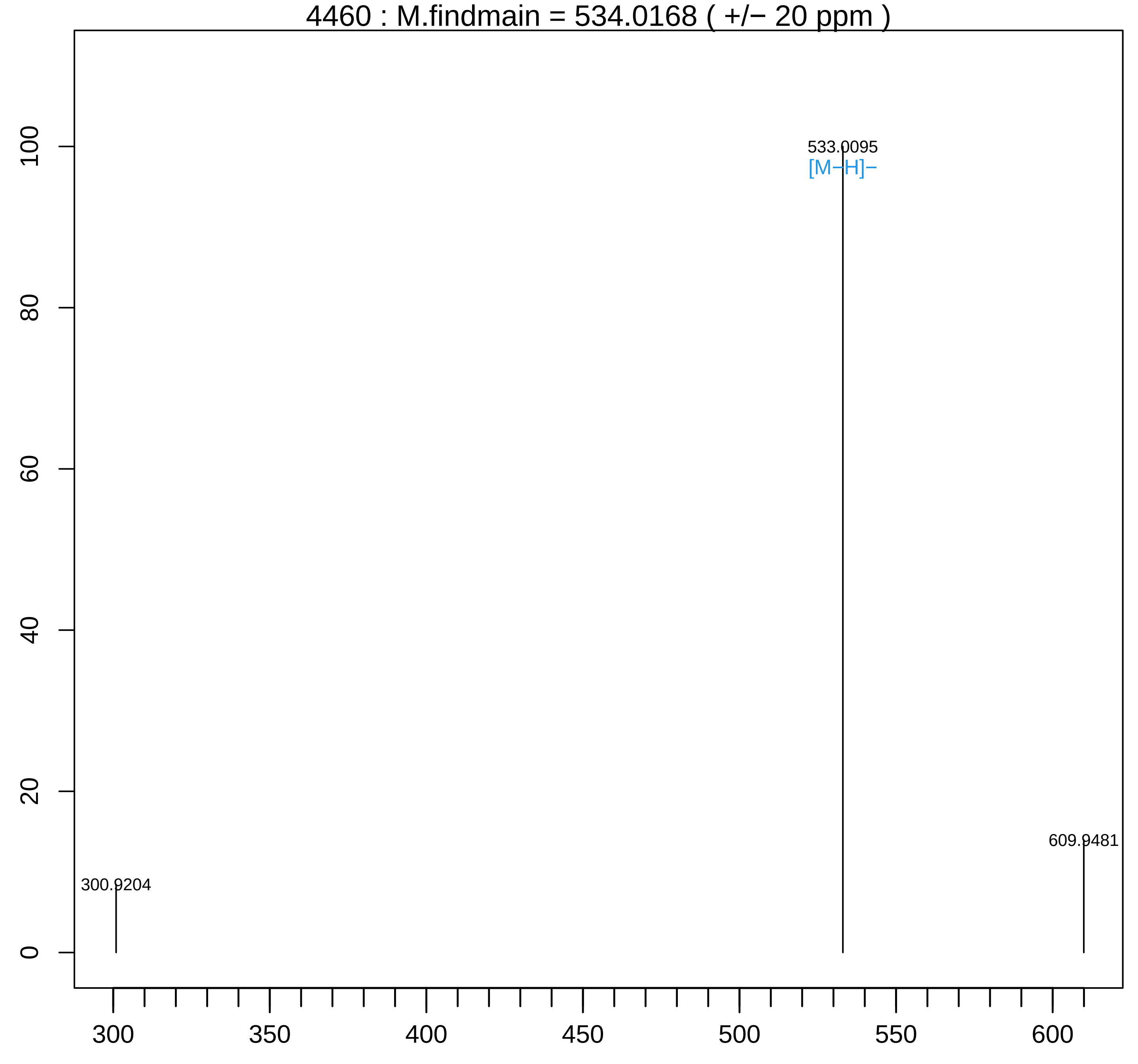
4460 : M.findmain = 534.0168 (+/− 20 ppm)
533.0095
[M−H]−
300.9204
609.9481
0
20
40
60
80
100
300
350
400
450
500
550
600

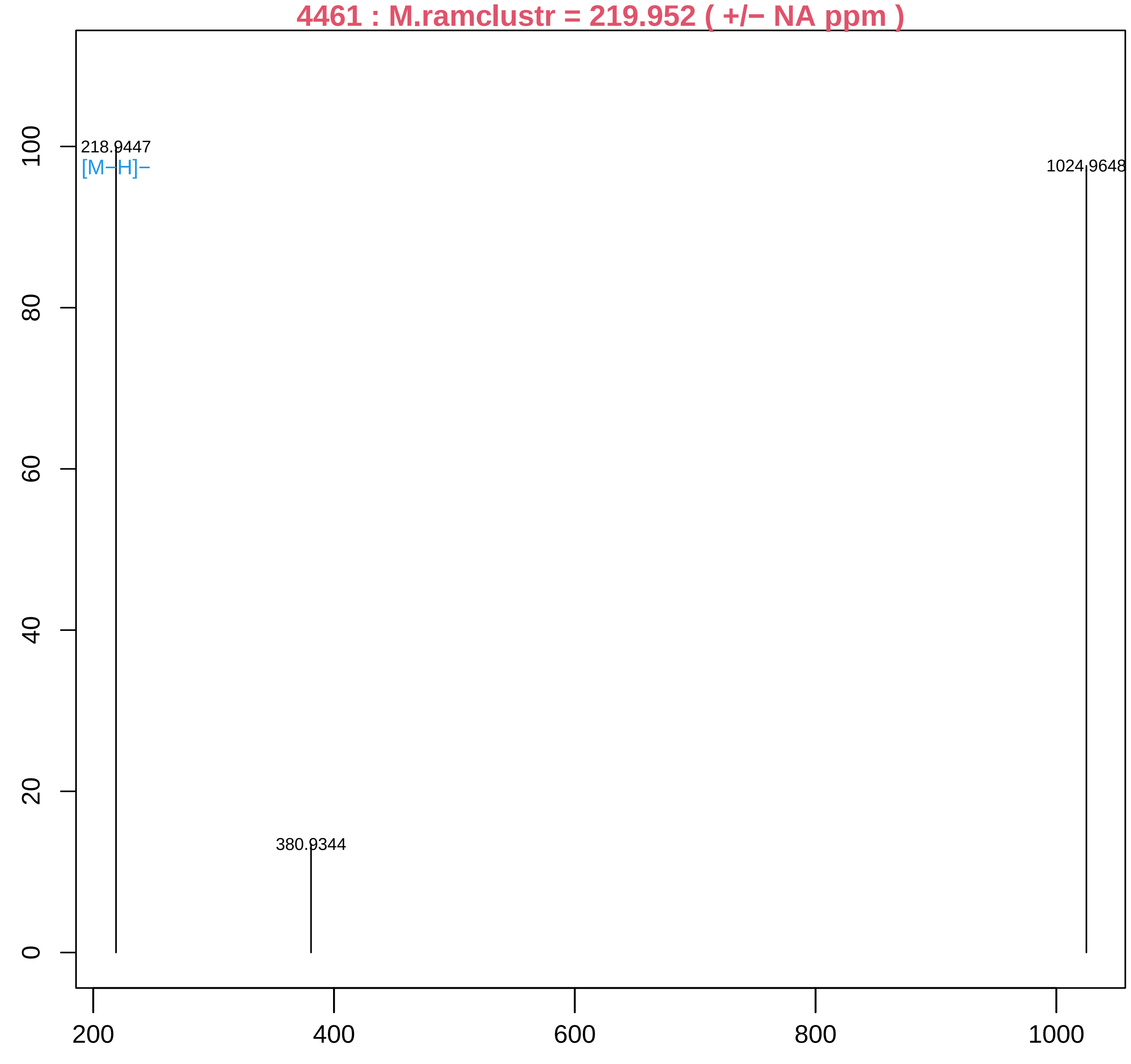
4461 : M.ramclustr = 219.952 (+/− NA ppm)
218.9447
[M−H]−
1024.9648
380.9344
0
20
40
60
80
100
200
400
600
800
1000

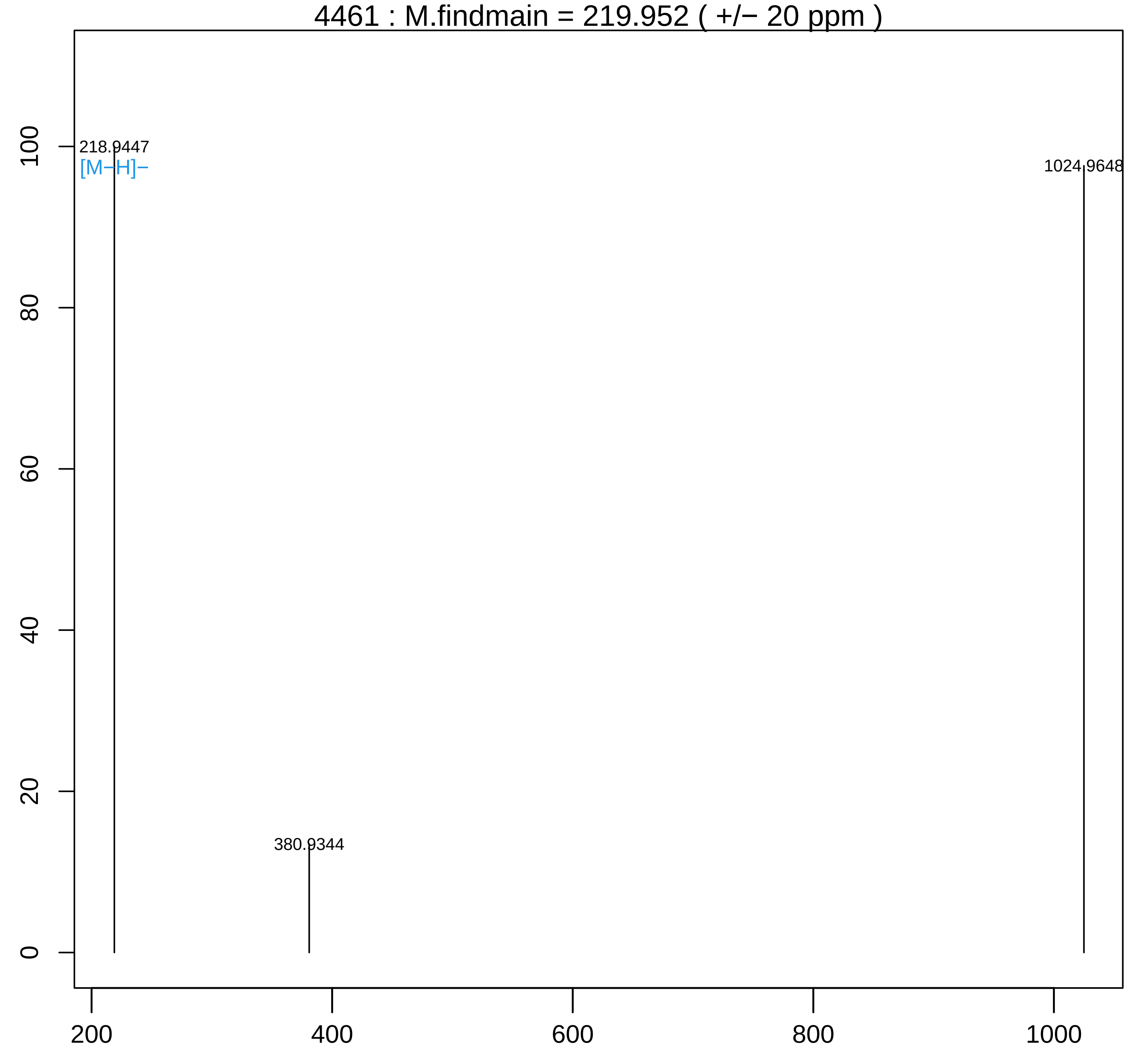
4461 : M.findmain = 219.952 (+/− 20 ppm)
218.9447
[M−H]−
1024.9648
380.9344
0
20
40
60
80
100
200
400
600
800
1000

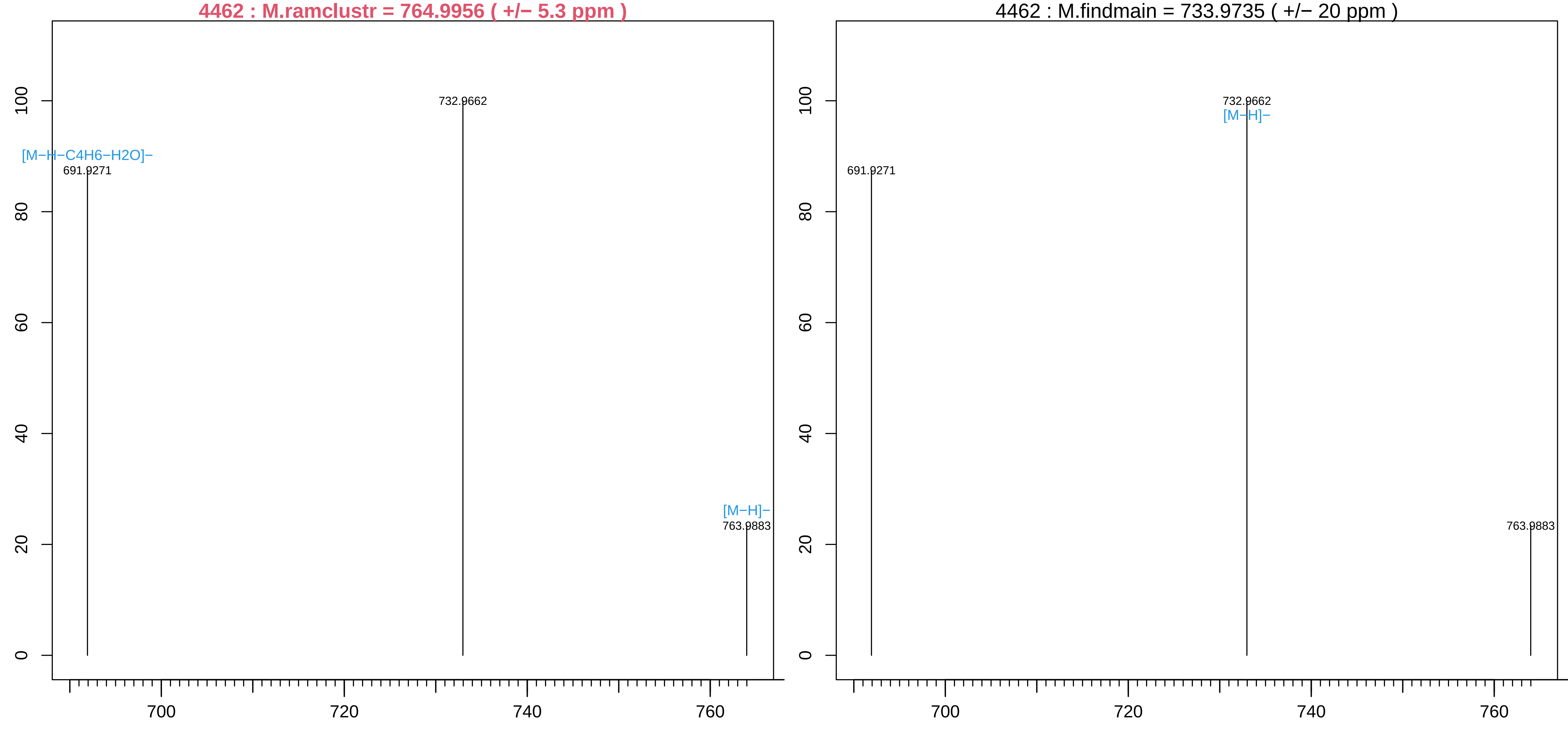
4462 : M.ramclustr = 764.9956 (+/− 5.3 ppm)
[M−H−C4H6−H2O]−
691.9271
732.9662
[M−H]−
763.9883
0
20
40
60
80
100
700
720
740
760
4462 : M.findmain = 733.9735 (+/− 20 ppm)
691.9271
732.9662
[M−H]−
763.9883
0
20
40
60
80
100
700
720
740
760

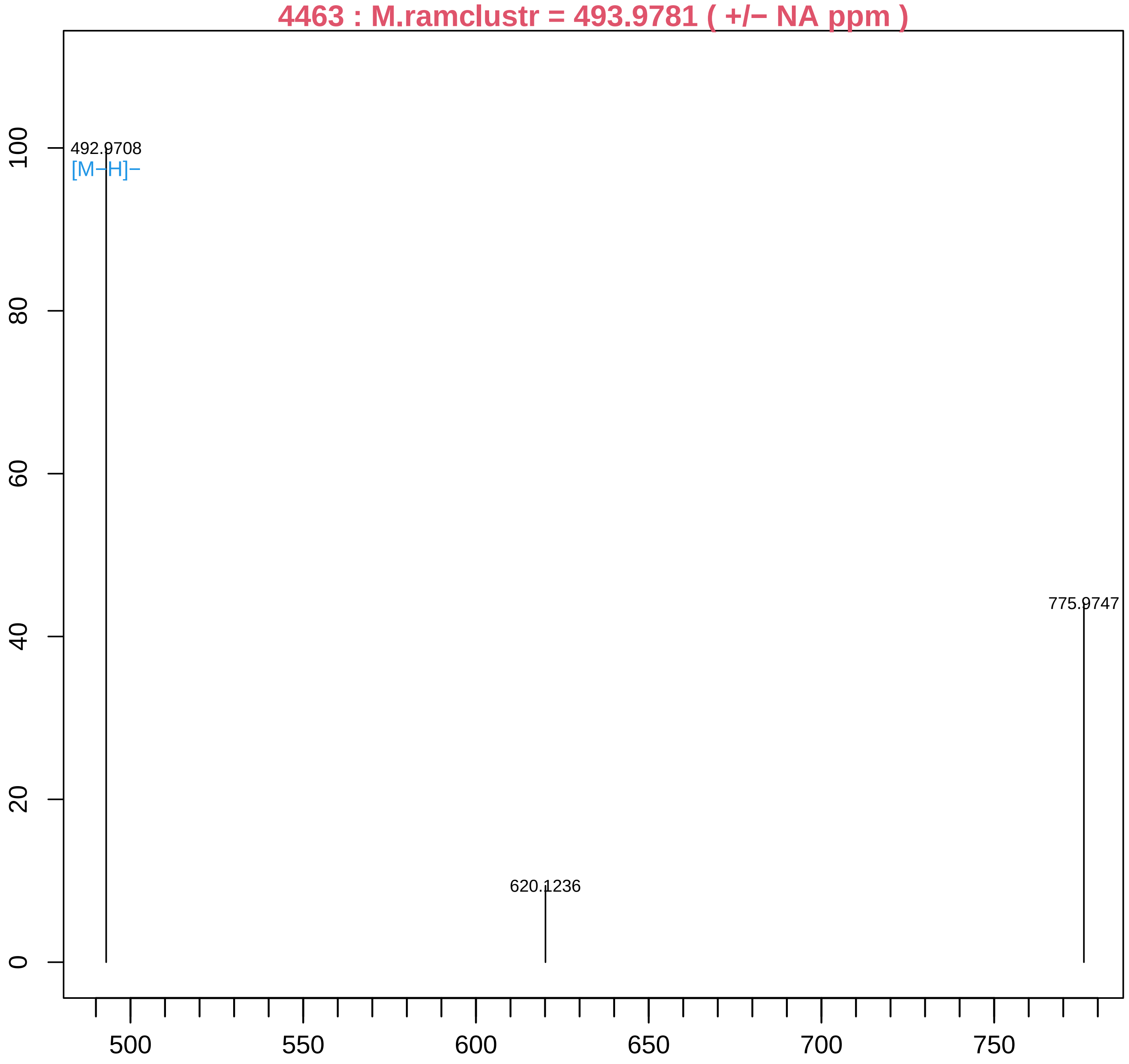
4463 : M.ramclustr = 493.9781 (+/− NA ppm)
492.9708
[M−H]−
620.1236
775.9747
0
20
40
60
80
100
500
550
600
650
700
750

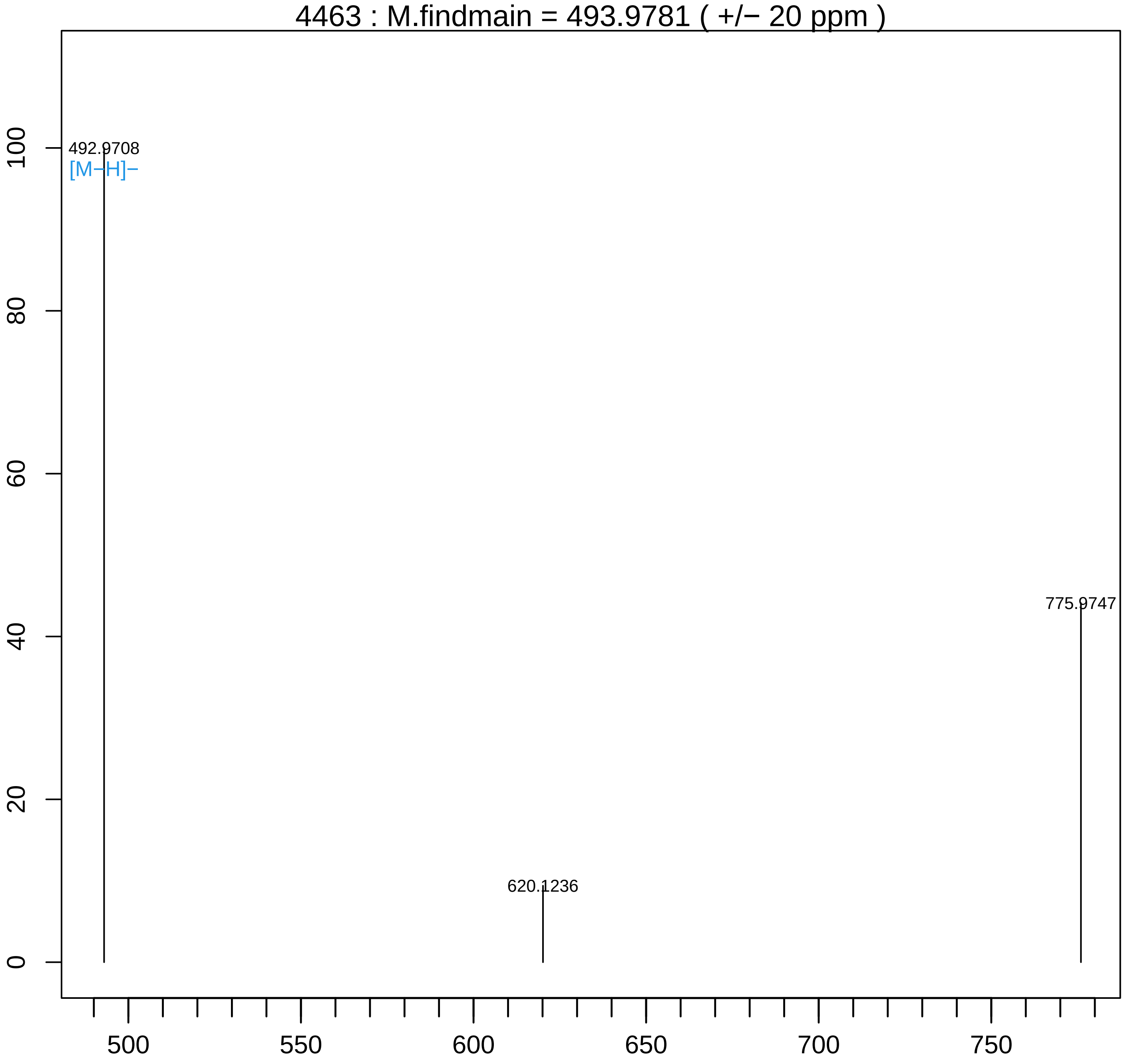
4463 : M.findmain = 493.9781 (+/− 20 ppm)
492.9708
[M−H]−
620.1236
775.9747
0
20
40
60
80
100
500
550
600
650
700
750

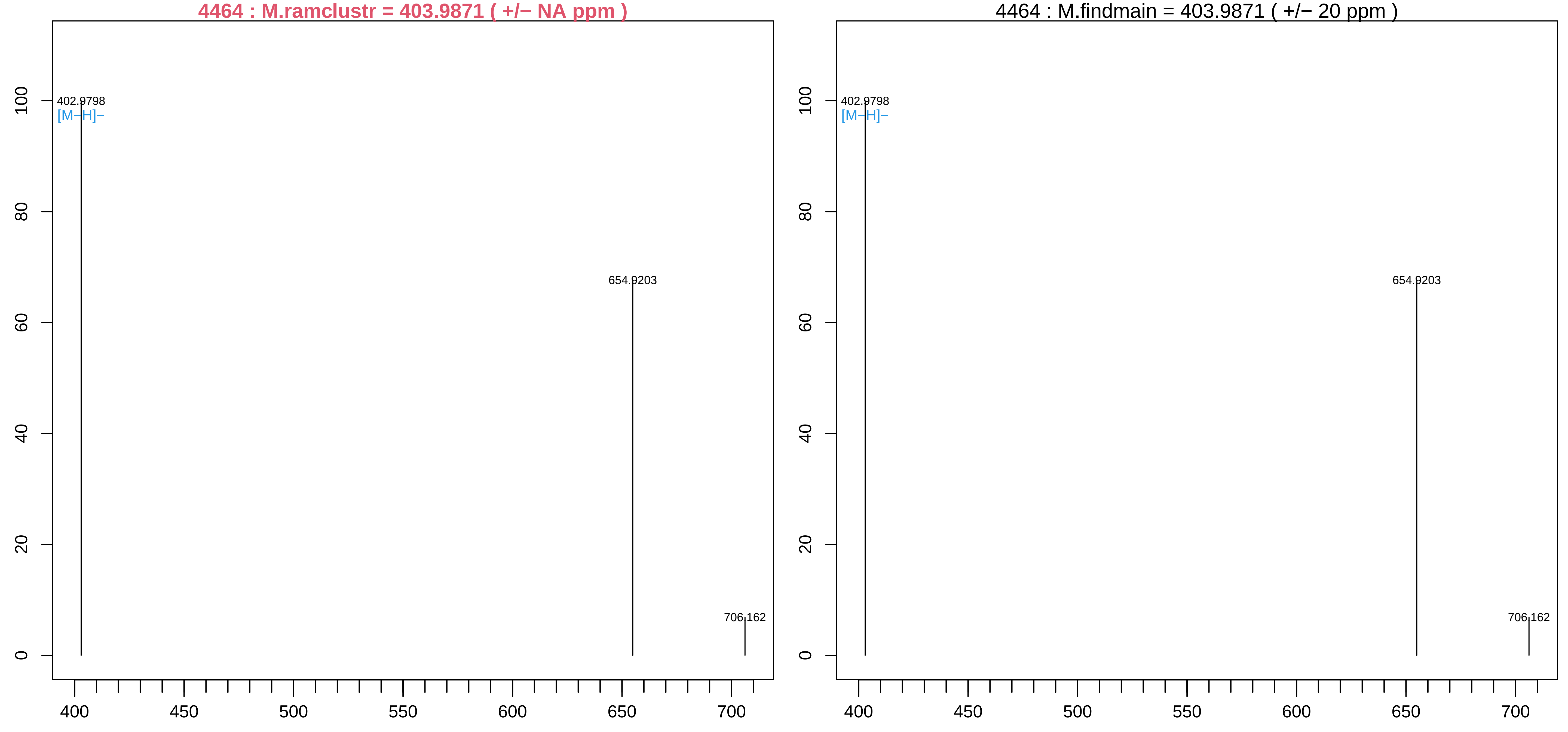

4464 : M.ramclustr = 403.9871 (+/− NA ppm)
402.9798
[M−H]−
654.9203
706.162
0
20
40
60
80
100
400
450
500
550
600
650
700
4464 : M.findmain = 403.9871 (+/− 20 ppm)
402.9798
[M−H]−
654.9203
706.162
0
20
40
60
80
100
400
450
500
550
600
650
700

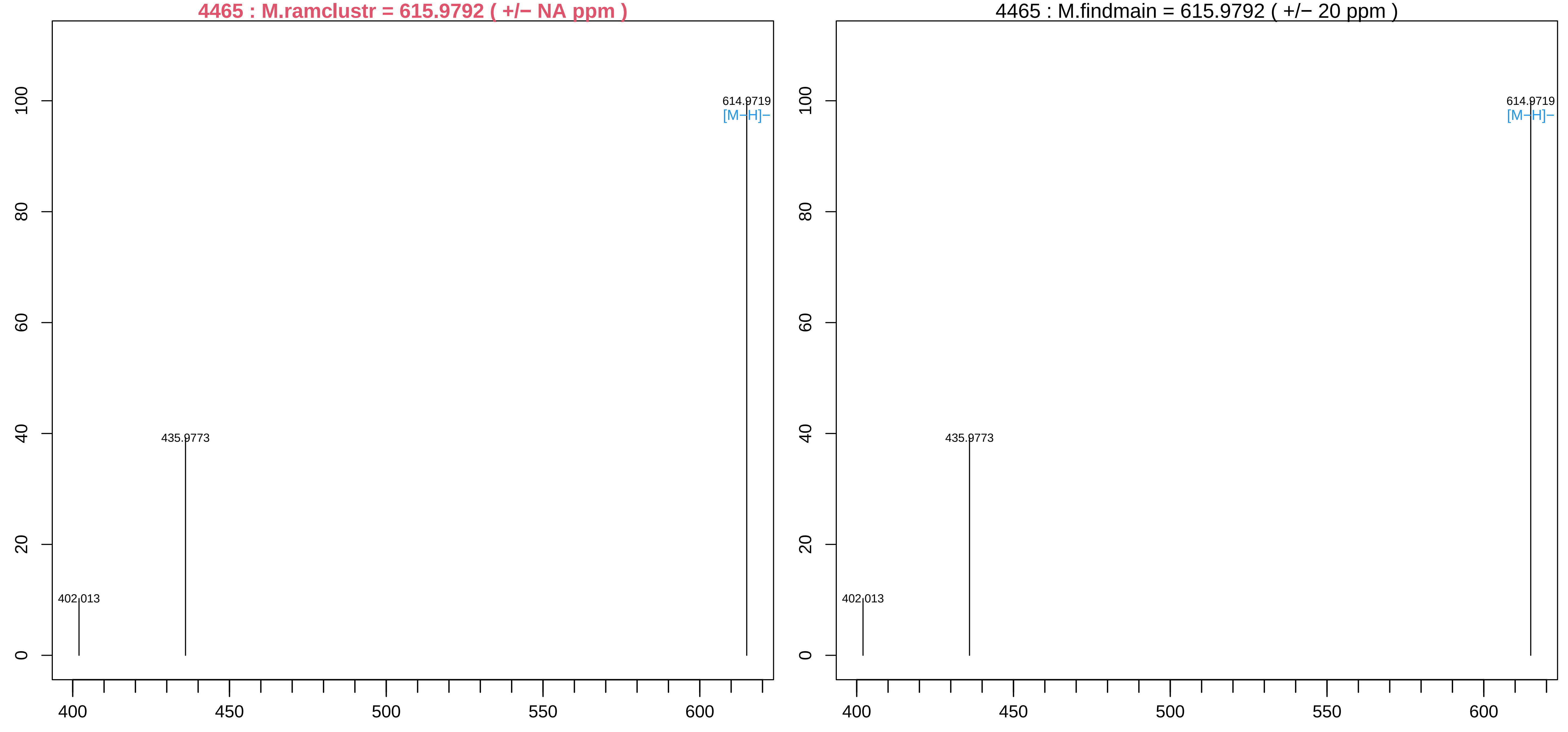
4465 : M.ramclustr = 615.9792 (+/− NA ppm)
614.9719
[M−H]−
435.9773
402.013
4465 : M.findmain = 615.9792 (+/− 20 ppm)
614.9719
[M−H]−
435.9773
402.013

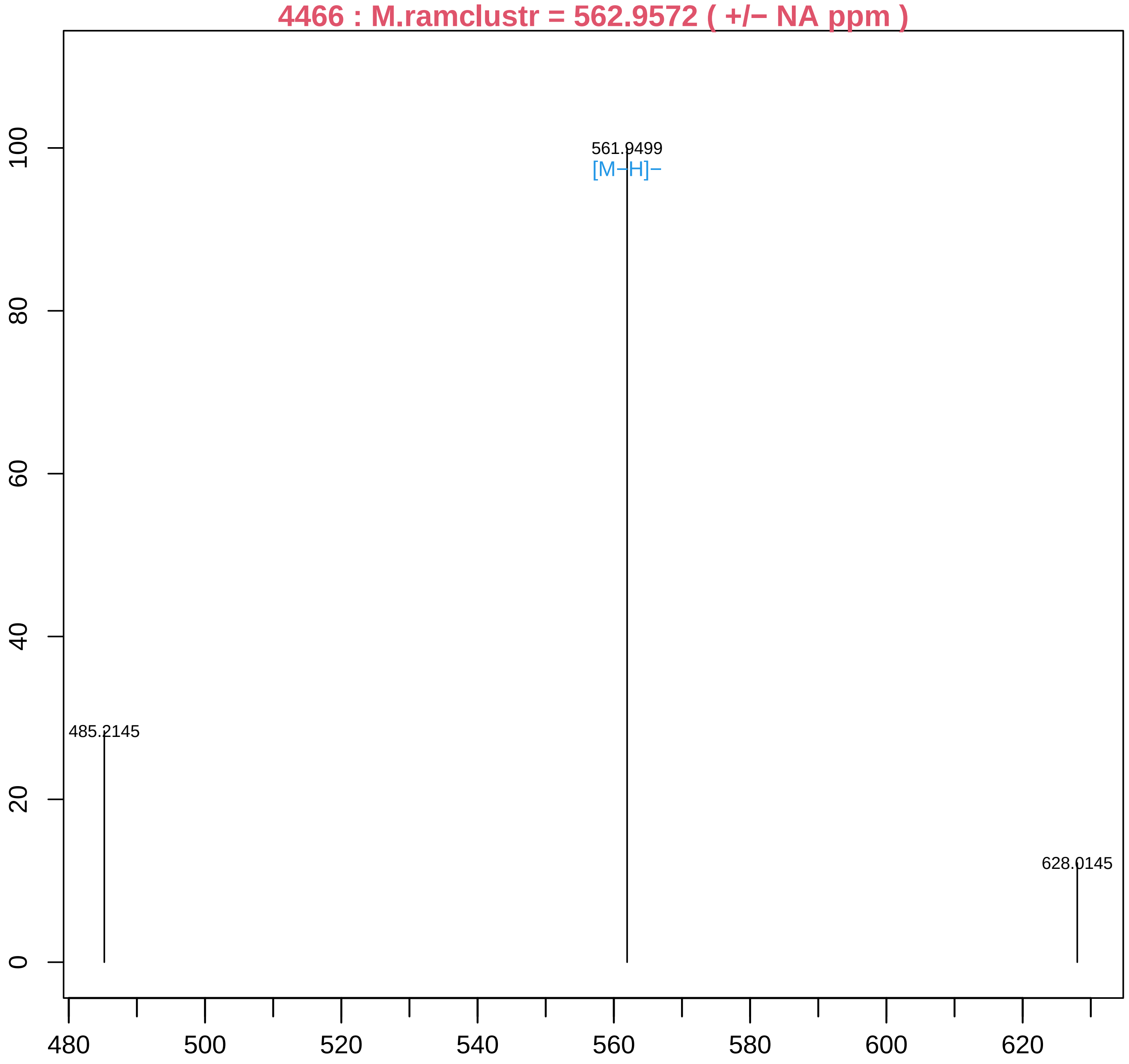
4466 : M.ramclustr = 562.9572 (+/− NA ppm)
561.9499
[M−H]−
485.2145
628.0145
0
20
40
60
80
100
480
500
520
540
560
580
600
620

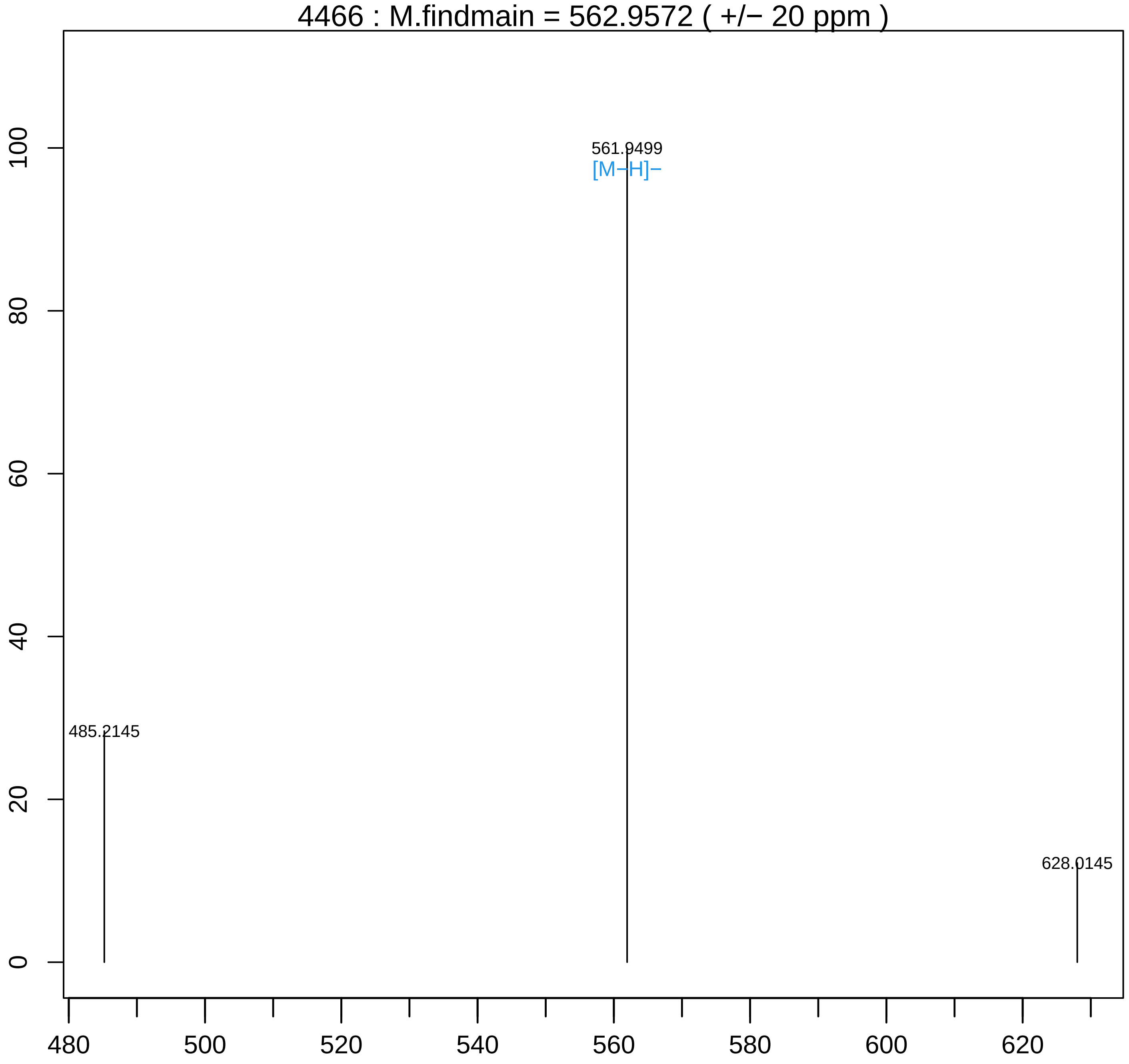
4466 : M.findmain = 562.9572 (+/− 20 ppm)
561.9499
[M−H]−
485.2145
628.0145
0
20
40
60
80
100
480
500
520
540
560
580
600
620

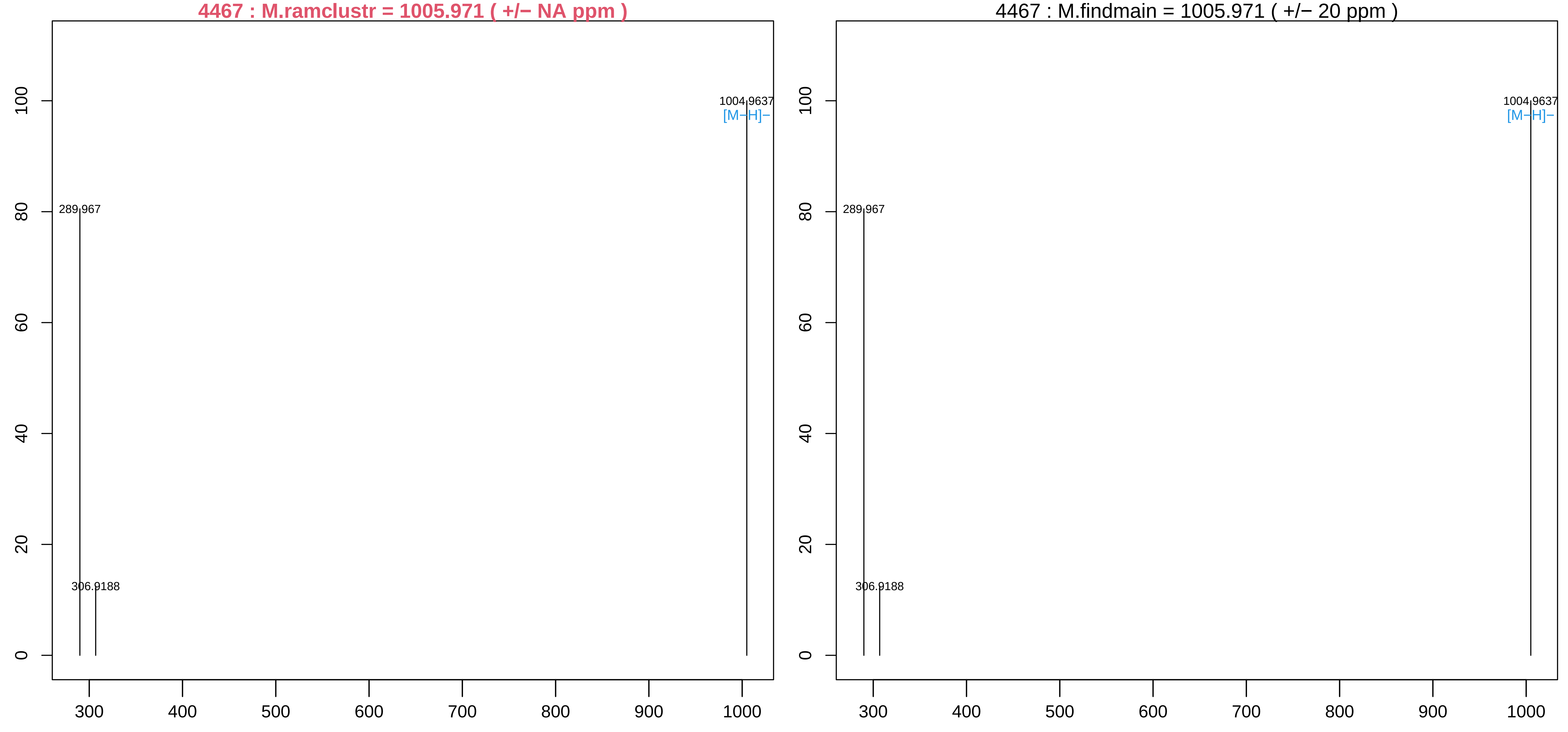
4467 : M.ramclustr = 1005.971 (+/− NA ppm)
4467 : M.findmain = 1005.971 (+/− 20 ppm)
1004.9637
[M−H]−
289.967
306.9188
0
20
40
60
80
100
300
400
500
600
700
800
900
1000

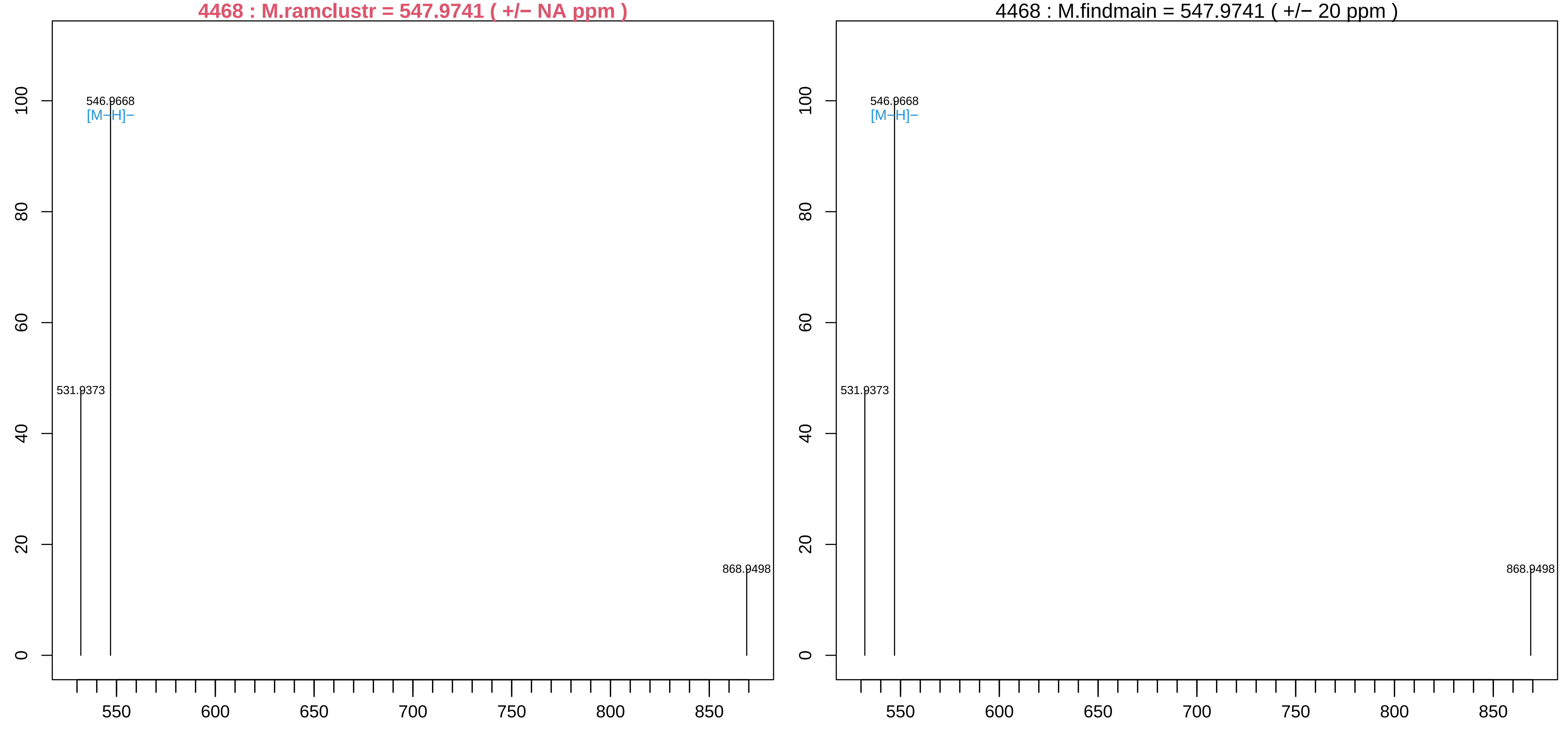
4468 : M.ramclustr = 547.9741 (+/− NA ppm)
546.9668
[M−H]−
531.9373
868.9498
0
20
40
60
80
100
550
600
650
700
750
800
850
4468 : M.findmain = 547.9741 (+/− 20 ppm)
546.9668
[M−H]−
531.9373
868.9498
0
20
40
60
80
100
550
600
650
700
750
800
850

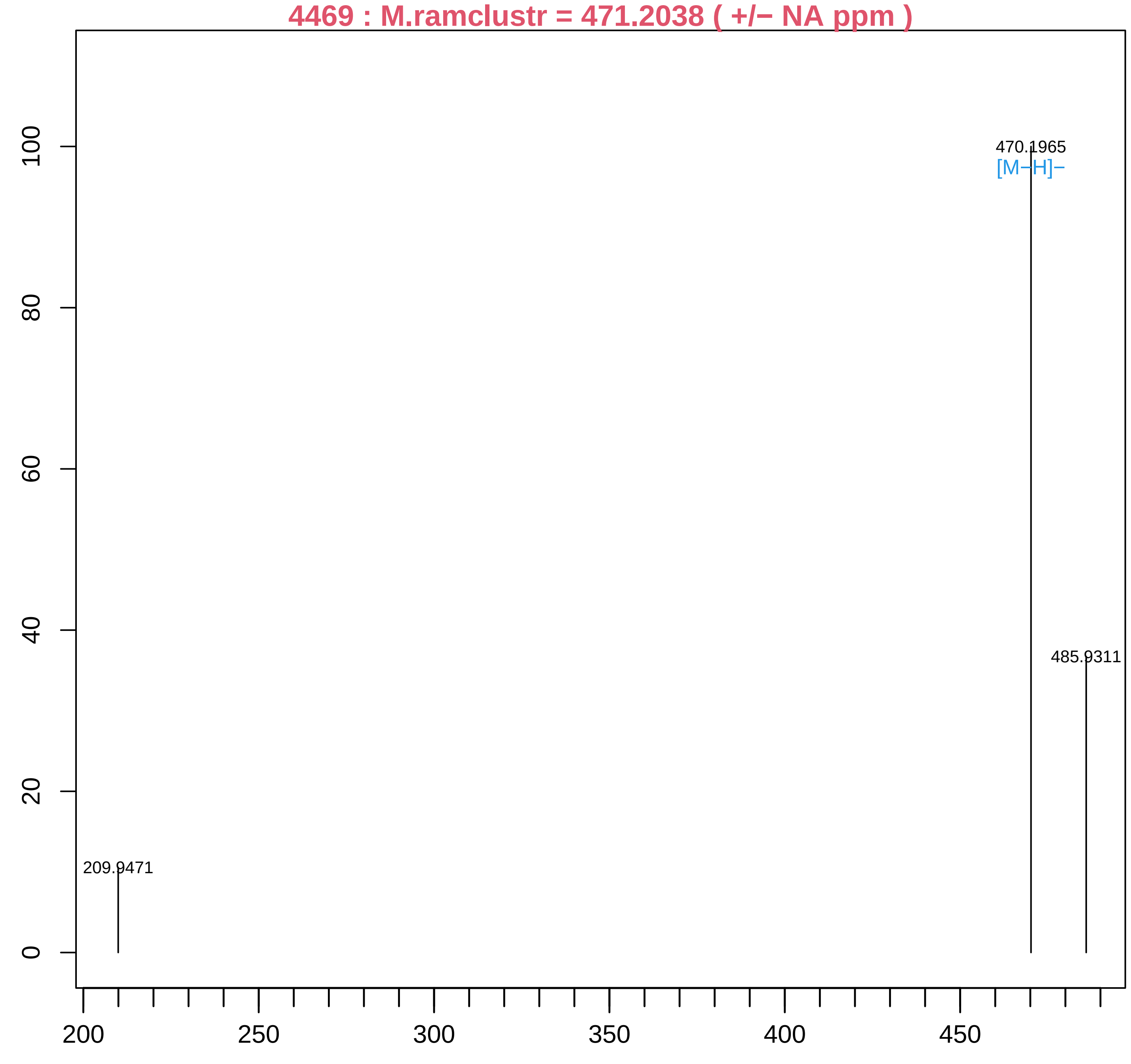

4469 : M.ramclustr = 471.2038 (+/− NA ppm)
470.1965
[M−H]−
485.9311
209.9471
0
20
40
60
80
100
200
250
300
350
400
450

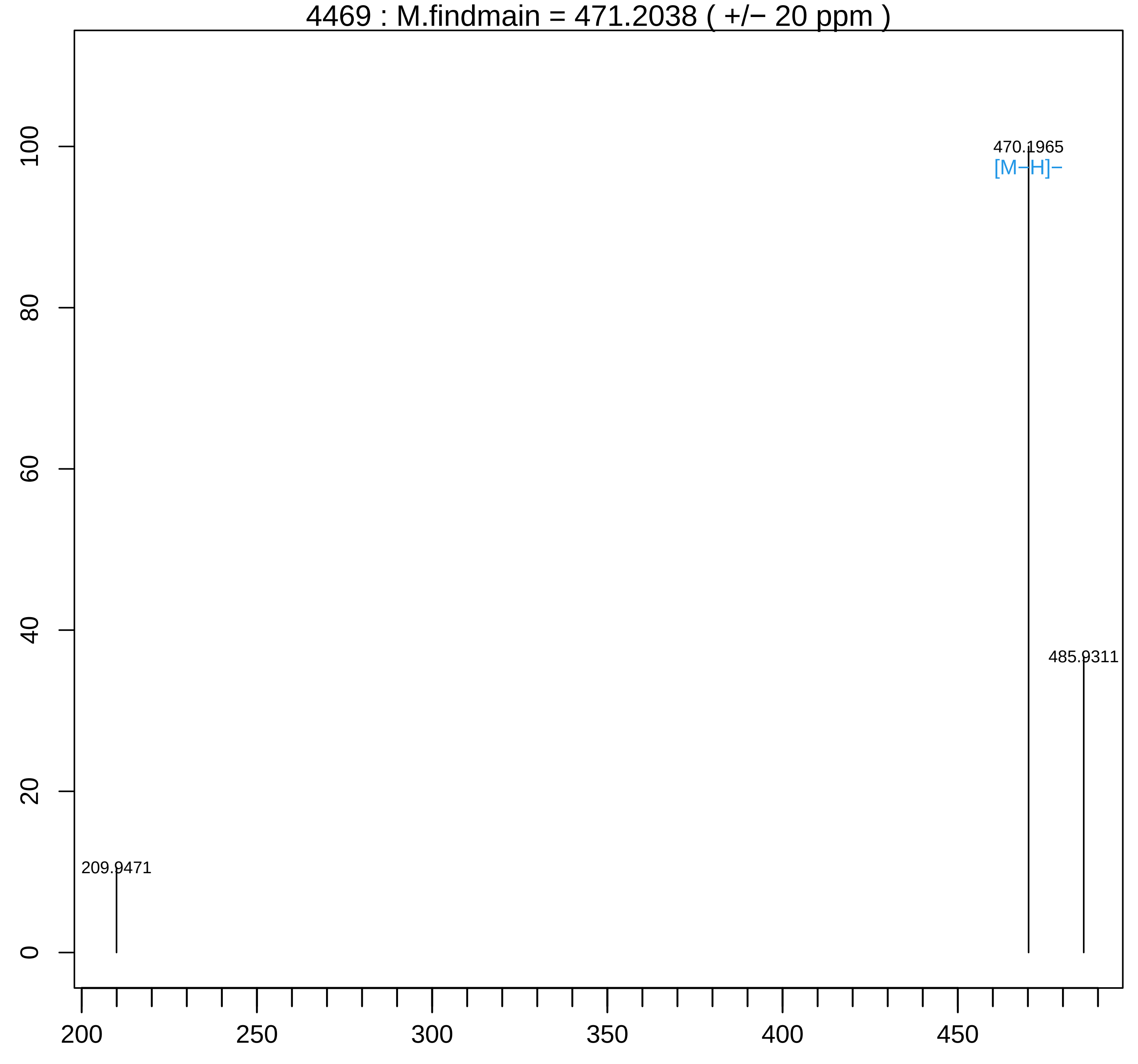

4469 : M.findmain = 471.2038 (+/− 20 ppm)
470.1965
[M−H]−
485.9311
209.9471
0
20
40
60
80
100
200
250
300
350
400
450

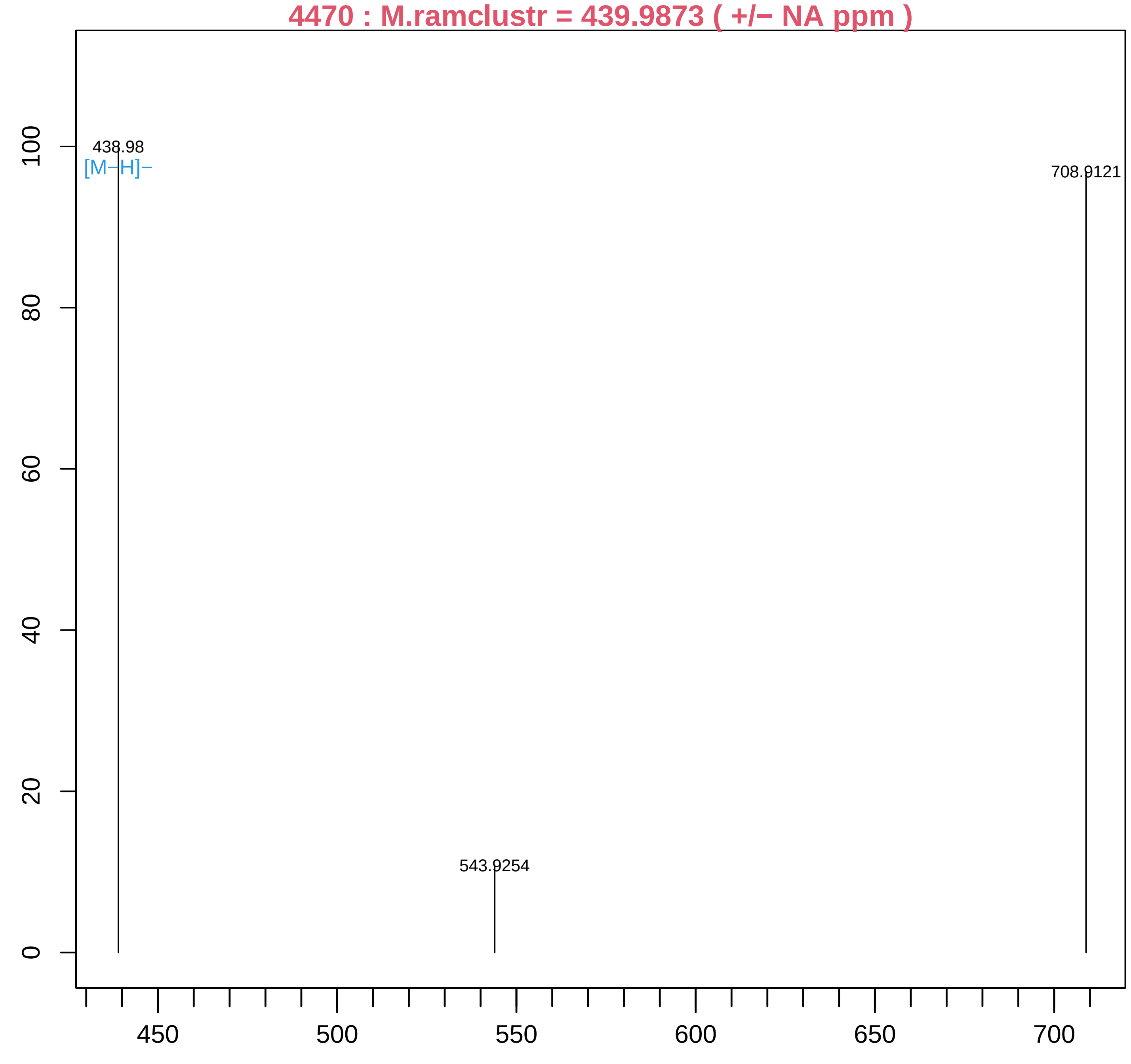
4470 : M.ramclustr = 439.9873 (+/− NA ppm)
438.98
[M−H]−
708.9121
543.9254
0
20
40
60
80
100
450
500
550
600
650
700

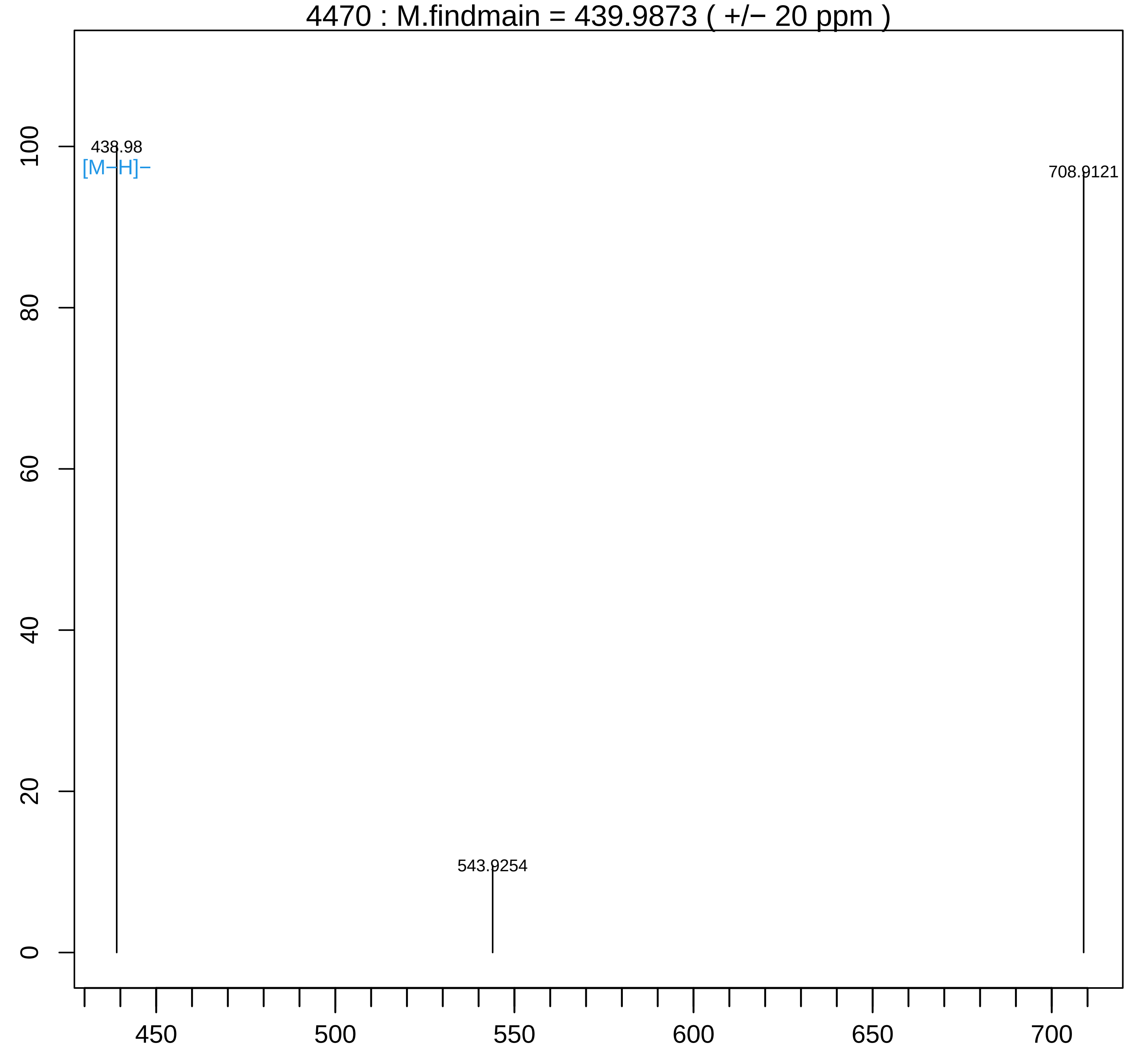
4470 : M.findmain = 439.9873 (+/− 20 ppm)
438.98
[M−H]−
708.9121
543.9254
0
20
40
60
80
100
450
500
550
600
650
700

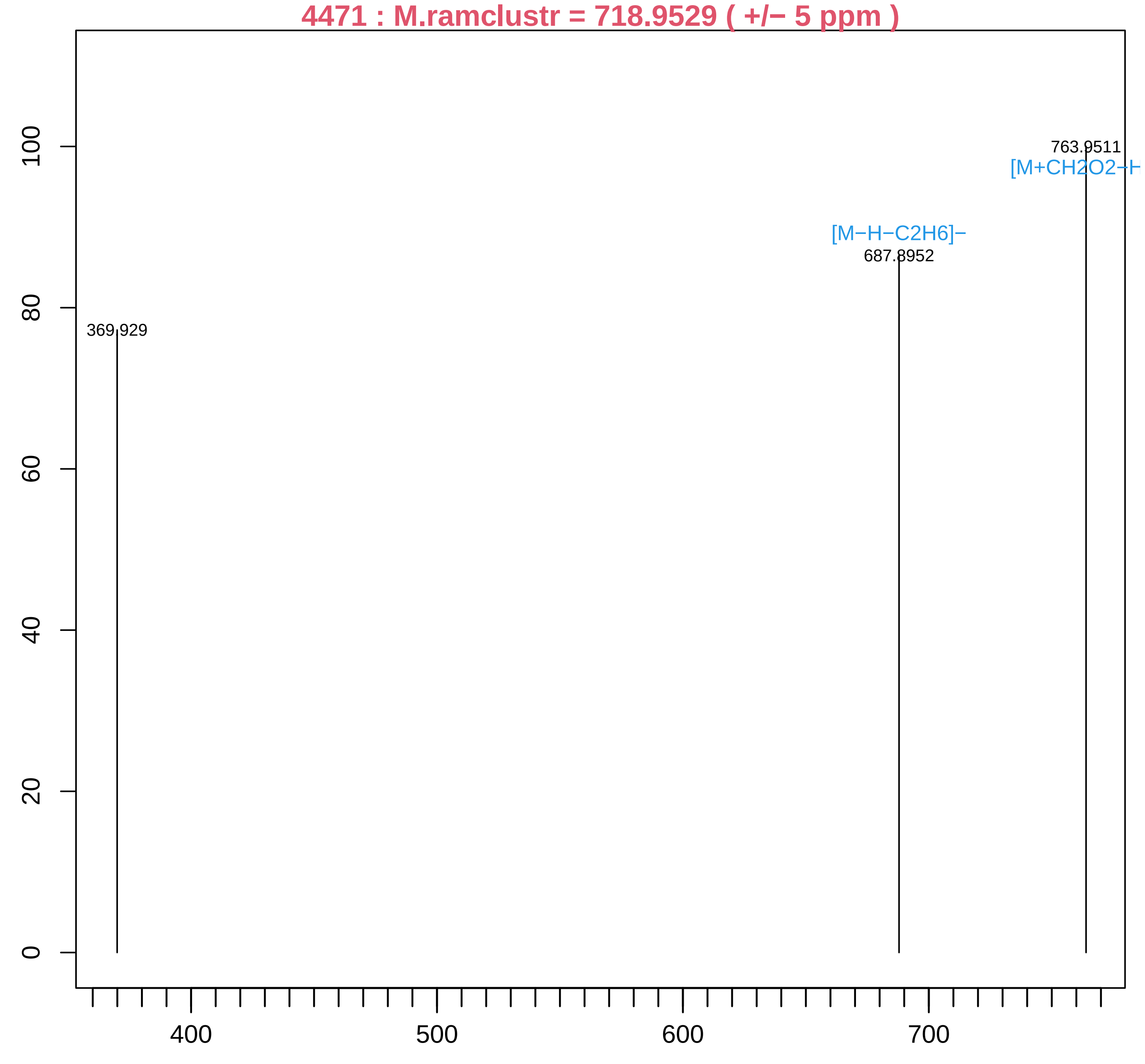
4471 : M.ramclustr = 718.9529 (+/− 5 ppm)
100
80
60
40
20
0
400
500
600
700
369.929
[M−H−C2H6]−
687.8952
763.9511
[M+CH2O2−H

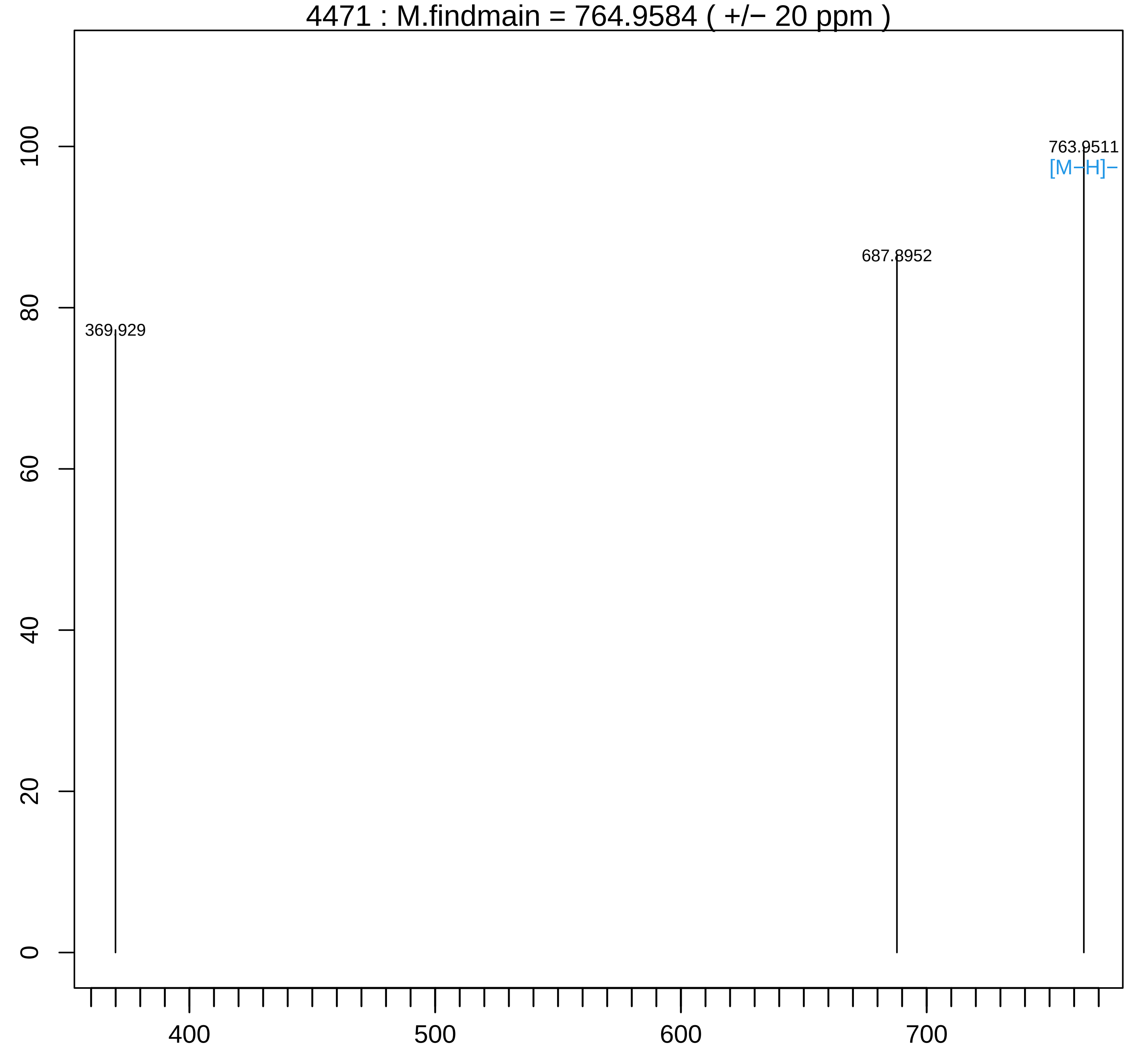
4471 : M.findmain = 764.9584 (+/− 20 ppm)
100
80
60
40
20
0
400
500
600
700
369.929
687.8952
763.9511
[M−H]−

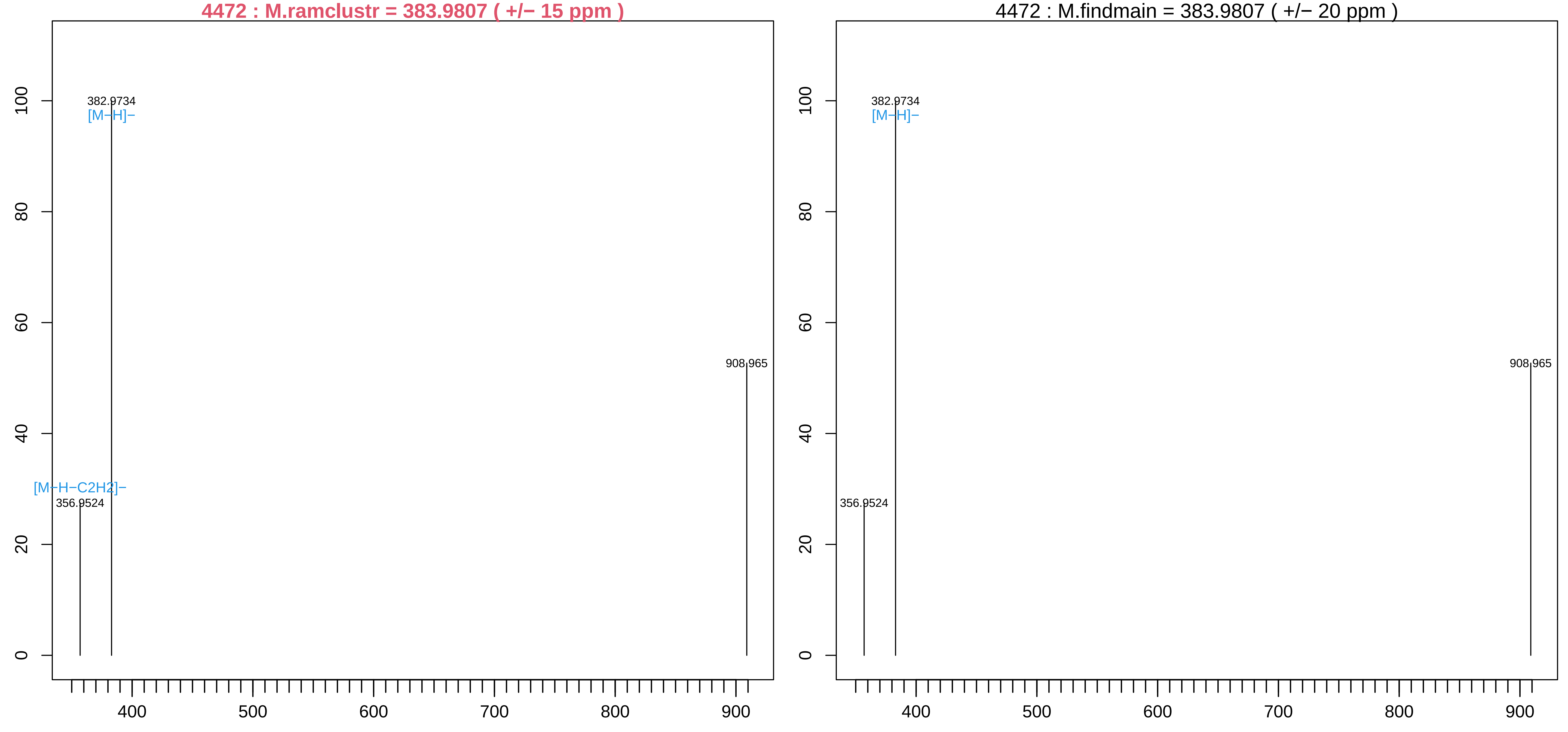
4472 : M.ramclustr = 383.9807 (+/− 15 ppm)
382.9734
[M−H]−
[M−H−C2H2]−
356.9524
908.965
4472 : M.findmain = 383.9807 (+/− 20 ppm)
382.9734
[M−H]−
356.9524
908.965
0
20
40
60
80
100
400
500
600
700
800
900

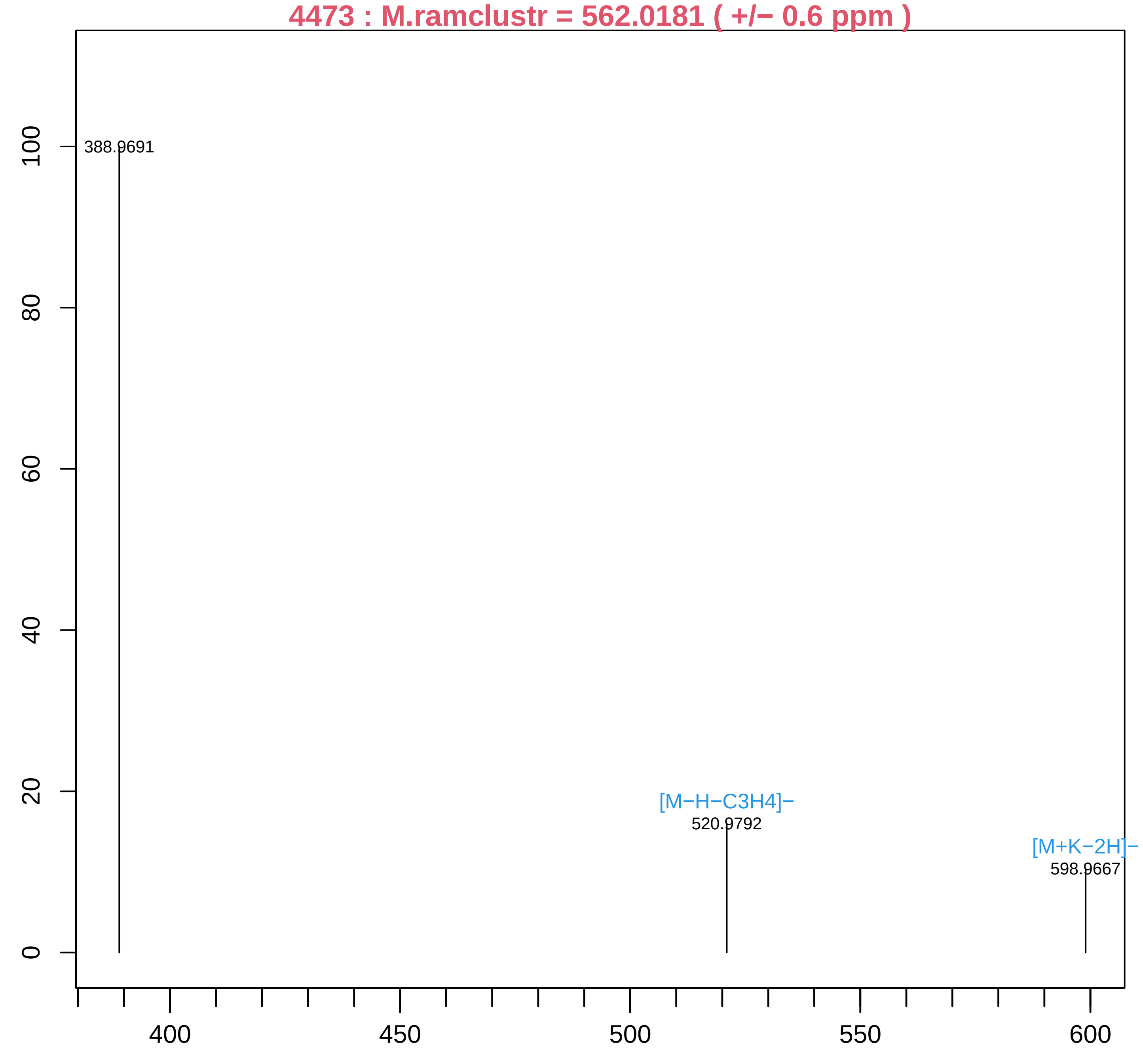
4473 : M.ramclustr = 562.0181 (+/− 0.6 ppm)
388.9691
[M−H−C3H4]−
520.9792
[M+K−2H]−
598.9667
0
20
40
60
80
100
400
450
500
550
600

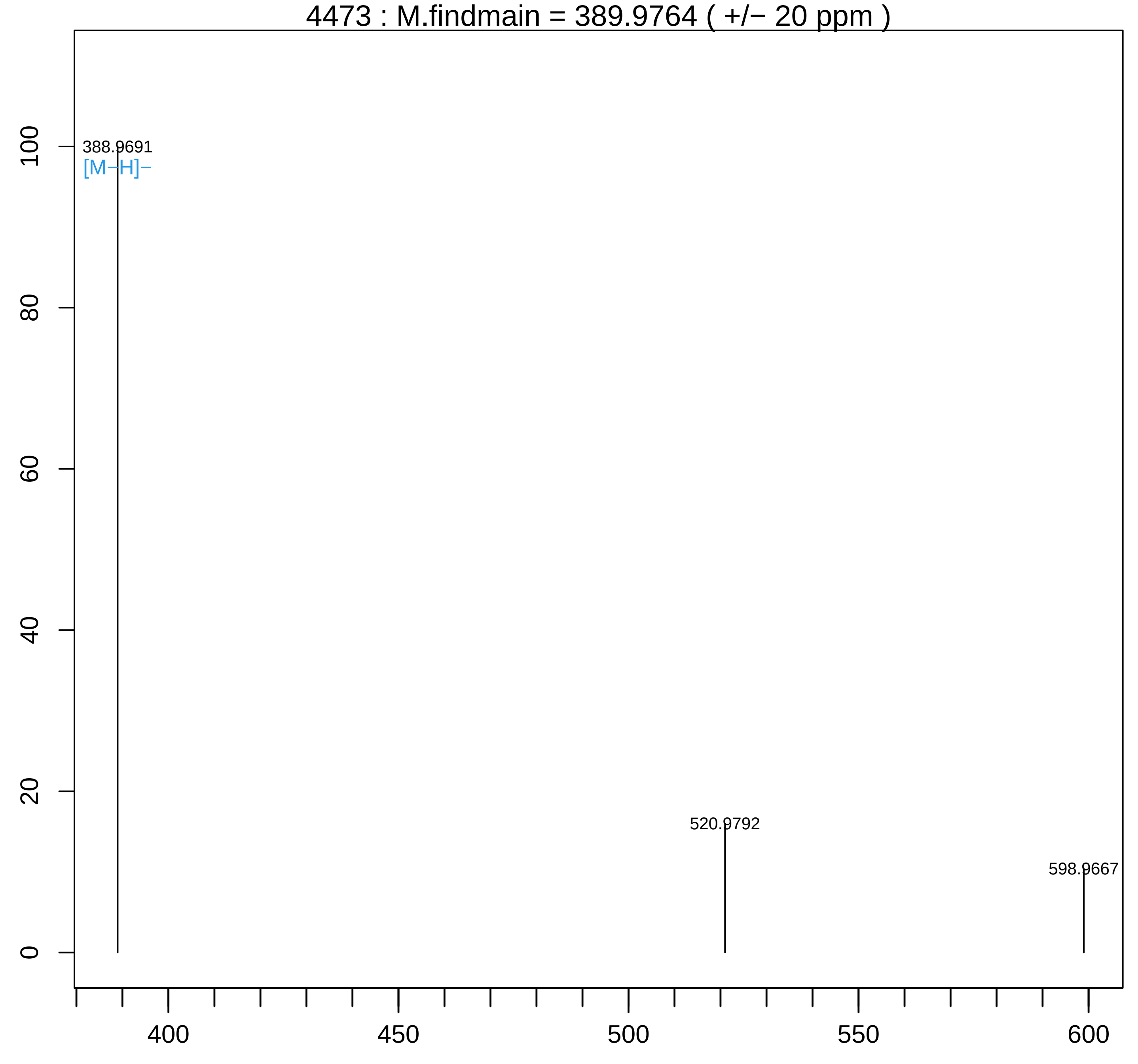
4473 : M.findmain = 389.9764 (+/− 20 ppm)
388.9691
[M−H]−
520.9792
598.9667
0
20
40
60
80
100
400
450
500
550
600

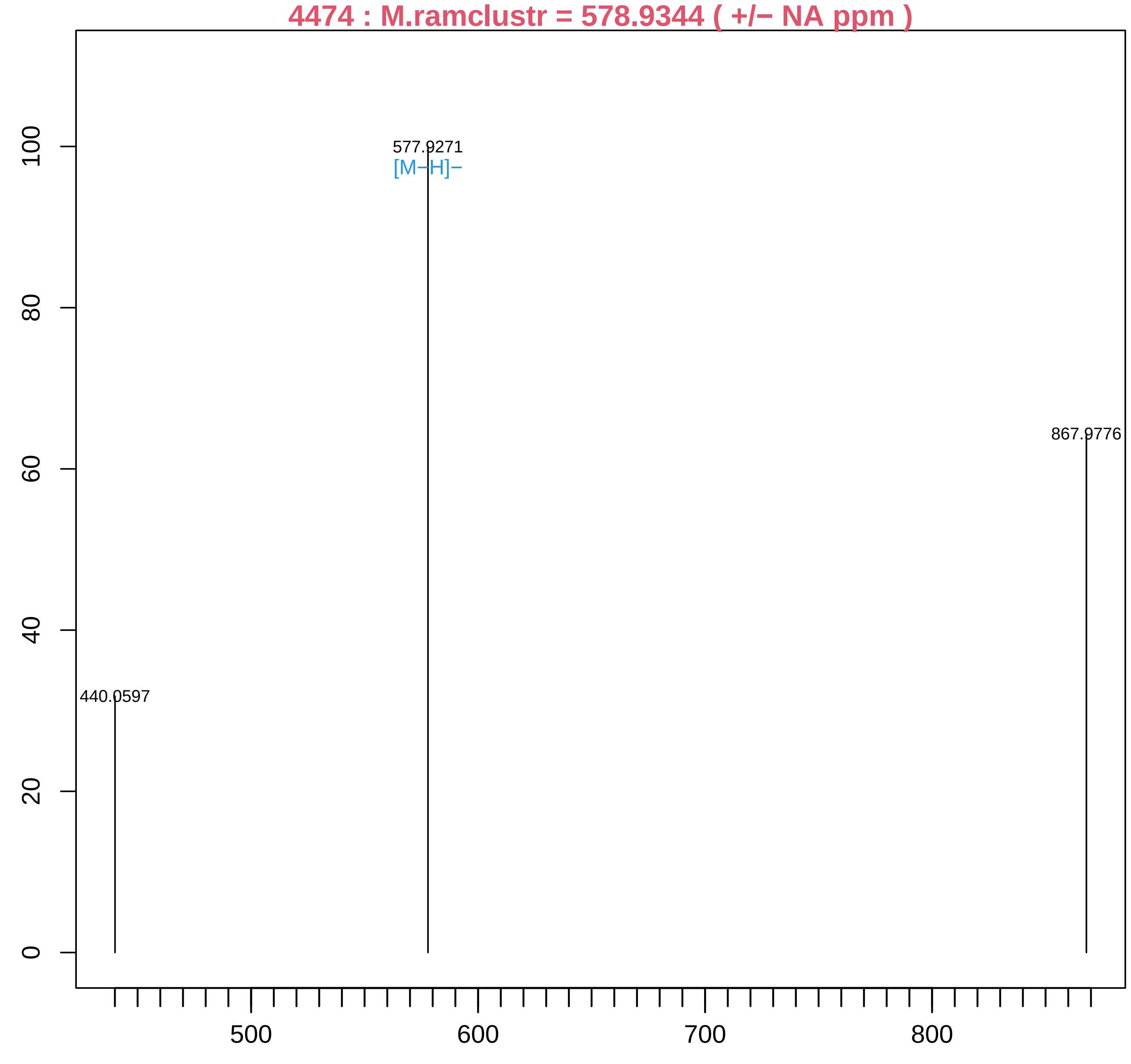
4474 : M.ramclustr = 578.9344 (+/− NA ppm)
577.9271
[M−H]−
867.9776
440.0597
0
20
40
60
80
100
500
600
700
800

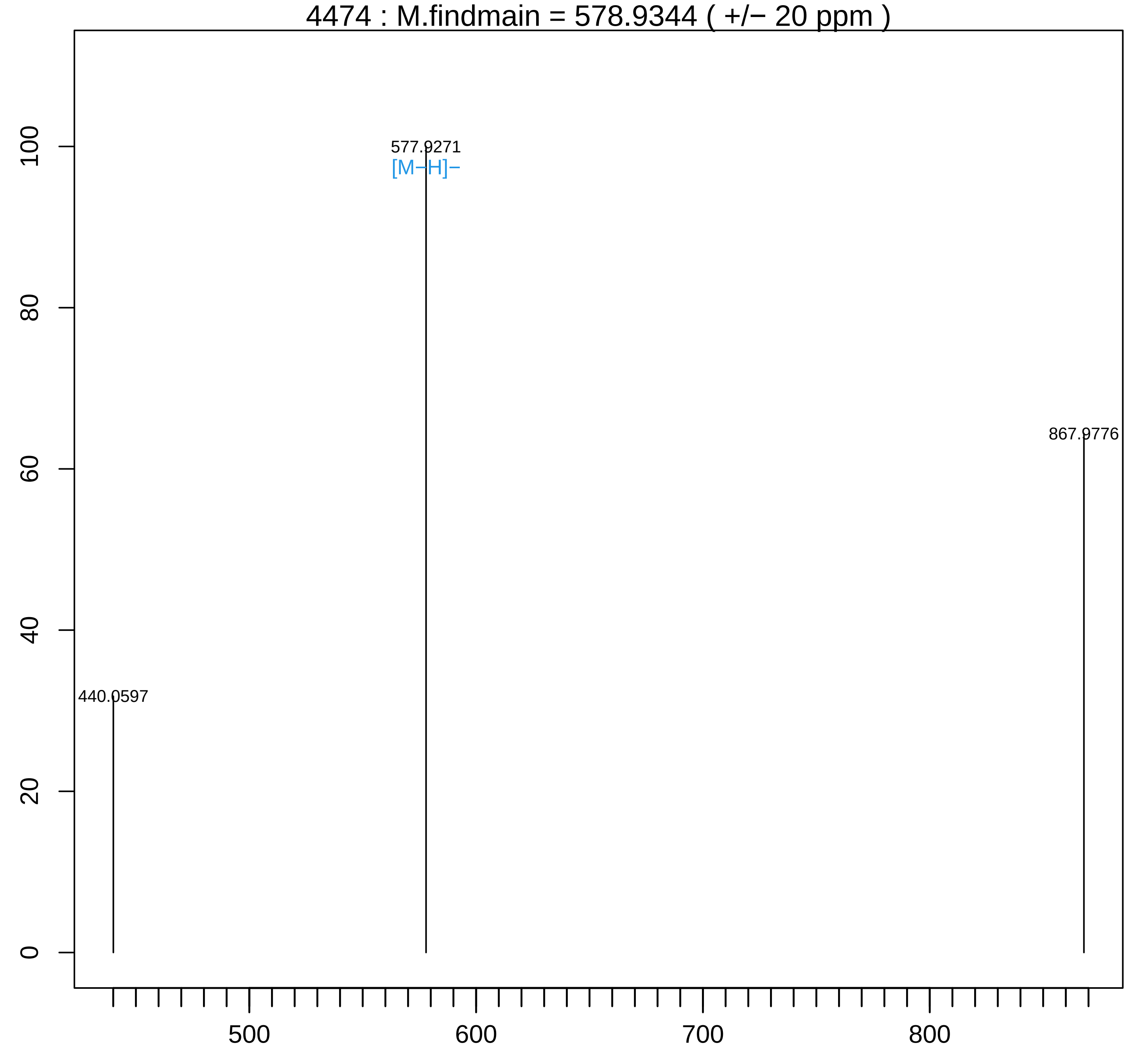
4474 : M.findmain = 578.9344 (+/− 20 ppm)
577.9271
[M−H]−
867.9776
440.0597
0
20
40
60
80
100
500
600
700
800

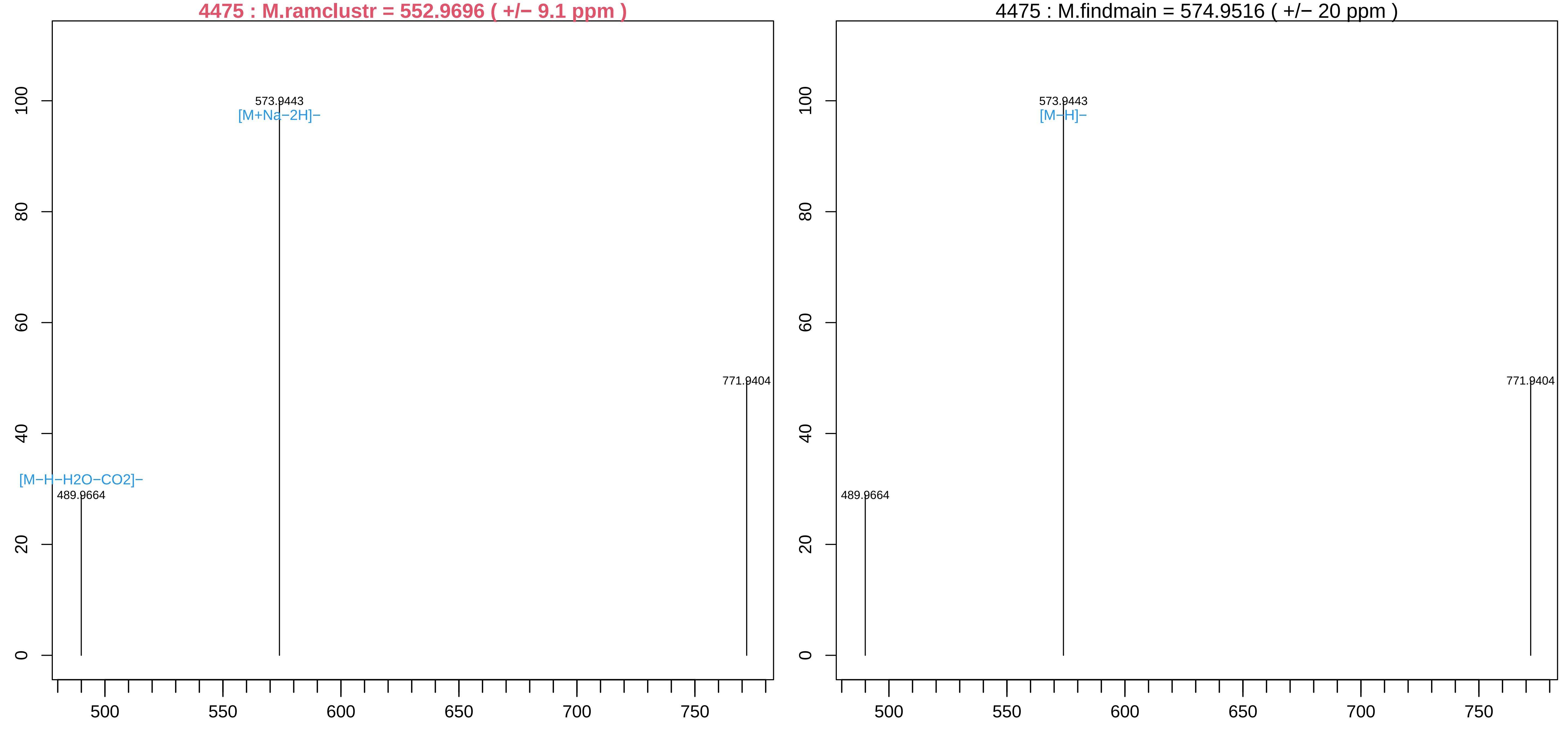

4475 : M.ramclustr = 552.9696 (+/− 9.1 ppm)
573.9443
[M+Na−2H]−
771.9404
[M−H−H2O−CO2]−
489.9664
4475 : M.findmain = 574.9516 (+/− 20 ppm)
573.9443
[M−H]−
771.9404
489.9664
0
20
40
60
80
100
500
550
600
650
700
750

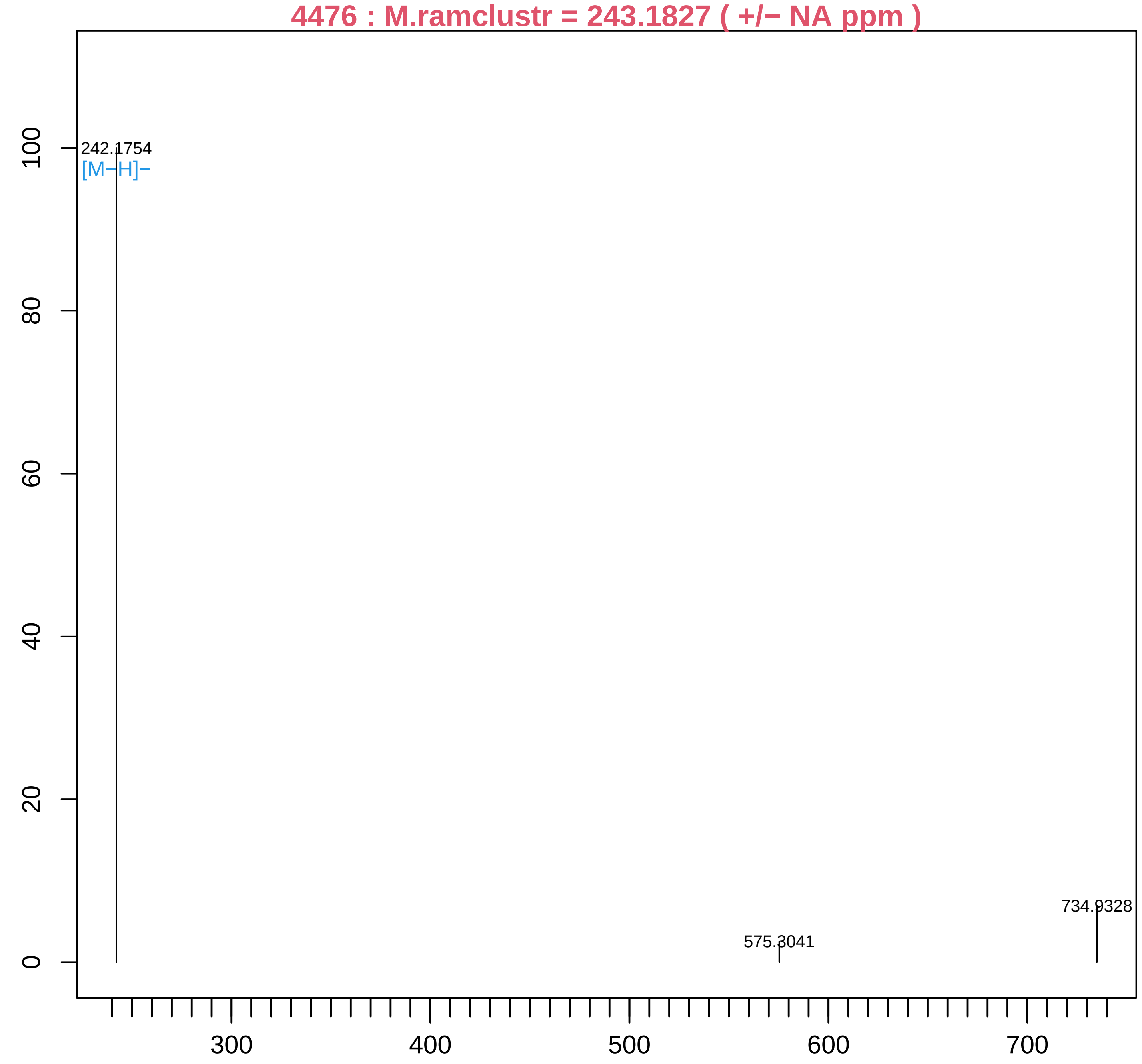
4476 : M.ramclustr = 243.1827 (+/− NA ppm)
242.1754
[M−H]−
575.3041
734.9328
0
20
40
60
80
100
300
400
500
600
700

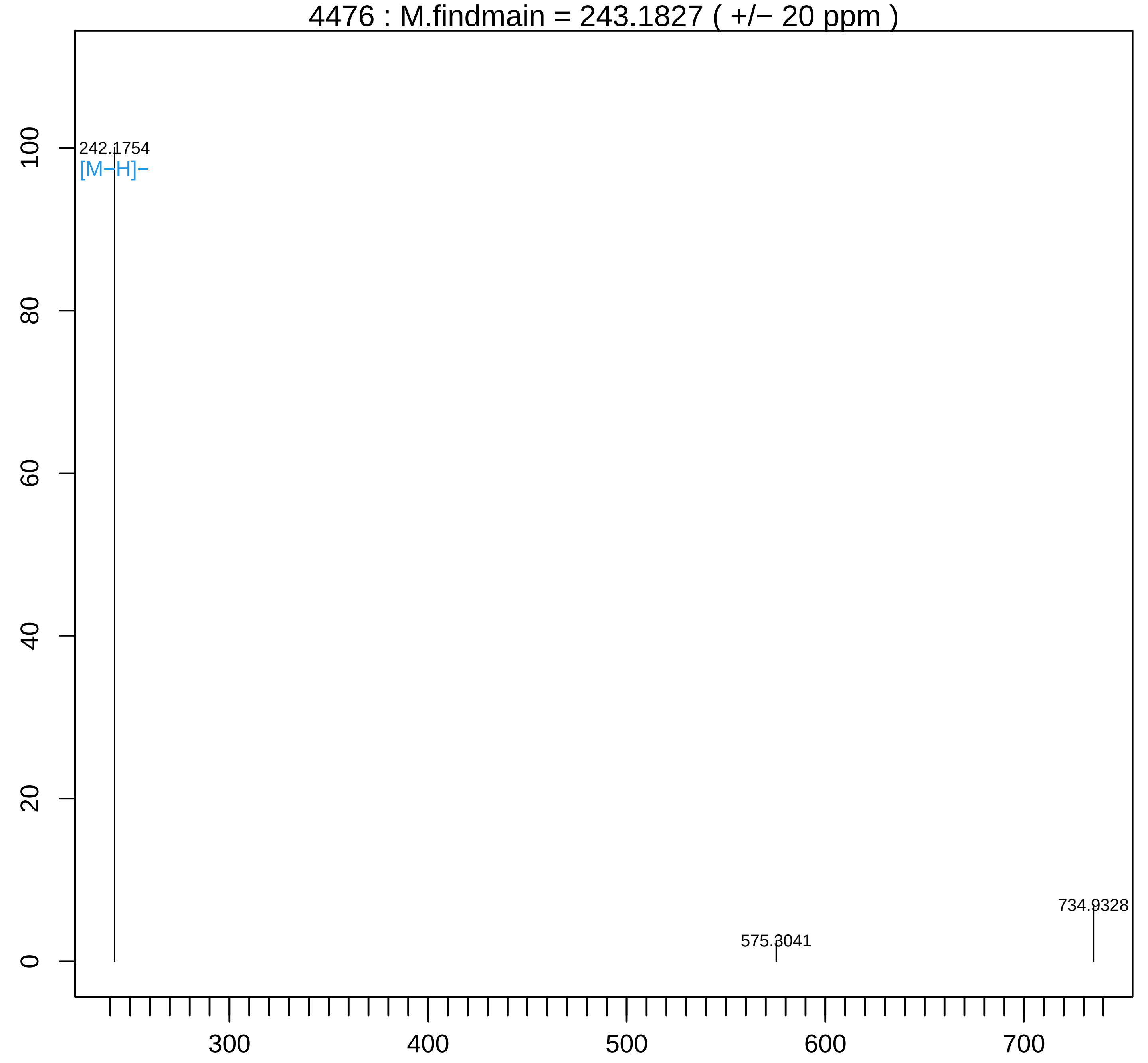
4476 : M.findmain = 243.1827 (+/− 20 ppm)
242.1754
[M−H]−
575.3041
734.9328
0
20
40
60
80
100
300
400
500
600
700

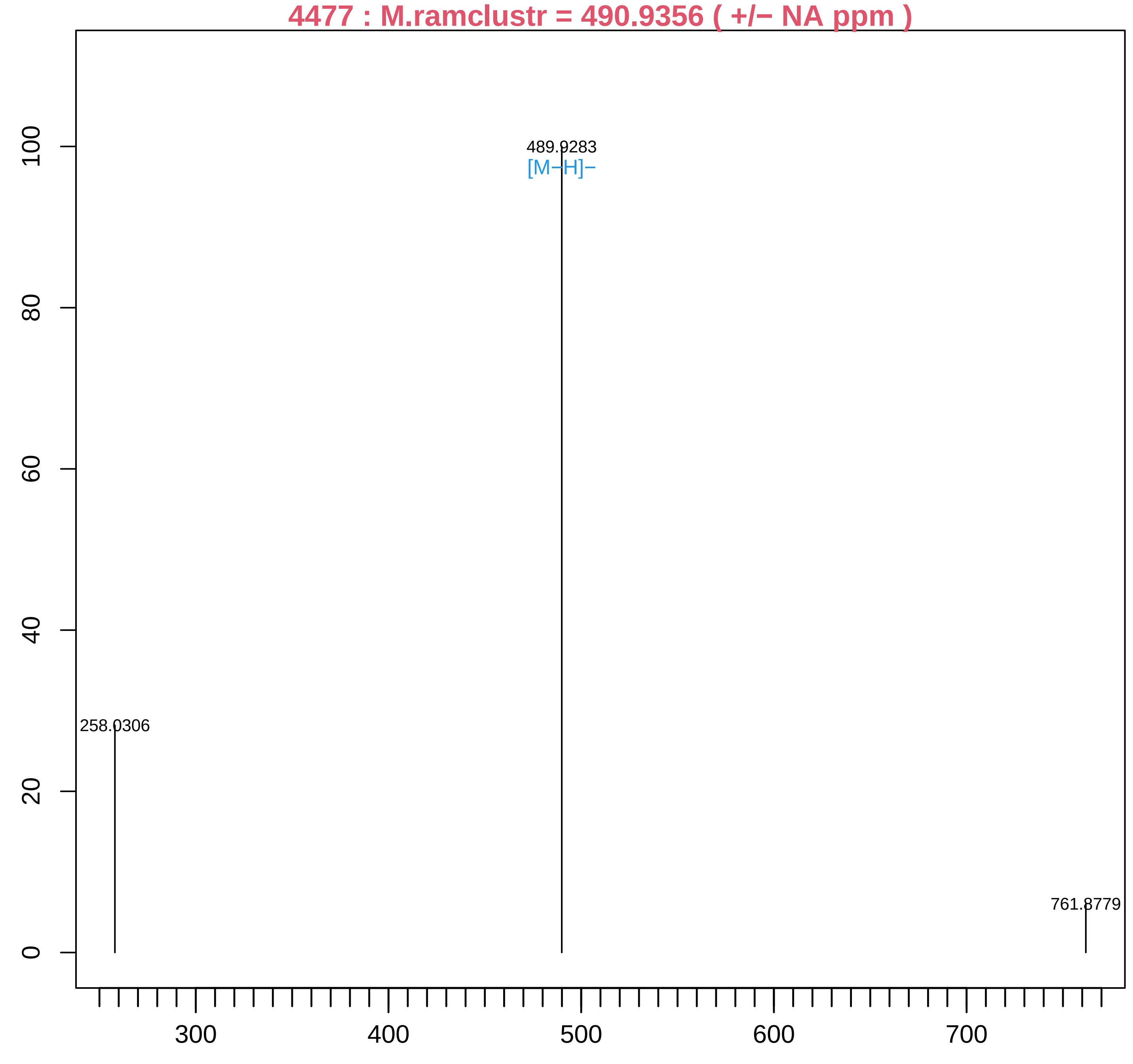
4477 : M.ramclustr = 490.9356 (+/− NA ppm)
489.9283
[M−H]−
258.0306
761.8779
0
20
40
60
80
100
300
400
500
600
700

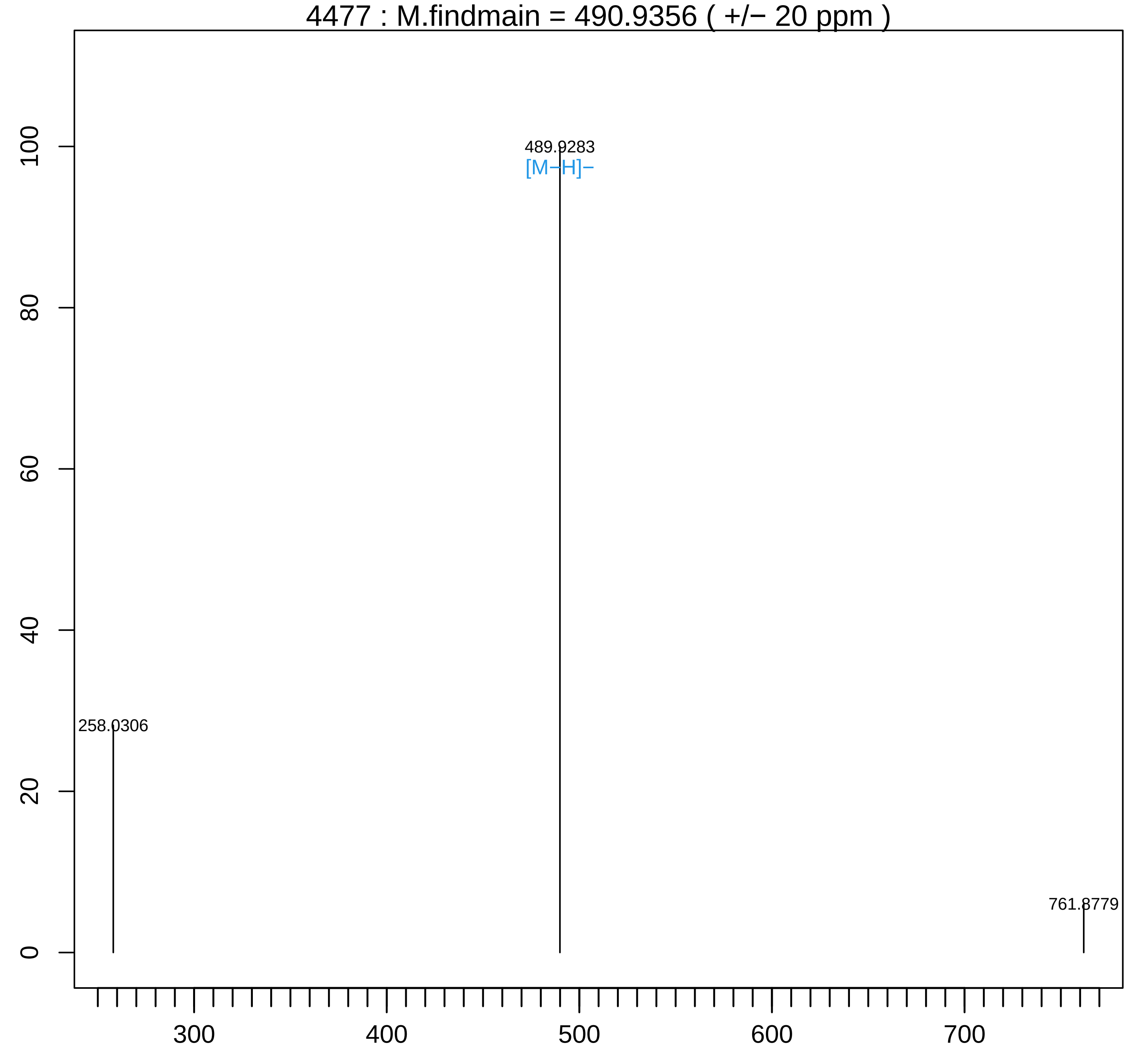
4477 : M.findmain = 490.9356 (+/− 20 ppm)
489.9283
[M−H]−
258.0306
761.8779
0
20
40
60
80
100
300
400
500
600
700

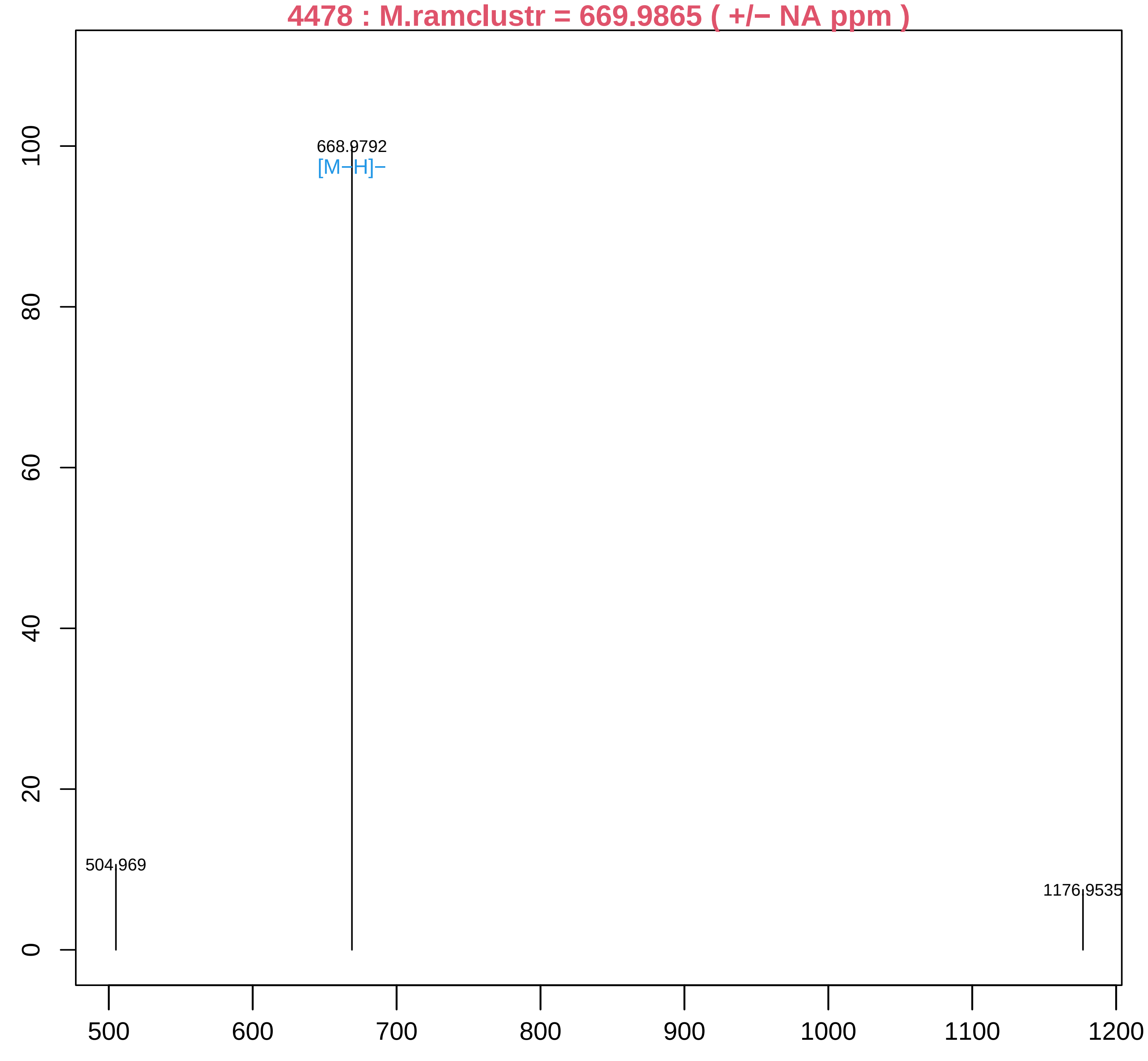
4478 : M.ramclustr = 669.9865 (+/− NA ppm)
668.9792
[M−H]−
504.969
1176.9535
0
20
40
60
80
100
500
600
700
800
900
1000
1100
1200

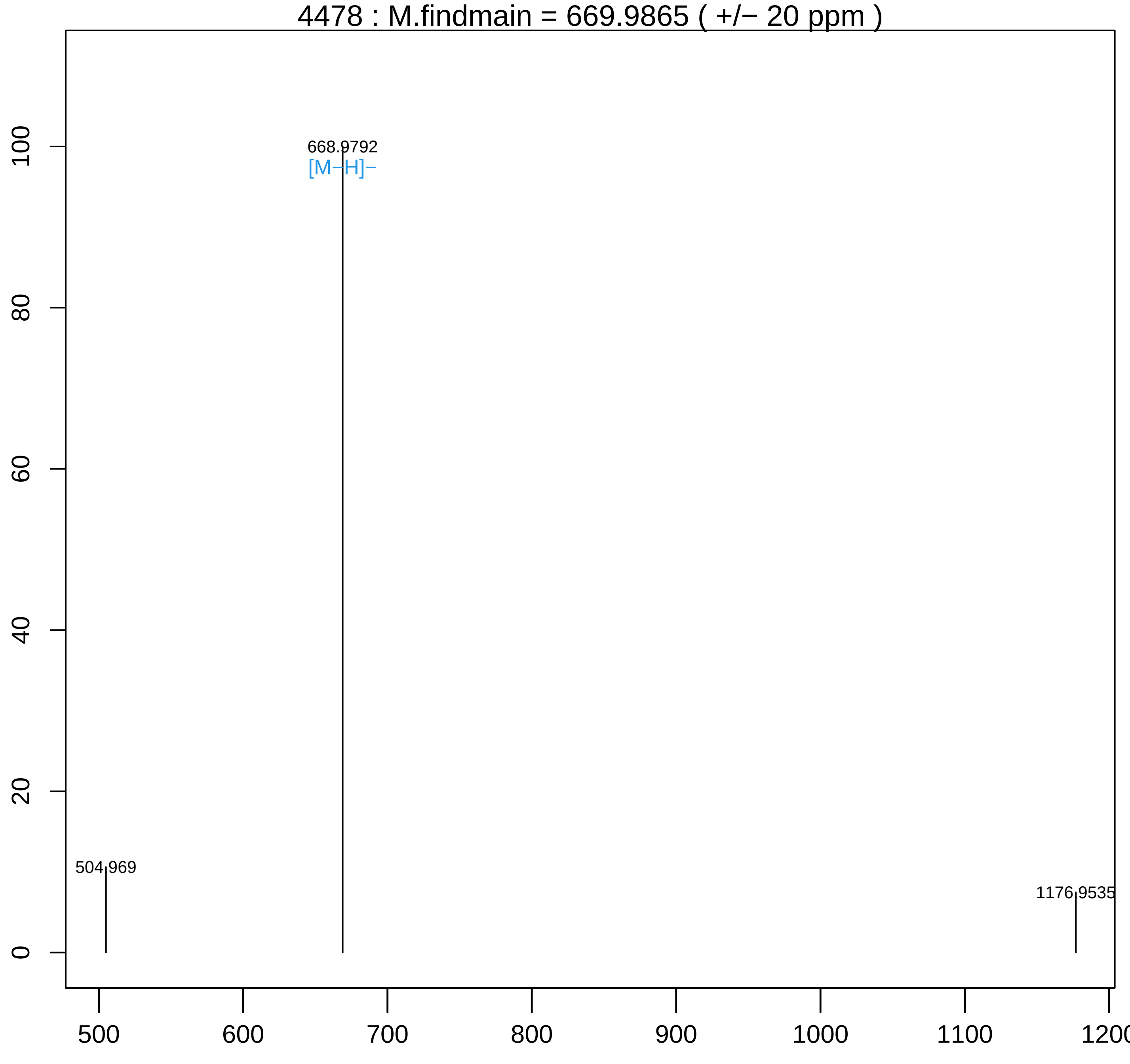
4478 : M.findmain = 669.9865 (+/− 20 ppm)
668.9792
[M−H]−
504.969
1176.9535
0
20
40
60
80
100
500
600
700
800
900
1000
1100
1200

4479 : M.ramclustr = 733.9463 (+/− NA ppm)

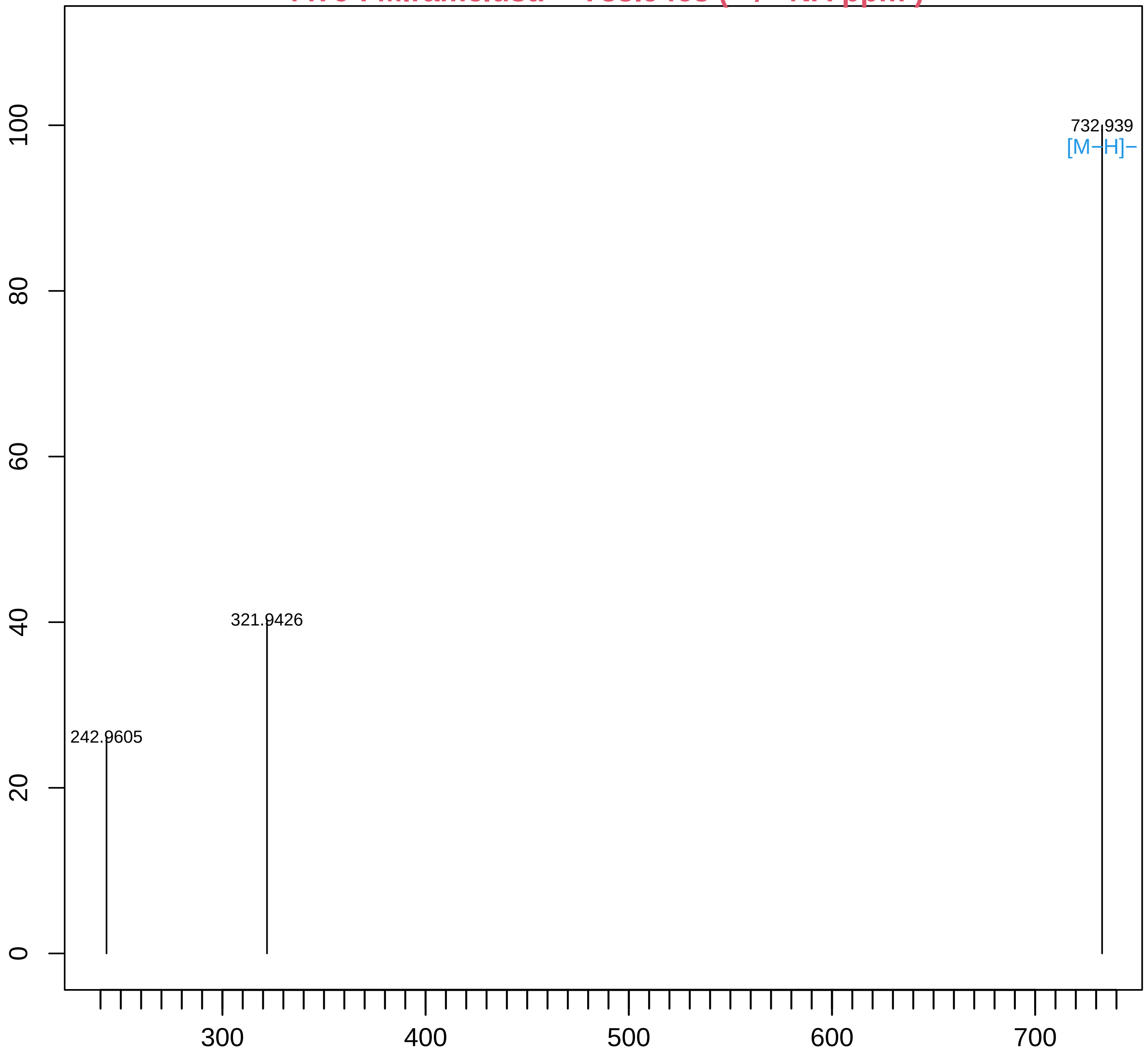

4479 : M.findmain = 733.9463 (+/− 20 ppm)

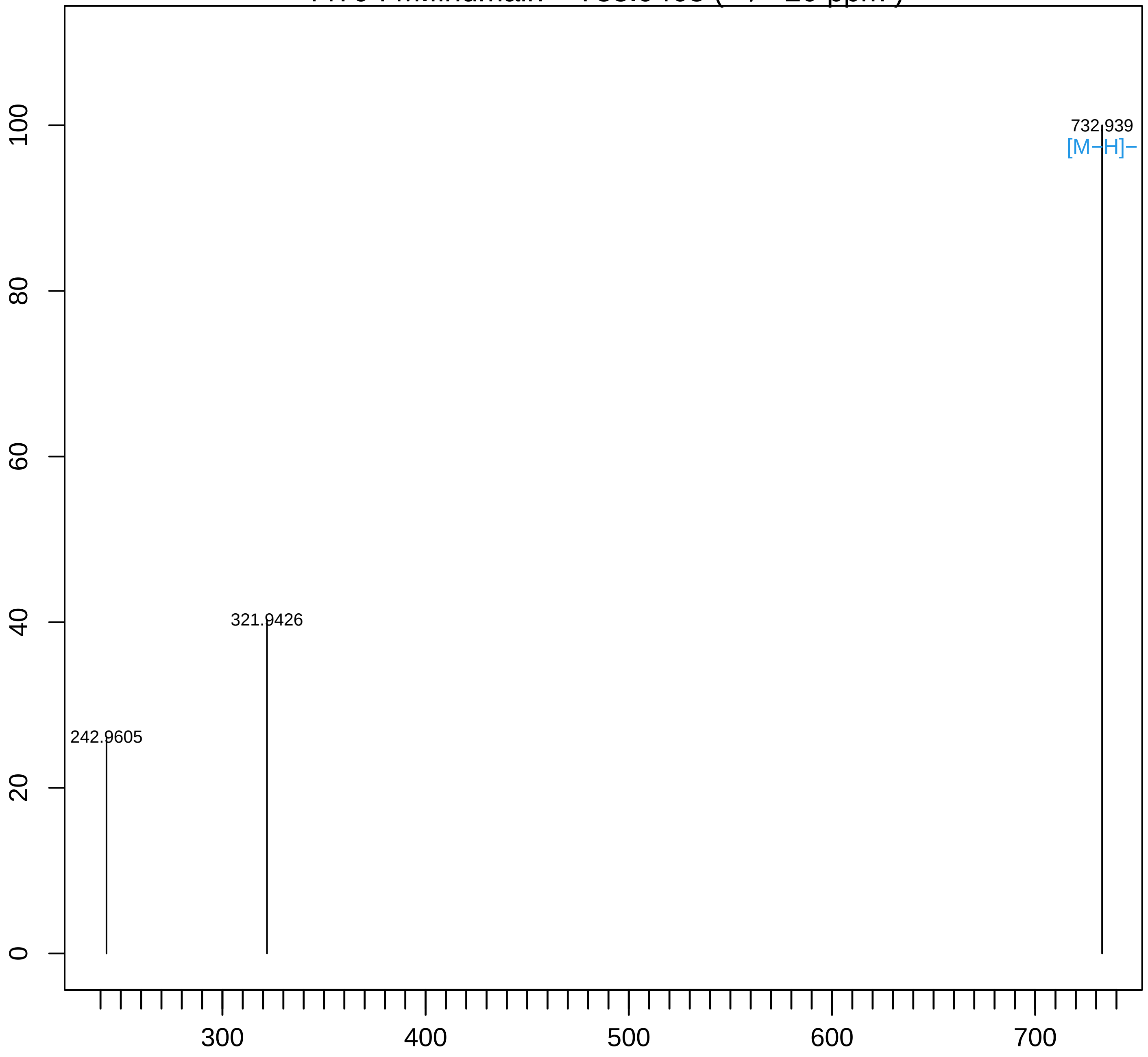

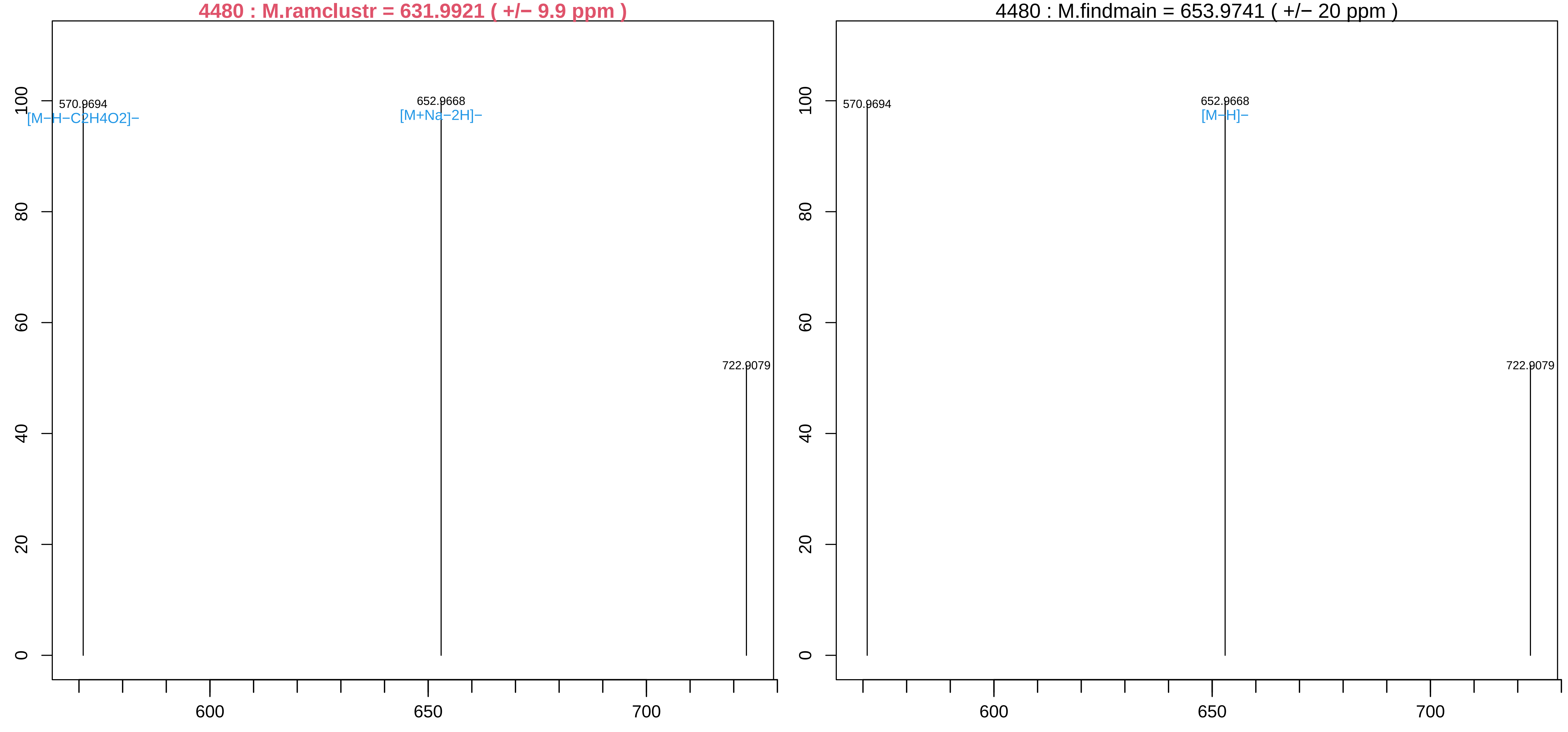
4480 : M.ramclustr = 631.9921 (+/− 9.9 ppm)
570.9694
[M−H−C2H4O2]−
652.9668
[M+Na−2H]−
722.9079
4480 : M.findmain = 653.9741 (+/− 20 ppm)
570.9694
652.9668
[M−H]−
722.9079
0
20
40
60
80
100
600
650
700

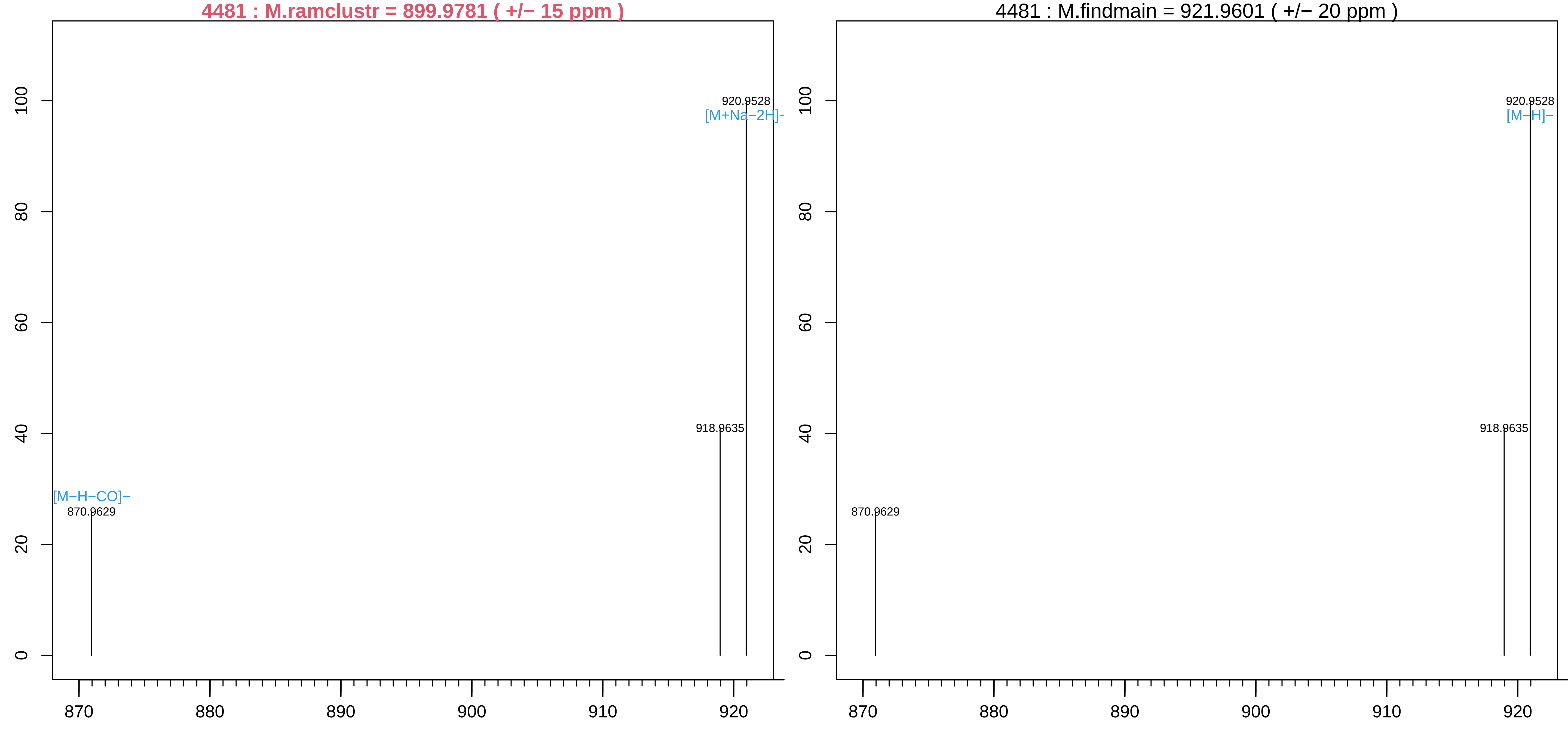
4481 : M.ramclustr = 899.9781 (+/− 15 ppm)
920.9528
[M+Na−2H]−
918.9635
[M−H−CO]−
870.9629
4481 : M.findmain = 921.9601 (+/− 20 ppm)
920.9528
[M−H]−
918.9635
870.9629
0
20
40
60
80
100
870
880
890
900
910
920

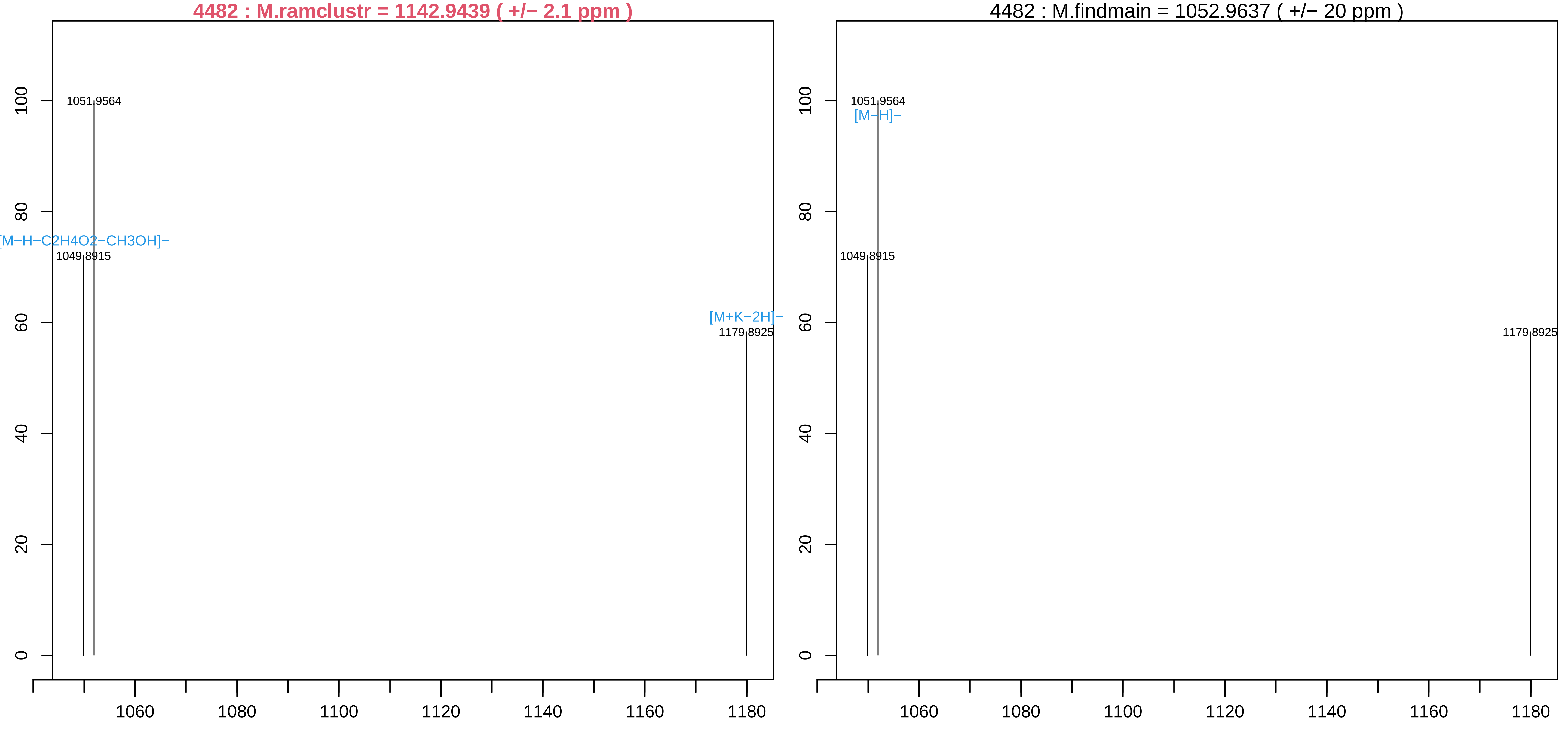

4482 : M.ramclustr = 1142.9439 (+/− 2.1 ppm)
1051.9564
[M−H−C2H4O2−CH3OH]−
1049.8915
[M+K−2H]−
1179.8925
4482 : M.findmain = 1052.9637 (+/− 20 ppm)
1051.9564
[M−H]−
1049.8915
1179.8925

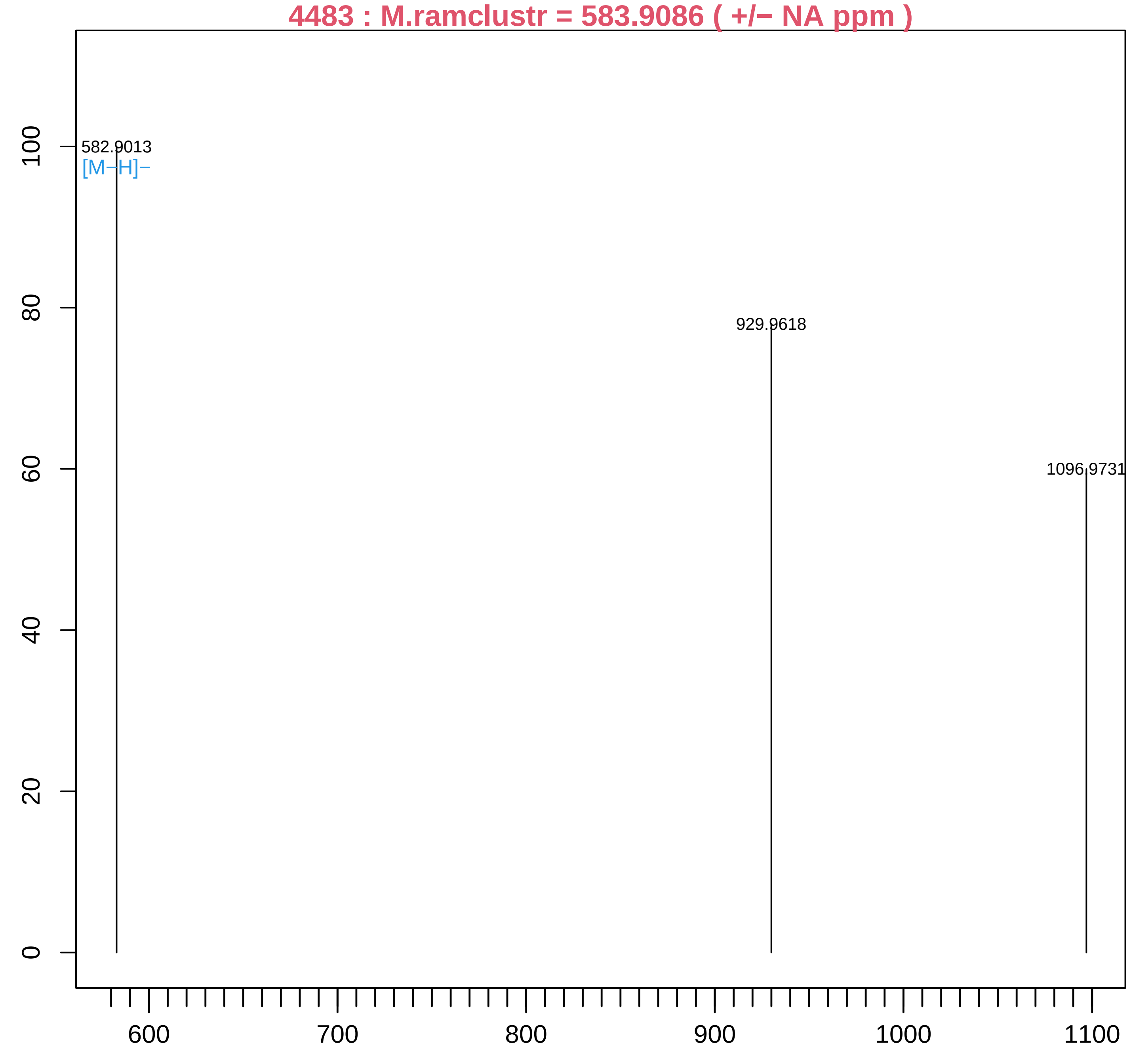
4483 : M.ramclustr = 583.9086 (+/− NA ppm)
582.9013
[M−H]−
929.9618
1096.9731
0
20
40
60
80
100
600
700
800
900
1000
1100

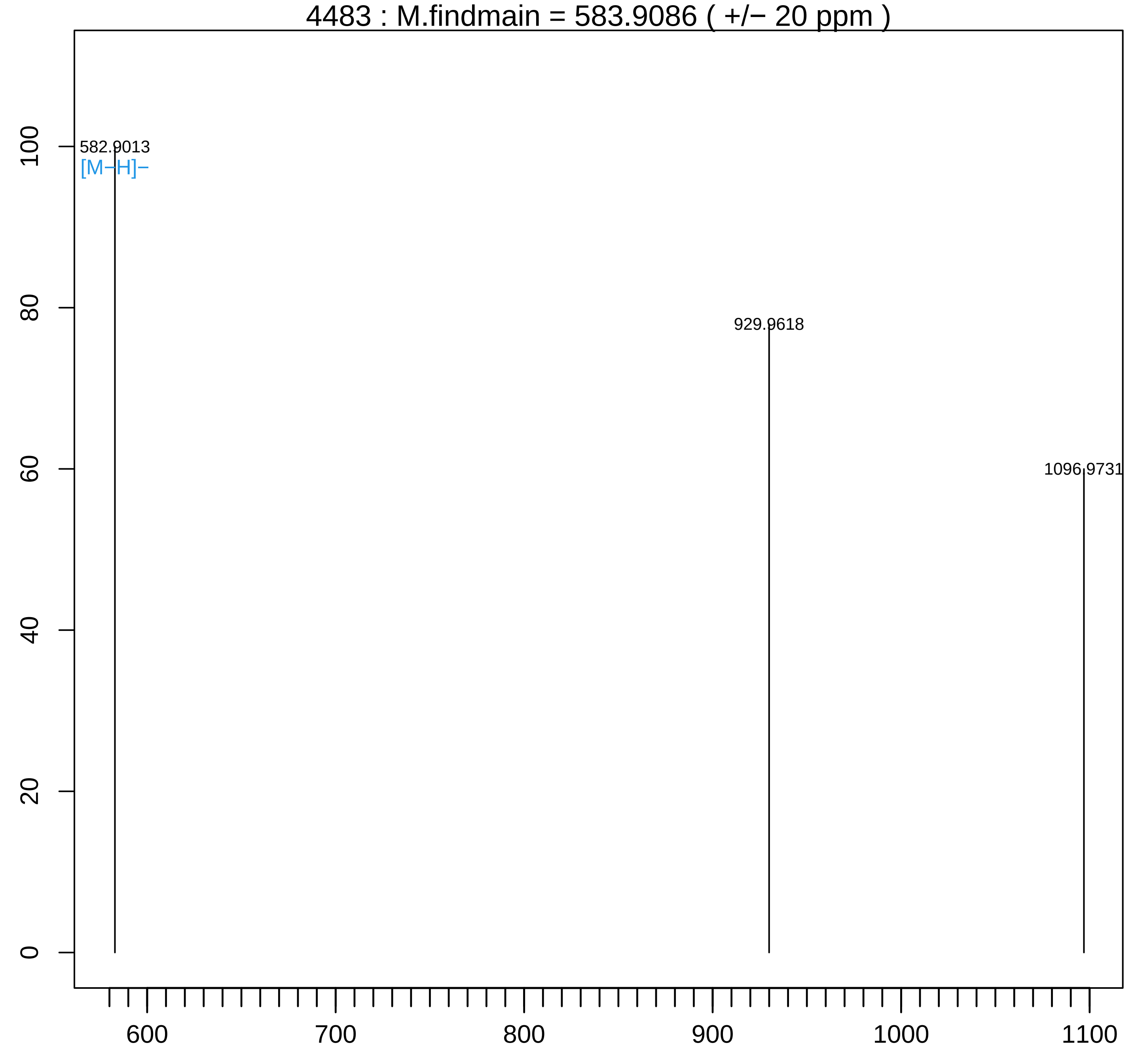
4483 : M.findmain = 583.9086 (+/− 20 ppm)
582.9013
[M−H]−
929.9618
1096.9731
0
20
40
60
80
100
600
700
800
900
1000
1100

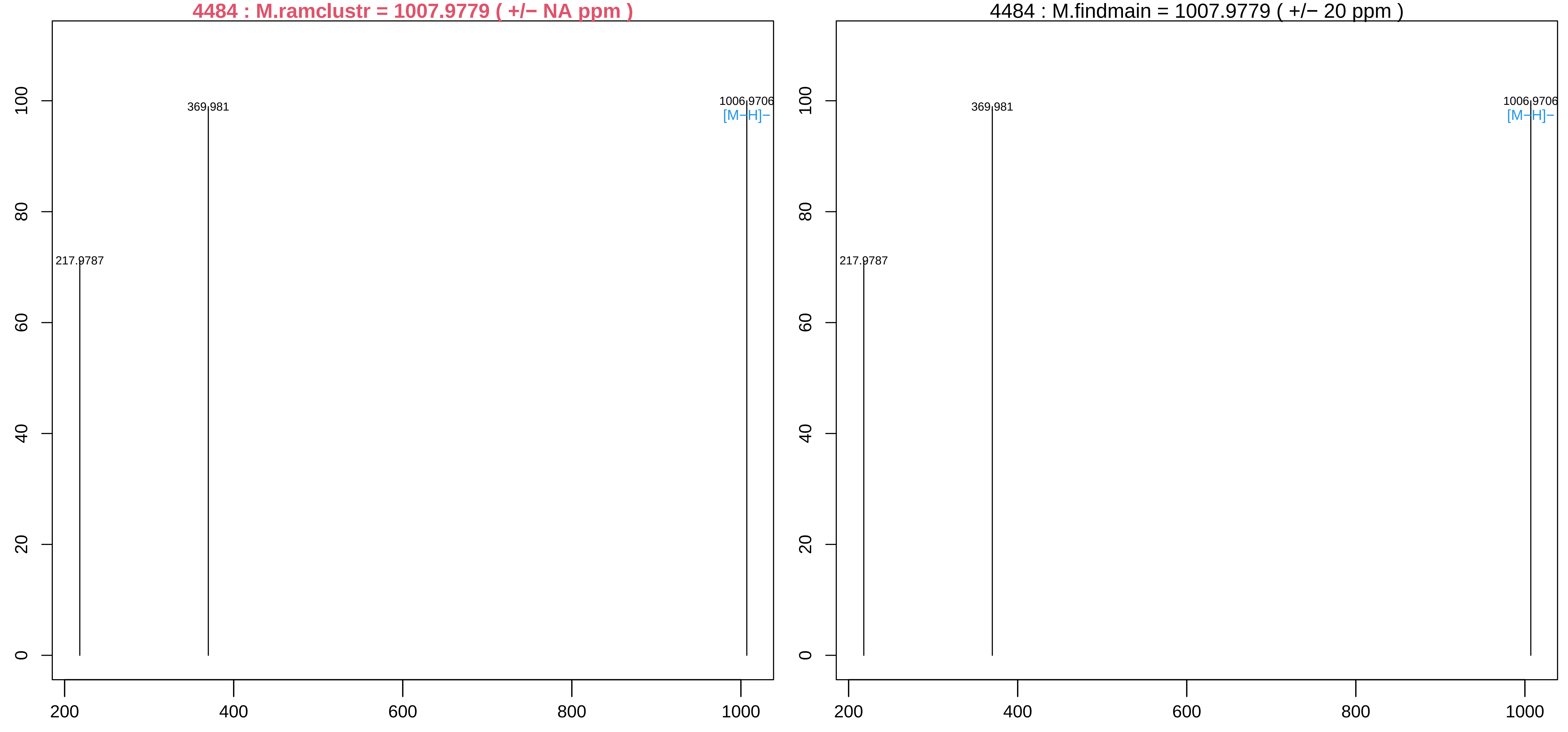

4484 : M.ramclustr = 1007.9779 (+/− NA ppm)
4484 : M.findmain = 1007.9779 (+/− 20 ppm)
217.9787
369.981
1006.9706
[M−H]−
217.9787
369.981
1006.9706
[M−H]−
0
20
40
60
80
100
200
400
600
800
1000

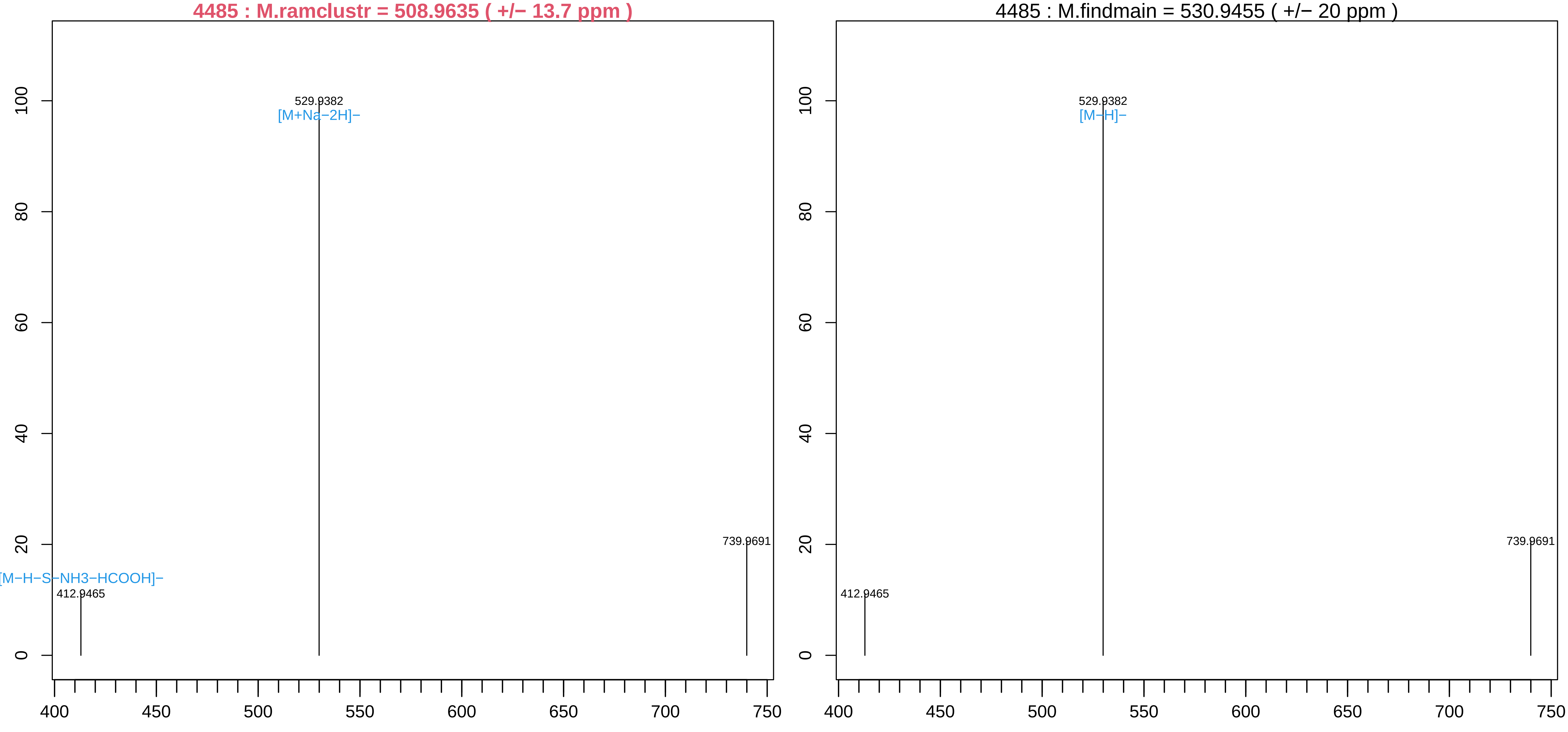

4485 : M.ramclustr = 508.9635 (+/− 13.7 ppm)
529.9382
[M+Na−2H]−
739.9691
[M−H−S−NH3−HCOOH]−
412.9465
0
20
40
60
80
100
400
450
500
550
600
650
700
750
4485 : M.findmain = 530.9455 (+/− 20 ppm)
529.9382
[M−H]−
739.9691
412.9465

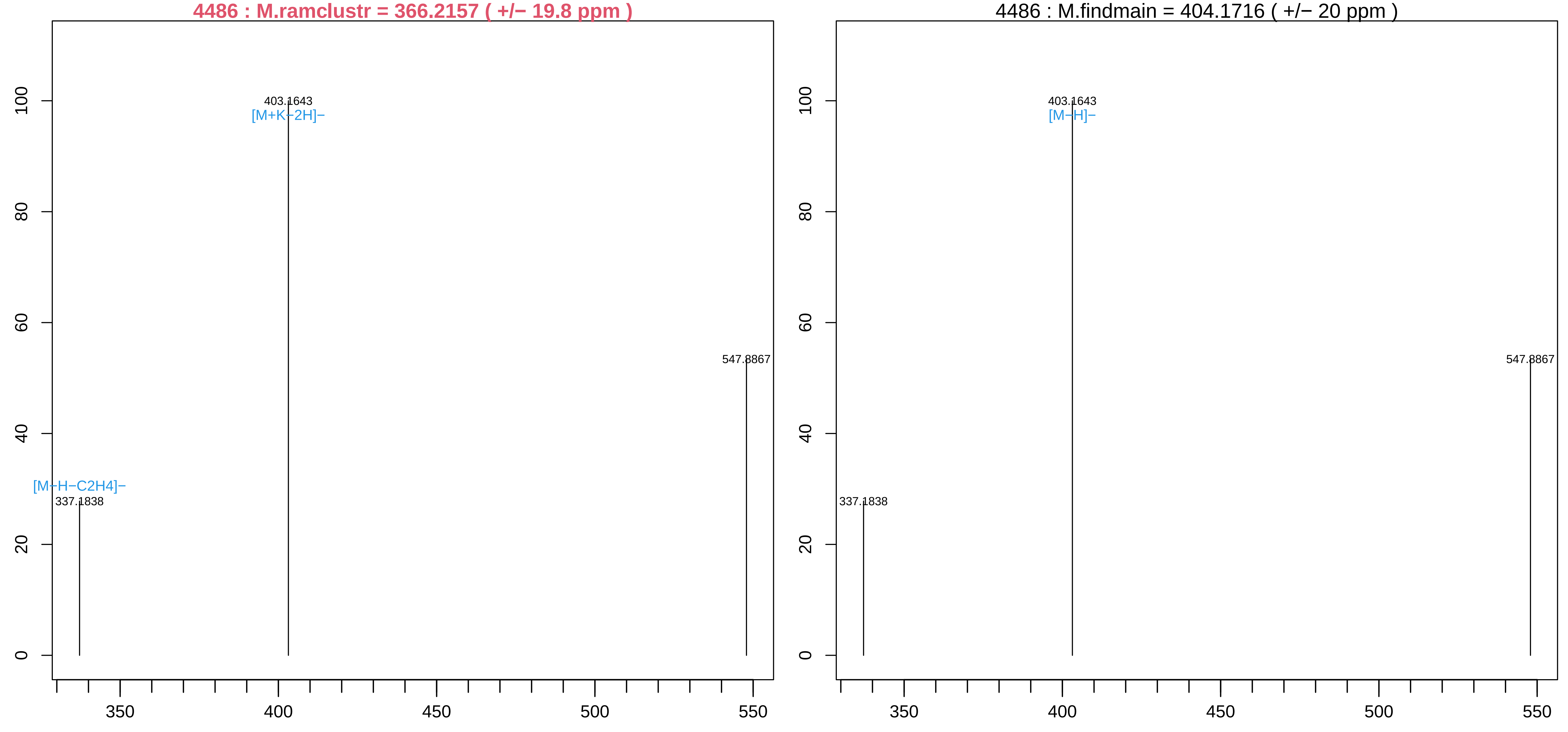
4486 : M.ramclustr = 366.2157 (+/− 19.8 ppm)
403.1643
[M+K−2H]−
547.8867
[M−H−C2H4]−
337.1838
4486 : M.findmain = 404.1716 (+/− 20 ppm)
403.1643
[M−H]−
547.8867
337.1838
0
20
40
60
80
100
350
400
450
500
550

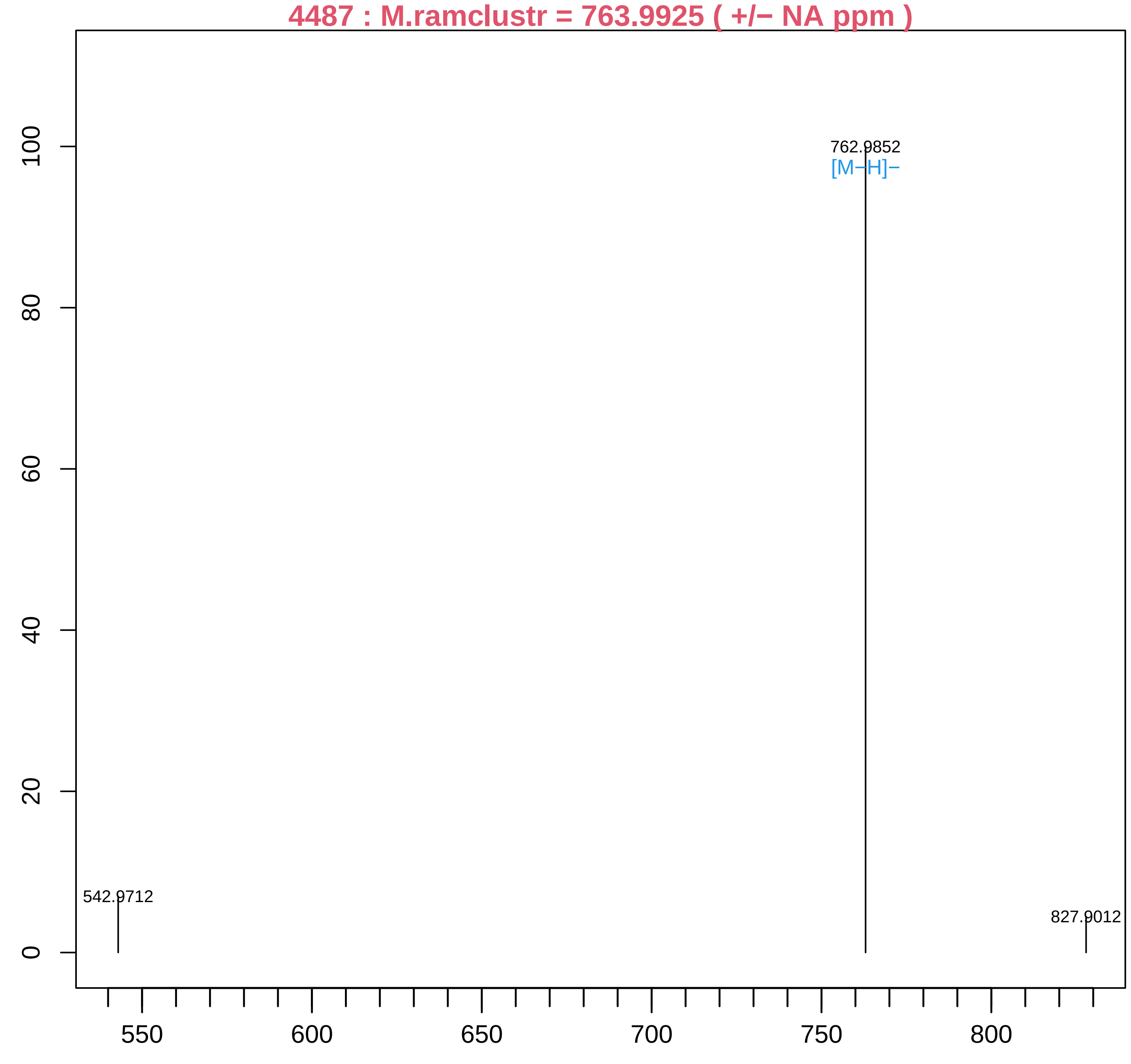
4487 : M.ramclustr = 763.9925 (+/− NA ppm)
762.9852
[M−H]−
542.9712
827.9012
0
20
40
60
80
100
550
600
650
700
750
800

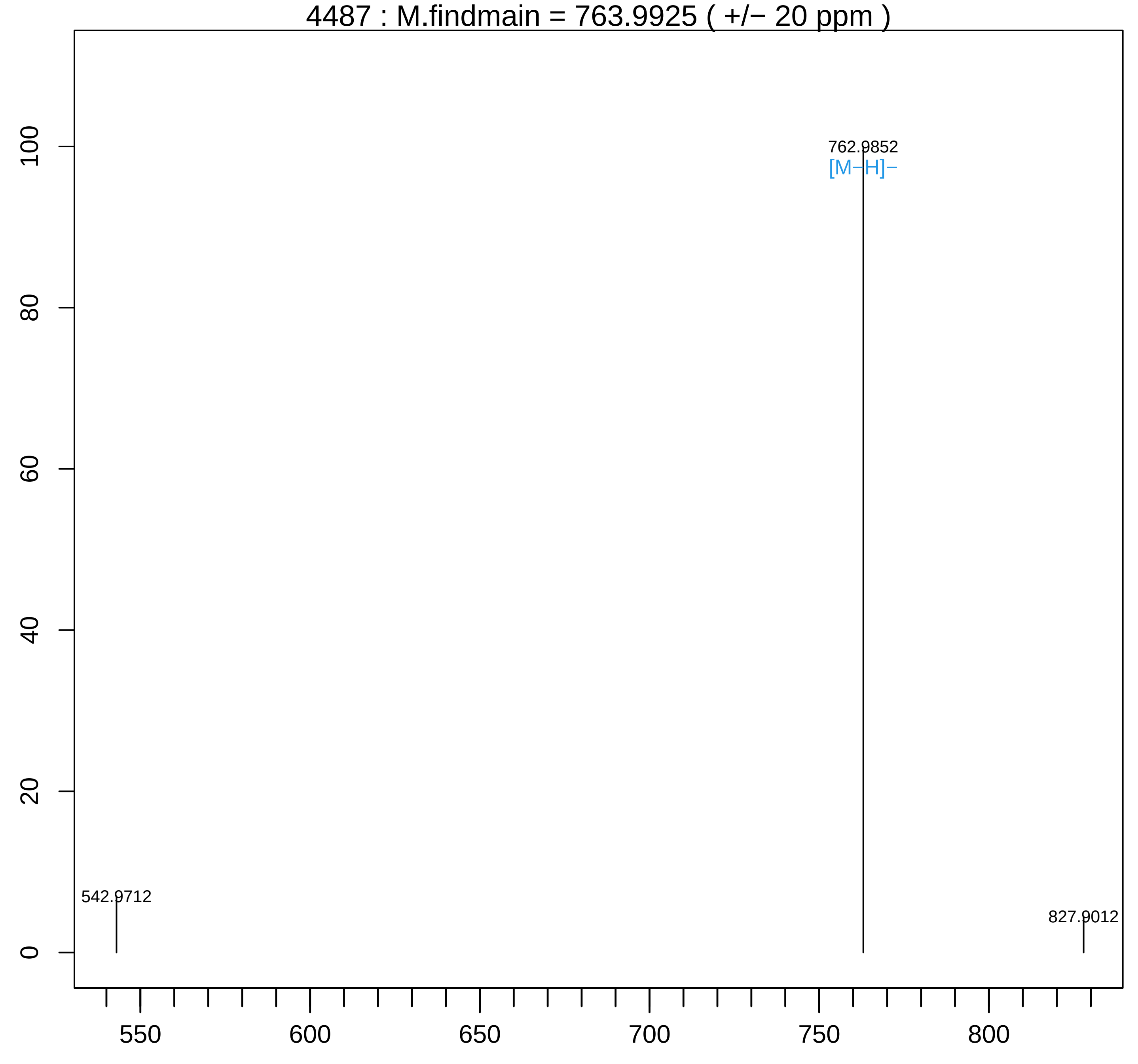
4487 : M.findmain = 763.9925 (+/− 20 ppm)
762.9852
[M−H]−
542.9712
827.9012
0
20
40
60
80
100
550
600
650
700
750
800

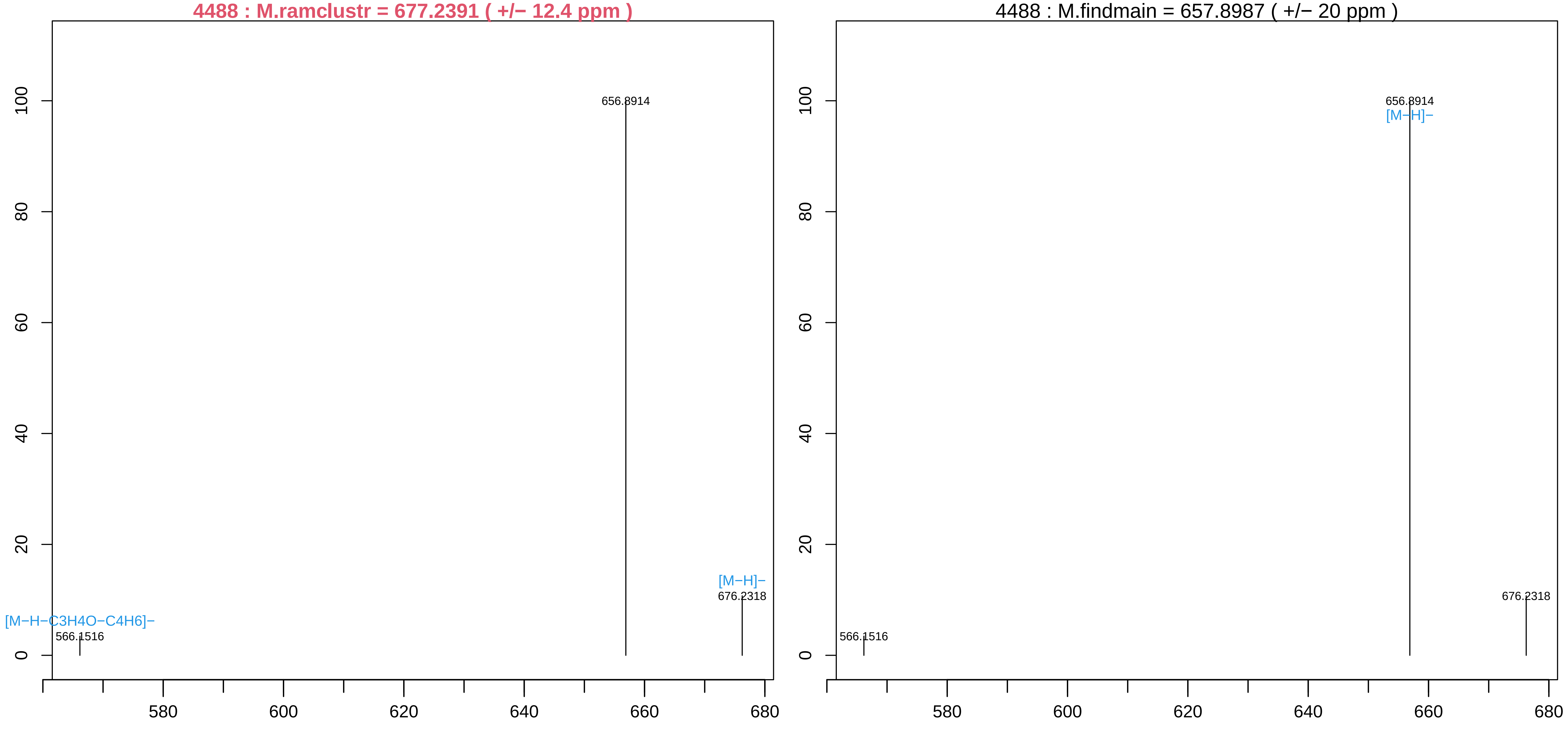

4488 : M.ramclustr = 677.2391 (+/− 12.4 ppm)
656.8914
[M−H]−
676.2318
[M−H−C3H4O−C4H6]−
566.1516
0
20
40
60
80
100
580
600
620
640
660
680
4488 : M.findmain = 657.8987 (+/− 20 ppm)
656.8914
[M−H]−
676.2318
566.1516

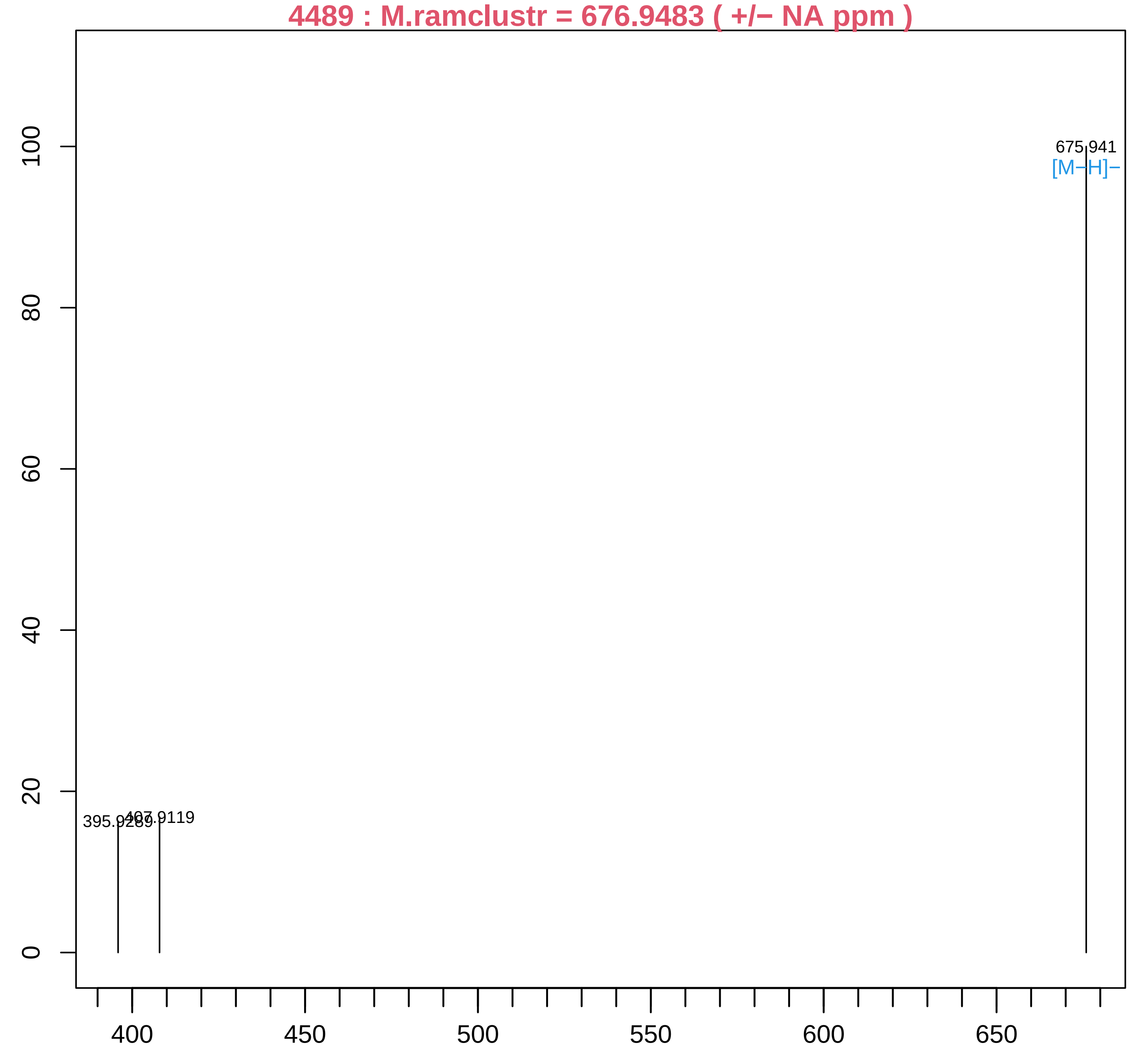
4489 : M.ramclustr = 676.9483 (+/− NA ppm)
675.941
[M−H]−
395.9289
407.9119
0
20
40
60
80
100
400
450
500
550
600
650

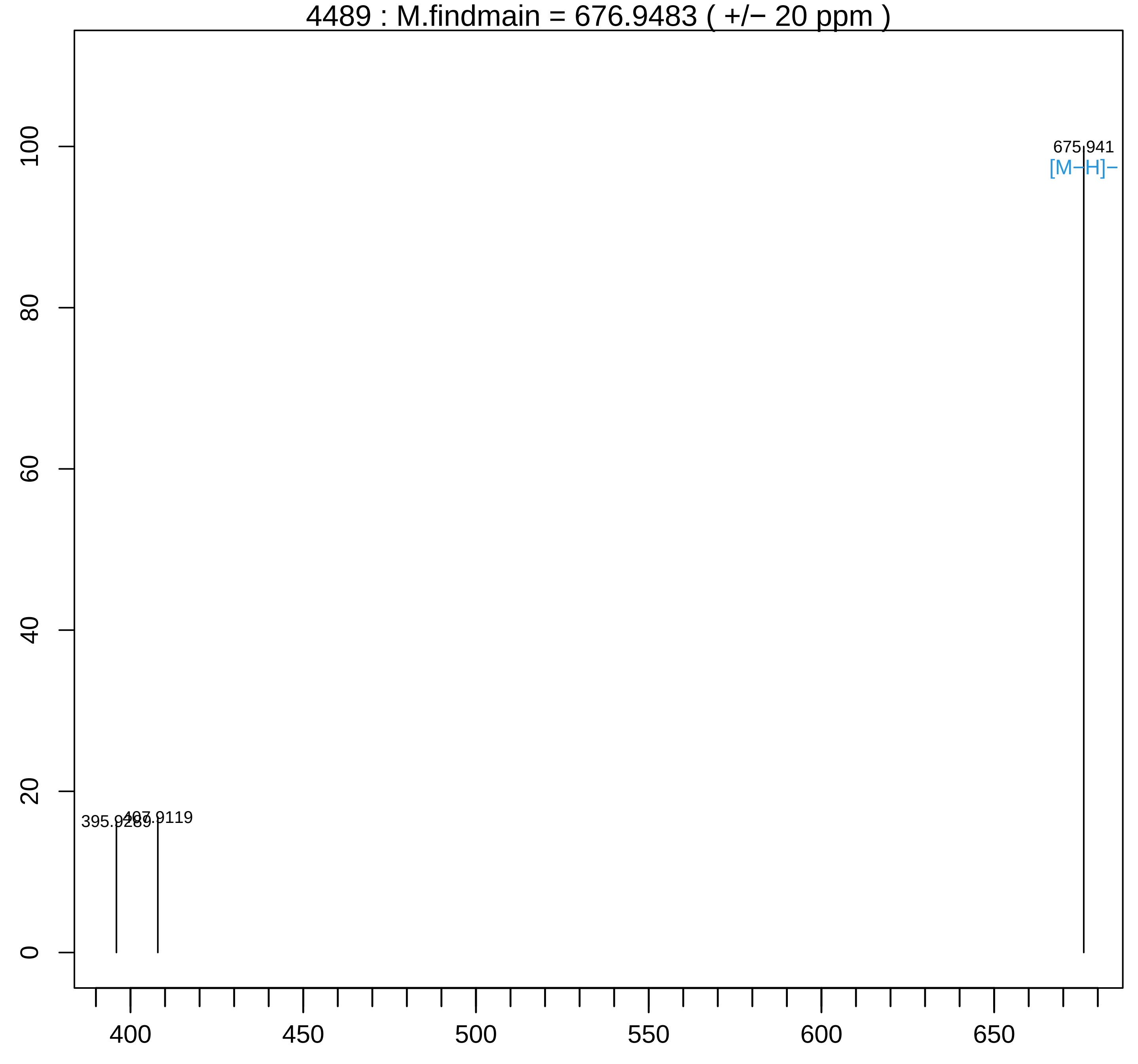
4489 : M.findmain = 676.9483 (+/− 20 ppm)
675.941
[M−H]−
395.9289
407.9119
0
20
40
60
80
100
400
450
500
550
600
650

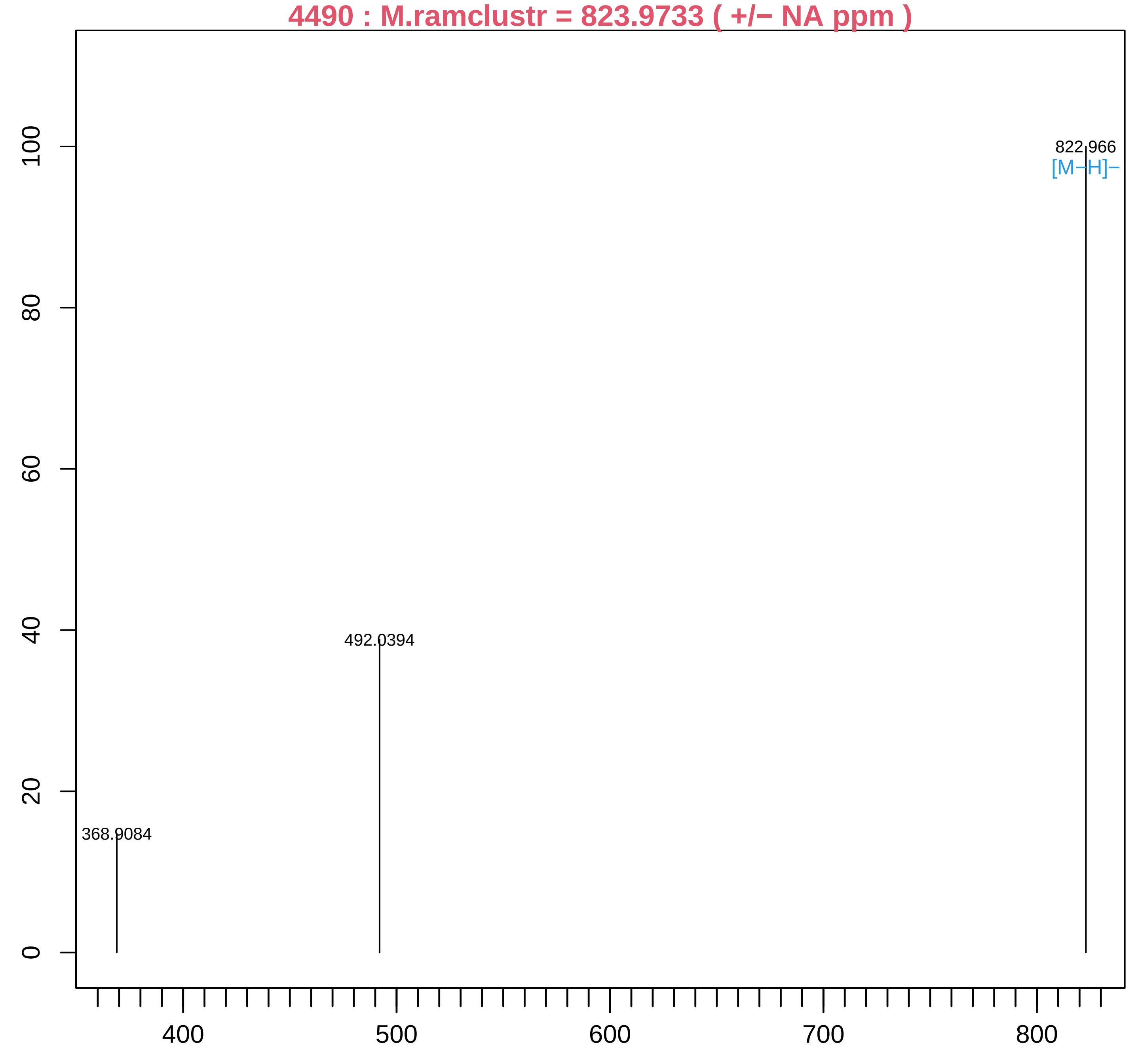
4490 : M.ramclustr = 823.9733 (+/− NA ppm)
822.966
[M−H]−
492.0394
368.9084
0
20
40
60
80
100
400
500
600
700
800

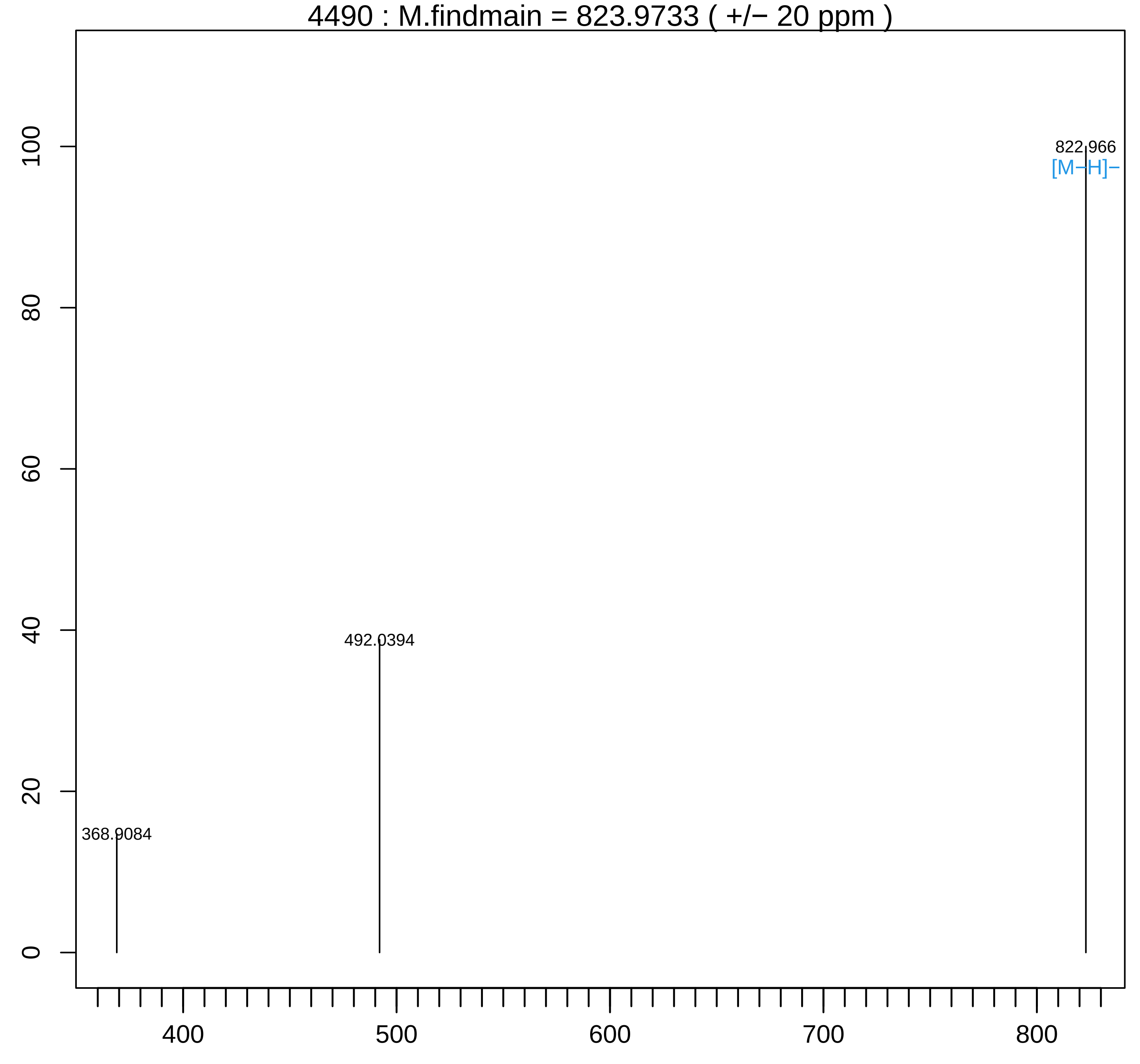
4490 : M.findmain = 823.9733 (+/− 20 ppm)
822.966
[M−H]−
492.0394
368.9084
0
20
40
60
80
100
400
500
600
700
800

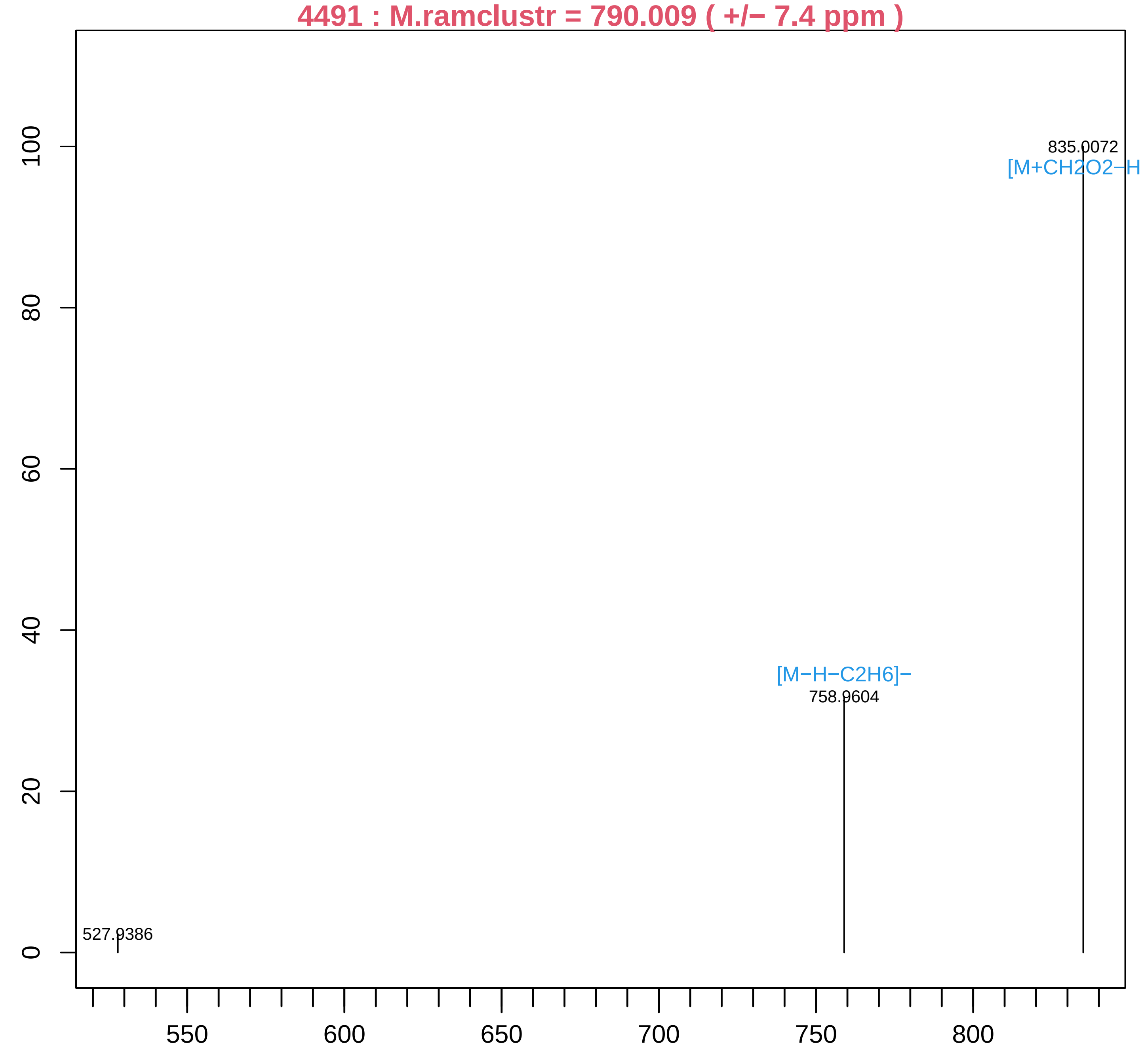
4491 : M.ramclustr = 790.009 (+/− 7.4 ppm)
835.0072
[M+CH2O2−H
[M−H−C2H6]−
758.9604
527.9386
0
20
40
60
80
100
550
600
650
700
750
800

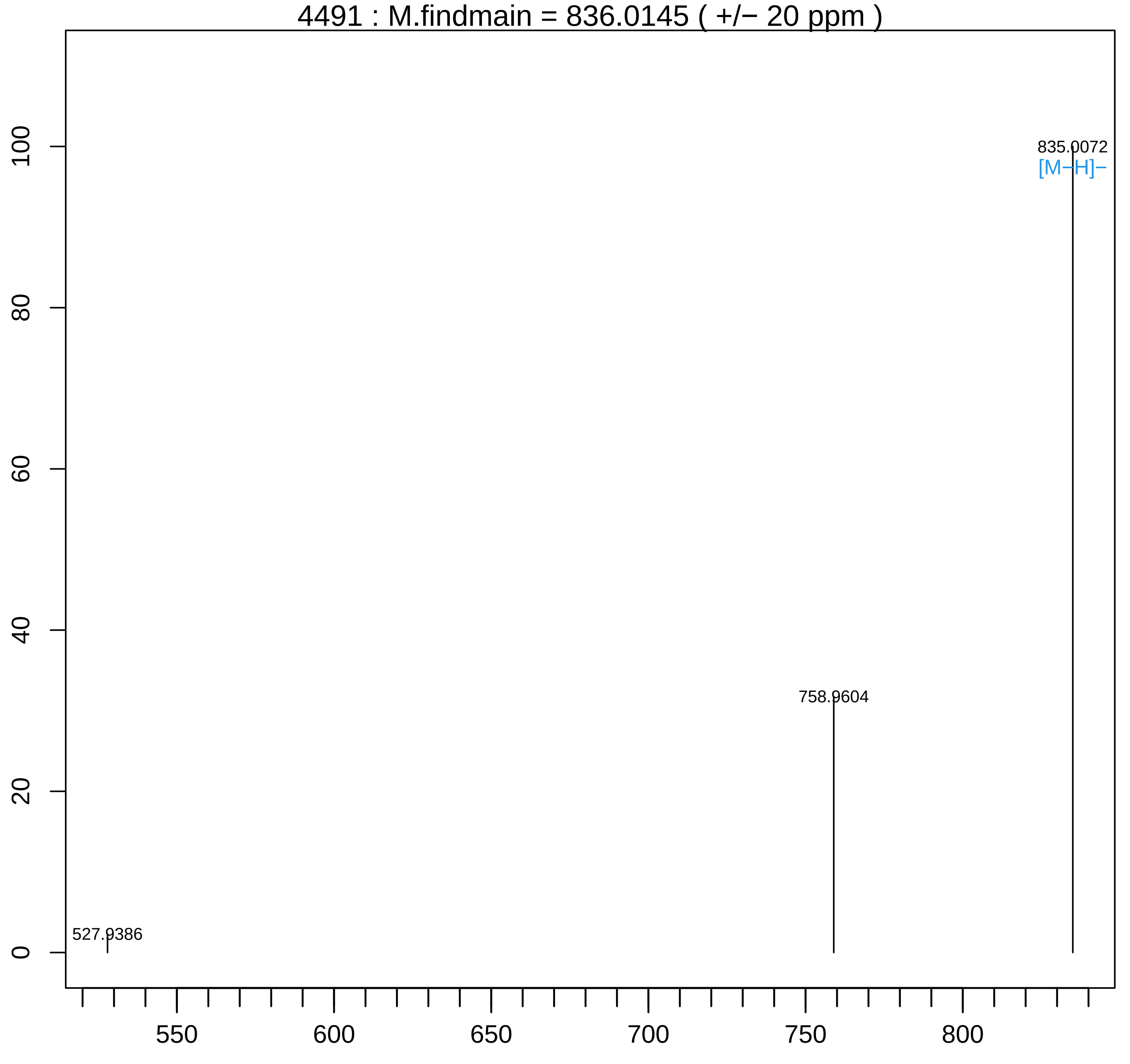
4491 : M.findmain = 836.0145 (+/− 20 ppm)
835.0072
[M−H]−
758.9604
527.9386
0
20
40
60
80
100
550
600
650
700
750
800

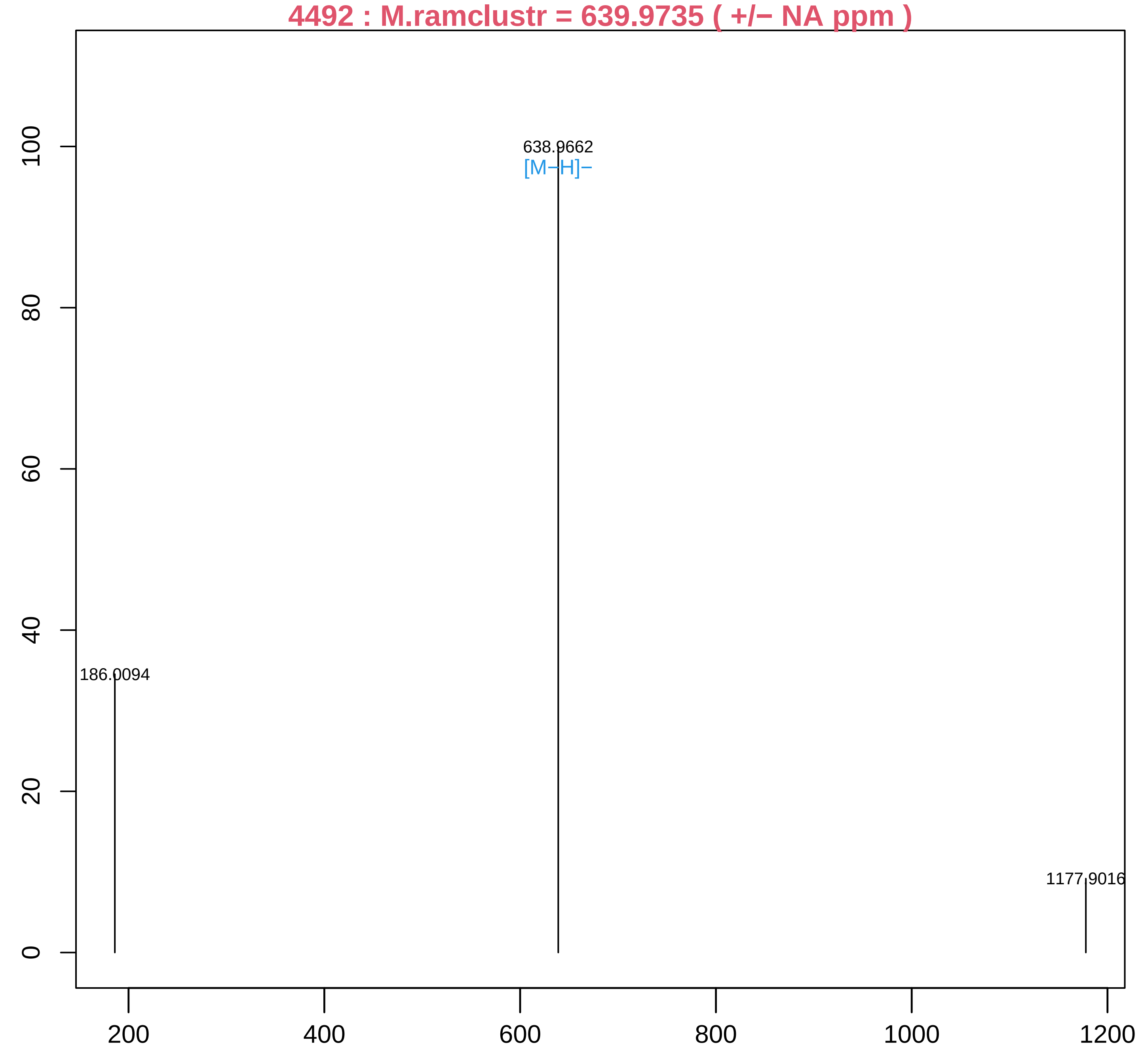
4492 : M.ramclustr = 639.9735 (+/− NA ppm)
638.9662
[M−H]−
186.0094
1177.9016
0
20
40
60
80
100
200
400
600
800
1000
1200

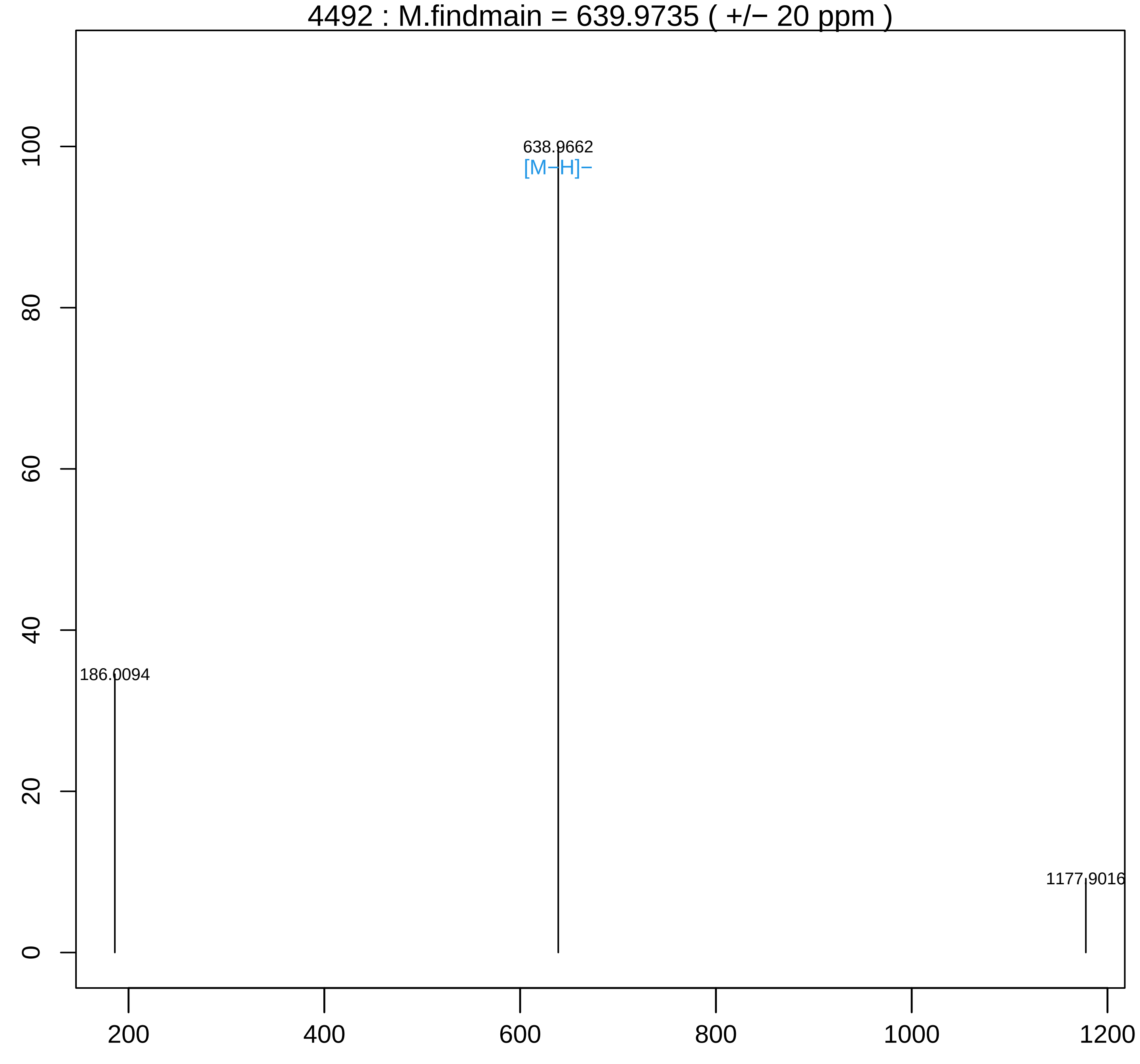
4492 : M.findmain = 639.9735 (+/− 20 ppm)
638.9662
[M−H]−
186.0094
1177.9016
0
20
40
60
80
100
200
400
600
800
1000
1200

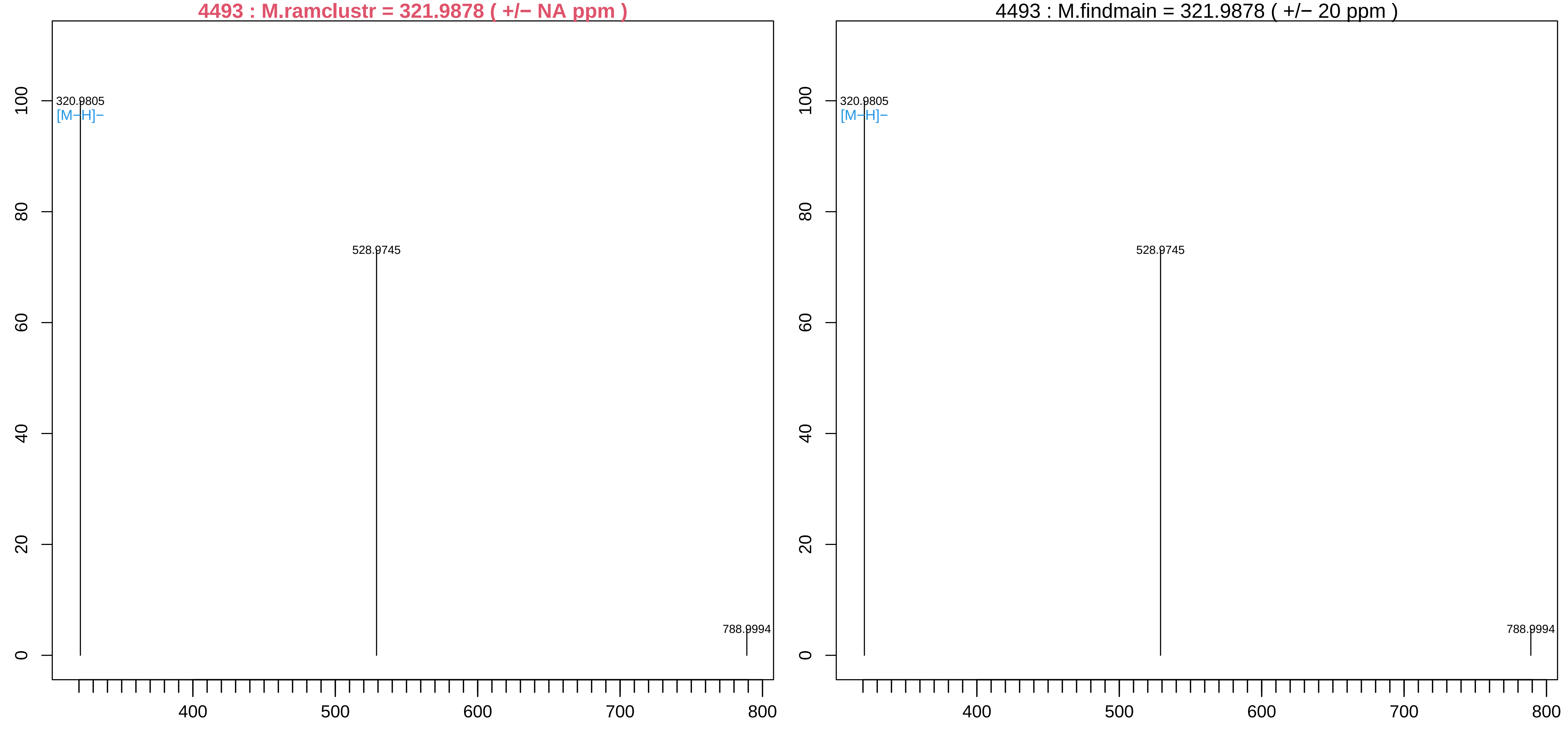
4493 : M.ramclustr = 321.9878 (+/− NA ppm)
320.9805
[M−H]−
528.9745
788.9994
0
20
40
60
80
100
400
500
600
700
800
4493 : M.findmain = 321.9878 (+/− 20 ppm)
320.9805
[M−H]−
528.9745
788.9994
0
20
40
60
80
100
400
500
600
700
800

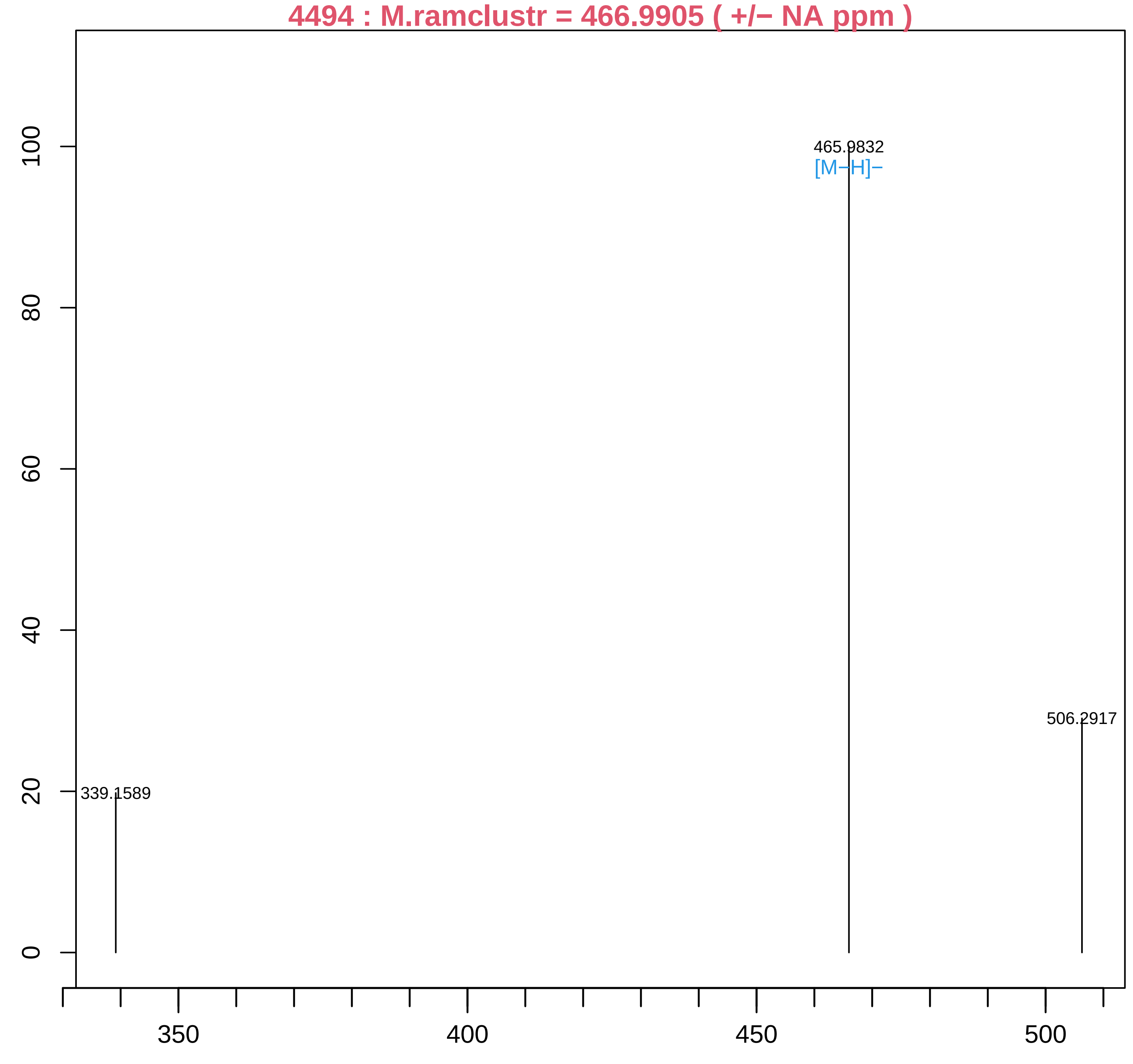
4494 : M.ramclustr = 466.9905 (+/− NA ppm)
465.9832
[M−H]−
506.2917
339.1589
0
20
40
60
80
100
350
400
450
500

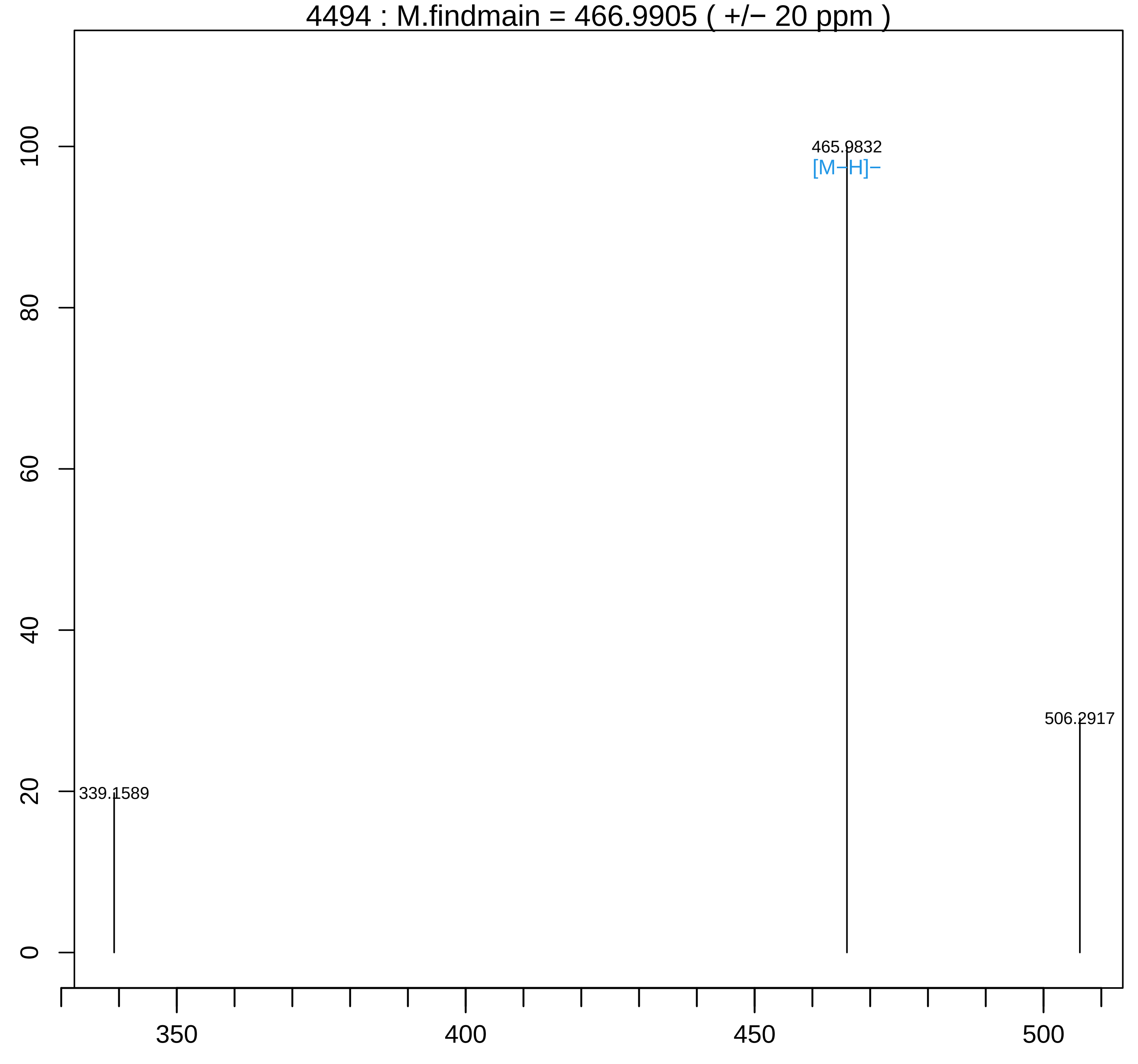
4494 : M.findmain = 466.9905 (+/− 20 ppm)
465.9832
[M−H]−
506.2917
339.1589
0
20
40
60
80
100
350
400
450
500

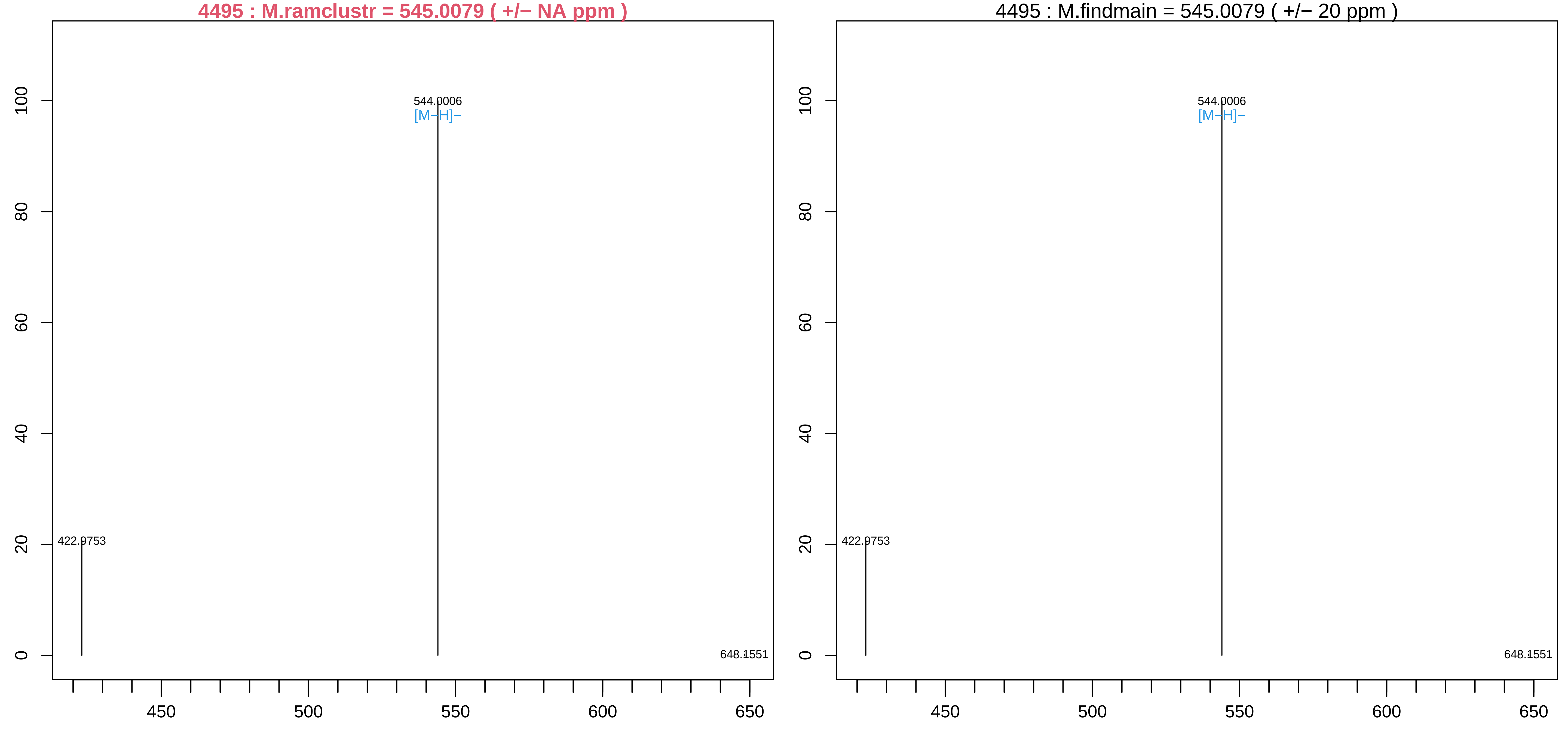
4495 : M.ramclustr = 545.0079 (+/− NA ppm)
544.0006
[M−H]−
422.9753
648.1551
0
20
40
60
80
100
450
500
550
600
650
4495 : M.findmain = 545.0079 (+/− 20 ppm)
544.0006
[M−H]−
422.9753
648.1551
0
20
40
60
80
100
450
500
550
600
650

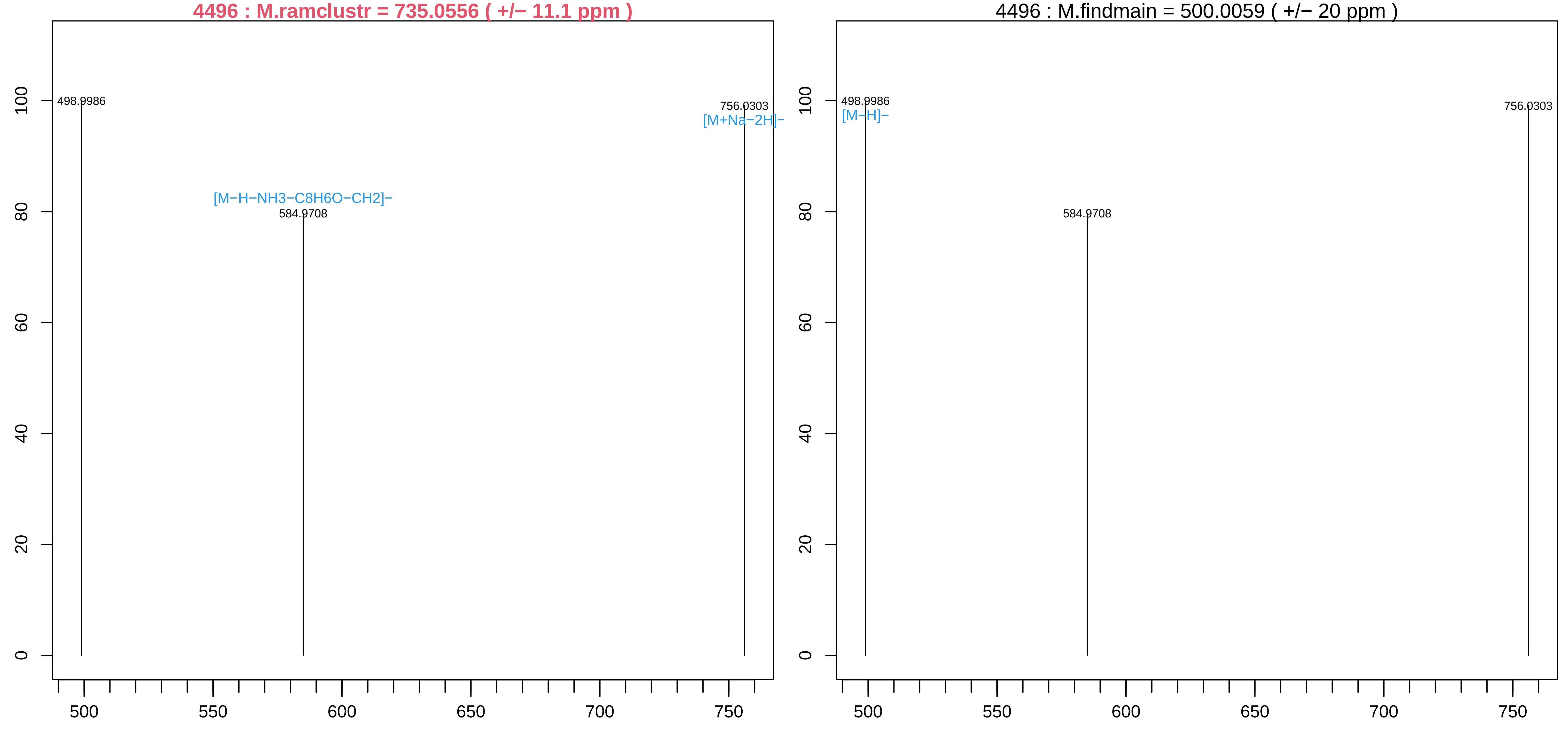
4496 : M.ramclustr = 735.0556 (+/− 11.1 ppm)
498.9986
[M−H−NH3−C8H6O−CH2]−
584.9708
756.0303
[M+Na−2H]−
0
20
40
60
80
100
500
550
600
650
700
750
4496 : M.findmain = 500.0059 (+/− 20 ppm)
498.9986
[M−H]−
584.9708
756.0303
0
20
40
60
80
100
500
550
600
650
700
750

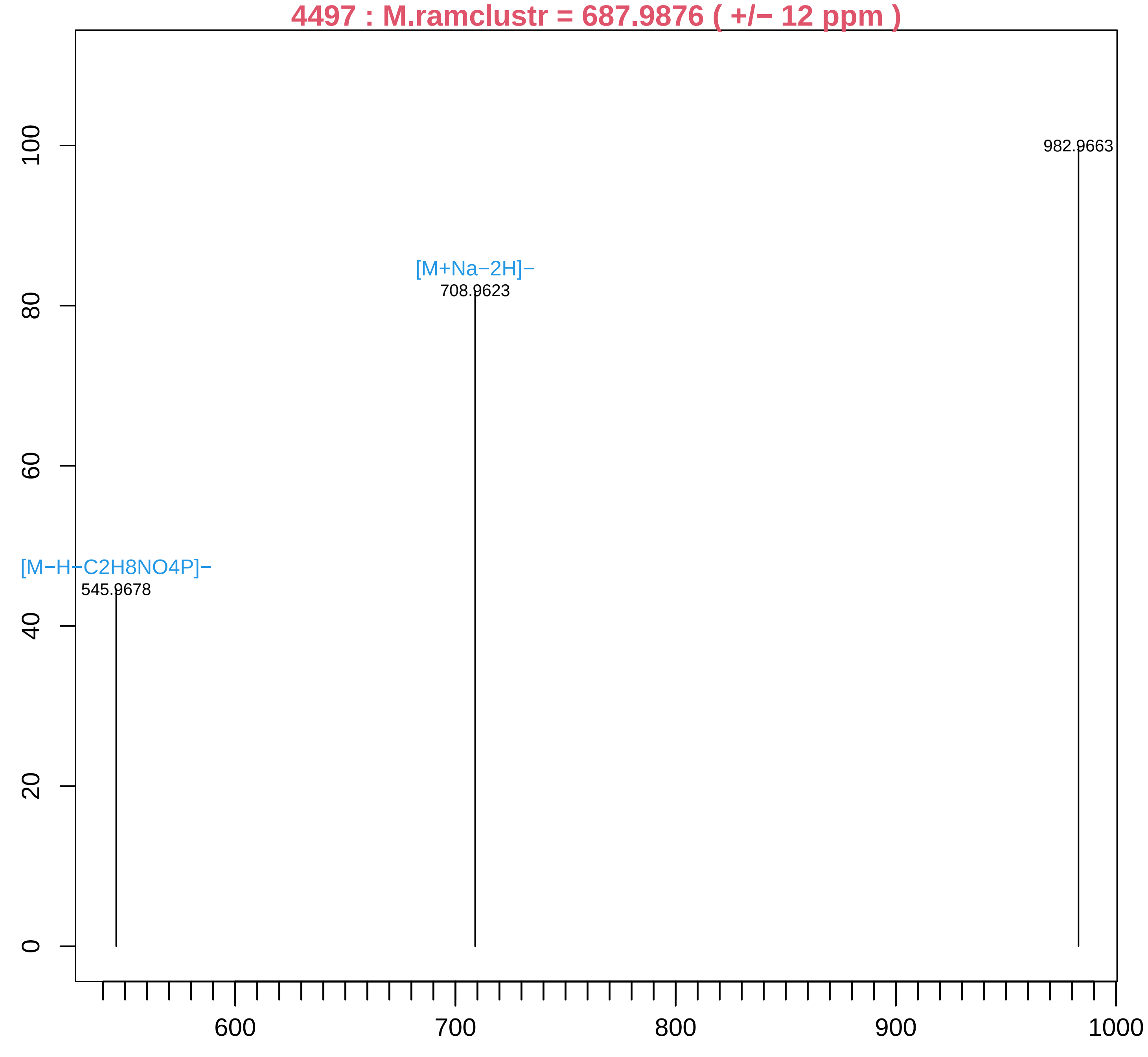
4497 : M.ramclustr = 687.9876 (+/− 12 ppm)
982.9663
[M+Na−2H]−
708.9623
[M−H−C2H8NO4P]−
545.9678
0
20
40
60
80
100
600
700
800
900
1000

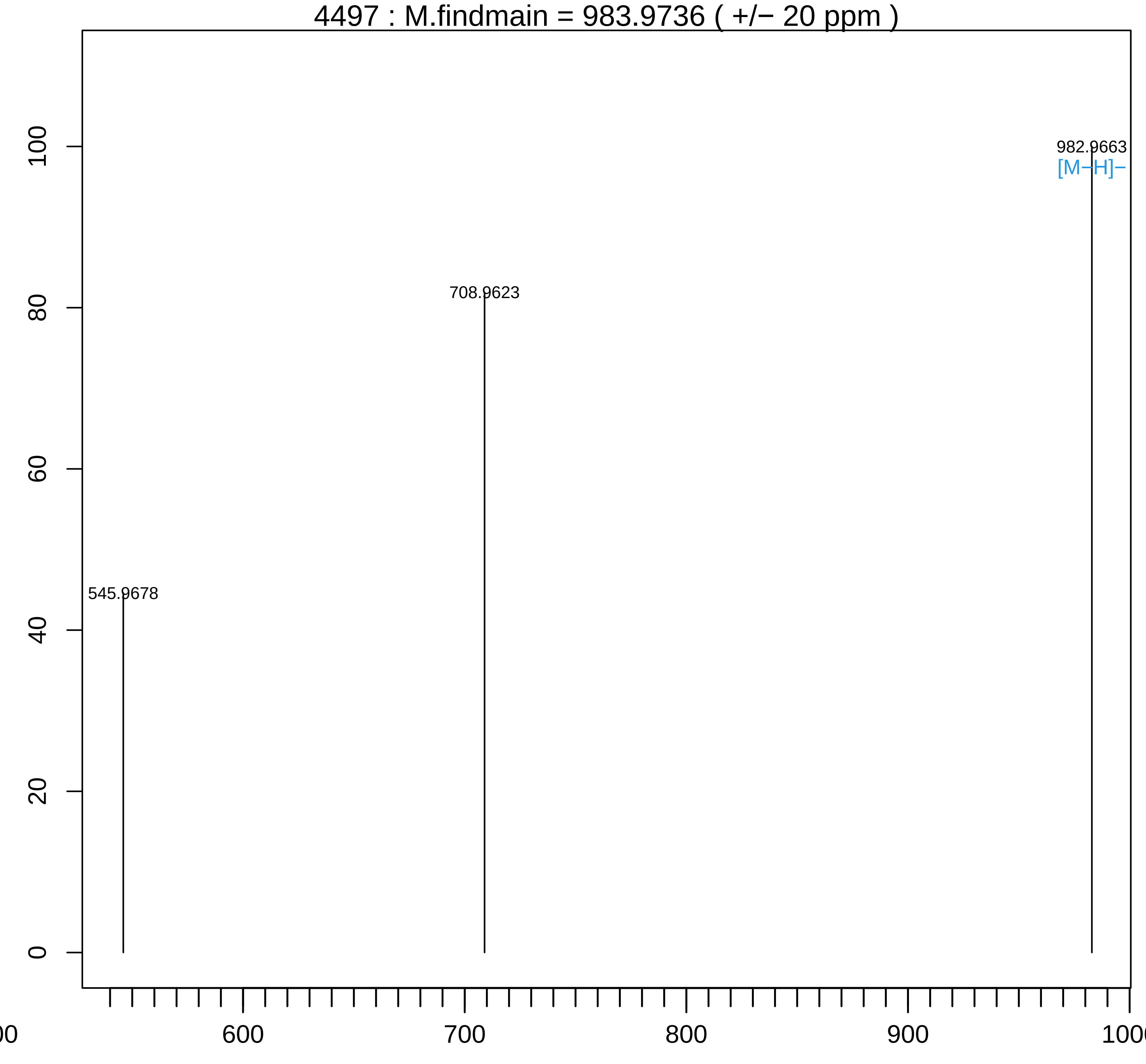
4497 : M.findmain = 983.9736 (+/− 20 ppm)
982.9663
[M−H]−
708.9623
545.9678
0
20
40
60
80
100
600
700
800
900
1000

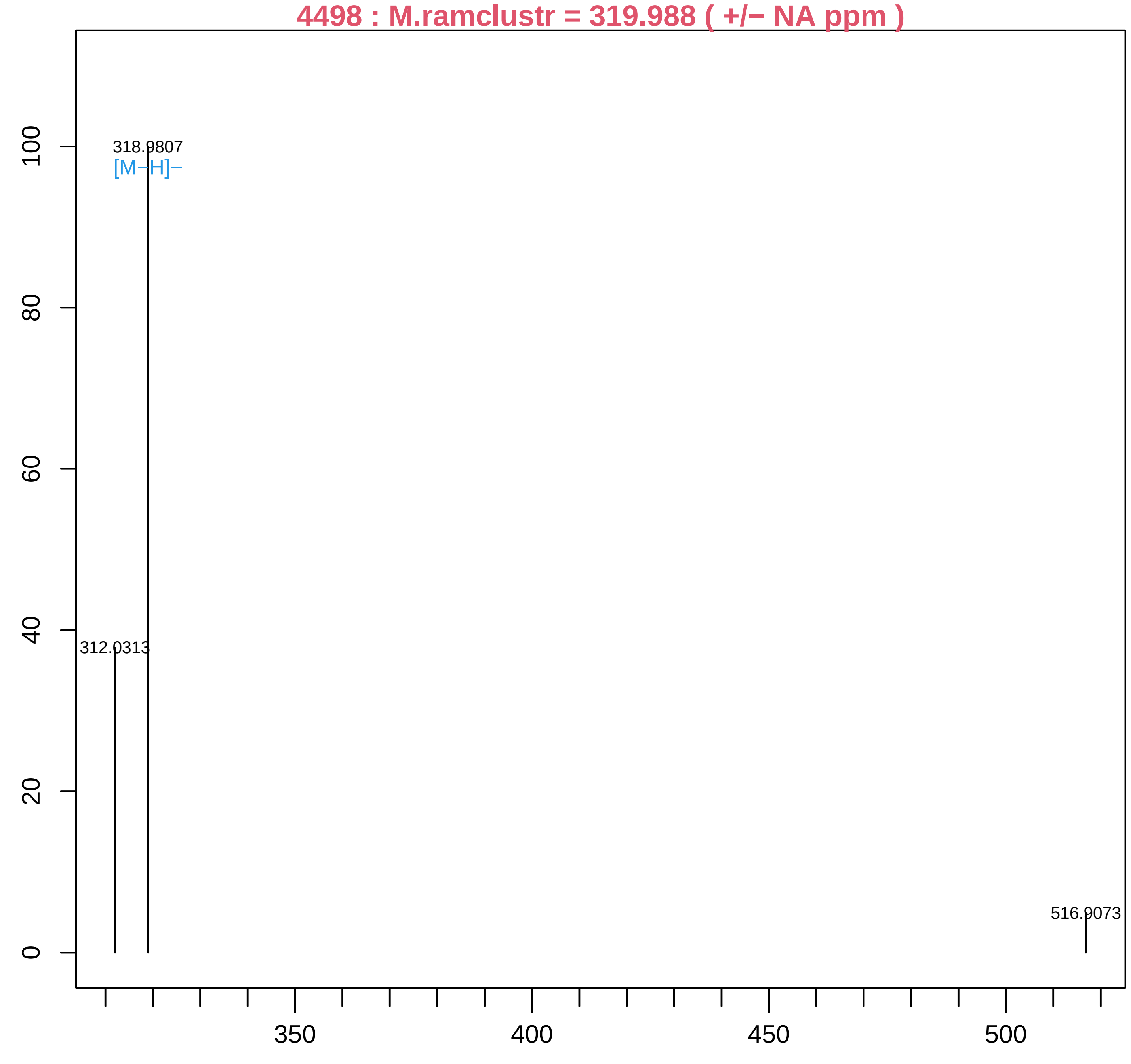
4498 : M.ramclustr = 319.988 (+/− NA ppm)
318.9807
[M−H]−
312.0313
516.9073
0
20
40
60
80
100
350
400
450
500
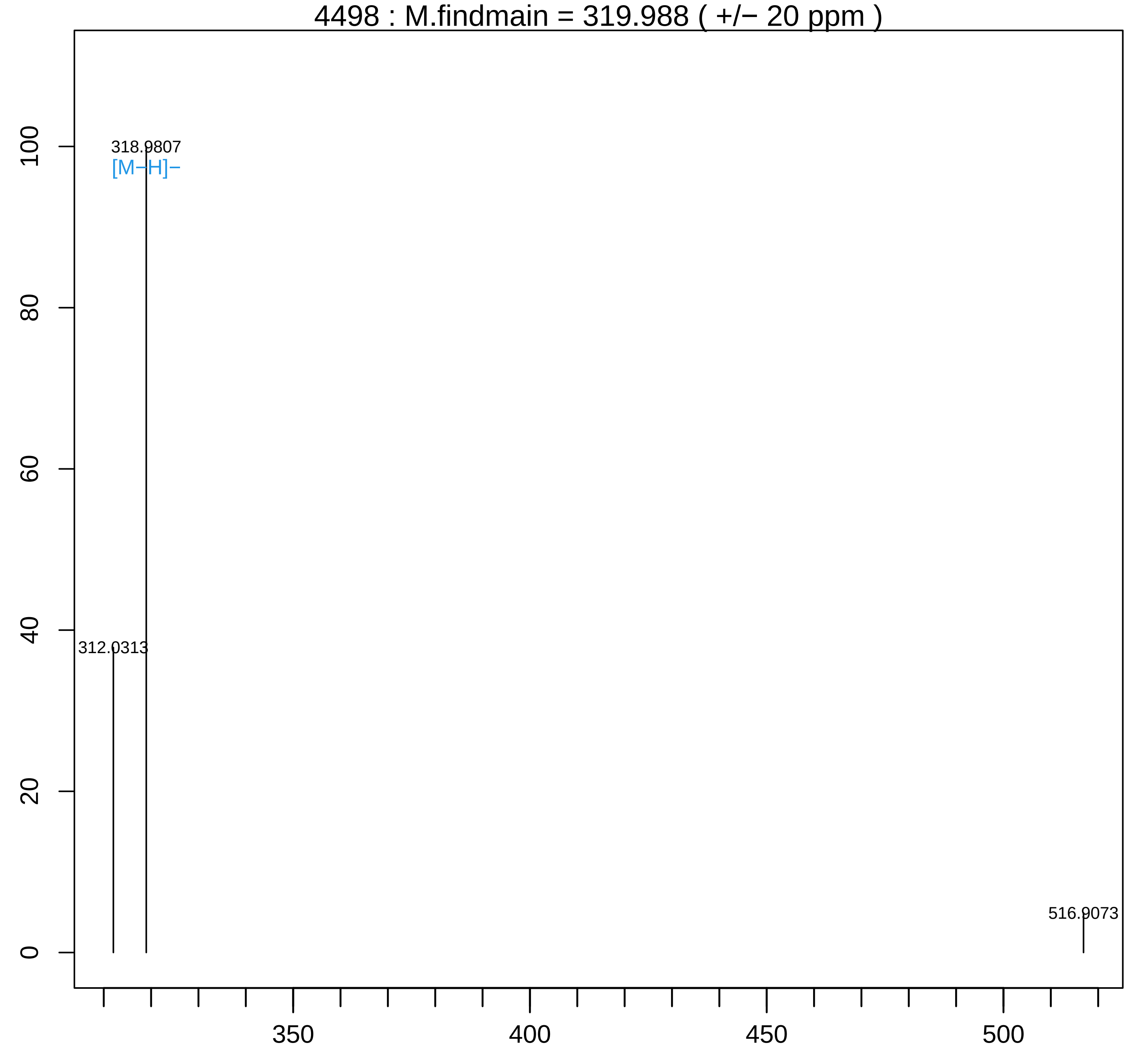
4498 : M.findmain = 319.988 (+/− 20 ppm)
318.9807
[M−H]−
312.0313
516.9073
0
20
40
60
80
100
350
400
450
500

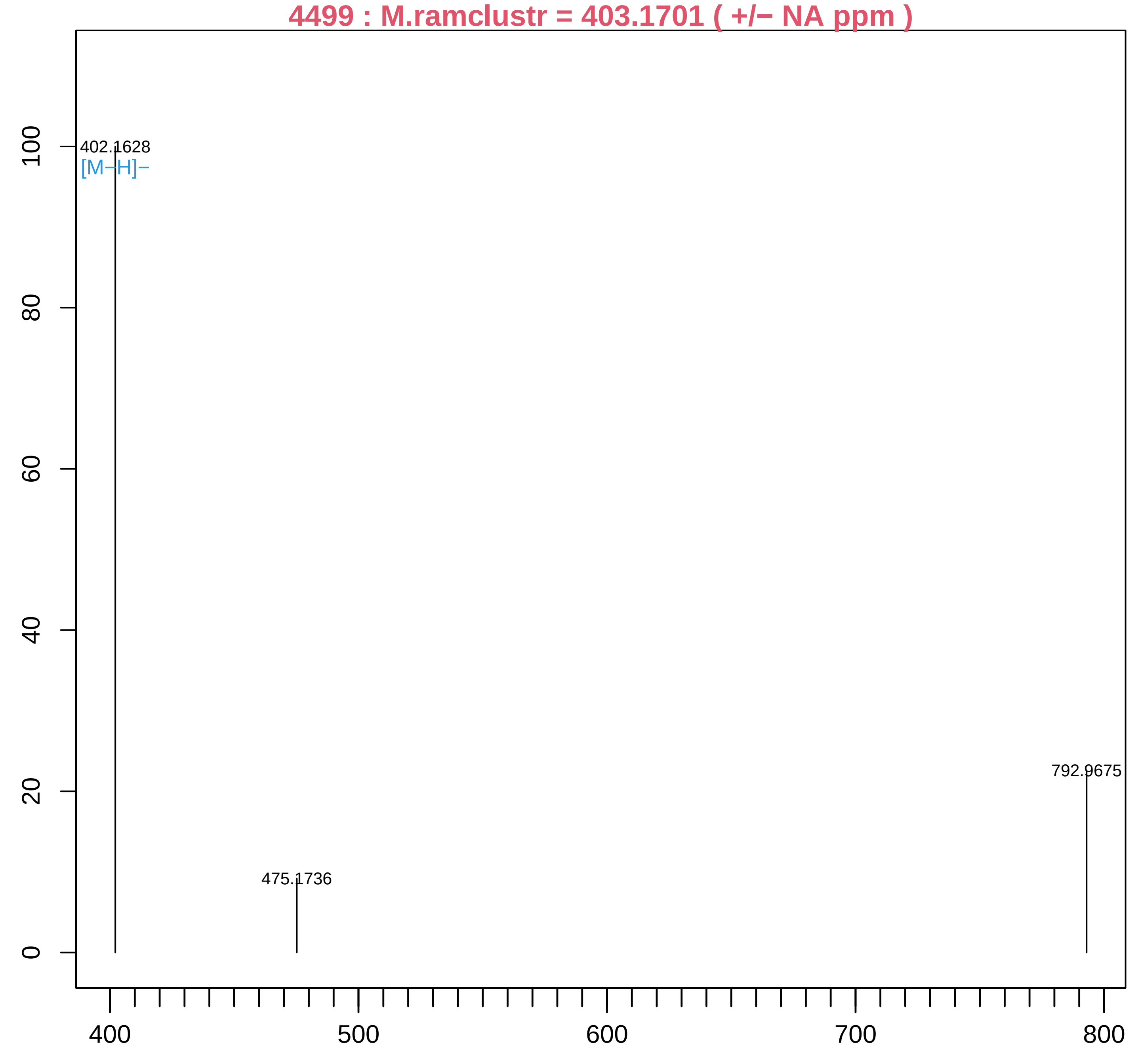

4499 : M.ramclustr = 403.1701 (+/− NA ppm)
402.1628
[M−H]−
475.1736
792.9675
0
20
40
60
80
100
400
500
600
700
800

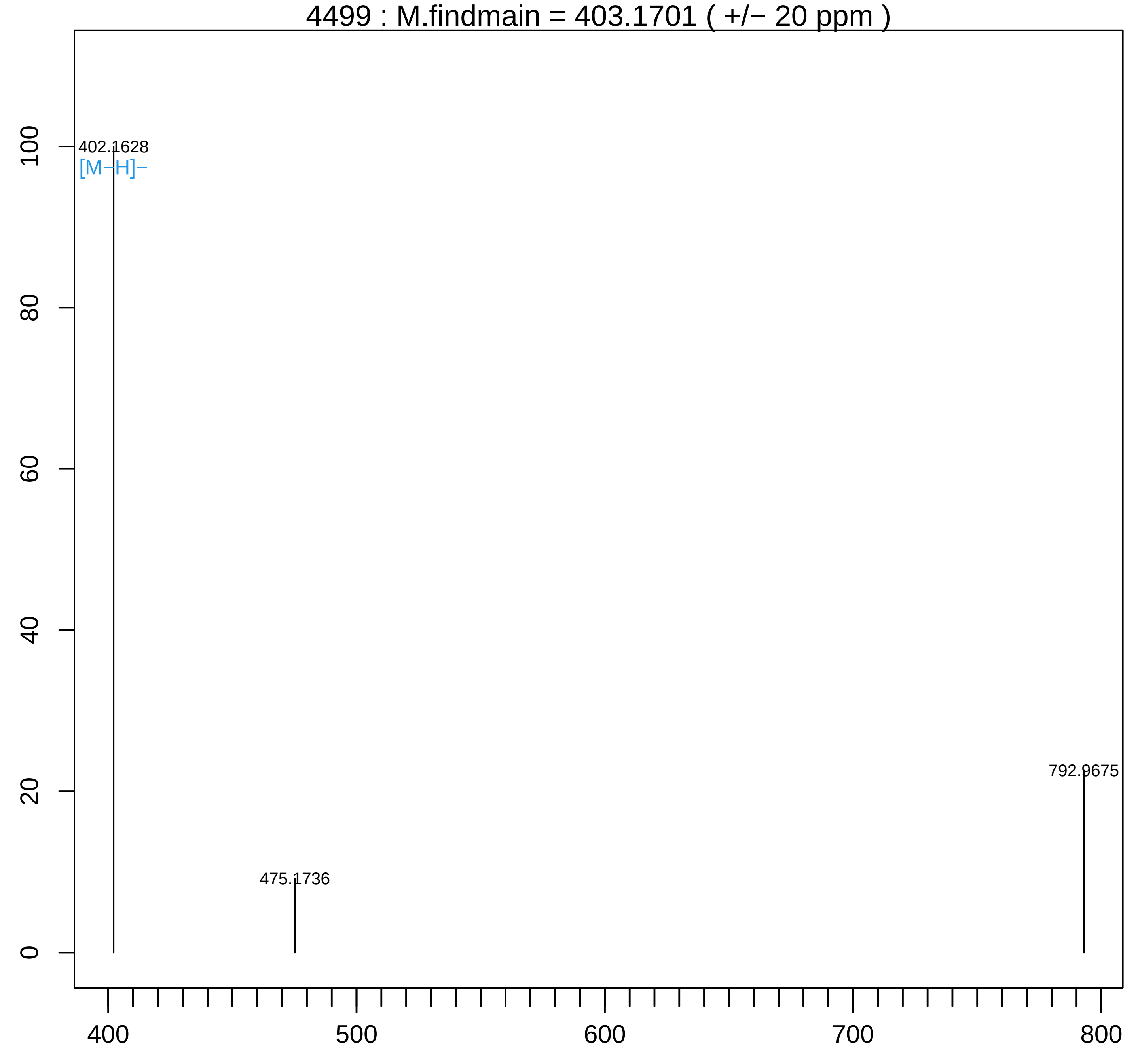

4499 : M.findmain = 403.1701 (+/− 20 ppm)
402.1628
[M−H]−
475.1736
792.9675
0
20
40
60
80
100
400
500
600
700
800

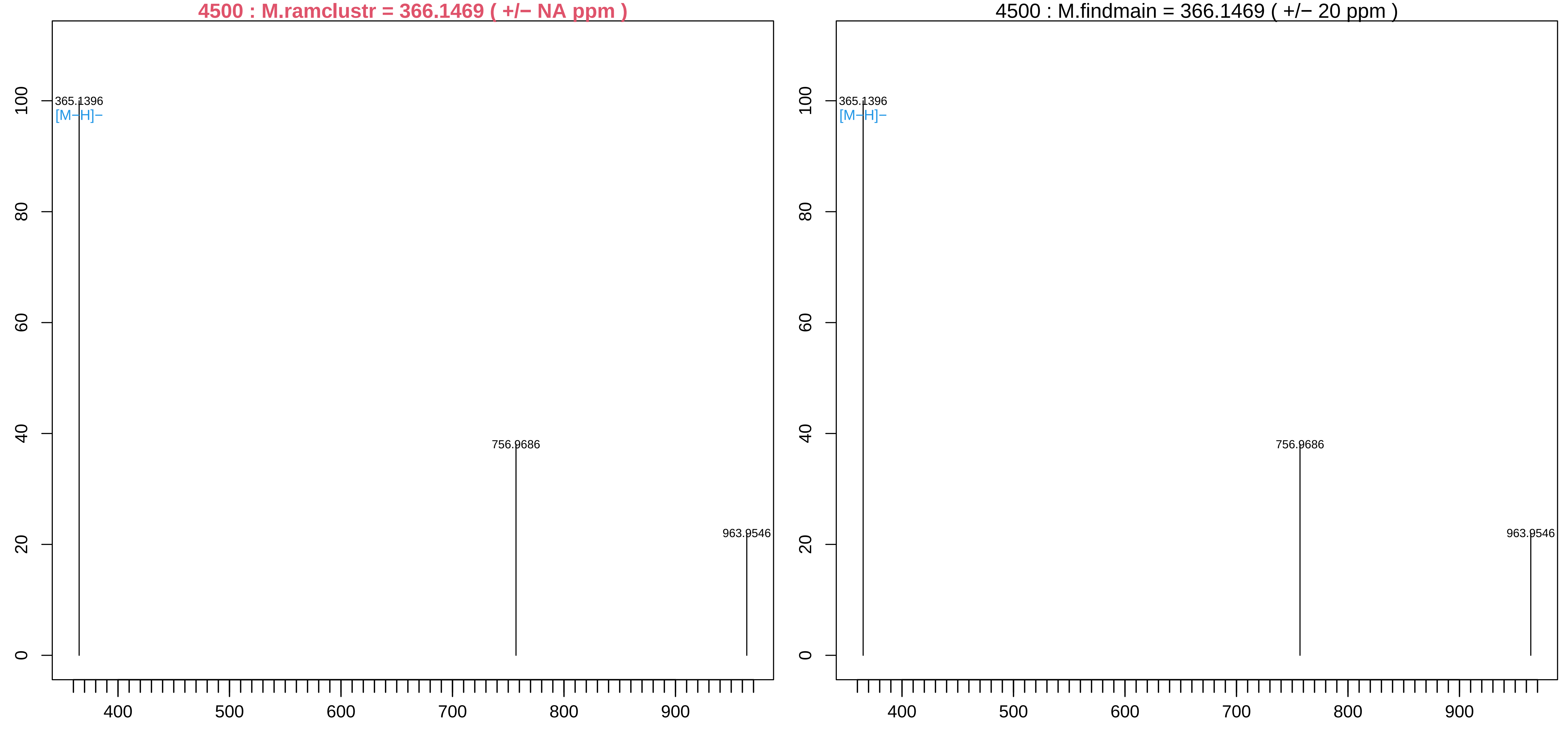
4500 : M.ramclustr = 366.1469 (+/− NA ppm)
365.1396
[M−H]−
756.9686
963.9546
4500 : M.findmain = 366.1469 (+/− 20 ppm)
365.1396
[M−H]−
756.9686
963.9546
0
20
40
60
80
100
400
500
600
700
800
900

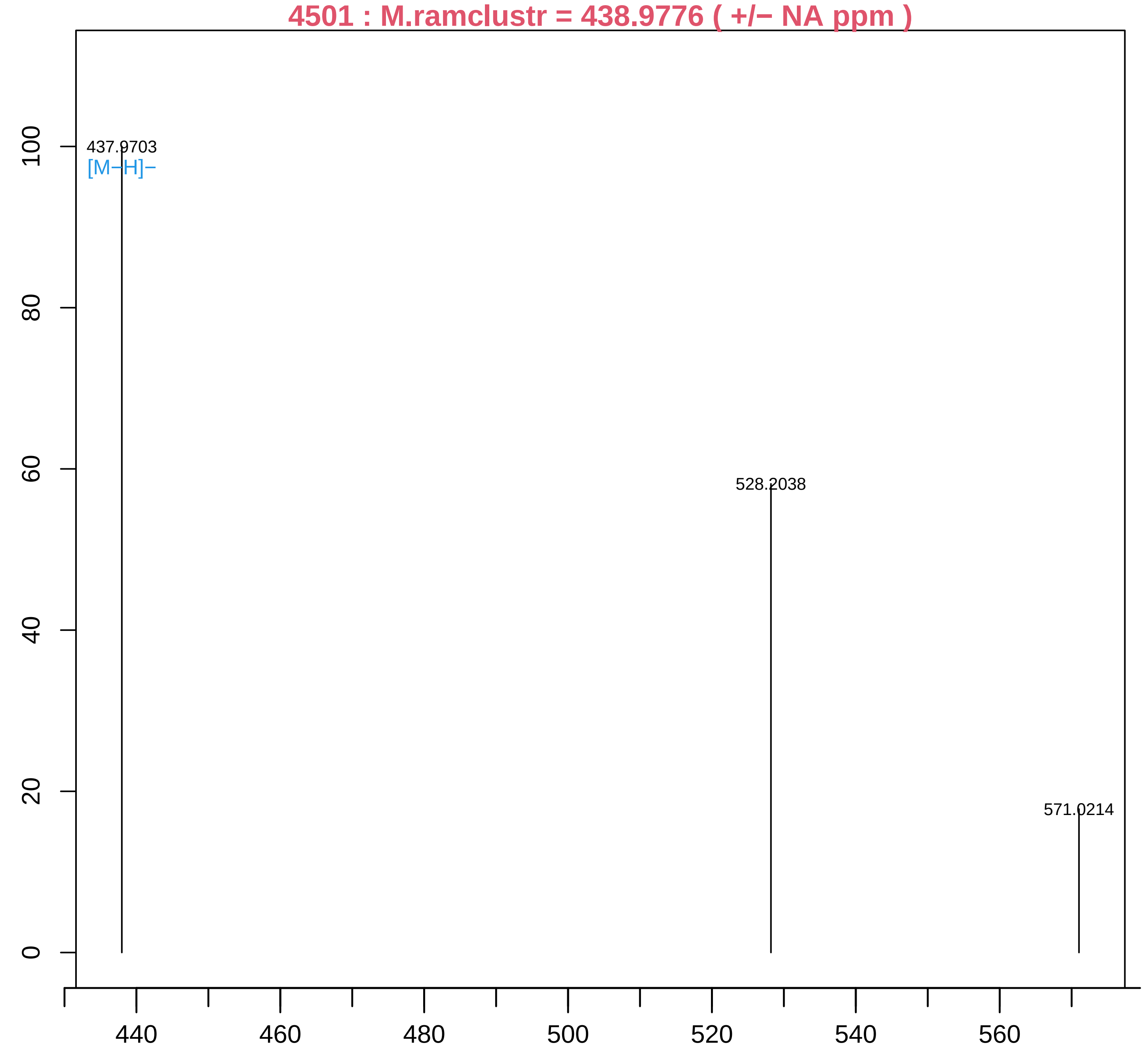
4501 : M.ramclustr = 438.9776 (+/− NA ppm)
437.9703
[M−H]−
528.2038
571.0214
0
20
40
60
80
100
440
460
480
500
520
540
560

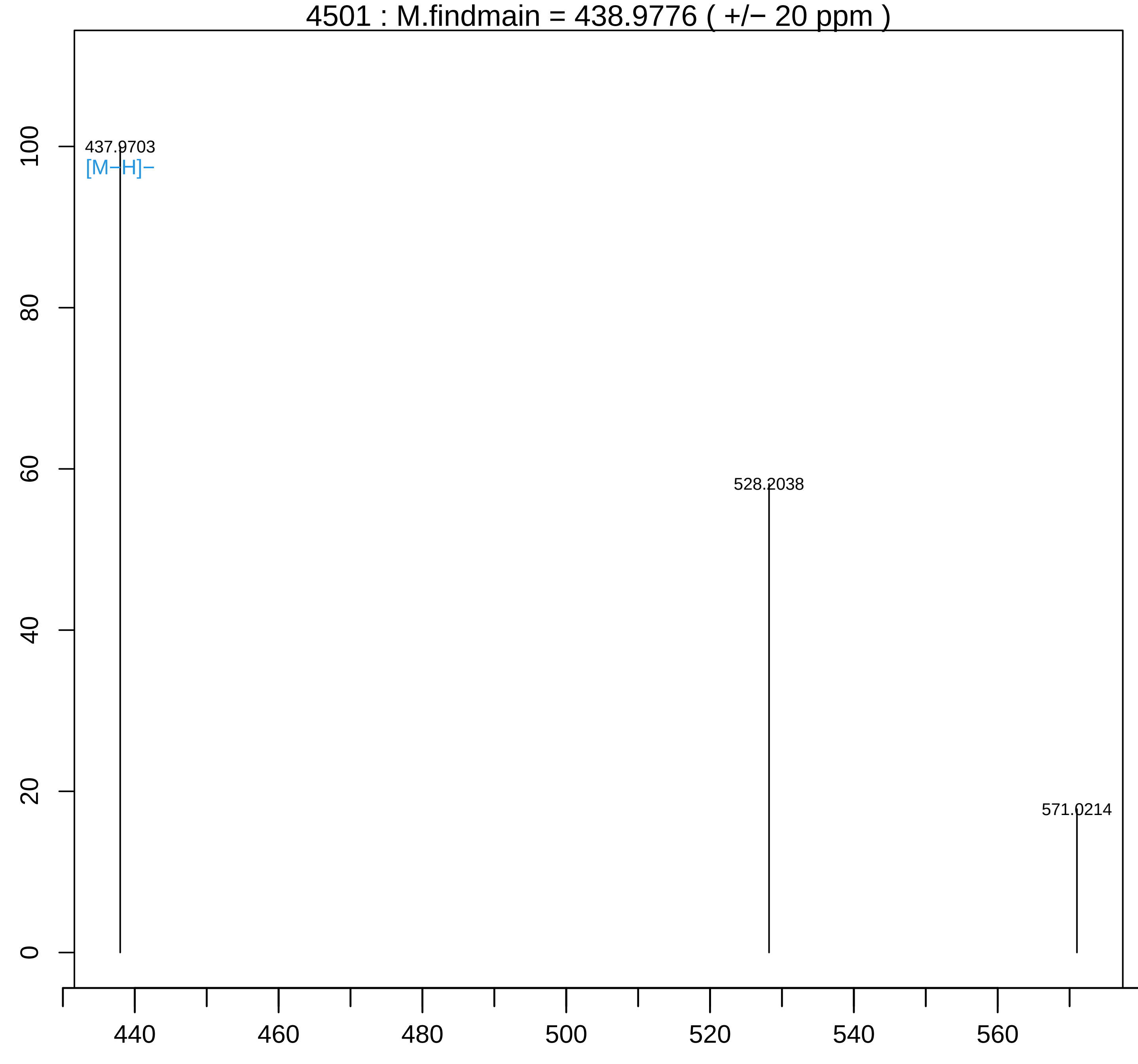
4501 : M.findmain = 438.9776 (+/− 20 ppm)
437.9703
[M−H]−
528.2038
571.0214
0
20
40
60
80
100
440
460
480
500
520
540
560

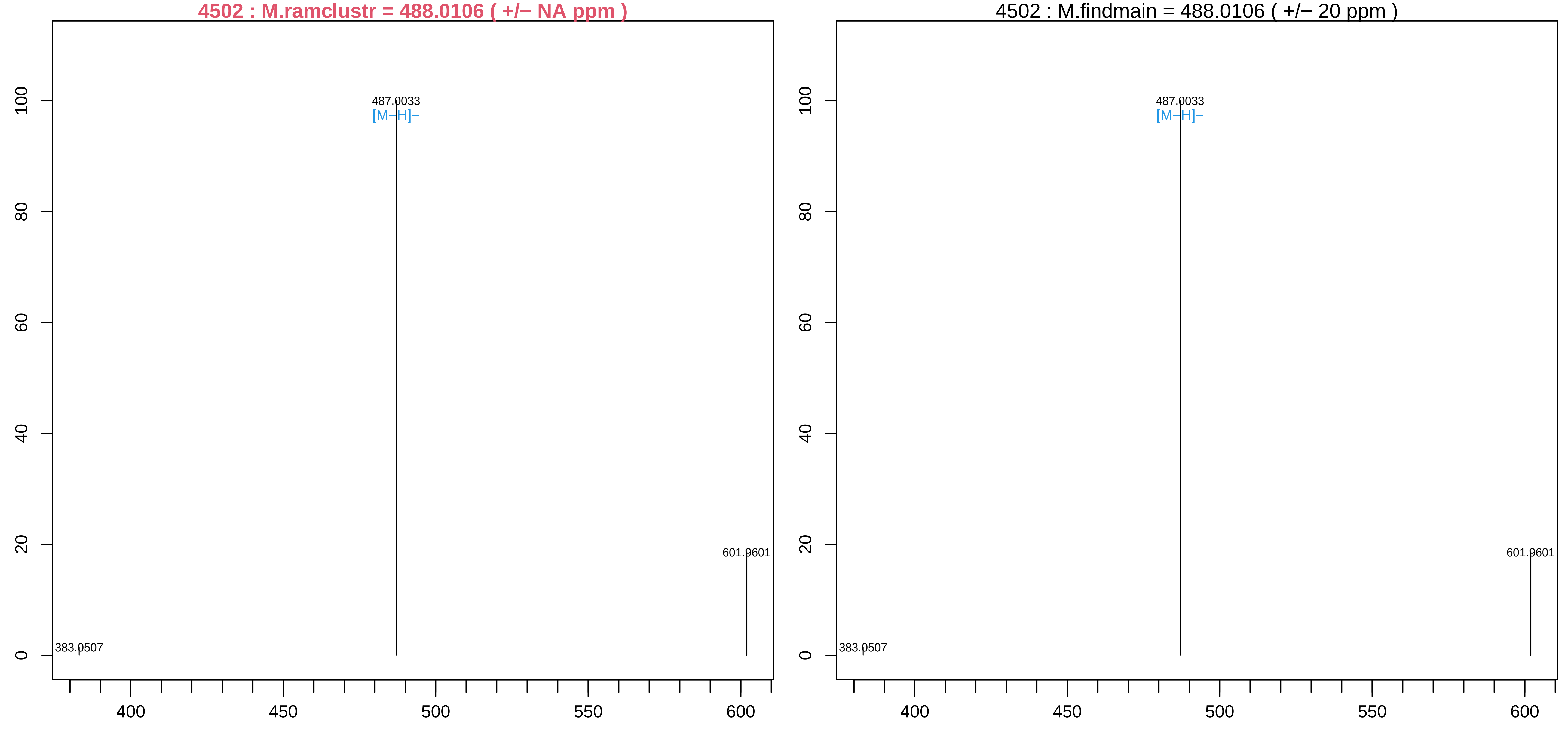
4502 : M.ramclustr = 488.0106 (+/− NA ppm)
487.0033
[M−H]−
601.9601
383.0507
4502 : M.findmain = 488.0106 (+/− 20 ppm)
487.0033
[M−H]−
601.9601
383.0507
0
20
40
60
80
100
400
450
500
550
600

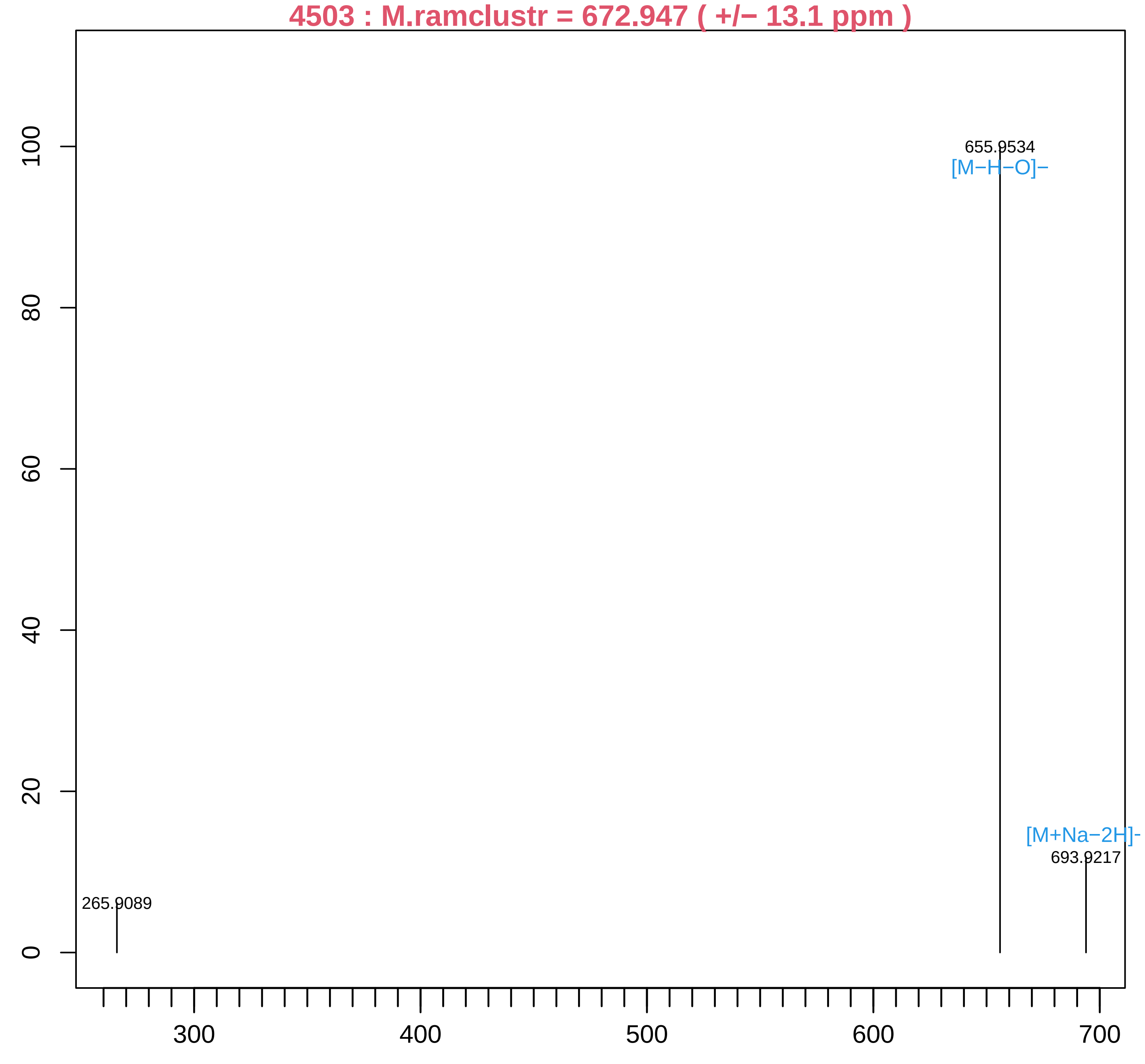

4503 : M.ramclustr = 672.947 (+/− 13.1 ppm)
655.9534
[M−H−O]−
[M+Na−2H]-
693.9217
265.9089
0
20
40
60
80
100
300
400
500
600
700

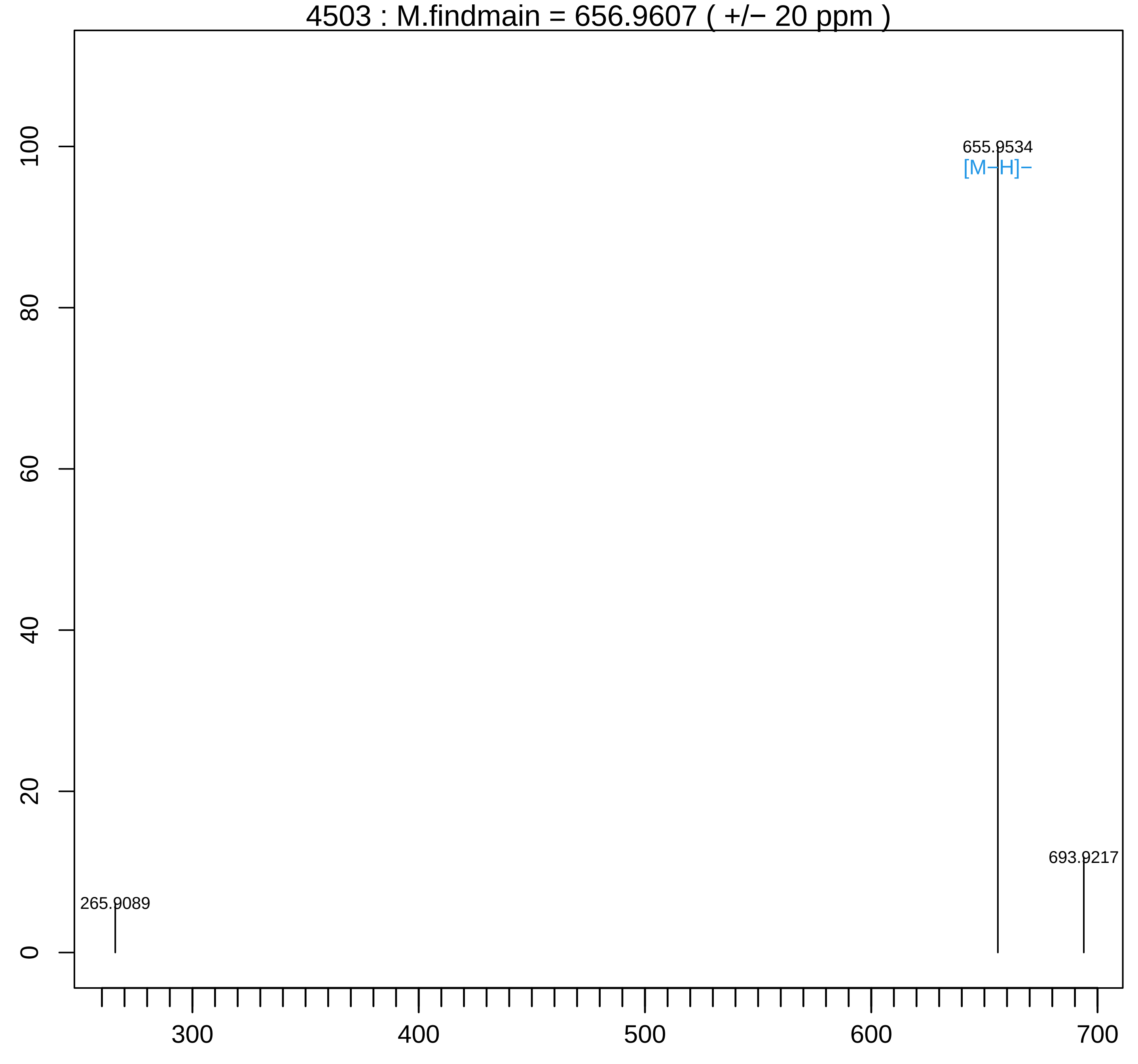

4503 : M.findmain = 656.9607 (+/− 20 ppm)
655.9534
[M−H]−
693.9217
265.9089
0
20
40
60
80
100
300
400
500
600
700

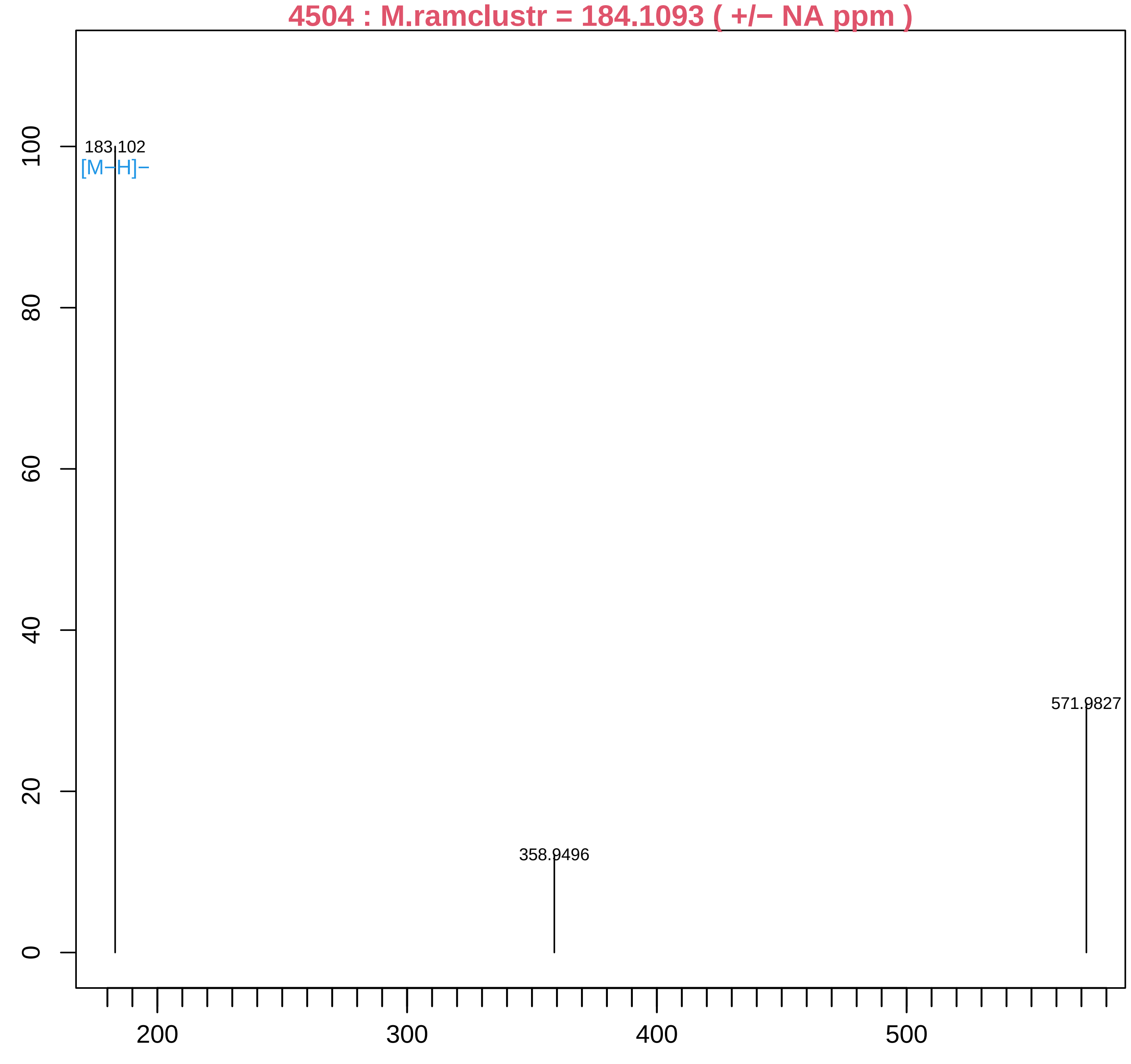
4504 : M.ramclustr = 184.1093 (+/− NA ppm)
183.102
[M−H]−
358.9496
571.9827
0
20
40
60
80
100
200
300
400
500

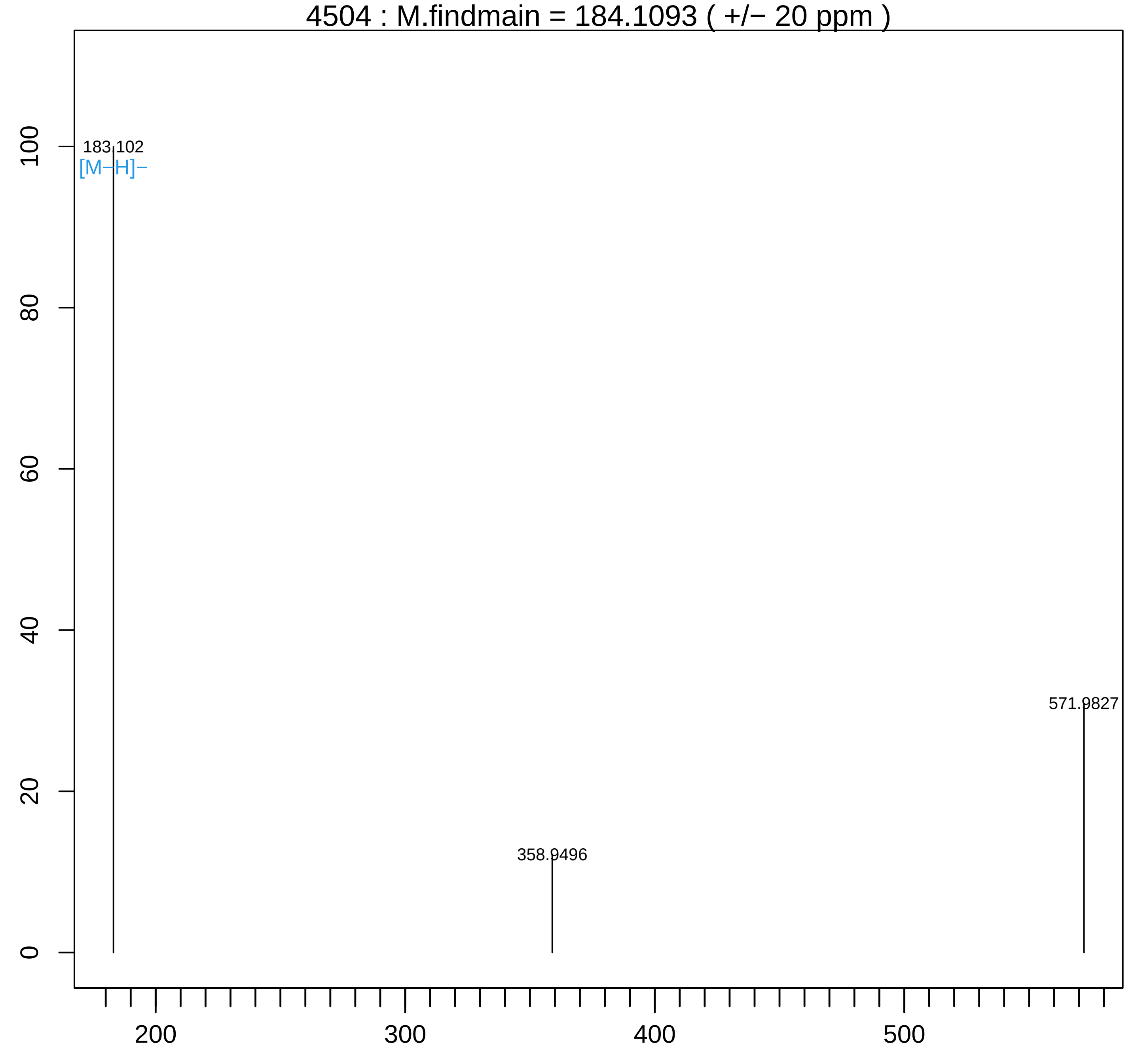
4504 : M.findmain = 184.1093 (+/− 20 ppm)
183.102
[M−H]−
358.9496
571.9827
0
20
40
60
80
100
200
300
400
500

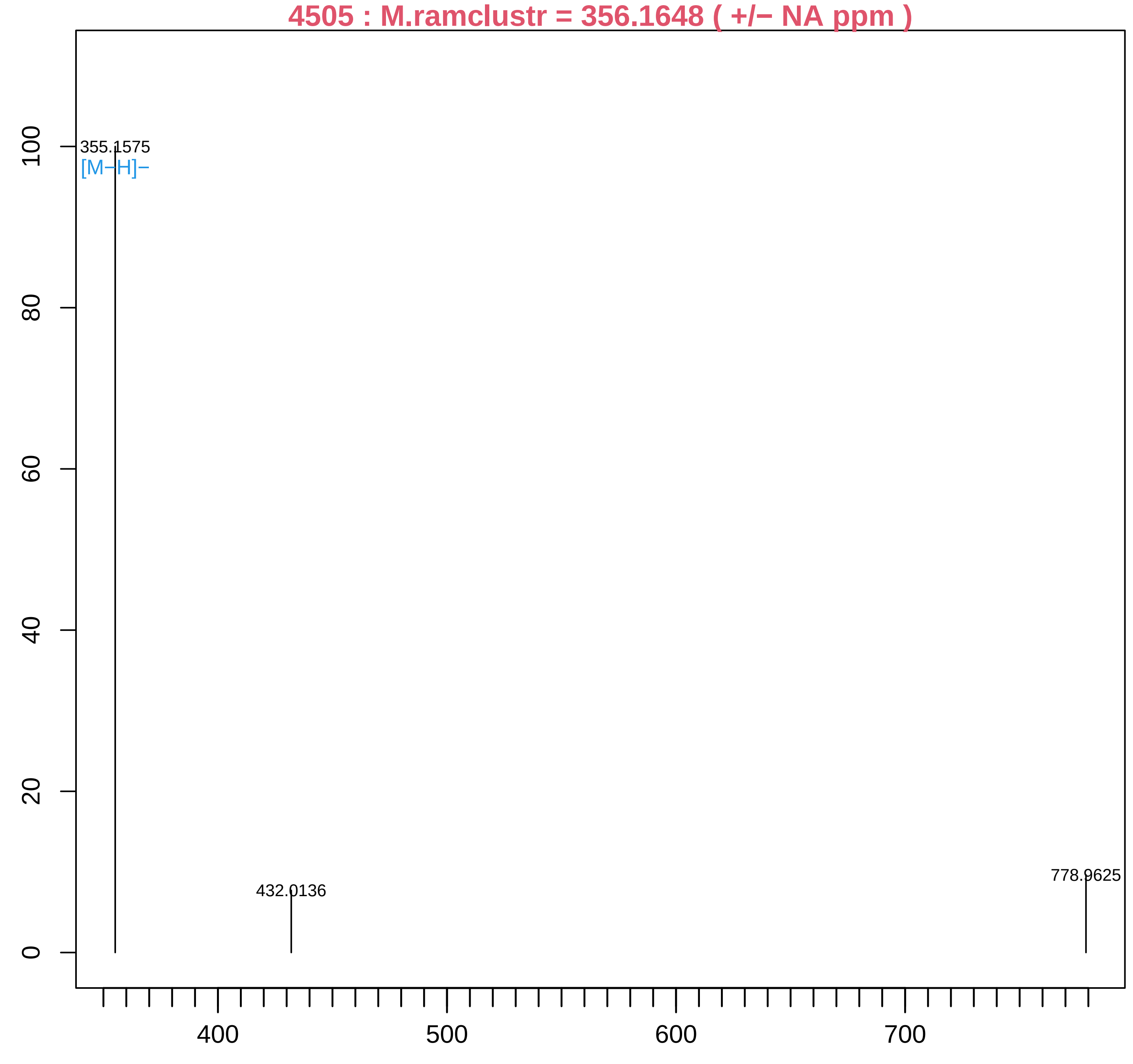

4505 : M.ramclustr = 356.1648 (+/− NA ppm)
355.1575
[M−H]−
432.0136
778.9625
0
20
40
60
80
100
400
500
600
700

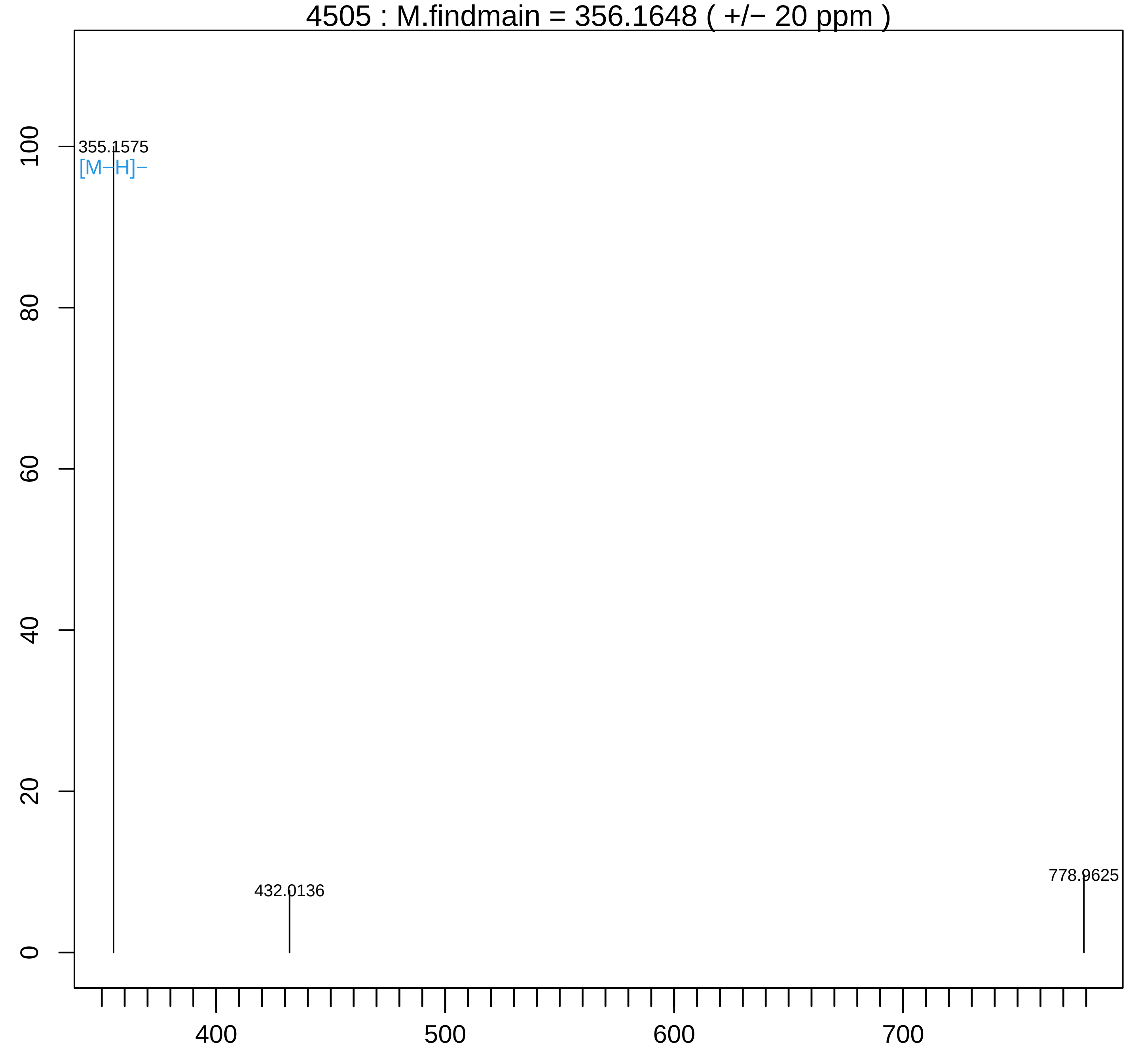

4505 : M.findmain = 356.1648 (+/− 20 ppm)
355.1575
[M−H]−
432.0136
778.9625
0
20
40
60
80
100
400
500
600
700

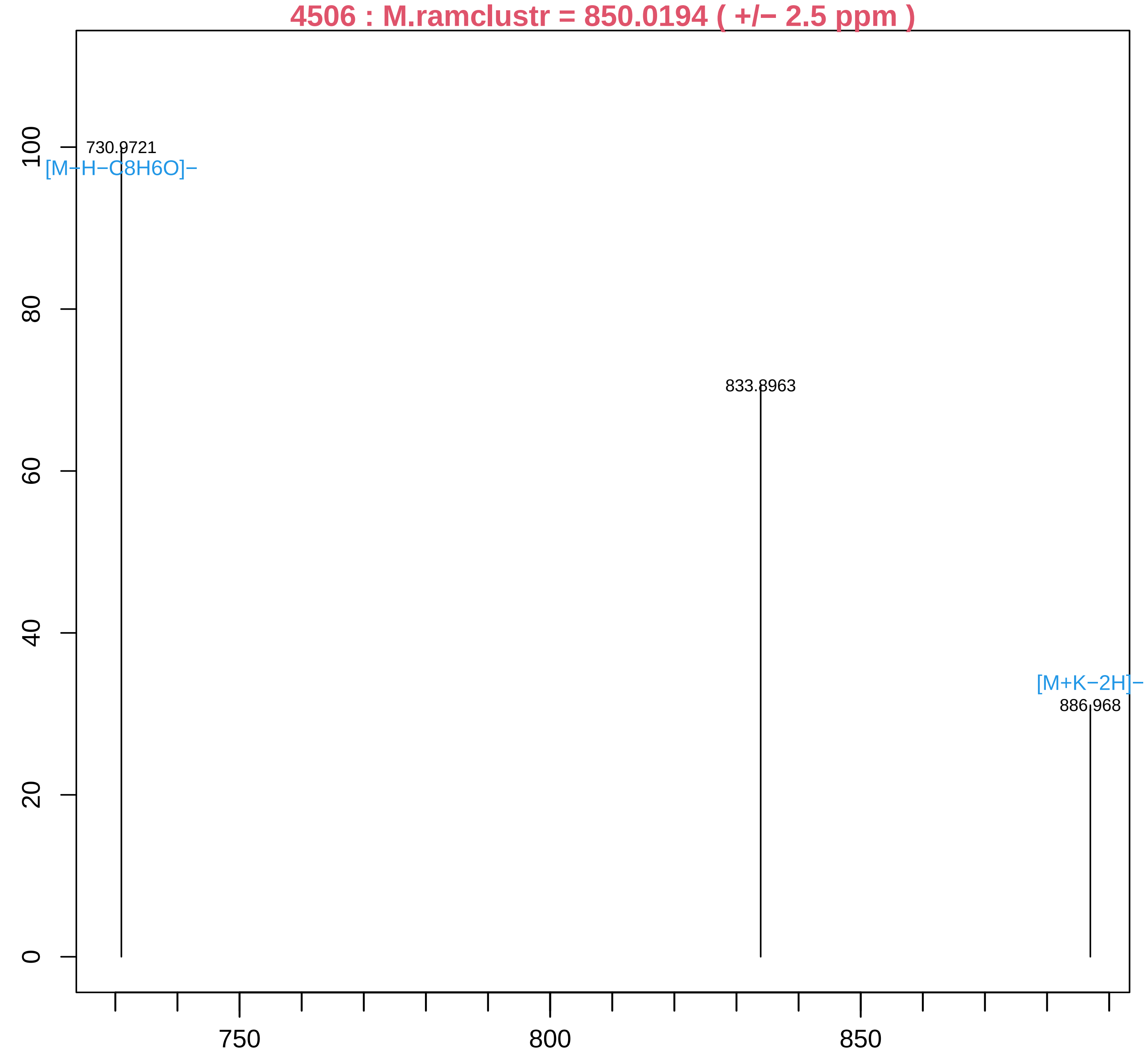
4506 : M.ramclustr = 850.0194 (+/− 2.5 ppm)
730.9721
[M−H−C8H6O]−
833.8963
[M+K−2H]−
886.968
0
20
40
60
80
100
750
800
850

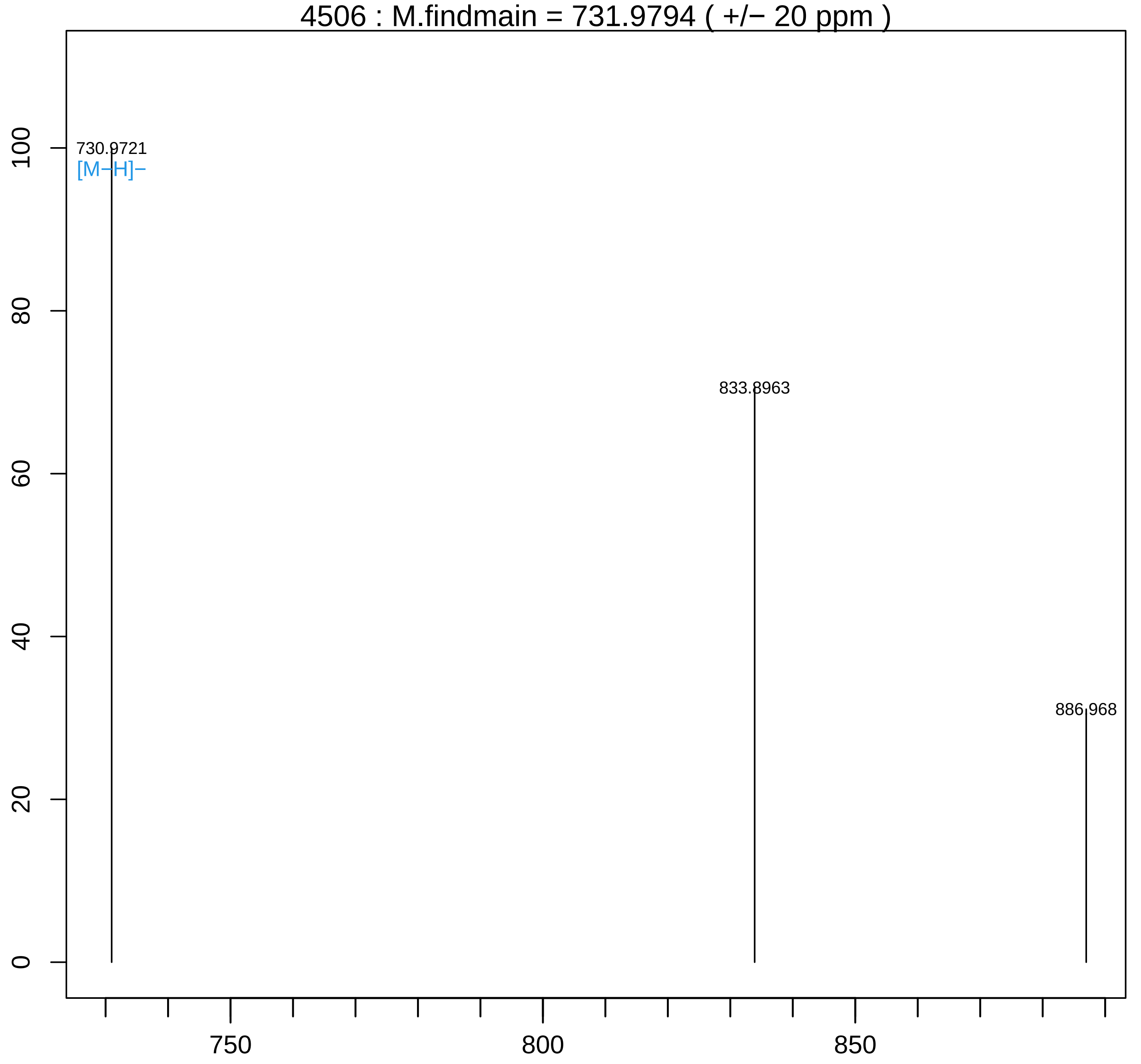
4506 : M.findmain = 731.9794 (+/− 20 ppm)
730.9721
[M−H]−
833.8963
886.968
0
20
40
60
80
100
750
800
850

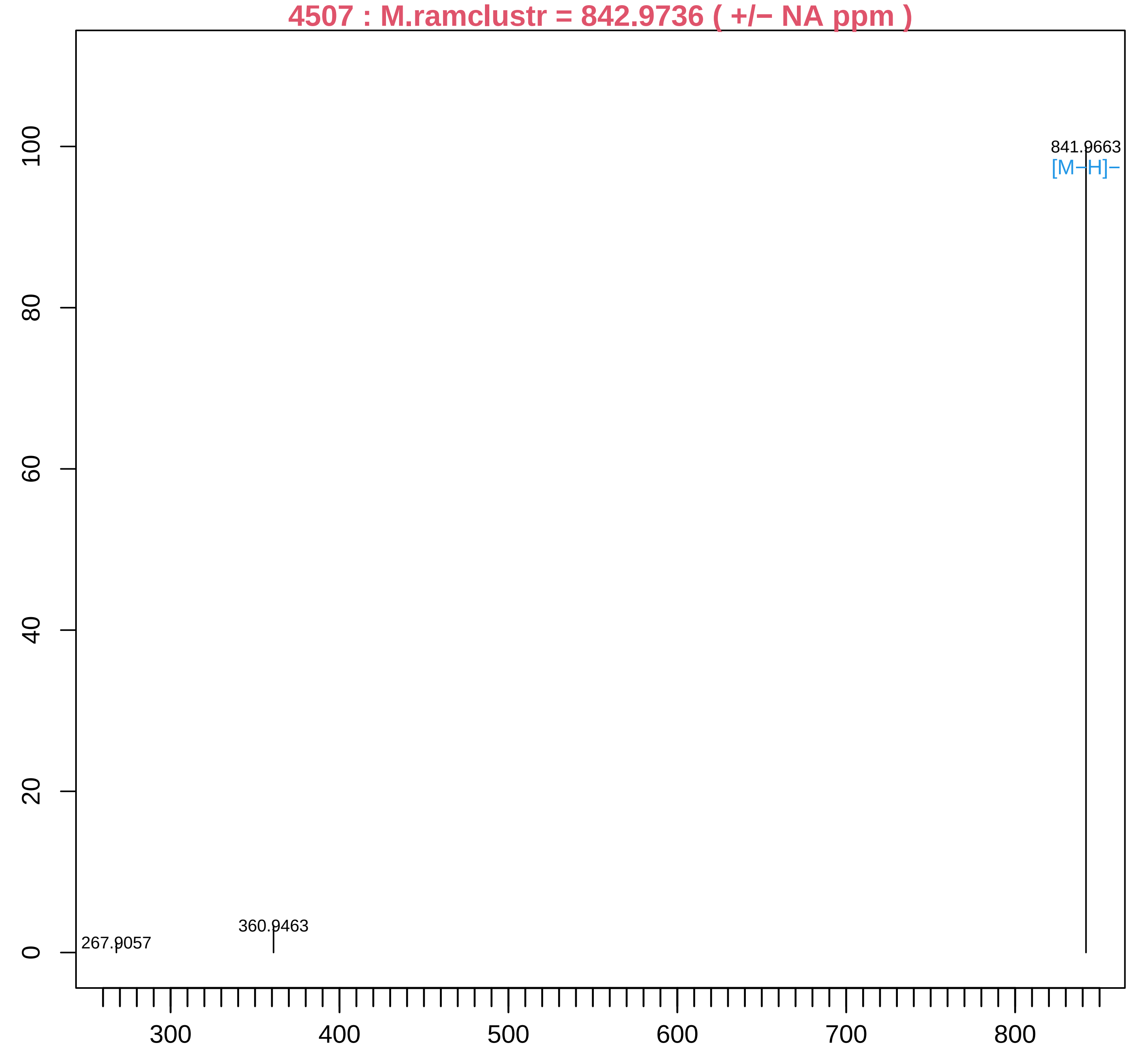
4507 : M.ramclustr = 842.9736 (+/− NA ppm)
841.9663
[M−H]−
360.9463
267.9057
0
20
40
60
80
100
300
400
500
600
700
800

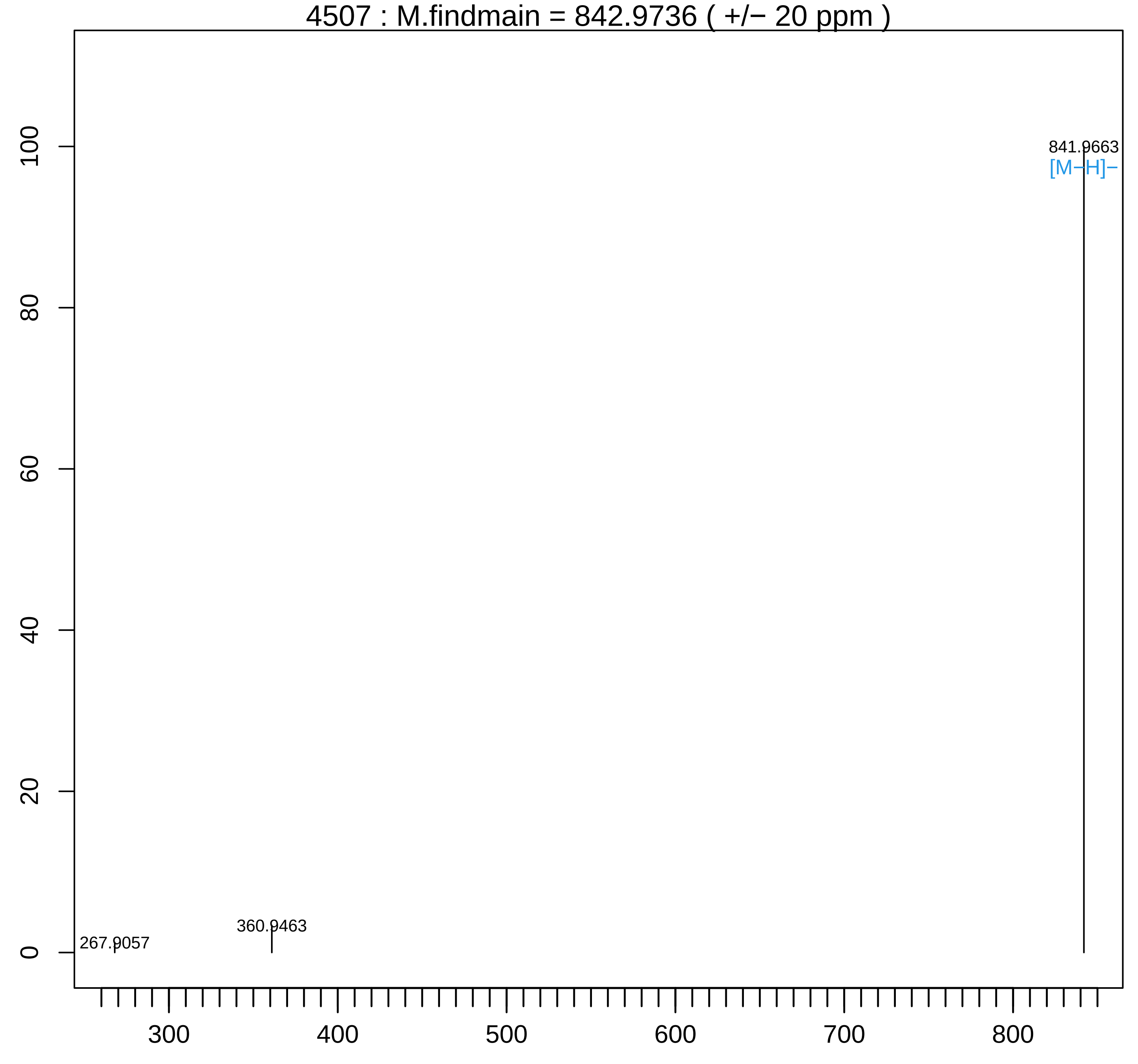
4507 : M.findmain = 842.9736 (+/− 20 ppm)
841.9663
[M−H]−
360.9463
267.9057
0
20
40
60
80
100
300
400
500
600
700
800

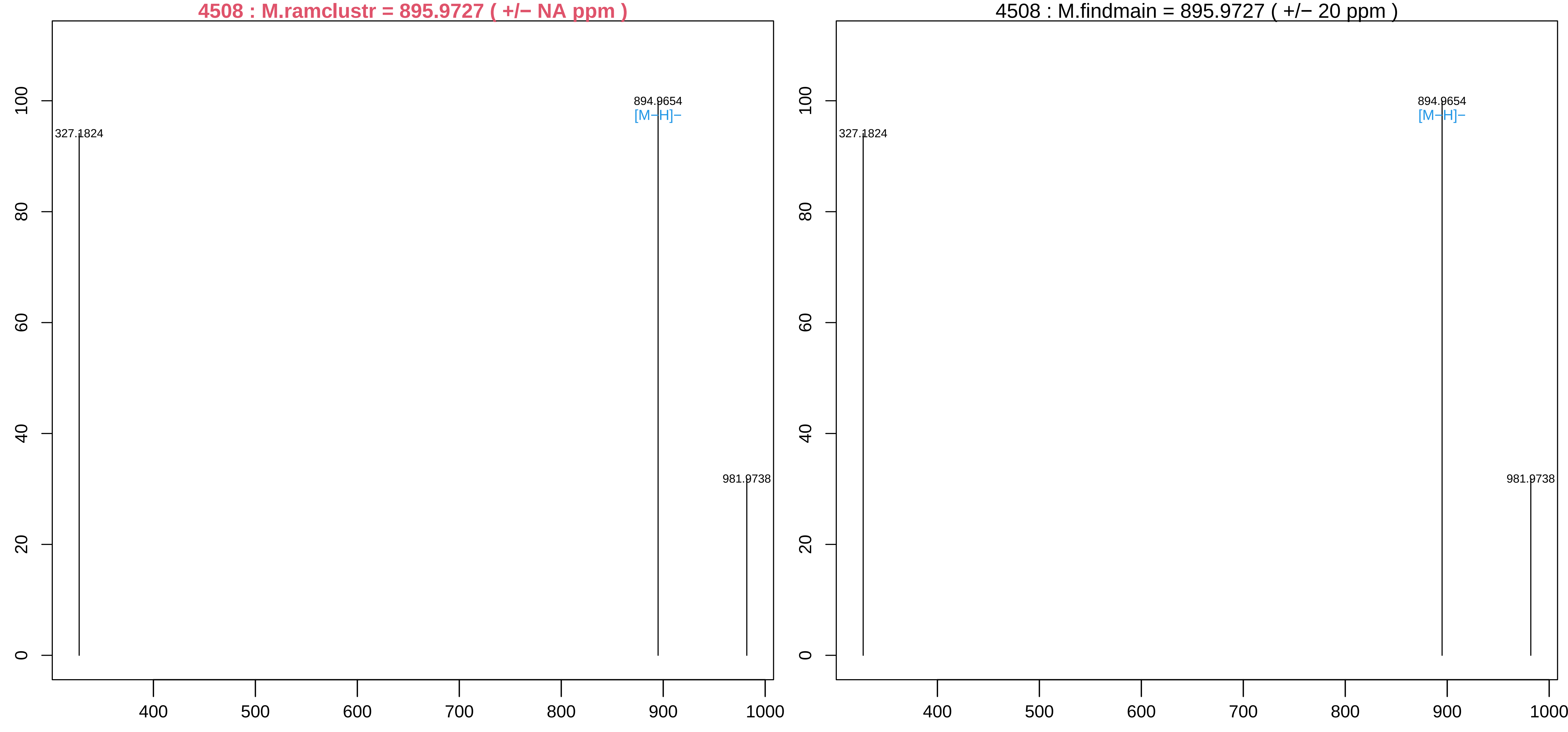
4508 : M.ramclustr = 895.9727 (+/− NA ppm)
327.1824
894.9654
[M−H]−
981.9738
4508 : M.findmain = 895.9727 (+/− 20 ppm)
327.1824
894.9654
[M−H]−
981.9738
0
20
40
60
80
100
400
500
600
700
800
900
1000

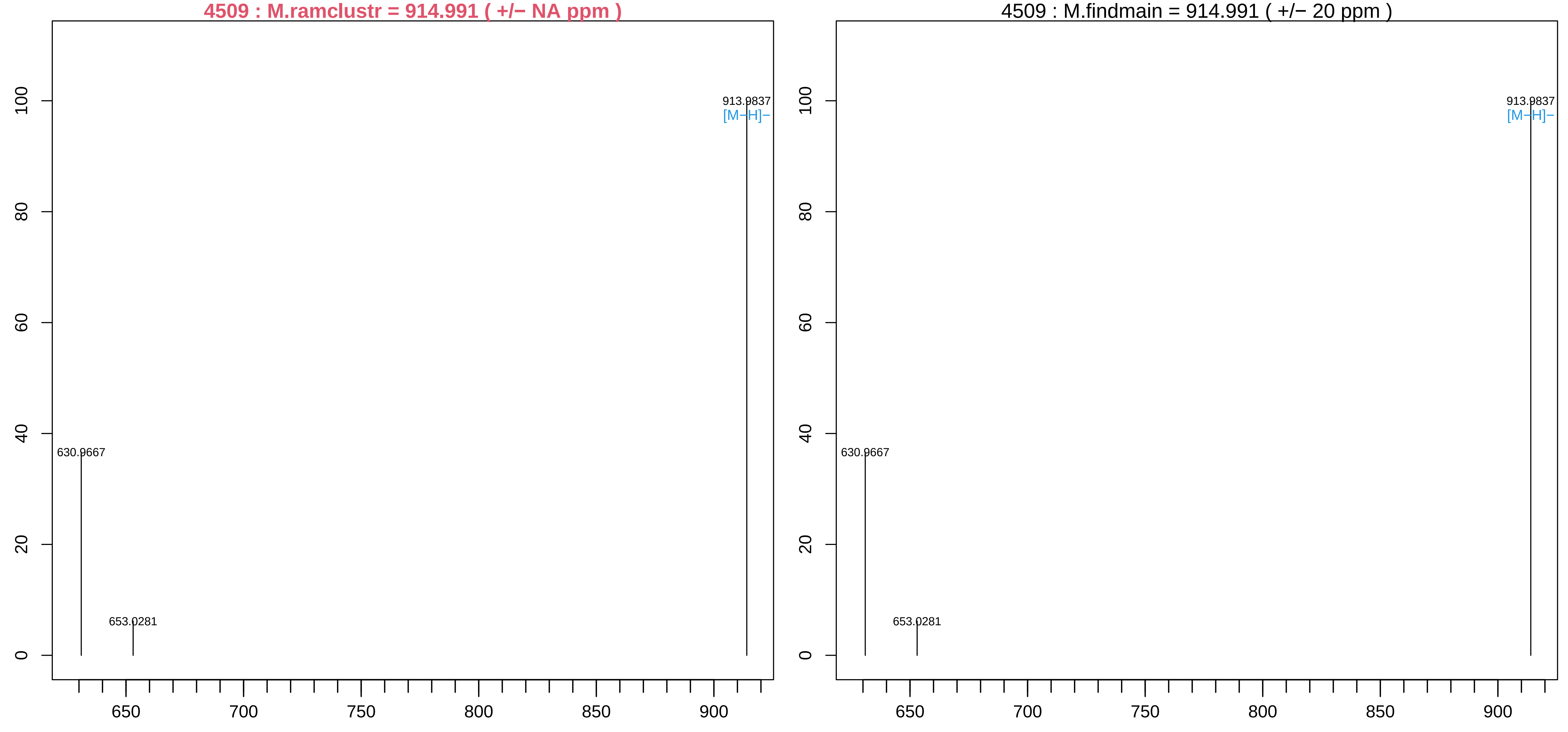
4509 : M.ramclustr = 914.991 (+/− NA ppm)
4509 : M.findmain = 914.991 (+/− 20 ppm)
913.9837
[M−H]−
630.9667
653.0281
913.9837
[M−H]−
630.9667
653.0281
0
20
40
60
80
100
650
700
750
800
850
900

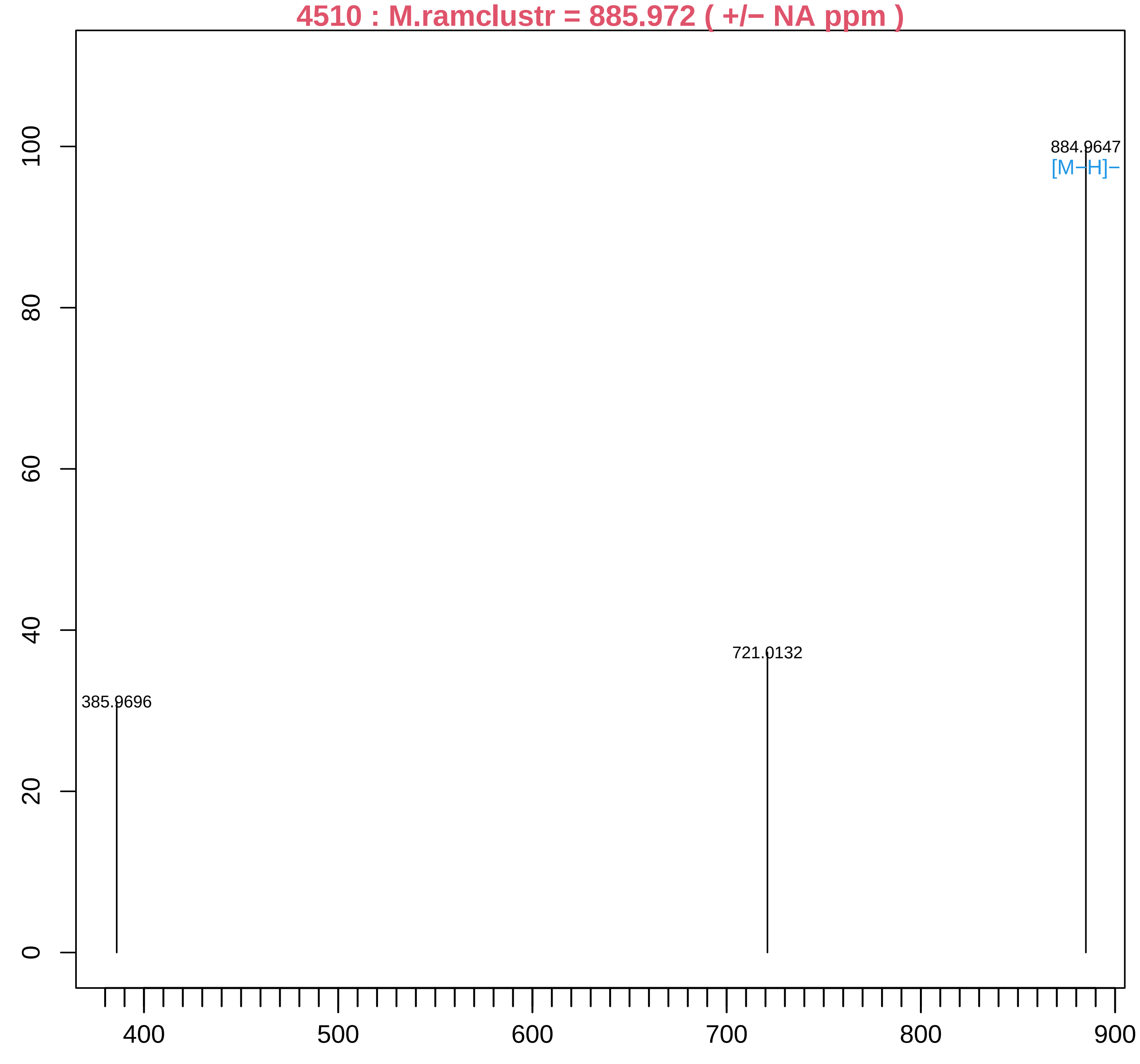

4510 : M.ramclustr = 885.972 (+/− NA ppm)
884.9647
[M−H]−
721.0132
385.9696
0
20
40
60
80
100
400
500
600
700
800
900

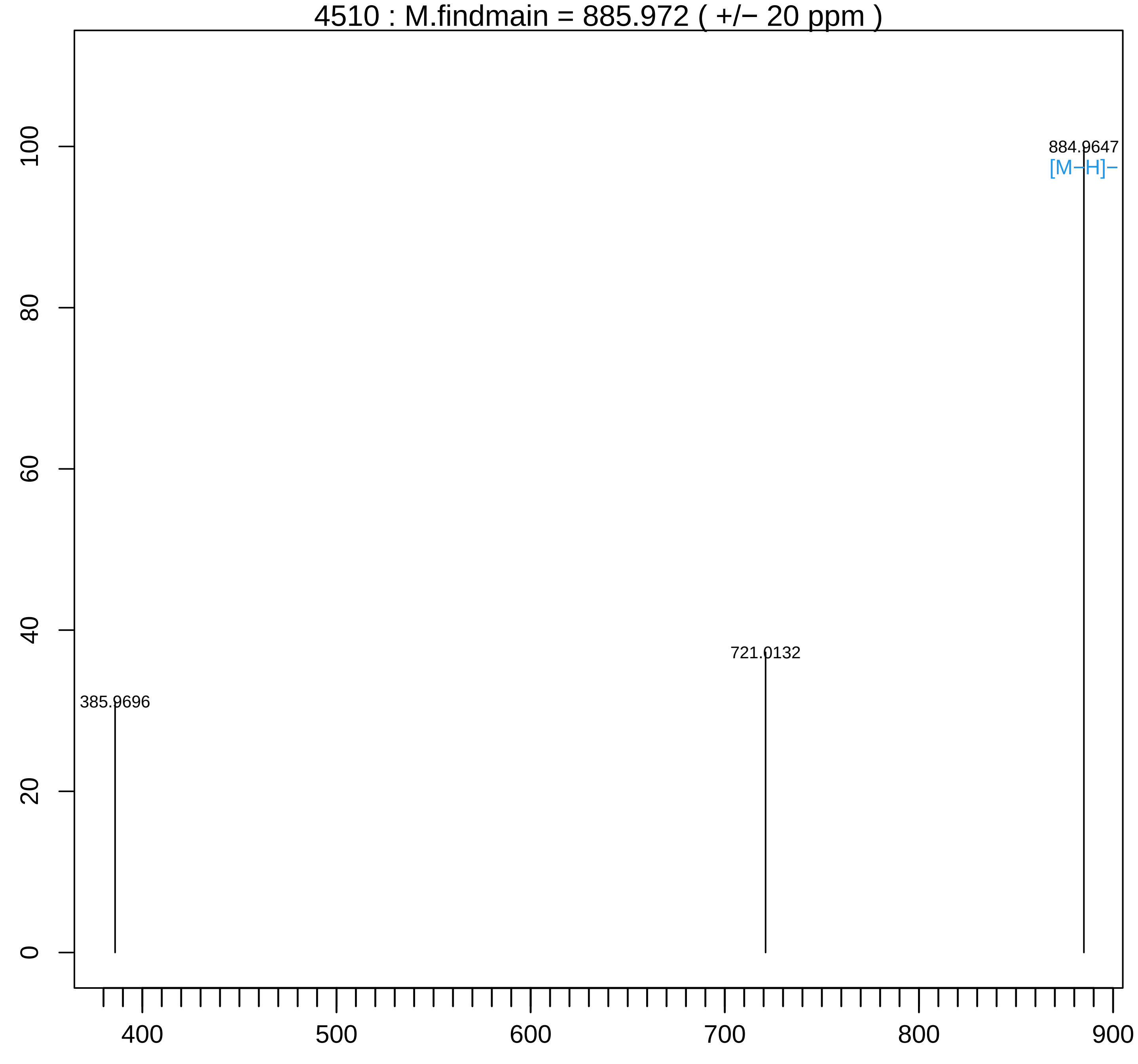

4510 : M.findmain = 885.972 (+/− 20 ppm)
884.9647
[M−H]−
721.0132
385.9696
0
20
40
60
80
100
400
500
600
700
800
900

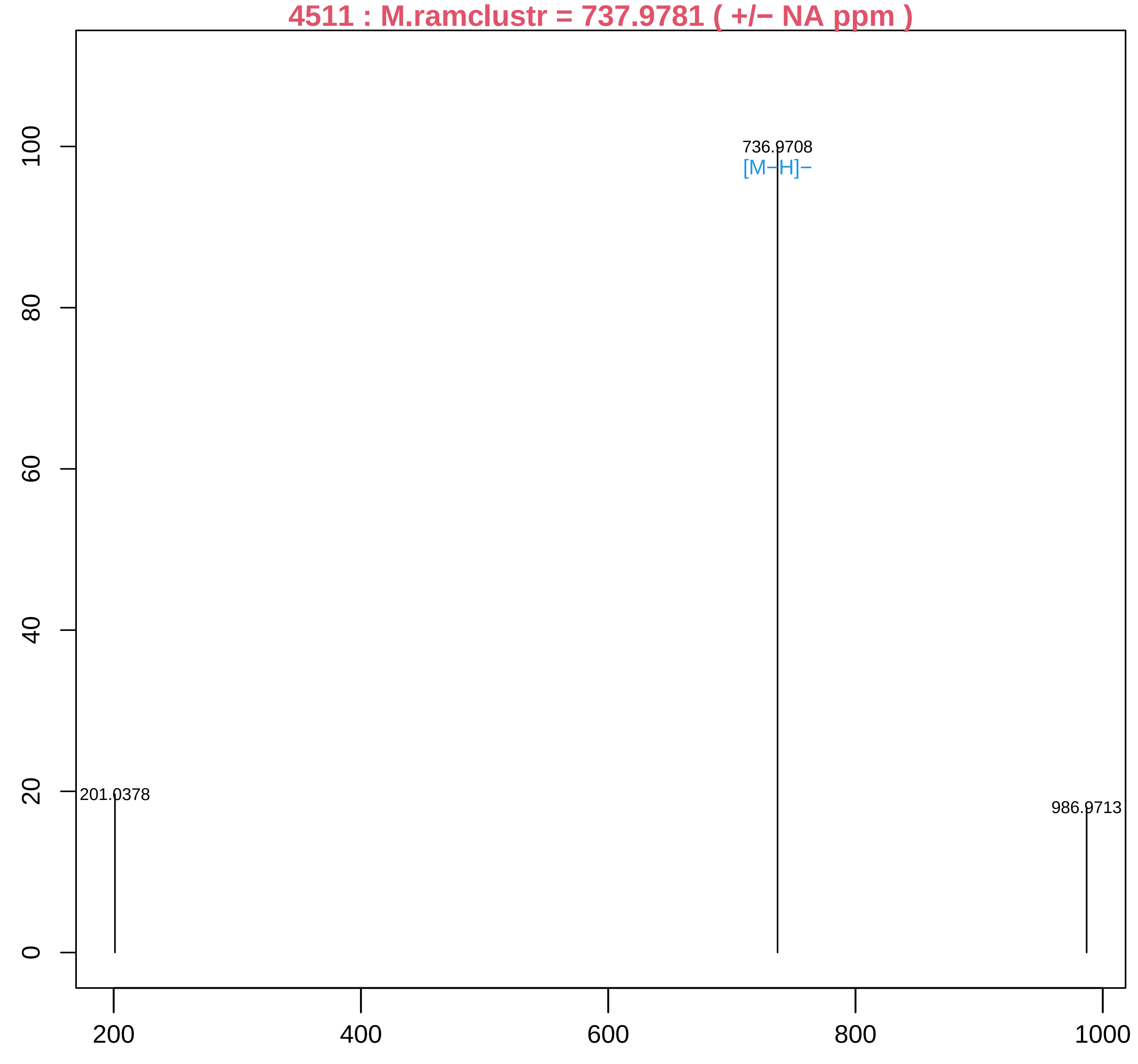
4511 : M.ramclustr = 737.9781 (+/− NA ppm)
736.9708
[M−H]−
201.0378
986.9713
0
20
40
60
80
100
200
400
600
800
1000
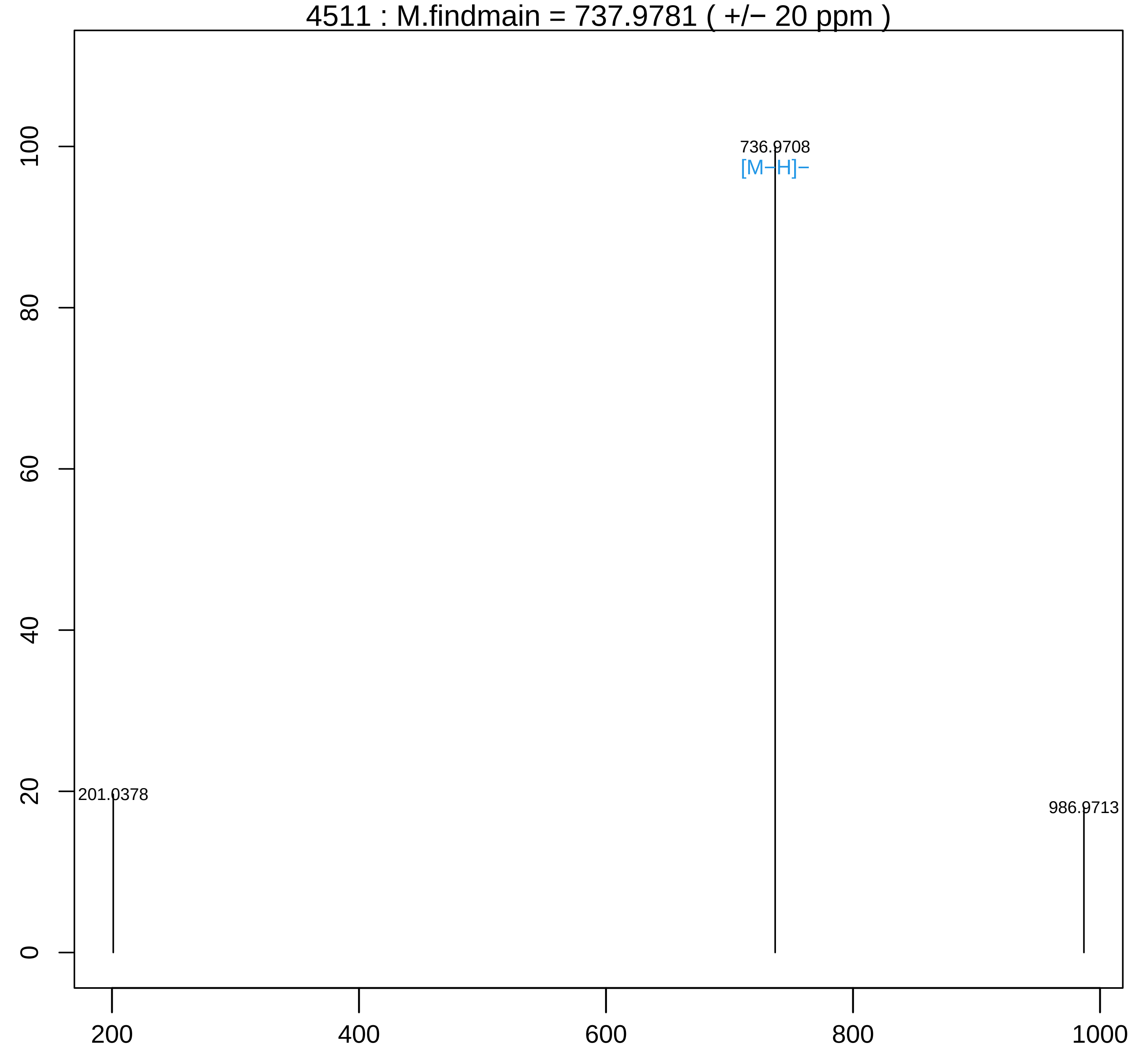
4511 : M.findmain = 737.9781 (+/− 20 ppm)
736.9708
[M−H]−
201.0378
986.9713
0
20
40
60
80
100
200
400
600
800
1000

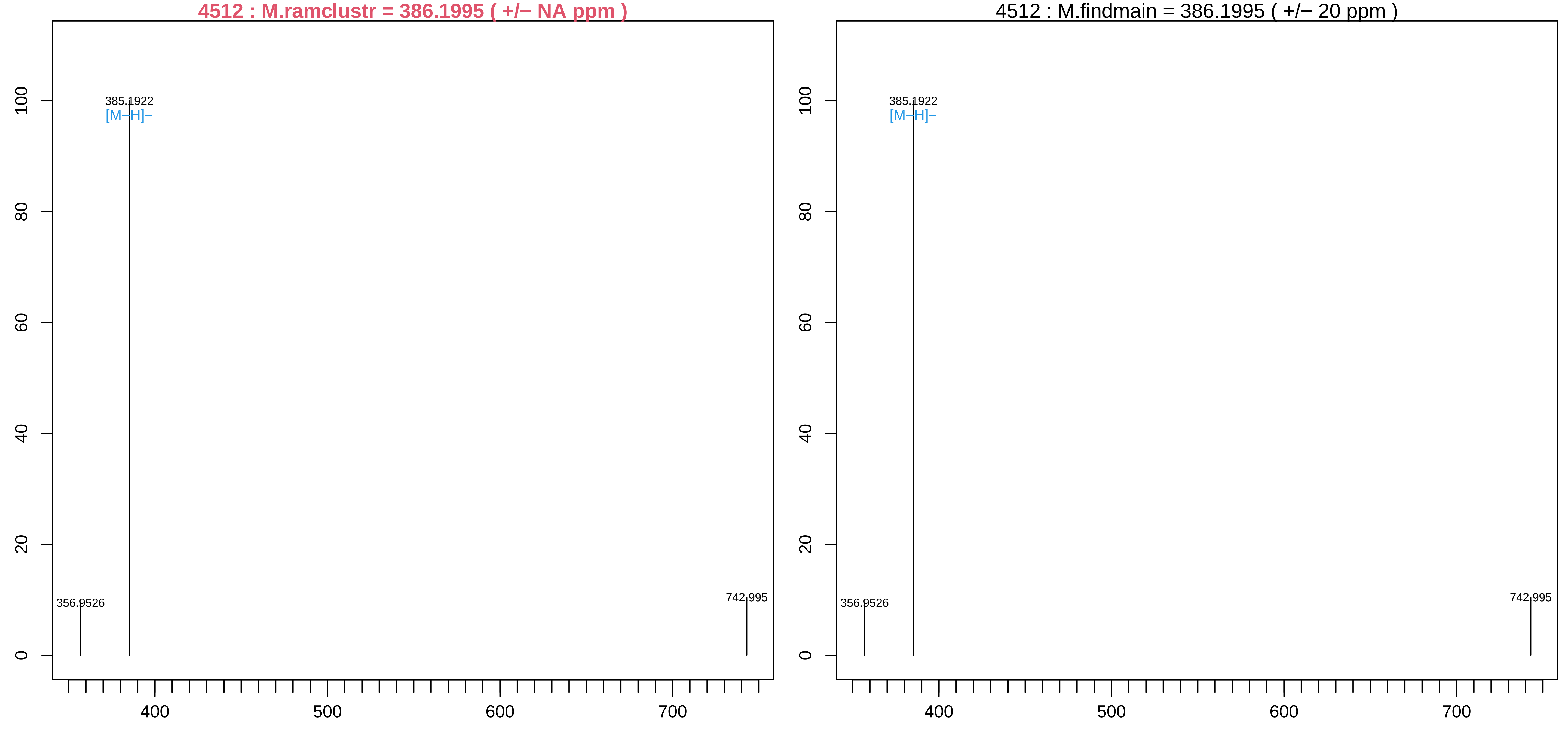
4512 : M.ramclustr = 386.1995 (+/− NA ppm)
4512 : M.findmain = 386.1995 (+/− 20 ppm)
385.1922
[M−H]−
356.9526
742.995
0
20
40
60
80
100
400
500
600
700

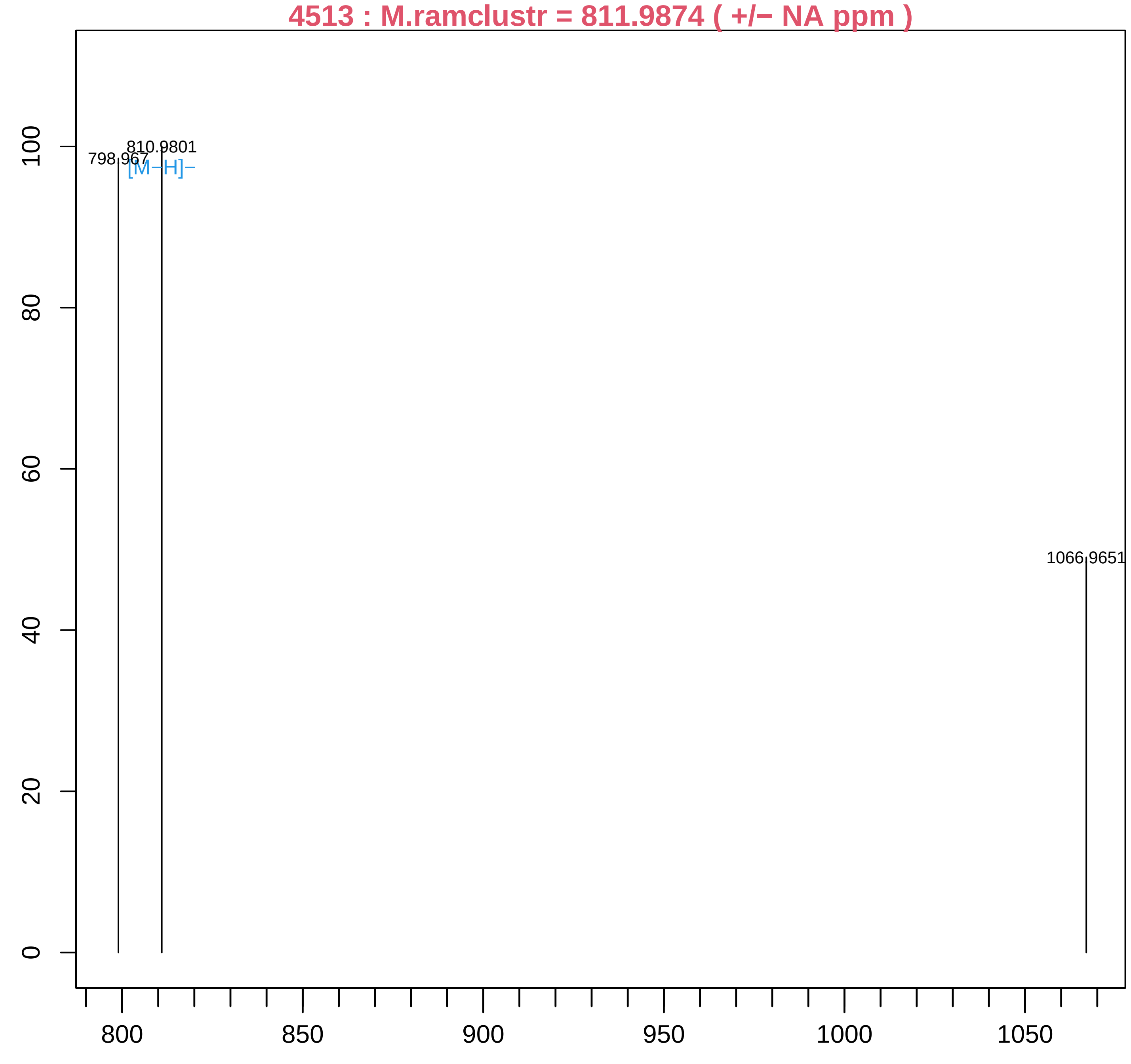

4513 : M.ramclustr = 811.9874 (+/− NA ppm)
798.967
810.9801
[M−H]−
1066.9651
0
20
40
60
80
100
800
850
900
950
1000
1050

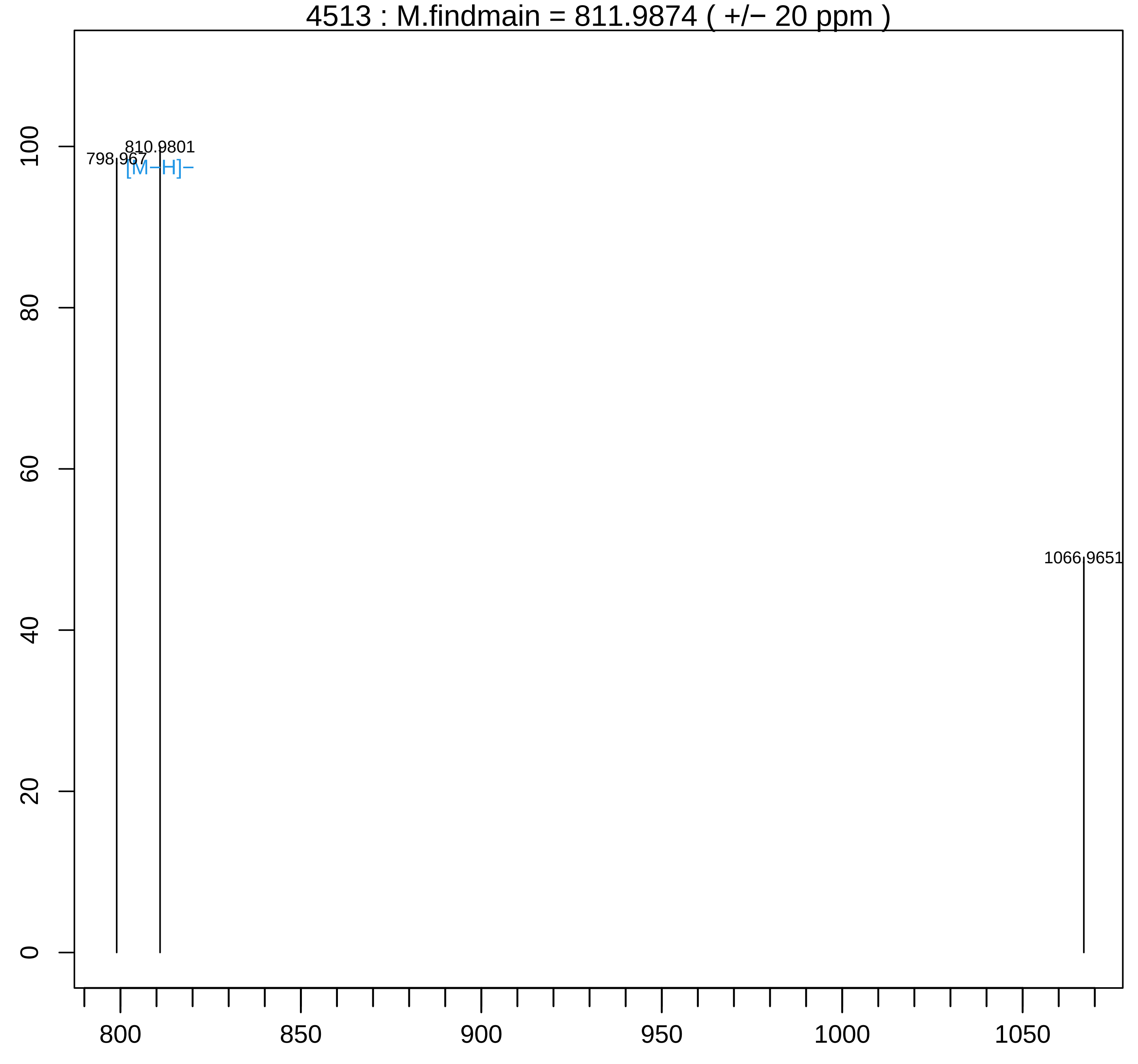

4513 : M.findmain = 811.9874 (+/− 20 ppm)
798.967
810.9801
[M−H]−
1066.9651
0
20
40
60
80
100
800
850
900
950
1000
1050

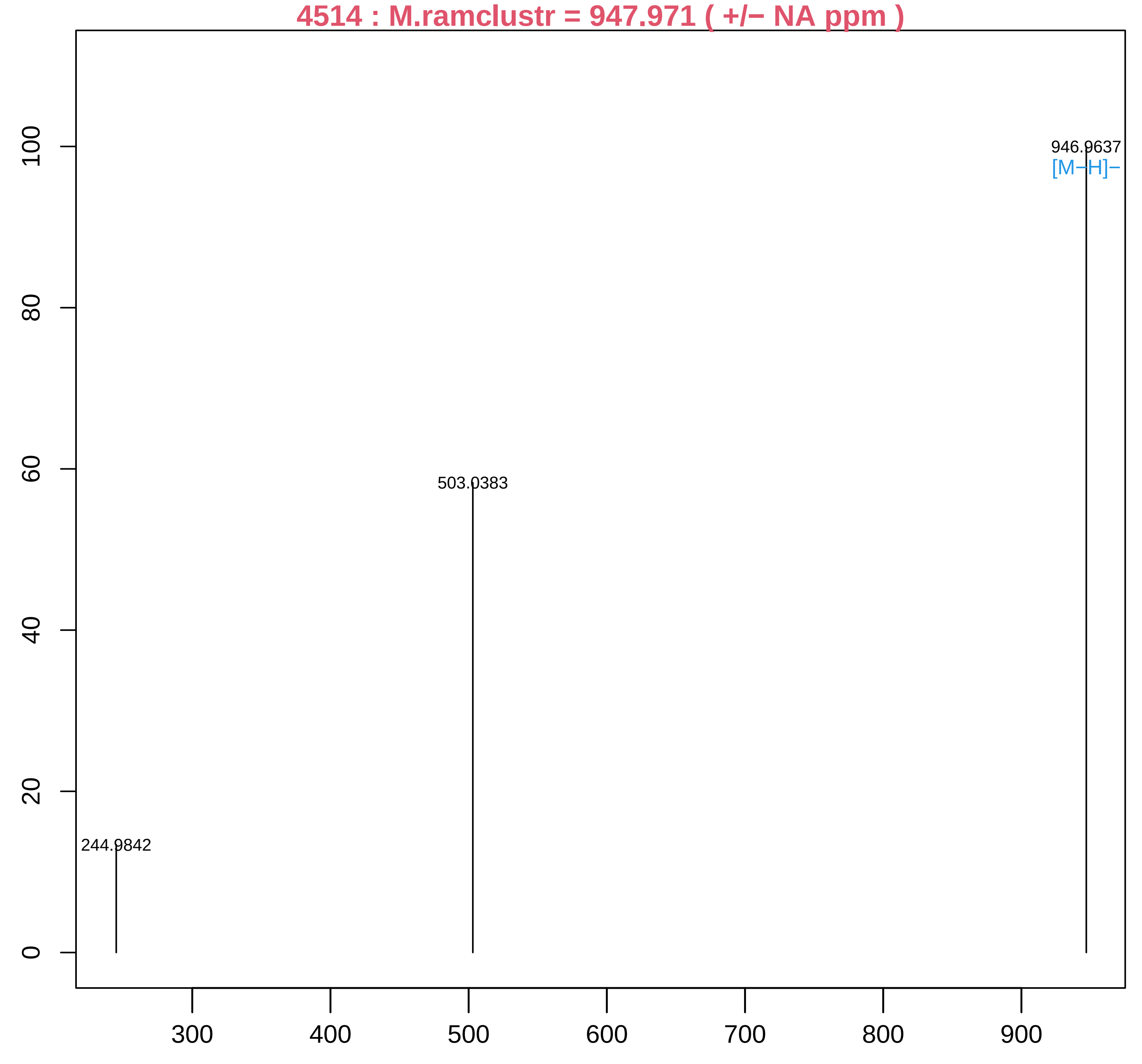

4514 : M.ramclustr = 947.971 (+/− NA ppm)
946.9637
[M−H]−
503.0383
244.9842
0
20
40
60
80
100
300
400
500
600
700
800
900

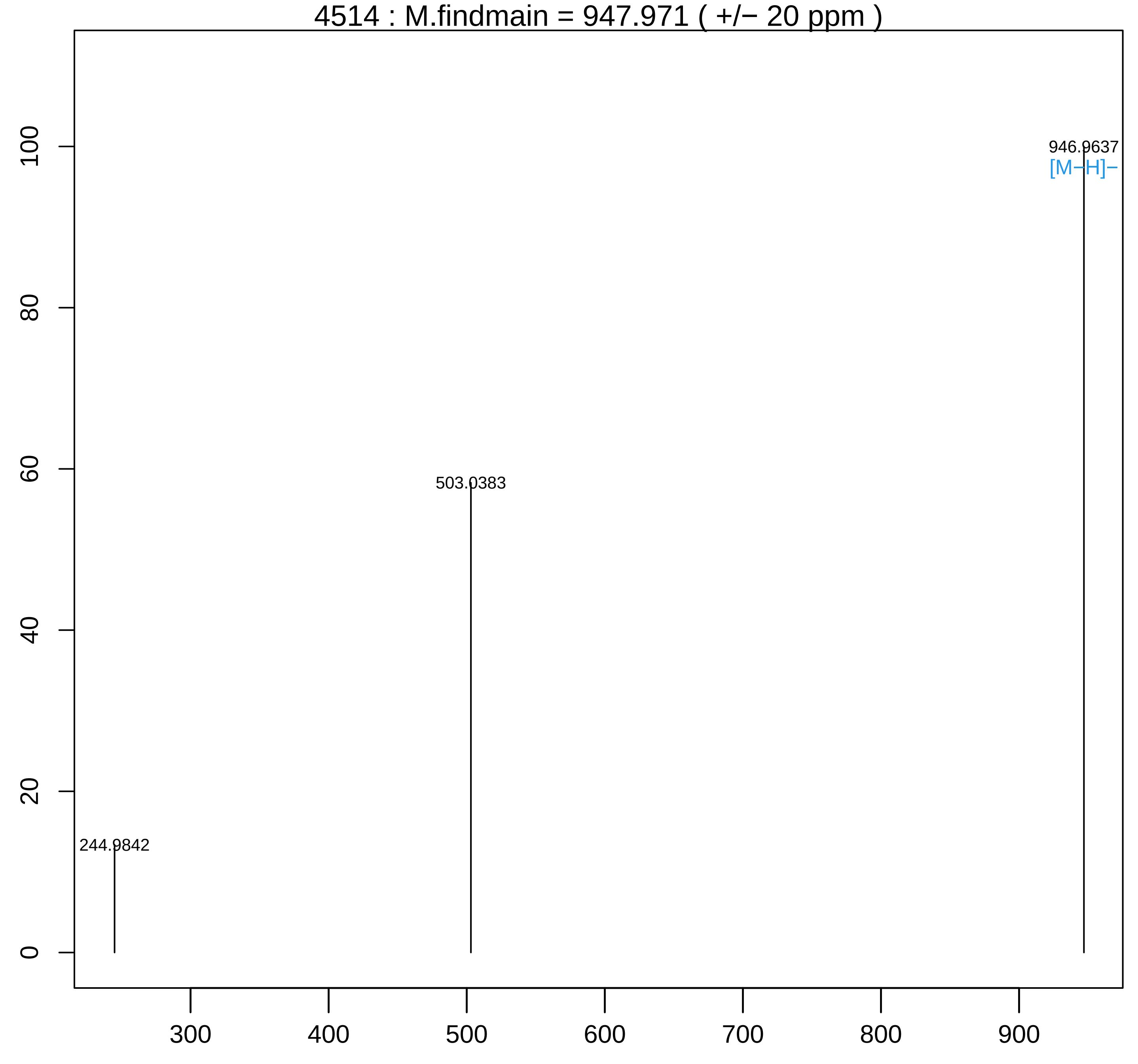

4514 : M.findmain = 947.971 (+/− 20 ppm)
946.9637
[M−H]−
503.0383
244.9842
0
20
40
60
80
100
300
400
500
600
700
800
900

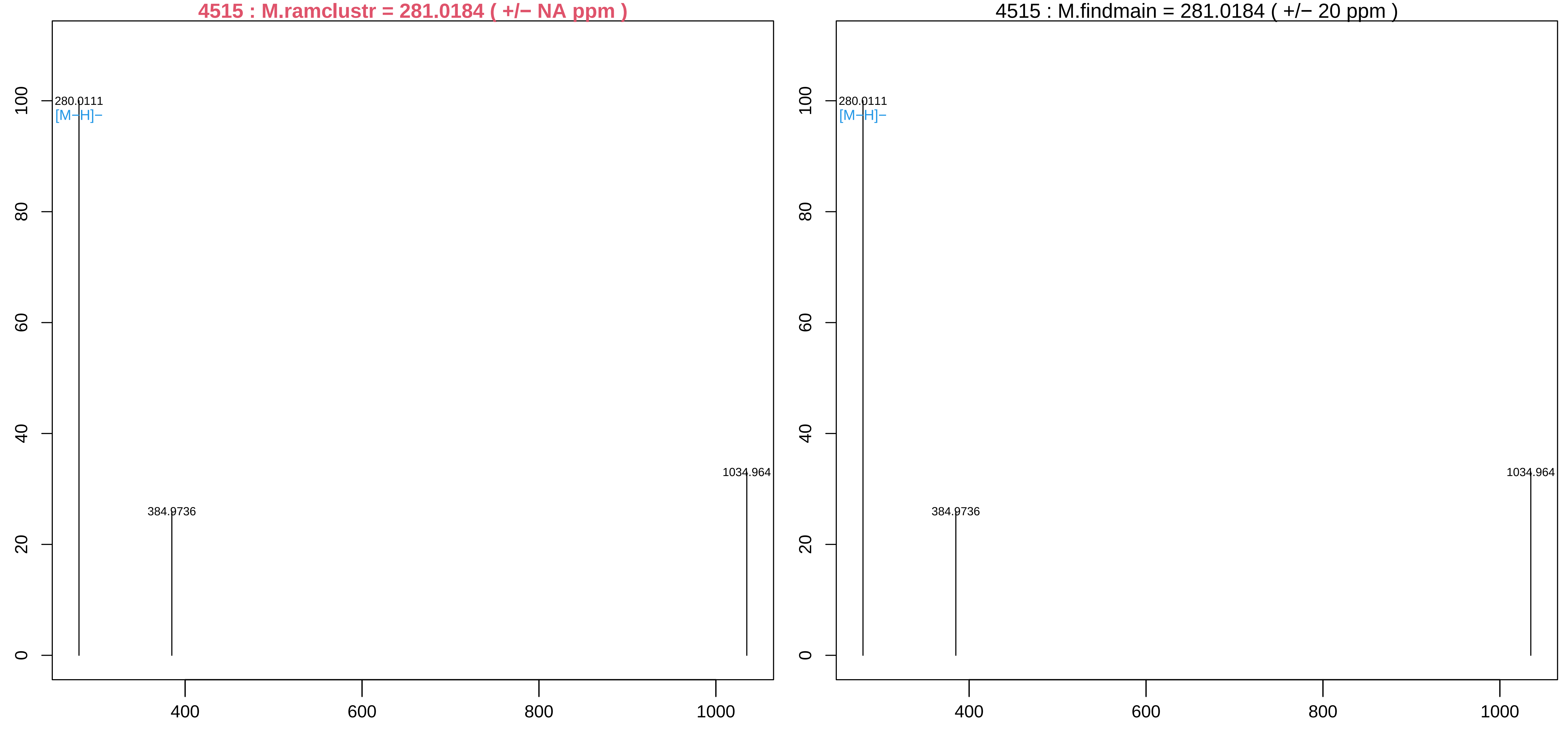

4515 : M.ramclustr = 281.0184 (+/− NA ppm)
280.0111
[M−H]−
384.9736
1034.964
0
20
40
60
80
100
400
600
800
1000
4515 : M.findmain = 281.0184 (+/− 20 ppm)
280.0111
[M−H]−
384.9736
1034.964
0
20
40
60
80
100
400
600
800
1000

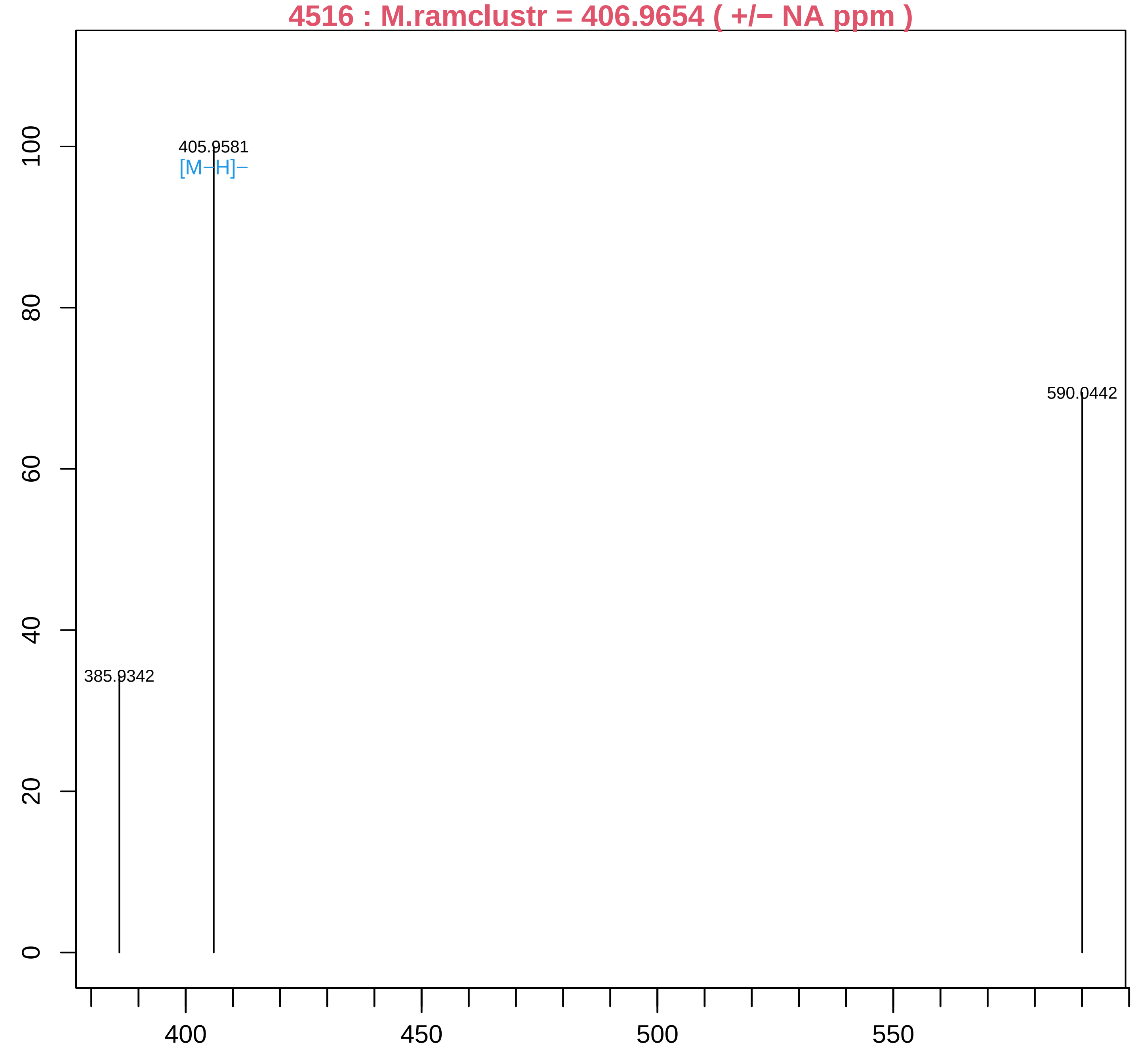
4516 : M.ramclustr = 406.9654 (+/− NA ppm)
405.9581
[M−H]−
590.0442
385.9342
0
20
40
60
80
100
400
450
500
550

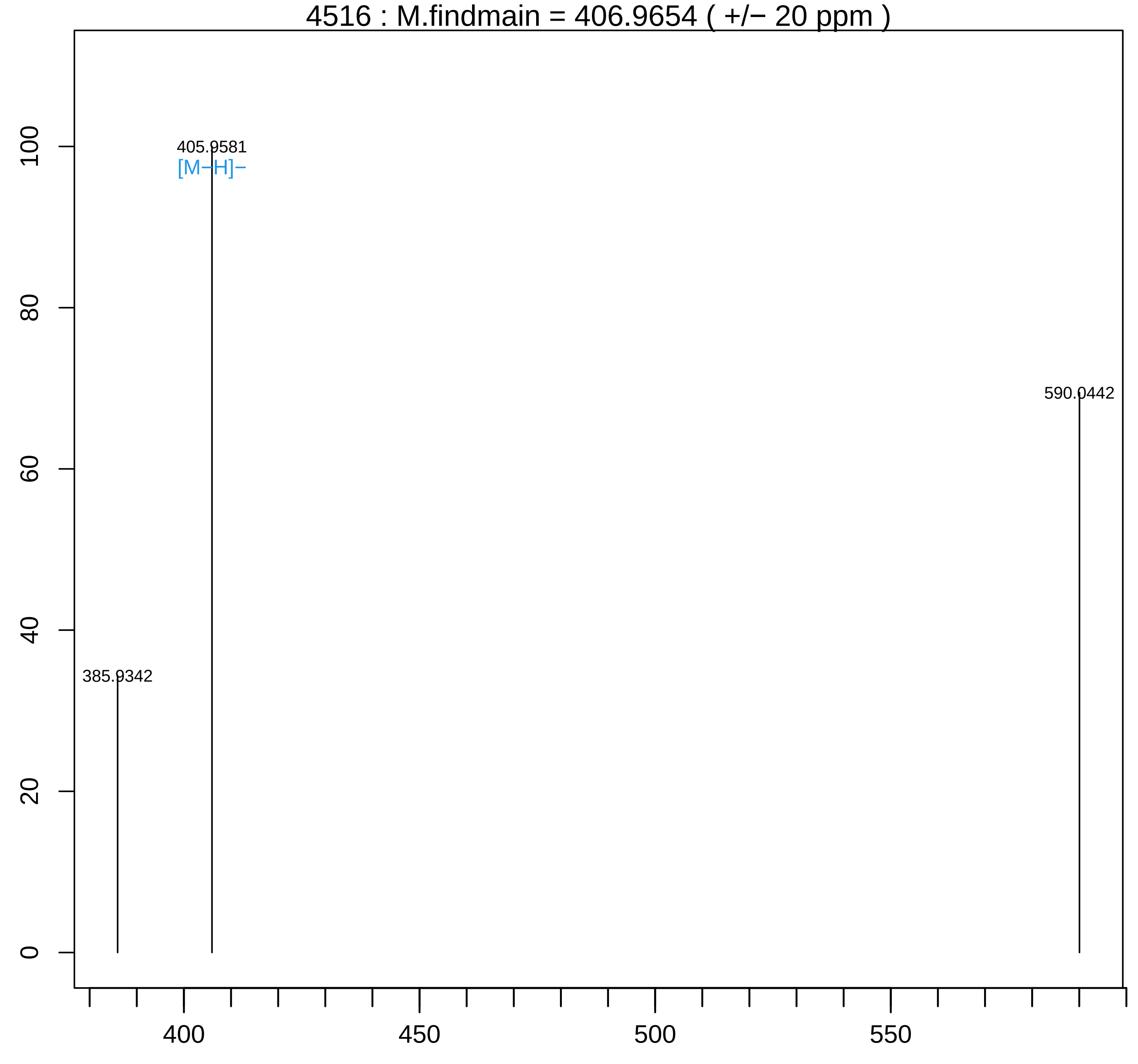
4516 : M.findmain = 406.9654 (+/− 20 ppm)
405.9581
[M−H]−
590.0442
385.9342
0
20
40
60
80
100
400
450
500
550

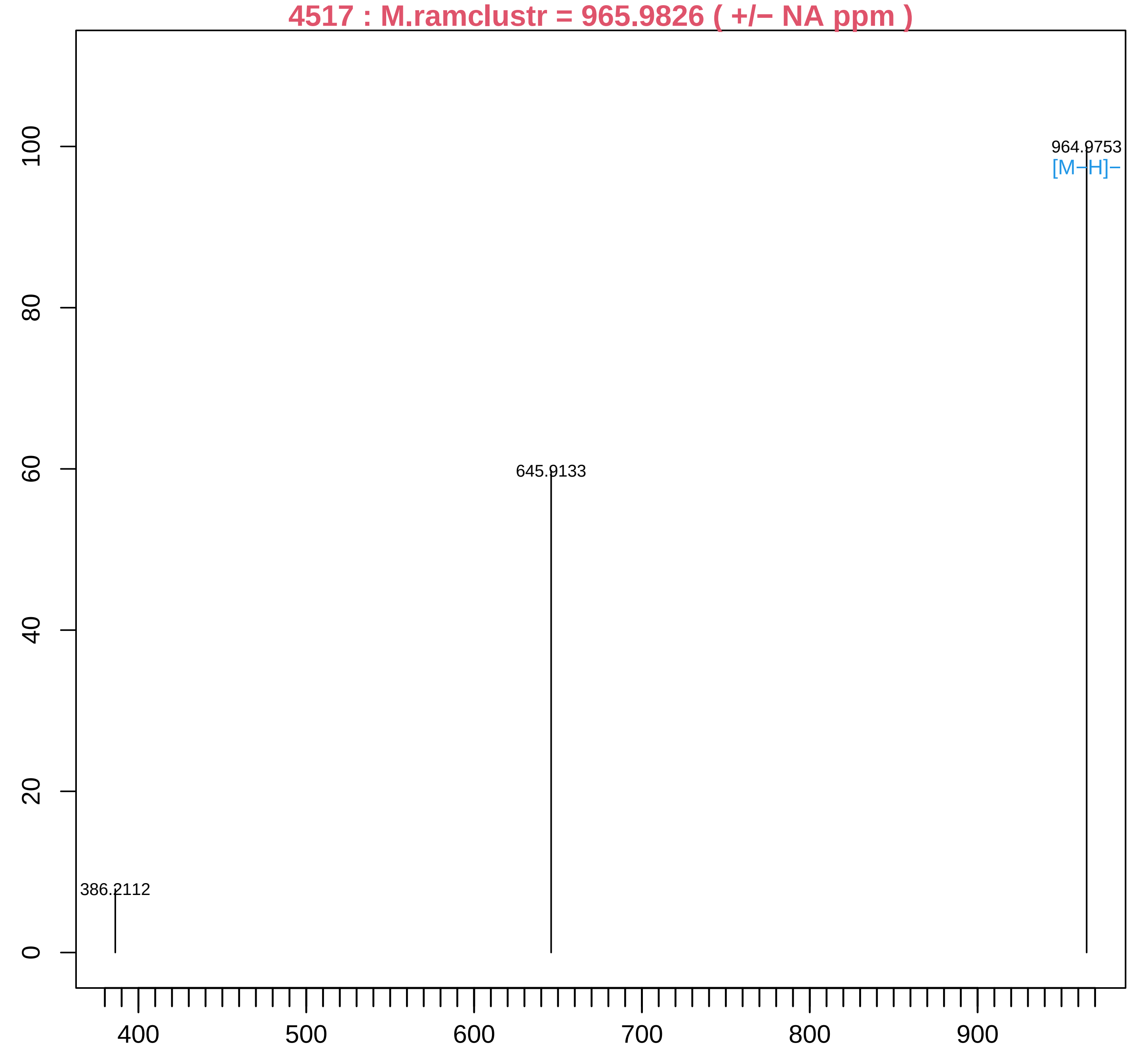

4517 : M.ramclustr = 965.9826 (+/− NA ppm)
964.9753
[M−H]−
645.9133
386.2112
0
20
40
60
80
100
400
500
600
700
800
900

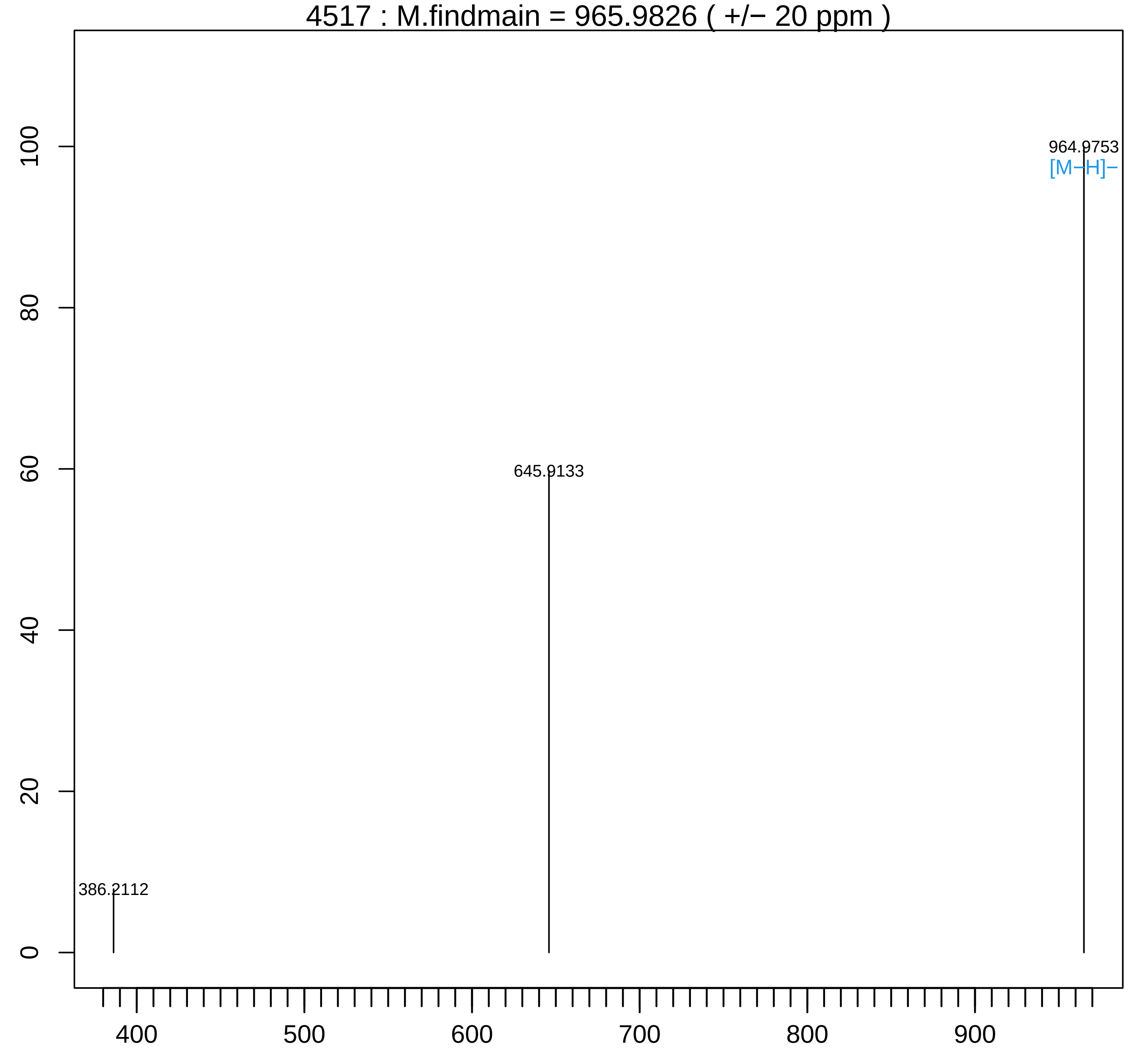

4517 : M.findmain = 965.9826 (+/− 20 ppm)
964.9753
[M−H]−
645.9133
386.2112
0
20
40
60
80
100
400
500
600
700
800
900

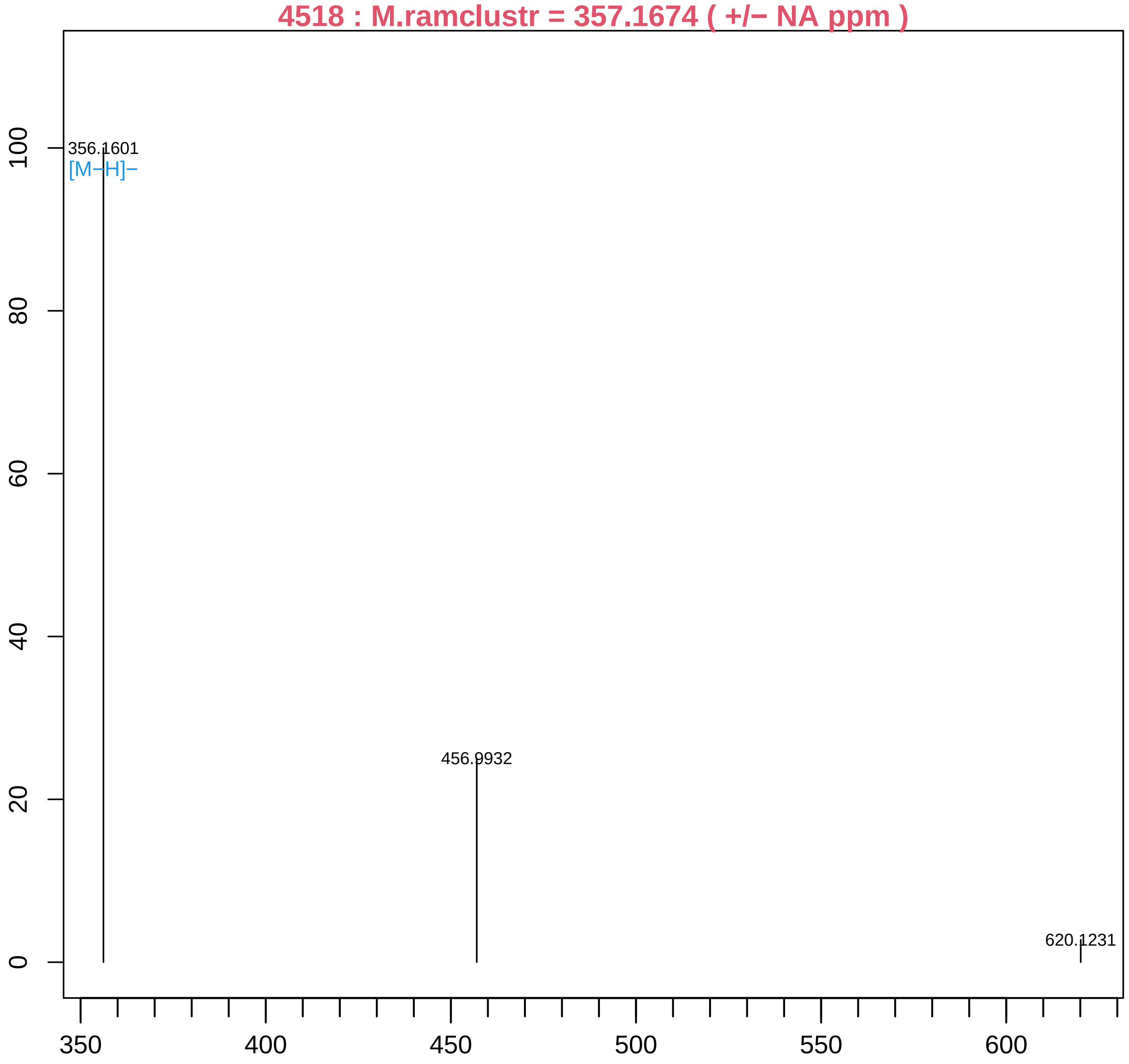
4518 : M.ramclustr = 357.1674 (+/− NA ppm)
356.1601
[M−H]−
456.9932
620.1231
0
20
40
60
80
100
350
400
450
500
550
600

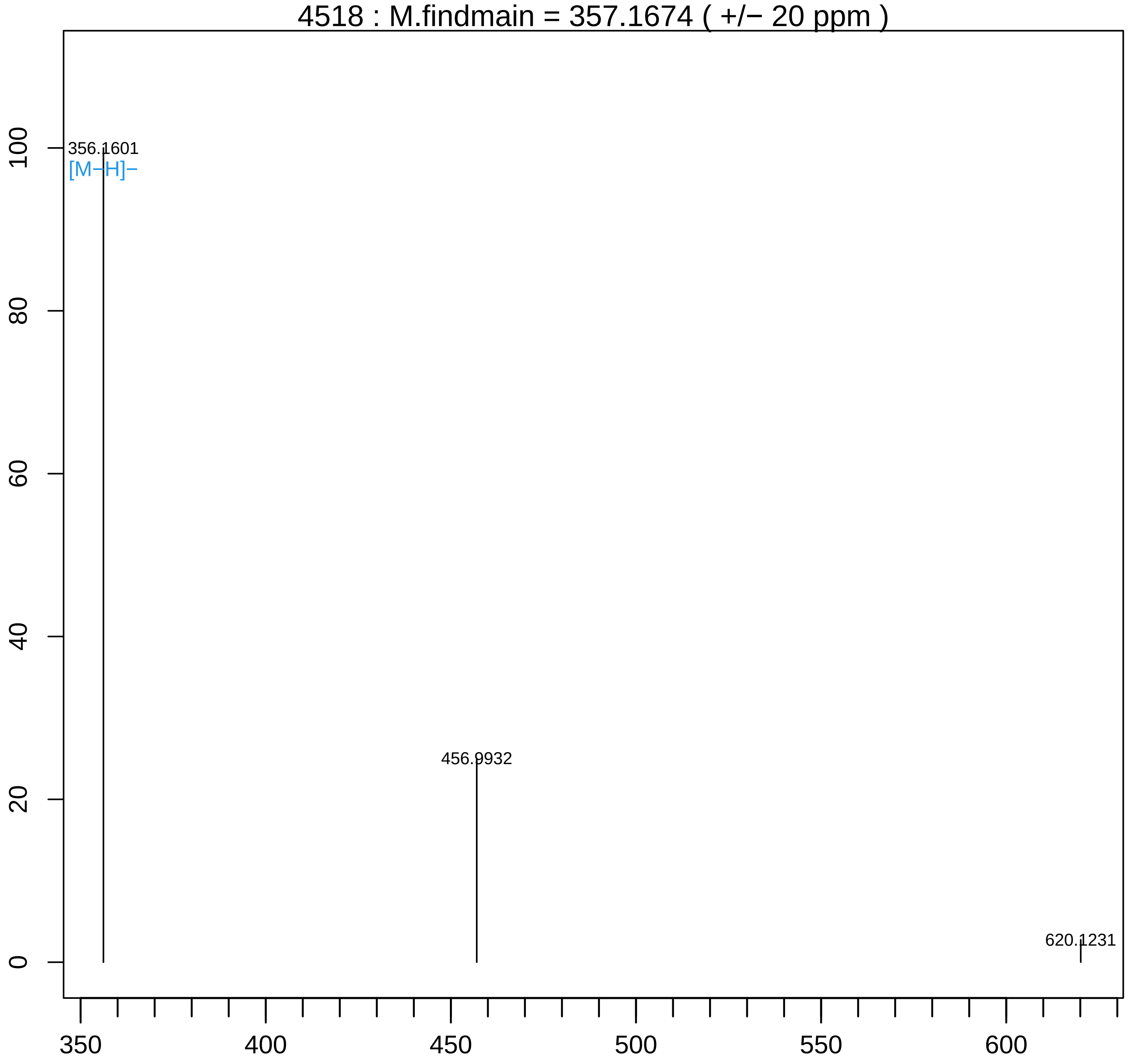
4518 : M.findmain = 357.1674 (+/− 20 ppm)
356.1601
[M−H]−
456.9932
620.1231
0
20
40
60
80
100
350
400
450
500
550
600

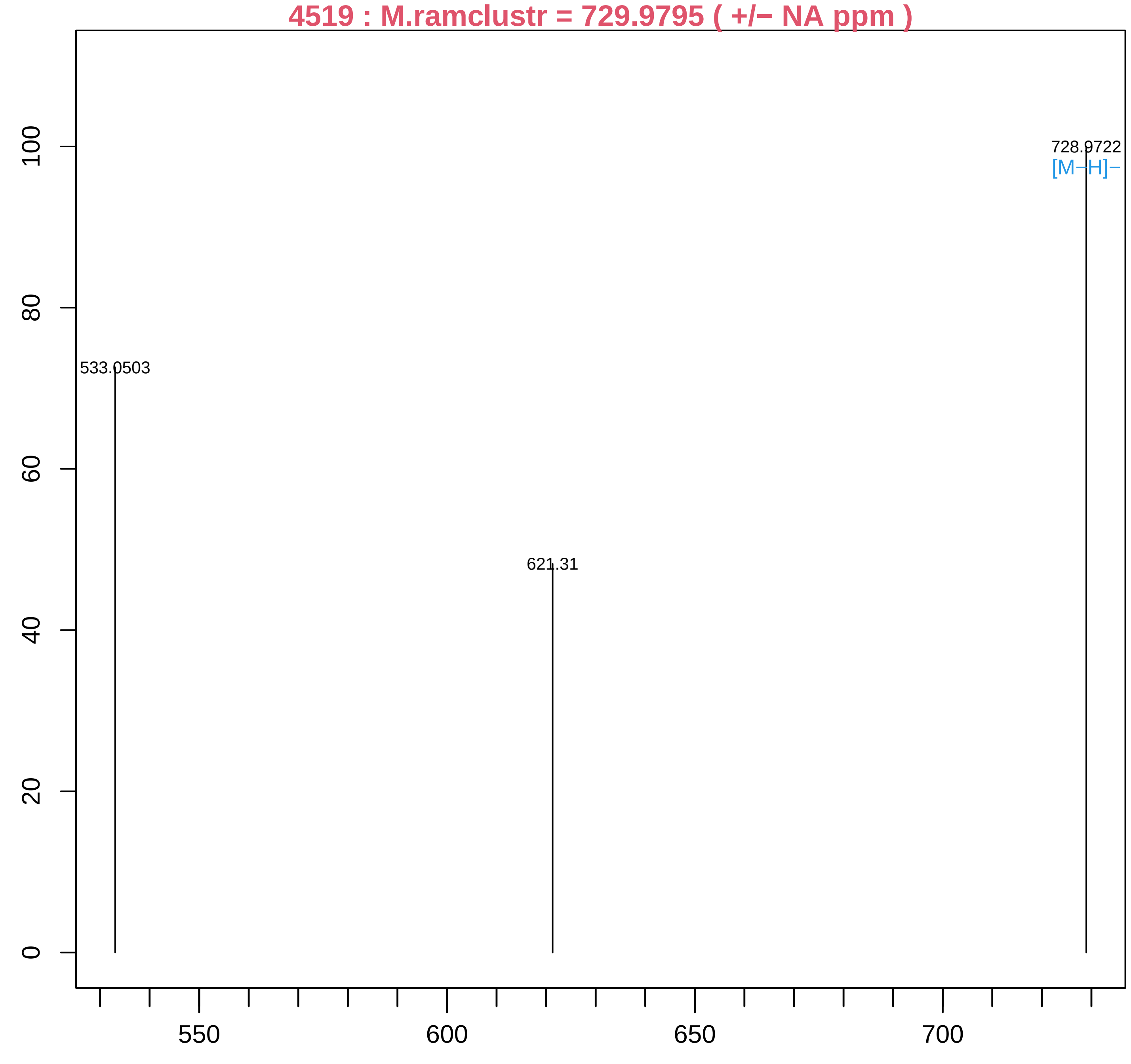

4519 : M.ramclustr = 729.9795 (+/− NA ppm)
728.9722
[M−H]−
533.0503
621.31
0
20
40
60
80
100
550
600
650
700

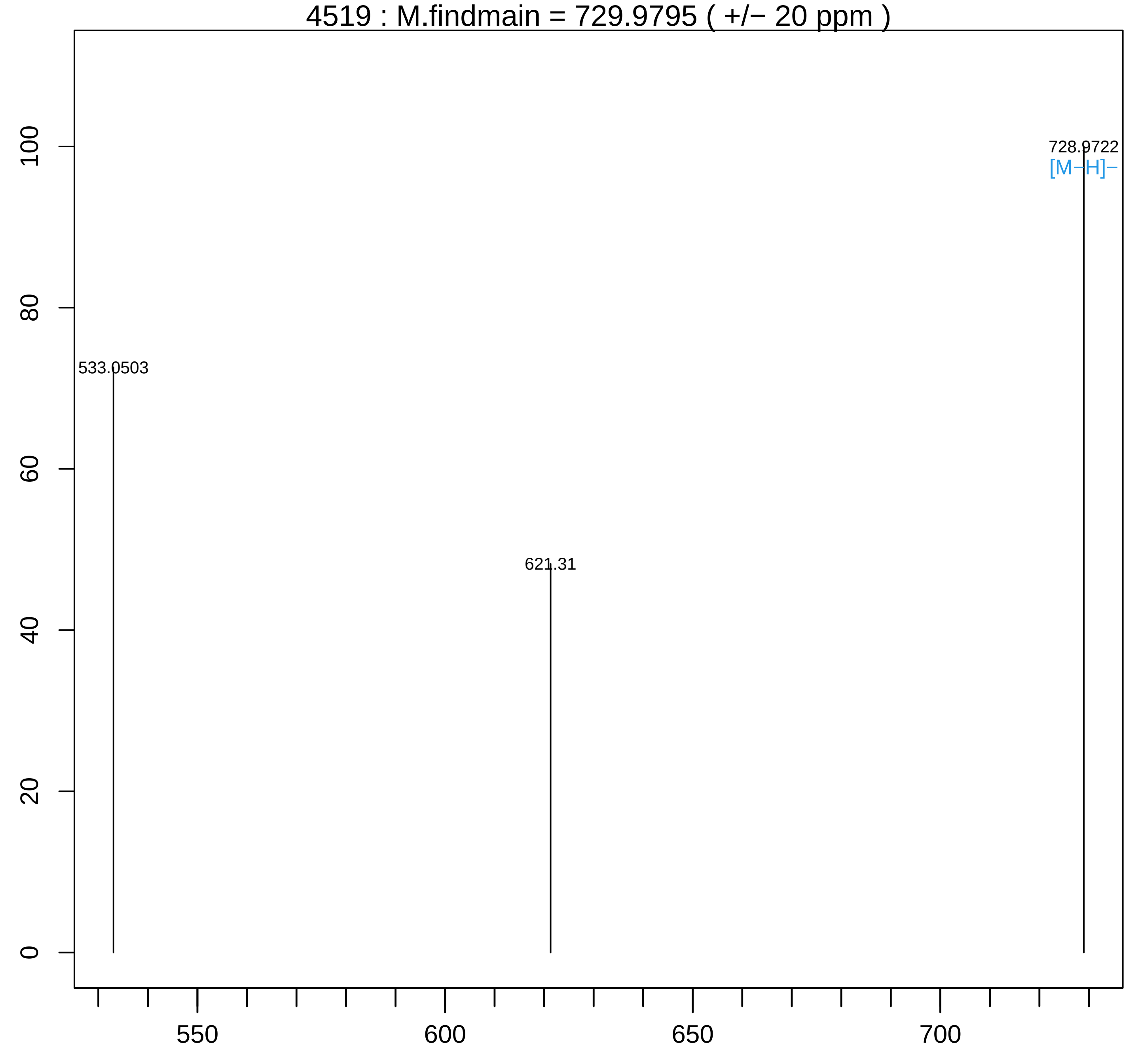

4519 : M.findmain = 729.9795 (+/− 20 ppm)
728.9722
[M−H]−
533.0503
621.31
0
20
40
60
80
100
550
600
650
700

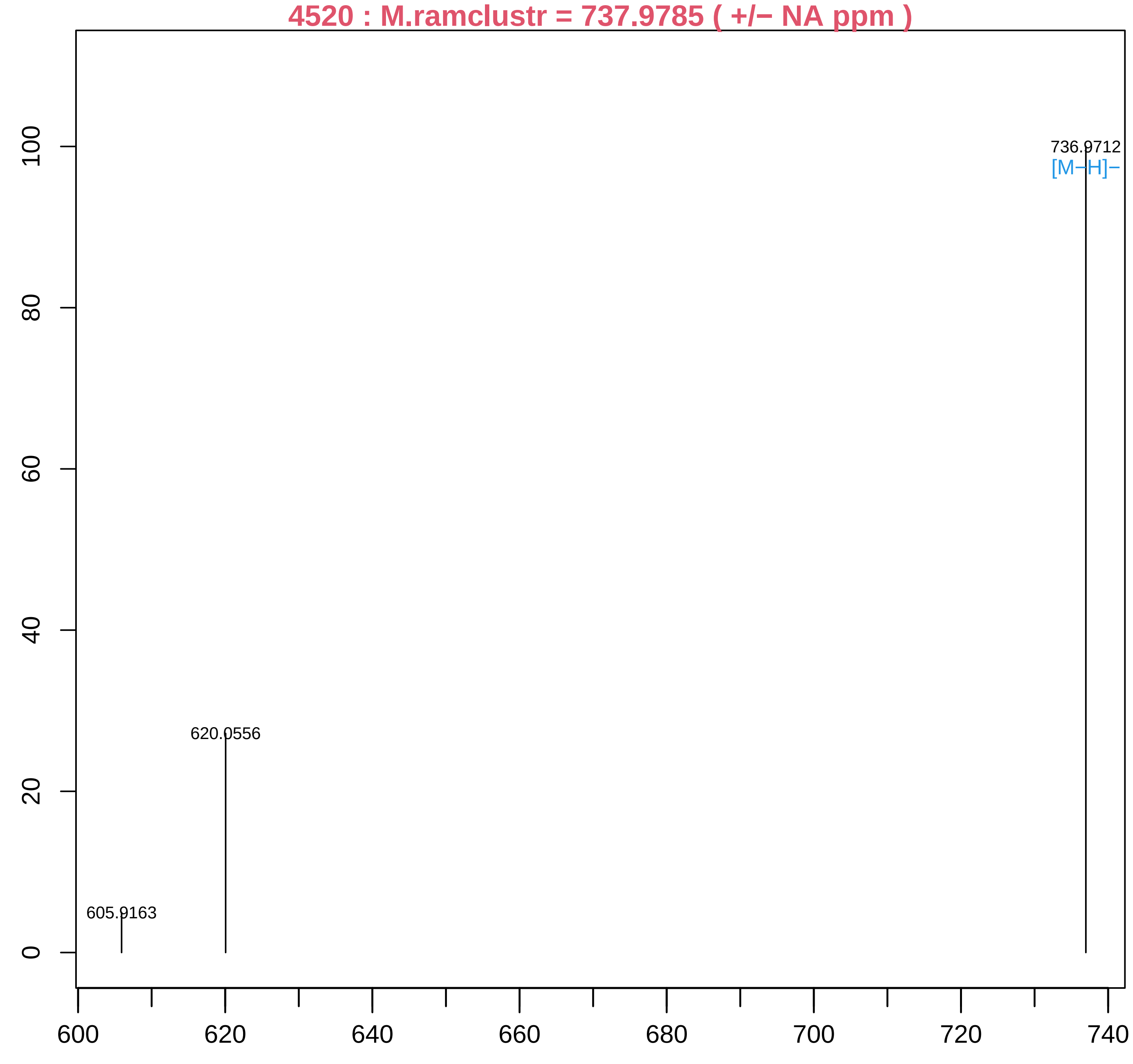
4520 : M.ramclustr = 737.9785 (+/− NA ppm)
736.9712
[M−H]−
620.0556
605.9163
0
20
40
60
80
100
600
620
640
660
680
700
720
740

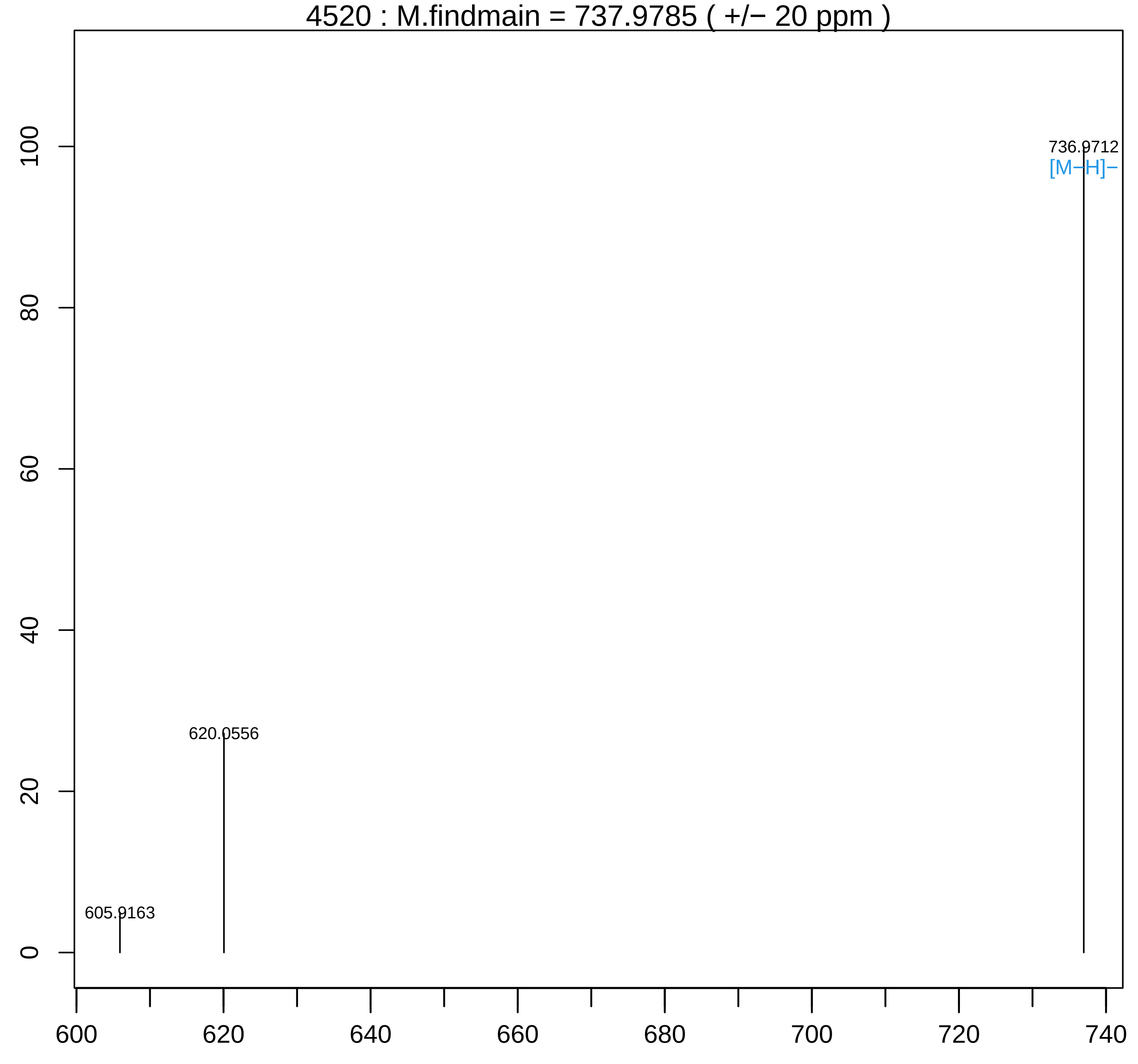
4520 : M.findmain = 737.9785 (+/− 20 ppm)
736.9712
[M−H]−
620.0556
605.9163
0
20
40
60
80
100
600
620
640
660
680
700
720
740

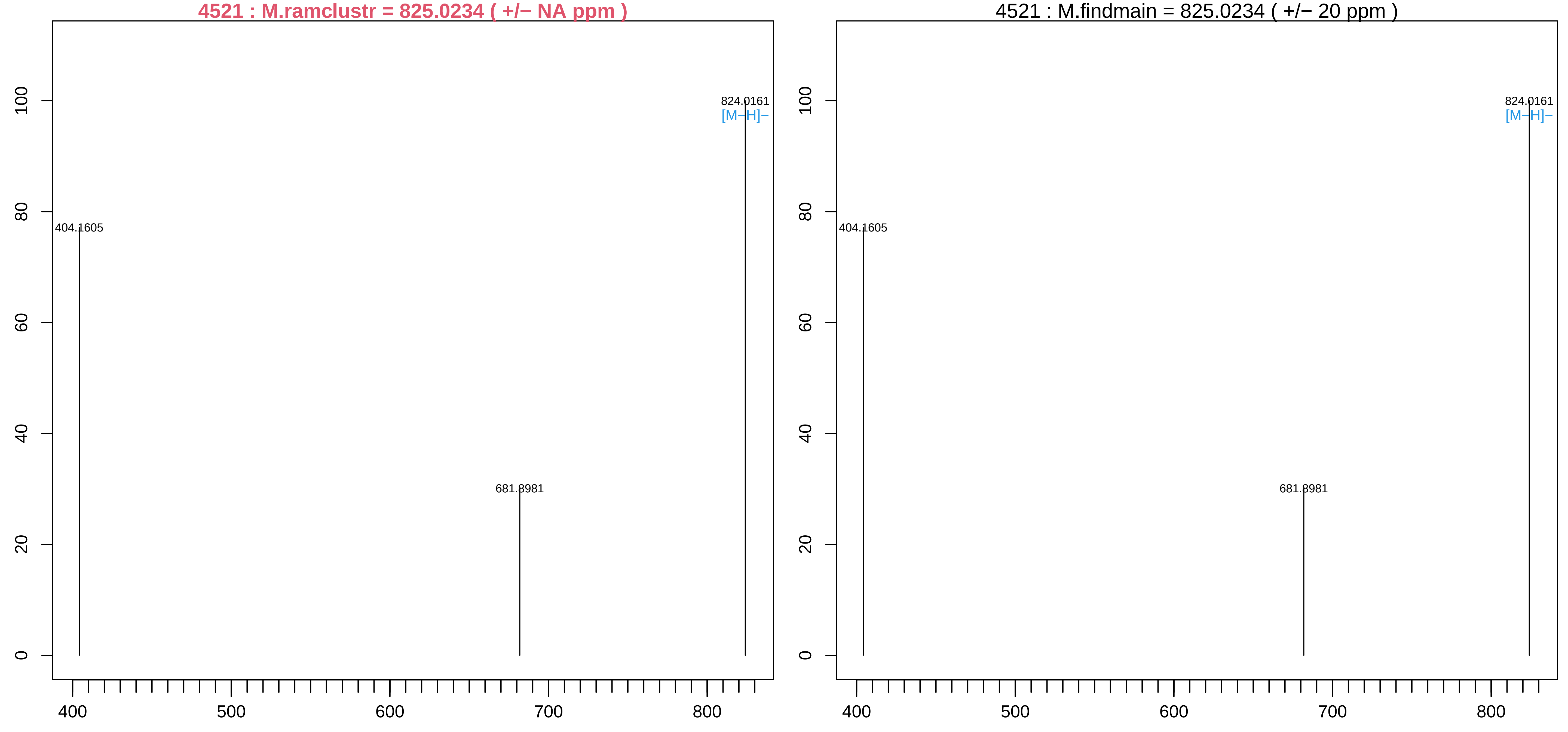
4521 : M.ramclustr = 825.0234 (+/− NA ppm)
404.1605
681.8981
824.0161
[M−H]−
4521 : M.findmain = 825.0234 (+/− 20 ppm)
404.1605
681.8981
824.0161
[M−H]−
0
20
40
60
80
100
400
500
600
700
800

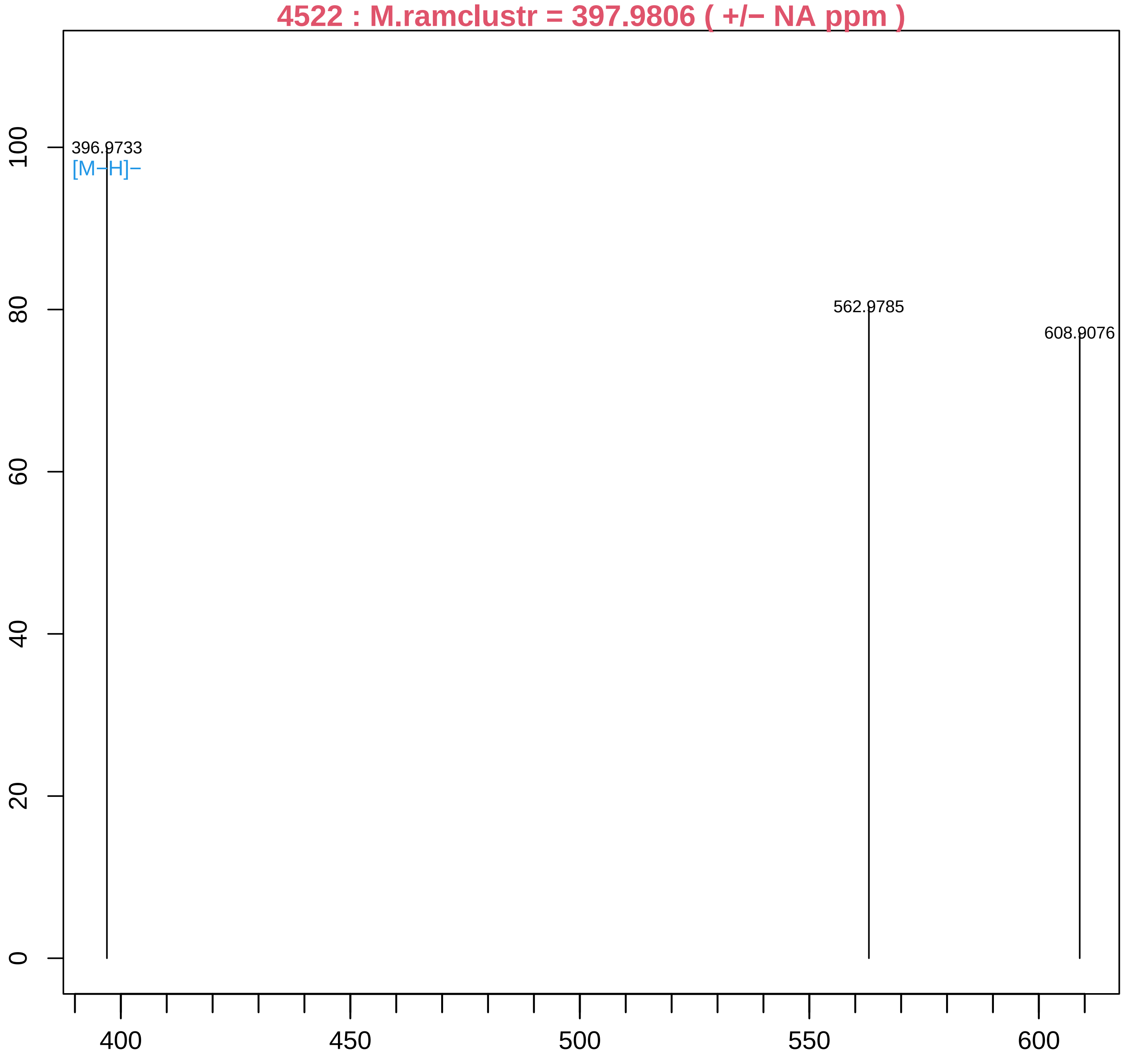
4522 : M.ramclustr = 397.9806 (+/− NA ppm)
396.9733
[M−H]−
562.9785
608.9076
0
20
40
60
80
100
400
450
500
550
600

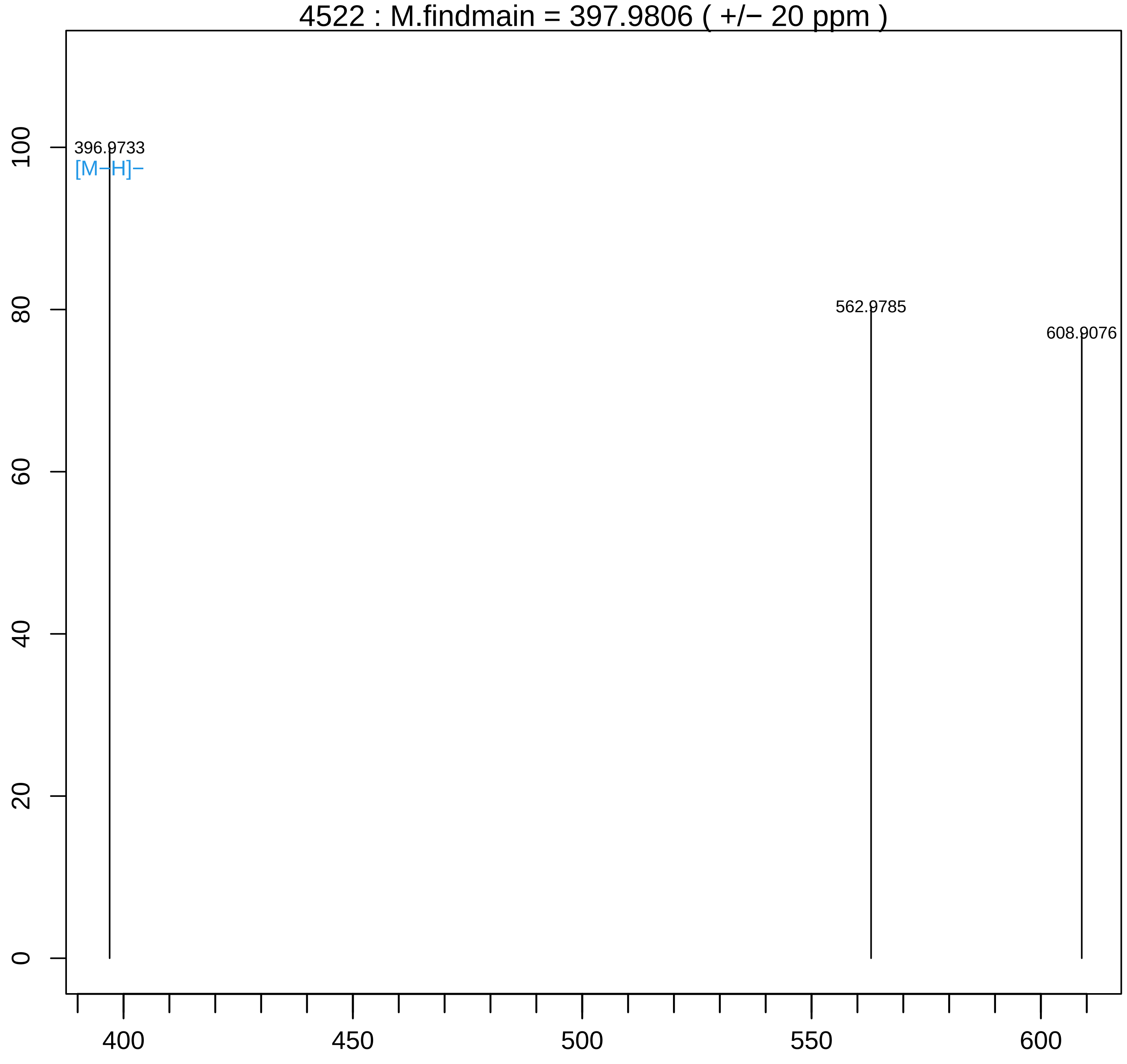
4522 : M.findmain = 397.9806 (+/− 20 ppm)
396.9733
[M−H]−
562.9785
608.9076
0
20
40
60
80
100
400
450
500
550
600

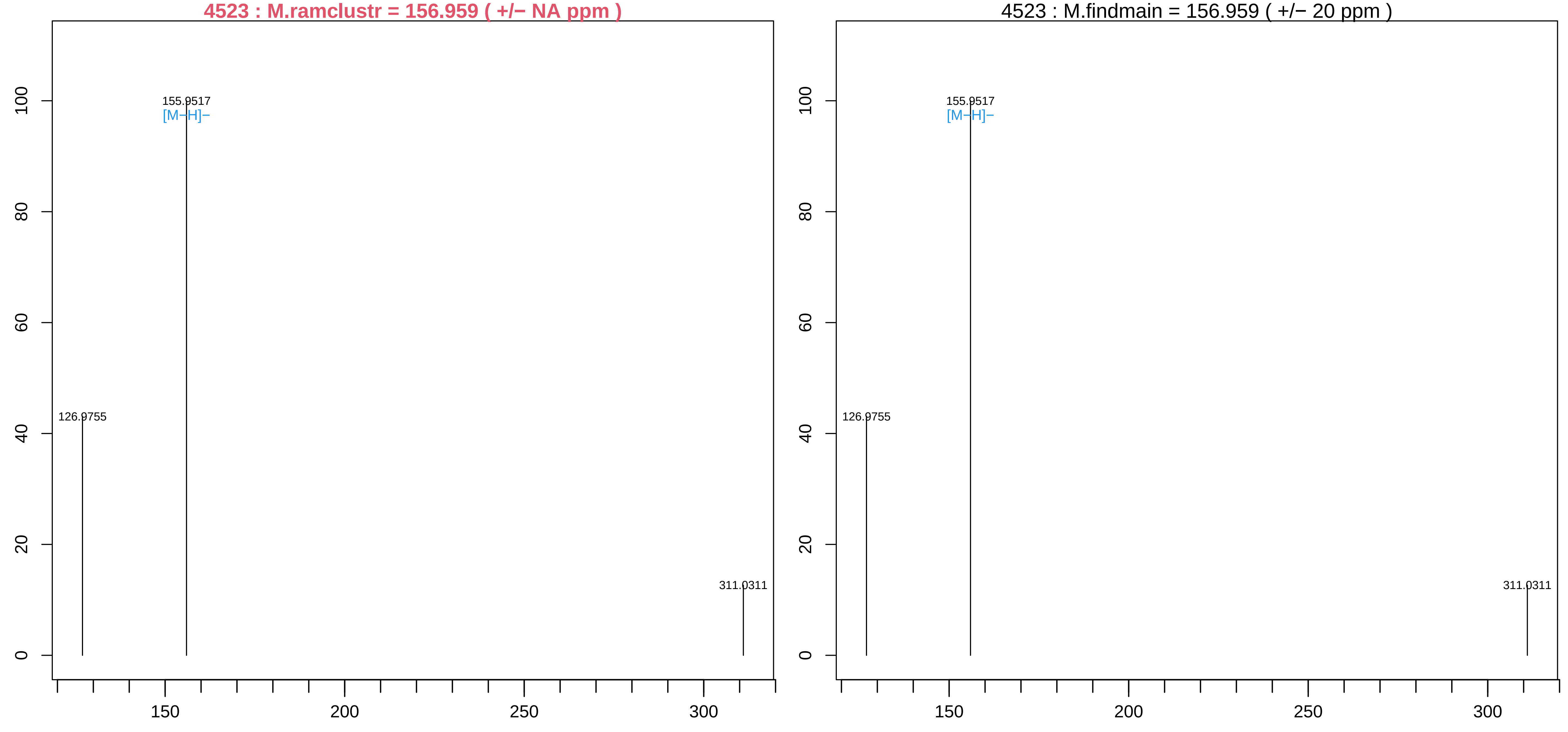
4523 : M.ramclustr = 156.959 (+/− NA ppm)
155.9517
[M−H]−
126.9755
311.0311
4523 : M.findmain = 156.959 (+/− 20 ppm)
155.9517
[M−H]−
126.9755
311.0311
0
20
40
60
80
100
150
200
250
300

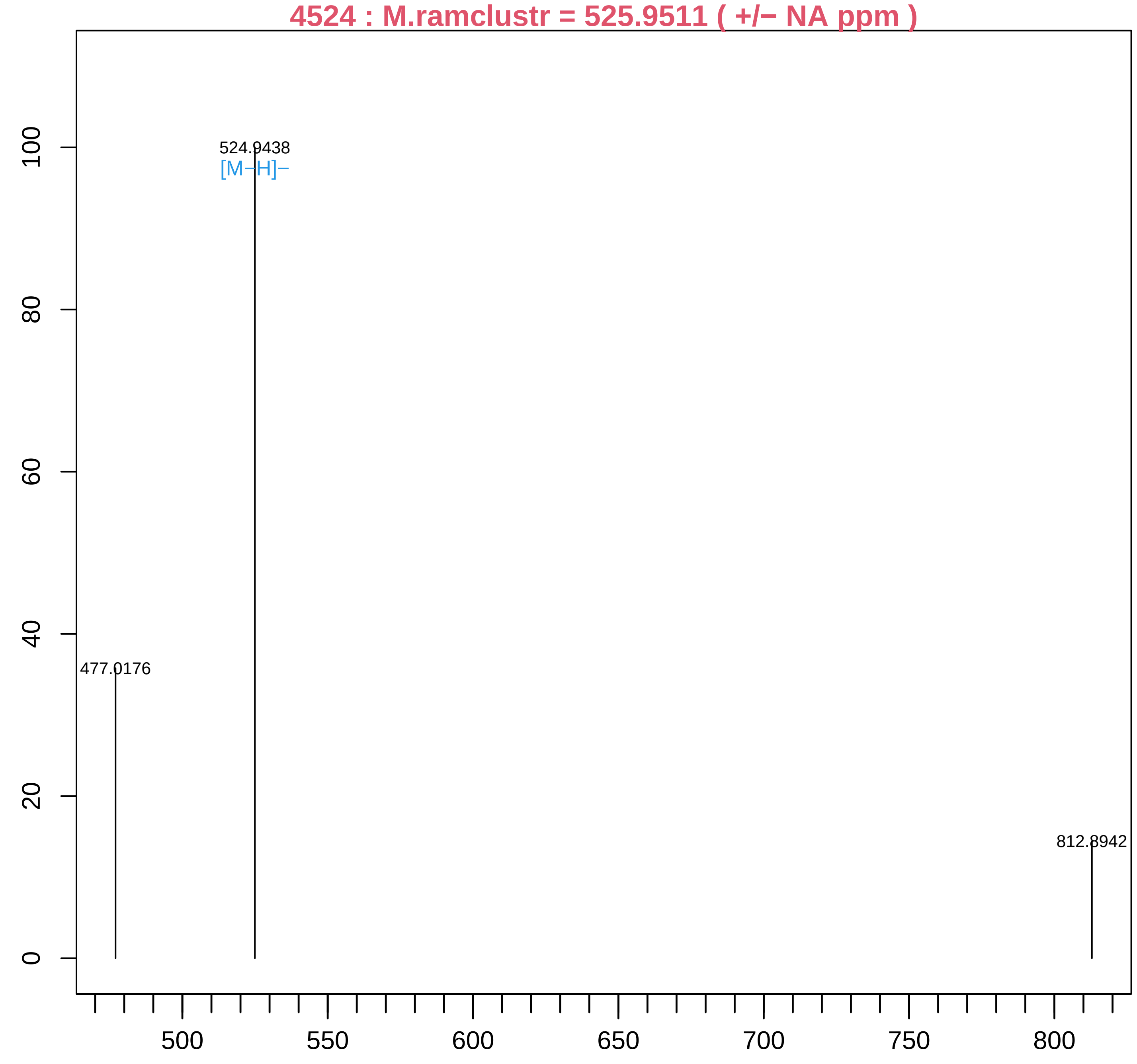
4524 : M.ramclustr = 525.9511 (+/− NA ppm)
524.9438
[M−H]−
477.0176
812.8942
0
20
40
60
80
100
500
550
600
650
700
750
800

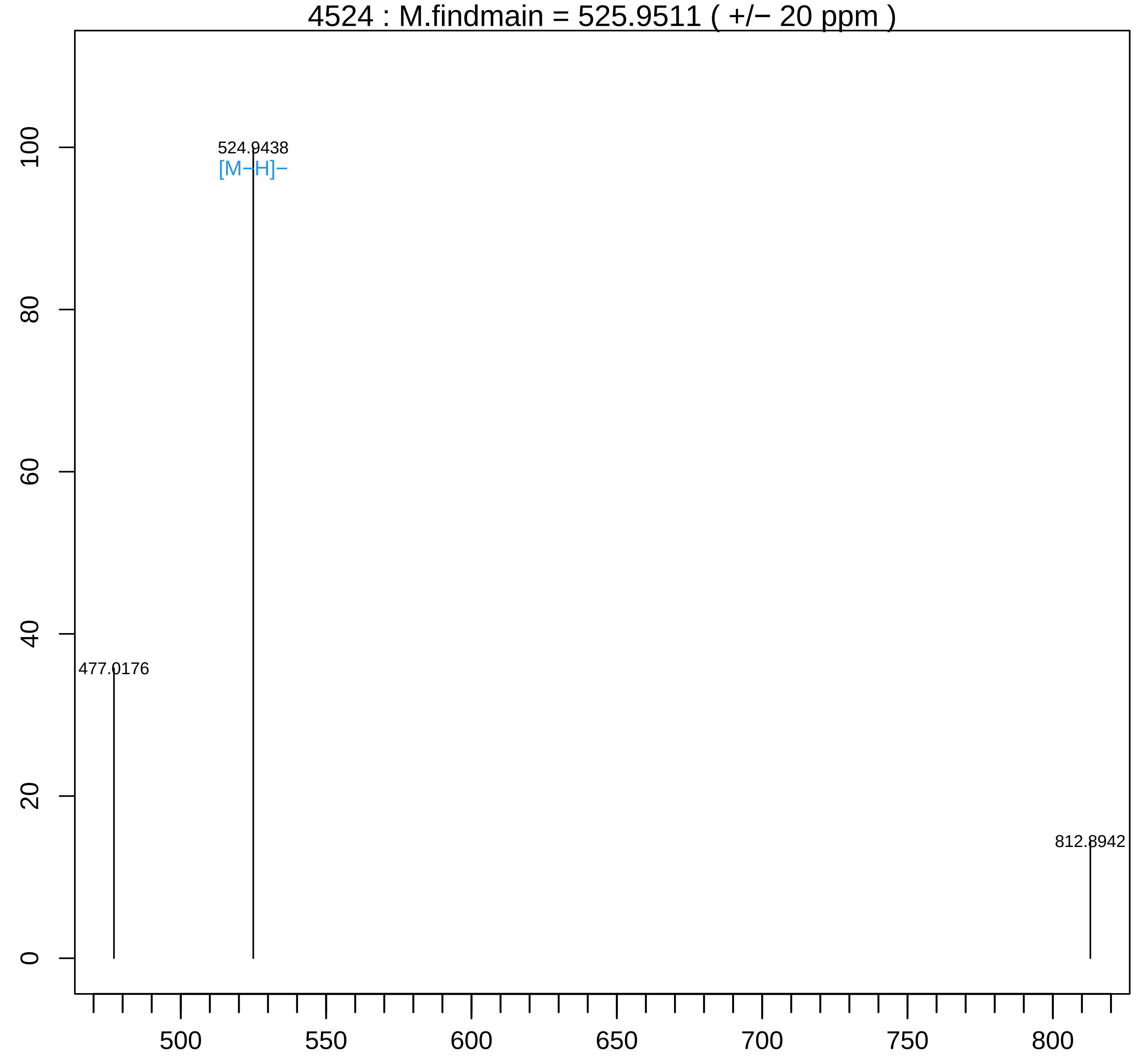
4524 : M.findmain = 525.9511 (+/− 20 ppm)
524.9438
[M−H]−
477.0176
812.8942
0
20
40
60
80
100
500
550
600
650
700
750
800

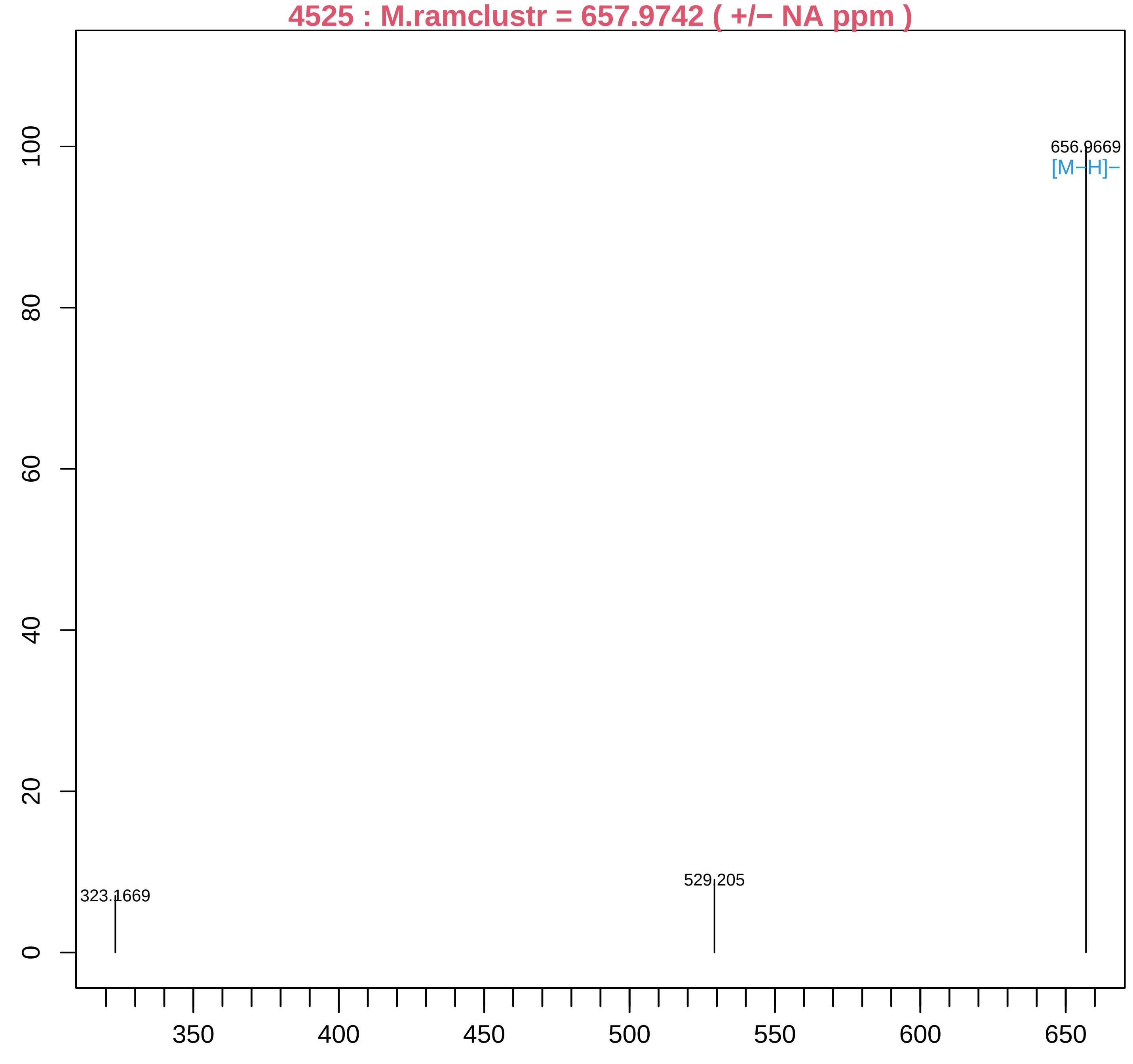
4525 : M.ramclustr = 657.9742 (+/− NA ppm)
656.9669
[M−H]−
529.205
323.1669
0
20
40
60
80
100
350
400
450
500
550
600
650

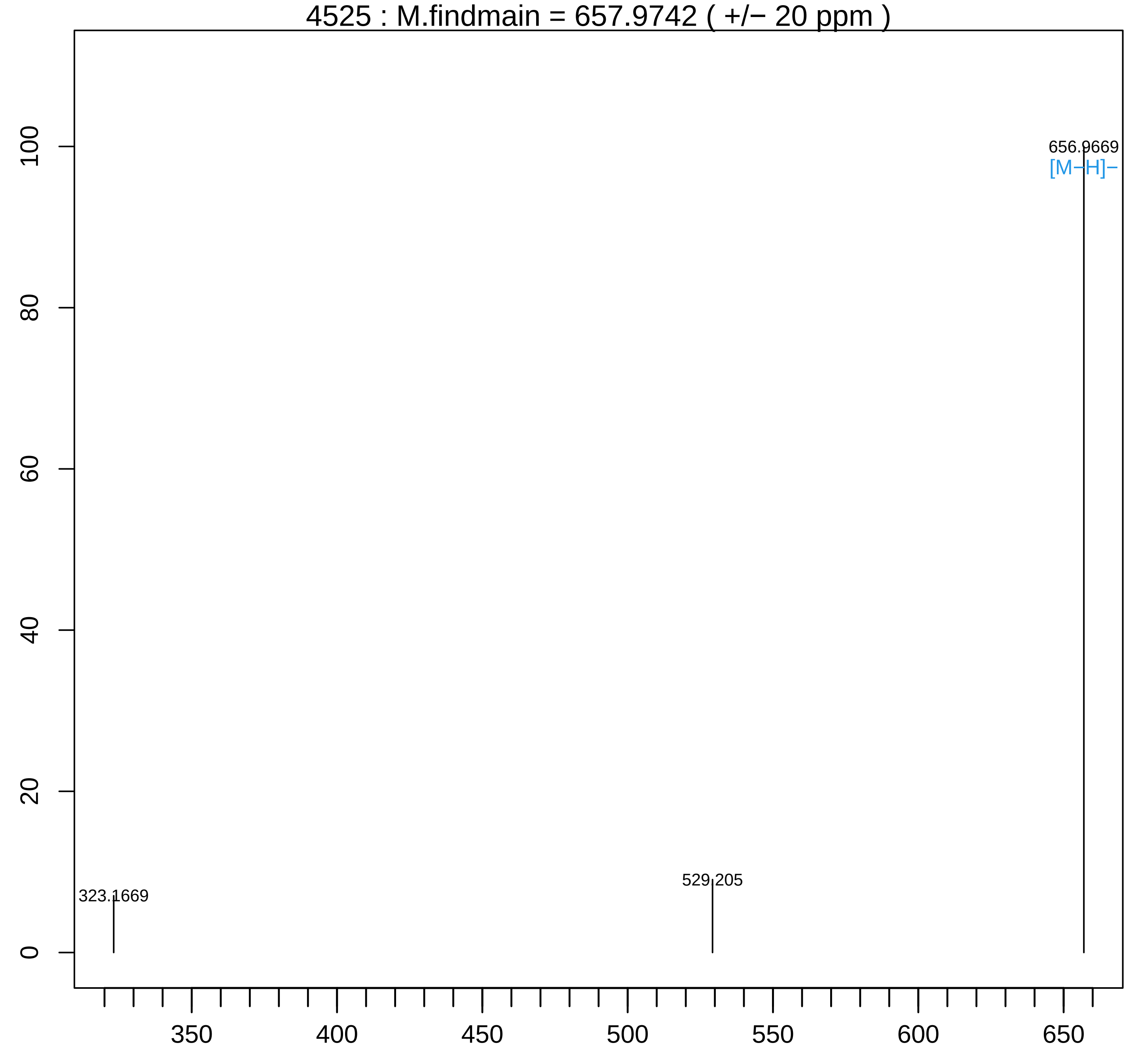
4525 : M.findmain = 657.9742 (+/− 20 ppm)
656.9669
[M−H]−
529.205
323.1669
0
20
40
60
80
100
350
400
450
500
550
600
650

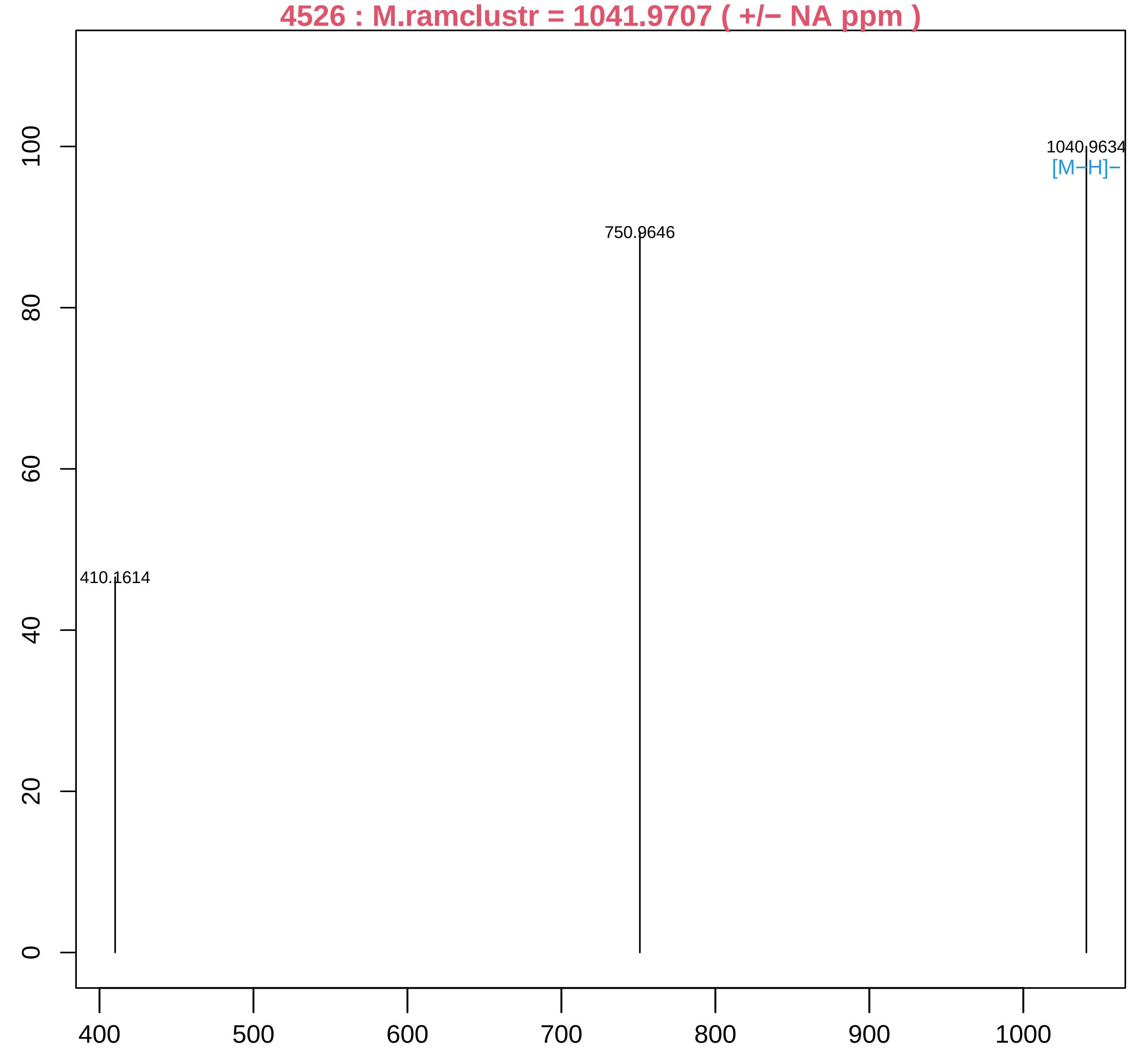
4526 : M.ramclustr = 1041.9707 (+/− NA ppm)
1040.9634
[M−H]−
750.9646
410.1614
0
20
40
60
80
100
400
500
600
700
800
900
1000

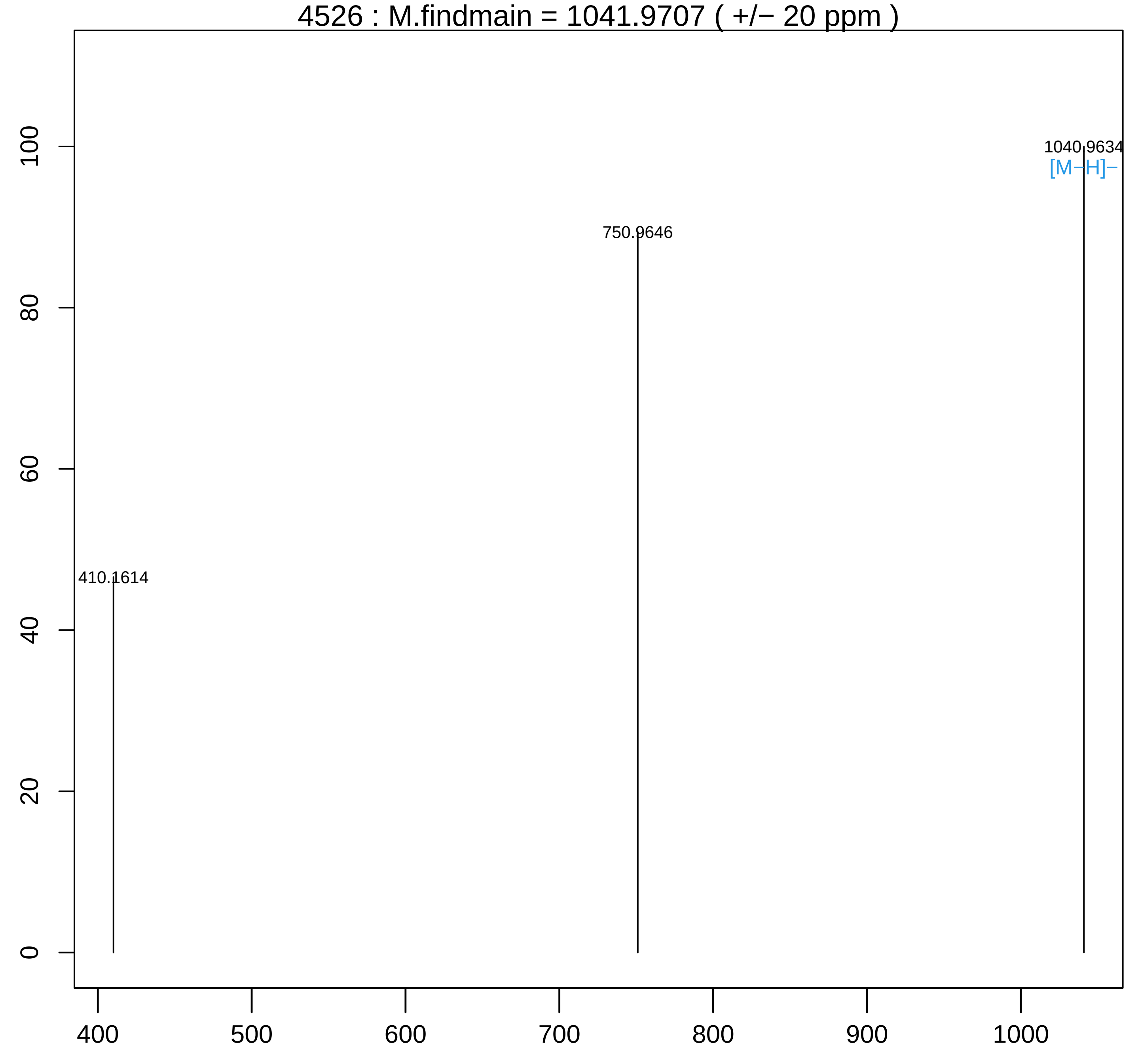
4526 : M.findmain = 1041.9707 (+/− 20 ppm)
1040.9634
[M−H]−
750.9646
410.1614
0
20
40
60
80
100
400
500
600
700
800
900
1000

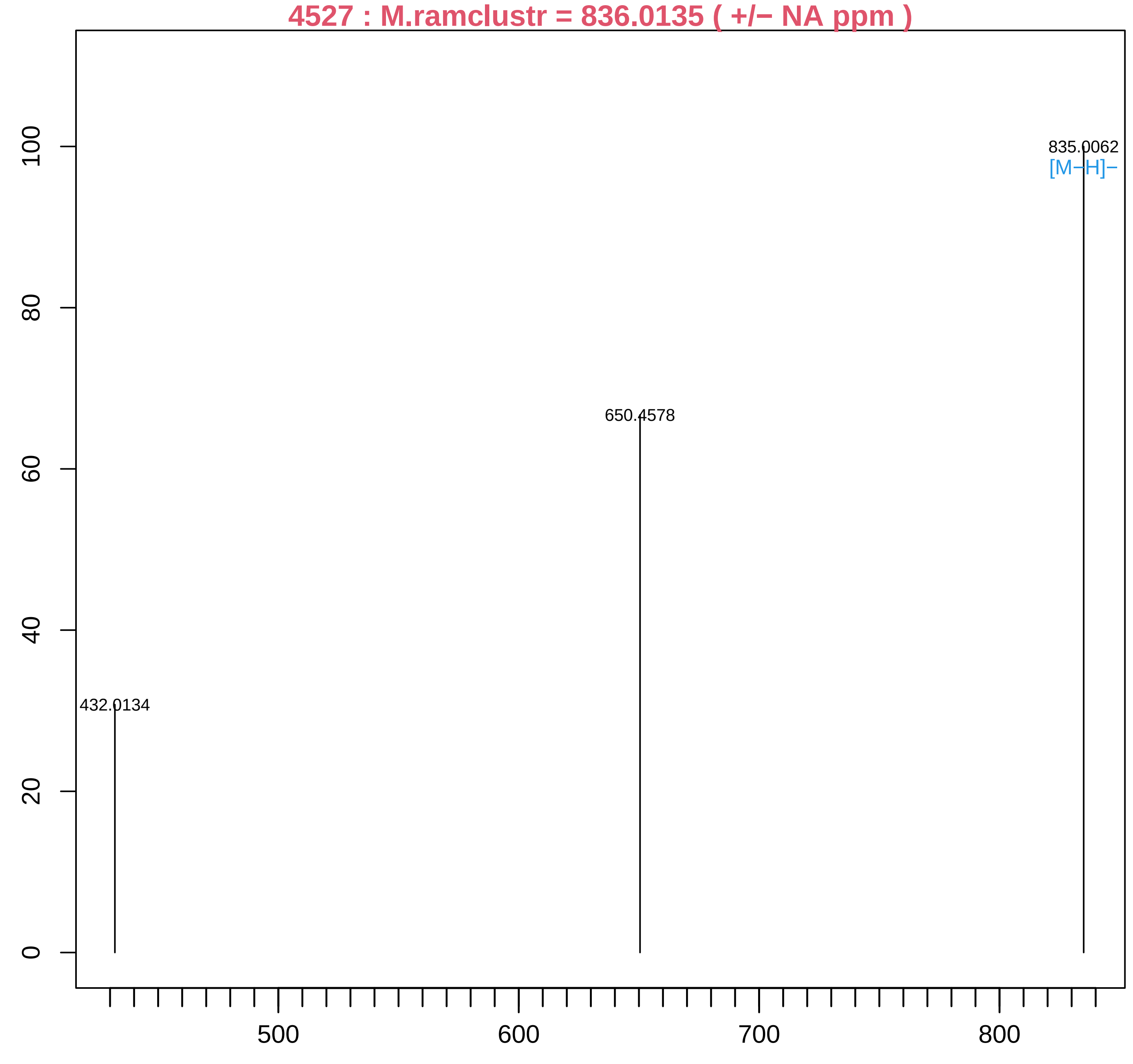

4527 : M.ramclustr = 836.0135 (+/− NA ppm)
835.0062
[M−H]−
650.4578
432.0134
0
20
40
60
80
100
500
600
700
800

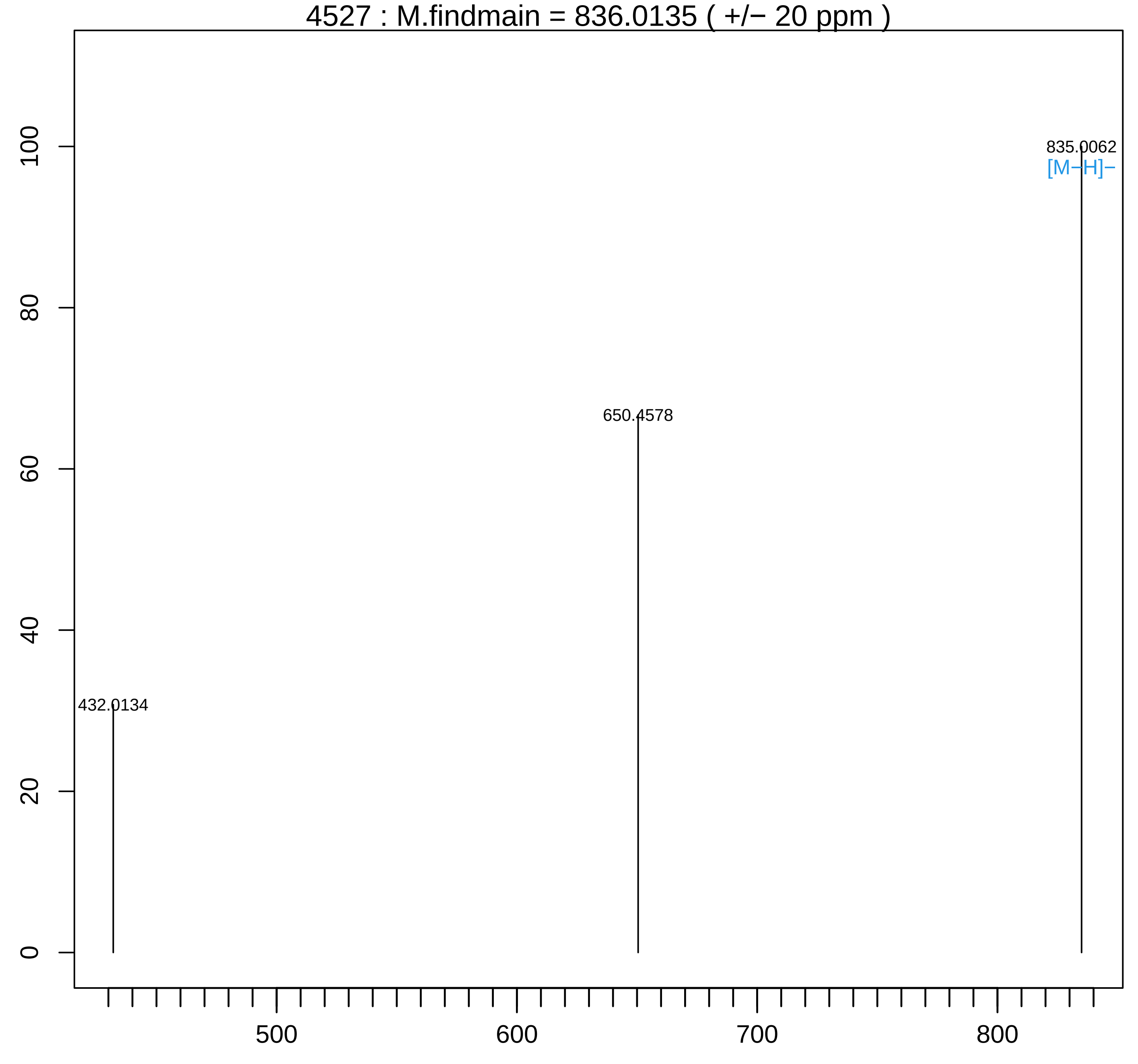

4527 : M.findmain = 836.0135 (+/− 20 ppm)
835.0062
[M−H]−
650.4578
432.0134
0
20
40
60
80
100
500
600
700
800

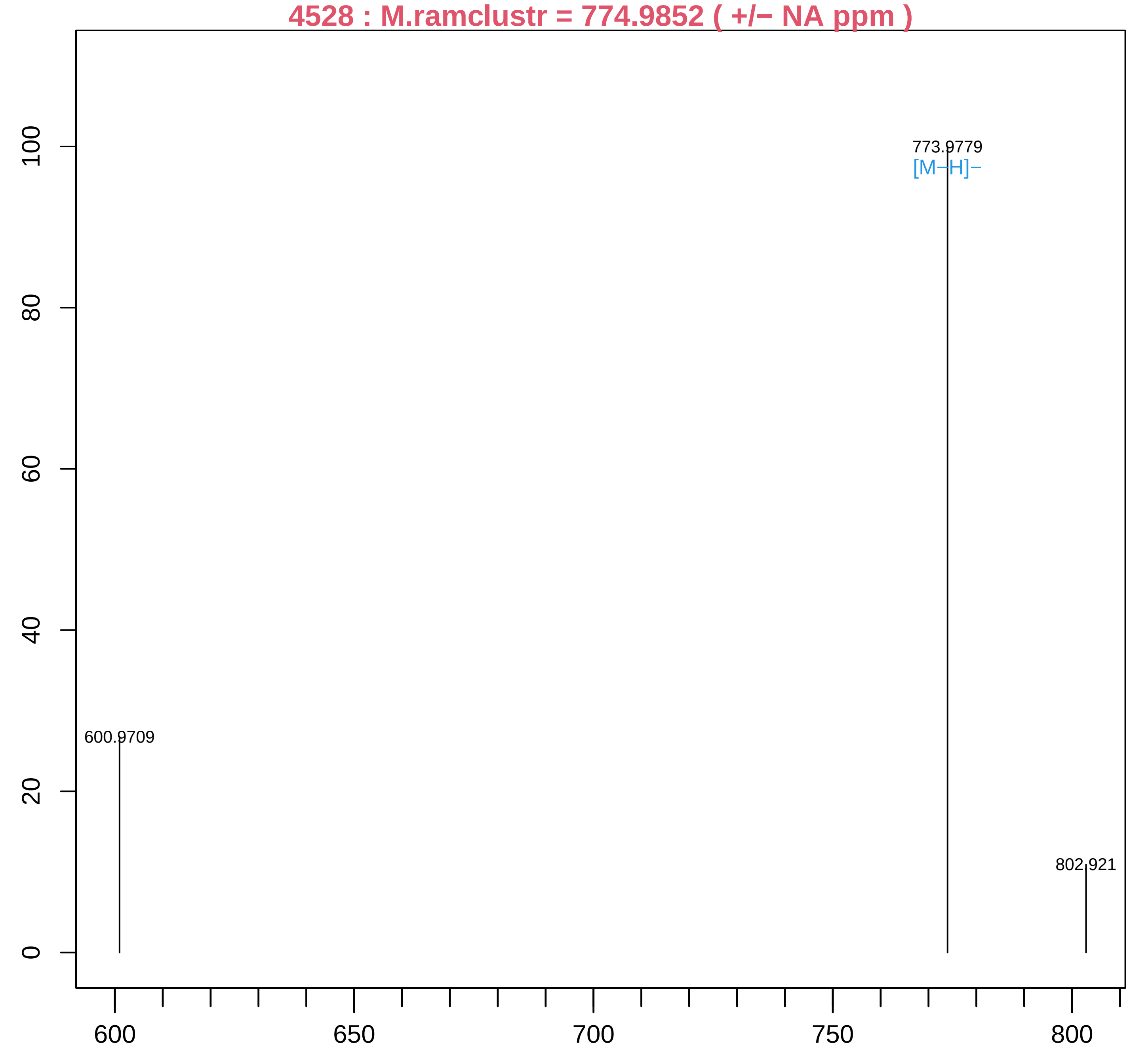

4528 : M.ramclustr = 774.9852 (+/− NA ppm)
773.9779
[M−H]−
600.9709
802.921
0
20
40
60
80
100
600
650
700
750
800

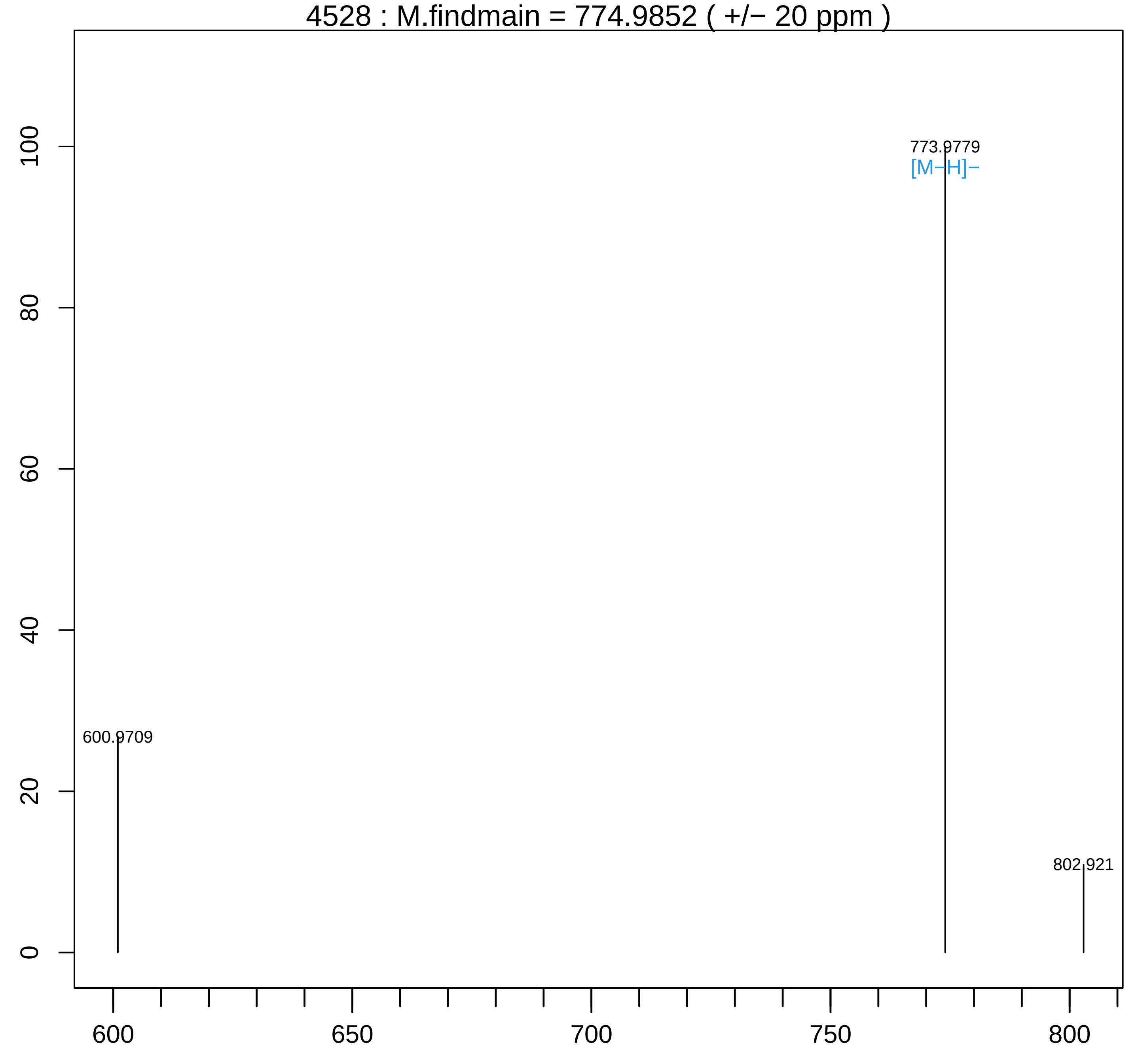

4528 : M.findmain = 774.9852 (+/− 20 ppm)
773.9779
[M−H]−
600.9709
802.921
0
20
40
60
80
100
600
650
700
750
800

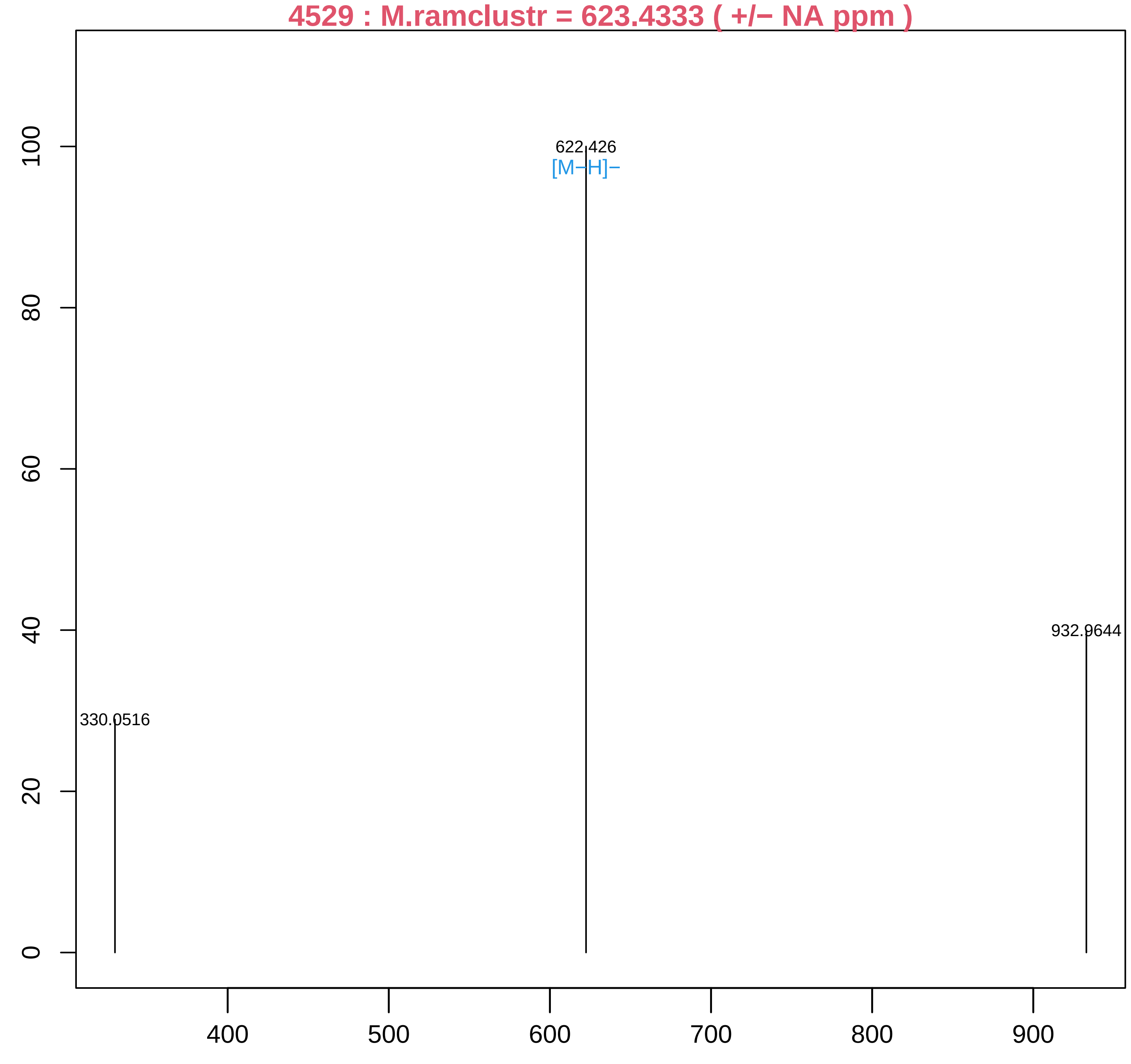
4529 : M.ramclustr = 623.4333 (+/− NA ppm)
622.426
[M−H]−
932.9644
330.0516
0
20
40
60
80
100
400
500
600
700
800
900

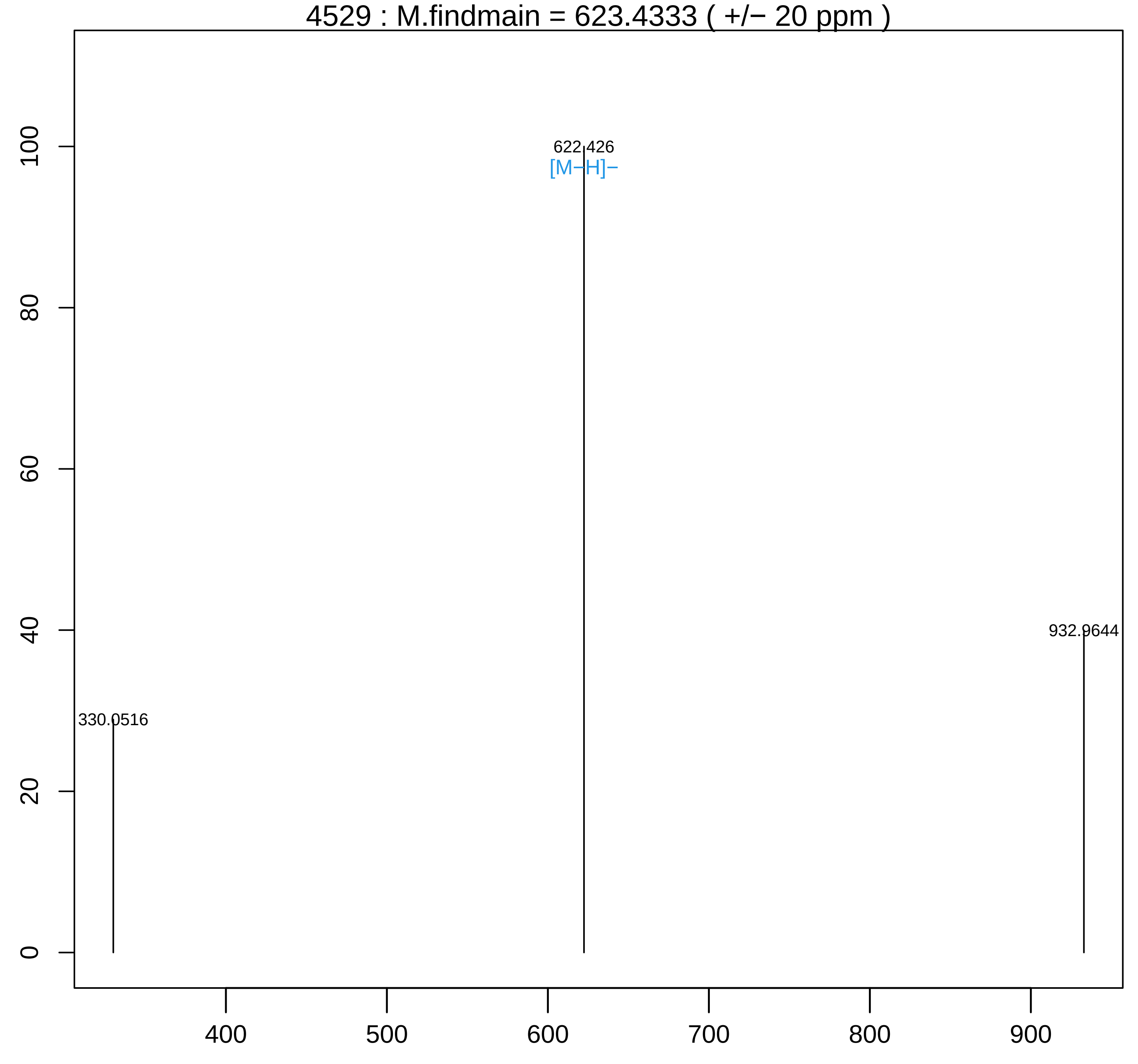
4529 : M.findmain = 623.4333 (+/− 20 ppm)
622.426
[M−H]−
932.9644
330.0516
0
20
40
60
80
100
400
500
600
700
800
900

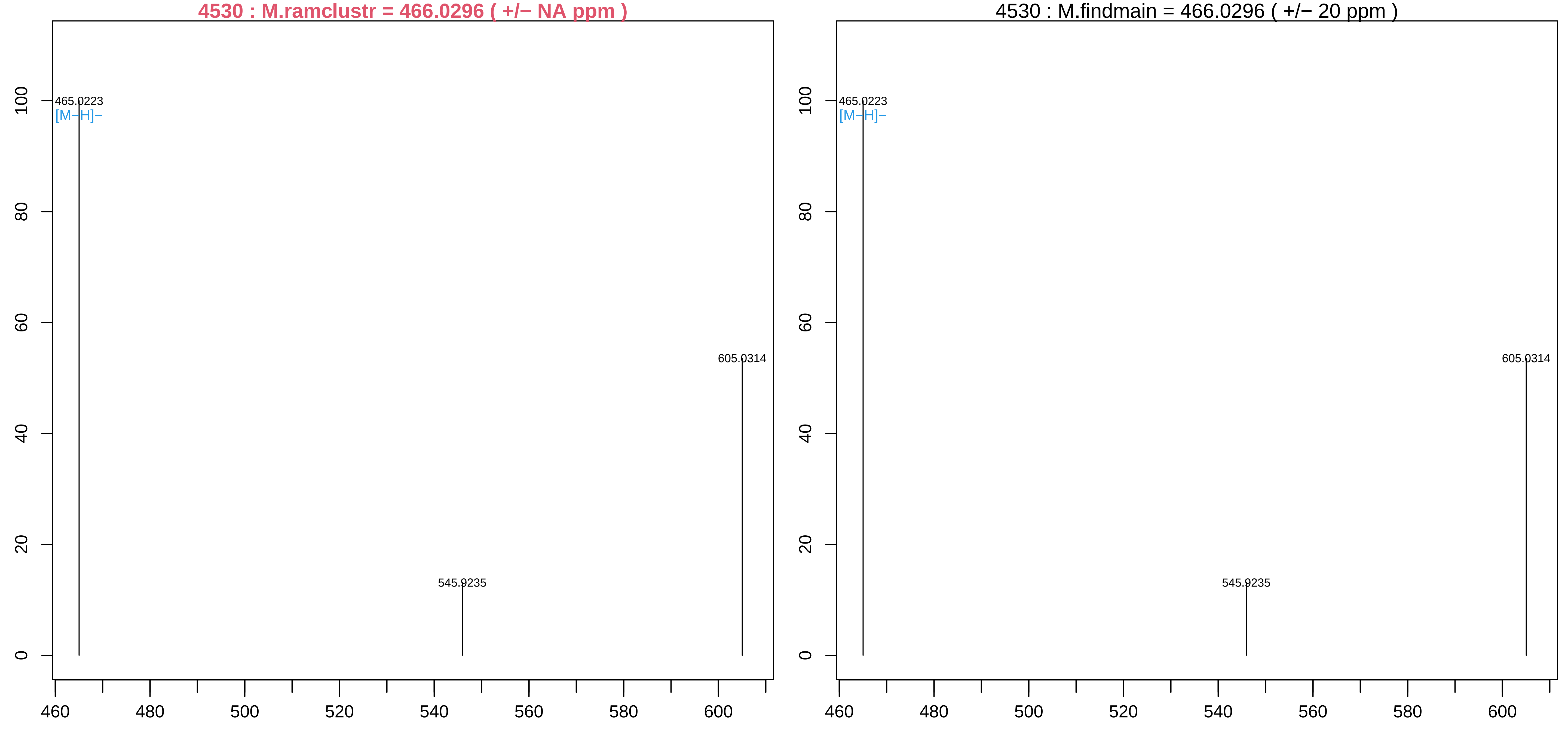

4530 : M.ramclustr = 466.0296 (+/− NA ppm)
465.0223
[M−H]−
545.9235
605.0314
4530 : M.findmain = 466.0296 (+/− 20 ppm)
465.0223
[M−H]−
545.9235
605.0314
0
20
40
60
80
100
460
480
500
520
540
560
580
600

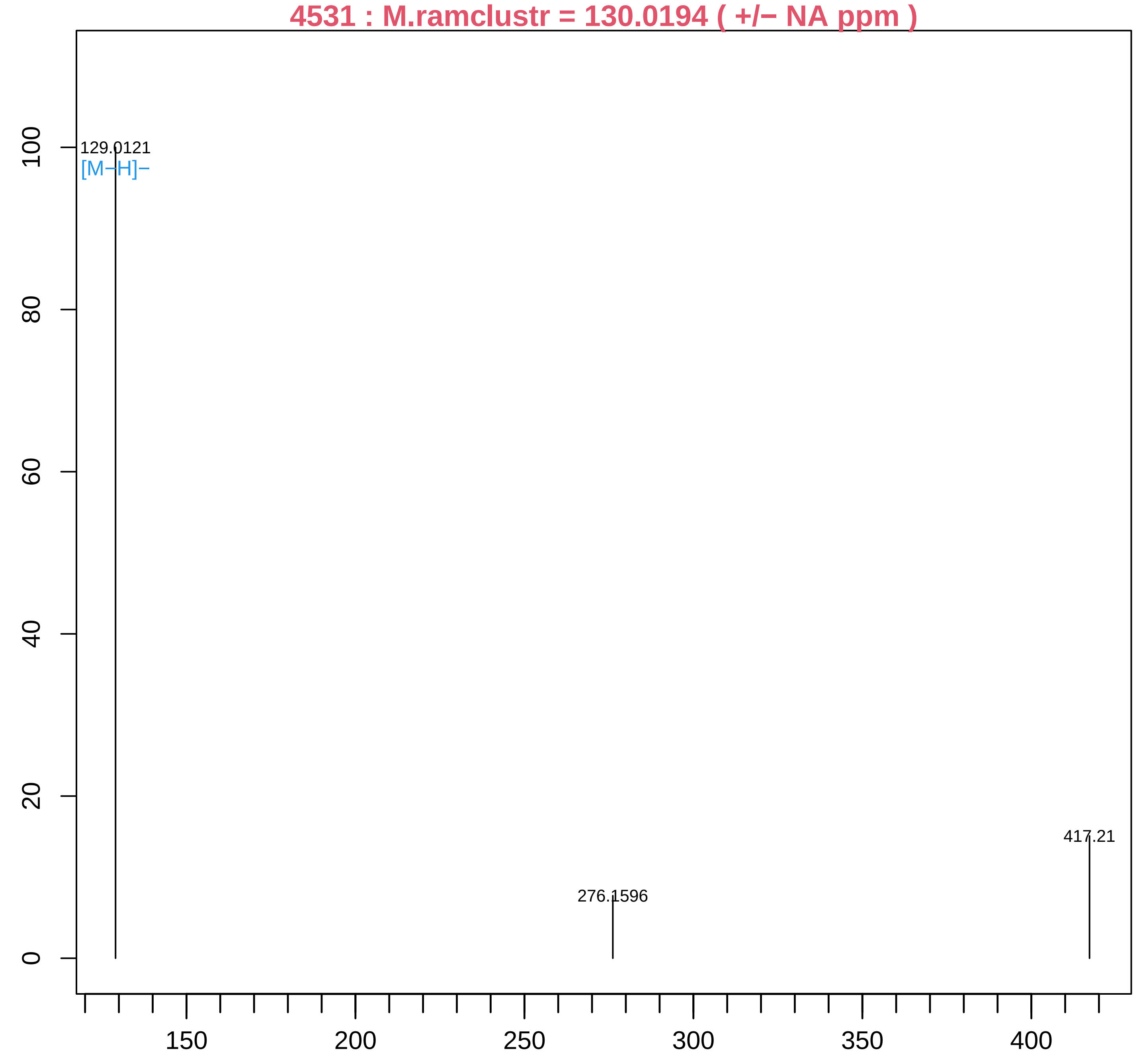
4531 : M.ramclustr = 130.0194 (+/− NA ppm)
129.0121
[M−H]−
276.1596
417.21
0
20
40
60
80
100
150
200
250
300
350
400

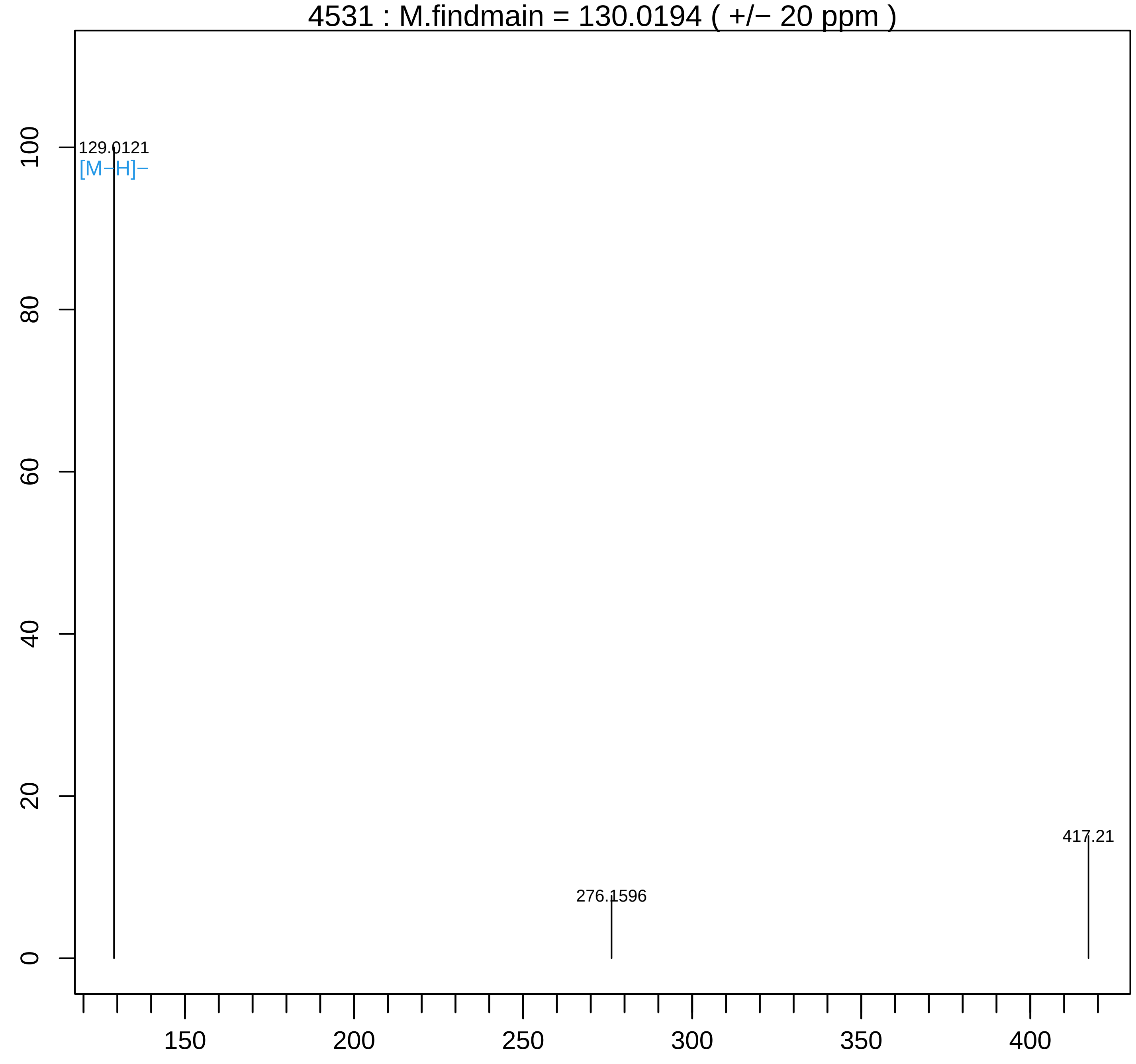
4531 : M.findmain = 130.0194 (+/− 20 ppm)
129.0121
[M−H]−
276.1596
417.21
0
20
40
60
80
100
150
200
250
300
350
400

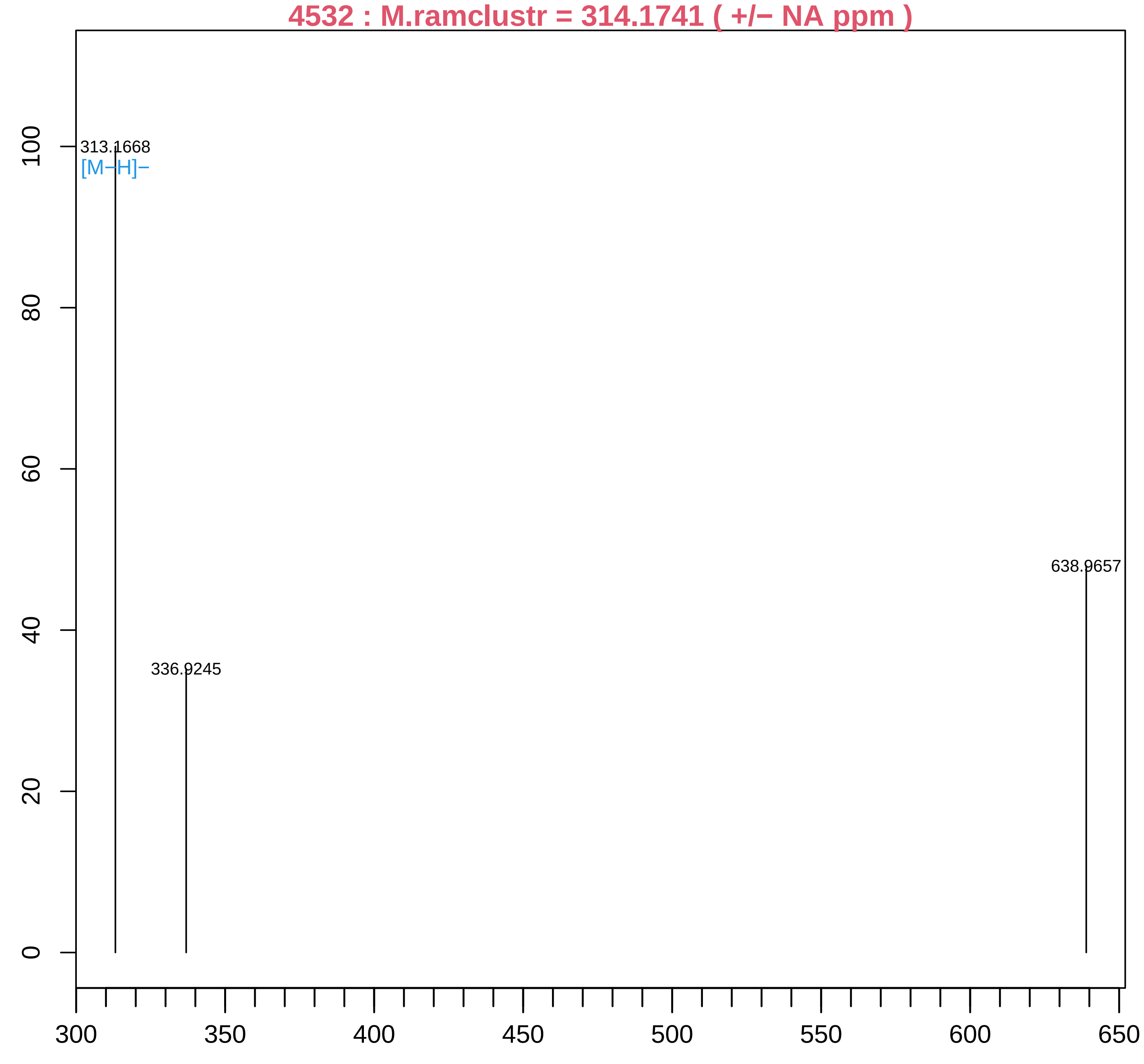
4532 : M.ramclustr = 314.1741 (+/− NA ppm)
313.1668
[M−H]−
336.9245
638.9657
0
20
40
60
80
100
300
350
400
450
500
550
600
650

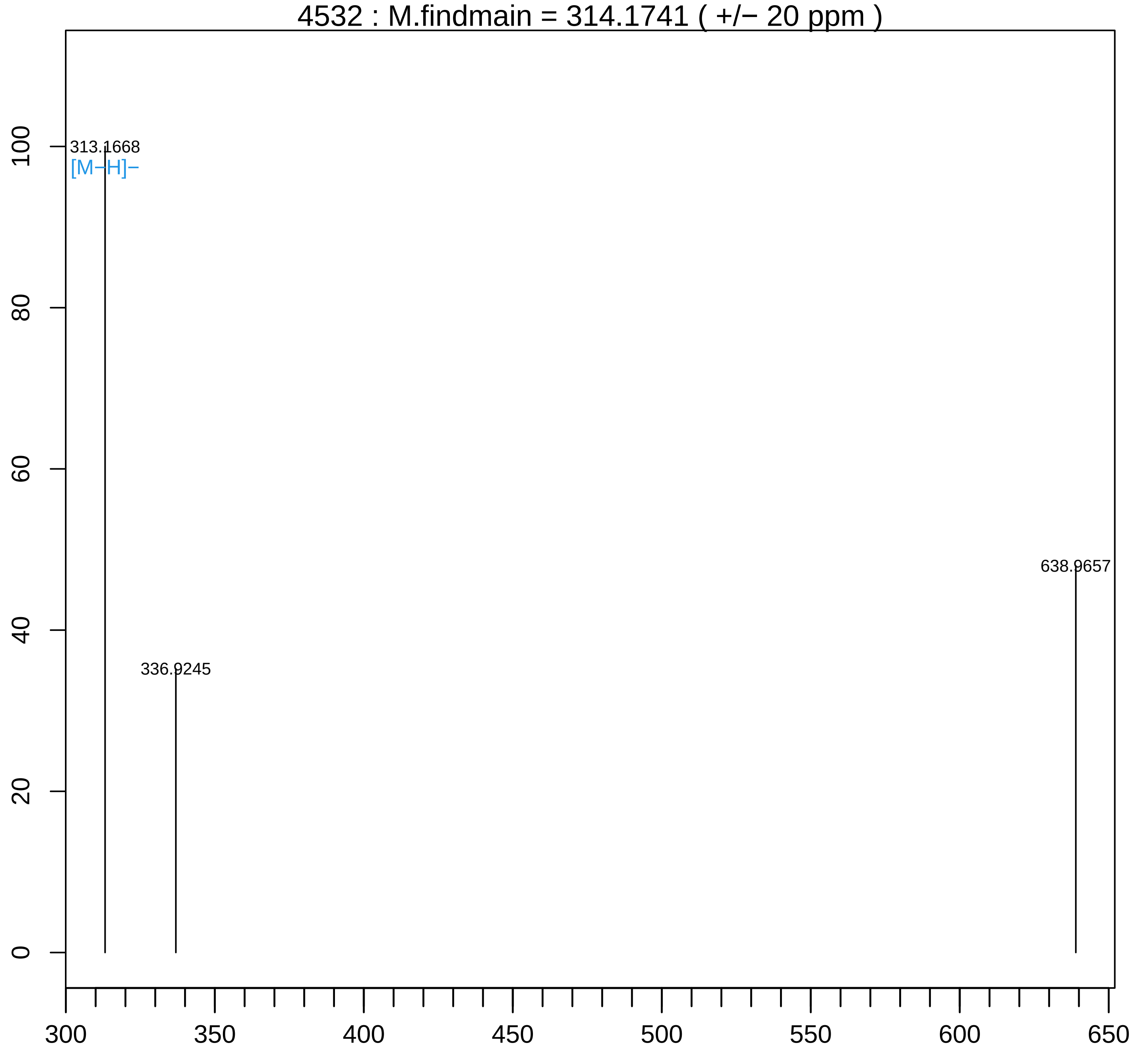
4532 : M.findmain = 314.1741 (+/− 20 ppm)
313.1668
[M−H]−
336.9245
638.9657
0
20
40
60
80
100
300
350
400
450
500
550
600
650

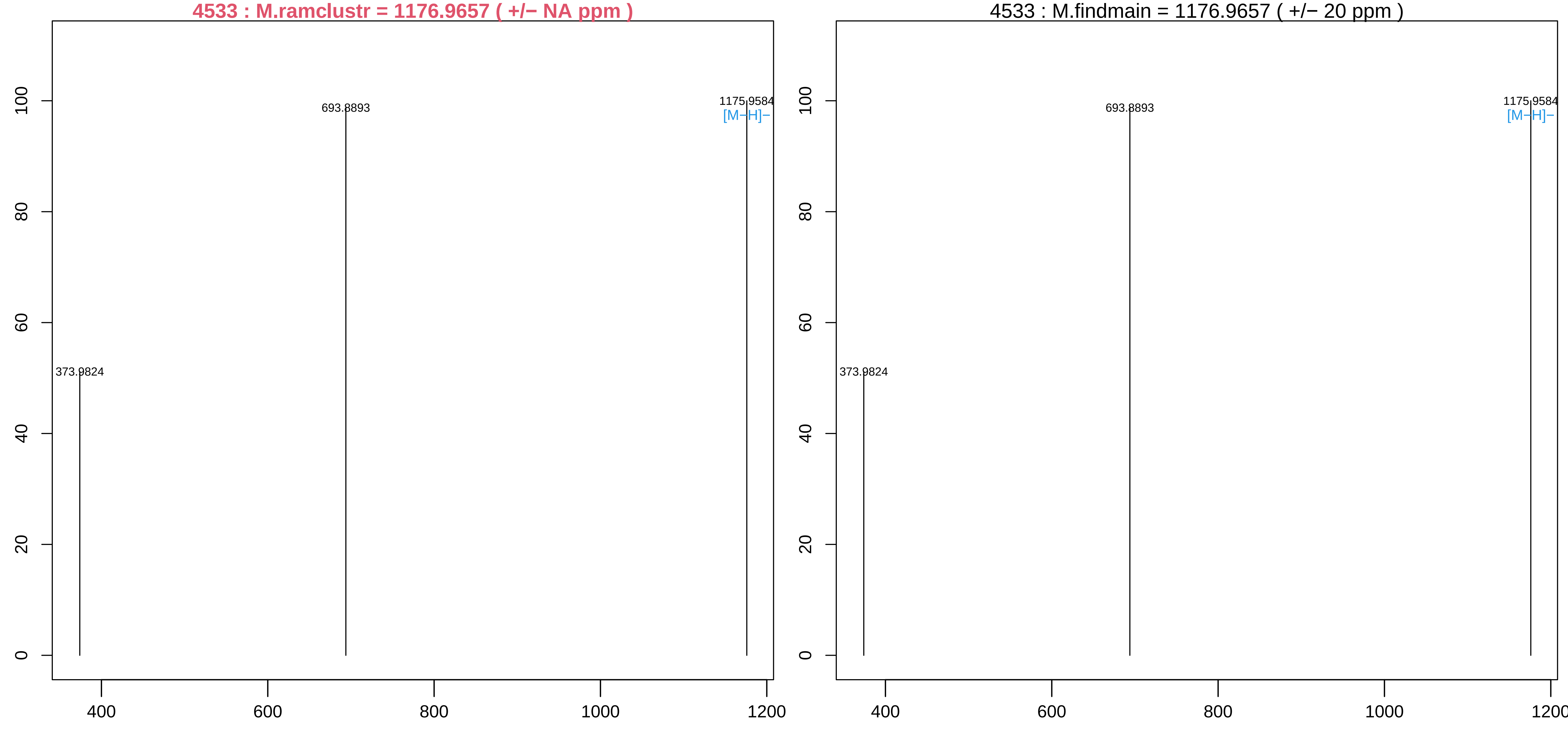
4533 : M.ramclustr = 1176.9657 (+/− NA ppm)
4533 : M.findmain = 1176.9657 (+/− 20 ppm)
373.9824
693.8893
1175.9584
[M−H]−
0
20
40
60
80
100
400
600
800
1000
1200

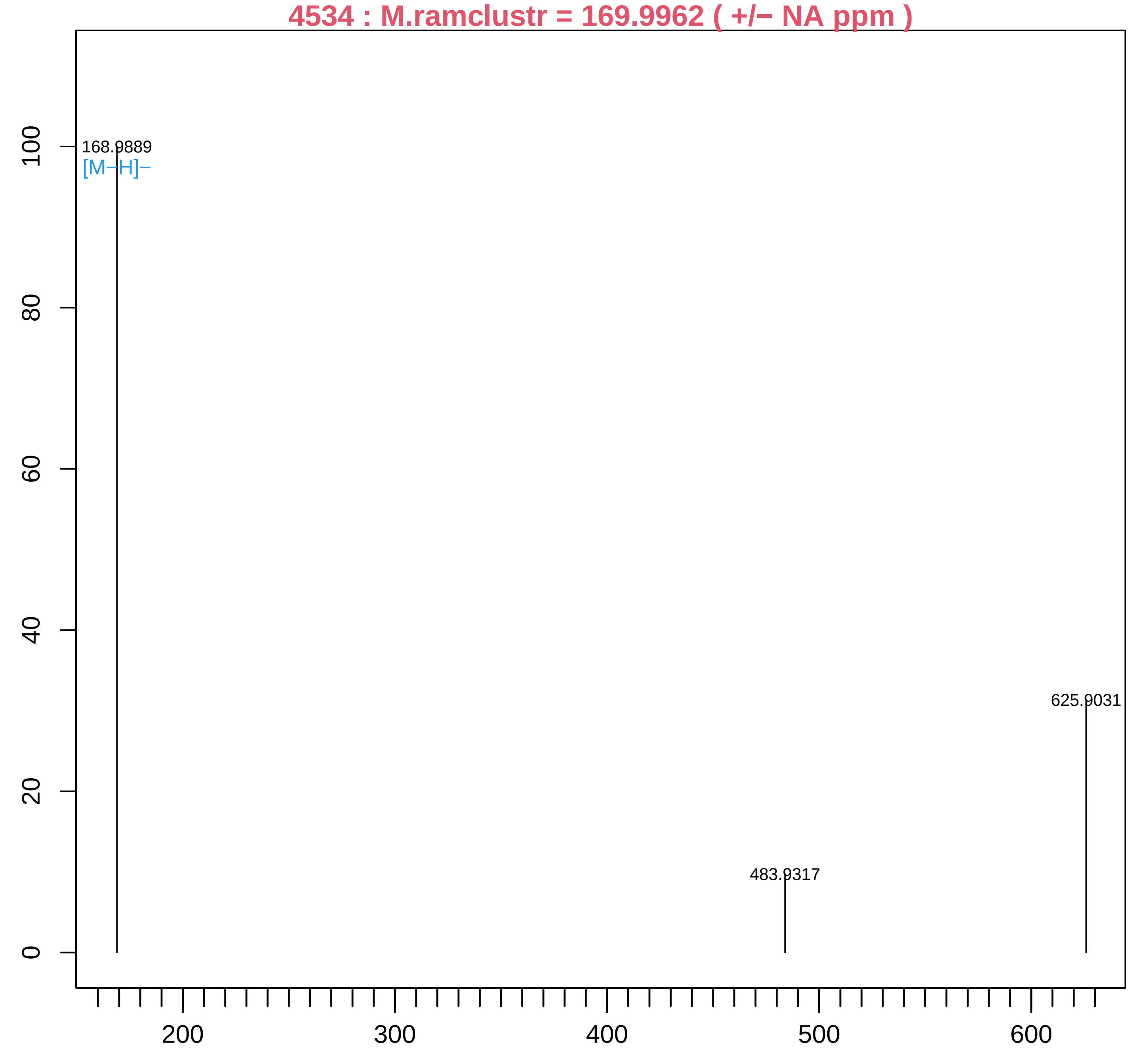
4534 : M.ramclustr = 169.9962 (+/− NA ppm)
168.9889
[M−H]−
483.9317
625.9031
0
20
40
60
80
100
200
300
400
500
600

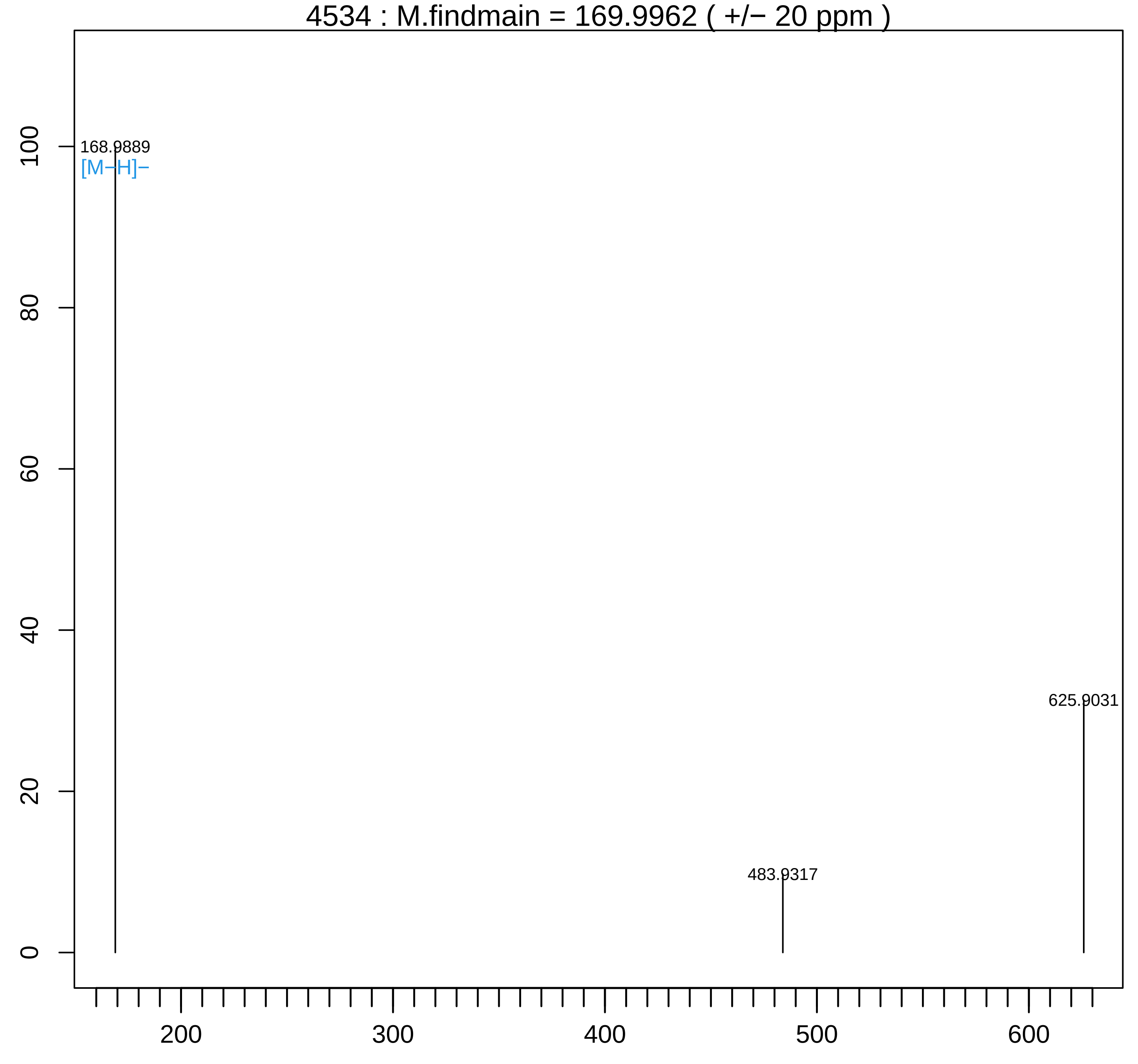
4534 : M.findmain = 169.9962 (+/− 20 ppm)
168.9889
[M−H]−
483.9317
625.9031
0
20
40
60
80
100
200
300
400
500
600

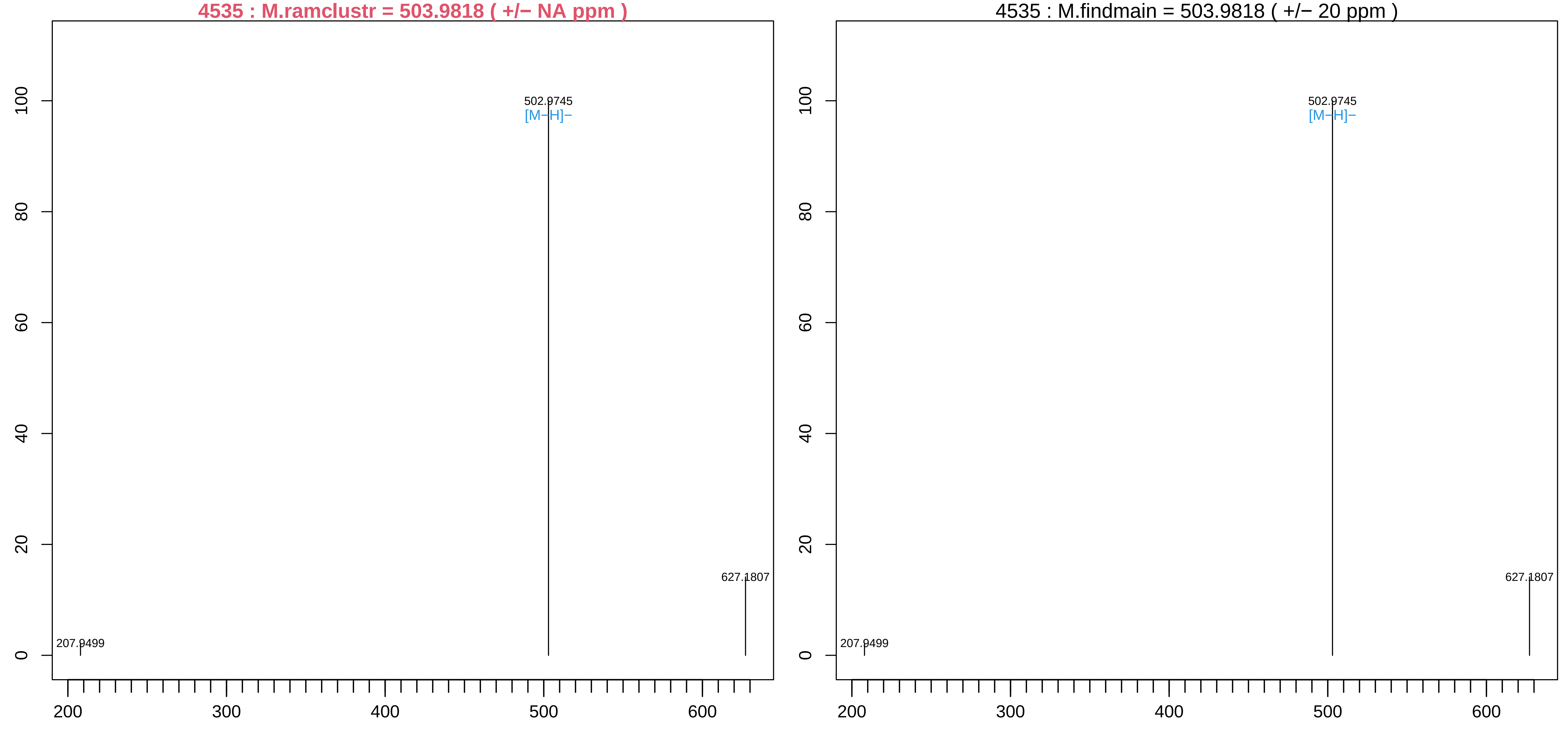
4535 : M.ramclustr = 503.9818 (+/− NA ppm)
502.9745
[M−H]−
627.1807
207.9499
4535 : M.findmain = 503.9818 (+/− 20 ppm)
502.9745
[M−H]−
627.1807
207.9499
0
20
40
60
80
100
200
300
400
500
600

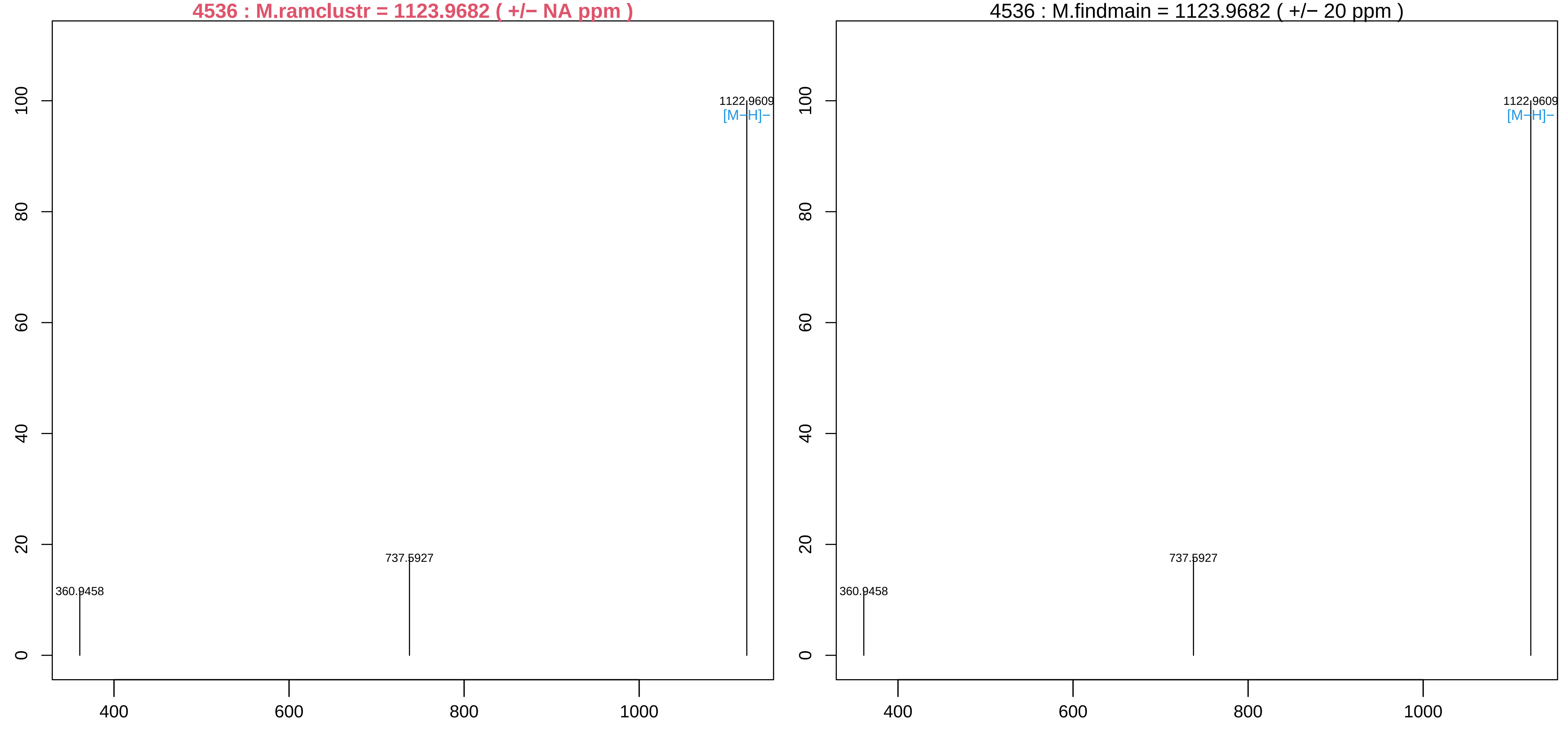
4536 : M.ramclustr = 1123.9682 (+/− NA ppm)
1122.9609
[M−H]−
737.5927
360.9458
0
20
40
60
80
100
400
600
800
1000
4536 : M.findmain = 1123.9682 (+/− 20 ppm)
1122.9609
[M−H]−
737.5927
360.9458
0
20
40
60
80
100
400
600
800
1000

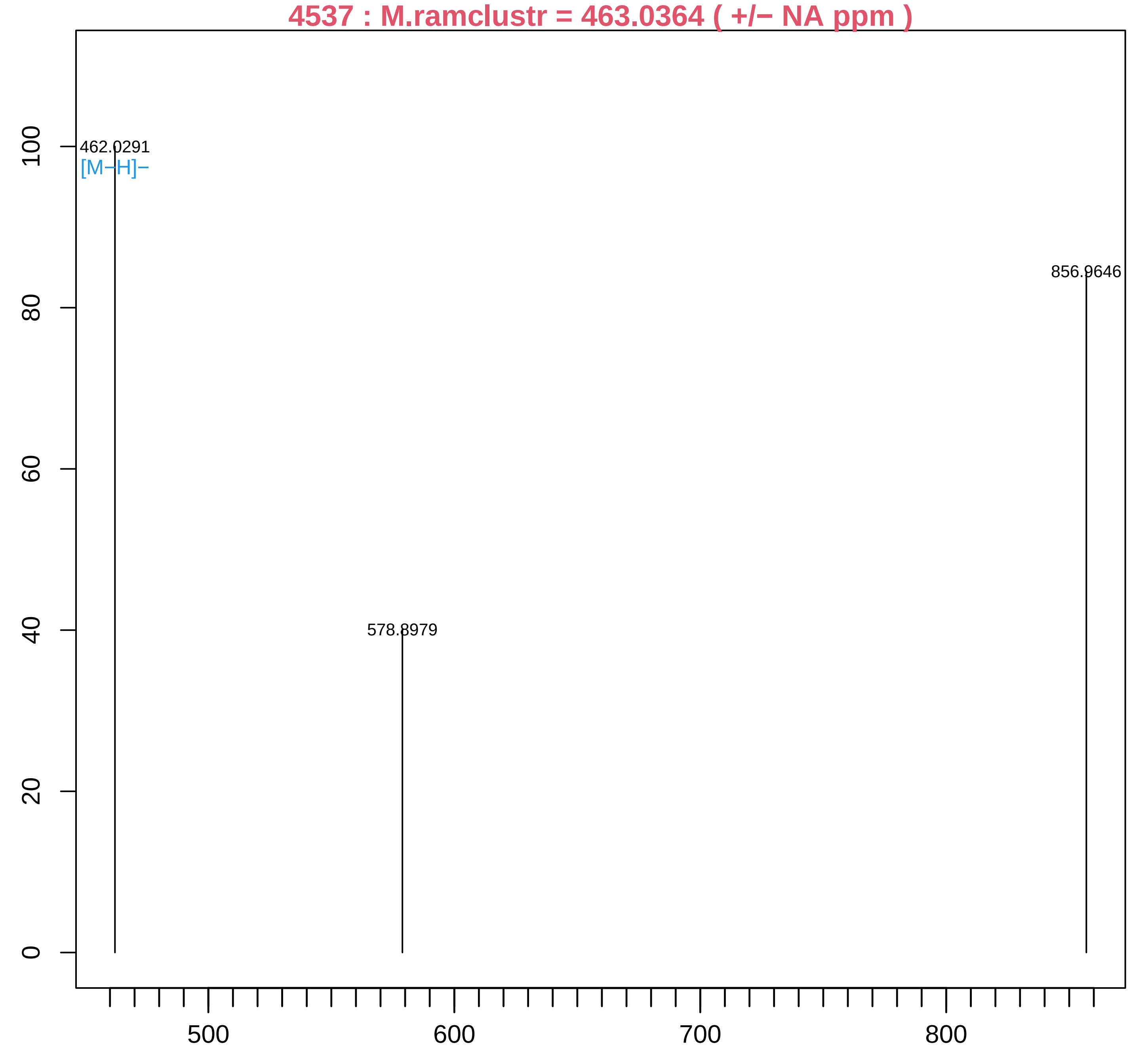
4537 : M.ramclustr = 463.0364 (+/− NA ppm)
462.0291
[M−H]−
578.8979
856.9646
0
20
40
60
80
100
500
600
700
800

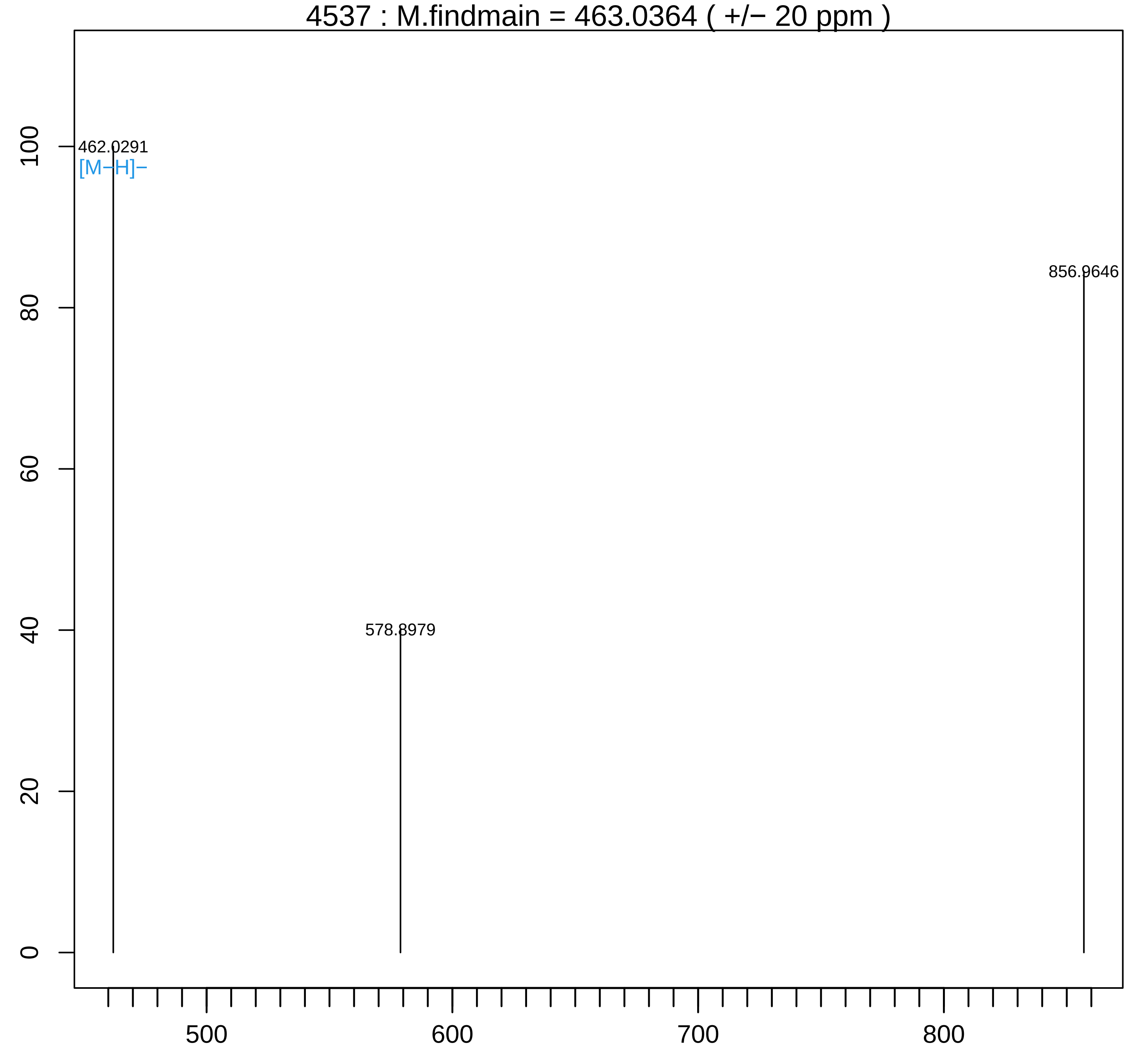
4537 : M.findmain = 463.0364 (+/− 20 ppm)
462.0291
[M−H]−
578.8979
856.9646
0
20
40
60
80
100
500
600
700
800

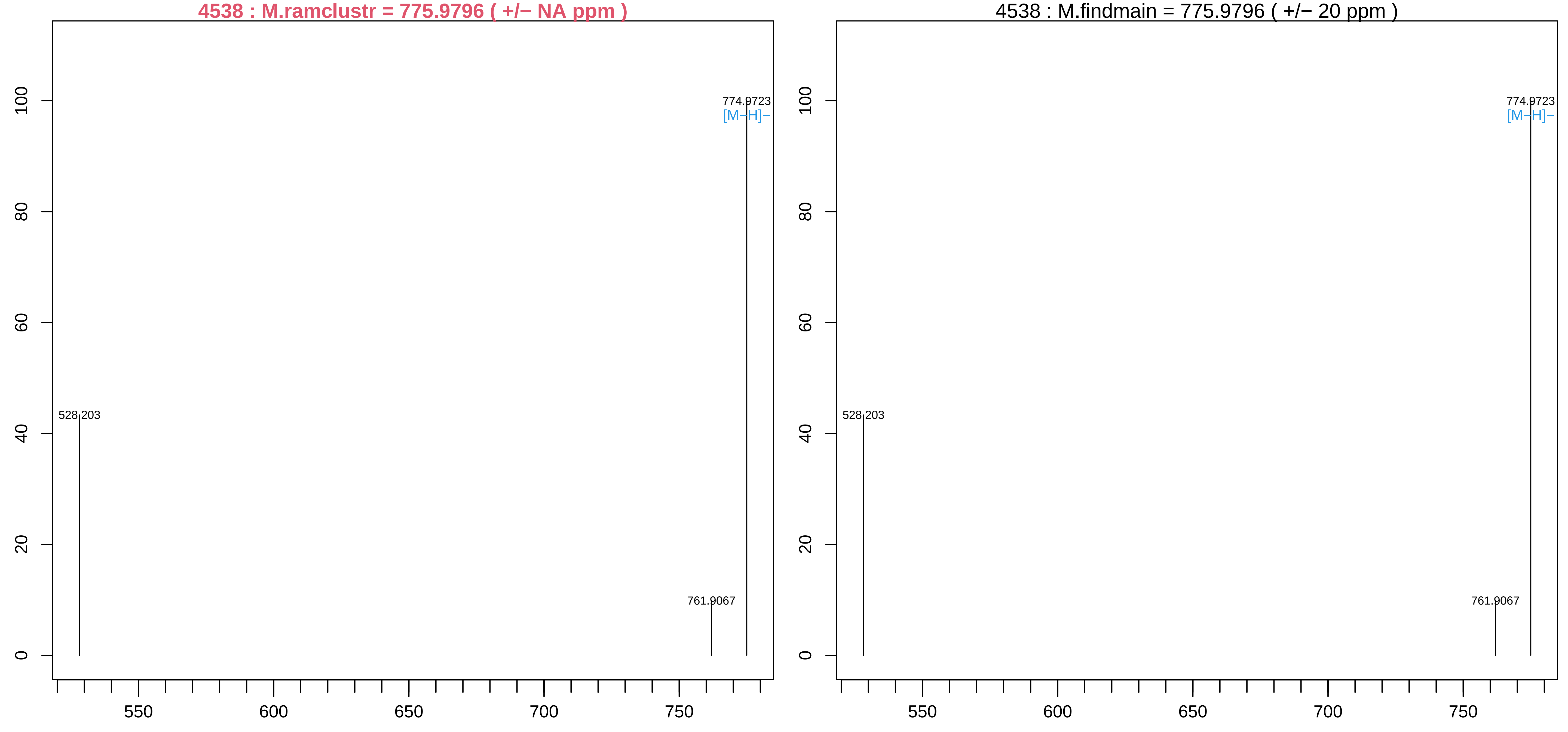
4538 : M.ramclustr = 775.9796 (+/− NA ppm)
774.9723
[M−H]−
528.203
761.9067
0
20
40
60
80
100
550
600
650
700
750
4538 : M.findmain = 775.9796 (+/− 20 ppm)
774.9723
[M−H]−
528.203
761.9067
0
20
40
60
80
100
550
600
650
700
750

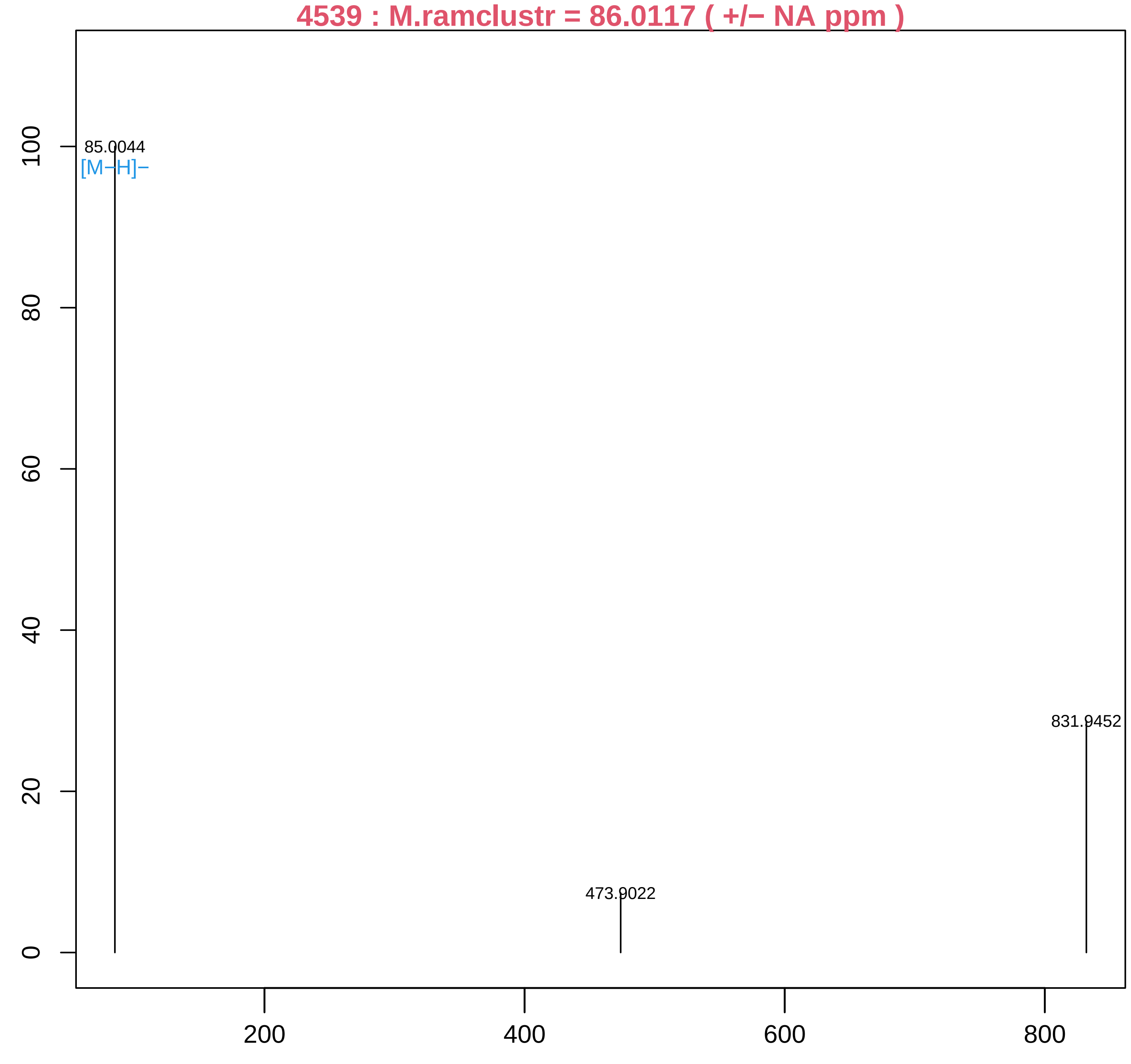
4539 : M.ramclustr = 86.0117 (+/− NA ppm)
85.0044
[M−H]−
473.9022
831.9452
0
20
40
60
80
100
200
400
600
800

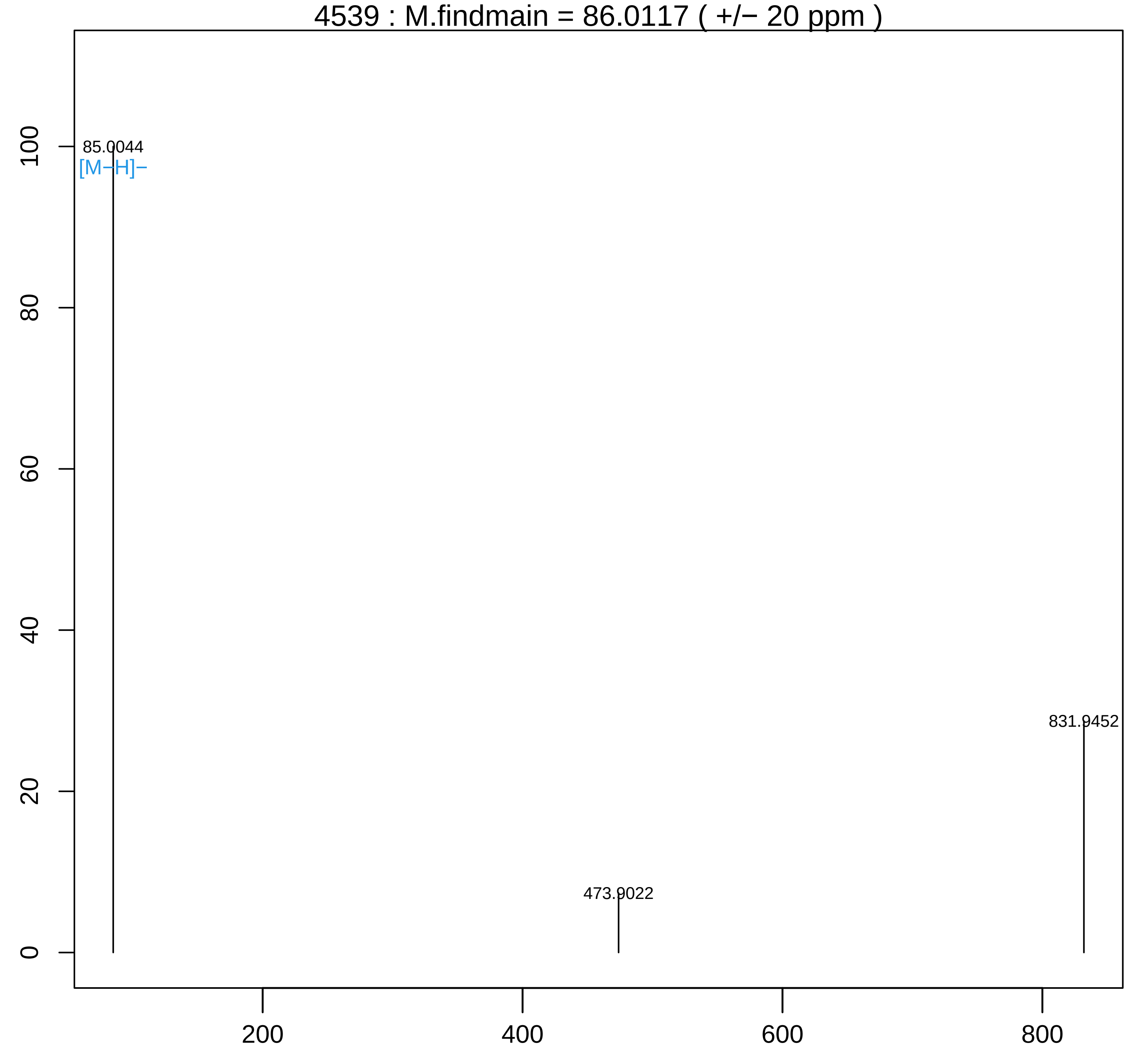
4539 : M.findmain = 86.0117 (+/− 20 ppm)
85.0044
[M−H]−
473.9022
831.9452
0
20
40
60
80
100
200
400
600
800

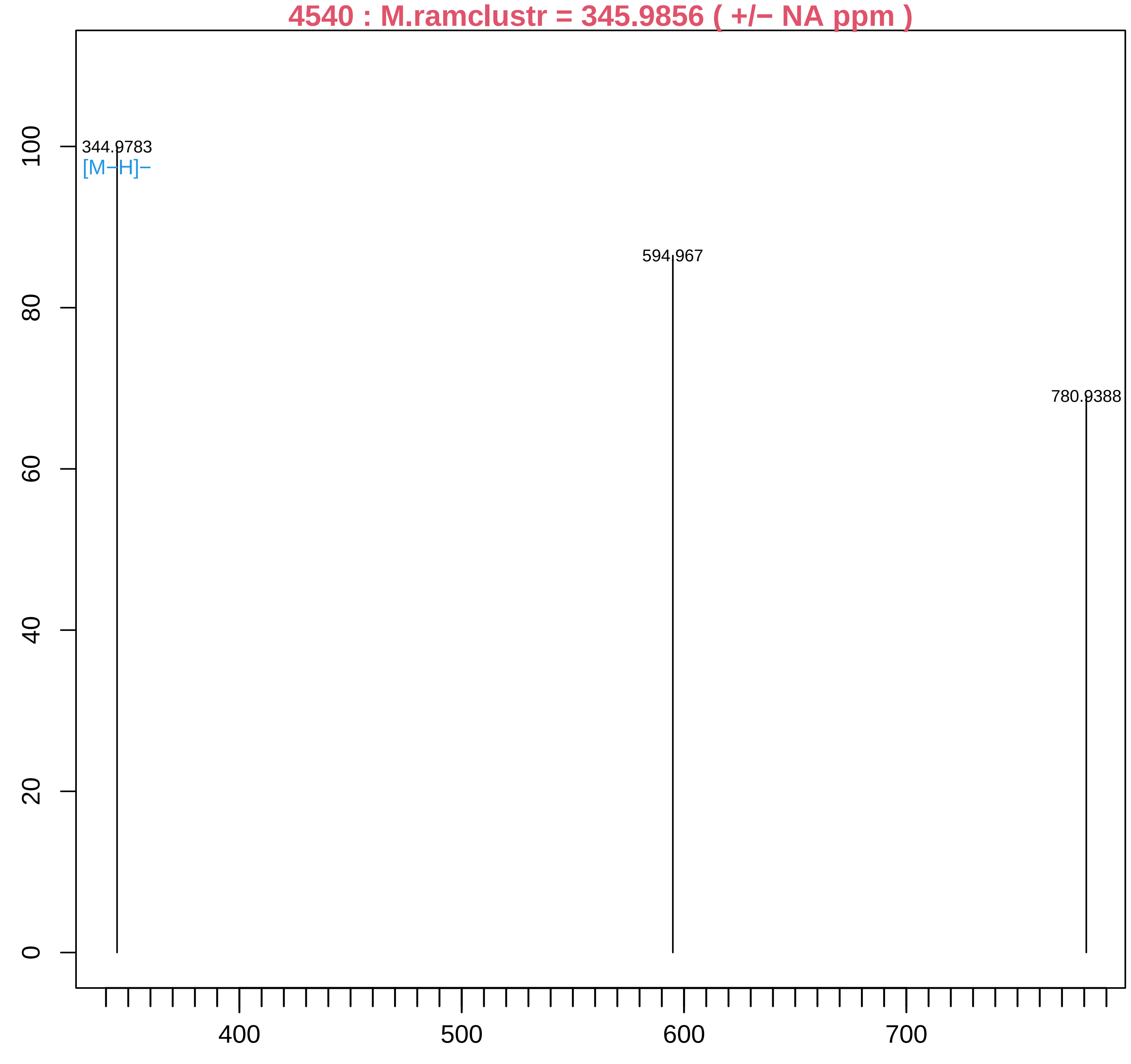
4540 : M.ramclustr = 345.9856 (+/− NA ppm)
344.9783
[M−H]−
594.967
780.9388
0
20
40
60
80
100
400
500
600
700

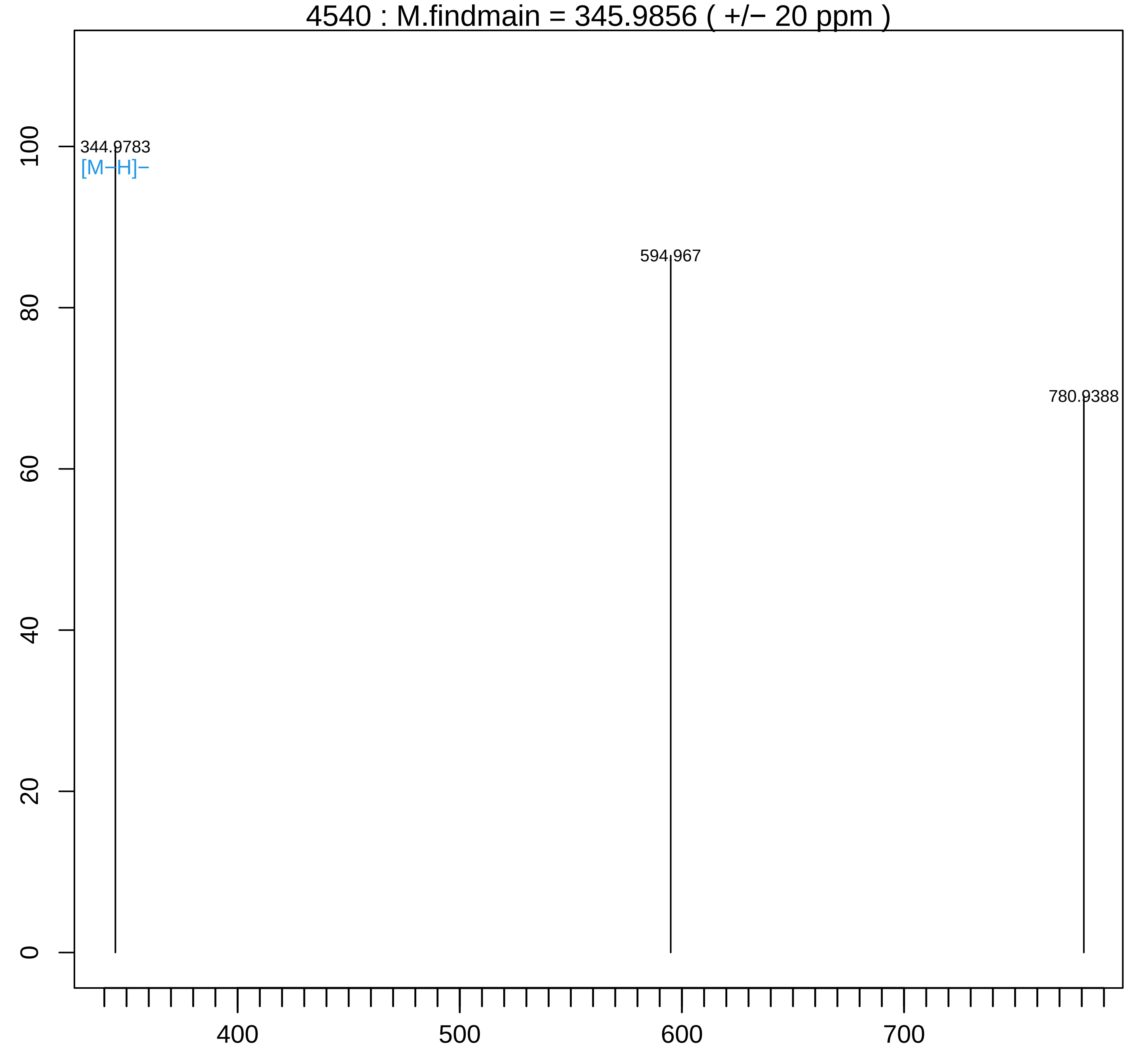
4540 : M.findmain = 345.9856 (+/− 20 ppm)
344.9783
[M−H]−
594.967
780.9388
0
20
40
60
80
100
400
500
600
700

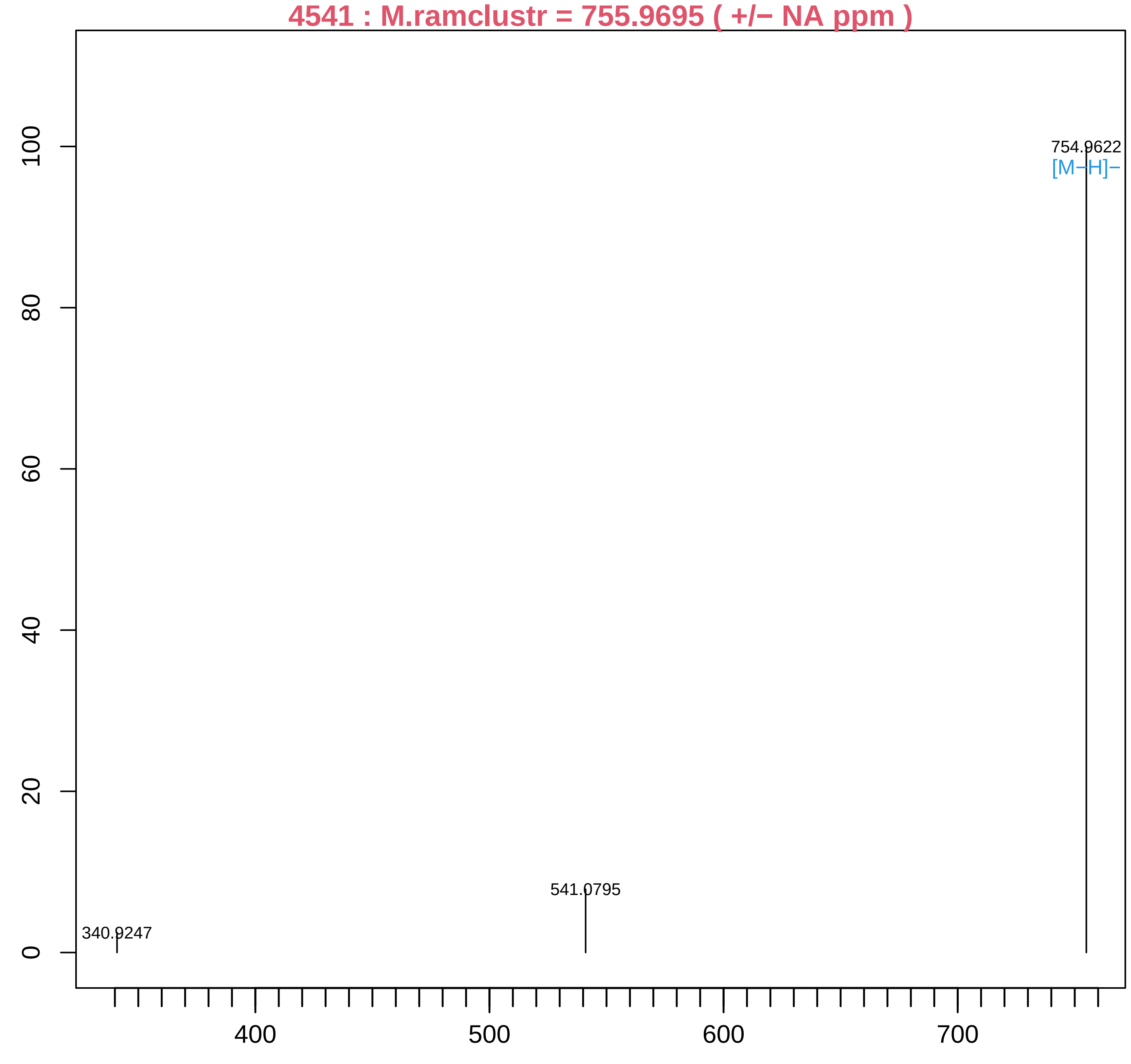
4541 : M.ramclustr = 755.9695 (+/− NA ppm)
754.9622
[M−H]−
541.0795
340.9247
0
20
40
60
80
100
400
500
600
700
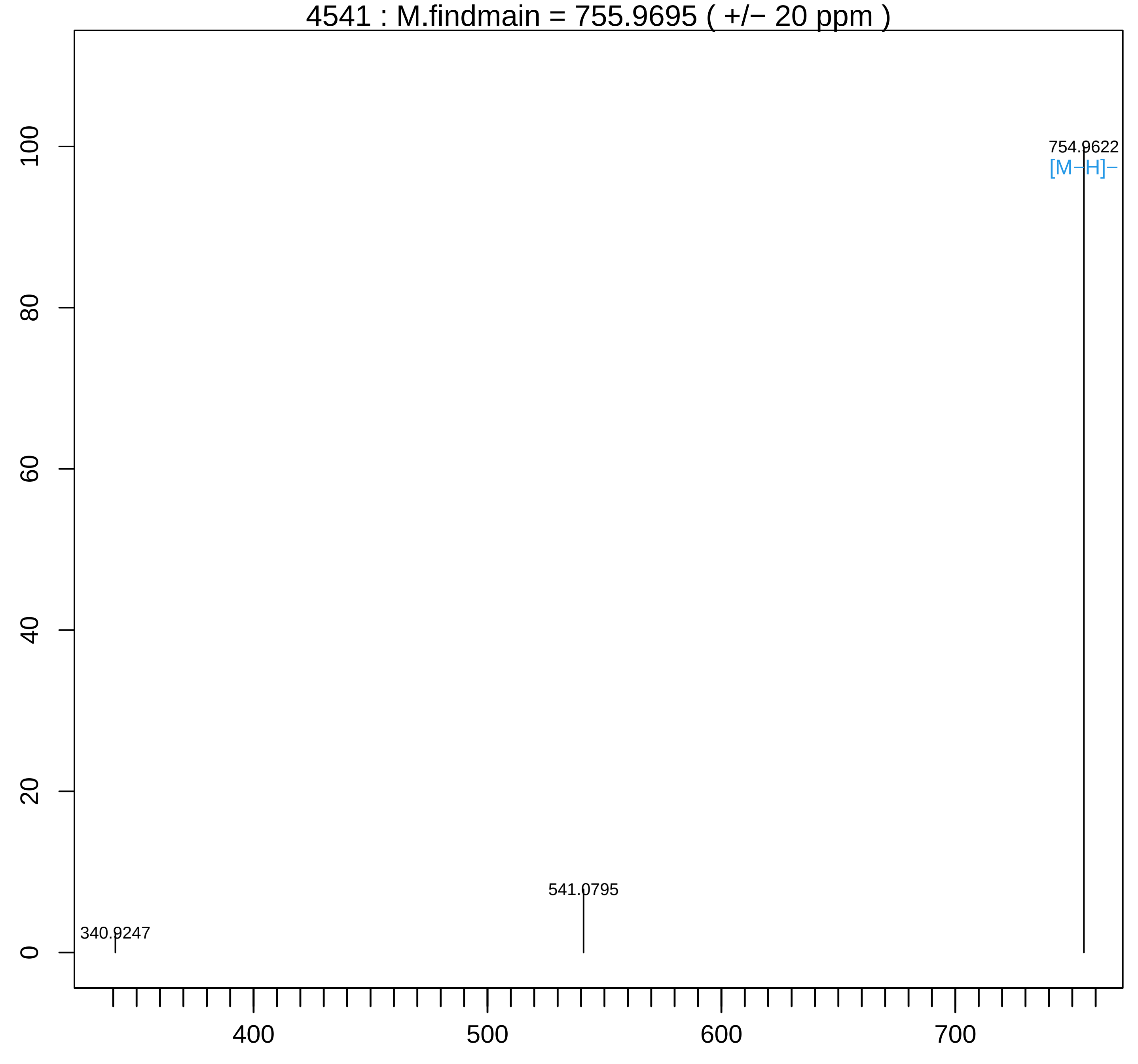
4541 : M.findmain = 755.9695 (+/− 20 ppm)
754.9622
[M−H]−
541.0795
340.9247
0
20
40
60
80
100
400
500
600
700

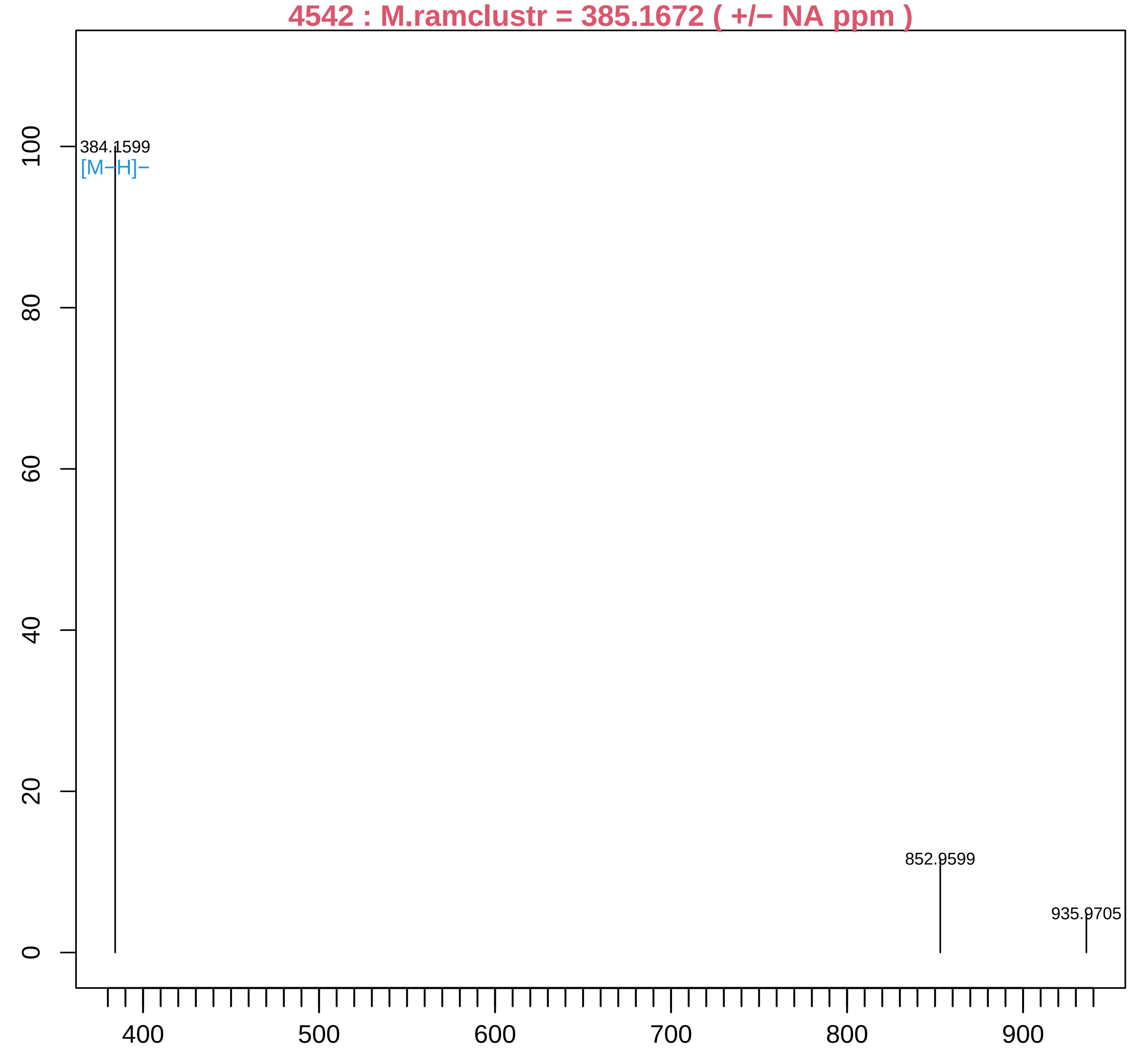
4542 : M.ramclustr = 385.1672 (+/− NA ppm)
384.1599
[M−H]−
852.9599
935.9705
0
20
40
60
80
100
400
500
600
700
800
900

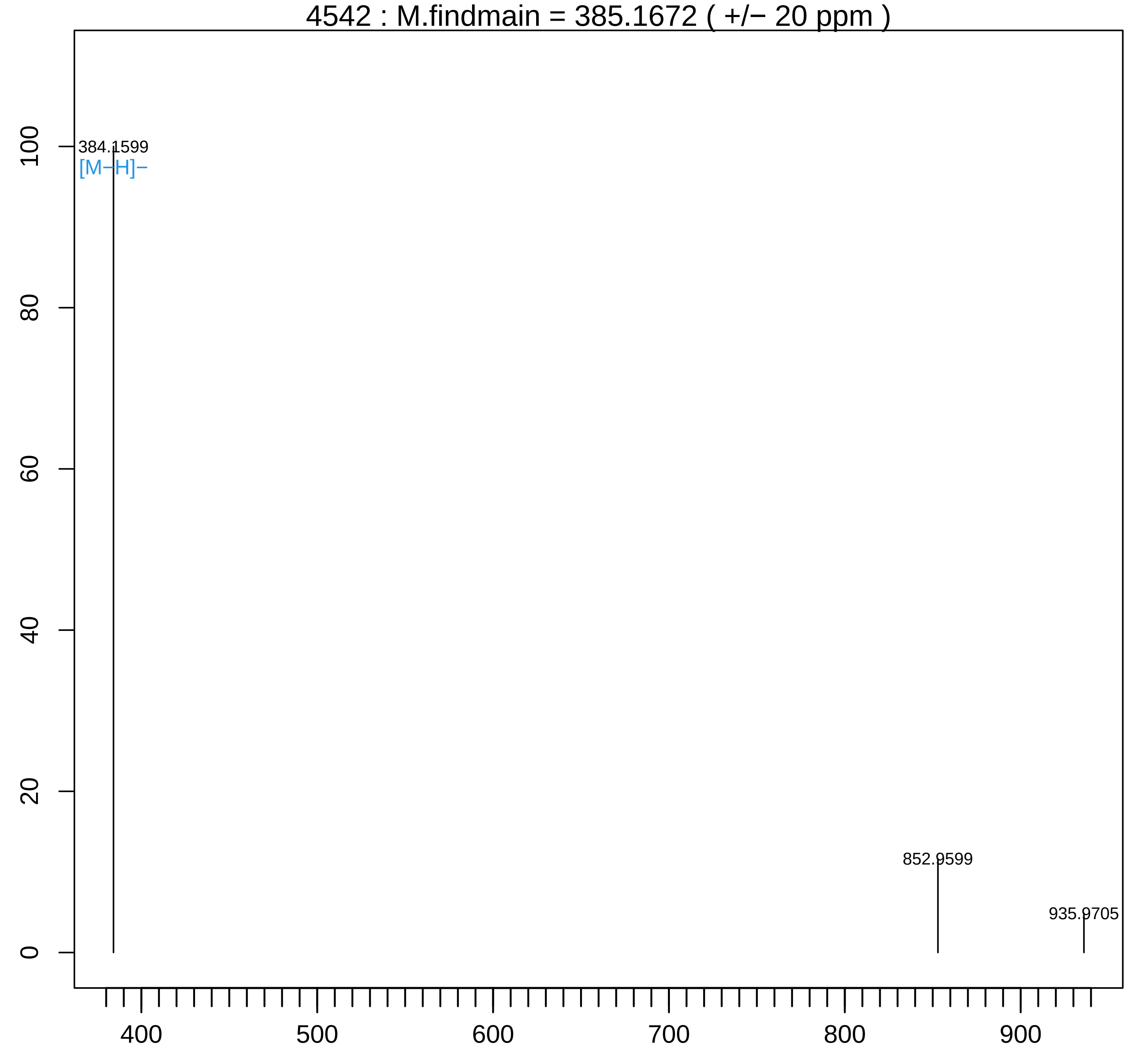
4542 : M.findmain = 385.1672 (+/− 20 ppm)
384.1599
[M−H]−
852.9599
935.9705
0
20
40
60
80
100
400
500
600
700
800
900

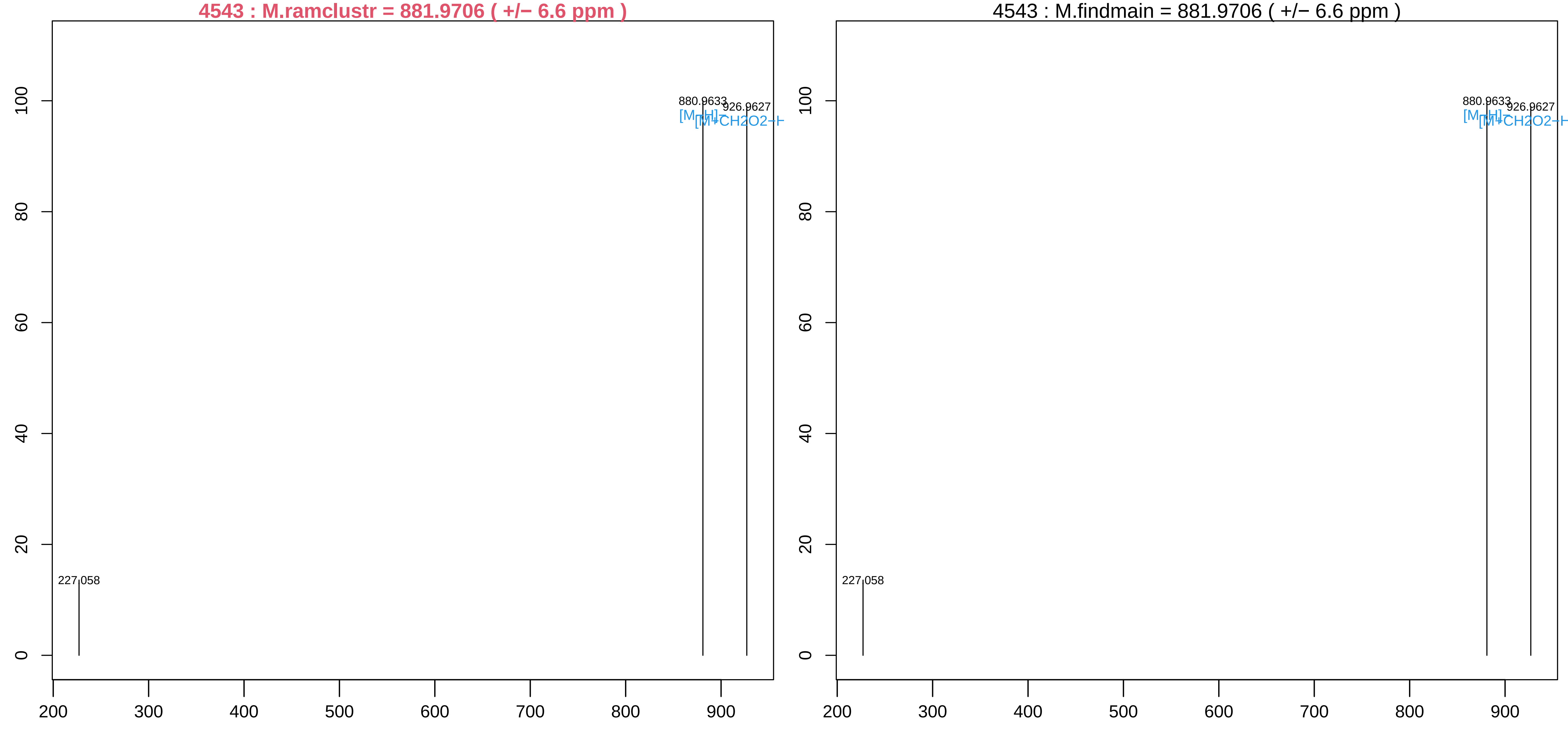
4543 : M.ramclustr = 881.9706 (+/− 6.6 ppm)
880.9633
[M−H]−
926.9627
[M+CH2O2−H
227.058
4543 : M.findmain = 881.9706 (+/− 6.6 ppm)
880.9633
[M−H]−
926.9627
[M+CH2O2−H
227.058
0
20
40
60
80
100
200
300
400
500
600
700
800
900

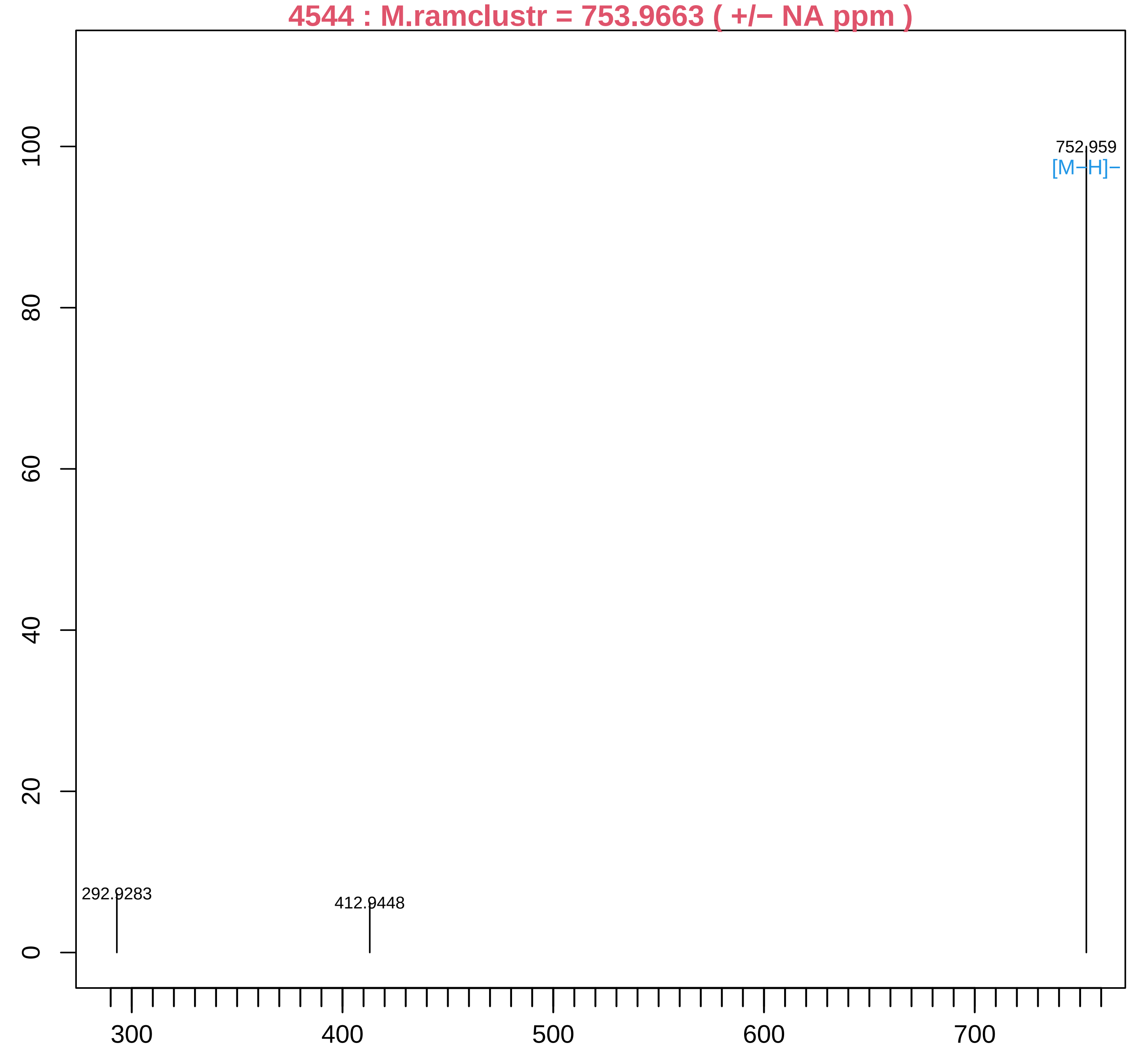
4544 : M.ramclustr = 753.9663 (+/− NA ppm)
752.959
[M−H]−
292.9283
412.9448
0
20
40
60
80
100
300
400
500
600
700

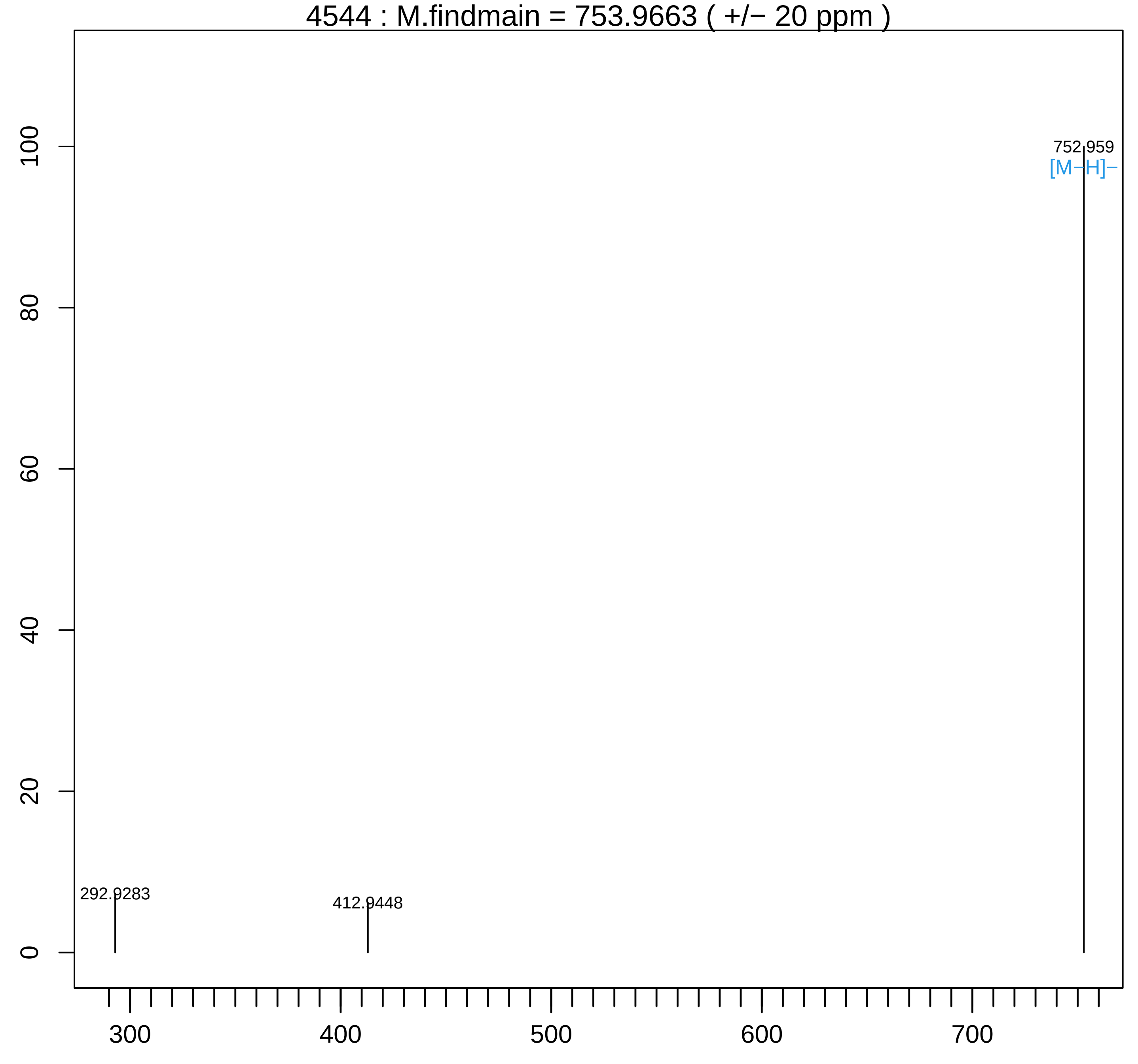
4544 : M.findmain = 753.9663 (+/− 20 ppm)
752.959
[M−H]−
292.9283
412.9448
0
20
40
60
80
100
300
400
500
600
700

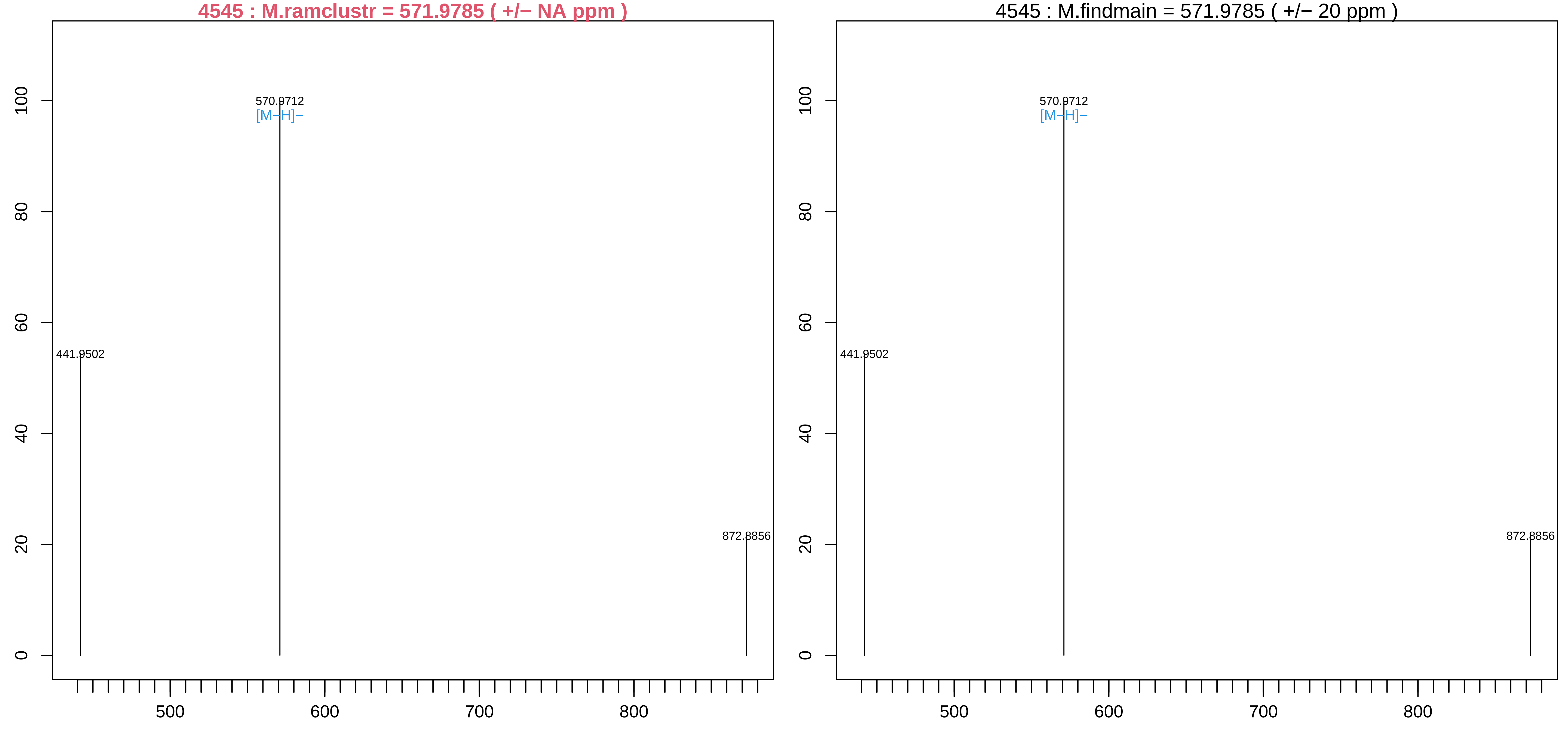
4545 : M.ramclustr = 571.9785 (+/− NA ppm)
570.9712
[M−H]−
441.9502
872.8856
4545 : M.findmain = 571.9785 (+/− 20 ppm)
570.9712
[M−H]−
441.9502
872.8856
0
20
40
60
80
100
500
600
700
800

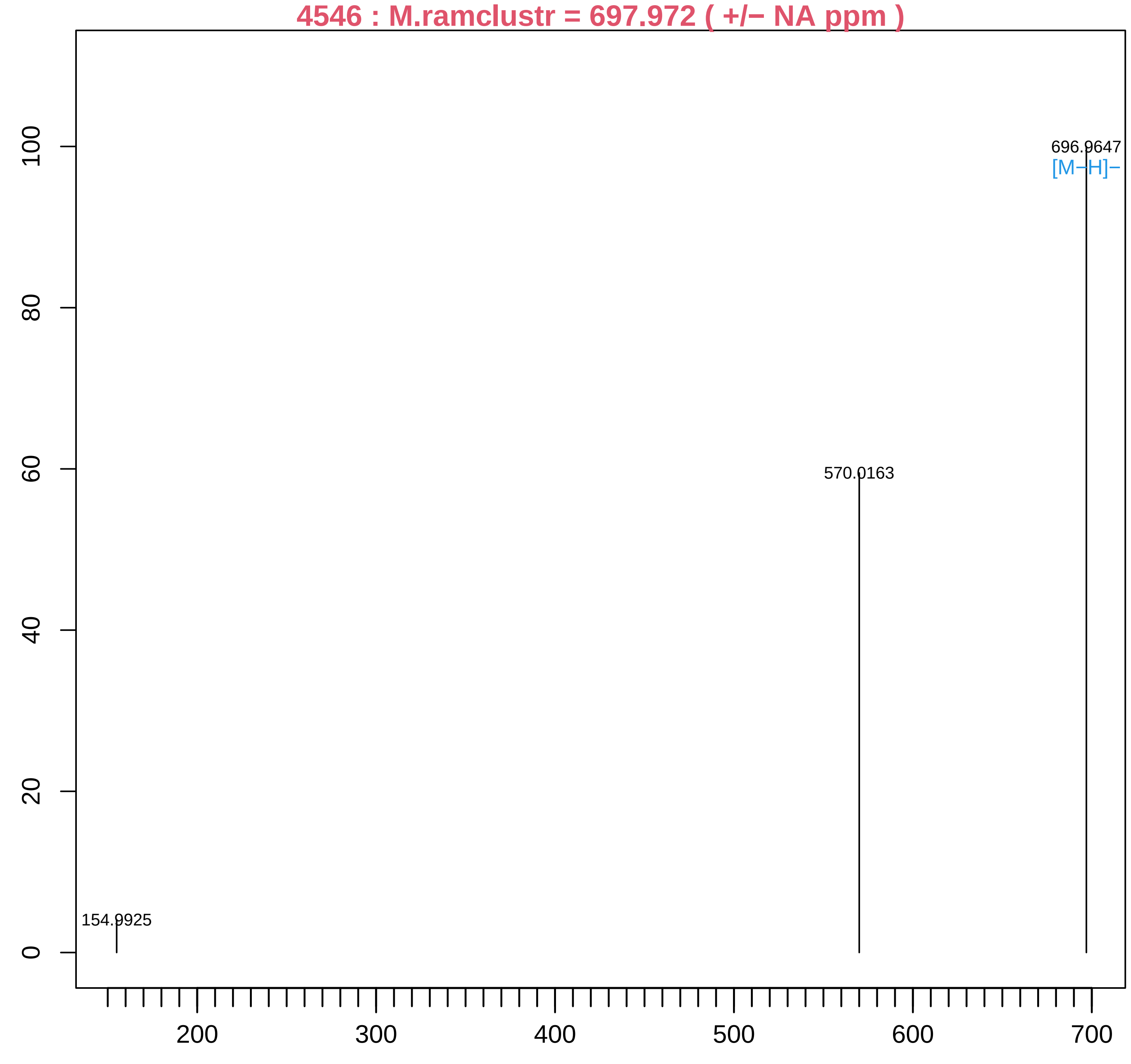

4546 : M.ramclustr = 697.972 (+/− NA ppm)
696.9647
[M−H]−
570.0163
154.9925
0
20
40
60
80
100
200
300
400
500
600
700

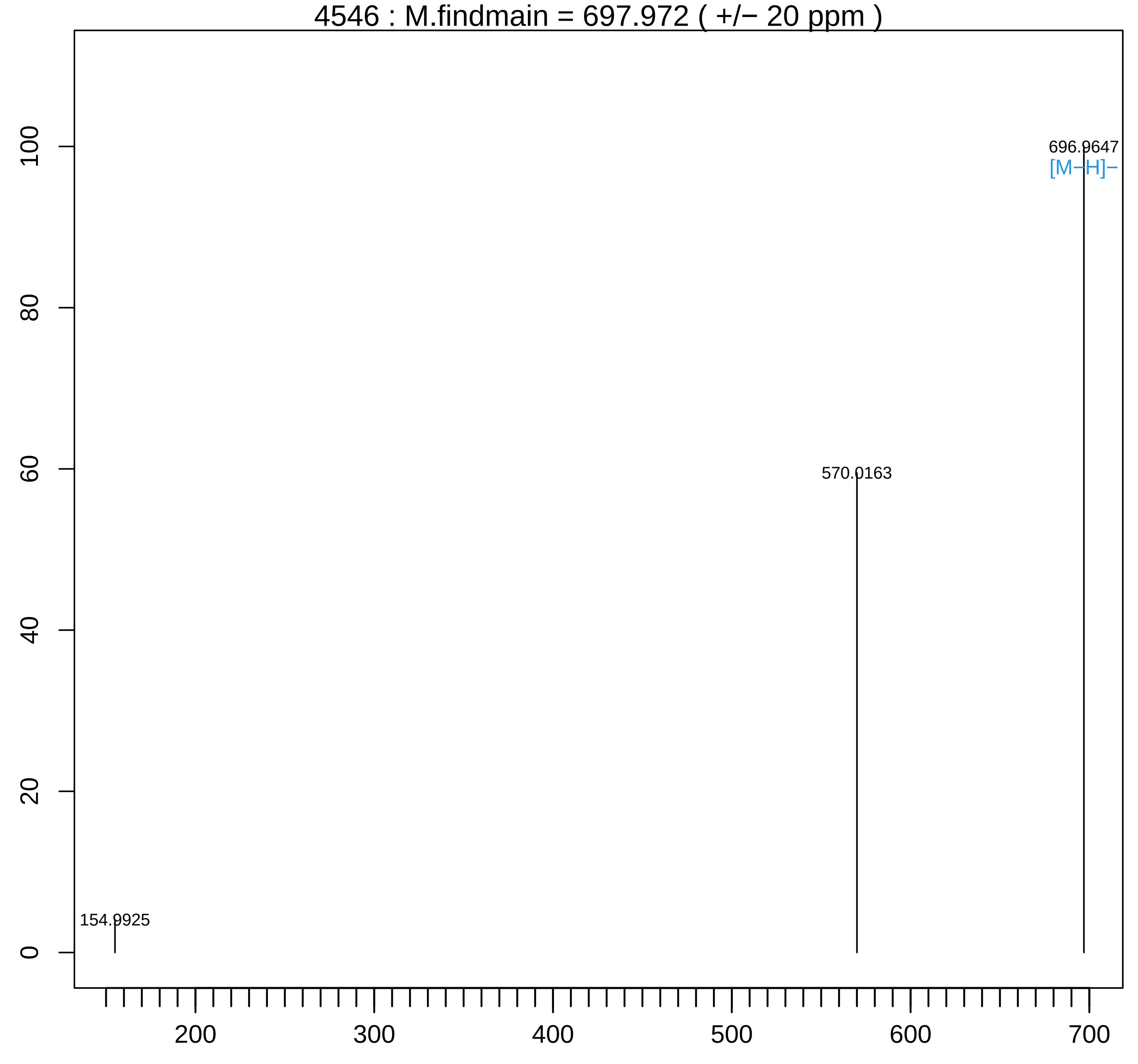

4546 : M.findmain = 697.972 (+/− 20 ppm)
696.9647
[M−H]−
570.0163
154.9925
0
20
40
60
80
100
200
300
400
500
600
700

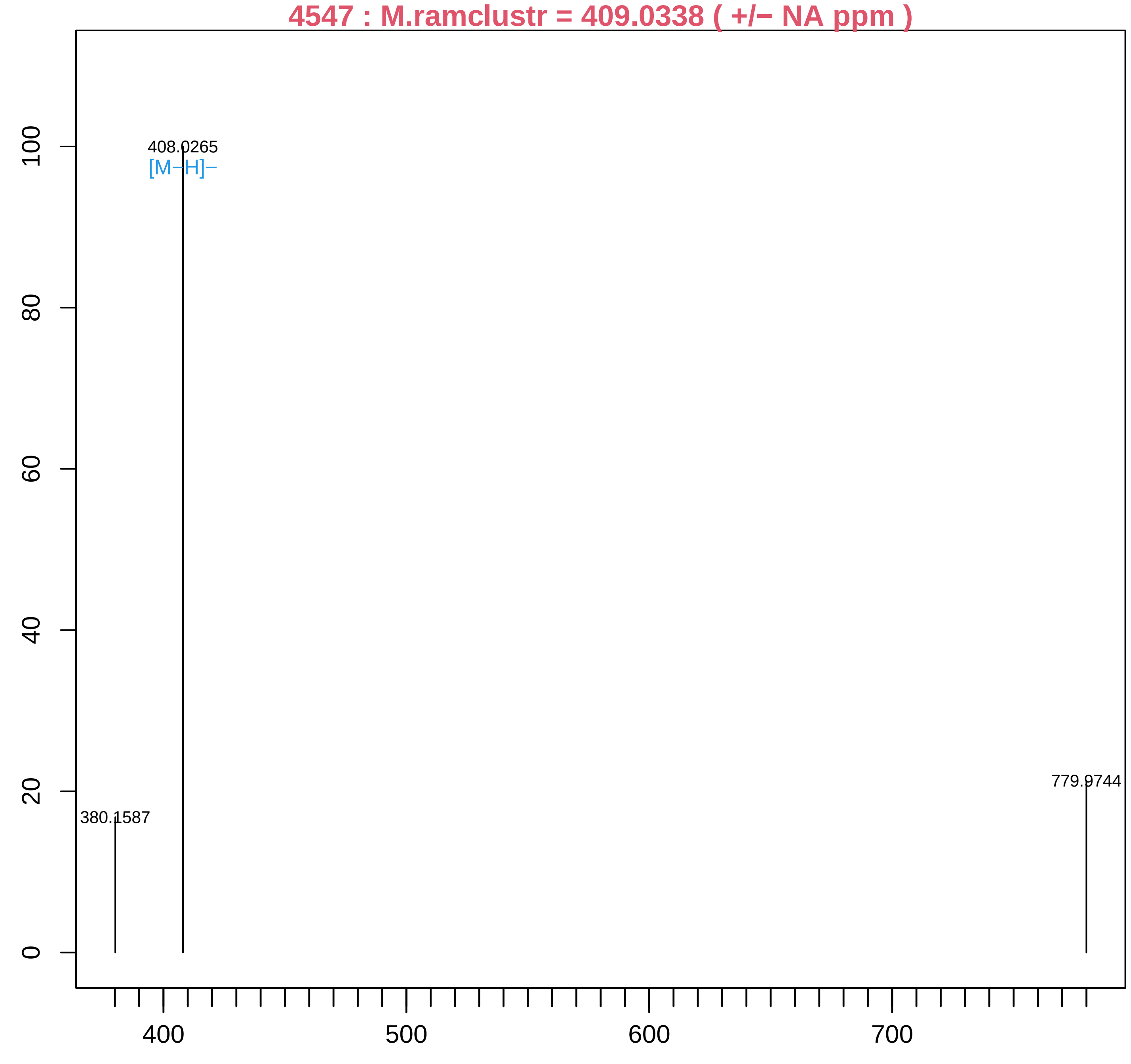
4547 : M.ramclustr = 409.0338 (+/− NA ppm)
408.0265
[M−H]−
380.1587
779.9744
0
20
40
60
80
100
400
500
600
700

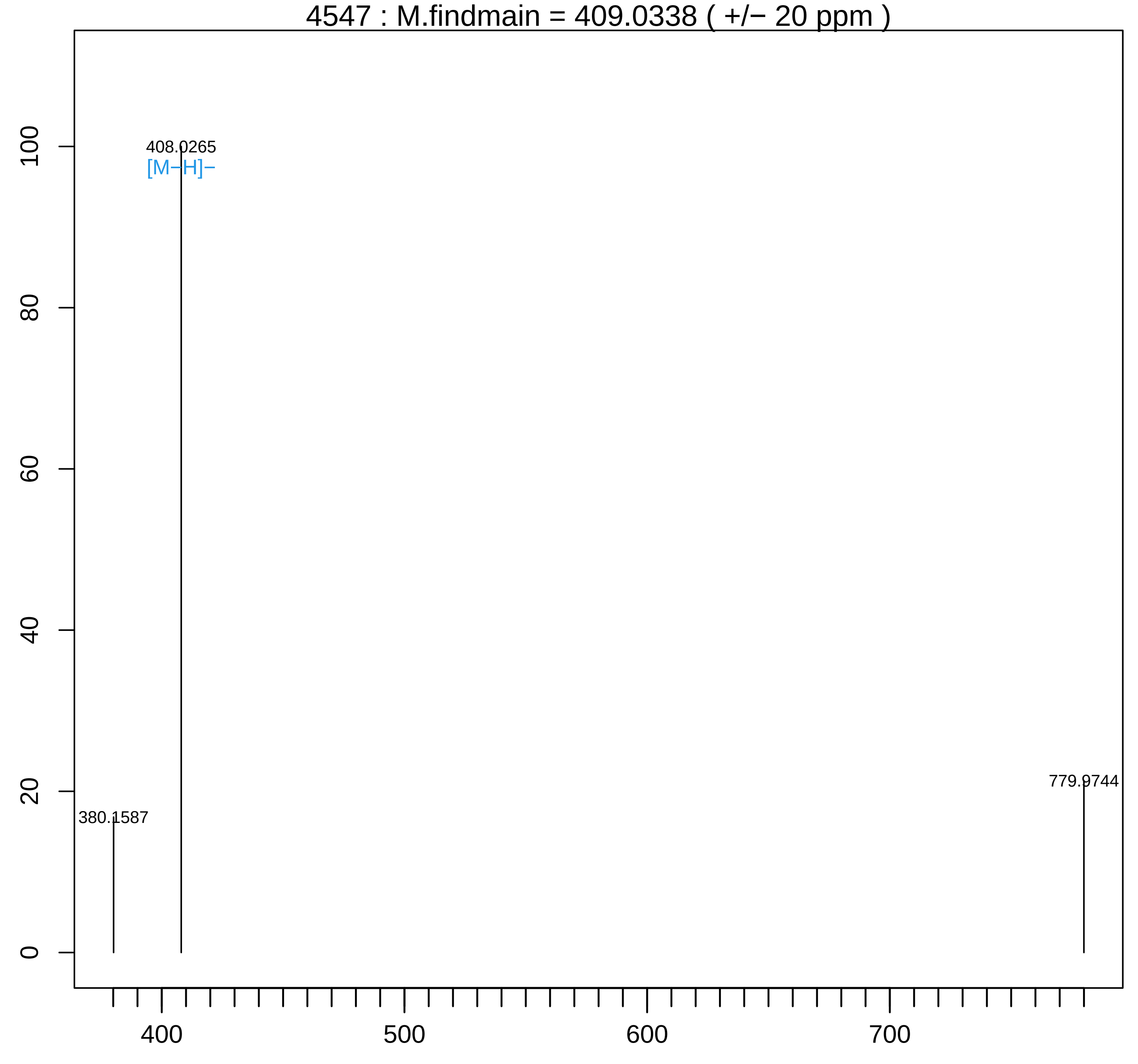
4547 : M.findmain = 409.0338 (+/− 20 ppm)
408.0265
[M−H]−
380.1587
779.9744
0
20
40
60
80
100
400
500
600
700

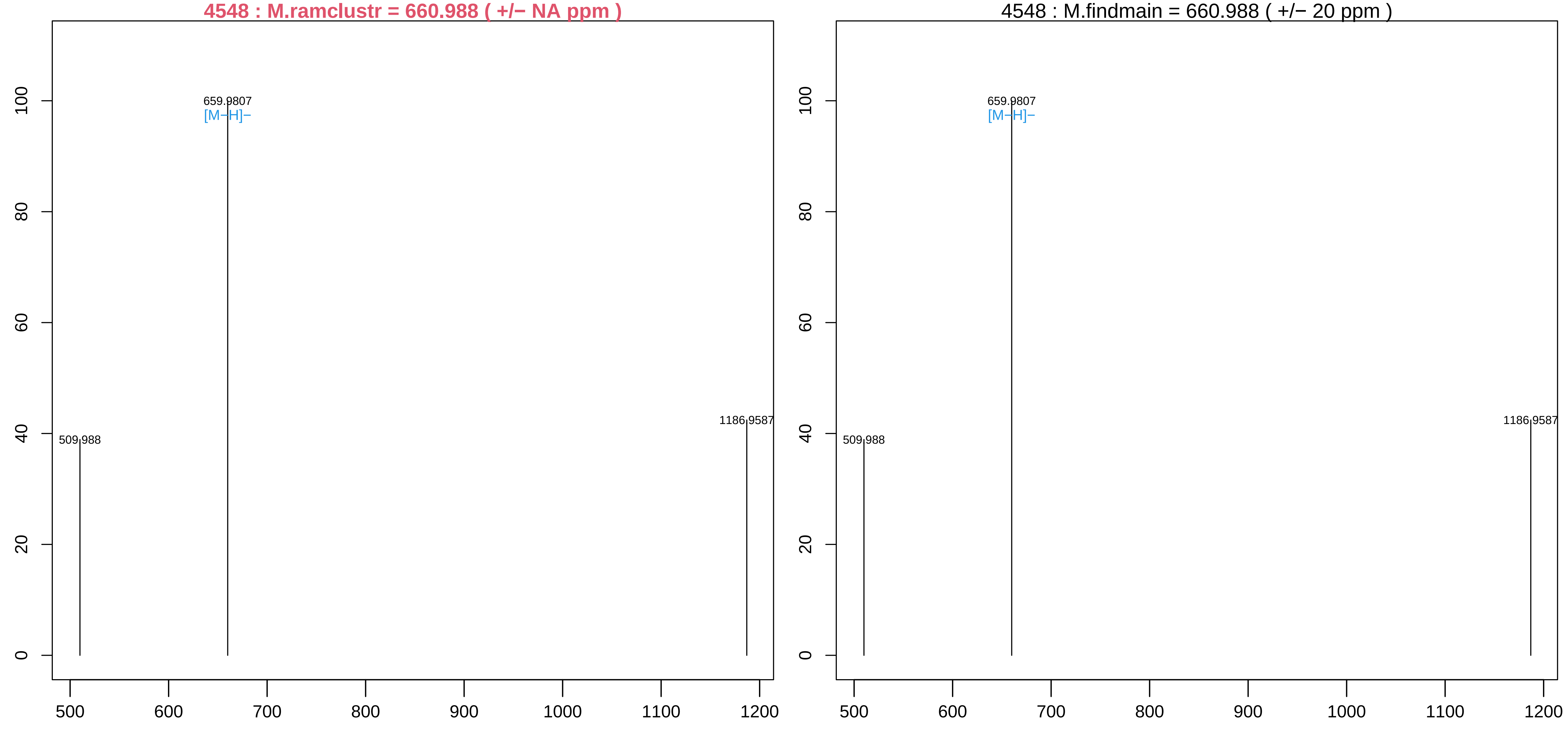
4548 : M.ramclustr = 660.988 (+/− NA ppm)
659.9807
[M−H]−
509.988
1186.9587
4548 : M.findmain = 660.988 (+/− 20 ppm)
659.9807
[M−H]−
509.988
1186.9587
0
20
40
60
80
100
500
600
700
800
900
1000
1100
1200

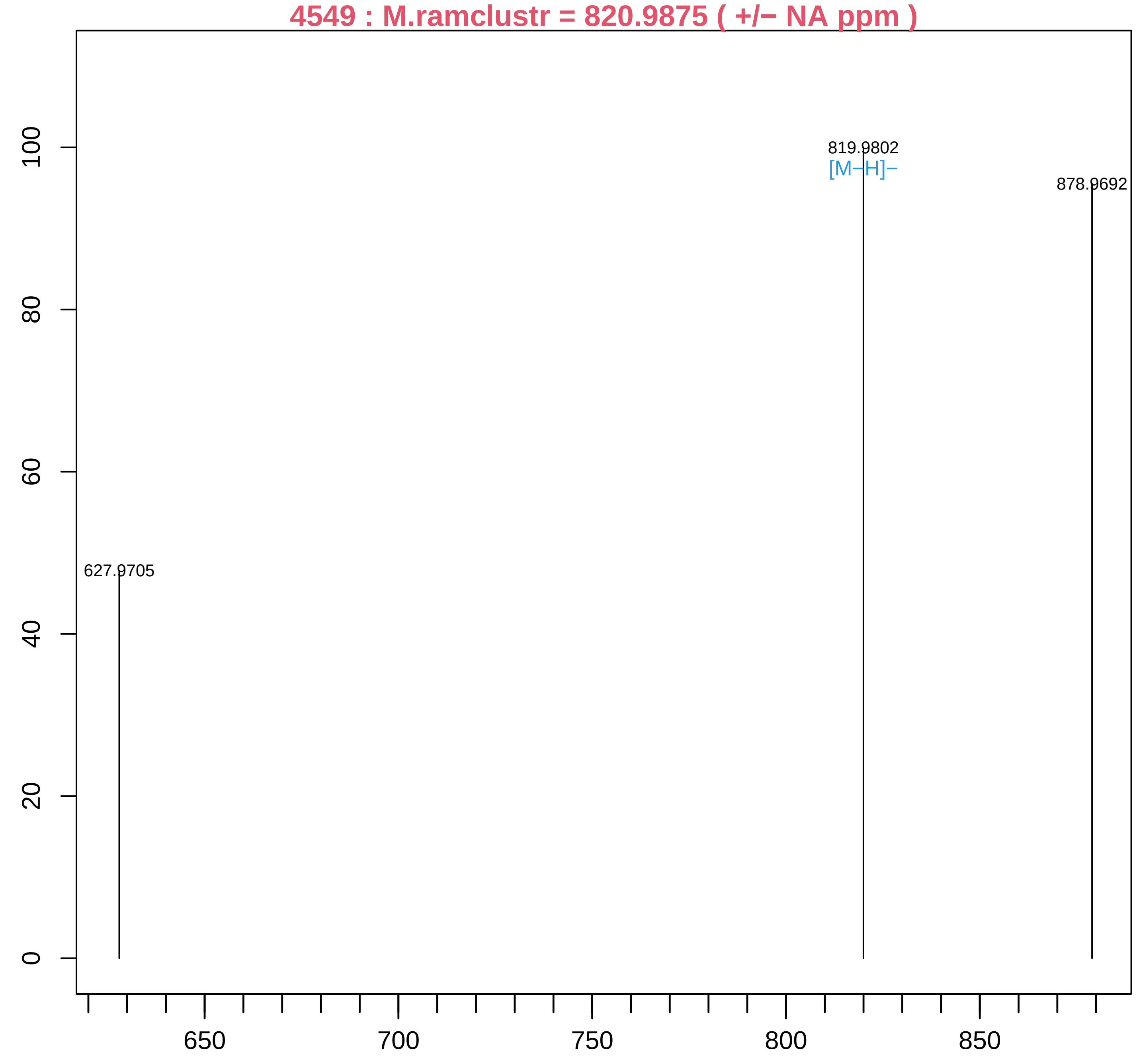
4549 : M.ramclustr = 820.9875 (+/− NA ppm)
627.9705
819.9802
[M−H]−
878.9692
0
20
40
60
80
100
650
700
750
800
850

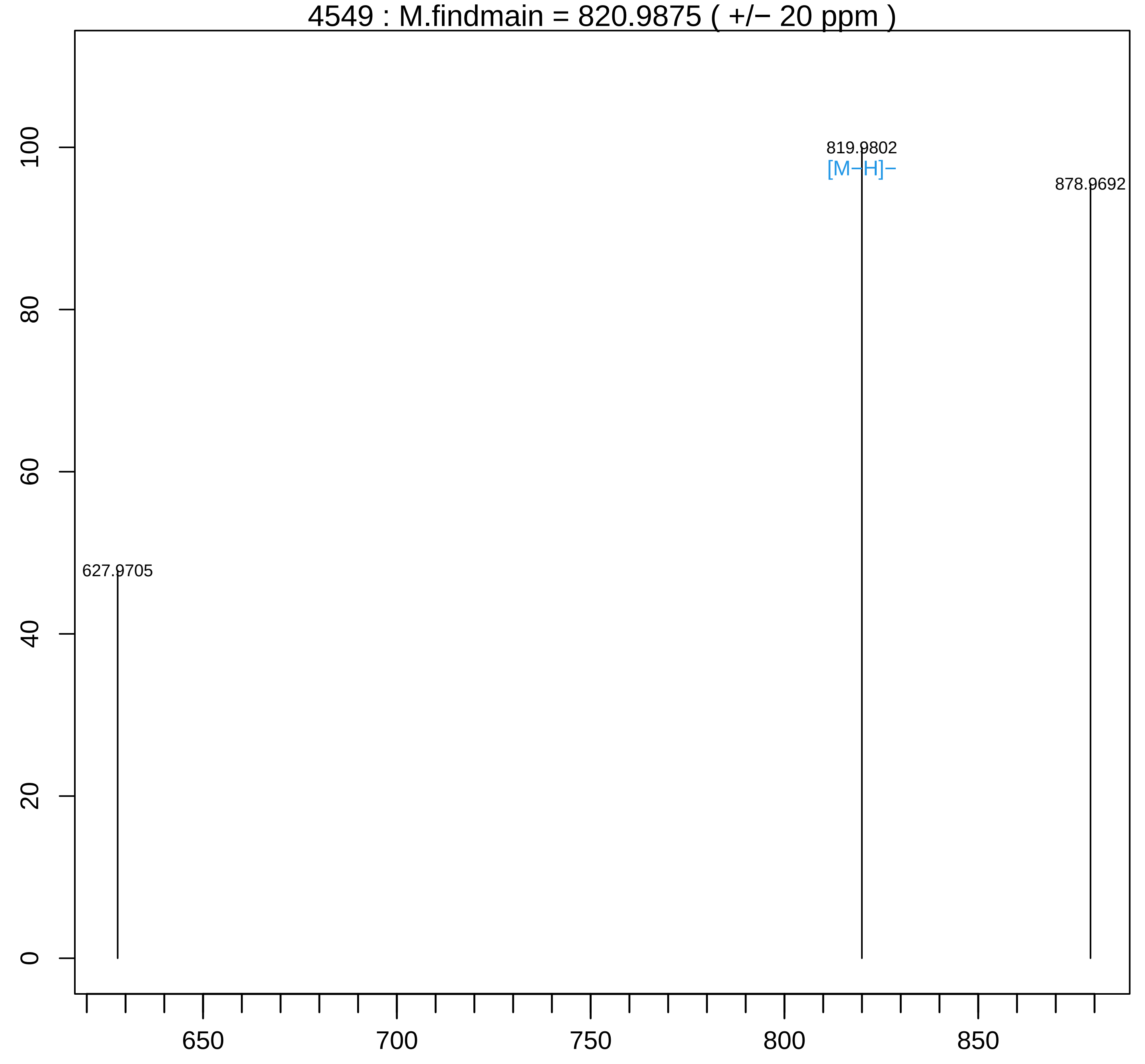
4549 : M.findmain = 820.9875 (+/− 20 ppm)
627.9705
819.9802
[M−H]−
878.9692
0
20
40
60
80
100
650
700
750
800
850

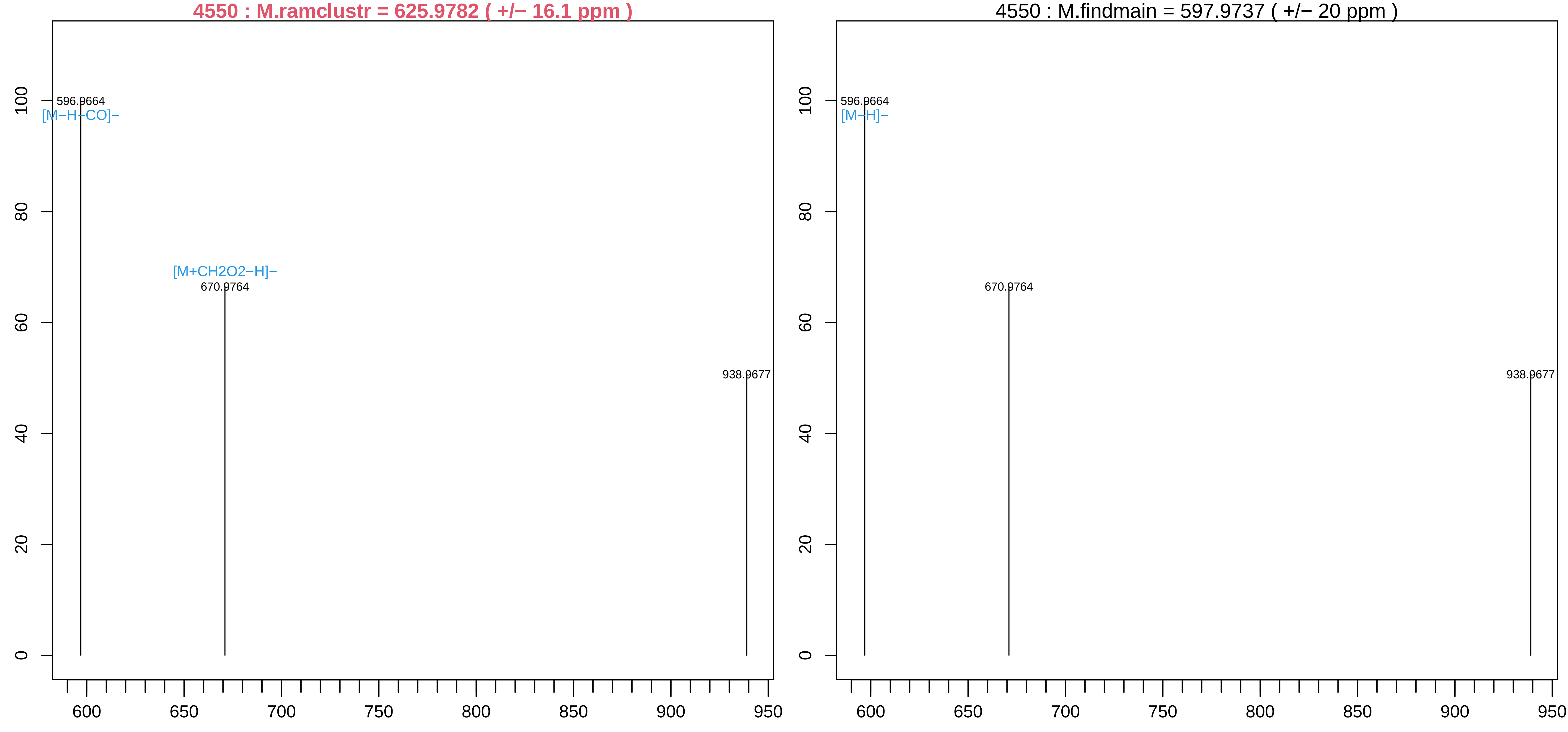

4550 : M.ramclustr = 625.9782 (+/− 16.1 ppm)
596.9664
[M−H−CO]−
[M+CH2O2−H]−
670.9764
938.9677
4550 : M.findmain = 597.9737 (+/− 20 ppm)
596.9664
[M−H]−
670.9764
938.9677

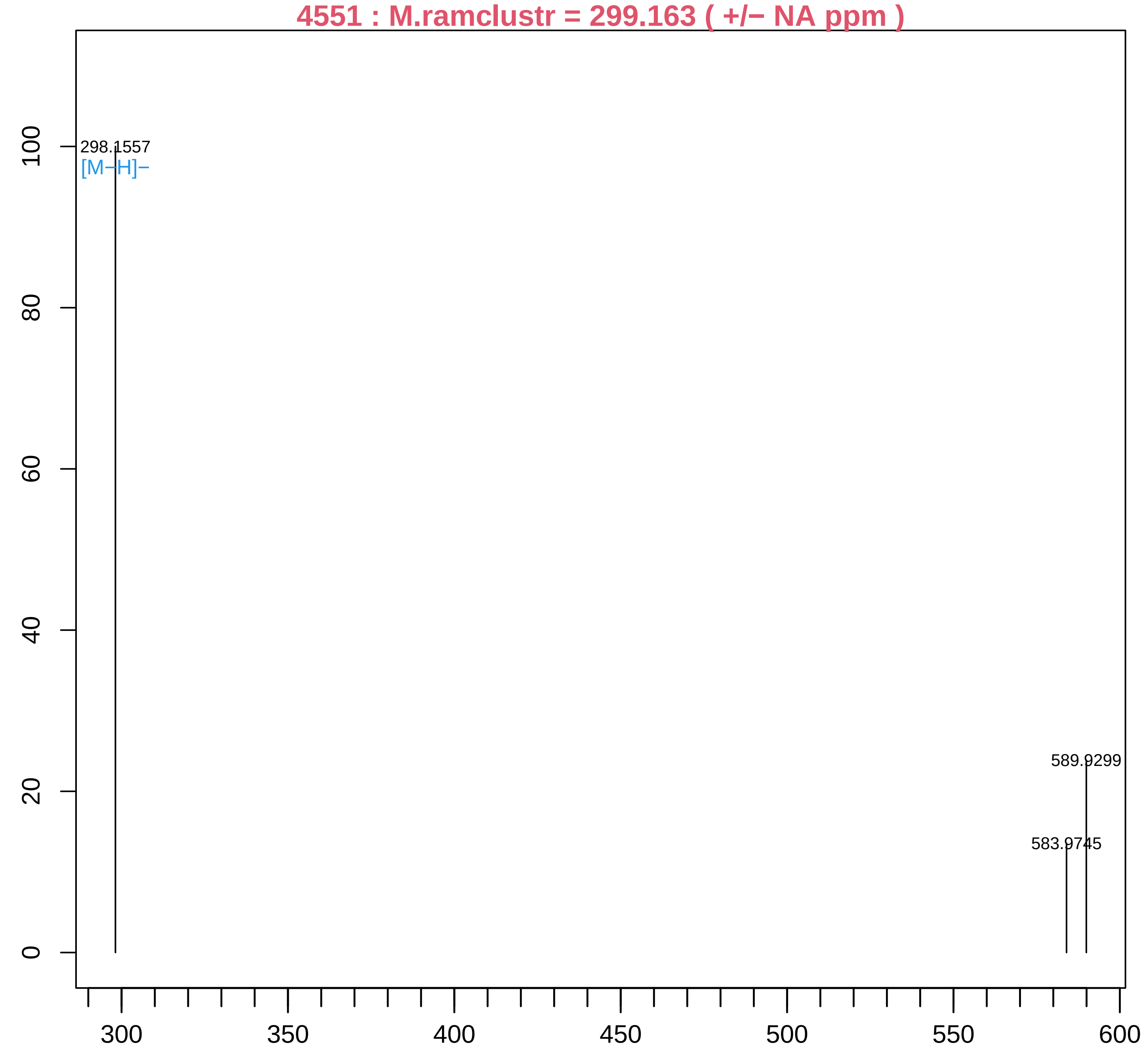
4551 : M.ramclustr = 299.163 (+/− NA ppm)
298.1557
[M−H]−
589.9299
583.9745
0
20
40
60
80
100
300
350
400
450
500
550
600

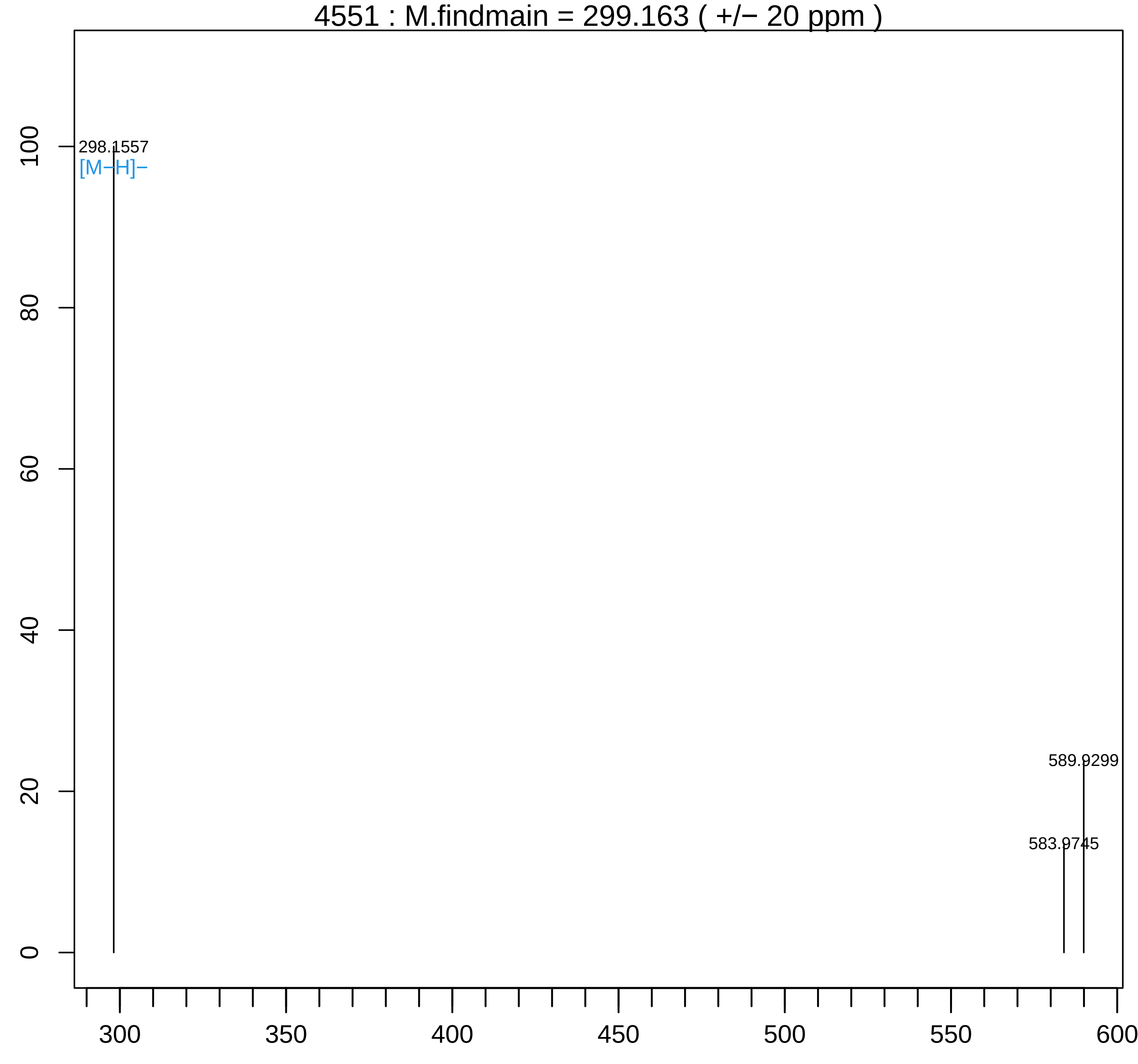
4551 : M.findmain = 299.163 (+/− 20 ppm)
298.1557
[M−H]−
589.9299
583.9745
0
20
40
60
80
100
300
350
400
450
500
550
600

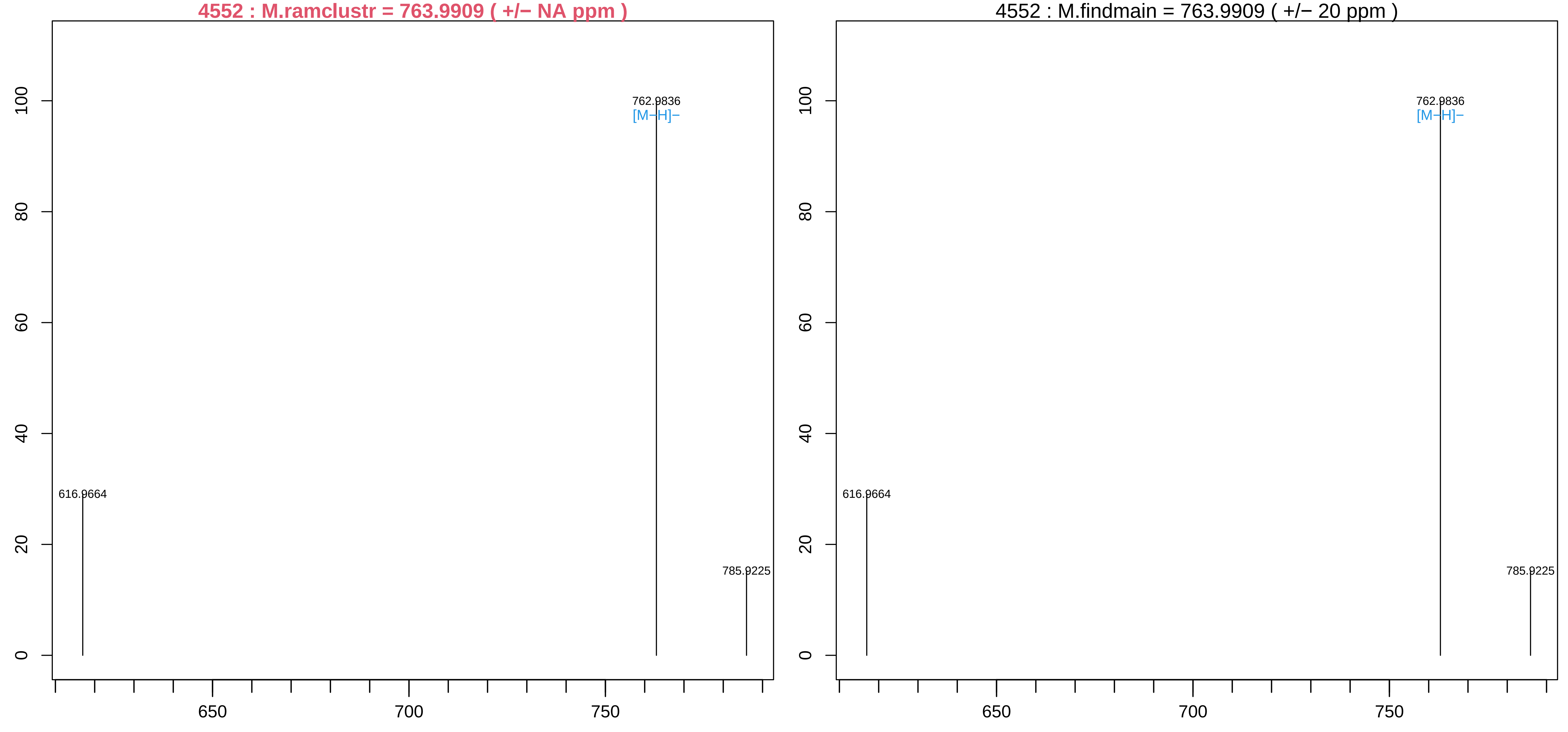

4552 : M.ramclustr = 763.9909 (+/− NA ppm)
762.9836
[M−H]−
616.9664
785.9225
0
20
40
60
80
100
650
700
750
4552 : M.findmain = 763.9909 (+/− 20 ppm)
762.9836
[M−H]−
616.9664
785.9225
0
20
40
60
80
100
650
700
750

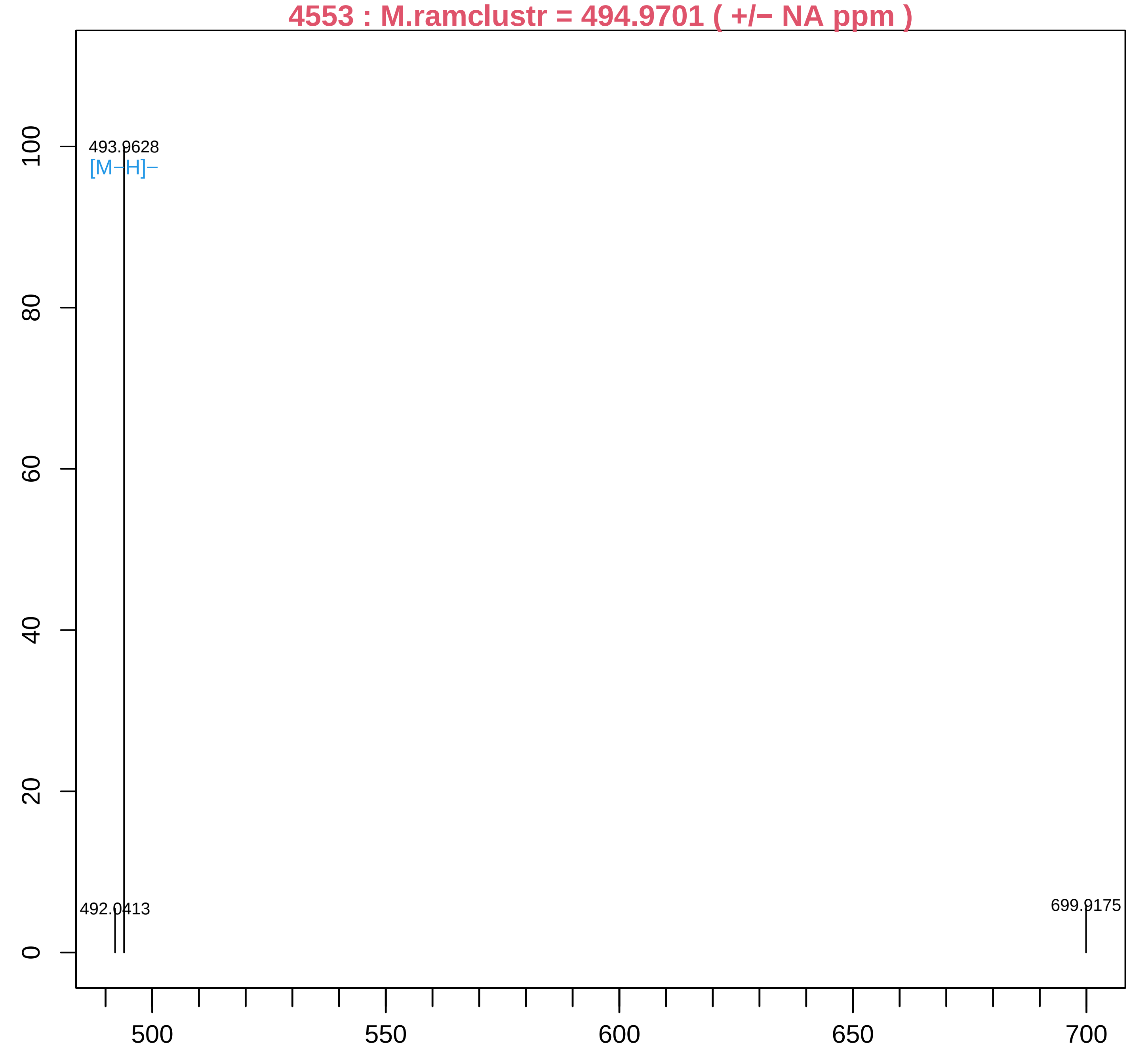

4553 : M.ramclustr = 494.9701 (+/− NA ppm)
493.9628
[M−H]−
492.0413
699.9175
0
20
40
60
80
100
500
550
600
650
700

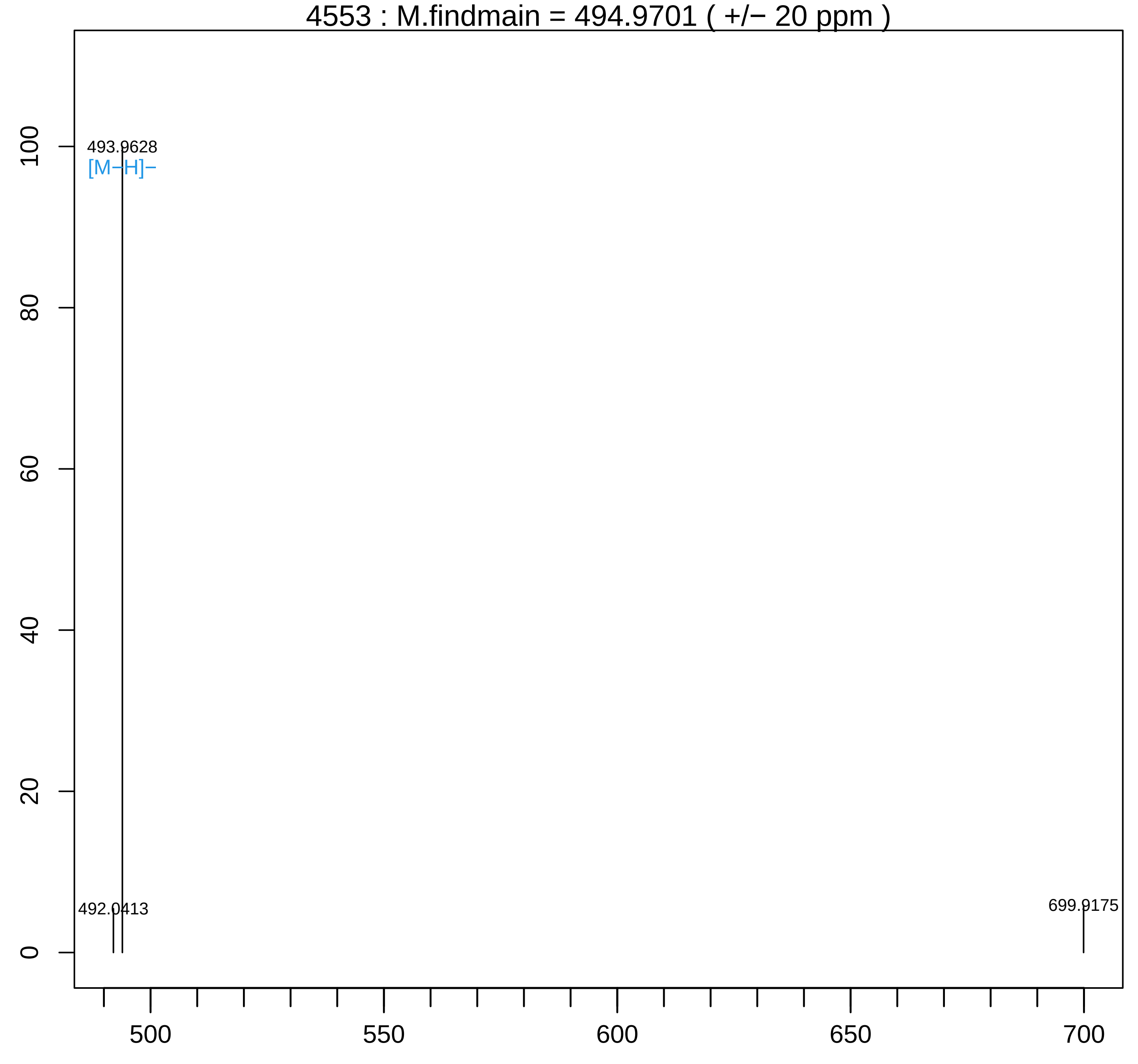

4553 : M.findmain = 494.9701 (+/− 20 ppm)
493.9628
[M−H]−
492.0413
699.9175
0
20
40
60
80
100
500
550
600
650
700

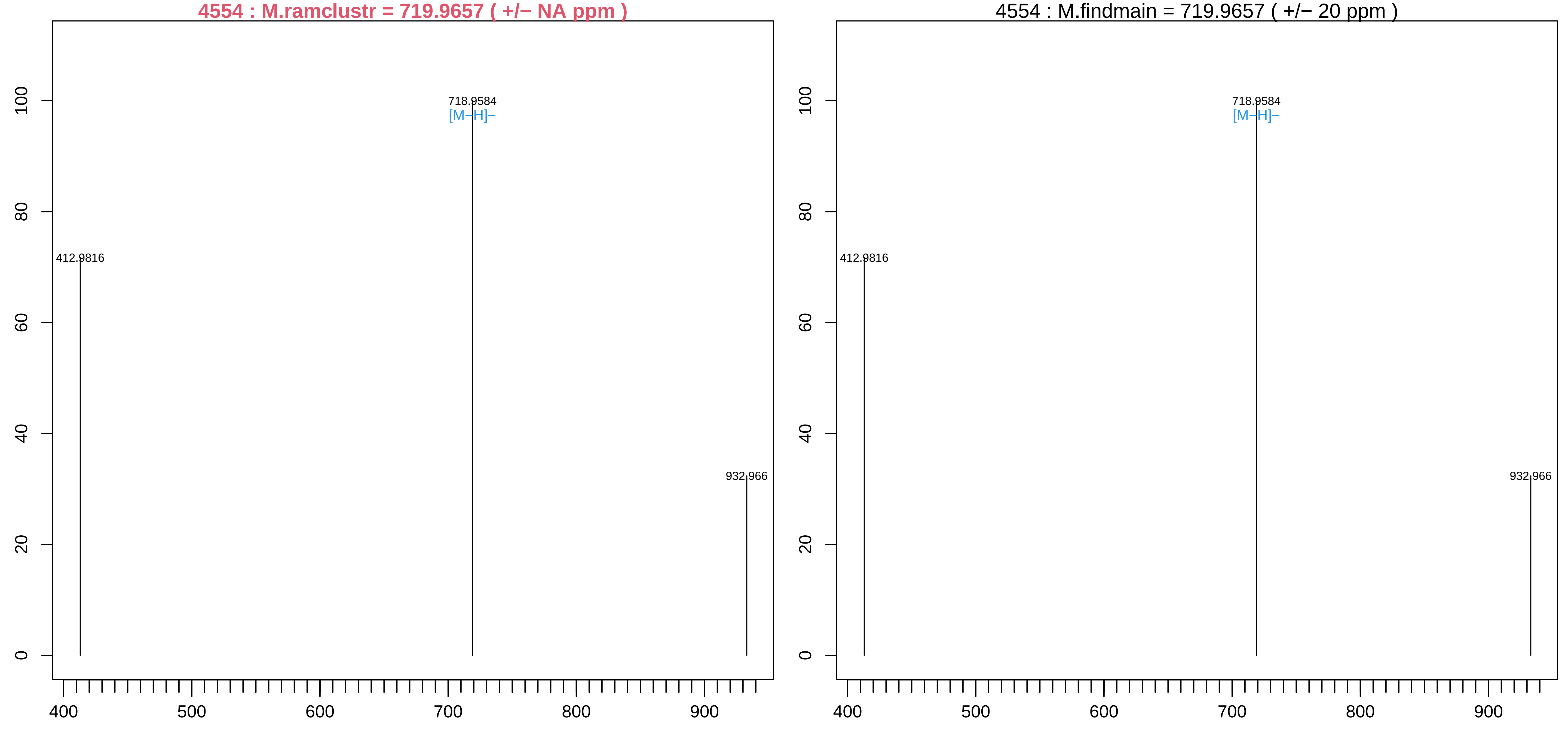
4554 : M.ramclustr = 719.9657 (+/− NA ppm)
412.9816
718.9584
[M−H]−
932.966
0
20
40
60
80
100
400
500
600
700
800
900
4554 : M.findmain = 719.9657 (+/− 20 ppm)
412.9816
718.9584
[M−H]−
932.966
0
20
40
60
80
100
400
500
600
700
800
900

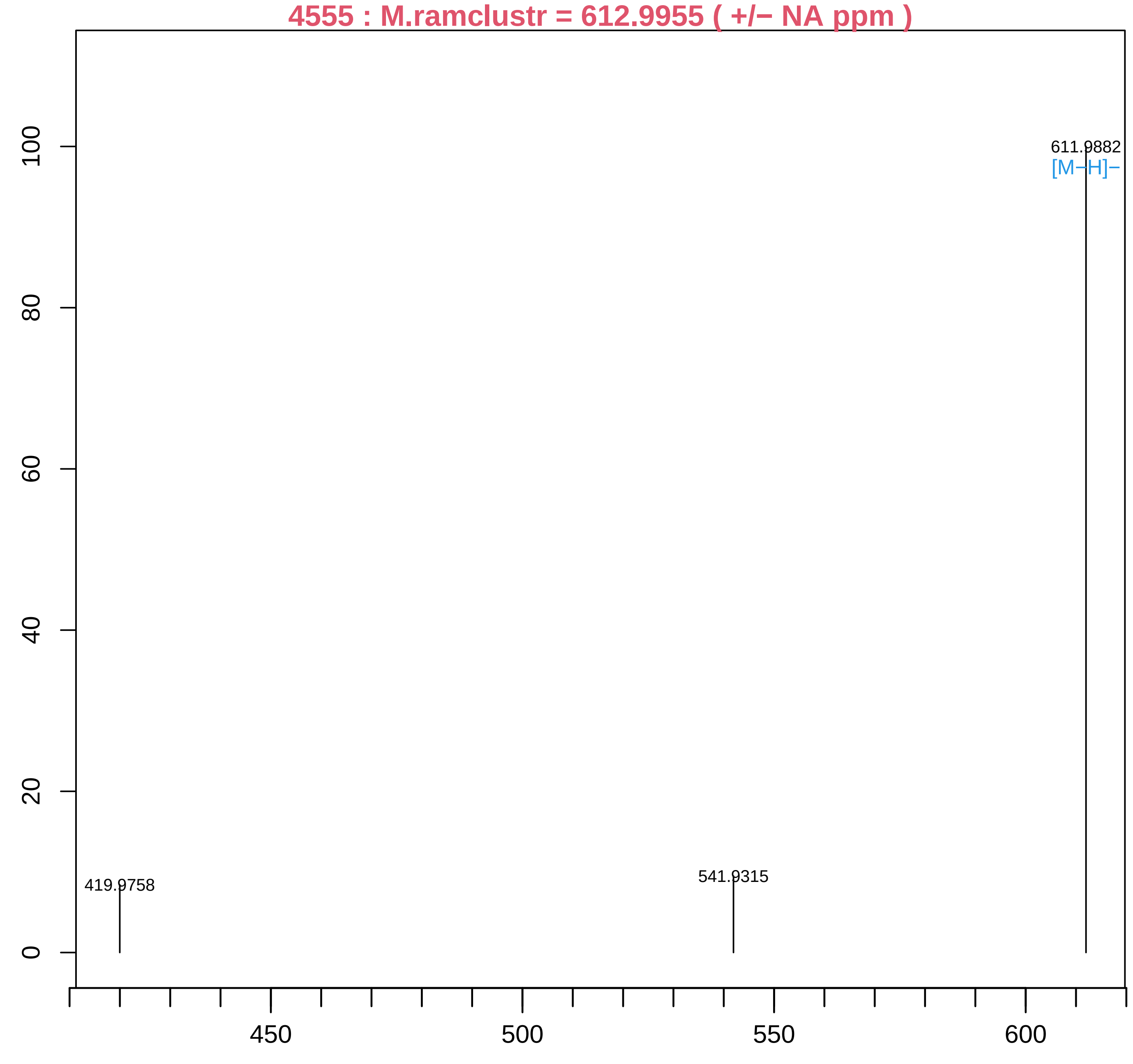
4555 : M.ramclustr = 612.9955 (+/− NA ppm)
611.9882
[M−H]−
541.9315
419.9758
0
20
40
60
80
100
450
500
550
600

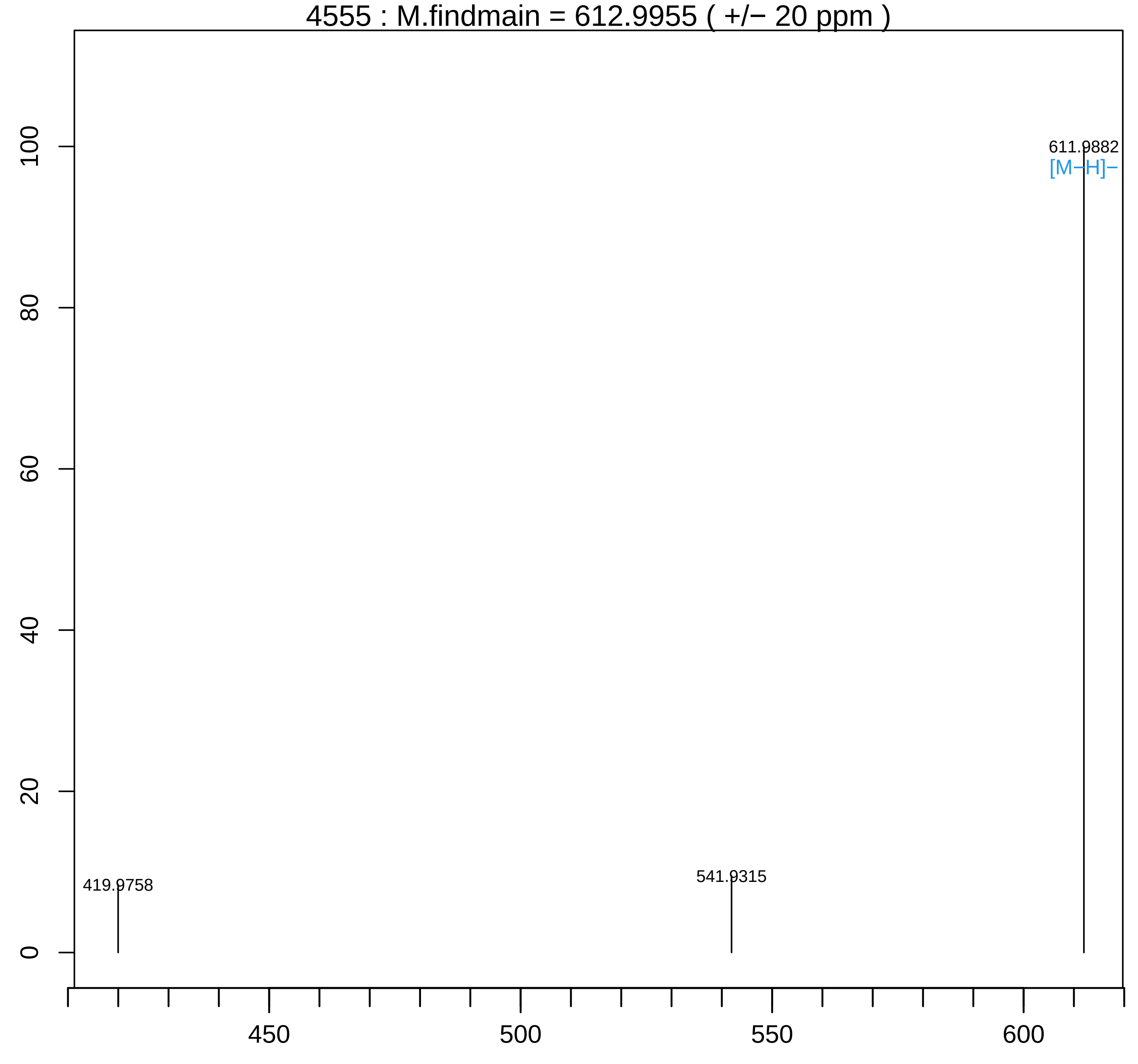
4555 : M.findmain = 612.9955 (+/− 20 ppm)
611.9882
[M−H]−
541.9315
419.9758
0
20
40
60
80
100
450
500
550
600

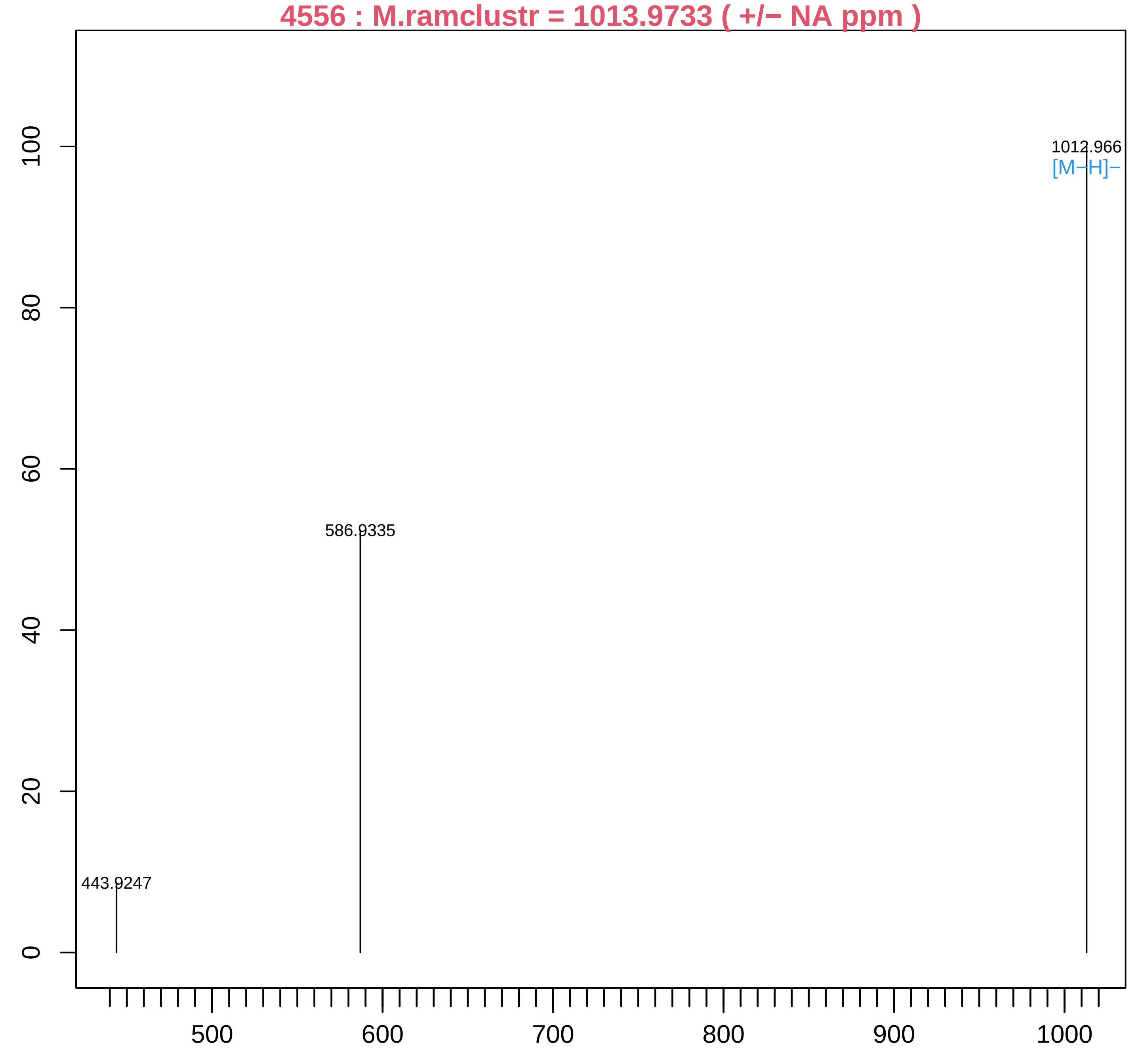
4556 : M.ramclustr = 1013.9733 (+/− NA ppm)
1012.966
[M−H]−
586.9335
443.9247
0
20
40
60
80
100
500
600
700
800
900
1000

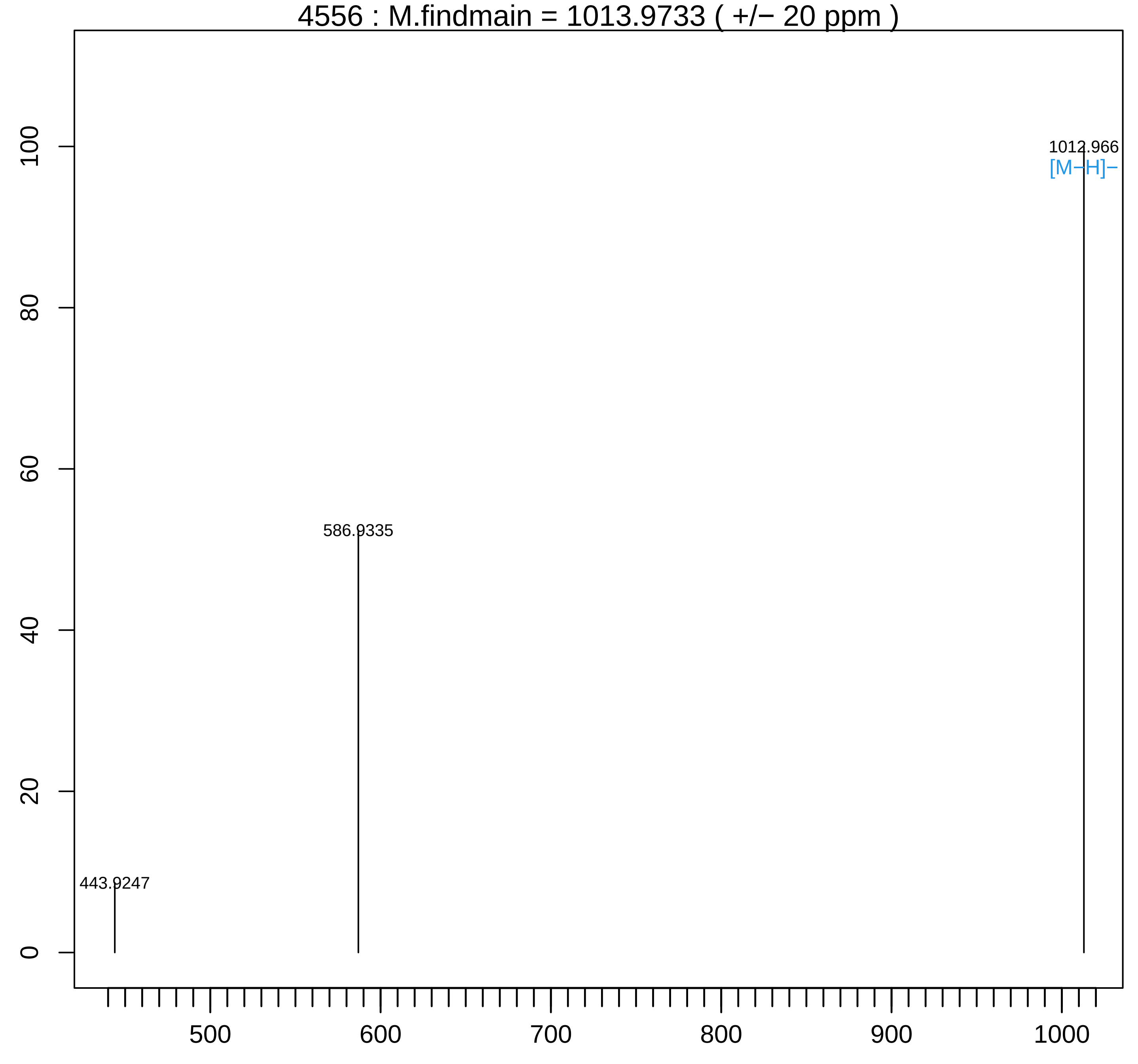
4556 : M.findmain = 1013.9733 (+/− 20 ppm)
1012.966
[M−H]−
586.9335
443.9247
0
20
40
60
80
100
500
600
700
800
900
1000

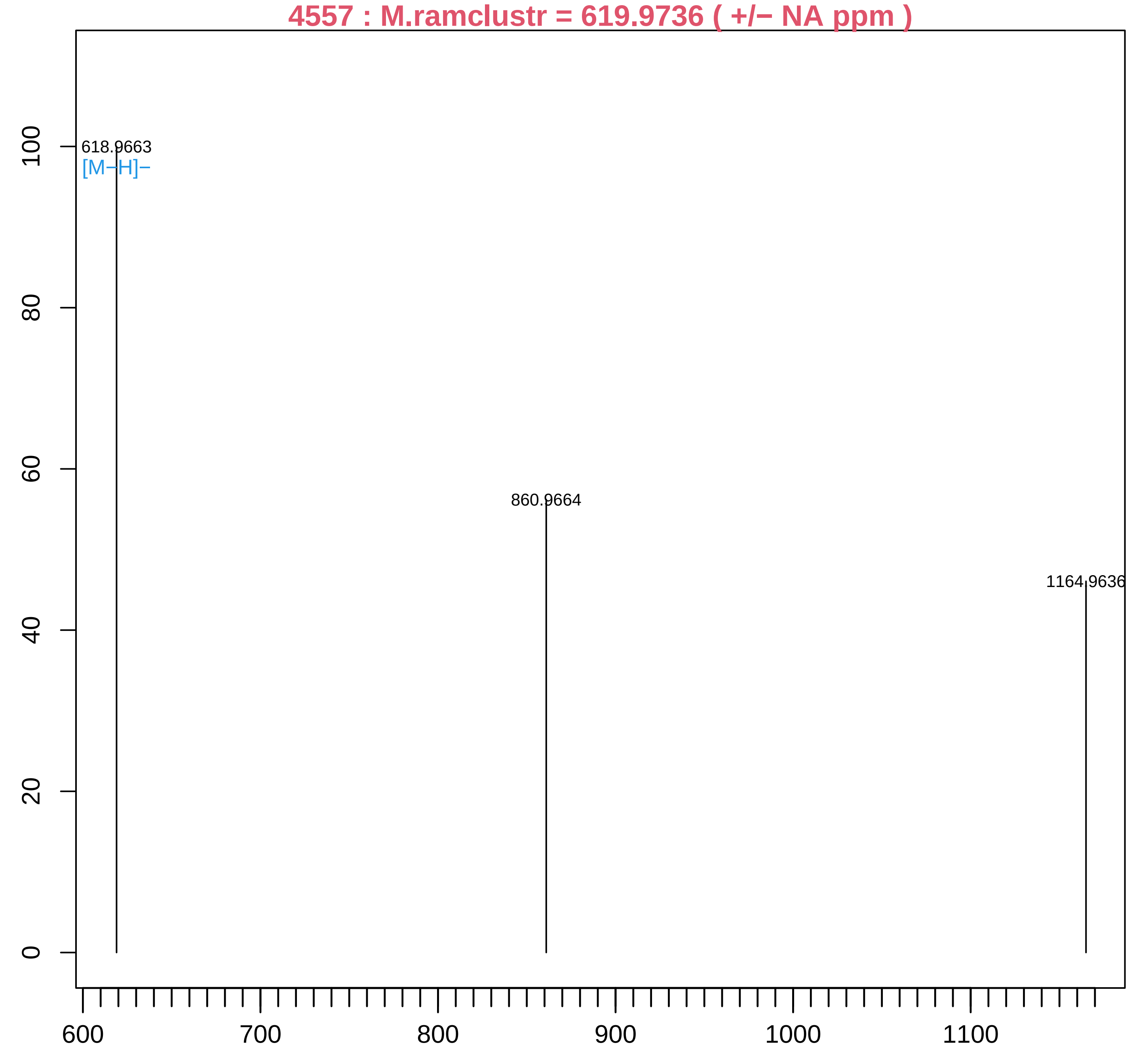

4557 : M.ramclustr = 619.9736 (+/− NA ppm)
618.9663
[M−H]−
860.9664
1164.9636
0
20
40
60
80
100
600
700
800
900
1000
1100

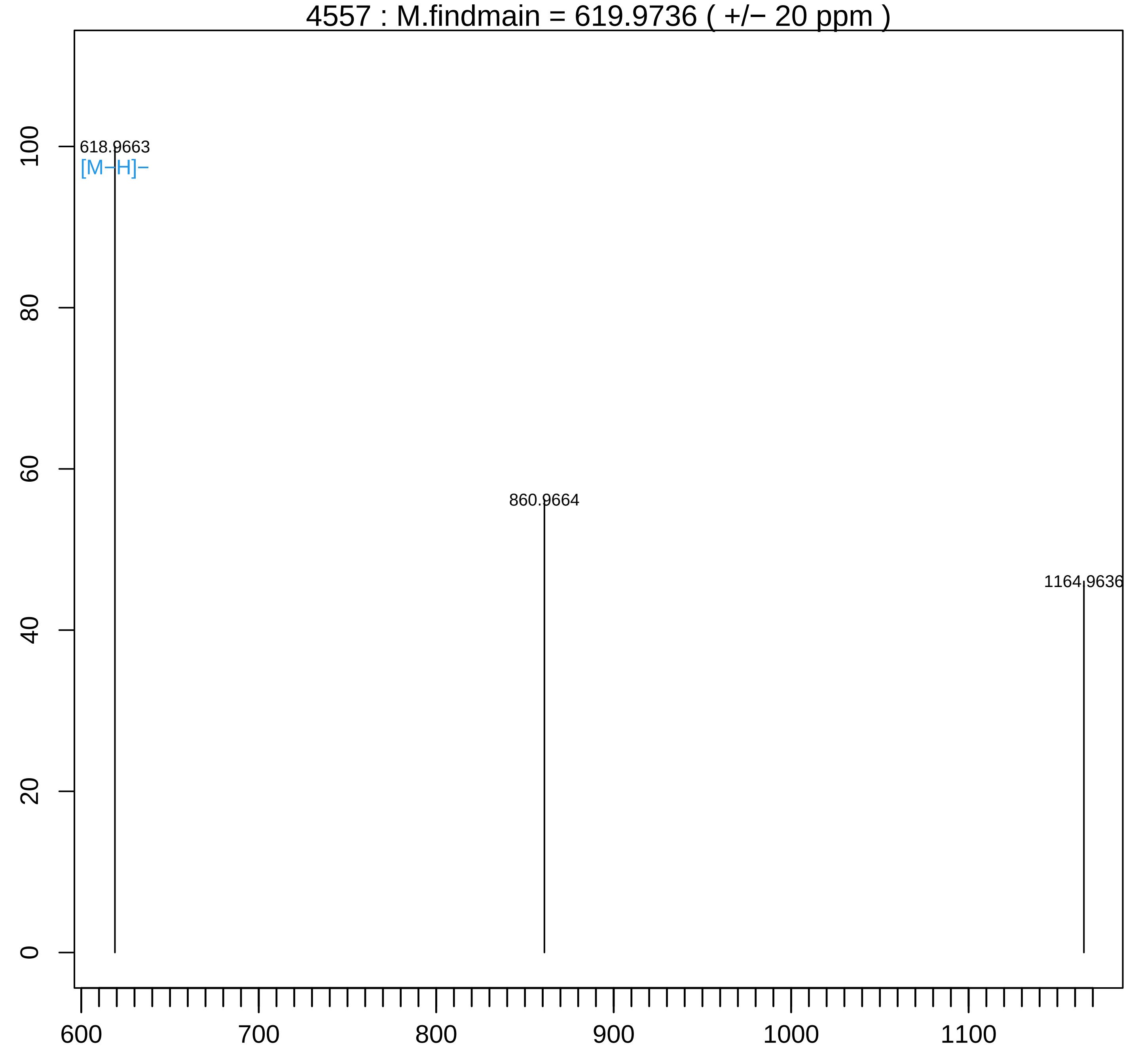

4557 : M.findmain = 619.9736 (+/− 20 ppm)
618.9663
[M−H]−
860.9664
1164.9636
0
20
40
60
80
100
600
700
800
900
1000
1100

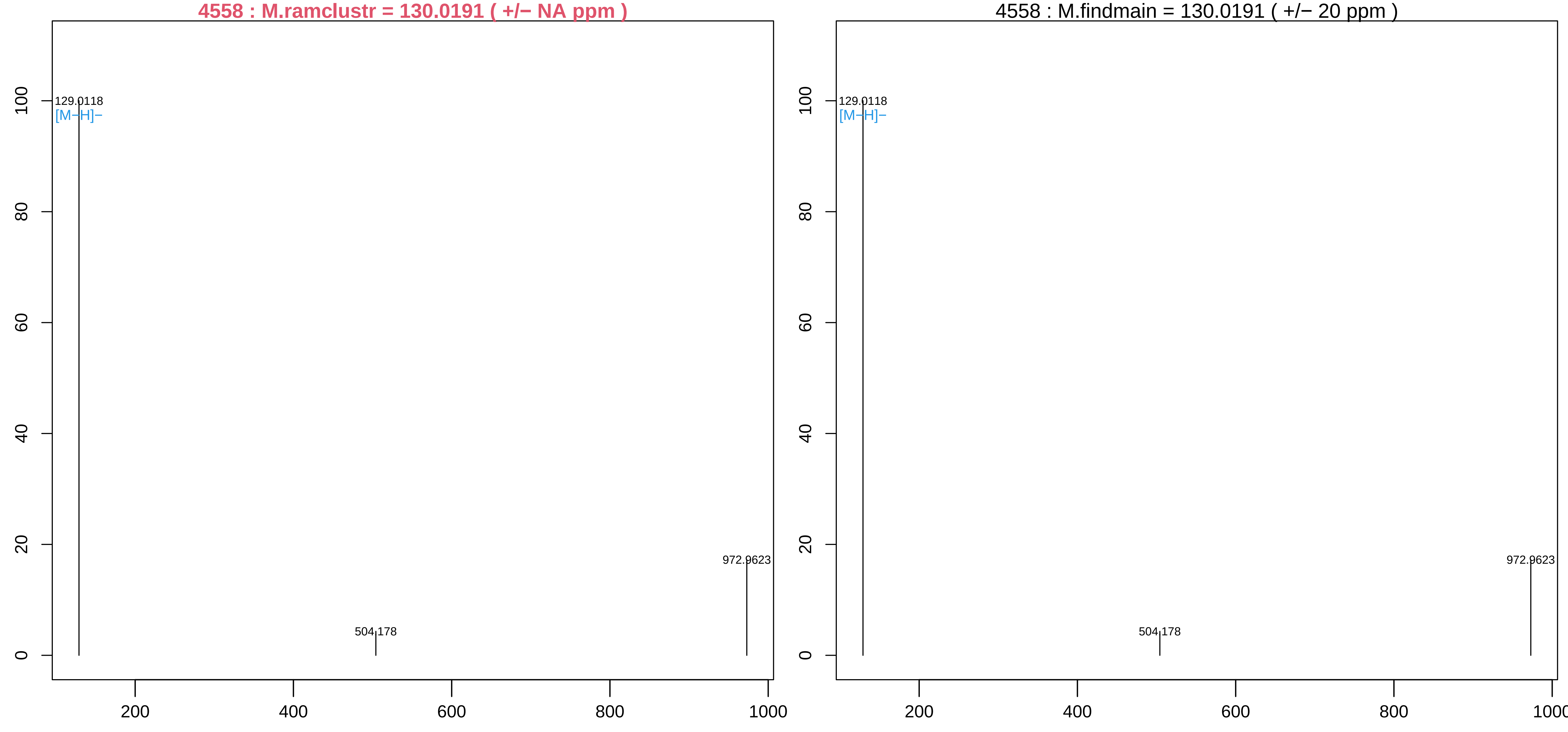

4558 : M.ramclustr = 130.0191 (+/− NA ppm)
129.0118
[M−H]−
504.178
972.9623
4558 : M.findmain = 130.0191 (+/− 20 ppm)
129.0118
[M−H]−
504.178
972.9623
0
20
40
60
80
100
200
400
600
800
1000

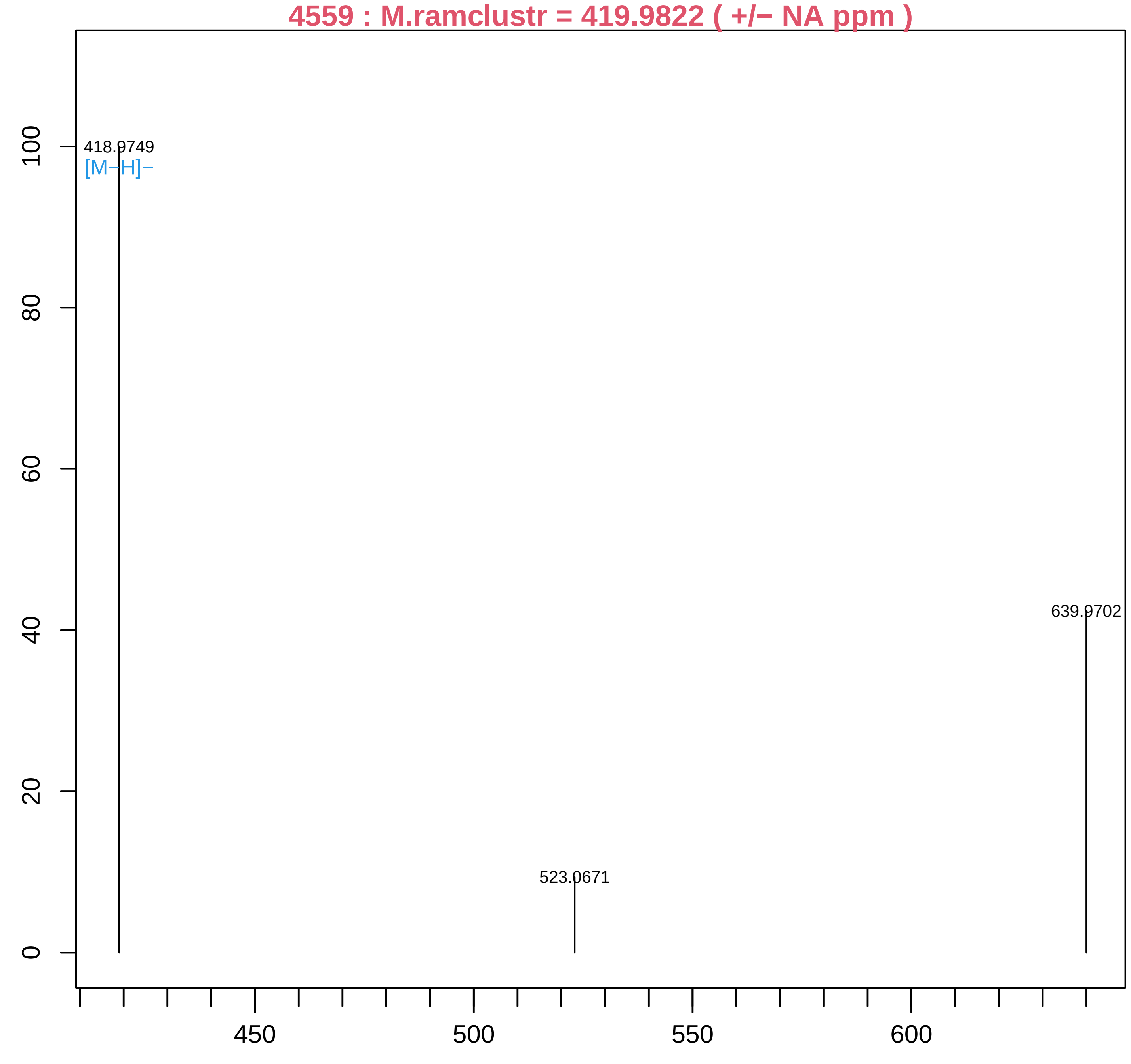
4559 : M.ramclustr = 419.9822 (+/− NA ppm)
418.9749
[M−H]−
523.0671
639.9702
0
20
40
60
80
100
450
500
550
600

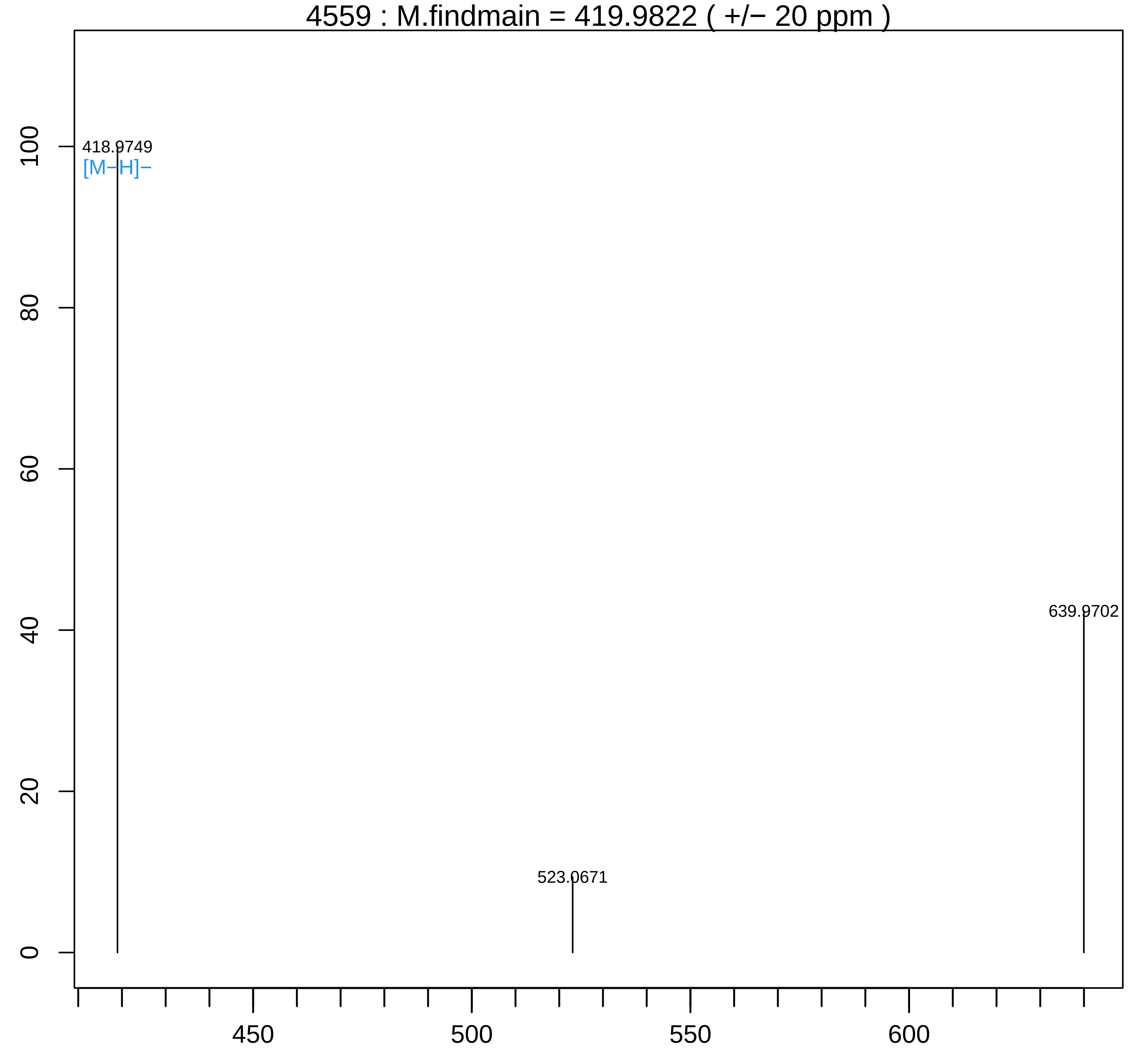
4559 : M.findmain = 419.9822 (+/− 20 ppm)
418.9749
[M−H]−
523.0671
639.9702
0
20
40
60
80
100
450
500
550
600

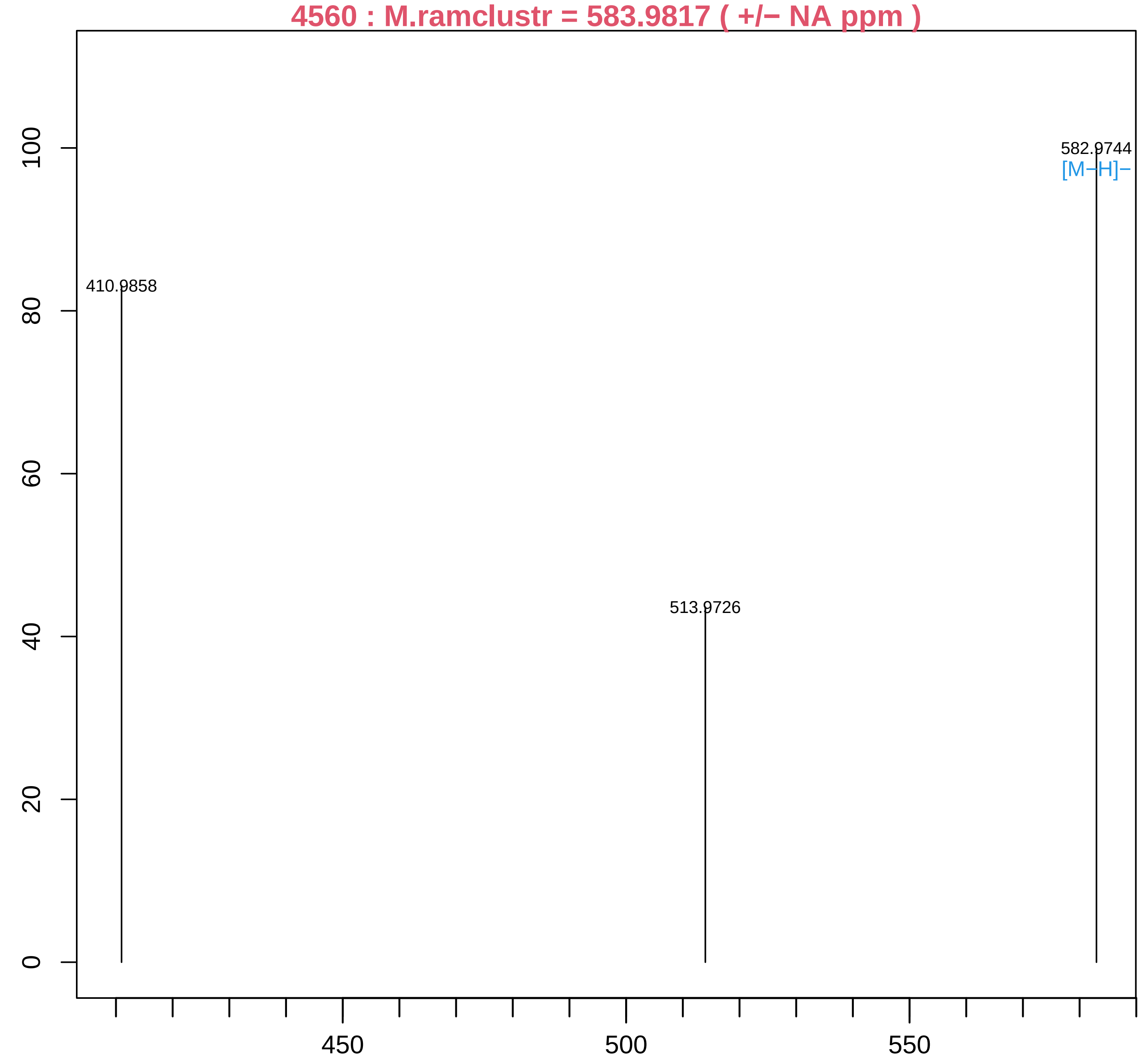
4560 : M.ramclustr = 583.9817 (+/− NA ppm)
582.9744
[M−H]−
410.9858
513.9726
0
20
40
60
80
100
450
500
550

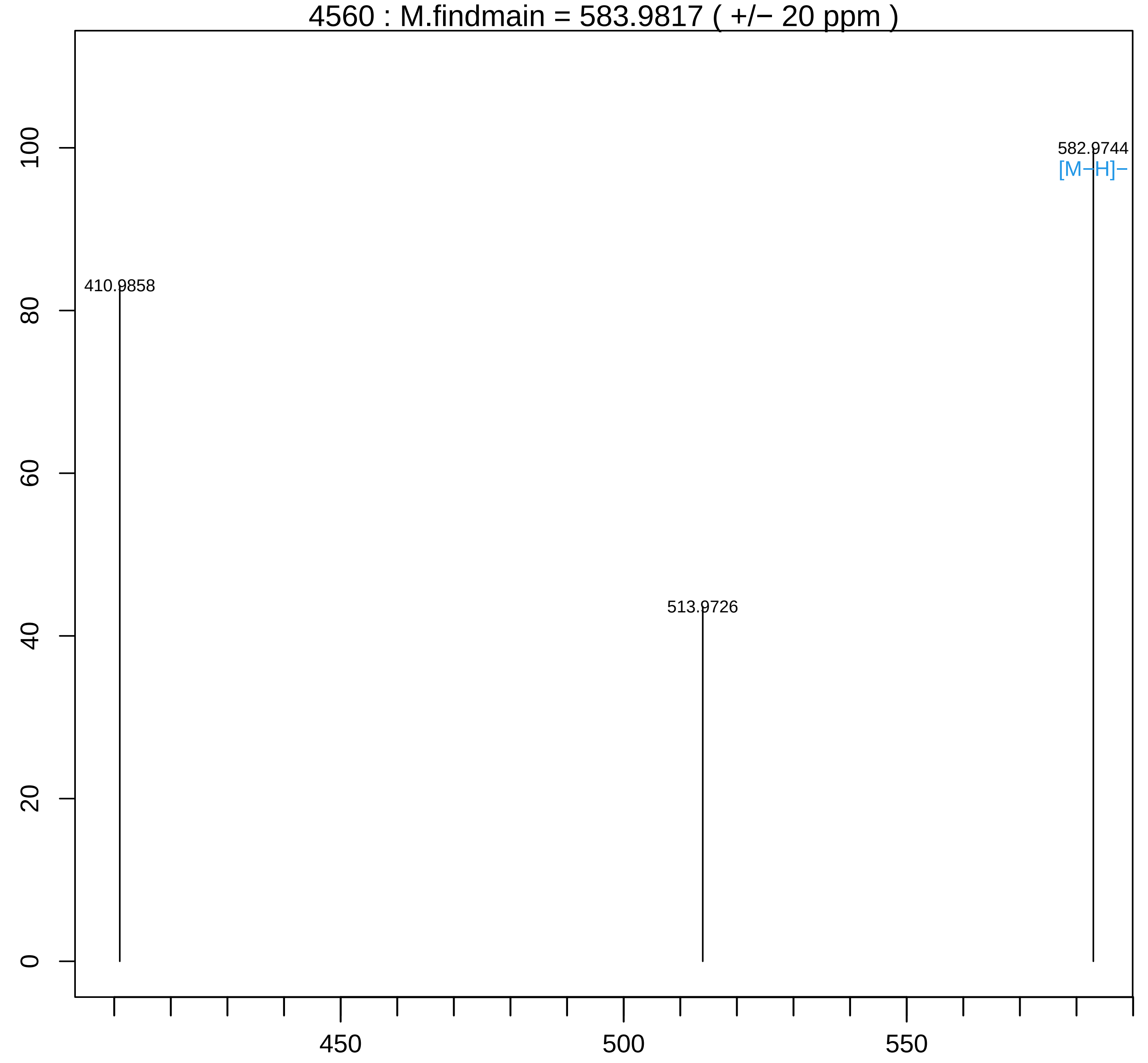
4560 : M.findmain = 583.9817 (+/− 20 ppm)
582.9744
[M−H]−
410.9858
513.9726
0
20
40
60
80
100
450
500
550

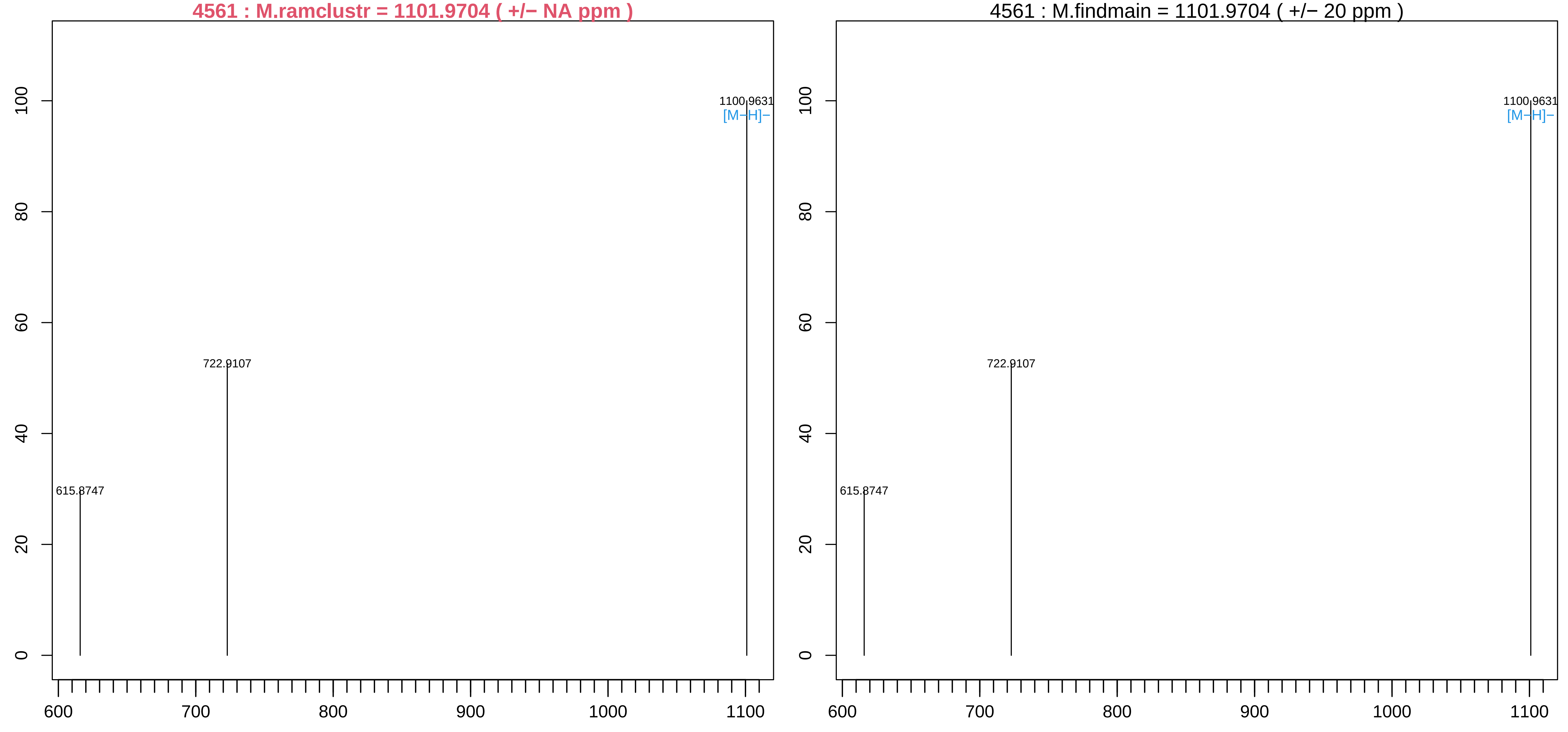
4561 : M.ramclustr = 1101.9704 (+/− NA ppm)
4561 : M.findmain = 1101.9704 (+/− 20 ppm)
1100.9631
[M−H]−
722.9107
615.8747
0
20
40
60
80
100
600
700
800
900
1000
1100

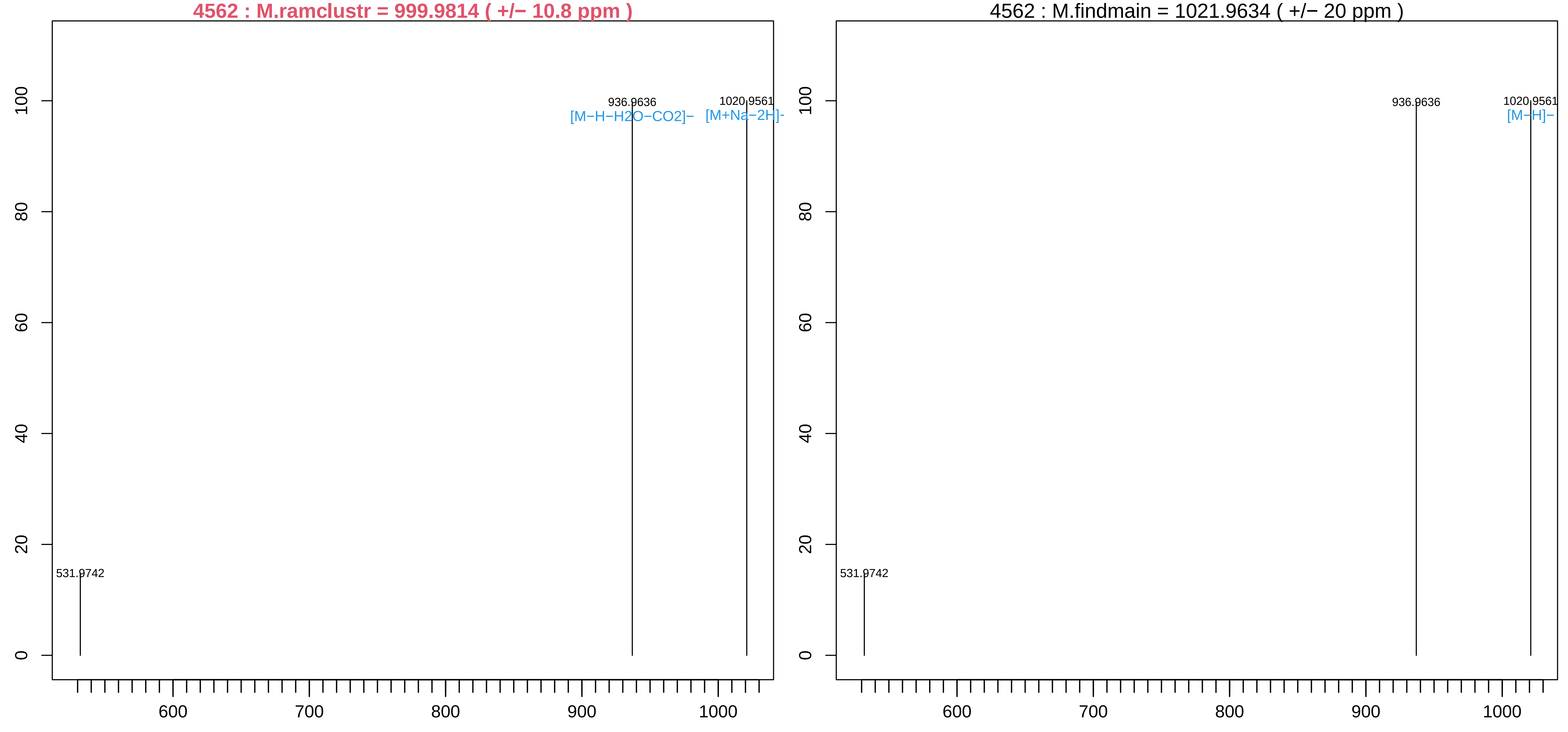

4562 : M.ramclustr = 999.9814 (+/− 10.8 ppm)
936.9636
[M−H−H2O−CO2]−
1020.9561
[M+Na−2H]-
531.9742
4562 : M.findmain = 1021.9634 (+/− 20 ppm)
936.9636
1020.9561
[M−H]−
531.9742
0
20
40
60
80
100
600
700
800
900
1000

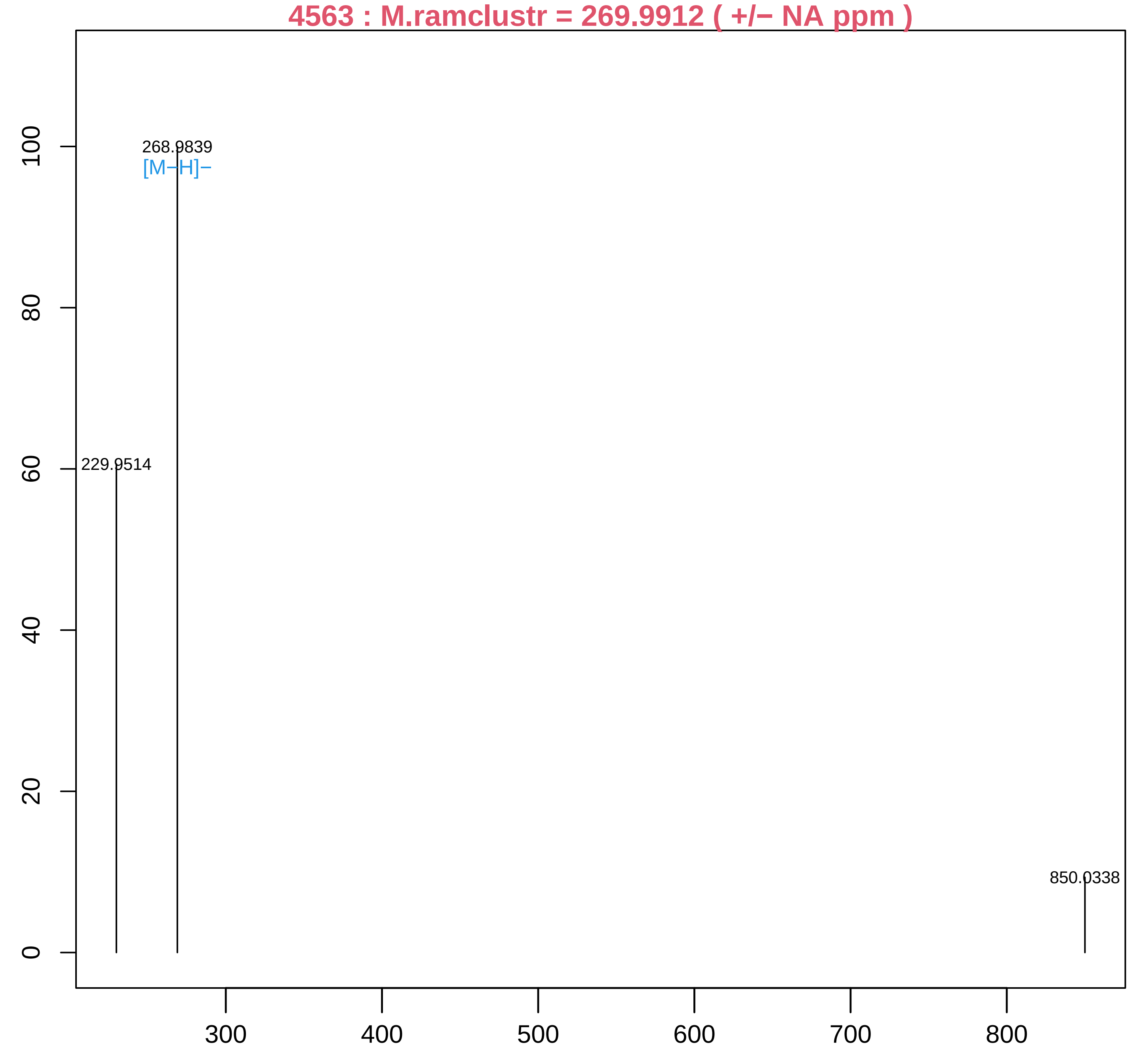
4563 : M.ramclustr = 269.9912 (+/− NA ppm)
268.9839
[M−H]−
229.9514
850.0338
0
20
40
60
80
100
300
400
500
600
700
800

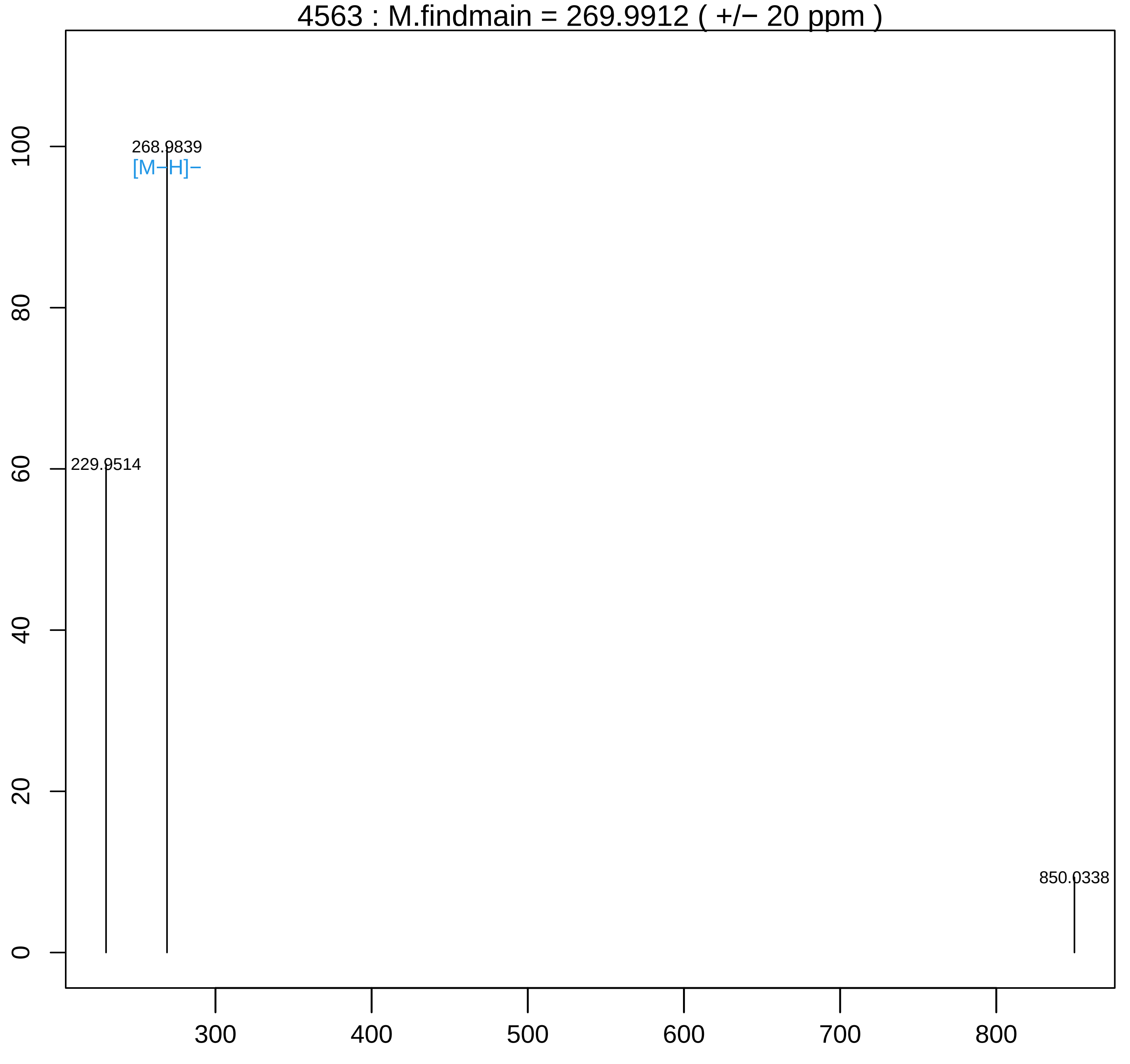
4563 : M.findmain = 269.9912 (+/− 20 ppm)
268.9839
[M−H]−
229.9514
850.0338
0
20
40
60
80
100
300
400
500
600
700
800

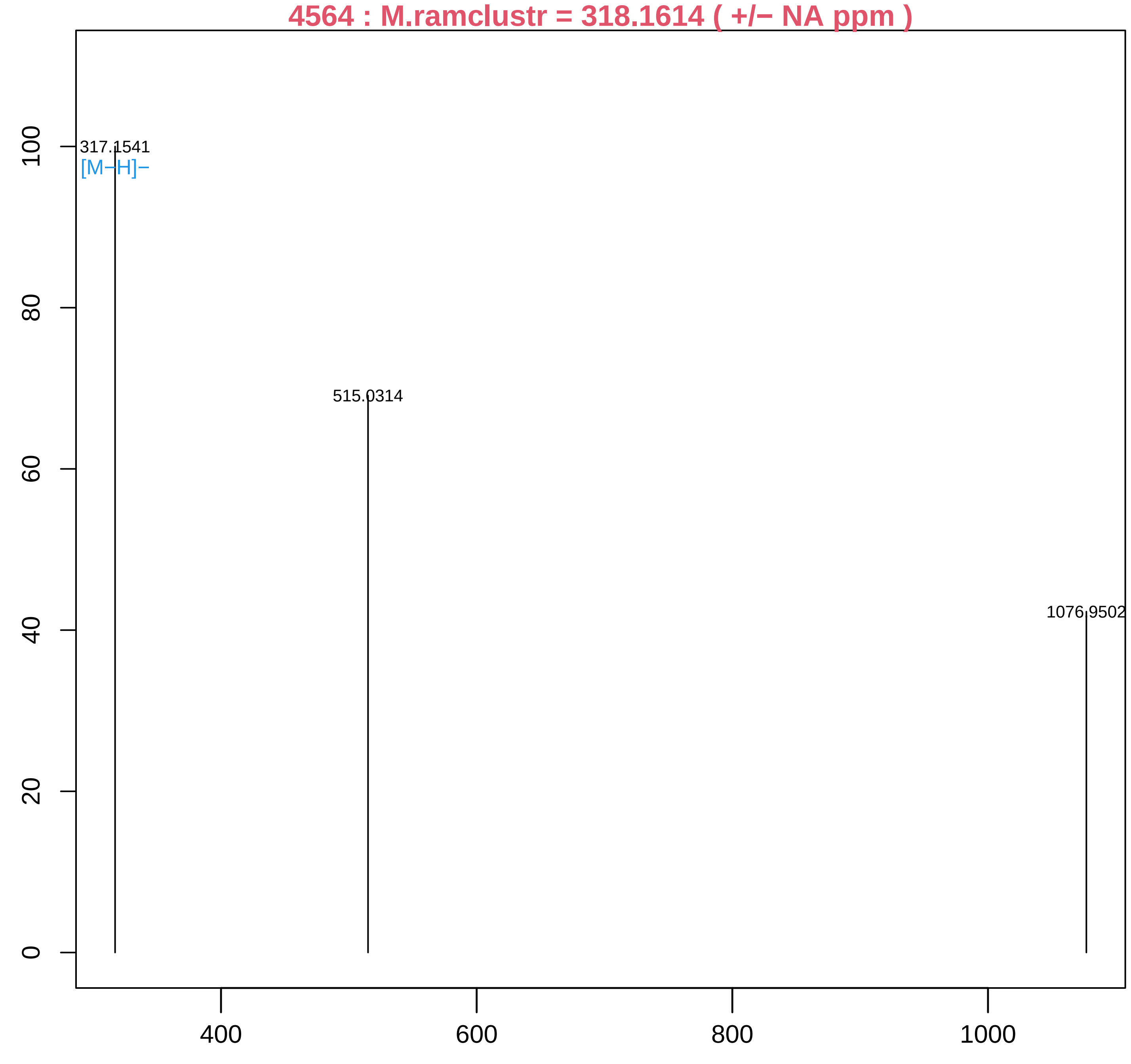
4564 : M.ramclustr = 318.1614 (+/− NA ppm)
317.1541
[M−H]−
515.0314
1076.9502
0
20
40
60
80
100
400
600
800
1000

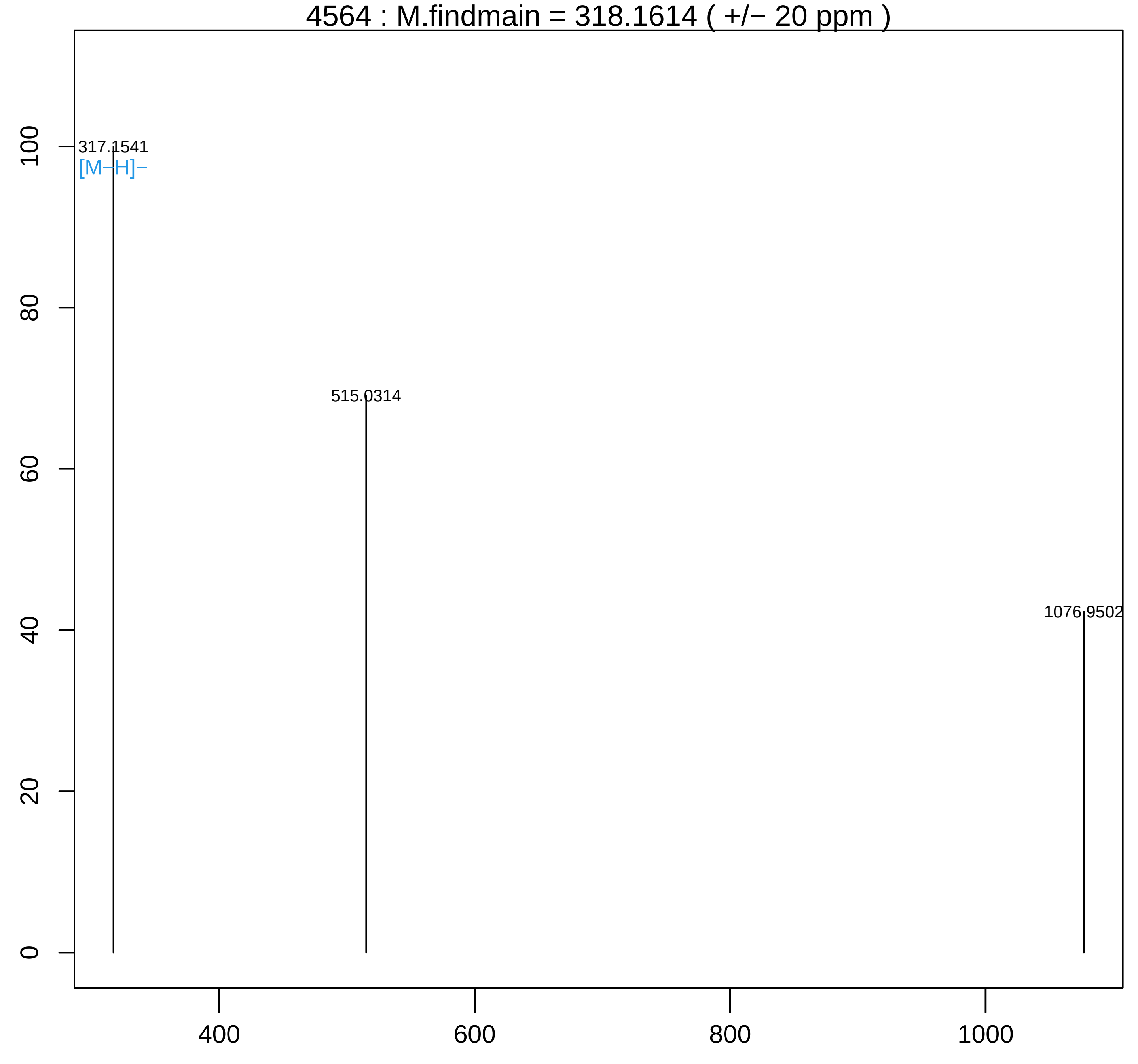
4564 : M.findmain = 318.1614 (+/− 20 ppm)
317.1541
[M−H]−
515.0314
1076.9502
0
20
40
60
80
100
400
600
800
1000

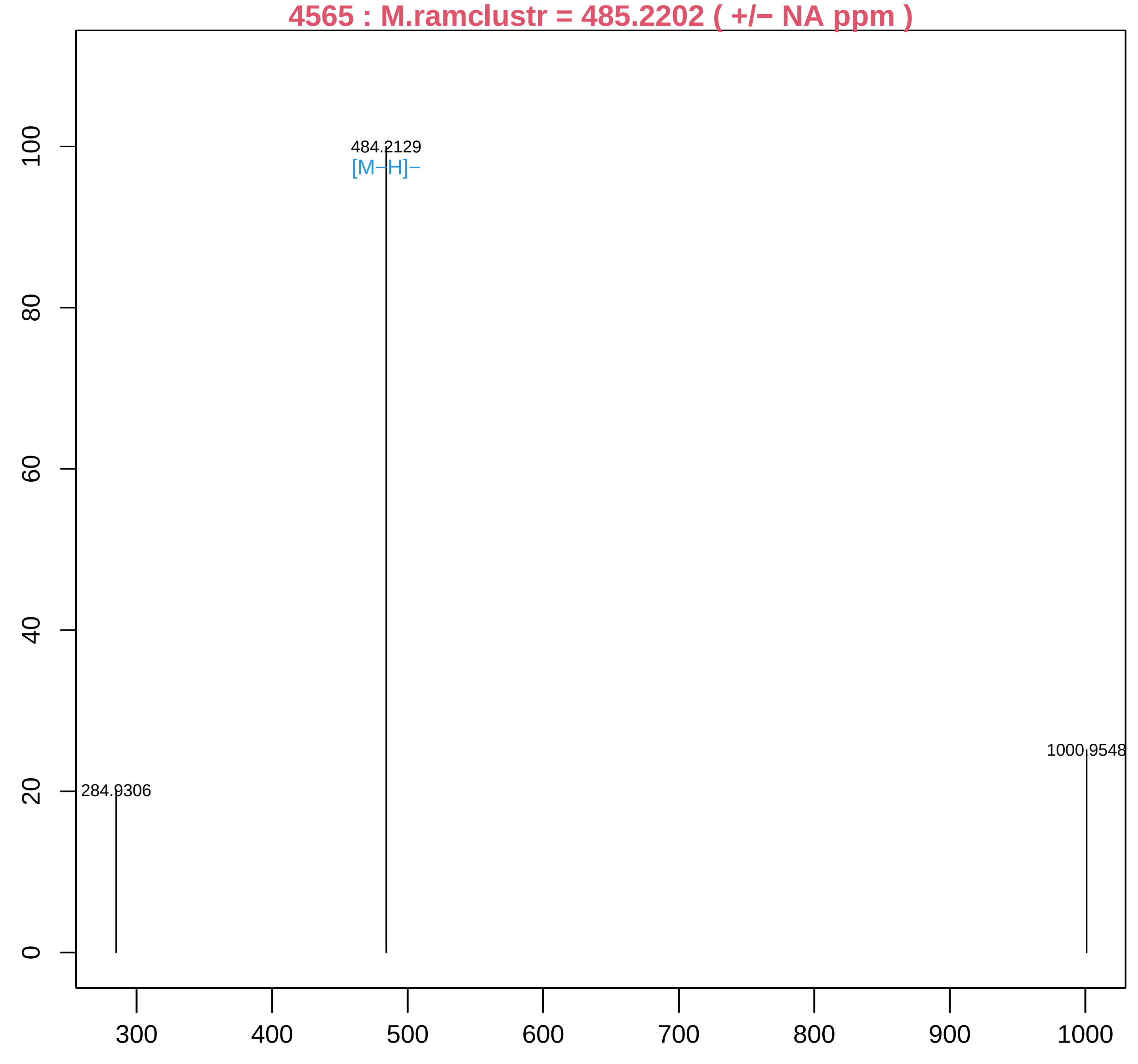

4565 : M.ramclustr = 485.2202 (+/− NA ppm)
484.2129
[M−H]−
284.9306
1000.9548
0
20
40
60
80
100
300
400
500
600
700
800
900
1000

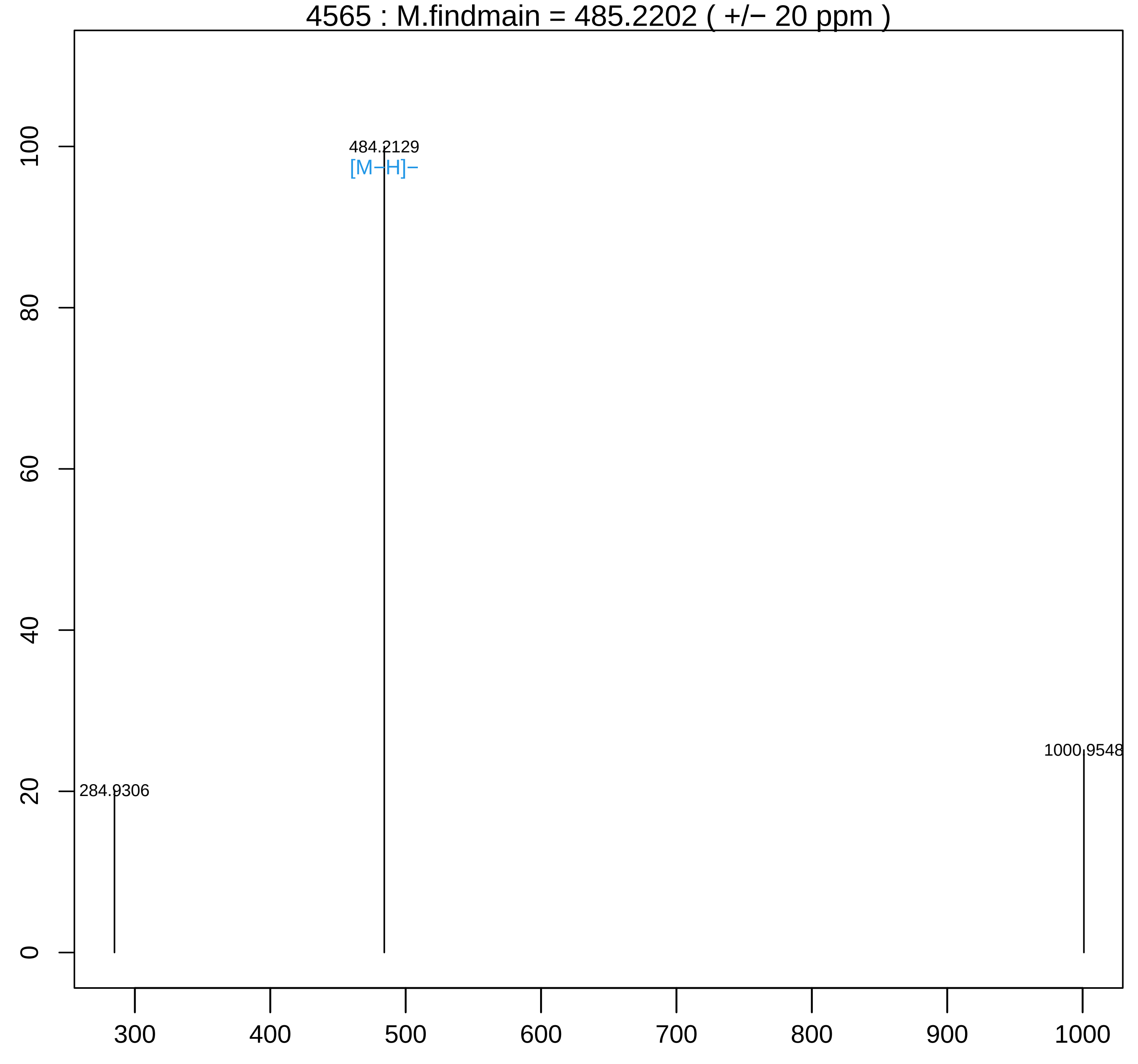

4565 : M.findmain = 485.2202 (+/− 20 ppm)
484.2129
[M−H]−
284.9306
1000.9548
0
20
40
60
80
100
300
400
500
600
700
800
900
1000

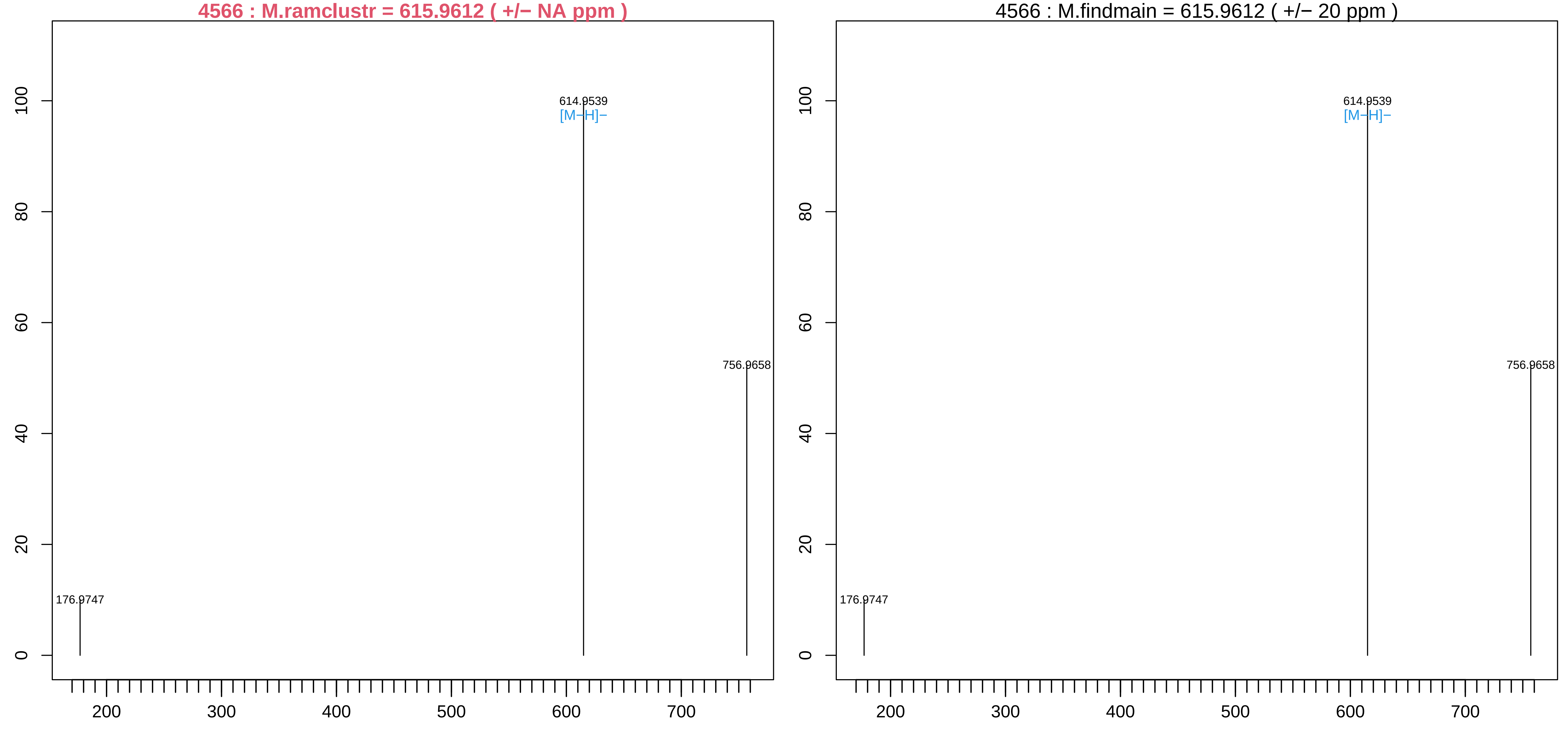

4566 : M.ramclustr = 615.9612 (+/− NA ppm)
4566 : M.findmain = 615.9612 (+/− 20 ppm)
614.9539
[M−H]−
756.9658
176.9747
614.9539
[M−H]−
756.9658
176.9747
0
20
40
60
80
100
200
300
400
500
600
700

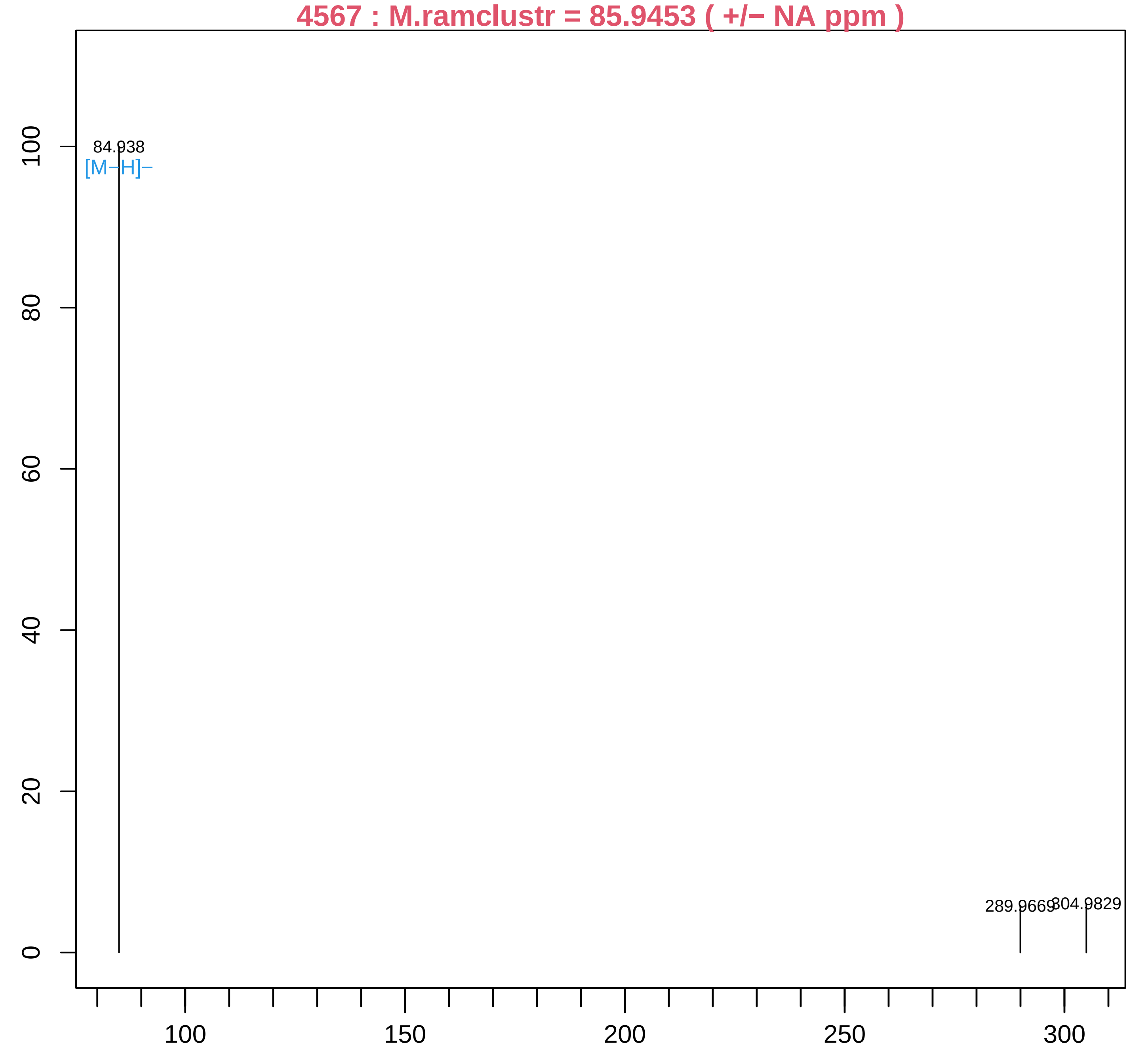
4567 : M.ramclustr = 85.9453 (+/− NA ppm)
84.938
[M−H]−
289.9669
304.9829
0
20
40
60
80
100
100
150
200
250
300

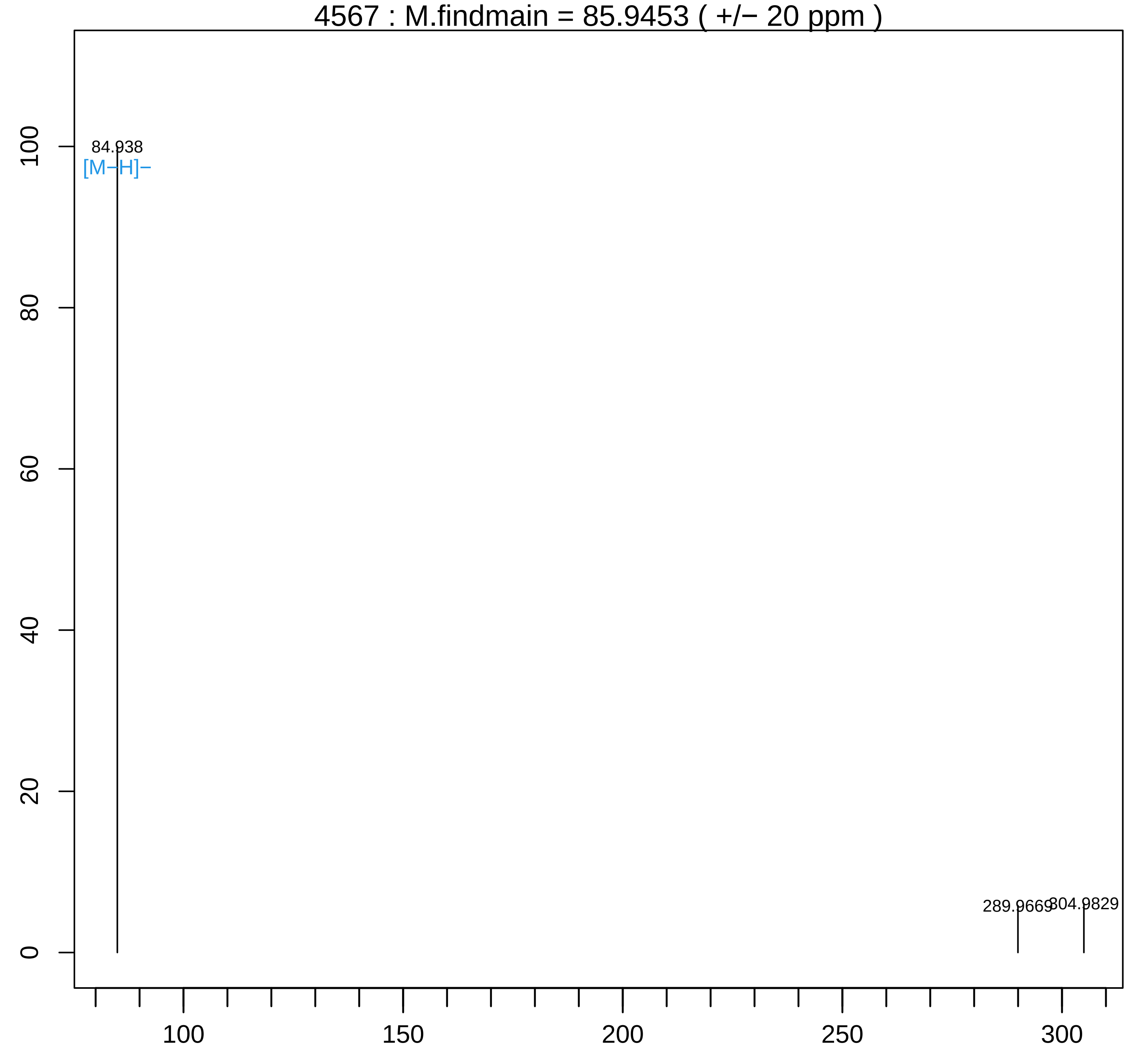
4567 : M.findmain = 85.9453 (+/− 20 ppm)
84.938
[M−H]−
289.9669
304.9829
0
20
40
60
80
100
100
150
200
250
300

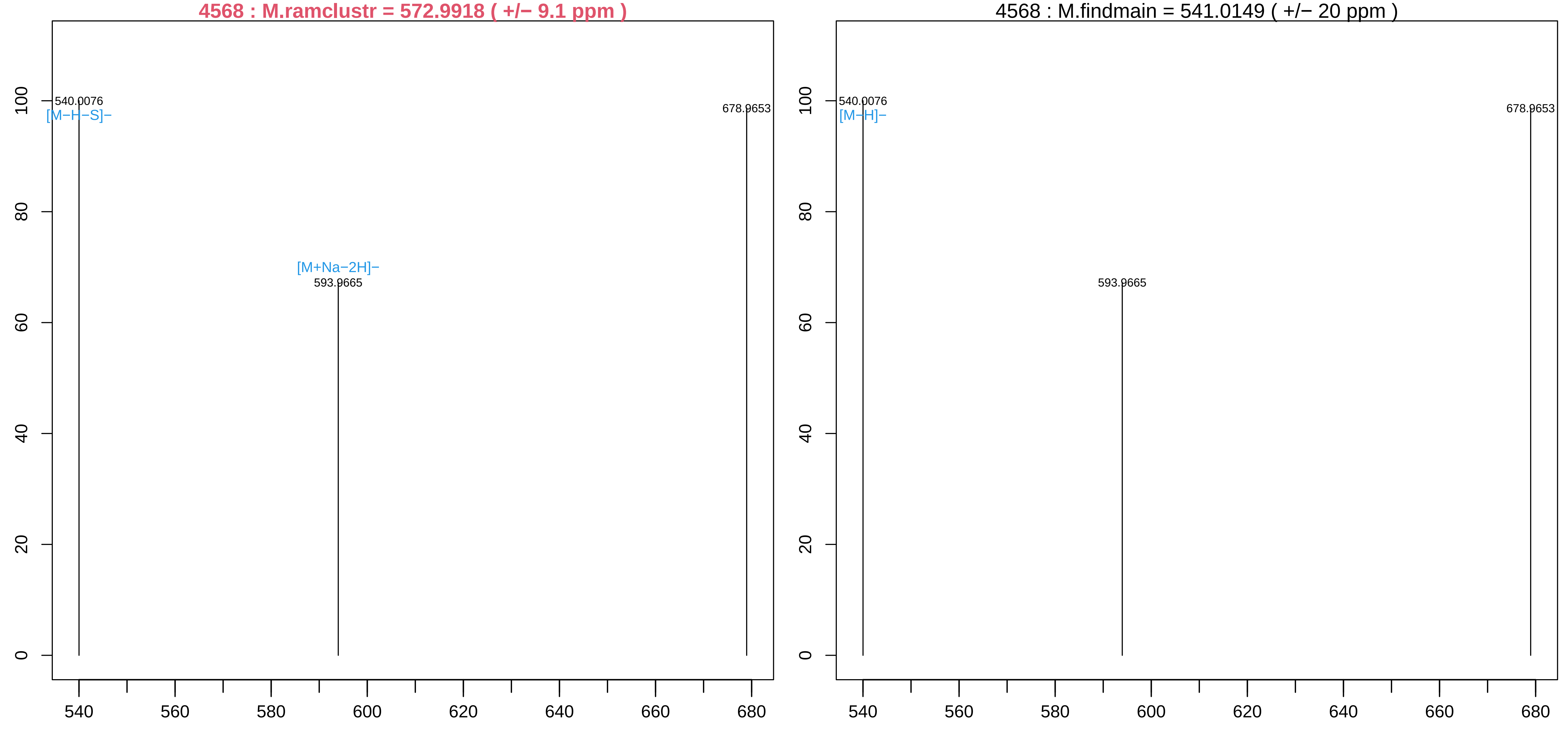
4568 : M.ramclustr = 572.9918 (+/− 9.1 ppm)
540.0076
[M−H−S]−
[M+Na−2H]−
593.9665
678.9653
4568 : M.findmain = 541.0149 (+/− 20 ppm)
540.0076
[M−H]−
593.9665
678.9653
0
20
40
60
80
100
540
560
580
600
620
640
660
680

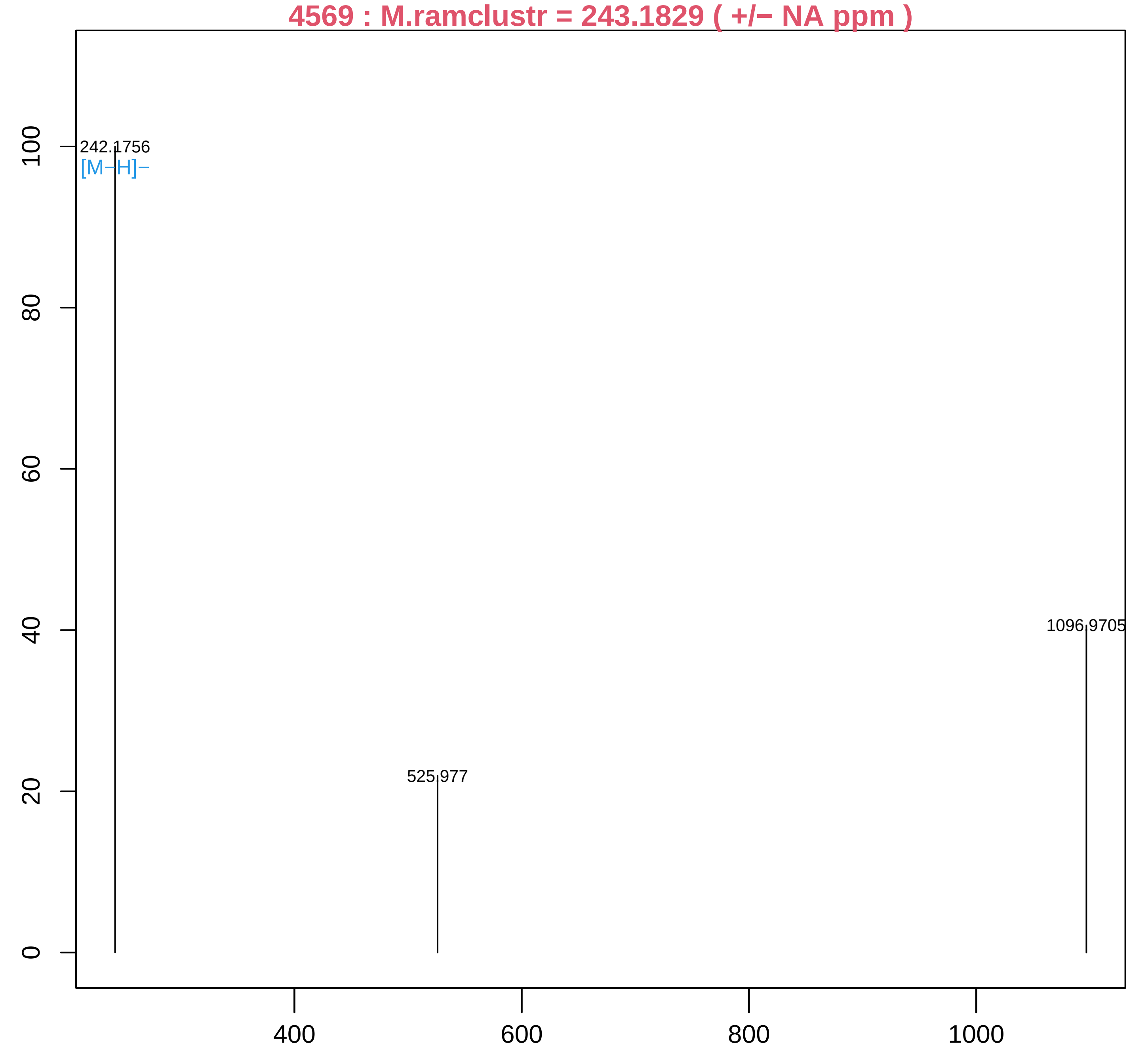
4569 : M.ramclustr = 243.1829 (+/− NA ppm)
242.1756
[M−H]−
525.977
1096.9705
0
20
40
60
80
100
400
600
800
1000

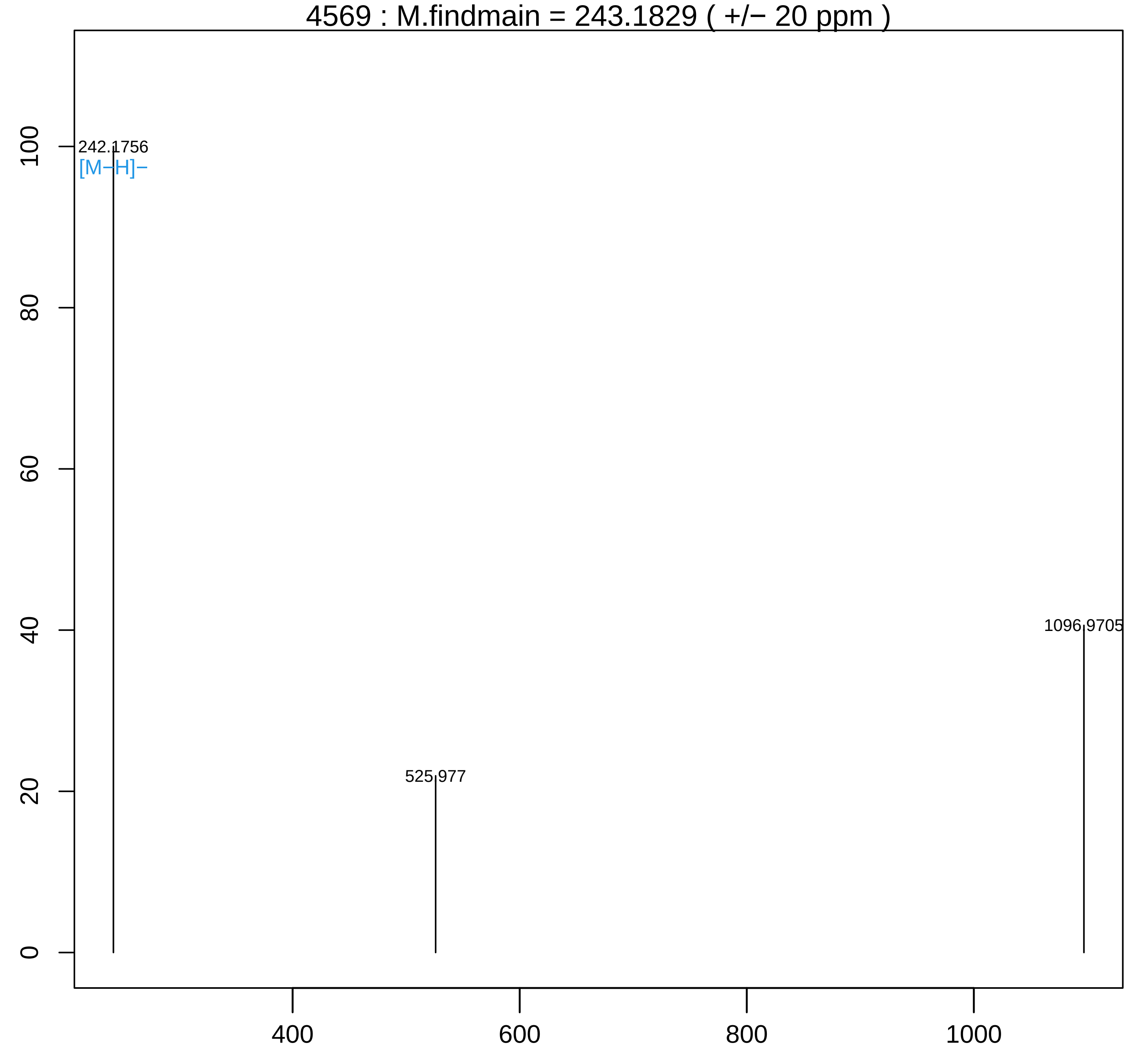
4569 : M.findmain = 243.1829 (+/− 20 ppm)
242.1756
[M−H]−
525.977
1096.9705
0
20
40
60
80
100
400
600
800
1000

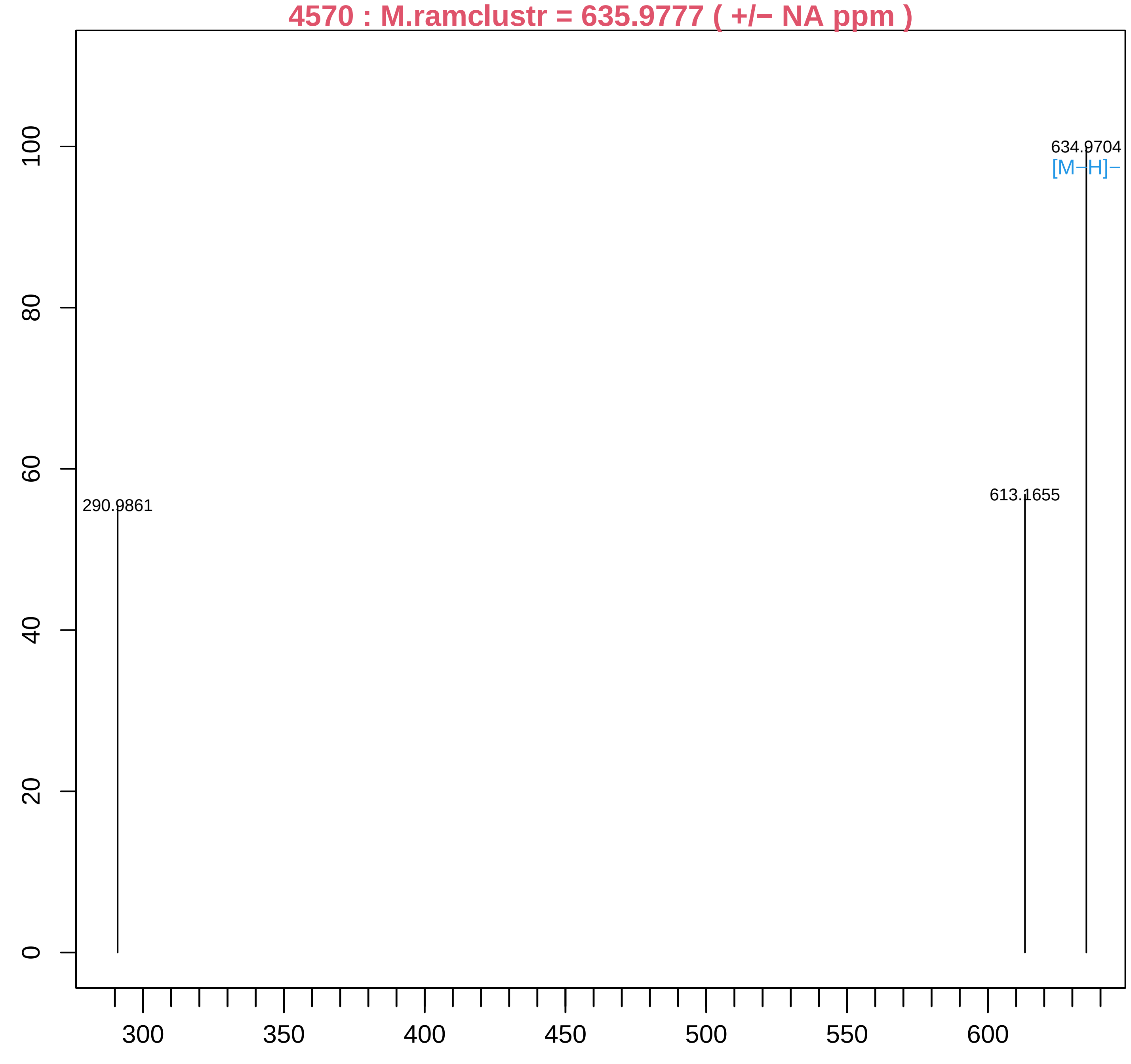
4570 : M.ramclustr = 635.9777 (+/− NA ppm)
634.9704
[M−H]−
613.1655
290.9861
0
20
40
60
80
100
300
350
400
450
500
550
600

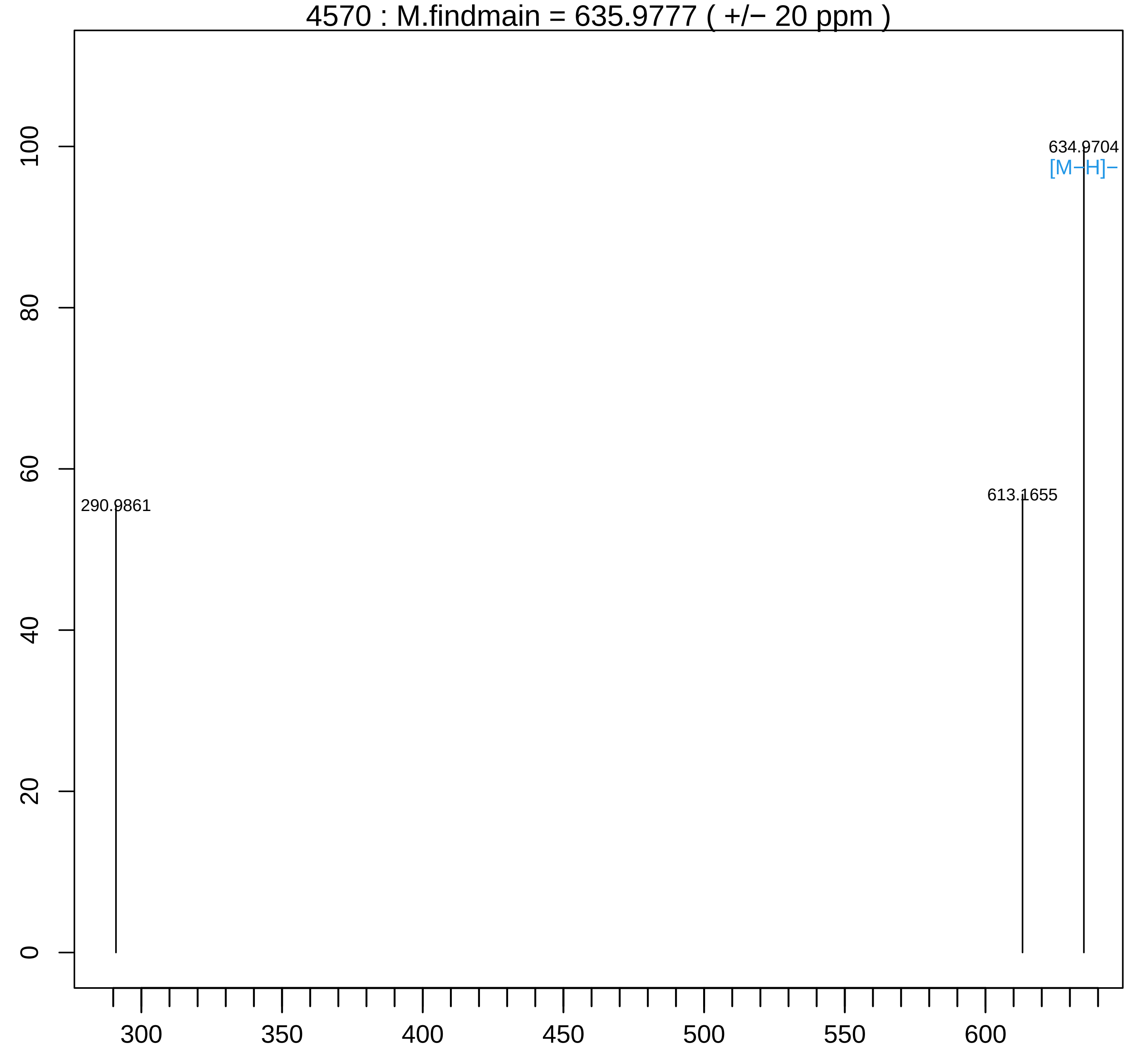
4570 : M.findmain = 635.9777 (+/− 20 ppm)
634.9704
[M−H]−
613.1655
290.9861
0
20
40
60
80
100
300
350
400
450
500
550
600

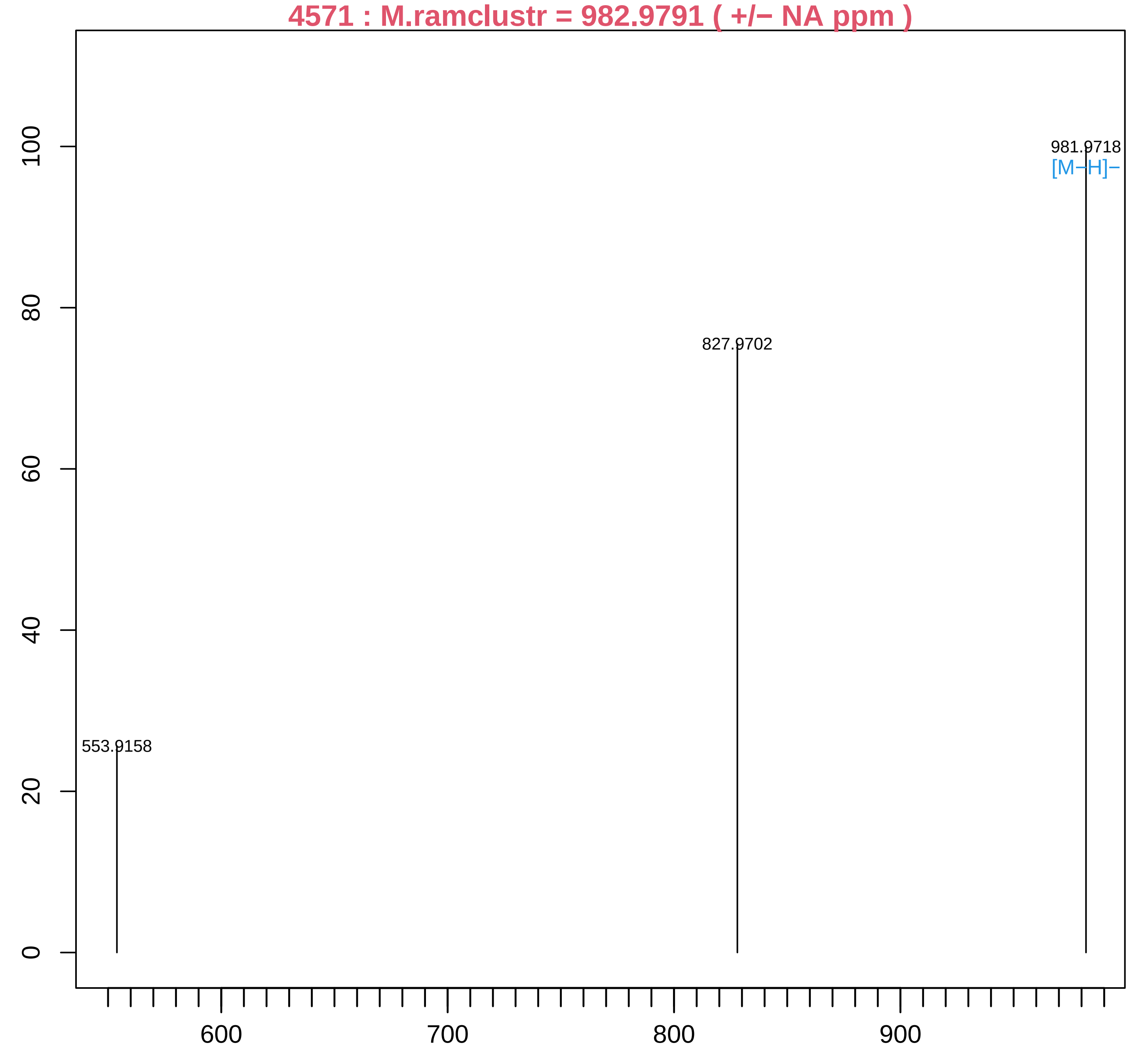
4571 : M.ramclustr = 982.9791 (+/− NA ppm)
981.9718
[M−H]−
827.9702
553.9158
0
20
40
60
80
100
600
700
800
900

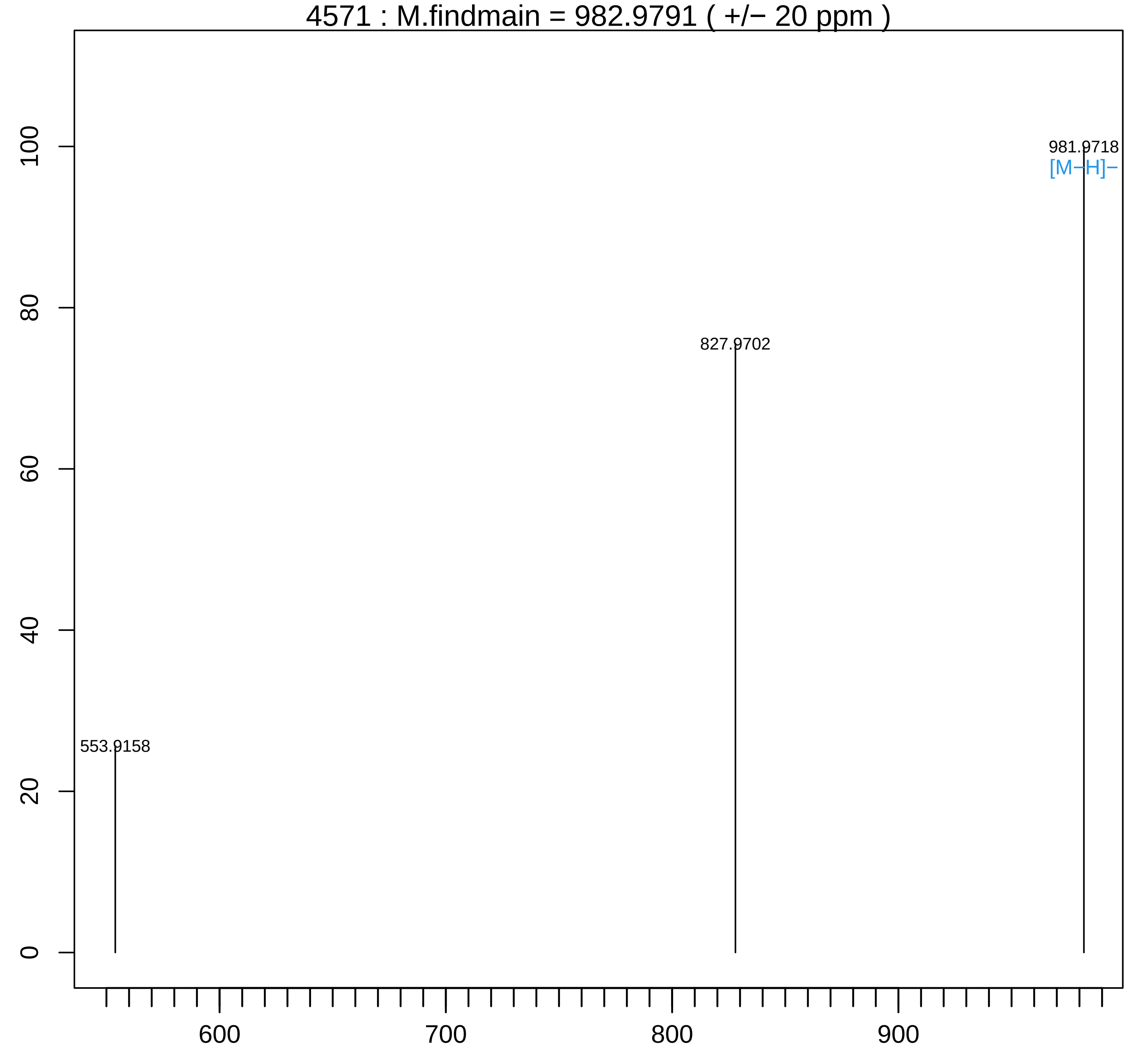
4571 : M.findmain = 982.9791 (+/− 20 ppm)
981.9718
[M−H]−
827.9702
553.9158
0
20
40
60
80
100
600
700
800
900

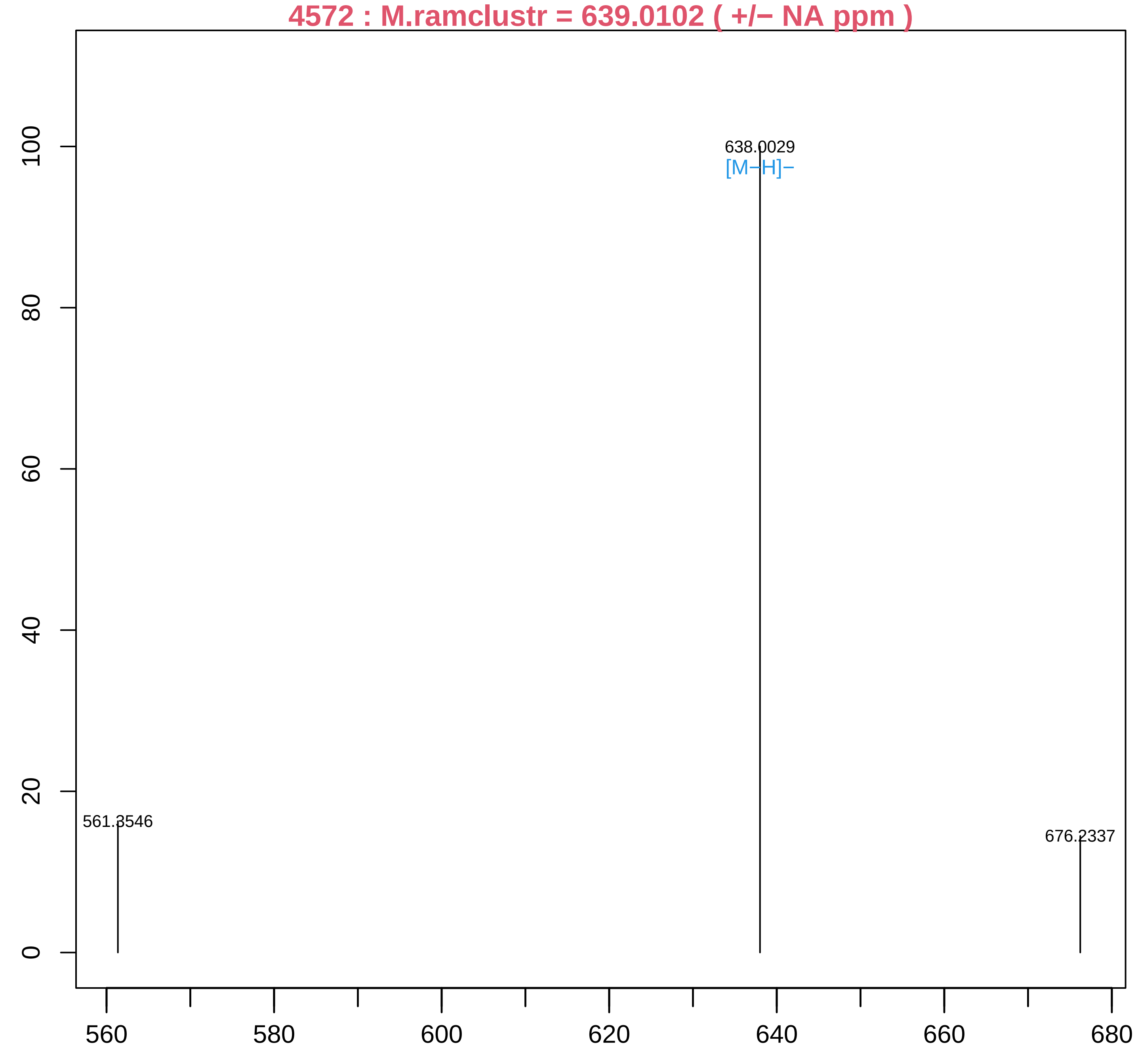
4572 : M.ramclustr = 639.0102 (+/− NA ppm)
638.0029
[M−H]−
561.3546
676.2337
0
20
40
60
80
100
560
580
600
620
640
660
680

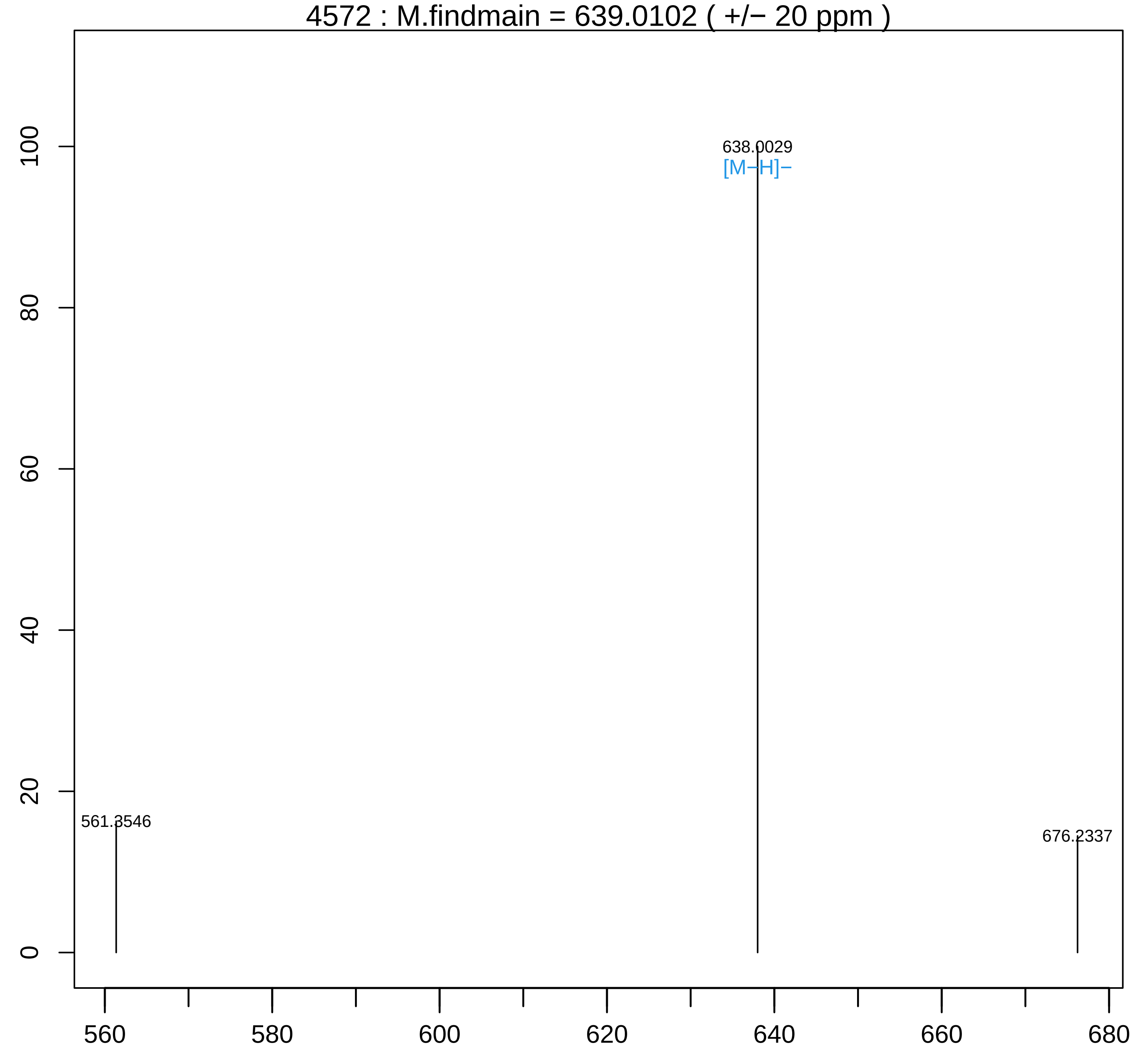
4572 : M.findmain = 639.0102 (+/− 20 ppm)
638.0029
[M−H]−
561.3546
676.2337
0
20
40
60
80
100
560
580
600
620
640
660
680

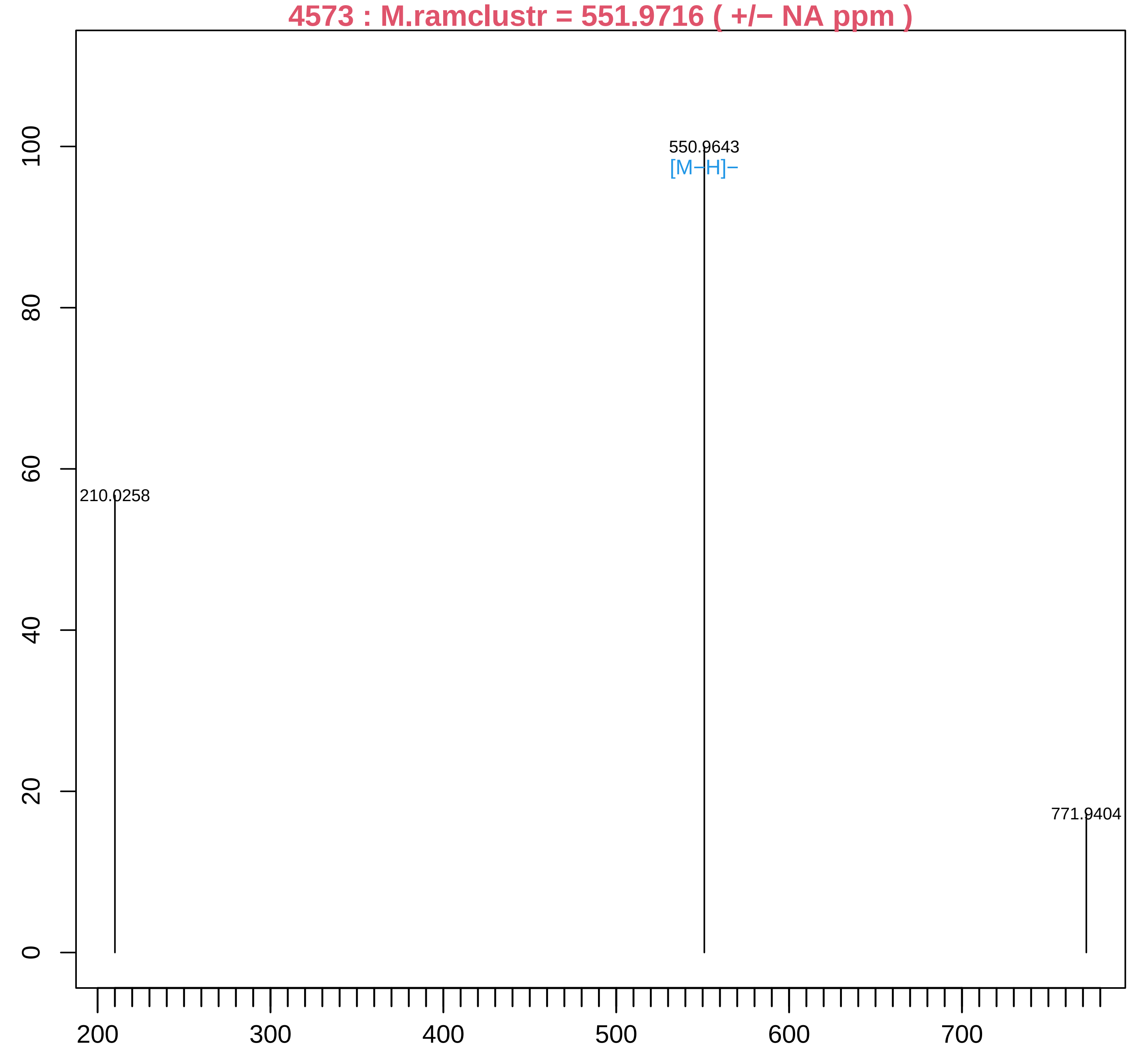
4573 : M.ramclustr = 551.9716 (+/− NA ppm)
550.9643
[M−H]−
210.0258
771.9404
0
20
40
60
80
100
200
300
400
500
600
700

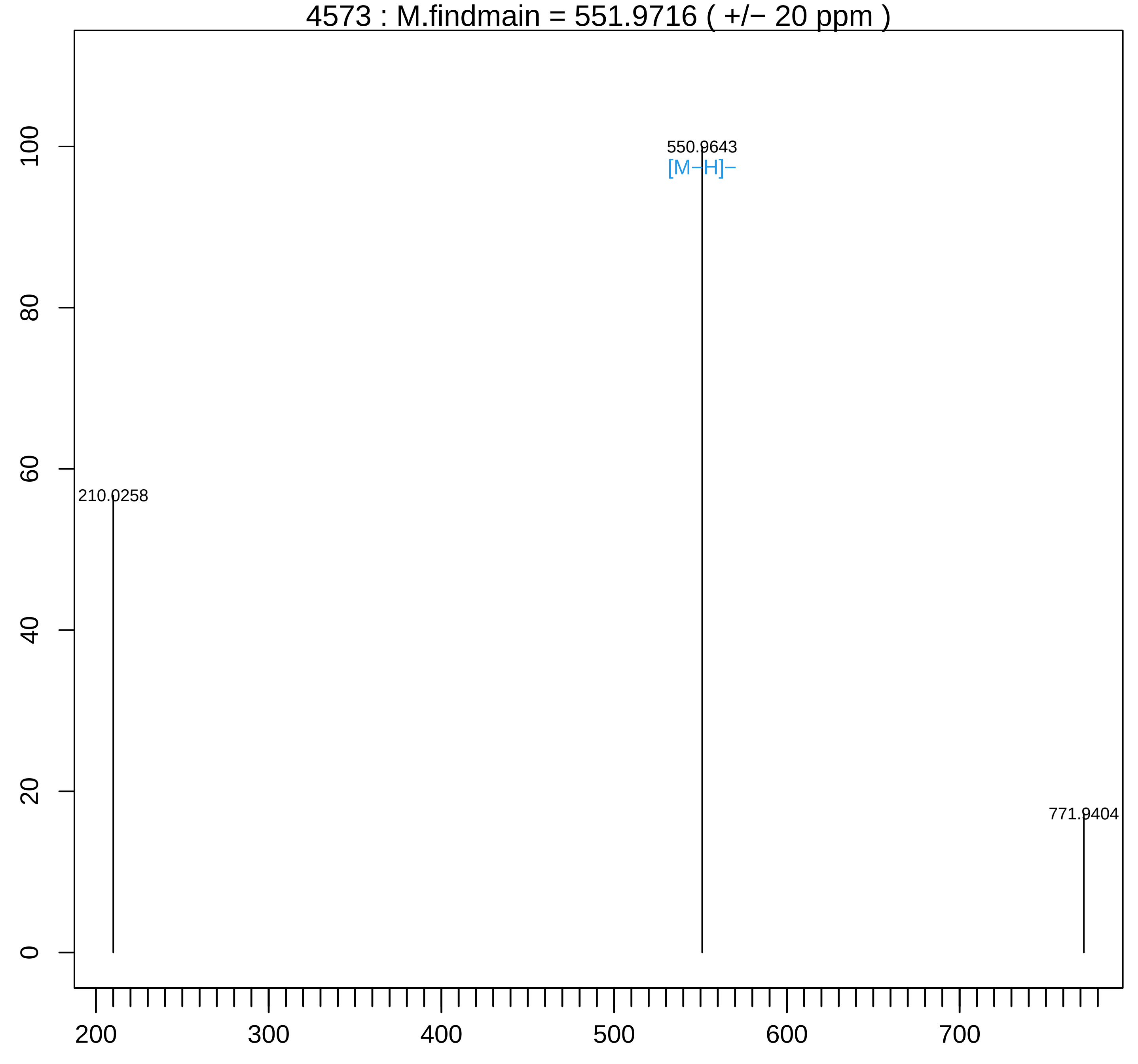
4573 : M.findmain = 551.9716 (+/− 20 ppm)
550.9643
[M−H]−
210.0258
771.9404
0
20
40
60
80
100
200
300
400
500
600
700

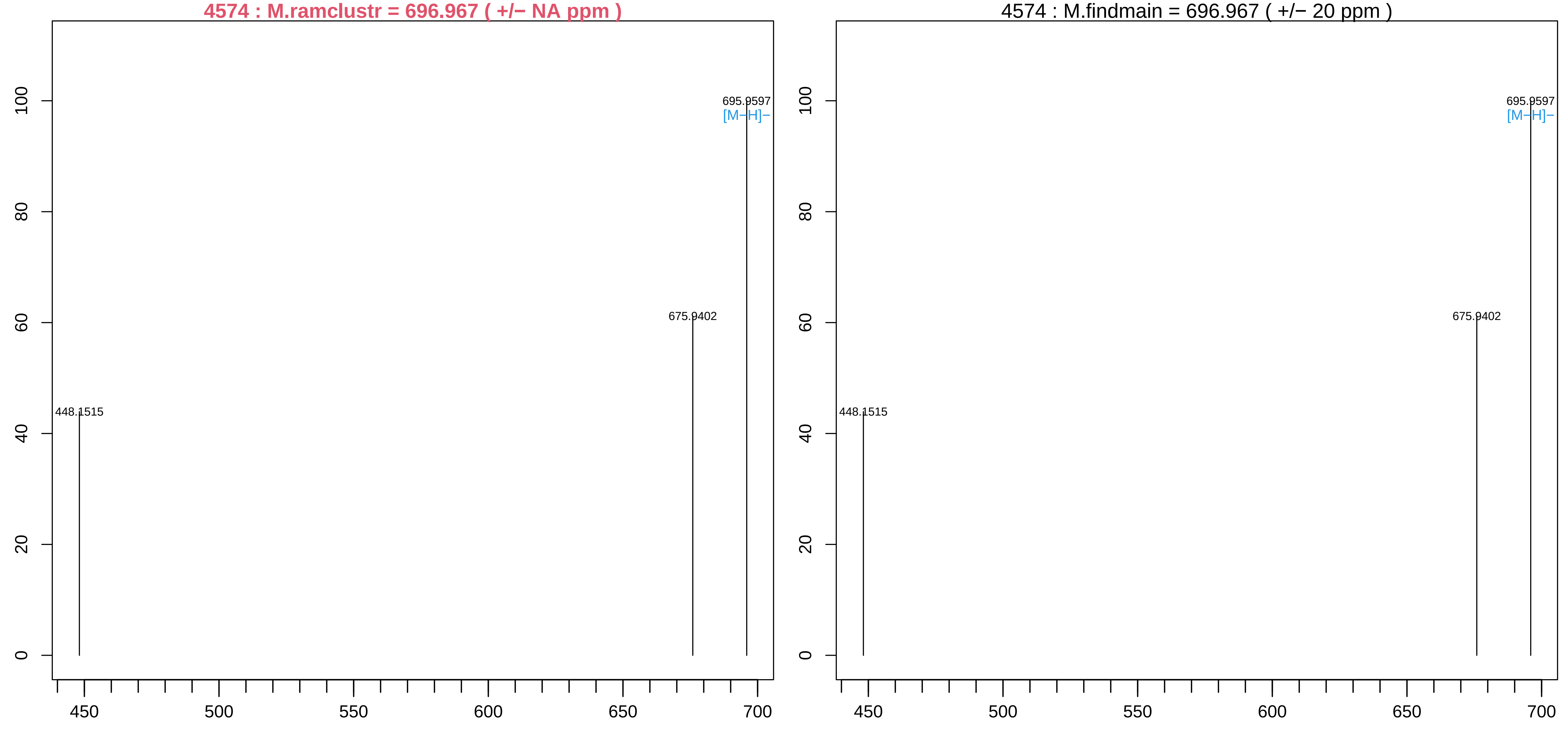
4574 : M.ramclustr = 696.967 (+/− NA ppm)
4574 : M.findmain = 696.967 (+/− 20 ppm)
695.9597
[M−H]−
675.9402
448.1515
0
20
40
60
80
100
450
500
550
600
650
700

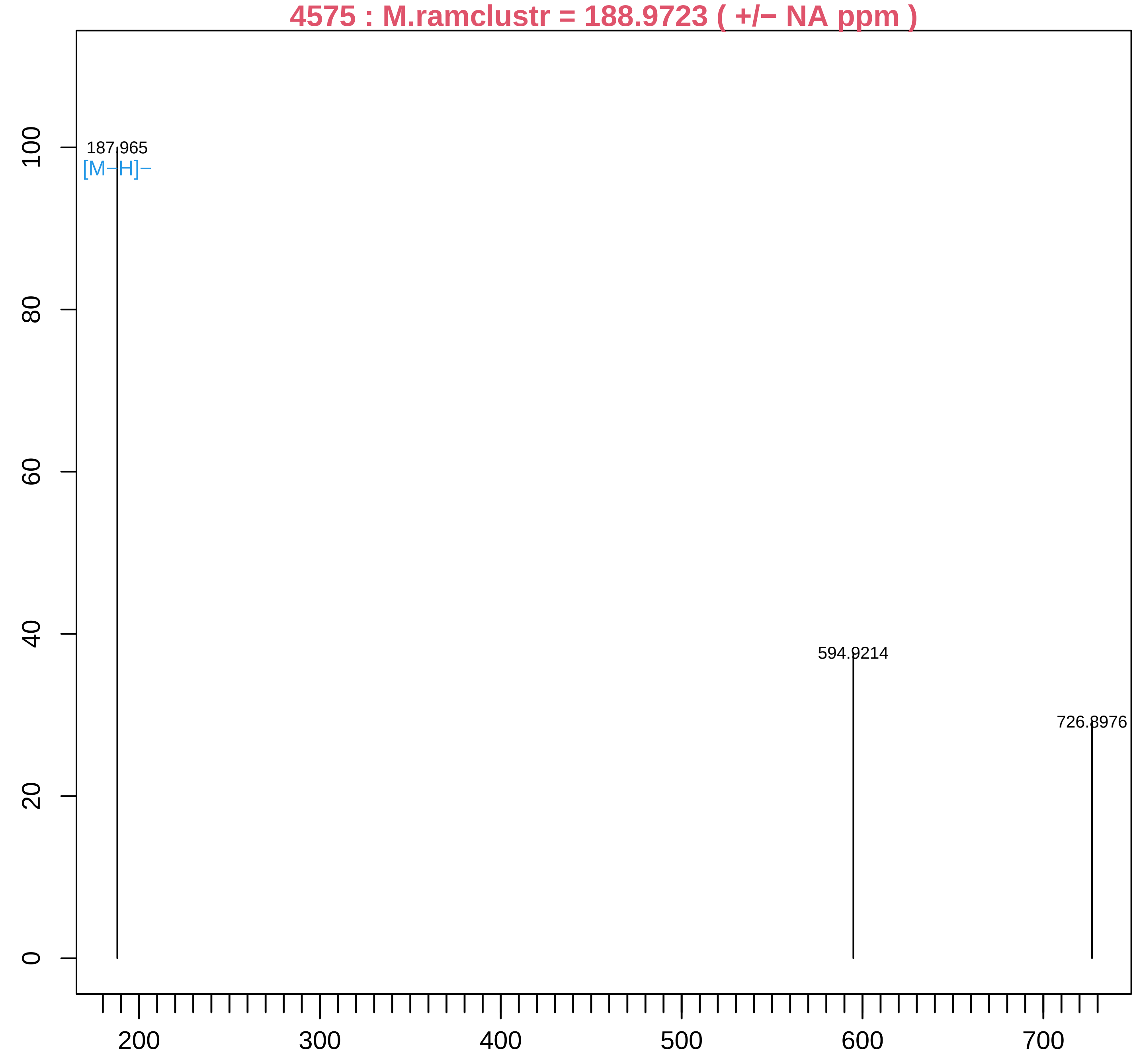

4575 : M.ramclustr = 188.9723 (+/− NA ppm)
187.965
[M−H]−
594.9214
726.8976
0
20
40
60
80
100
200
300
400
500
600
700

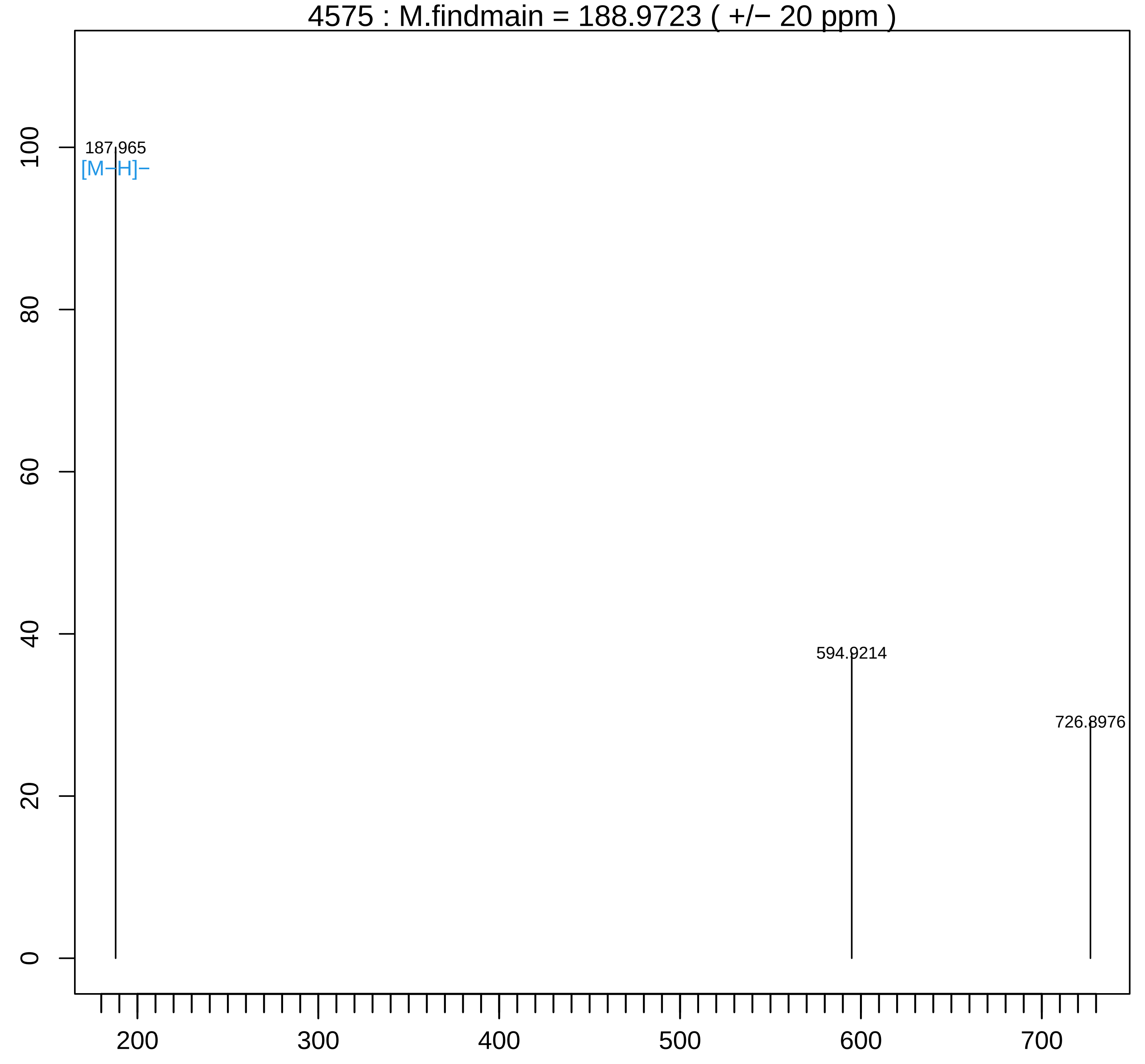

4575 : M.findmain = 188.9723 (+/− 20 ppm)
187.965
[M−H]−
594.9214
726.8976
0
20
40
60
80
100
200
300
400
500
600
700

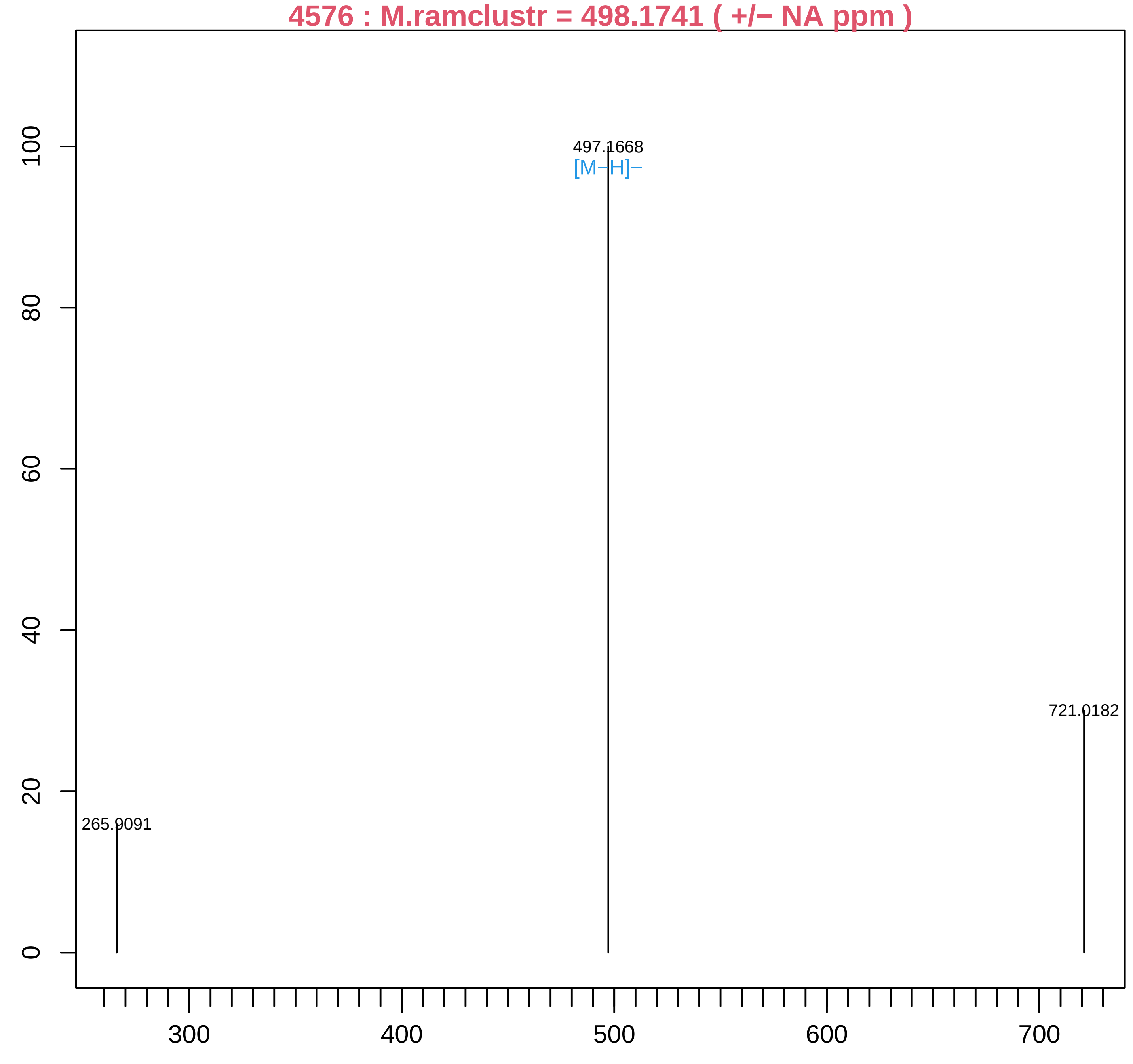
4576 : M.ramclustr = 498.1741 (+/− NA ppm)
497.1668
[M−H]−
265.9091
721.0182
0
20
40
60
80
100
300
400
500
600
700

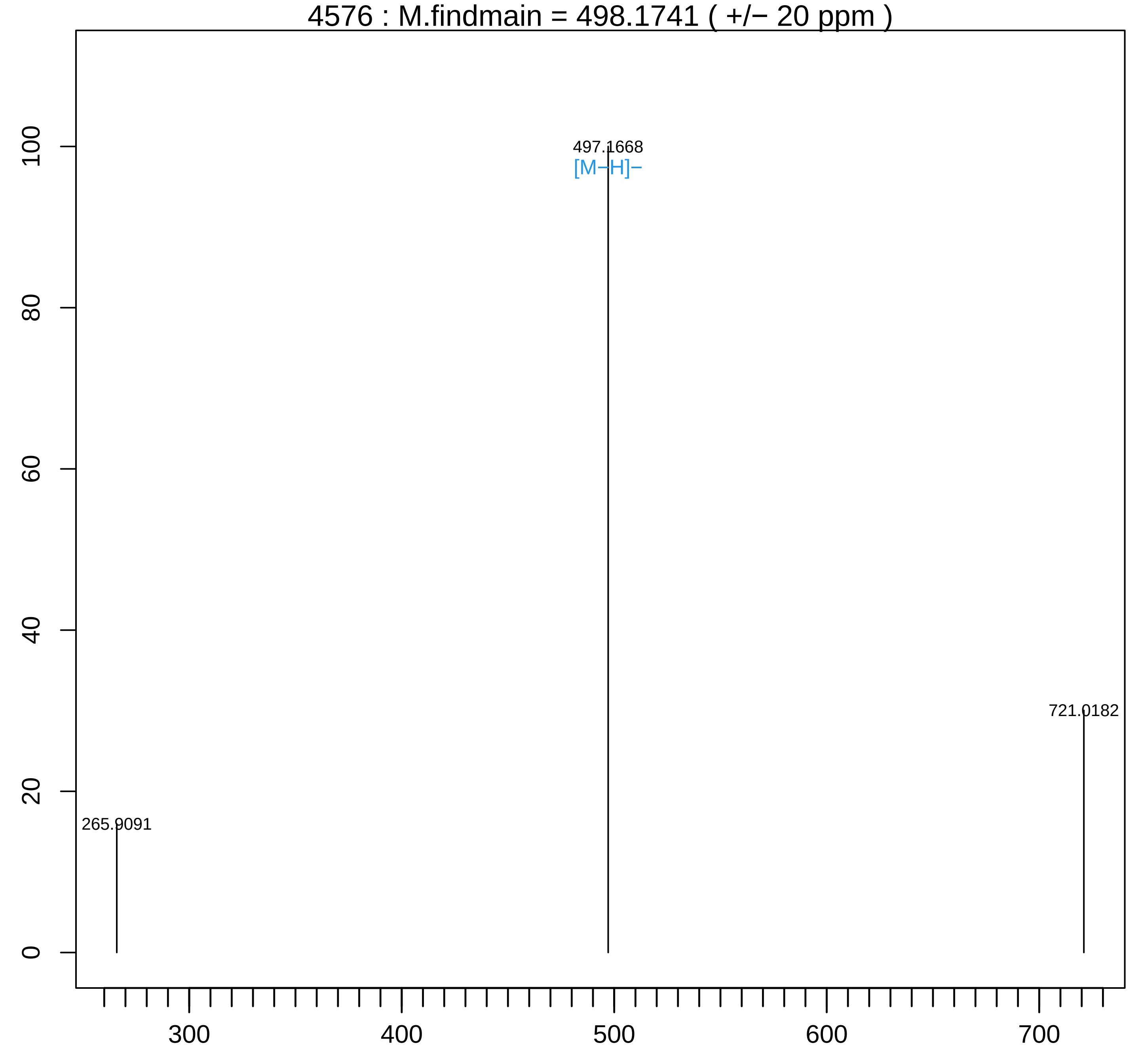
4576 : M.findmain = 498.1741 (+/− 20 ppm)
497.1668
[M−H]−
265.9091
721.0182
0
20
40
60
80
100
300
400
500
600
700

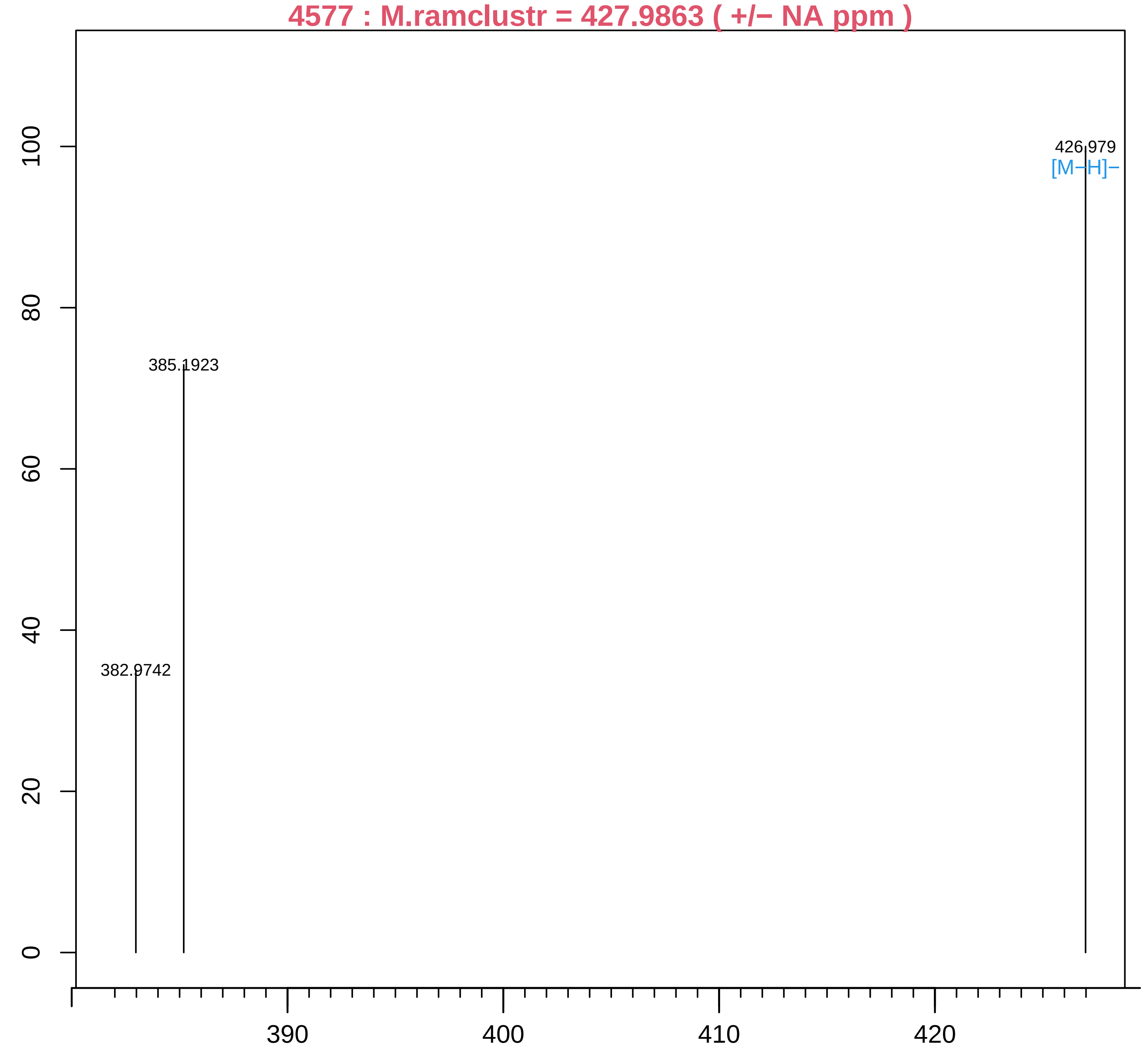
4577 : M.ramclustr = 427.9863 (+/− NA ppm)
426.979
[M−H]−
385.1923
382.9742
0
20
40
60
80
100
390
400
410
420

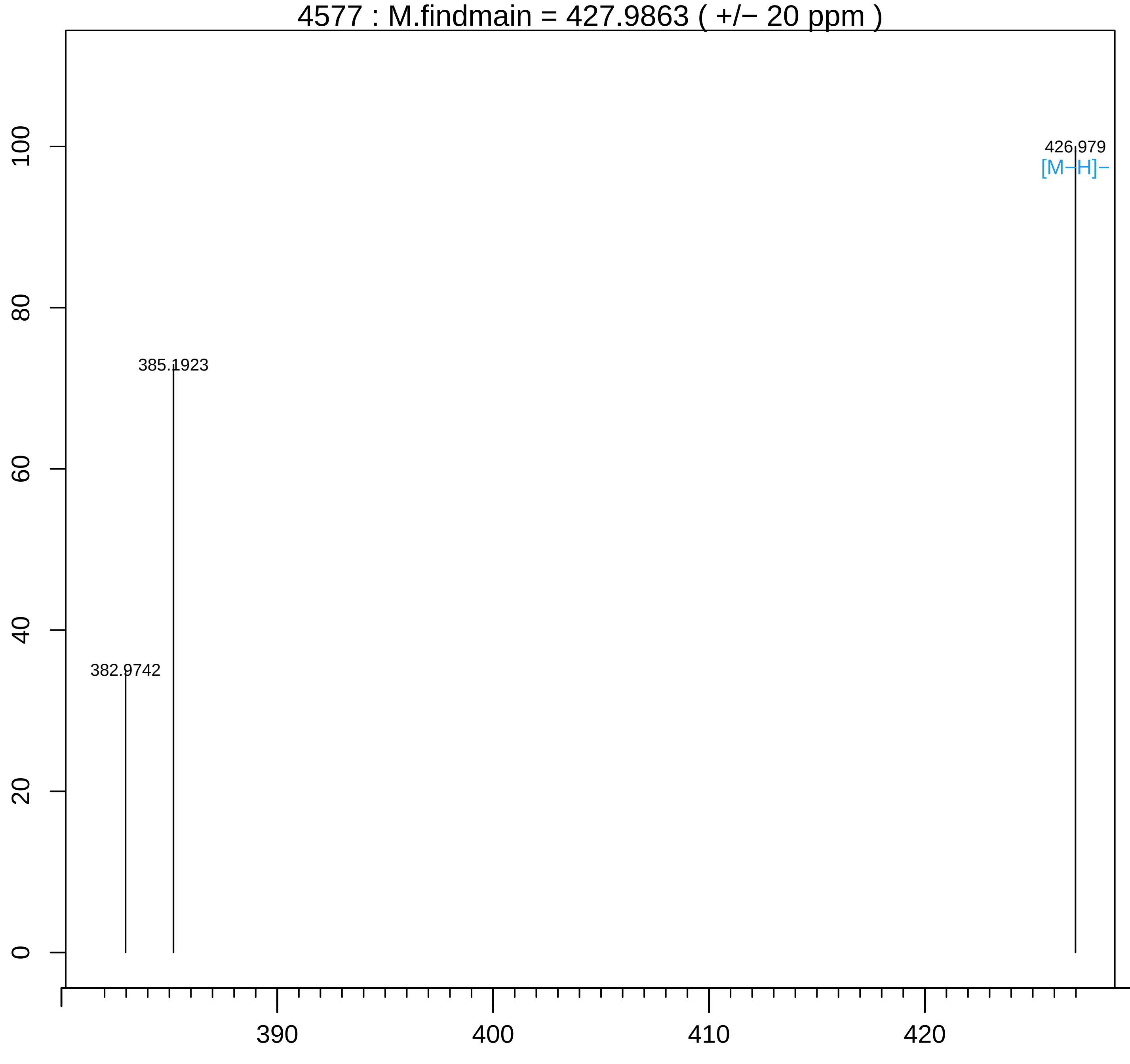
4577 : M.findmain = 427.9863 (+/− 20 ppm)
426.979
[M−H]−
385.1923
382.9742
0
20
40
60
80
100
390
400
410
420

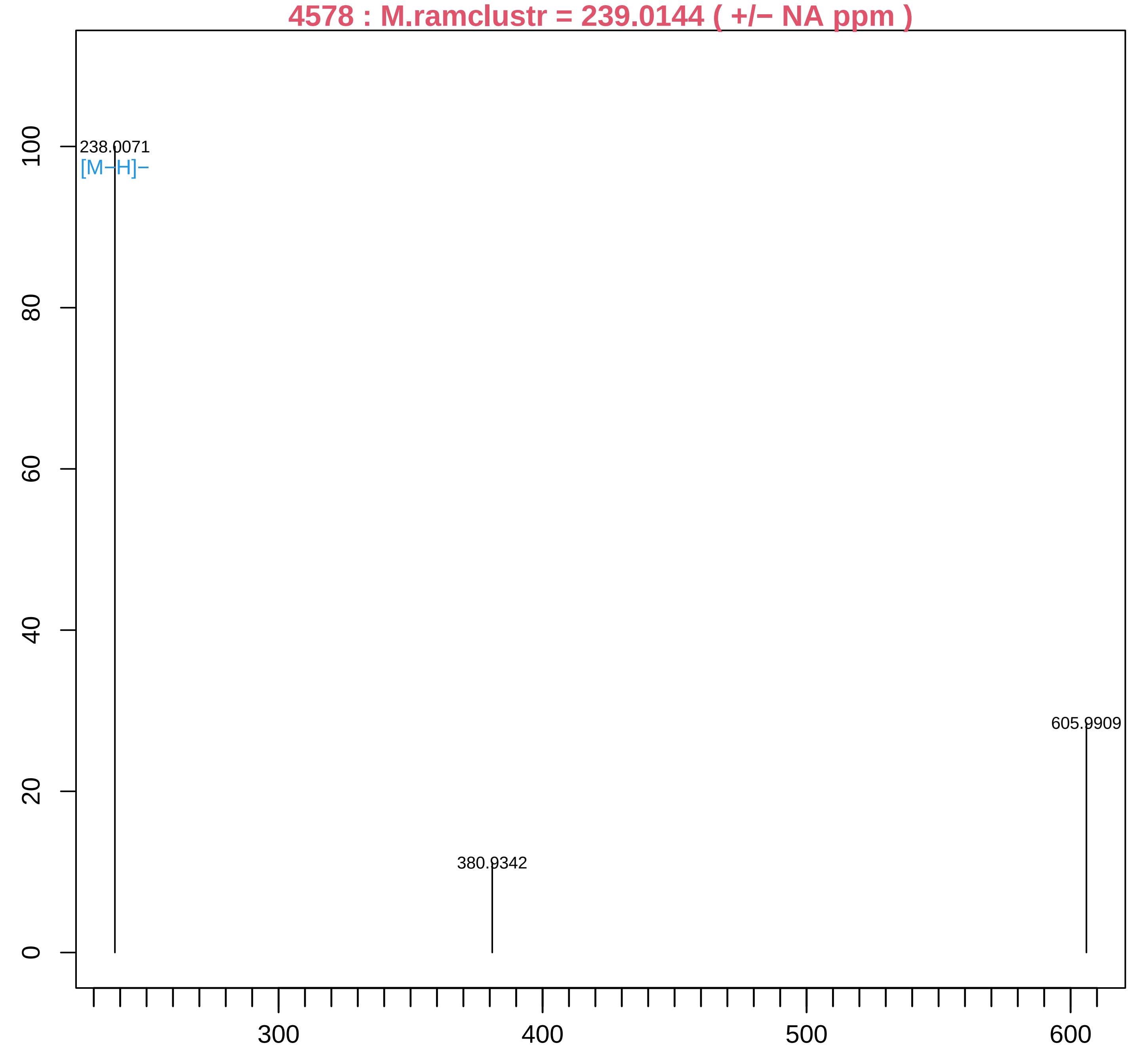
4578 : M.ramclustr = 239.0144 (+/− NA ppm)
238.0071
[M−H]−
380.9342
605.9909
0
20
40
60
80
100
300
400
500
600

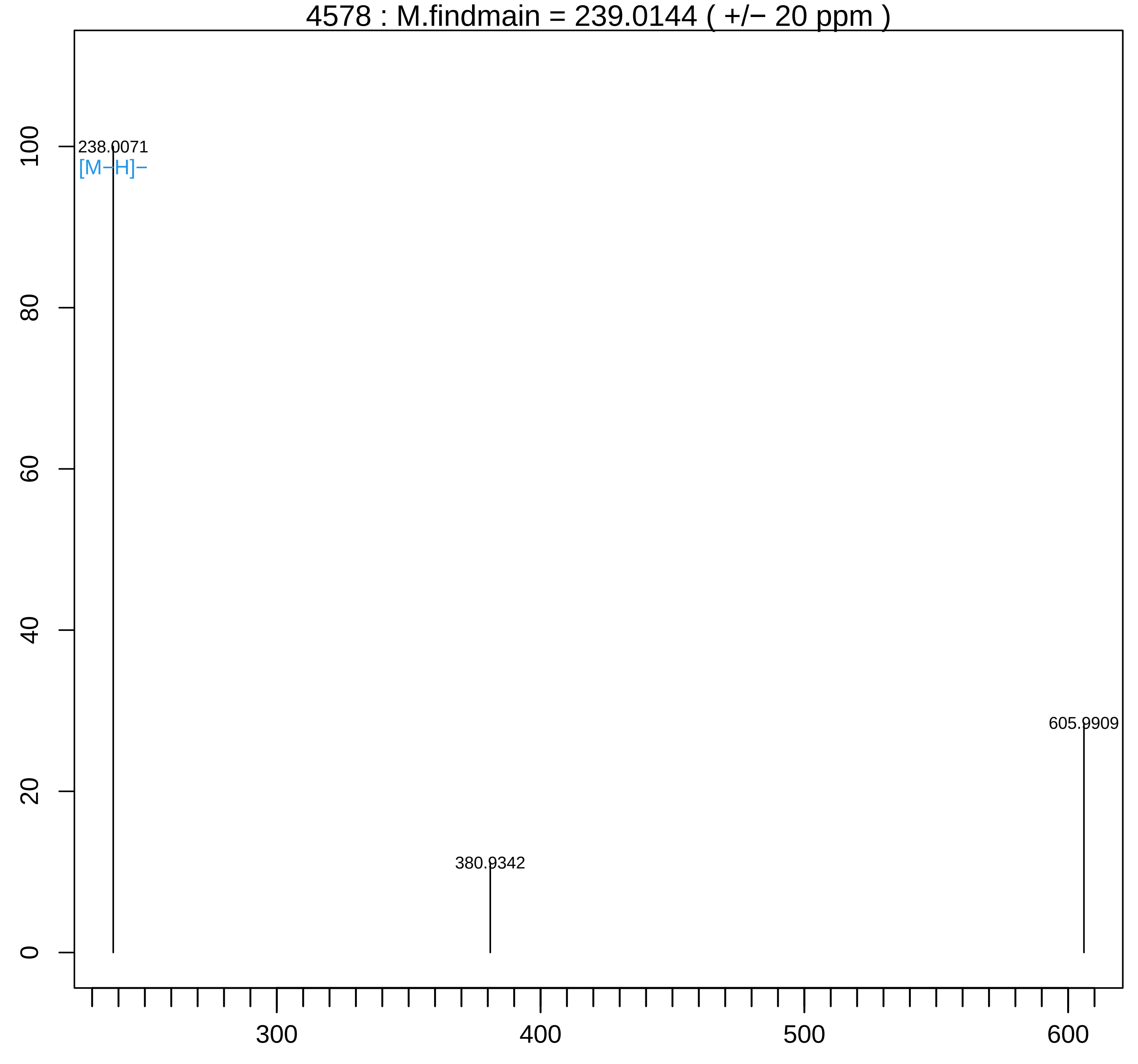
4578 : M.findmain = 239.0144 (+/− 20 ppm)
238.0071
[M−H]−
380.9342
605.9909
0
20
40
60
80
100
300
400
500
600

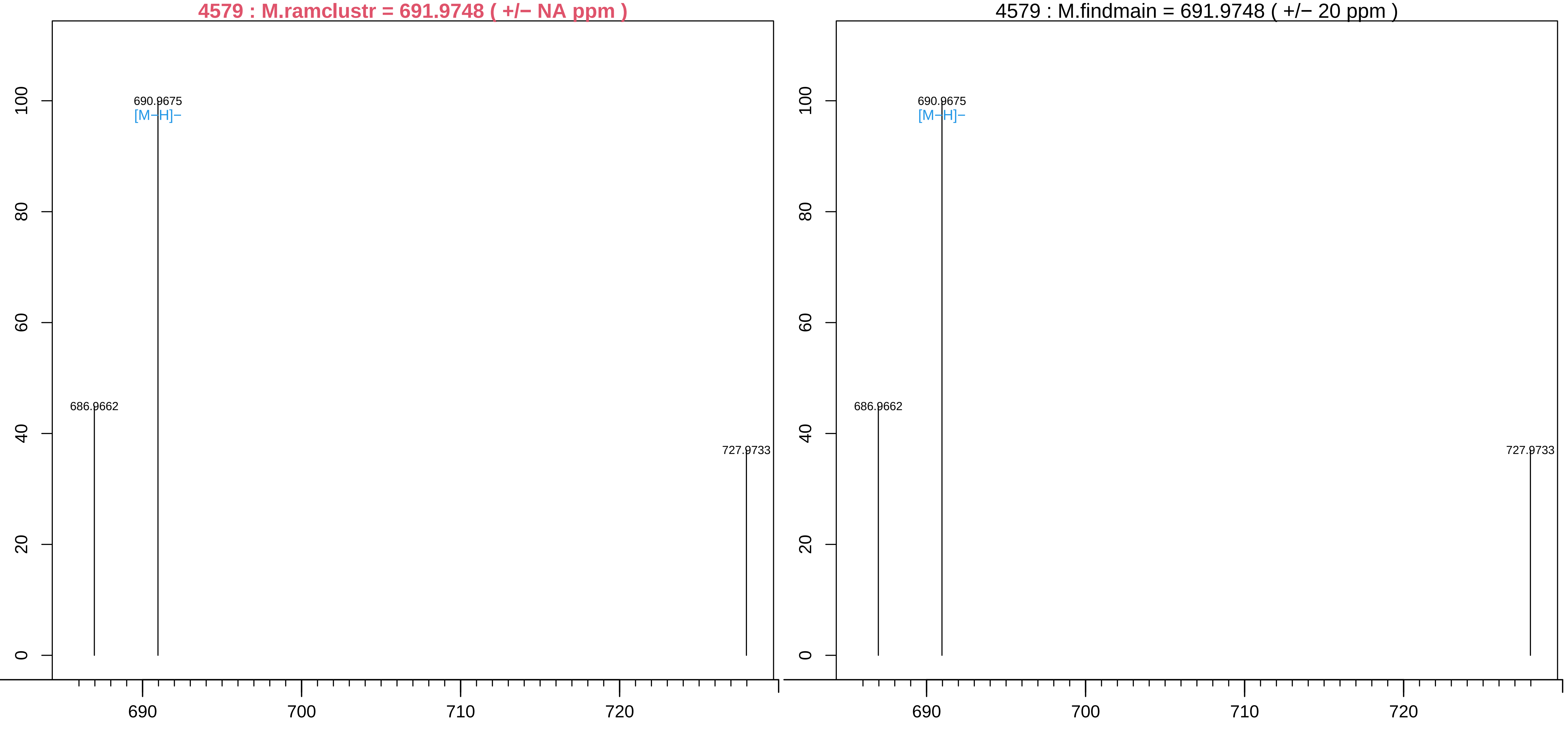

4579 : M.ramclustr = 691.9748 (+/− NA ppm)
690.9675
[M−H]−
686.9662
727.9733
4579 : M.findmain = 691.9748 (+/− 20 ppm)
690.9675
[M−H]−
686.9662
727.9733
0
20
40
60
80
100
690
700
710
720

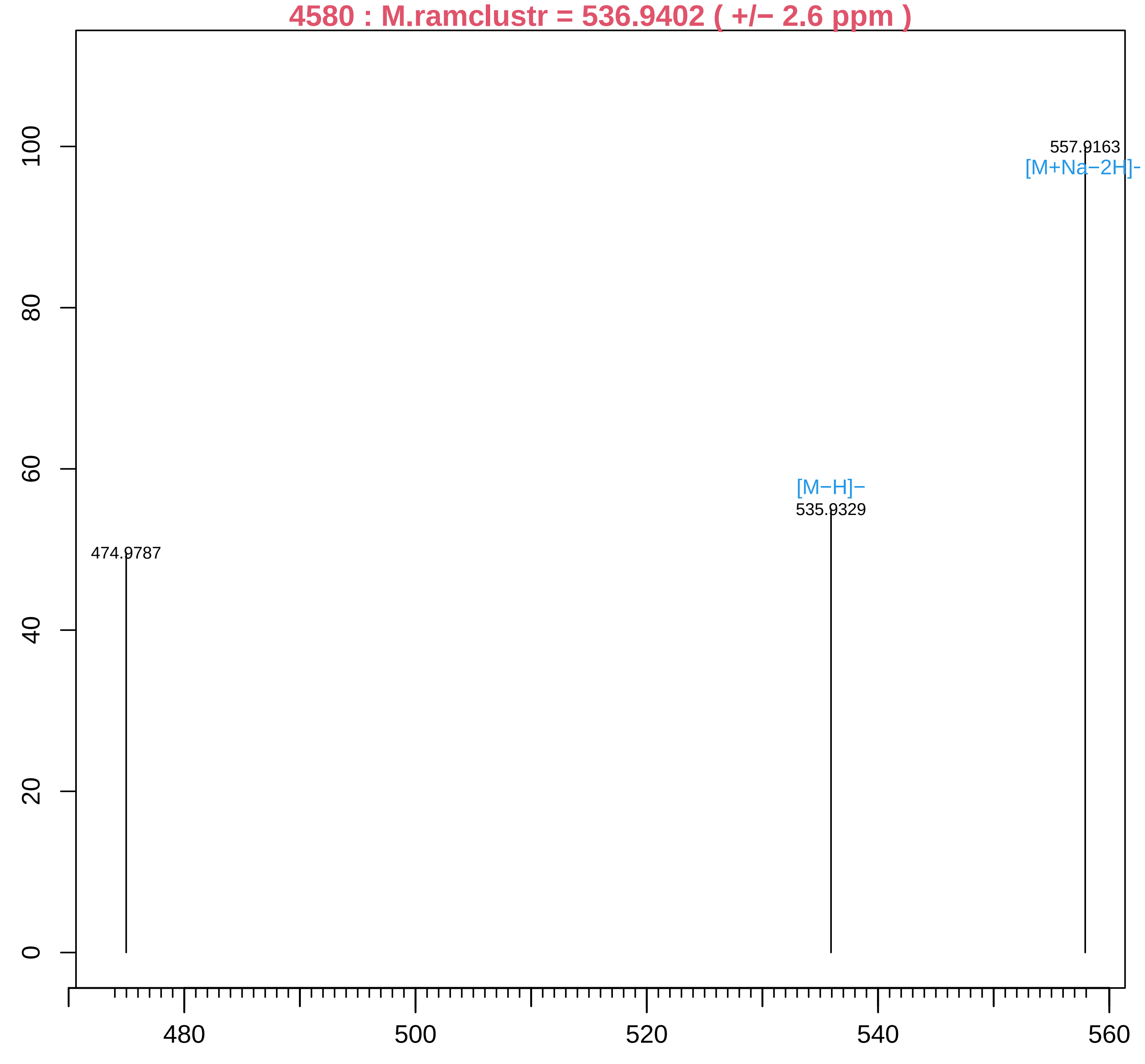
4580 : M.ramclustr = 536.9402 (+/− 2.6 ppm)
557.9163
[M+Na−2H]−
[M−H]−
535.9329
474.9787
0
20
40
60
80
100
480
500
520
540
560

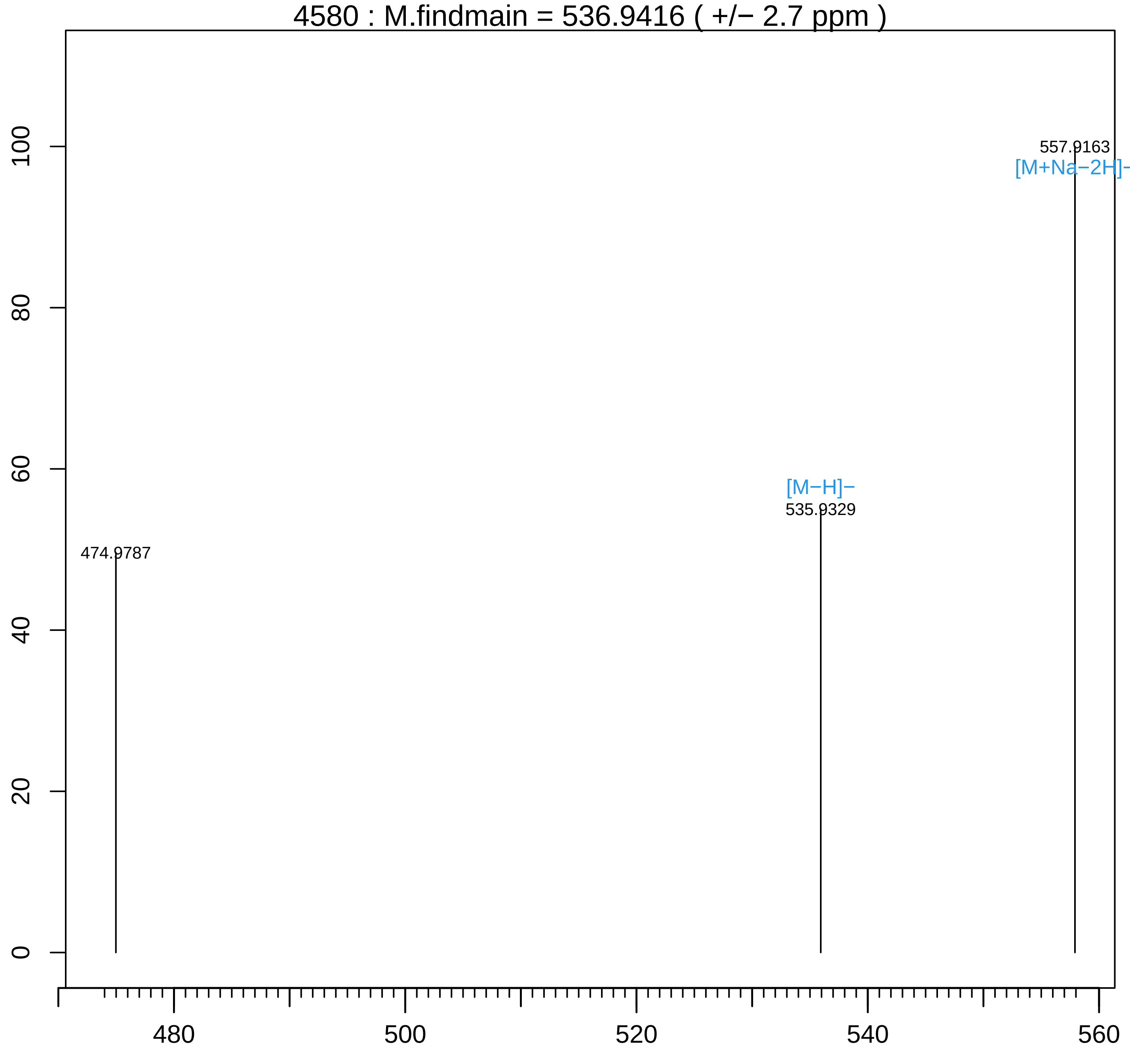
4580 : M.findmain = 536.9416 (+/− 2.7 ppm)
557.9163
[M+Na−2H]−
[M−H]−
535.9329
474.9787
0
20
40
60
80
100
480
500
520
540
560

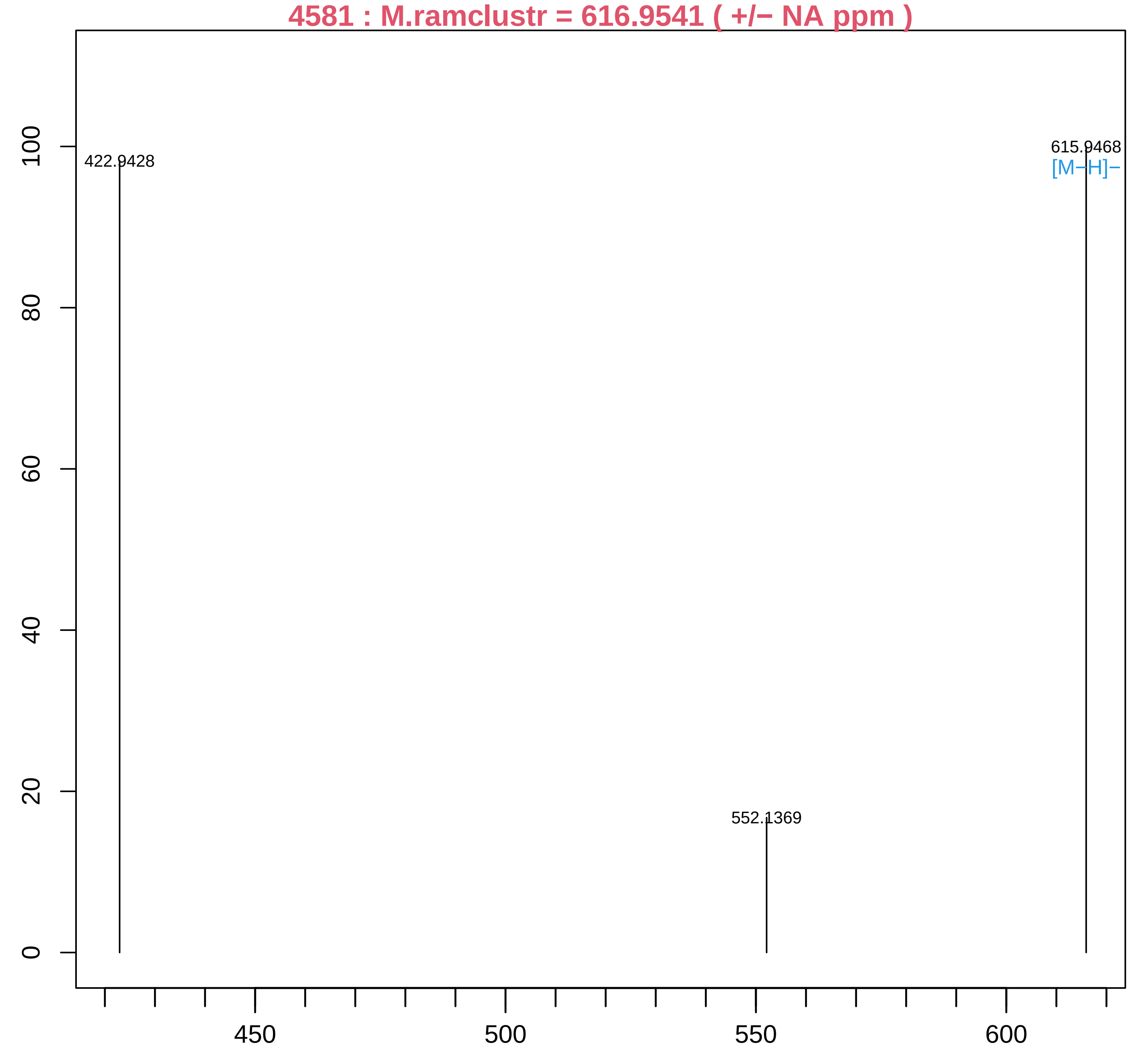
4581 : M.ramclustr = 616.9541 (+/− NA ppm)
422.9428
552.1369
615.9468
[M−H]−
0
20
40
60
80
100
450
500
550
600

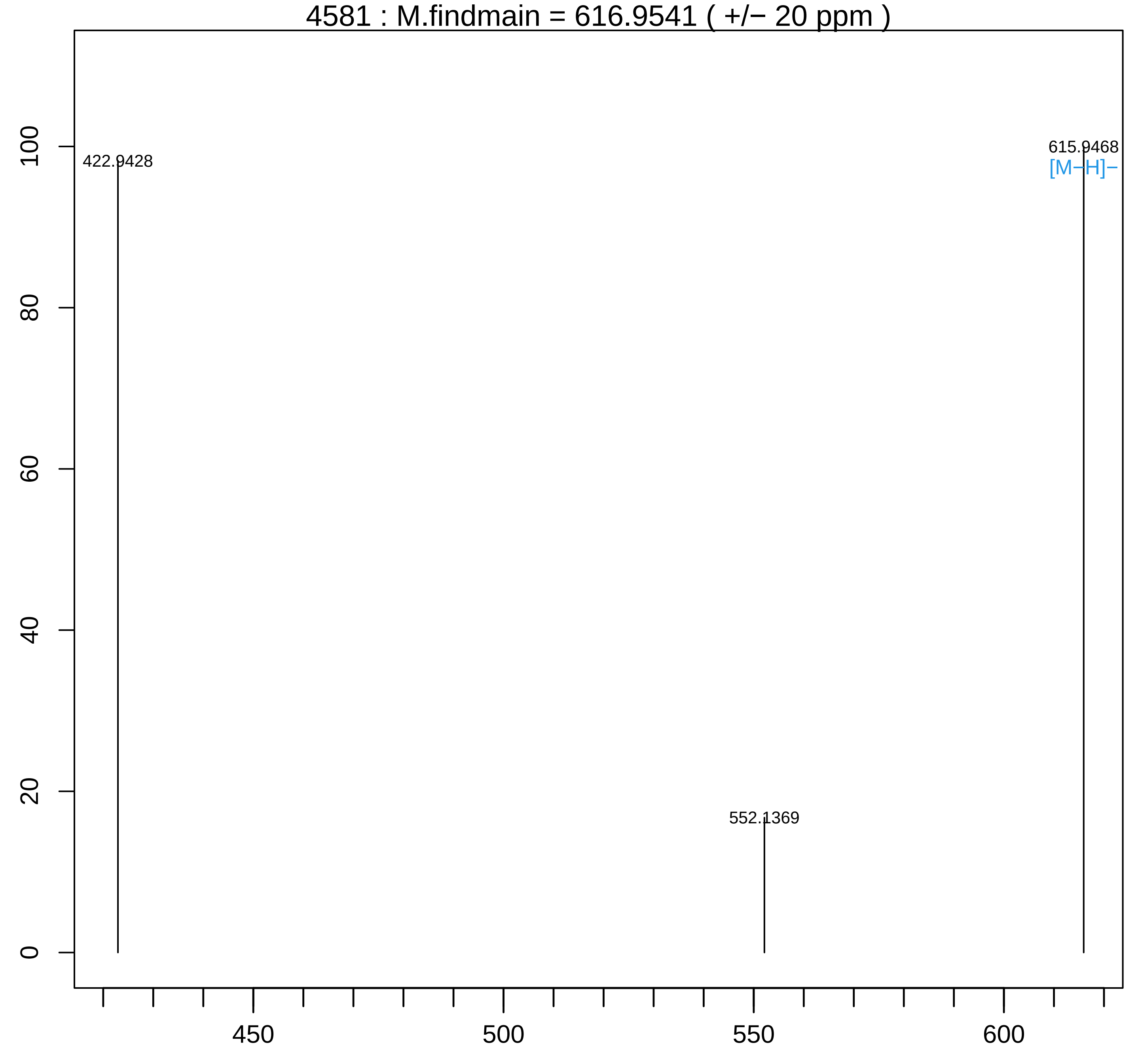
4581 : M.findmain = 616.9541 (+/− 20 ppm)
422.9428
552.1369
615.9468
[M−H]−
0
20
40
60
80
100
450
500
550
600

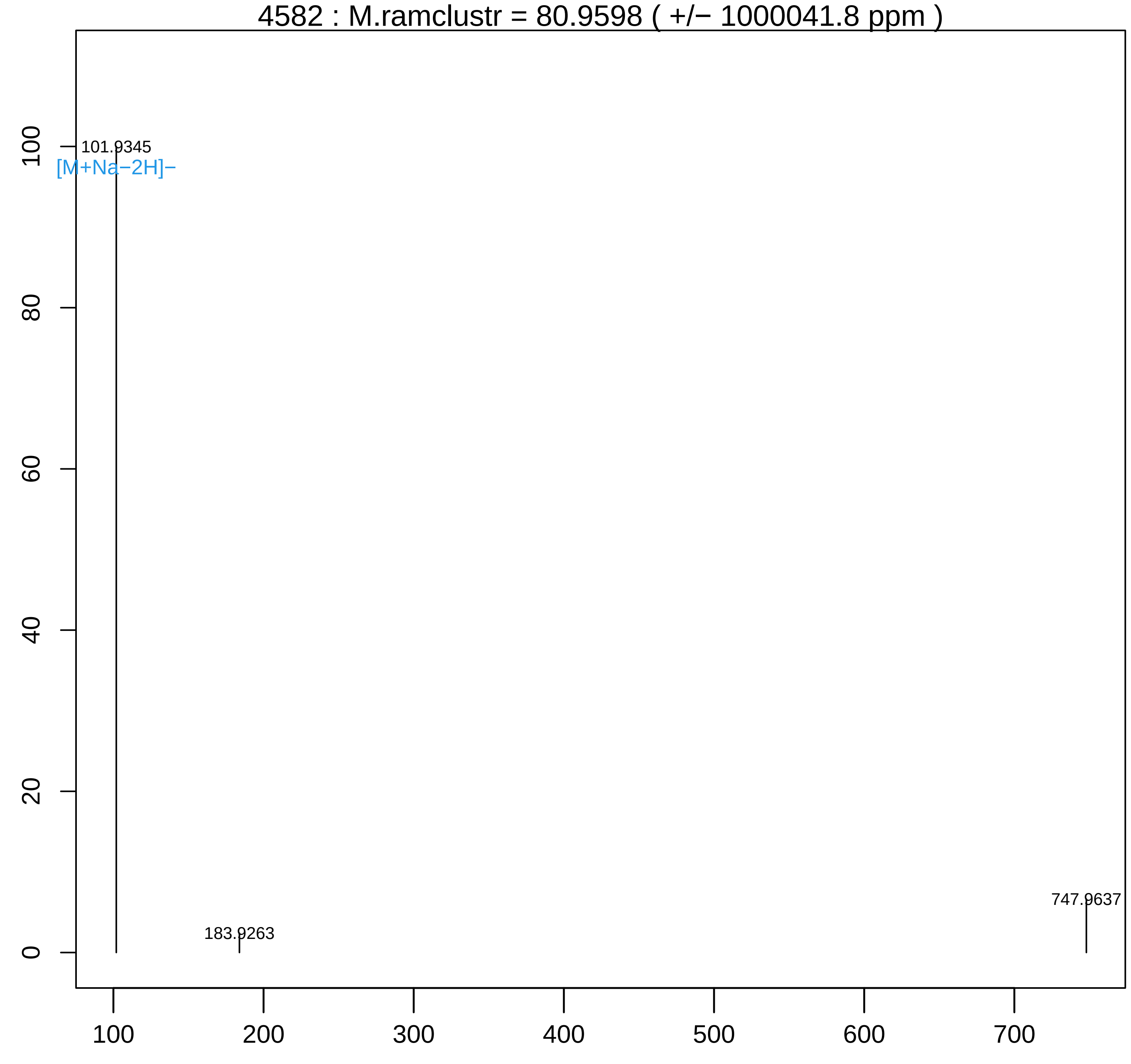

4582 : M.ramclustr = 80.9598 (+/− 1000041.8 ppm)
101.9345
[M+Na−2H]−
183.9263
747.9637
0
20
40
60
80
100
100
200
300
400
500
600
700

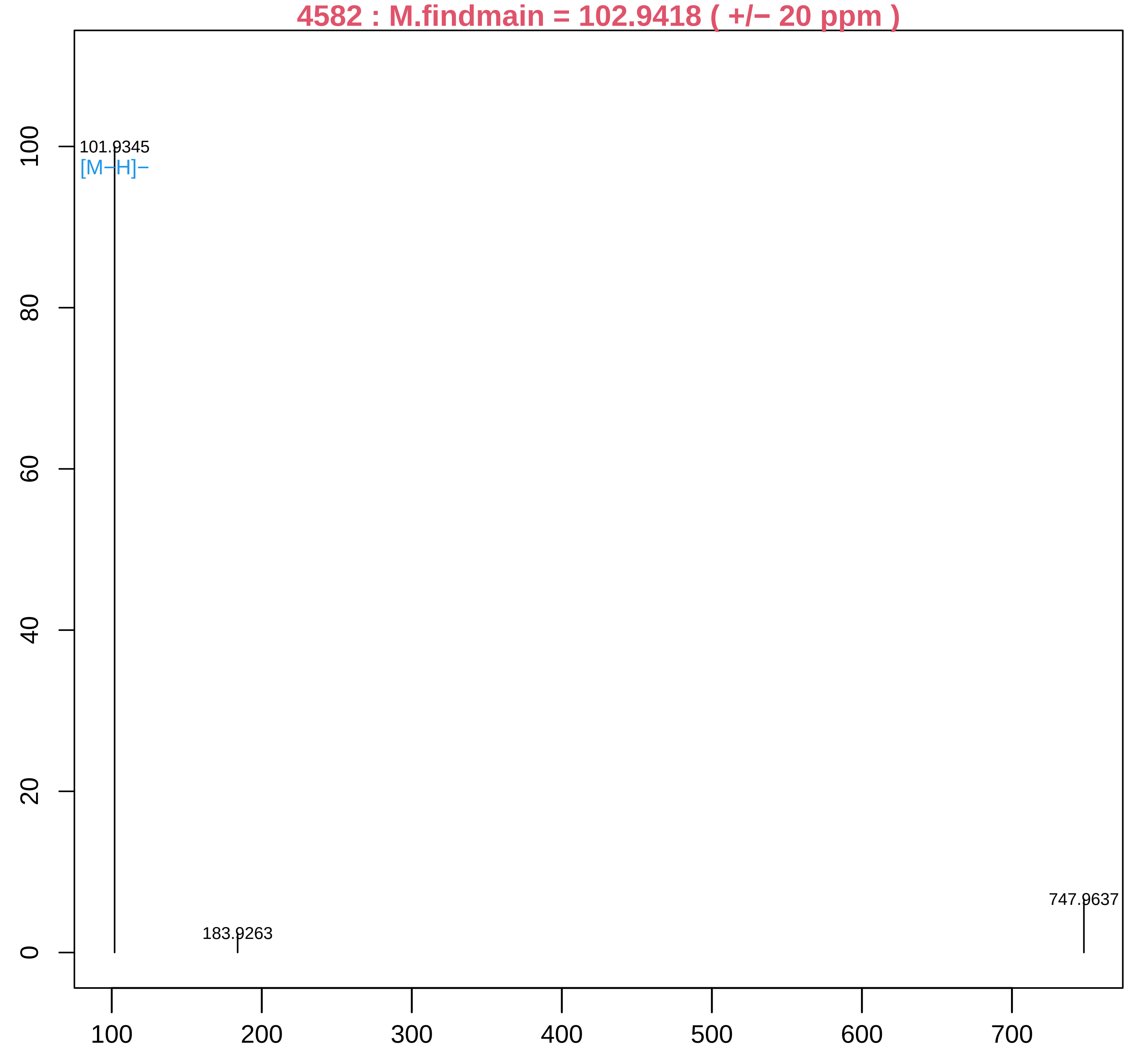

4582 : M.findmain = 102.9418 (+/− 20 ppm)
101.9345
[M−H]−
183.9263
747.9637
0
20
40
60
80
100
100
200
300
400
500
600
700

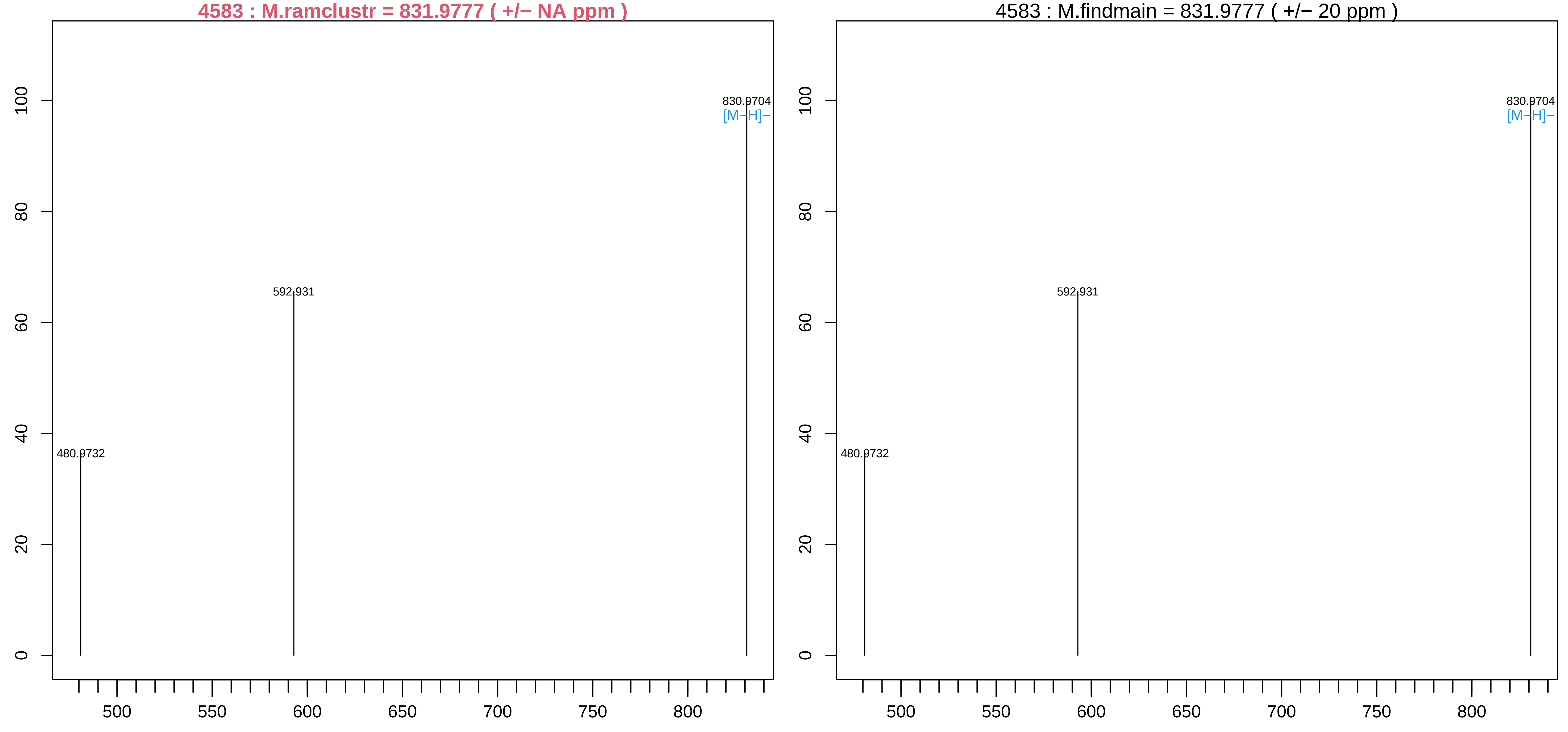
4583 : M.ramclustr = 831.9777 (+/− NA ppm)
830.9704
[M−H]−
592.931
480.9732
4583 : M.findmain = 831.9777 (+/− 20 ppm)
830.9704
[M−H]−
592.931
480.9732
0
20
40
60
80
100
500
550
600
650
700
750
800

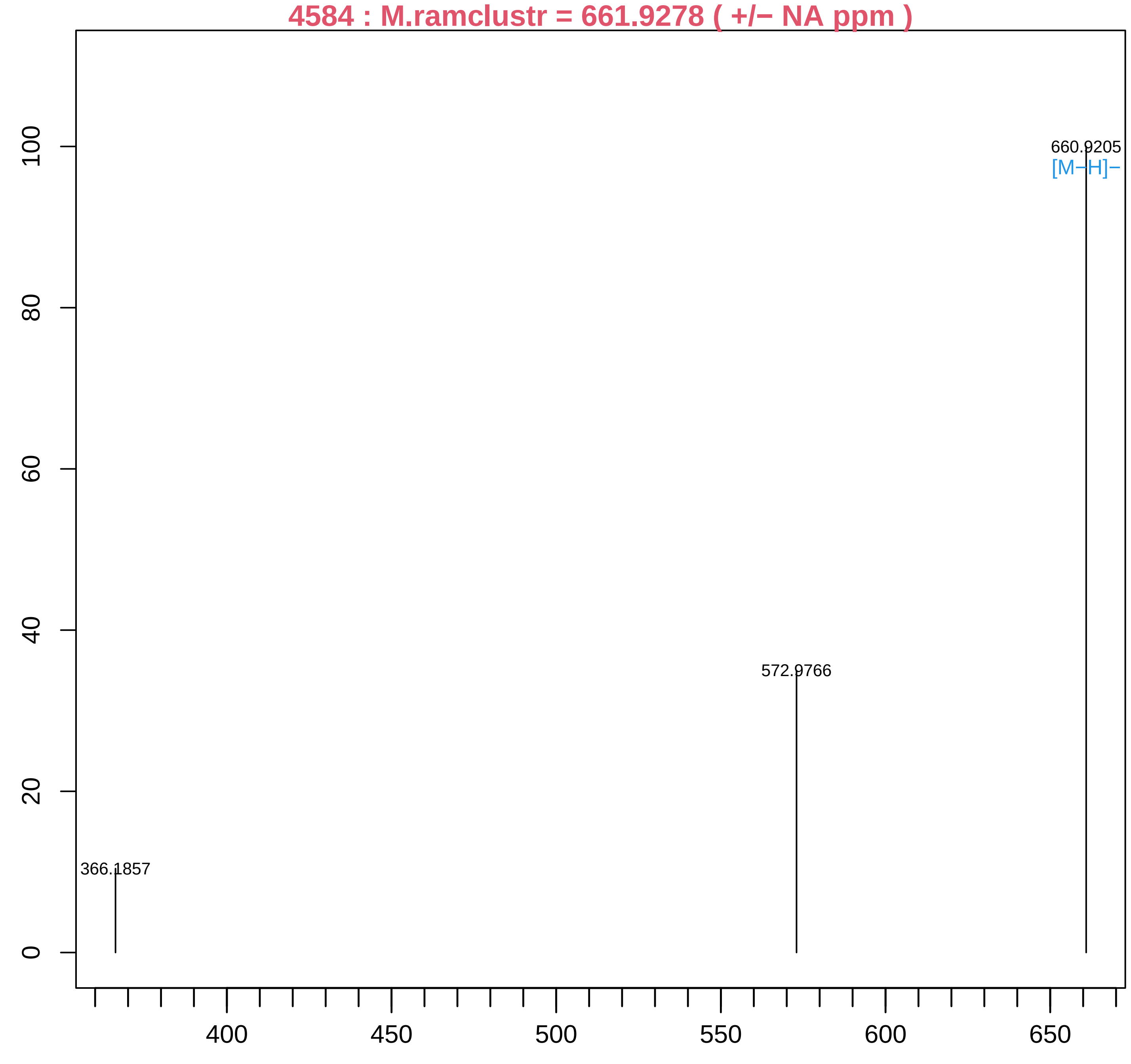

4584 : M.ramclustr = 661.9278 (+/− NA ppm)
660.9205
[M−H]−
572.9766
366.1857
0
20
40
60
80
100
400
450
500
550
600
650

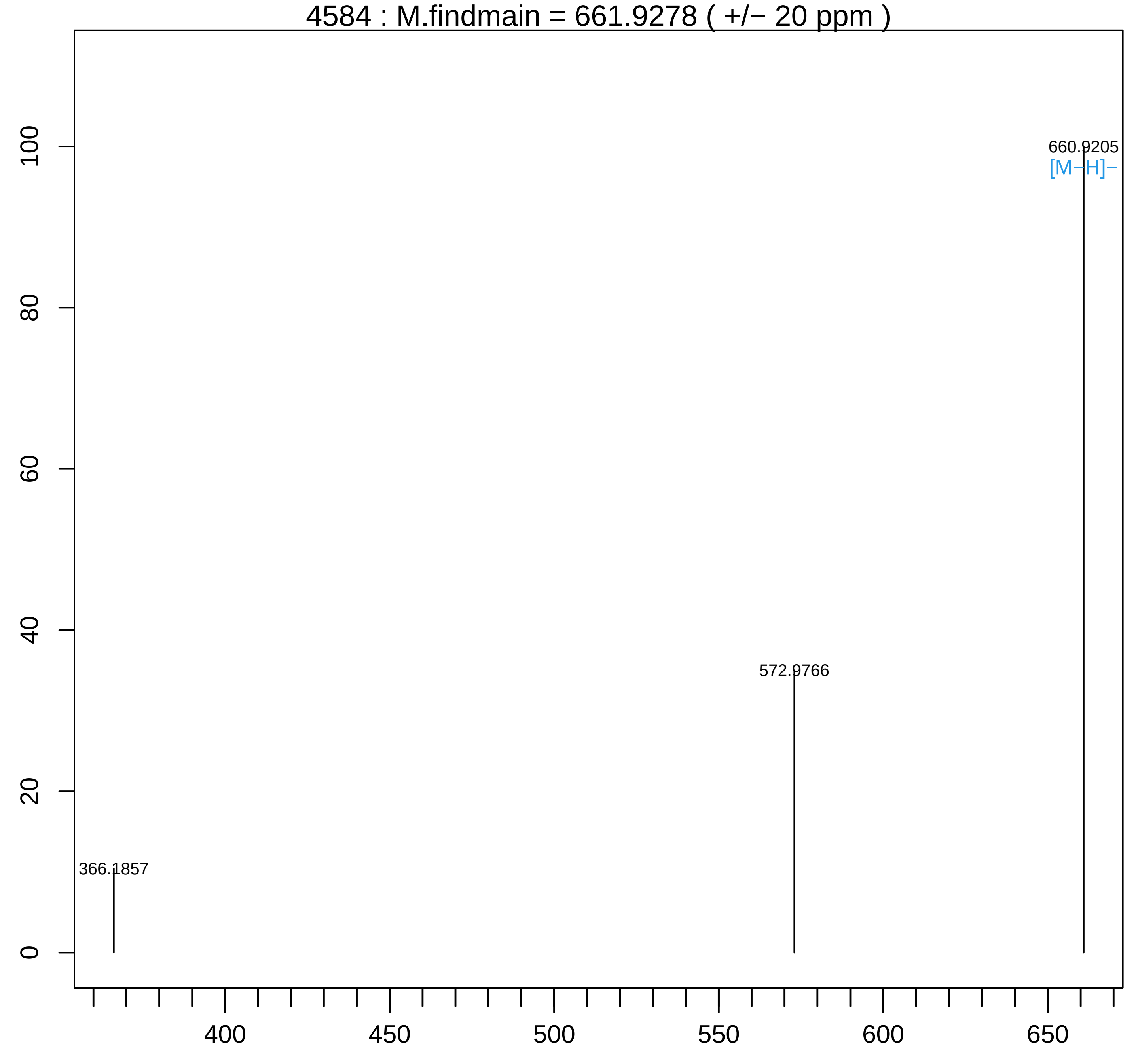

4584 : M.findmain = 661.9278 (+/− 20 ppm)
660.9205
[M−H]−
572.9766
366.1857
0
20
40
60
80
100
400
450
500
550
600
650

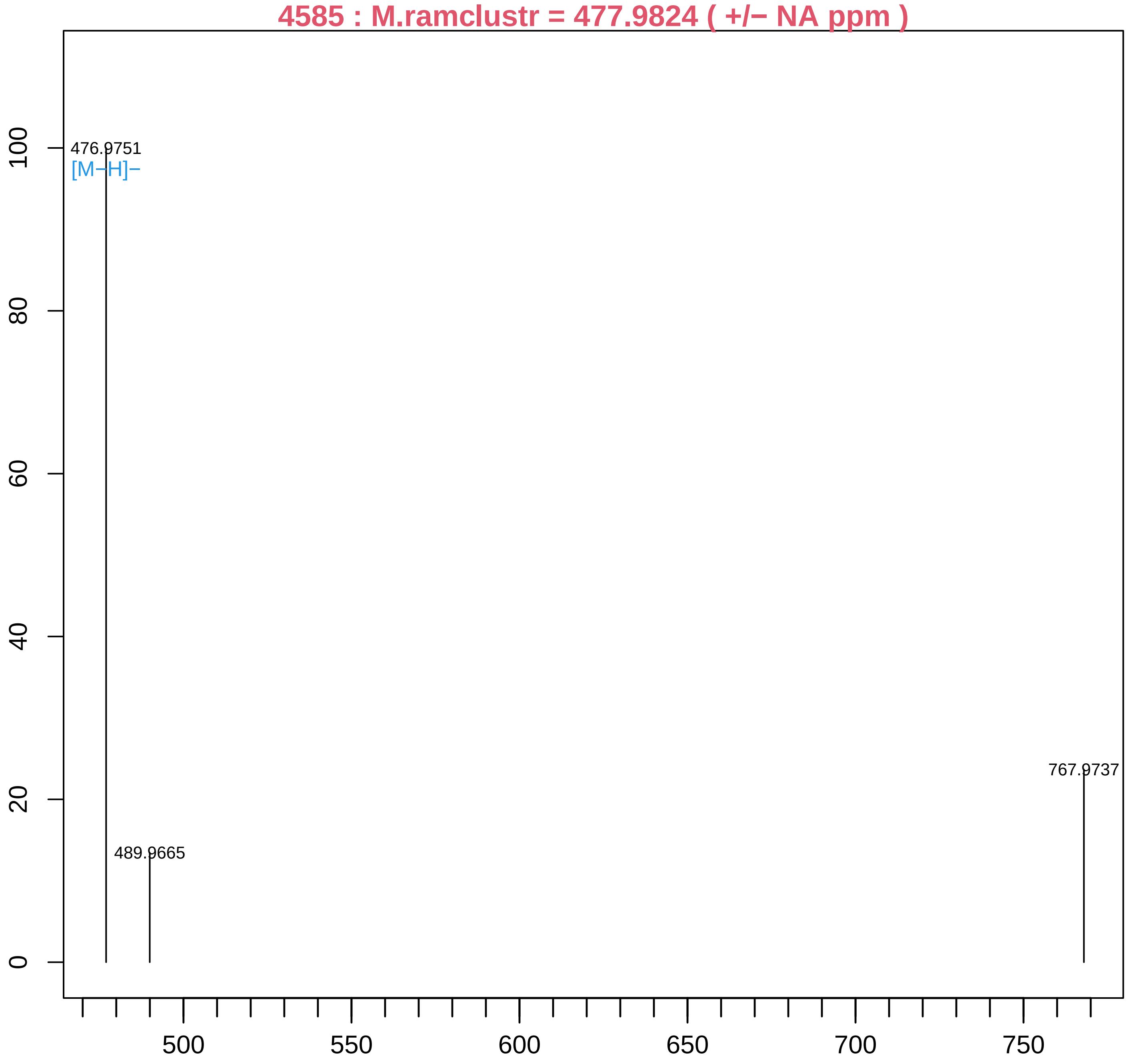
4585 : M.ramclustr = 477.9824 (+/− NA ppm)
476.9751
[M−H]−
489.9665
767.9737
0
20
40
60
80
100
500
550
600
650
700
750

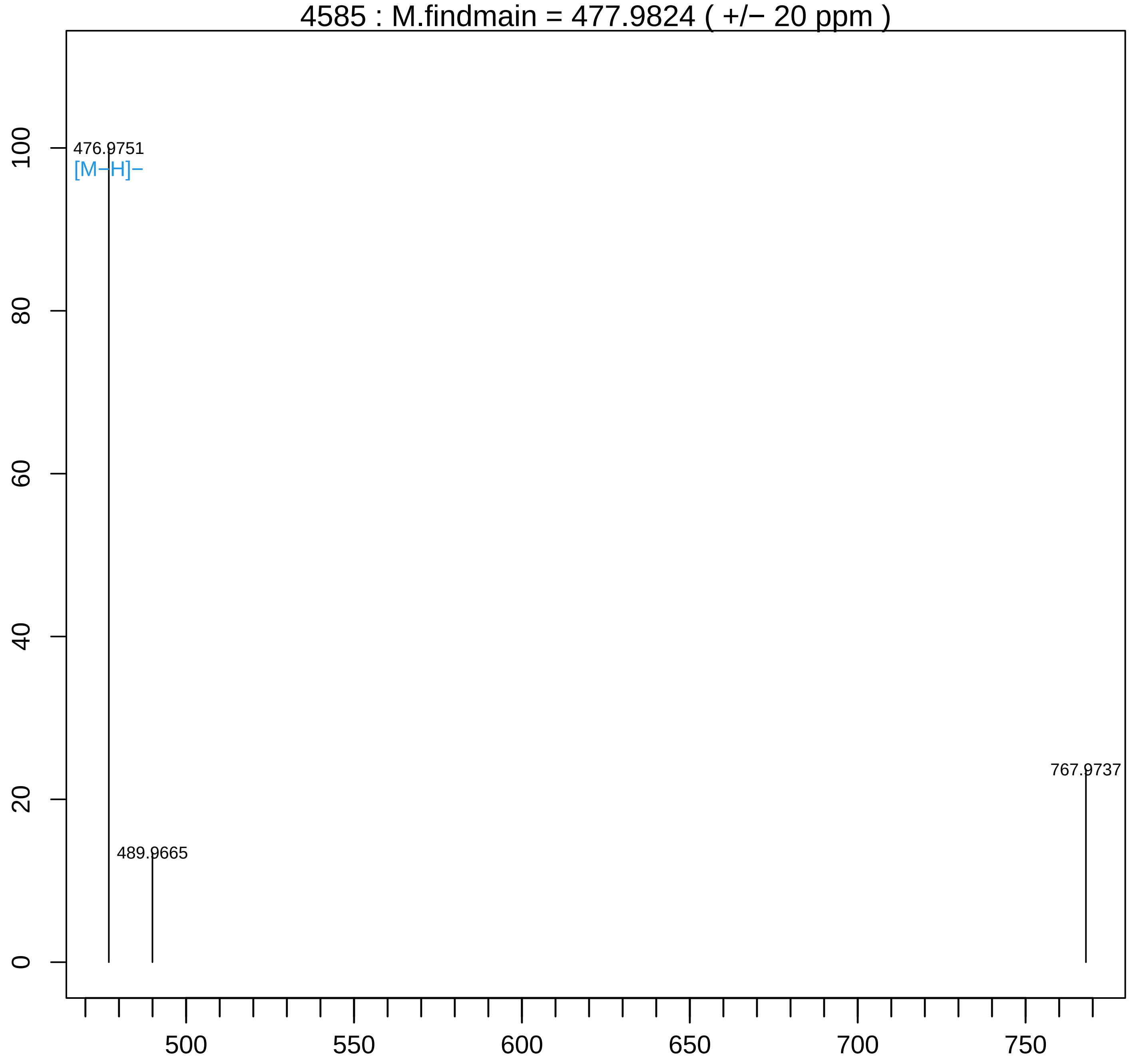
4585 : M.findmain = 477.9824 (+/− 20 ppm)
476.9751
[M−H]−
489.9665
767.9737
0
20
40
60
80
100
500
550
600
650
700
750

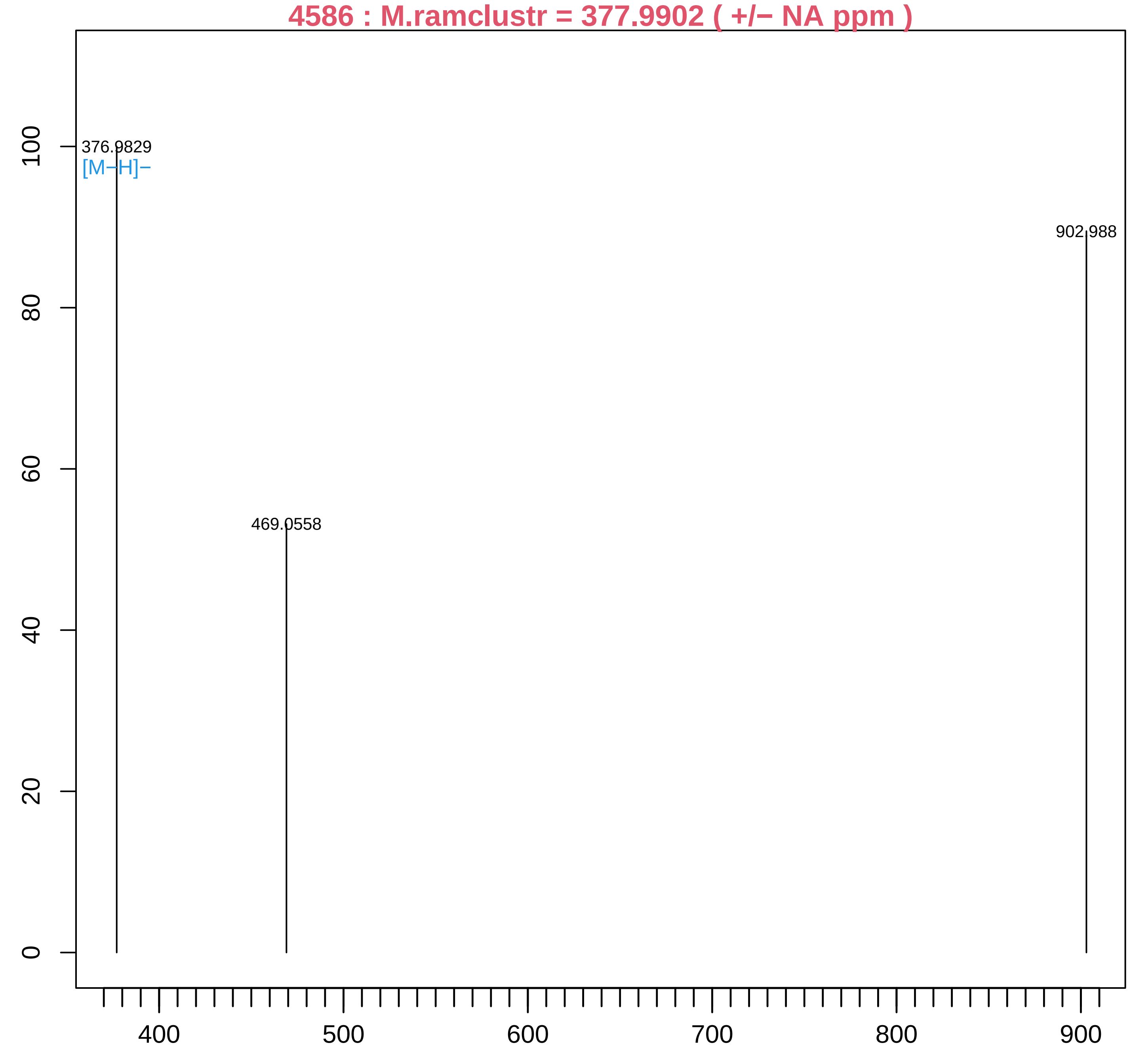
4586 : M.ramclustr = 377.9902 (+/− NA ppm)
376.9829
[M−H]−
469.0558
902.988
0
20
40
60
80
100
400
500
600
700
800
900

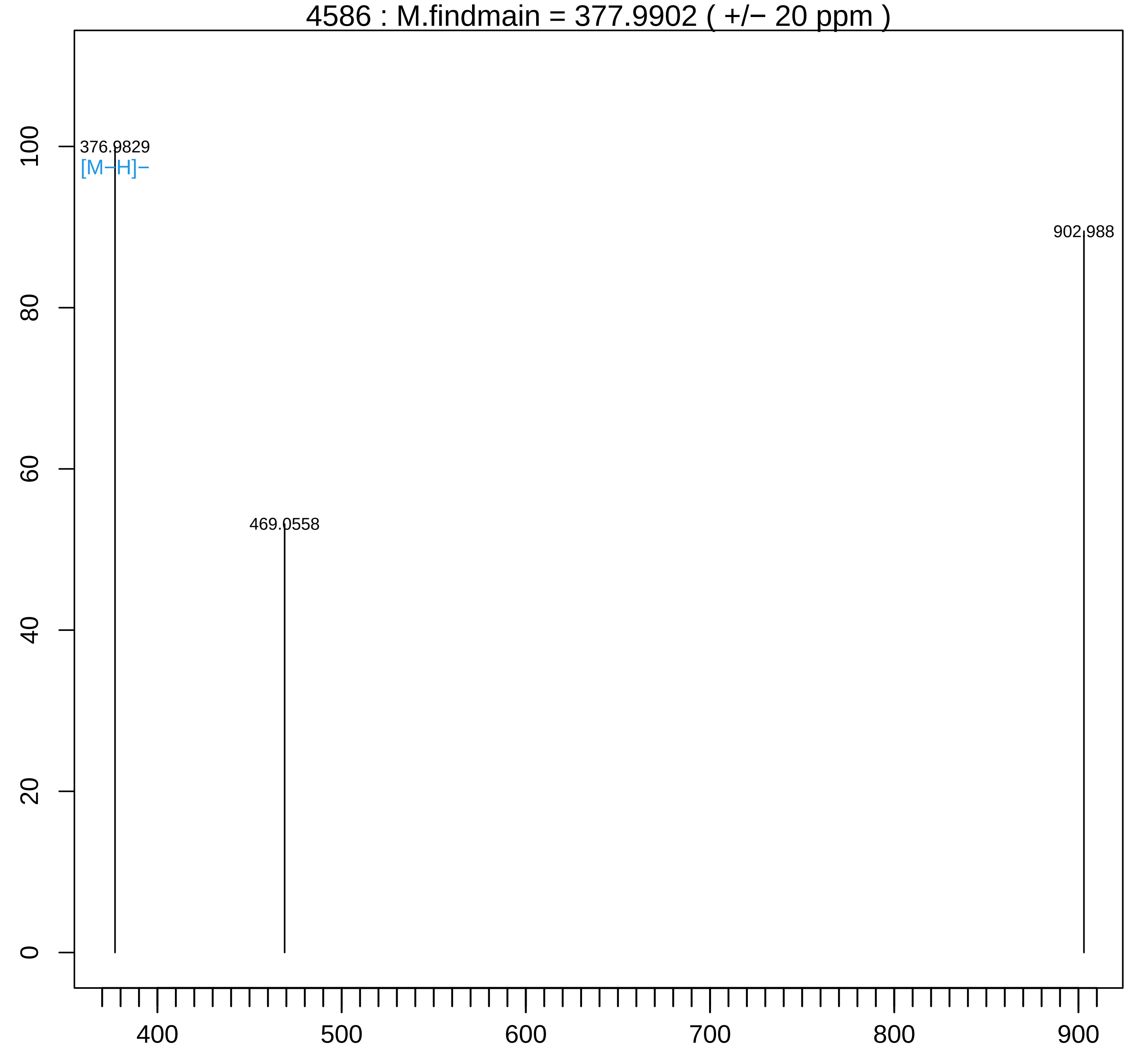
4586 : M.findmain = 377.9902 (+/− 20 ppm)
376.9829
[M−H]−
469.0558
902.988
0
20
40
60
80
100
400
500
600
700
800
900

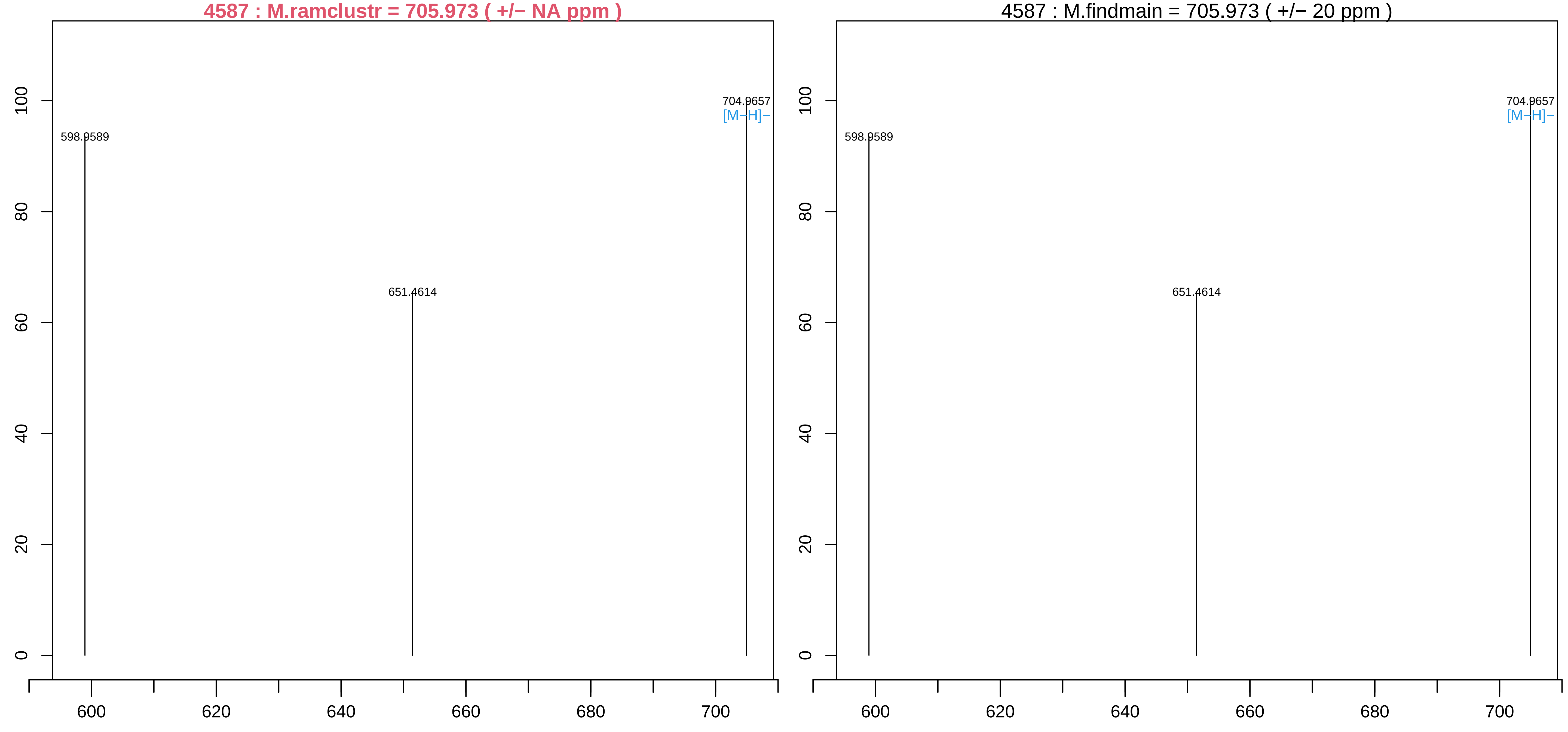
4587 : M.ramclustr = 705.973 (+/− NA ppm)
598.9589
651.4614
704.9657
[M−H]−
4587 : M.findmain = 705.973 (+/− 20 ppm)
598.9589
651.4614
704.9657
[M−H]−
0
20
40
60
80
100
600
620
640
660
680
700

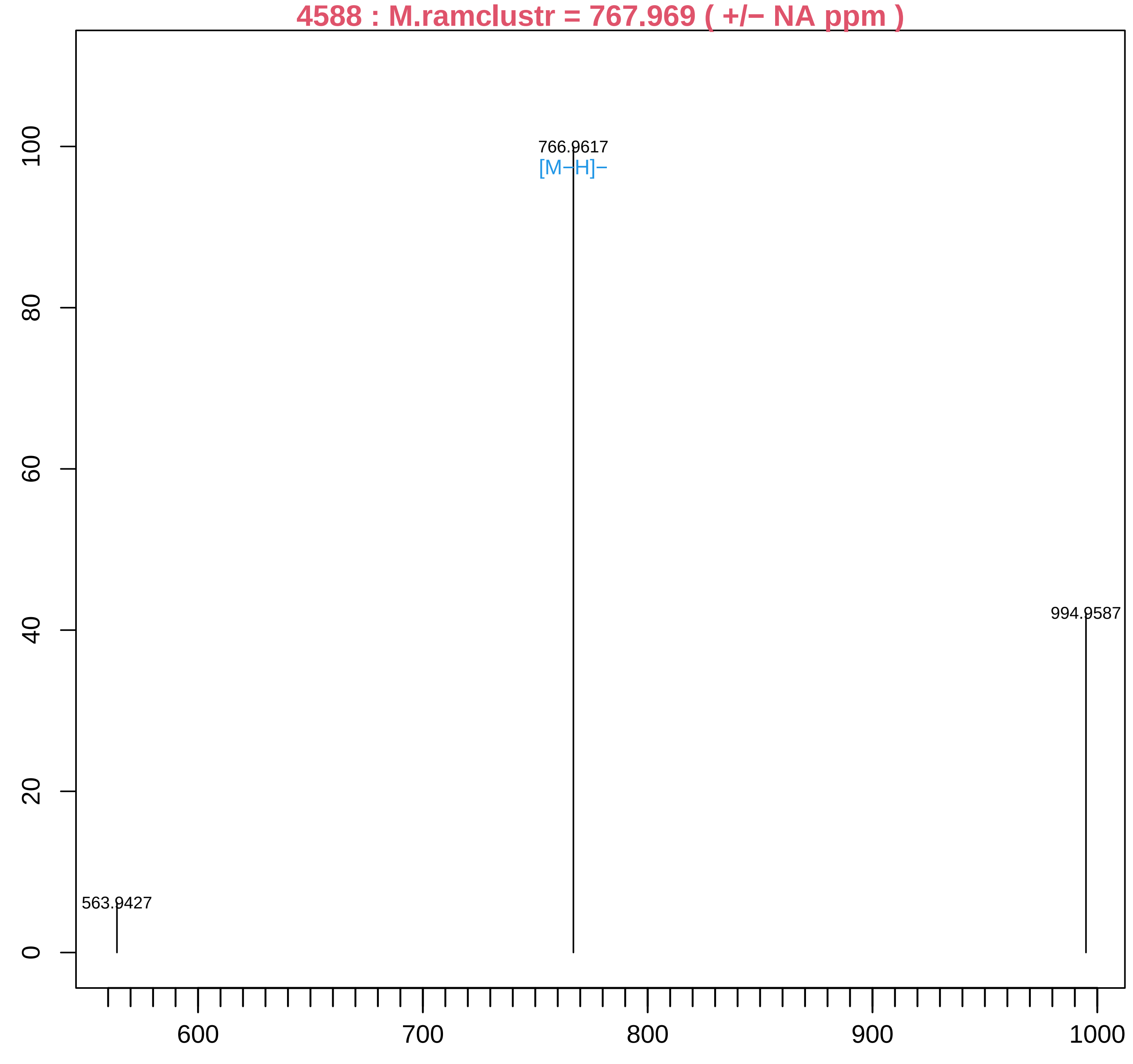
4588 : M.ramclustr = 767.969 (+/− NA ppm)
766.9617
[M−H]−
994.9587
563.9427
0
20
40
60
80
100
600
700
800
900
1000

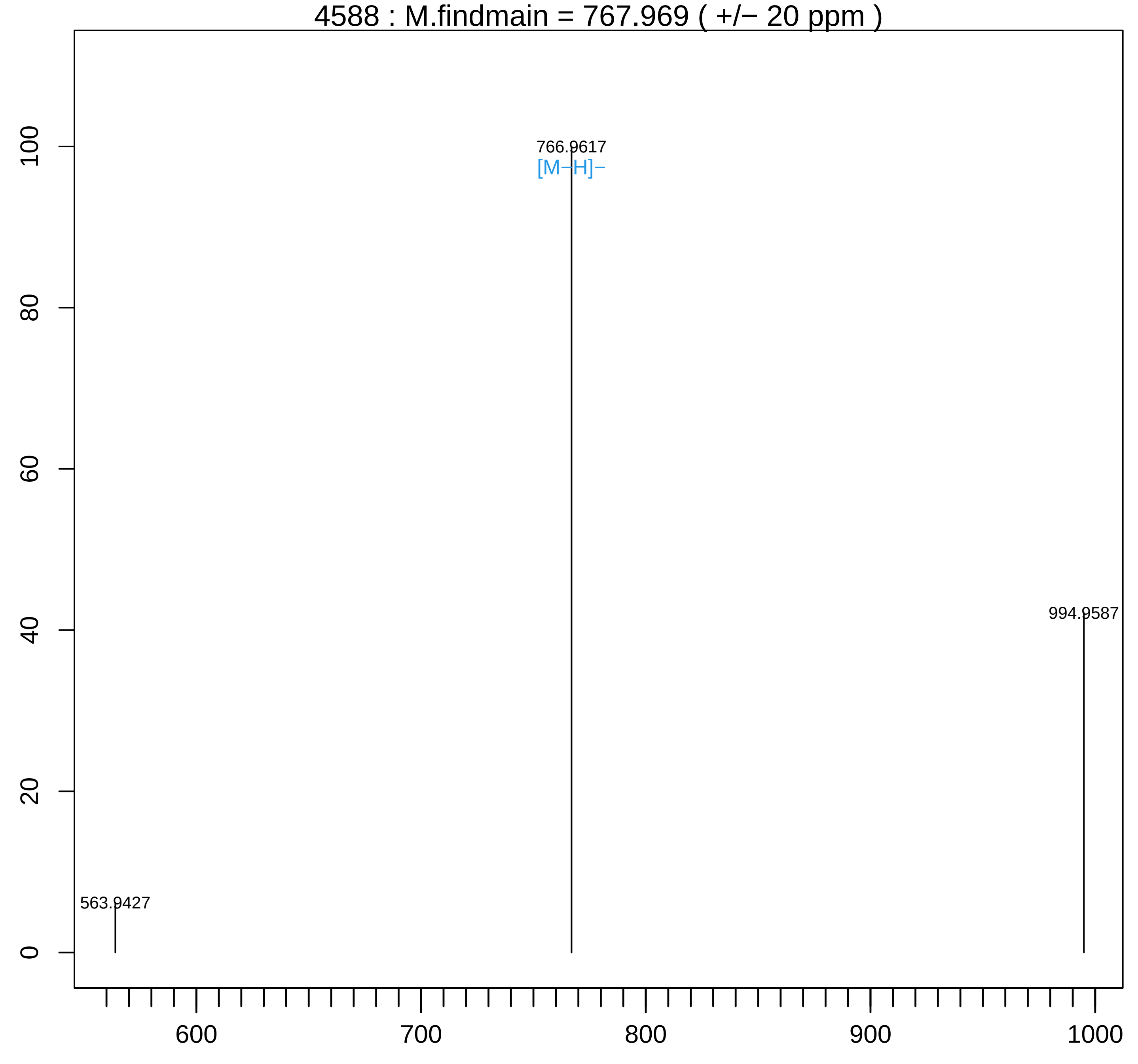
4588 : M.findmain = 767.969 (+/− 20 ppm)
766.9617
[M−H]−
994.9587
563.9427
0
20
40
60
80
100
600
700
800
900
1000

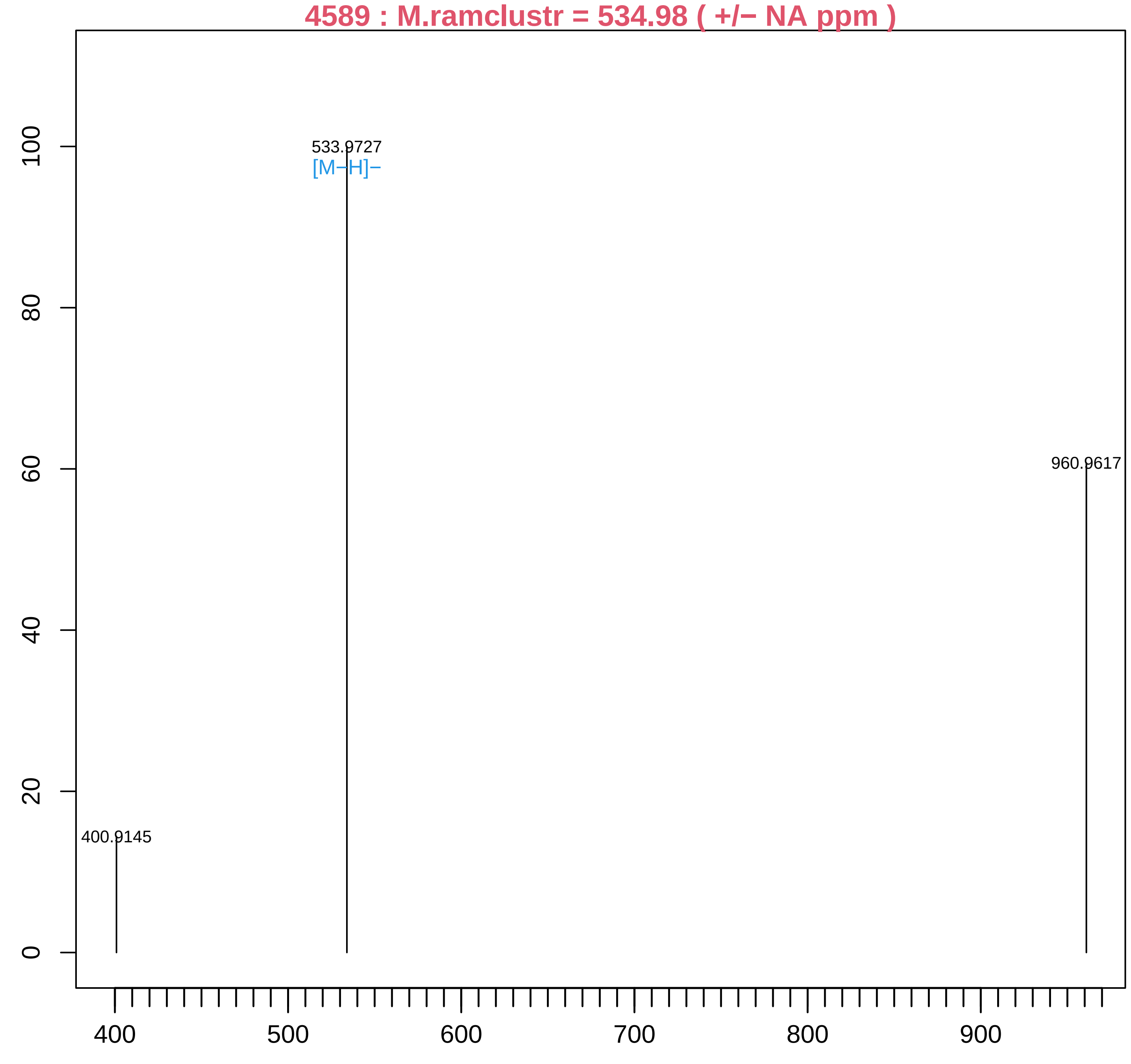

4589 : M.ramclustr = 534.98 (+/− NA ppm)
533.9727
[M−H]−
960.9617
400.9145
0
20
40
60
80
100
400
500
600
700
800
900

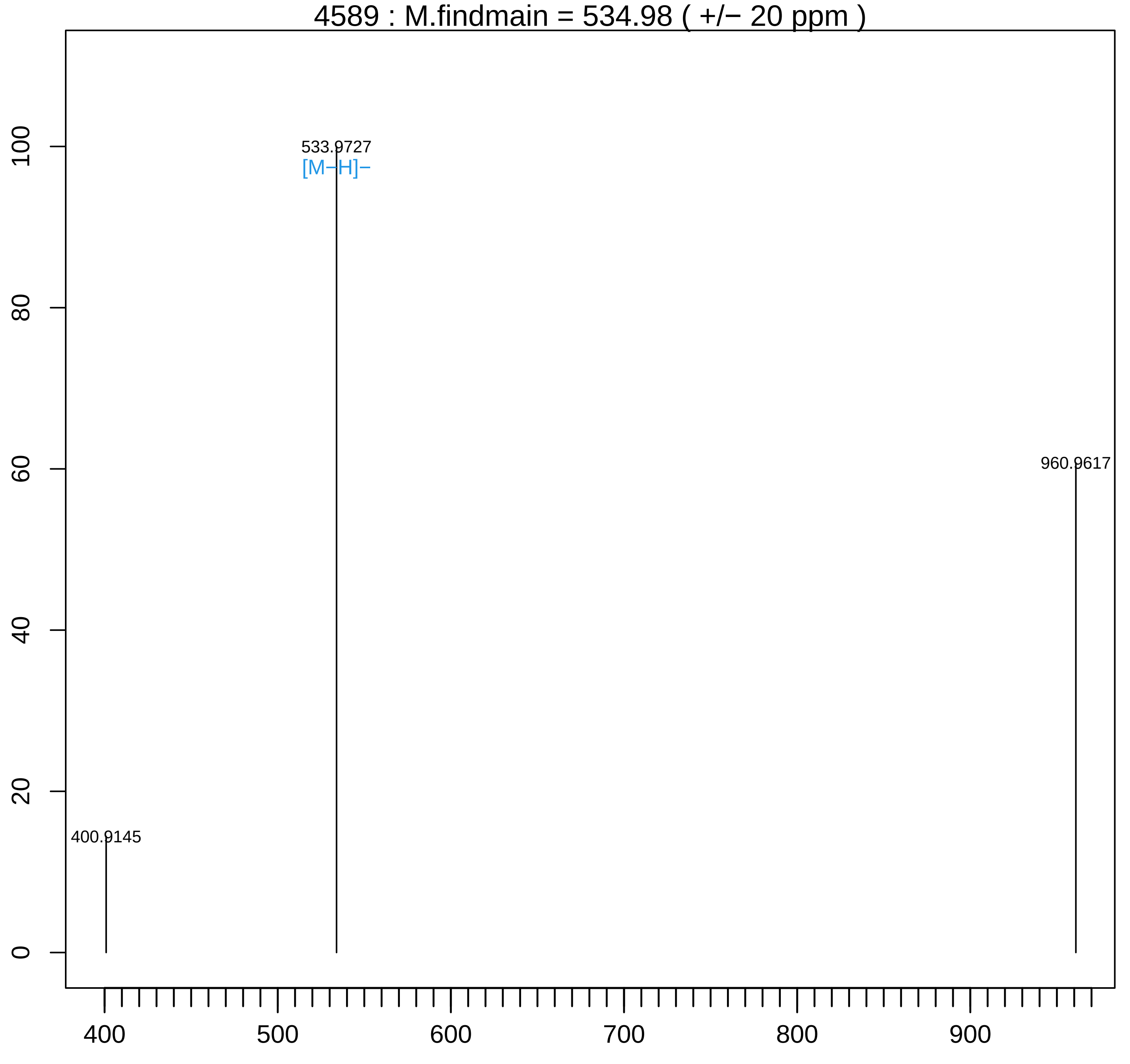

4589 : M.findmain = 534.98 (+/− 20 ppm)
533.9727
[M−H]−
960.9617
400.9145
0
20
40
60
80
100
400
500
600
700
800
900

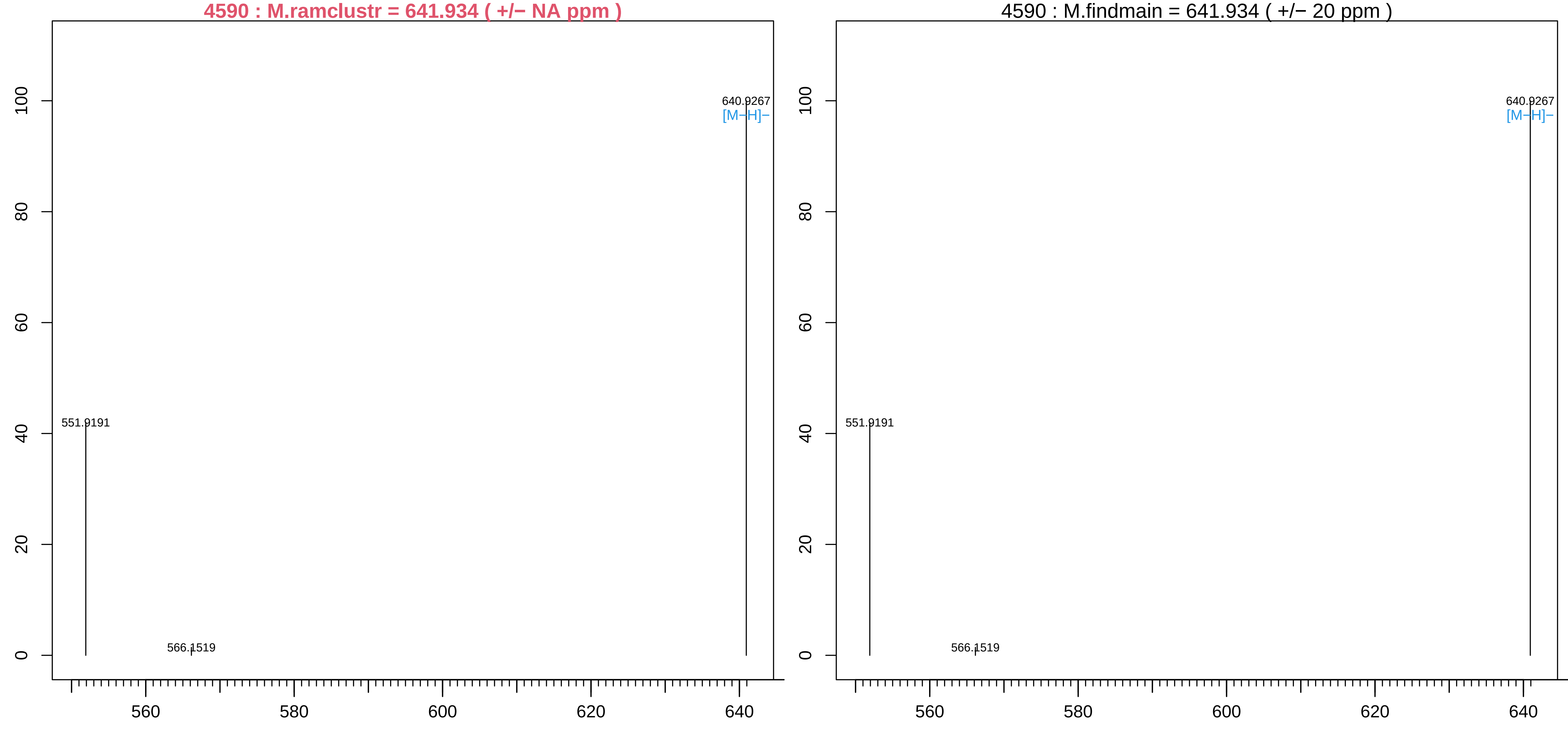
4590 : M.ramclustr = 641.934 (+/− NA ppm)
4590 : M.findmain = 641.934 (+/− 20 ppm)
640.9267
[M−H]−
551.9191
566.1519
0
20
40
60
80
100
560
580
600
620
640

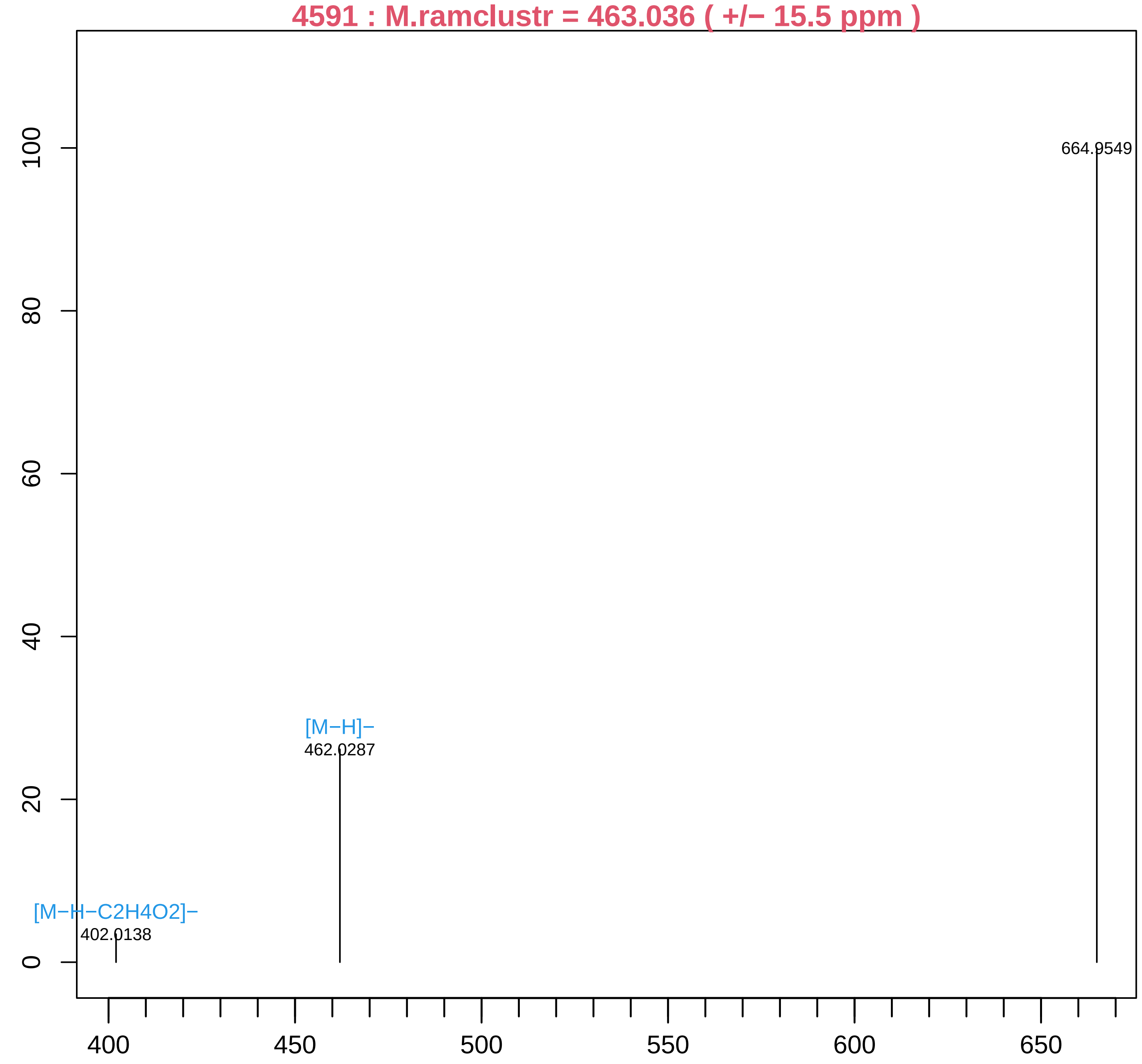
4591 : M.ramclustr = 463.036 (+/− 15.5 ppm)
664.9549
[M−H]−
462.0287
[M−H−C2H4O2]−
402.0138
0
20
40
60
80
100
400
450
500
550
600
650

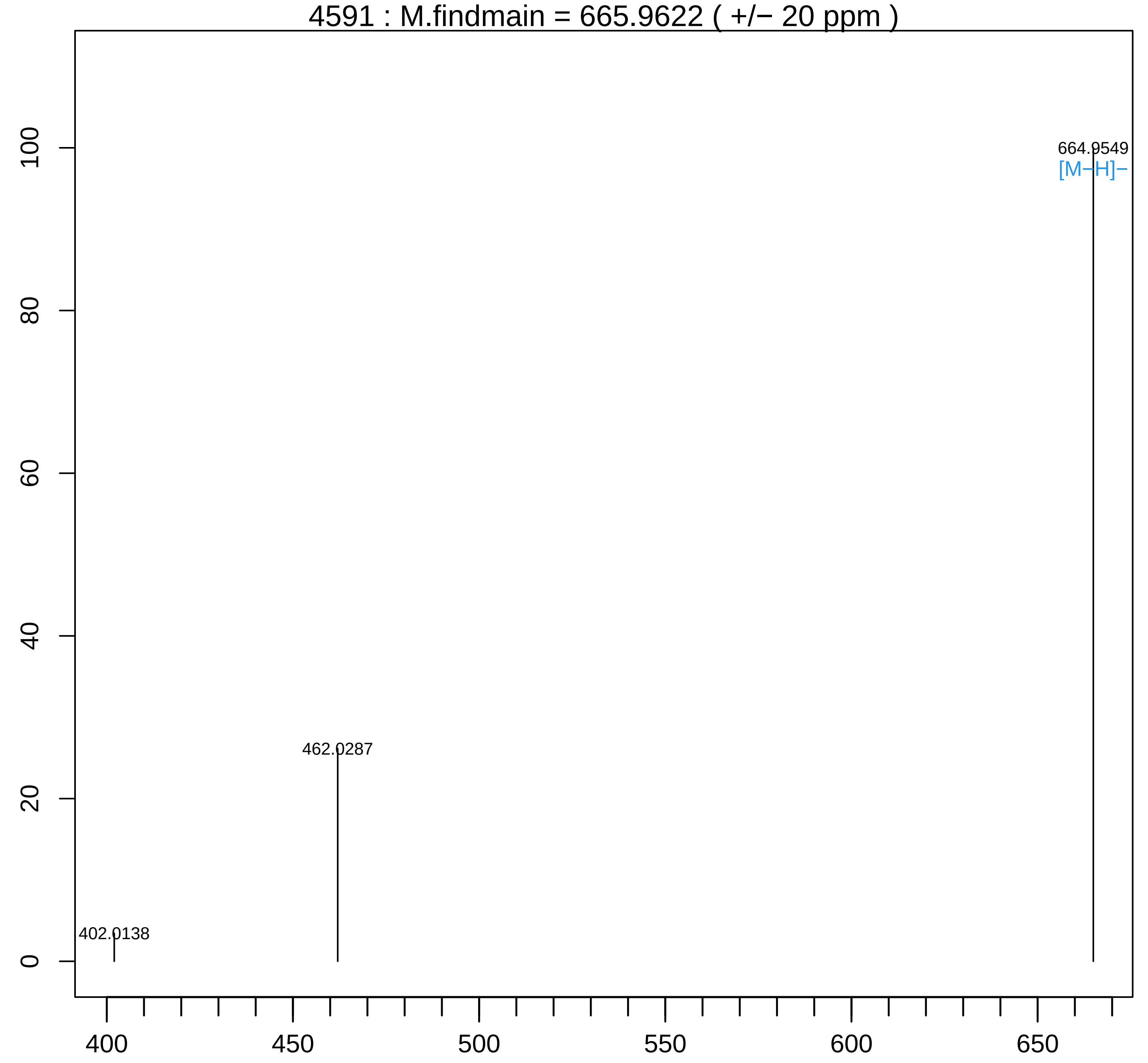
4591 : M.findmain = 665.9622 (+/− 20 ppm)
664.9549
[M−H]−
462.0287
402.0138
0
20
40
60
80
100
400
450
500
550
600
650

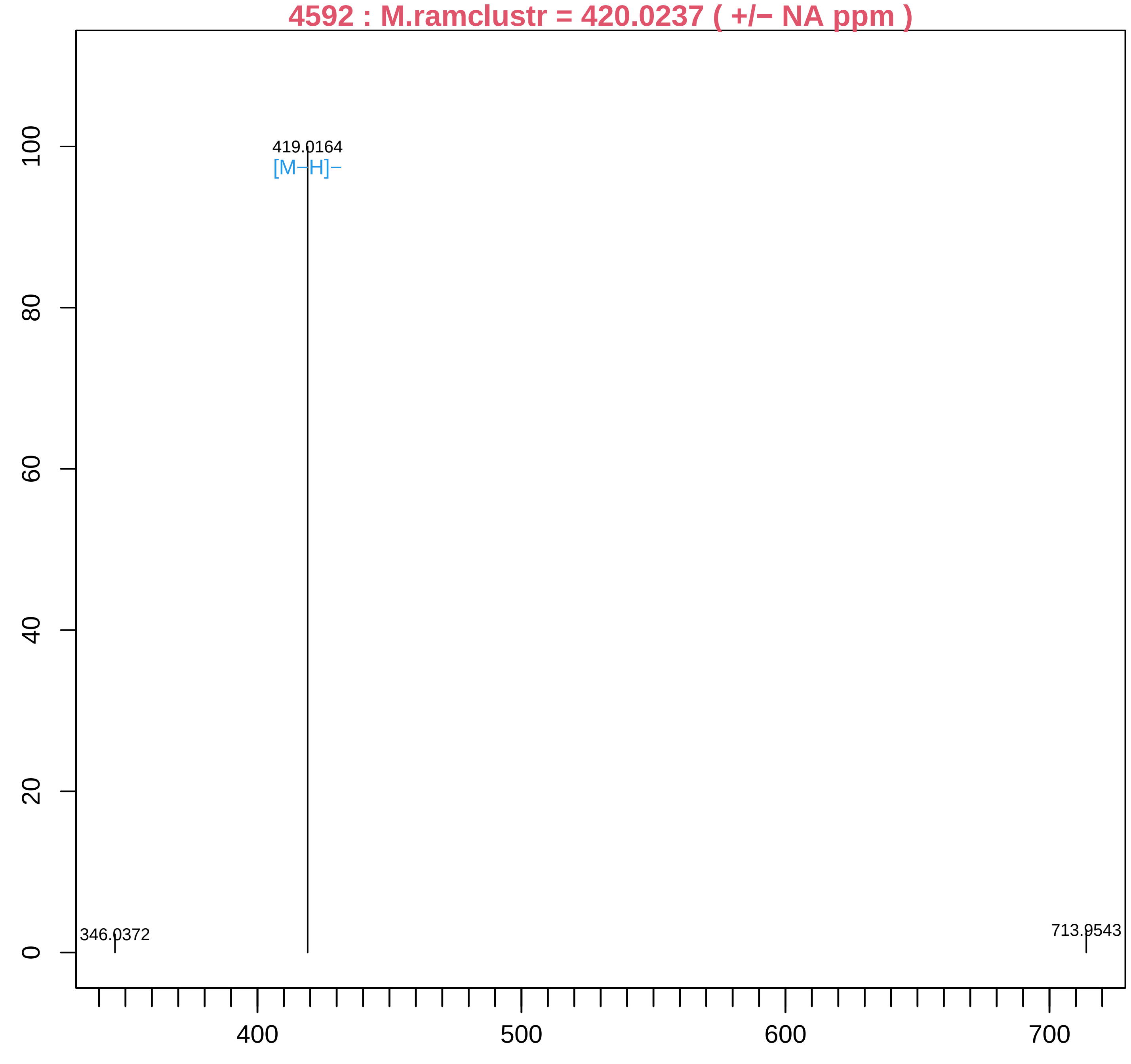

4592 : M.ramclustr = 420.0237 (+/− NA ppm)
419.0164
[M−H]−
346.0372
713.9543
0
20
40
60
80
100
400
500
600
700

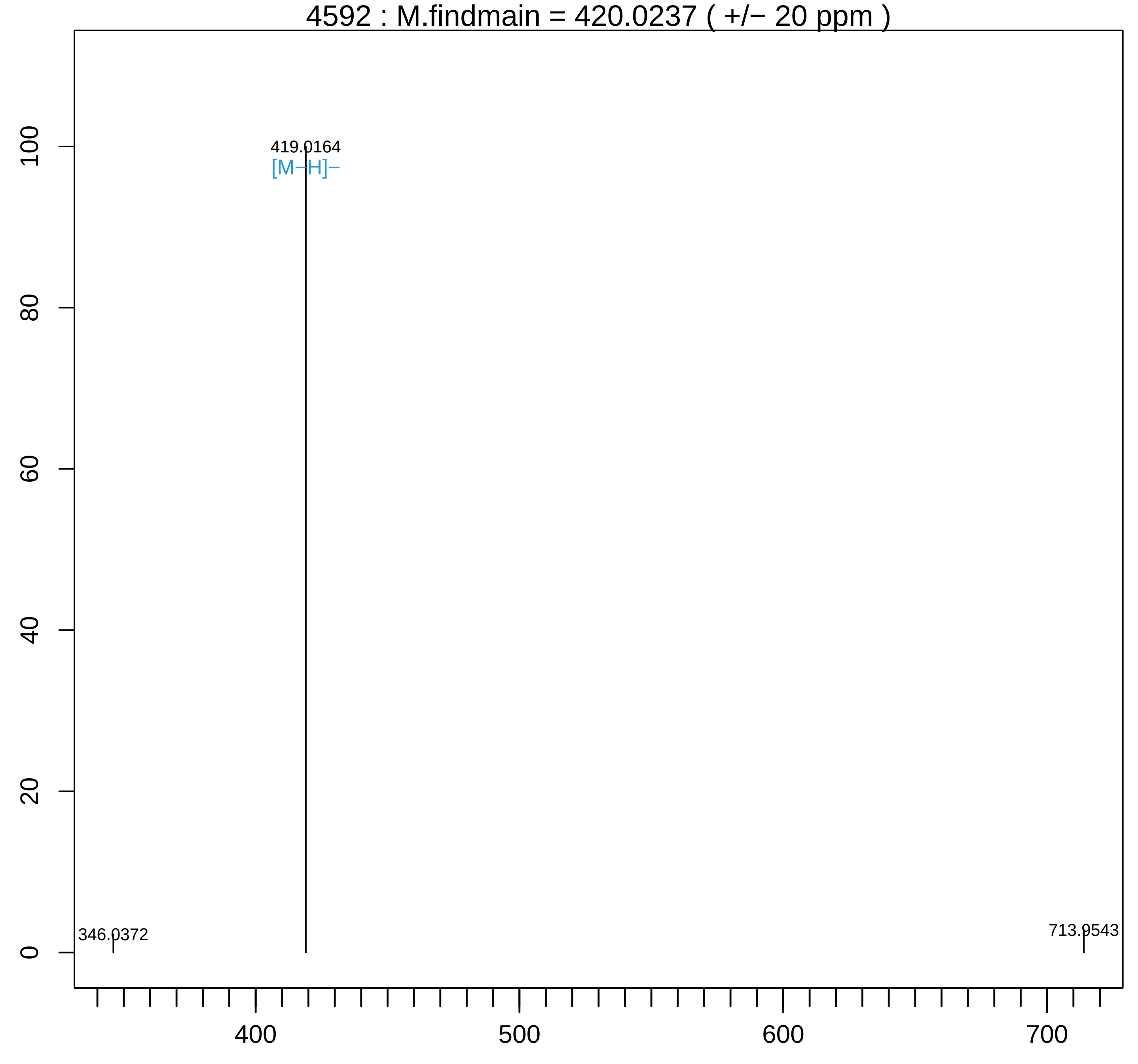

4592 : M.findmain = 420.0237 (+/− 20 ppm)
419.0164
[M−H]−
346.0372
713.9543
0
20
40
60
80
100
400
500
600
700

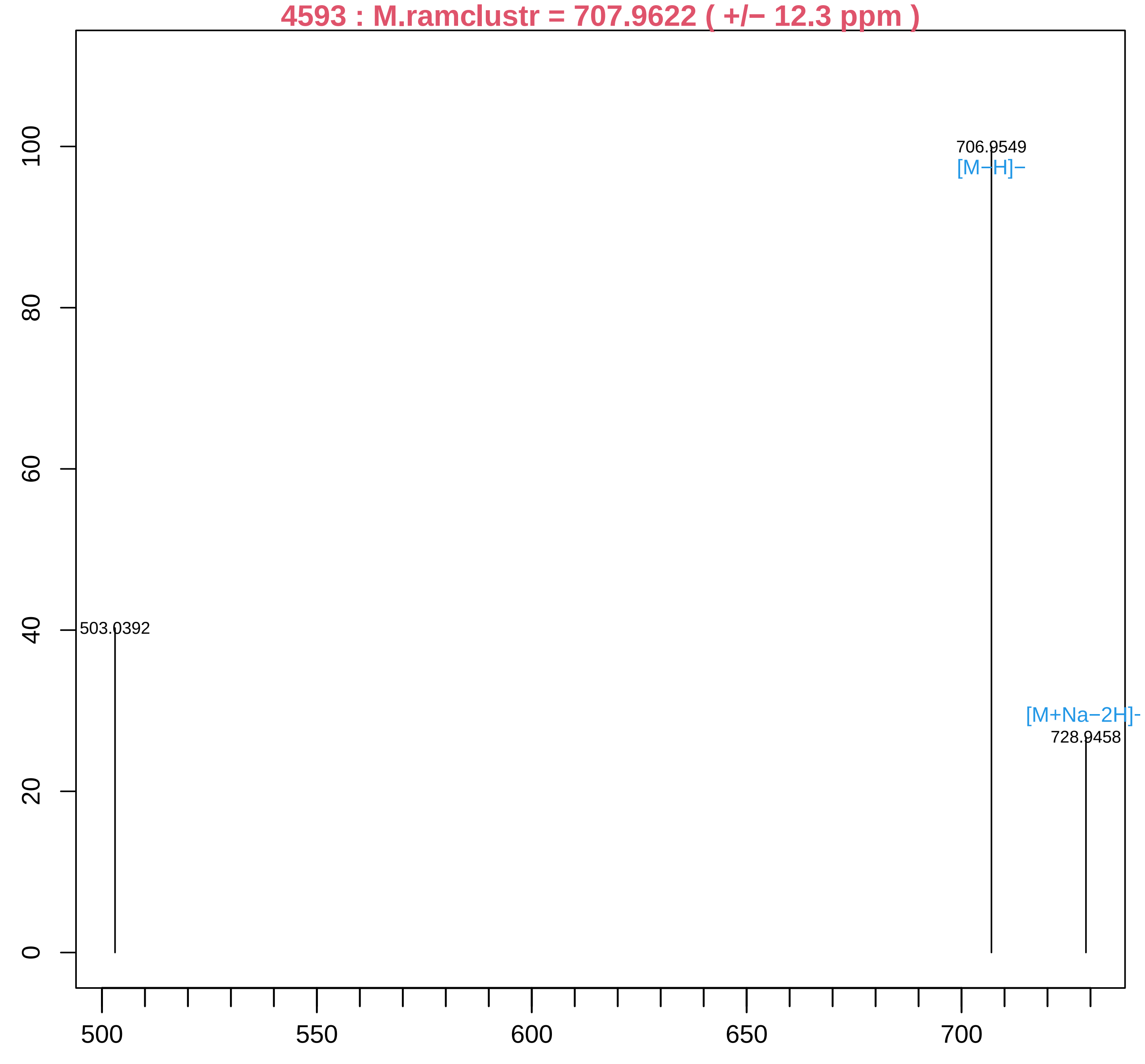

4593 : M.ramclustr = 707.9622 (+/− 12.3 ppm)
706.9549
[M−H]−
503.0392
[M+Na−2H]-
728.9458
0
20
40
60
80
100
500
550
600
650
700

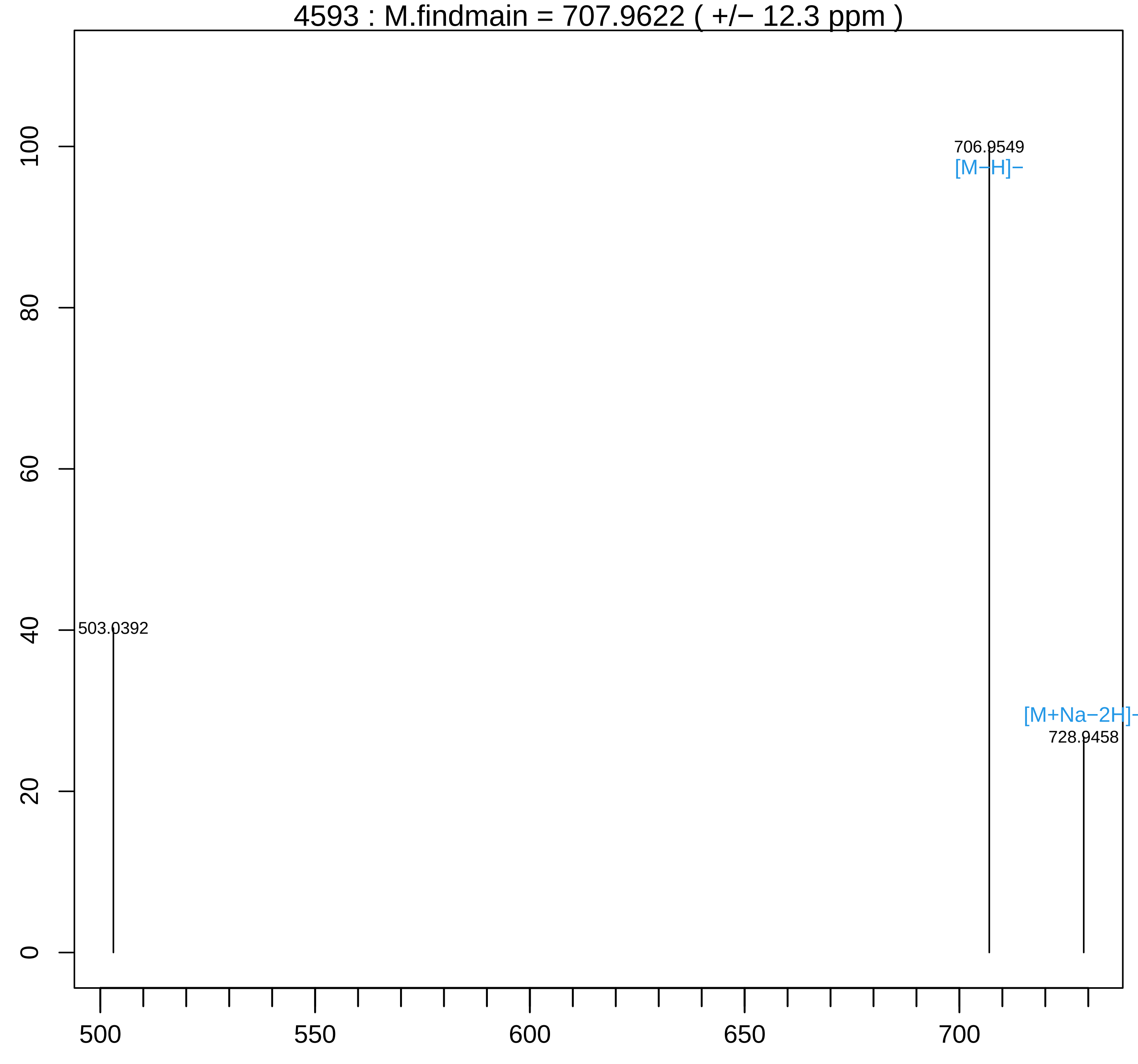

4593 : M.findmain = 707.9622 (+/− 12.3 ppm)
706.9549
[M−H]−
503.0392
[M+Na−2H]-
728.9458
0
20
40
60
80
100
500
550
600
650
700

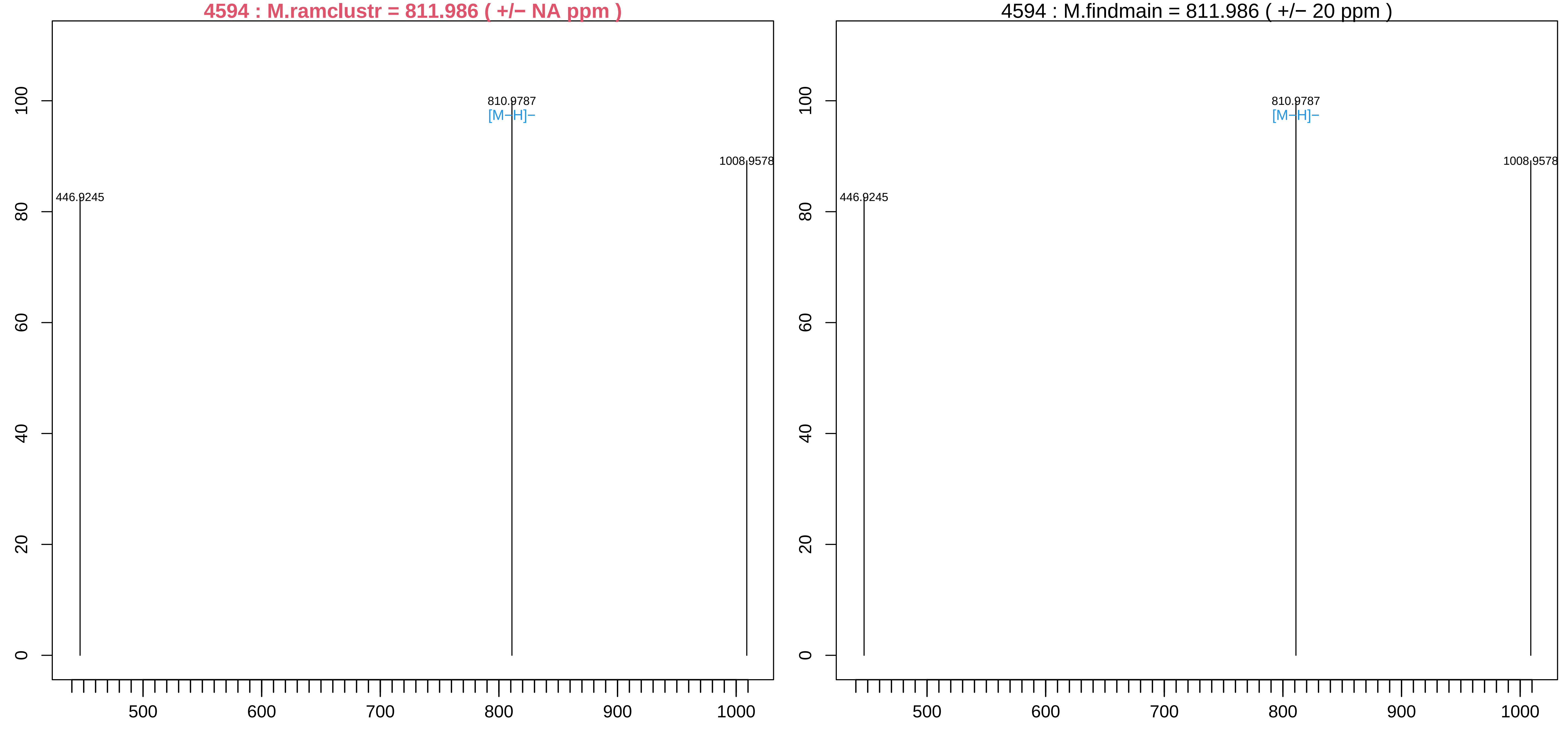
4594 : M.ramclustr = 811.986 (+/− NA ppm)
446.9245
810.9787
[M−H]−
1008.9578
4594 : M.findmain = 811.986 (+/− 20 ppm)
446.9245
810.9787
[M−H]−
1008.9578

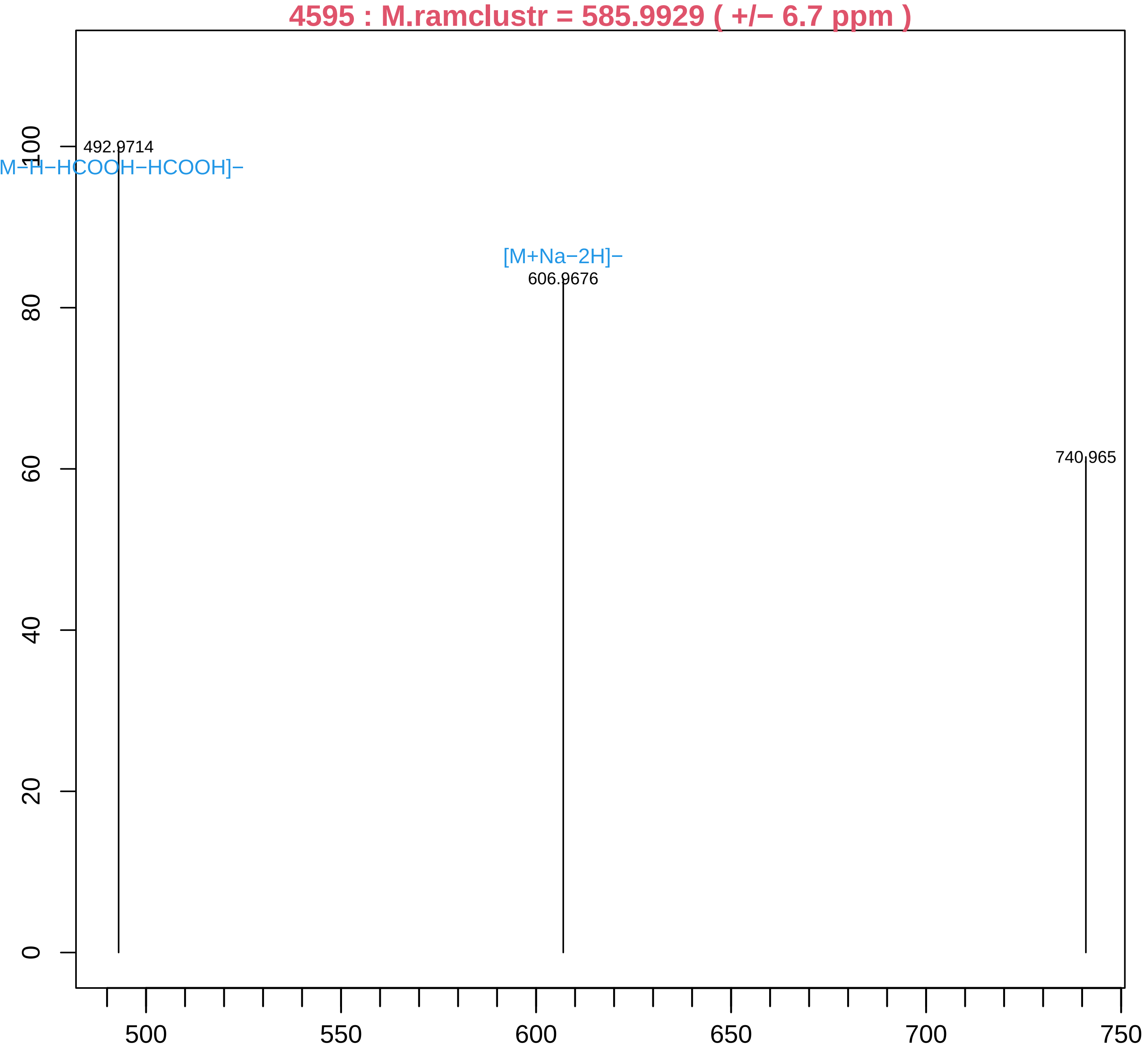

4595 : M.ramclustr = 585.9929 (+/− 6.7 ppm)
492.9714
[M−H−HCOOH−HCOOH]−
[M+Na−2H]−
606.9676
740.965
0
20
40
60
80
100
500
550
600
650
700
750

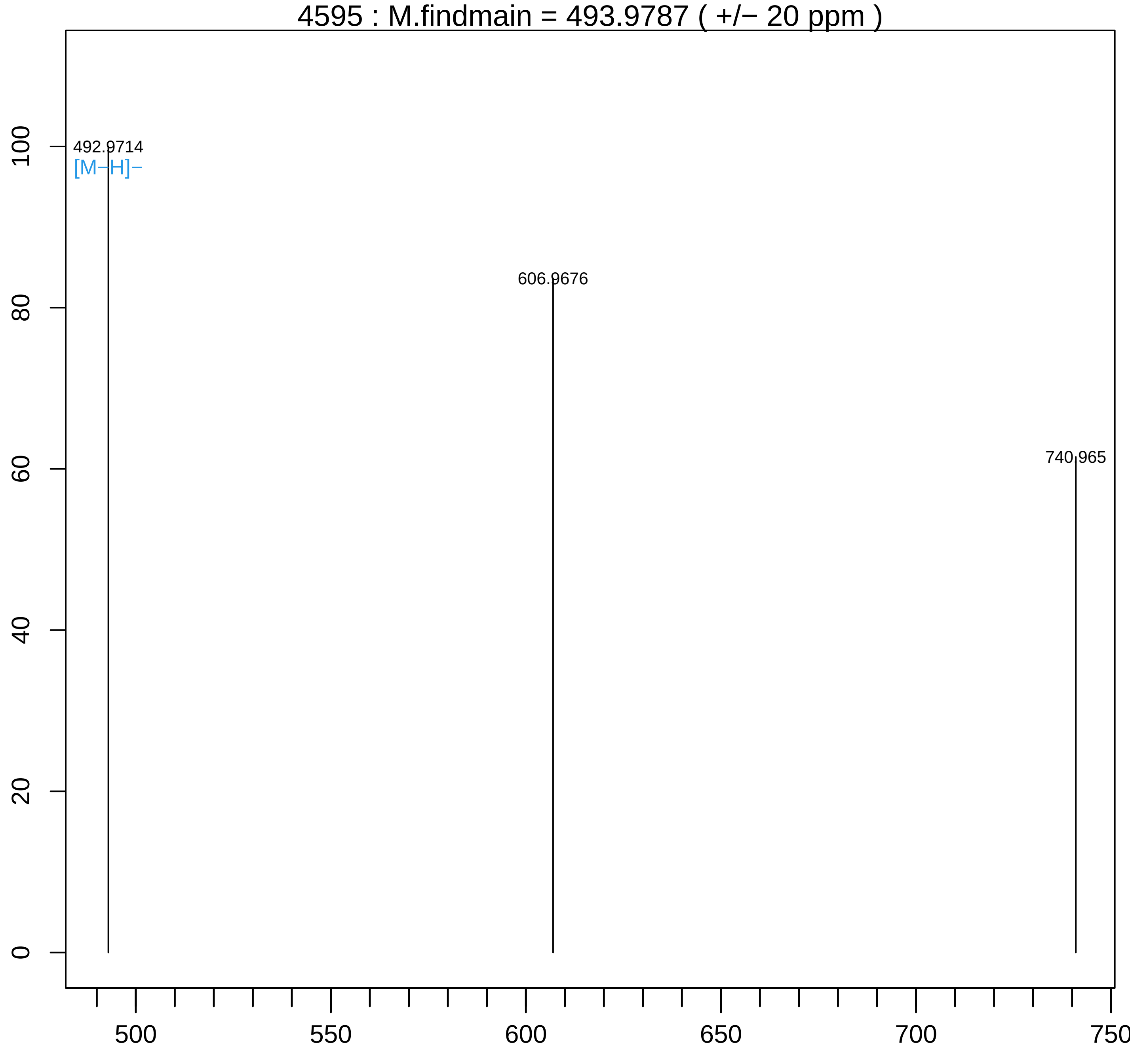

4595 : M.findmain = 493.9787 (+/− 20 ppm)
492.9714
[M−H]−
606.9676
740.965
0
20
40
60
80
100
500
550
600
650
700
750

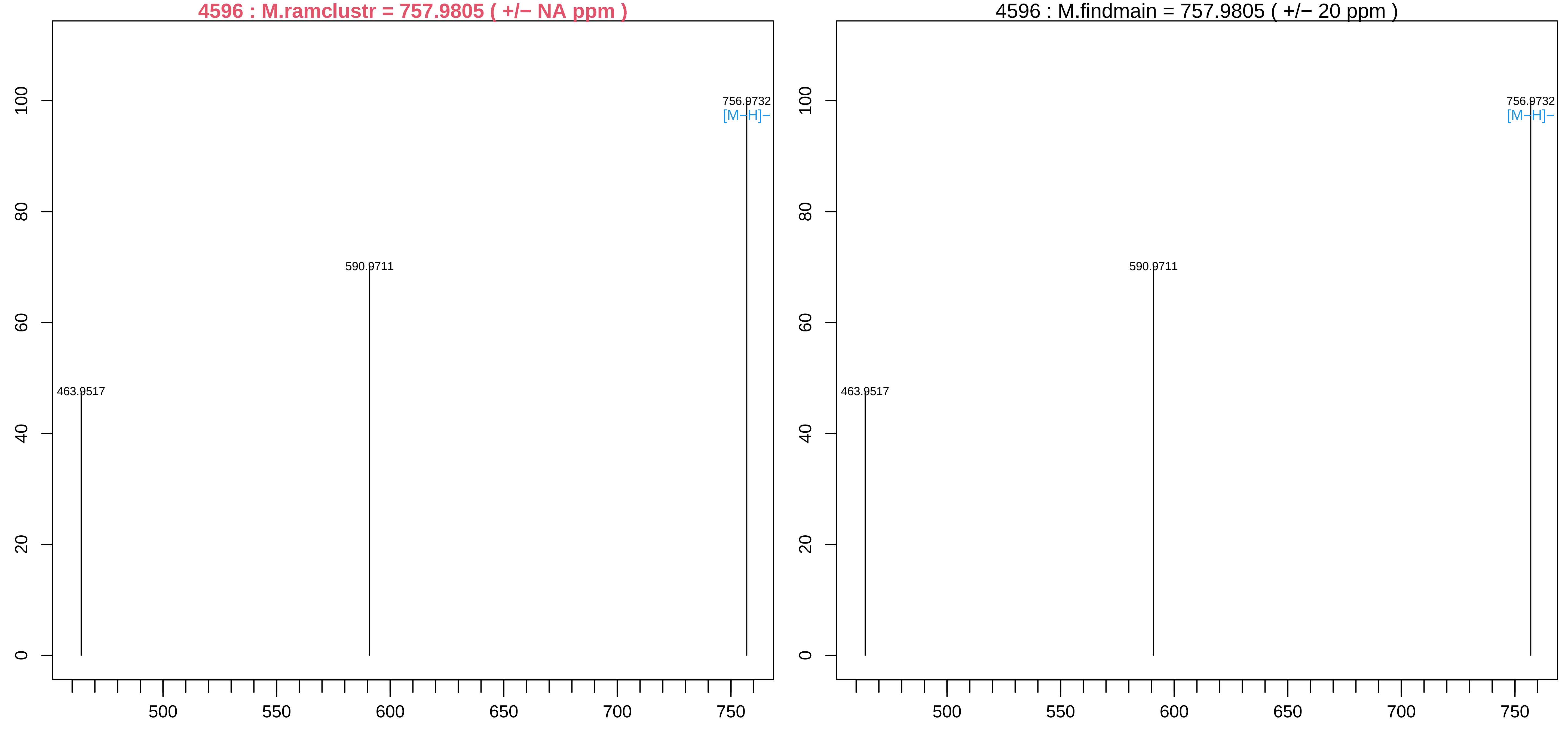

4596 : M.ramclustr = 757.9805 (+/− NA ppm)
4596 : M.findmain = 757.9805 (+/− 20 ppm)
756.9732
[M−H]−
590.9711
463.9517
756.9732
[M−H]−
590.9711
463.9517
0
20
40
60
80
100
500
550
600
650
700
750

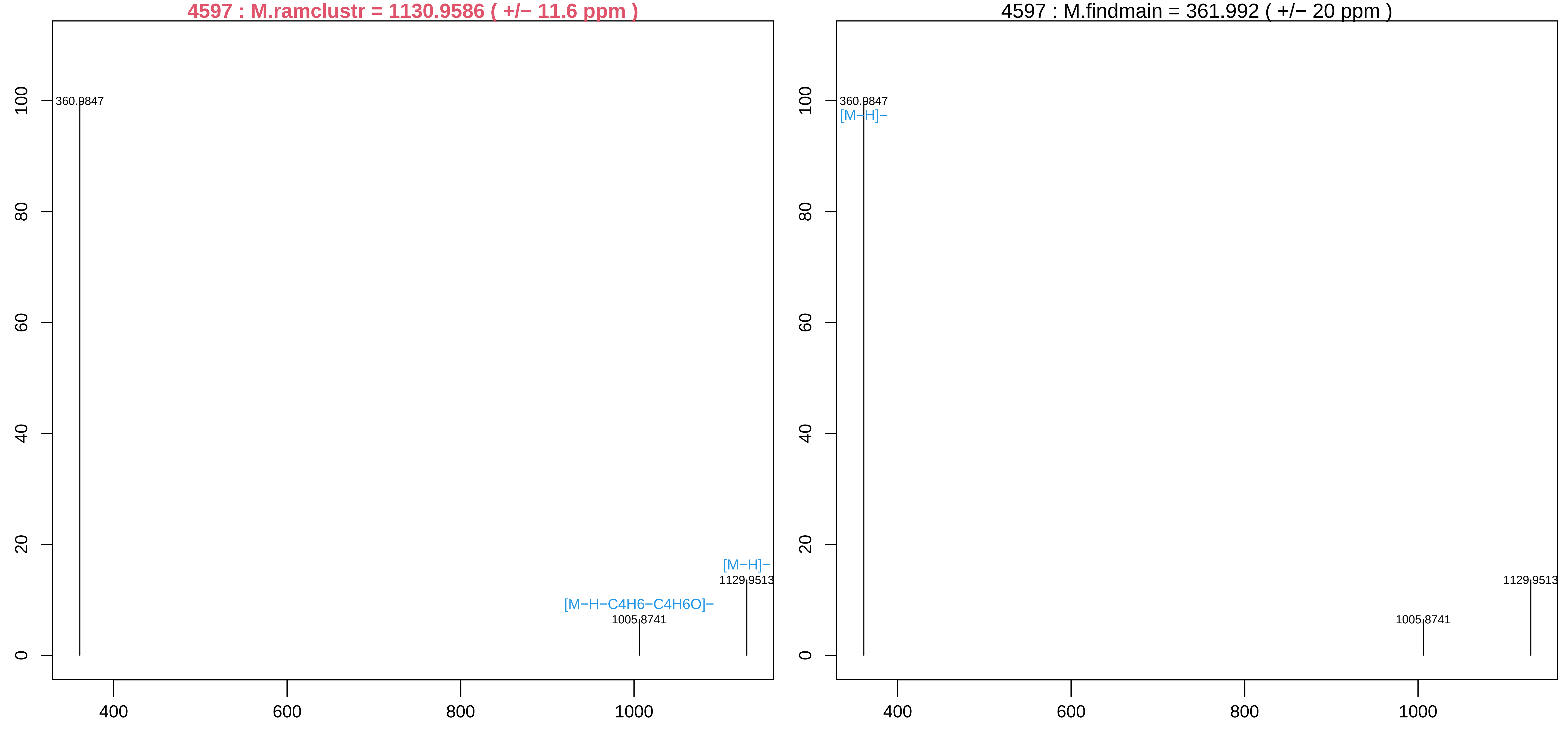
4597 : M.ramclustr = 1130.9586 (+/− 11.6 ppm)
360.9847
[M−H−C4H6−C4H6O]−
1005.8741
[M−H]−
1129.9513
4597 : M.findmain = 361.992 (+/− 20 ppm)
360.9847
[M−H]−
1005.8741
1129.9513
0
20
40
60
80
100
400
600
800
1000

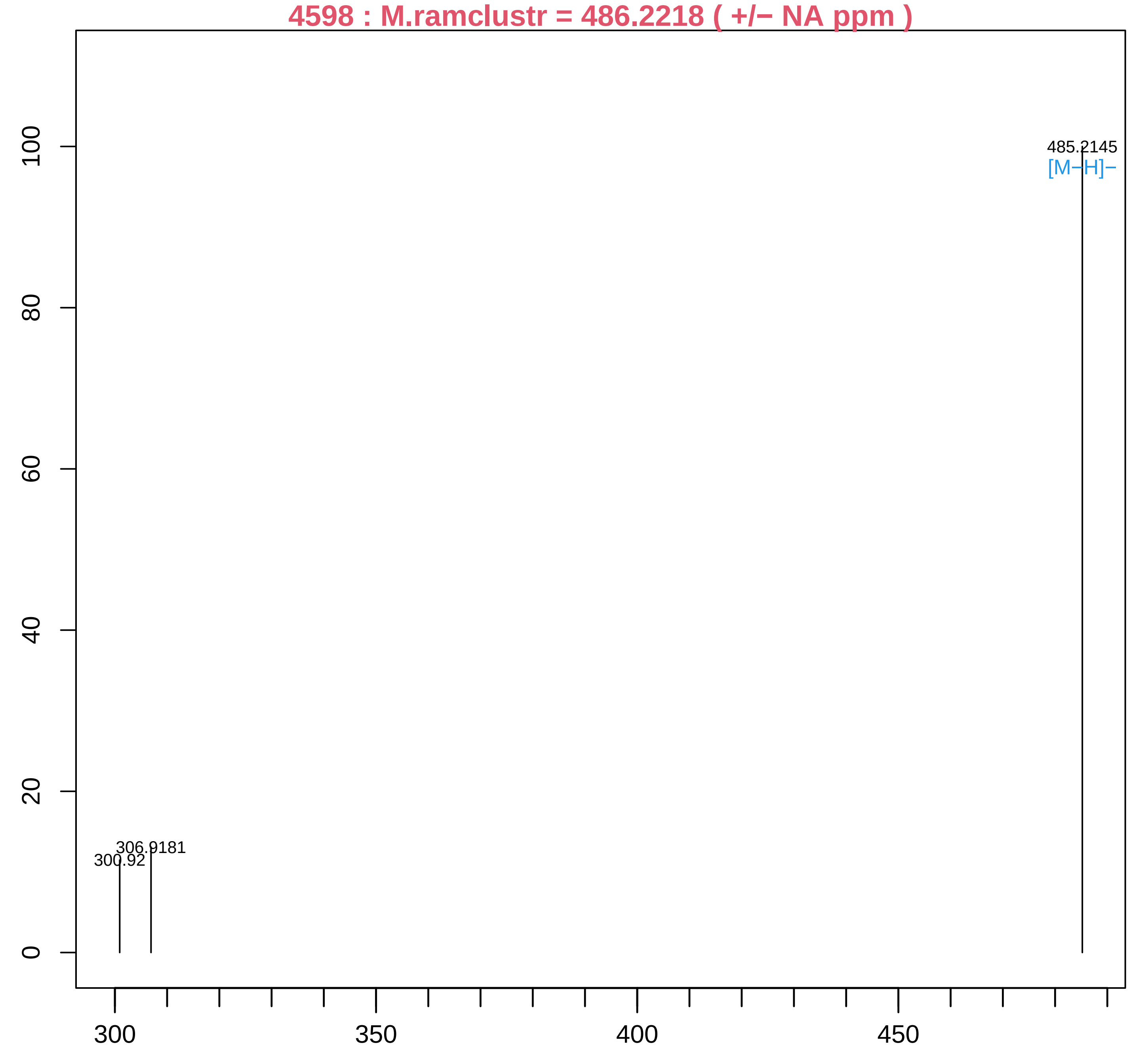

4598 : M.ramclustr = 486.2218 (+/− NA ppm)
485.2145
[M−H]−
306.9181
300.92
0
20
40
60
80
100
300
350
400
450

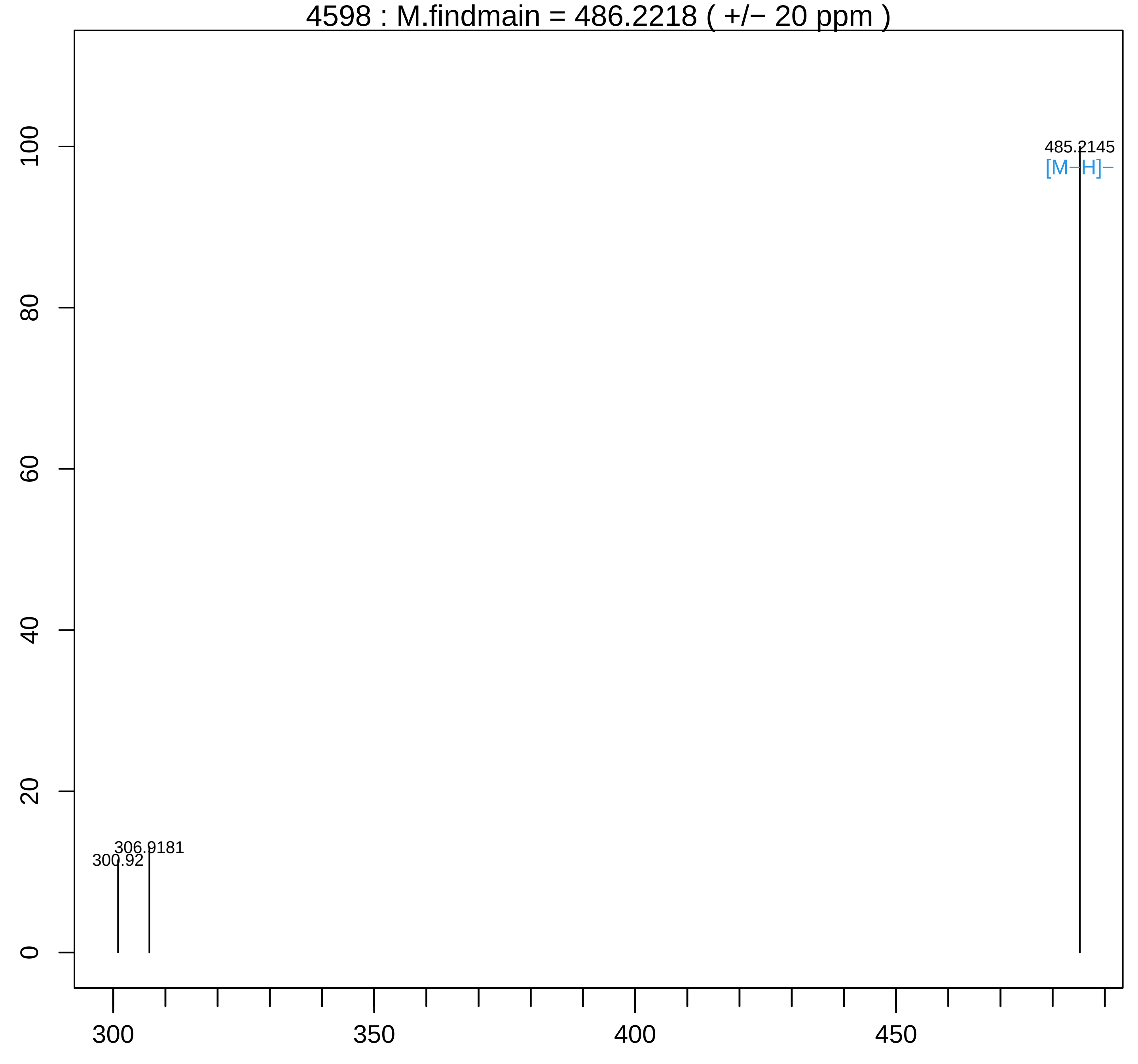

4598 : M.findmain = 486.2218 (+/− 20 ppm)
485.2145
[M−H]−
306.9181
300.92
0
20
40
60
80
100
300
350
400
450

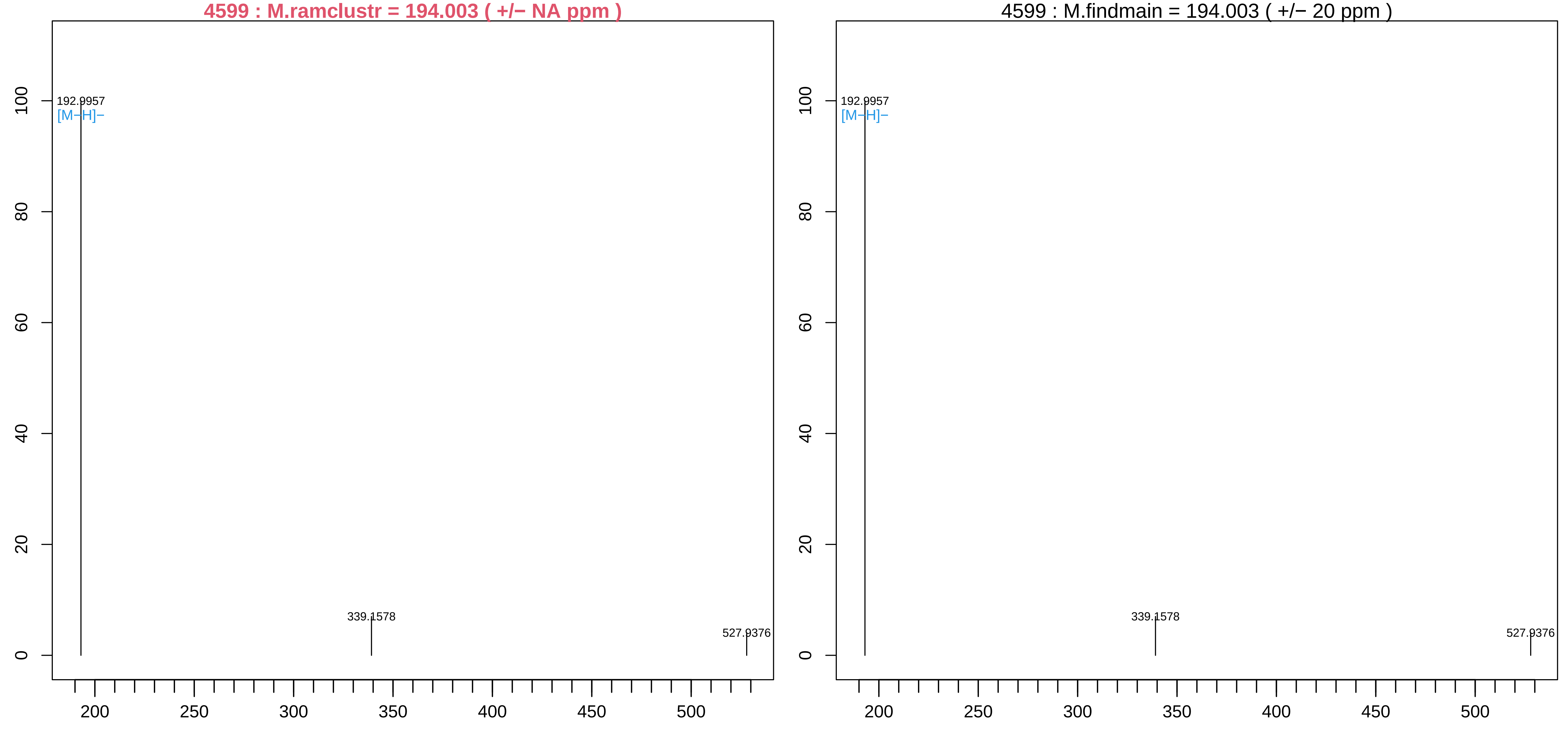
4599 : M.ramclustr = 194.003 (+/− NA ppm)
192.9957
[M−H]−
339.1578
527.9376
0
20
40
60
80
100
200
250
300
350
400
450
500
4599 : M.findmain = 194.003 (+/− 20 ppm)
192.9957
[M−H]−
339.1578
527.9376

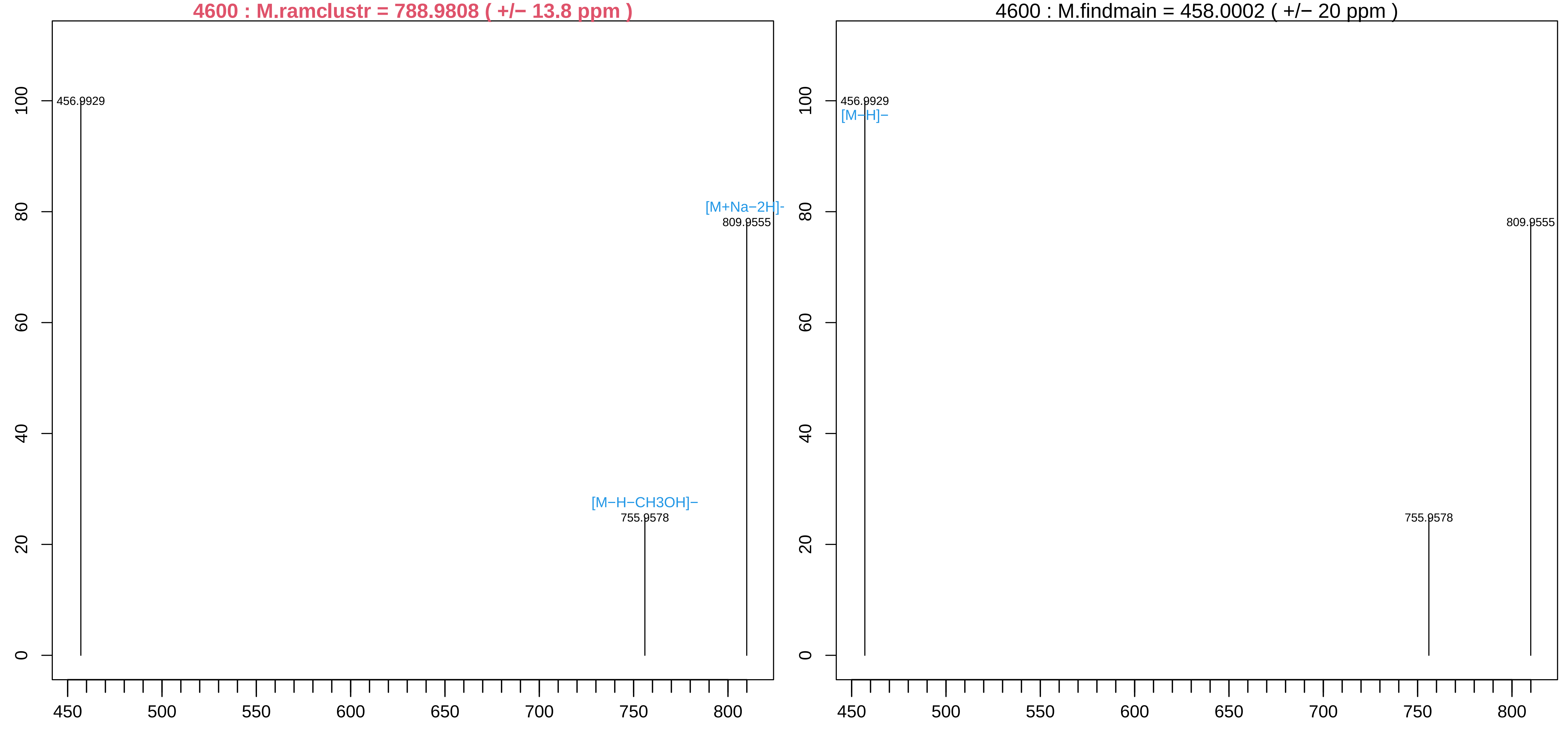

4600 : M.ramclustr = 788.9808 (+/− 13.8 ppm)
456.9929
[M+Na−2H]-
809.9555
[M−H−CH3OH]−
755.9578
4600 : M.findmain = 458.0002 (+/− 20 ppm)
456.9929
[M−H]−
809.9555
755.9578
0
20
40
60
80
100
450
500
550
600
650
700
750
800

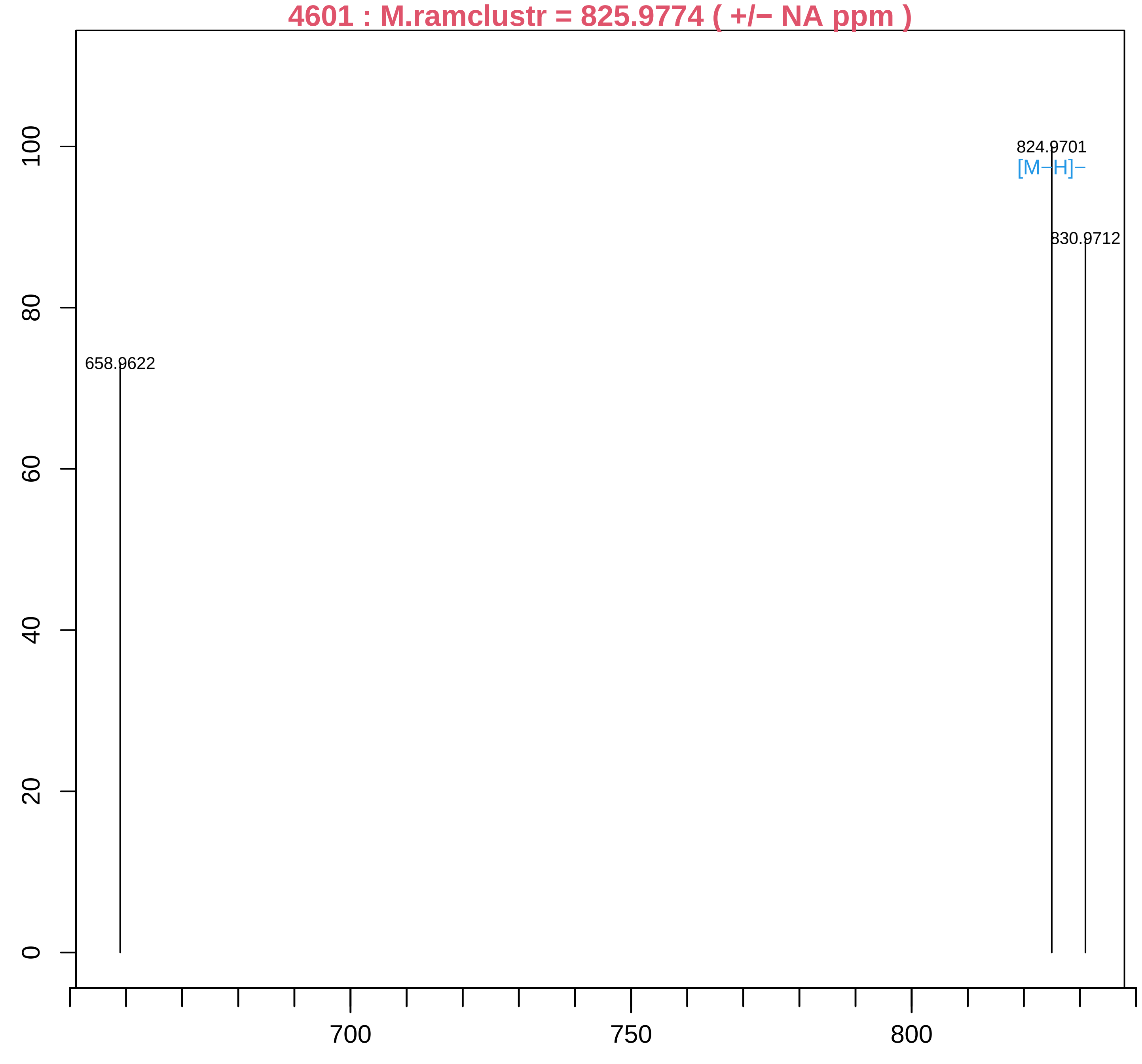
4601 : M.ramclustr = 825.9774 (+/− NA ppm)
824.9701
[M−H]−
830.9712
658.9622
0
20
40
60
80
100
700
750
800

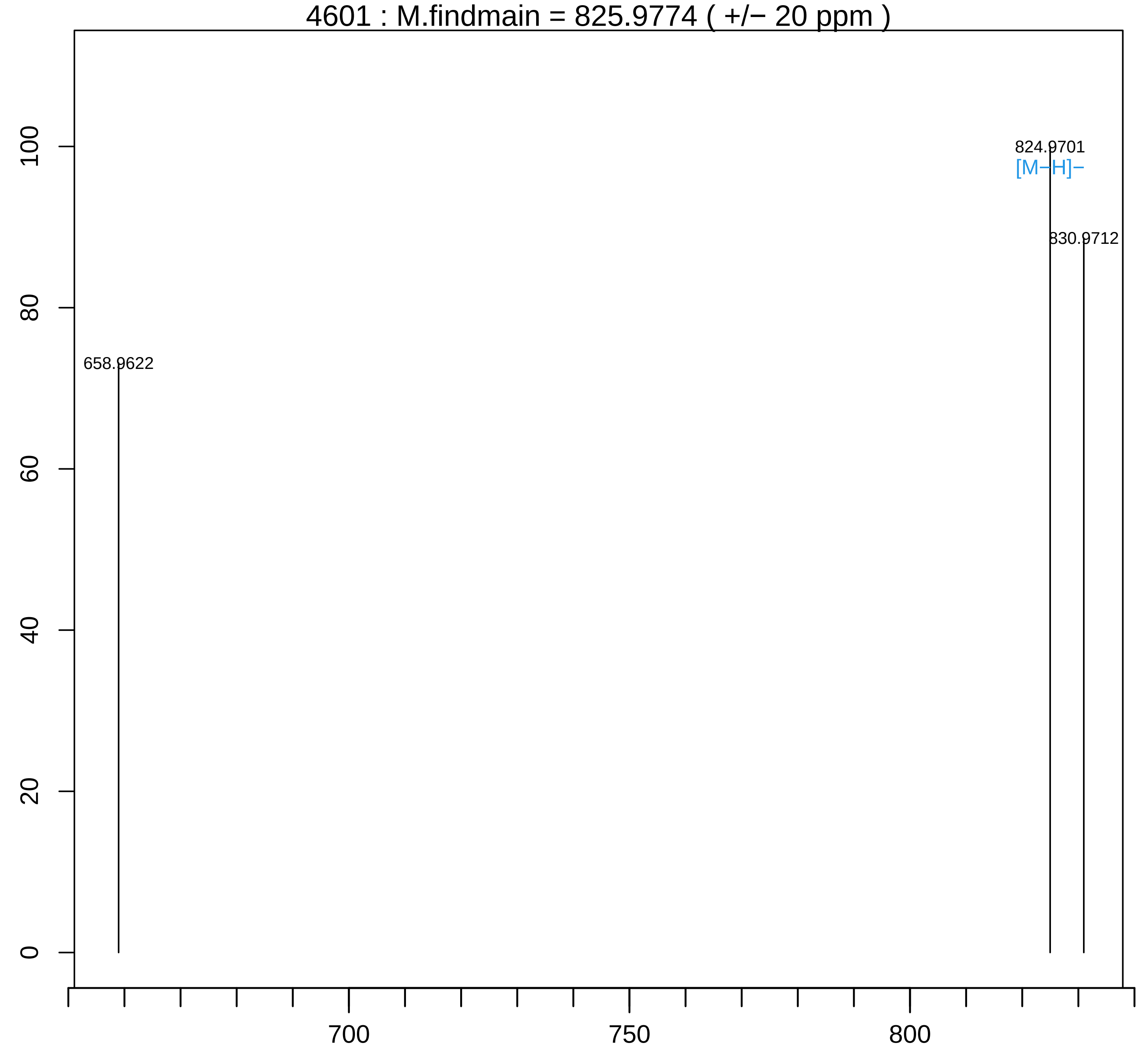
4601 : M.findmain = 825.9774 (+/− 20 ppm)
824.9701
[M−H]−
830.9712
658.9622
0
20
40
60
80
100
700
750
800

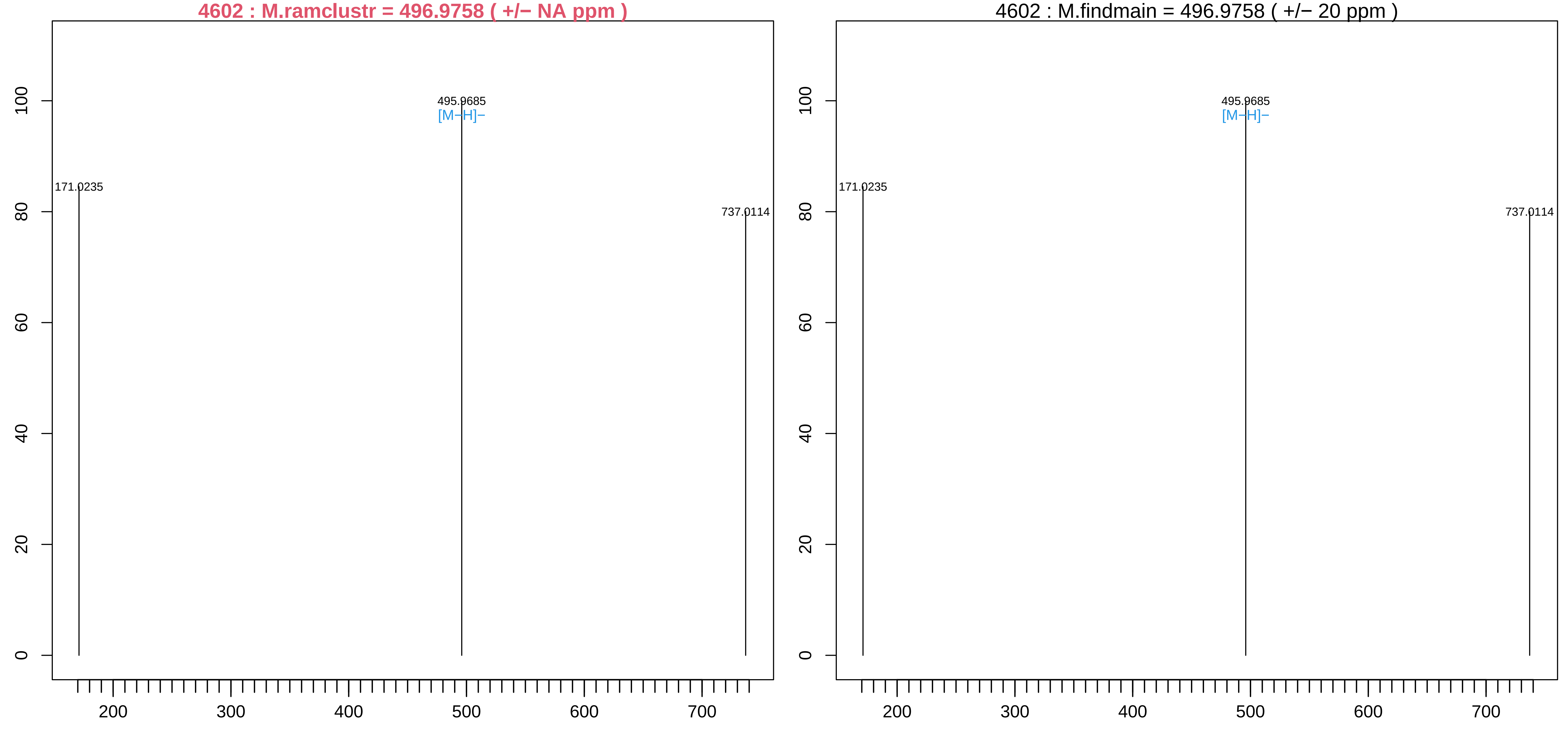
4602 : M.ramclustr = 496.9758 (+/− NA ppm)
4602 : M.findmain = 496.9758 (+/− 20 ppm)
171.0235
495.9685
[M−H]−
737.0114
0
20
40
60
80
100
200
300
400
500
600
700

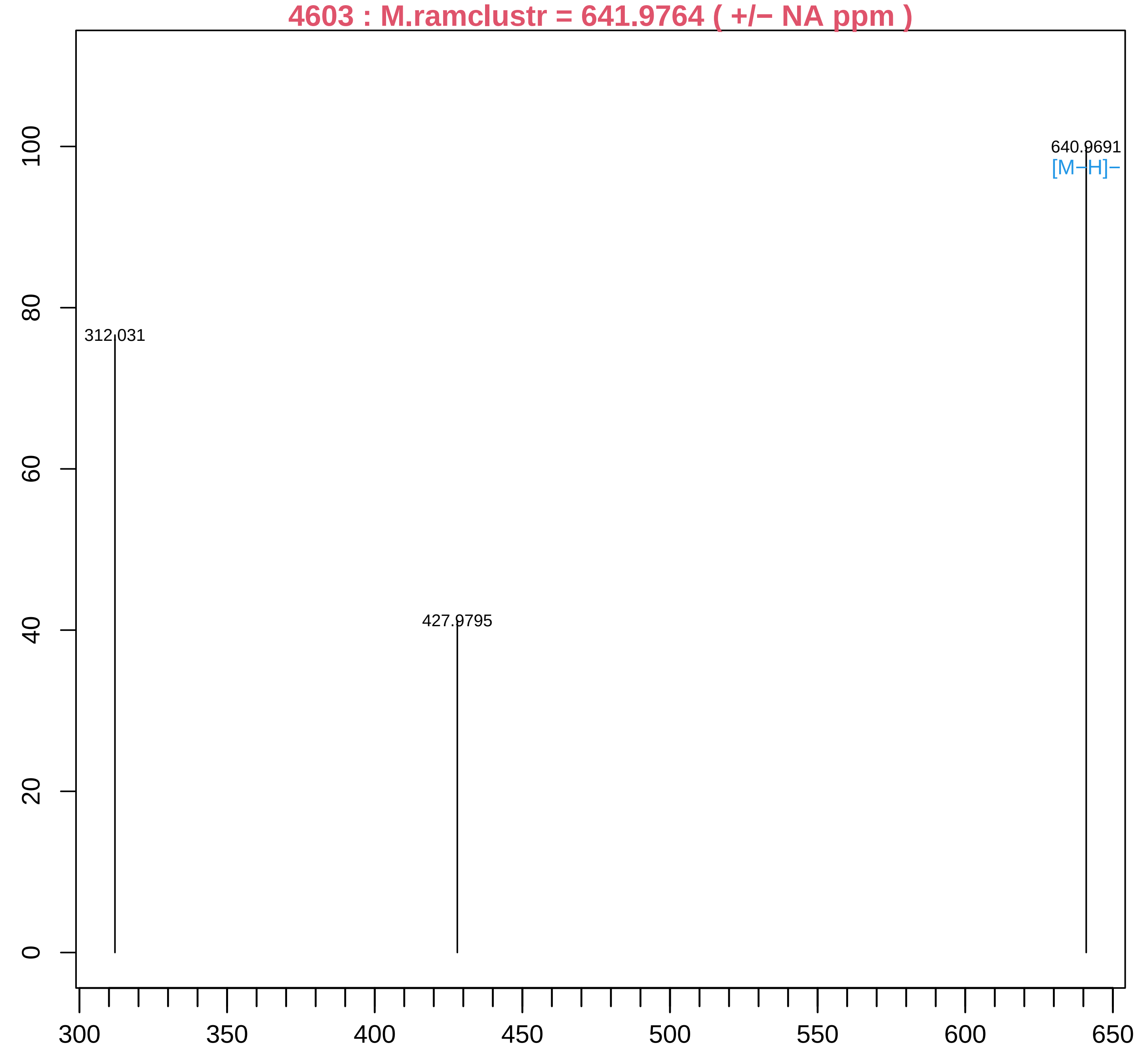
4603 : M.ramclustr = 641.9764 (+/− NA ppm)
640.9691
[M−H]−
312.031
427.9795
0
20
40
60
80
100
300
350
400
450
500
550
600
650

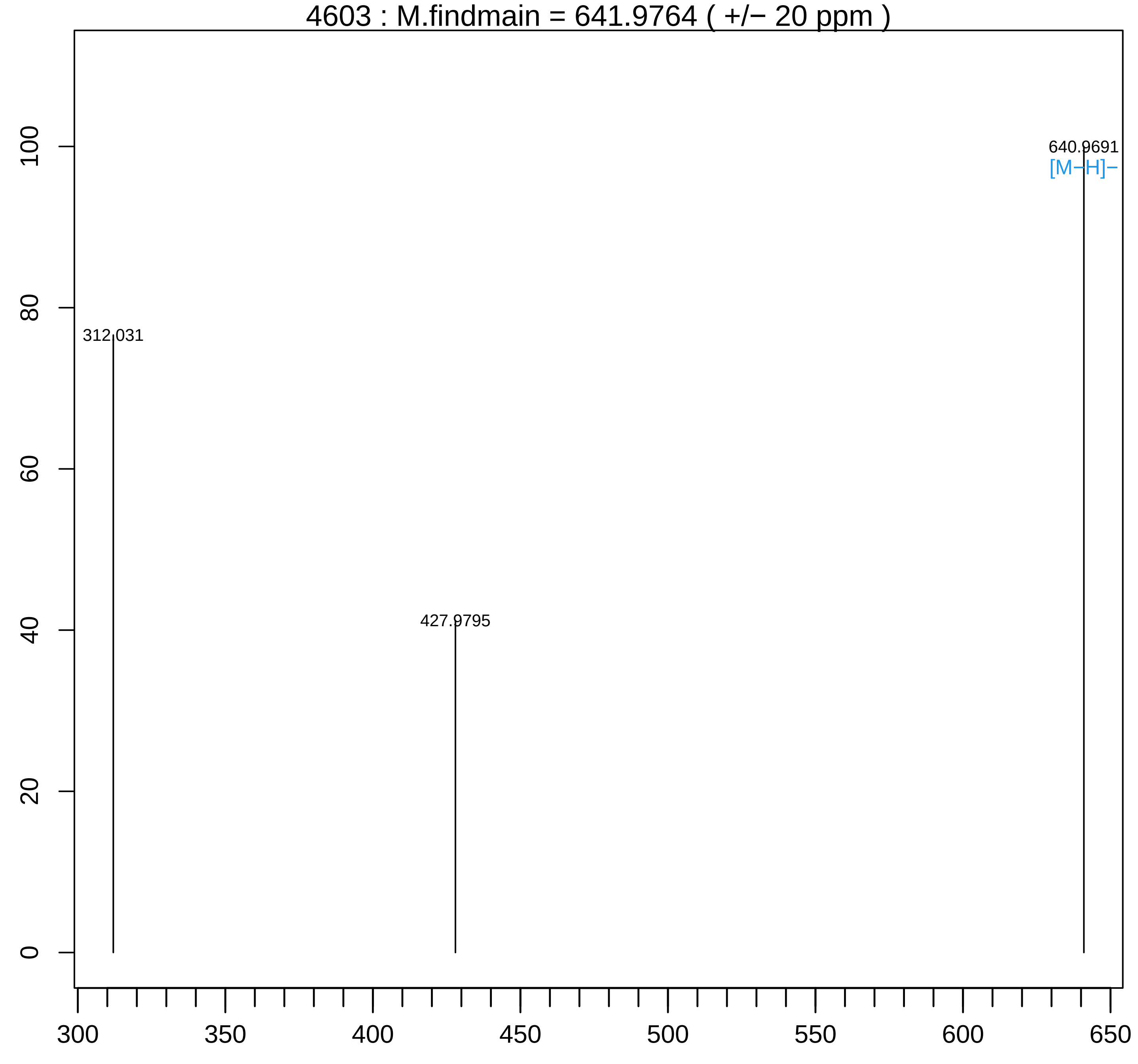
4603 : M.findmain = 641.9764 (+/− 20 ppm)
640.9691
[M−H]−
312.031
427.9795
0
20
40
60
80
100
300
350
400
450
500
550
600
650

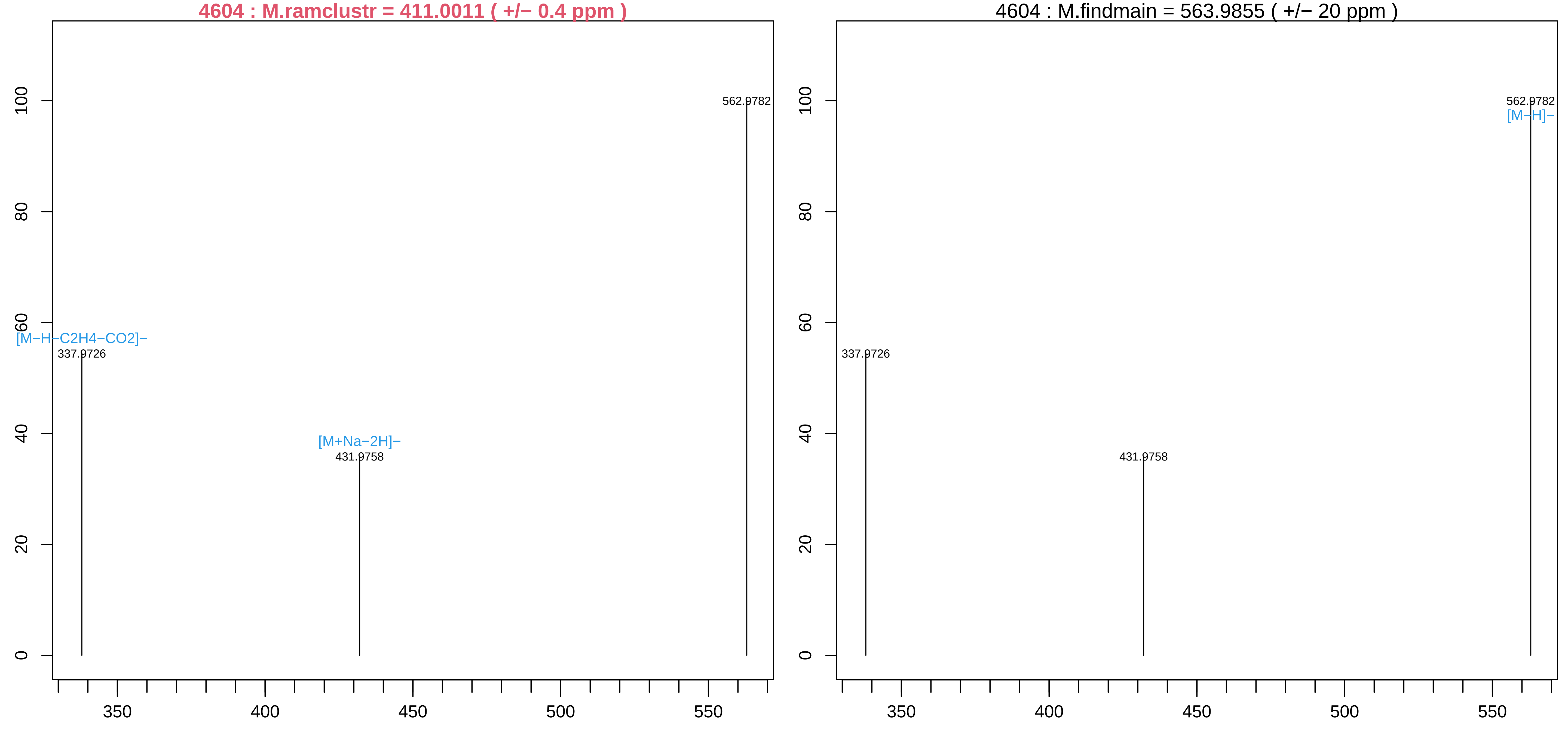
4604 : M.ramclustr = 411.0011 (+/− 0.4 ppm)
562.9782
[M−H−C2H4−CO2]−
337.9726
[M+Na−2H]−
431.9758
4604 : M.findmain = 563.9855 (+/− 20 ppm)
562.9782
[M−H]−
337.9726
431.9758
0
20
40
60
80
100
350
400
450
500
550

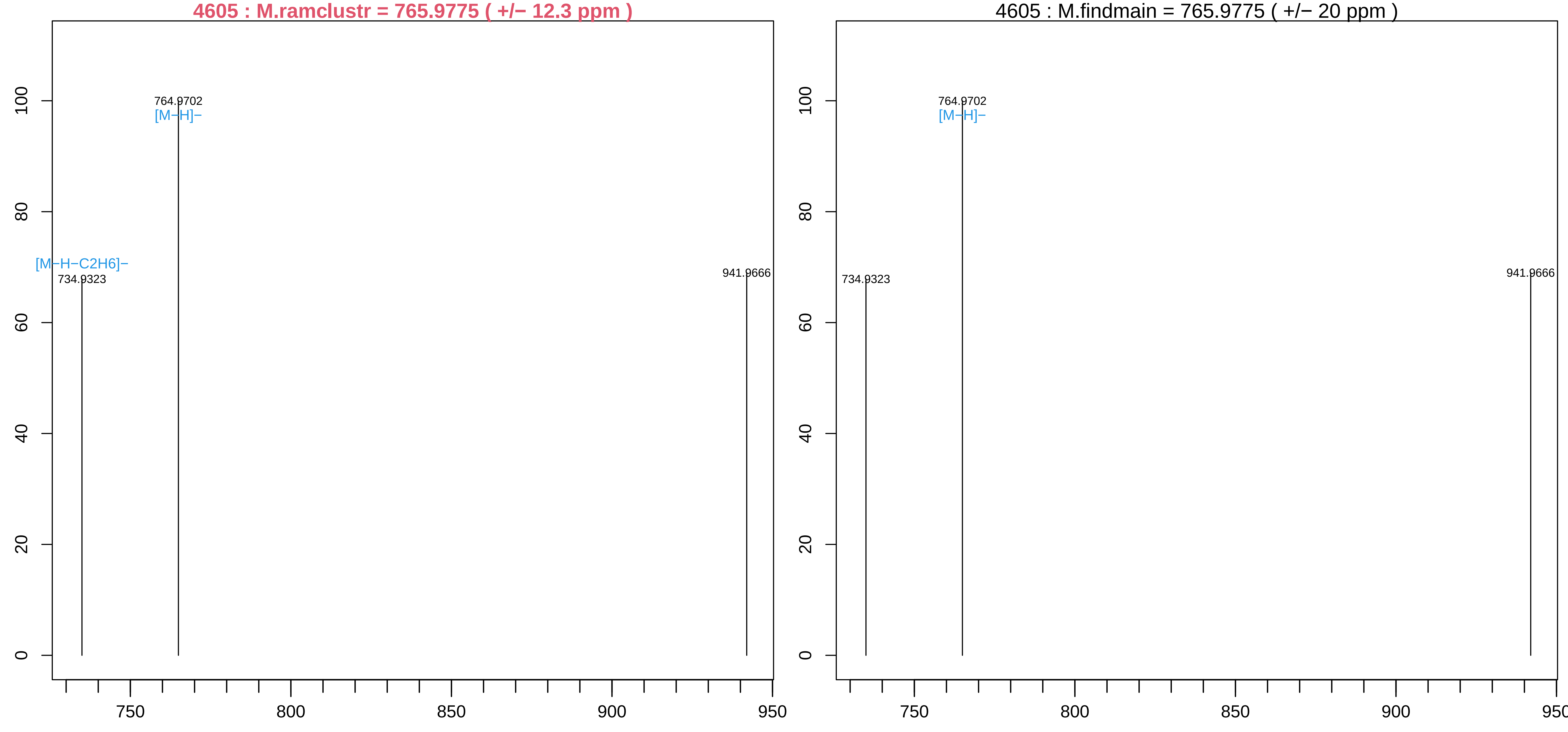

4605 : M.ramclustr = 765.9775 (+/− 12.3 ppm)
764.9702
[M−H]−
[M−H−C2H6]−
734.9323
941.9666
0
20
40
60
80
100
750
800
850
900
950
4605 : M.findmain = 765.9775 (+/− 20 ppm)
764.9702
[M−H]−
734.9323
941.9666

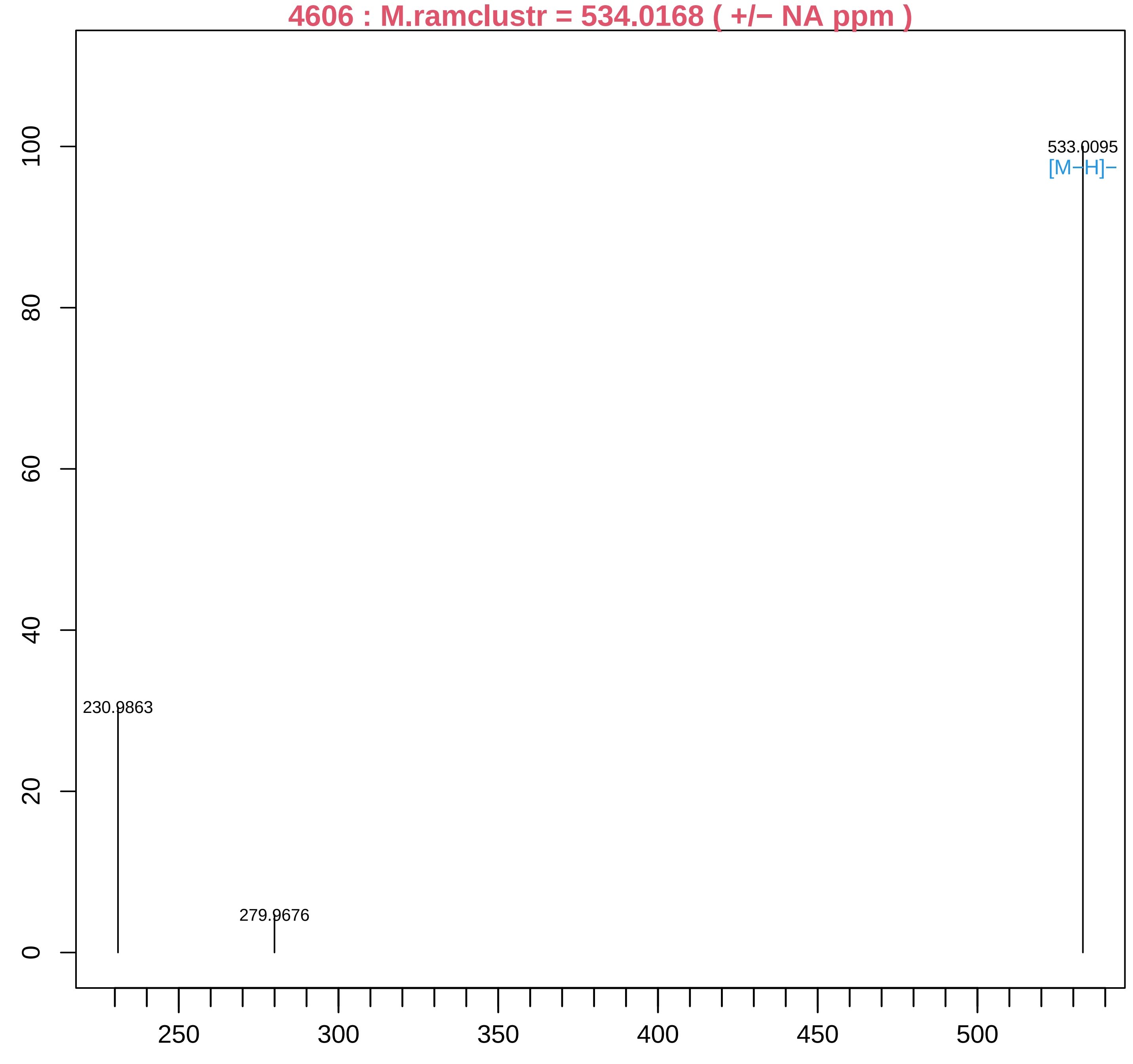
4606 : M.ramclustr = 534.0168 (+/− NA ppm)
533.0095
[M−H]−
230.9863
279.9676
0
20
40
60
80
100
250
300
350
400
450
500

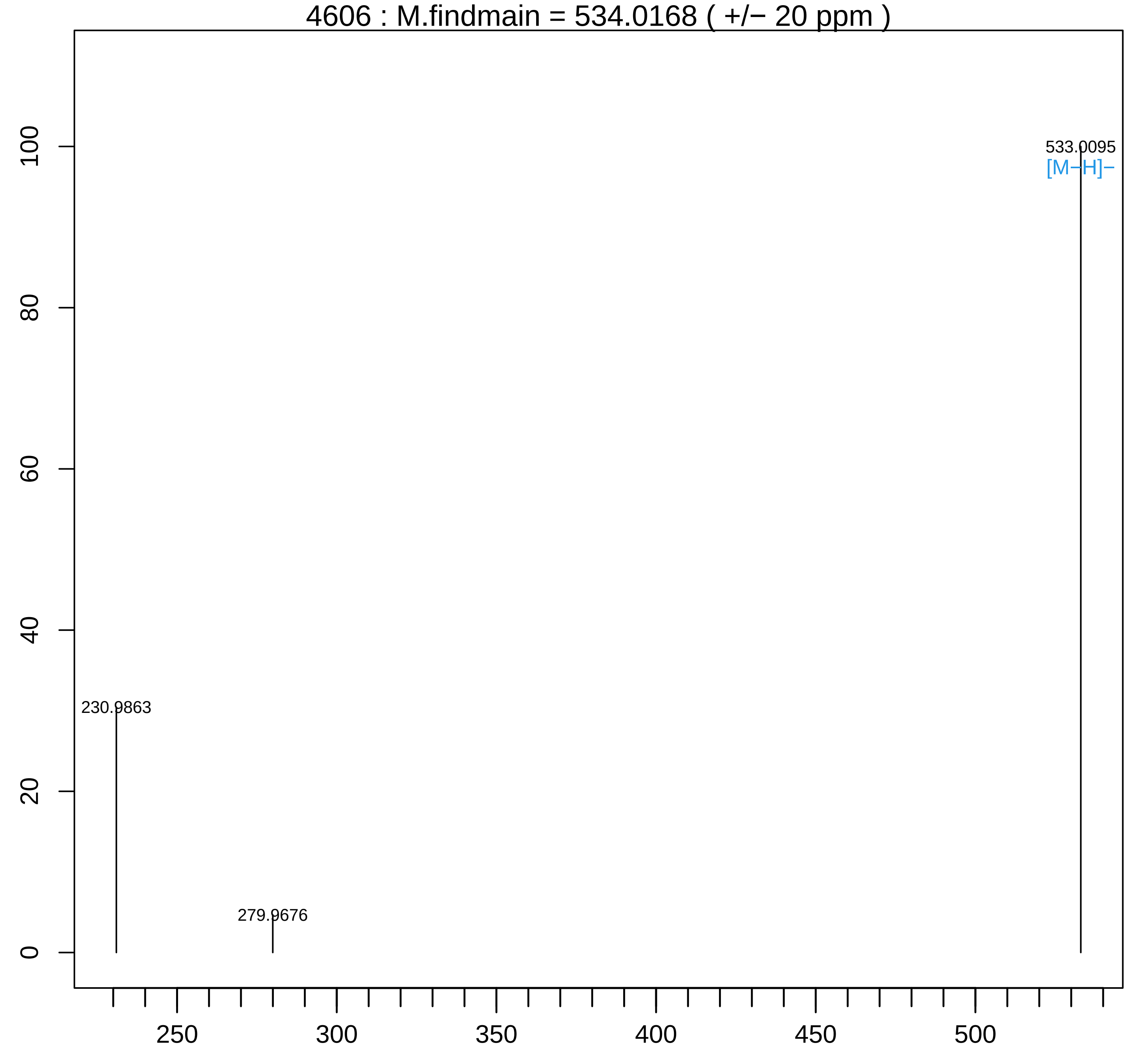
4606 : M.findmain = 534.0168 (+/− 20 ppm)
533.0095
[M−H]−
230.9863
279.9676
0
20
40
60
80
100
250
300
350
400
450
500

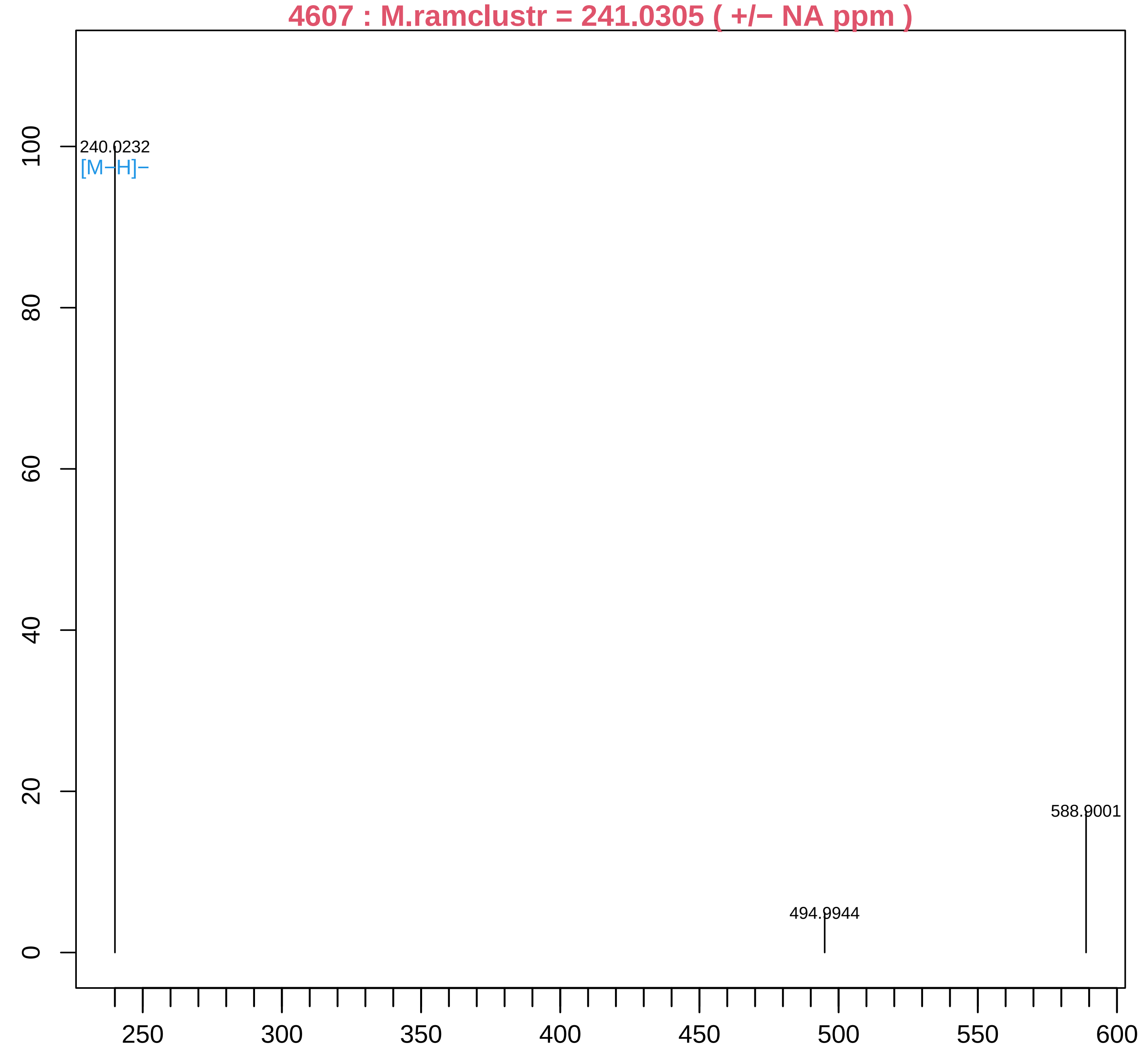
4607 : M.ramclustr = 241.0305 (+/− NA ppm)
240.0232
[M−H]−
494.9944
588.9001
0
20
40
60
80
100
250
300
350
400
450
500
550
600

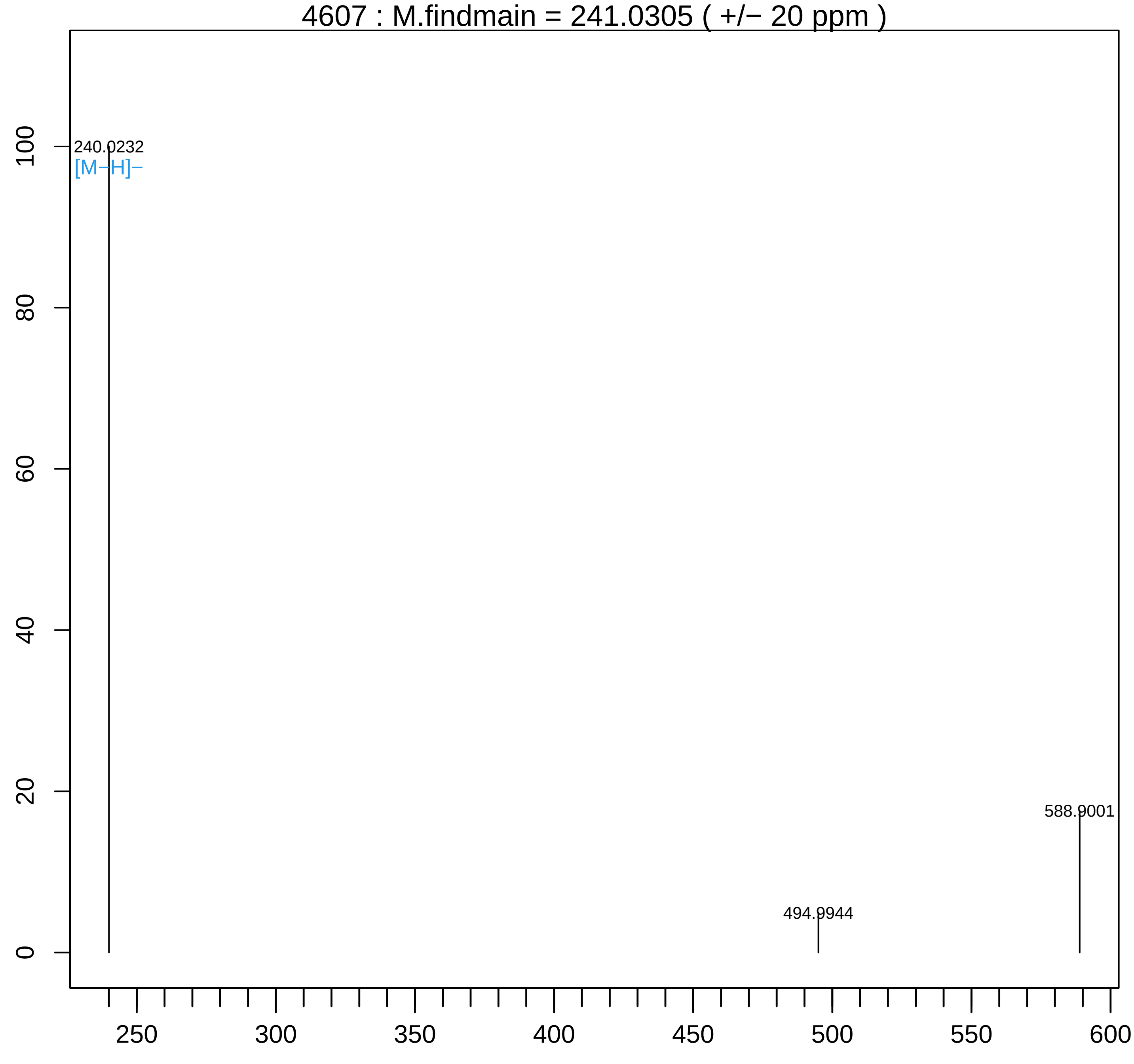
4607 : M.findmain = 241.0305 (+/− 20 ppm)
240.0232
[M−H]−
494.9944
588.9001
0
20
40
60
80
100
250
300
350
400
450
500
550
600

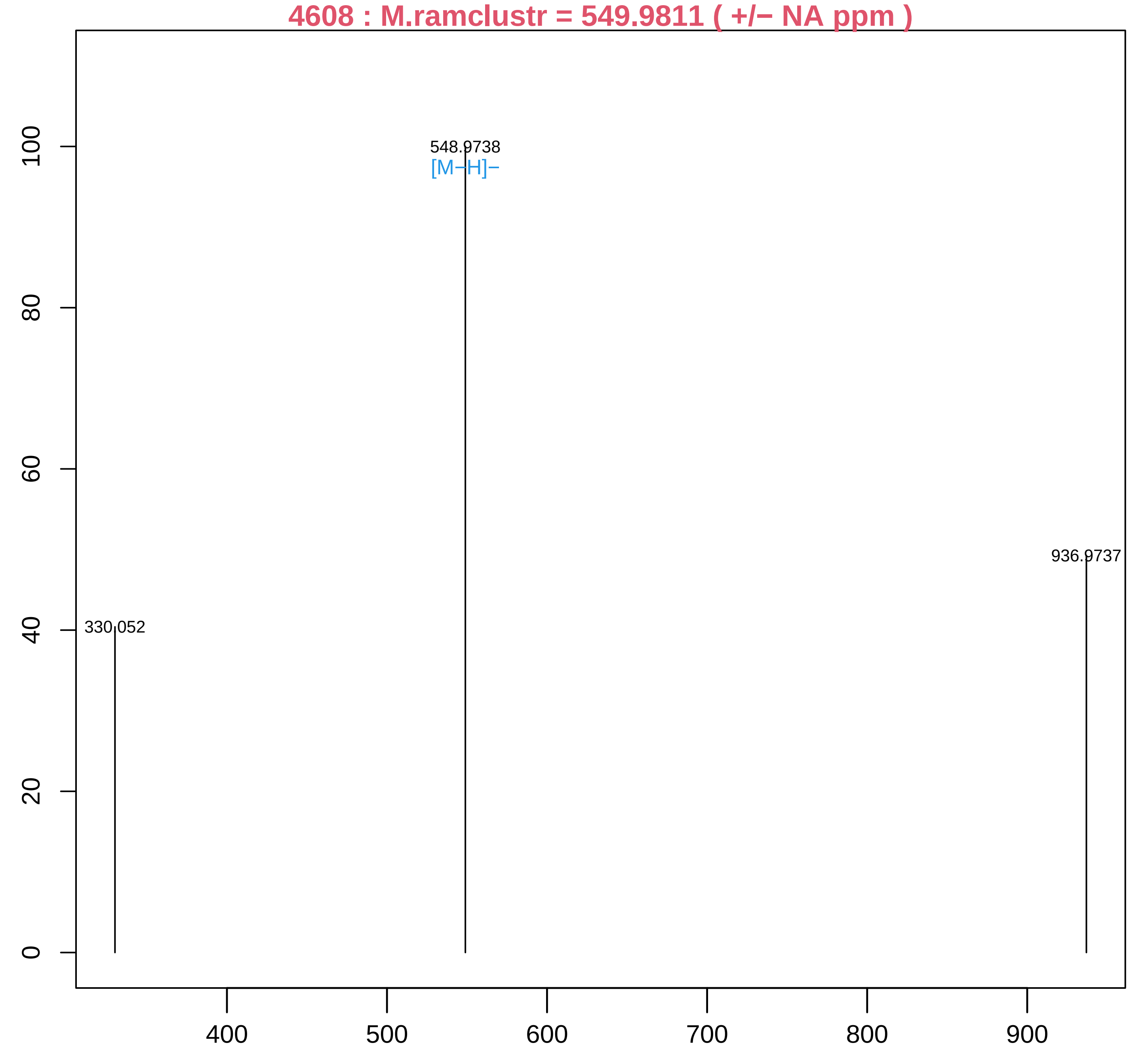

4608 : M.ramclustr = 549.9811 (+/− NA ppm)
548.9738
[M−H]−
936.9737
330.052
0
20
40
60
80
100
400
500
600
700
800
900

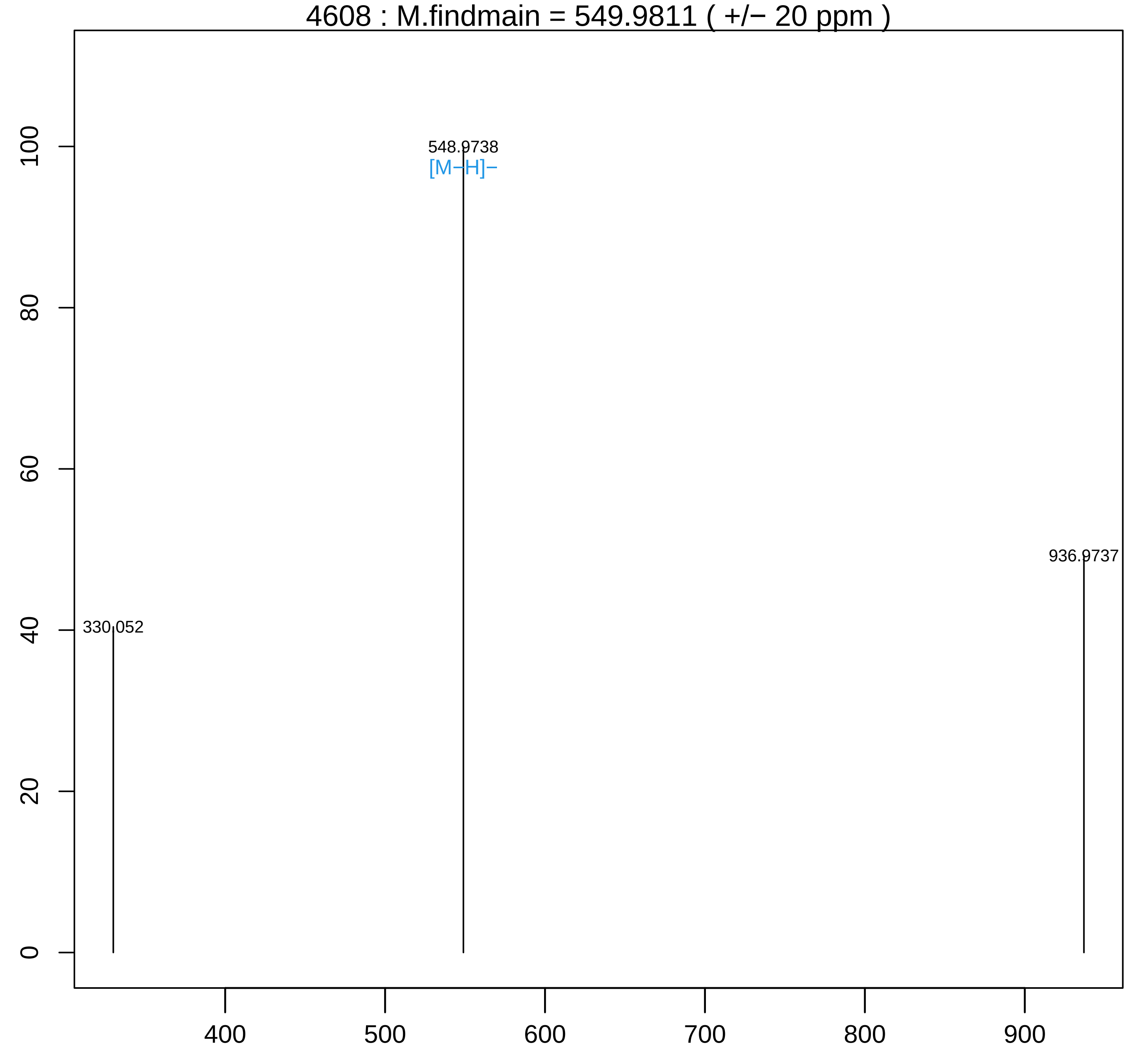

4608 : M.findmain = 549.9811 (+/− 20 ppm)
548.9738
[M−H]−
936.9737
330.052
0
20
40
60
80
100
400
500
600
700
800
900

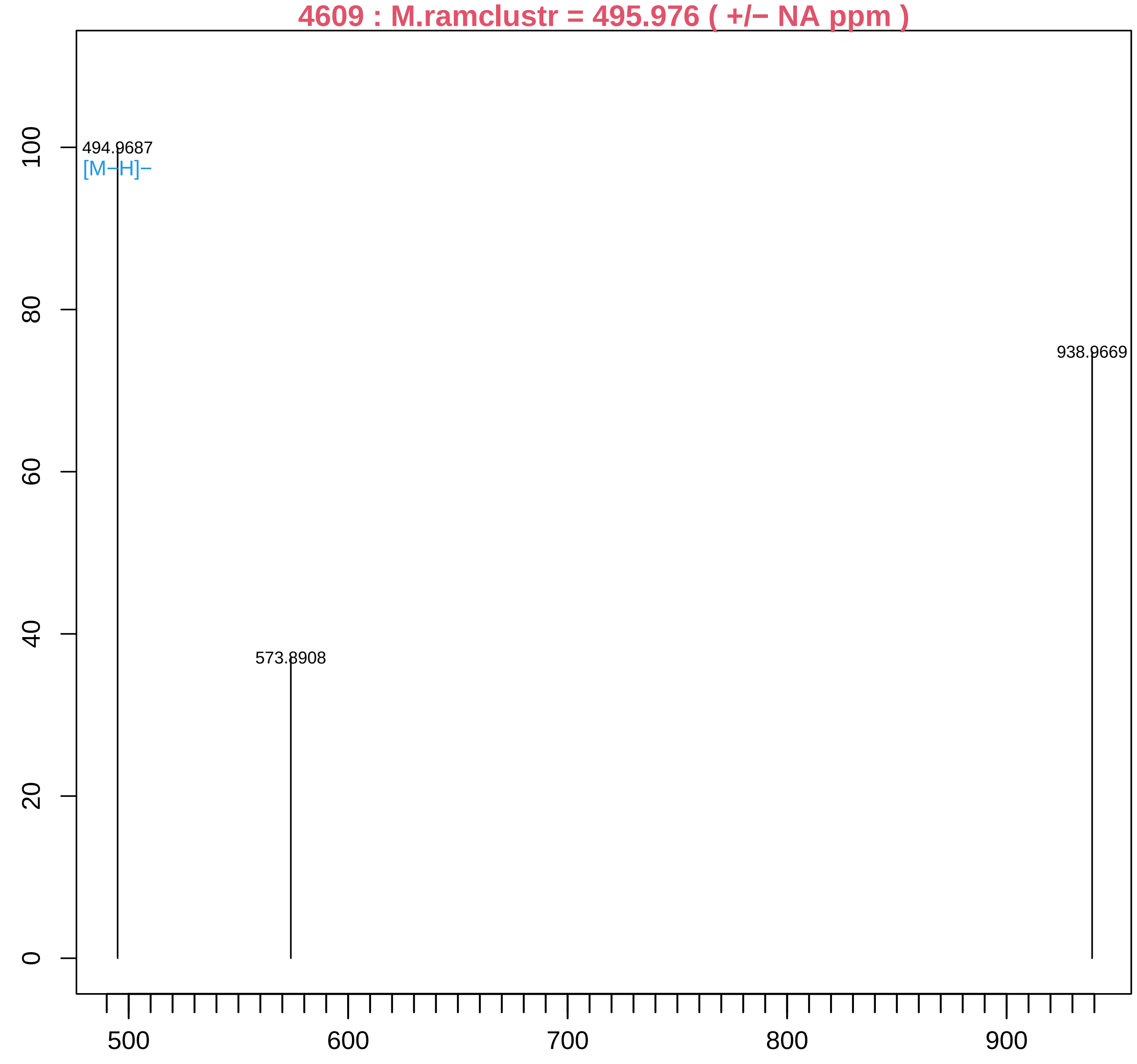
4609 : M.ramclustr = 495.976 (+/− NA ppm)
494.9687
[M−H]−
573.8908
938.9669
0
20
40
60
80
100
500
600
700
800
900

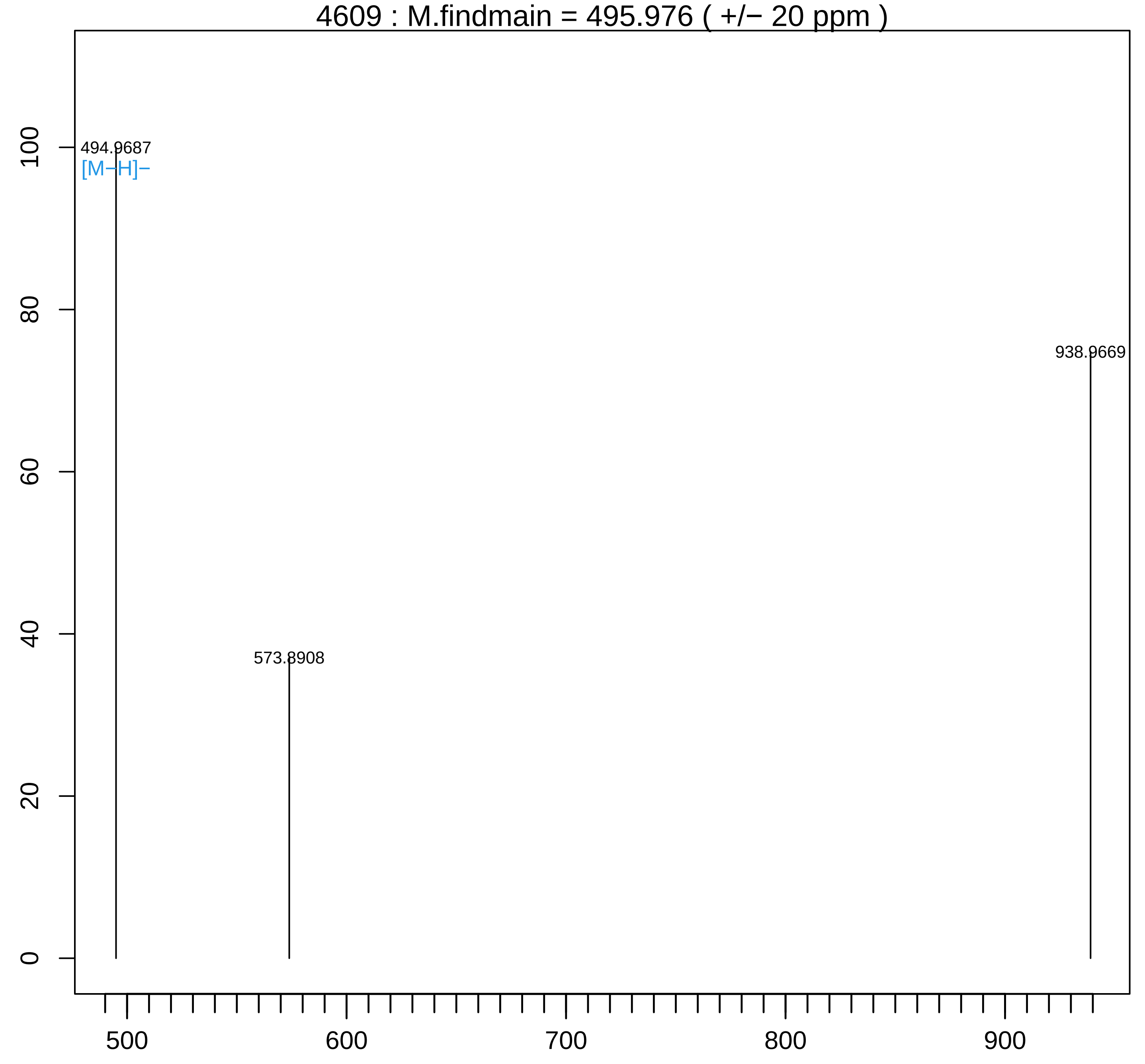
4609 : M.findmain = 495.976 (+/− 20 ppm)
494.9687
[M−H]−
573.8908
938.9669
0
20
40
60
80
100
500
600
700
800
900

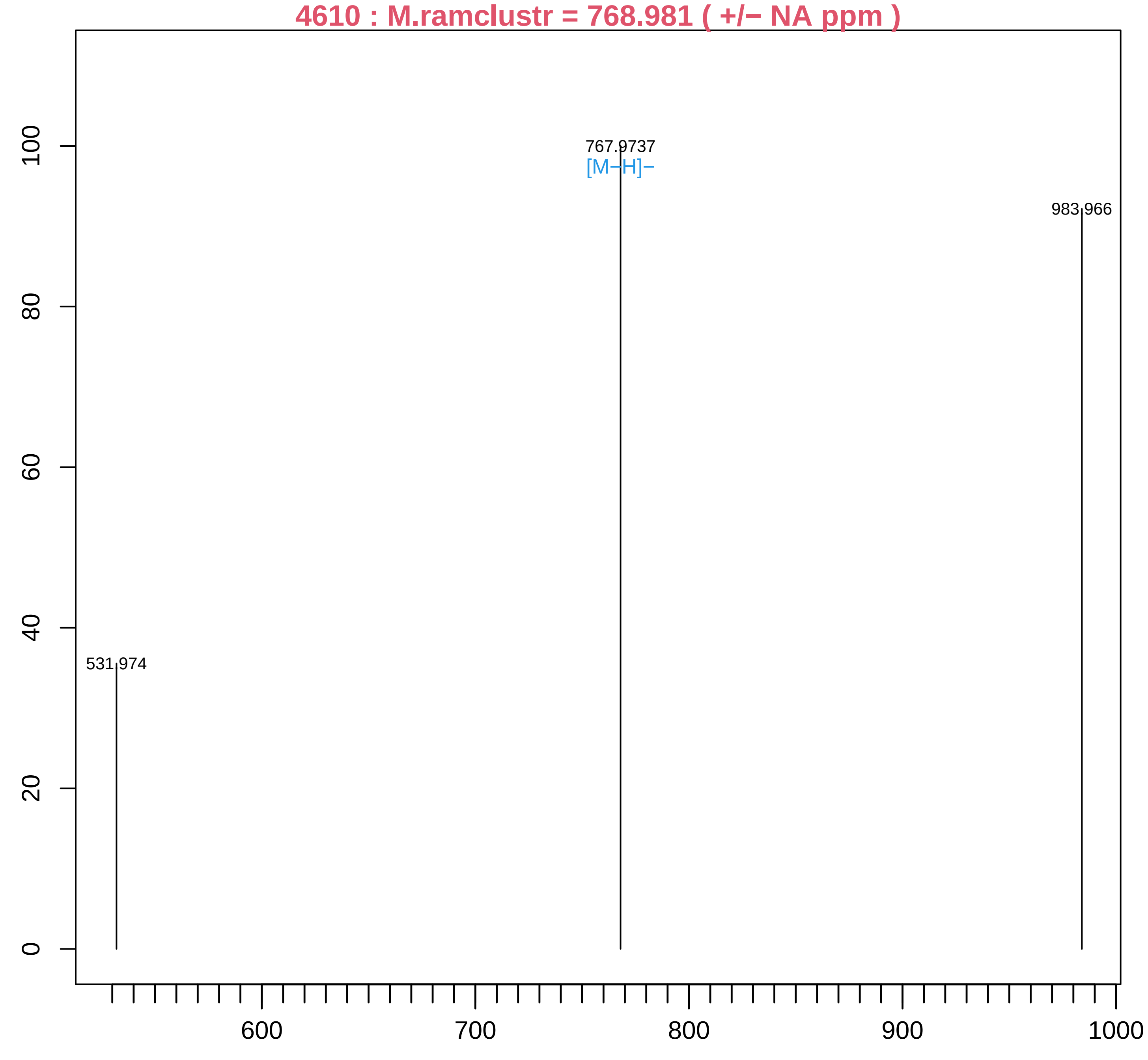
4610 : M.ramclustr = 768.981 (+/− NA ppm)
767.9737
[M−H]−
983.966
531.974
0
20
40
60
80
100
600
700
800
900
1000

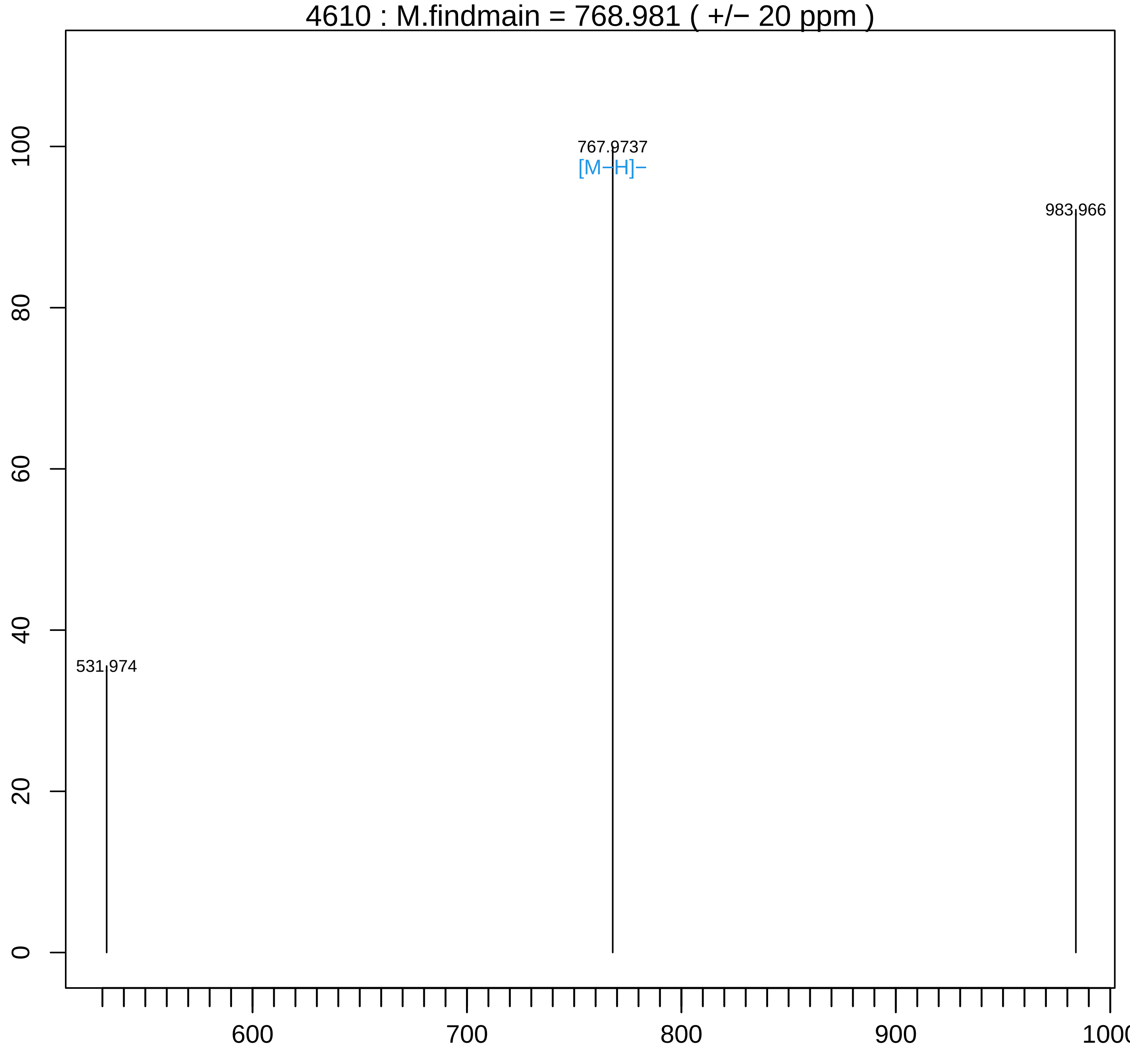
4610 : M.findmain = 768.981 (+/− 20 ppm)
767.9737
[M−H]−
983.966
531.974
0
20
40
60
80
100
600
700
800
900
1000

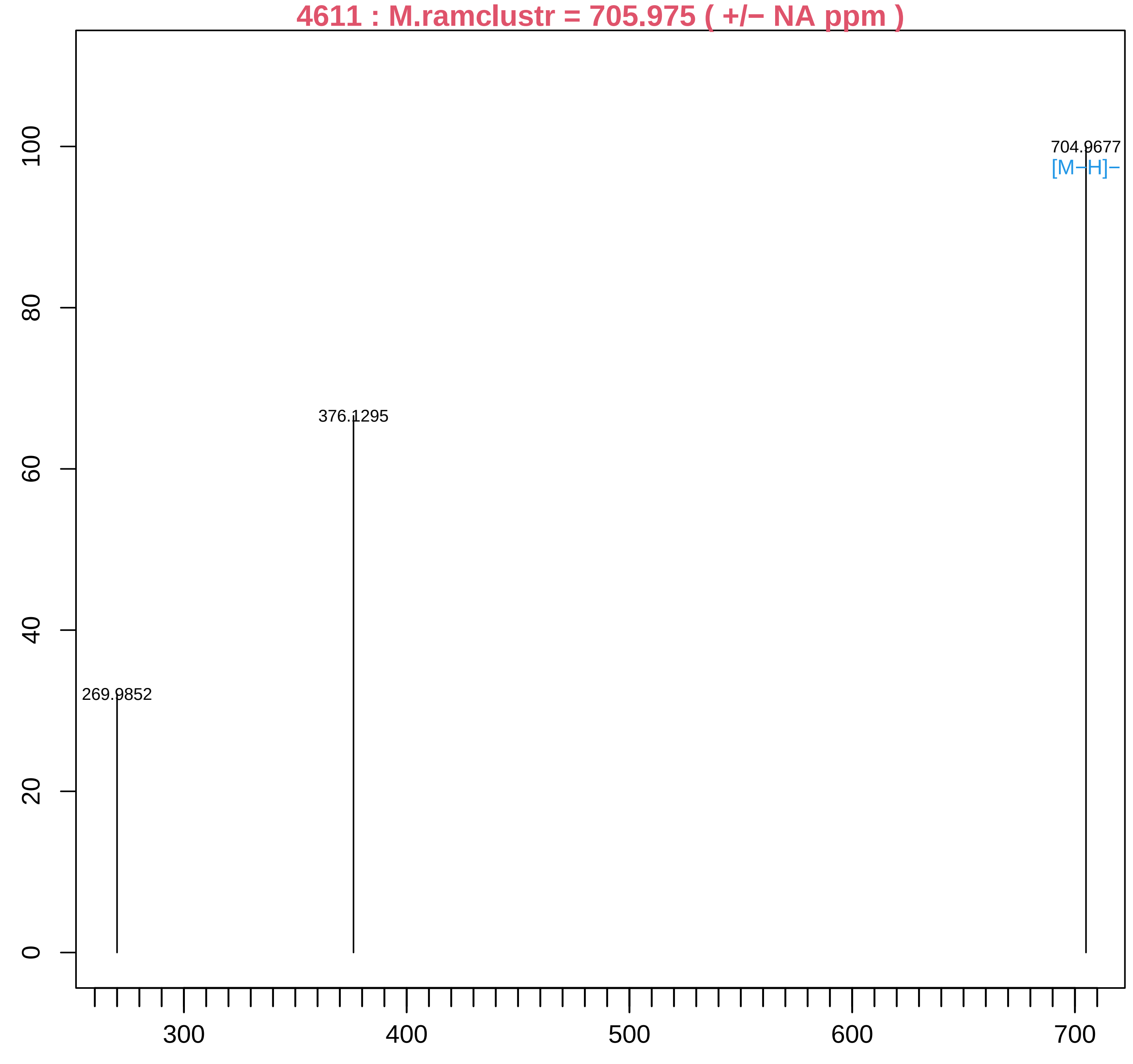

4611 : M.ramclustr = 705.975 (+/− NA ppm)
704.9677
[M−H]−
376.1295
269.9852
0
20
40
60
80
100
300
400
500
600
700

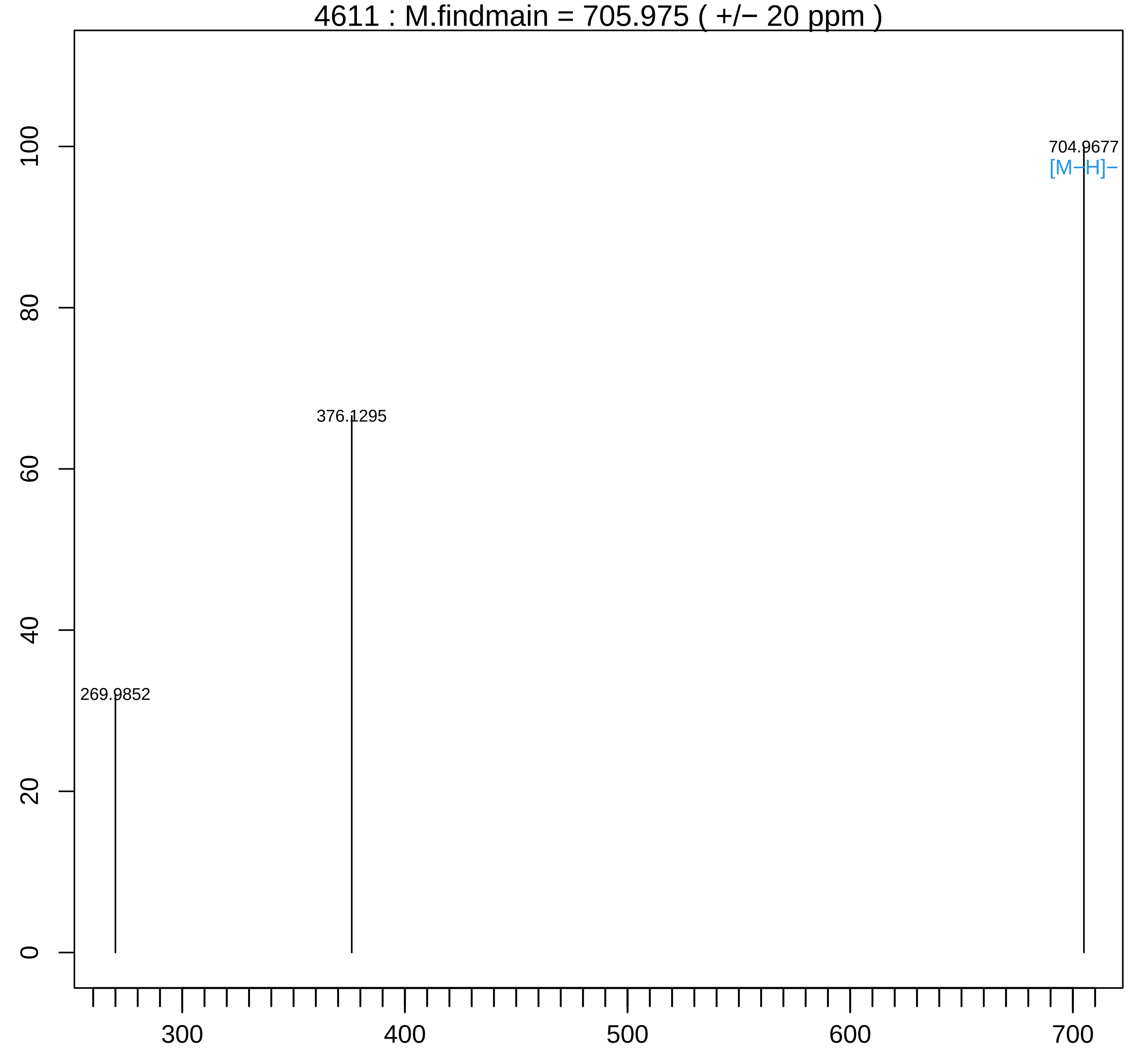

4611 : M.findmain = 705.975 (+/− 20 ppm)
704.9677
[M−H]−
376.1295
269.9852
0
20
40
60
80
100
300
400
500
600
700

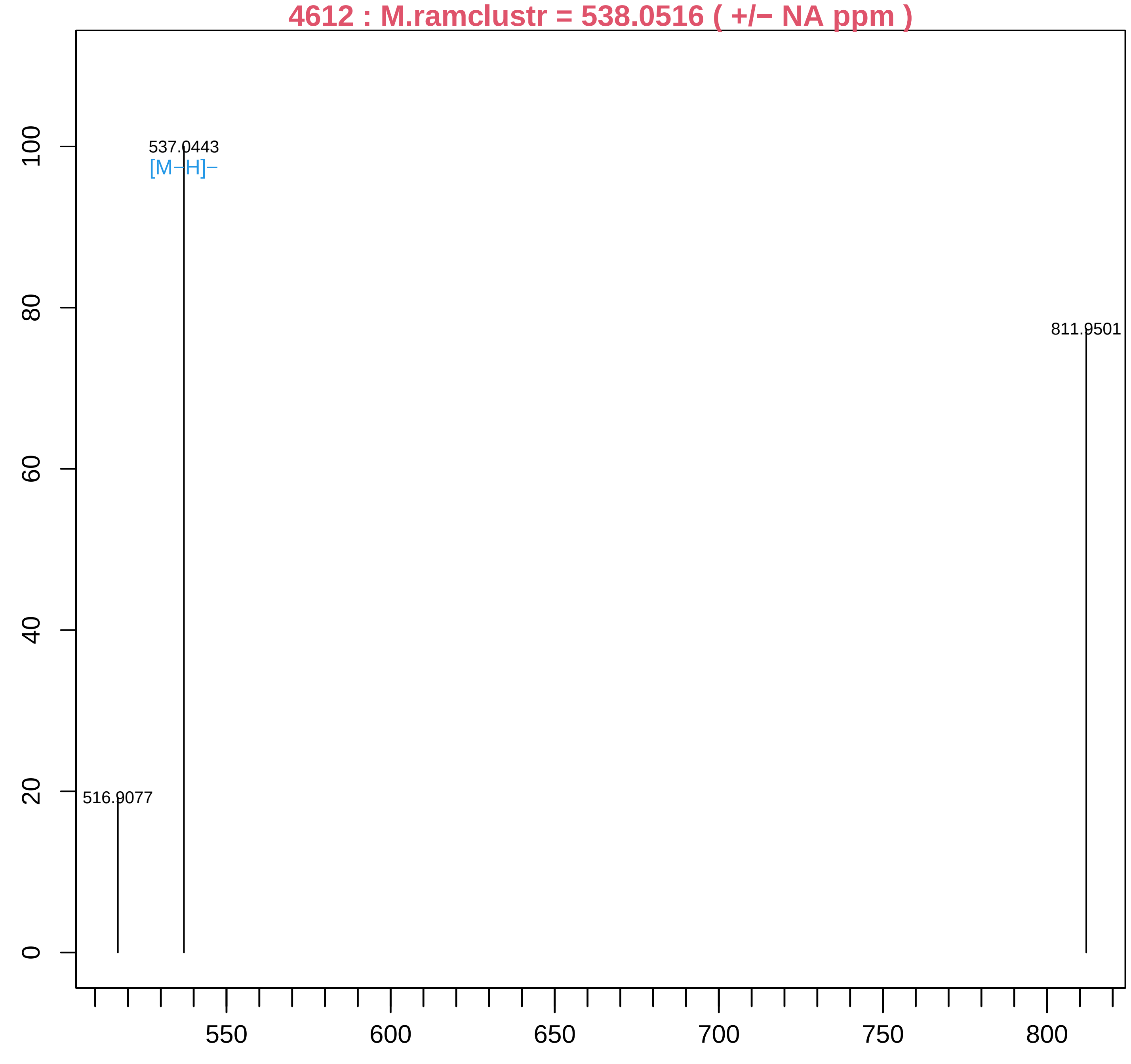
4612 : M.ramclustr = 538.0516 (+/− NA ppm)
537.0443
[M−H]−
811.9501
516.9077
0
20
40
60
80
100
550
600
650
700
750
800

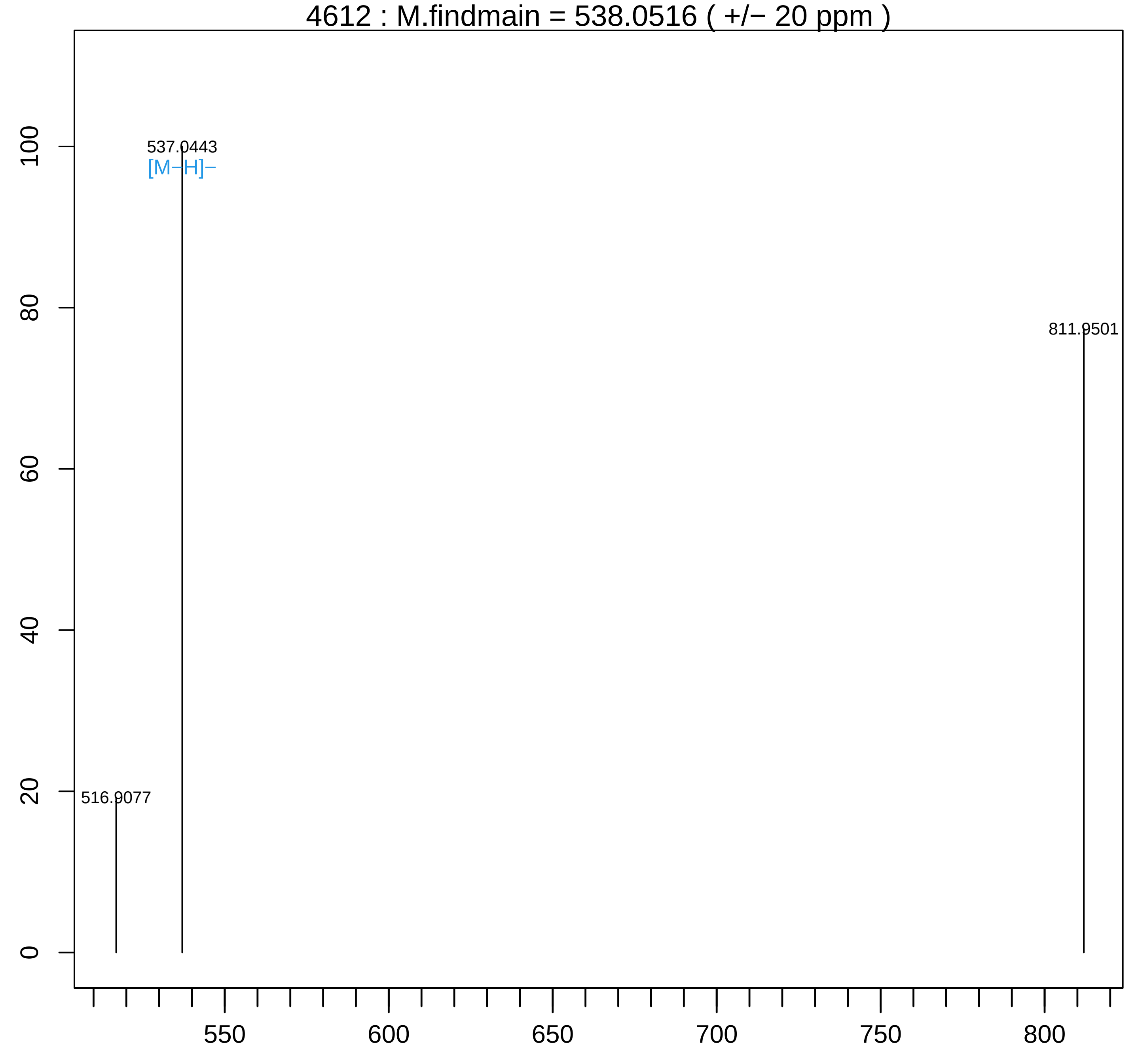
4612 : M.findmain = 538.0516 (+/− 20 ppm)
537.0443
[M−H]−
811.9501
516.9077
0
20
40
60
80
100
550
600
650
700
750
800

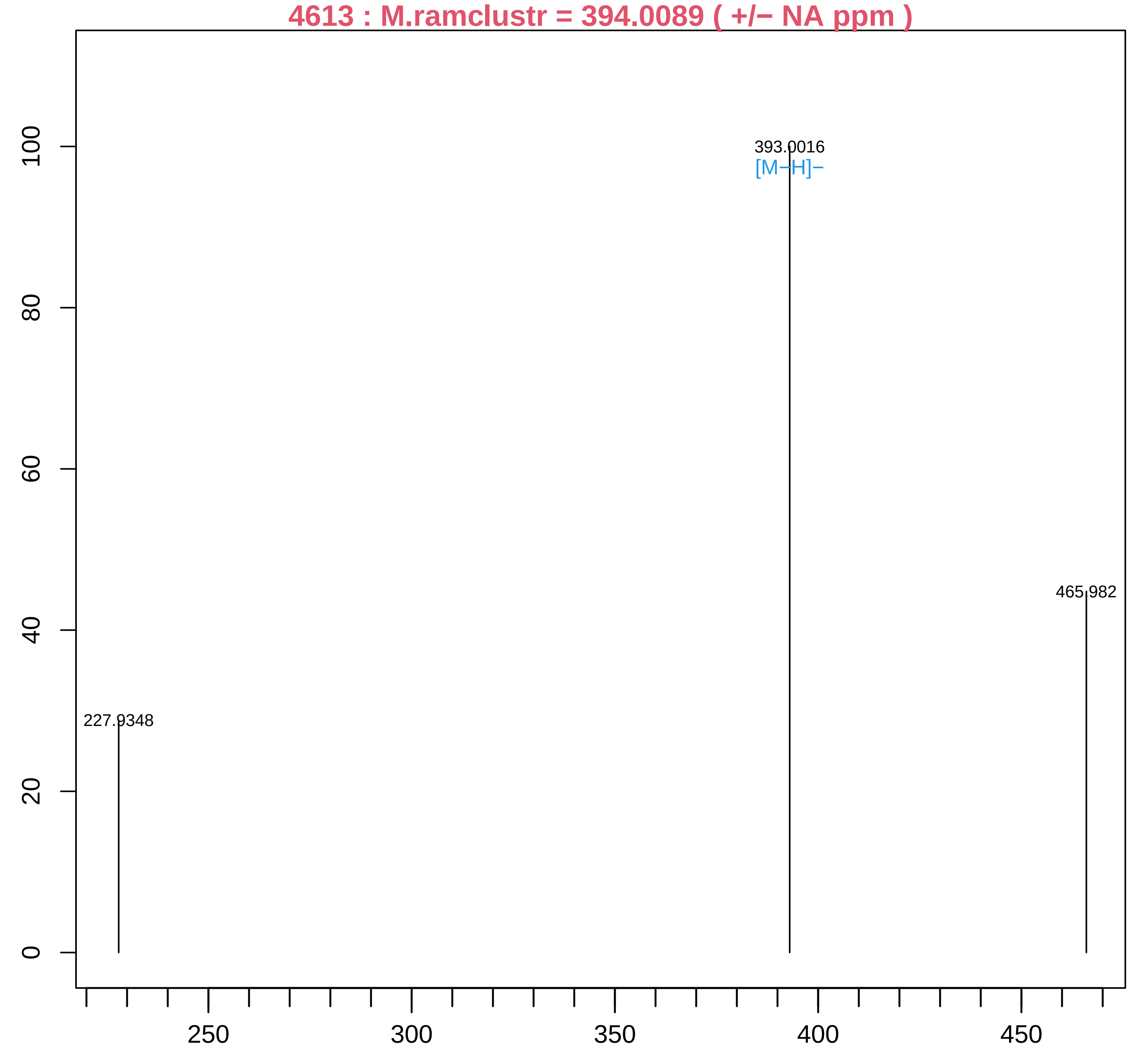

4613 : M.ramclustr = 394.0089 (+/− NA ppm)
393.0016
[M−H]−
465.982
227.9348
0
20
40
60
80
100
250
300
350
400
450

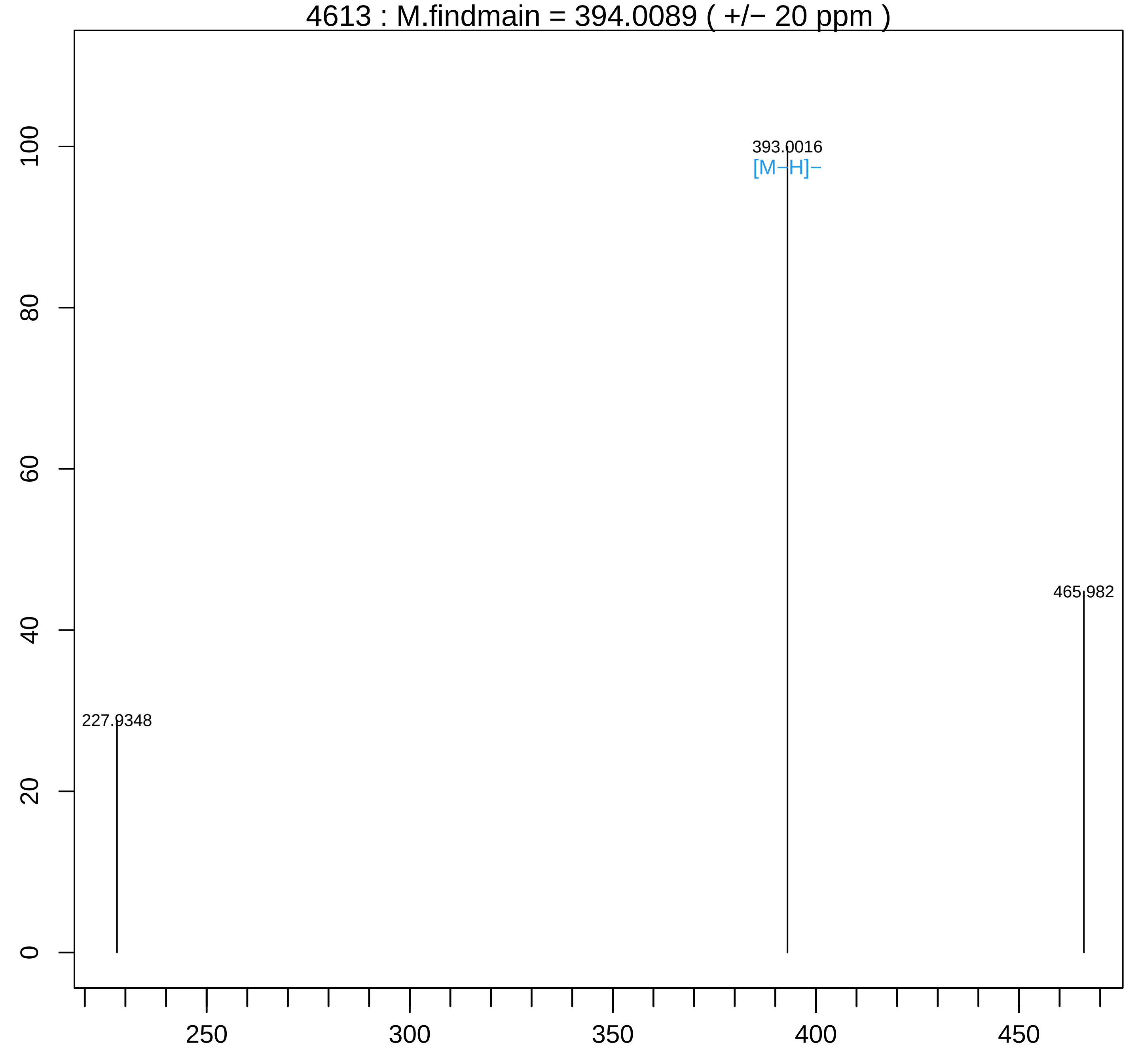

4613 : M.findmain = 394.0089 (+/− 20 ppm)
393.0016
[M−H]−
465.982
227.9348
0
20
40
60
80
100
250
300
350
400
450

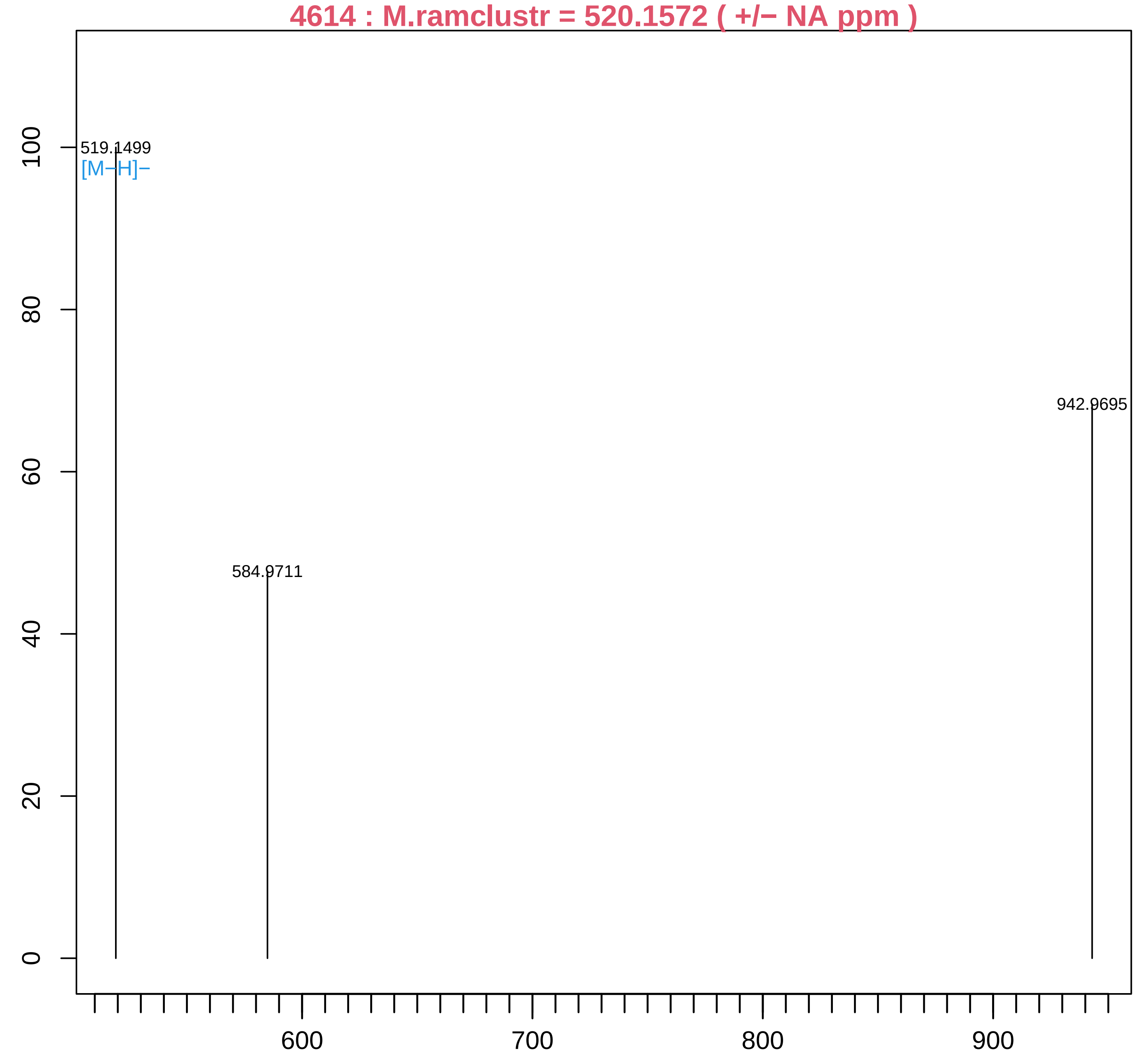
4614 : M.ramclustr = 520.1572 (+/− NA ppm)
519.1499
[M−H]−
584.9711
942.9695
0
20
40
60
80
100
600
700
800
900

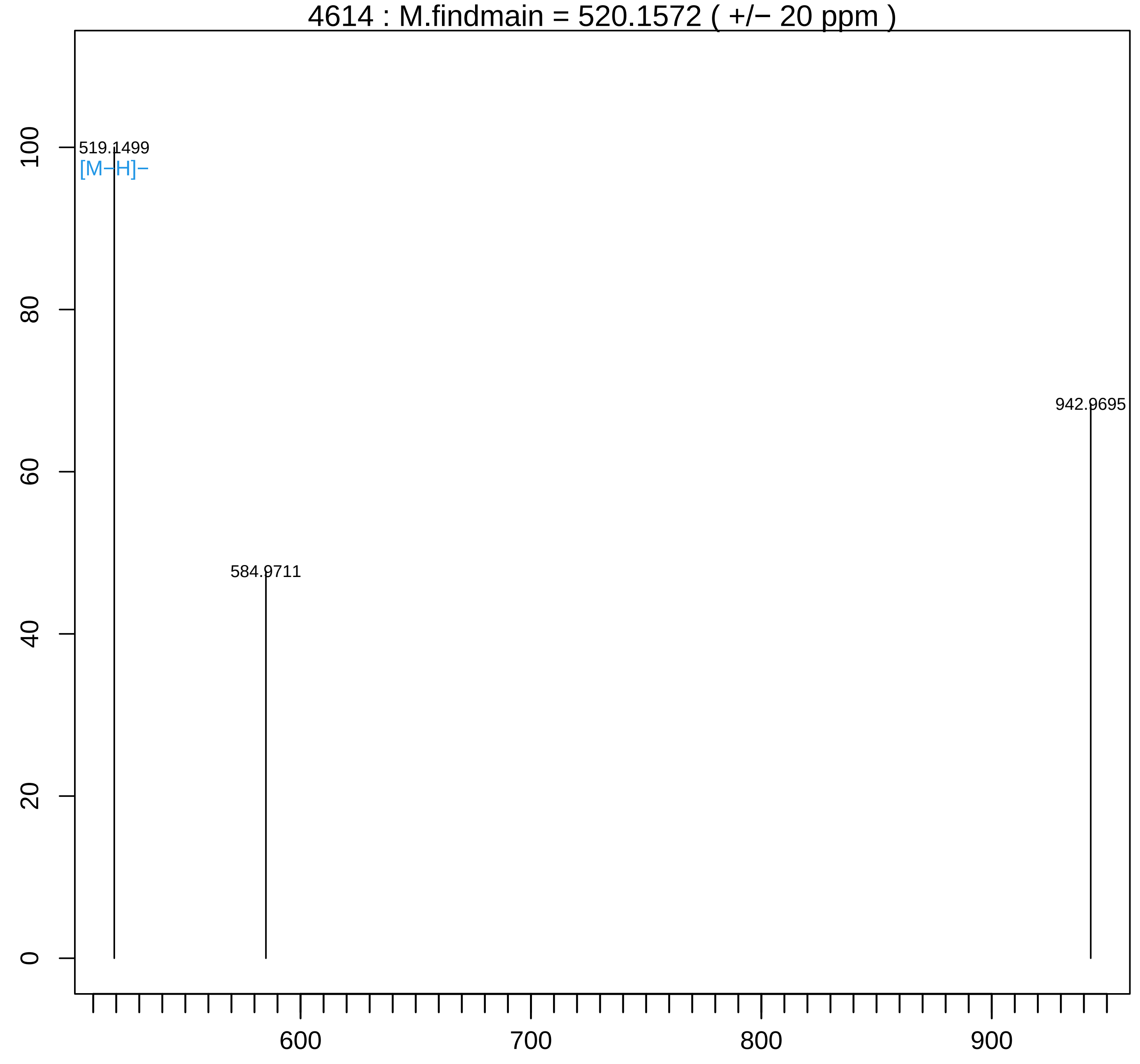
4614 : M.findmain = 520.1572 (+/− 20 ppm)
519.1499
[M−H]−
584.9711
942.9695
0
20
40
60
80
100
600
700
800
900

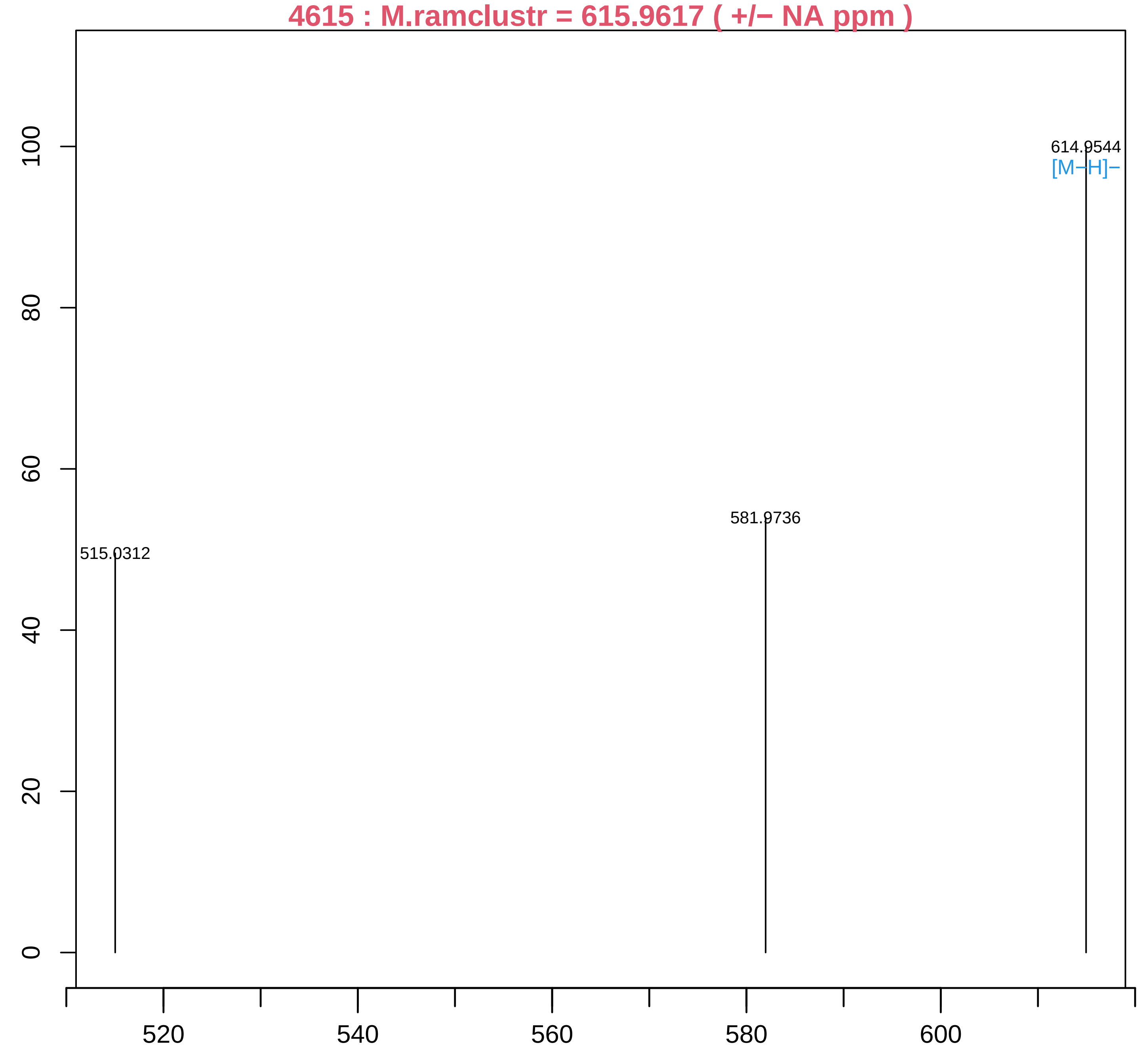

4615 : M.ramclustr = 615.9617 (+/− NA ppm)
614.9544
[M−H]−
581.9736
515.0312
0
20
40
60
80
100
520
540
560
580
600

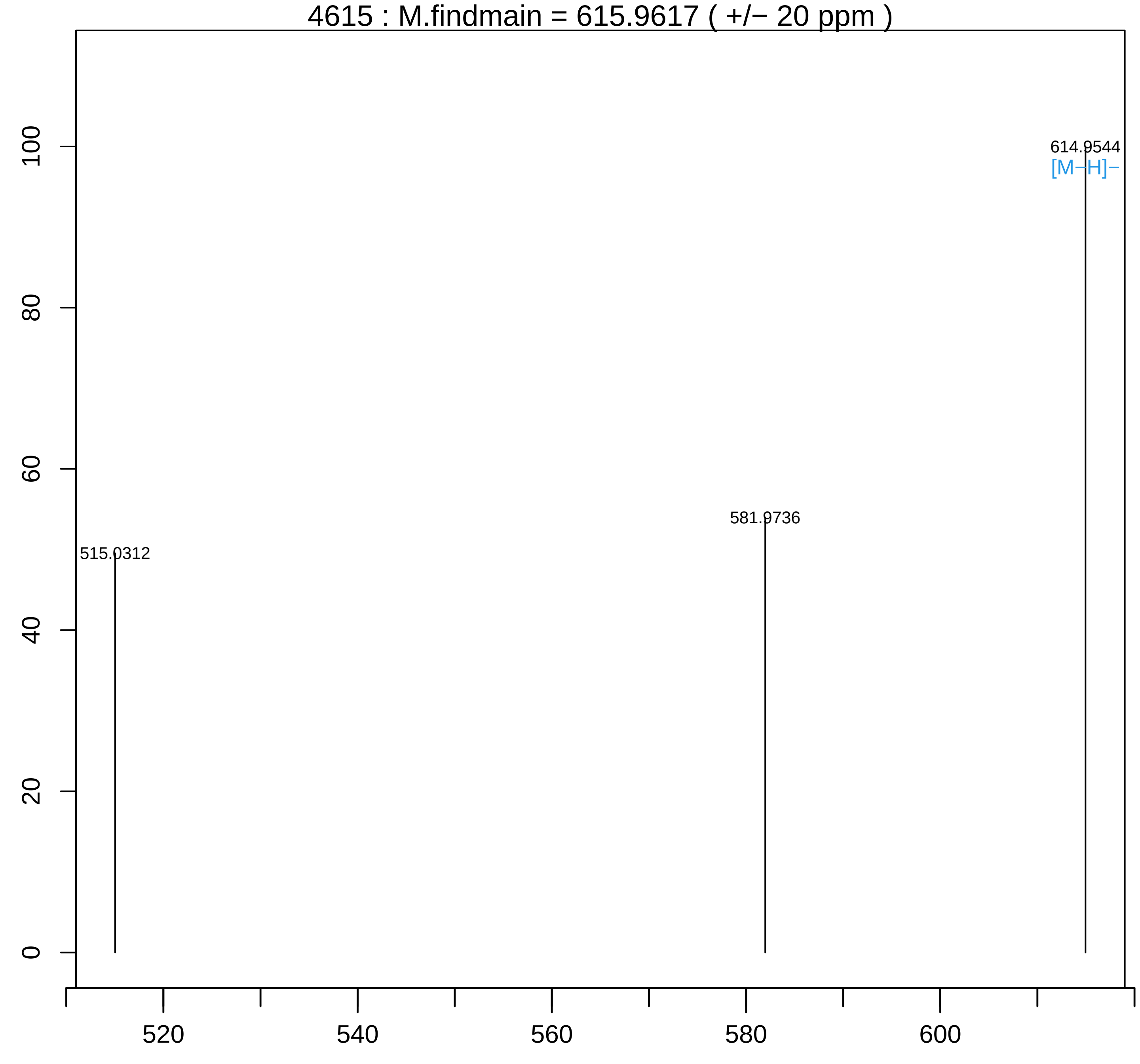

4615 : M.findmain = 615.9617 (+/− 20 ppm)
614.9544
[M−H]−
581.9736
515.0312
0
20
40
60
80
100
520
540
560
580
600

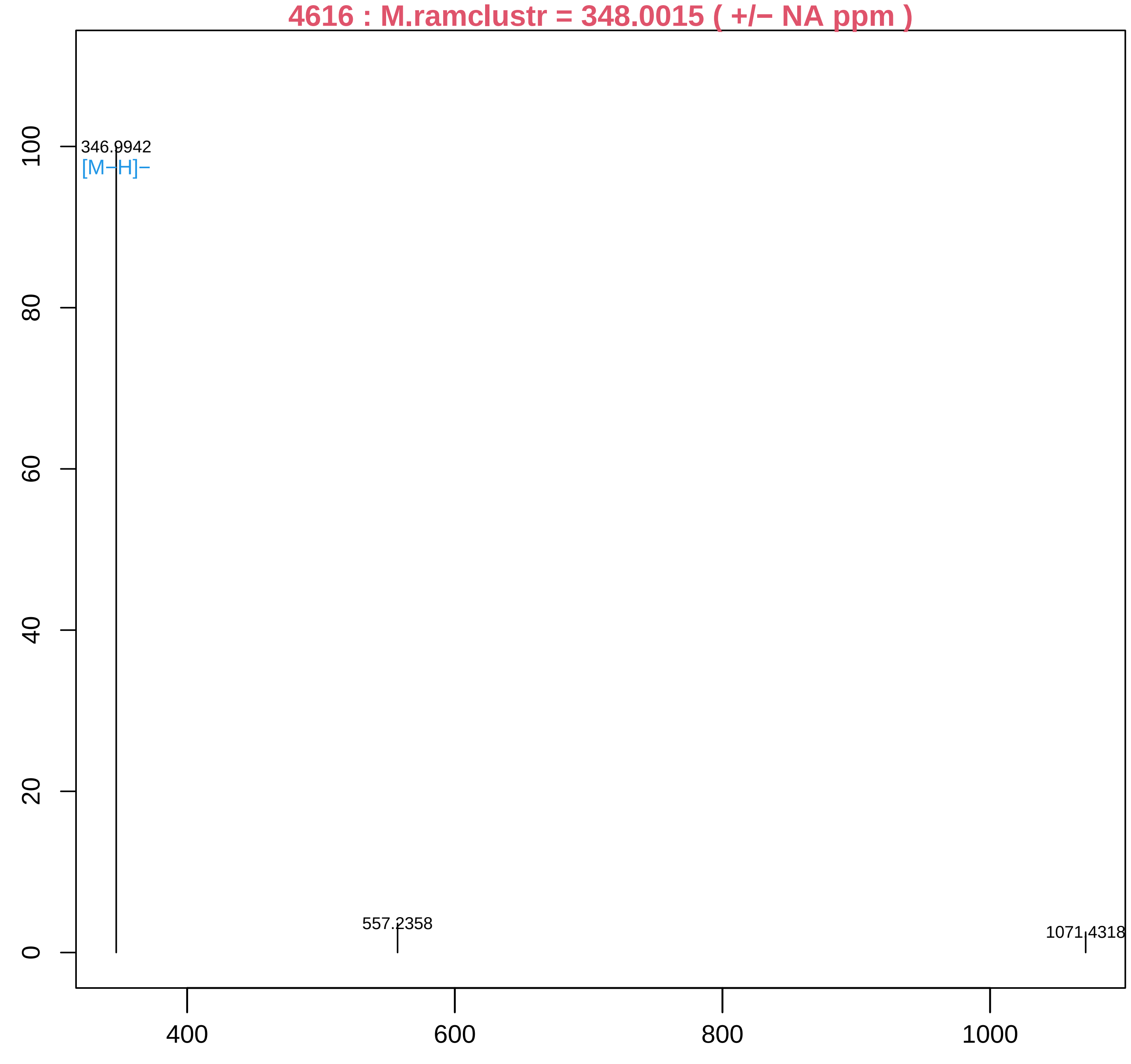
4616 : M.ramclustr = 348.0015 (+/− NA ppm)
346.9942
[M−H]−
557.2358
1071.4318
0
20
40
60
80
100
400
600
800
1000

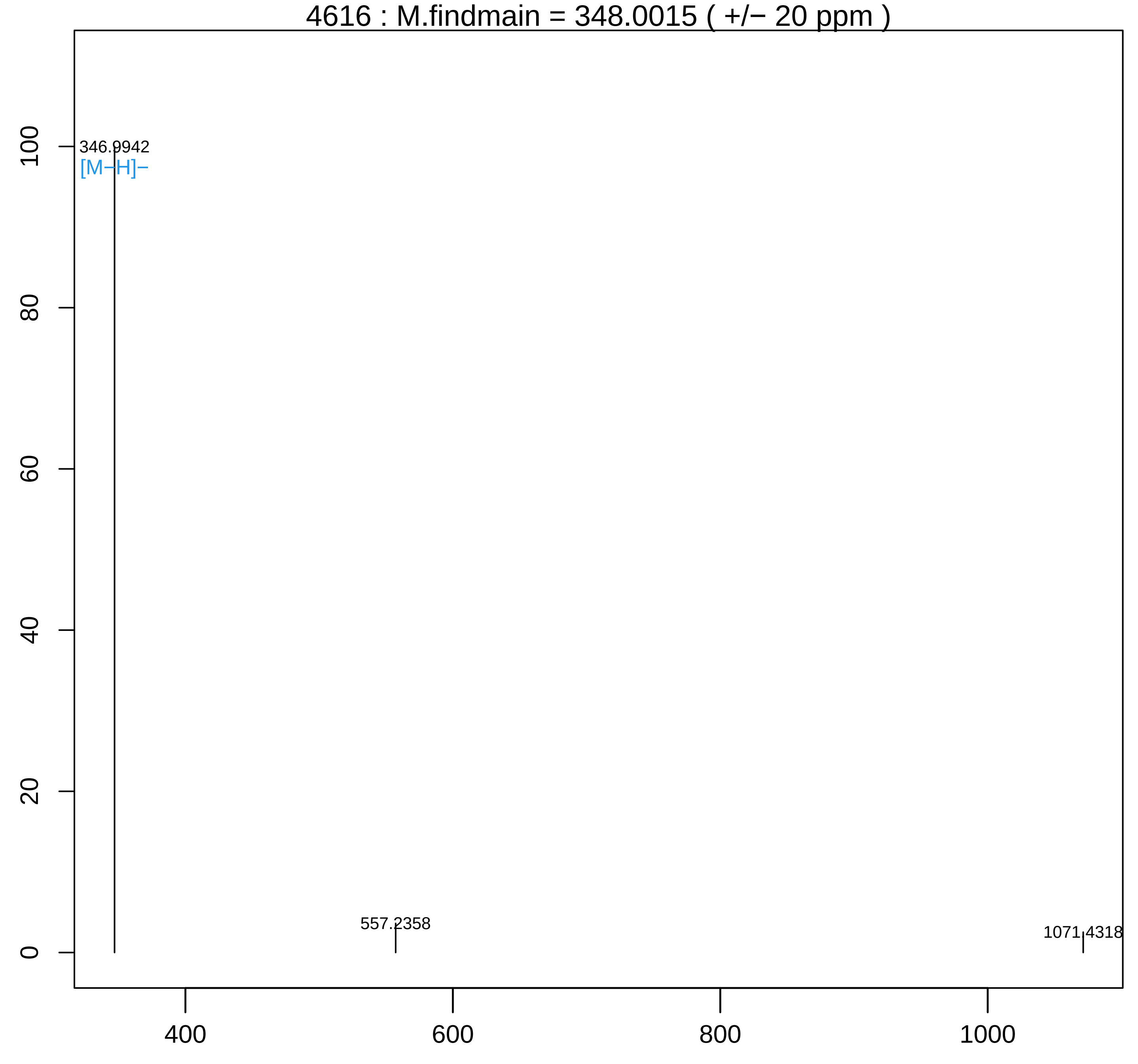
4616 : M.findmain = 348.0015 (+/− 20 ppm)
346.9942
[M−H]−
557.2358
1071.4318
0
20
40
60
80
100
400
600
800
1000

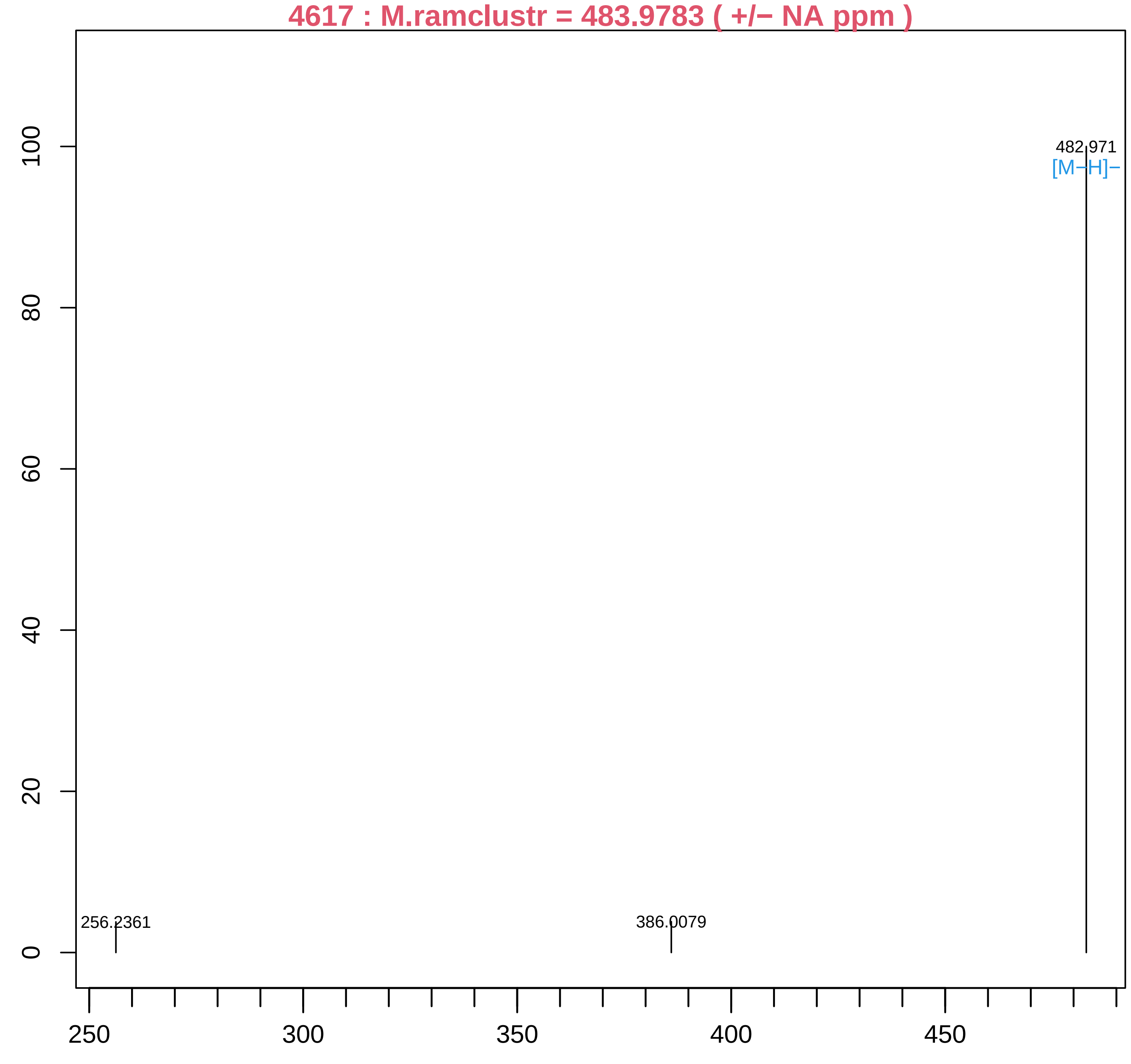

4617 : M.ramclustr = 483.9783 (+/− NA ppm)
482.971
[M−H]−
256.2361
386.0079
0
20
40
60
80
100
250
300
350
400
450

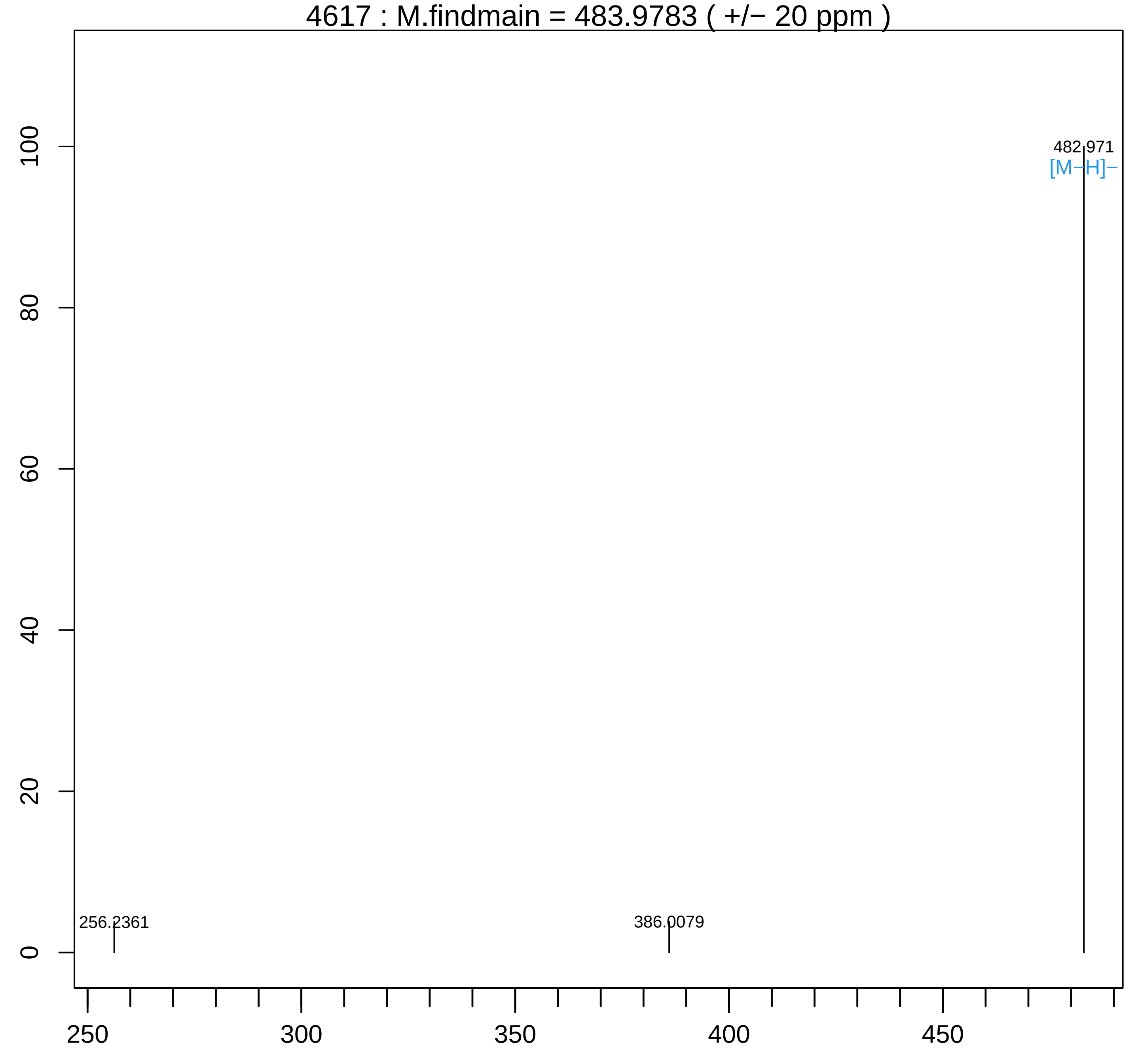

4617 : M.findmain = 483.9783 (+/− 20 ppm)
482.971
[M−H]−
256.2361
386.0079
0
20
40
60
80
100
250
300
350
400
450

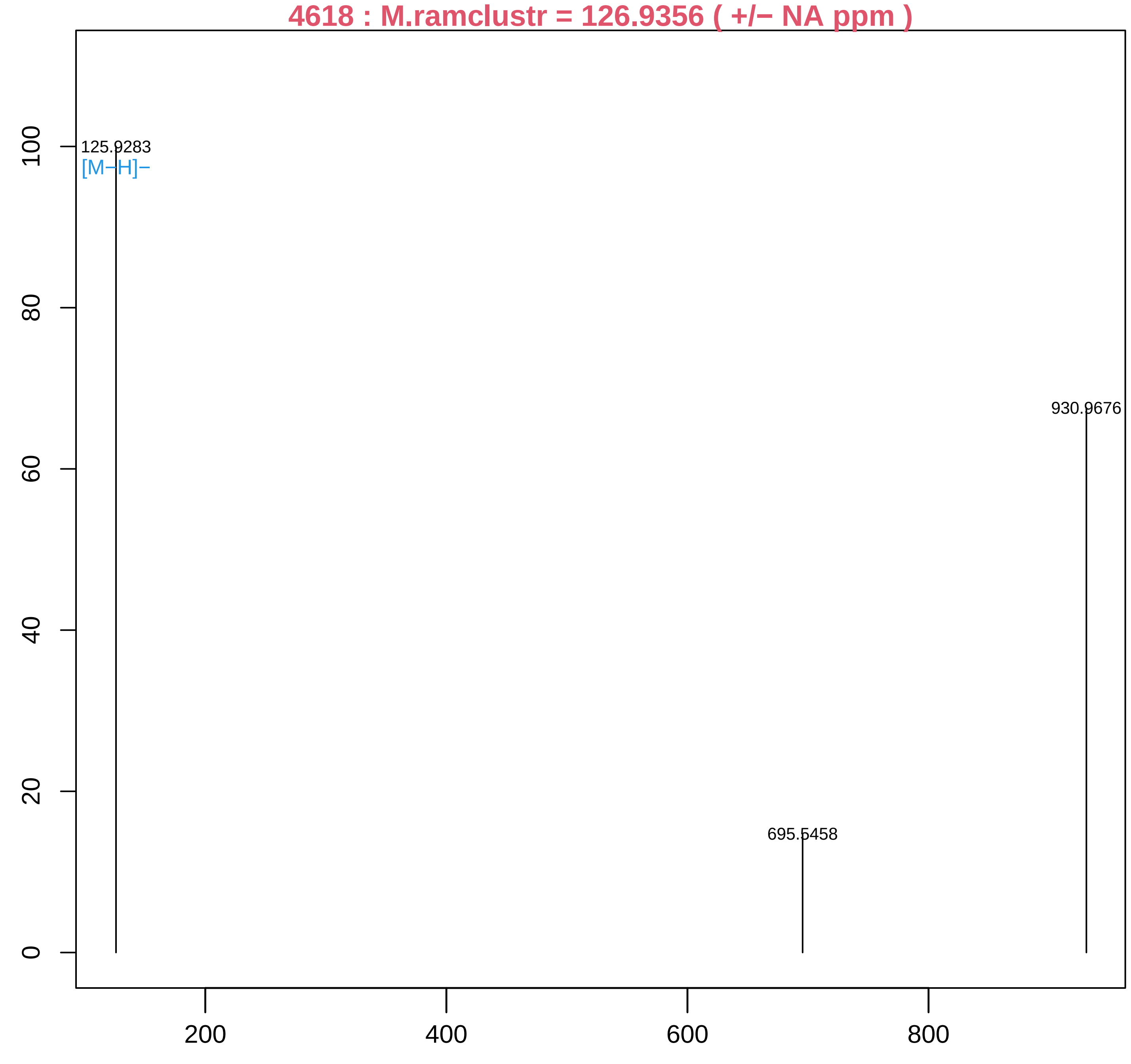
4618 : M.ramclustr = 126.9356 (+/− NA ppm)
125.9283
[M−H]−
930.9676
695.5458
0
20
40
60
80
100
200
400
600
800

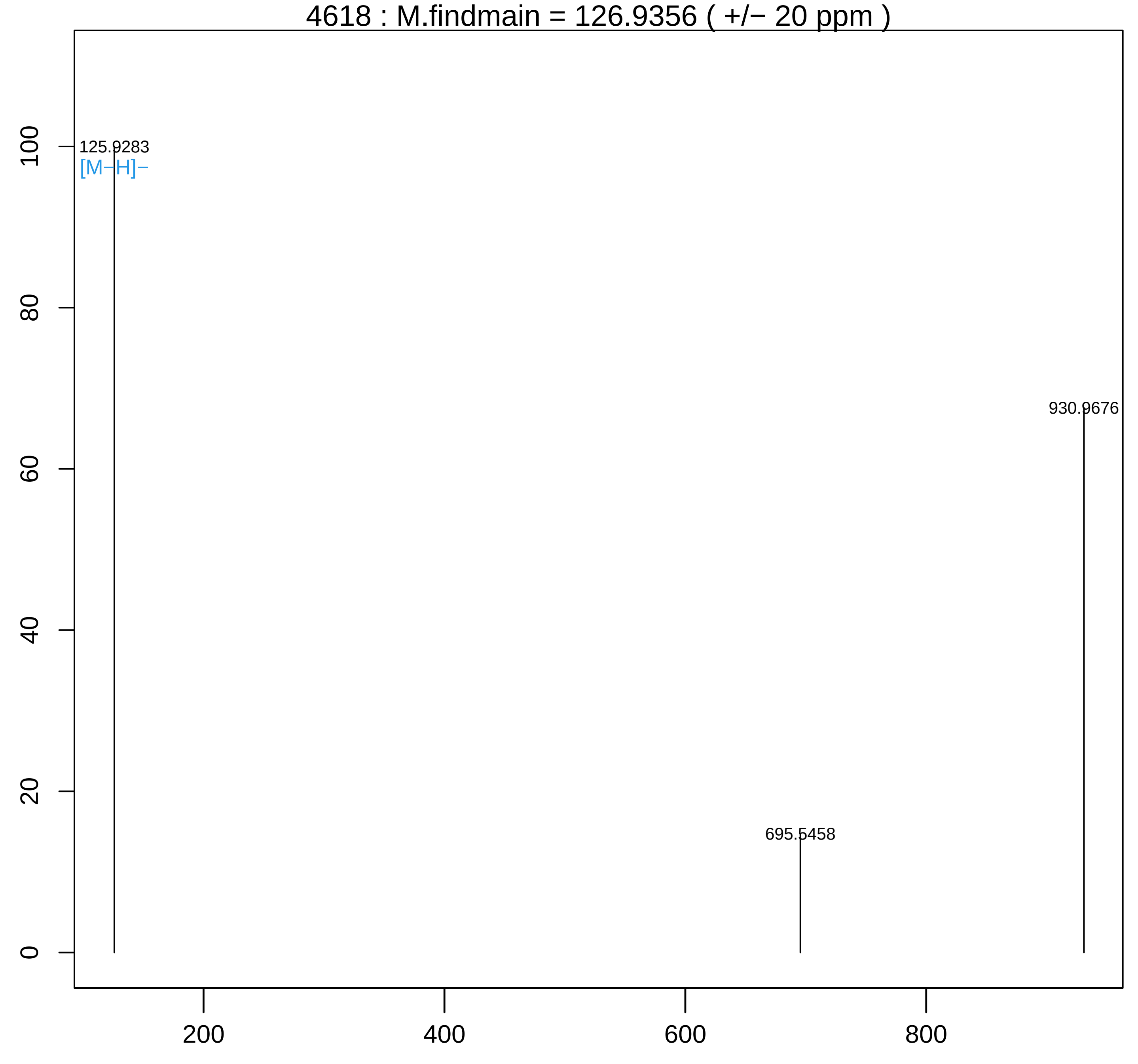
4618 : M.findmain = 126.9356 (+/− 20 ppm)
125.9283
[M−H]−
930.9676
695.5458
0
20
40
60
80
100
200
400
600
800

4619 : M.ramclustr = 209.079 (+/− NA ppm)

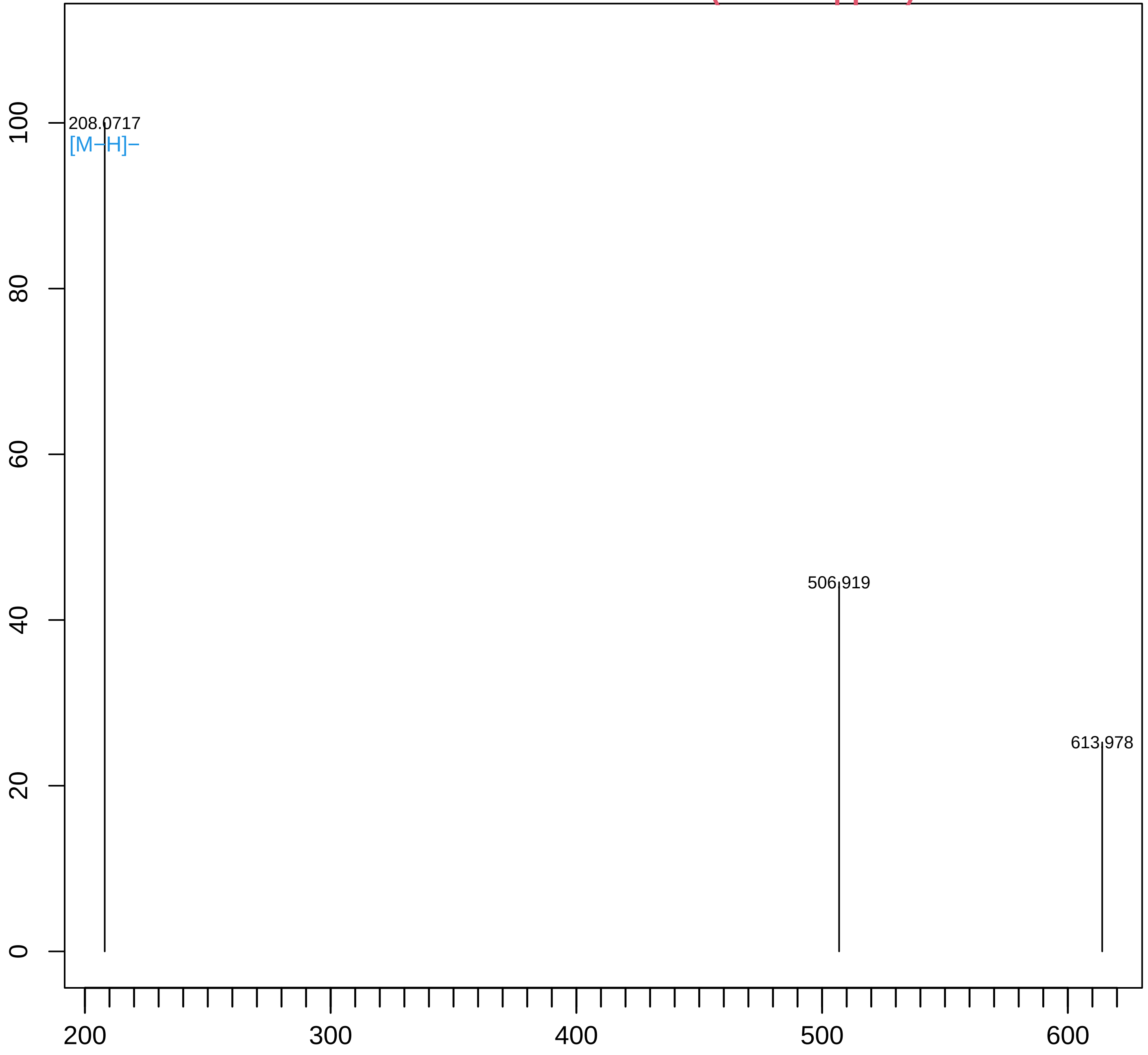

4619 : M.findmain = 209.079 (+/− 20 ppm)

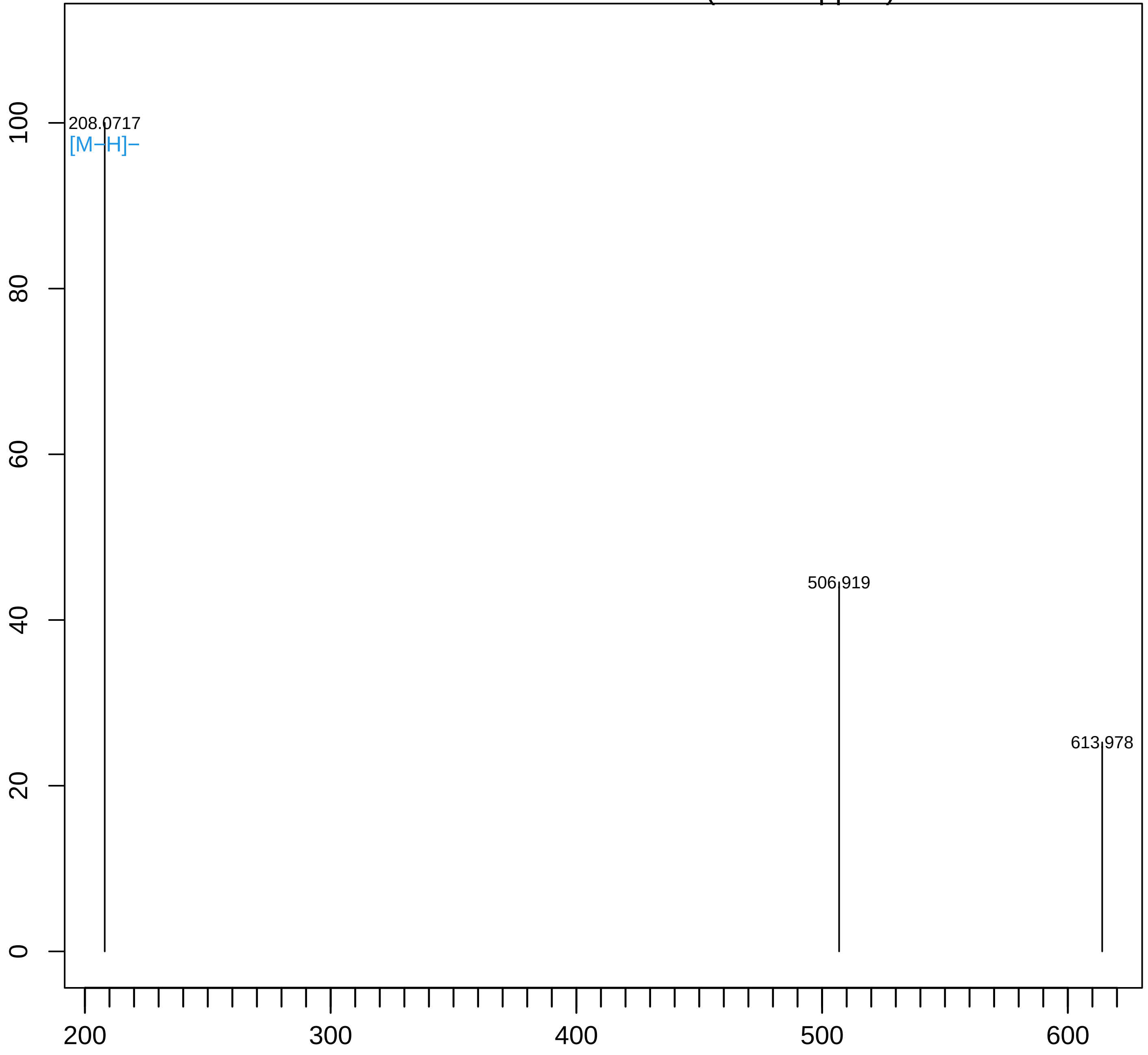

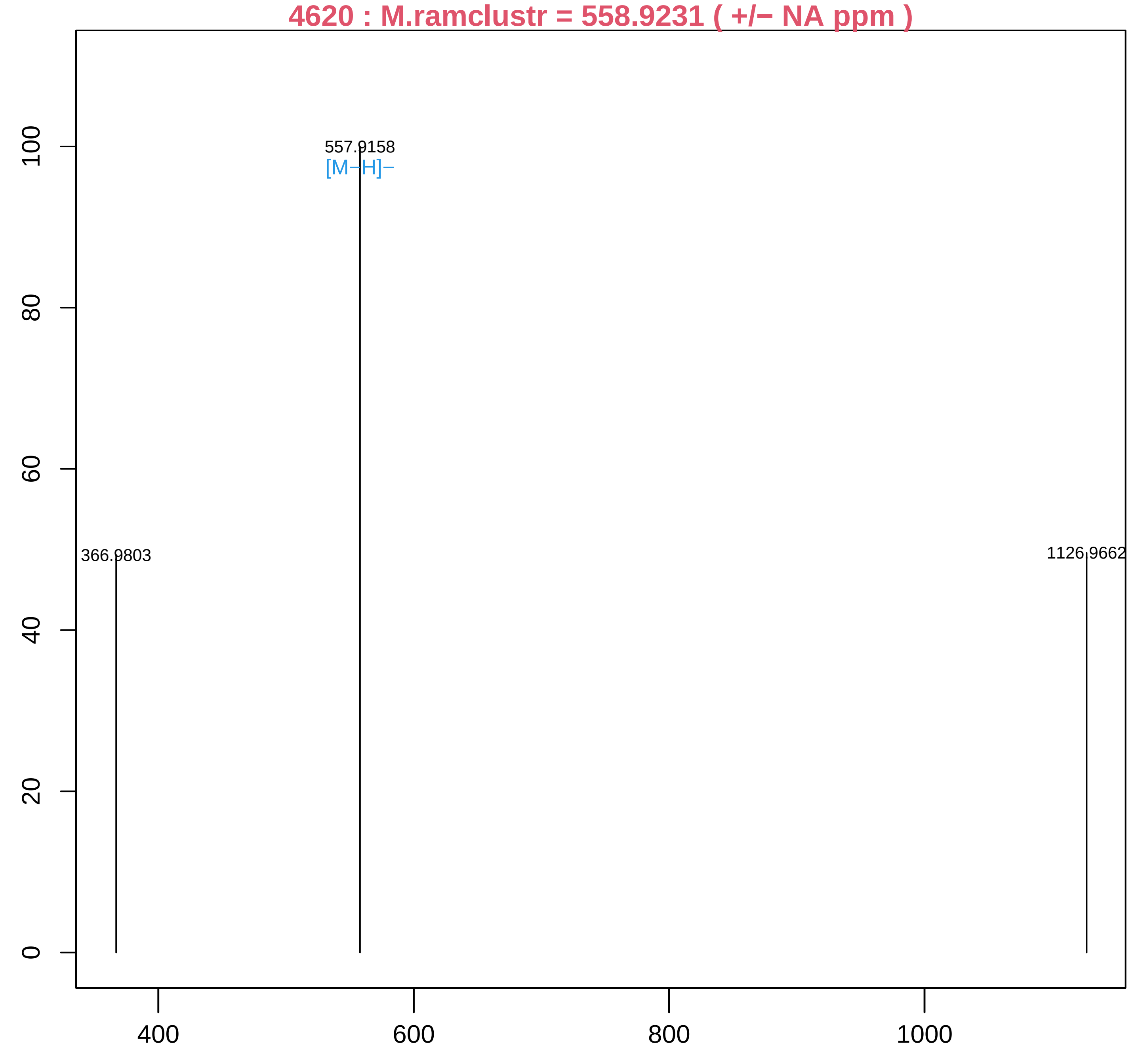

4620 : M.ramclustr = 558.9231 (+/− NA ppm)
557.9158
[M−H]−
366.9803
1126.9662
0
20
40
60
80
100
400
600
800
1000

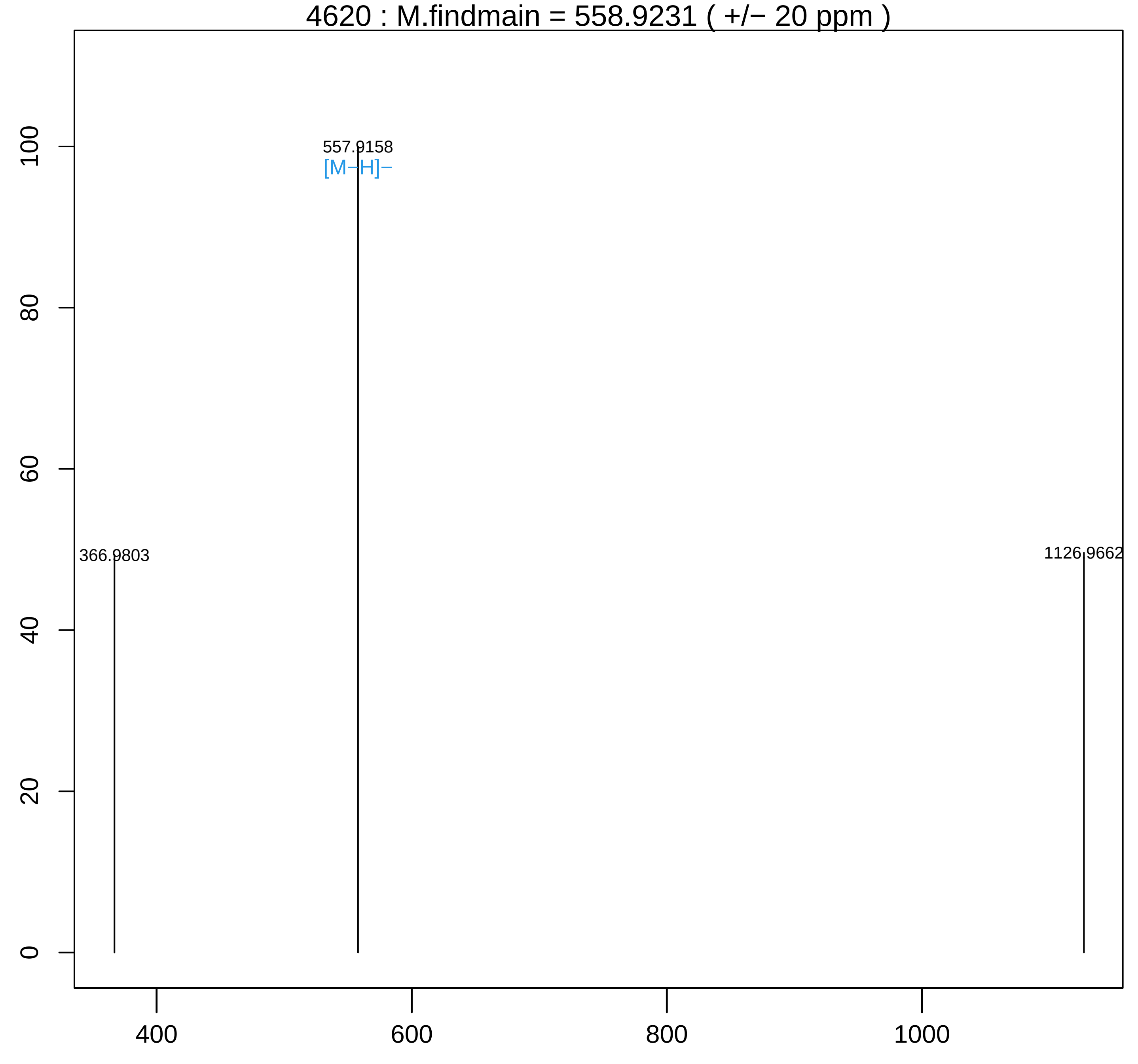

4620 : M.findmain = 558.9231 (+/− 20 ppm)
557.9158
[M−H]−
366.9803
1126.9662
0
20
40
60
80
100
400
600
800
1000

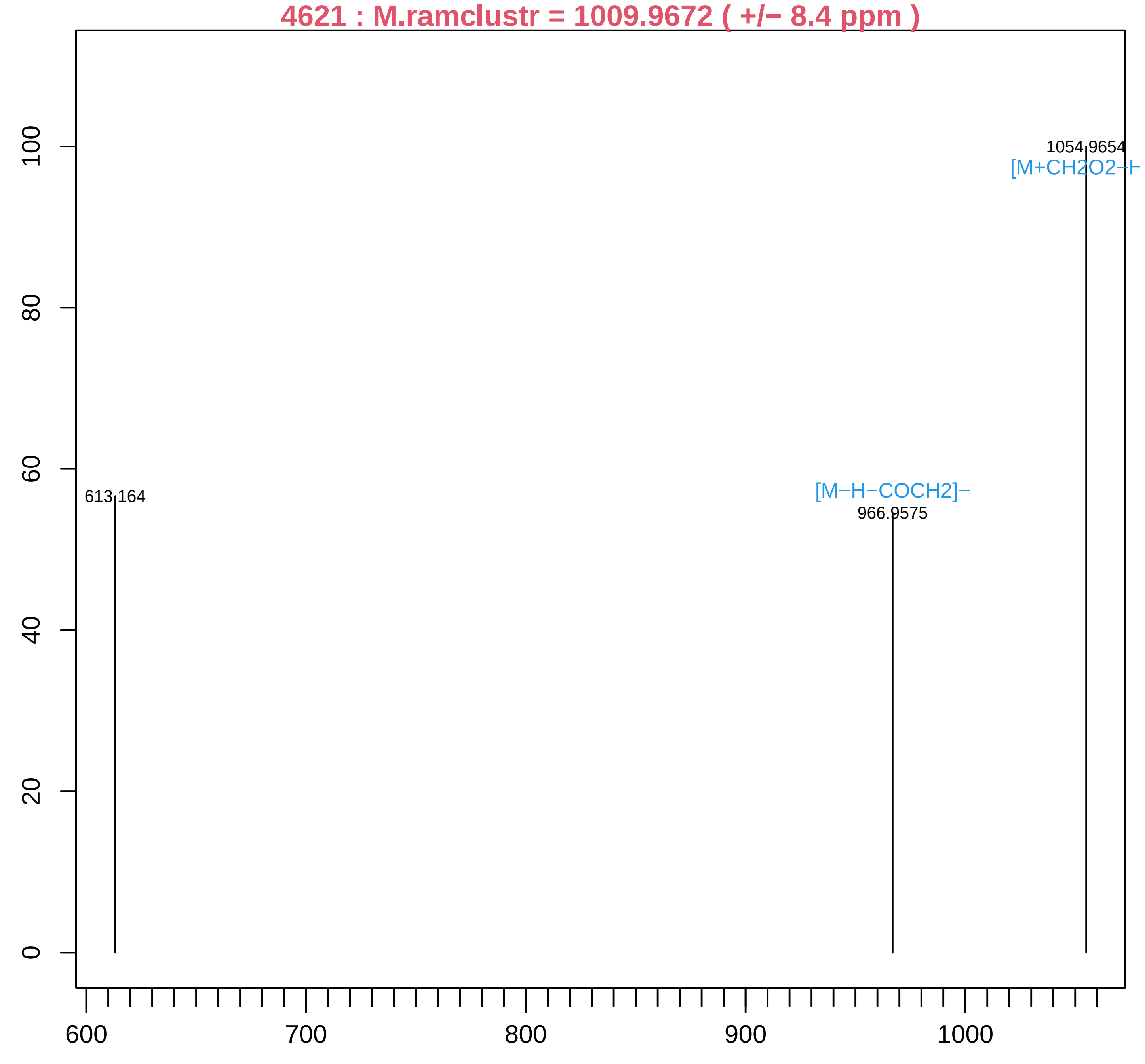
4621 : M.ramclustr = 1009.9672 (+/− 8.4 ppm)
1054.9654
[M+CH2O2−H
[M−H−COCH2]−
966.9575
613.164
0
20
40
60
80
100
600
700
800
900
1000

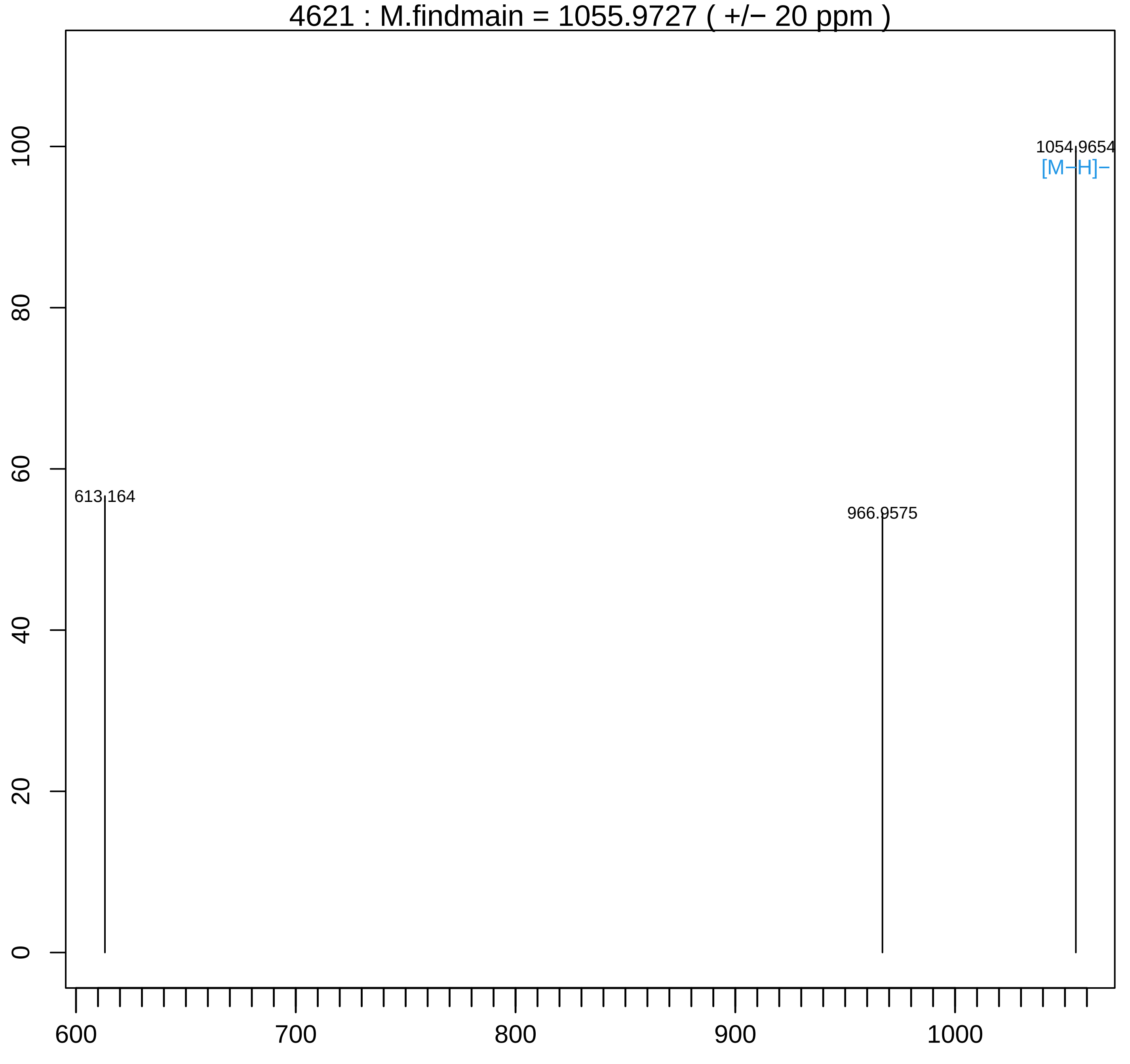
4621 : M.findmain = 1055.9727 (+/− 20 ppm)
1054.9654
[M−H]−
966.9575
613.164
0
20
40
60
80
100
600
700
800
900
1000

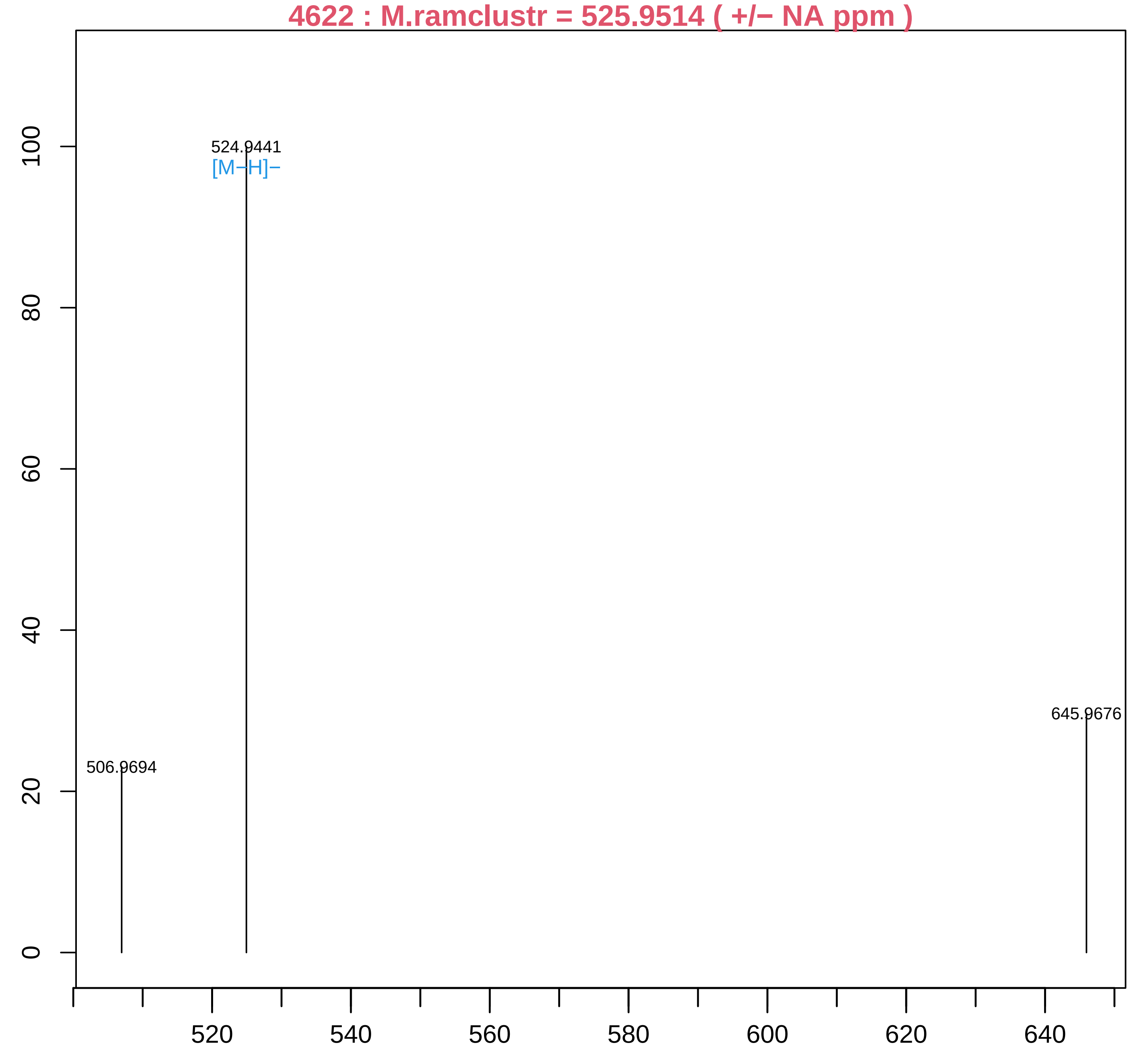
4622 : M.ramclustr = 525.9514 (+/− NA ppm)
524.9441
[M−H]−
506.9694
645.9676
0
20
40
60
80
100
520
540
560
580
600
620
640

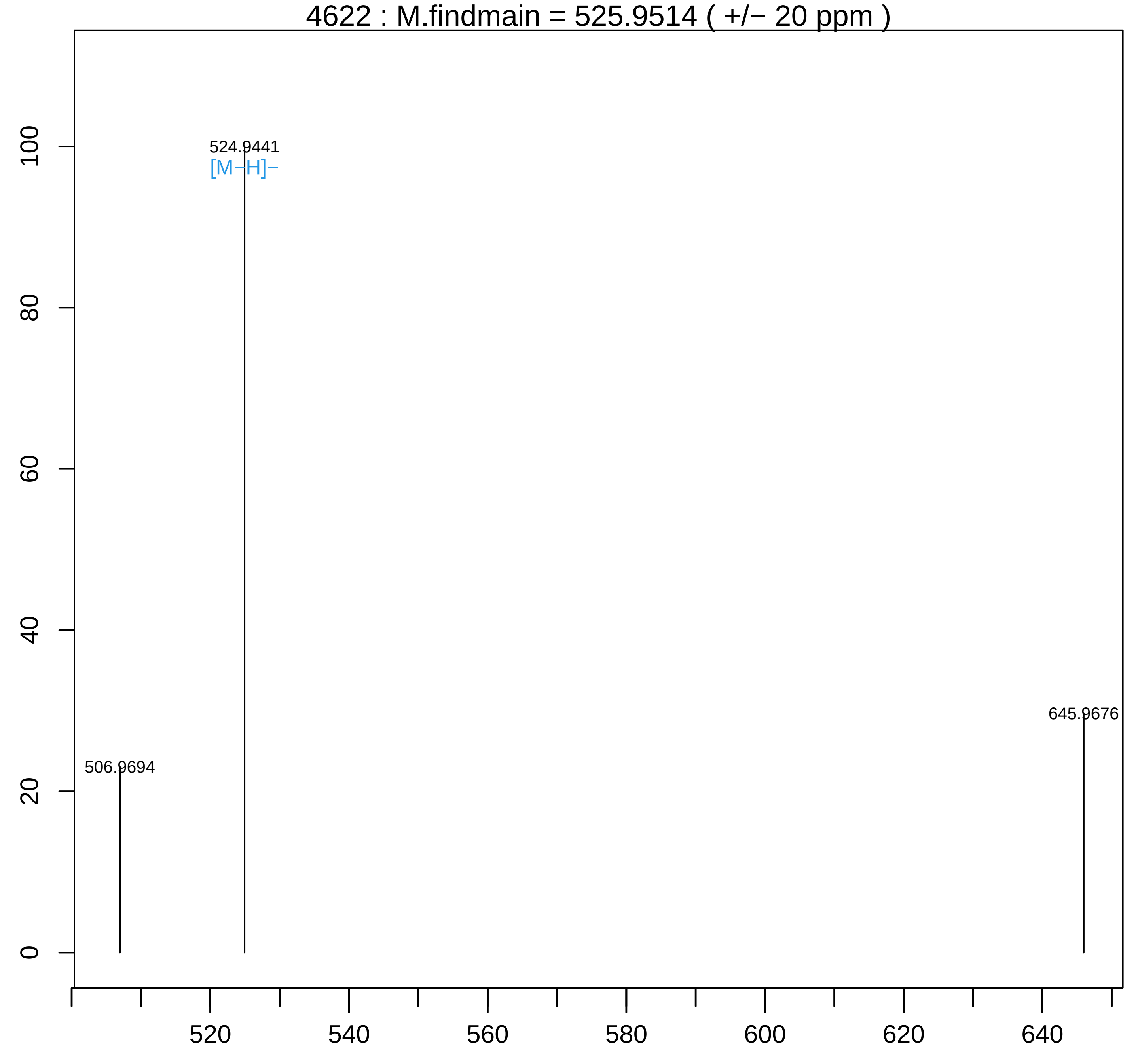
4622 : M.findmain = 525.9514 (+/− 20 ppm)
524.9441
[M−H]−
506.9694
645.9676
0
20
40
60
80
100
520
540
560
580
600
620
640

4623 : M.ramclustr = 814.9919 (+/− 1.1 ppm)

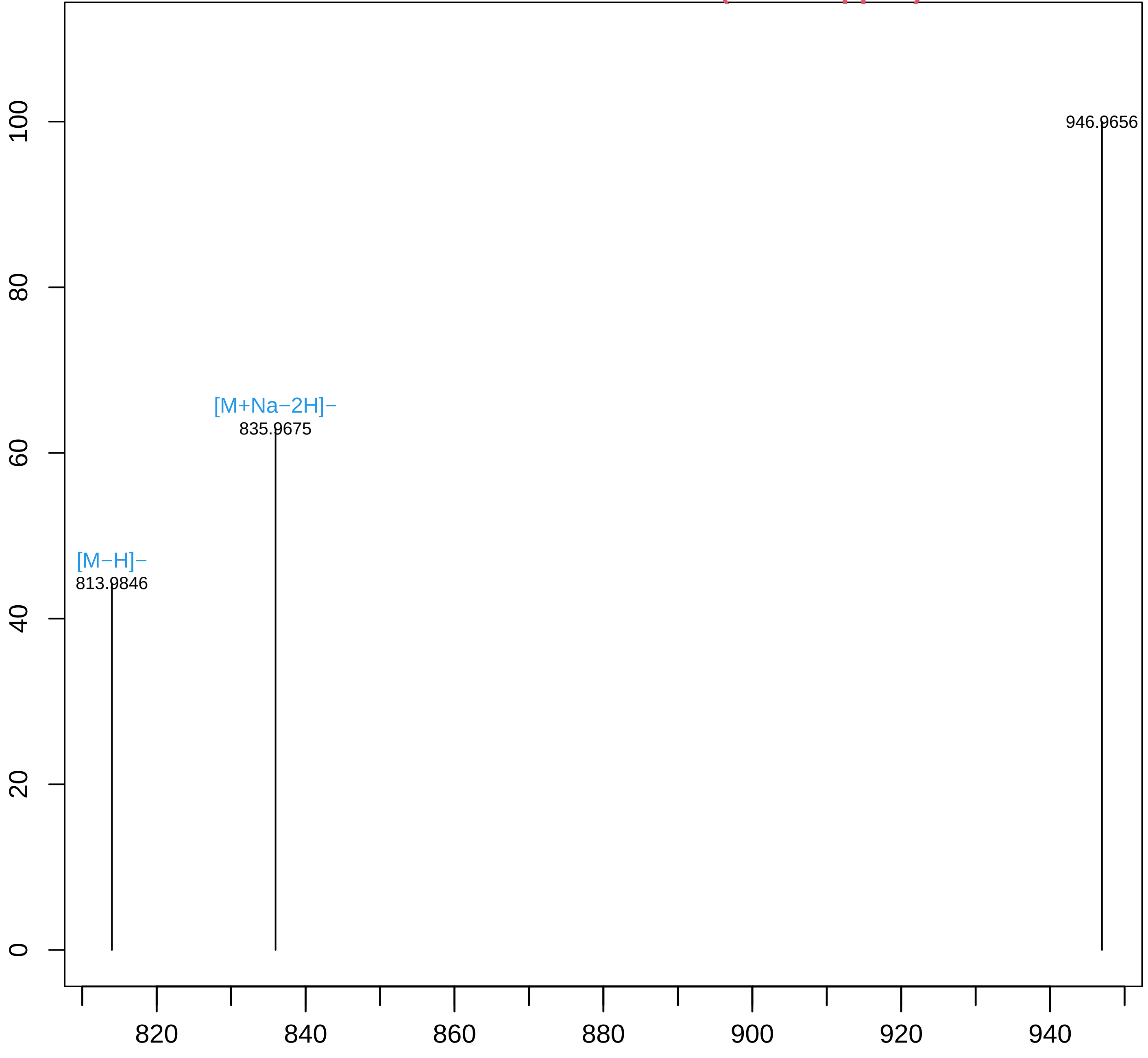

4623 : M.findmain = 814.9928 (+/− 1.2 ppm)

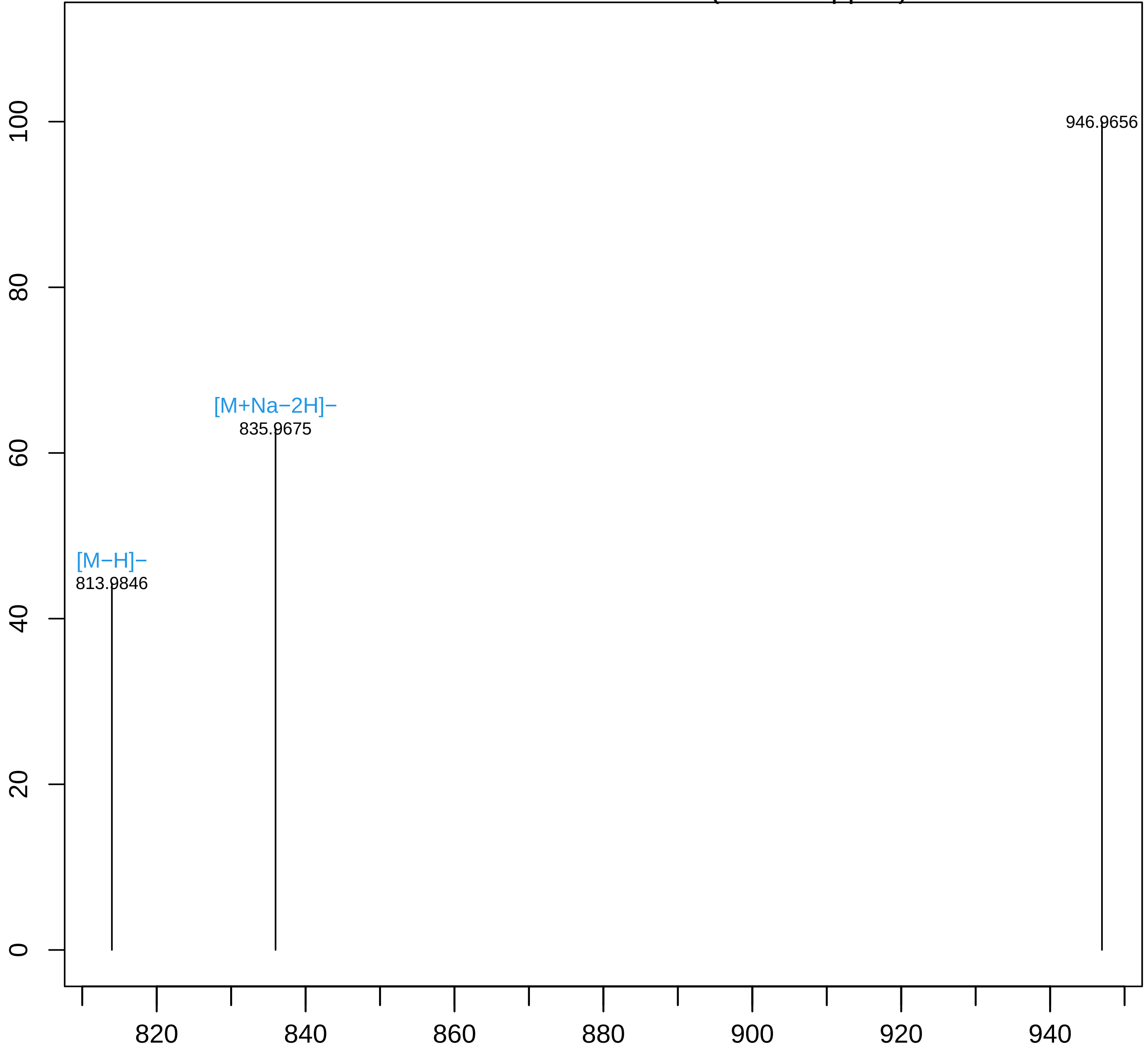

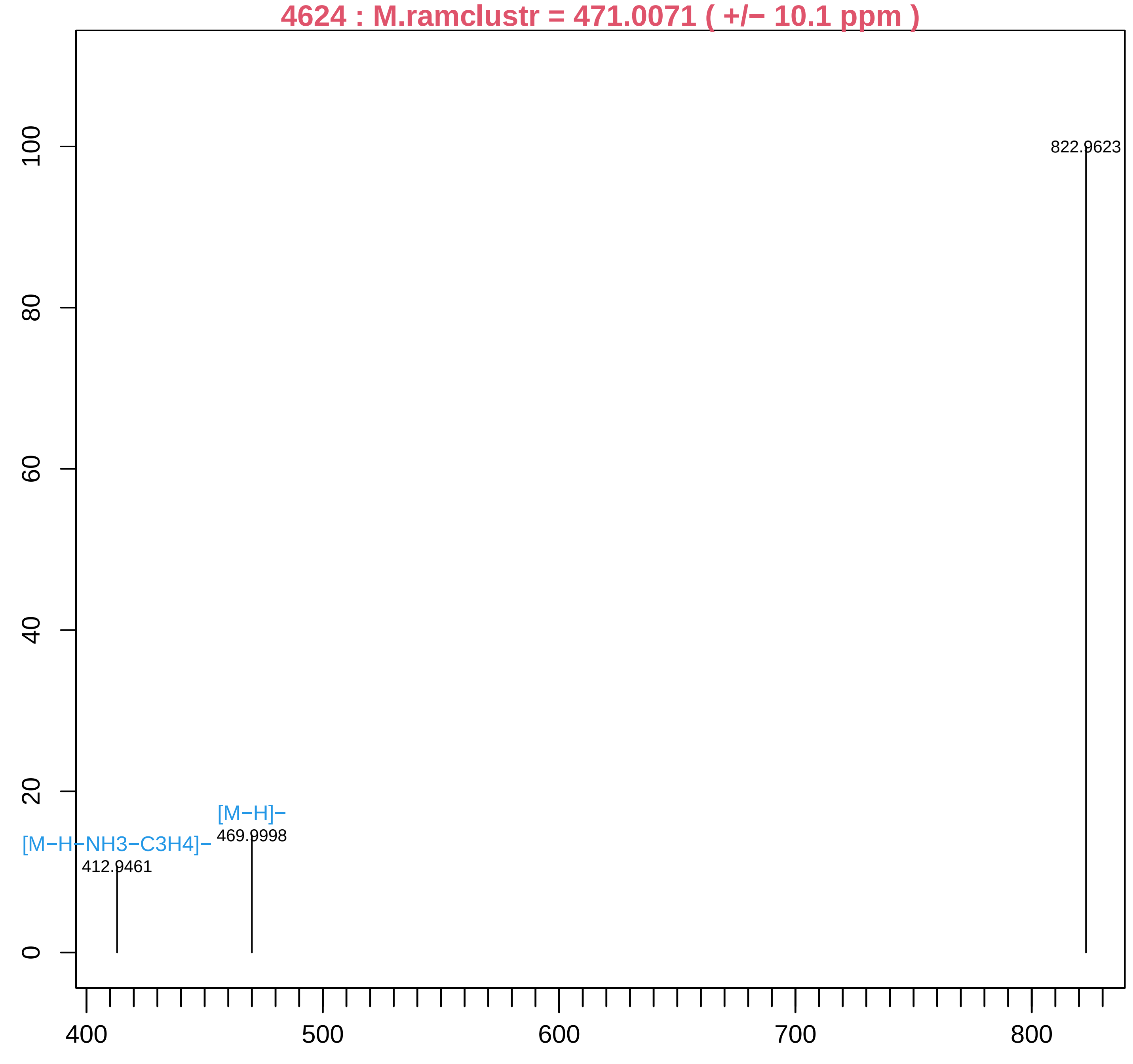
4624 : M.ramclustr = 471.0071 (+/− 10.1 ppm)
822.9623
[M−H]−
469.9998
[M−H−NH3−C3H4]−
412.9461
0
20
40
60
80
100
400
500
600
700
800

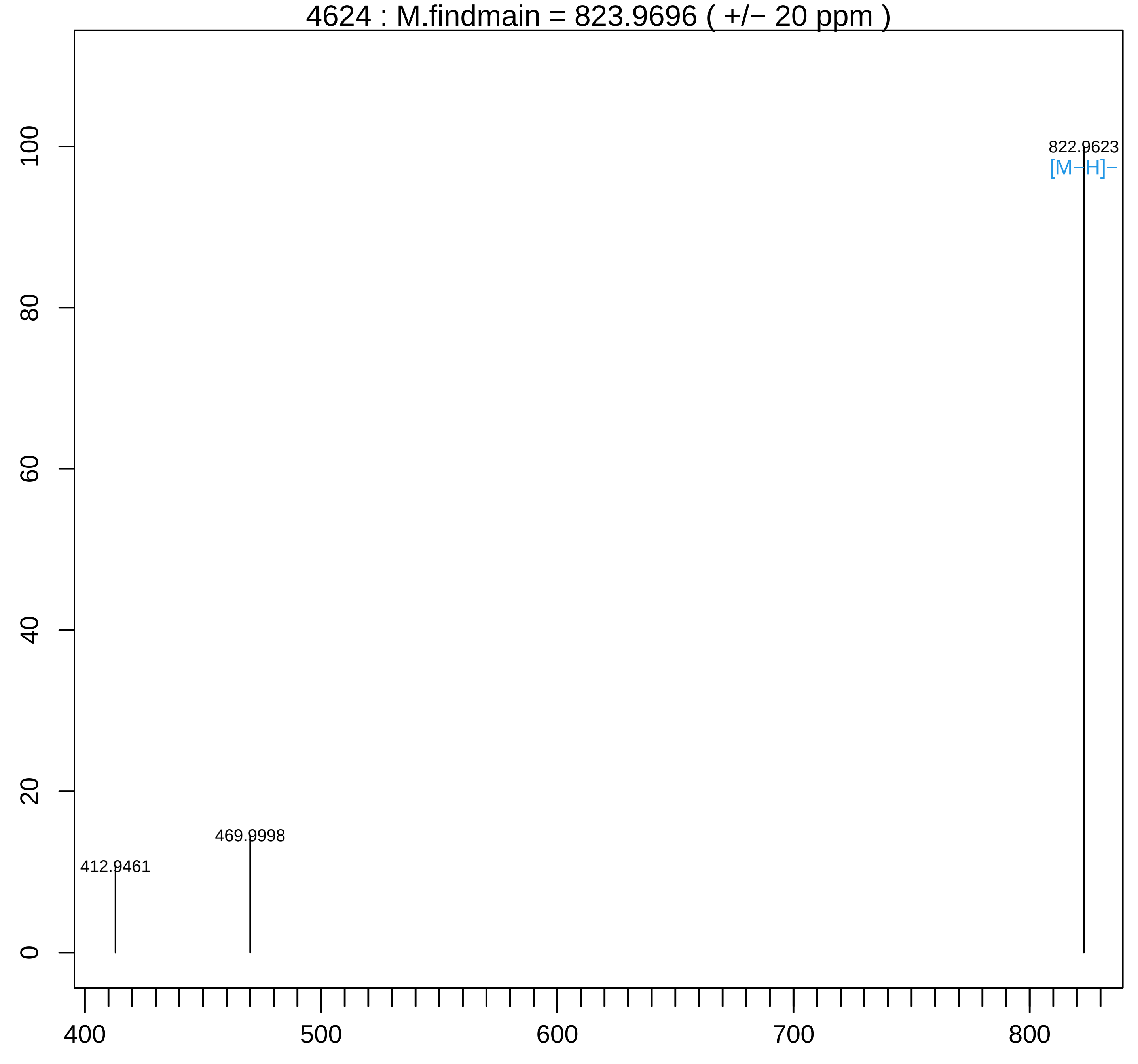
4624 : M.findmain = 823.9696 (+/− 20 ppm)
822.9623
[M−H]−
469.9998
412.9461
0
20
40
60
80
100
400
500
600
700
800

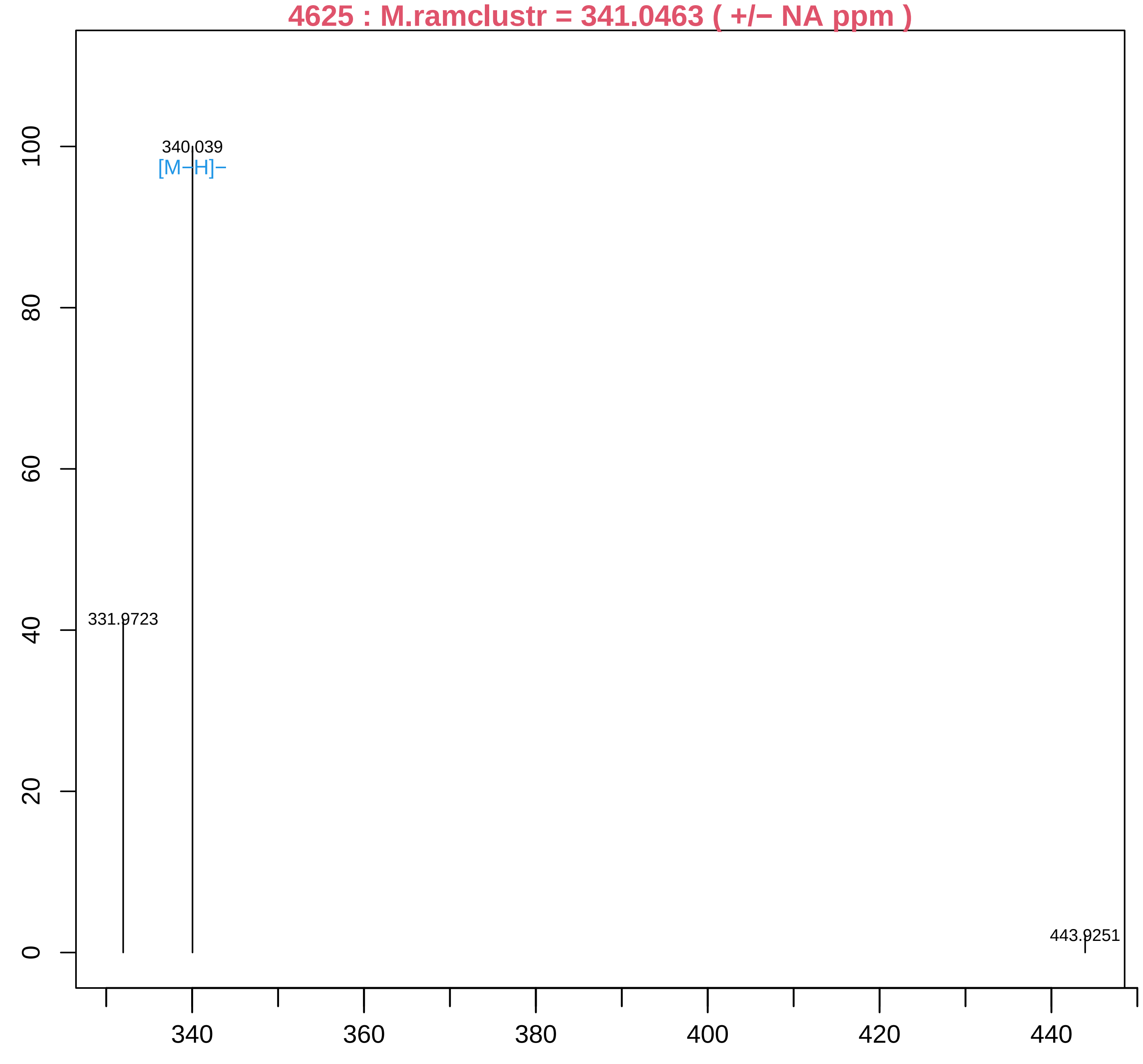

4625 : M.ramclustr = 341.0463 (+/− NA ppm)
340.039
[M−H]−
331.9723
443.9251
0
20
40
60
80
100
340
360
380
400
420
440

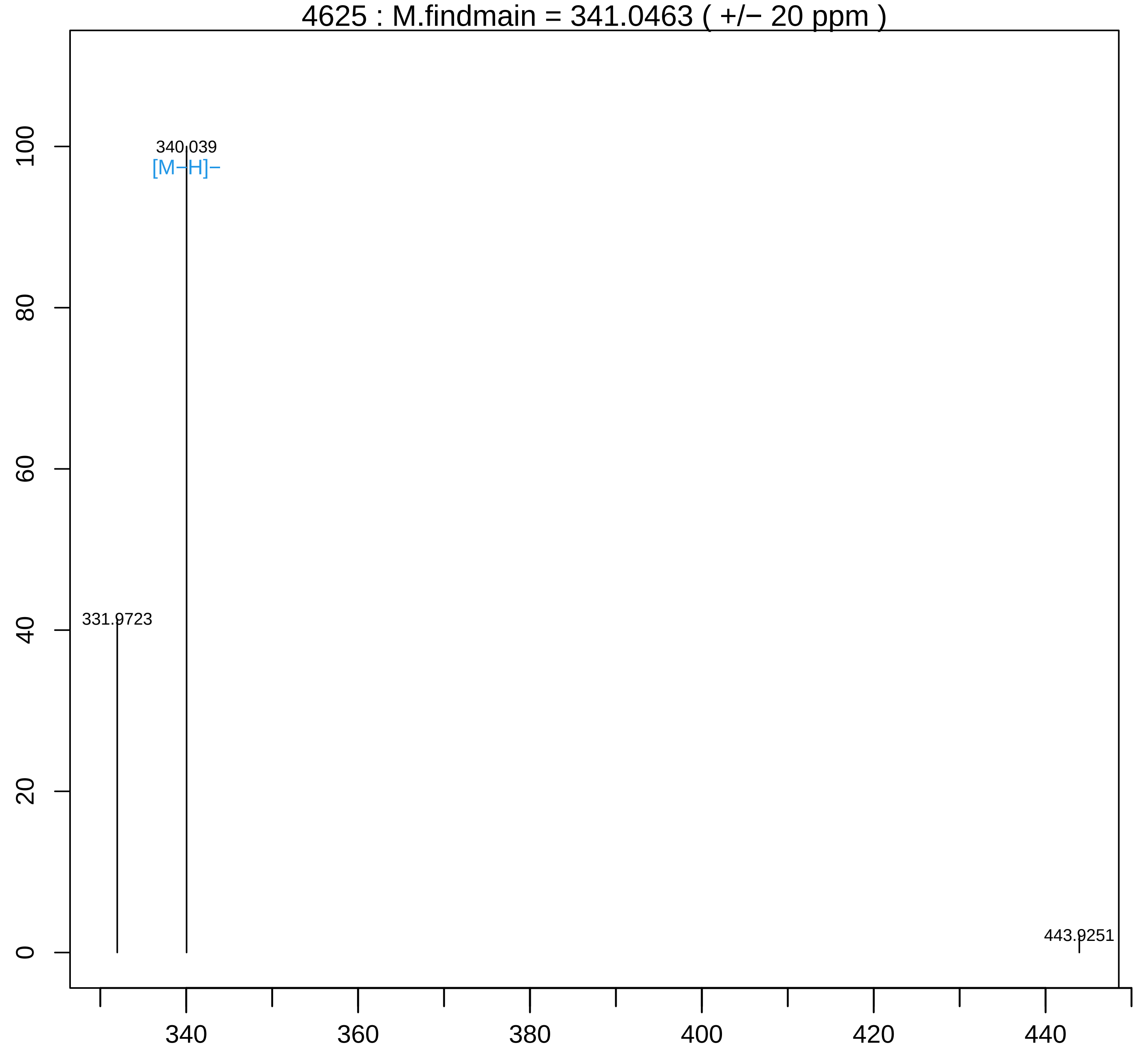

4625 : M.findmain = 341.0463 (+/− 20 ppm)
340.039
[M−H]−
331.9723
443.9251
0
20
40
60
80
100
340
360
380
400
420
440

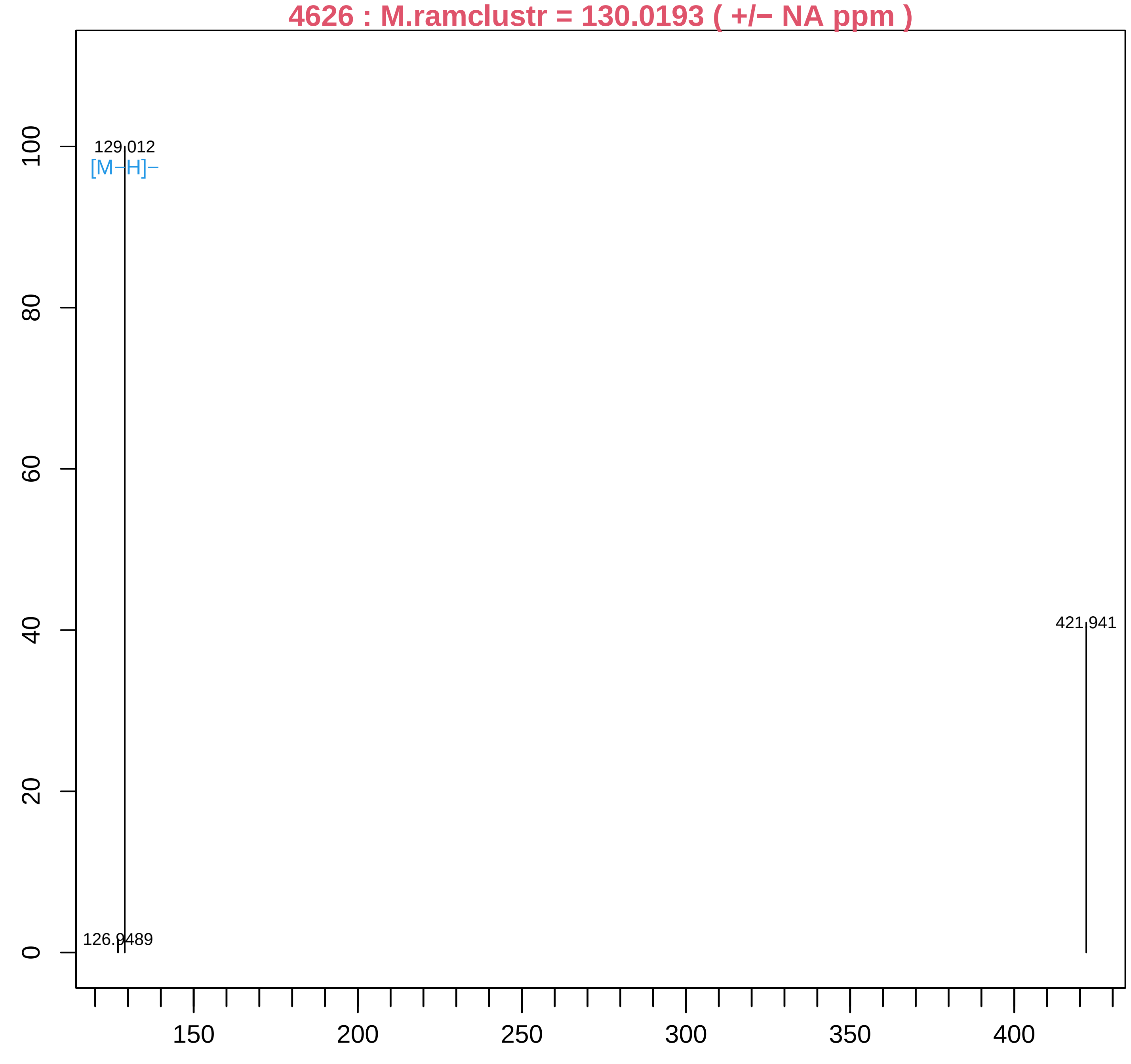
4626 : M.ramclustr = 130.0193 (+/− NA ppm)
129.012
[M−H]−
421.941
126.9489
0
20
40
60
80
100
150
200
250
300
350
400

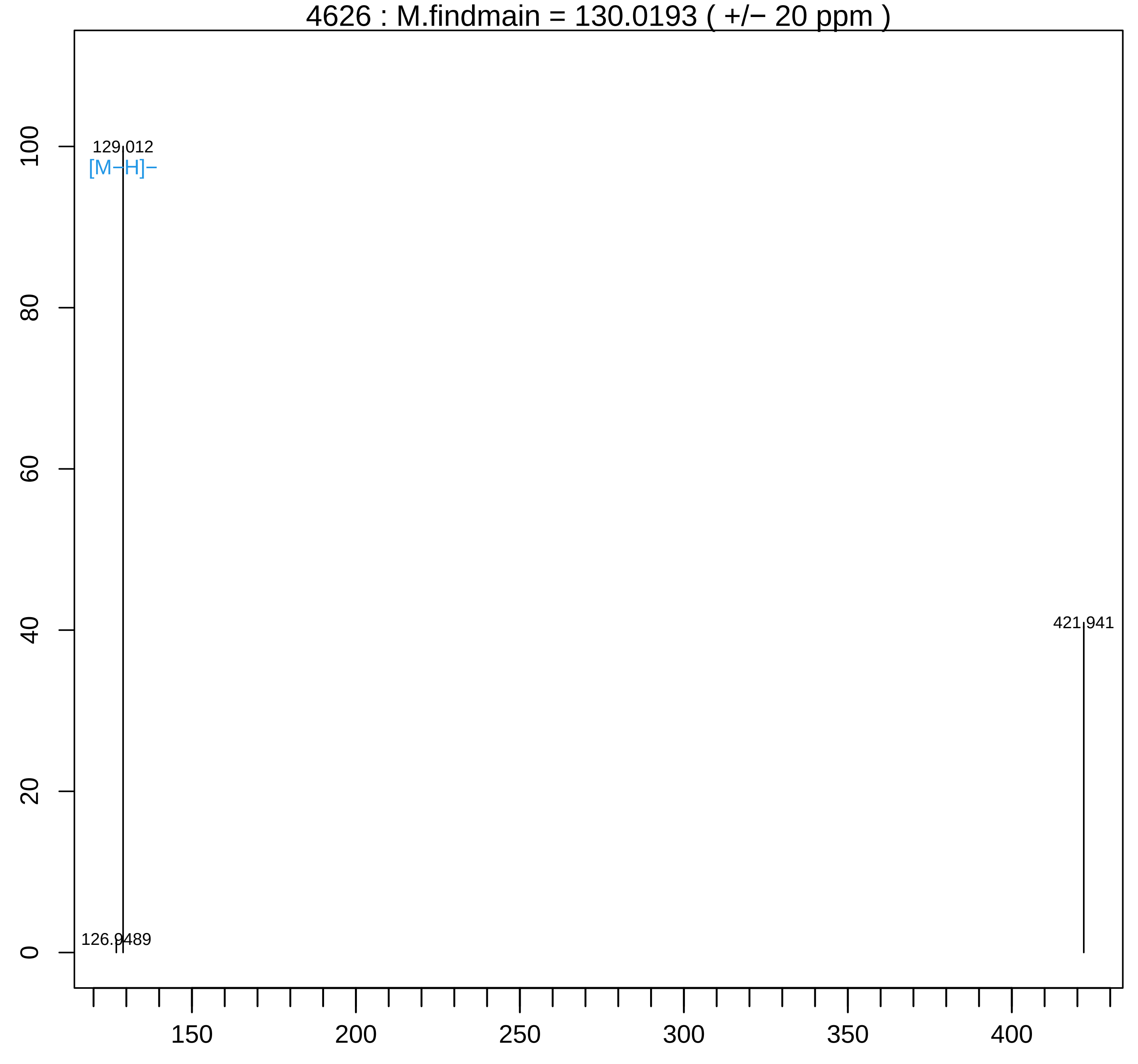
4626 : M.findmain = 130.0193 (+/− 20 ppm)
129.012
[M−H]−
421.941
126.9489
0
20
40
60
80
100
150
200
250
300
350
400

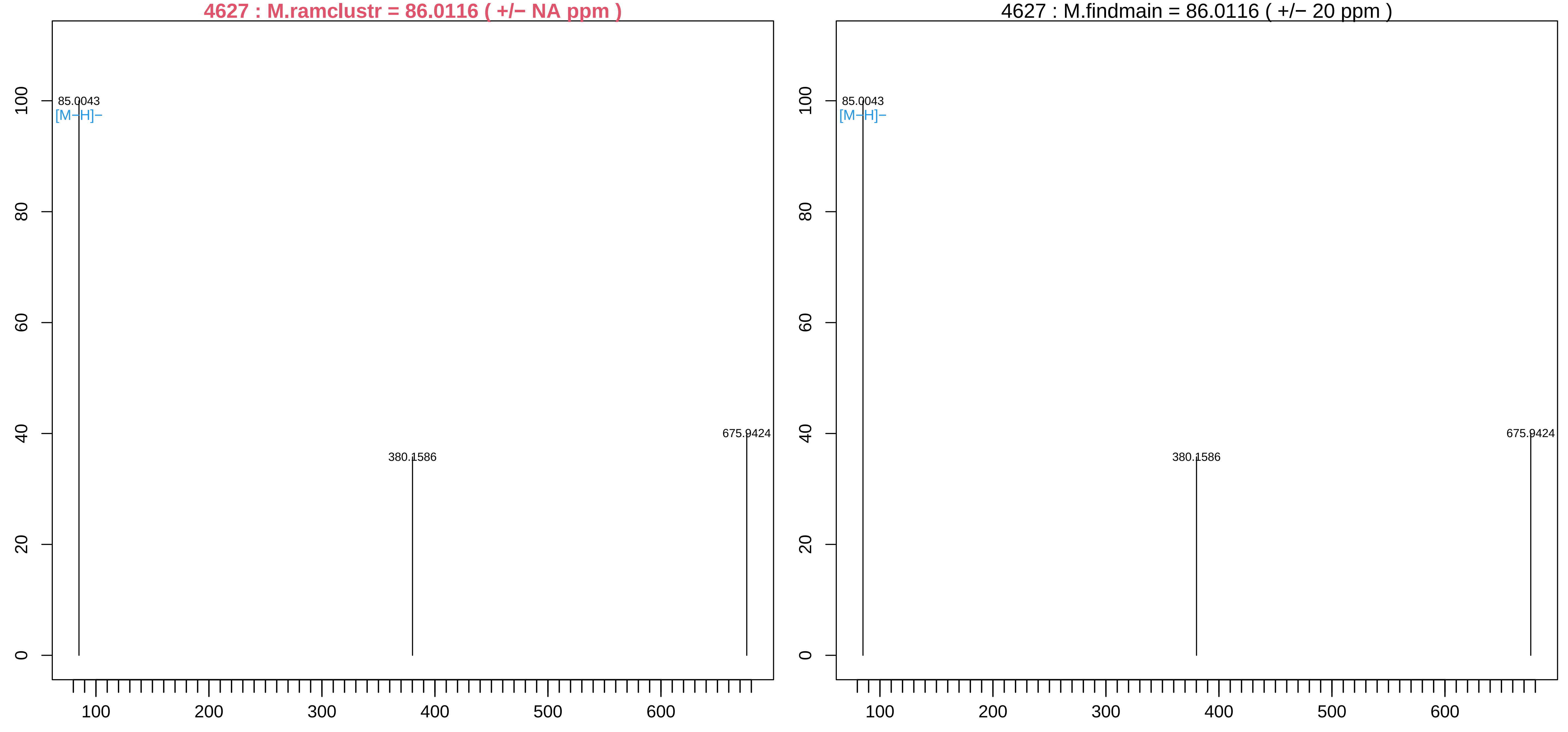
4627 : M.ramclustr = 86.0116 (+/− NA ppm)
85.0043
[M−H]−
380.1586
675.9424
4627 : M.findmain = 86.0116 (+/− 20 ppm)
85.0043
[M−H]−
380.1586
675.9424

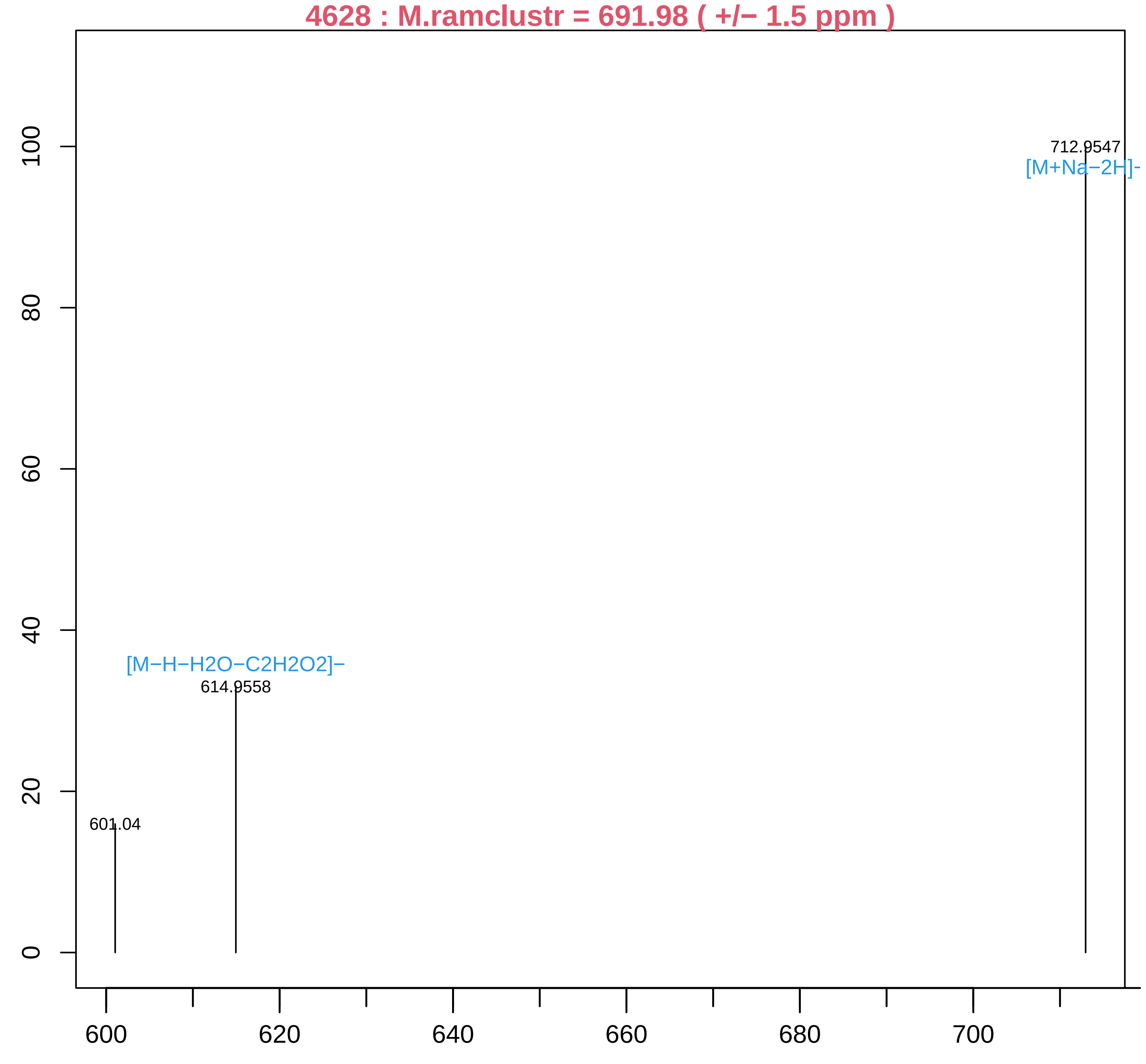
4628 : M.ramclustr = 691.98 (+/− 1.5 ppm)
712.9547
[M+Na−2H]−
[M−H−H2O−C2H2O2]−
614.9558
601.04
0
20
40
60
80
100
600
620
640
660
680
700

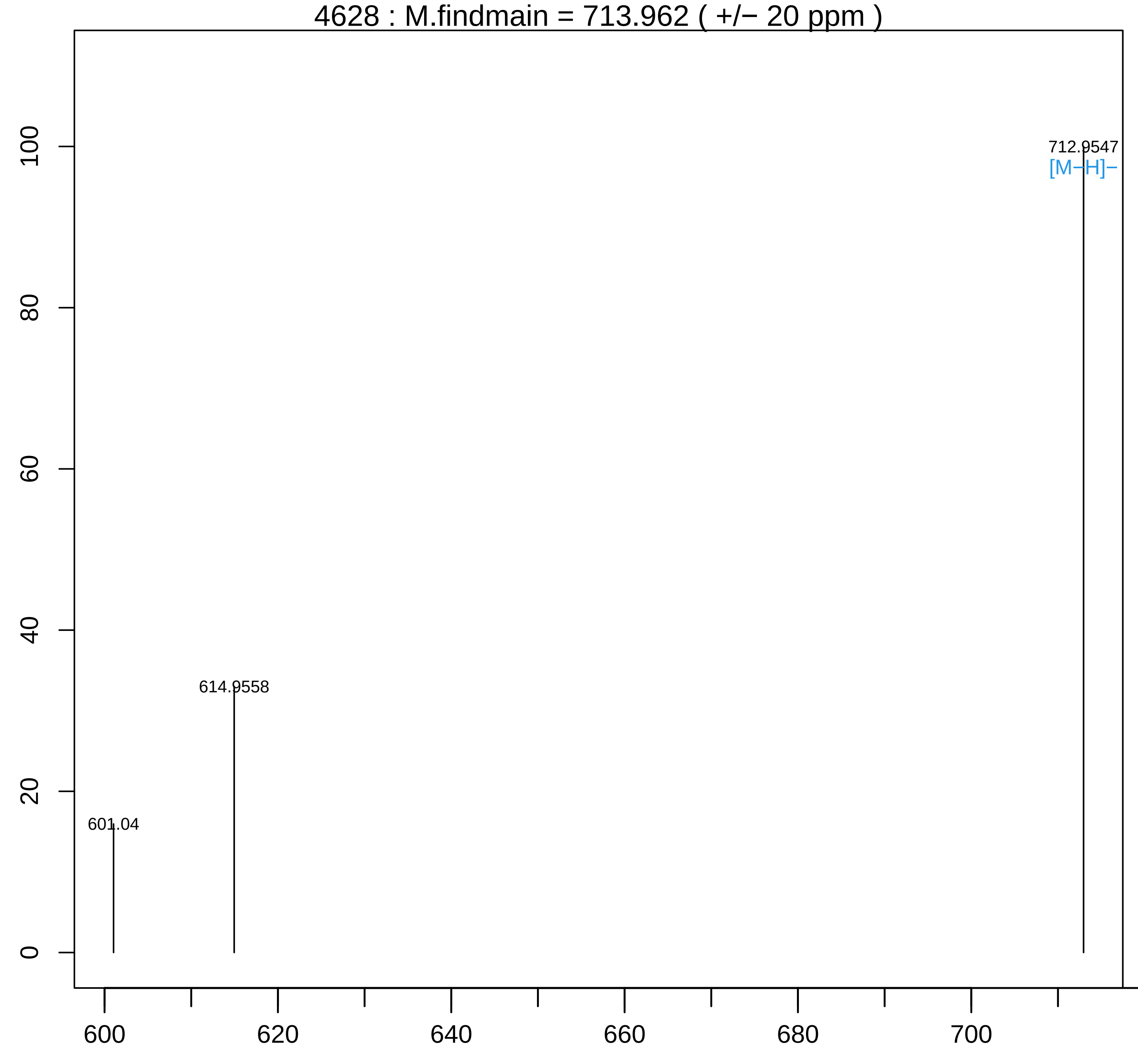
4628 : M.findmain = 713.962 (+/− 20 ppm)
712.9547
[M−H]−
614.9558
601.04
0
20
40
60
80
100
600
620
640
660
680
700

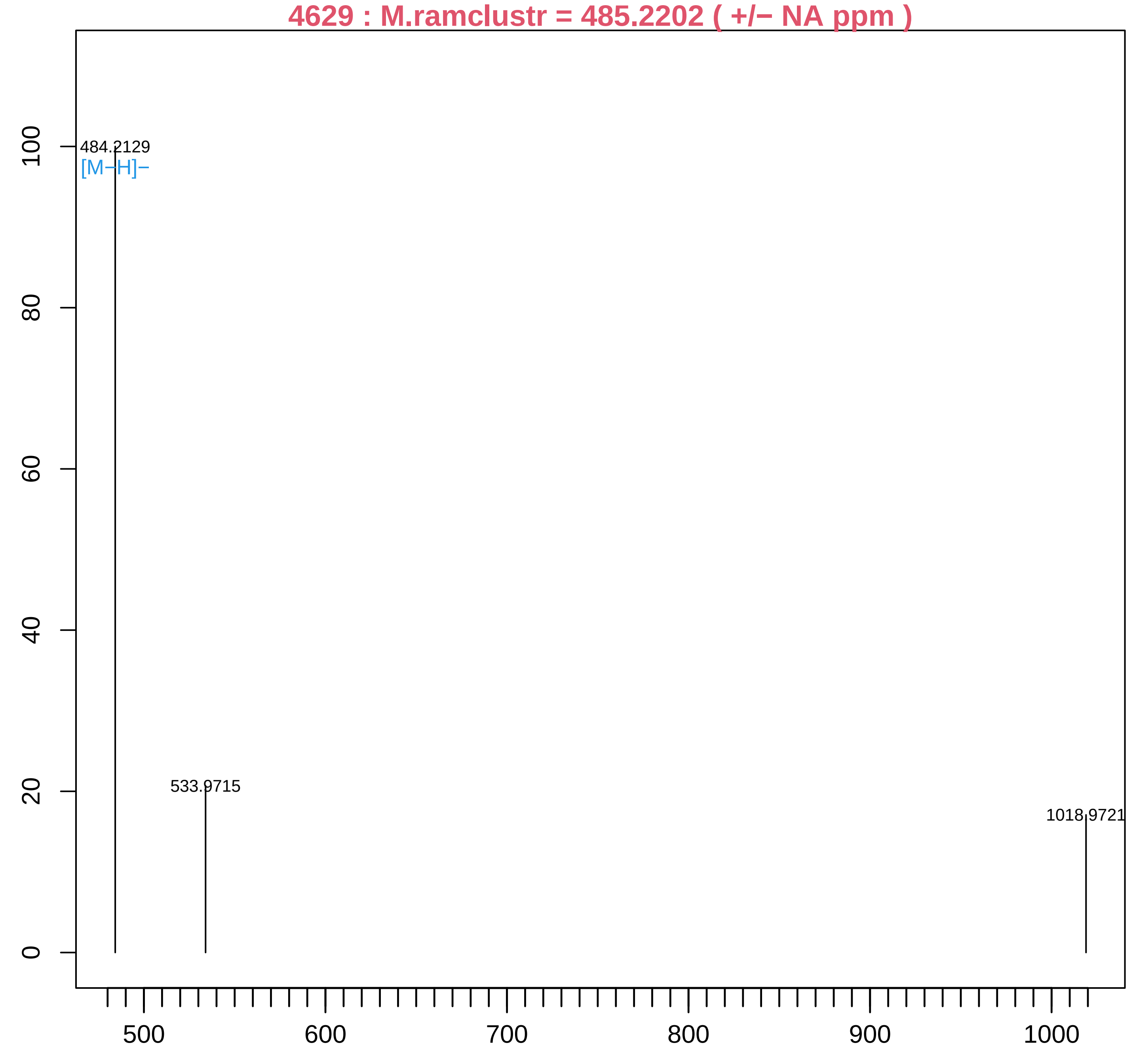
4629 : M.ramclustr = 485.2202 (+/− NA ppm)
484.2129
[M−H]−
533.9715
1018.9721
0
20
40
60
80
100
500
600
700
800
900
1000

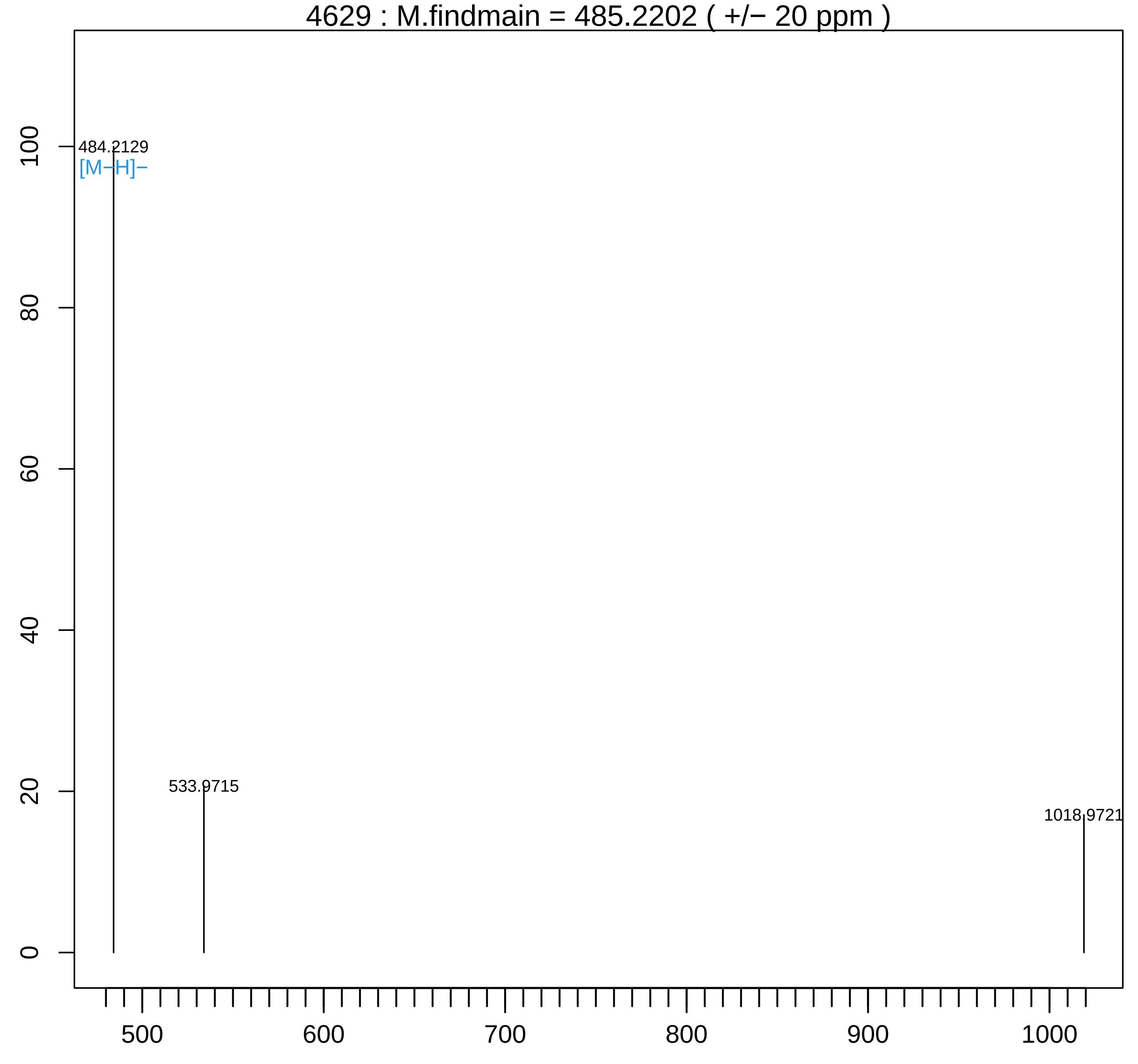
4629 : M.findmain = 485.2202 (+/− 20 ppm)
484.2129
[M−H]−
533.9715
1018.9721
0
20
40
60
80
100
500
600
700
800
900
1000

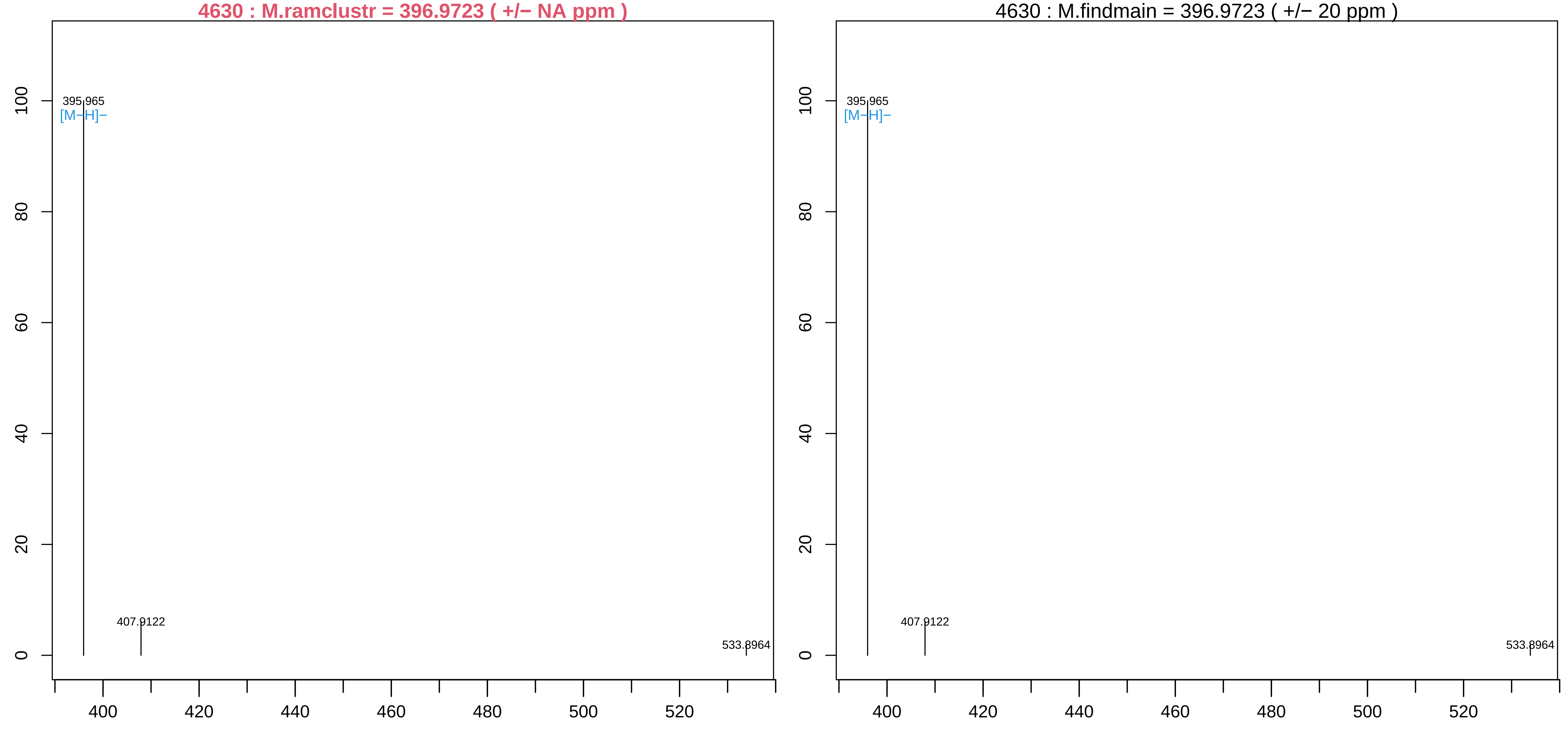
4630 : M.ramclustr = 396.9723 (+/− NA ppm)
395.965
[M−H]−
407.9122
533.8964
4630 : M.findmain = 396.9723 (+/− 20 ppm)
395.965
[M−H]−
407.9122
533.8964
0
20
40
60
80
100
400
420
440
460
480
500
520

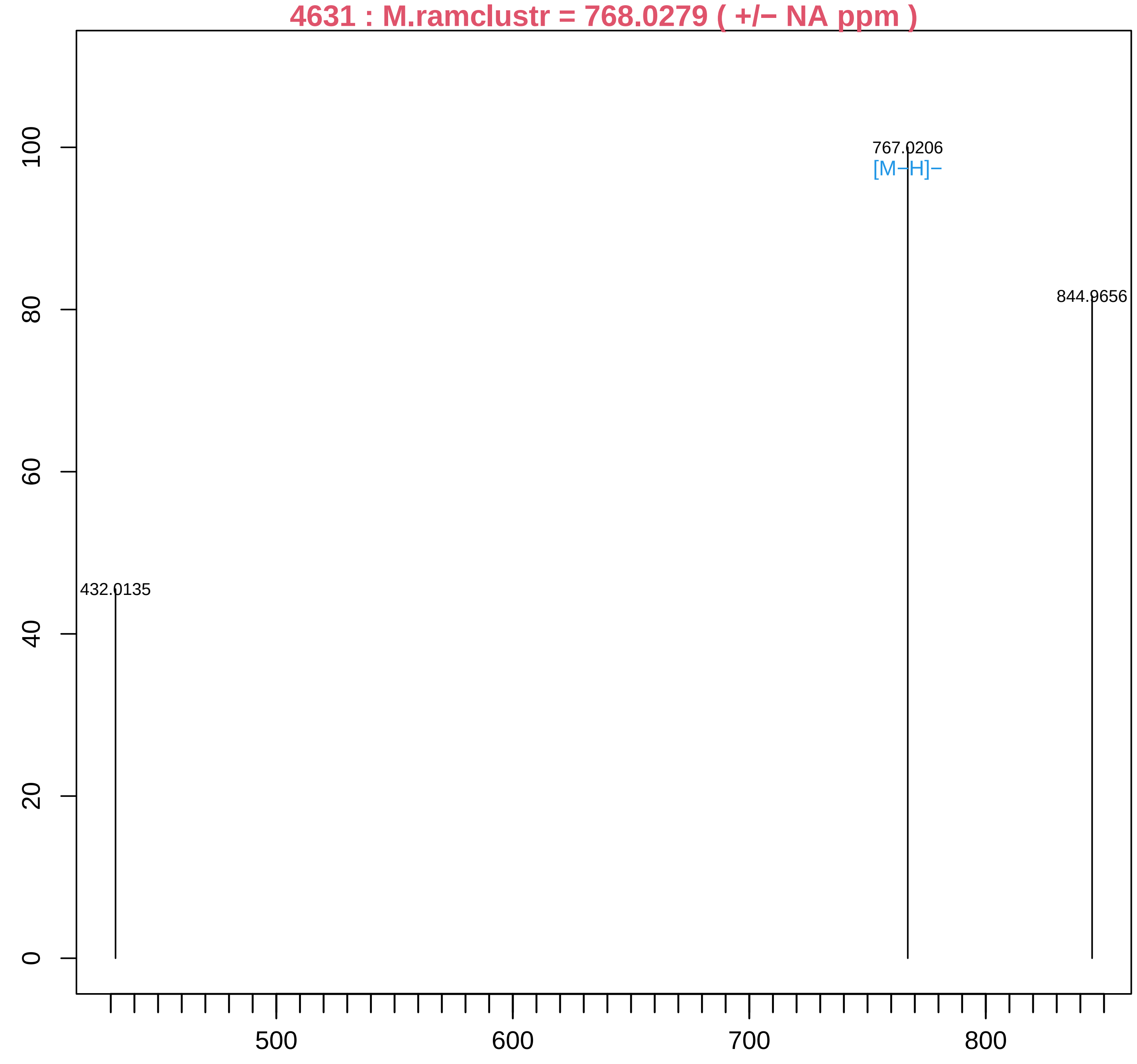
4631 : M.ramclustr = 768.0279 (+/− NA ppm)
767.0206
[M−H]−
844.9656
432.0135
0
20
40
60
80
100
500
600
700
800

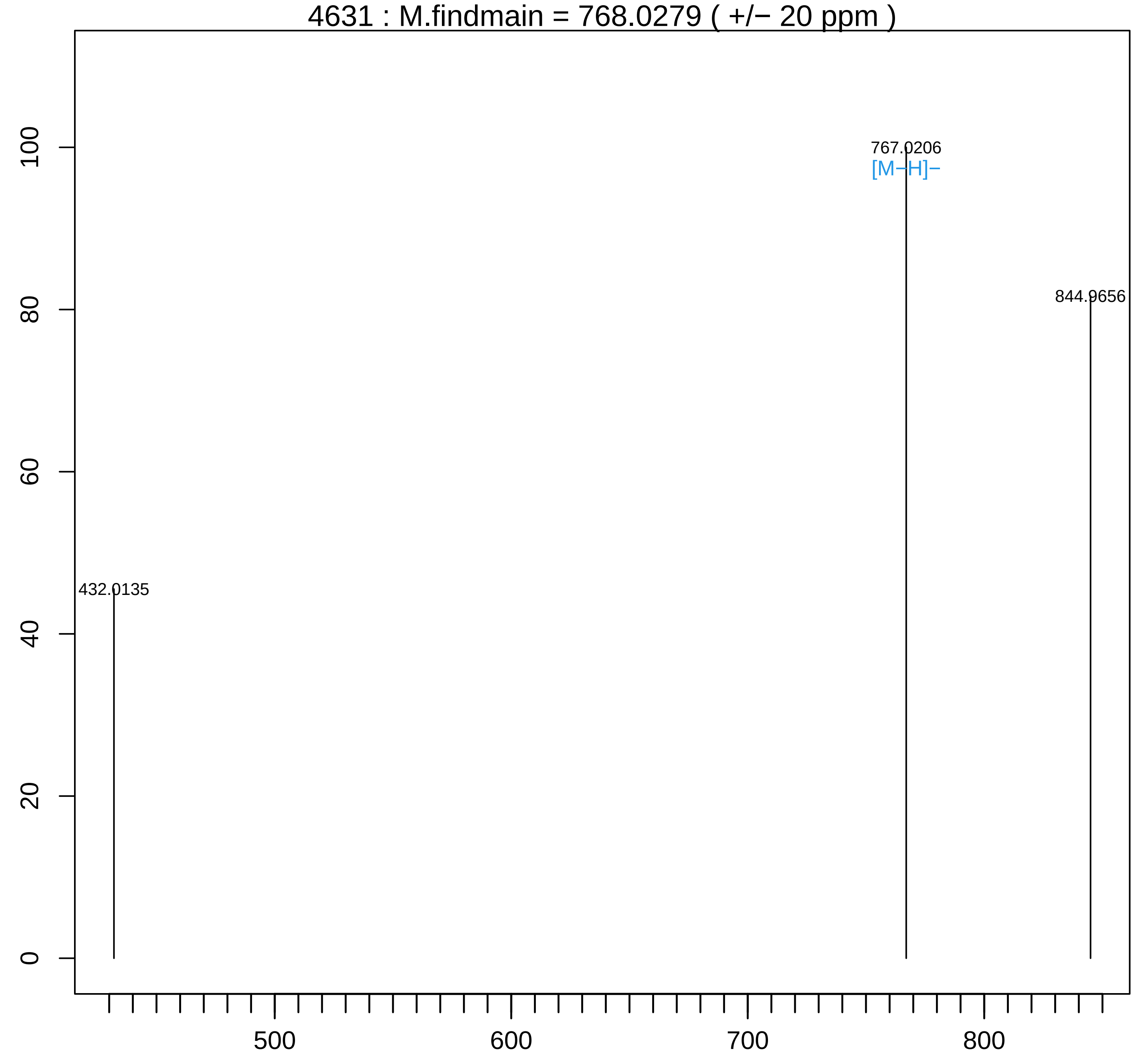
4631 : M.findmain = 768.0279 (+/− 20 ppm)
767.0206
[M−H]−
844.9656
432.0135
0
20
40
60
80
100
500
600
700
800

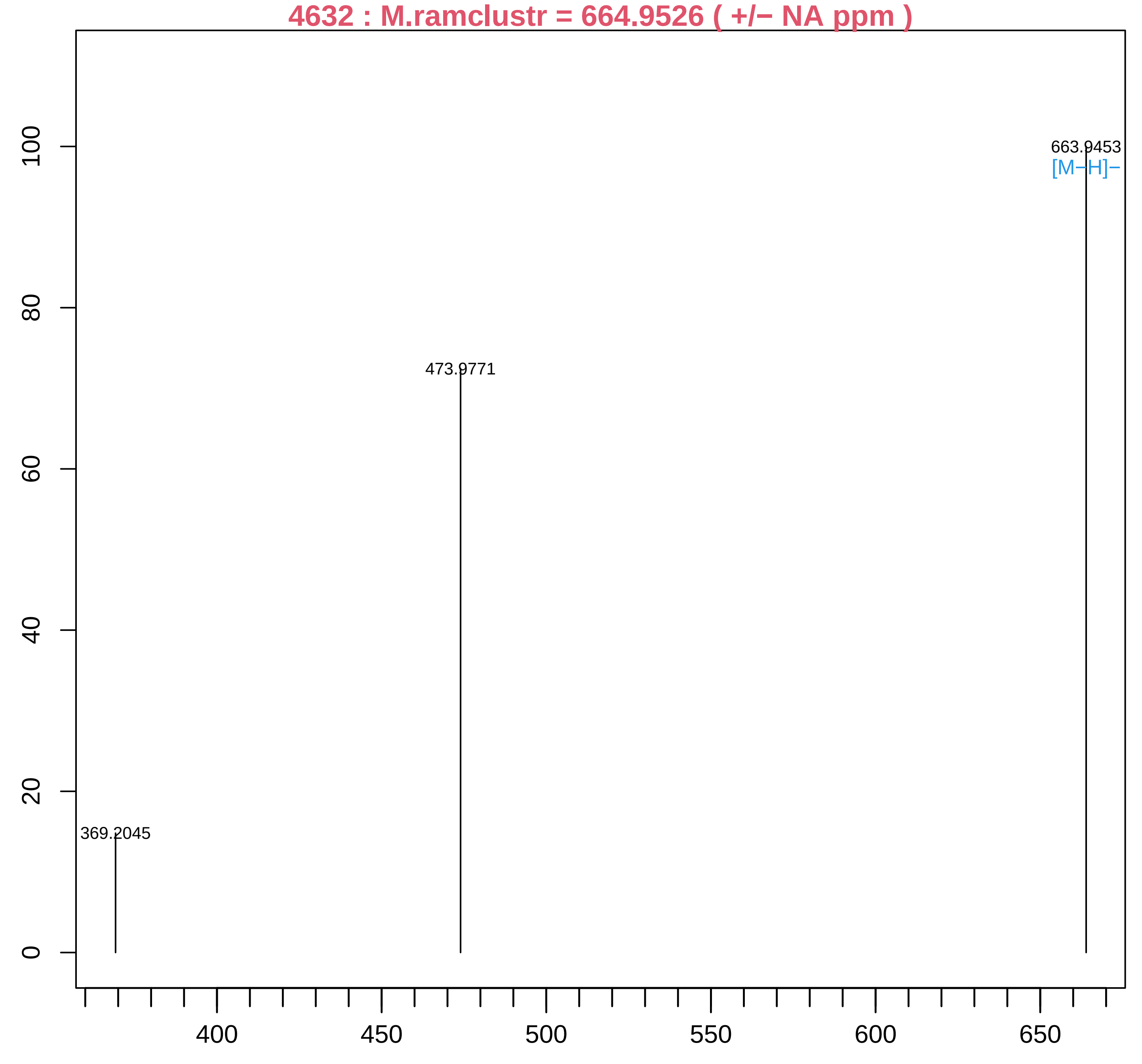
4632 : M.ramclustr = 664.9526 (+/− NA ppm)
663.9453
[M−H]−
473.9771
369.2045
0
20
40
60
80
100
400
450
500
550
600
650

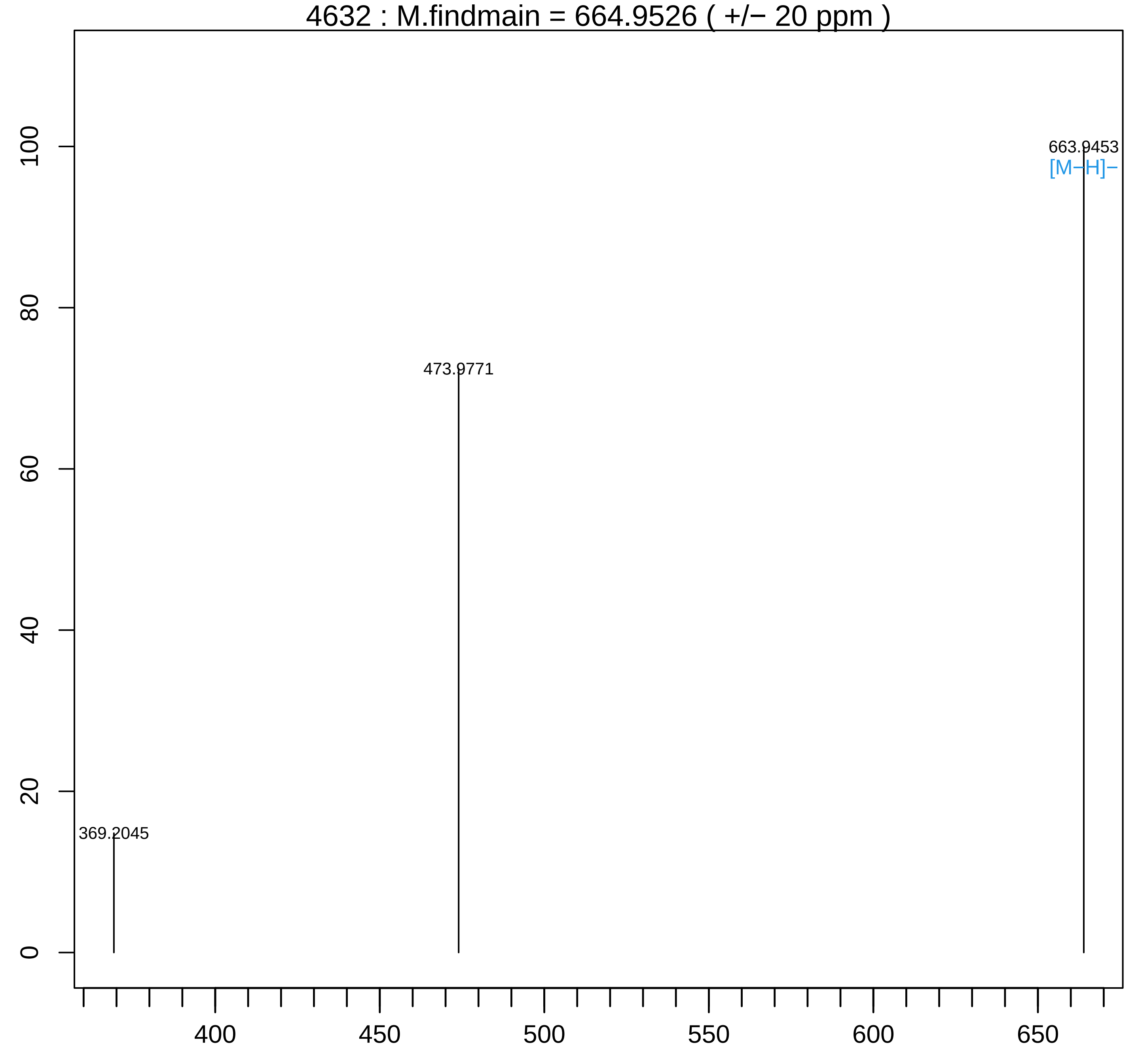
4632 : M.findmain = 664.9526 (+/− 20 ppm)
663.9453
[M−H]−
473.9771
369.2045
0
20
40
60
80
100
400
450
500
550
600
650

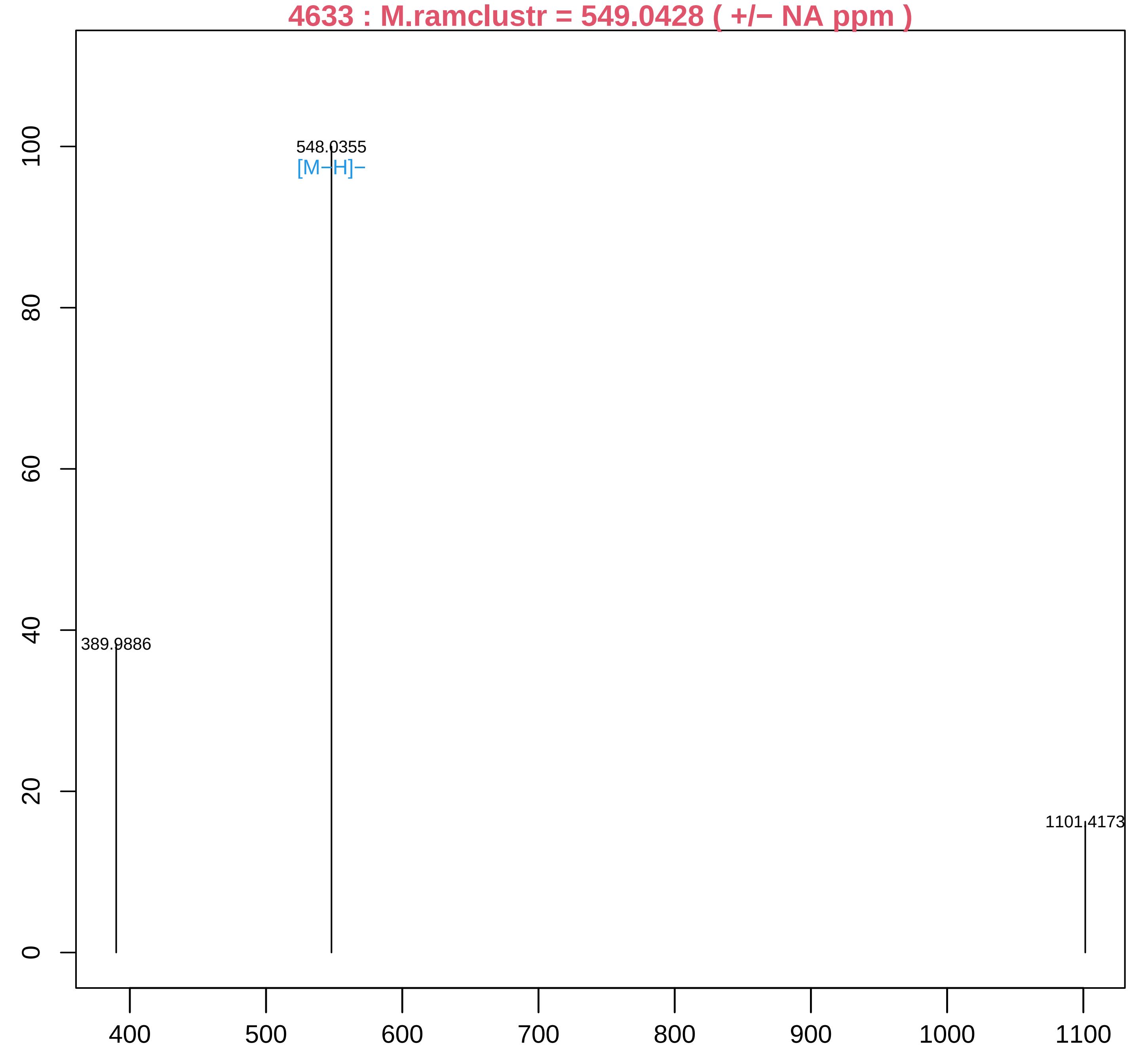
4633 : M.ramclustr = 549.0428 (+/− NA ppm)
548.0355
[M−H]−
389.9886
1101.4173
0
20
40
60
80
100
400
500
600
700
800
900
1000
1100

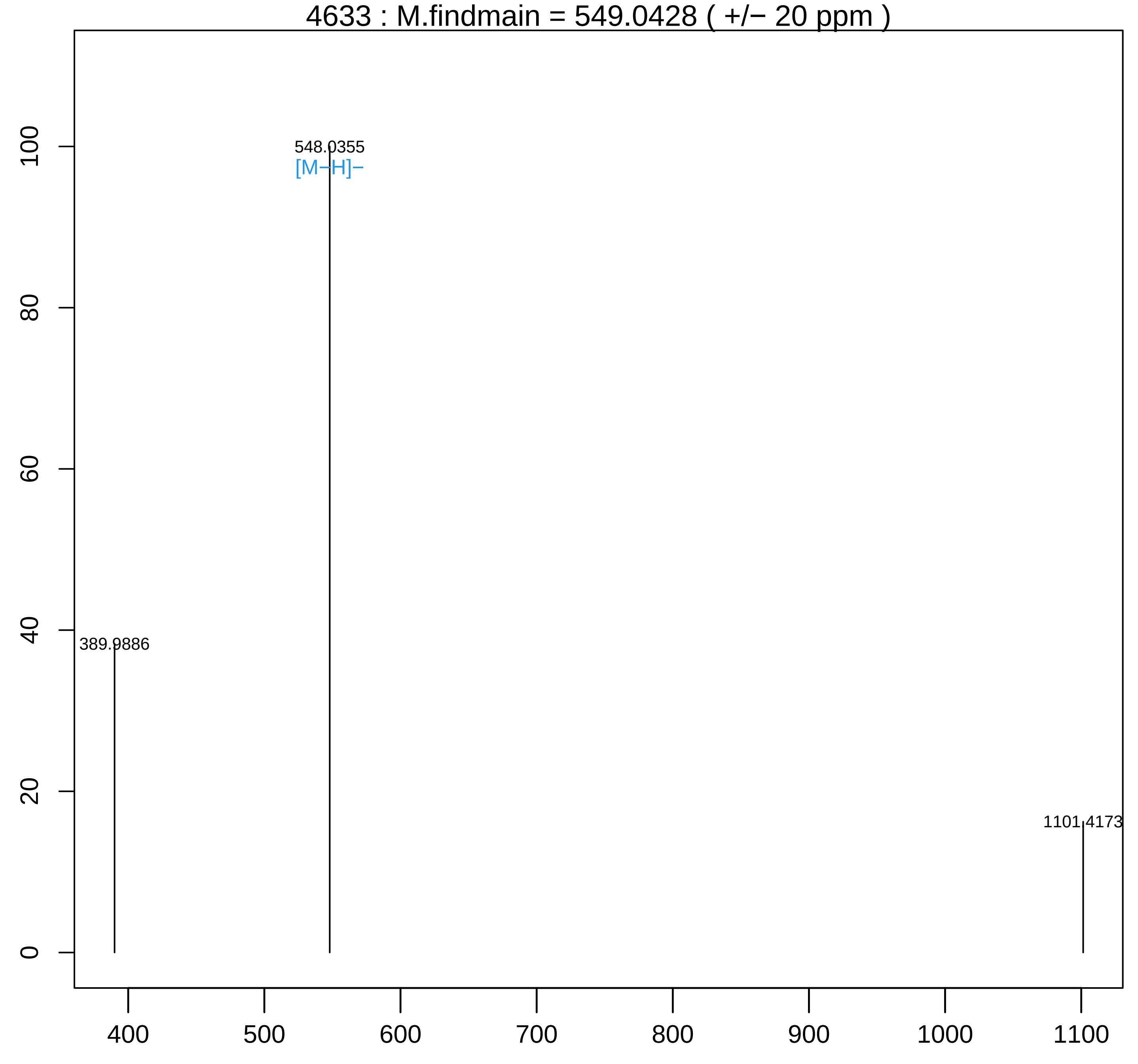
4633 : M.findmain = 549.0428 (+/− 20 ppm)
548.0355
[M−H]−
389.9886
1101.4173
0
20
40
60
80
100
400
500
600
700
800
900
1000
1100

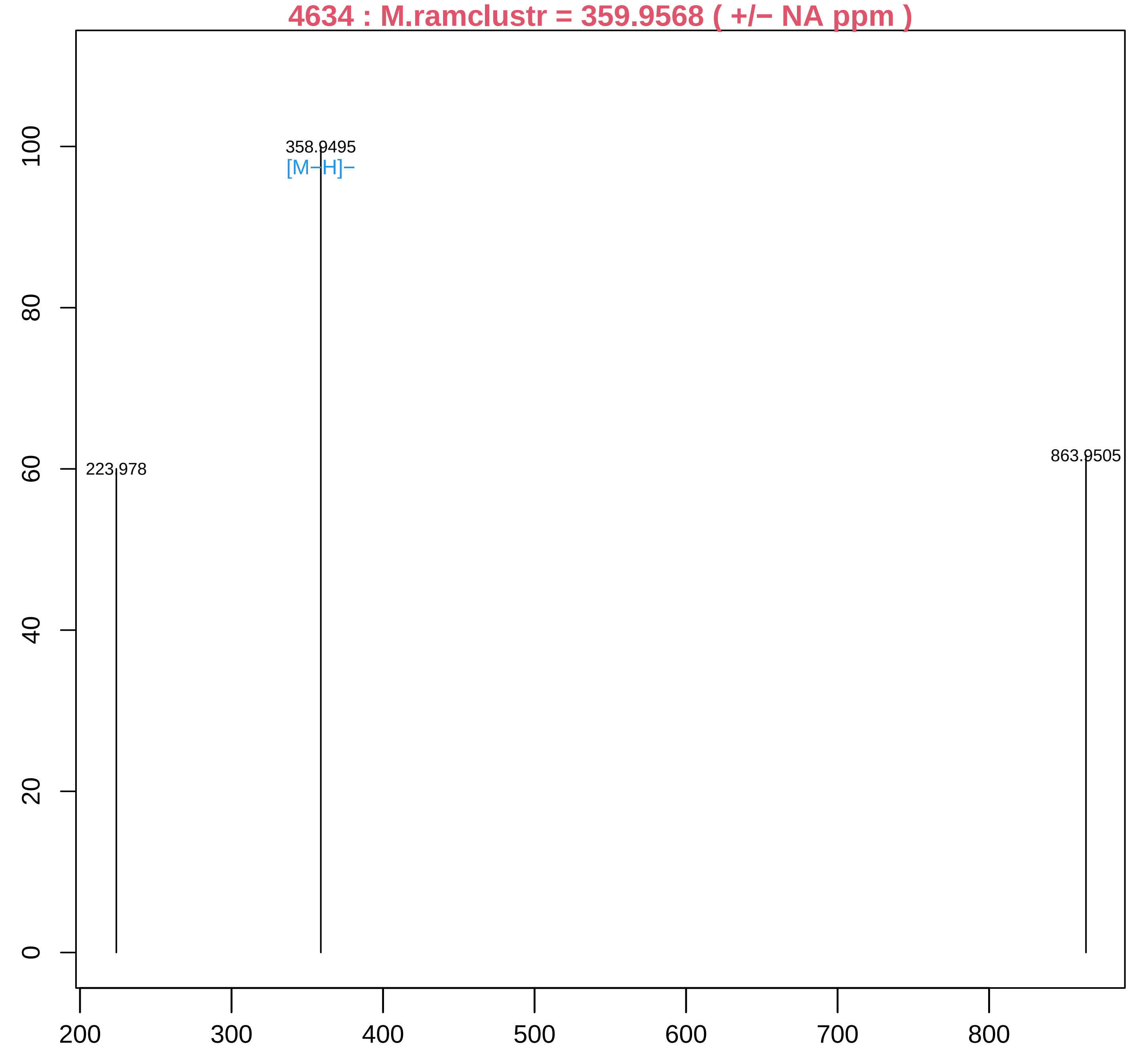

4634 : M.ramclustr = 359.9568 (+/− NA ppm)
358.9495
[M−H]−
223.978
863.9505
0
20
40
60
80
100
200
300
400
500
600
700
800

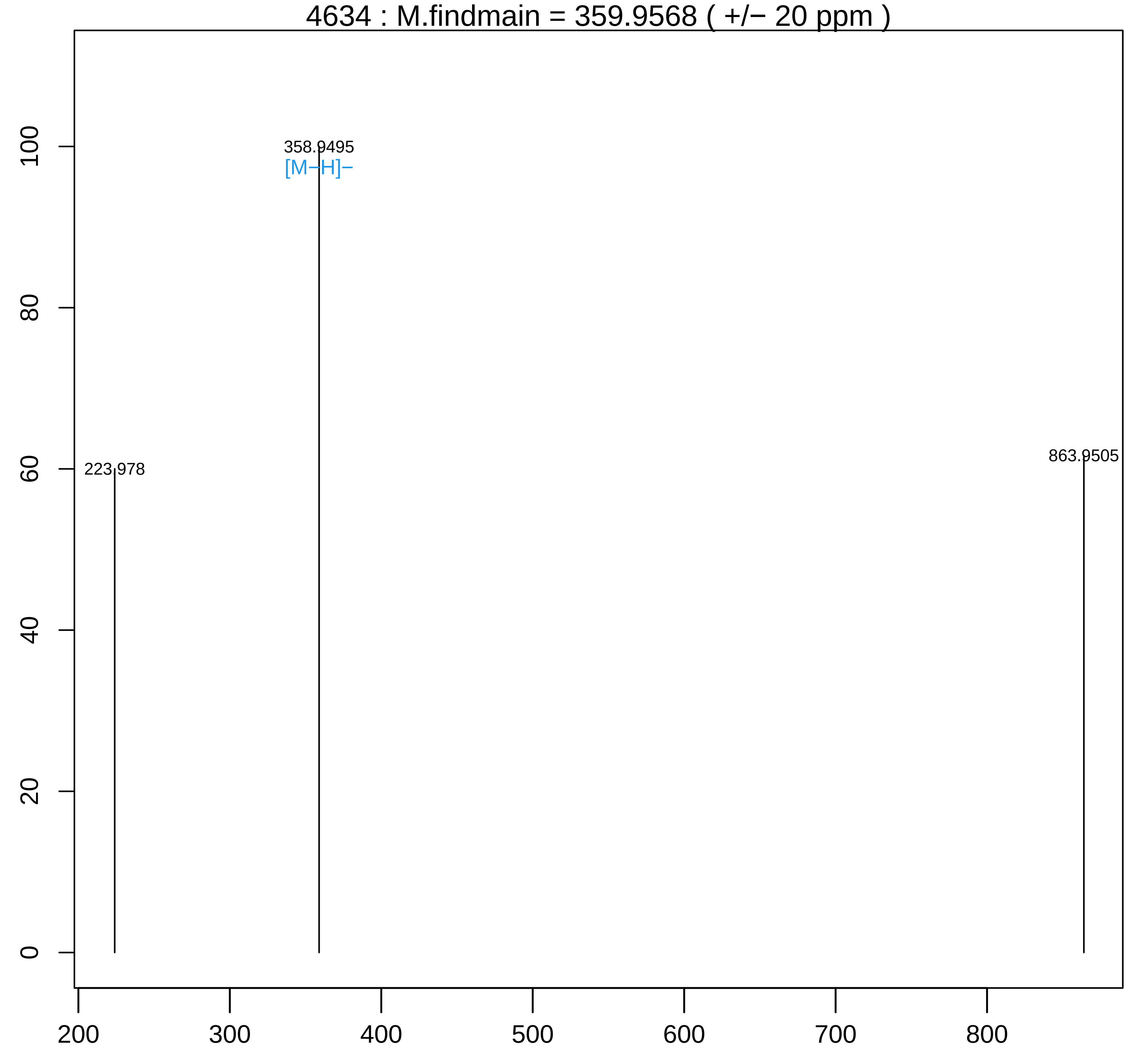

4634 : M.findmain = 359.9568 (+/− 20 ppm)
358.9495
[M−H]−
223.978
863.9505
0
20
40
60
80
100
200
300
400
500
600
700
800

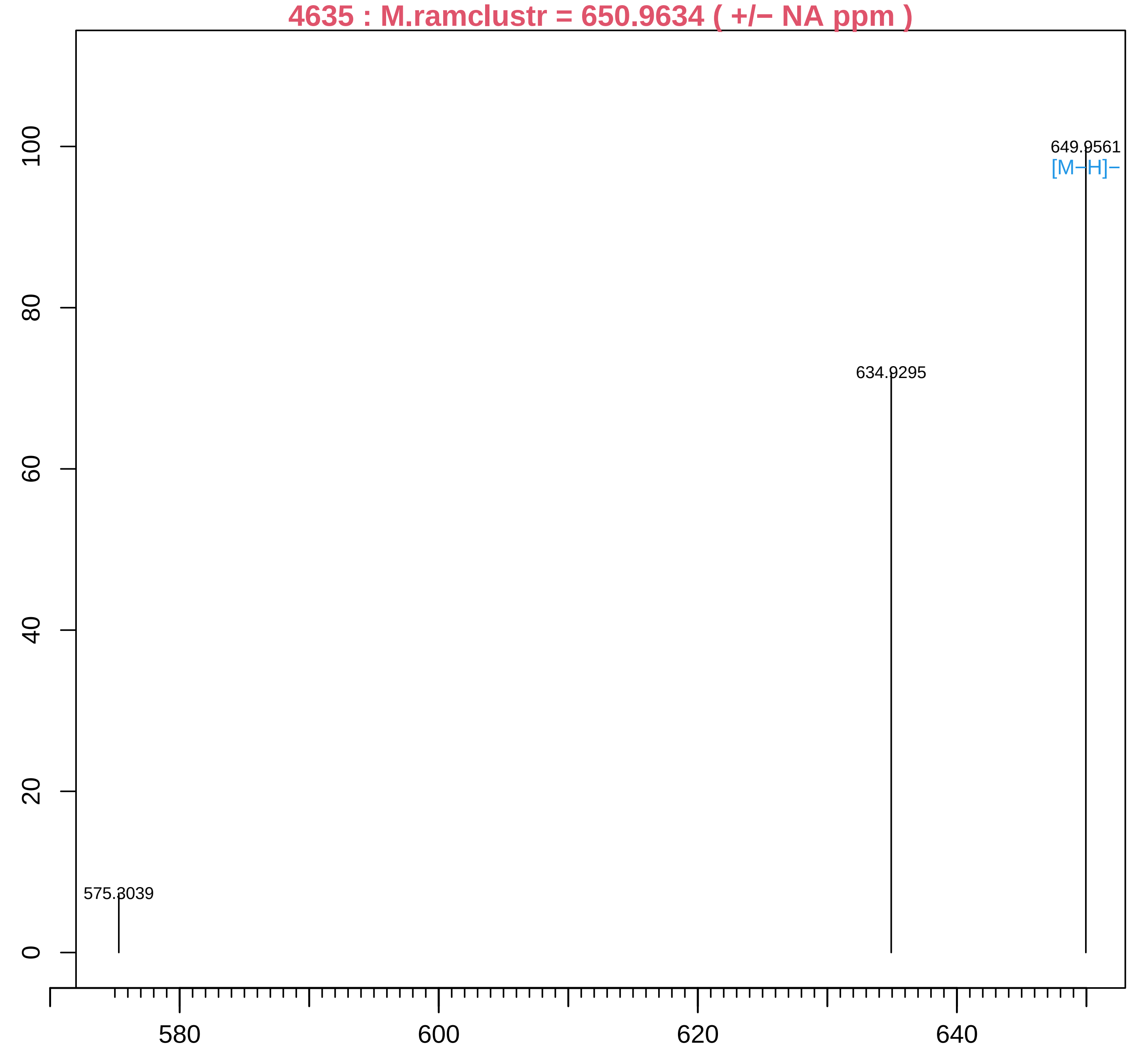
4635 : M.ramclustr = 650.9634 (+/− NA ppm)
649.9561
[M−H]−
634.9295
575.3039
0
20
40
60
80
100
580
600
620
640

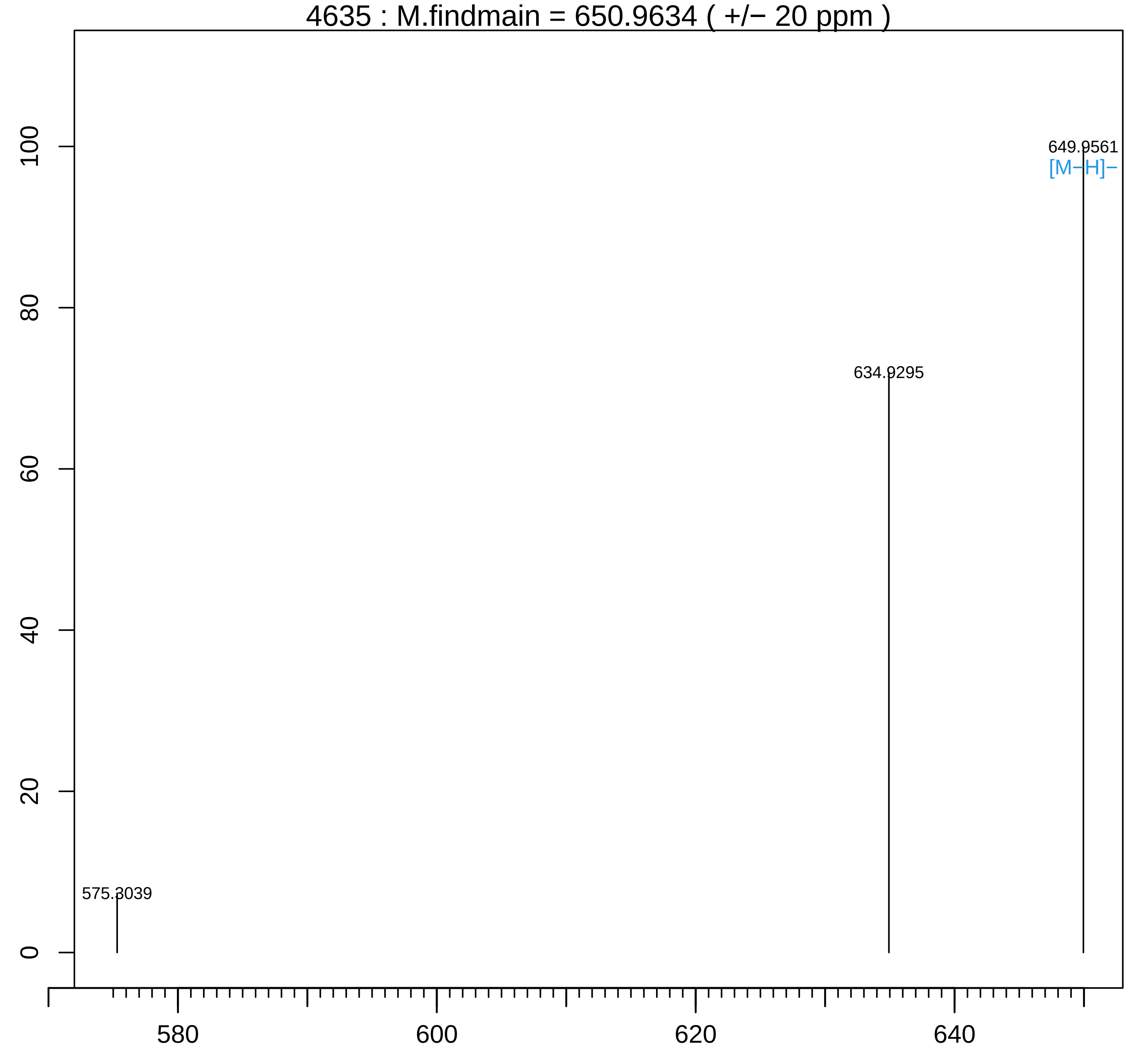
4635 : M.findmain = 650.9634 (+/− 20 ppm)
649.9561
[M−H]−
634.9295
575.3039
0
20
40
60
80
100
580
600
620
640

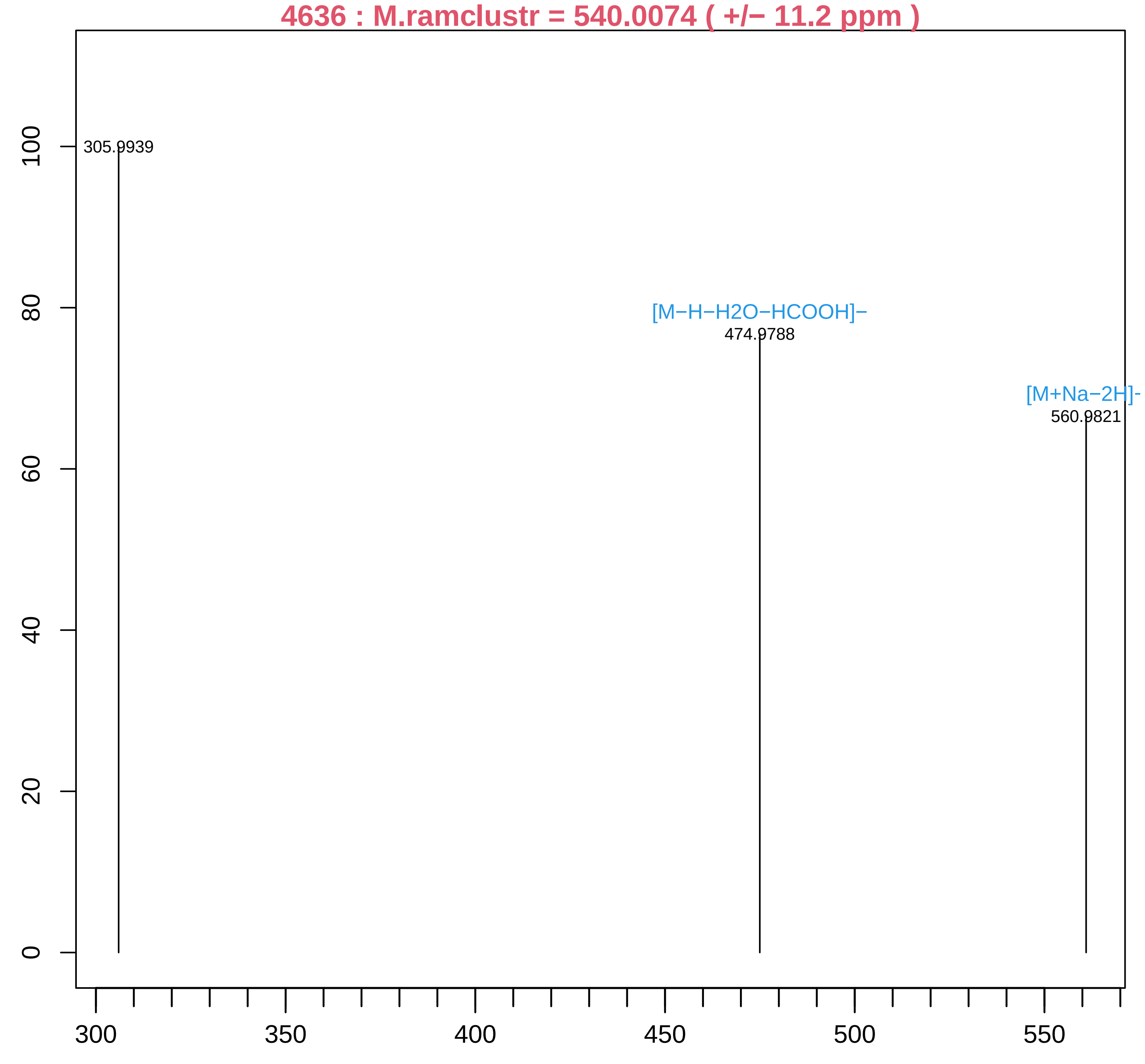
4636 : M.ramclustr = 540.0074 (+/− 11.2 ppm)
305.9939
[M−H−H2O−HCOOH]−
474.9788
[M+Na−2H]-
560.9821
0
20
40
60
80
100
300
350
400
450
500
550

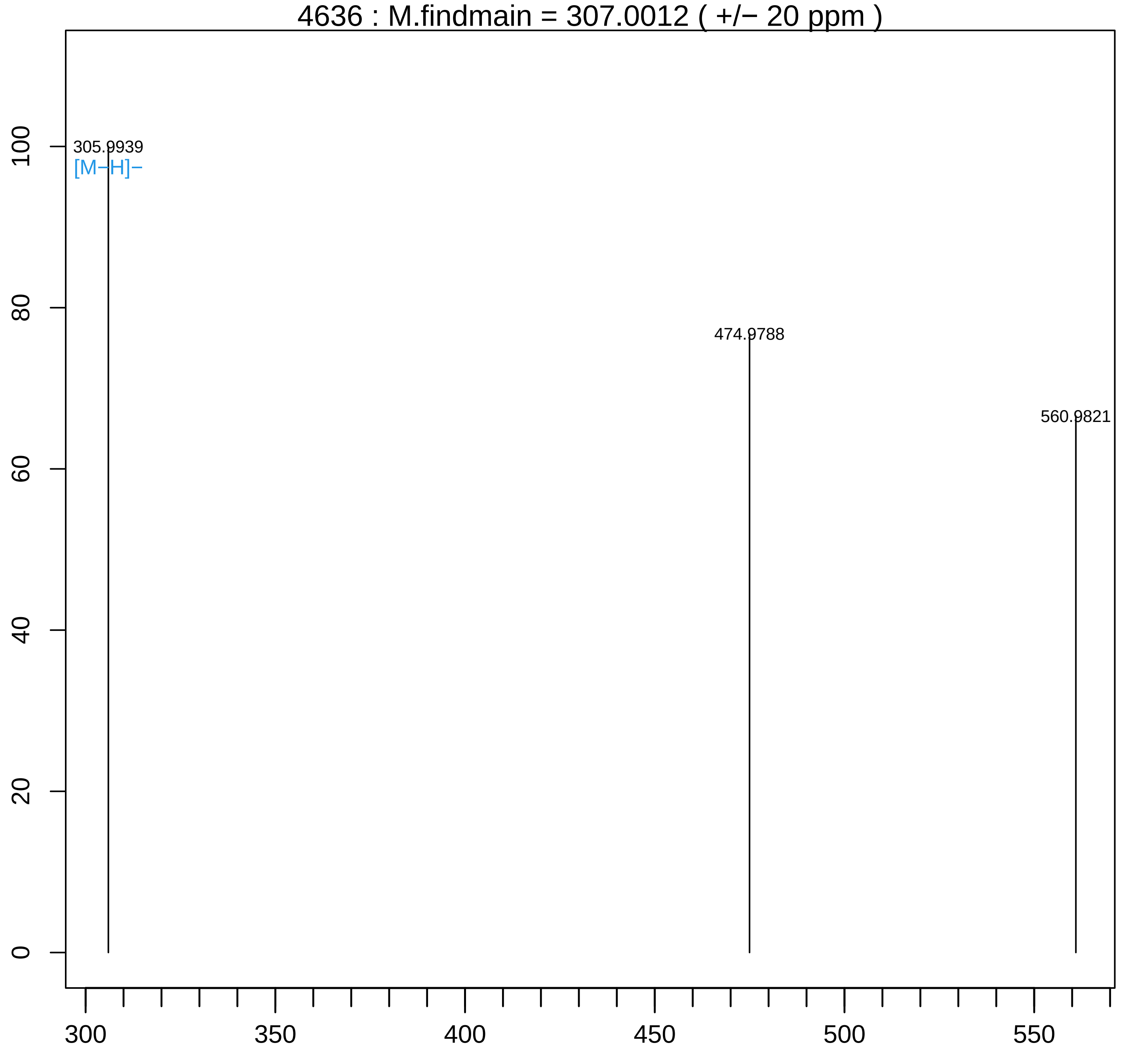
4636 : M.findmain = 307.0012 (+/− 20 ppm)
305.9939
[M−H]−
474.9788
560.9821
0
20
40
60
80
100
300
350
400
450
500
550

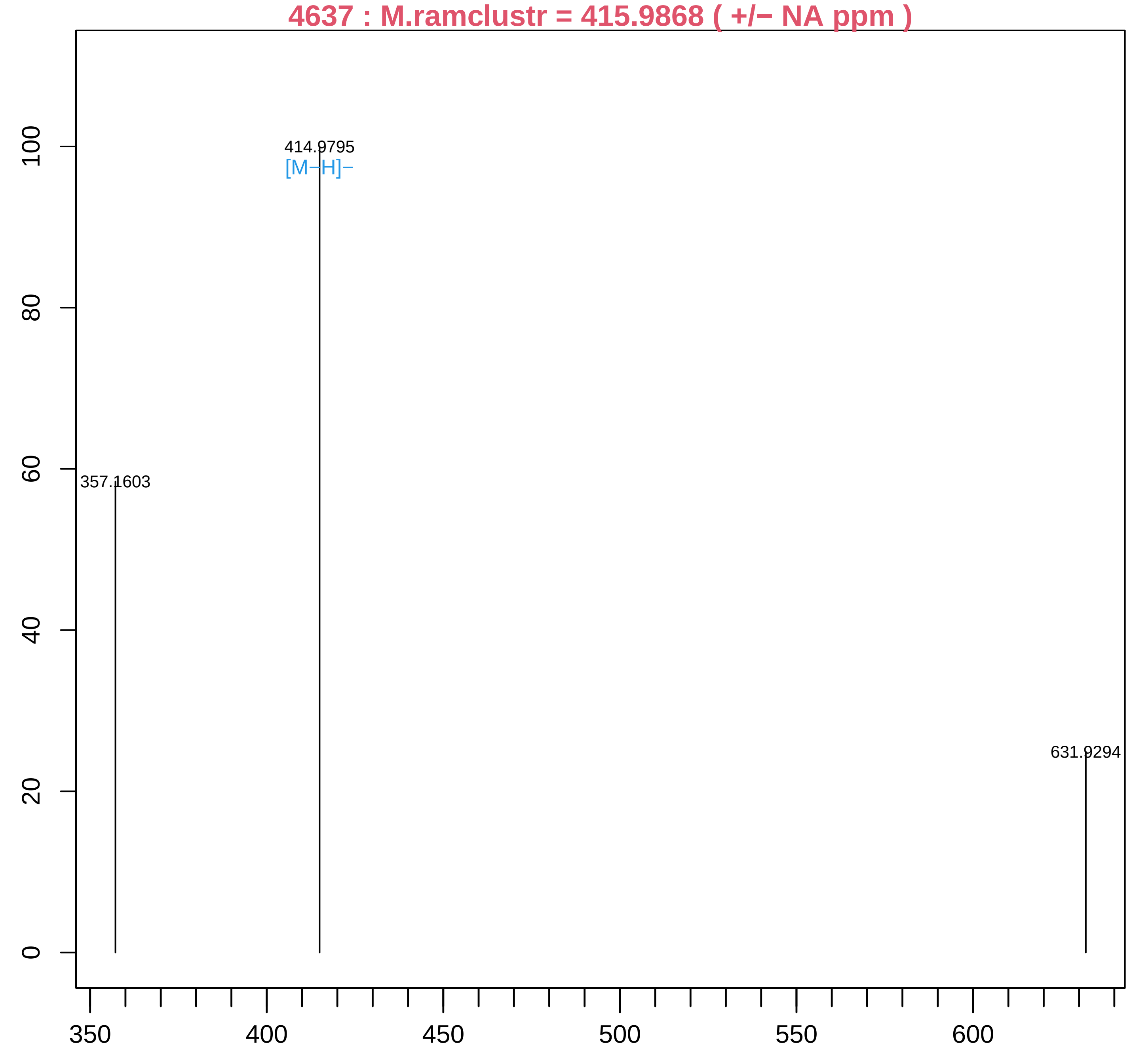
4637 : M.ramclustr = 415.9868 (+/− NA ppm)
414.9795
[M−H]−
357.1603
631.9294
0
20
40
60
80
100
350
400
450
500
550
600
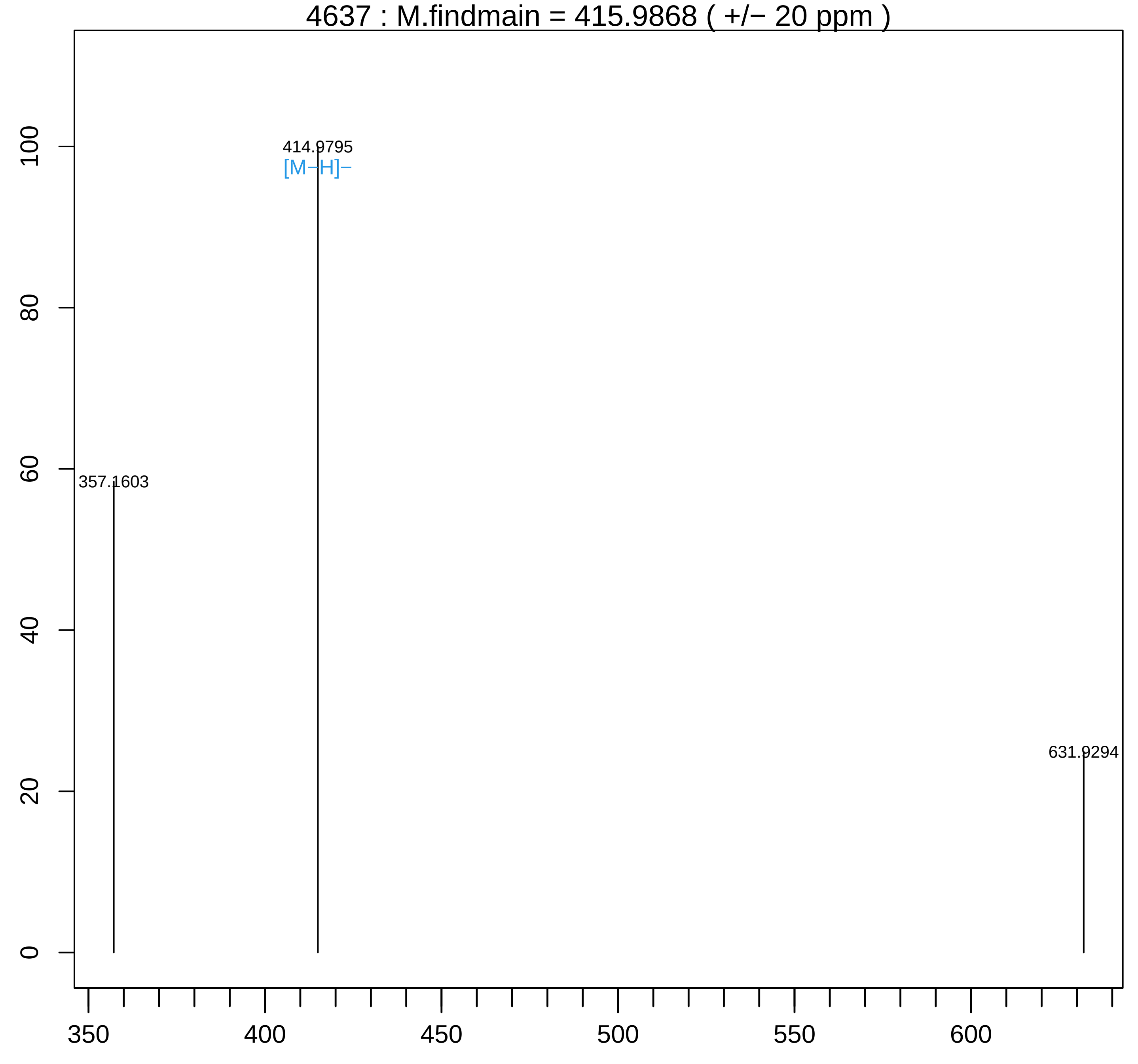
4637 : M.findmain = 415.9868 (+/− 20 ppm)
414.9795
[M−H]−
357.1603
631.9294
0
20
40
60
80
100
350
400
450
500
550
600

4638 : M.ramclustr = 85.9454 (+/− NA ppm)

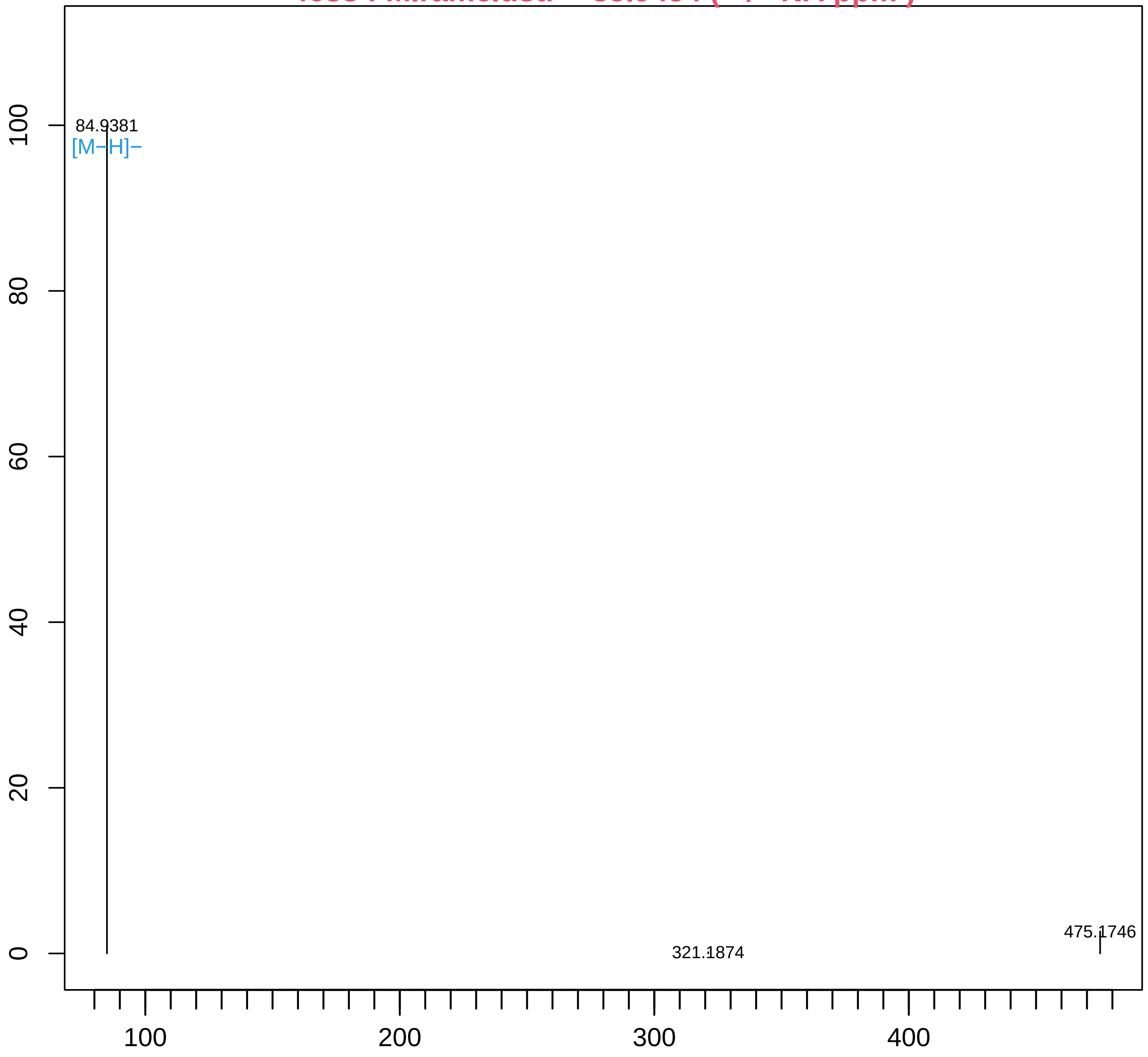

4638 : M.findmain = 85.9454 (+/− 20 ppm)

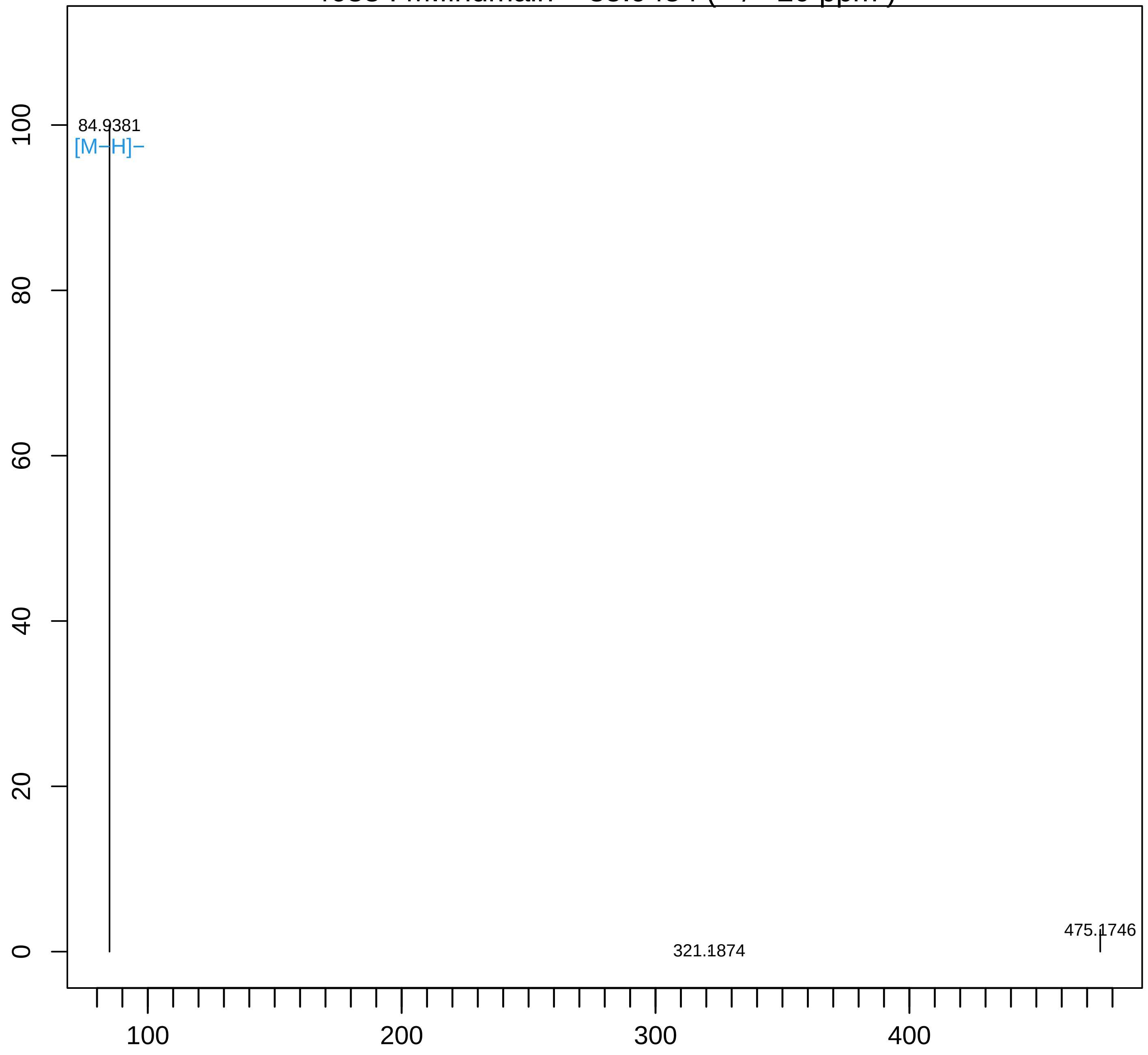

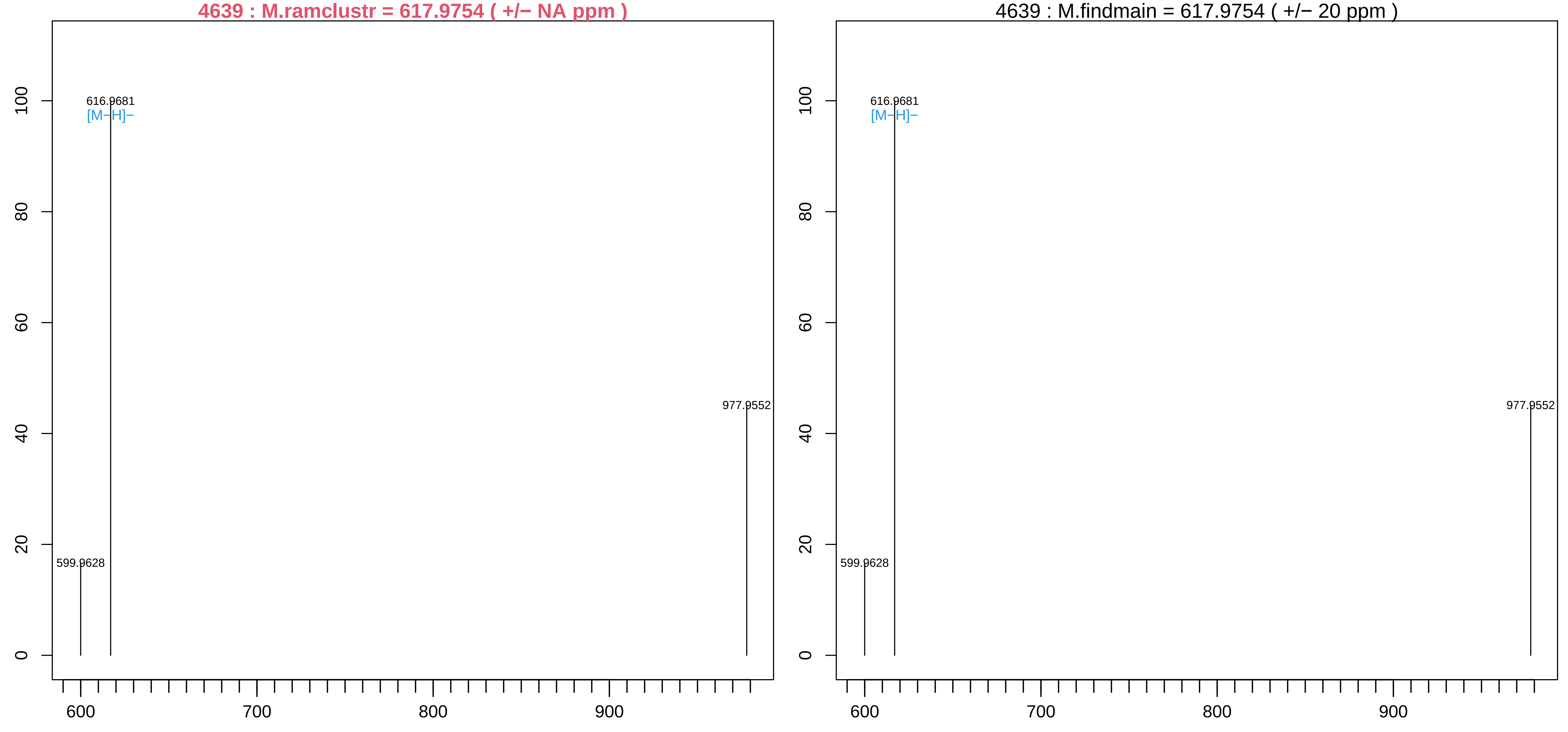

4639 : M.ramclustr = 617.9754 (+/− NA ppm)
616.9681
[M−H]−
977.9552
599.9628
4639 : M.findmain = 617.9754 (+/− 20 ppm)
616.9681
[M−H]−
977.9552
599.9628
0
20
40
60
80
100
600
700
800
900

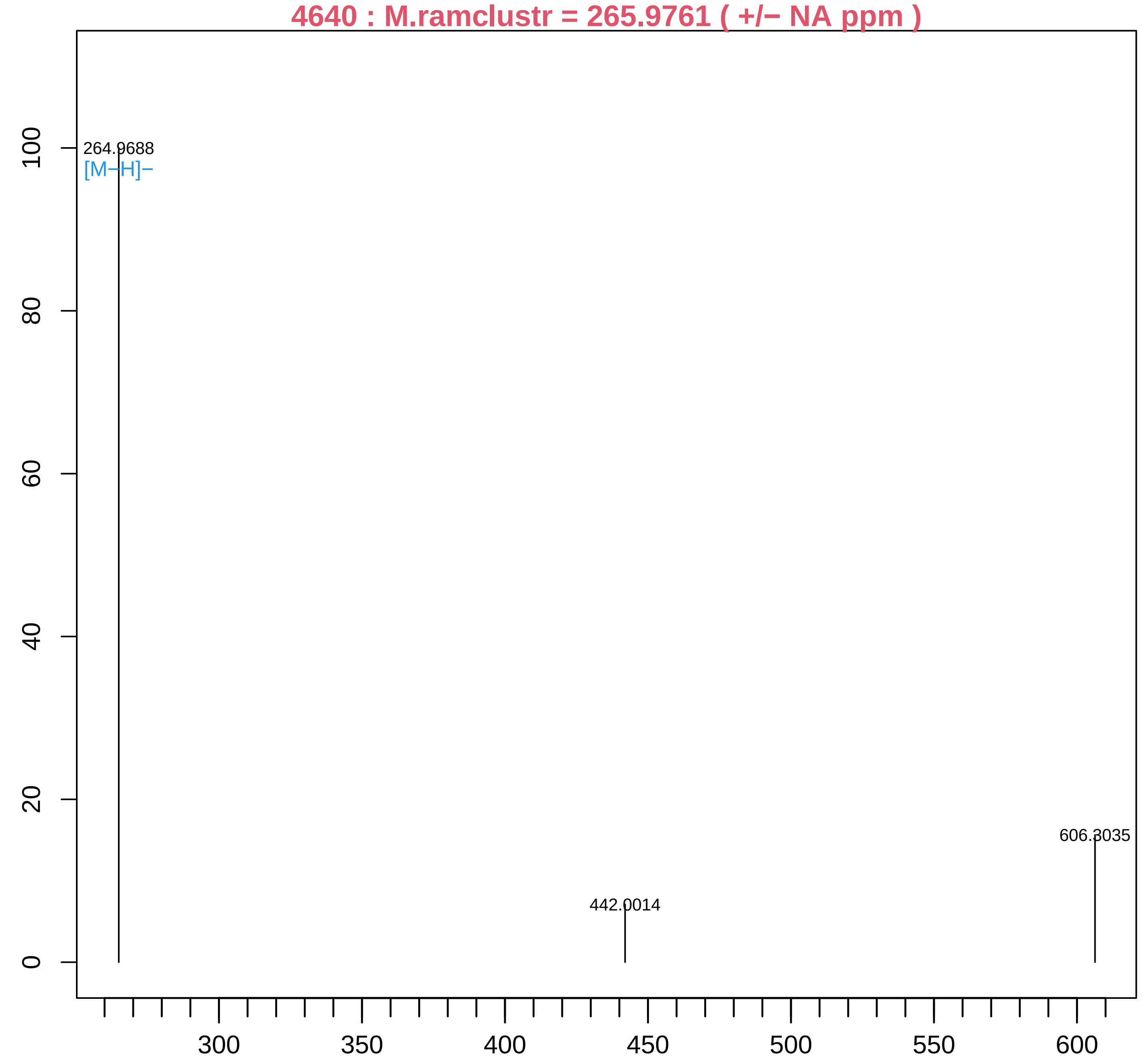
4640 : M.ramclustr = 265.9761 (+/− NA ppm)
264.9688
[M−H]−
442.0014
606.3035
0
20
40
60
80
100
300
350
400
450
500
550
600

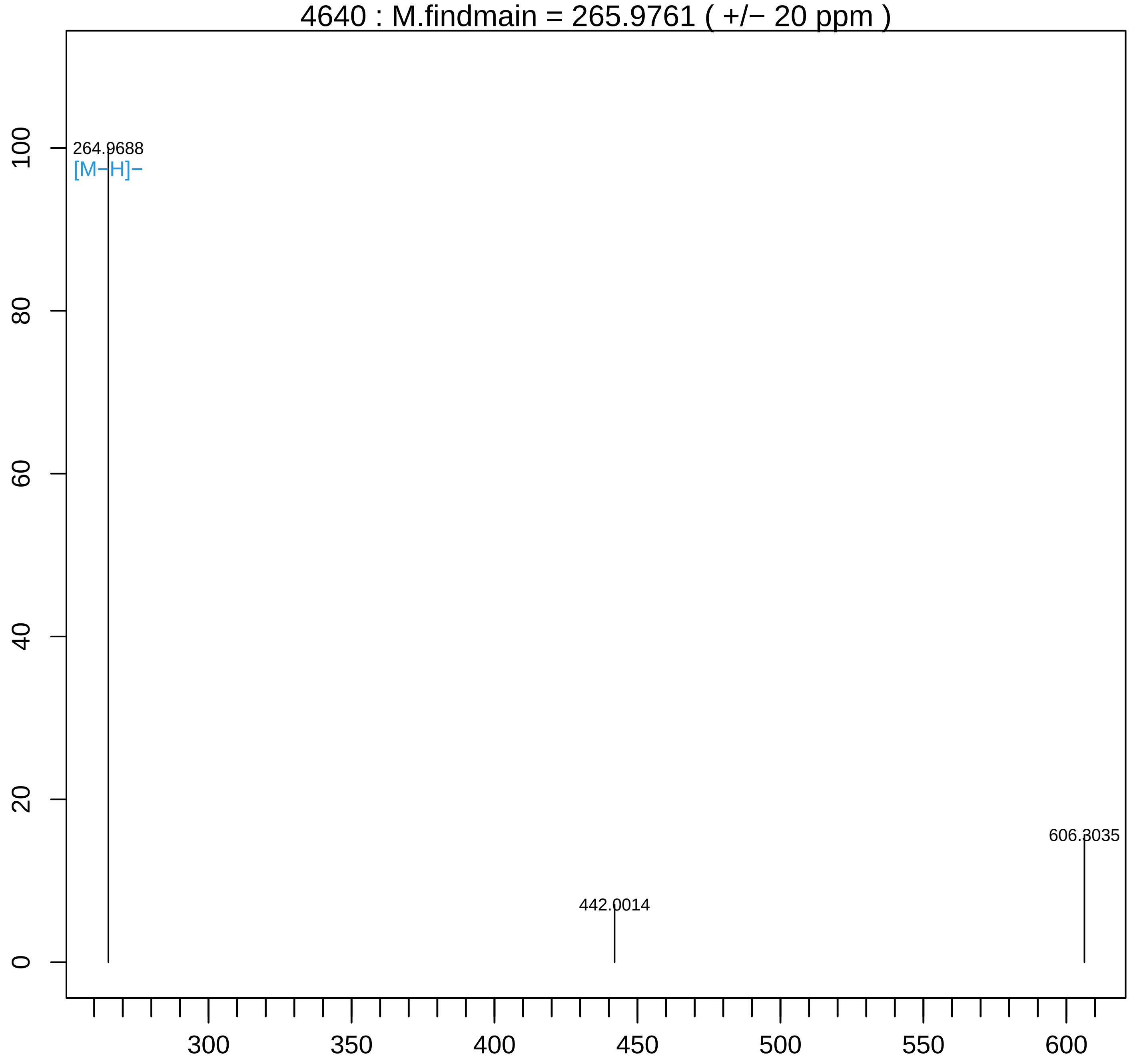
4640 : M.findmain = 265.9761 (+/− 20 ppm)
264.9688
[M−H]−
442.0014
606.3035
0
20
40
60
80
100
300
350
400
450
500
550
600

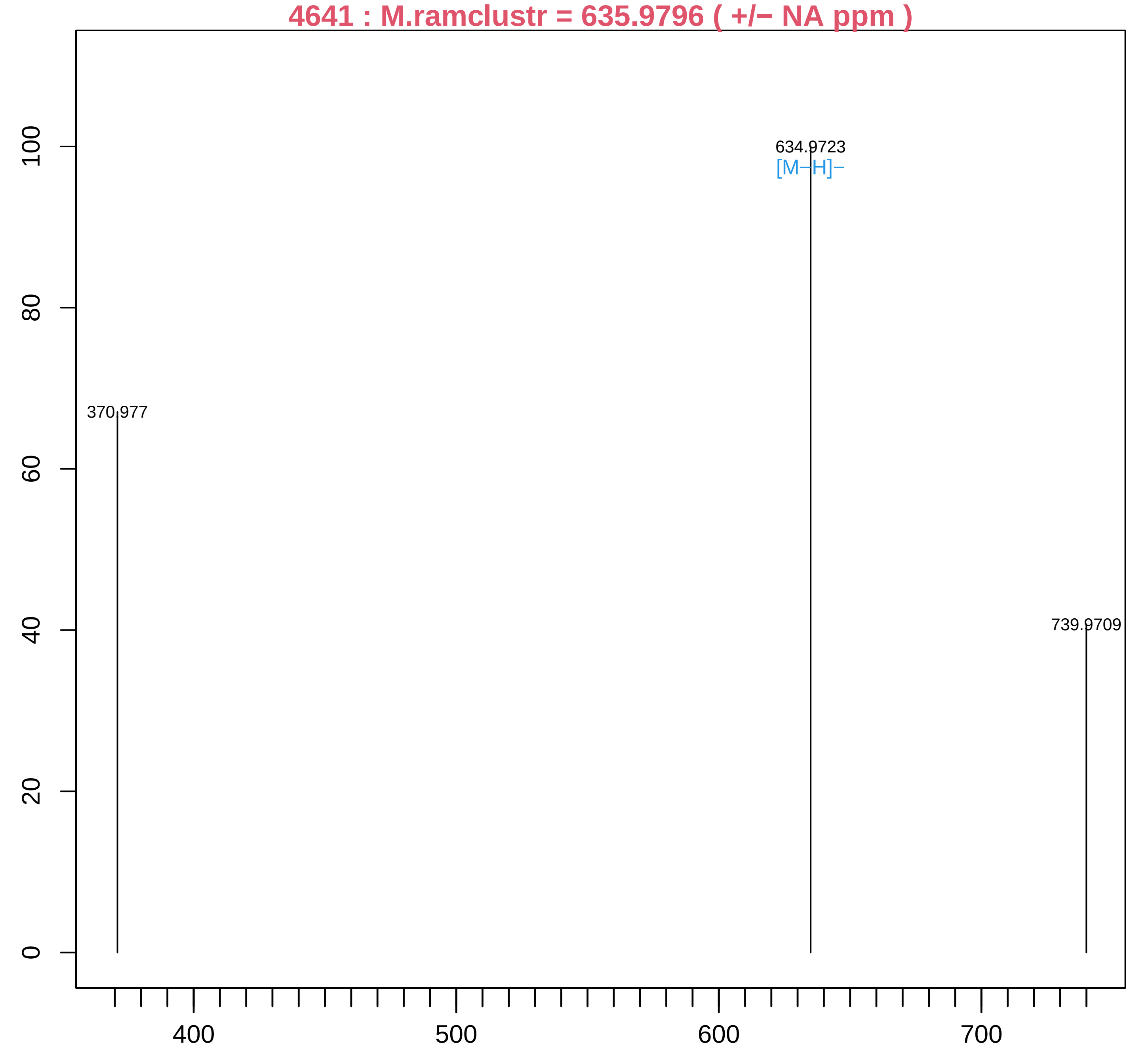
4641 : M.ramclustr = 635.9796 (+/− NA ppm)
634.9723
[M−H]−
370.977
739.9709
0
20
40
60
80
100
400
500
600
700

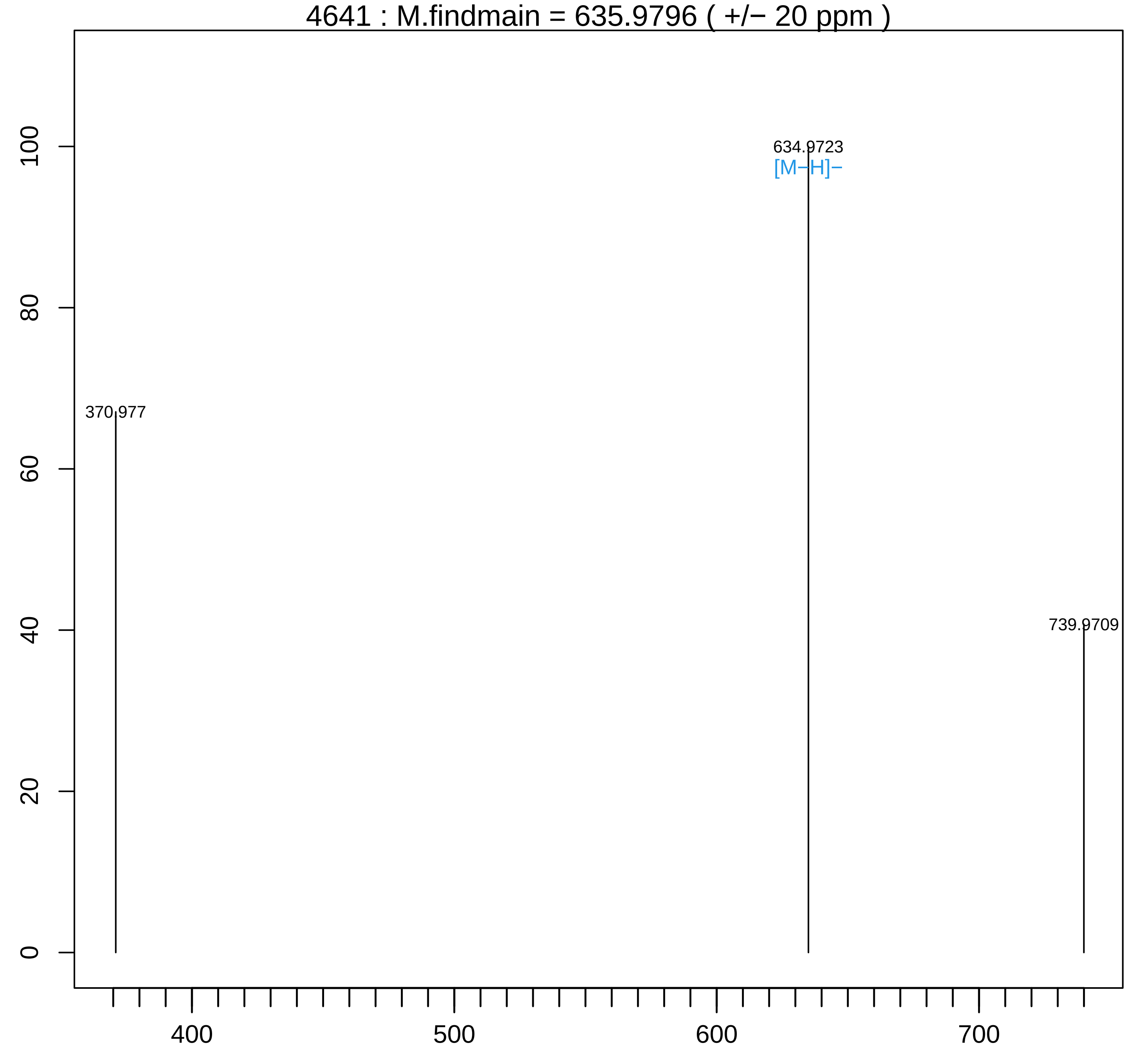
4641 : M.findmain = 635.9796 (+/− 20 ppm)
634.9723
[M−H]−
370.977
739.9709
0
20
40
60
80
100
400
500
600
700

4642 : M.ramclustr = 1067.9773 (+/− NA ppm)

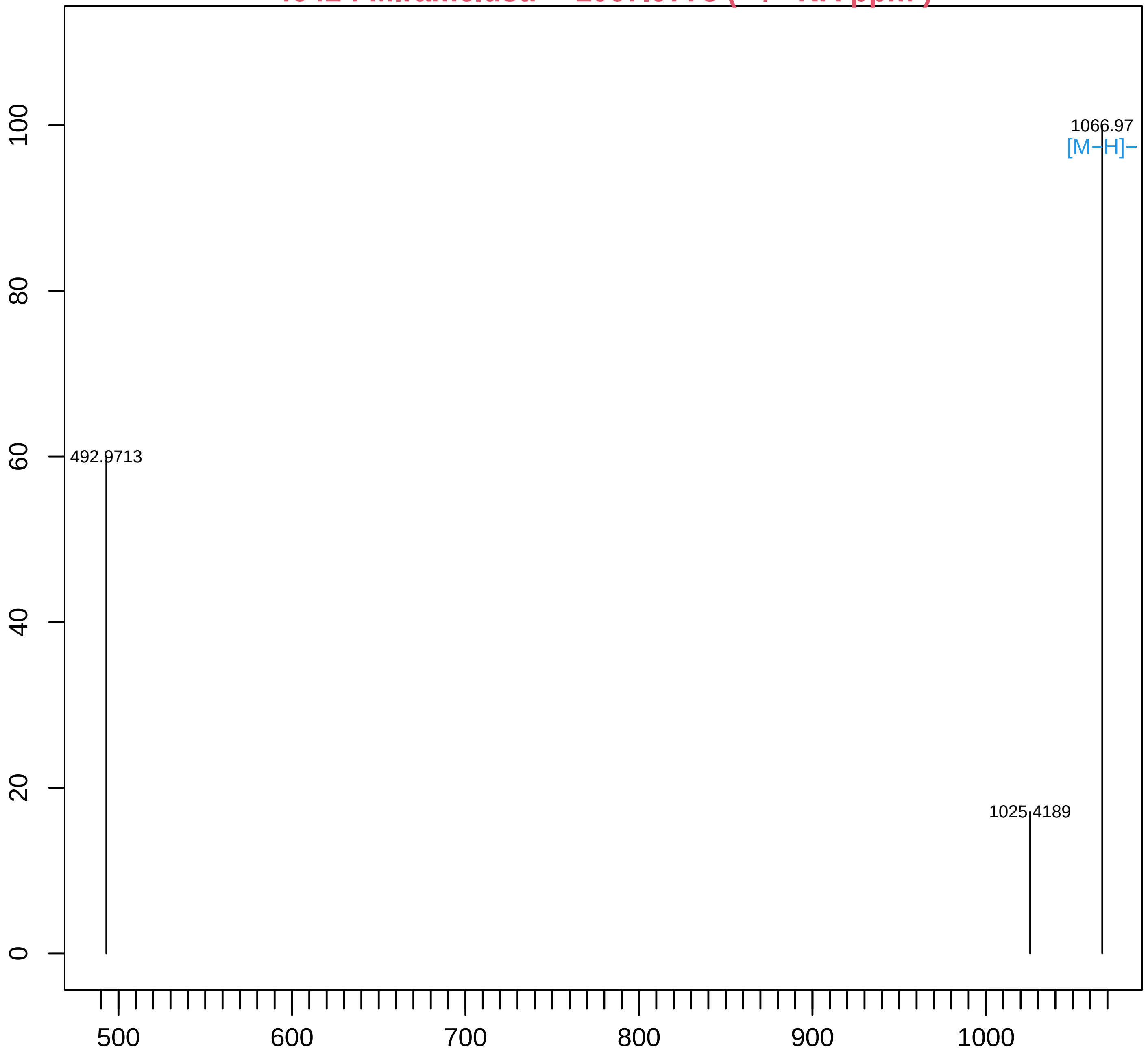

4642 : M.findmain = 1067.9773 (+/− 20 ppm)

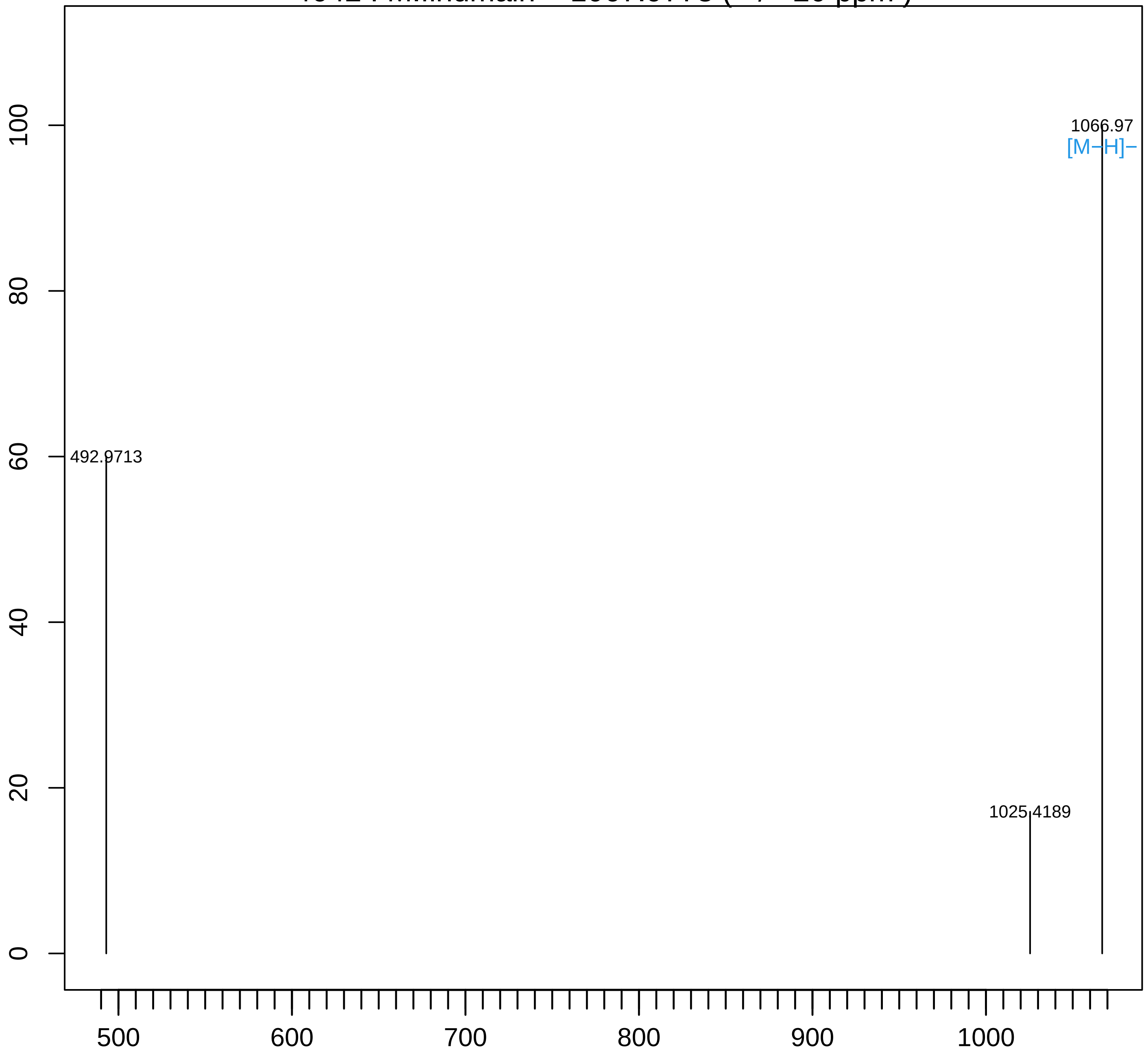

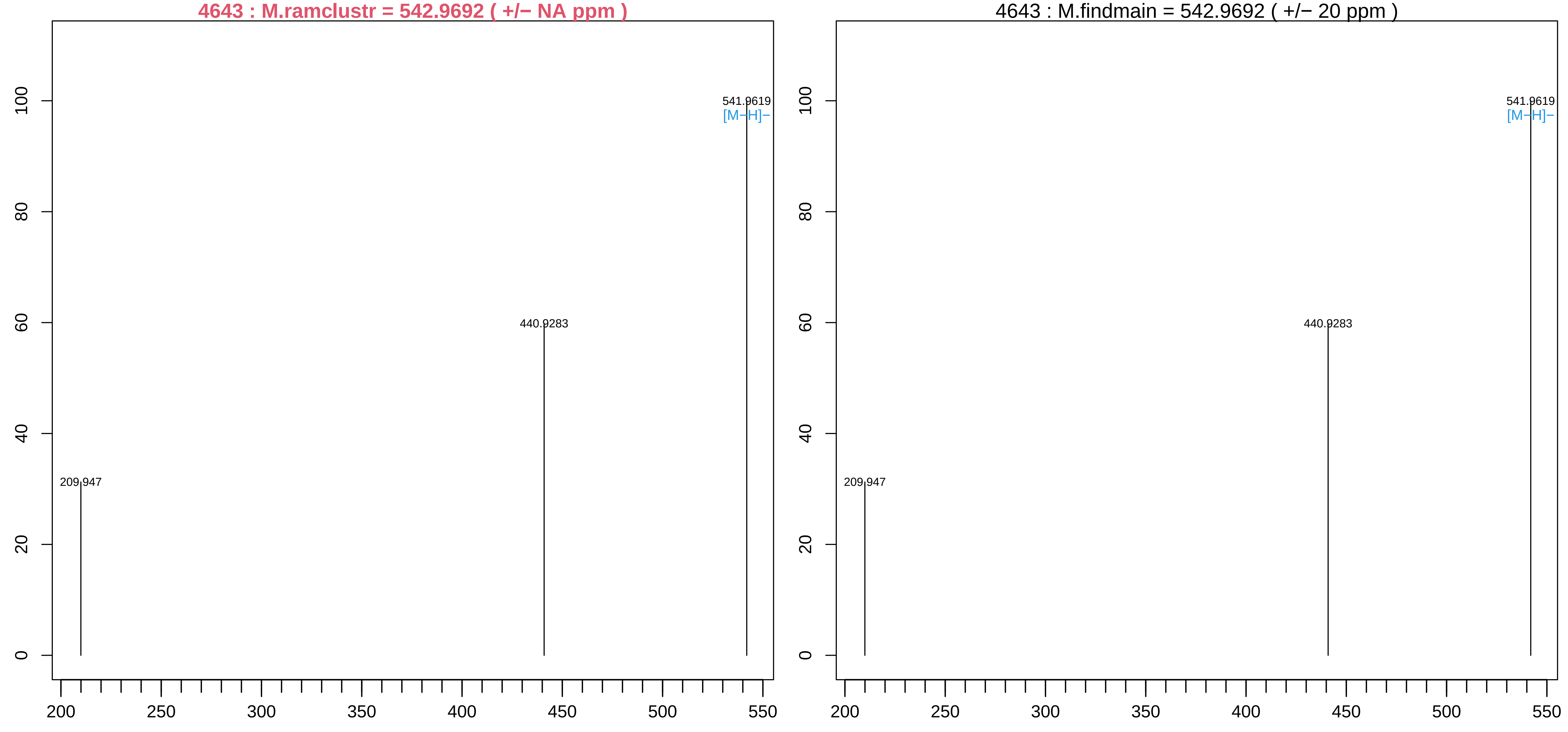
4643 : M.ramclustr = 542.9692 (+/− NA ppm)
4643 : M.findmain = 542.9692 (+/− 20 ppm)
541.9619
[M−H]−
440.9283
209.947
541.9619
[M−H]−
440.9283
209.947
0
20
40
60
80
100
200
250
300
350
400
450
500
550

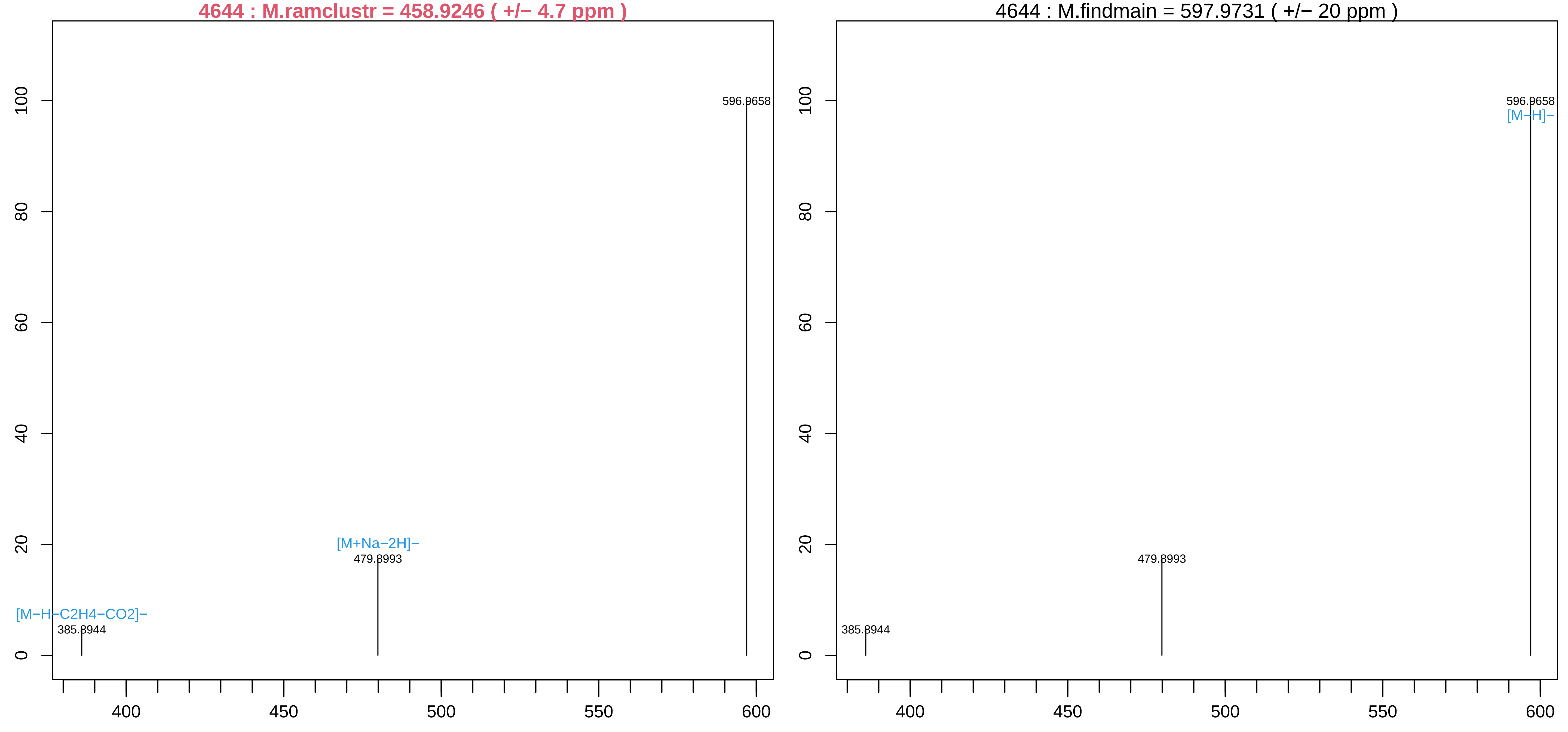

4644 : M.ramclustr = 458.9246 (+/− 4.7 ppm)
596.9658
[M+Na−2H]−
479.8993
[M−H−C2H4−CO2]−
385.8944
0
20
40
60
80
100
400
450
500
550
600
4644 : M.findmain = 597.9731 (+/− 20 ppm)
596.9658
[M−H]−
479.8993
385.8944

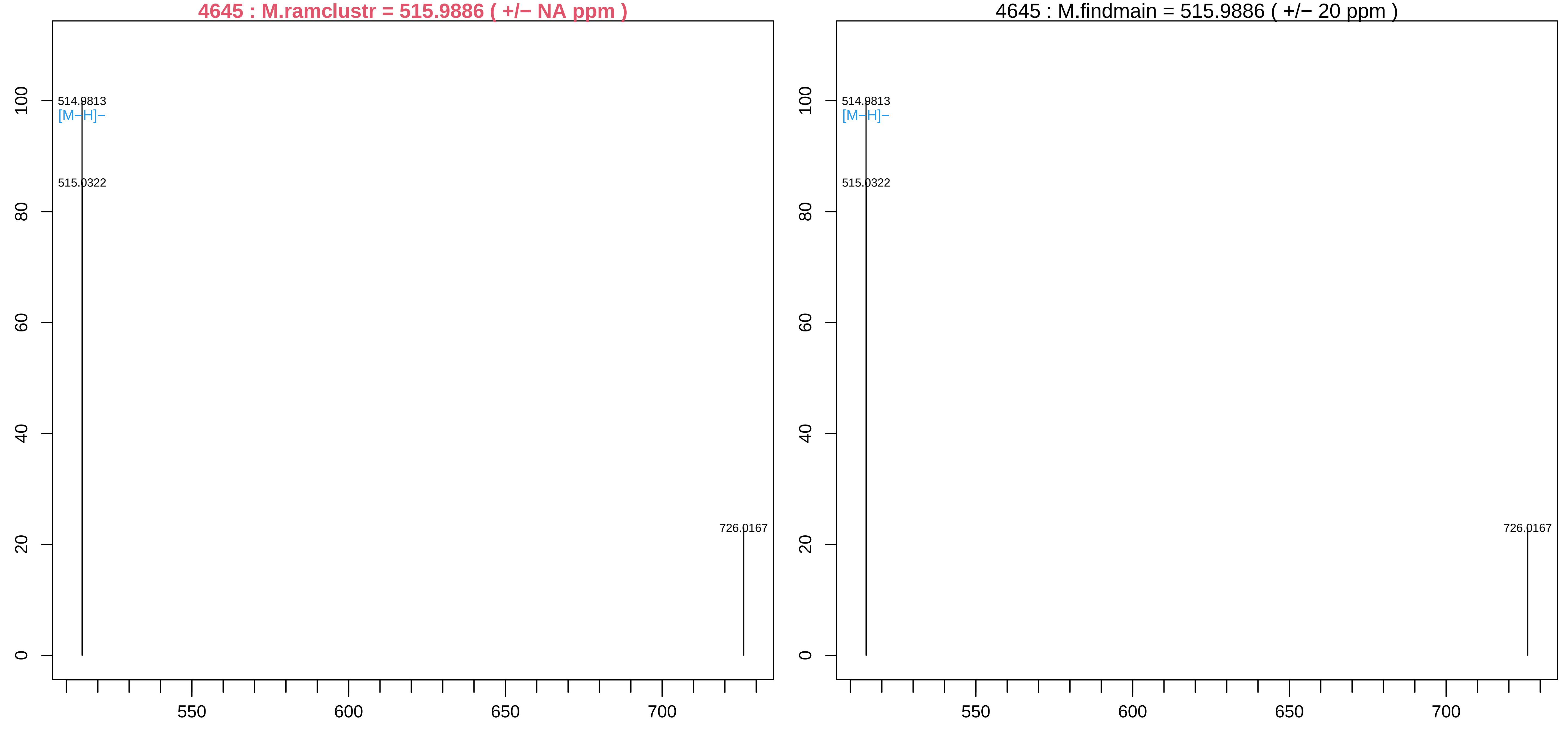

4645 : M.ramclustr = 515.9886 (+/− NA ppm)
514.9813
[M−H]−
515.0322
726.0167
0
20
40
60
80
100
550
600
650
700
4645 : M.findmain = 515.9886 (+/− 20 ppm)
514.9813
[M−H]−
515.0322
726.0167
0
20
40
60
80
100
550
600
650
700

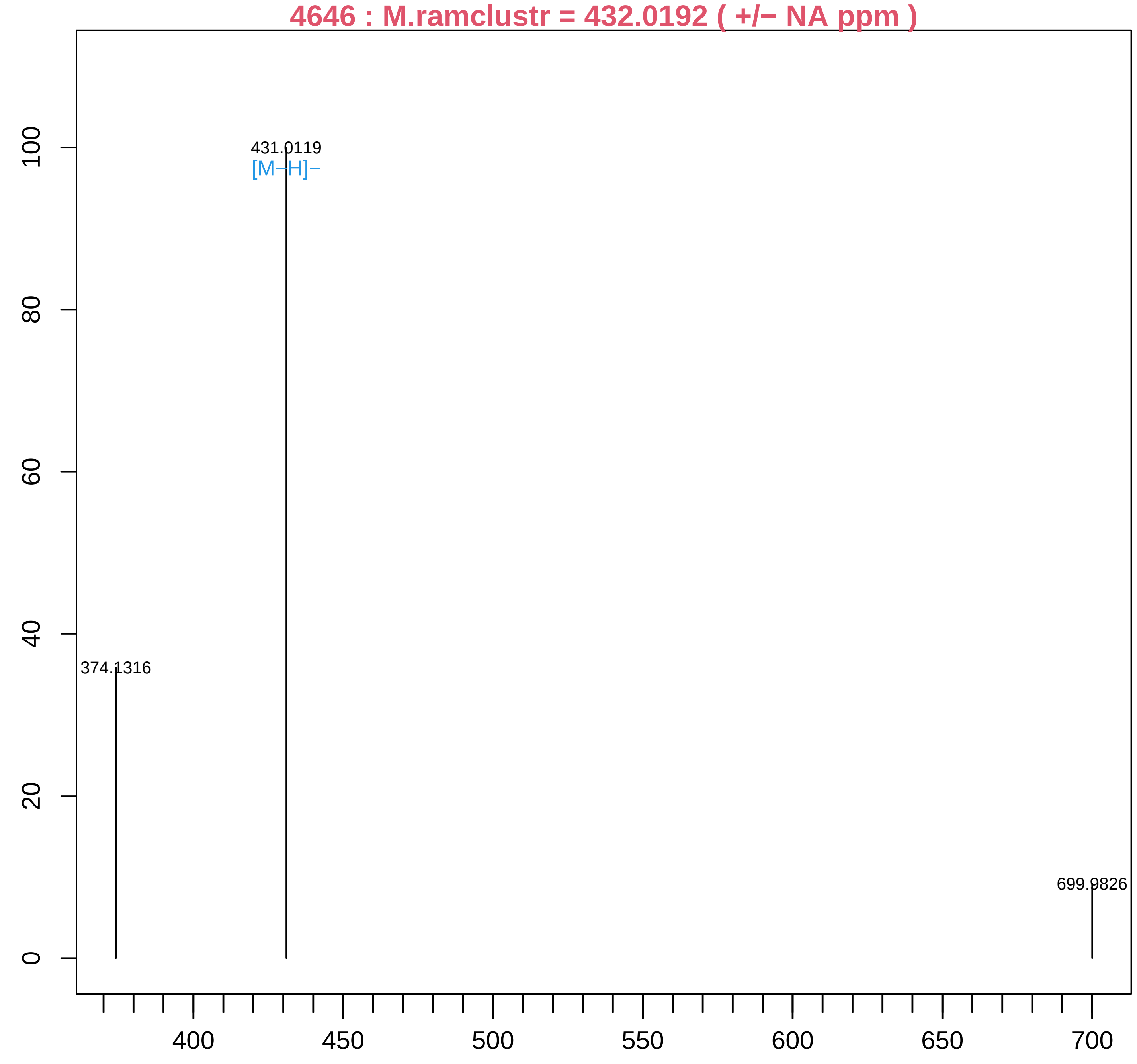

4646 : M.ramclustr = 432.0192 (+/− NA ppm)
431.0119
[M−H]−
374.1316
699.9826
0
20
40
60
80
100
400
450
500
550
600
650
700

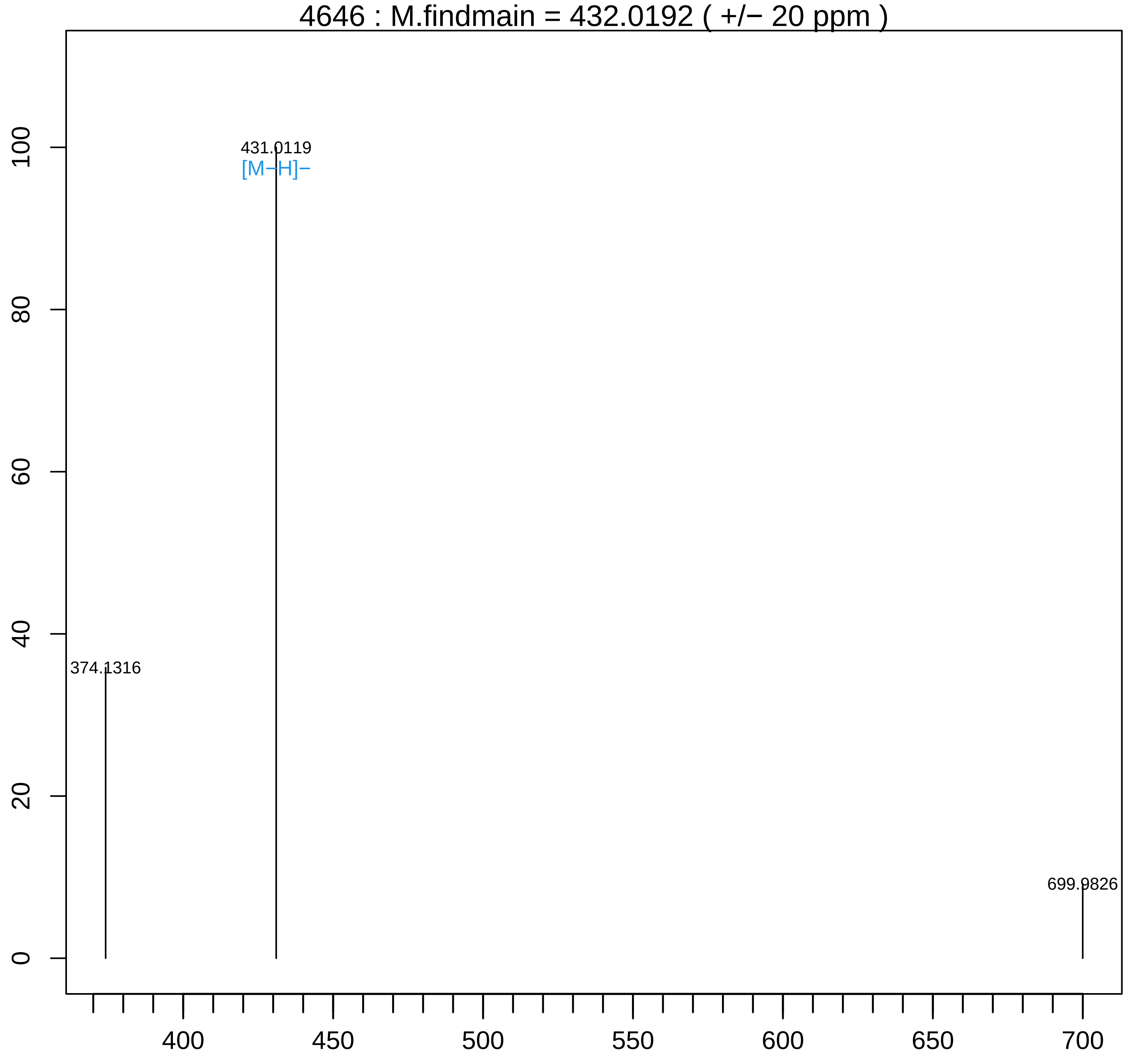

4646 : M.findmain = 432.0192 (+/− 20 ppm)
431.0119
[M−H]−
374.1316
699.9826
0
20
40
60
80
100
400
450
500
550
600
650
700

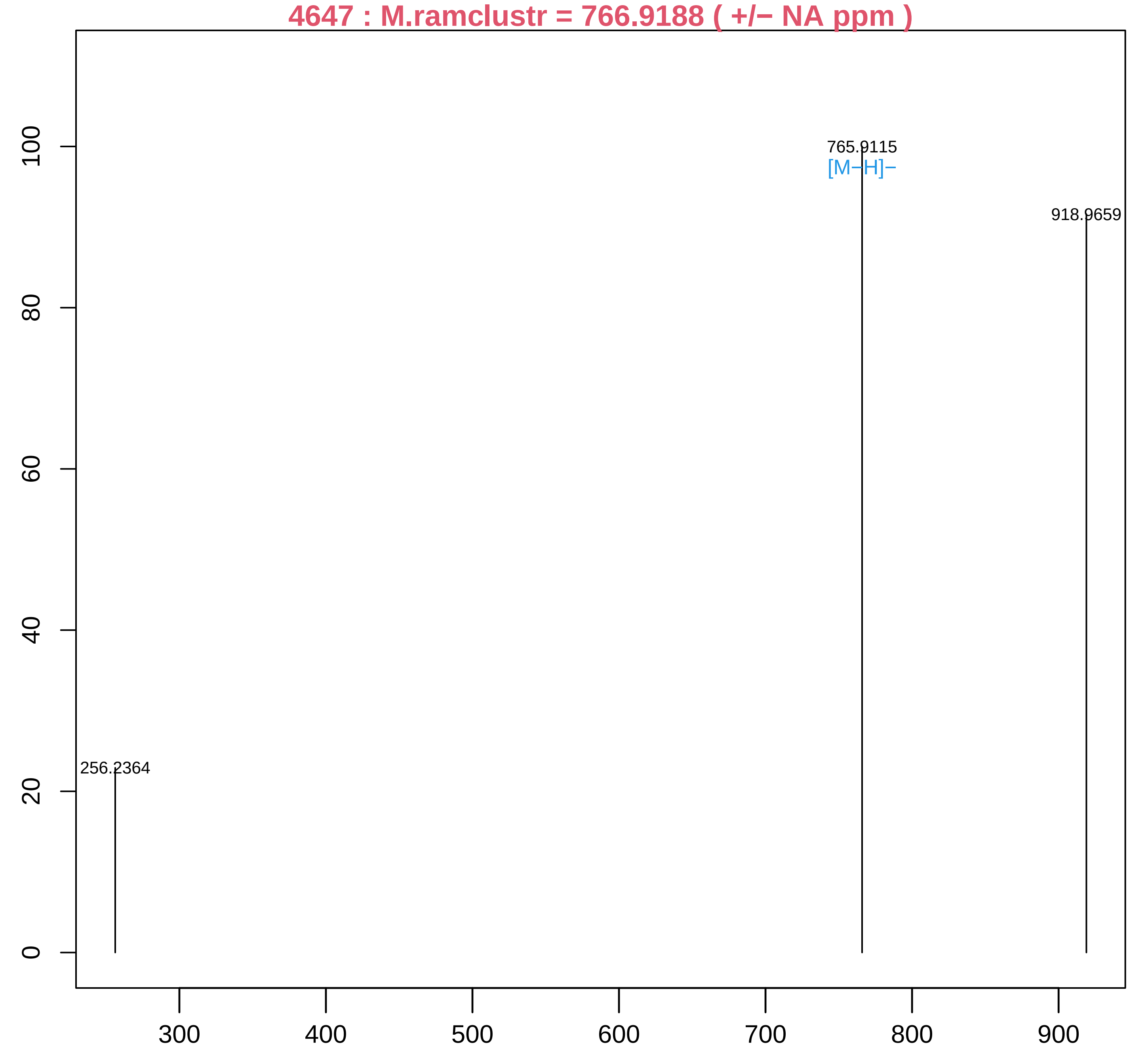
4647 : M.ramclustr = 766.9188 (+/− NA ppm)
765.9115
[M−H]−
918.9659
256.2364
0
20
40
60
80
100
300
400
500
600
700
800
900

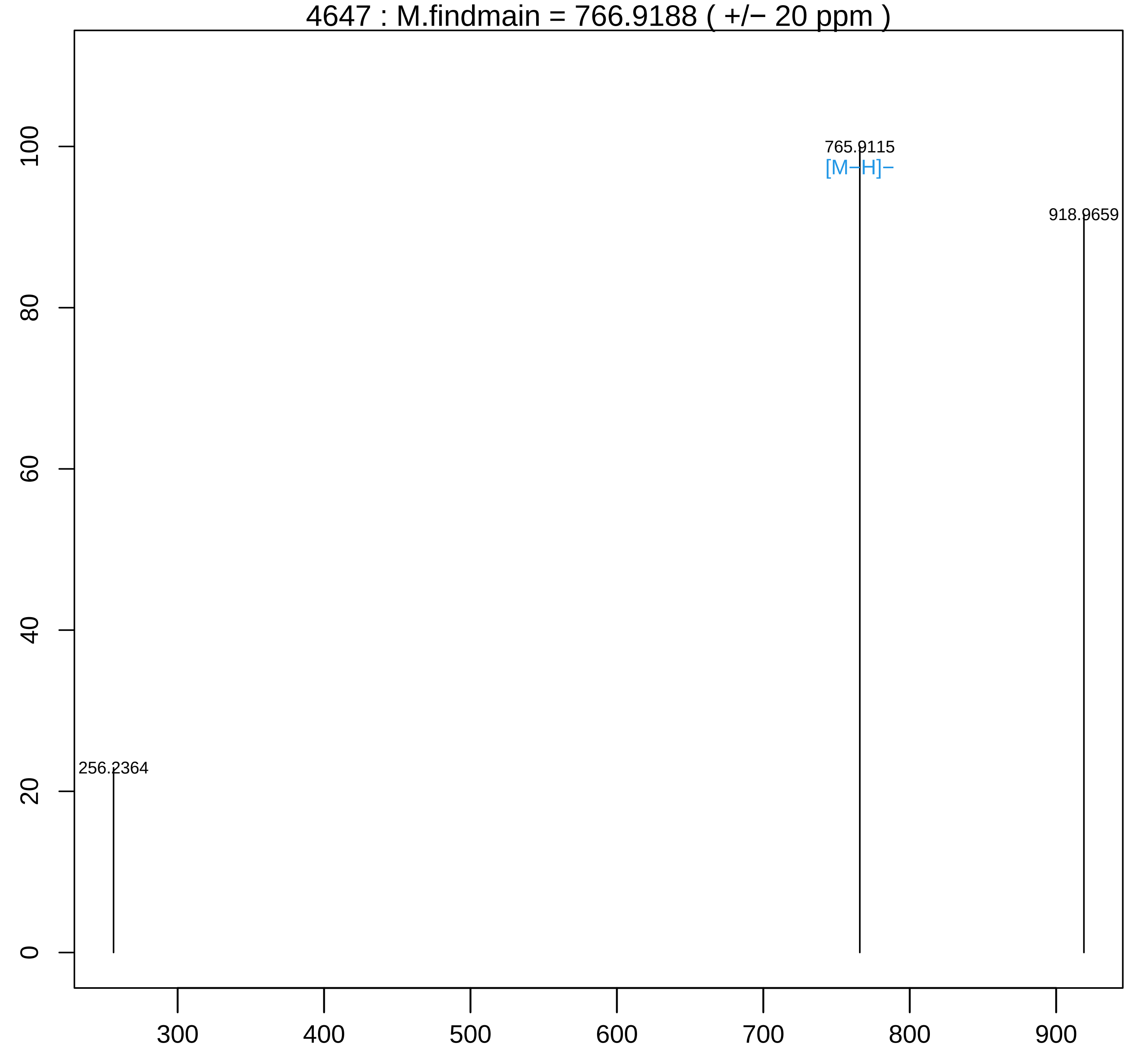
4647 : M.findmain = 766.9188 (+/− 20 ppm)
765.9115
[M−H]−
918.9659
256.2364
0
20
40
60
80
100
300
400
500
600
700
800
900

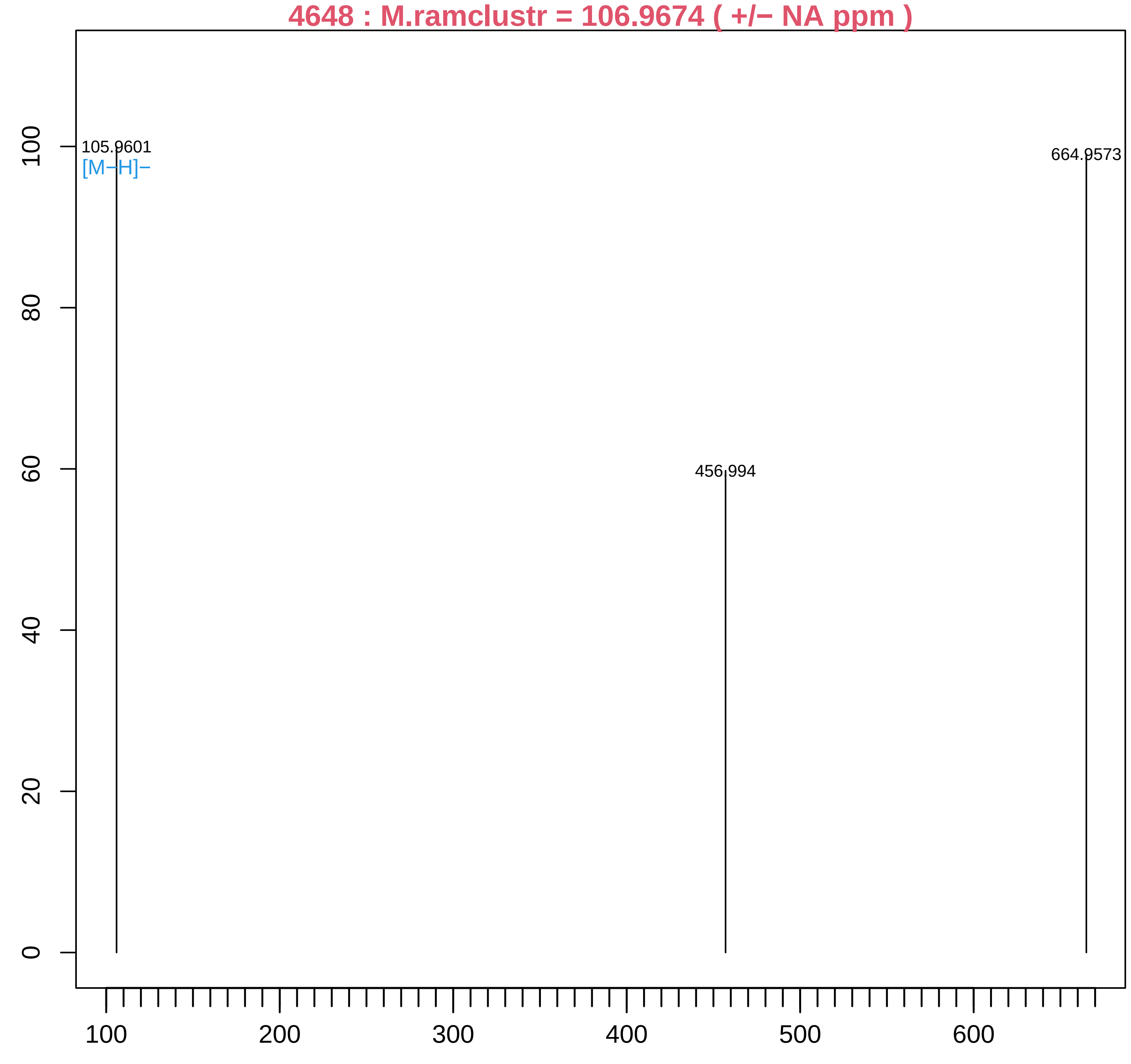

4648 : M.ramclustr = 106.9674 (+/− NA ppm)
105.9601
[M−H]−
664.9573
456.994
0
20
40
60
80
100
100
200
300
400
500
600

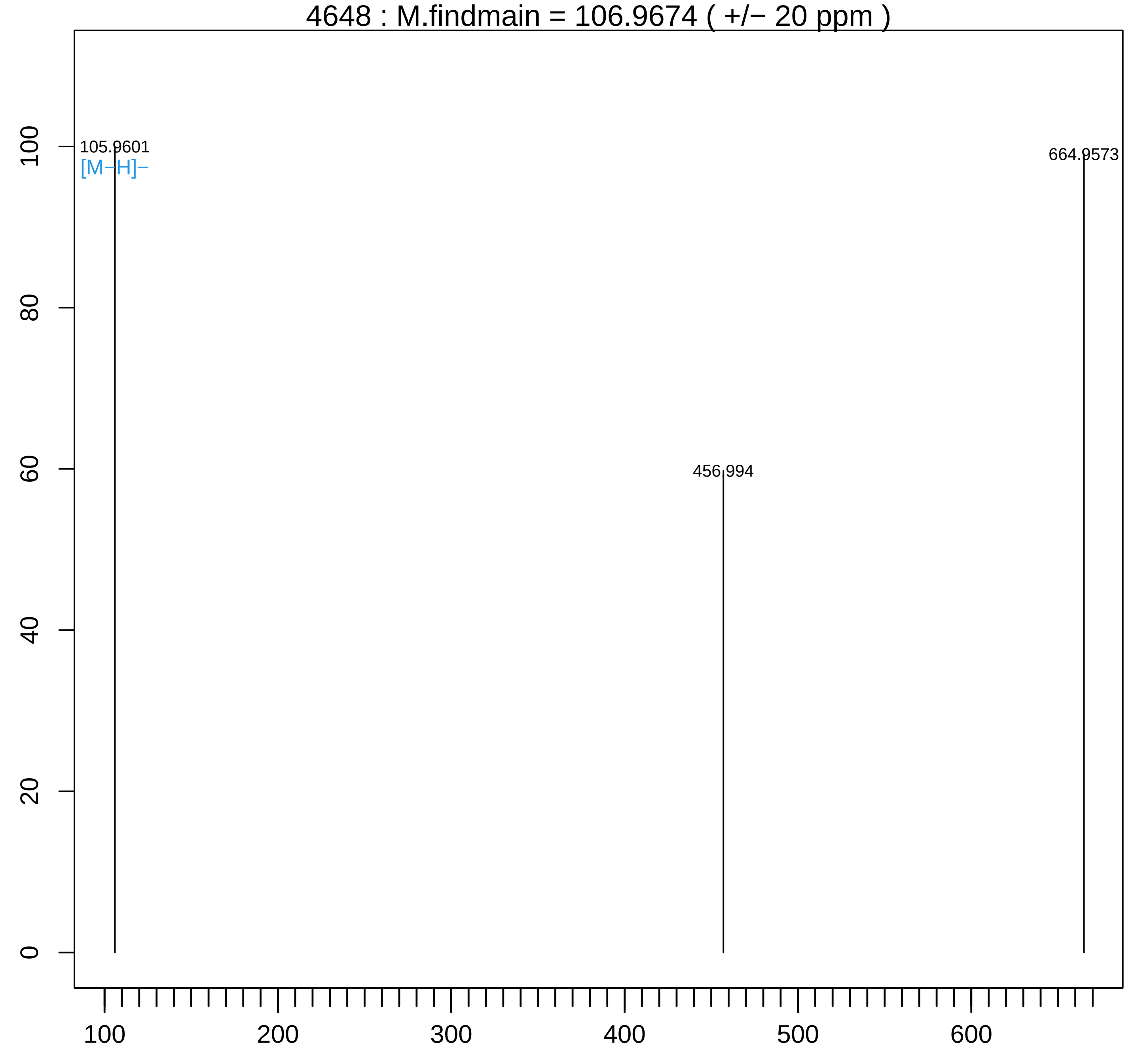

4648 : M.findmain = 106.9674 (+/− 20 ppm)
105.9601
[M−H]−
664.9573
456.994
0
20
40
60
80
100
100
200
300
400
500
600

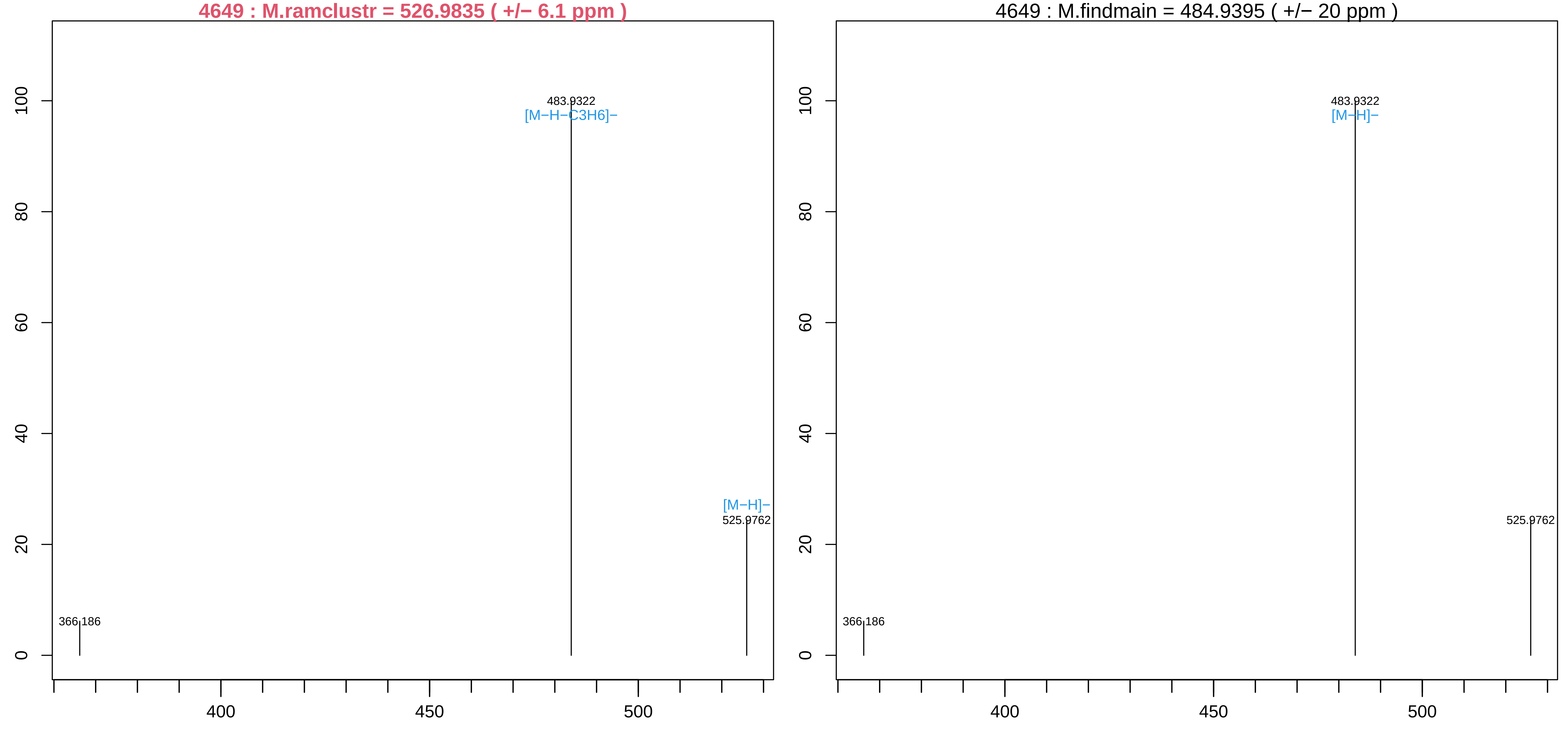
4649 : M.ramclustr = 526.9835 (+/− 6.1 ppm)
483.9322
[M−H−C3H6]−
[M−H]−
525.9762
366.186
4649 : M.findmain = 484.9395 (+/− 20 ppm)
483.9322
[M−H]−
525.9762
366.186
0
20
40
60
80
100
400
450
500

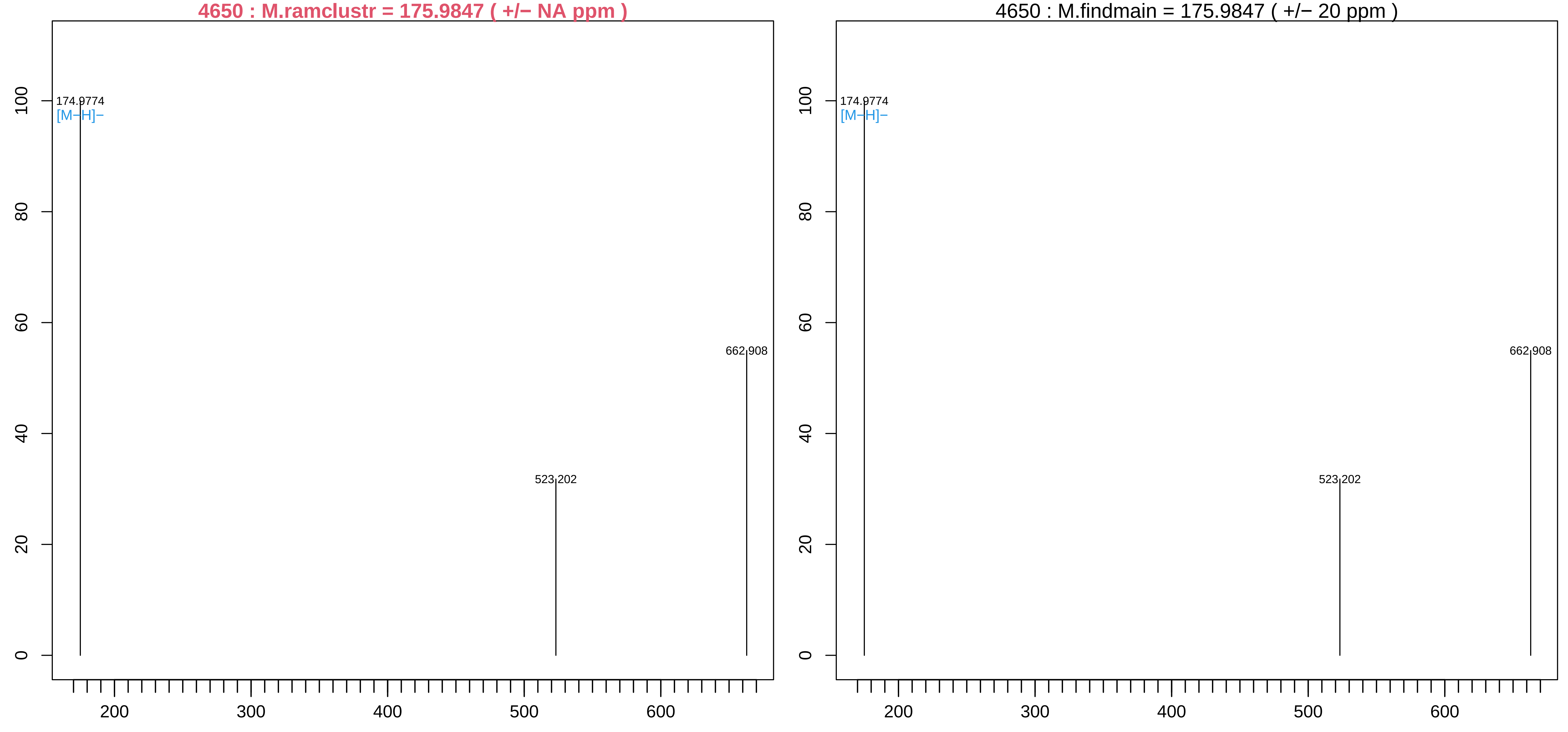

4650 : M.ramclustr = 175.9847 (+/− NA ppm)
174.9774
[M−H]−
523.202
662.908
4650 : M.findmain = 175.9847 (+/− 20 ppm)
174.9774
[M−H]−
523.202
662.908
0
20
40
60
80
100
200
300
400
500
600

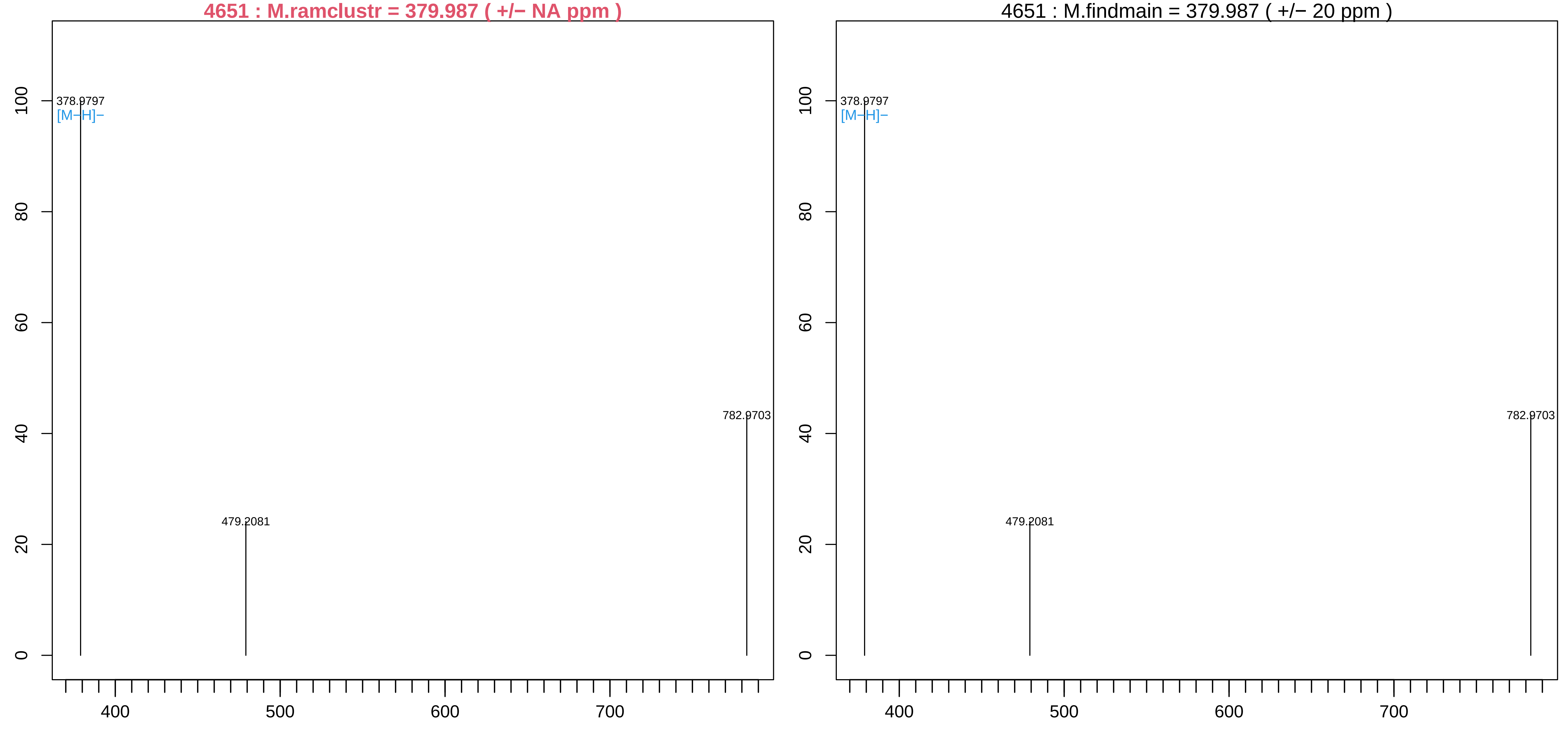
4651 : M.ramclustr = 379.987 (+/− NA ppm)
378.9797
[M−H]−
479.2081
782.9703
0
20
40
60
80
100
400
500
600
700
4651 : M.findmain = 379.987 (+/− 20 ppm)
378.9797
[M−H]−
479.2081
782.9703
0
20
40
60
80
100
400
500
600
700

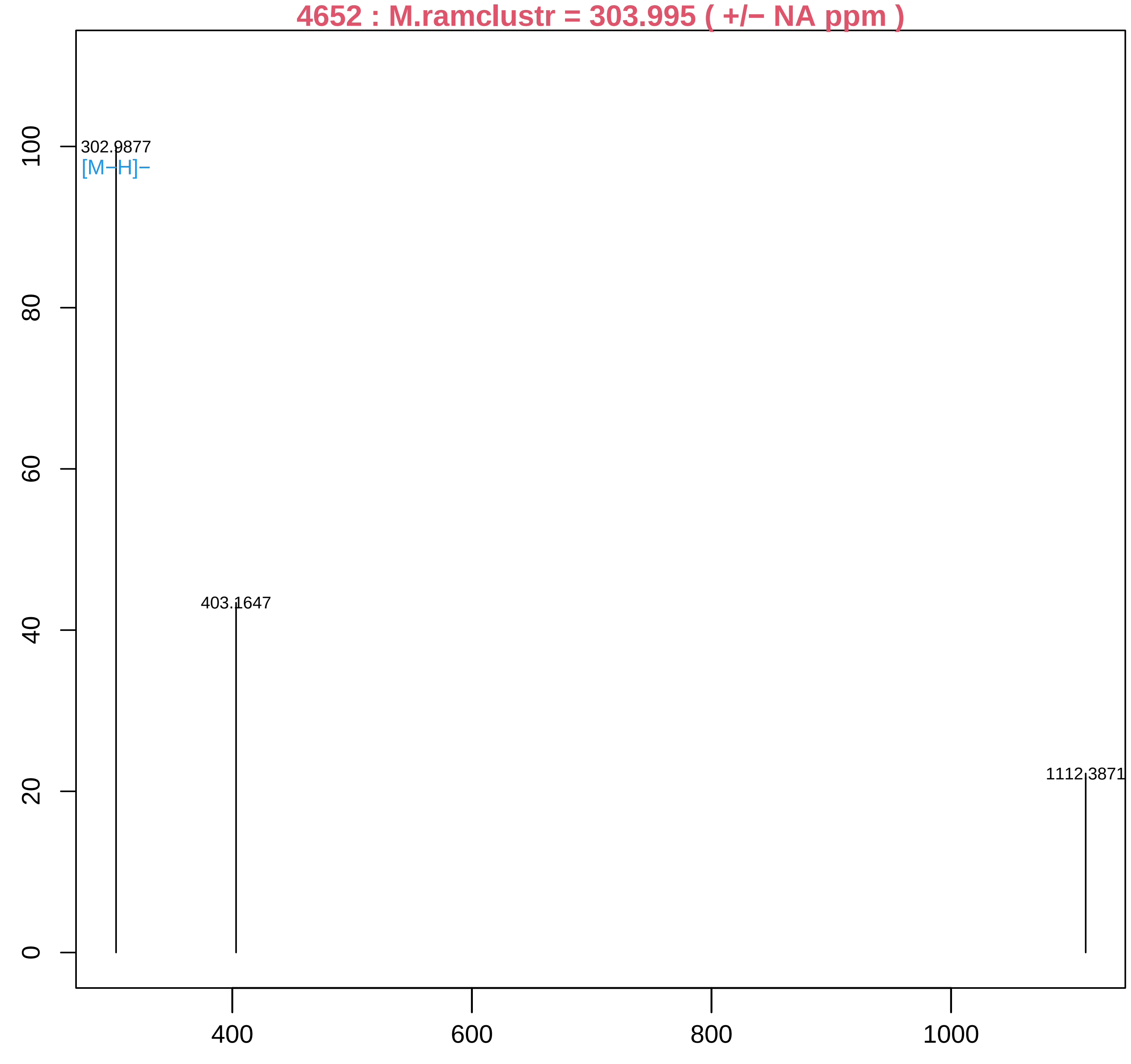
4652 : M.ramclustr = 303.995 (+/− NA ppm)
302.9877
[M−H]−
403.1647
1112.3871
0
20
40
60
80
100
400
600
800
1000

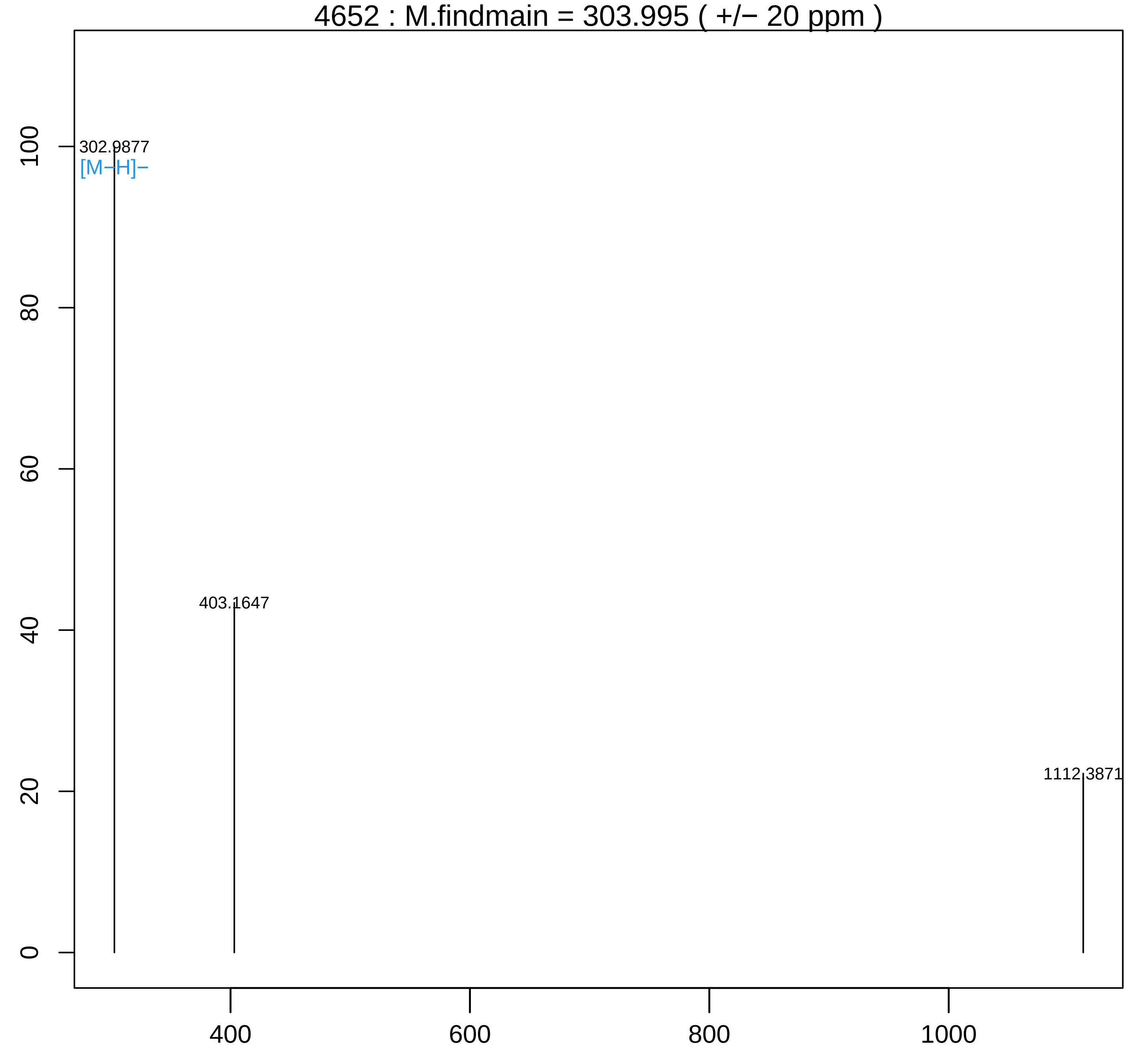
4652 : M.findmain = 303.995 (+/− 20 ppm)
302.9877
[M−H]−
403.1647
1112.3871
0
20
40
60
80
100
400
600
800
1000

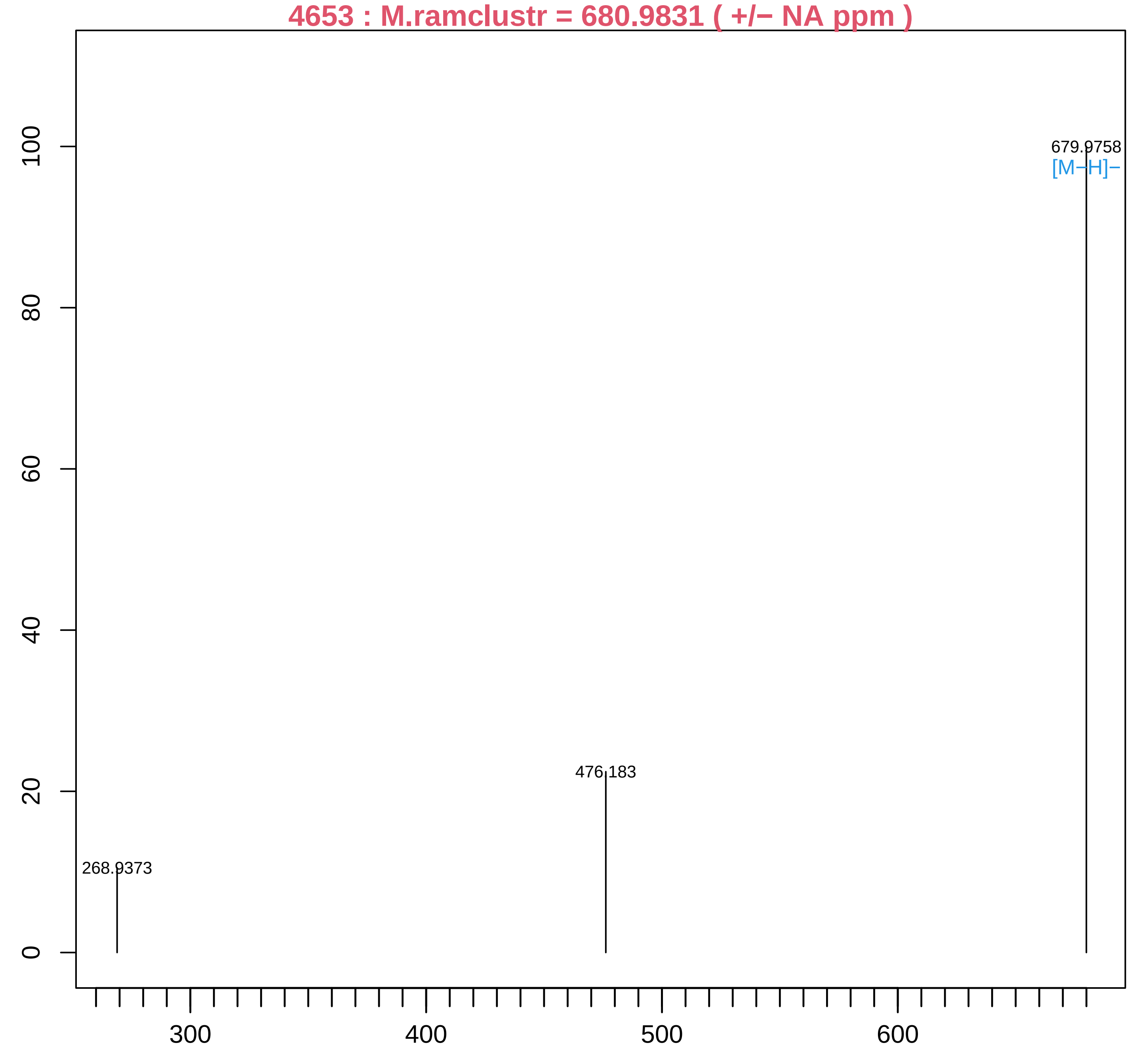

4653 : M.ramclustr = 680.9831 (+/− NA ppm)
679.9758
[M−H]−
476.183
268.9373
0
20
40
60
80
100
300
400
500
600

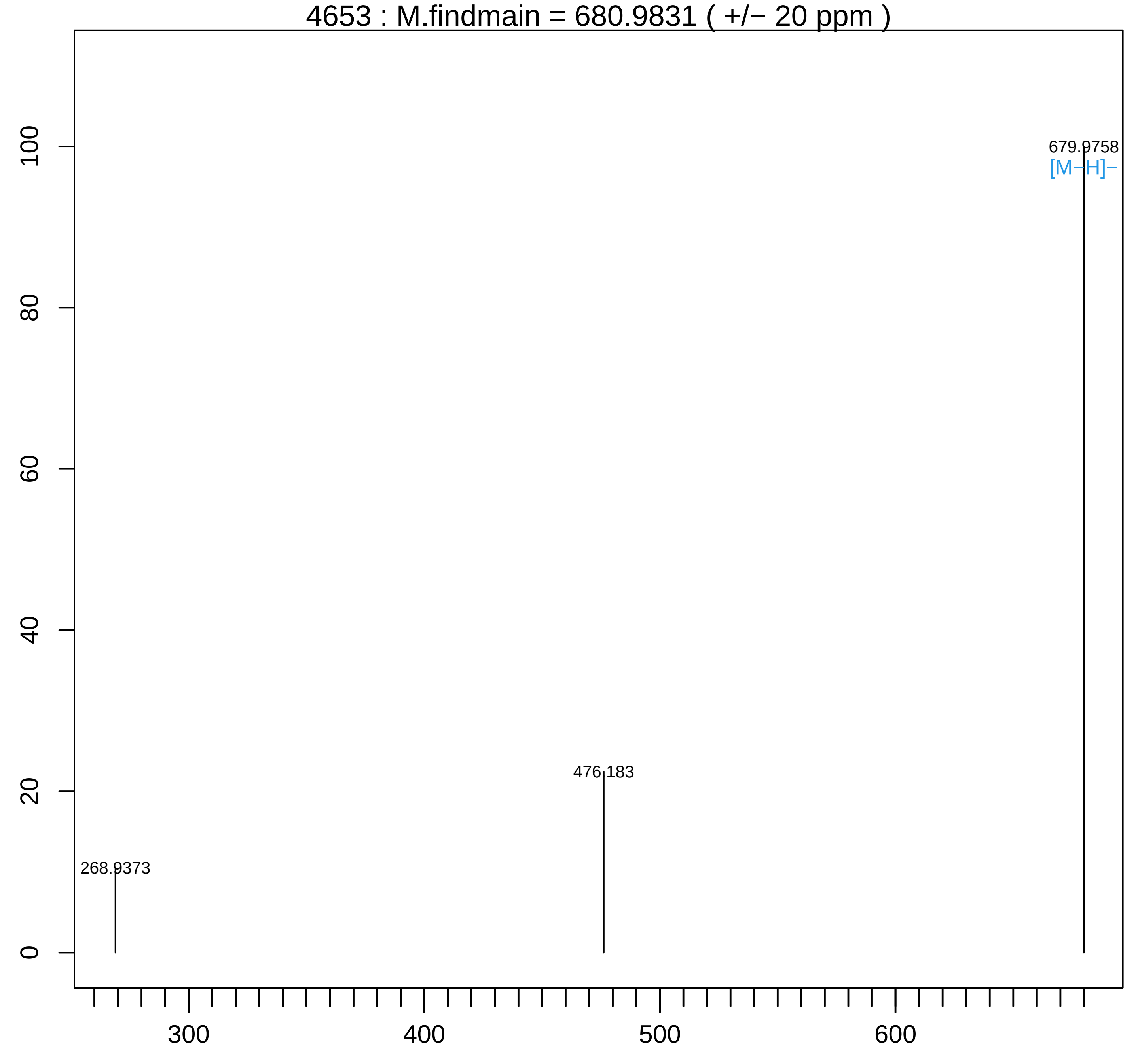

4653 : M.findmain = 680.9831 (+/− 20 ppm)
679.9758
[M−H]−
476.183
268.9373
0
20
40
60
80
100
300
400
500
600

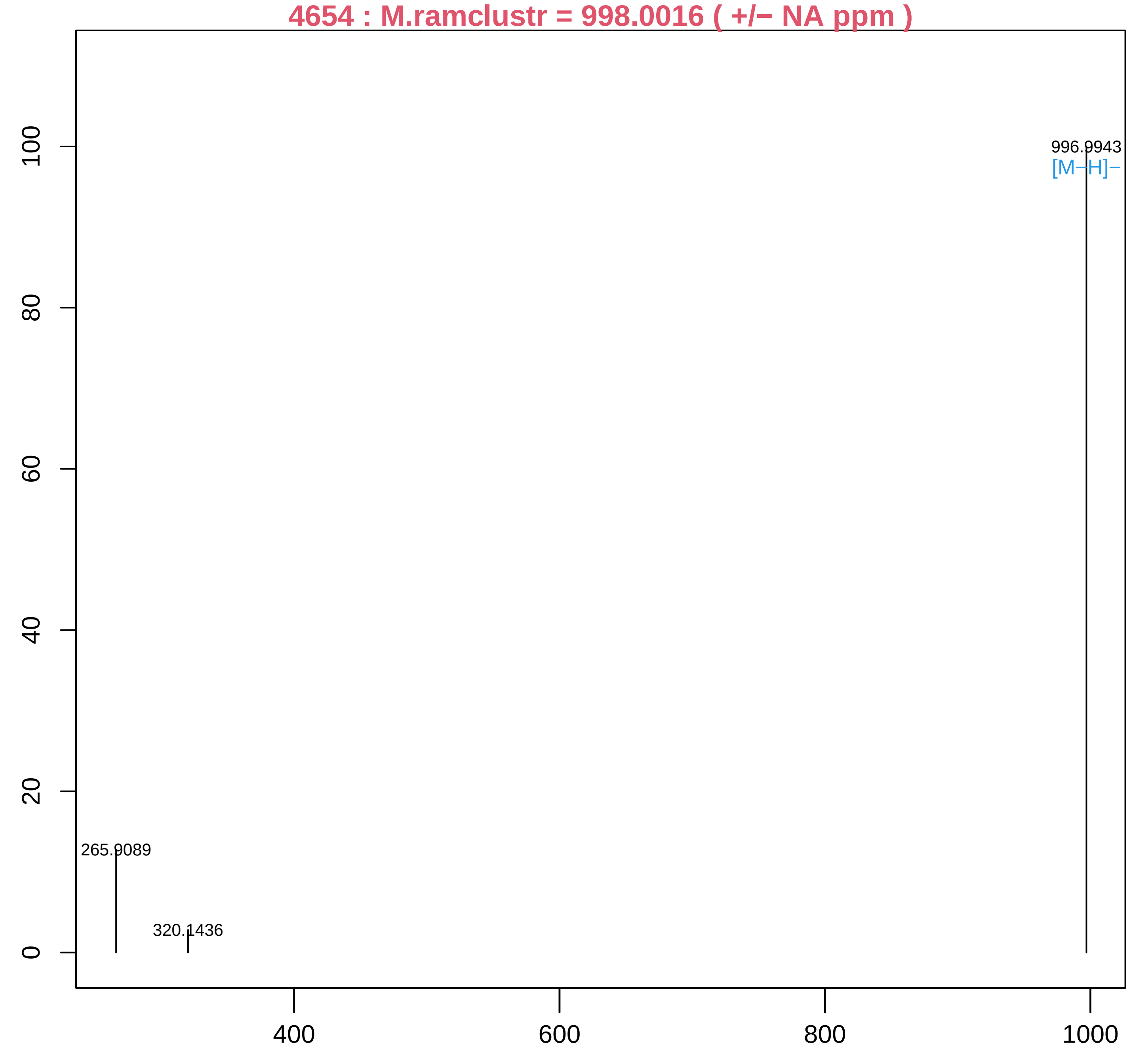
4654 : M.ramclustr = 998.0016 (+/− NA ppm)
996.9943
[M−H]−
265.9089
320.1436
0
20
40
60
80
100
400
600
800
1000

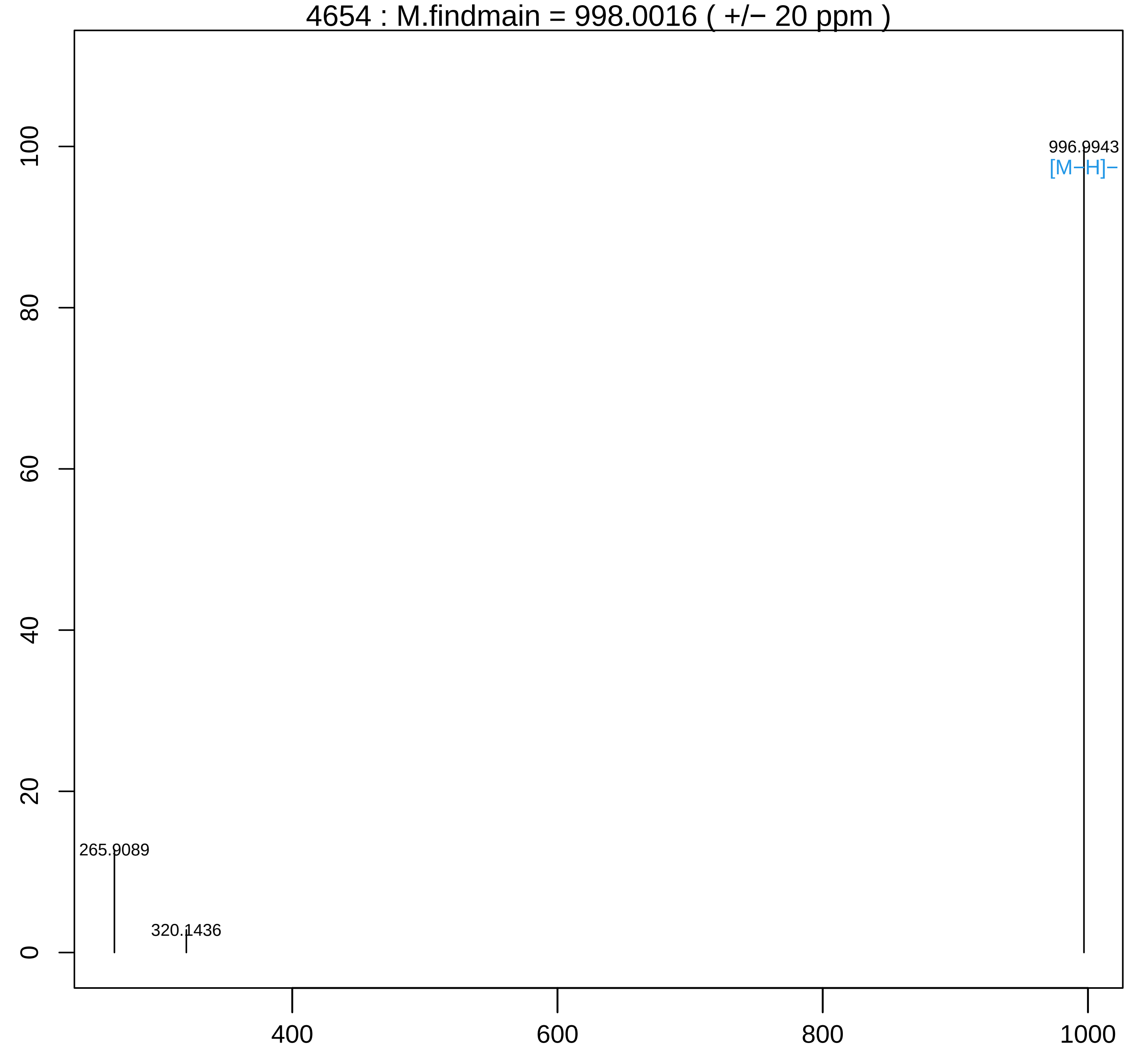
4654 : M.findmain = 998.0016 (+/− 20 ppm)
996.9943
[M−H]−
265.9089
320.1436
0
20
40
60
80
100
400
600
800
1000

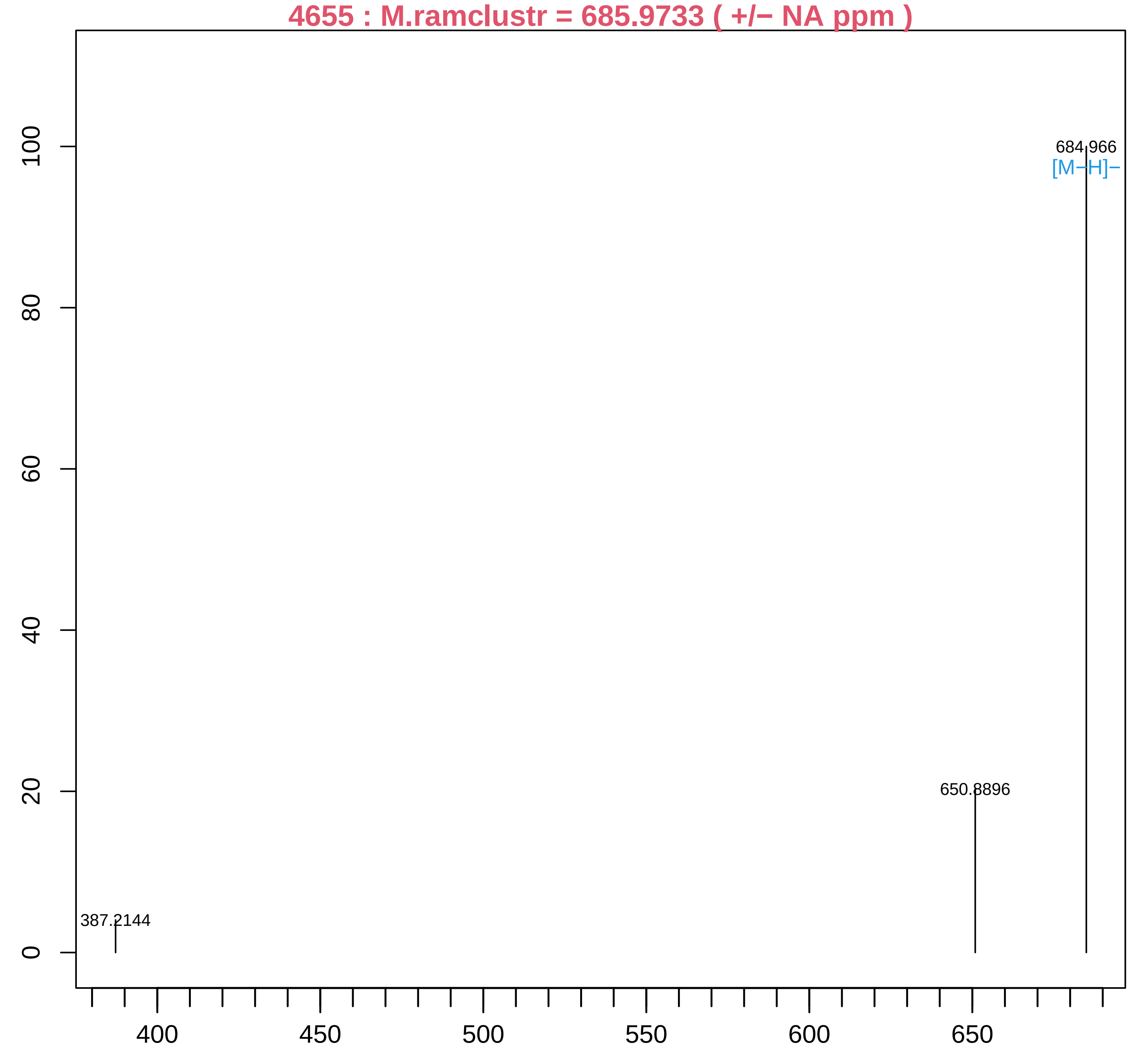

4655 : M.ramclustr = 685.9733 (+/− NA ppm)
684.966
[M−H]−
650.8896
387.2144
0
20
40
60
80
100
400
450
500
550
600
650

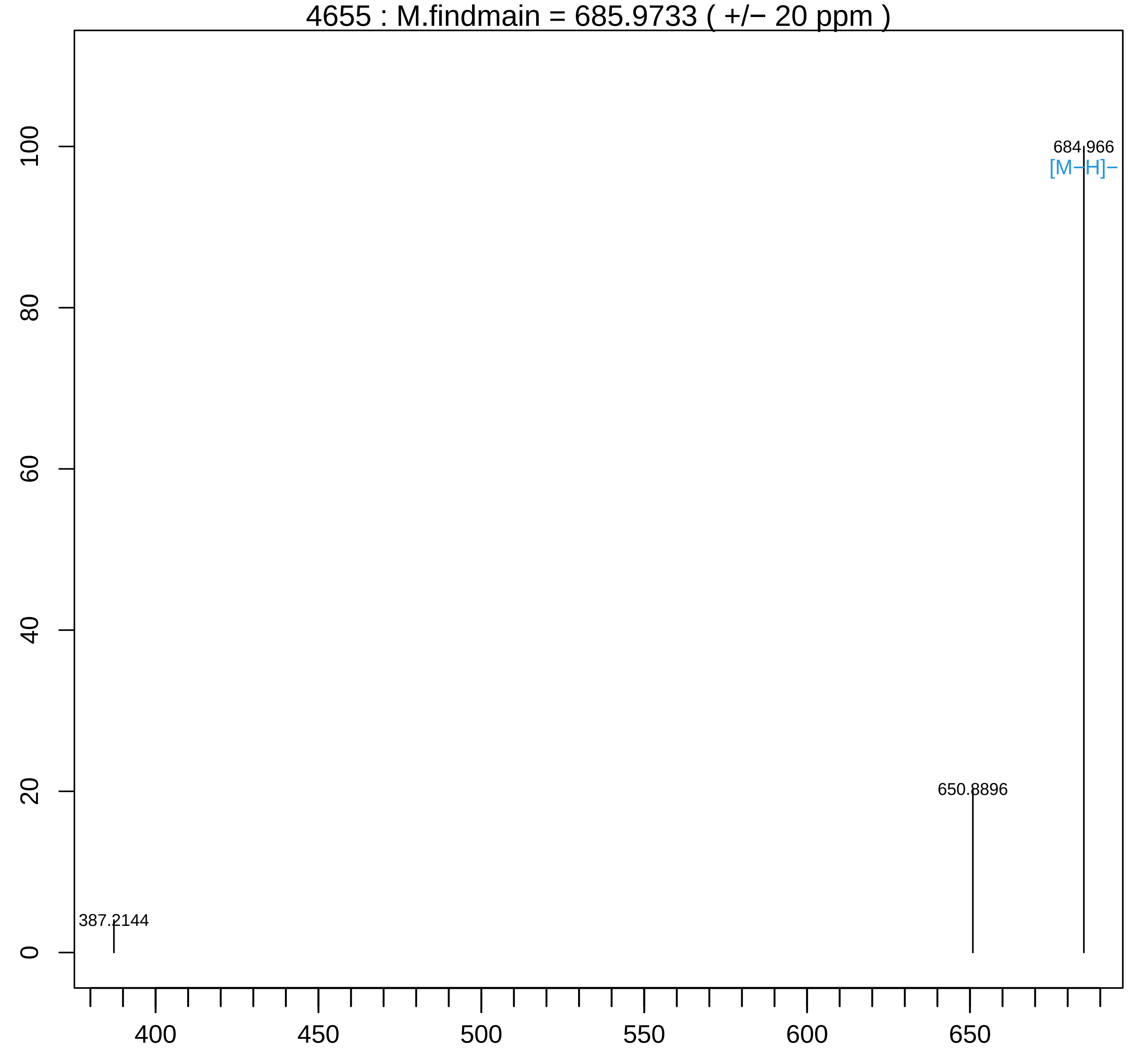

4655 : M.findmain = 685.9733 (+/− 20 ppm)
684.966
[M−H]−
650.8896
387.2144
0
20
40
60
80
100
400
450
500
550
600
650

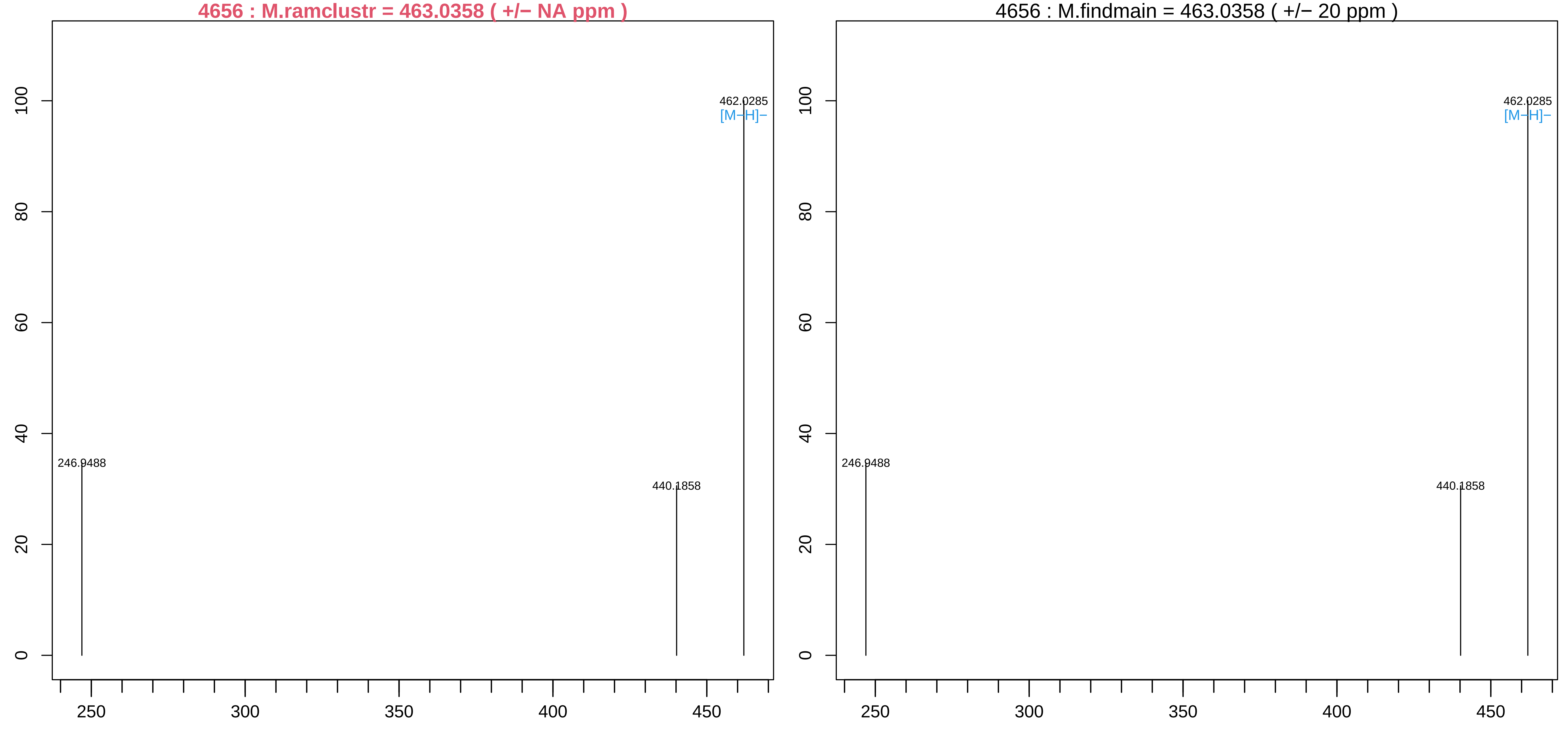
4656 : M.ramclustr = 463.0358 (+/− NA ppm)
246.9488
440.1858
462.0285
[M−H]−
4656 : M.findmain = 463.0358 (+/− 20 ppm)
246.9488
440.1858
462.0285
[M−H]−
0
20
40
60
80
100
250
300
350
400
450

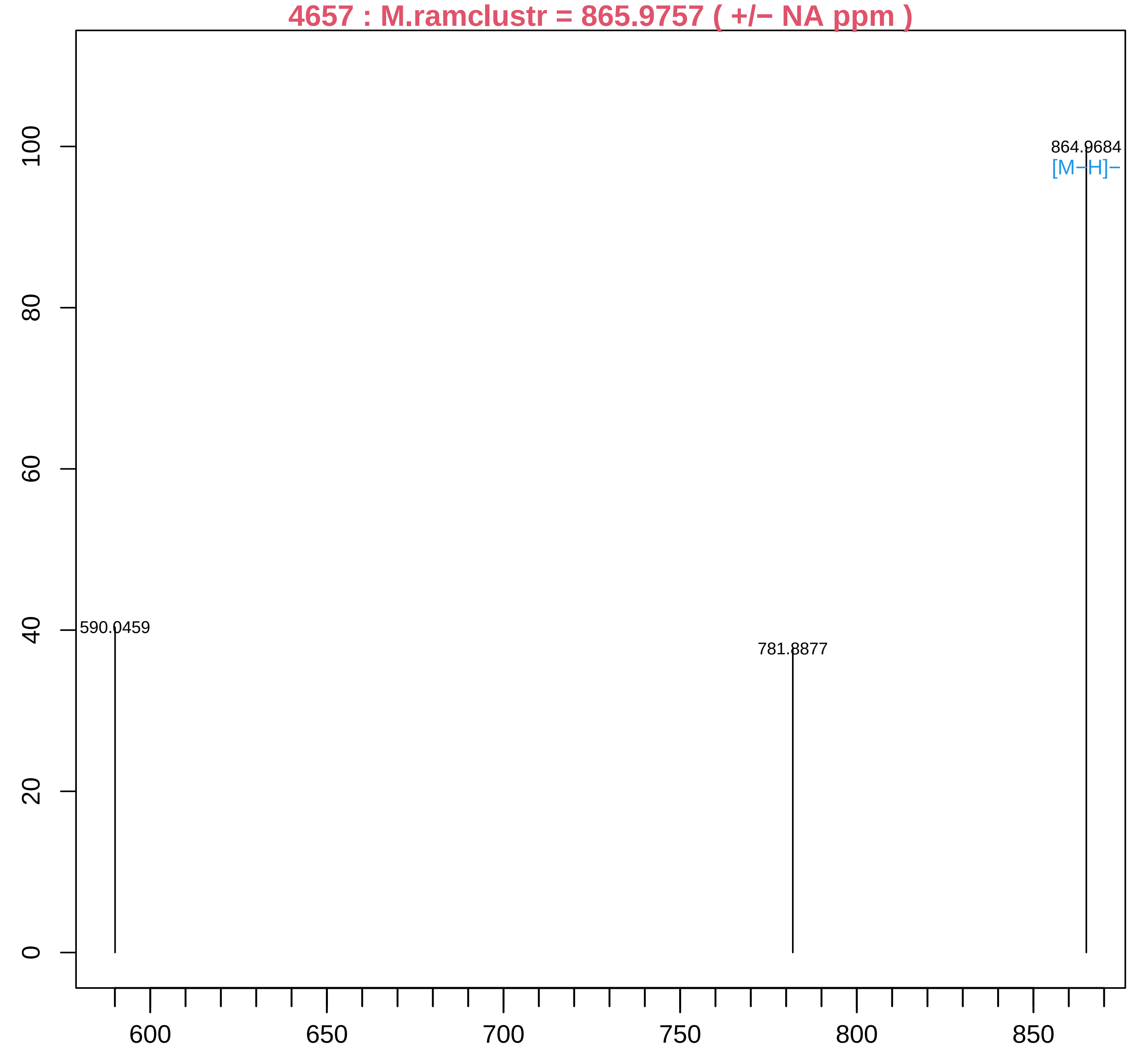
4657 : M.ramclustr = 865.9757 (+/− NA ppm)
864.9684
[M−H]−
590.0459
781.8877

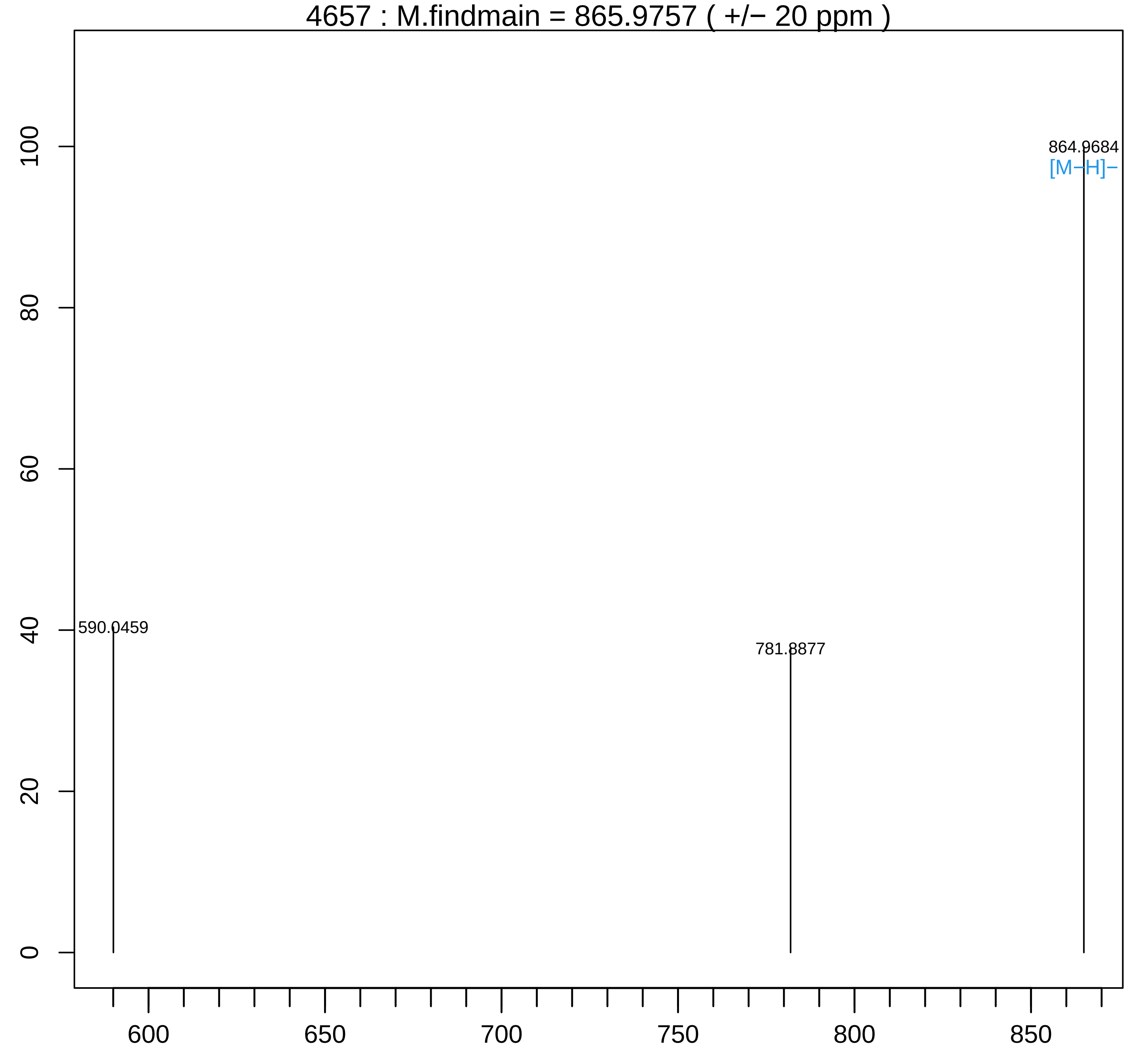
4657 : M.findmain = 865.9757 (+/− 20 ppm)
864.9684
[M−H]−
590.0459
781.8877

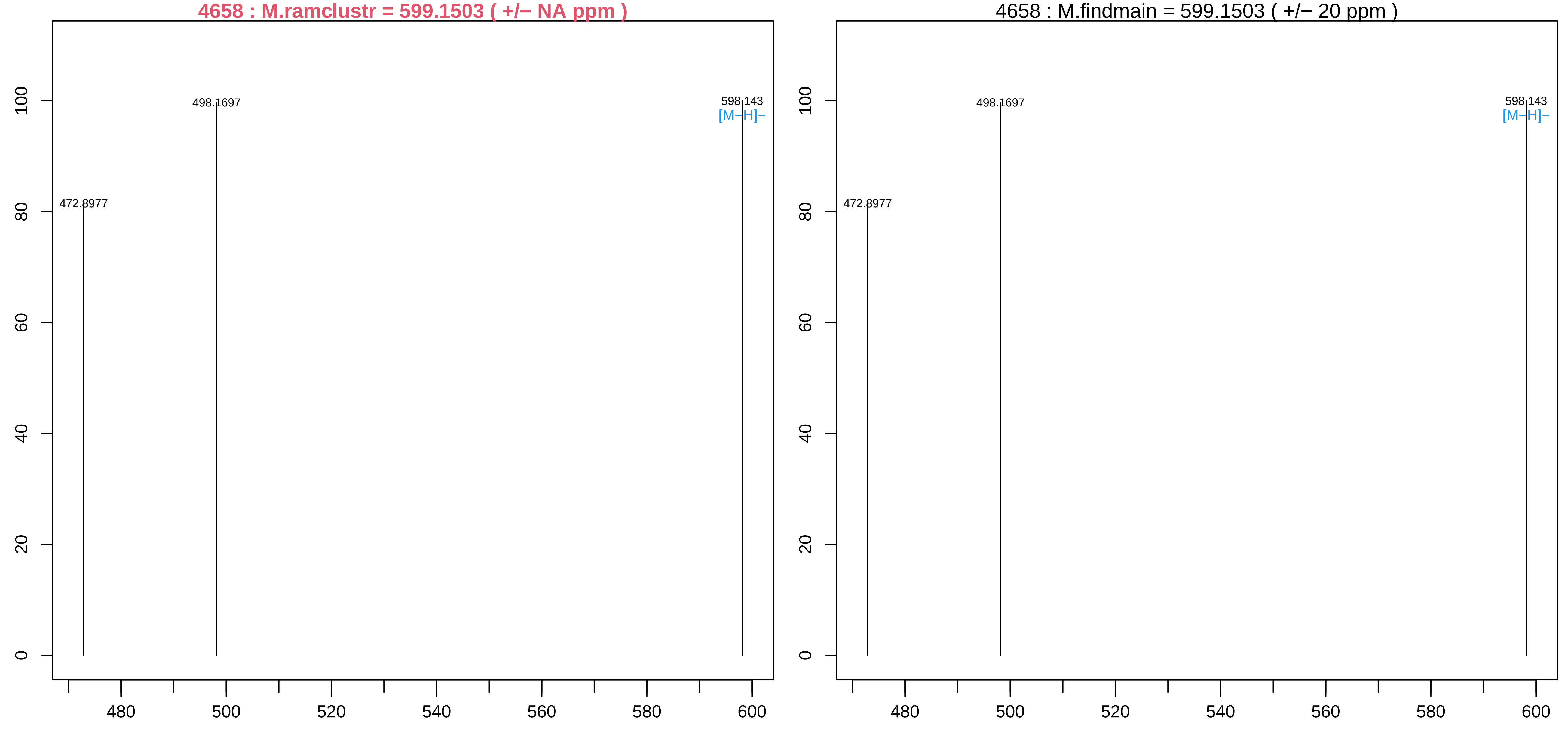
4658 : M.ramclustr = 599.1503 (+/− NA ppm)
472.8977
498.1697
598.143
[M−H]−
0
20
40
60
80
100
480
500
520
540
560
580
600
4658 : M.findmain = 599.1503 (+/− 20 ppm)
472.8977
498.1697
598.143
[M−H]−
0
20
40
60
80
100
480
500
520
540
560
580
600

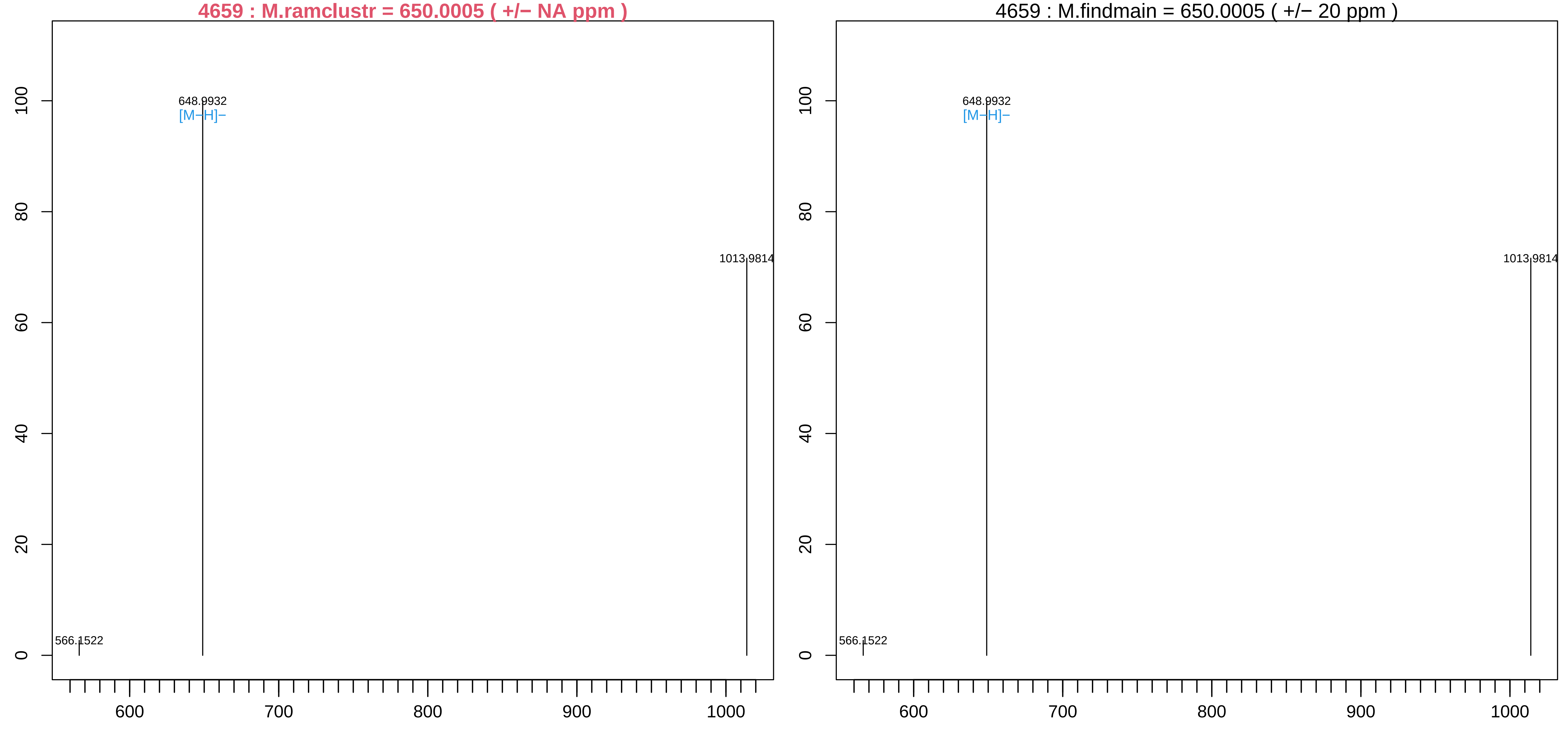
4659 : M.ramclustr = 650.0005 (+/− NA ppm)
4659 : M.findmain = 650.0005 (+/− 20 ppm)
648.9932
[M−H]−
1013.9814
566.1522
0
20
40
60
80
100
600
700
800
900
1000

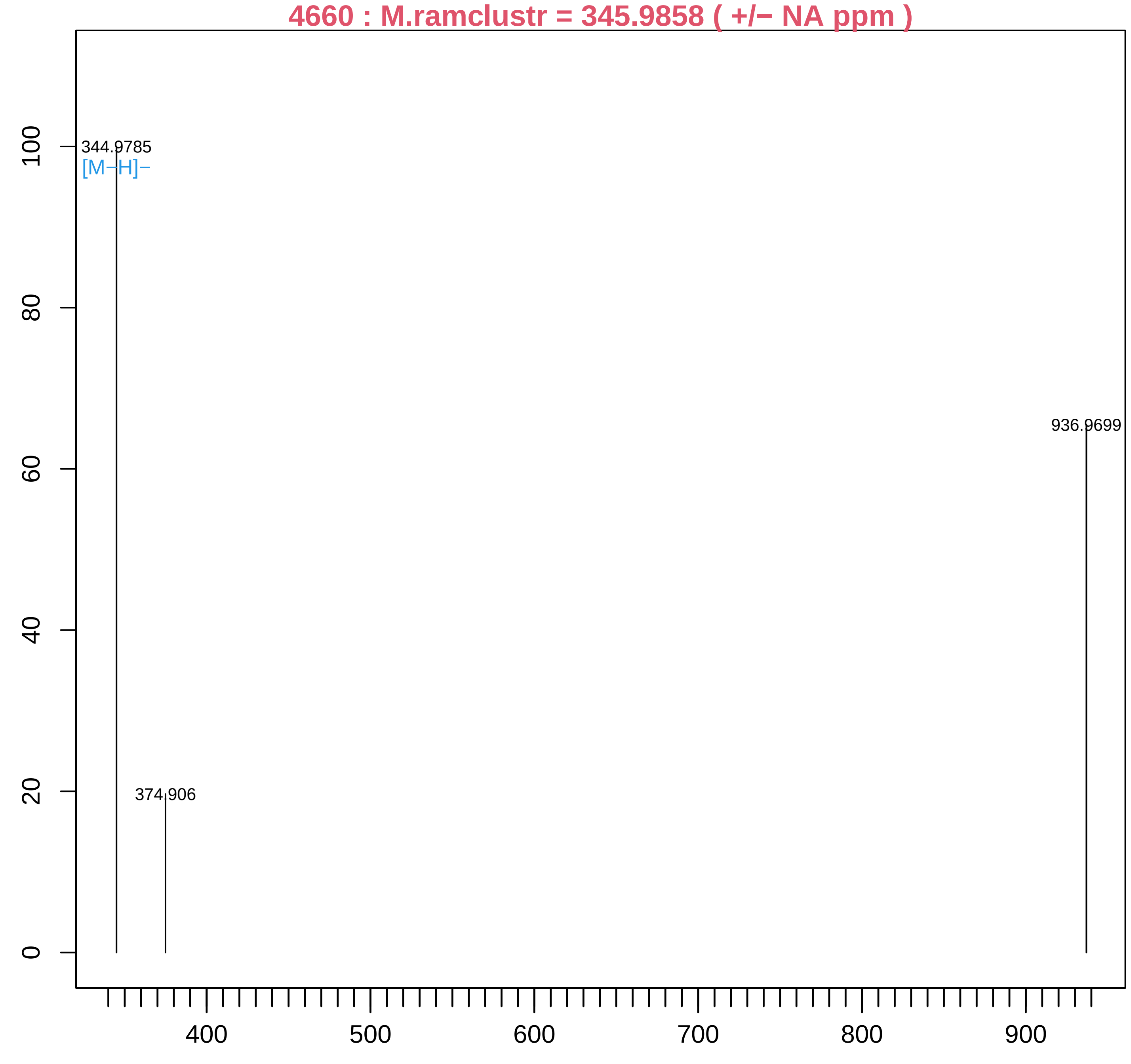
4660 : M.ramclustr = 345.9858 (+/− NA ppm)
344.9785
[M−H]−
374.906
936.9699
0
20
40
60
80
100
400
500
600
700
800
900

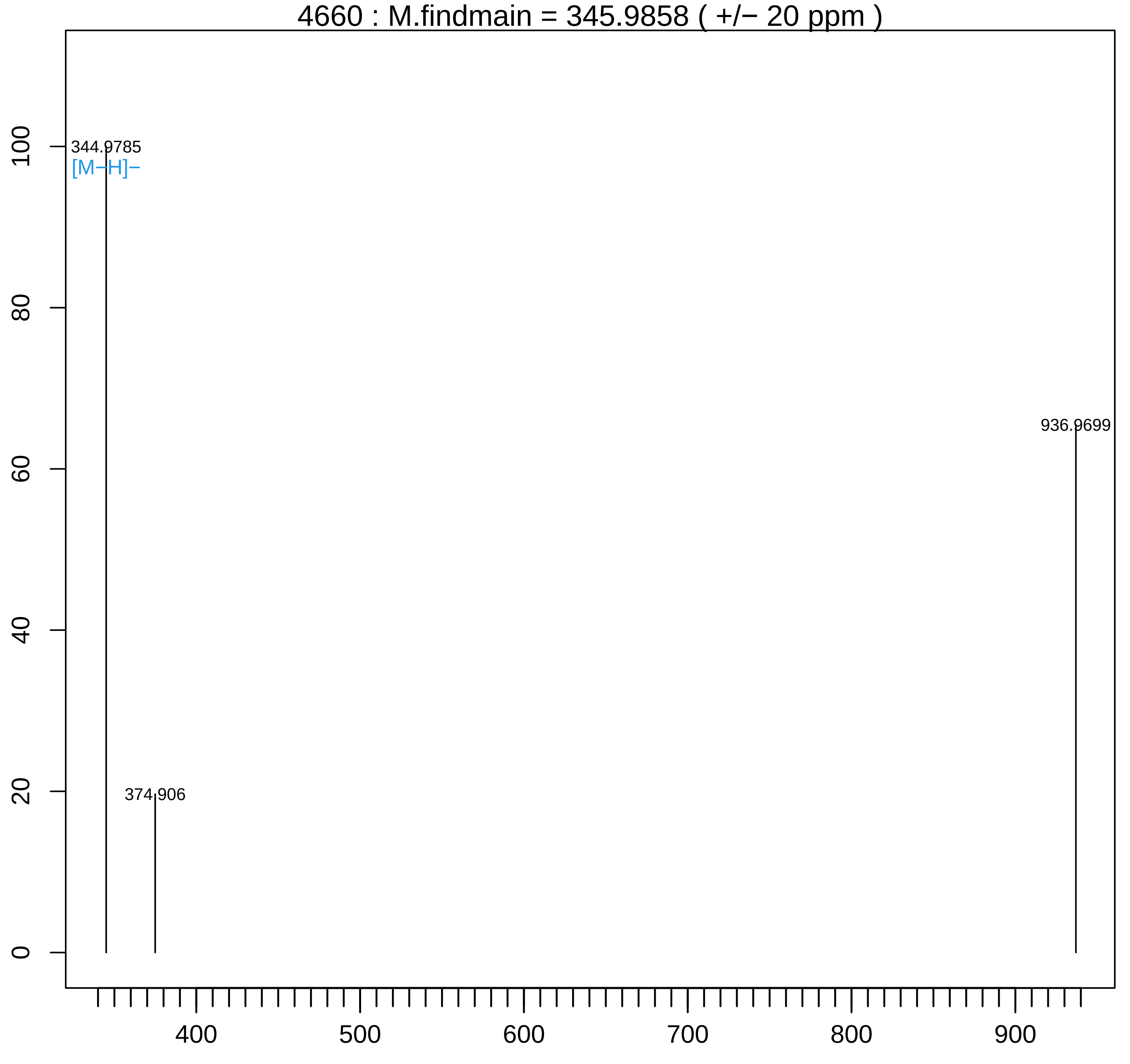
4660 : M.findmain = 345.9858 (+/− 20 ppm)
344.9785
[M−H]−
374.906
936.9699
0
20
40
60
80
100
400
500
600
700
800
900

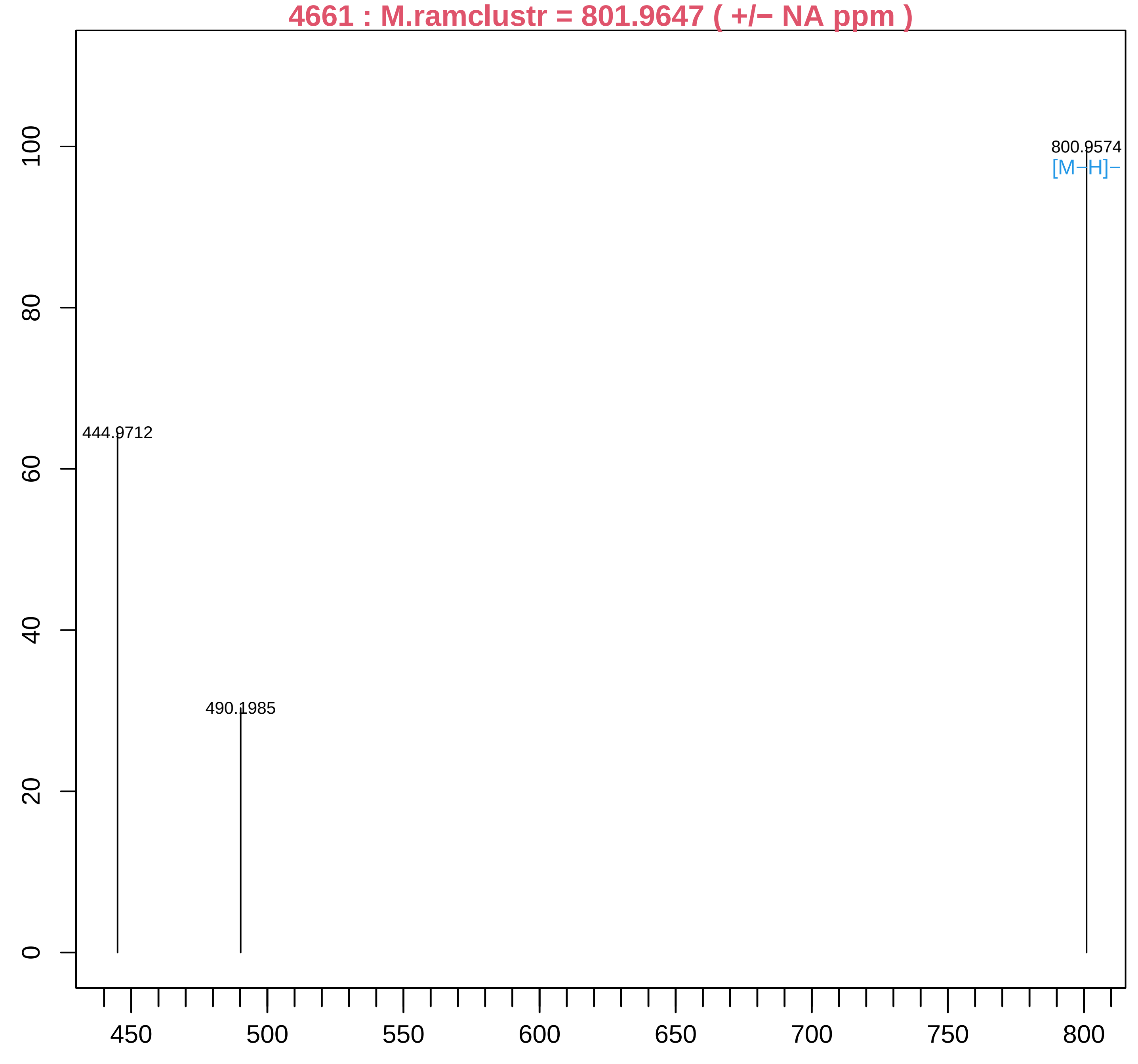
4661 : M.ramclustr = 801.9647 (+/− NA ppm)
800.9574
[M−H]−
444.9712
490.1985
0
20
40
60
80
100
450
500
550
600
650
700
750
800

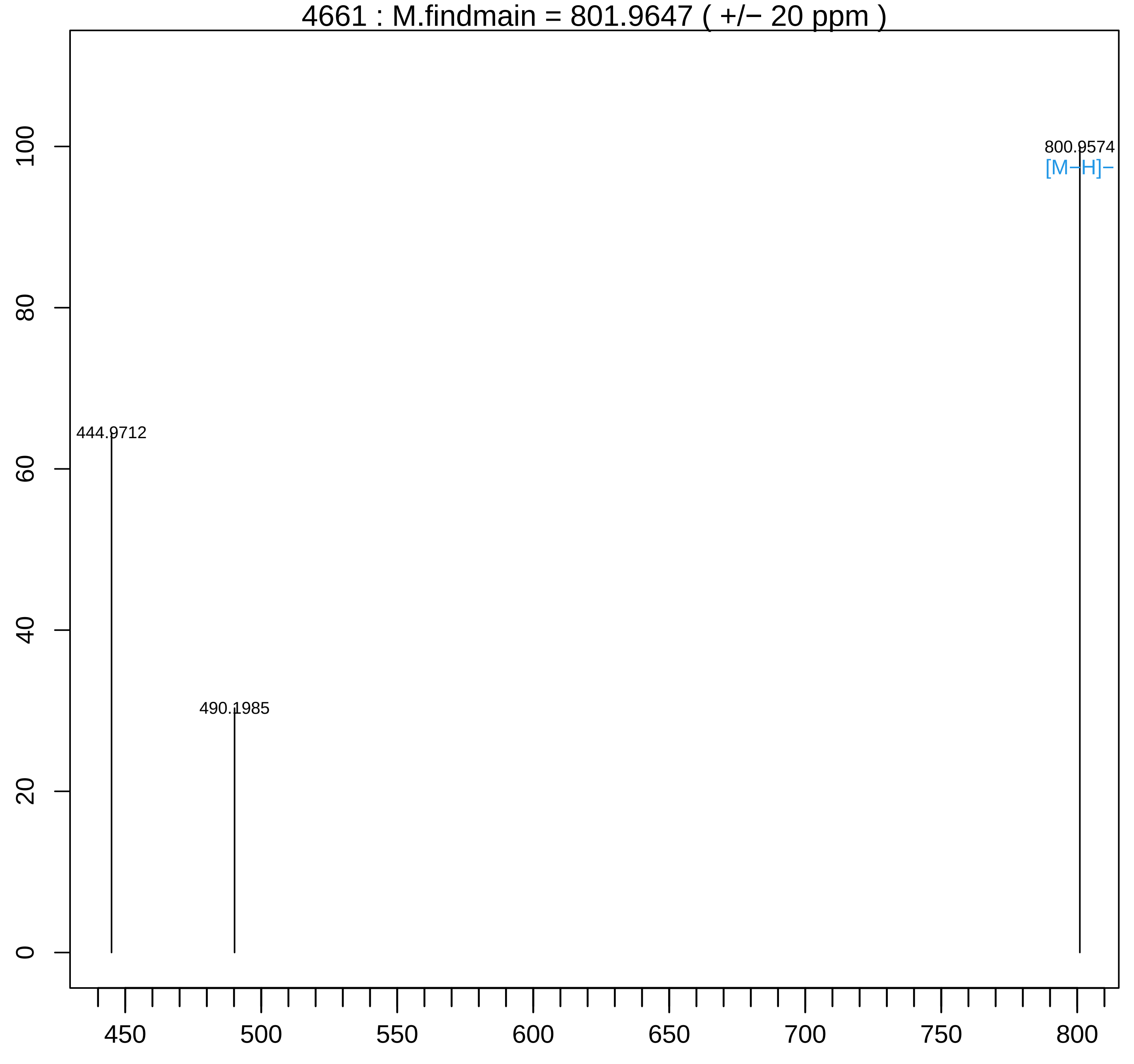
4661 : M.findmain = 801.9647 (+/− 20 ppm)
800.9574
[M−H]−
444.9712
490.1985
0
20
40
60
80
100
450
500
550
600
650
700
750
800

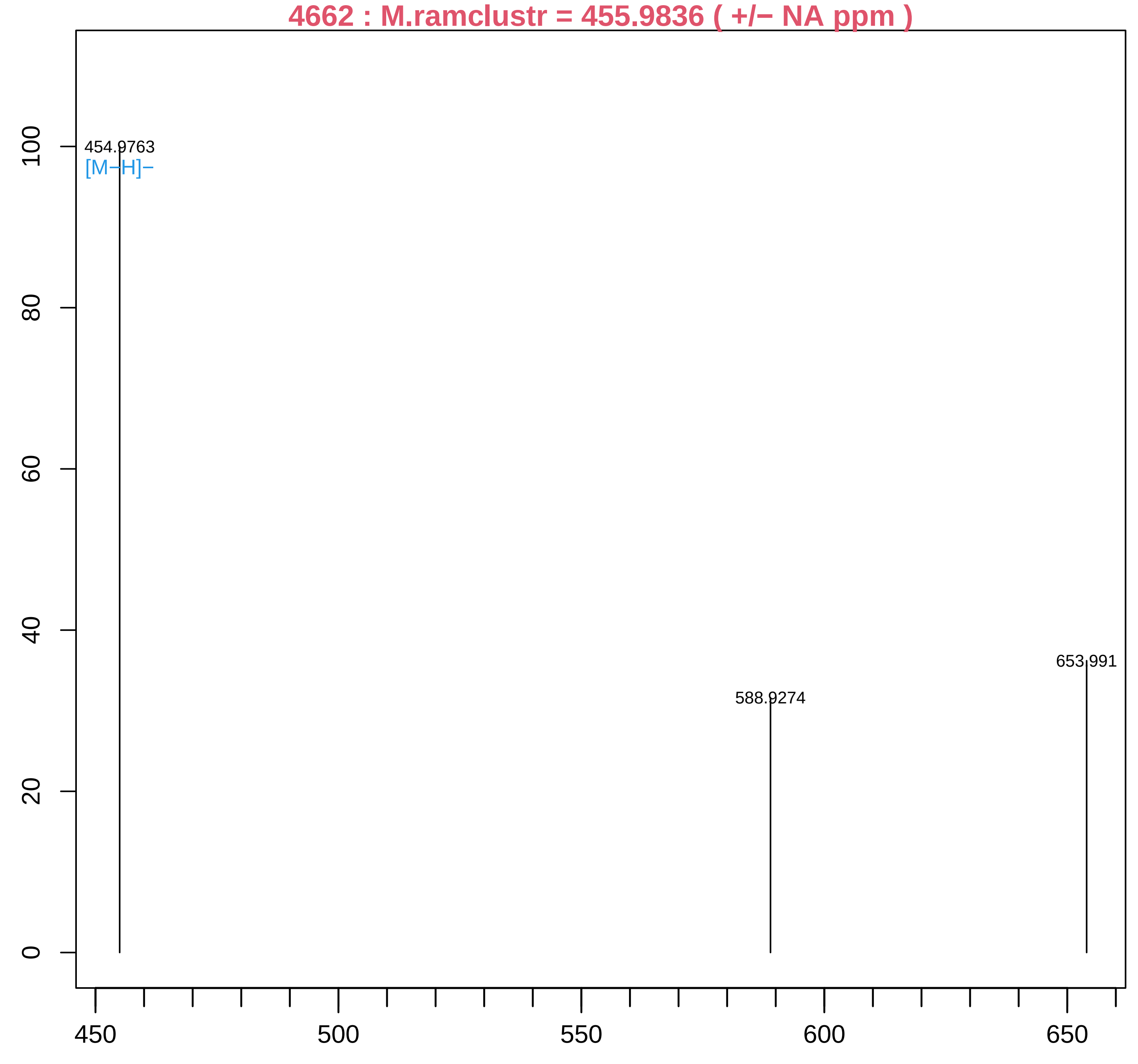
4662 : M.ramclustr = 455.9836 (+/− NA ppm)
454.9763
[M−H]−
588.9274
653.991
0
20
40
60
80
100
450
500
550
600
650

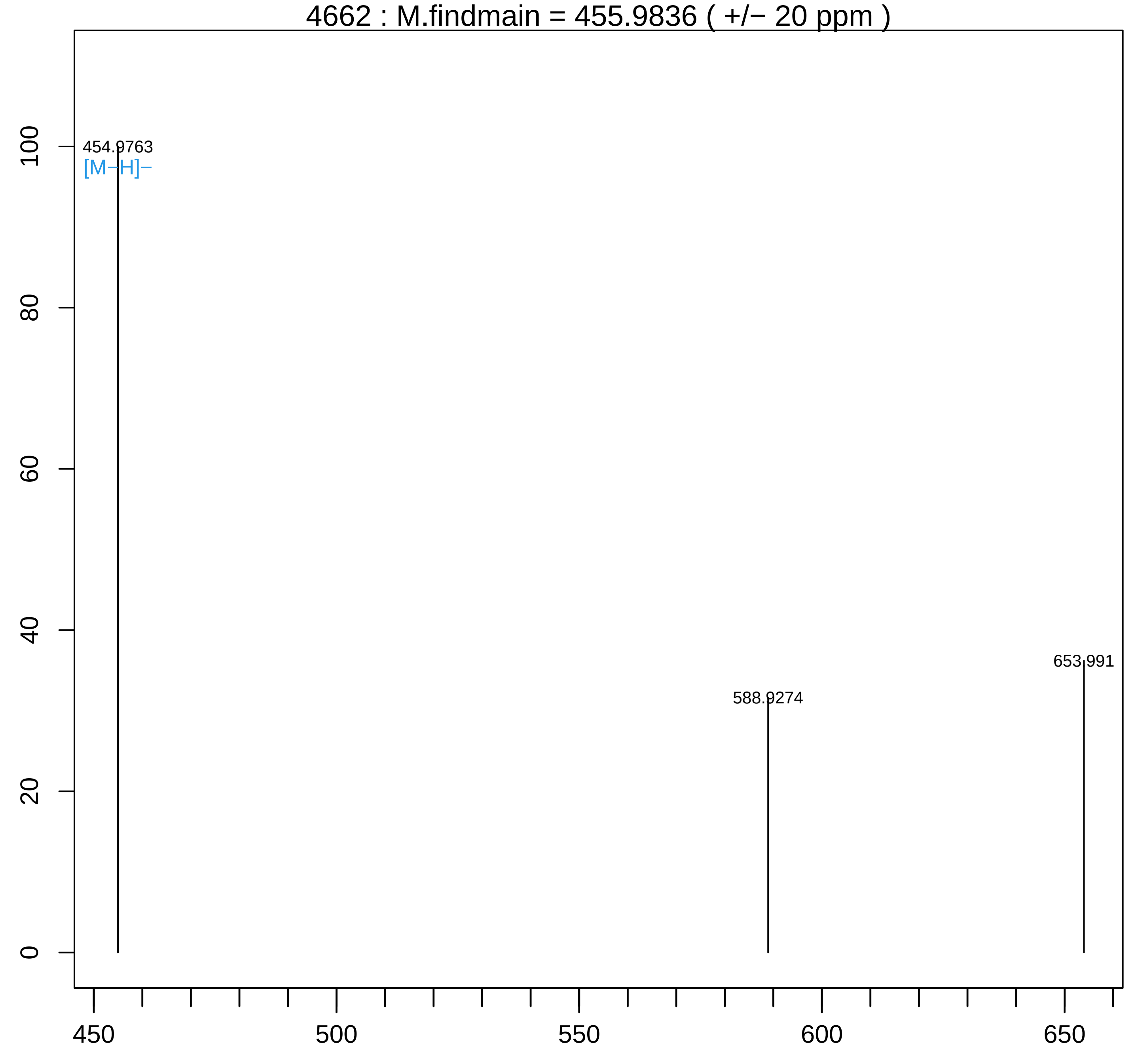
4662 : M.findmain = 455.9836 (+/− 20 ppm)
454.9763
[M−H]−
588.9274
653.991
0
20
40
60
80
100
450
500
550
600
650

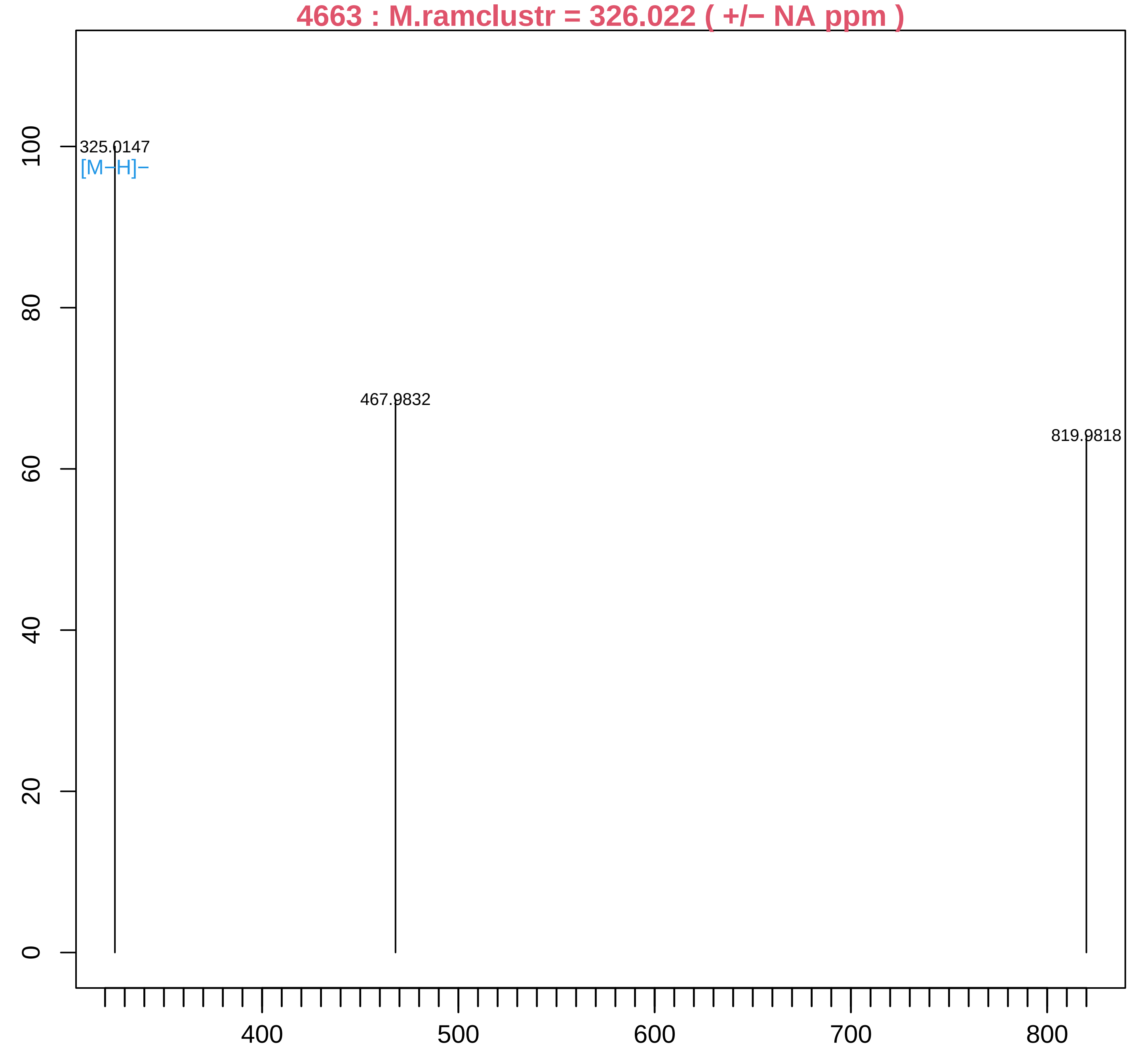
4663 : M.ramclustr = 326.022 (+/− NA ppm)
325.0147
[M−H]−
467.9832
819.9818
0
20
40
60
80
100
400
500
600
700
800

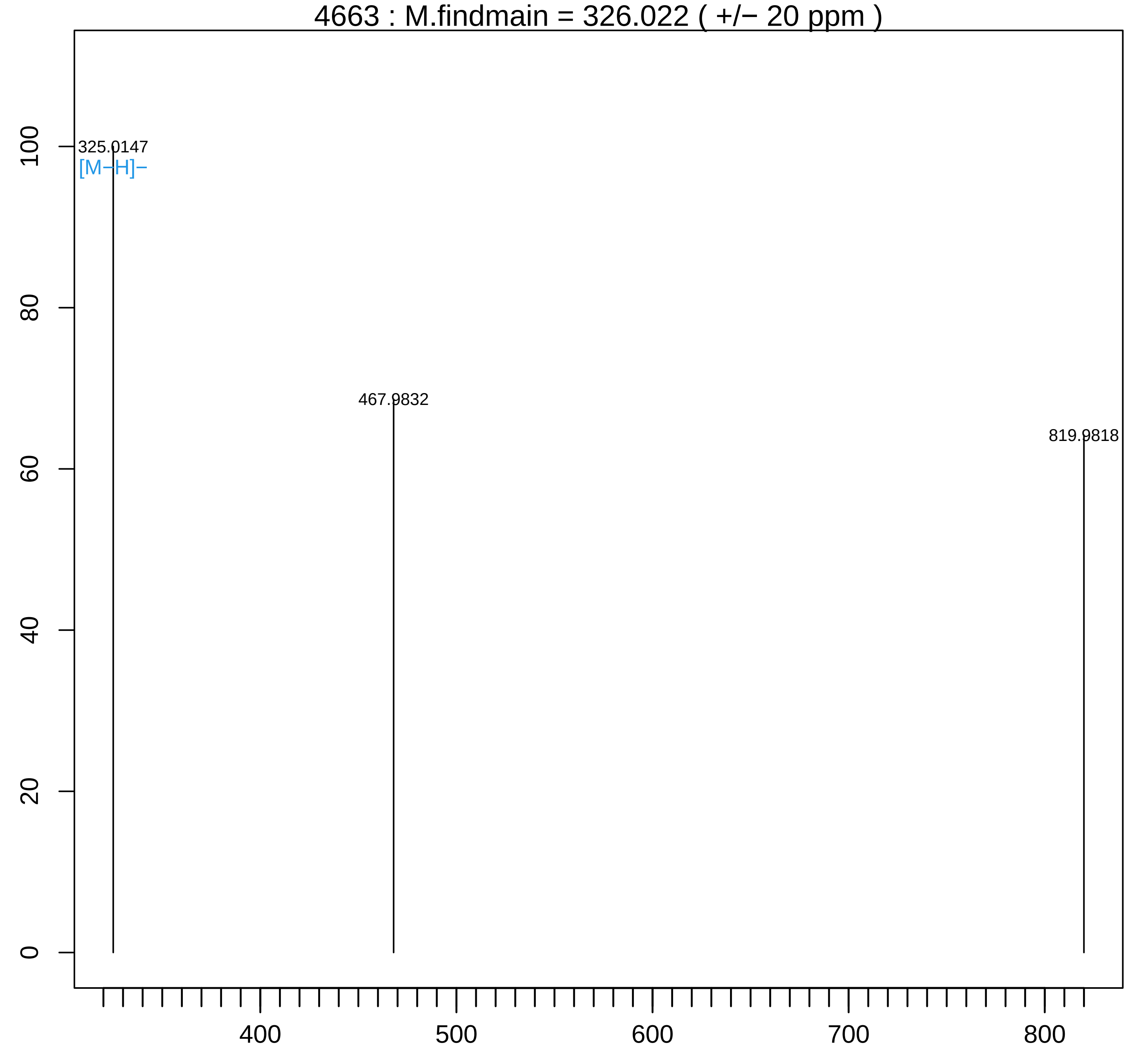
4663 : M.findmain = 326.022 (+/− 20 ppm)
325.0147
[M−H]−
467.9832
819.9818
0
20
40
60
80
100
400
500
600
700
800

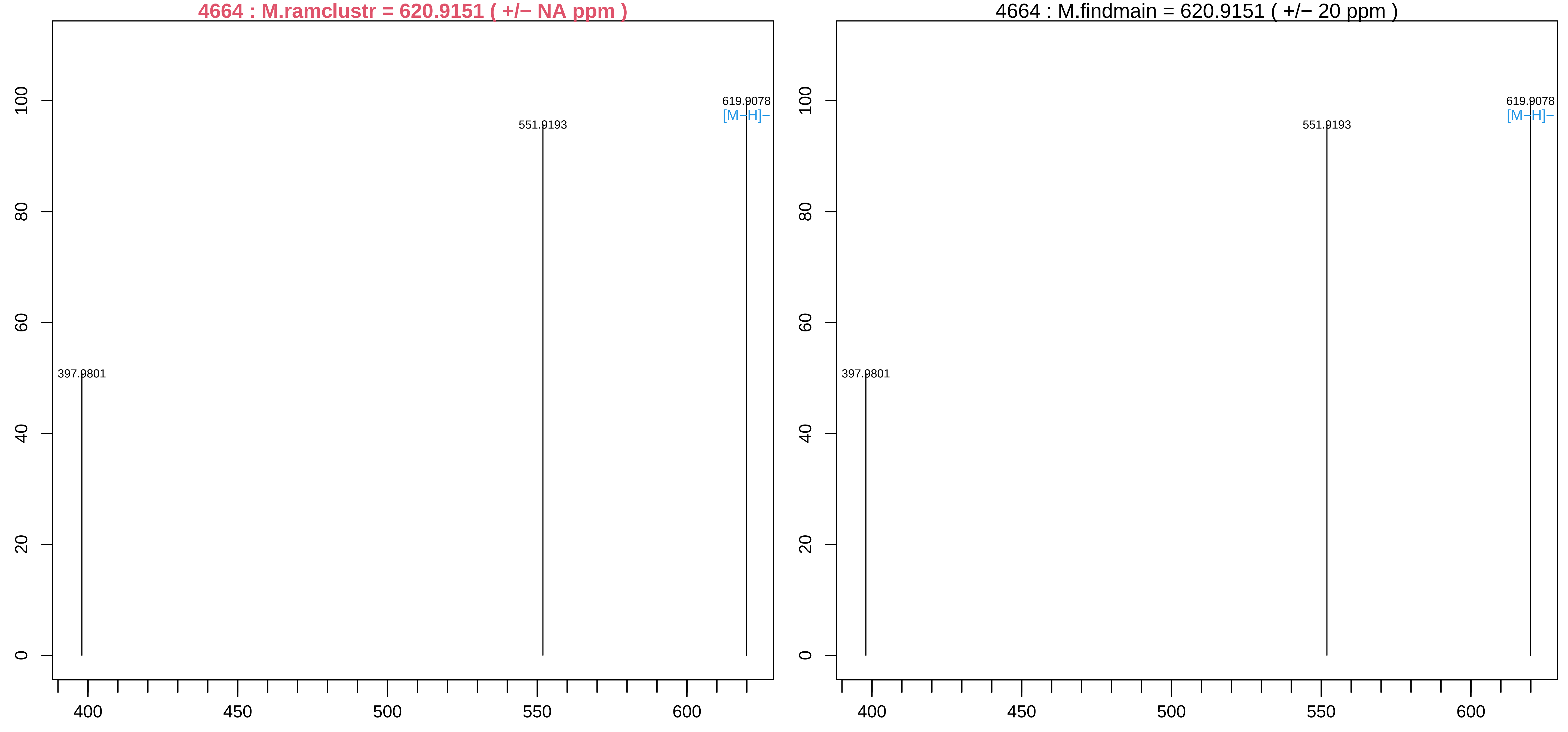
4664 : M.ramclustr = 620.9151 (+/− NA ppm)
397.9801
551.9193
619.9078
[M−H]−
0
20
40
60
80
100
400
450
500
550
600
4664 : M.findmain = 620.9151 (+/− 20 ppm)
397.9801
551.9193
619.9078
[M−H]−
0
20
40
60
80
100
400
450
500
550
600

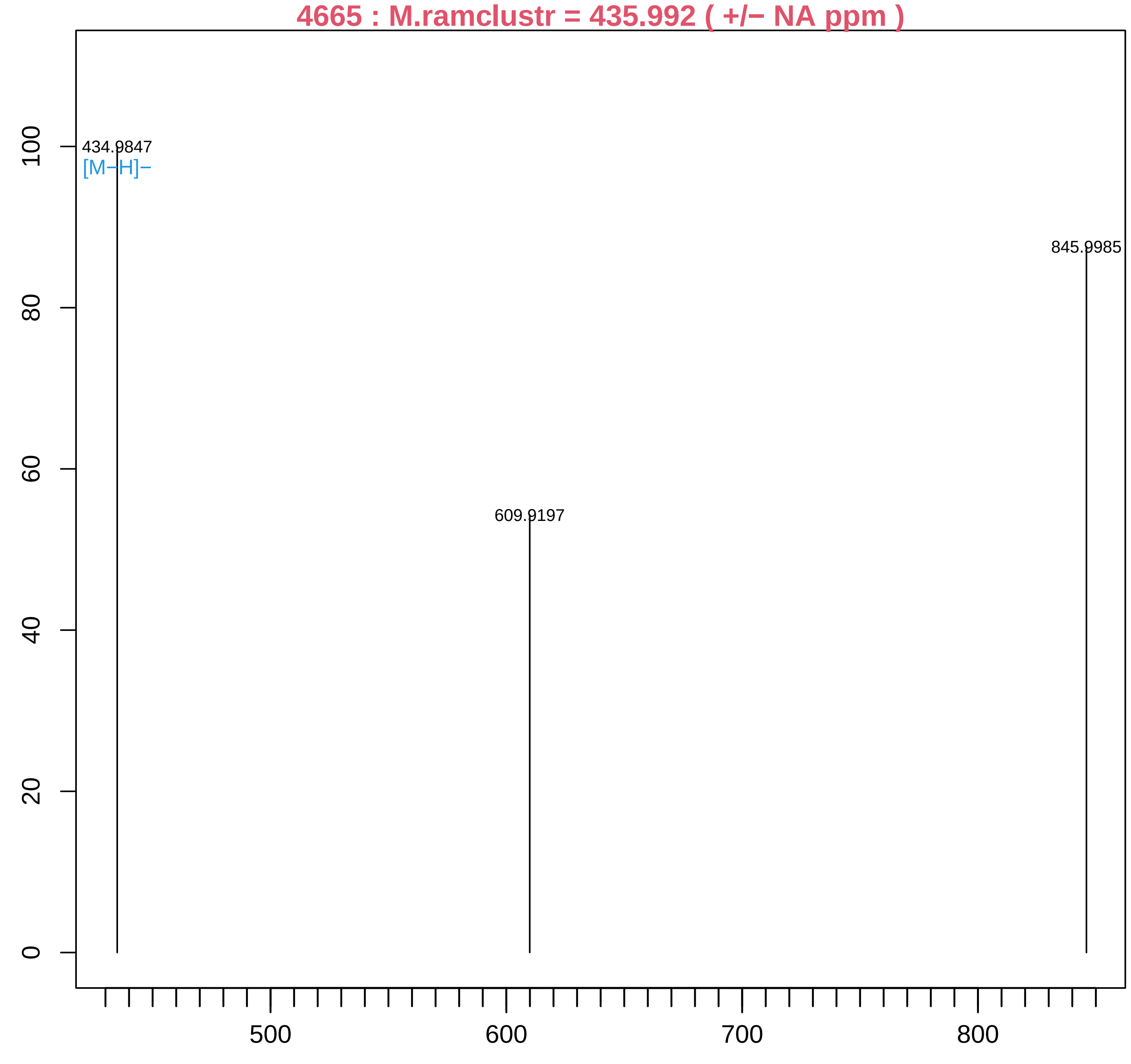
4665 : M.ramclustr = 435.992 (+/− NA ppm)
434.9847
[M−H]−
609.9197
845.9985
0
20
40
60
80
100
500
600
700
800

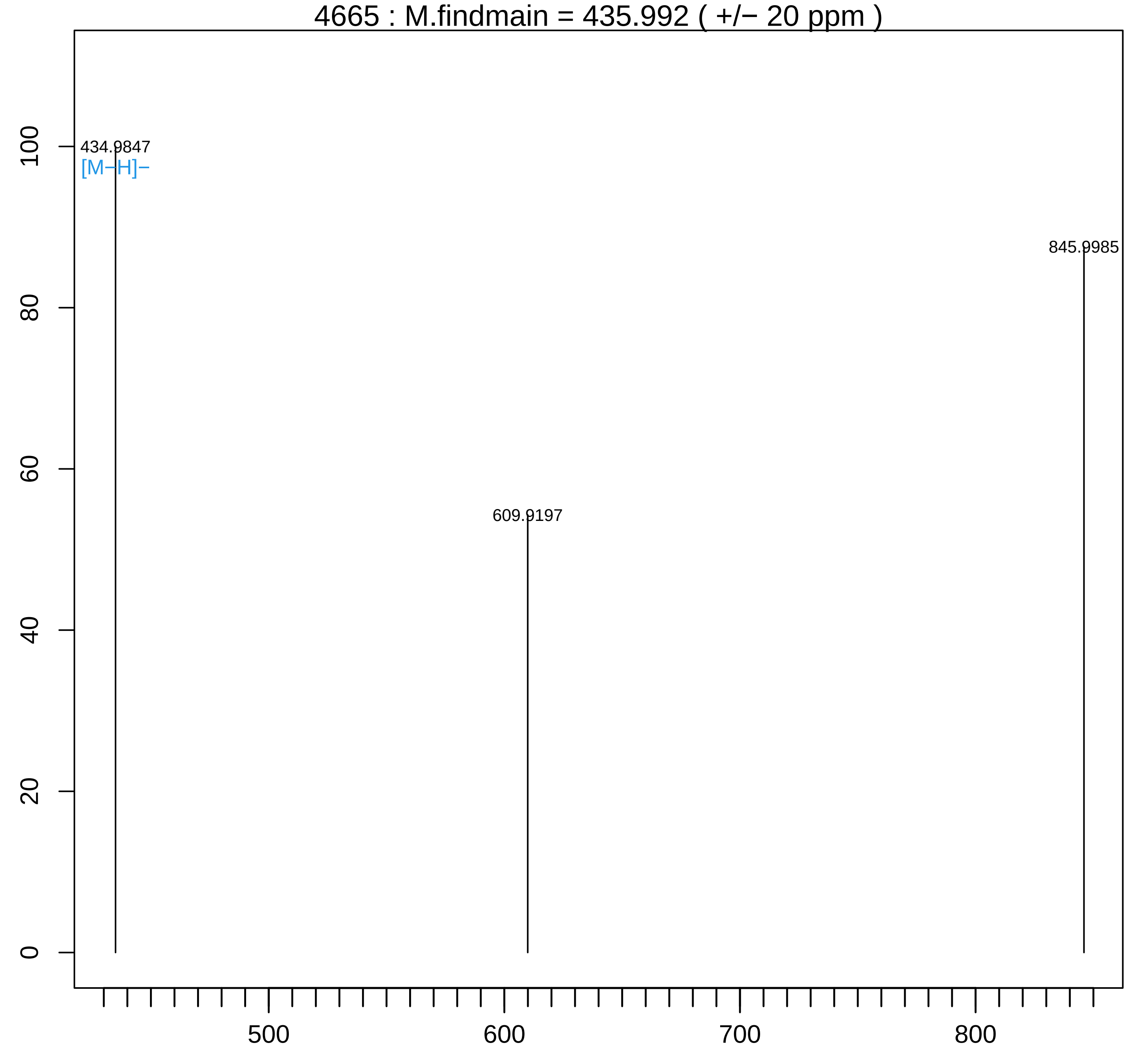
4665 : M.findmain = 435.992 (+/− 20 ppm)
434.9847
[M−H]−
609.9197
845.9985
0
20
40
60
80
100
500
600
700
800

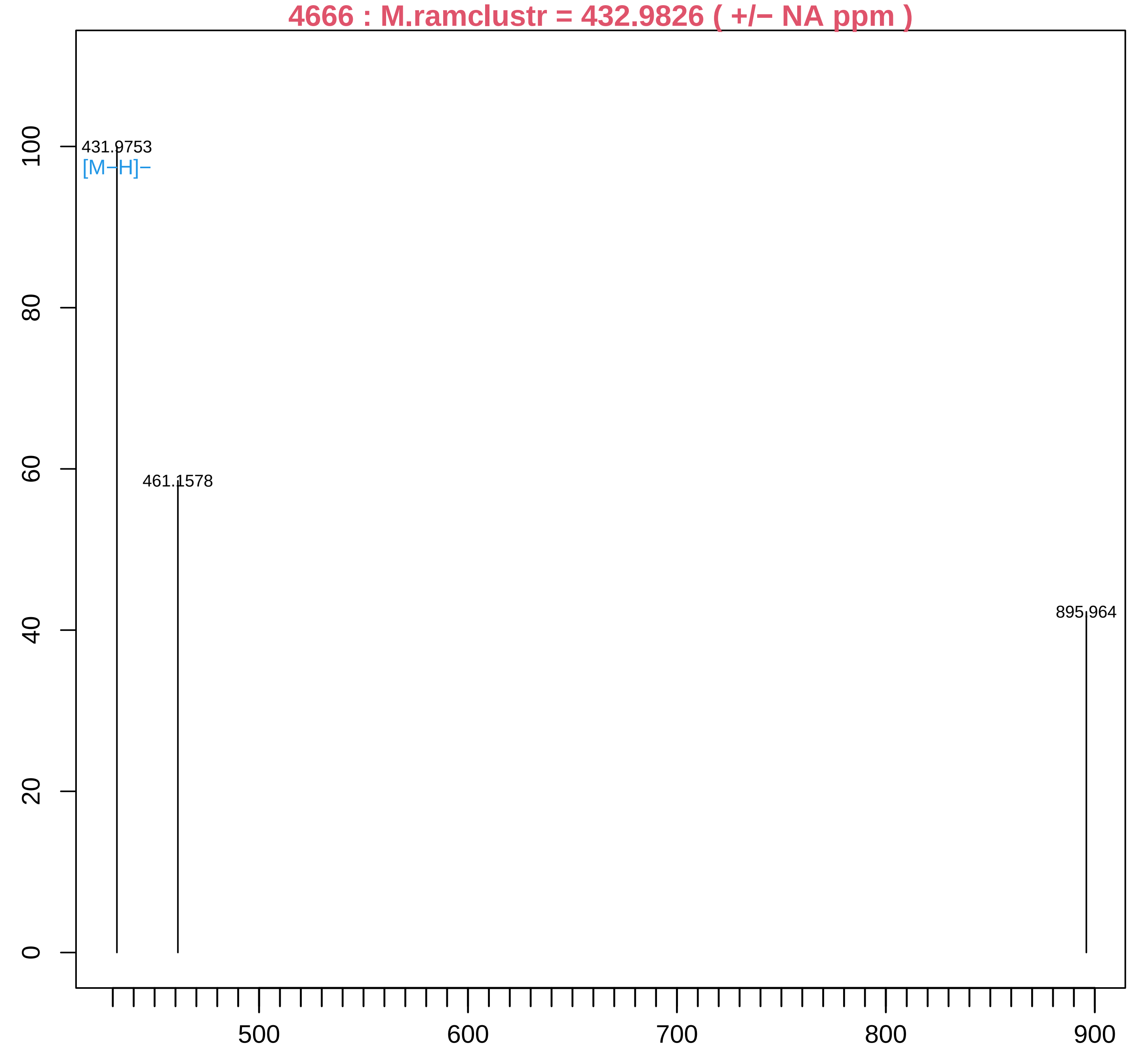
4666 : M.ramclustr = 432.9826 (+/− NA ppm)
431.9753
[M−H]−
461.1578
895.964
0
20
40
60
80
100
500
600
700
800
900

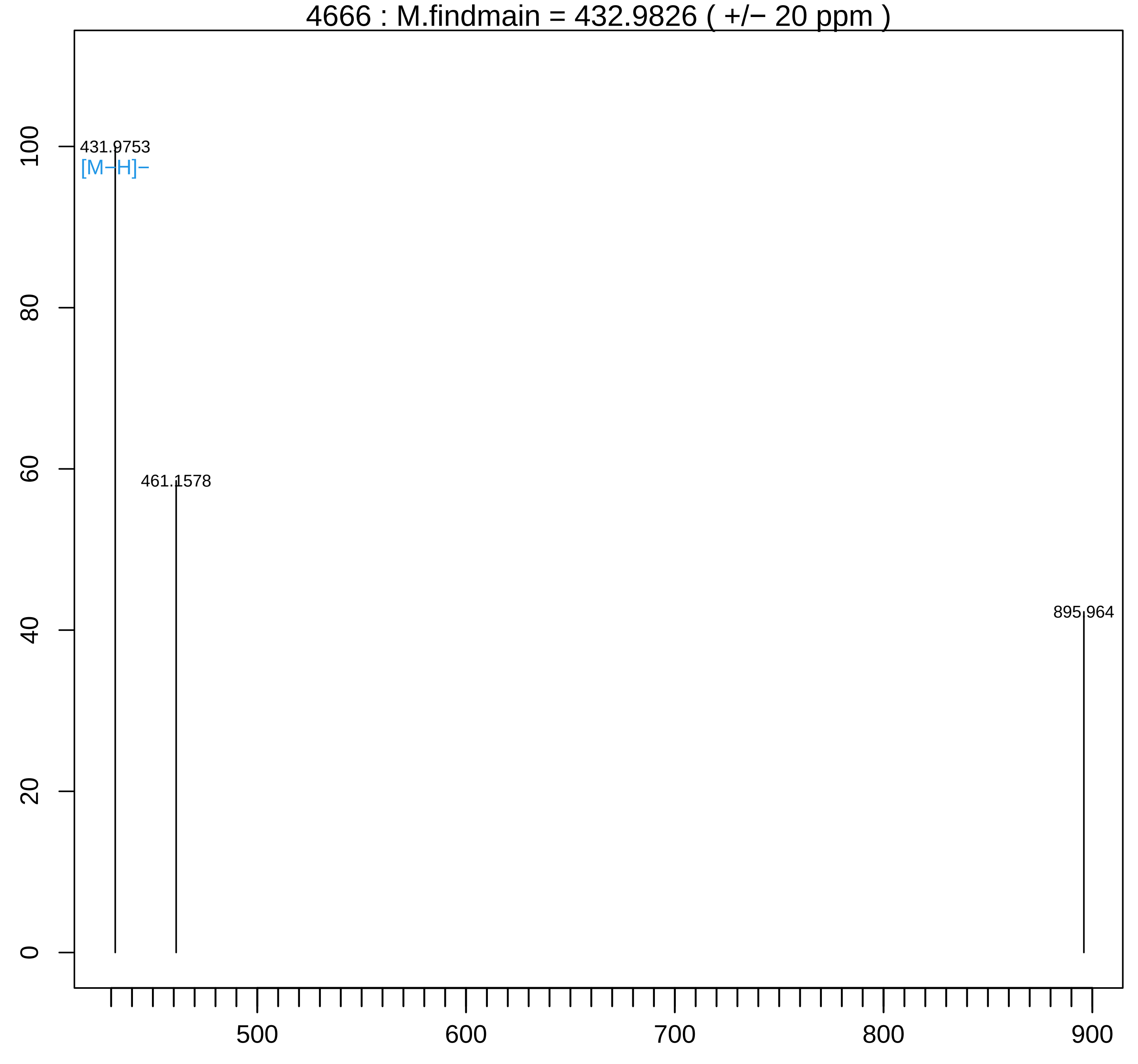
4666 : M.findmain = 432.9826 (+/− 20 ppm)
431.9753
[M−H]−
461.1578
895.964
0
20
40
60
80
100
500
600
700
800
900

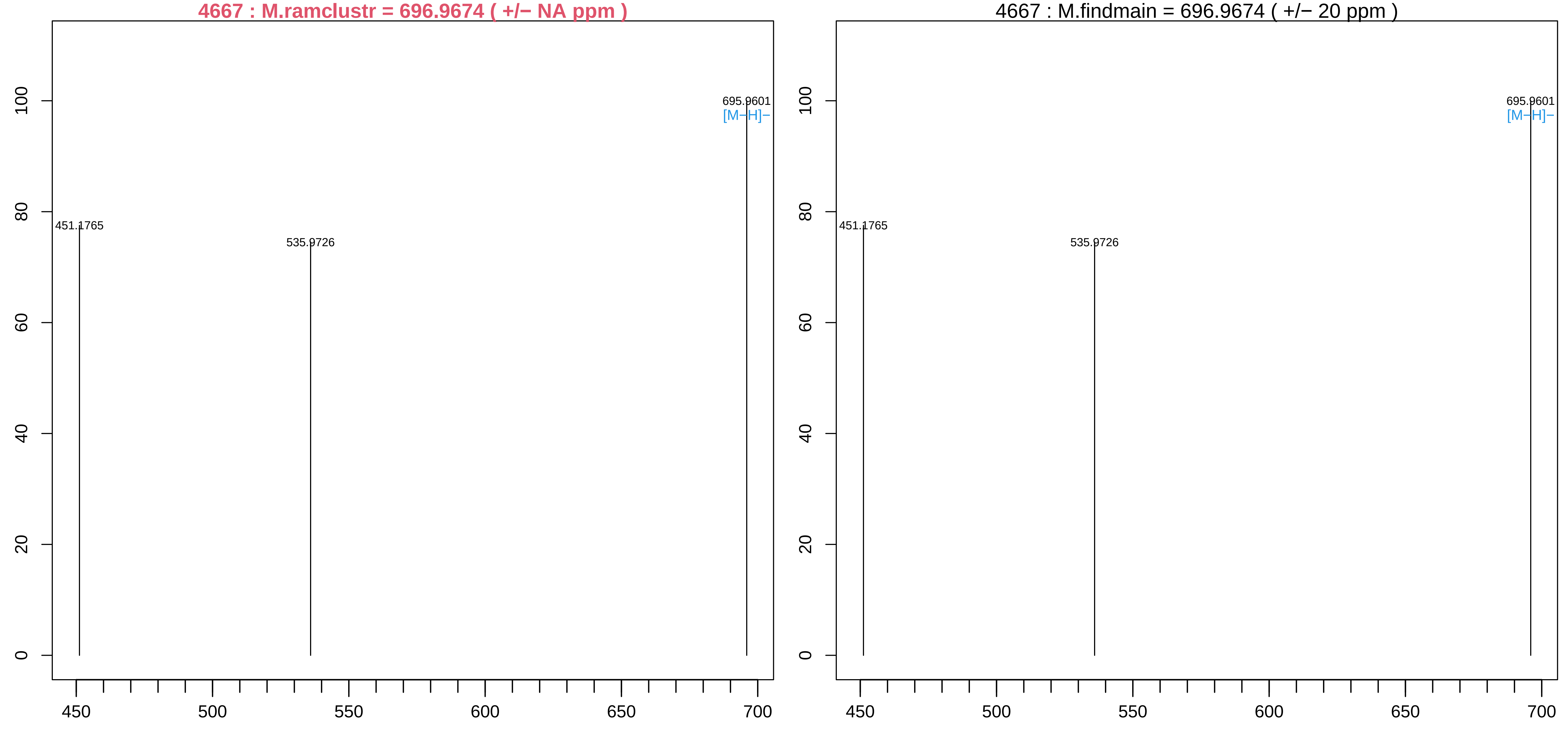
4667 : M.ramclustr = 696.9674 (+/− NA ppm)
4667 : M.findmain = 696.9674 (+/− 20 ppm)
451.1765
535.9726
695.9601
[M−H]−
0
20
40
60
80
100
450
500
550
600
650
700

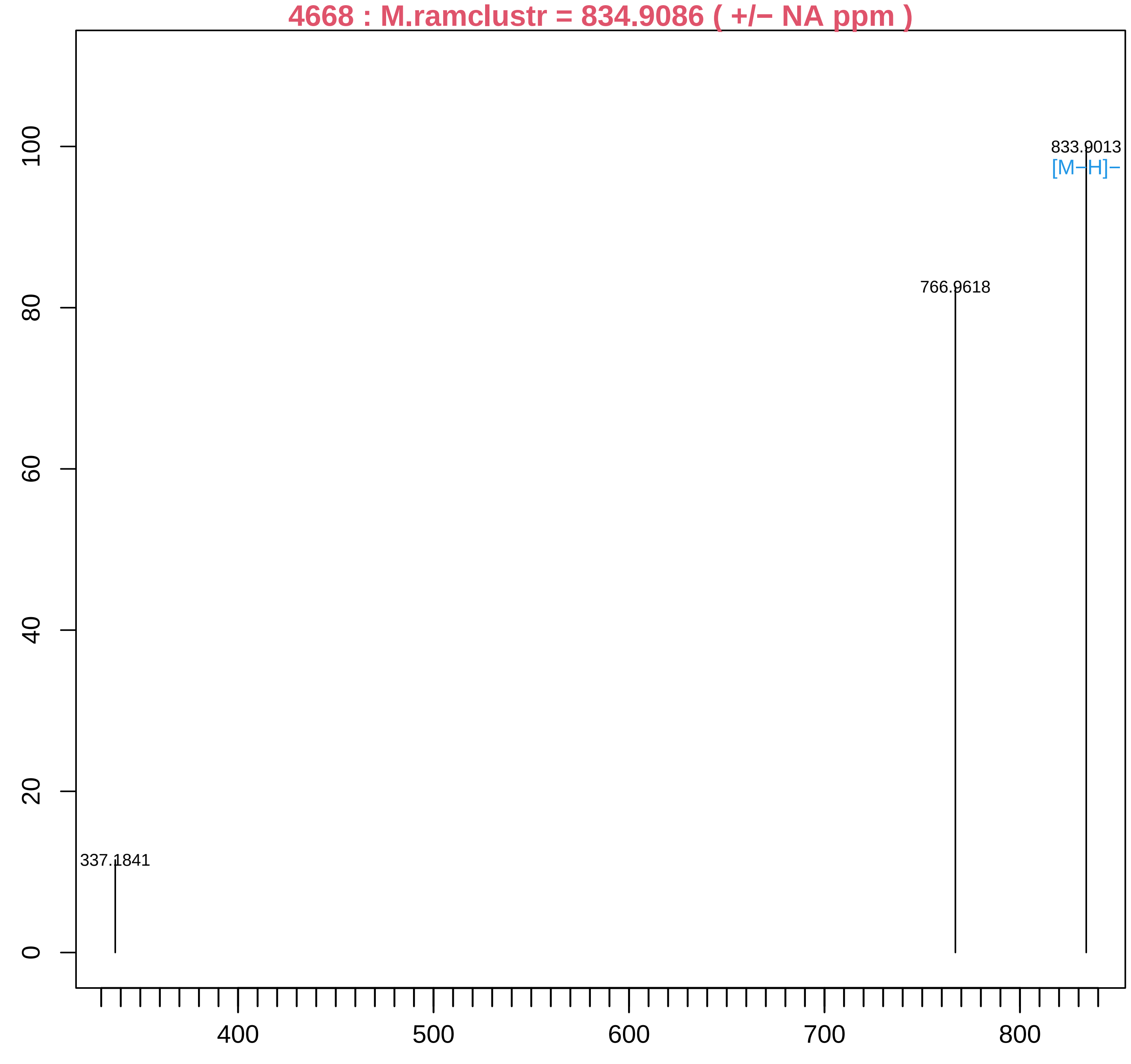

4668 : M.ramclustr = 834.9086 (+/− NA ppm)
100
80
60
40
20
0
400
500
600
700
800
833.9013
[M−H]−
766.9618
337.1841

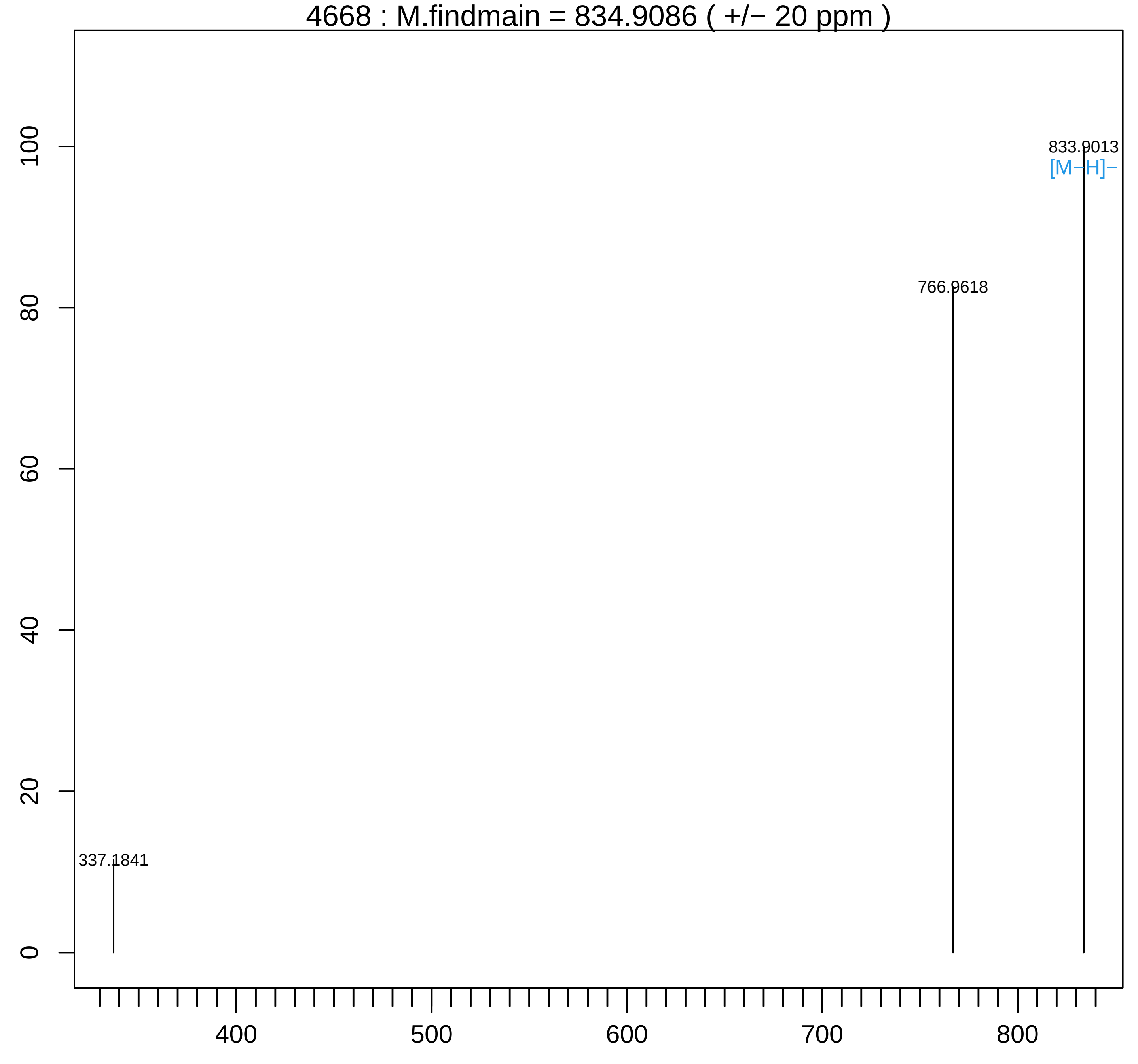

4668 : M.findmain = 834.9086 (+/− 20 ppm)
100
80
60
40
20
0
400
500
600
700
800
833.9013
[M−H]−
766.9618
337.1841

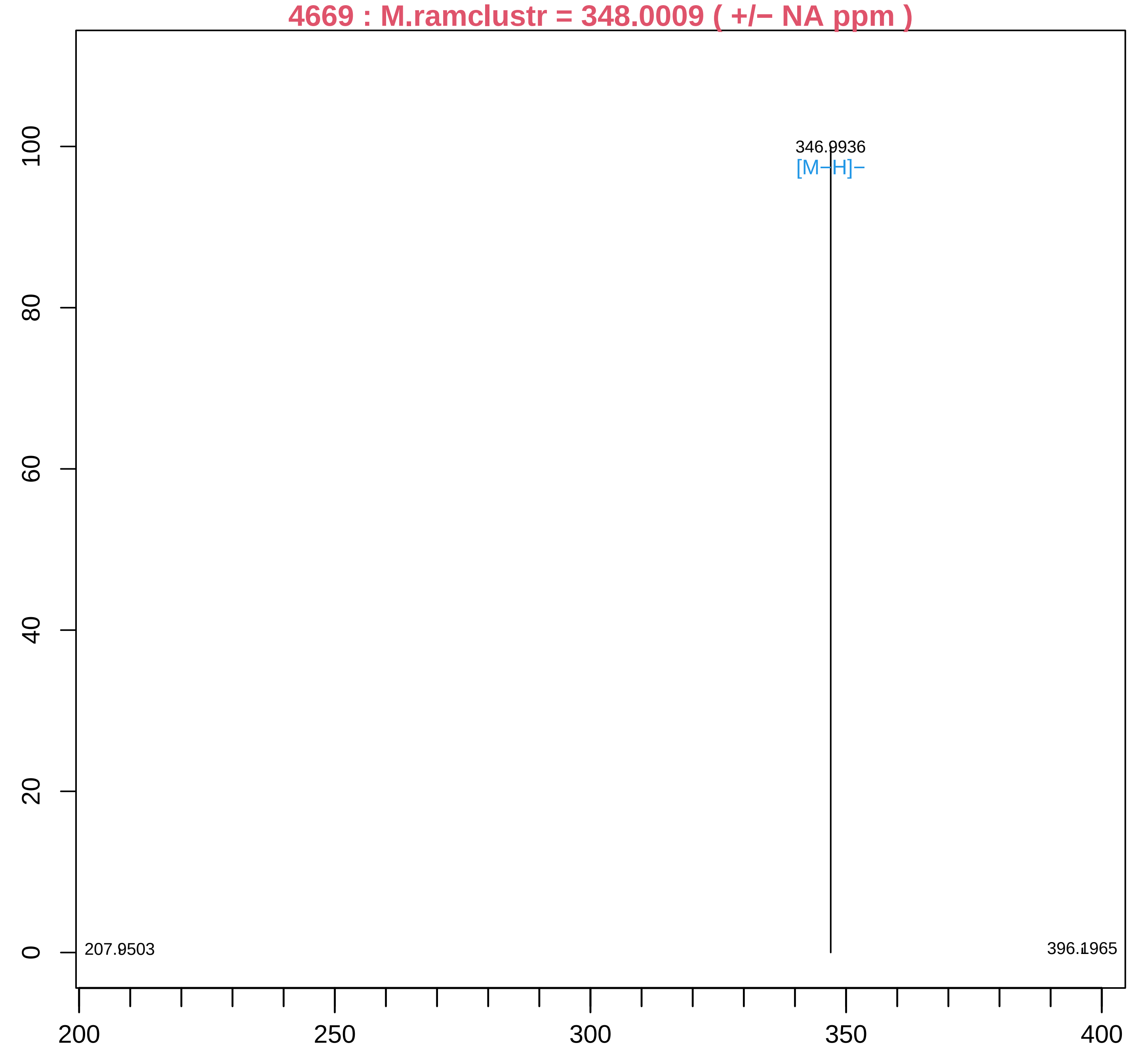
4669 : M.ramclustr = 348.0009 (+/− NA ppm)
346.9936
[M−H]−
207.9503
396.1965

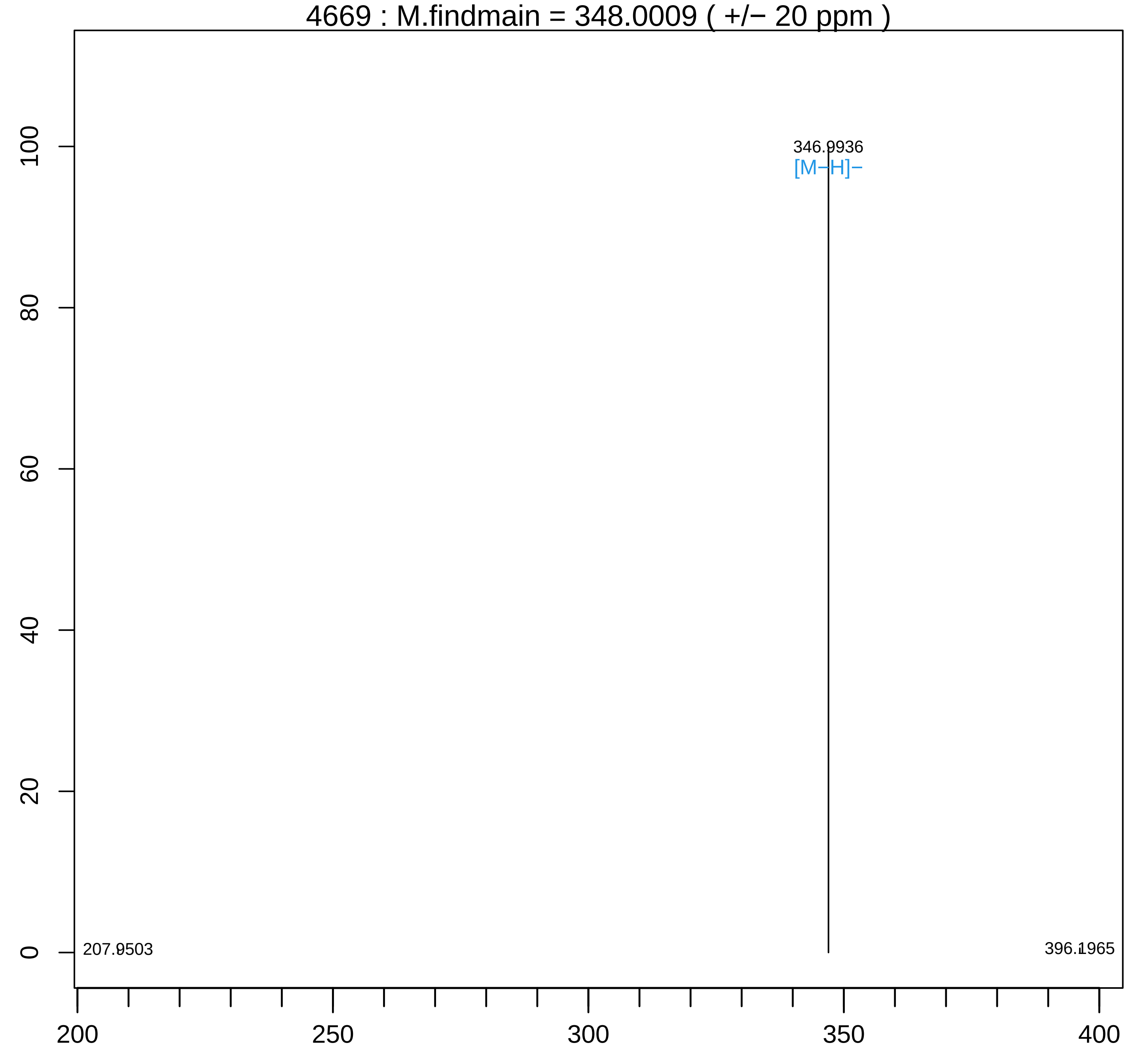
4669 : M.findmain = 348.0009 (+/− 20 ppm)
346.9936
[M−H]−
207.9503
396.1965

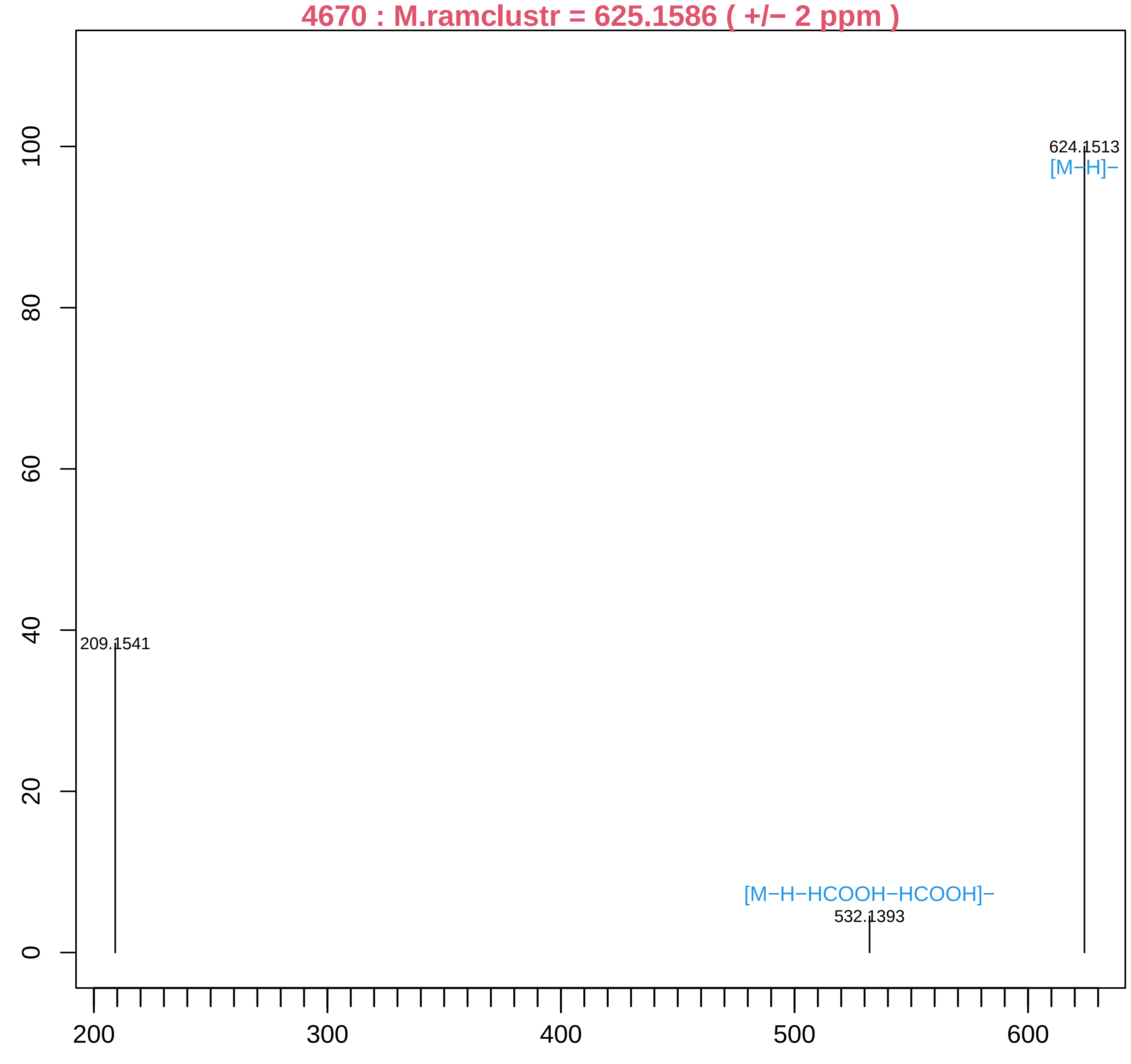
4670 : M.ramclustr = 625.1586 (+/− 2 ppm)
624.1513
[M−H]−
209.1541
[M−H−HCOOH−HCOOH]−
532.1393
0
20
40
60
80
100
200
300
400
500
600

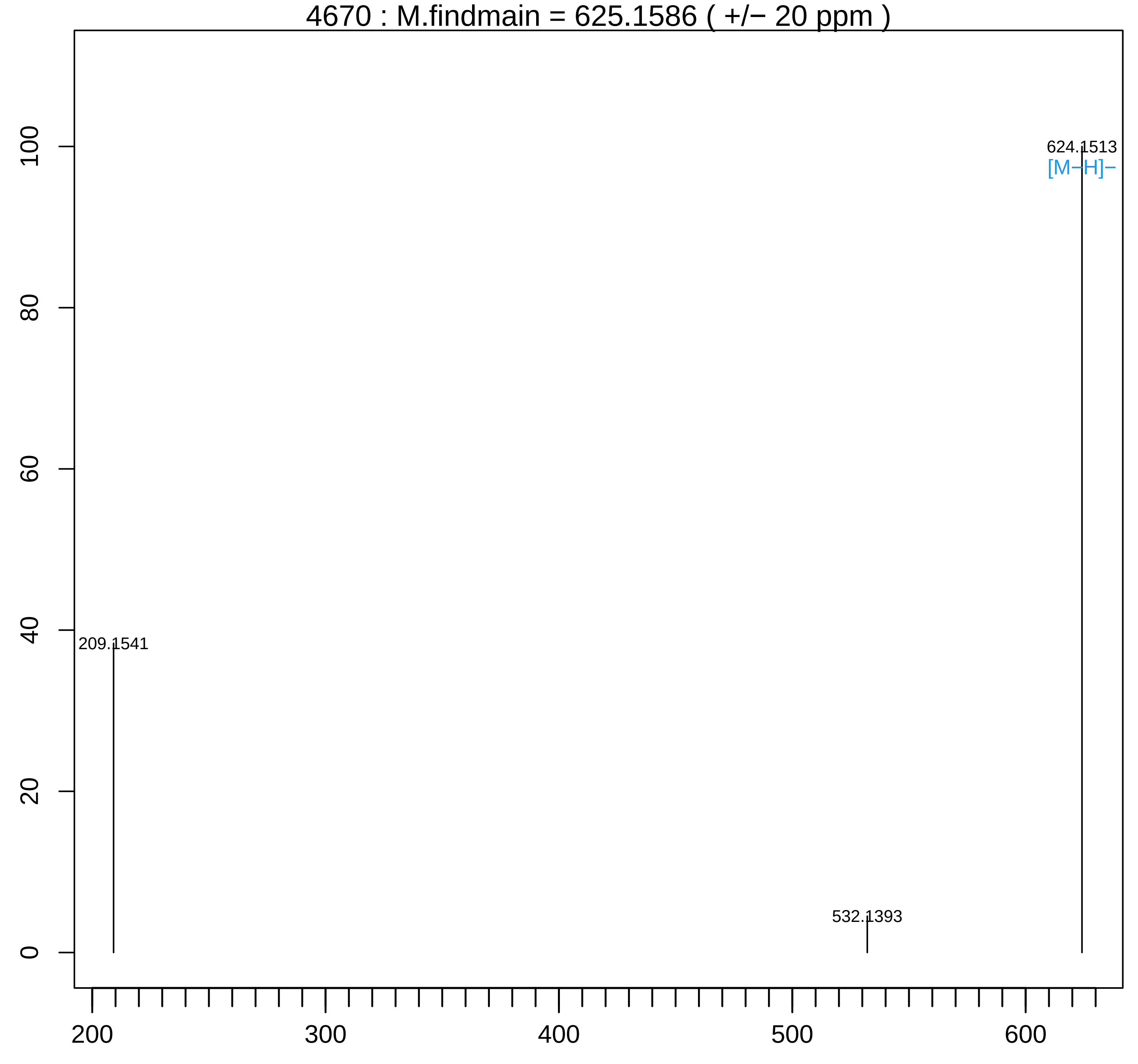
4670 : M.findmain = 625.1586 (+/− 20 ppm)
624.1513
[M−H]−
209.1541
532.1393
0
20
40
60
80
100
200
300
400
500
600

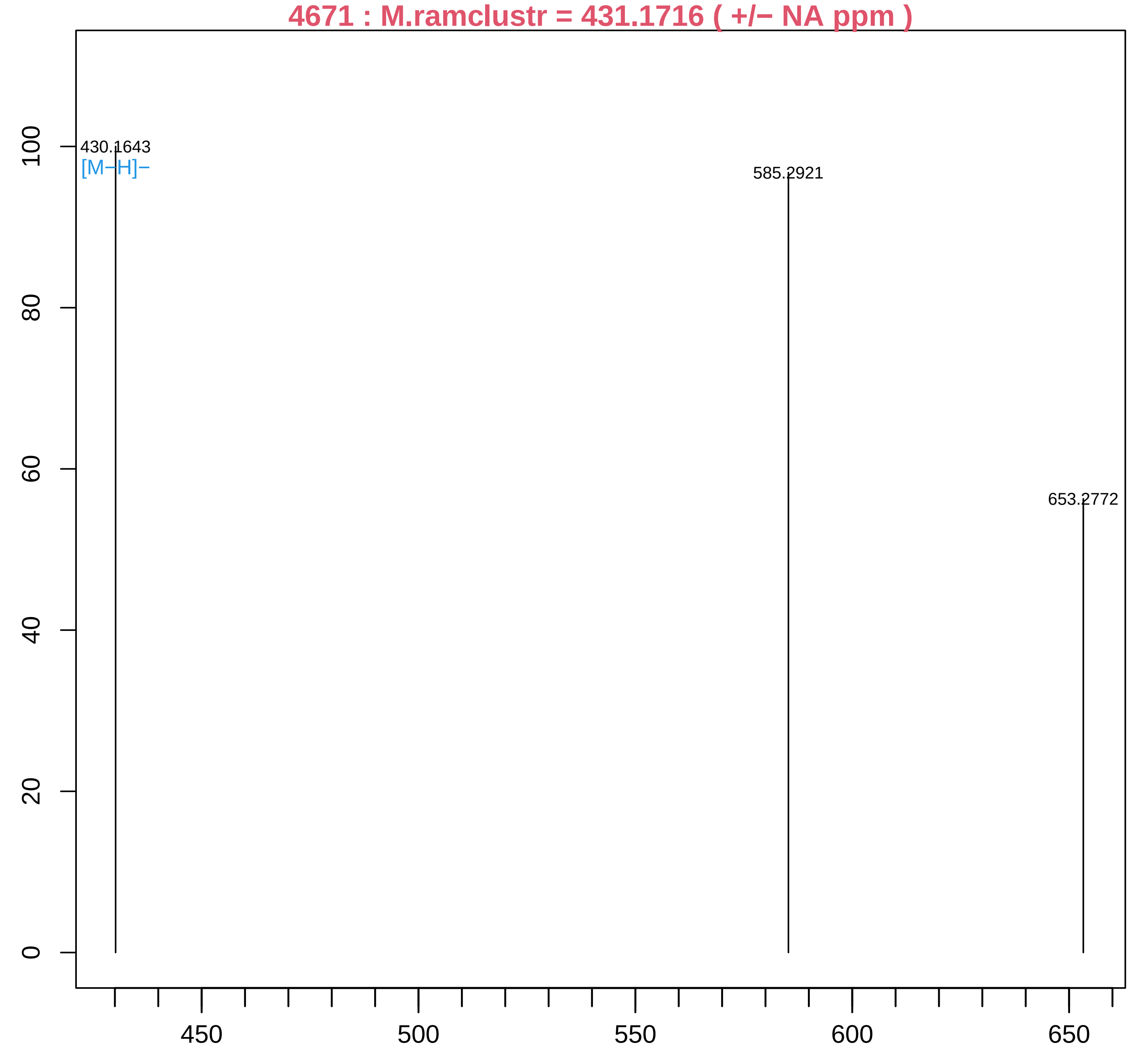
4671 : M.ramclustr = 431.1716 (+/− NA ppm)
430.1643
[M−H]−
585.2921
653.2772
0
20
40
60
80
100
450
500
550
600
650

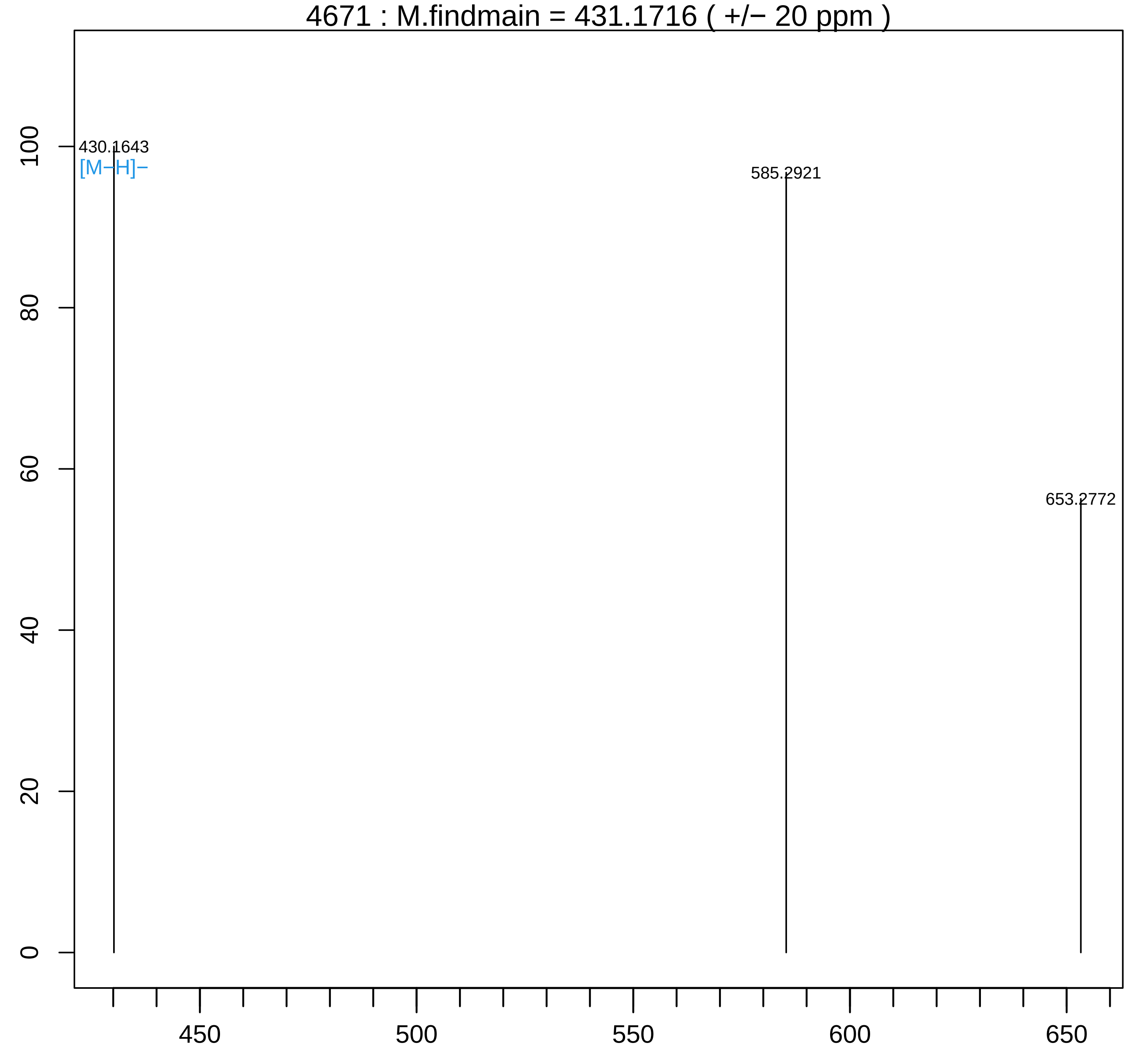
4671 : M.findmain = 431.1716 (+/− 20 ppm)
430.1643
[M−H]−
585.2921
653.2772
0
20
40
60
80
100
450
500
550
600
650

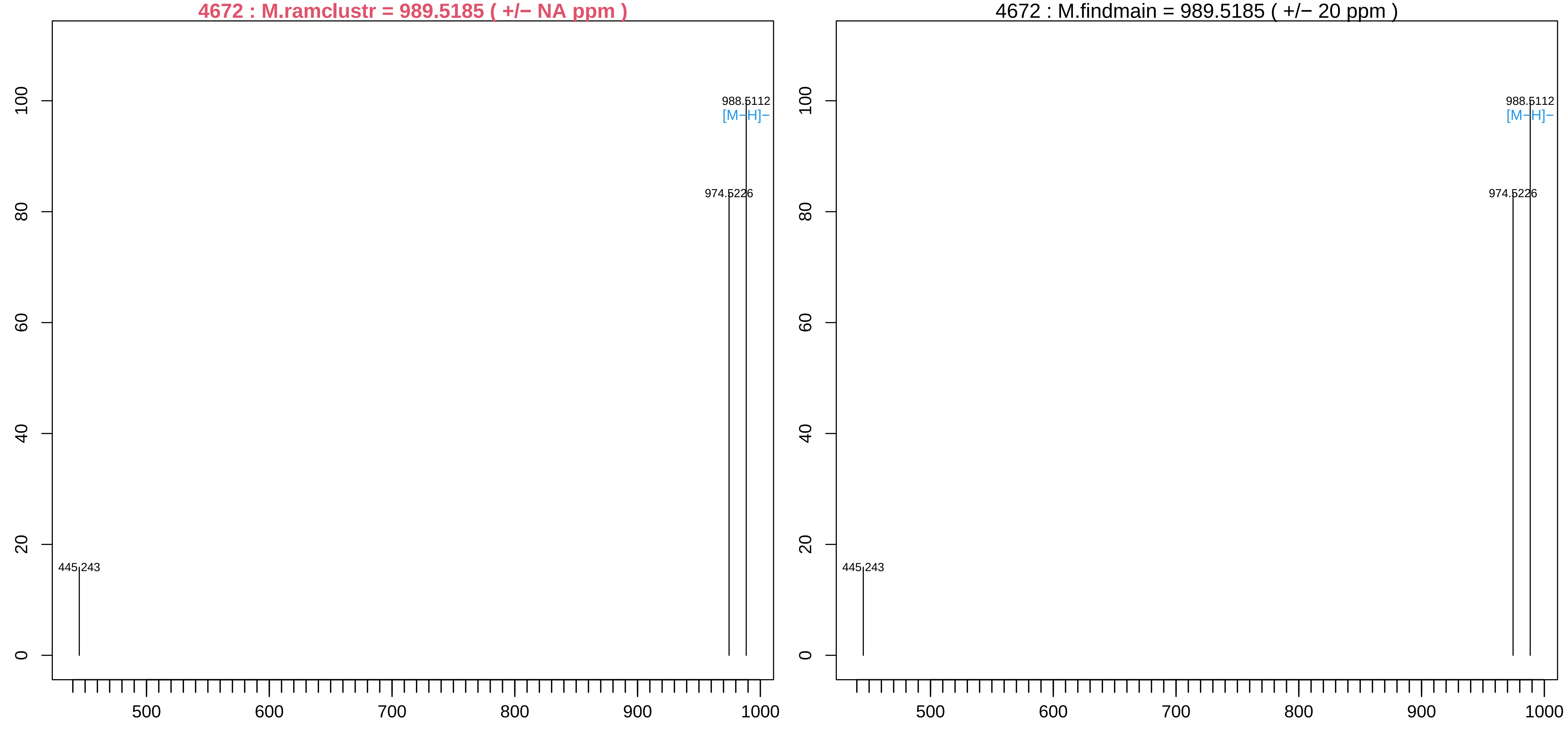

4672 : M.ramclustr = 989.5185 (+/− NA ppm)
988.5112
[M−H]−
974.5226
445.243
0
20
40
60
80
100
500
600
700
800
900
1000
4672 : M.findmain = 989.5185 (+/− 20 ppm)
988.5112
[M−H]−
974.5226
445.243
0
20
40
60
80
100
500
600
700
800
900
1000

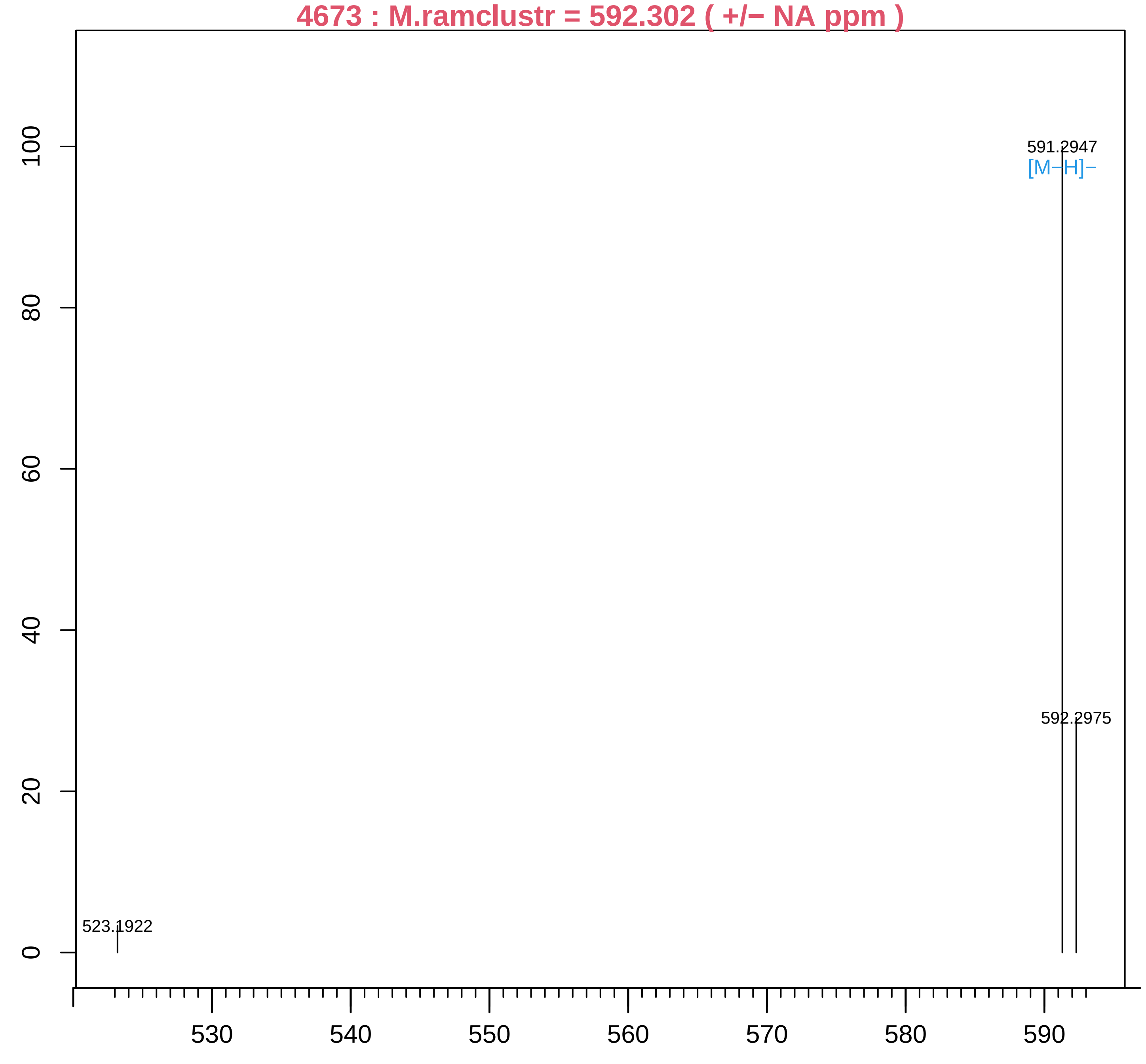
4673 : M.ramclustr = 592.302 (+/− NA ppm)
591.2947
[M−H]−
592.2975
523.1922

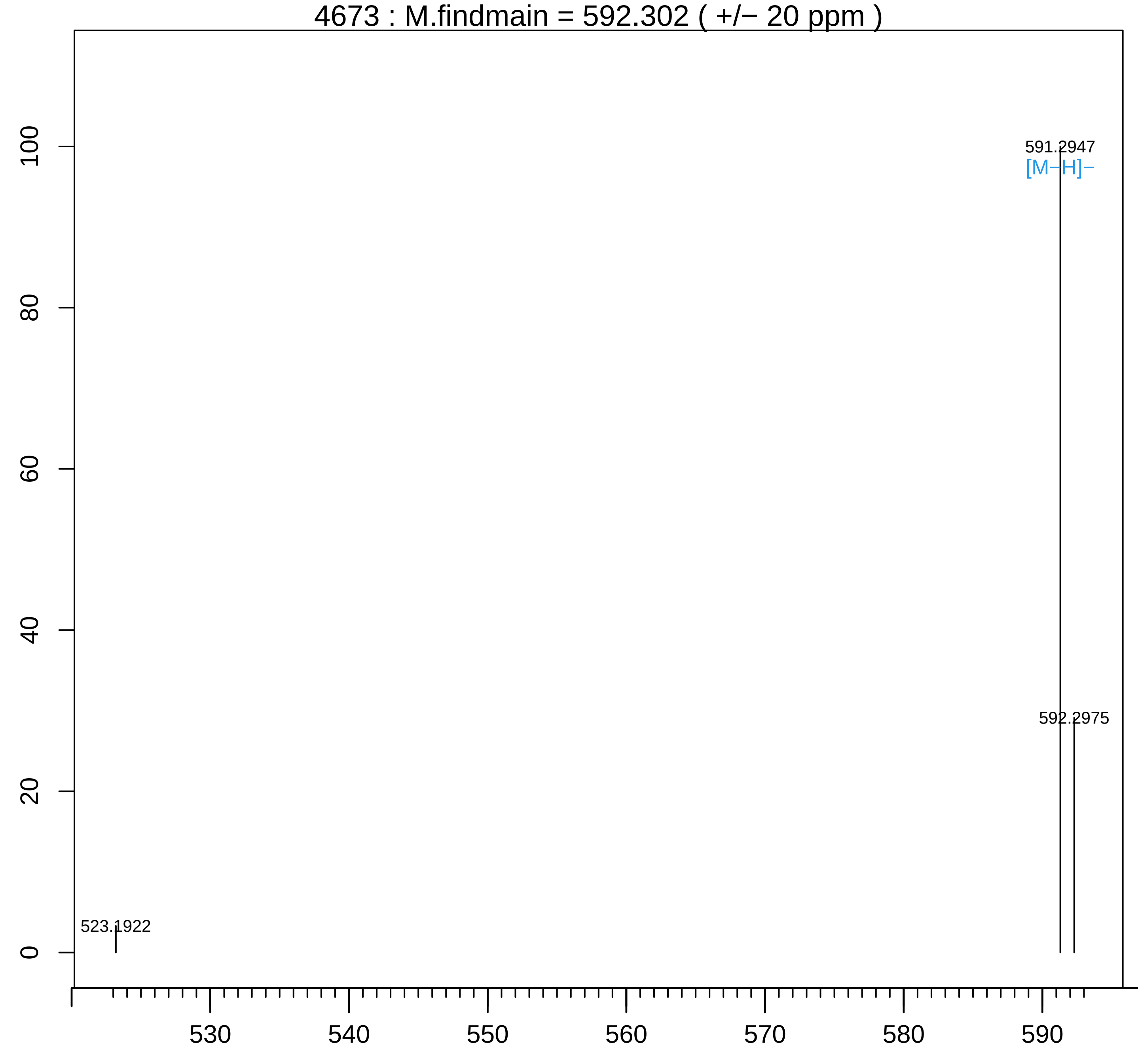
4673 : M.findmain = 592.302 (+/− 20 ppm)
591.2947
[M−H]−
592.2975
523.1922

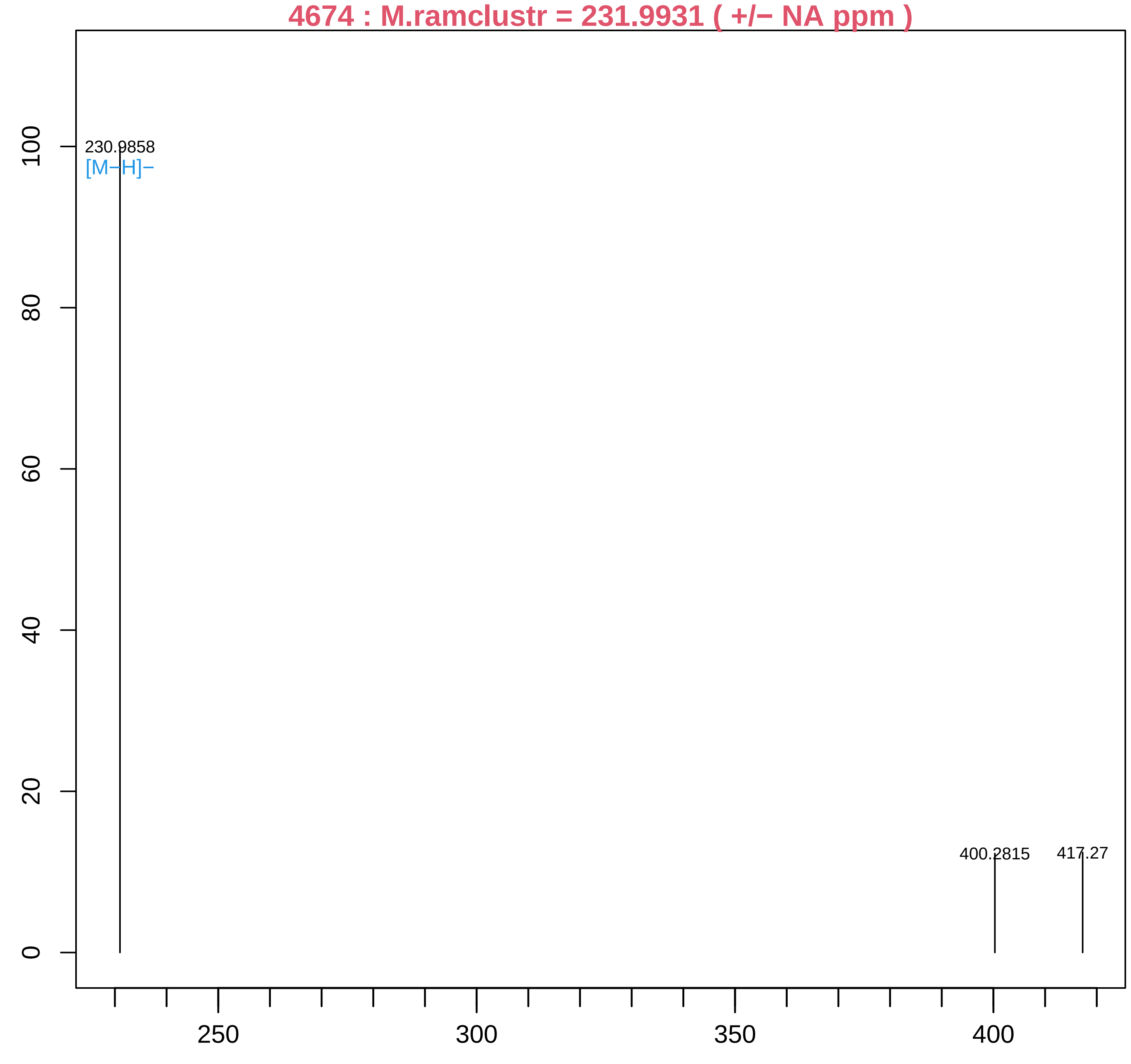
4674 : M.ramclustr = 231.9931 (+/− NA ppm)
230.9858
[M−H]−
400.2815
417.27
0
20
40
60
80
100
250
300
350
400

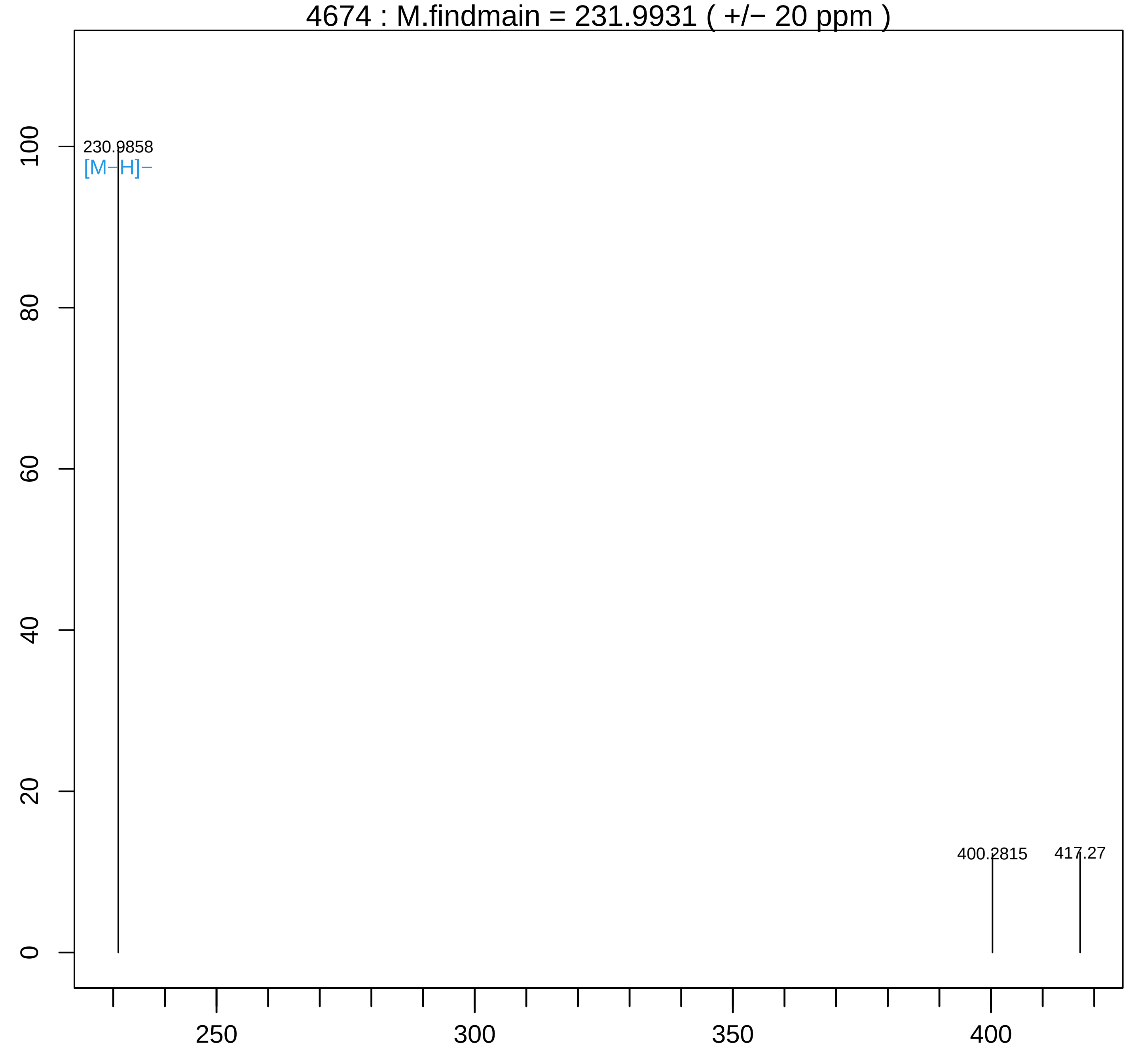
4674 : M.findmain = 231.9931 (+/− 20 ppm)
230.9858
[M−H]−
400.2815
417.27
0
20
40
60
80
100
250
300
350
400

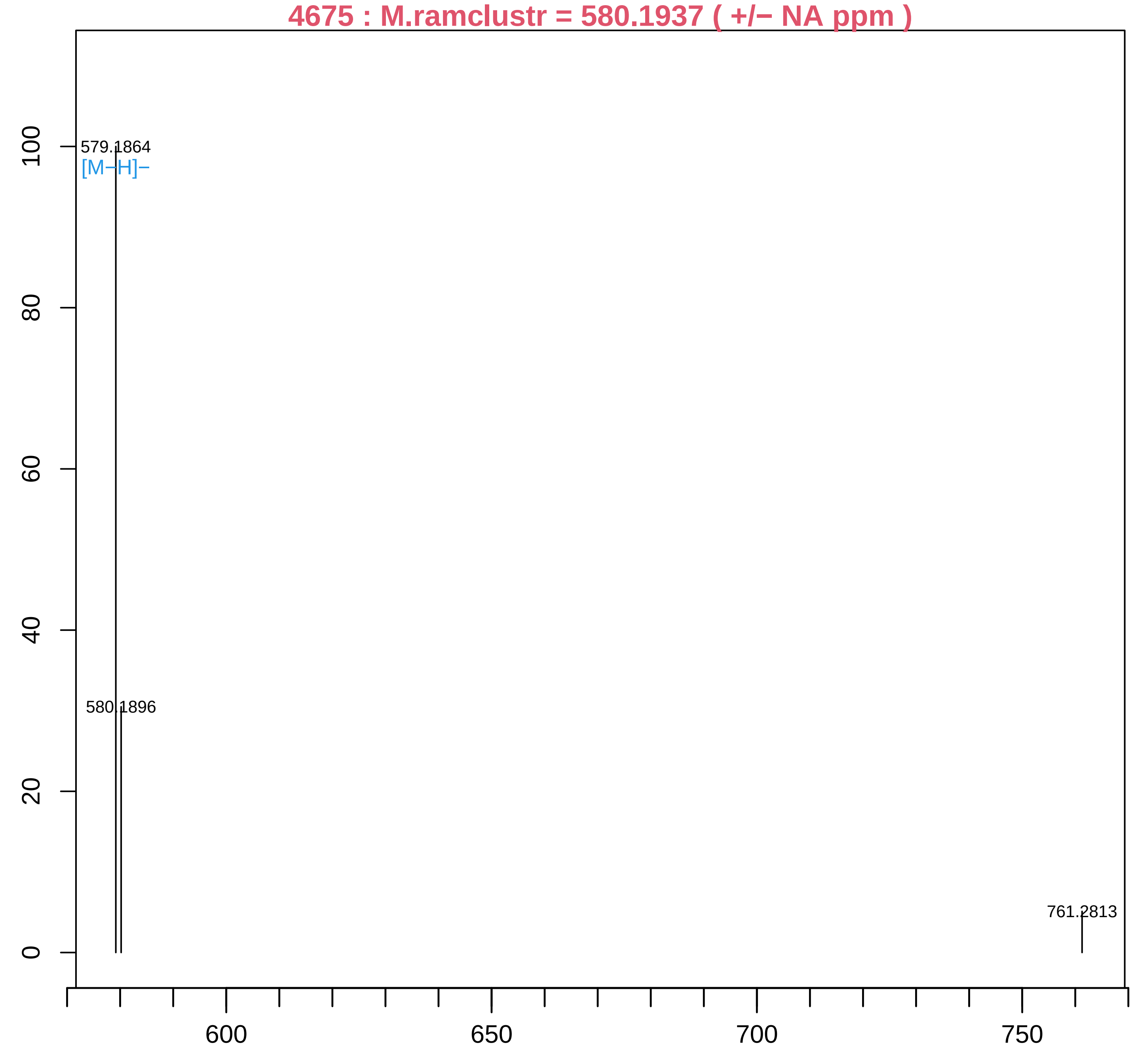
4675 : M.ramclustr = 580.1937 (+/− NA ppm)
579.1864
[M−H]−
580.1896
761.2813
0
20
40
60
80
100
600
650
700
750

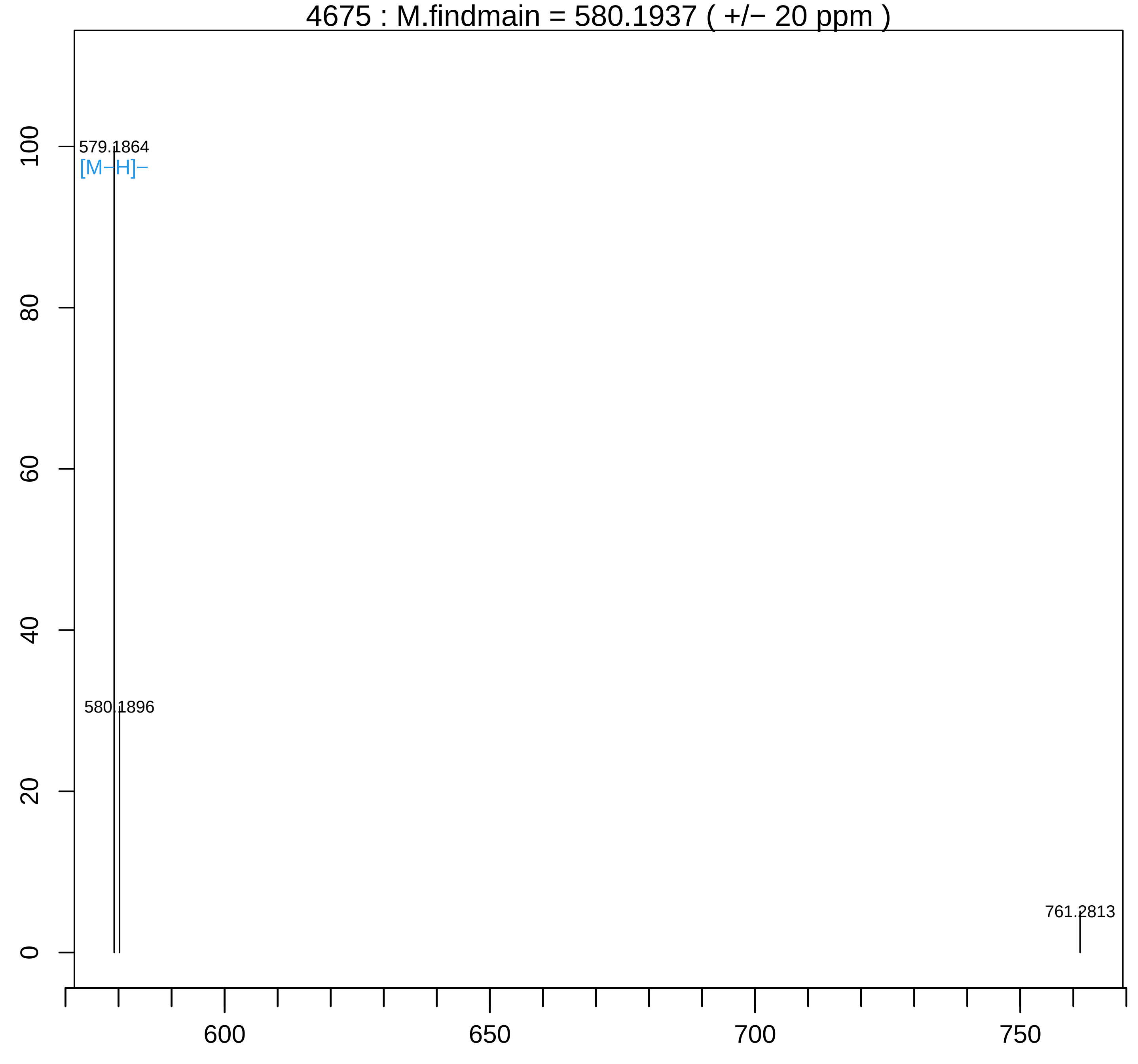
4675 : M.findmain = 580.1937 (+/− 20 ppm)
579.1864
[M−H]−
580.1896
761.2813
0
20
40
60
80
100
600
650
700
750

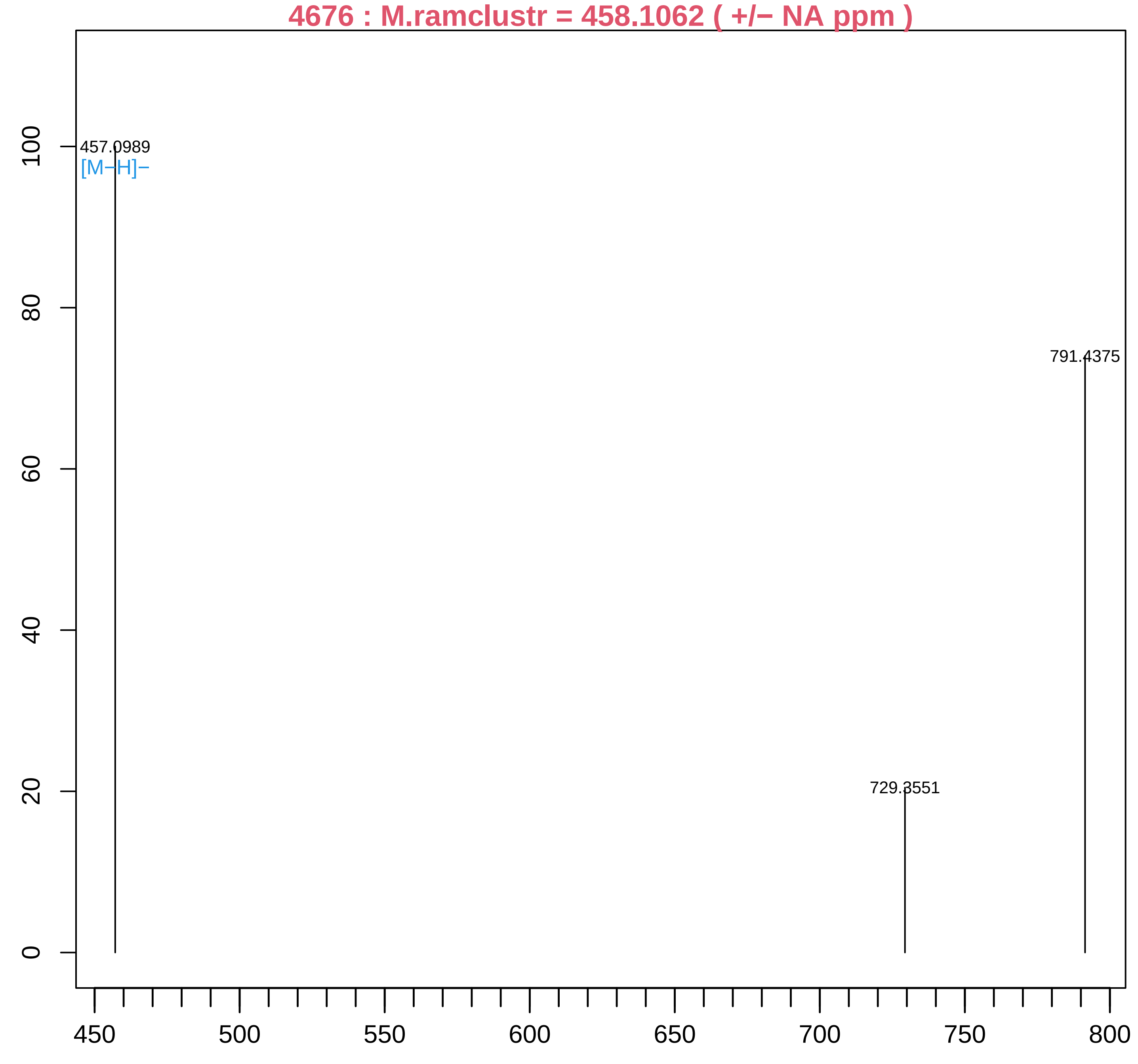

4676 : M.ramclustr = 458.1062 (+/− NA ppm)
457.0989
[M−H]−
791.4375
729.3551
0
20
40
60
80
100
450
500
550
600
650
700
750
800

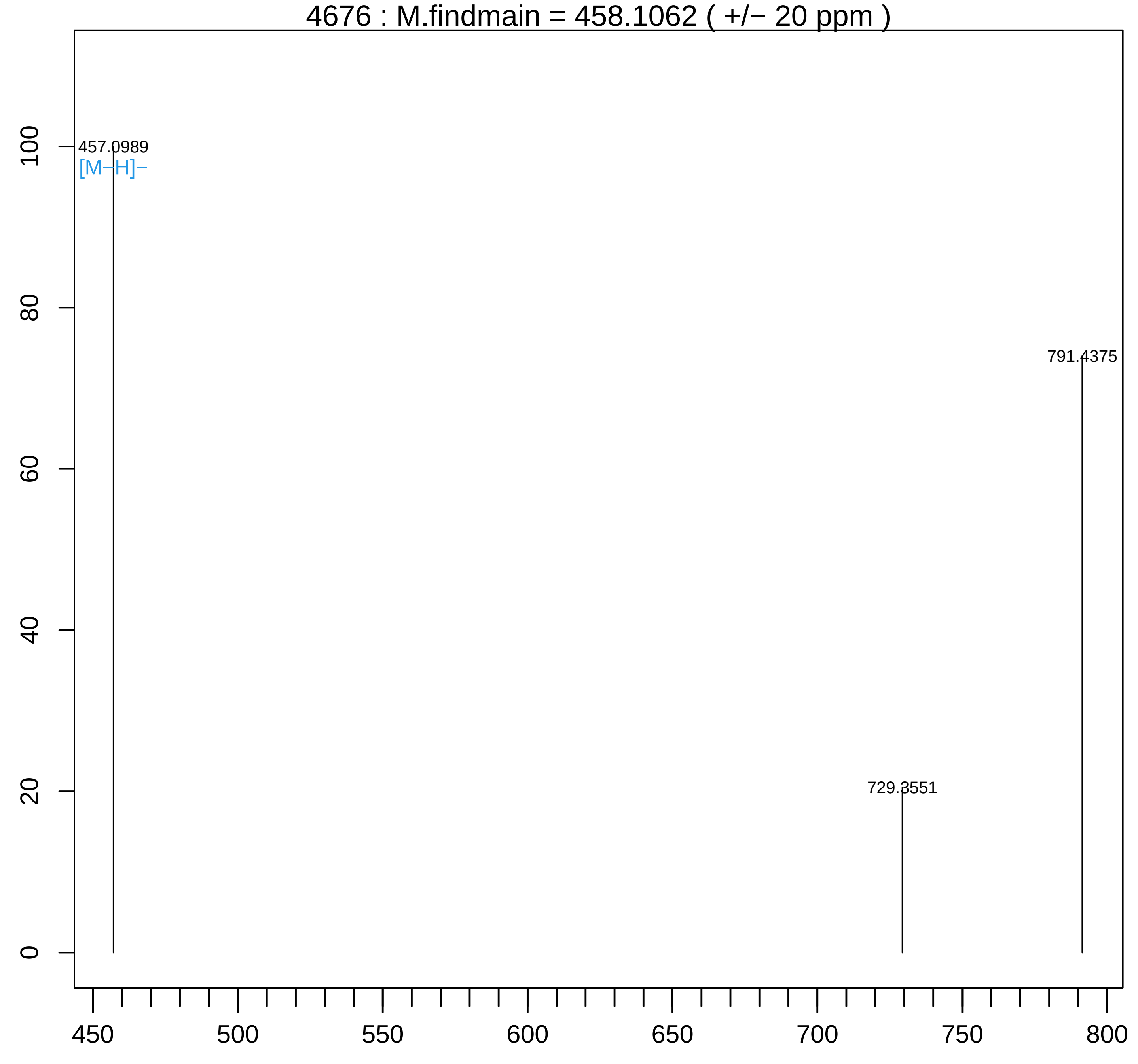

4676 : M.findmain = 458.1062 (+/− 20 ppm)
457.0989
[M−H]−
791.4375
729.3551
0
20
40
60
80
100
450
500
550
600
650
700
750
800

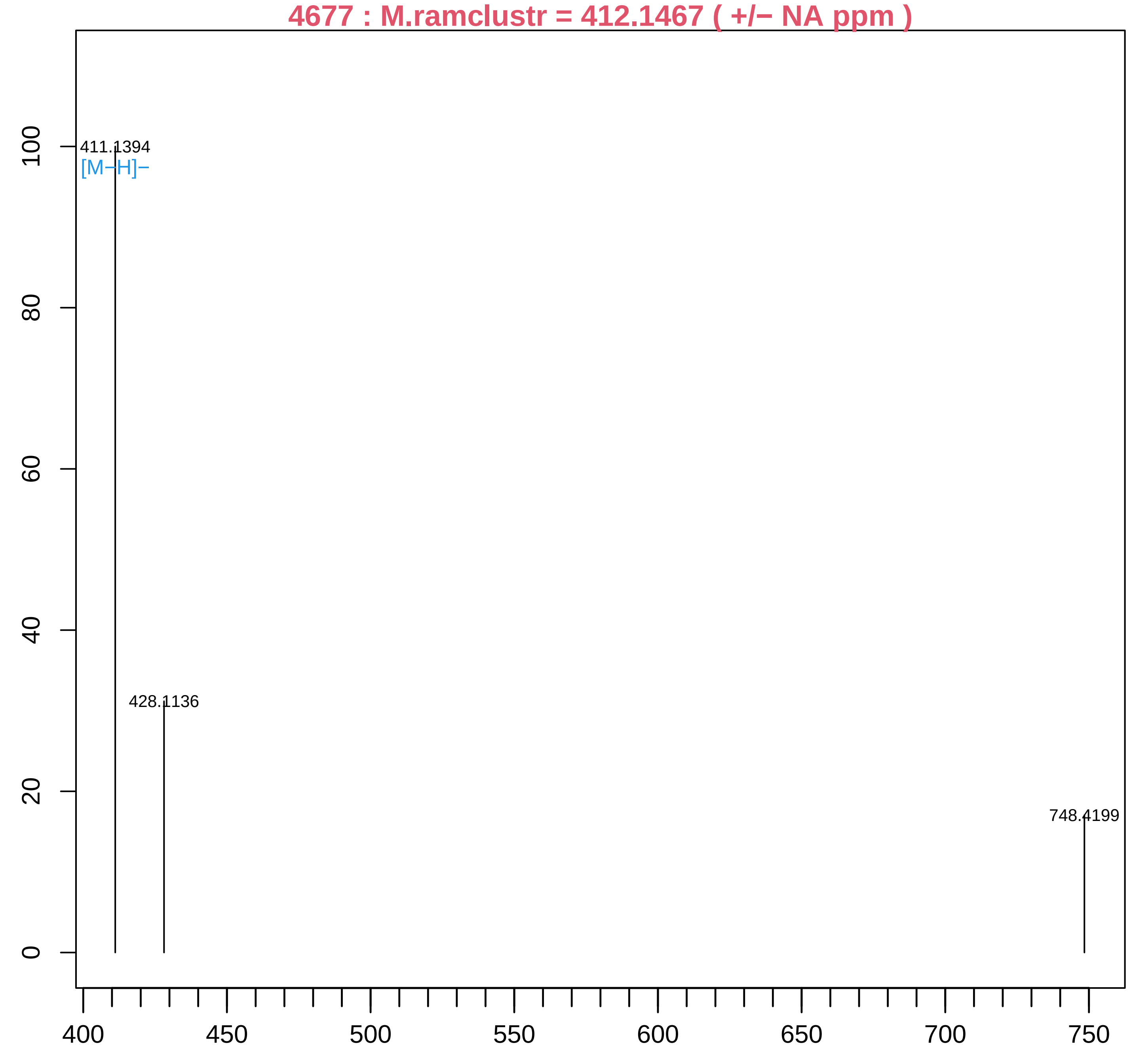
4677 : M.ramclustr = 412.1467 (+/− NA ppm)
411.1394
[M−H]−
428.1136
748.4199
0
20
40
60
80
100
400
450
500
550
600
650
700
750
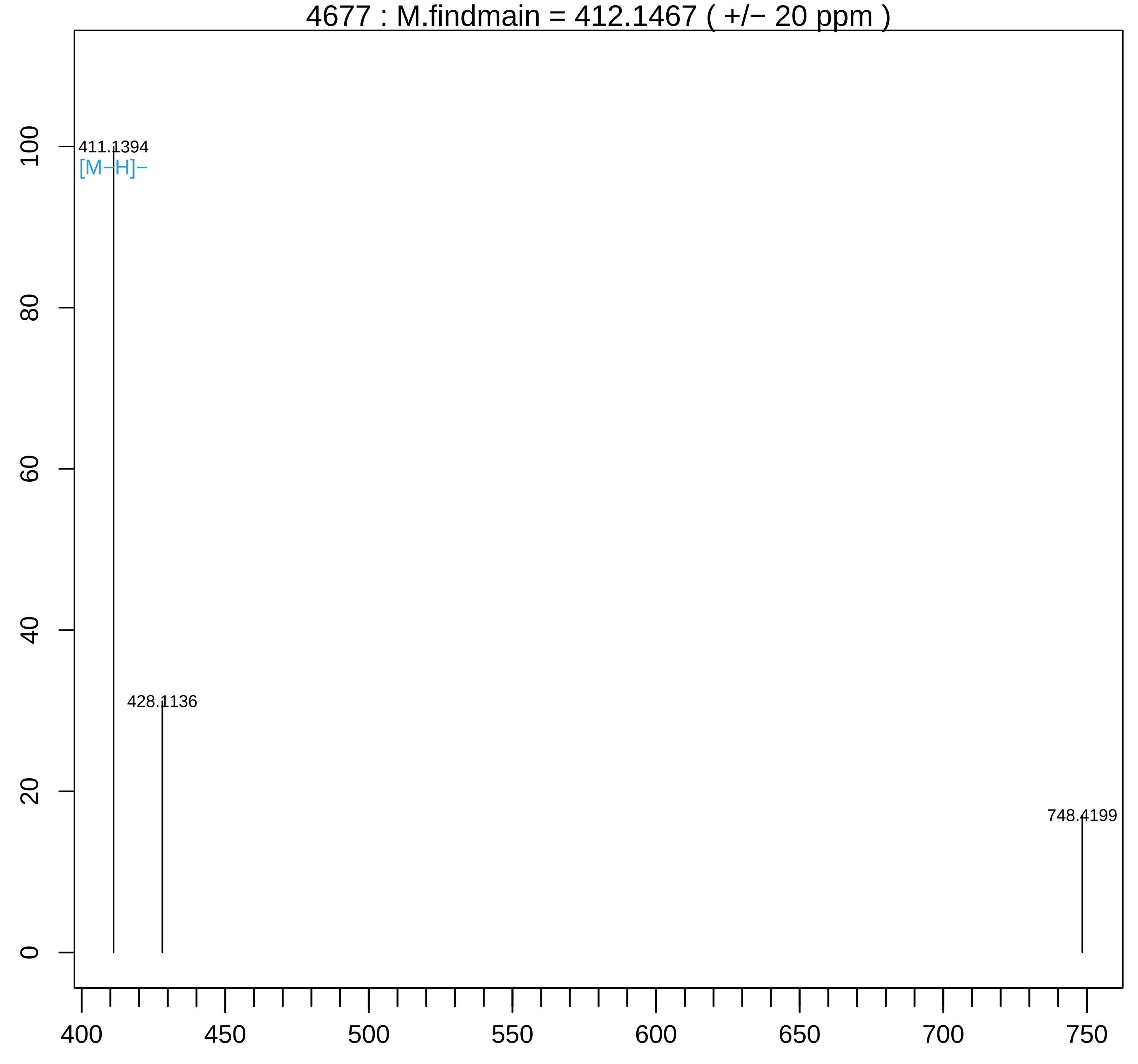
4677 : M.findmain = 412.1467 (+/− 20 ppm)
411.1394
[M−H]−
428.1136
748.4199
0
20
40
60
80
100
400
450
500
550
600
650
700
750

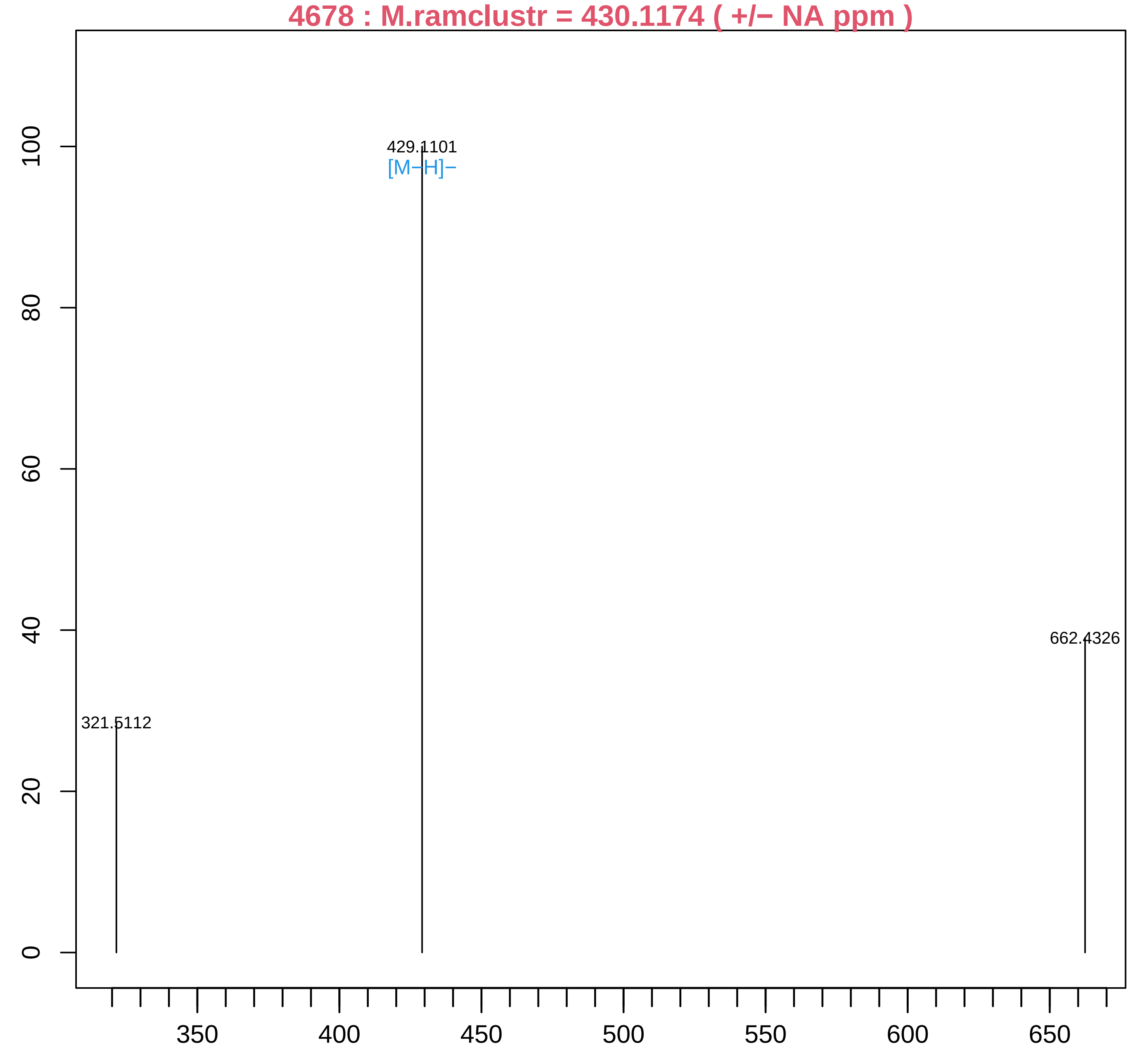
4678 : M.ramclustr = 430.1174 (+/− NA ppm)
429.1101
[M−H]−
662.4326
321.5112
0
20
40
60
80
100
350
400
450
500
550
600
650

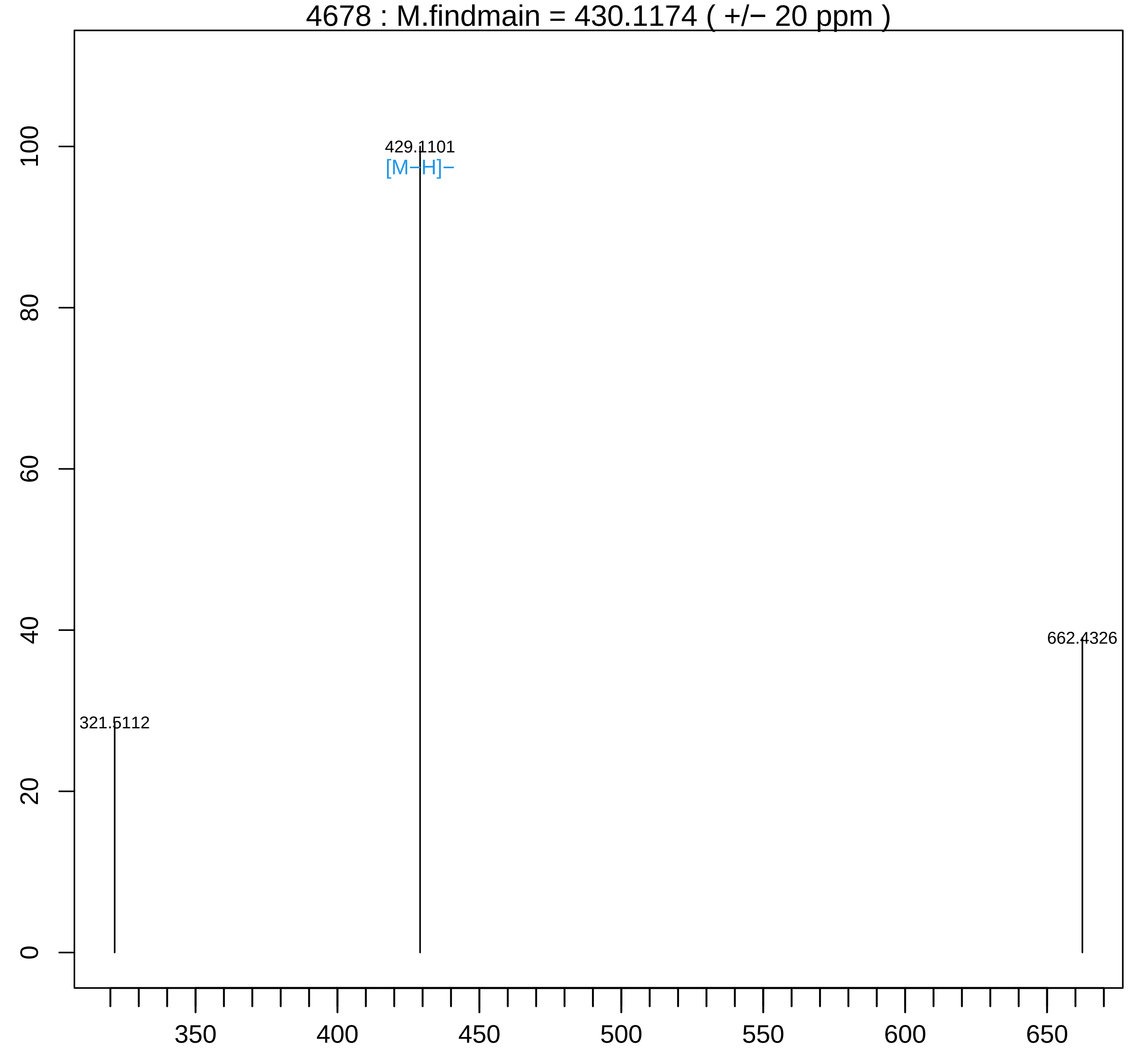
4678 : M.findmain = 430.1174 (+/− 20 ppm)
429.1101
[M−H]−
662.4326
321.5112
0
20
40
60
80
100
350
400
450
500
550
600
650

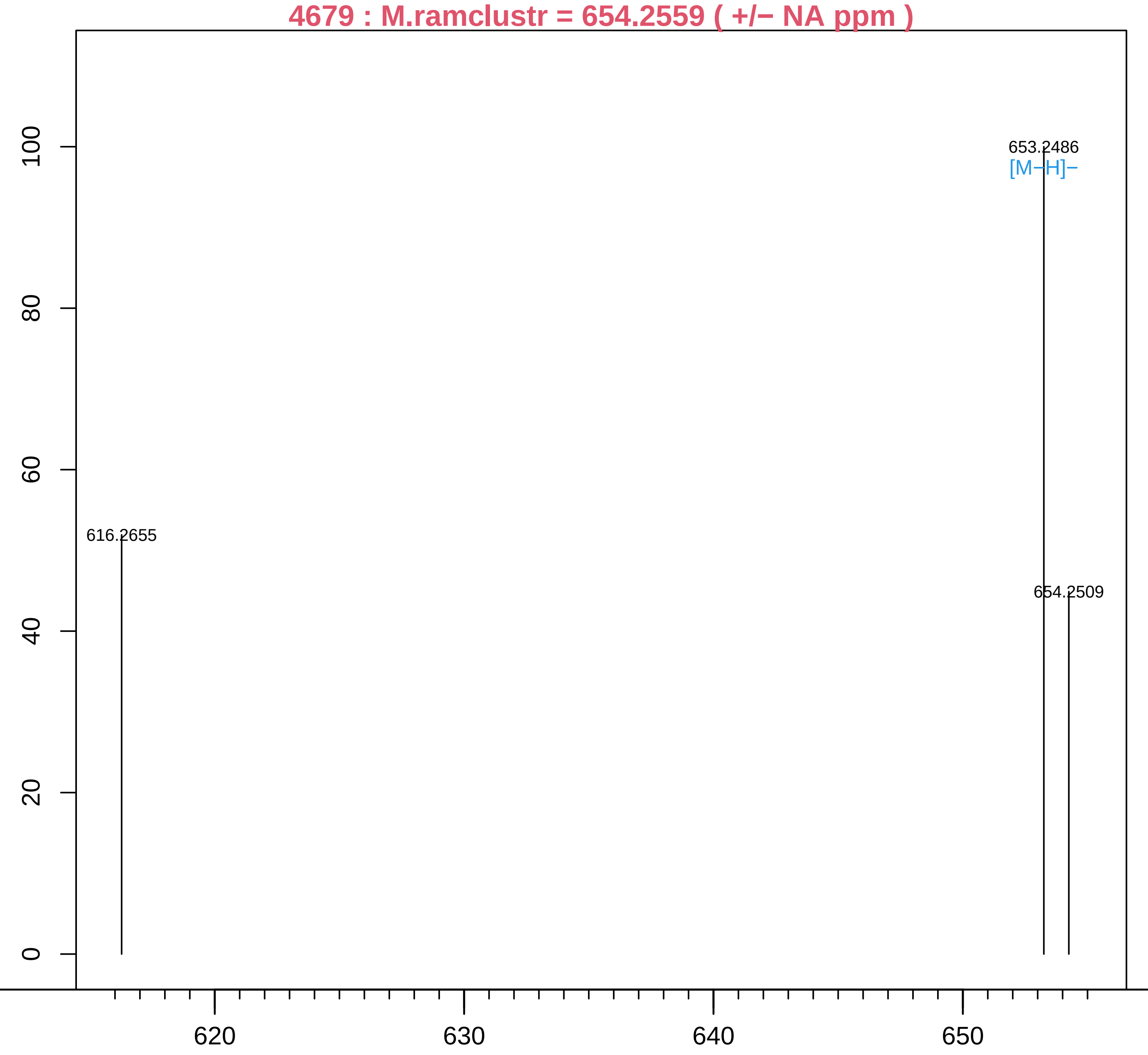
4679 : M.ramclustr = 654.2559 (+/− NA ppm)
653.2486
[M−H]−
616.2655
654.2509
0
20
40
60
80
100
620
630
640
650

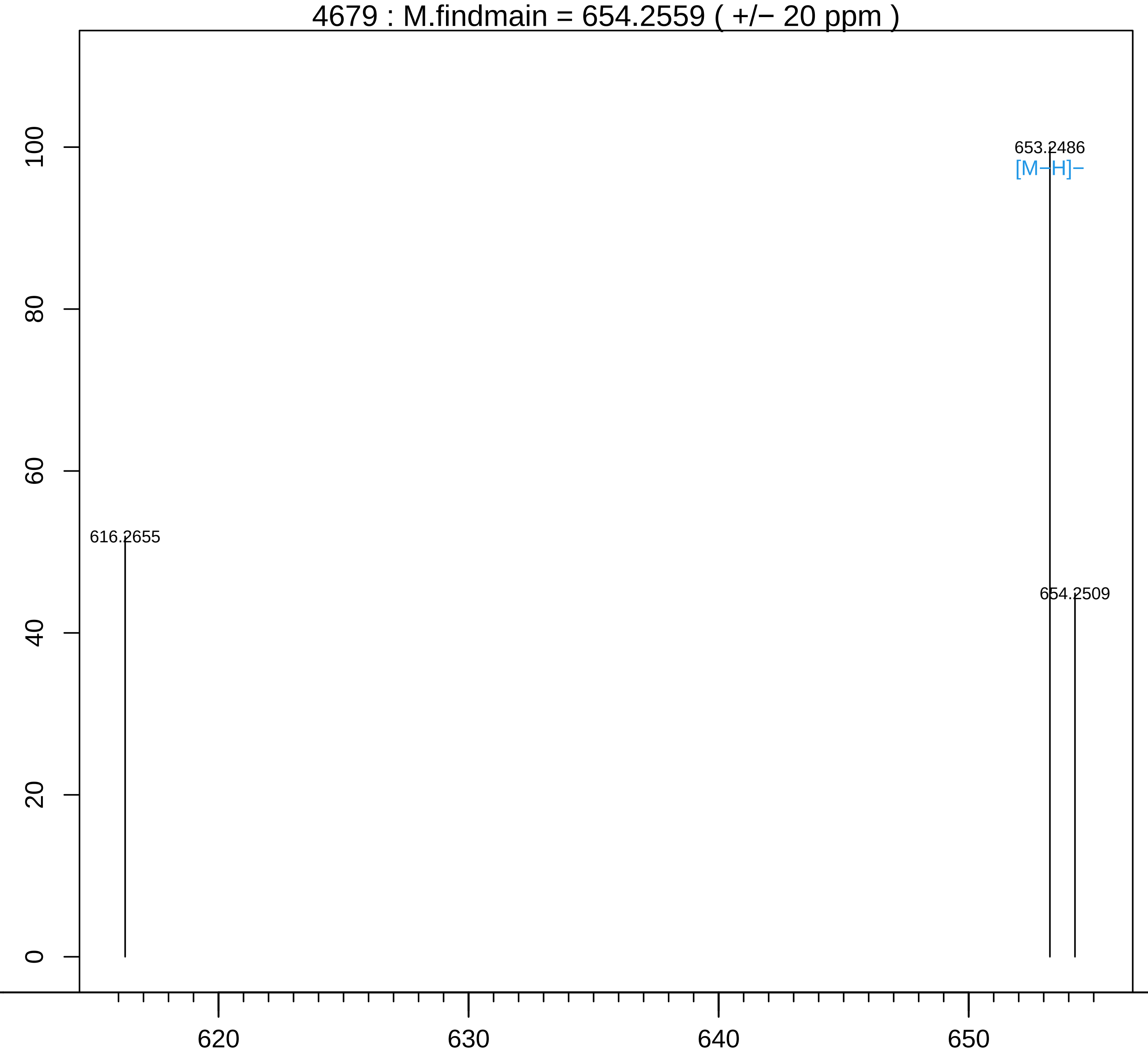
4679 : M.findmain = 654.2559 (+/− 20 ppm)
653.2486
[M−H]−
616.2655
654.2509
0
20
40
60
80
100
620
630
640
650

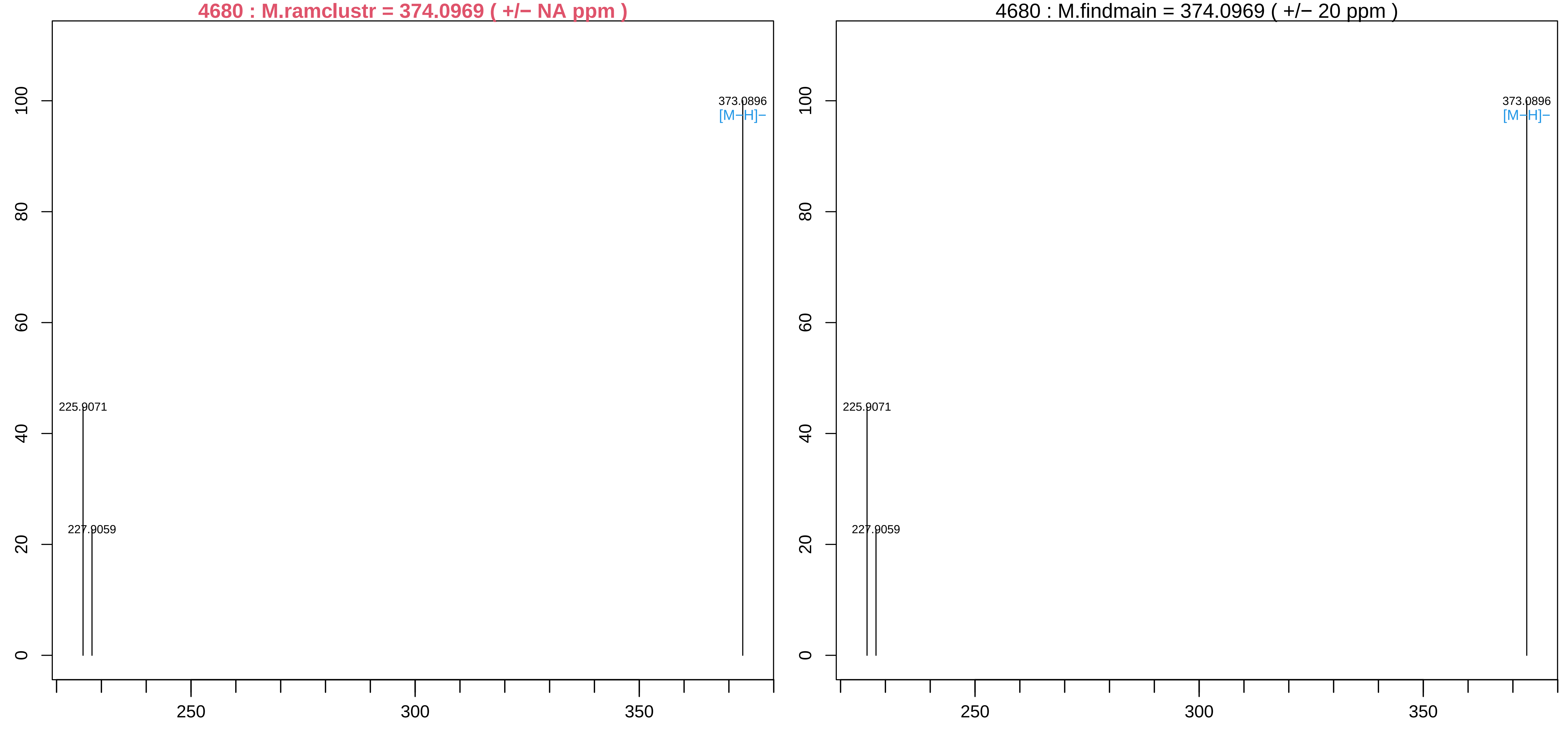
4680 : M.ramclustr = 374.0969 (+/− NA ppm)
373.0896
[M−H]−
225.9071
227.9059
4680 : M.findmain = 374.0969 (+/− 20 ppm)
373.0896
[M−H]−
225.9071
227.9059
0
20
40
60
80
100
250
300
350

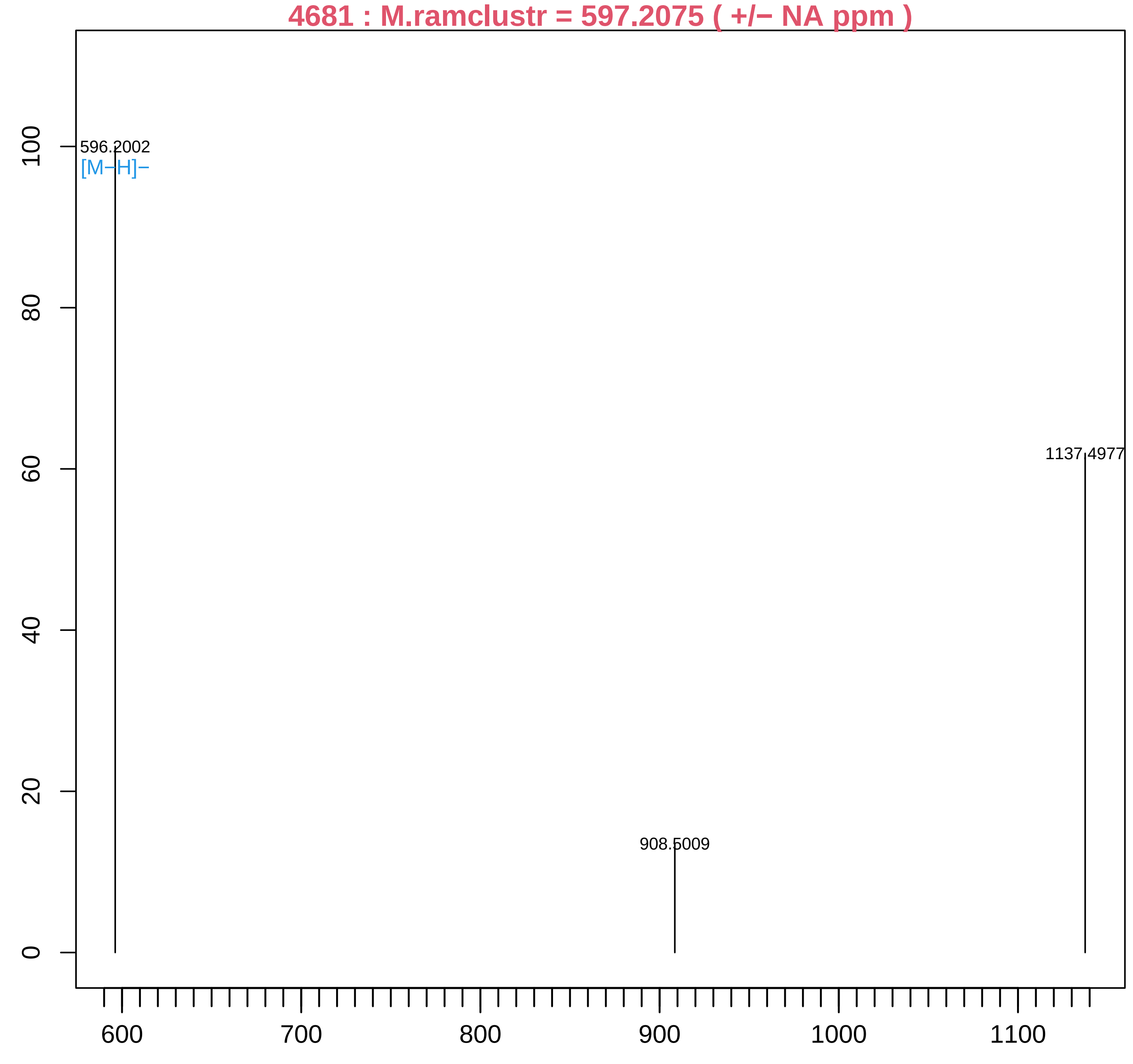
4681 : M.ramclustr = 597.2075 (+/− NA ppm)
596.2002
[M−H]−
1137.4977
908.5009
0
20
40
60
80
100
600
700
800
900
1000
1100

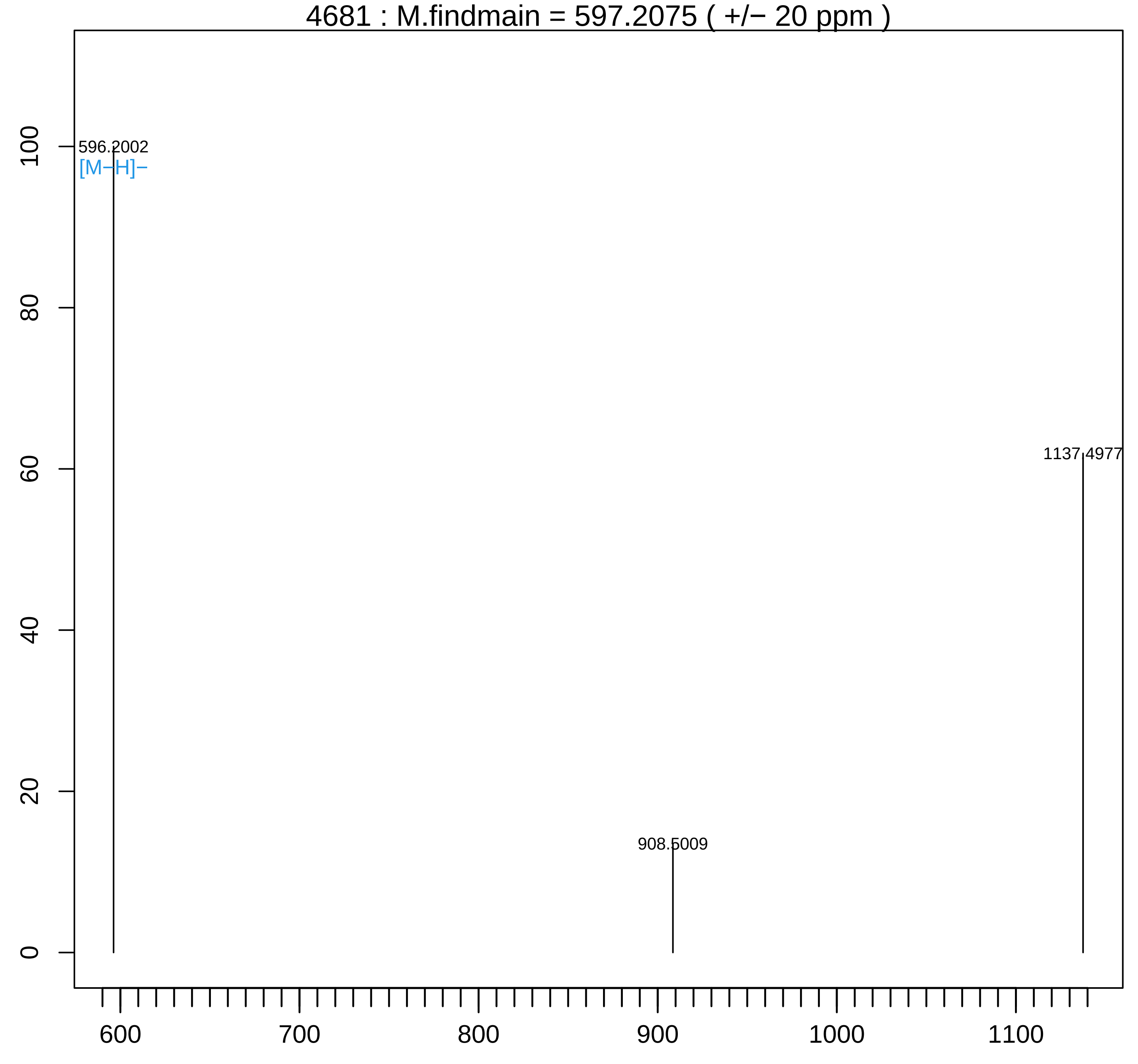
4681 : M.findmain = 597.2075 (+/− 20 ppm)
596.2002
[M−H]−
1137.4977
908.5009
0
20
40
60
80
100
600
700
800
900
1000
1100

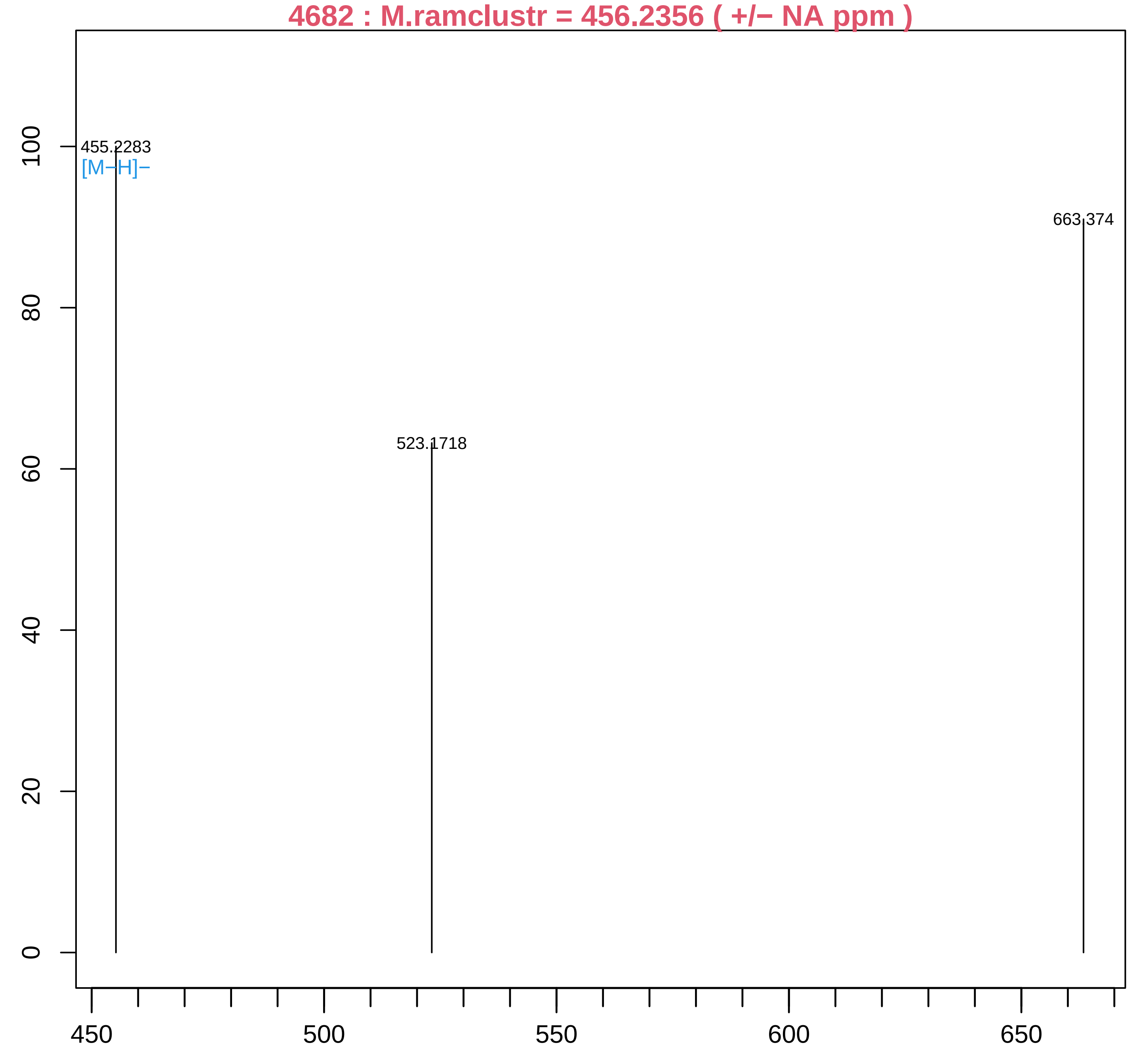
4682 : M.ramclustr = 456.2356 (+/− NA ppm)
455.2283
[M−H]−
523.1718
663.374
0
20
40
60
80
100
450
500
550
600
650

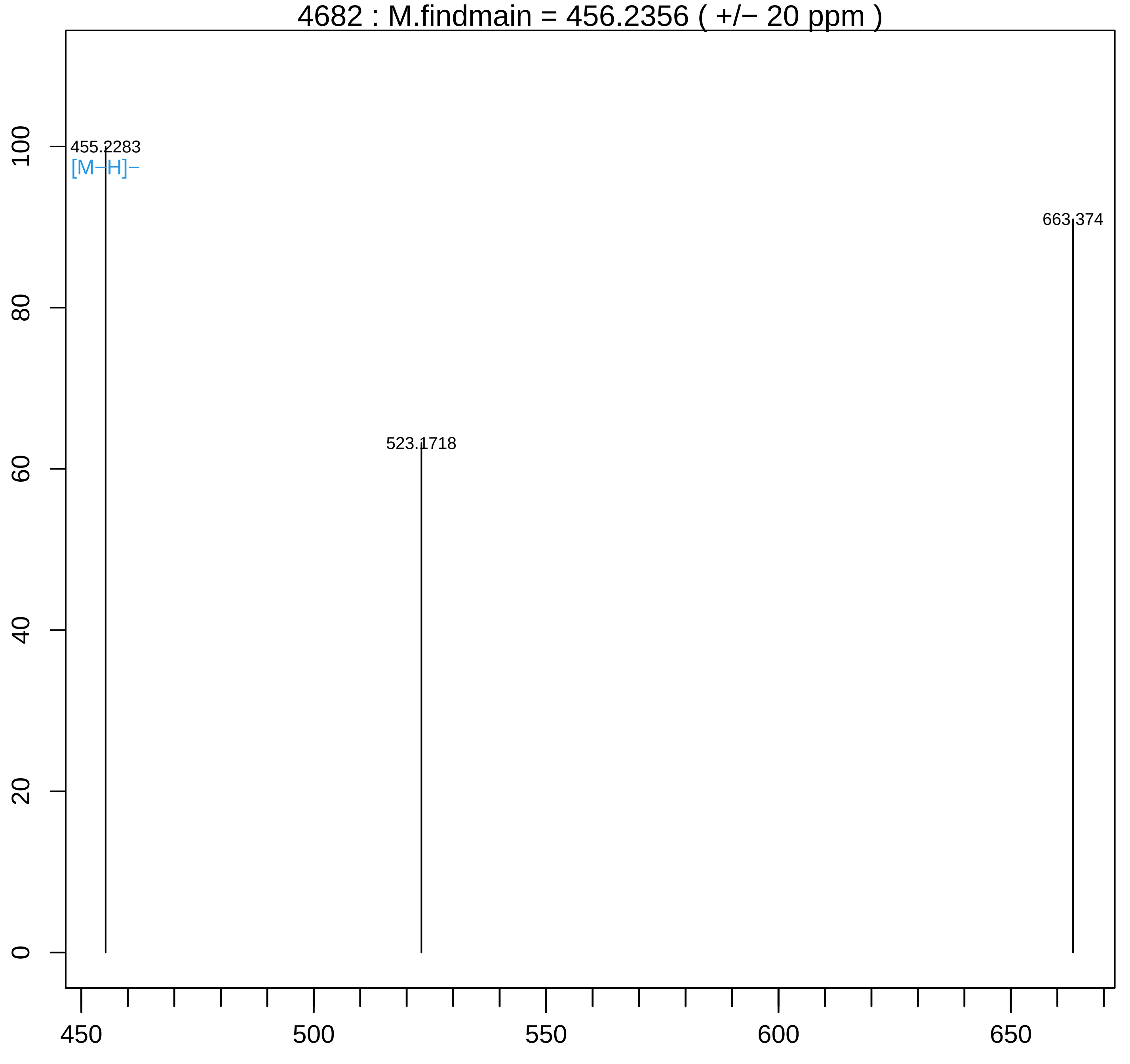
4682 : M.findmain = 456.2356 (+/− 20 ppm)
455.2283
[M−H]−
523.1718
663.374
0
20
40
60
80
100
450
500
550
600
650

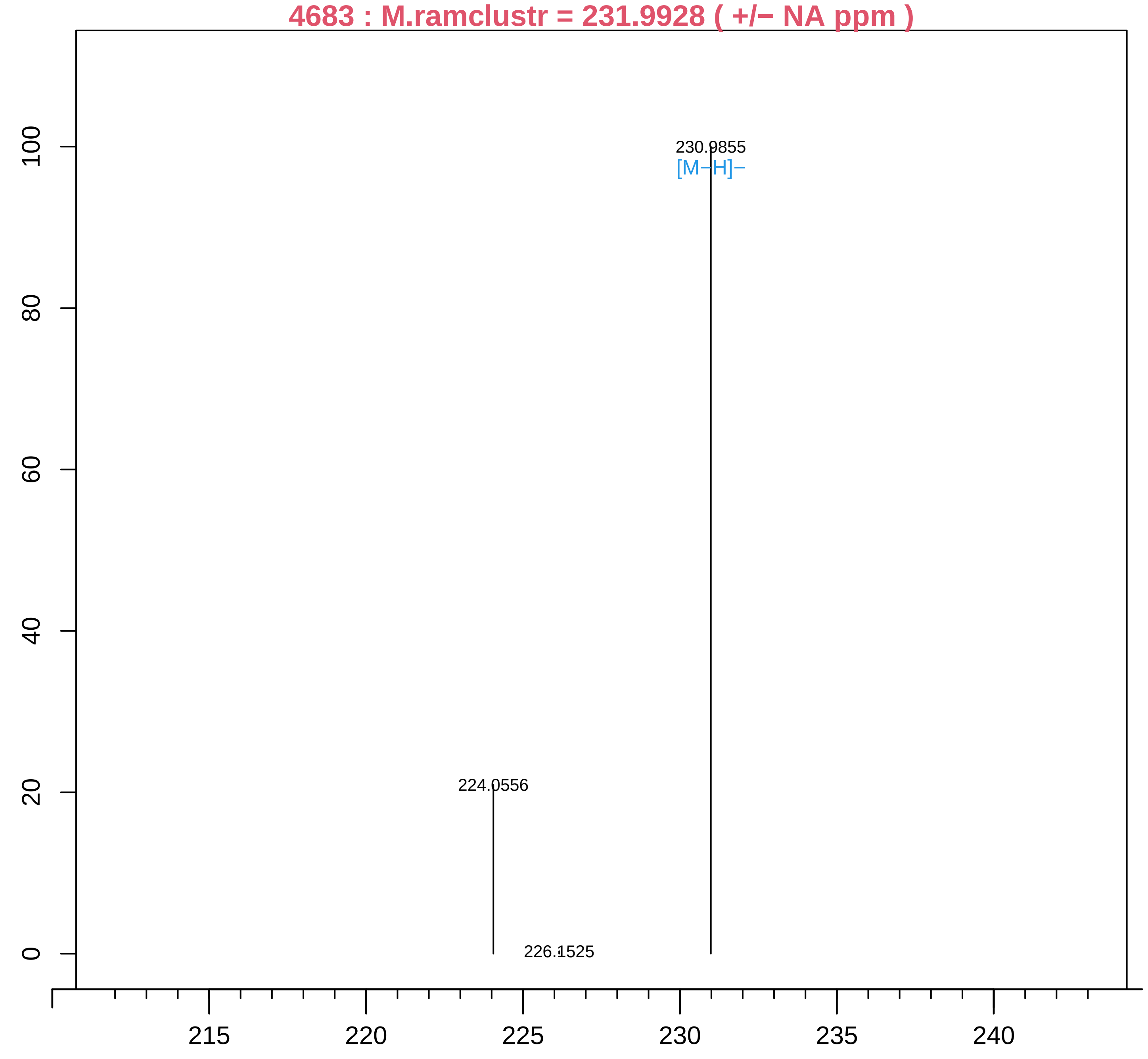

4683 : M.ramclustr = 231.9928 (+/− NA ppm)
230.9855
[M−H]−
224.0556
226.1525
0
20
40
60
80
100
215
220
225
230
235
240

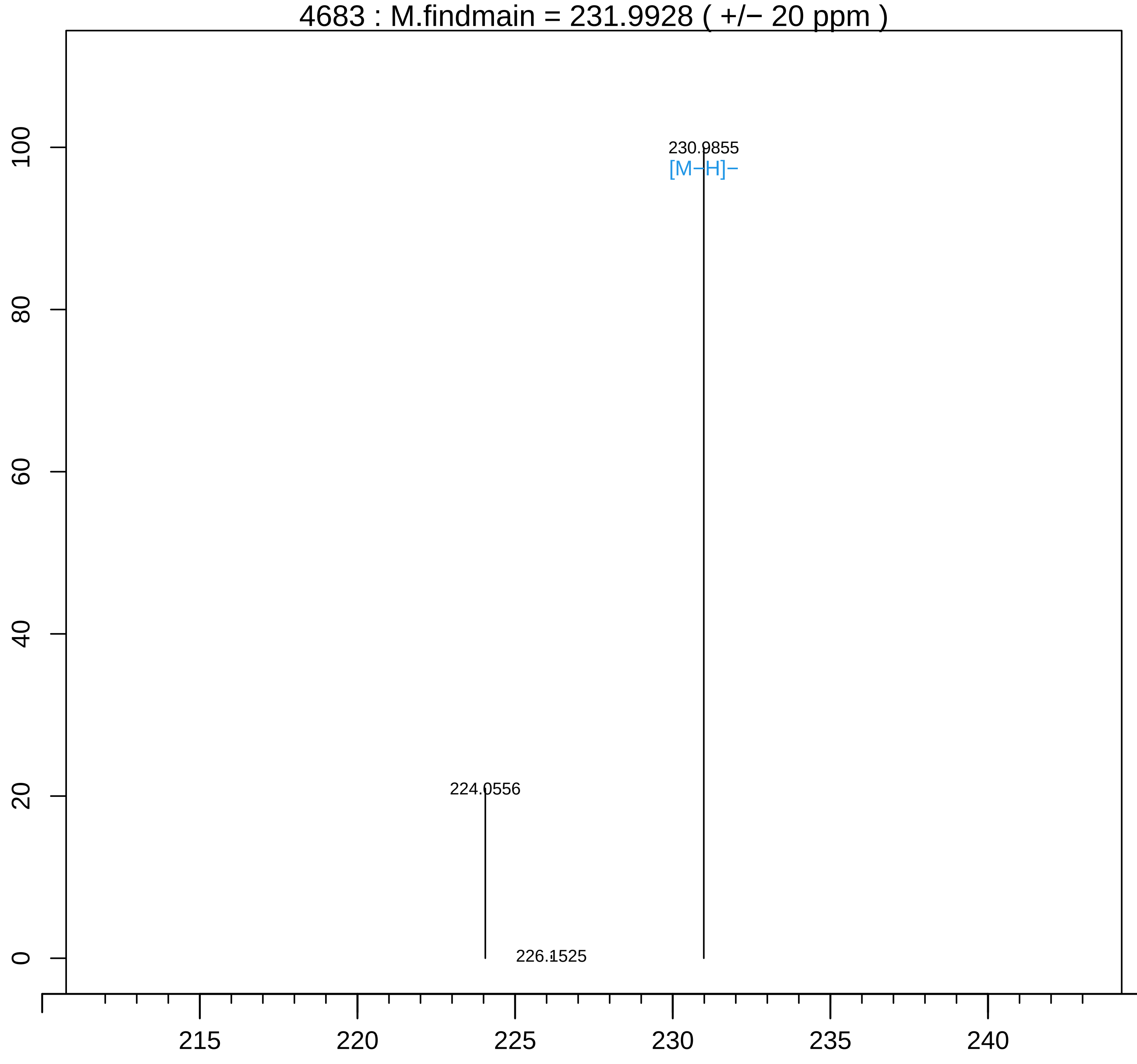

4683 : M.findmain = 231.9928 (+/− 20 ppm)
230.9855
[M−H]−
224.0556
226.1525
0
20
40
60
80
100
215
220
225
230
235
240

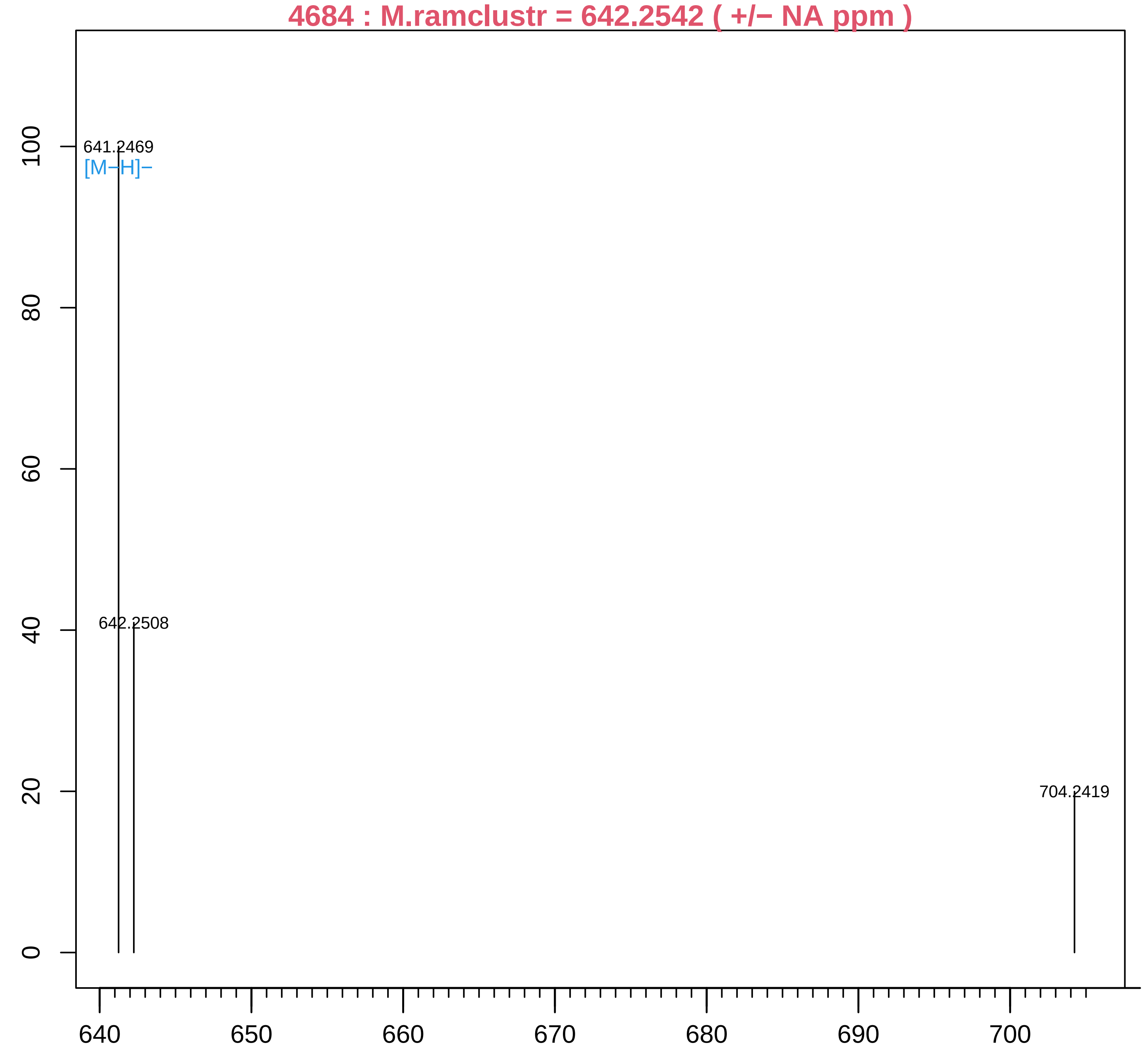

4684 : M.ramclustr = 642.2542 (+/− NA ppm)
641.2469
[M−H]−
642.2508
704.2419
0
20
40
60
80
100
640
650
660
670
680
690
700

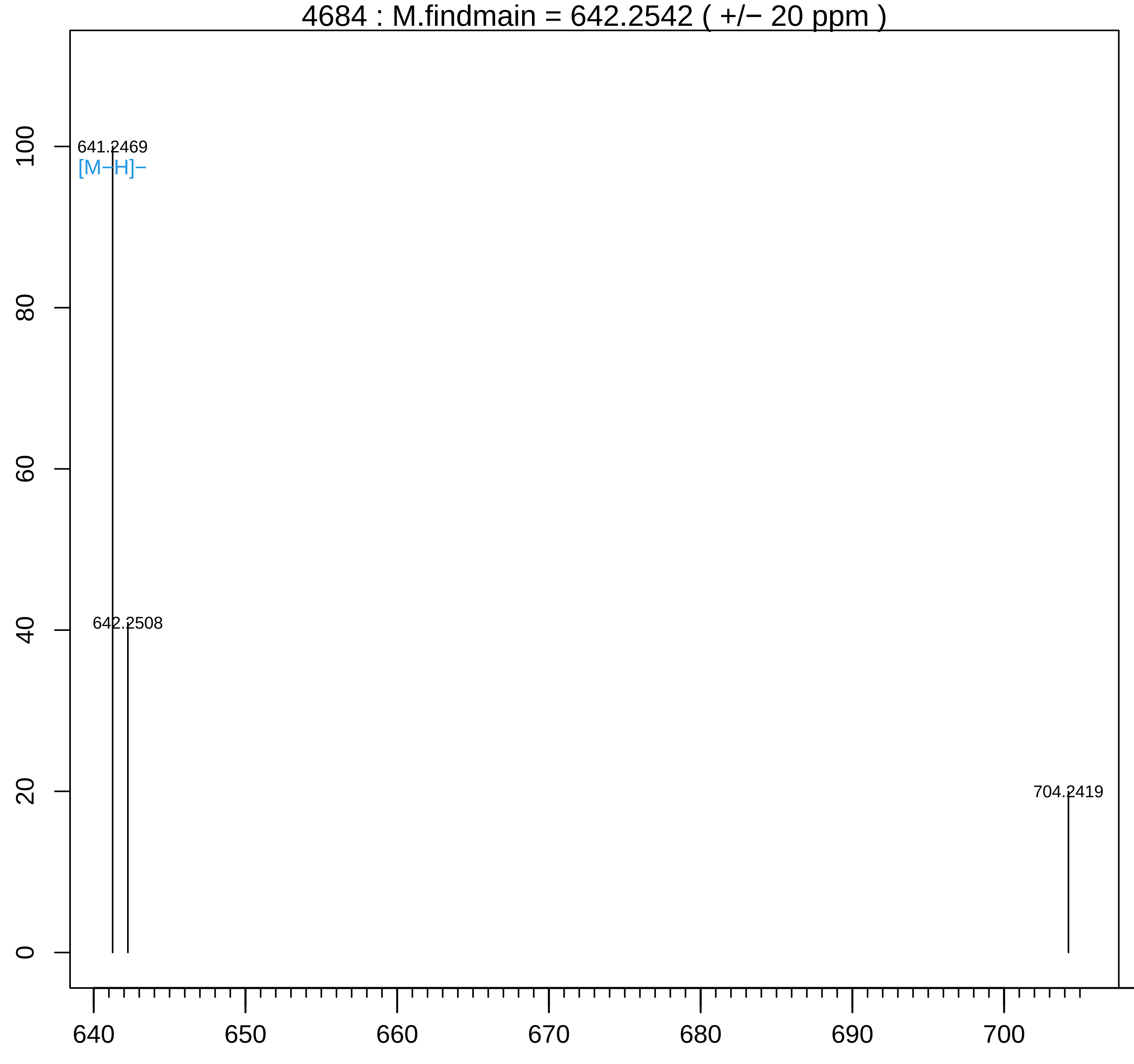

4684 : M.findmain = 642.2542 (+/− 20 ppm)
641.2469
[M−H]−
642.2508
704.2419
0
20
40
60
80
100
640
650
660
670
680
690
700

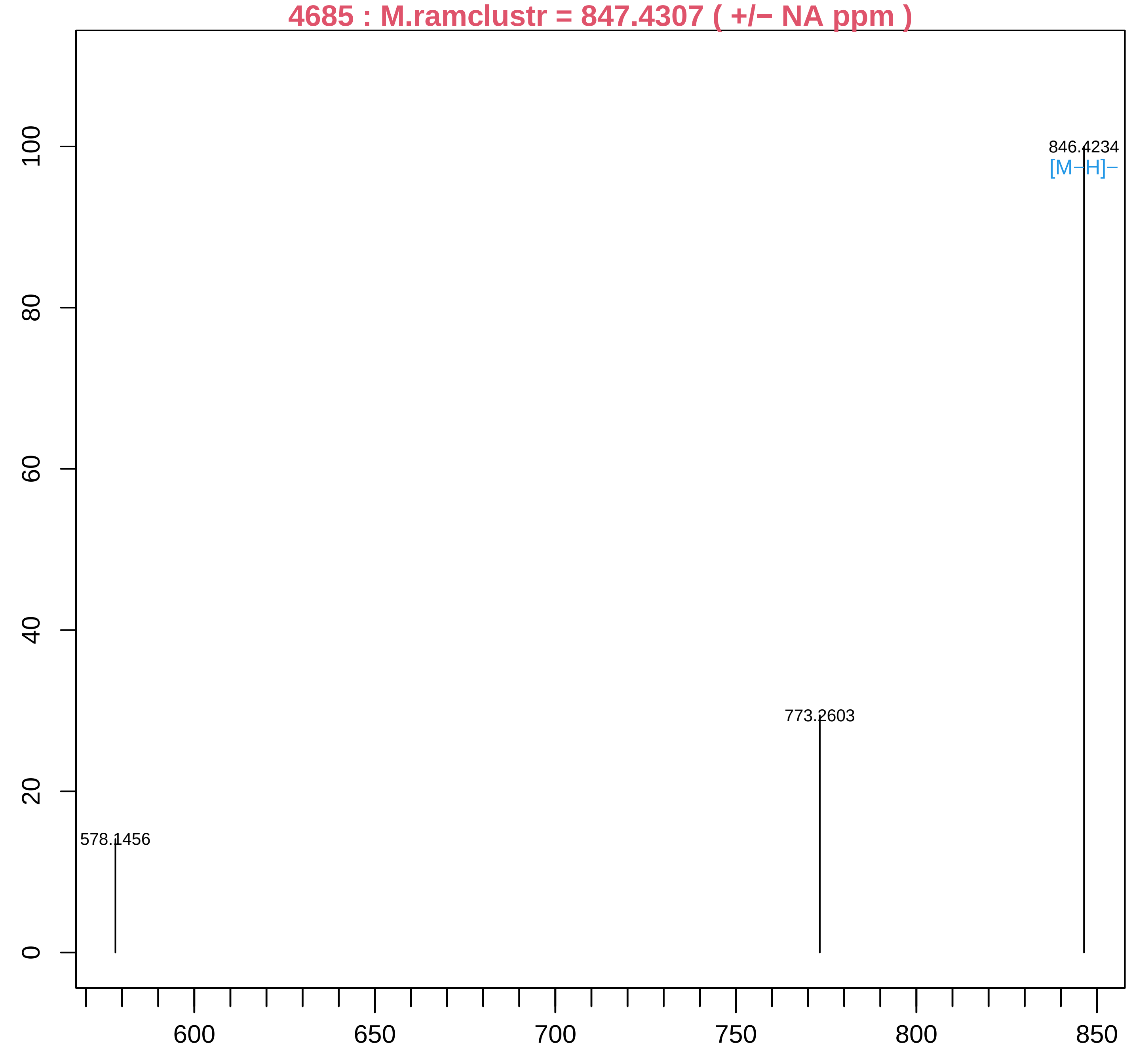

4685 : M.ramclustr = 847.4307 (+/− NA ppm)
846.4234
[M−H]−
773.2603
578.1456
0
20
40
60
80
100
600
650
700
750
800
850

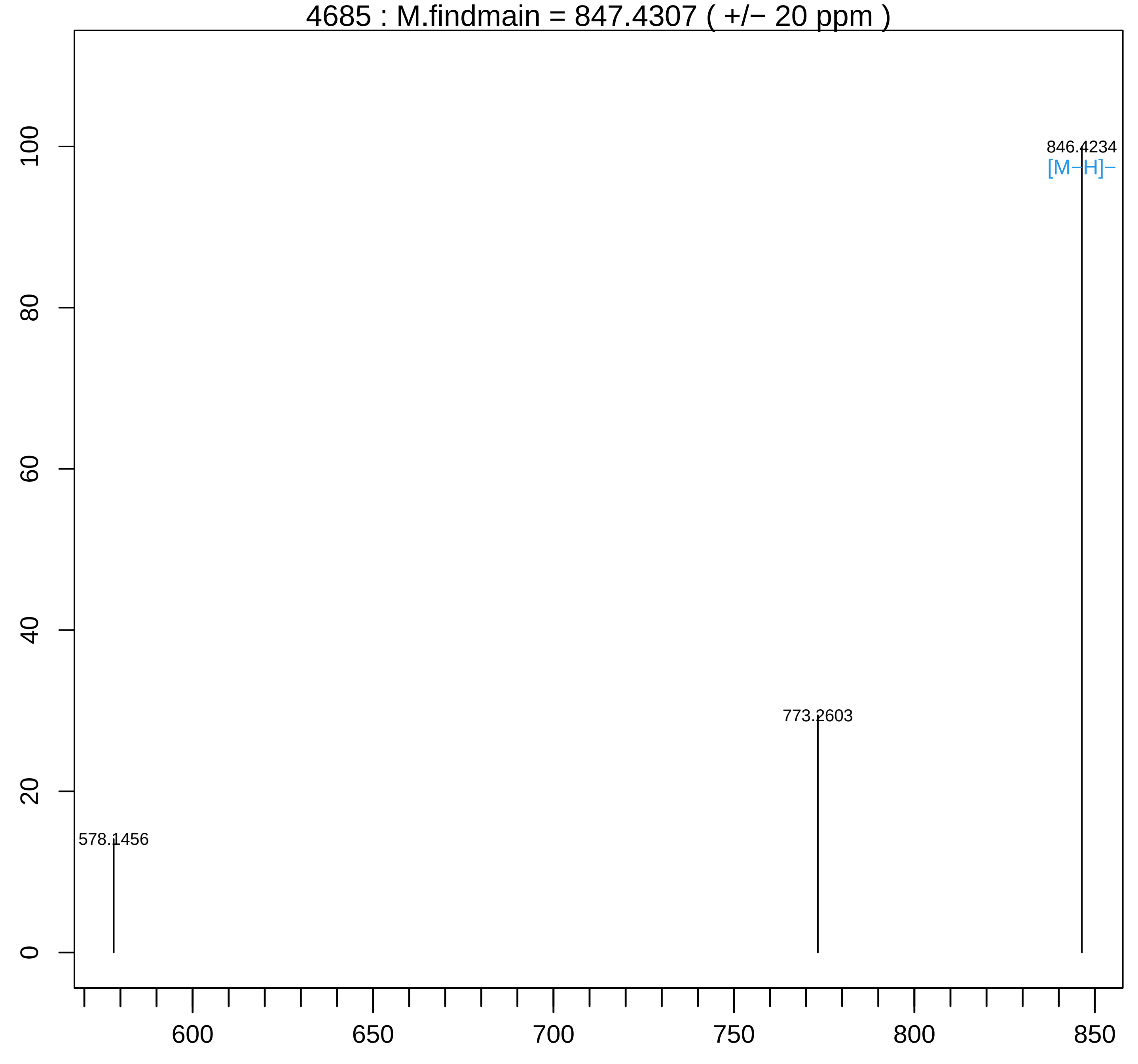

4685 : M.findmain = 847.4307 (+/− 20 ppm)
846.4234
[M−H]−
773.2603
578.1456
0
20
40
60
80
100
600
650
700
750
800
850

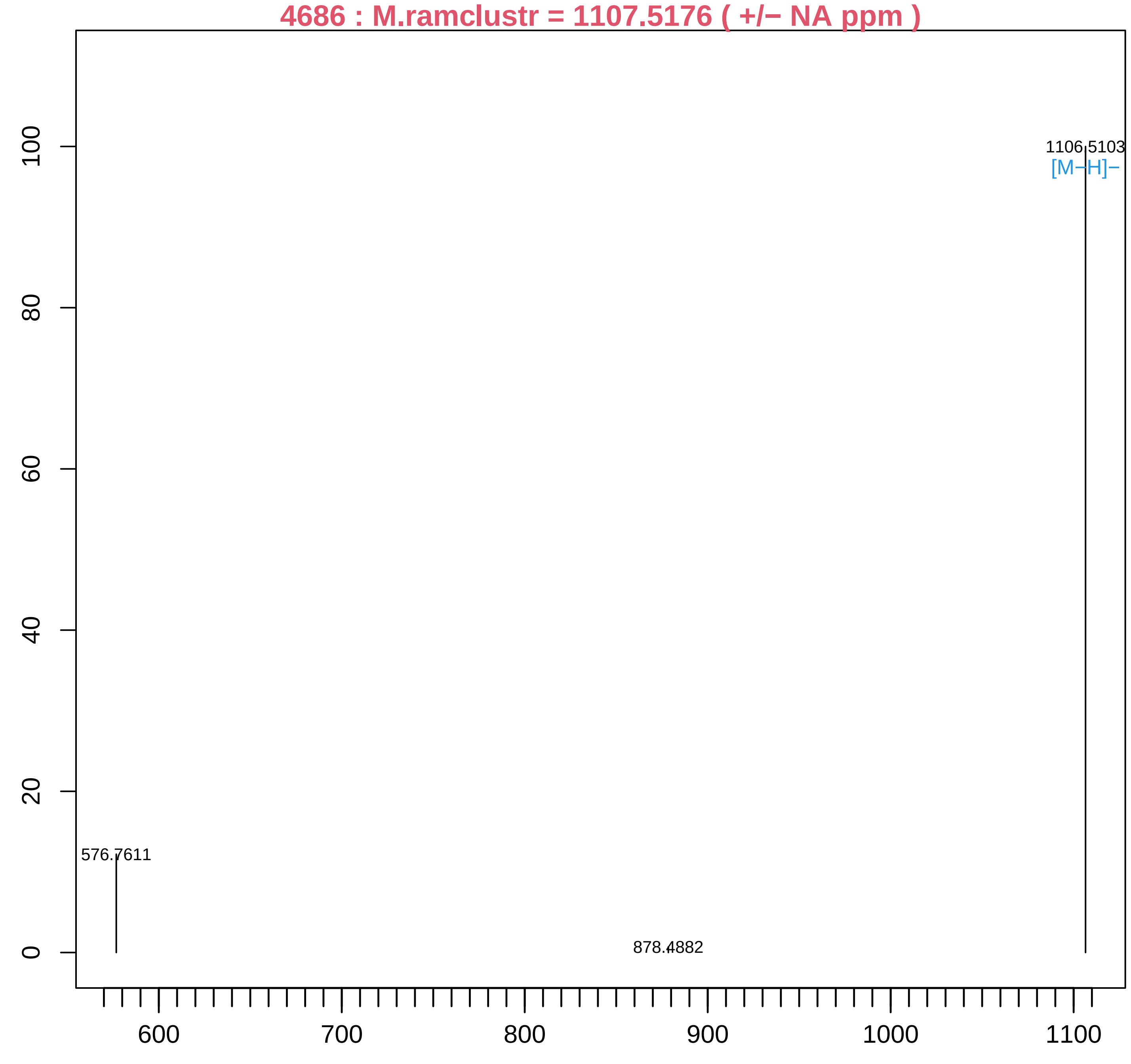
4686 : M.ramclustr = 1107.5176 (+/− NA ppm)
1106.5103
[M−H]−
576.7611
878.4882
0
20
40
60
80
100
600
700
800
900
1000
1100
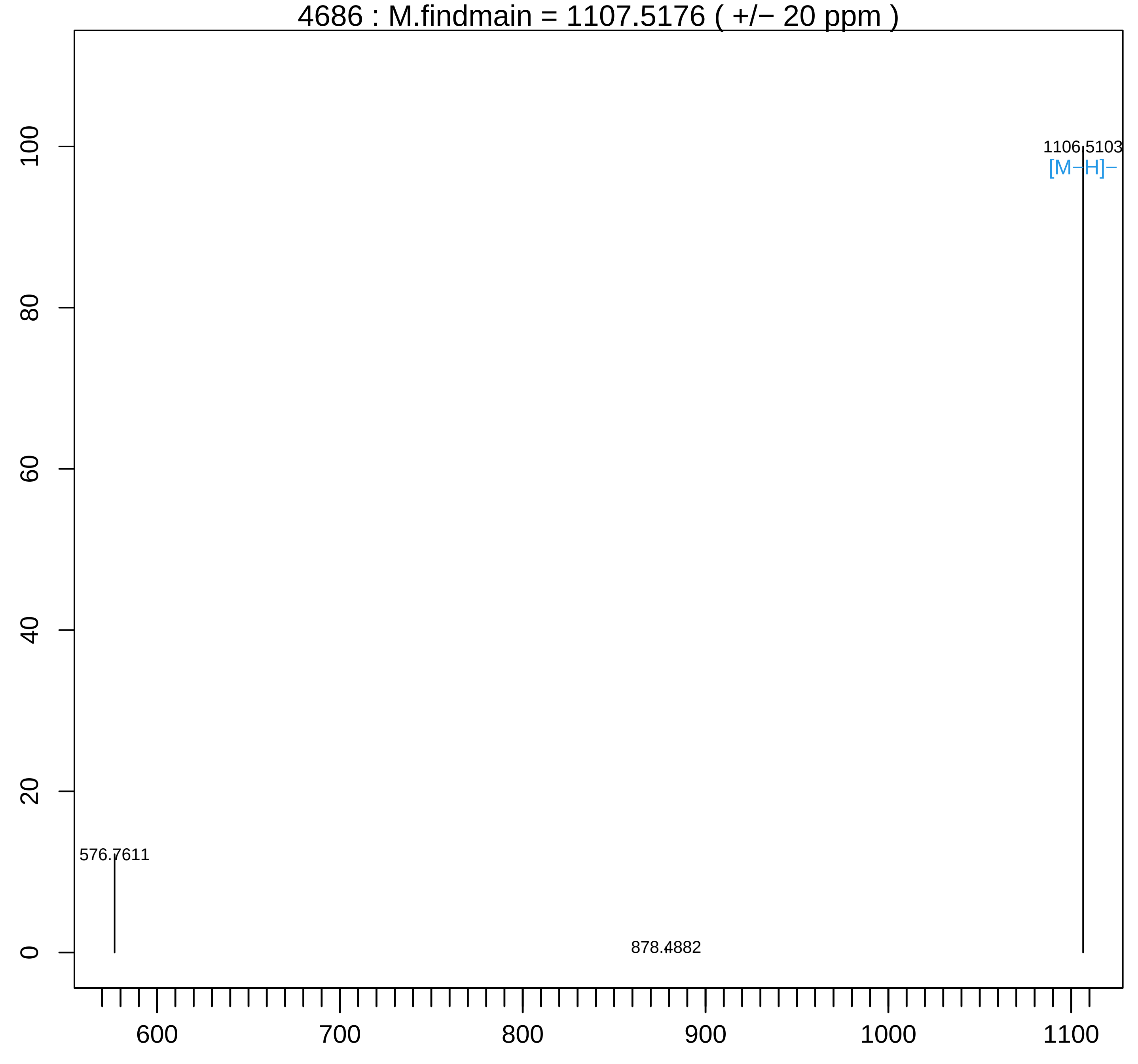
4686 : M.findmain = 1107.5176 (+/− 20 ppm)
1106.5103
[M−H]−
576.7611
878.4882
0
20
40
60
80
100
600
700
800
900
1000
1100

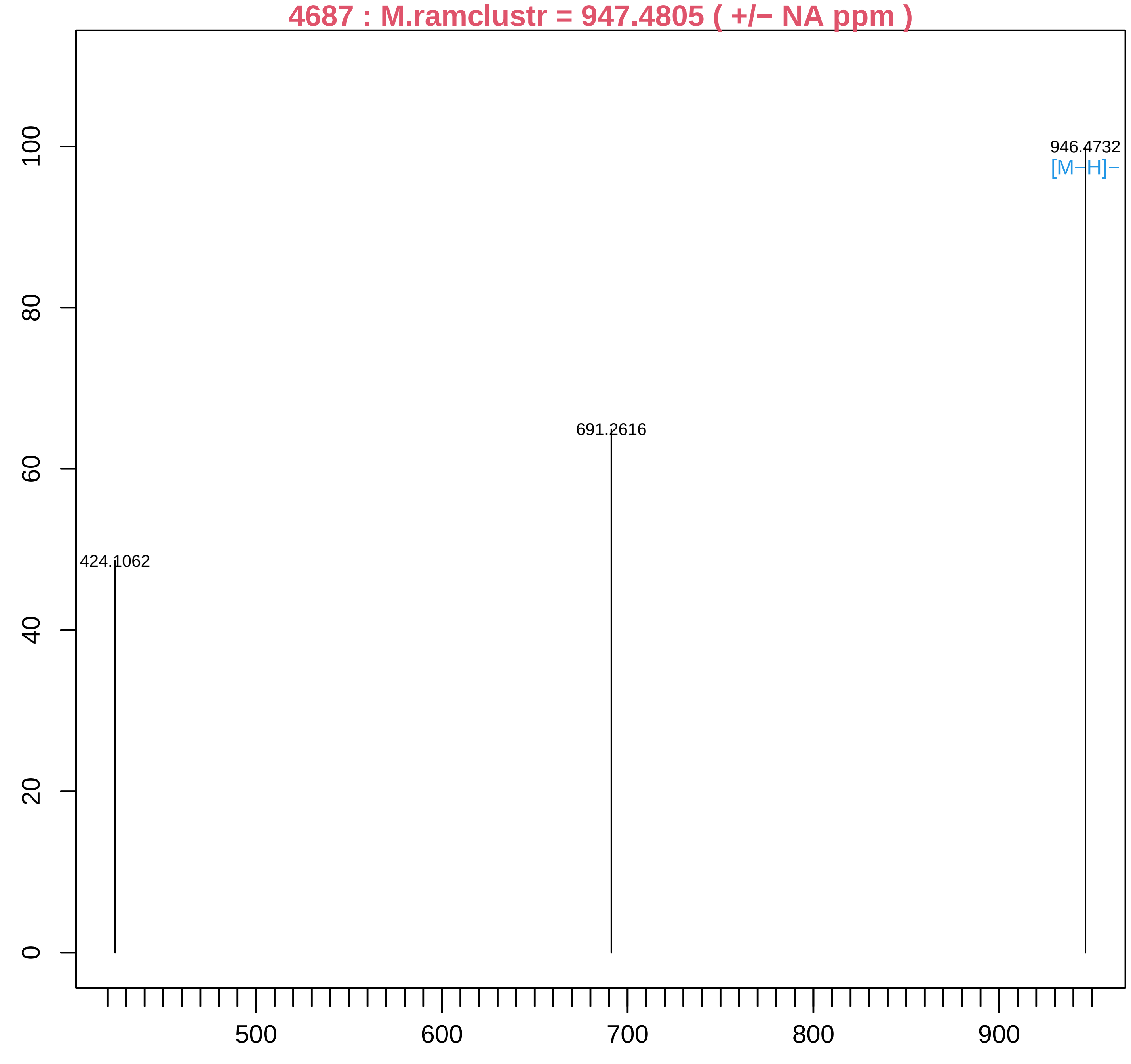
4687 : M.ramclustr = 947.4805 (+/− NA ppm)
946.4732
[M−H]−
691.2616
424.1062
0
20
40
60
80
100
500
600
700
800
900

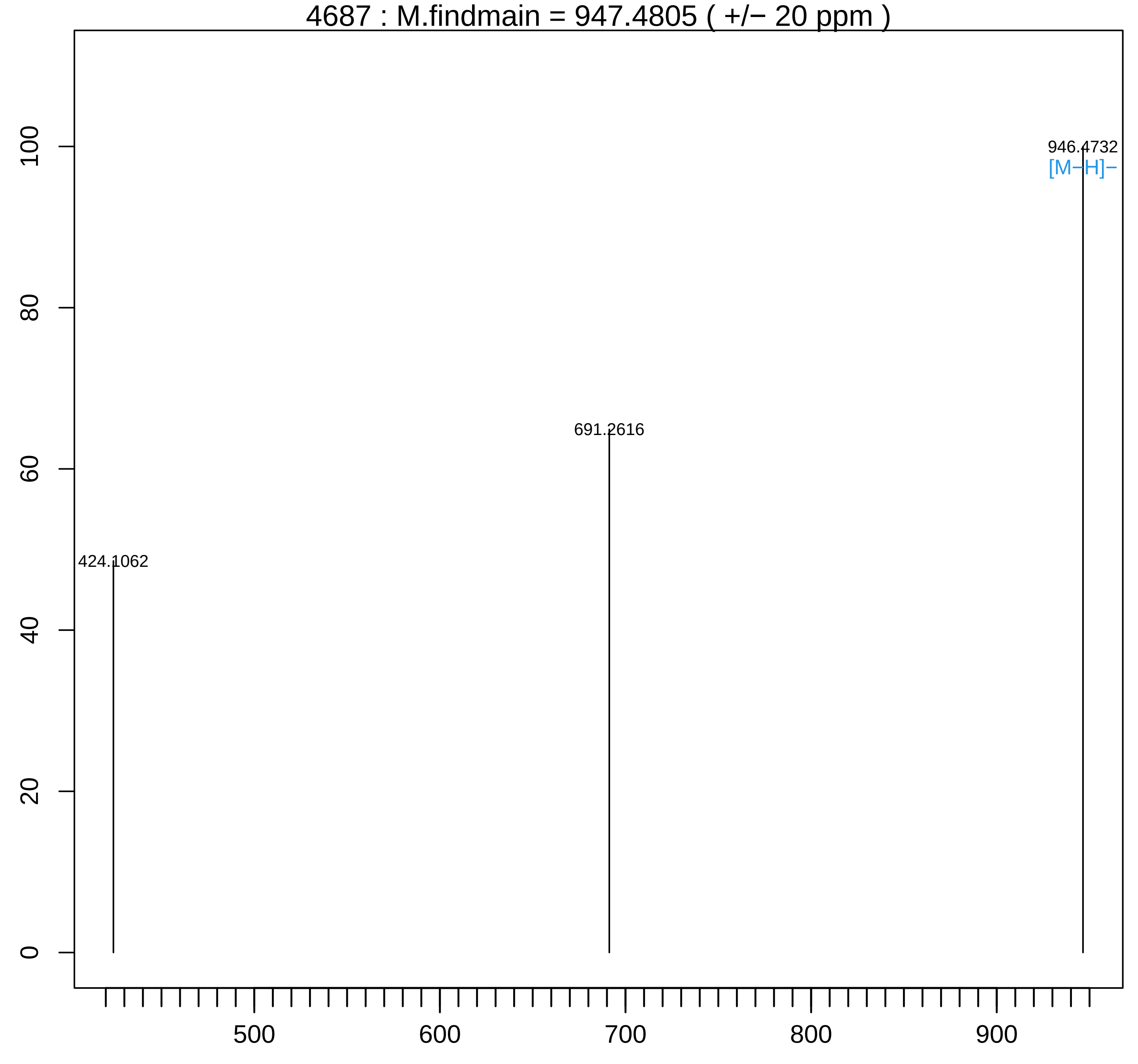
4687 : M.findmain = 947.4805 (+/− 20 ppm)
946.4732
[M−H]−
691.2616
424.1062
0
20
40
60
80
100
500
600
700
800
900

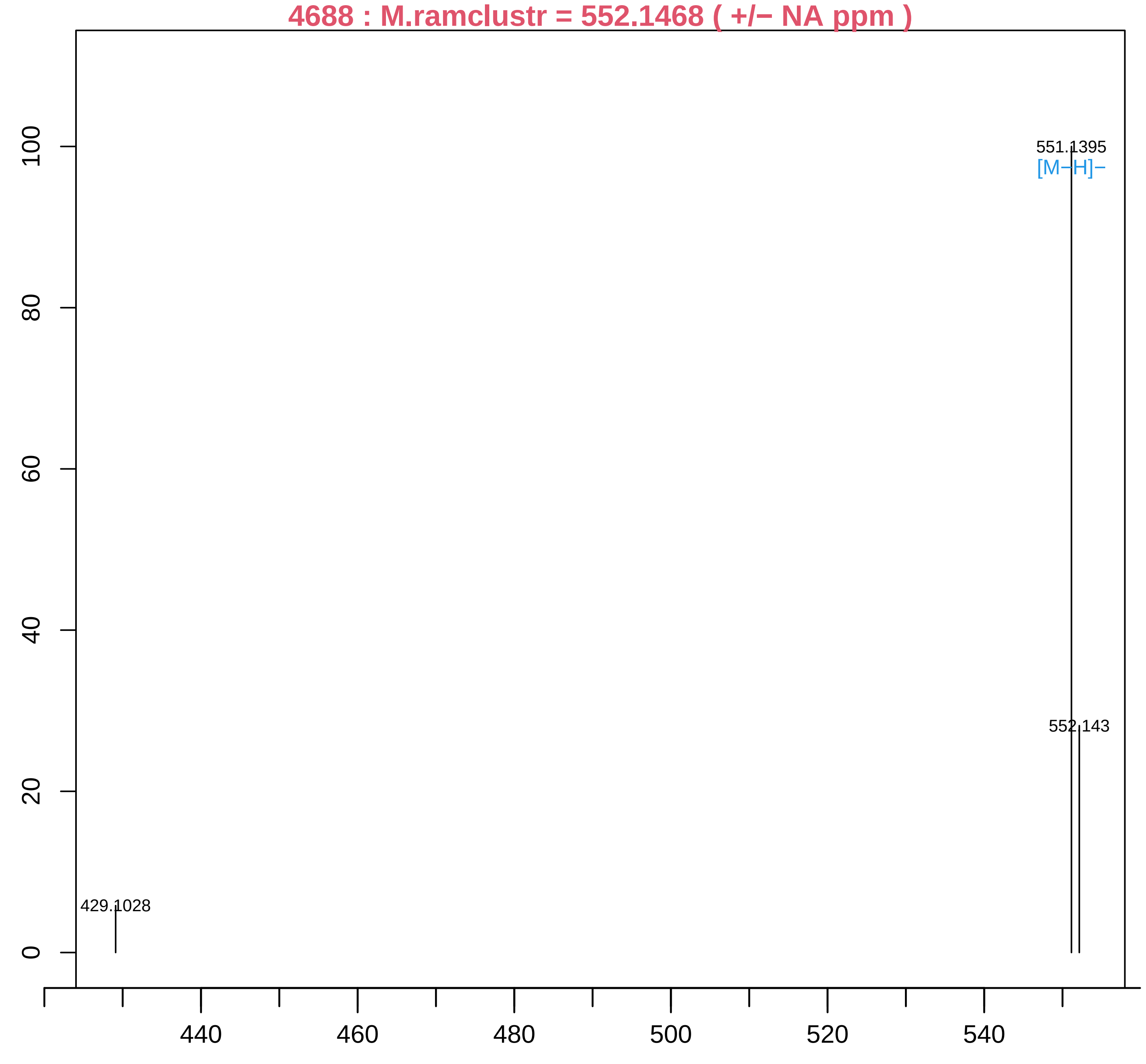

4688 : M.ramclustr = 552.1468 (+/− NA ppm)
551.1395
[M−H]−
552.143
429.1028
0
20
40
60
80
100
440
460
480
500
520
540

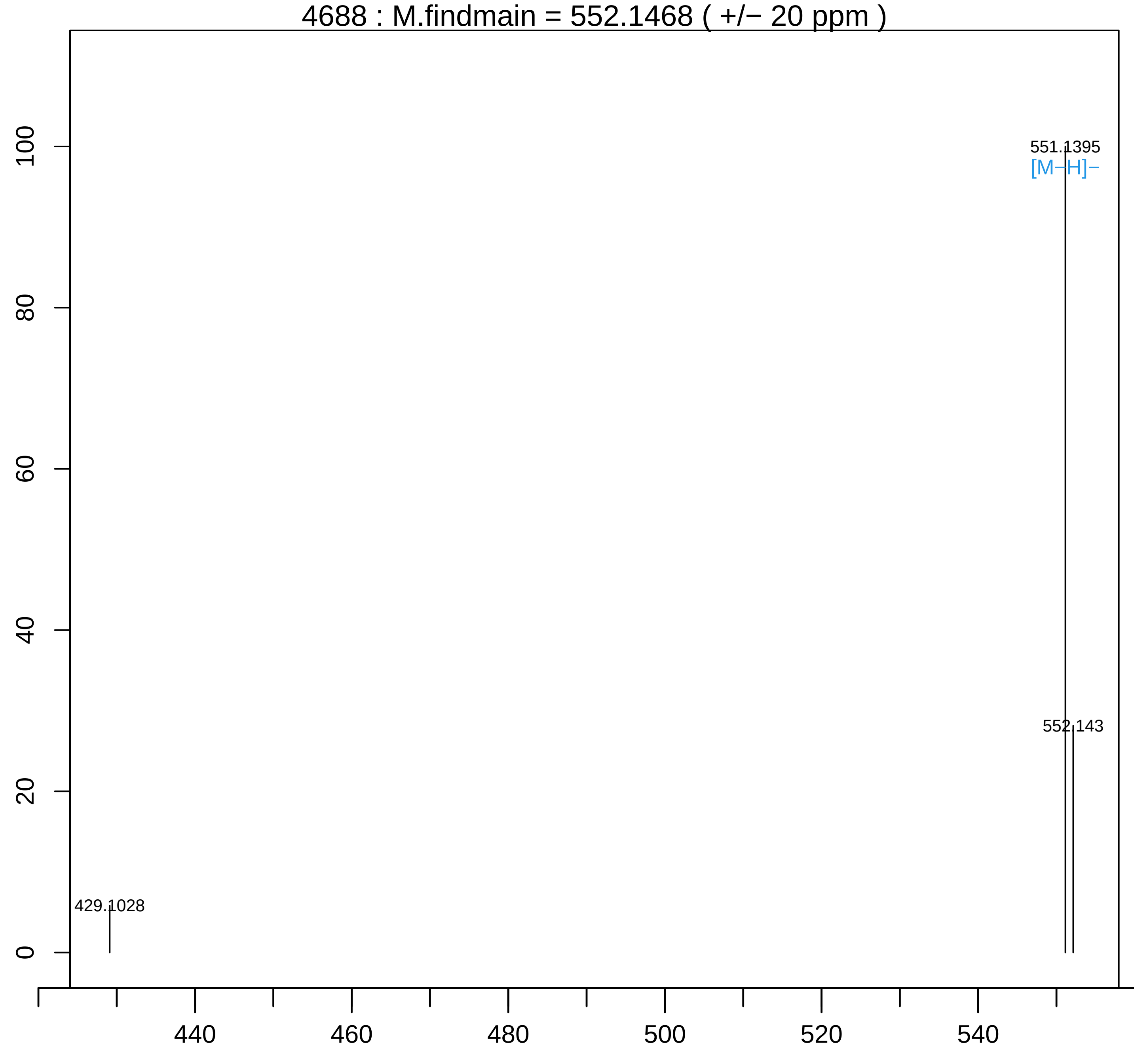

4688 : M.findmain = 552.1468 (+/− 20 ppm)
551.1395
[M−H]−
552.143
429.1028
0
20
40
60
80
100
440
460
480
500
520
540

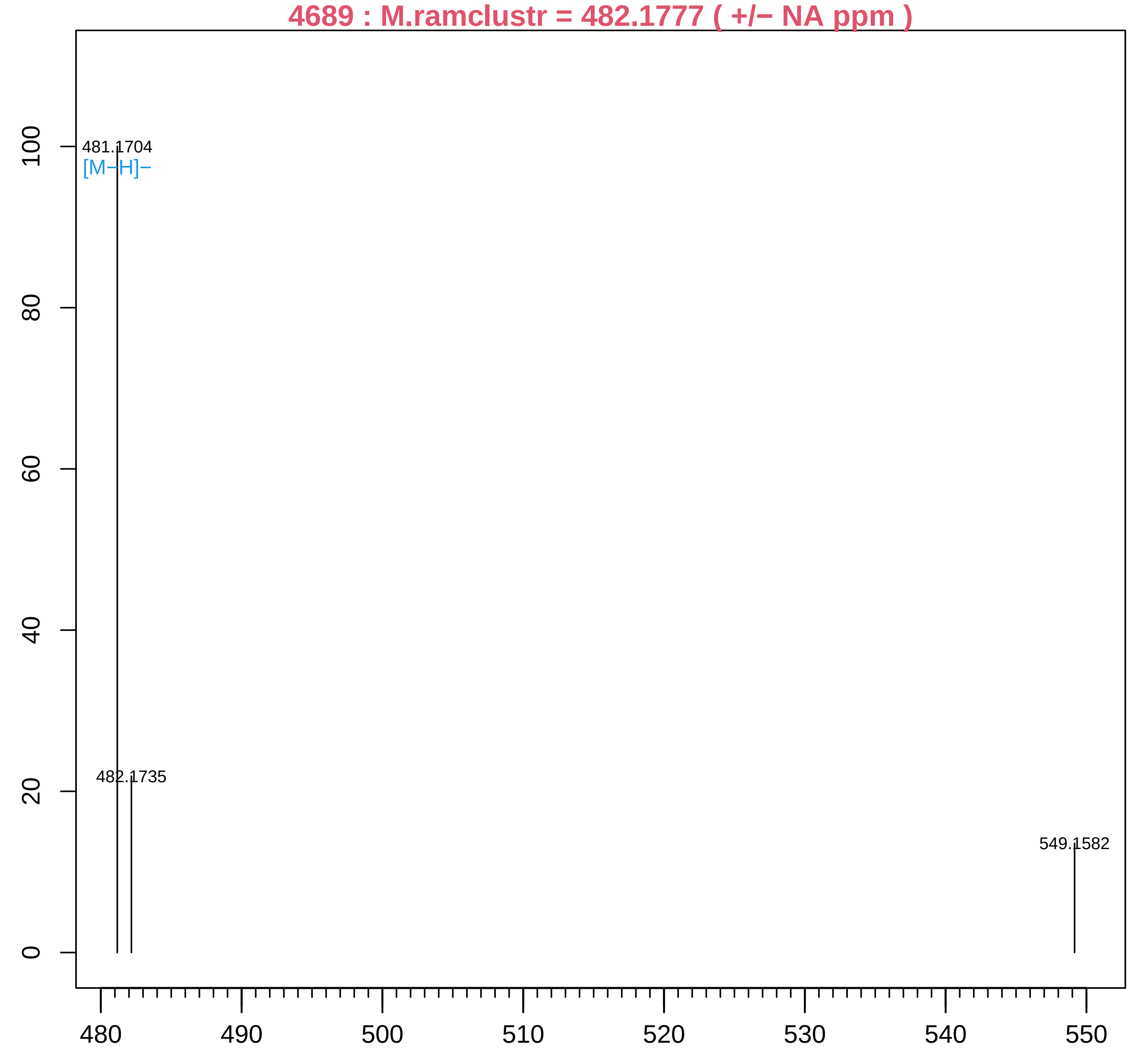

4689 : M.ramclustr = 482.1777 (+/− NA ppm)
481.1704
[M−H]−
482.1735
549.1582
0
20
40
60
80
100
480
490
500
510
520
530
540
550

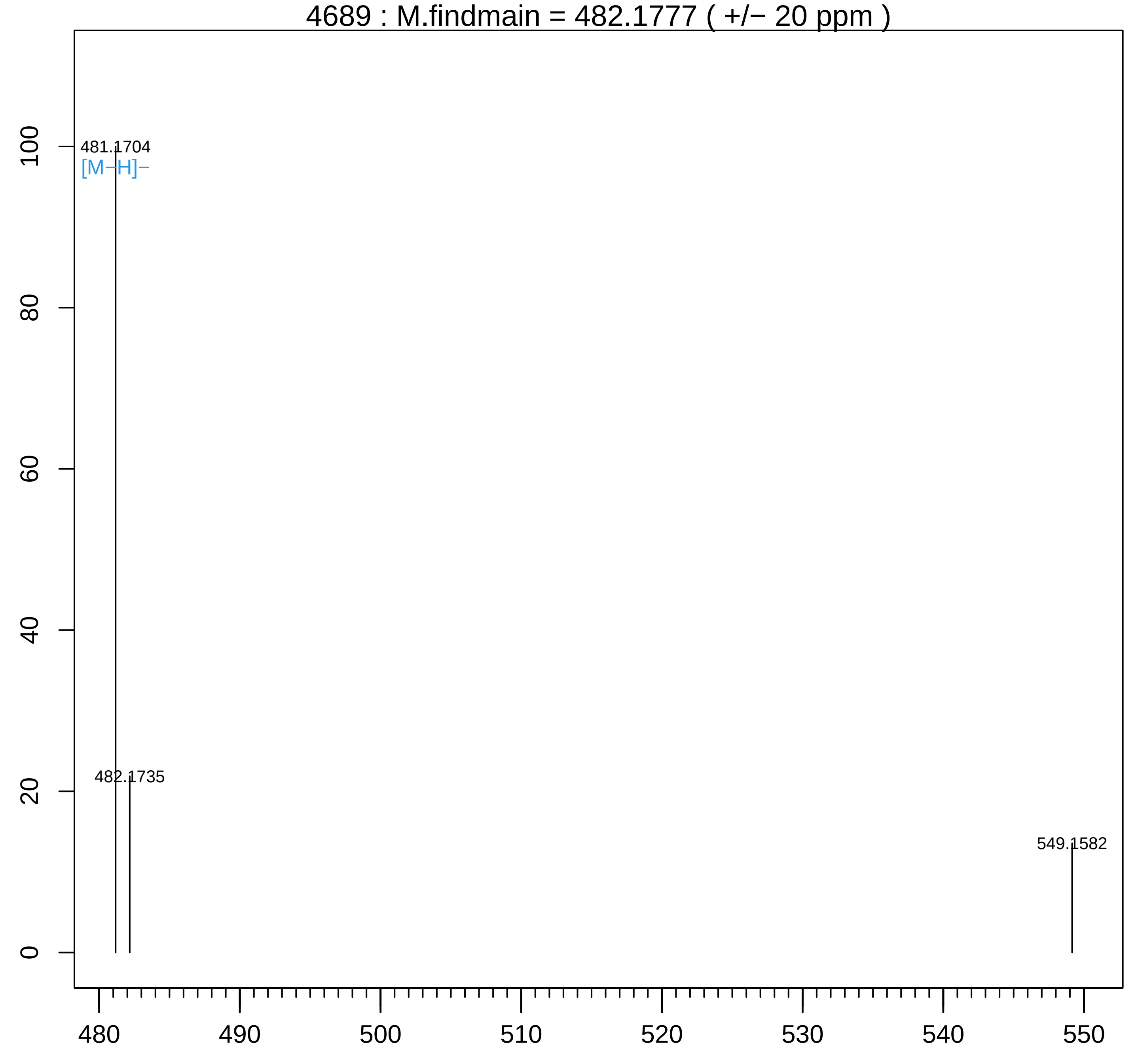

4689 : M.findmain = 482.1777 (+/− 20 ppm)
481.1704
[M−H]−
482.1735
549.1582
0
20
40
60
80
100
480
490
500
510
520
530
540
550

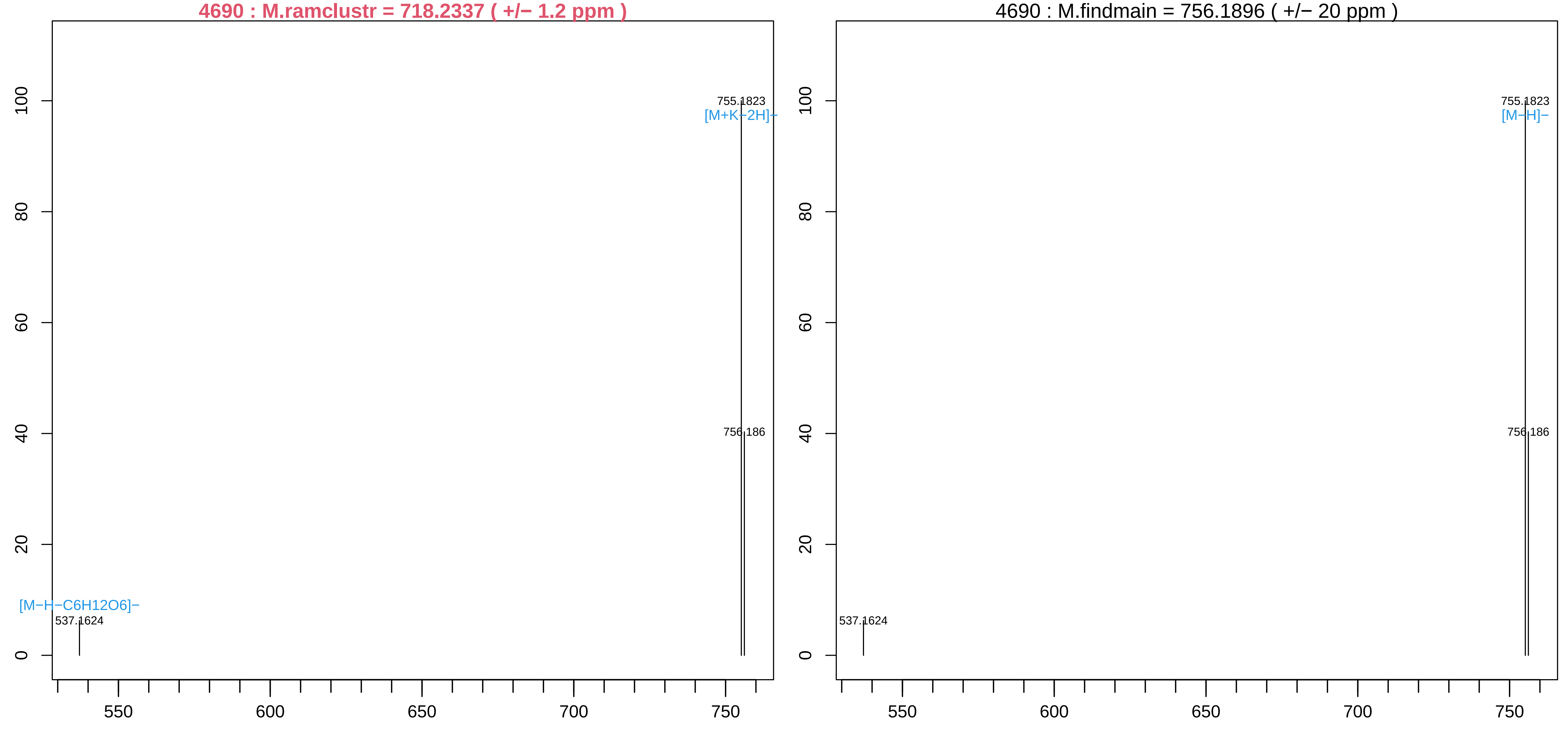
4690 : M.ramclustr = 718.2337 (+/− 1.2 ppm)
755.1823
[M+K−2H]−
756.186
[M−H−C6H12O6]−
537.1624
0
20
40
60
80
100
550
600
650
700
750
4690 : M.findmain = 756.1896 (+/− 20 ppm)
755.1823
[M−H]−
756.186
537.1624
0
20
40
60
80
100
550
600
650
700
750

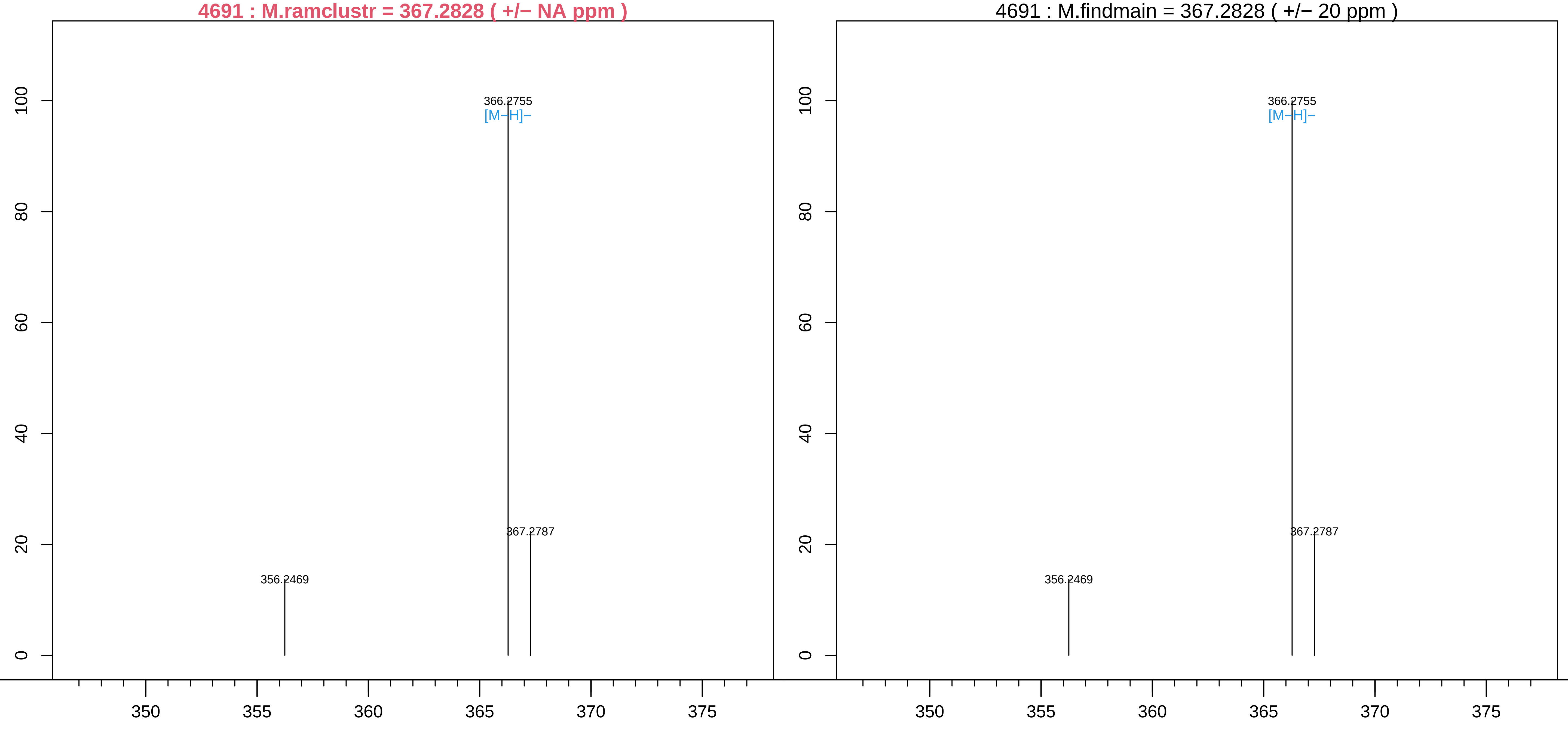
4691 : M.ramclustr = 367.2828 (+/− NA ppm)
4691 : M.findmain = 367.2828 (+/− 20 ppm)
366.2755
[M−H]−
367.2787
356.2469
0
20
40
60
80
100
350
355
360
365
370
375

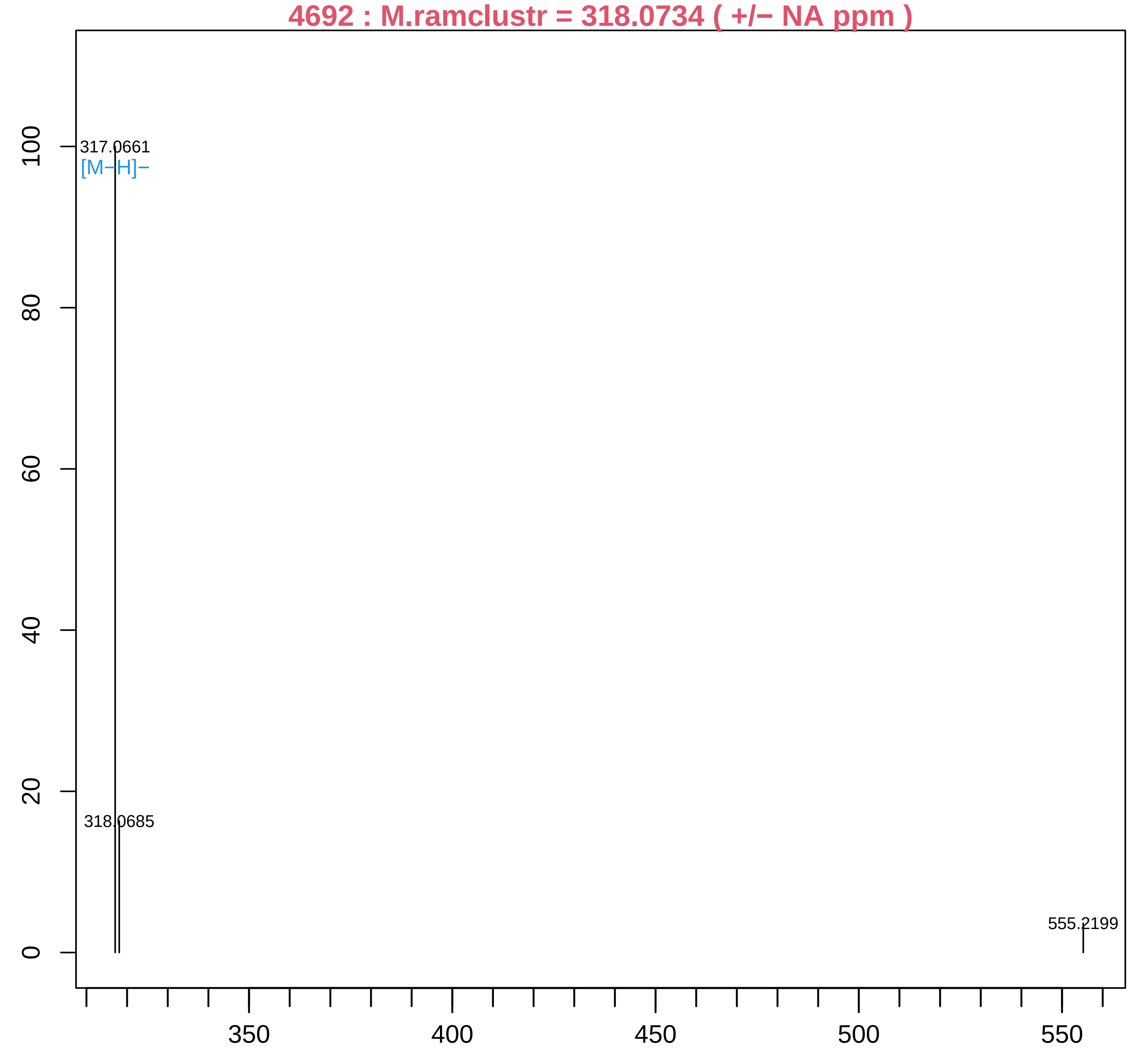
4692 : M.ramclustr = 318.0734 (+/− NA ppm)
317.0661
[M−H]−
318.0685
555.2199
0
20
40
60
80
100
350
400
450
500
550

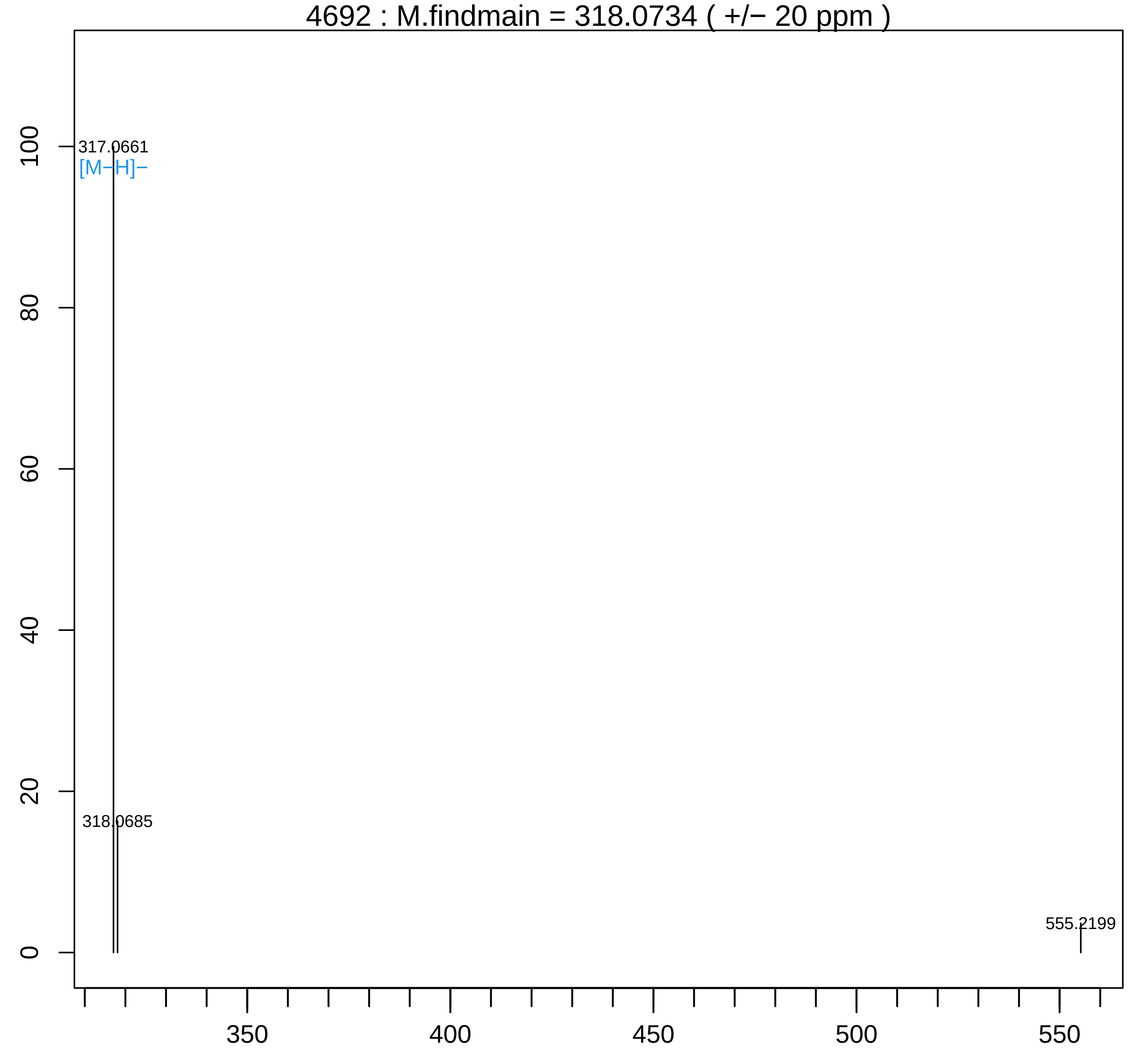
4692 : M.findmain = 318.0734 (+/− 20 ppm)
317.0661
[M−H]−
318.0685
555.2199
0
20
40
60
80
100
350
400
450
500
550

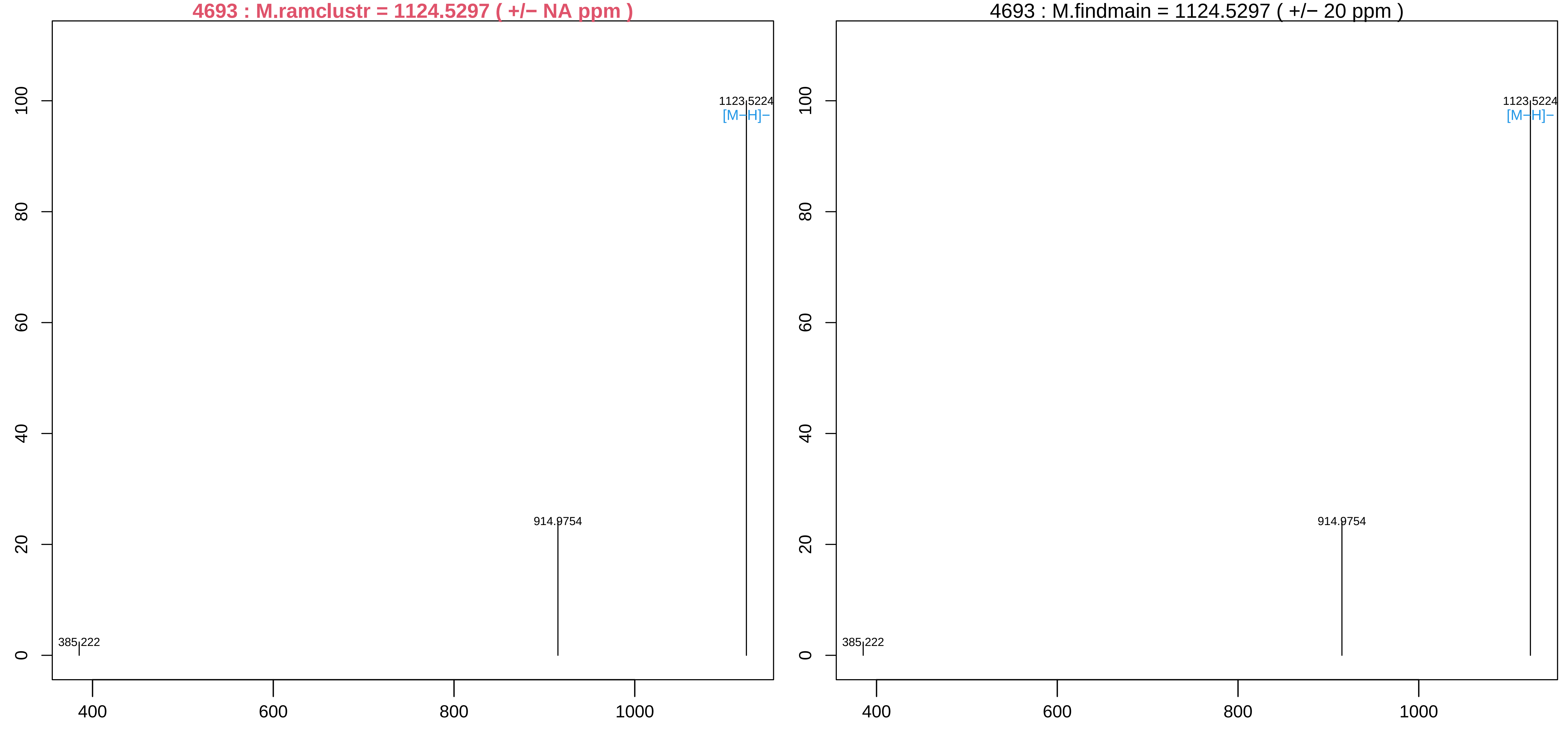
4693 : M.ramclustr = 1124.5297 (+/− NA ppm)
4693 : M.findmain = 1124.5297 (+/− 20 ppm)
1123.5224
[M−H]−
914.9754
385.222
0
20
40
60
80
100
400
600
800
1000

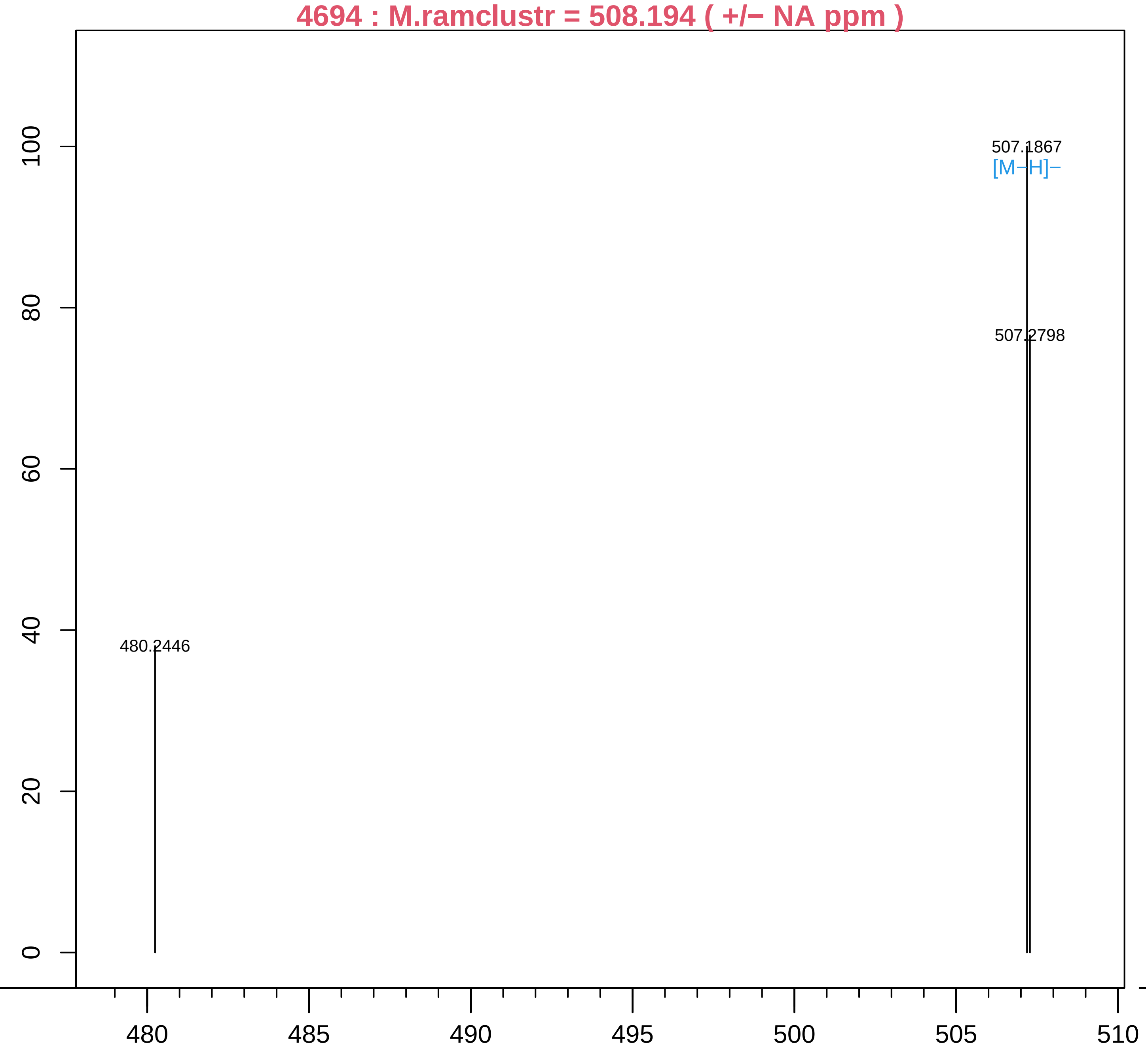
4694 : M.ramclustr = 508.194 (+/− NA ppm)
507.1867
[M−H]−
507.2798
480.2446
0
20
40
60
80
100
480
485
490
495
500
505
510

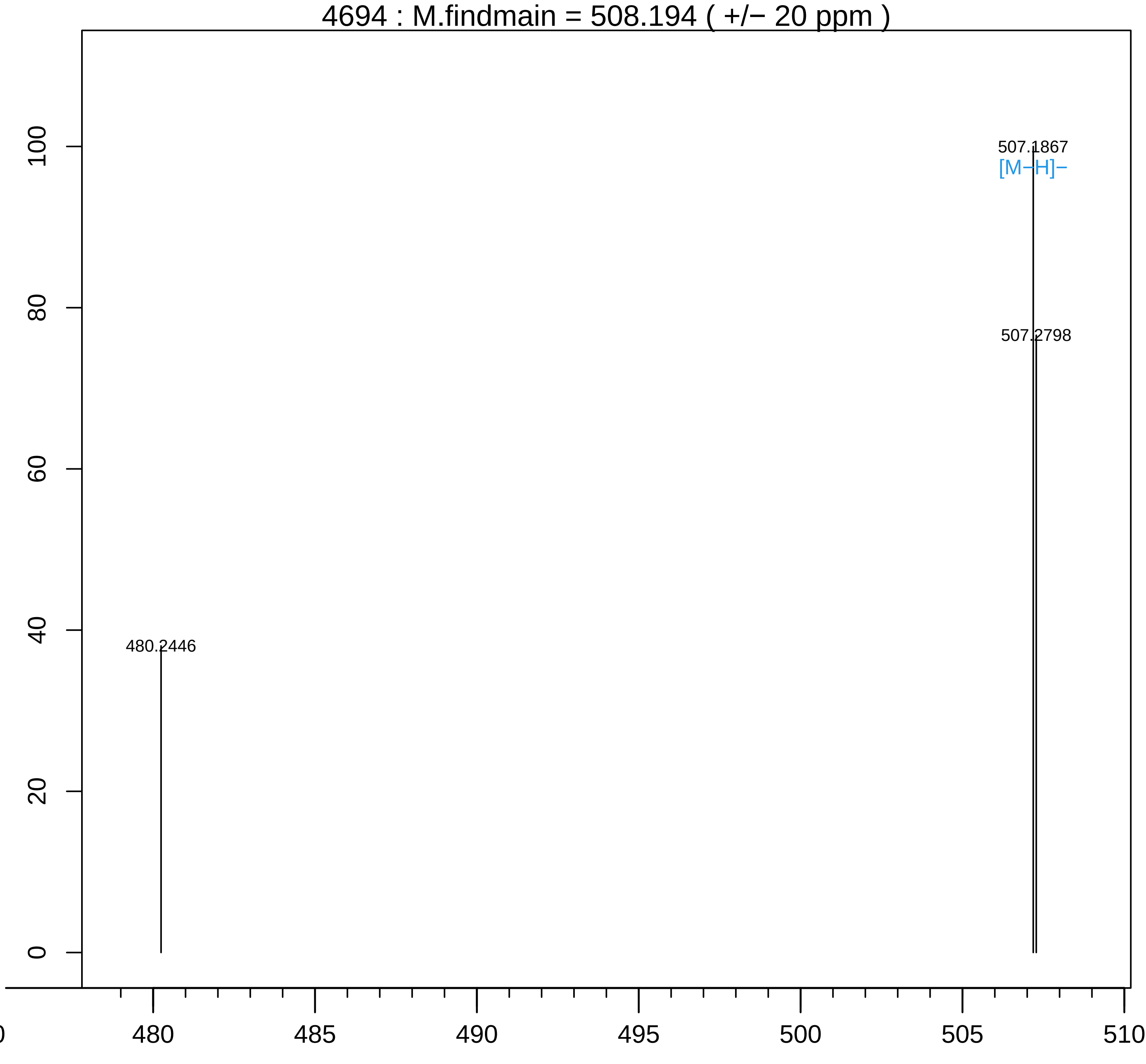
4694 : M.findmain = 508.194 (+/− 20 ppm)
507.1867
[M−H]−
507.2798
480.2446
0
20
40
60
80
100
480
485
490
495
500
505
510

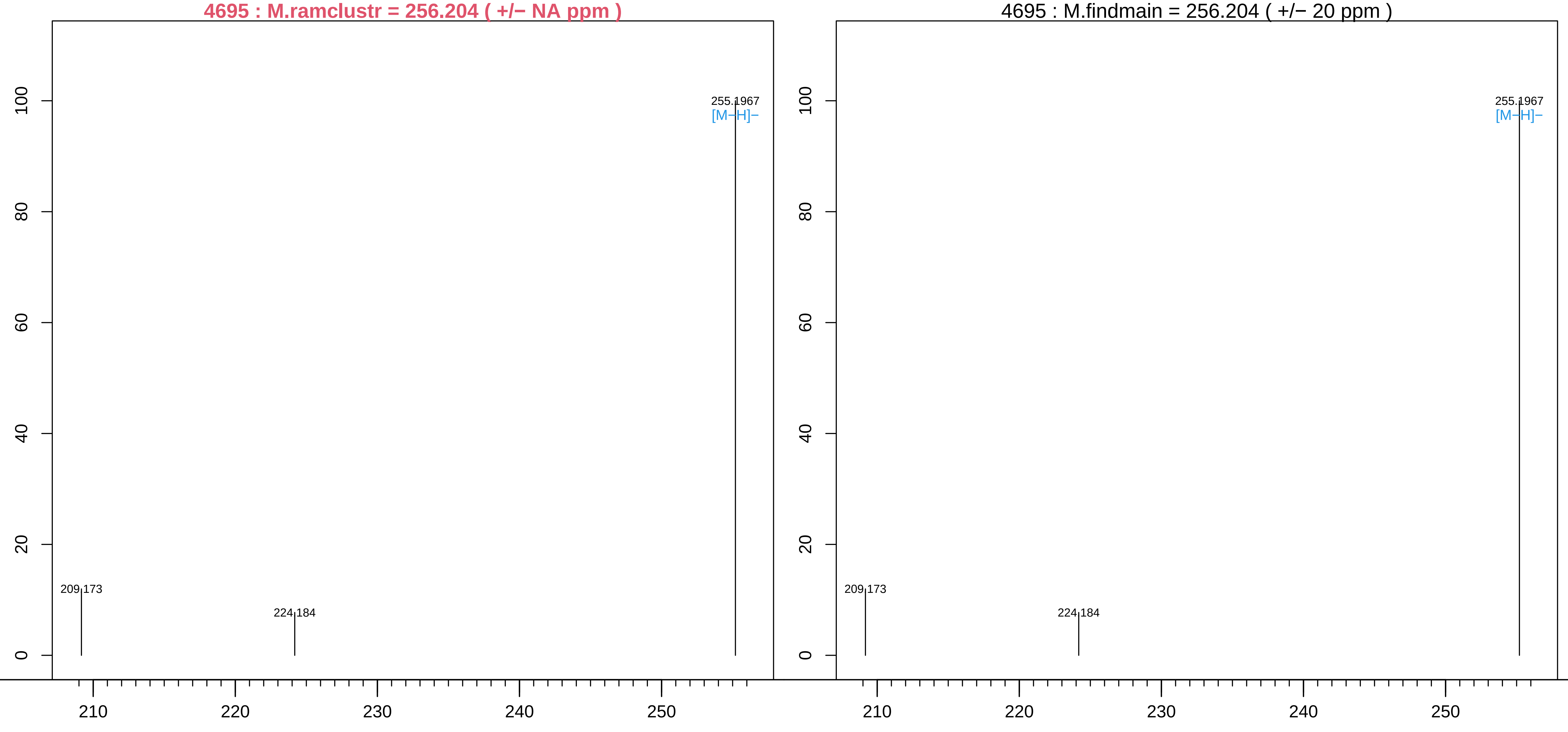
4695 : M.ramclustr = 256.204 (+/− NA ppm)
4695 : M.findmain = 256.204 (+/− 20 ppm)
255.1967
[M−H]−
209.173
224.184
0
20
40
60
80
100
210
220
230
240
250

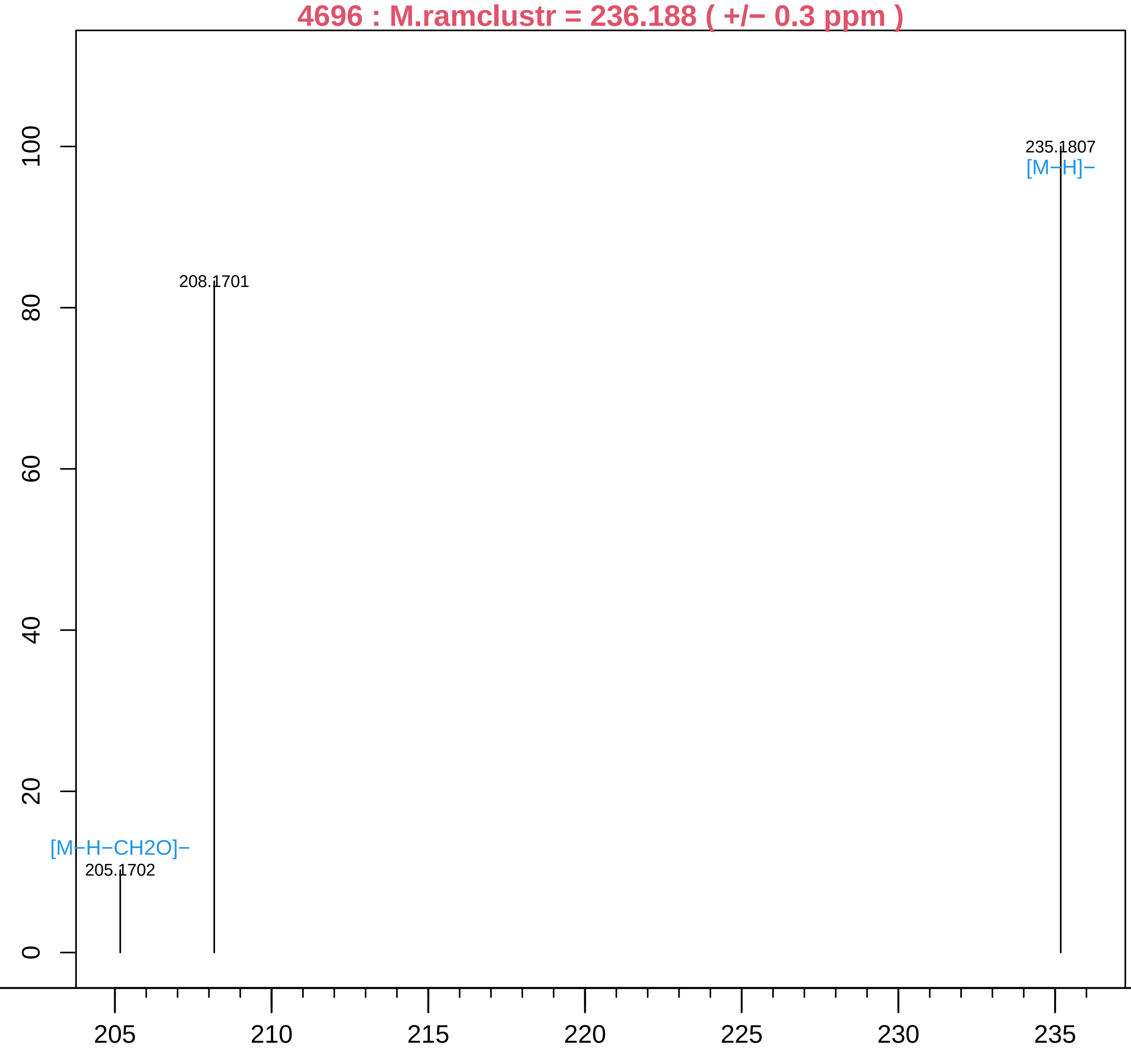

4696 : M.ramclustr = 236.188 (+/− 0.3 ppm)
235.1807
[M−H]−
208.1701
[M−H−CH2O]−
205.1702
0
20
40
60
80
100
205
210
215
220
225
230
235

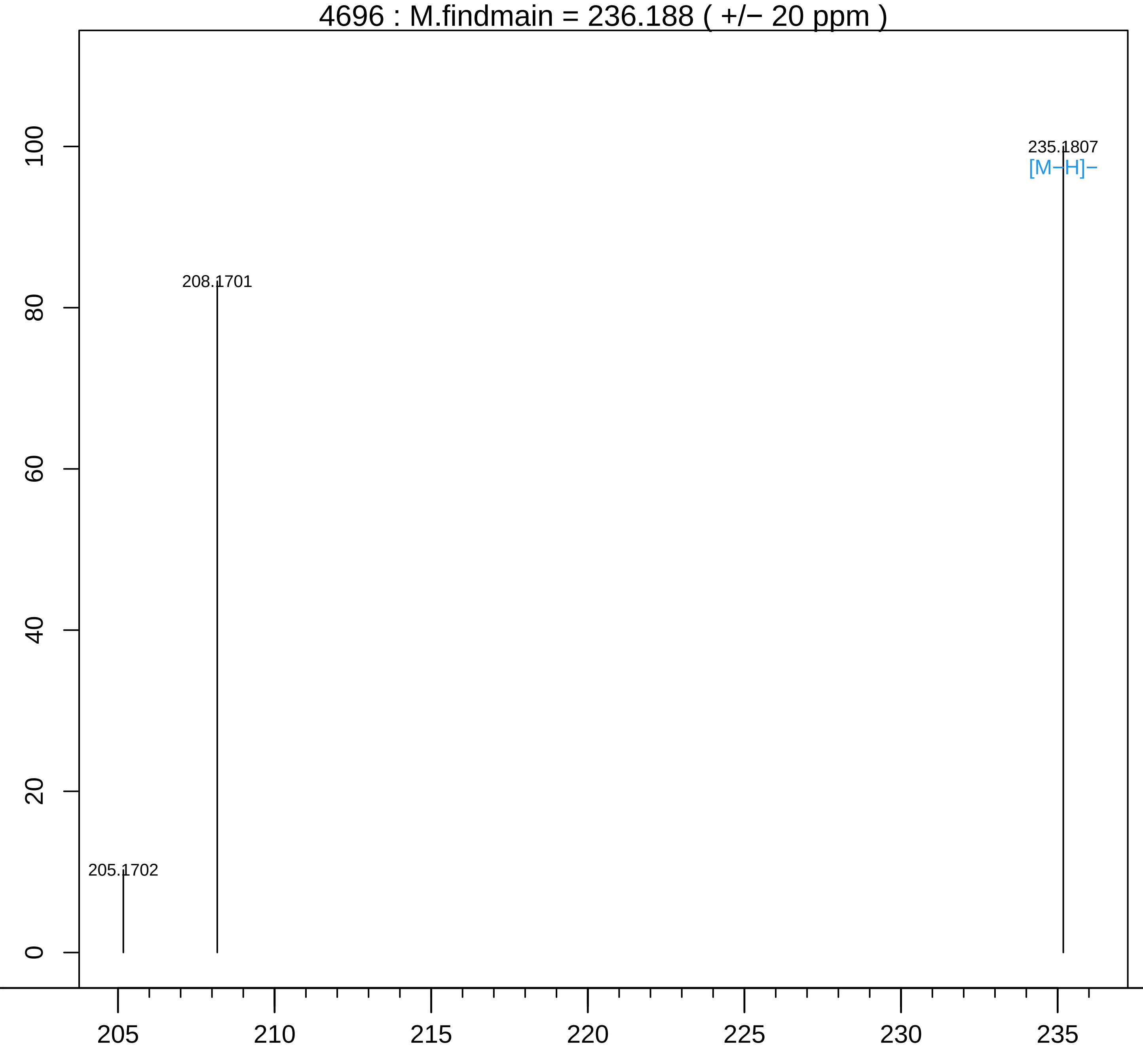

4696 : M.findmain = 236.188 (+/− 20 ppm)
235.1807
[M−H]−
208.1701
205.1702
0
20
40
60
80
100
205
210
215
220
225
230
235

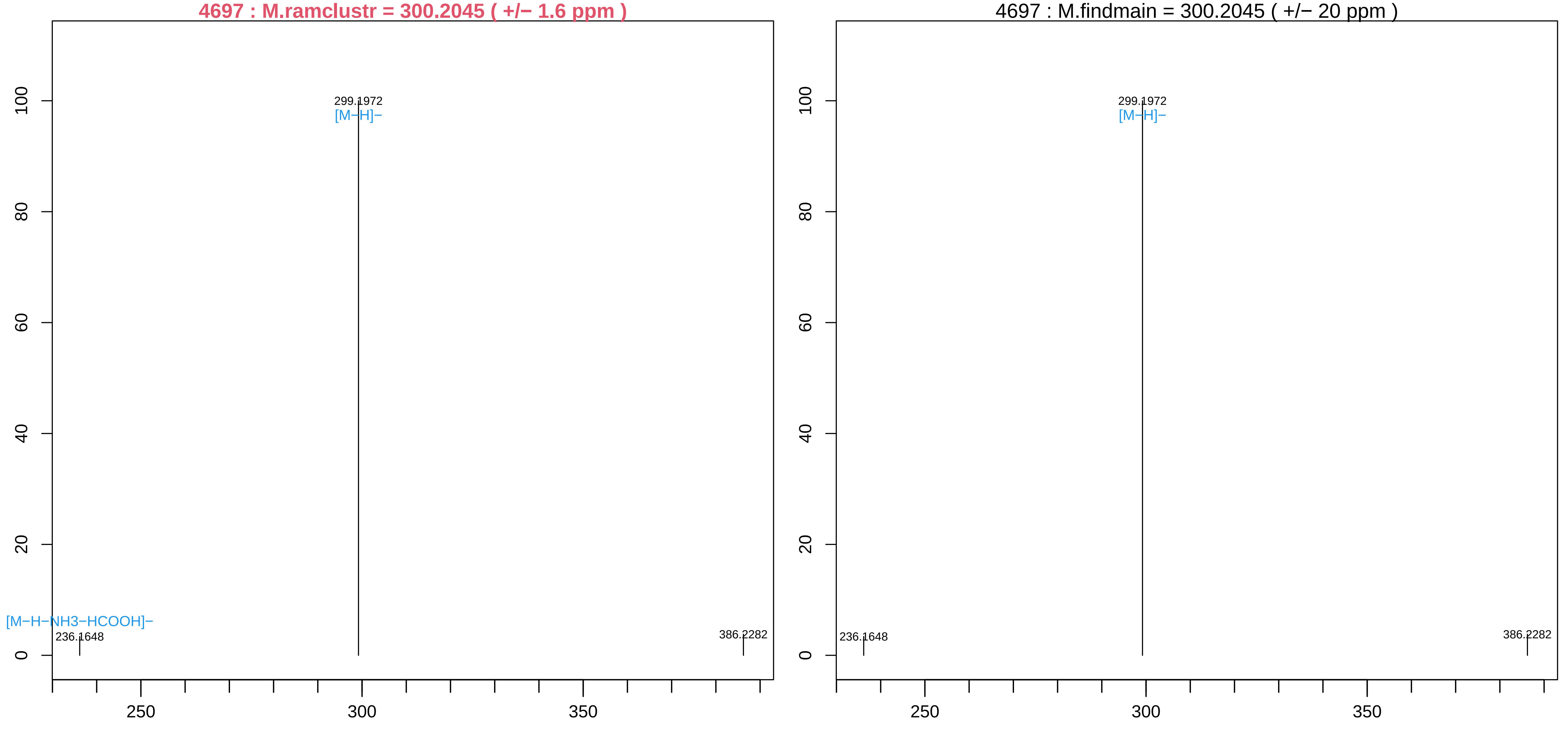

4697 : M.ramclustr = 300.2045 (+/− 1.6 ppm)
299.1972
[M−H]−
[M−H−NH3−HCOOH]−
236.1648
386.2282
0
20
40
60
80
100
250
300
350
4697 : M.findmain = 300.2045 (+/− 20 ppm)
299.1972
[M−H]−
236.1648
386.2282
0
20
40
60
80
100
250
300
350

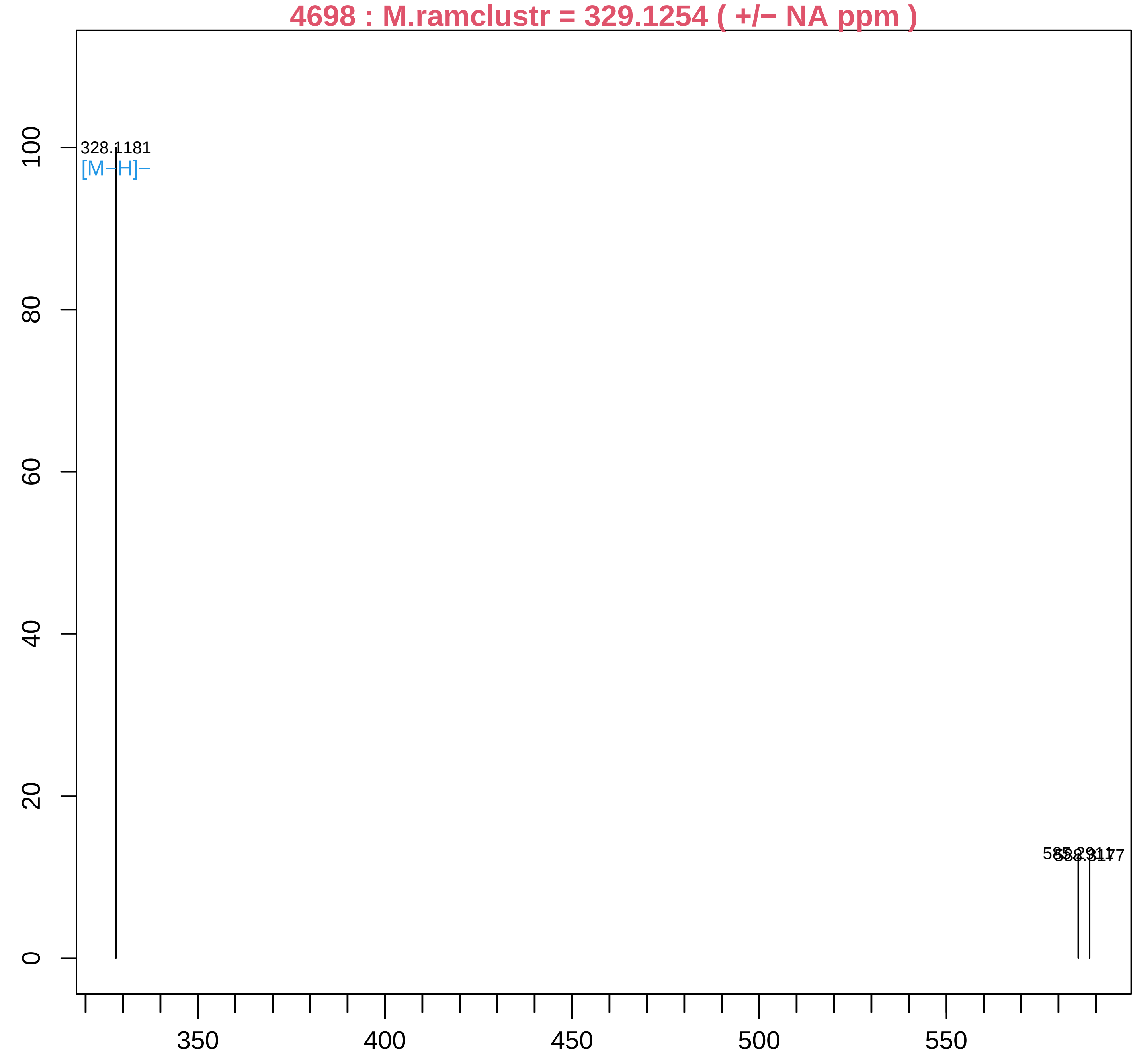
4698 : M.ramclustr = 329.1254 (+/− NA ppm)
328.1181
[M−H]−
0
20
40
60
80
100
350
400
450
500
550

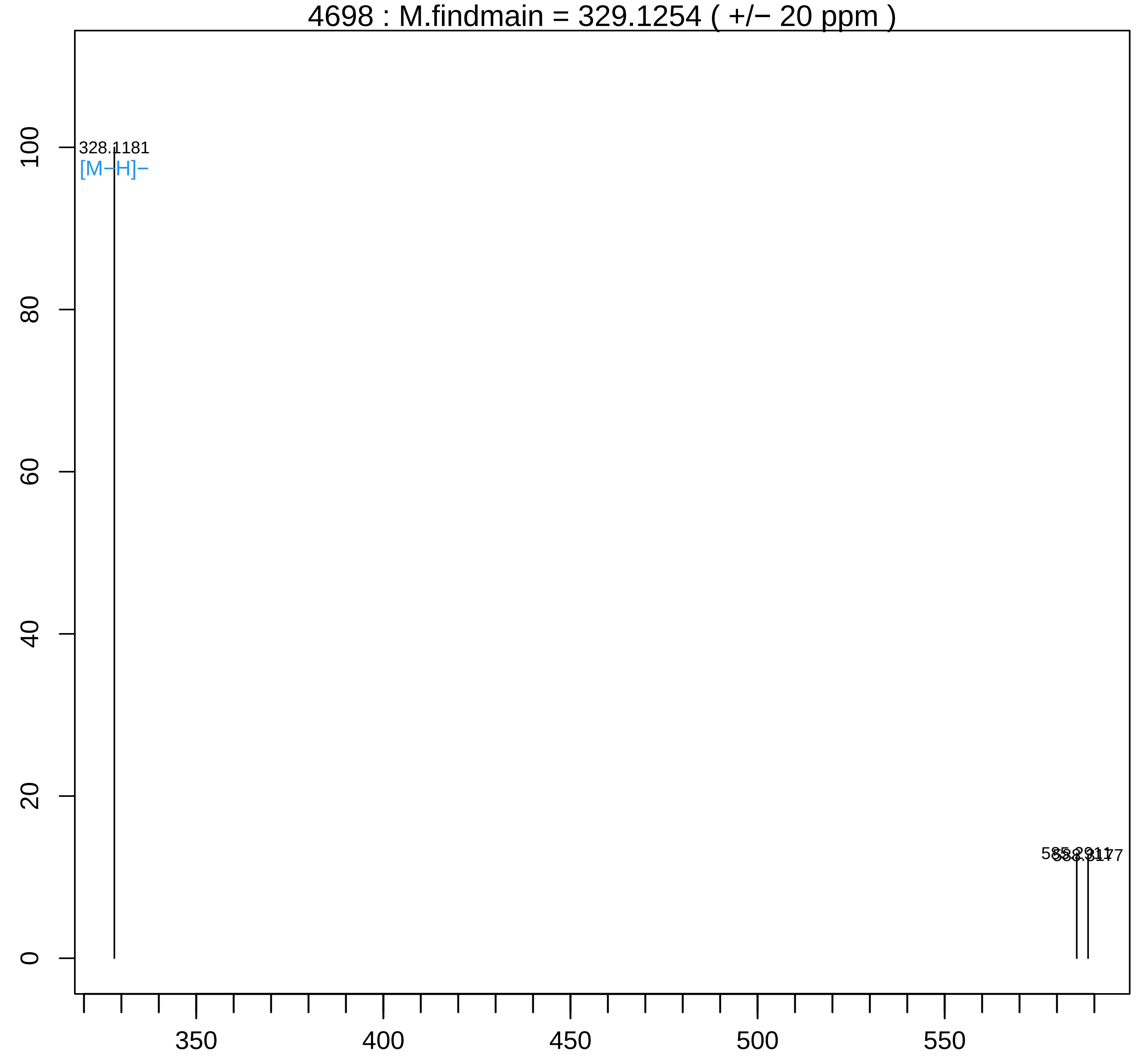
4698 : M.findmain = 329.1254 (+/− 20 ppm)
328.1181
[M−H]−
0
20
40
60
80
100
350
400
450
500
550

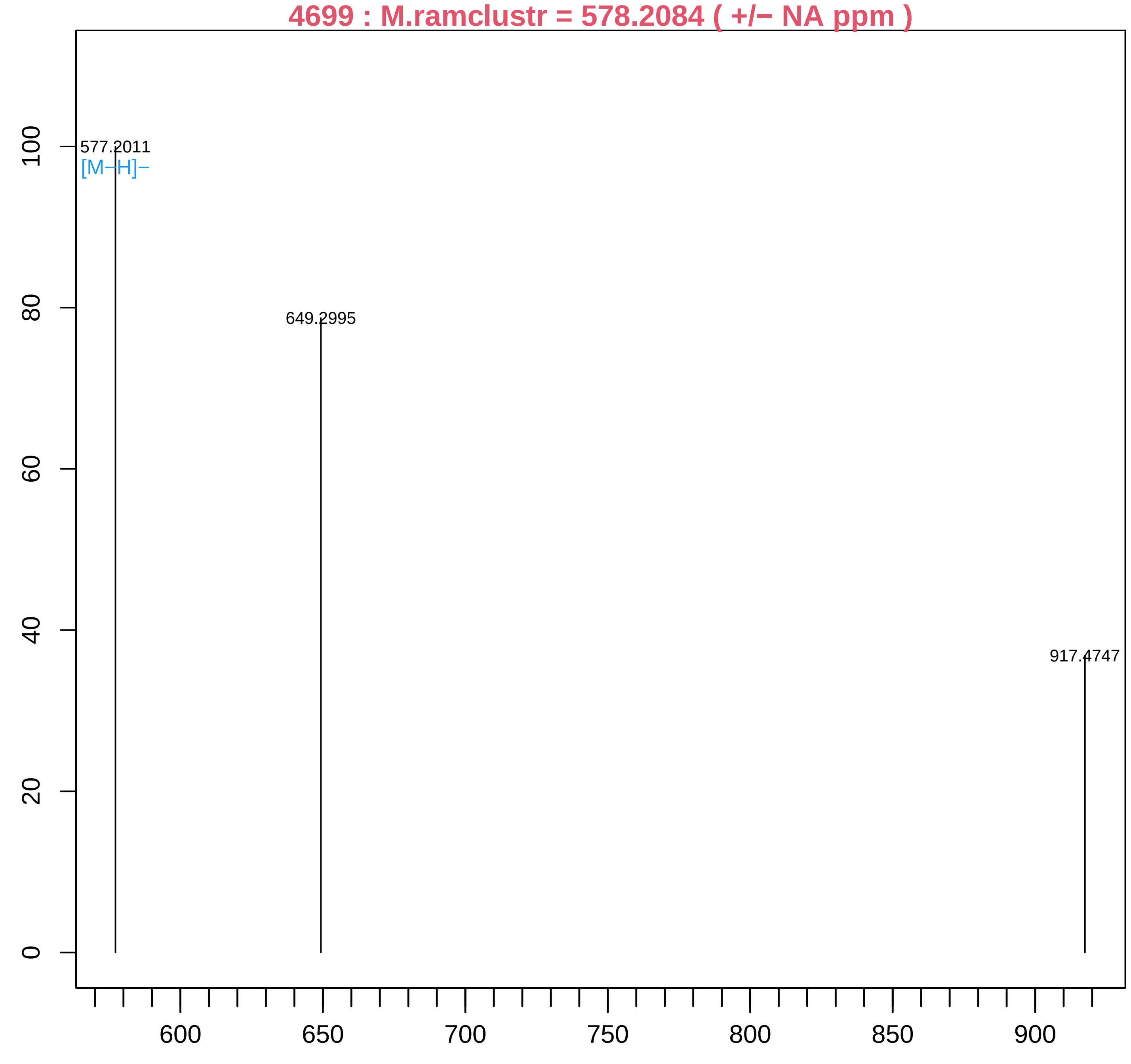
4699 : M.ramclustr = 578.2084 (+/− NA ppm)
577.2011
[M−H]−
649.2995
917.4747
0
20
40
60
80
100
600
650
700
750
800
850
900

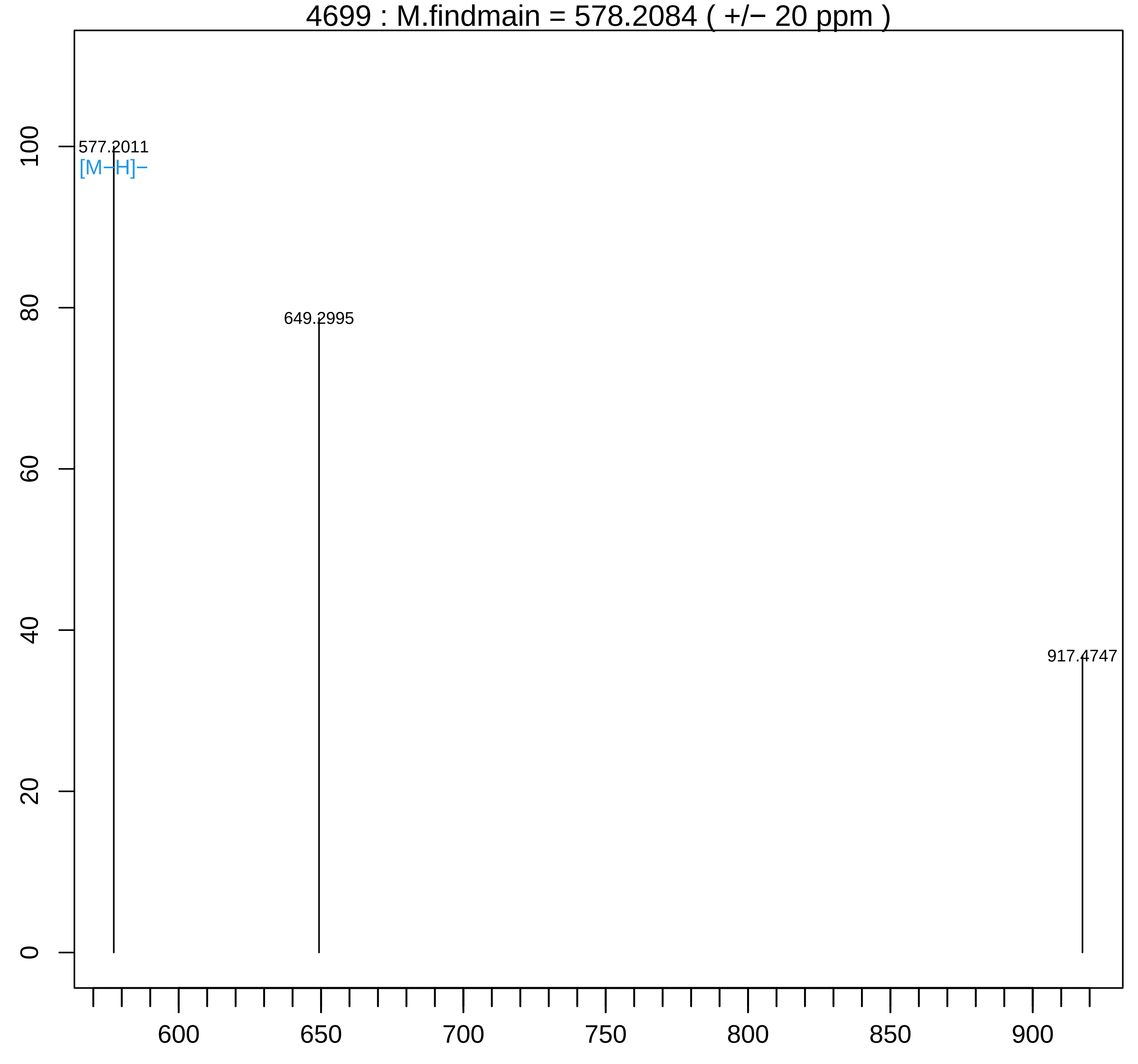
4699 : M.findmain = 578.2084 (+/− 20 ppm)
577.2011
[M−H]−
649.2995
917.4747
0
20
40
60
80
100
600
650
700
750
800
850
900

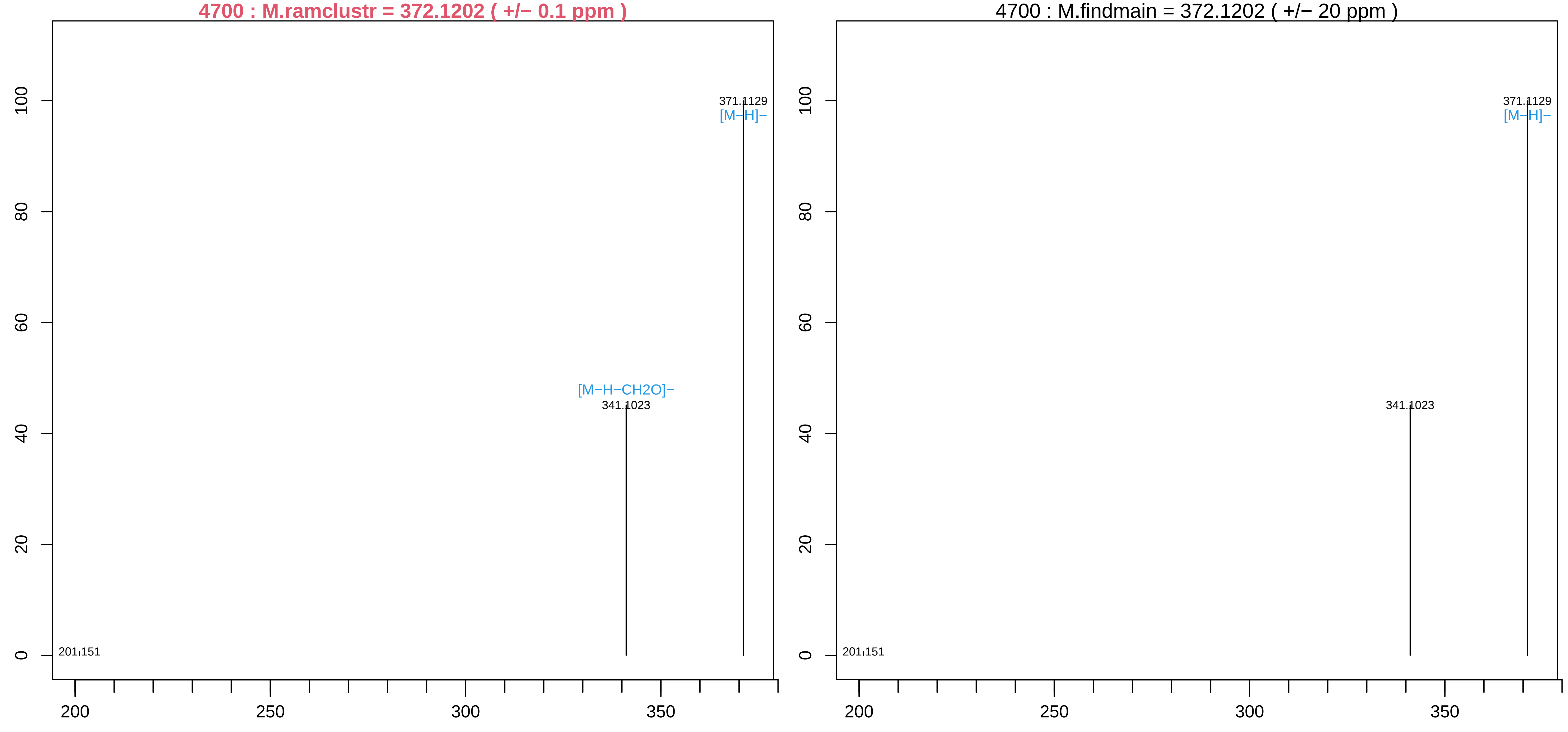
4700 : M.ramclustr = 372.1202 (+/− 0.1 ppm)
371.1129
[M−H]−
[M−H−CH2O]−
341.1023
201.151
4700 : M.findmain = 372.1202 (+/− 20 ppm)
371.1129
[M−H]−
341.1023
201.151

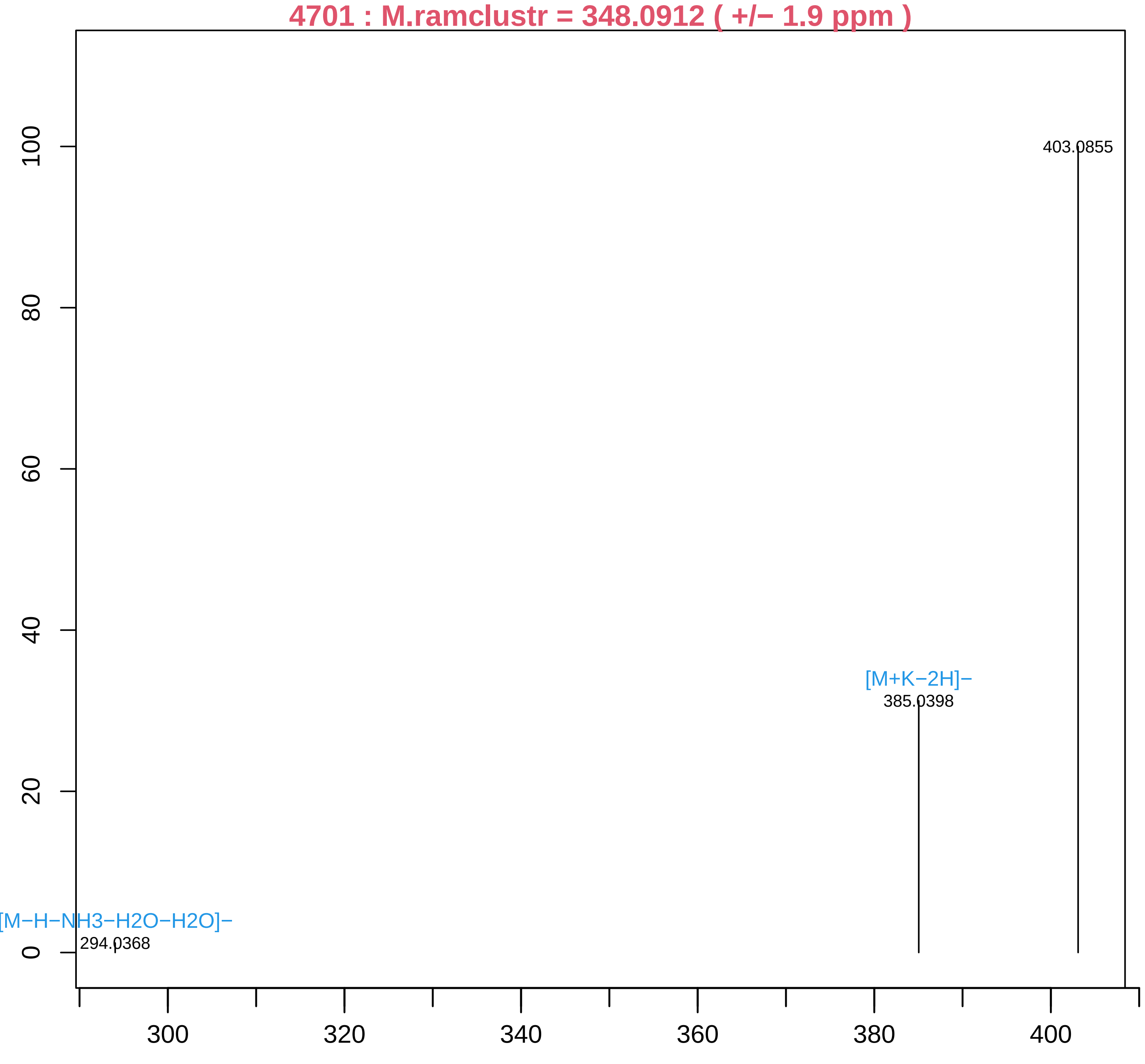
4701 : M.ramclustr = 348.0912 (+/− 1.9 ppm)
403.0855
[M+K−2H]−
385.0398
[M−H−NH3−H2O−H2O]−
294.0368
0
20
40
60
80
100
300
320
340
360
380
400

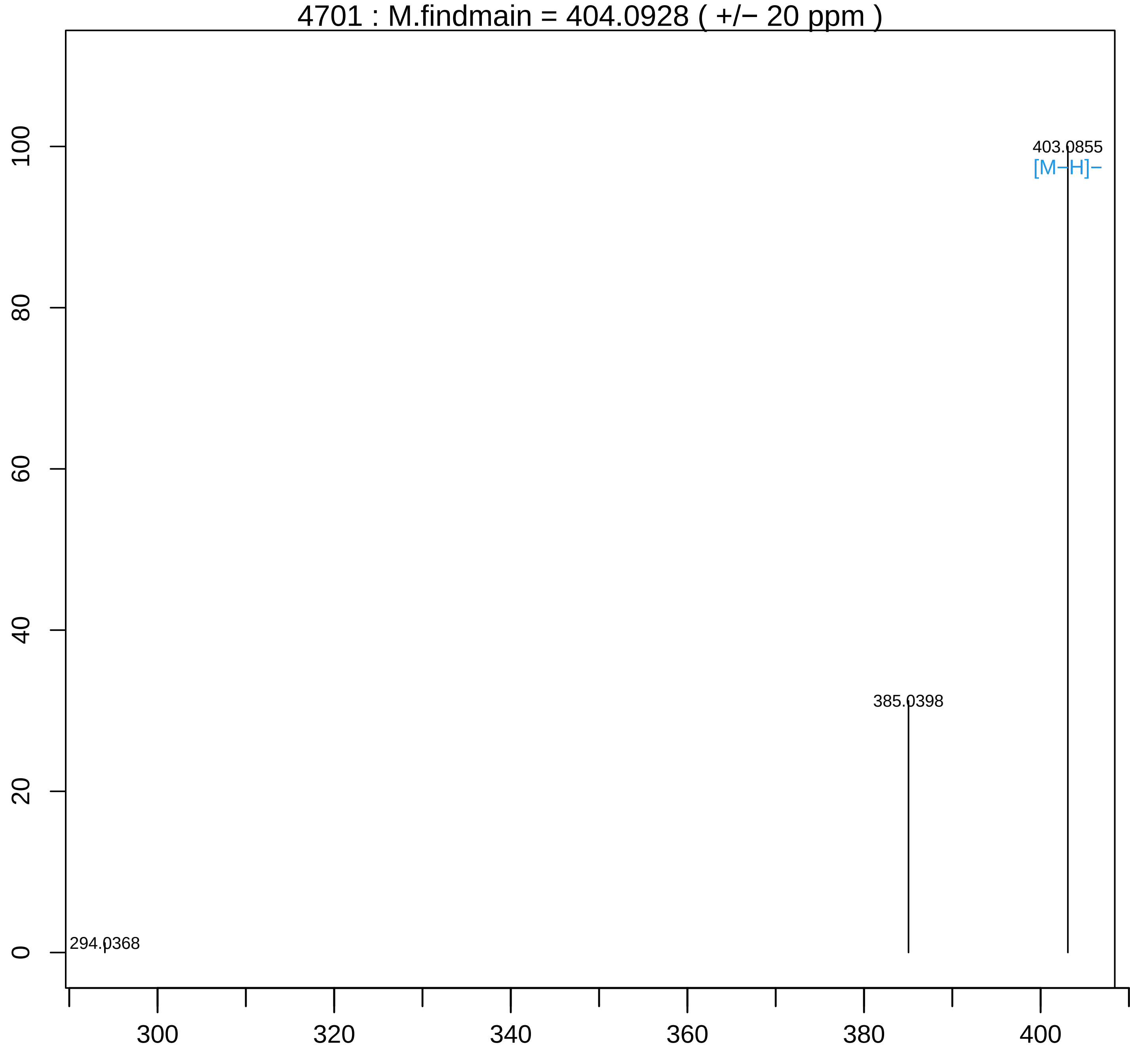
4701 : M.findmain = 404.0928 (+/− 20 ppm)
403.0855
[M−H]−
385.0398
294.0368
0
20
40
60
80
100
300
320
340
360
380
400

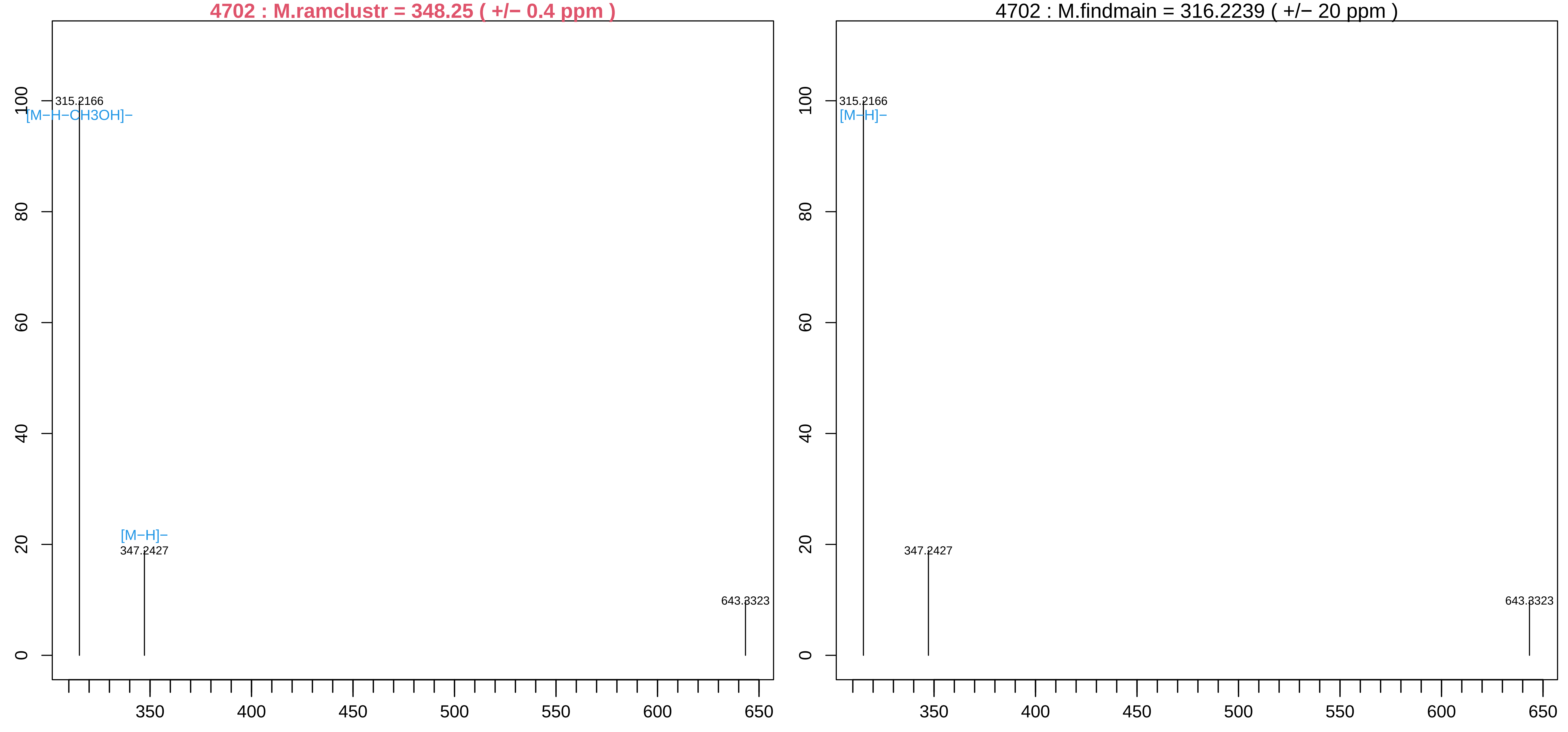

4702 : M.ramclustr = 348.25 (+/− 0.4 ppm)
315.2166
[M−H−CH3OH]−
[M−H]−
347.2427
643.3323
0
20
40
60
80
100
350
400
450
500
550
600
650
4702 : M.findmain = 316.2239 (+/− 20 ppm)
315.2166
[M−H]−
347.2427
643.3323

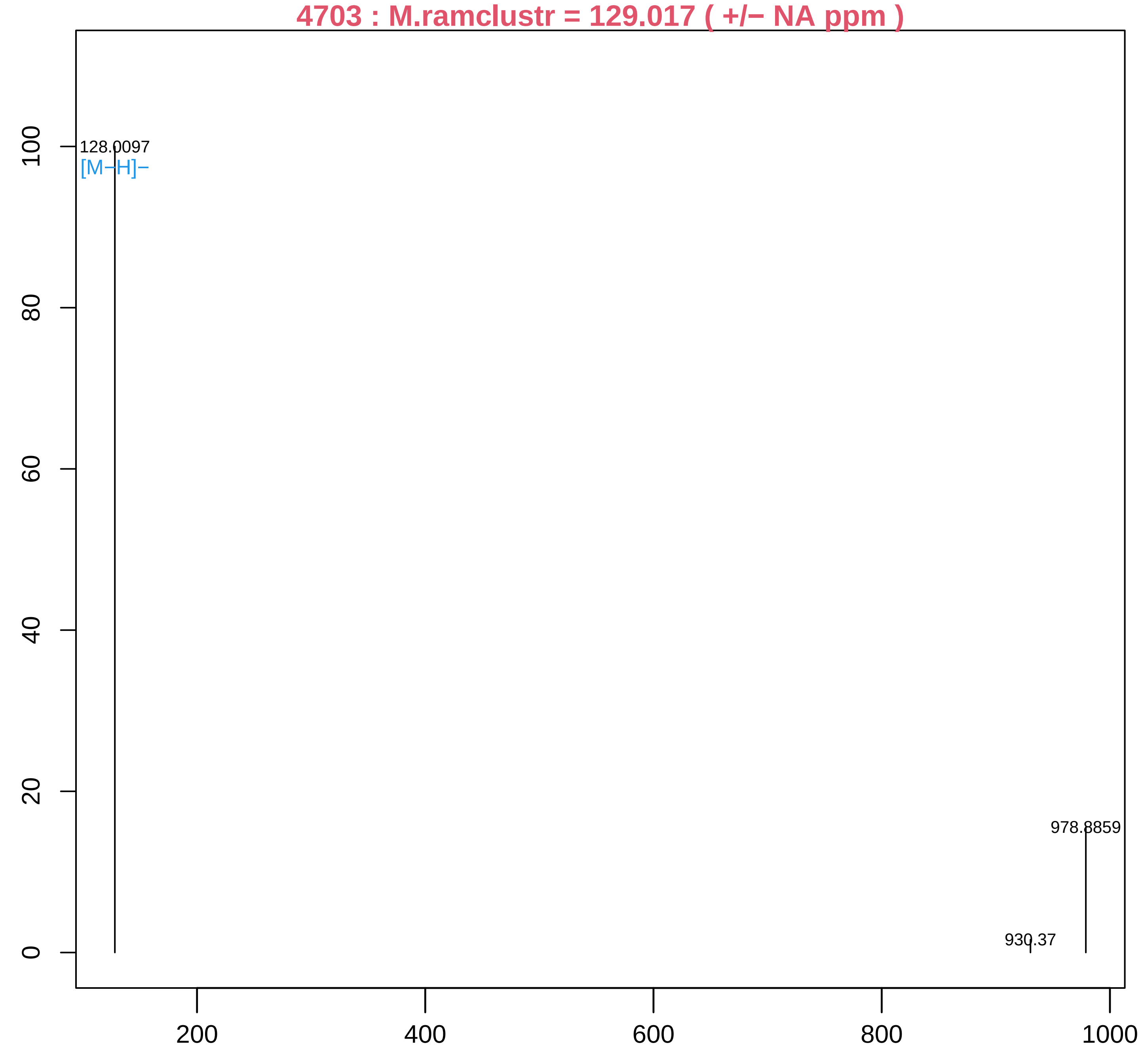
4703 : M.ramclustr = 129.017 (+/− NA ppm)
128.0097
[M−H]−
978.8859
930.37
0
20
40
60
80
100
200
400
600
800
1000

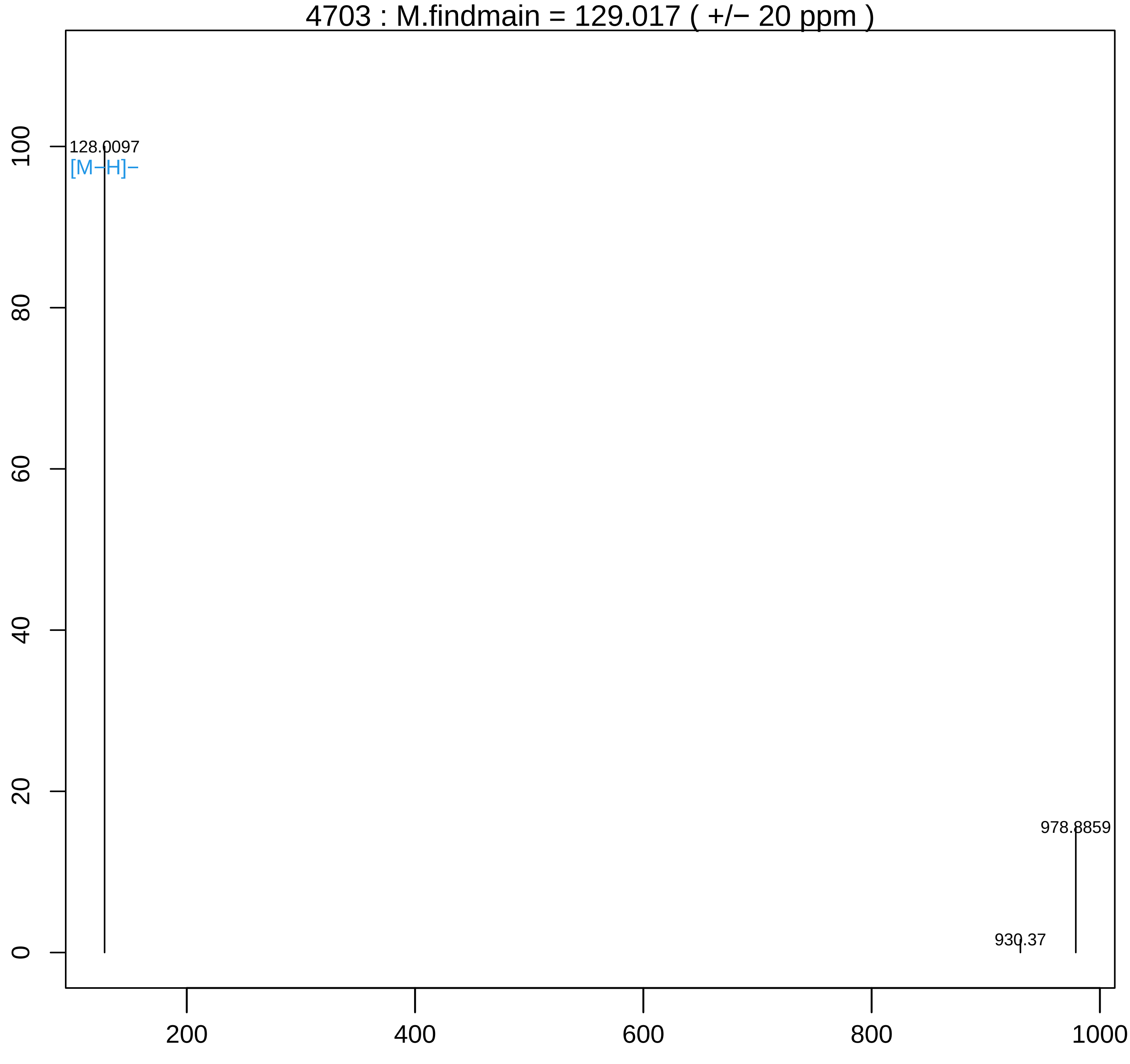
4703 : M.findmain = 129.017 (+/− 20 ppm)
128.0097
[M−H]−
978.8859
930.37
0
20
40
60
80
100
200
400
600
800
1000

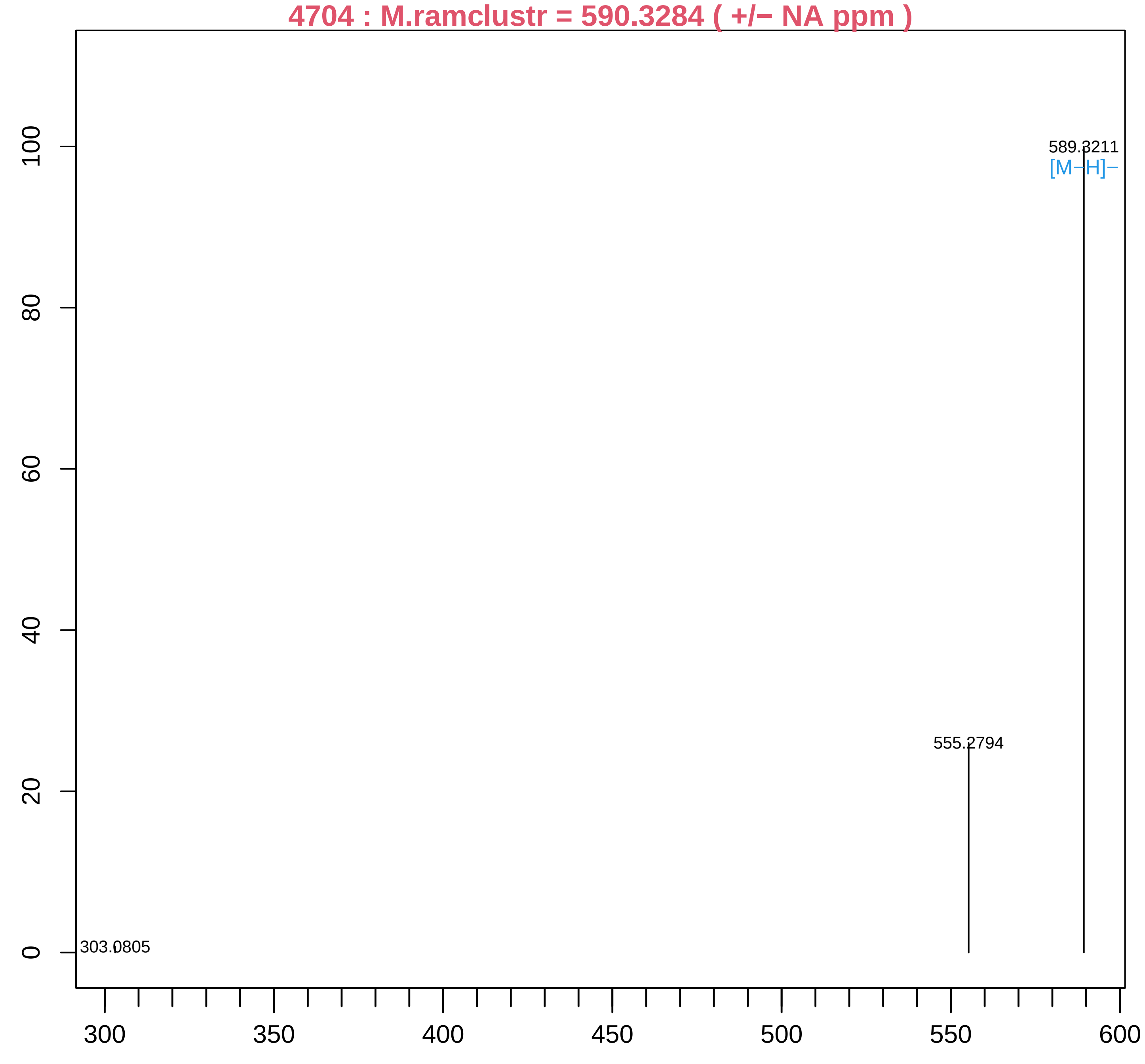

4704 : M.ramclustr = 590.3284 (+/− NA ppm)
589.3211
[M−H]−
555.2794
303.0805
0
20
40
60
80
100
300
350
400
450
500
550
600

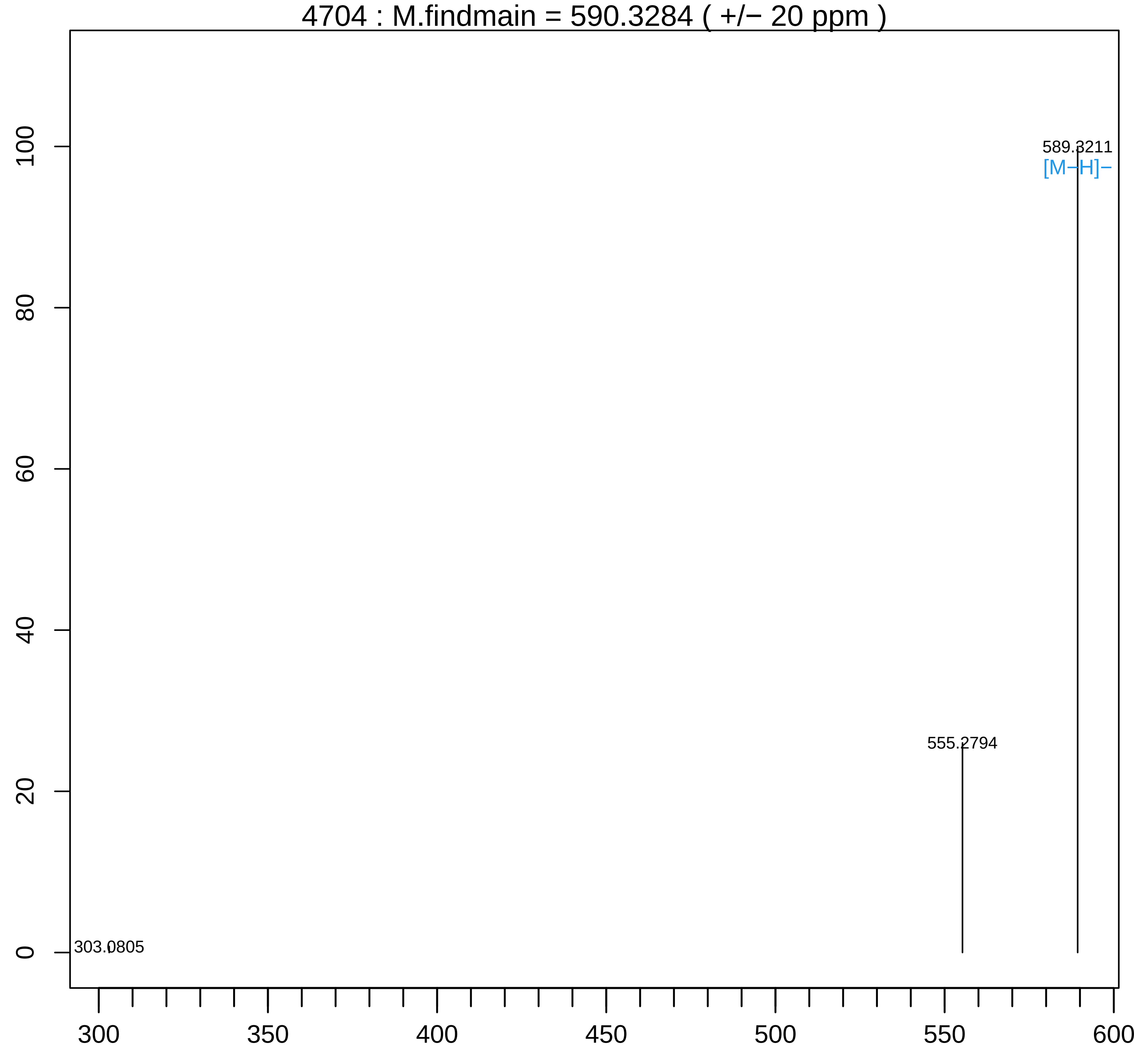

4704 : M.findmain = 590.3284 (+/− 20 ppm)
589.3211
[M−H]−
555.2794
303.0805
0
20
40
60
80
100
300
350
400
450
500
550
600

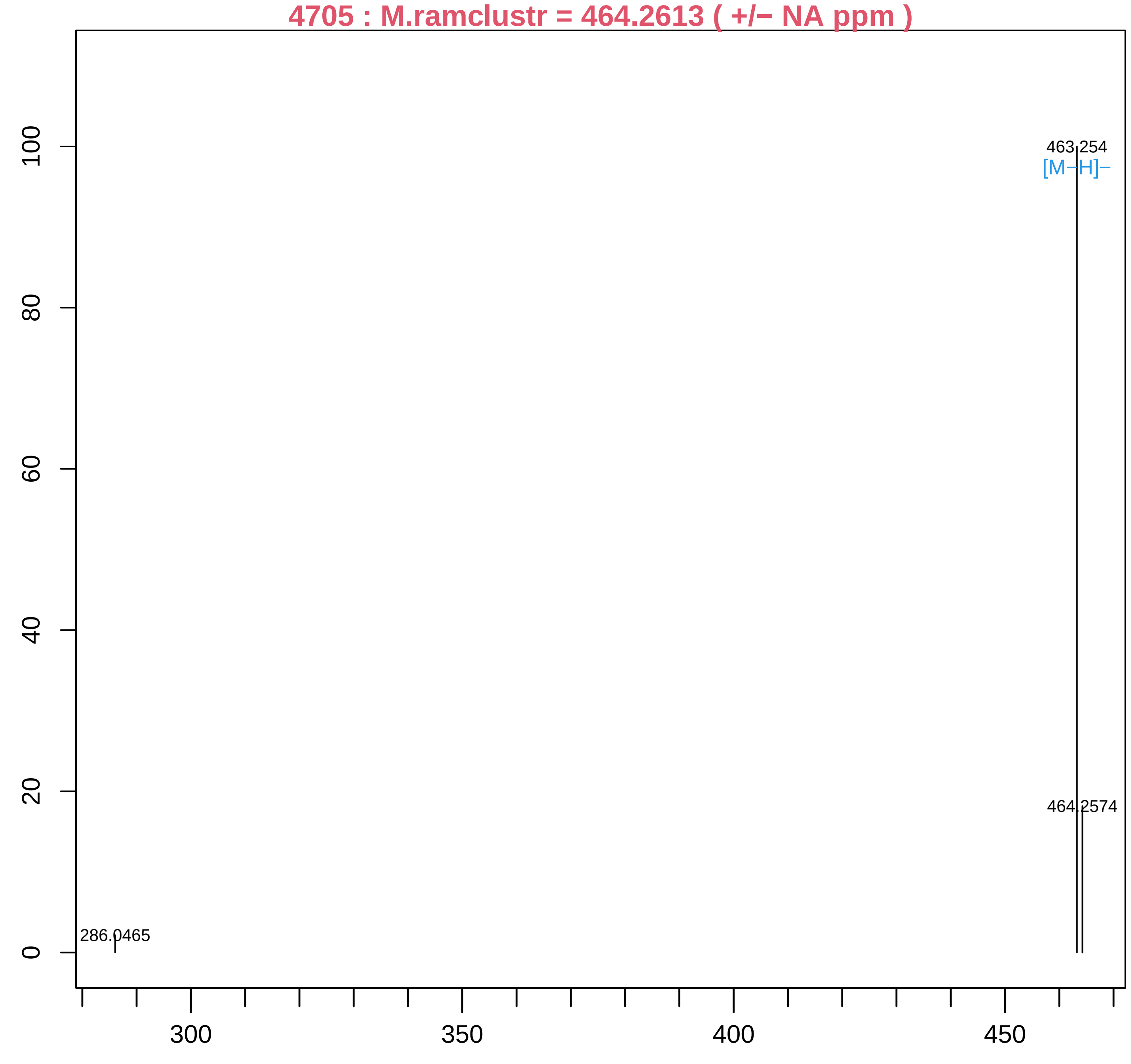
4705 : M.ramclustr = 464.2613 (+/− NA ppm)
463.254
[M−H]−
464.2574
286.0465
0
20
40
60
80
100
300
350
400
450

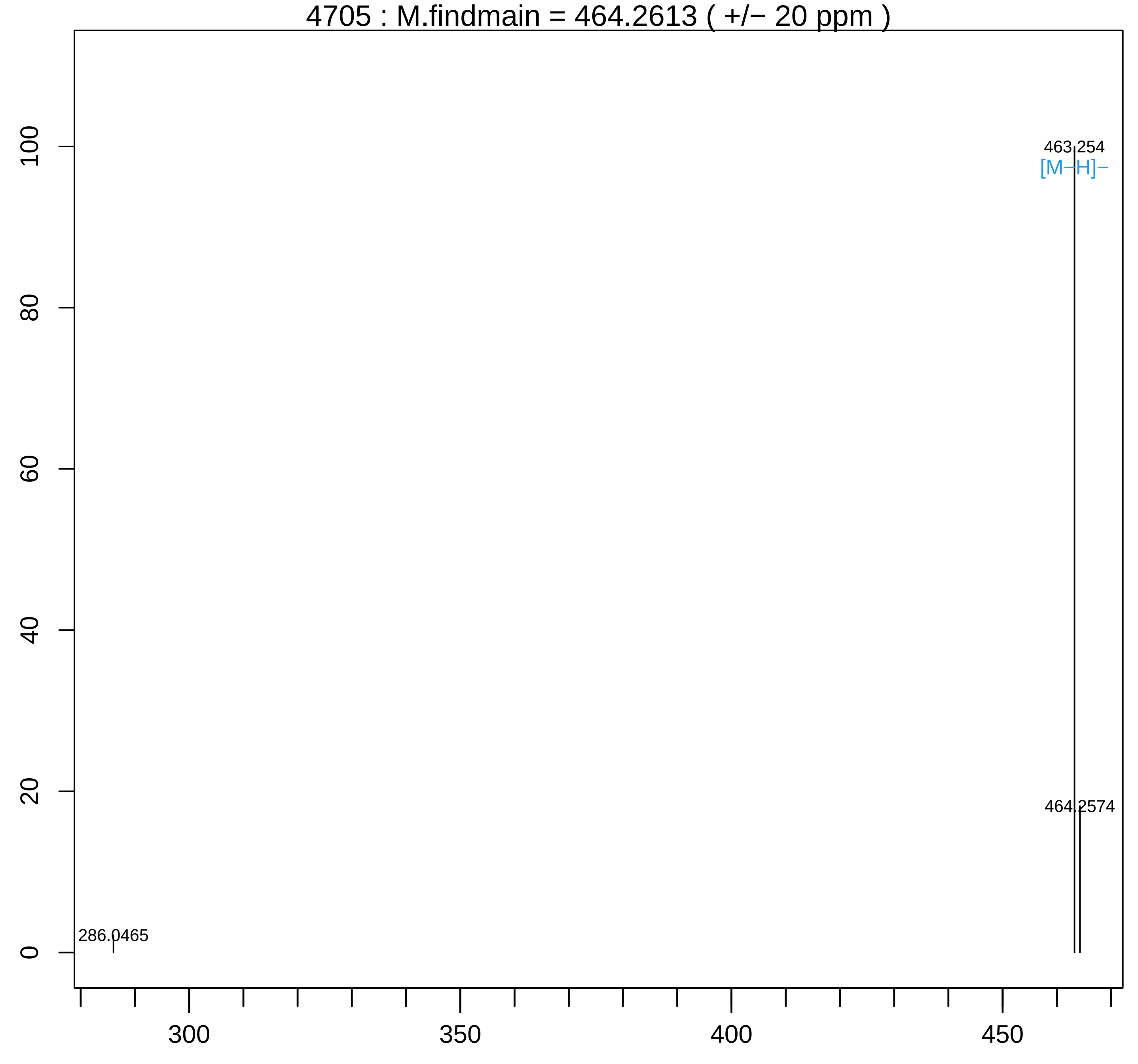
4705 : M.findmain = 464.2613 (+/− 20 ppm)
463.254
[M−H]−
464.2574
286.0465
0
20
40
60
80
100
300
350
400
450

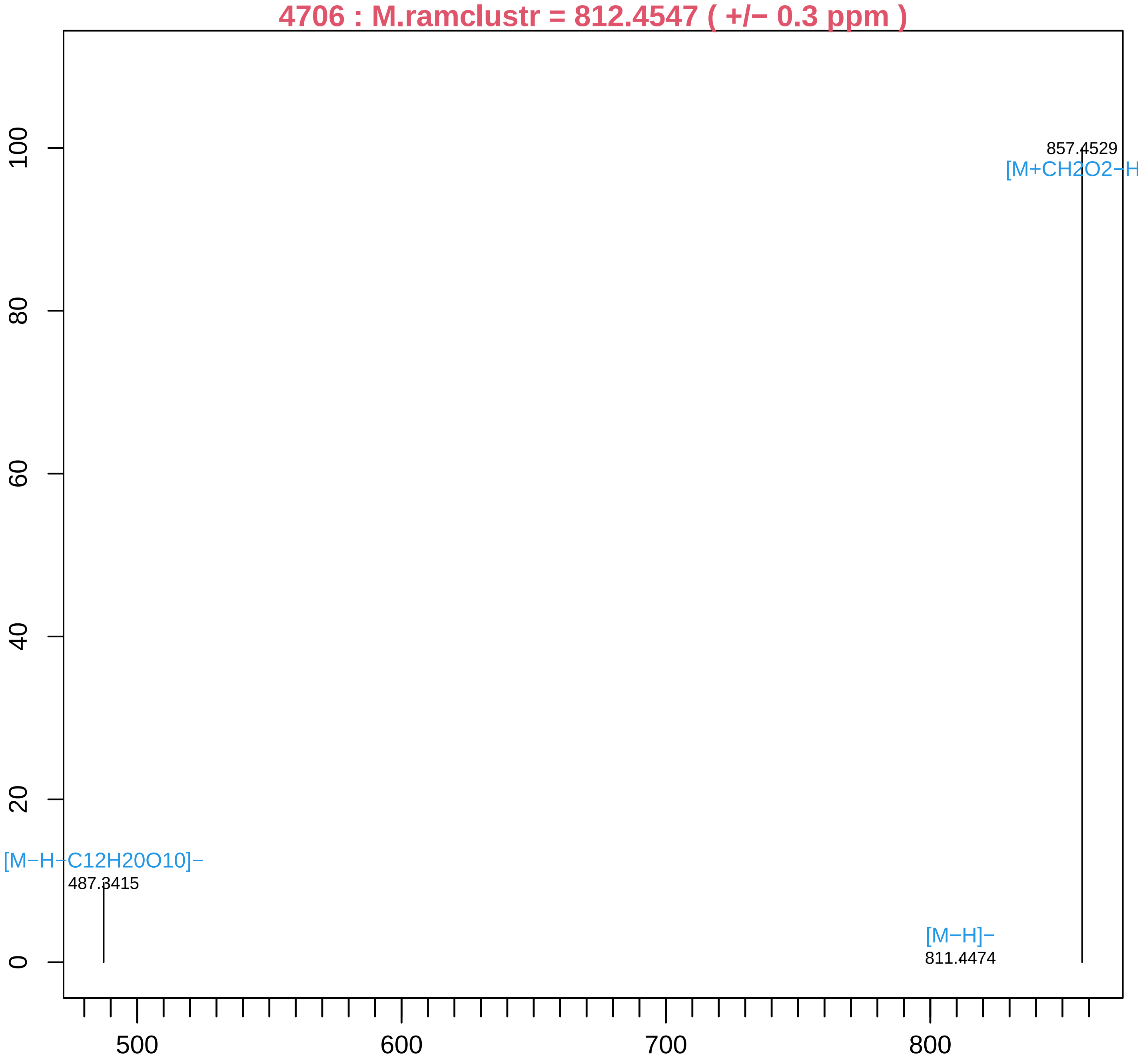
4706 : M.ramclustr = 812.4547 (+/− 0.3 ppm)
857.4529
[M+CH2O2−H
[M−H−C12H20O10]−
487.3415
[M−H]−
811.4474
0
20
40
60
80
100
500
600
700
800

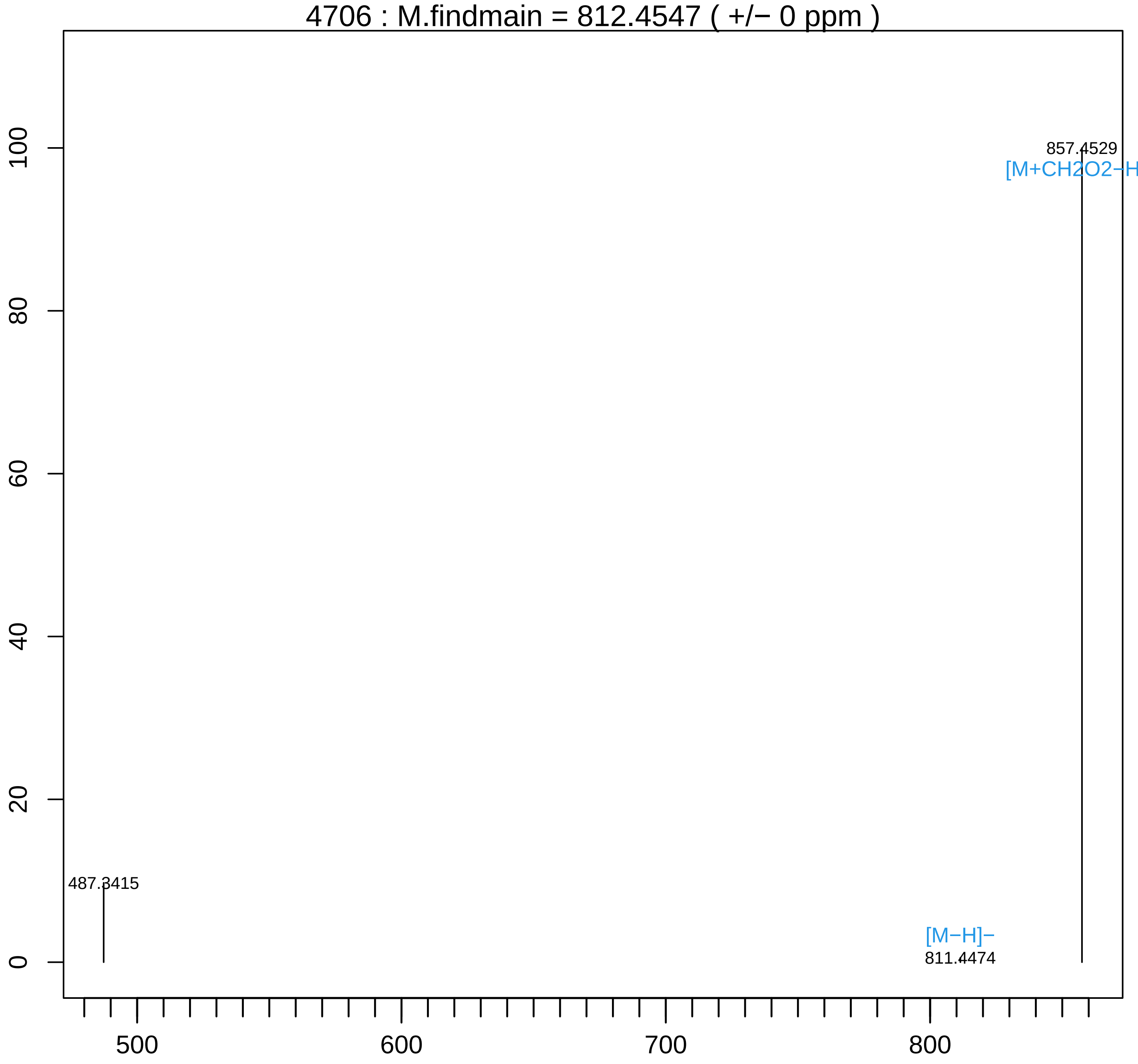
4706 : M.findmain = 812.4547 (+/− 0 ppm)
857.4529
[M+CH2O2−H
487.3415
[M−H]−
811.4474
0
20
40
60
80
100
500
600
700
800

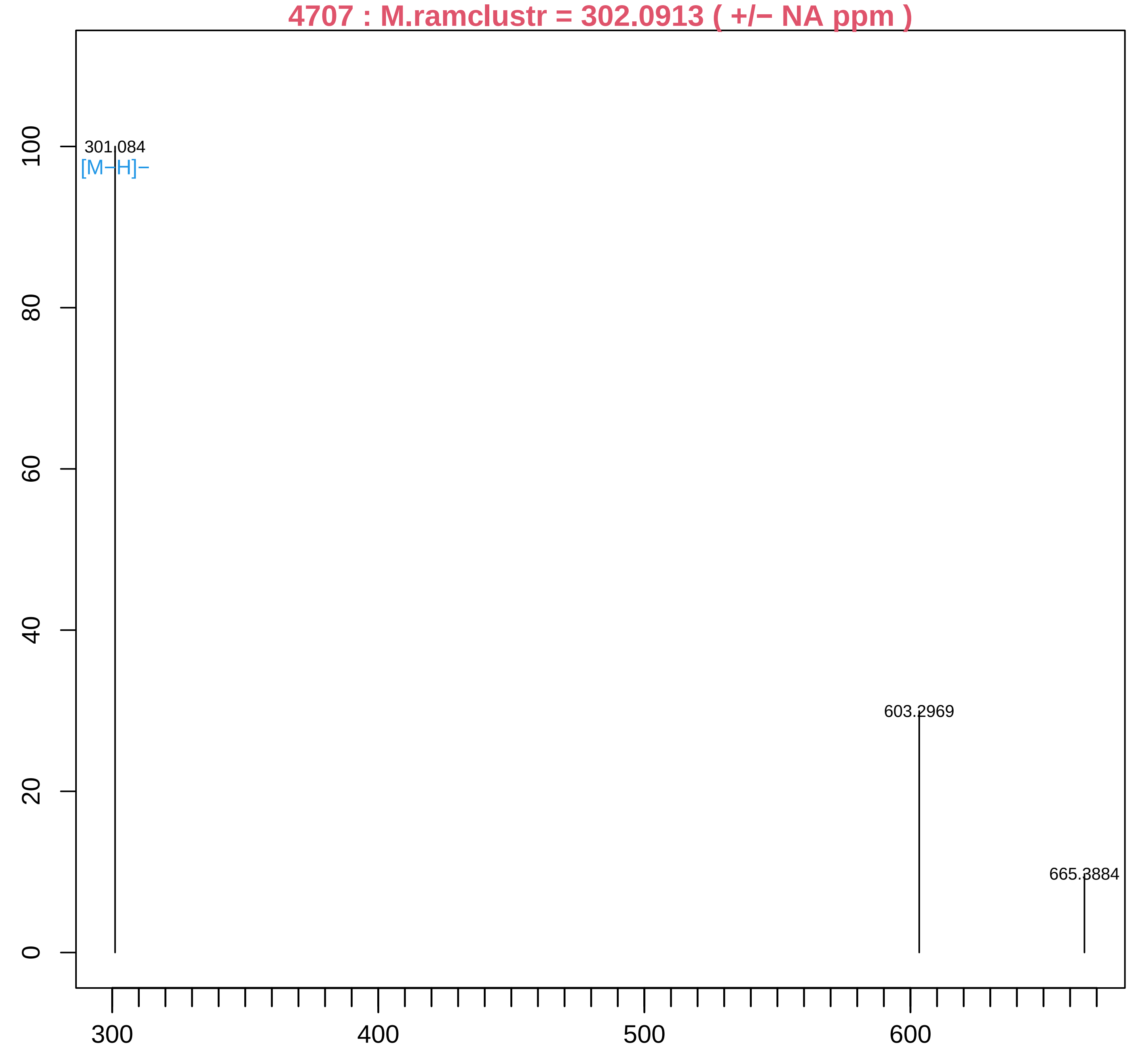
4707 : M.ramclustr = 302.0913 (+/− NA ppm)
301.084
[M−H]−
603.2969
665.3884
0
20
40
60
80
100
300
400
500
600
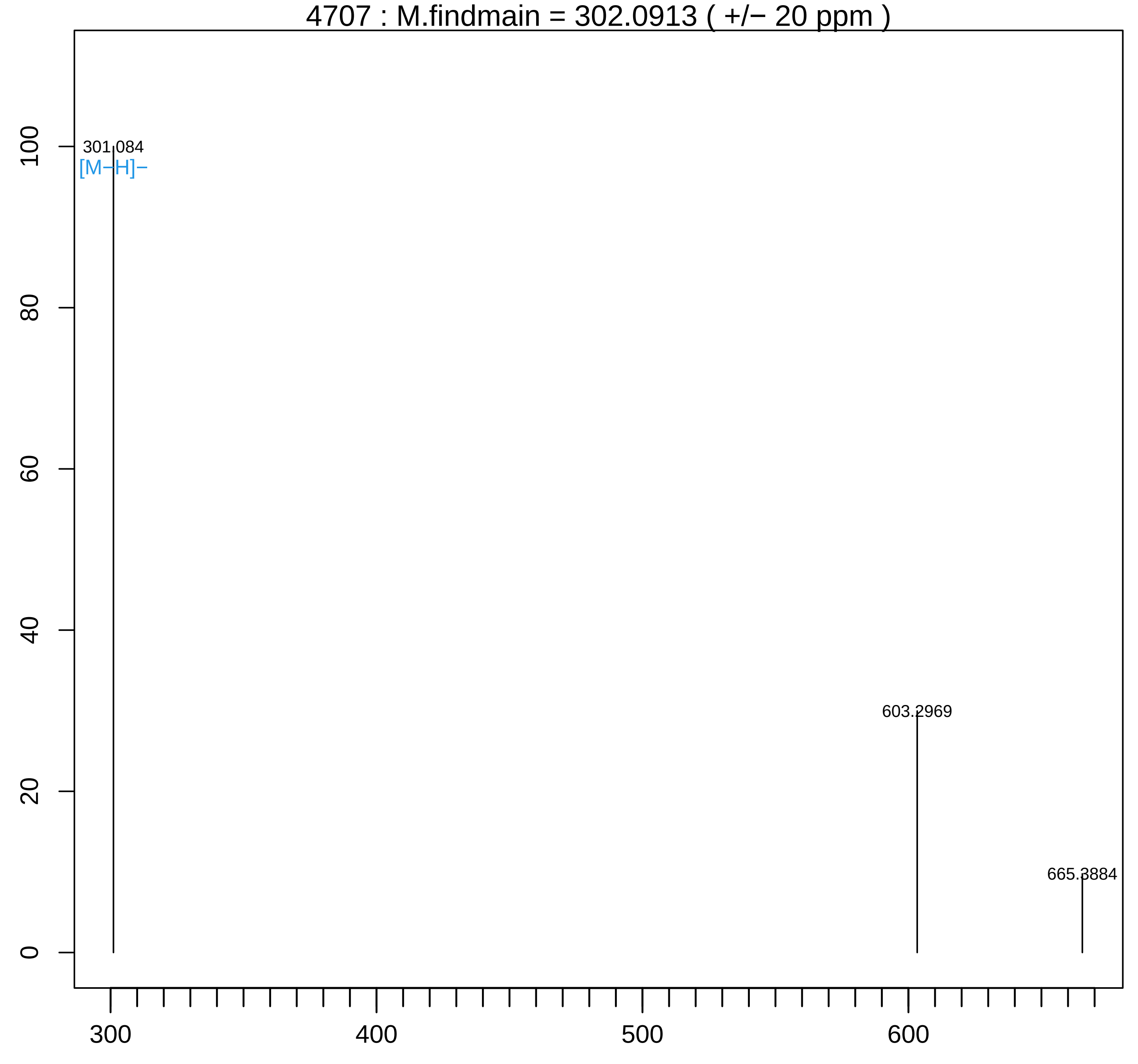
4707 : M.findmain = 302.0913 (+/− 20 ppm)
301.084
[M−H]−
603.2969
665.3884
0
20
40
60
80
100
300
400
500
600

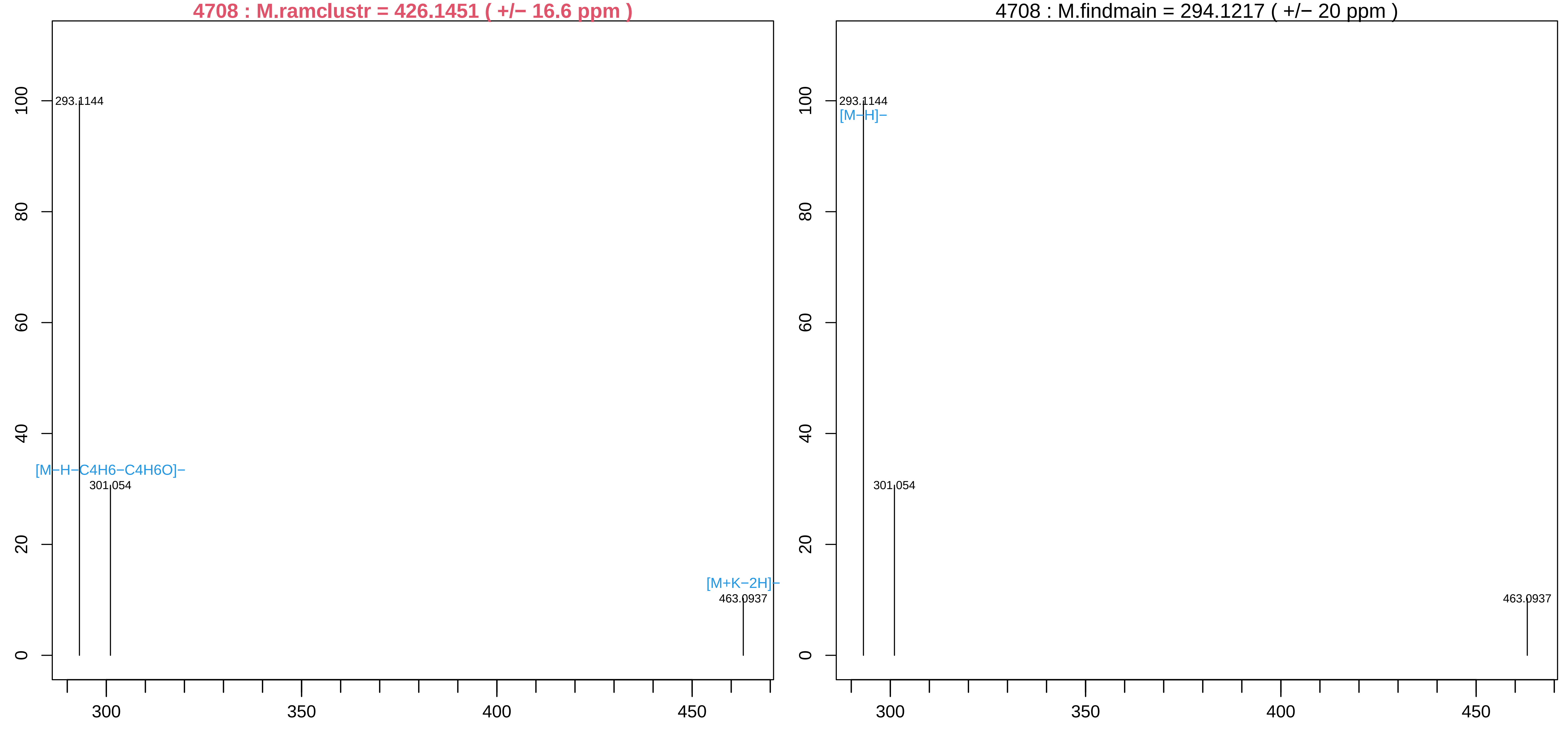

4708 : M.ramclustr = 426.1451 (+/− 16.6 ppm)
293.1144
[M−H−C4H6−C4H6O]−
301.054
[M+K−2H]−
463.0937
4708 : M.findmain = 294.1217 (+/− 20 ppm)
293.1144
[M−H]−
301.054
463.0937
0
20
40
60
80
100
300
350
400
450

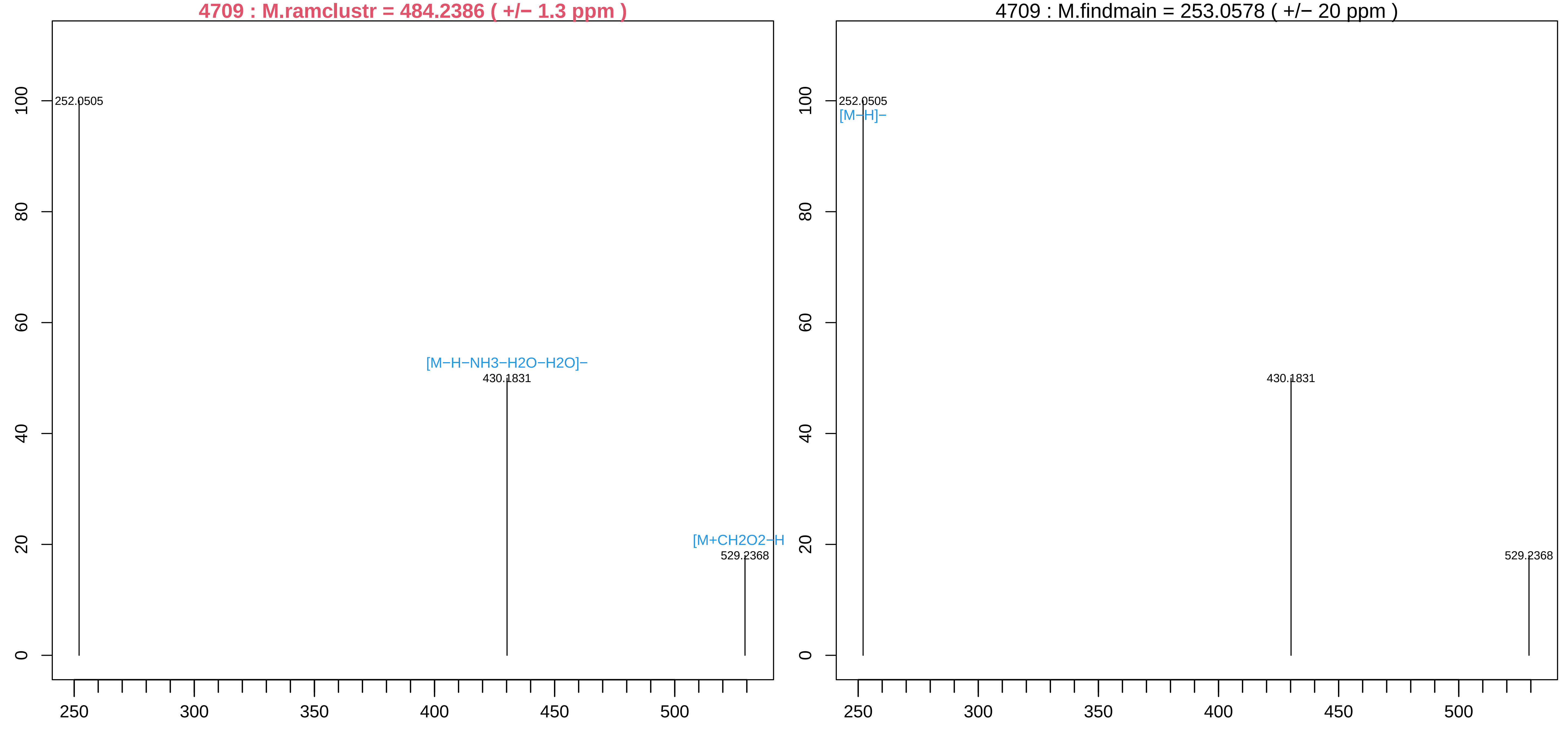

4709 : M.ramclustr = 484.2386 (+/− 1.3 ppm)
252.0505
[M−H−NH3−H2O−H2O]−
430.1831
[M+CH2O2−H
529.2368
4709 : M.findmain = 253.0578 (+/− 20 ppm)
252.0505
[M−H]−
430.1831
529.2368

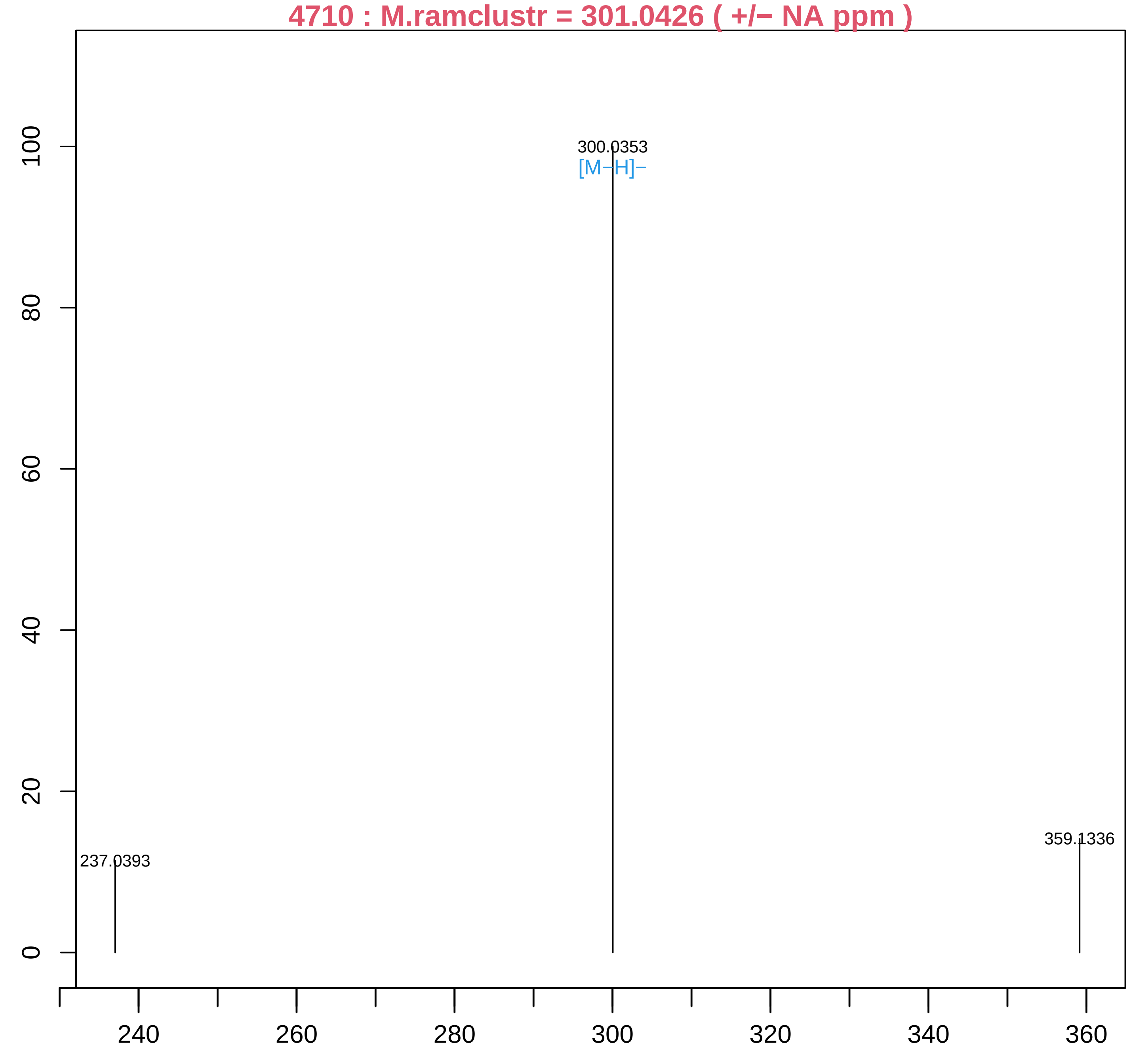
4710 : M.ramclustr = 301.0426 (+/− NA ppm)
300.0353
[M−H]−
237.0393
359.1336
0
20
40
60
80
100
240
260
280
300
320
340
360

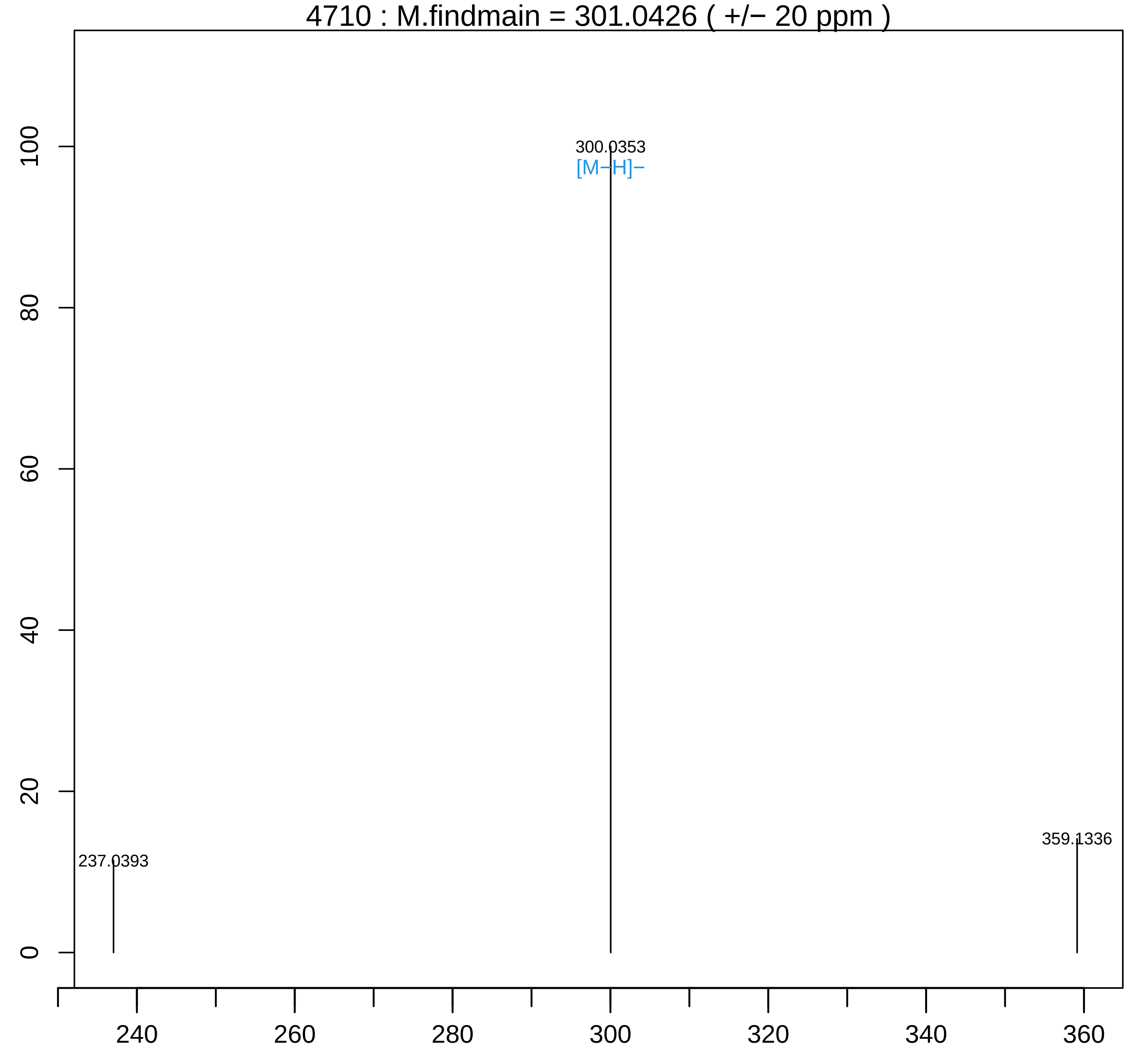
4710 : M.findmain = 301.0426 (+/− 20 ppm)
300.0353
[M−H]−
237.0393
359.1336
0
20
40
60
80
100
240
260
280
300
320
340
360

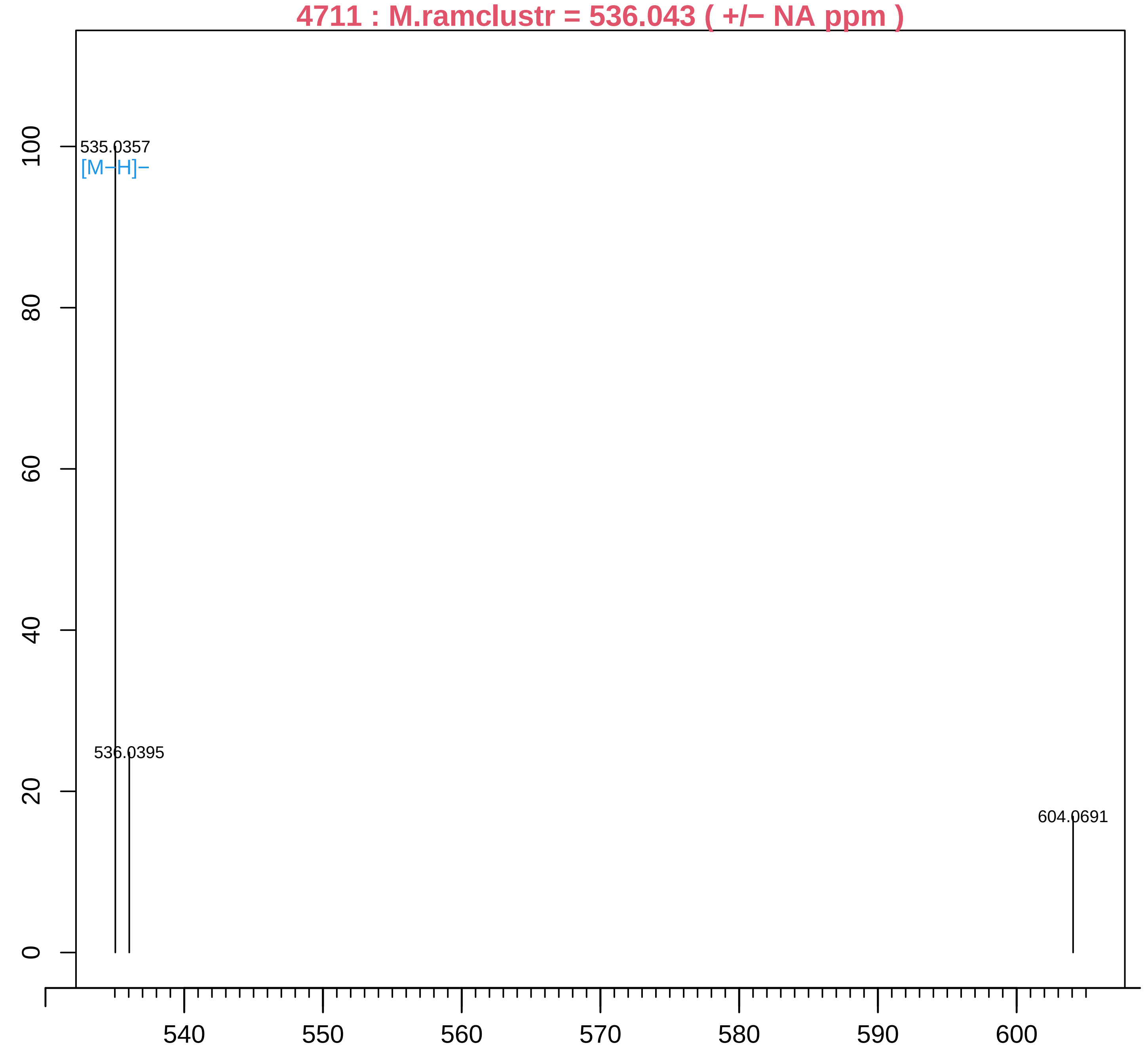

4711 : M.ramclustr = 536.043 (+/− NA ppm)
535.0357
[M−H]−
536.0395
604.0691
0
20
40
60
80
100
540
550
560
570
580
590
600

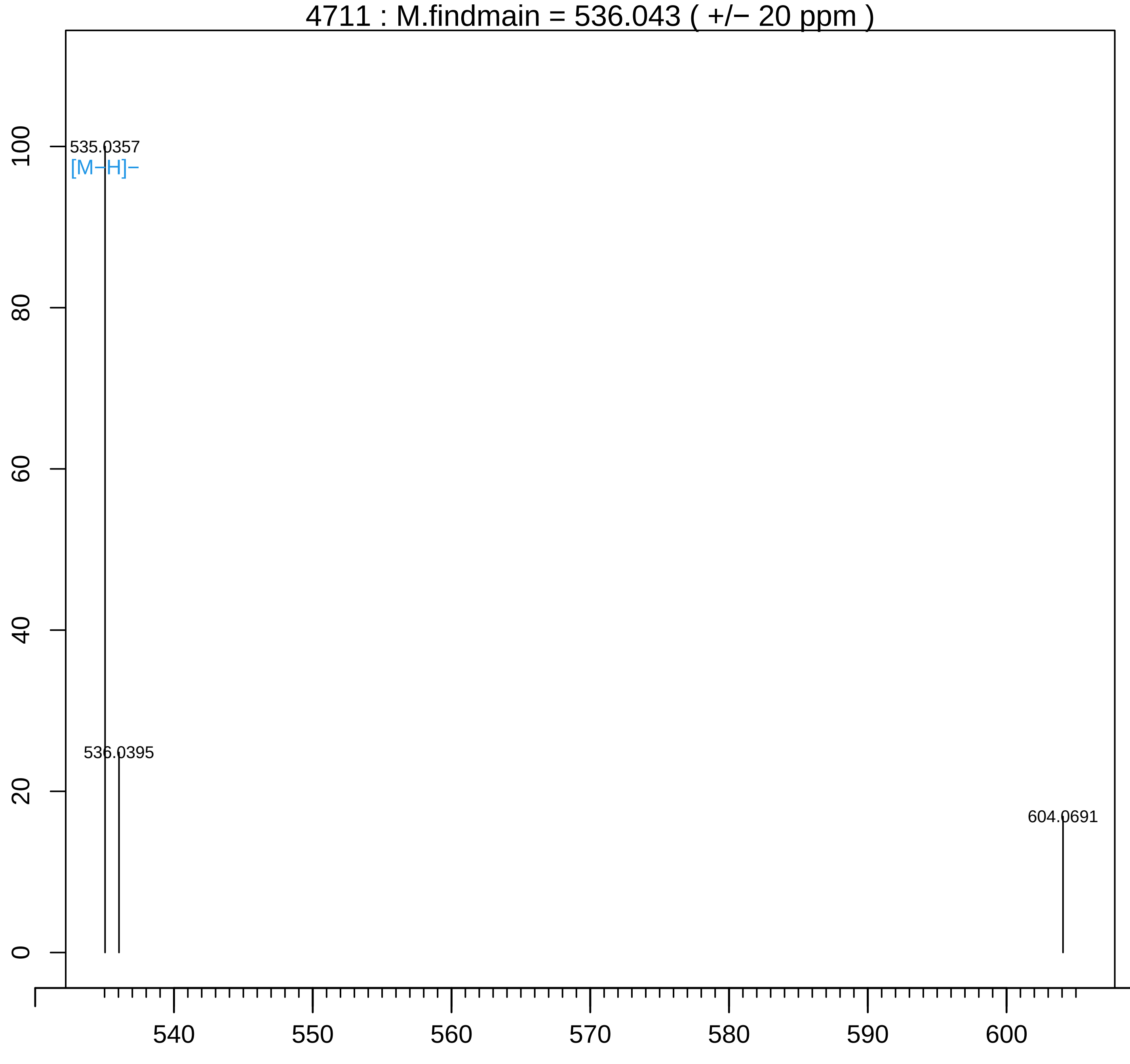

4711 : M.findmain = 536.043 (+/− 20 ppm)
535.0357
[M−H]−
536.0395
604.0691
0
20
40
60
80
100
540
550
560
570
580
590
600

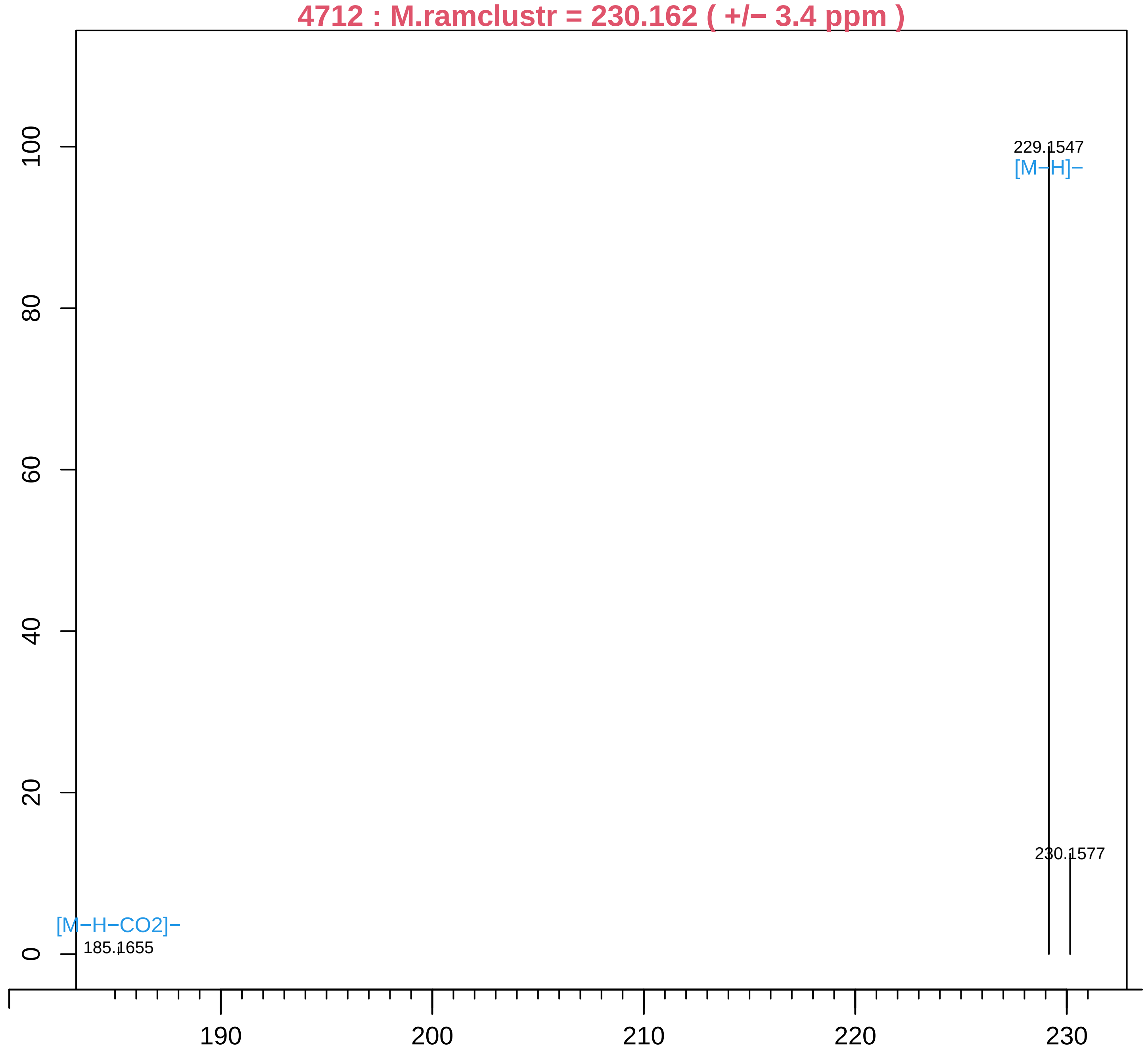

4712 : M.ramclustr = 230.162 (+/− 3.4 ppm)
229.1547
[M−H]−
230.1577
[M−H−CO2]−
185.1655
0
20
40
60
80
100
190
200
210
220
230

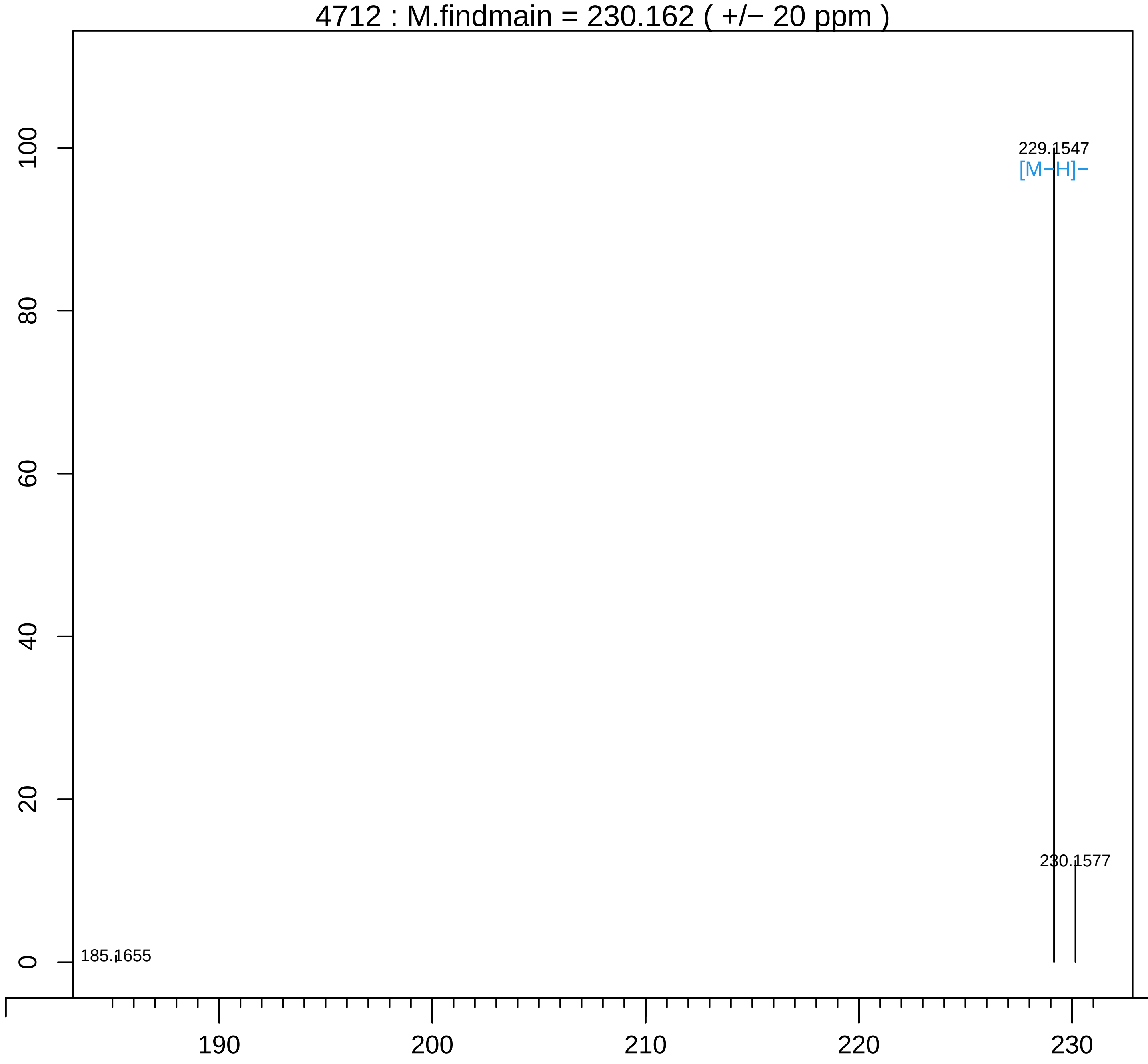

4712 : M.findmain = 230.162 (+/− 20 ppm)
229.1547
[M−H]−
230.1577
185.1655
0
20
40
60
80
100
190
200
210
220
230

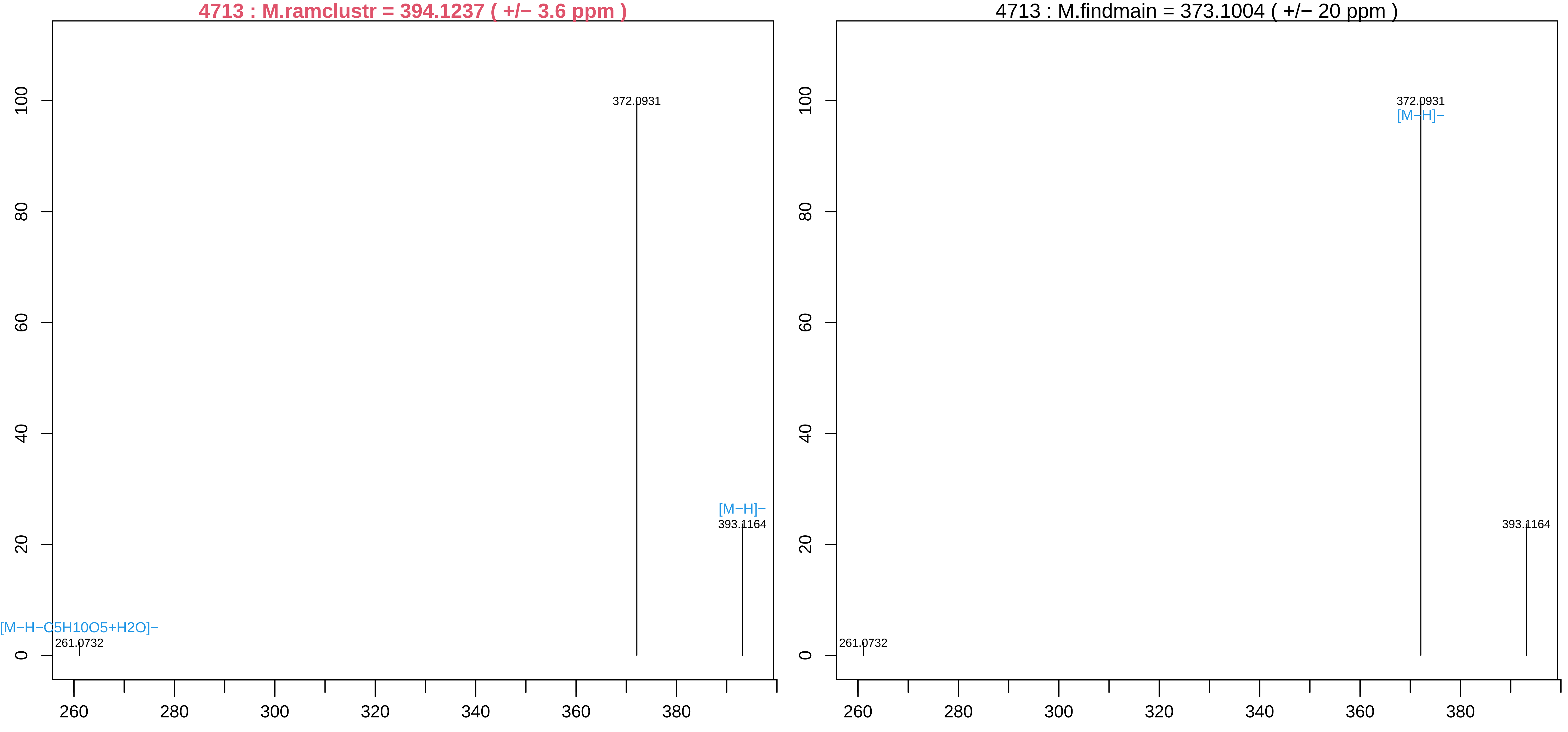
4713 : M.ramclustr = 394.1237 (+/− 3.6 ppm)
4713 : M.findmain = 373.1004 (+/− 20 ppm)
372.0931
[M−H]−
393.1164
[M−H−C5H10O5+H2O]−
261.0732
0
20
40
60
80
100
260
280
300
320
340
360
380

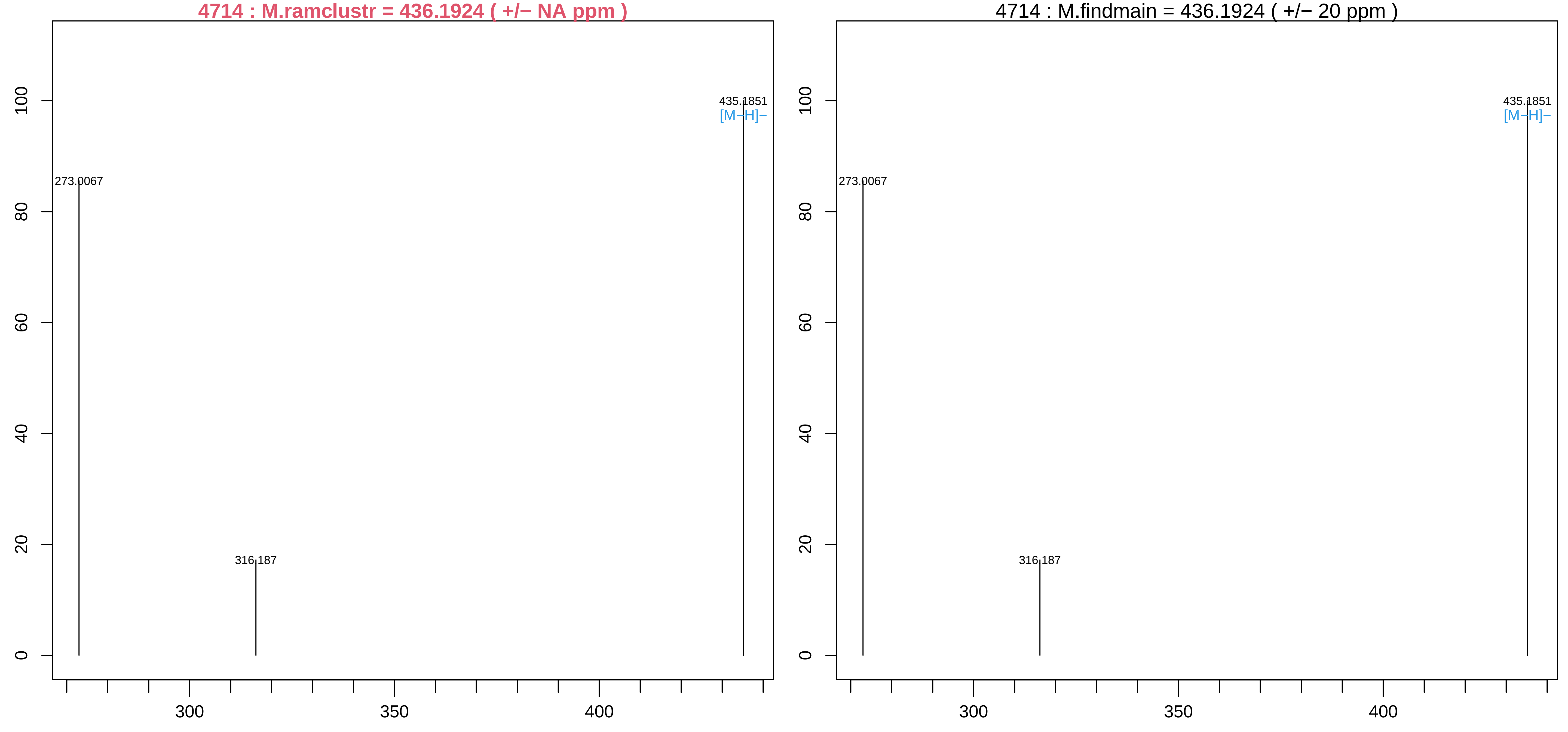

4714 : M.ramclustr = 436.1924 (+/− NA ppm)
435.1851
[M−H]−
273.0067
316.187
0
20
40
60
80
100
300
350
400
4714 : M.findmain = 436.1924 (+/− 20 ppm)
435.1851
[M−H]−
273.0067
316.187
0
20
40
60
80
100
300
350
400

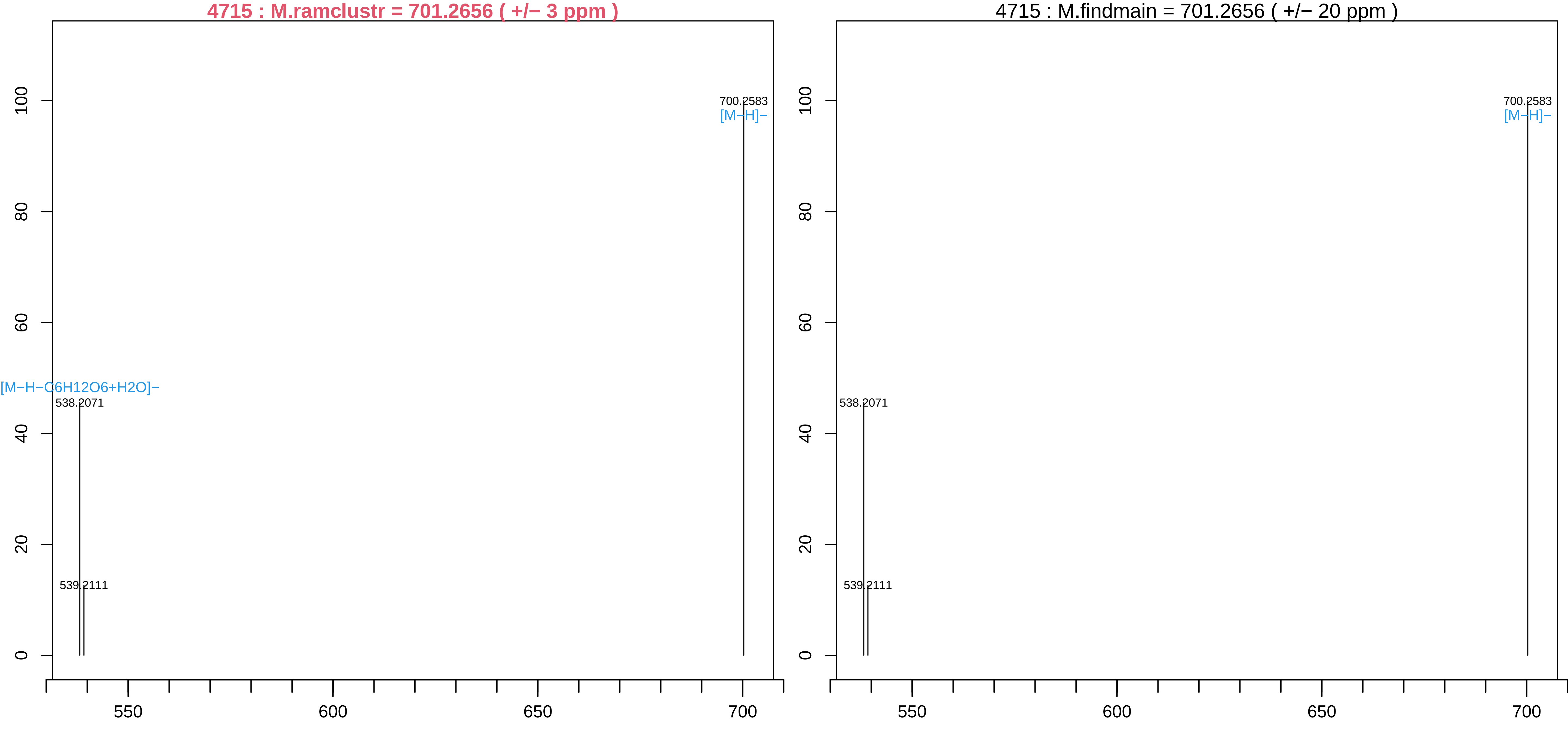
4715 : M.ramclustr = 701.2656 (+/− 3 ppm)
700.2583
[M−H]−
[M−H−C6H12O6+H2O]−
538.2071
539.2111
4715 : M.findmain = 701.2656 (+/− 20 ppm)
700.2583
[M−H]−
538.2071
539.2111
0
20
40
60
80
100
550
600
650
700

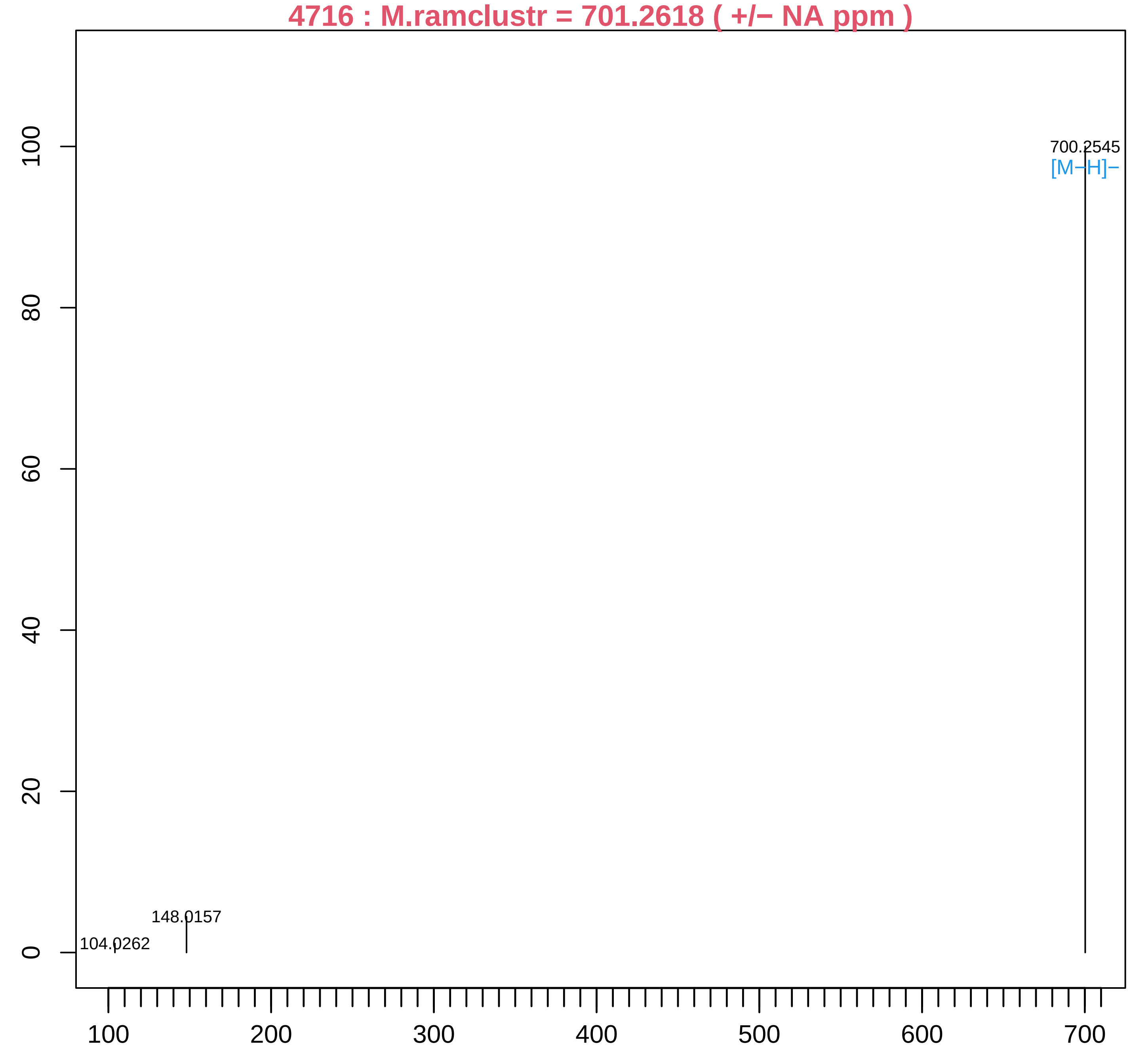
4716 : M.ramclustr = 701.2618 (+/− NA ppm)
700.2545
[M−H]−
148.0157
104.0262
0
20
40
60
80
100
100
200
300
400
500
600
700

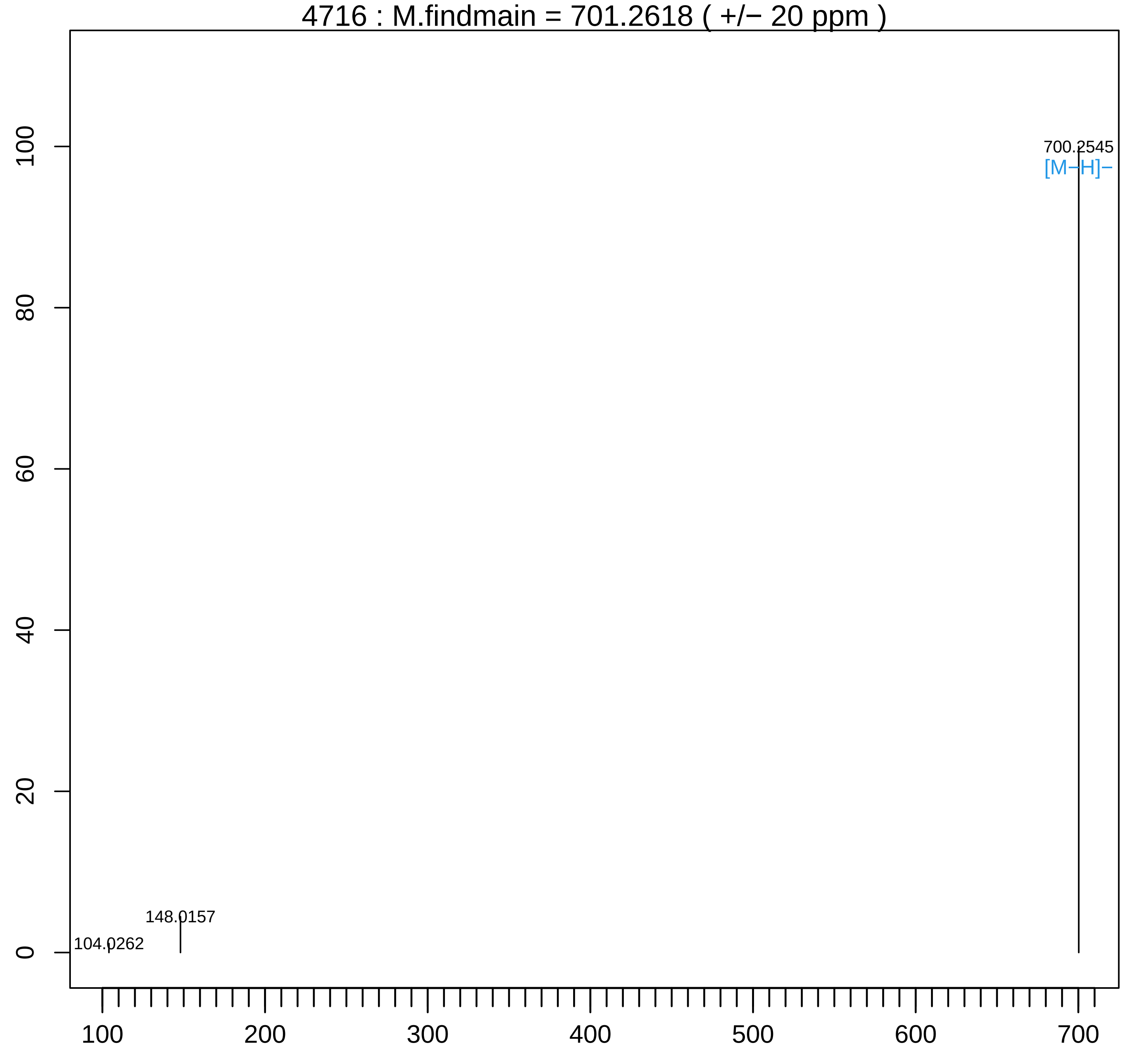
4716 : M.findmain = 701.2618 (+/− 20 ppm)
700.2545
[M−H]−
148.0157
104.0262
0
20
40
60
80
100
100
200
300
400
500
600
700

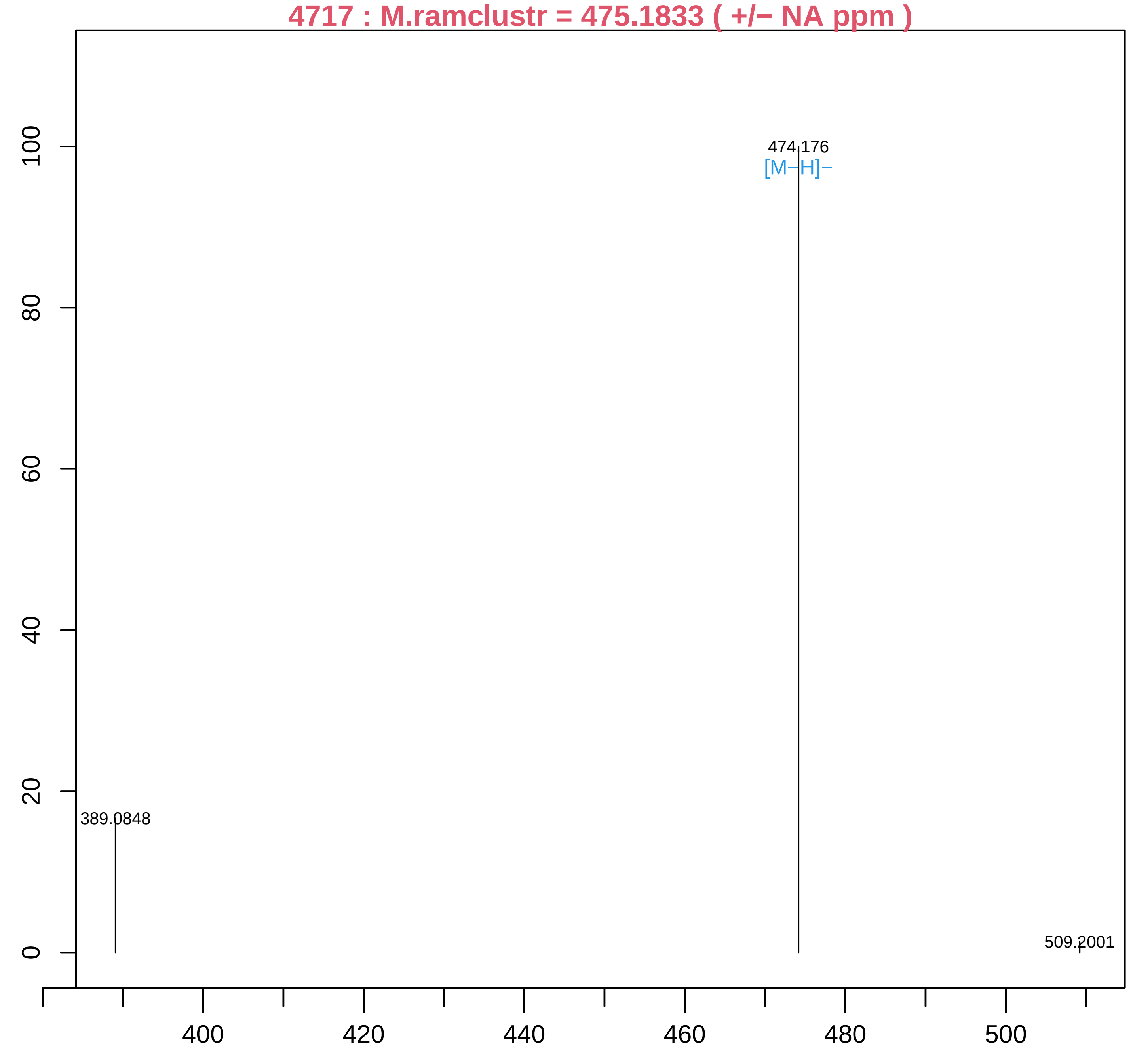

4717 : M.ramclustr = 475.1833 (+/− NA ppm)
474.176
[M−H]−
389.0848
509.2001
0
20
40
60
80
100
400
420
440
460
480
500

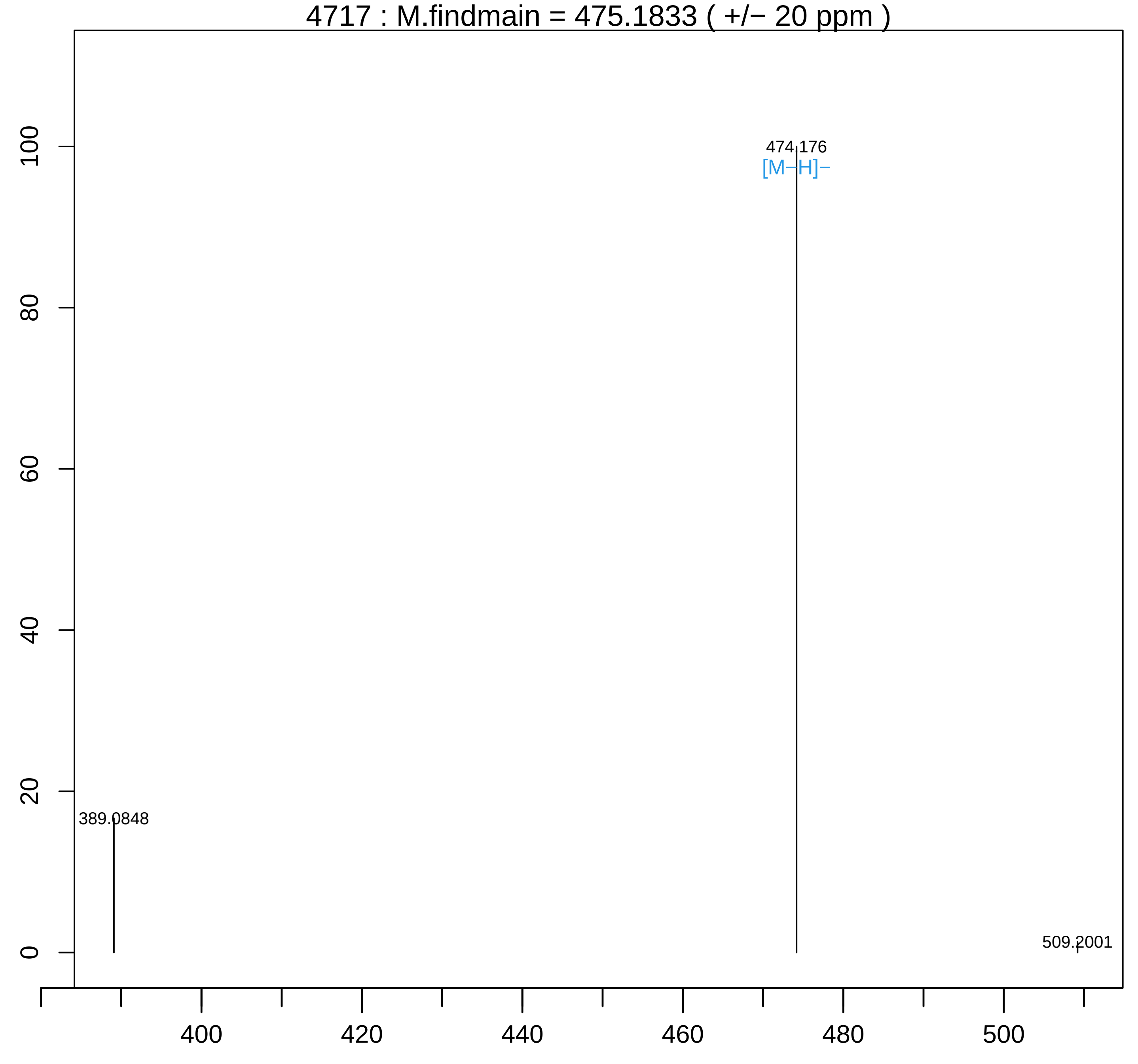

4717 : M.findmain = 475.1833 (+/− 20 ppm)
474.176
[M−H]−
389.0848
509.2001
0
20
40
60
80
100
400
420
440
460
480
500

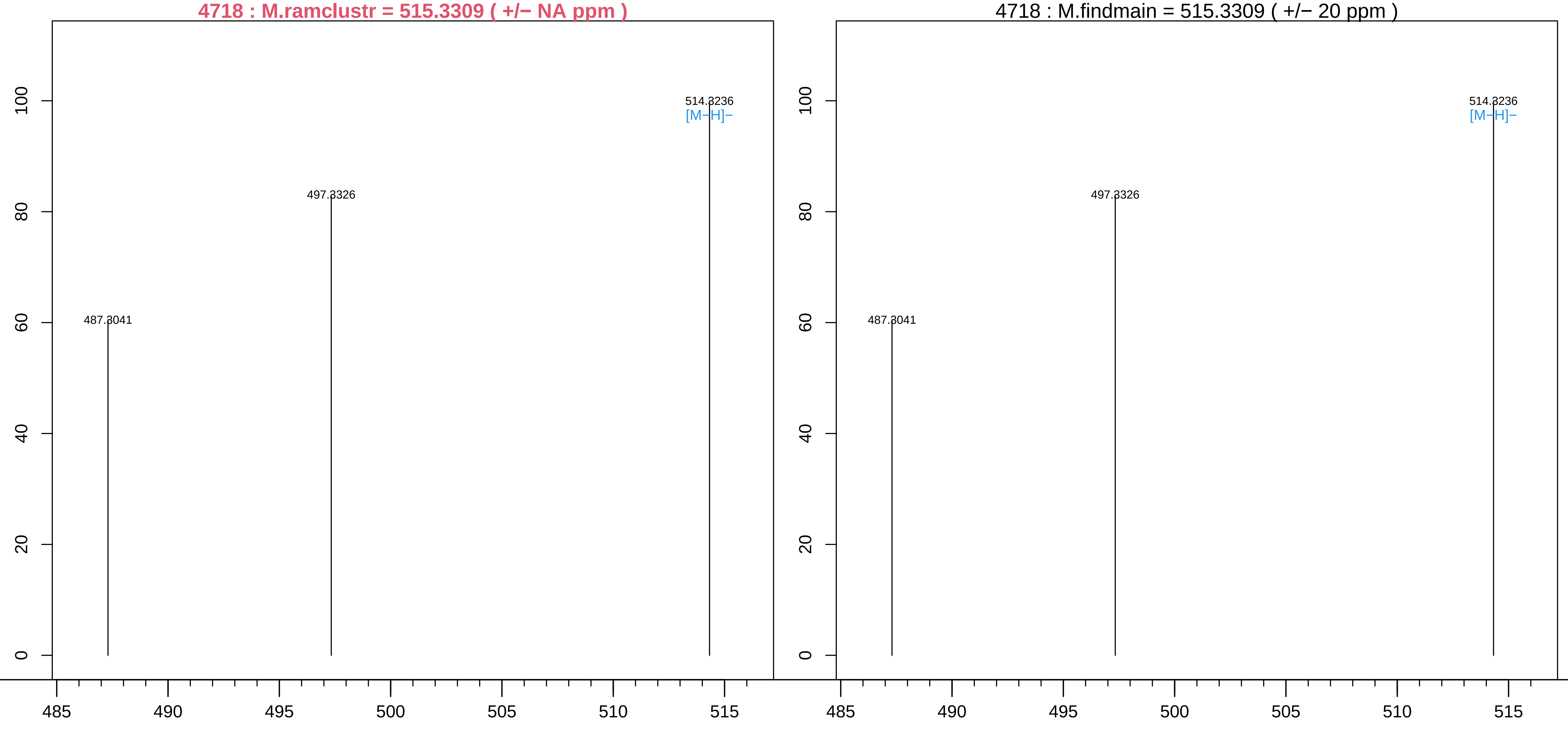
4718 : M.ramclustr = 515.3309 (+/− NA ppm)
4718 : M.findmain = 515.3309 (+/− 20 ppm)
514.3236
[M−H]−
497.3326
487.3041
0
20
40
60
80
100
485
490
495
500
505
510
515

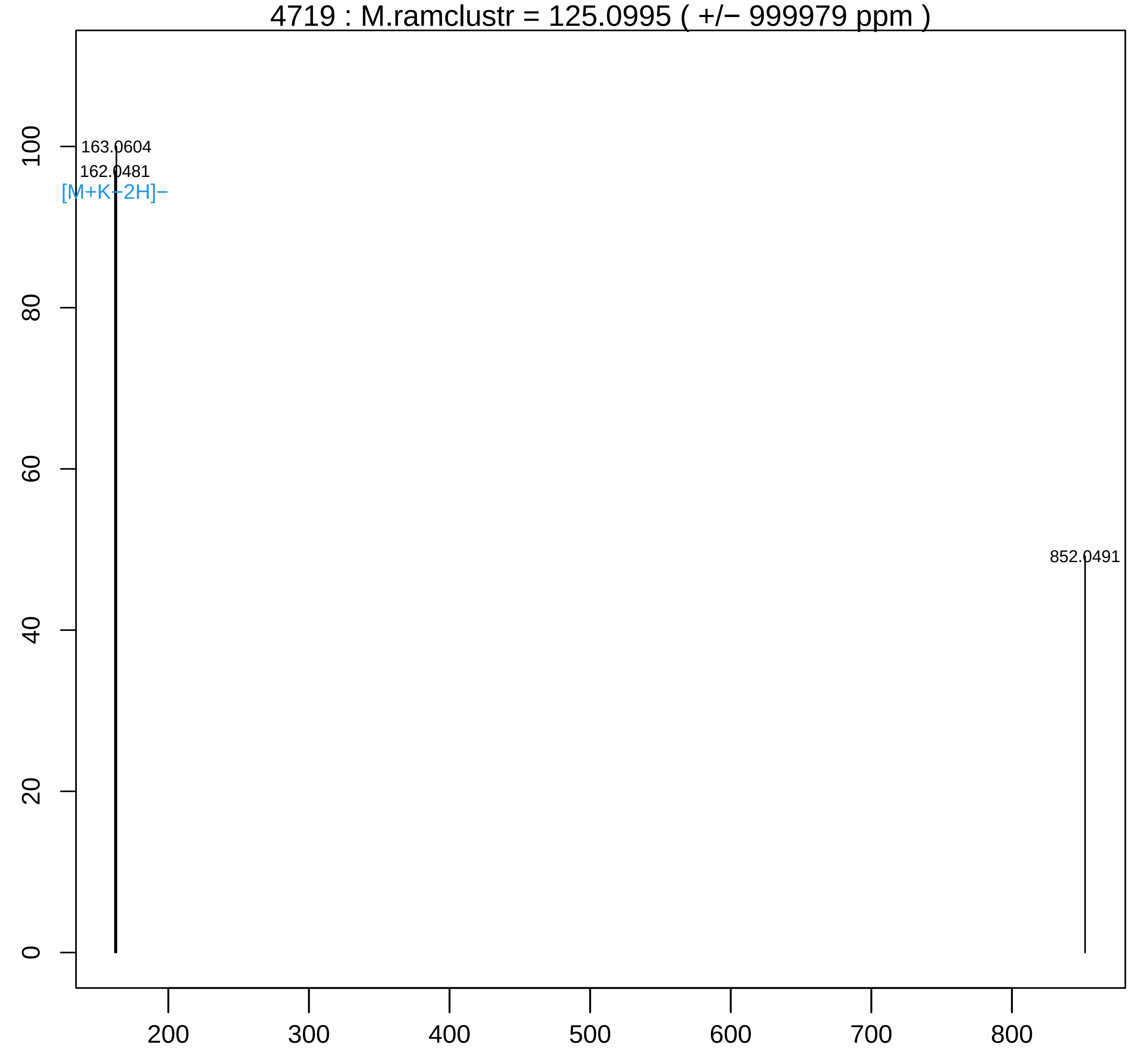
4719 : M.ramclustr = 125.0995 (+/− 999979 ppm)
163.0604
162.0481
[M+K−2H]−
852.0491
0
20
40
60
80
100
200
300
400
500
600
700
800

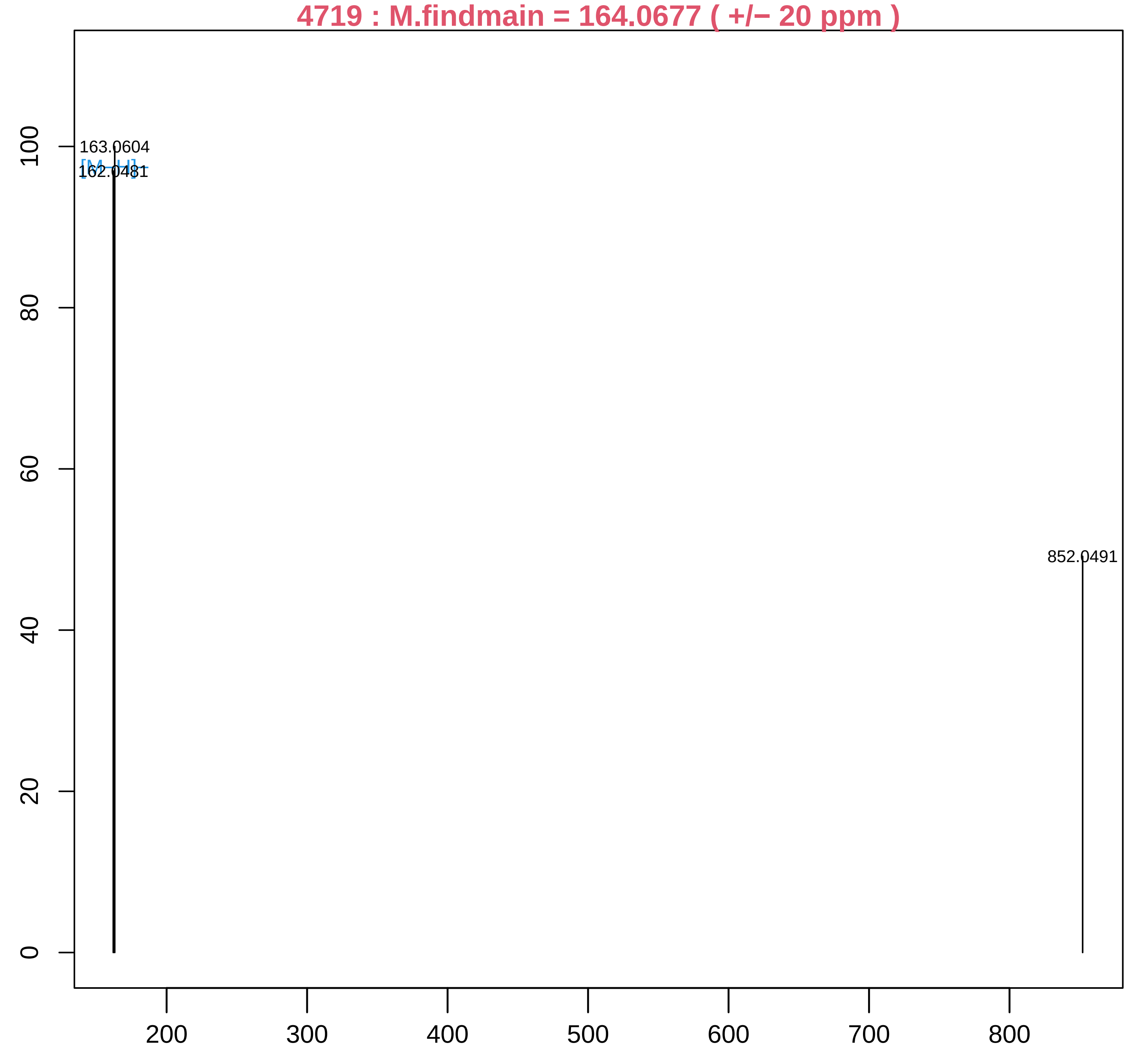
4719 : M.findmain = 164.0677 (+/− 20 ppm)
163.0604
162.0481
852.0491
0
20
40
60
80
100
200
300
400
500
600
700
800

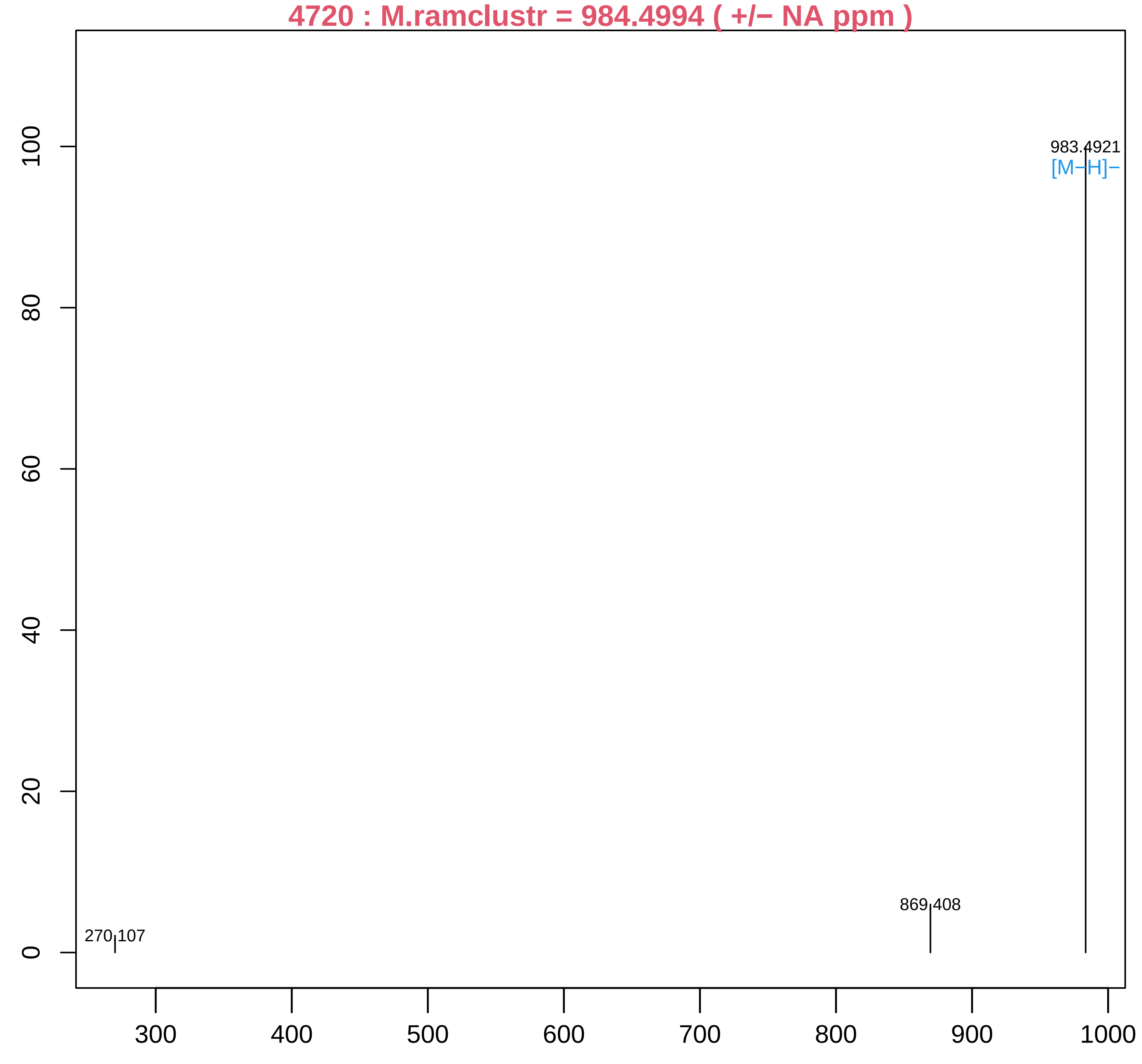
4720 : M.ramclustr = 984.4994 (+/− NA ppm)
983.4921
[M−H]−
869.408
270.107
0
20
40
60
80
100
300
400
500
600
700
800
900
1000

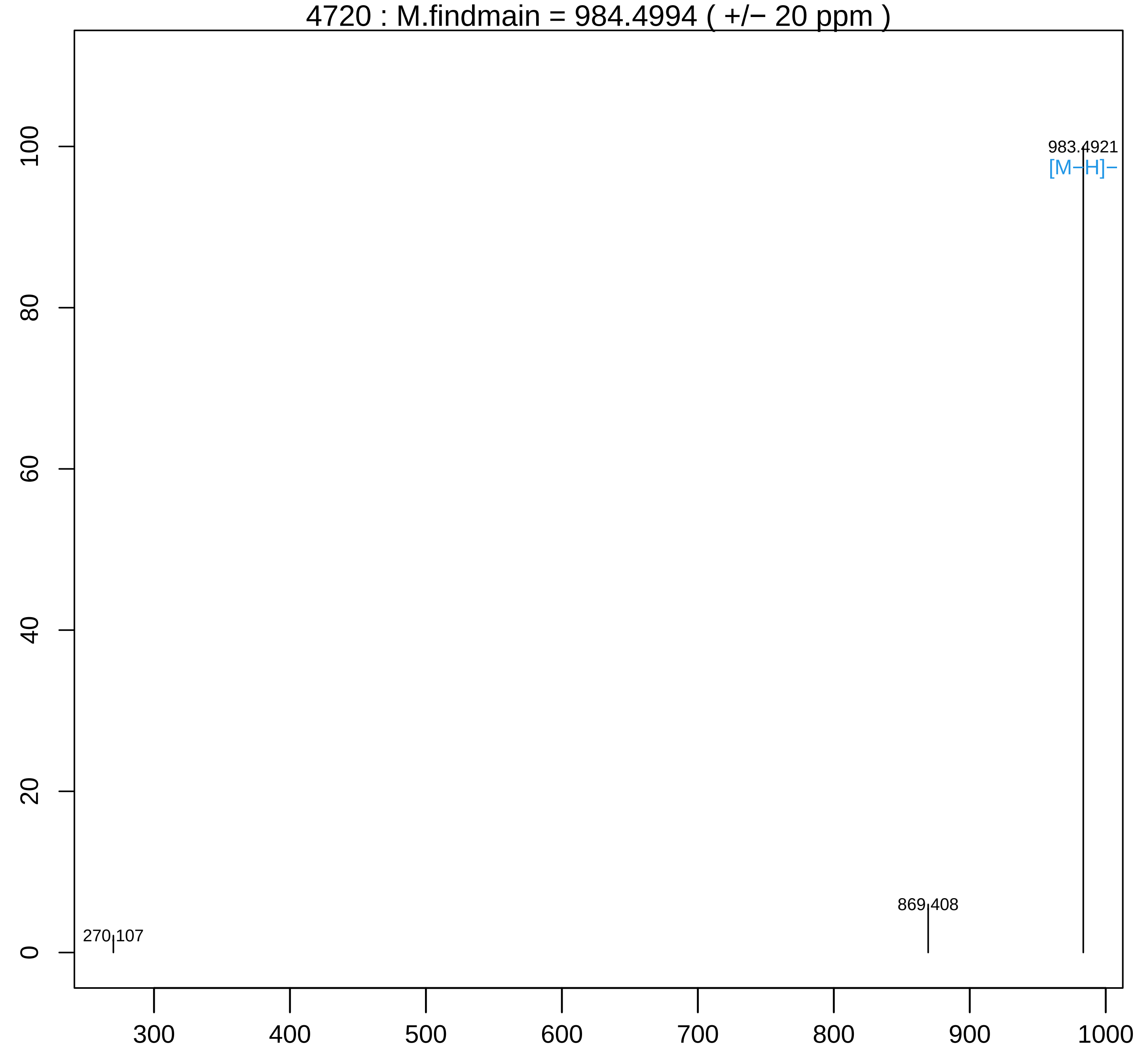
4720 : M.findmain = 984.4994 (+/− 20 ppm)
983.4921
[M−H]−
869.408
270.107
0
20
40
60
80
100
300
400
500
600
700
800
900
1000

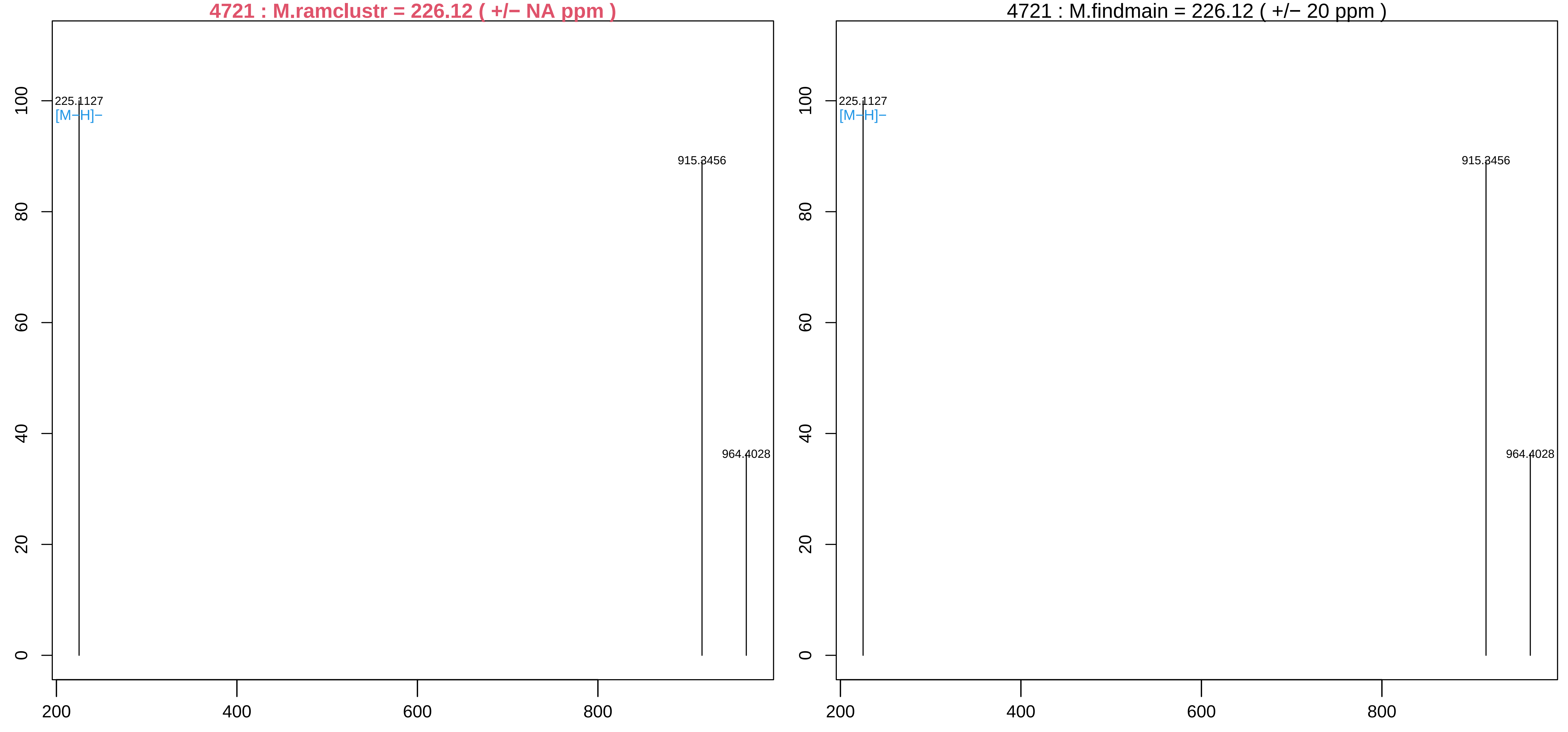

4721 : M.ramclustr = 226.12 (+/− NA ppm)
225.1127
[M−H]−
915.3456
964.4028
4721 : M.findmain = 226.12 (+/− 20 ppm)
225.1127
[M−H]−
915.3456
964.4028
0
20
40
60
80
100
200
400
600
800

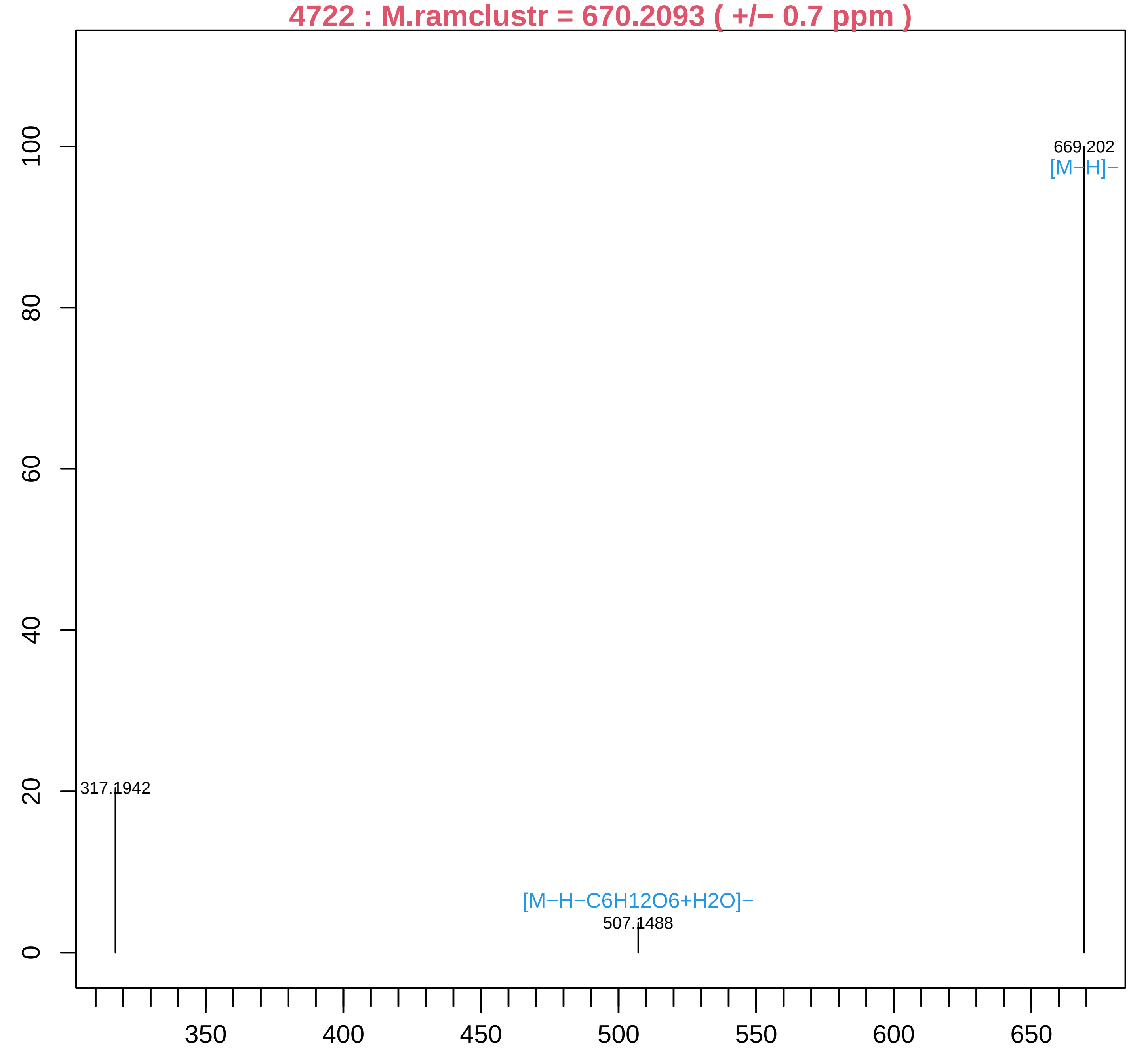
4722 : M.ramclustr = 670.2093 (+/− 0.7 ppm)
669.202
[M−H]−
317.1942
[M−H−C6H12O6+H2O]−
507.1488
0
20
40
60
80
100
350
400
450
500
550
600
650

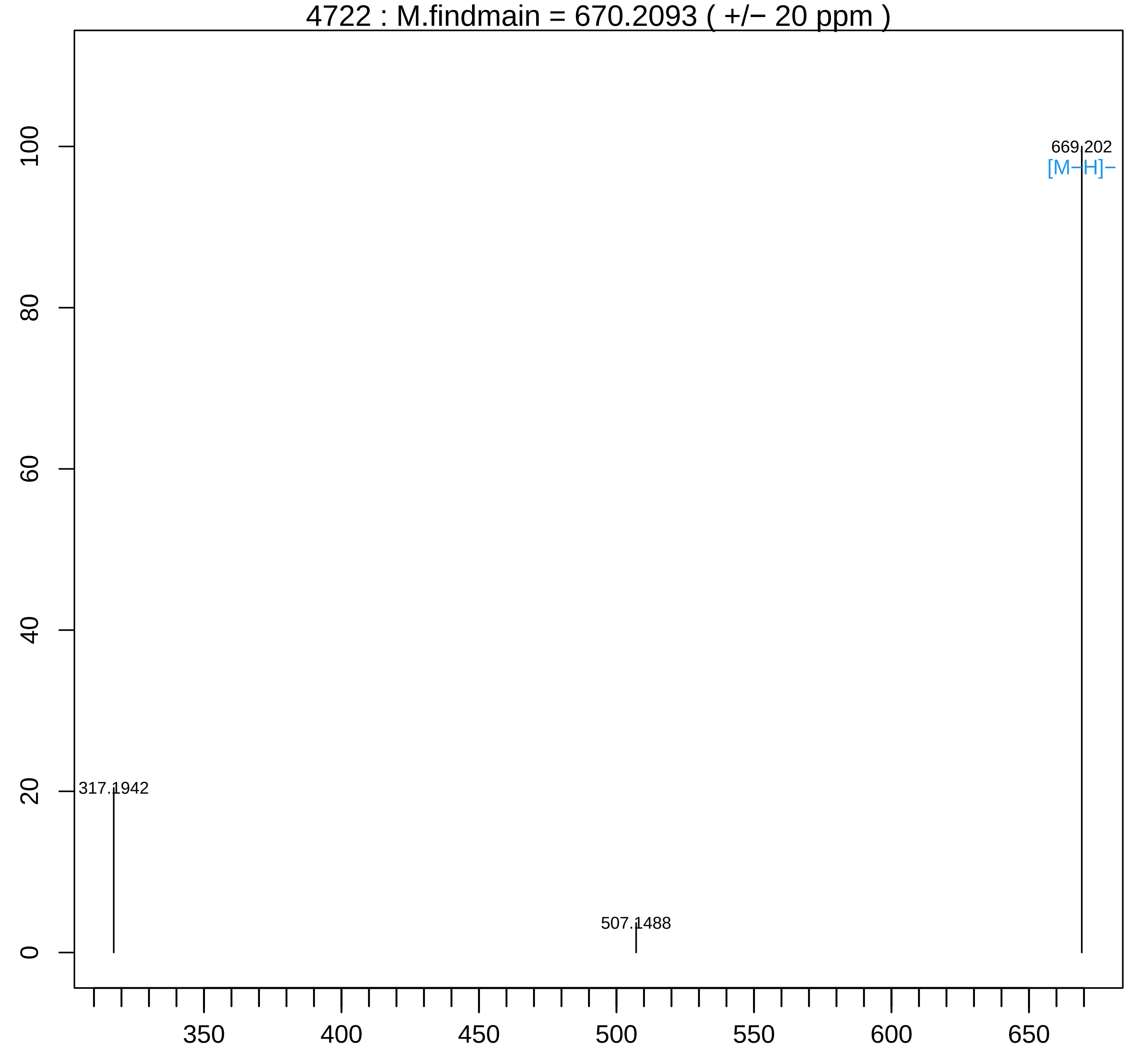
4722 : M.findmain = 670.2093 (+/− 20 ppm)
669.202
[M−H]−
317.1942
507.1488
0
20
40
60
80
100
350
400
450
500
550
600
650

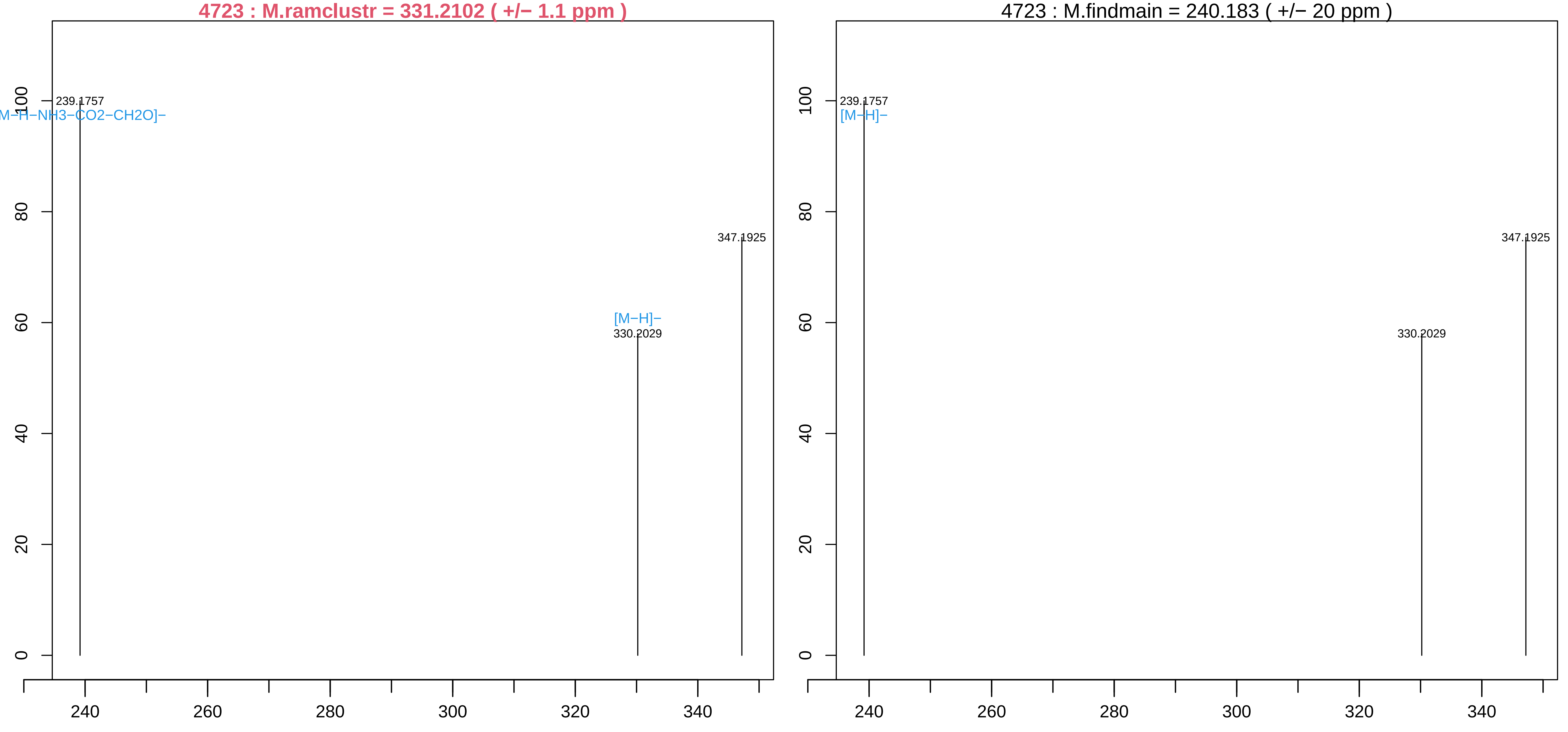
4723 : M.ramclustr = 331.2102 (+/− 1.1 ppm)
239.1757
M−H−NH3−CO2−CH2O]−
[M−H]−
330.2029
347.1925
4723 : M.findmain = 240.183 (+/− 20 ppm)
239.1757
[M−H]−
330.2029
347.1925
0
20
40
60
80
100
240
260
280
300
320
340

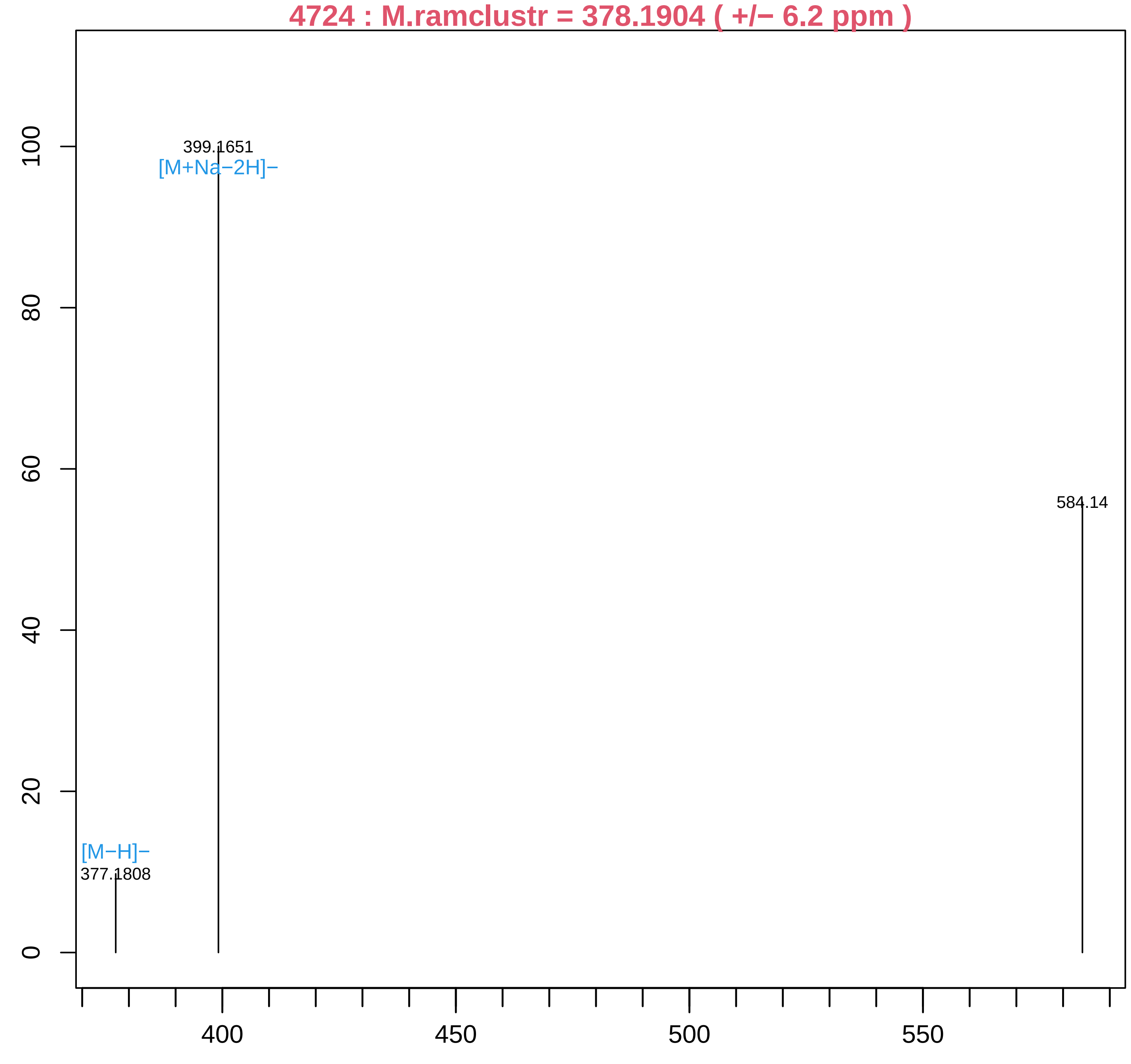
4724 : M.ramclustr = 378.1904 (+/− 6.2 ppm)
399.1651
[M+Na−2H]−
584.14
[M−H]−
377.1808
0
20
40
60
80
100
400
450
500
550

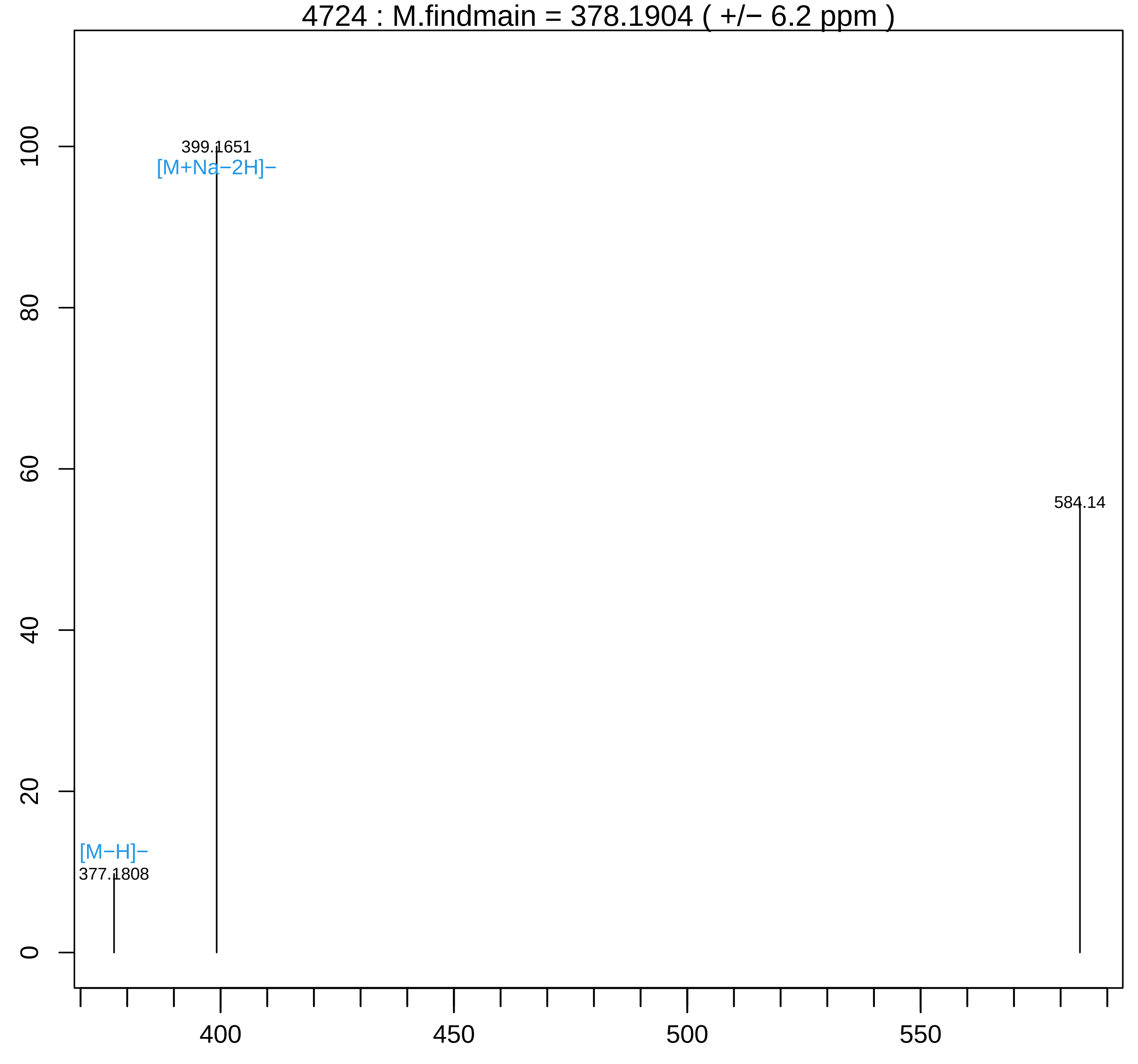
4724 : M.findmain = 378.1904 (+/− 6.2 ppm)
399.1651
[M+Na−2H]−
584.14
[M−H]−
377.1808
0
20
40
60
80
100
400
450
500
550

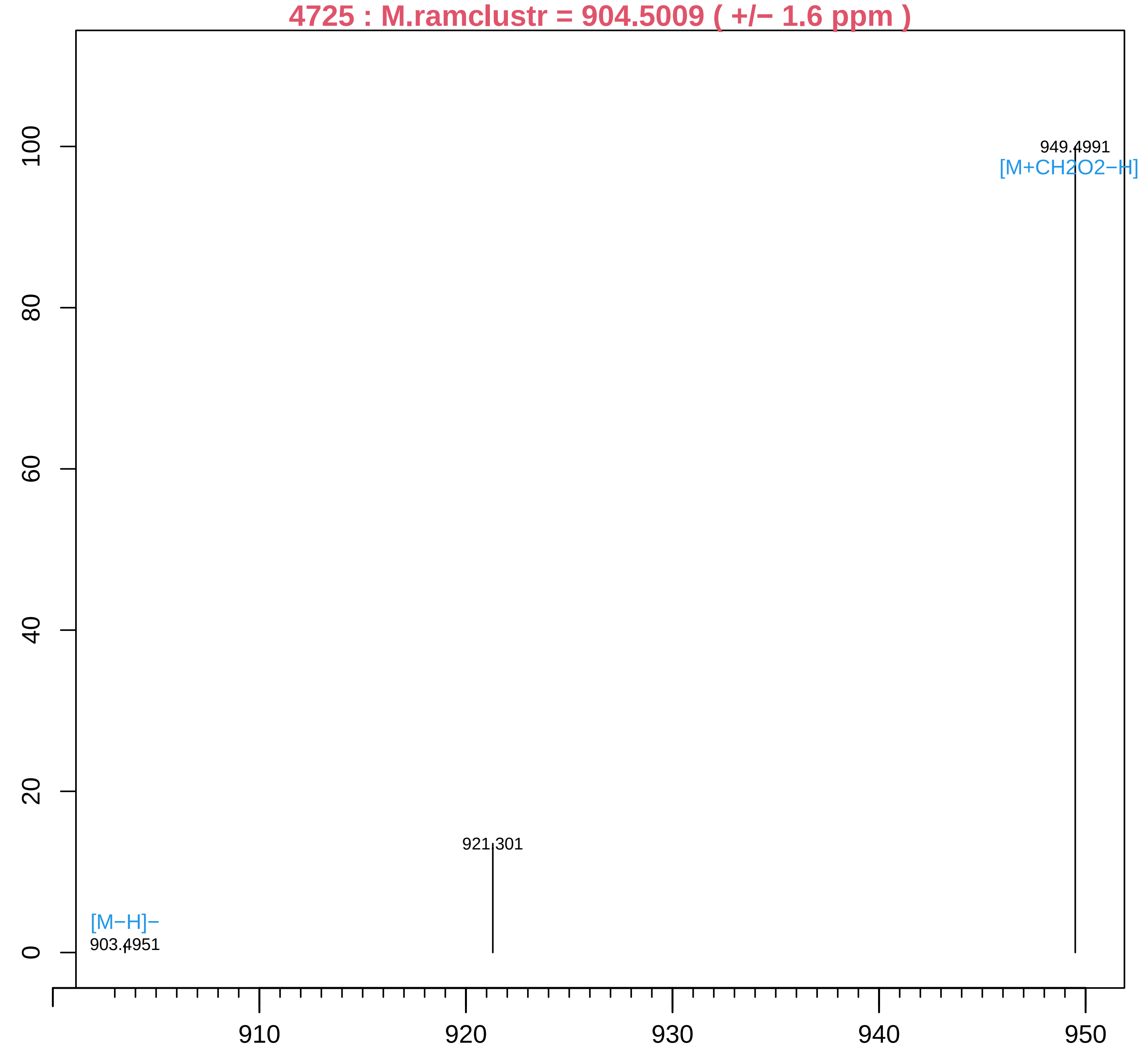
4725 : M.ramclustr = 904.5009 (+/− 1.6 ppm)
949.4991
[M+CH2O2−H]
921.301
[M−H]−
903.4951
0
20
40
60
80
100
910
920
930
940
950

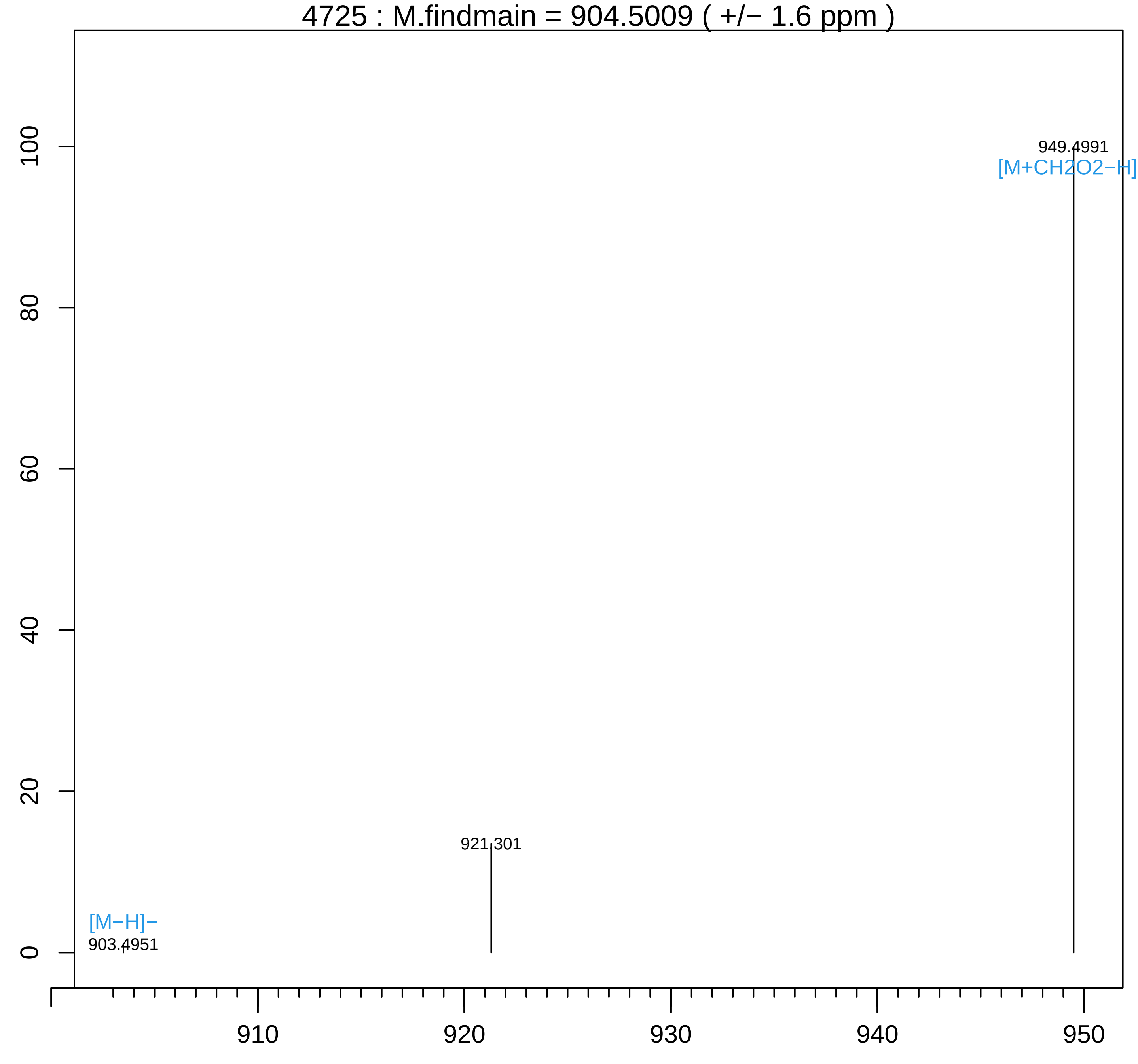
4725 : M.findmain = 904.5009 (+/− 1.6 ppm)
949.4991
[M+CH2O2−H]
921.301
[M−H]−
903.4951
0
20
40
60
80
100
910
920
930
940
950

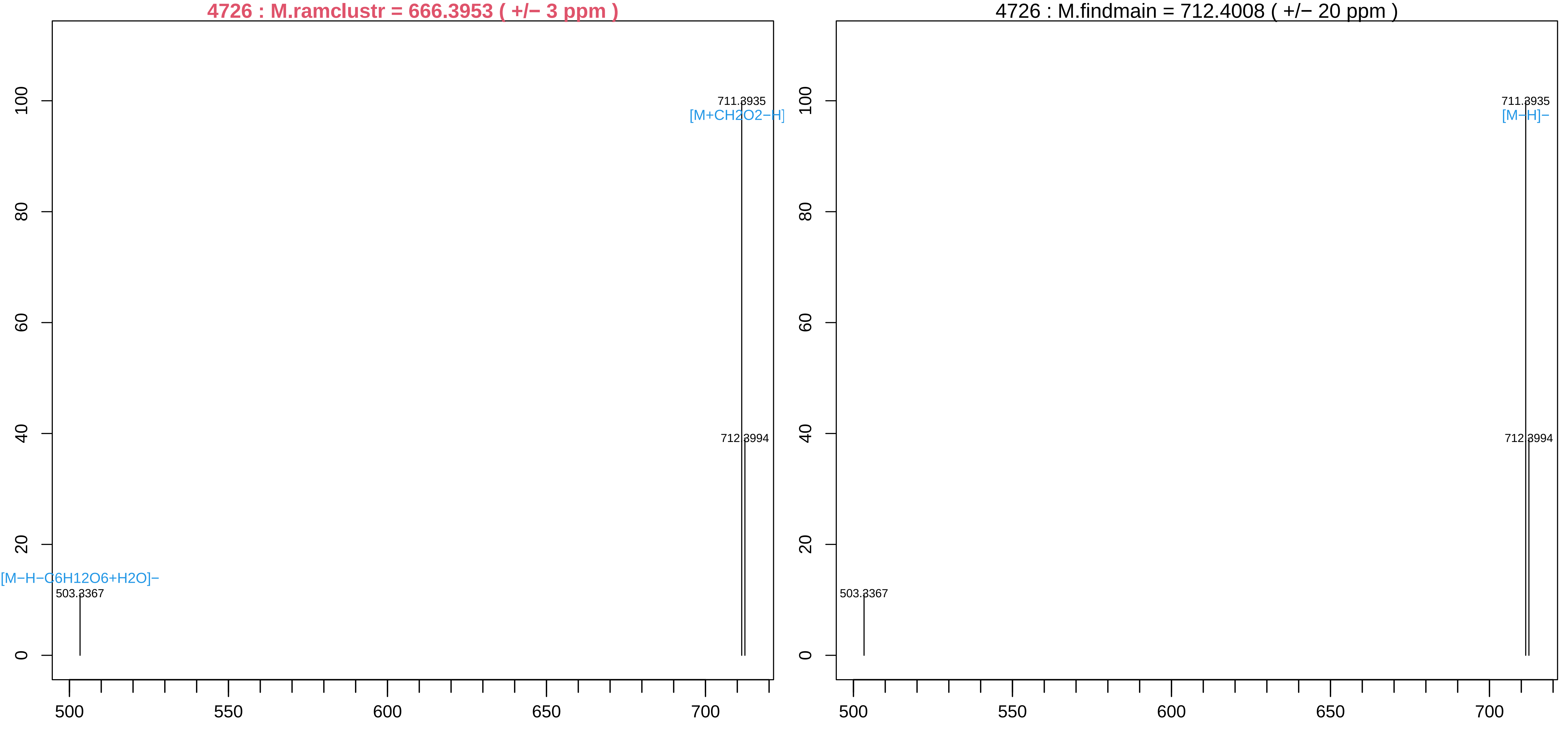
4726 : M.ramclustr = 666.3953 (+/− 3 ppm)
711.3935
[M+CH2O2−H]
712.3994
[M−H−C6H12O6+H2O]−
503.3367
4726 : M.findmain = 712.4008 (+/− 20 ppm)
711.3935
[M−H]−
712.3994
503.3367
0
20
40
60
80
100
500
550
600
650
700

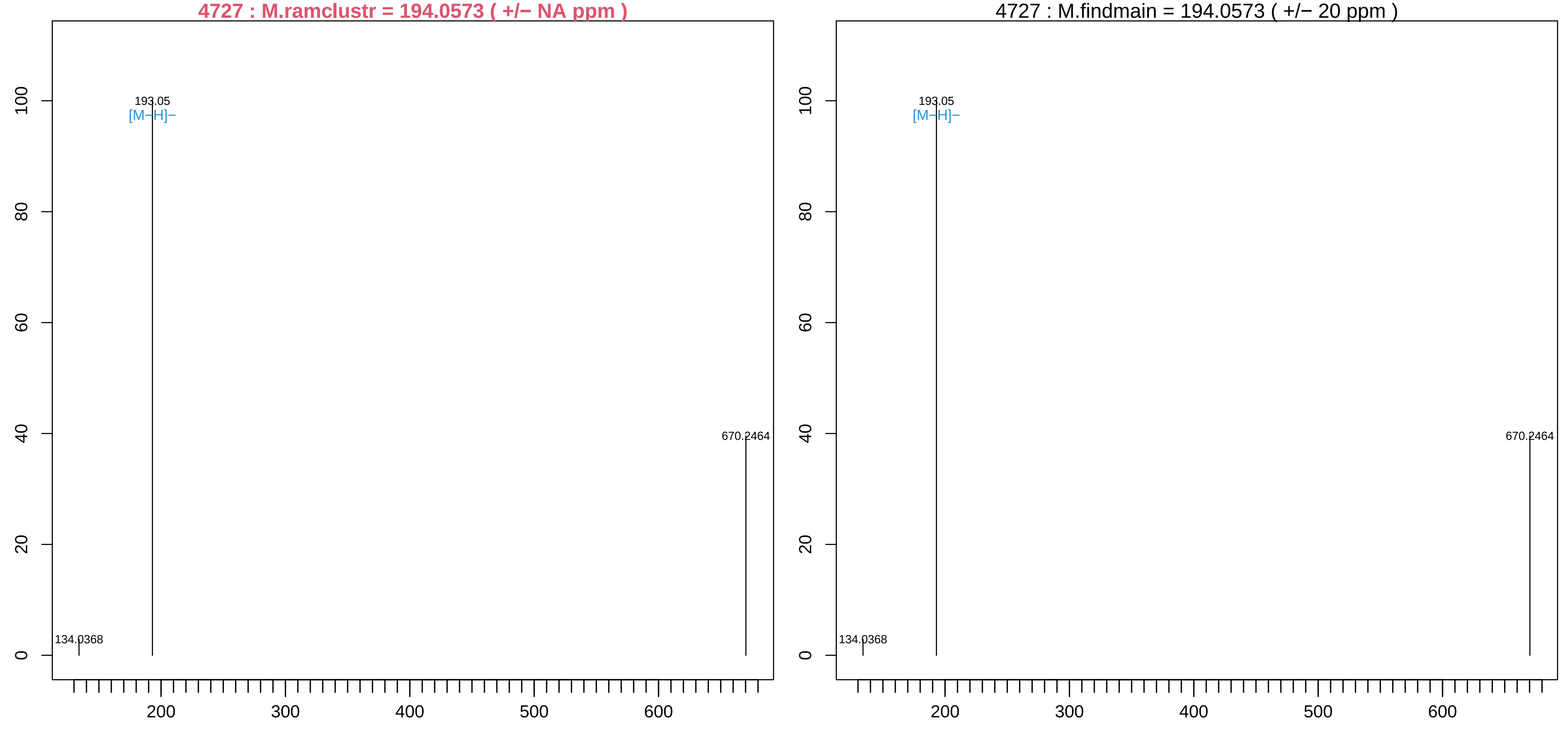
4727 : M.ramclustr = 194.0573 (+/− NA ppm)
193.05
[M−H]−
670.2464
134.0368
0
20
40
60
80
100
200
300
400
500
600
4727 : M.findmain = 194.0573 (+/− 20 ppm)
193.05
[M−H]−
670.2464
134.0368

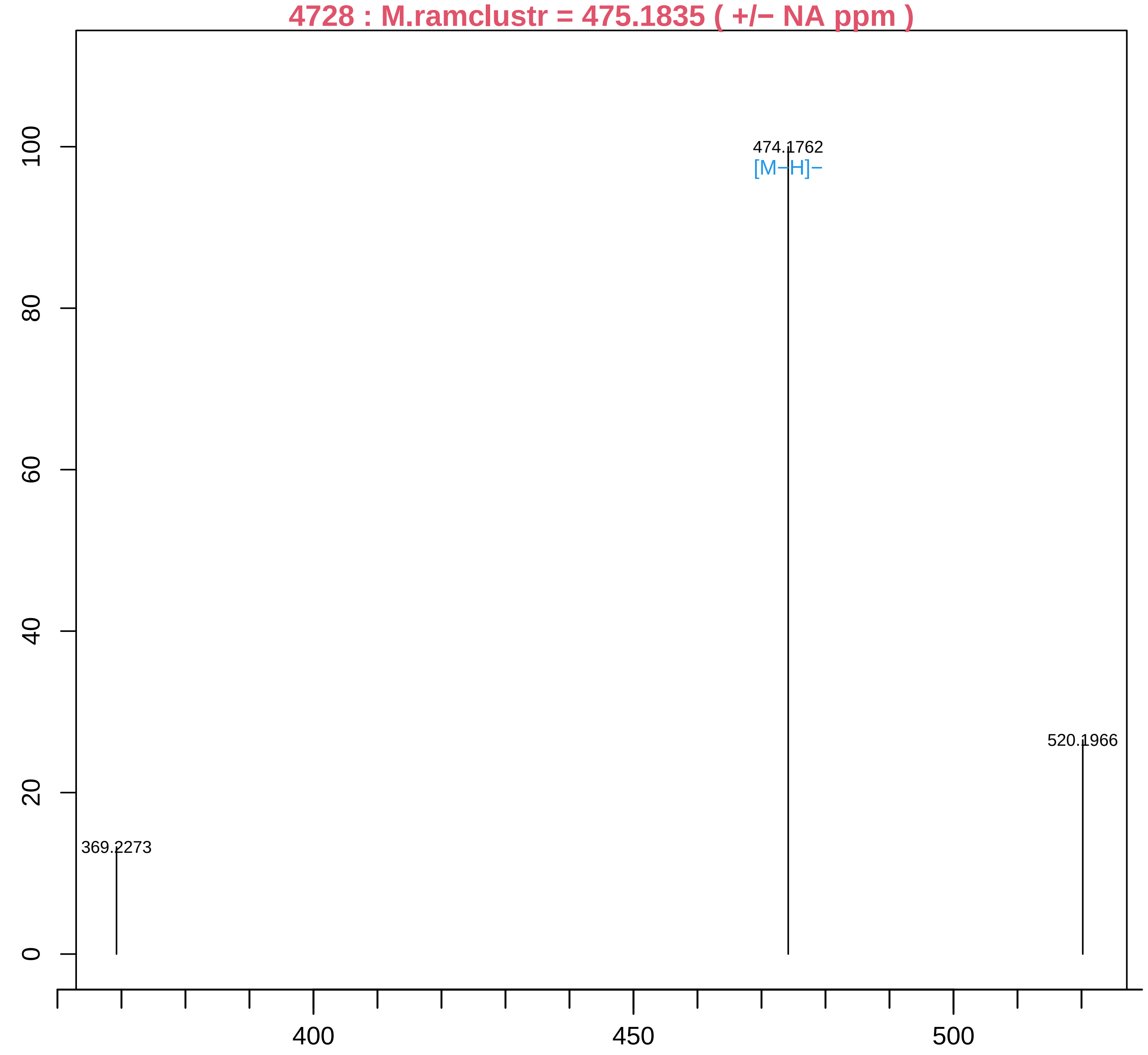
4728 : M.ramclustr = 475.1835 (+/− NA ppm)
474.1762
[M−H]−
520.1966
369.2273
0
20
40
60
80
100
400
450
500

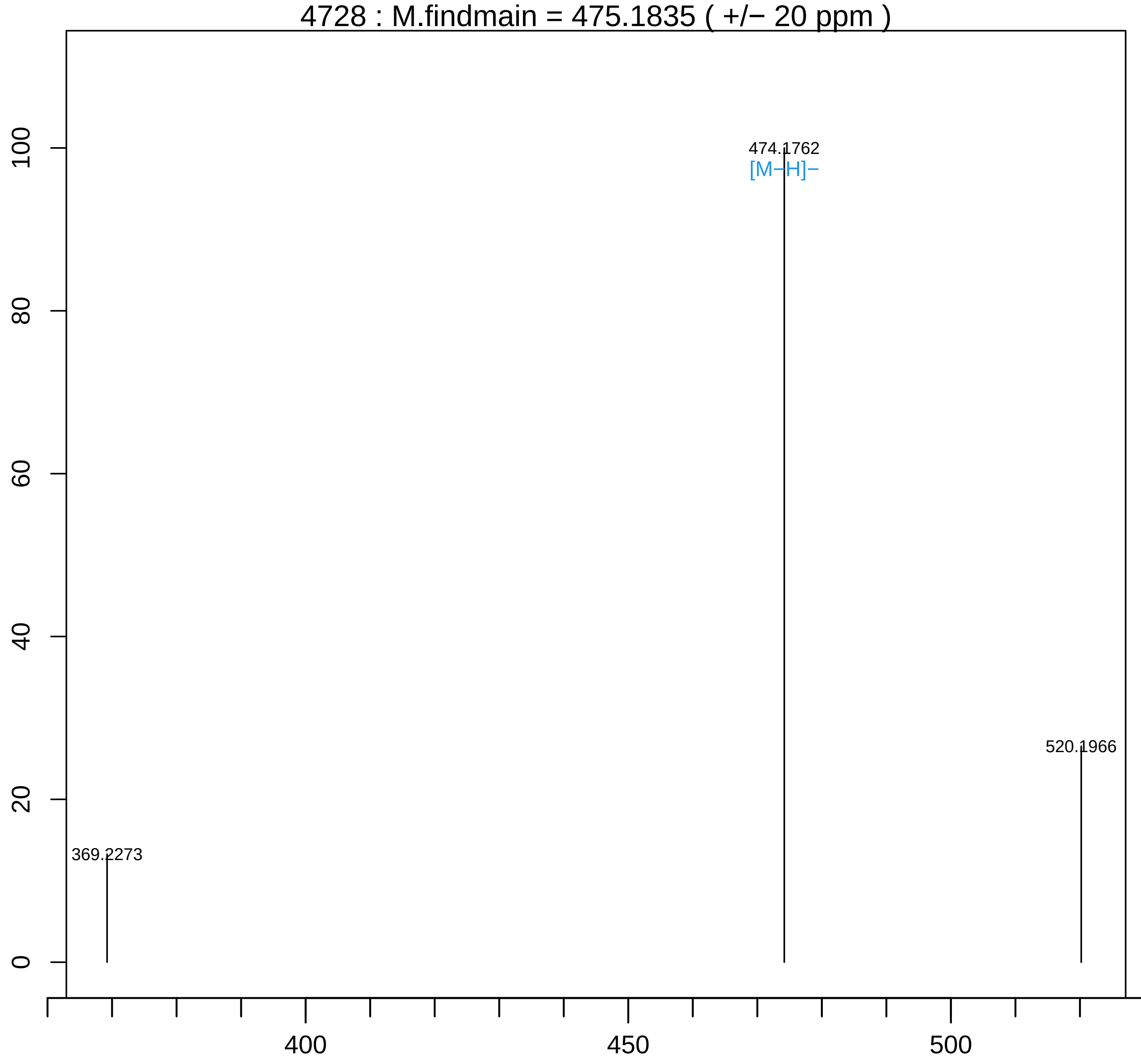
4728 : M.findmain = 475.1835 (+/− 20 ppm)
474.1762
[M−H]−
520.1966
369.2273
0
20
40
60
80
100
400
450
500

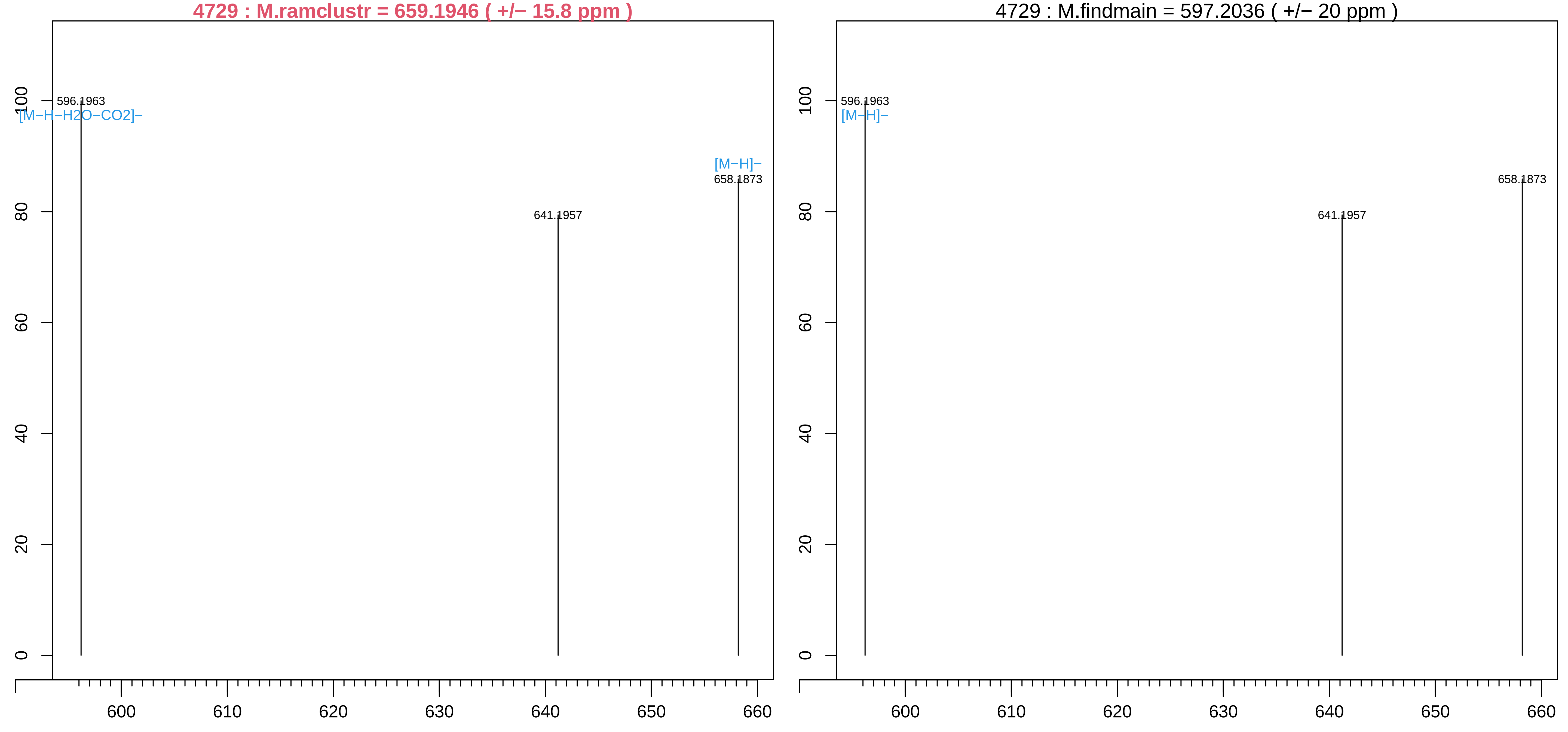

4729 : M.ramclustr = 659.1946 (+/− 15.8 ppm)
596.1963
[M−H−H2O−CO2]−
641.1957
[M−H]−
658.1873
0
20
40
60
80
100
600
610
620
630
640
650
660
4729 : M.findmain = 597.2036 (+/− 20 ppm)
596.1963
[M−H]−
641.1957
658.1873
0
20
40
60
80
100
600
610
620
630
640
650
660

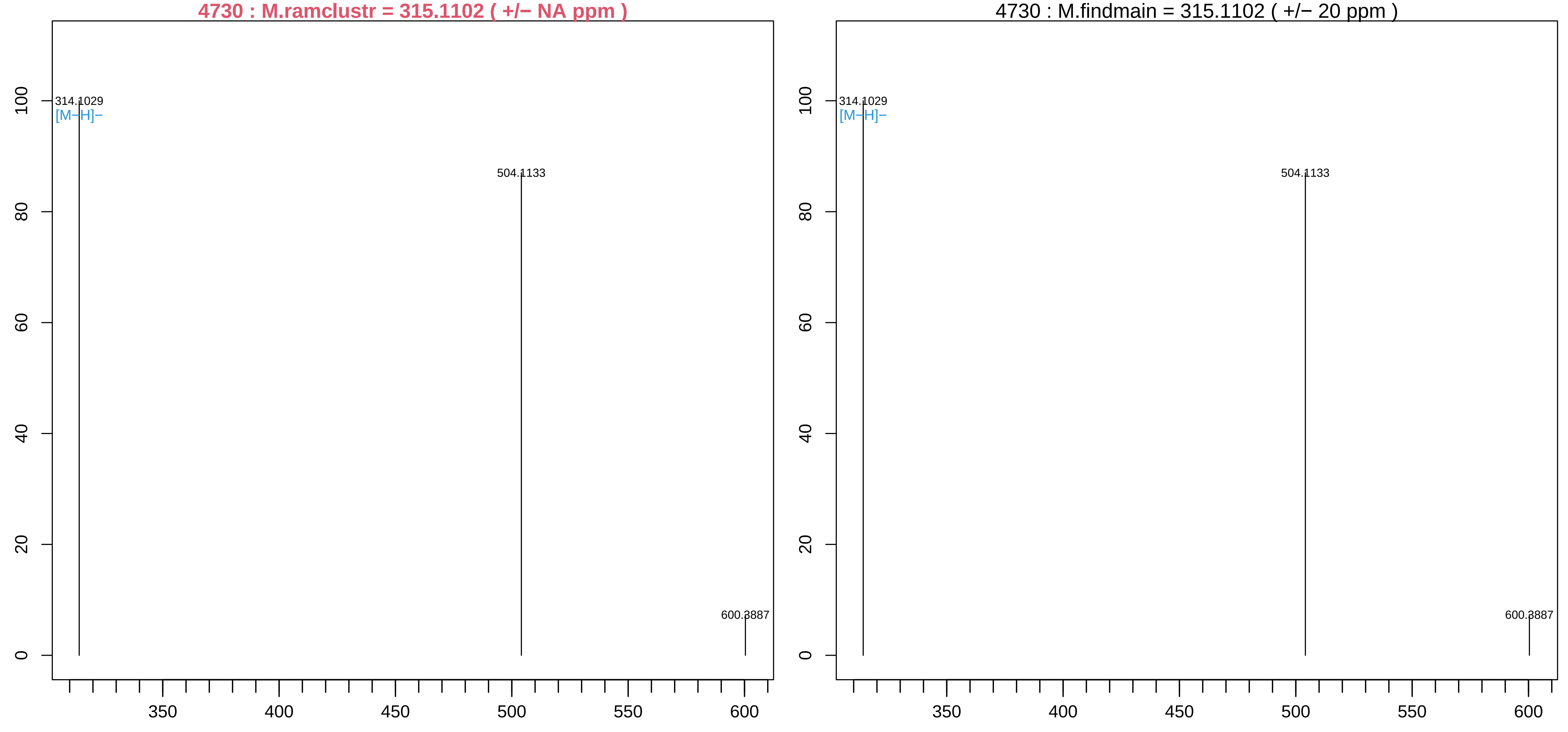

4730 : M.ramclustr = 315.1102 (+/− NA ppm)
314.1029
[M−H]−
504.1133
600.3887
0
20
40
60
80
100
350
400
450
500
550
600
4730 : M.findmain = 315.1102 (+/− 20 ppm)
314.1029
[M−H]−
504.1133
600.3887
0
20
40
60
80
100
350
400
450
500
550
600

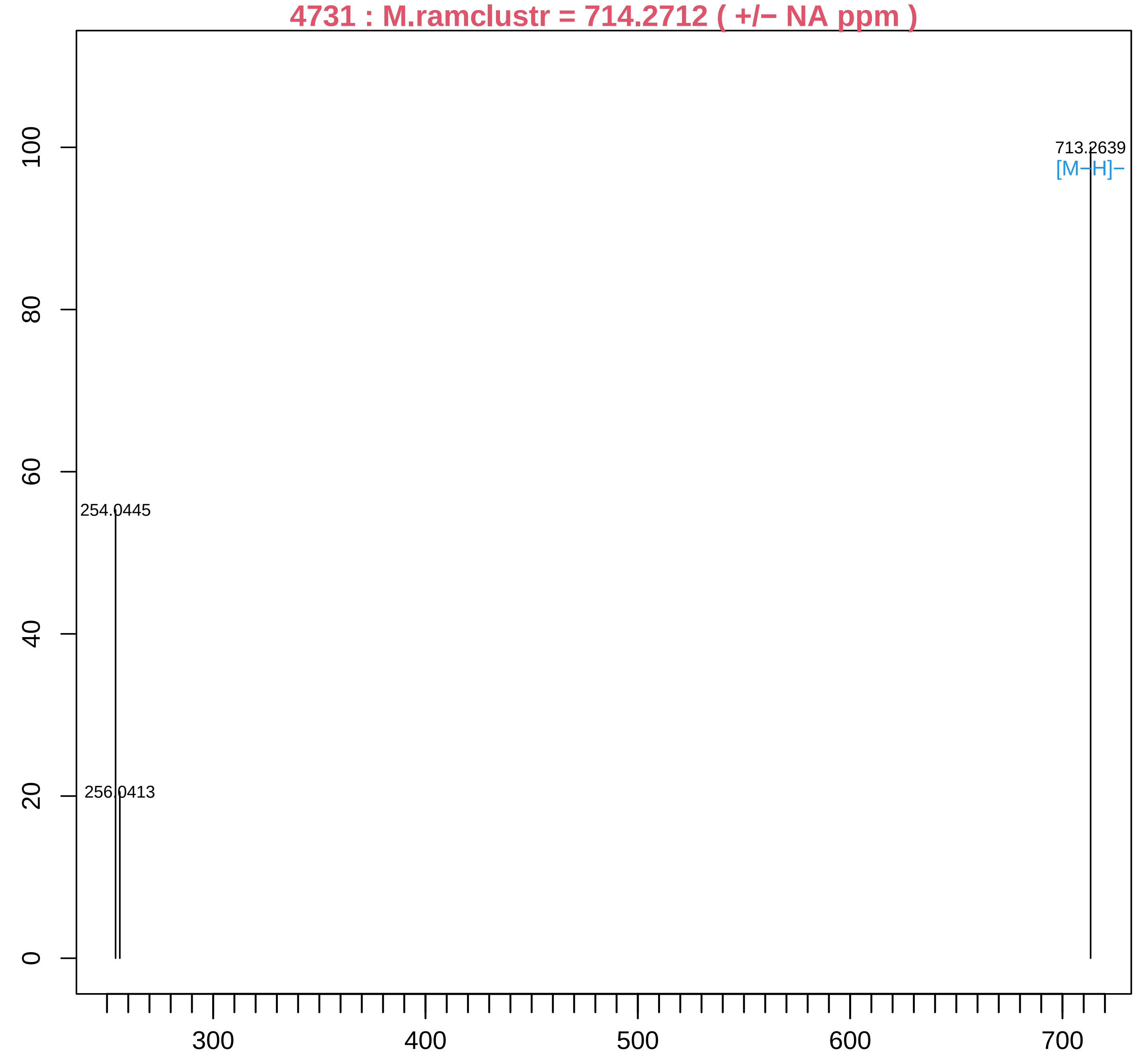
4731 : M.ramclustr = 714.2712 (+/− NA ppm)
713.2639
[M−H]−
254.0445
256.0413
0
20
40
60
80
100
300
400
500
600
700

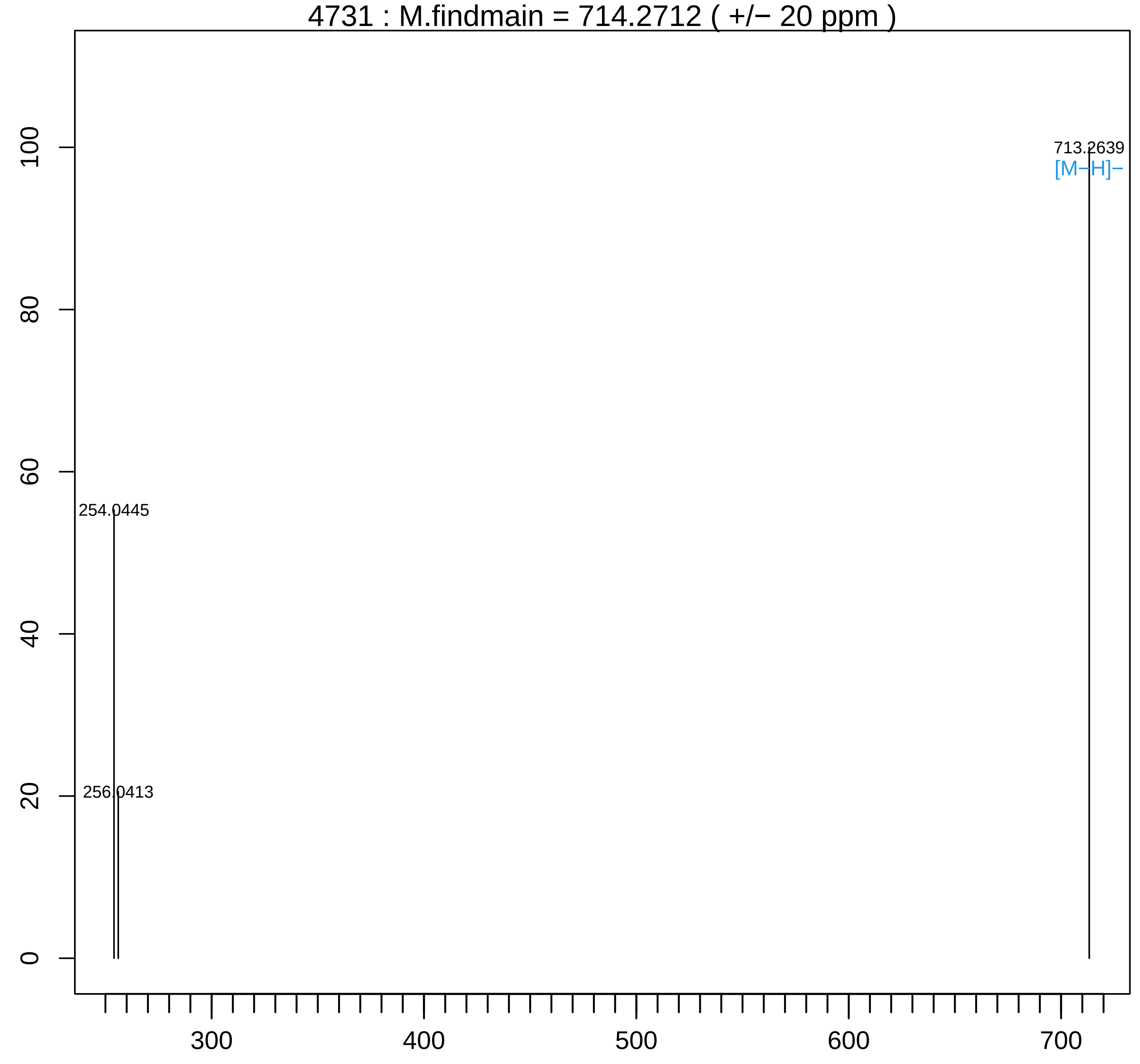
4731 : M.findmain = 714.2712 (+/− 20 ppm)
713.2639
[M−H]−
254.0445
256.0413
0
20
40
60
80
100
300
400
500
600
700

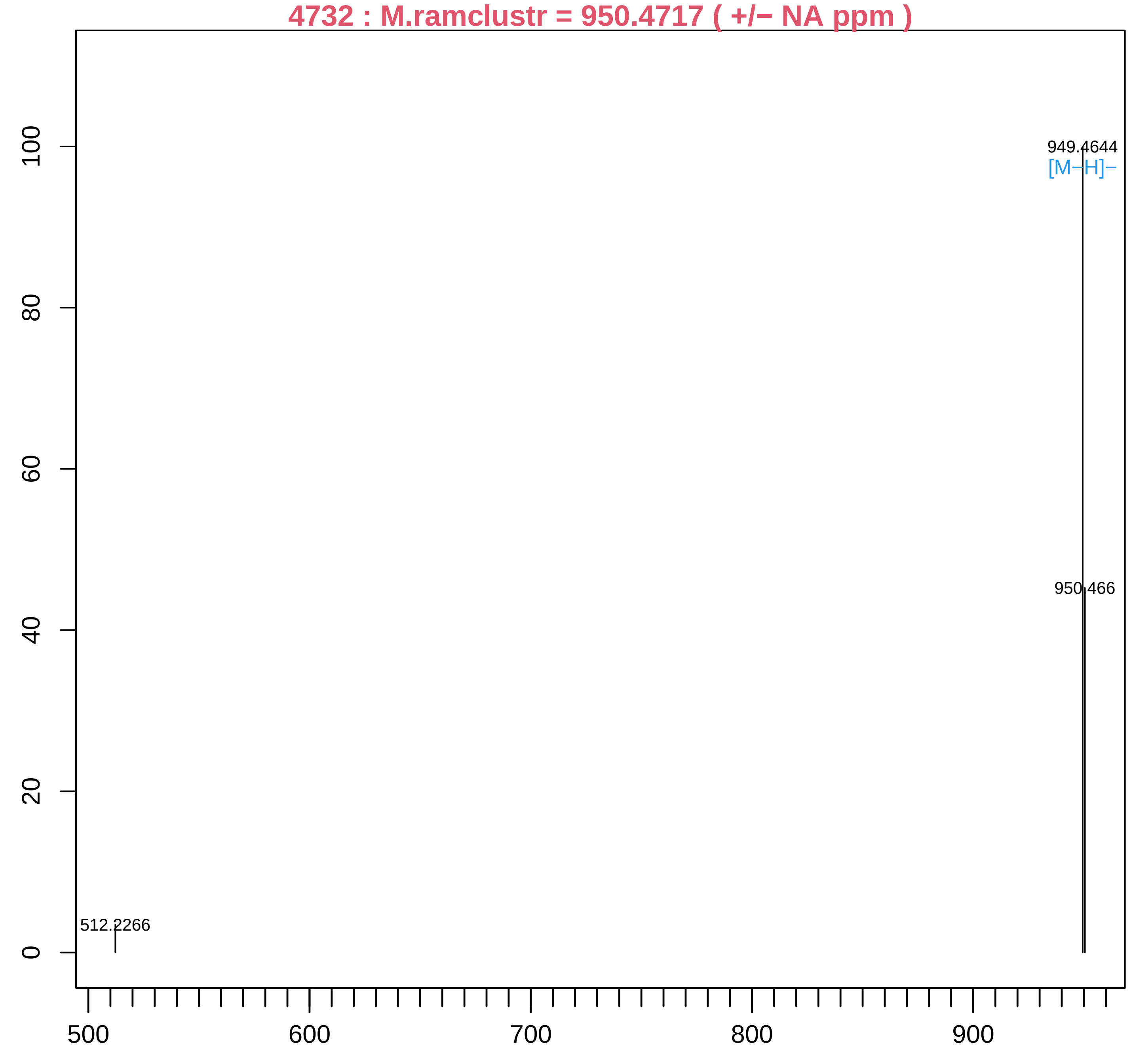

4732 : M.ramclustr = 950.4717 (+/− NA ppm)
949.4644
[M−H]−
950.466
512.2266
0
20
40
60
80
100
500
600
700
800
900

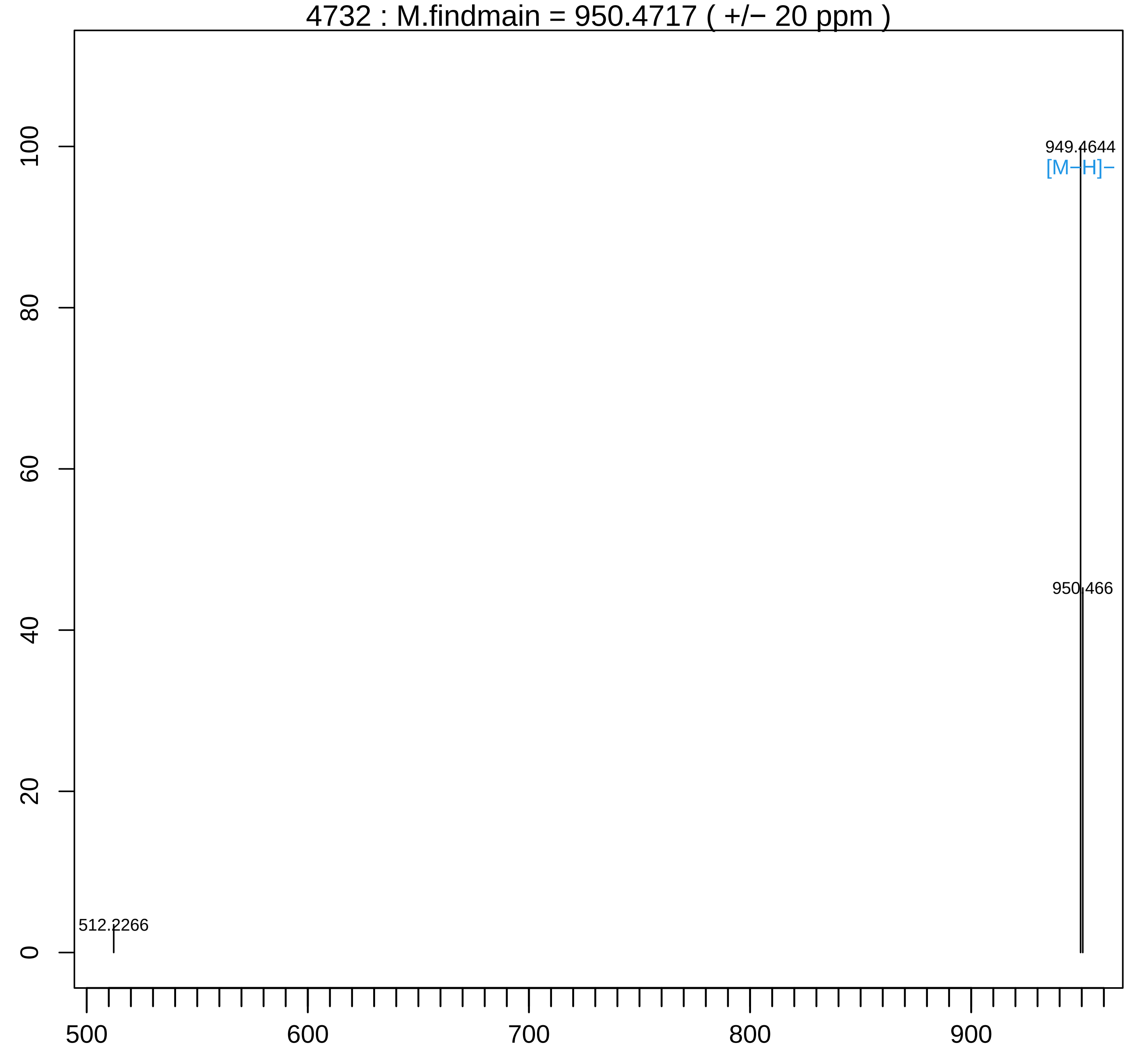

4732 : M.findmain = 950.4717 (+/− 20 ppm)
949.4644
[M−H]−
950.466
512.2266
0
20
40
60
80
100
500
600
700
800
900

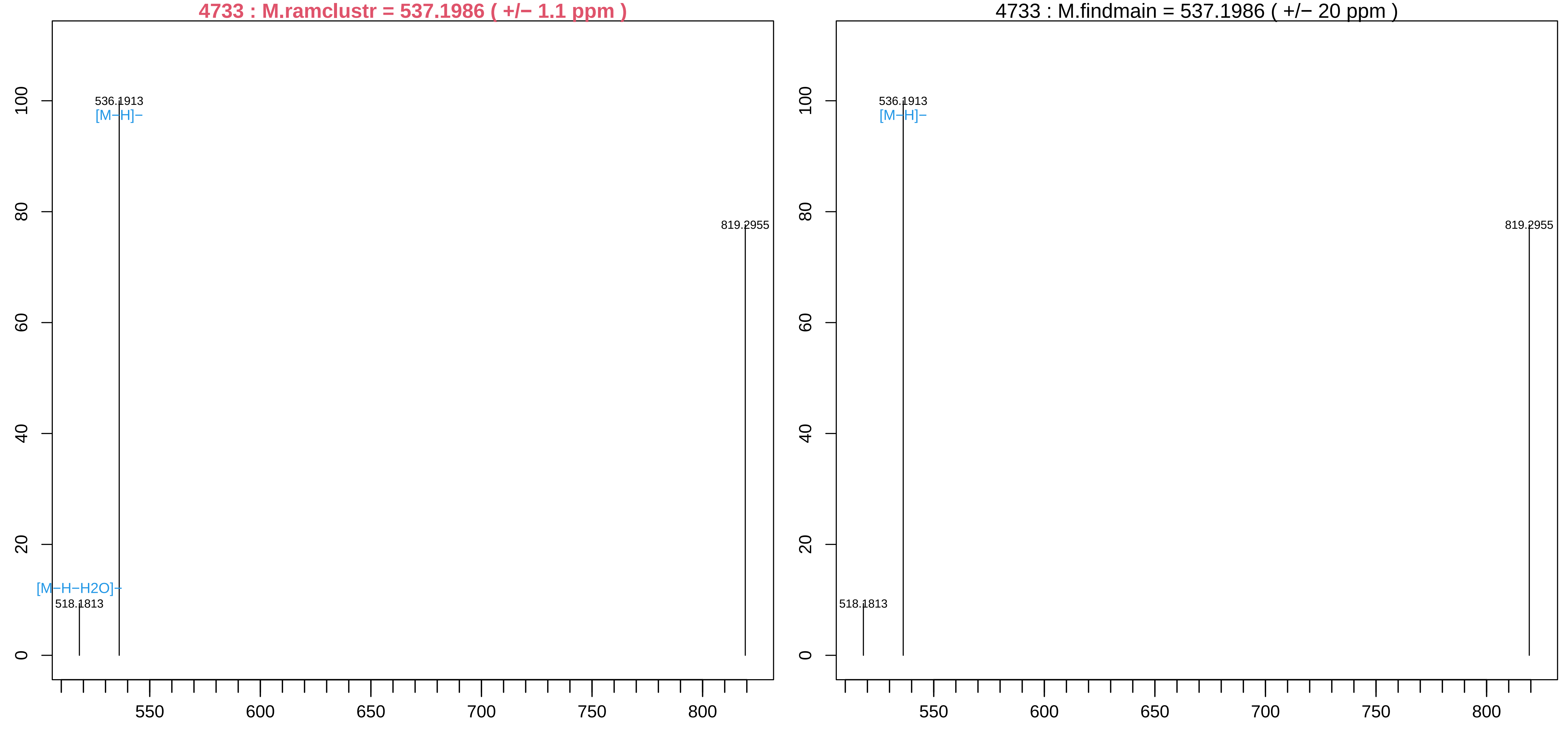
4733 : M.ramclustr = 537.1986 (+/− 1.1 ppm)
536.1913
[M−H]−
819.2955
[M−H−H2O]−
518.1813
0
20
40
60
80
100
550
600
650
700
750
800
4733 : M.findmain = 537.1986 (+/− 20 ppm)
536.1913
[M−H]−
819.2955
518.1813
0
20
40
60
80
100
550
600
650
700
750
800

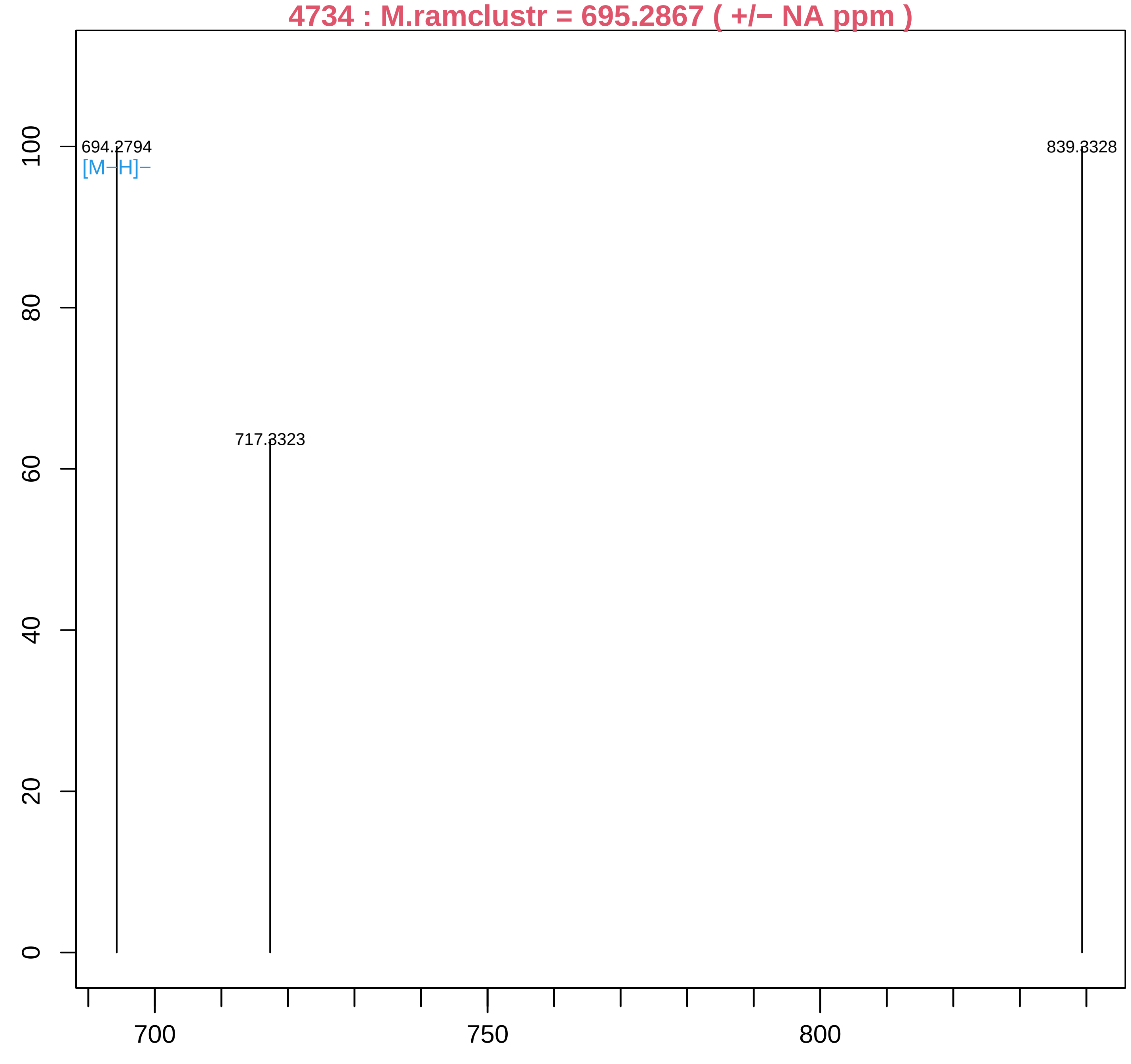
4734 : M.ramclustr = 695.2867 (+/− NA ppm)
694.2794
[M−H]−
717.3323
839.3328
0
20
40
60
80
100
700
750
800

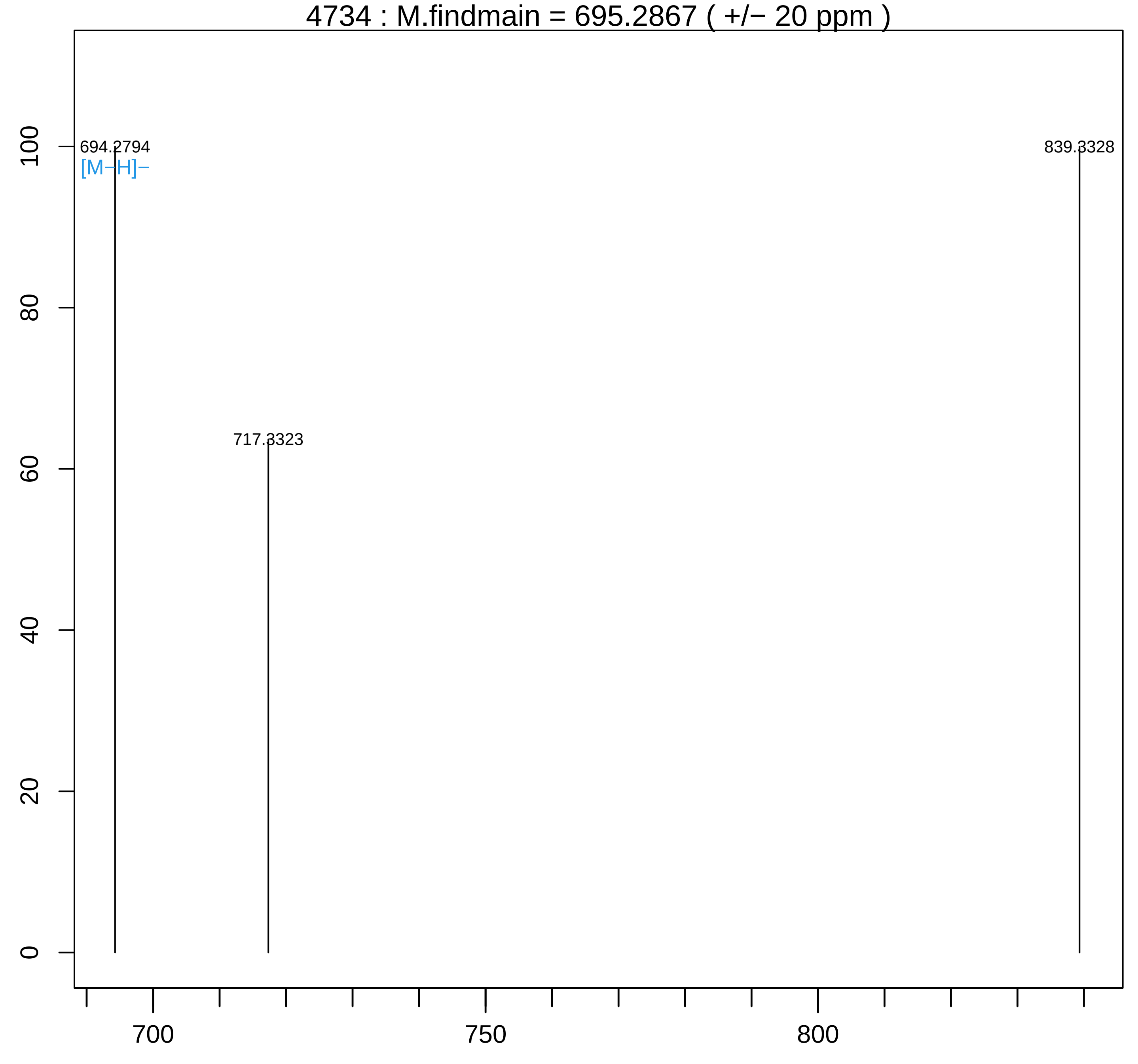
4734 : M.findmain = 695.2867 (+/− 20 ppm)
694.2794
[M−H]−
717.3323
839.3328
0
20
40
60
80
100
700
750
800

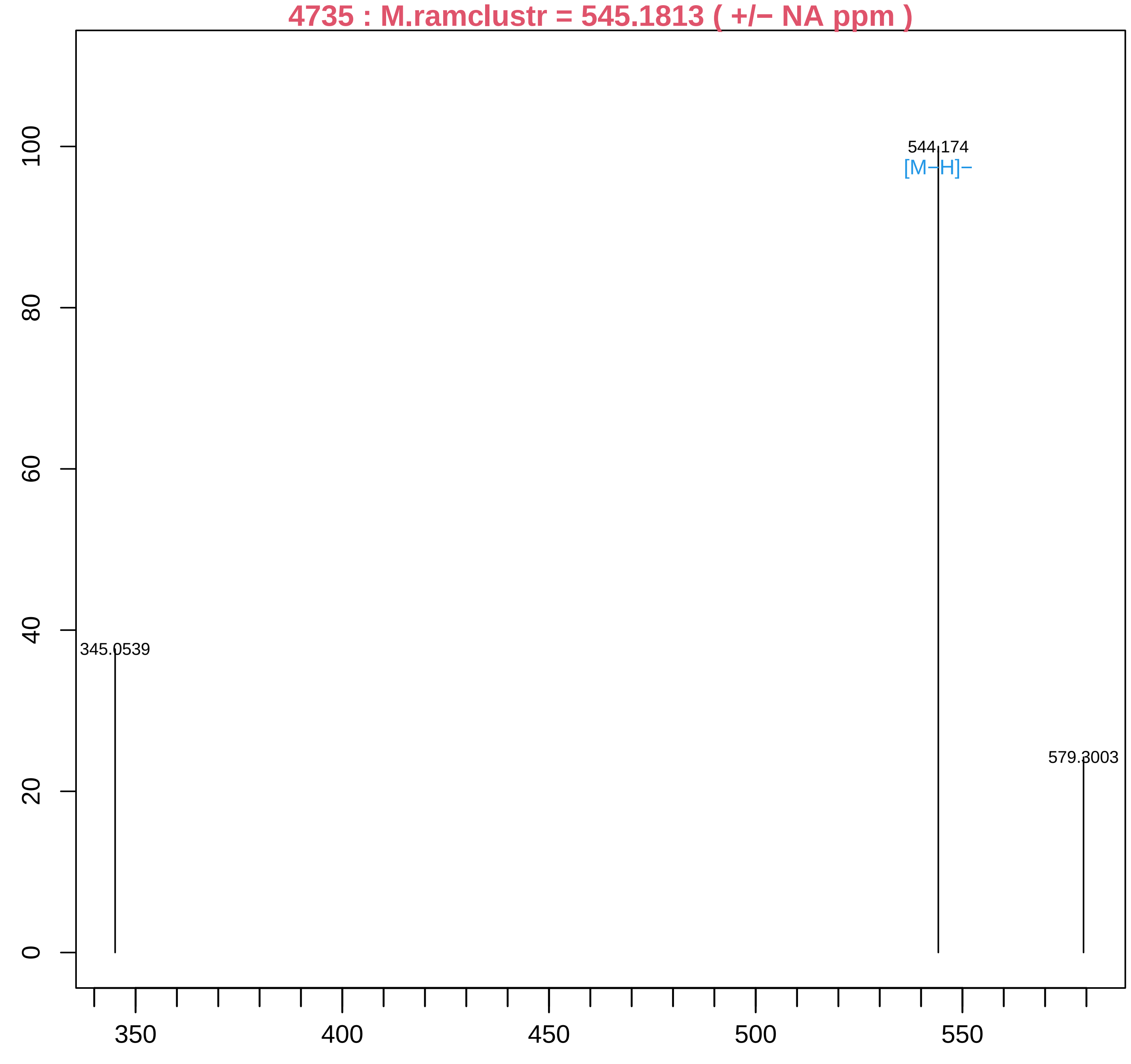
4735 : M.ramclustr = 545.1813 (+/− NA ppm)
544.174
[M−H]−
345.0539
579.3003
0
20
40
60
80
100
350
400
450
500
550

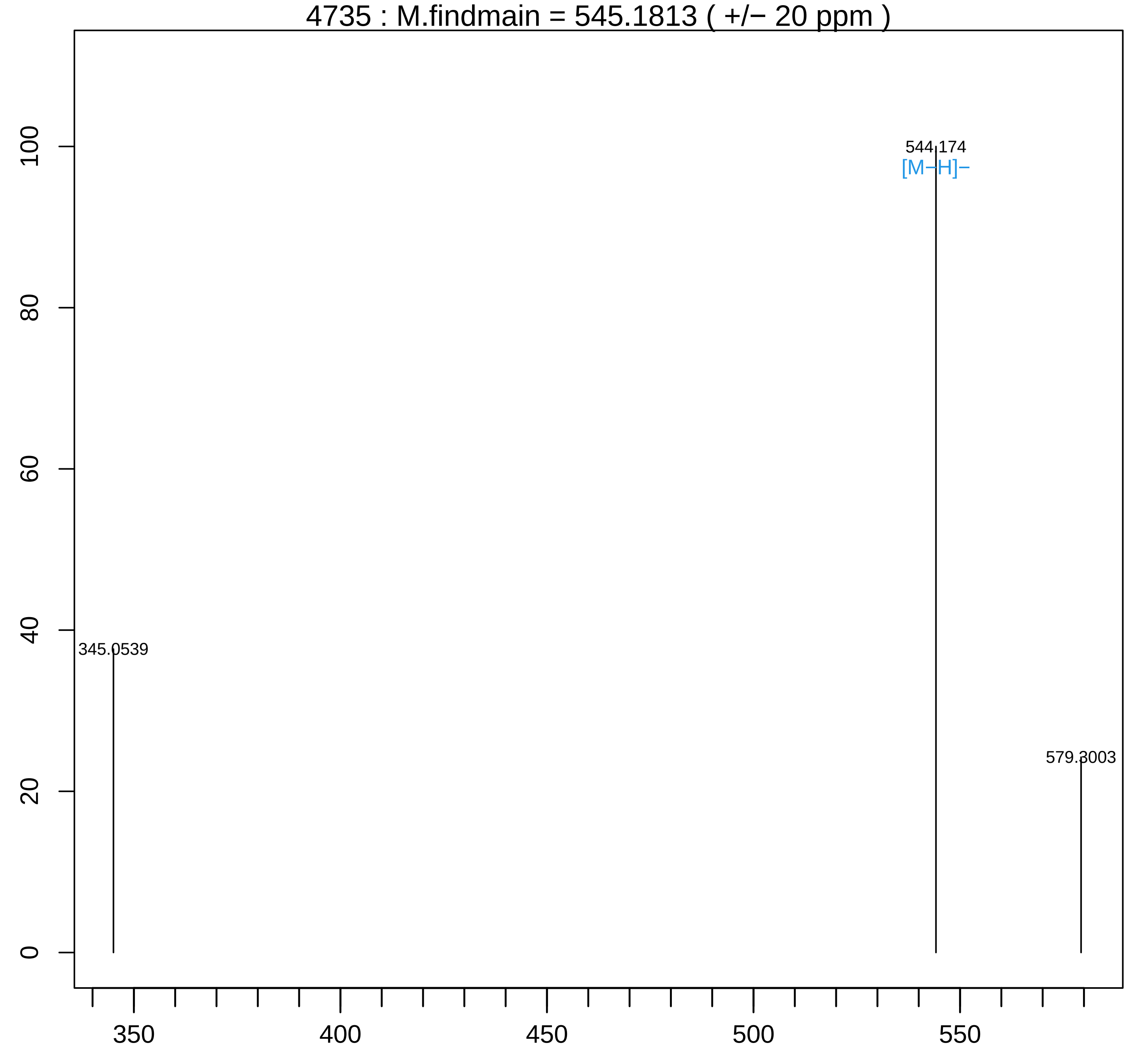
4735 : M.findmain = 545.1813 (+/− 20 ppm)
544.174
[M−H]−
345.0539
579.3003
0
20
40
60
80
100
350
400
450
500
550

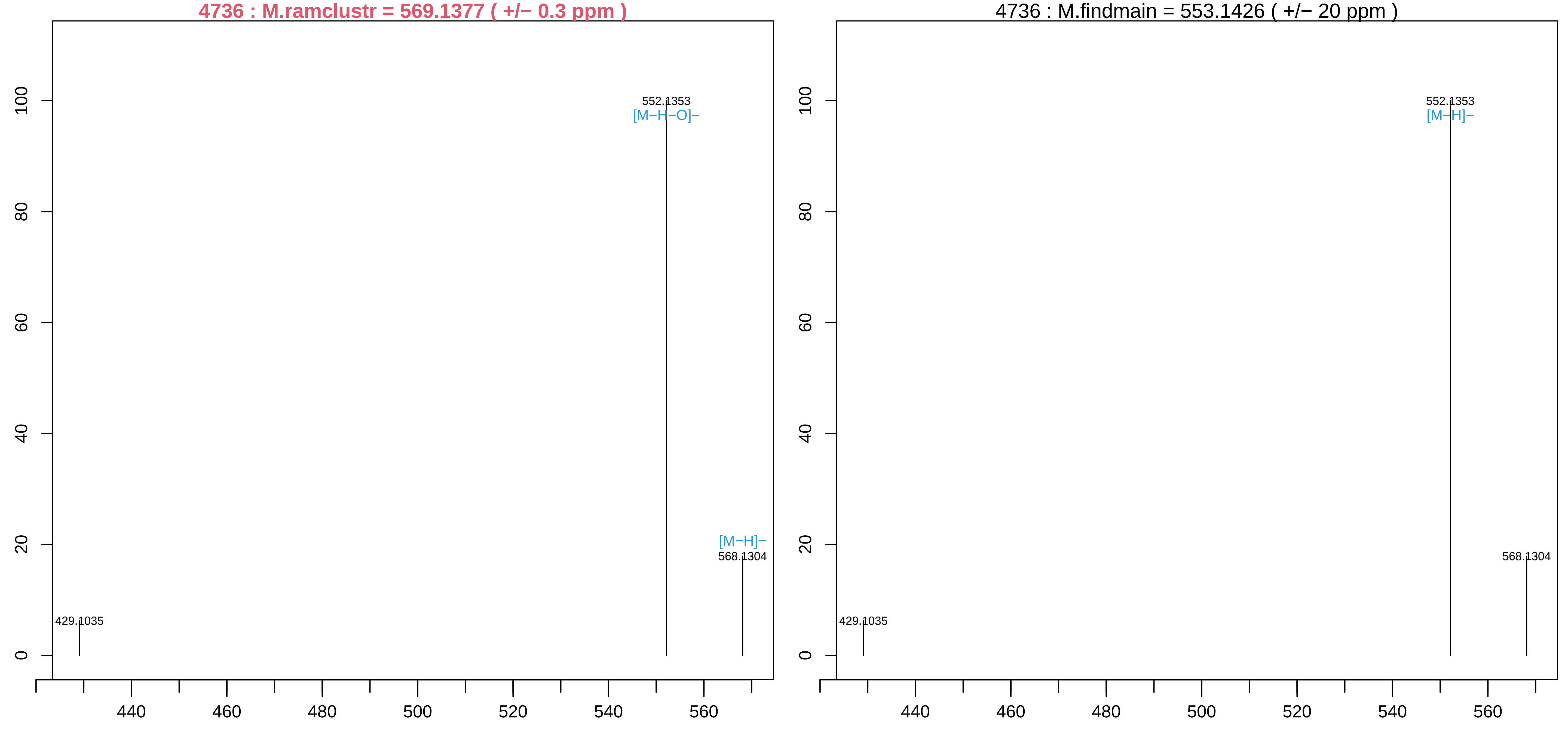
4736 : M.ramclustr = 569.1377 (+/− 0.3 ppm)
552.1353
[M−H−O]−
[M−H]−
568.1304
429.1035
4736 : M.findmain = 553.1426 (+/− 20 ppm)
552.1353
[M−H]−
568.1304
429.1035
0
20
40
60
80
100
440
460
480
500
520
540
560

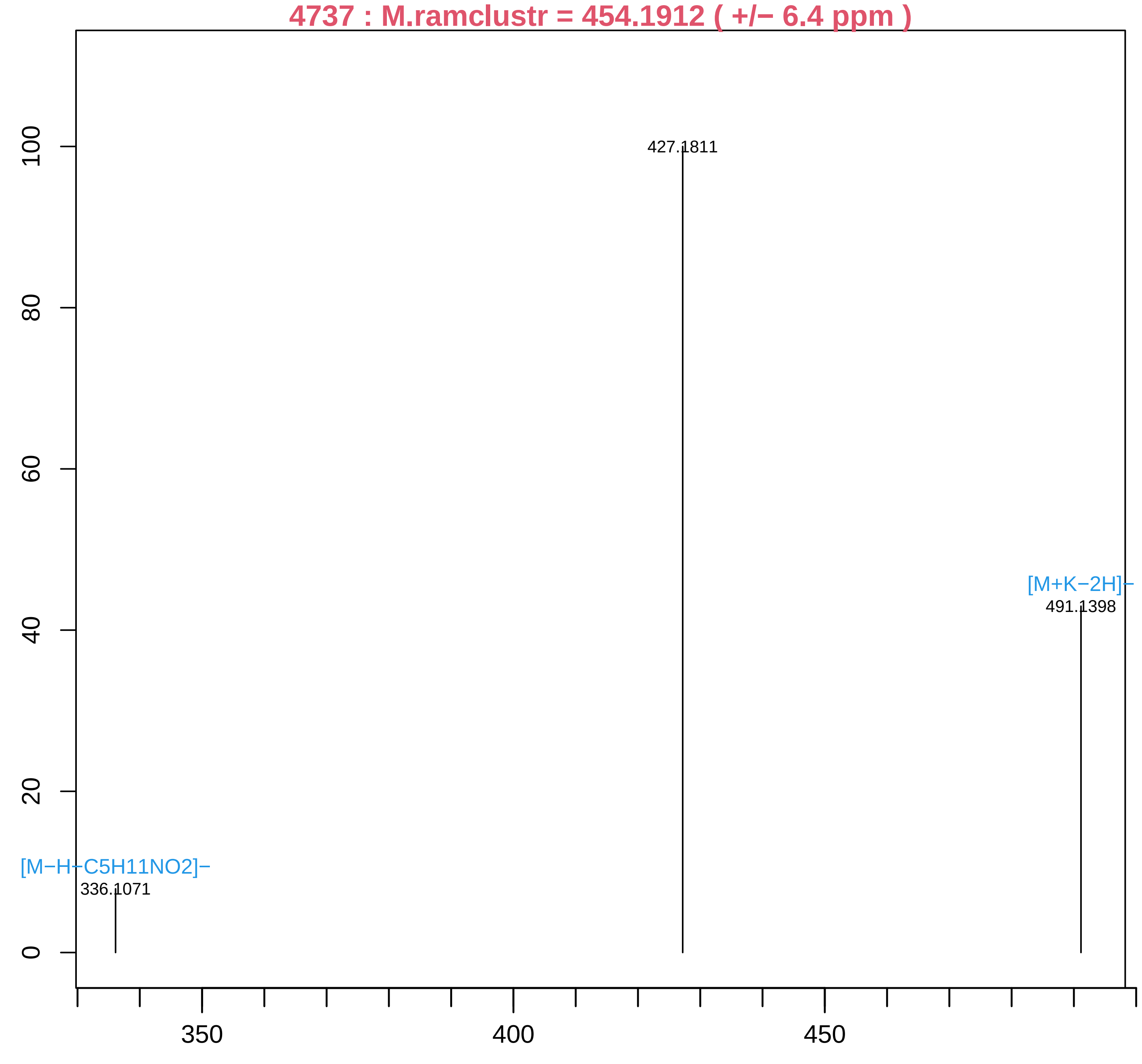
4737 : M.ramclustr = 454.1912 (+/− 6.4 ppm)
427.1811
[M+K−2H]−
491.1398
[M−H−C5H11NO2]−
336.1071
0
20
40
60
80
100
350
400
450

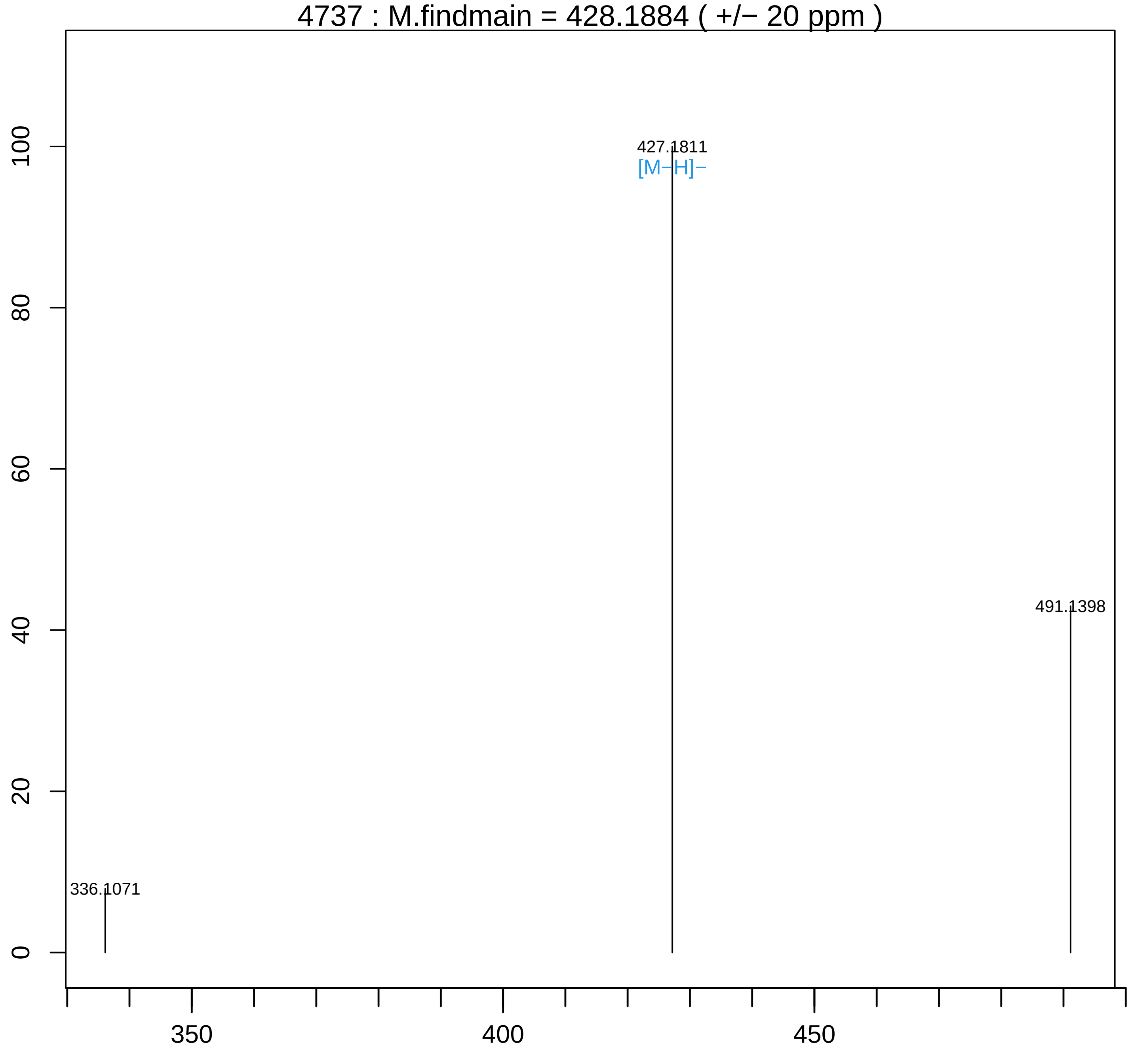
4737 : M.findmain = 428.1884 (+/− 20 ppm)
427.1811
[M−H]−
491.1398
336.1071
0
20
40
60
80
100
350
400
450

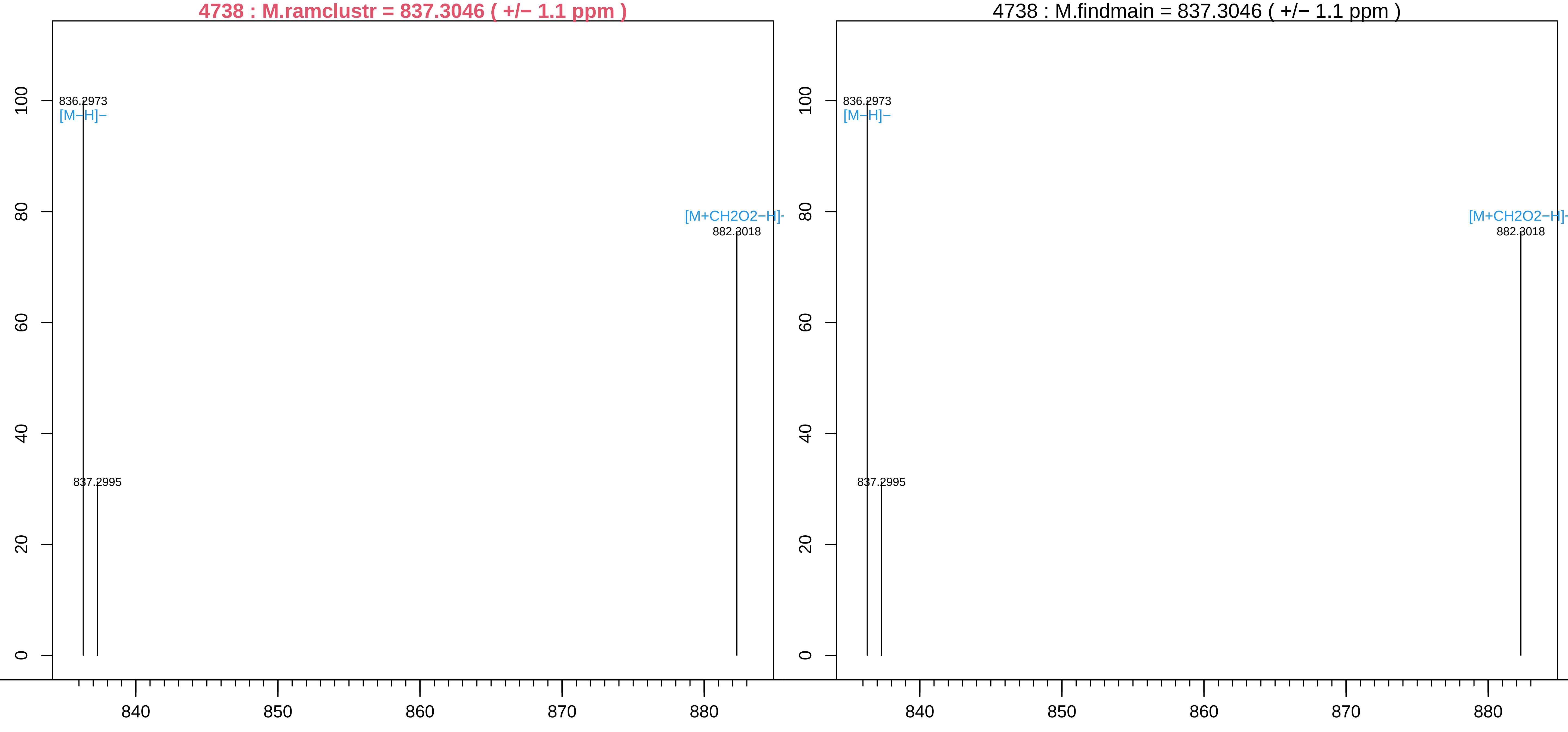
4738 : M.ramclustr = 837.3046 (+/− 1.1 ppm)
836.2973
[M−H]−
837.2995
[M+CH2O2−H]-
882.3018
4738 : M.findmain = 837.3046 (+/− 1.1 ppm)
836.2973
[M−H]−
837.2995
[M+CH2O2−H]-
882.3018
0
20
40
60
80
100
840
850
860
870
880

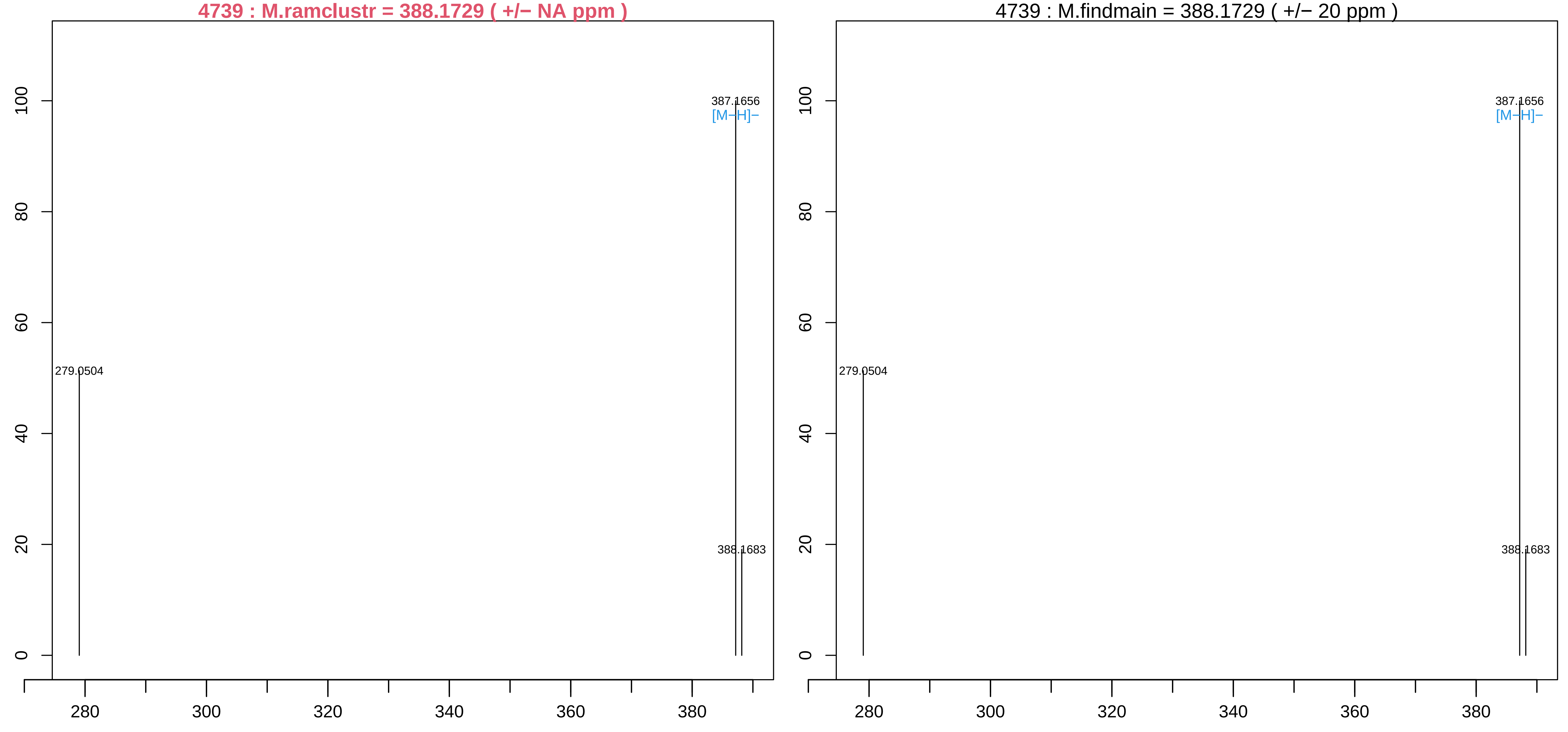
4739 : M.ramclustr = 388.1729 (+/− NA ppm)
387.1656
[M−H]−
279.0504
388.1683
4739 : M.findmain = 388.1729 (+/− 20 ppm)
387.1656
[M−H]−
279.0504
388.1683
0
20
40
60
80
100
280
300
320
340
360
380

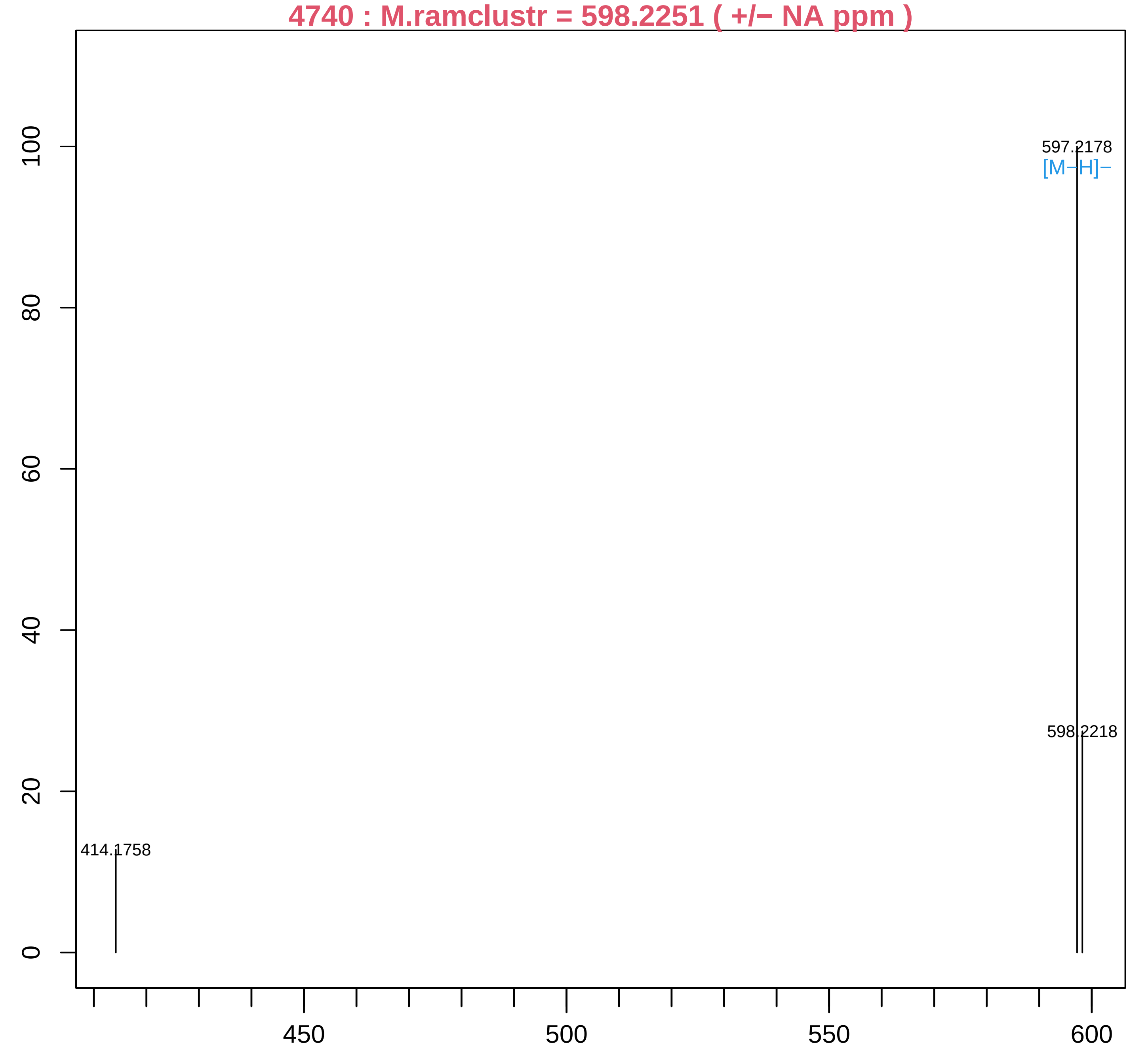

4740 : M.ramclustr = 598.2251 (+/− NA ppm)
597.2178
[M−H]−
598.2218
414.1758
0
20
40
60
80
100
450
500
550
600

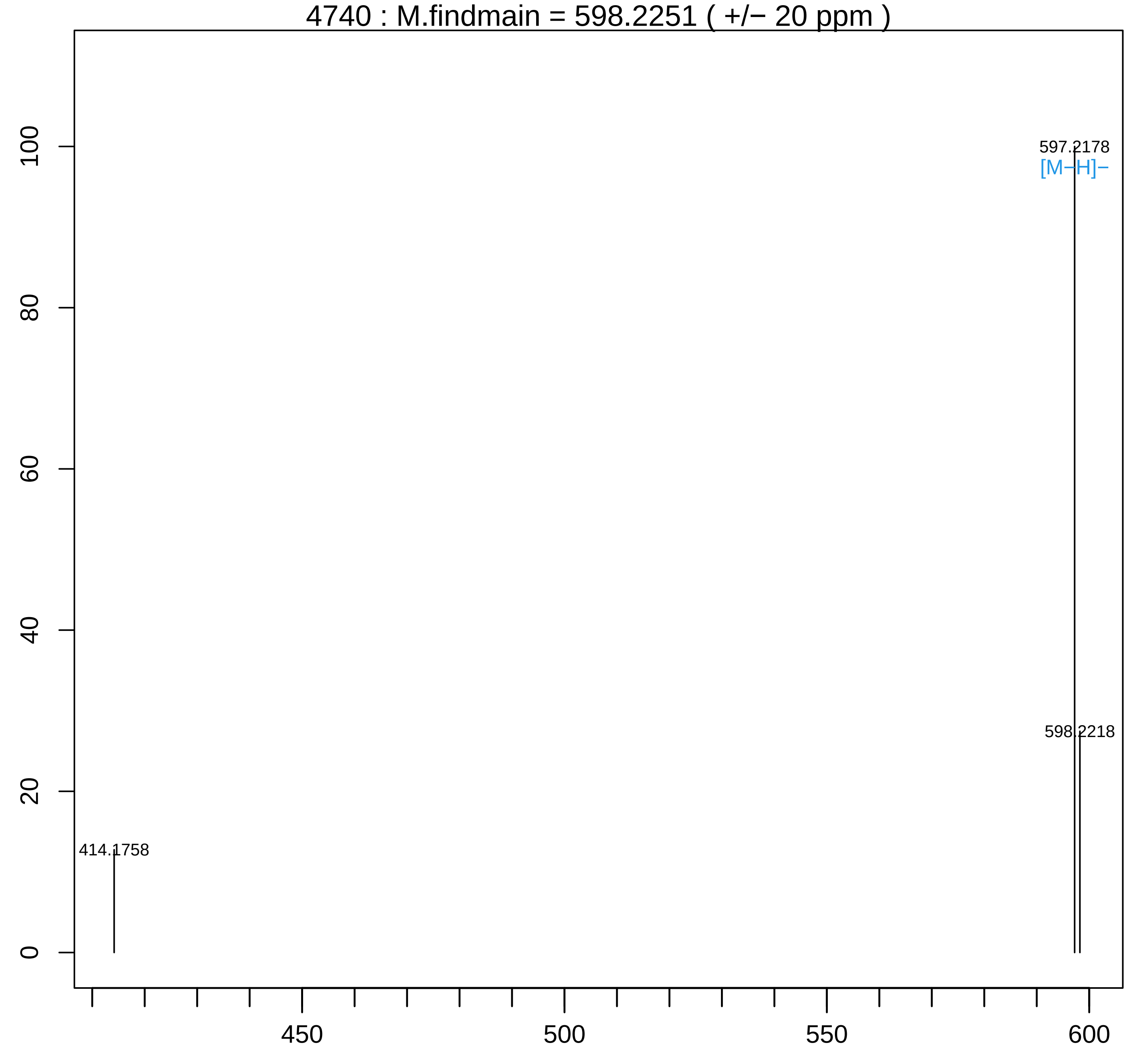

4740 : M.findmain = 598.2251 (+/− 20 ppm)
597.2178
[M−H]−
598.2218
414.1758
0
20
40
60
80
100
450
500
550
600

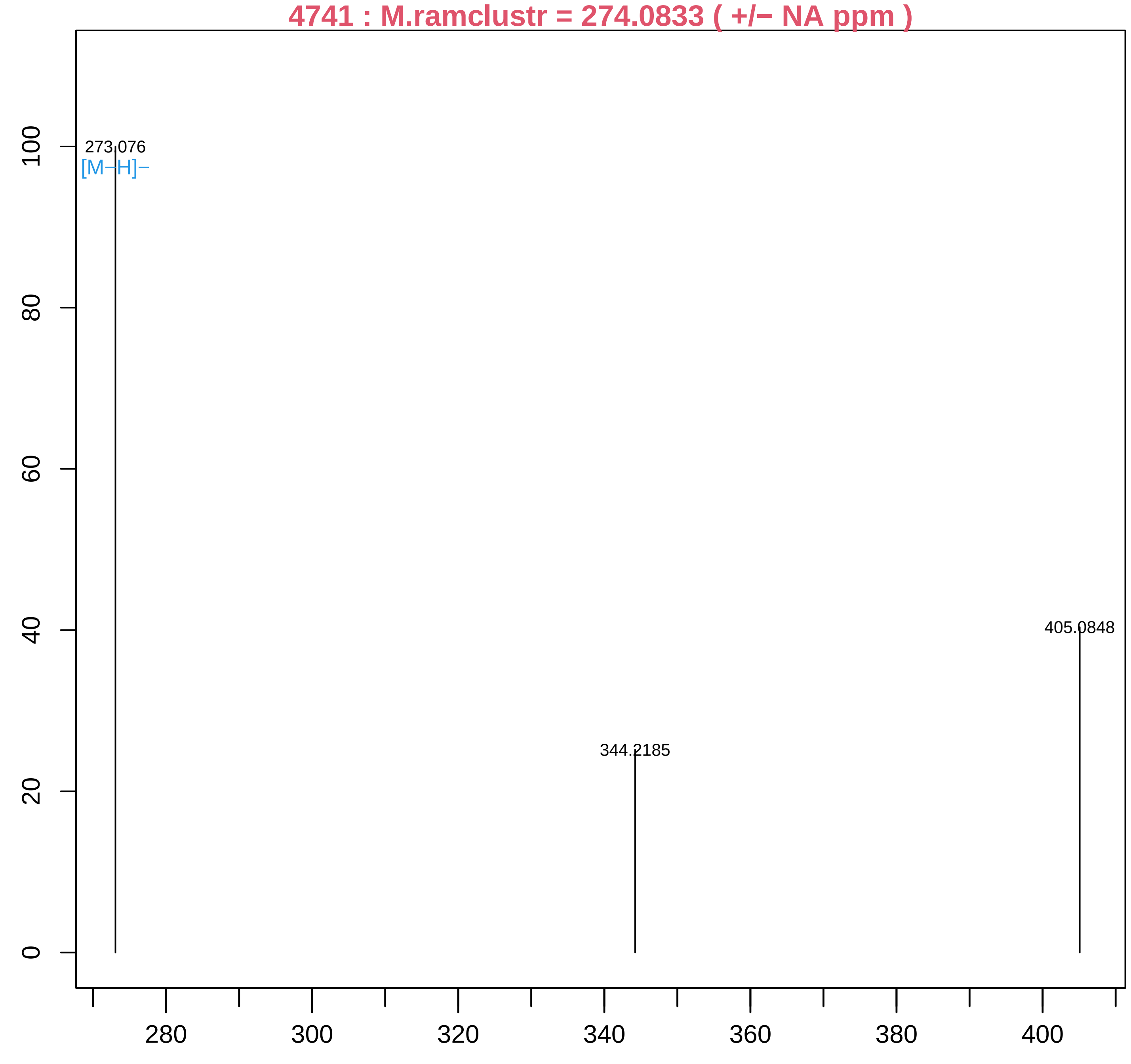
4741 : M.ramclustr = 274.0833 (+/− NA ppm)
273.076
[M−H]−
344.2185
405.0848
0
20
40
60
80
100
280
300
320
340
360
380
400

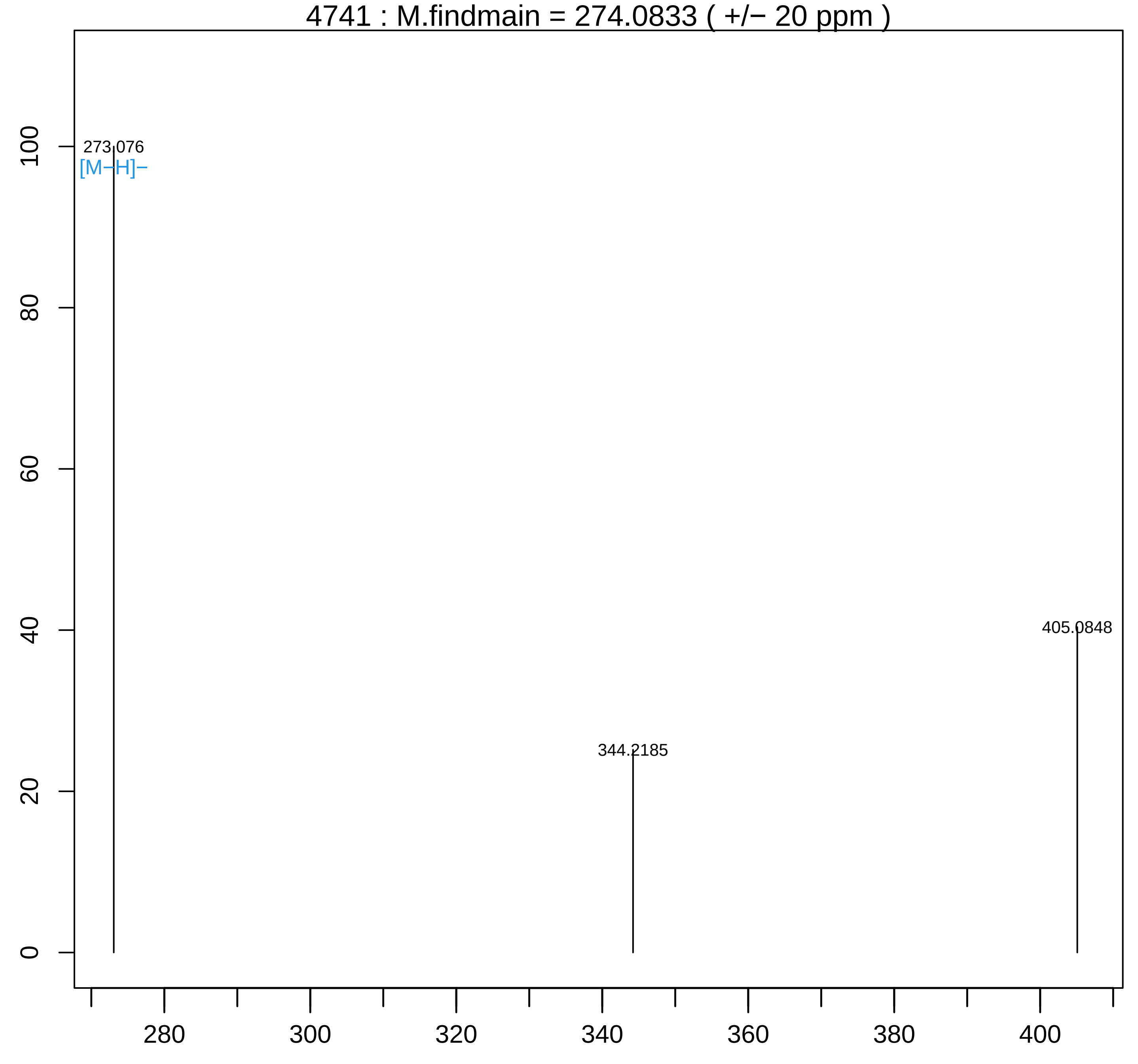
4741 : M.findmain = 274.0833 (+/− 20 ppm)
273.076
[M−H]−
344.2185
405.0848
0
20
40
60
80
100
280
300
320
340
360
380
400

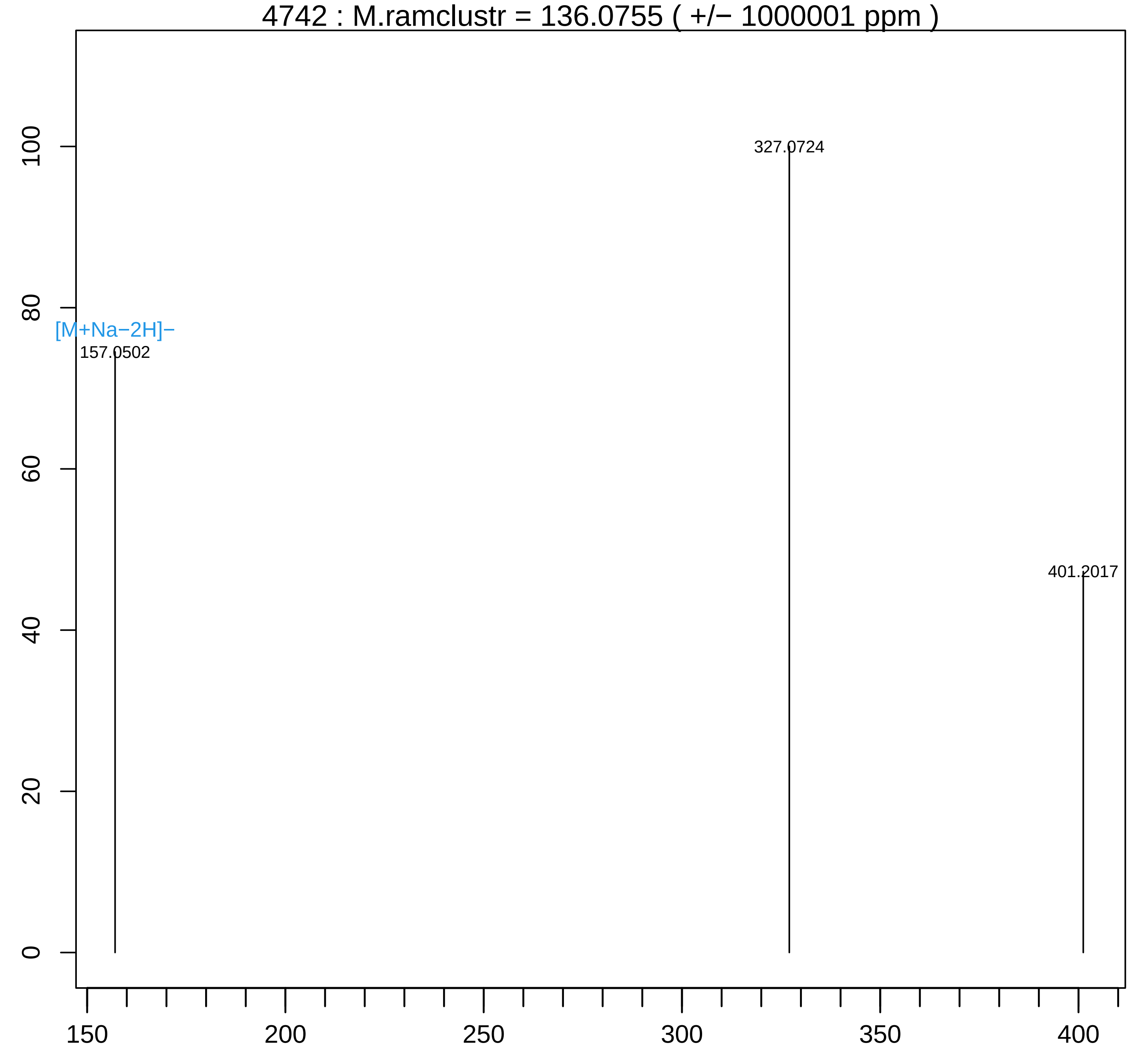
4742 : M.ramclustr = 136.0755 (+/− 1000001 ppm)
[M+Na−2H]−
157.0502
327.0724
401.2017
0
20
40
60
80
100
150
200
250
300
350
400

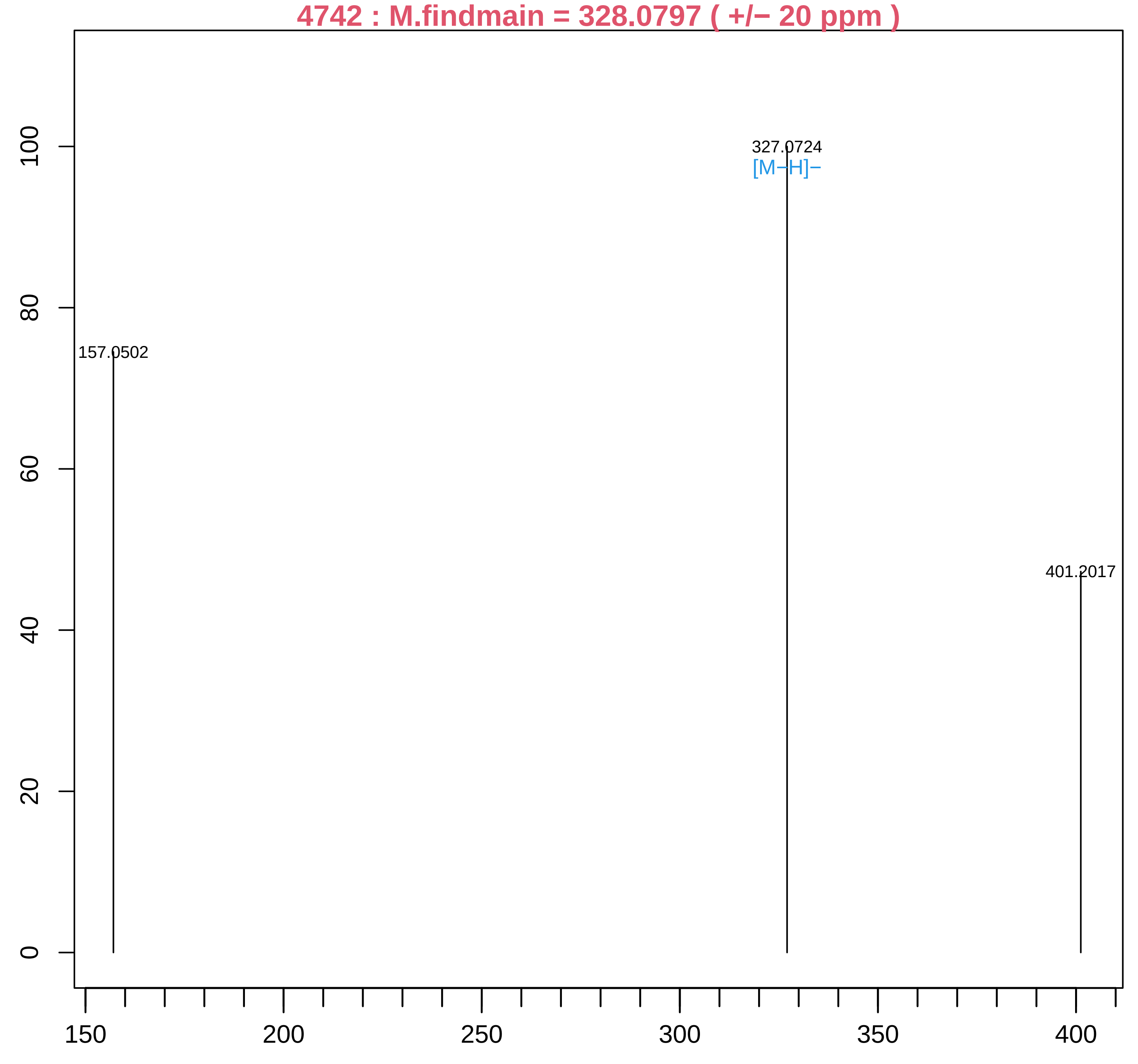
4742 : M.findmain = 328.0797 (+/− 20 ppm)
157.0502
327.0724
[M−H]−
401.2017
0
20
40
60
80
100
150
200
250
300
350
400

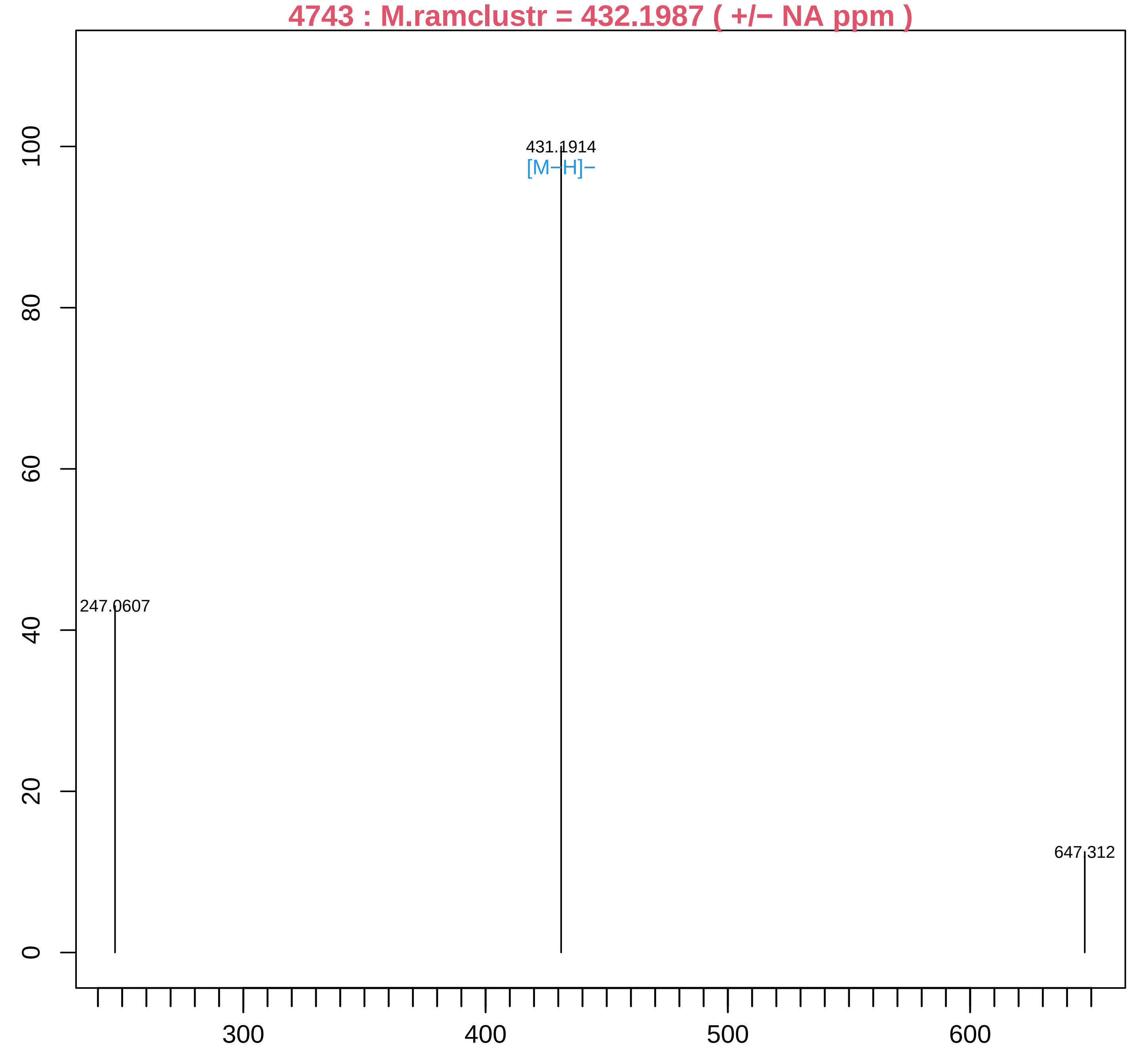

4743 : M.ramclustr = 432.1987 (+/− NA ppm)
431.1914
[M−H]−
247.0607
647.312
0
20
40
60
80
100
300
400
500
600

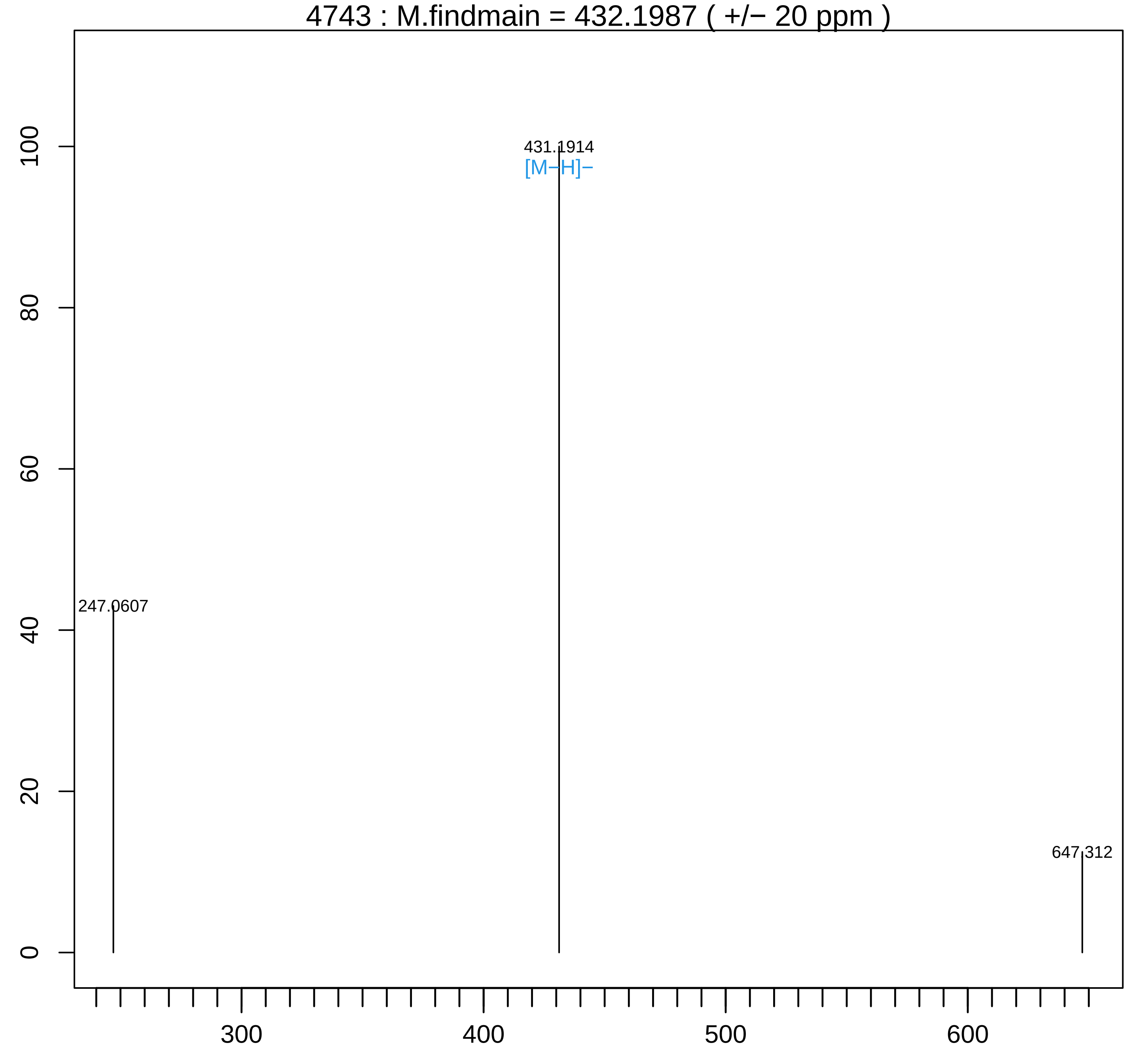

4743 : M.findmain = 432.1987 (+/− 20 ppm)
431.1914
[M−H]−
247.0607
647.312
0
20
40
60
80
100
300
400
500
600

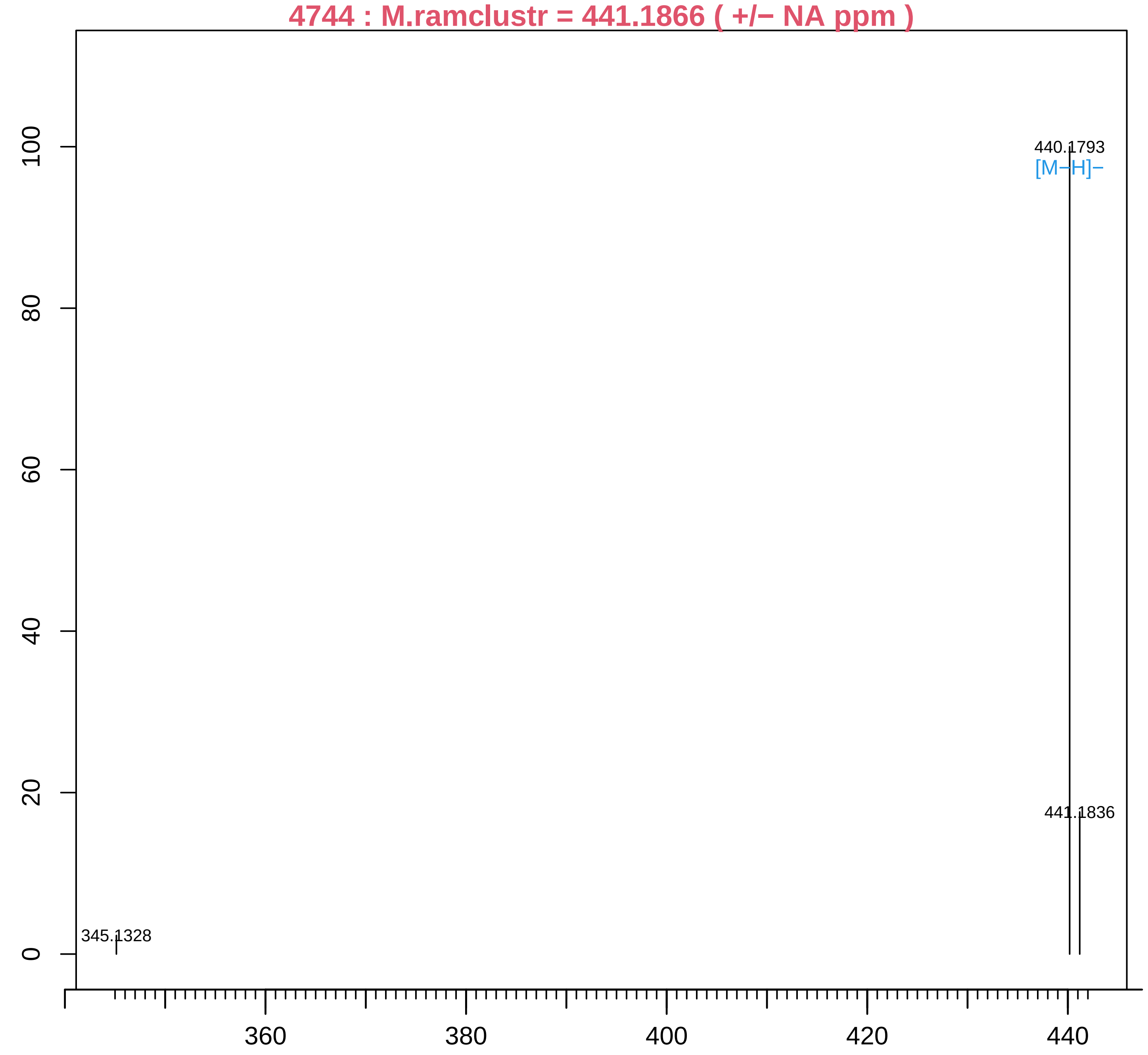

4744 : M.ramclustr = 441.1866 (+/− NA ppm)
440.1793
[M−H]−
441.1836
345.1328
0
20
40
60
80
100
360
380
400
420
440

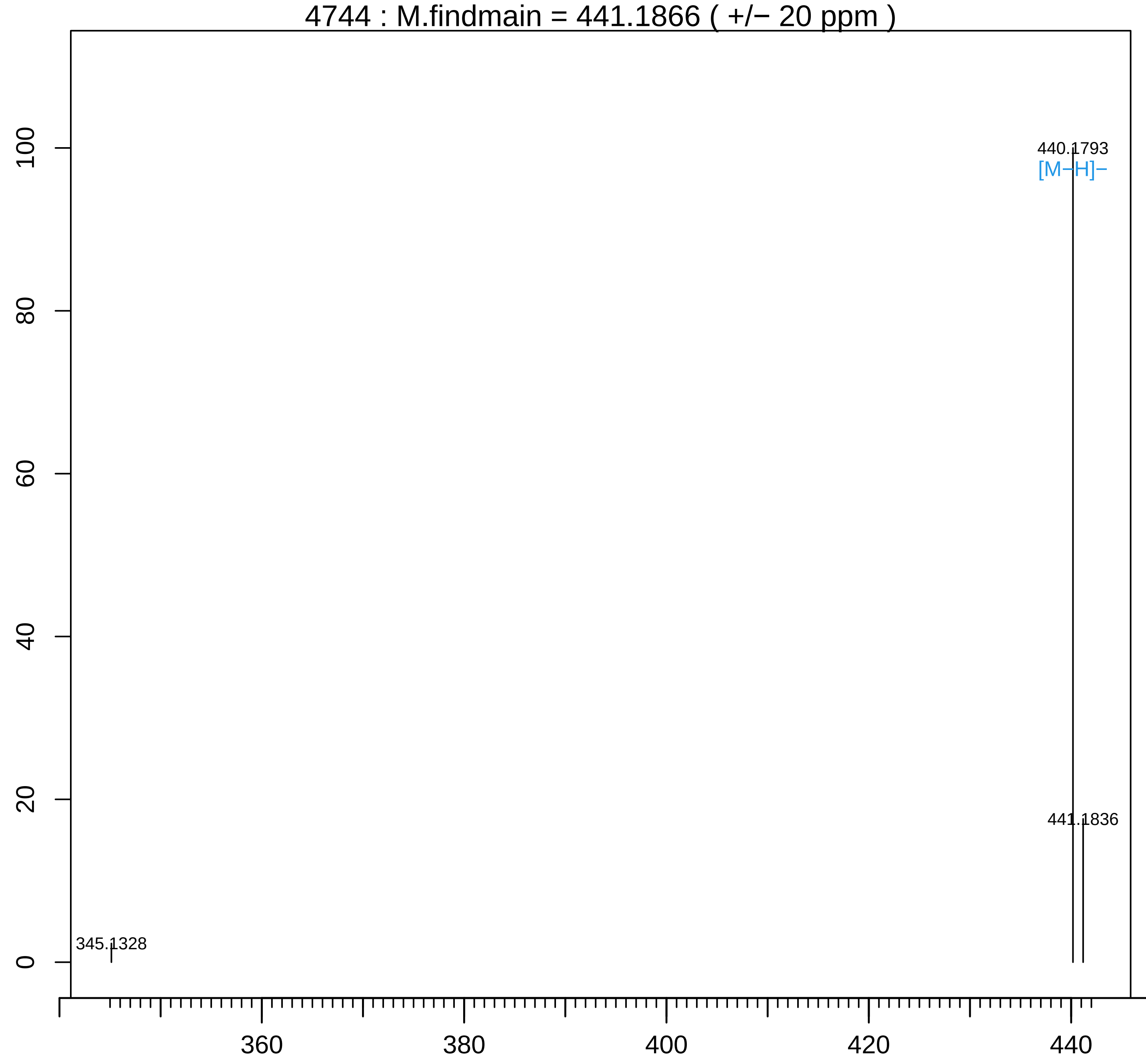

4744 : M.findmain = 441.1866 (+/− 20 ppm)
440.1793
[M−H]−
441.1836
345.1328
0
20
40
60
80
100
360
380
400
420
440

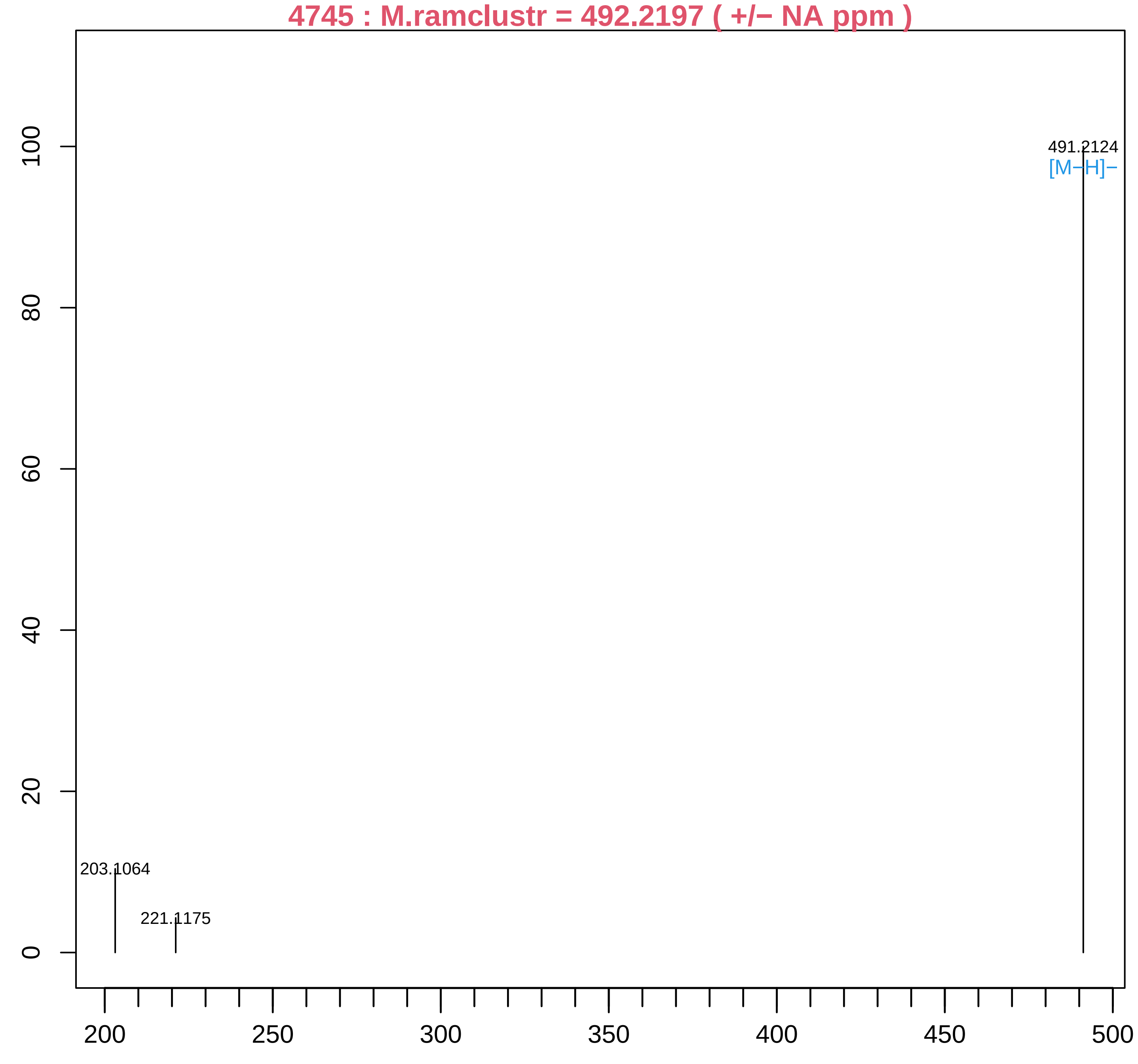
4745 : M.ramclustr = 492.2197 (+/− NA ppm)
491.2124
[M−H]−
203.1064
221.1175
0
20
40
60
80
100
200
250
300
350
400
450
500

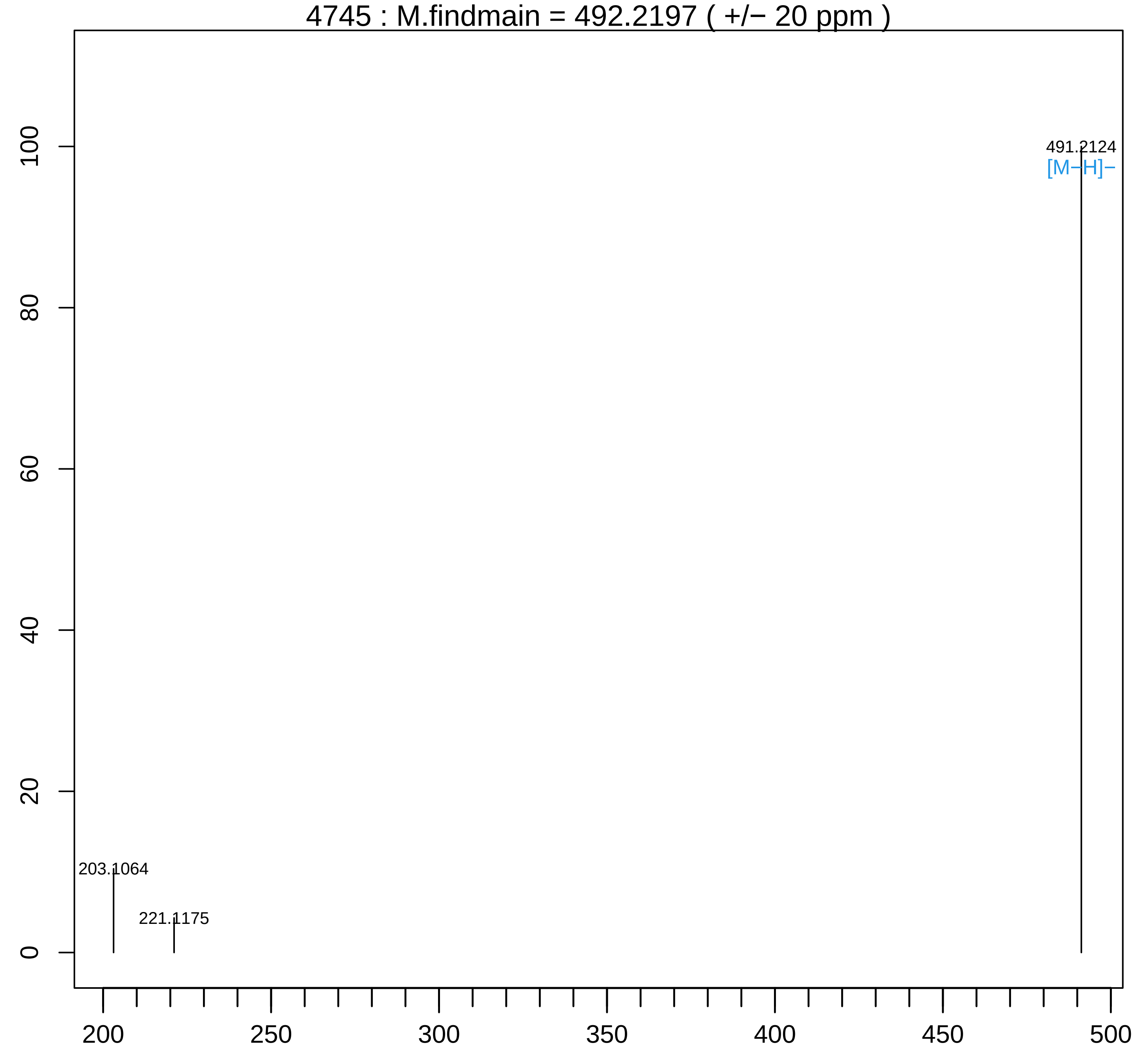
4745 : M.findmain = 492.2197 (+/− 20 ppm)
491.2124
[M−H]−
203.1064
221.1175
0
20
40
60
80
100
200
250
300
350
400
450
500

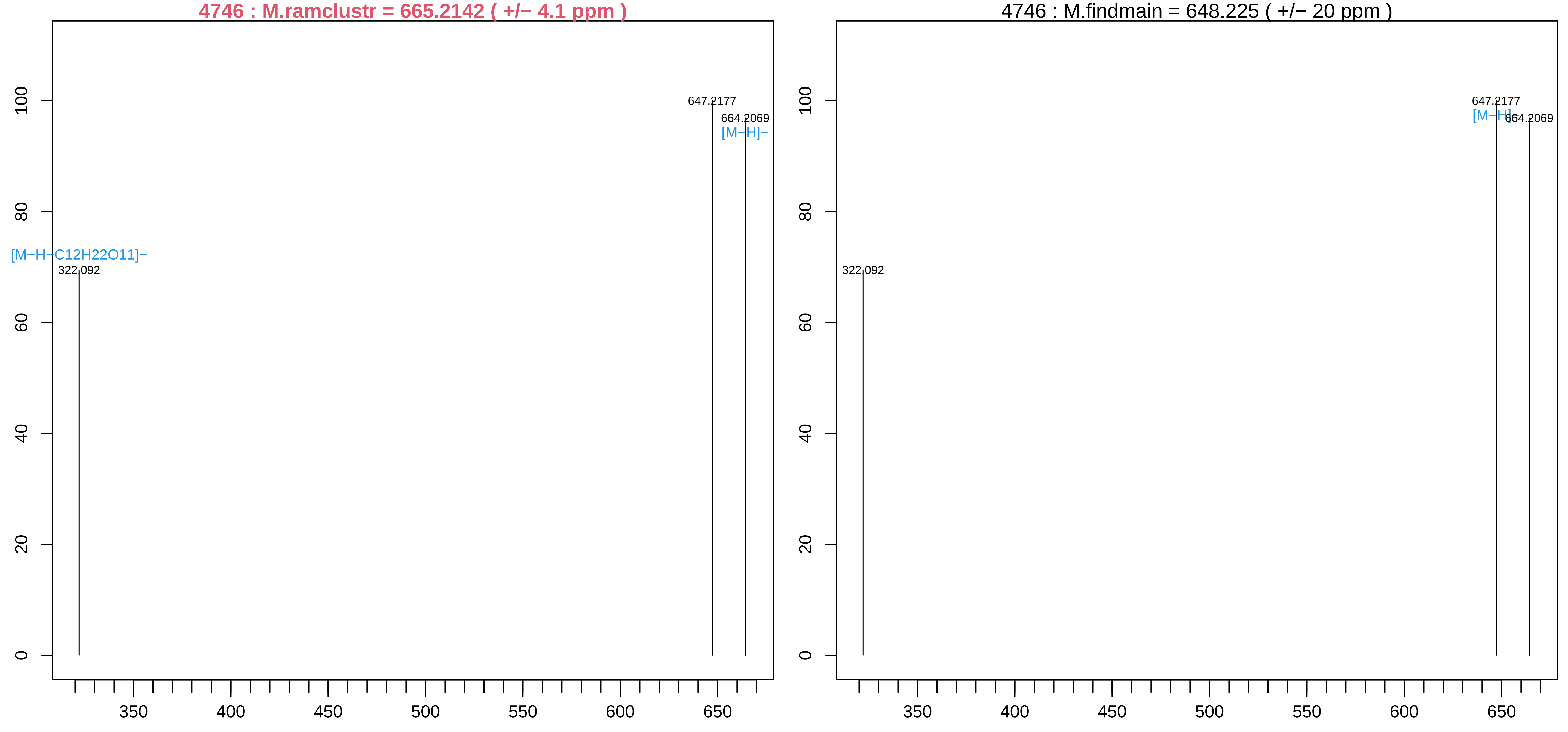
4746 : M.ramclustr = 665.2142 (+/− 4.1 ppm)
647.2177
664.2069
[M−H]−
[M−H−C12H22O11]−
322.092
4746 : M.findmain = 648.225 (+/− 20 ppm)
647.2177
[M−H]−
664.2069
322.092

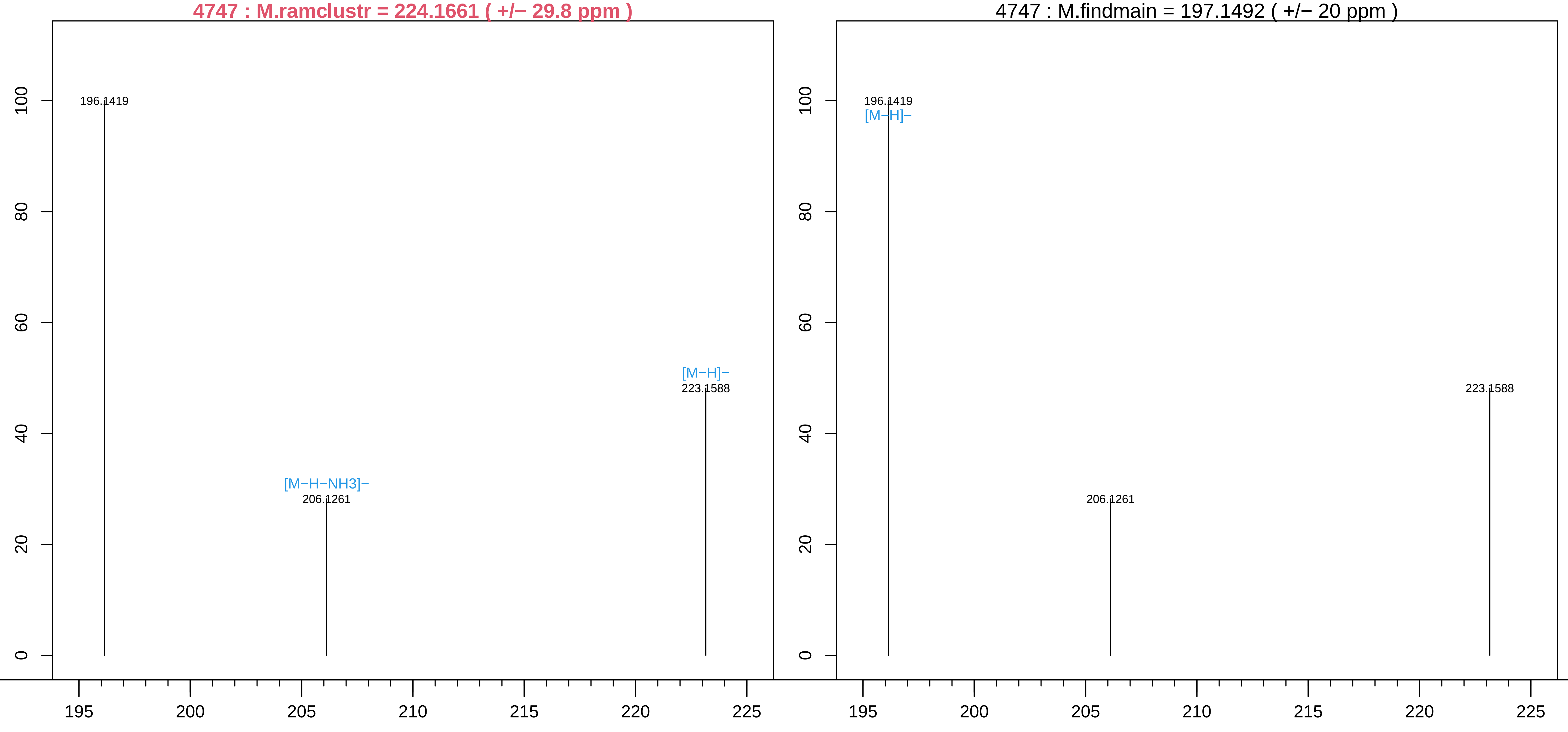
4747 : M.ramclustr = 224.1661 (+/− 29.8 ppm)
196.1419
[M−H−NH3]−
206.1261
[M−H]−
223.1588
4747 : M.findmain = 197.1492 (+/− 20 ppm)
196.1419
[M−H]−
206.1261
223.1588
0
20
40
60
80
100
195
200
205
210
215
220
225

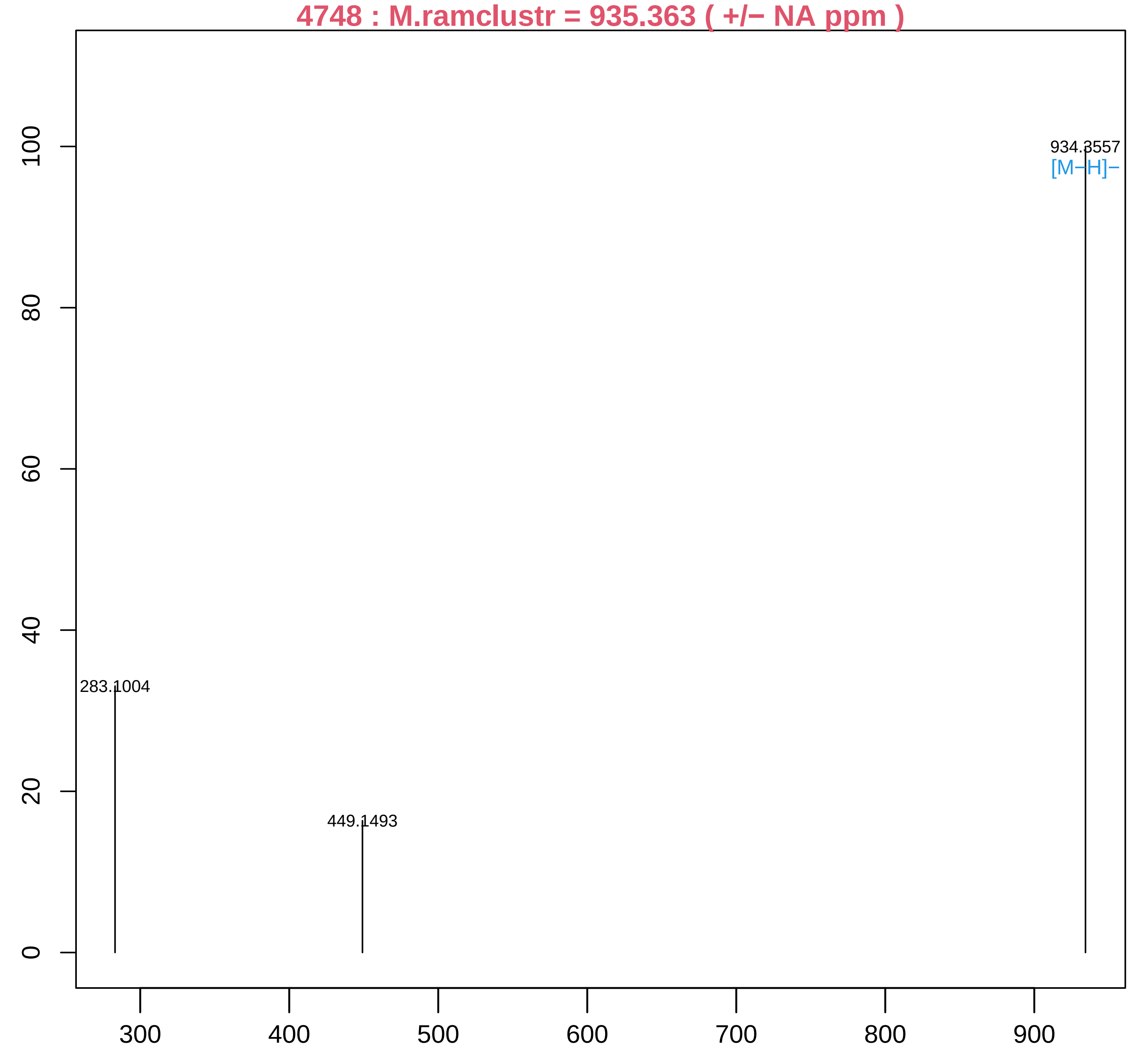

4748 : M.ramclustr = 935.363 (+/− NA ppm)
934.3557
[M−H]−
283.1004
449.1493
0
20
40
60
80
100
300
400
500
600
700
800
900

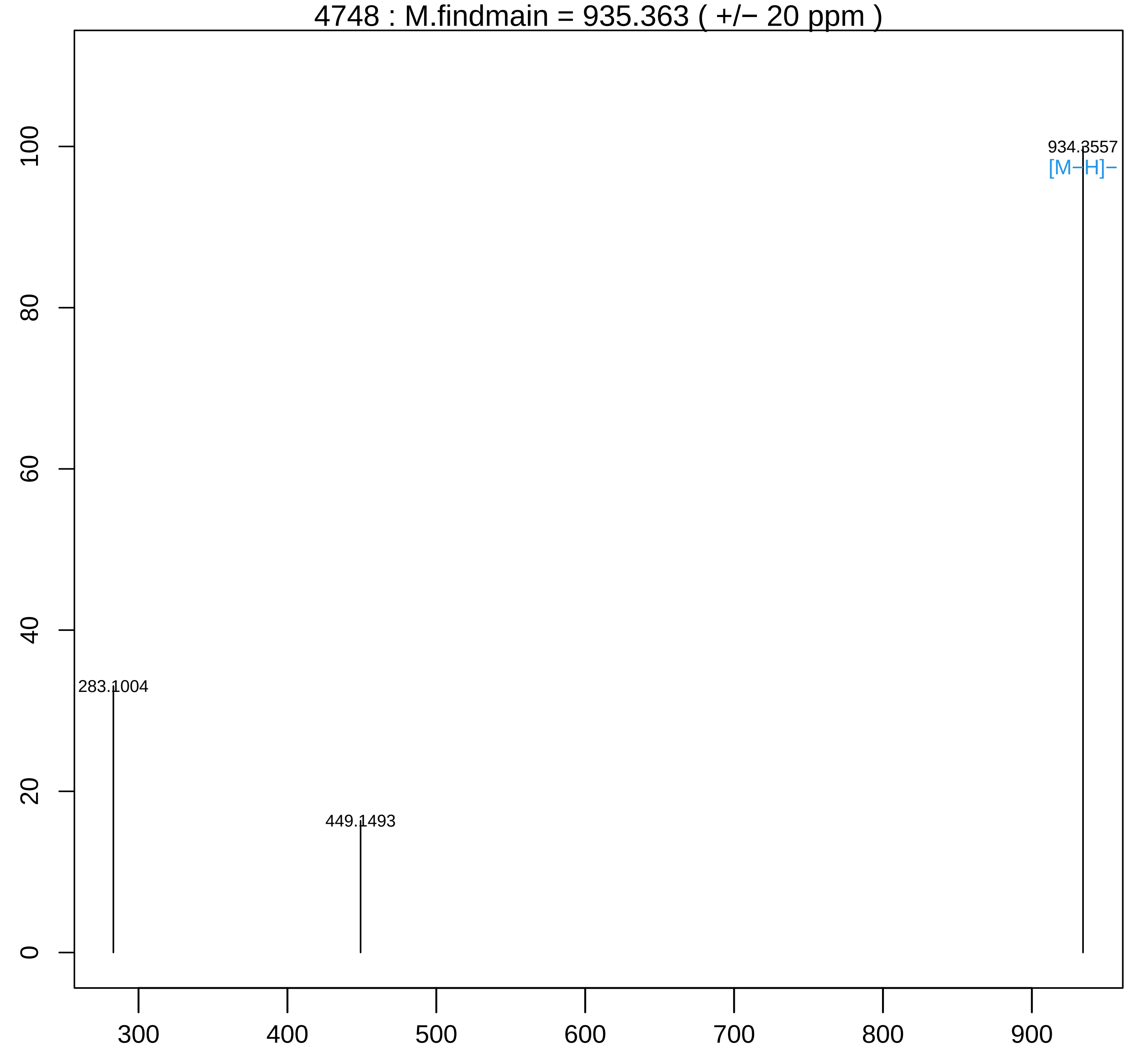

4748 : M.findmain = 935.363 (+/− 20 ppm)
934.3557
[M−H]−
283.1004
449.1493
0
20
40
60
80
100
300
400
500
600
700
800
900

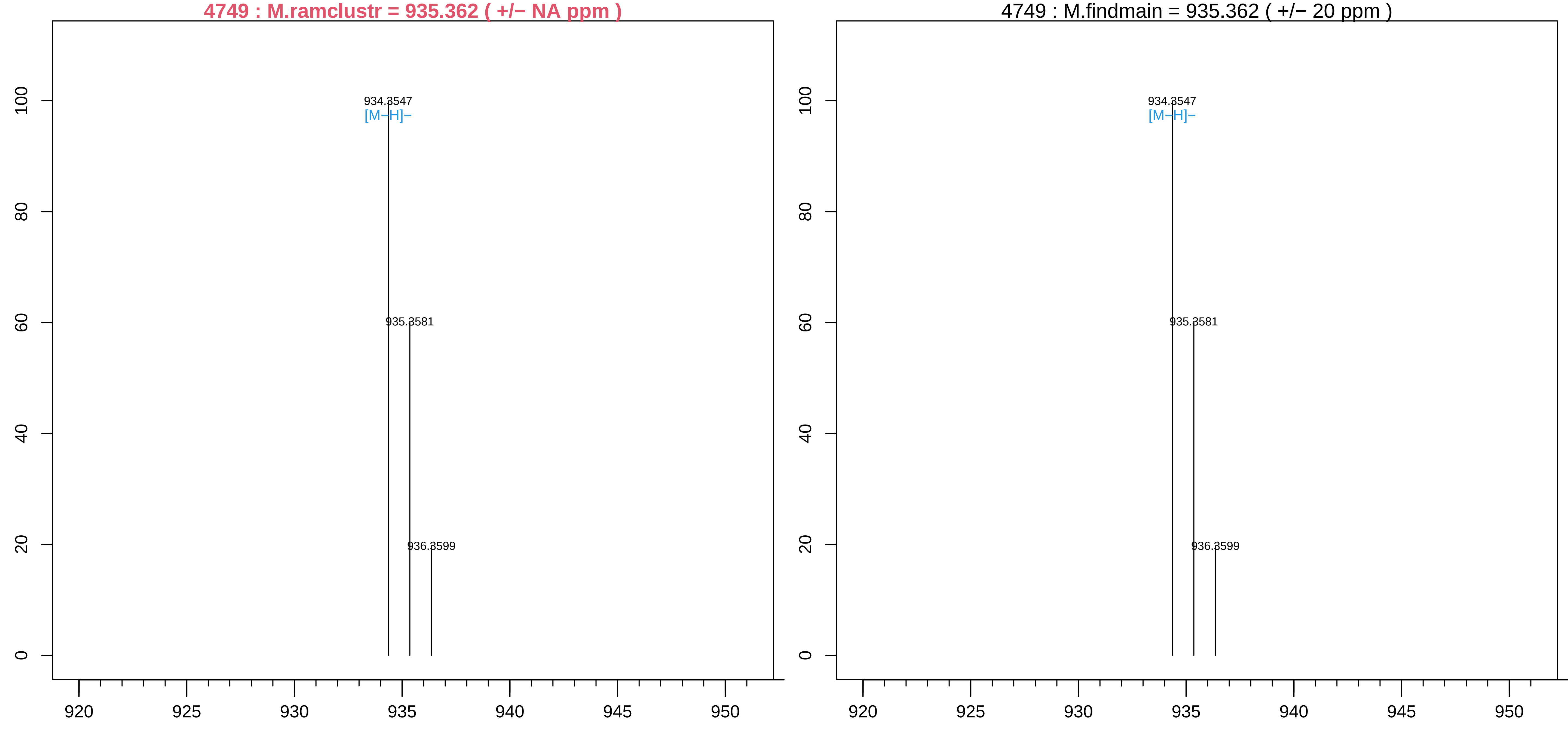
4749 : M.ramclustr = 935.362 (+/− NA ppm)
934.3547
[M−H]−
935.3581
936.3599
0
20
40
60
80
100
920
925
930
935
940
945
950
4749 : M.findmain = 935.362 (+/− 20 ppm)
934.3547
[M−H]−
935.3581
936.3599
0
20
40
60
80
100
920
925
930
935
940
945
950

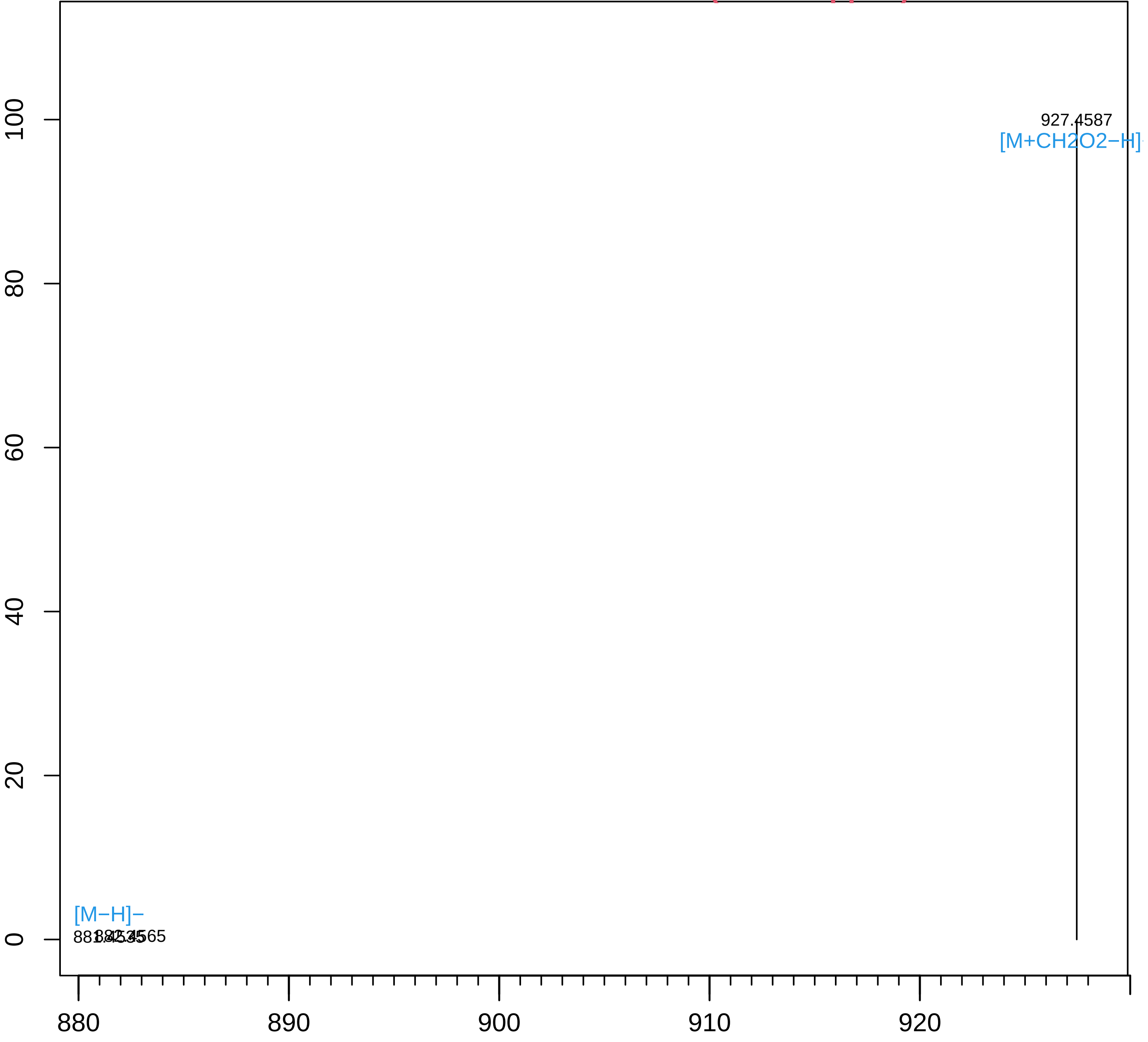

4750 : M.ramclustr = 882.4605 (+/− 0.3 ppm)

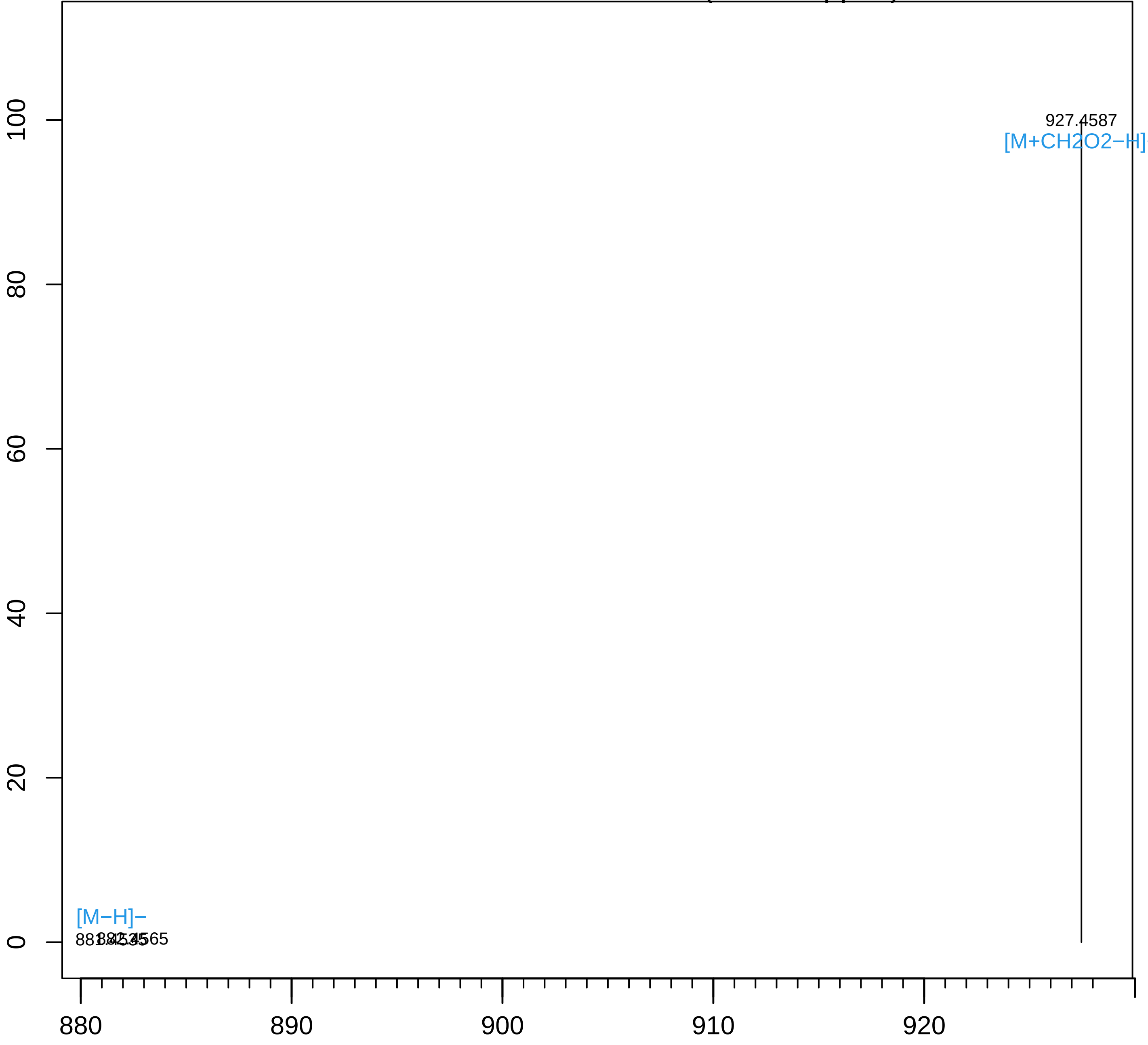

4750 : M.findmain = 882.4605 (+/− 0.3 ppm)

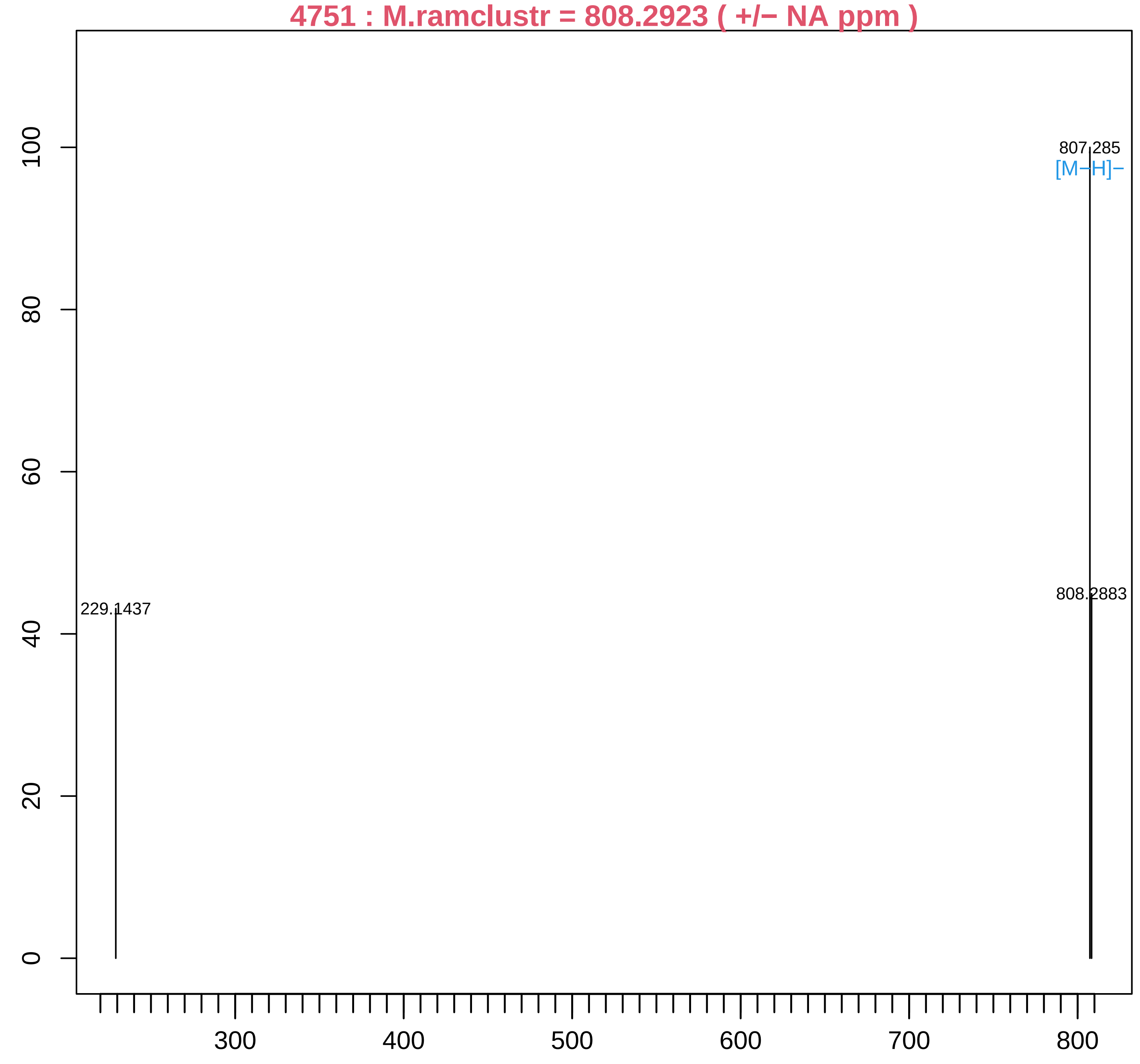
4751 : M.ramclustr = 808.2923 (+/− NA ppm)
807.285
[M−H]−
808.2883
229.1437
0
20
40
60
80
100
300
400
500
600
700
800

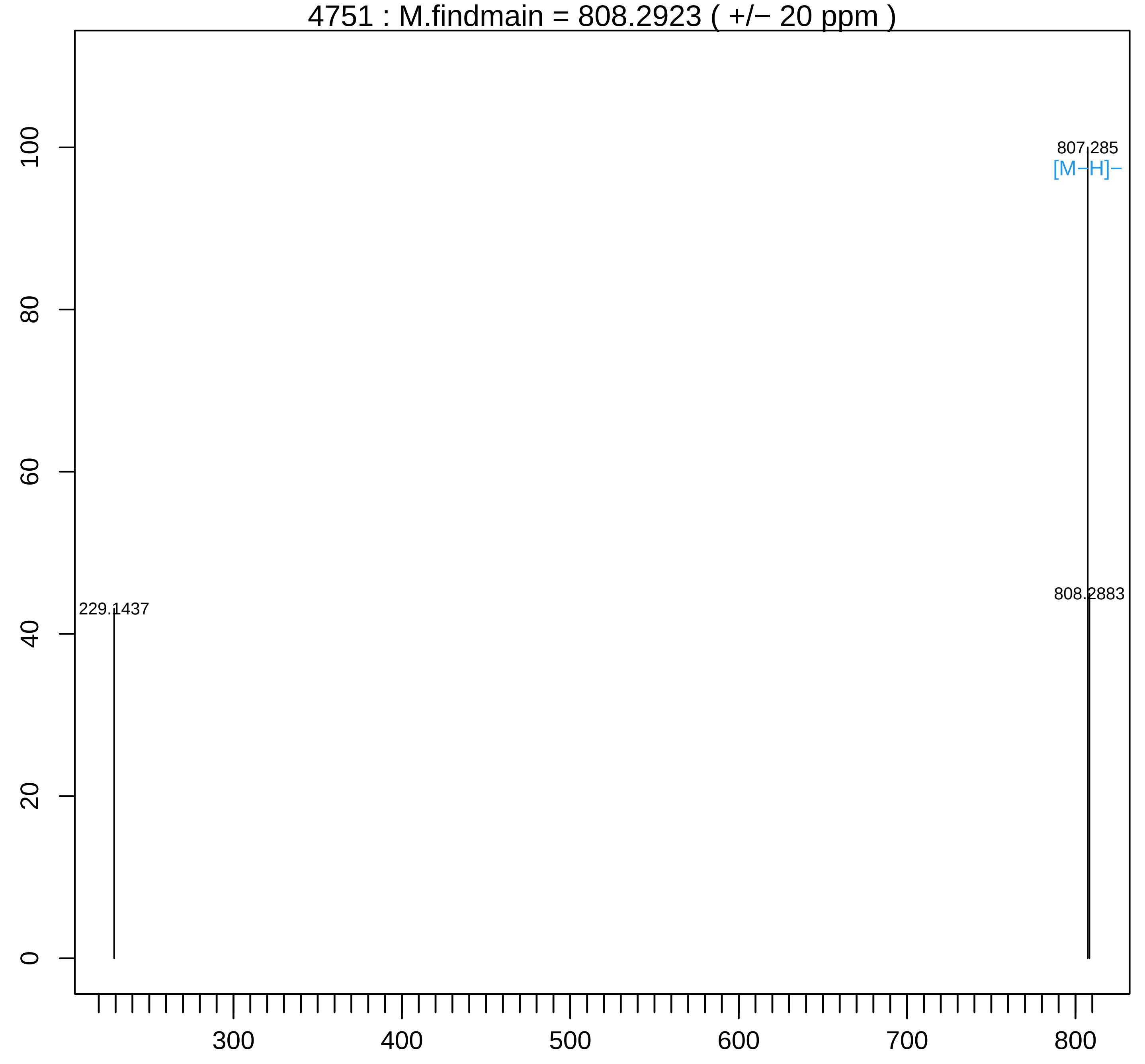
4751 : M.findmain = 808.2923 (+/− 20 ppm)
807.285
[M−H]−
808.2883
229.1437
0
20
40
60
80
100
300
400
500
600
700
800

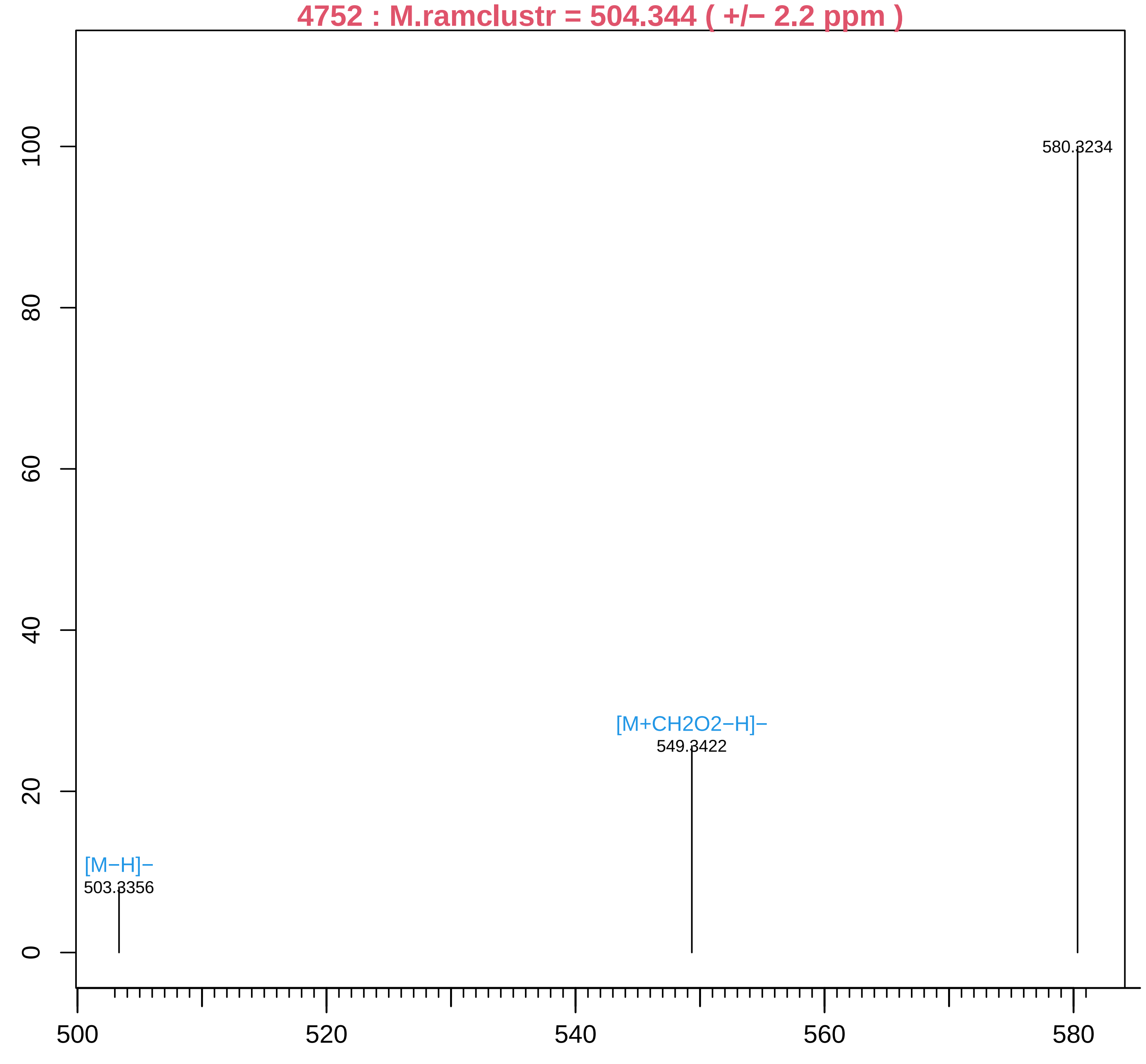
4752 : M.ramclustr = 504.344 (+/− 2.2 ppm)
580.3234
[M+CH2O2−H]−
549.3422
[M−H]−
503.3356
0
20
40
60
80
100
500
520
540
560
580

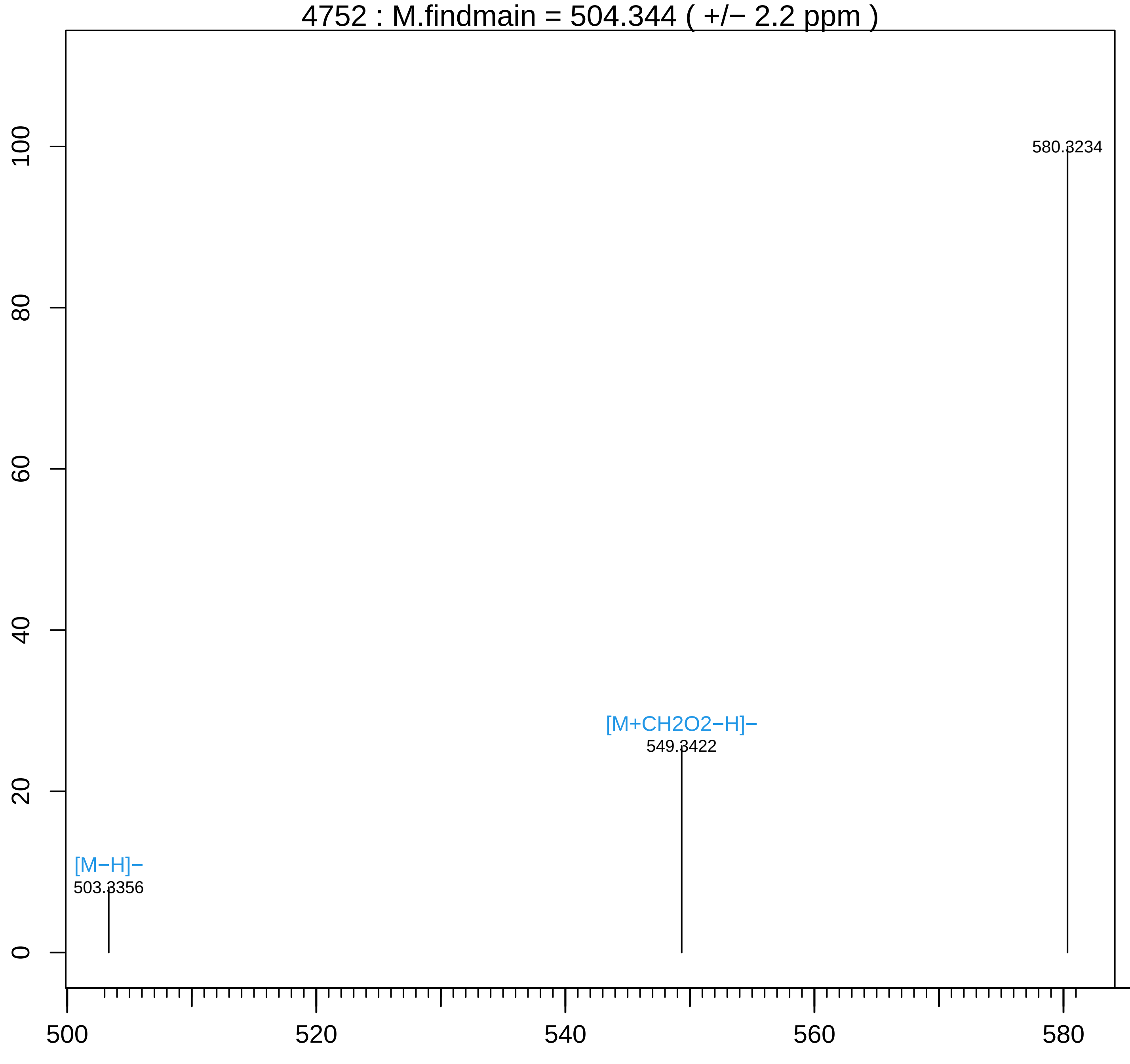
4752 : M.findmain = 504.344 (+/− 2.2 ppm)
580.3234
[M+CH2O2−H]−
549.3422
[M−H]−
503.3356
0
20
40
60
80
100
500
520
540
560
580

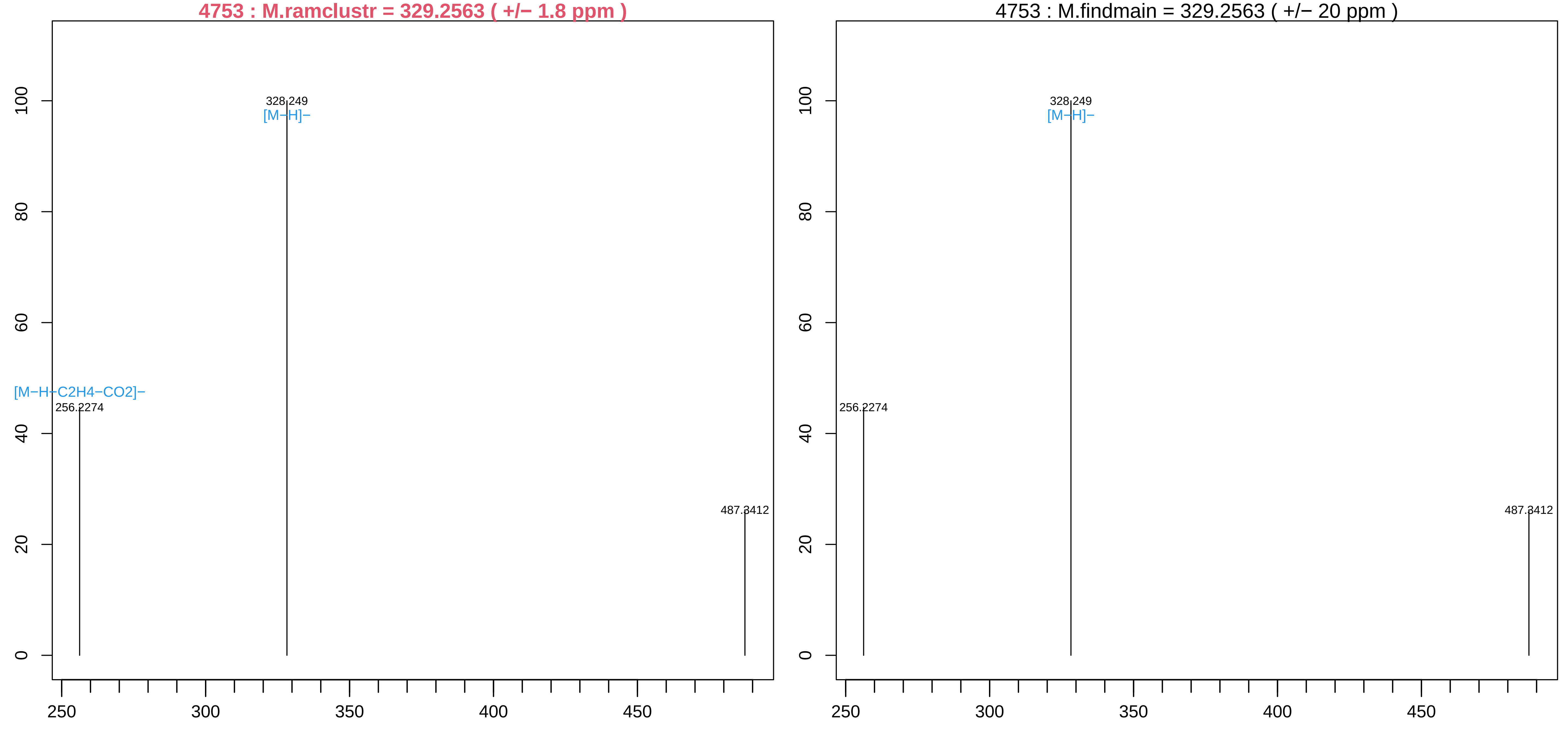
4753 : M.ramclustr = 329.2563 (+/− 1.8 ppm)
328.249
[M−H]−
[M−H−C2H4−CO2]−
256.2274
487.3412
4753 : M.findmain = 329.2563 (+/− 20 ppm)
328.249
[M−H]−
256.2274
487.3412
0
20
40
60
80
100
250
300
350
400
450

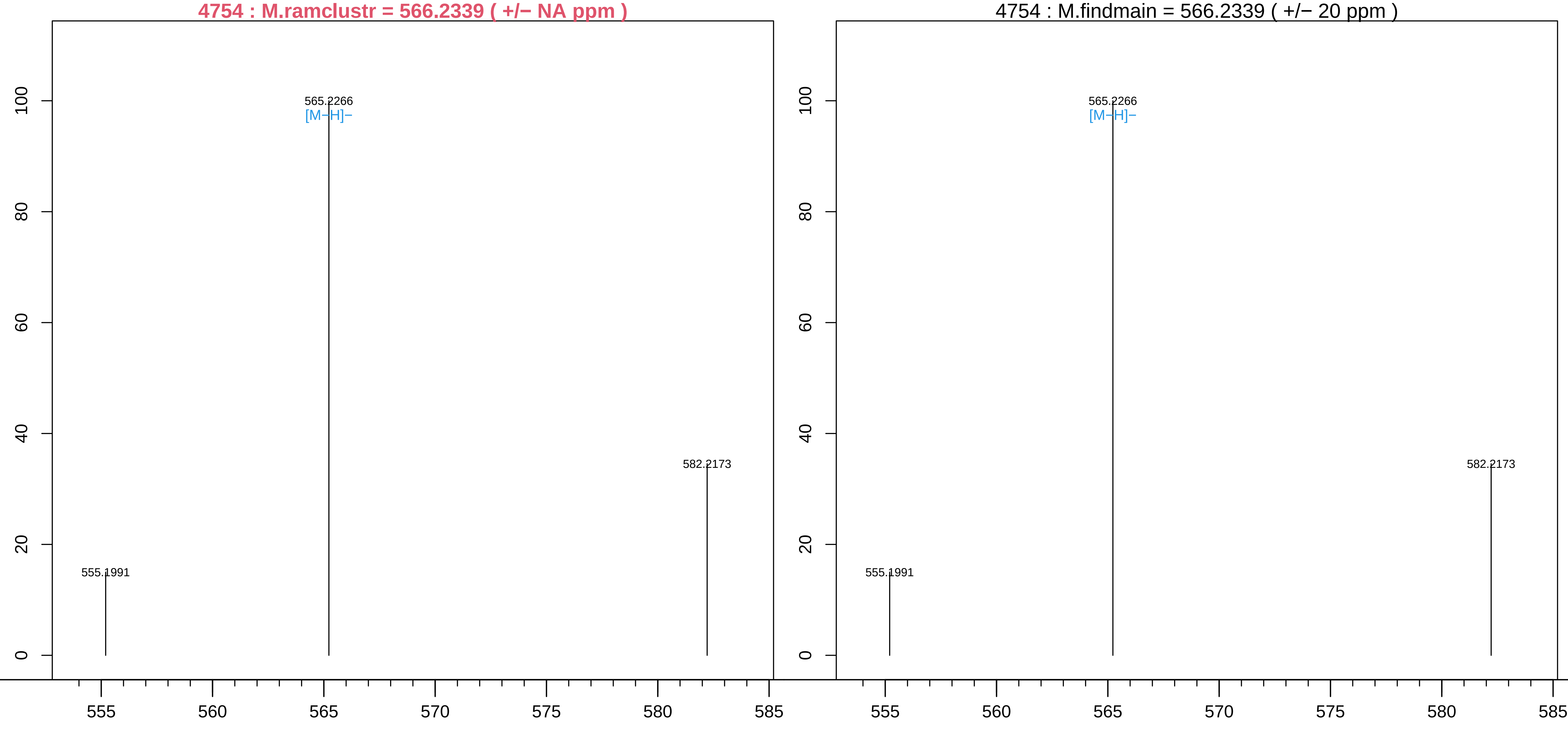

4754 : M.ramclustr = 566.2339 (+/− NA ppm)
565.2266
[M−H]−
582.2173
555.1991
0
20
40
60
80
100
555
560
565
570
575
580
585
4754 : M.findmain = 566.2339 (+/− 20 ppm)
565.2266
[M−H]−
582.2173
555.1991
0
20
40
60
80
100
555
560
565
570
575
580
585

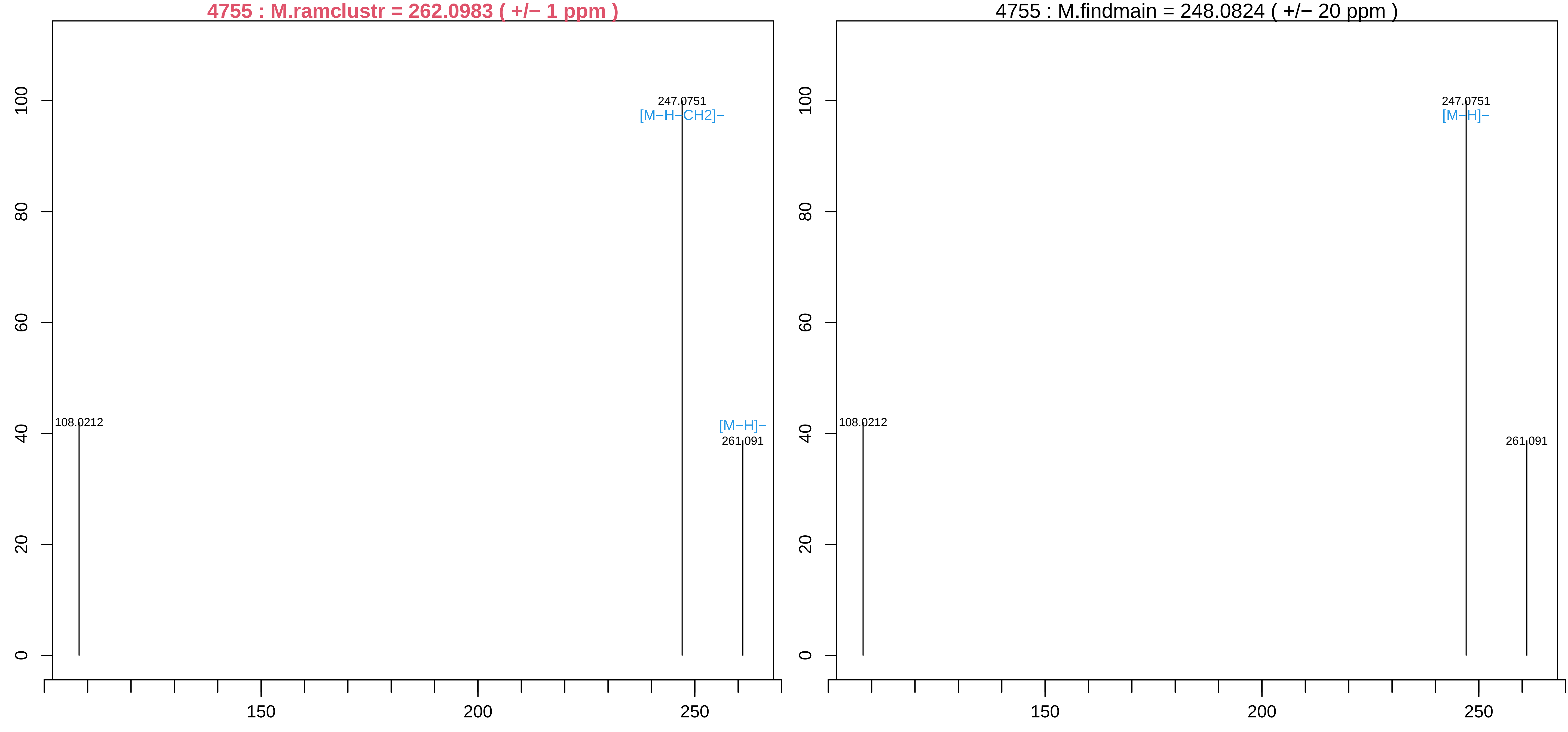

4755 : M.ramclustr = 262.0983 (+/− 1 ppm)
247.0751
[M−H−CH2]−
108.0212
[M−H]−
261.091
4755 : M.findmain = 248.0824 (+/− 20 ppm)
247.0751
[M−H]−
108.0212
261.091
0
20
40
60
80
100
150
200
250

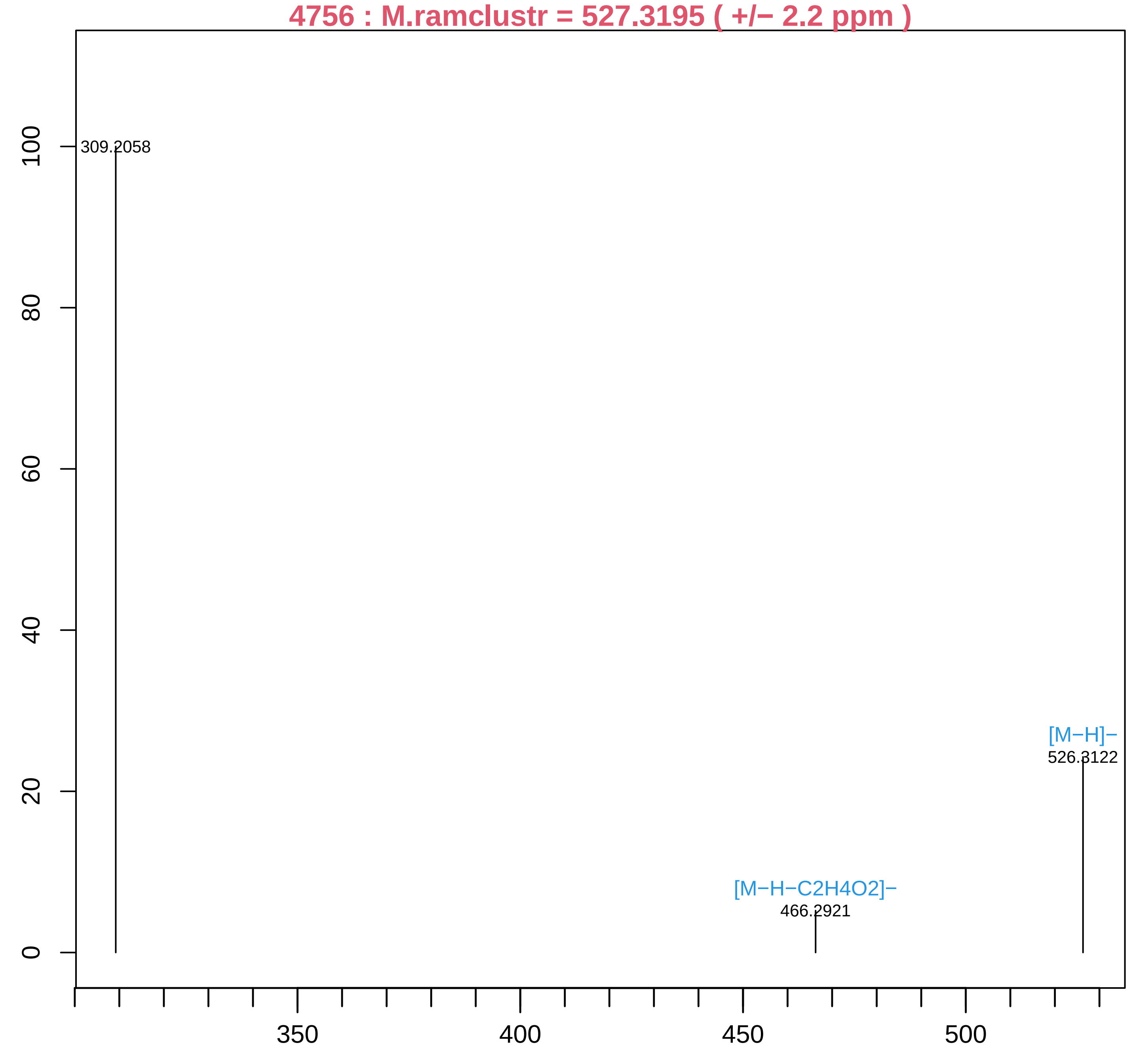
4756 : M.ramclustr = 527.3195 (+/− 2.2 ppm)
309.2058
[M−H−C2H4O2]−
466.2921
[M−H]−
526.3122
0
20
40
60
80
100
350
400
450
500

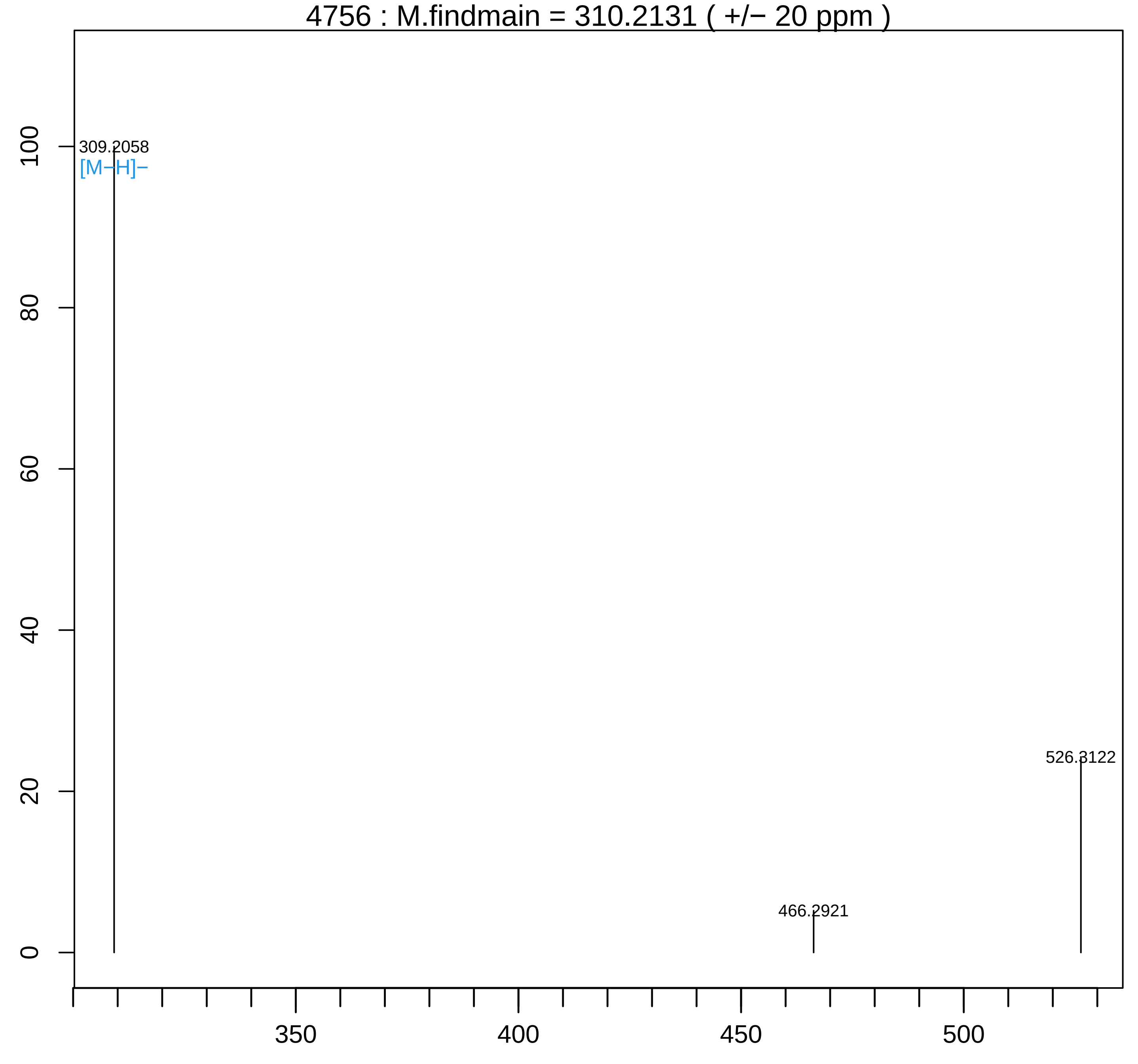
4756 : M.findmain = 310.2131 (+/− 20 ppm)
309.2058
[M−H]−
466.2921
526.3122
0
20
40
60
80
100
350
400
450
500

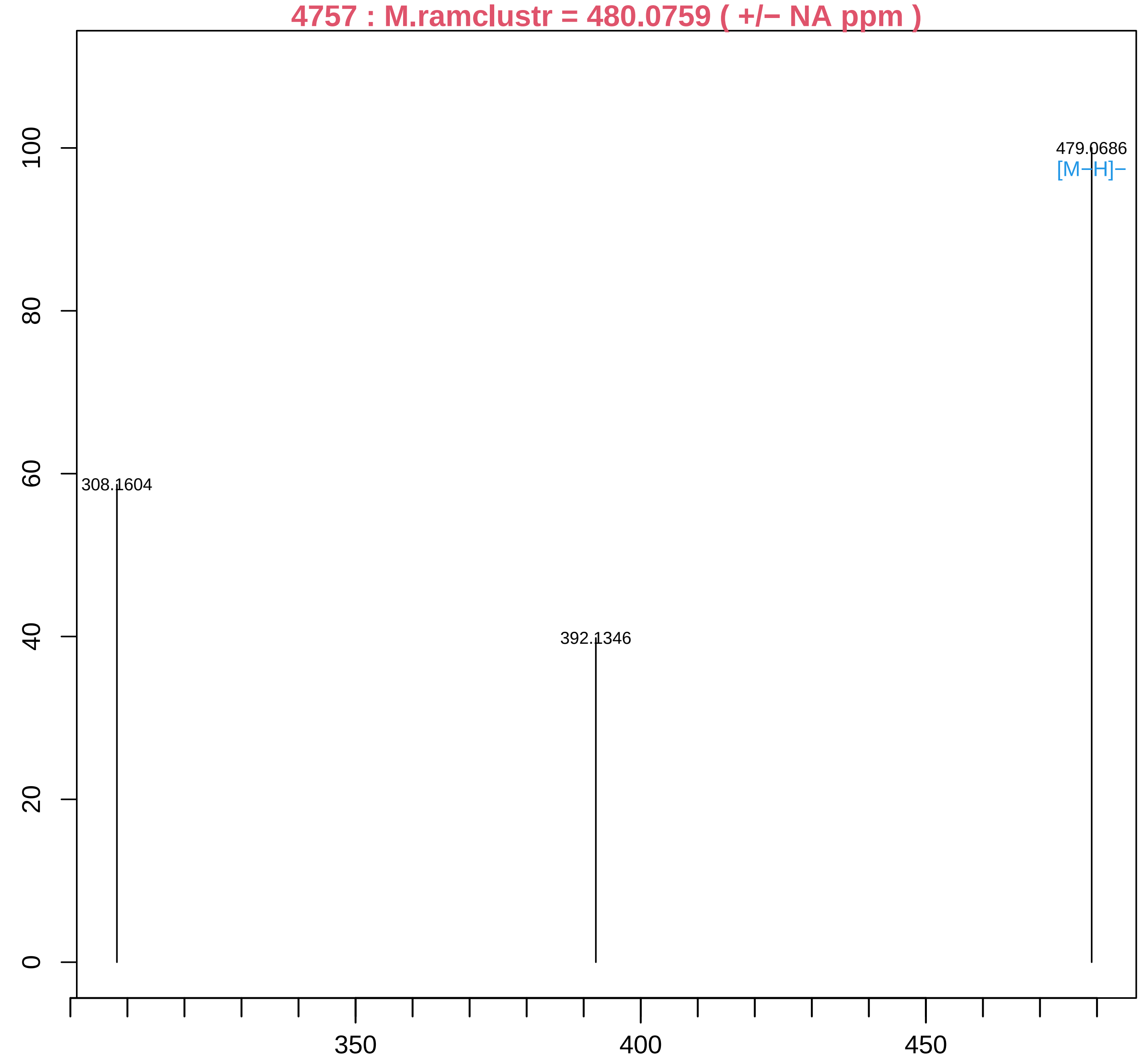

4757 : M.ramclustr = 480.0759 (+/− NA ppm)
479.0686
[M−H]−
308.1604
392.1346
0
20
40
60
80
100
350
400
450

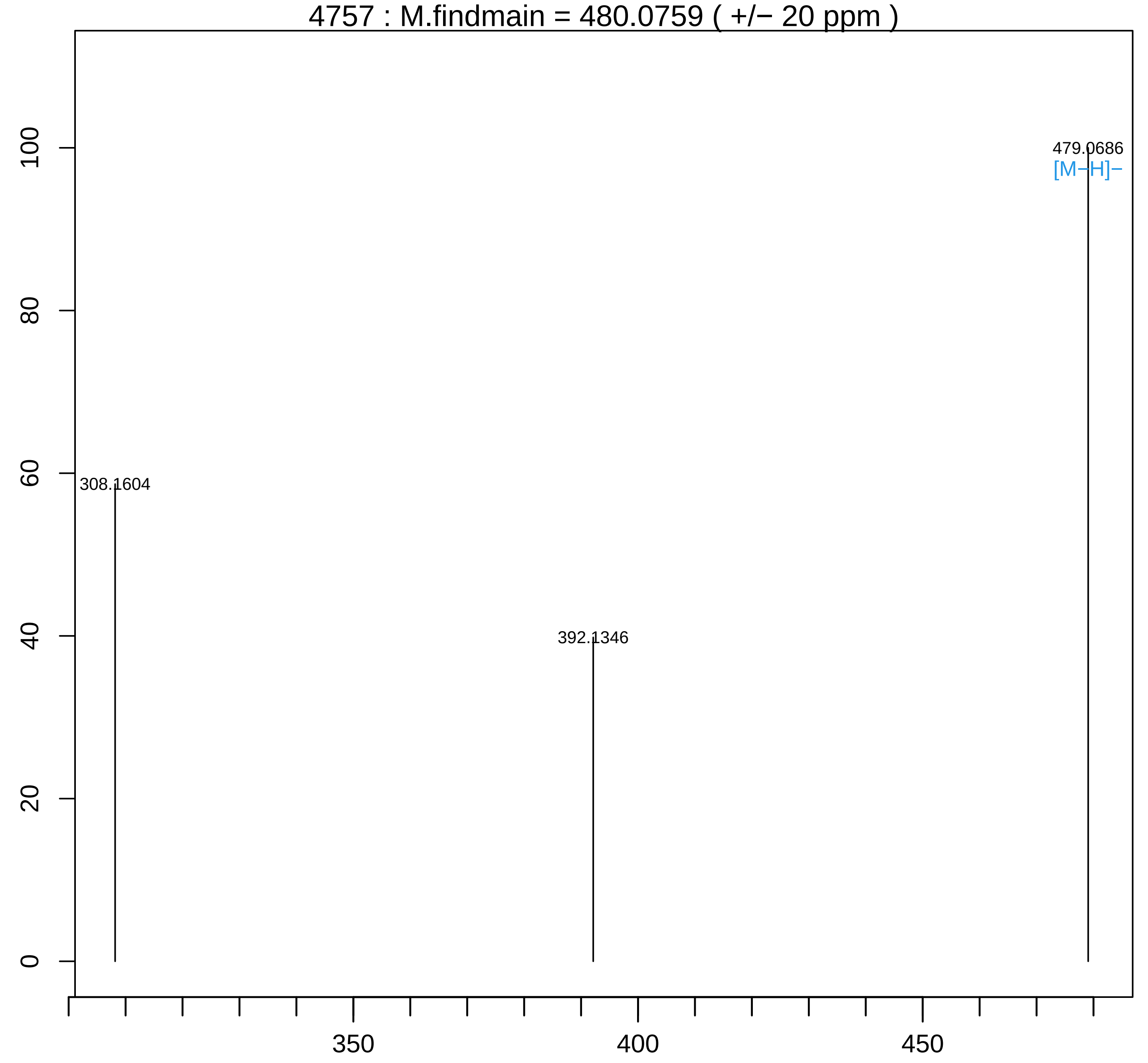

4757 : M.findmain = 480.0759 (+/− 20 ppm)
479.0686
[M−H]−
308.1604
392.1346
0
20
40
60
80
100
350
400
450

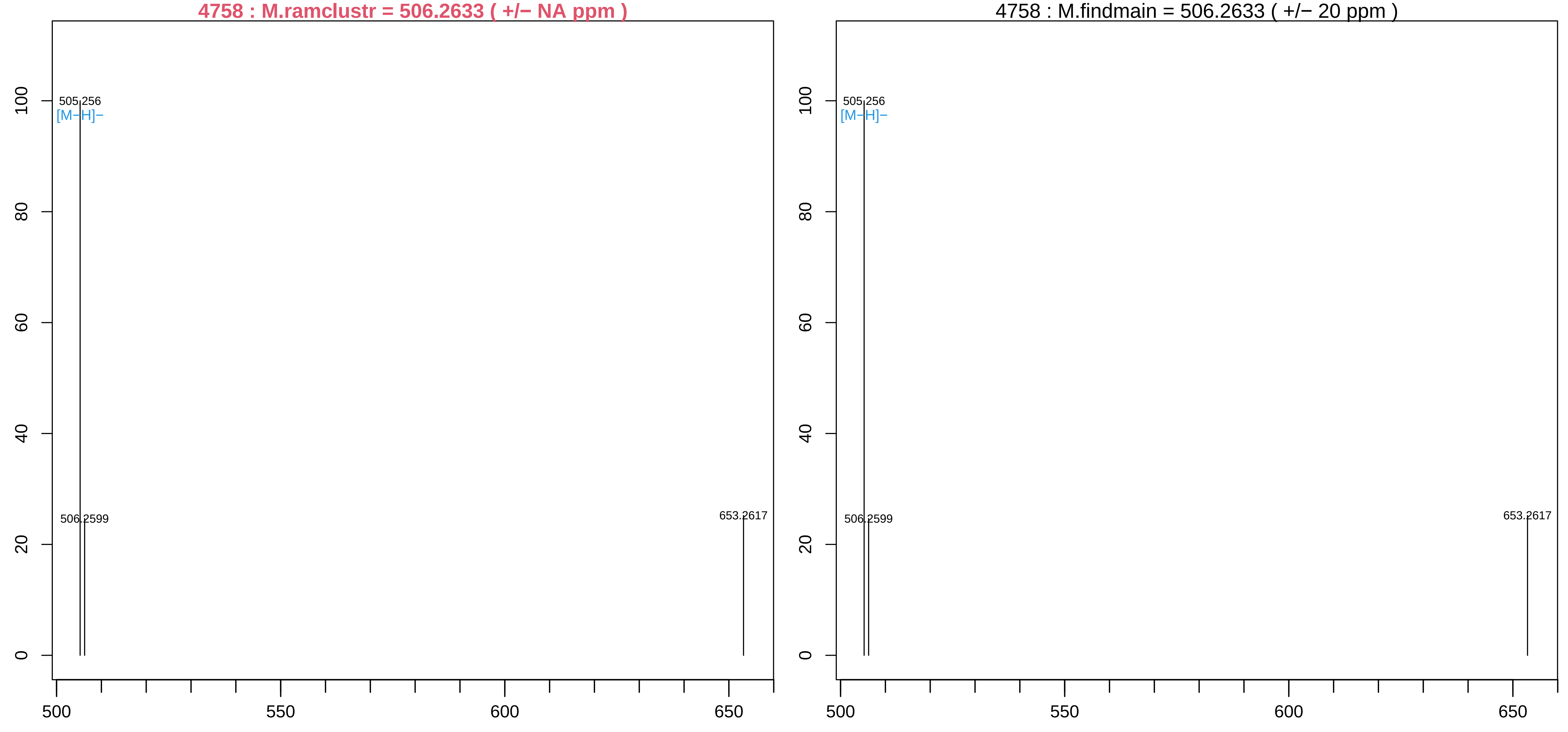

4758 : M.ramclustr = 506.2633 (+/− NA ppm)
505.256
[M−H]−
506.2599
653.2617
4758 : M.findmain = 506.2633 (+/− 20 ppm)
505.256
[M−H]−
506.2599
653.2617

4759 : M.ramclustr = 332.1981 (+/− NA ppm)

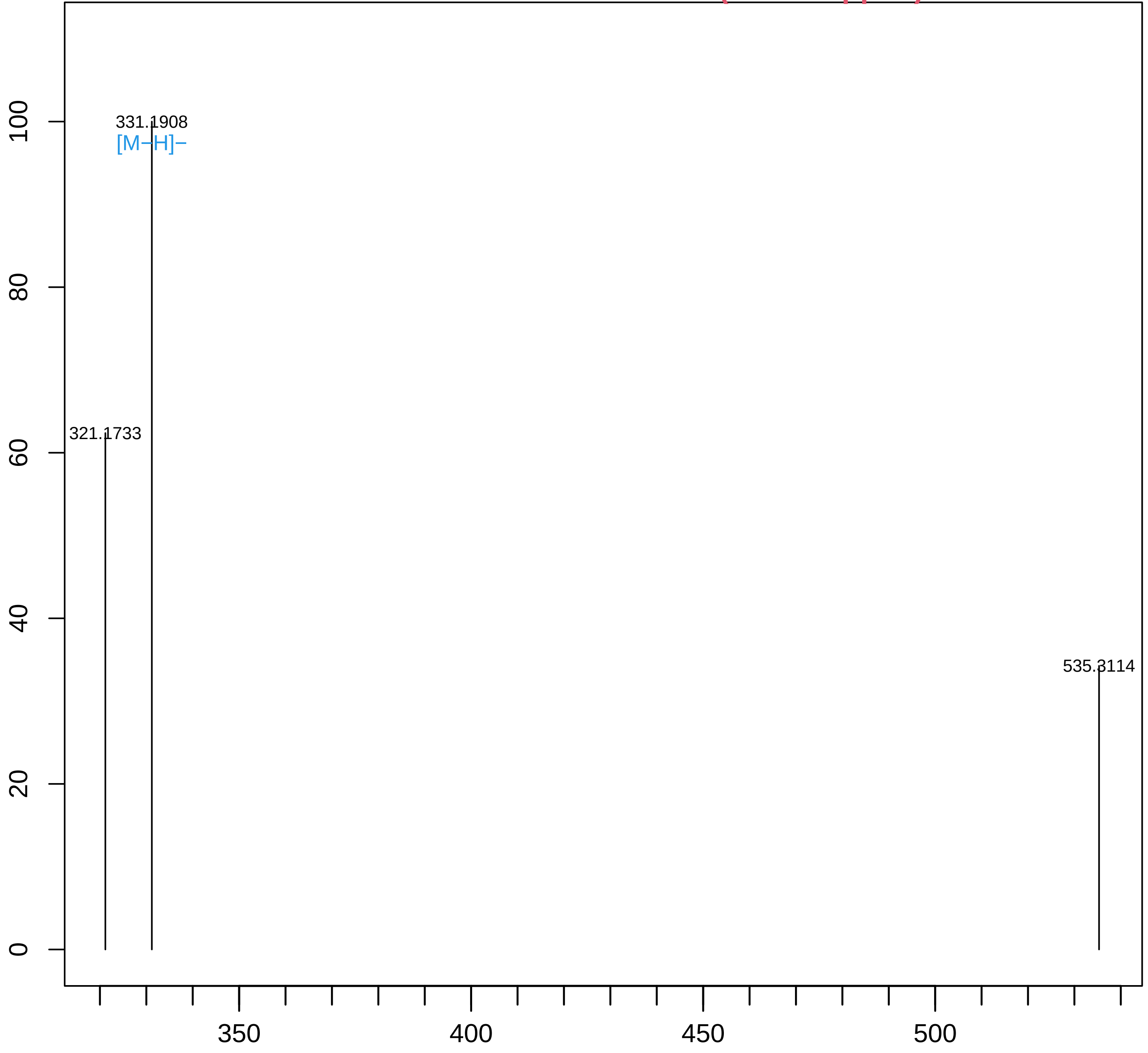

4759 : M.findmain = 332.1981 (+/− 20 ppm)

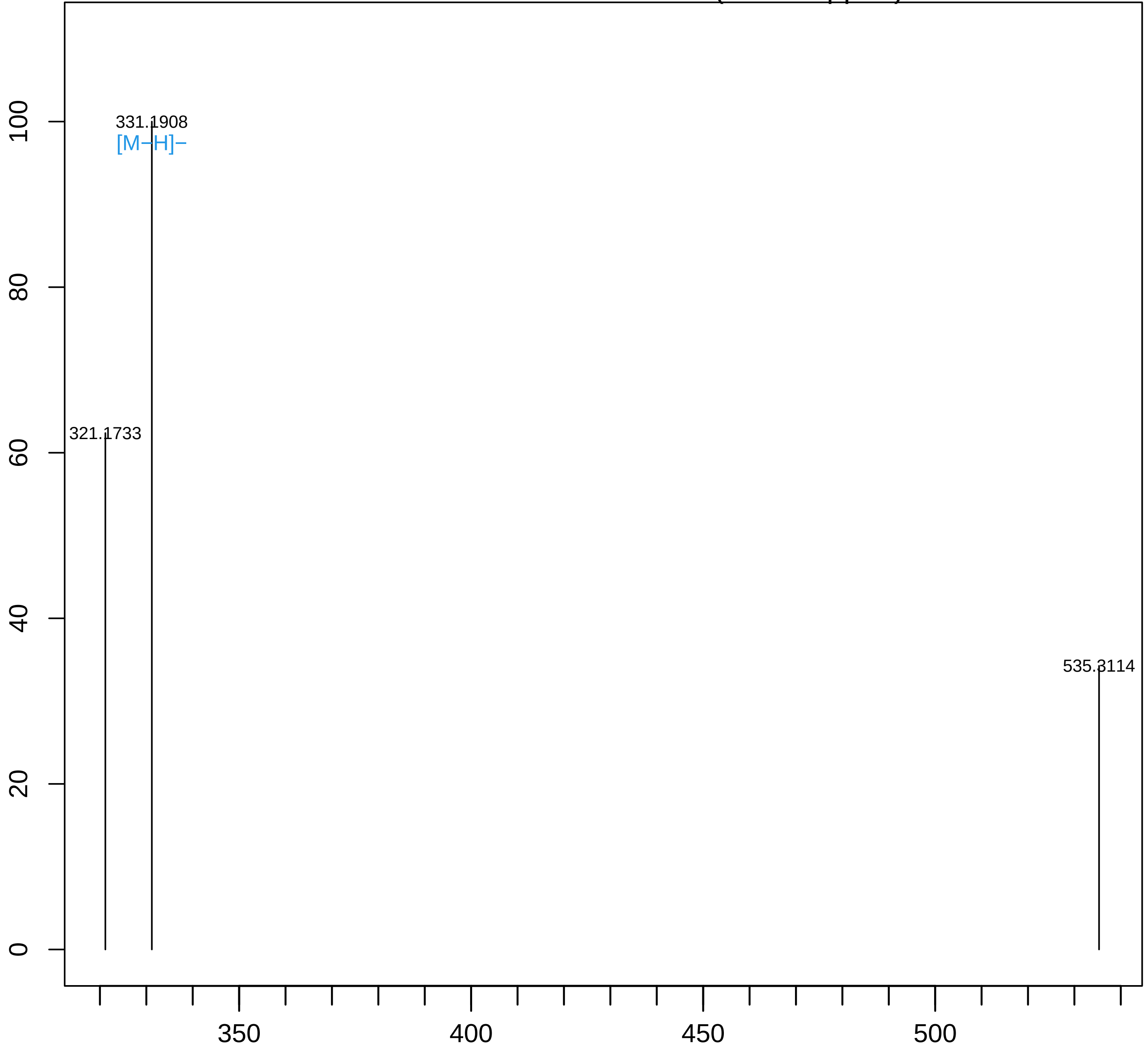

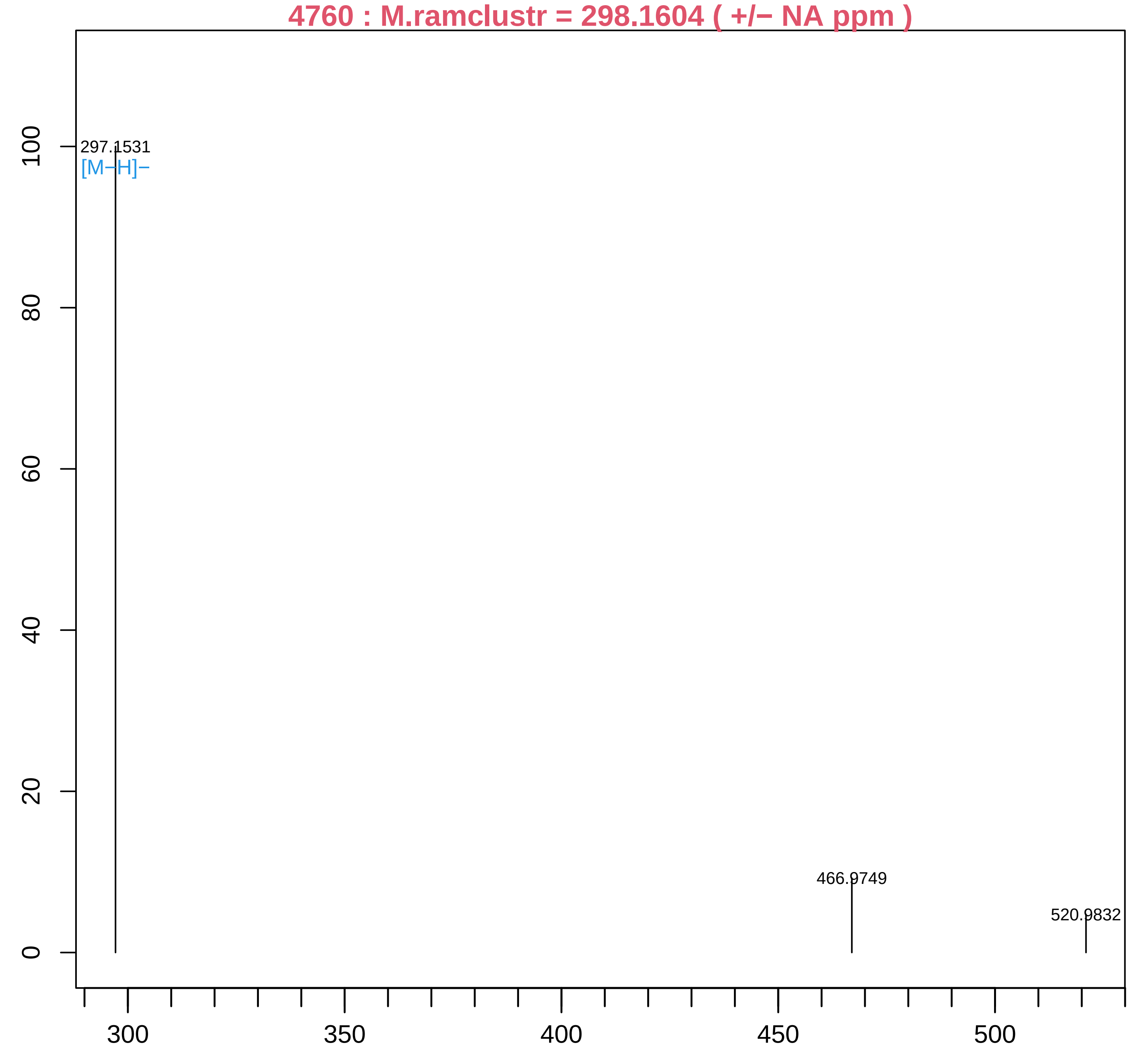
4760 : M.ramclustr = 298.1604 (+/− NA ppm)
297.1531
[M−H]−
466.9749
520.9832
0
20
40
60
80
100
300
350
400
450
500

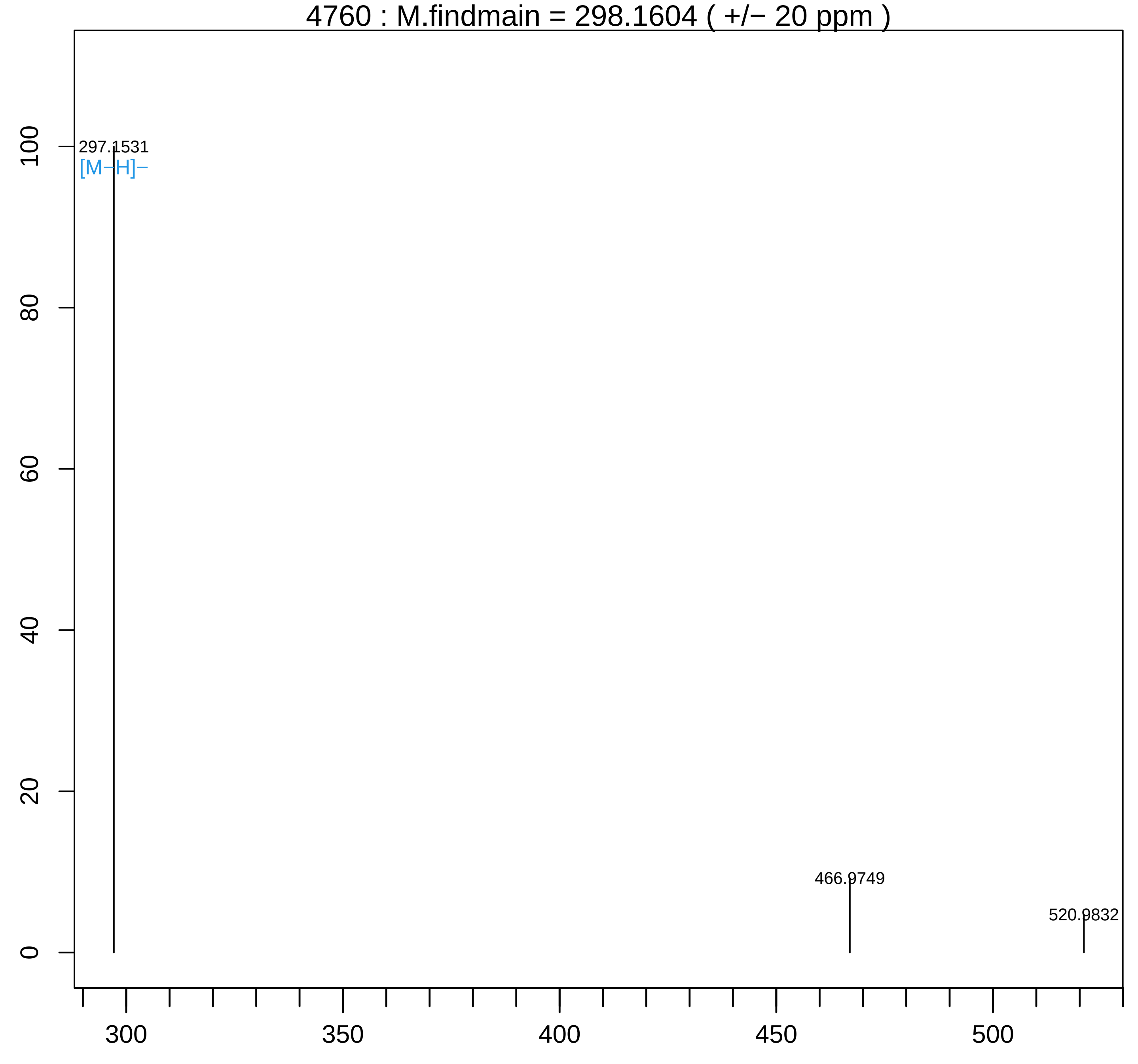
4760 : M.findmain = 298.1604 (+/− 20 ppm)
297.1531
[M−H]−
466.9749
520.9832
0
20
40
60
80
100
300
350
400
450
500

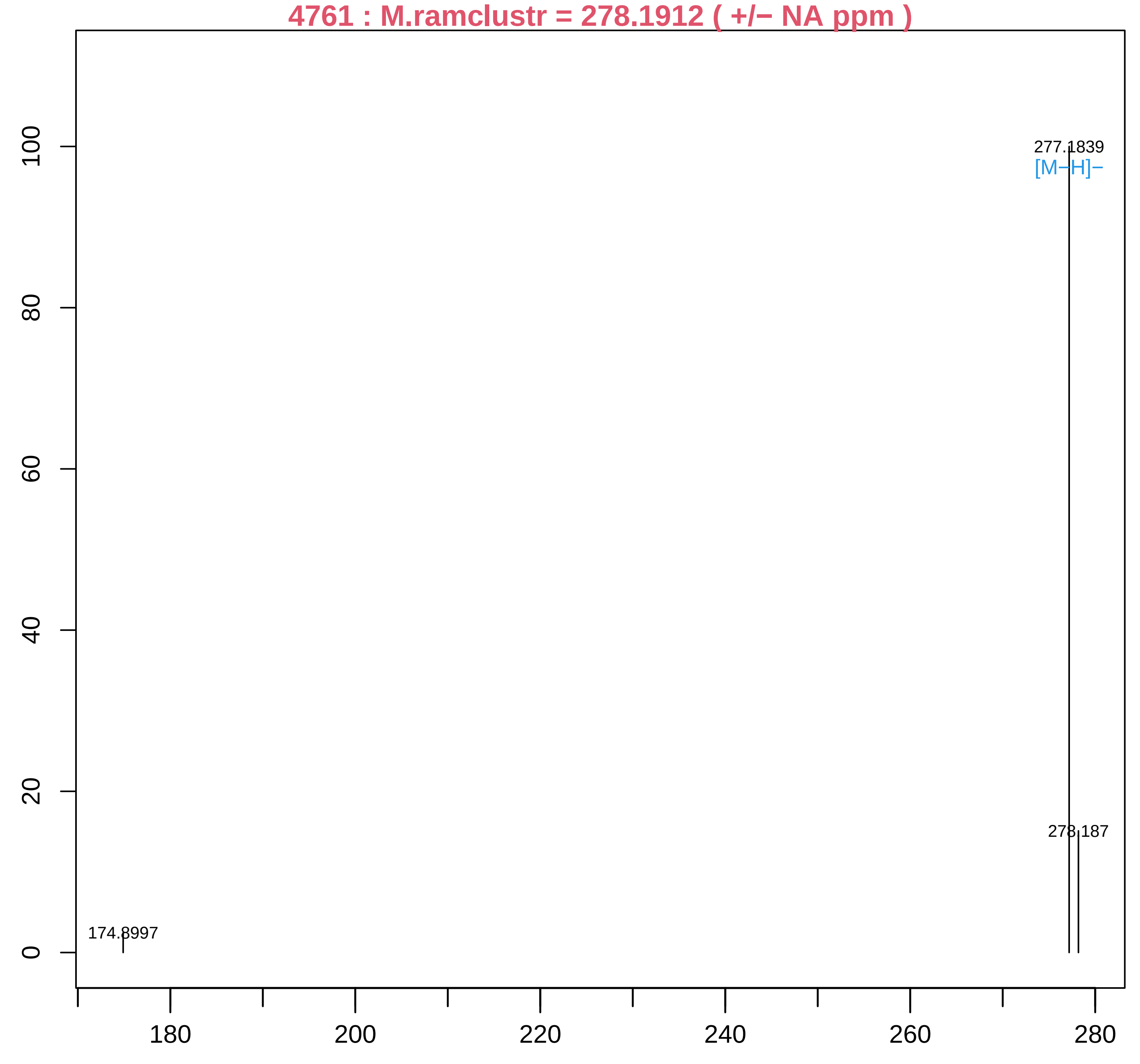
4761 : M.ramclustr = 278.1912 (+/− NA ppm)
277.1839
[M−H]−
278.187
174.8997
0
20
40
60
80
100
180
200
220
240
260
280

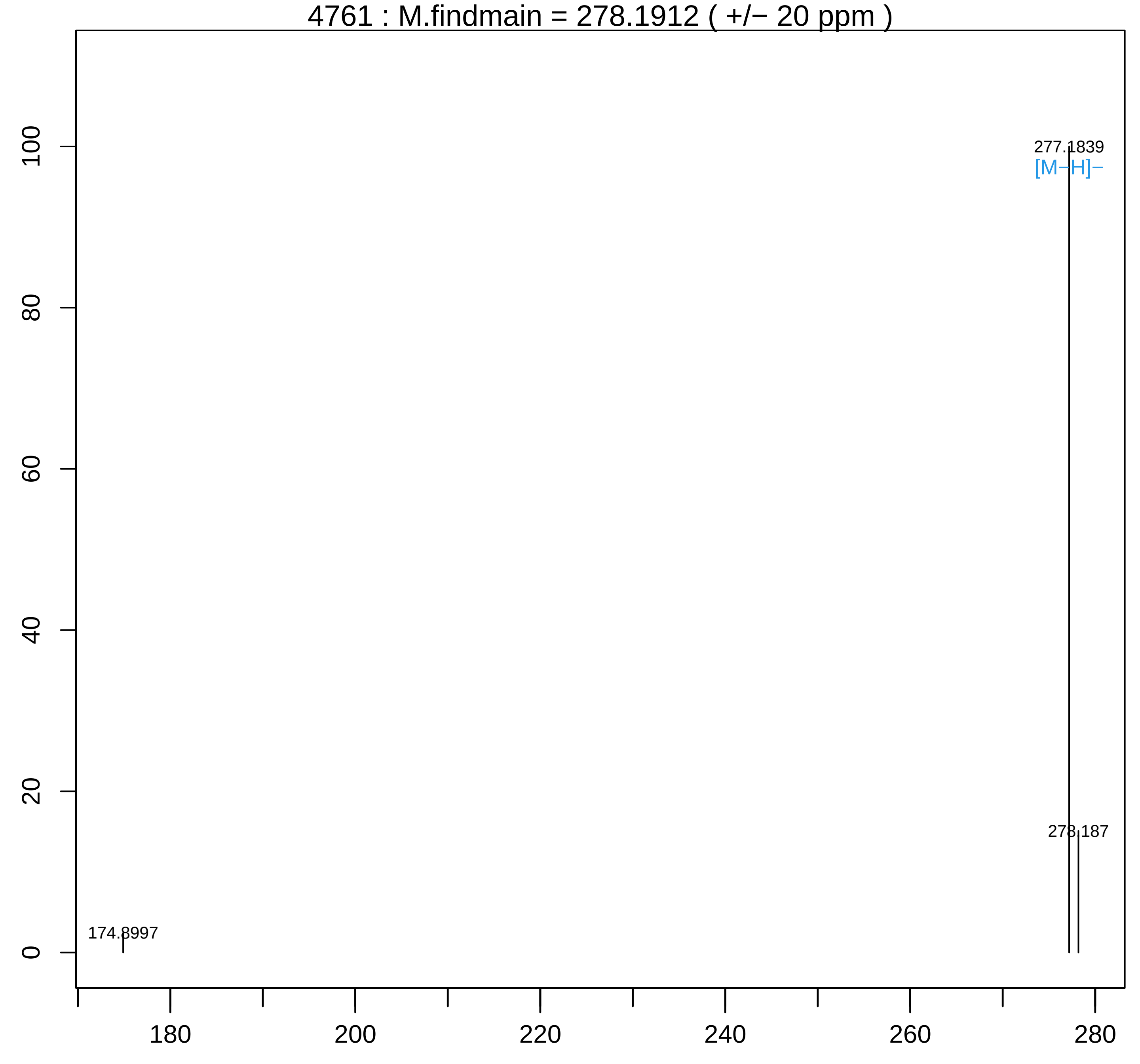
4761 : M.findmain = 278.1912 (+/− 20 ppm)
277.1839
[M−H]−
278.187
174.8997
0
20
40
60
80
100
180
200
220
240
260
280

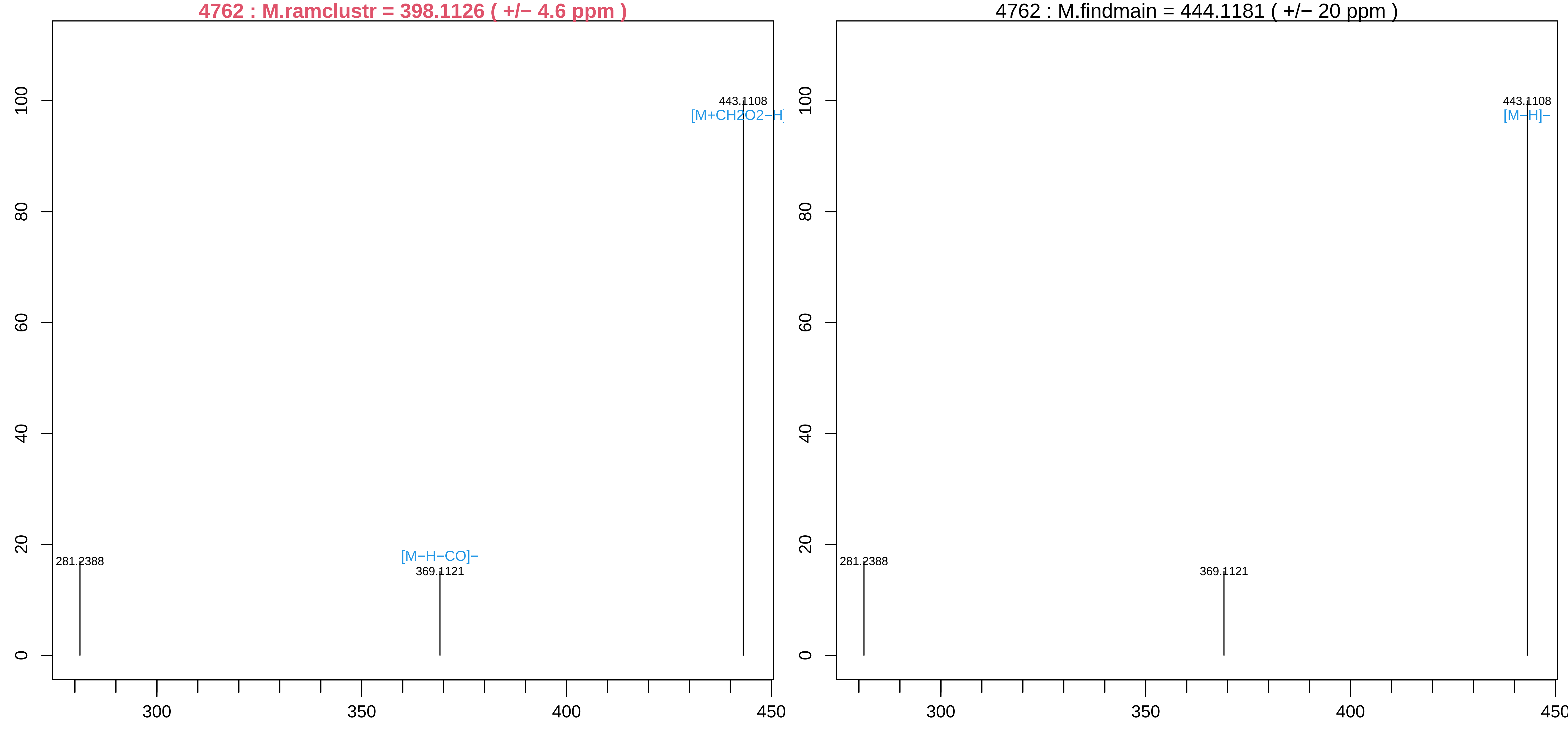

4762 : M.ramclustr = 398.1126 (+/− 4.6 ppm)
443.1108
[M+CH2O2−H]
[M−H−CO]−
369.1121
281.2388
4762 : M.findmain = 444.1181 (+/− 20 ppm)
443.1108
[M−H]−
369.1121
281.2388
0
20
40
60
80
100
300
350
400
450

4763 : M.ramclustr = 280.2397 (+/− NA ppm)

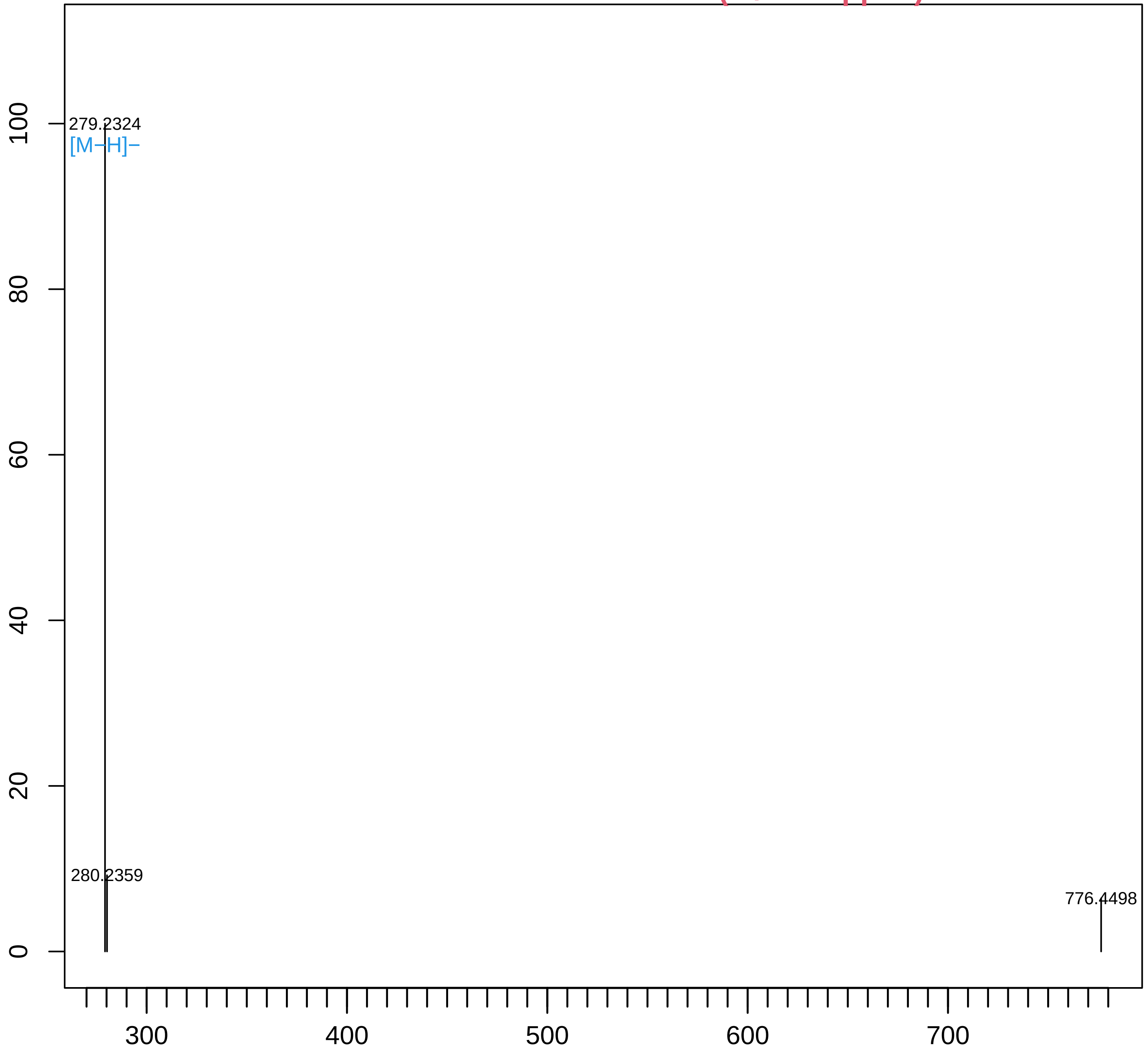

4763 : M.findmain = 280.2397 (+/− 20 ppm)

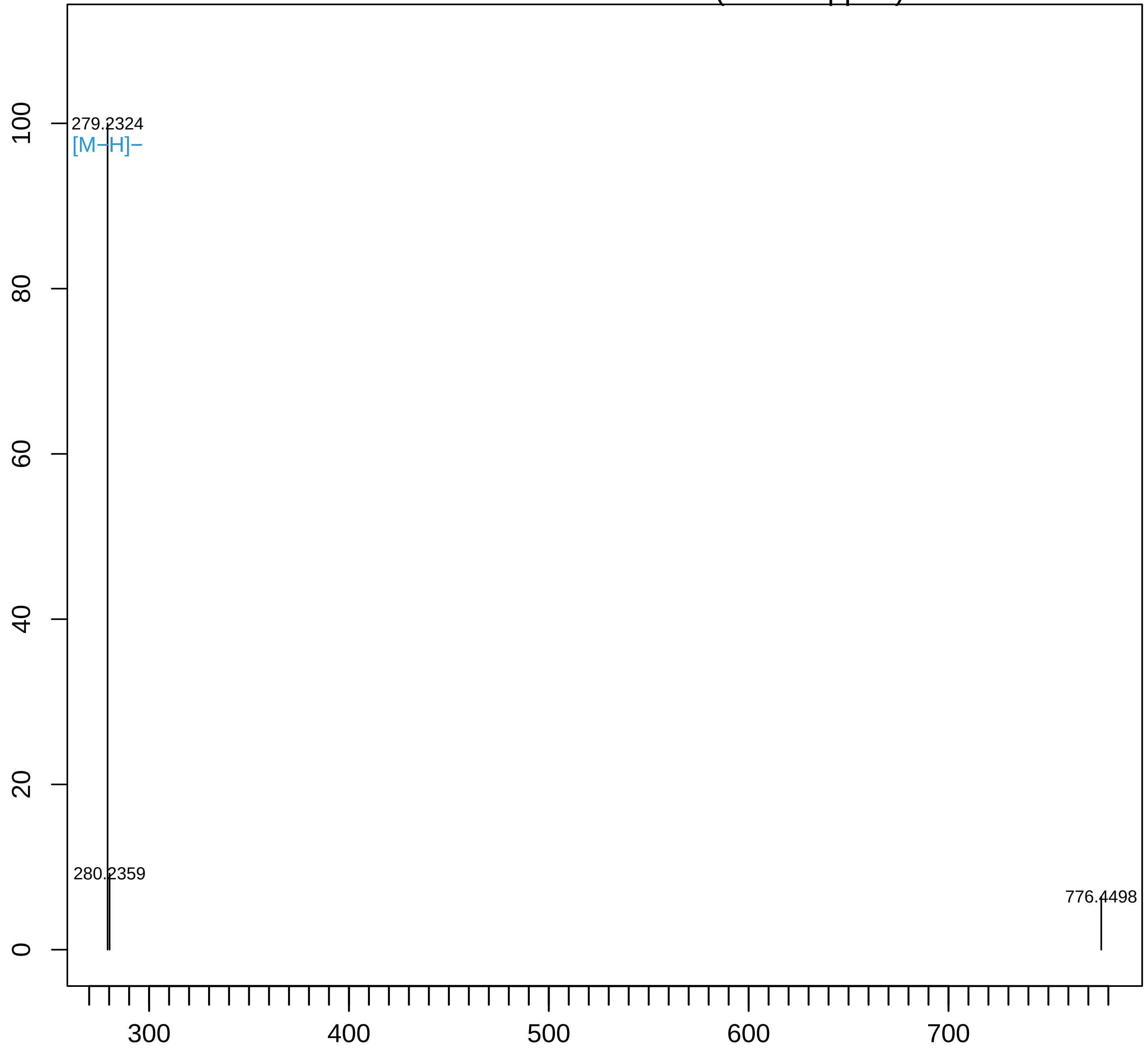

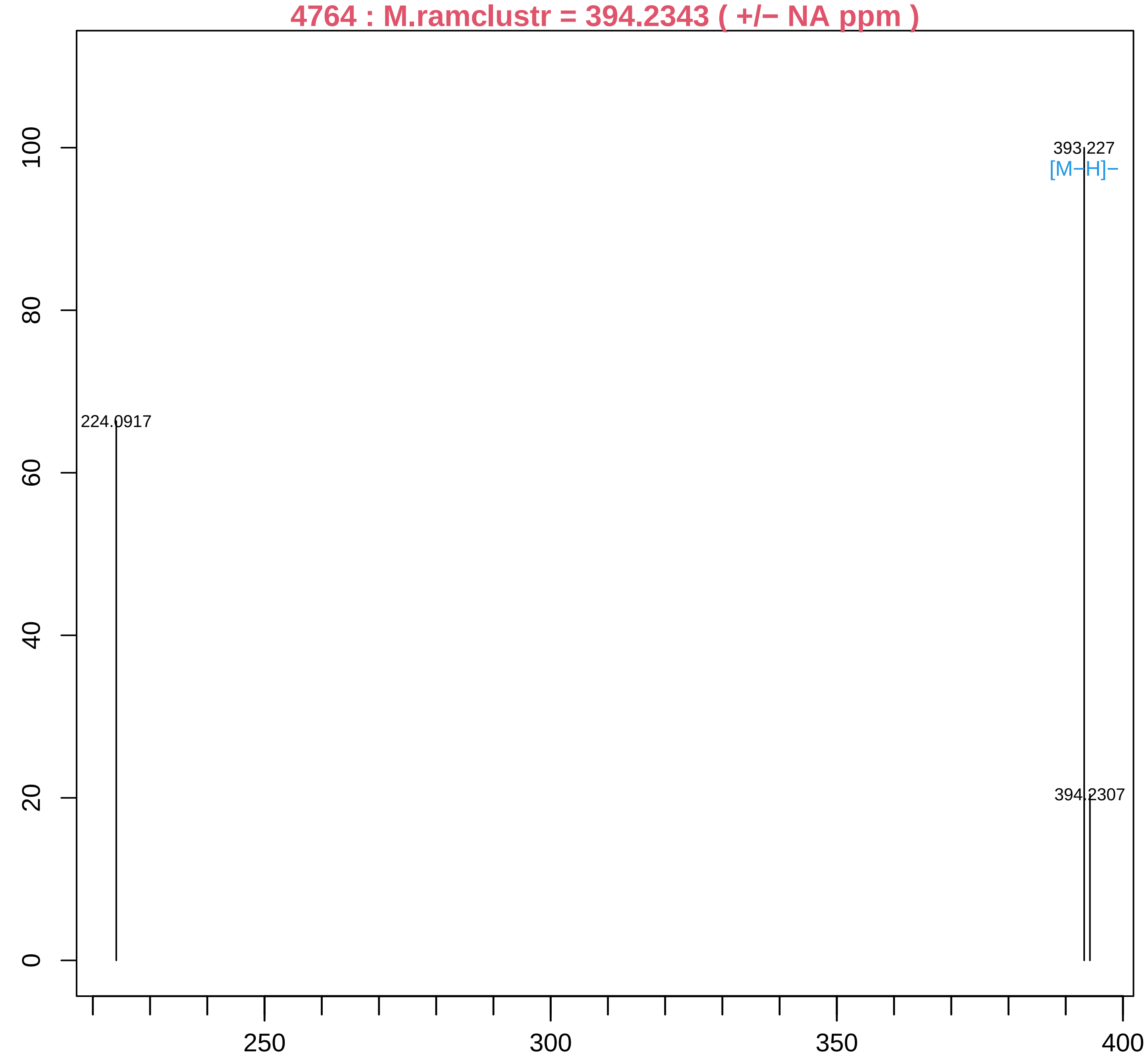
4764 : M.ramclustr = 394.2343 (+/− NA ppm)
393.227
[M−H]−
224.0917
394.2307
0
20
40
60
80
100
250
300
350
400

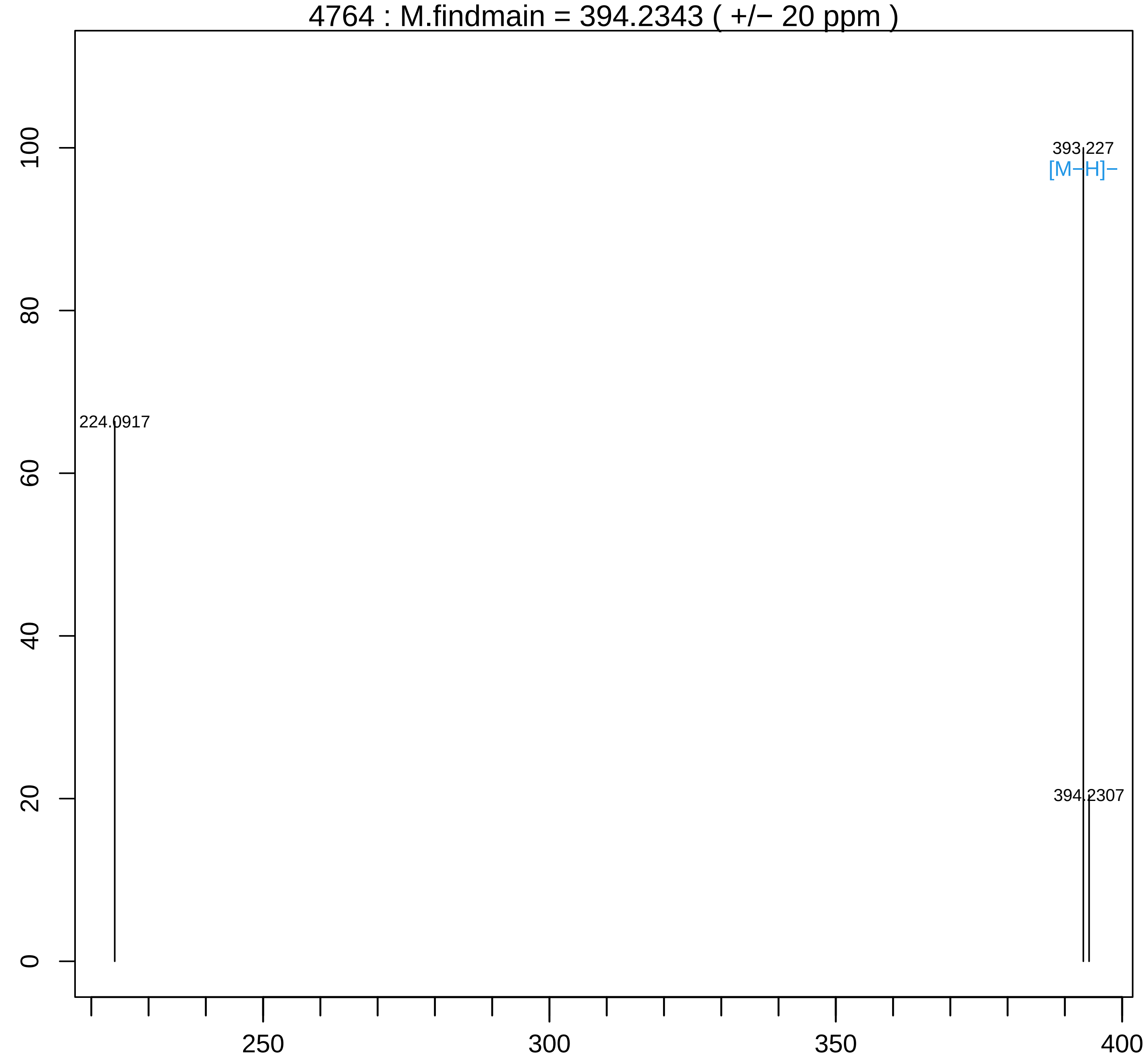
4764 : M.findmain = 394.2343 (+/− 20 ppm)
393.227
[M−H]−
224.0917
394.2307
0
20
40
60
80
100
250
300
350
400

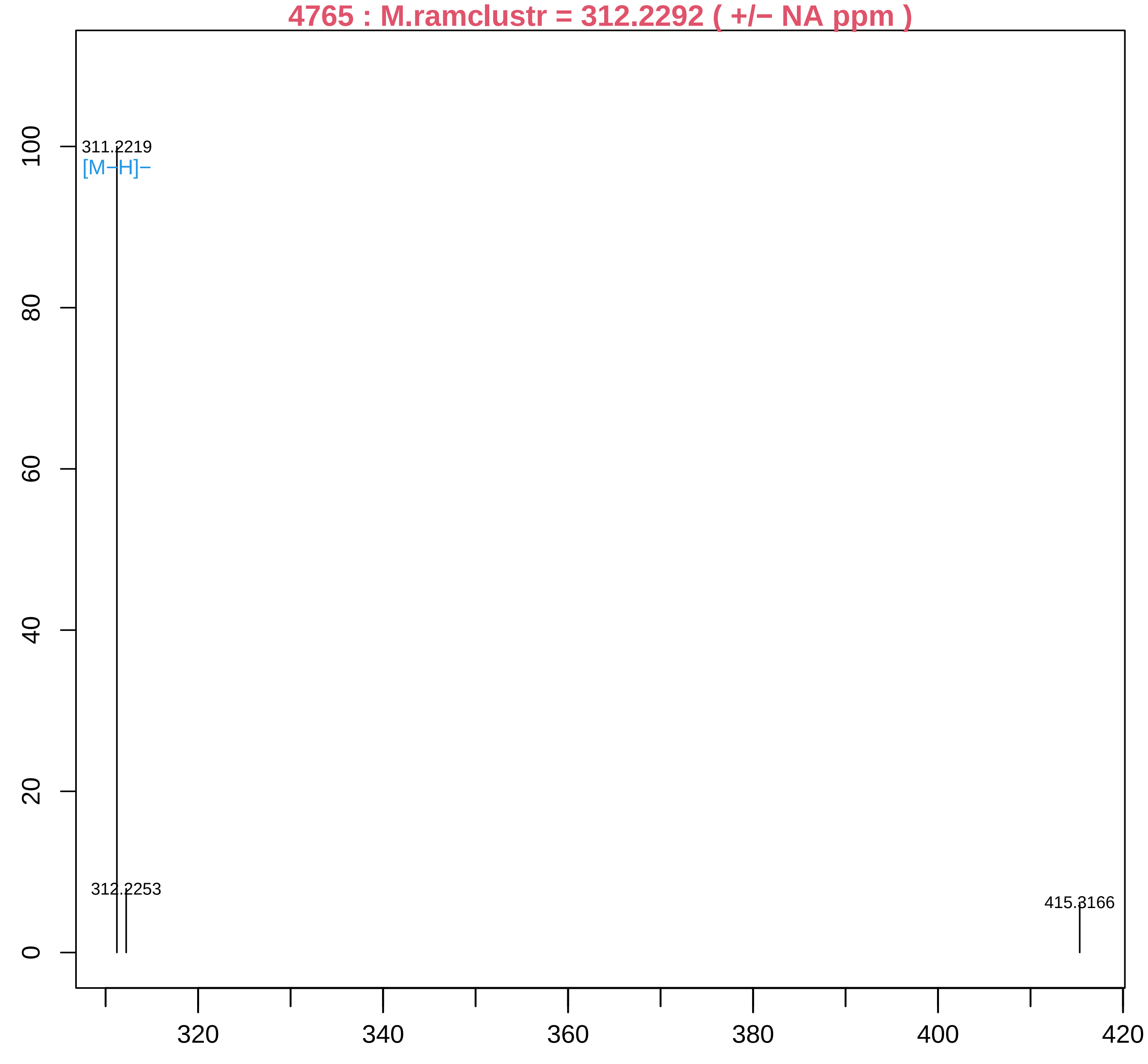
4765 : M.ramclustr = 312.2292 (+/− NA ppm)
311.2219
[M−H]−
312.2253
415.3166
0
20
40
60
80
100
320
340
360
380
400
420

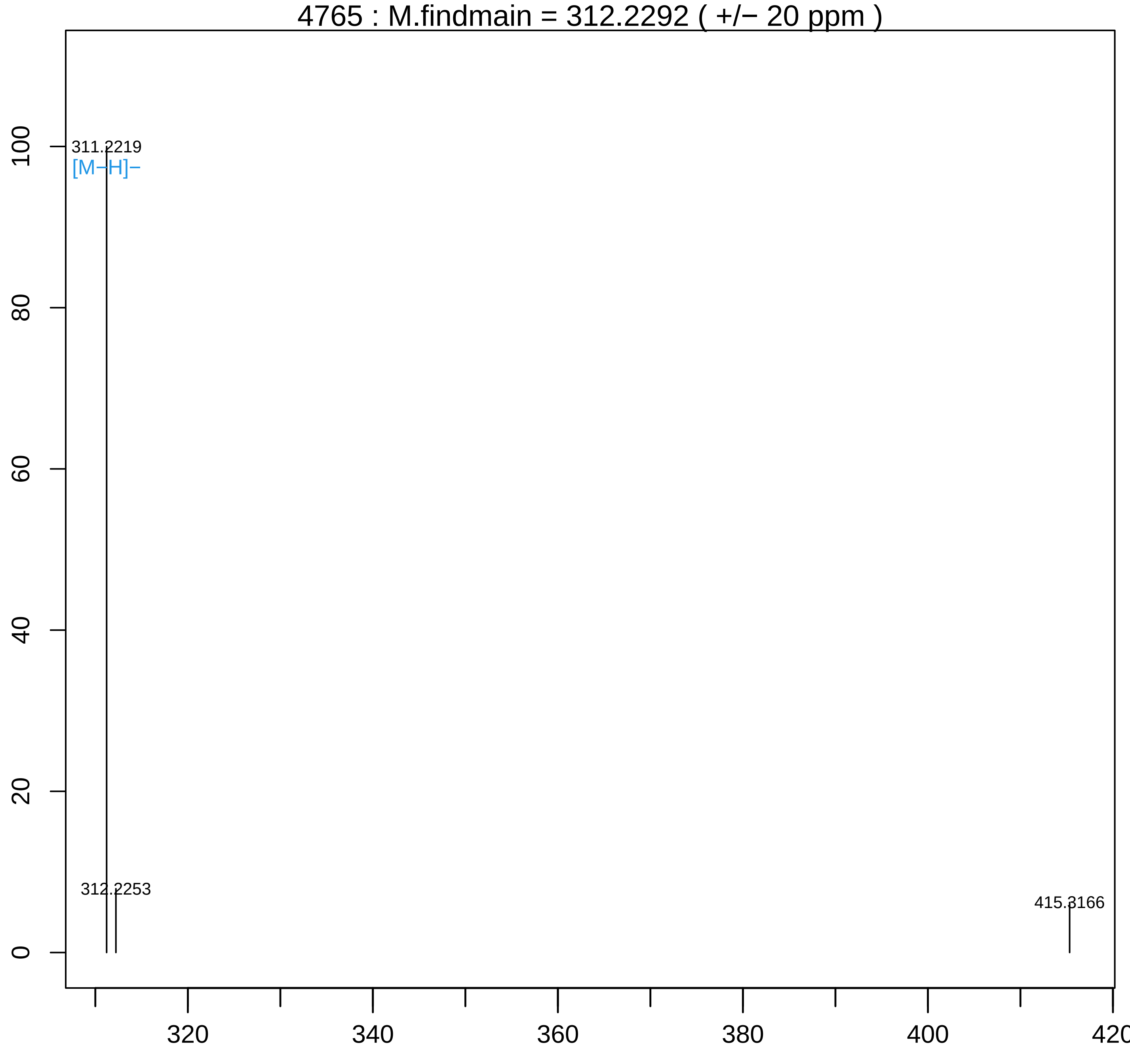
4765 : M.findmain = 312.2292 (+/− 20 ppm)
311.2219
[M−H]−
312.2253
415.3166
0
20
40
60
80
100
320
340
360
380
400
420

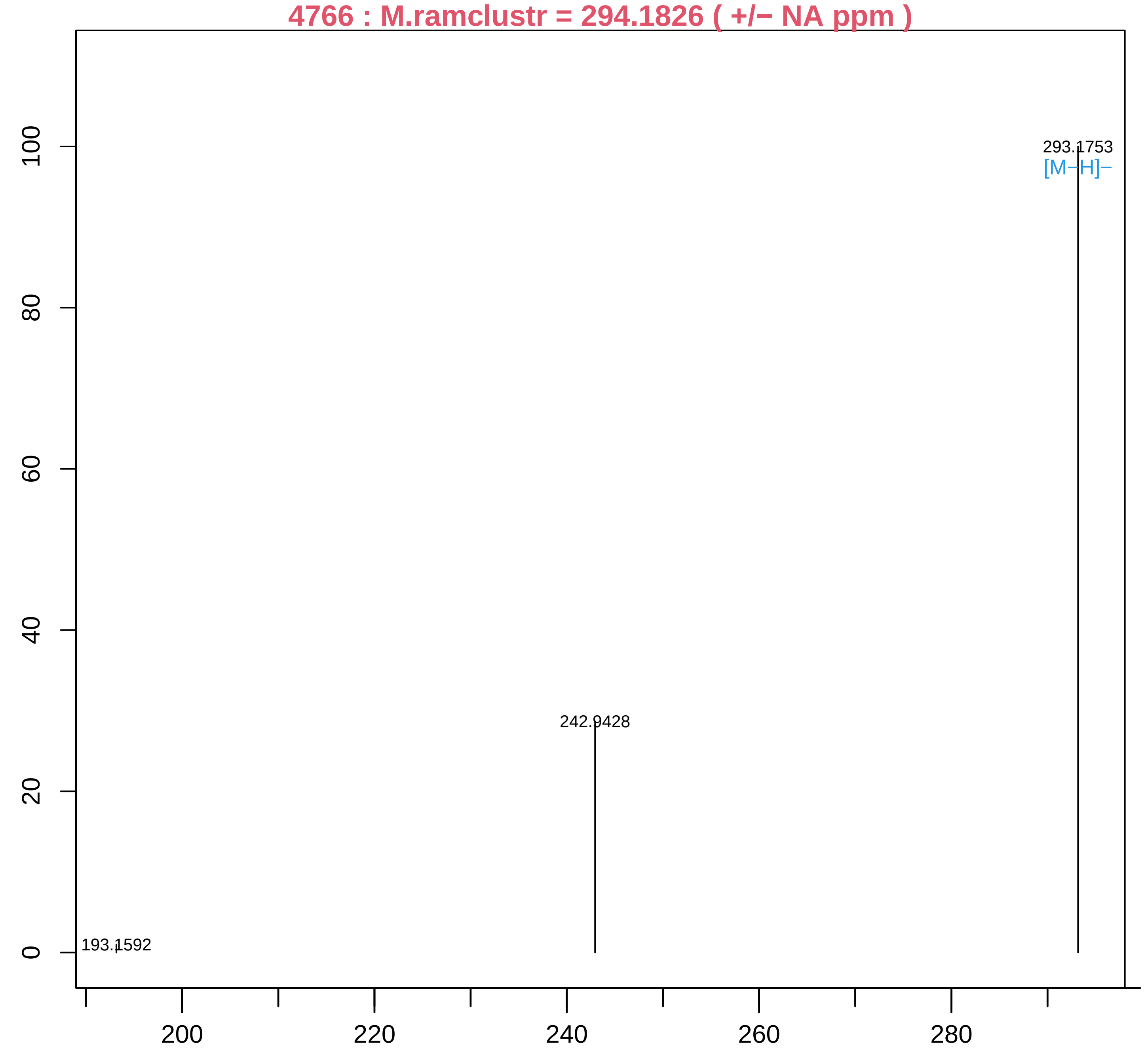
4766 : M.ramclustr = 294.1826 (+/− NA ppm)
293.1753
[M−H]−
242.9428
193.1592
0
20
40
60
80
100
200
220
240
260
280

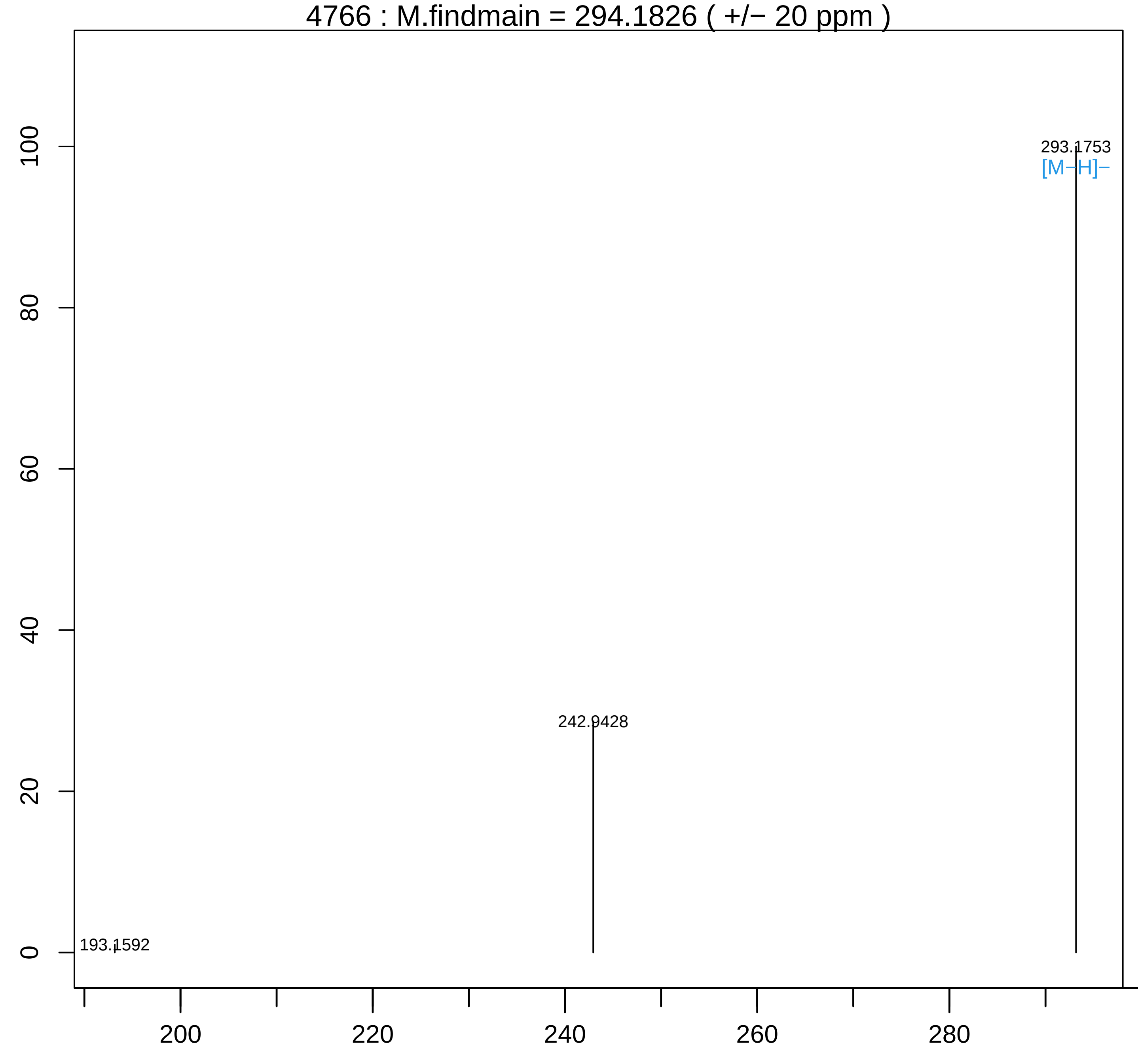
4766 : M.findmain = 294.1826 (+/− 20 ppm)
293.1753
[M−H]−
242.9428
193.1592
0
20
40
60
80
100
200
220
240
260
280

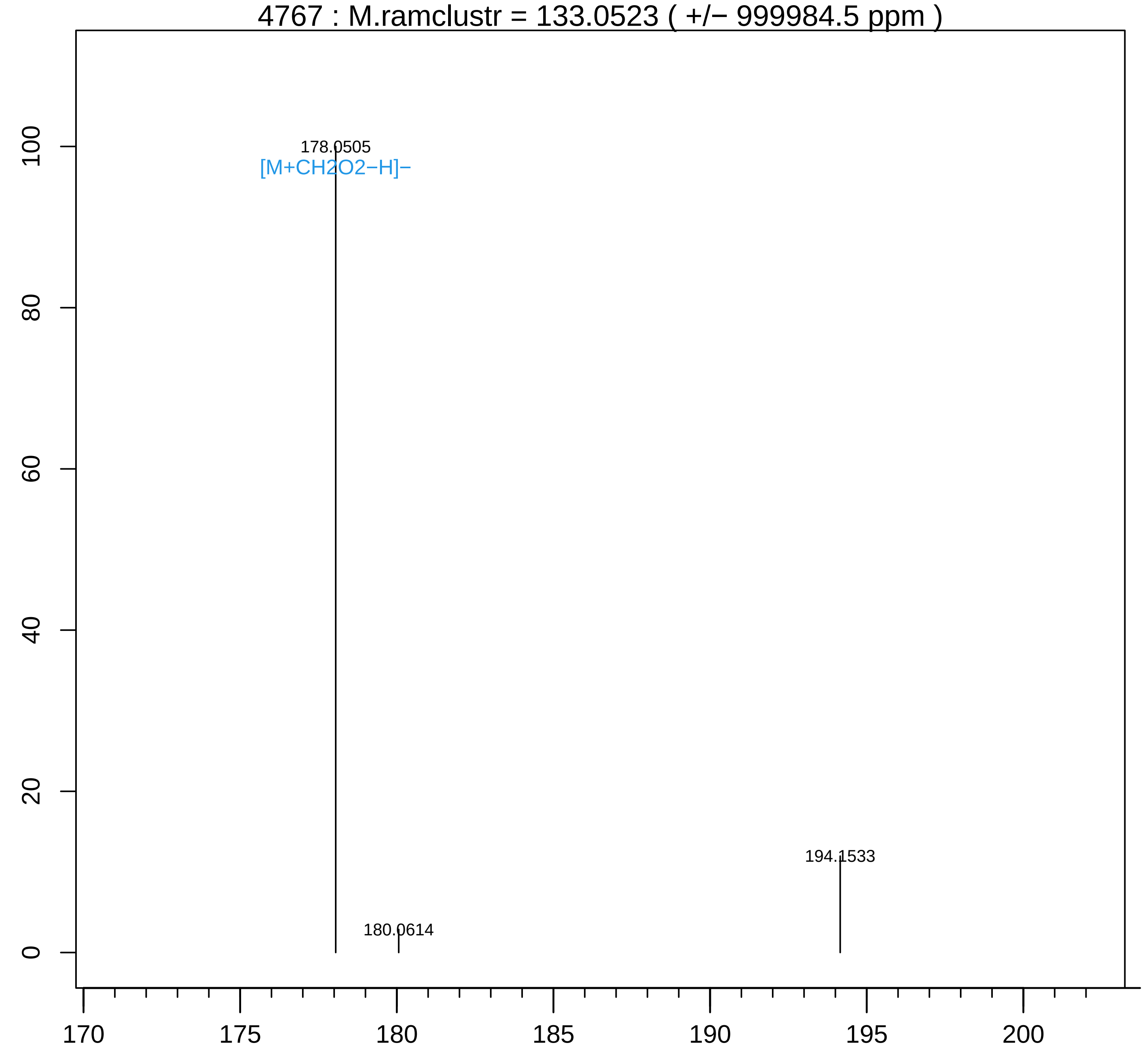
4767 : M.ramclustr = 133.0523 (+/− 999984.5 ppm)
178.0505
[M+CH2O2−H]−
180.0614
194.1533
0
20
40
60
80
100
170
175
180
185
190
195
200

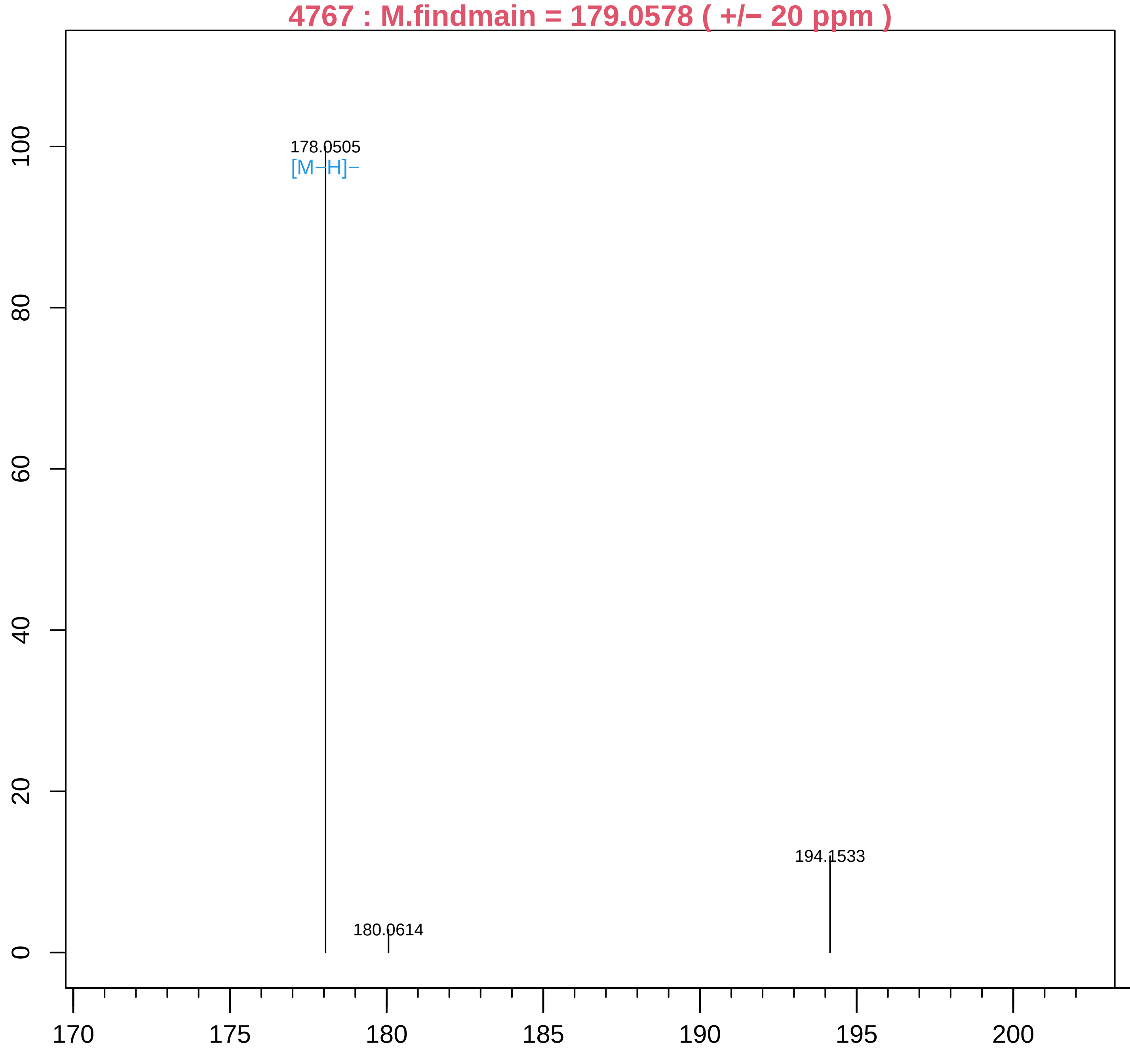
4767 : M.findmain = 179.0578 (+/− 20 ppm)
178.0505
[M−H]−
180.0614
194.1533
0
20
40
60
80
100
170
175
180
185
190
195
200

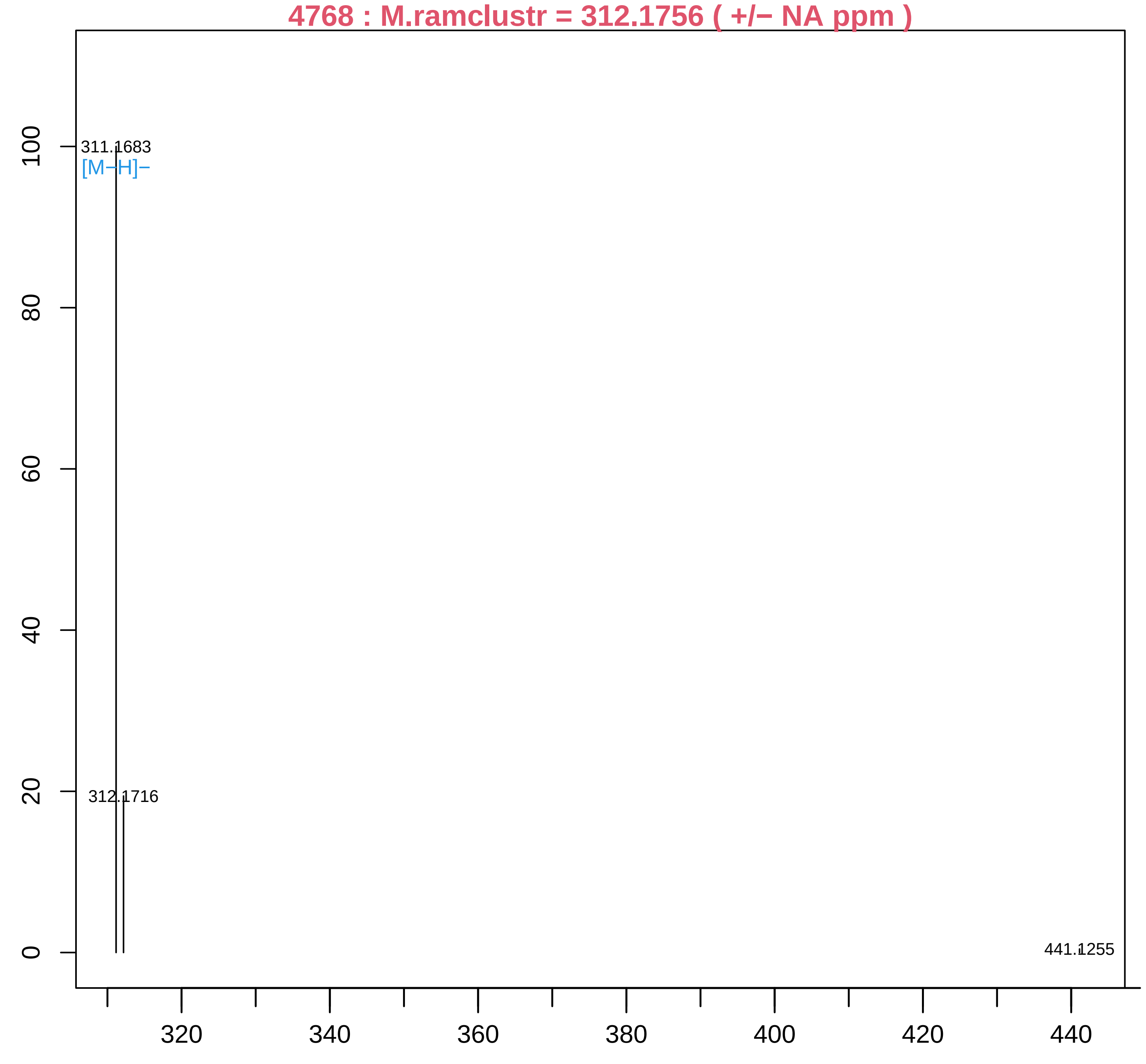
4768 : M.ramclustr = 312.1756 (+/− NA ppm)
311.1683
[M−H]−
312.1716
441.1255
0
20
40
60
80
100
320
340
360
380
400
420
440

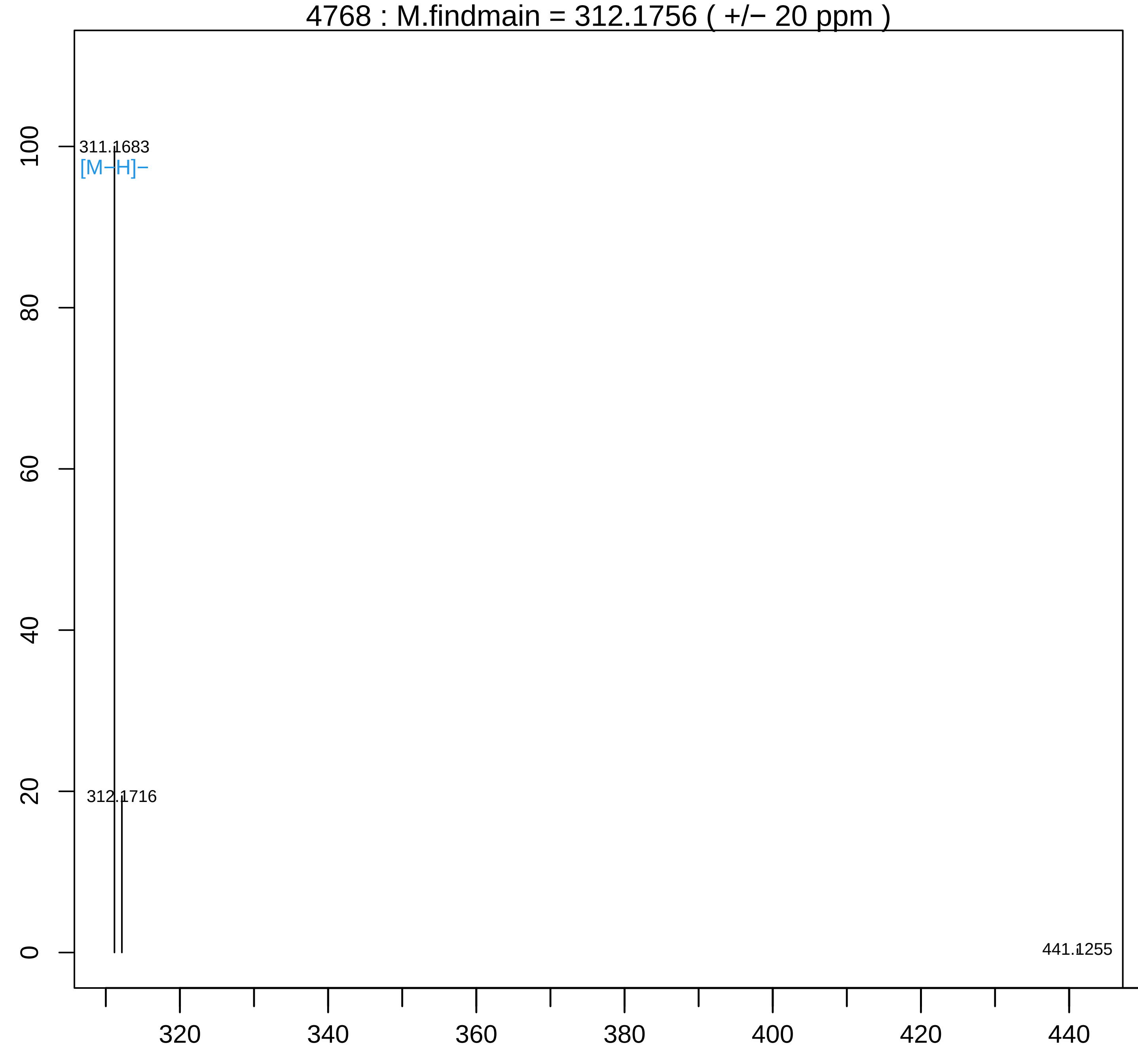
4768 : M.findmain = 312.1756 (+/− 20 ppm)
311.1683
[M−H]−
312.1716
441.1255
0
20
40
60
80
100
320
340
360
380
400
420
440

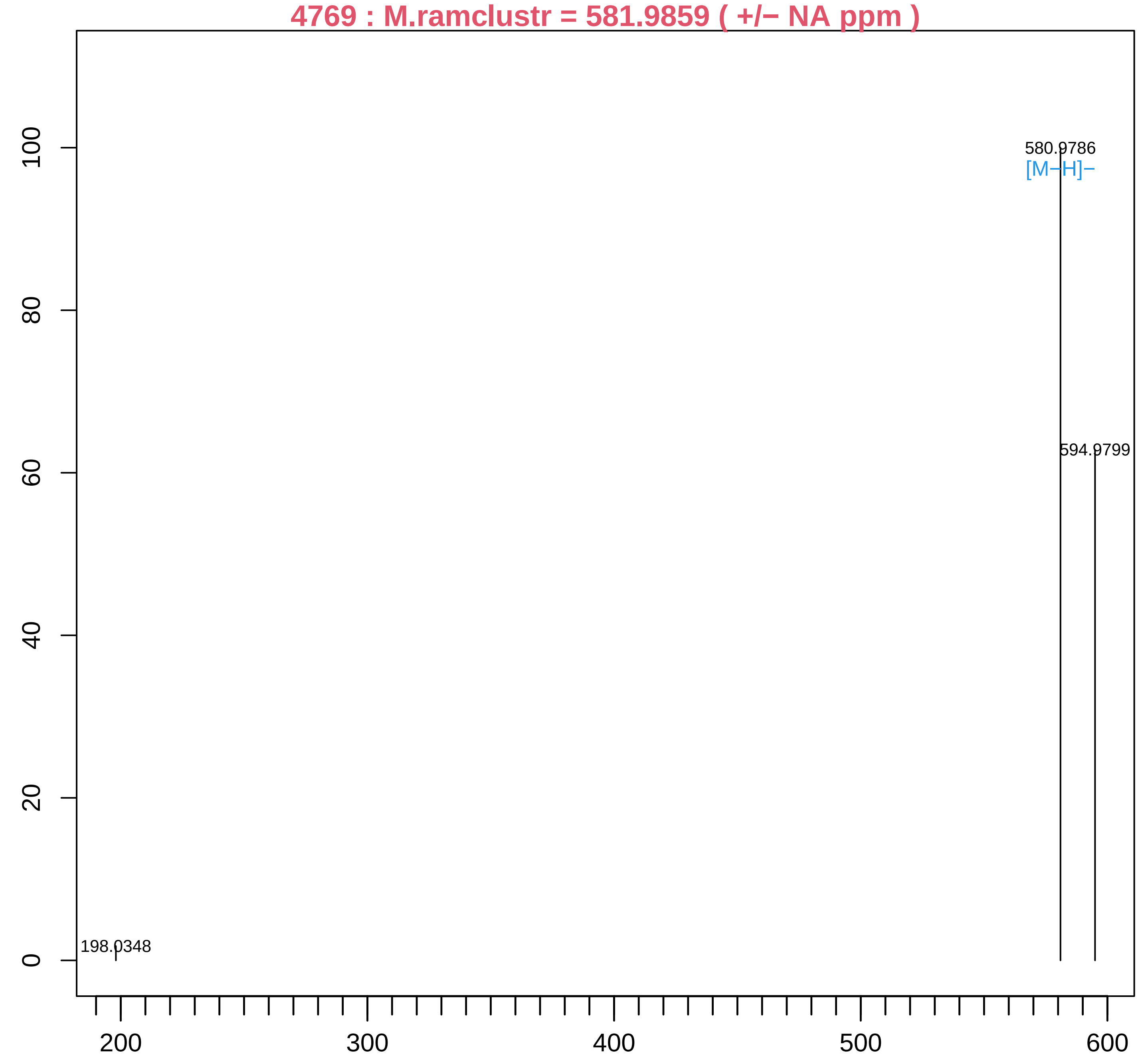
4769 : M.ramclustr = 581.9859 (+/− NA ppm)
580.9786
[M−H]−
594.9799
198.0348
0
20
40
60
80
100
200
300
400
500
600

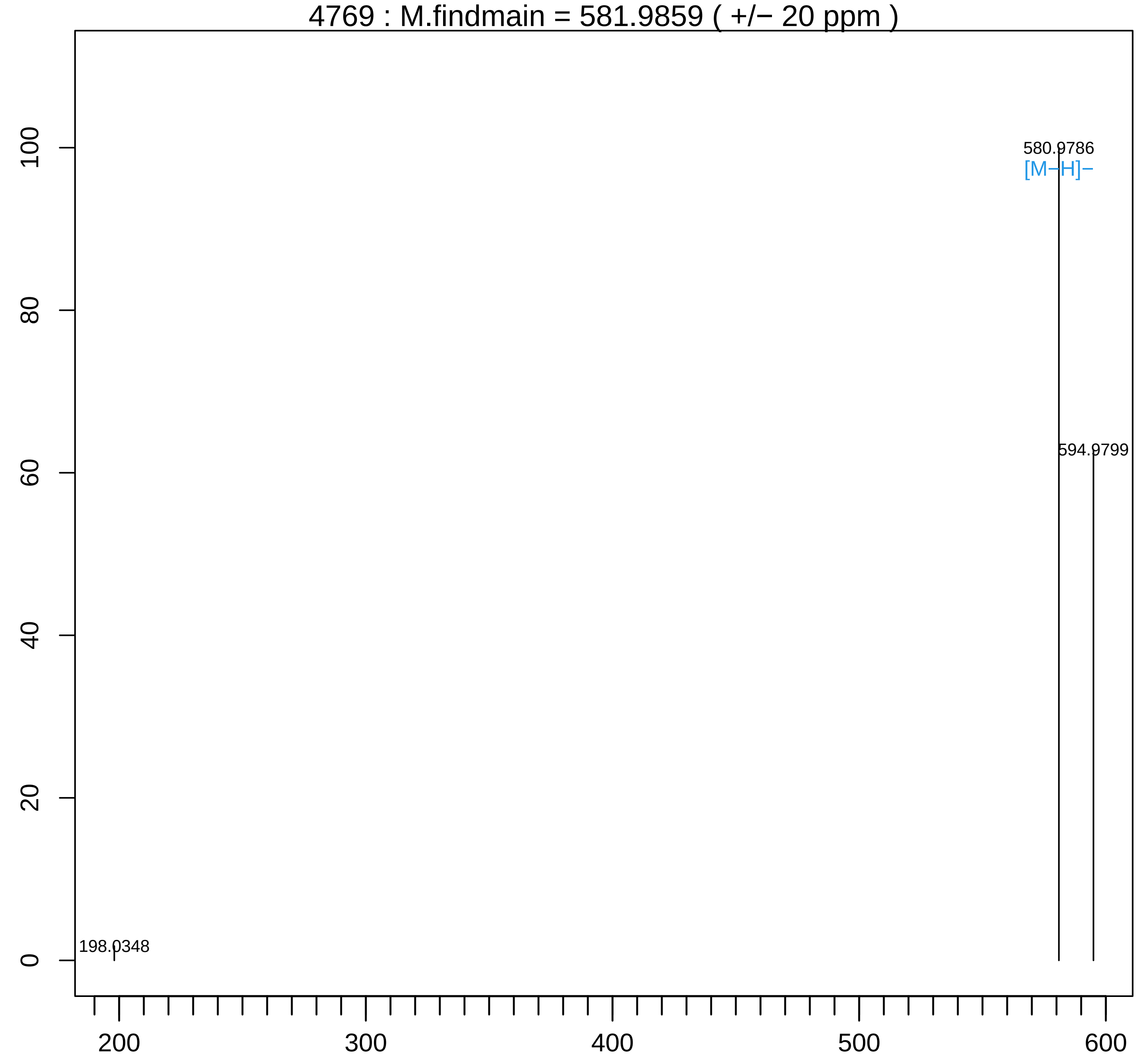
4769 : M.findmain = 581.9859 (+/− 20 ppm)
580.9786
[M−H]−
594.9799
198.0348
0
20
40
60
80
100
200
300
400
500
600

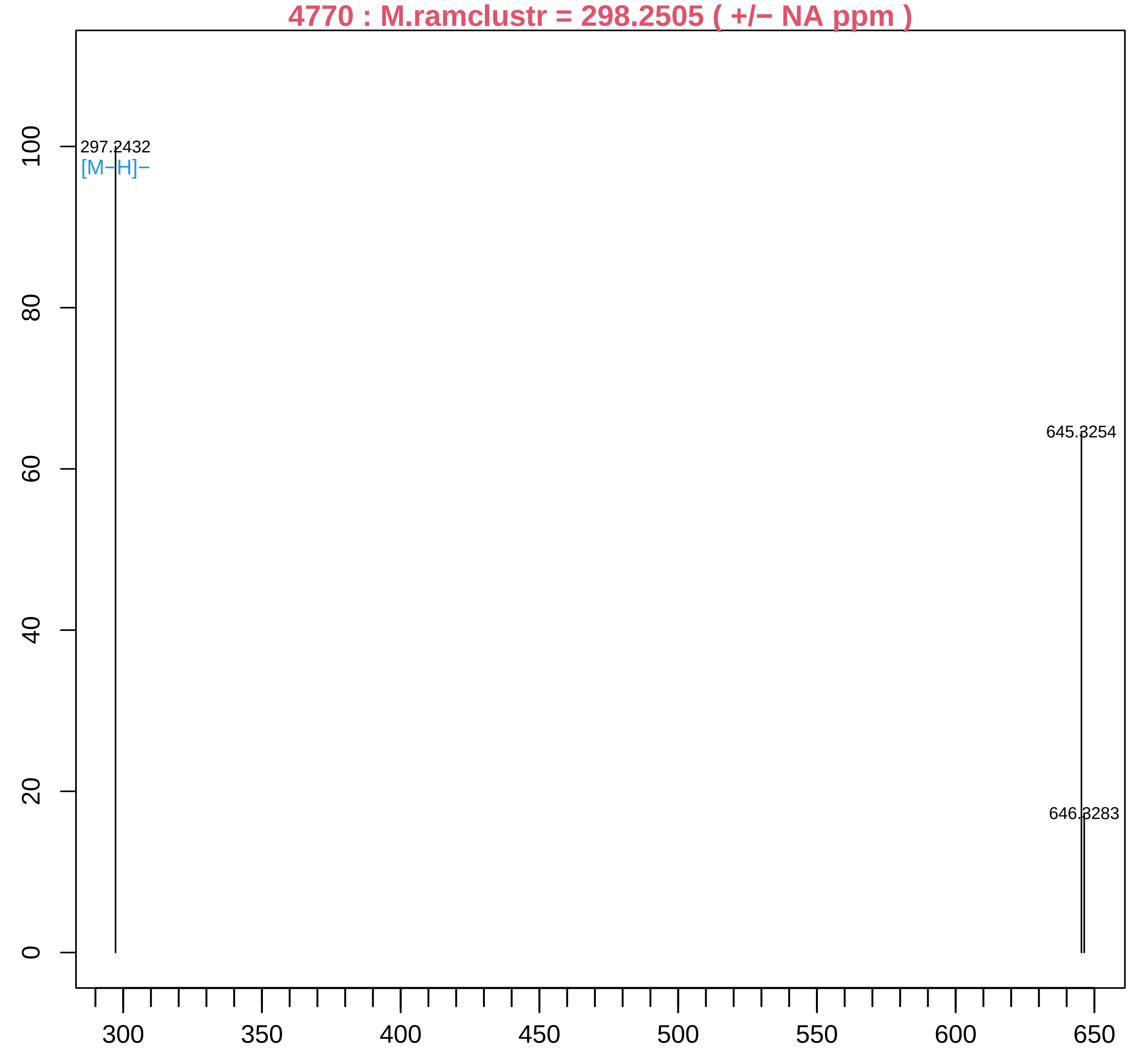
4770 : M.ramclustr = 298.2505 (+/− NA ppm)
297.2432
[M−H]−
645.3254
646.3283
0
20
40
60
80
100
300
350
400
450
500
550
600
650

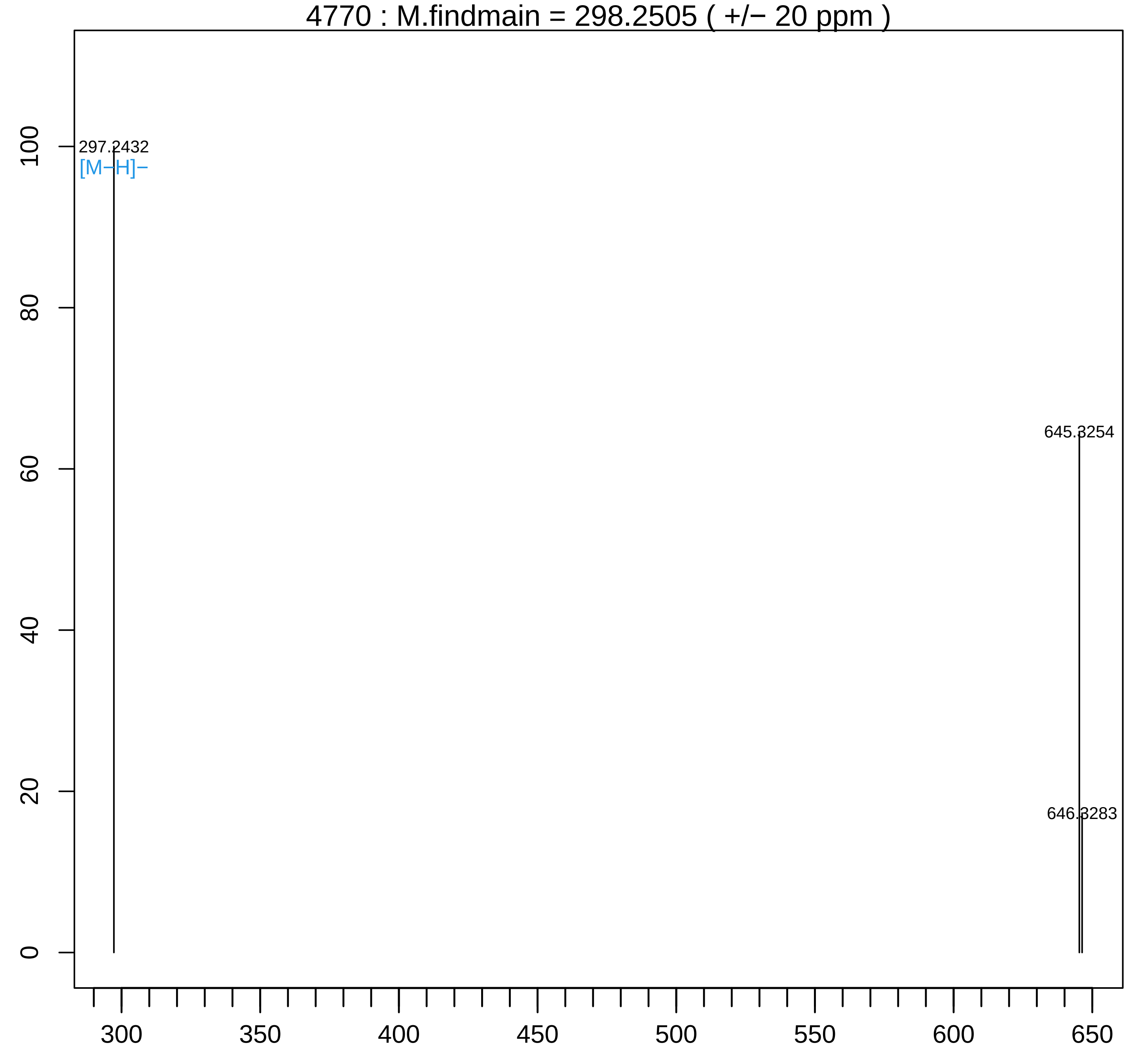
4770 : M.findmain = 298.2505 (+/− 20 ppm)
297.2432
[M−H]−
645.3254
646.3283
0
20
40
60
80
100
300
350
400
450
500
550
600
650

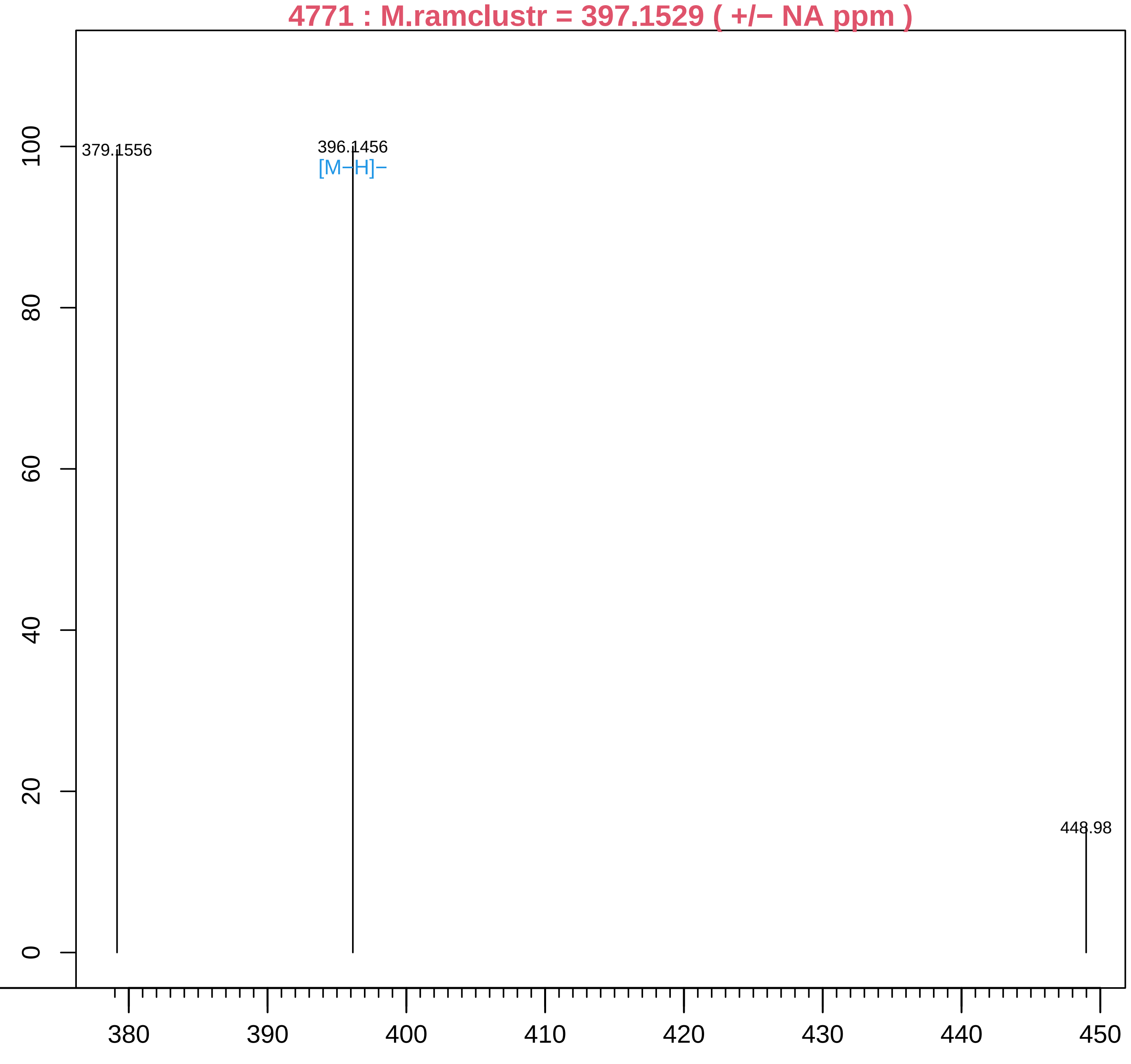
4771 : M.ramclustr = 397.1529 (+/− NA ppm)
379.1556
396.1456
[M−H]−
448.98
0
20
40
60
80
100
380
390
400
410
420
430
440
450

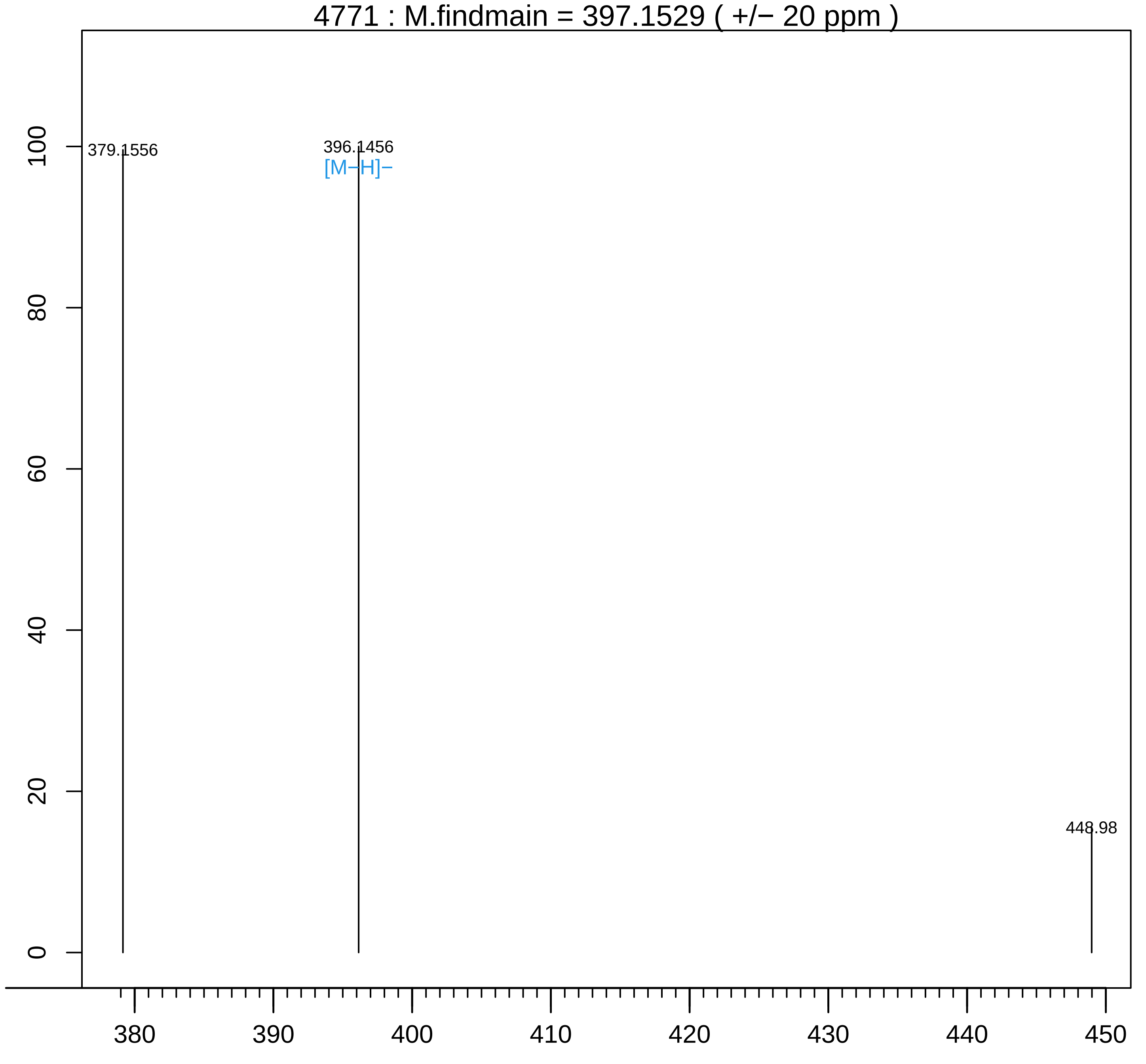
4771 : M.findmain = 397.1529 (+/− 20 ppm)
379.1556
396.1456
[M−H]−
448.98
0
20
40
60
80
100
380
390
400
410
420
430
440
450

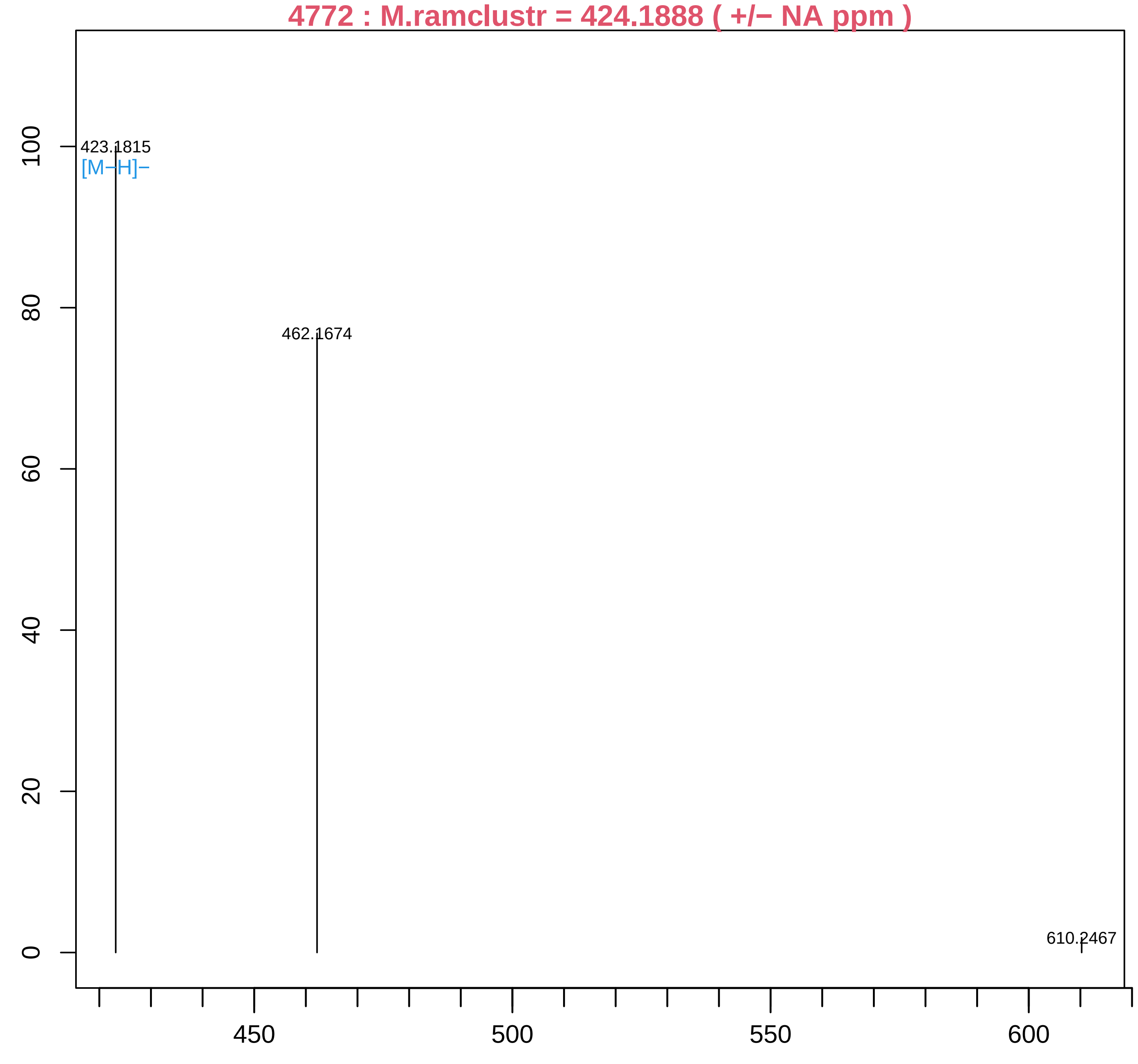

4772 : M.ramclustr = 424.1888 (+/− NA ppm)
423.1815
[M−H]−
462.1674
610.2467
0
20
40
60
80
100
450
500
550
600

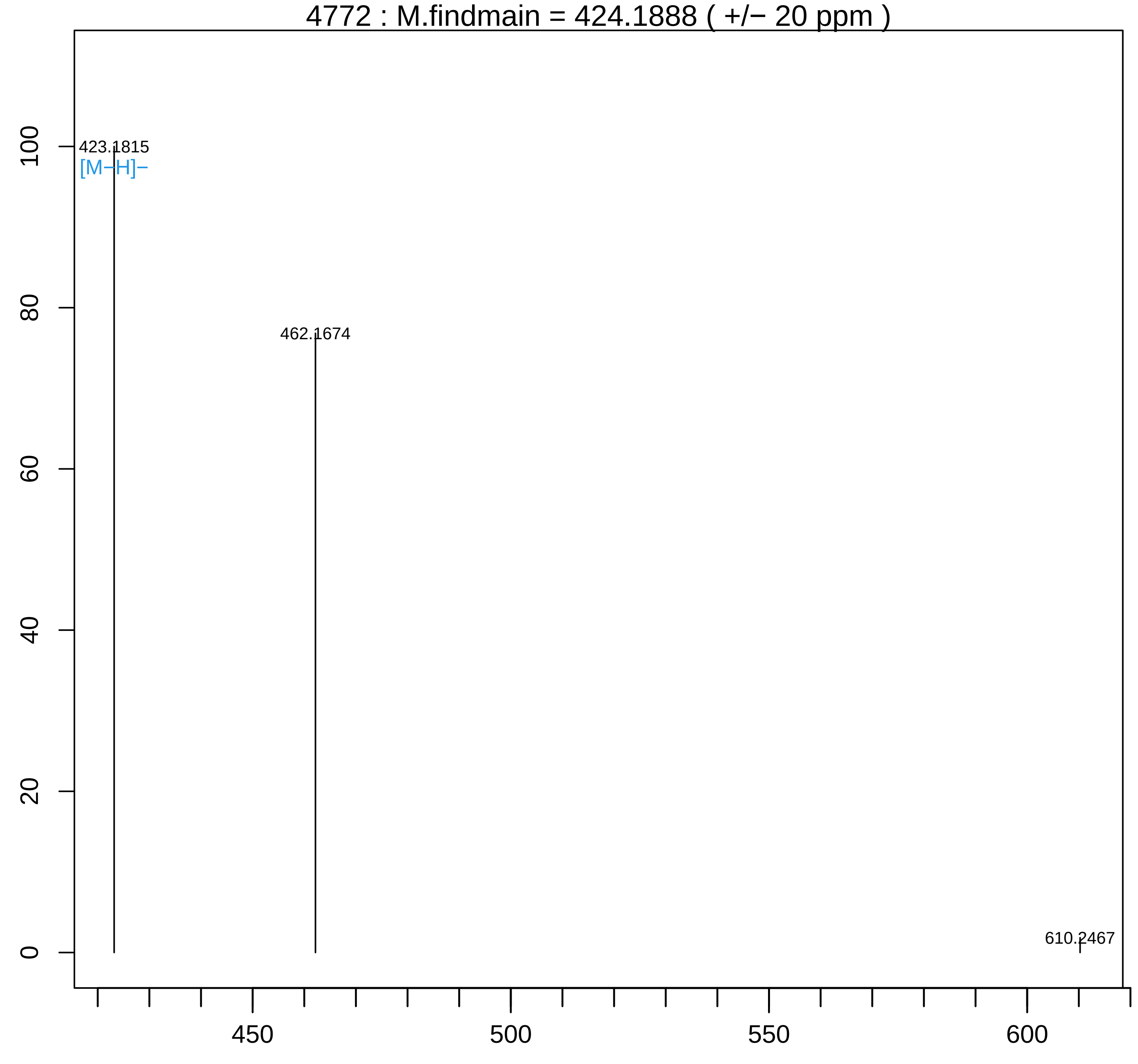

4772 : M.findmain = 424.1888 (+/− 20 ppm)
423.1815
[M−H]−
462.1674
610.2467
0
20
40
60
80
100
450
500
550
600

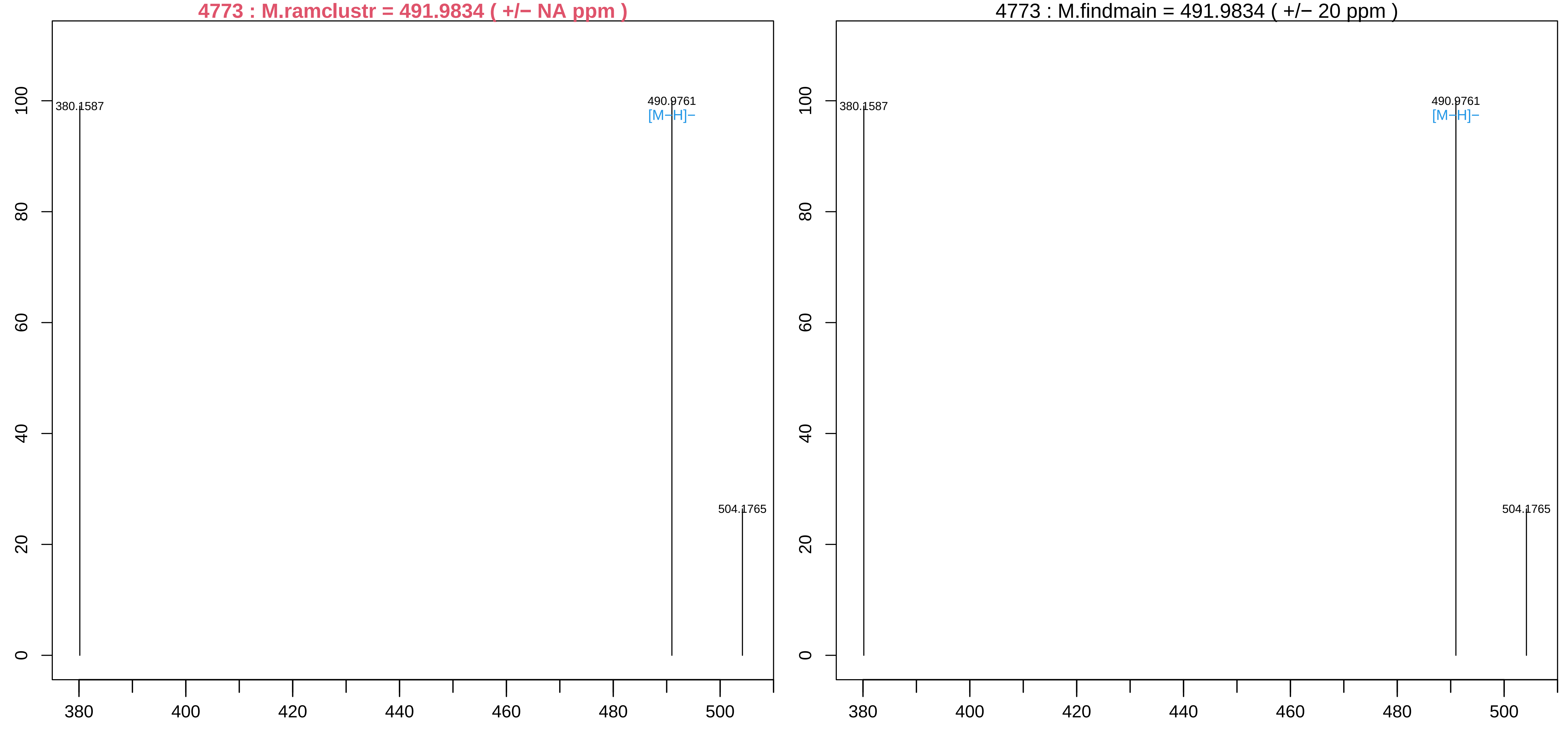

4773 : M.ramclustr = 491.9834 (+/− NA ppm)
380.1587
490.9761
[M−H]−
504.1765
4773 : M.findmain = 491.9834 (+/− 20 ppm)
380.1587
490.9761
[M−H]−
504.1765
0
20
40
60
80
100
380
400
420
440
460
480
500

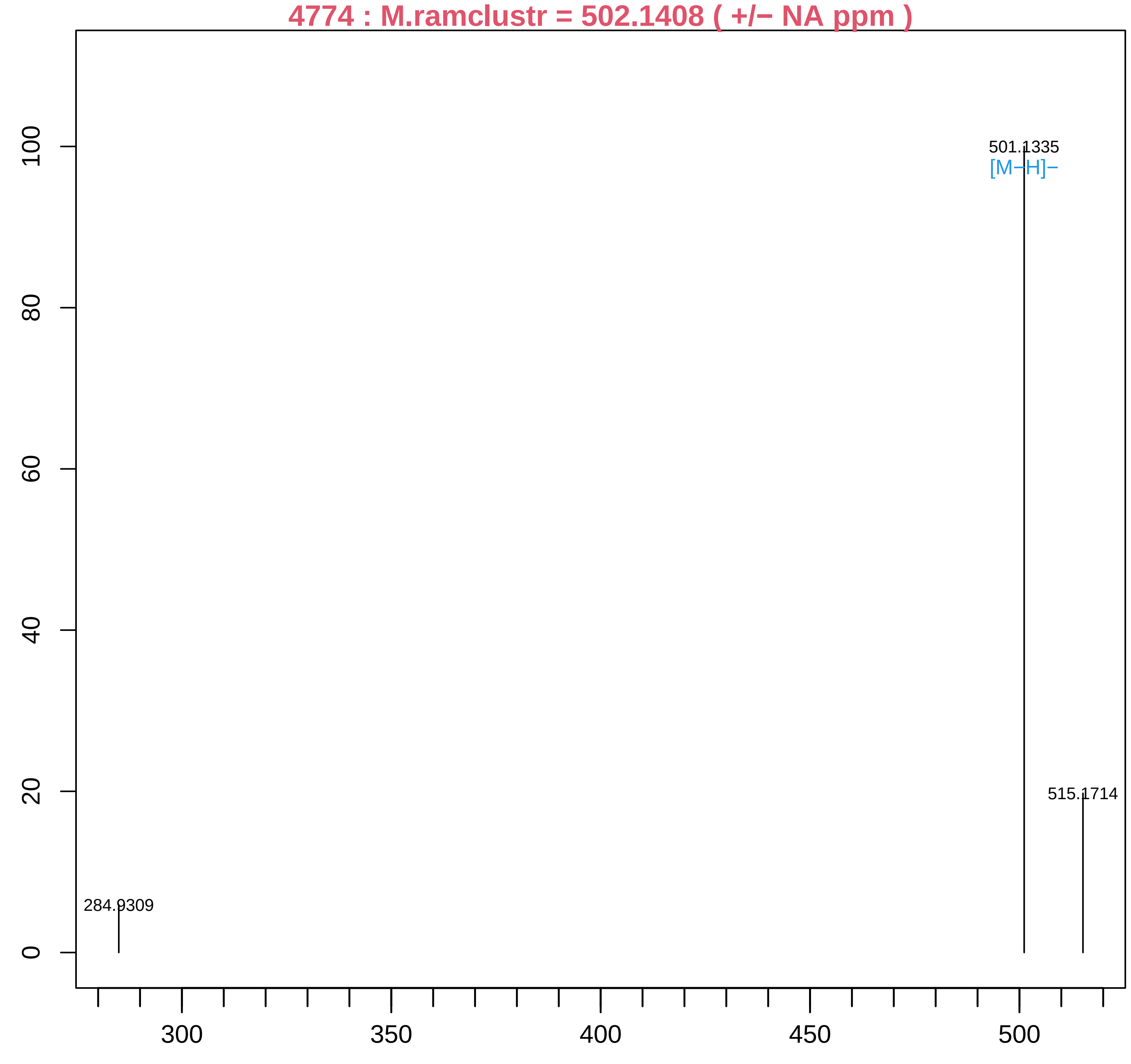
4774 : M.ramclustr = 502.1408 (+/− NA ppm)
501.1335
[M−H]−
515.1714
284.9309
0
20
40
60
80
100
300
350
400
450
500

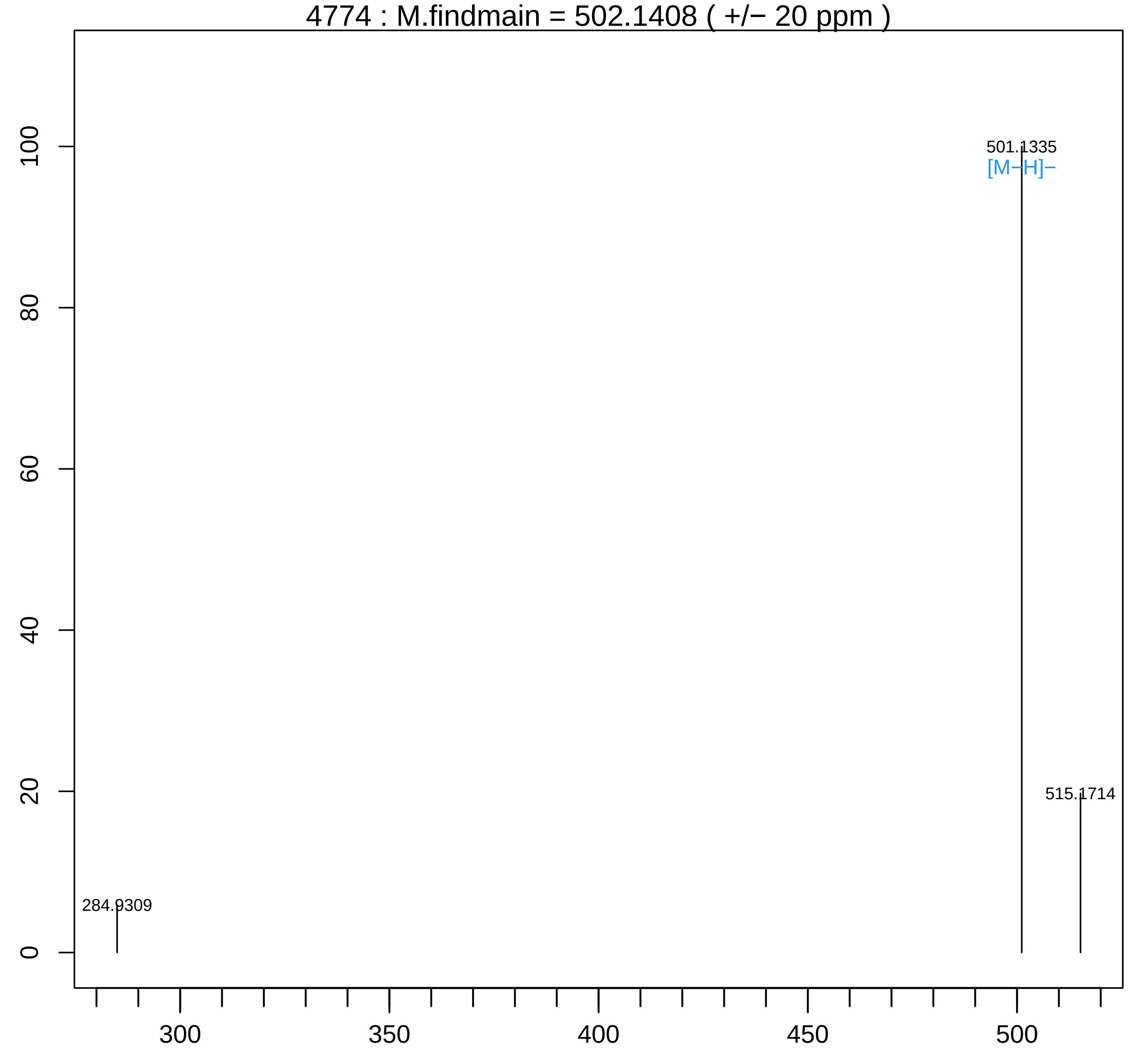
4774 : M.findmain = 502.1408 (+/− 20 ppm)
501.1335
[M−H]−
515.1714
284.9309
0
20
40
60
80
100
300
350
400
450
500

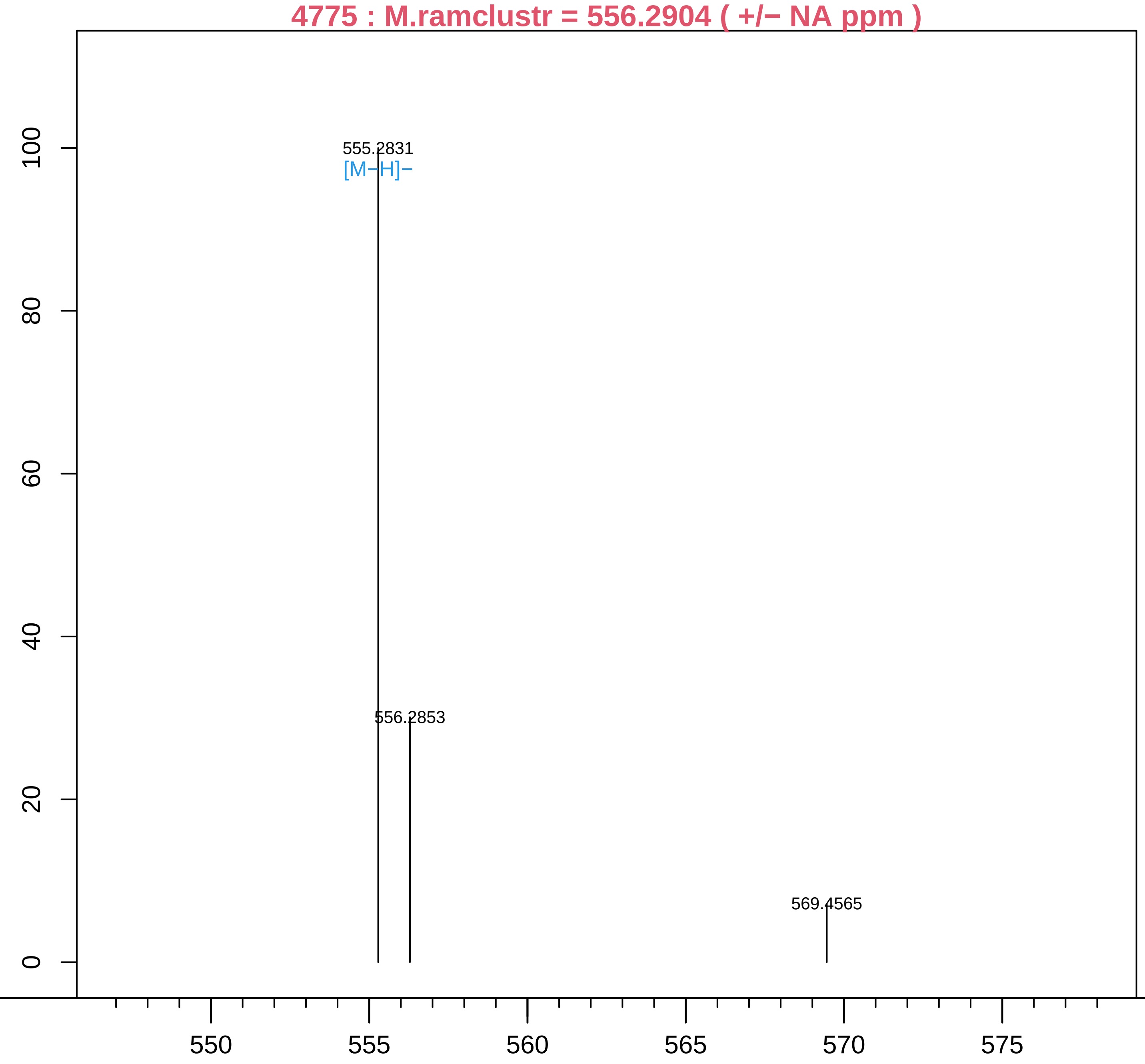
4775 : M.ramclustr = 556.2904 (+/− NA ppm)
555.2831
[M−H]−
556.2853
569.4565
0
20
40
60
80
100
550
555
560
565
570
575

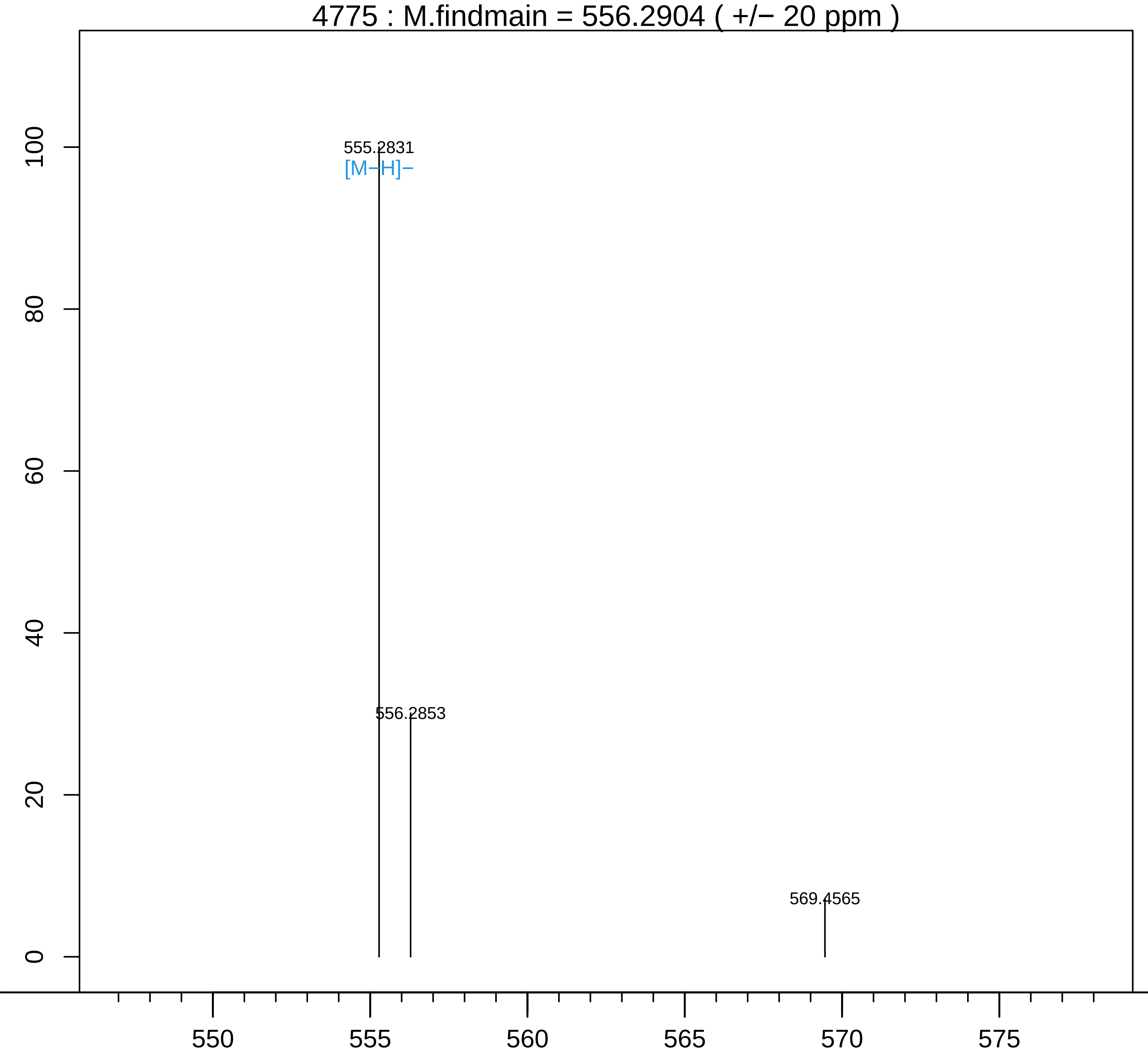
4775 : M.findmain = 556.2904 (+/− 20 ppm)
555.2831
[M−H]−
556.2853
569.4565
0
20
40
60
80
100
550
555
560
565
570
575

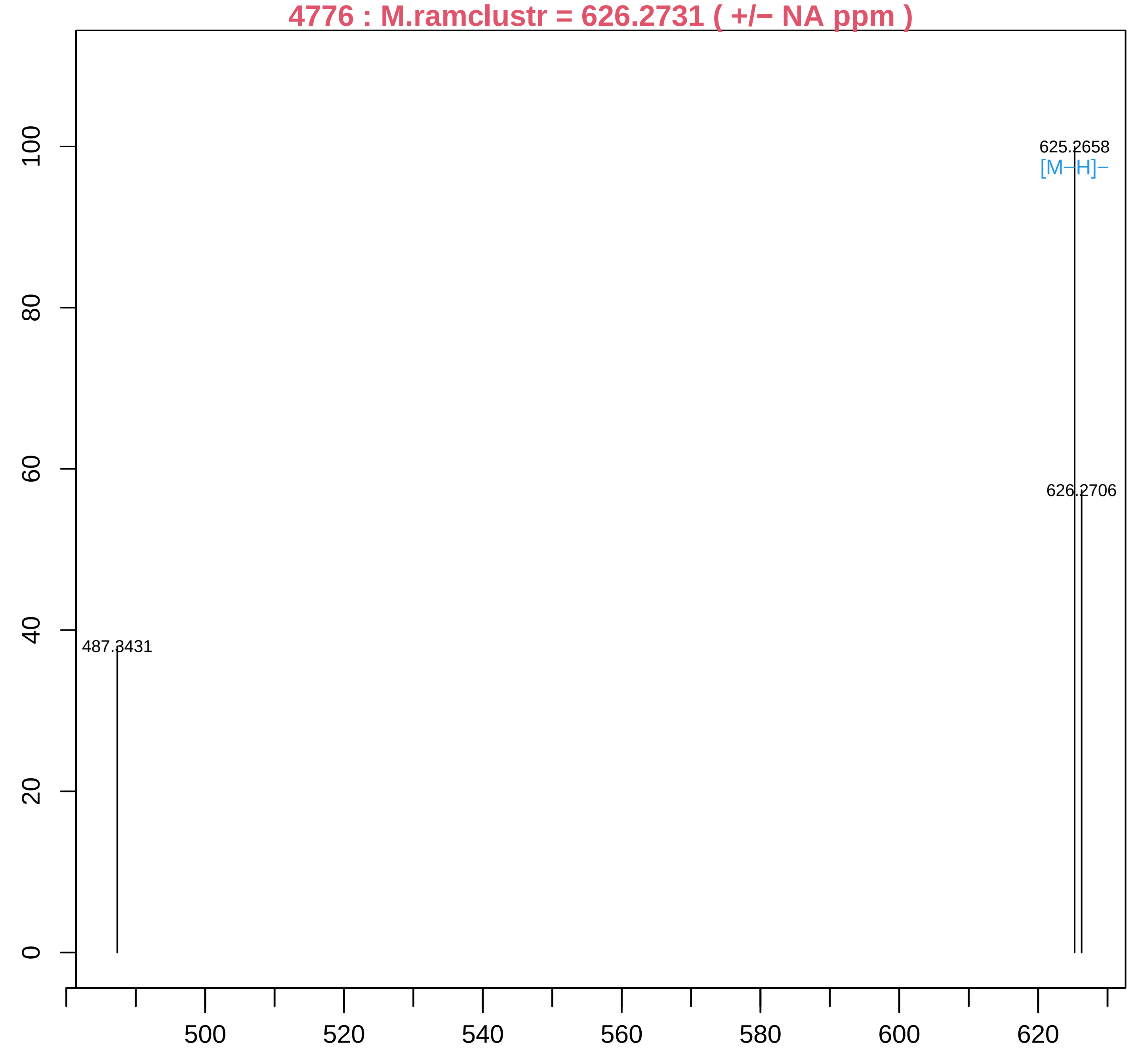
4776 : M.ramclustr = 626.2731 (+/− NA ppm)
625.2658
[M−H]−
626.2706
487.3431
0
20
40
60
80
100
500
520
540
560
580
600
620

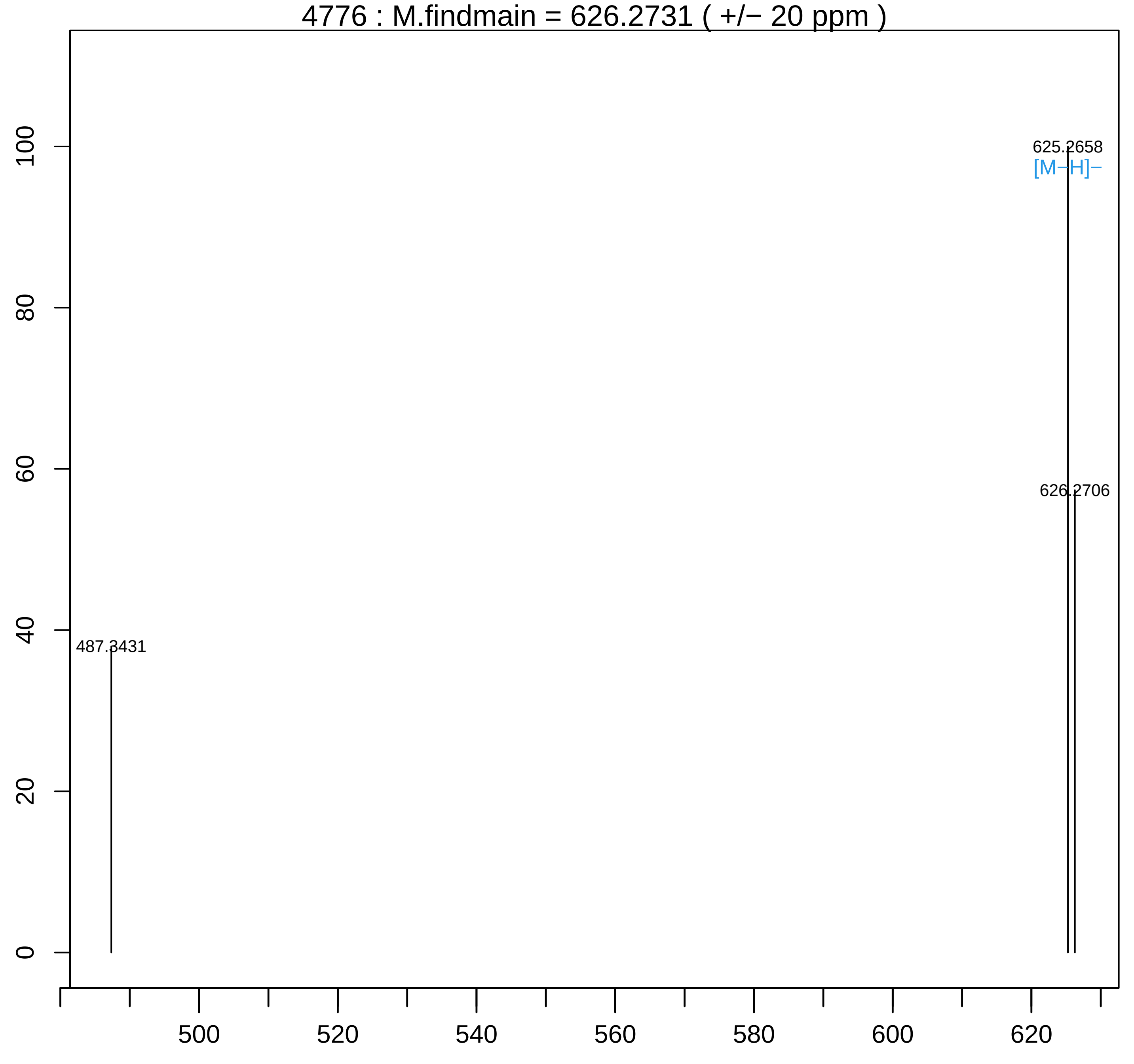
4776 : M.findmain = 626.2731 (+/− 20 ppm)
625.2658
[M−H]−
626.2706
487.3431
0
20
40
60
80
100
500
520
540
560
580
600
620

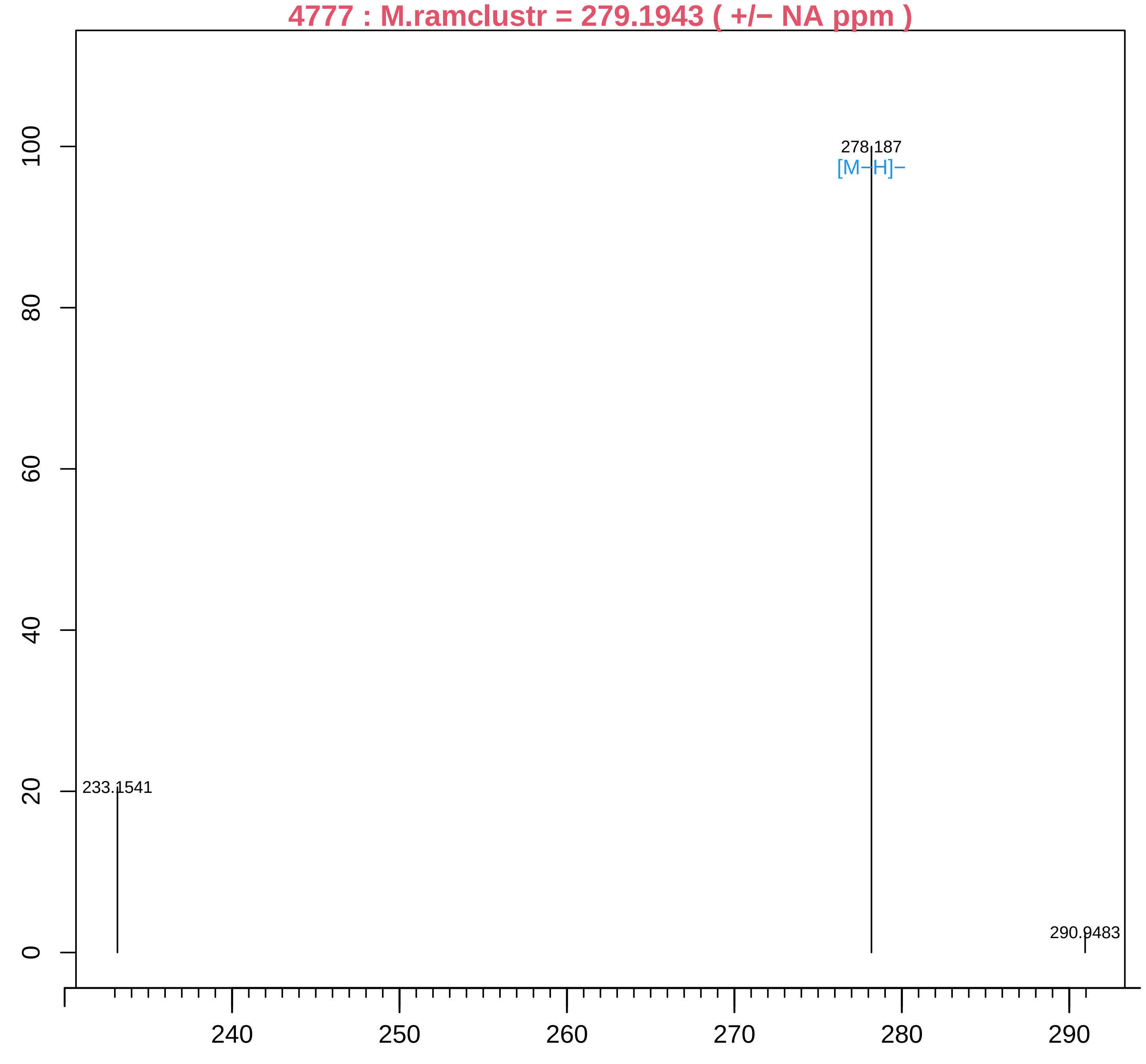
4777 : M.ramclustr = 279.1943 (+/− NA ppm)
278.187
[M−H]−
233.1541
290.9483
0
20
40
60
80
100
240
250
260
270
280
290

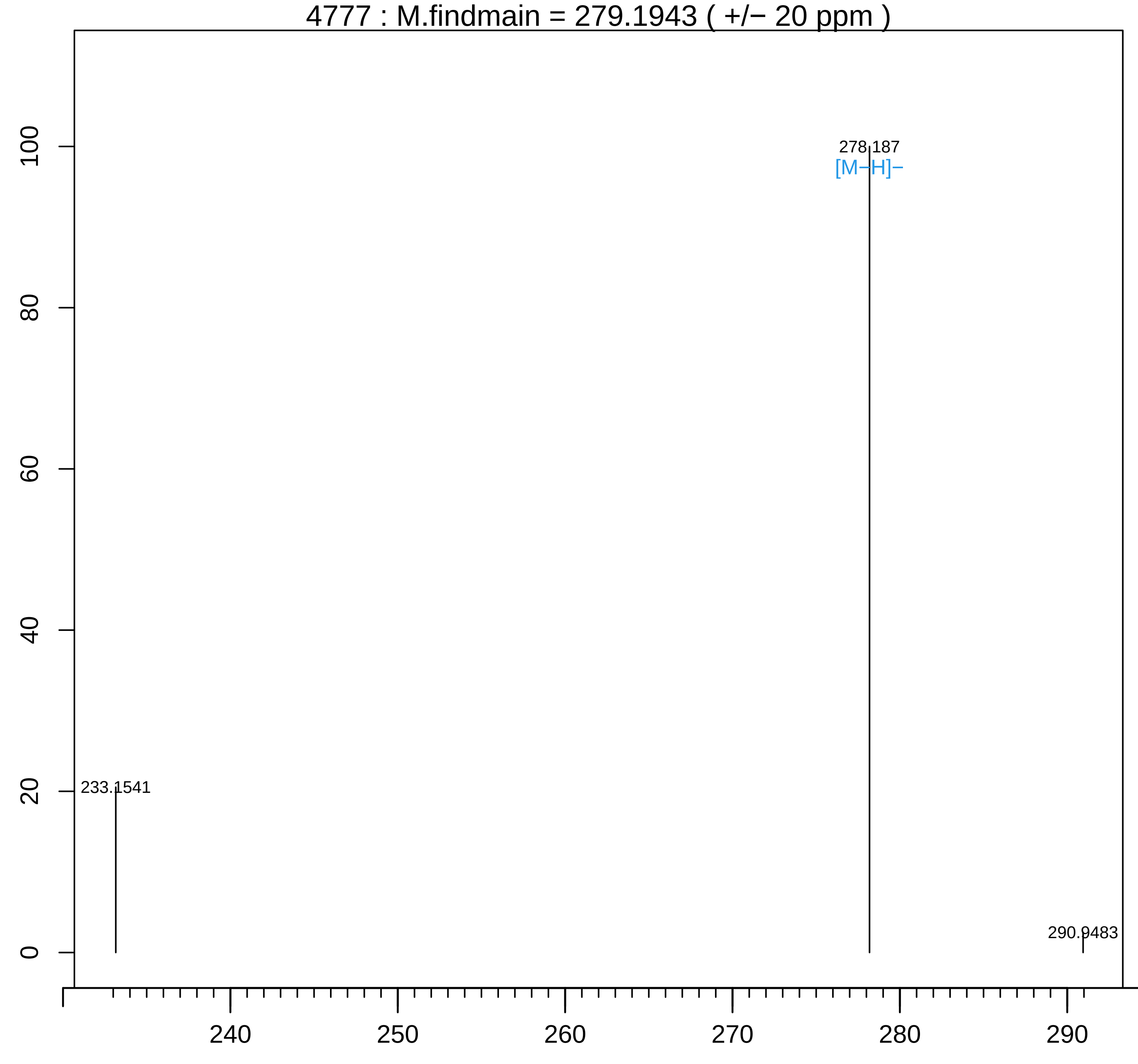
4777 : M.findmain = 279.1943 (+/− 20 ppm)
278.187
[M−H]−
233.1541
290.9483
0
20
40
60
80
100
240
250
260
270
280
290

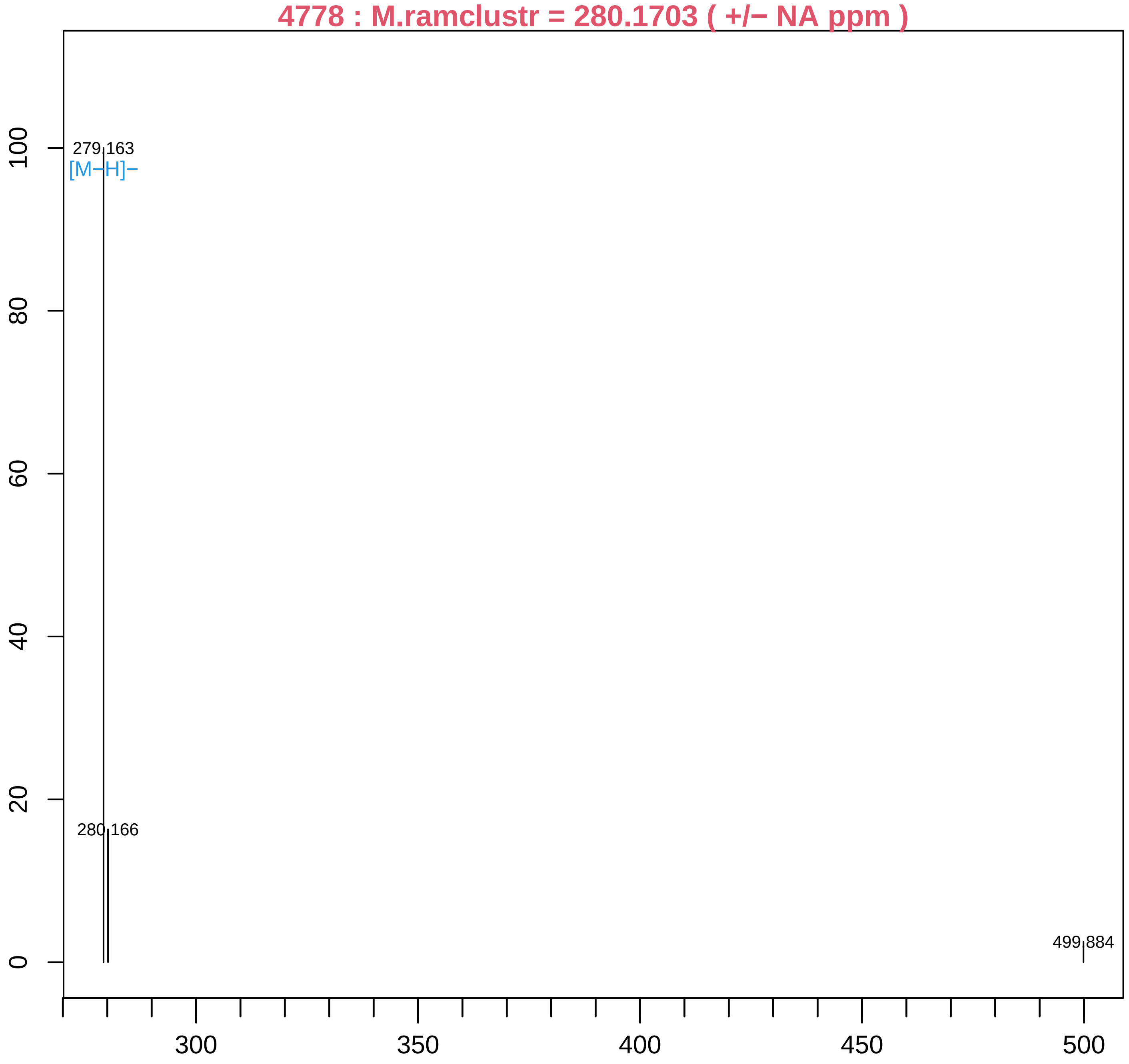
4778 : M.ramclustr = 280.1703 (+/− NA ppm)
279.163
[M−H]−
280.166
499.884
0
20
40
60
80
100
300
350
400
450
500

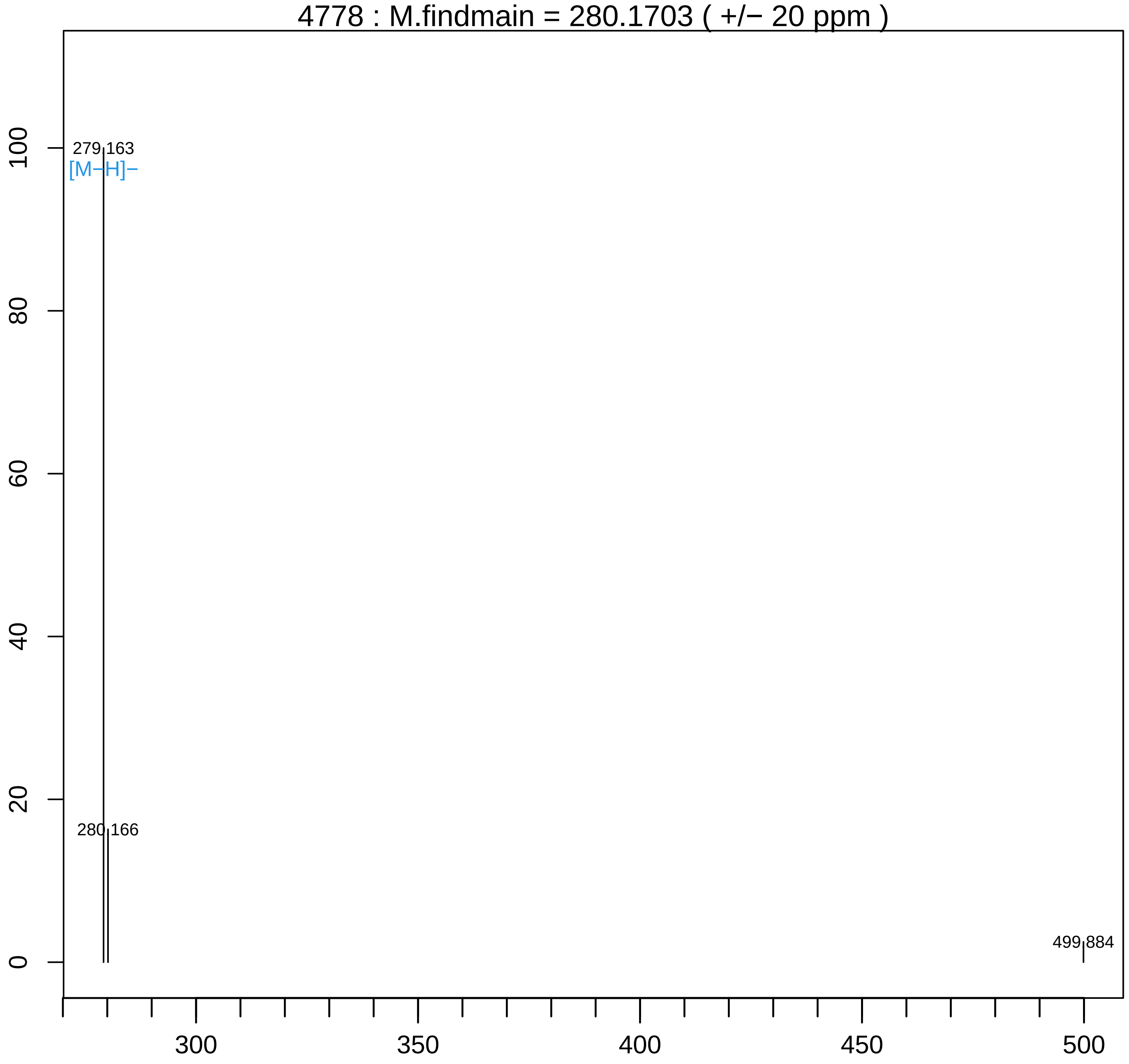
4778 : M.findmain = 280.1703 (+/− 20 ppm)
279.163
[M−H]−
280.166
499.884
0
20
40
60
80
100
300
350
400
450
500

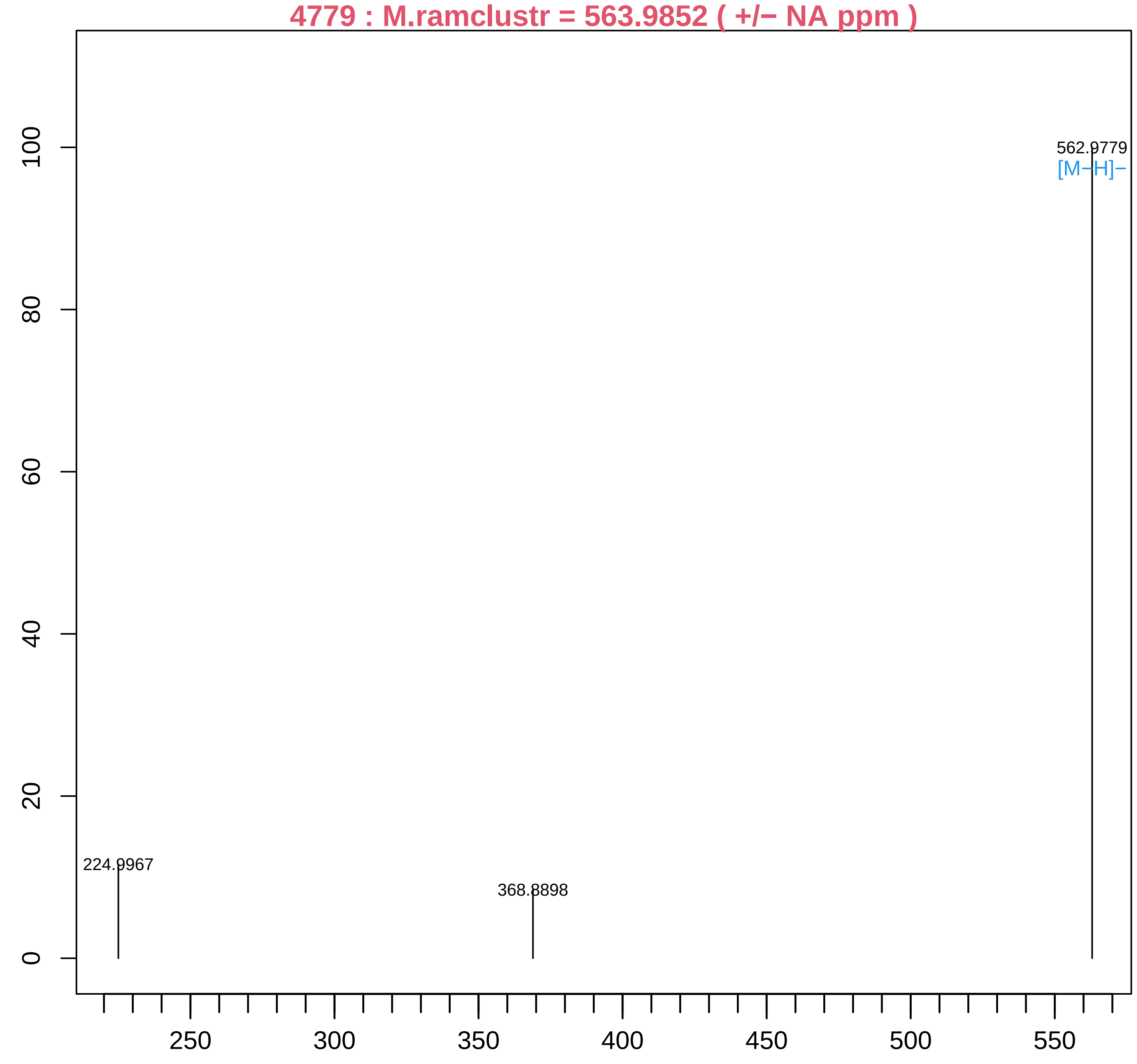
4779 : M.ramclustr = 563.9852 (+/− NA ppm)
562.9779
[M−H]−
224.9967
368.8898
0
20
40
60
80
100
250
300
350
400
450
500
550
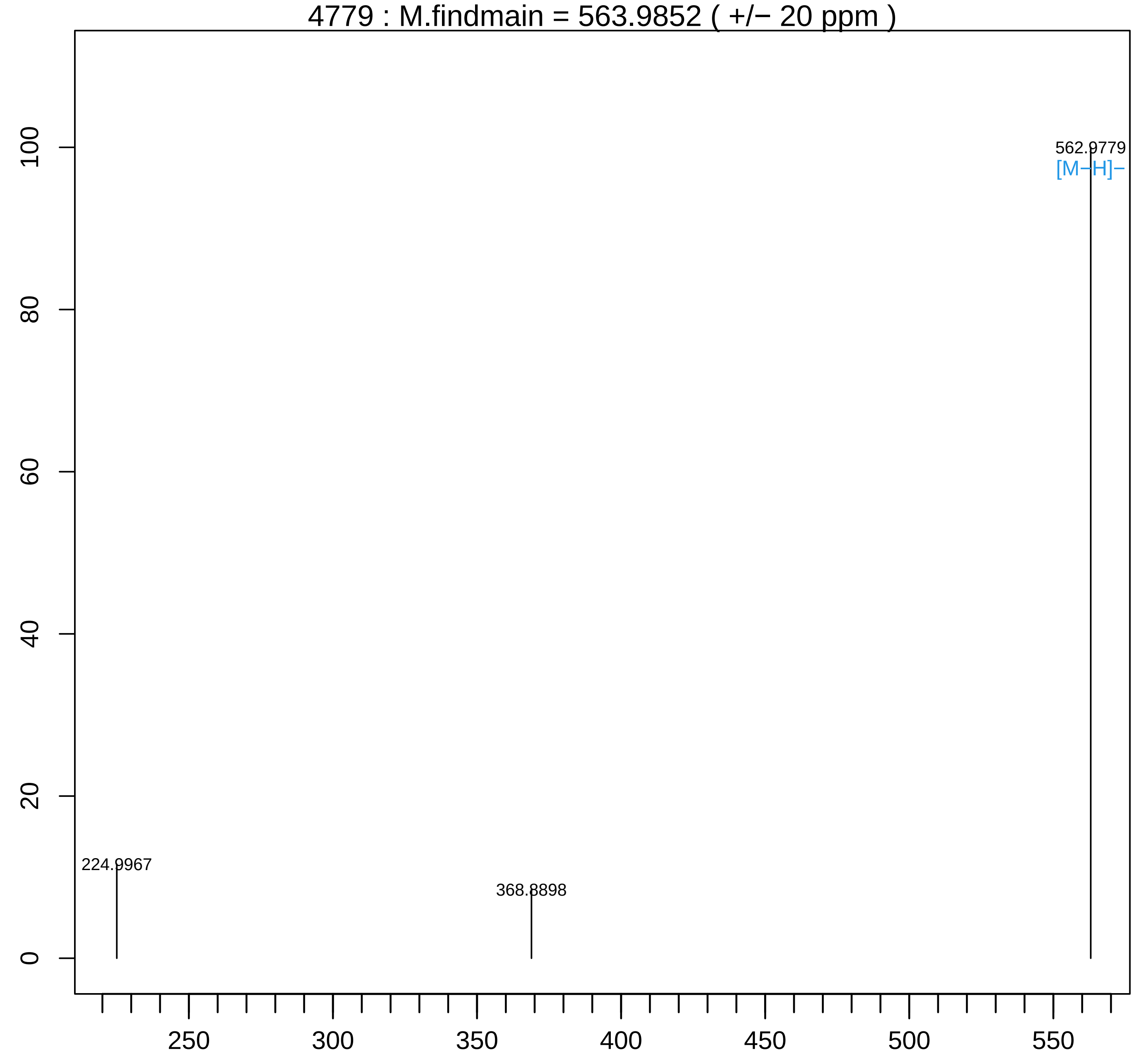
4779 : M.findmain = 563.9852 (+/− 20 ppm)
562.9779
[M−H]−
224.9967
368.8898
0
20
40
60
80
100
250
300
350
400
450
500
550

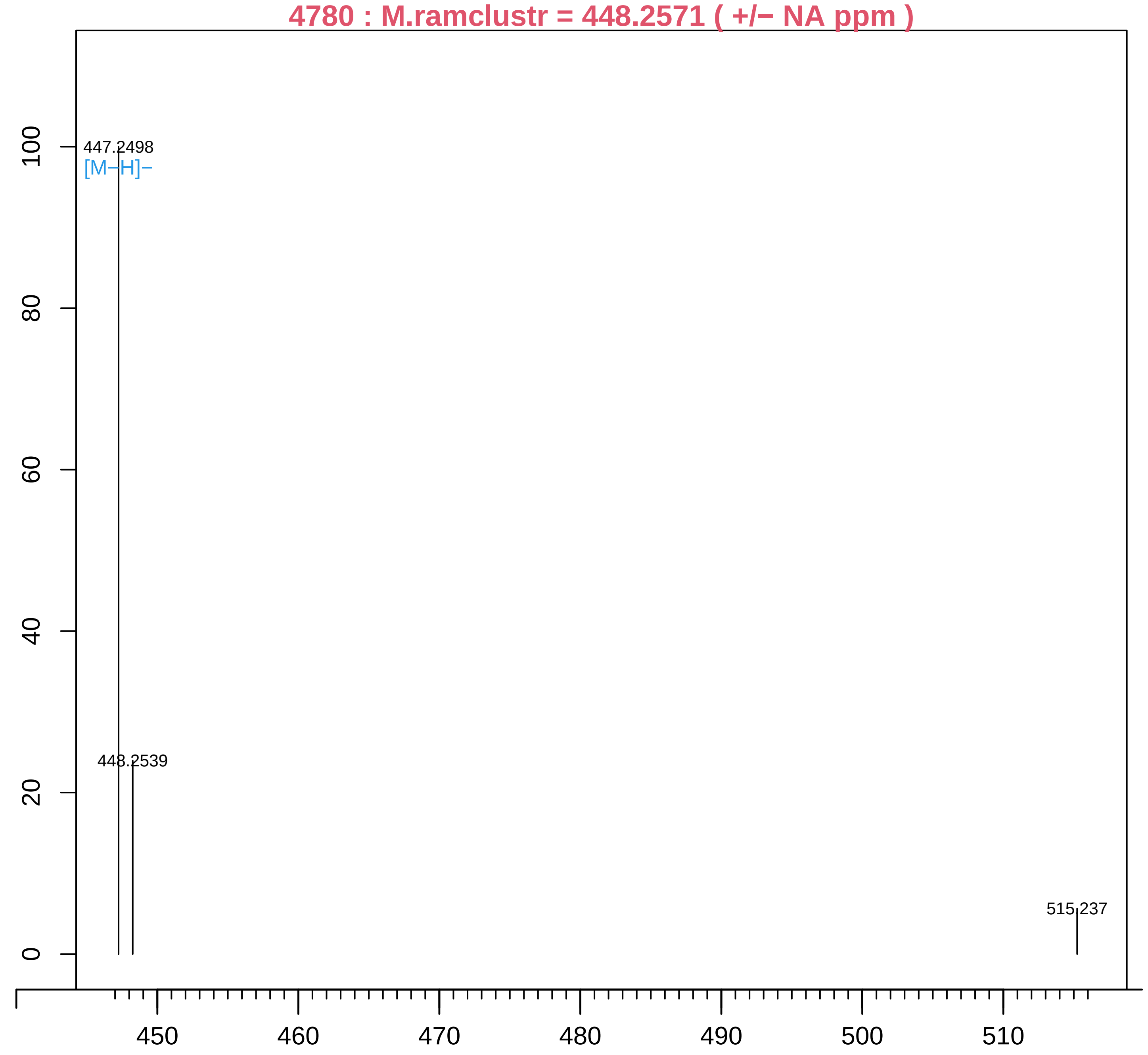

4780 : M.ramclustr = 448.2571 (+/− NA ppm)
447.2498
[M−H]−
448.2539
515.237
0
20
40
60
80
100
450
460
470
480
490
500
510

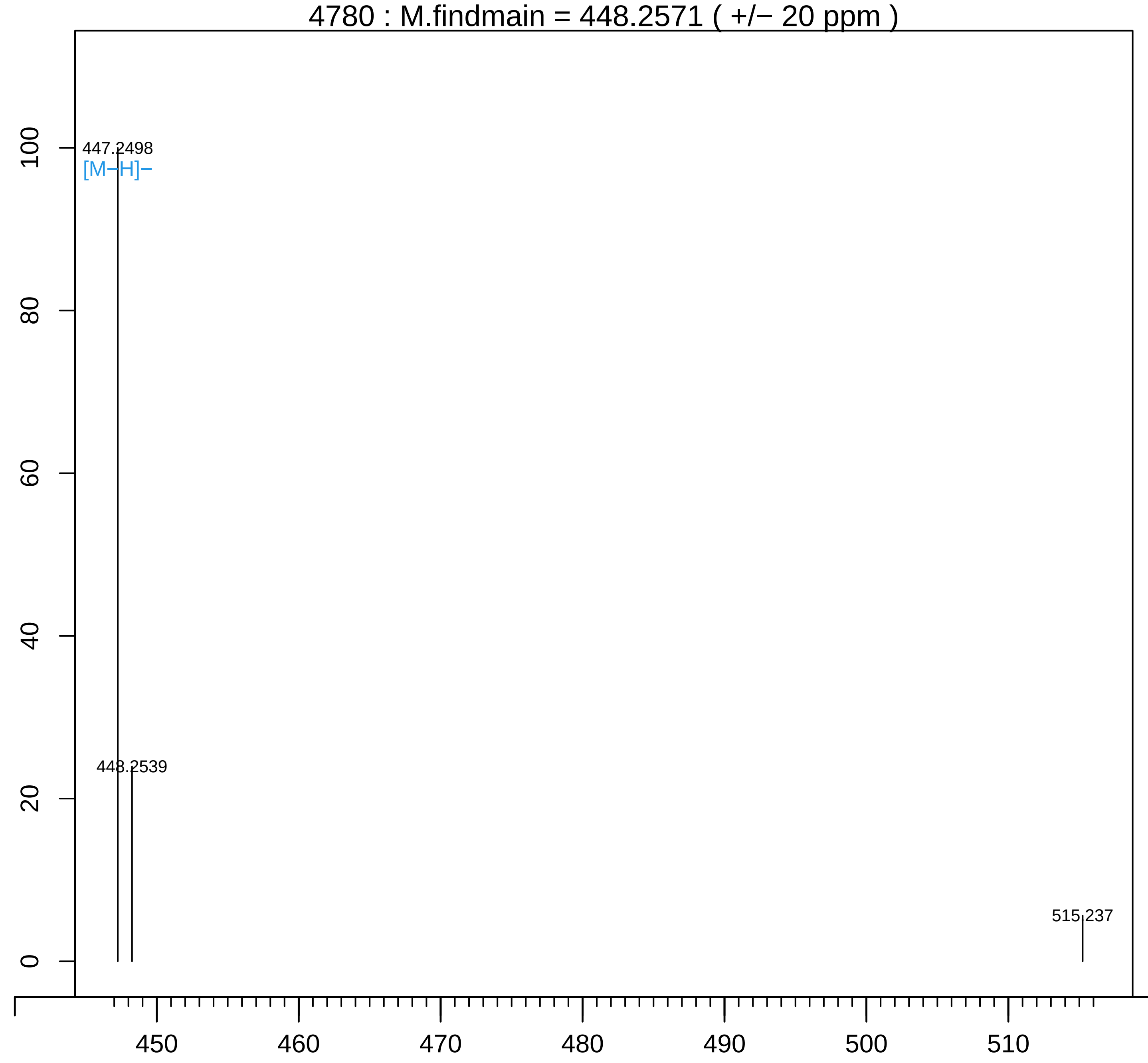

4780 : M.findmain = 448.2571 (+/− 20 ppm)
447.2498
[M−H]−
448.2539
515.237
0
20
40
60
80
100
450
460
470
480
490
500
510

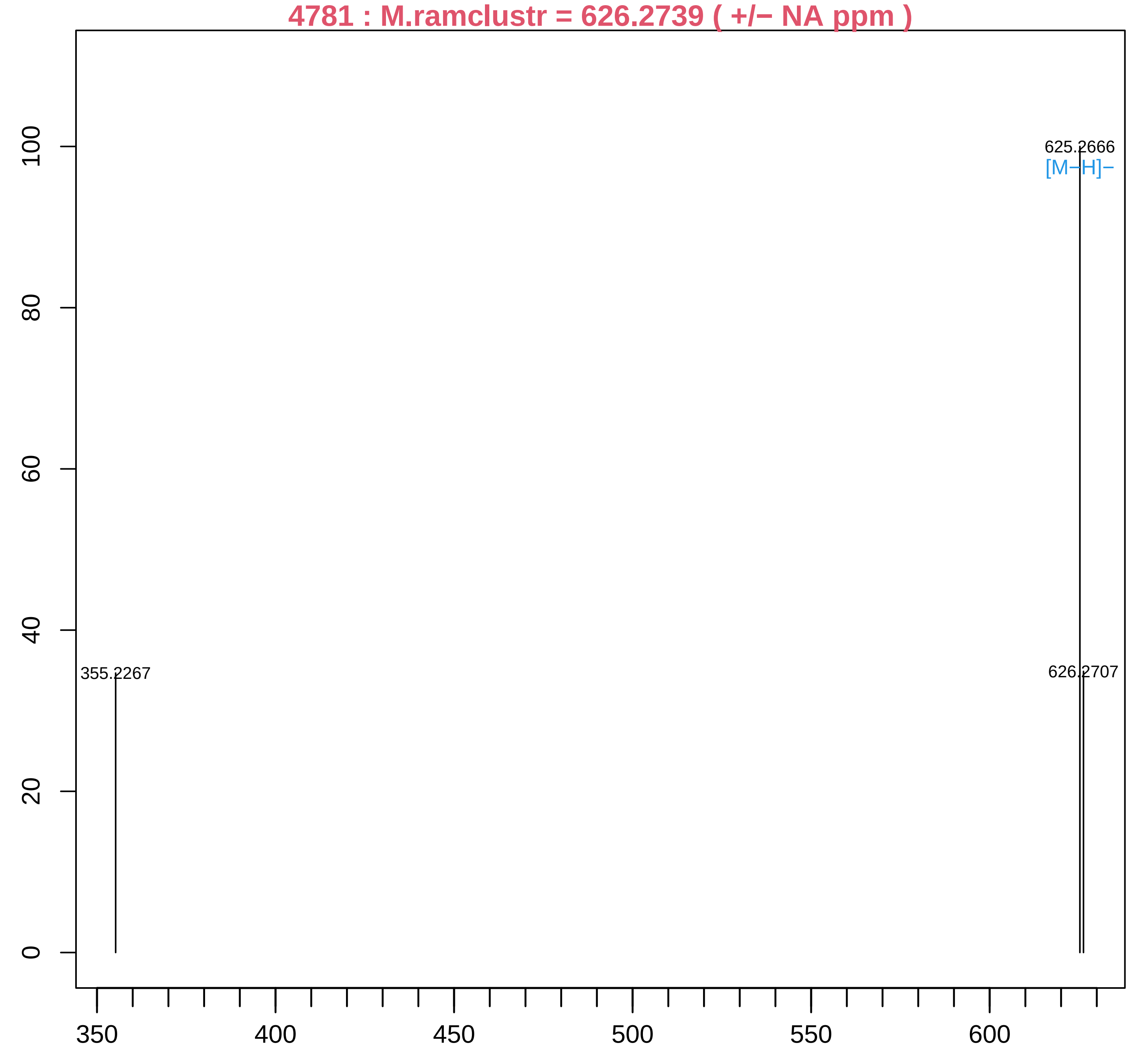
4781 : M.ramclustr = 626.2739 (+/− NA ppm)
625.2666
[M−H]−
355.2267
626.2707
0
20
40
60
80
100
350
400
450
500
550
600

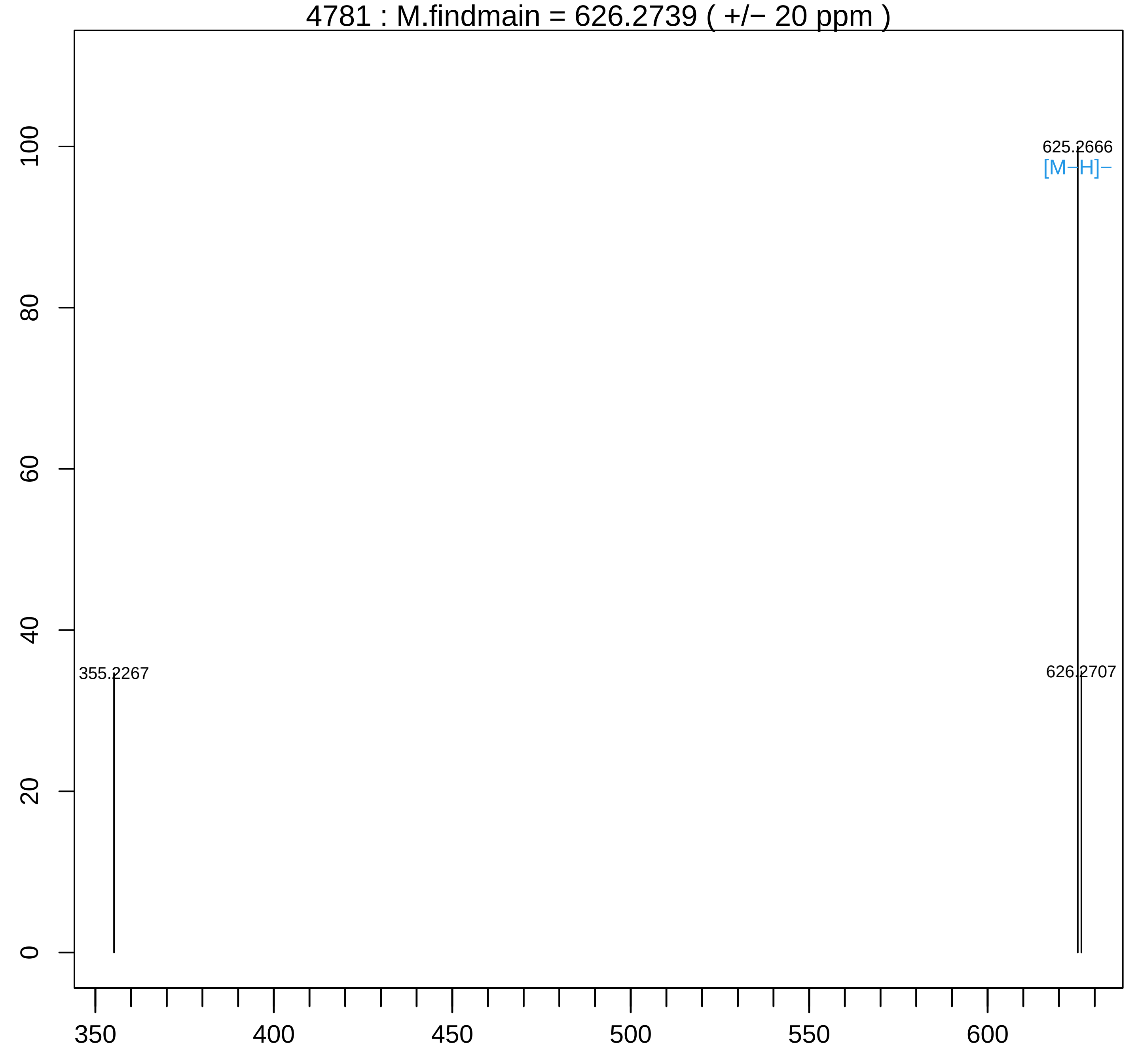
4781 : M.findmain = 626.2739 (+/− 20 ppm)
625.2666
[M−H]−
355.2267
626.2707
0
20
40
60
80
100
350
400
450
500
550
600

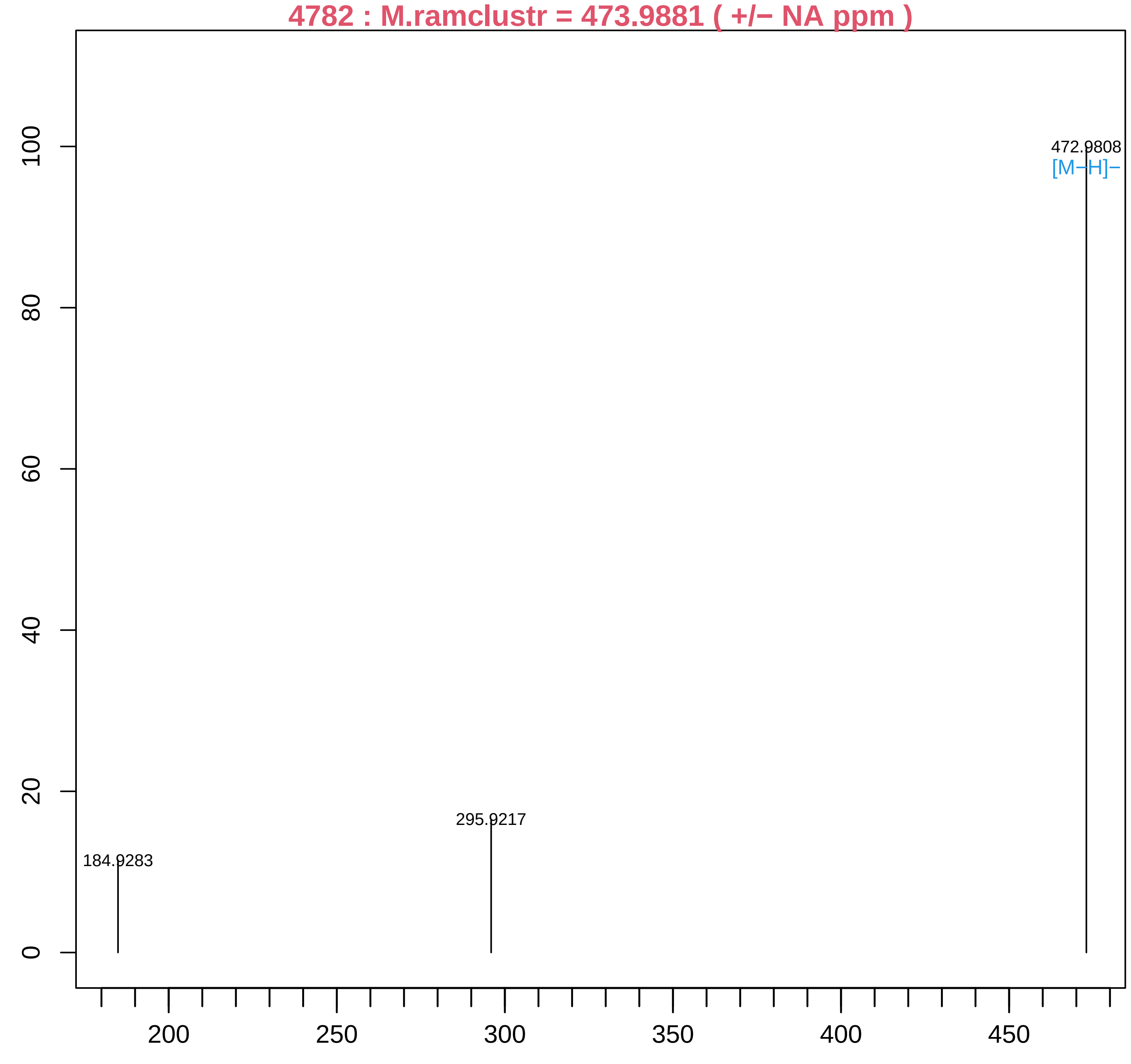
4782 : M.ramclustr = 473.9881 (+/− NA ppm)
472.9808
[M−H]−
295.9217
184.9283
0
20
40
60
80
100
200
250
300
350
400
450

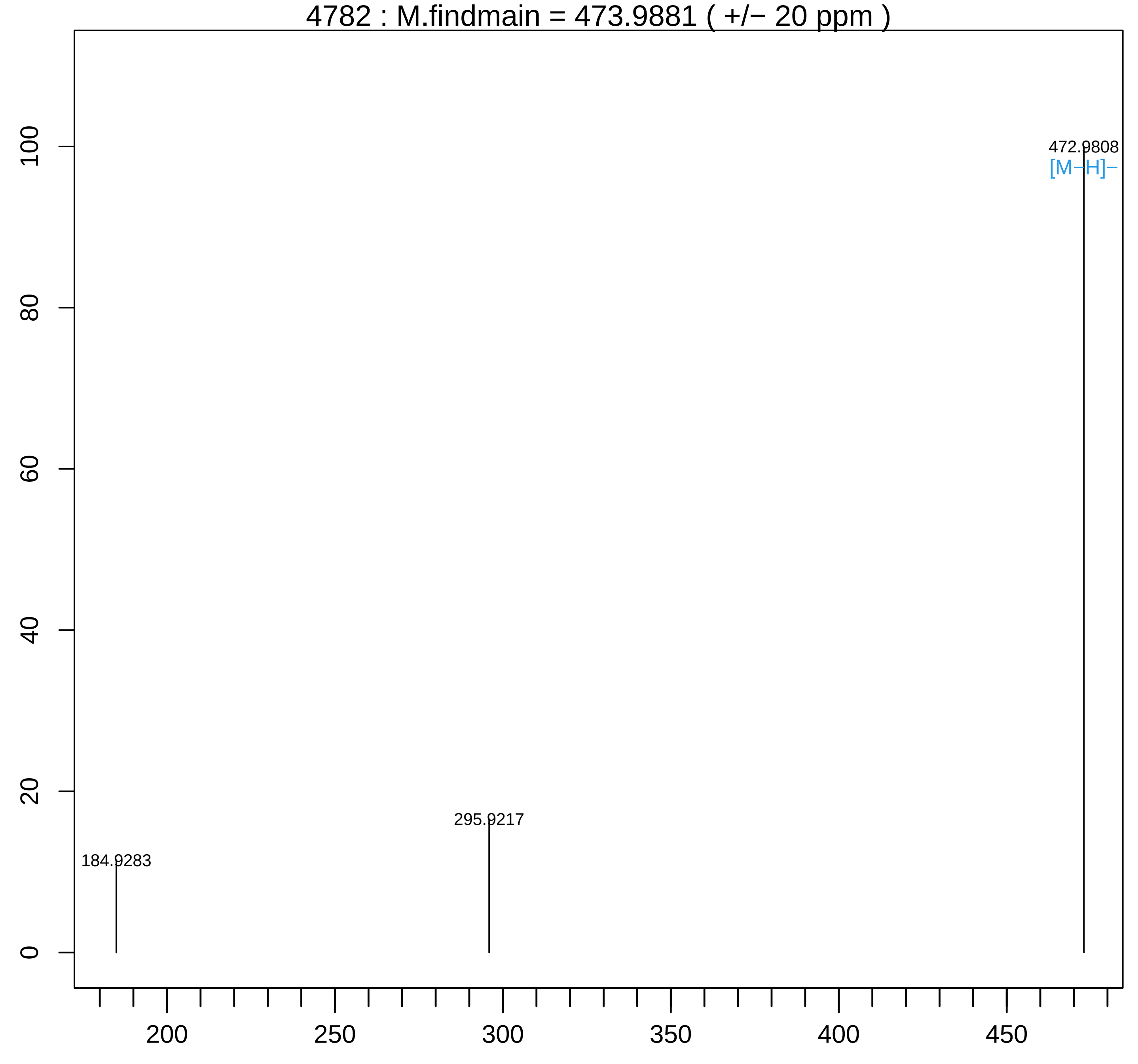
4782 : M.findmain = 473.9881 (+/− 20 ppm)
472.9808
[M−H]−
295.9217
184.9283
0
20
40
60
80
100
200
250
300
350
400
450

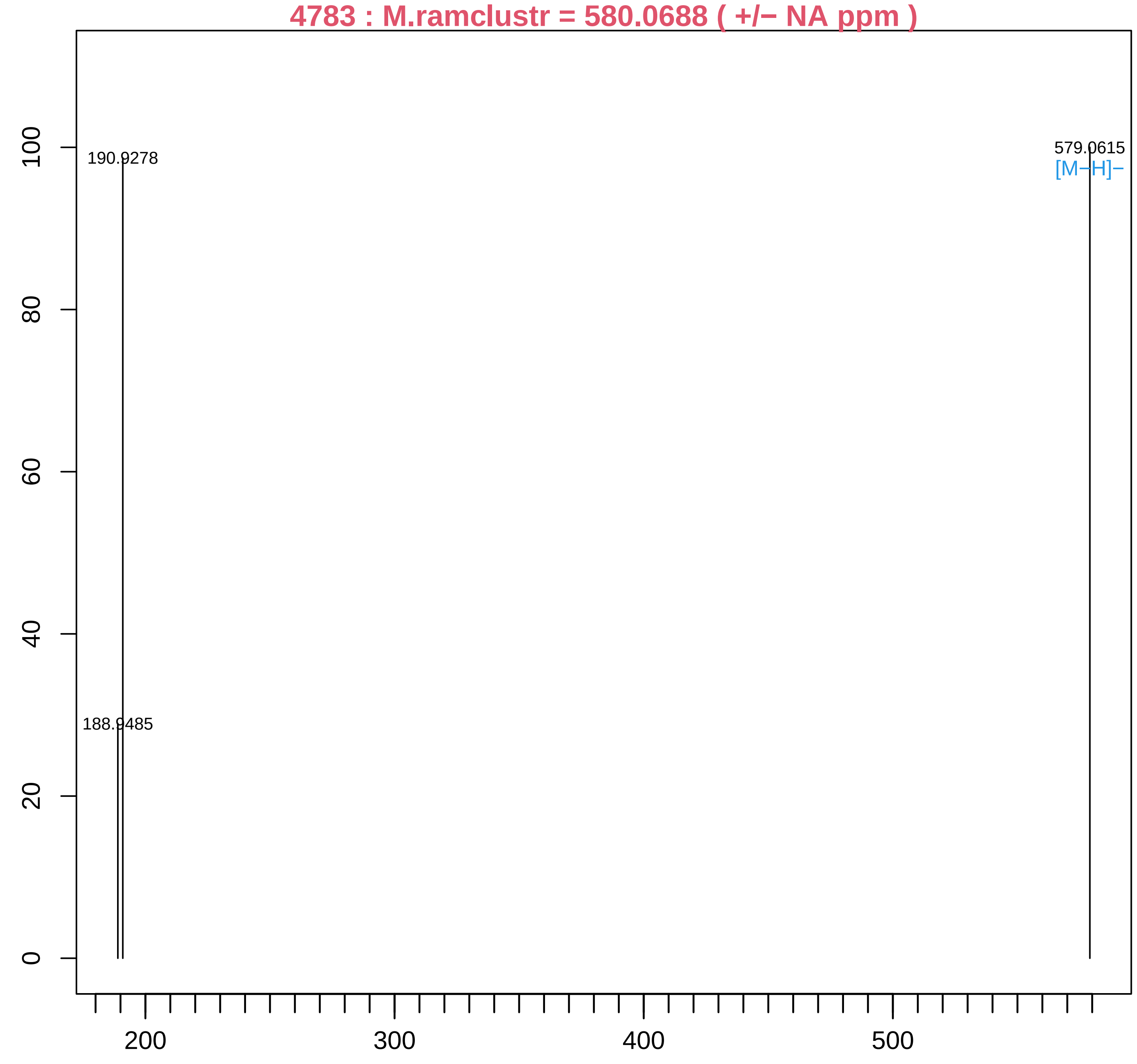
4783 : M.ramclustr = 580.0688 (+/− NA ppm)
190.9278
188.9485
579.0615
[M−H]−
0
20
40
60
80
100
200
300
400
500

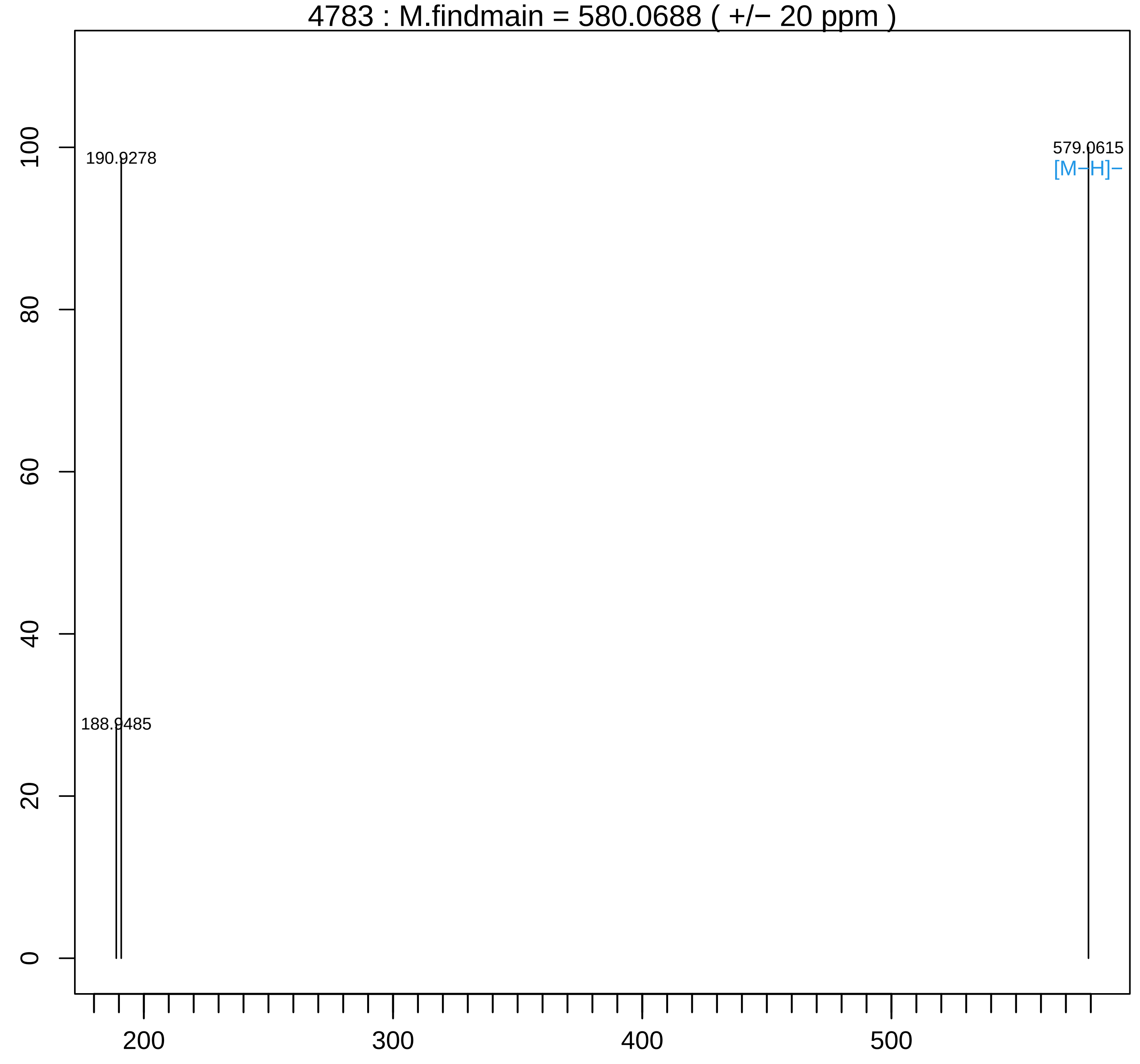
4783 : M.findmain = 580.0688 (+/− 20 ppm)
190.9278
188.9485
579.0615
[M−H]−
0
20
40
60
80
100
200
300
400
500

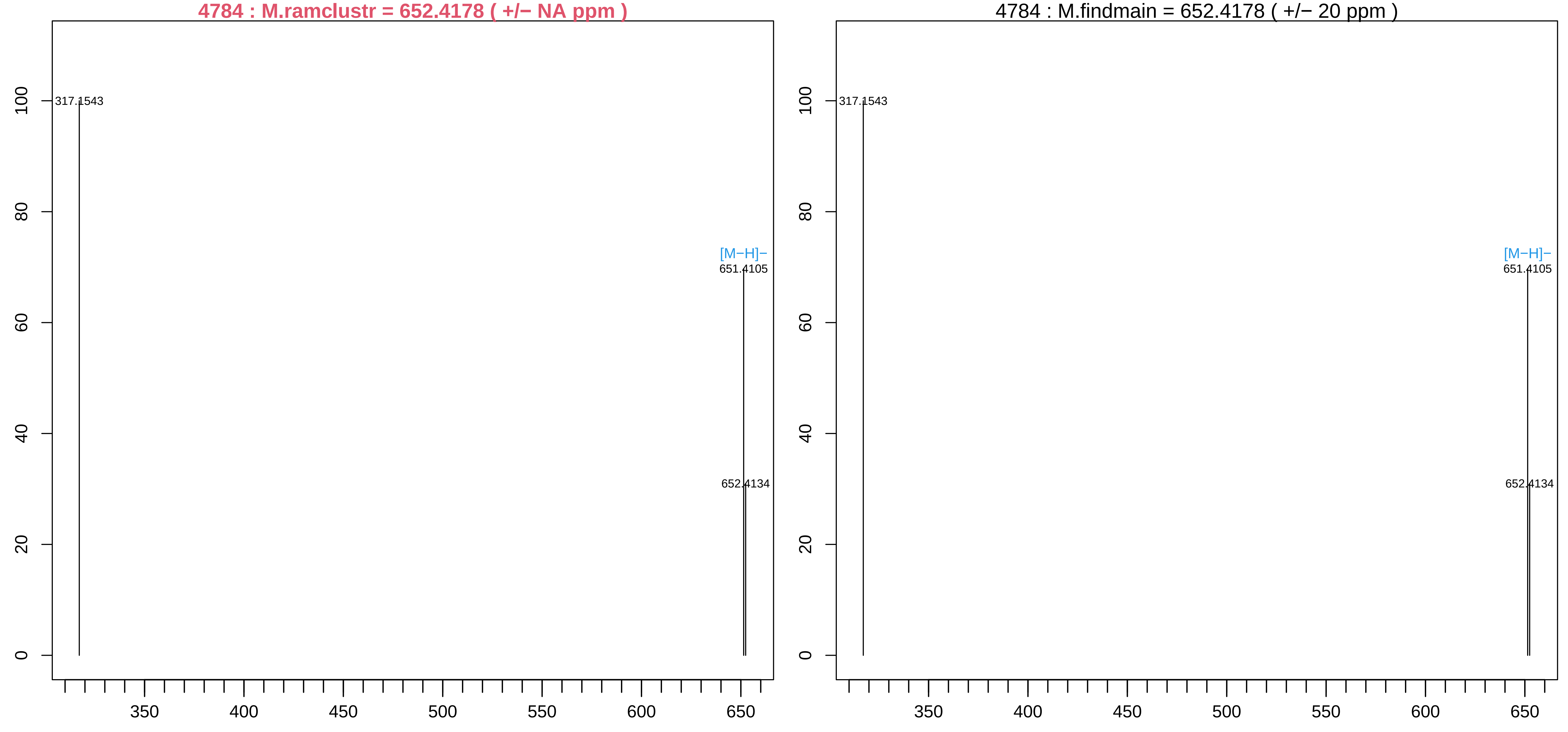

4784 : M.ramclustr = 652.4178 (+/− NA ppm)
317.1543
[M−H]−
651.4105
652.4134
4784 : M.findmain = 652.4178 (+/− 20 ppm)
317.1543
[M−H]−
651.4105
652.4134

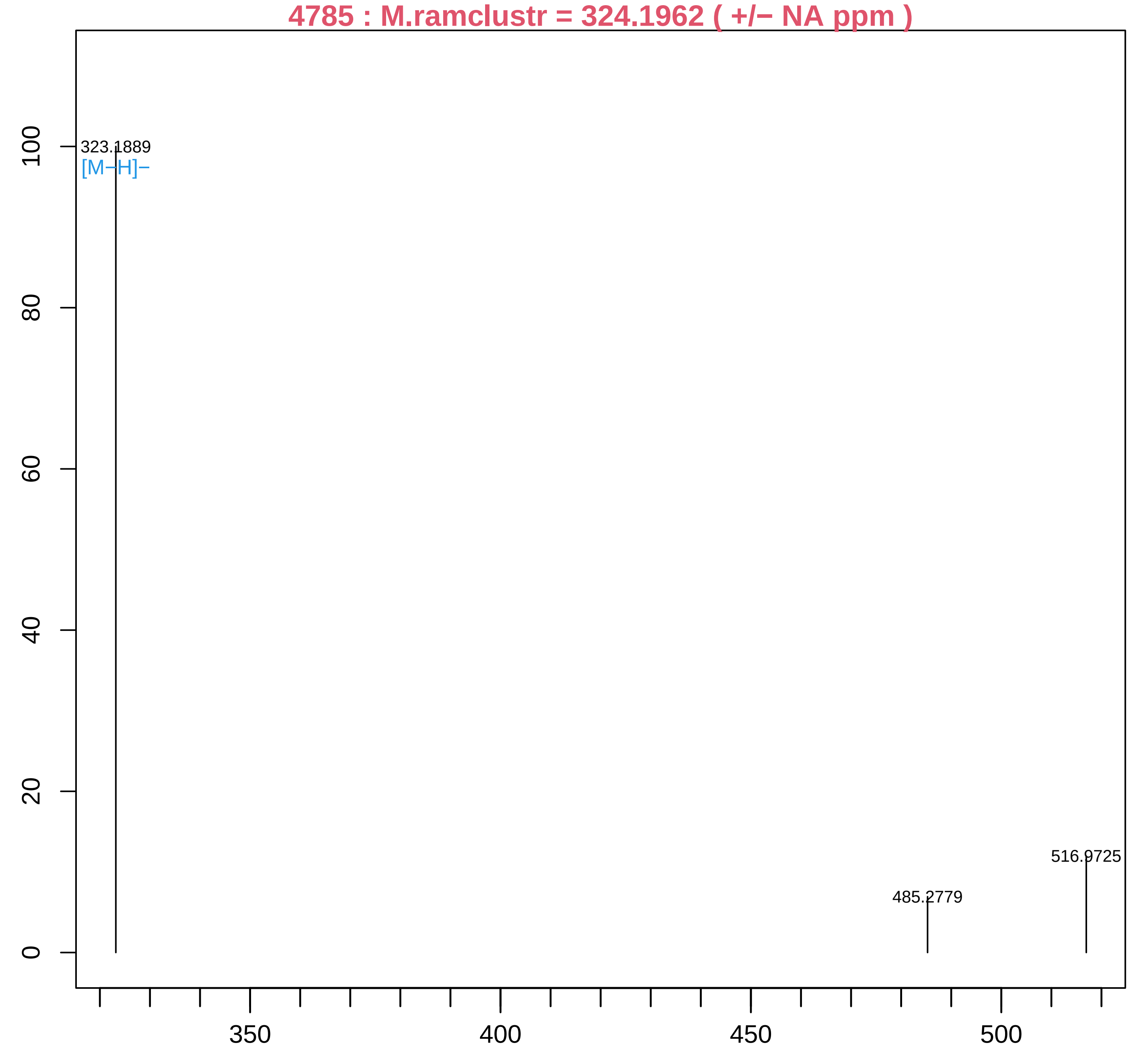
4785 : M.ramclustr = 324.1962 (+/− NA ppm)
323.1889
[M−H]−
485.2779
516.9725
0
20
40
60
80
100
350
400
450
500

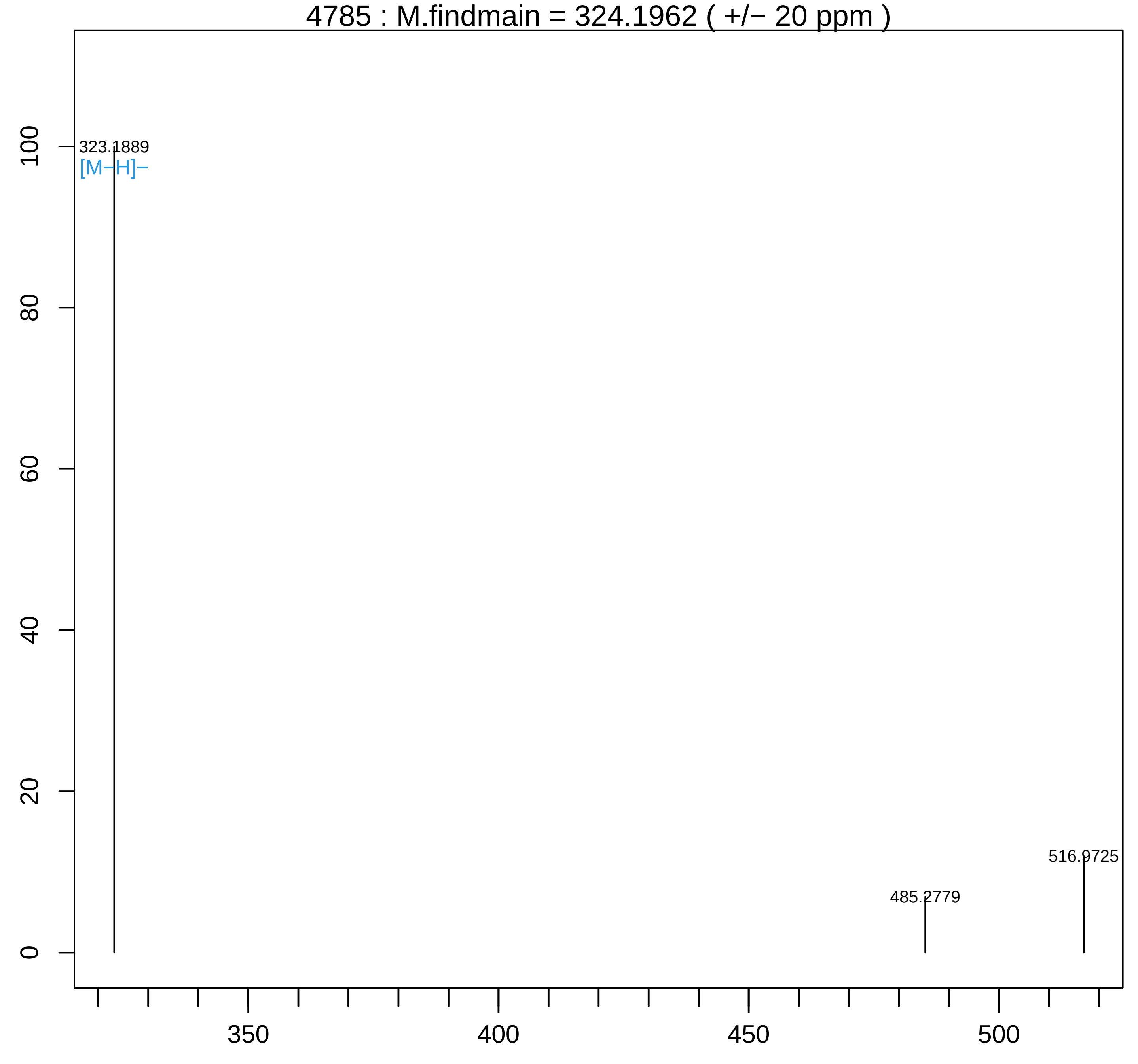
4785 : M.findmain = 324.1962 (+/− 20 ppm)
323.1889
[M−H]−
485.2779
516.9725
0
20
40
60
80
100
350
400
450
500

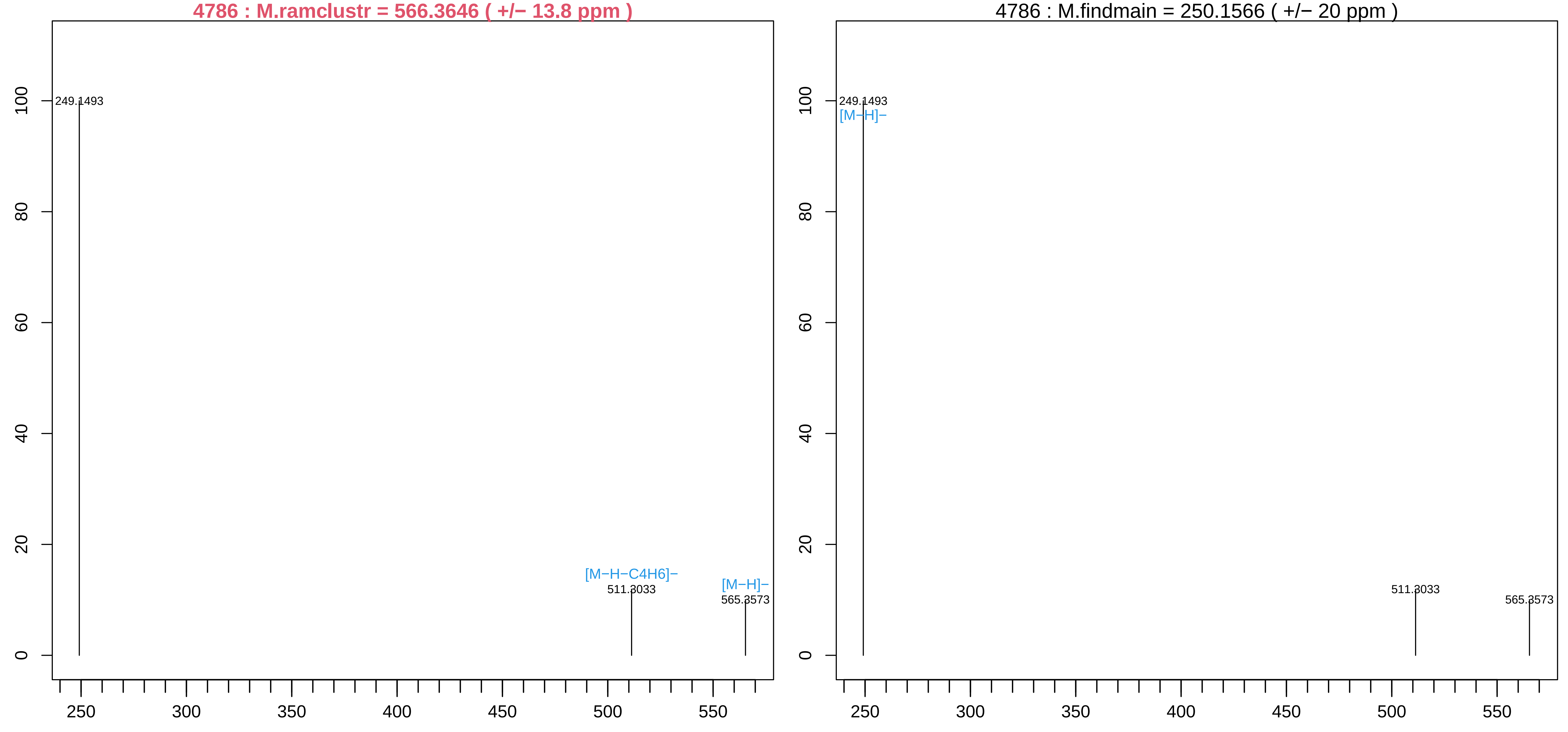

4786 : M.ramclustr = 566.3646 (+/− 13.8 ppm)
249.1493
[M−H−C4H6]−
511.3033
[M−H]−
565.3573
4786 : M.findmain = 250.1566 (+/− 20 ppm)
249.1493
[M−H]−
511.3033
565.3573
0
20
40
60
80
100
250
300
350
400
450
500
550

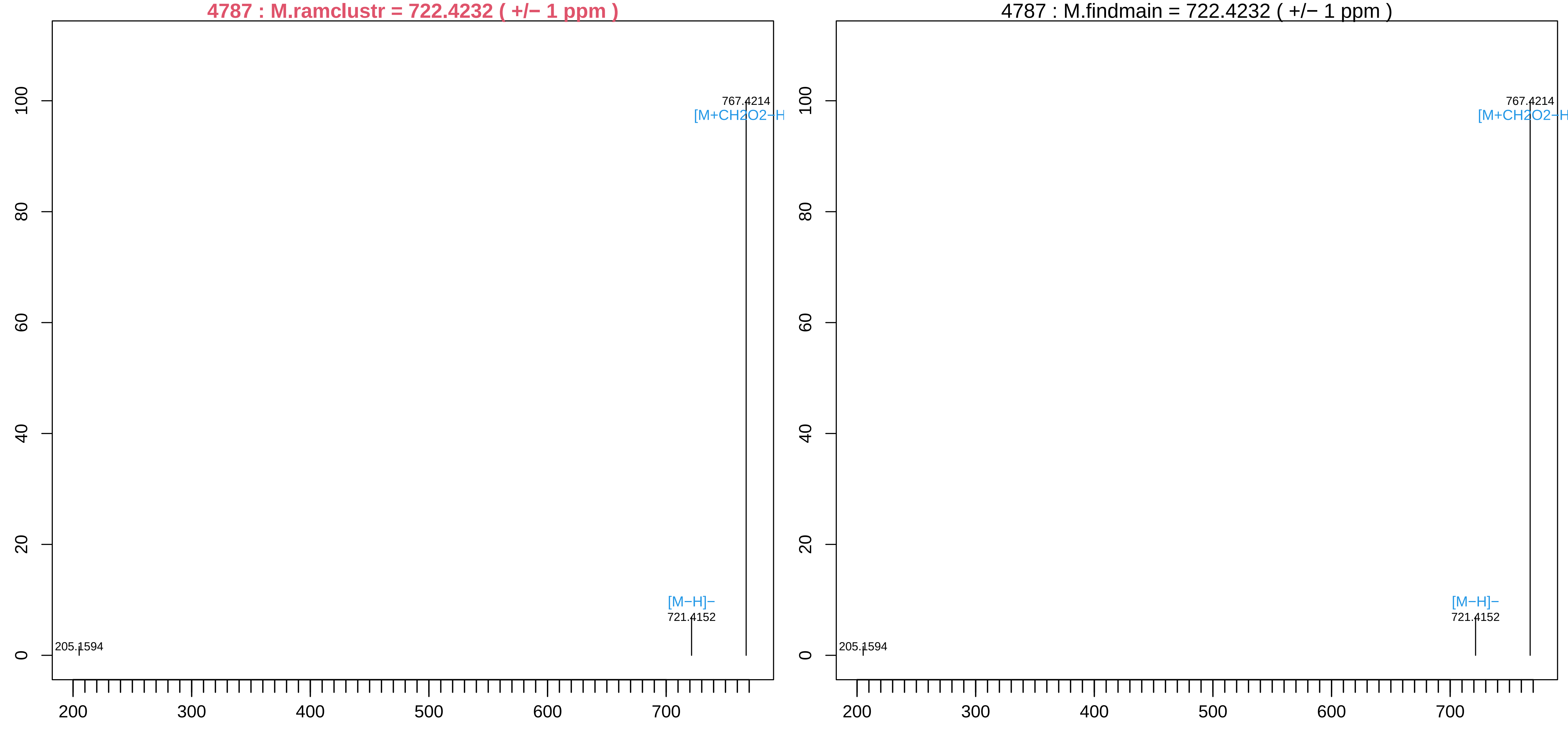
4787 : M.ramclustr = 722.4232 (+/− 1 ppm)
767.4214
[M+CH2O2−H
[M−H]−
721.4152
205.1594
4787 : M.findmain = 722.4232 (+/− 1 ppm)
767.4214
[M+CH2O2−H
[M−H]−
721.4152
205.1594
0
20
40
60
80
100
200
300
400
500
600
700

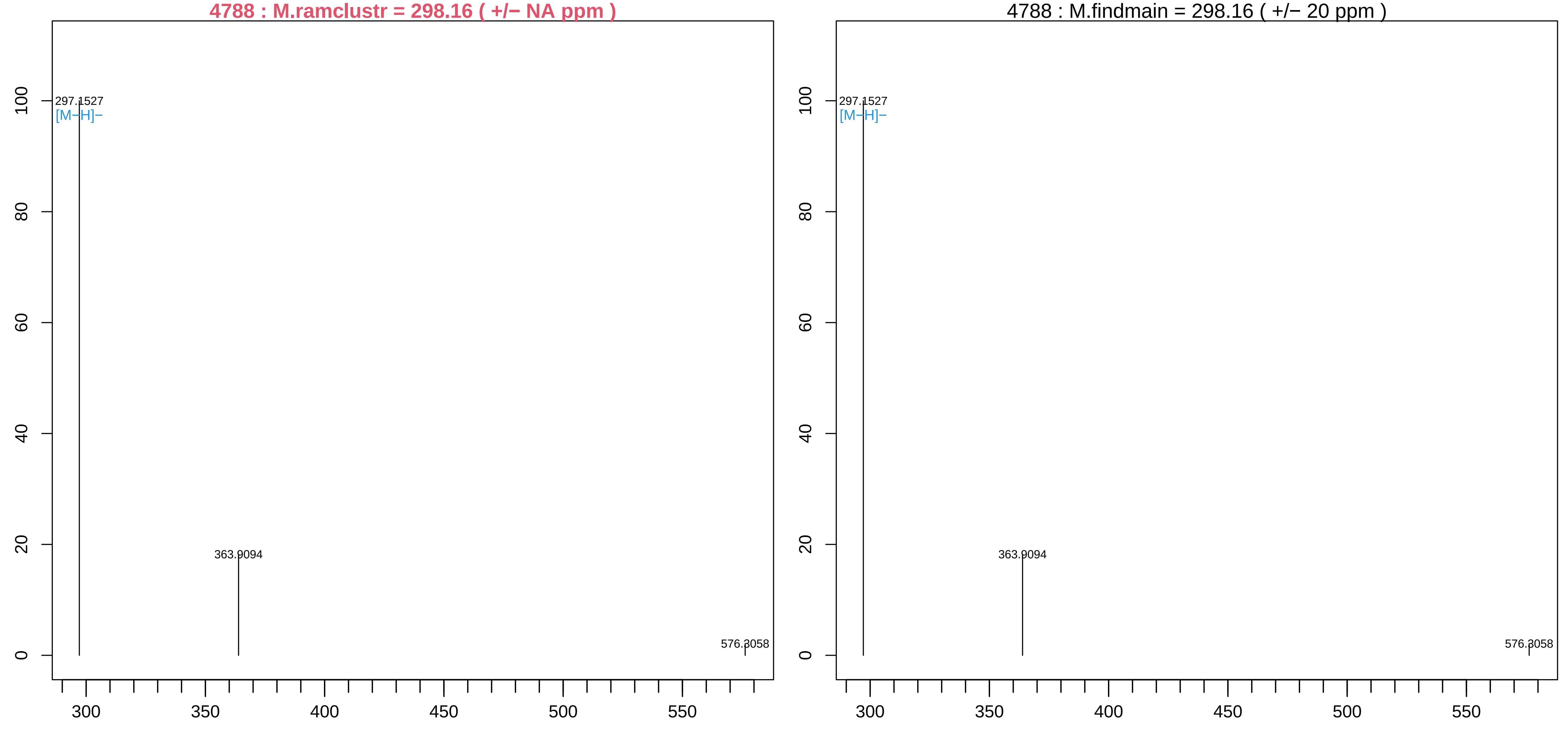
4788 : M.ramclustr = 298.16 (+/− NA ppm)
297.1527
[M−H]−
363.9094
576.3058
0
20
40
60
80
100
300
350
400
450
500
550
4788 : M.findmain = 298.16 (+/− 20 ppm)
297.1527
[M−H]−
363.9094
576.3058
0
20
40
60
80
100
300
350
400
450
500
550

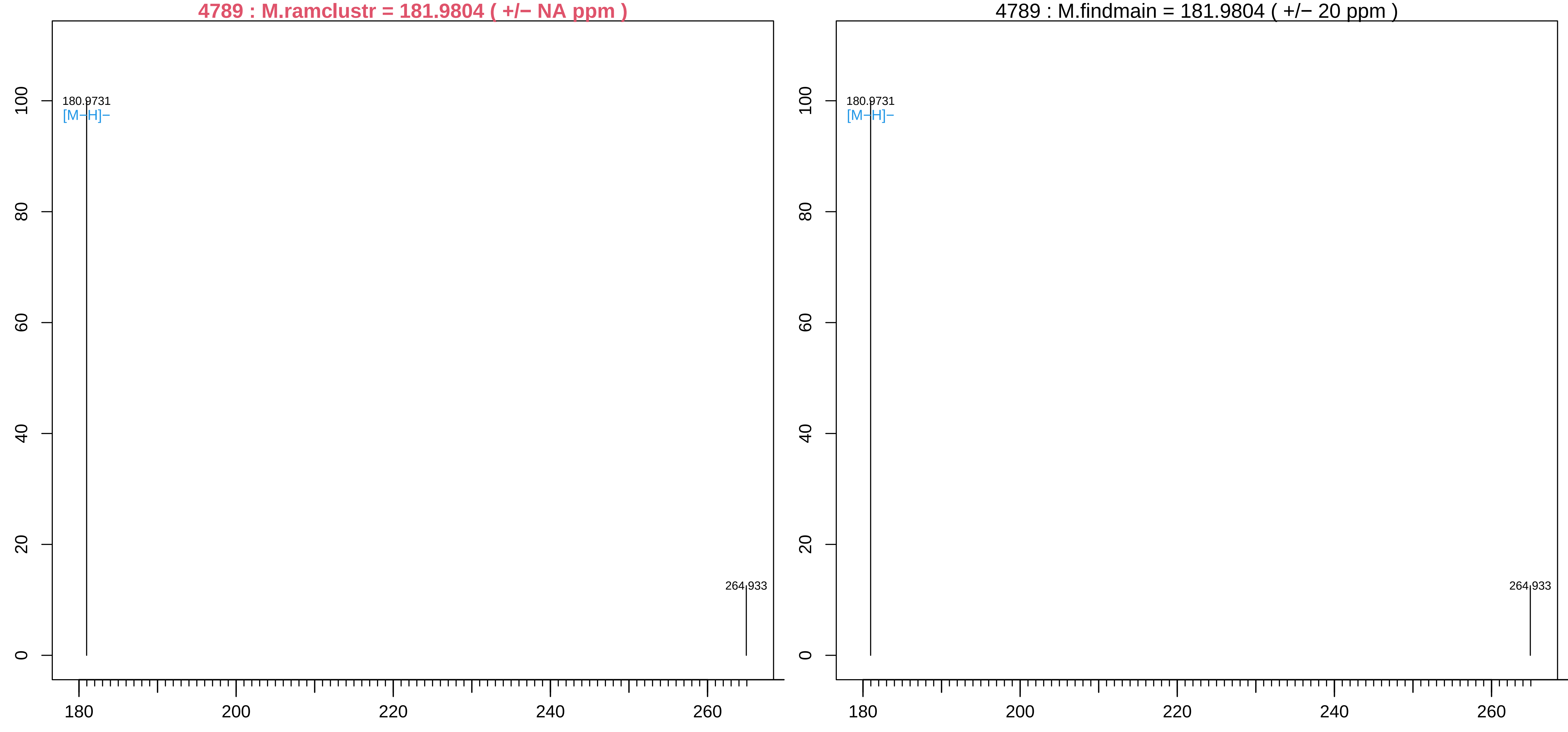

4789 : M.ramclustr = 181.9804 (+/− NA ppm)
180.9731
[M−H]−
264.933
0
20
40
60
80
100
180
200
220
240
260
4789 : M.findmain = 181.9804 (+/− 20 ppm)
180.9731
[M−H]−
264.933
0
20
40
60
80
100
180
200
220
240
260

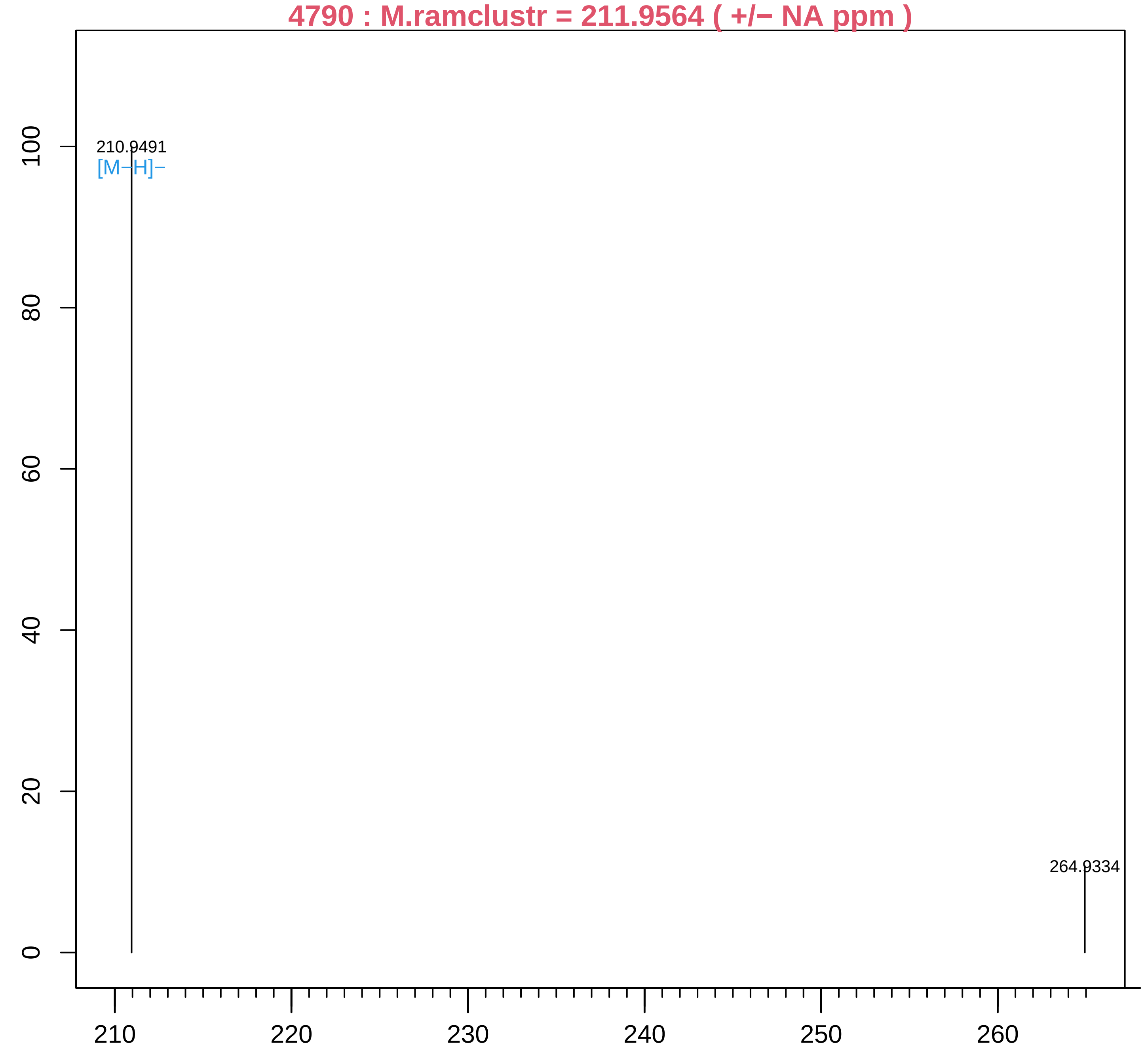
4790 : M.ramclustr = 211.9564 (+/− NA ppm)
210.9491
[M−H]−
264.9334
0
20
40
60
80
100
210
220
230
240
250
260
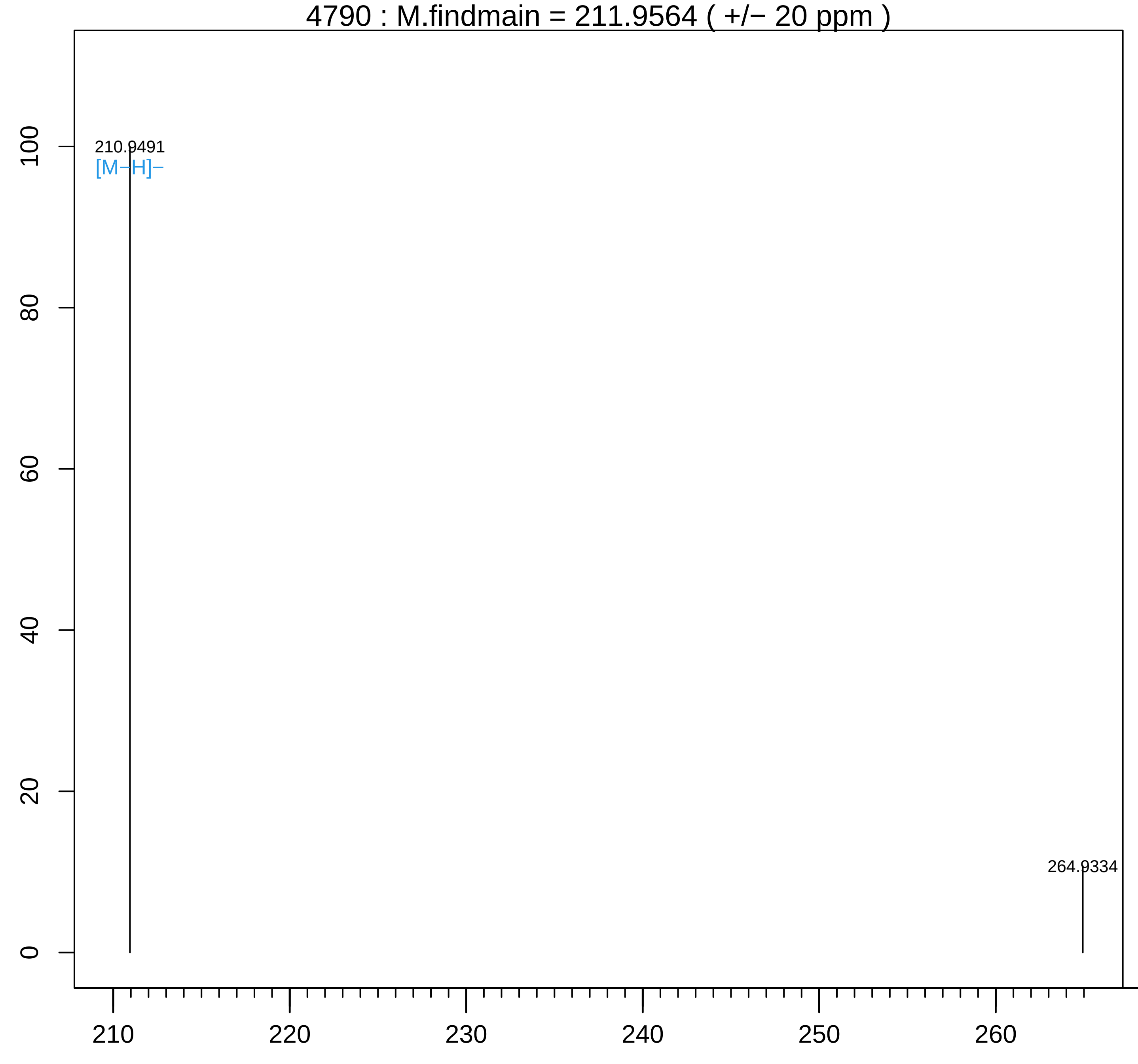
4790 : M.findmain = 211.9564 (+/− 20 ppm)
210.9491
[M−H]−
264.9334
0
20
40
60
80
100
210
220
230
240
250
260

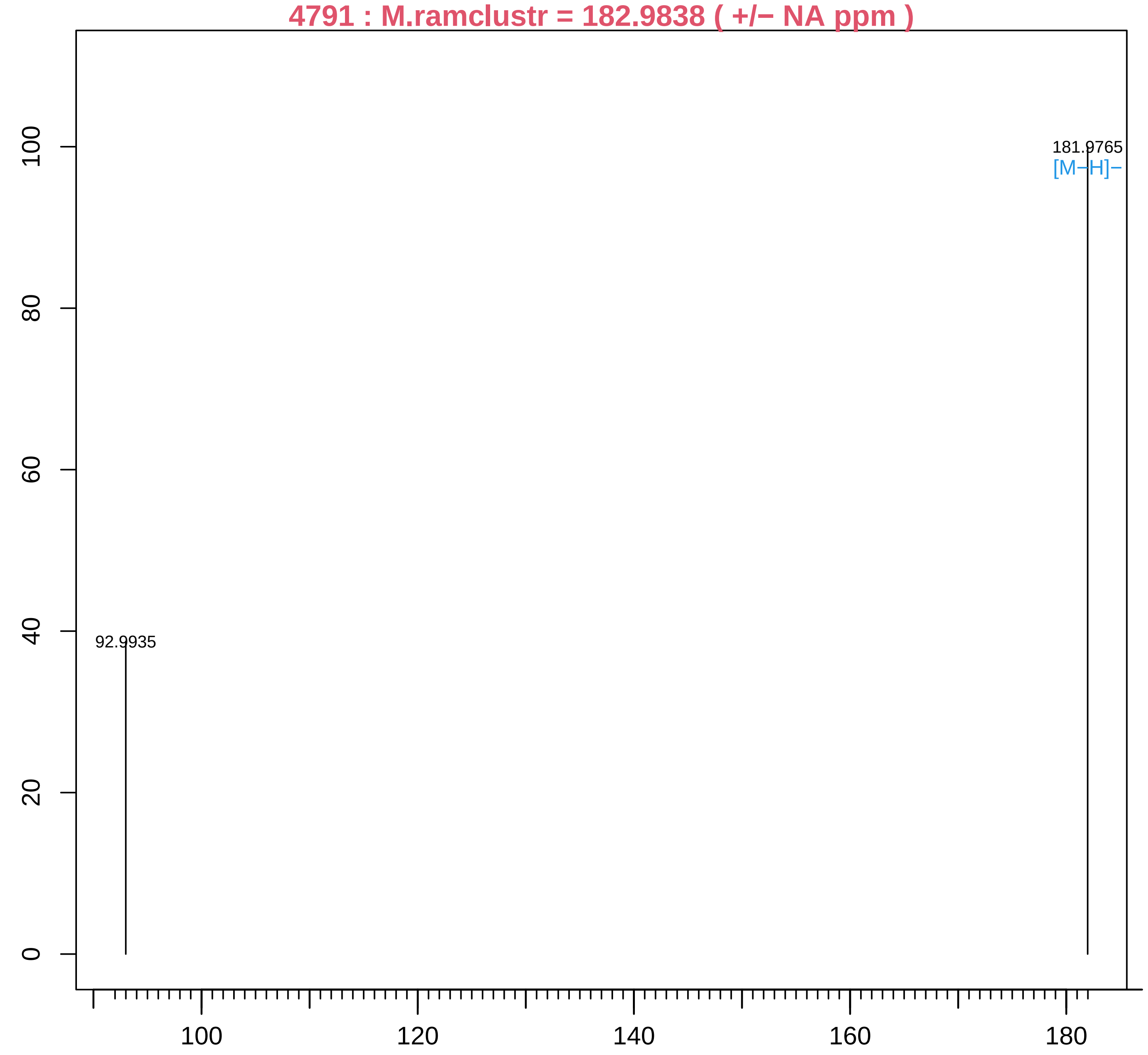

4791 : M.ramclustr = 182.9838 (+/− NA ppm)
181.9765
[M−H]−
92.9935
0
20
40
60
80
100
100
120
140
160
180

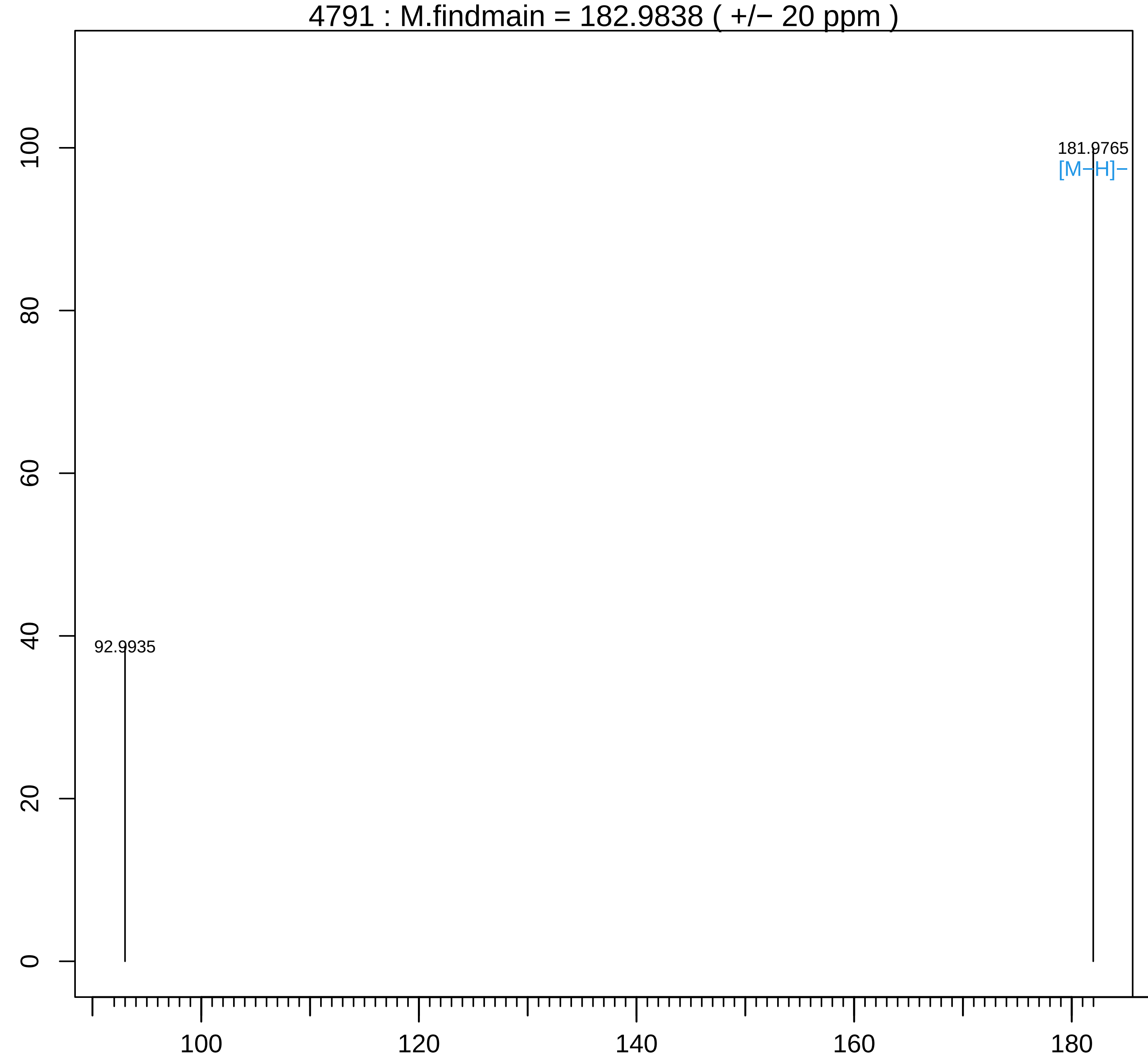

4791 : M.findmain = 182.9838 (+/− 20 ppm)
181.9765
[M−H]−
92.9935
0
20
40
60
80
100
100
120
140
160
180

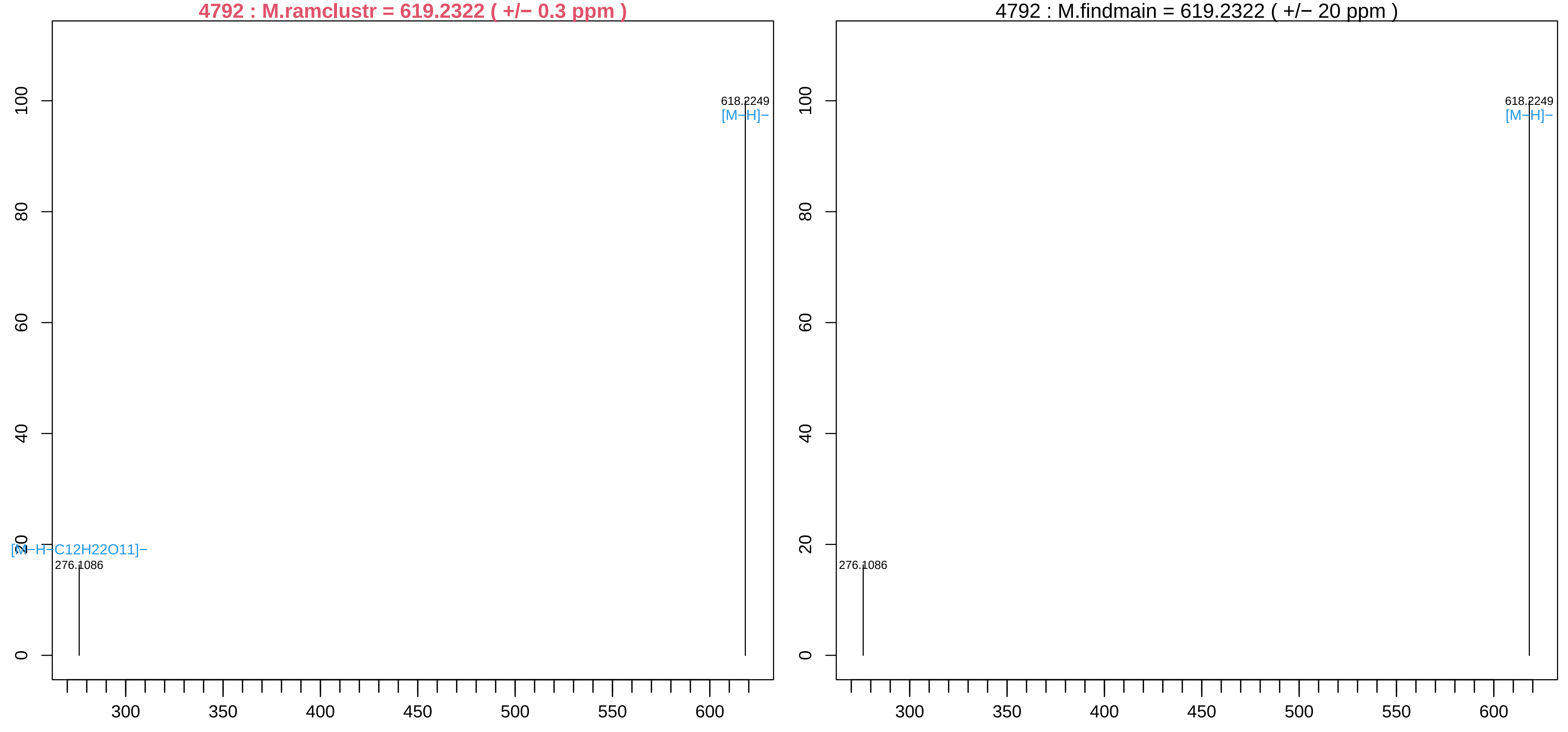
4792 : M.ramclustr = 619.2322 (+/− 0.3 ppm)
618.2249
[M−H]−
[M−H−C12H22O11]−
276.1086
4792 : M.findmain = 619.2322 (+/− 20 ppm)
618.2249
[M−H]−
276.1086
0
20
40
60
80
100
300
350
400
450
500
550
600

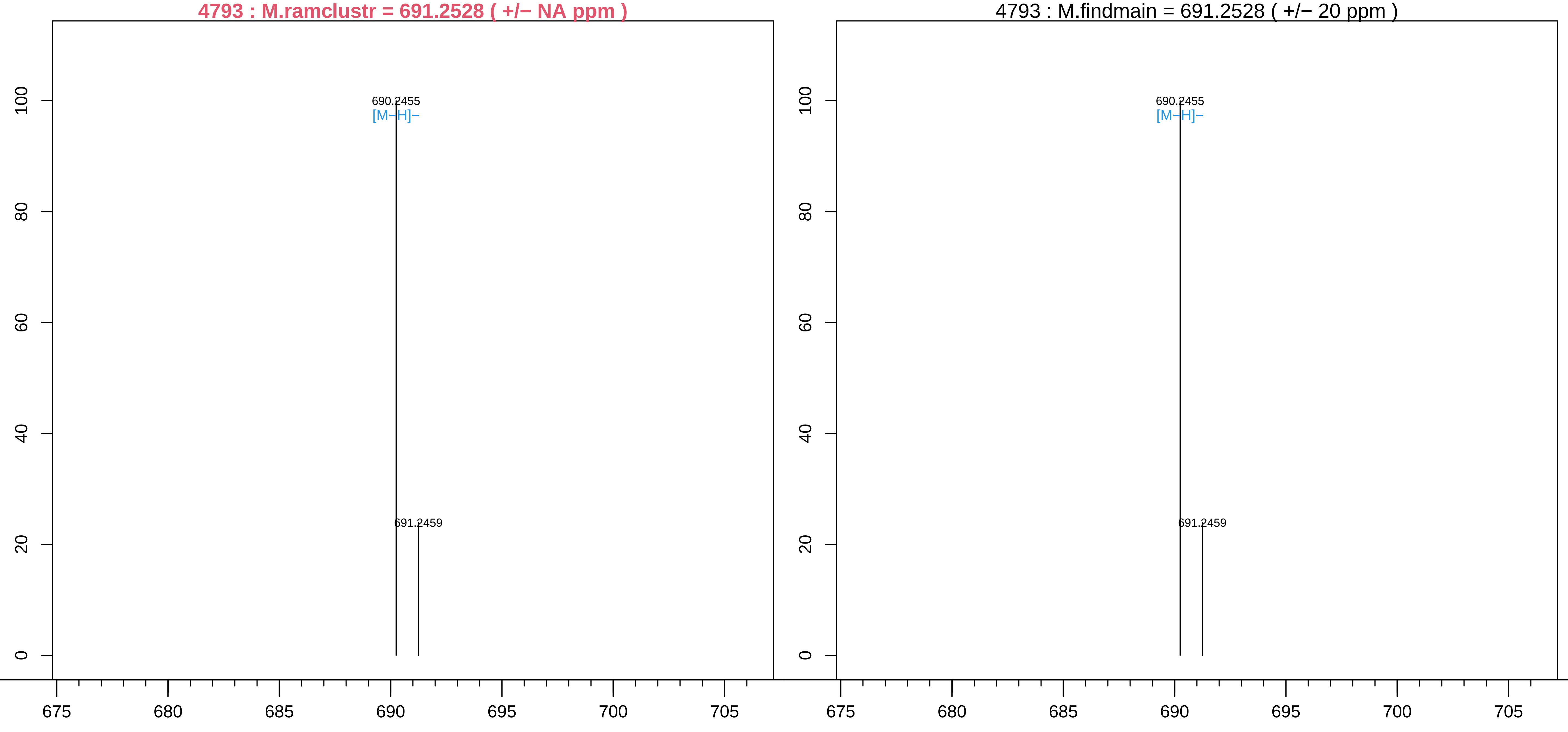
4793 : M.ramclustr = 691.2528 (+/− NA ppm)
690.2455
[M−H]−
691.2459
4793 : M.findmain = 691.2528 (+/− 20 ppm)
690.2455
[M−H]−
691.2459
0
20
40
60
80
100
675
680
685
690
695
700
705

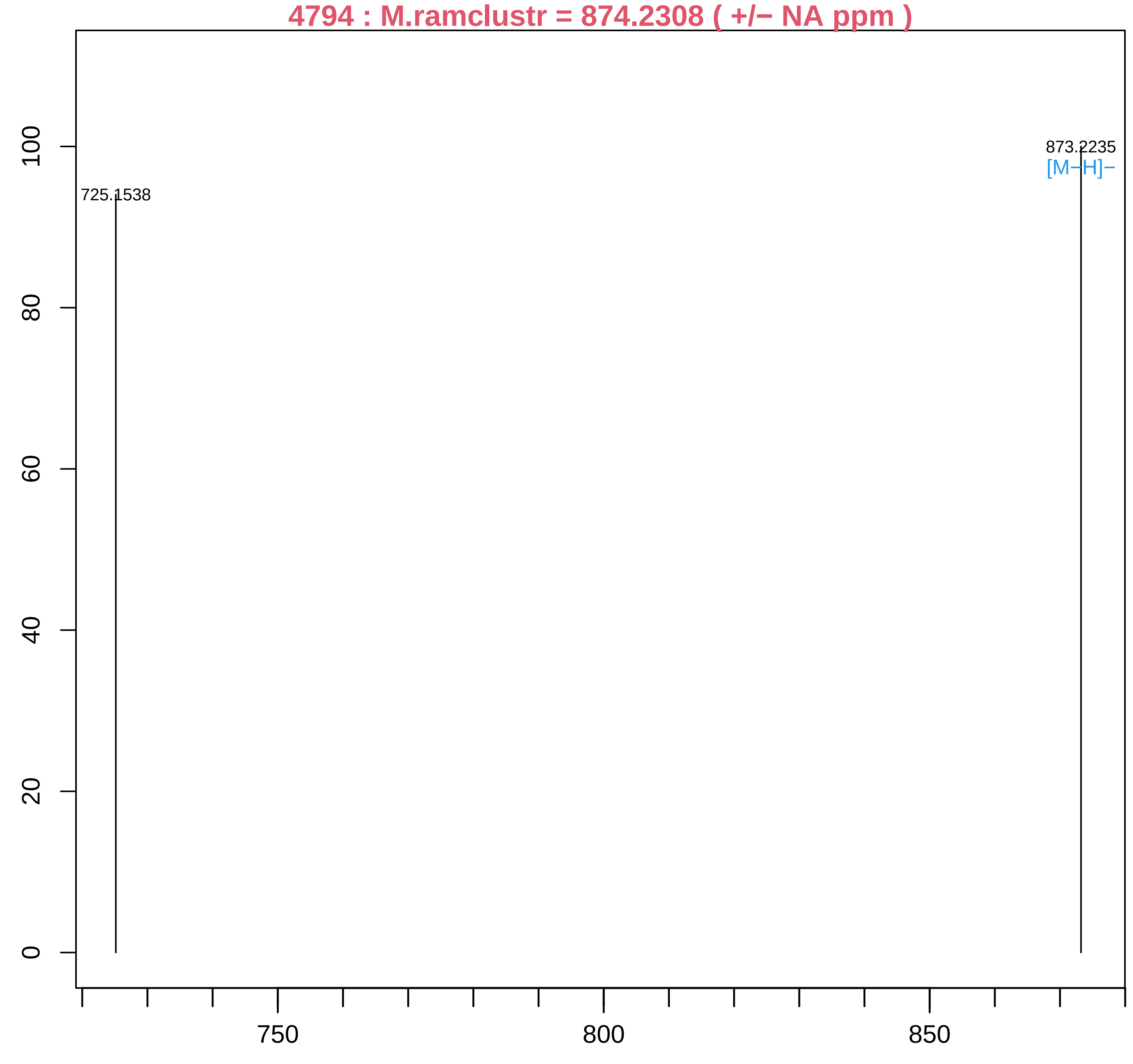
4794 : M.ramclustr = 874.2308 (+/− NA ppm)
873.2235
[M−H]−
725.1538
0
20
40
60
80
100
750
800
850

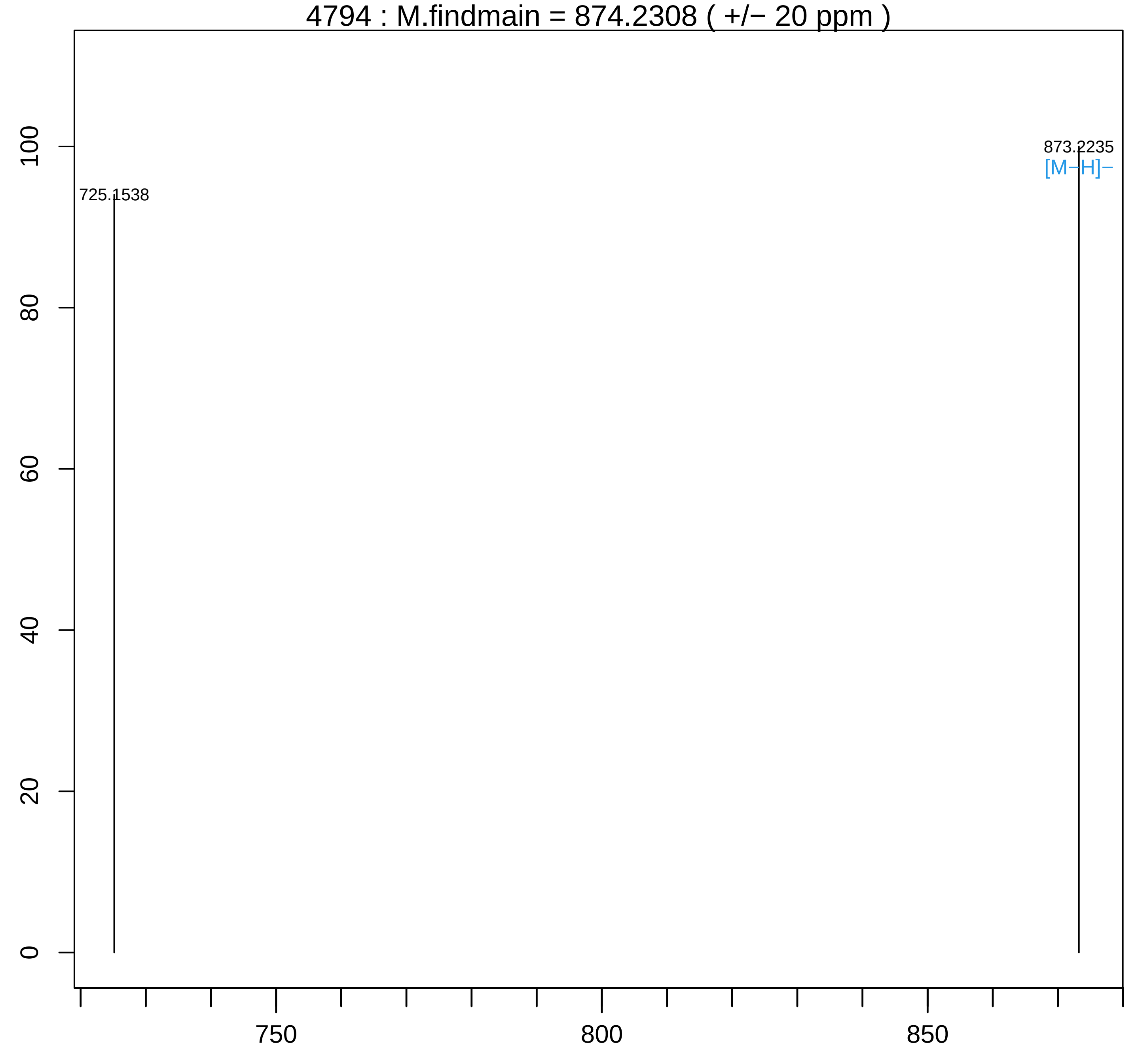
4794 : M.findmain = 874.2308 (+/− 20 ppm)
873.2235
[M−H]−
725.1538
0
20
40
60
80
100
750
800
850

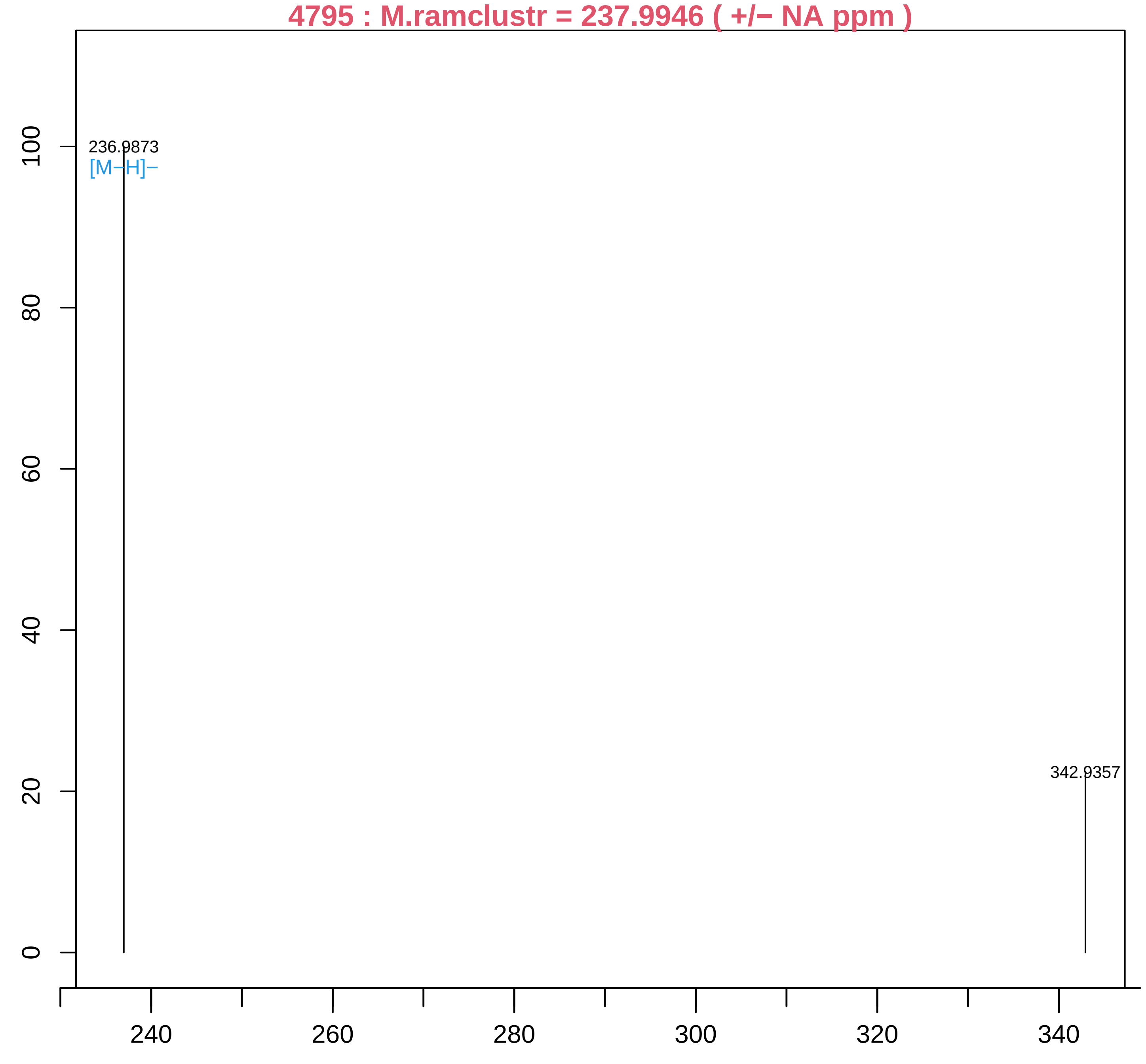
4795 : M.ramclustr = 237.9946 (+/− NA ppm)
236.9873
[M−H]−
342.9357
0
20
40
60
80
100
240
260
280
300
320
340

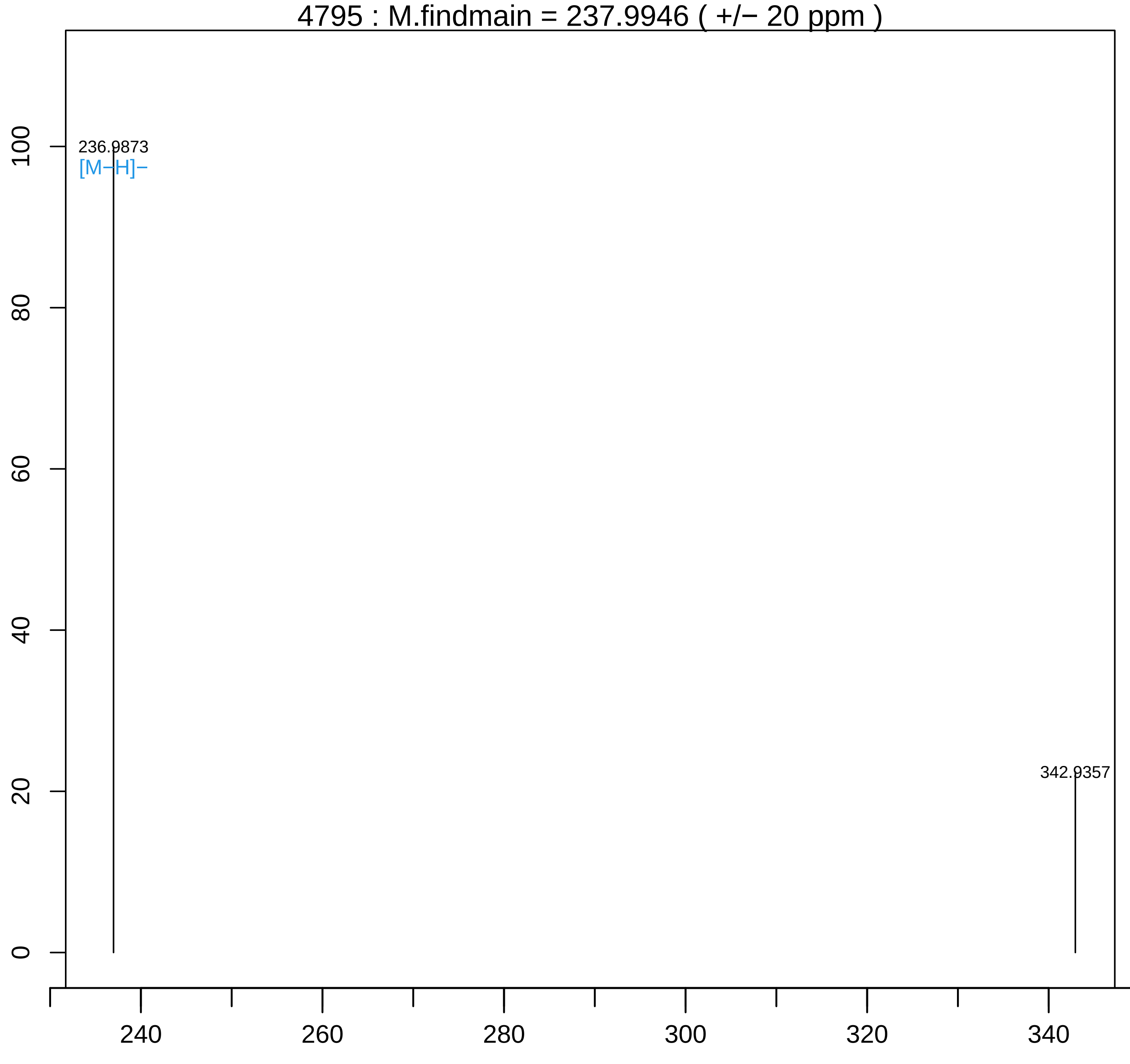
4795 : M.findmain = 237.9946 (+/− 20 ppm)
236.9873
[M−H]−
342.9357
0
20
40
60
80
100
240
260
280
300
320
340

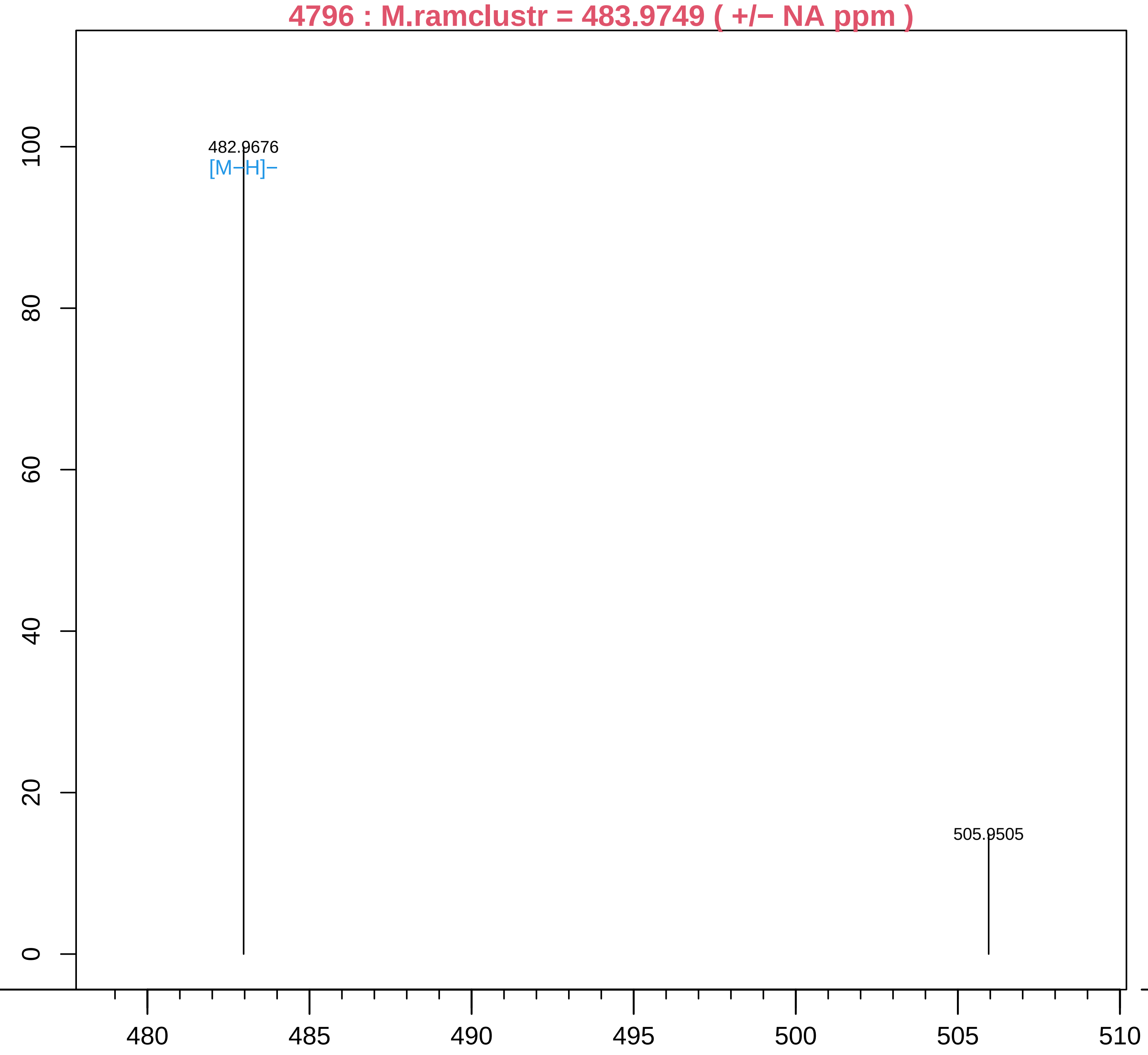
4796 : M.ramclustr = 483.9749 (+/− NA ppm)
482.9676
[M−H]−
505.9505
0
20
40
60
80
100
480
485
490
495
500
505
510

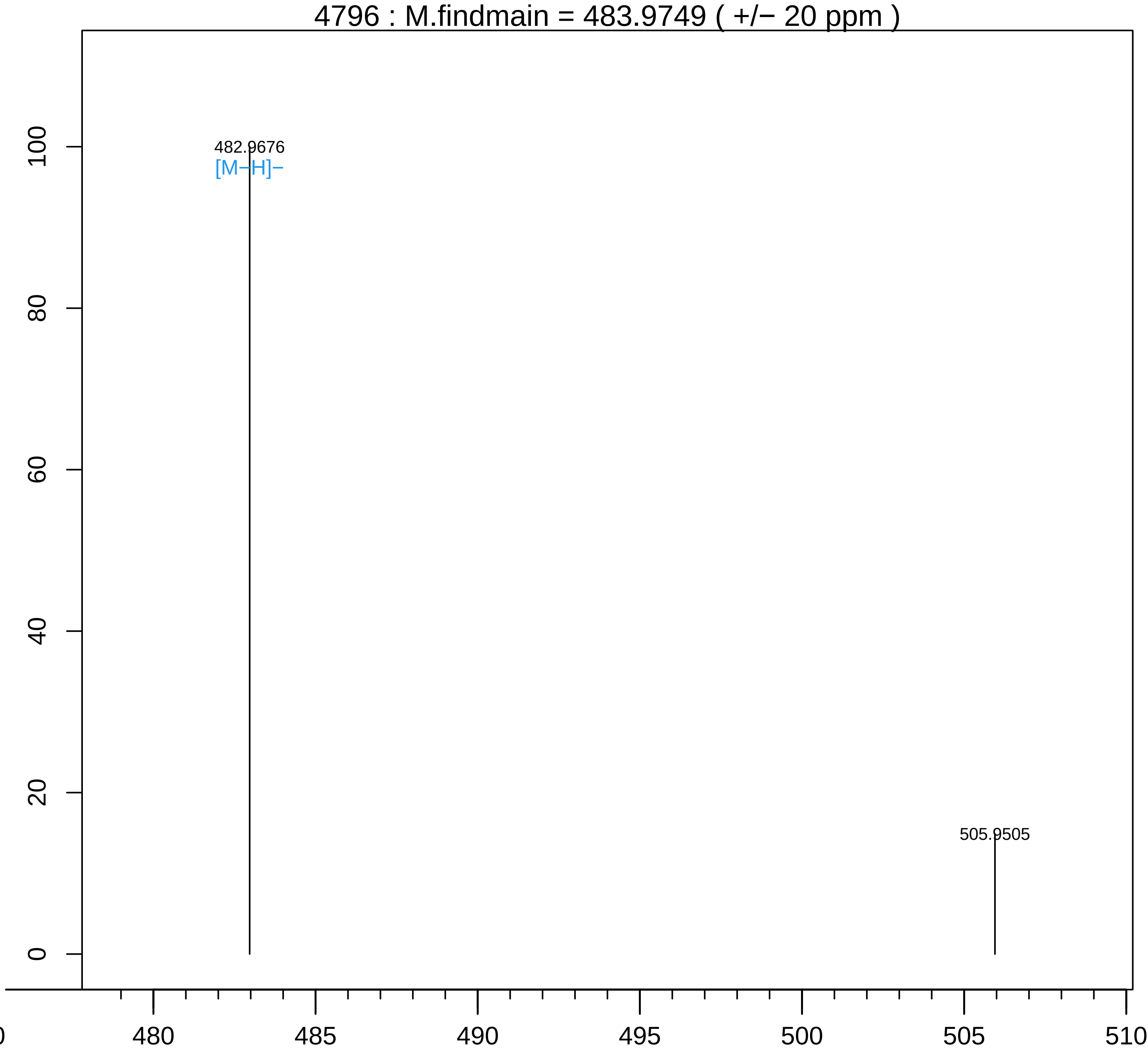
4796 : M.findmain = 483.9749 (+/− 20 ppm)
482.9676
[M−H]−
505.9505
0
20
40
60
80
100
480
485
490
495
500
505
510

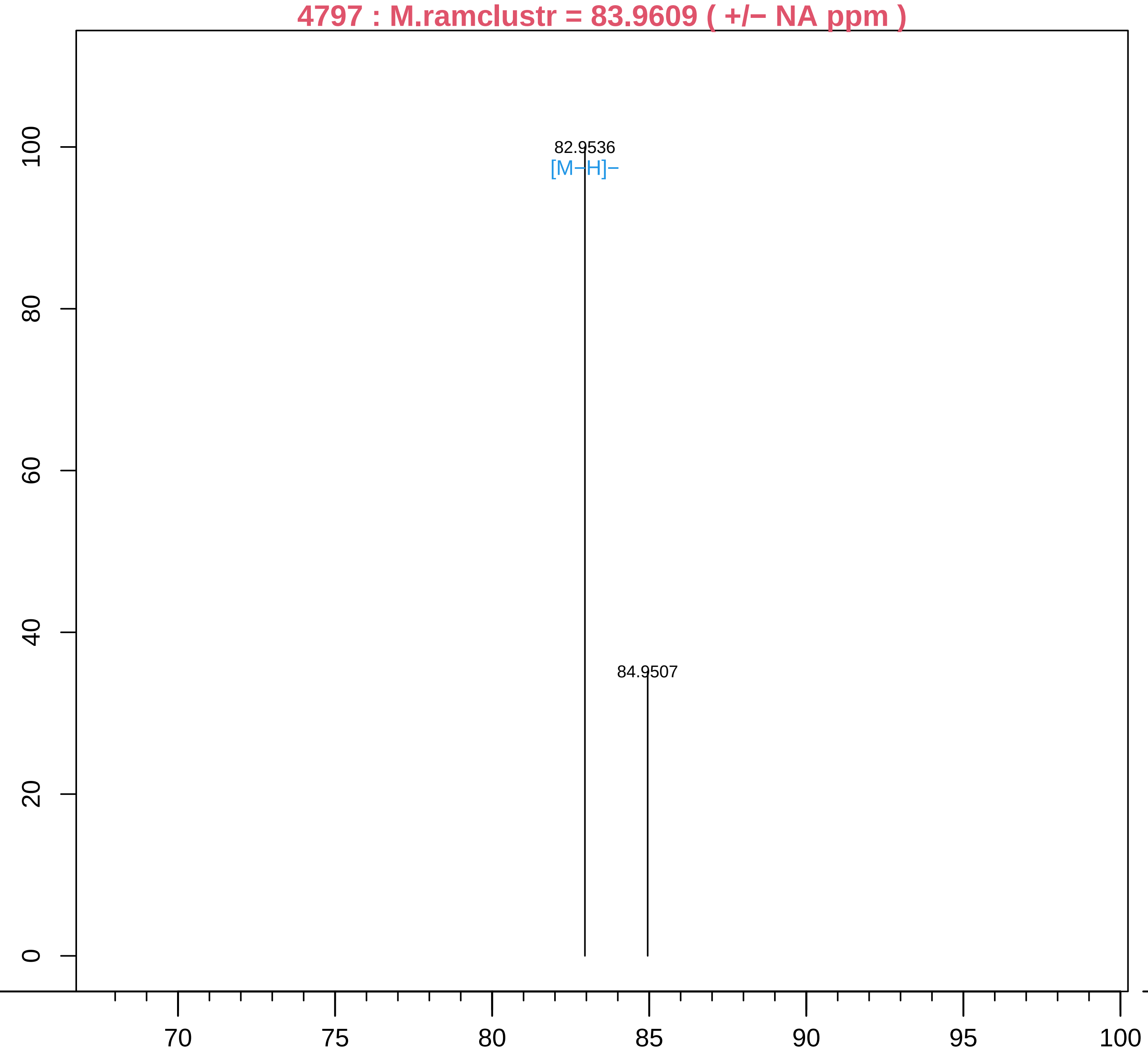
4797 : M.ramclustr = 83.9609 (+/− NA ppm)
82.9536
[M−H]−
84.9507
0
20
40
60
80
100
70
75
80
85
90
95
100

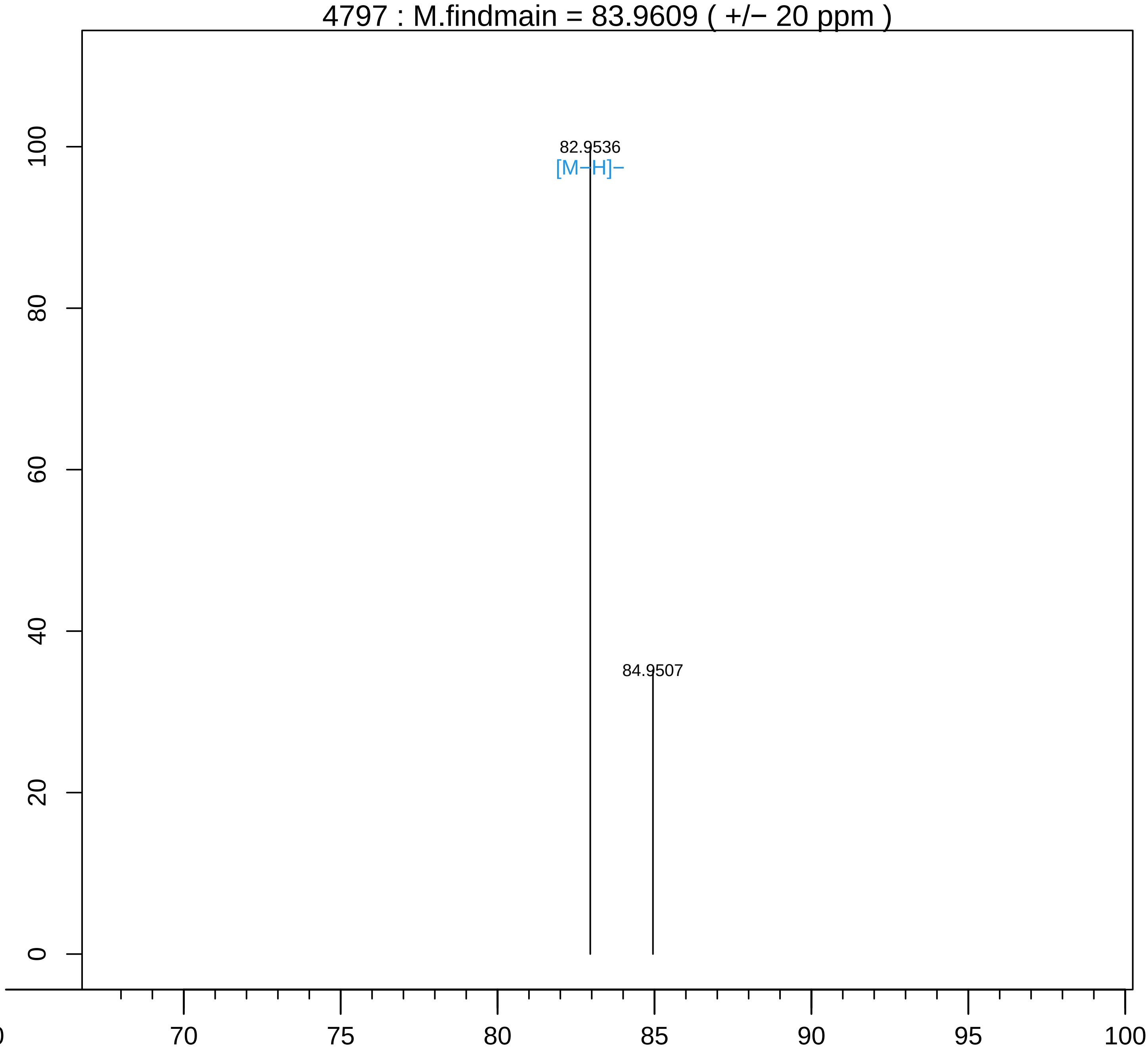
4797 : M.findmain = 83.9609 (+/− 20 ppm)
82.9536
[M−H]−
84.9507
0
20
40
60
80
100
70
75
80
85
90
95
100

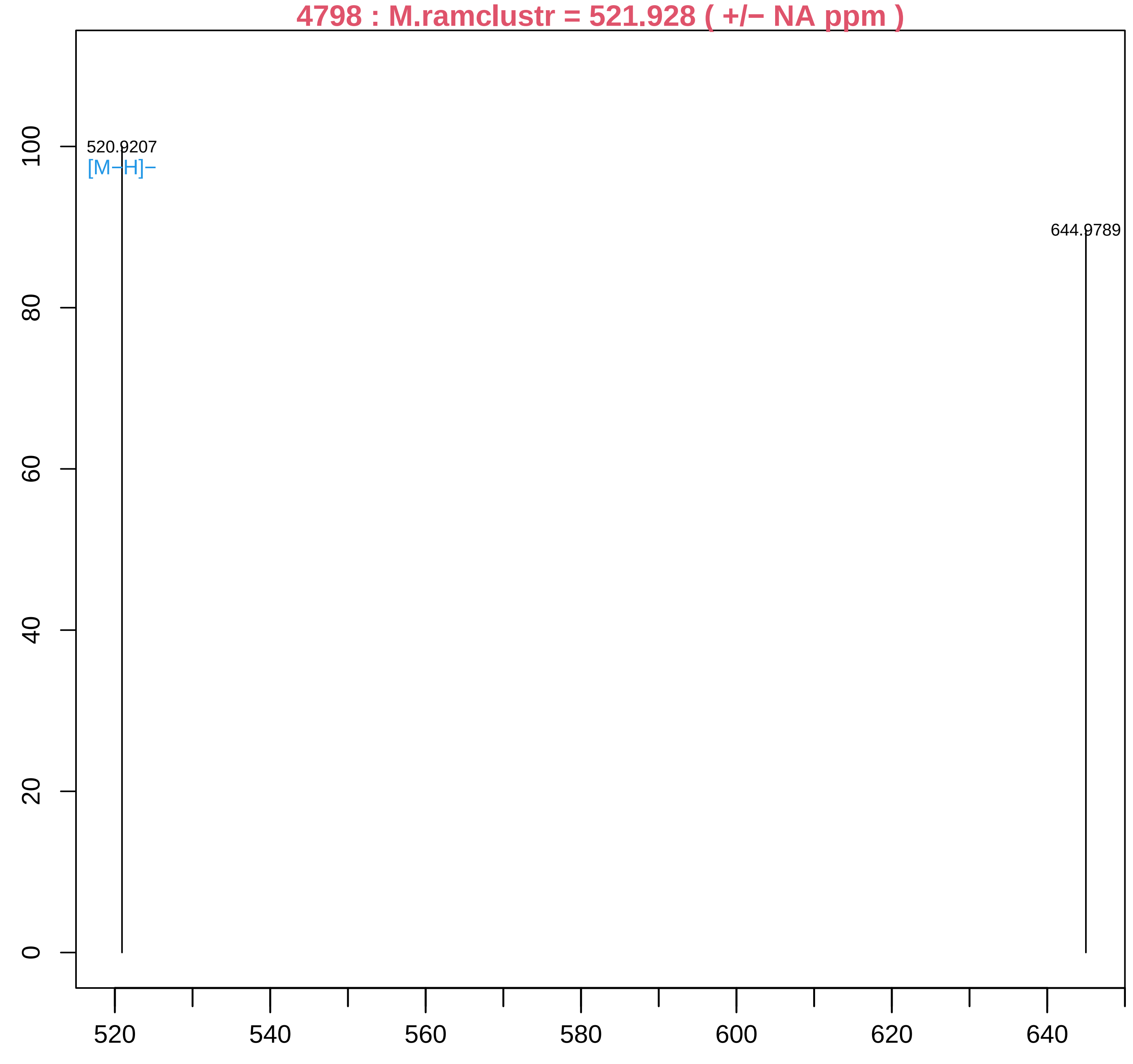

4798 : M.ramclustr = 521.928 (+/− NA ppm)
520.9207
[M−H]−
644.9789
0
20
40
60
80
100
520
540
560
580
600
620
640

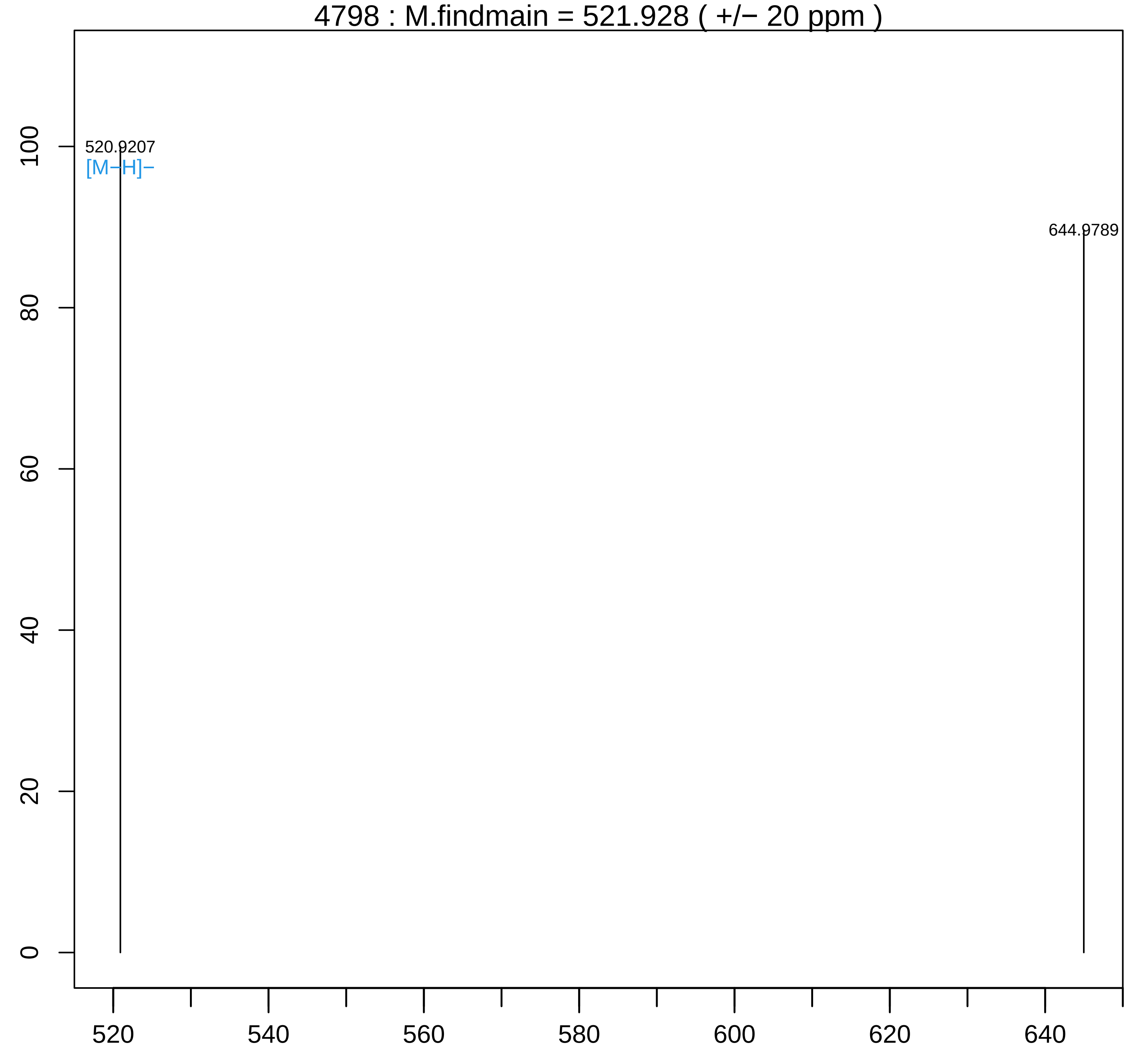

4798 : M.findmain = 521.928 (+/− 20 ppm)
520.9207
[M−H]−
644.9789
0
20
40
60
80
100
520
540
560
580
600
620
640

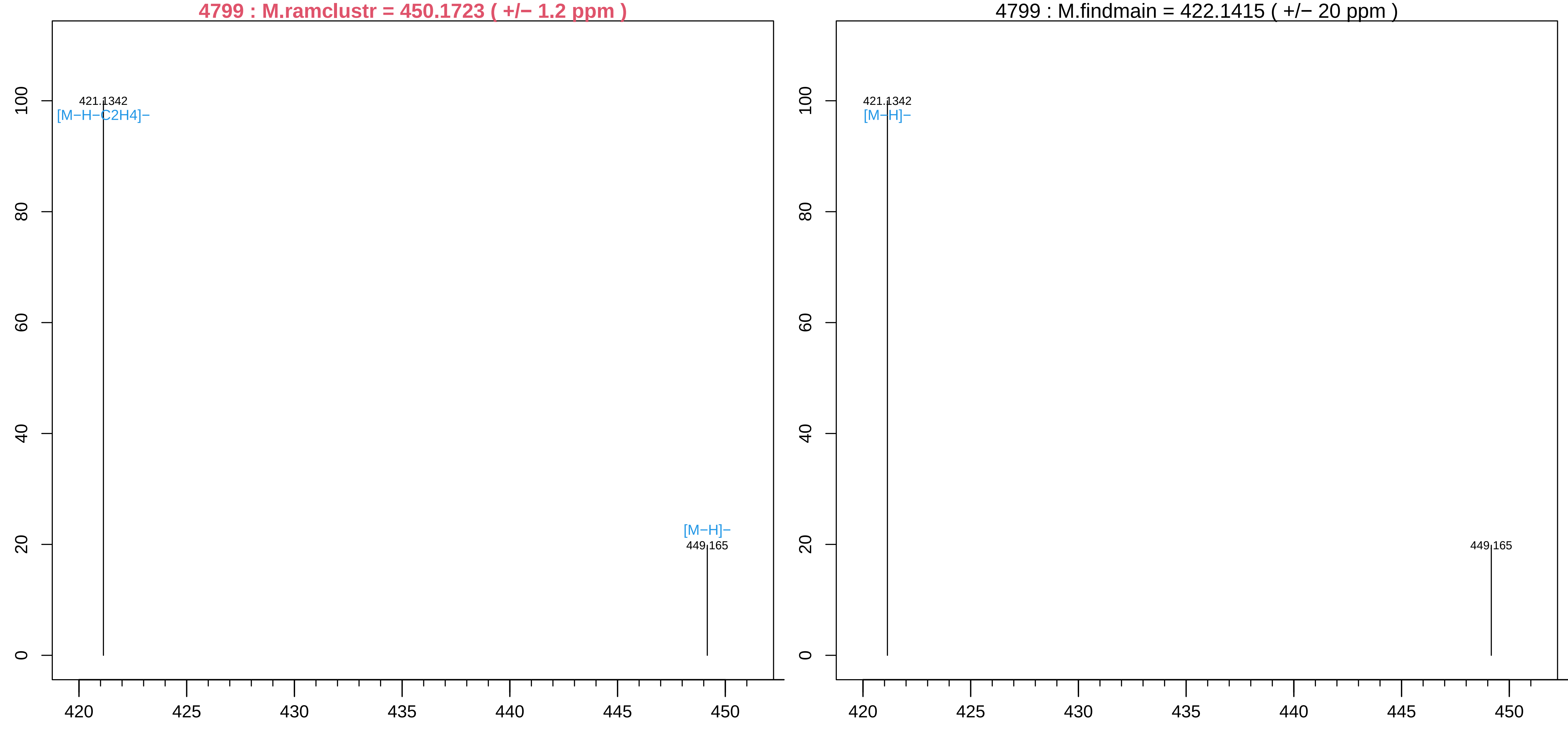

4799 : M.ramclustr = 450.1723 (+/− 1.2 ppm)
421.1342
[M−H−C2H4]−
[M−H]−
449.165
4799 : M.findmain = 422.1415 (+/− 20 ppm)
421.1342
[M−H]−
449.165

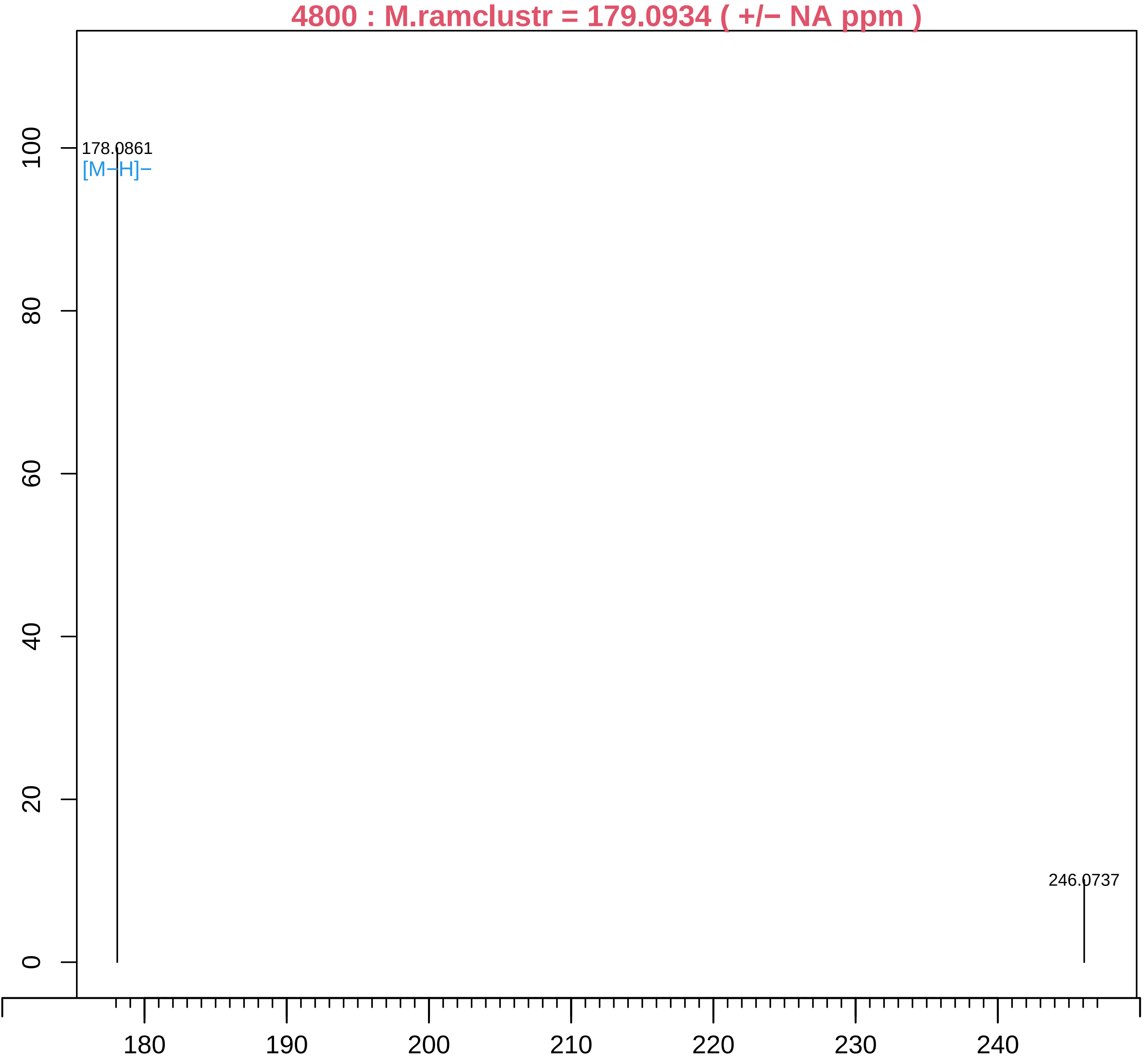
4800 : M.ramclustr = 179.0934 (+/− NA ppm)
178.0861
[M−H]−
246.0737
0
20
40
60
80
100
180
190
200
210
220
230
240

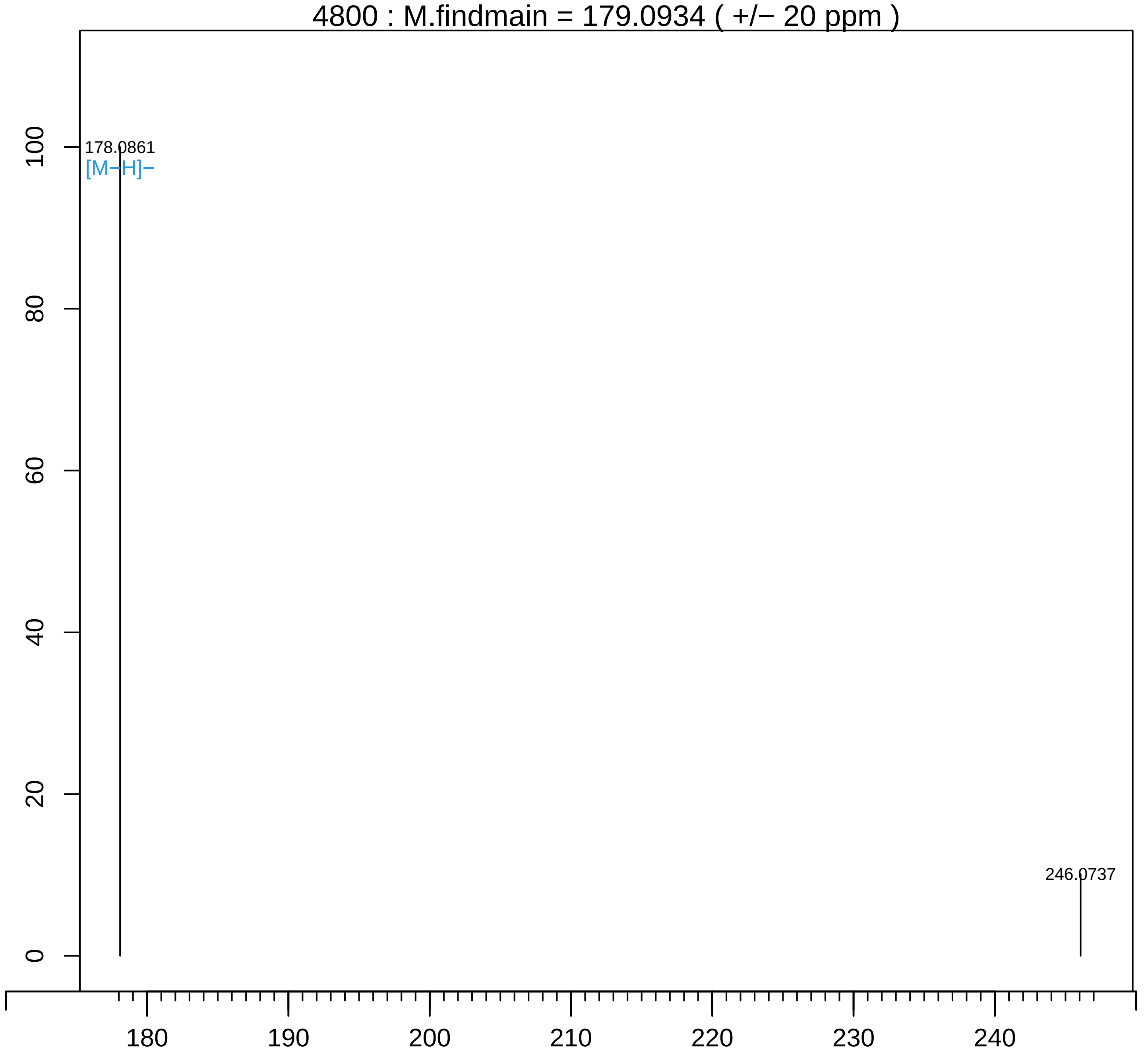
4800 : M.findmain = 179.0934 (+/− 20 ppm)
178.0861
[M−H]−
246.0737
0
20
40
60
80
100
180
190
200
210
220
230
240

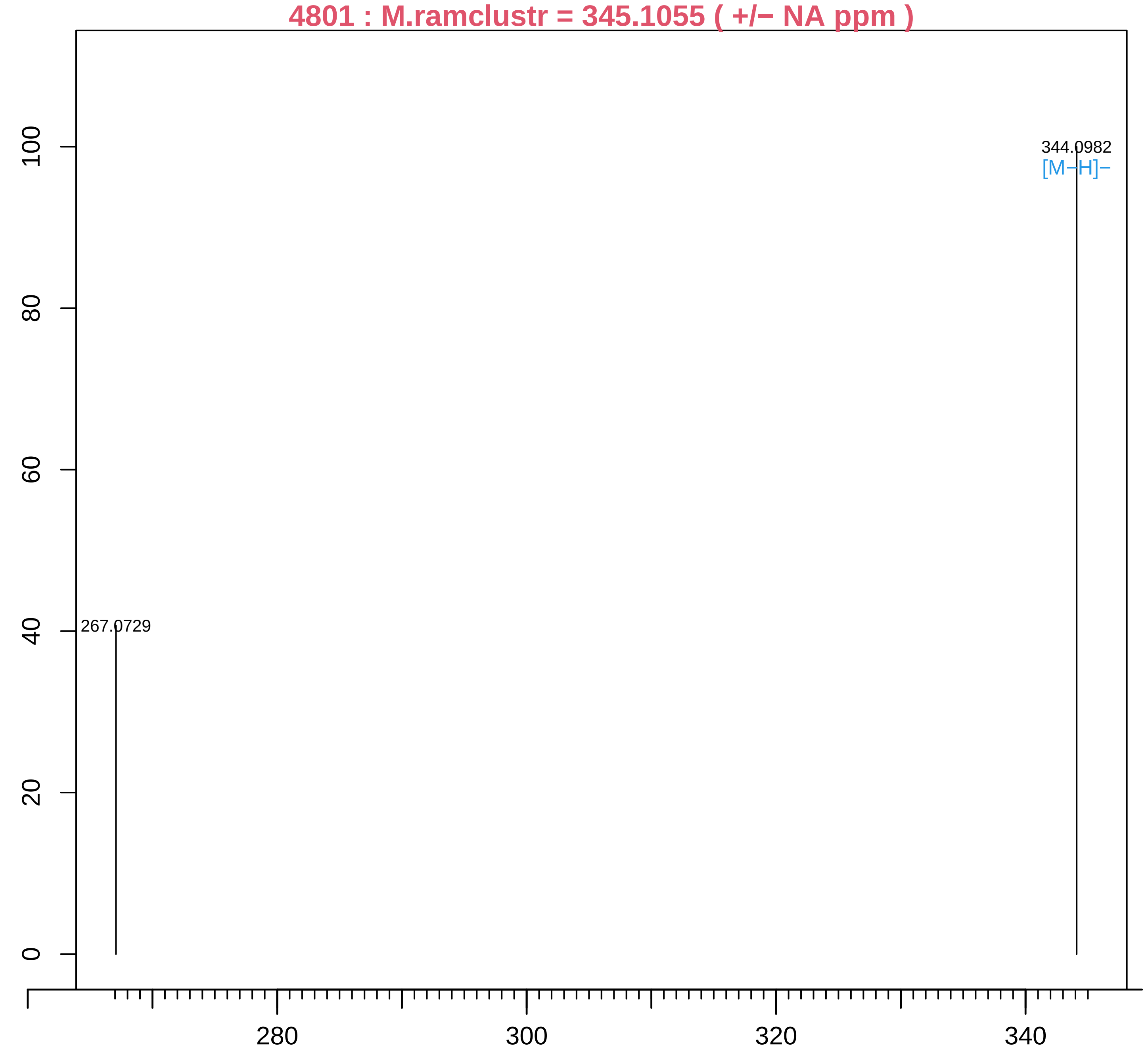
4801 : M.ramclustr = 345.1055 (+/− NA ppm)
344.0982
[M−H]−
267.0729
0
20
40
60
80
100
280
300
320
340

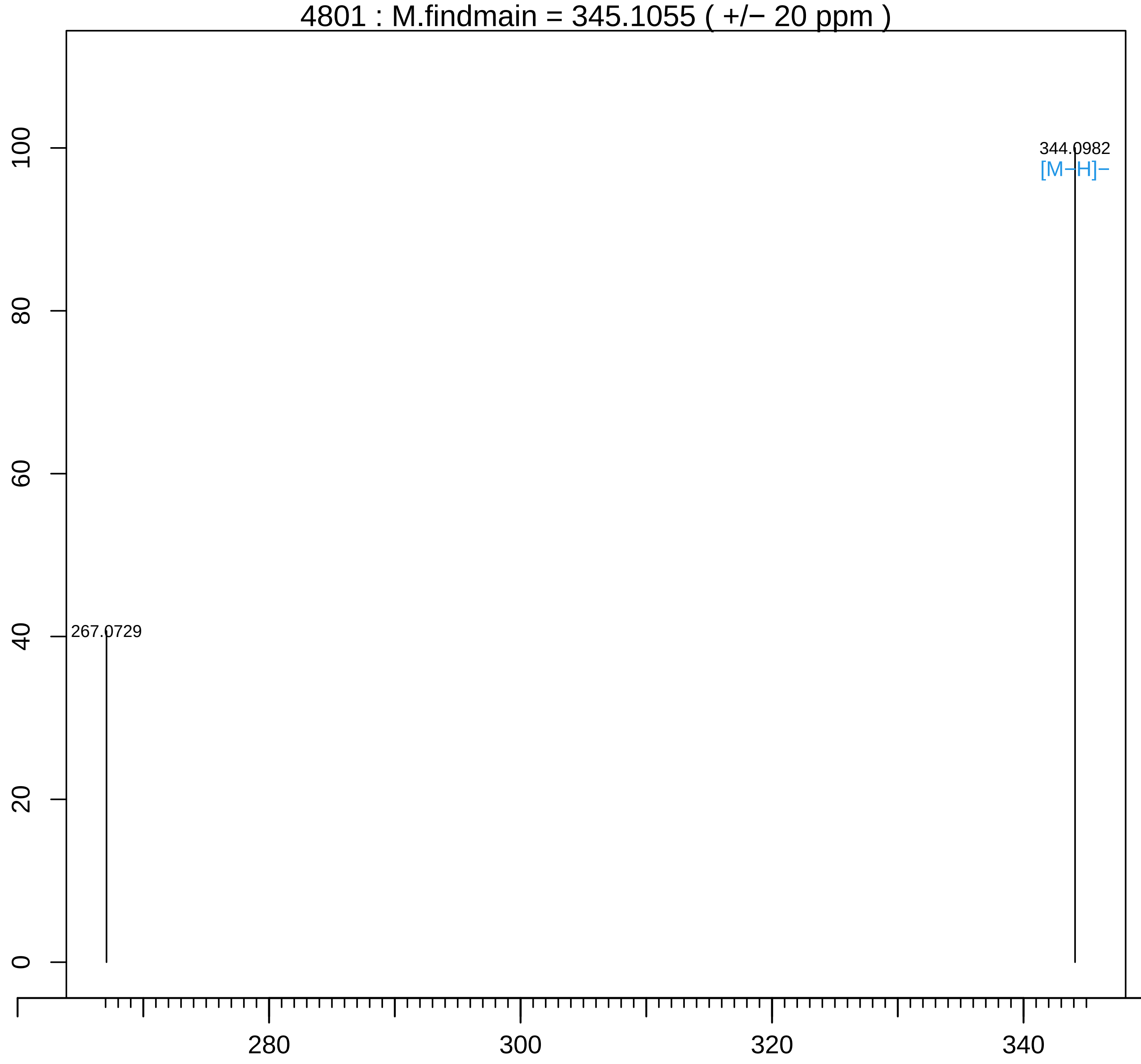
4801 : M.findmain = 345.1055 (+/− 20 ppm)
344.0982
[M−H]−
267.0729
0
20
40
60
80
100
280
300
320
340

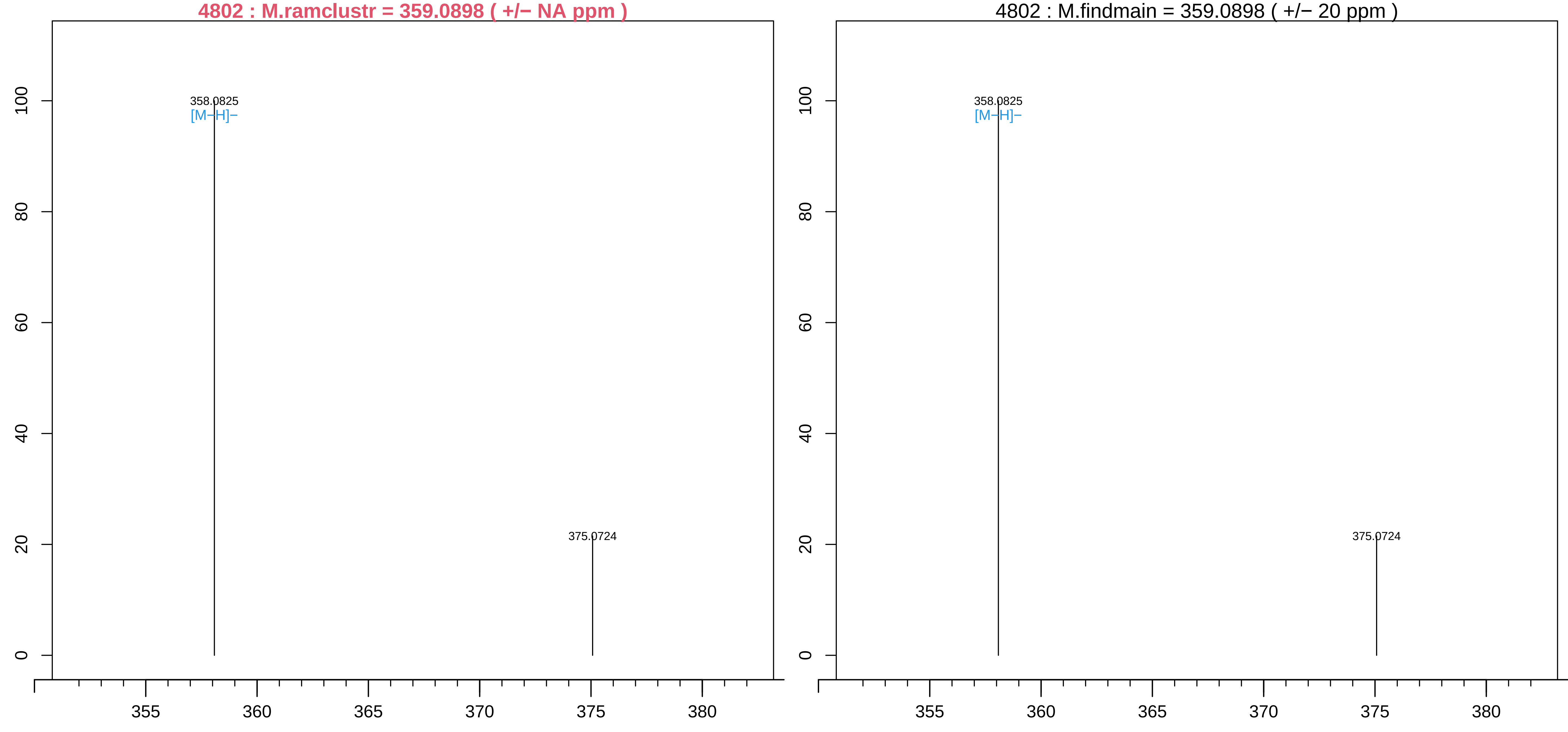
4802 : M.ramclustr = 359.0898 (+/− NA ppm)
358.0825
[M−H]−
375.0724
0
20
40
60
80
100
355
360
365
370
375
380
4802 : M.findmain = 359.0898 (+/− 20 ppm)
358.0825
[M−H]−
375.0724
0
20
40
60
80
100
355
360
365
370
375
380

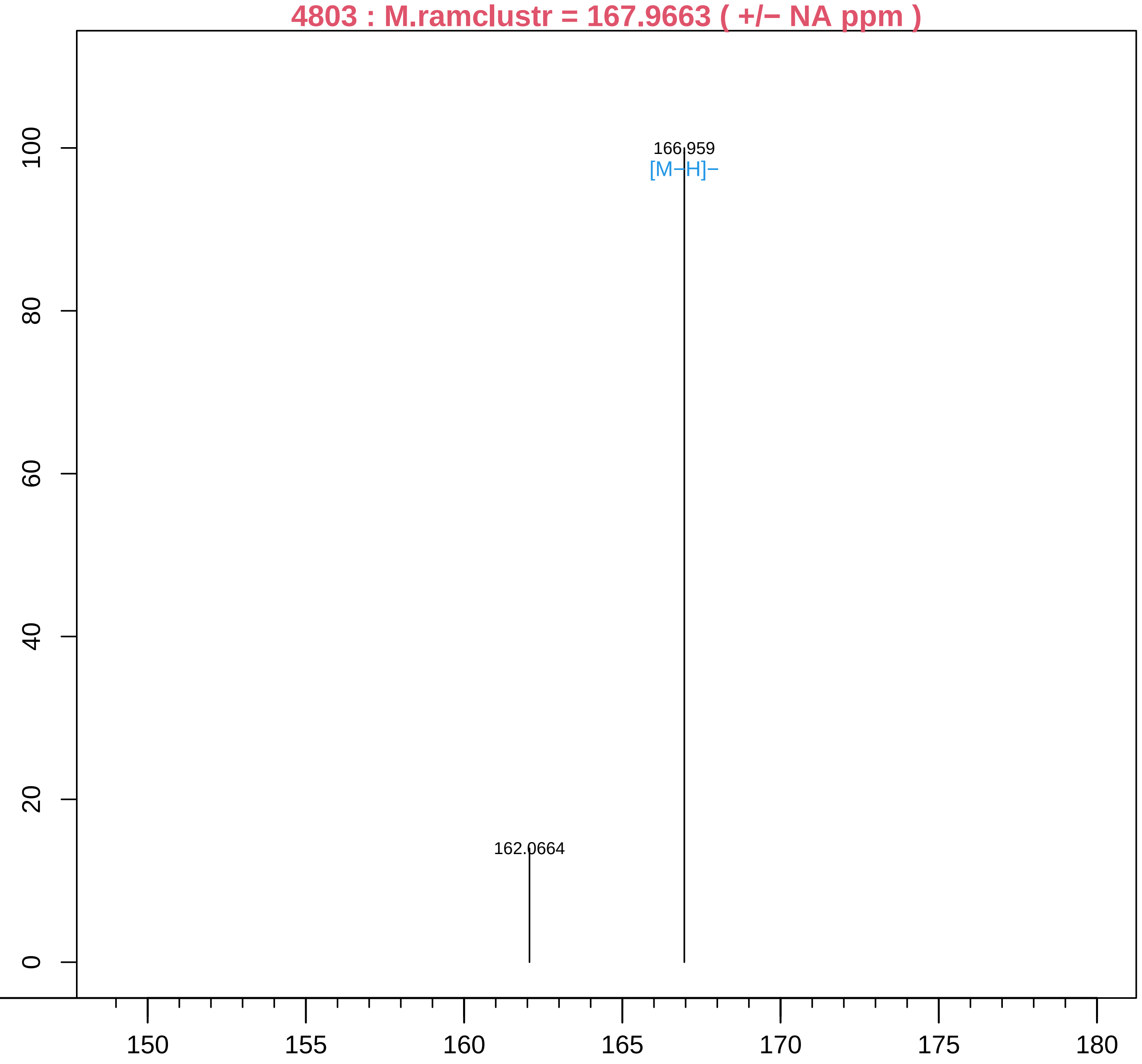
4803 : M.ramclustr = 167.9663 (+/− NA ppm)
166.959
[M−H]−
162.0664
0
20
40
60
80
100
150
155
160
165
170
175
180
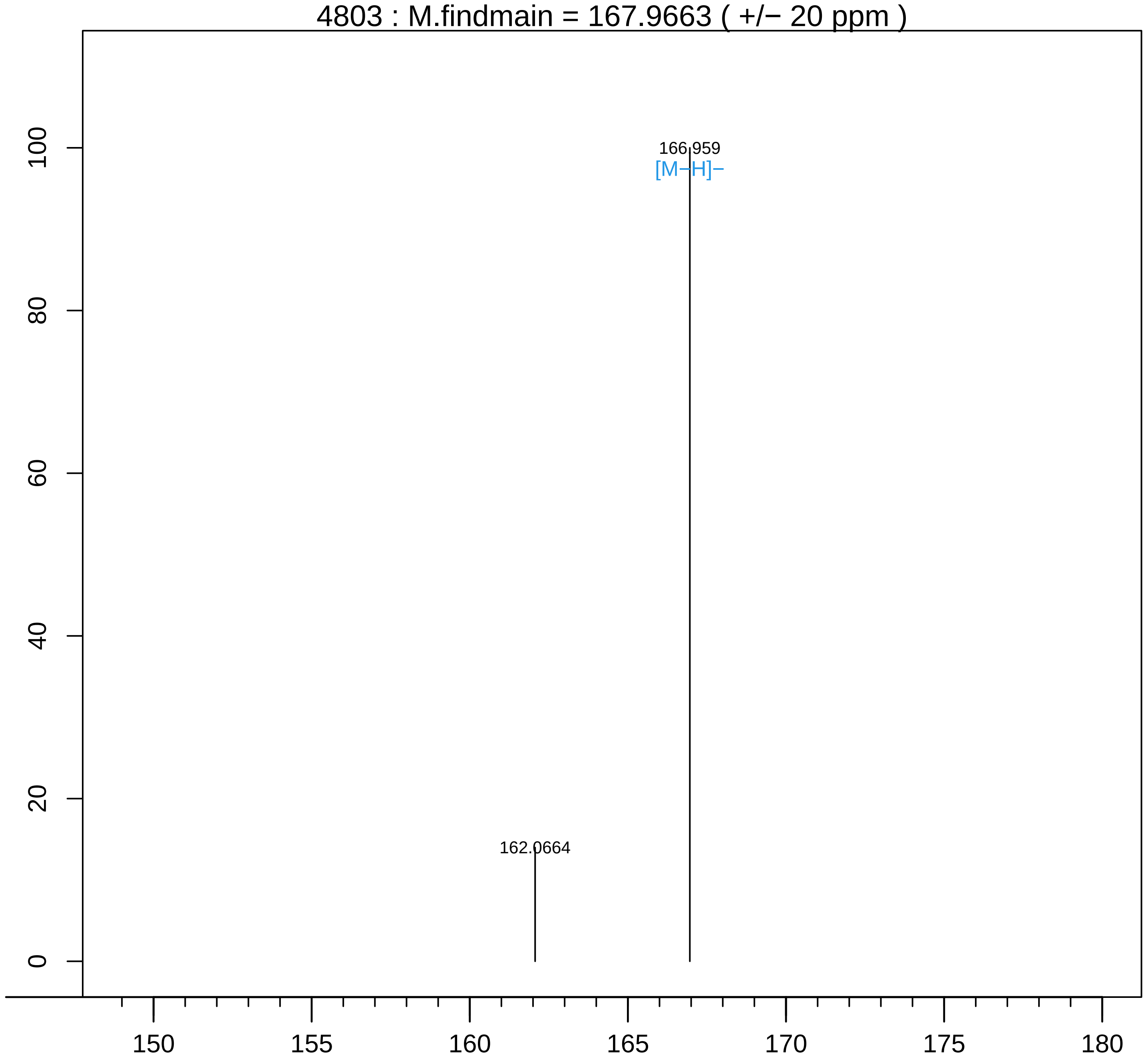
4803 : M.findmain = 167.9663 (+/− 20 ppm)
166.959
[M−H]−
162.0664
0
20
40
60
80
100
150
155
160
165
170
175
180

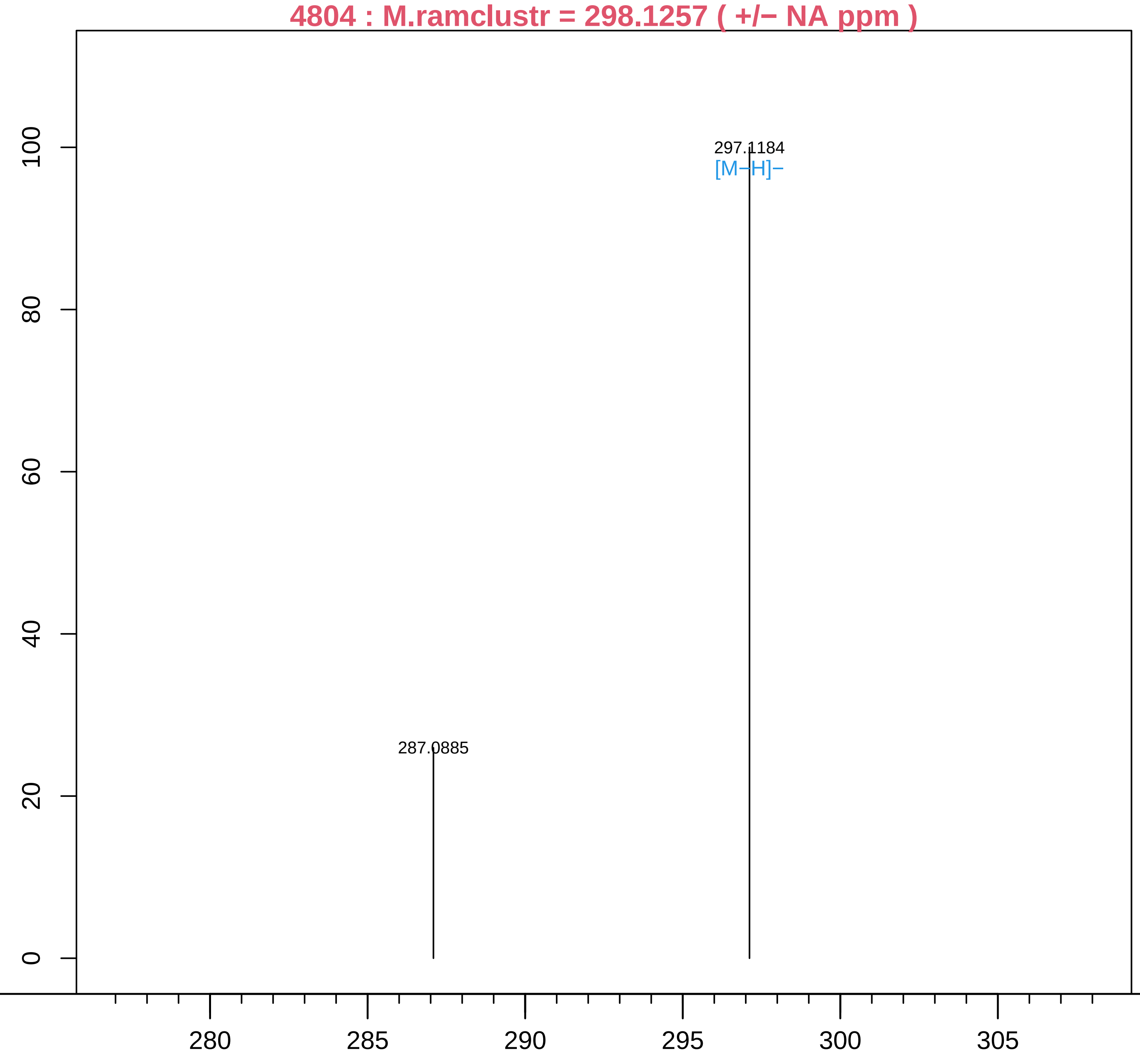
4804 : M.ramclustr = 298.1257 (+/− NA ppm)
297.1184
[M−H]−
287.0885
0
20
40
60
80
100
280
285
290
295
300
305

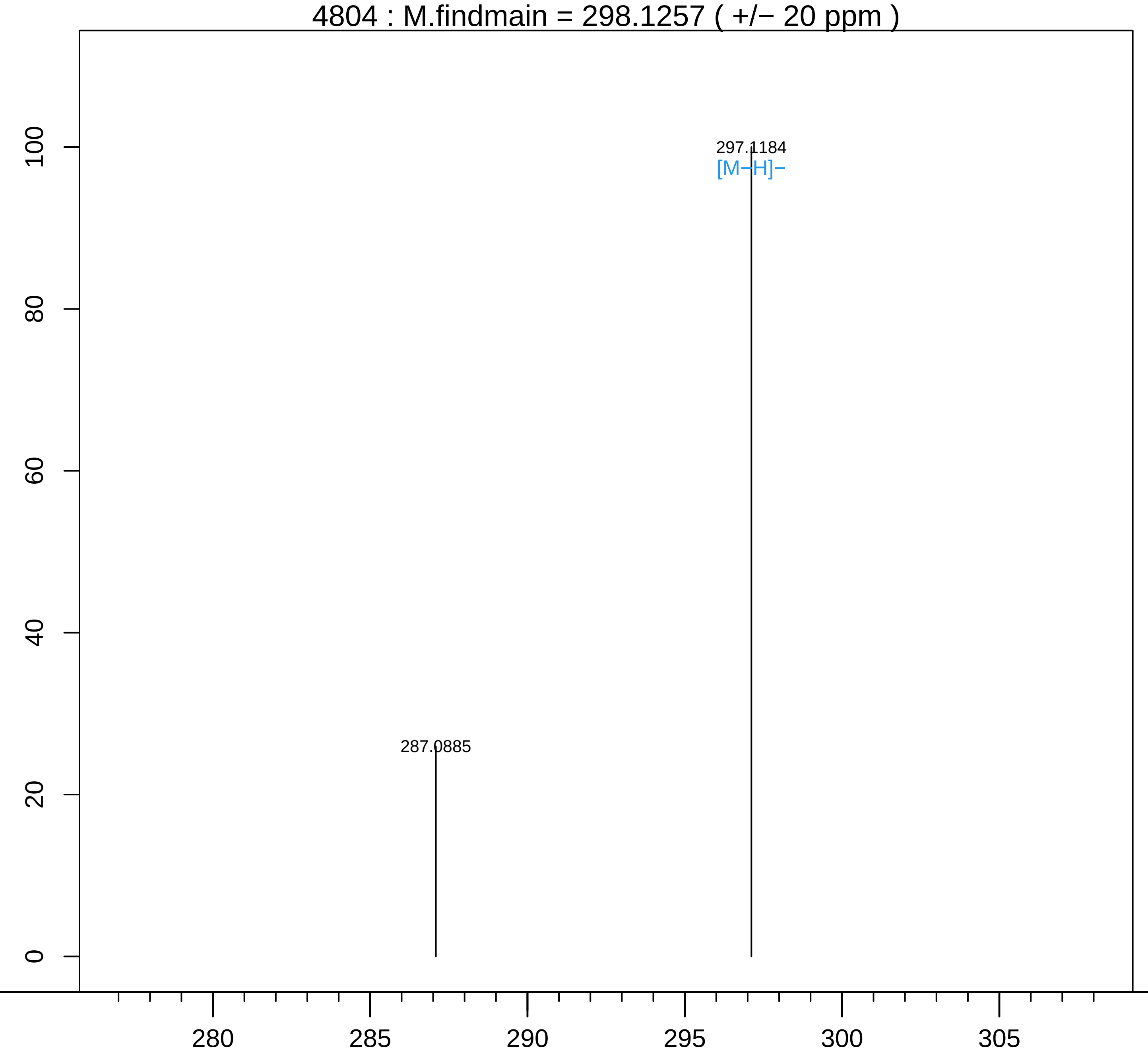
4804 : M.findmain = 298.1257 (+/− 20 ppm)
297.1184
[M−H]−
287.0885
0
20
40
60
80
100
280
285
290
295
300
305

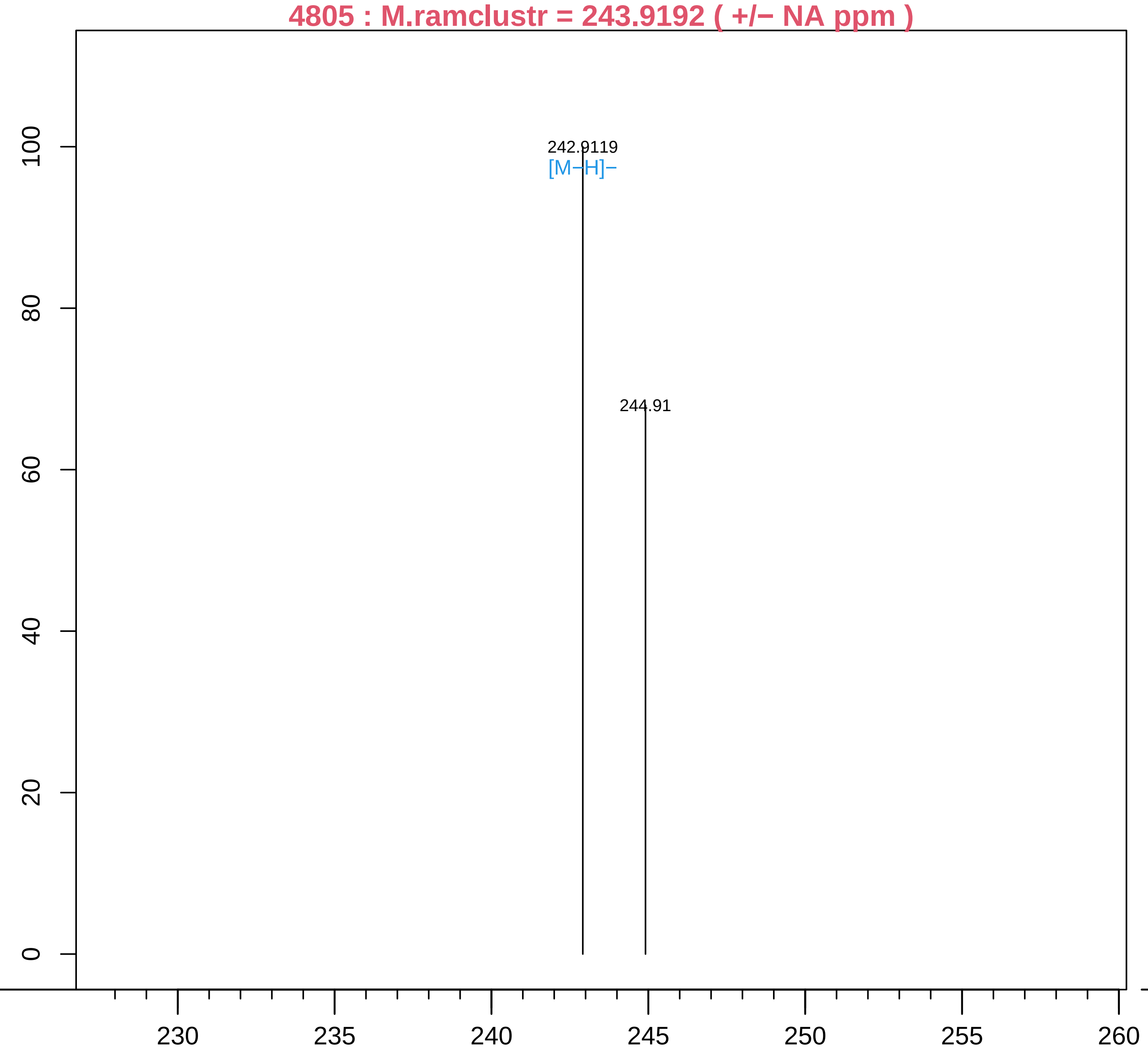
4805 : M.ramclustr = 243.9192 (+/− NA ppm)
242.9119
[M−H]−
244.91
0
20
40
60
80
100
230
235
240
245
250
255
260

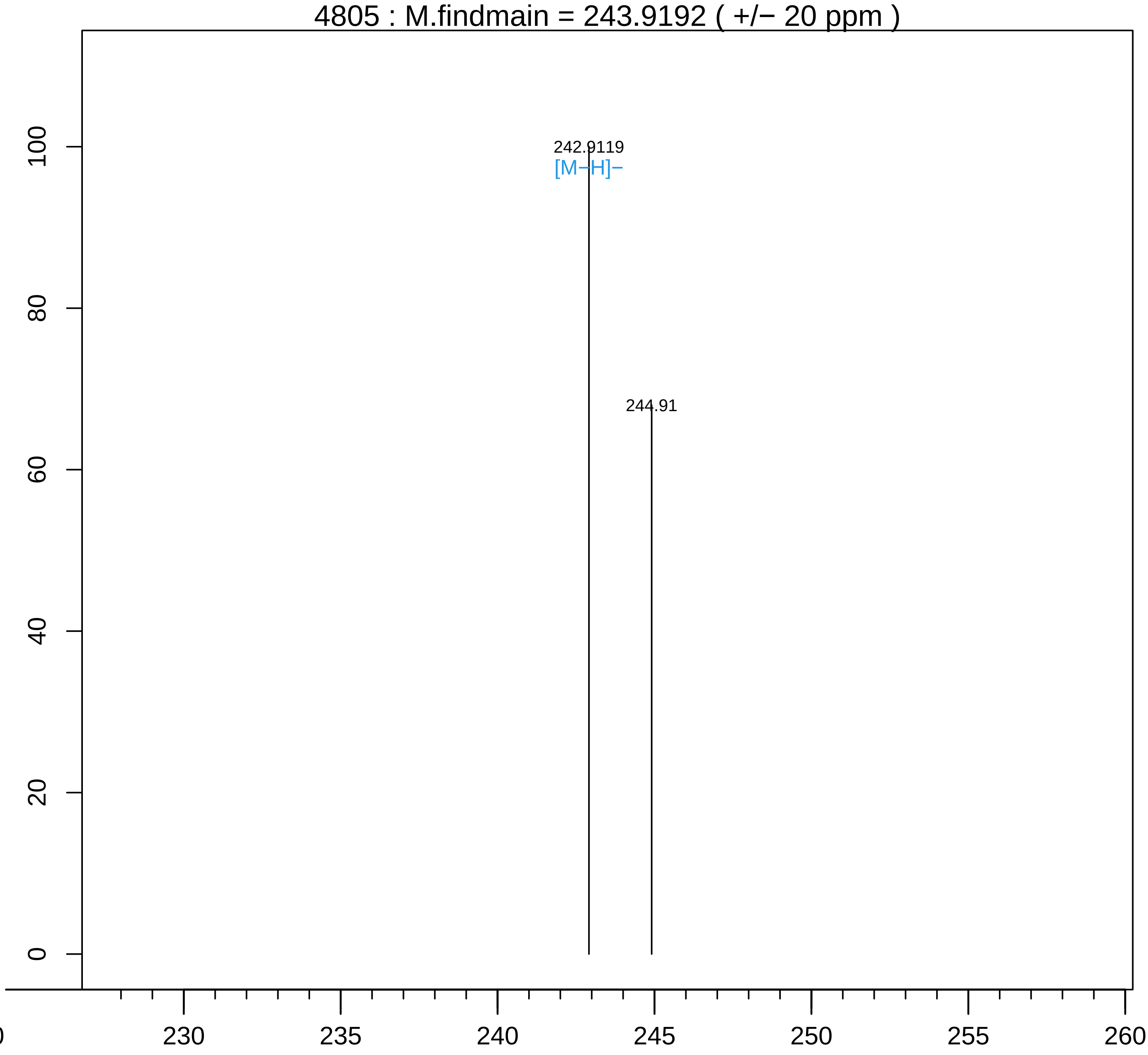
4805 : M.findmain = 243.9192 (+/− 20 ppm)
242.9119
[M−H]−
244.91
0
20
40
60
80
100
230
235
240
245
250
255
260

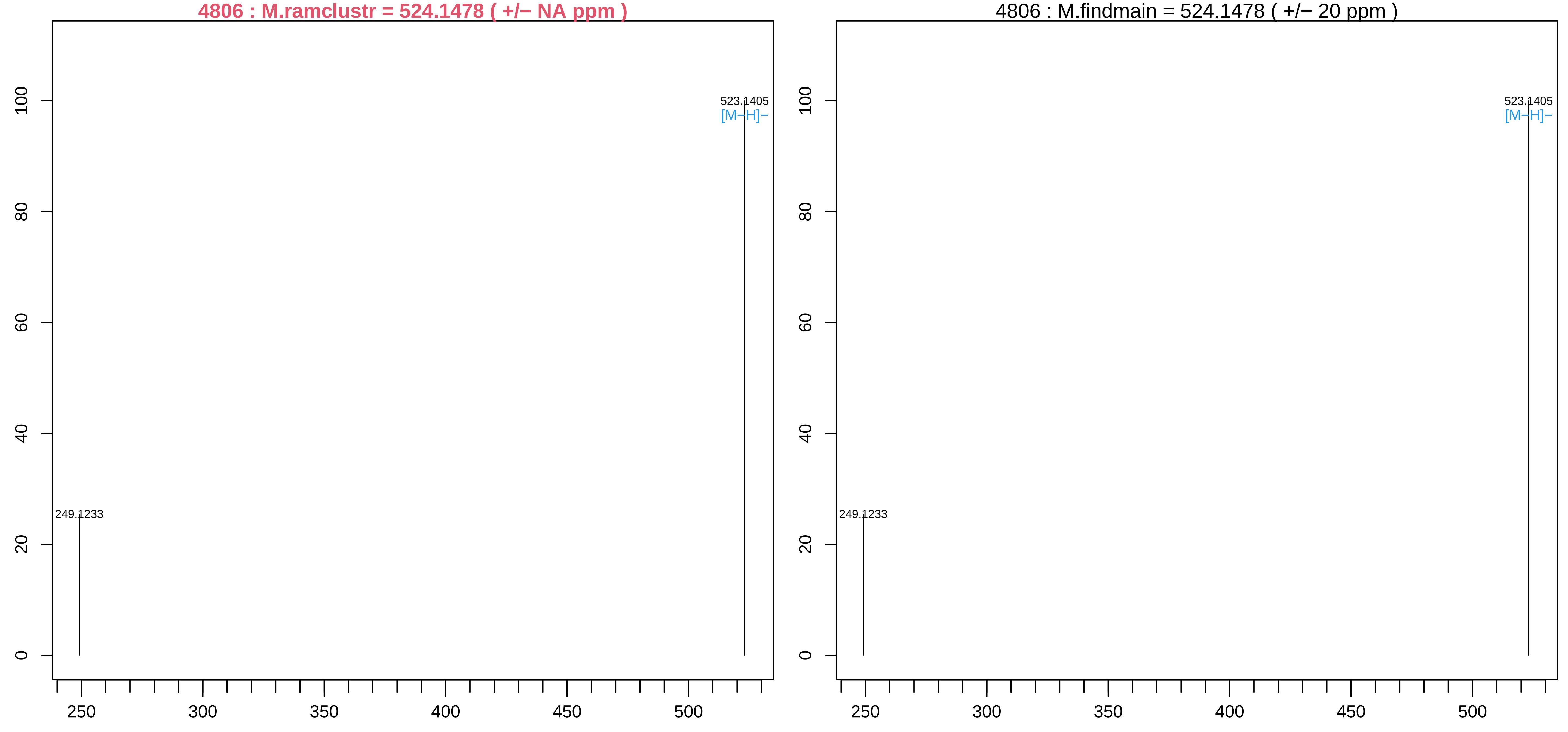

4806 : M.ramclustr = 524.1478 (+/− NA ppm)
523.1405
[M−H]−
249.1233
4806 : M.findmain = 524.1478 (+/− 20 ppm)
523.1405
[M−H]−
249.1233
0
20
40
60
80
100
250
300
350
400
450
500

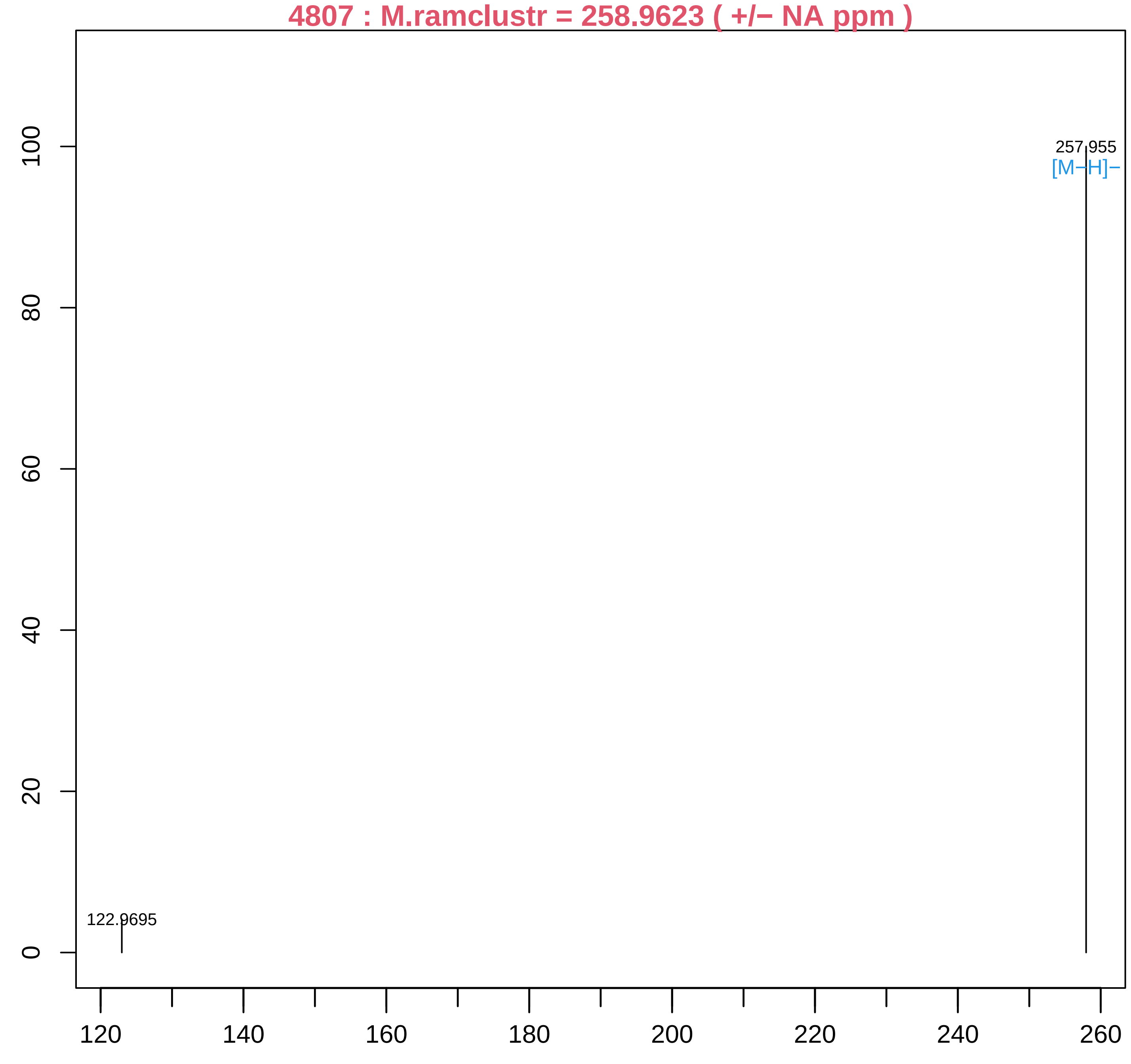
4807 : M.ramclustr = 258.9623 (+/− NA ppm)
257.955
[M−H]−
122.9695
0
20
40
60
80
100
120
140
160
180
200
220
240
260

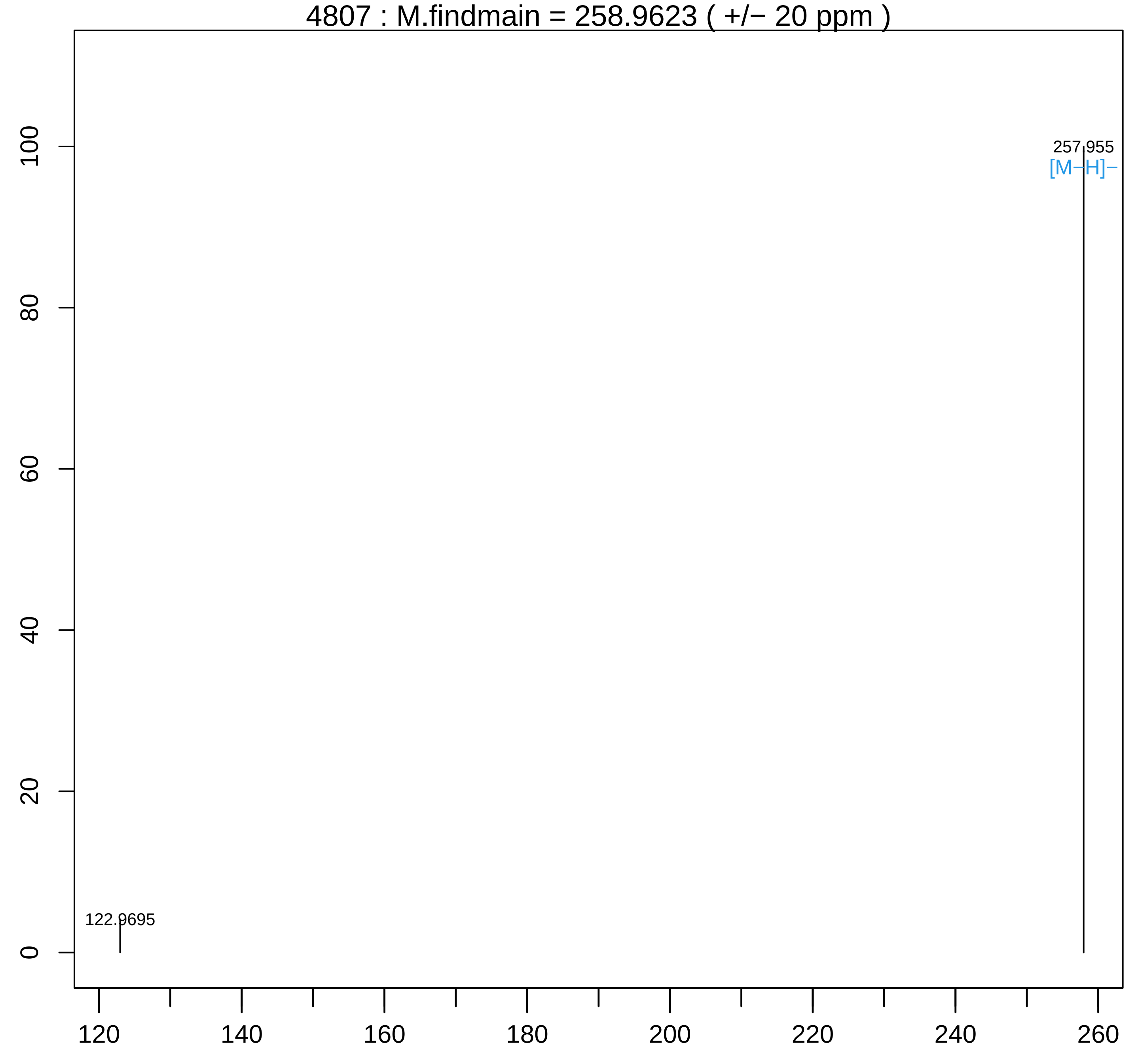
4807 : M.findmain = 258.9623 (+/− 20 ppm)
257.955
[M−H]−
122.9695
0
20
40
60
80
100
120
140
160
180
200
220
240
260

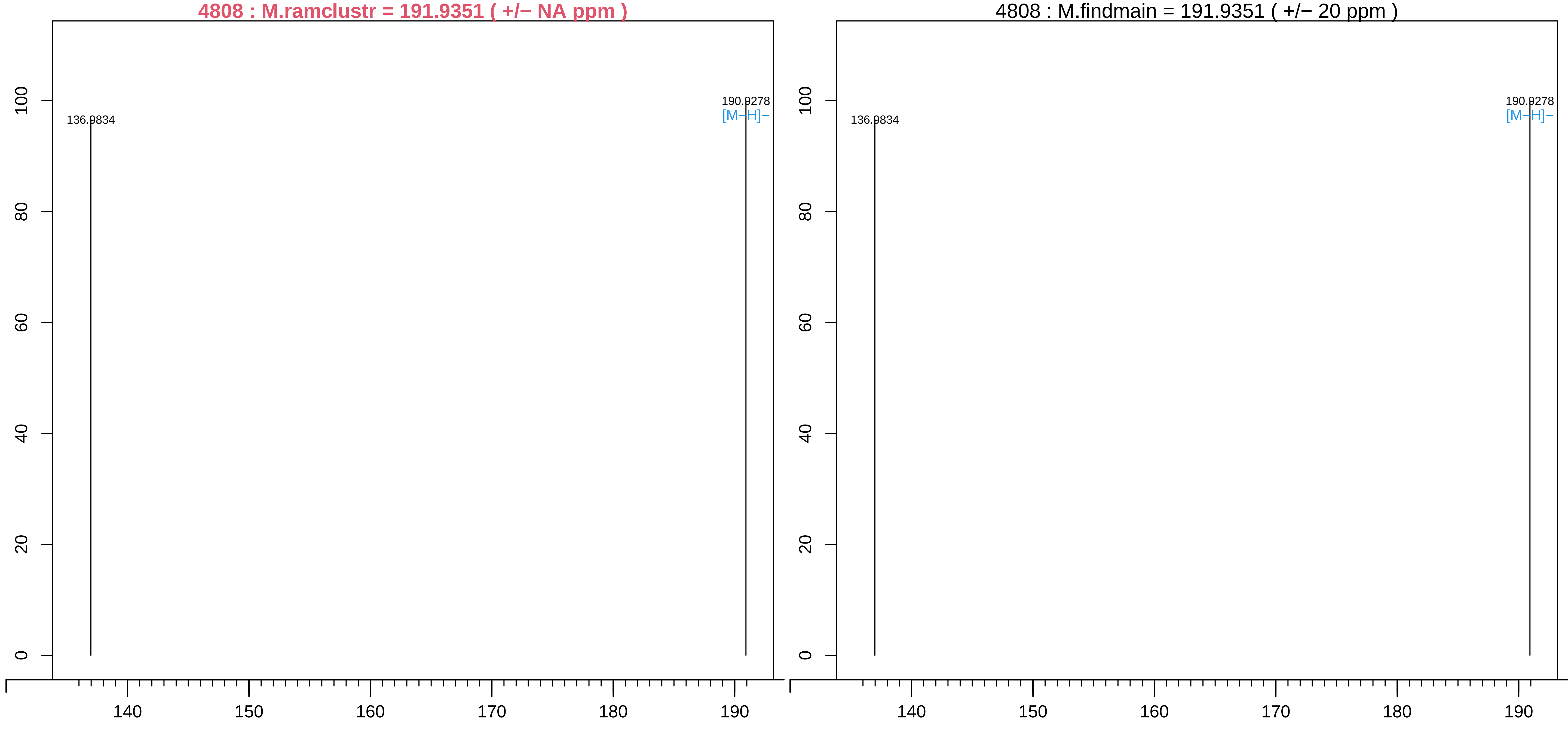

4808 : M.ramclustr = 191.9351 (+/− NA ppm)
136.9834
190.9278
[M−H]−
0
20
40
60
80
100
140
150
160
170
180
190
4808 : M.findmain = 191.9351 (+/− 20 ppm)
136.9834
190.9278
[M−H]−
0
20
40
60
80
100
140
150
160
170
180
190

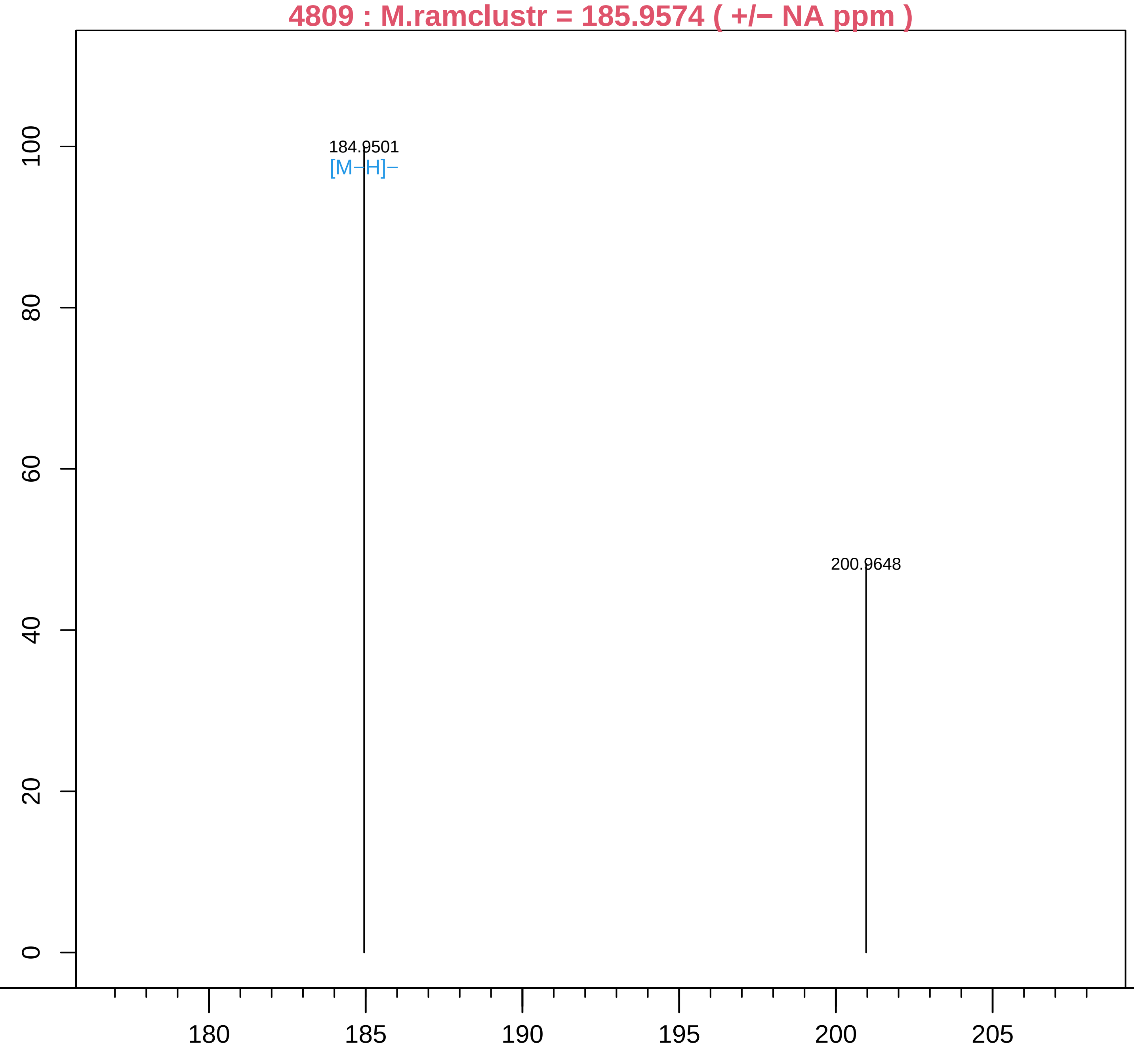

4809 : M.ramclustr = 185.9574 (+/− NA ppm)
184.9501
[M−H]−
200.9648
0
20
40
60
80
100
180
185
190
195
200
205

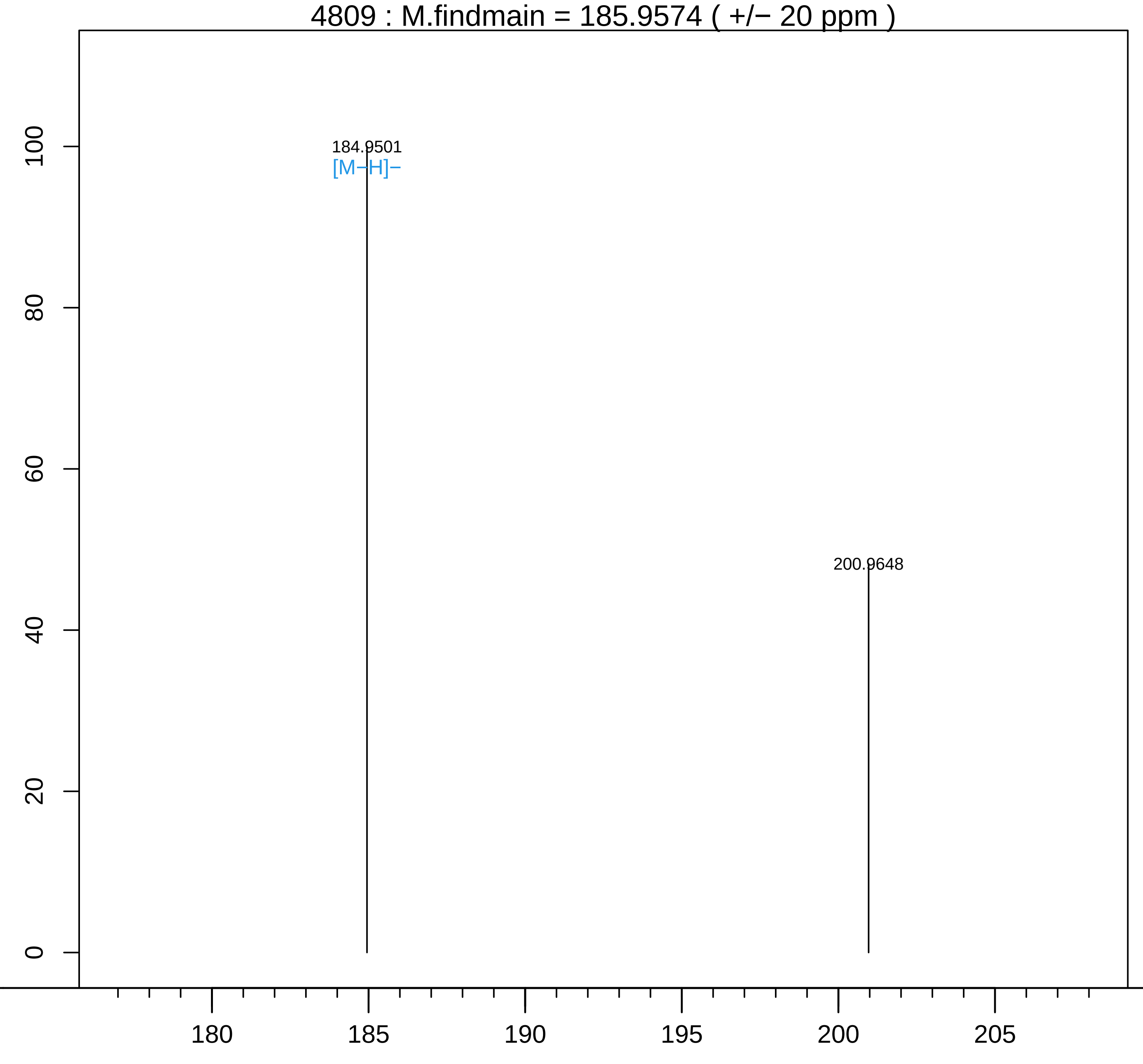

4809 : M.findmain = 185.9574 (+/− 20 ppm)
184.9501
[M−H]−
200.9648
0
20
40
60
80
100
180
185
190
195
200
205

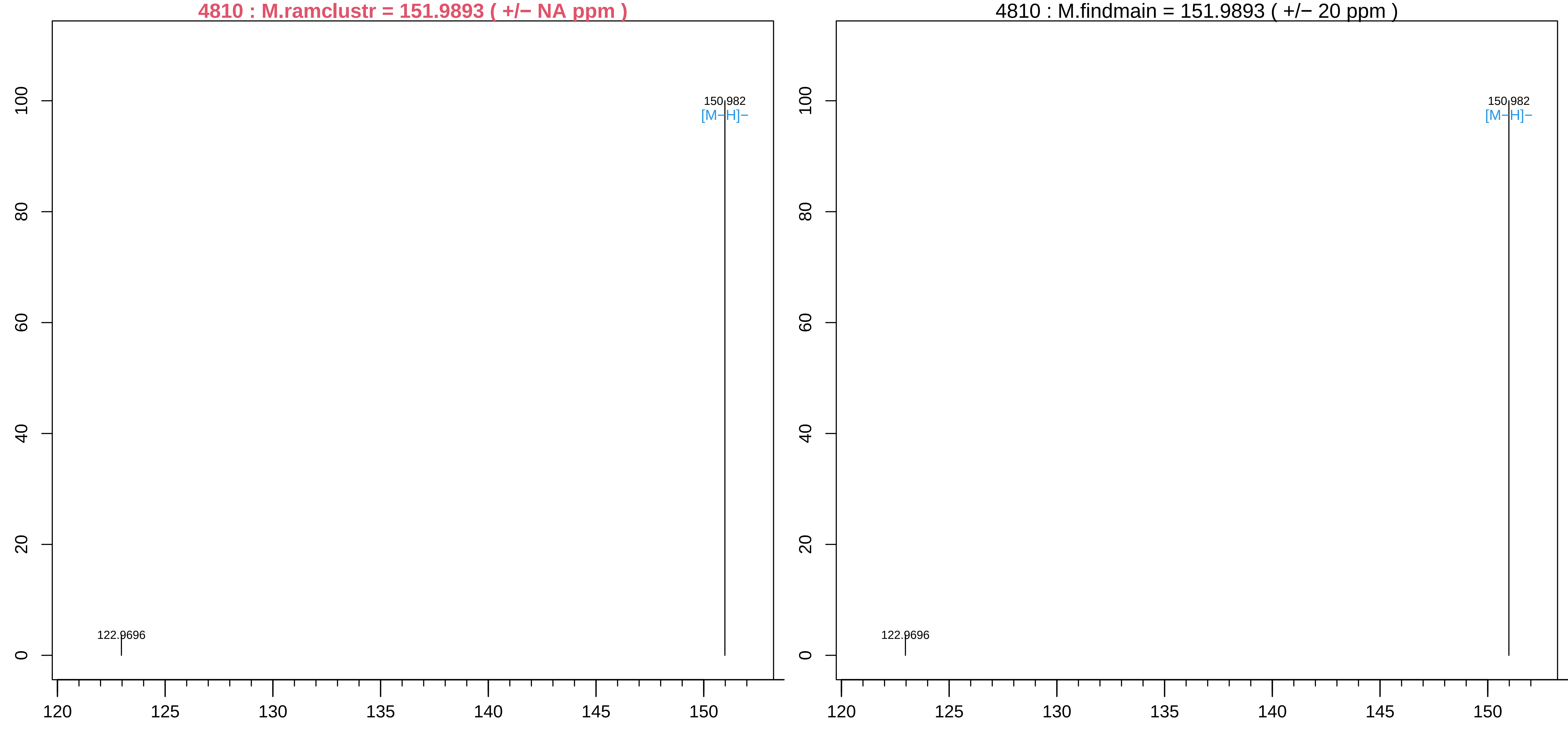
4810 : M.ramclustr = 151.9893 (+/− NA ppm)
4810 : M.findmain = 151.9893 (+/− 20 ppm)
150.982
[M−H]−
122.9696
0
20
40
60
80
100
120
125
130
135
140
145
150

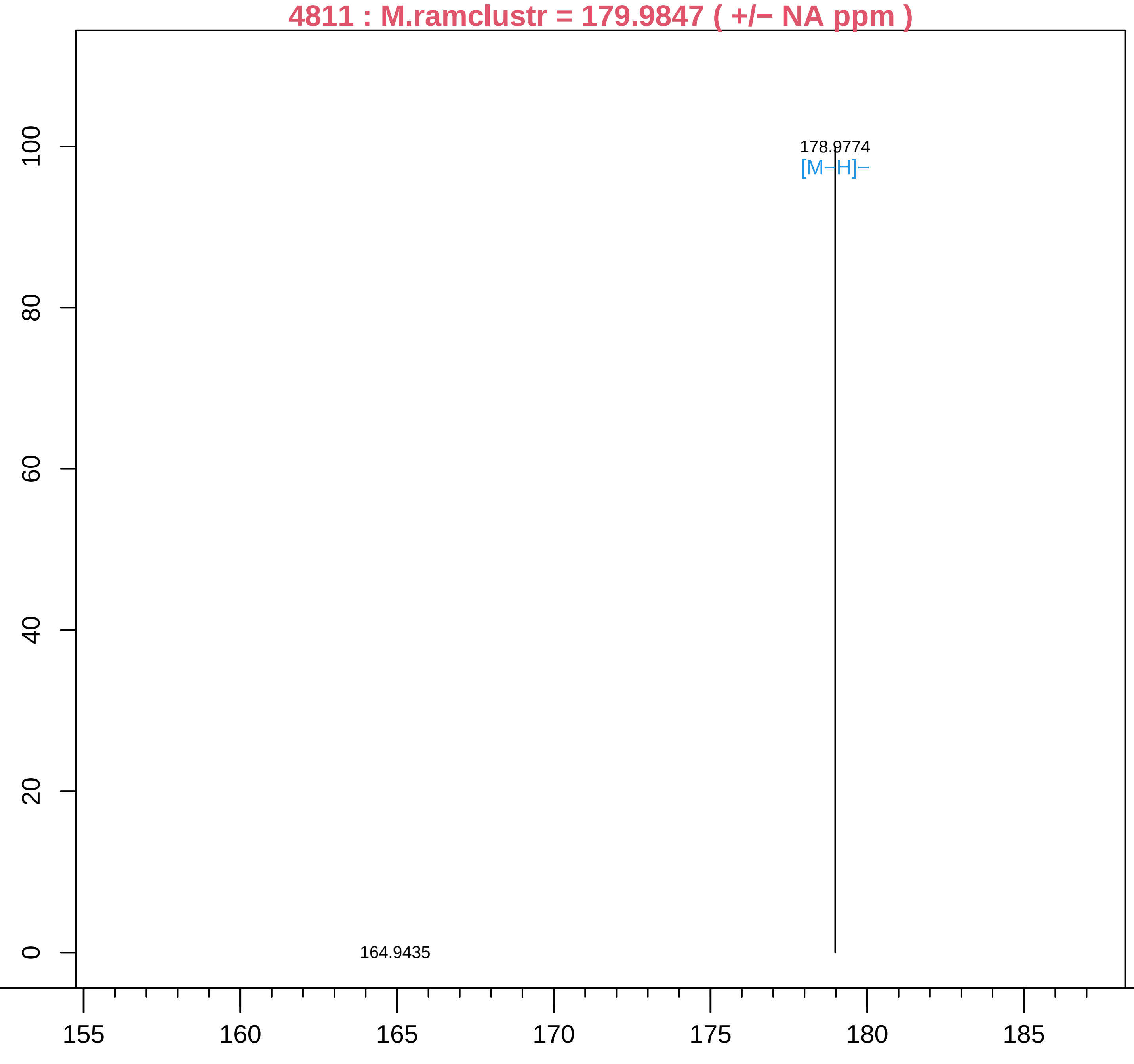
4811 : M.ramclustr = 179.9847 (+/− NA ppm)
178.9774
[M−H]−
164.9435
0
20
40
60
80
100
155
160
165
170
175
180
185

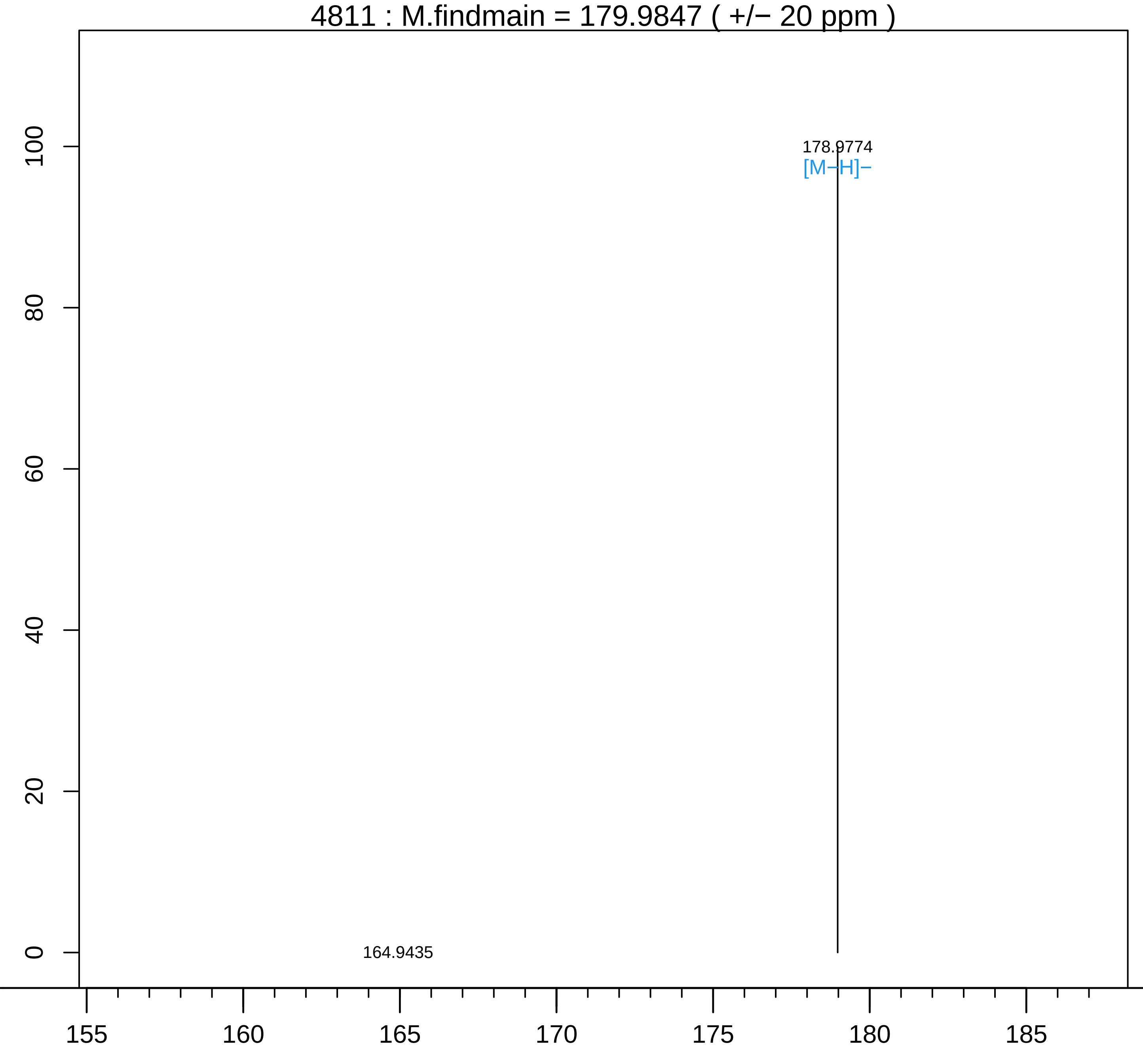
4811 : M.findmain = 179.9847 (+/− 20 ppm)
178.9774
[M−H]−
164.9435
0
20
40
60
80
100
155
160
165
170
175
180
185

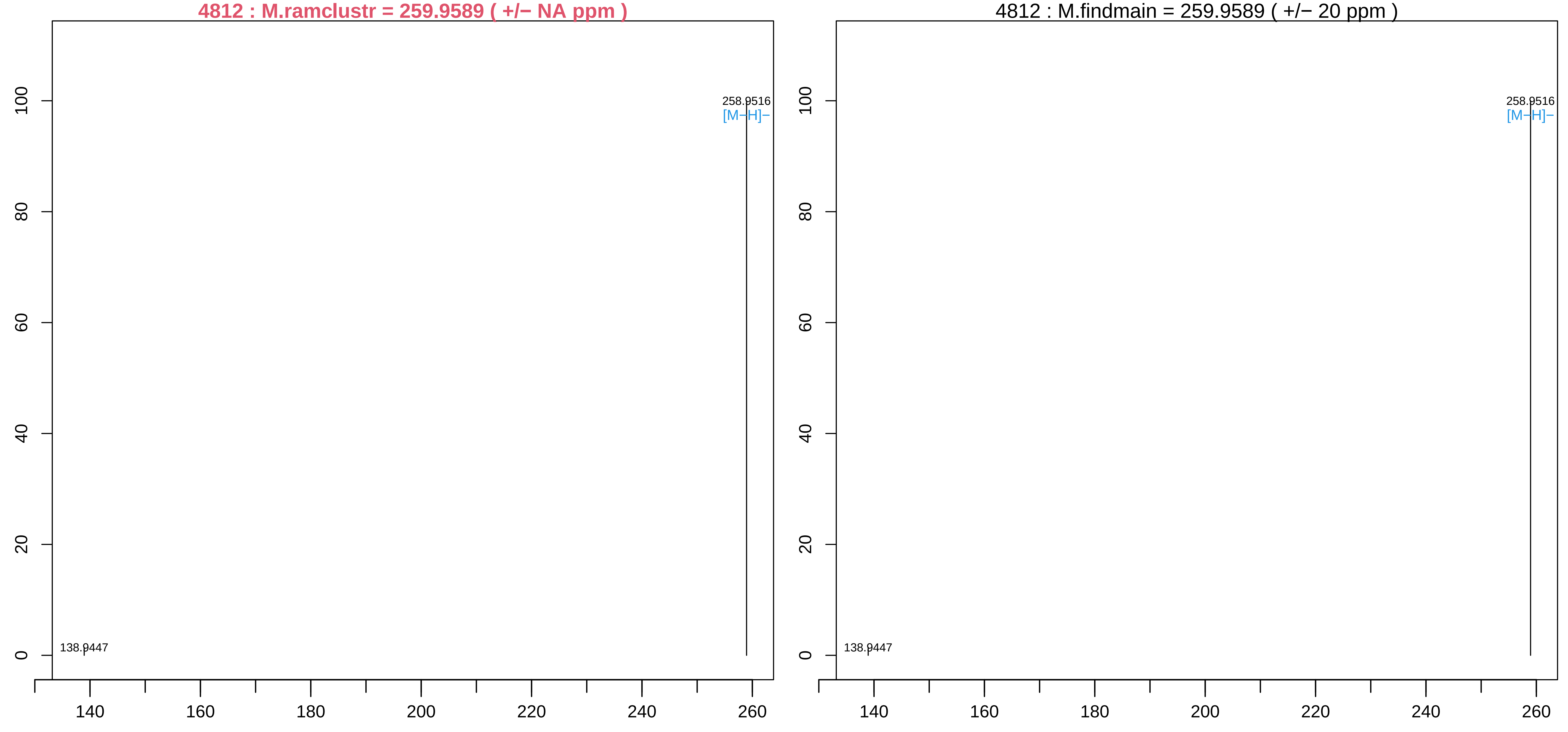
4812 : M.ramclustr = 259.9589 (+/− NA ppm)
258.9516
[M−H]−
138.9447
4812 : M.findmain = 259.9589 (+/− 20 ppm)
258.9516
[M−H]−
138.9447
0
20
40
60
80
100
140
160
180
200
220
240
260

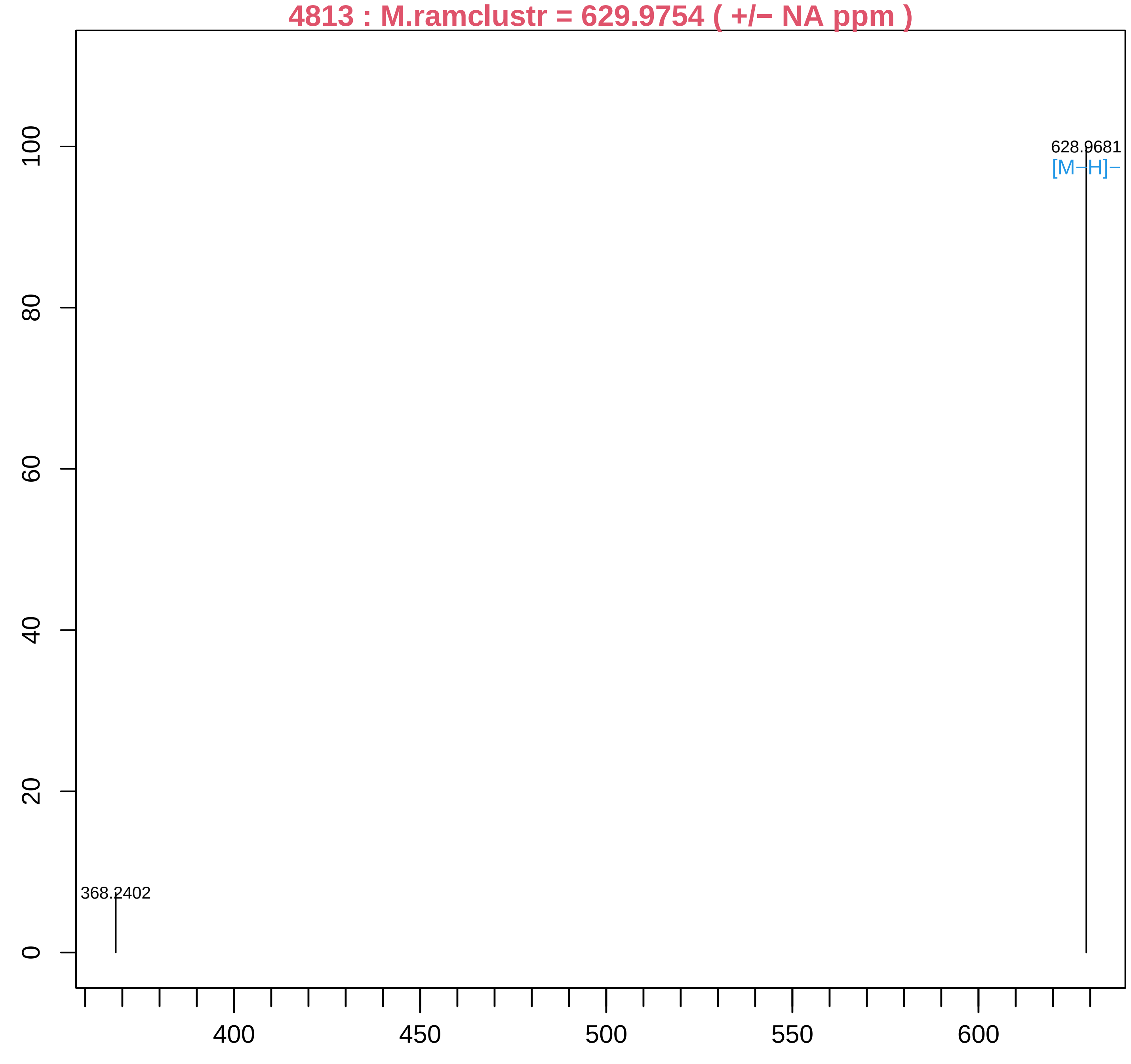
4813 : M.ramclustr = 629.9754 (+/− NA ppm)
628.9681
[M−H]−
368.2402
0
20
40
60
80
100
400
450
500
550
600

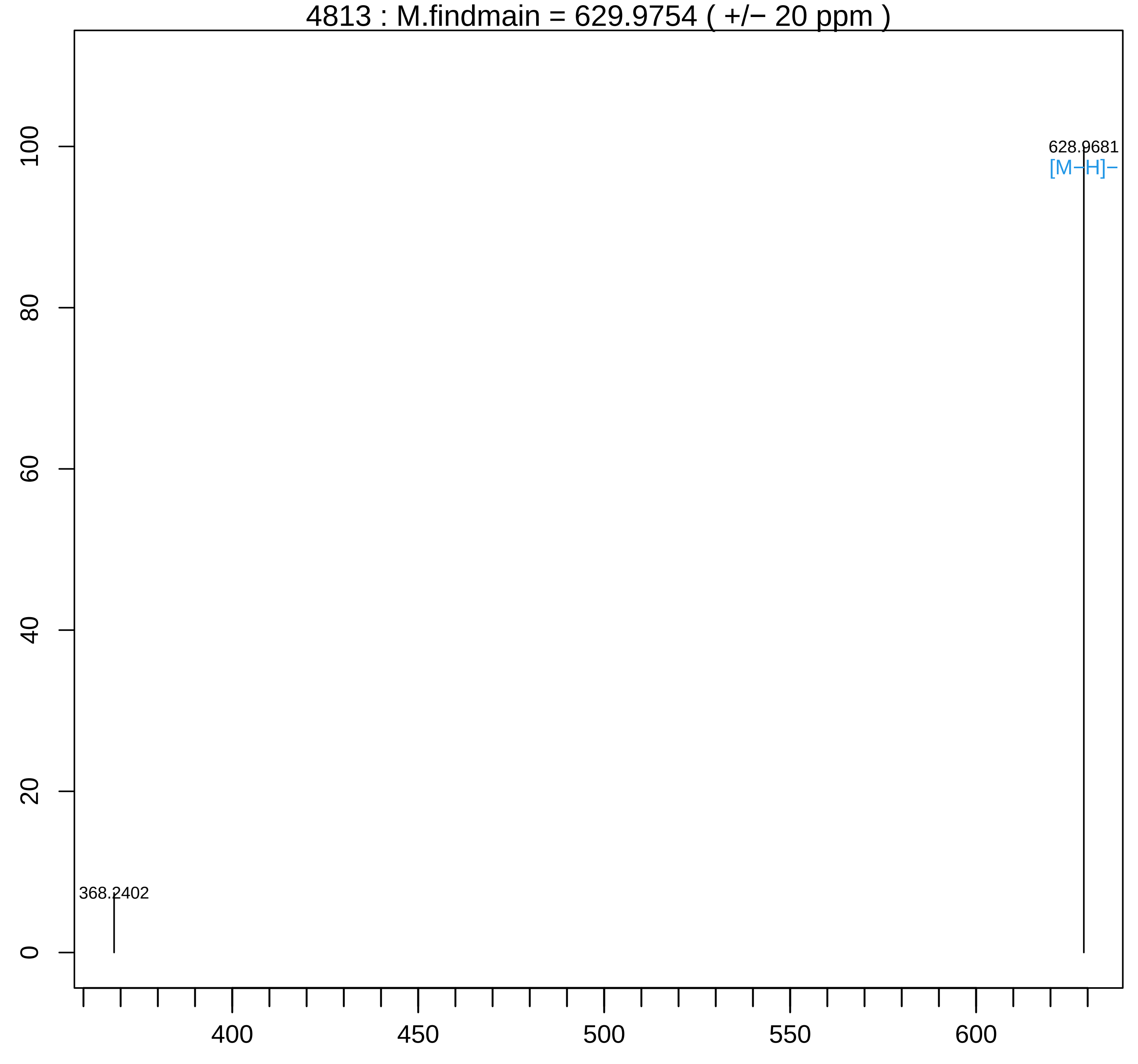
4813 : M.findmain = 629.9754 (+/− 20 ppm)
628.9681
[M−H]−
368.2402
0
20
40
60
80
100
400
450
500
550
600

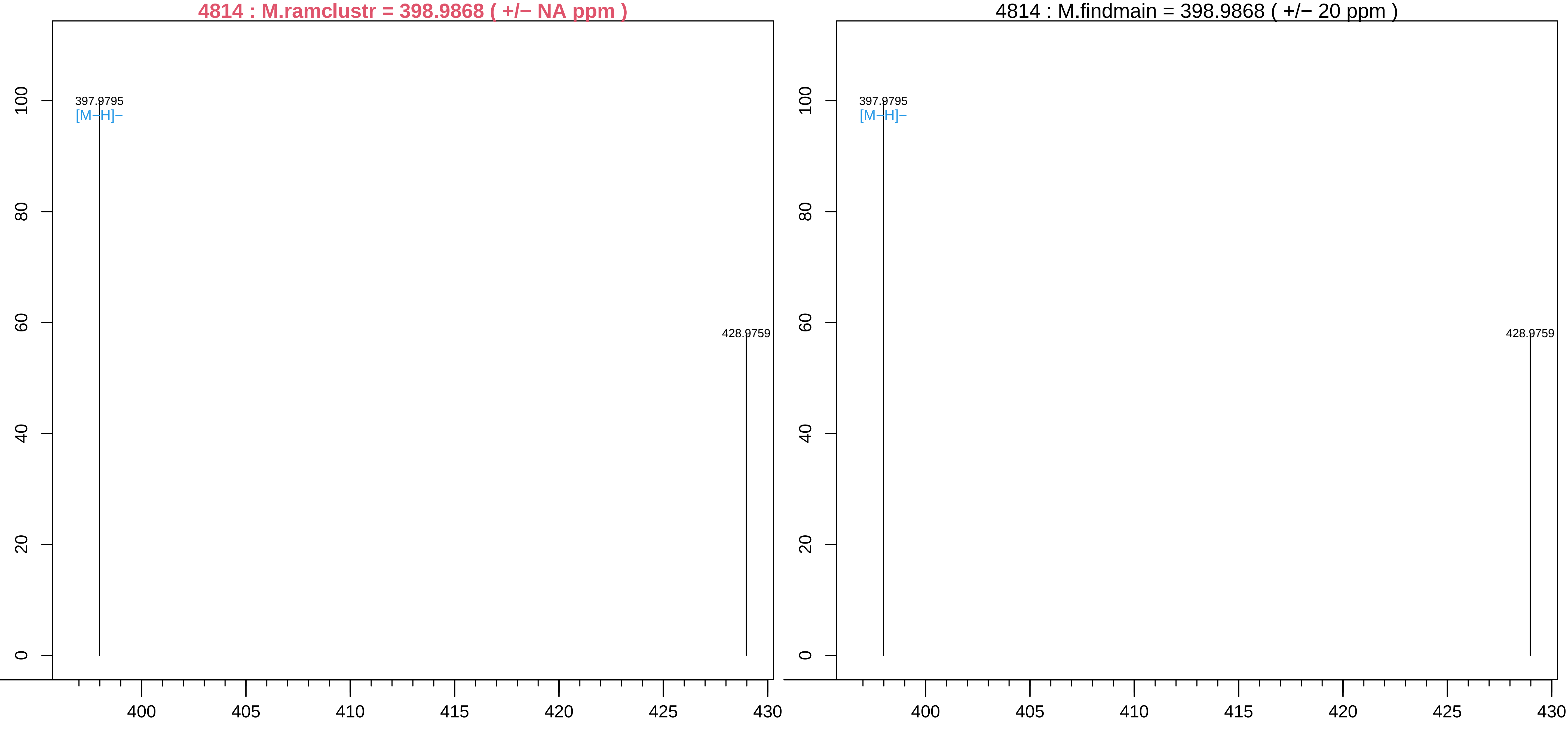
4814 : M.ramclustr = 398.9868 (+/− NA ppm)
397.9795
[M−H]−
428.9759
0
20
40
60
80
100
400
405
410
415
420
425
430
4814 : M.findmain = 398.9868 (+/− 20 ppm)
397.9795
[M−H]−
428.9759
0
20
40
60
80
100
400
405
410
415
420
425
430

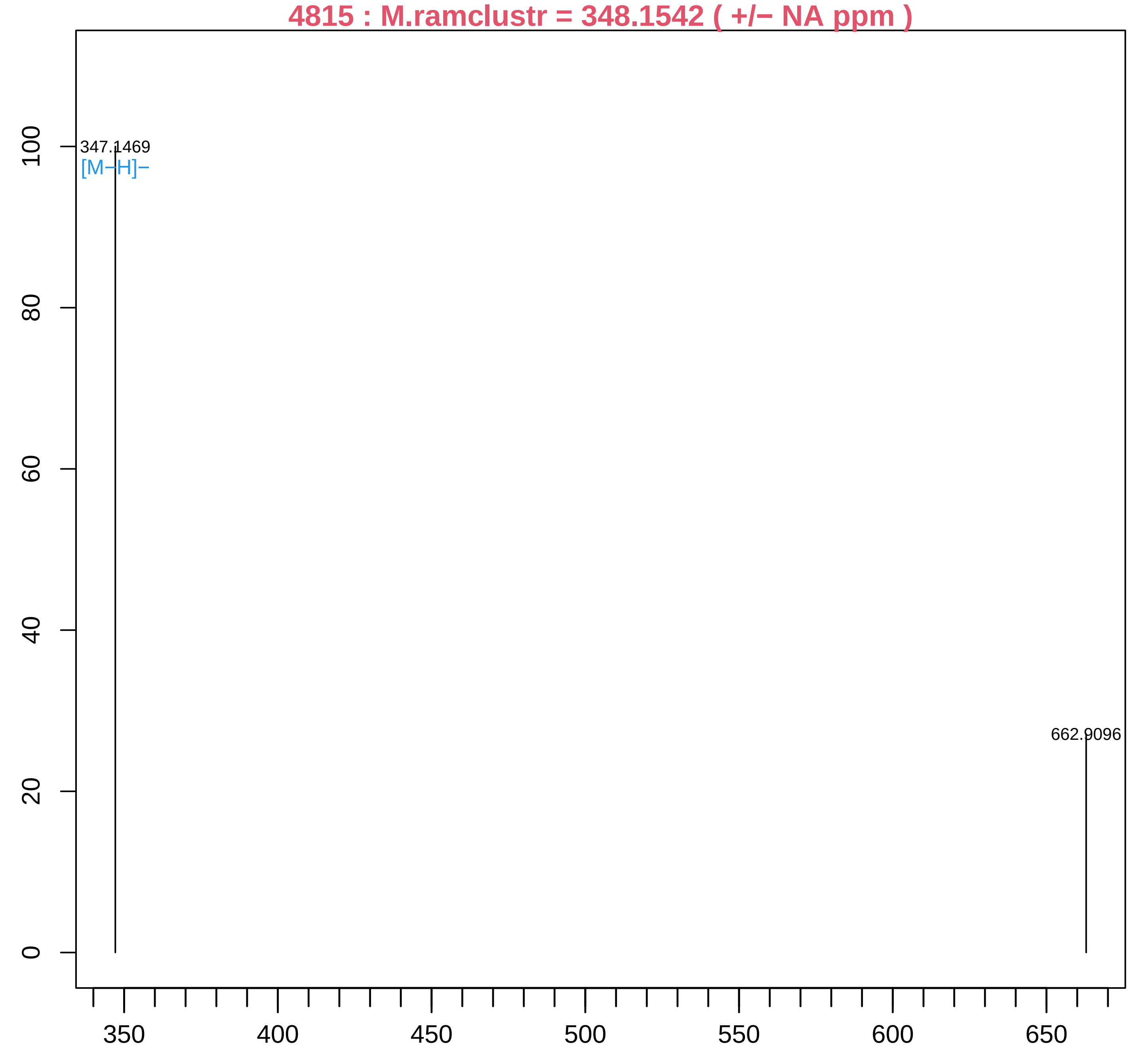
4815 : M.ramclustr = 348.1542 (+/− NA ppm)
347.1469
[M−H]−
662.9096
0
20
40
60
80
100
350
400
450
500
550
600
650

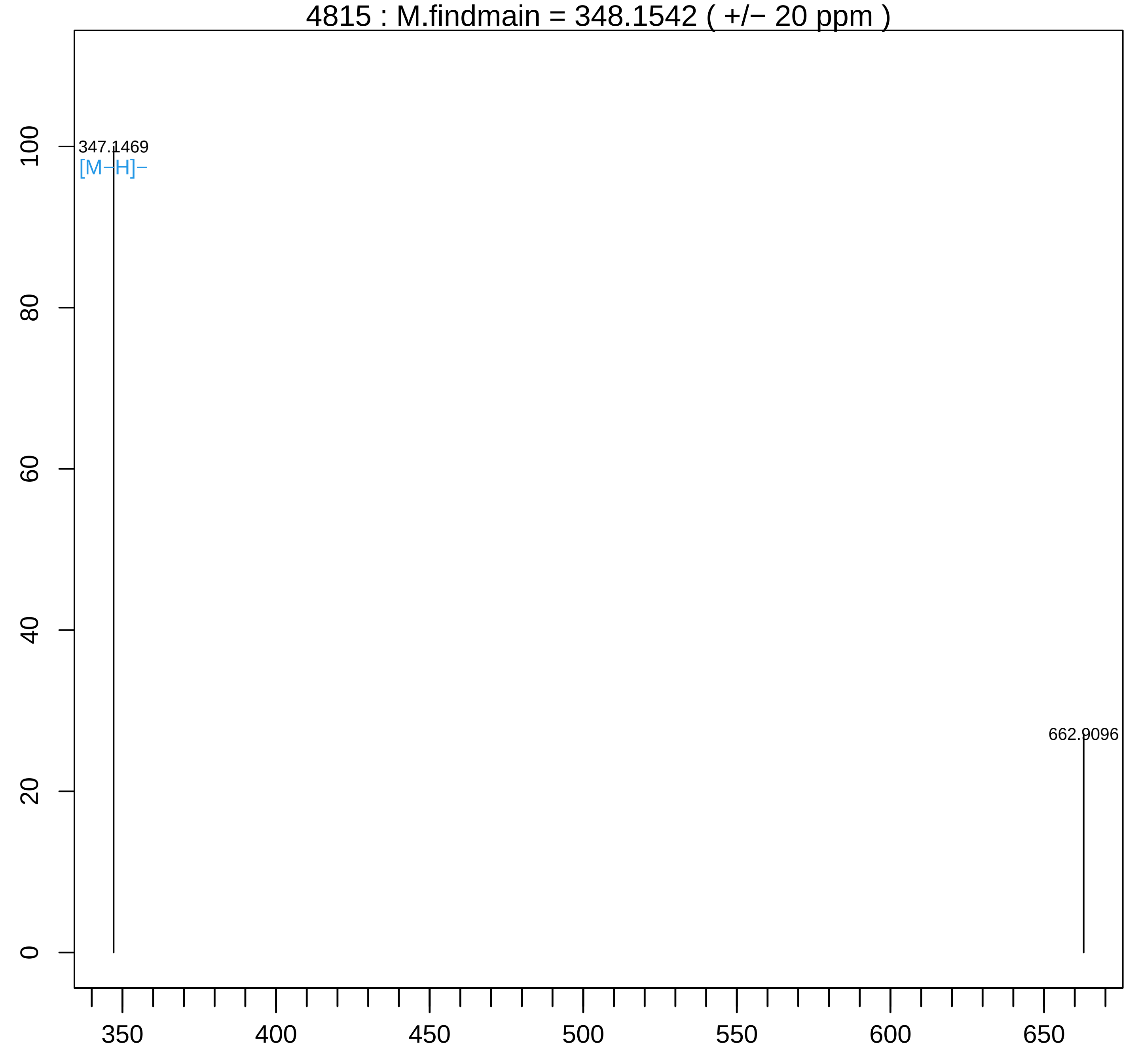
4815 : M.findmain = 348.1542 (+/− 20 ppm)
347.1469
[M−H]−
662.9096
0
20
40
60
80
100
350
400
450
500
550
600
650

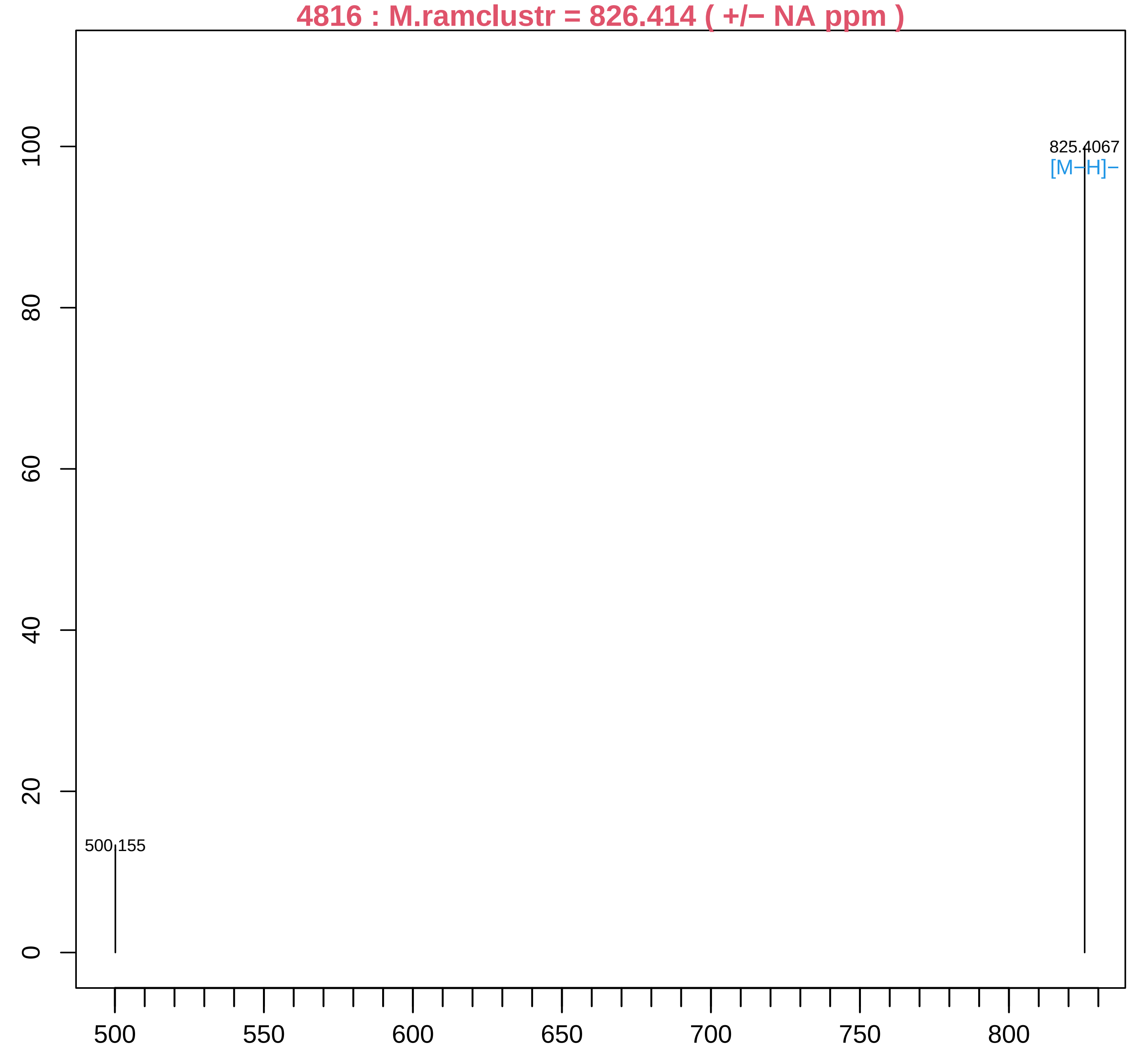
4816 : M.ramclustr = 826.414 (+/− NA ppm)
825.4067
[M−H]−
500.155
0
20
40
60
80
100
500
550
600
650
700
750
800

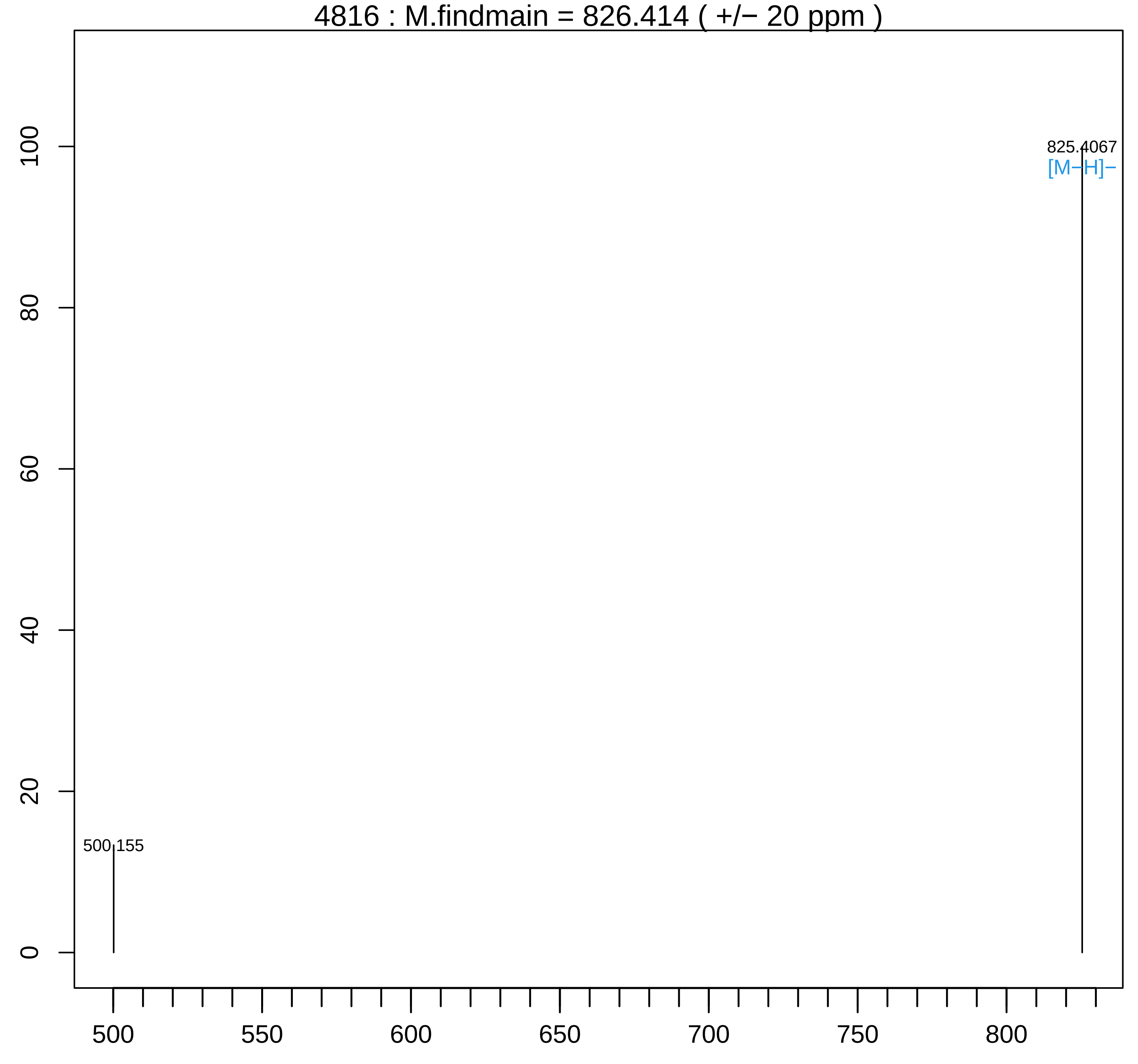
4816 : M.findmain = 826.414 (+/− 20 ppm)
825.4067
[M−H]−
500.155
0
20
40
60
80
100
500
550
600
650
700
750
800

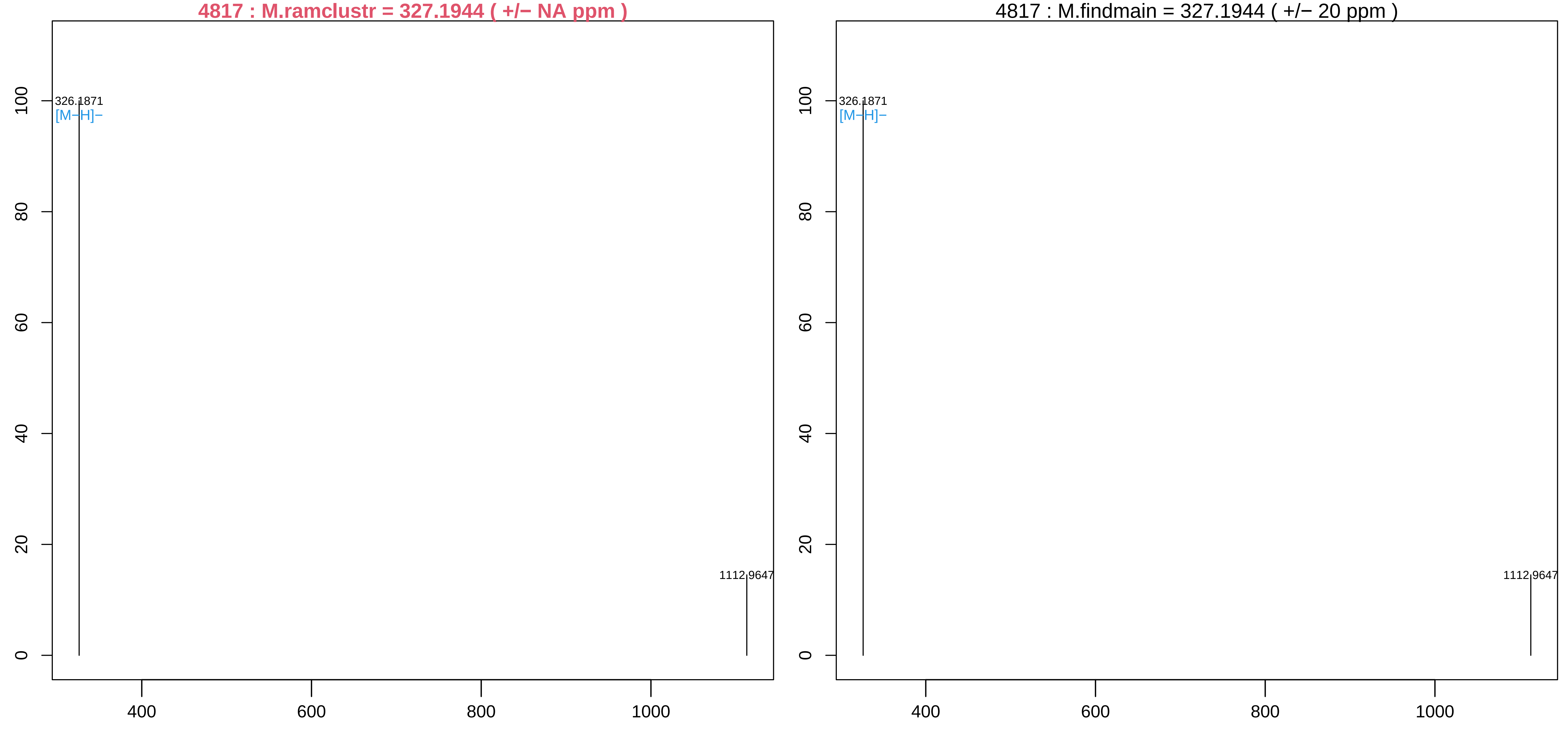
4817 : M.ramclustr = 327.1944 (+/− NA ppm)
326.1871
[M−H]−
1112.9647
0
20
40
60
80
100
400
600
800
1000
4817 : M.findmain = 327.1944 (+/− 20 ppm)
326.1871
[M−H]−
1112.9647
0
20
40
60
80
100
400
600
800
1000

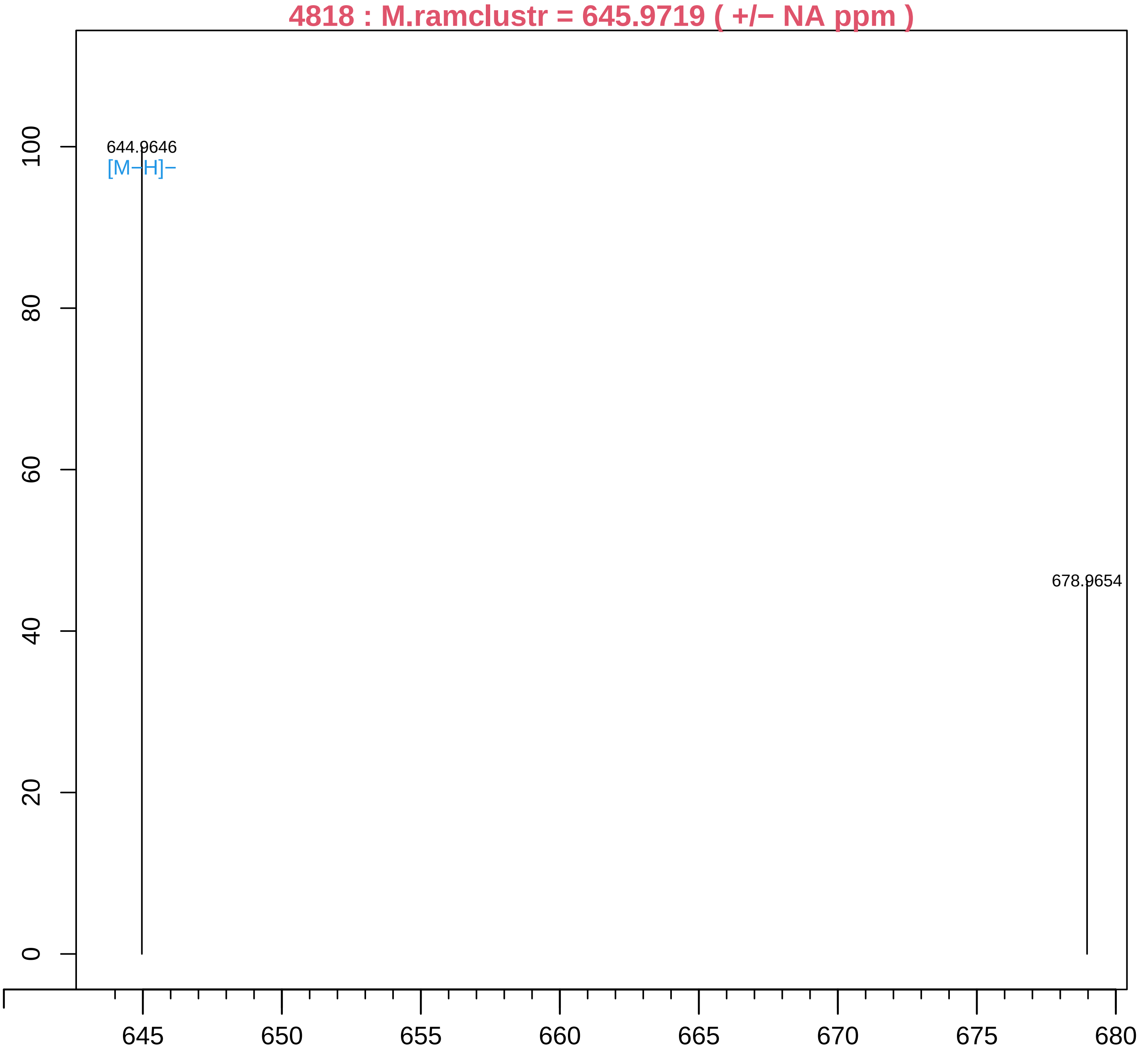
4818 : M.ramclustr = 645.9719 (+/− NA ppm)
644.9646
[M−H]−
678.9654
0
20
40
60
80
100
645
650
655
660
665
670
675
680

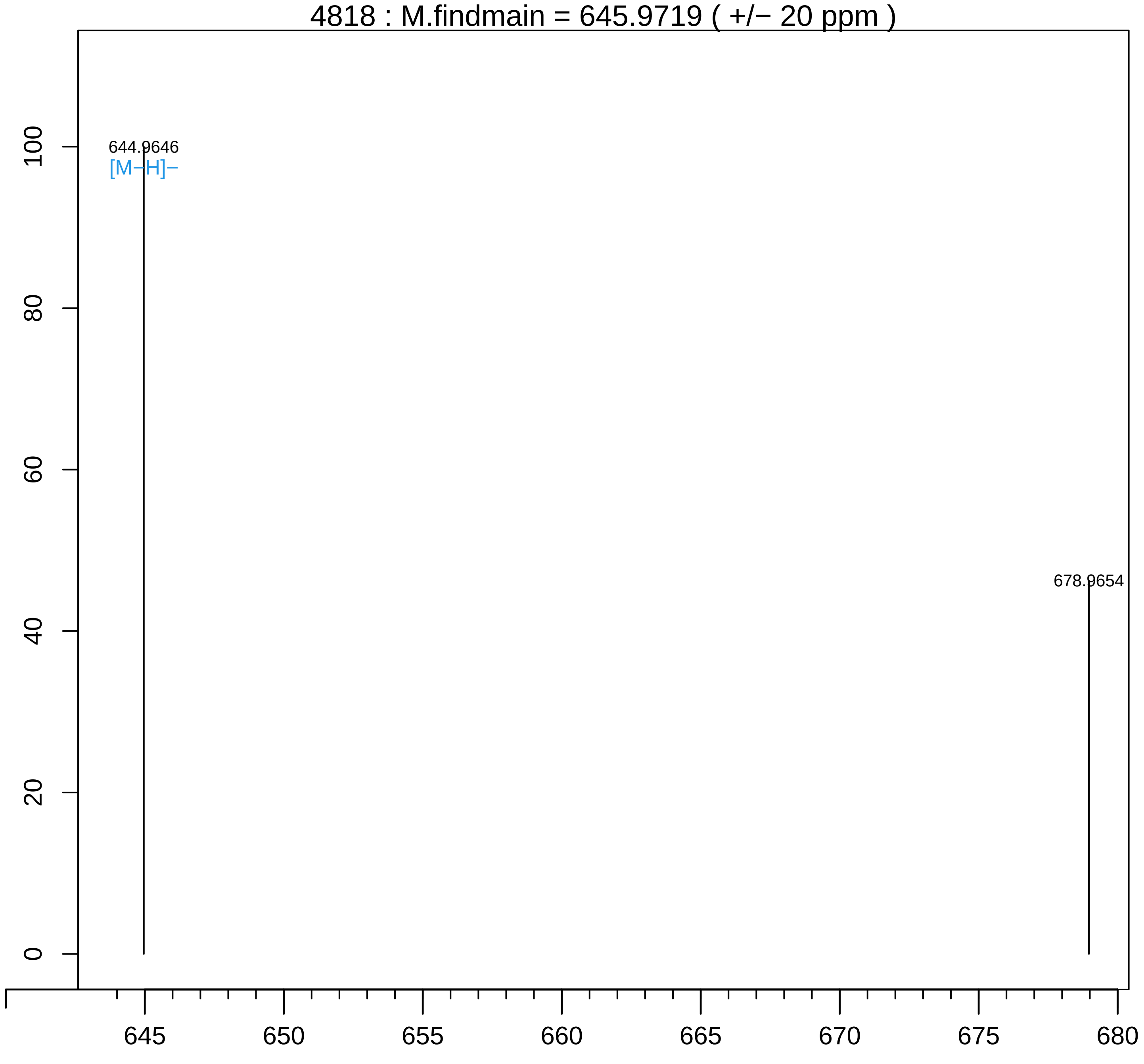
4818 : M.findmain = 645.9719 (+/− 20 ppm)
644.9646
[M−H]−
678.9654
0
20
40
60
80
100
645
650
655
660
665
670
675
680

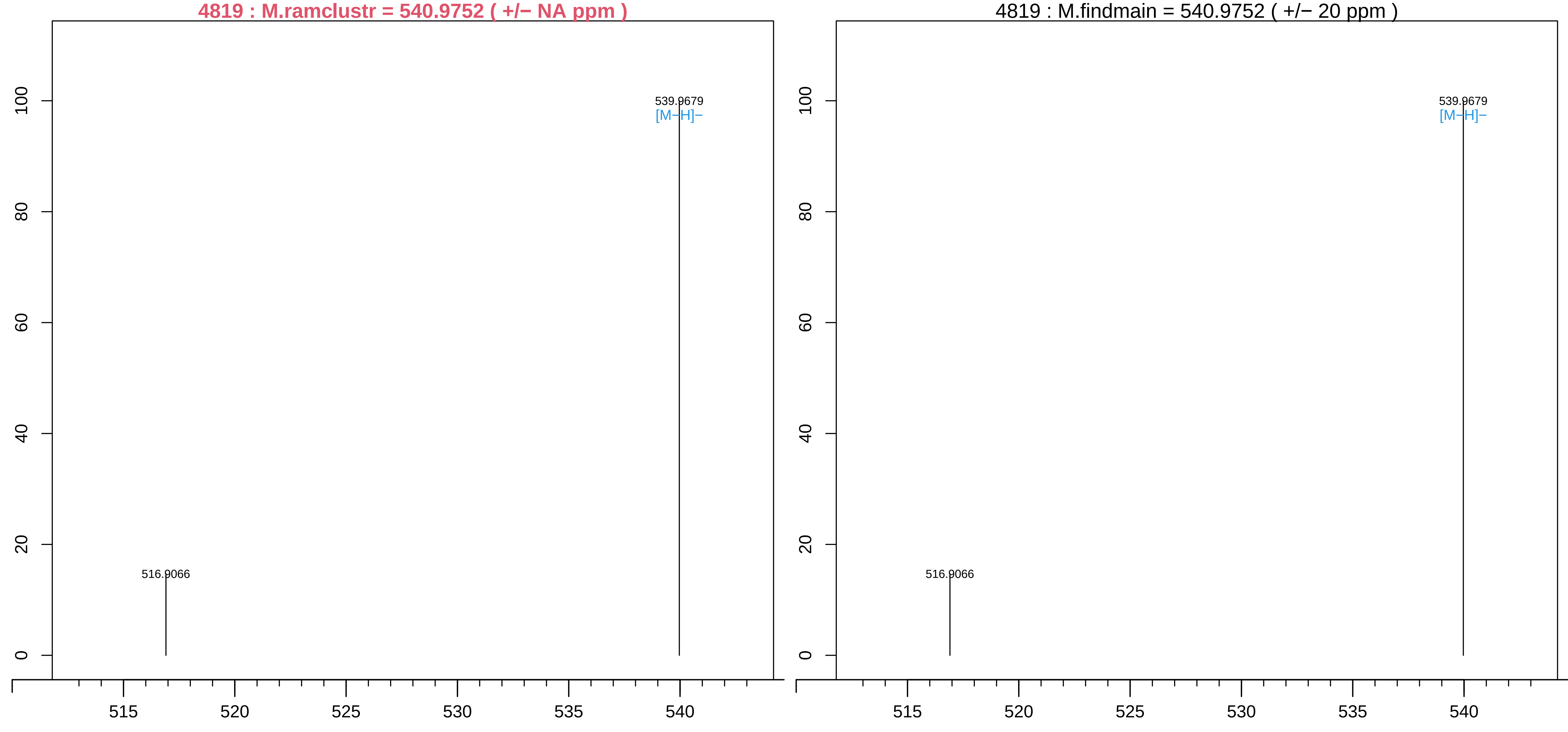
4819 : M.ramclustr = 540.9752 (+/− NA ppm)
4819 : M.findmain = 540.9752 (+/− 20 ppm)
539.9679
[M−H]−
516.9066
0
20
40
60
80
100
515
520
525
530
535
540

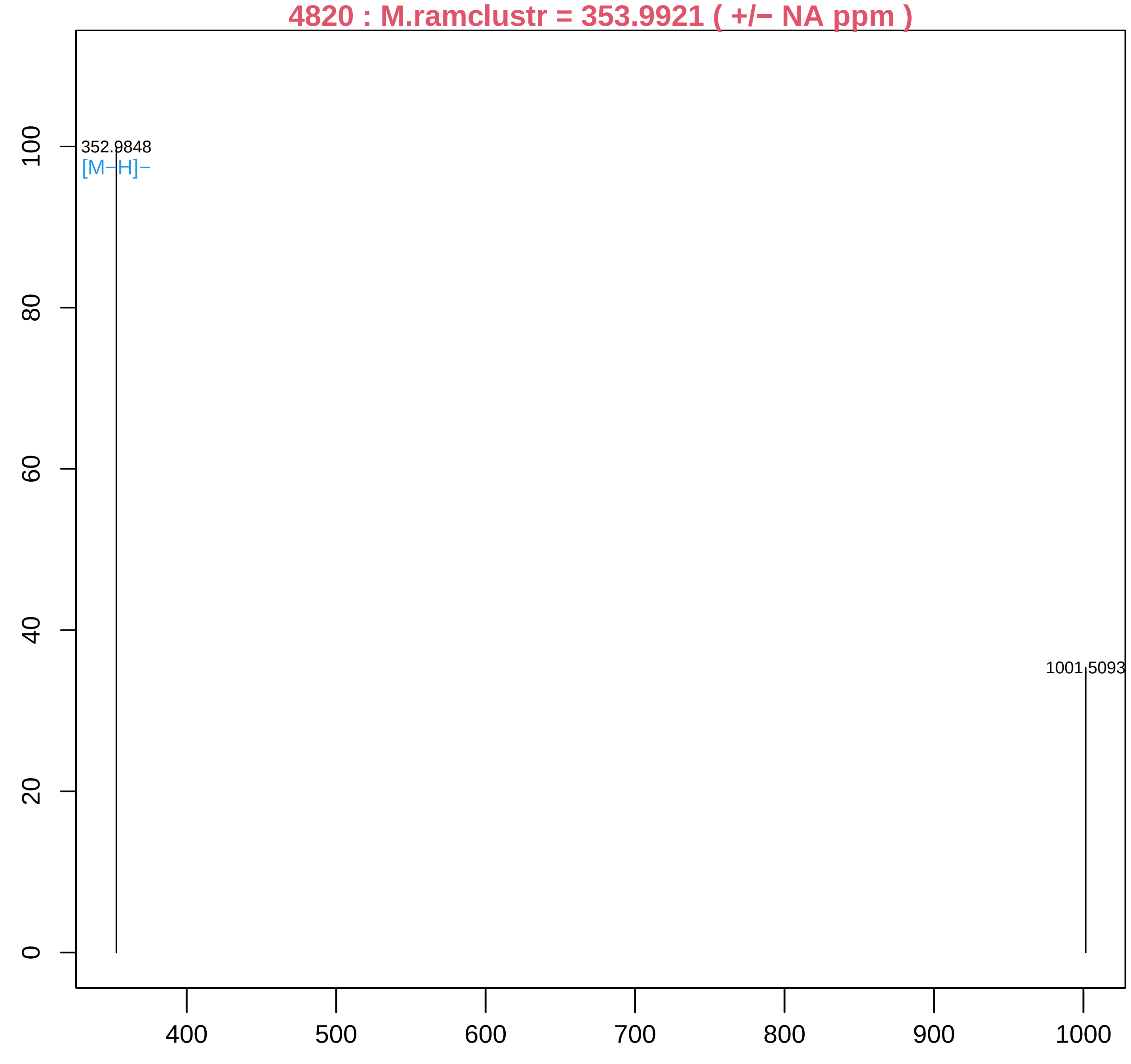
4820 : M.ramclustr = 353.9921 (+/− NA ppm)
352.9848
[M−H]−
1001.5093
0
20
40
60
80
100
400
500
600
700
800
900
1000
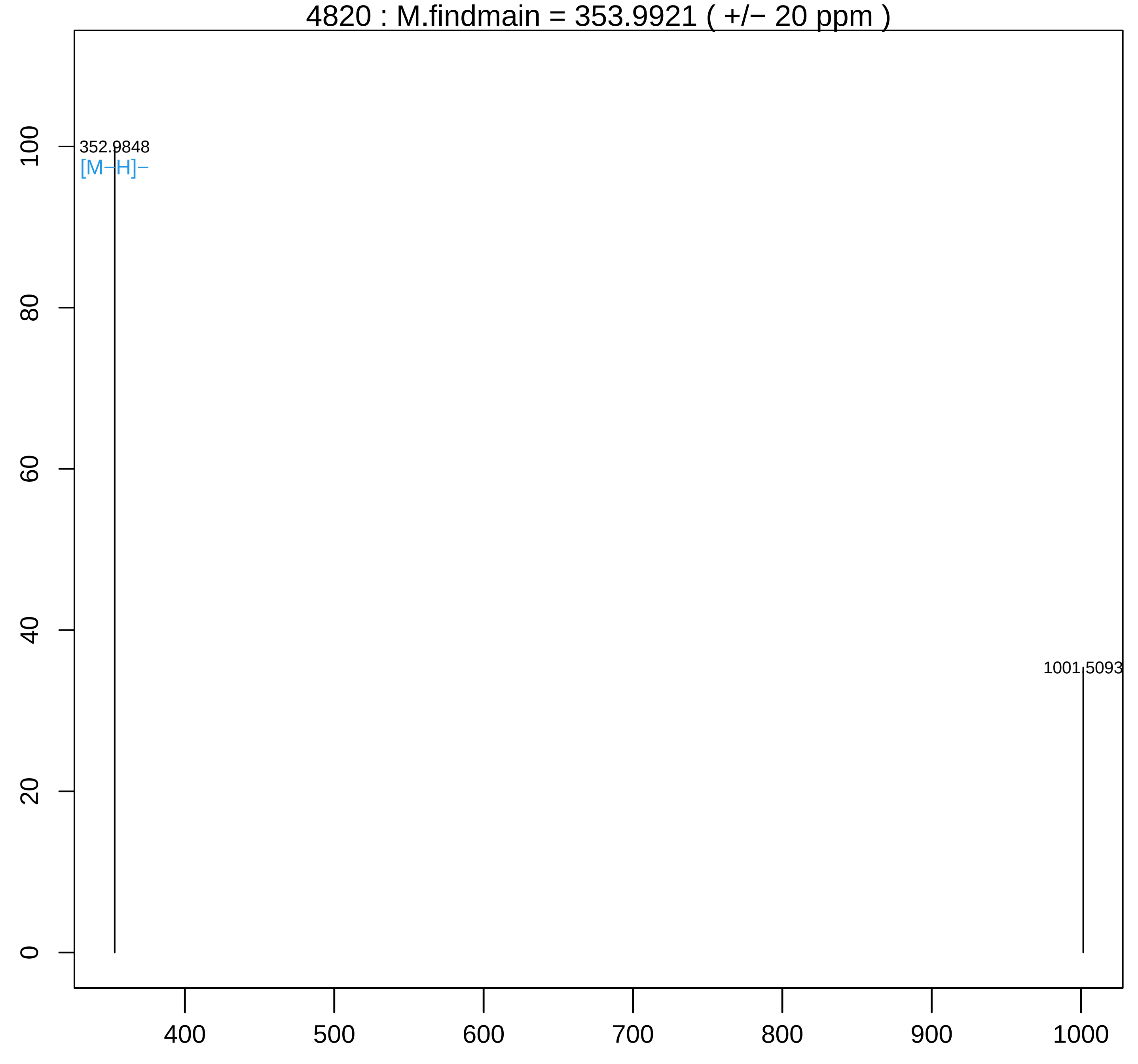
4820 : M.findmain = 353.9921 (+/− 20 ppm)
352.9848
[M−H]−
1001.5093
0
20
40
60
80
100
400
500
600
700
800
900
1000

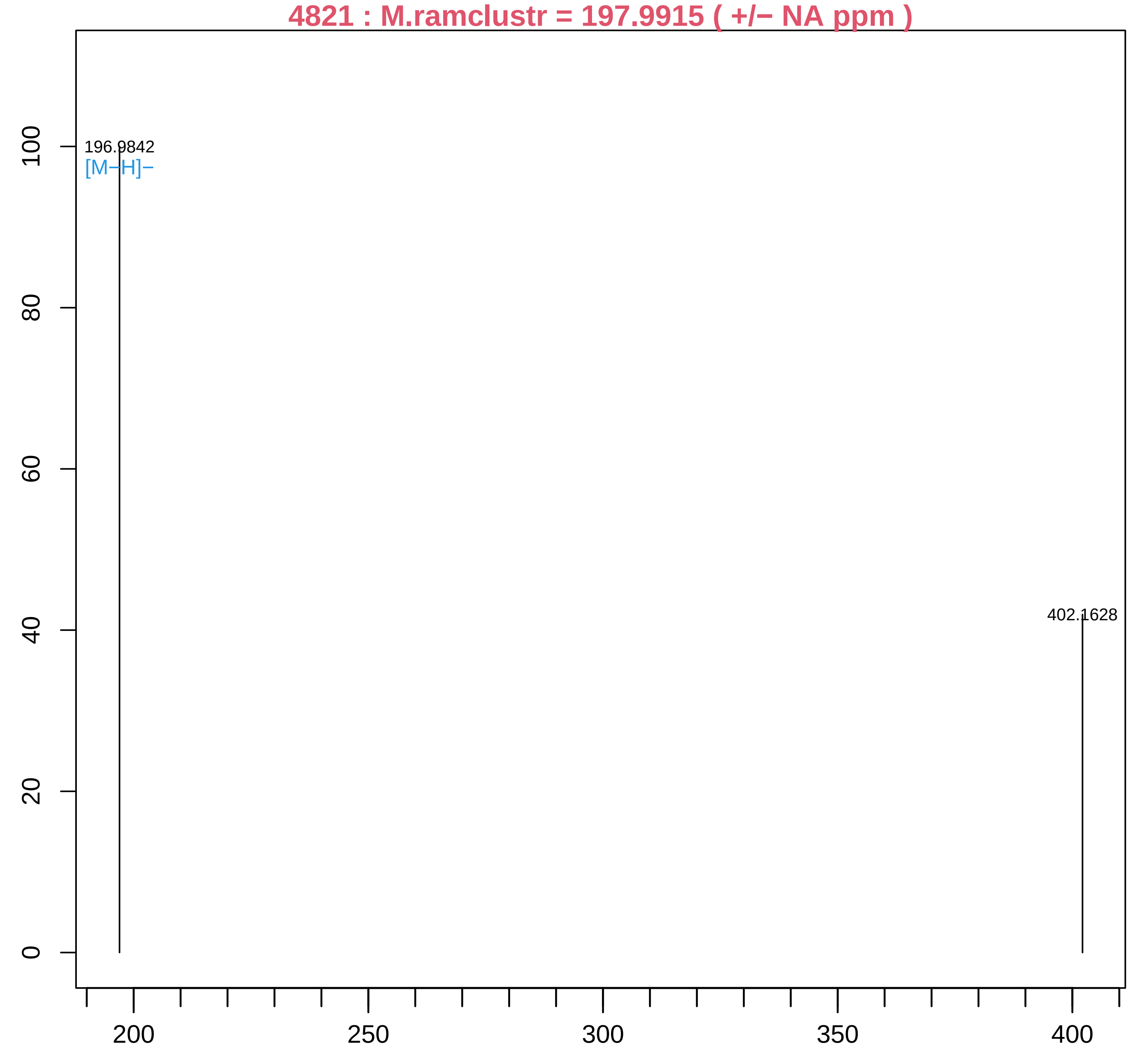

4821 : M.ramclustr = 197.9915 (+/− NA ppm)
196.9842
[M−H]−
402.1628
0
20
40
60
80
100
200
250
300
350
400

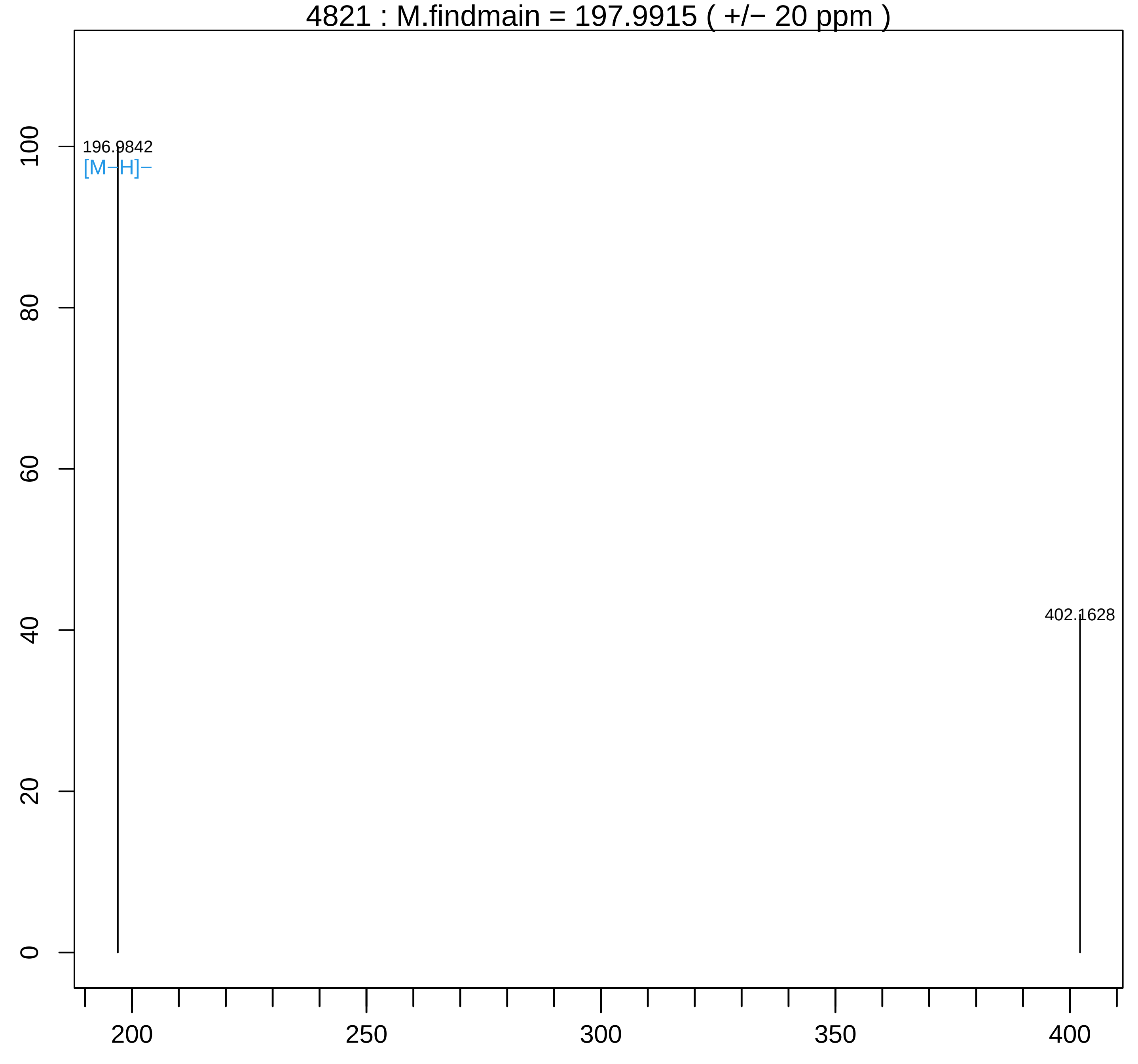

4821 : M.findmain = 197.9915 (+/− 20 ppm)
196.9842
[M−H]−
402.1628
0
20
40
60
80
100
200
250
300
350
400

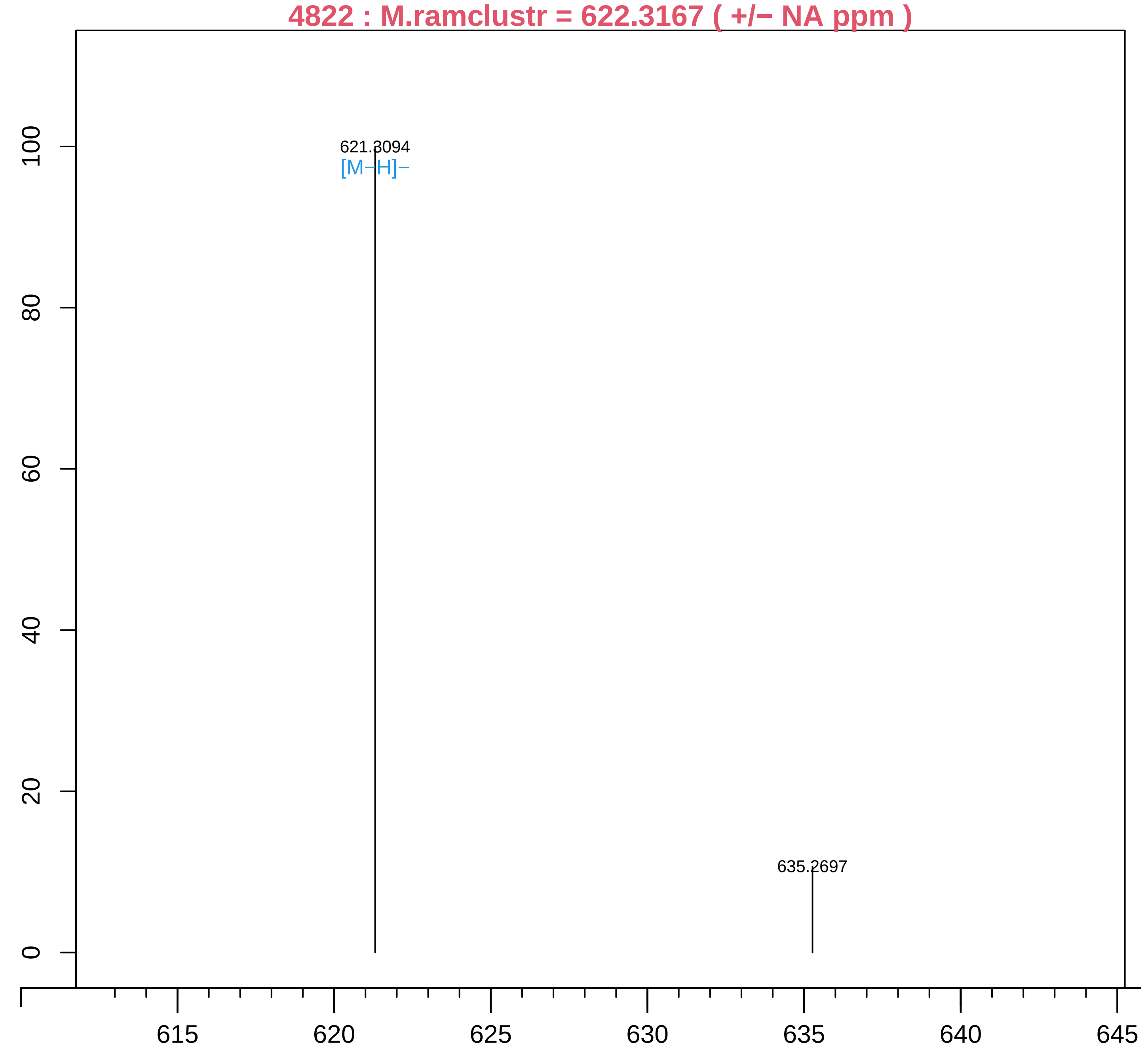

4822 : M.ramclustr = 622.3167 (+/− NA ppm)
621.3094
[M−H]−
635.2697
0
20
40
60
80
100
615
620
625
630
635
640
645

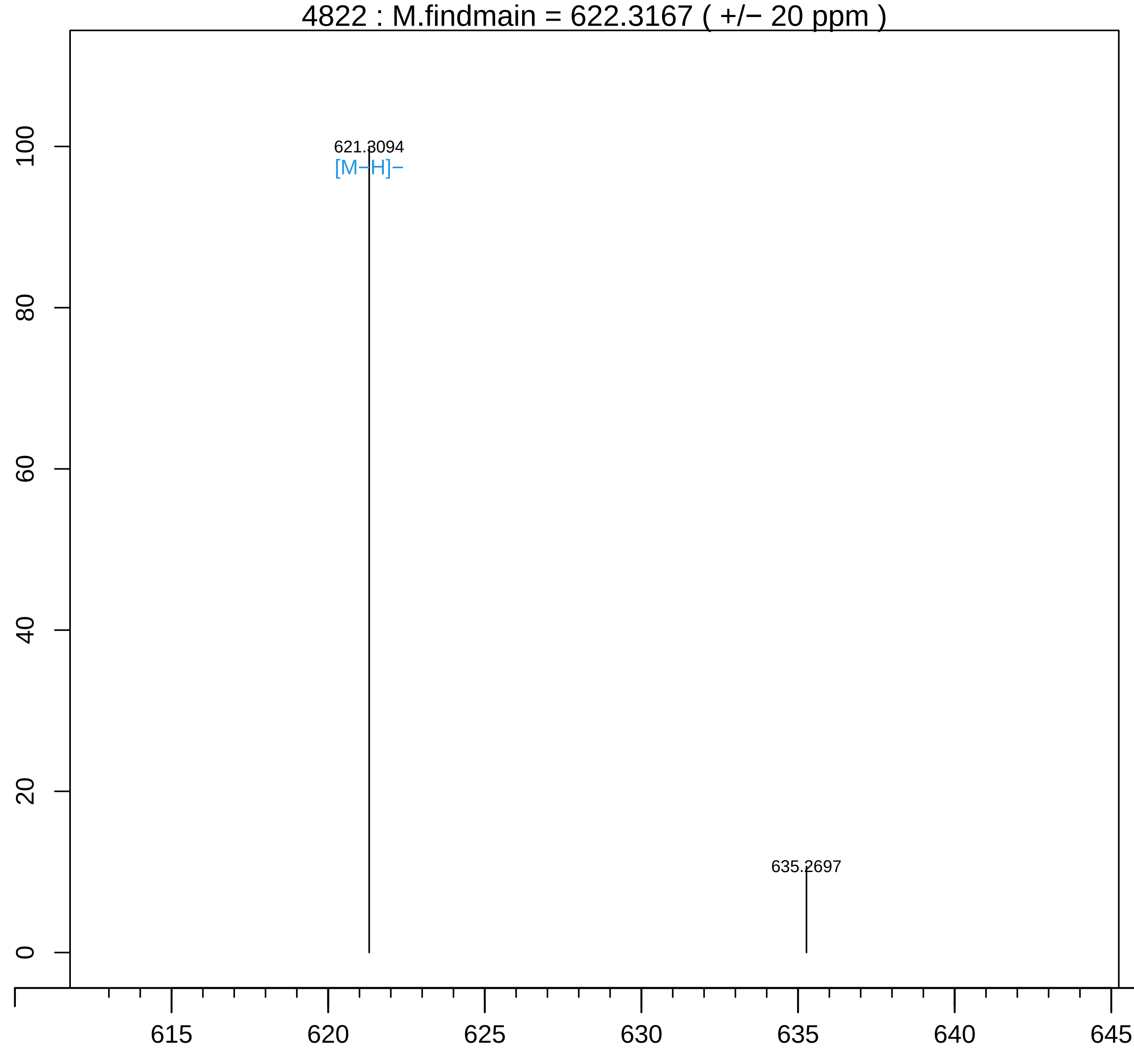

4822 : M.findmain = 622.3167 (+/− 20 ppm)
621.3094
[M−H]−
635.2697
0
20
40
60
80
100
615
620
625
630
635
640
645

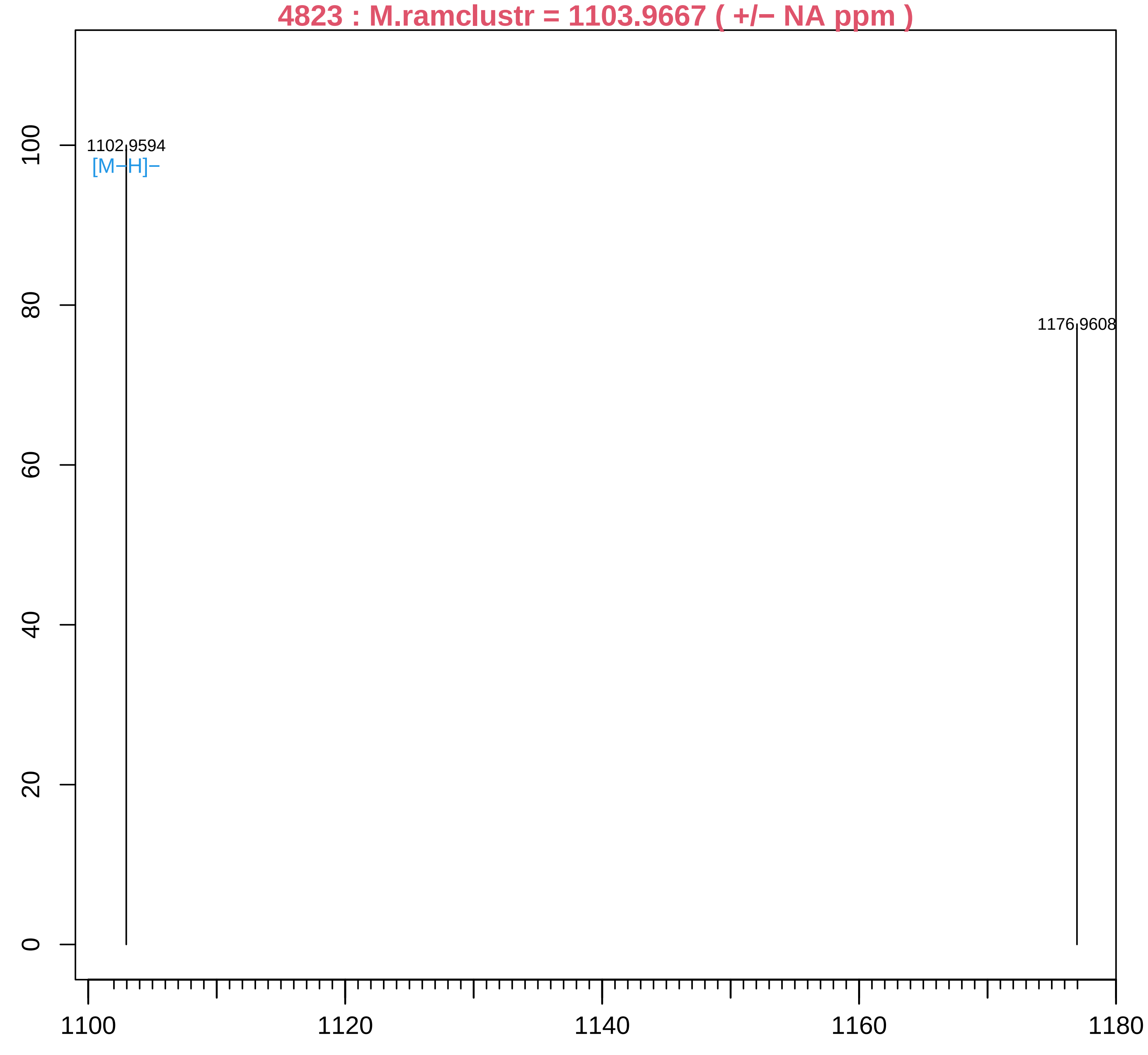
4823 : M.ramclustr = 1103.9667 (+/− NA ppm)
1102.9594
[M−H]−
1176.9608
0
20
40
60
80
100
1100
1120
1140
1160
1180

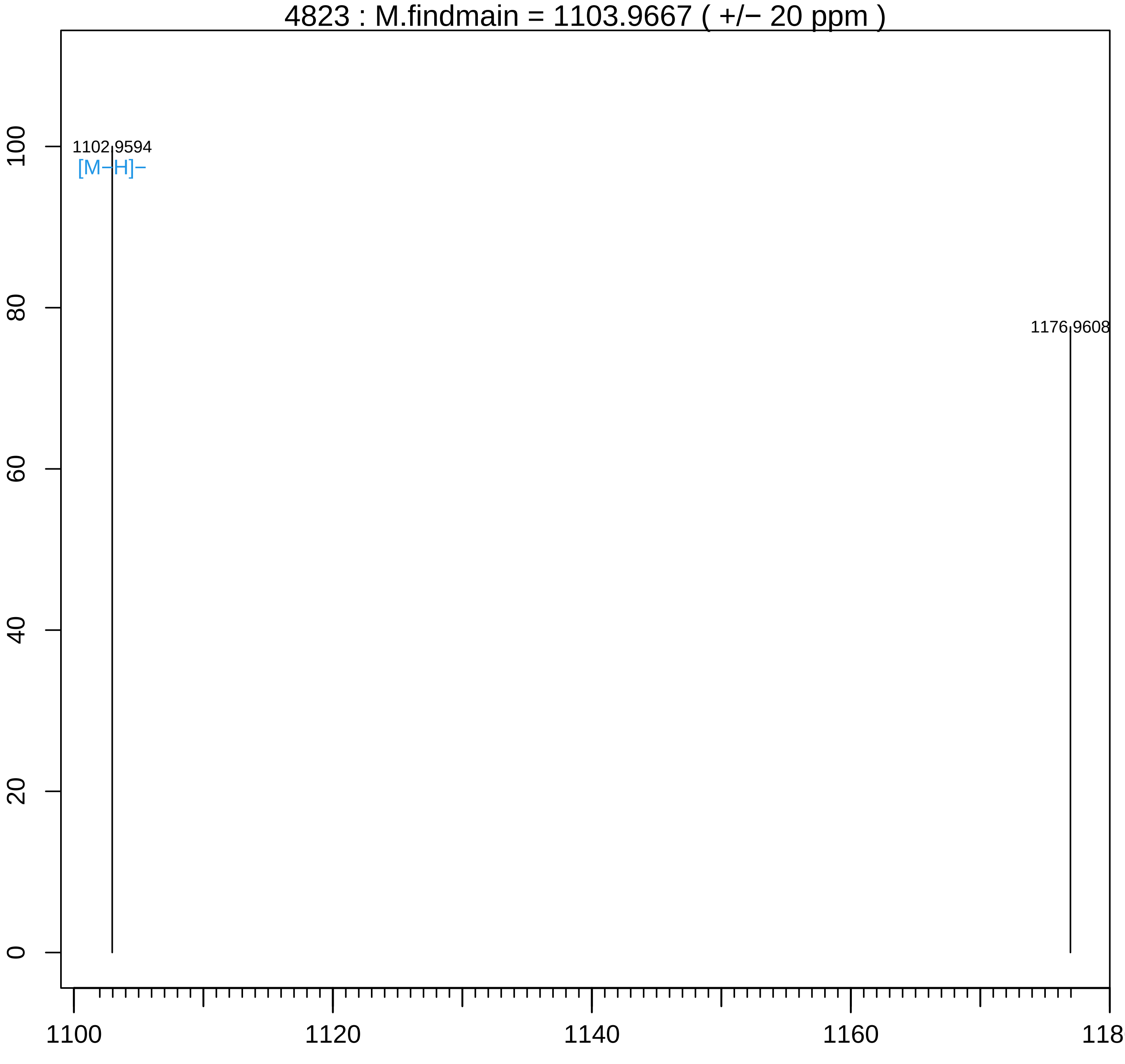
4823 : M.findmain = 1103.9667 (+/− 20 ppm)
1102.9594
[M−H]−
1176.9608
0
20
40
60
80
100
1100
1120
1140
1160
118

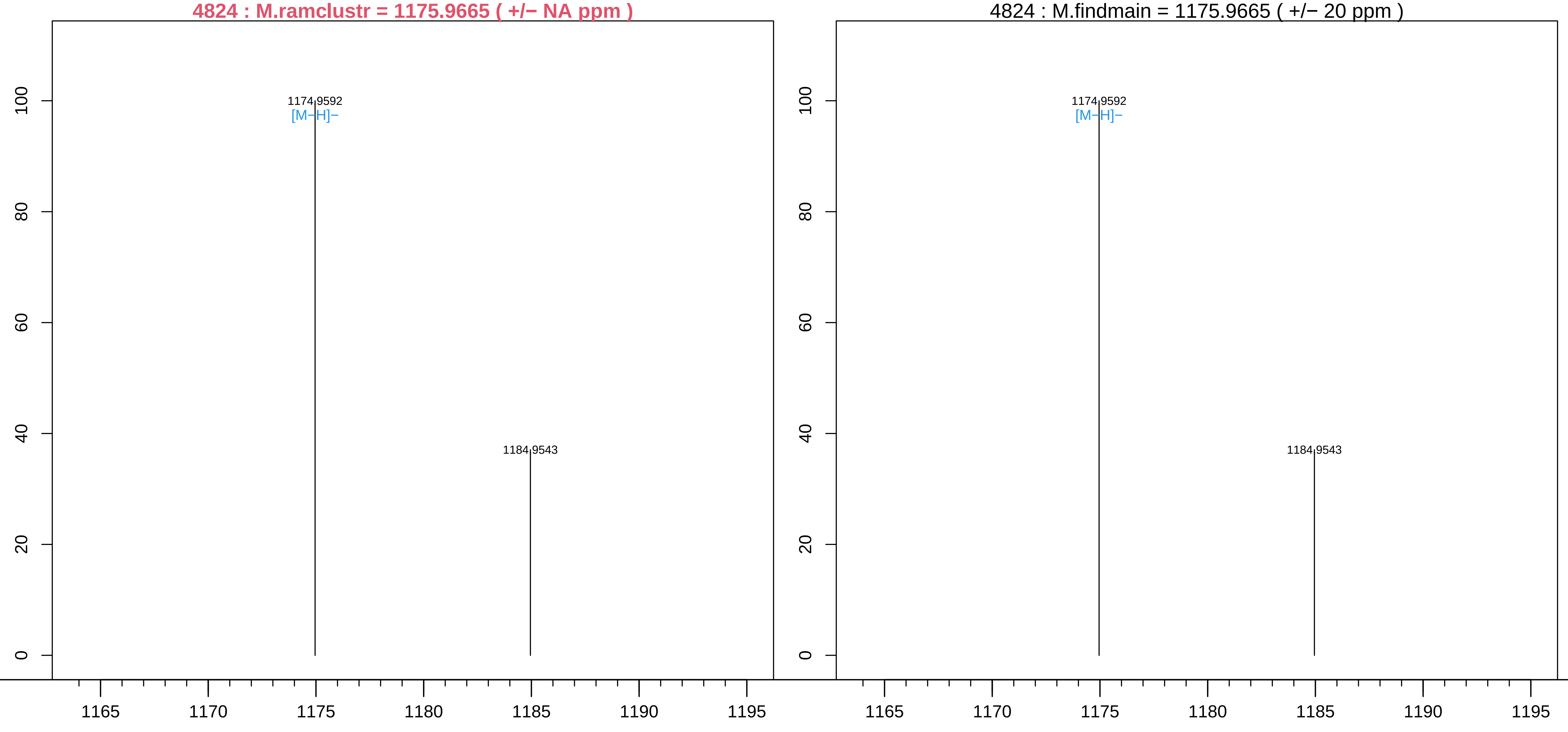

4824 : M.ramclustr = 1175.9665 (+/− NA ppm)
1174.9592
[M−H]−
1184.9543
4824 : M.findmain = 1175.9665 (+/− 20 ppm)
1174.9592
[M−H]−
1184.9543

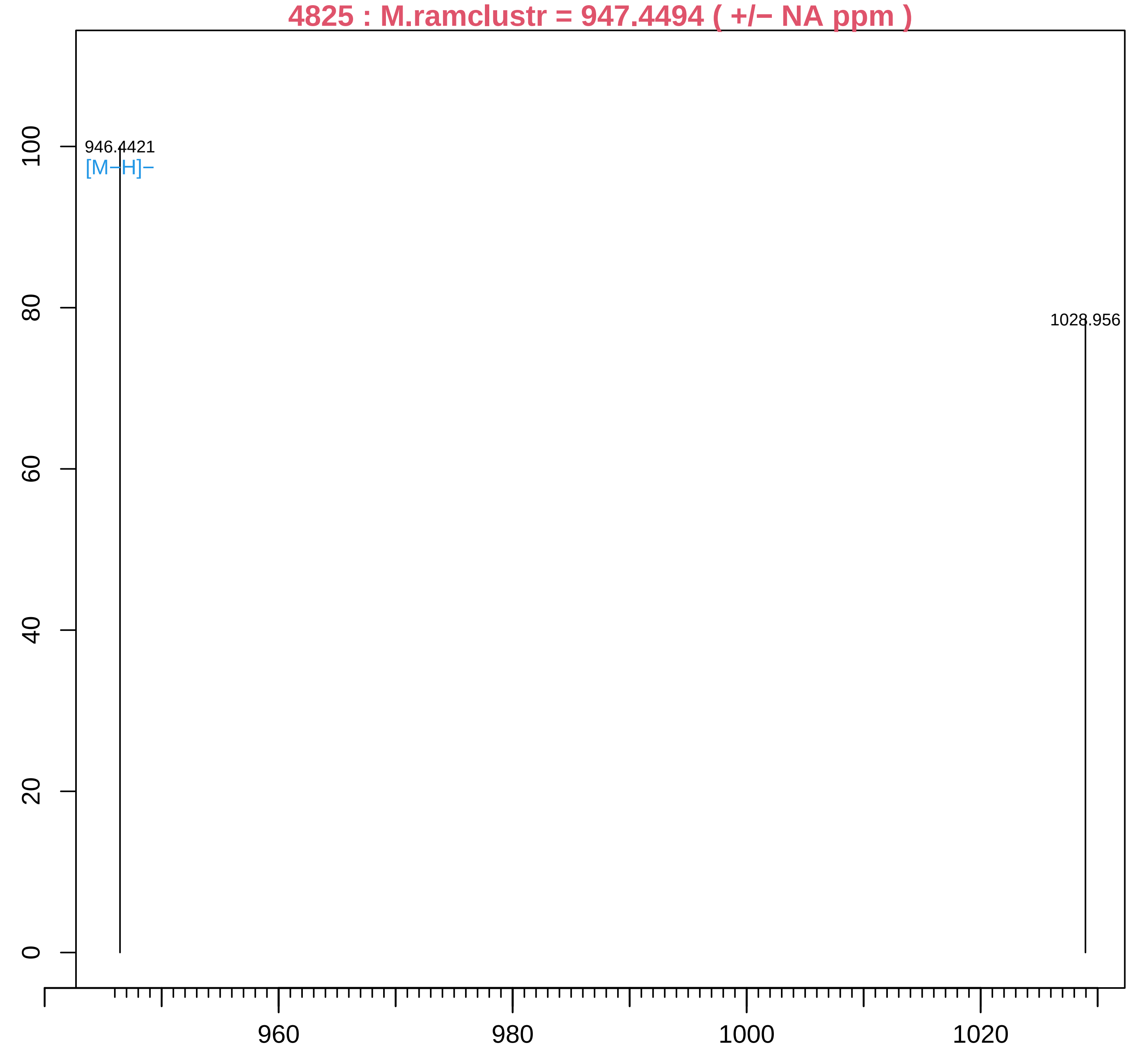
4825 : M.ramclustr = 947.4494 (+/− NA ppm)
946.4421
[M−H]−
1028.956
0
20
40
60
80
100
960
980
1000
1020

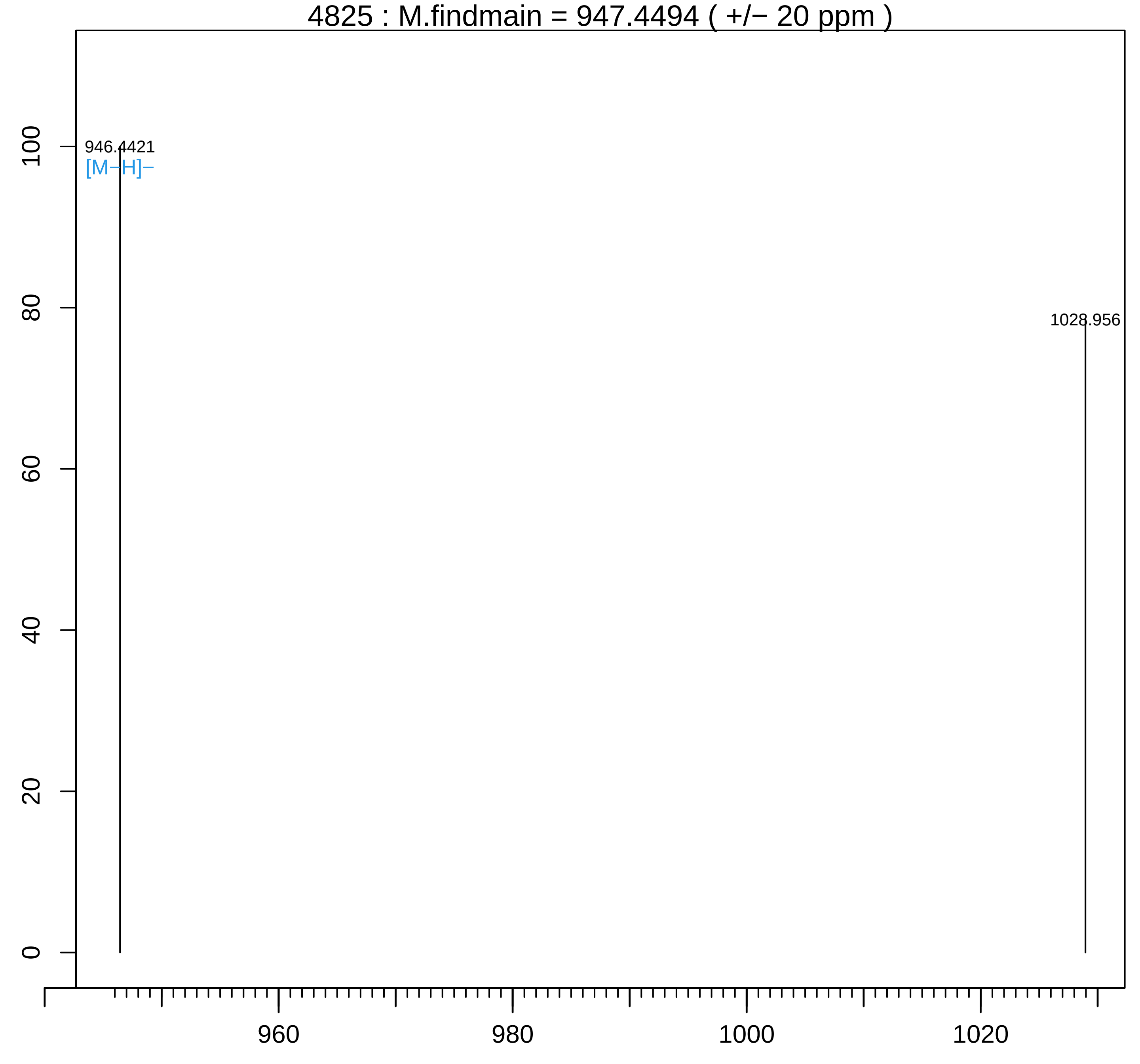
4825 : M.findmain = 947.4494 (+/− 20 ppm)
946.4421
[M−H]−
1028.956
0
20
40
60
80
100
960
980
1000
1020

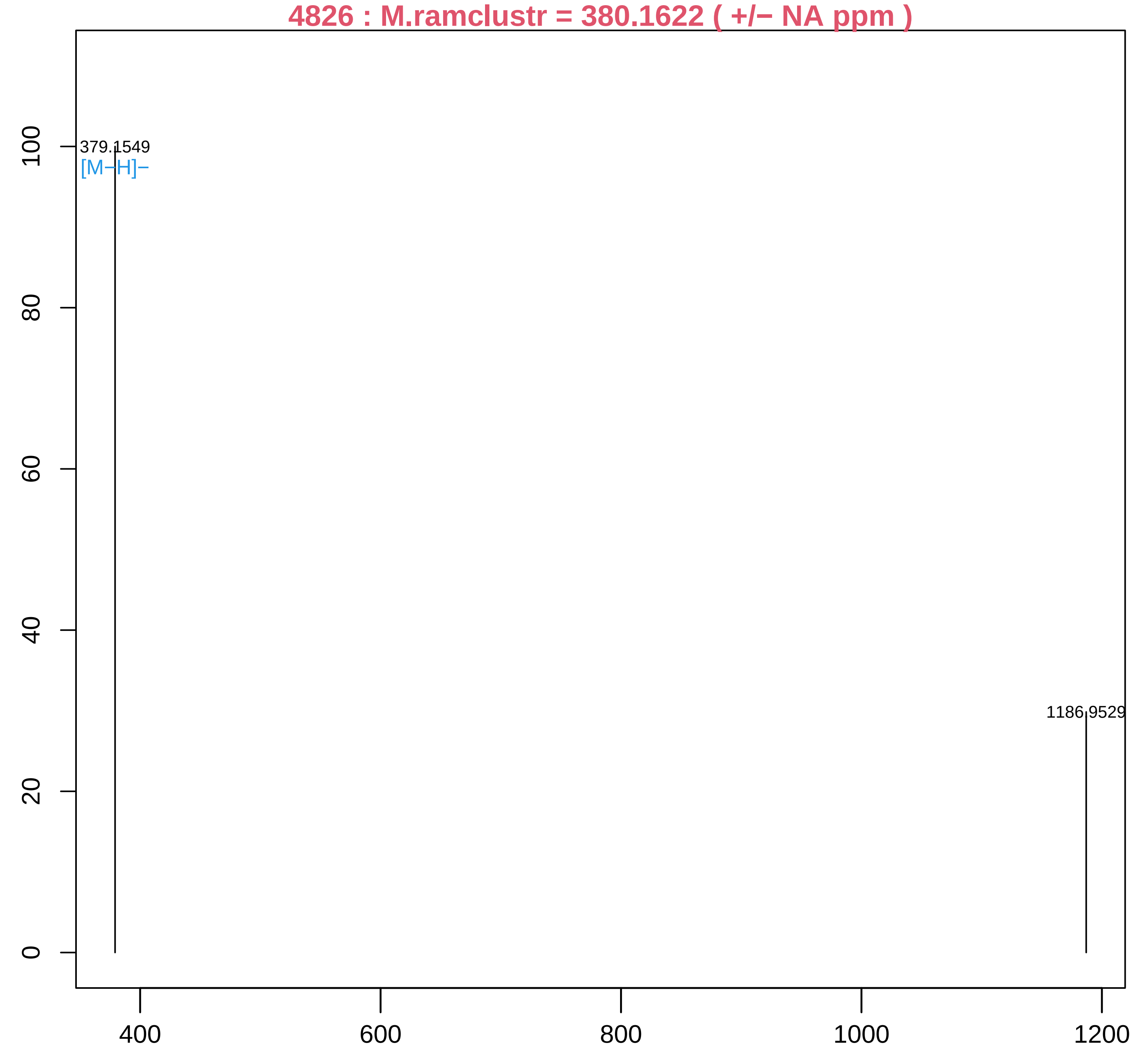
4826 : M.ramclustr = 380.1622 (+/− NA ppm)
379.1549
[M−H]−
1186.9529
0
20
40
60
80
100
400
600
800
1000
1200

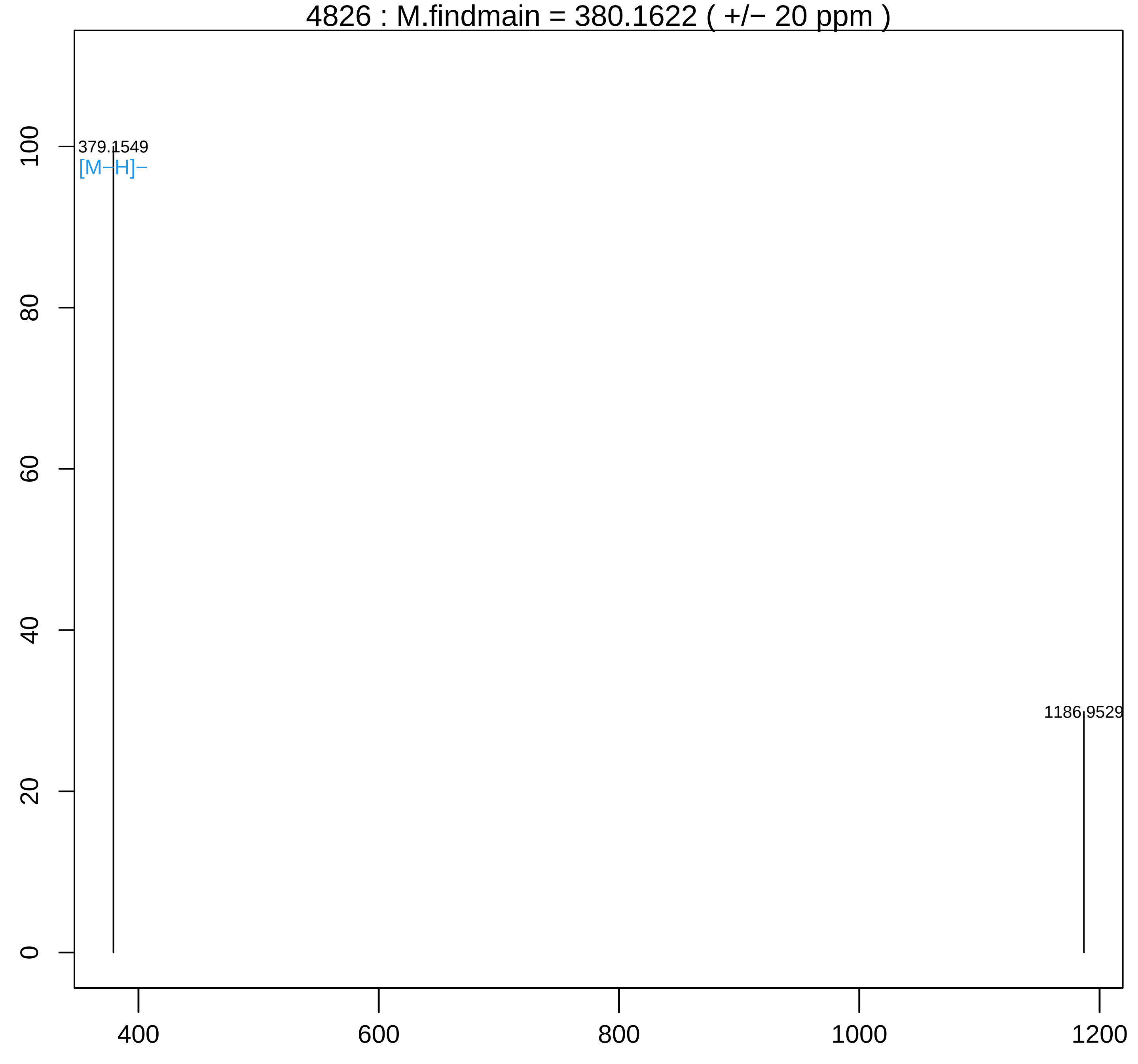
4826 : M.findmain = 380.1622 (+/− 20 ppm)
379.1549
[M−H]−
1186.9529
0
20
40
60
80
100
400
600
800
1000
1200

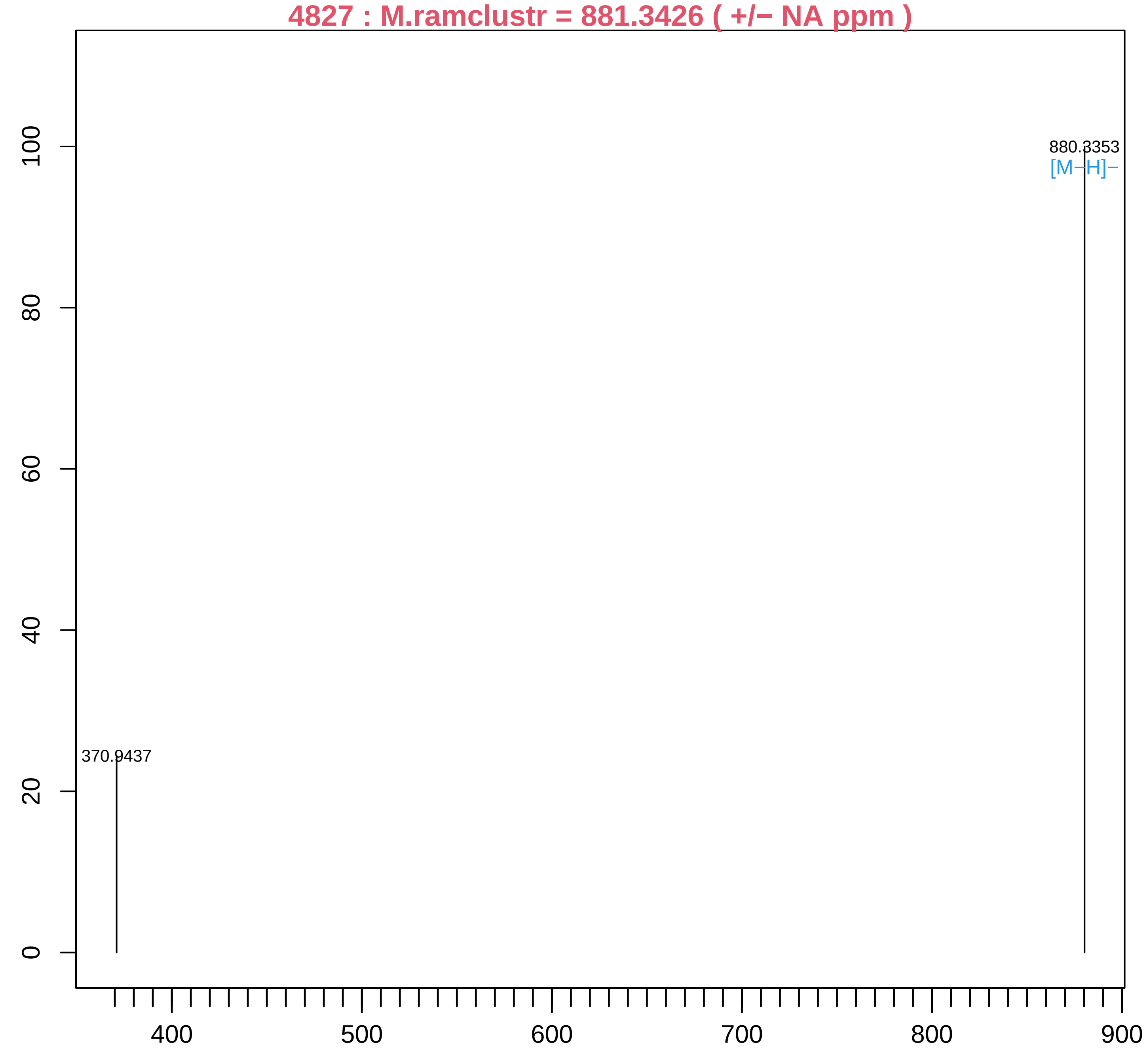
4827 : M.ramclustr = 881.3426 (+/− NA ppm)
880.3353
[M−H]−
370.9437
0
20
40
60
80
100
400
500
600
700
800
900

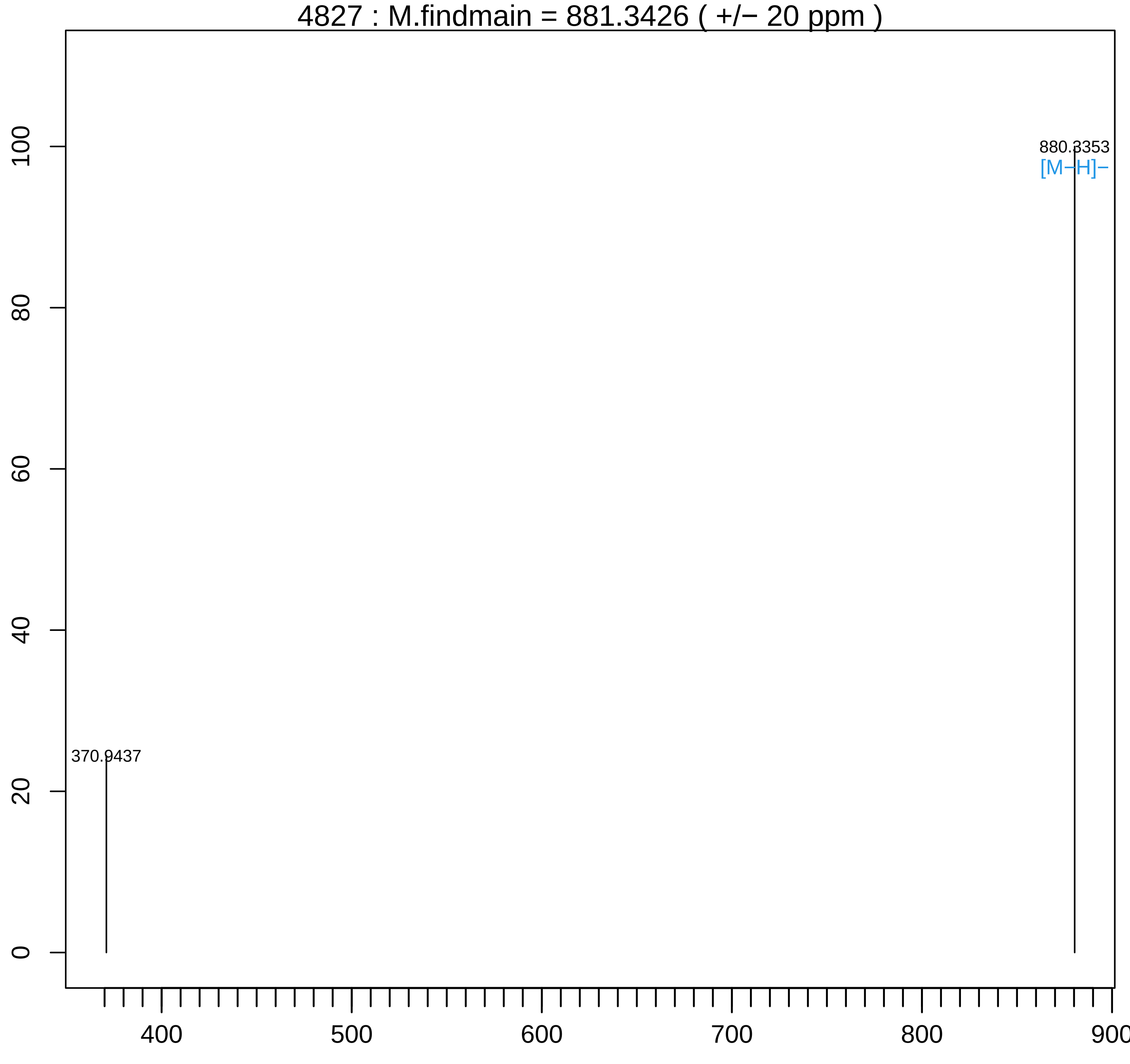
4827 : M.findmain = 881.3426 (+/− 20 ppm)
880.3353
[M−H]−
370.9437
0
20
40
60
80
100
400
500
600
700
800
900

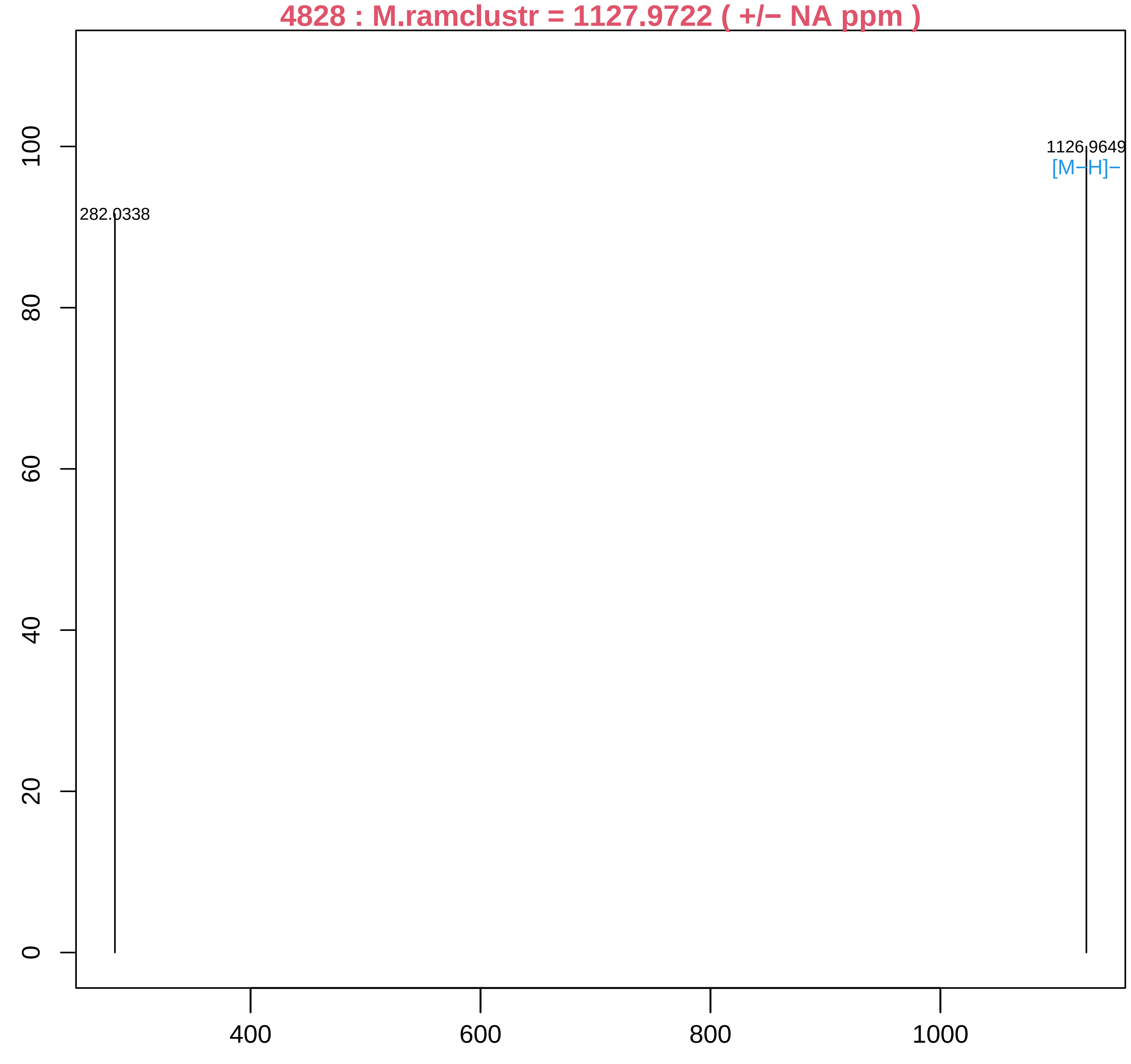
4828 : M.ramclustr = 1127.9722 (+/− NA ppm)
1126.9649
[M−H]−
282.0338
0
20
40
60
80
100
400
600
800
1000

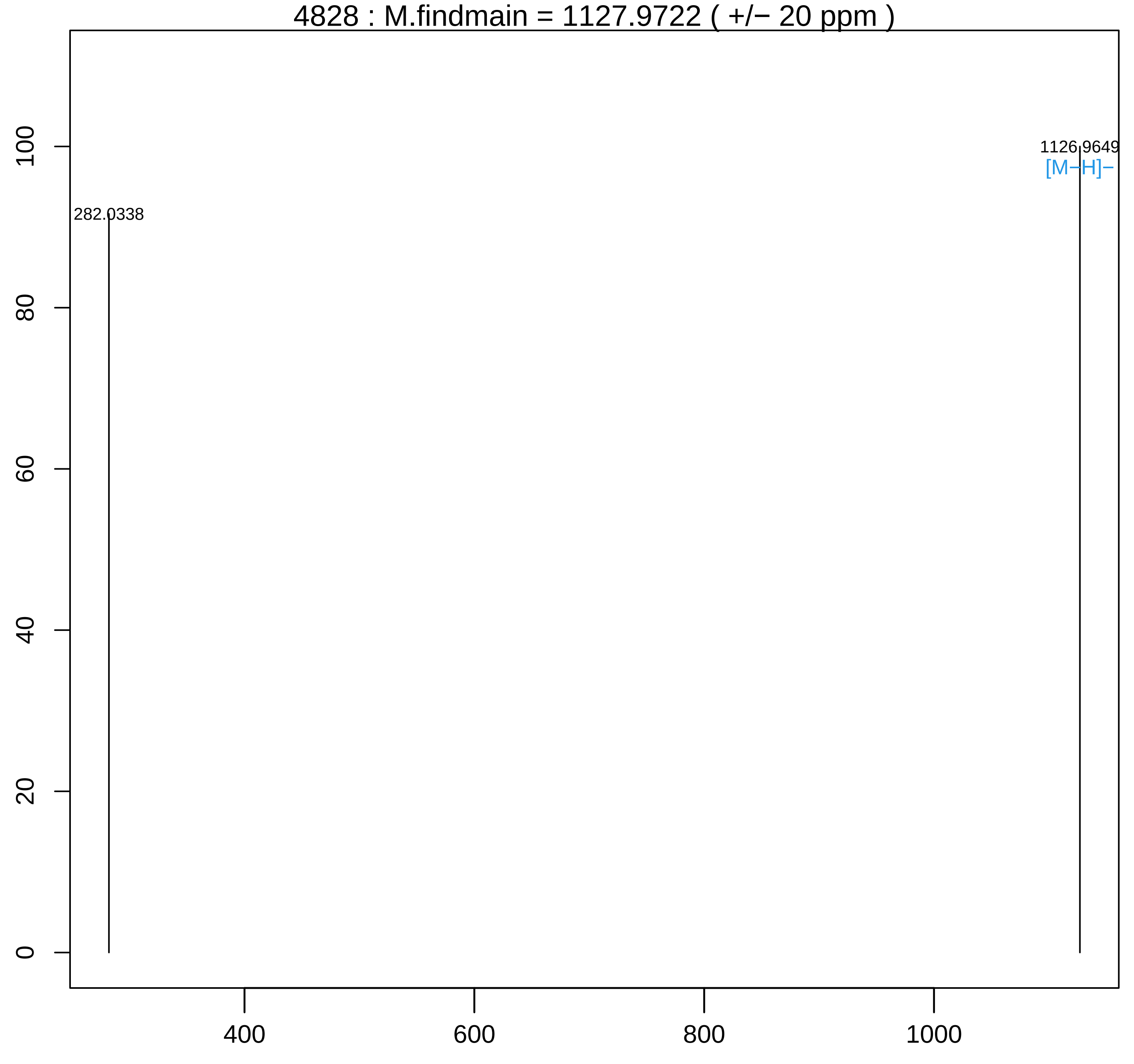
4828 : M.findmain = 1127.9722 (+/− 20 ppm)
1126.9649
[M−H]−
282.0338
0
20
40
60
80
100
400
600
800
1000

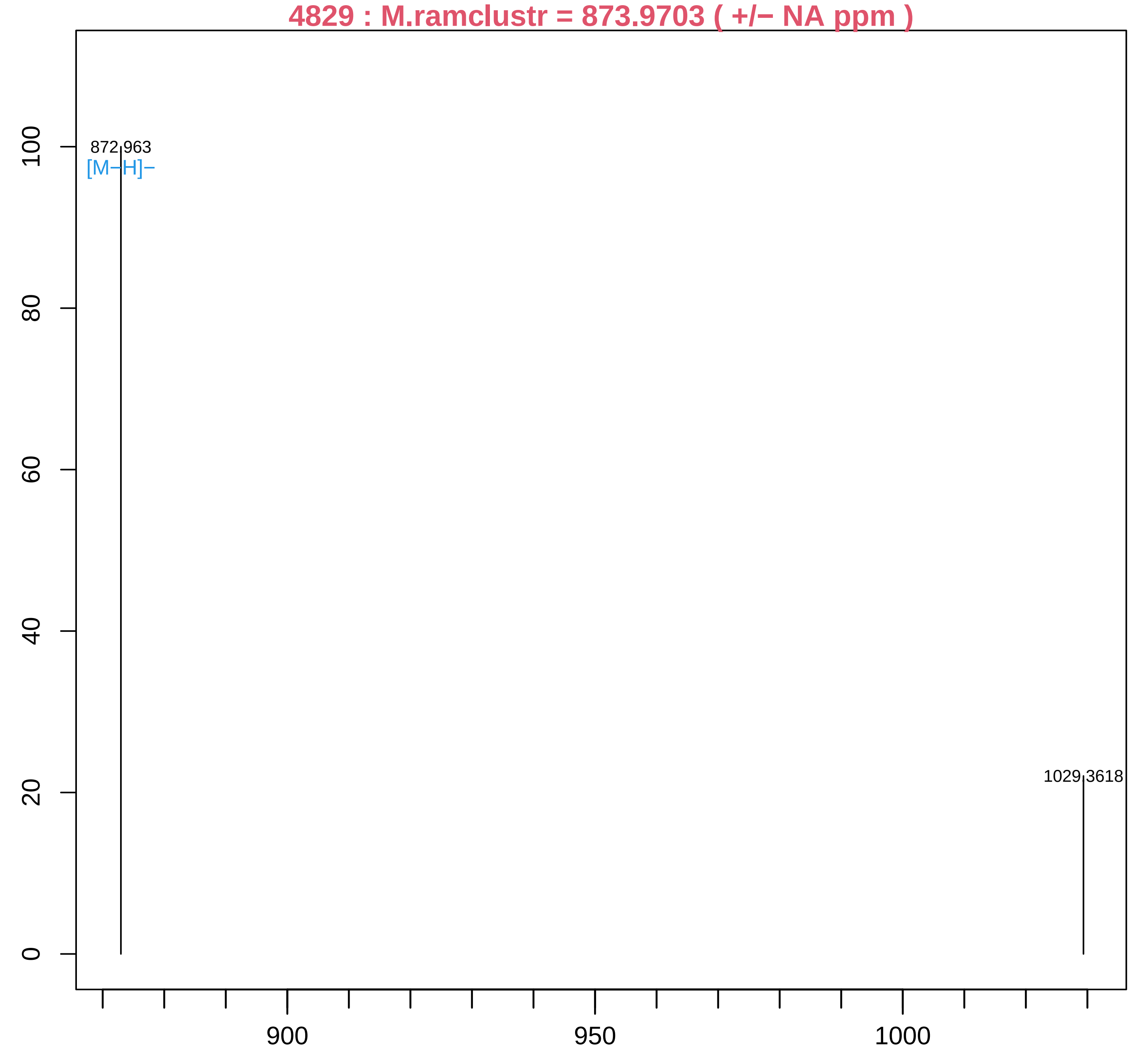
4829 : M.ramclustr = 873.9703 (+/− NA ppm)
872.963
[M−H]−
1029.3618
0
20
40
60
80
100
900
950
1000

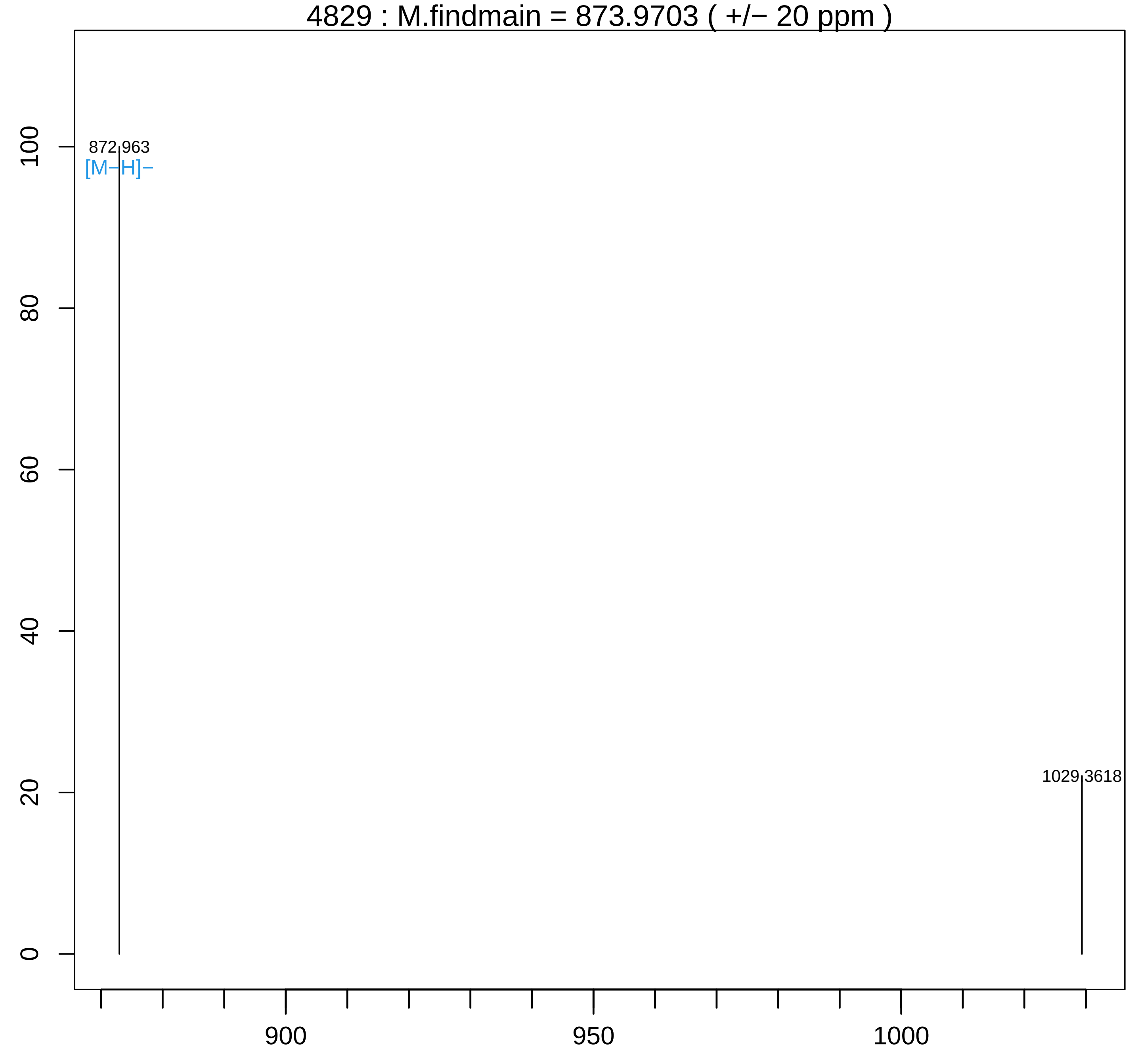
4829 : M.findmain = 873.9703 (+/− 20 ppm)
872.963
[M−H]−
1029.3618
0
20
40
60
80
100
900
950
1000

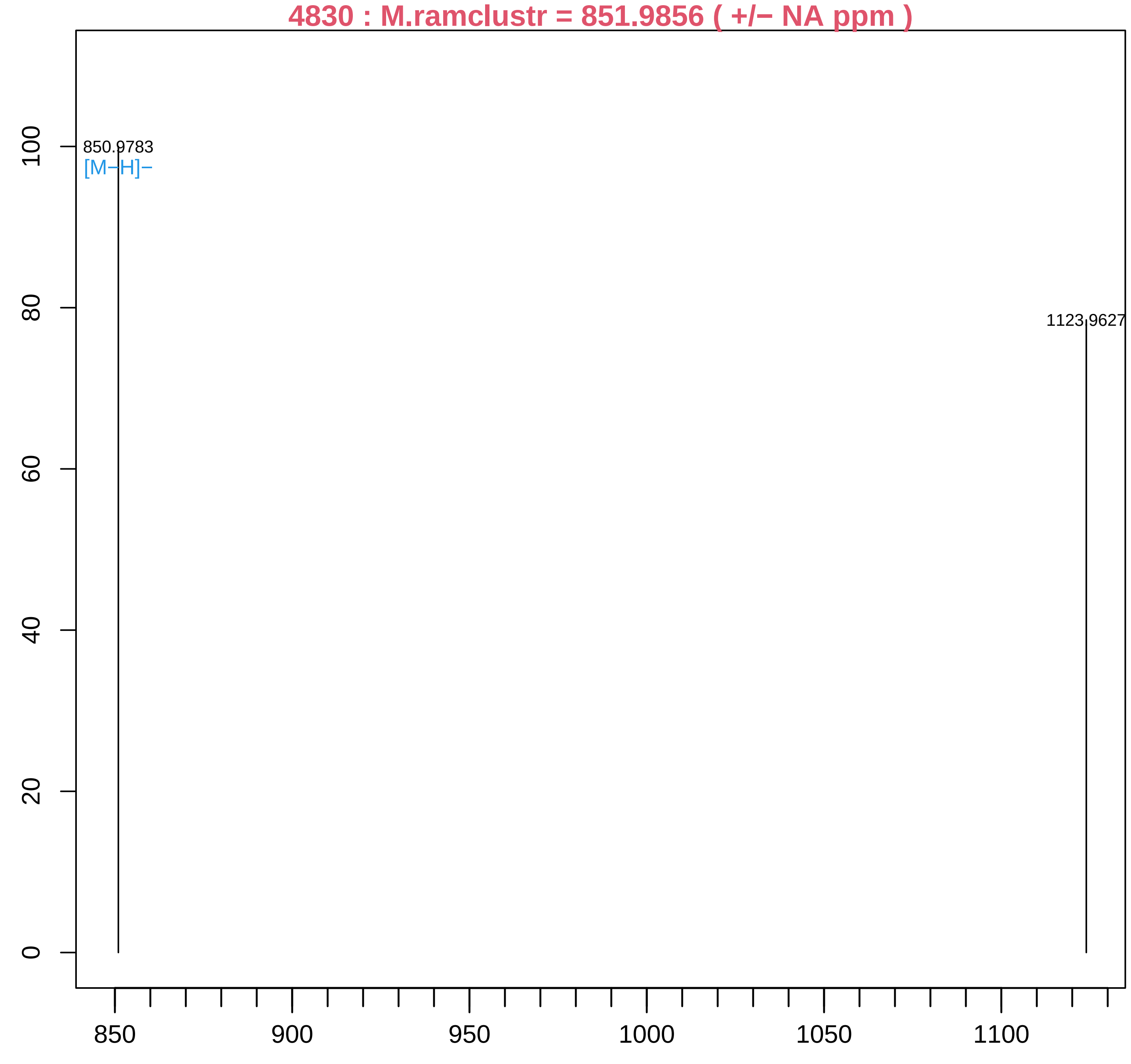
4830 : M.ramclustr = 851.9856 (+/− NA ppm)
850.9783
[M−H]−
1123.9627
0
20
40
60
80
100
850
900
950
1000
1050
1100

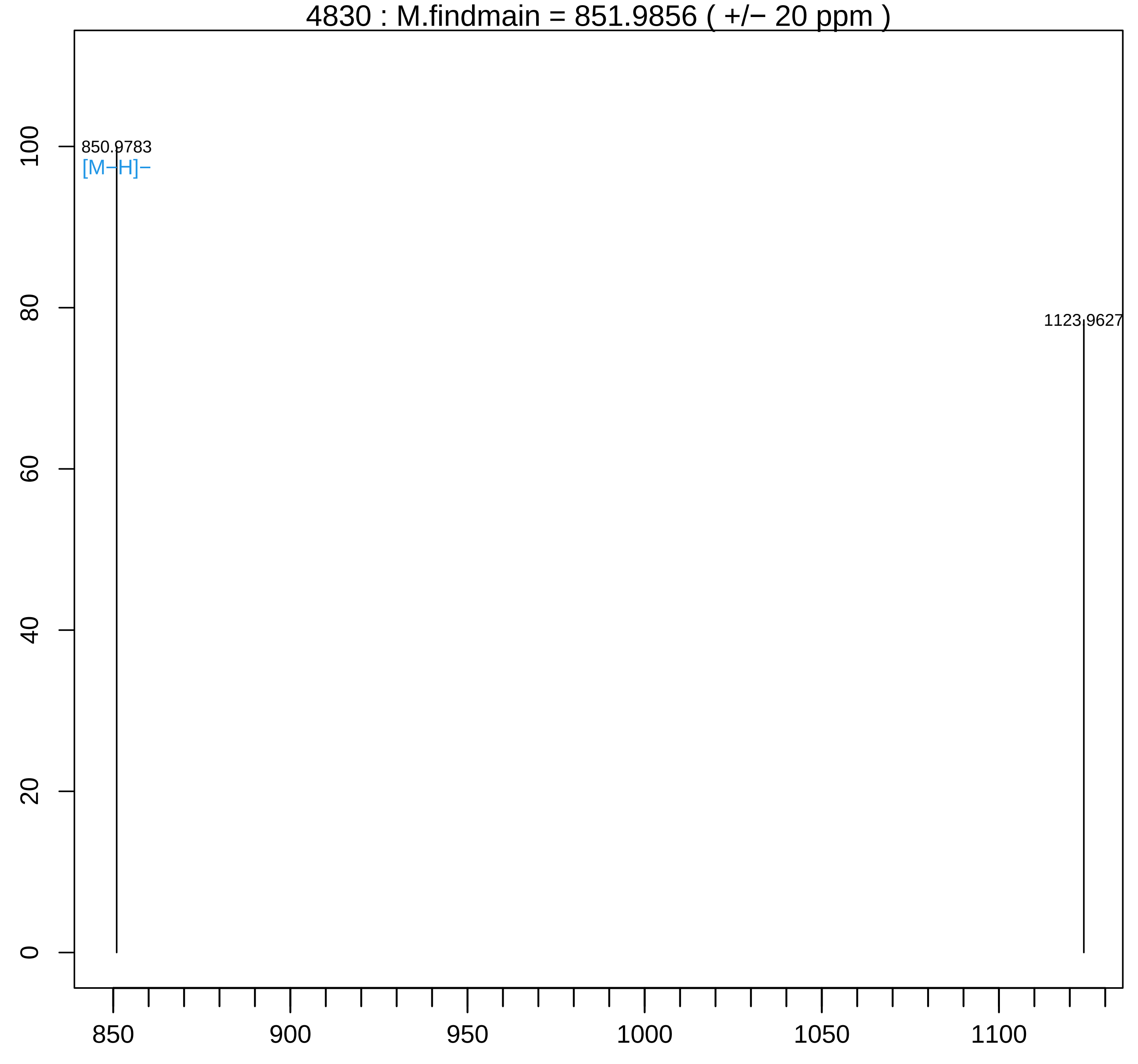
4830 : M.findmain = 851.9856 (+/− 20 ppm)
850.9783
[M−H]−
1123.9627
0
20
40
60
80
100
850
900
950
1000
1050
1100

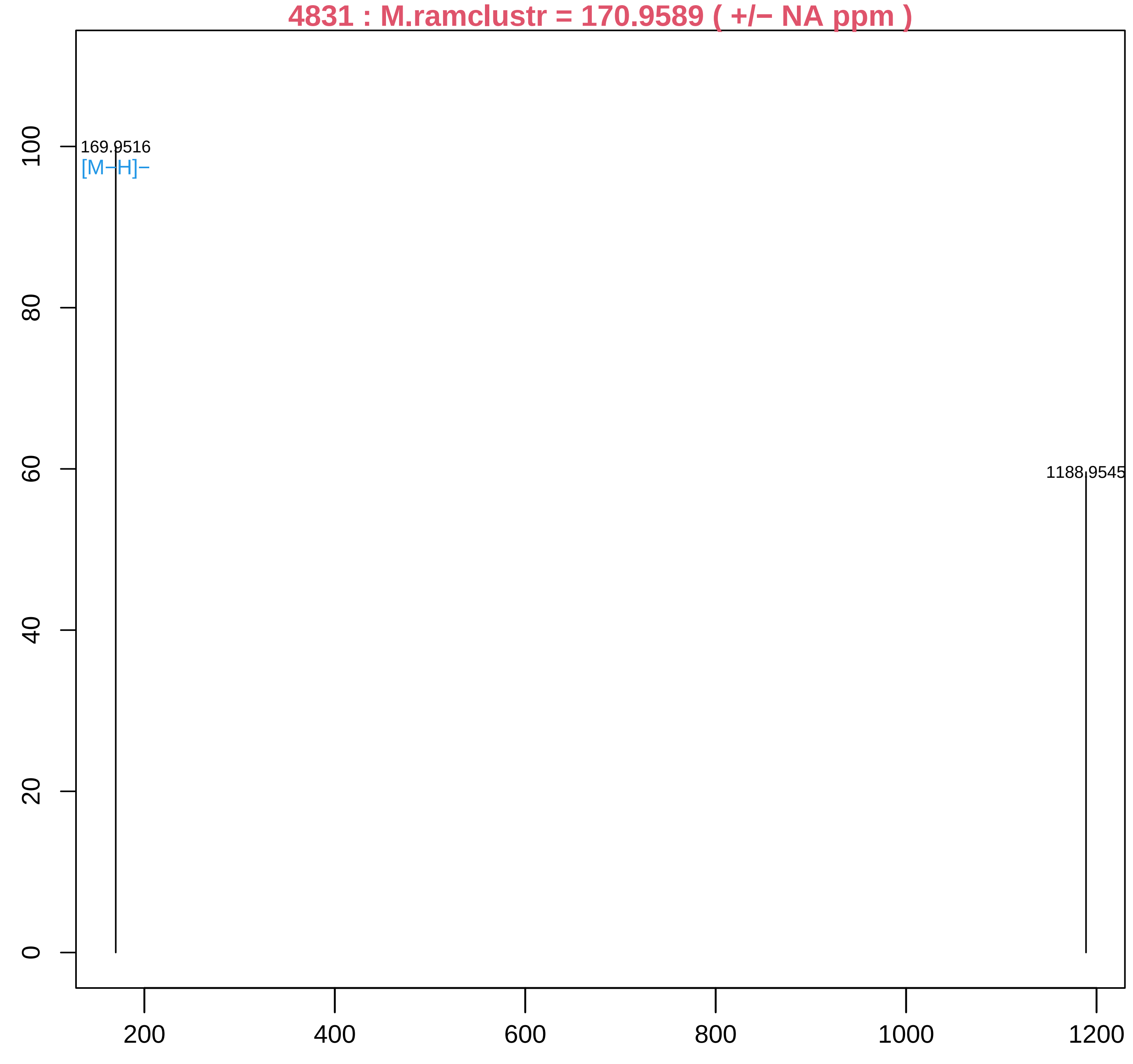
4831 : M.ramclustr = 170.9589 (+/− NA ppm)
169.9516
[M−H]−
1188.9545
0
20
40
60
80
100
200
400
600
800
1000
1200

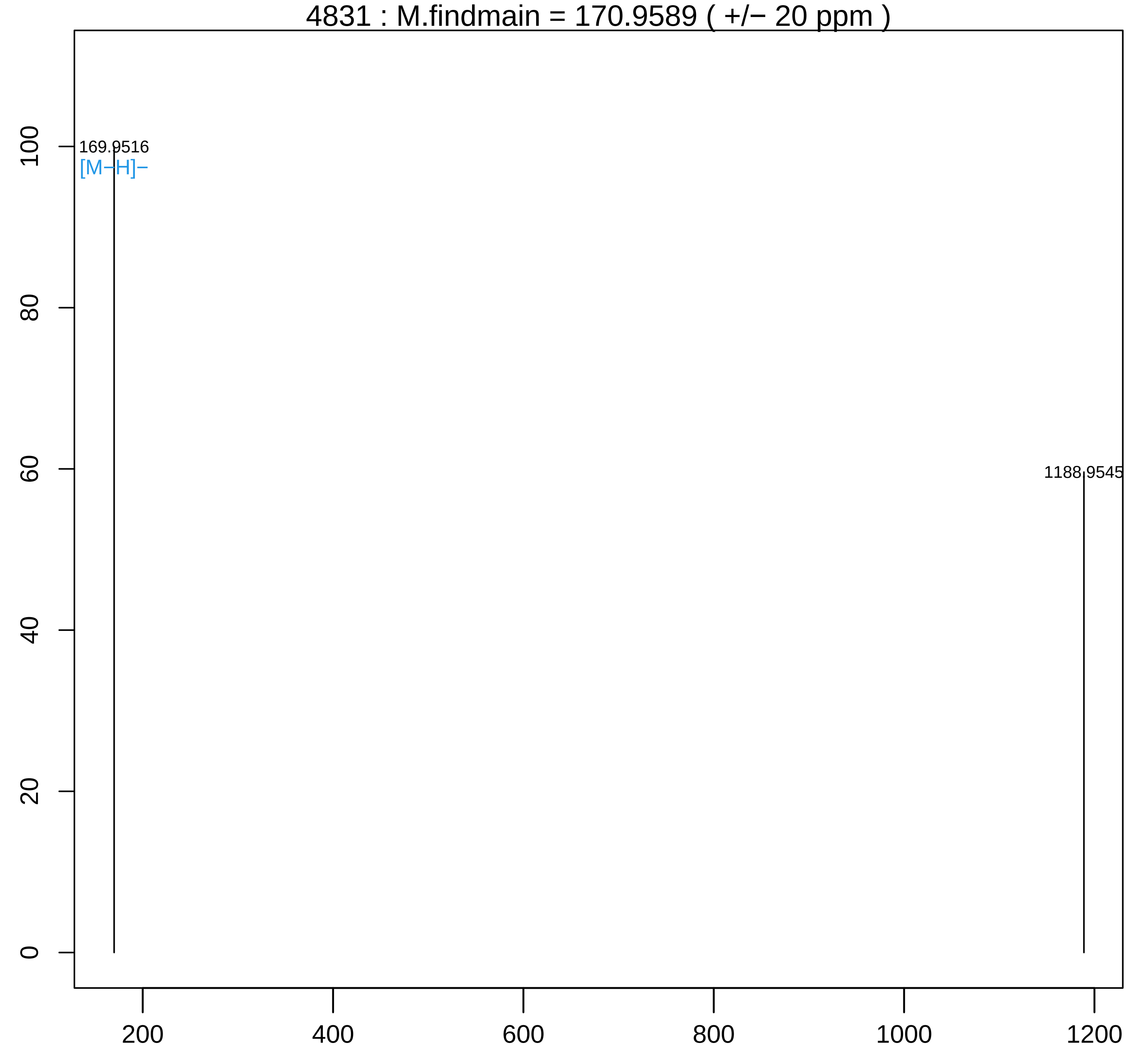
4831 : M.findmain = 170.9589 (+/− 20 ppm)
169.9516
[M−H]−
1188.9545
0
20
40
60
80
100
200
400
600
800
1000
1200

4832 : M.ramclustr = 440.2583 (+/− NA ppm)

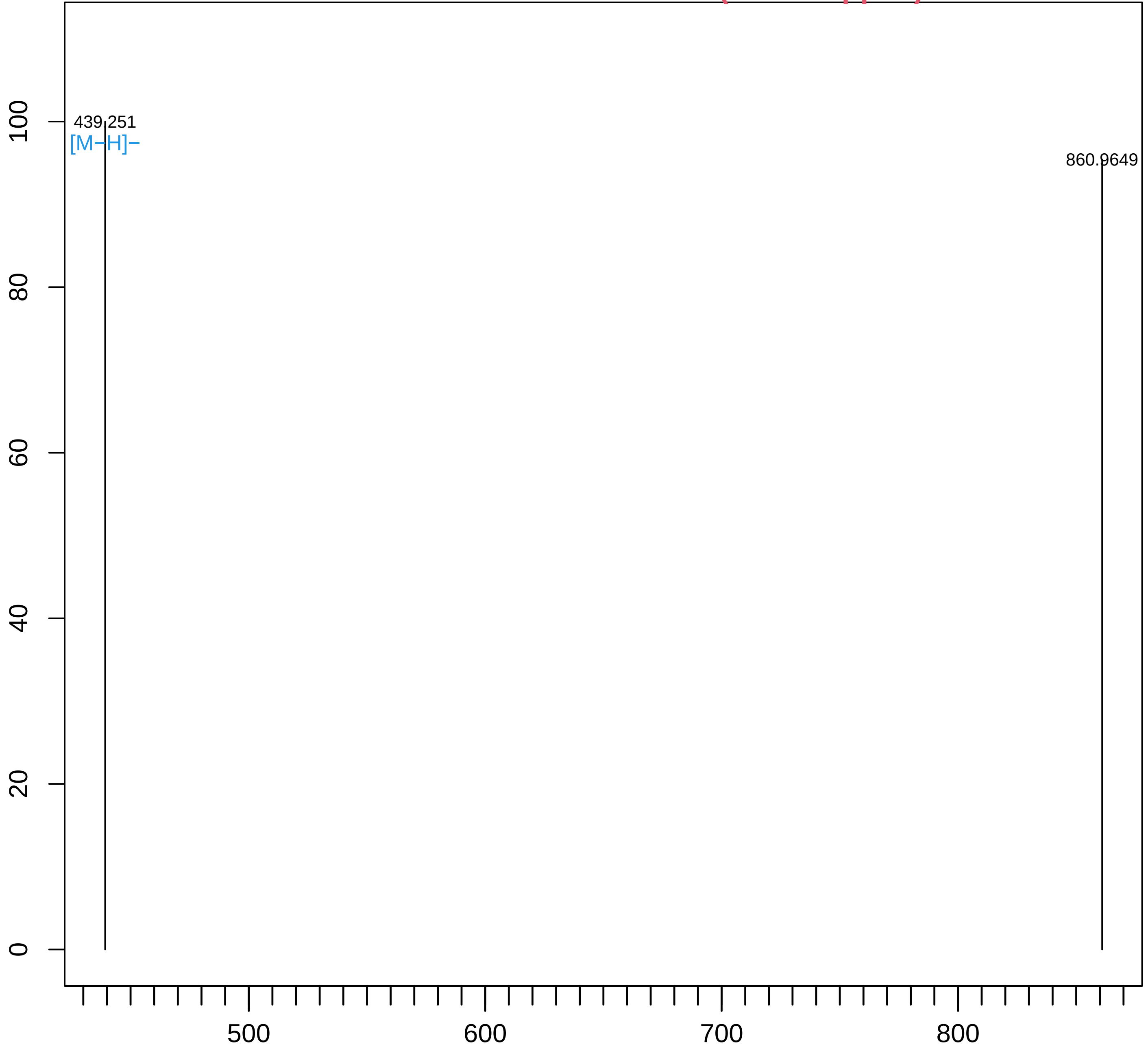

4832 : M.findmain = 440.2583 (+/− 20 ppm)

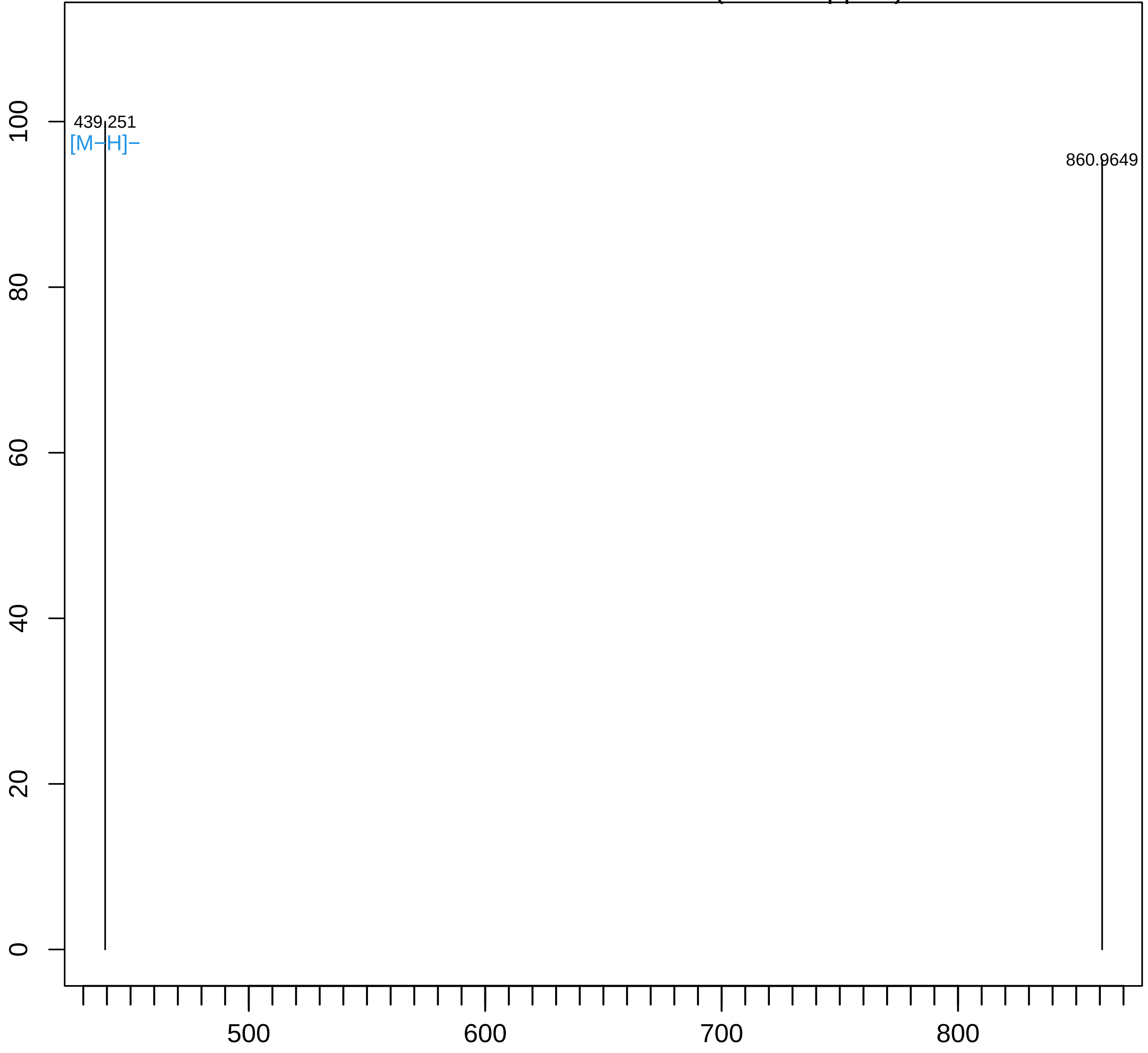

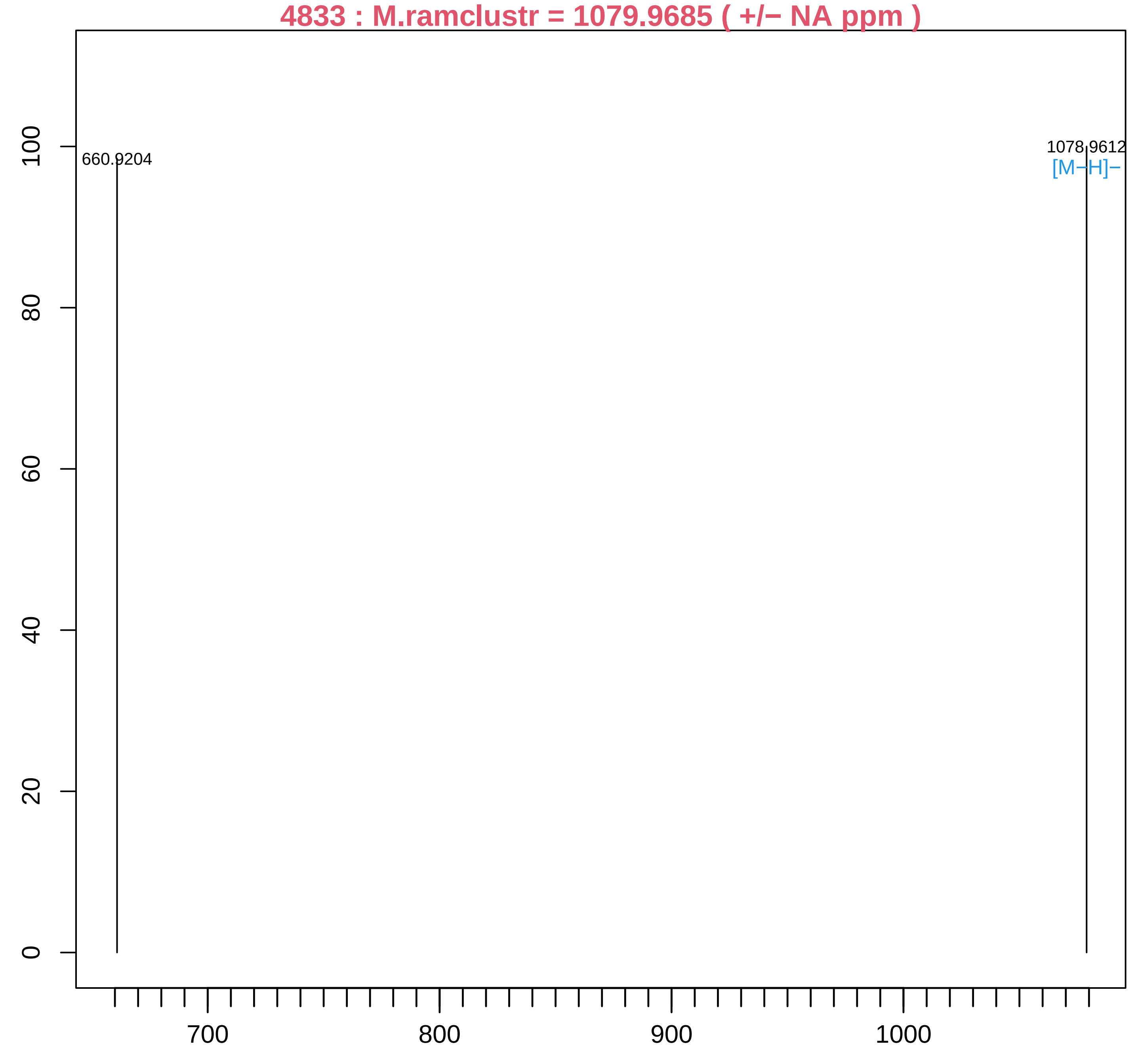

4833 : M.ramclustr = 1079.9685 (+/− NA ppm)
660.9204
1078.9612
[M−H]−
0
20
40
60
80
100
700
800
900
1000

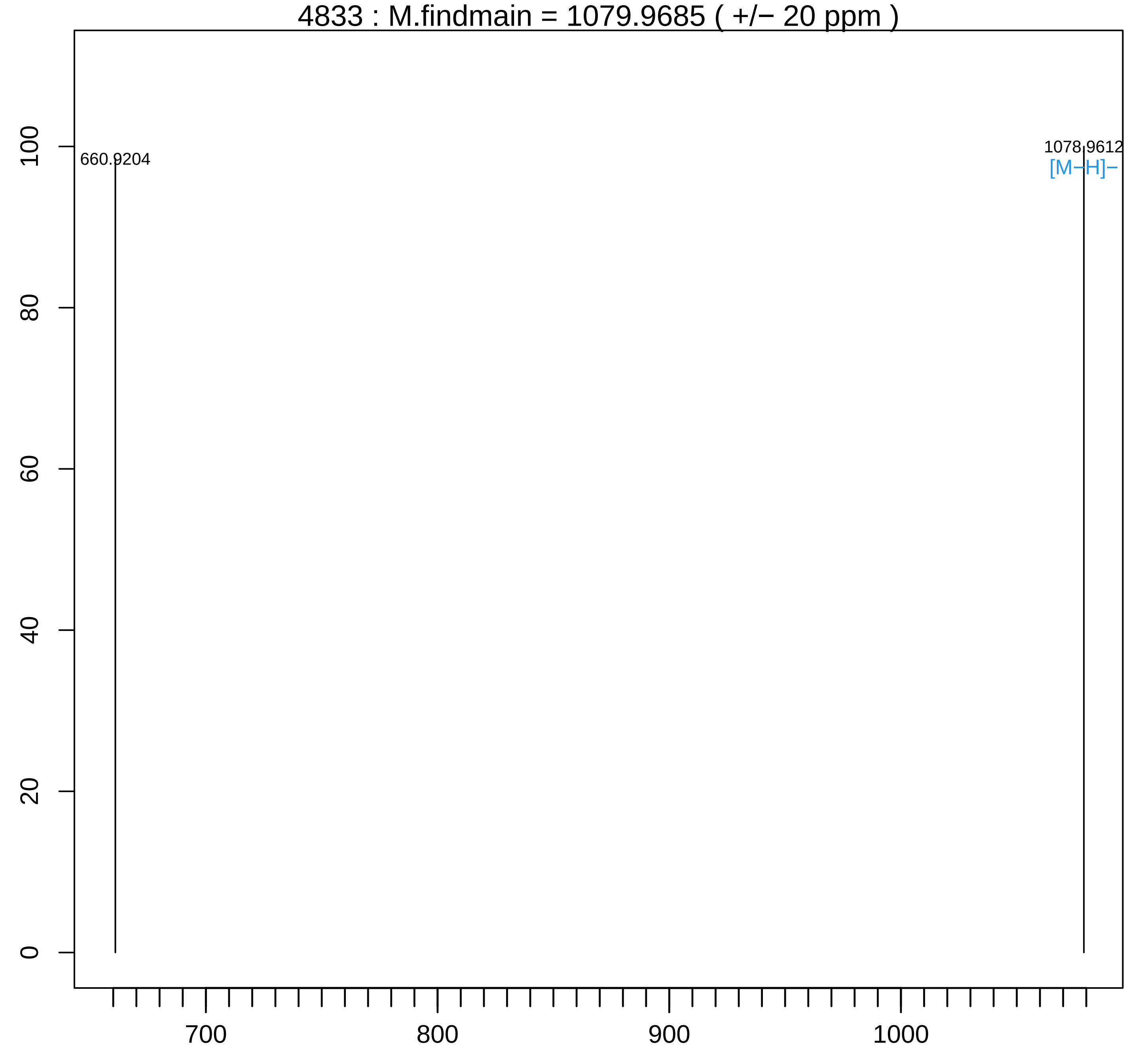

4833 : M.findmain = 1079.9685 (+/− 20 ppm)
660.9204
1078.9612
[M−H]−
0
20
40
60
80
100
700
800
900
1000

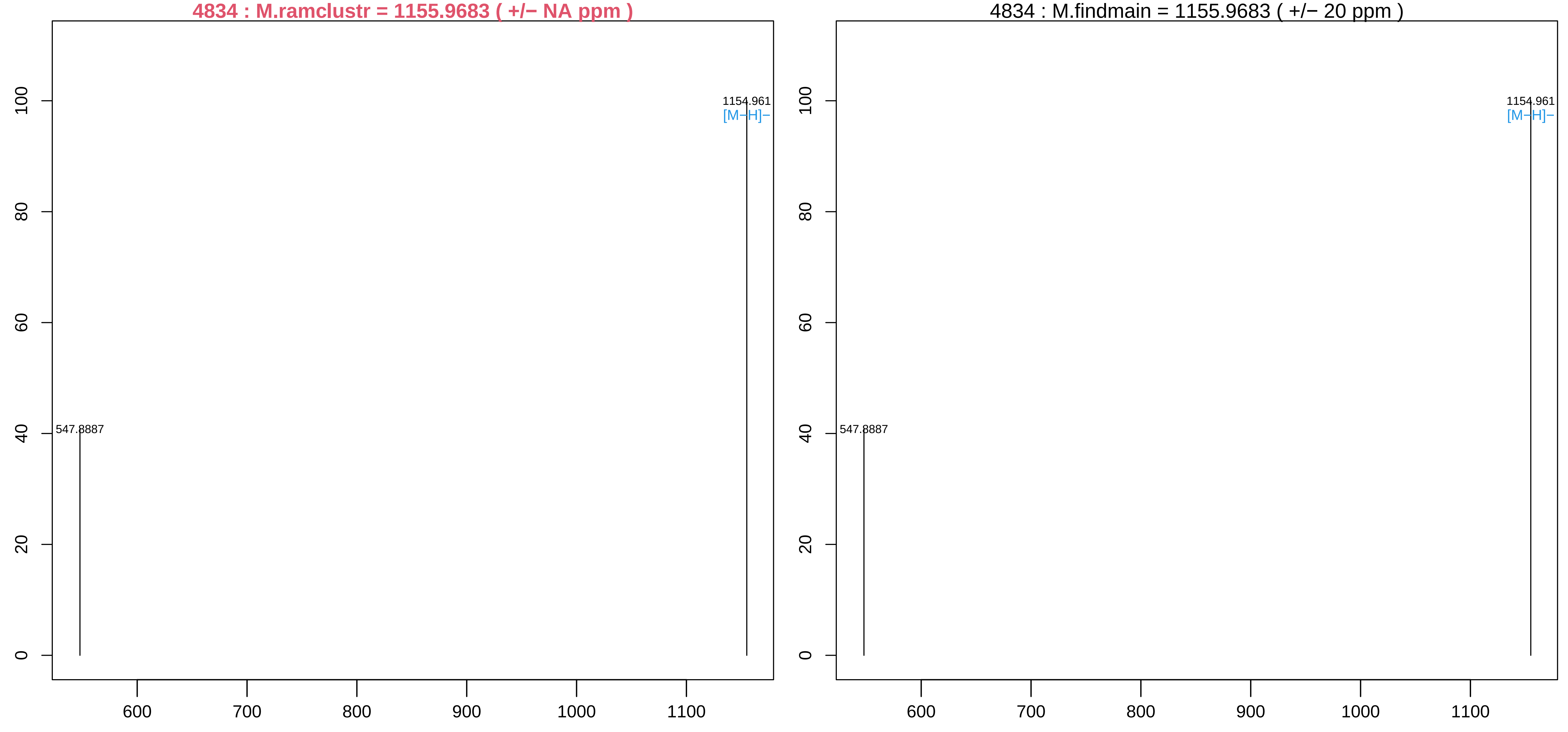
4834 : M.ramclustr = 1155.9683 (+/− NA ppm)
4834 : M.findmain = 1155.9683 (+/− 20 ppm)
1154.961
[M−H]−
547.8887
1154.961
[M−H]−
547.8887
0
20
40
60
80
100
600
700
800
900
1000
1100

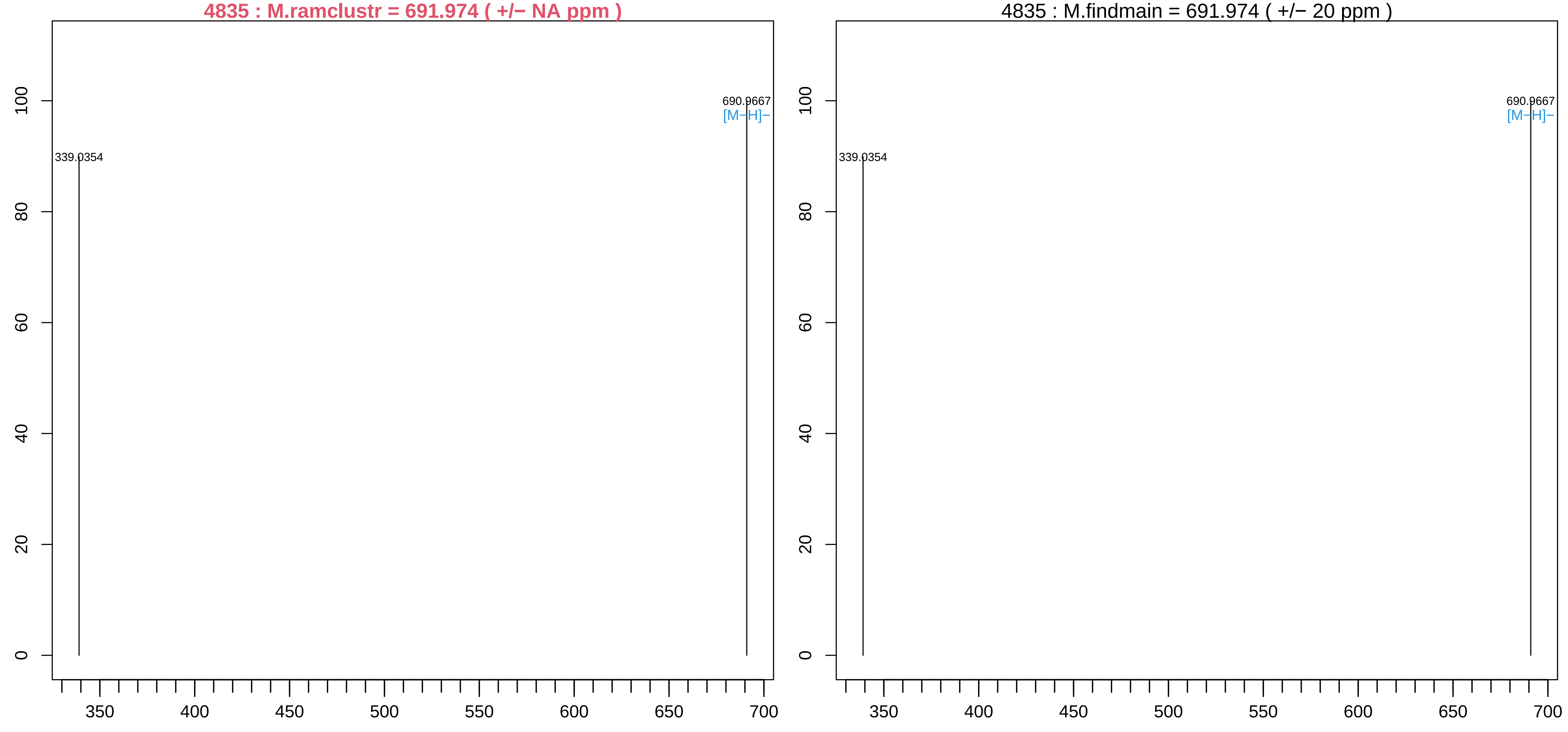
4835 : M.ramclustr = 691.974 (+/− NA ppm)
4835 : M.findmain = 691.974 (+/− 20 ppm)
339.0354
690.9667
[M−H]−
0
20
40
60
80
100
350
400
450
500
550
600
650
700

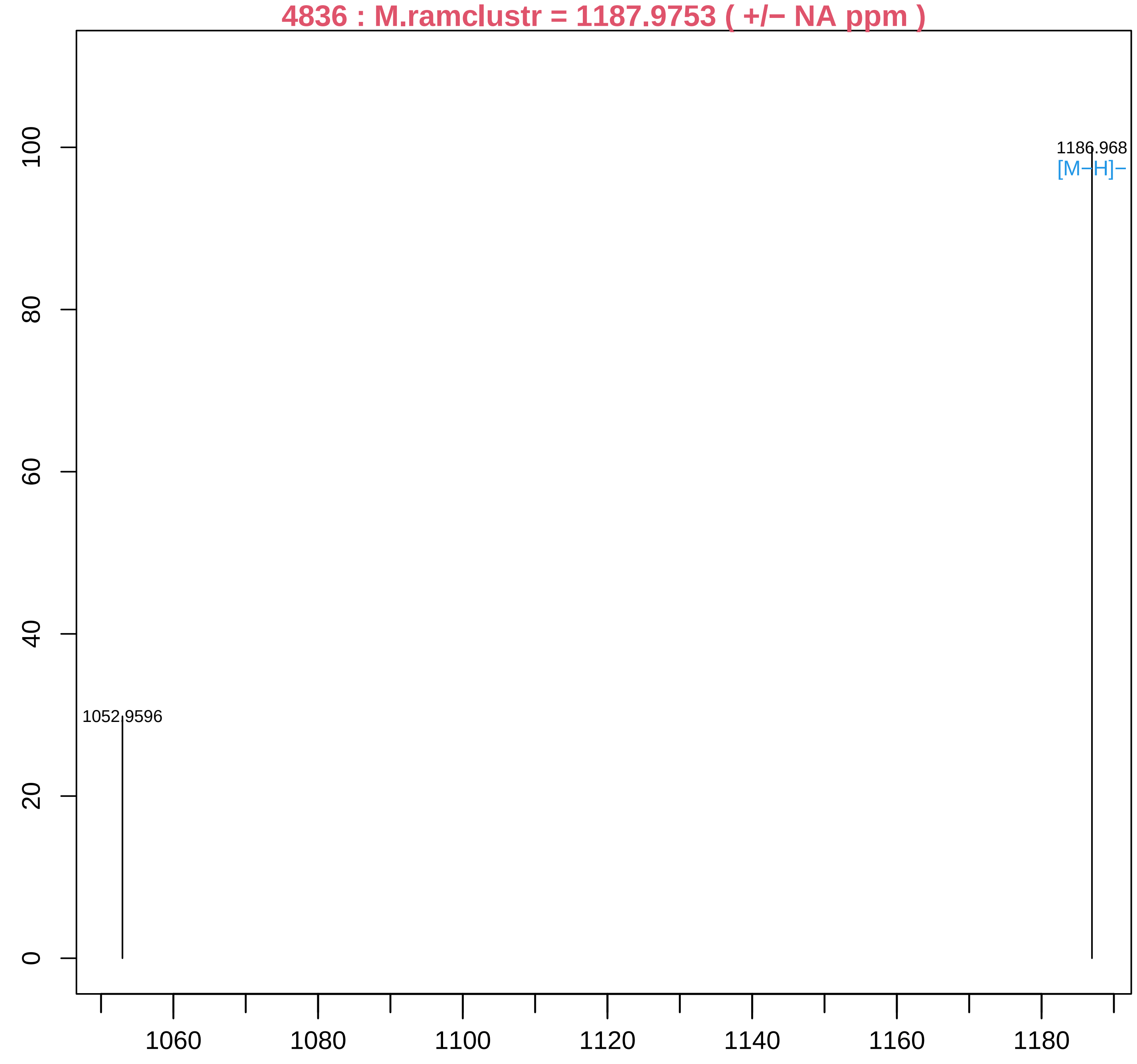
4836 : M.ramclustr = 1187.9753 (+/− NA ppm)
1186.968
[M−H]−
1052.9596
0
20
40
60
80
100
1060
1080
1100
1120
1140
1160
1180

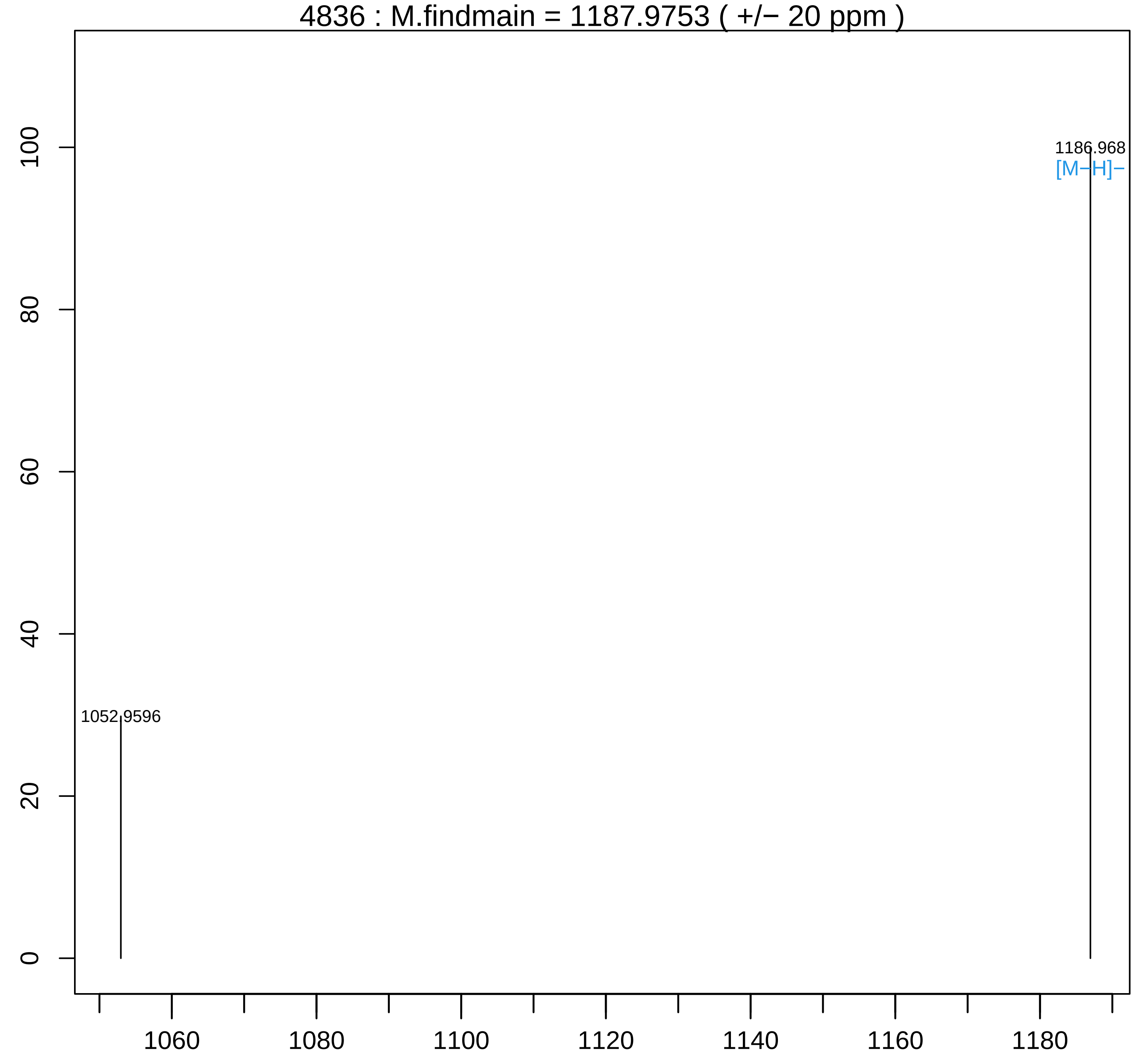
4836 : M.findmain = 1187.9753 (+/− 20 ppm)
1186.968
[M−H]−
1052.9596
0
20
40
60
80
100
1060
1080
1100
1120
1140
1160
1180

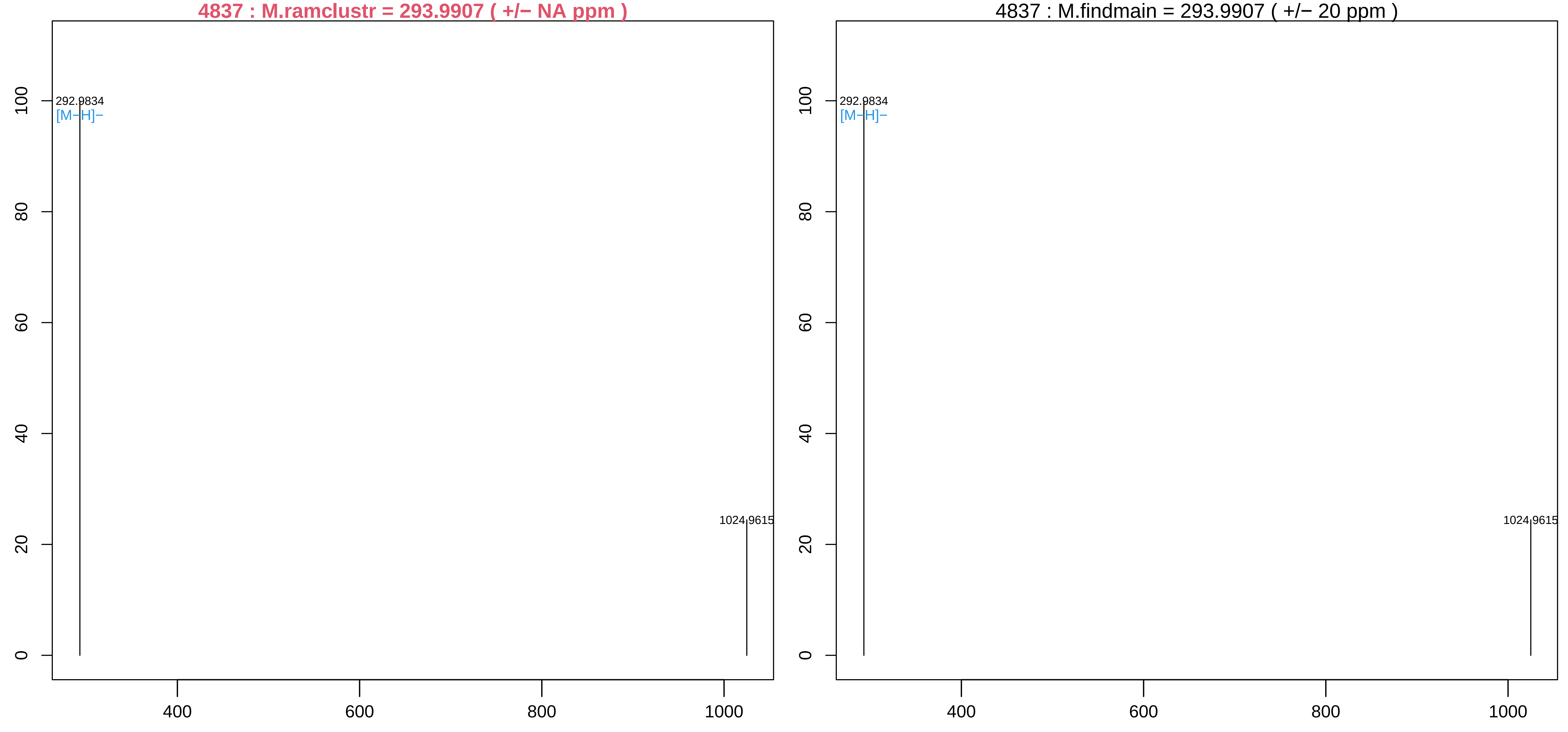
4837 : M.ramclustr = 293.9907 (+/− NA ppm)
292.9834
[M−H]−
1024.9615
4837 : M.findmain = 293.9907 (+/− 20 ppm)
292.9834
[M−H]−
1024.9615
0
20
40
60
80
100
400
600
800
1000

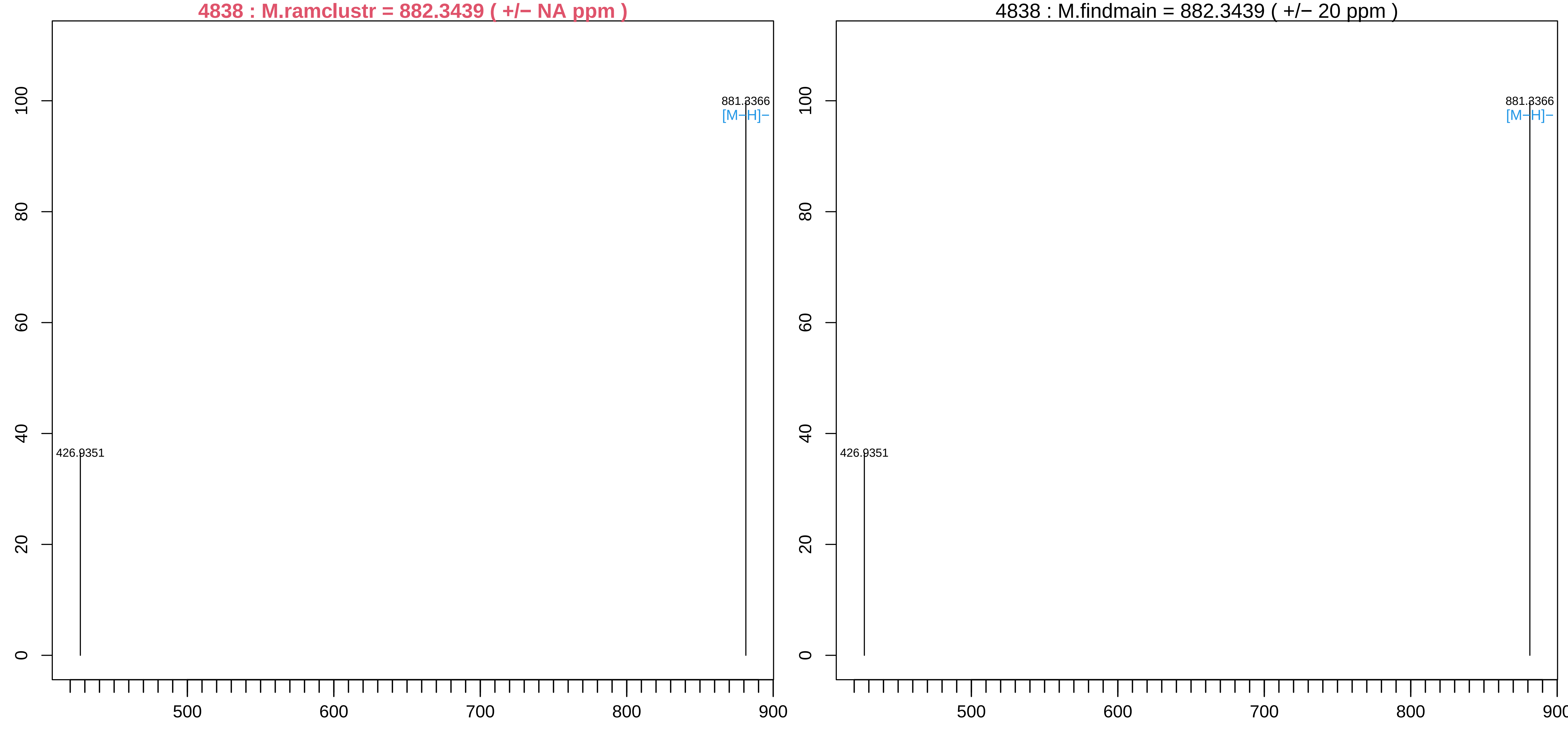
4838 : M.ramclustr = 882.3439 (+/− NA ppm)
881.3366
[M−H]−
426.9351
0
20
40
60
80
100
500
600
700
800
900
4838 : M.findmain = 882.3439 (+/− 20 ppm)
881.3366
[M−H]−
426.9351
0
20
40
60
80
100
500
600
700
800
900

4839 : M.ramclustr = 593.9382 (+/− NA ppm)

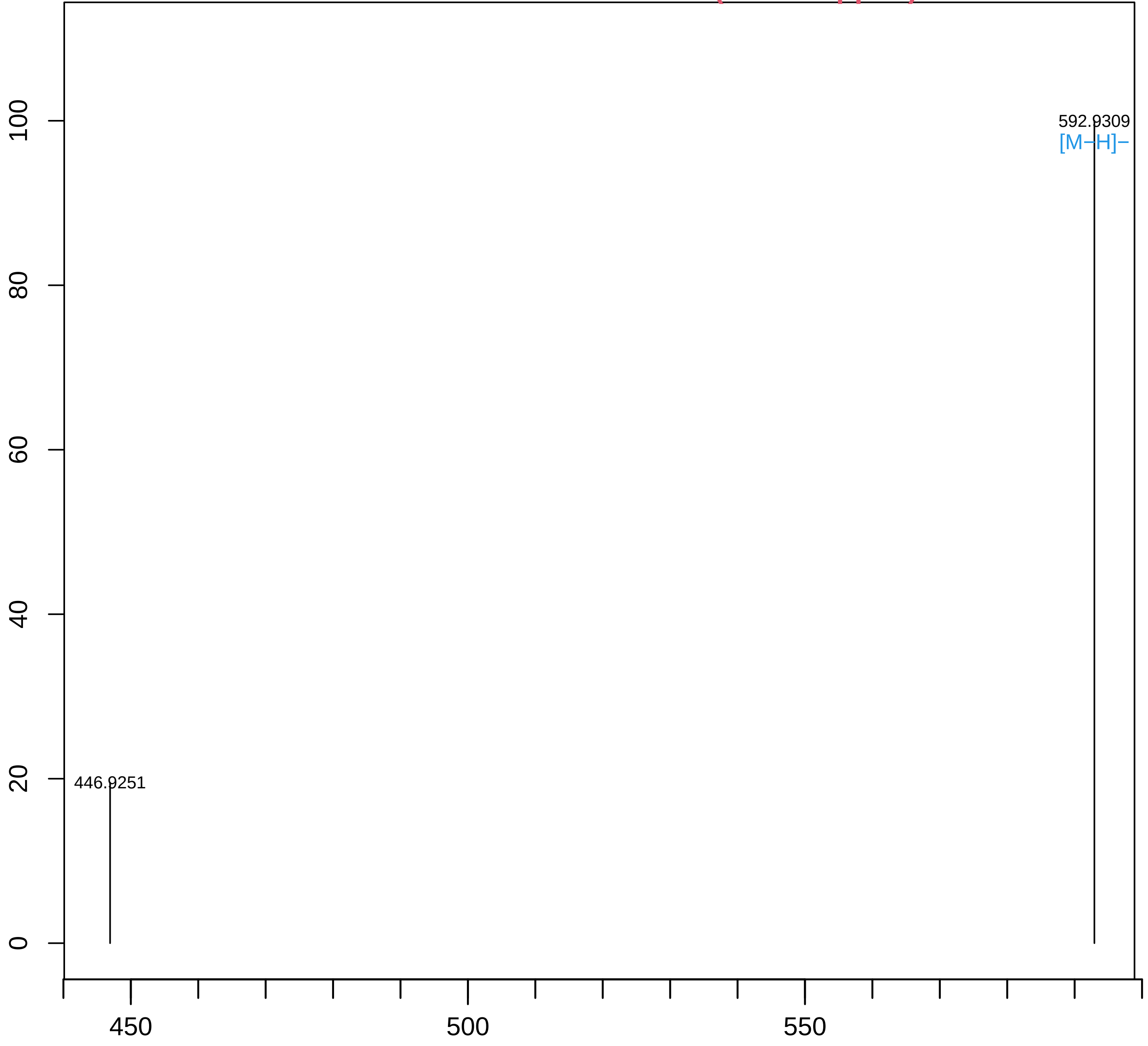

4839 : M.findmain = 593.9382 (+/− 20 ppm)

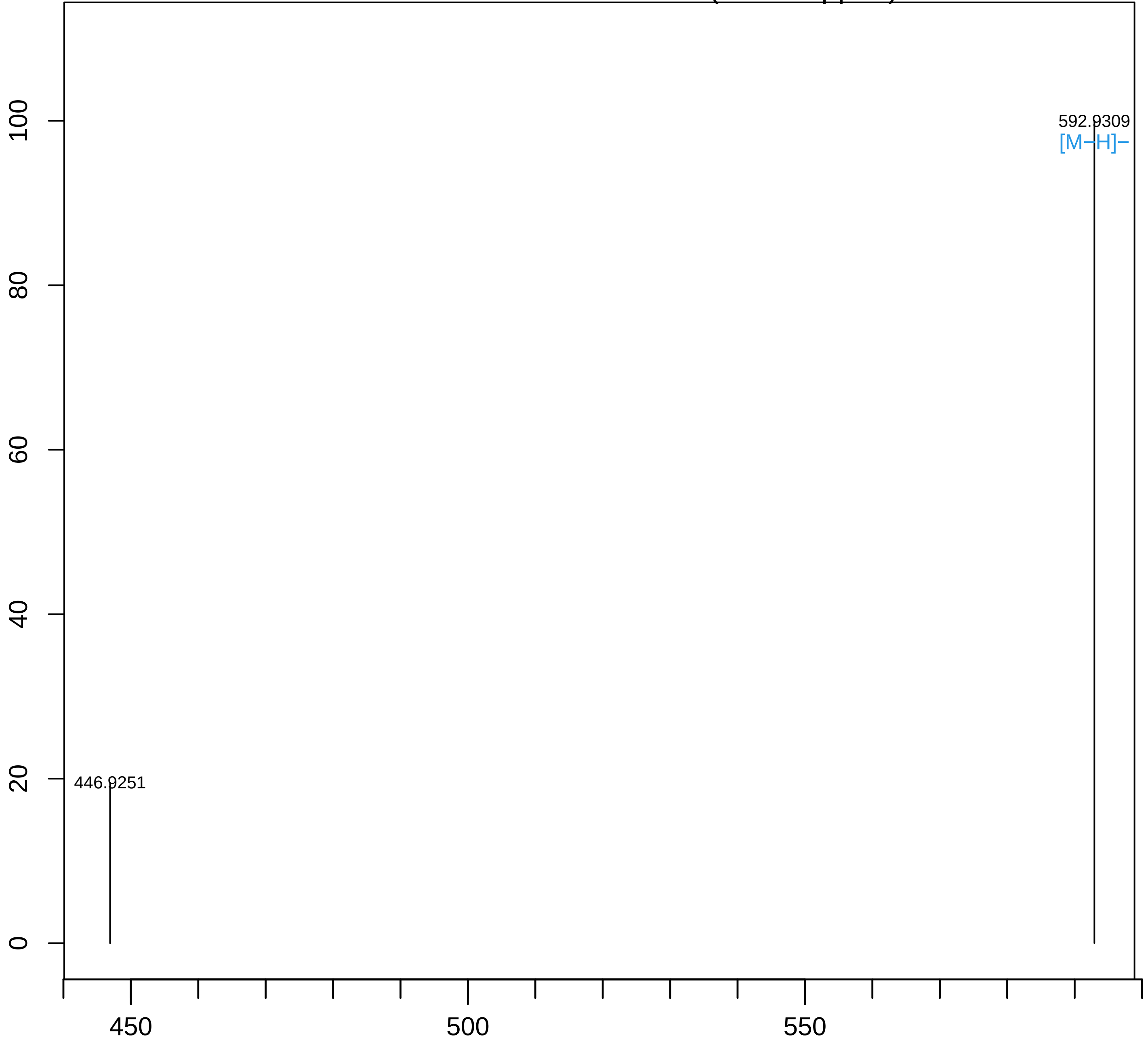

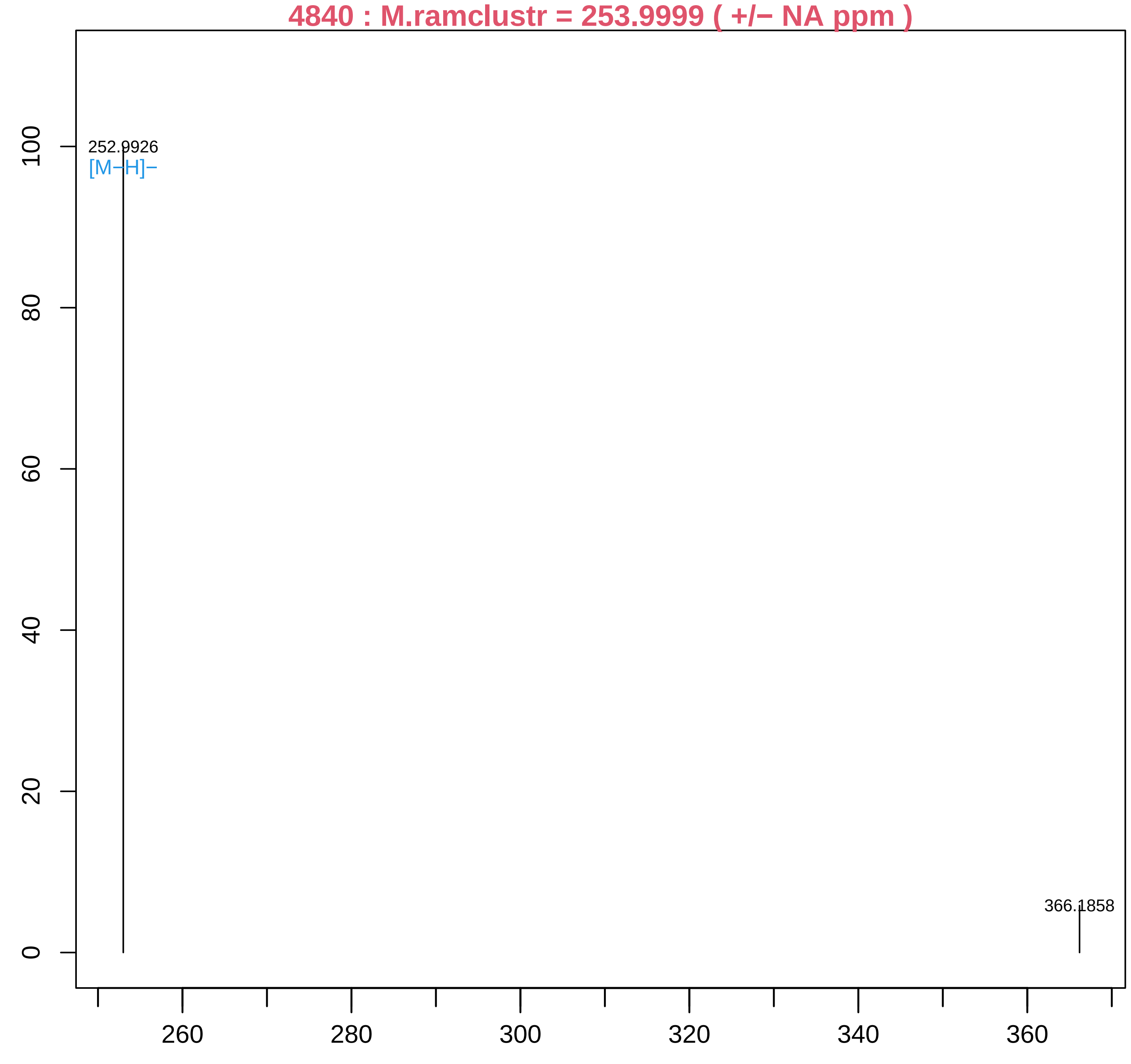
4840 : M.ramclustr = 253.9999 (+/− NA ppm)
252.9926
[M−H]−
366.1858
0
20
40
60
80
100
260
280
300
320
340
360

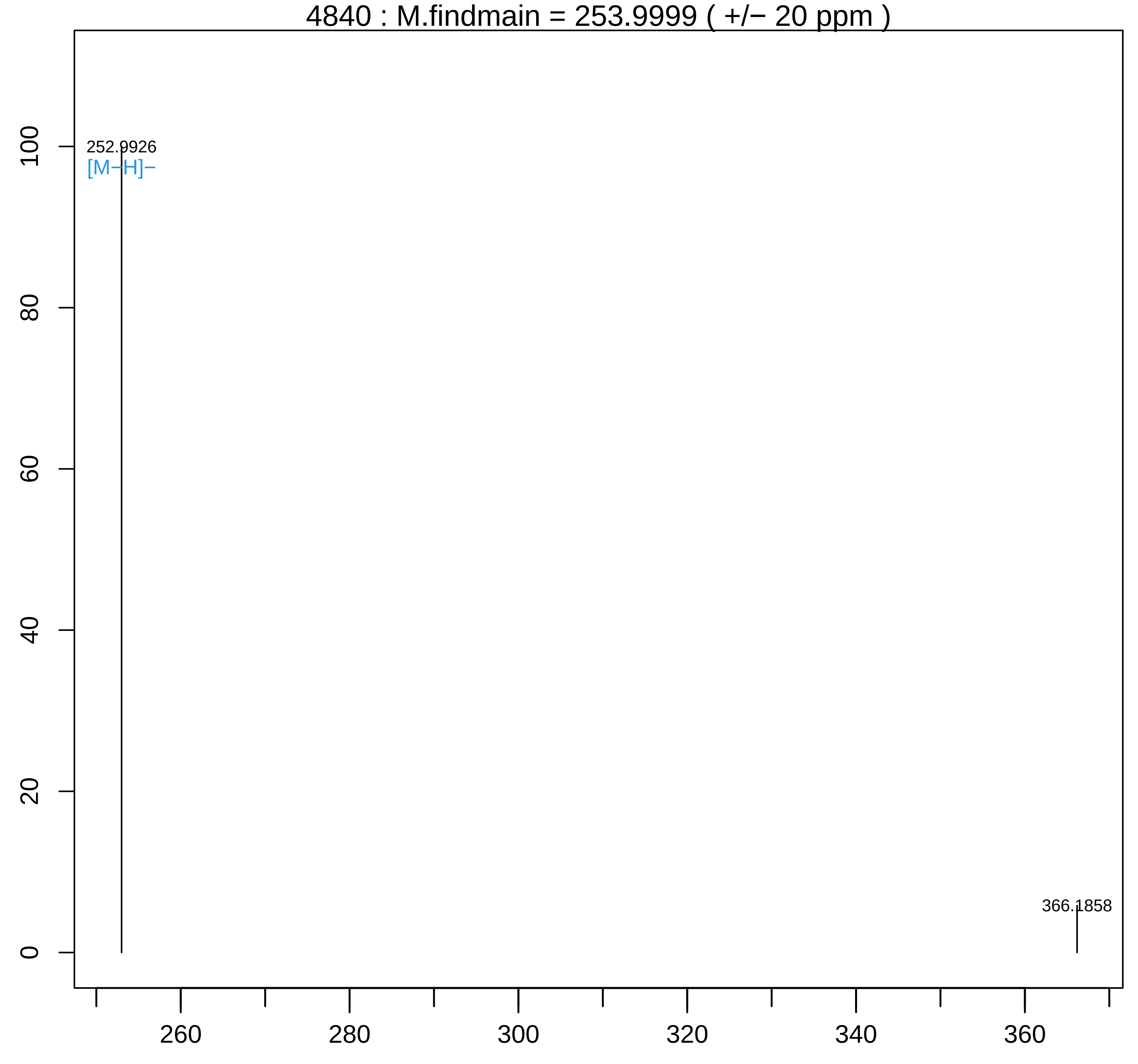
4840 : M.findmain = 253.9999 (+/− 20 ppm)
252.9926
[M−H]−
366.1858
0
20
40
60
80
100
260
280
300
320
340
360

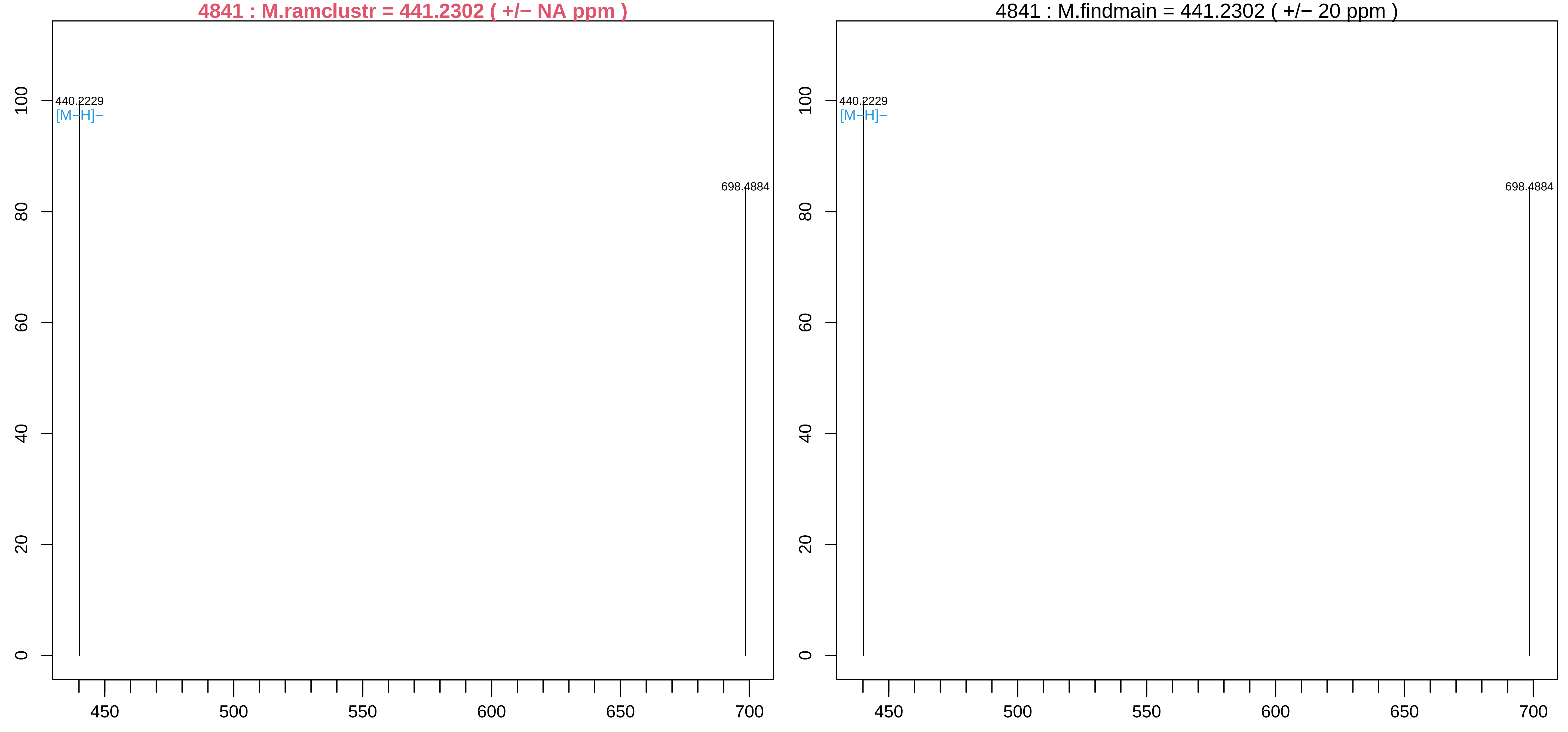
4841 : M.ramclustr = 441.2302 (+/− NA ppm)
440.2229
[M−H]−
698.4884
4841 : M.findmain = 441.2302 (+/− 20 ppm)
440.2229
[M−H]−
698.4884
0
20
40
60
80
100
450
500
550
600
650
700

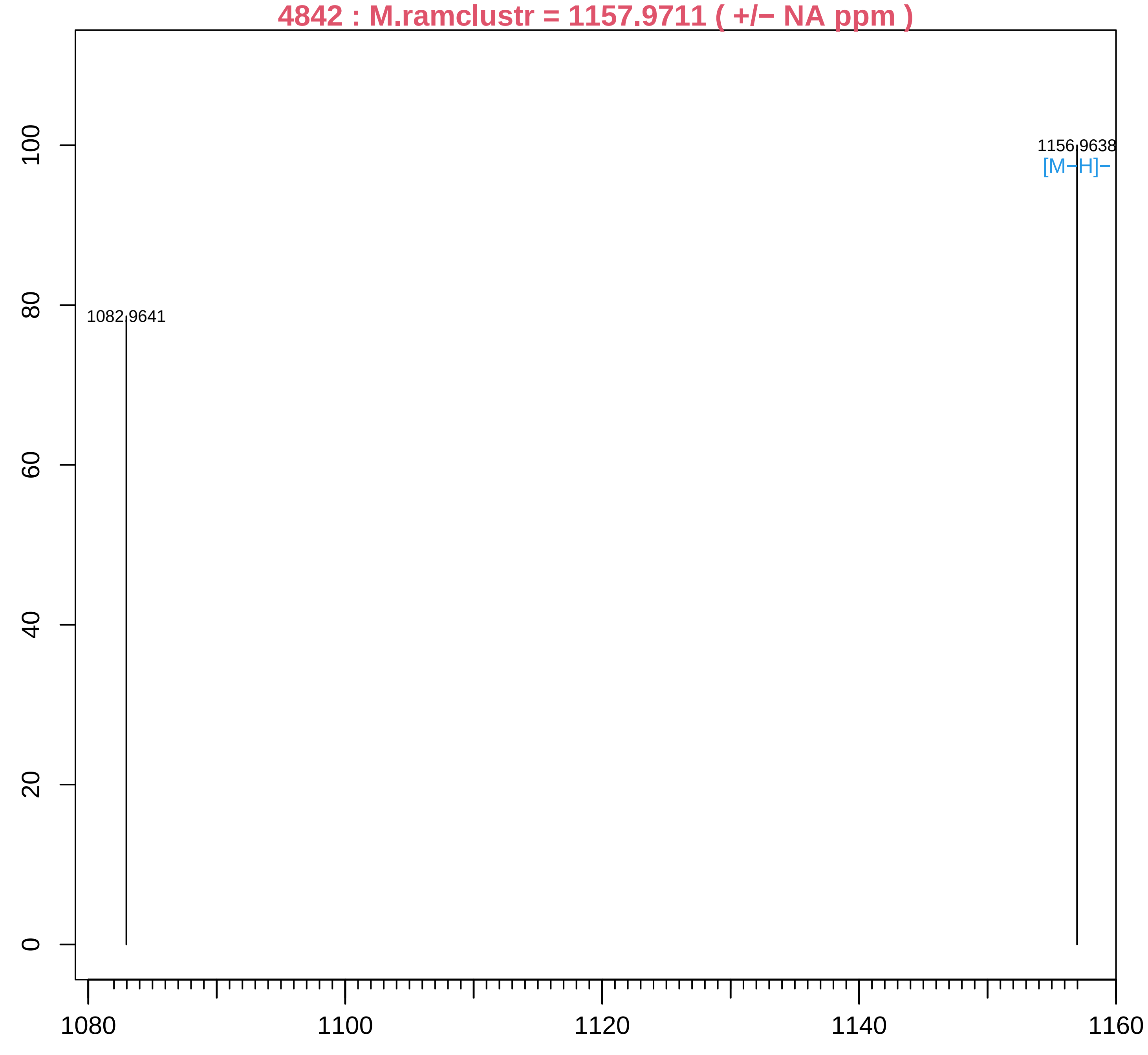
4842 : M.ramclustr = 1157.9711 (+/− NA ppm)
1156.9638
[M−H]−
1082.9641
0
20
40
60
80
100
1080
1100
1120
1140
1160

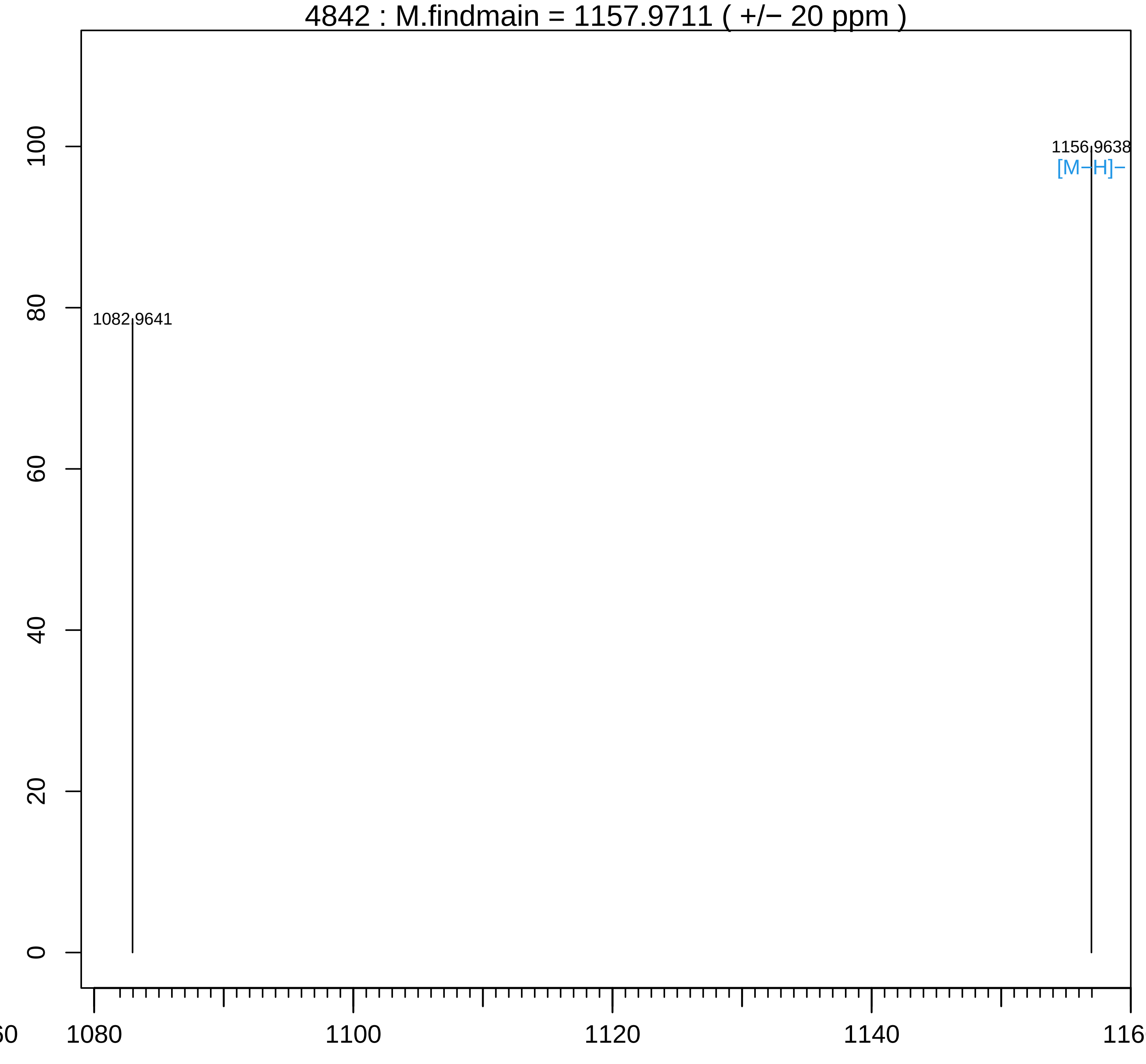
4842 : M.findmain = 1157.9711 (+/− 20 ppm)
1156.9638
[M−H]−
1082.9641
0
20
40
60
80
100
1080
1100
1120
1140

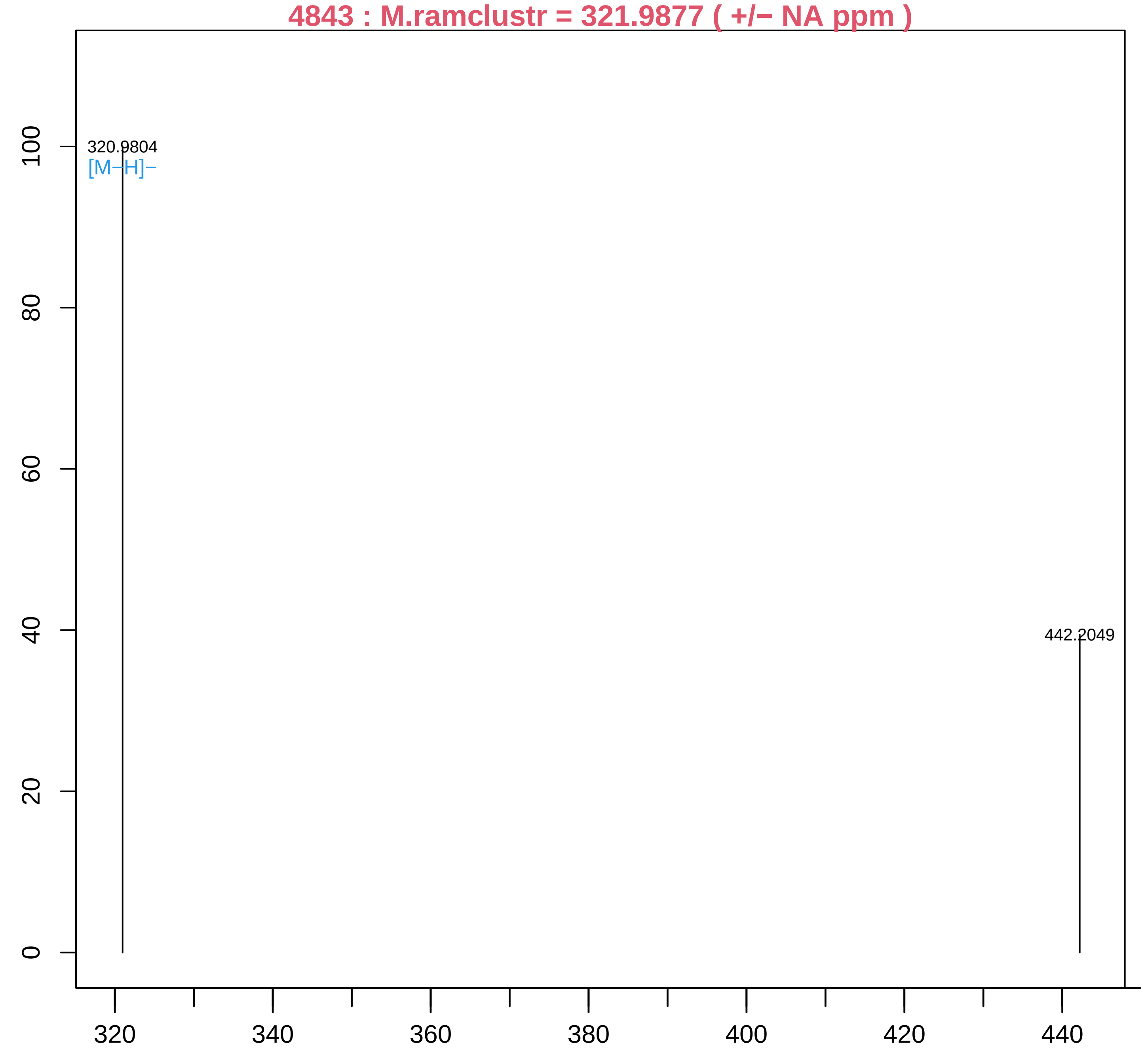
4843 : M.ramclustr = 321.9877 (+/− NA ppm)
320.9804
[M−H]−
442.2049
0
20
40
60
80
100
320
340
360
380
400
420
440

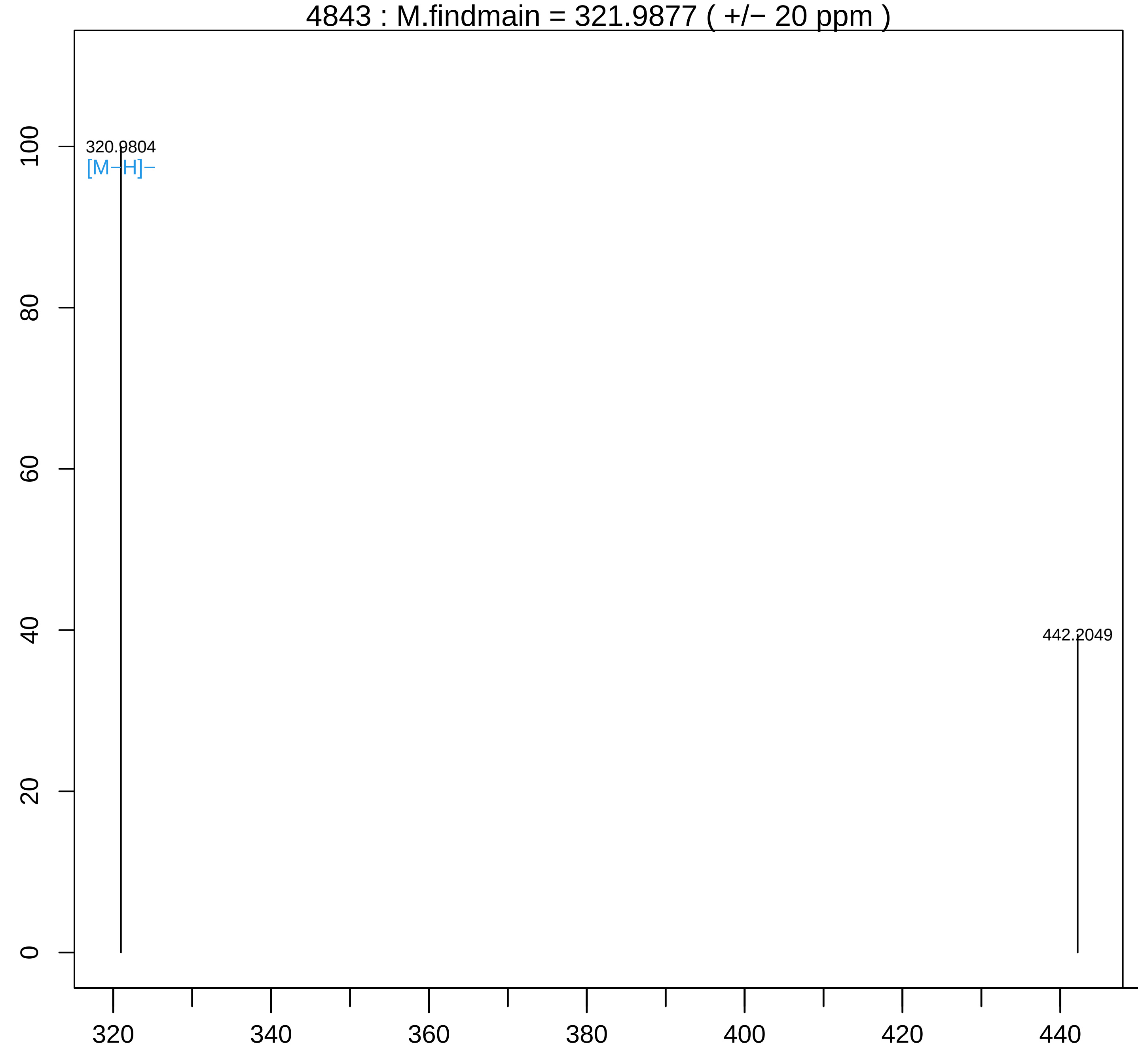
4843 : M.findmain = 321.9877 (+/− 20 ppm)
320.9804
[M−H]−
442.2049
0
20
40
60
80
100
320
340
360
380
400
420
440

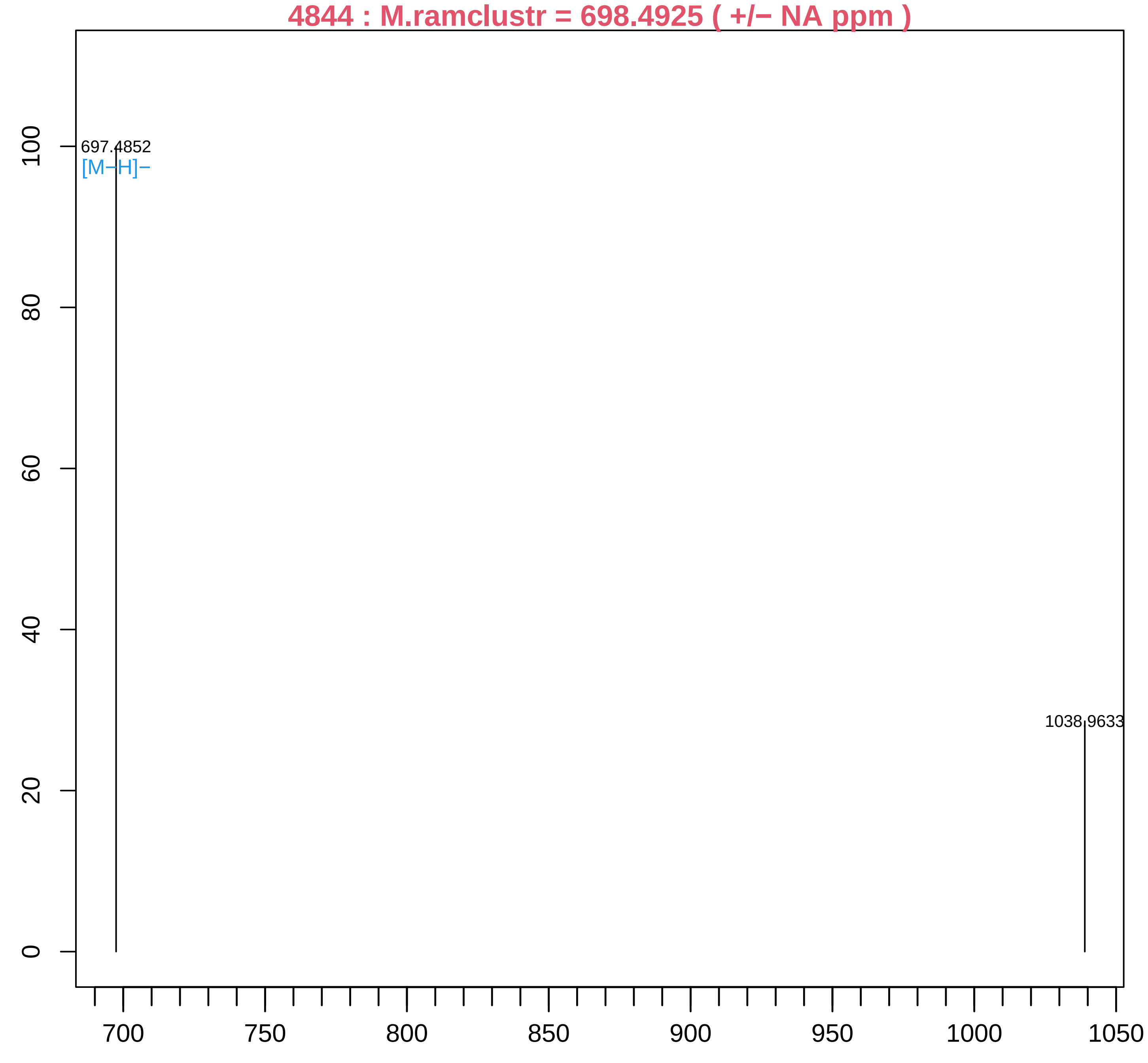
4844 : M.ramclustr = 698.4925 (+/− NA ppm)
697.4852
[M−H]−
1038.9633
0
20
40
60
80
100
700
750
800
850
900
950
1000
1050

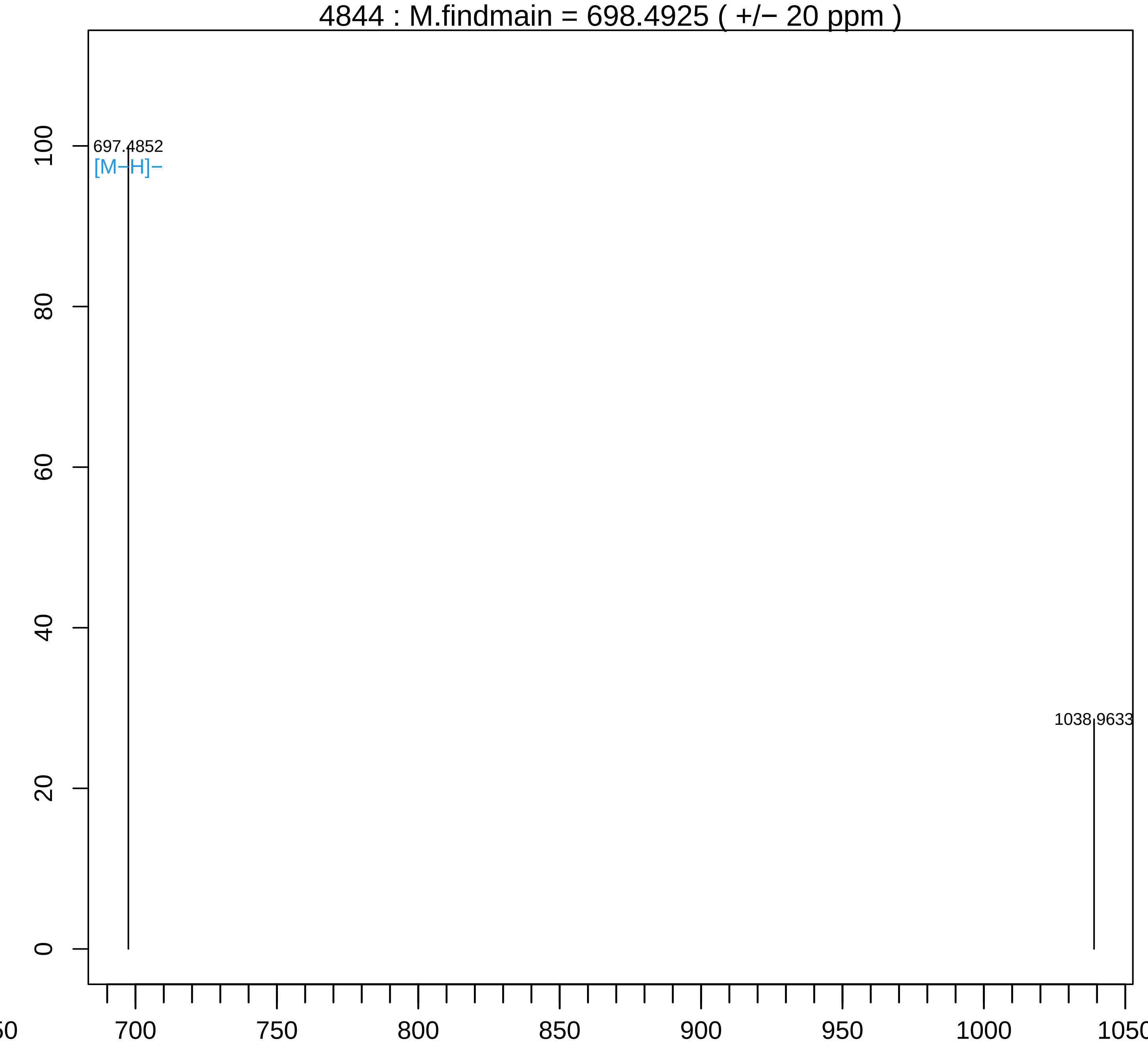
4844 : M.findmain = 698.4925 (+/− 20 ppm)
697.4852
[M−H]−
1038.9633
0
20
40
60
80
100
700
750
800
850
900
950
1000
1050

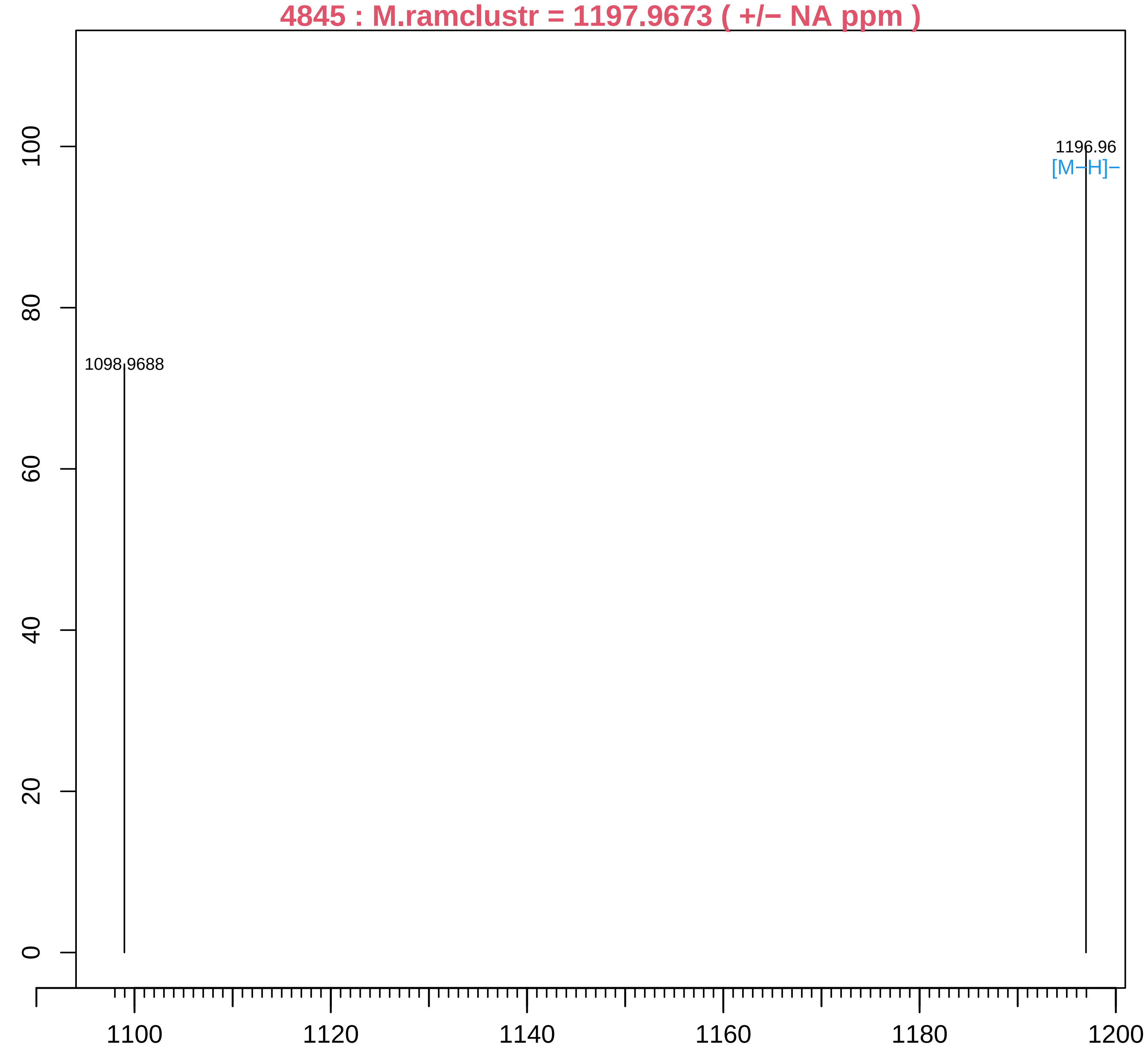
4845 : M.ramclustr = 1197.9673 (+/− NA ppm)
1196.96
[M−H]−
1098.9688
0
20
40
60
80
100
1100
1120
1140
1160
1180
1200

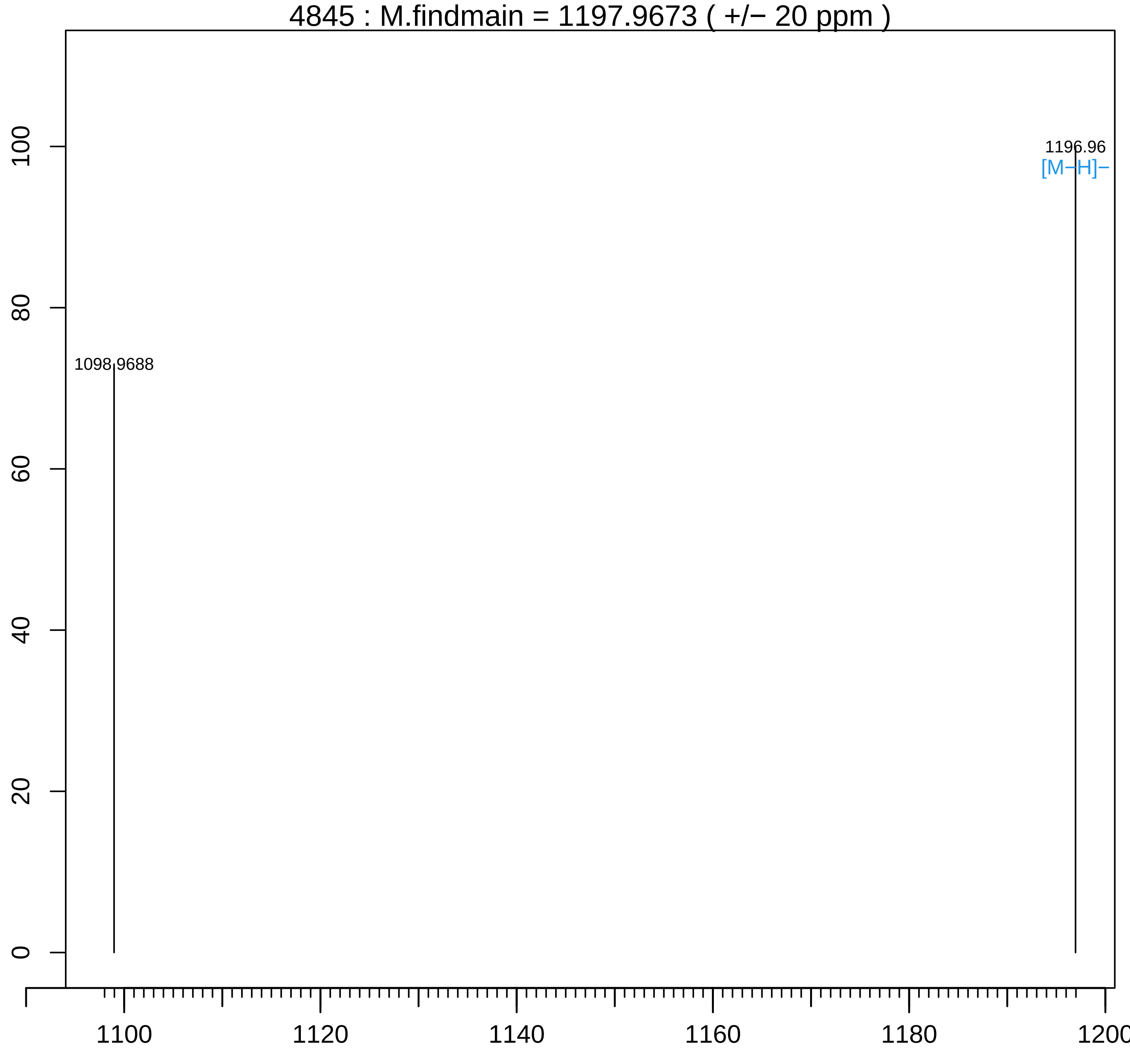
4845 : M.findmain = 1197.9673 (+/− 20 ppm)
1196.96
[M−H]−
1098.9688
0
20
40
60
80
100
1100
1120
1140
1160
1180
1200

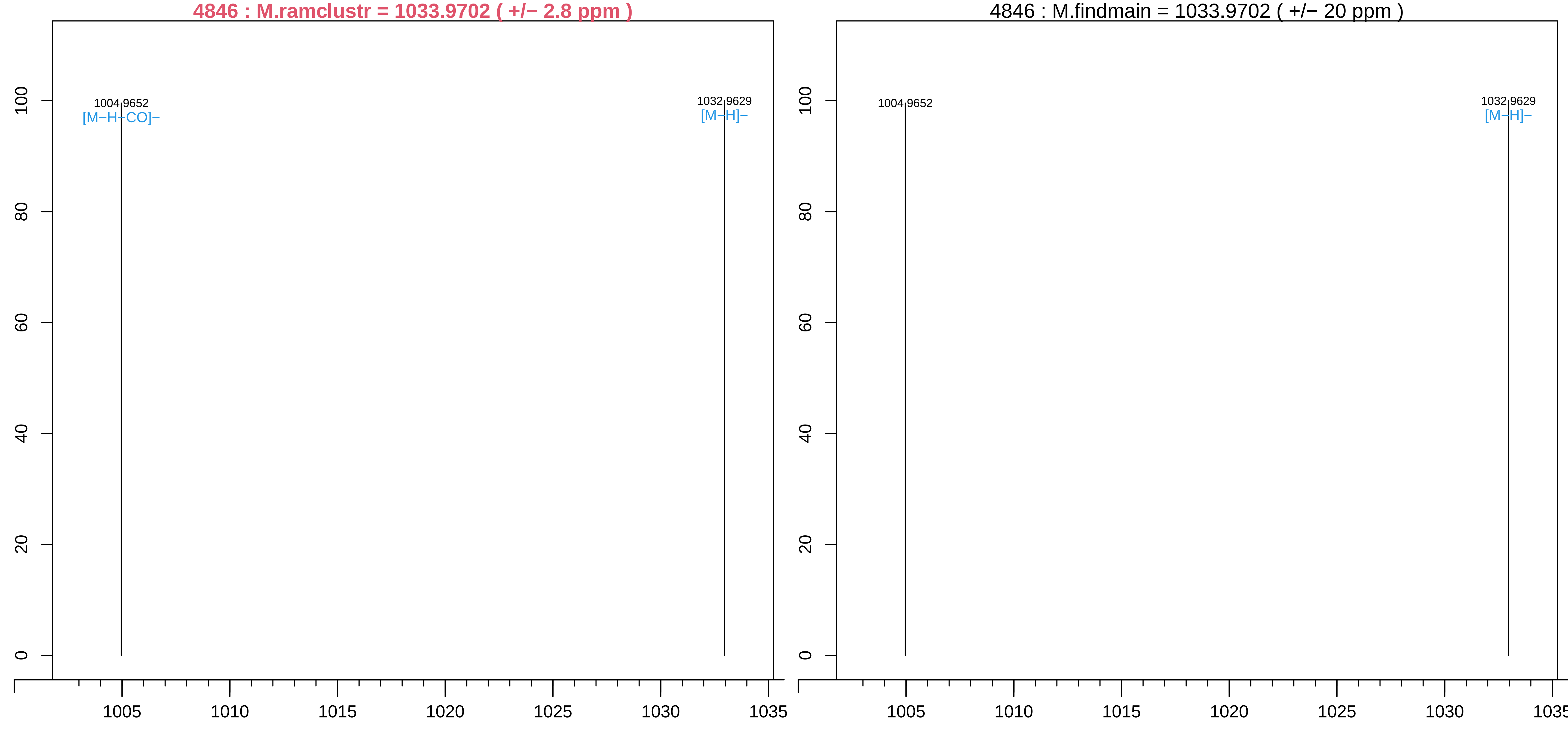
4846 : M.ramclustr = 1033.9702 (+/− 2.8 ppm)
1004.9652
[M−H−CO]−
1032.9629
[M−H]−
4846 : M.findmain = 1033.9702 (+/− 20 ppm)
1004.9652
1032.9629
[M−H]−
0
20
40
60
80
100
1005
1010
1015
1020
1025
1030
1035

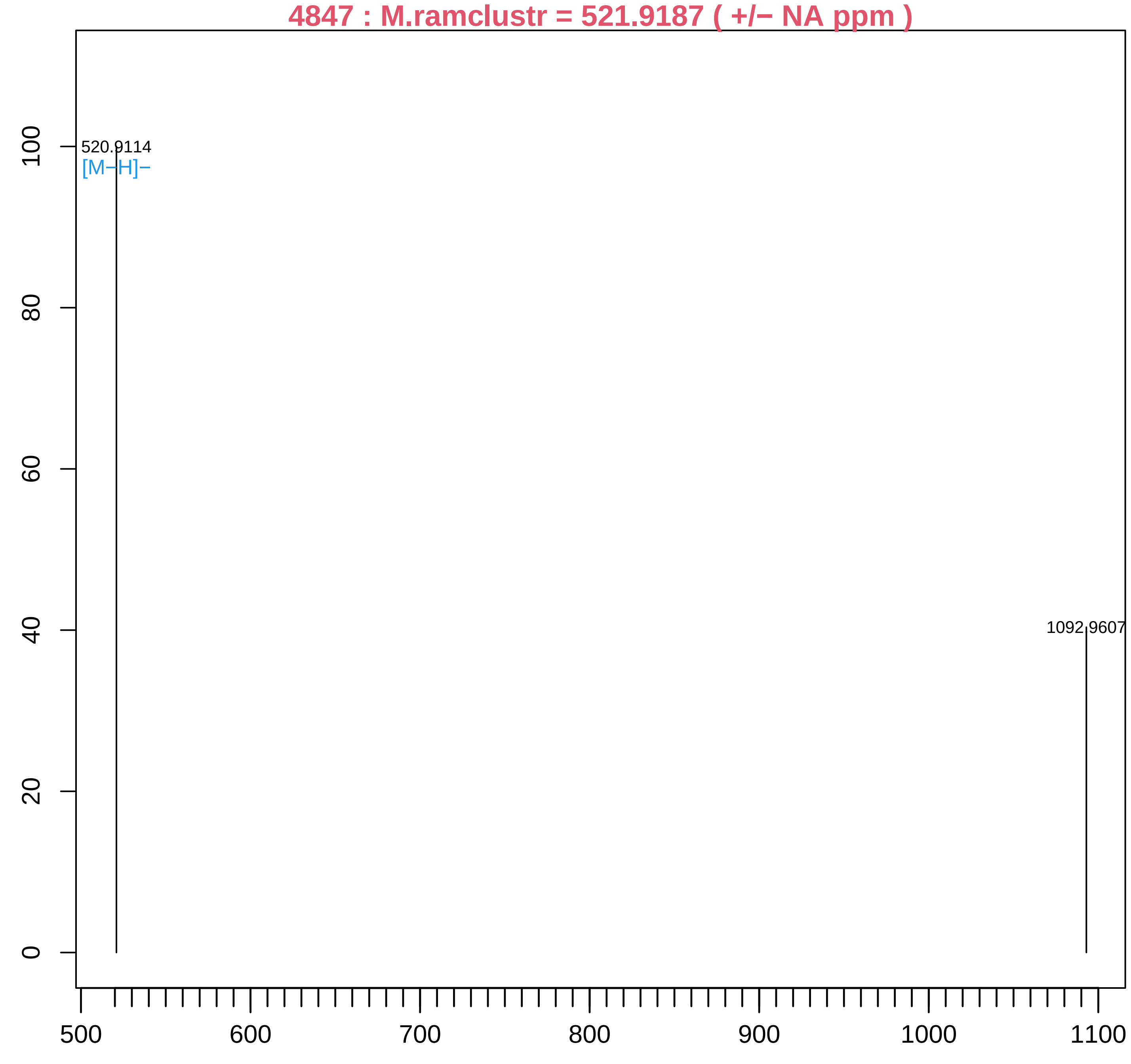
4847 : M.ramclustr = 521.9187 (+/− NA ppm)
520.9114
[M−H]−
1092.9607
0
20
40
60
80
100
500
600
700
800
900
1000
1100

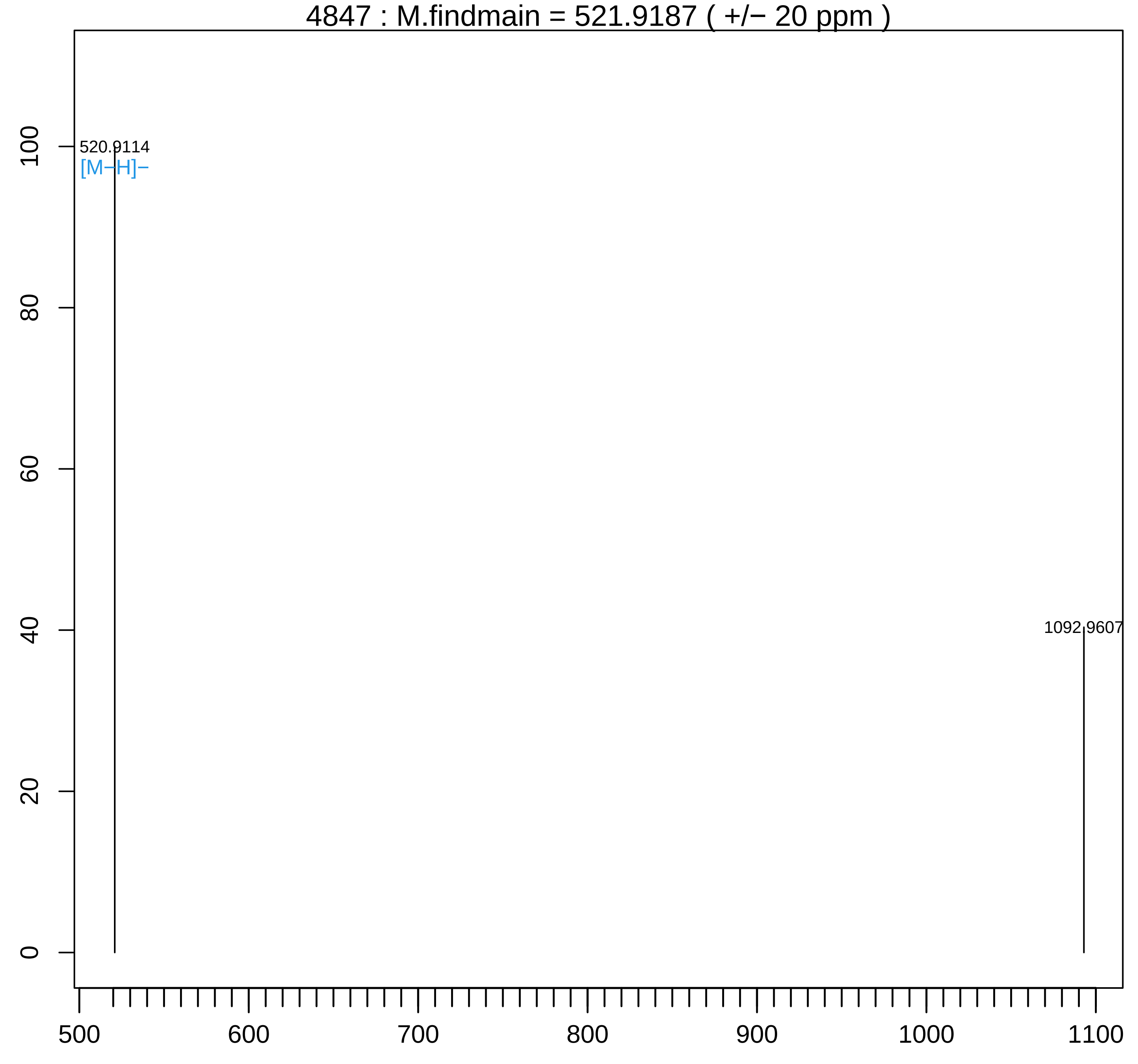
4847 : M.findmain = 521.9187 (+/− 20 ppm)
520.9114
[M−H]−
1092.9607
0
20
40
60
80
100
500
600
700
800
900
1000
1100

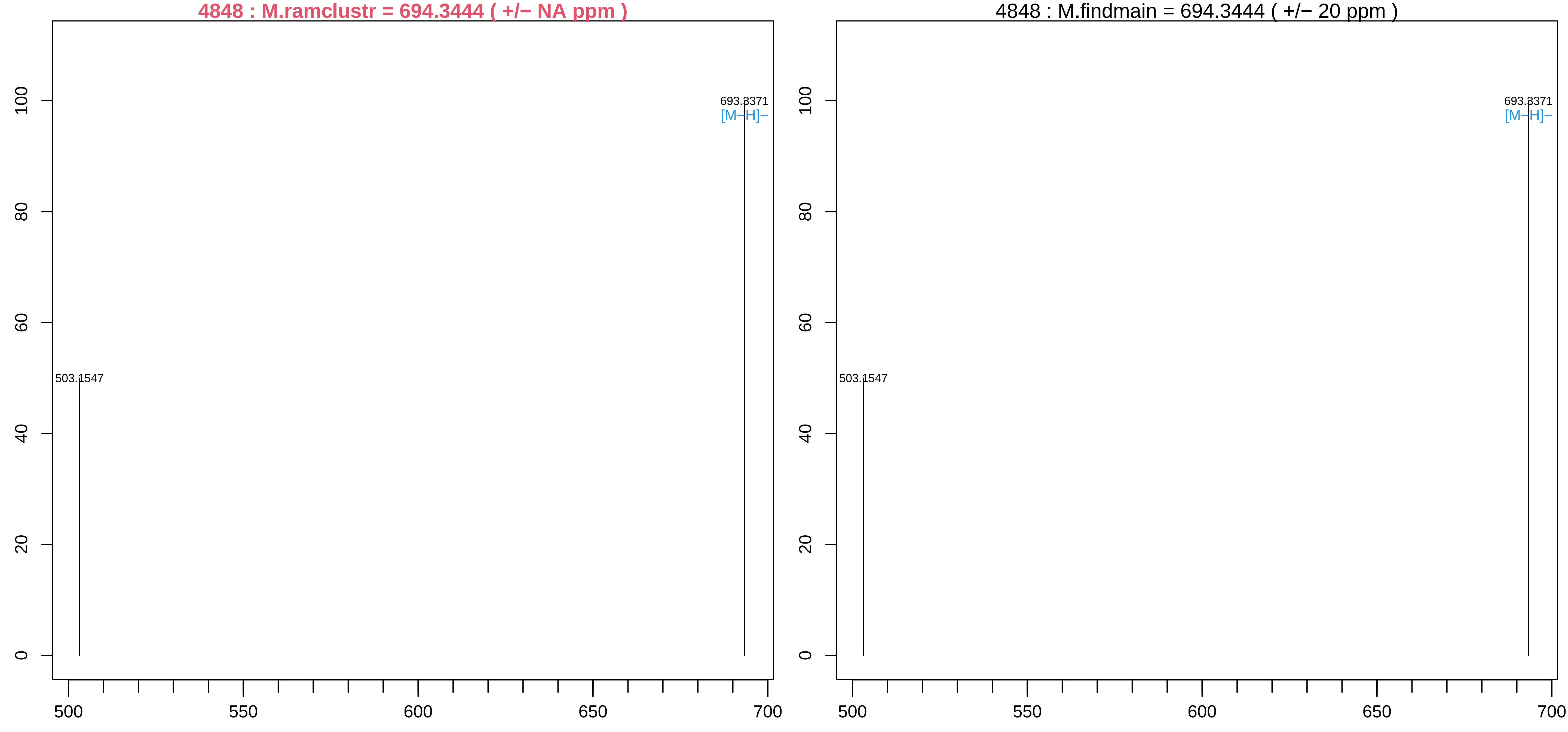
4848 : M.ramclustr = 694.3444 (+/− NA ppm)
693.3371
[M−H]−
503.1547
4848 : M.findmain = 694.3444 (+/− 20 ppm)
693.3371
[M−H]−
503.1547
0
20
40
60
80
100
500
550
600
650
700

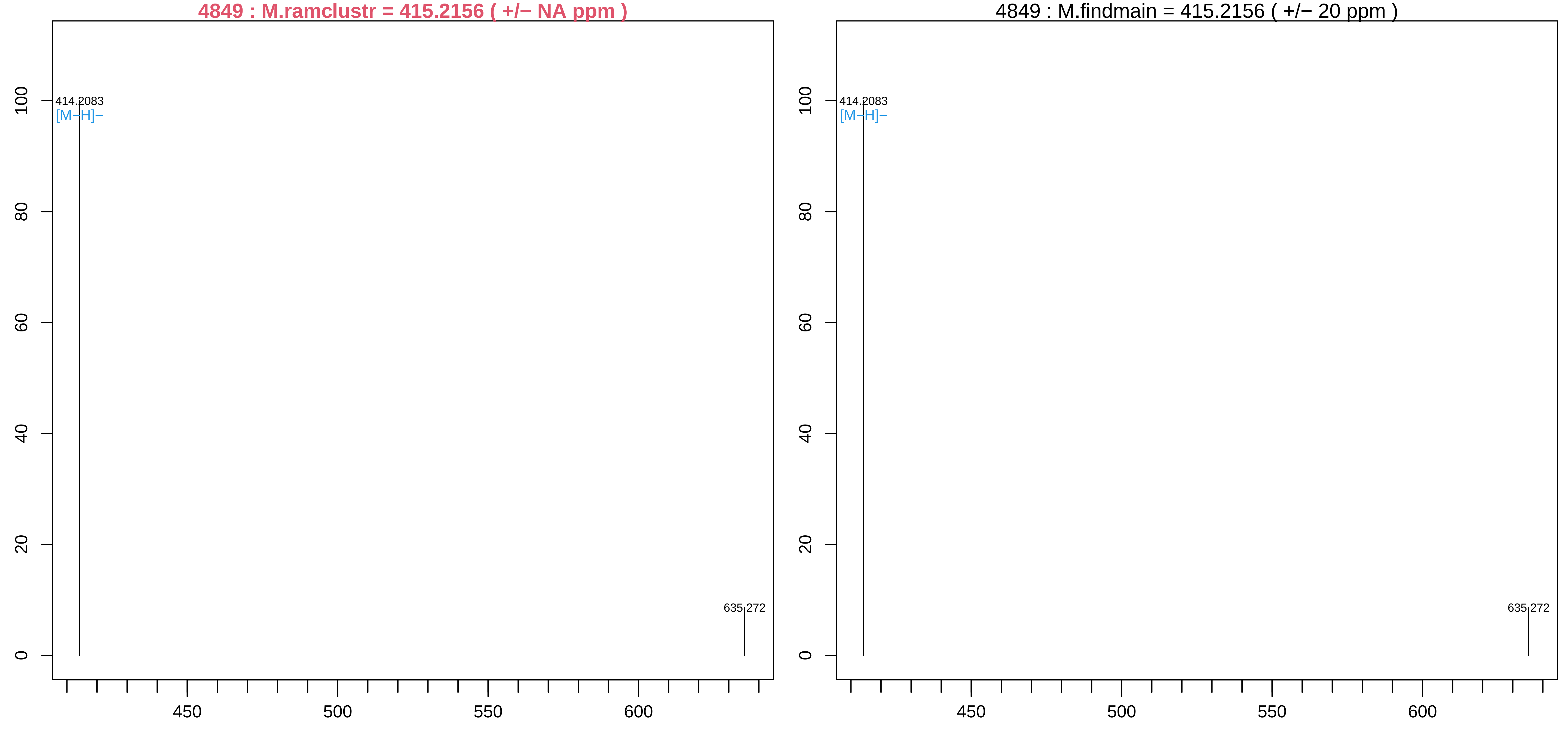
4849 : M.ramclustr = 415.2156 (+/− NA ppm)
414.2083
[M−H]−
635.272
4849 : M.findmain = 415.2156 (+/− 20 ppm)
414.2083
[M−H]−
635.272
0
20
40
60
80
100
450
500
550
600

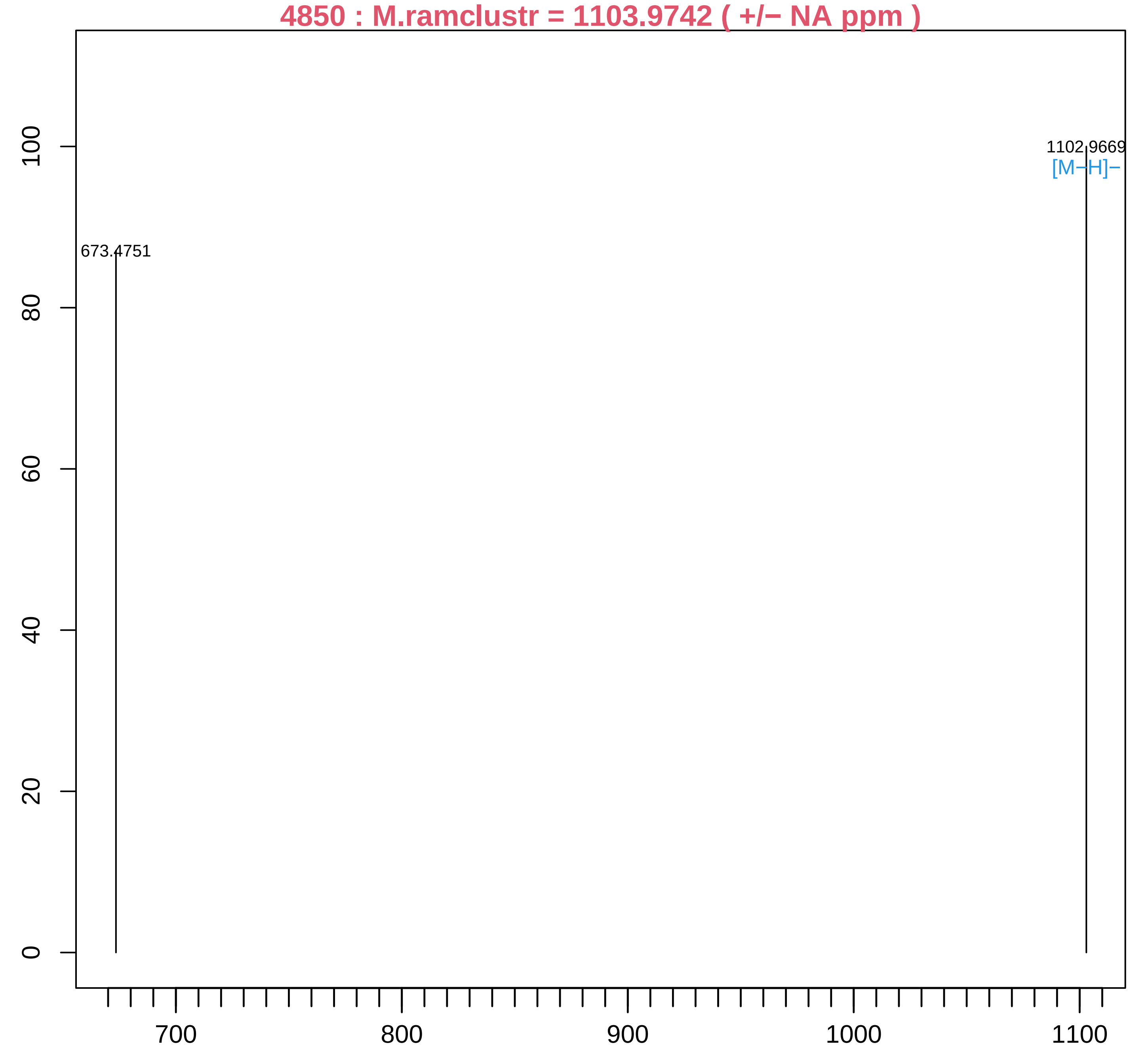

4850 : M.ramclustr = 1103.9742 (+/− NA ppm)
673.4751
1102.9669
[M−H]−
0
20
40
60
80
100
700
800
900
1000
1100

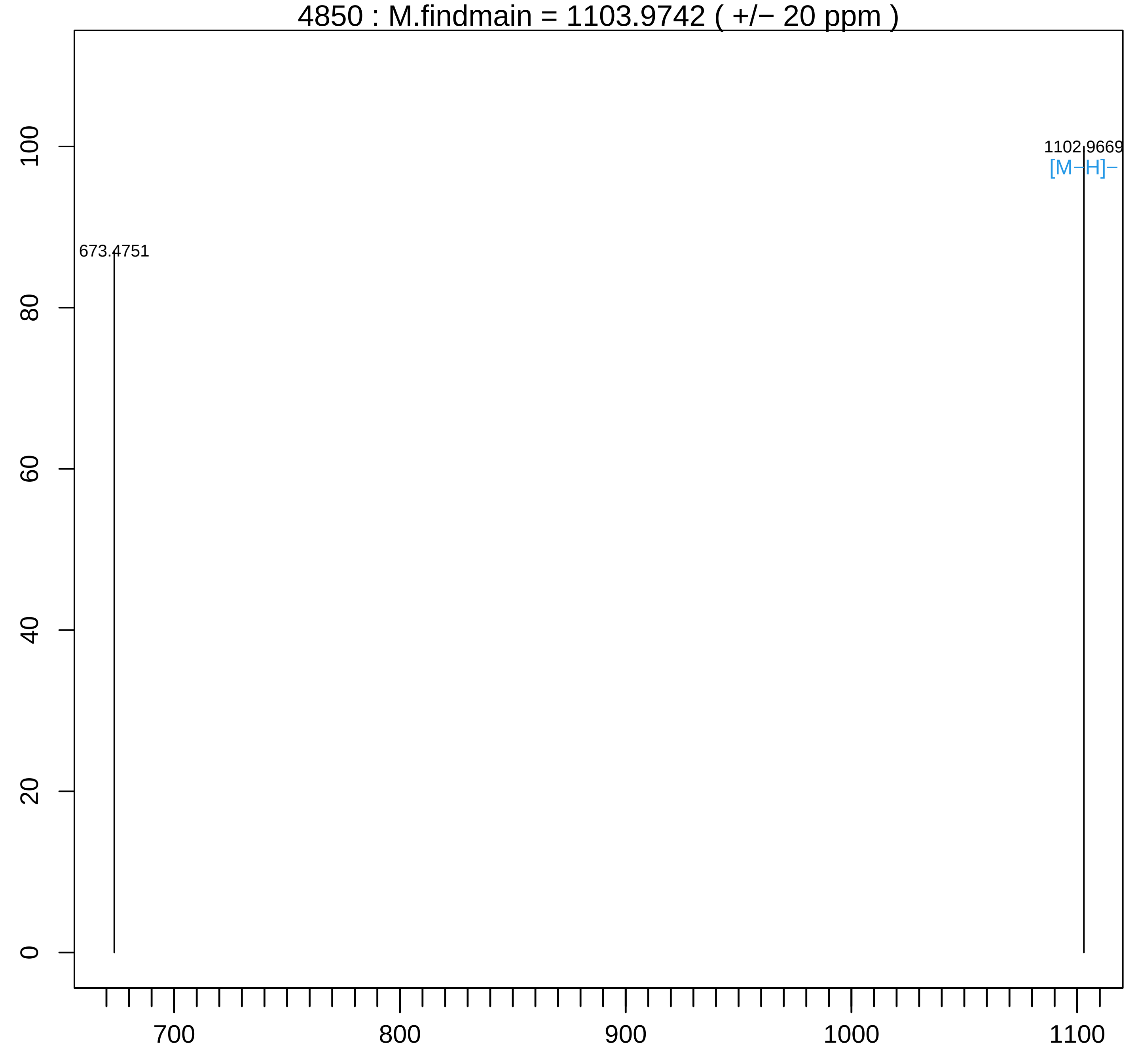

4850 : M.findmain = 1103.9742 (+/− 20 ppm)
673.4751
1102.9669
[M−H]−
0
20
40
60
80
100
700
800
900
1000
1100

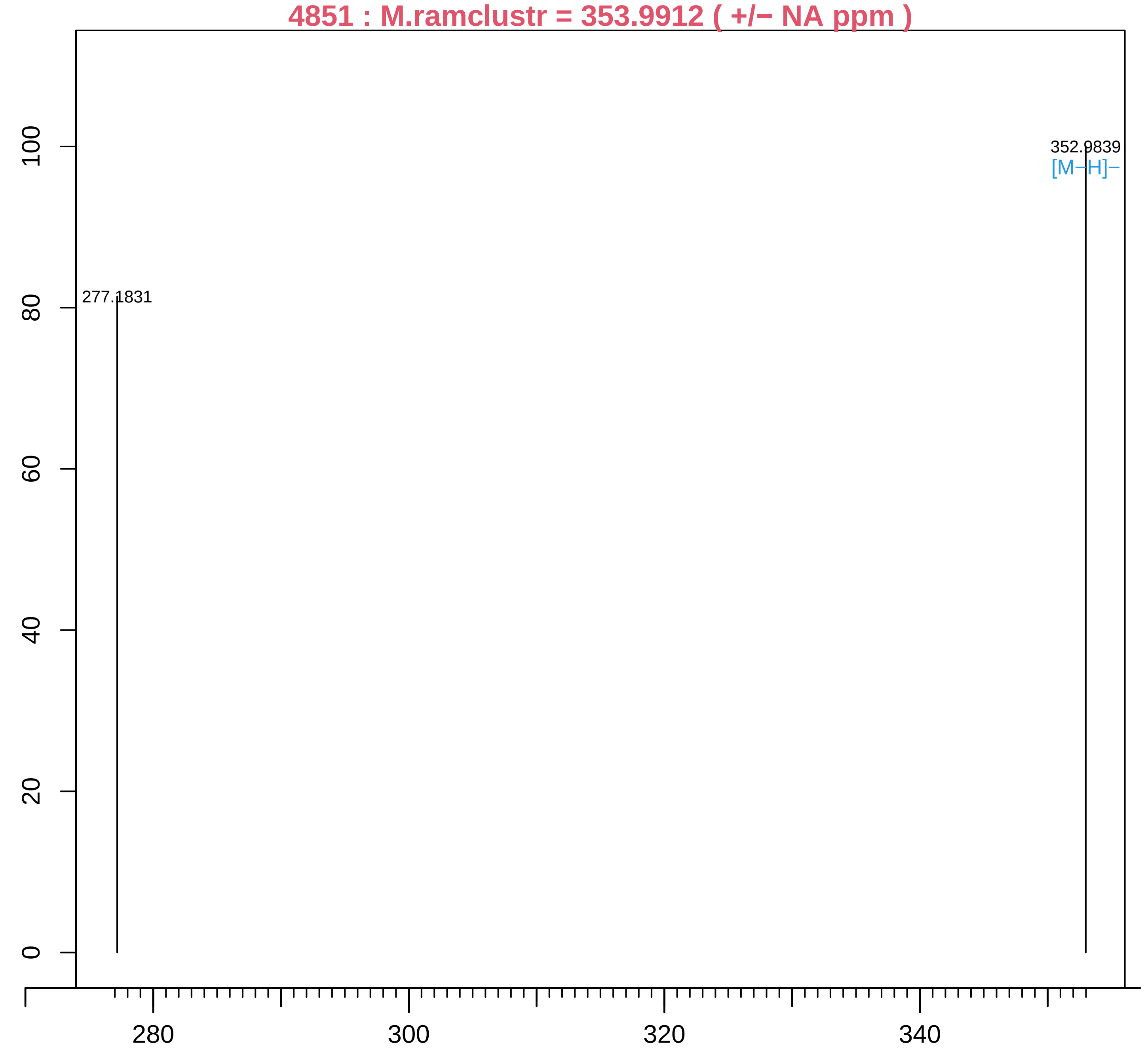

4851 : M.ramclustr = 353.9912 (+/− NA ppm)
277.1831
352.9839
[M−H]−
0
20
40
60
80
100
280
300
320
340

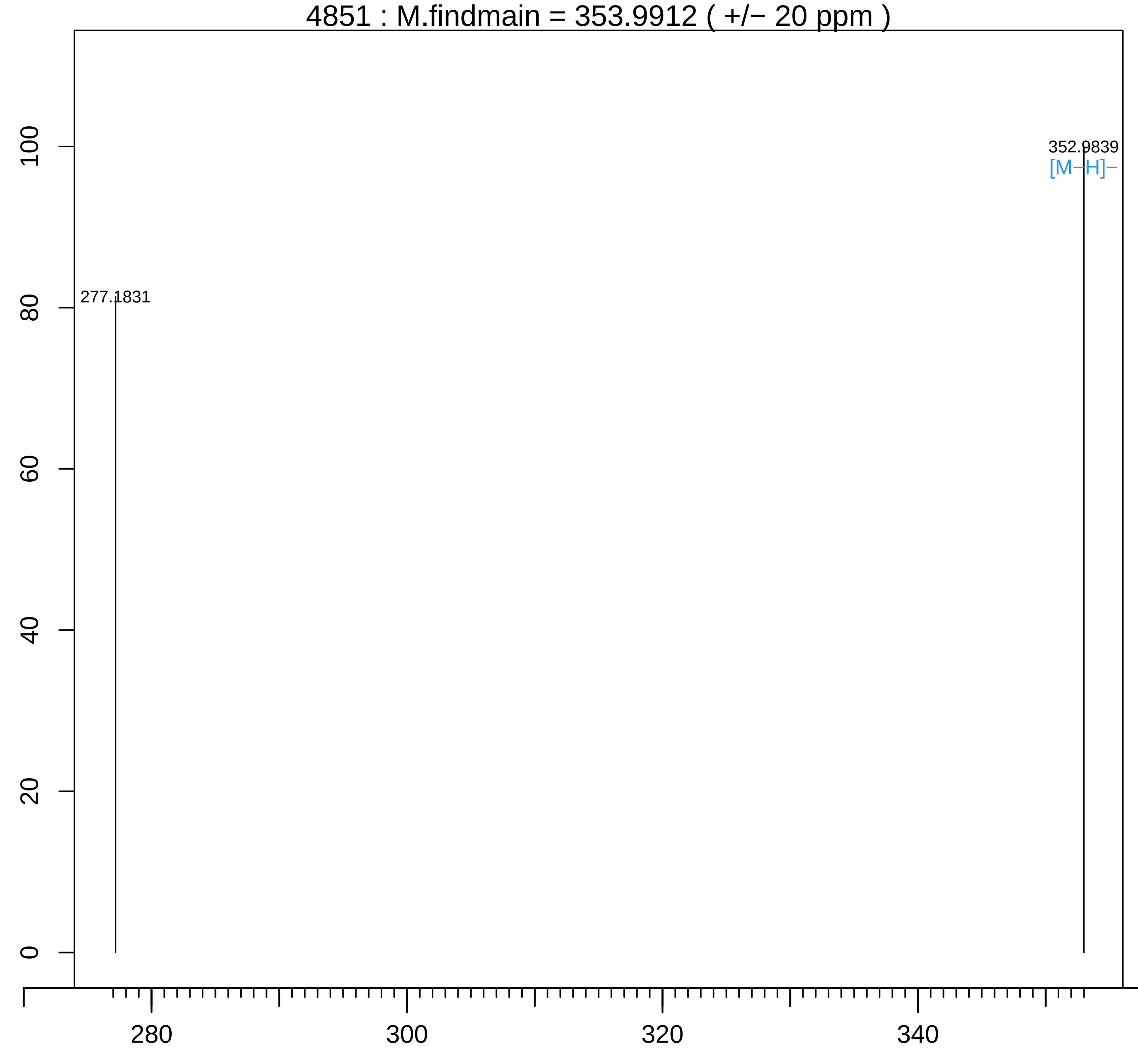

4851 : M.findmain = 353.9912 (+/− 20 ppm)
277.1831
352.9839
[M−H]−
0
20
40
60
80
100
280
300
320
340

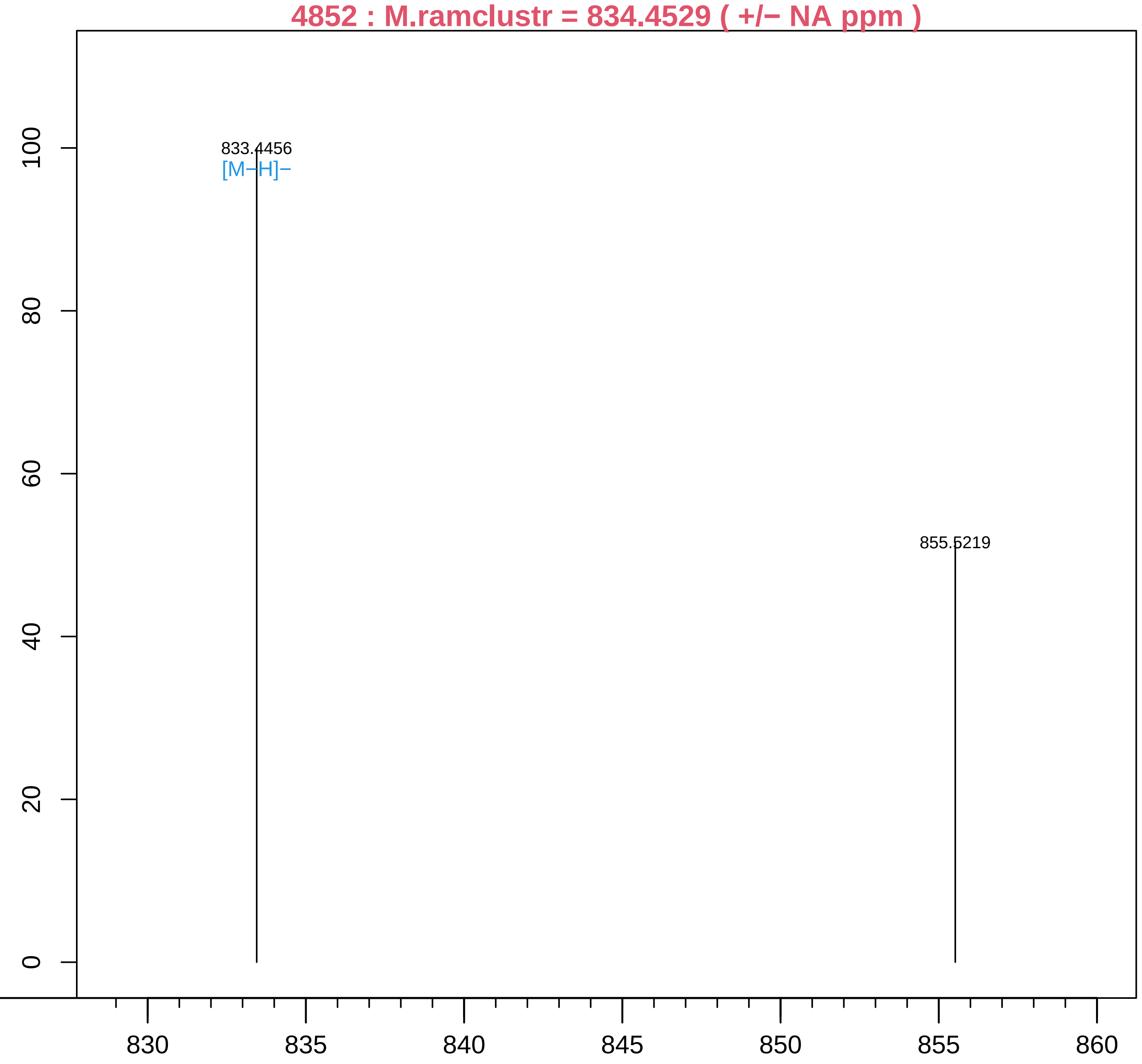
4852 : M.ramclustr = 834.4529 (+/− NA ppm)
833.4456
[M−H]−
855.5219
0
20
40
60
80
100
830
835
840
845
850
855
860

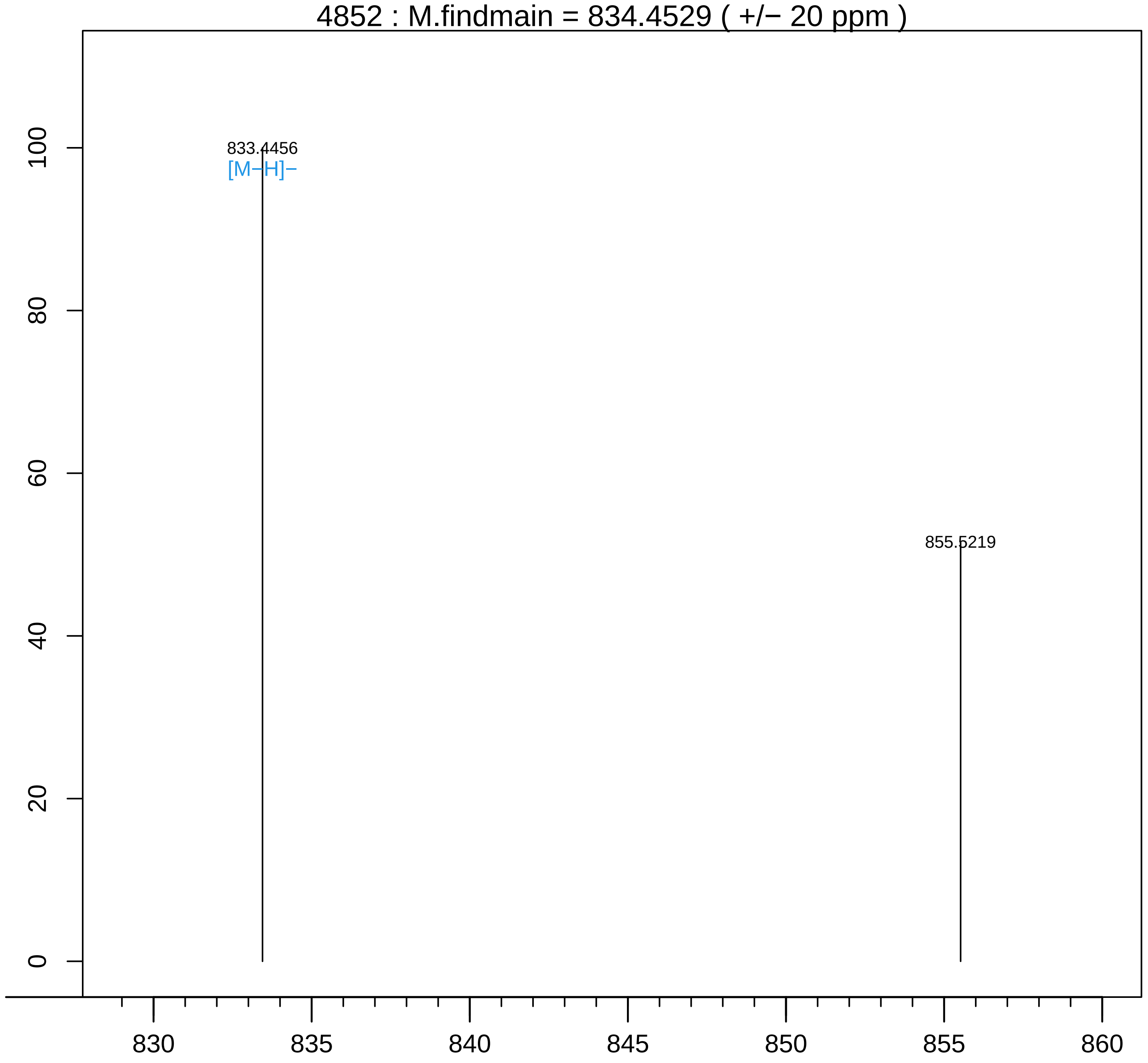
4852 : M.findmain = 834.4529 (+/− 20 ppm)
833.4456
[M−H]−
855.5219
0
20
40
60
80
100
830
835
840
845
850
855
860

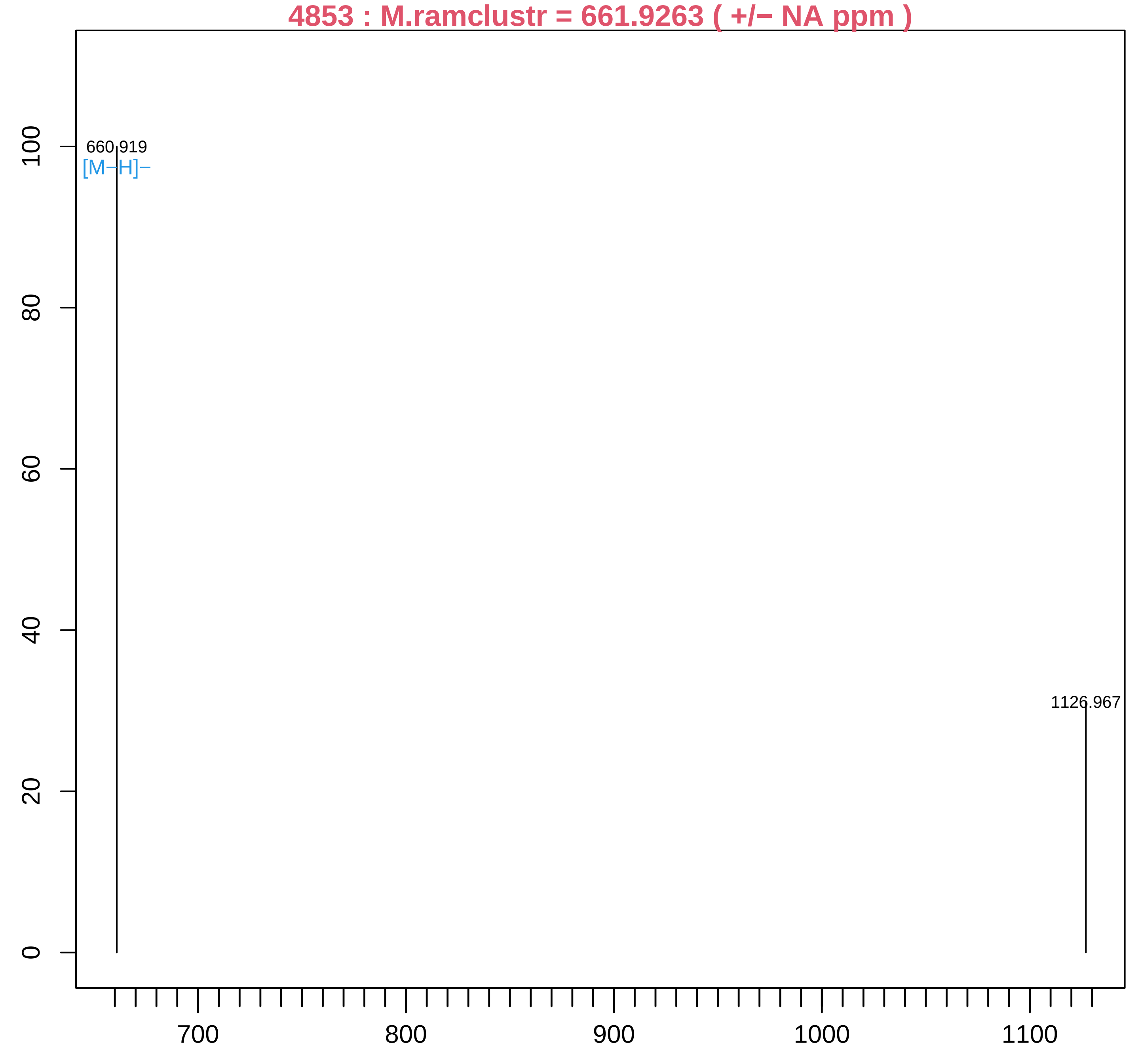
4853 : M.ramclustr = 661.9263 (+/− NA ppm)
660.919
[M−H]−
1126.967
0
20
40
60
80
100
700
800
900
1000
1100

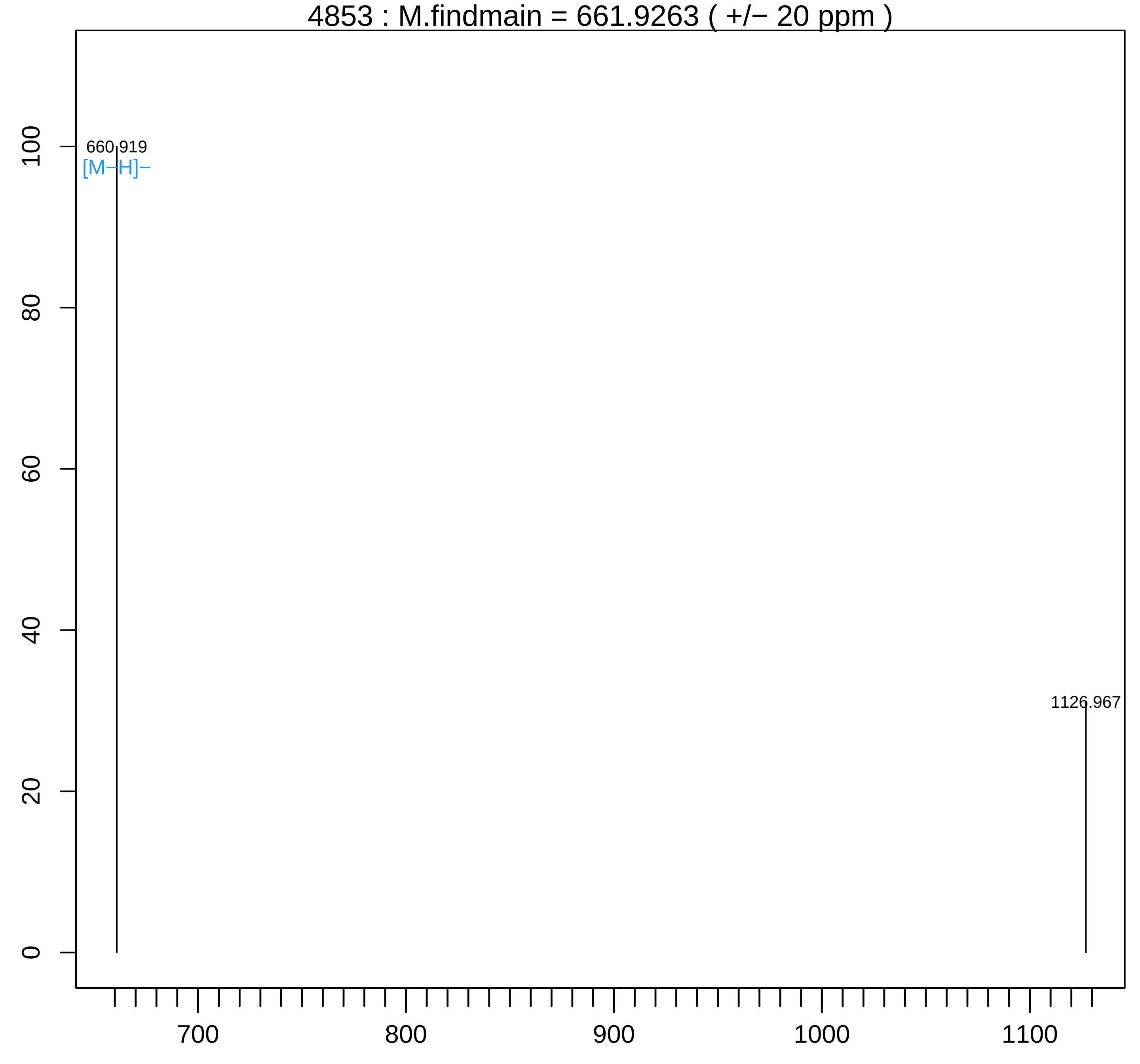
4853 : M.findmain = 661.9263 (+/− 20 ppm)
660.919
[M−H]−
1126.967
0
20
40
60
80
100
700
800
900
1000
1100

4854 : M.ramclustr = 469.225 (+/− NA ppm)

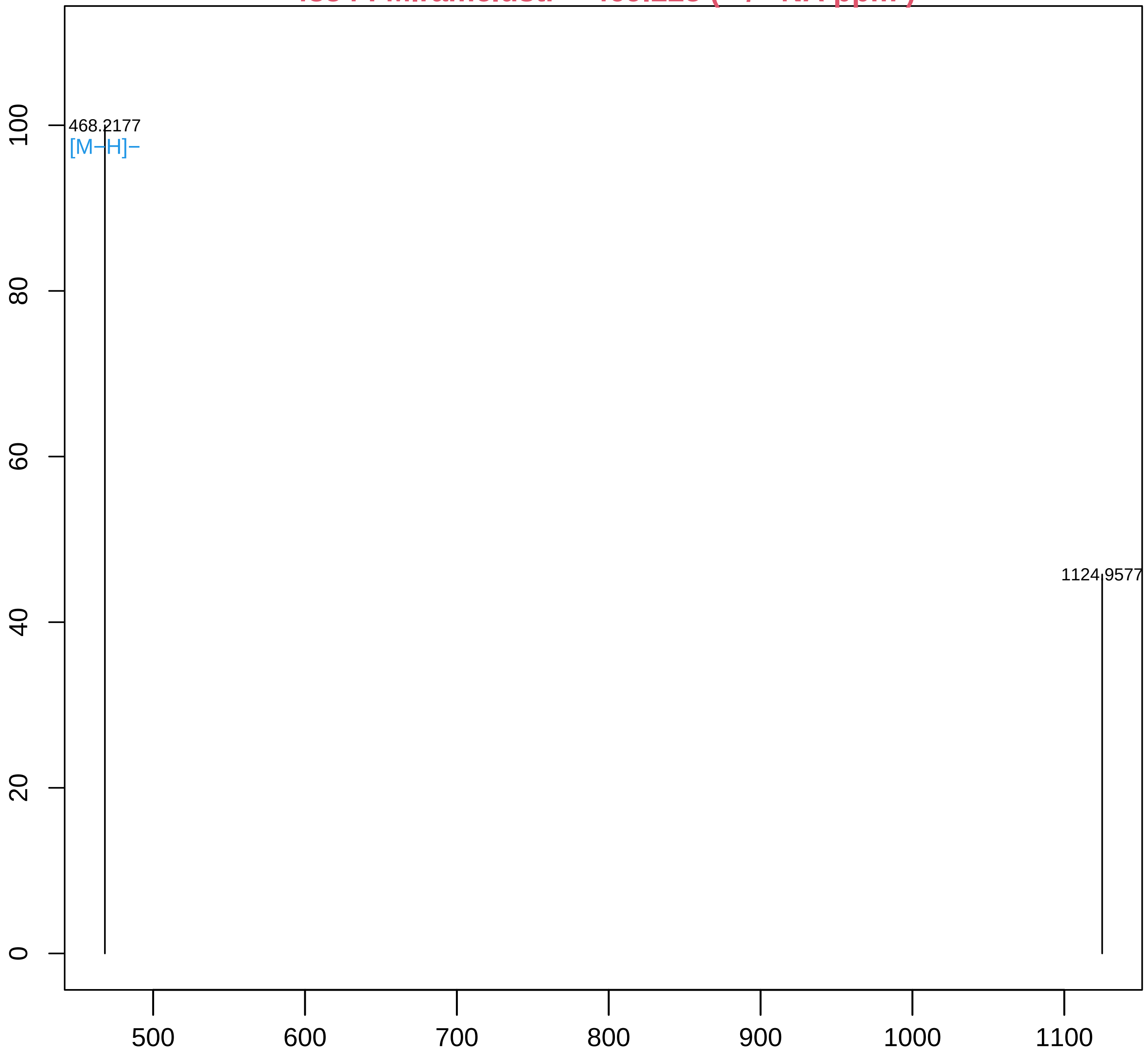

4854 : M.findmain = 469.225 (+/− 20 ppm)

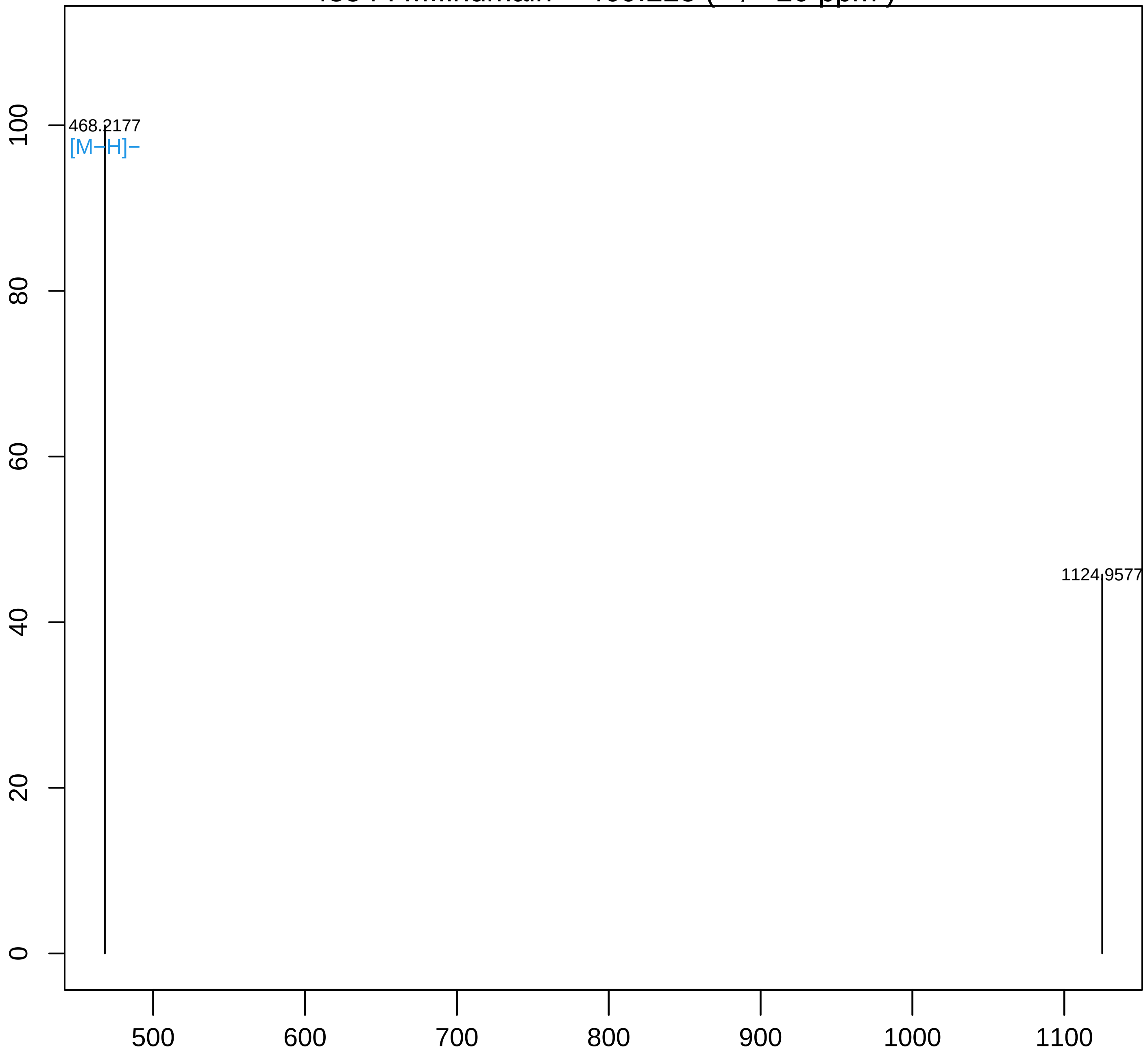

4855 : M.ramclustr = 933.9748 (+/− NA ppm)

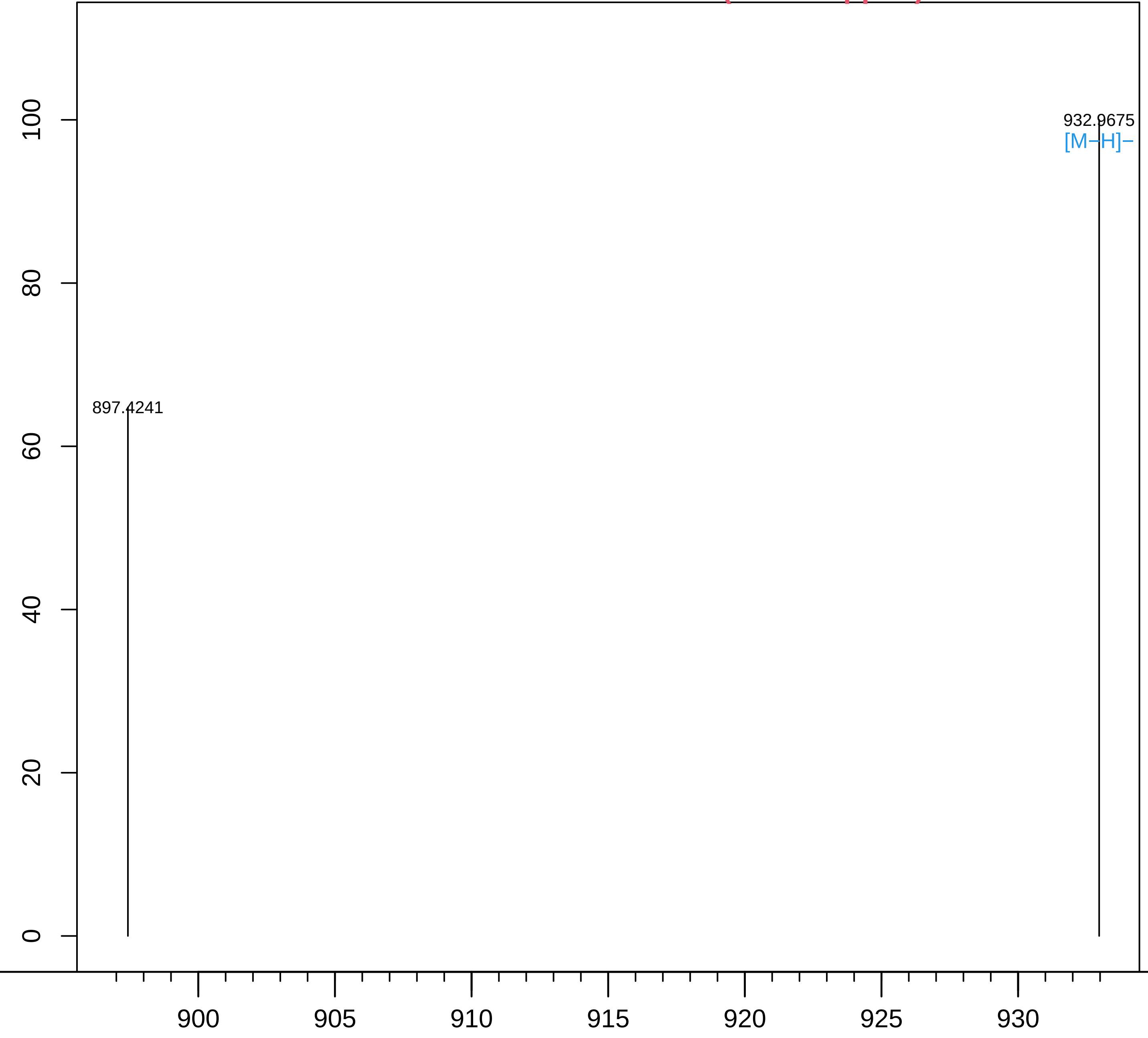

4855 : M.findmain = 933.9748 (+/− 20 ppm)

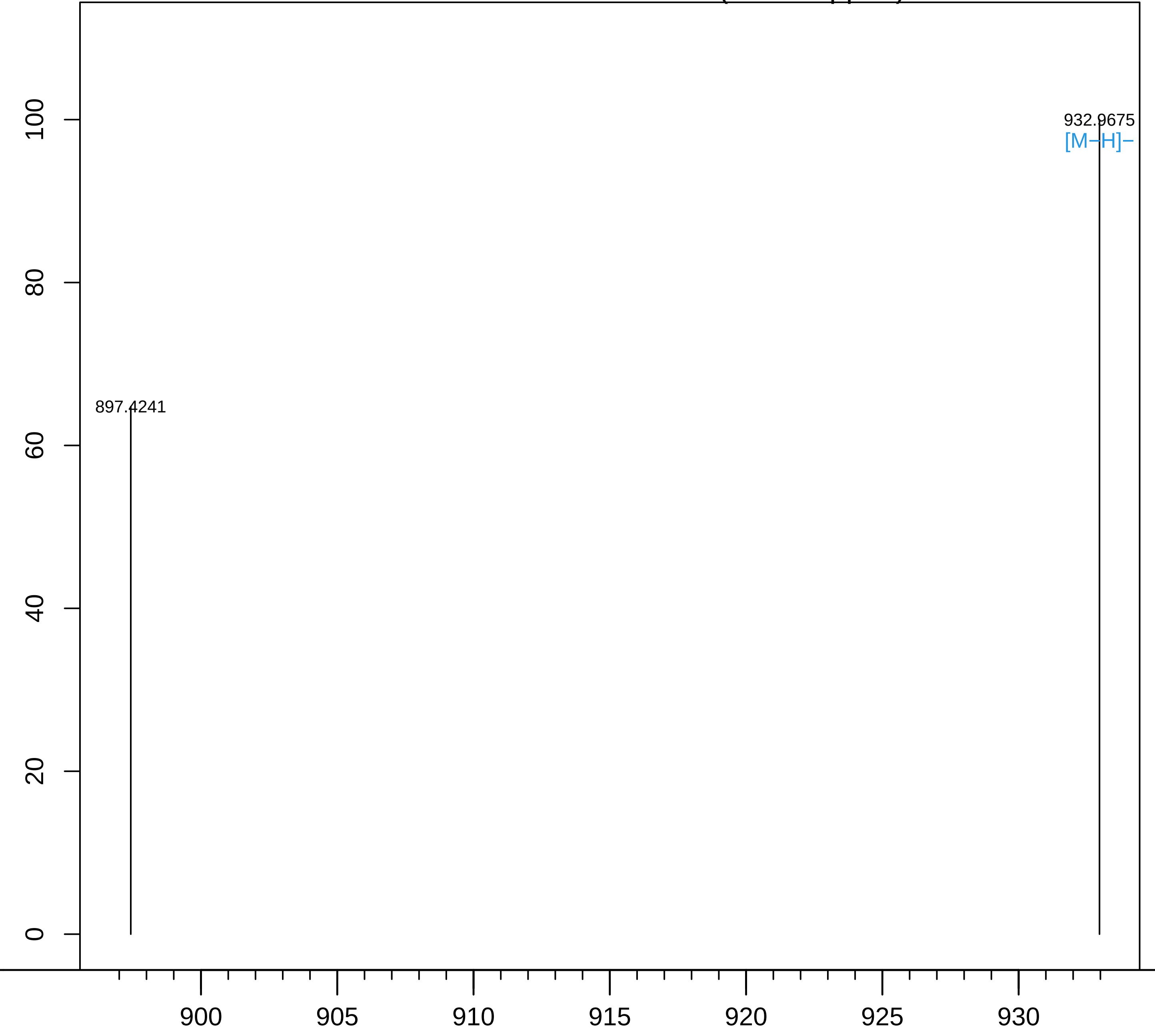

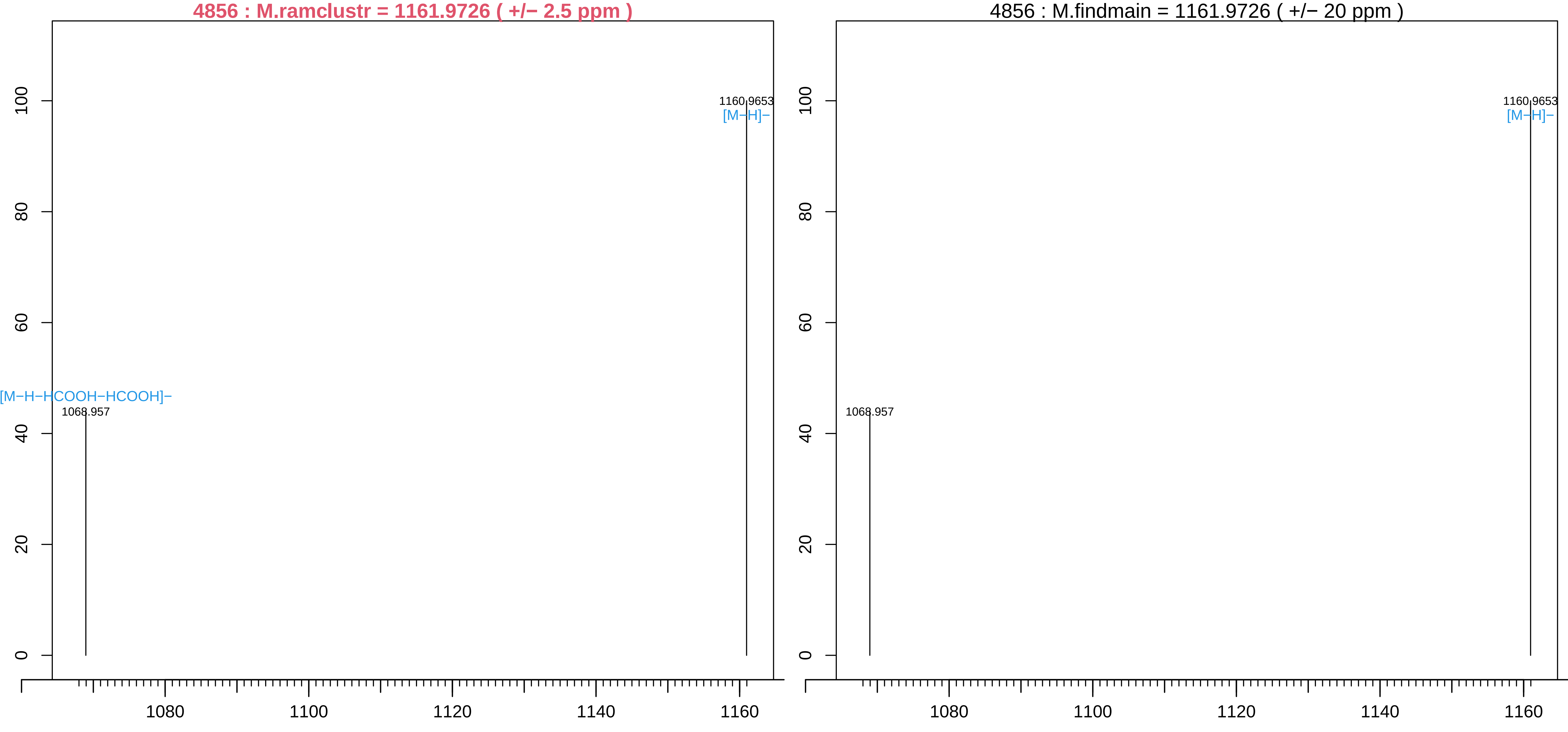
4856 : M.ramclustr = 1161.9726 (+/− 2.5 ppm)
1160.9653
[M−H]−
[M−H−HCOOH−HCOOH]−
1068.957
4856 : M.findmain = 1161.9726 (+/− 20 ppm)
1160.9653
[M−H]−
1068.957
0
20
40
60
80
100
1080
1100
1120
1140
1160

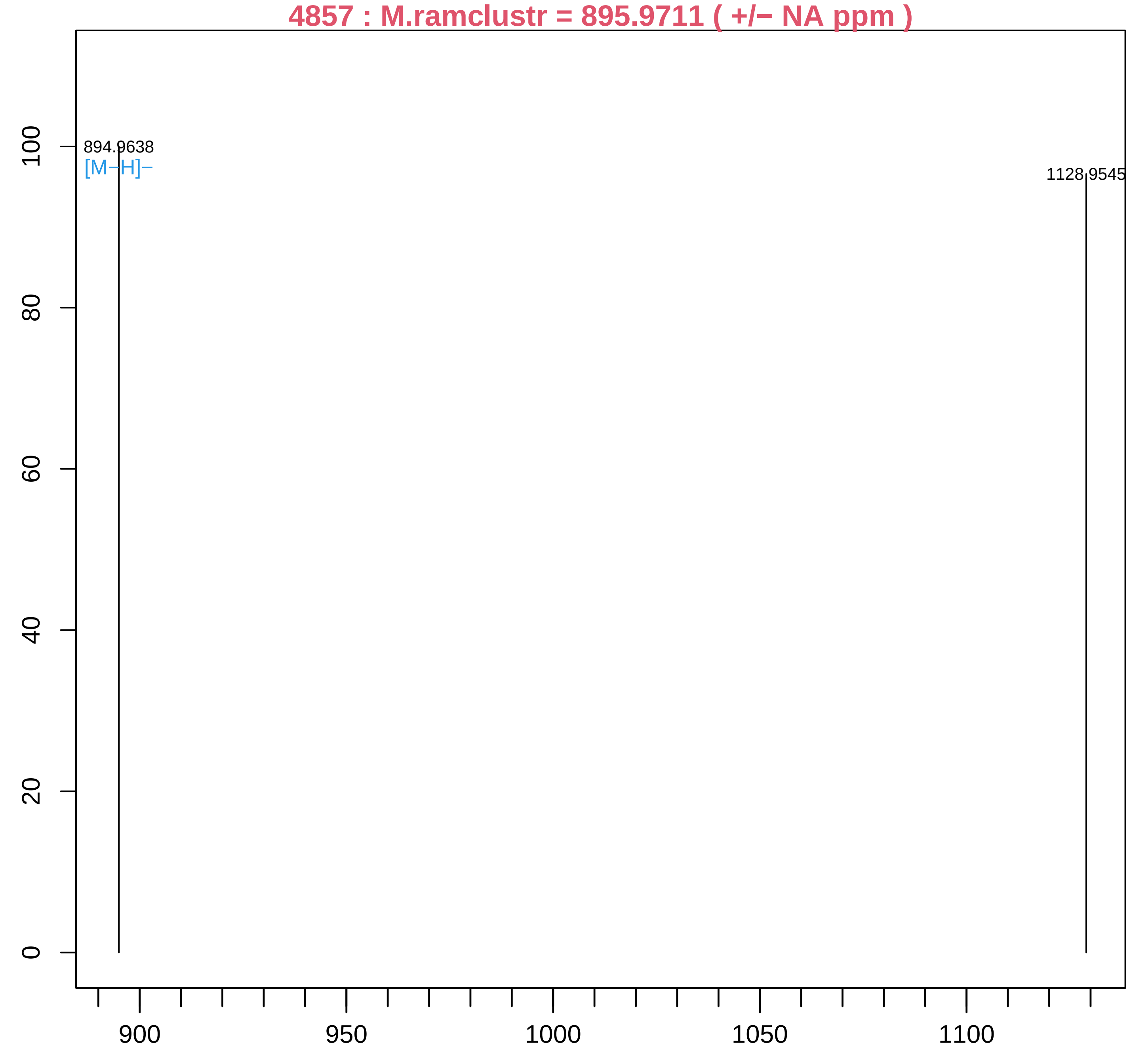
4857 : M.ramclustr = 895.9711 (+/− NA ppm)
894.9638
[M−H]−
1128.9545
0
20
40
60
80
100
900
950
1000
1050
1100

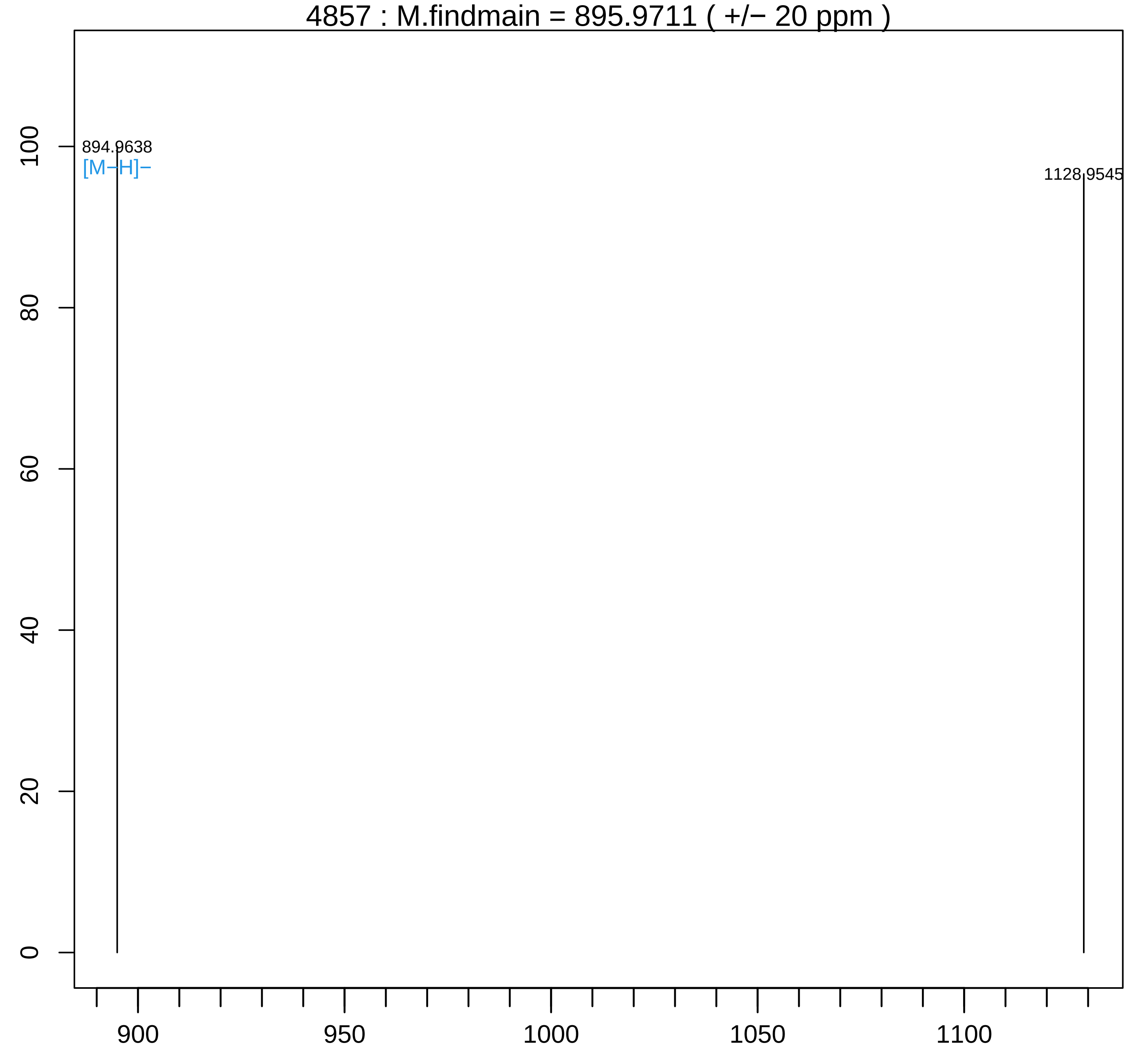
4857 : M.findmain = 895.9711 (+/− 20 ppm)
894.9638
[M−H]−
1128.9545
0
20
40
60
80
100
900
950
1000
1050
1100

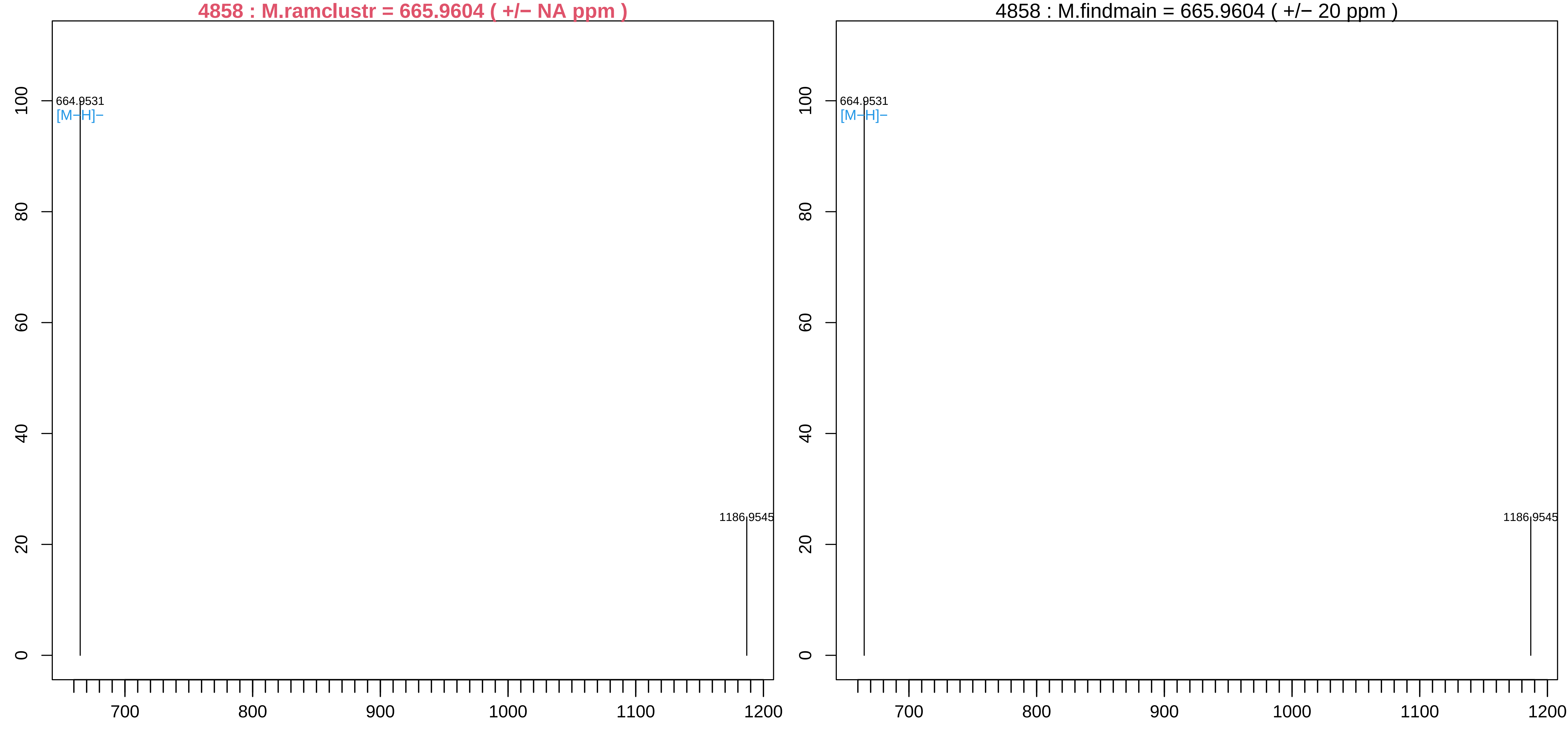

4858 : M.ramclustr = 665.9604 (+/− NA ppm)
664.9531
[M−H]−
1186.9545
4858 : M.findmain = 665.9604 (+/− 20 ppm)
664.9531
[M−H]−
1186.9545
0
20
40
60
80
100
700
800
900
1000
1100
1200

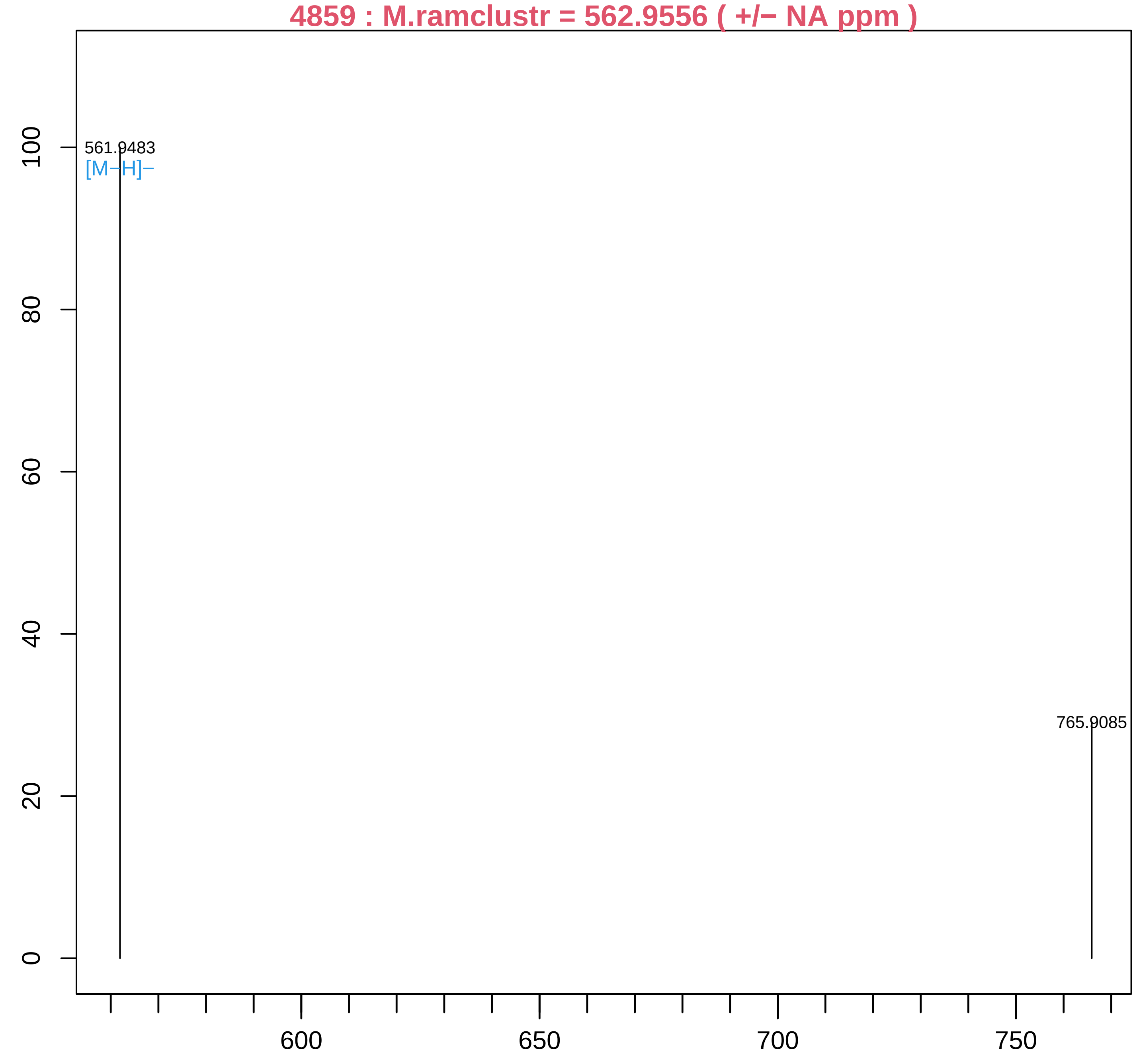
4859 : M.ramclustr = 562.9556 (+/− NA ppm)
561.9483
[M−H]−
765.9085
0
20
40
60
80
100
600
650
700
750

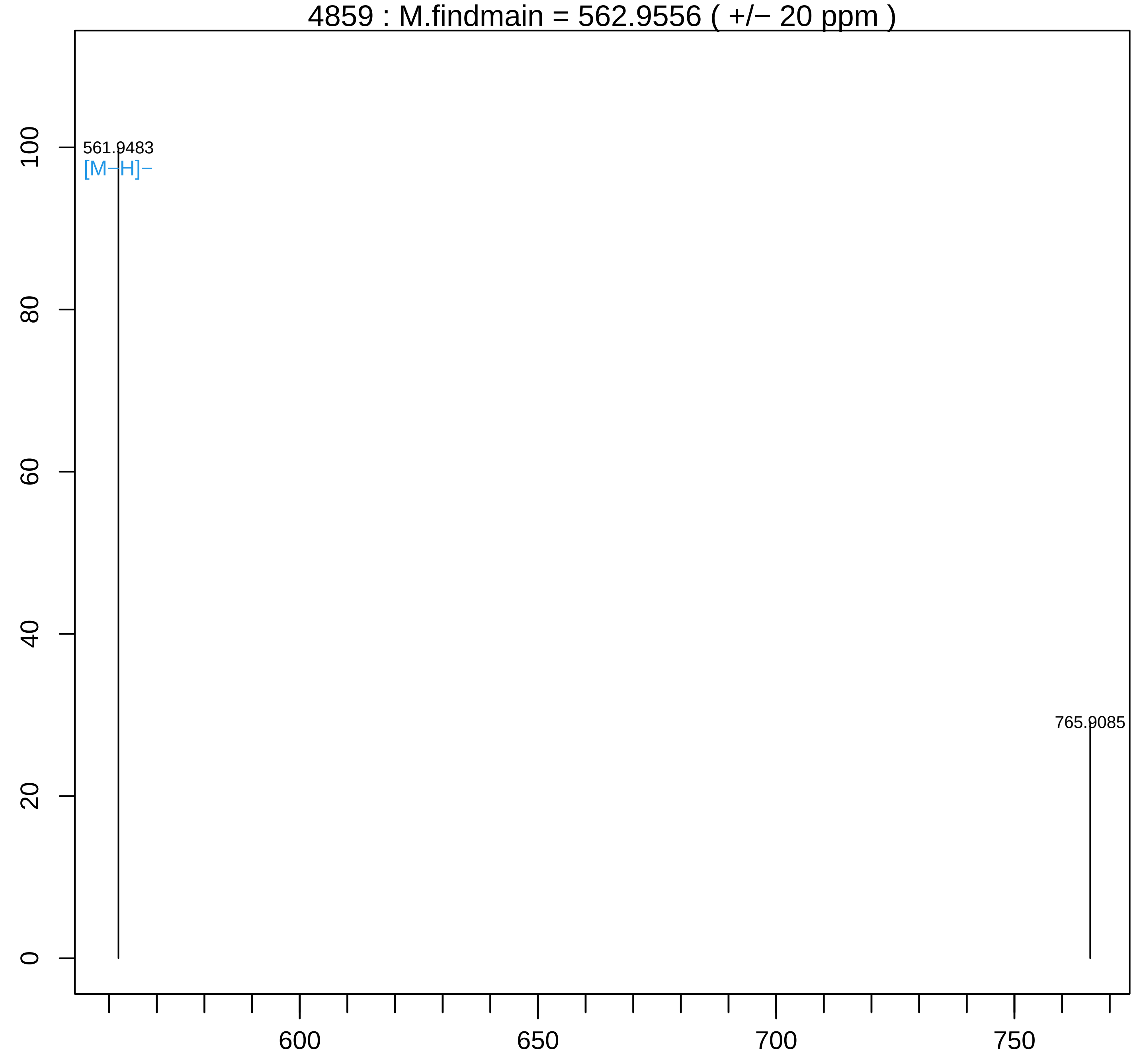
4859 : M.findmain = 562.9556 (+/− 20 ppm)
561.9483
[M−H]−
765.9085
0
20
40
60
80
100
600
650
700
750

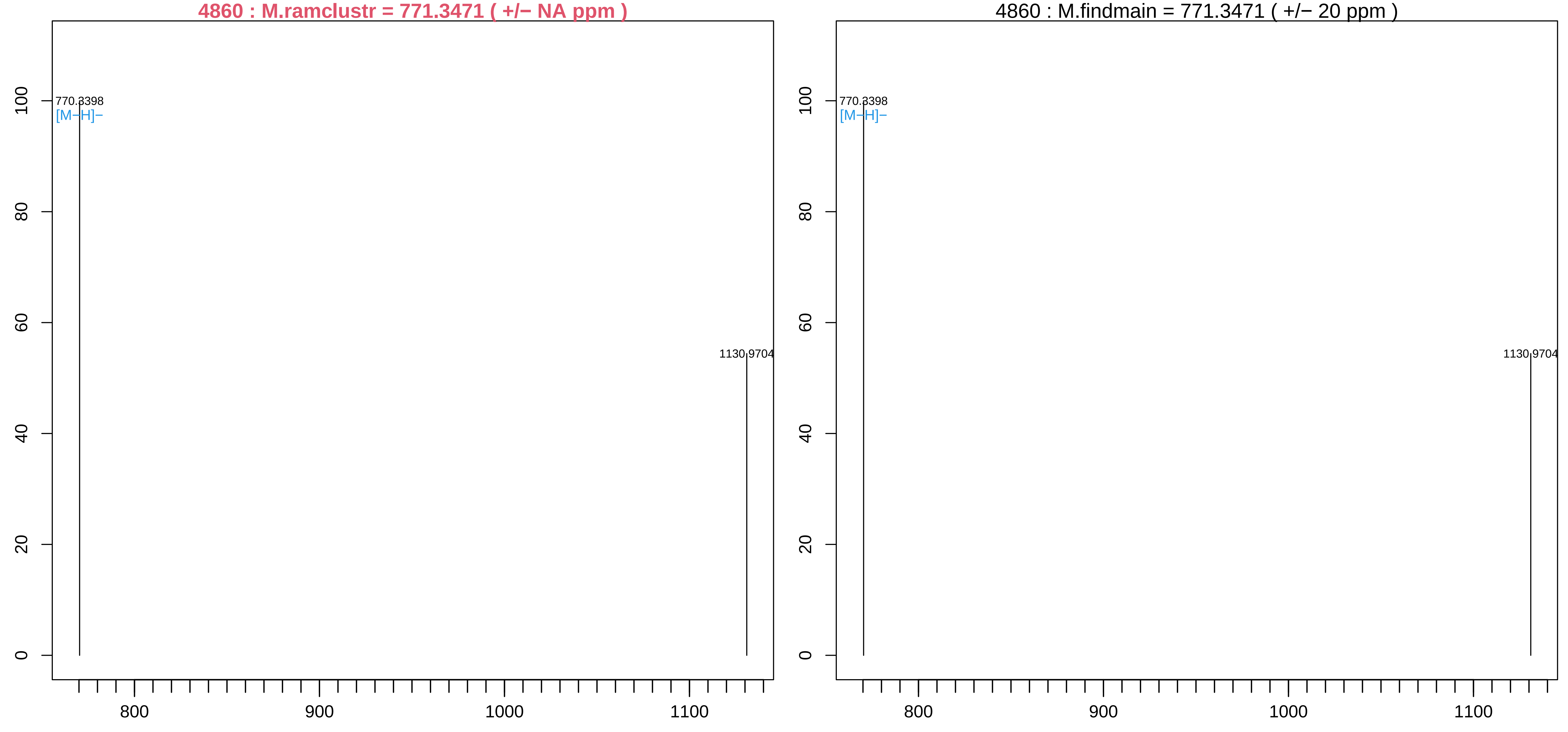
4860 : M.ramclustr = 771.3471 (+/− NA ppm)
770.3398
[M−H]−
1130.9704
0
20
40
60
80
100
800
900
1000
1100
4860 : M.findmain = 771.3471 (+/− 20 ppm)
770.3398
[M−H]−
1130.9704
0
20
40
60
80
100
800
900
1000
1100

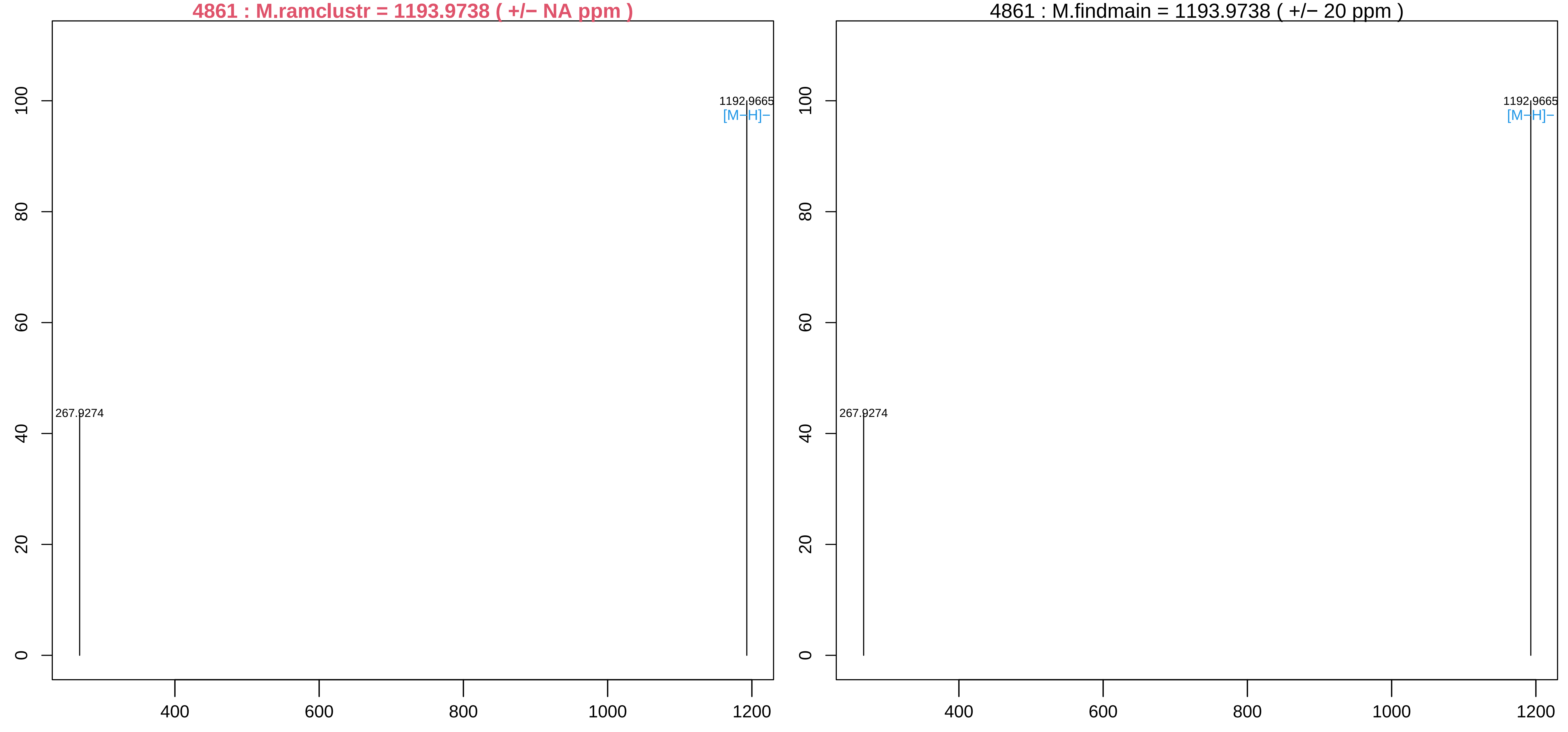
4861 : M.ramclustr = 1193.9738 (+/− NA ppm)
1192.9665
[M−H]−
267.9274
0
20
40
60
80
100
400
600
800
1000
1200
4861 : M.findmain = 1193.9738 (+/− 20 ppm)
1192.9665
[M−H]−
267.9274
0
20
40
60
80
100
400
600
800
1000
1200

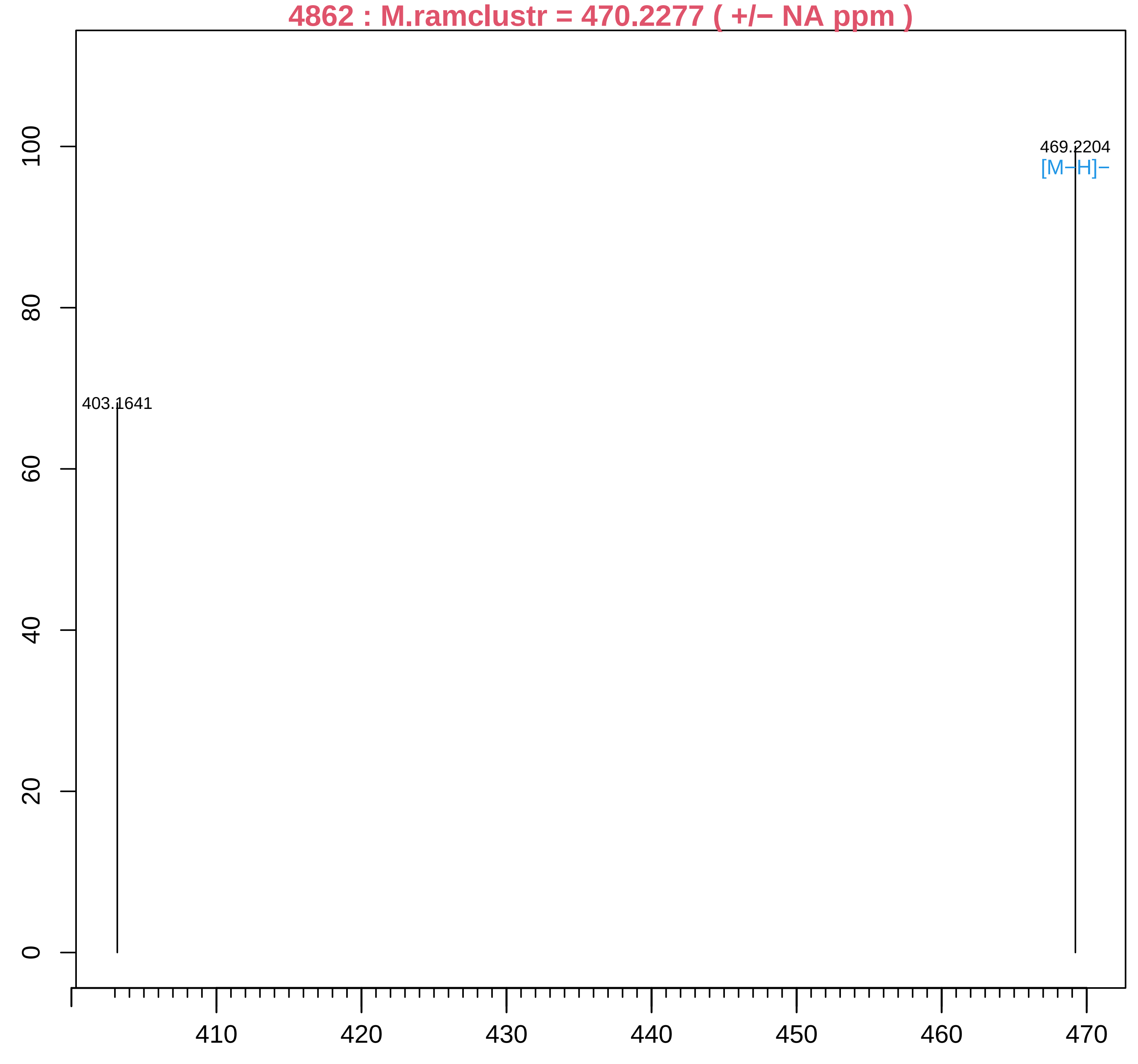

4862 : M.ramclustr = 470.2277 (+/− NA ppm)
403.1641
469.2204
[M−H]−
0
20
40
60
80
100
410
420
430
440
450
460
470

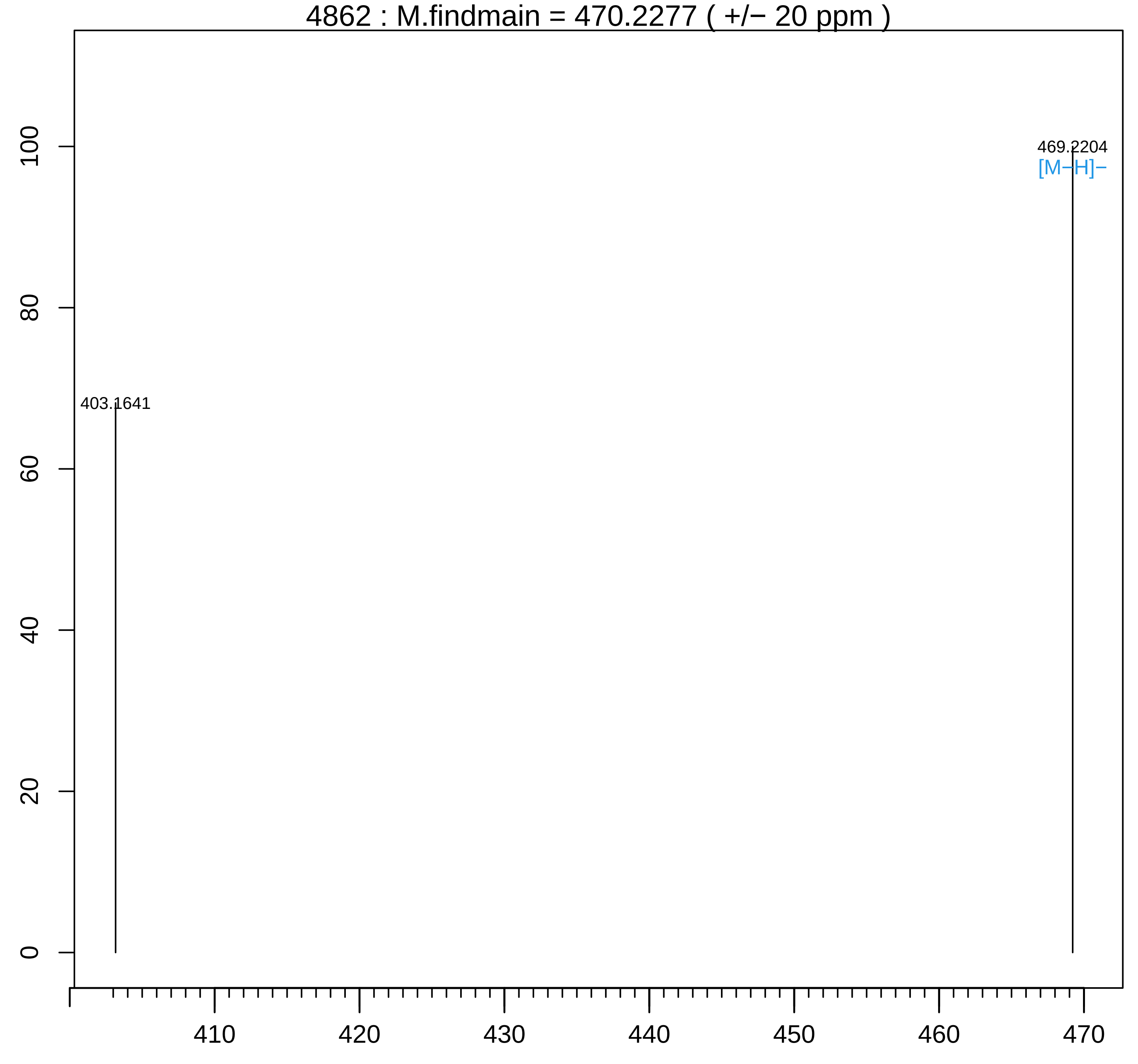

4862 : M.findmain = 470.2277 (+/− 20 ppm)
403.1641
469.2204
[M−H]−
0
20
40
60
80
100
410
420
430
440
450
460
470

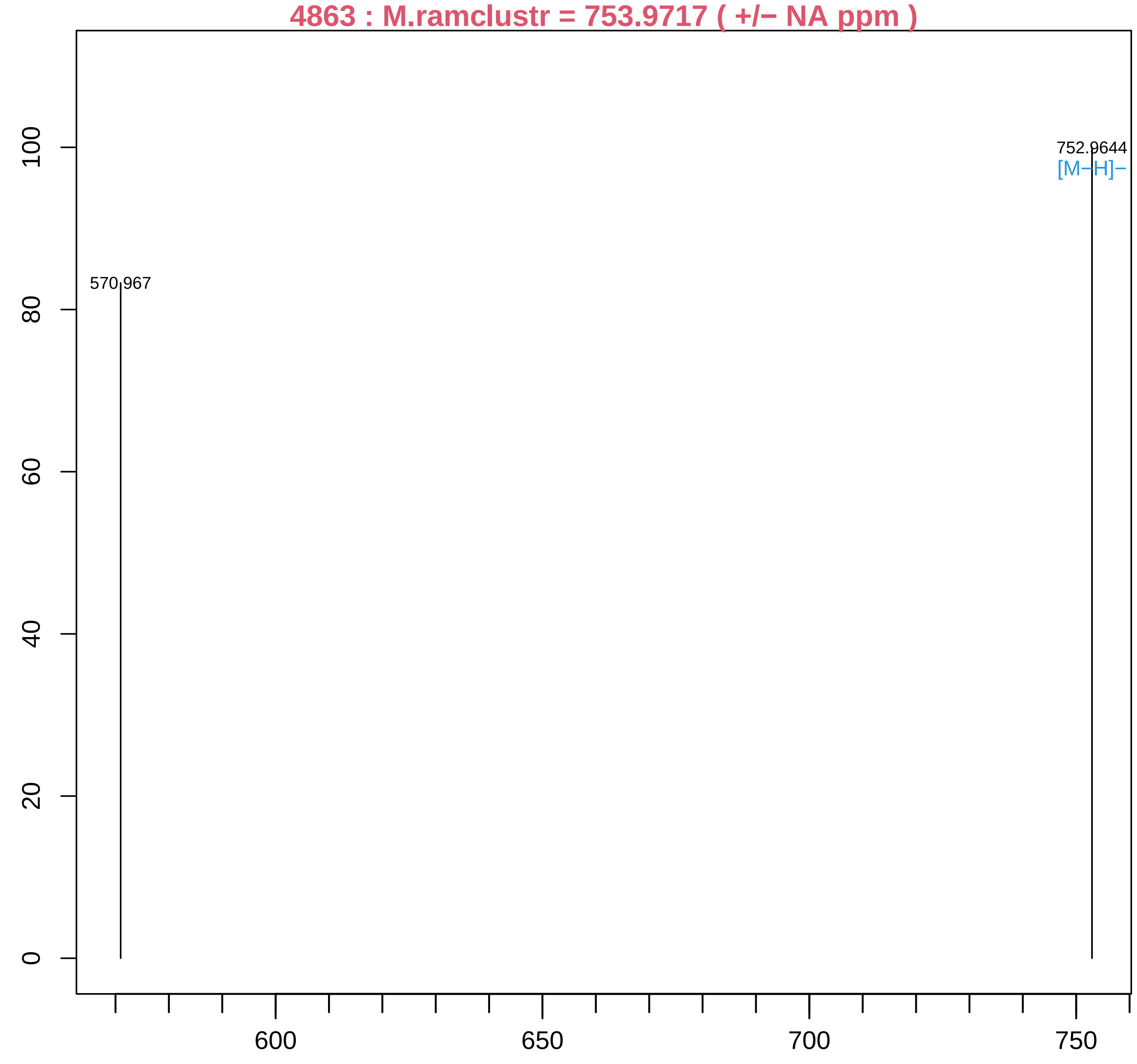
4863 : M.ramclustr = 753.9717 (+/− NA ppm)
752.9644
[M−H]−
570.967
0
20
40
60
80
100
600
650
700
750

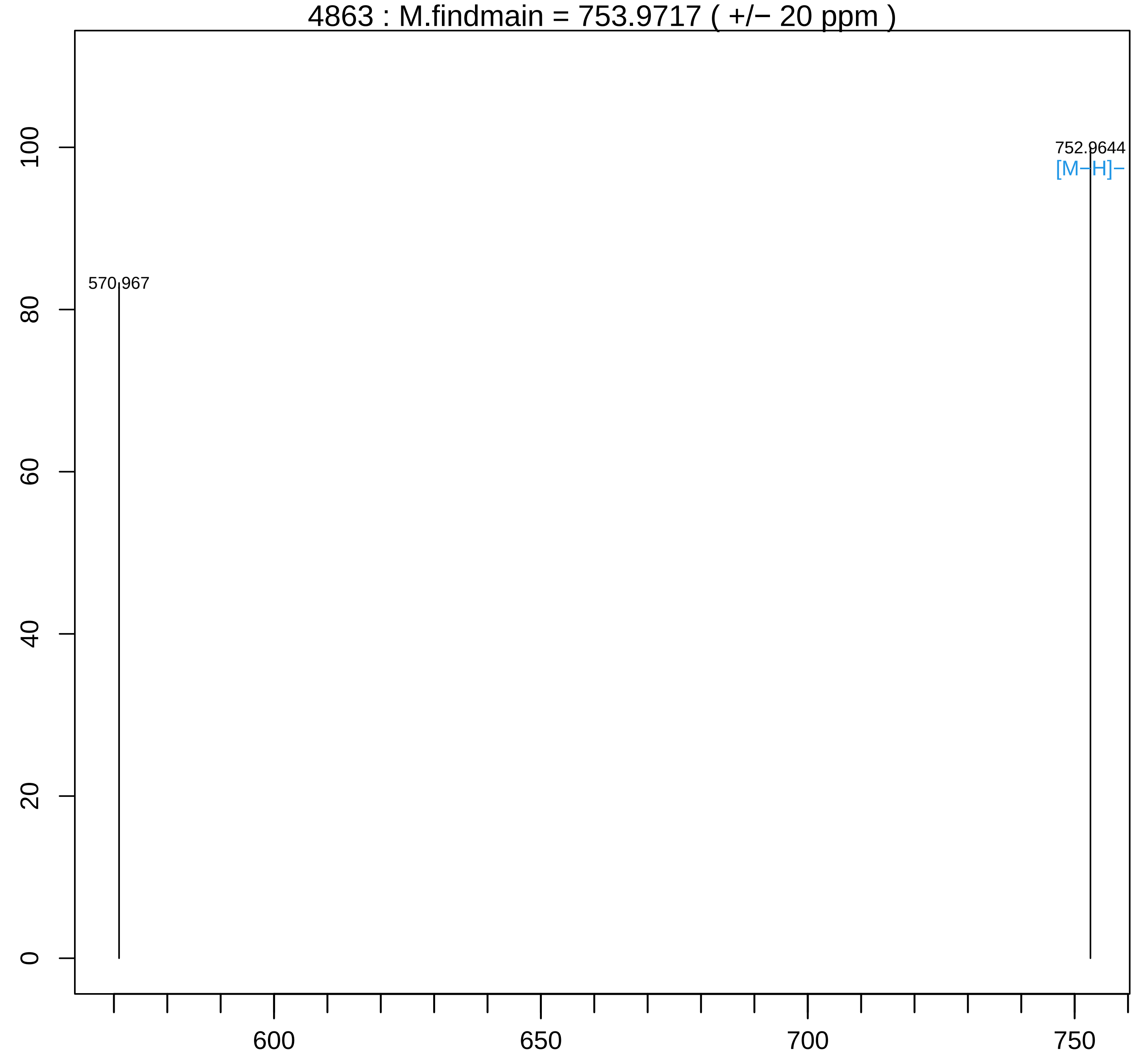
4863 : M.findmain = 753.9717 (+/− 20 ppm)
752.9644
[M−H]−
570.967
0
20
40
60
80
100
600
650
700
750

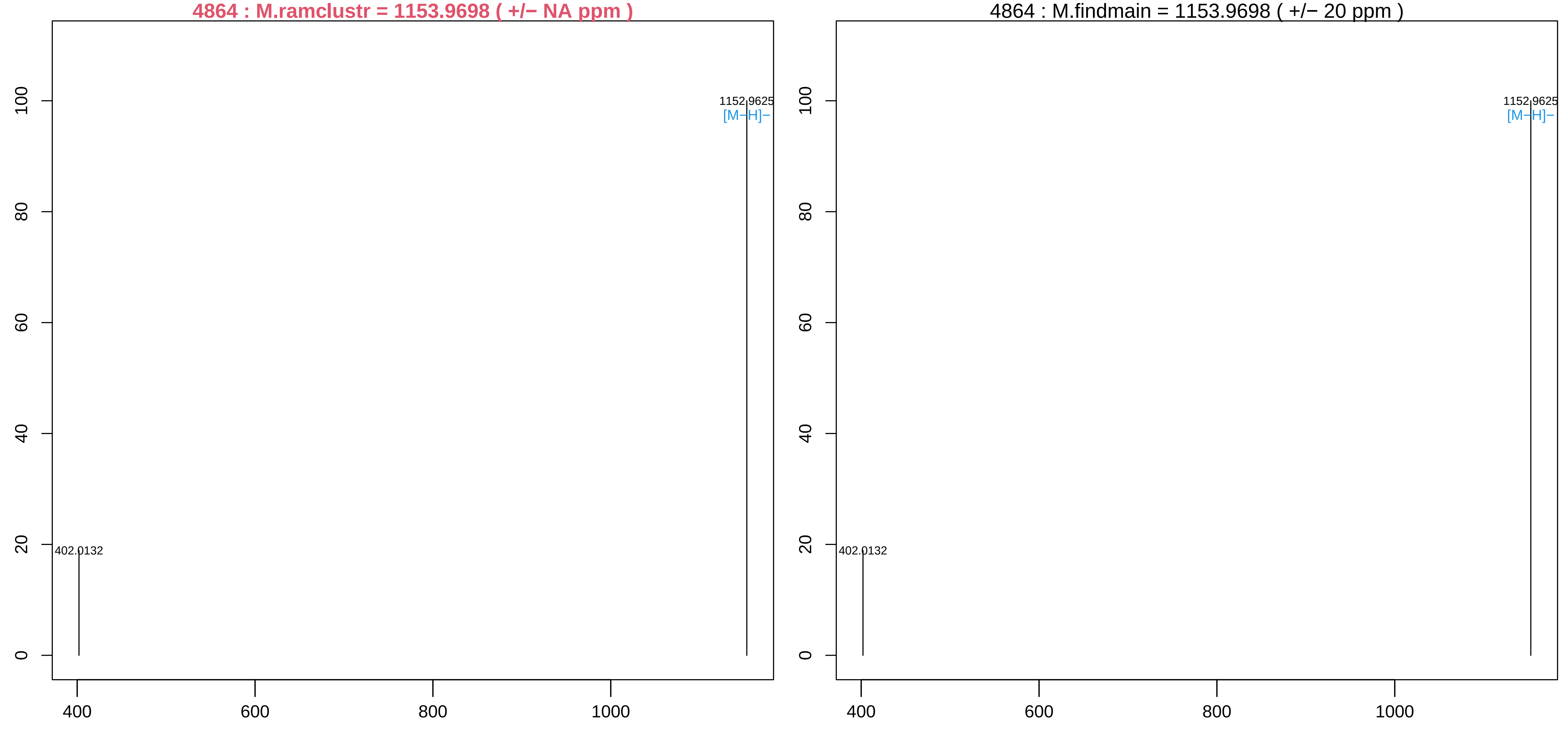
4864 : M.ramclustr = 1153.9698 (+/− NA ppm)
4864 : M.findmain = 1153.9698 (+/− 20 ppm)
1152.9625
[M−H]−
402.0132
0
20
40
60
80
100
400
600
800
1000

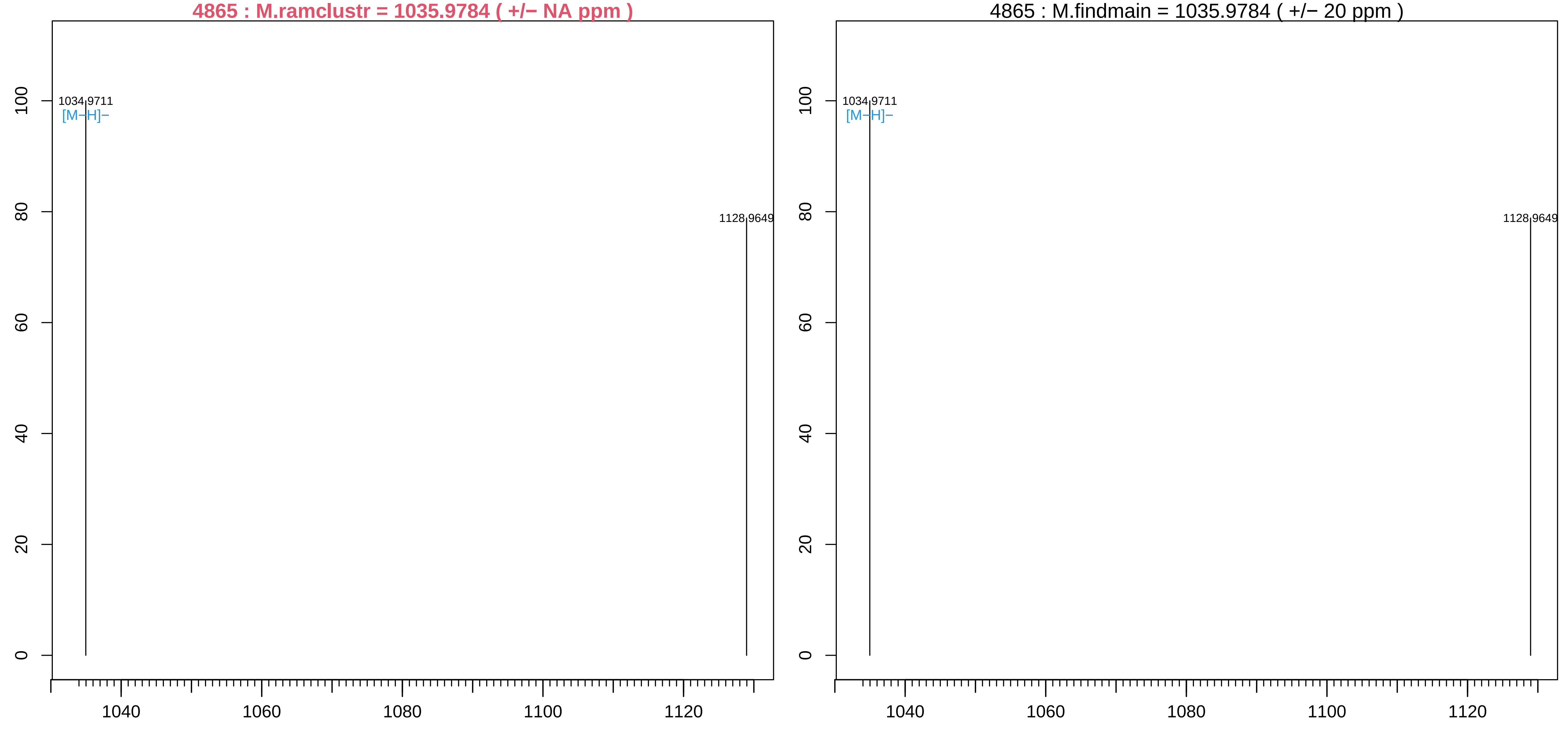
4865 : M.ramclustr = 1035.9784 (+/− NA ppm)
1034.9711
[M−H]−
1128.9649
4865 : M.findmain = 1035.9784 (+/− 20 ppm)
1034.9711
[M−H]−
1128.9649
0
20
40
60
80
100
1040
1060
1080
1100
1120

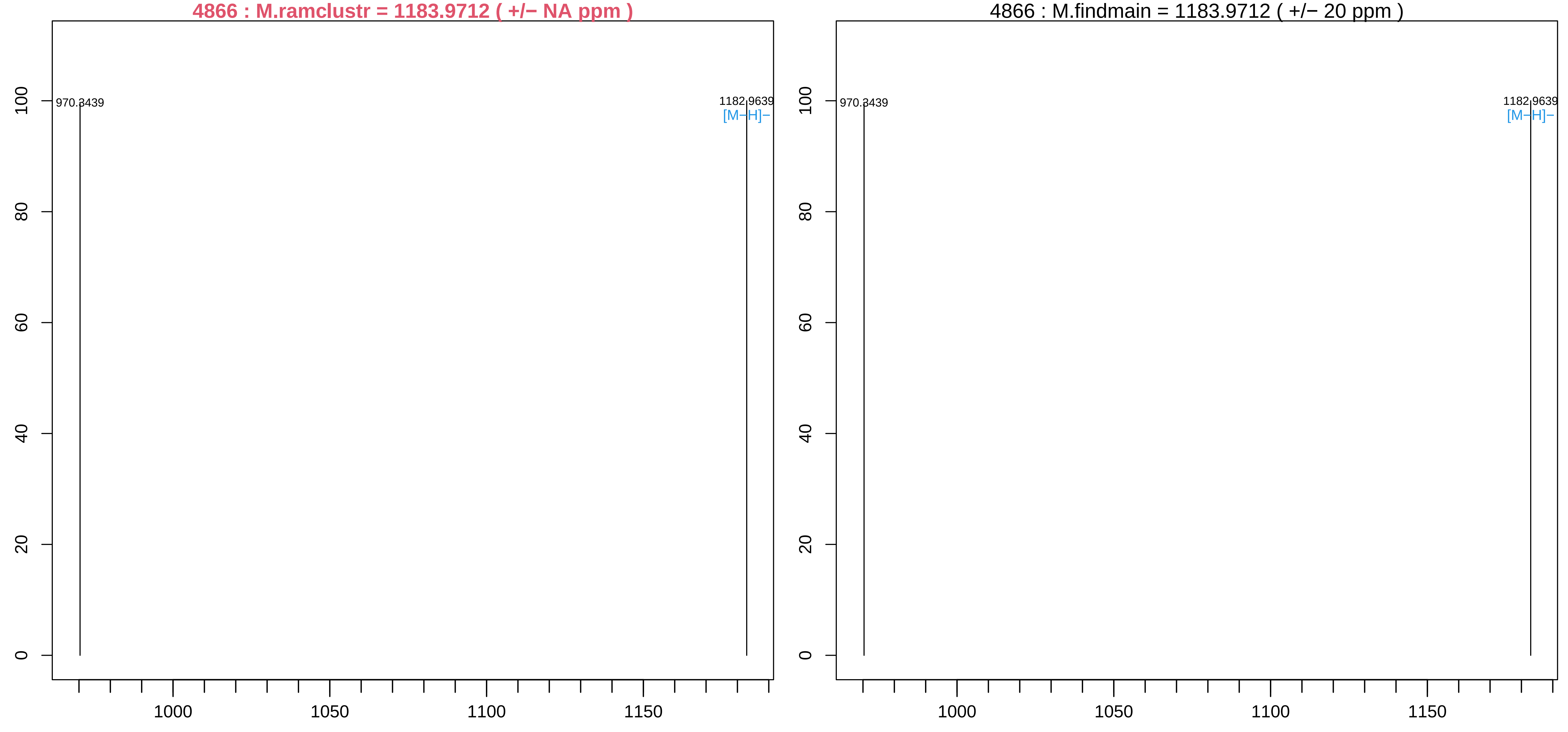

4866 : M.ramclustr = 1183.9712 (+/− NA ppm)
970.3439
1182.9639
[M−H]−
4866 : M.findmain = 1183.9712 (+/− 20 ppm)
970.3439
1182.9639
[M−H]−
0
20
40
60
80
100
1000
1050
1100
1150

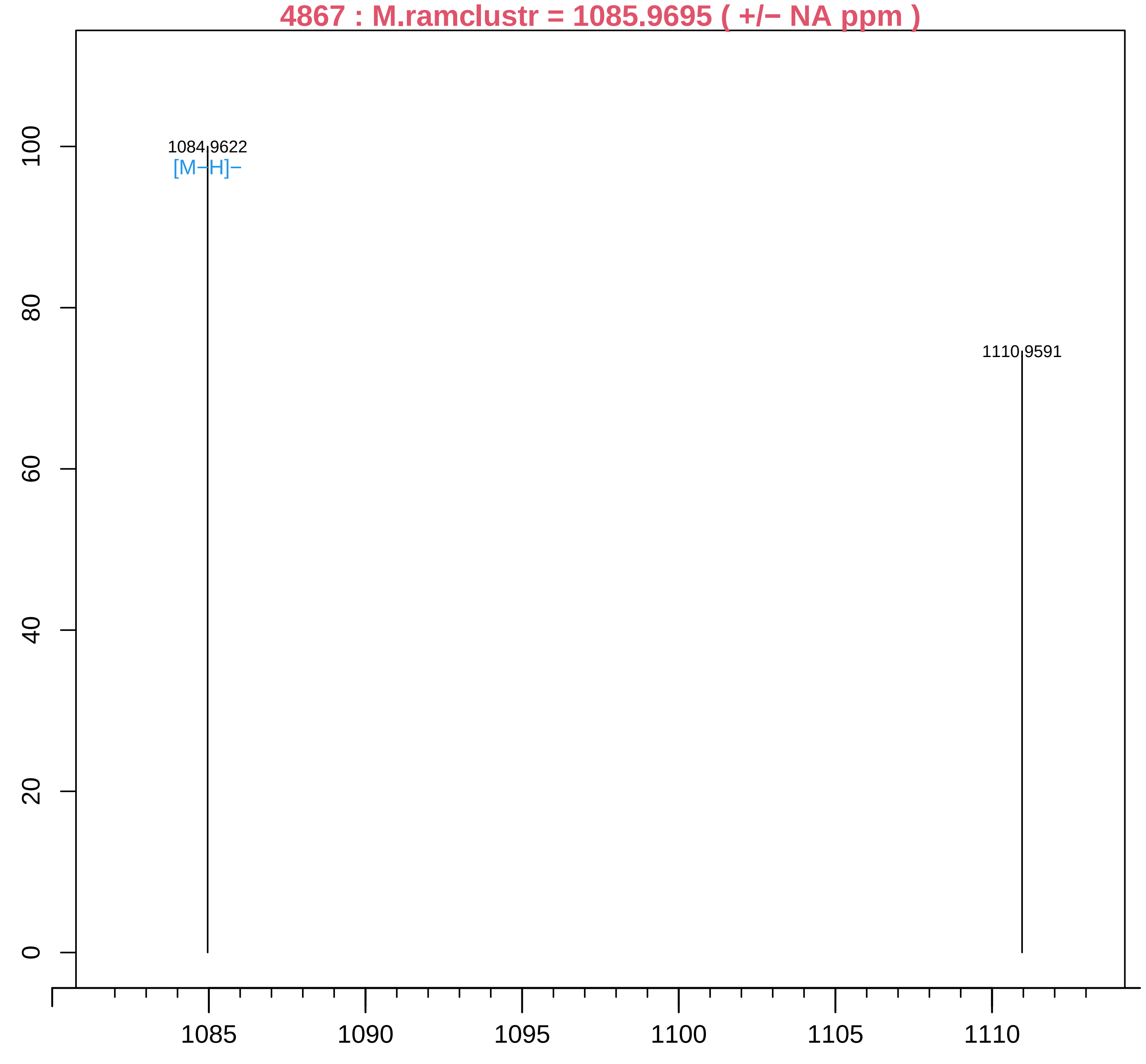

4867 : M.ramclustr = 1085.9695 (+/− NA ppm)
1084.9622
[M−H]−
1110.9591
0
20
40
60
80
100
1085
1090
1095
1100
1105
1110

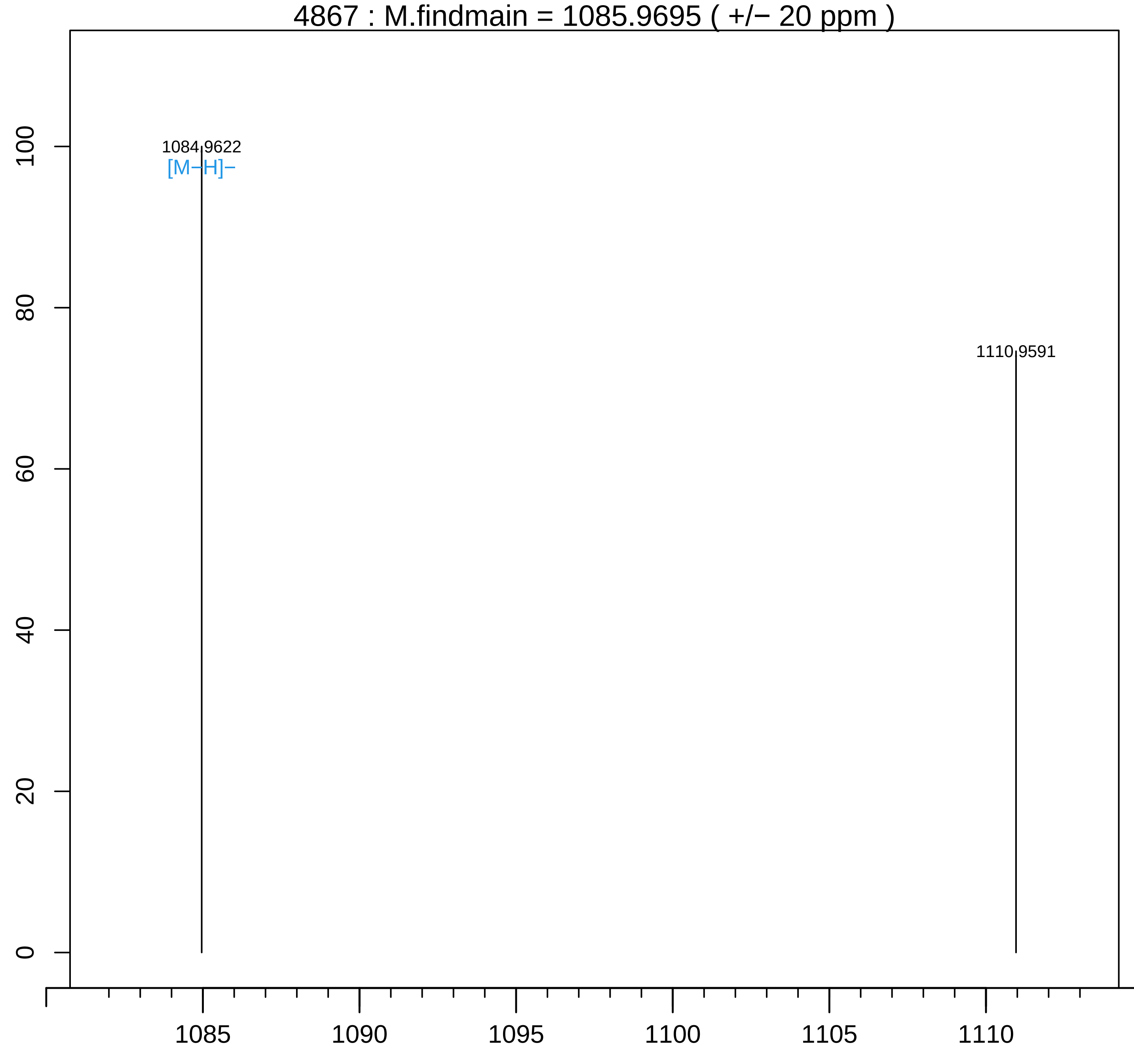

4867 : M.findmain = 1085.9695 (+/− 20 ppm)
1084.9622
[M−H]−
1110.9591
0
20
40
60
80
100
1085
1090
1095
1100
1105
1110

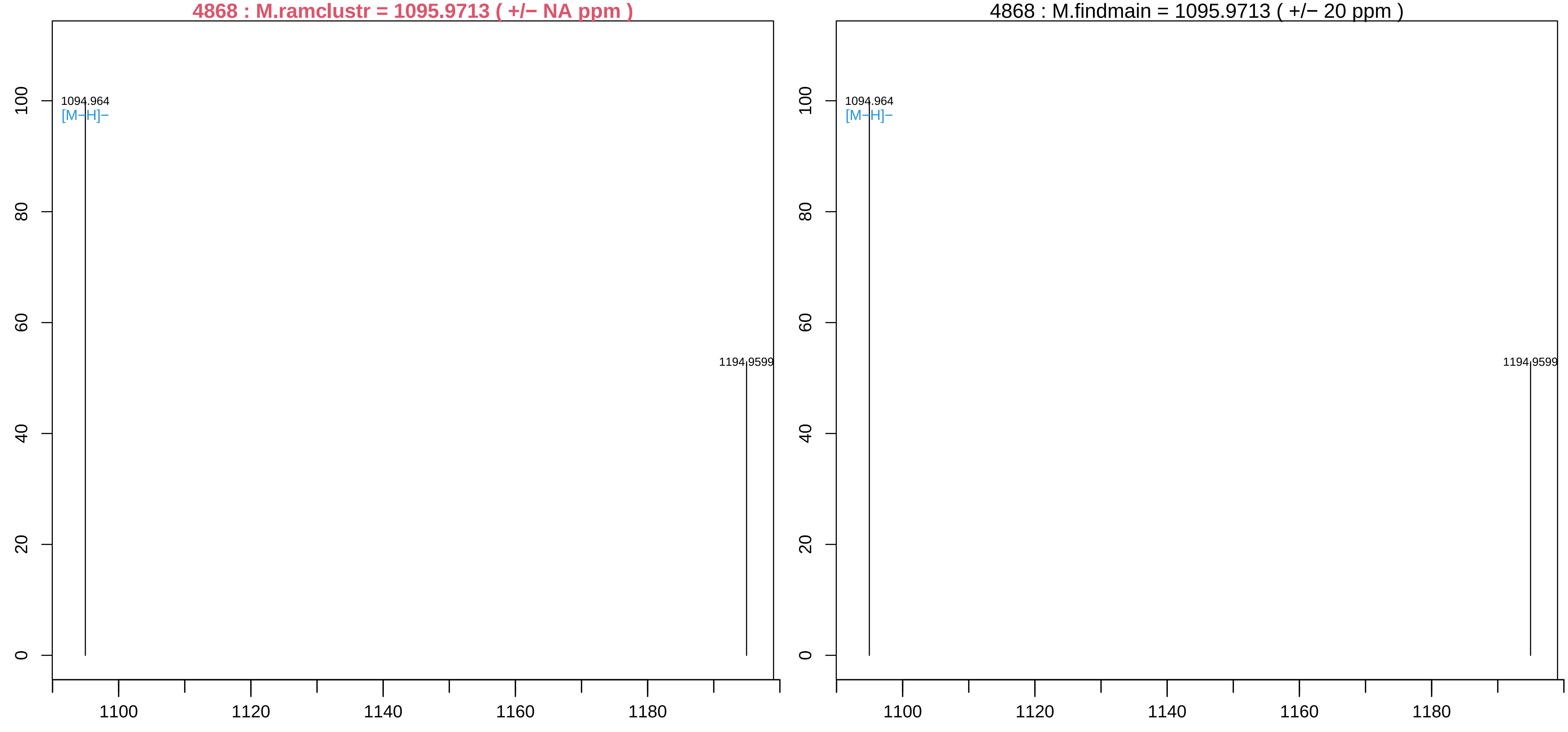

4868 : M.ramclustr = 1095.9713 (+/− NA ppm)
1094.964
[M−H]−
1194.9599
4868 : M.findmain = 1095.9713 (+/− 20 ppm)
1094.964
[M−H]−
1194.9599
0
20
40
60
80
100
1100
1120
1140
1160
1180

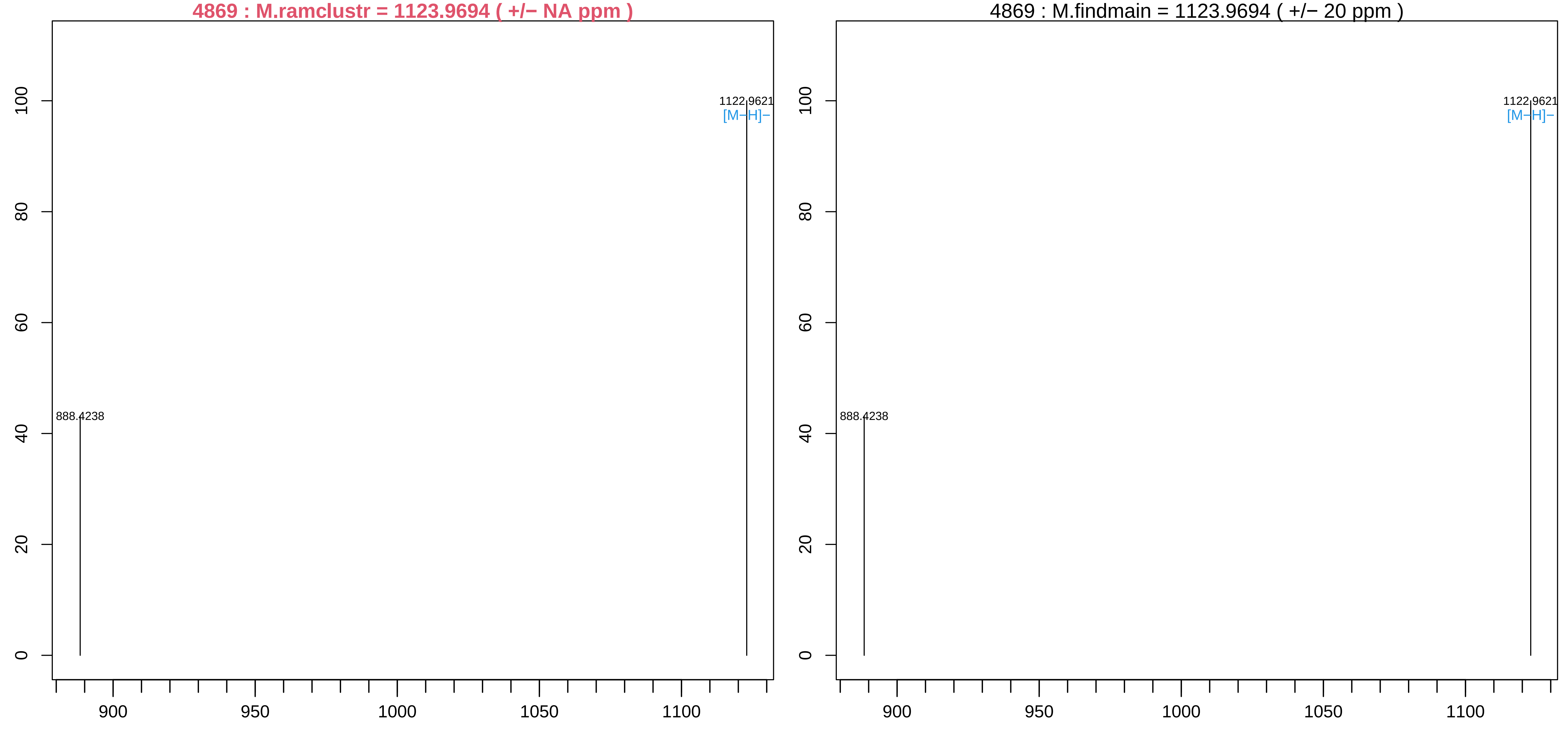
4869 : M.ramclustr = 1123.9694 (+/− NA ppm)
4869 : M.findmain = 1123.9694 (+/− 20 ppm)
1122.9621
[M−H]−
888.4238
0
20
40
60
80
100
900
950
1000
1050
1100

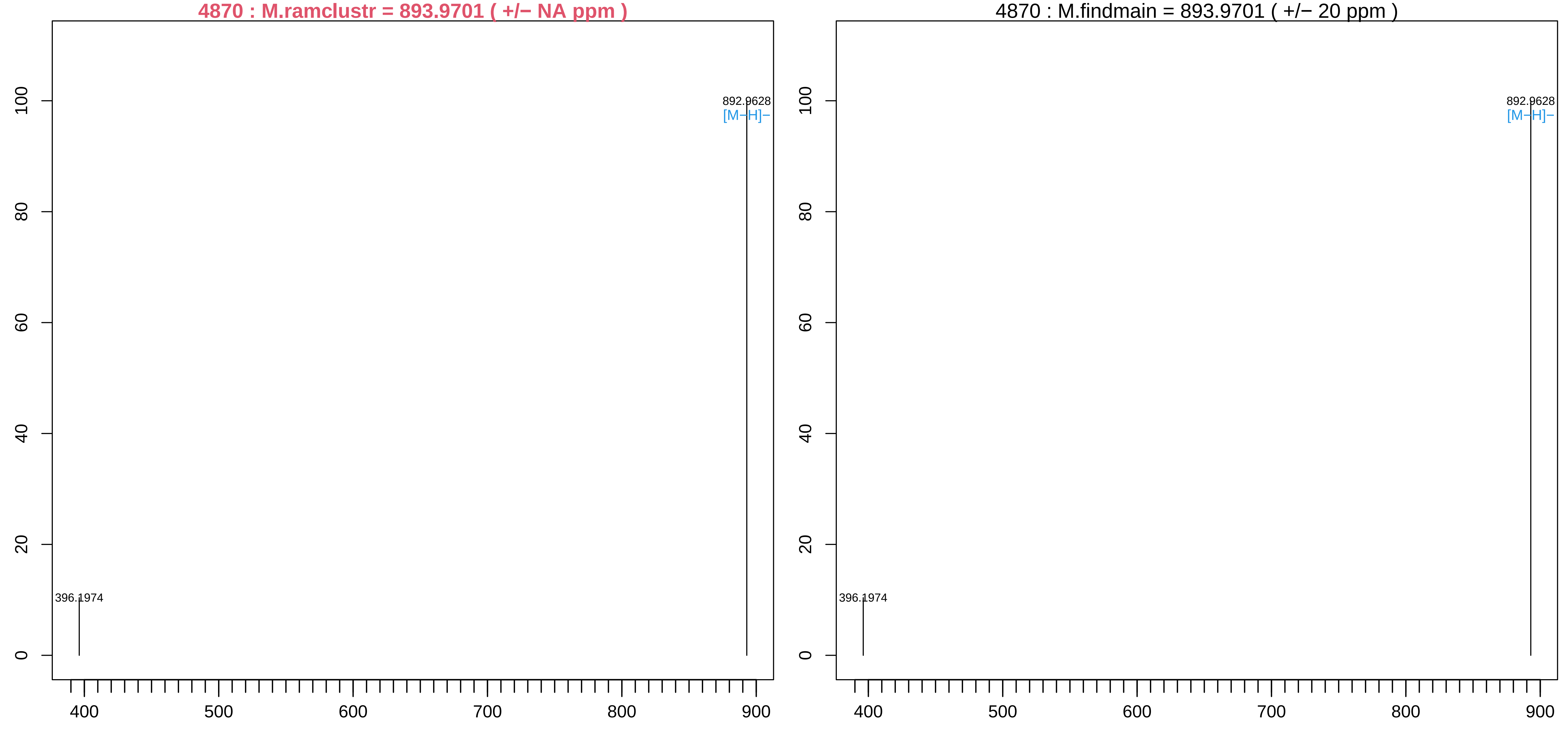
4870 : M.ramclustr = 893.9701 (+/− NA ppm)
892.9628
[M−H]−
396.1974
4870 : M.findmain = 893.9701 (+/− 20 ppm)
892.9628
[M−H]−
396.1974
0
20
40
60
80
100
400
500
600
700
800
900

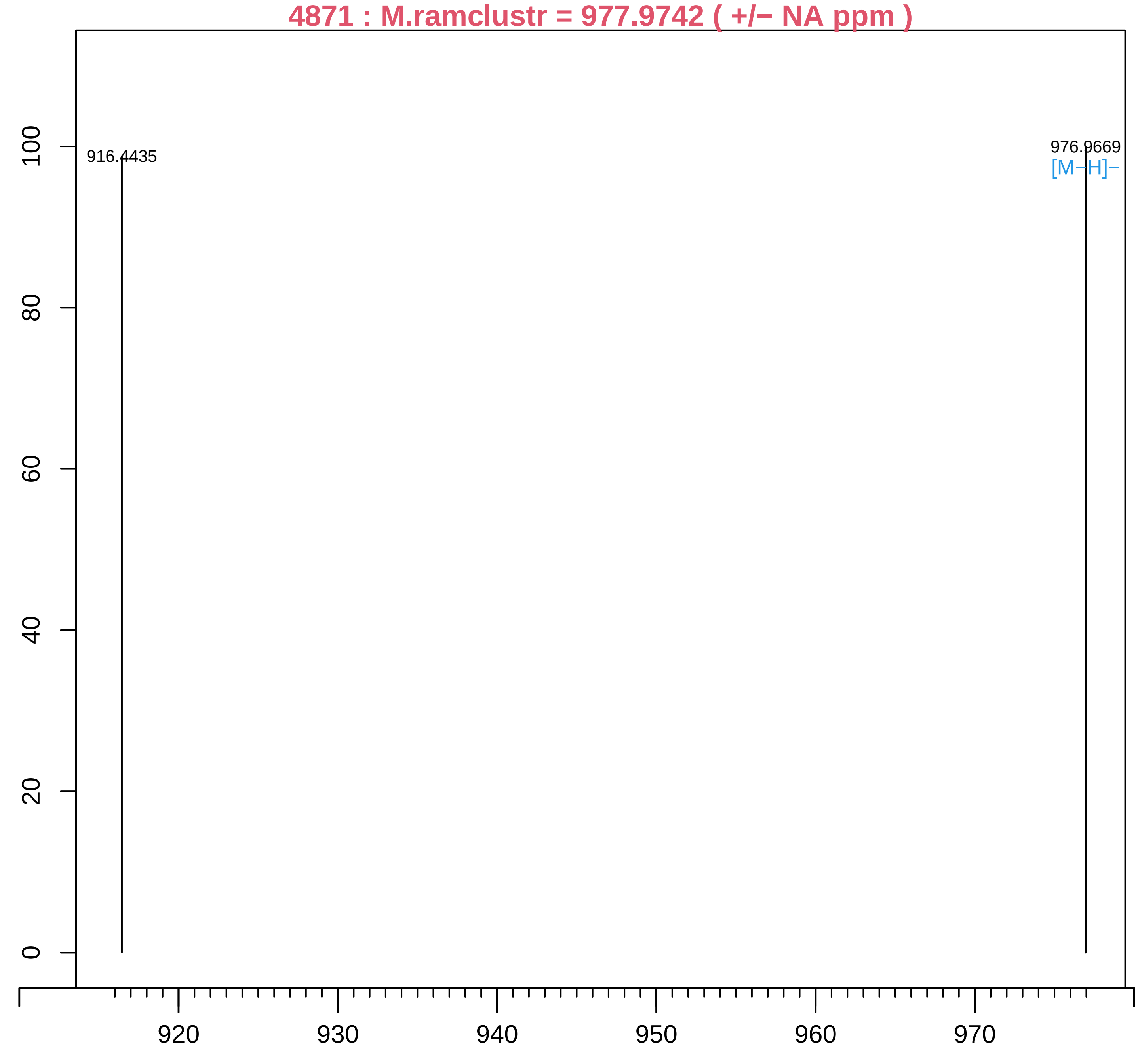
4871 : M.ramclustr = 977.9742 (+/− NA ppm)
916.4435
976.9669
[M−H]−
0
20
40
60
80
100
920
930
940
950
960
970

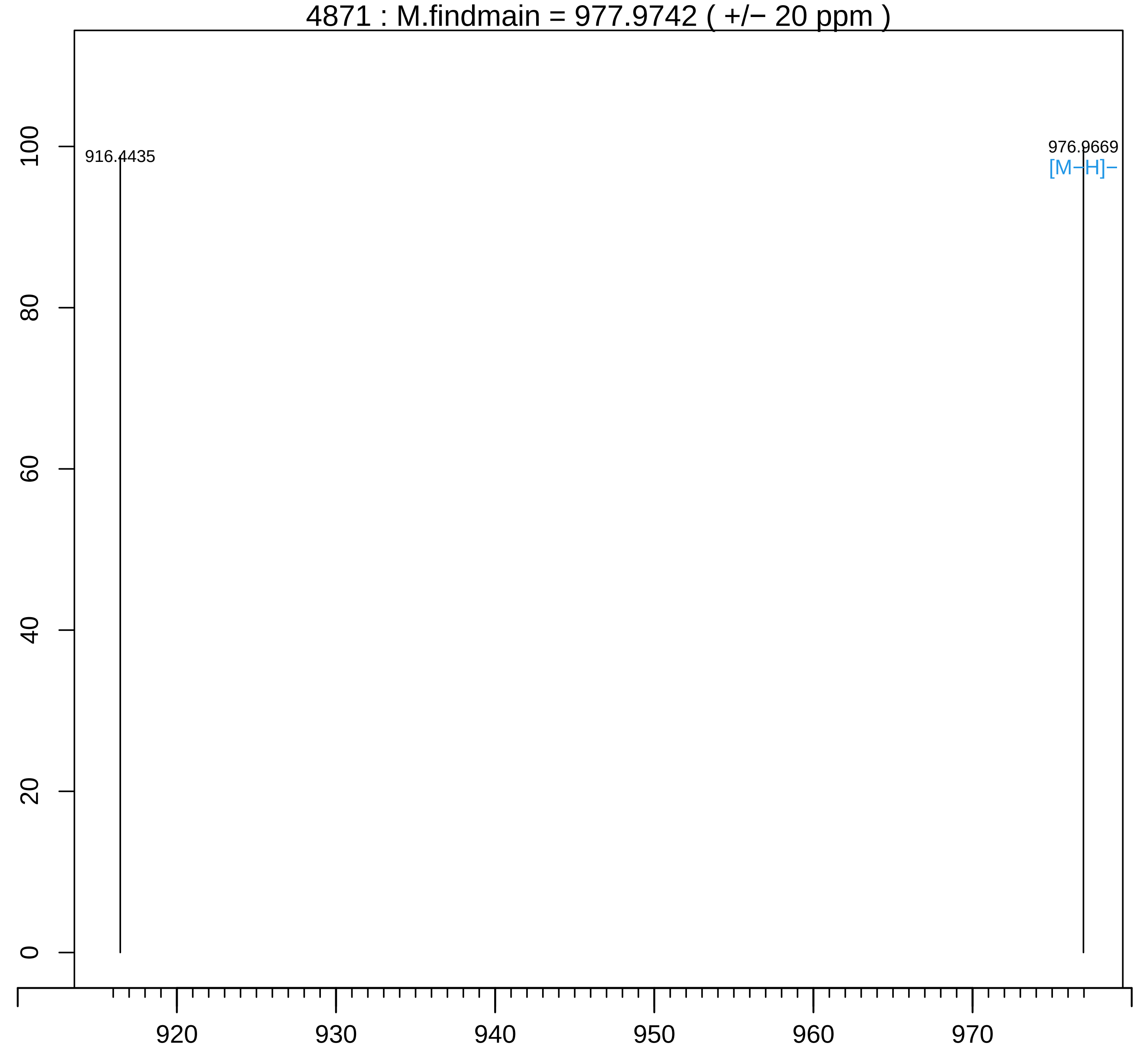
4871 : M.findmain = 977.9742 (+/− 20 ppm)
916.4435
976.9669
[M−H]−
0
20
40
60
80
100
920
930
940
950
960
970

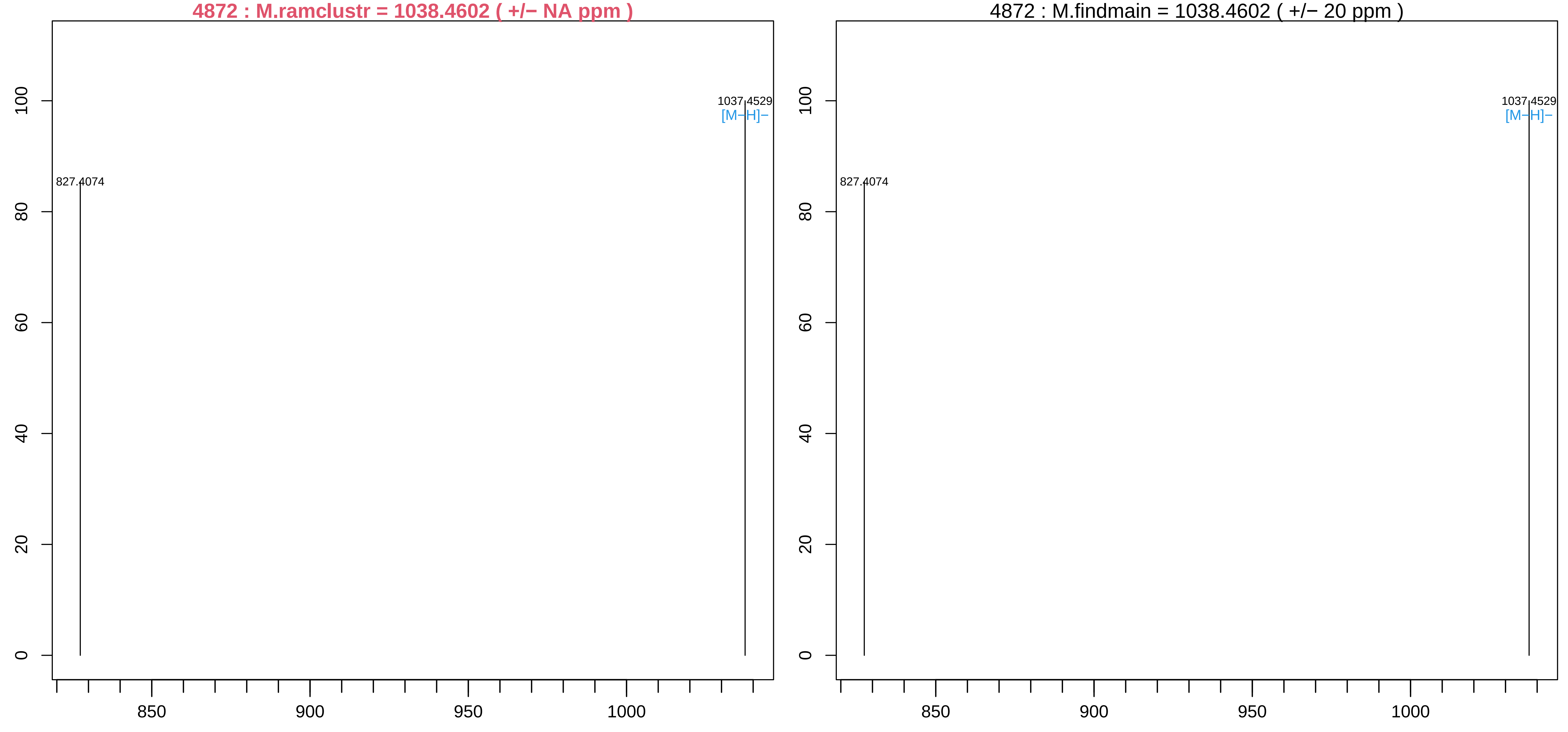

4872 : M.ramclustr = 1038.4602 (+/− NA ppm)
4872 : M.findmain = 1038.4602 (+/− 20 ppm)
827.4074
1037.4529
[M−H]−
827.4074
1037.4529
[M−H]−
0
20
40
60
80
100
850
900
950
1000

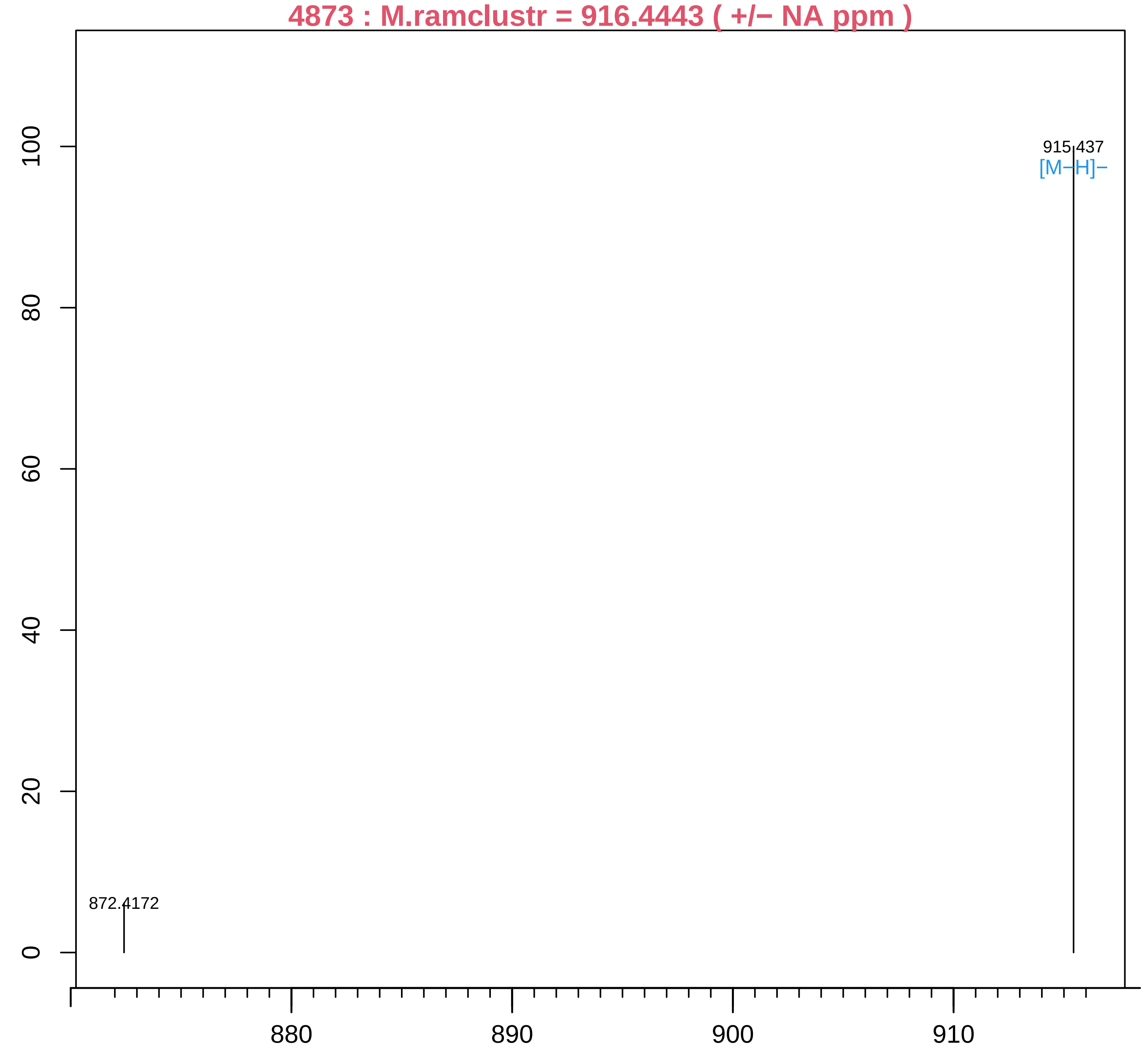
4873 : M.ramclustr = 916.4443 (+/− NA ppm)
915.437
[M−H]−
872.4172
0
20
40
60
80
100
880
890
900
910

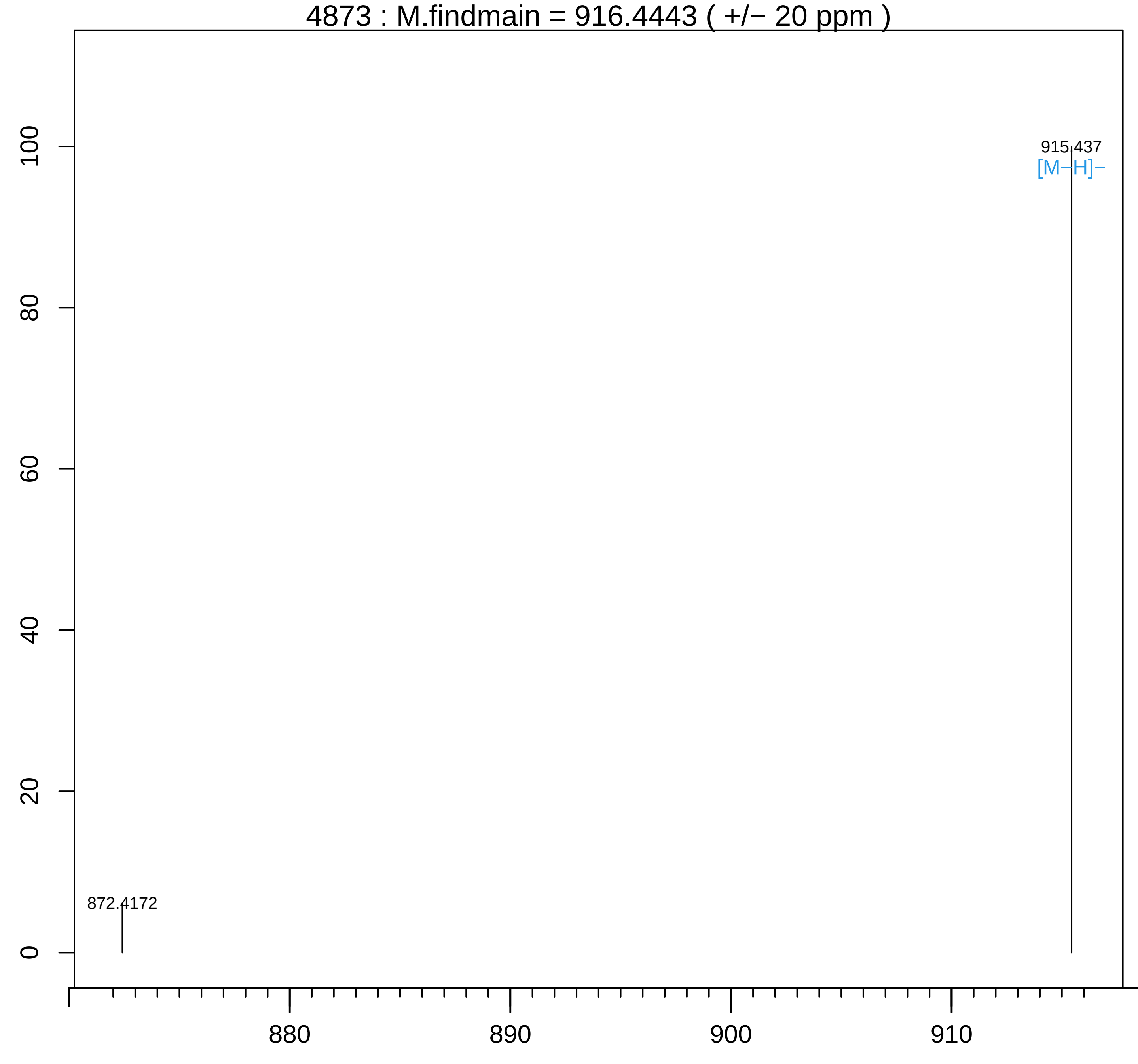
4873 : M.findmain = 916.4443 (+/− 20 ppm)
915.437
[M−H]−
872.4172
0
20
40
60
80
100
880
890
900
910

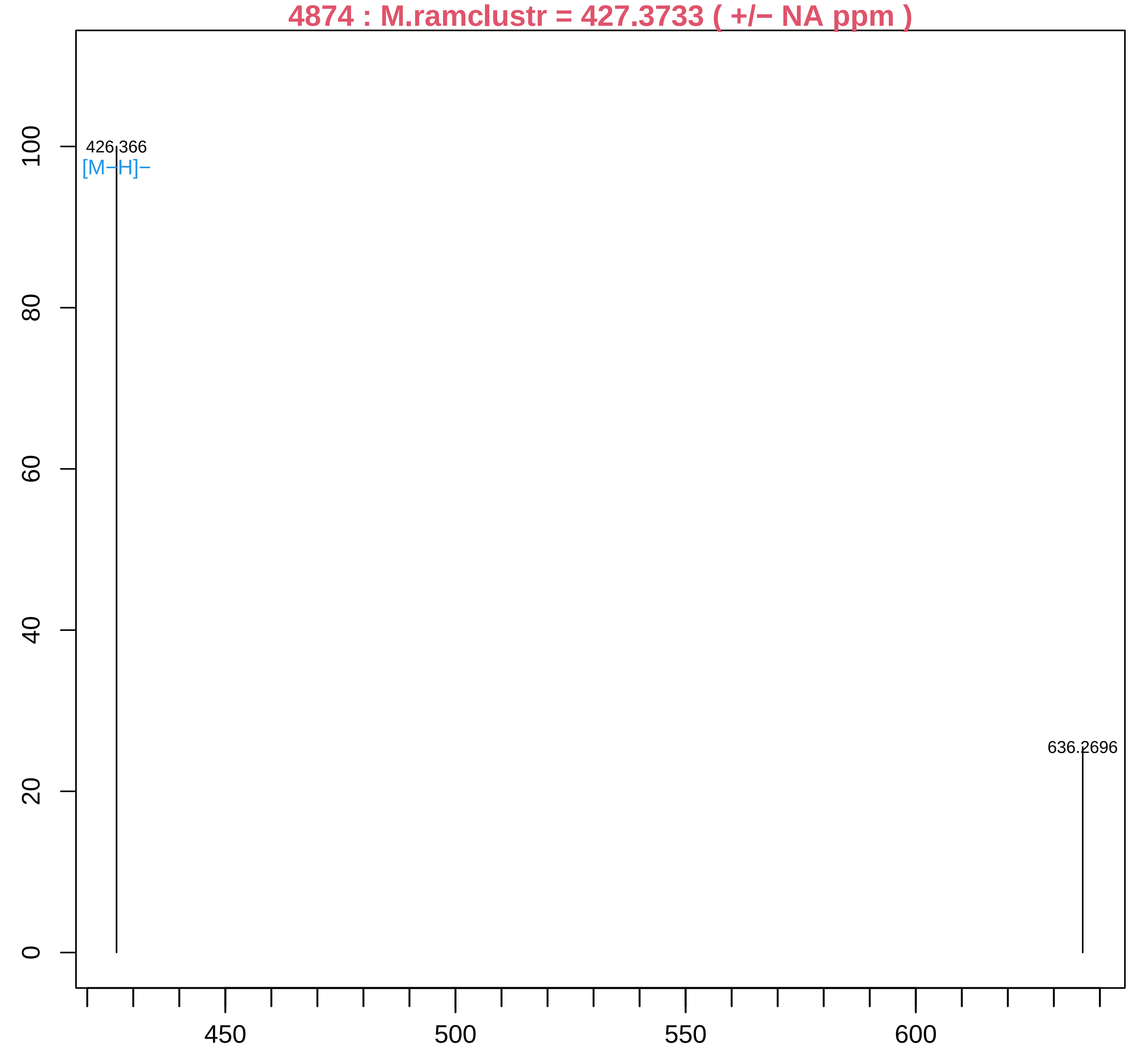
4874 : M.ramclustr = 427.3733 (+/− NA ppm)
426.366
[M−H]−
636.2696
0
20
40
60
80
100
450
500
550
600

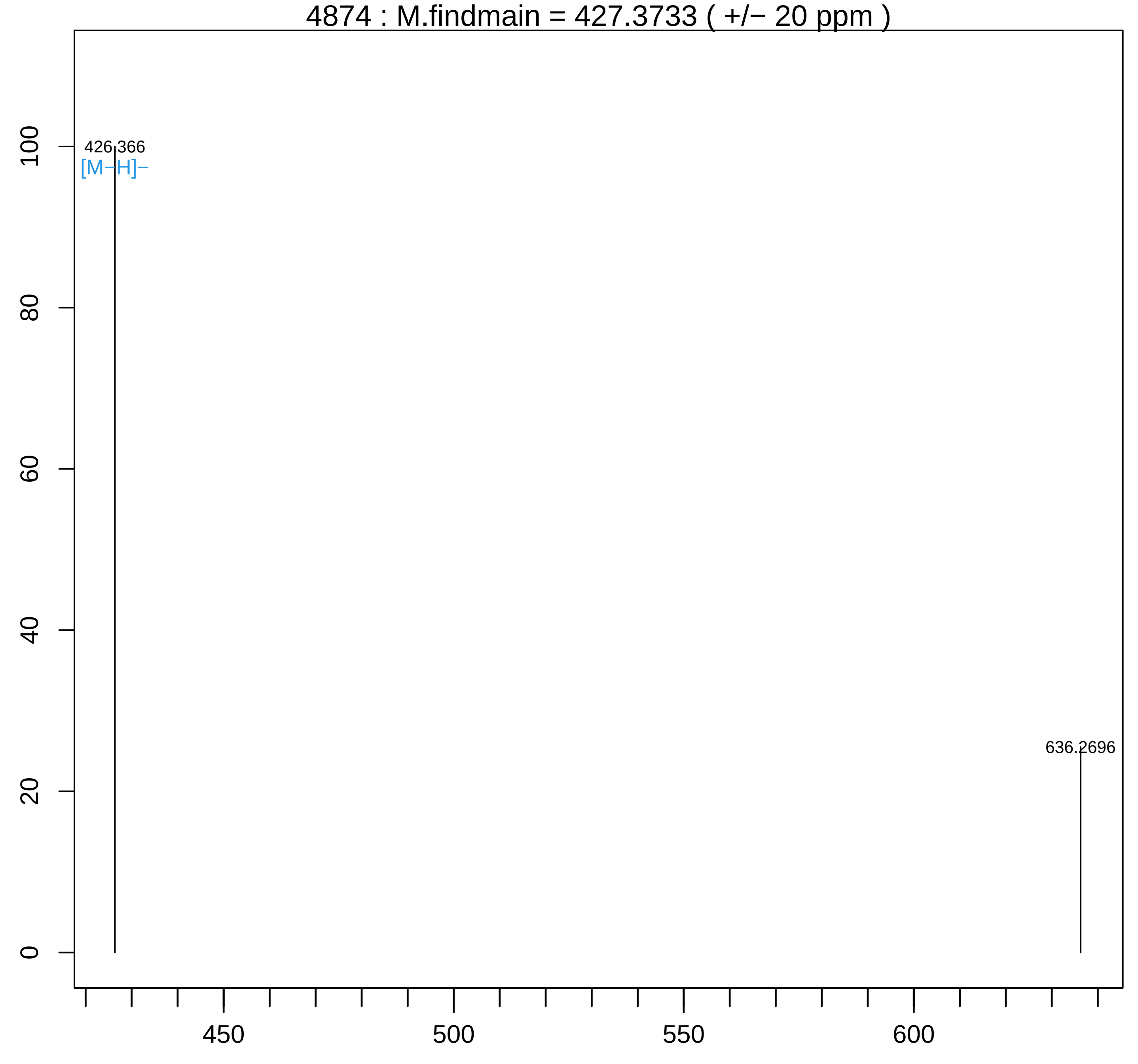
4874 : M.findmain = 427.3733 (+/− 20 ppm)
426.366
[M−H]−
636.2696
0
20
40
60
80
100
450
500
550
600

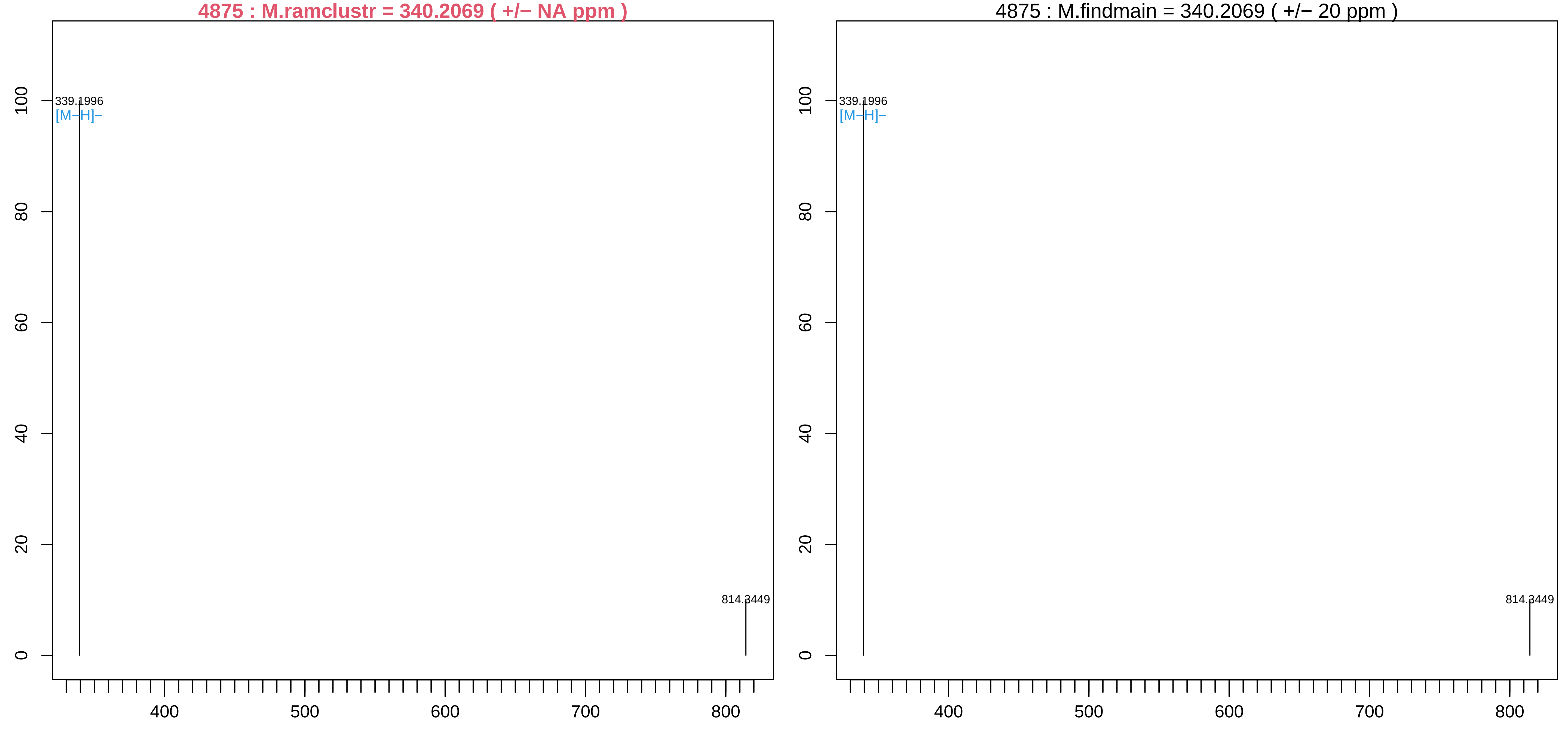
4875 : M.ramclustr = 340.2069 (+/− NA ppm)
339.1996
[M−H]−
814.3449
4875 : M.findmain = 340.2069 (+/− 20 ppm)
339.1996
[M−H]−
814.3449
0
20
40
60
80
100
400
500
600
700
800

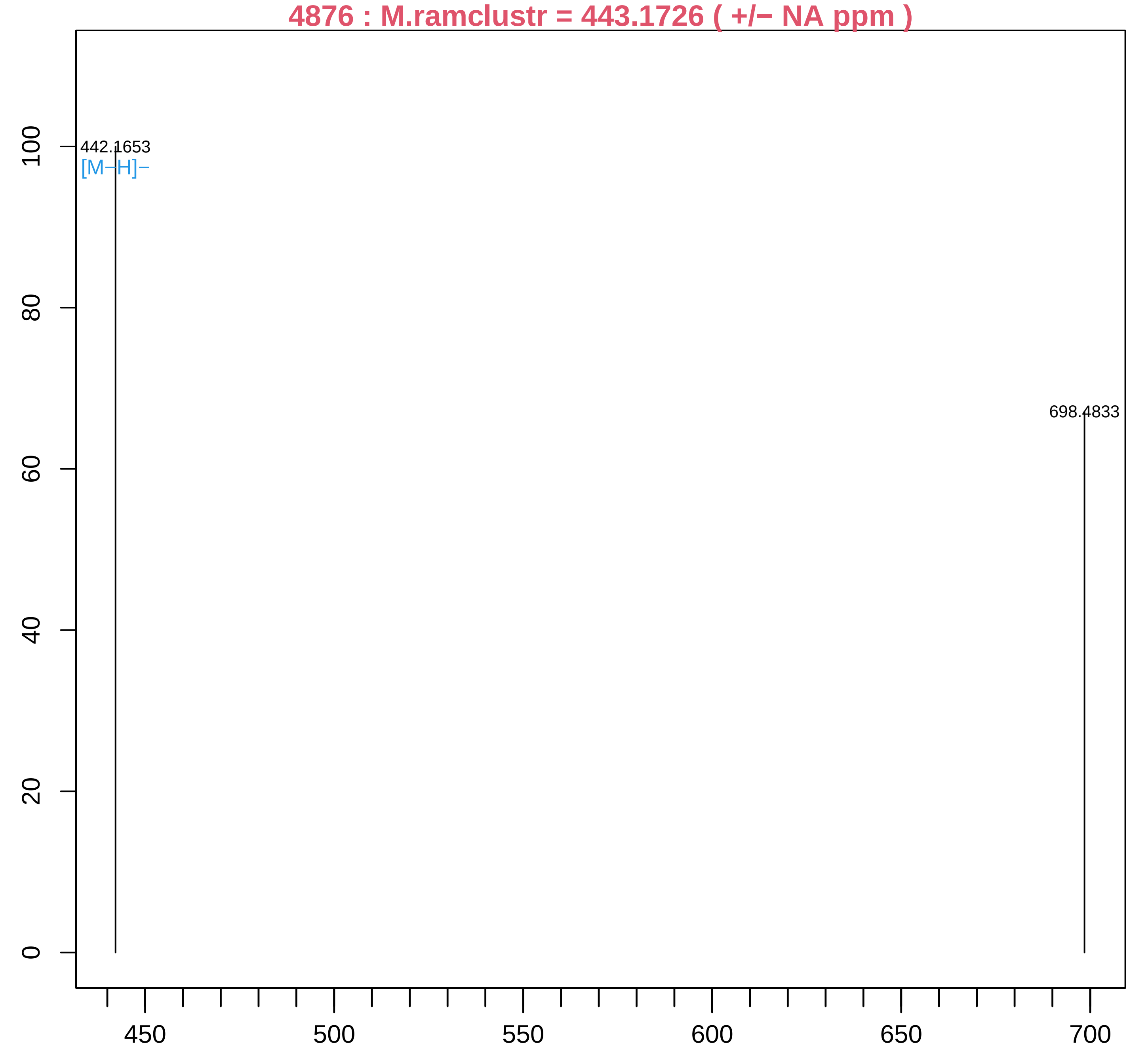
4876 : M.ramclustr = 443.1726 (+/− NA ppm)
442.1653
[M−H]−
698.4833
0
20
40
60
80
100
450
500
550
600
650
700

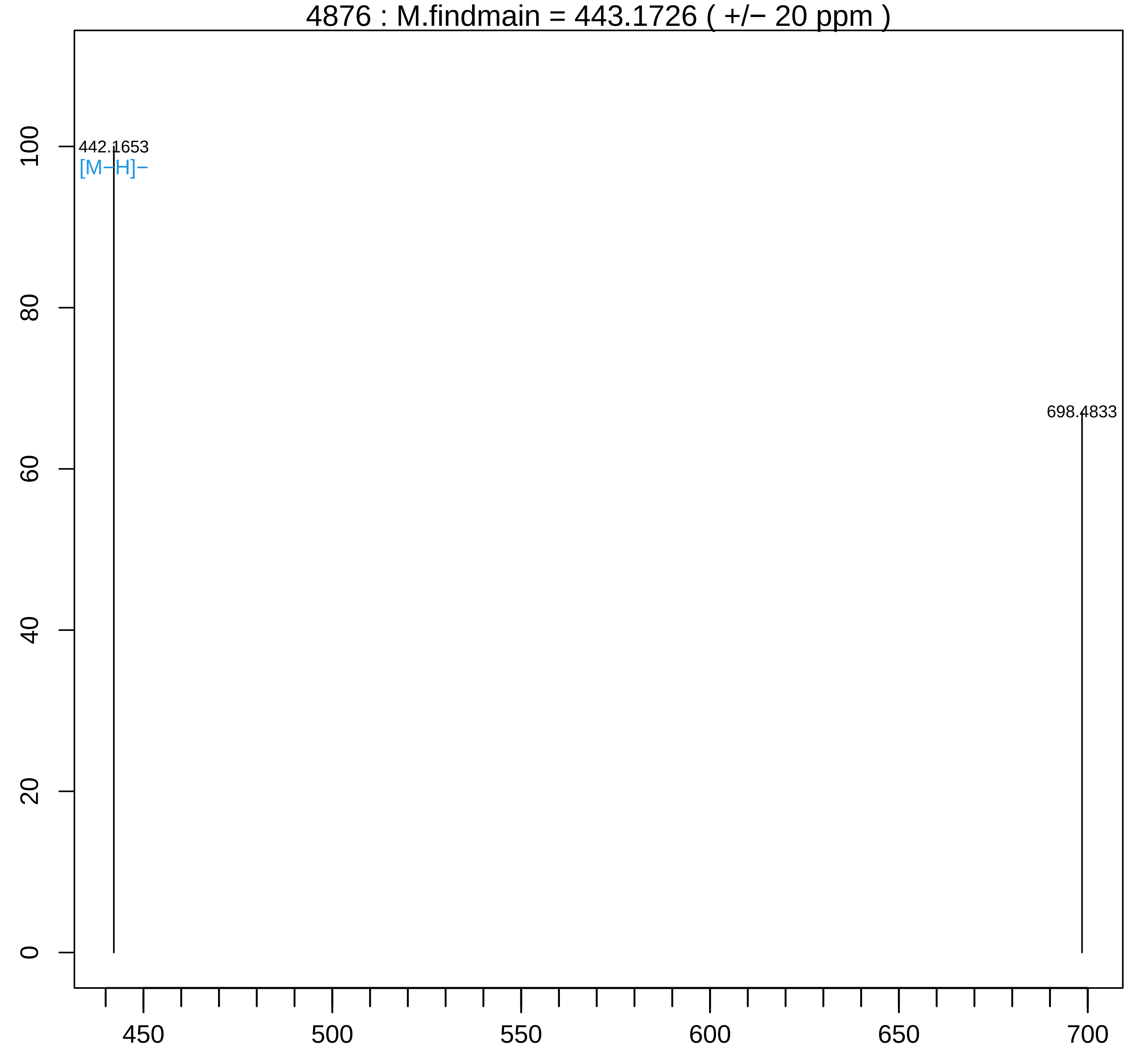
4876 : M.findmain = 443.1726 (+/− 20 ppm)
442.1653
[M−H]−
698.4833
0
20
40
60
80
100
450
500
550
600
650
700

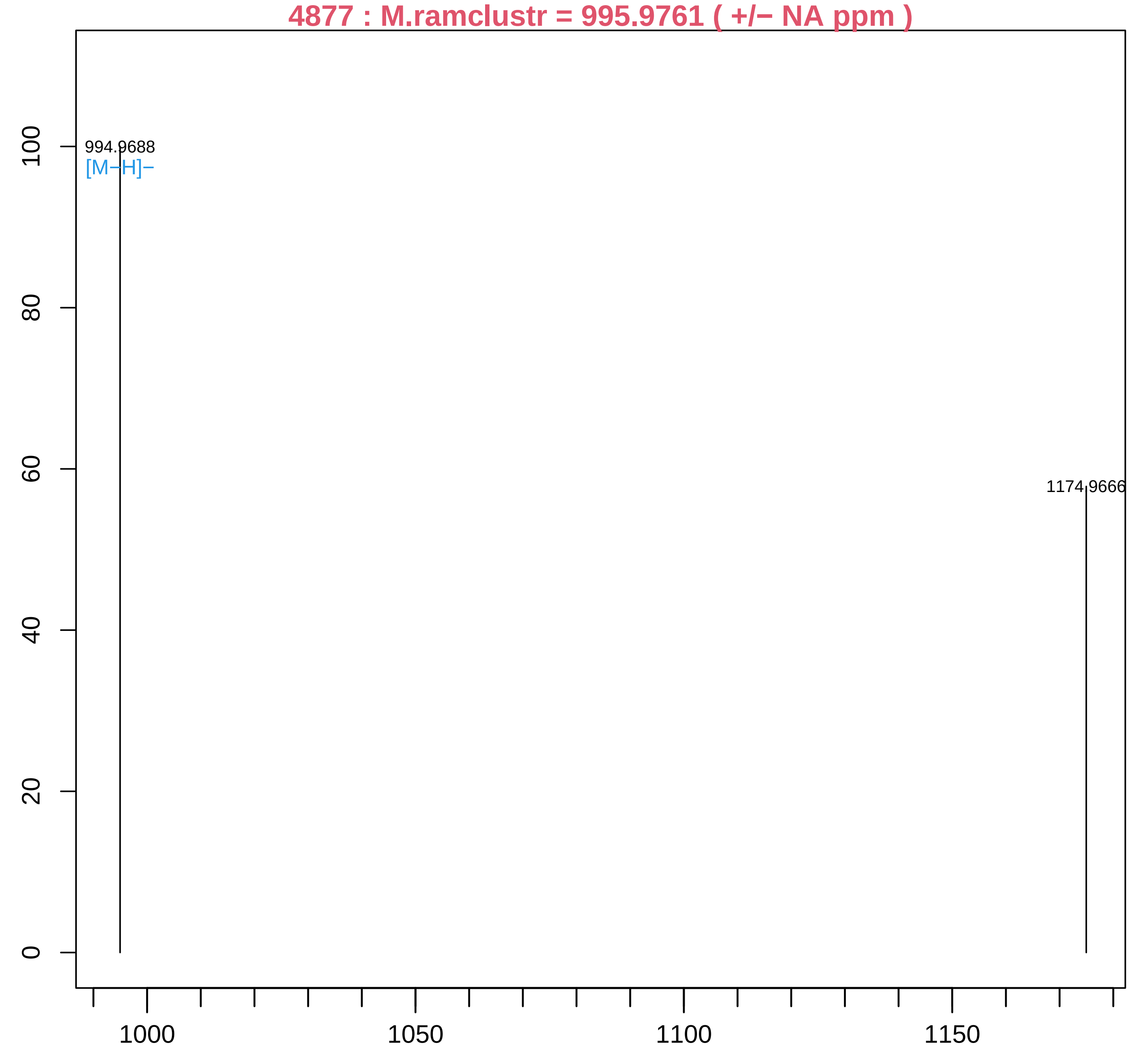

4877 : M.ramclustr = 995.9761 (+/− NA ppm)
994.9688
[M−H]−
1174.9666
0
20
40
60
80
100
1000
1050
1100
1150

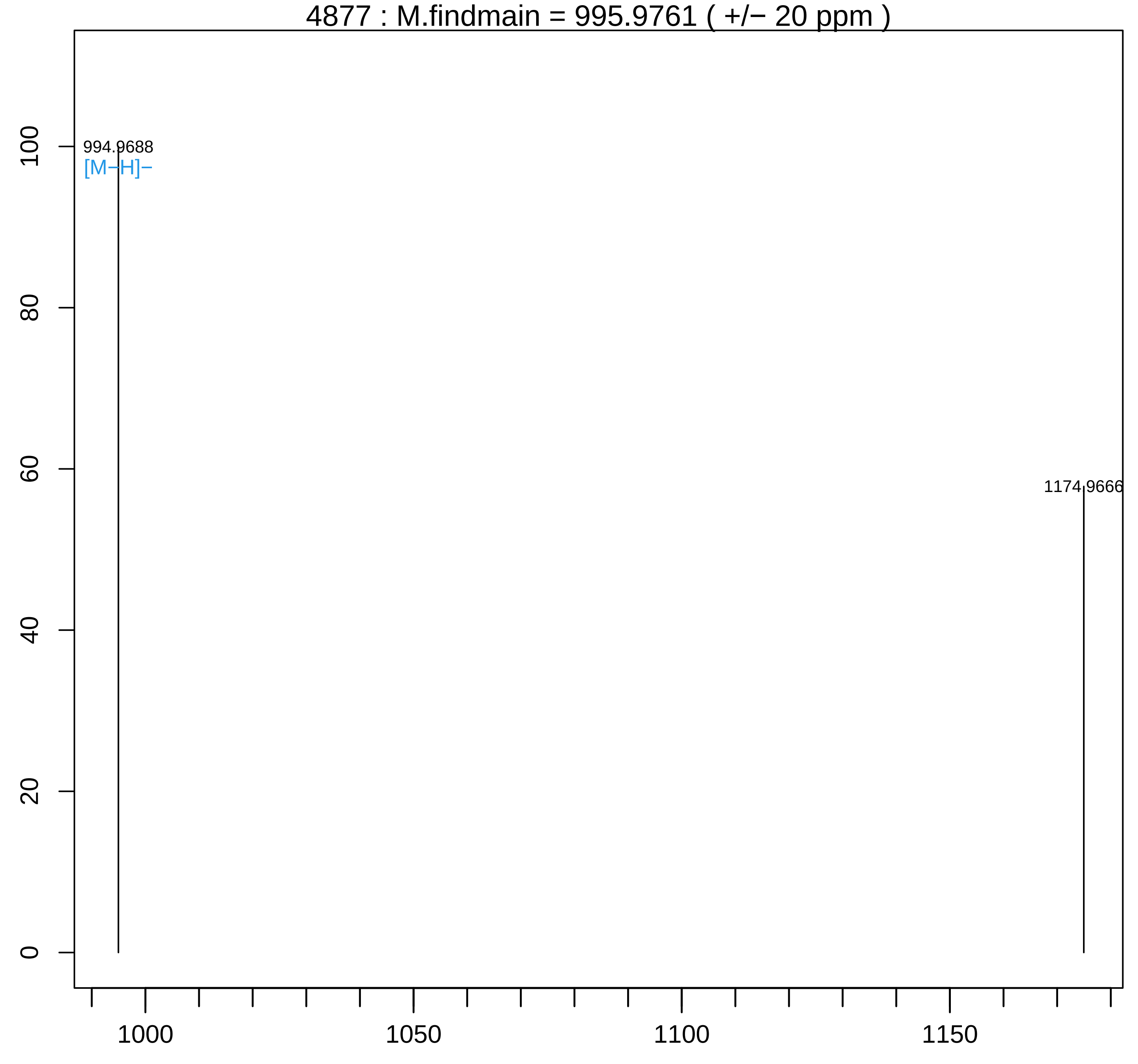

4877 : M.findmain = 995.9761 (+/− 20 ppm)
994.9688
[M−H]−
1174.9666
0
20
40
60
80
100
1000
1050
1100
1150

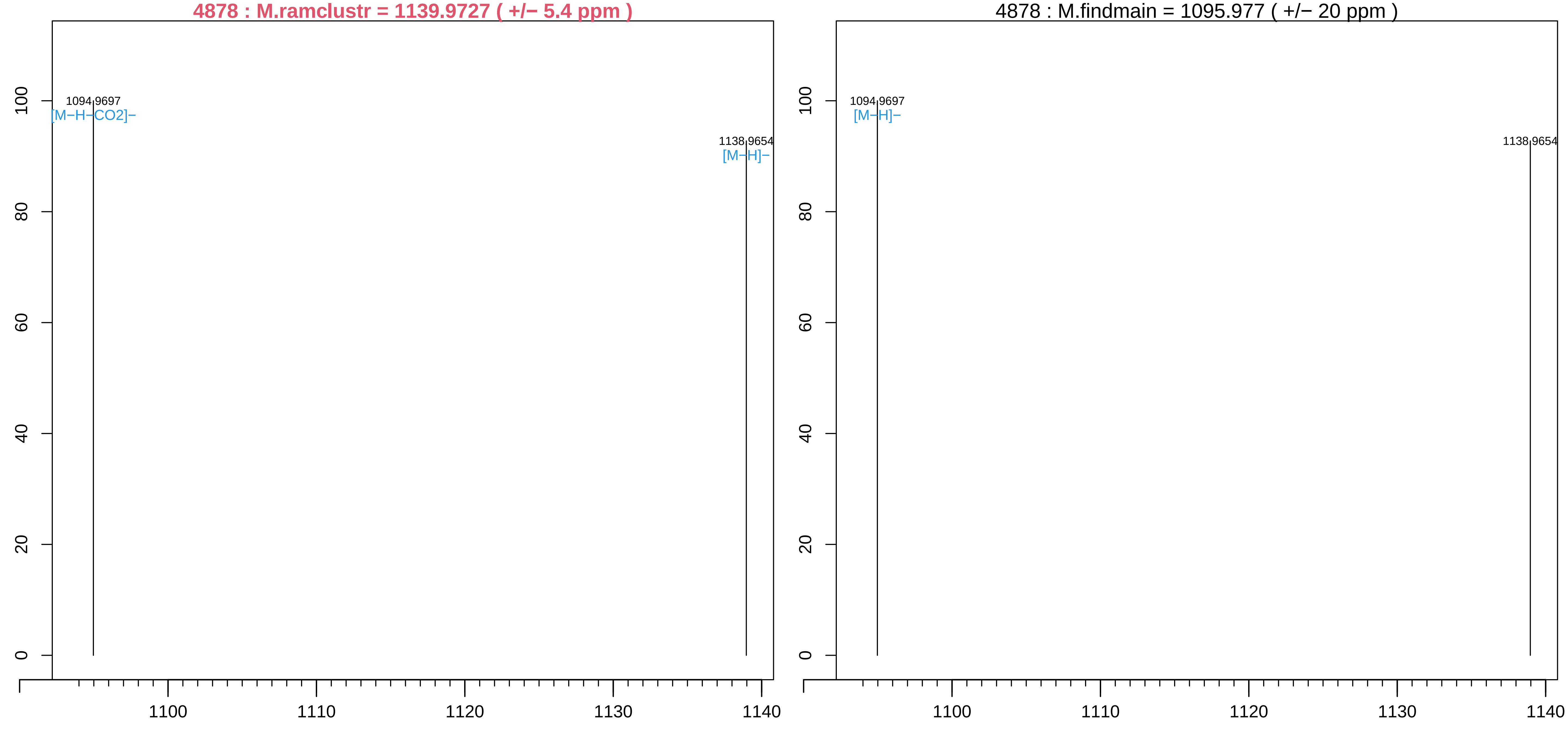

4878 : M.ramclustr = 1139.9727 (+/− 5.4 ppm)
1094.9697
[M−H−CO2]−
1138.9654
[M−H]−
0
20
40
60
80
100
1100
1110
1120
1130
1140
4878 : M.findmain = 1095.977 (+/− 20 ppm)
1094.9697
[M−H]−
1138.9654
0
20
40
60
80
100
1100
1110
1120
1130
1140

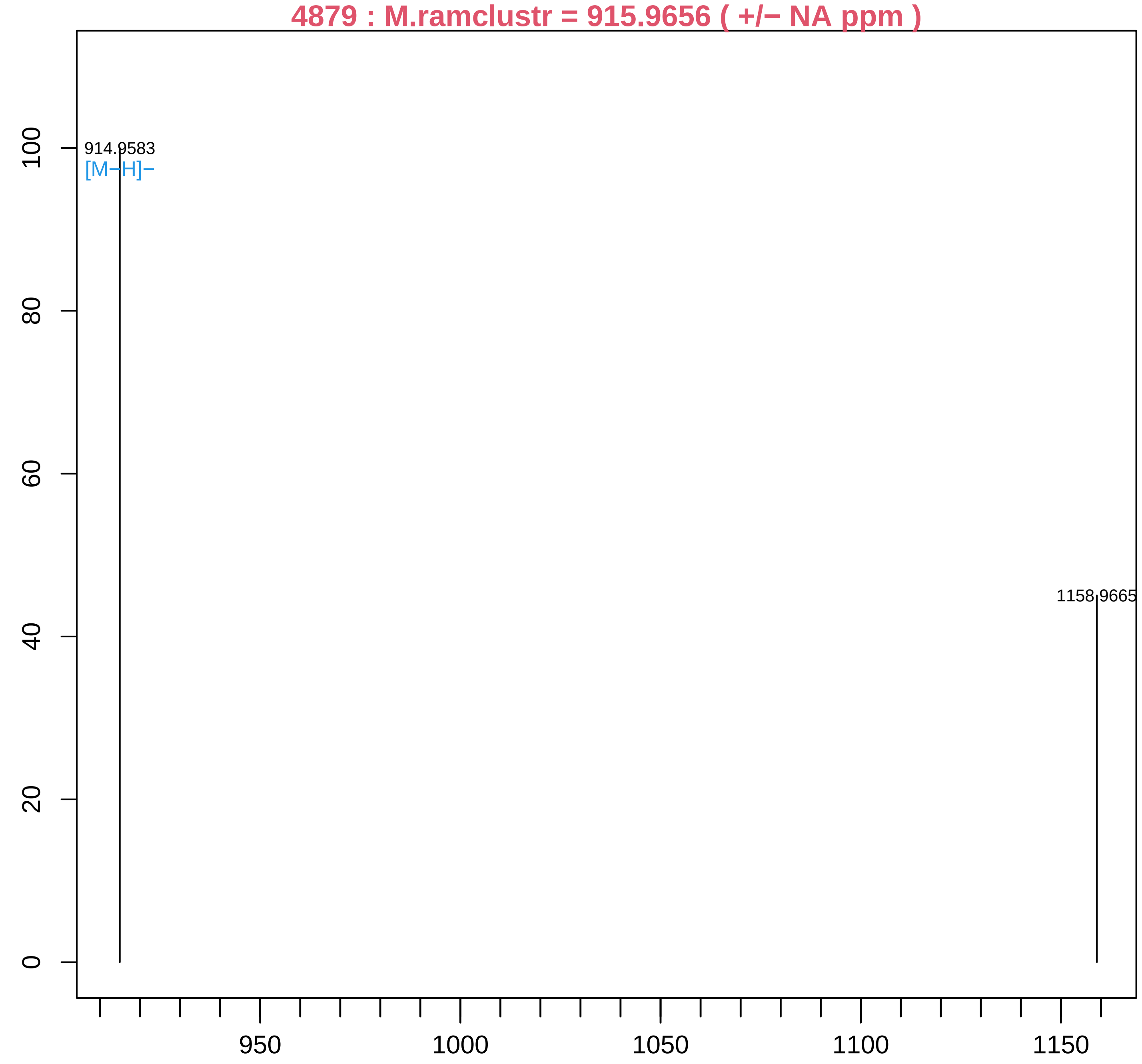
4879 : M.ramclustr = 915.9656 (+/− NA ppm)
914.9583
[M−H]−
1158.9665
0
20
40
60
80
100
950
1000
1050
1100
1150

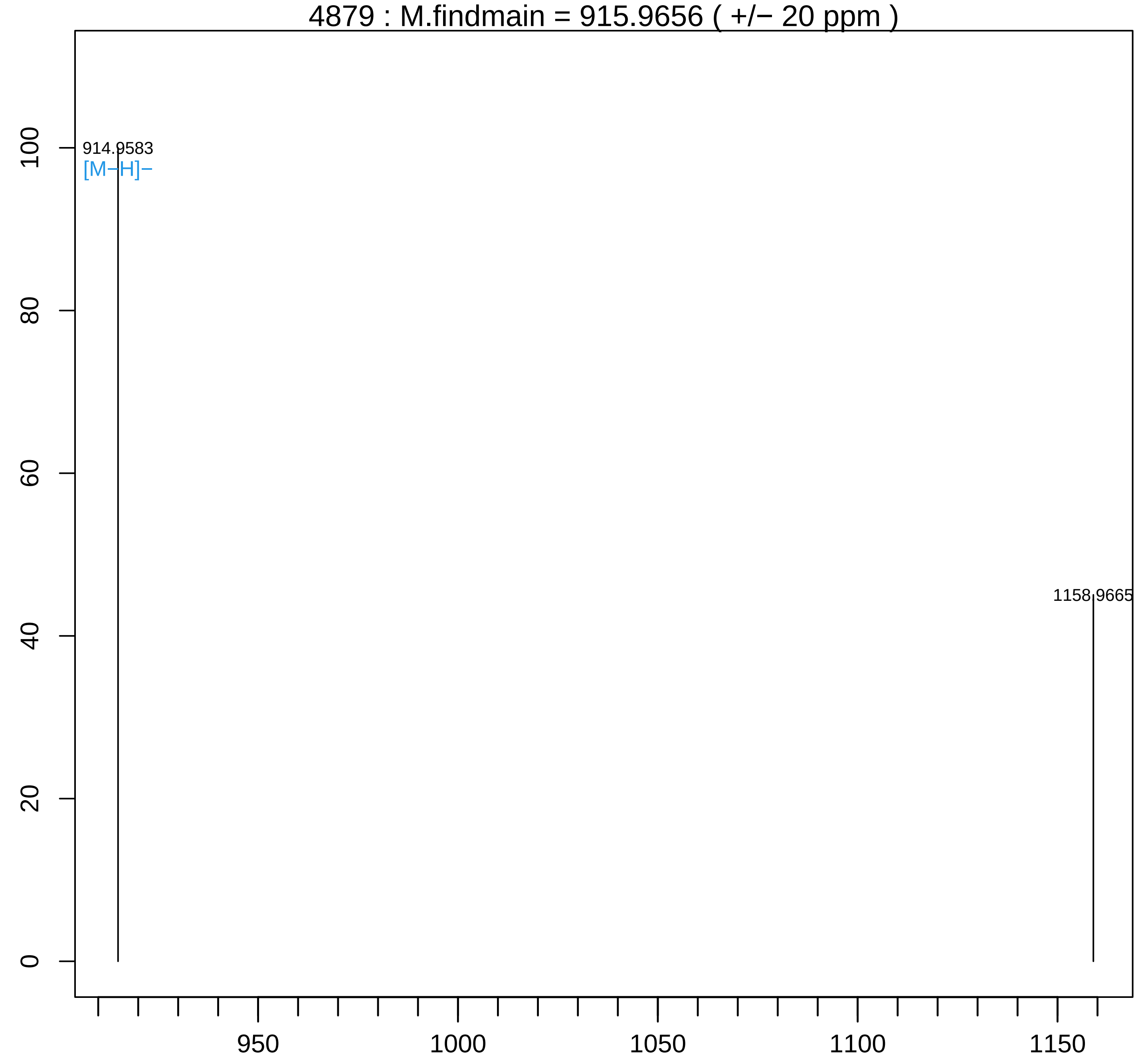
4879 : M.findmain = 915.9656 (+/− 20 ppm)
914.9583
[M−H]−
1158.9665
0
20
40
60
80
100
950
1000
1050
1100
1150

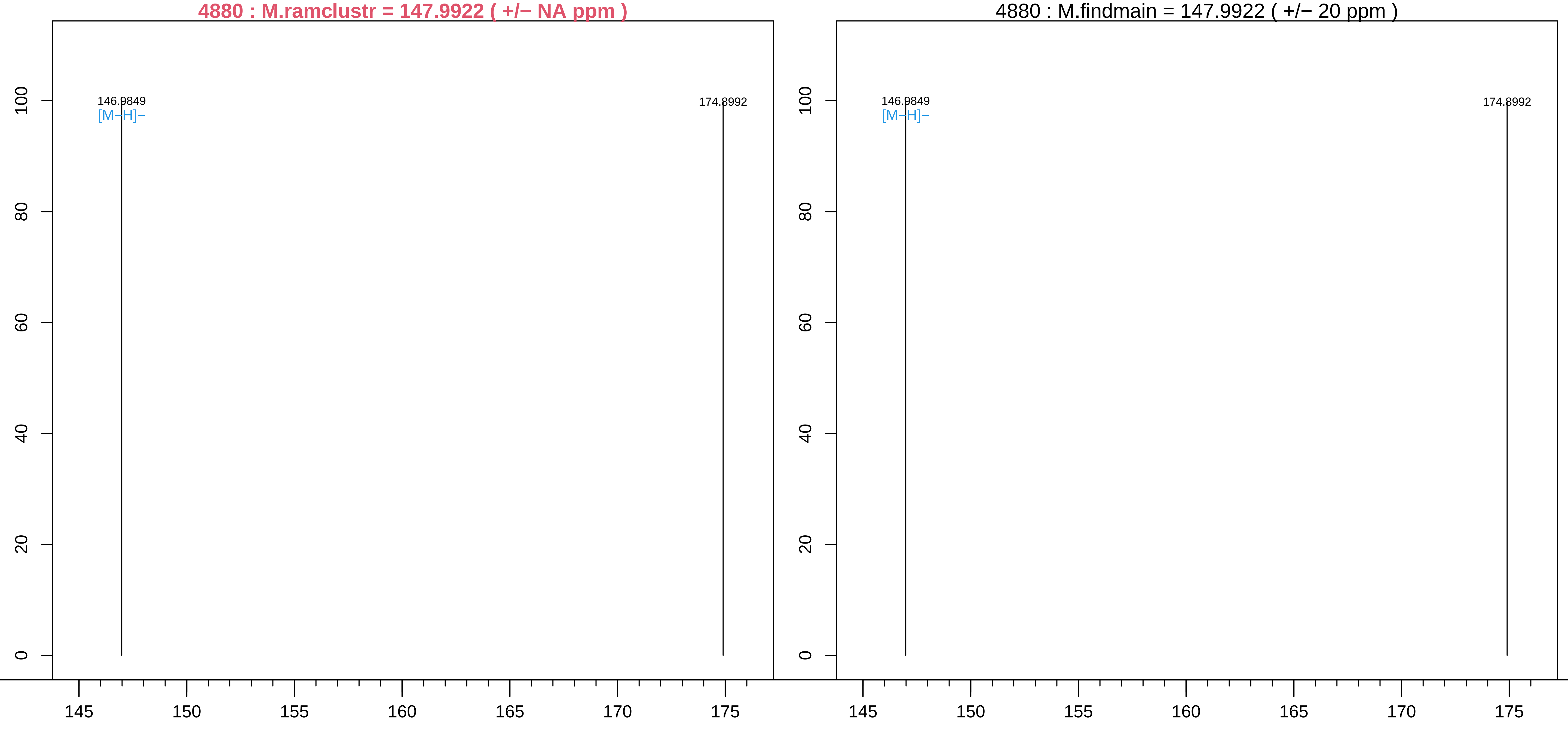
4880 : M.ramclustr = 147.9922 (+/− NA ppm)
146.9849
[M−H]−
174.8992
4880 : M.findmain = 147.9922 (+/− 20 ppm)
146.9849
[M−H]−
174.8992
0
20
40
60
80
100
145
150
155
160
165
170
175

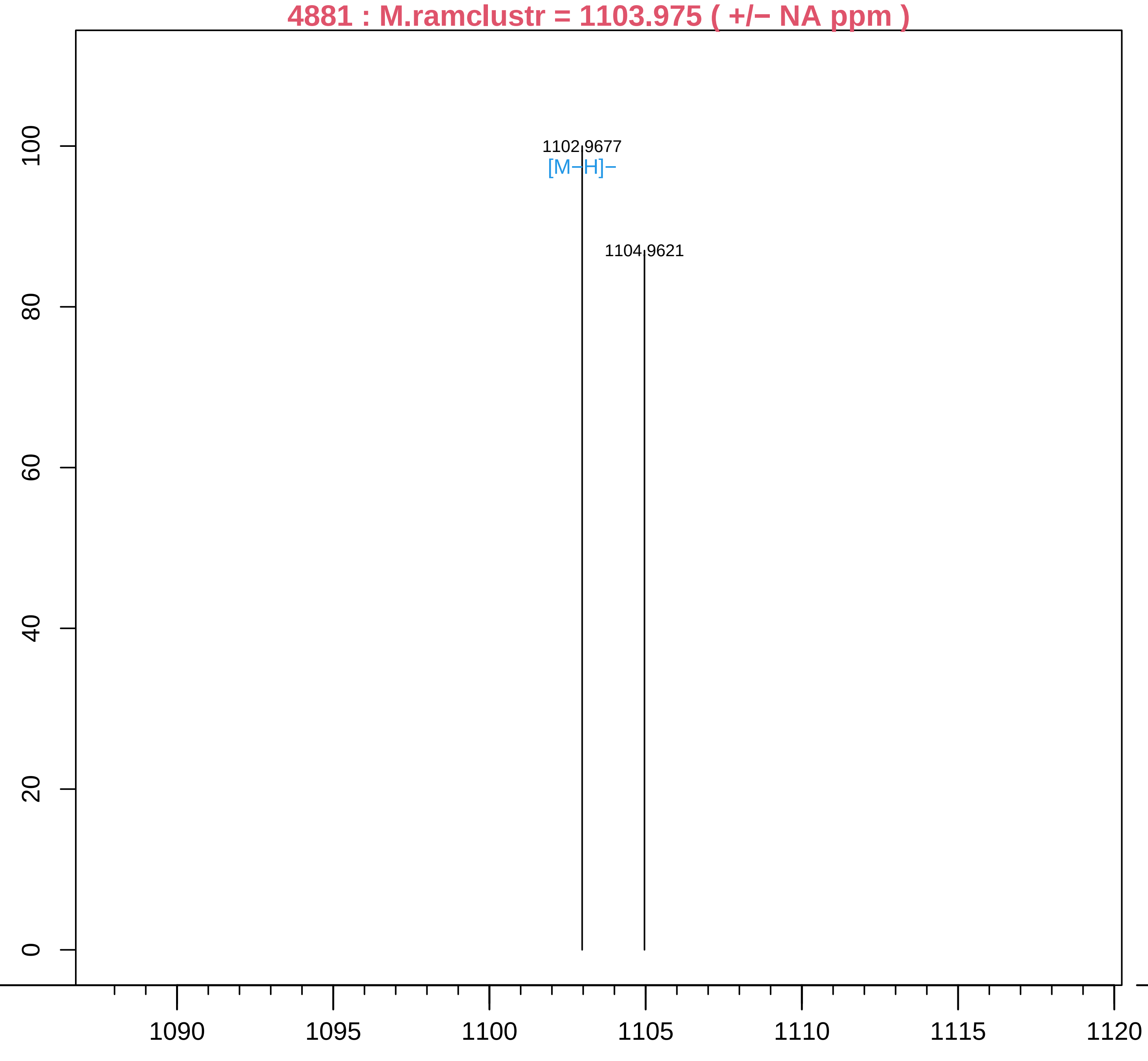
4881 : M.ramclustr = 1103.975 (+/− NA ppm)
1102.9677
[M−H]−
1104.9621
0
20
40
60
80
100
1090
1095
1100
1105
1110
1115
1120

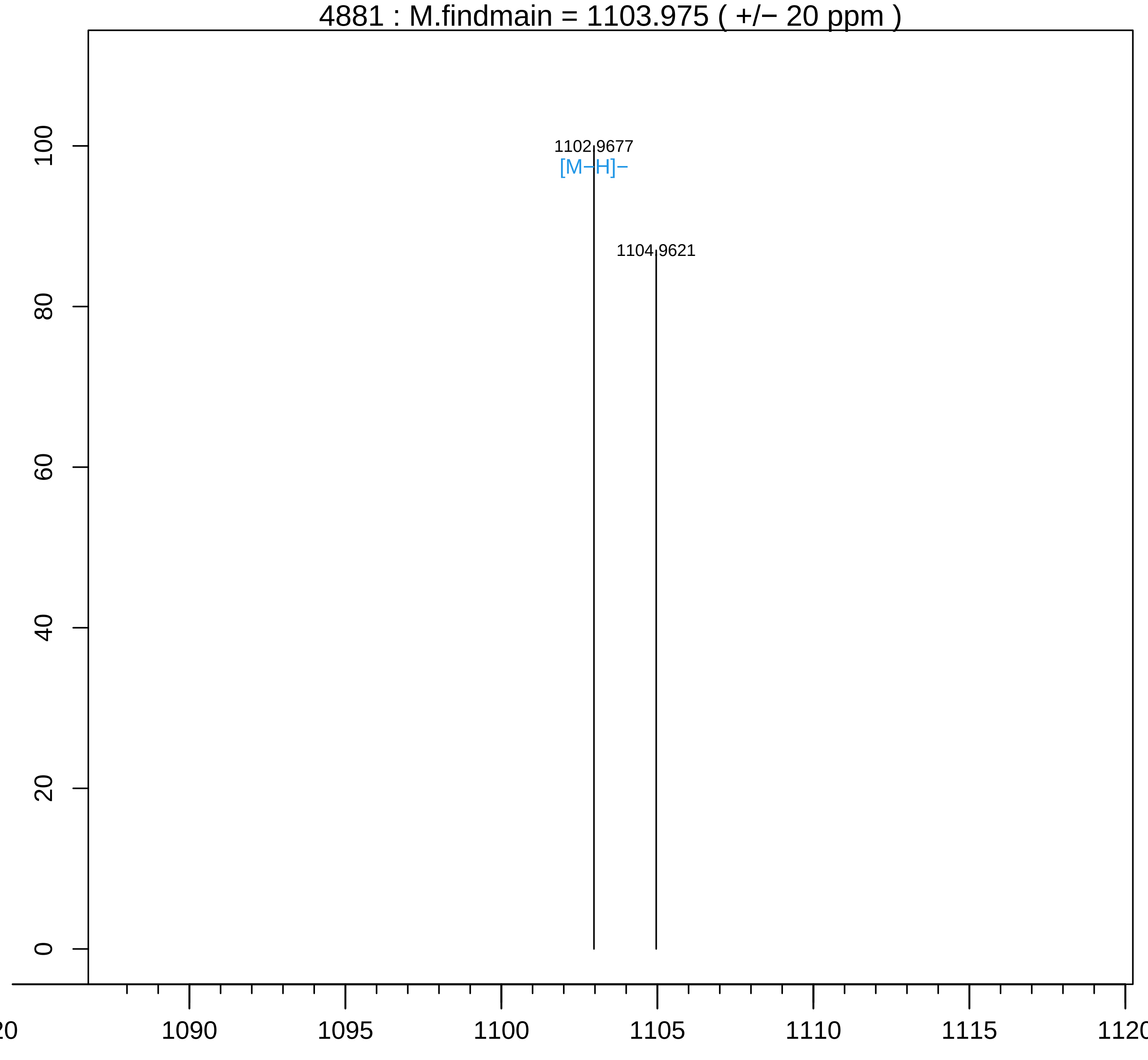
4881 : M.findmain = 1103.975 (+/− 20 ppm)
1102.9677
[M−H]−
1104.9621
0
20
40
60
80
100
1090
1095
1100
1105
1110
1115
1120

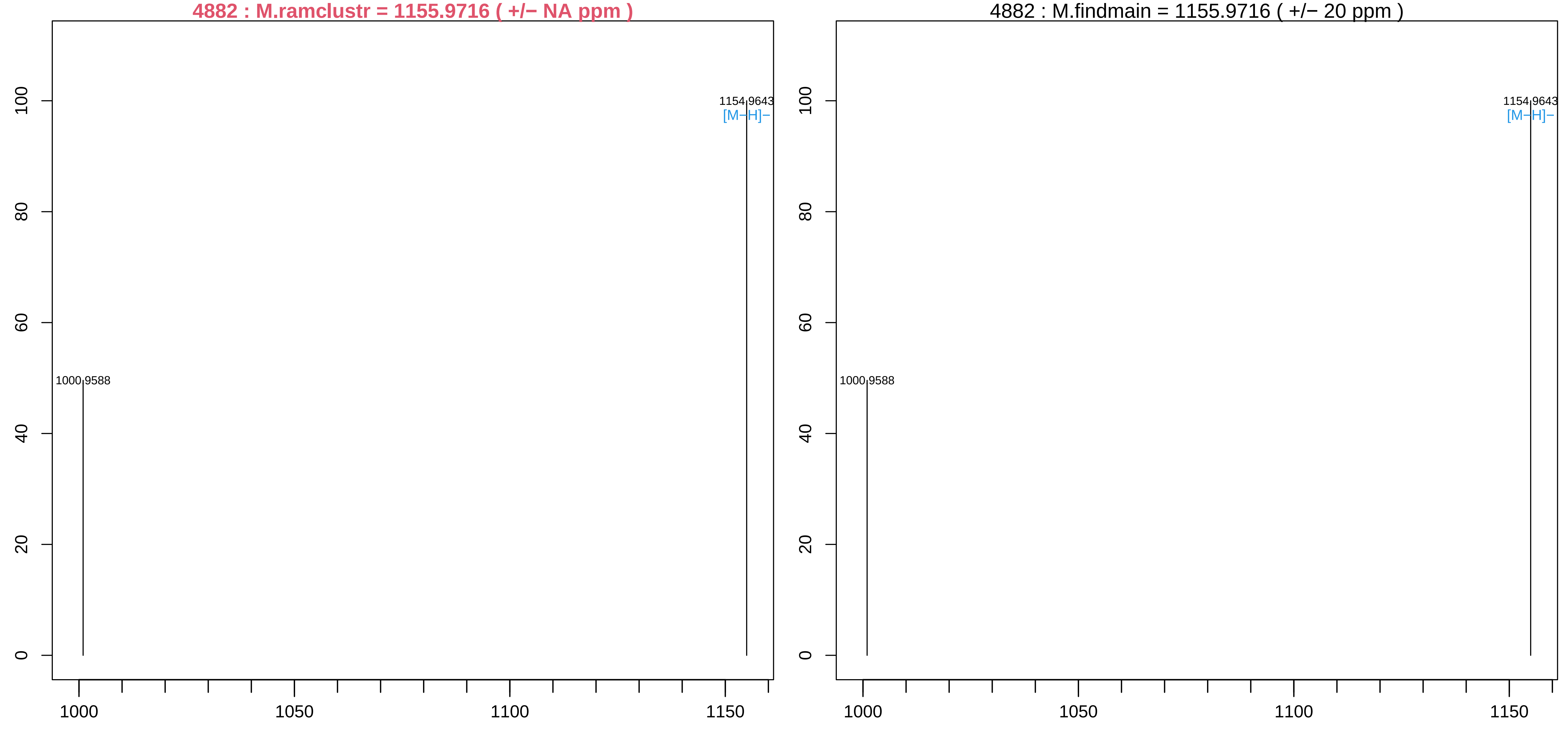
4882 : M.ramclustr = 1155.9716 (+/− NA ppm)
4882 : M.findmain = 1155.9716 (+/− 20 ppm)
1154.9643
[M−H]−
1000.9588
0
20
40
60
80
100
1000
1050
1100
1150

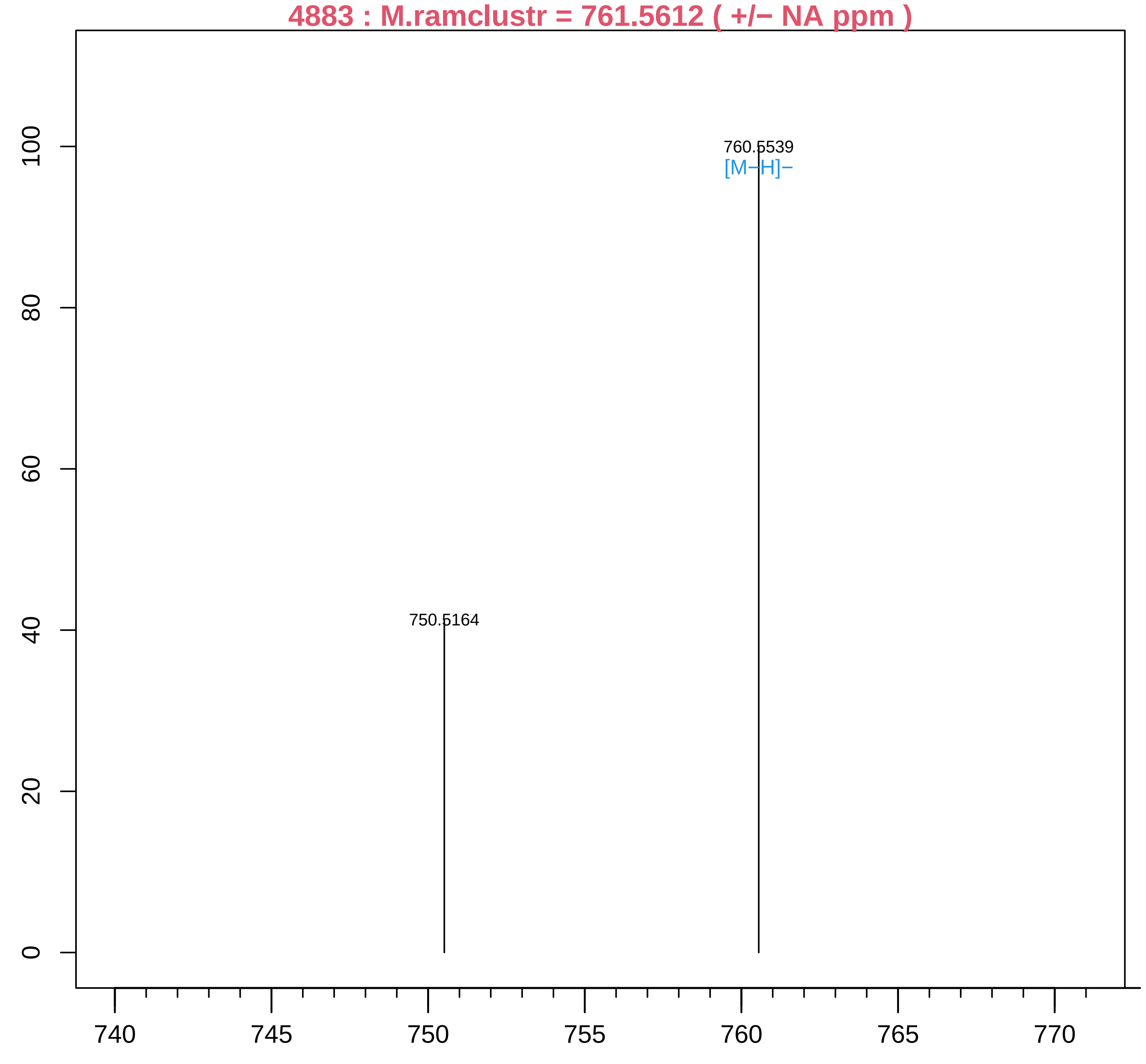
4883 : M.ramclustr = 761.5612 (+/− NA ppm)
760.5539
[M−H]−
750.5164
0
20
40
60
80
100
740
745
750
755
760
765
770

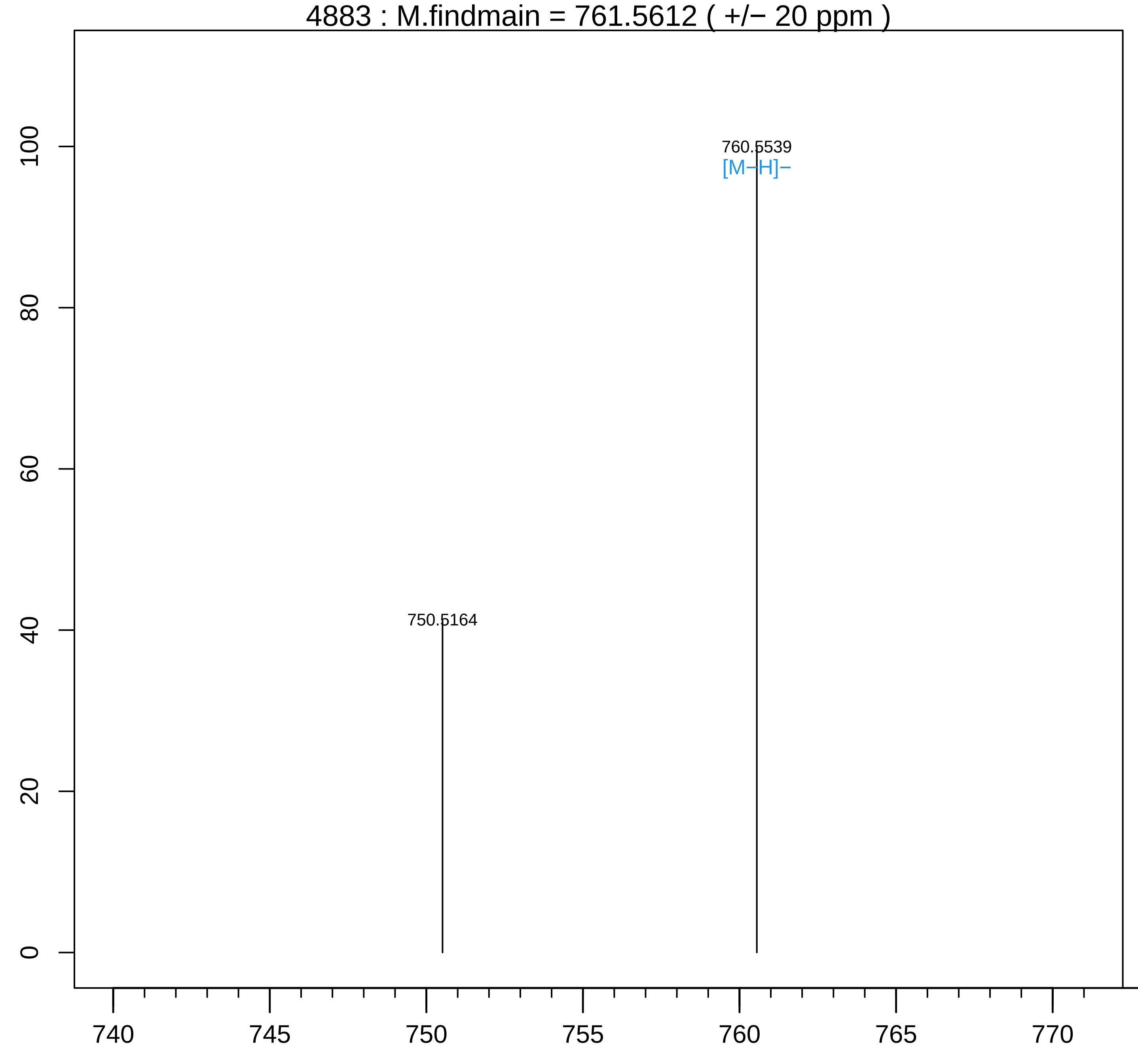
4883 : M.findmain = 761.5612 (+/− 20 ppm)
760.5539
[M−H]−
750.5164
0
20
40
60
80
100
740
745
750
755
760
765
770

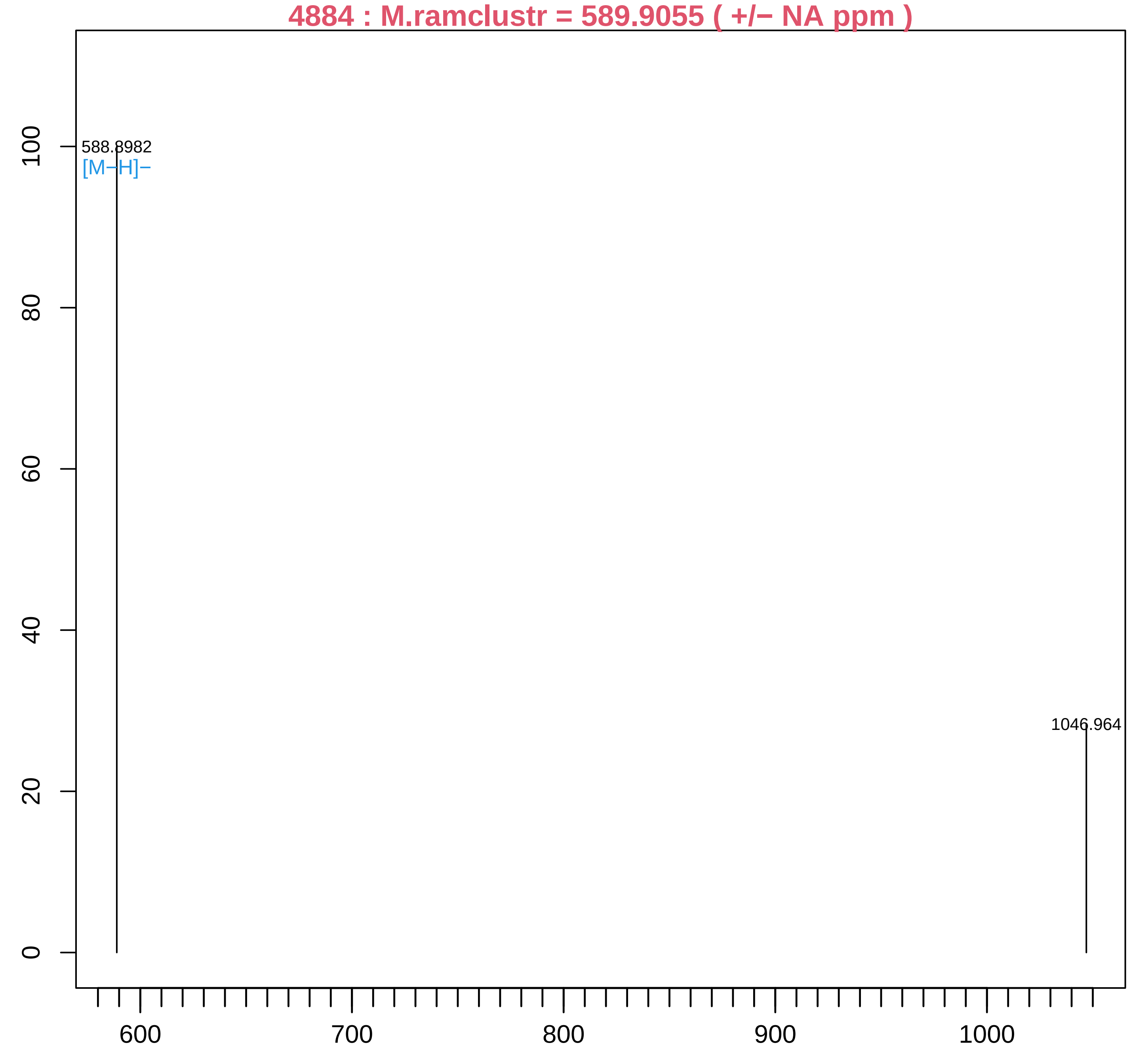
4884 : M.ramclustr = 589.9055 (+/− NA ppm)
588.8982
[M−H]−
1046.964
0
20
40
60
80
100
600
700
800
900
1000

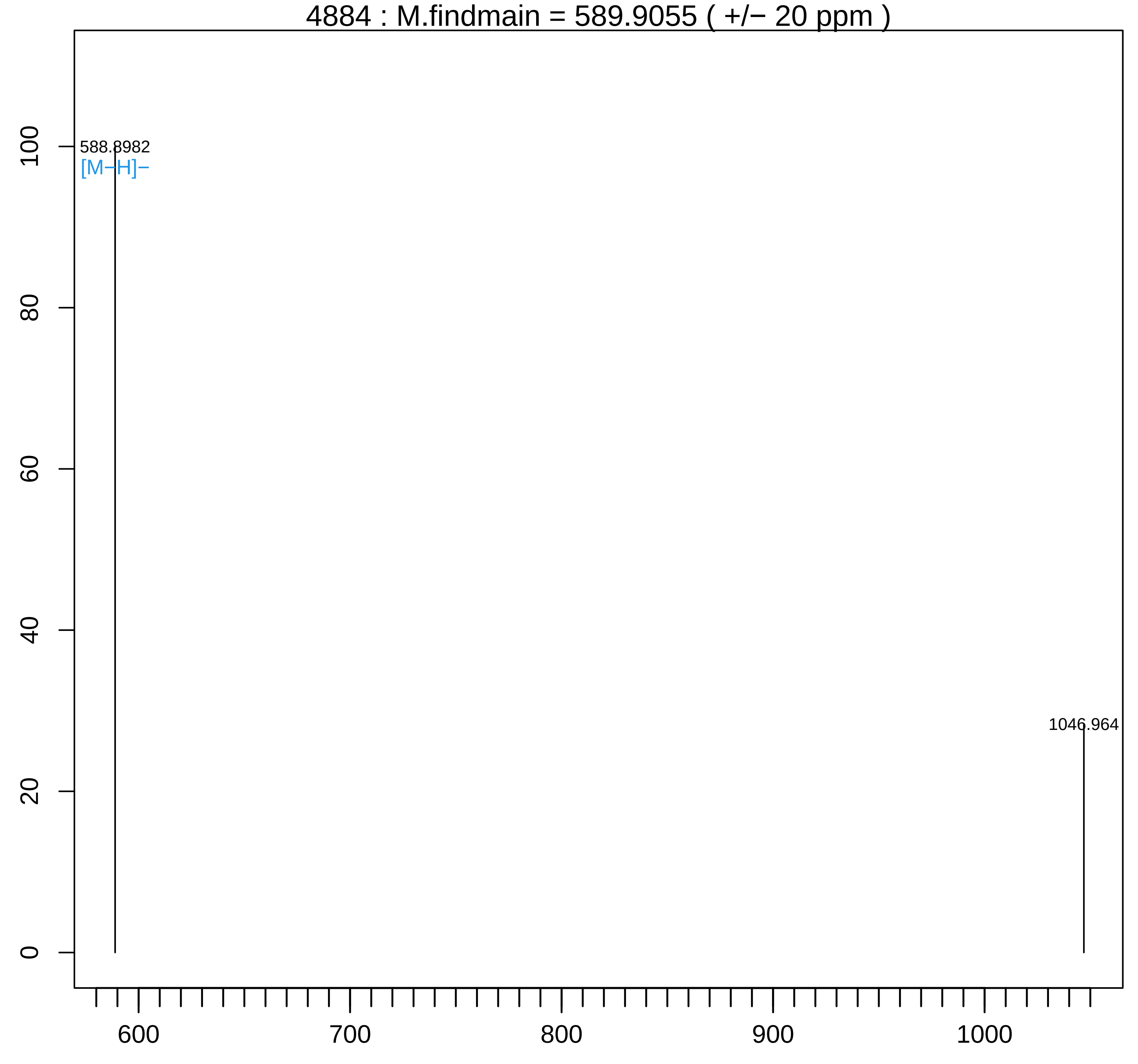
4884 : M.findmain = 589.9055 (+/− 20 ppm)
588.8982
[M−H]−
1046.964
0
20
40
60
80
100
600
700
800
900
1000

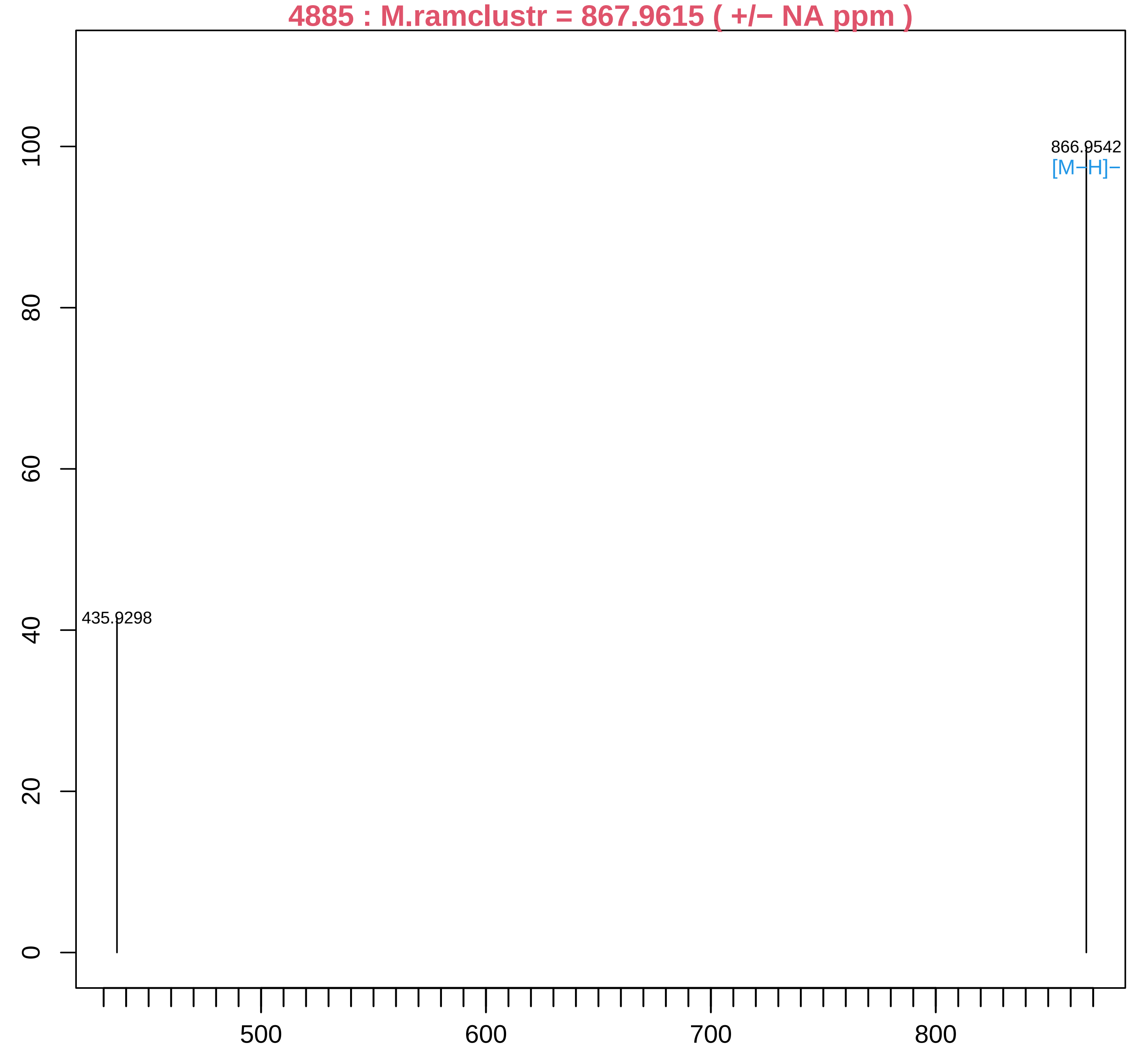
4885 : M.ramclustr = 867.9615 (+/− NA ppm)
866.9542
[M−H]−
435.9298
0
20
40
60
80
100
500
600
700
800

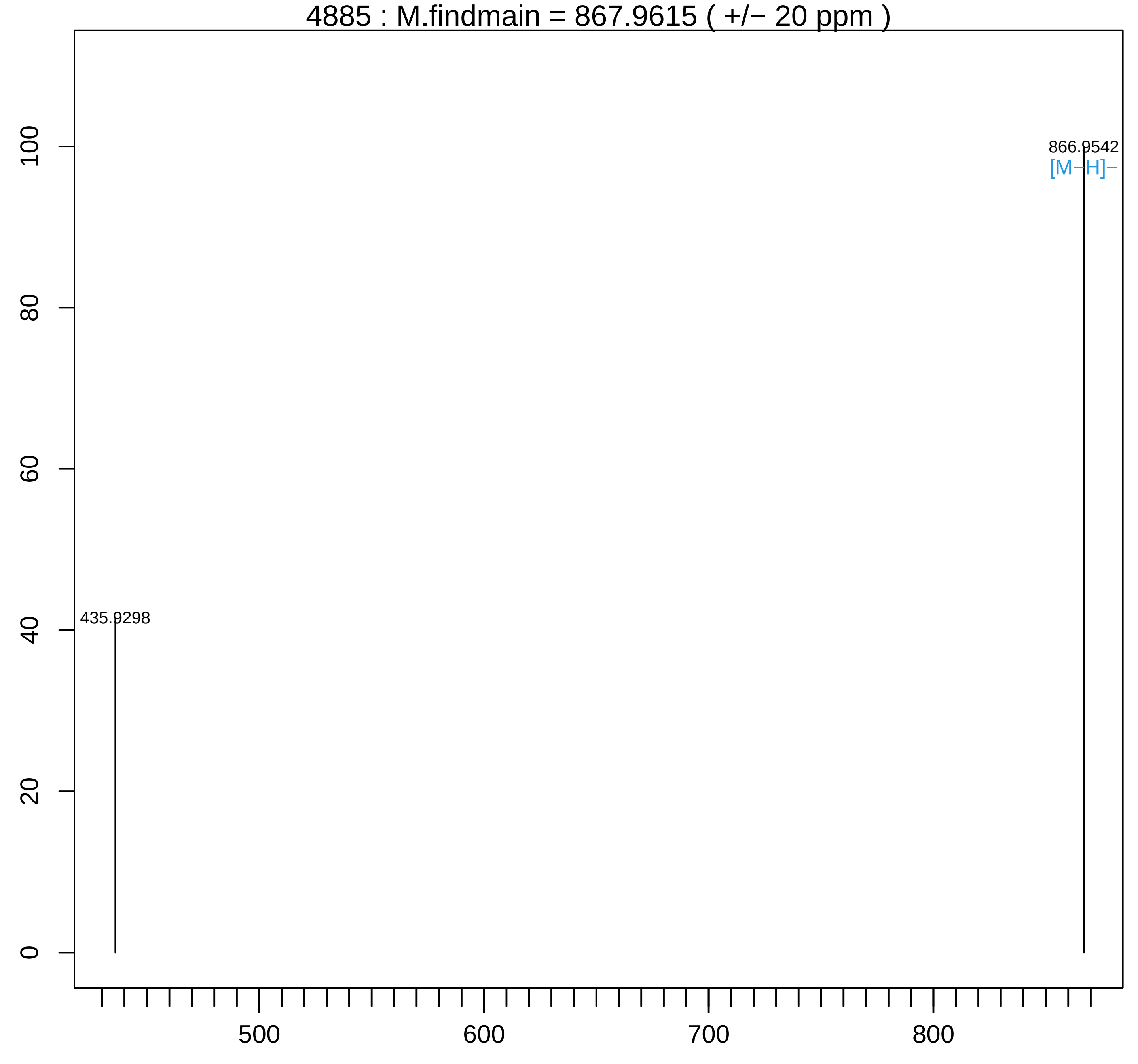
4885 : M.findmain = 867.9615 (+/− 20 ppm)
866.9542
[M−H]−
435.9298
0
20
40
60
80
100
500
600
700
800

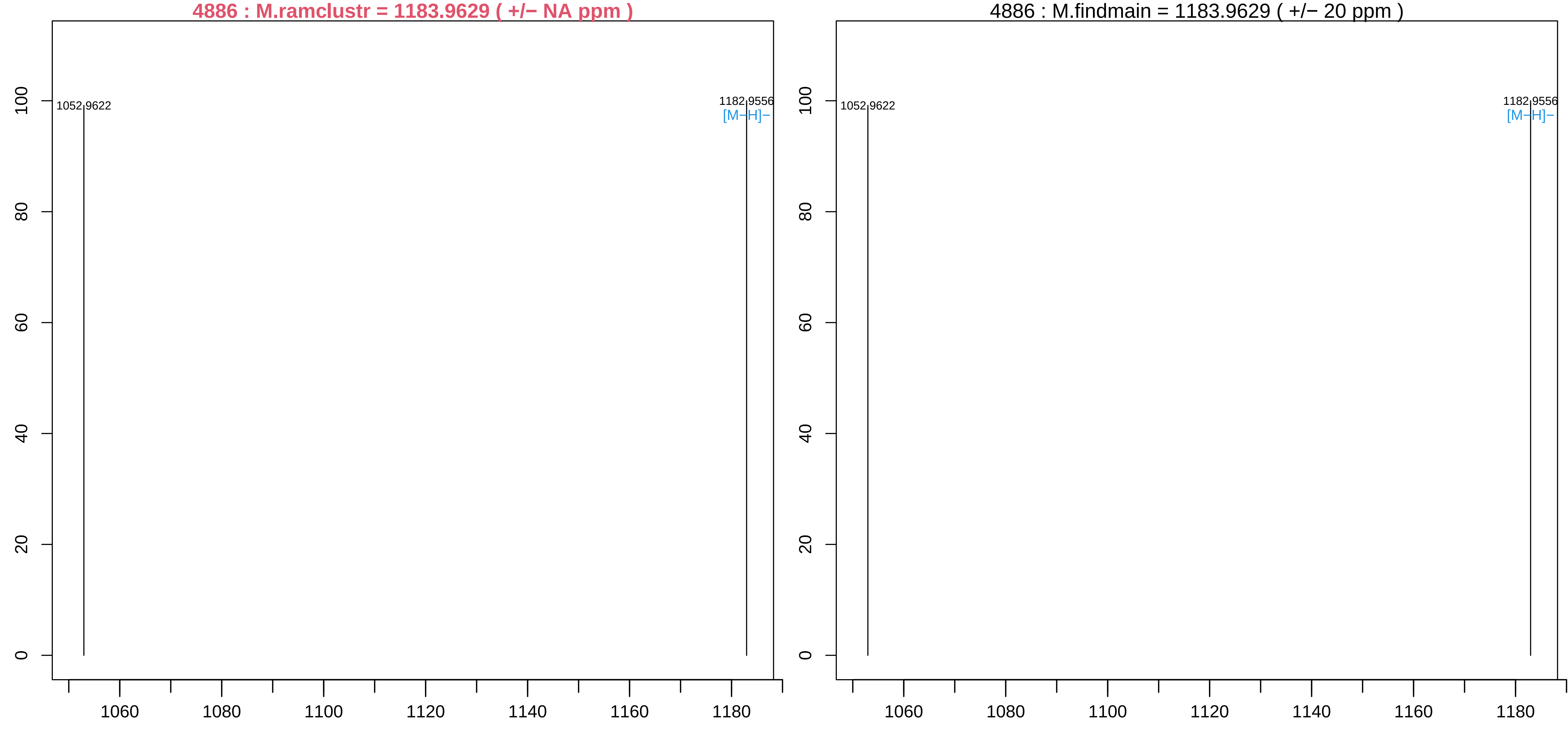
4886 : M.ramclustr = 1183.9629 (+/− NA ppm)
1052.9622
1182.9556
[M−H]−
4886 : M.findmain = 1183.9629 (+/− 20 ppm)
1052.9622
1182.9556
[M−H]−
0
20
40
60
80
100
1060
1080
1100
1120
1140
1160
1180

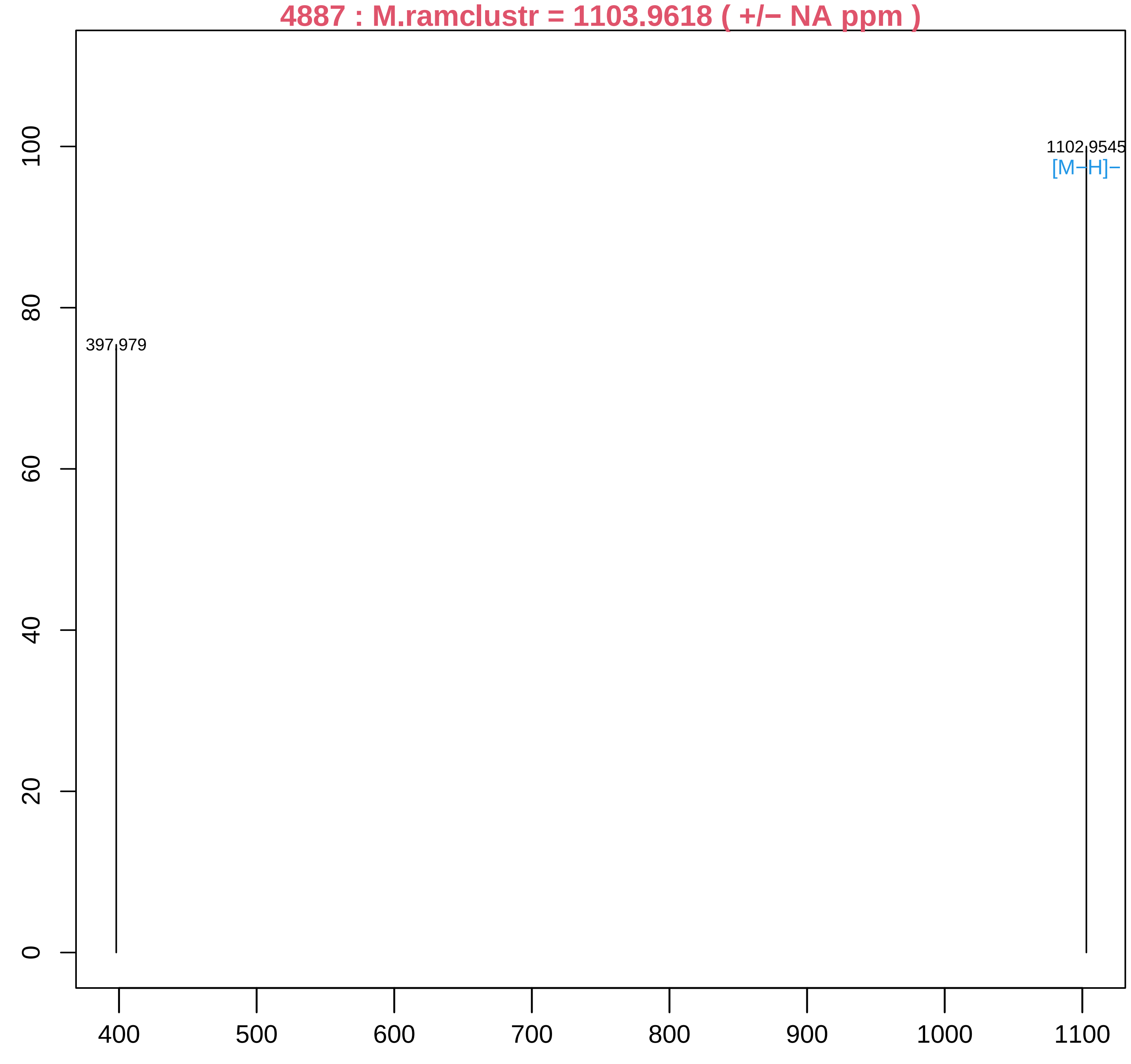
4887 : M.ramclustr = 1103.9618 (+/− NA ppm)
1102.9545
[M−H]−
397.979
0
20
40
60
80
100
400
500
600
700
800
900
1000
1100

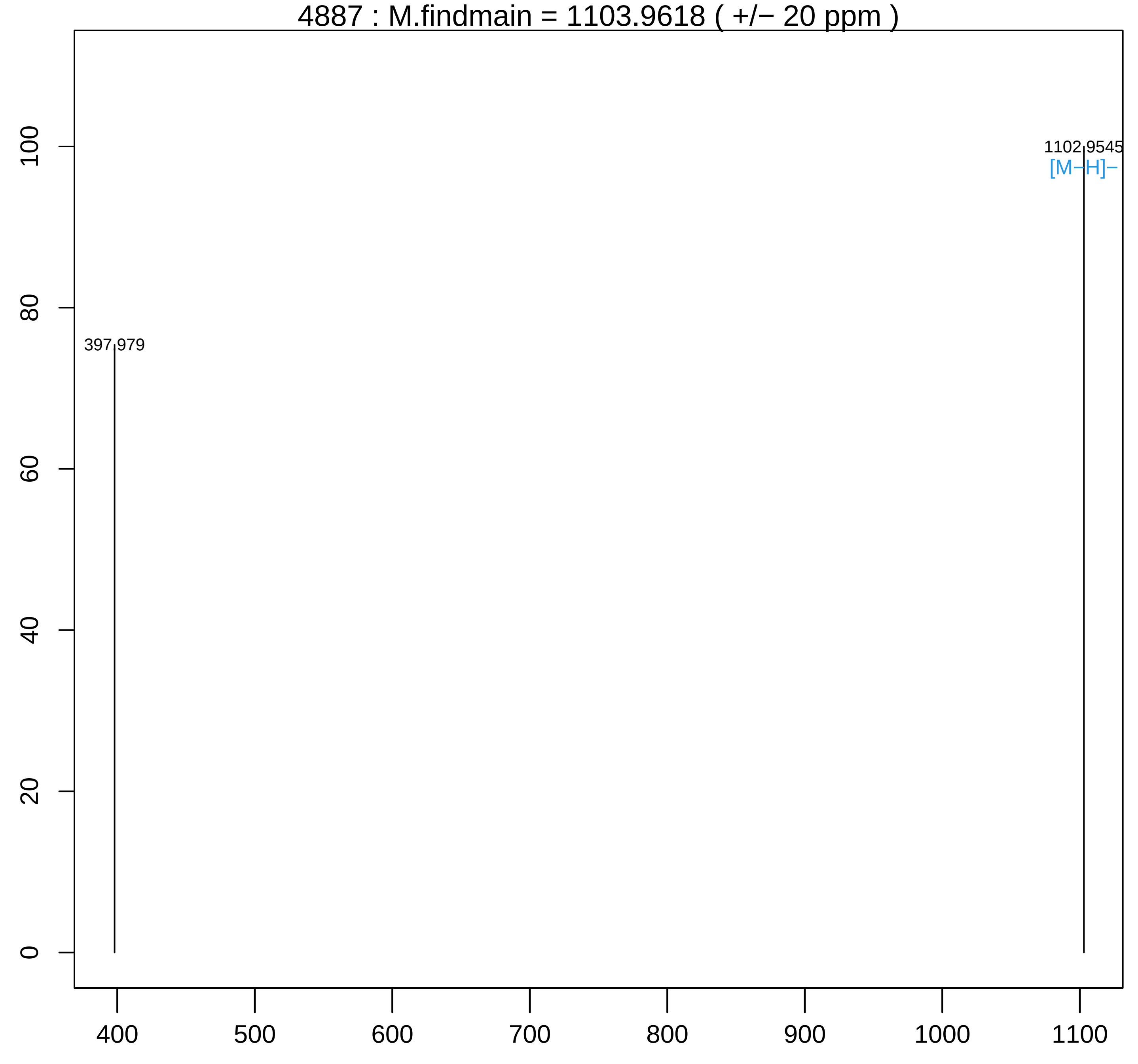
4887 : M.findmain = 1103.9618 (+/− 20 ppm)
1102.9545
[M−H]−
397.979
0
20
40
60
80
100
400
500
600
700
800
900
1000
1100

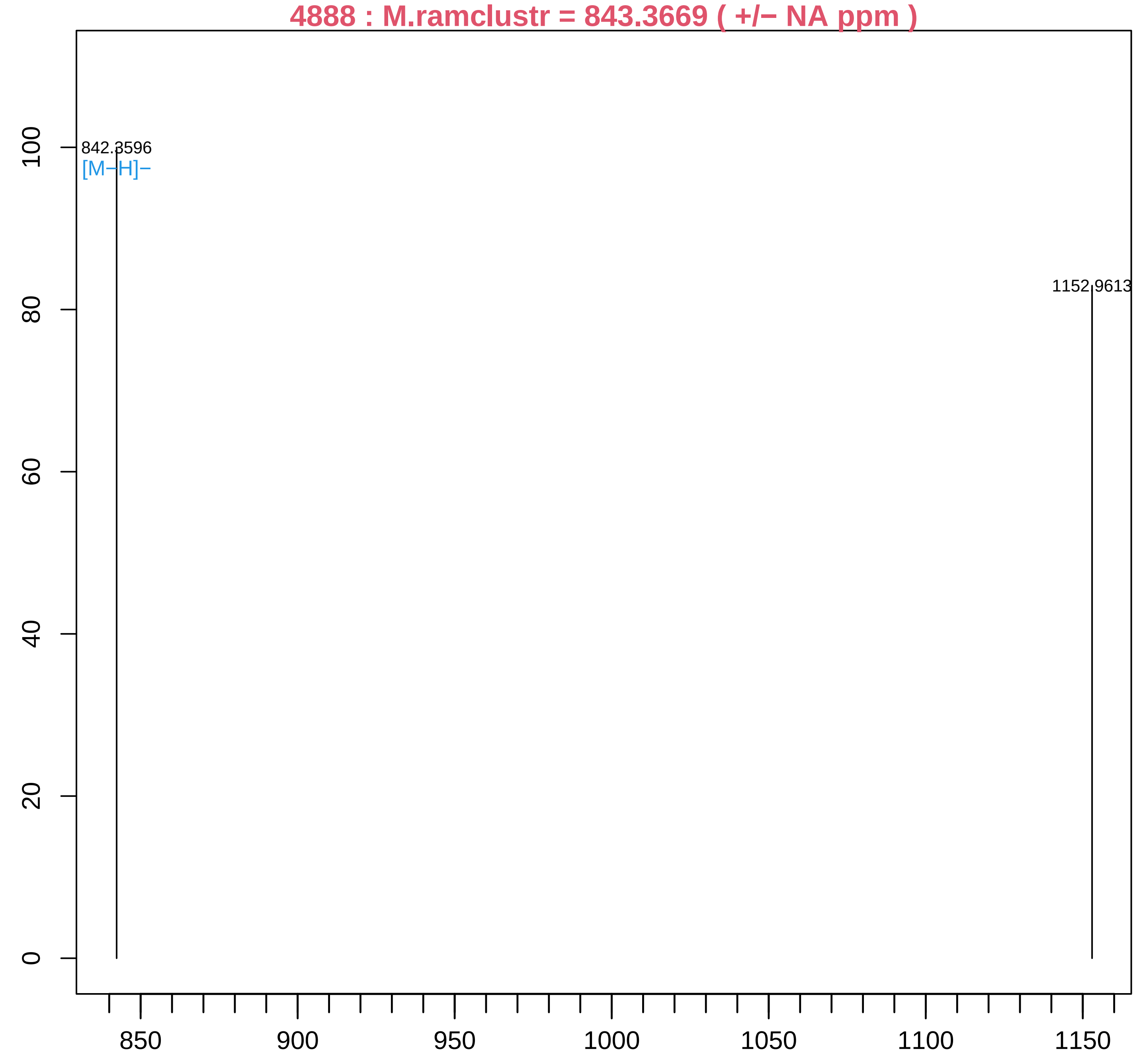

4888 : M.ramclustr = 843.3669 (+/− NA ppm)
842.3596
[M−H]−
1152.9613
0
20
40
60
80
100
850
900
950
1000
1050
1100
1150

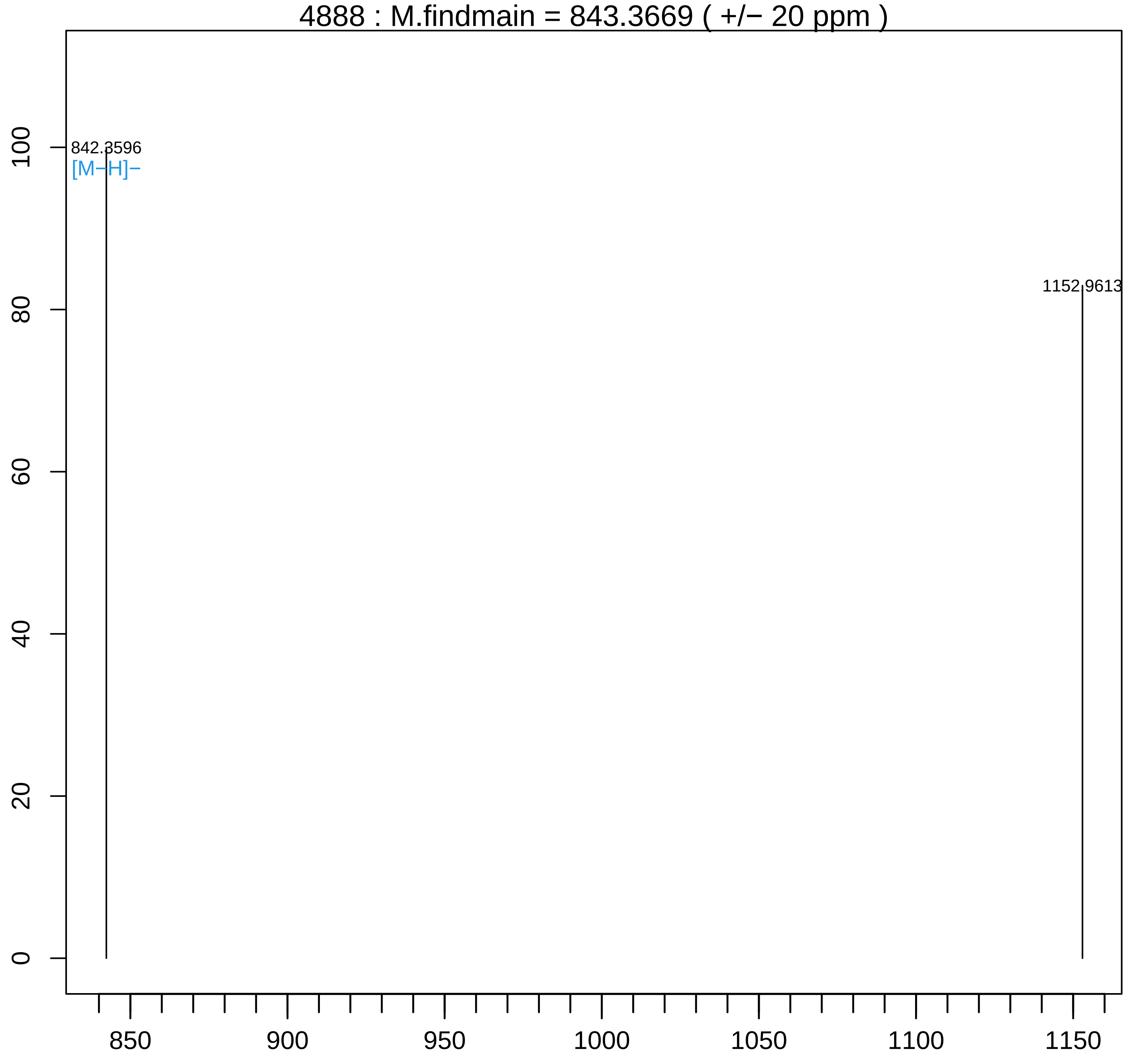

4888 : M.findmain = 843.3669 (+/− 20 ppm)
842.3596
[M−H]−
1152.9613
0
20
40
60
80
100
850
900
950
1000
1050
1100
1150

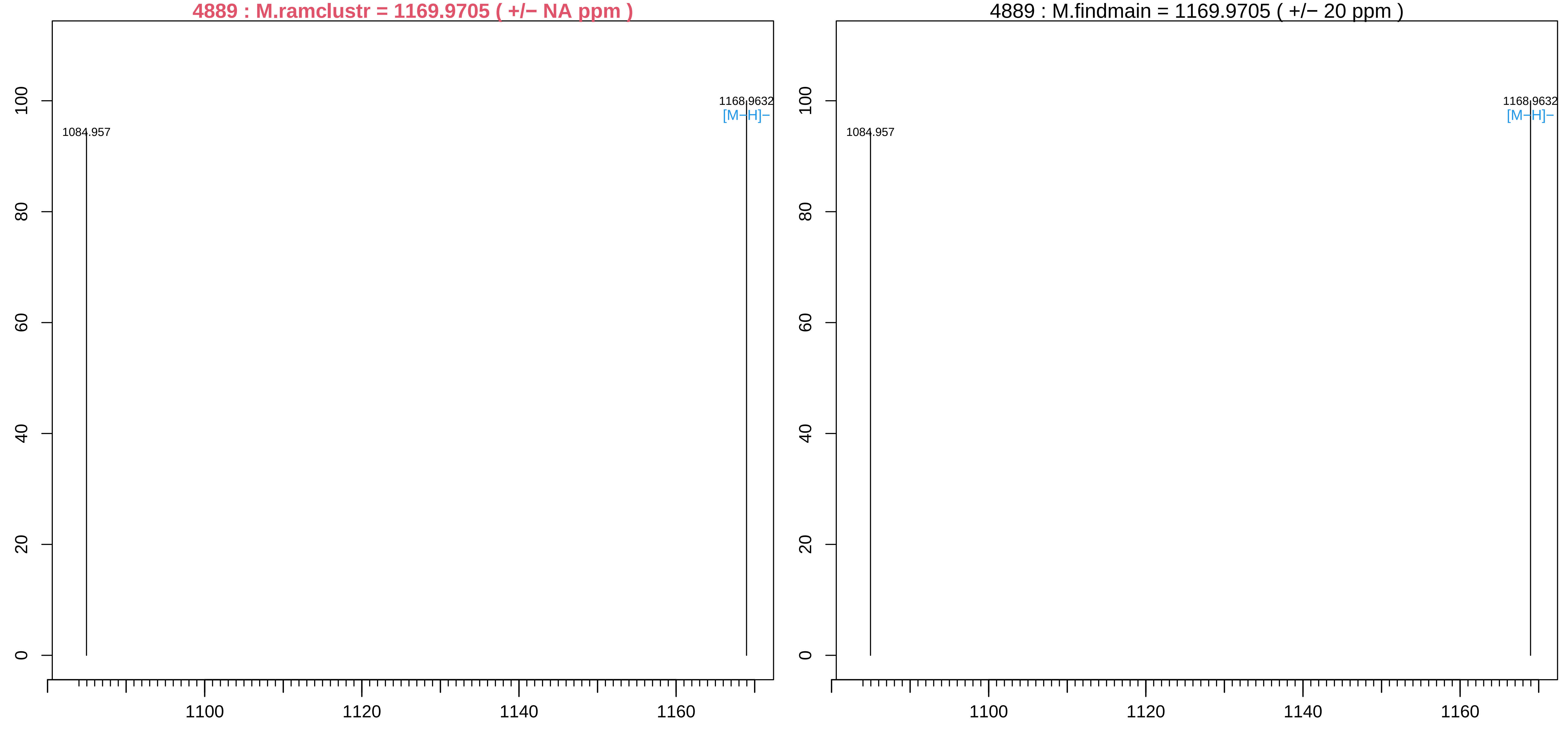
4889 : M.ramclustr = 1169.9705 (+/− NA ppm)
1168.9632
[M−H]−
1084.957
0
20
40
60
80
100
1100
1120
1140
1160
4889 : M.findmain = 1169.9705 (+/− 20 ppm)
1168.9632
[M−H]−
1084.957
0
20
40
60
80
100
1100
1120
1140
1160

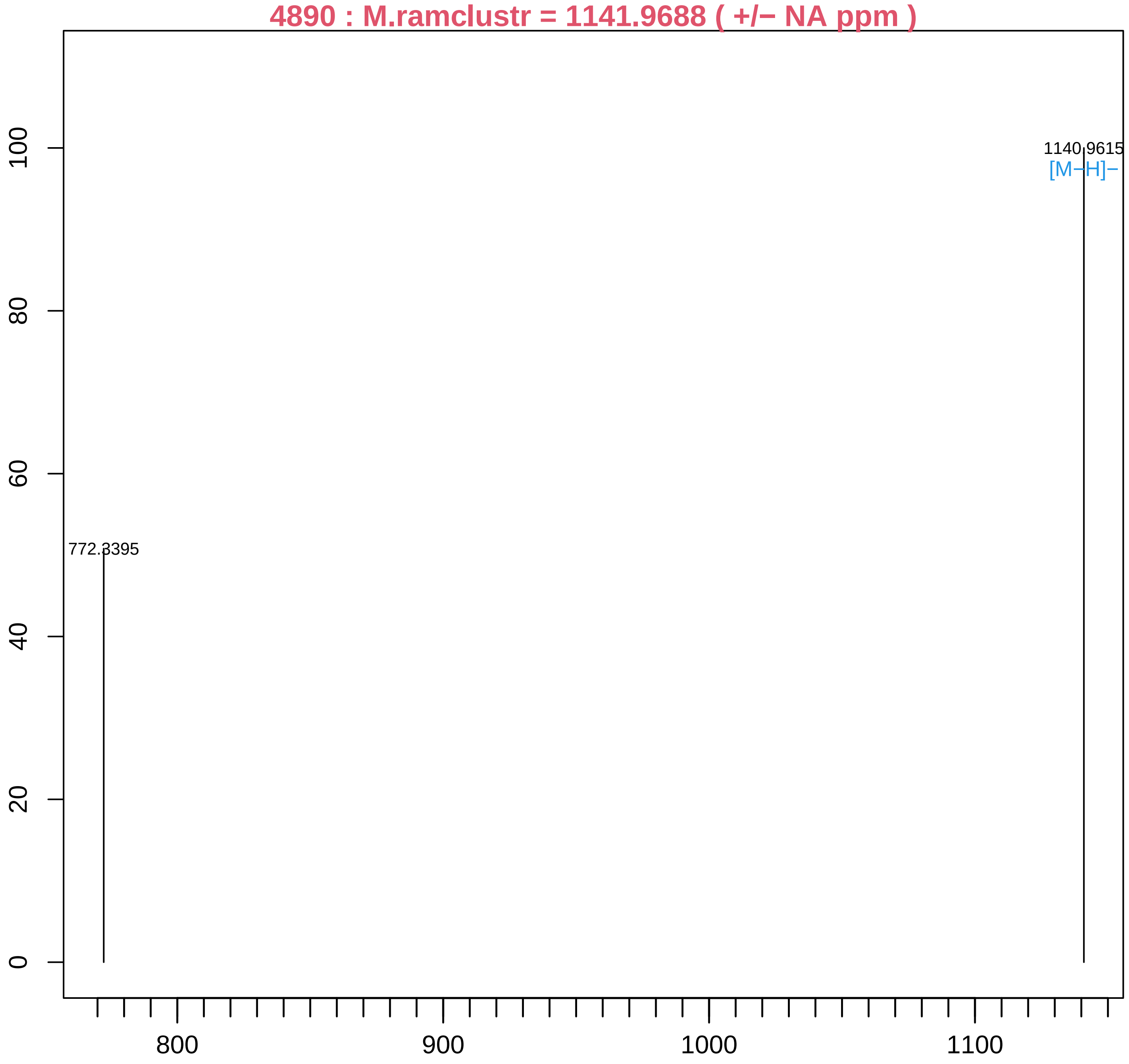
4890 : M.ramclustr = 1141.9688 (+/− NA ppm)
1140.9615
[M−H]−
772.3395
0
20
40
60
80
100
800
900
1000
1100
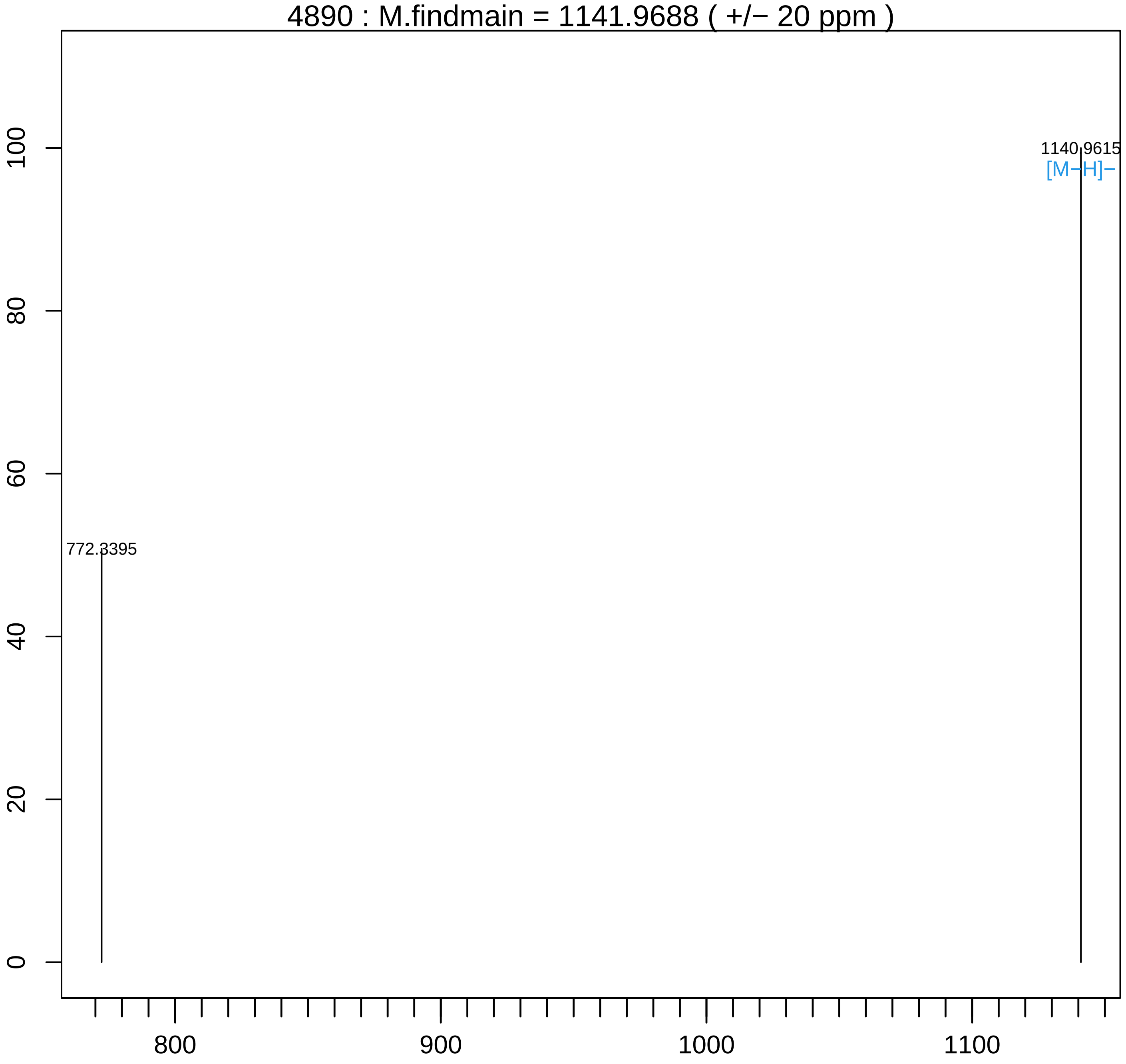
4890 : M.findmain = 1141.9688 (+/− 20 ppm)
1140.9615
[M−H]−
772.3395
0
20
40
60
80
100
800
900
1000
1100

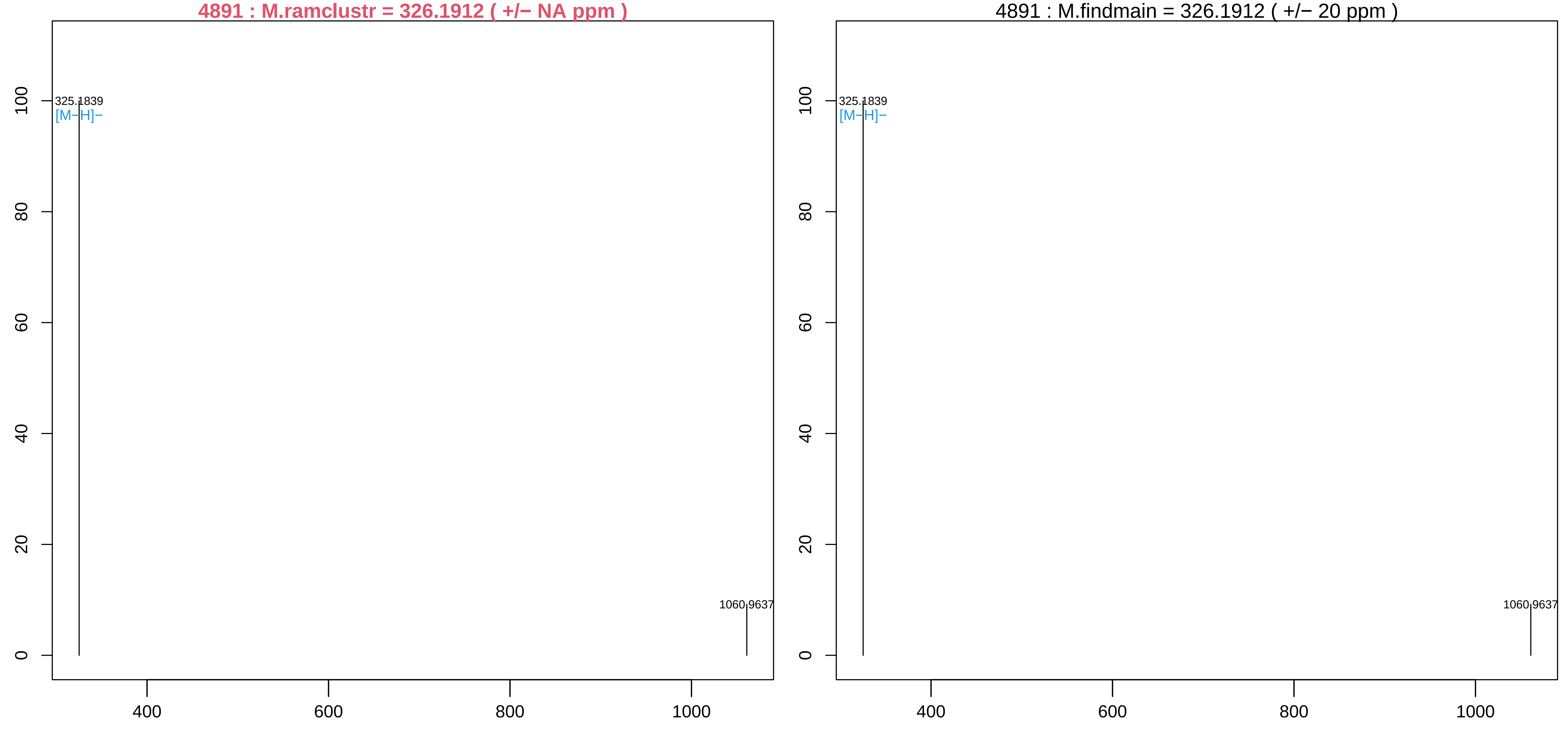
4891 : M.ramclustr = 326.1912 (+/− NA ppm)
325.1839
[M−H]−
1060.9637
0
20
40
60
80
100
400
600
800
1000
4891 : M.findmain = 326.1912 (+/− 20 ppm)
325.1839
[M−H]−
1060.9637
0
20
40
60
80
100
400
600
800
1000

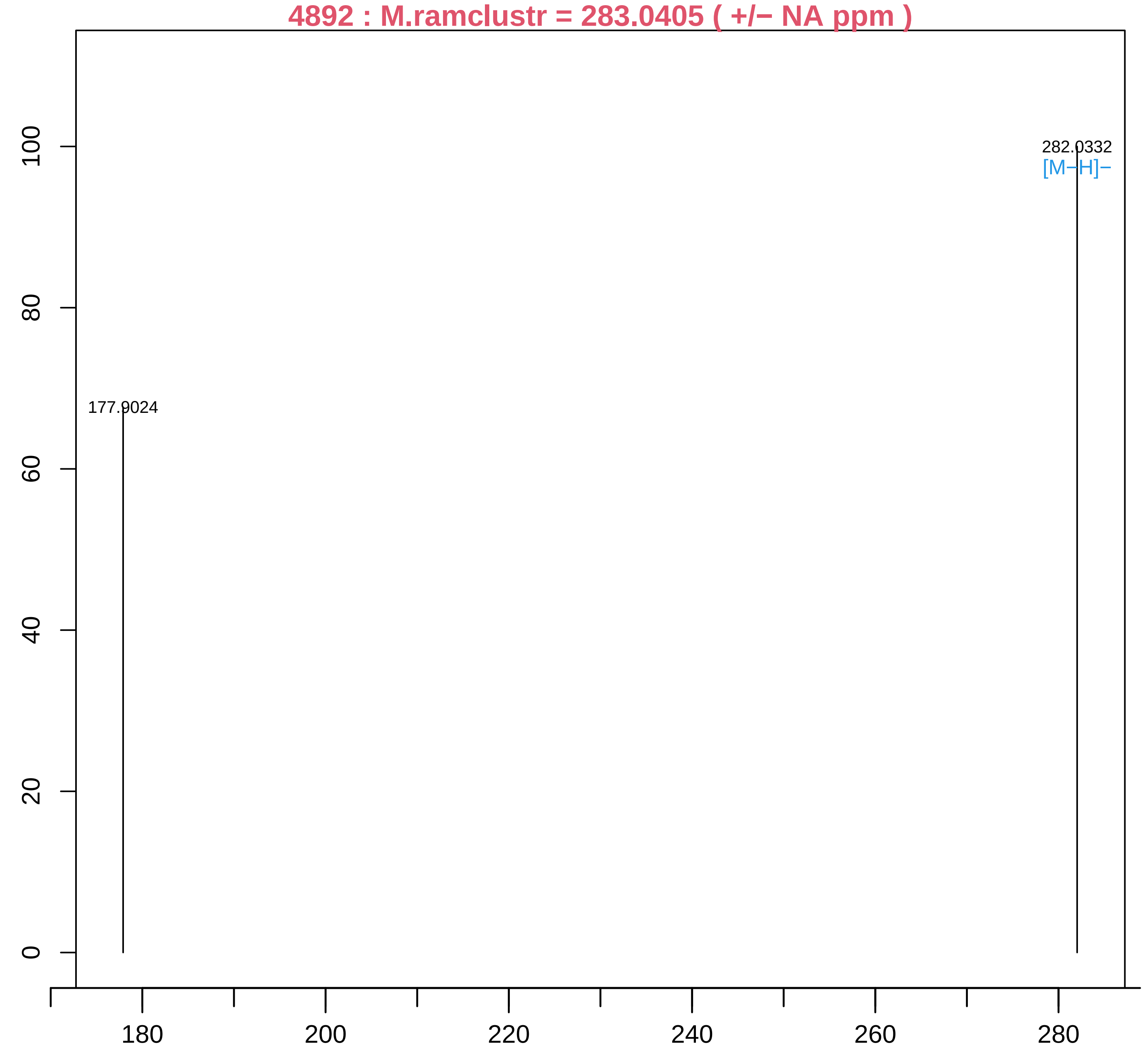
4892 : M.ramclustr = 283.0405 (+/− NA ppm)
282.0332
[M−H]−
177.9024
0
20
40
60
80
100
180
200
220
240
260
280

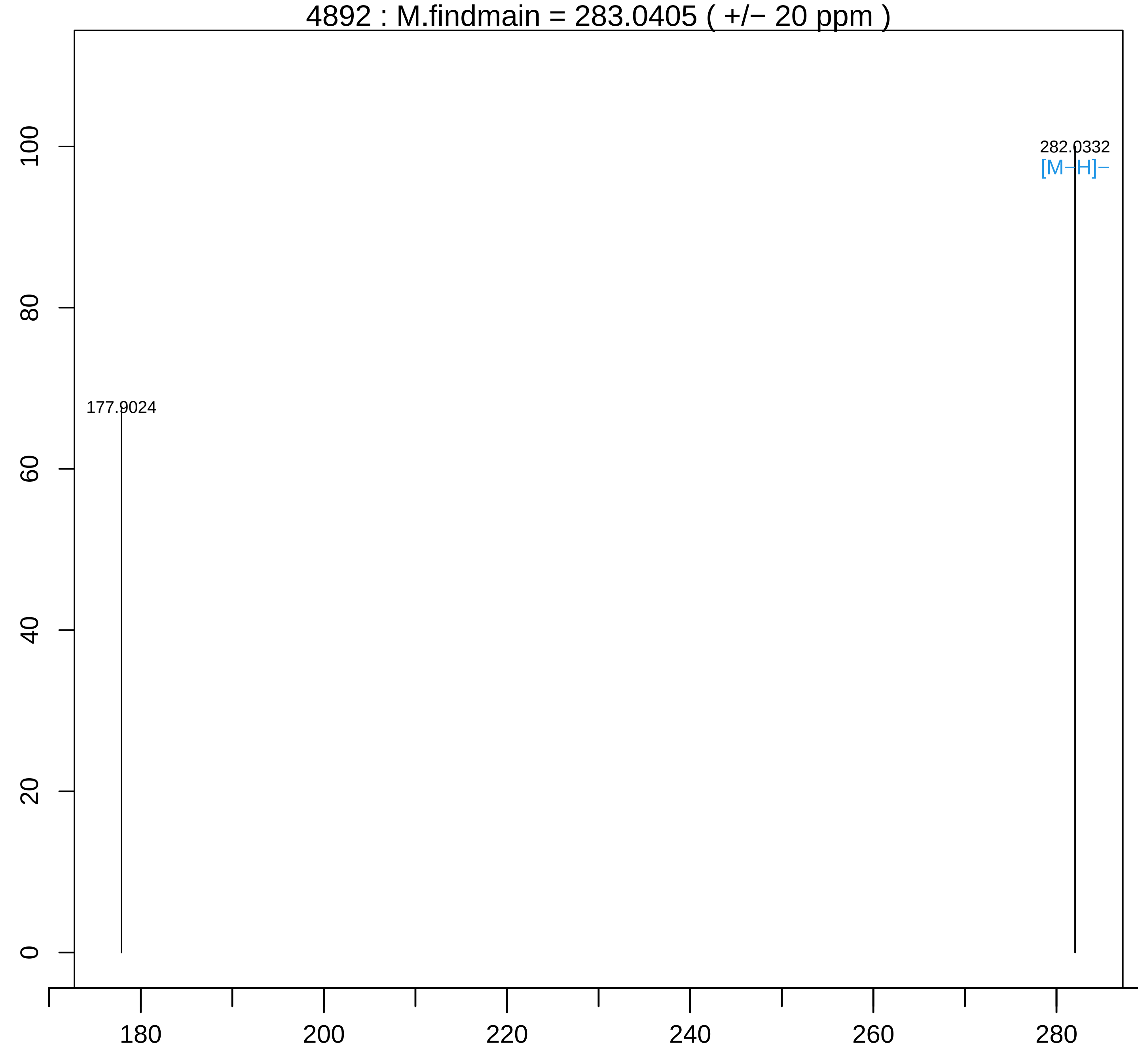
4892 : M.findmain = 283.0405 (+/− 20 ppm)
282.0332
[M−H]−
177.9024
0
20
40
60
80
100
180
200
220
240
260
280

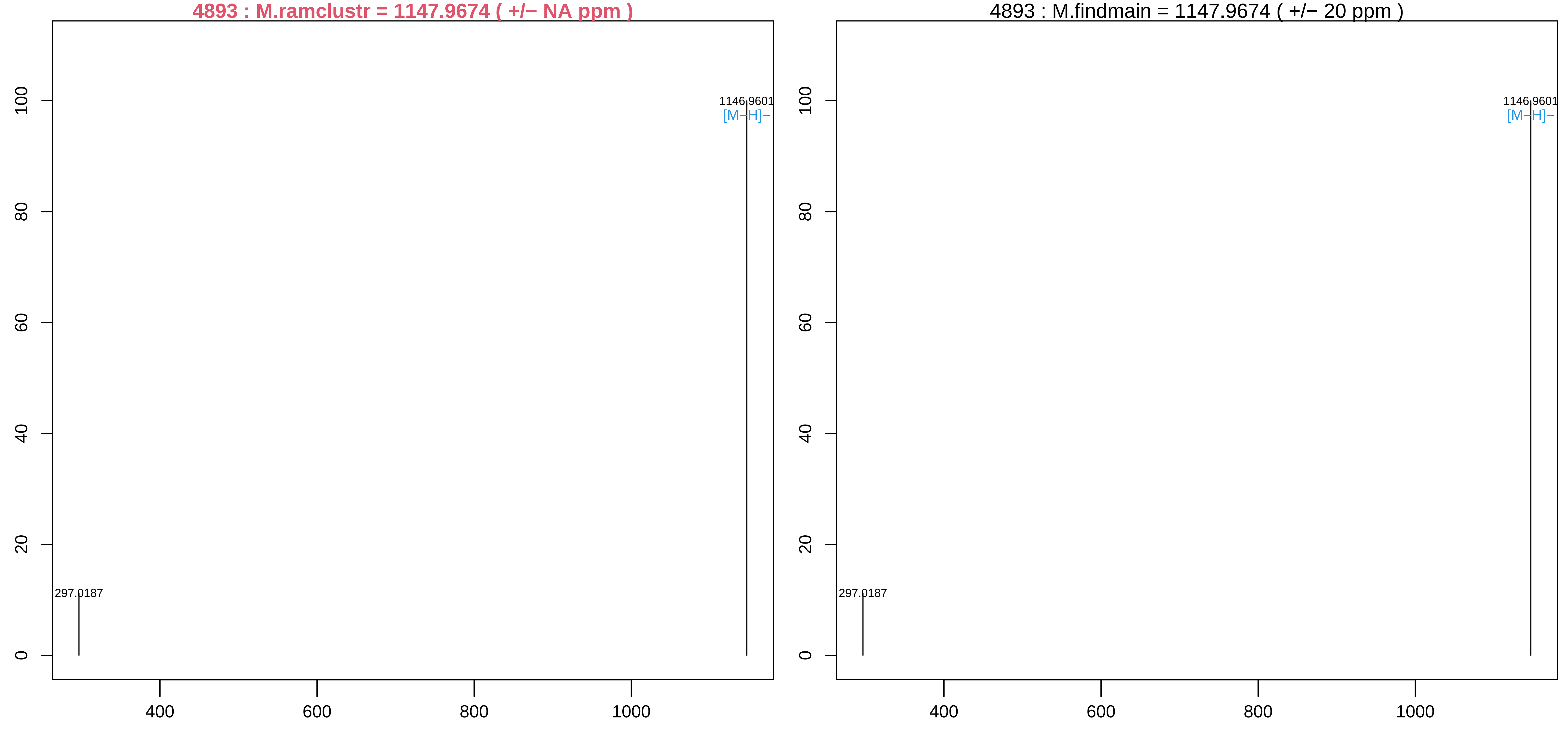
4893 : M.ramclustr = 1147.9674 (+/− NA ppm)
1146.9601
[M−H]−
297.0187
0
20
40
60
80
100
400
600
800
1000
4893 : M.findmain = 1147.9674 (+/− 20 ppm)
1146.9601
[M−H]−
297.0187
0
20
40
60
80
100
400
600
800
1000

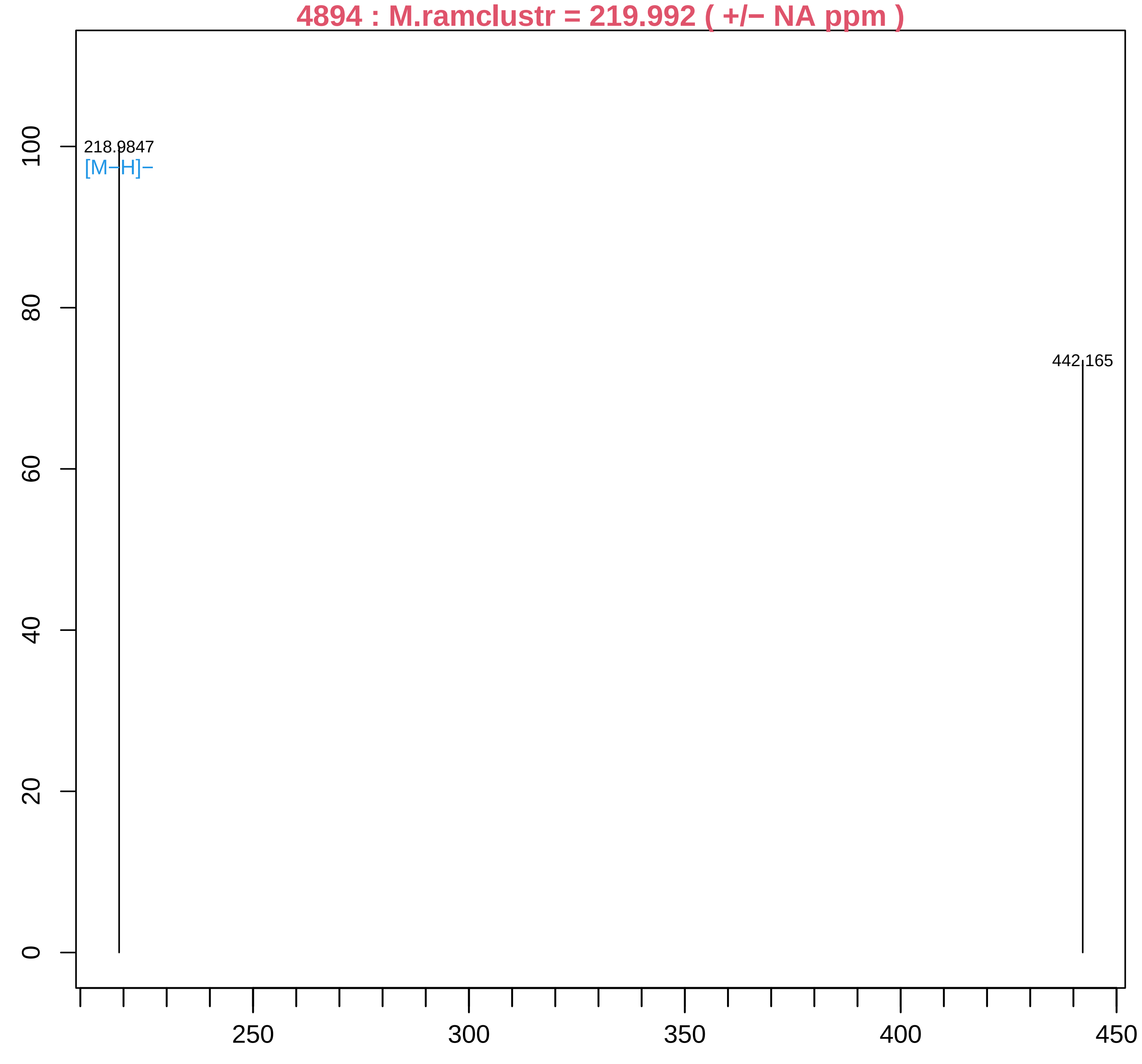
4894 : M.ramclustr = 219.992 (+/− NA ppm)
218.9847
[M−H]−
442.165
0
20
40
60
80
100
250
300
350
400
450

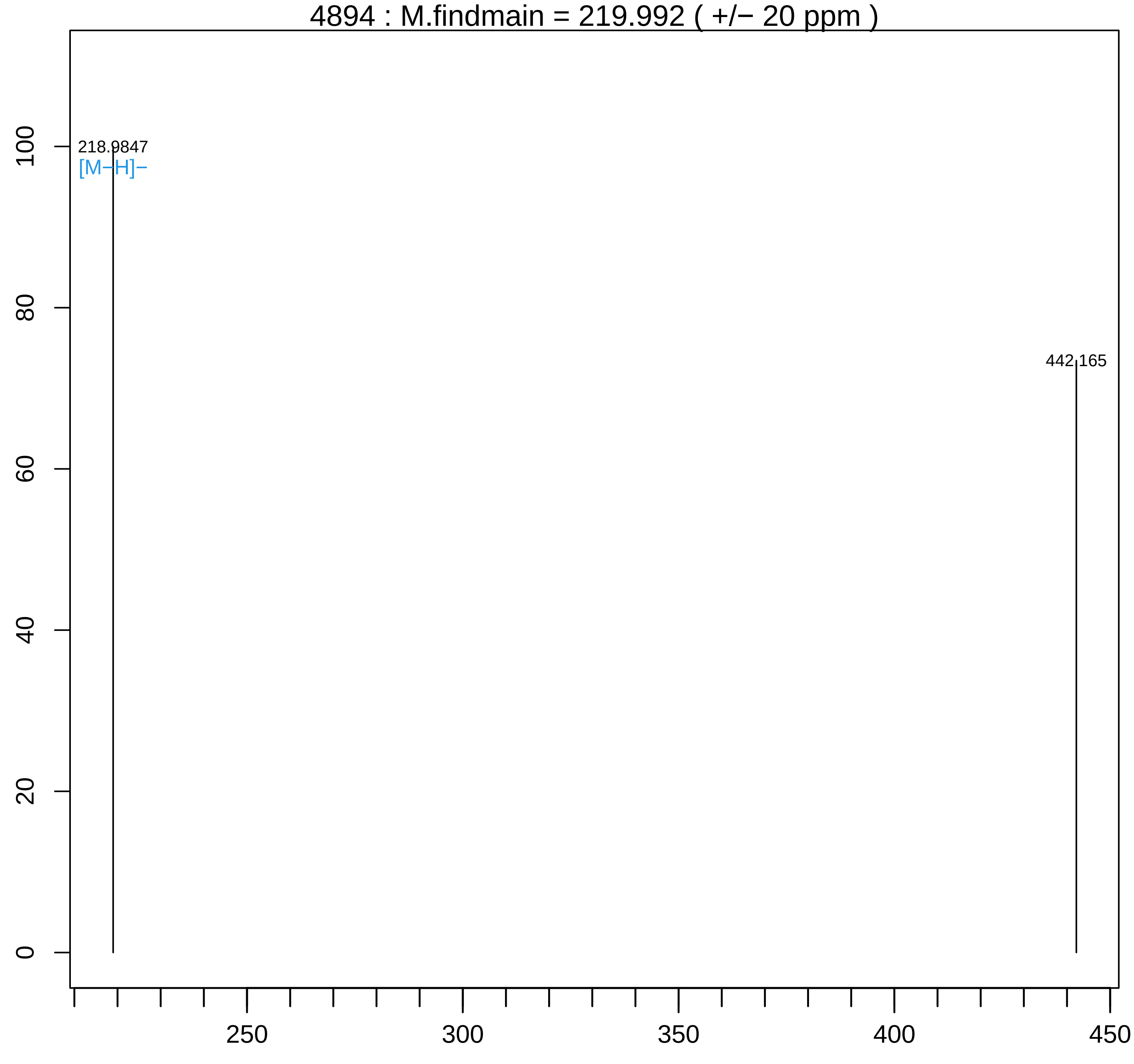
4894 : M.findmain = 219.992 (+/− 20 ppm)
218.9847
[M−H]−
442.165
0
20
40
60
80
100
250
300
350
400
450

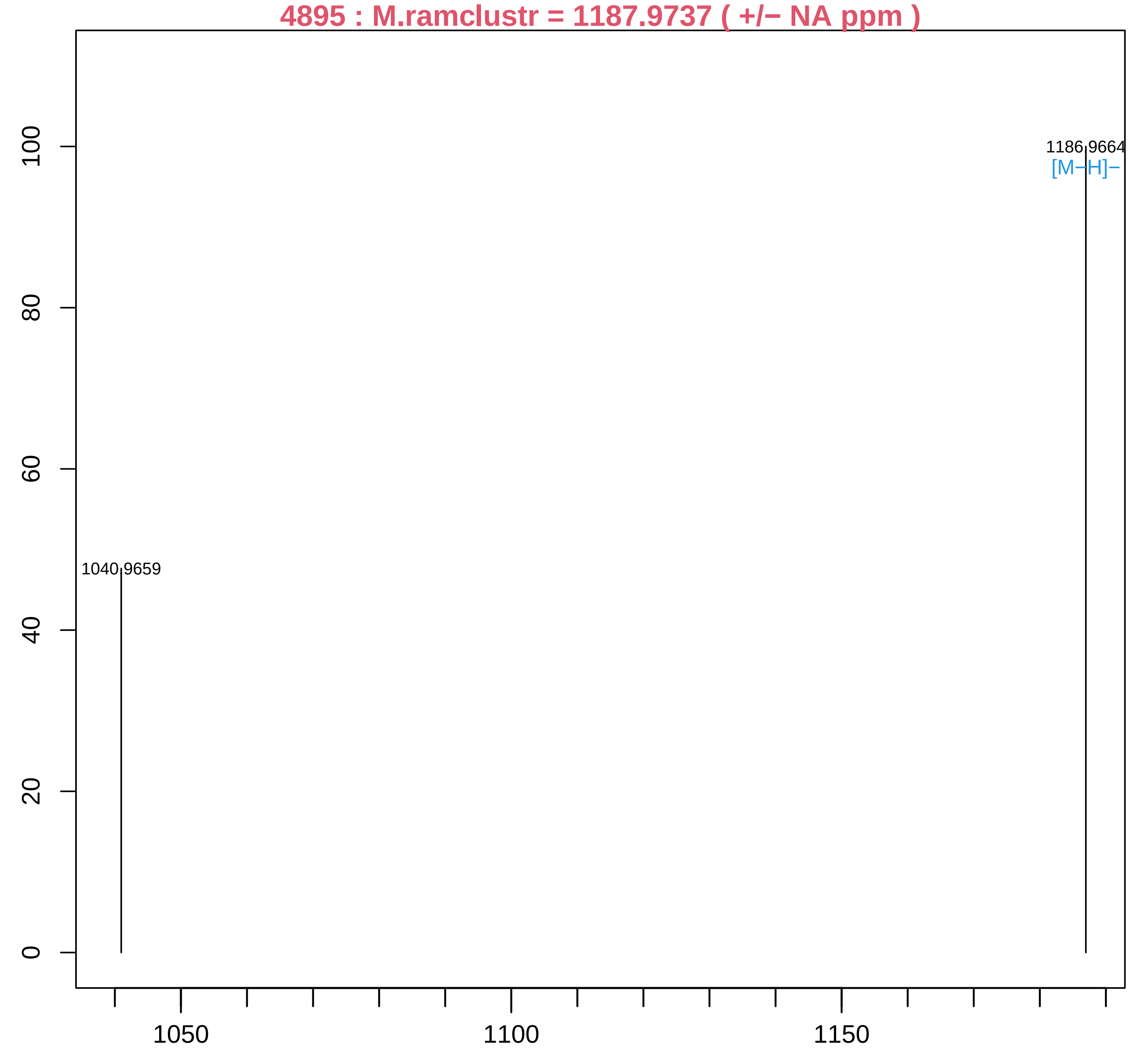
4895 : M.ramclustr = 1187.9737 (+/− NA ppm)
1186.9664
[M−H]−
1040.9659
0
20
40
60
80
100
1050
1100
1150

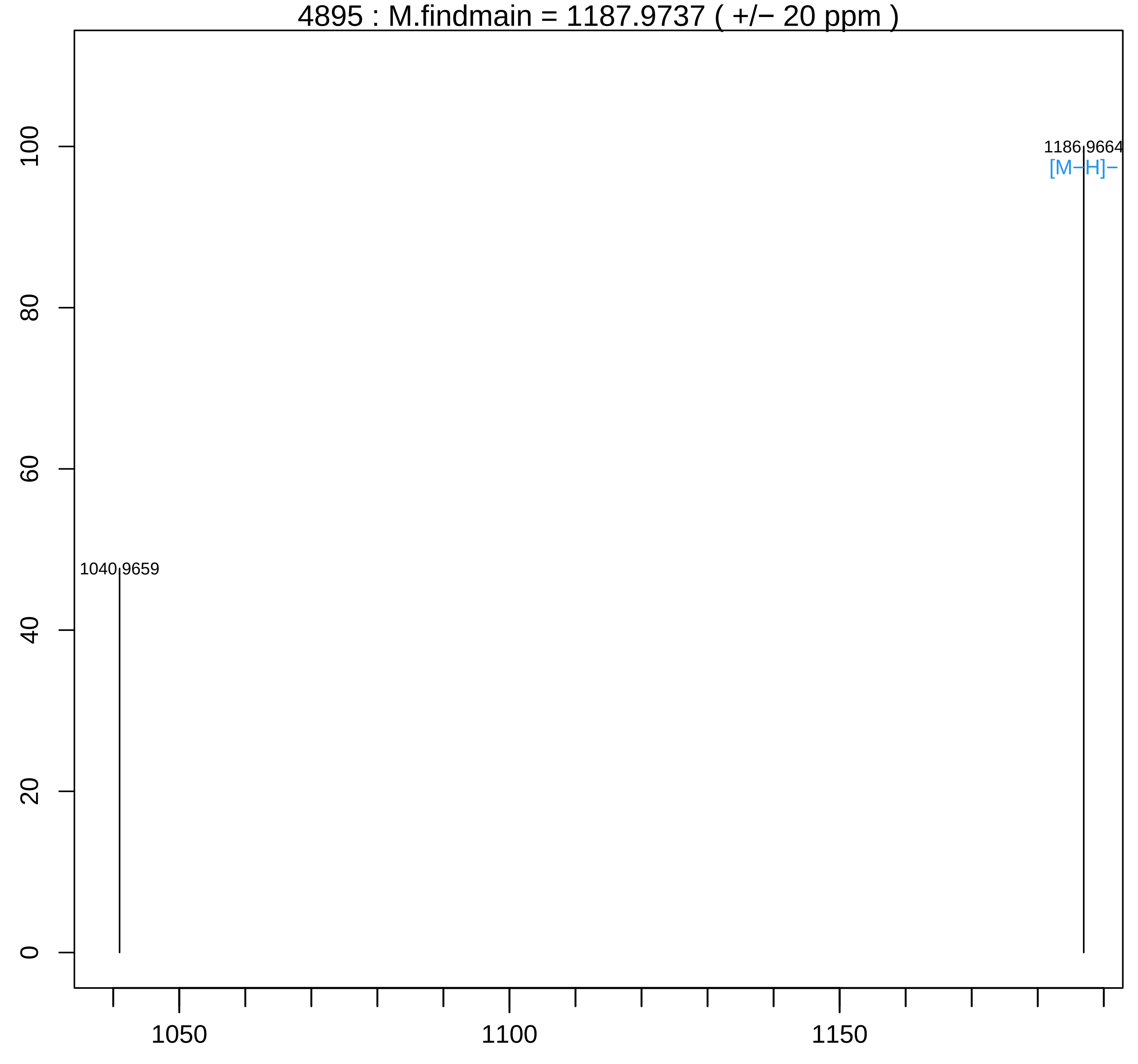
4895 : M.findmain = 1187.9737 (+/− 20 ppm)
1186.9664
[M−H]−
1040.9659
0
20
40
60
80
100
1050
1100
1150

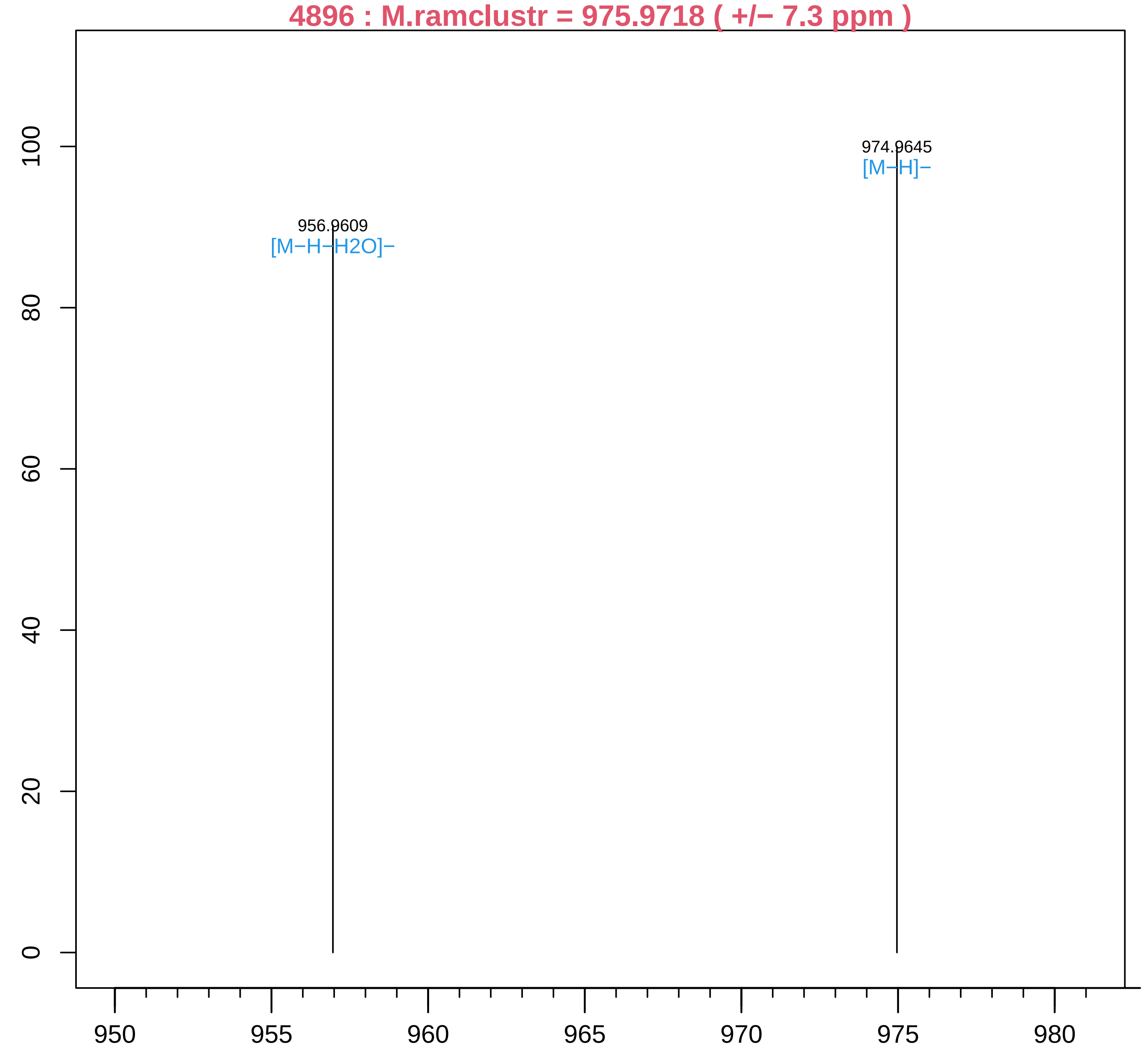
4896 : M.ramclustr = 975.9718 (+/− 7.3 ppm)
974.9645
[M−H]−
956.9609
[M−H−H2O]−
0
20
40
60
80
100
950
955
960
965
970
975
980
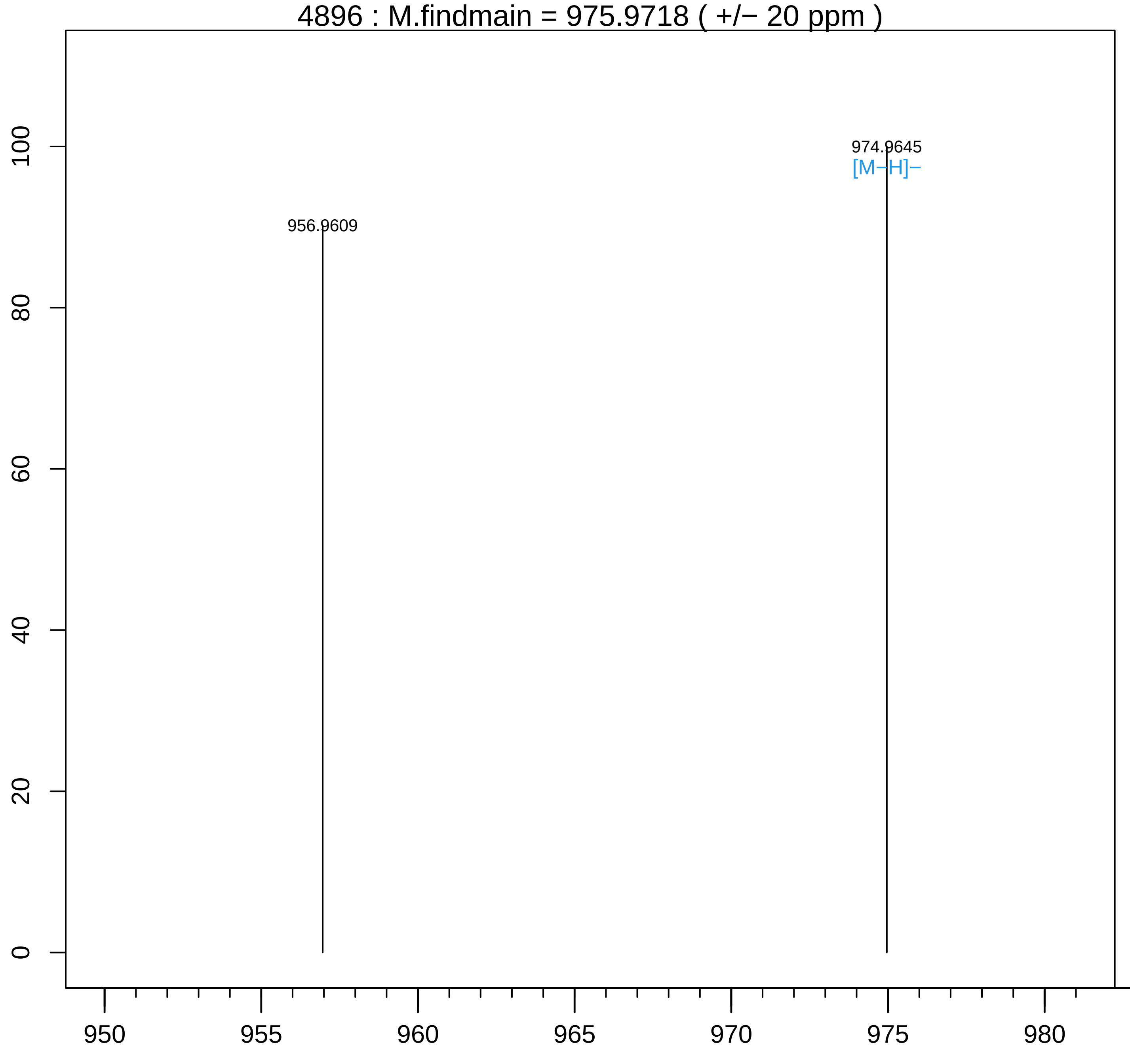
4896 : M.findmain = 975.9718 (+/− 20 ppm)
974.9645
[M−H]−
956.9609
0
20
40
60
80
100
950
955
960
965
970
975
980

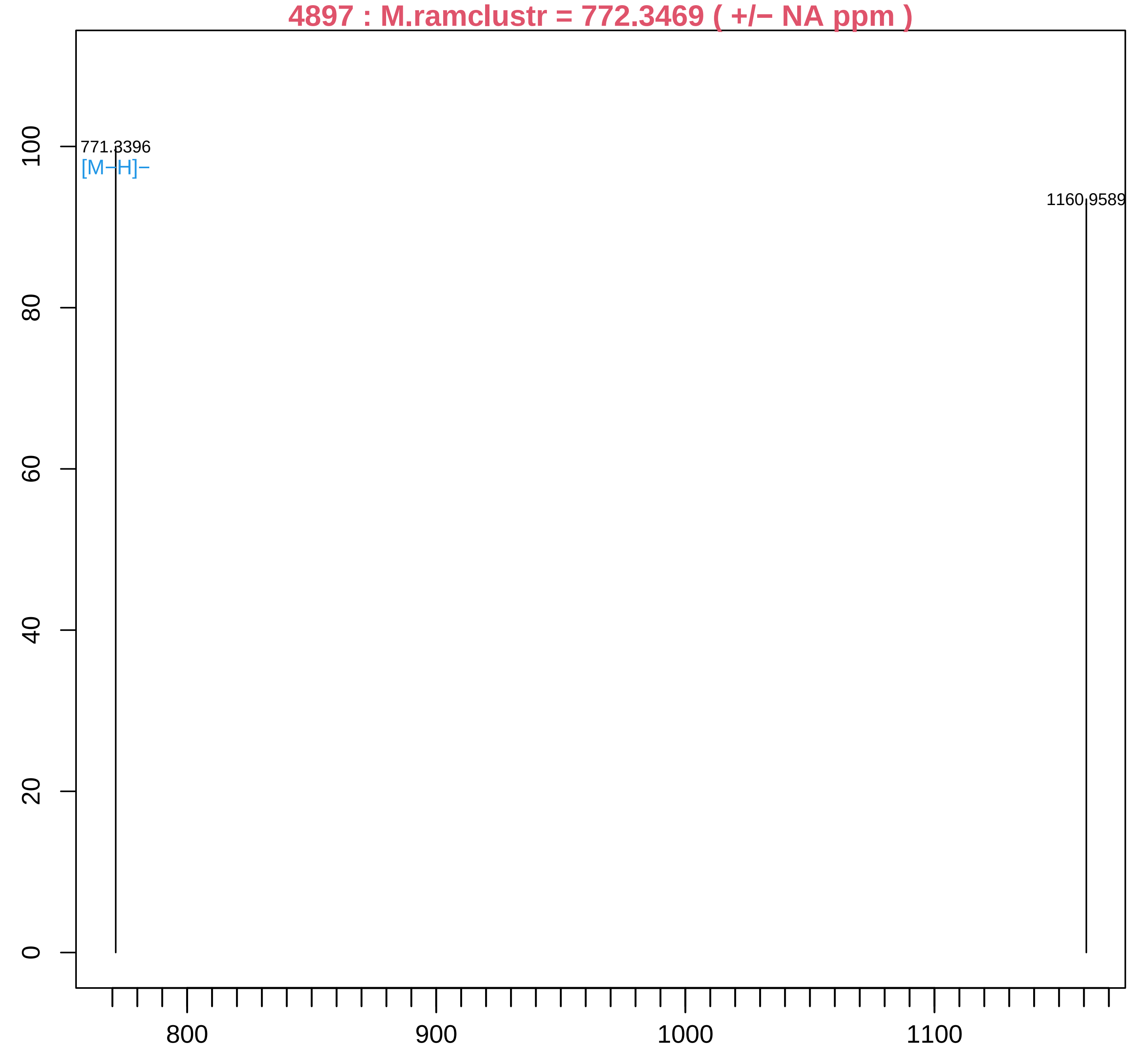
4897 : M.ramclustr = 772.3469 (+/− NA ppm)
771.3396
[M−H]−
1160.9589
0
20
40
60
80
100
800
900
1000
1100

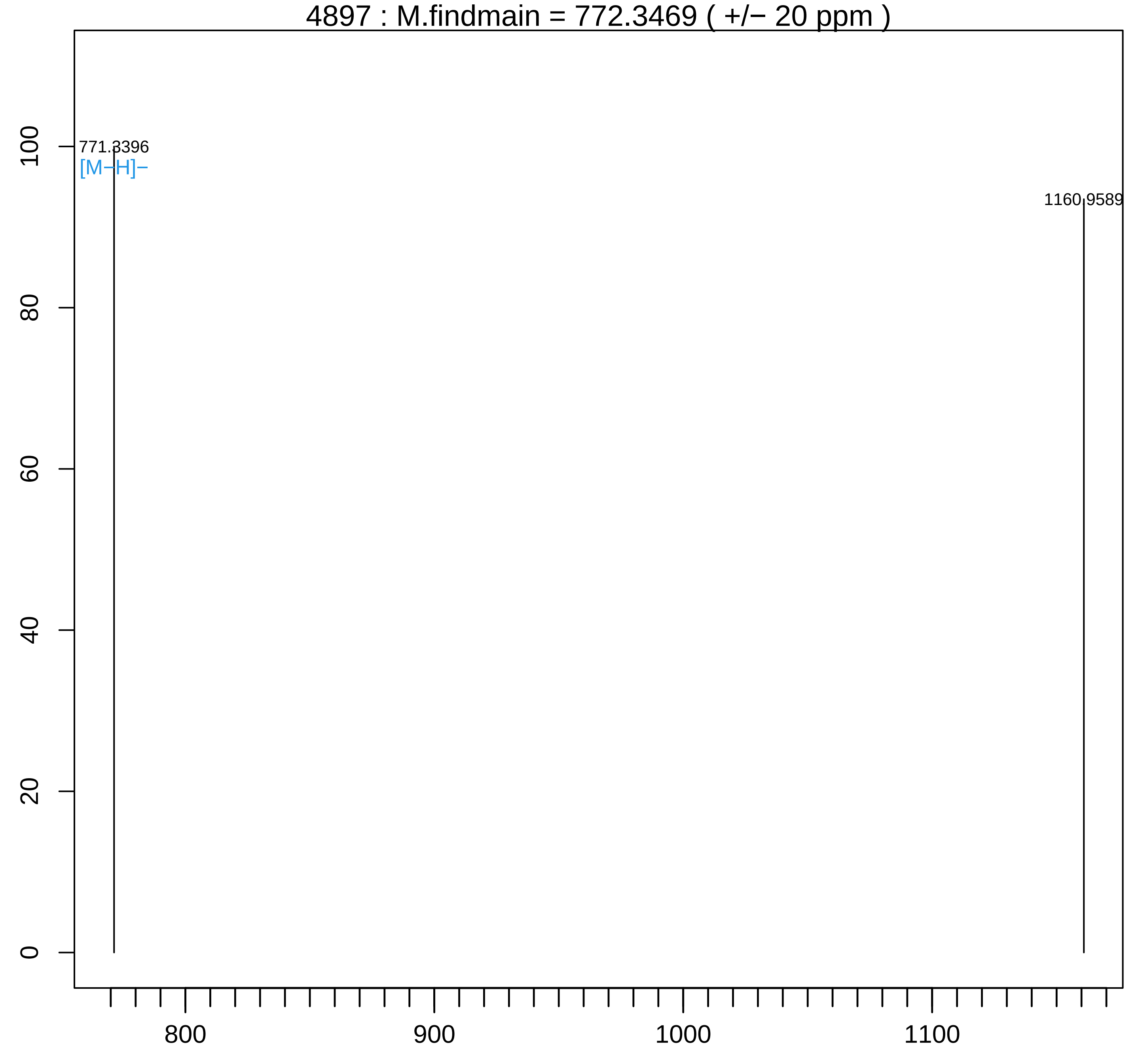
4897 : M.findmain = 772.3469 (+/− 20 ppm)
771.3396
[M−H]−
1160.9589
0
20
40
60
80
100
800
900
1000
1100

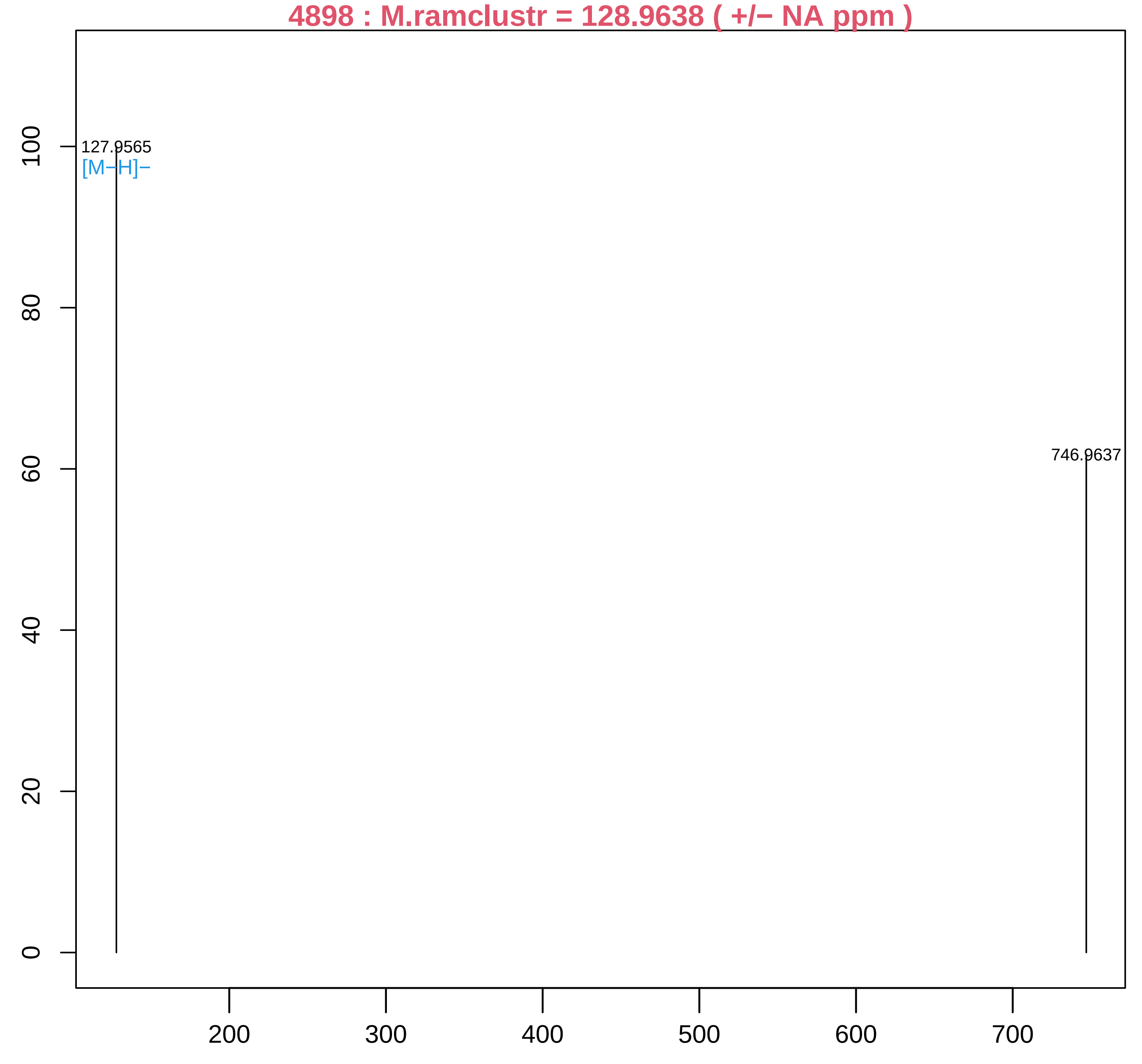

4898 : M.ramclustr = 128.9638 (+/− NA ppm)
127.9565
[M−H]−
746.9637
0
20
40
60
80
100
200
300
400
500
600
700

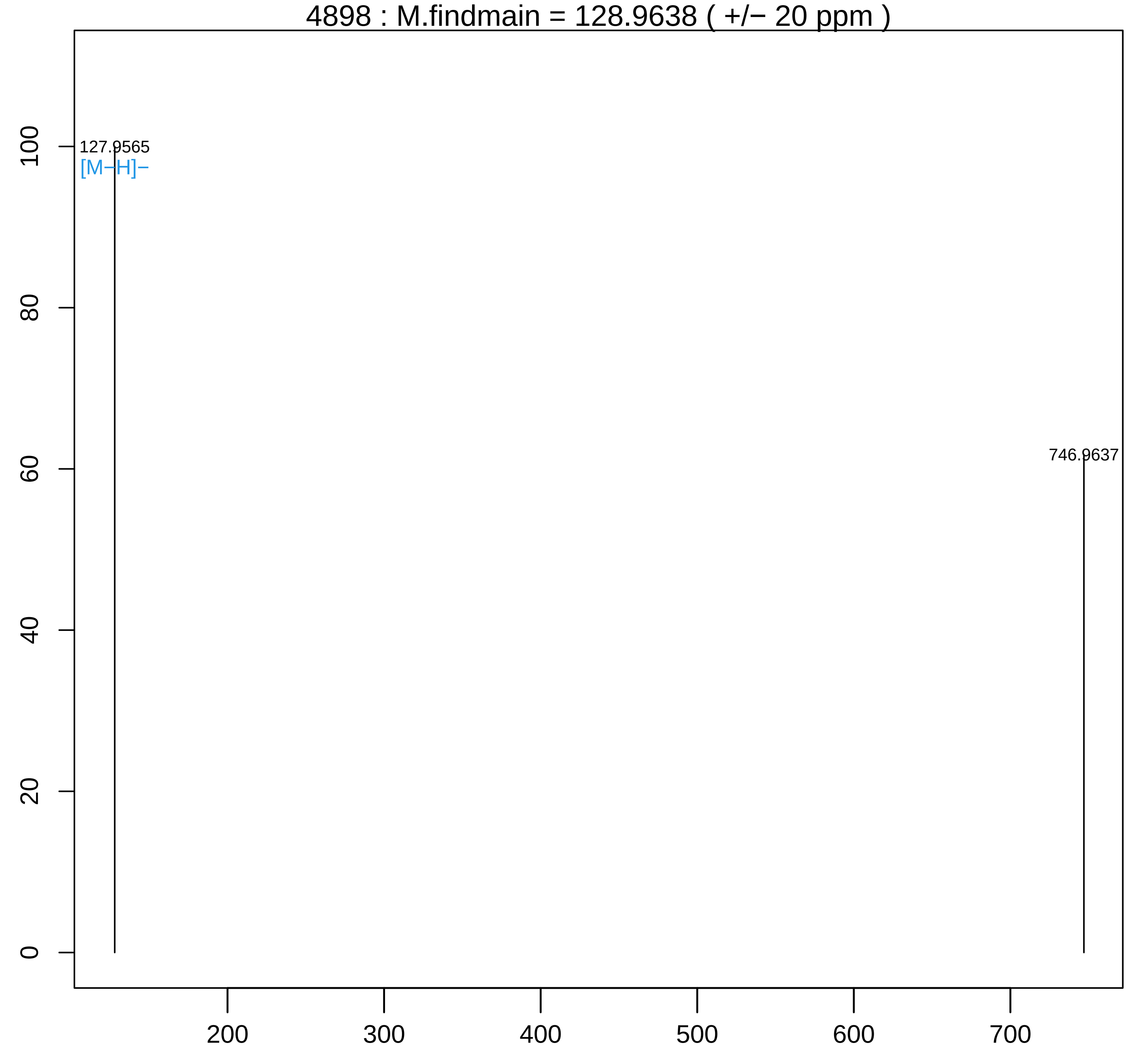

4898 : M.findmain = 128.9638 (+/− 20 ppm)
127.9565
[M−H]−
746.9637
0
20
40
60
80
100
200
300
400
500
600
700

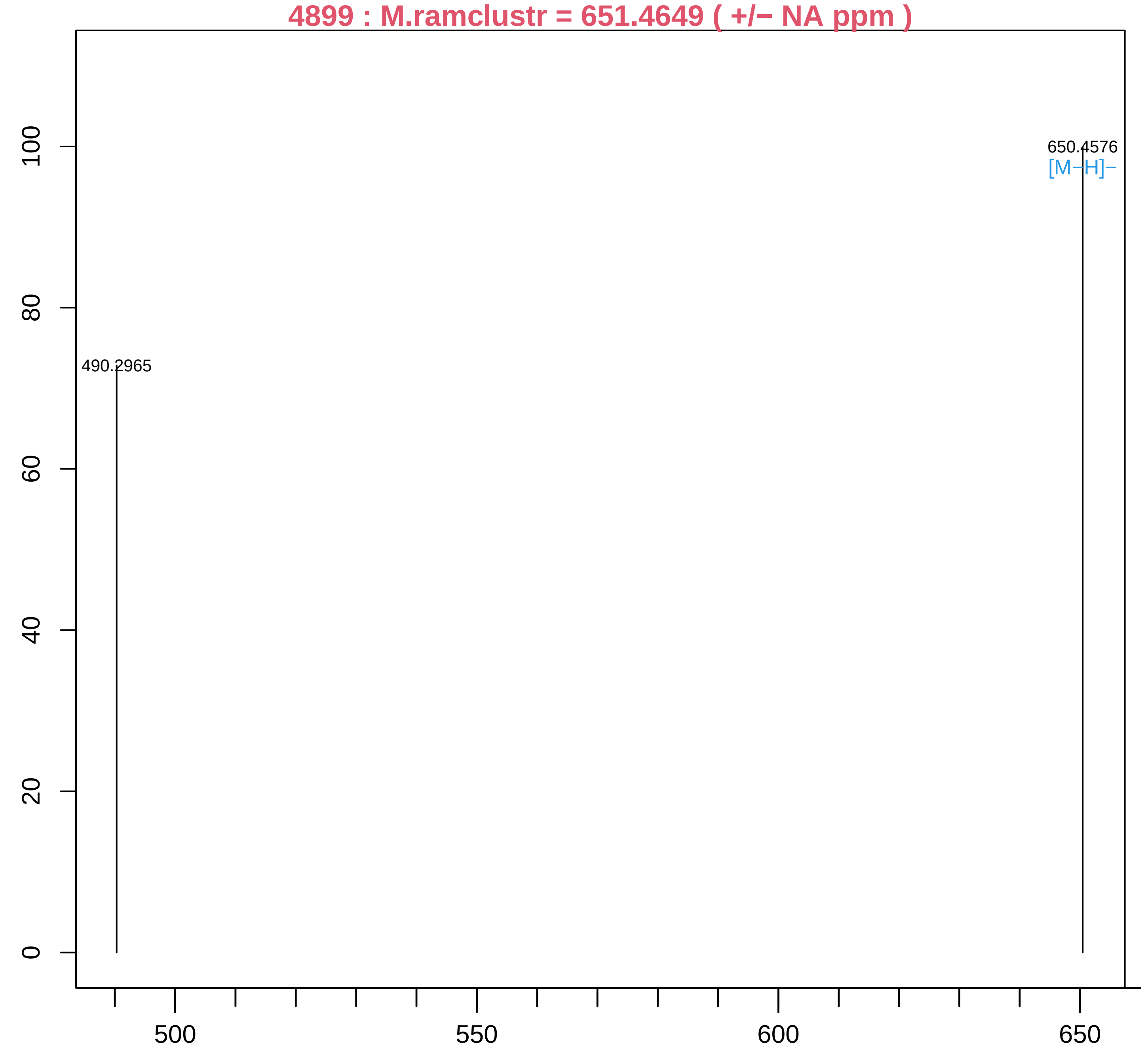

4899 : M.ramclustr = 651.4649 (+/− NA ppm)
650.4576
[M−H]−
490.2965
0
20
40
60
80
100
500
550
600
650

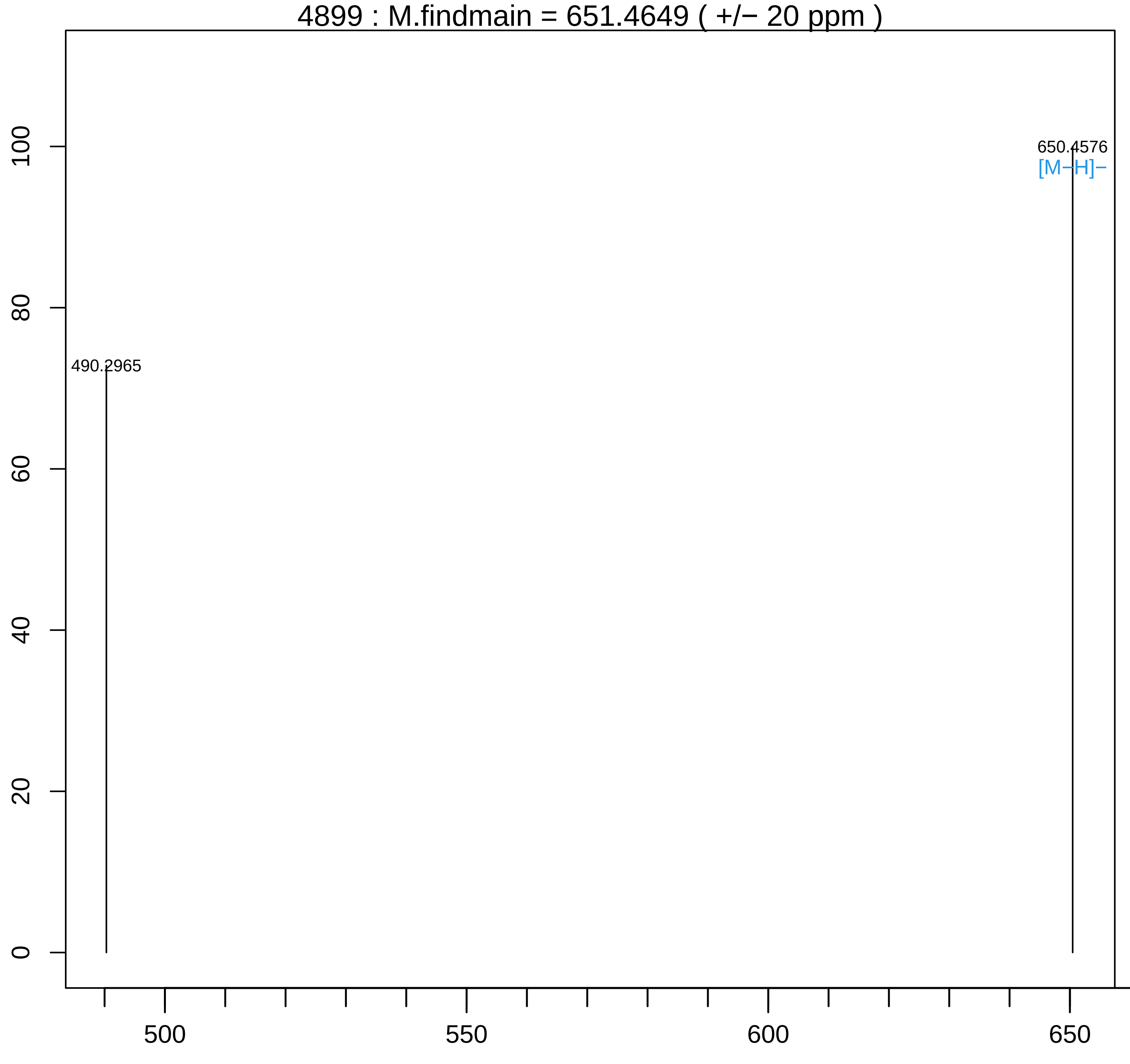

4899 : M.findmain = 651.4649 (+/− 20 ppm)
650.4576
[M−H]−
490.2965
0
20
40
60
80
100
500
550
600
650

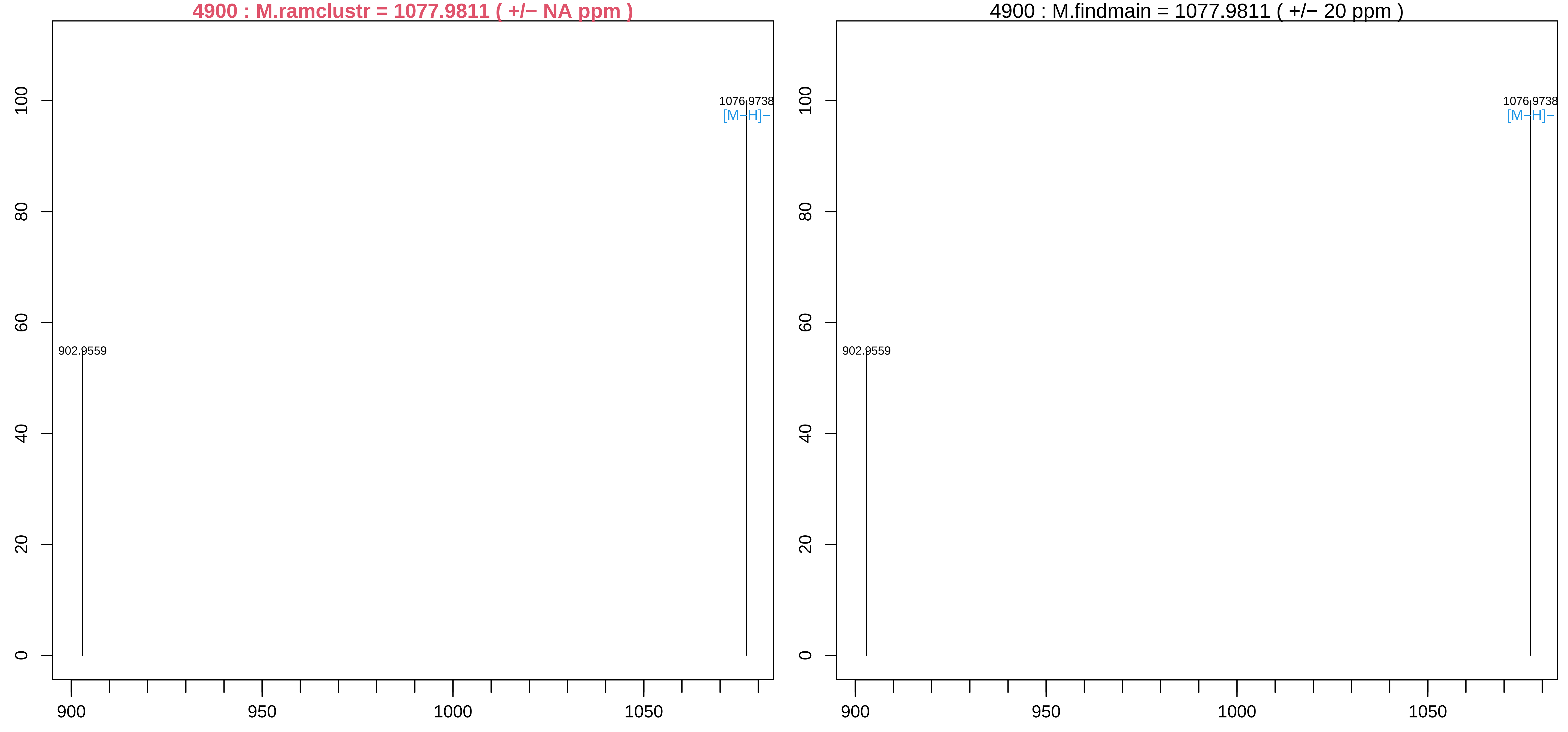
4900 : M.ramclustr = 1077.9811 (+/− NA ppm)
4900 : M.findmain = 1077.9811 (+/− 20 ppm)
1076.9738
[M−H]−
902.9559
0
20
40
60
80
100
900
950
1000
1050

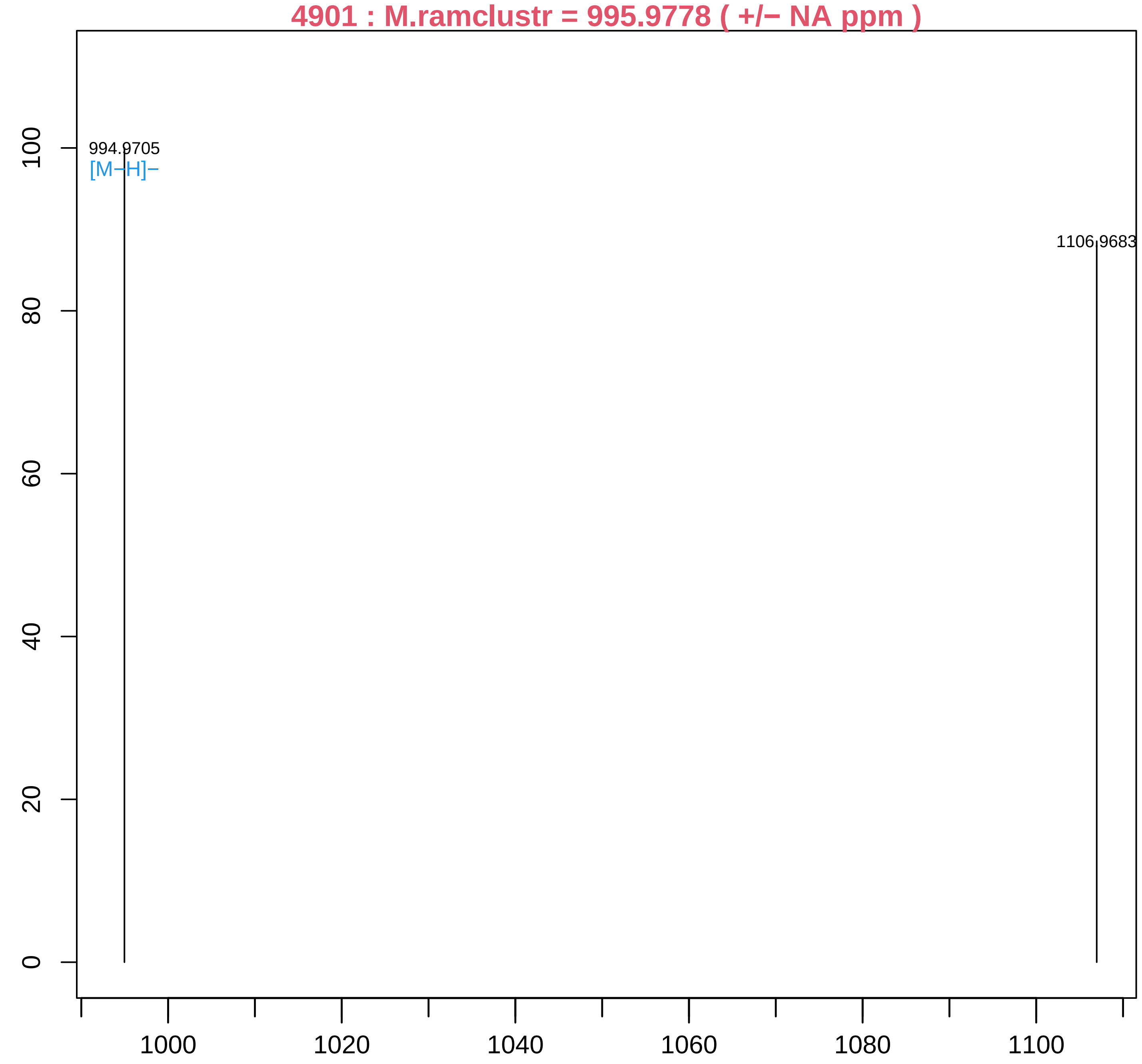
4901 : M.ramclustr = 995.9778 (+/− NA ppm)
994.9705
[M−H]−
1106.9683
0
20
40
60
80
100
1000
1020
1040
1060
1080
1100
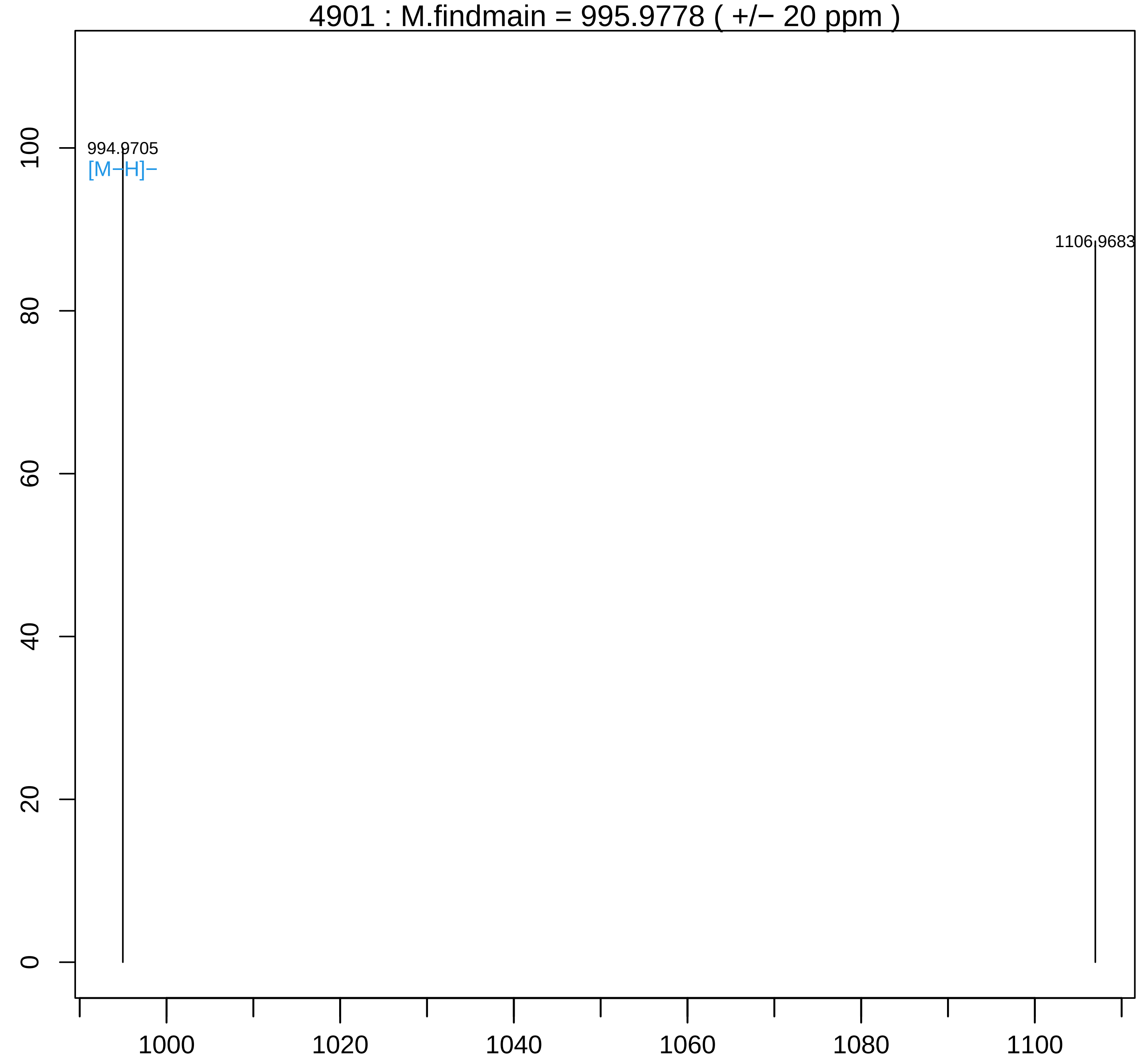
4901 : M.findmain = 995.9778 (+/− 20 ppm)
994.9705
[M−H]−
1106.9683
0
20
40
60
80
100
1000
1020
1040
1060
1080
1100

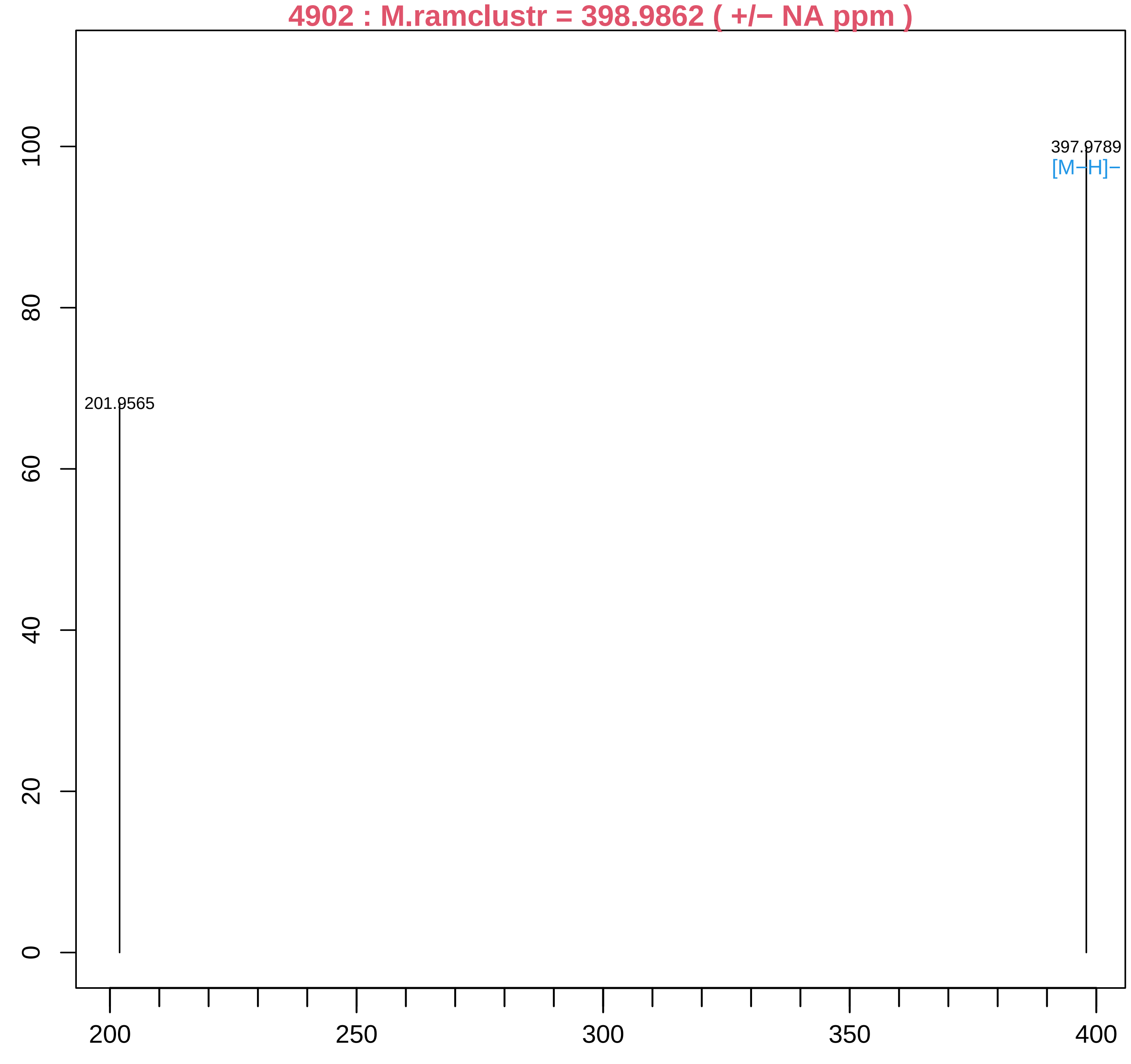
4902 : M.ramclustr = 398.9862 (+/− NA ppm)
397.9789
[M−H]−
201.9565
0
20
40
60
80
100
200
250
300
350
400

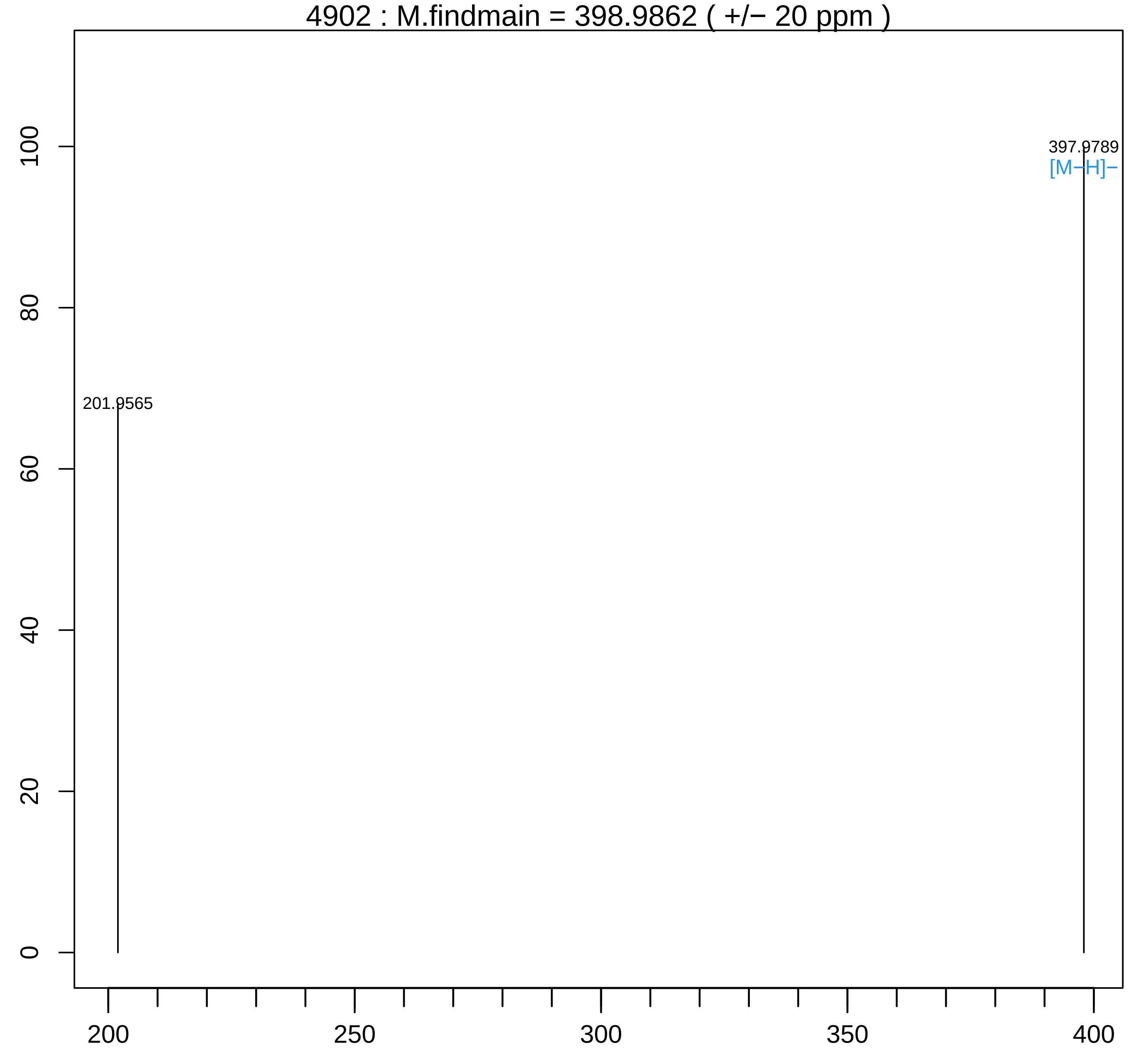
4902 : M.findmain = 398.9862 (+/− 20 ppm)
397.9789
[M−H]−
201.9565
0
20
40
60
80
100
200
250
300
350
400

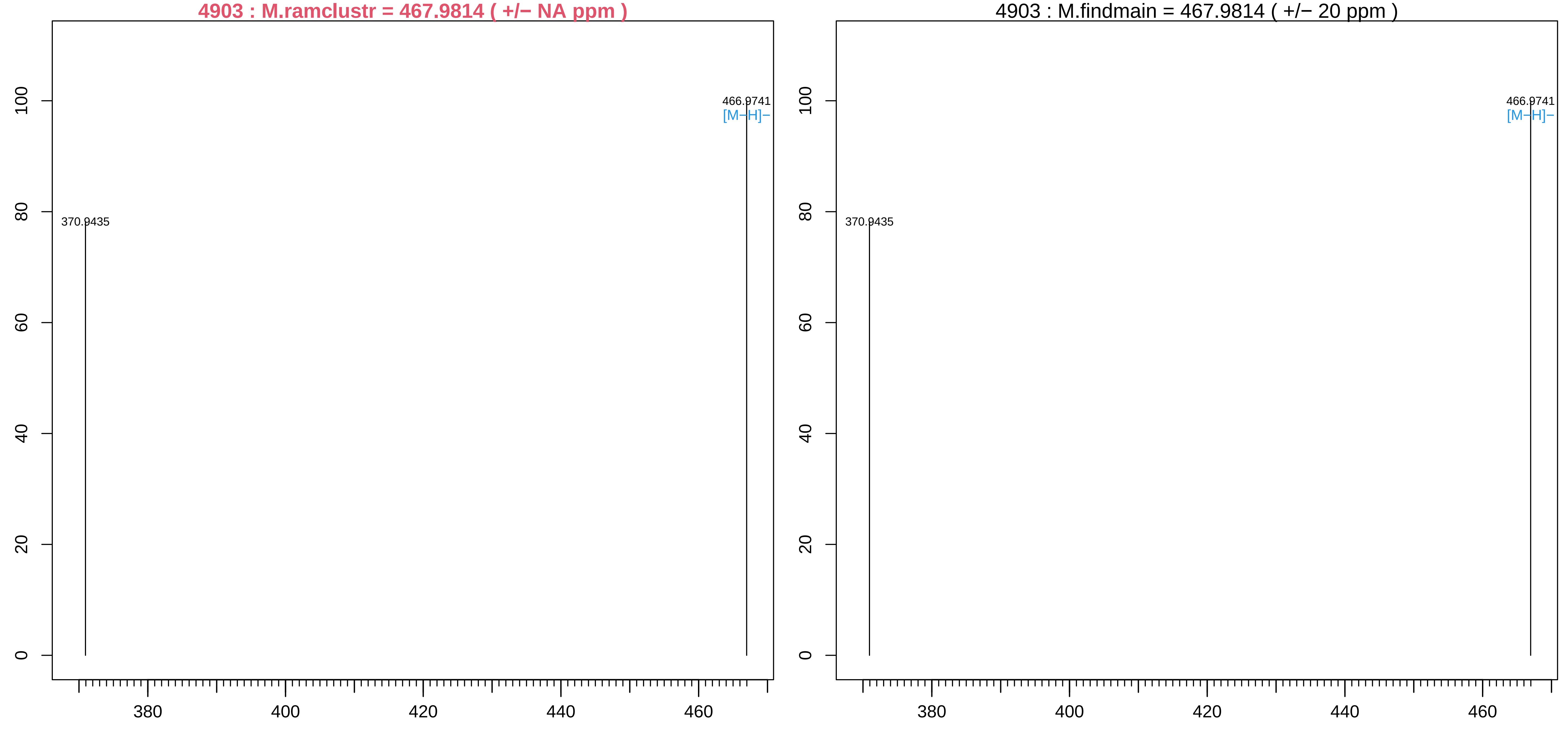
4903 : M.ramclustr = 467.9814 (+/− NA ppm)
4903 : M.findmain = 467.9814 (+/− 20 ppm)
370.9435
466.9741
[M−H]−
0
20
40
60
80
100
380
400
420
440
460

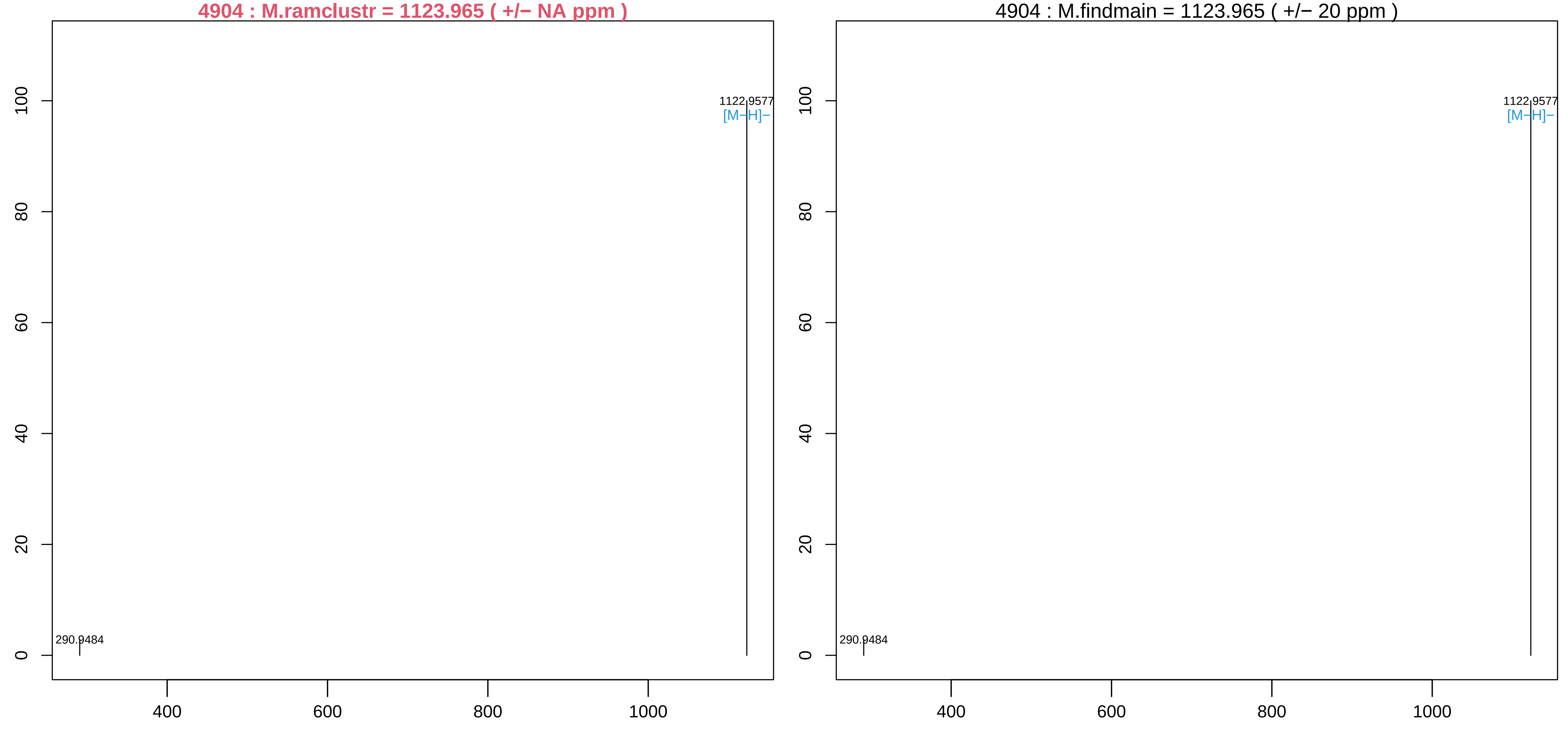

4904 : M.ramclustr = 1123.965 (+/− NA ppm)
1122.9577
[M−H]−
290.9484
0
20
40
60
80
100
400
600
800
1000
4904 : M.findmain = 1123.965 (+/− 20 ppm)
1122.9577
[M−H]−
290.9484
0
20
40
60
80
100
400
600
800
1000

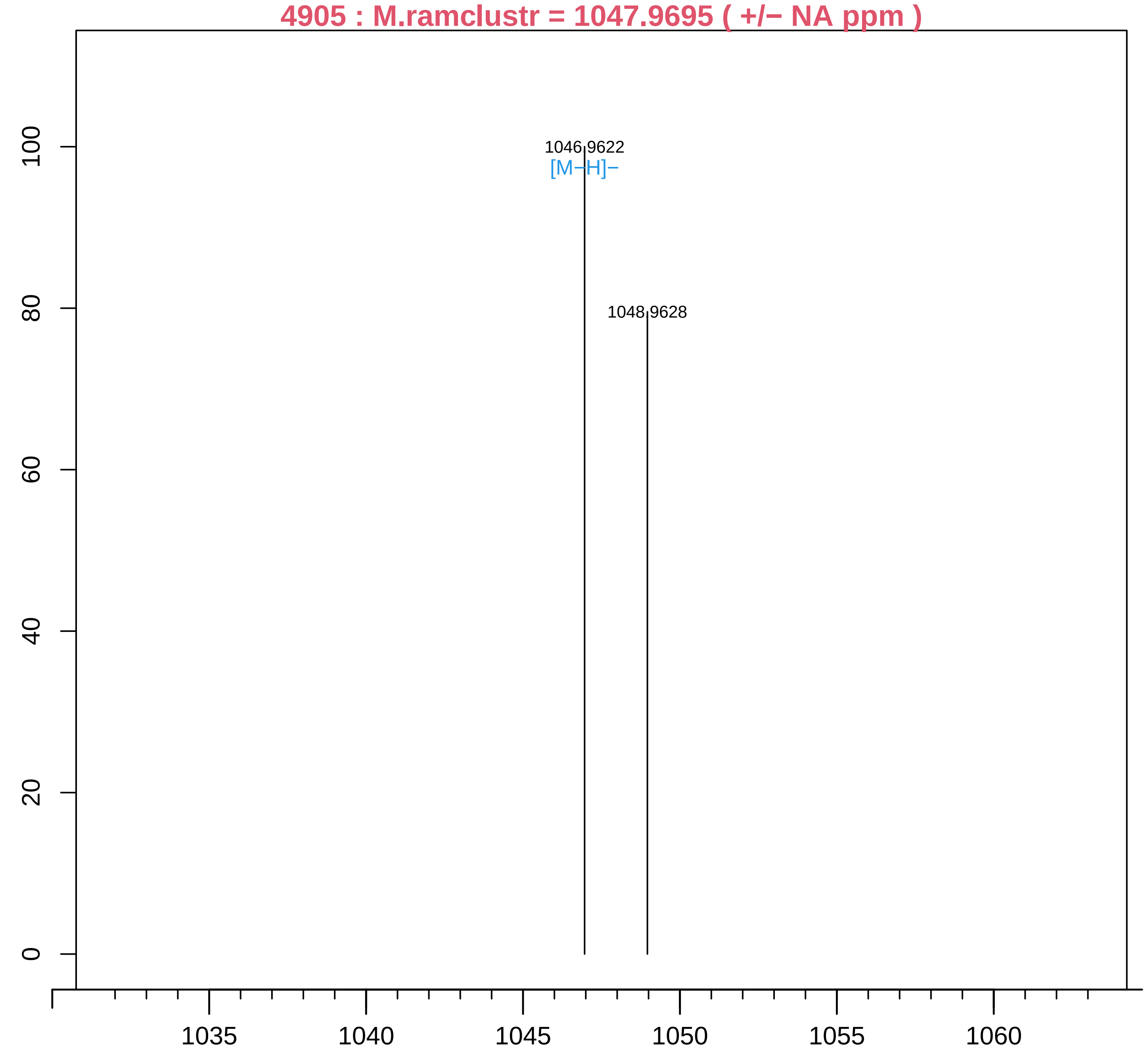
4905 : M.ramclustr = 1047.9695 (+/− NA ppm)
1046.9622
[M−H]−
1048.9628
0
20
40
60
80
100
1035
1040
1045
1050
1055
1060

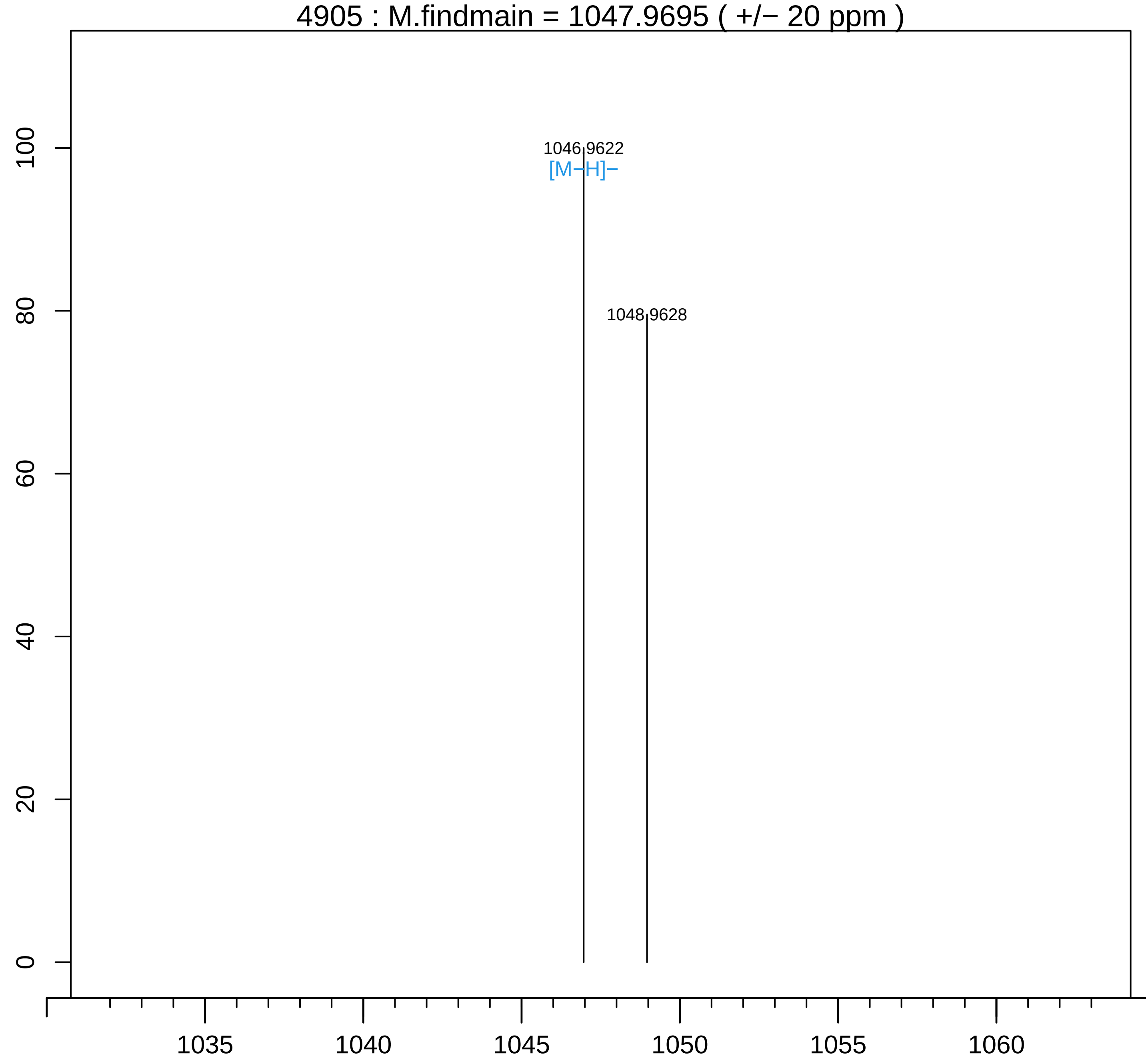
4905 : M.findmain = 1047.9695 (+/− 20 ppm)
1046.9622
[M−H]−
1048.9628
0
20
40
60
80
100
1035
1040
1045
1050
1055
1060

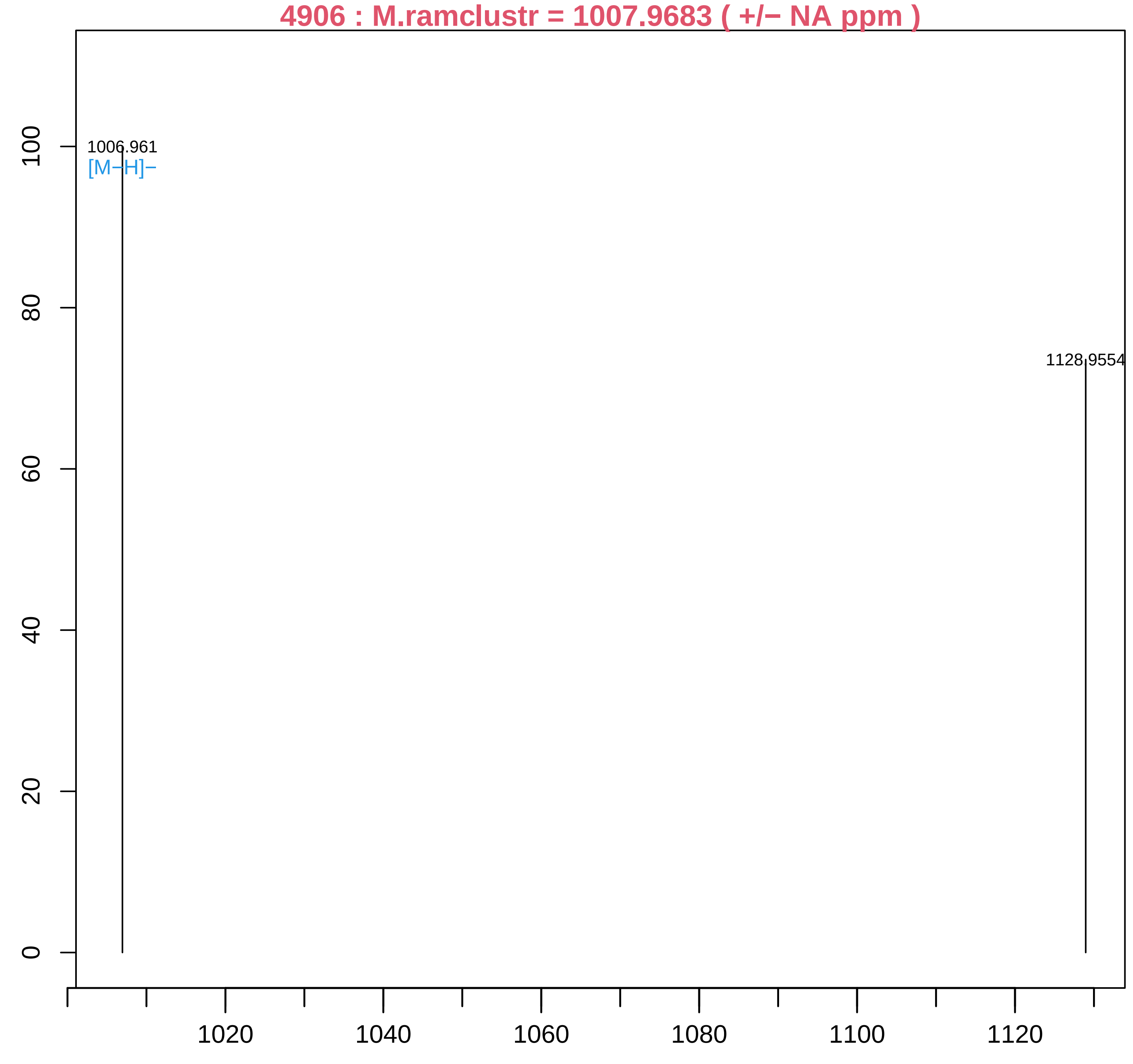
4906 : M.ramclustr = 1007.9683 (+/− NA ppm)
1006.961
[M−H]−
1128.9554
0
20
40
60
80
100
1020
1040
1060
1080
1100
1120

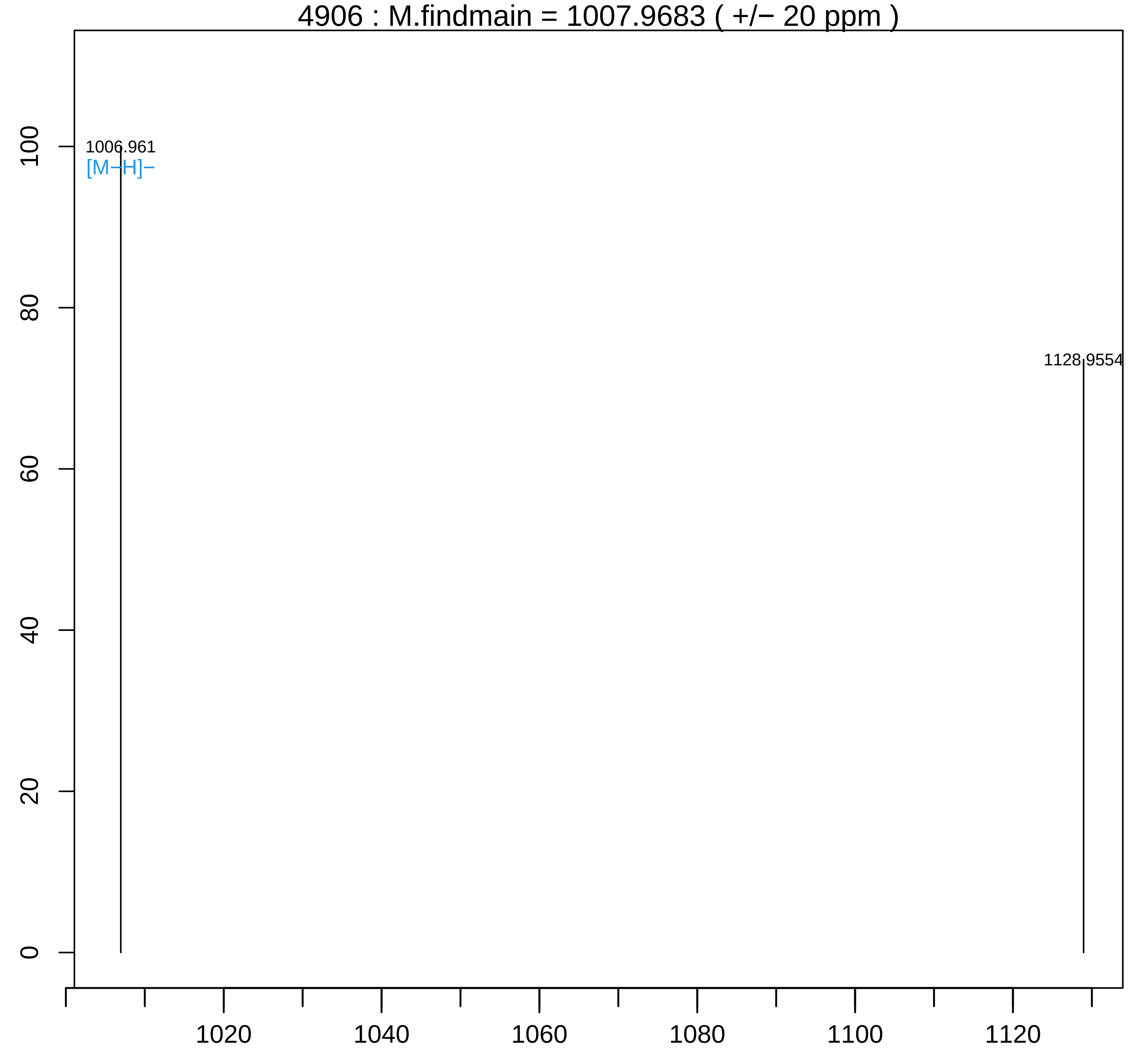
4906 : M.findmain = 1007.9683 (+/− 20 ppm)
1006.961
[M−H]−
1128.9554
0
20
40
60
80
100
1020
1040
1060
1080
1100
1120

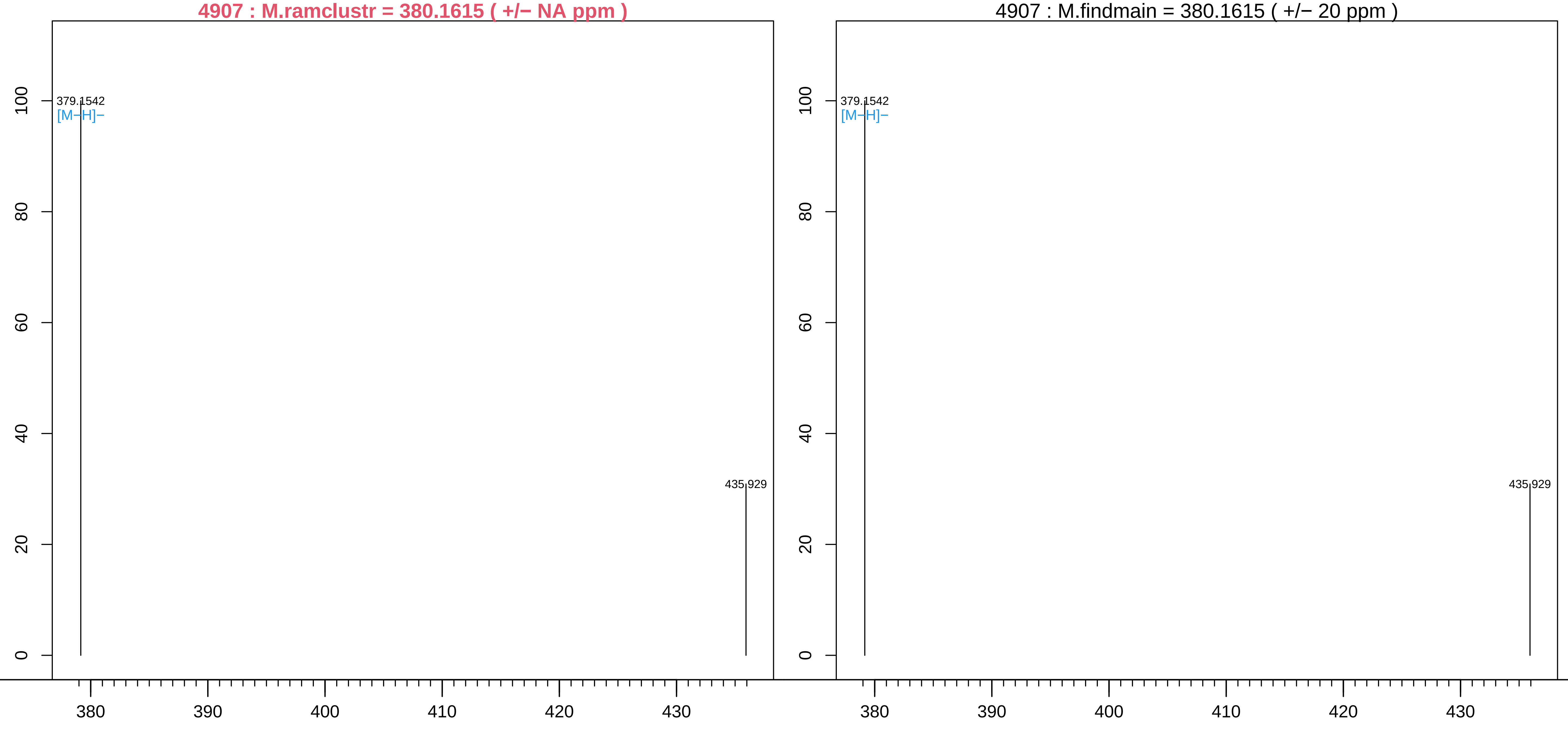
4907 : M.ramclustr = 380.1615 (+/− NA ppm)
379.1542
[M−H]−
435.929
4907 : M.findmain = 380.1615 (+/− 20 ppm)
379.1542
[M−H]−
435.929
0
20
40
60
80
100
380
390
400
410
420
430

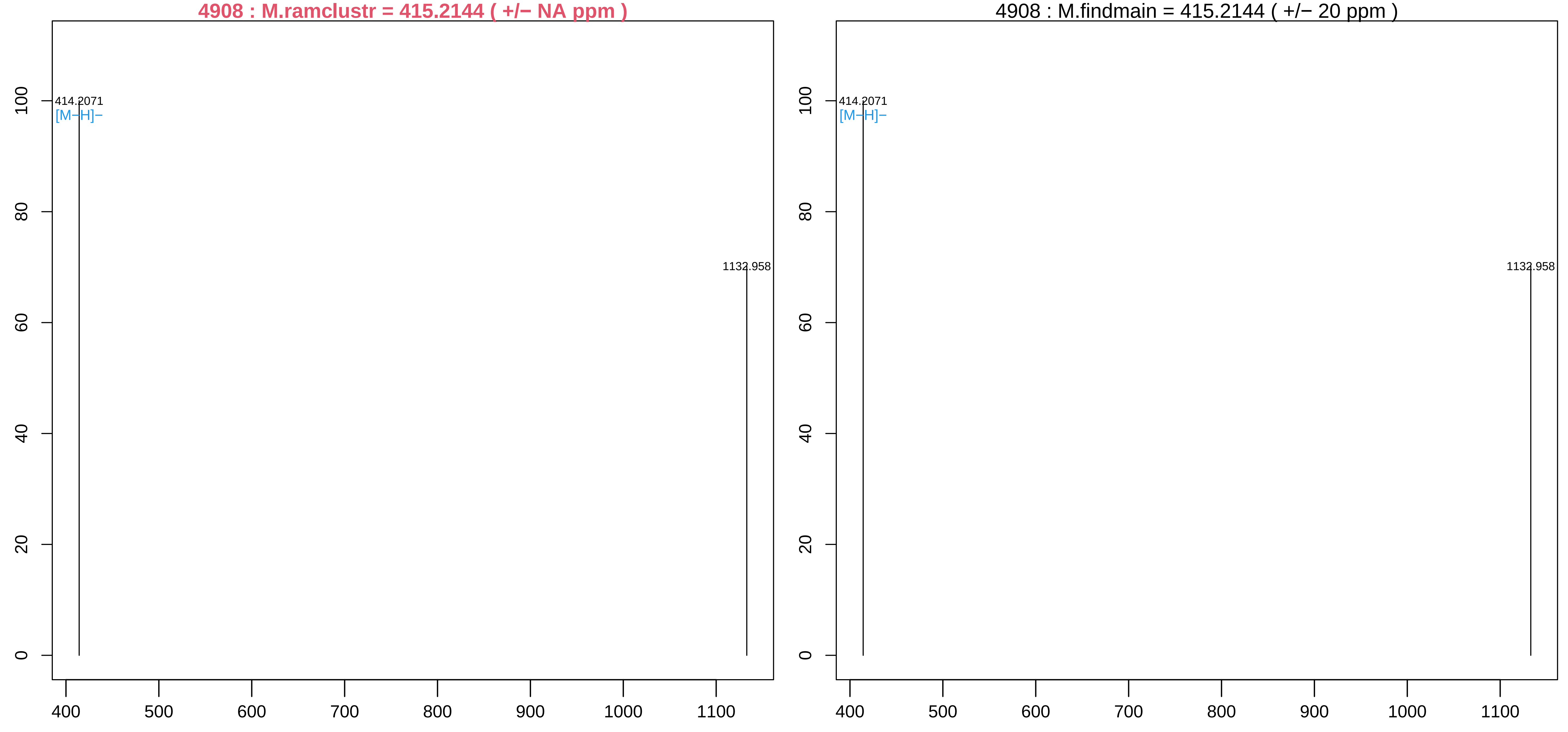
4908 : M.ramclustr = 415.2144 (+/− NA ppm)
414.2071
[M−H]−
1132.958
4908 : M.findmain = 415.2144 (+/− 20 ppm)
414.2071
[M−H]−
1132.958
0
20
40
60
80
100
400
500
600
700
800
900
1000
1100

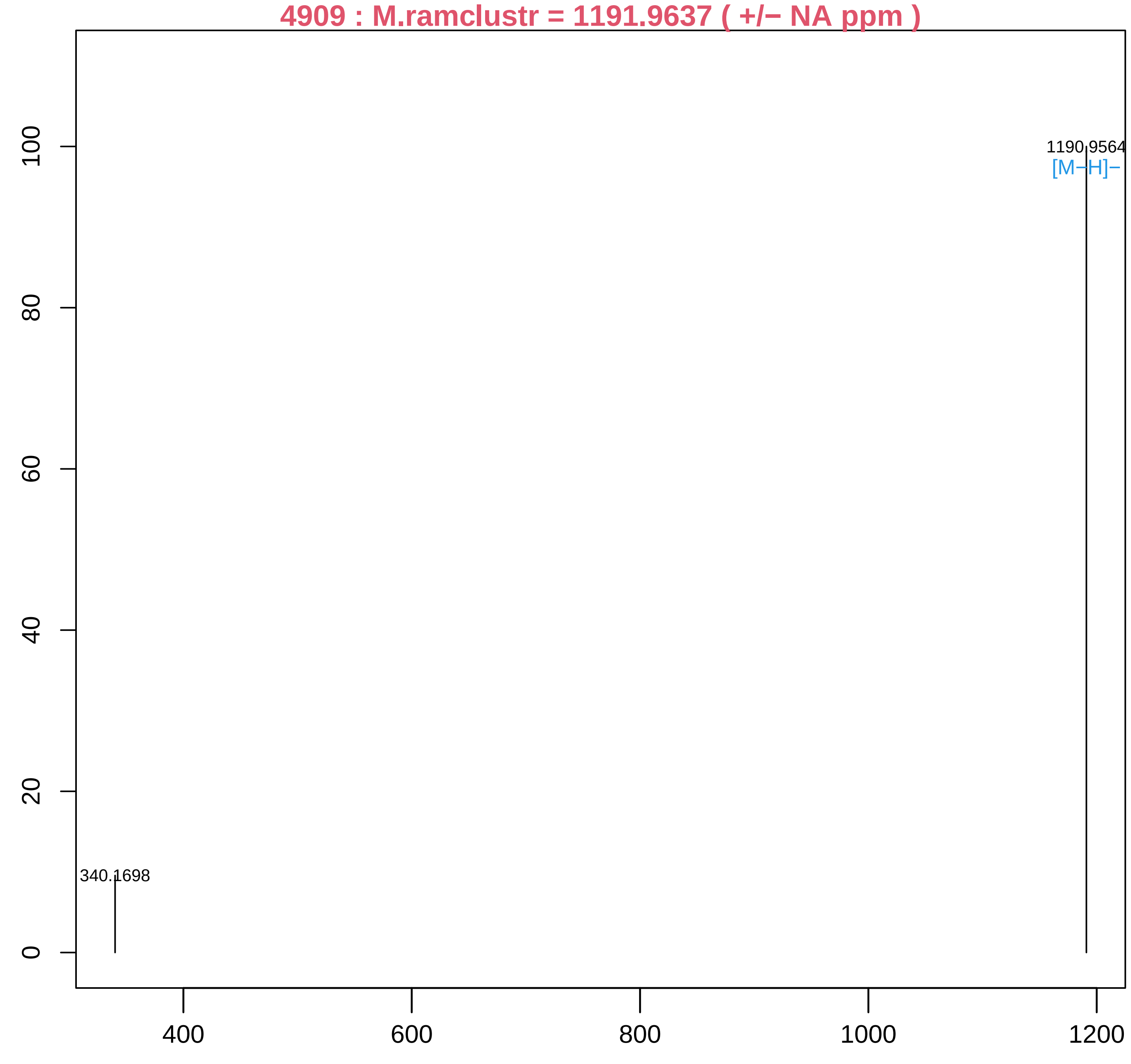

4909 : M.ramclustr = 1191.9637 (+/− NA ppm)
1190.9564
[M−H]−
340.1698
0
20
40
60
80
100
400
600
800
1000
1200

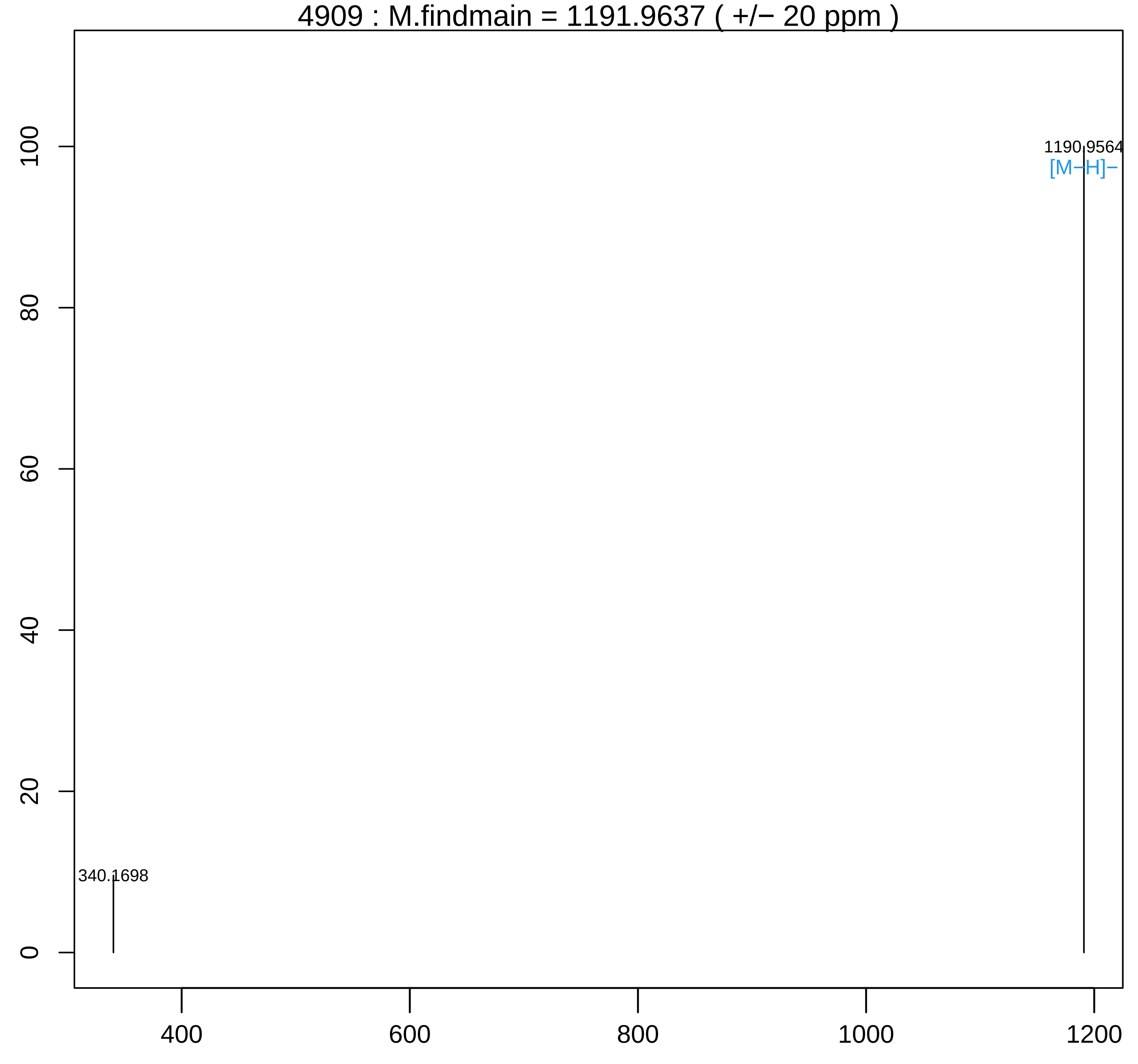

4909 : M.findmain = 1191.9637 (+/− 20 ppm)
1190.9564
[M−H]−
340.1698
0
20
40
60
80
100
400
600
800
1000
1200

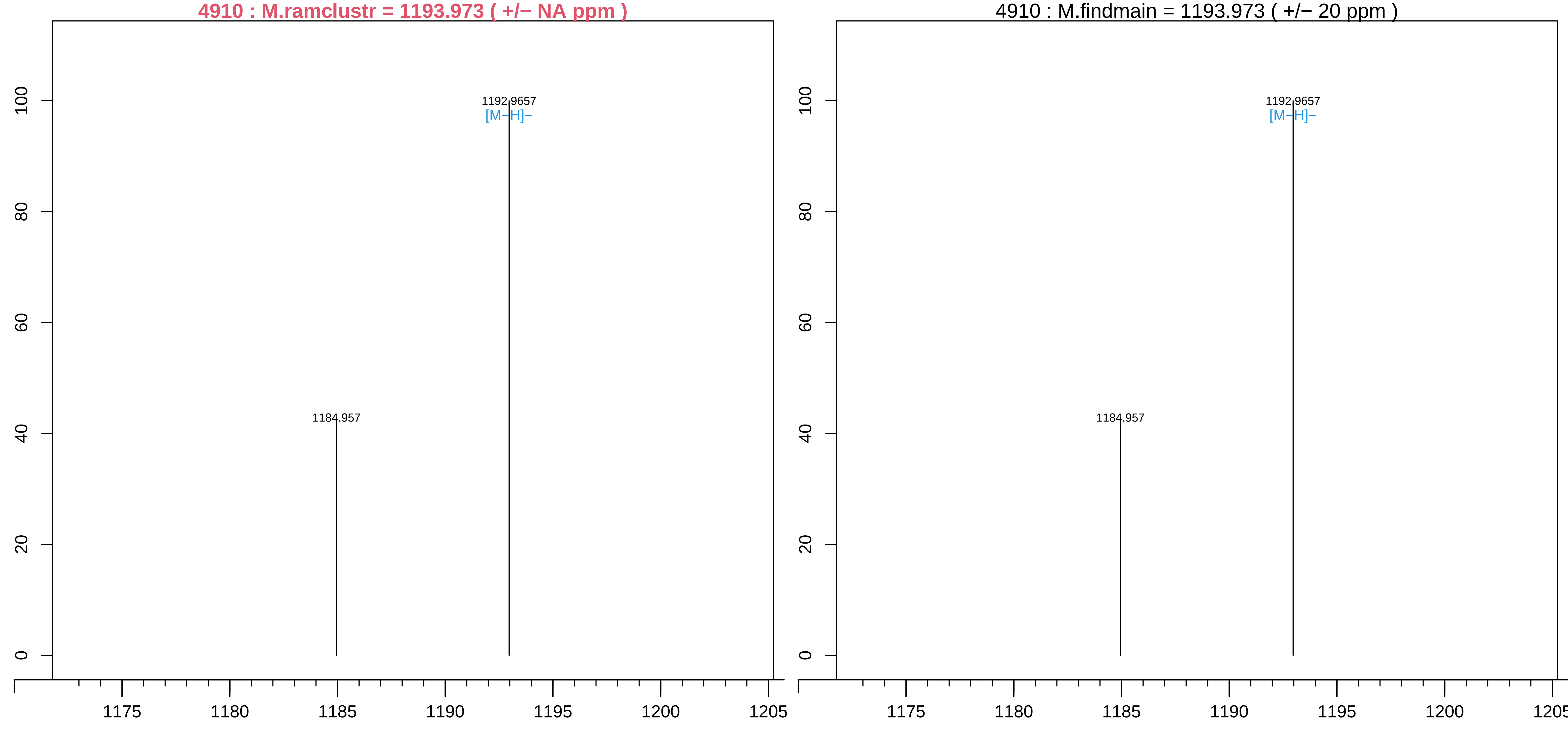
4910 : M.ramclustr = 1193.973 (+/− NA ppm)
1192.9657
[M−H]−
1184.957
4910 : M.findmain = 1193.973 (+/− 20 ppm)
1192.9657
[M−H]−
1184.957
0
20
40
60
80
100
1175
1180
1185
1190
1195
1200
1205

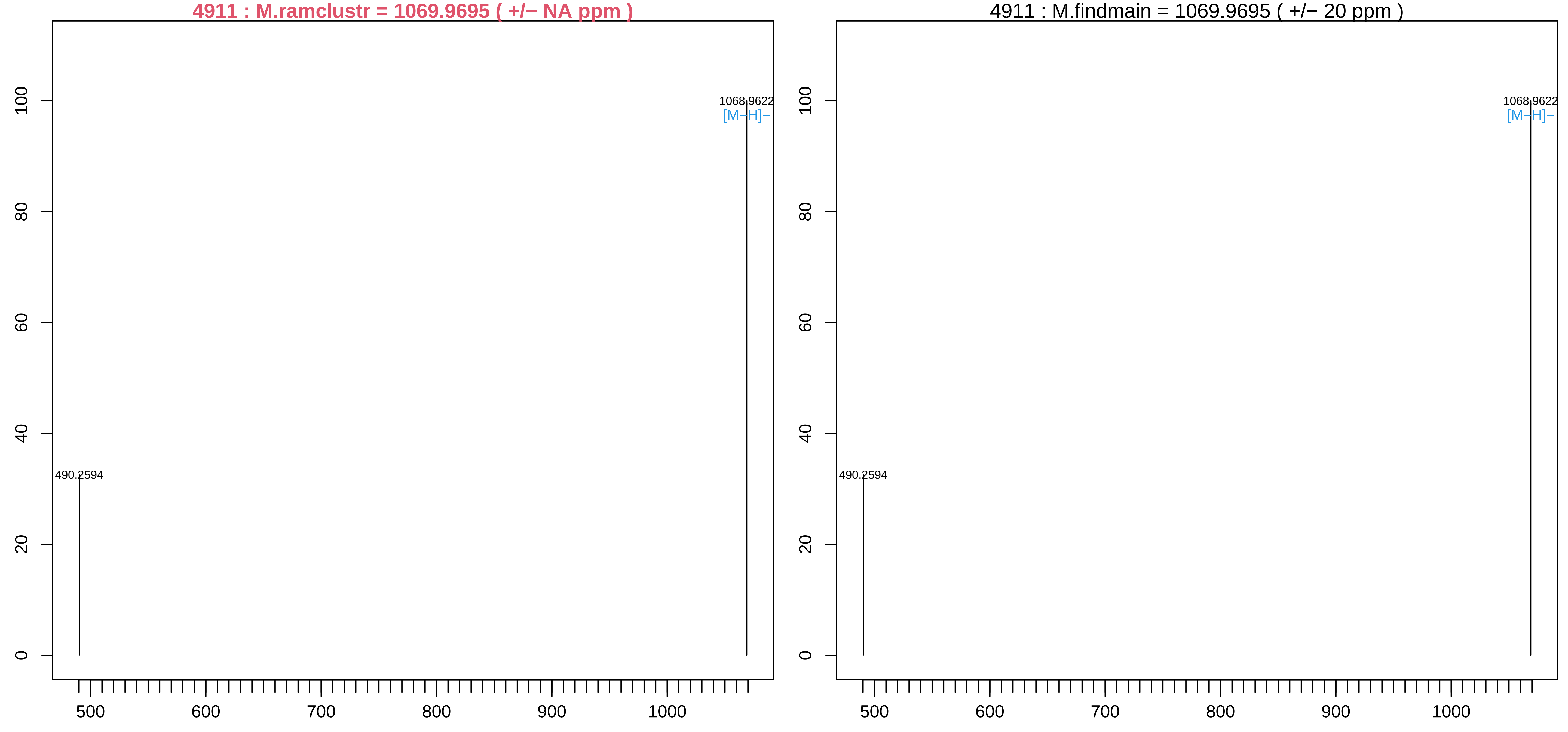

4911 : M.ramclustr = 1069.9695 (+/− NA ppm)
1068.9622
[M−H]−
490.2594
4911 : M.findmain = 1069.9695 (+/− 20 ppm)
1068.9622
[M−H]−
490.2594
0
20
40
60
80
100
500
600
700
800
900
1000

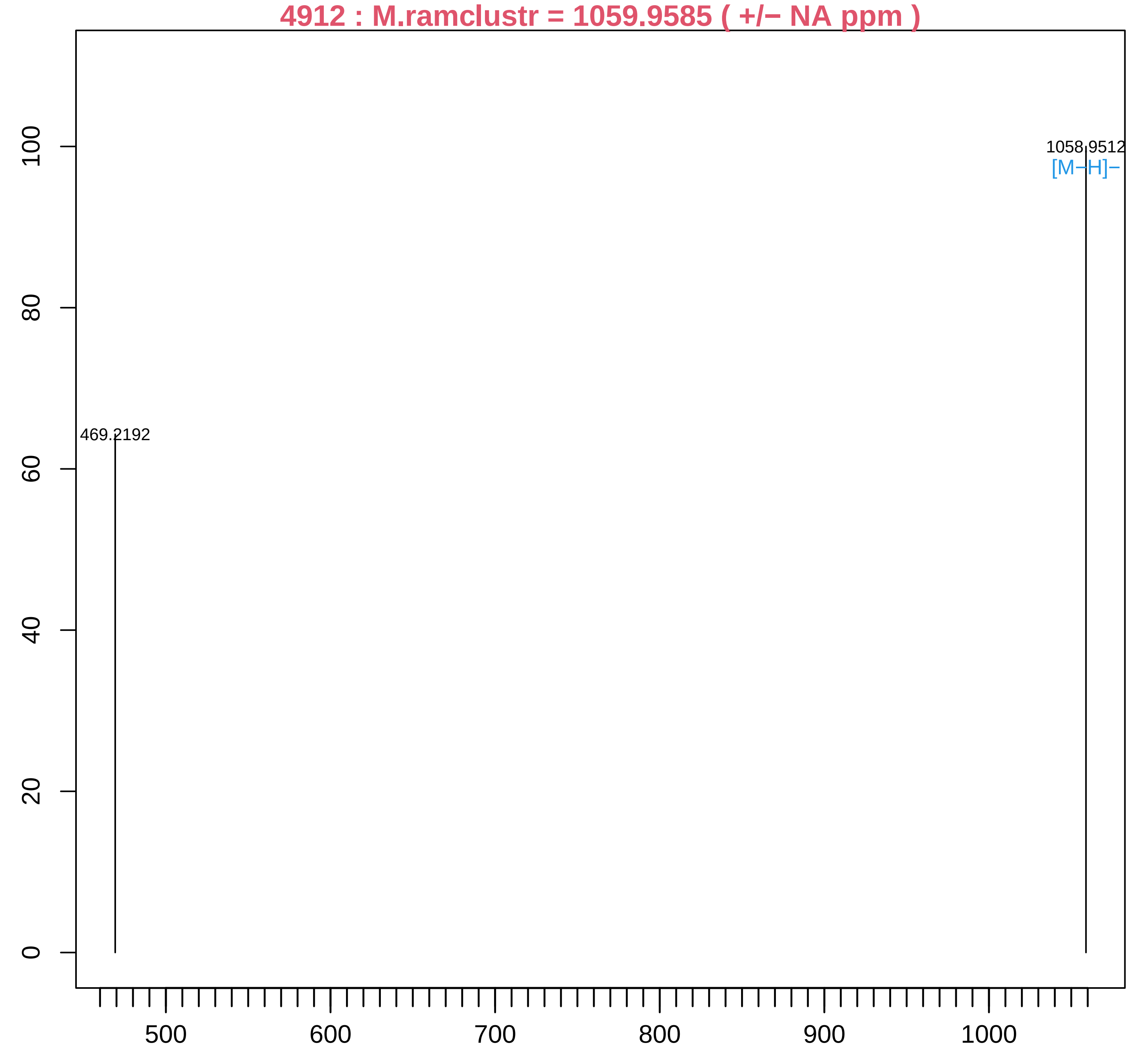
4912 : M.ramclustr = 1059.9585 (+/− NA ppm)
1058.9512
[M−H]−
469.2192
0
20
40
60
80
100
500
600
700
800
900
1000

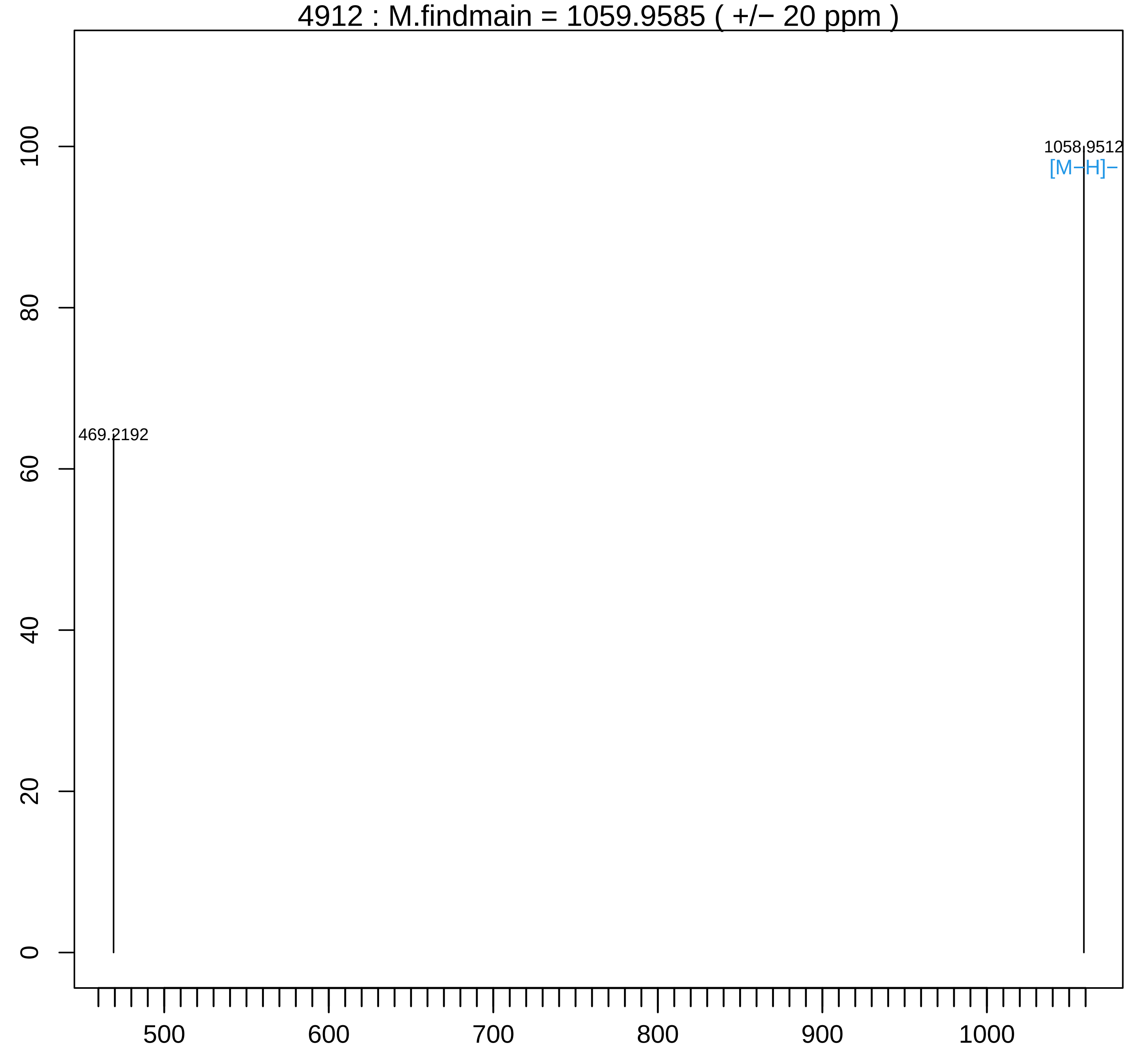
4912 : M.findmain = 1059.9585 (+/− 20 ppm)
1058.9512
[M−H]−
469.2192
0
20
40
60
80
100
500
600
700
800
900
1000

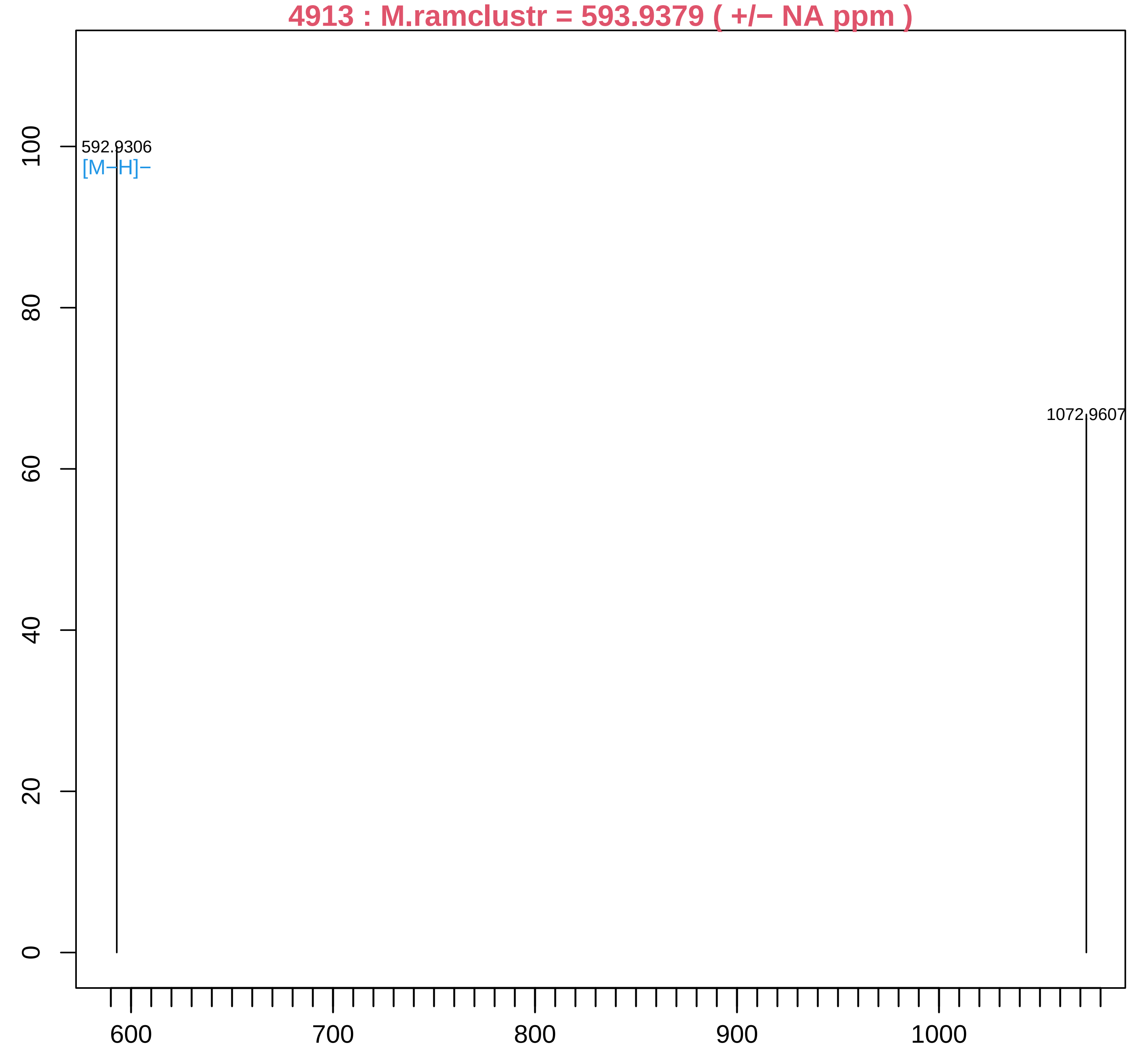
4913 : M.ramclustr = 593.9379 (+/− NA ppm)
592.9306
[M−H]−
1072.9607
0
20
40
60
80
100
600
700
800
900
1000

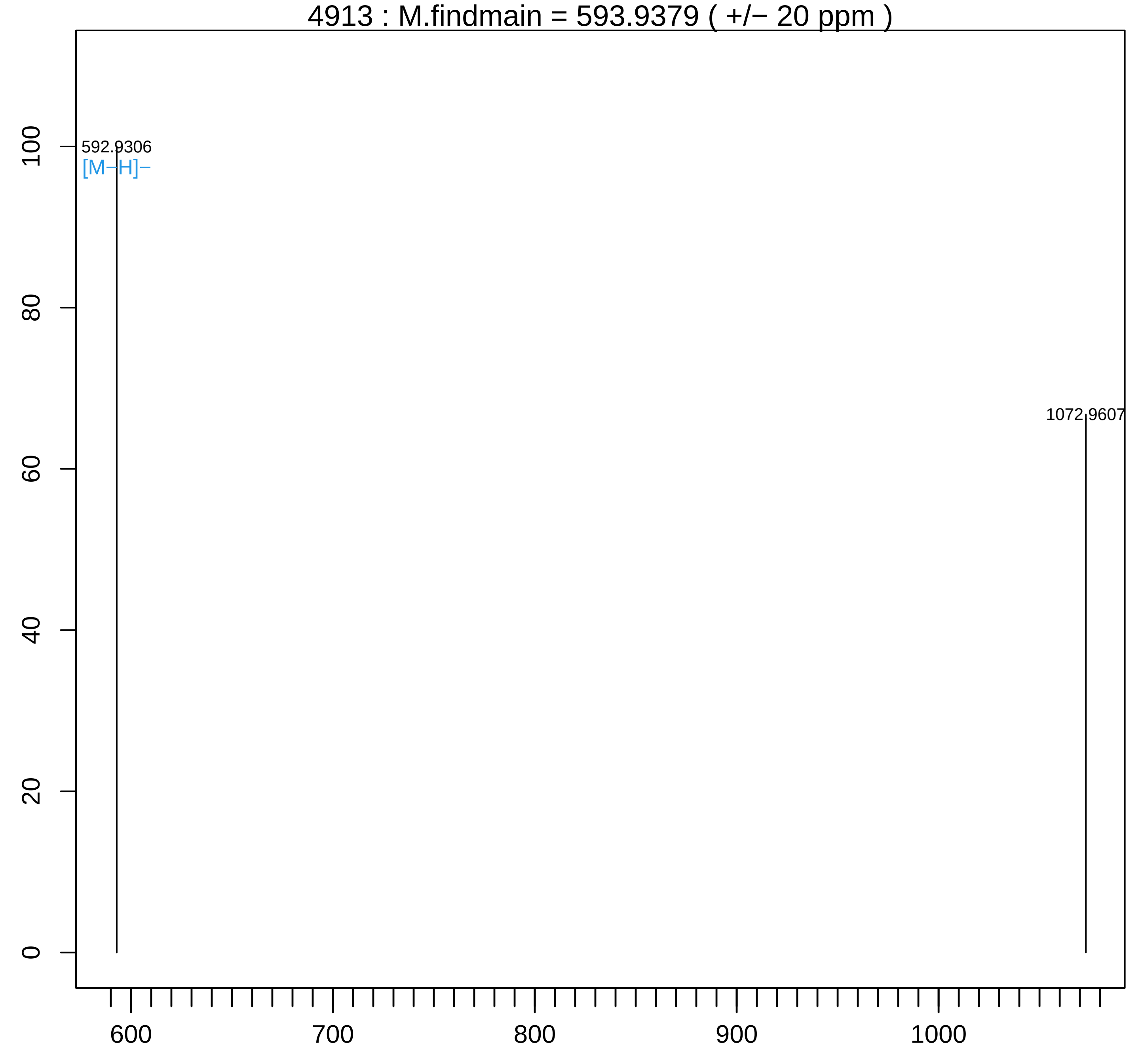
4913 : M.findmain = 593.9379 (+/− 20 ppm)
592.9306
[M−H]−
1072.9607
0
20
40
60
80
100
600
700
800
900
1000

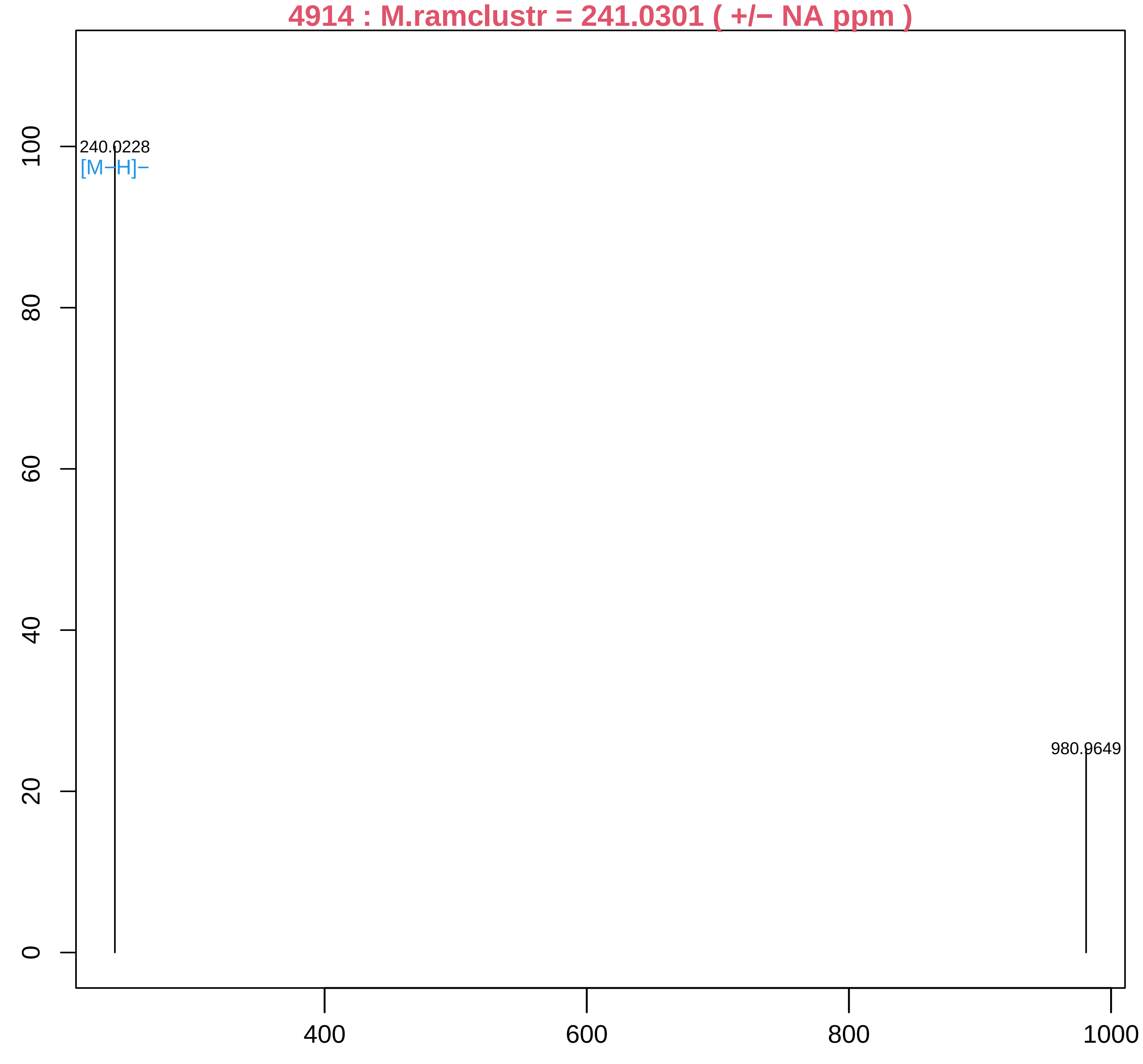
4914 : M.ramclustr = 241.0301 (+/− NA ppm)
240.0228
[M−H]−
980.9649
0
20
40
60
80
100
400
600
800
1000

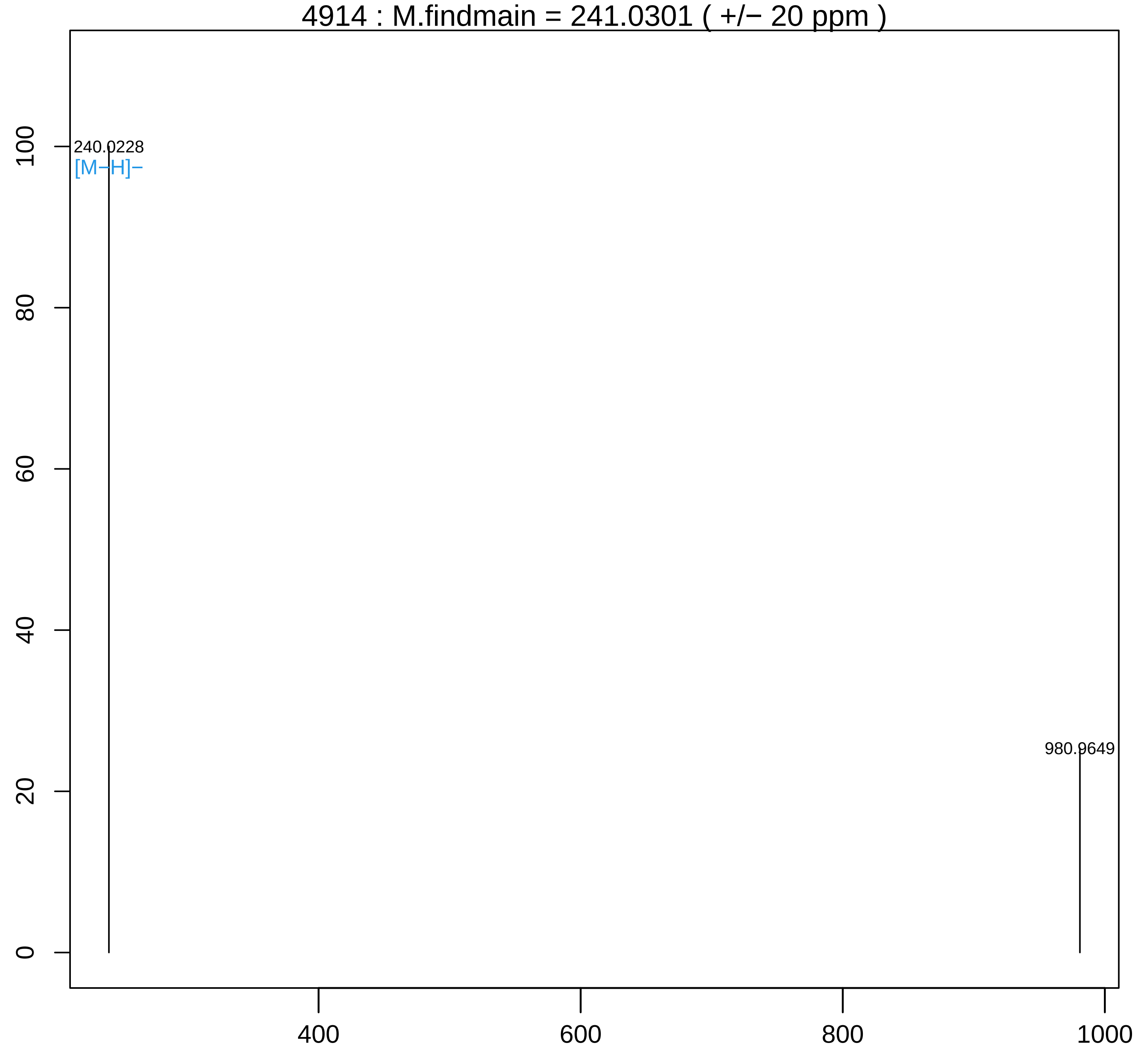
4914 : M.findmain = 241.0301 (+/− 20 ppm)
240.0228
[M−H]−
980.9649
0
20
40
60
80
100
400
600
800
1000

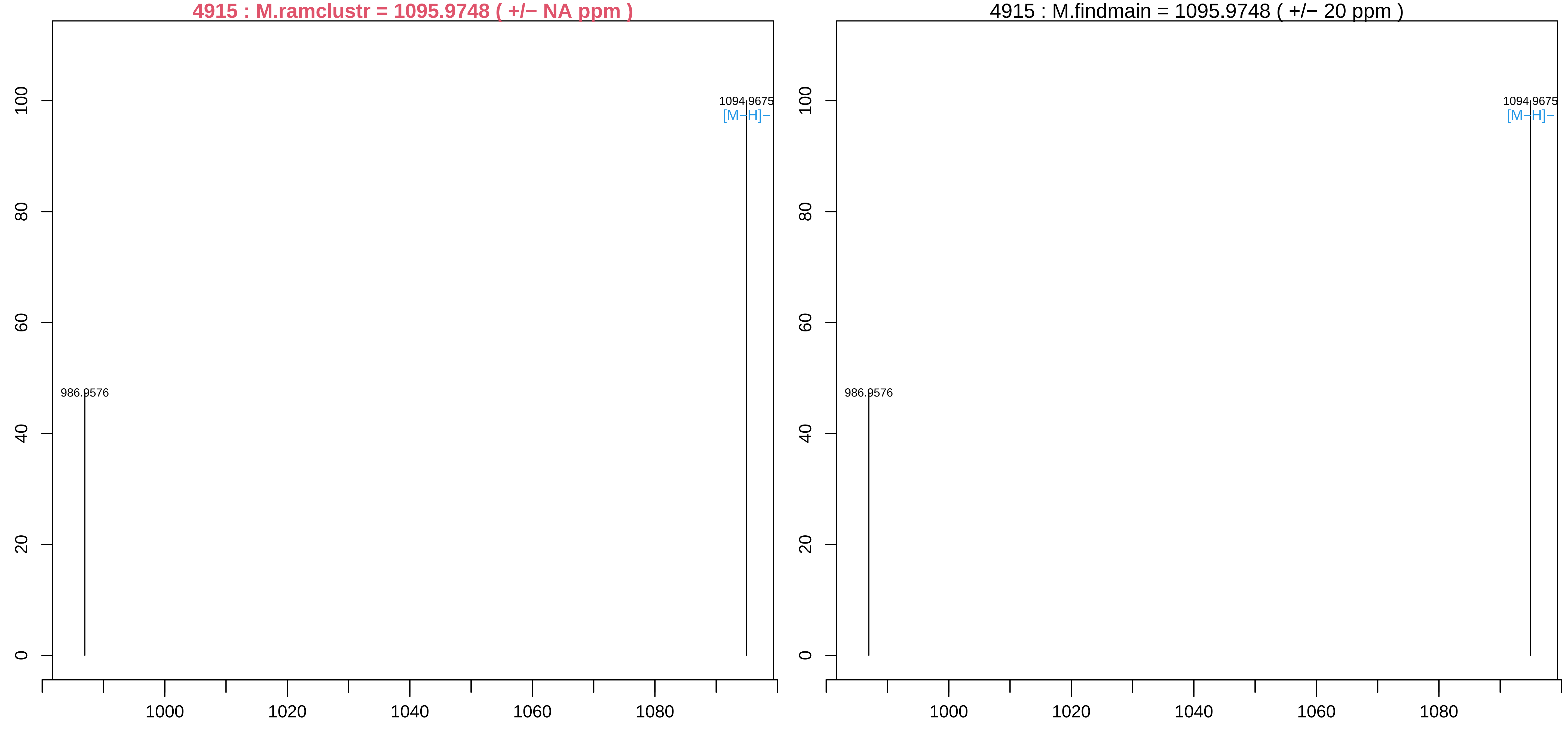

4915 : M.ramclustr = 1095.9748 (+/− NA ppm)
1094.9675
[M−H]−
986.9576
4915 : M.findmain = 1095.9748 (+/− 20 ppm)
1094.9675
[M−H]−
986.9576
0
20
40
60
80
100
1000
1020
1040
1060
1080

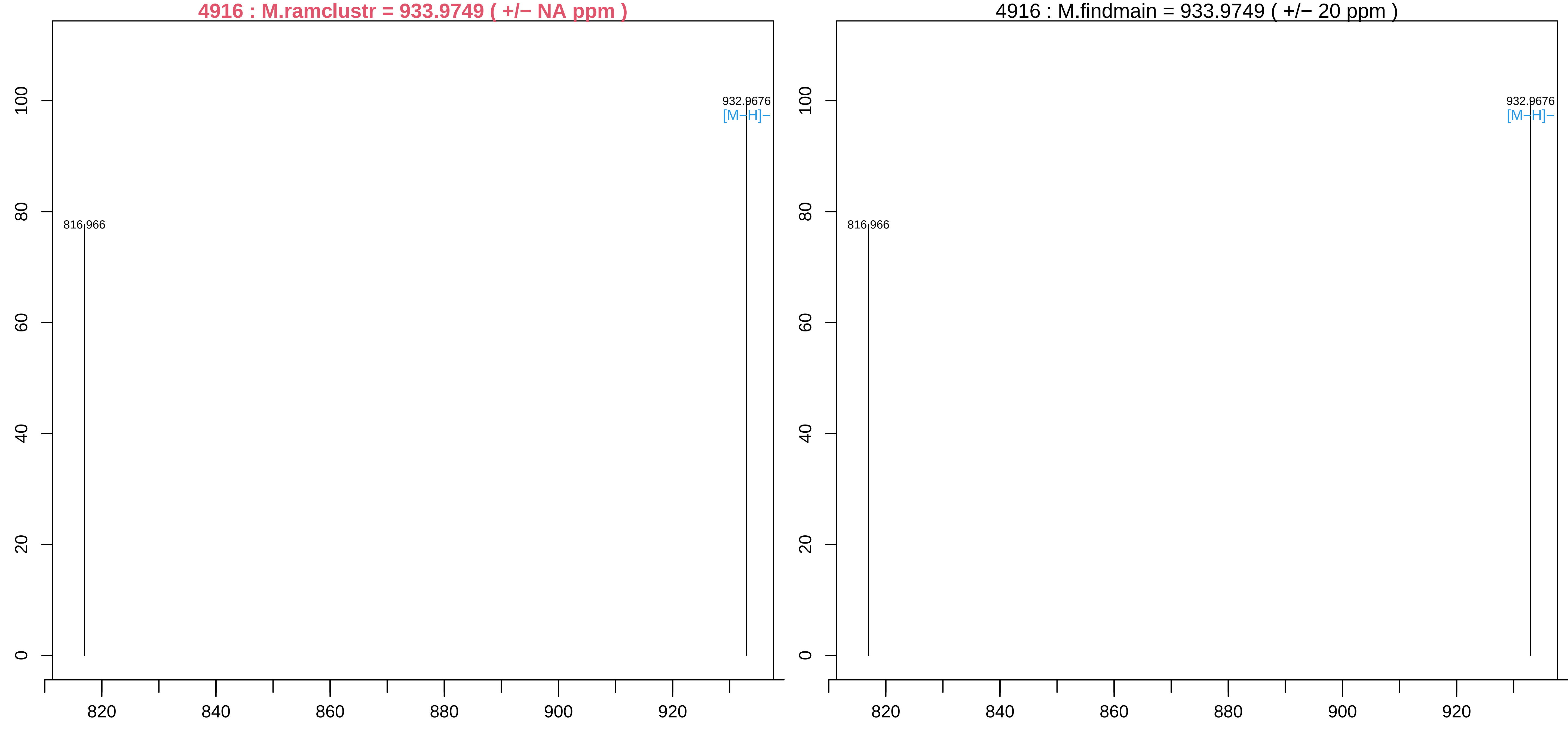

4916 : M.ramclustr = 933.9749 (+/− NA ppm)
816.966
932.9676
[M−H]−
4916 : M.findmain = 933.9749 (+/− 20 ppm)
816.966
932.9676
[M−H]−
0
20
40
60
80
100
820
840
860
880
900
920

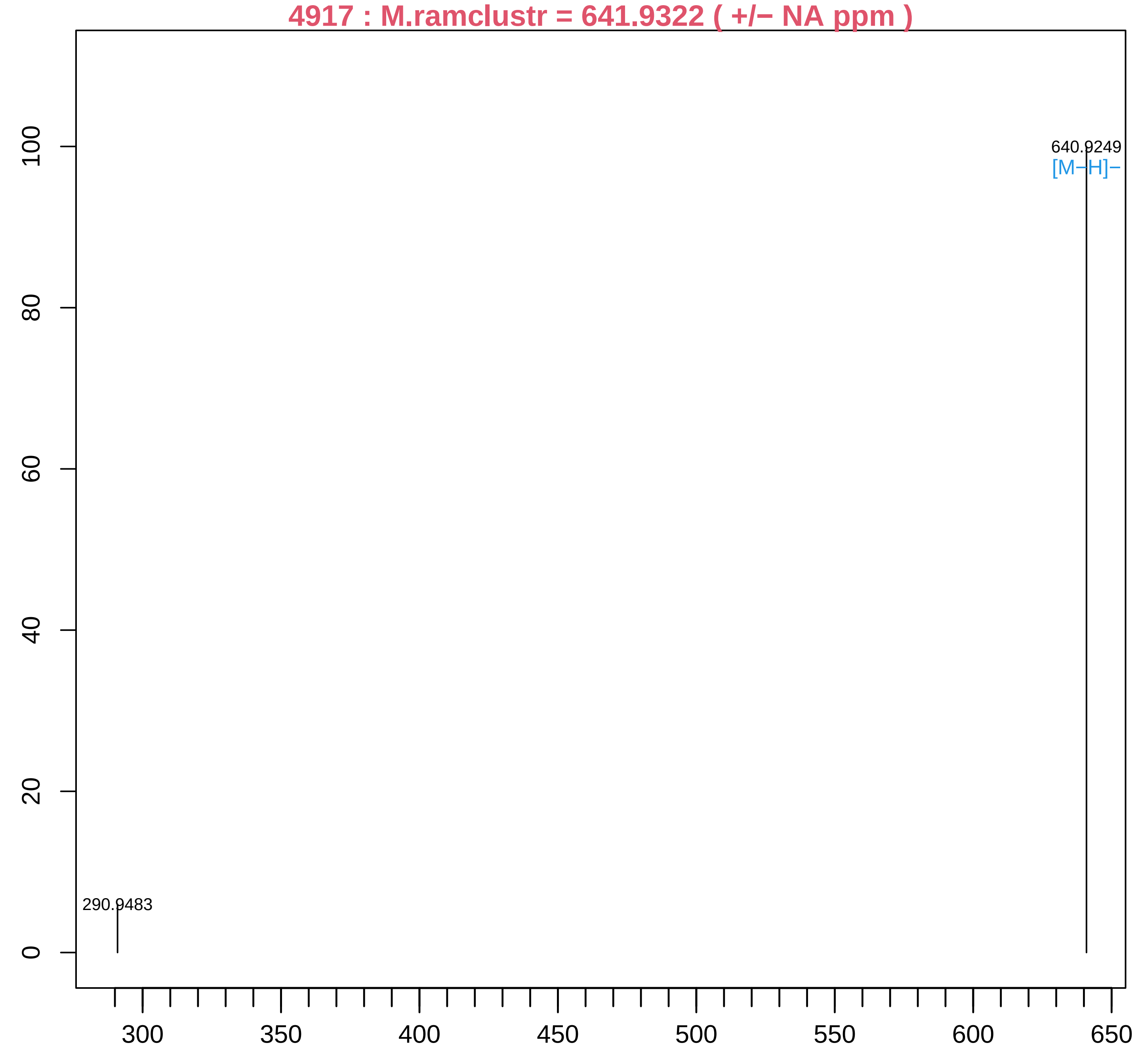
4917 : M.ramclustr = 641.9322 (+/− NA ppm)
640.9249
[M−H]−
290.9483
0
20
40
60
80
100
300
350
400
450
500
550
600
650

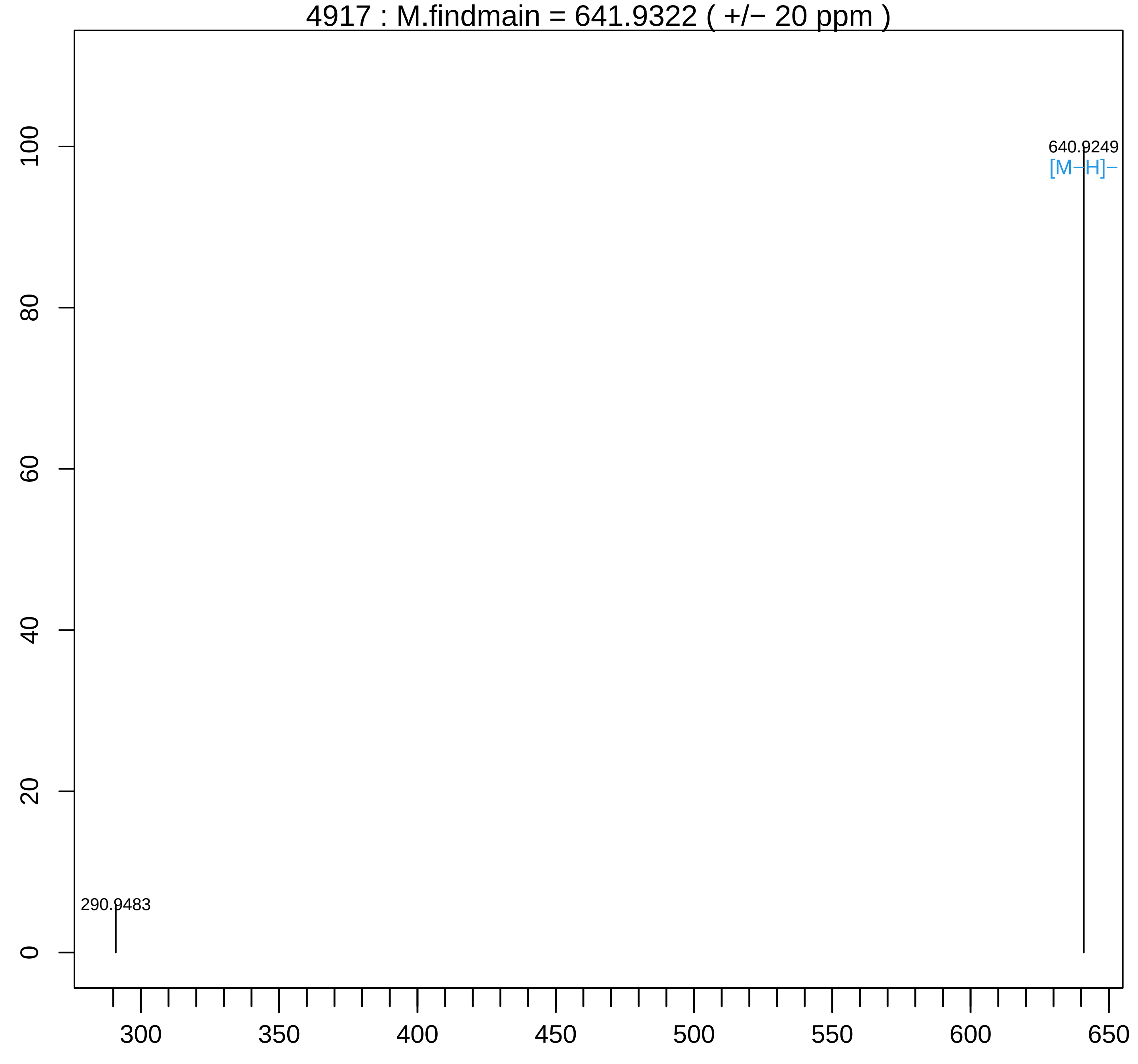
4917 : M.findmain = 641.9322 (+/− 20 ppm)
640.9249
[M−H]−
290.9483
0
20
40
60
80
100
300
350
400
450
500
550
600
650

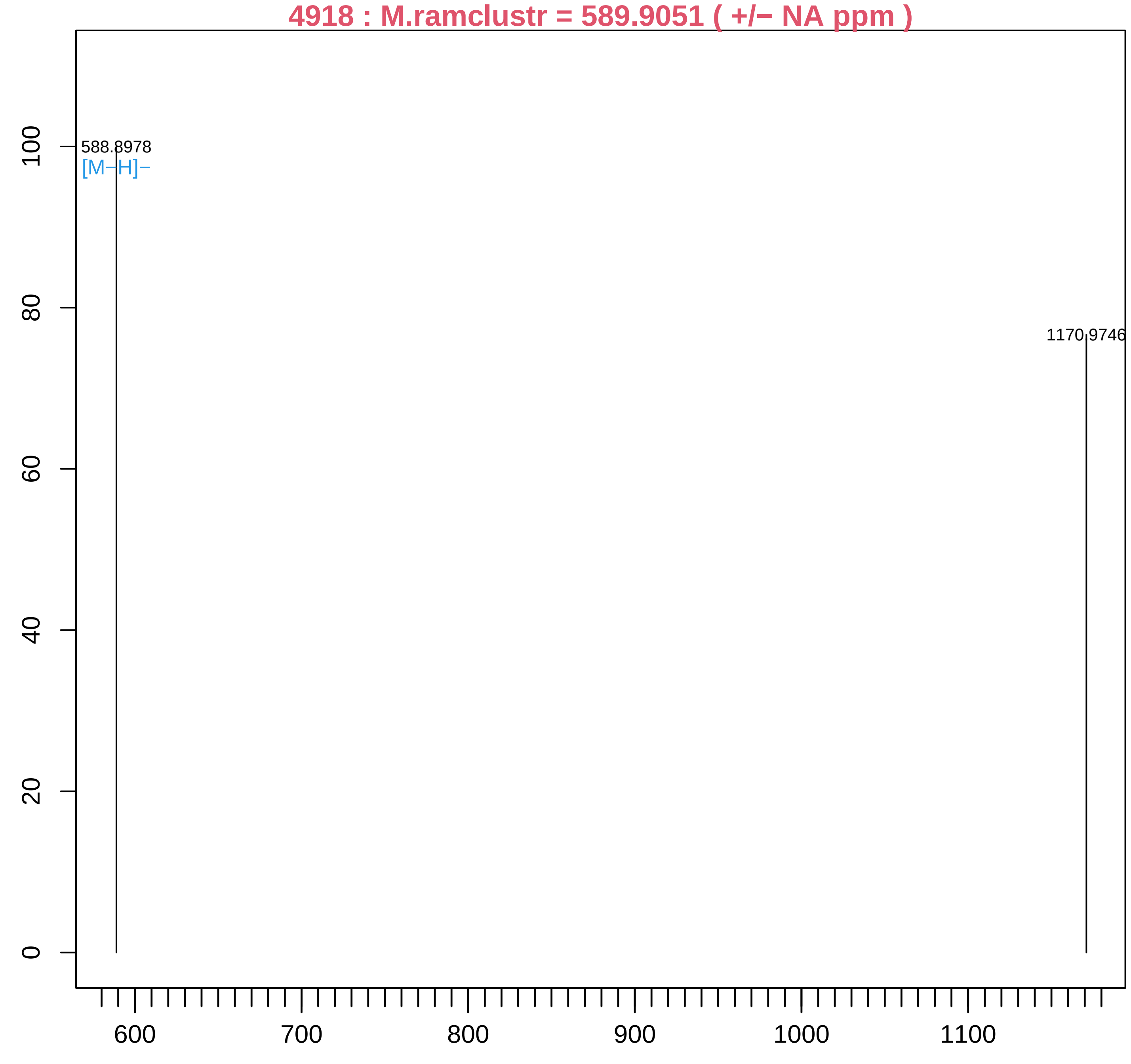
4918 : M.ramclustr = 589.9051 (+/− NA ppm)
588.8978
[M−H]−
1170.9746
0
20
40
60
80
100
600
700
800
900
1000
1100

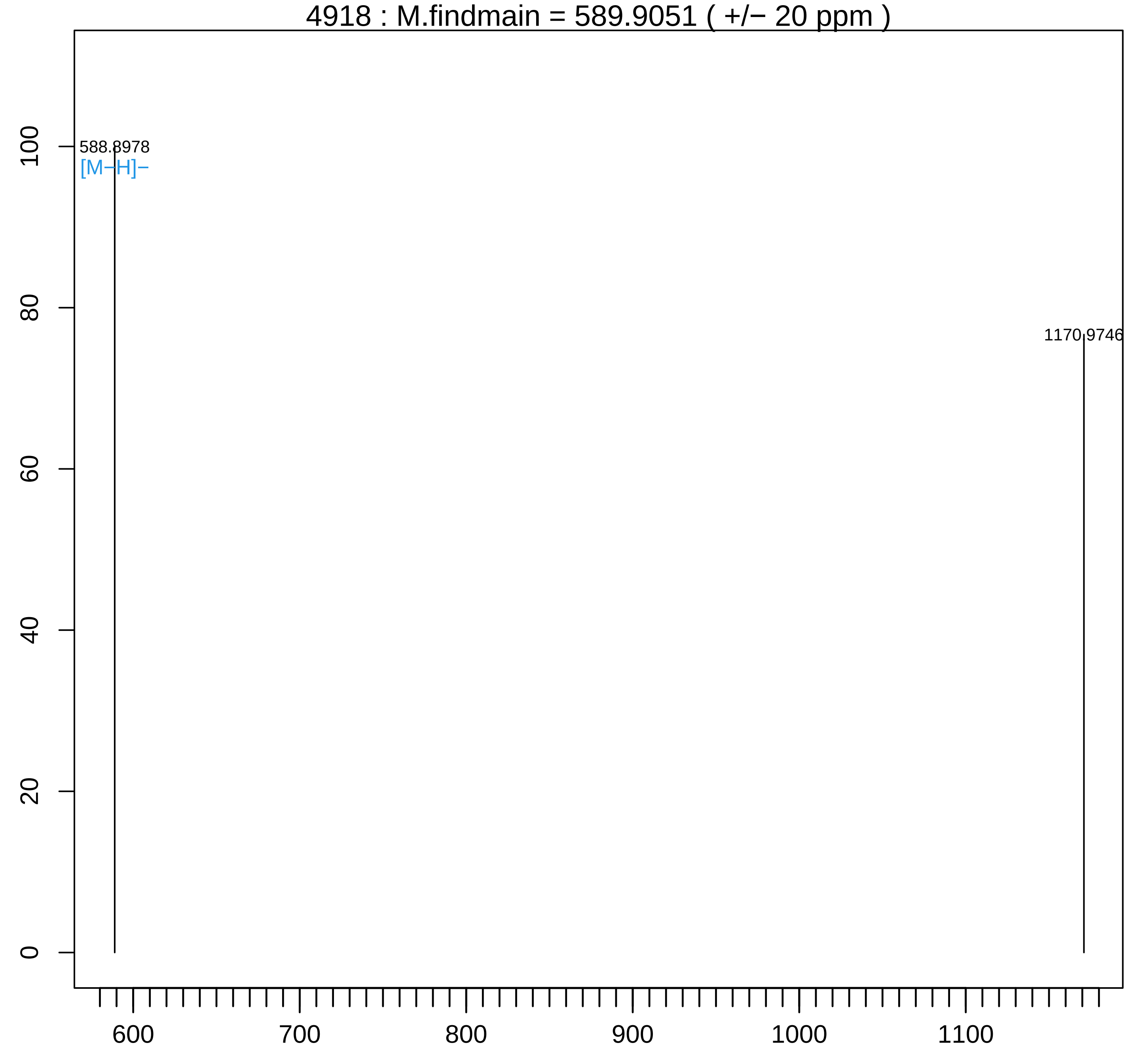
4918 : M.findmain = 589.9051 (+/− 20 ppm)
588.8978
[M−H]−
1170.9746
0
20
40
60
80
100
600
700
800
900
1000
1100

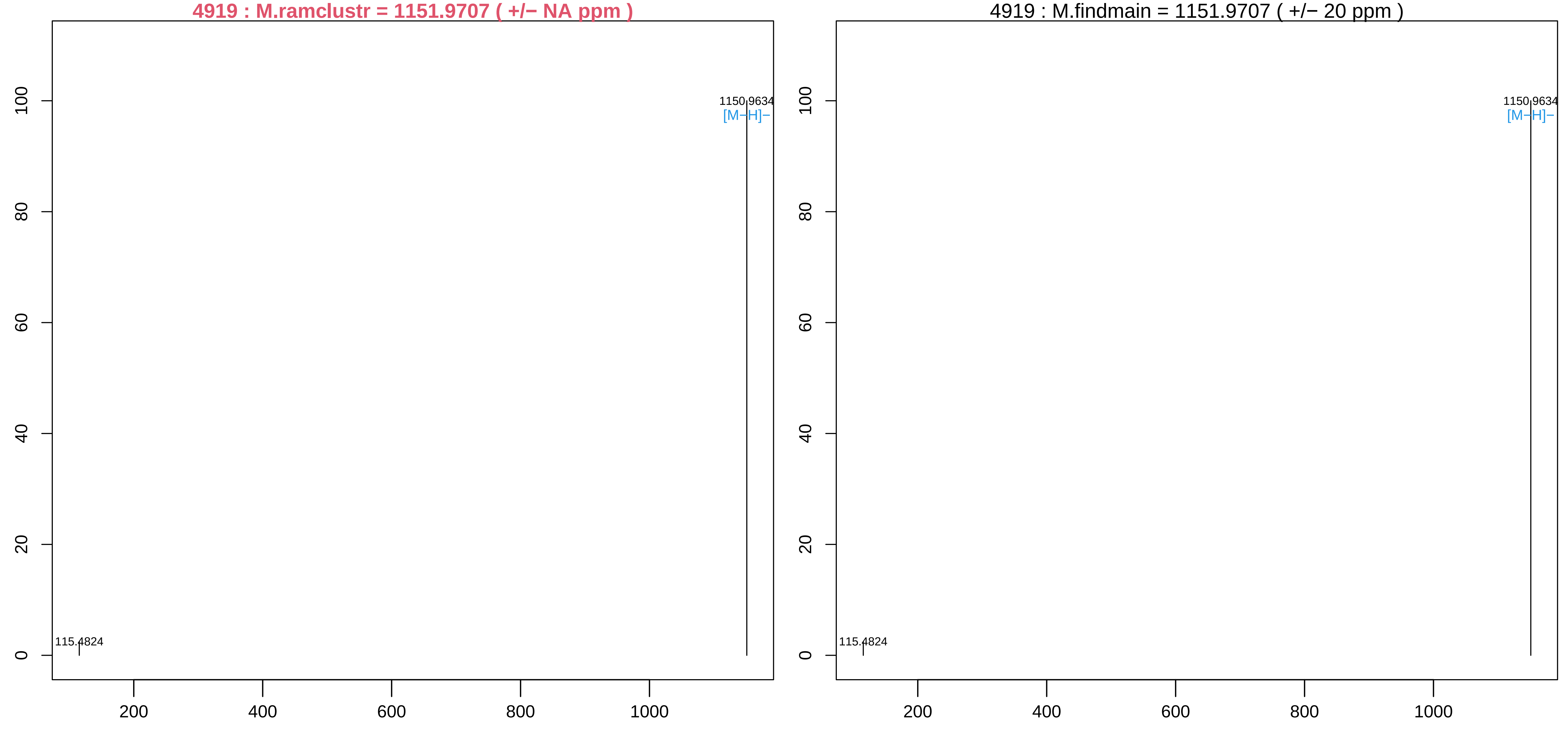

4919 : M.ramclustr = 1151.9707 (+/− NA ppm)
1150.9634
[M−H]−
115.4824
0
20
40
60
80
100
200
400
600
800
1000
4919 : M.findmain = 1151.9707 (+/− 20 ppm)
1150.9634
[M−H]−
115.4824
0
20
40
60
80
100
200
400
600
800
1000

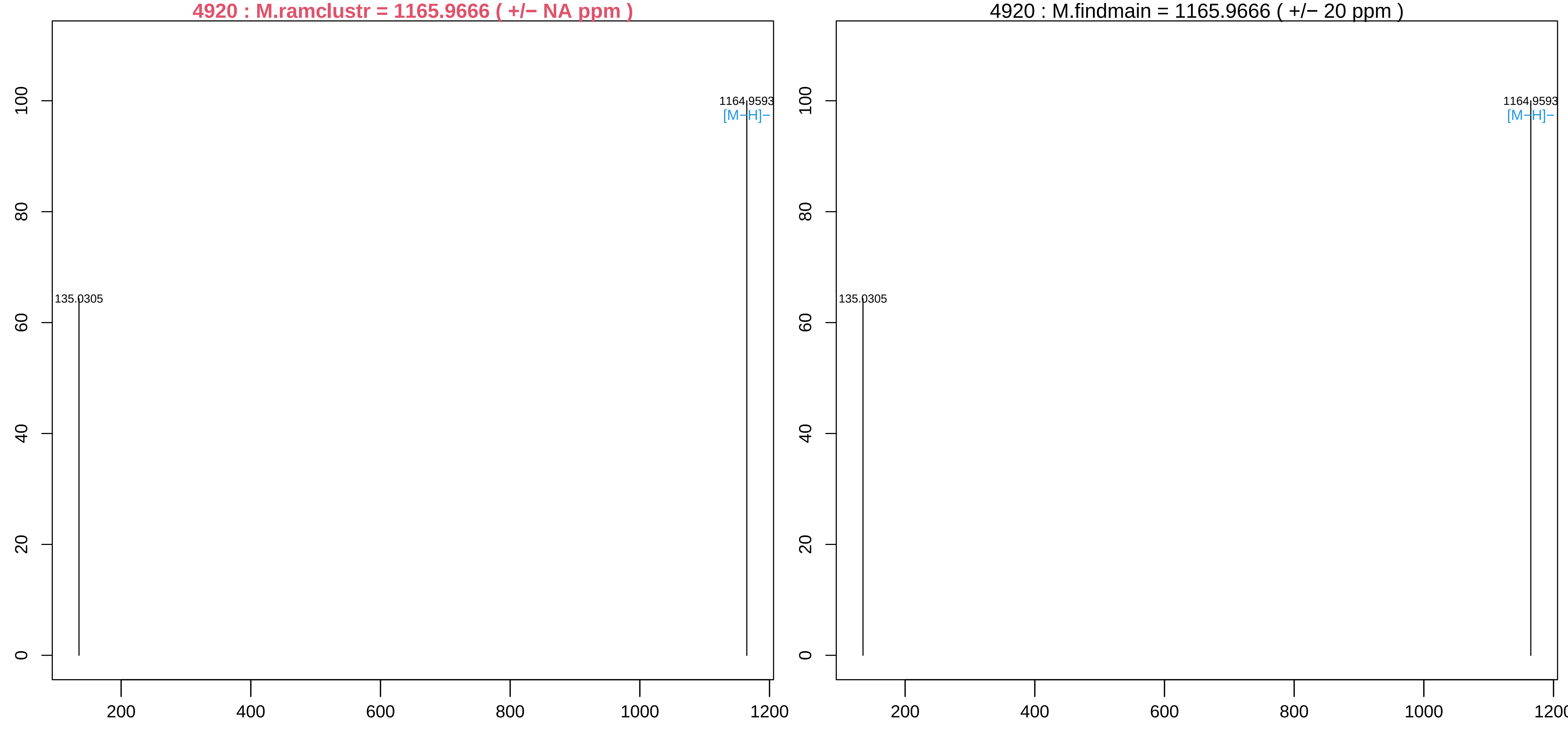
4920 : M.ramclustr = 1165.9666 (+/− NA ppm)
4920 : M.findmain = 1165.9666 (+/− 20 ppm)
1164.9593
[M−H]−
135.0305
0
20
40
60
80
100
200
400
600
800
1000
1200

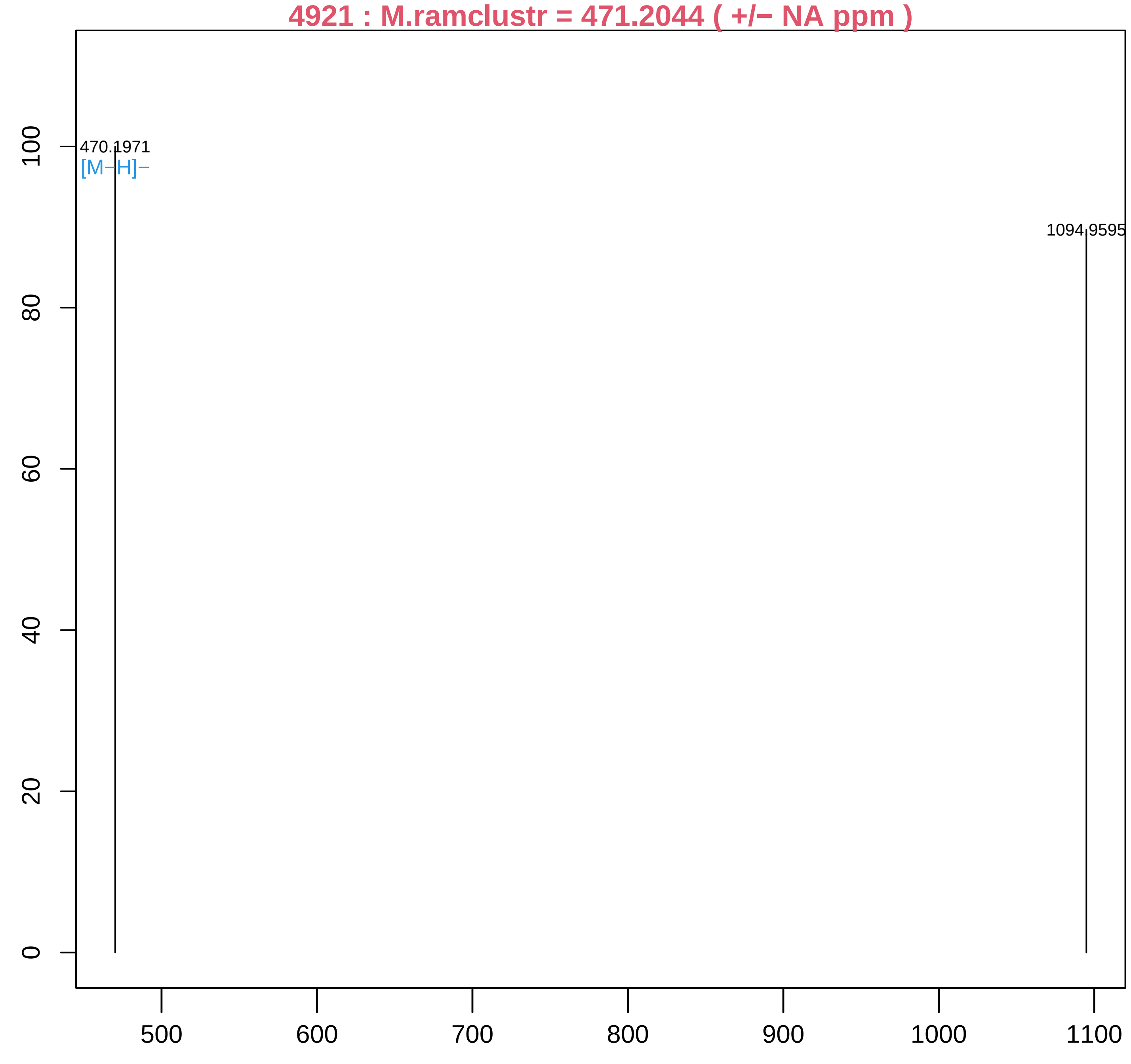
4921 : M.ramclustr = 471.2044 (+/− NA ppm)
470.1971
[M−H]−
1094.9595
0
20
40
60
80
100
500
600
700
800
900
1000
1100

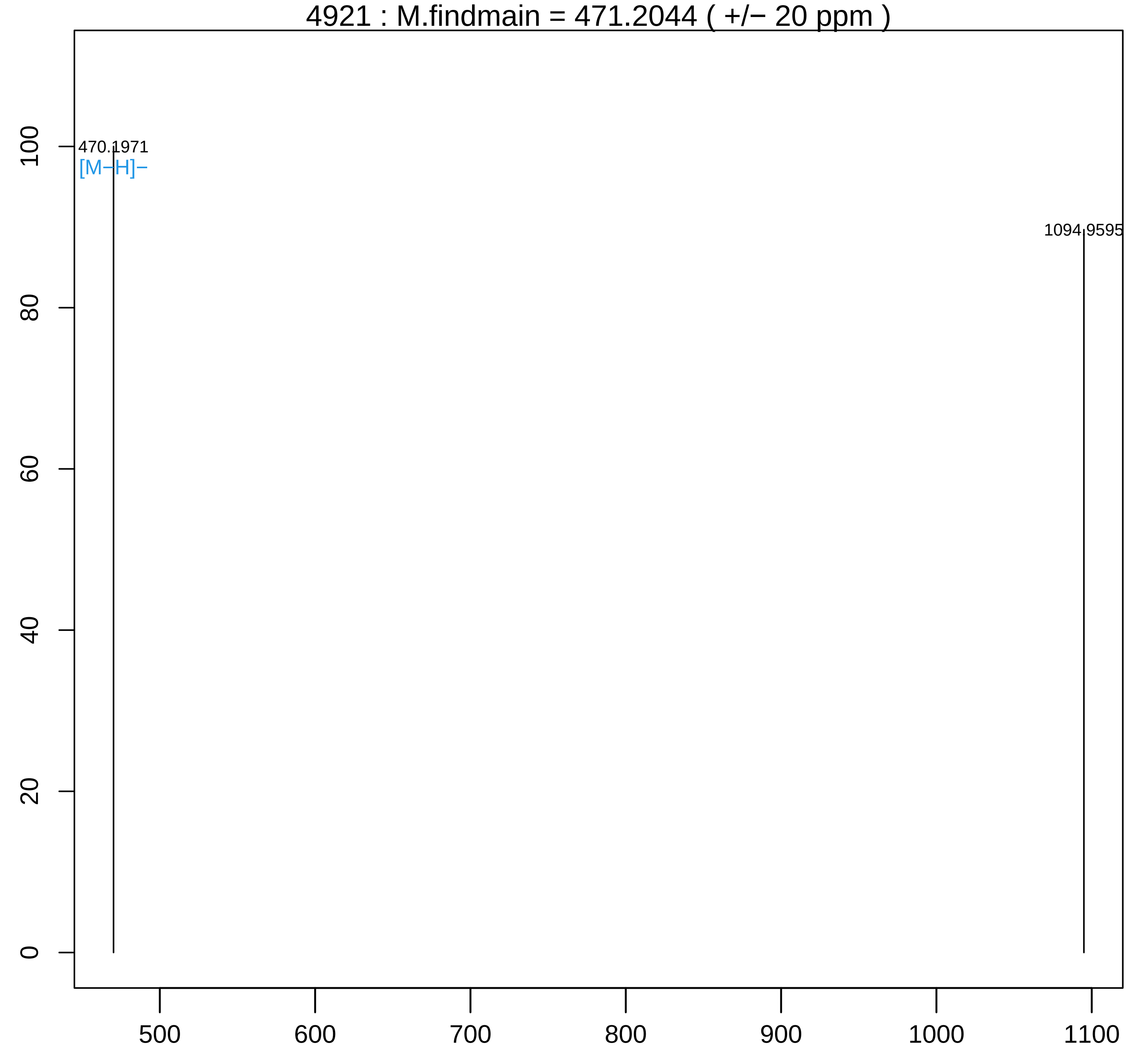
4921 : M.findmain = 471.2044 (+/− 20 ppm)
470.1971
[M−H]−
1094.9595
0
20
40
60
80
100
500
600
700
800
900
1000
1100

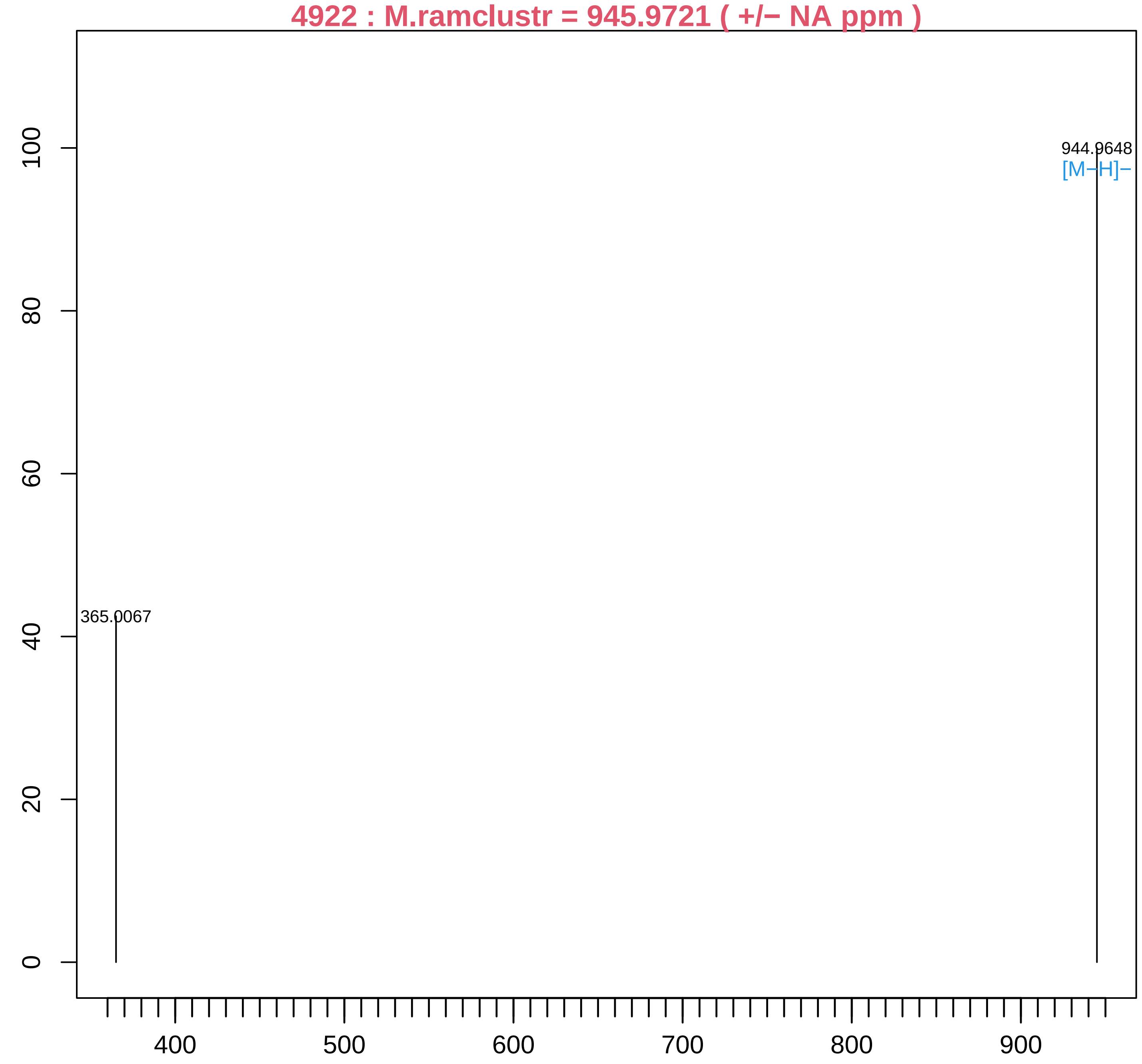
4922 : M.ramclustr = 945.9721 (+/− NA ppm)
944.9648
[M−H]−
365.0067
0
20
40
60
80
100
400
500
600
700
800
900

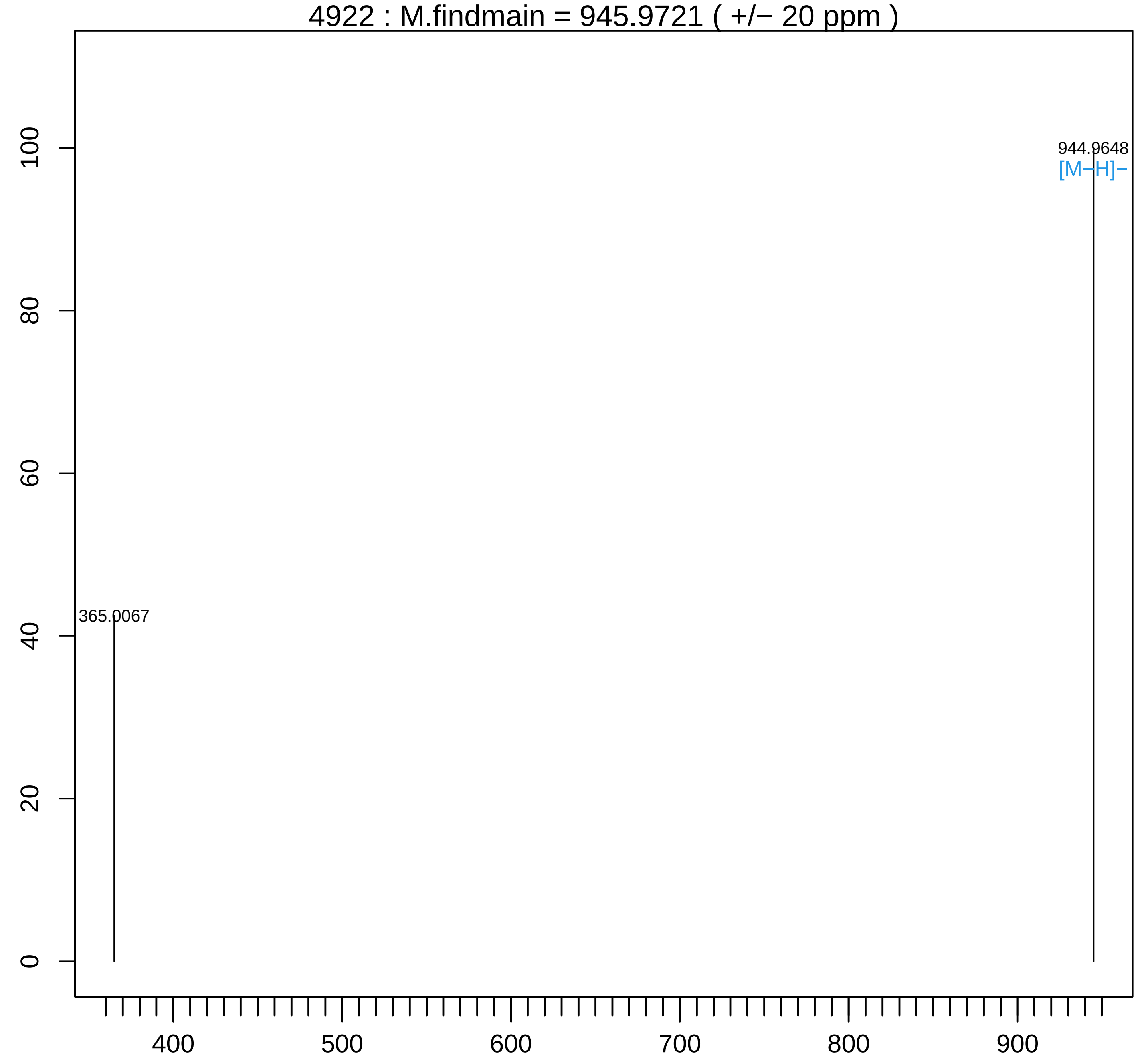
4922 : M.findmain = 945.9721 (+/− 20 ppm)
944.9648
[M−H]−
365.0067
0
20
40
60
80
100
400
500
600
700
800
900

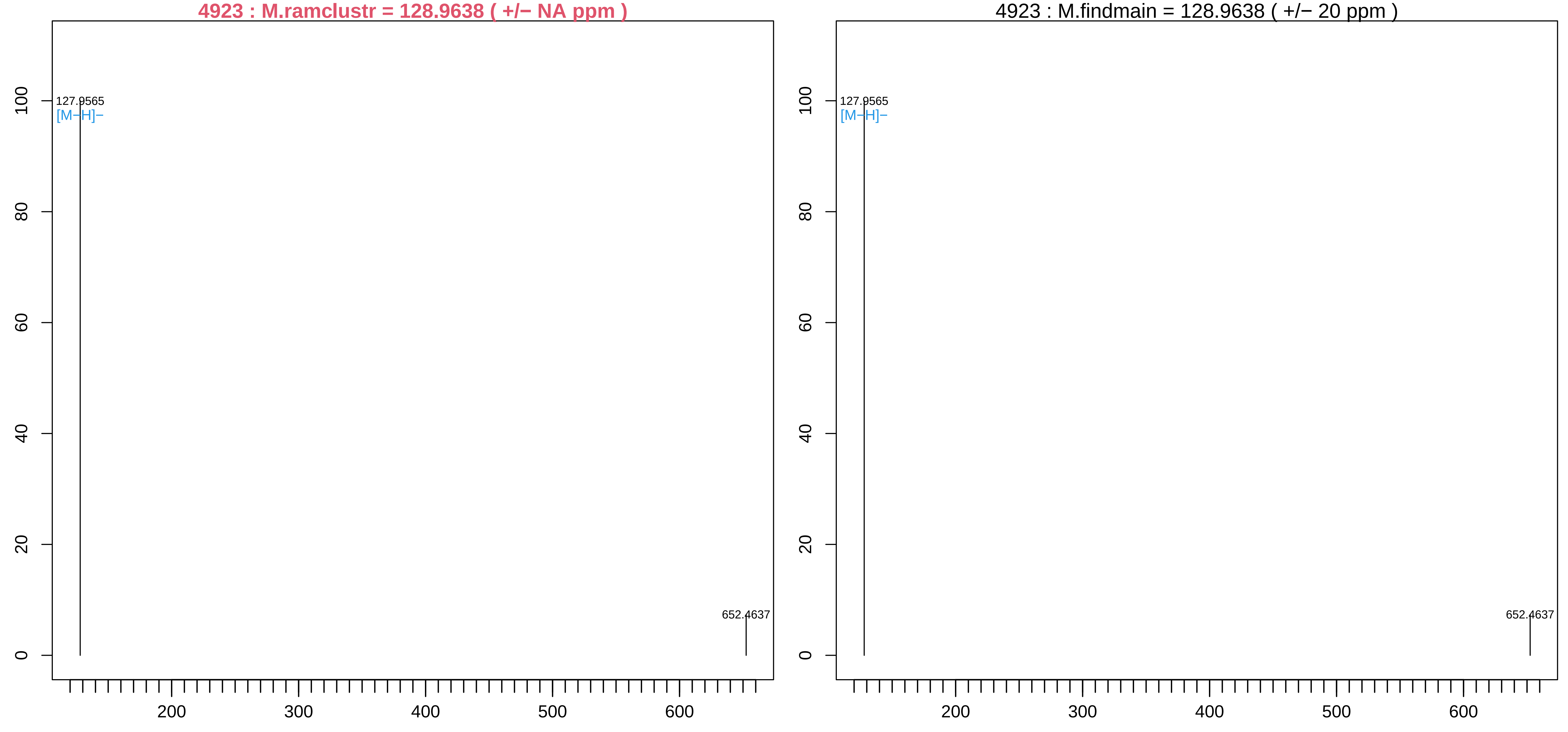
4923 : M.ramclustr = 128.9638 (+/− NA ppm)
127.9565
[M−H]−
652.4637
4923 : M.findmain = 128.9638 (+/− 20 ppm)
127.9565
[M−H]−
652.4637
0
20
40
60
80
100
200
300
400
500
600

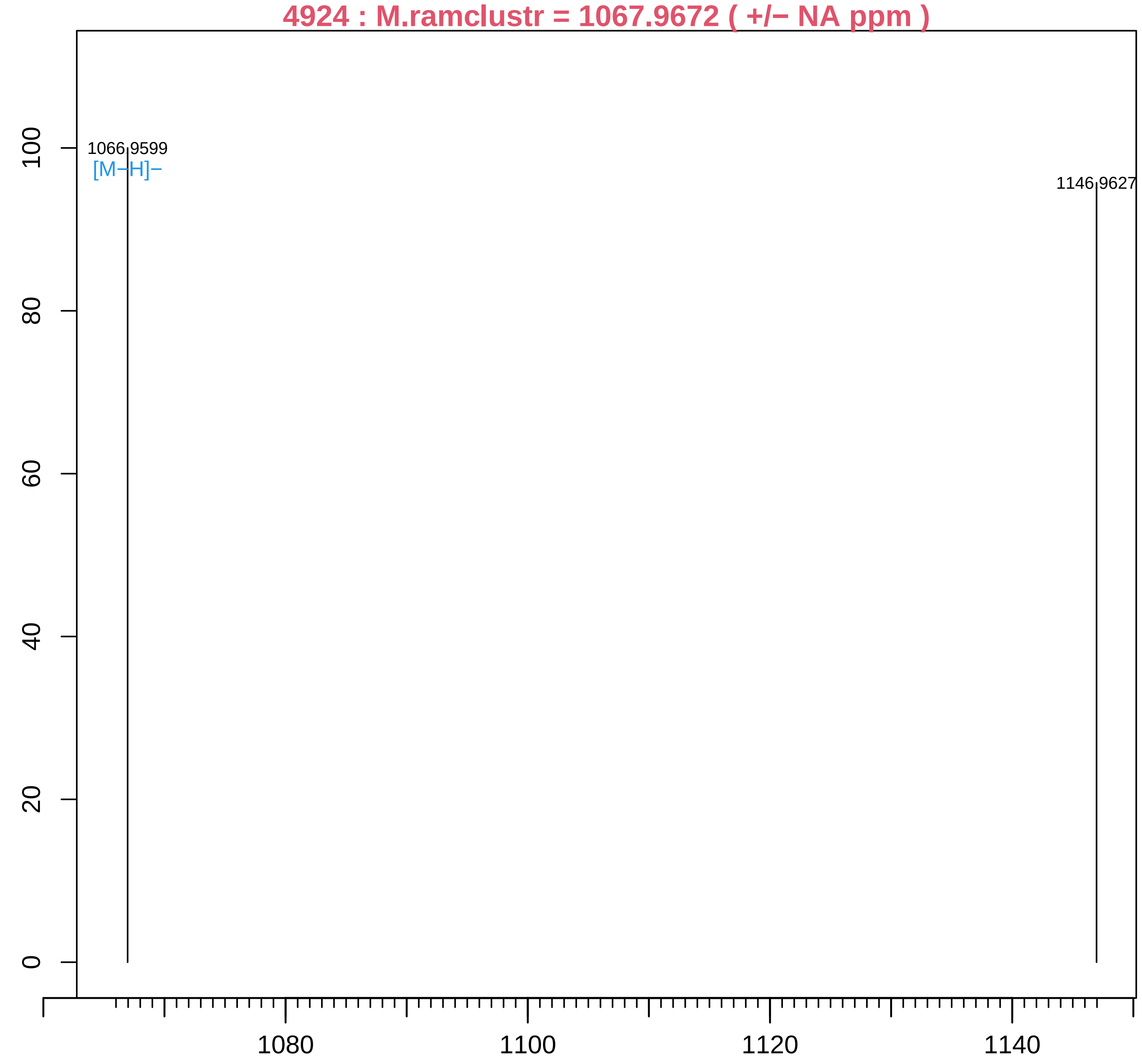
4924 : M.ramclustr = 1067.9672 (+/− NA ppm)
1066.9599
[M−H]−
1146.9627
0
20
40
60
80
100
1080
1100
1120
1140

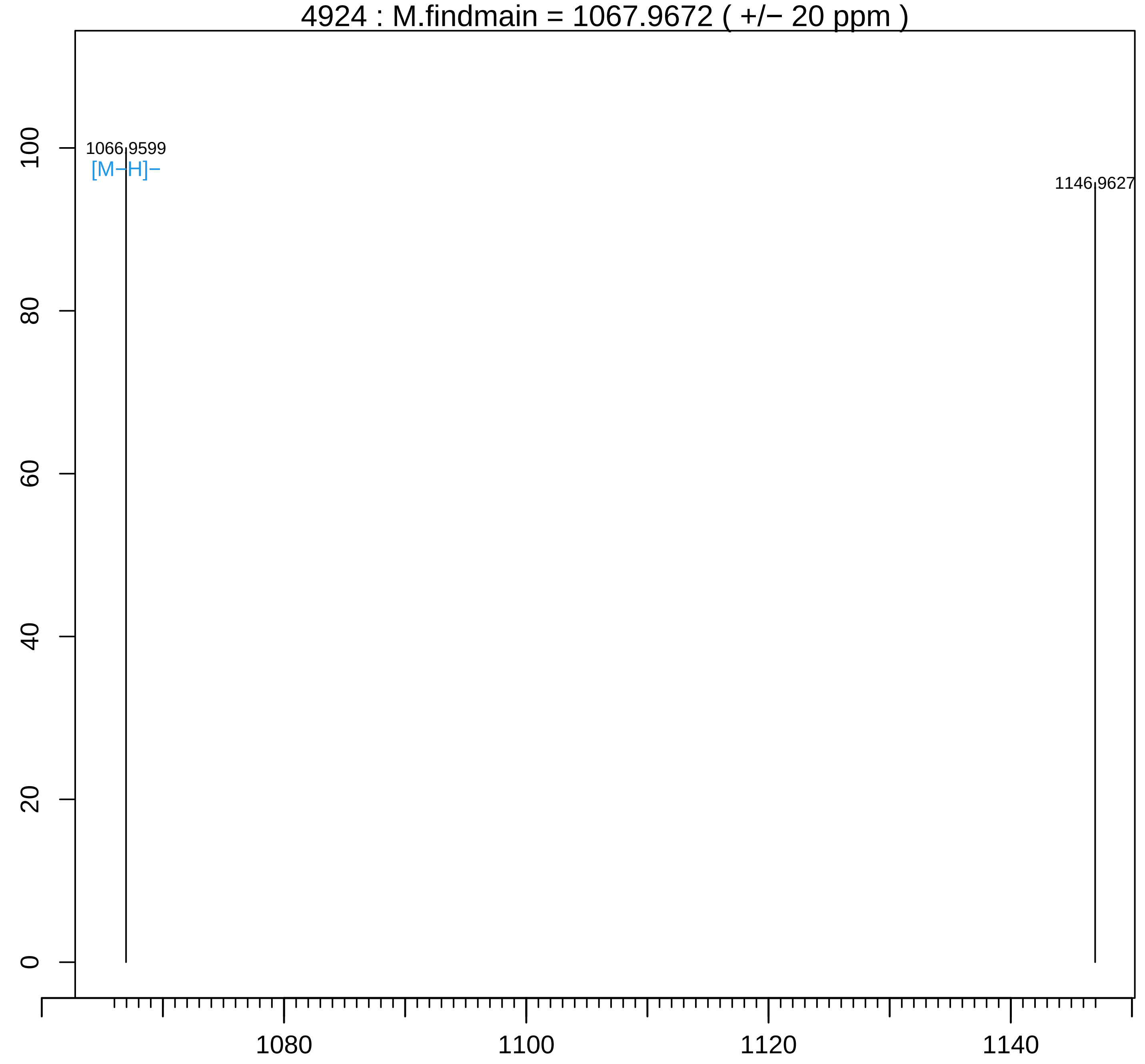
4924 : M.findmain = 1067.9672 (+/− 20 ppm)
1066.9599
[M−H]−
1146.9627
0
20
40
60
80
100
1080
1100
1120
1140

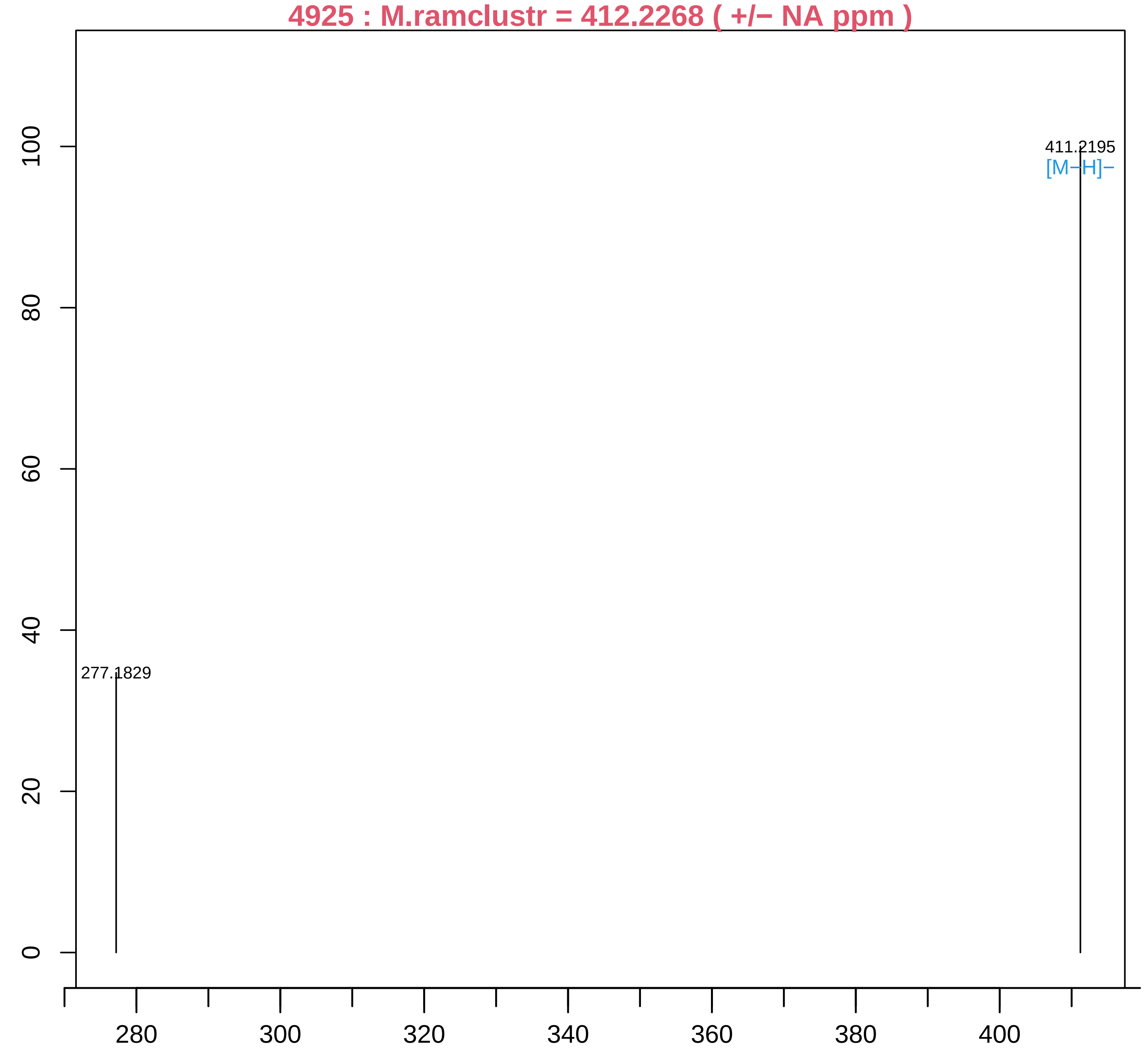
4925 : M.ramclustr = 412.2268 (+/− NA ppm)
411.2195
[M−H]−
277.1829
0
20
40
60
80
100
280
300
320
340
360
380
400

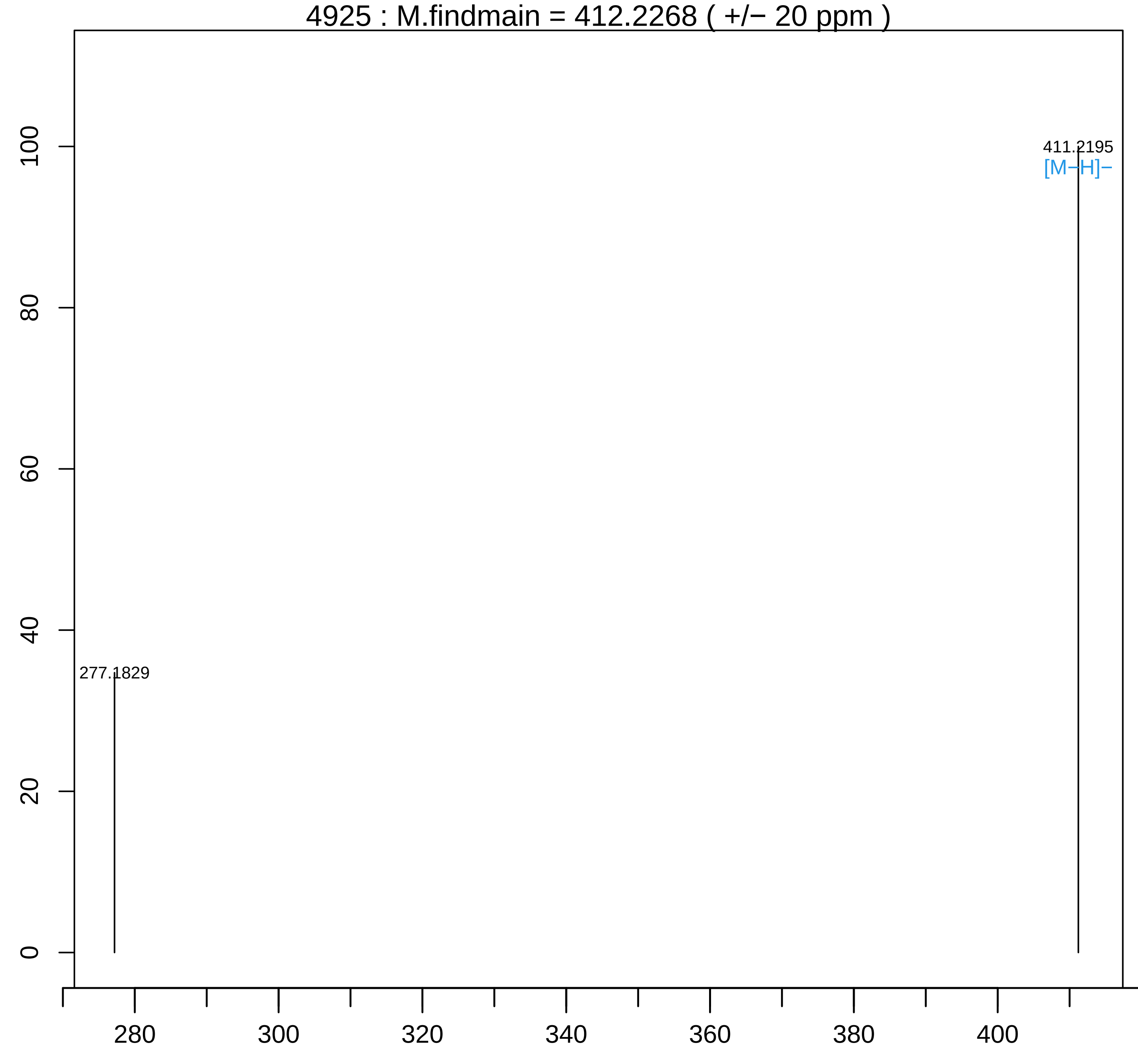
4925 : M.findmain = 412.2268 (+/− 20 ppm)
411.2195
[M−H]−
277.1829
0
20
40
60
80
100
280
300
320
340
360
380
400

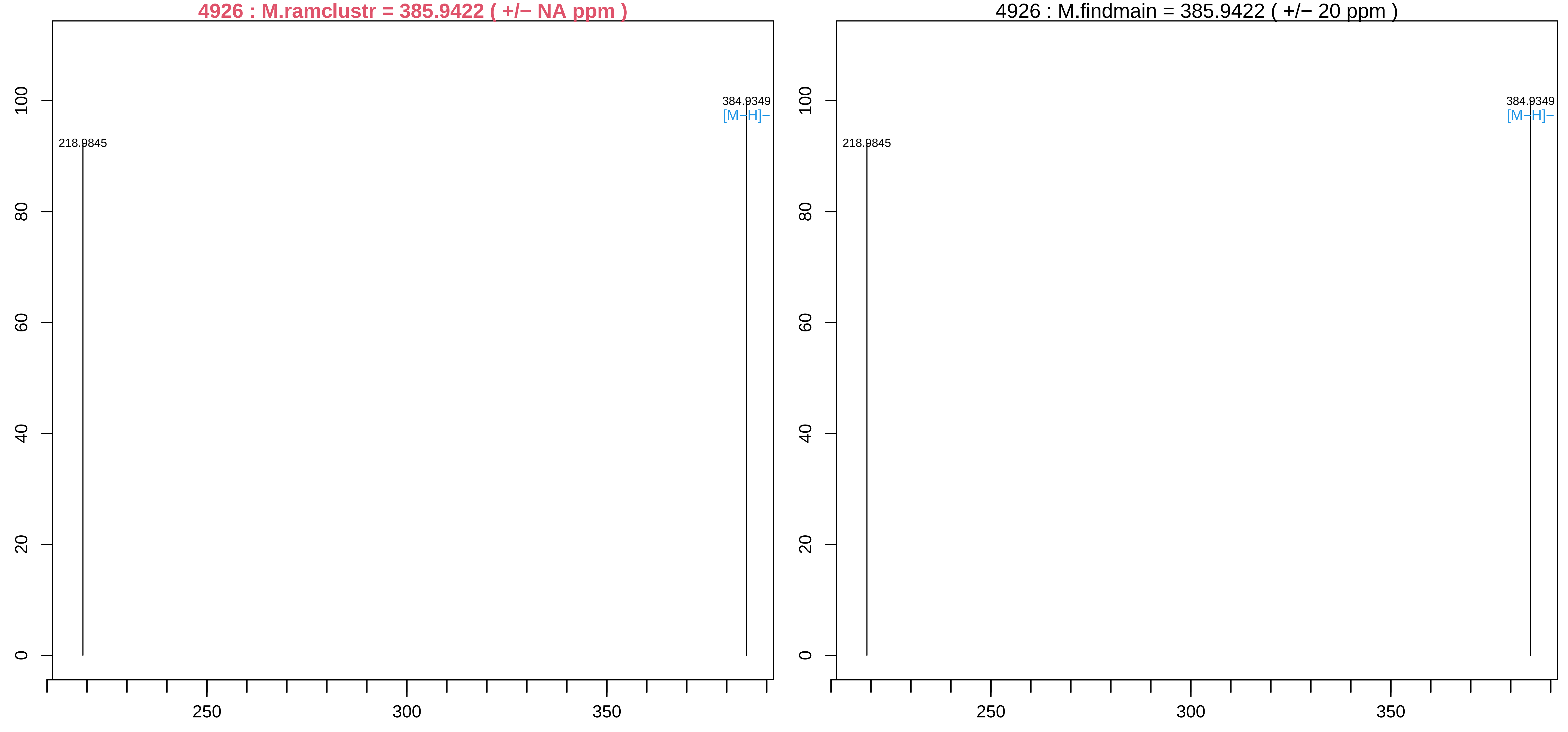
4926 : M.ramclustr = 385.9422 (+/− NA ppm)
4926 : M.findmain = 385.9422 (+/− 20 ppm)
384.9349
[M−H]−
218.9845
384.9349
[M−H]−
218.9845
0
20
40
60
80
100
250
300
350

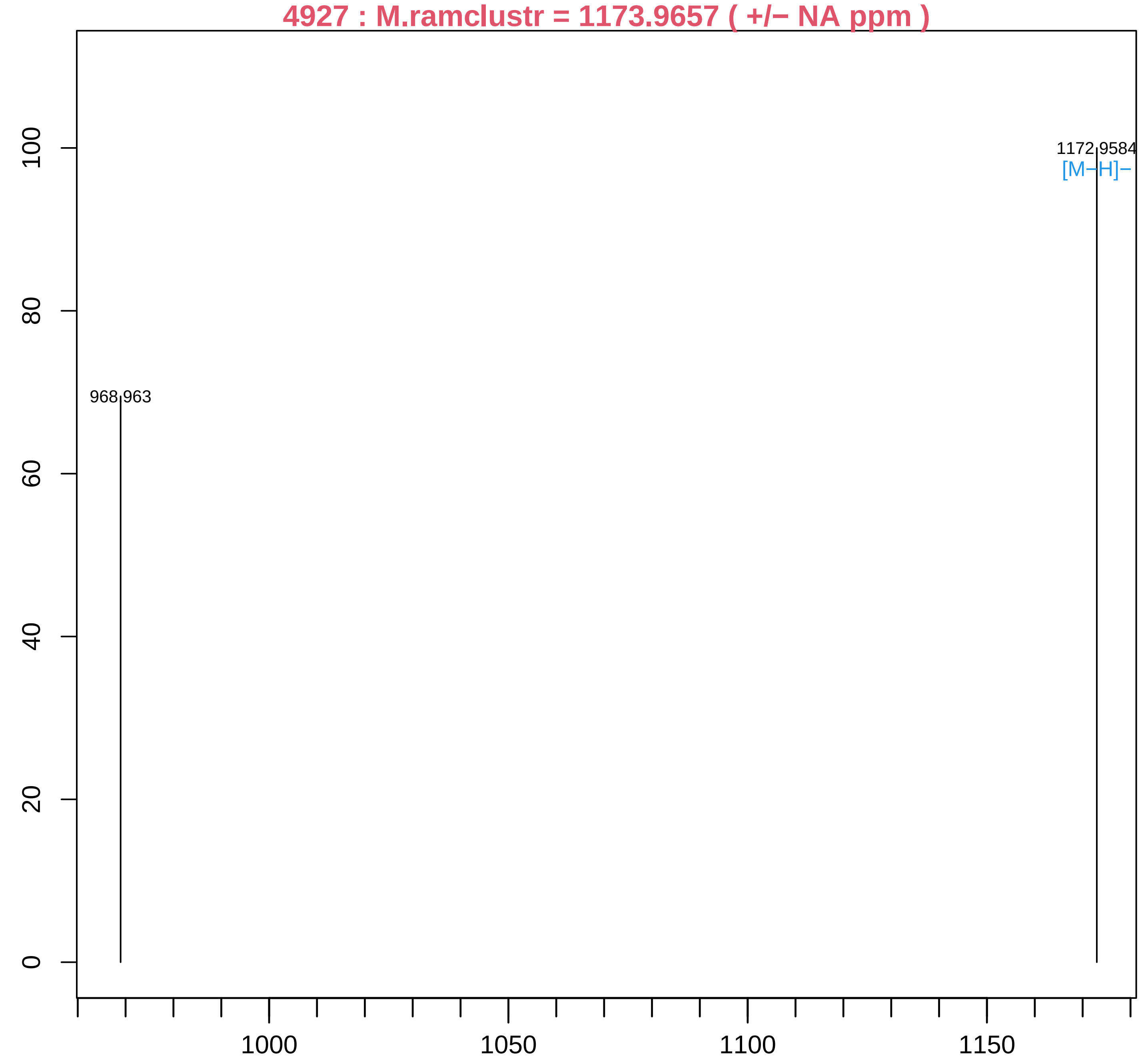
4927 : M.ramclustr = 1173.9657 (+/− NA ppm)
968.963
1172.9584
[M−H]−
0
20
40
60
80
100
1000
1050
1100
1150

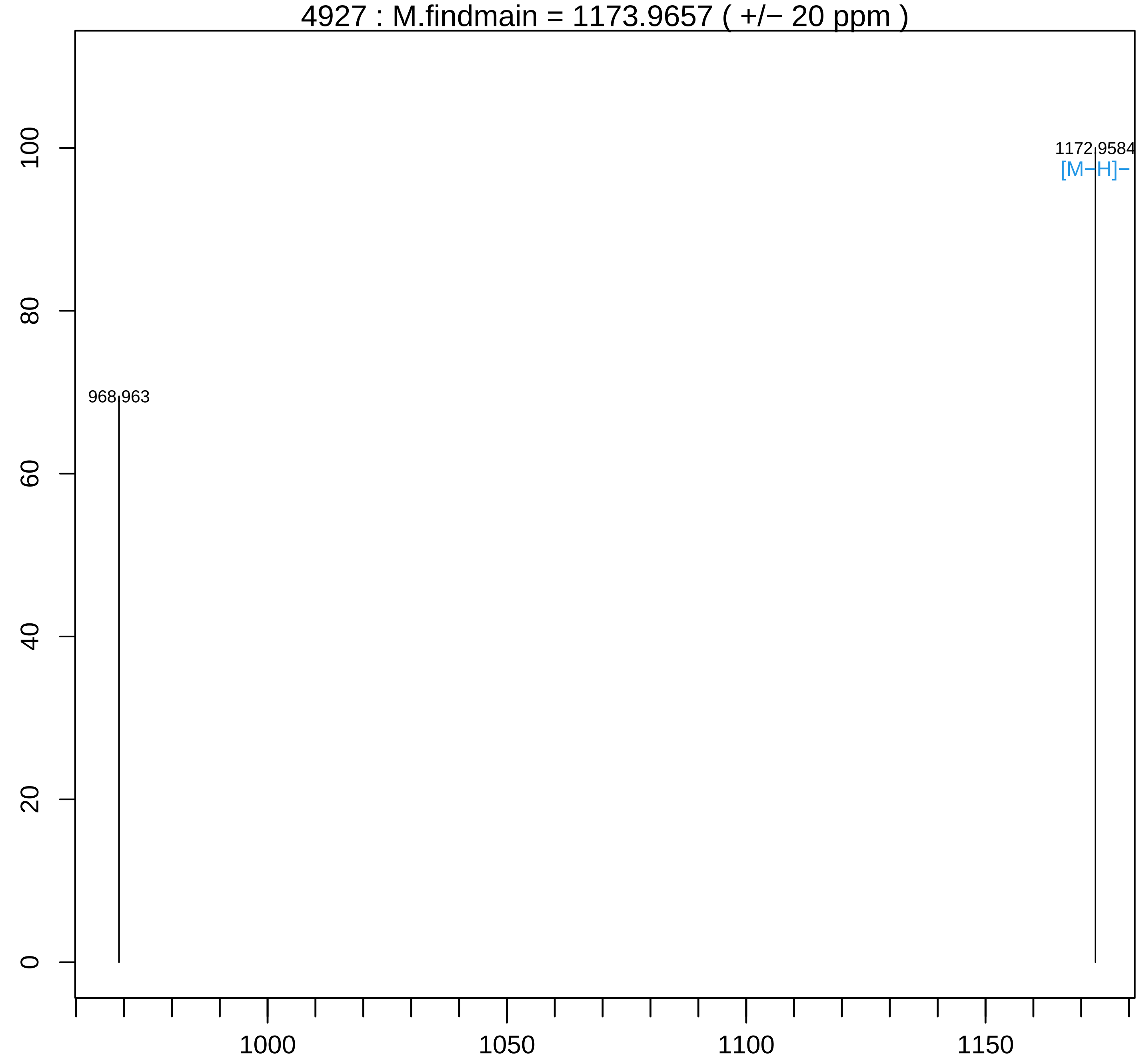
4927 : M.findmain = 1173.9657 (+/− 20 ppm)
968.963
1172.9584
[M−H]−
0
20
40
60
80
100
1000
1050
1100
1150

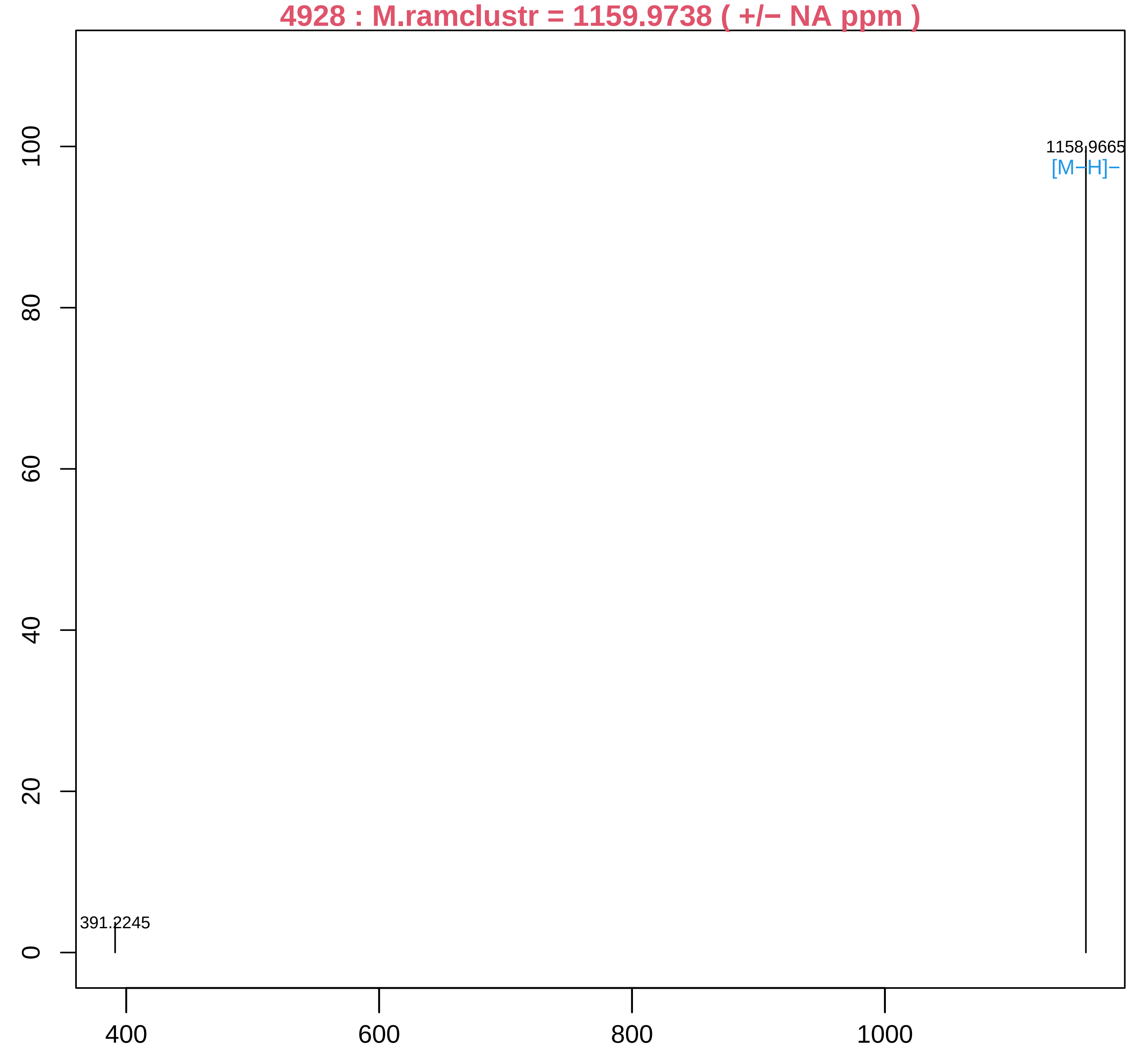
4928 : M.ramclustr = 1159.9738 (+/− NA ppm)
1158.9665
[M−H]−
391.2245
0
20
40
60
80
100
400
600
800
1000

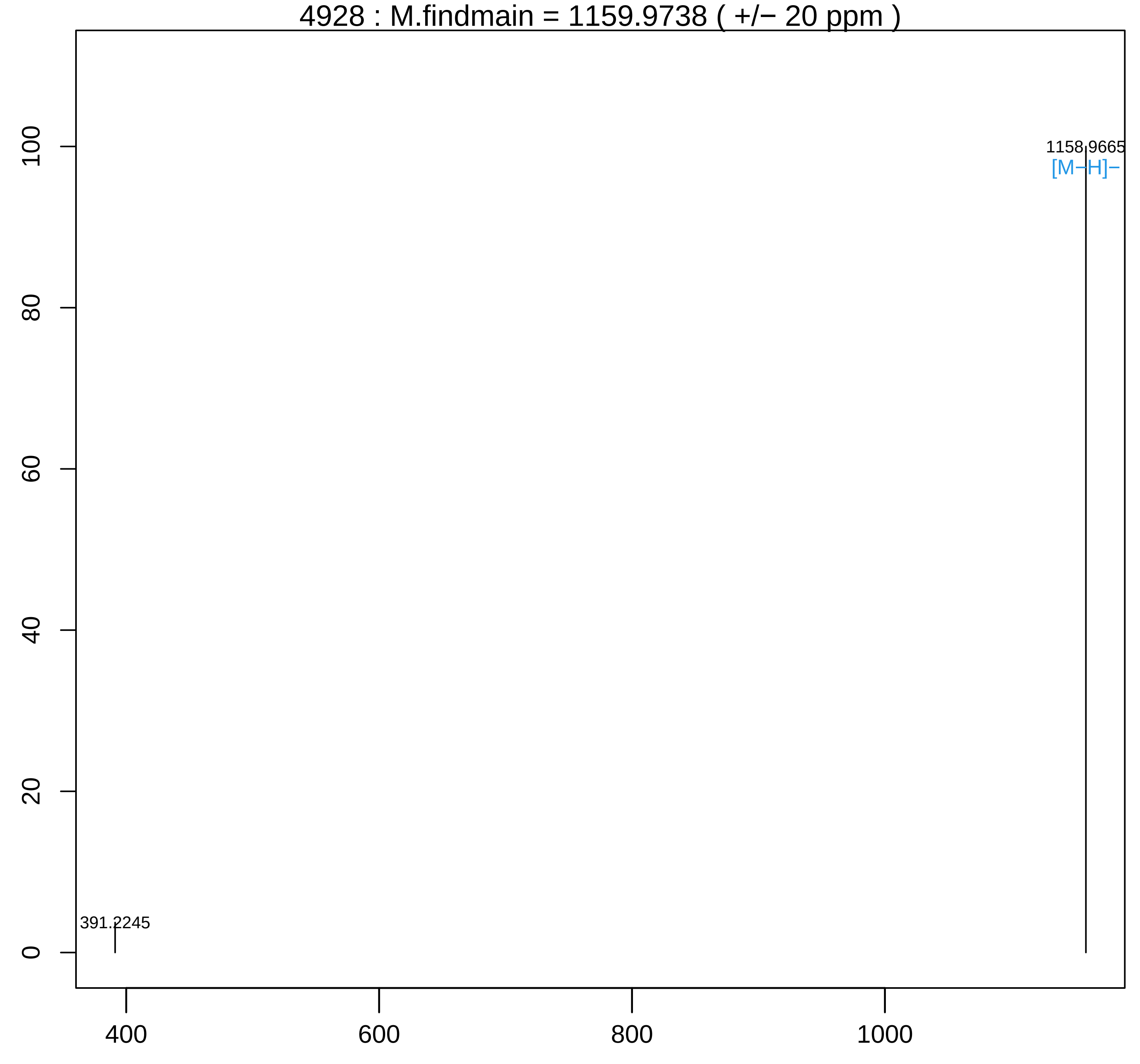
4928 : M.findmain = 1159.9738 (+/− 20 ppm)
1158.9665
[M−H]−
391.2245
0
20
40
60
80
100
400
600
800
1000

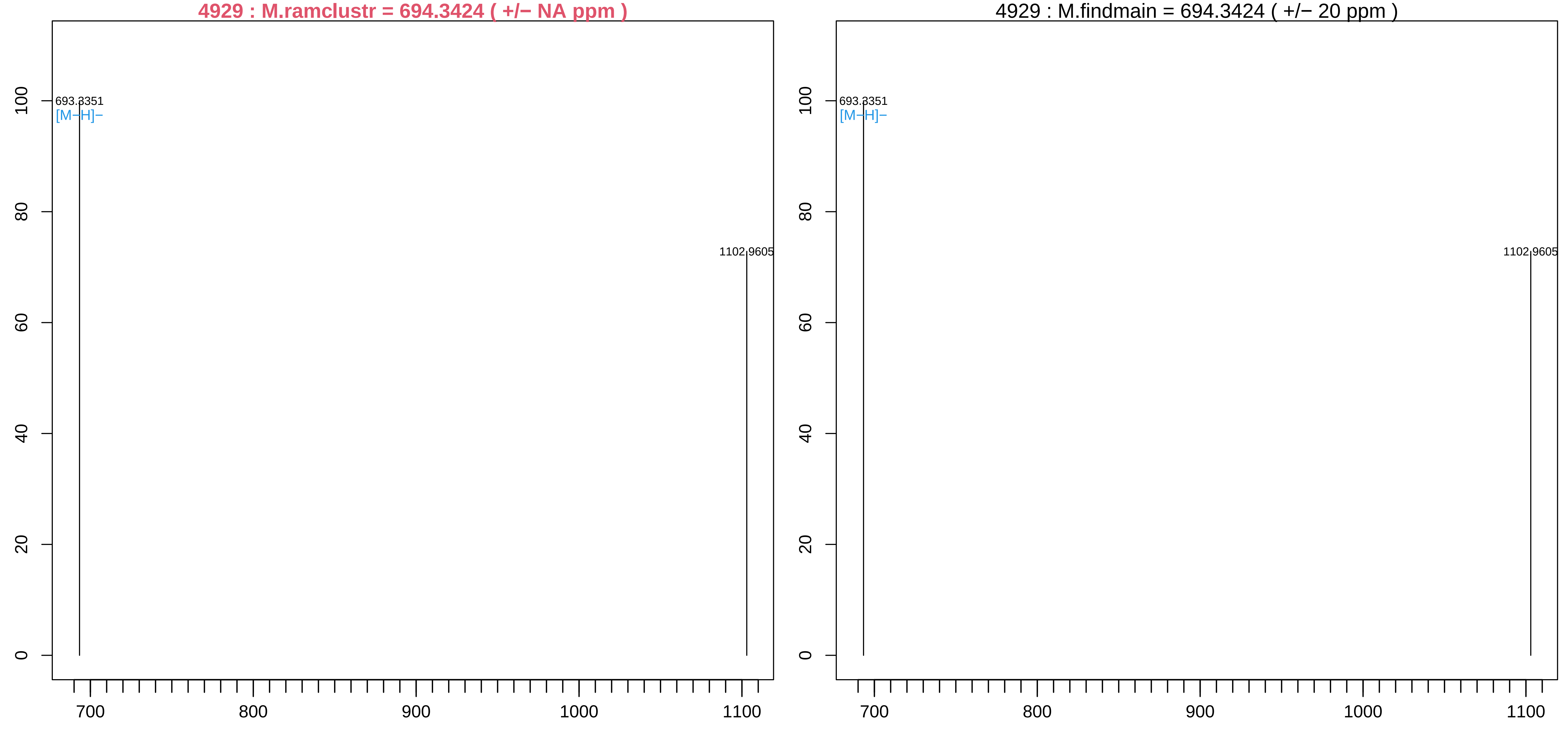

4929 : M.ramclustr = 694.3424 (+/− NA ppm)
693.3351
[M−H]−
1102.9605
0
20
40
60
80
100
700
800
900
1000
1100
4929 : M.findmain = 694.3424 (+/− 20 ppm)
693.3351
[M−H]−
1102.9605
0
20
40
60
80
100
700
800
900
1000
1100

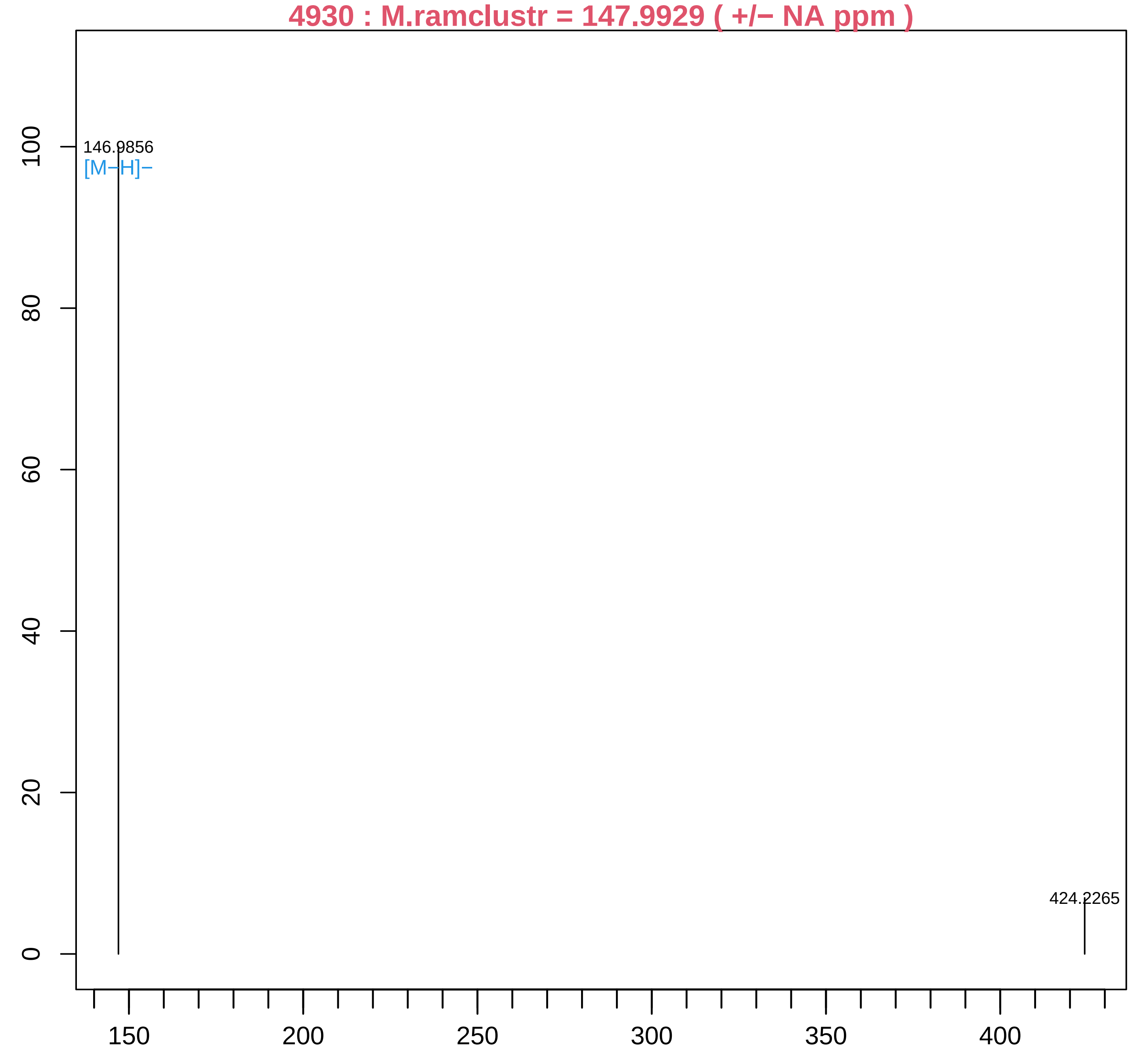
4930 : M.ramclustr = 147.9929 (+/− NA ppm)
146.9856
[M−H]−
424.2265
0
20
40
60
80
100
150
200
250
300
350
400

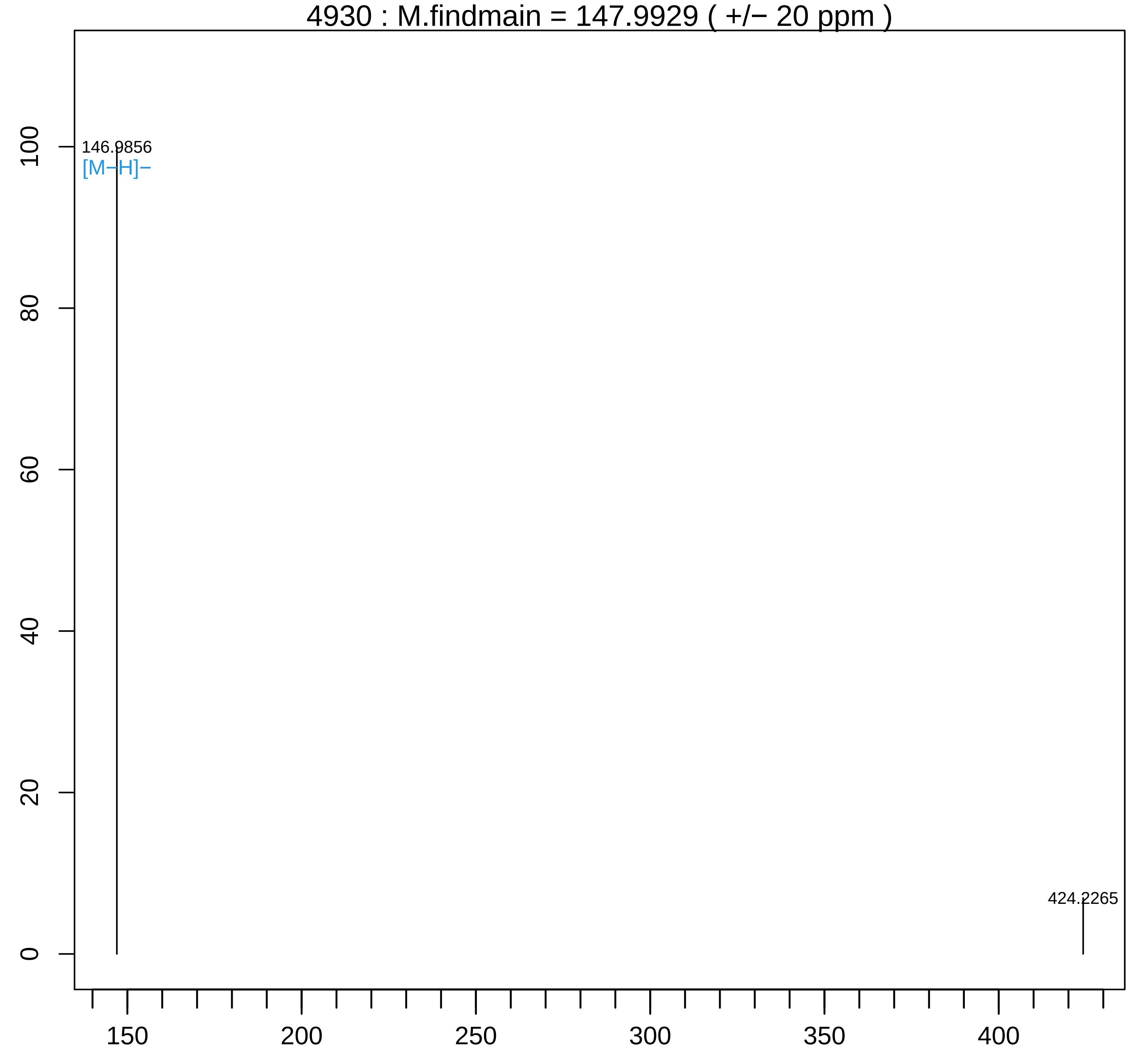
4930 : M.findmain = 147.9929 (+/− 20 ppm)
146.9856
[M−H]−
424.2265
0
20
40
60
80
100
150
200
250
300
350
400

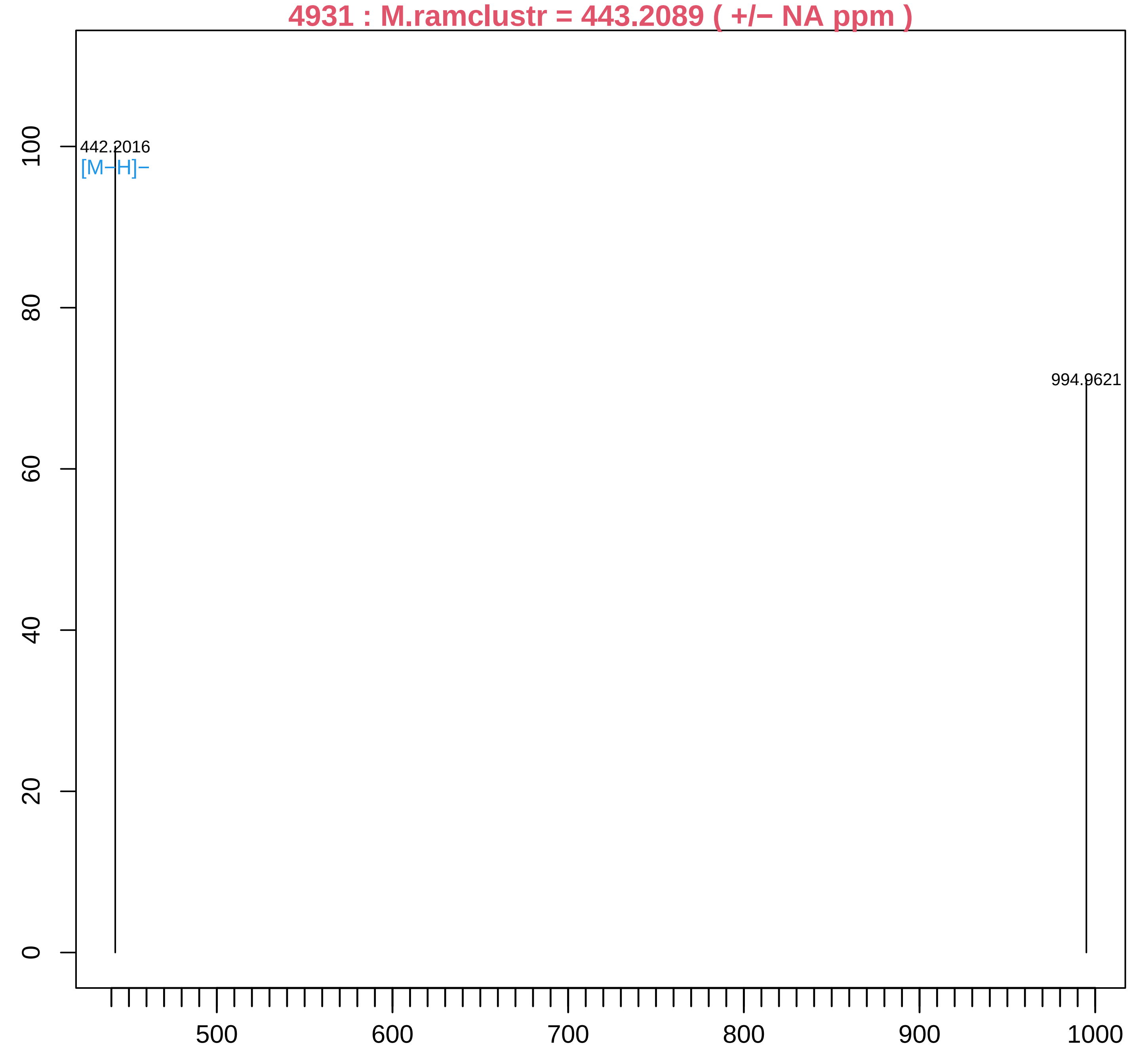
4931 : M.ramclustr = 443.2089 (+/− NA ppm)
442.2016
[M−H]−
994.9621
0
20
40
60
80
100
500
600
700
800
900
1000

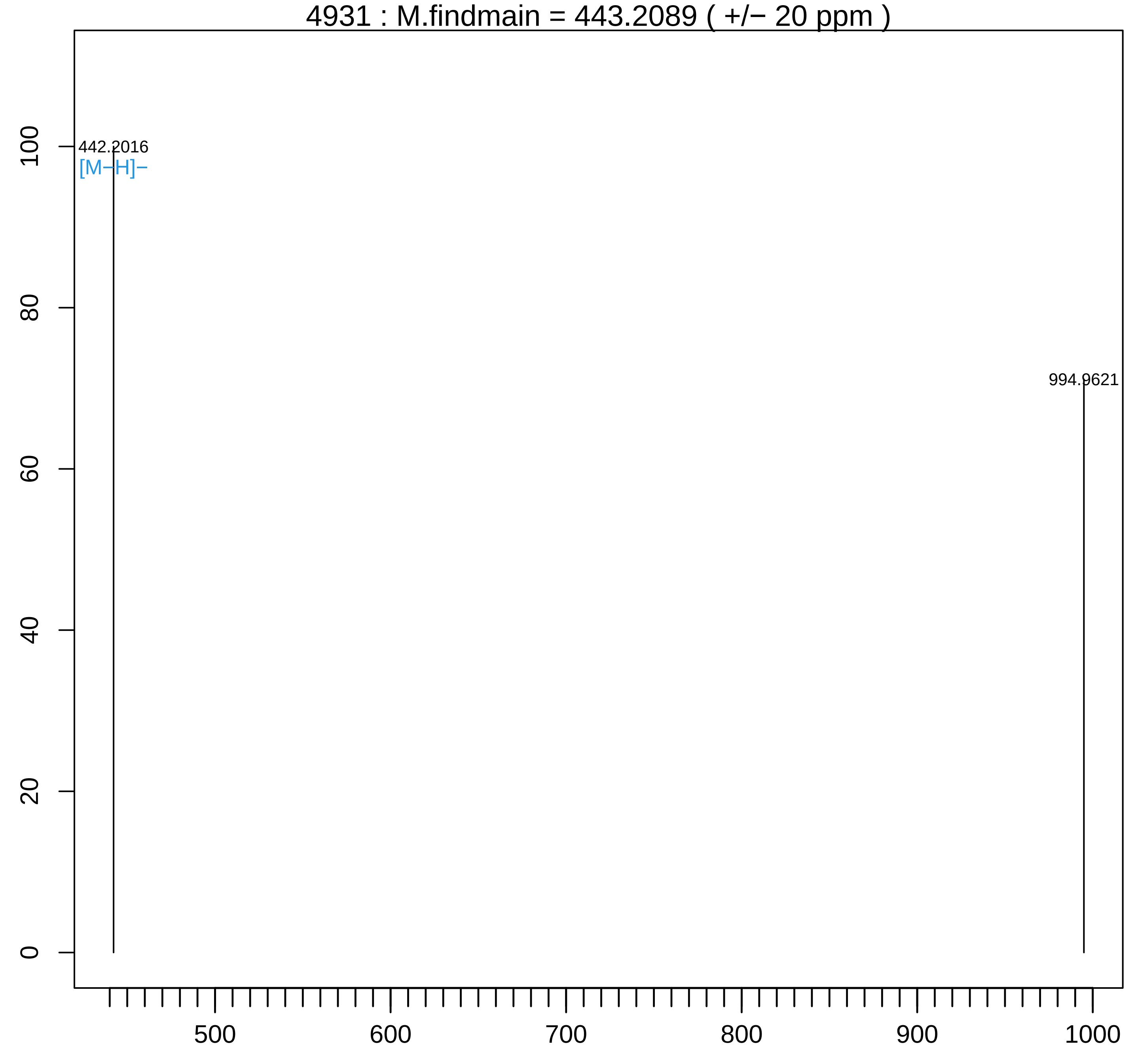
4931 : M.findmain = 443.2089 (+/− 20 ppm)
442.2016
[M−H]−
994.9621
0
20
40
60
80
100
500
600
700
800
900
1000

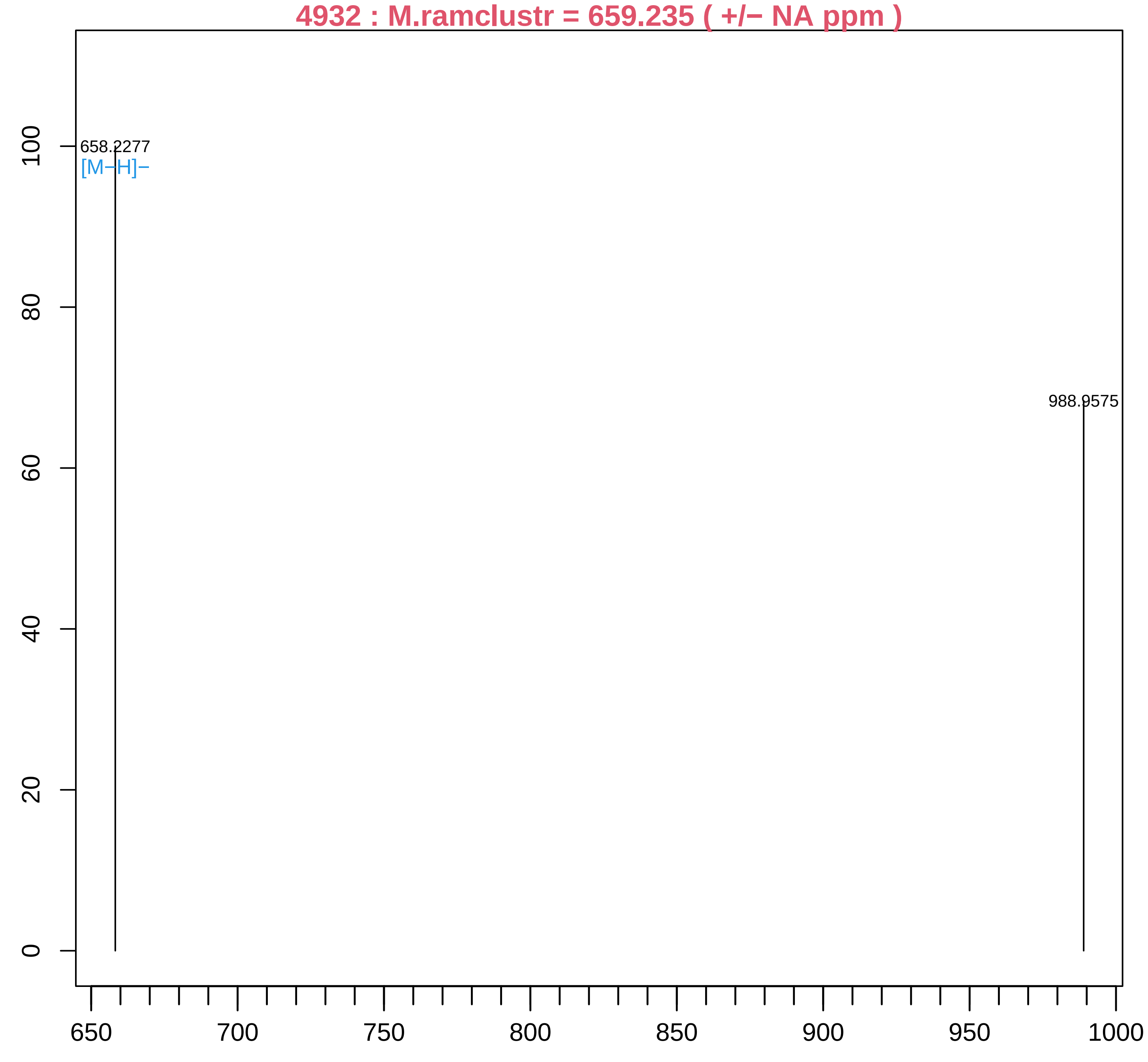
4932 : M.ramclustr = 659.235 (+/− NA ppm)
658.2277
[M−H]−
988.9575
0
20
40
60
80
100
650
700
750
800
850
900
950
1000

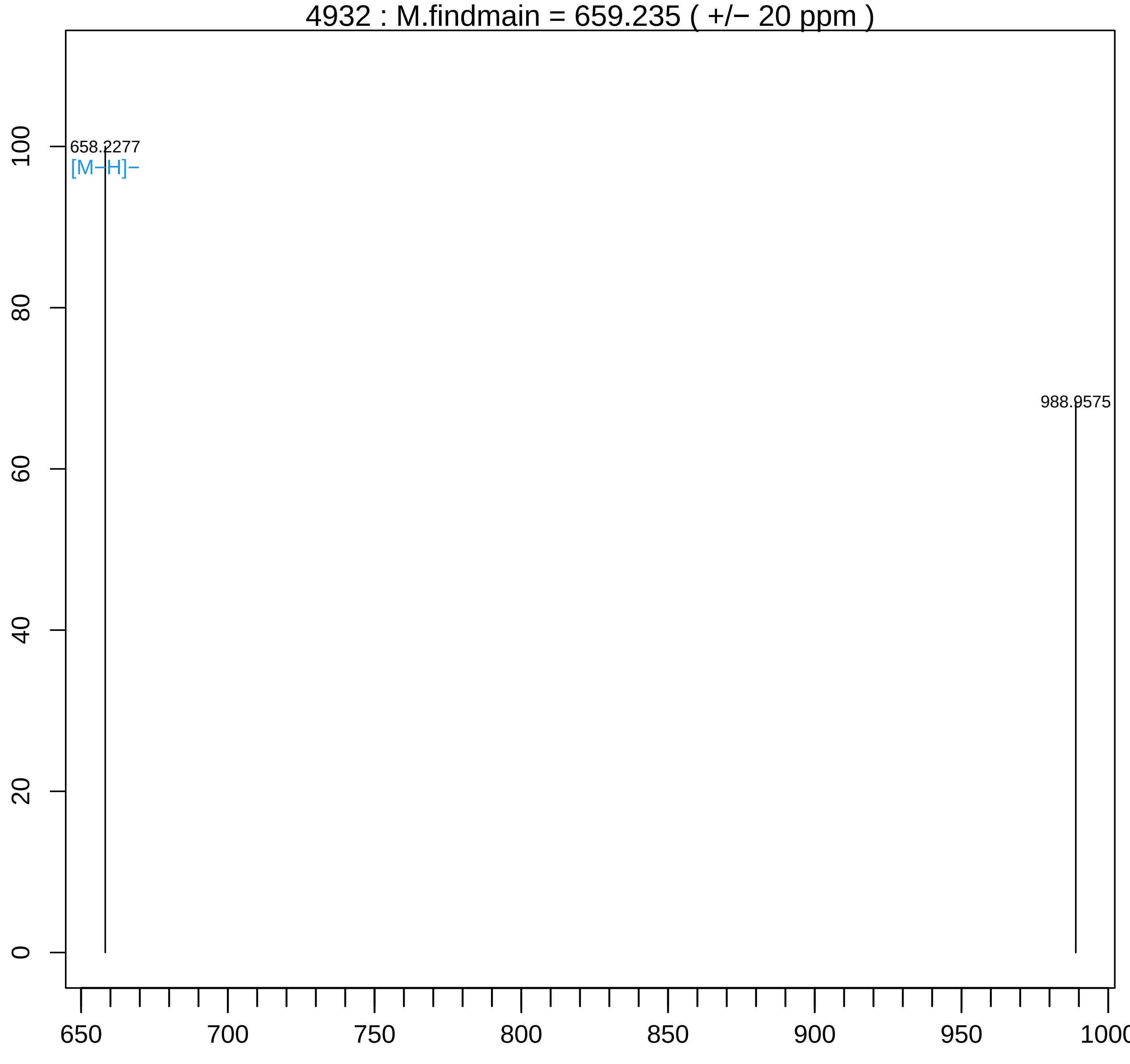
4932 : M.findmain = 659.235 (+/− 20 ppm)
658.2277
[M−H]−
988.9575
0
20
40
60
80
100
650
700
750
800
850
900
950
1000

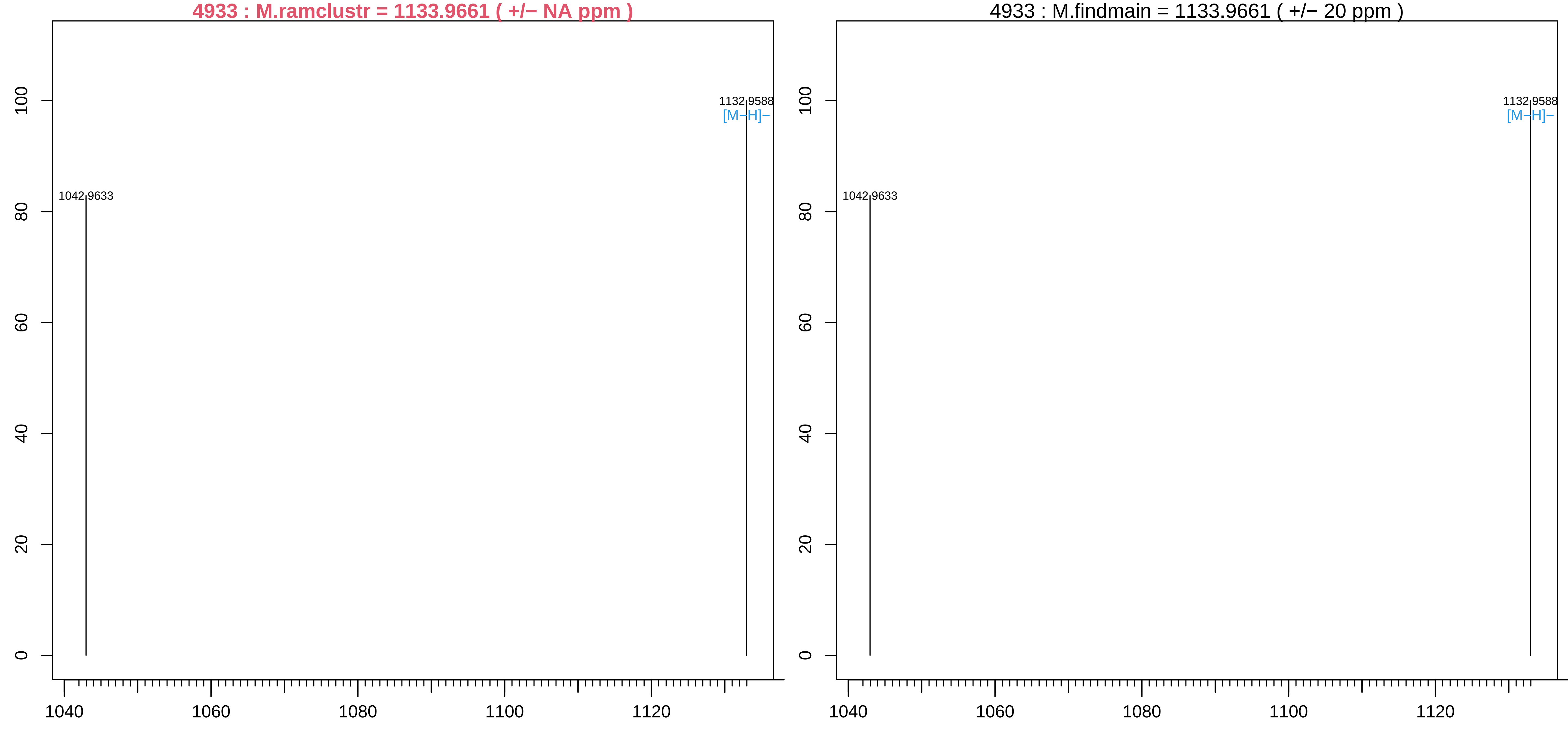
4933 : M.ramclustr = 1133.9661 (+/− NA ppm)
1132.9588
[M−H]−
1042.9633
4933 : M.findmain = 1133.9661 (+/− 20 ppm)
1132.9588
[M−H]−
1042.9633
0
20
40
60
80
100
1040
1060
1080
1100
1120

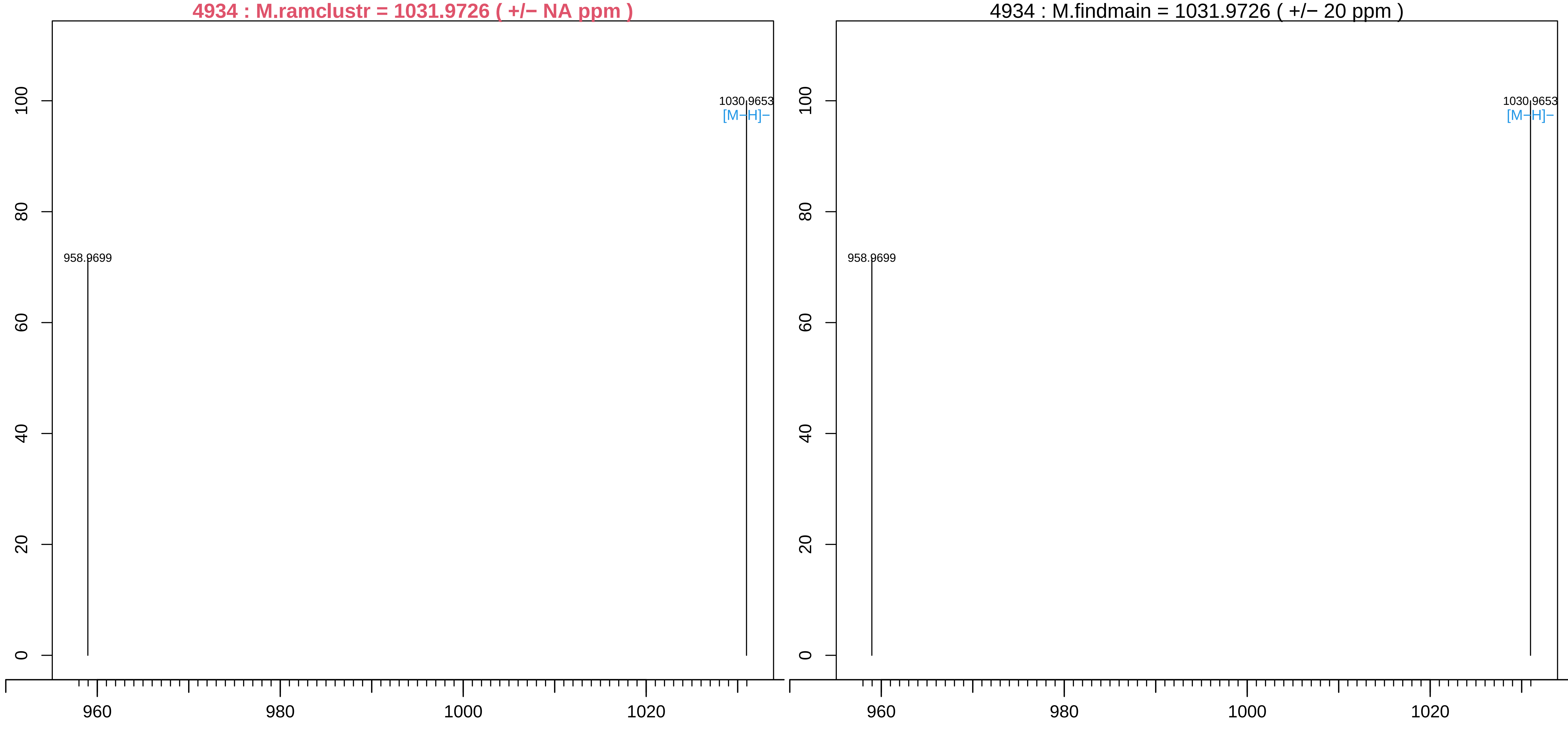

4934 : M.ramclustr = 1031.9726 (+/− NA ppm)
4934 : M.findmain = 1031.9726 (+/− 20 ppm)
958.9699
1030.9653
[M−H]−
958.9699
1030.9653
[M−H]−
0
20
40
60
80
100
960
980
1000
1020

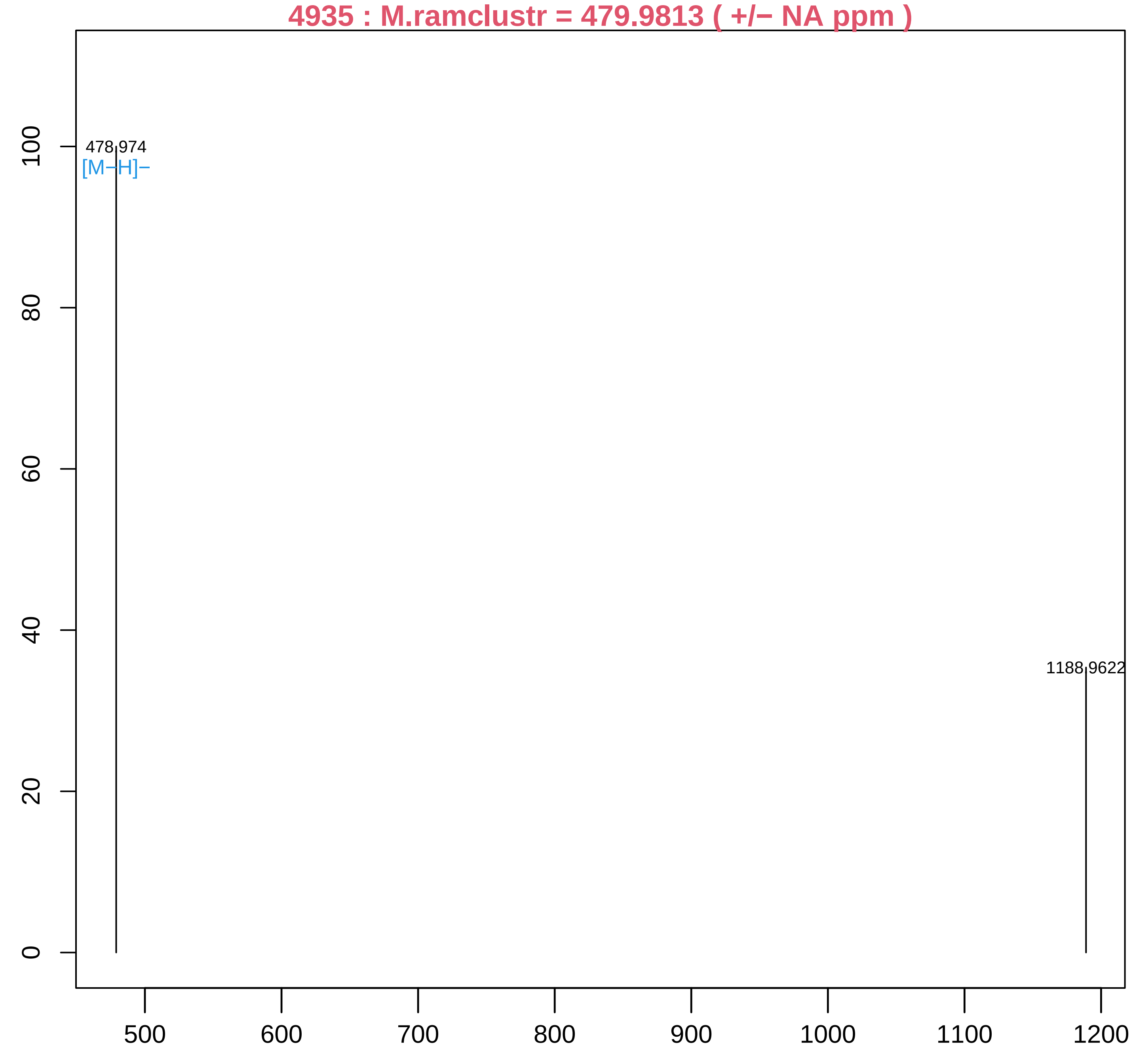
4935 : M.ramclustr = 479.9813 (+/− NA ppm)
478.974
[M−H]−
1188.9622
0
20
40
60
80
100
500
600
700
800
900
1000
1100
1200

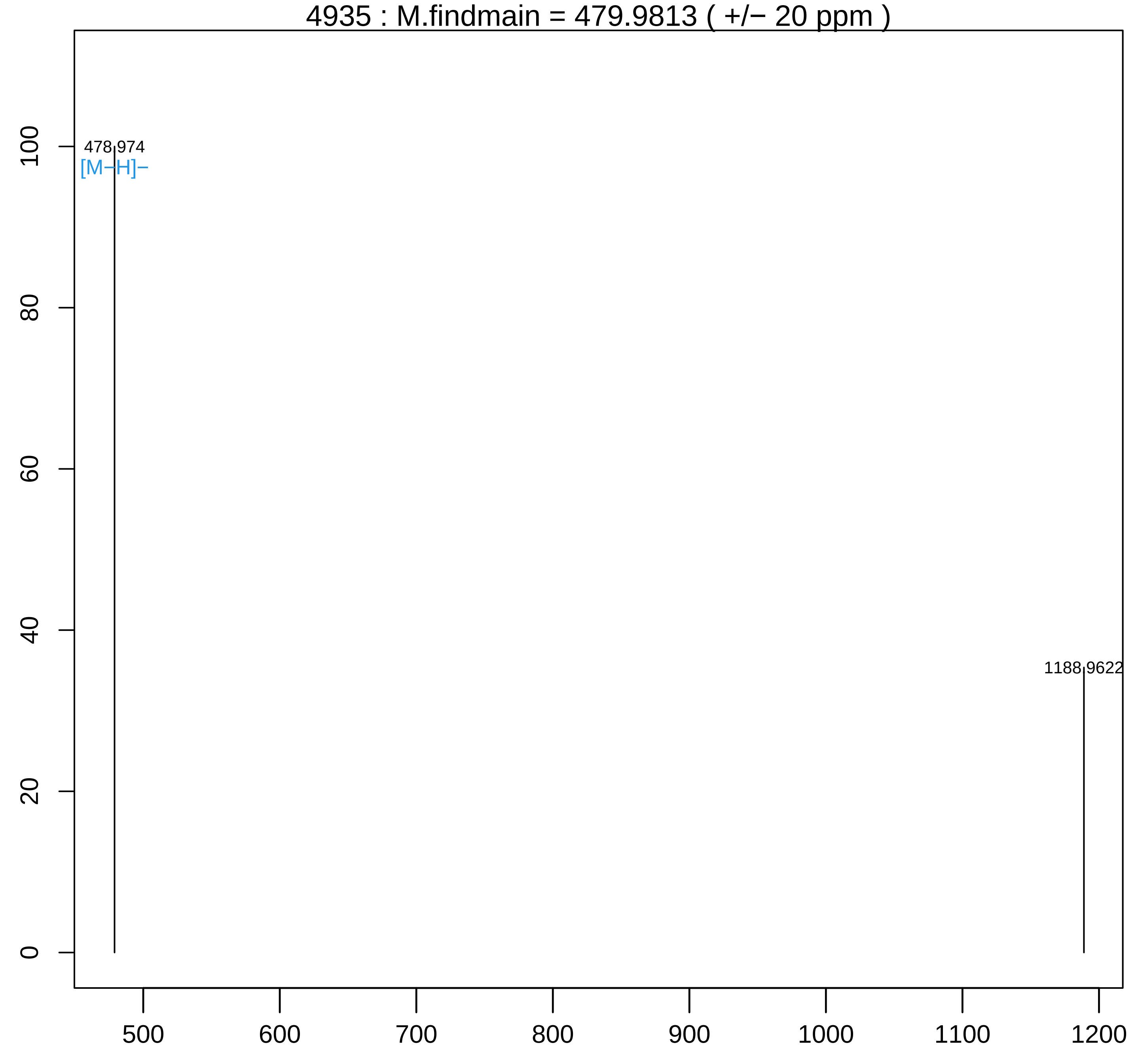
4935 : M.findmain = 479.9813 (+/− 20 ppm)
478.974
[M−H]−
1188.9622
0
20
40
60
80
100
500
600
700
800
900
1000
1100
1200

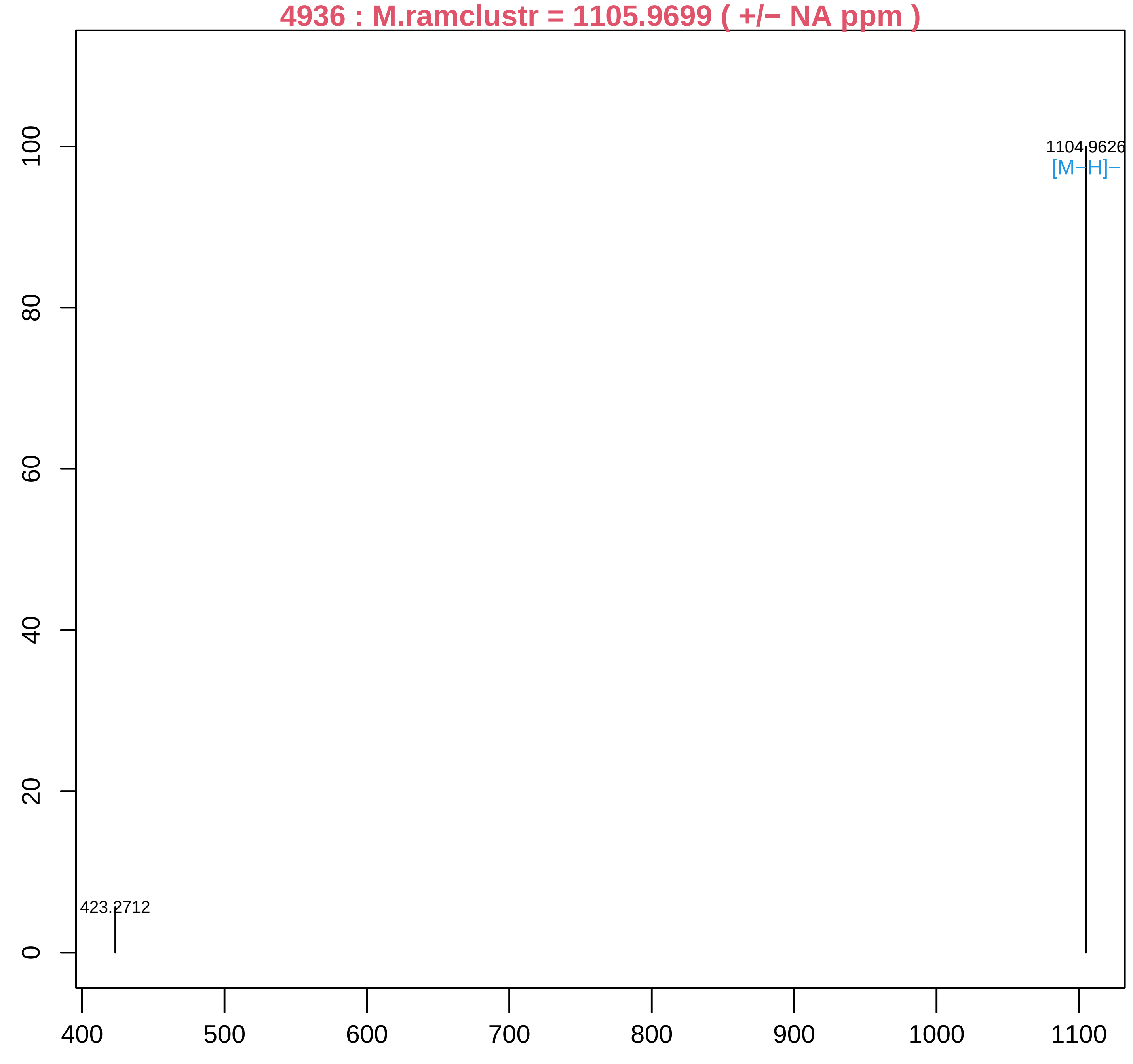
4936 : M.ramclustr = 1105.9699 (+/− NA ppm)
1104.9626
[M−H]−
423.2712
0
20
40
60
80
100
400
500
600
700
800
900
1000
1100
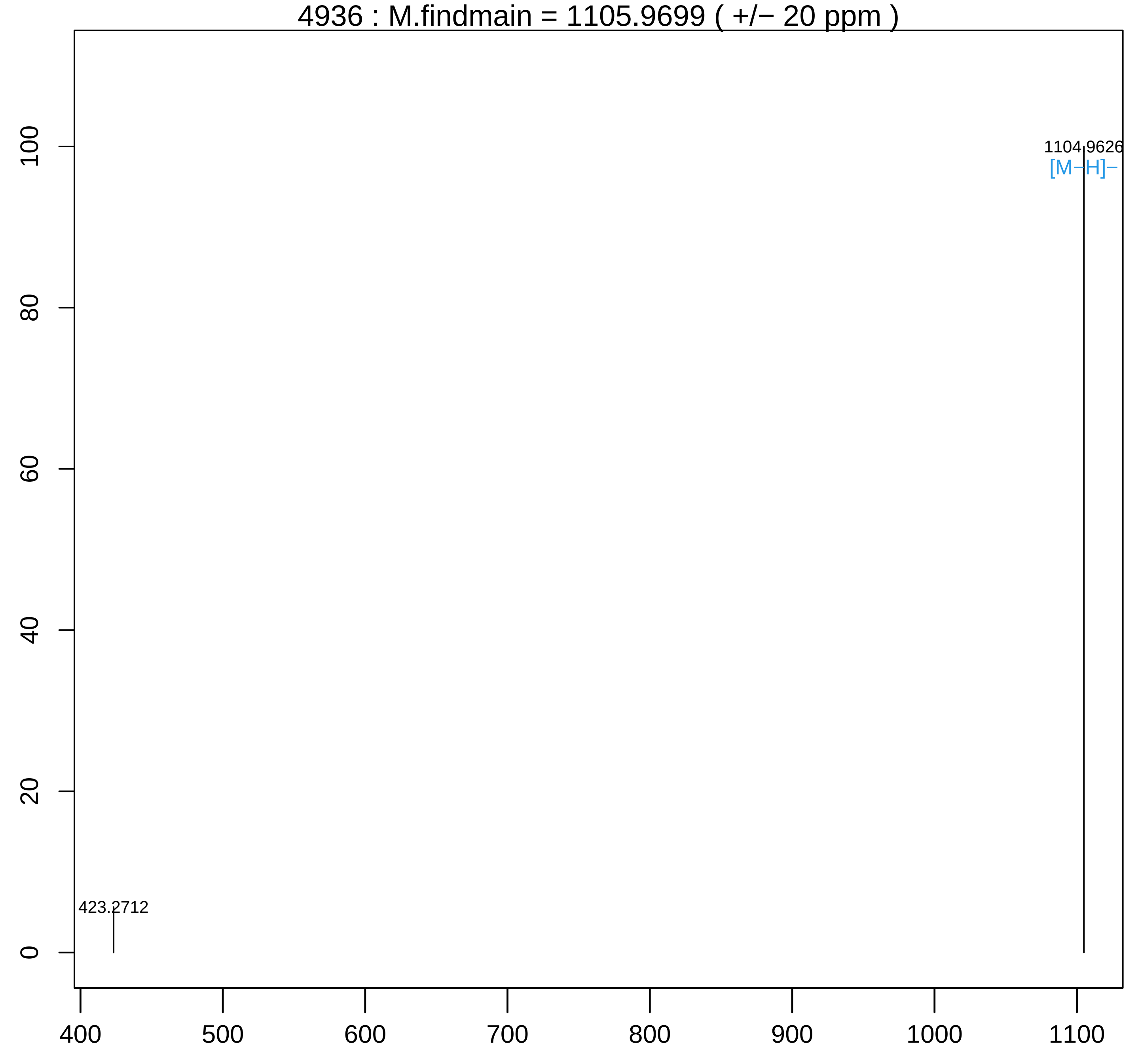
4936 : M.findmain = 1105.9699 (+/− 20 ppm)
1104.9626
[M−H]−
423.2712
0
20
40
60
80
100
400
500
600
700
800
900
1000
1100

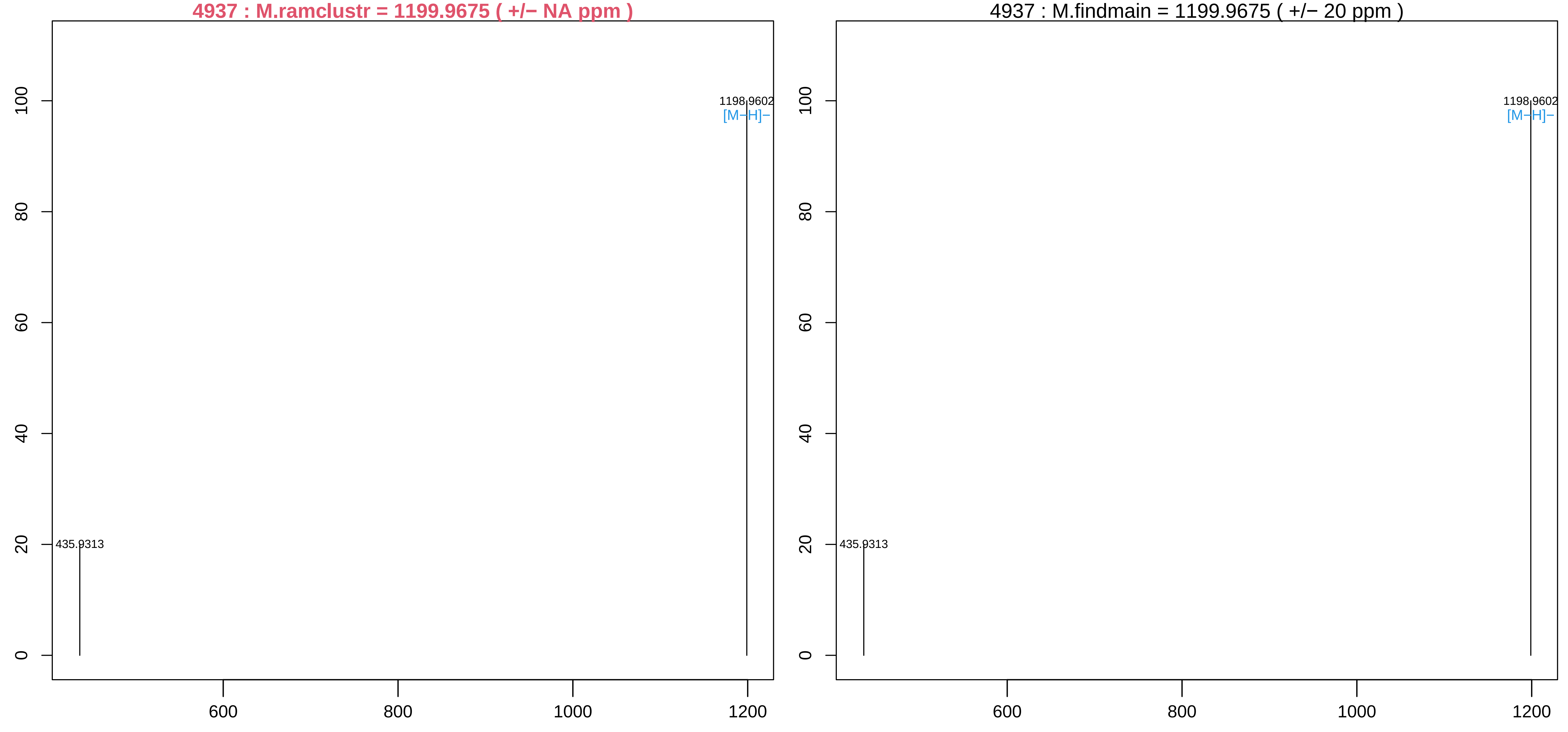
4937 : M.ramclustr = 1199.9675 (+/− NA ppm)
4937 : M.findmain = 1199.9675 (+/− 20 ppm)
1198.9602
[M−H]−
435.9313
0
20
40
60
80
100
600
800
1000
1200

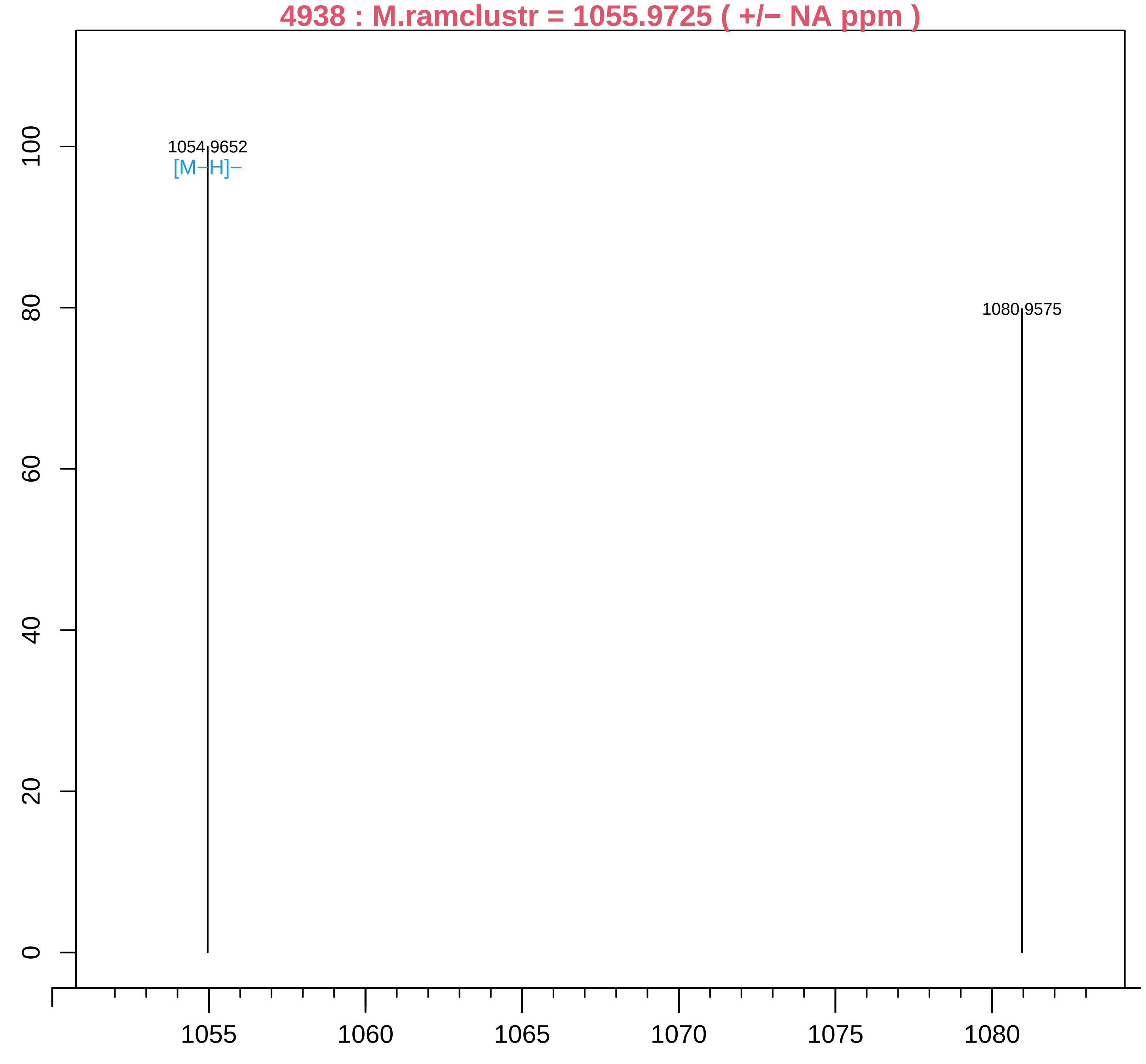
4938 : M.ramclustr = 1055.9725 (+/− NA ppm)
1054.9652
[M−H]−
1080.9575
0
20
40
60
80
100
1055
1060
1065
1070
1075
1080

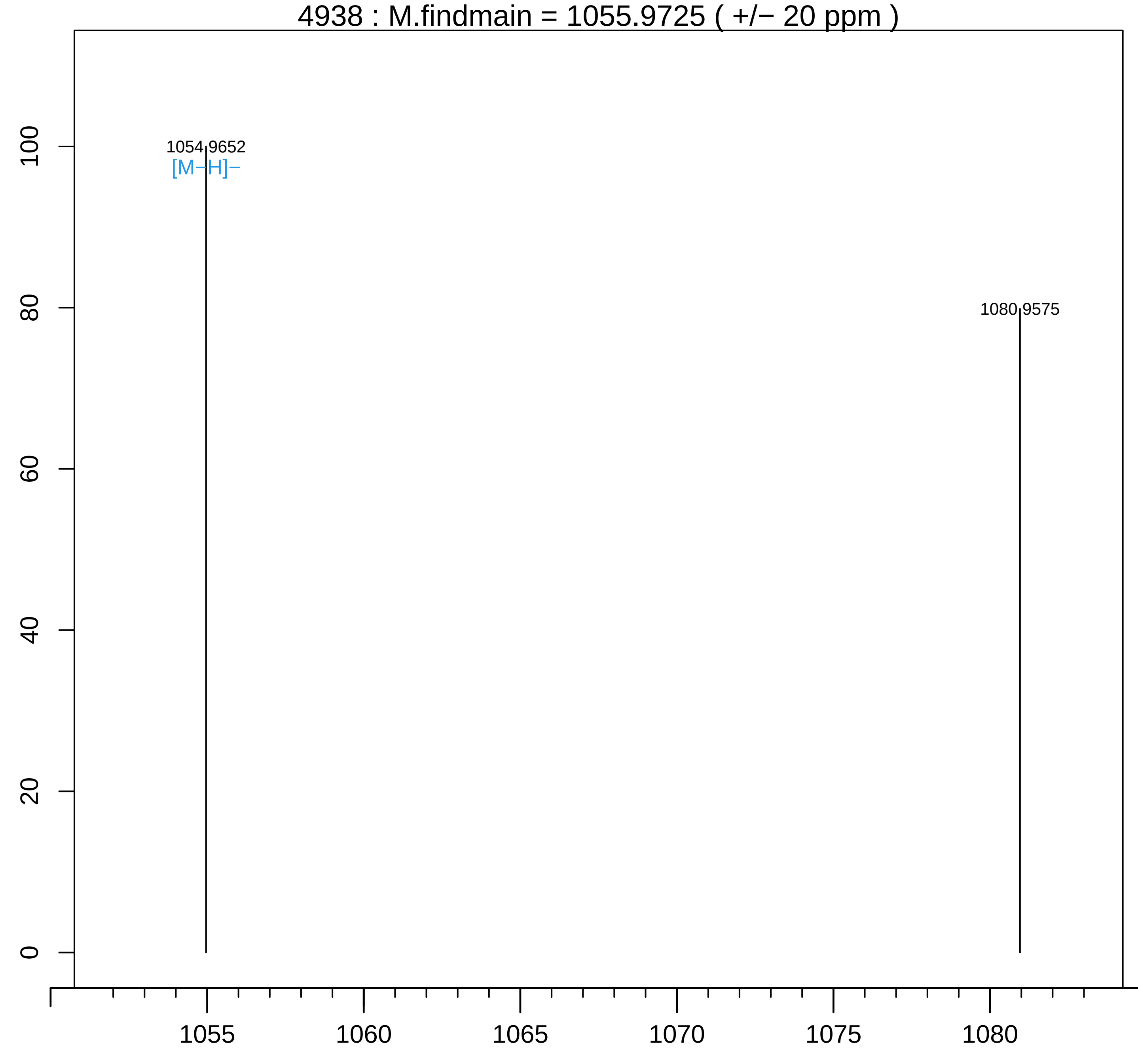
4938 : M.findmain = 1055.9725 (+/− 20 ppm)
1054.9652
[M−H]−
1080.9575
0
20
40
60
80
100
1055
1060
1065
1070
1075
1080

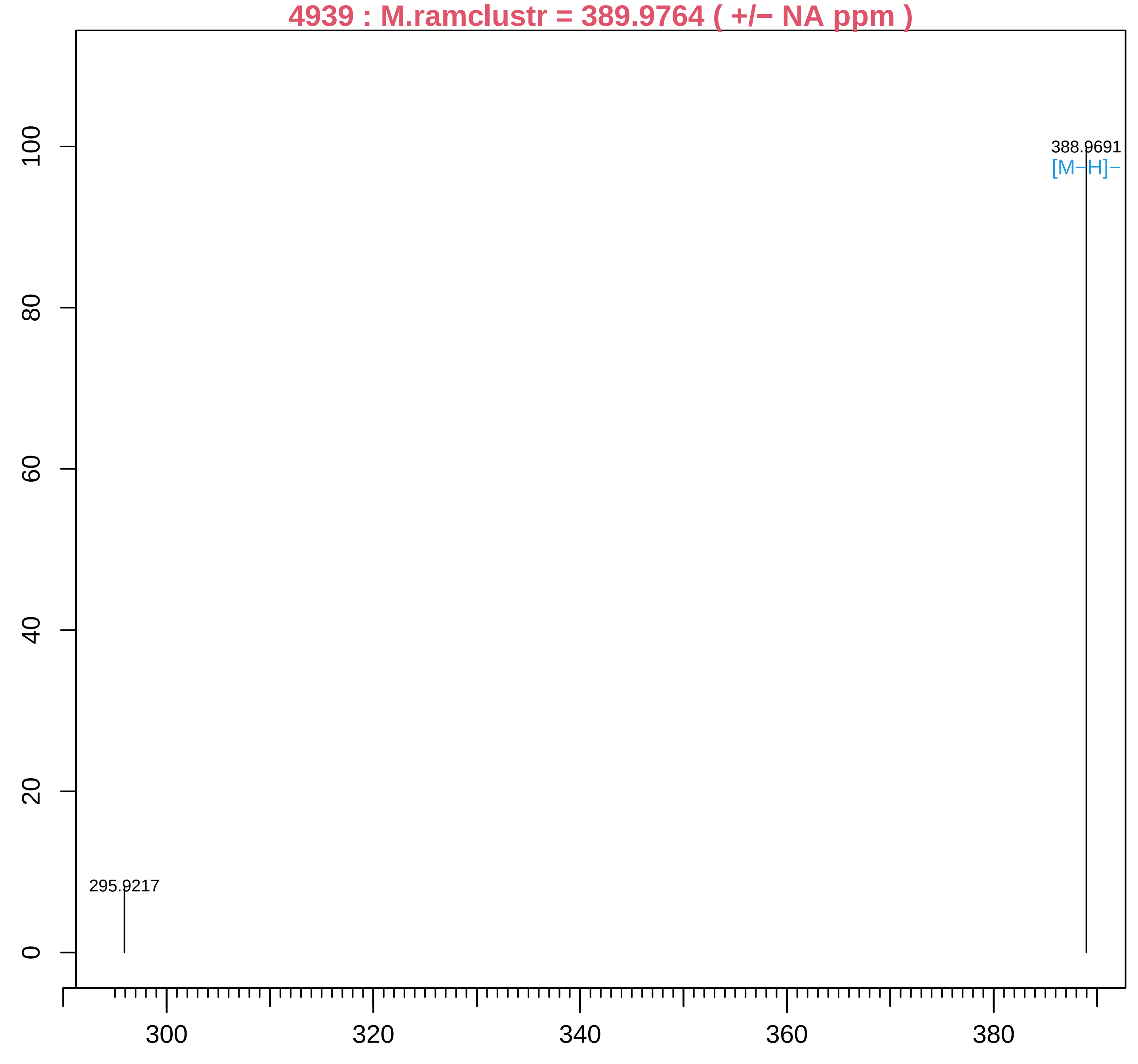
4939 : M.ramclustr = 389.9764 (+/− NA ppm)
388.9691
[M−H]−
295.9217
0
20
40
60
80
100
300
320
340
360
380

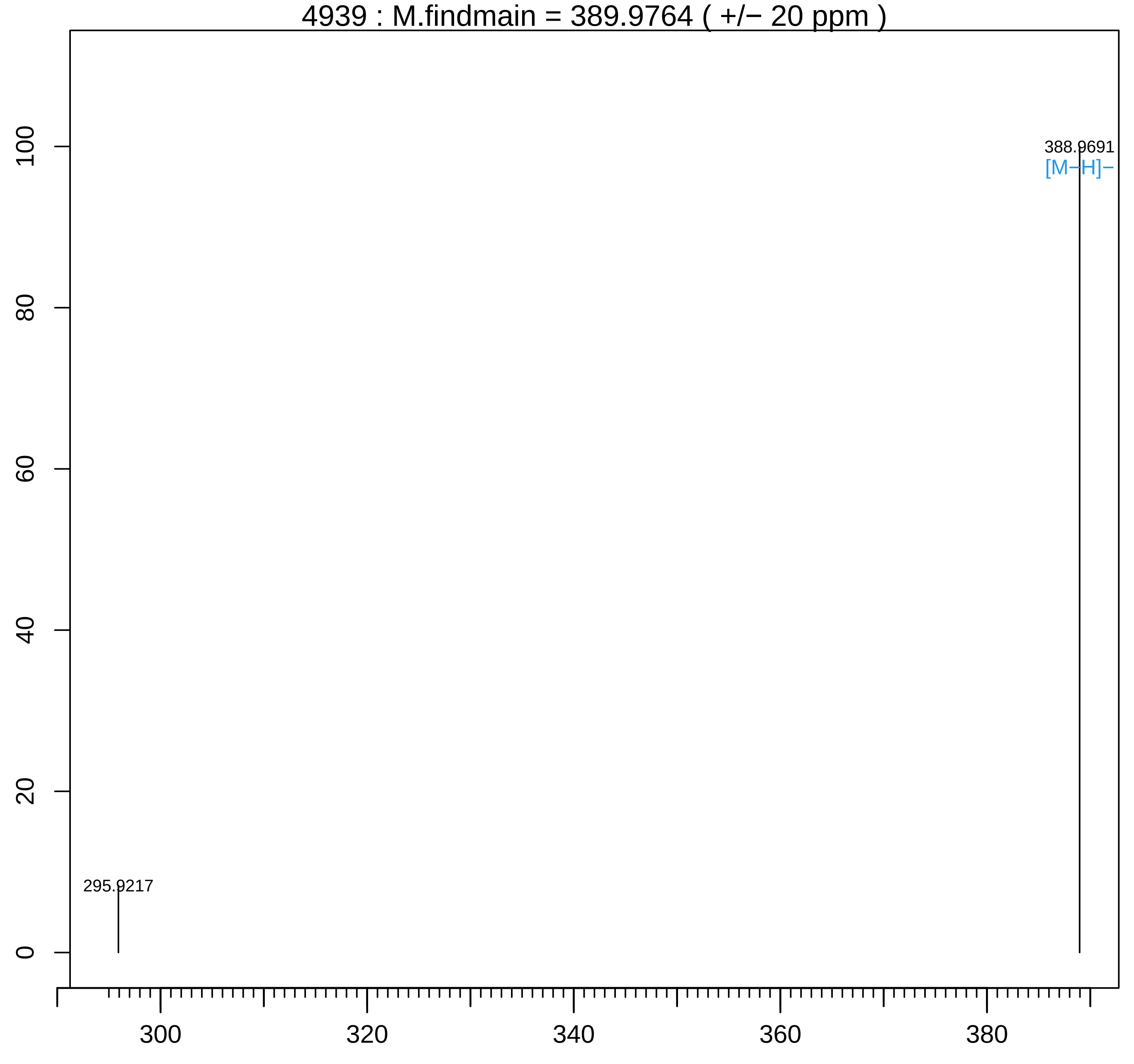
4939 : M.findmain = 389.9764 (+/− 20 ppm)
388.9691
[M−H]−
295.9217
0
20
40
60
80
100
300
320
340
360
380

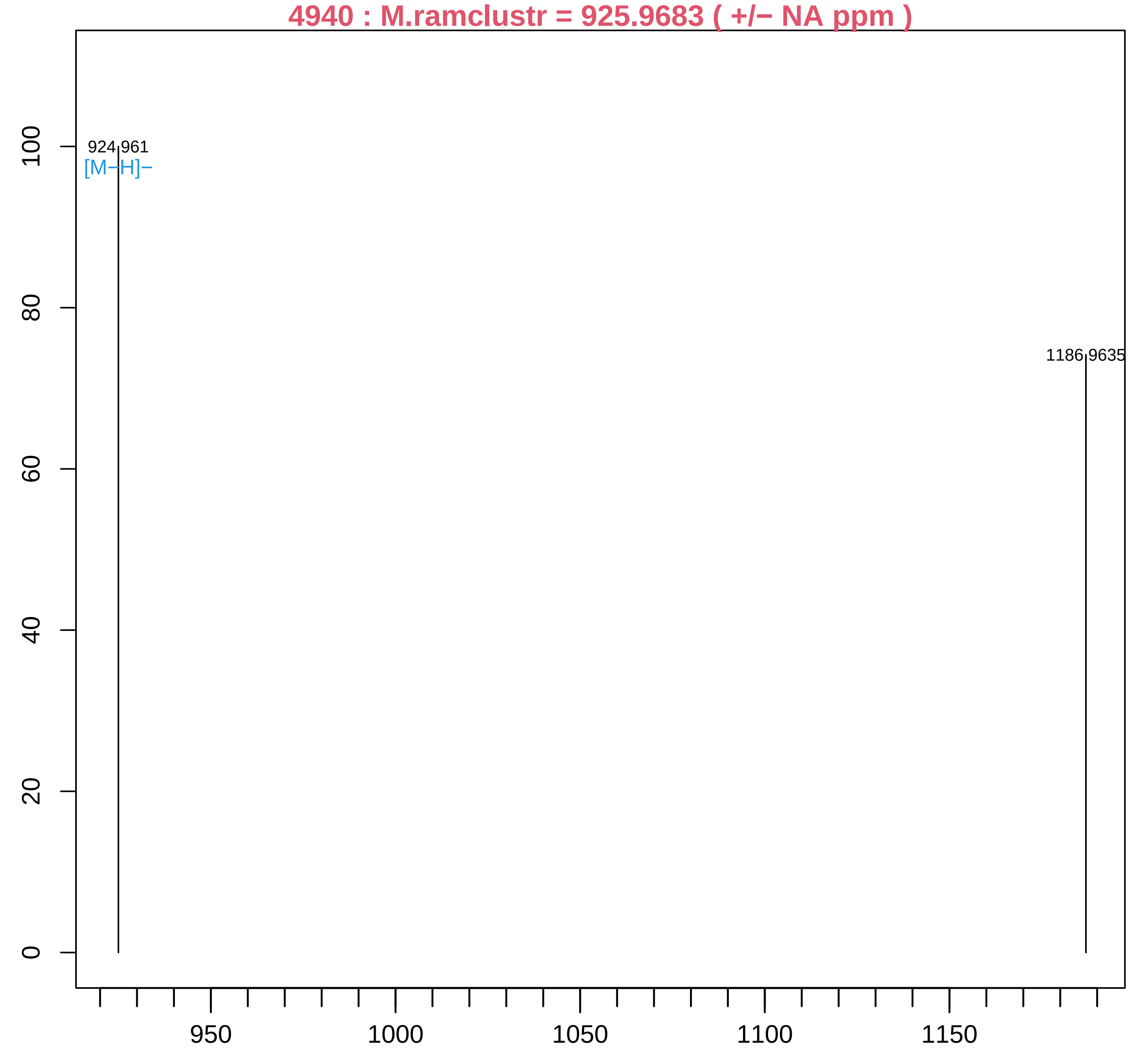

4940 : M.ramclustr = 925.9683 (+/− NA ppm)
924.961
[M−H]−
1186.9635
0
20
40
60
80
100
950
1000
1050
1100
1150

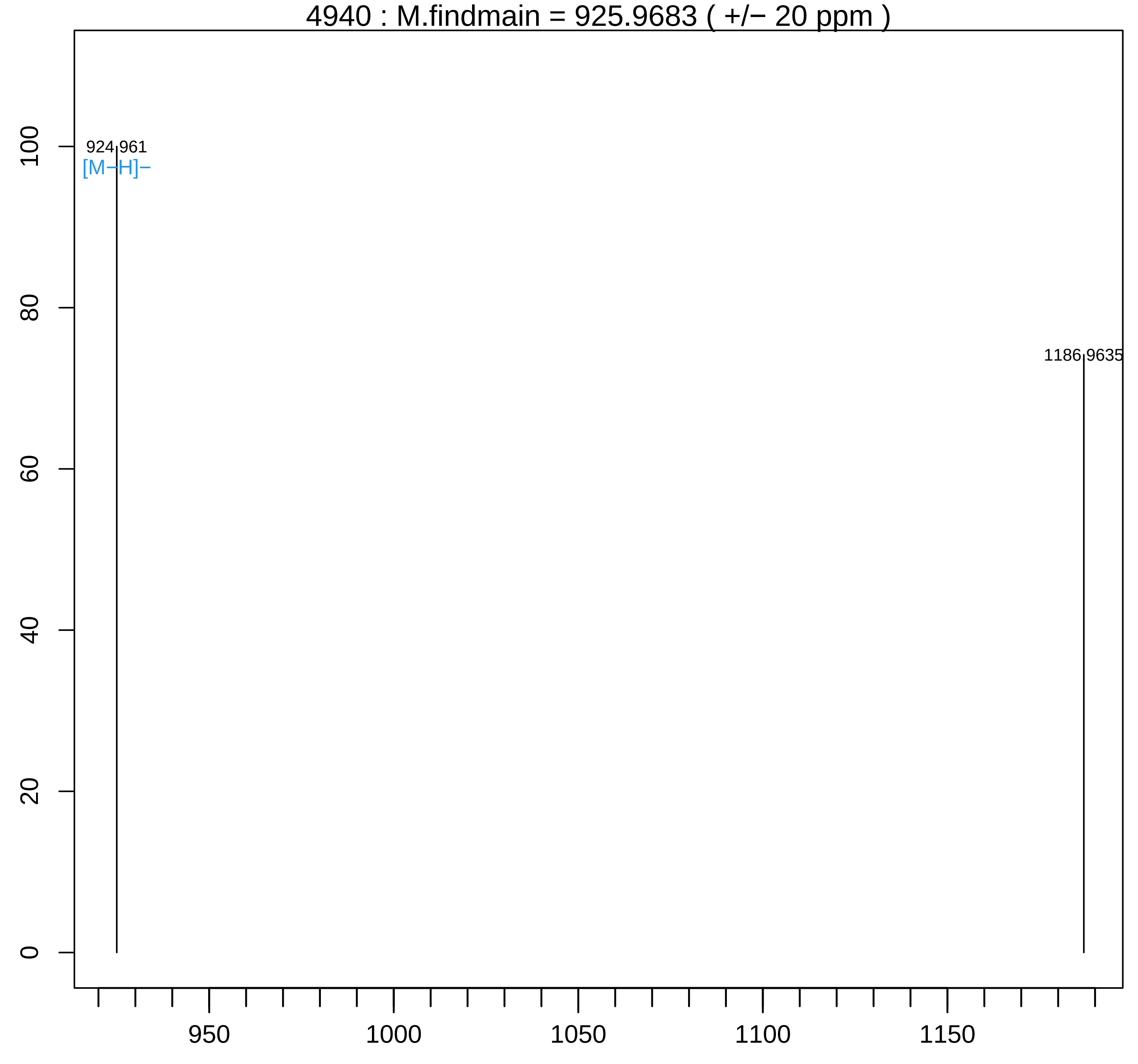

4940 : M.findmain = 925.9683 (+/− 20 ppm)
924.961
[M−H]−
1186.9635
0
20
40
60
80
100
950
1000
1050
1100
1150

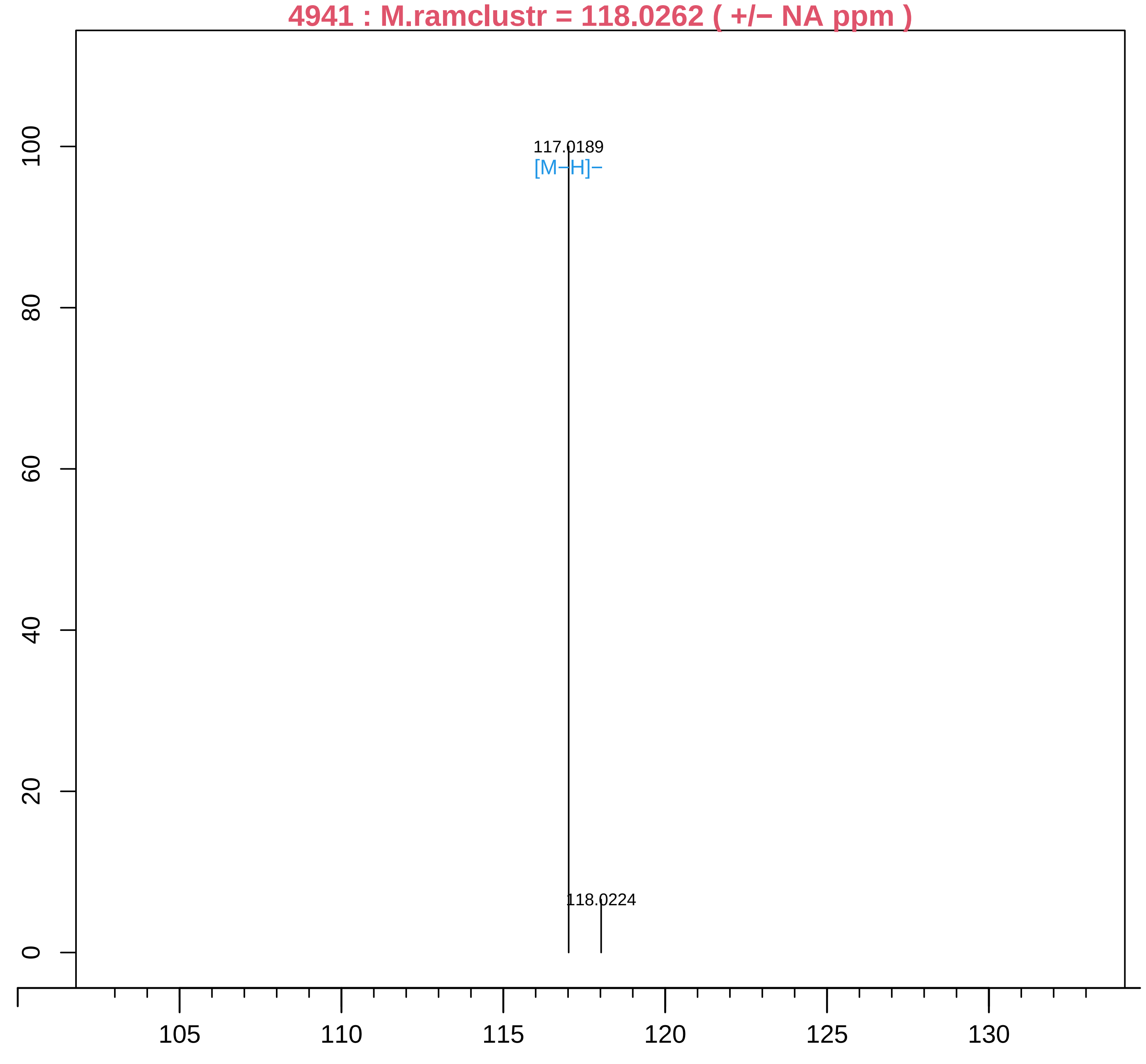
4941 : M.ramclustr = 118.0262 (+/− NA ppm)
117.0189
[M−H]−
118.0224
0
20
40
60
80
100
105
110
115
120
125
130

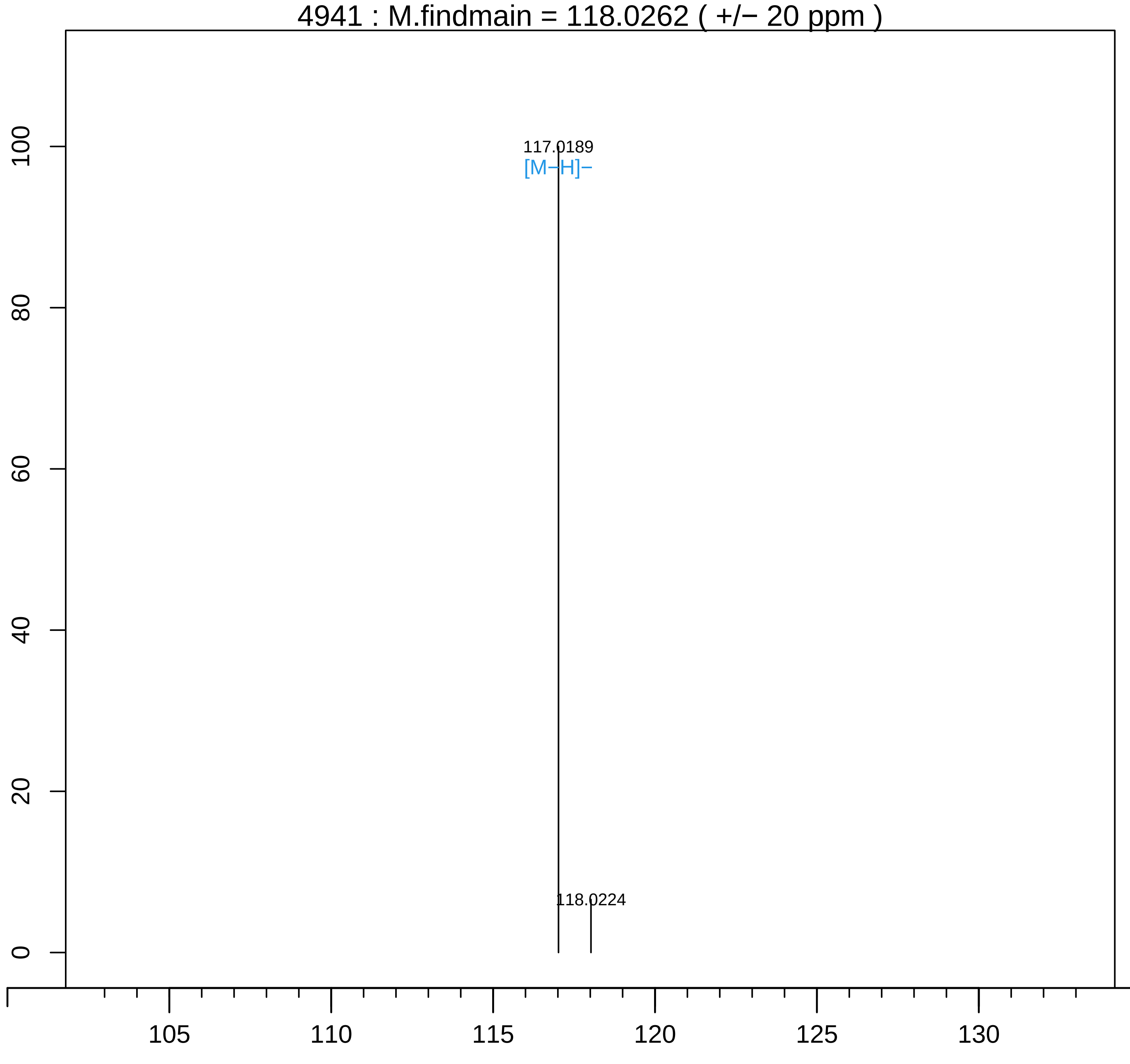
4941 : M.findmain = 118.0262 (+/− 20 ppm)
117.0189
[M−H]−
118.0224
0
20
40
60
80
100
105
110
115
120
125
130

4942 : M.ramclustr = 965.9724 (+/− NA ppm)

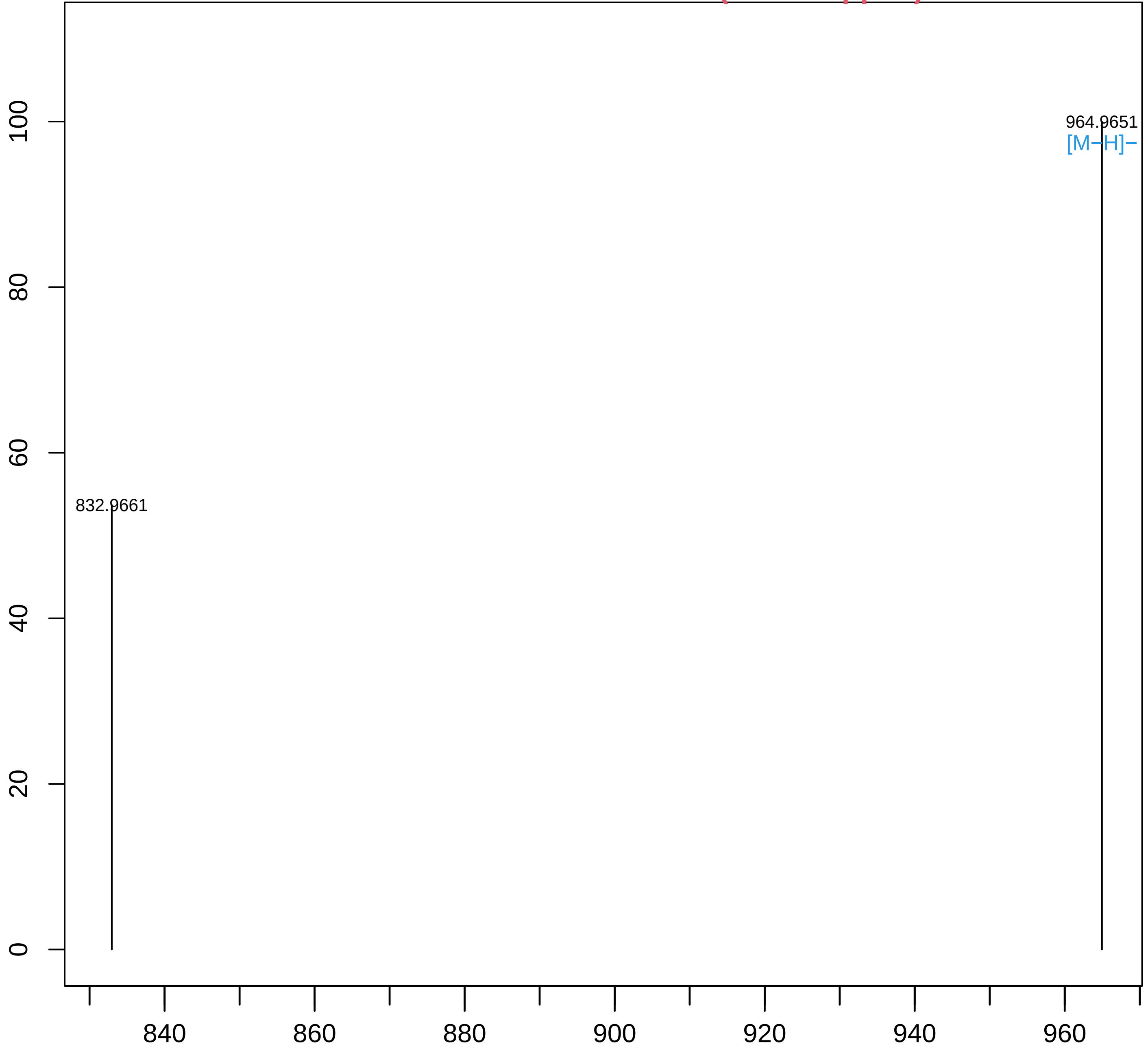

4942 : M.findmain = 965.9724 (+/− 20 ppm)

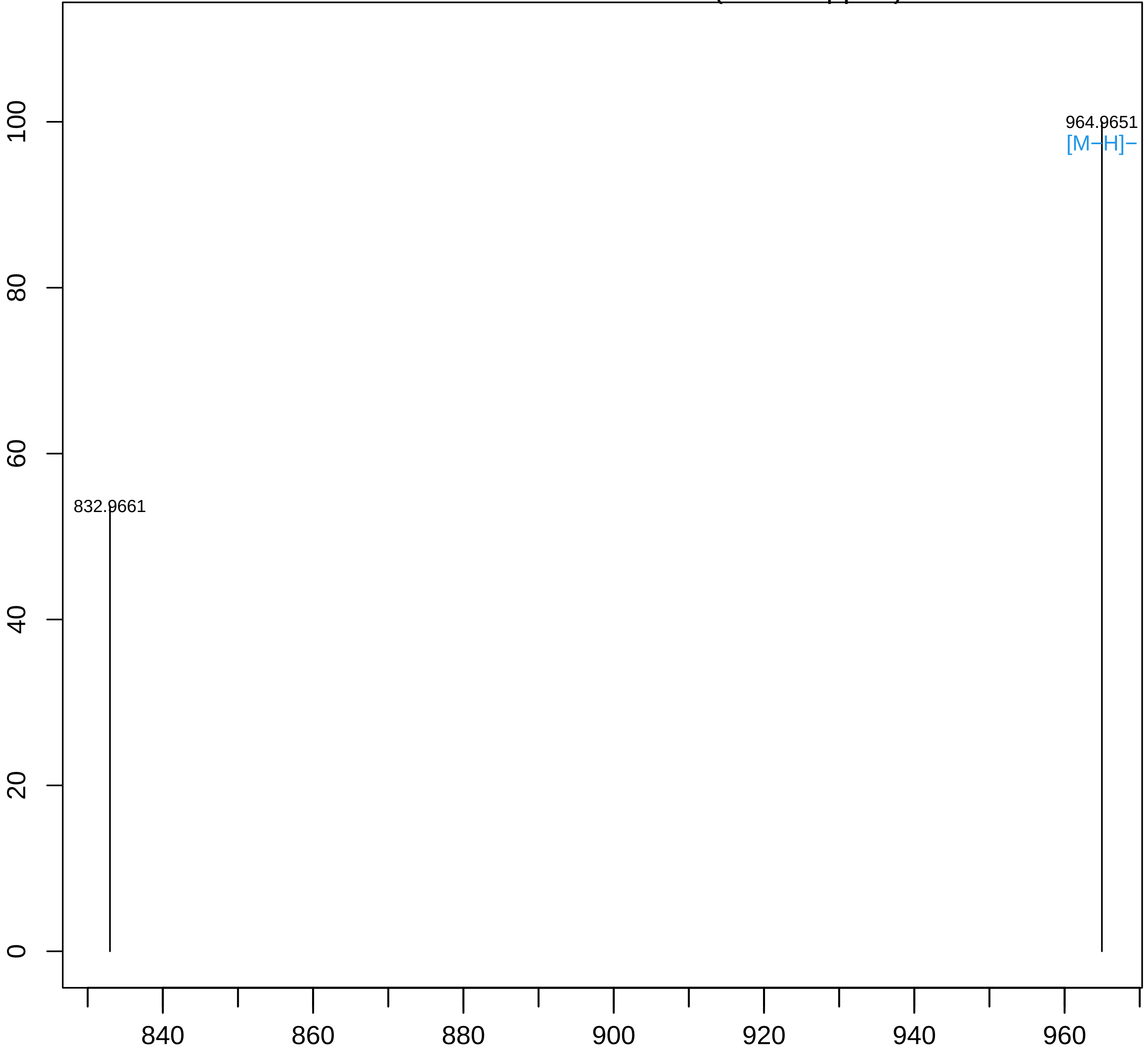

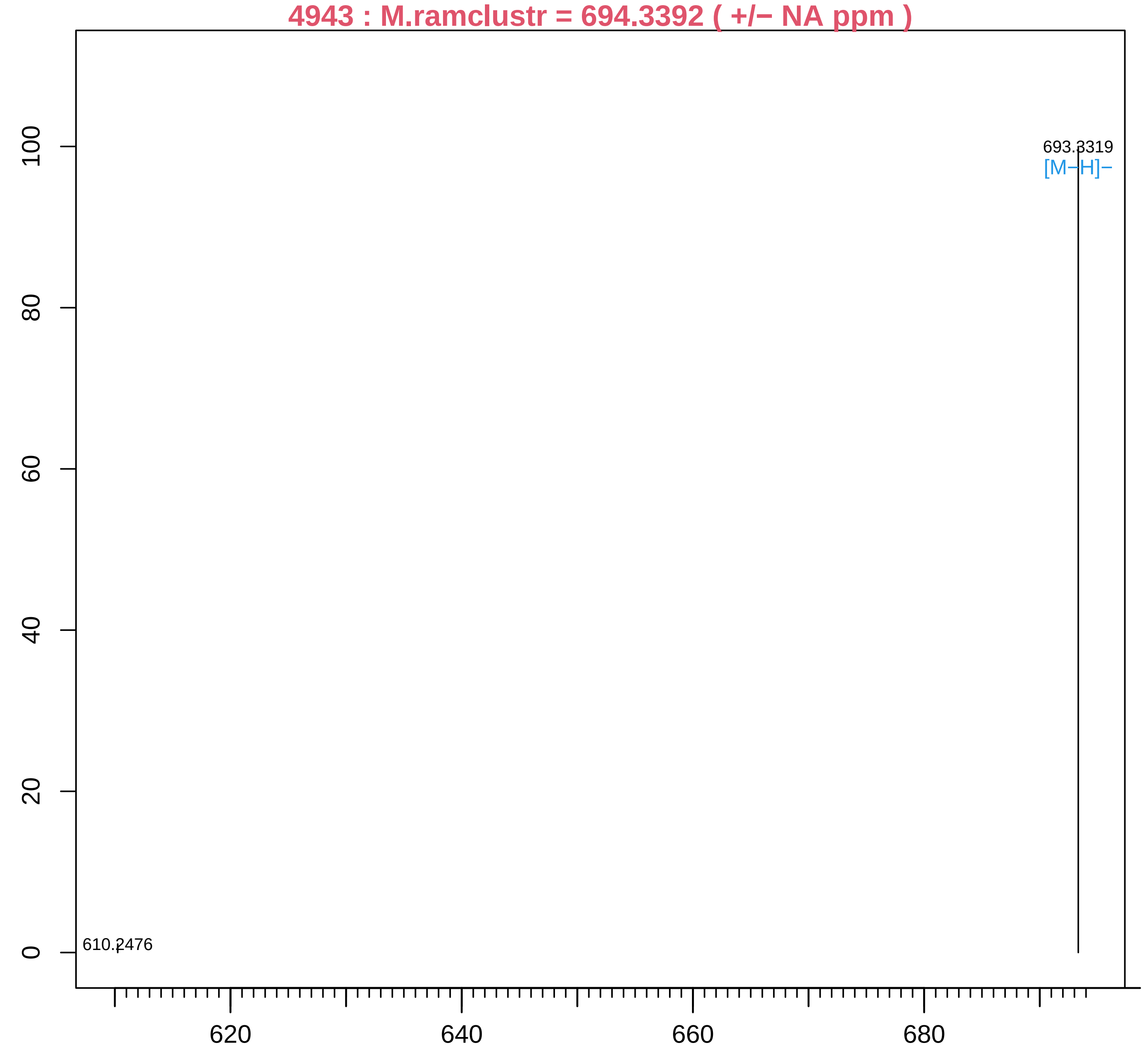
4943 : M.ramclustr = 694.3392 (+/− NA ppm)
693.3319
[M−H]−
610.2476
0
20
40
60
80
100
620
640
660
680

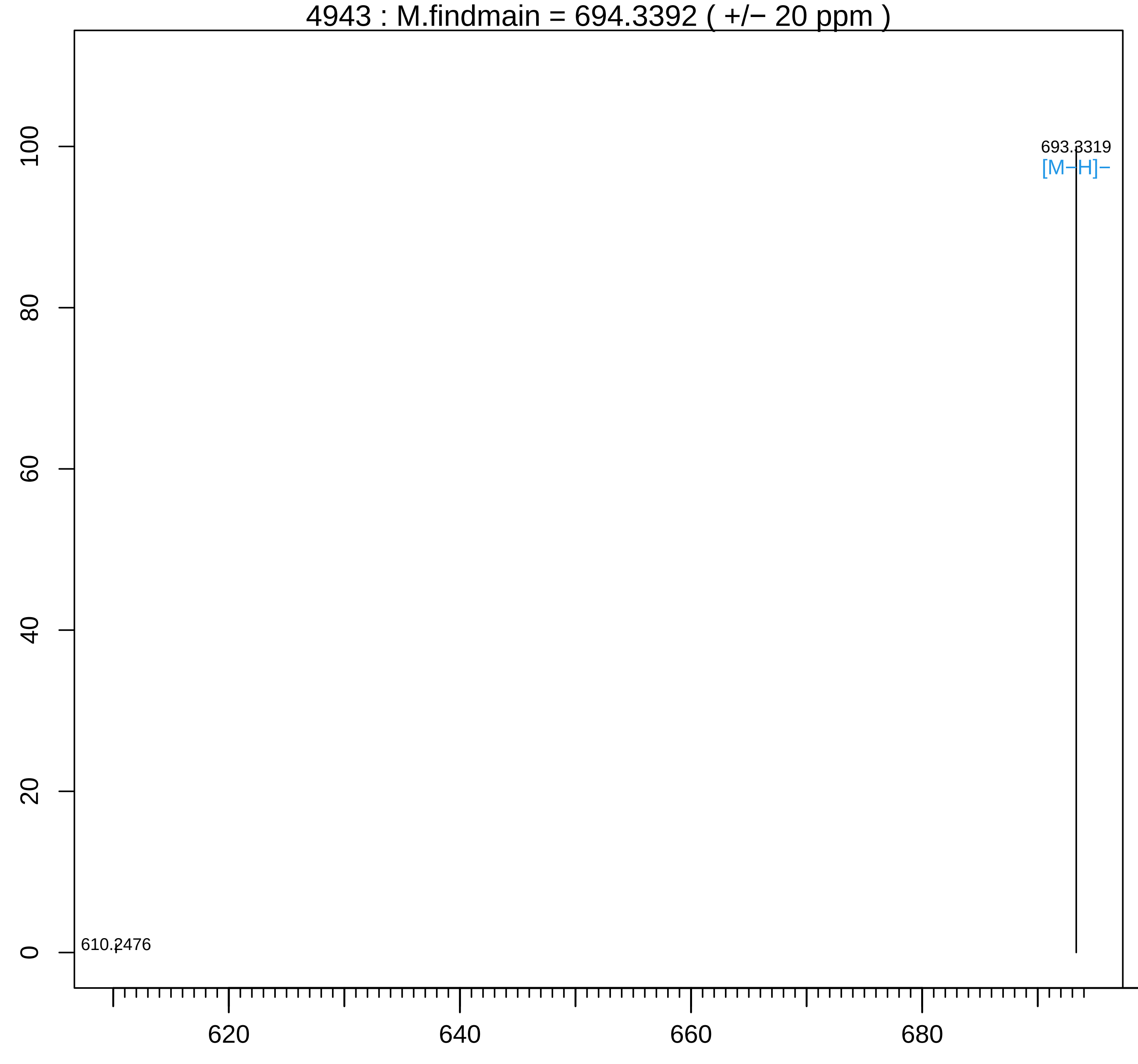
4943 : M.findmain = 694.3392 (+/− 20 ppm)
693.3319
[M−H]−
610.2476
0
20
40
60
80
100
620
640
660
680

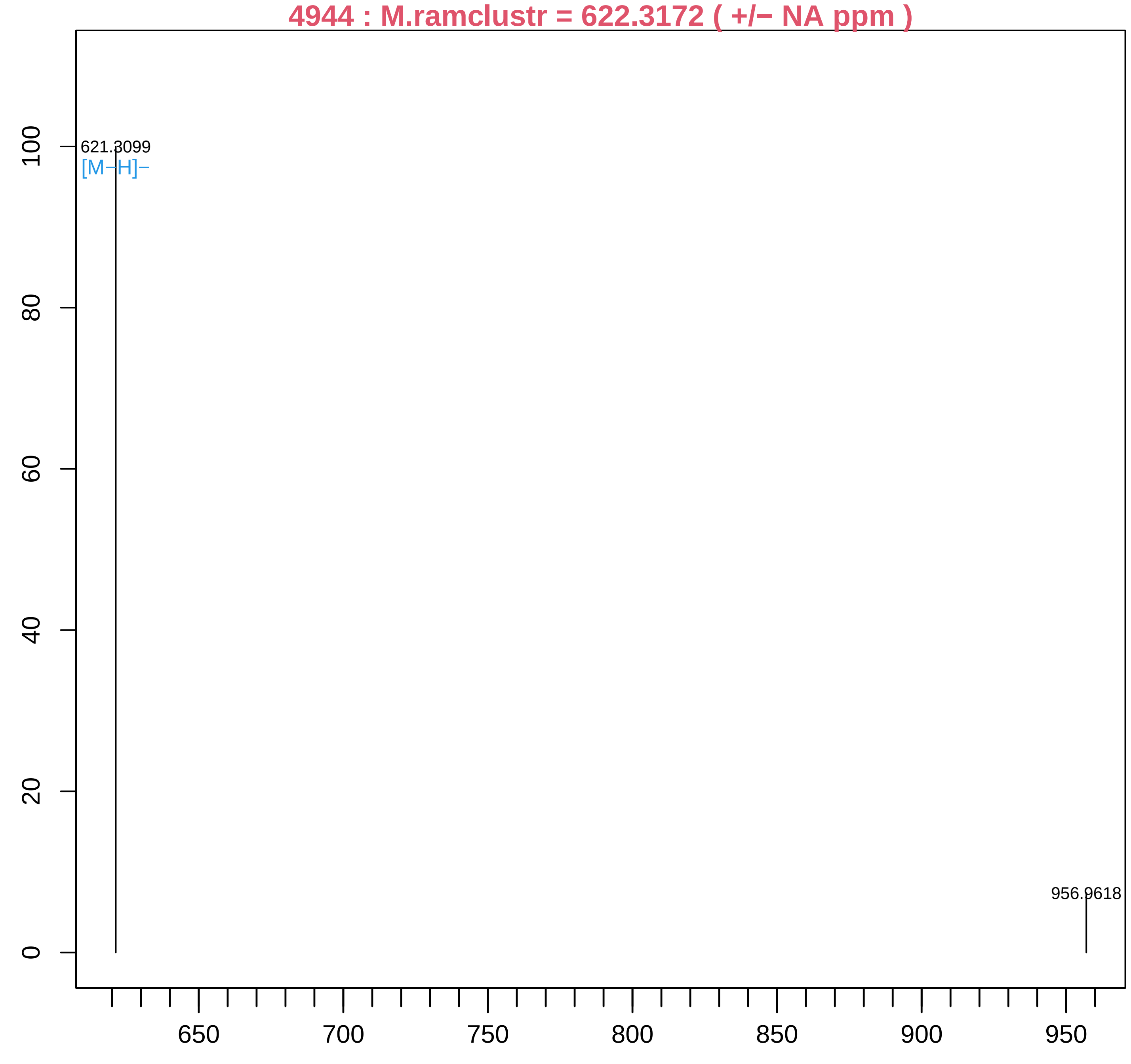
4944 : M.ramclustr = 622.3172 (+/− NA ppm)
621.3099
[M−H]−
956.9618
0
20
40
60
80
100
650
700
750
800
850
900
950

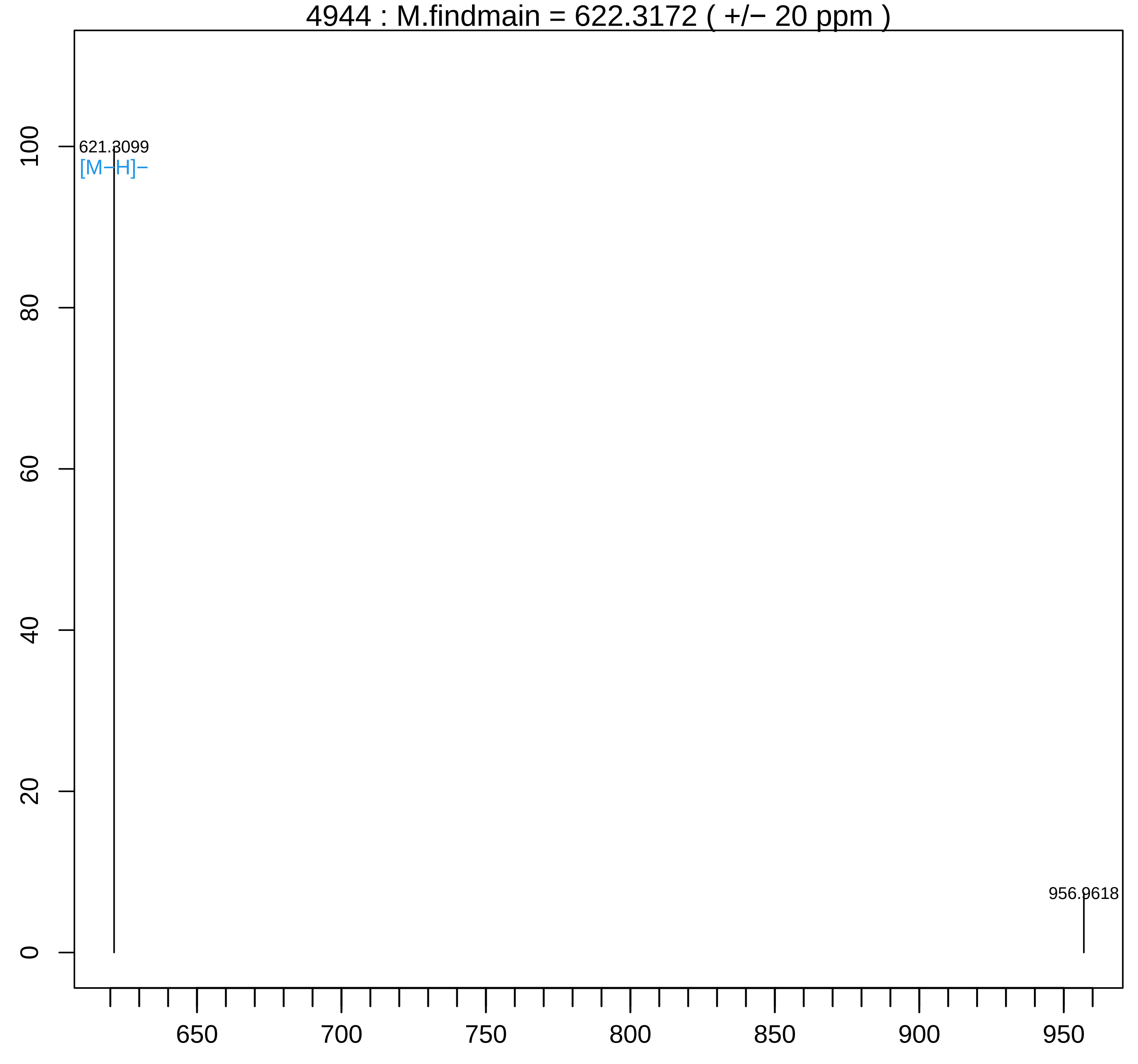
4944 : M.findmain = 622.3172 (+/− 20 ppm)
621.3099
[M−H]−
956.9618
0
20
40
60
80
100
650
700
750
800
850
900
950

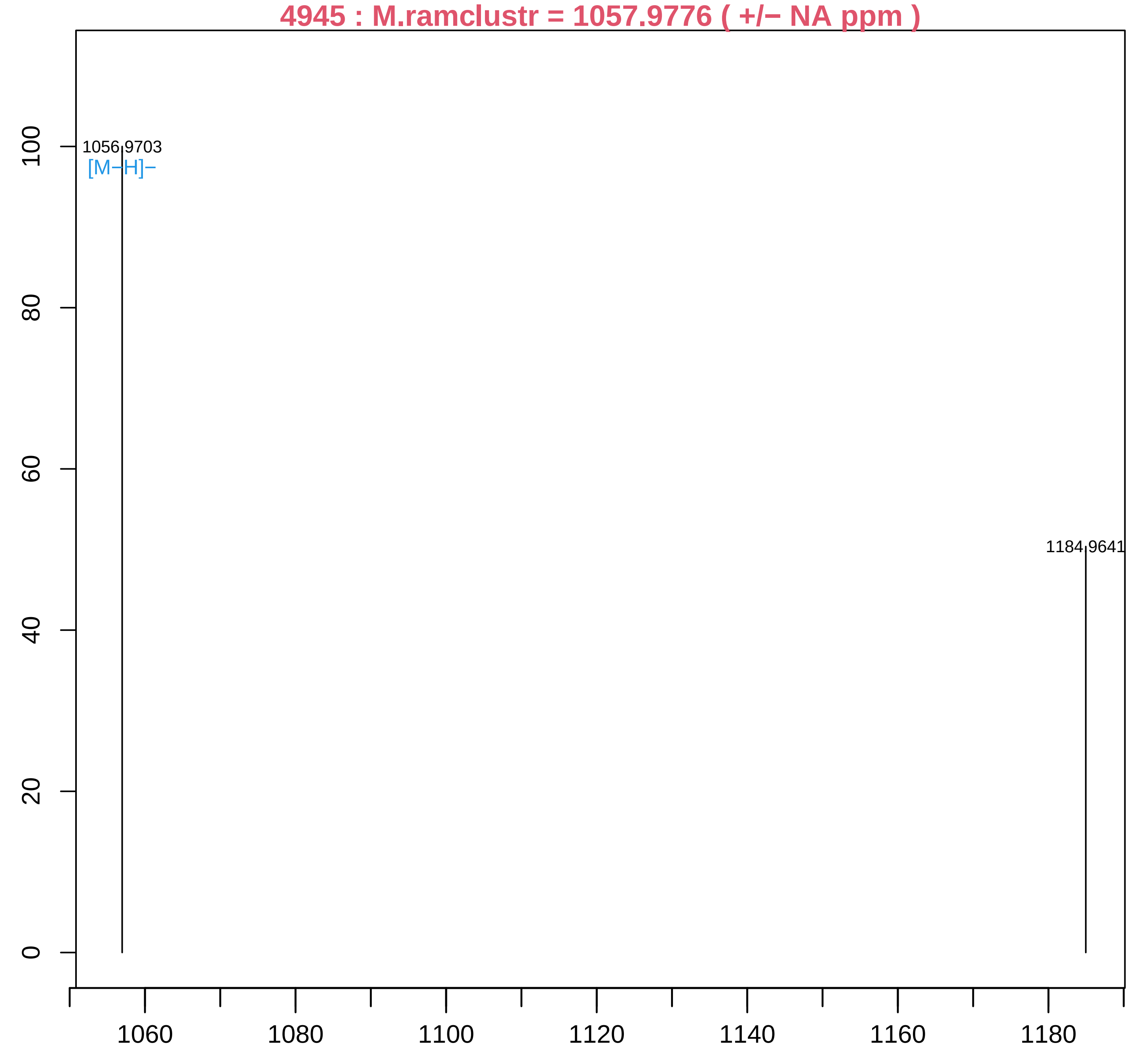
4945 : M.ramclustr = 1057.9776 (+/− NA ppm)
1056.9703
[M−H]−
1184.9641
0
20
40
60
80
100
1060
1080
1100
1120
1140
1160
1180

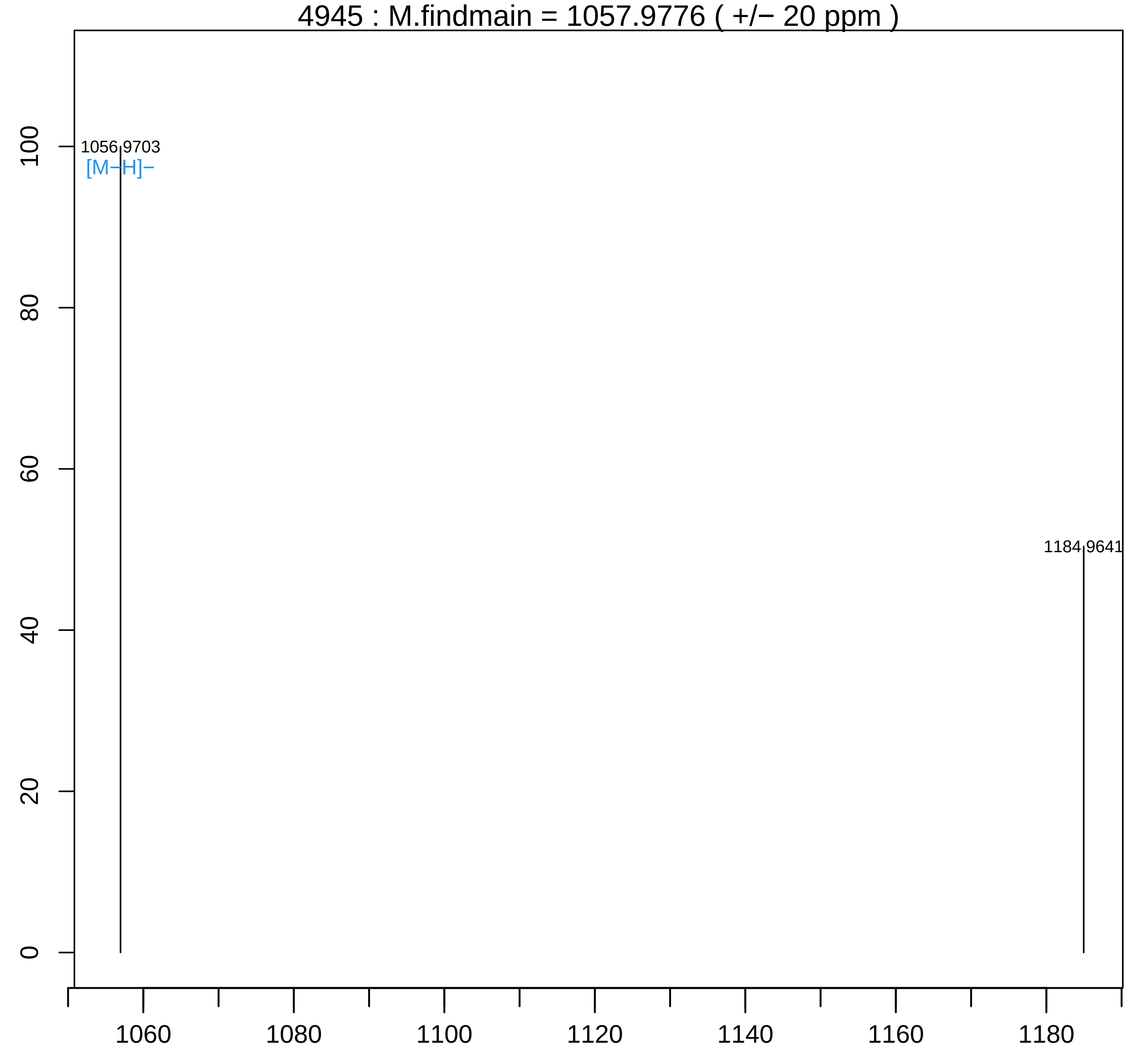
4945 : M.findmain = 1057.9776 (+/− 20 ppm)
1056.9703
[M−H]−
1184.9641
0
20
40
60
80
100
1060
1080
1100
1120
1140
1160
1180

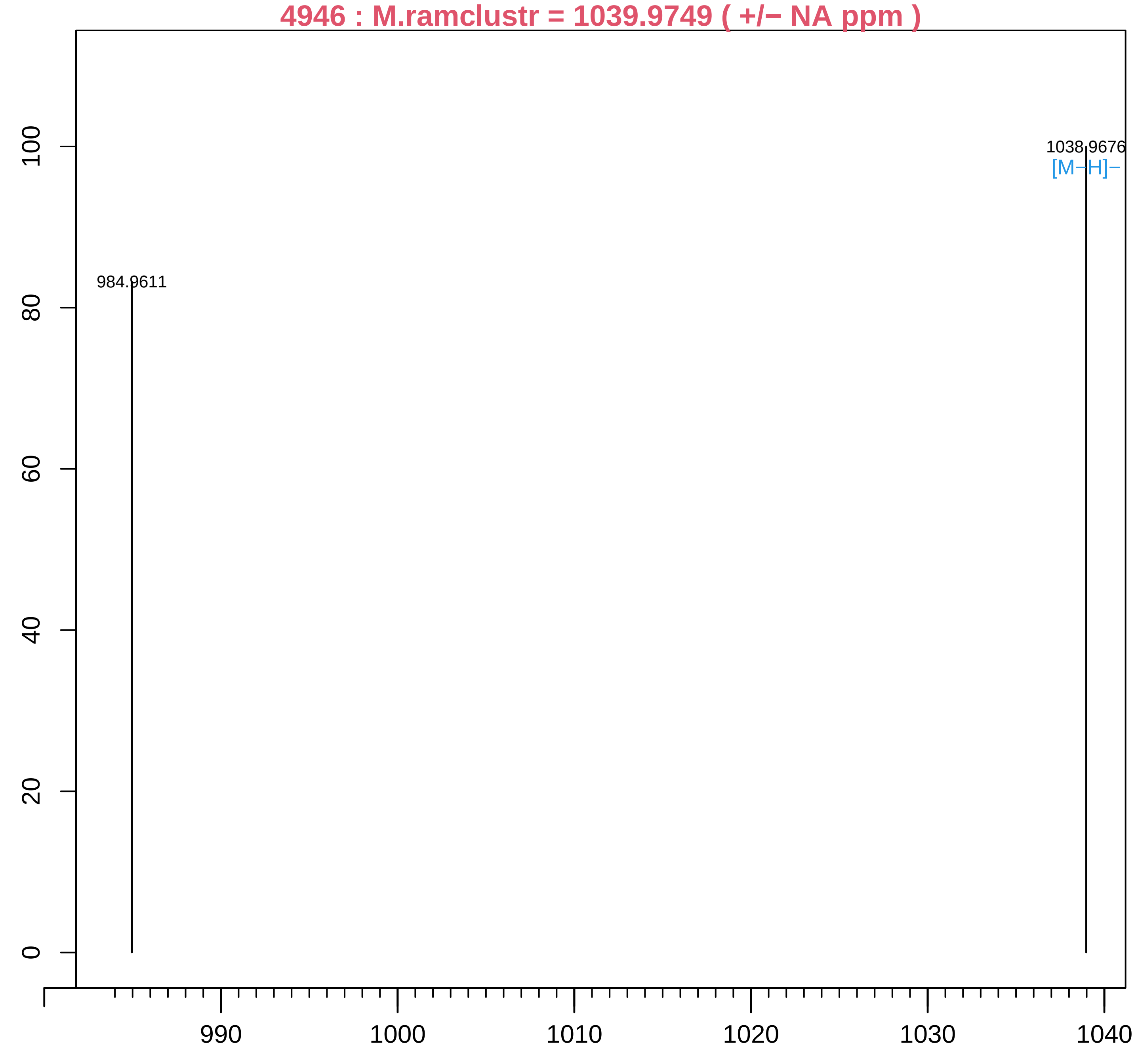

4946 : M.ramclustr = 1039.9749 (+/− NA ppm)
1038.9676
[M−H]−
984.9611
0
20
40
60
80
100
990
1000
1010
1020
1030
1040

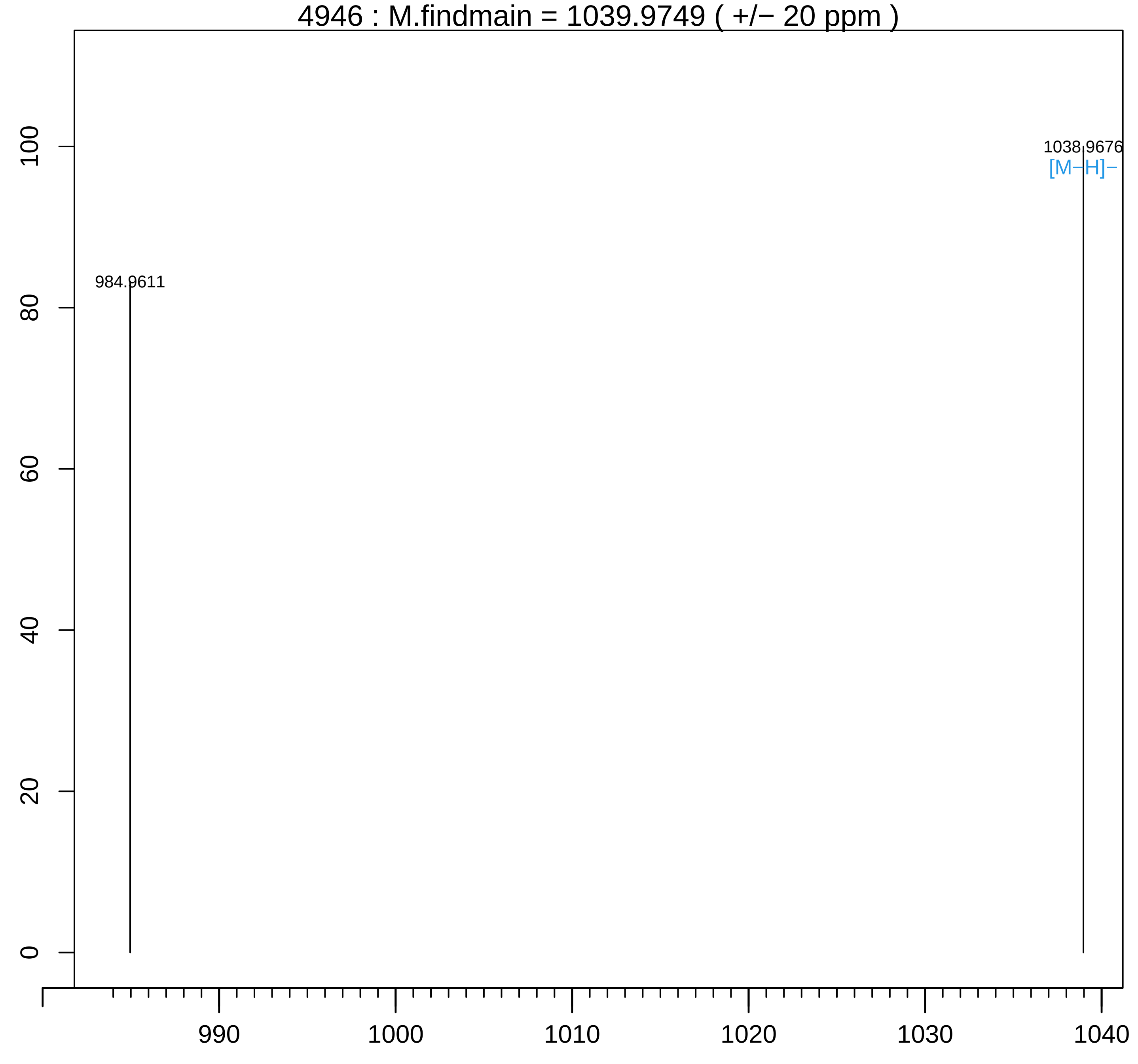

4946 : M.findmain = 1039.9749 (+/− 20 ppm)
1038.9676
[M−H]−
984.9611
0
20
40
60
80
100
990
1000
1010
1020
1030
1040

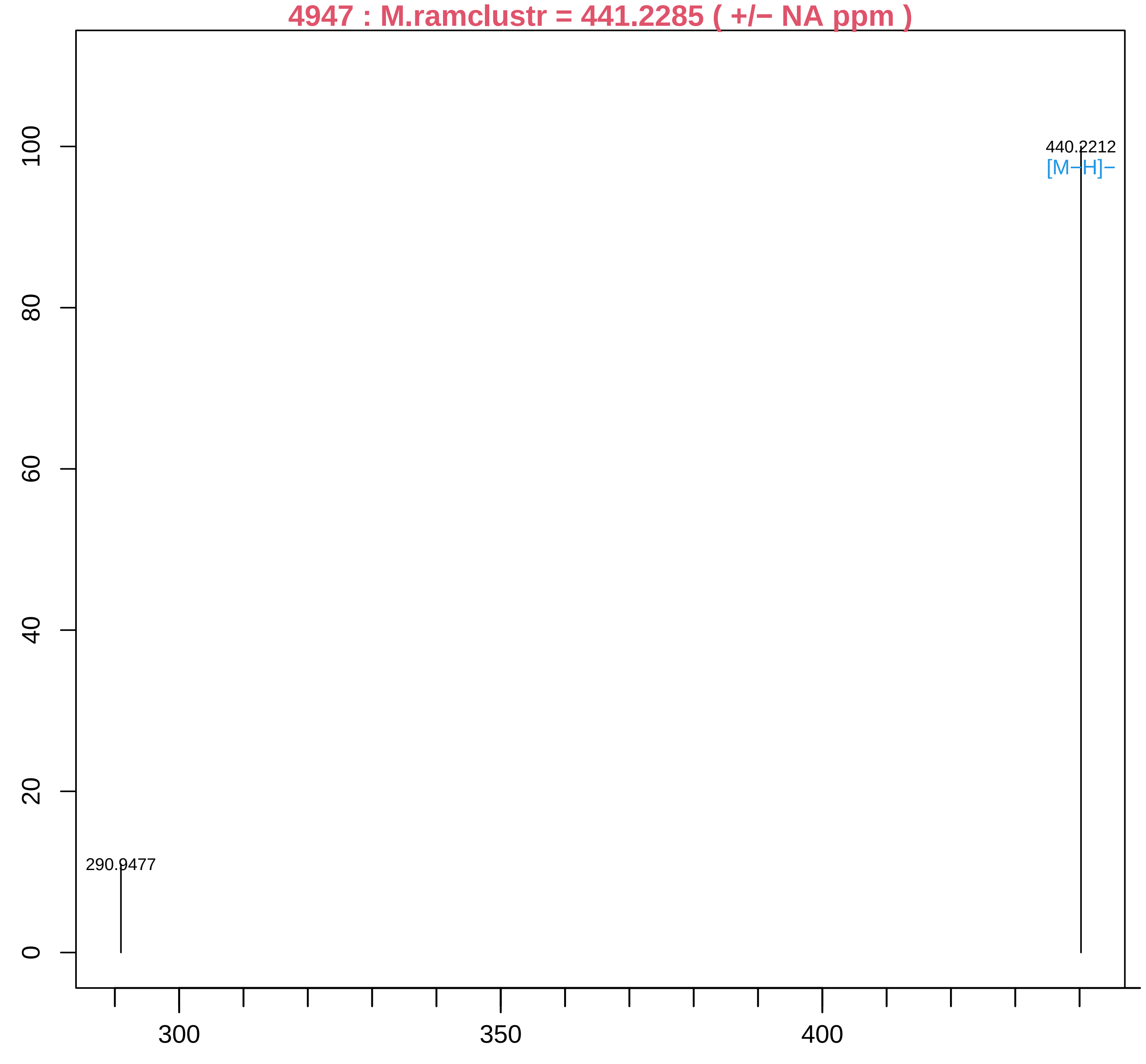
4947 : M.ramclustr = 441.2285 (+/− NA ppm)
440.2212
[M−H]−
290.9477
0
20
40
60
80
100
300
350
400

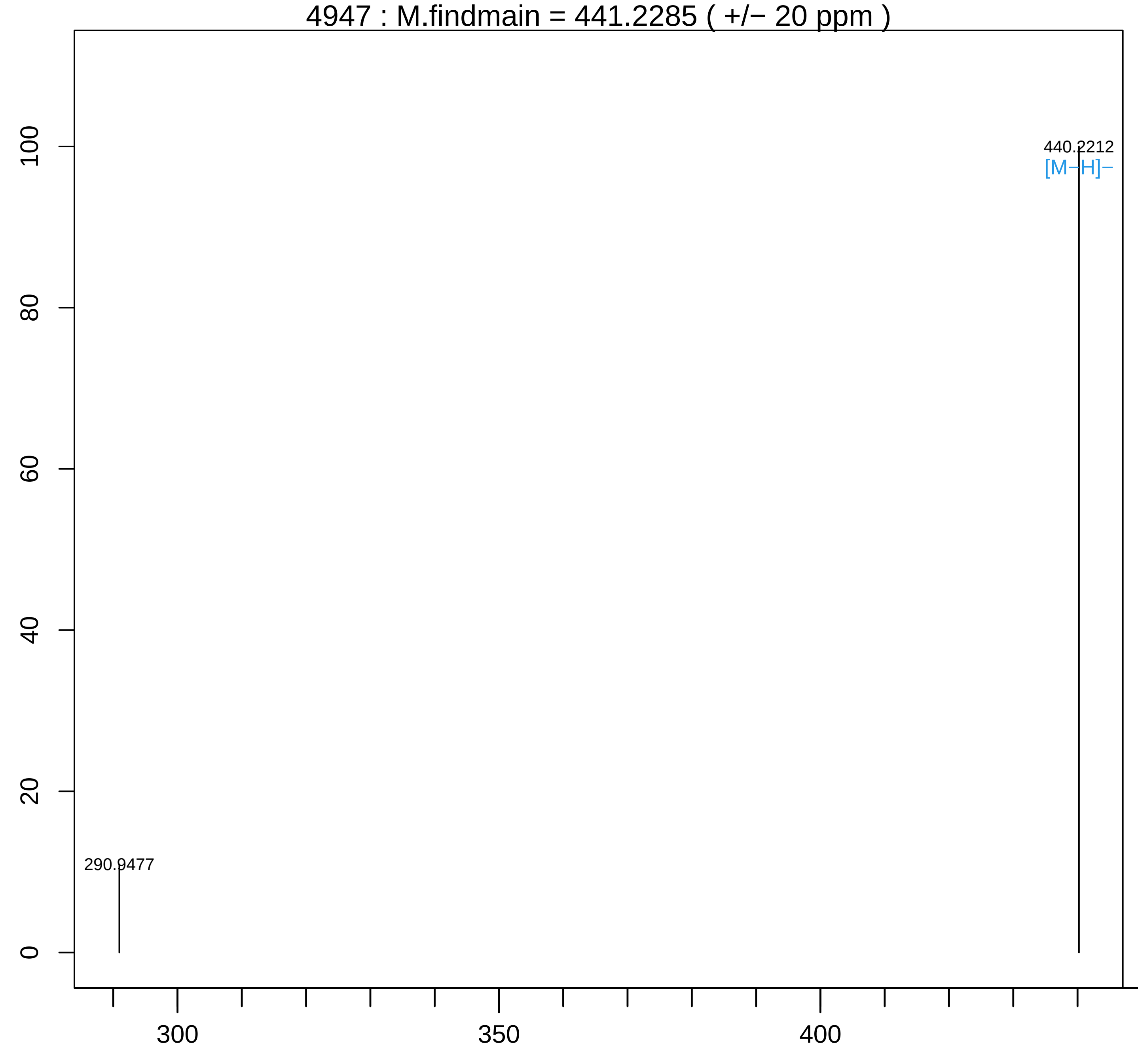
4947 : M.findmain = 441.2285 (+/− 20 ppm)
440.2212
[M−H]−
290.9477
0
20
40
60
80
100
300
350
400

4948 : M.ramclustr = 660.2351 (+/− NA ppm)

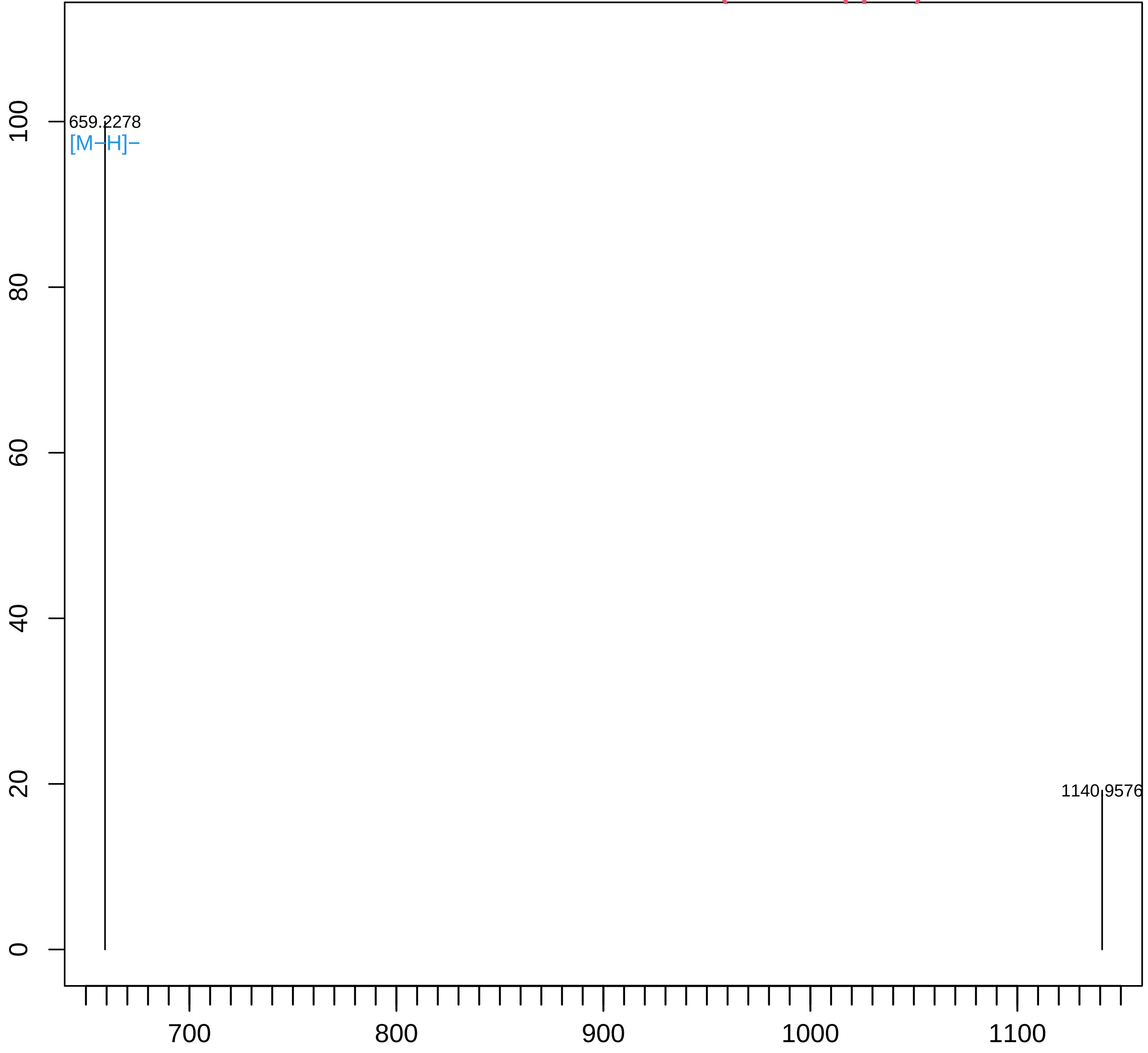

4948 : M.findmain = 660.2351 (+/− 20 ppm)

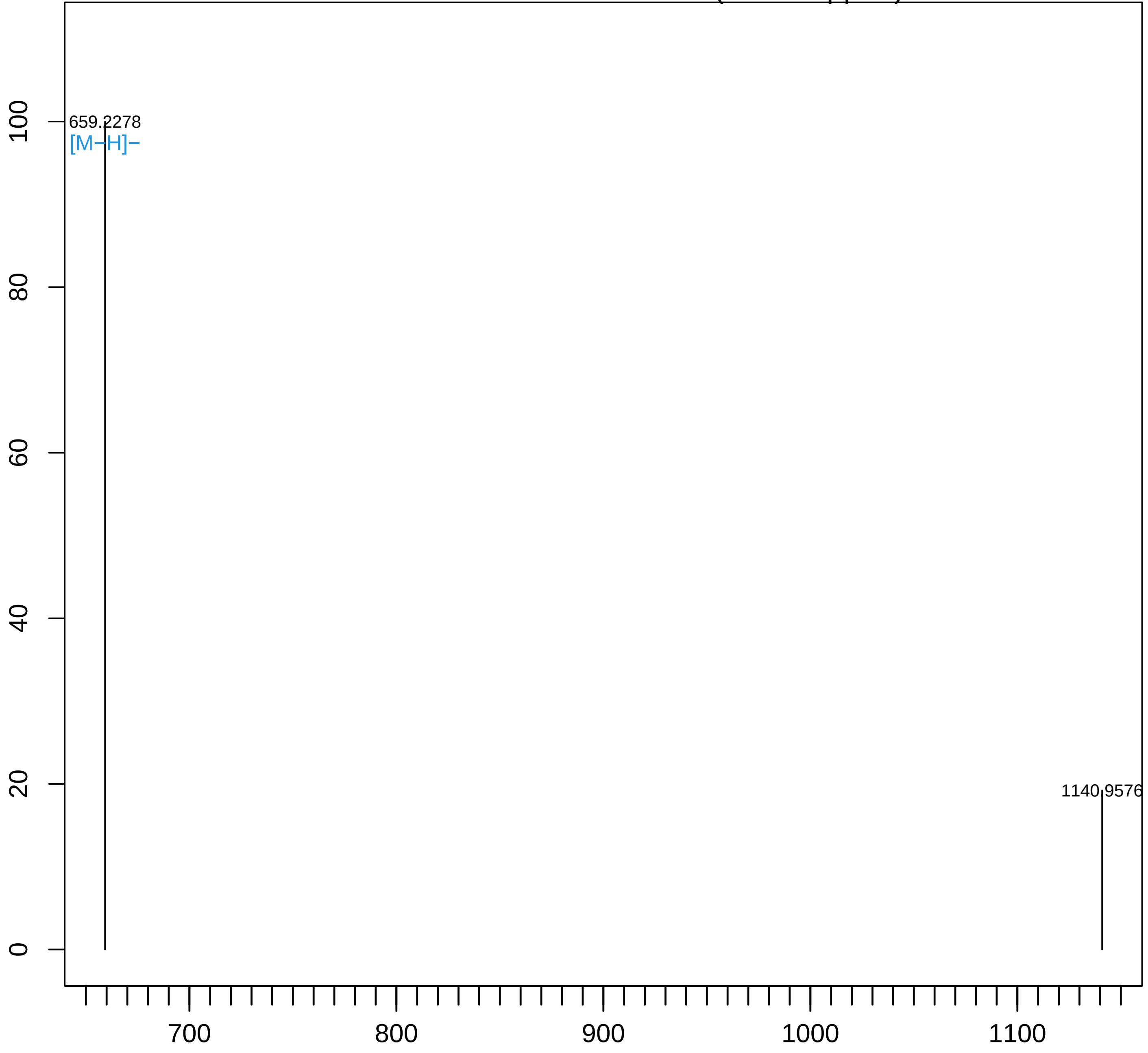

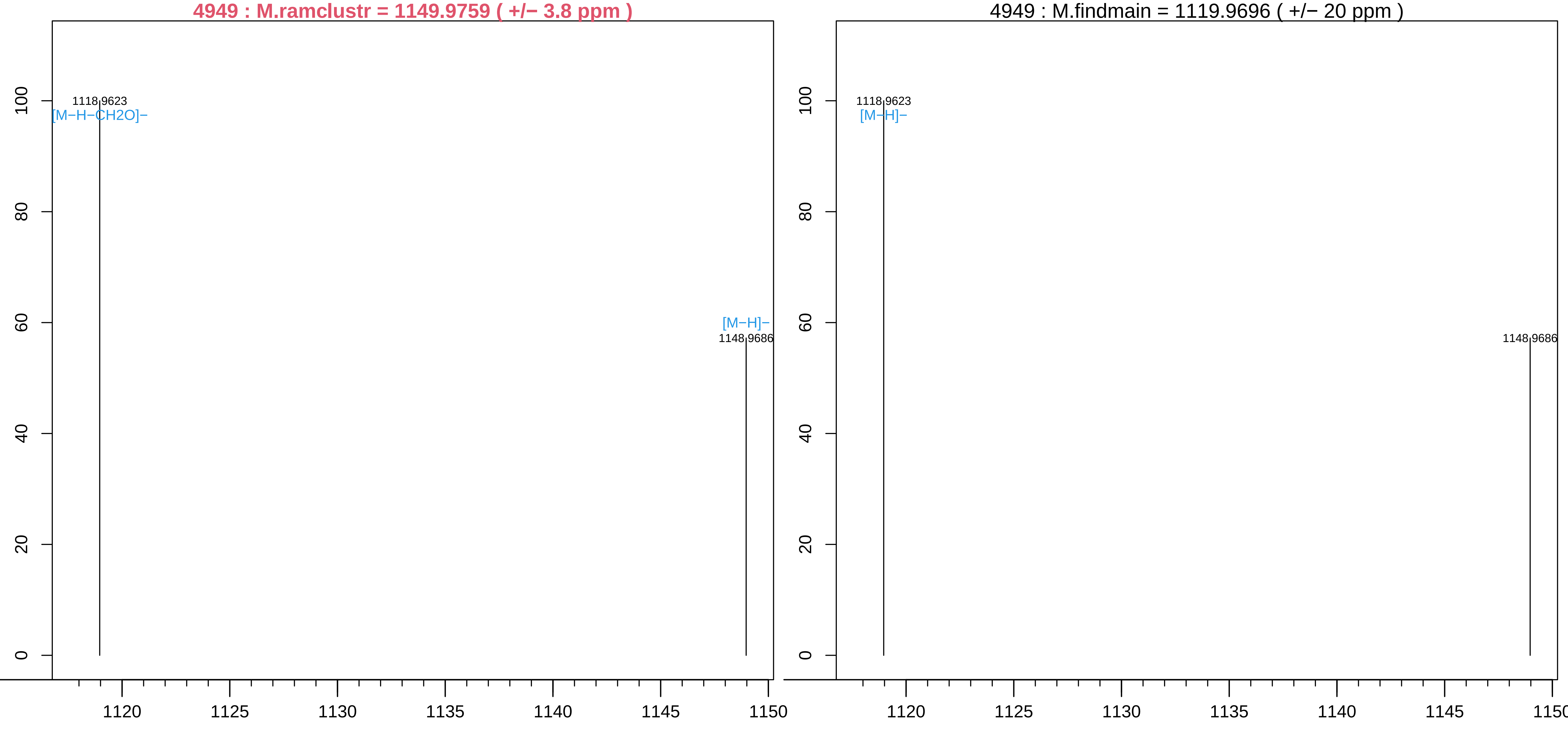
4949 : M.ramclustr = 1149.9759 (+/− 3.8 ppm)
1118.9623
[M−H−CH2O]−
[M−H]−
1148.9686
4949 : M.findmain = 1119.9696 (+/− 20 ppm)
1118.9623
[M−H]−
1148.9686
0
20
40
60
80
100
1120
1125
1130
1135
1140
1145
1150

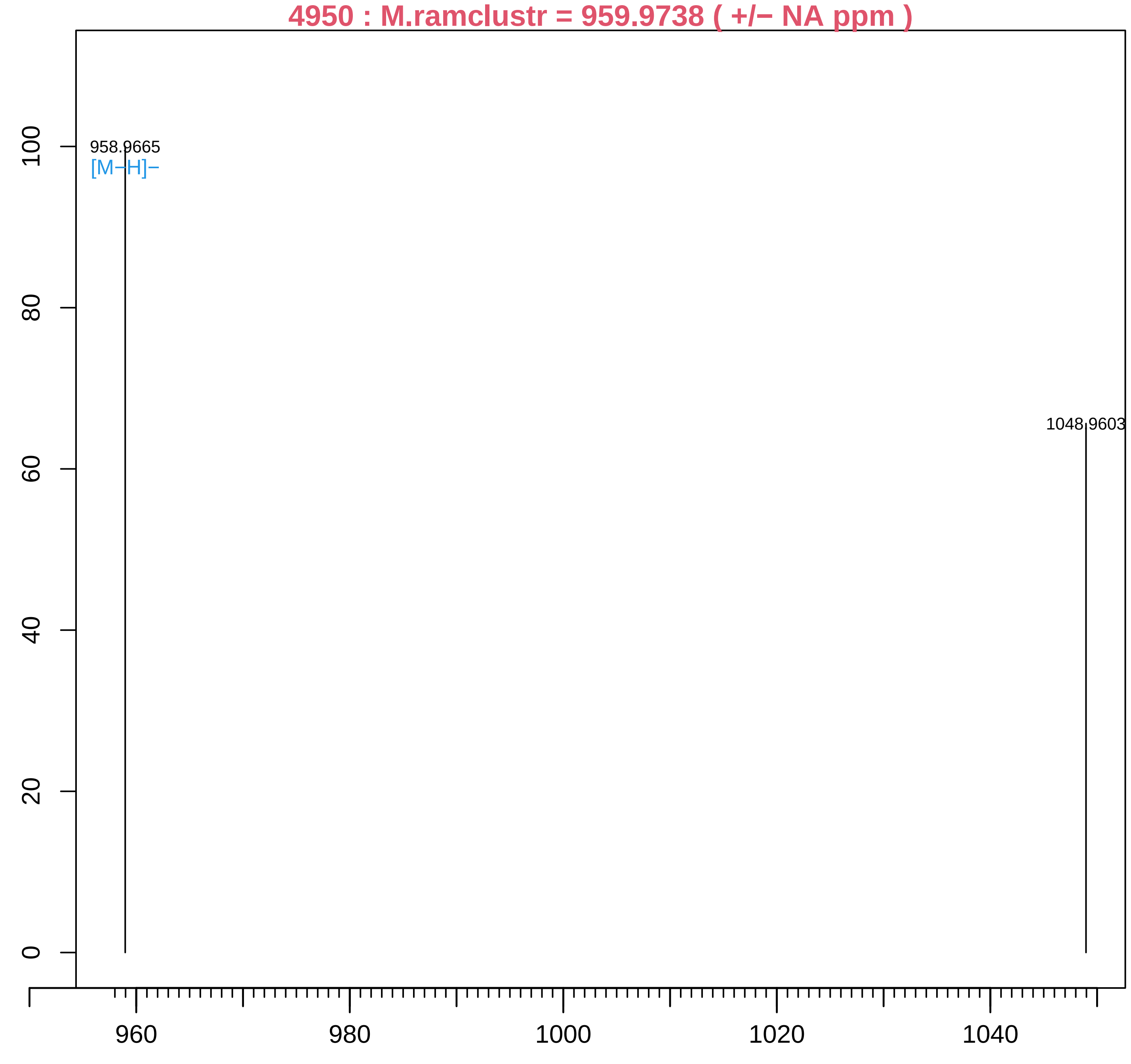
4950 : M.ramclustr = 959.9738 (+/− NA ppm)
958.9665
[M−H]−
1048.9603
0
20
40
60
80
100
960
980
1000
1020
1040

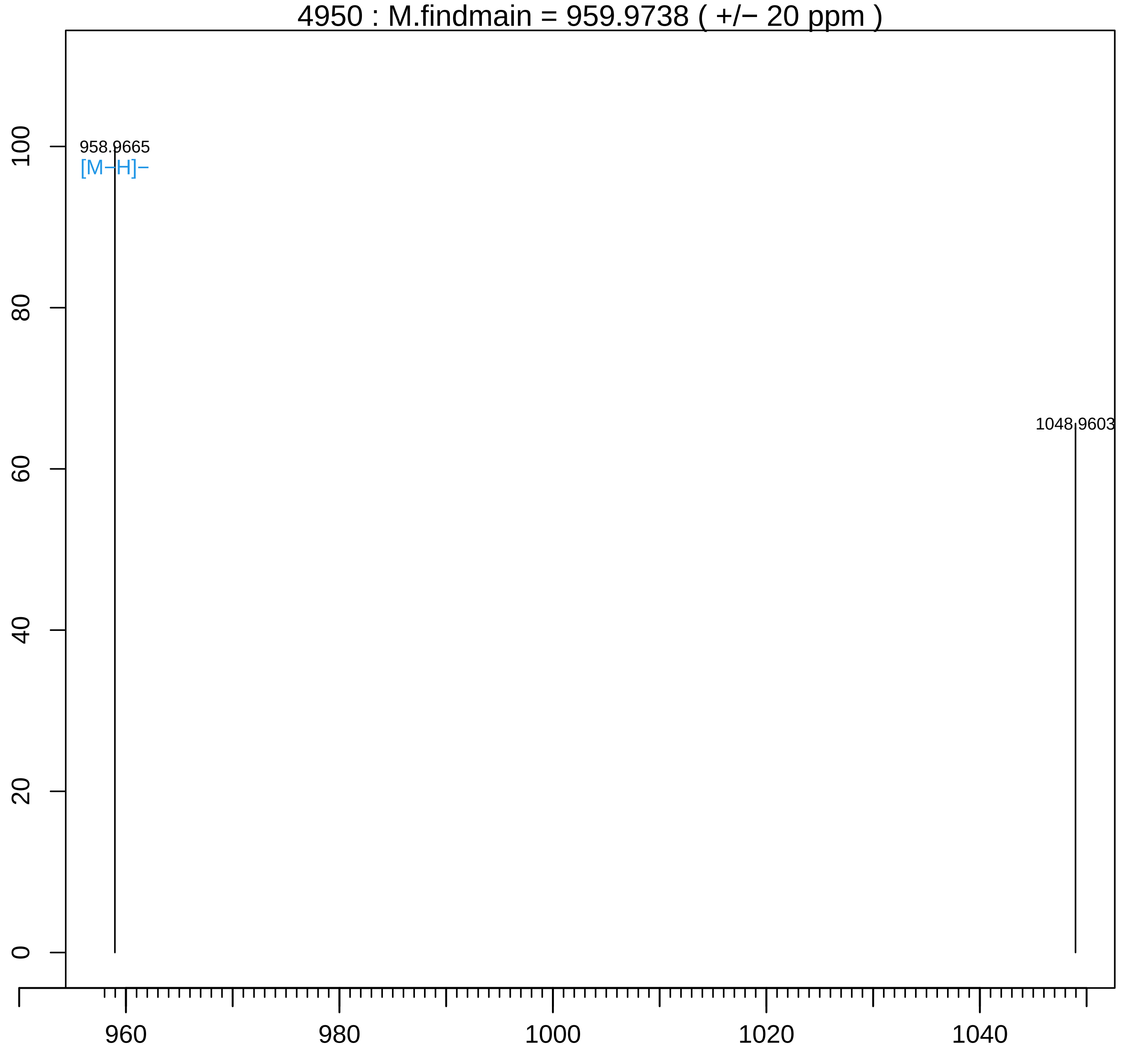
4950 : M.findmain = 959.9738 (+/− 20 ppm)
958.9665
[M−H]−
1048.9603
0
20
40
60
80
100
960
980
1000
1020
1040

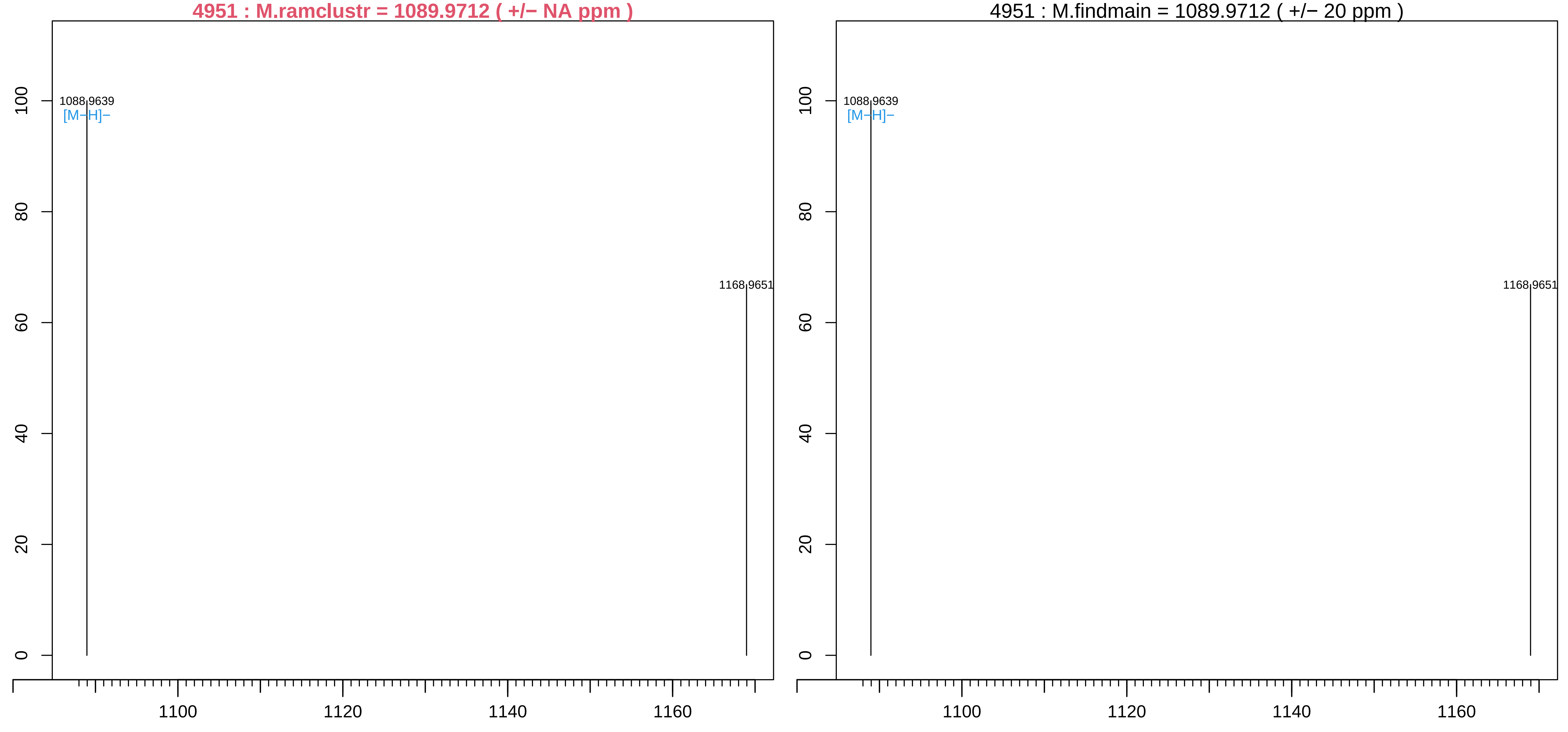

4951 : M.ramclustr = 1089.9712 (+/− NA ppm)
1088.9639
[M−H]−
1168.9651
0
20
40
60
80
100
1100
1120
1140
1160
4951 : M.findmain = 1089.9712 (+/− 20 ppm)
1088.9639
[M−H]−
1168.9651
0
20
40
60
80
100
1100
1120
1140
1160

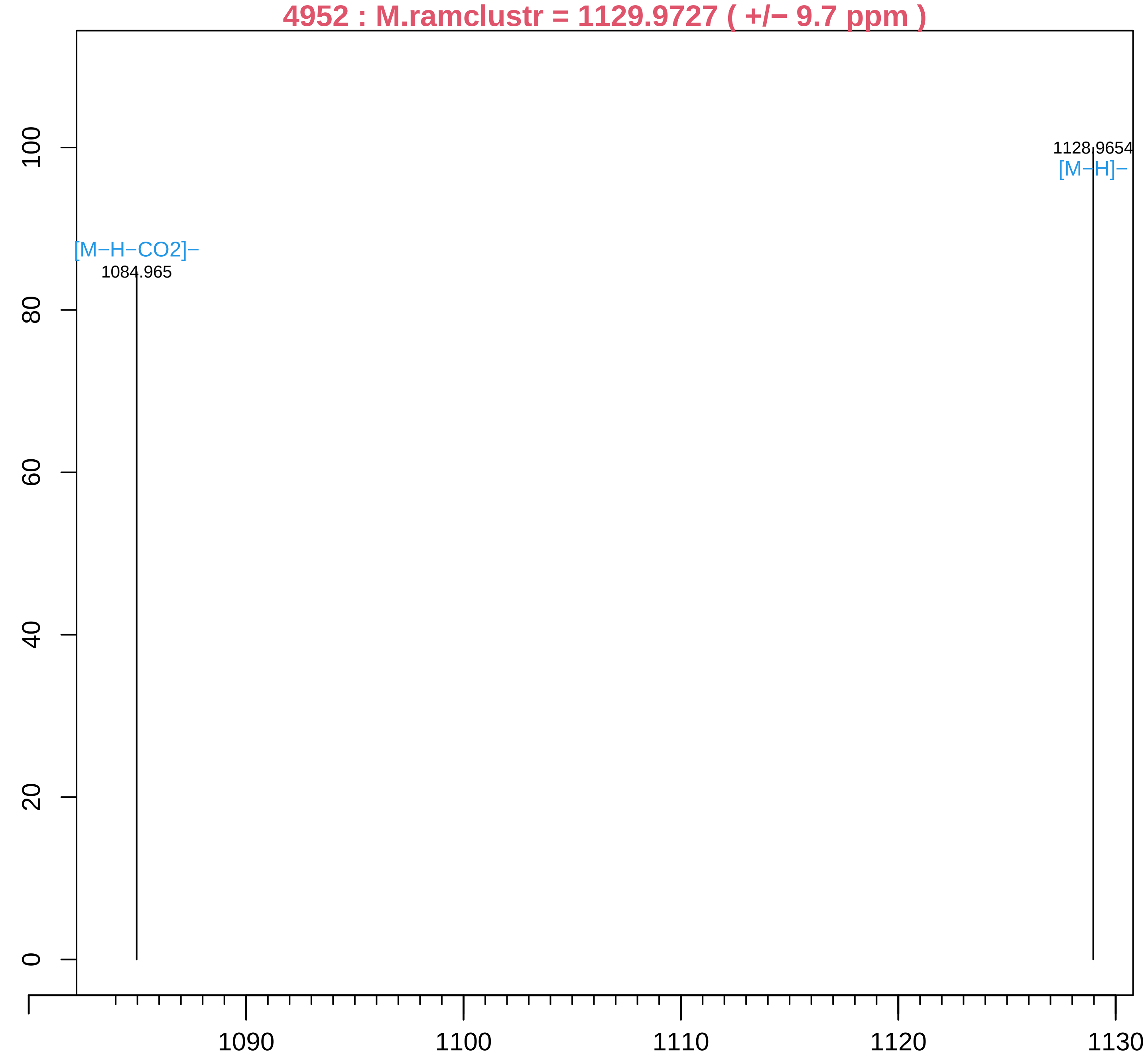

4952 : M.ramclustr = 1129.9727 (+/− 9.7 ppm)
1128.9654
[M−H]−
[M−H−CO2]−
1084.965
0
20
40
60
80
100
1090
1100
1110
1120
1130

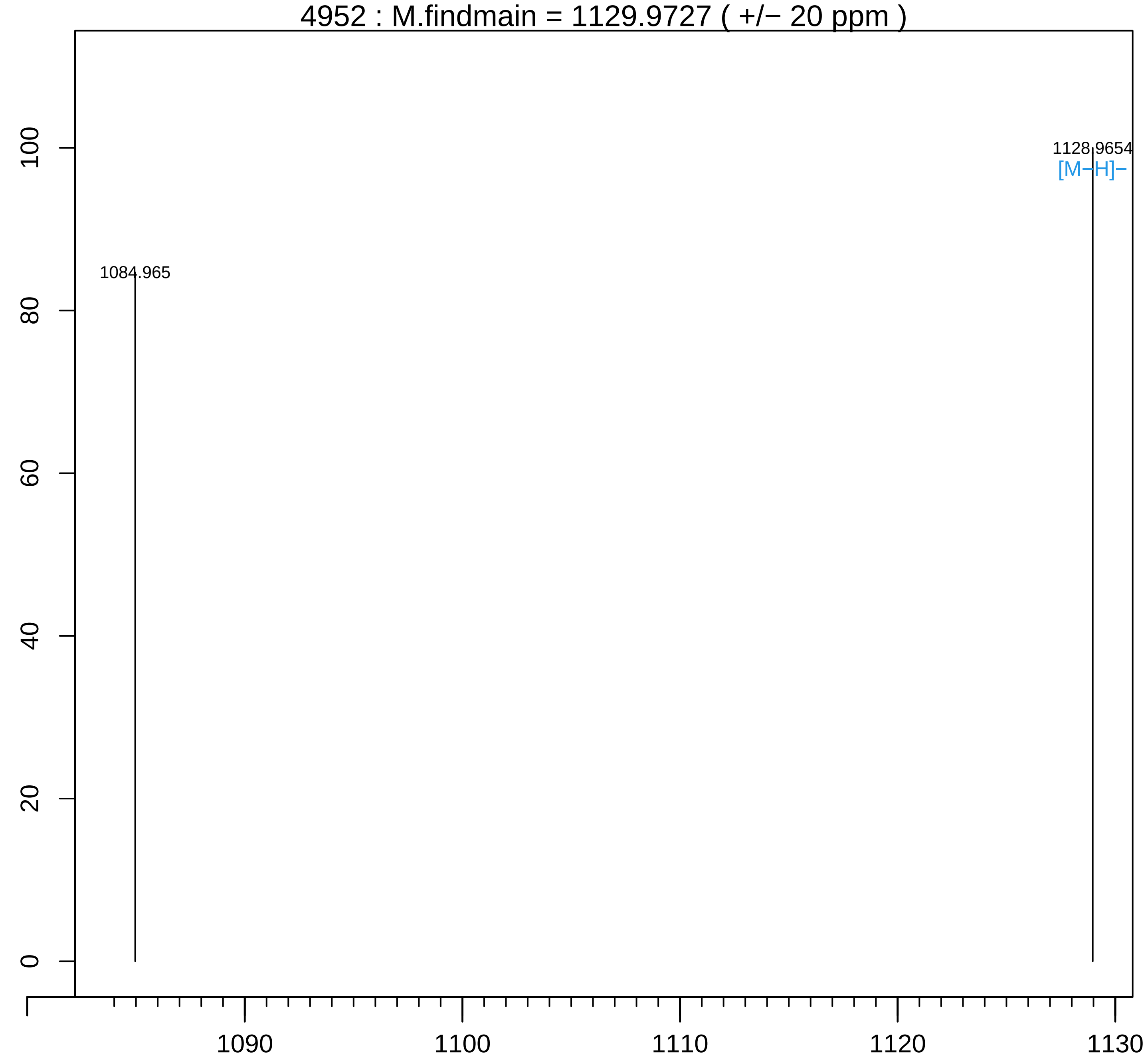

4952 : M.findmain = 1129.9727 (+/− 20 ppm)
1128.9654
[M−H]−
1084.965
0
20
40
60
80
100
1090
1100
1110
1120
1130

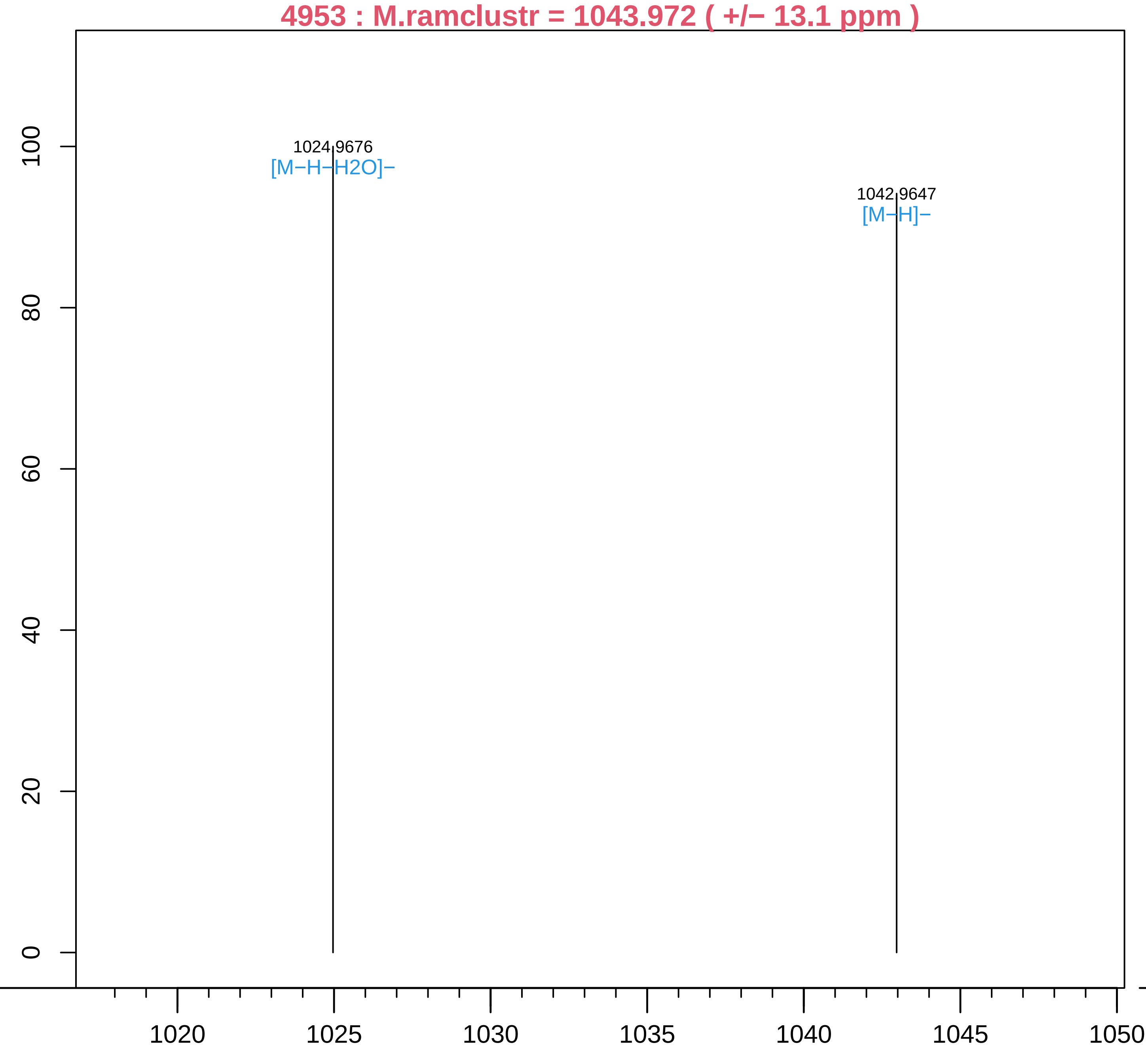
4953 : M.ramclustr = 1043.972 (+/− 13.1 ppm)
1024.9676
[M−H−H2O]−
1042.9647
[M−H]−
0
20
40
60
80
100
1020
1025
1030
1035
1040
1045
1050

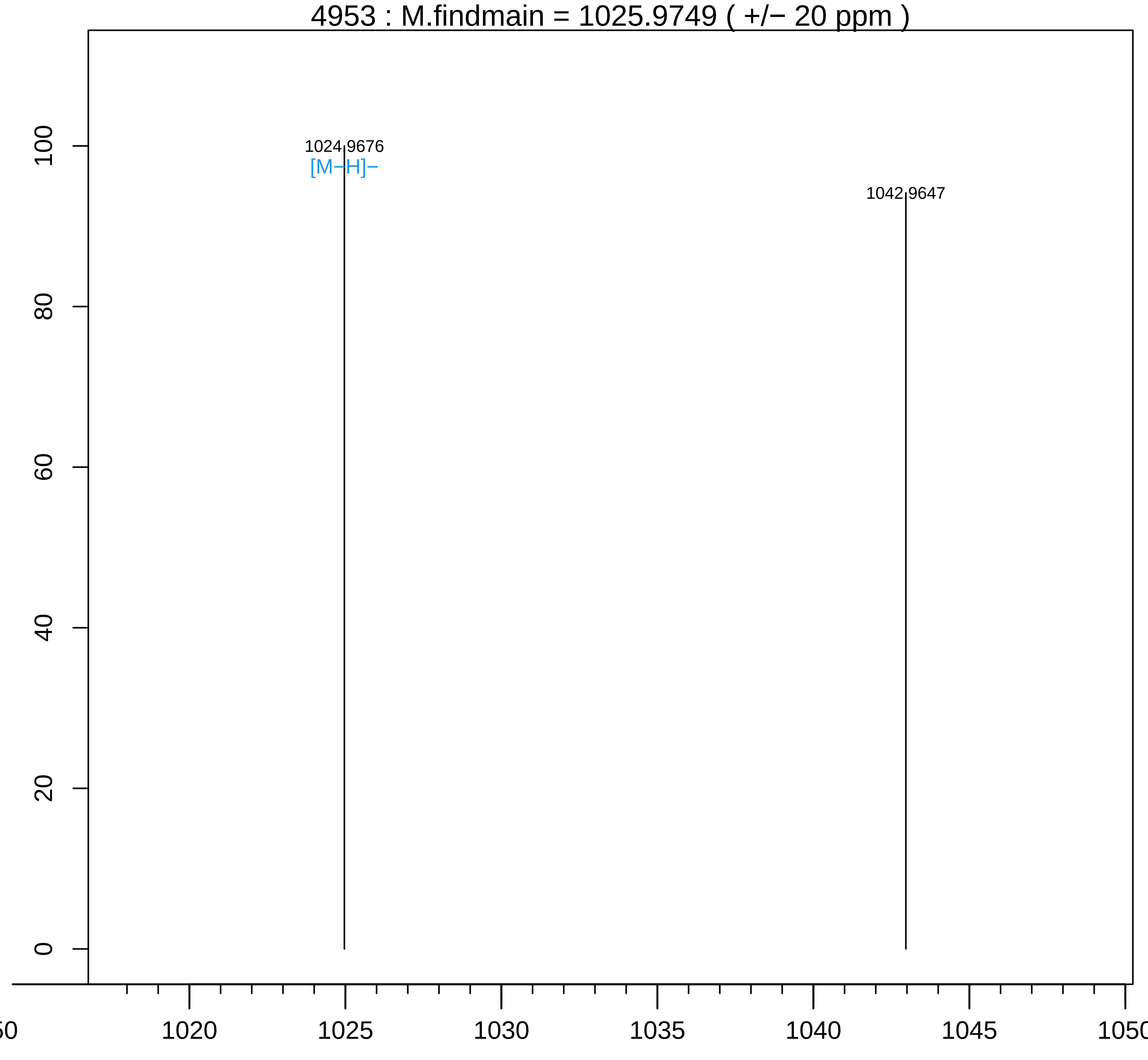
4953 : M.findmain = 1025.9749 (+/− 20 ppm)
1024.9676
[M−H]−
1042.9647
0
20
40
60
80
100
1020
1025
1030
1035
1040
1045
1050

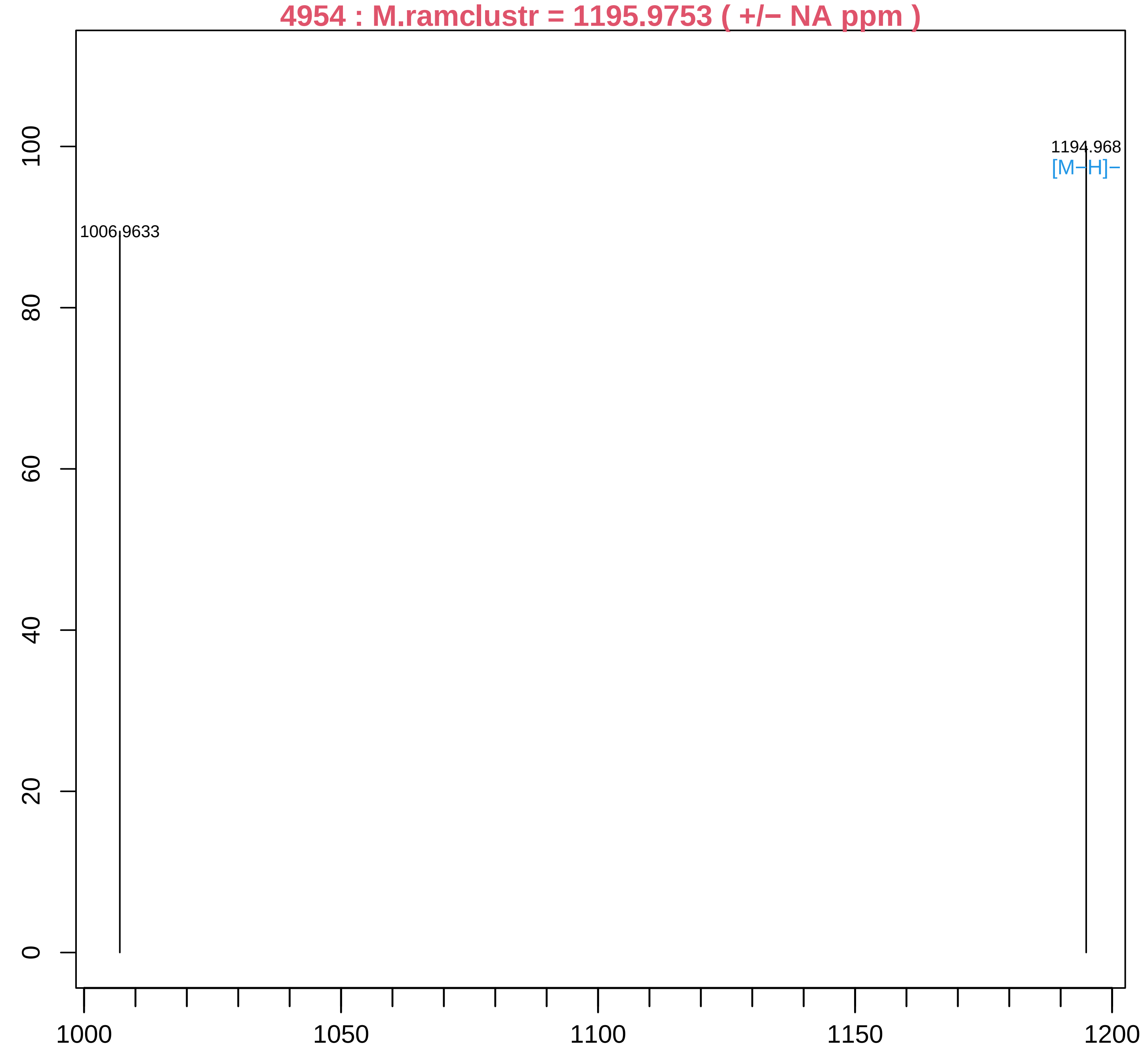

4954 : M.ramclustr = 1195.9753 (+/− NA ppm)
1194.968
[M−H]−
1006.9633

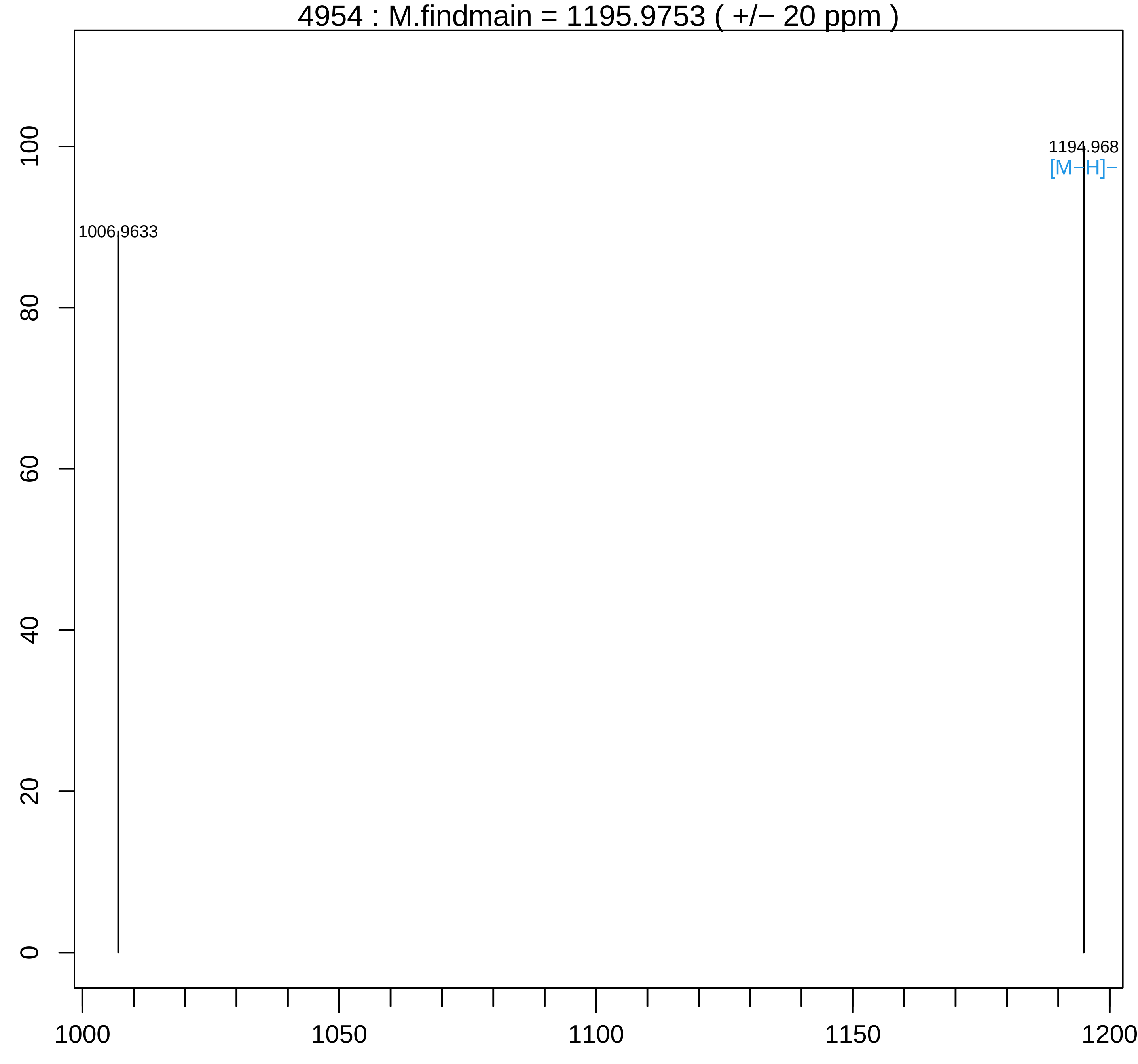

4954 : M.findmain = 1195.9753 (+/− 20 ppm)
1194.968
[M−H]−
1006.9633

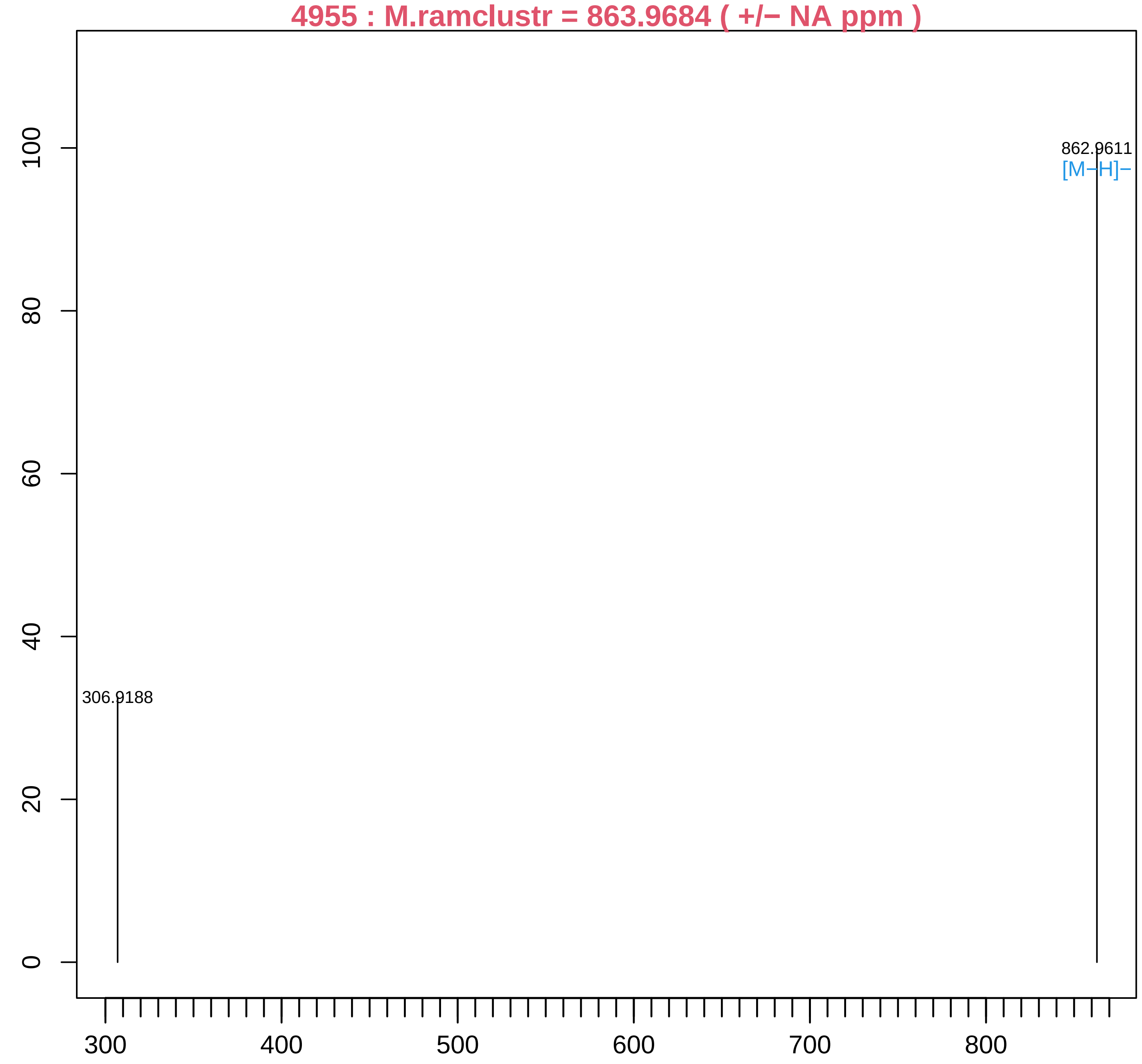
4955 : M.ramclustr = 863.9684 (+/− NA ppm)
862.9611
[M−H]−
306.9188
0
20
40
60
80
100
300
400
500
600
700
800

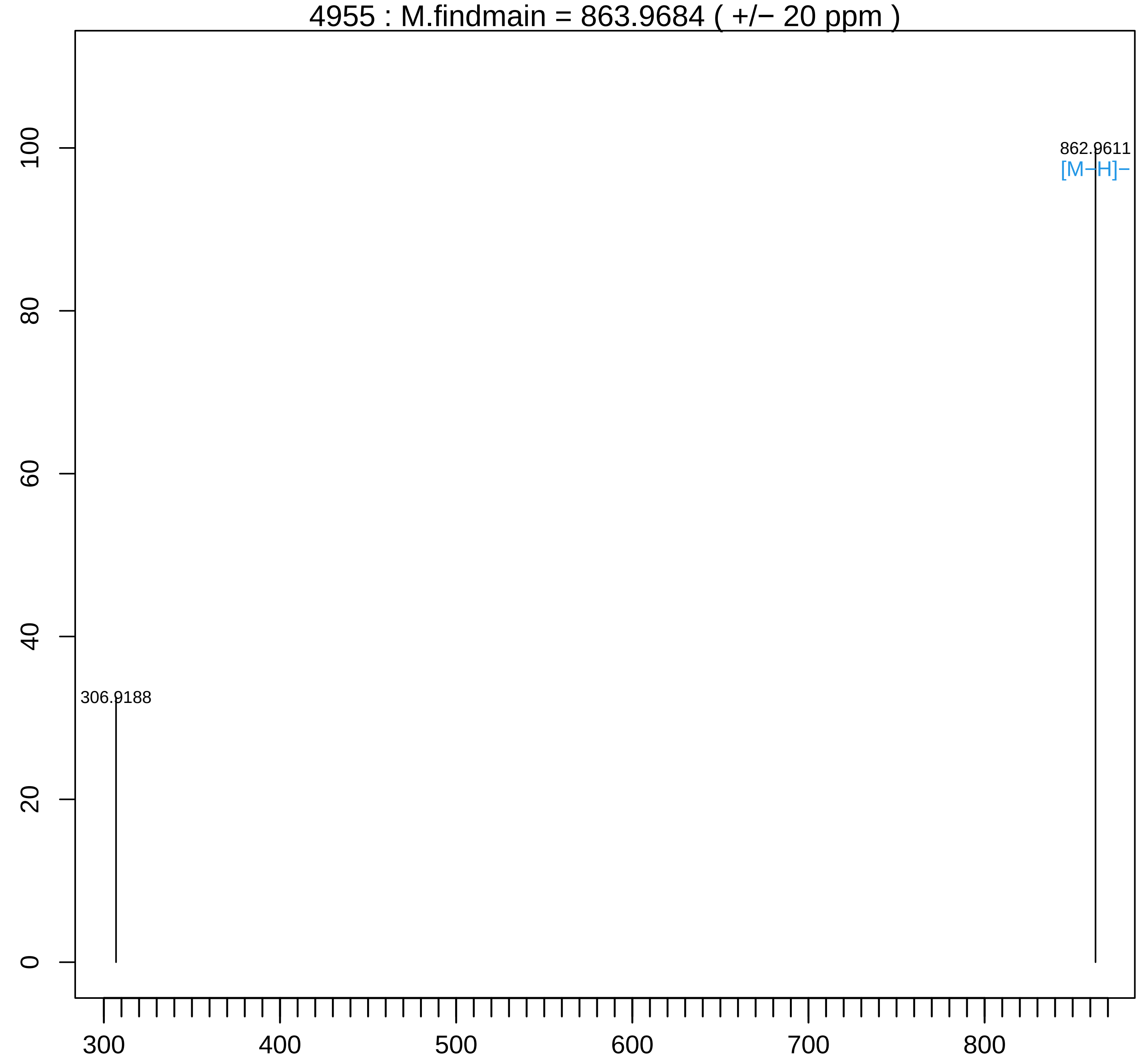
4955 : M.findmain = 863.9684 (+/− 20 ppm)
862.9611
[M−H]−
306.9188
0
20
40
60
80
100
300
400
500
600
700
800

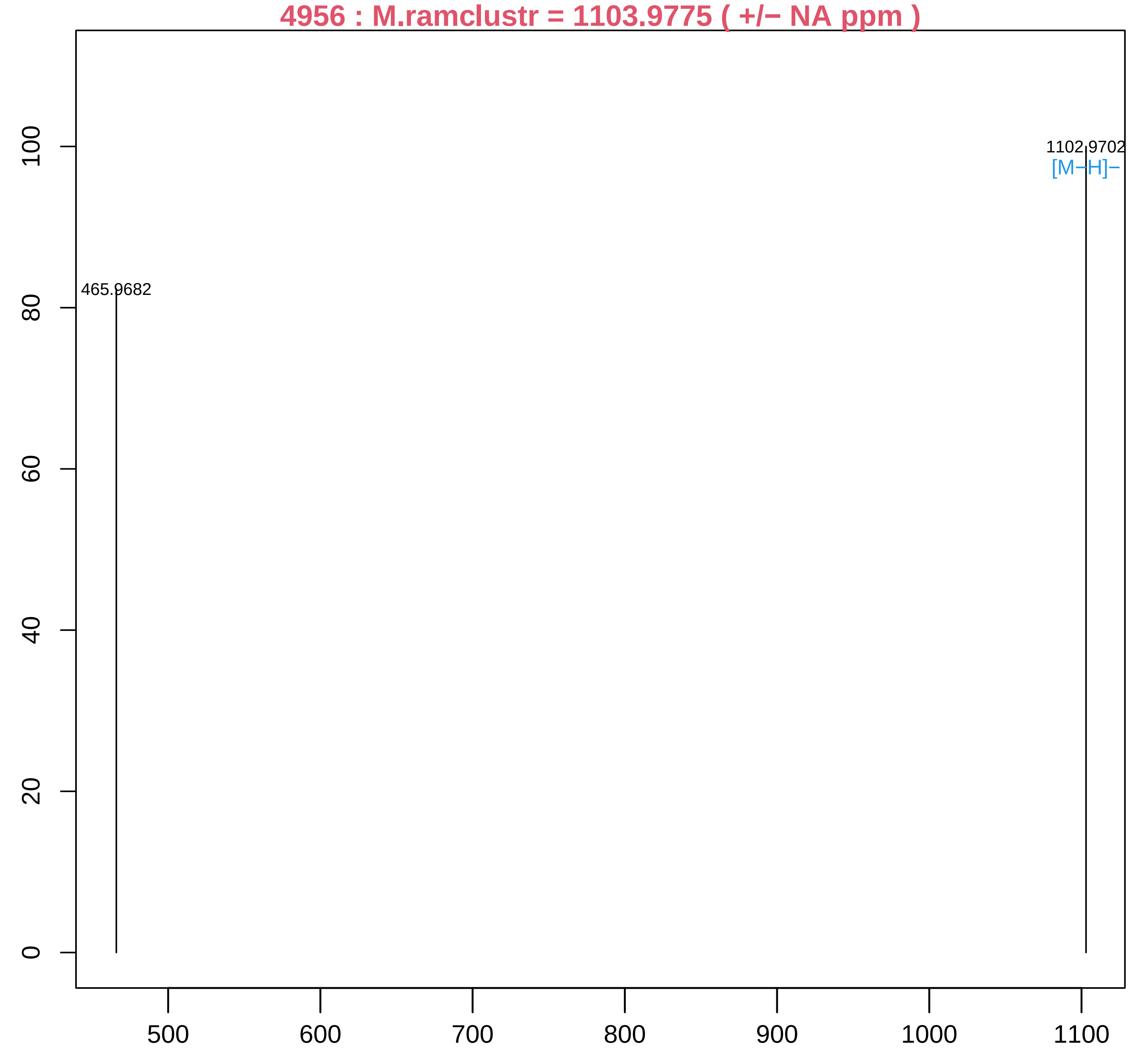
4956 : M.ramclustr = 1103.9775 (+/− NA ppm)
465.9682
1102.9702
[M−H]−
0
20
40
60
80
100
500
600
700
800
900
1000
1100

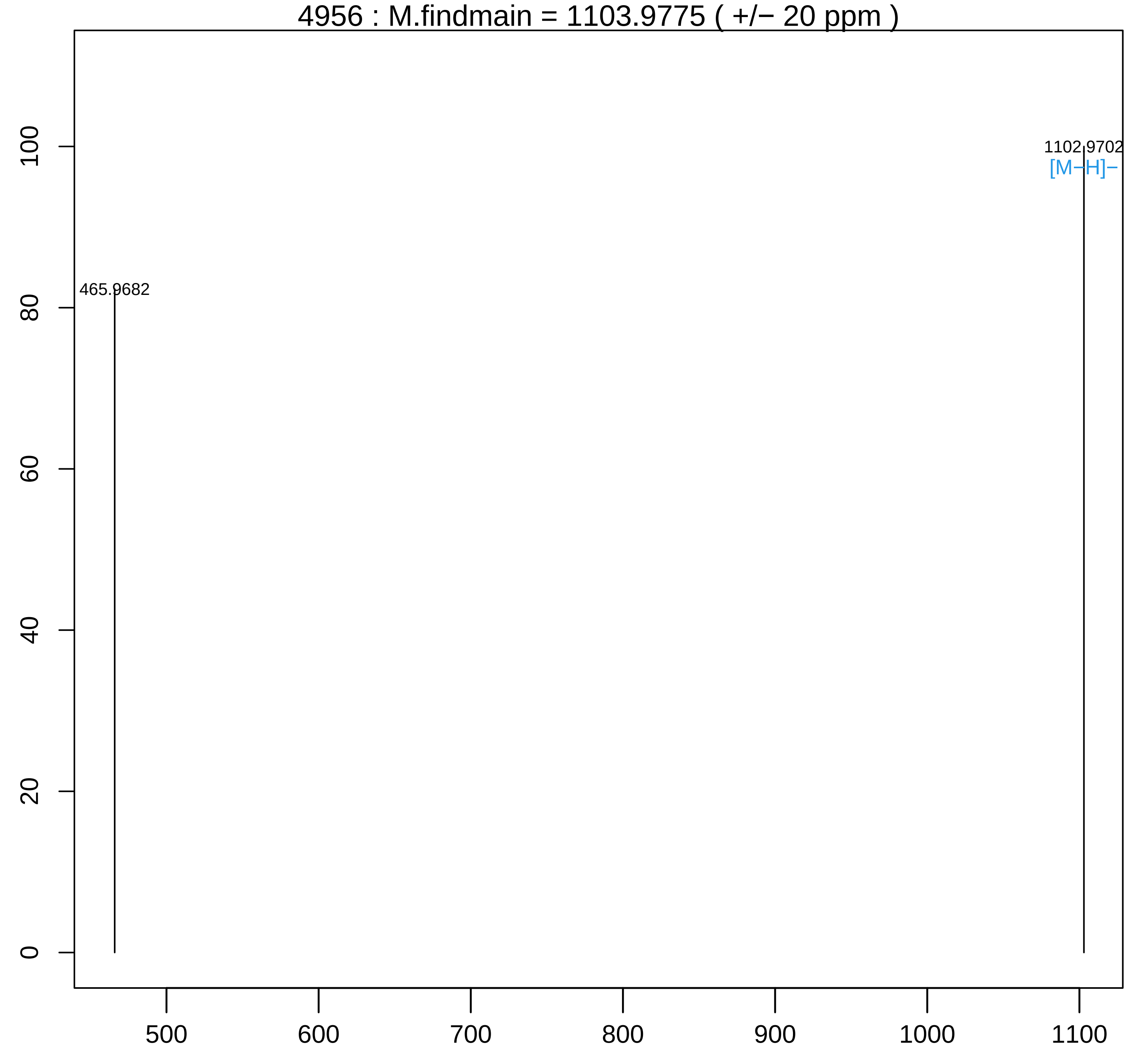
4956 : M.findmain = 1103.9775 (+/− 20 ppm)
465.9682
1102.9702
[M−H]−
0
20
40
60
80
100
500
600
700
800
900
1000
1100

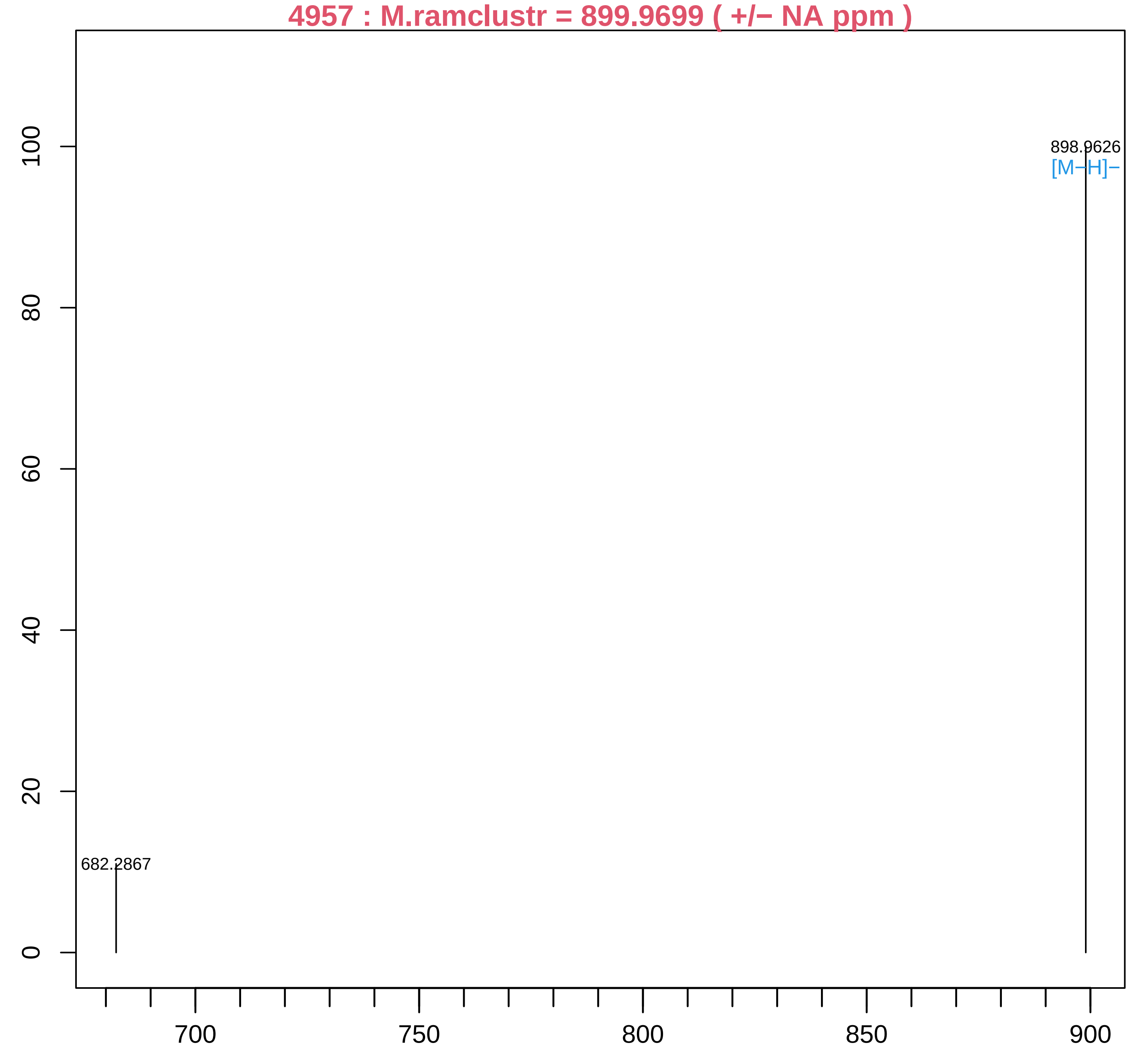
4957 : M.ramclustr = 899.9699 (+/− NA ppm)
898.9626
[M−H]−
682.2867
0
20
40
60
80
100
700
750
800
850
900

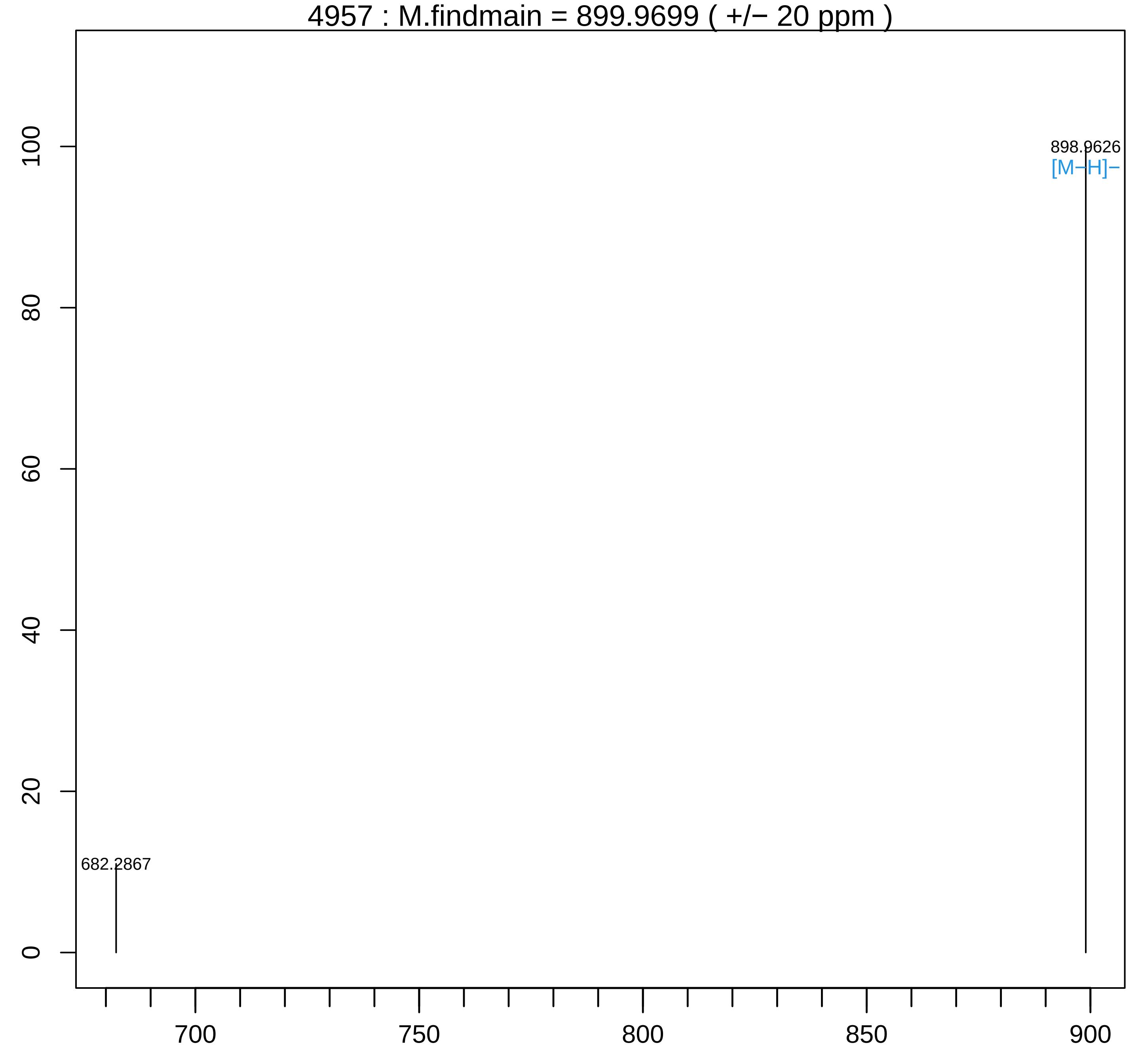
4957 : M.findmain = 899.9699 (+/− 20 ppm)
898.9626
[M−H]−
682.2867
0
20
40
60
80
100
700
750
800
850
900

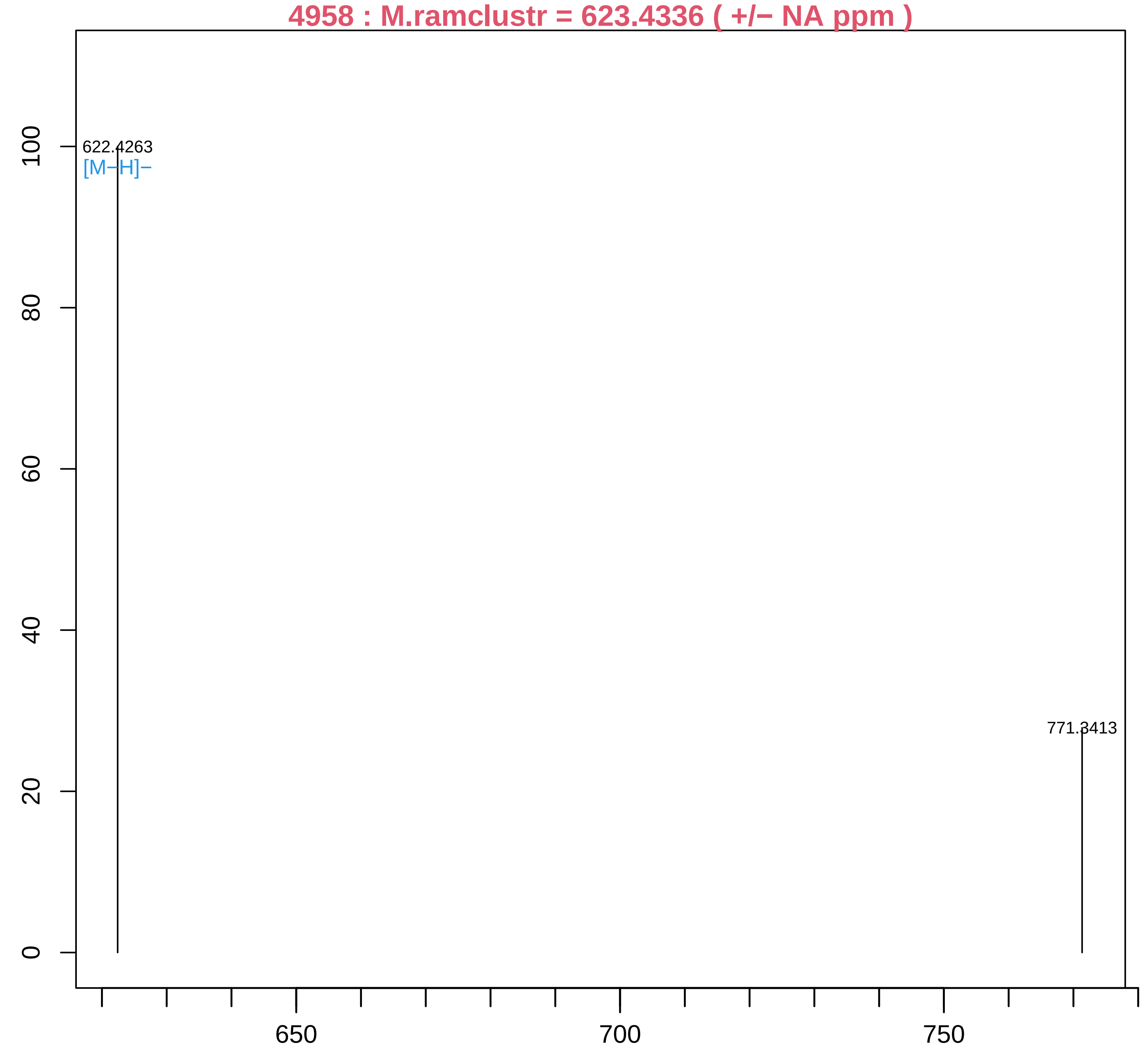
4958 : M.ramclustr = 623.4336 (+/− NA ppm)
622.4263
[M−H]−
771.3413
0
20
40
60
80
100
650
700
750

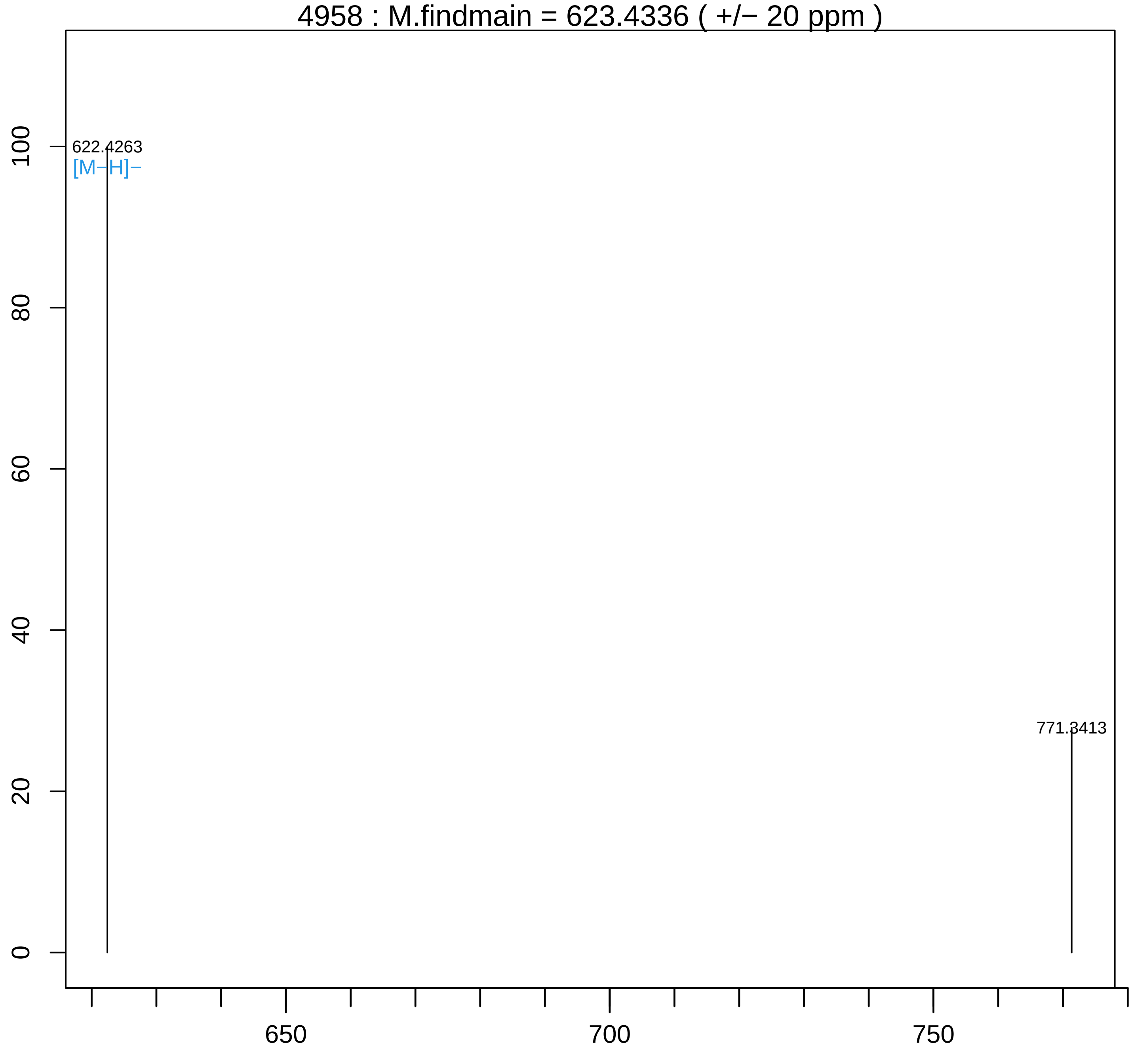
4958 : M.findmain = 623.4336 (+/− 20 ppm)
622.4263
[M−H]−
771.3413
0
20
40
60
80
100
650
700
750

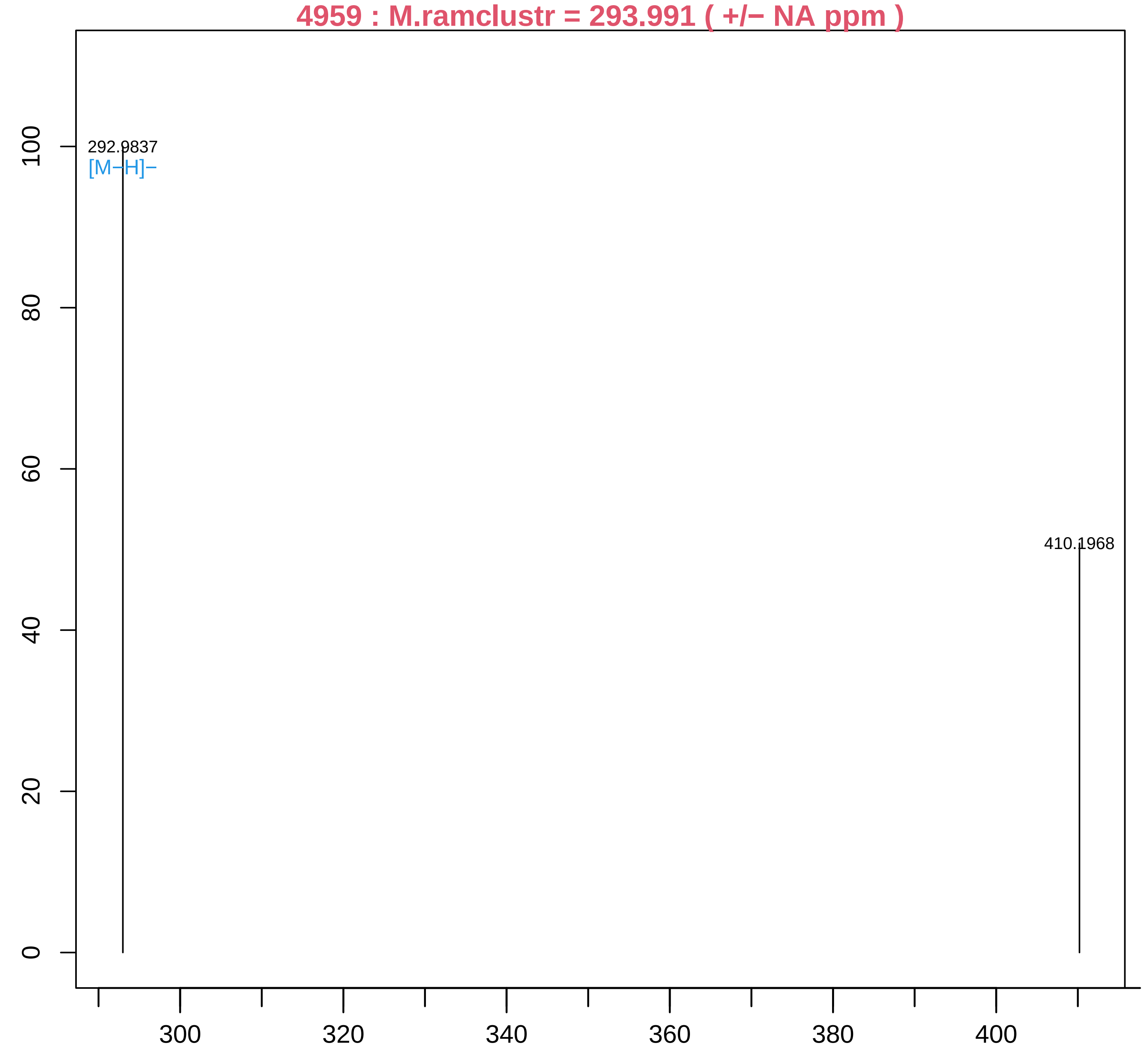
4959 : M.ramclustr = 293.991 (+/− NA ppm)
292.9837
[M−H]−
410.1968
0
20
40
60
80
100
300
320
340
360
380
400

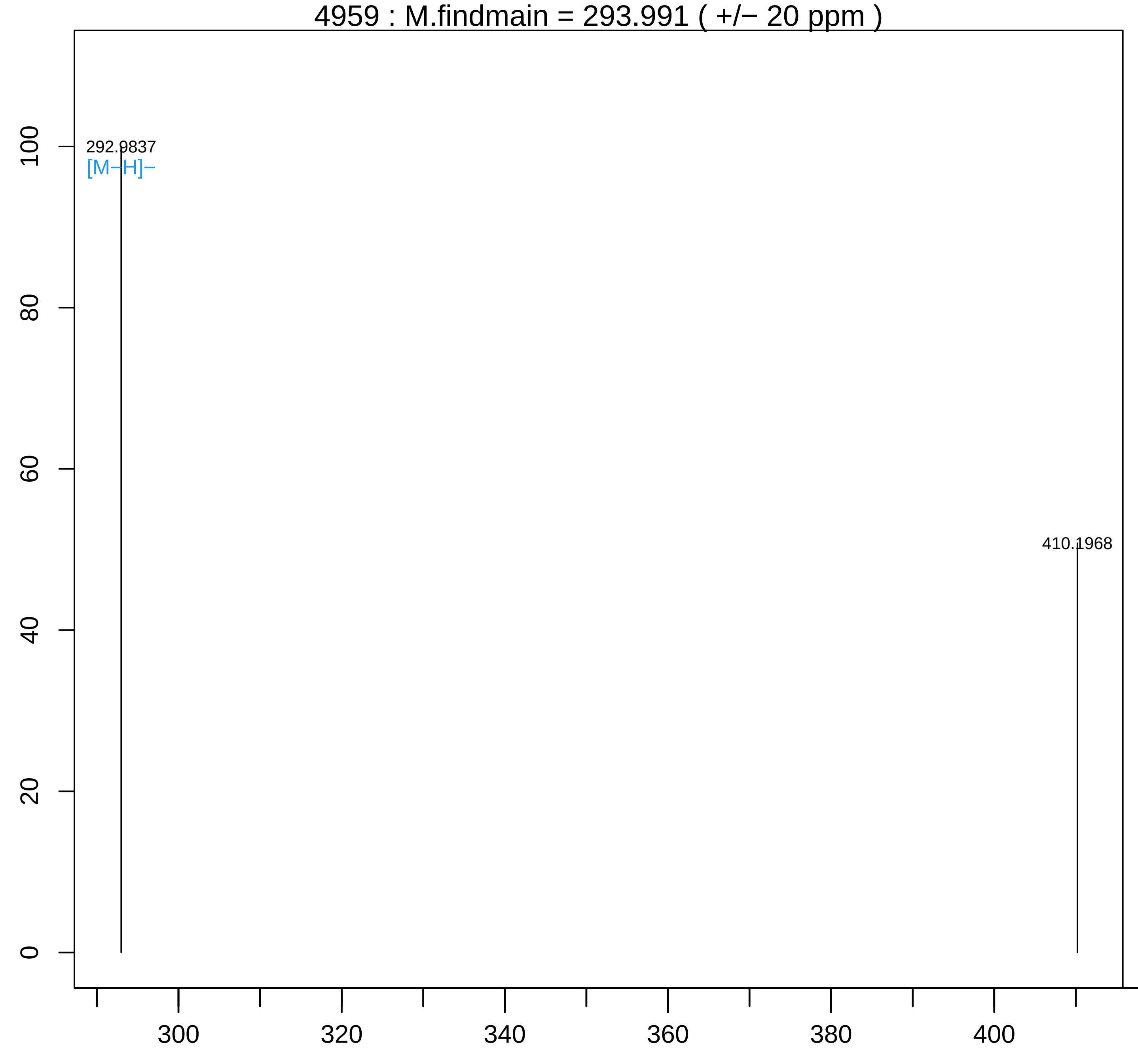
4959 : M.findmain = 293.991 (+/− 20 ppm)
292.9837
[M−H]−
410.1968
0
20
40
60
80
100
300
320
340
360
380
400

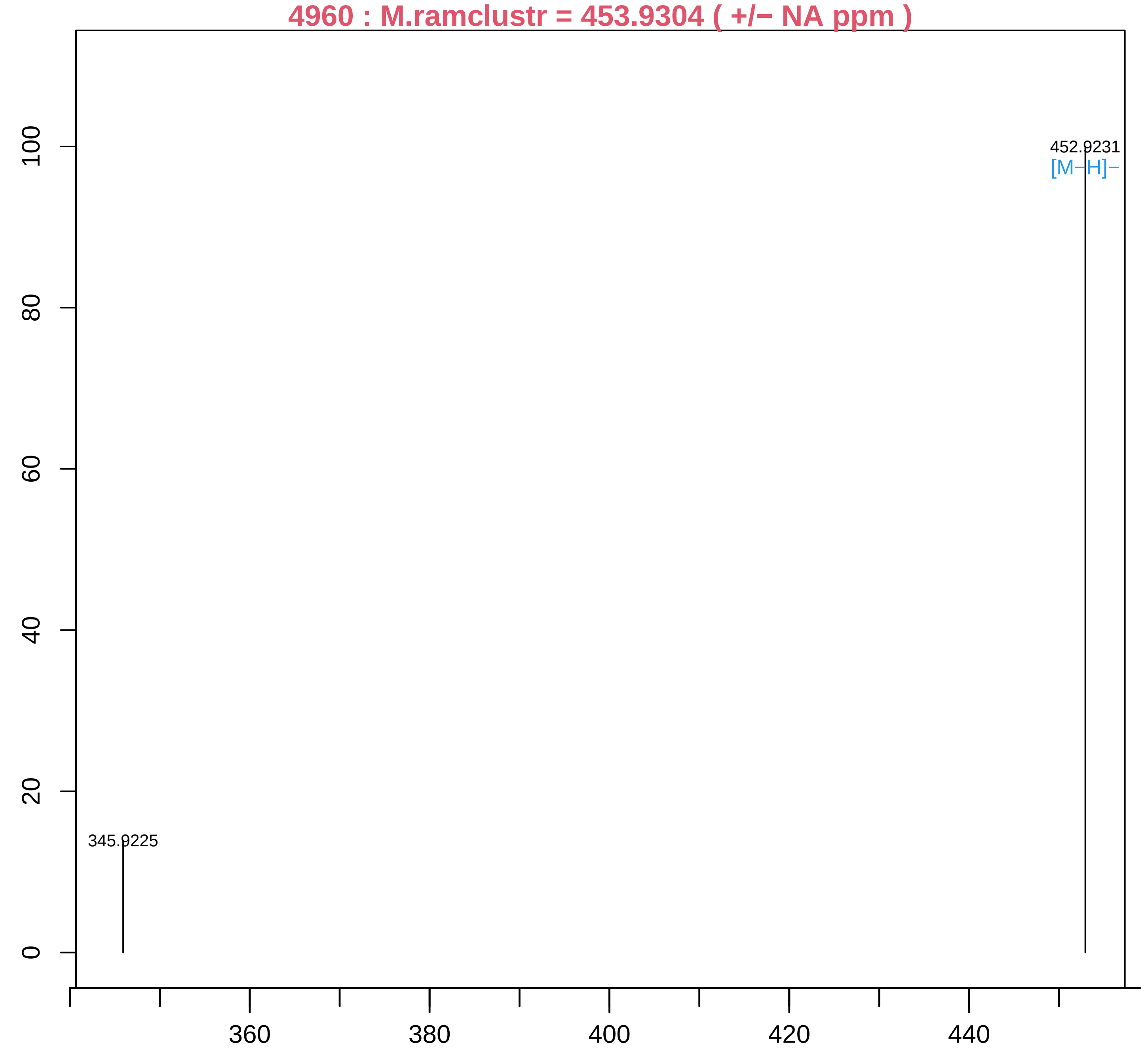
4960 : M.ramclustr = 453.9304 (+/− NA ppm)
452.9231
[M−H]−
345.9225
0
20
40
60
80
100
360
380
400
420
440

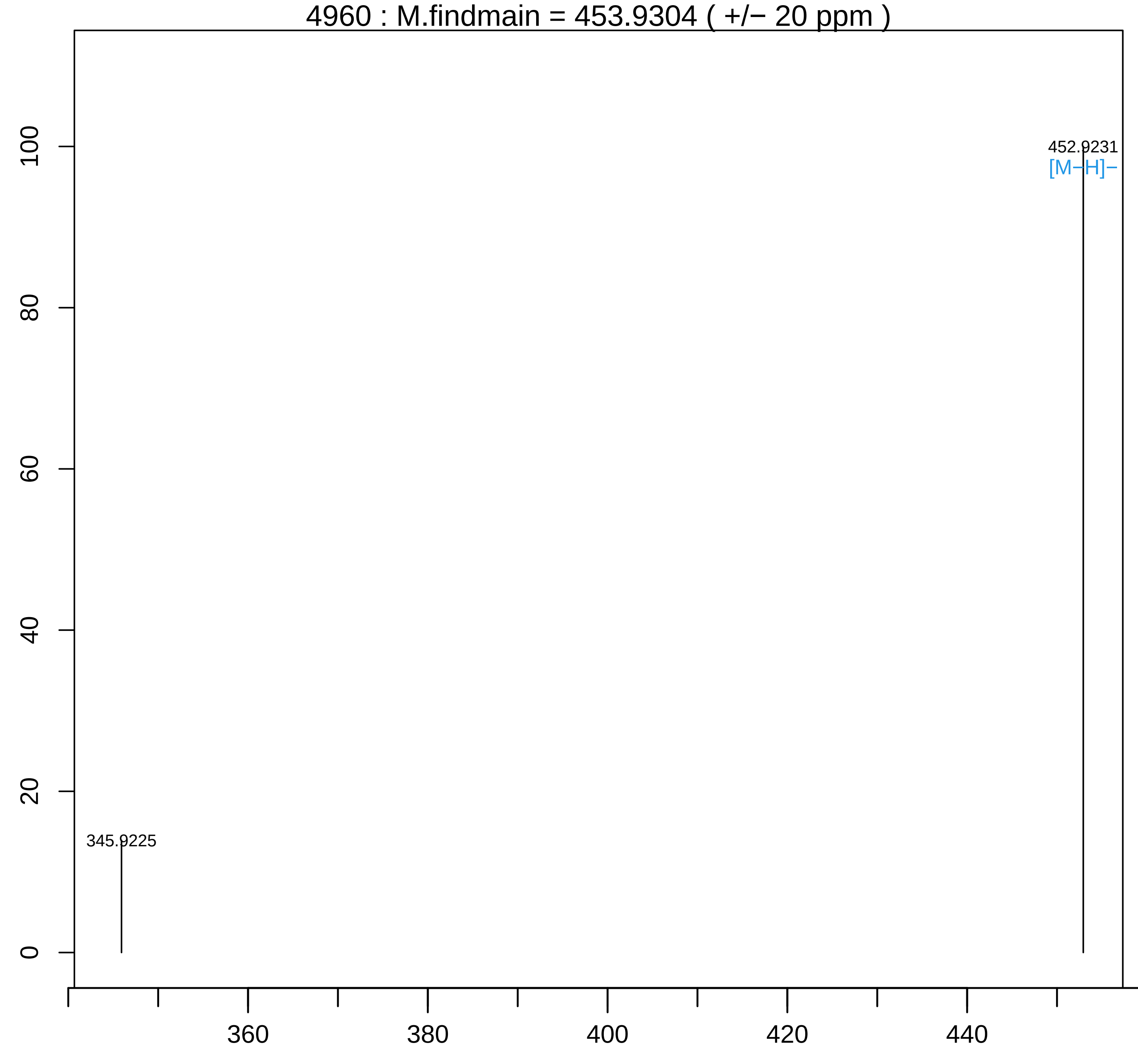
4960 : M.findmain = 453.9304 (+/− 20 ppm)
452.9231
[M−H]−
345.9225
0
20
40
60
80
100
360
380
400
420
440

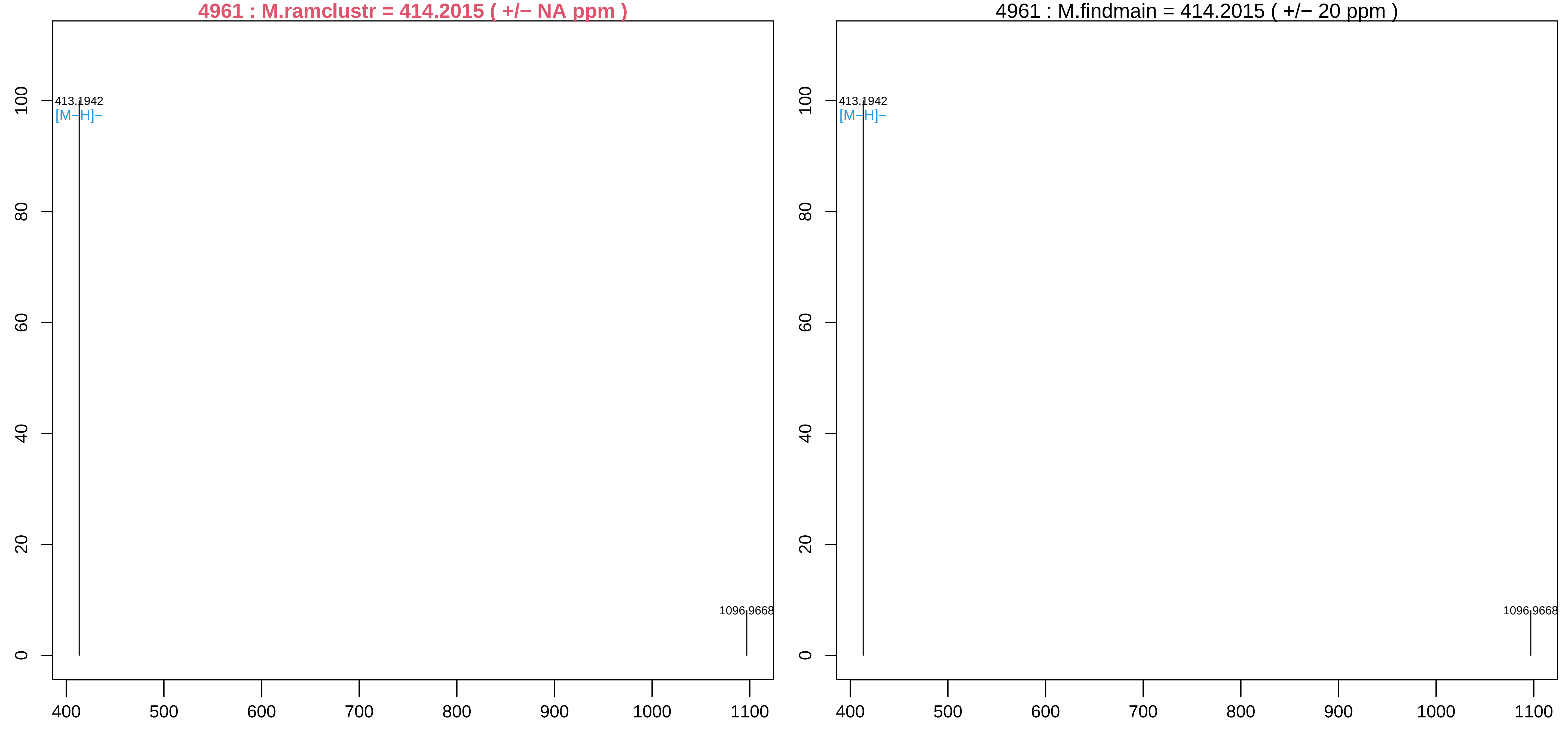
4961 : M.ramclustr = 414.2015 (+/− NA ppm)
413.1942
[M−H]−
1096.9668
0
20
40
60
80
100
400
500
600
700
800
900
1000
1100
4961 : M.findmain = 414.2015 (+/− 20 ppm)
413.1942
[M−H]−
1096.9668
0
20
40
60
80
100
400
500
600
700
800
900
1000
1100

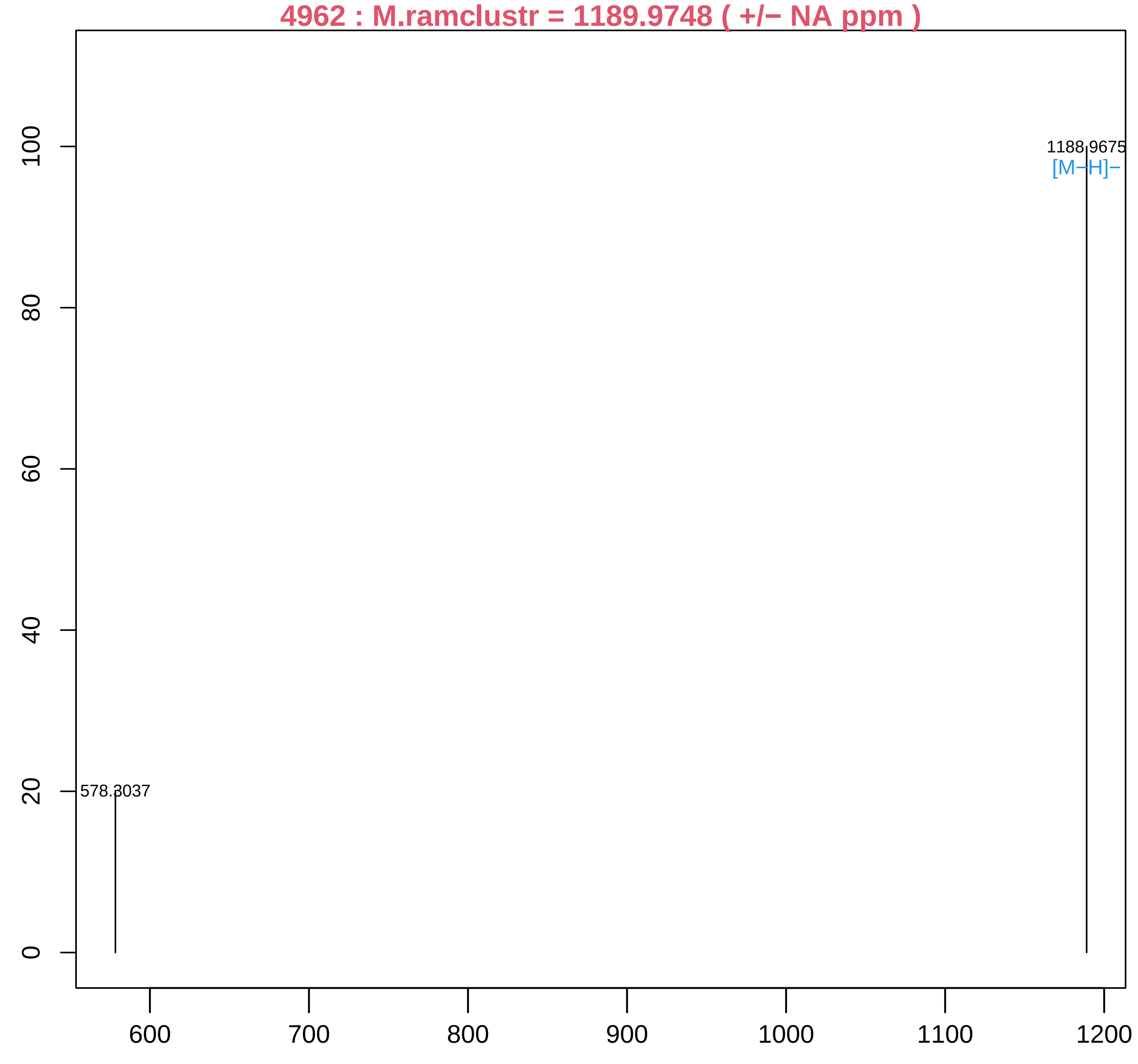
4962 : M.ramclustr = 1189.9748 (+/− NA ppm)
1188.9675
[M−H]−
578.3037
0
20
40
60
80
100
600
700
800
900
1000
1100
1200

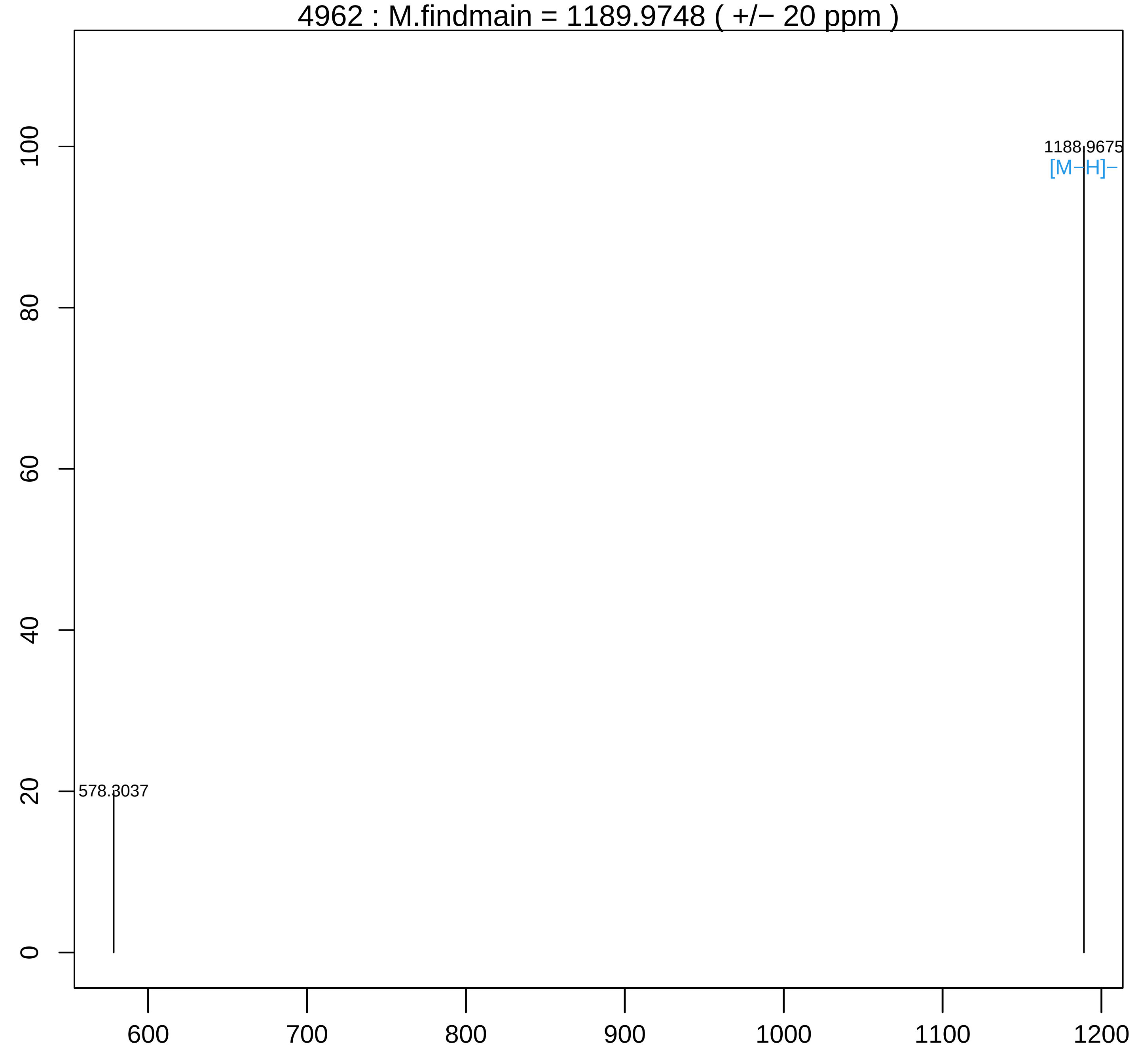
4962 : M.findmain = 1189.9748 (+/− 20 ppm)
1188.9675
[M−H]−
578.3037
0
20
40
60
80
100
600
700
800
900
1000
1100
1200

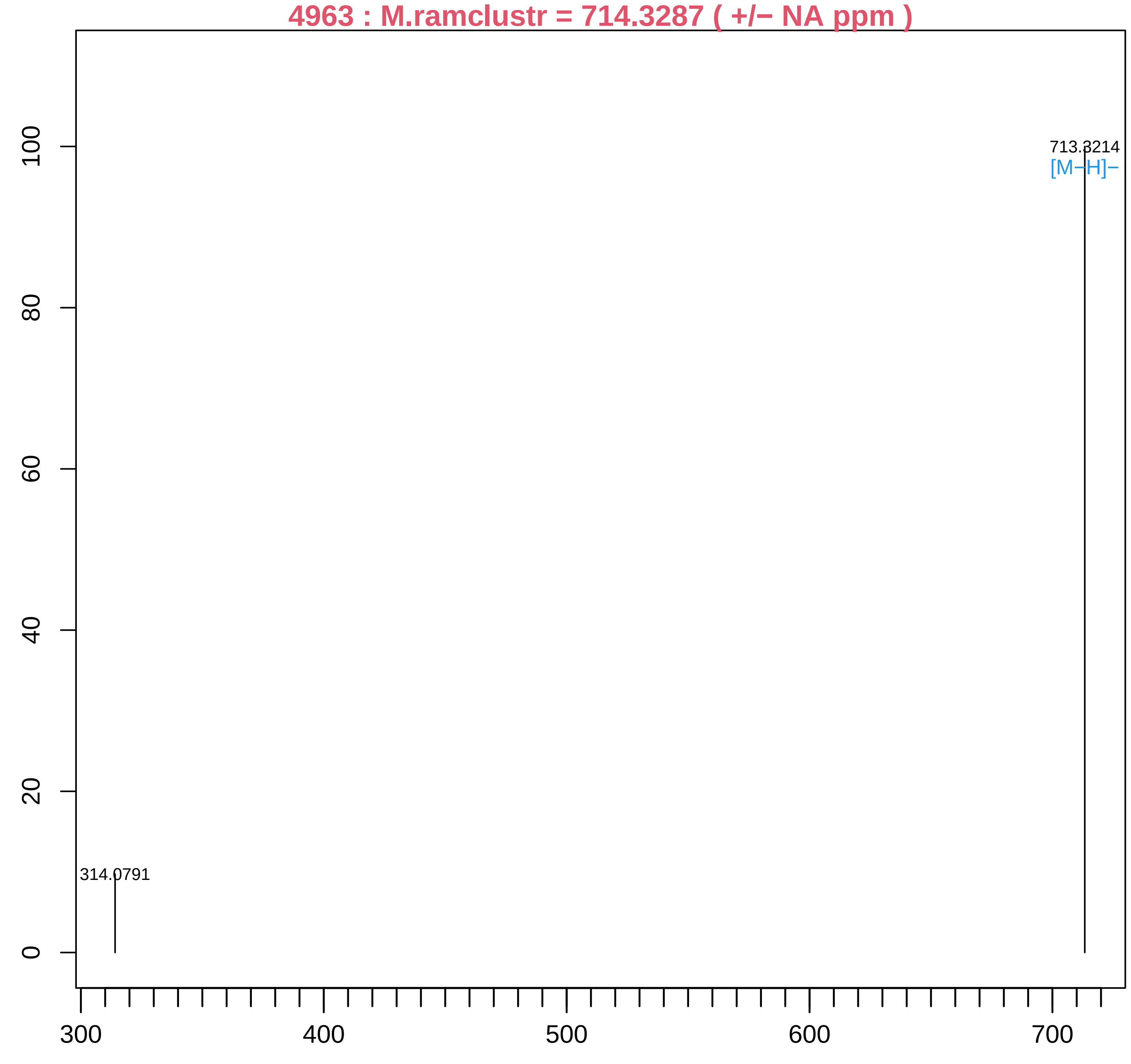

4963 : M.ramclustr = 714.3287 (+/− NA ppm)
713.3214
[M−H]−
314.0791
0
20
40
60
80
100
300
400
500
600
700

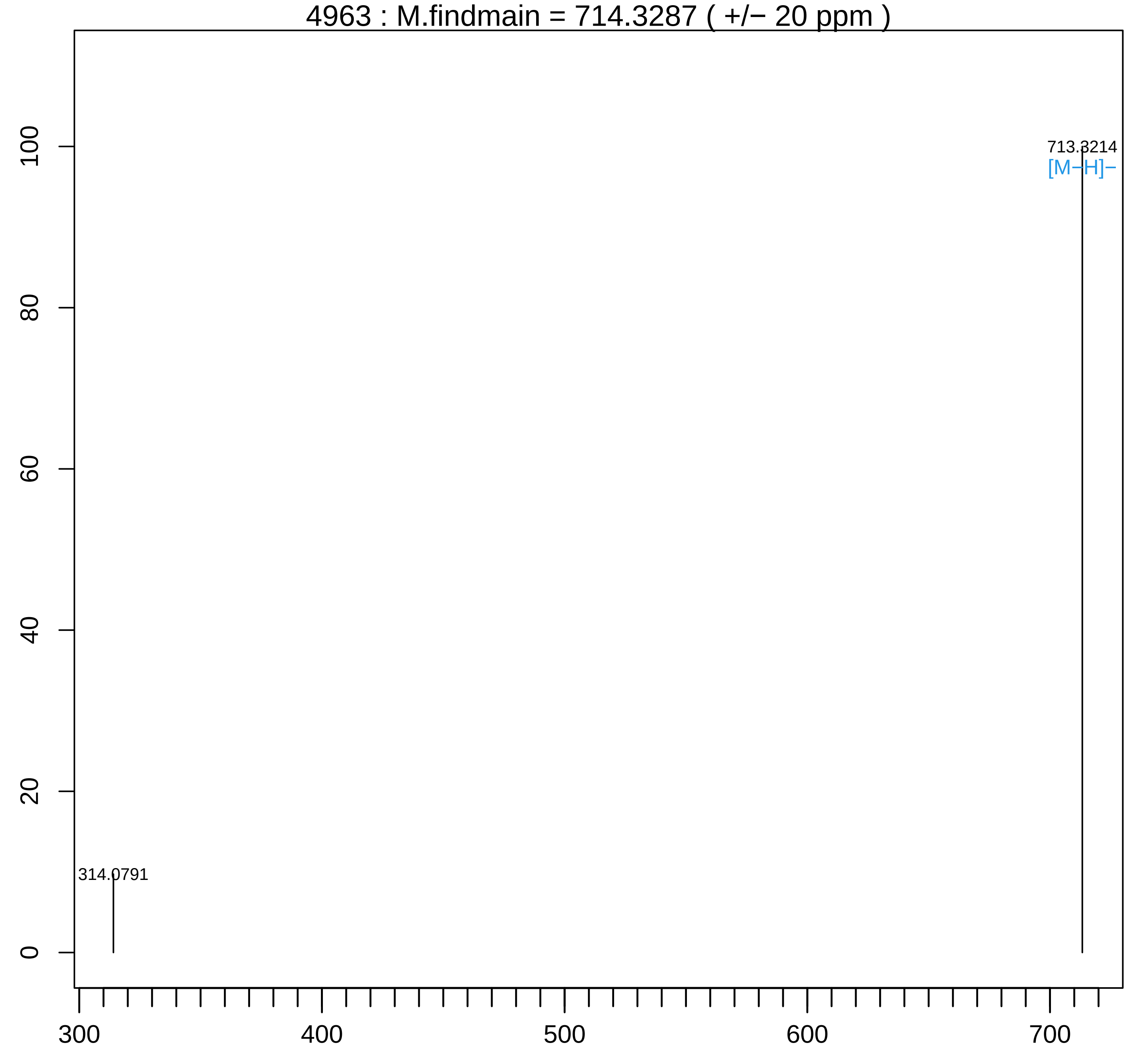

4963 : M.findmain = 714.3287 (+/− 20 ppm)
713.3214
[M−H]−
314.0791
0
20
40
60
80
100
300
400
500
600
700

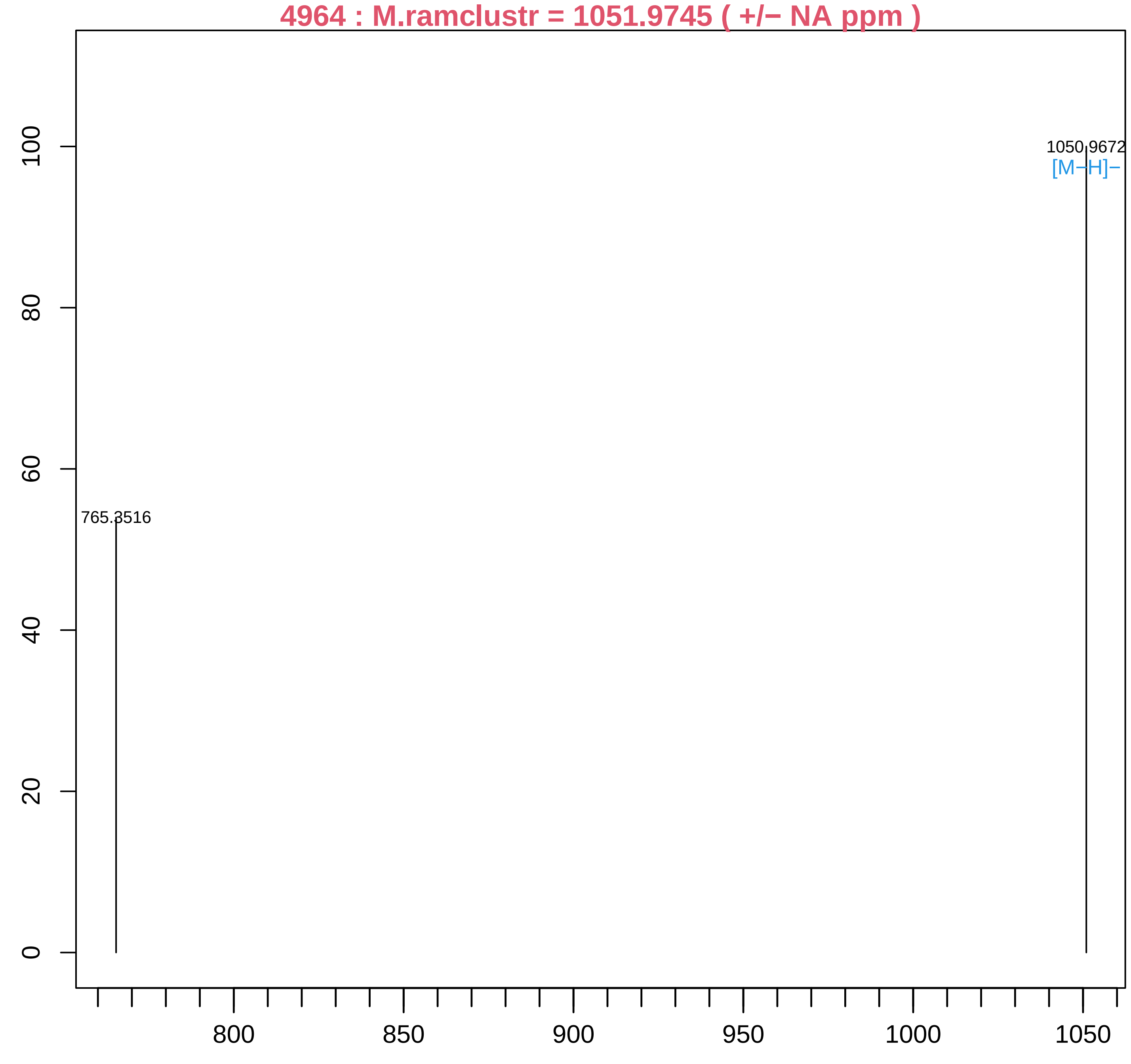
4964 : M.ramclustr = 1051.9745 (+/− NA ppm)
1050.9672
[M−H]−
765.3516
0
20
40
60
80
100
800
850
900
950
1000
1050

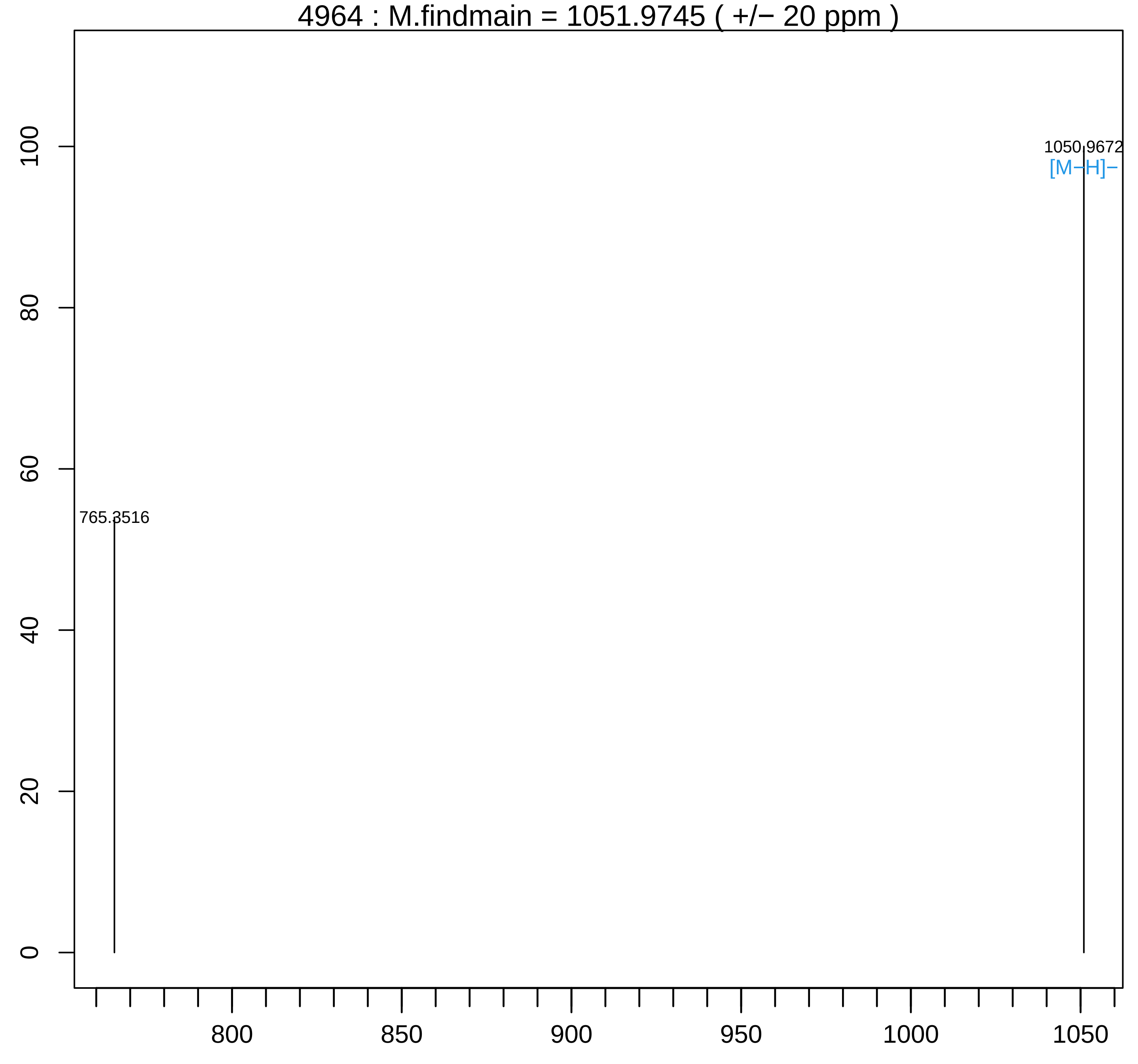
4964 : M.findmain = 1051.9745 (+/− 20 ppm)
1050.9672
[M−H]−
765.3516
0
20
40
60
80
100
800
850
900
950
1000
1050

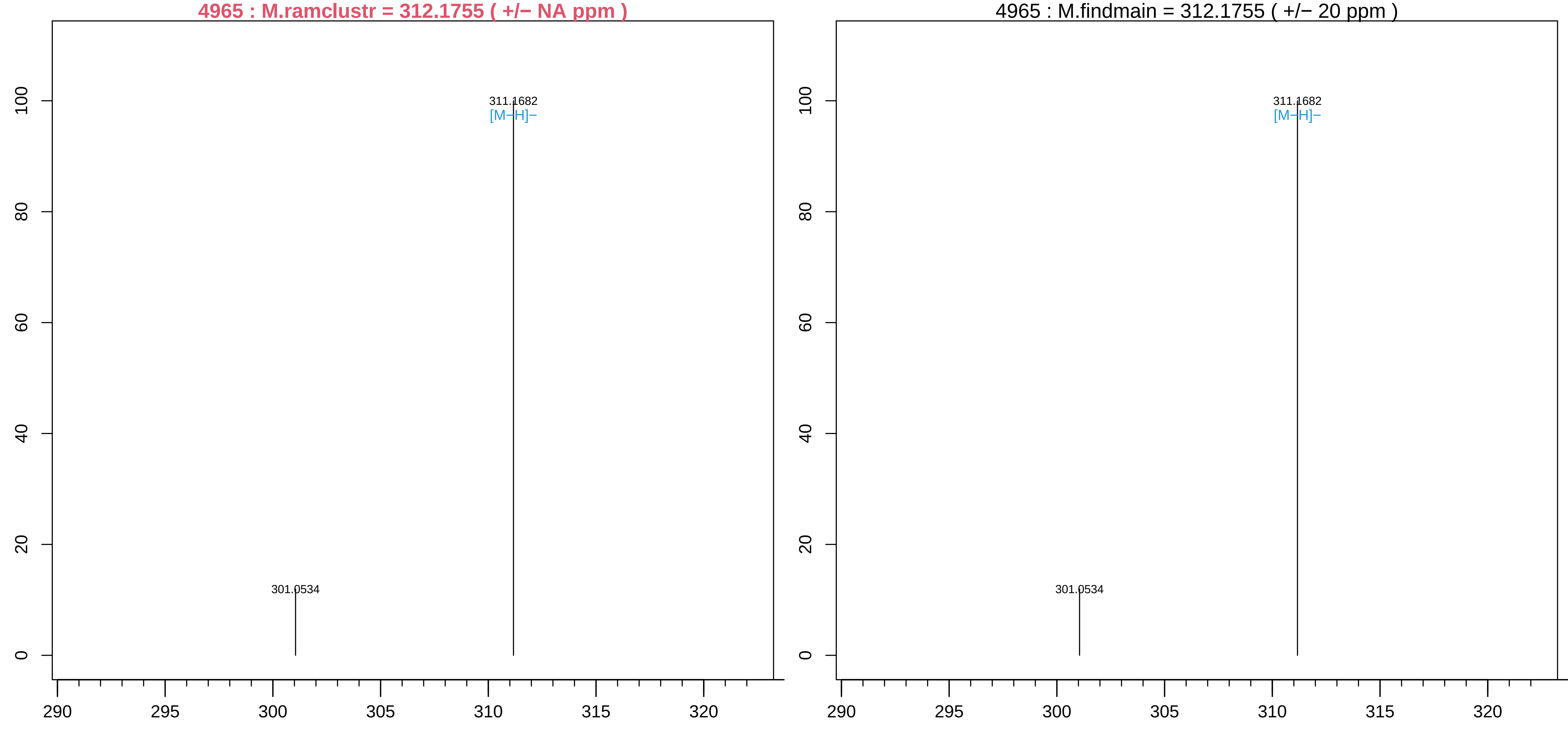

4965 : M.ramclustr = 312.1755 (+/− NA ppm)
311.1682
[M−H]−
301.0534
4965 : M.findmain = 312.1755 (+/− 20 ppm)
311.1682
[M−H]−
301.0534
0
20
40
60
80
100
290
295
300
305
310
315
320

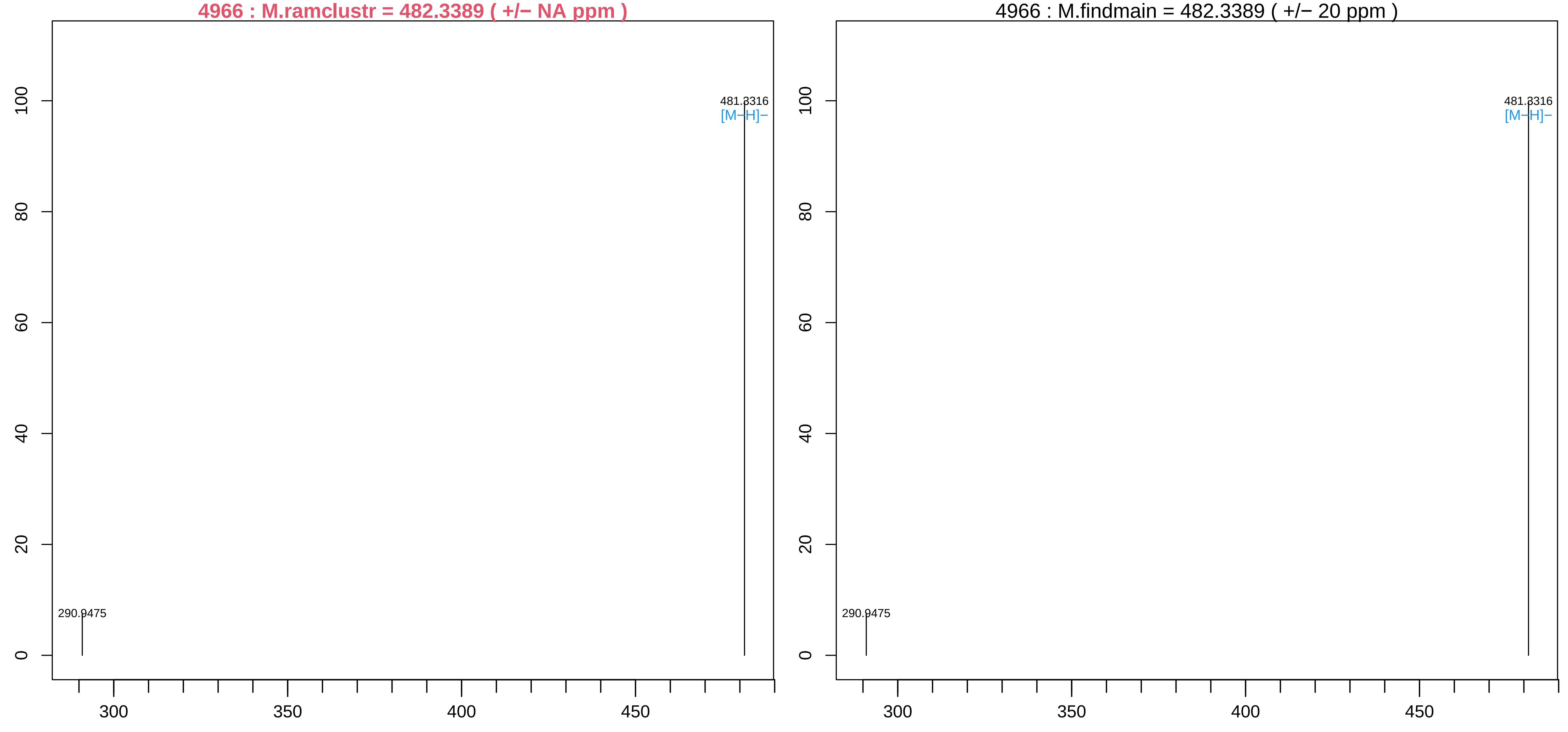
4966 : M.ramclustr = 482.3389 (+/− NA ppm)
481.3316
[M−H]−
290.9475
0
20
40
60
80
100
300
350
400
450
4966 : M.findmain = 482.3389 (+/− 20 ppm)
481.3316
[M−H]−
290.9475
0
20
40
60
80
100
300
350
400
450

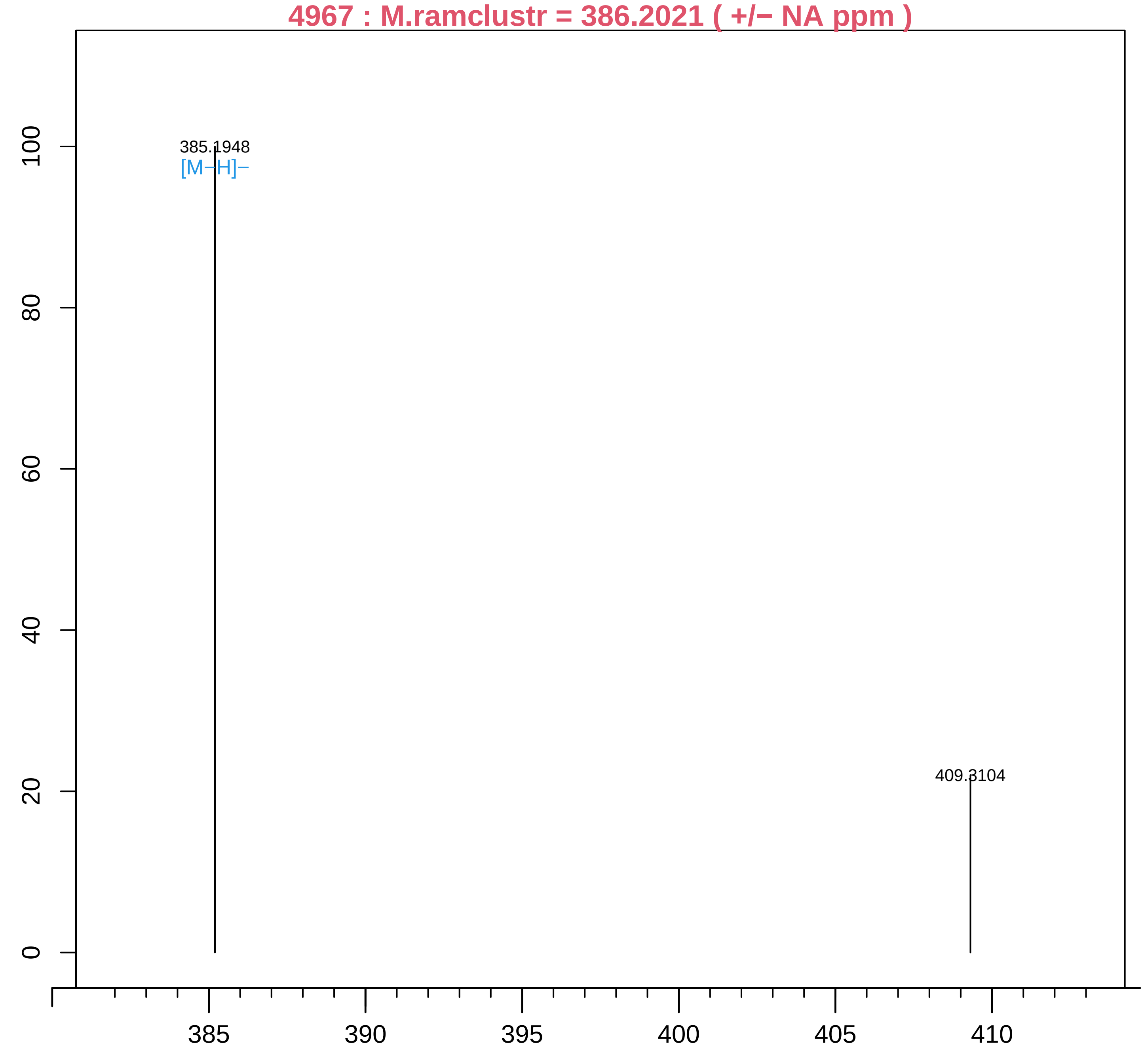
4967 : M.ramclustr = 386.2021 (+/− NA ppm)
385.1948
[M−H]−
409.3104
0
20
40
60
80
100
385
390
395
400
405
410

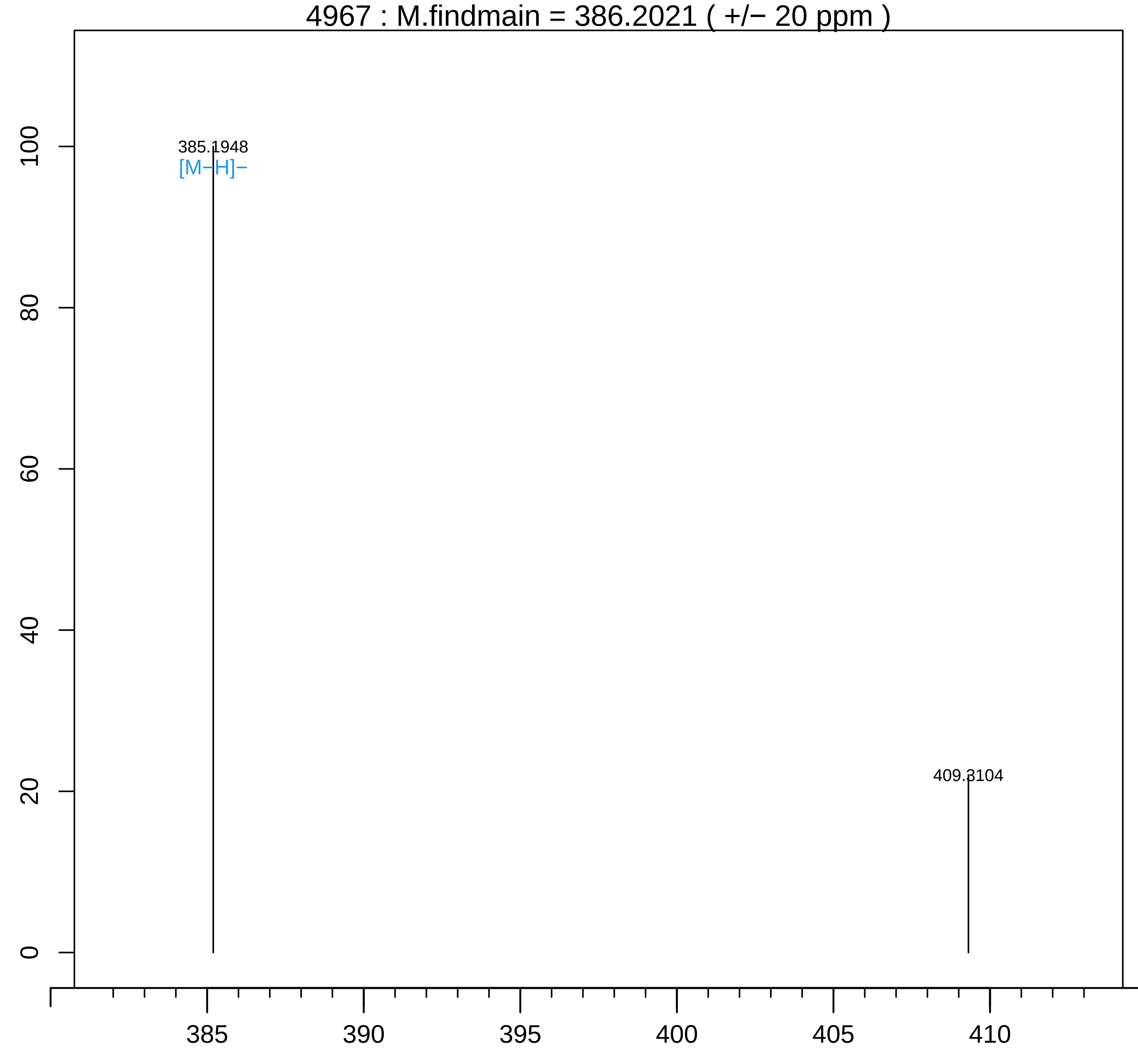
4967 : M.findmain = 386.2021 (+/− 20 ppm)
385.1948
[M−H]−
409.3104
0
20
40
60
80
100
385
390
395
400
405
410

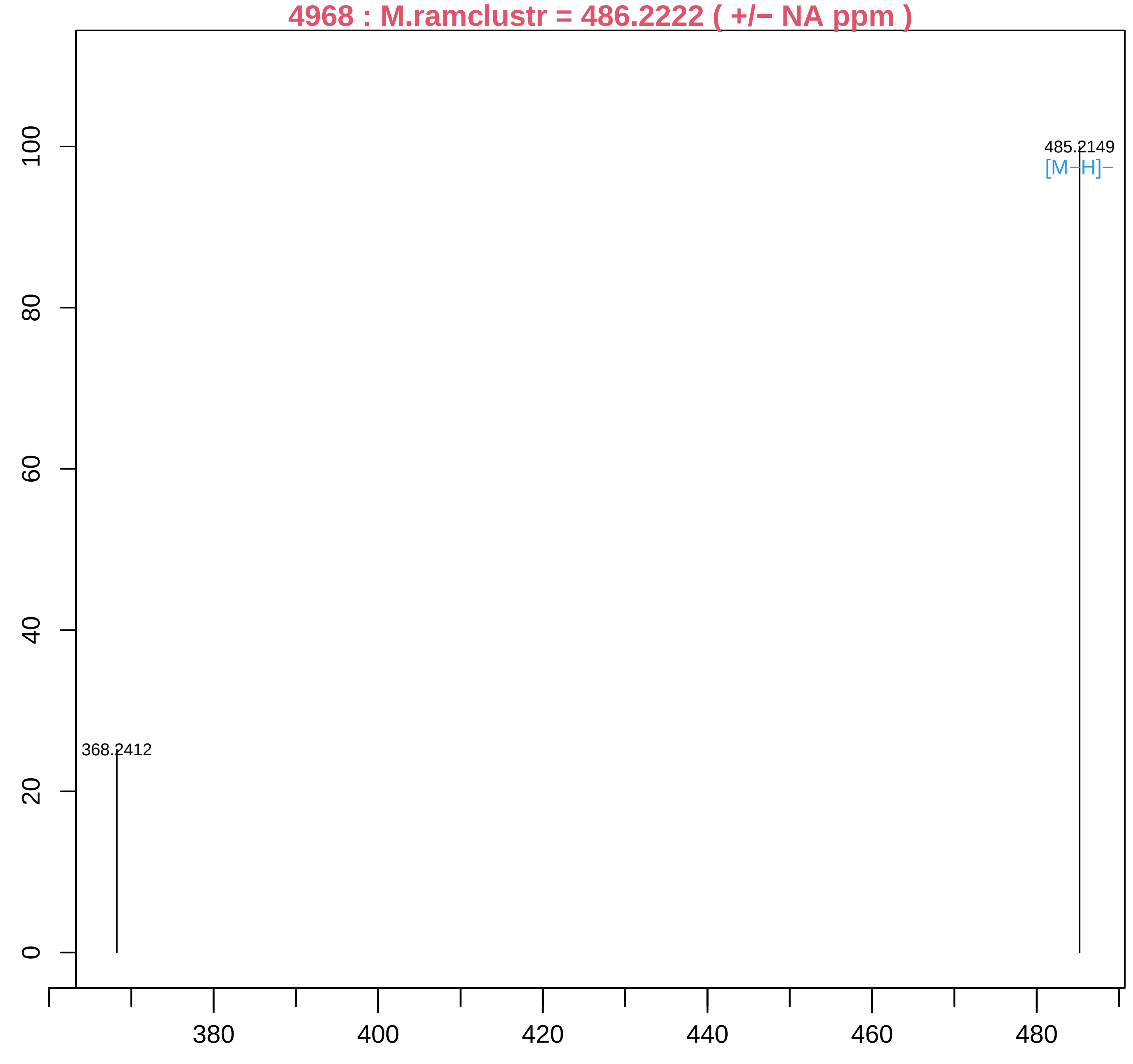
4968 : M.ramclustr = 486.2222 (+/− NA ppm)
485.2149
[M−H]−
368.2412
0
20
40
60
80
100
380
400
420
440
460
480

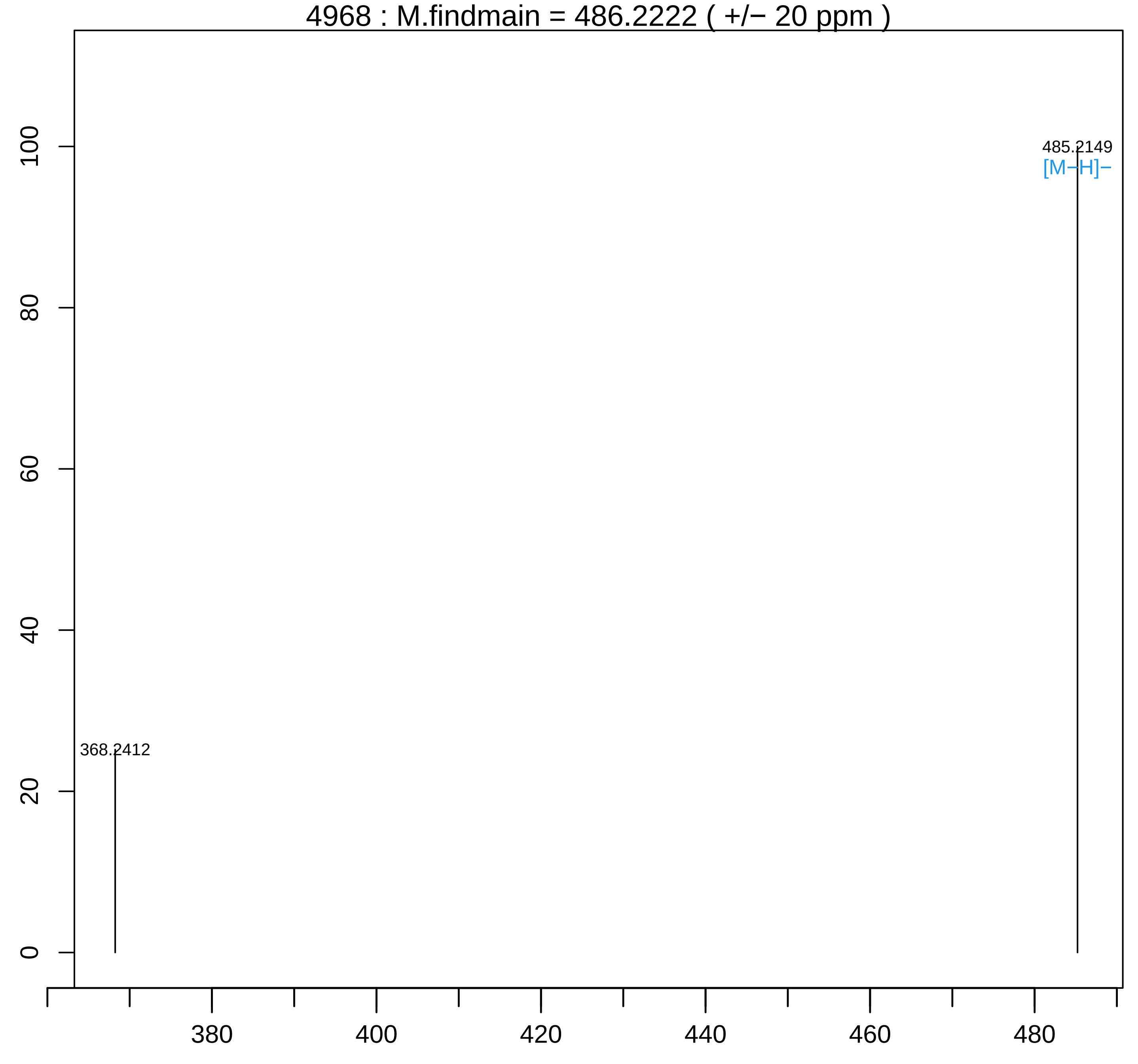
4968 : M.findmain = 486.2222 (+/− 20 ppm)
485.2149
[M−H]−
368.2412
0
20
40
60
80
100
380
400
420
440
460
480

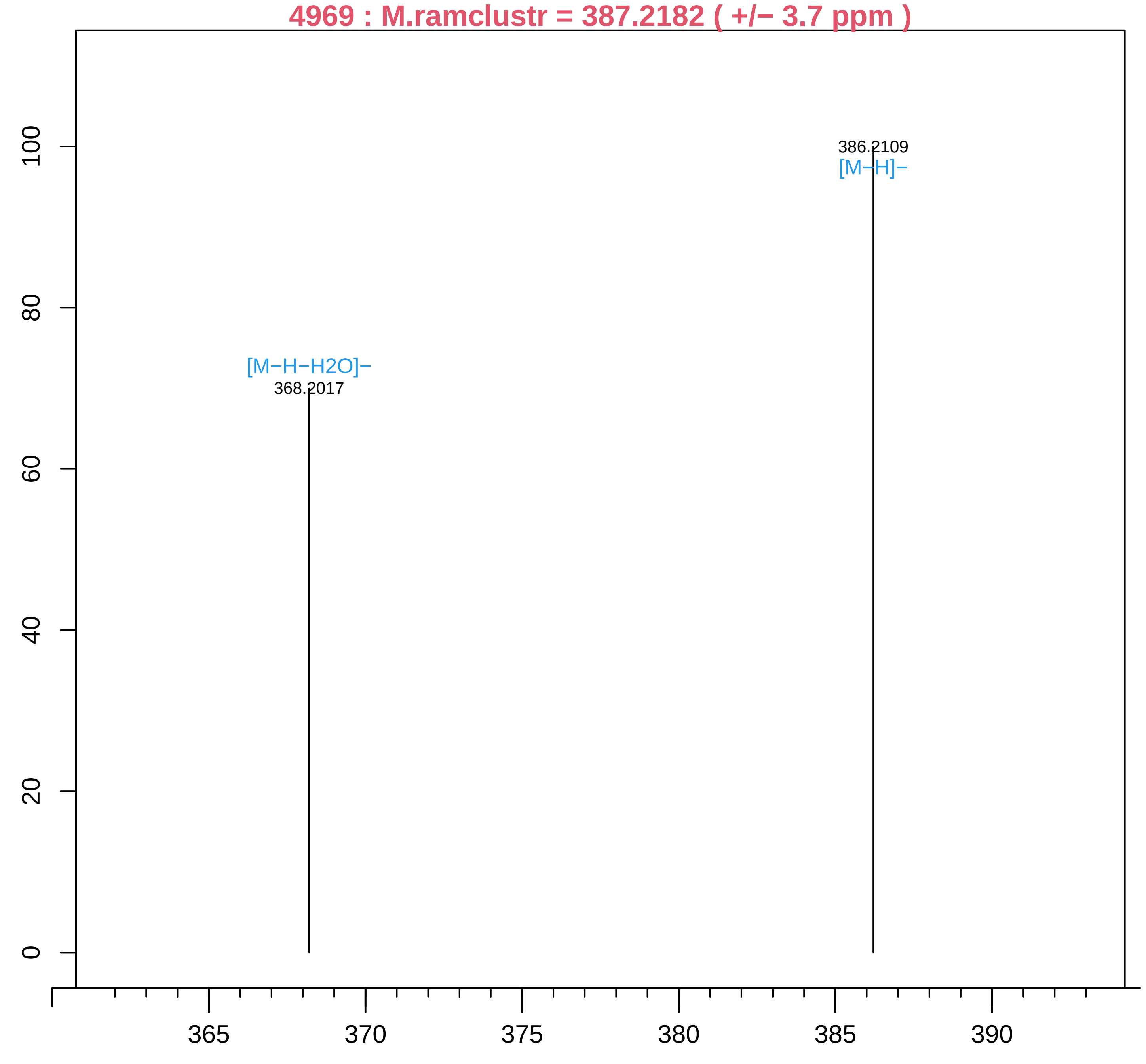

4969 : M.ramclustr = 387.2182 (+/− 3.7 ppm)
386.2109
[M−H]−
[M−H−H2O]−
368.2017
0
20
40
60
80
100
365
370
375
380
385
390

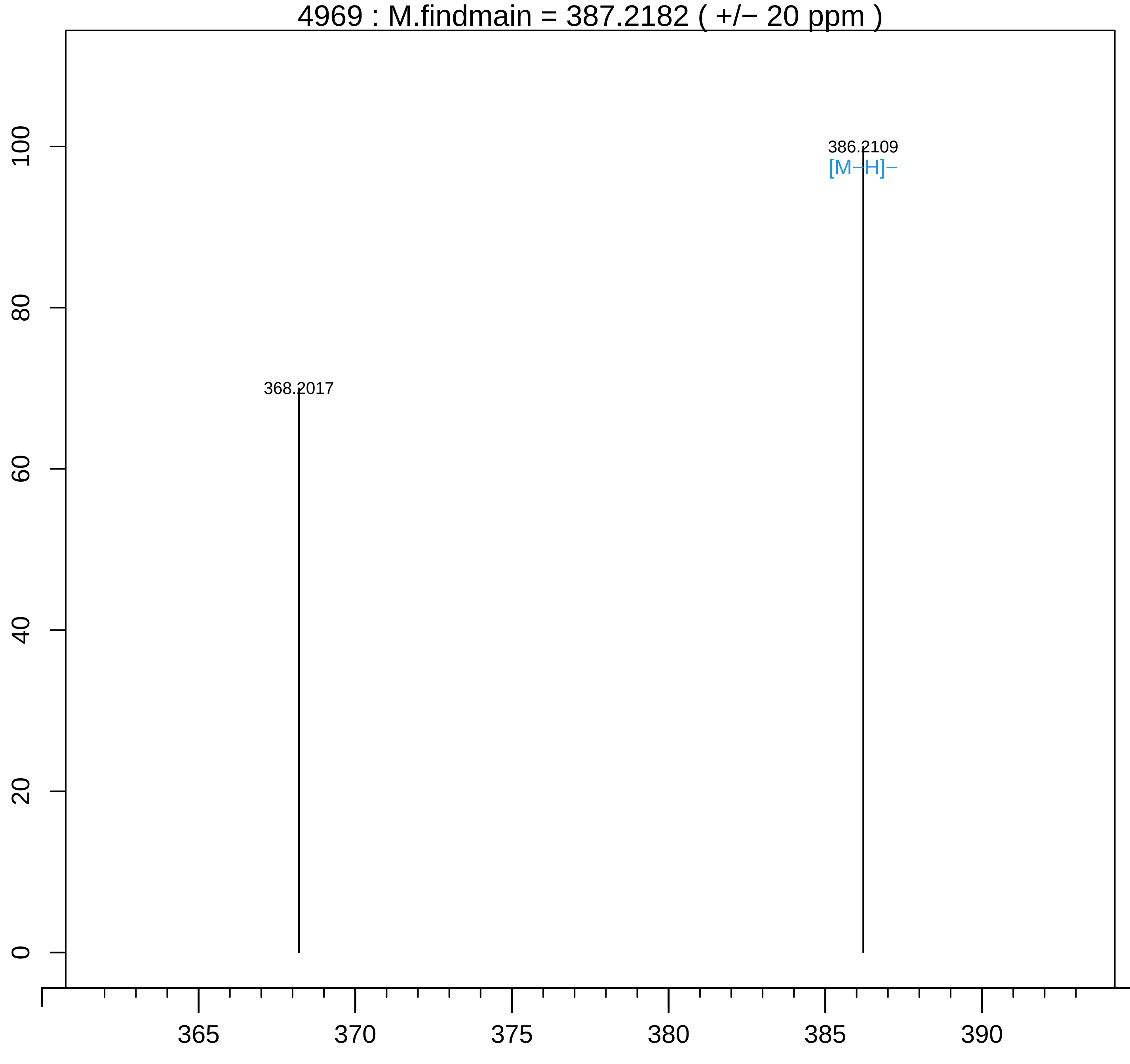

4969 : M.findmain = 387.2182 (+/− 20 ppm)
386.2109
[M−H]−
368.2017
0
20
40
60
80
100
365
370
375
380
385
390

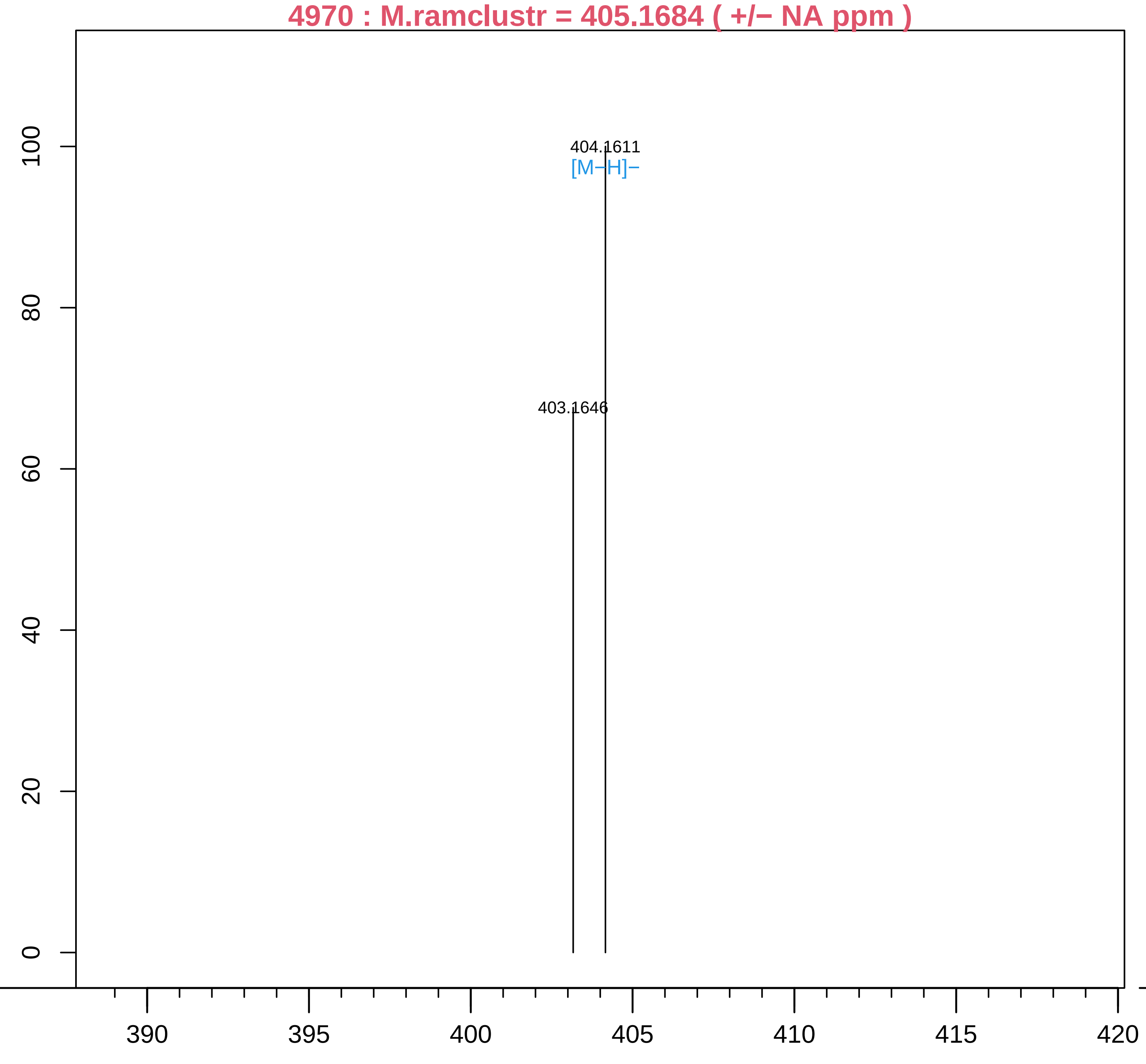
4970 : M.ramclustr = 405.1684 (+/− NA ppm)
404.1611
[M−H]−
403.1646
0
20
40
60
80
100
390
395
400
405
410
415
420

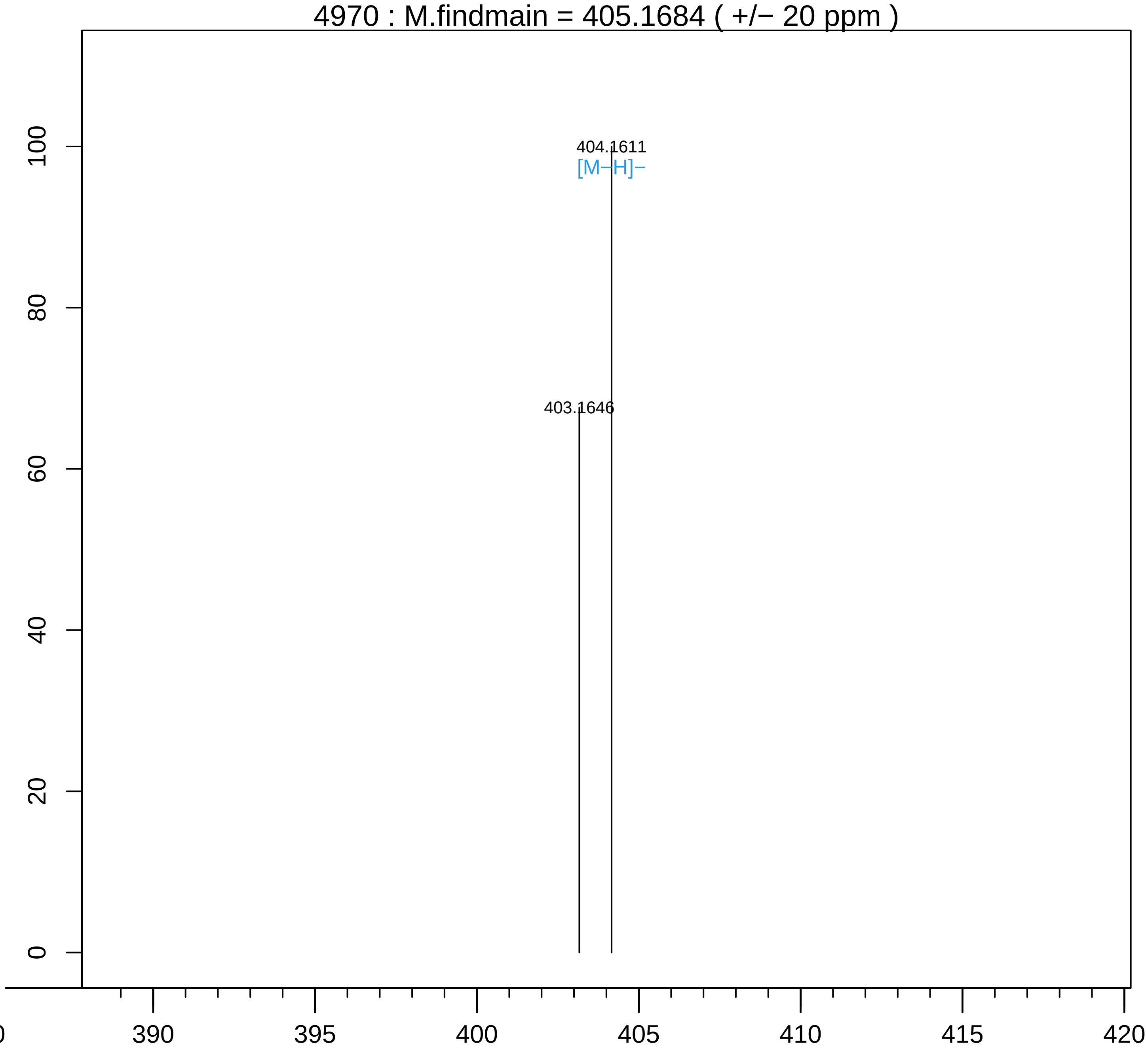
4970 : M.findmain = 405.1684 (+/− 20 ppm)
404.1611
[M−H]−
403.1646
0
20
40
60
80
100
390
395
400
405
410
415
420

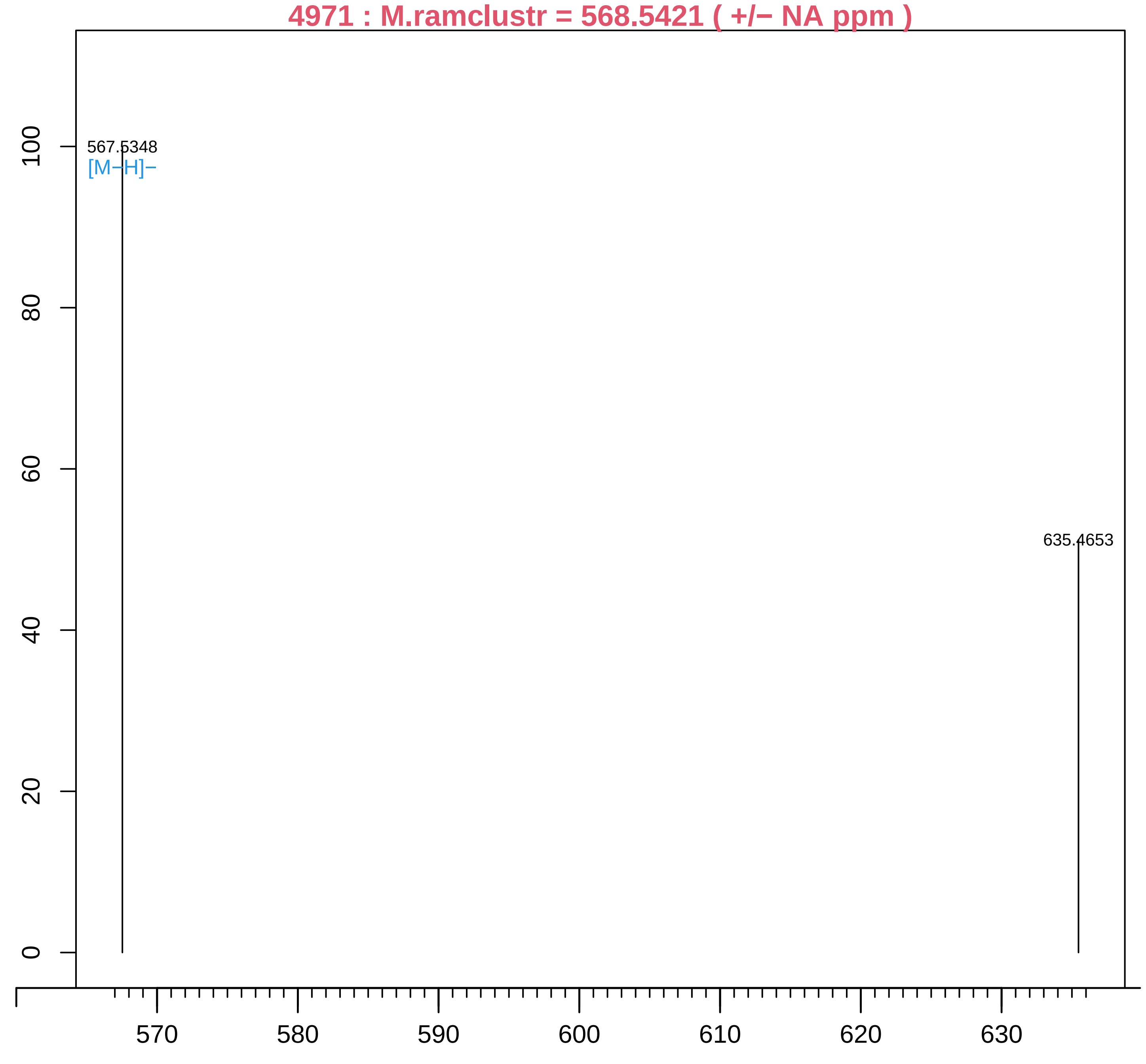
4971 : M.ramclustr = 568.5421 (+/− NA ppm)
567.5348
[M−H]−
635.4653
0
20
40
60
80
100
570
580
590
600
610
620
630

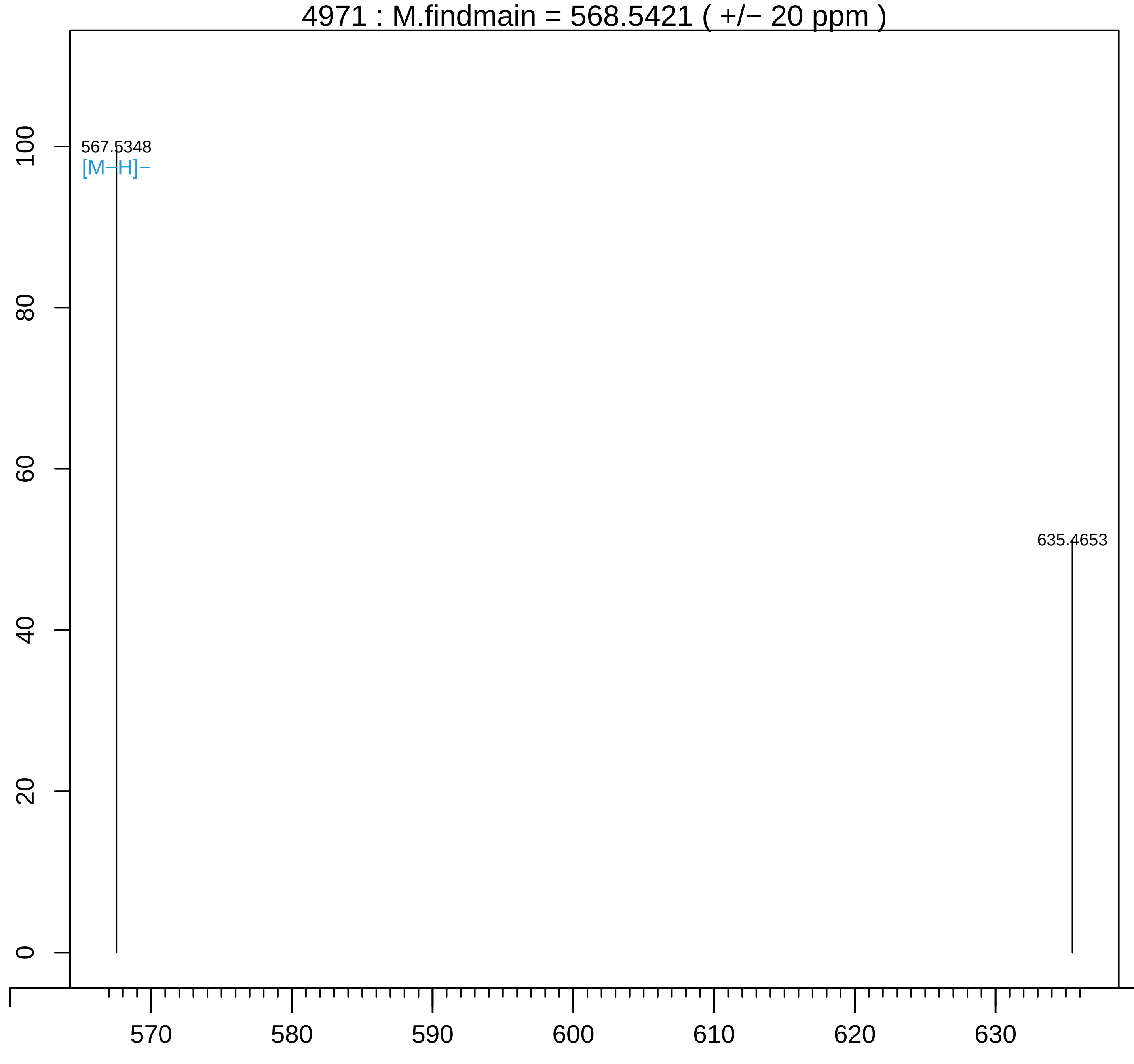
4971 : M.findmain = 568.5421 (+/− 20 ppm)
567.5348
[M−H]−
635.4653
0
20
40
60
80
100
570
580
590
600
610
620
630

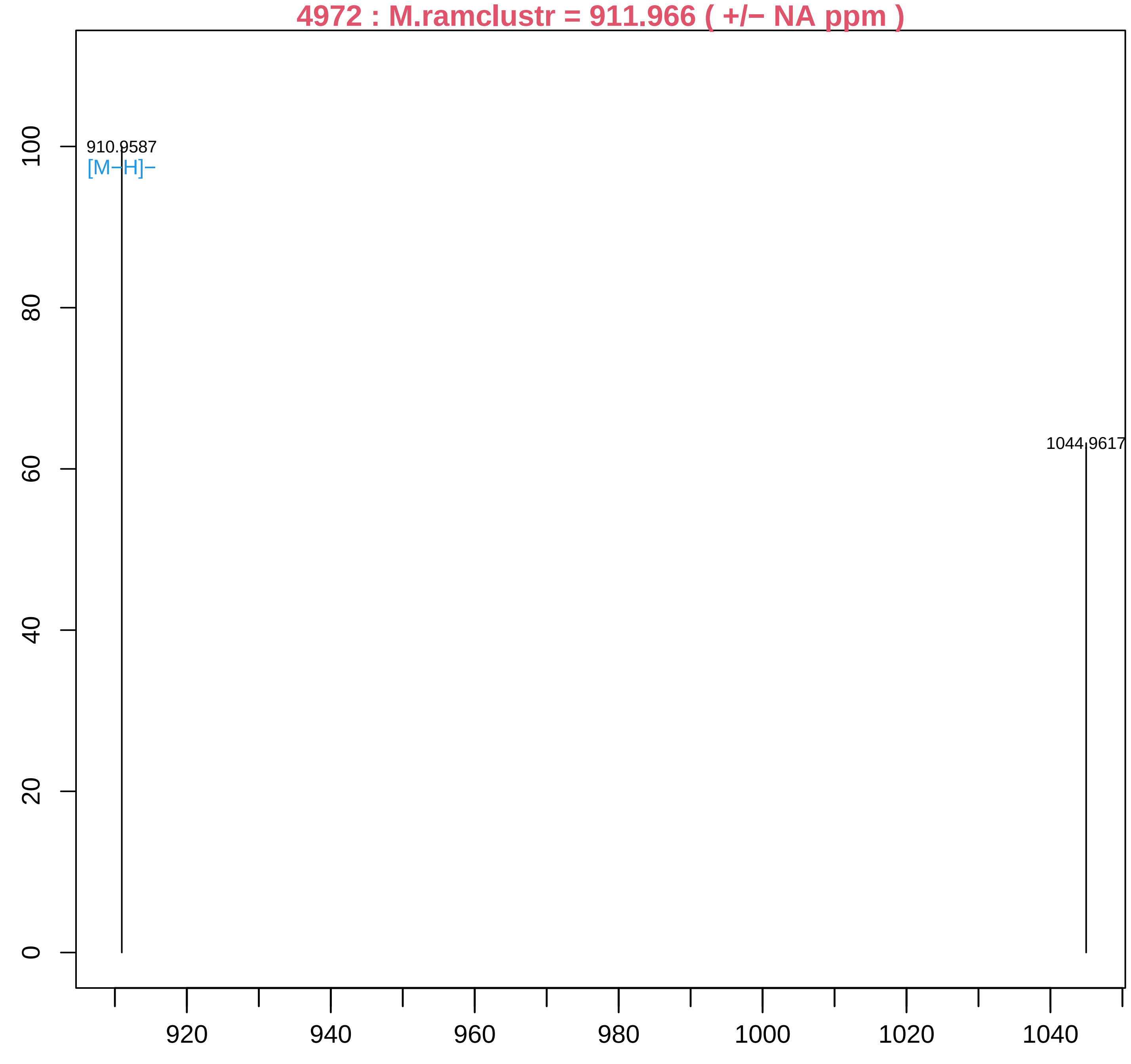

4972 : M.ramclustr = 911.966 (+/− NA ppm)
910.9587
[M−H]−
1044.9617
0
20
40
60
80
100
920
940
960
980
1000
1020
1040

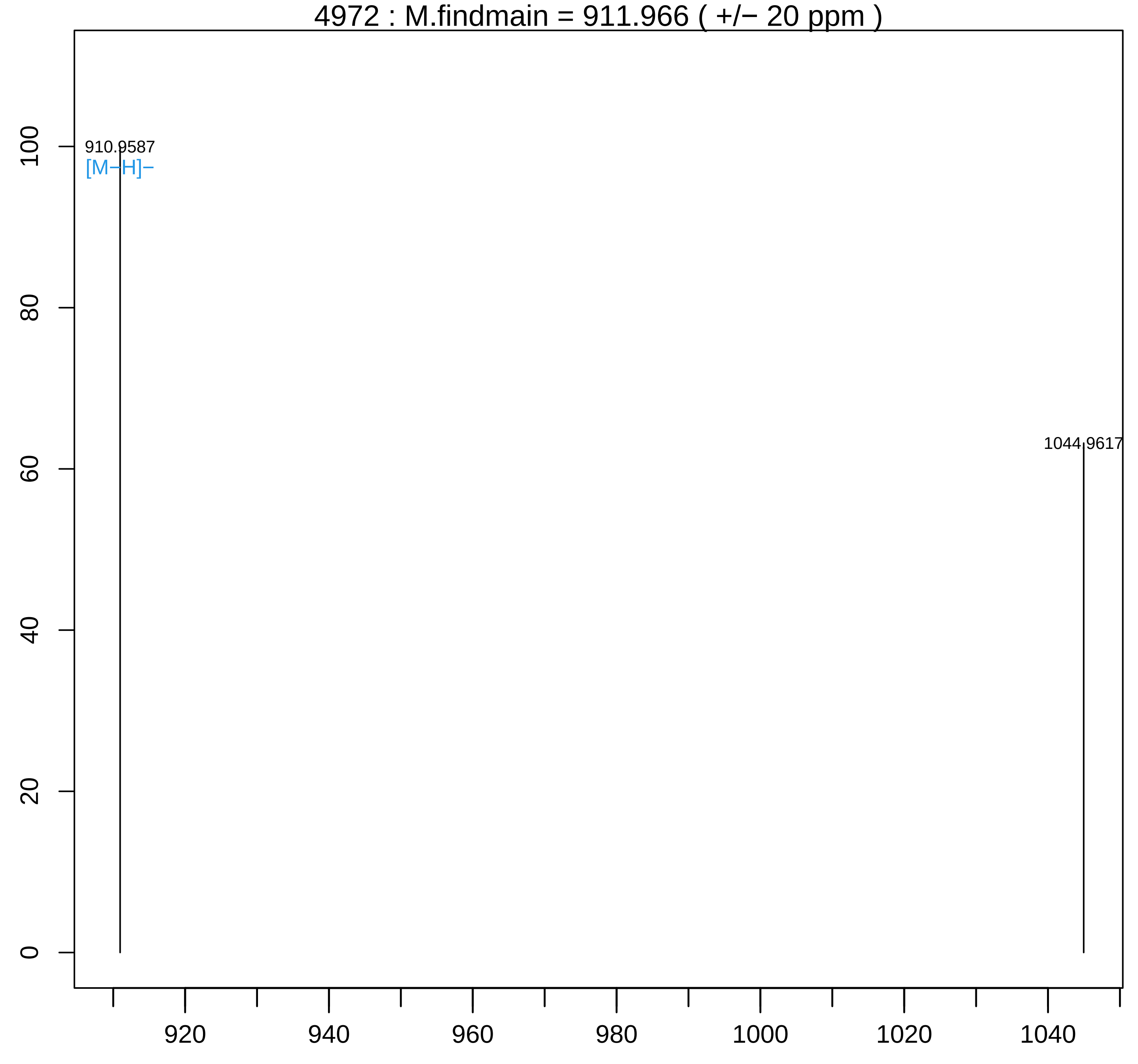

4972 : M.findmain = 911.966 (+/− 20 ppm)
910.9587
[M−H]−
1044.9617
0
20
40
60
80
100
920
940
960
980
1000
1020
1040

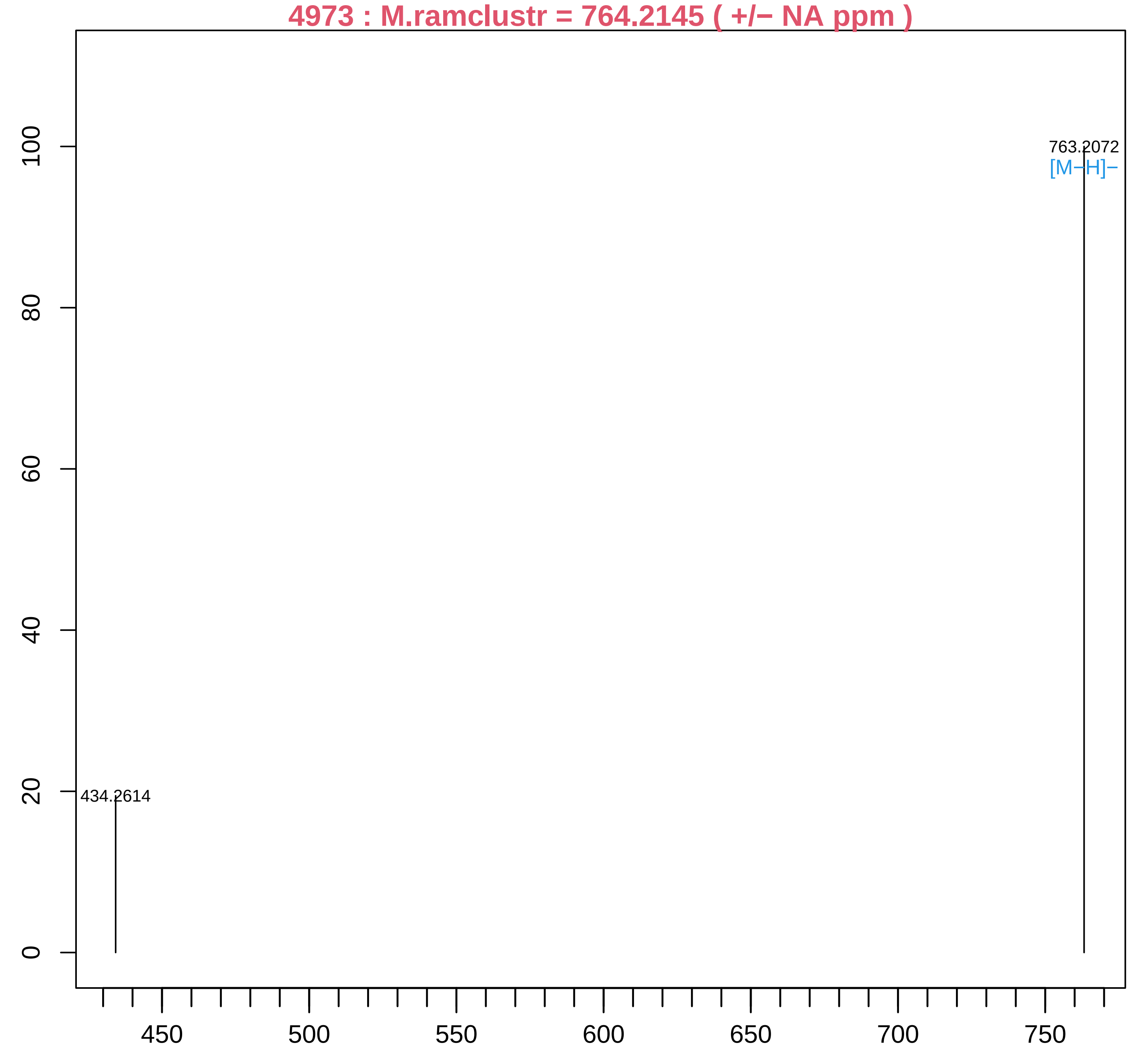
4973 : M.ramclustr = 764.2145 (+/− NA ppm)
763.2072
[M−H]−
434.2614
0
20
40
60
80
100
450
500
550
600
650
700
750

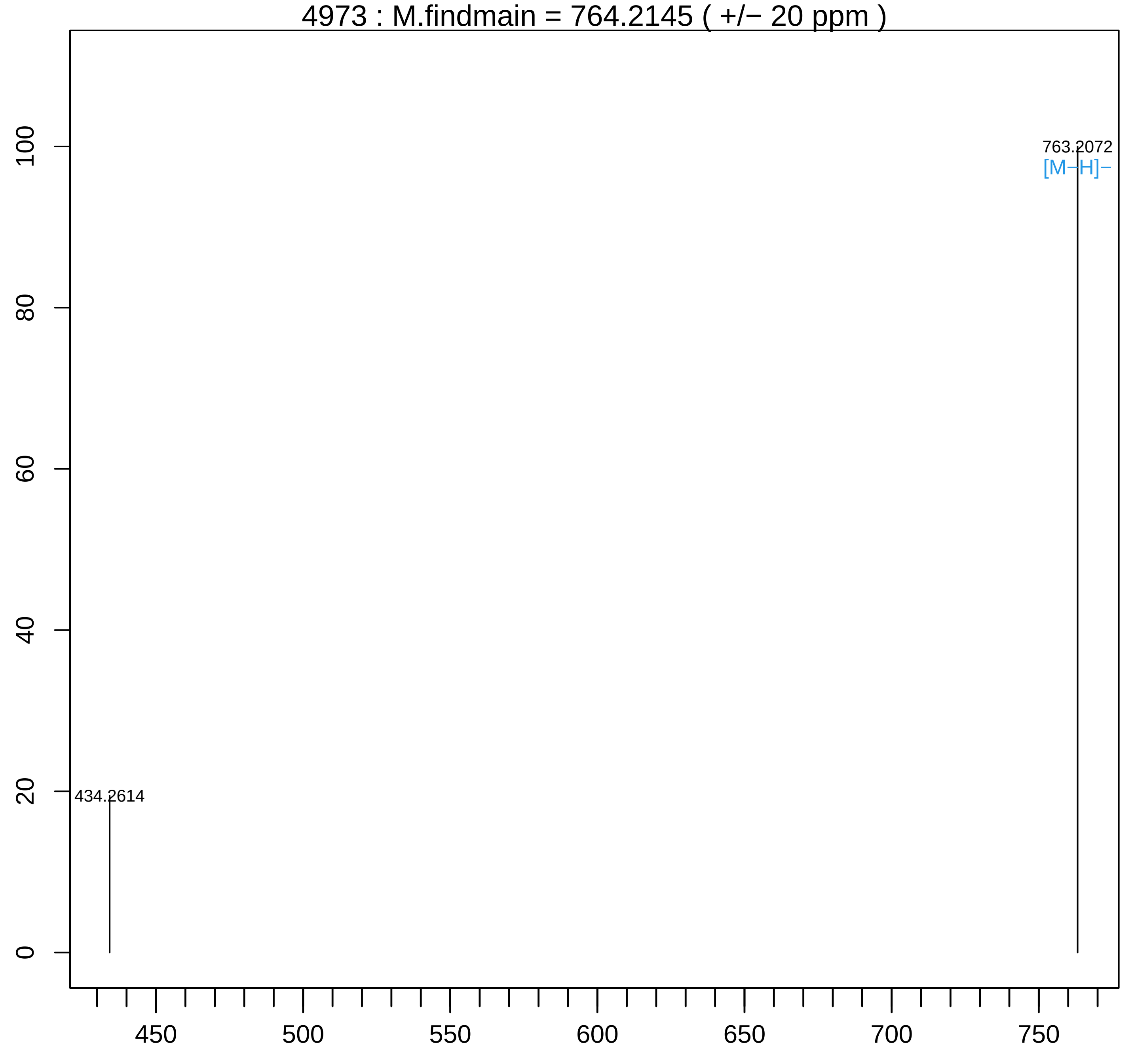
4973 : M.findmain = 764.2145 (+/− 20 ppm)
763.2072
[M−H]−
434.2614
0
20
40
60
80
100
450
500
550
600
650
700
750

4974 : M.ramclustr = 326.1911 (+/− NA ppm)

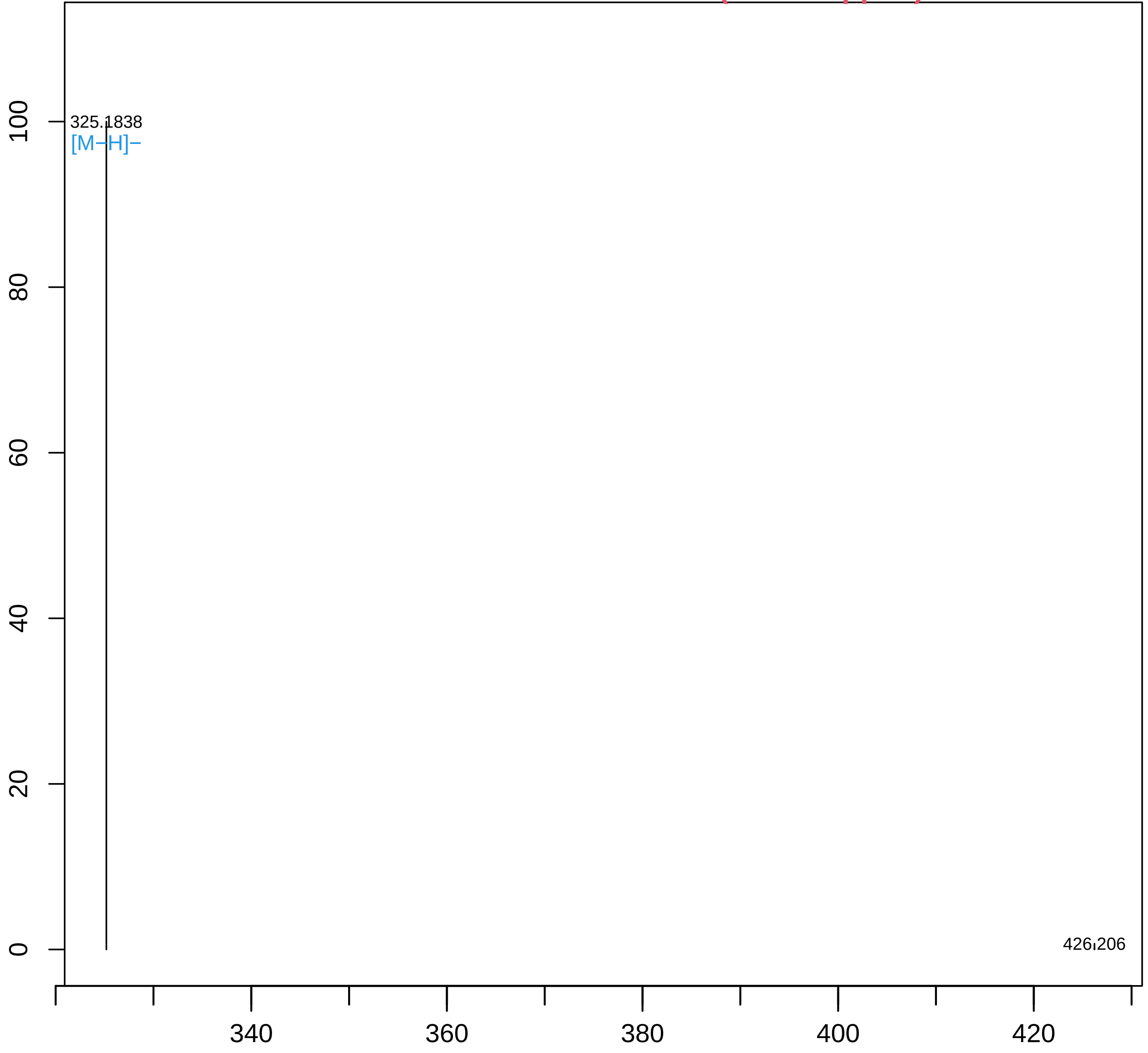

4974 : M.findmain = 326.1911 (+/− 20 ppm)

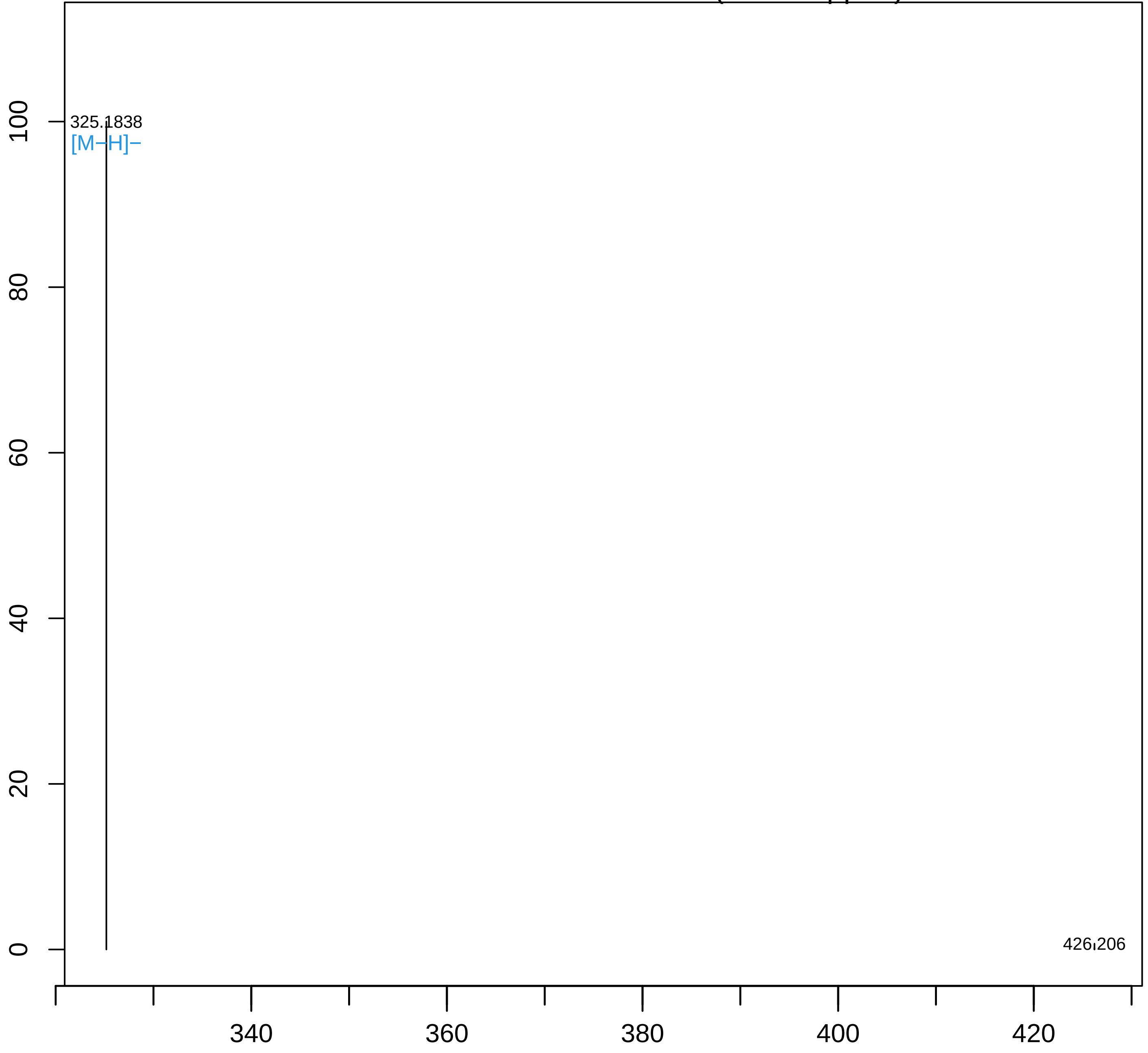

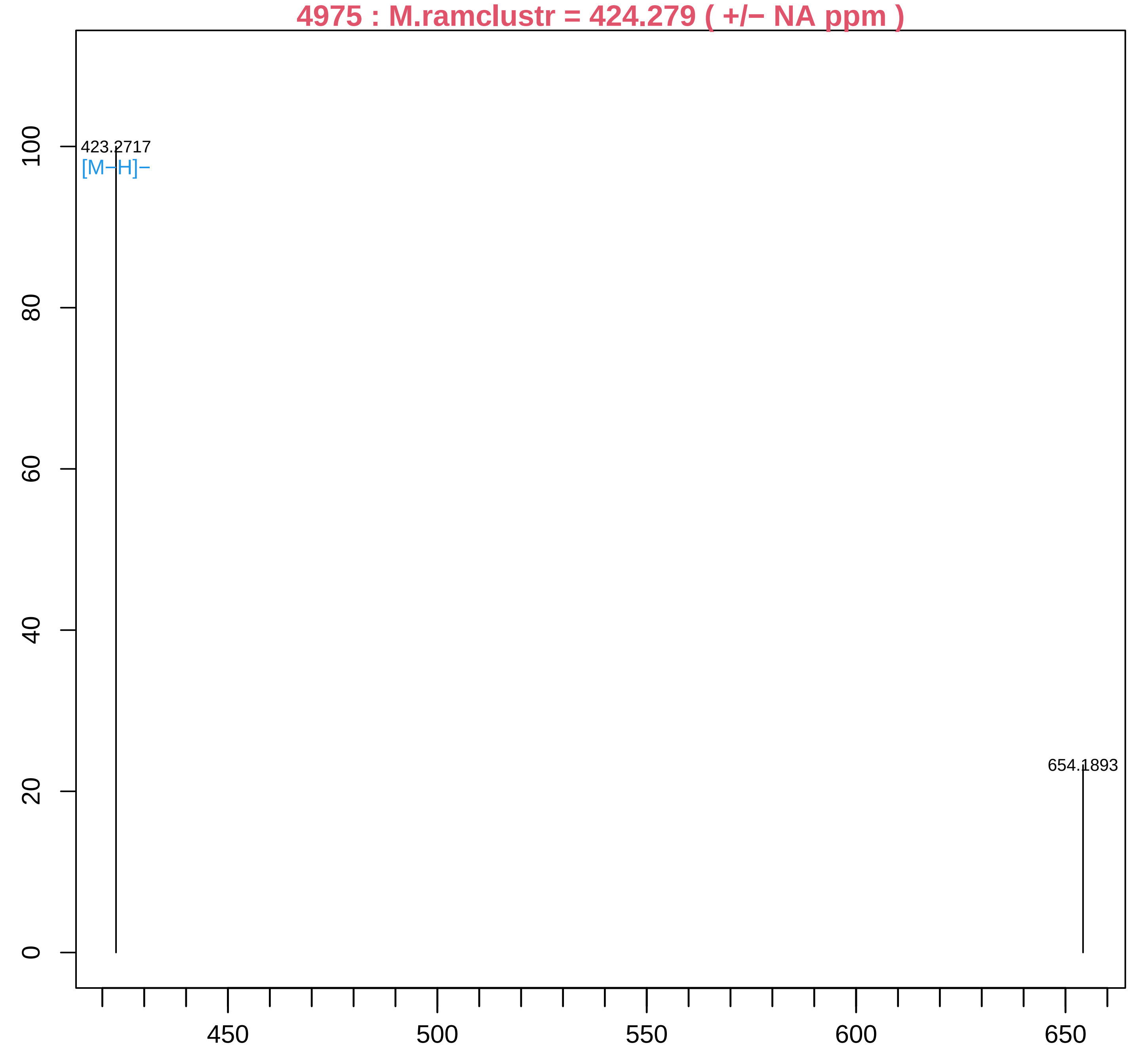
4975 : M.ramclustr = 424.279 (+/− NA ppm)
423.2717
[M−H]−
654.1893
0
20
40
60
80
100
450
500
550
600
650

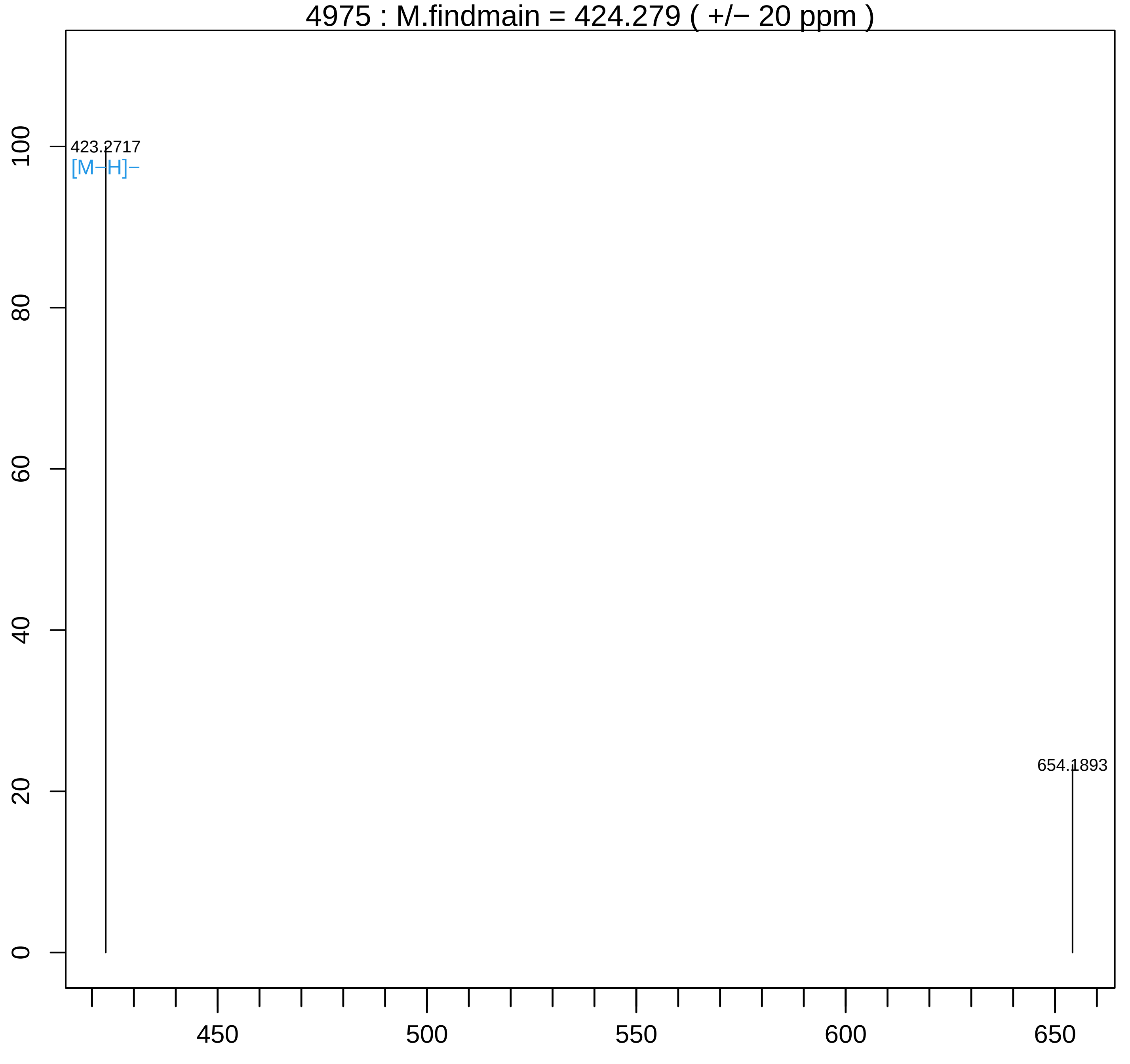
4975 : M.findmain = 424.279 (+/− 20 ppm)
423.2717
[M−H]−
654.1893
0
20
40
60
80
100
450
500
550
600
650

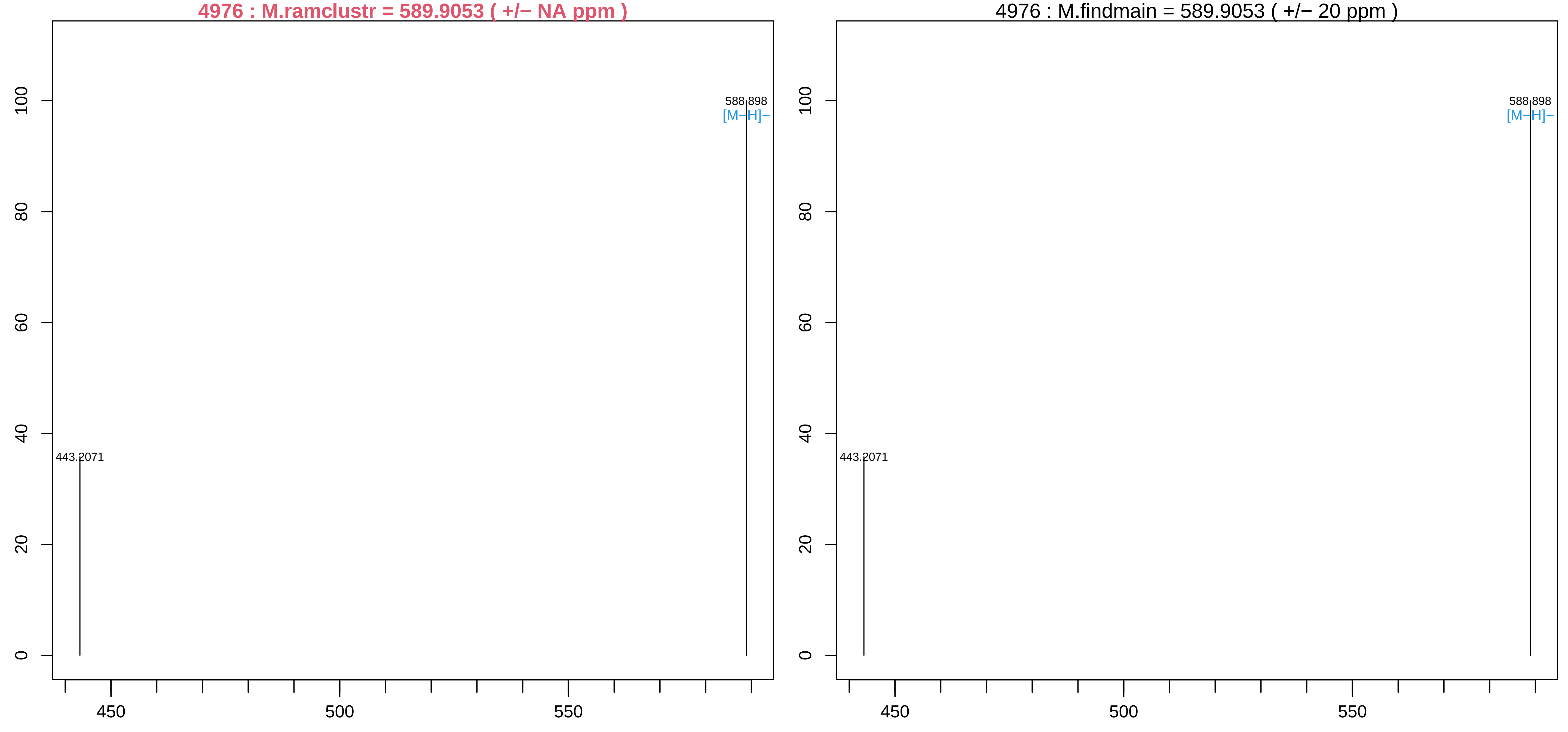

4976 : M.ramclustr = 589.9053 (+/− NA ppm)
588.898
[M−H]−
443.2071
0
20
40
60
80
100
450
500
550
4976 : M.findmain = 589.9053 (+/− 20 ppm)
588.898
[M−H]−
443.2071
0
20
40
60
80
100
450
500
550

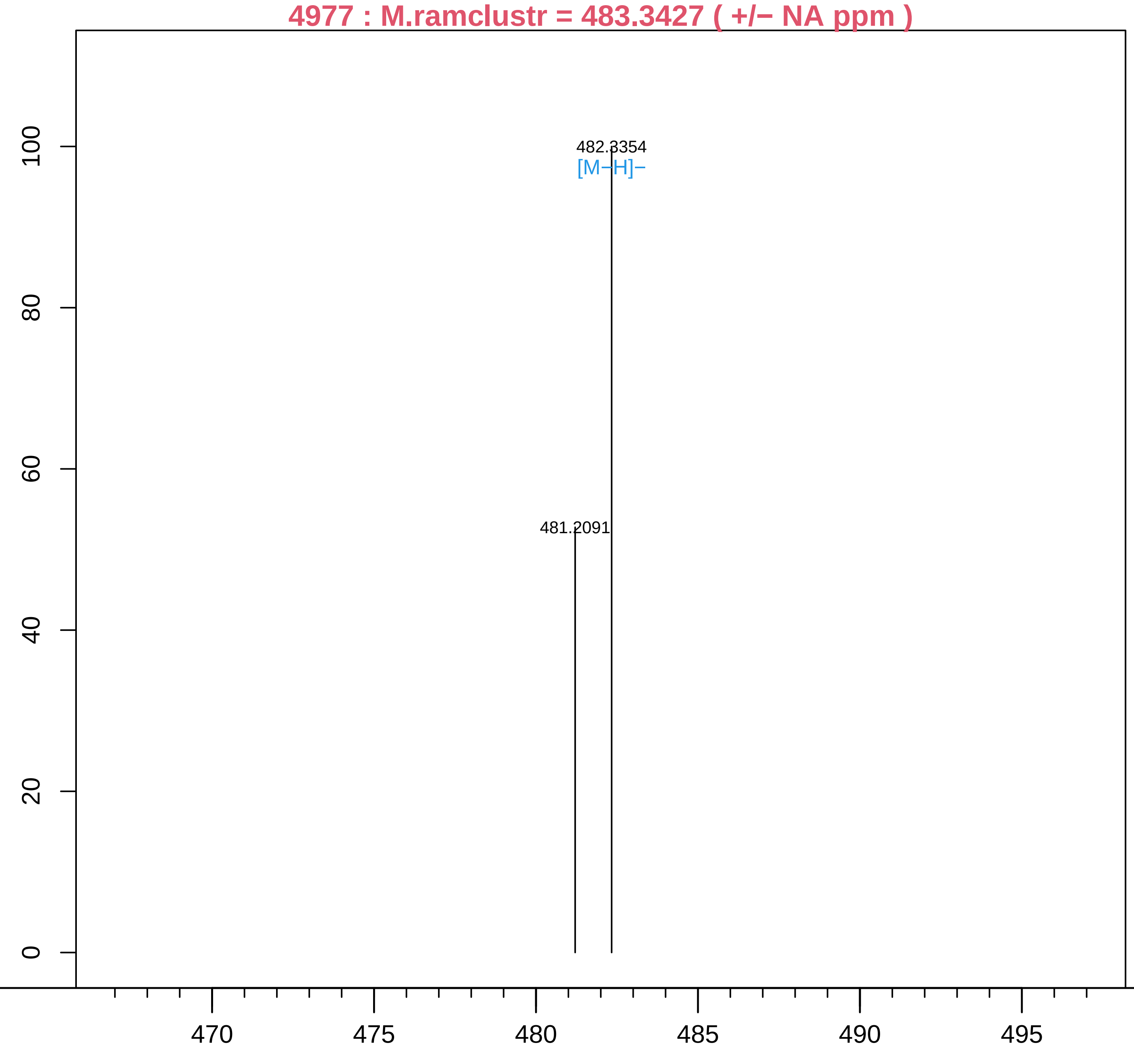
4977 : M.ramclustr = 483.3427 (+/− NA ppm)
482.3354
[M−H]−
481.2091
0
20
40
60
80
100
470
475
480
485
490
495

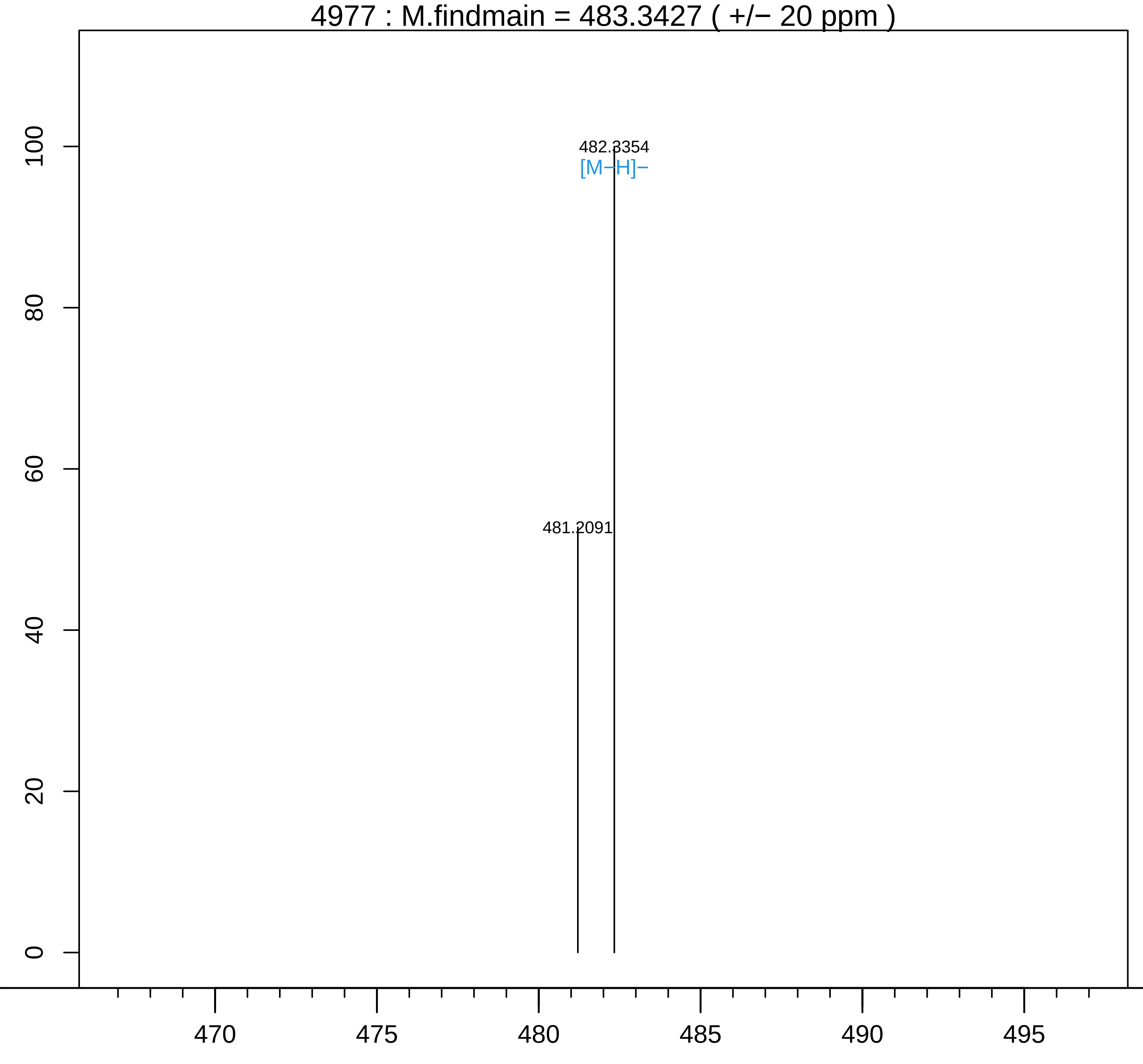
4977 : M.findmain = 483.3427 (+/− 20 ppm)
482.3354
[M−H]−
481.2091
0
20
40
60
80
100
470
475
480
485
490
495

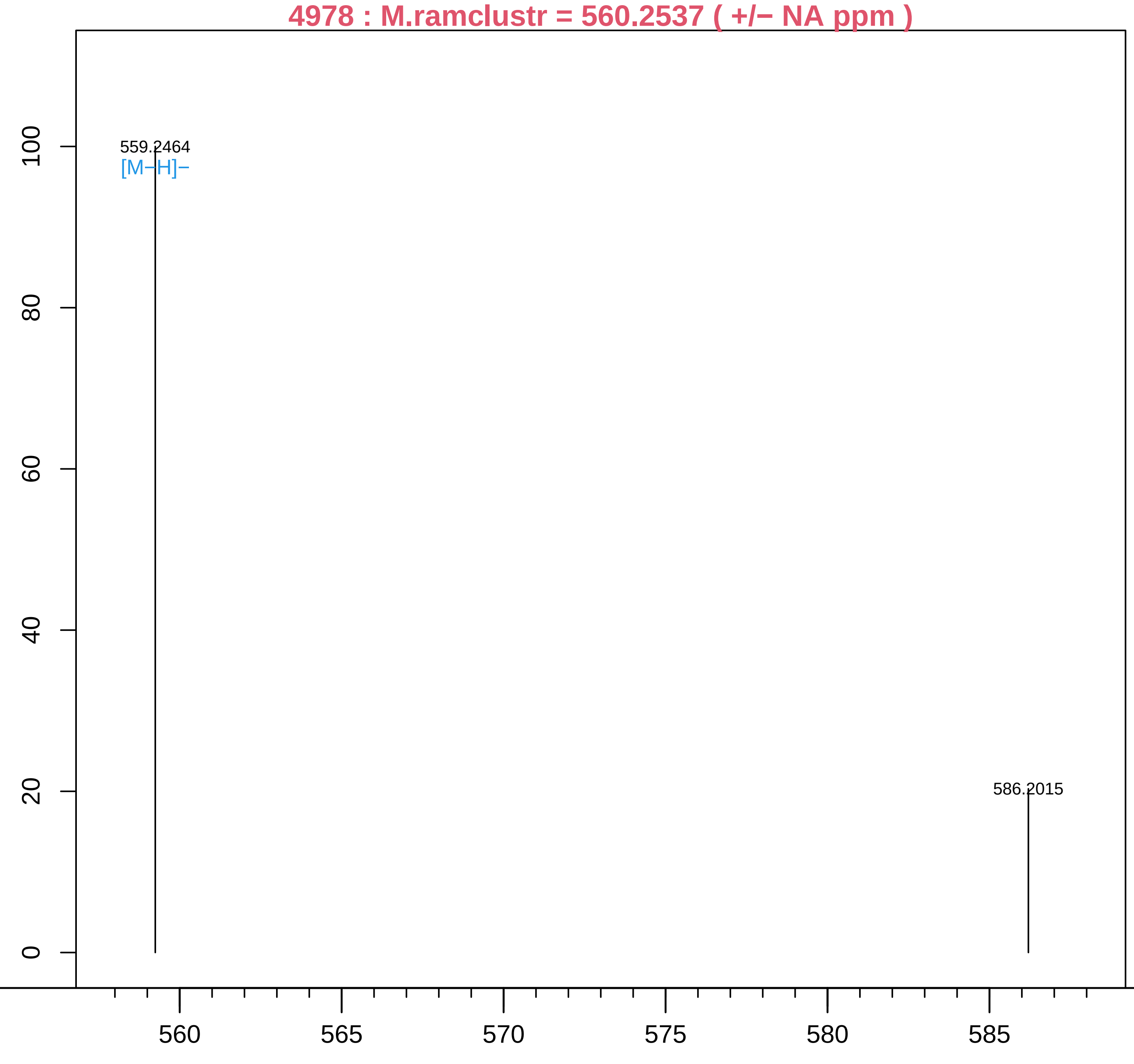
4978 : M.ramclustr = 560.2537 (+/− NA ppm)
559.2464
[M−H]−
586.2015
0
20
40
60
80
100
560
565
570
575
580
585

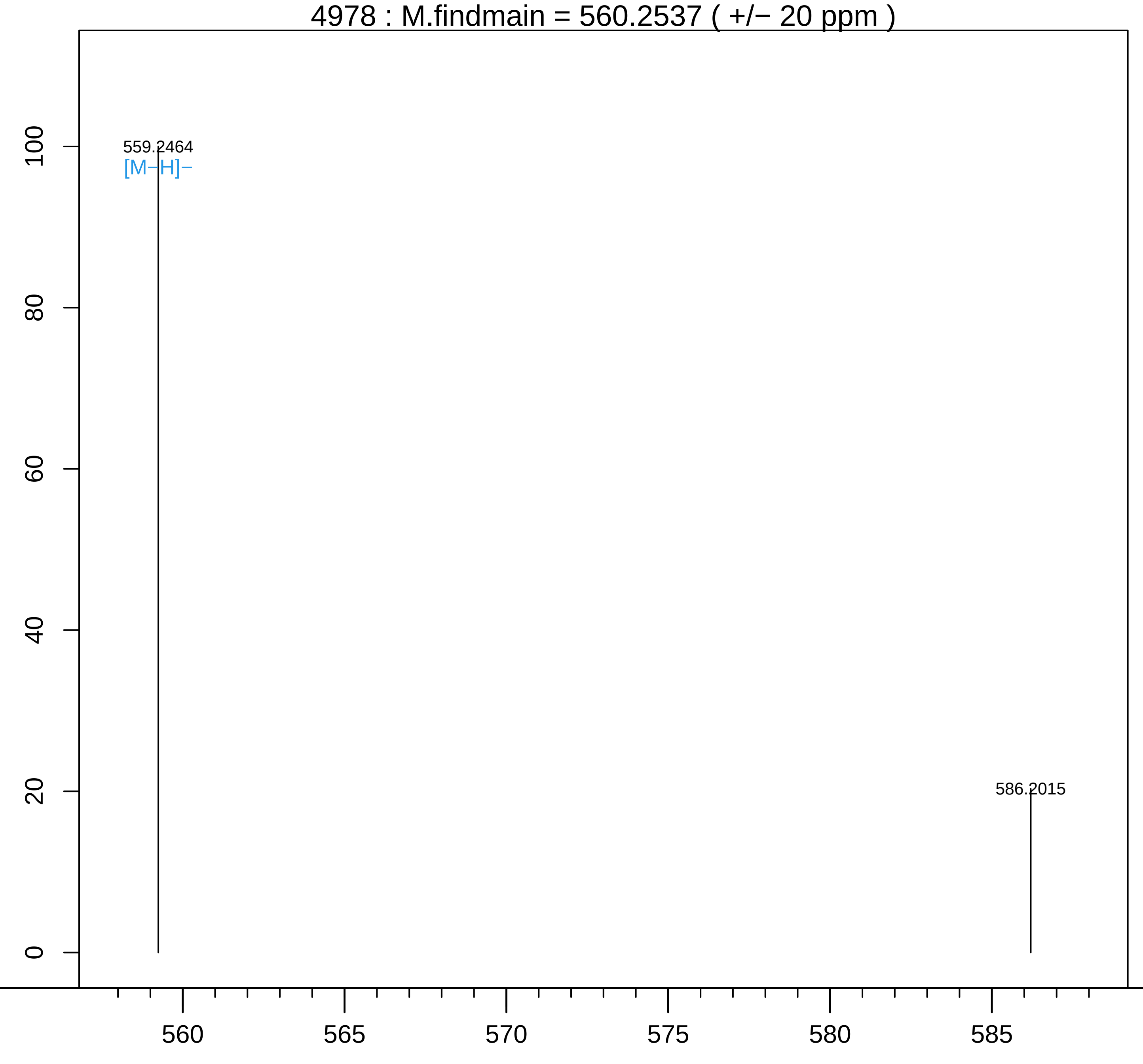
4978 : M.findmain = 560.2537 (+/− 20 ppm)
559.2464
[M−H]−
586.2015
0
20
40
60
80
100
560
565
570
575
580
585

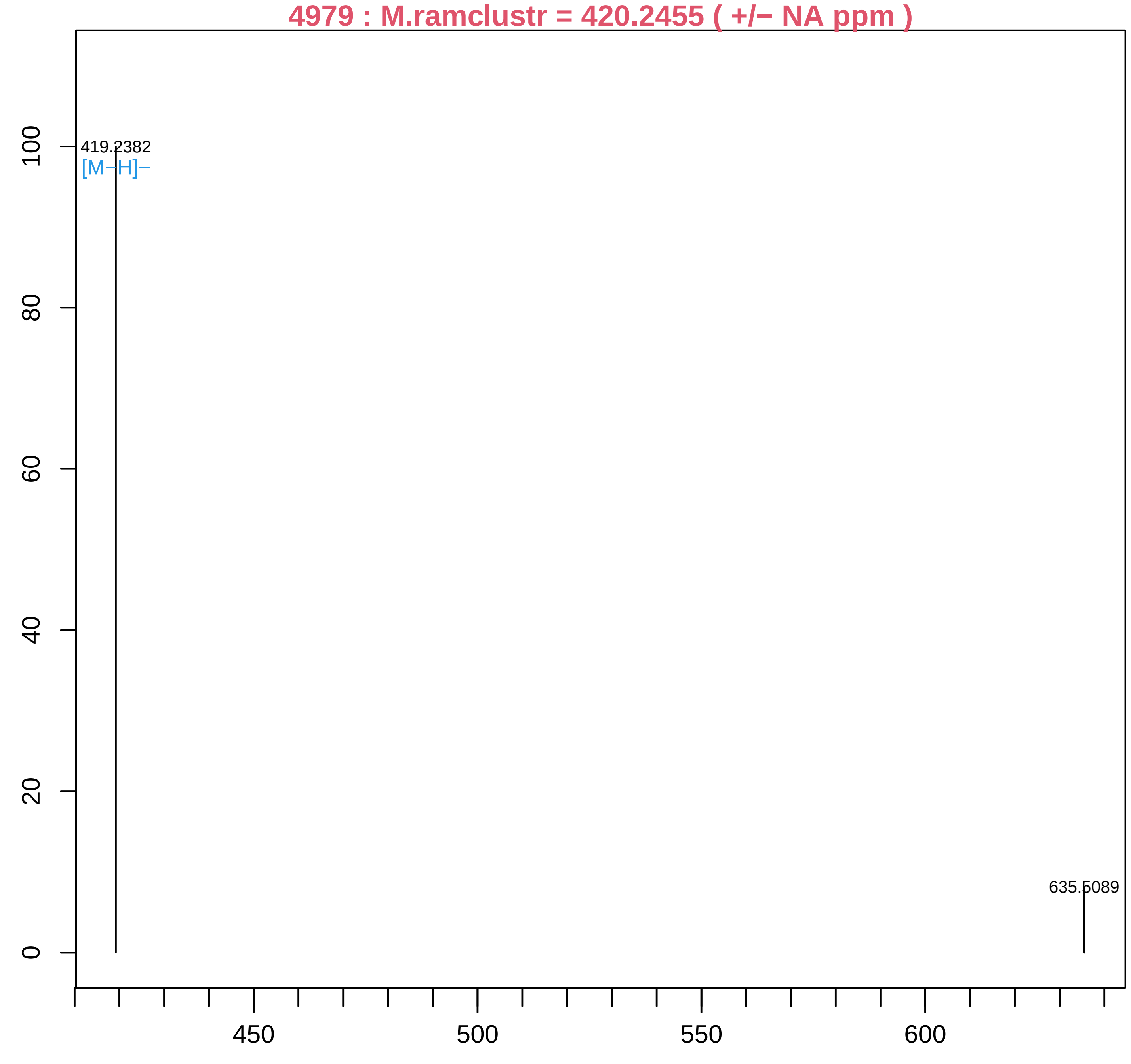
4979 : M.ramclustr = 420.2455 (+/− NA ppm)
419.2382
[M−H]−
635.5089
0
20
40
60
80
100
450
500
550
600

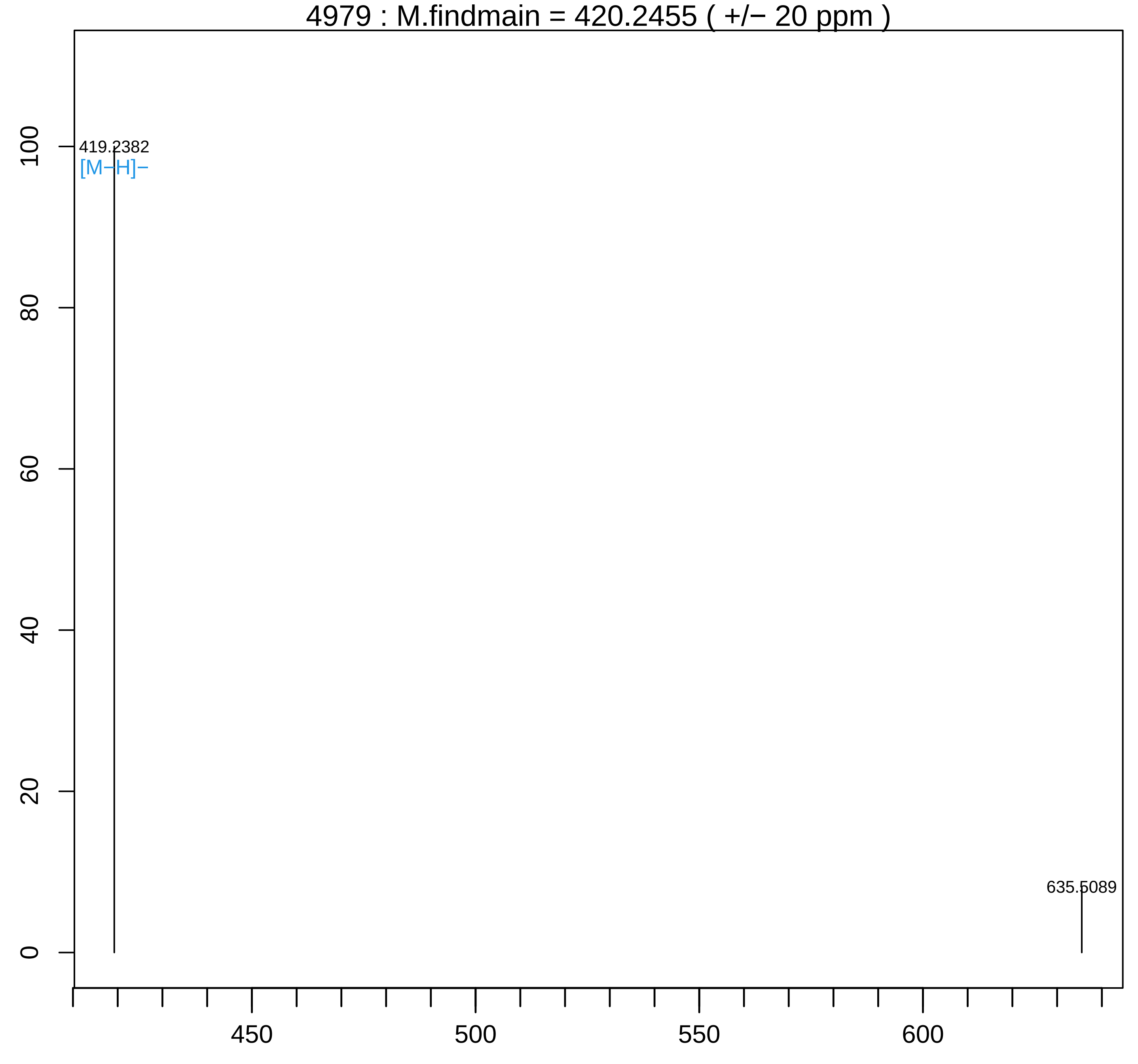
4979 : M.findmain = 420.2455 (+/− 20 ppm)
419.2382
[M−H]−
635.5089
0
20
40
60
80
100
450
500
550
600

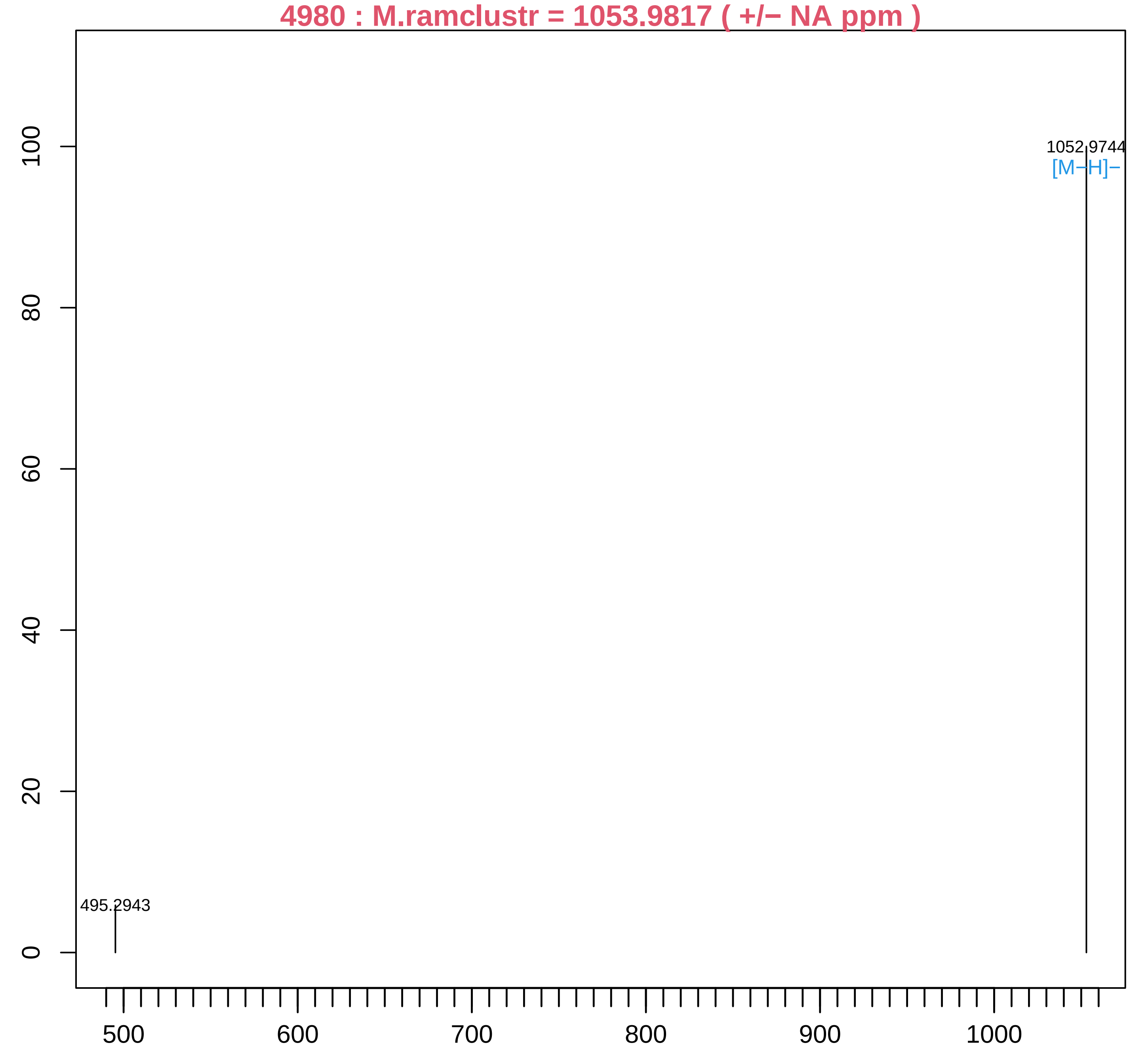
4980 : M.ramclustr = 1053.9817 (+/− NA ppm)
1052.9744
[M−H]−
495.2943
0
20
40
60
80
100
500
600
700
800
900
1000

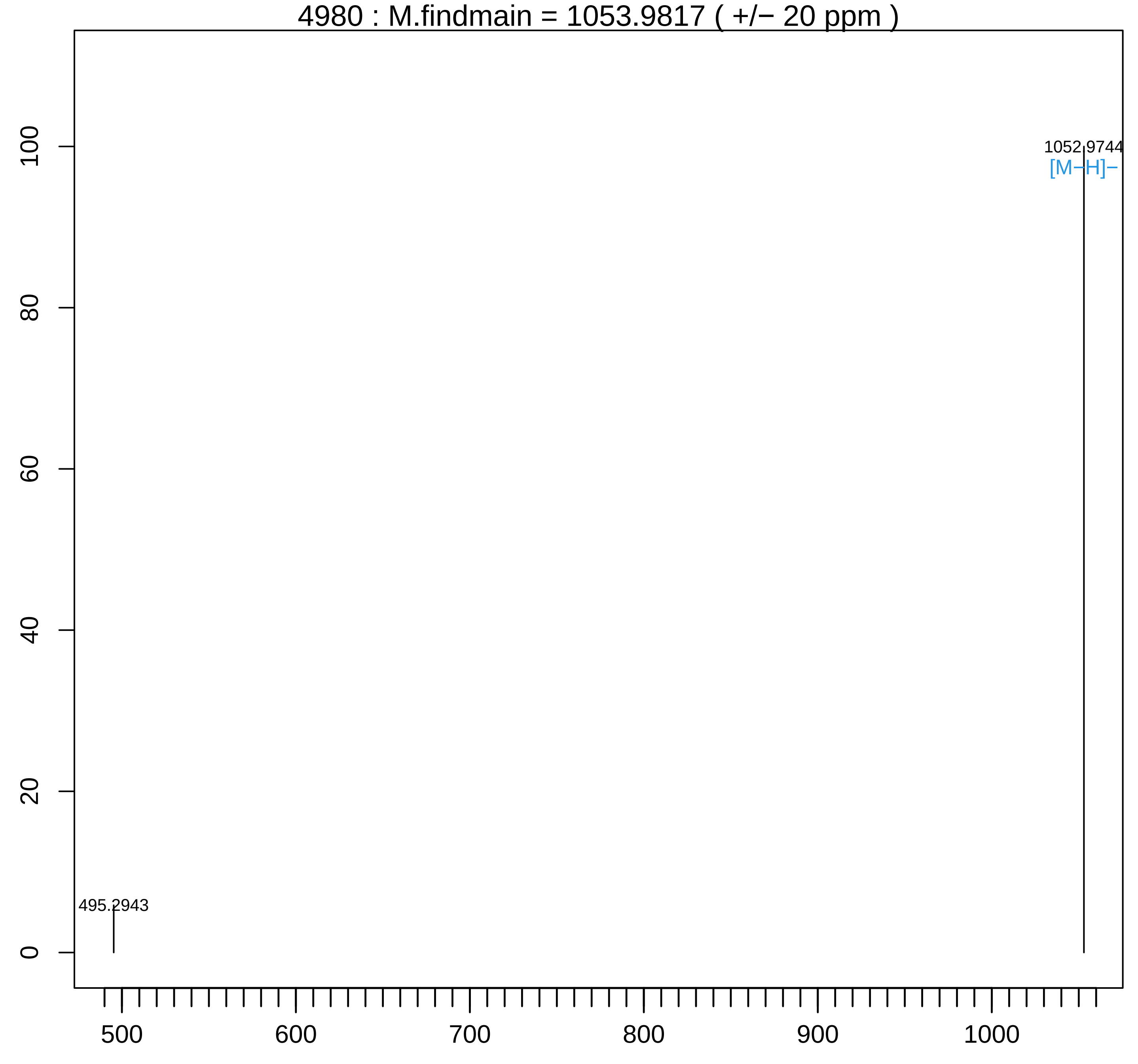
4980 : M.findmain = 1053.9817 (+/− 20 ppm)
1052.9744
[M−H]−
495.2943
0
20
40
60
80
100
500
600
700
800
900
1000

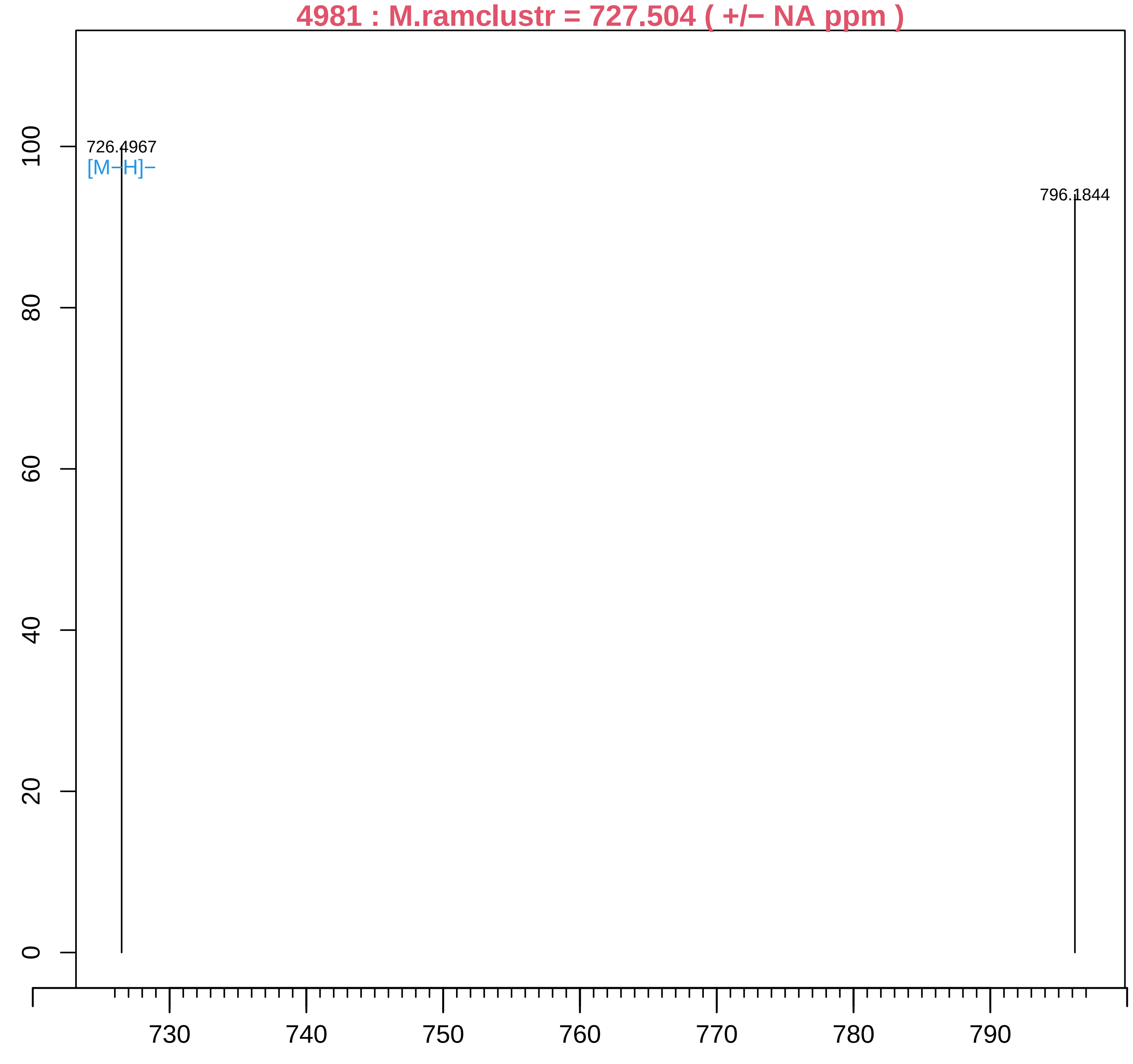
4981 : M.ramclustr = 727.504 (+/− NA ppm)
726.4967
[M−H]−
796.1844
0
20
40
60
80
100
730
740
750
760
770
780
790

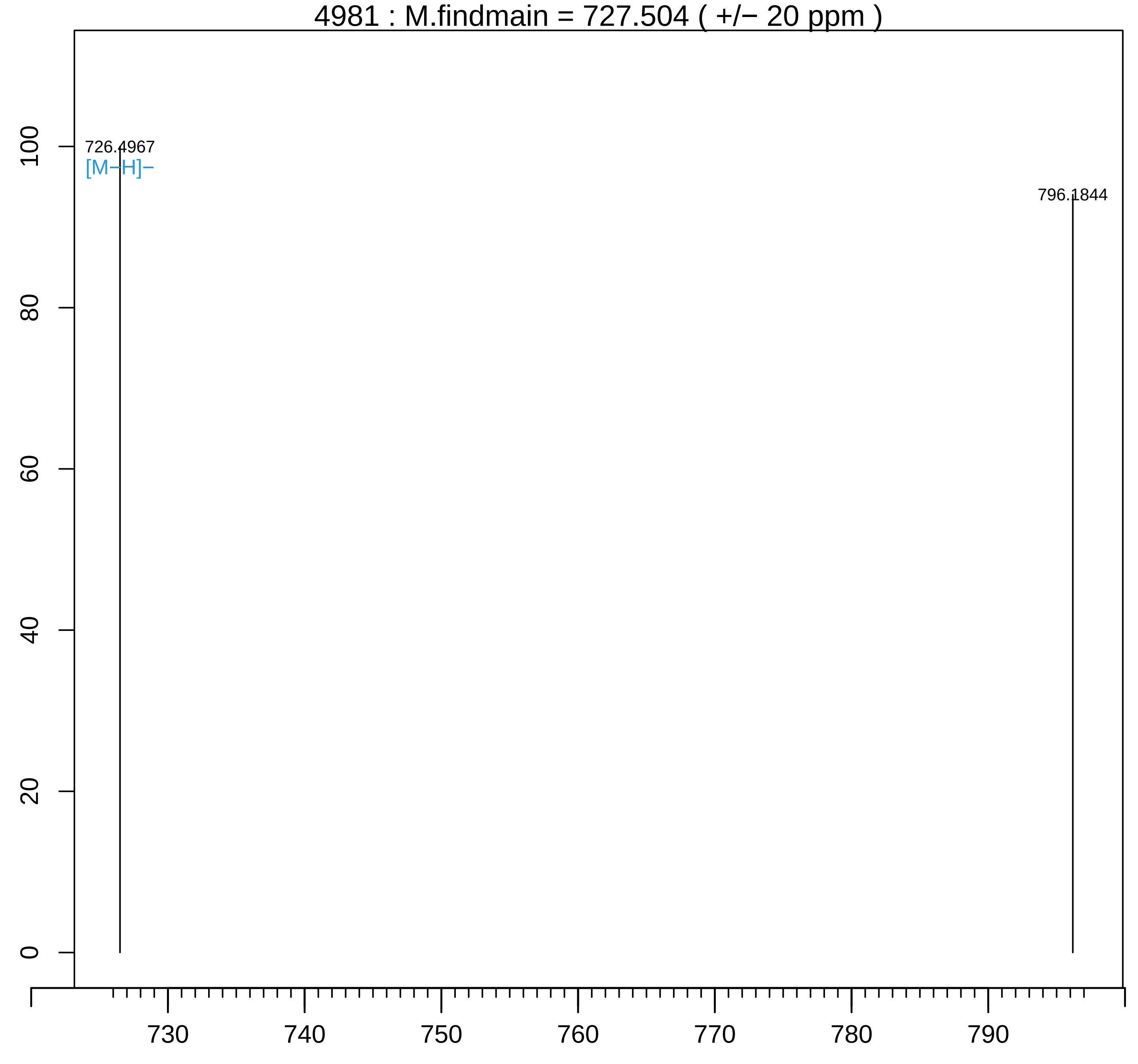
4981 : M.findmain = 727.504 (+/− 20 ppm)
726.4967
[M−H]−
796.1844
0
20
40
60
80
100
730
740
750
760
770
780
790

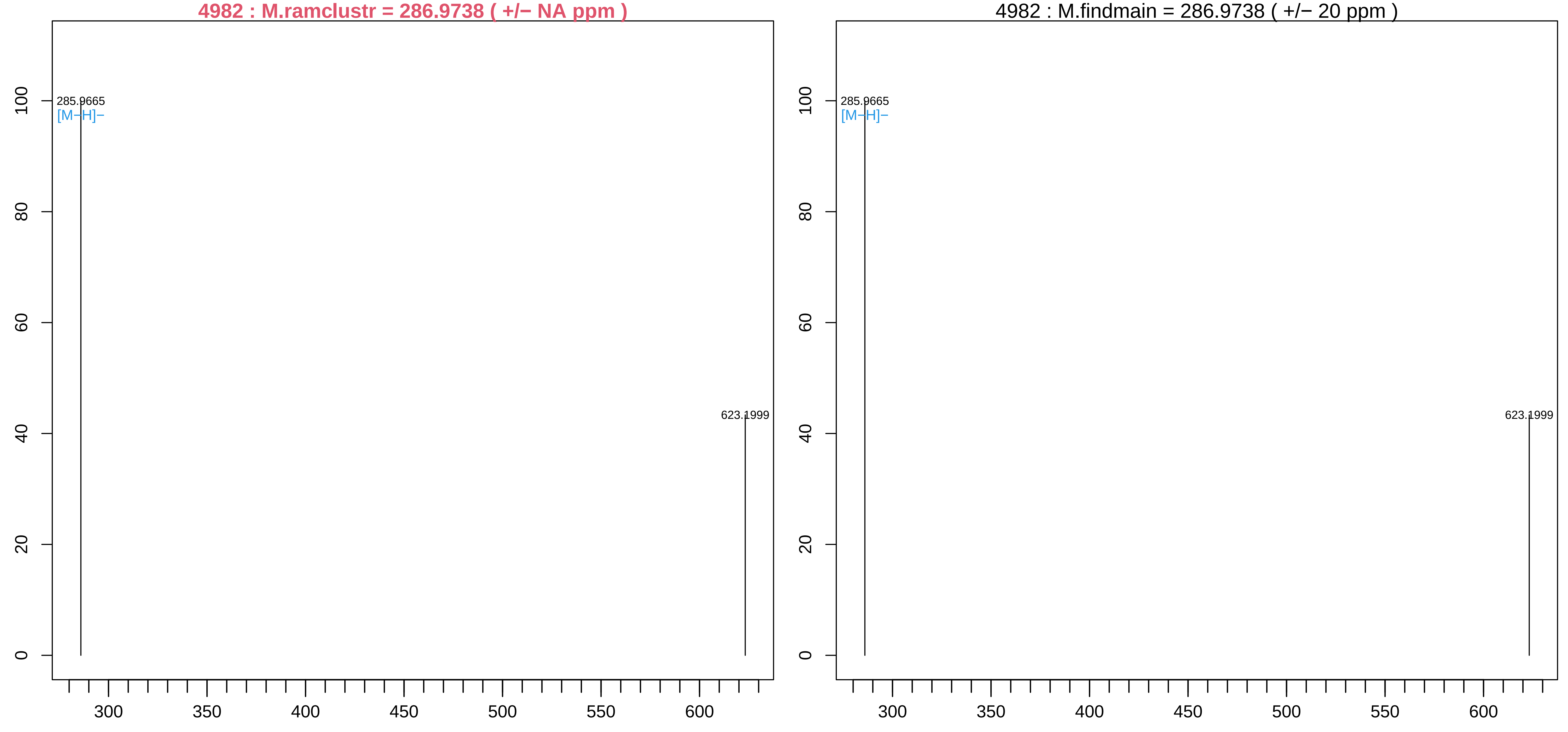

4982 : M.ramclustr = 286.9738 (+/− NA ppm)
285.9665
[M−H]−
623.1999
4982 : M.findmain = 286.9738 (+/− 20 ppm)
285.9665
[M−H]−
623.1999
0
20
40
60
80
100
300
350
400
450
500
550
600

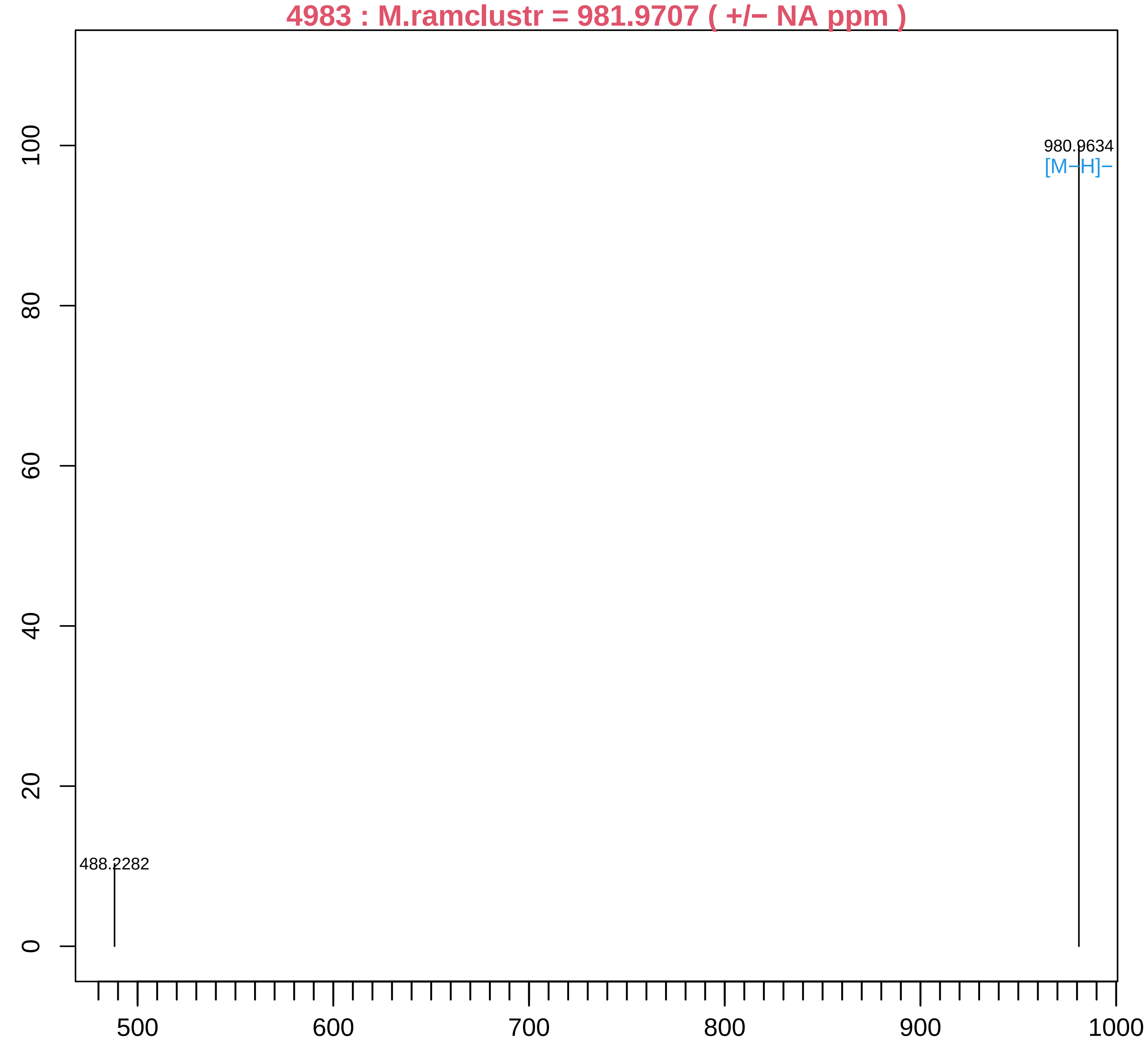
4983 : M.ramclustr = 981.9707 (+/− NA ppm)
980.9634
[M−H]−
488.2282
0
20
40
60
80
100
500
600
700
800
900
1000

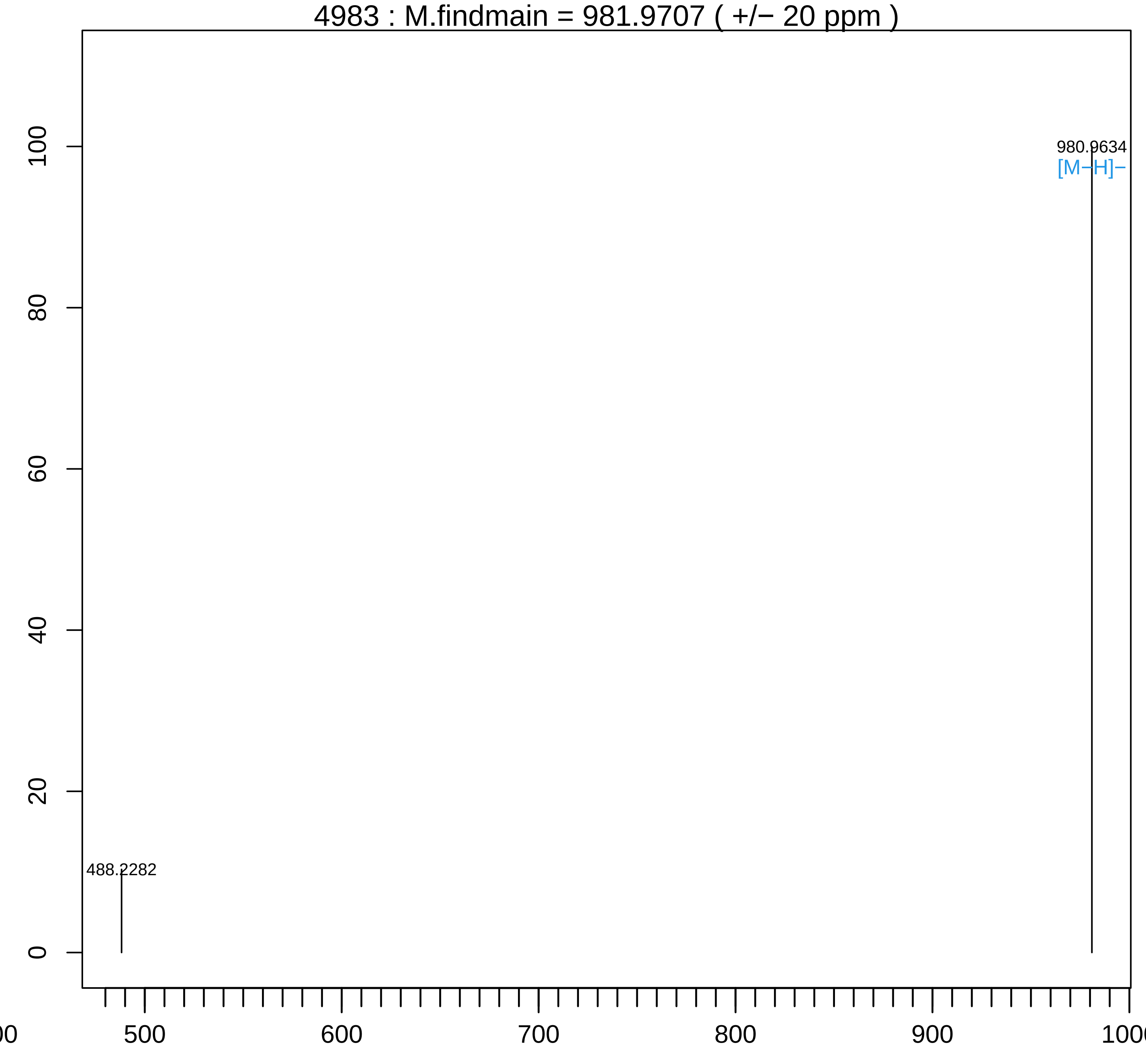
4983 : M.findmain = 981.9707 (+/− 20 ppm)
980.9634
[M−H]−
488.2282
0
20
40
60
80
100
500
600
700
800
900

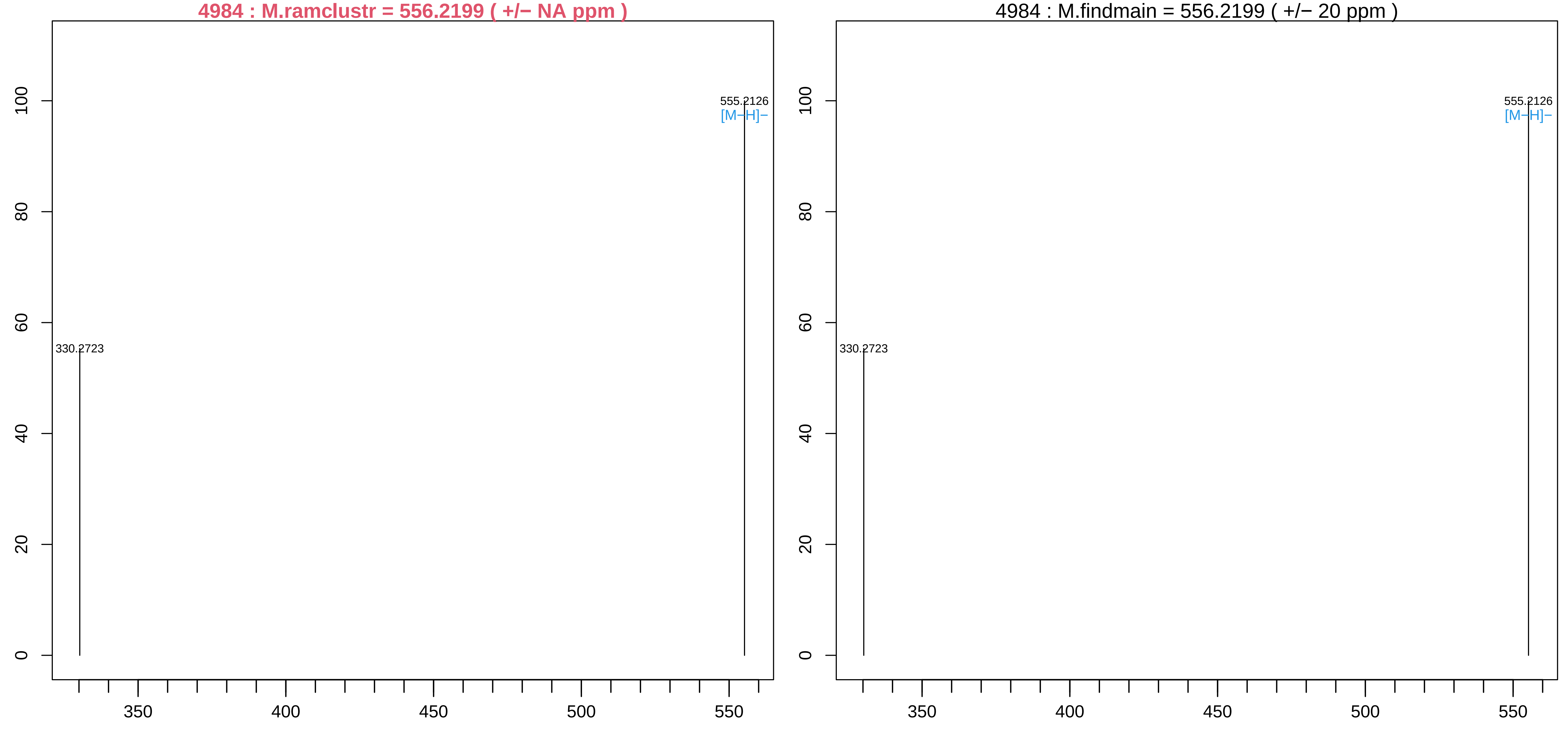
4984 : M.ramclustr = 556.2199 (+/− NA ppm)
555.2126
[M−H]−
330.2723
4984 : M.findmain = 556.2199 (+/− 20 ppm)
555.2126
[M−H]−
330.2723
0
20
40
60
80
100
350
400
450
500
550

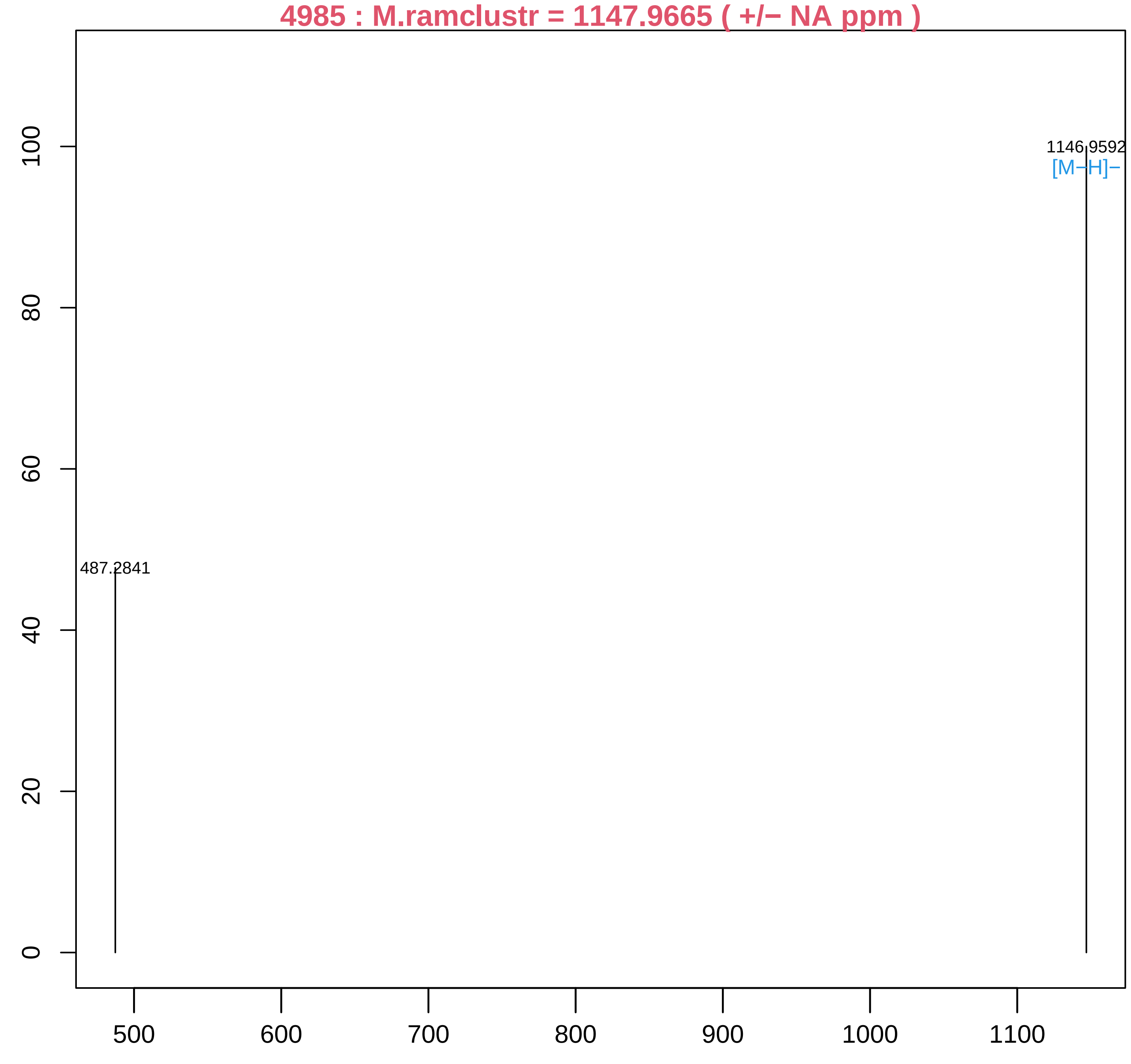
4985 : M.ramclustr = 1147.9665 (+/− NA ppm)
1146.9592
[M−H]−
487.2841
0
20
40
60
80
100
500
600
700
800
900
1000
1100

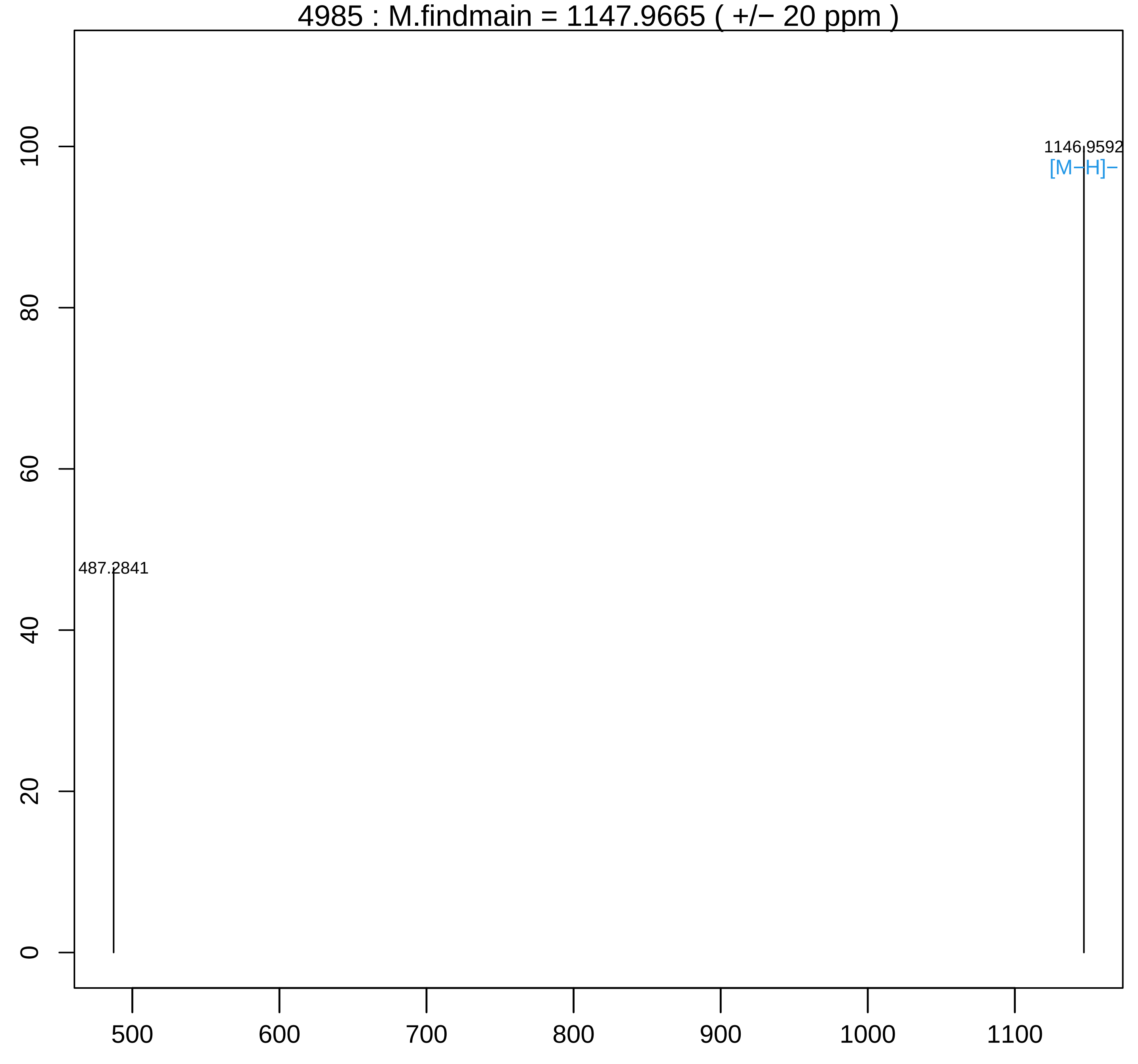
4985 : M.findmain = 1147.9665 (+/− 20 ppm)
1146.9592
[M−H]−
487.2841
0
20
40
60
80
100
500
600
700
800
900
1000
1100

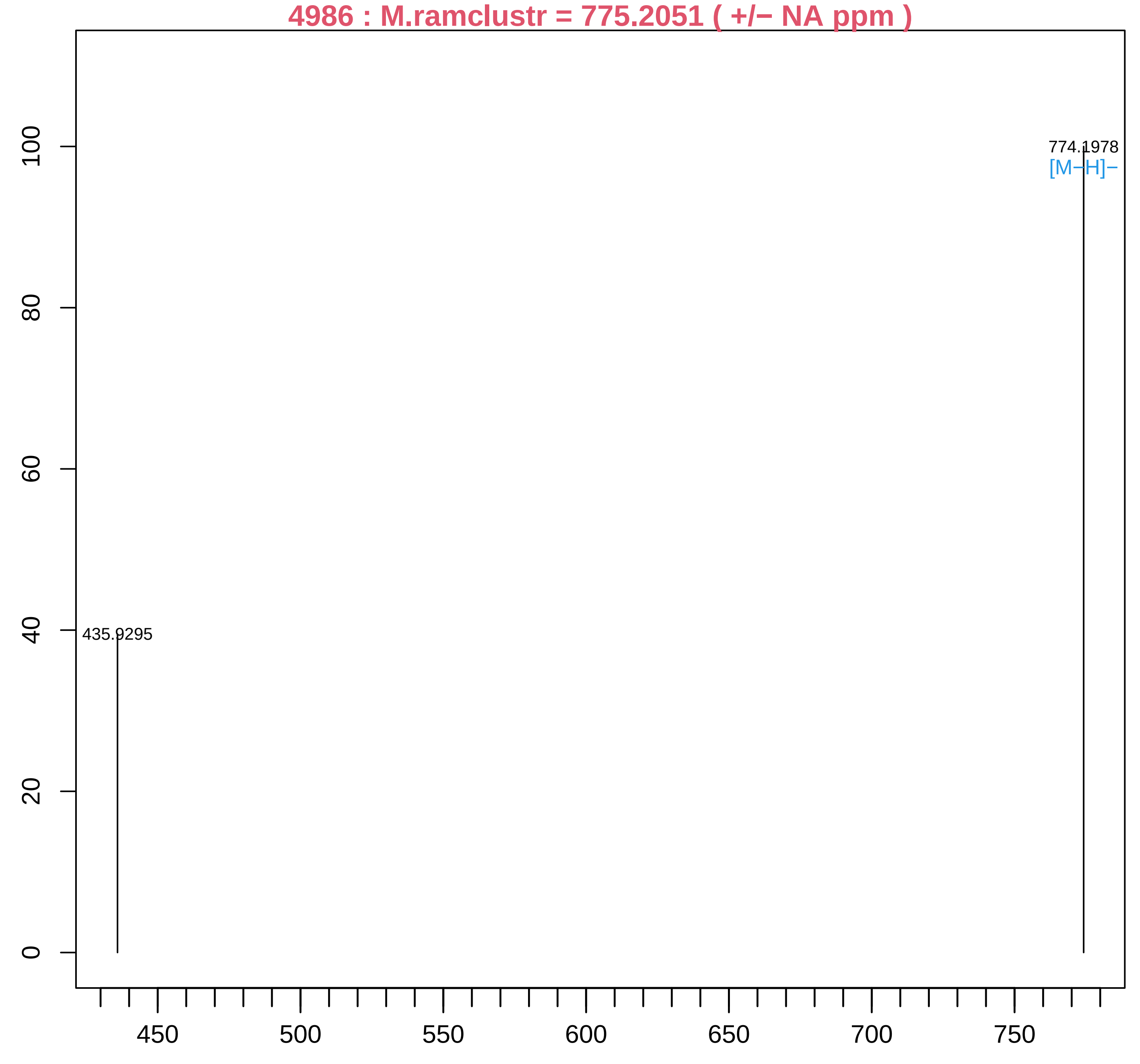

4986 : M.ramclustr = 775.2051 (+/− NA ppm)
774.1978
[M−H]−
435.9295
0
20
40
60
80
100
450
500
550
600
650
700
750

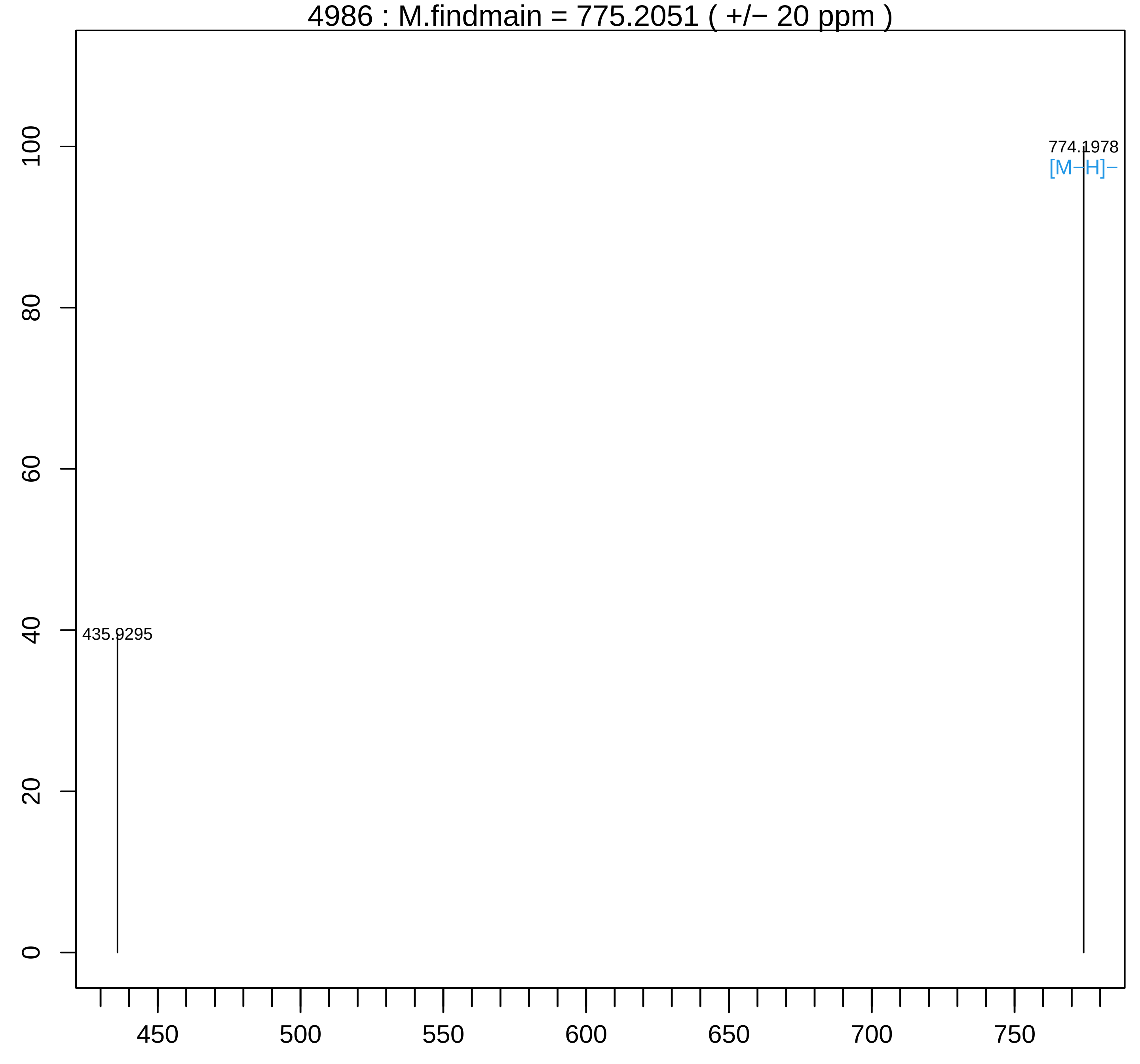

4986 : M.findmain = 775.2051 (+/− 20 ppm)
774.1978
[M−H]−
435.9295
0
20
40
60
80
100
450
500
550
600
650
700
750

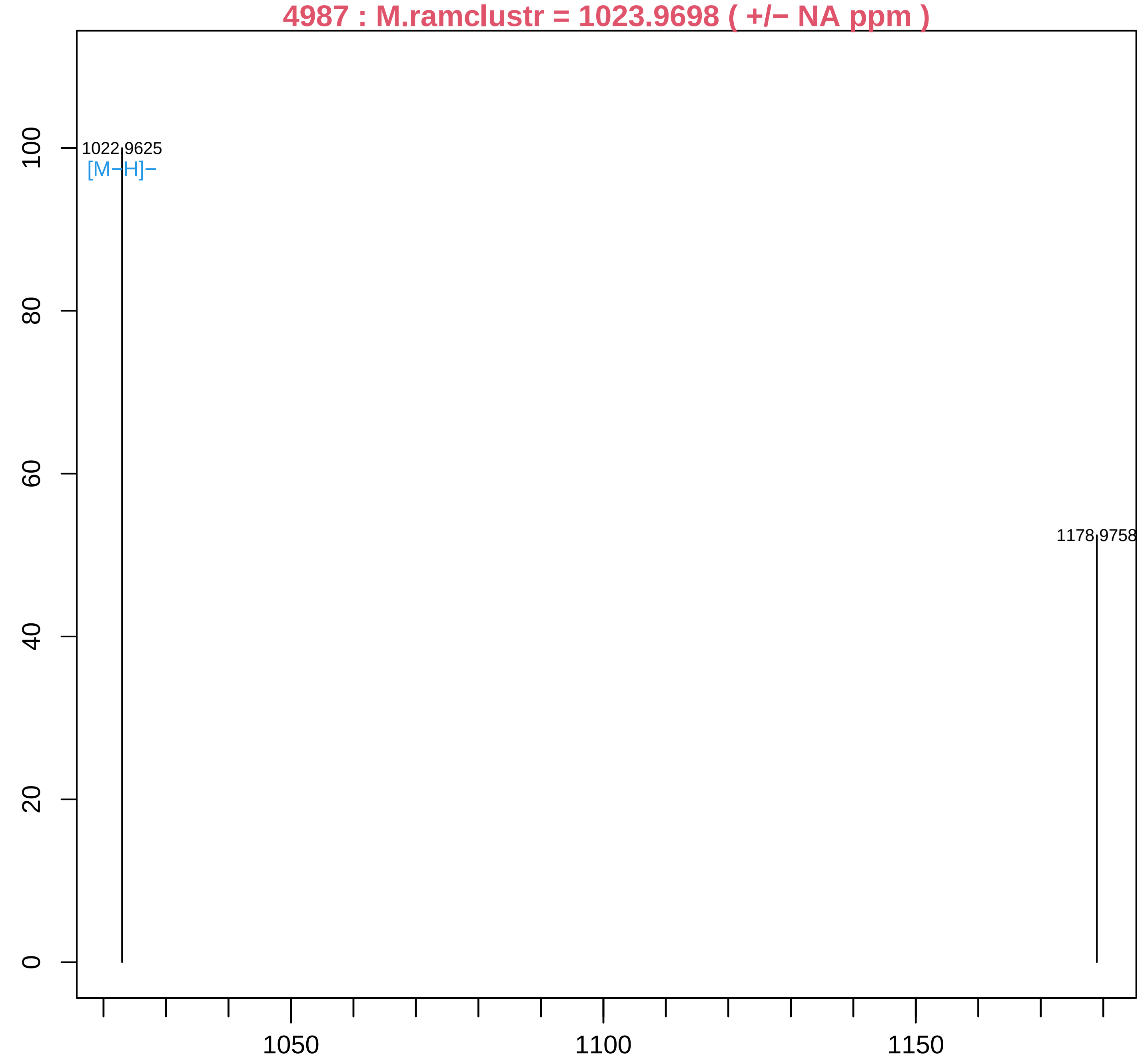
4987 : M.ramclustr = 1023.9698 (+/− NA ppm)
1022.9625
[M−H]−
1178.9758
0
20
40
60
80
100
1050
1100
1150

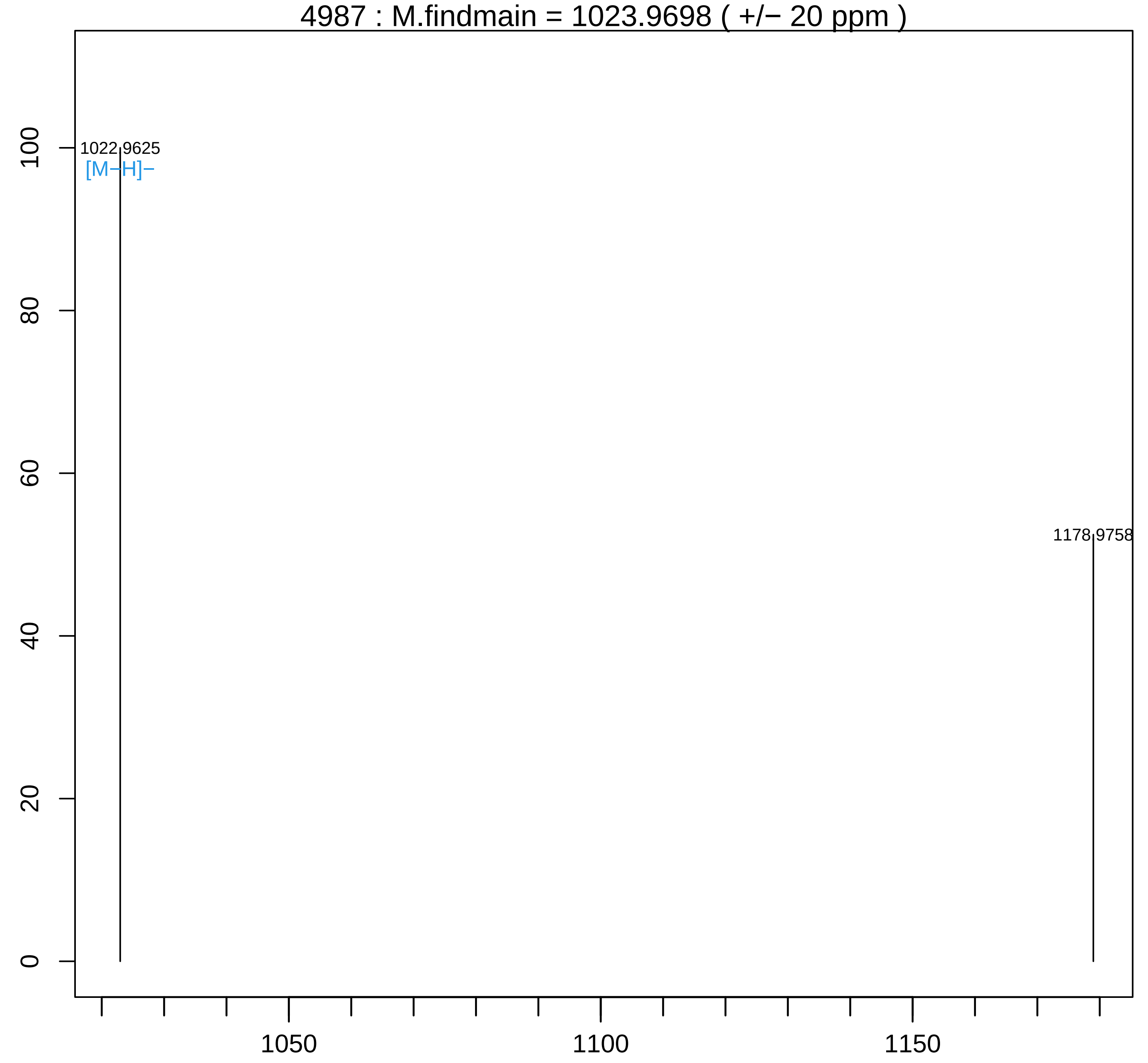
4987 : M.findmain = 1023.9698 (+/− 20 ppm)
1022.9625
[M−H]−
1178.9758
0
20
40
60
80
100
1050
1100
1150

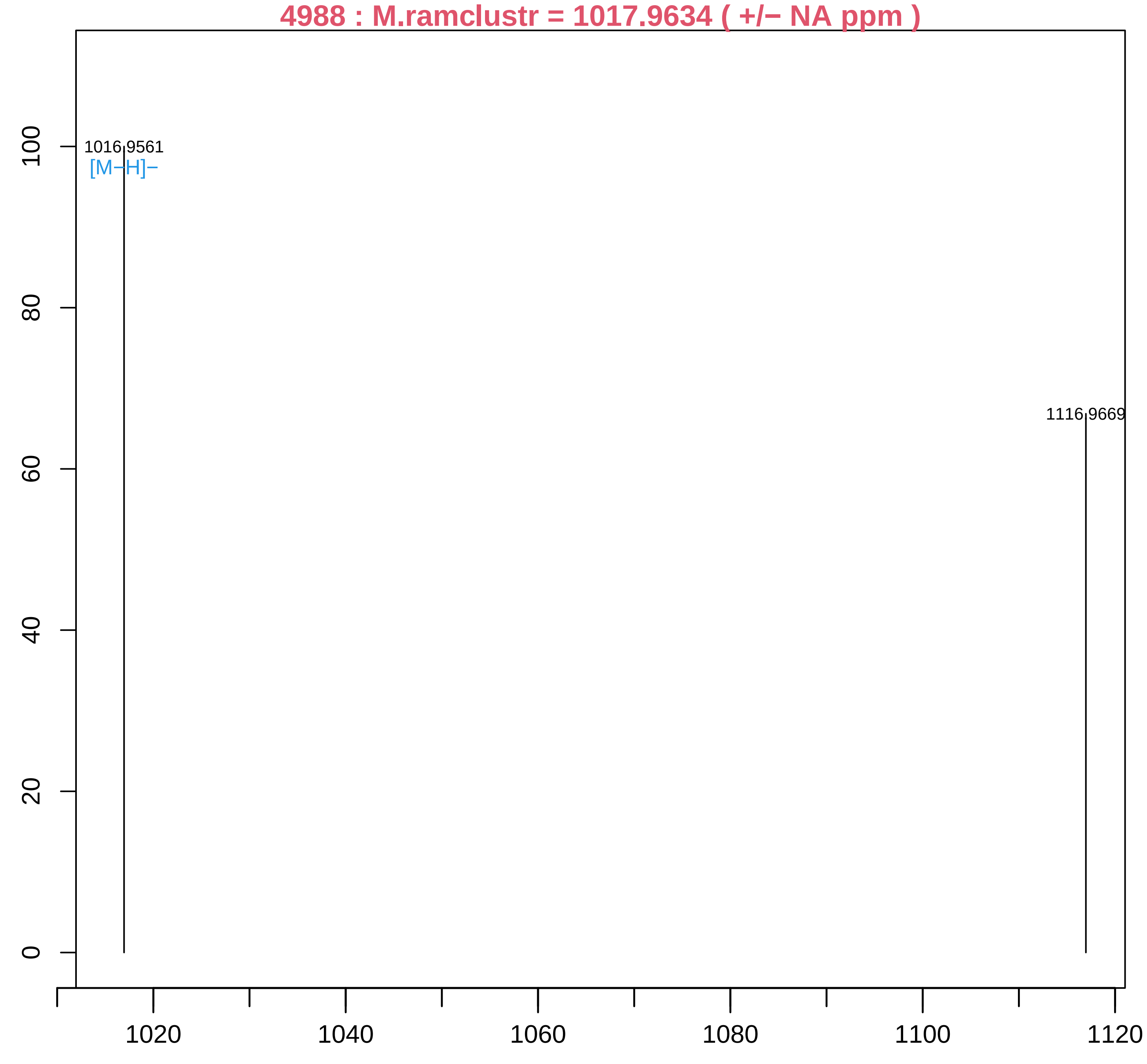
4988 : M.ramclustr = 1017.9634 (+/− NA ppm)
1016.9561
[M−H]−
1116.9669
0
20
40
60
80
100
1020
1040
1060
1080
1100
1120

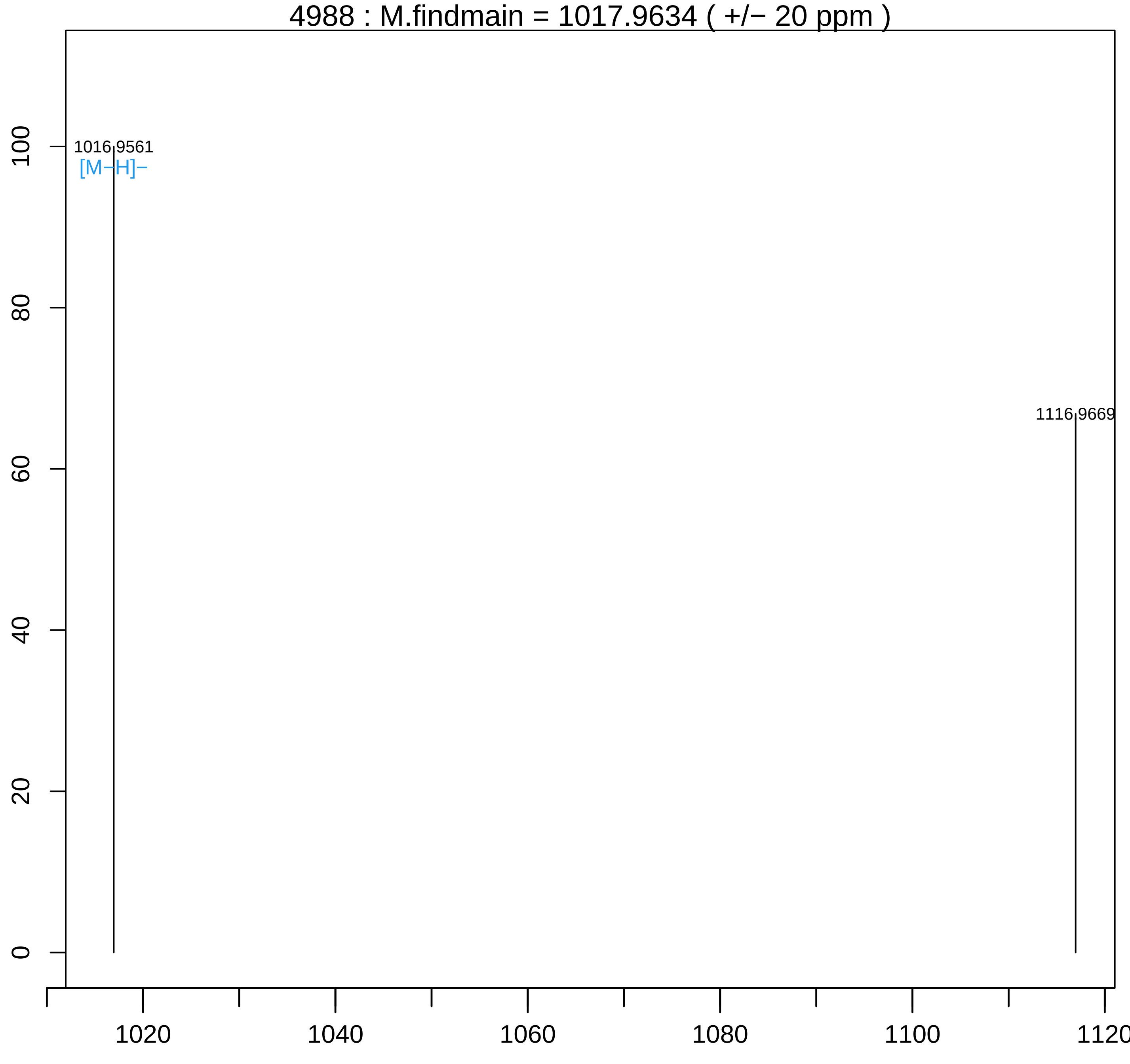
4988 : M.findmain = 1017.9634 (+/− 20 ppm)
1016.9561
[M−H]−
1116.9669
0
20
40
60
80
100
1020
1040
1060
1080
1100
1120

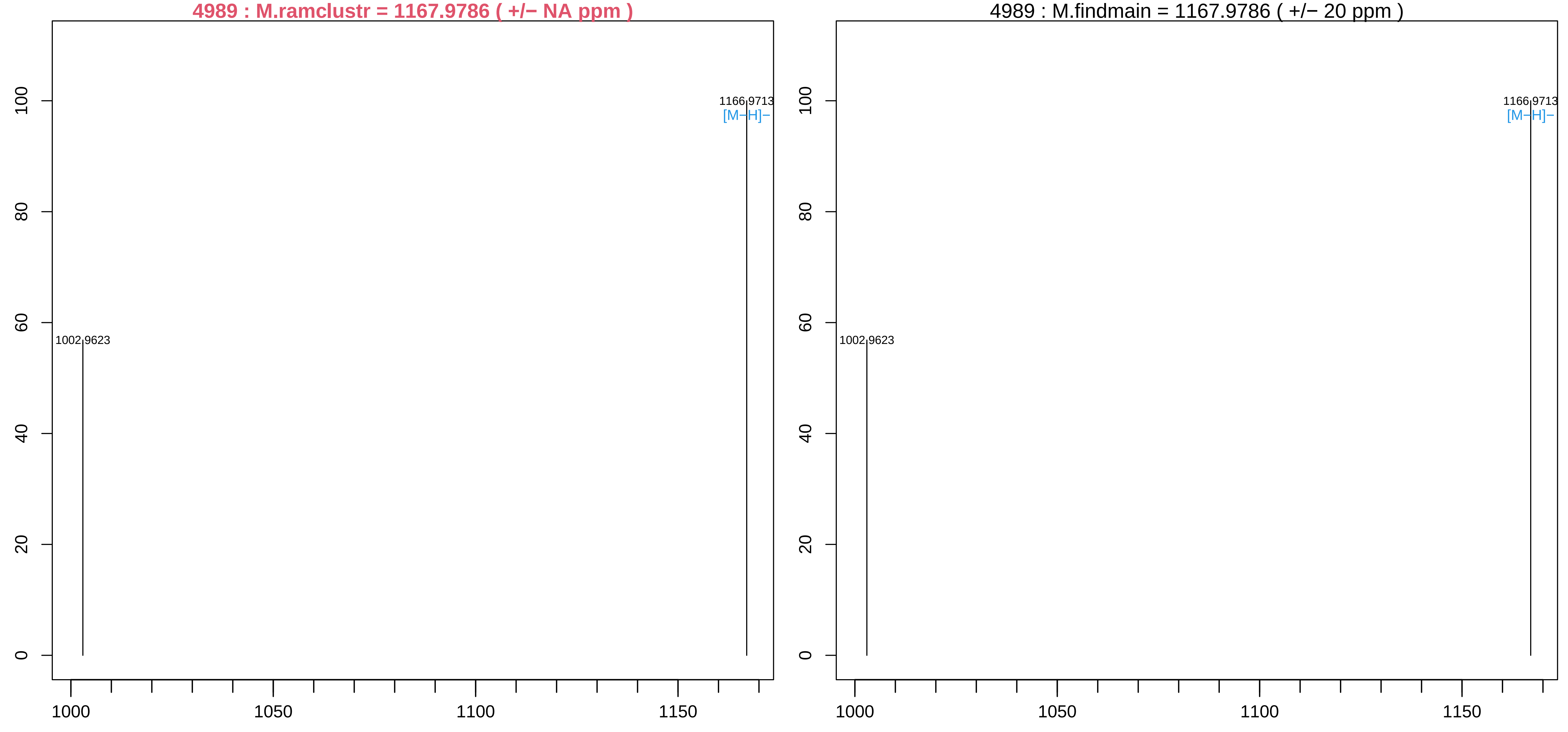
4989 : M.ramclustr = 1167.9786 (+/− NA ppm)
1166.9713
[M−H]−
1002.9623
4989 : M.findmain = 1167.9786 (+/− 20 ppm)
1166.9713
[M−H]−
1002.9623
0
20
40
60
80
100
1000
1050
1100
1150

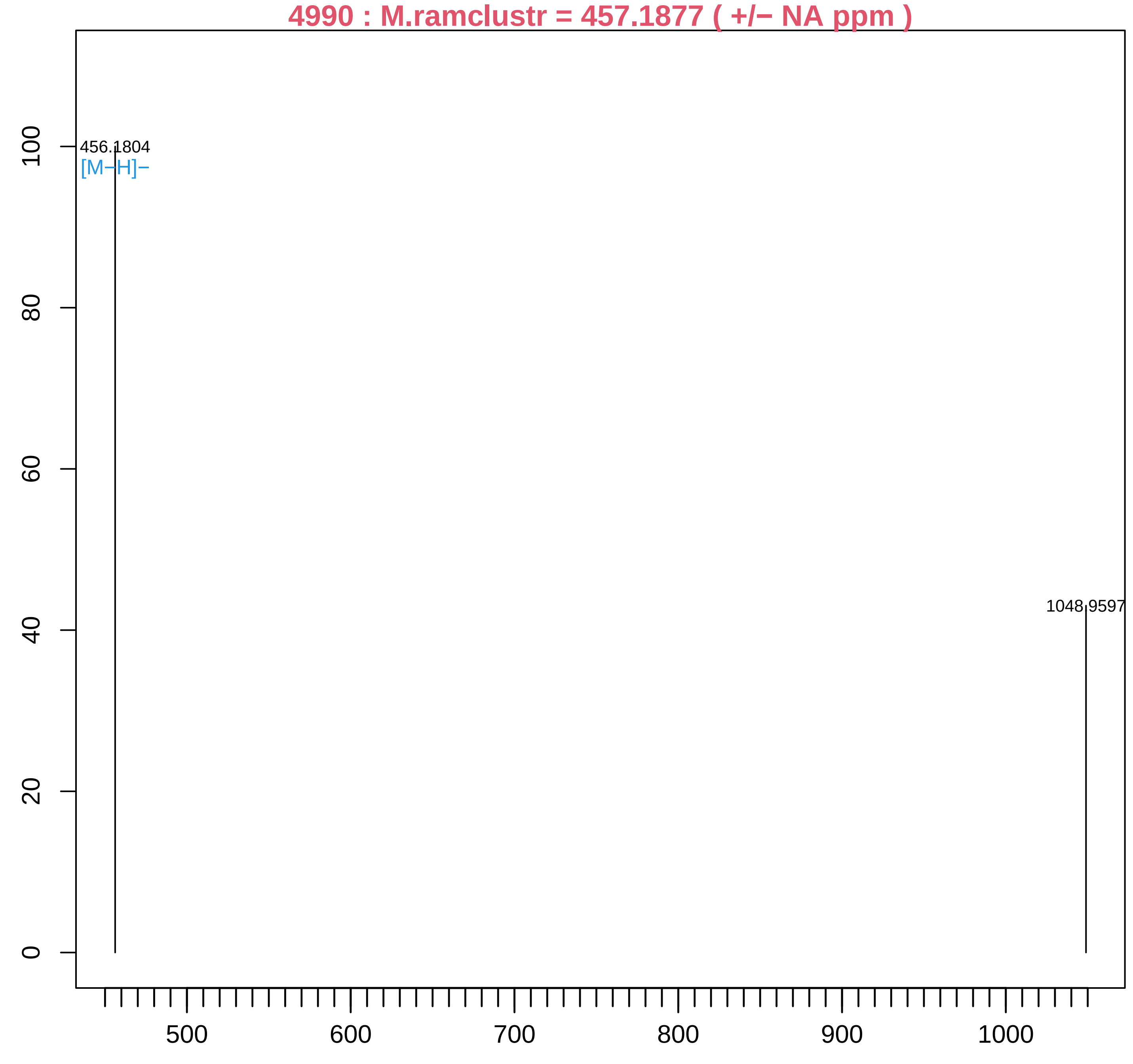
4990 : M.ramclustr = 457.1877 (+/− NA ppm)
456.1804
[M−H]−
1048.9597
0
20
40
60
80
100
500
600
700
800
900
1000
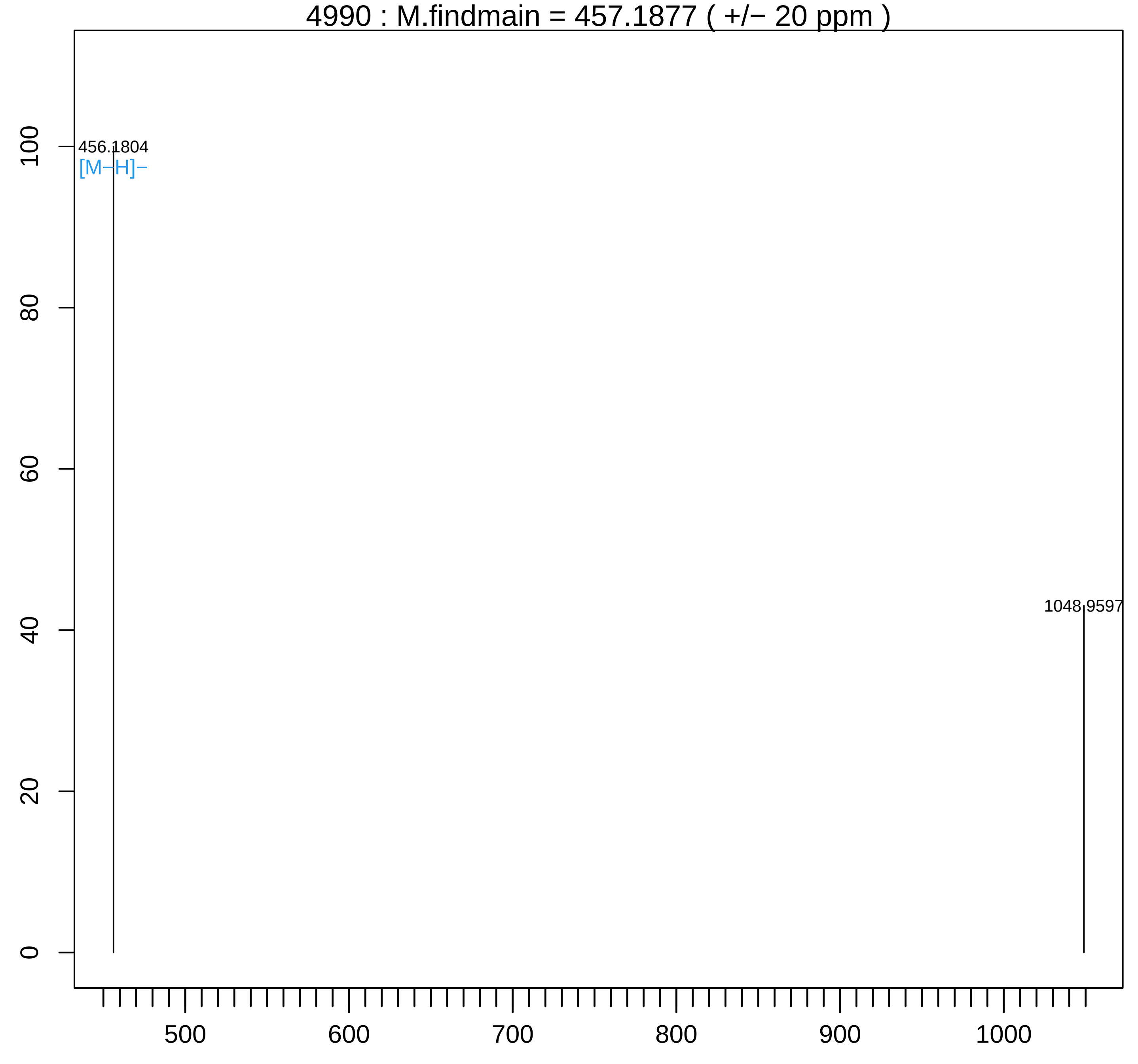
4990 : M.findmain = 457.1877 (+/− 20 ppm)
456.1804
[M−H]−
1048.9597
0
20
40
60
80
100
500
600
700
800
900
1000

4991 : M.ramclustr = 937.9685 (+/− NA ppm)

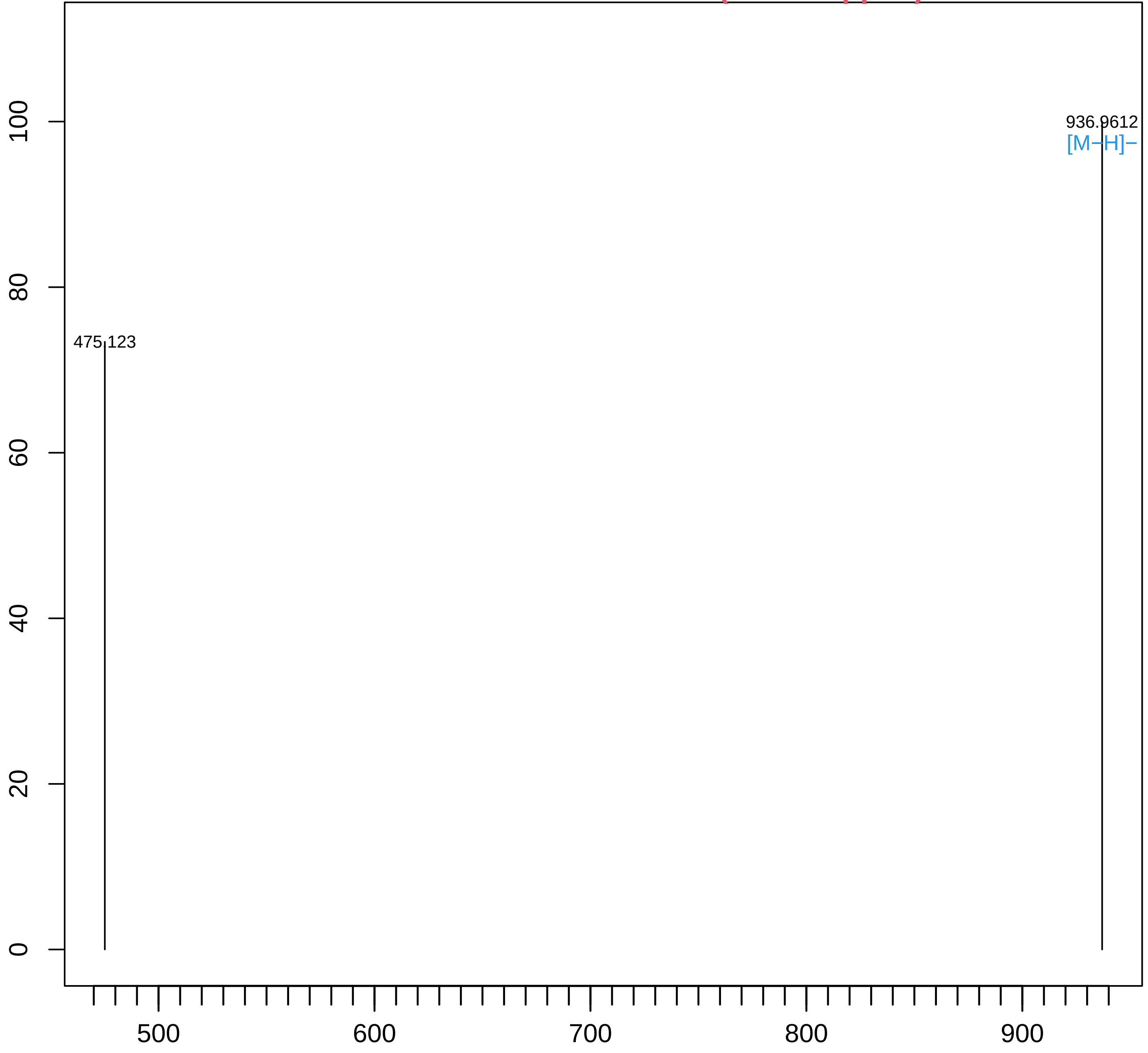

4991 : M.findmain = 937.9685 (+/− 20 ppm)

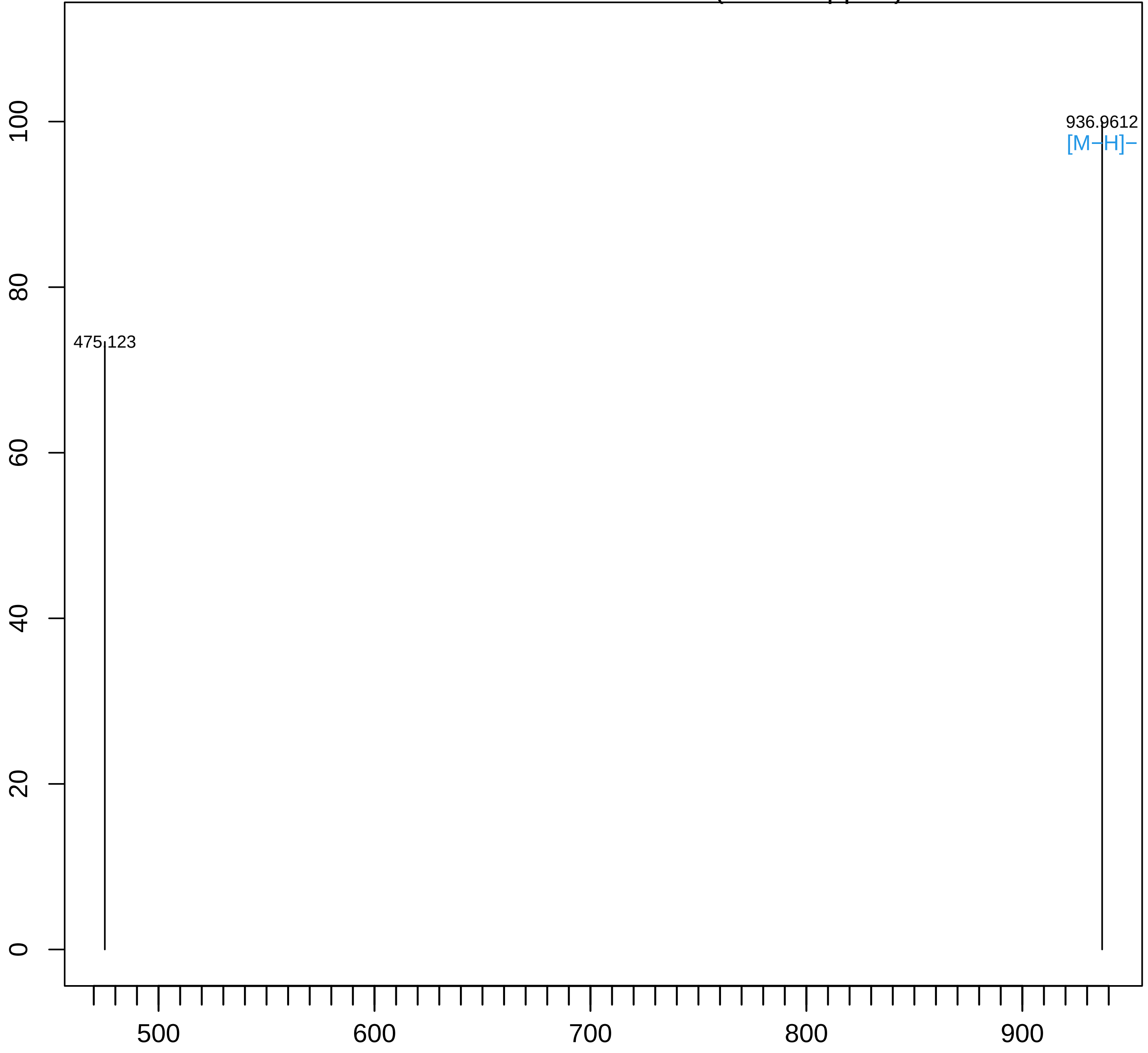

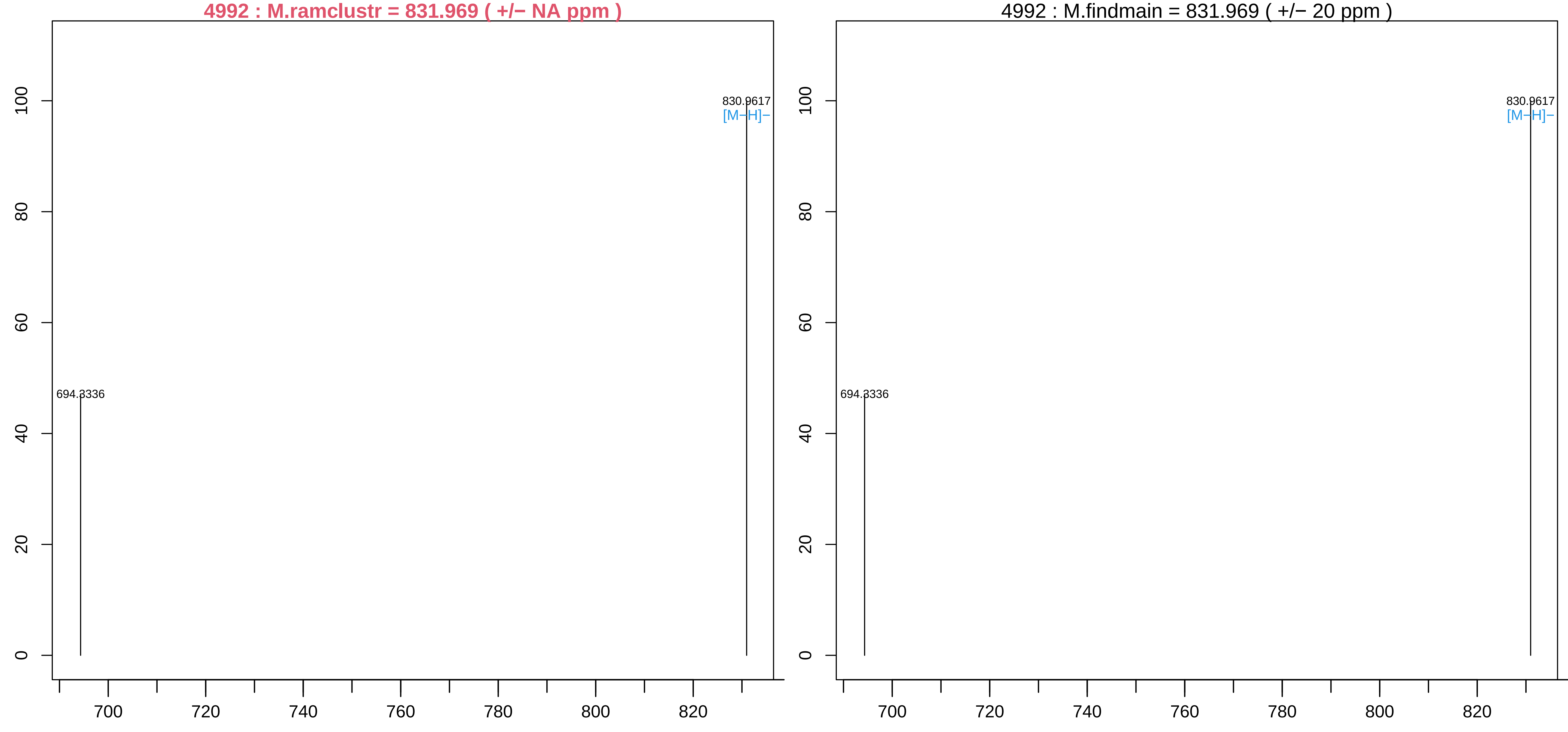

4992 : M.ramclustr = 831.969 (+/− NA ppm)
830.9617
[M−H]−
694.3336
0
20
40
60
80
100
700
720
740
760
780
800
820
4992 : M.findmain = 831.969 (+/− 20 ppm)
830.9617
[M−H]−
694.3336
0
20
40
60
80
100
700
720
740
760
780
800
820

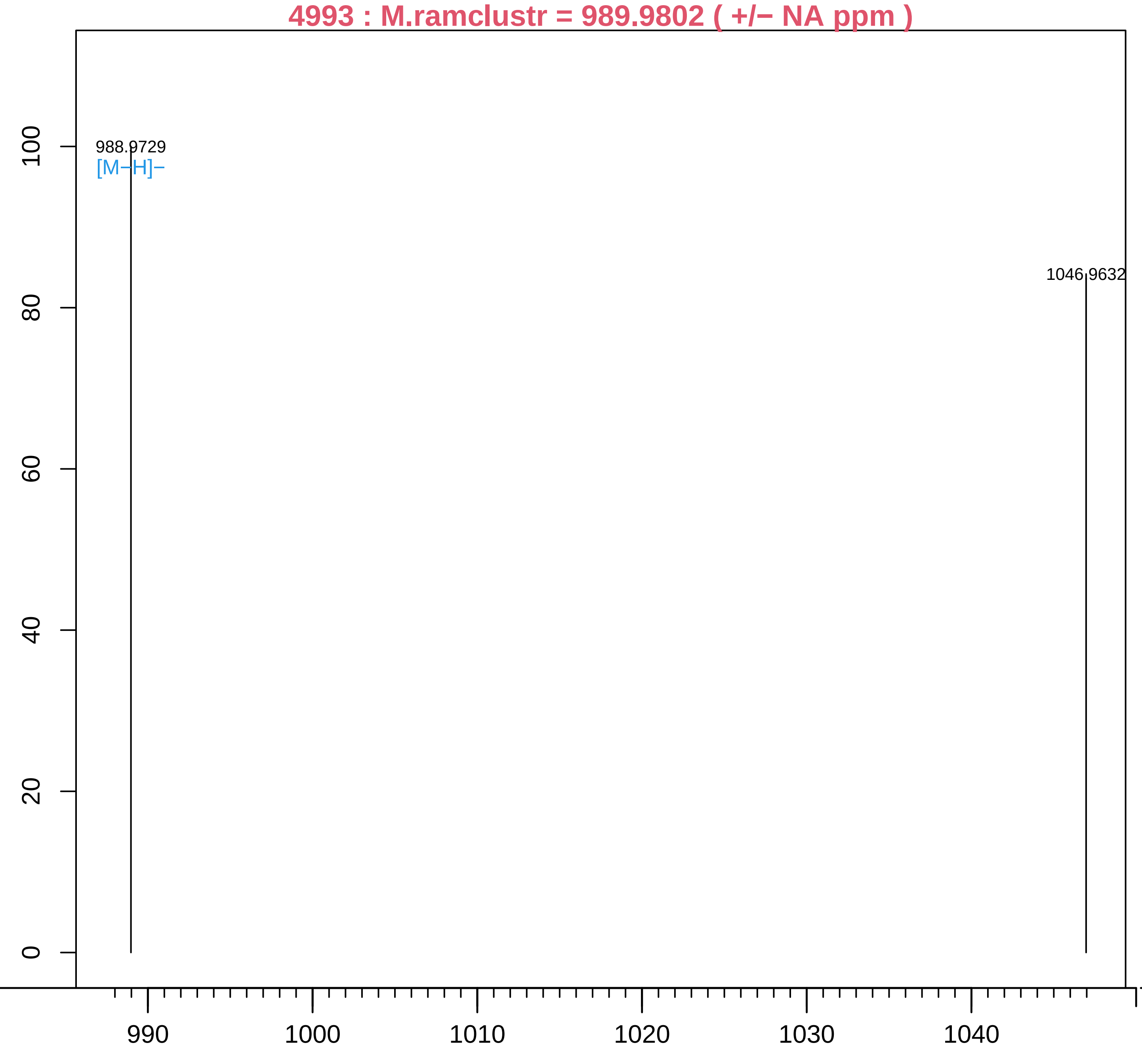
4993 : M.ramclustr = 989.9802 (+/− NA ppm)
988.9729
[M−H]−
1046.9632
0
20
40
60
80
100
990
1000
1010
1020
1030
1040

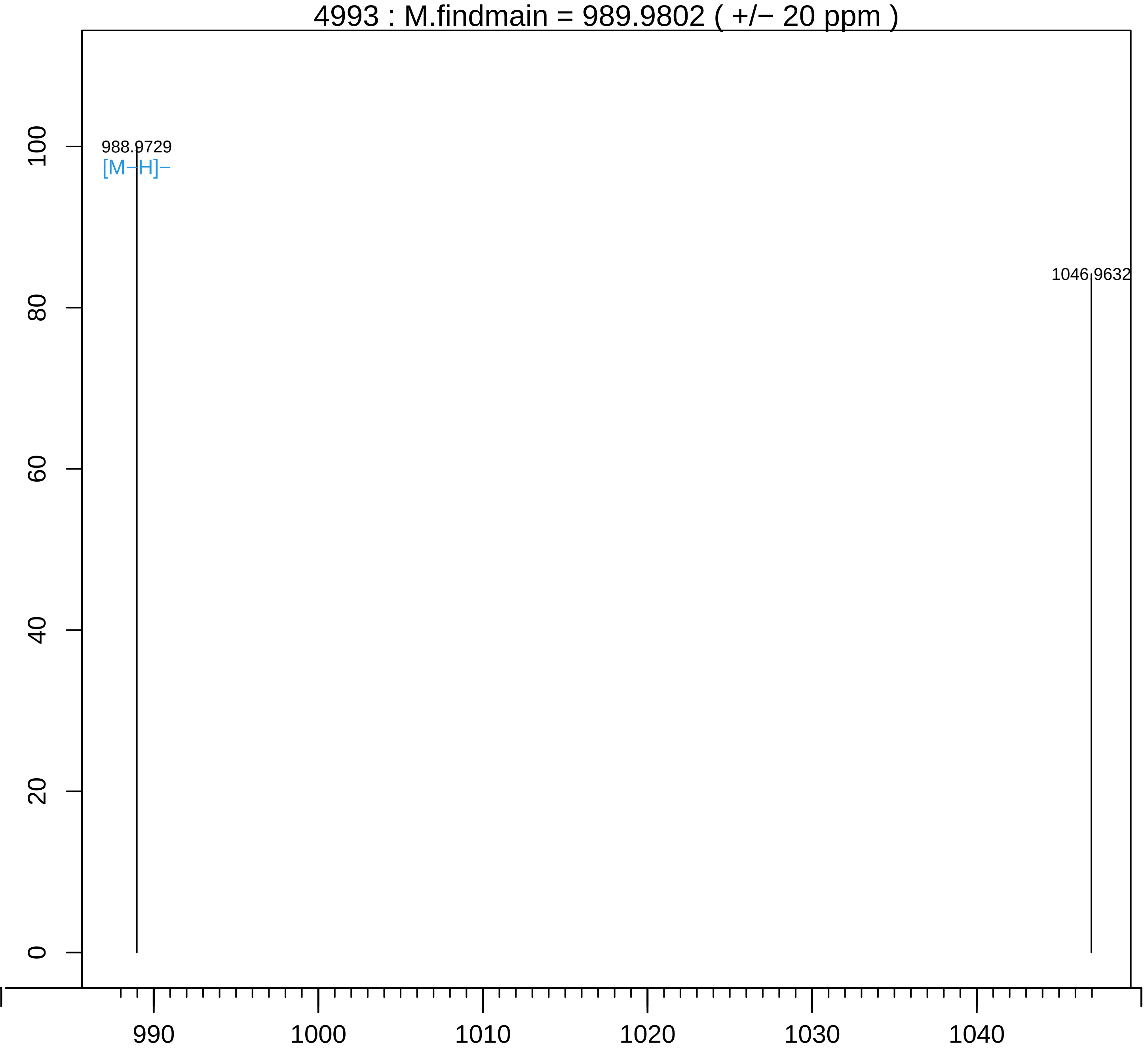
4993 : M.findmain = 989.9802 (+/− 20 ppm)
988.9729
[M−H]−
1046.9632
0
20
40
60
80
100
990
1000
1010
1020
1030
1040

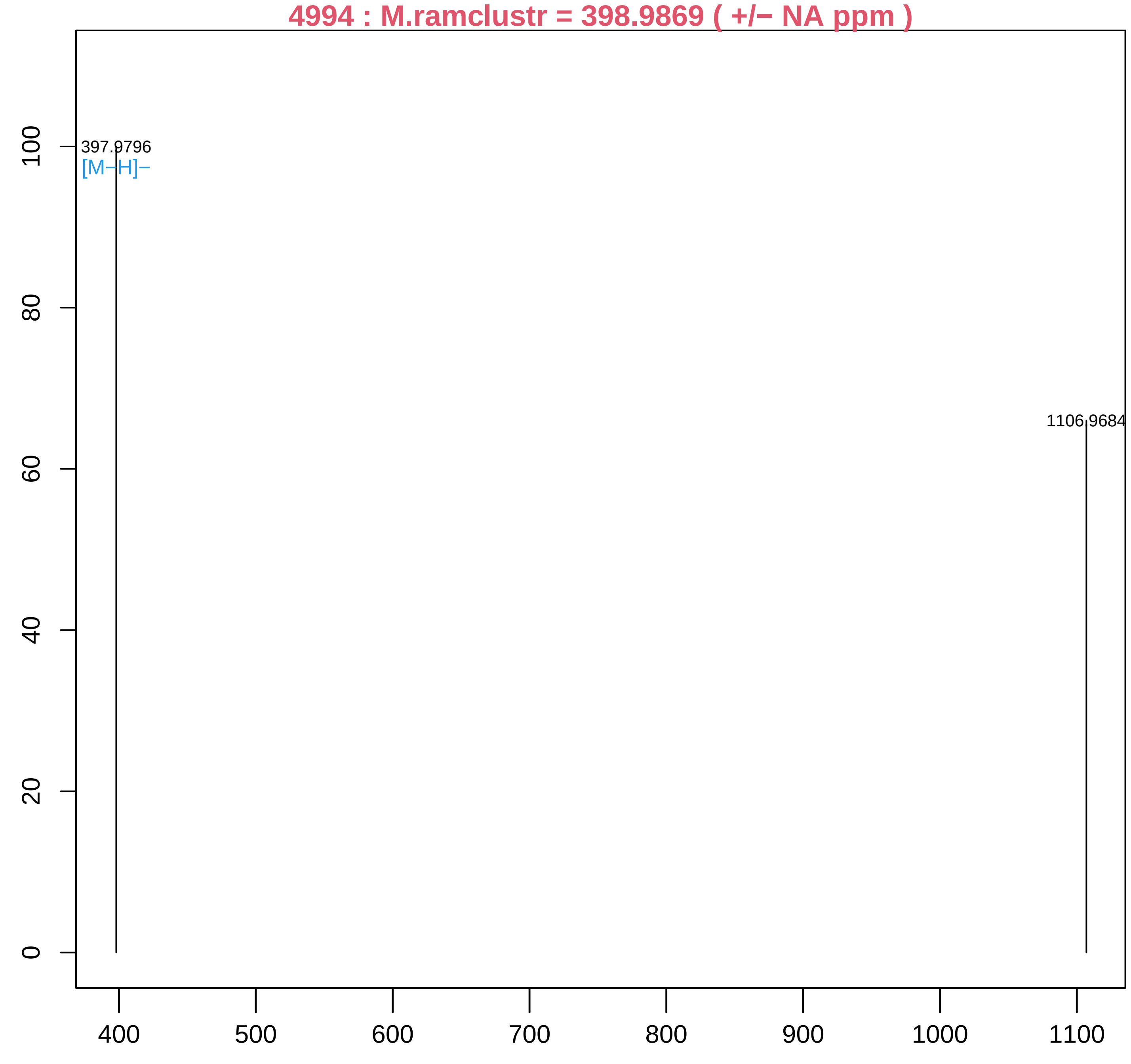
4994 : M.ramclustr = 398.9869 (+/− NA ppm)
397.9796
[M−H]−
1106.9684
0
20
40
60
80
100
400
500
600
700
800
900
1000
1100

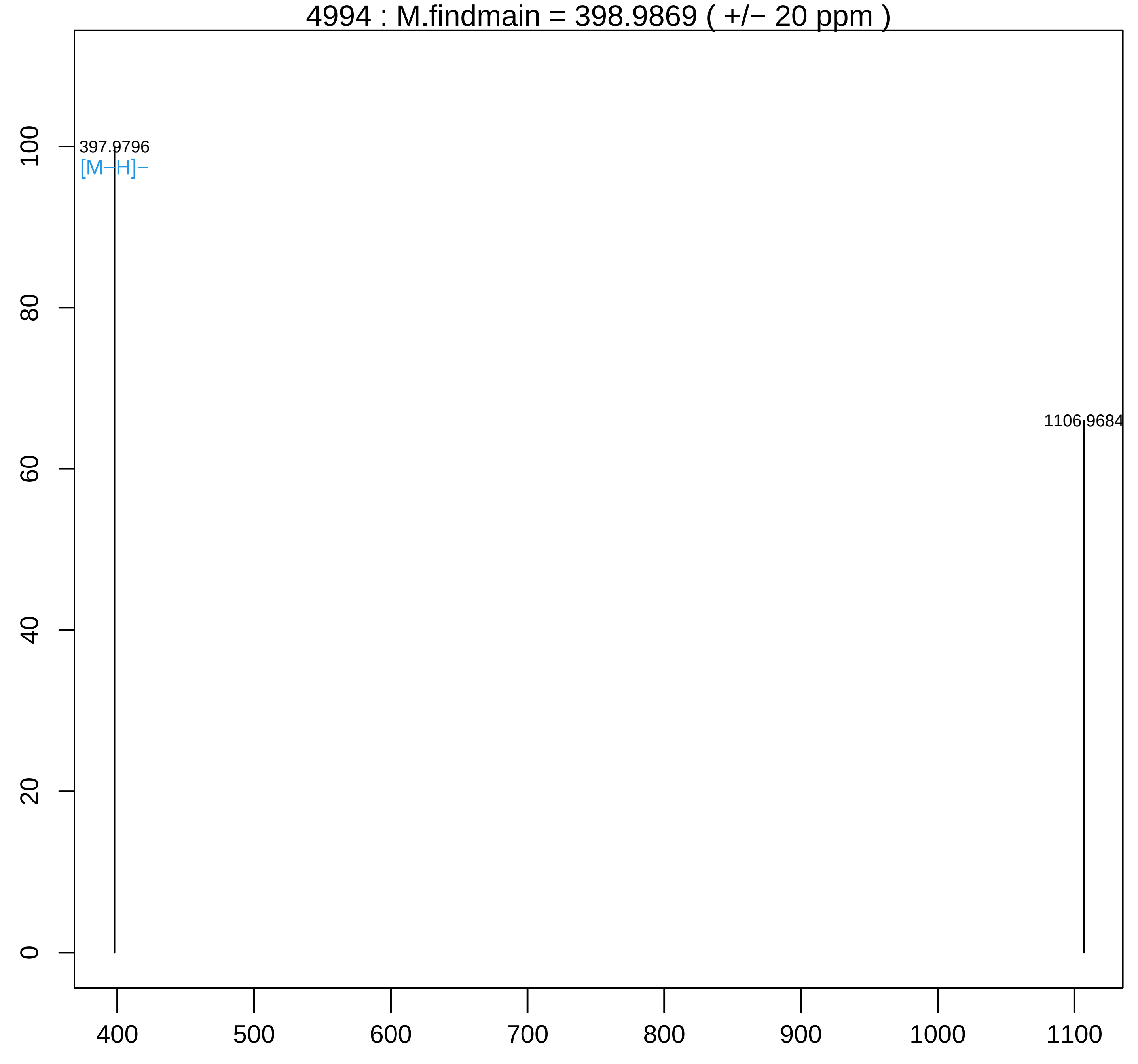
4994 : M.findmain = 398.9869 (+/− 20 ppm)
397.9796
[M−H]−
1106.9684
0
20
40
60
80
100
400
500
600
700
800
900
1000
1100

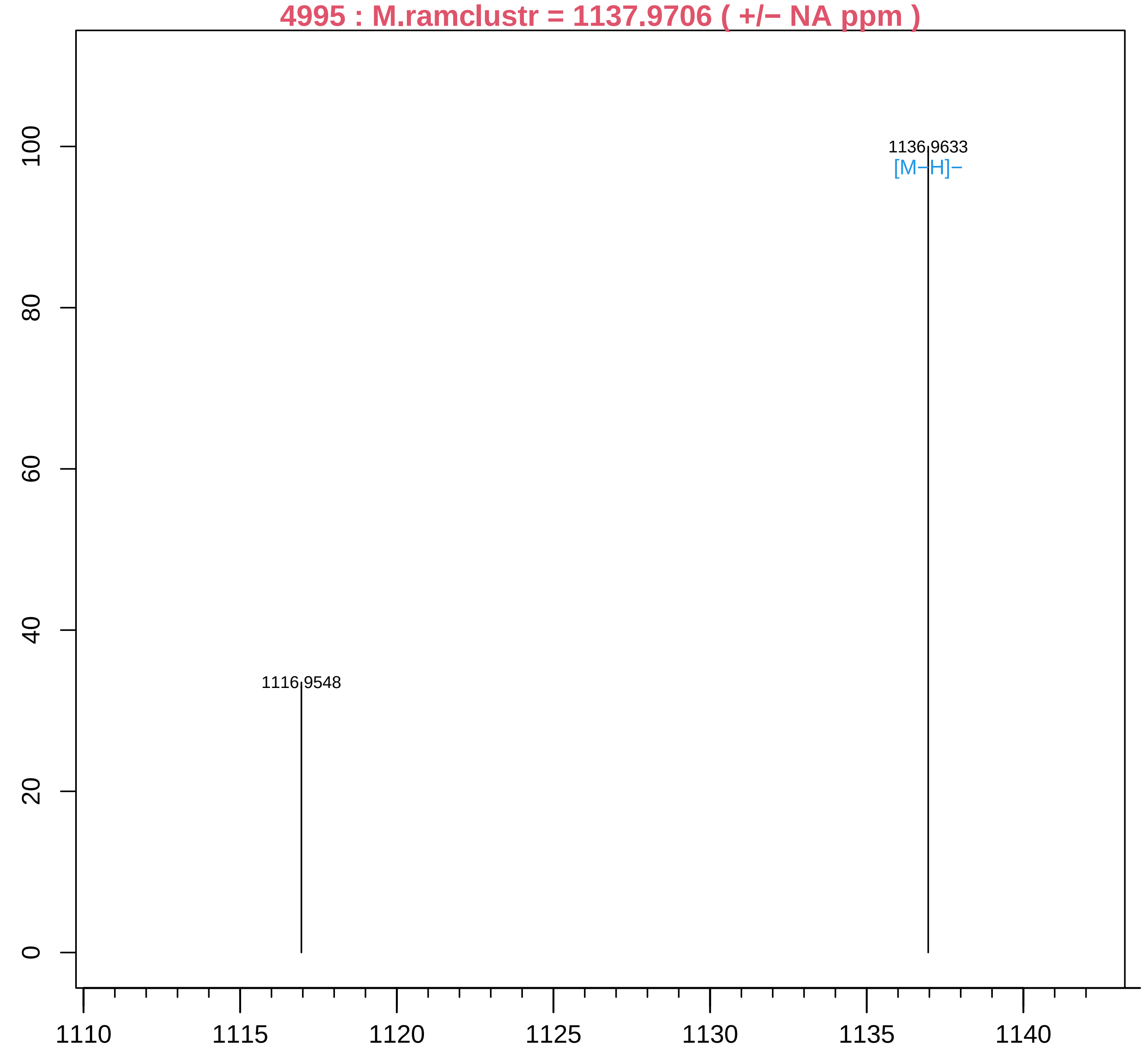

4995 : M.ramclustr = 1137.9706 (+/− NA ppm)
1136.9633
[M−H]−
1116.9548
0
20
40
60
80
100
1110
1115
1120
1125
1130
1135
1140

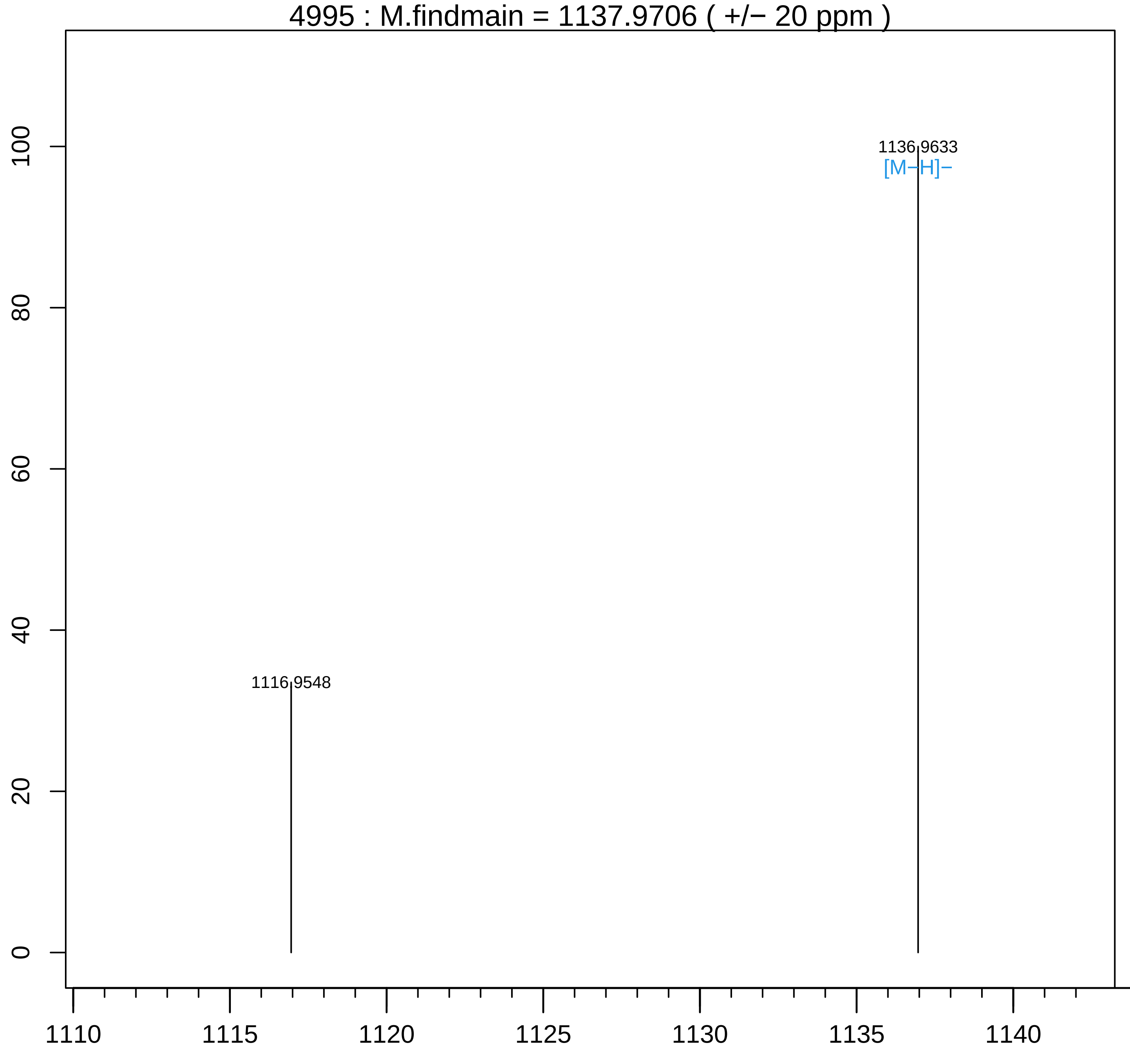

4995 : M.findmain = 1137.9706 (+/− 20 ppm)
1136.9633
[M−H]−
1116.9548
0
20
40
60
80
100
1110
1115
1120
1125
1130
1135
1140

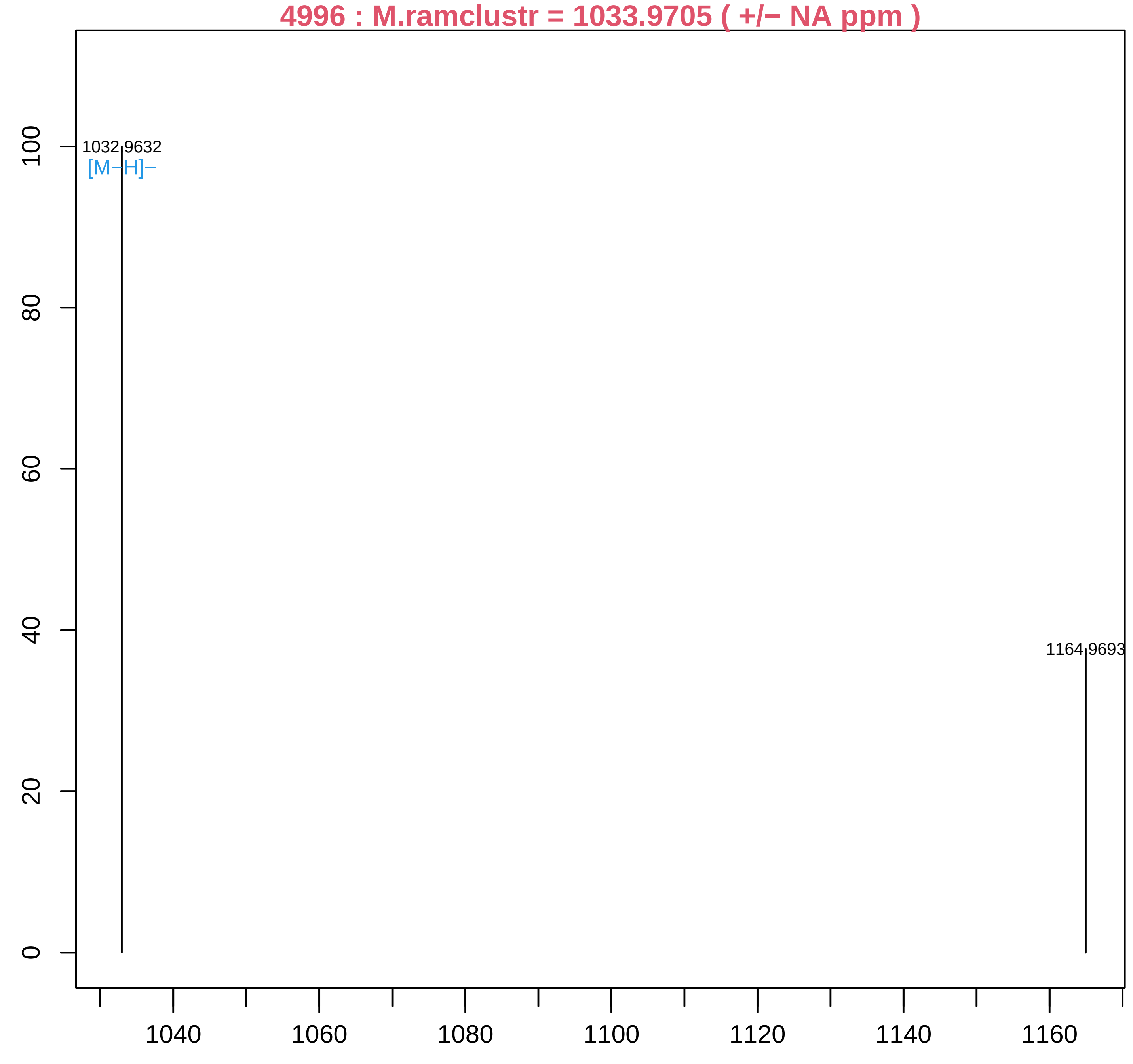
4996 : M.ramclustr = 1033.9705 (+/− NA ppm)
1032.9632
[M−H]−
1164.9693
0
20
40
60
80
100
1040
1060
1080
1100
1120
1140
1160

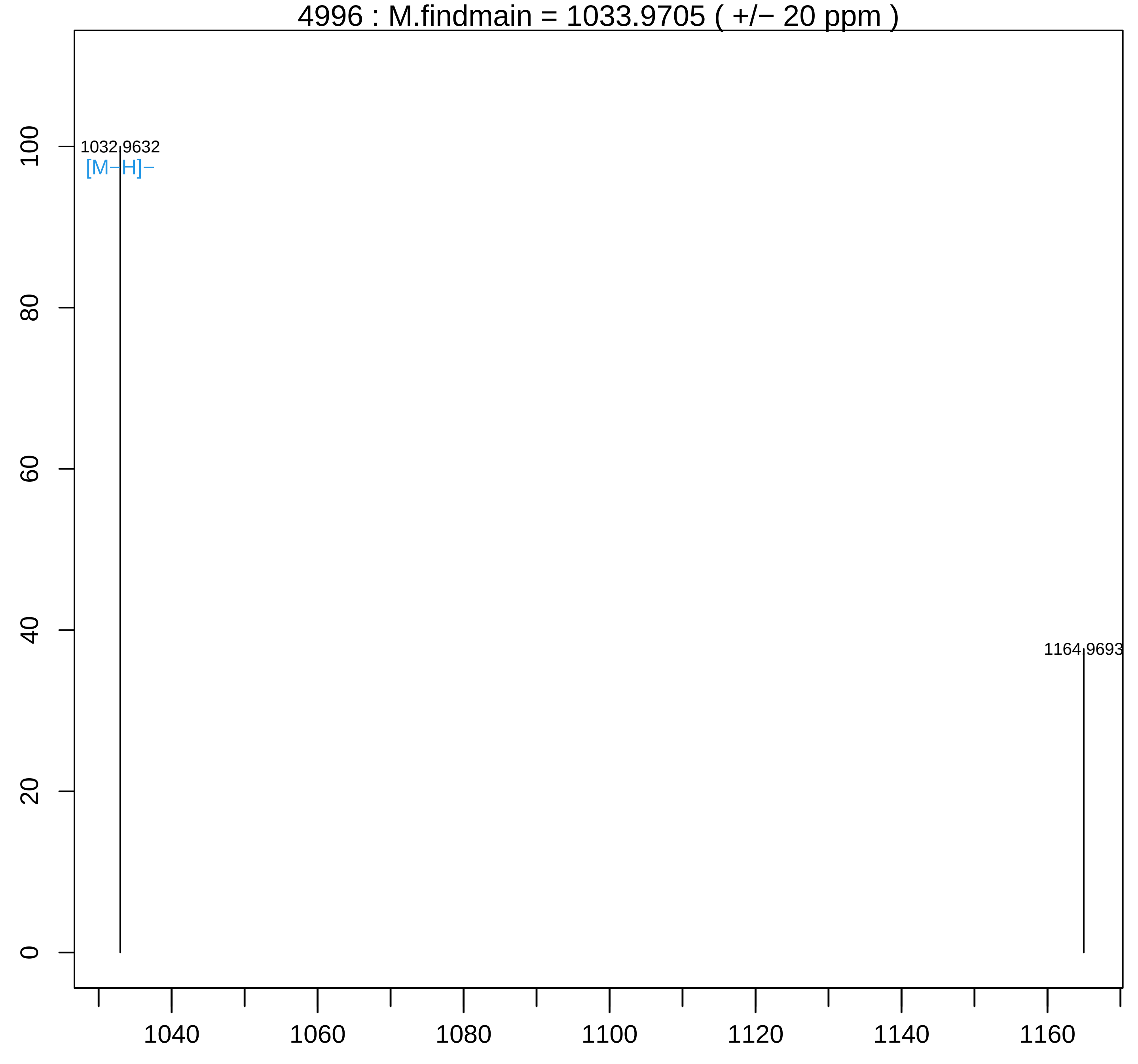
4996 : M.findmain = 1033.9705 (+/− 20 ppm)
1032.9632
[M−H]−
1164.9693
0
20
40
60
80
100
1040
1060
1080
1100
1120
1140
1160

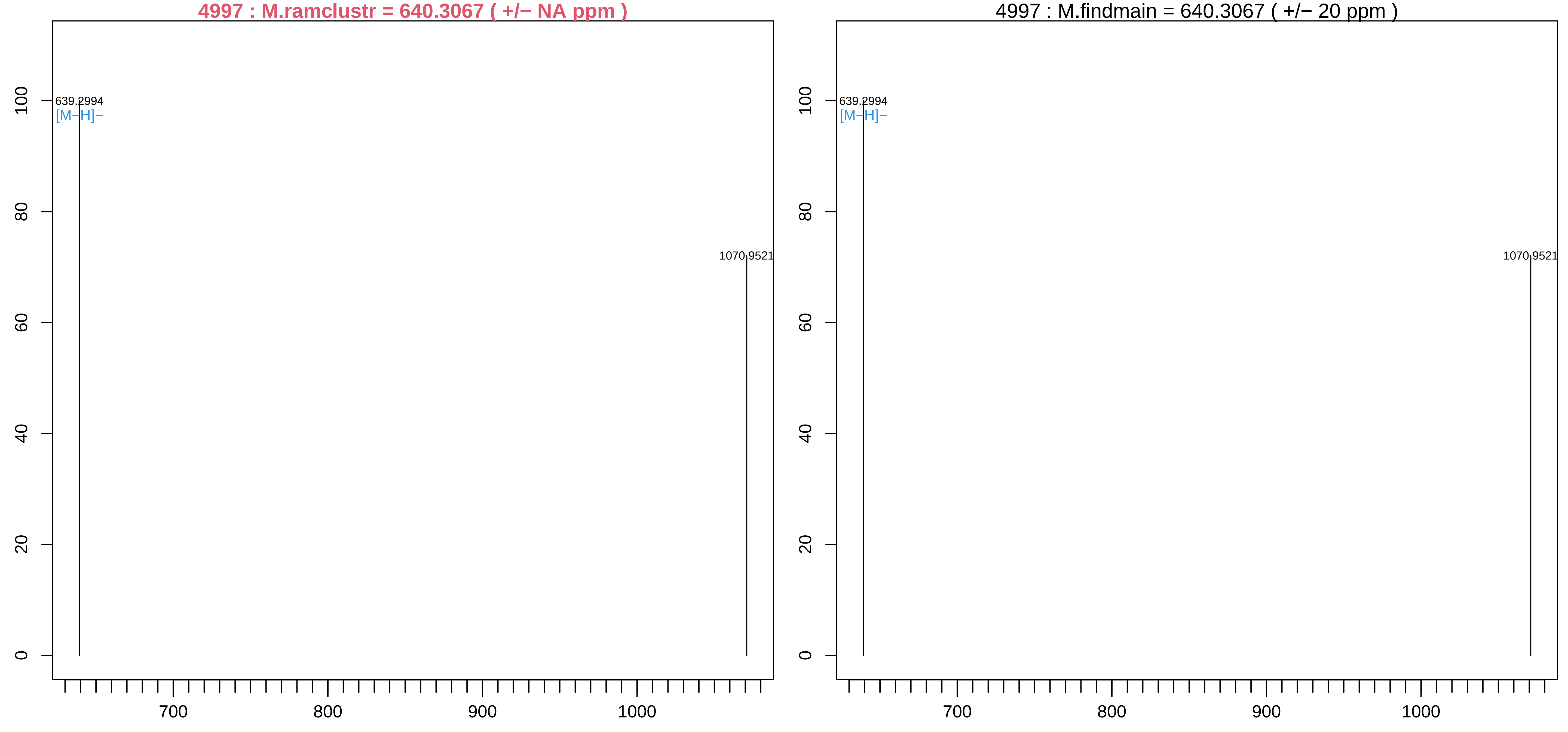
4997 : M.ramclustr = 640.3067 (+/− NA ppm)
639.2994
[M−H]−
1070.9521
4997 : M.findmain = 640.3067 (+/− 20 ppm)
639.2994
[M−H]−
1070.9521
0
20
40
60
80
100
700
800
900
1000

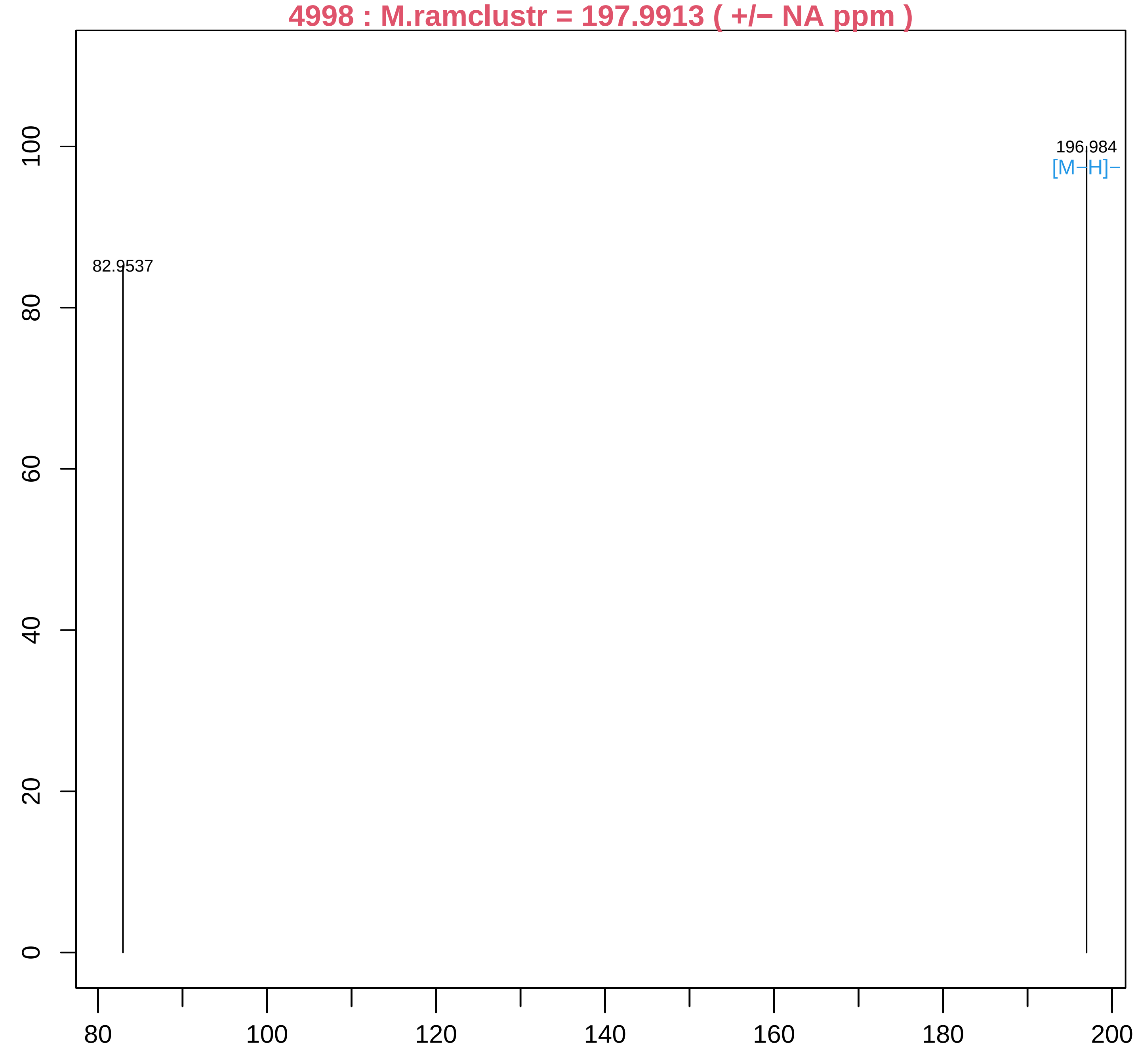
4998 : M.ramclustr = 197.9913 (+/− NA ppm)
196.984
[M−H]−
82.9537

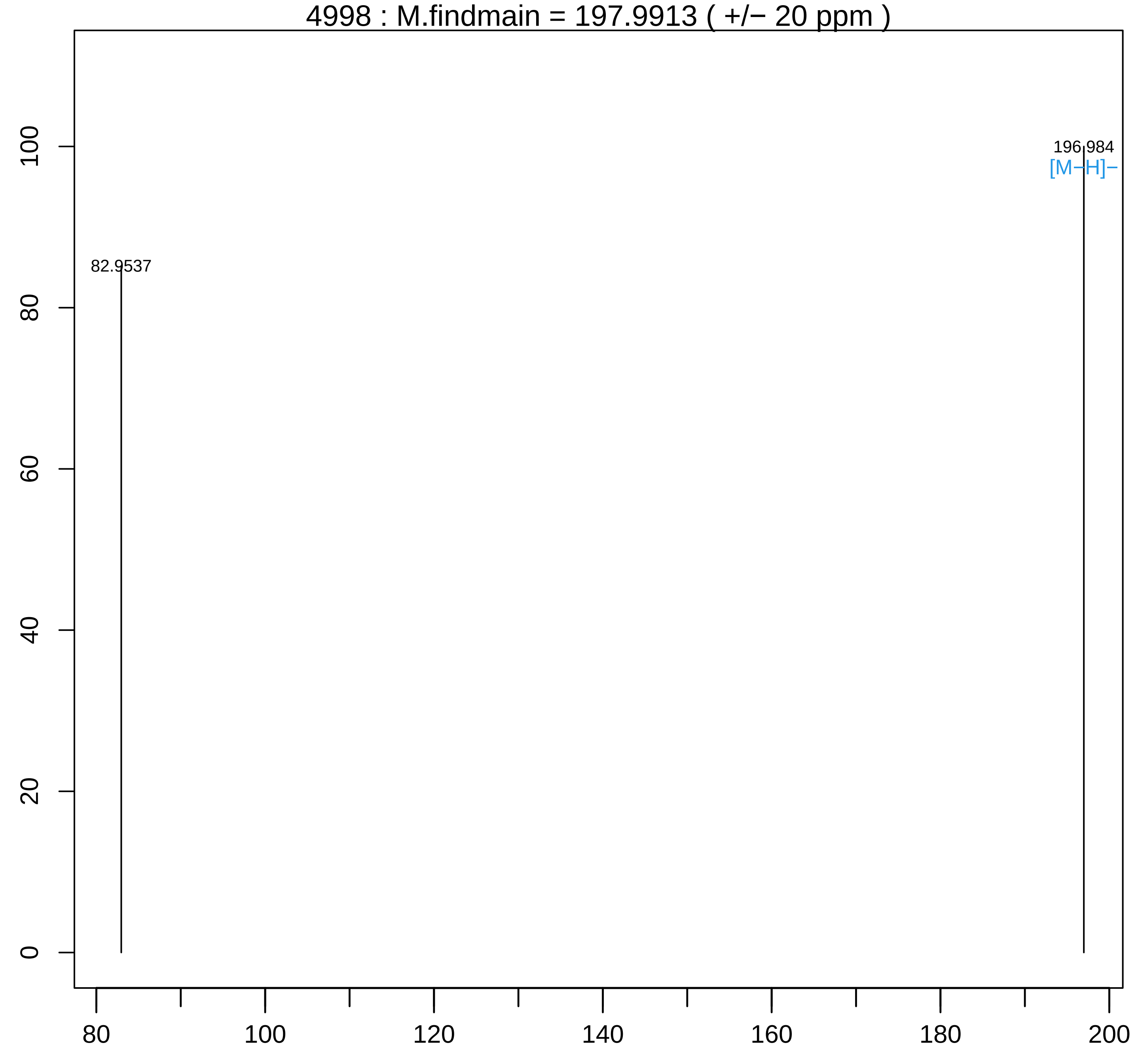
4998 : M.findmain = 197.9913 (+/− 20 ppm)
196.984
[M−H]−
82.9537

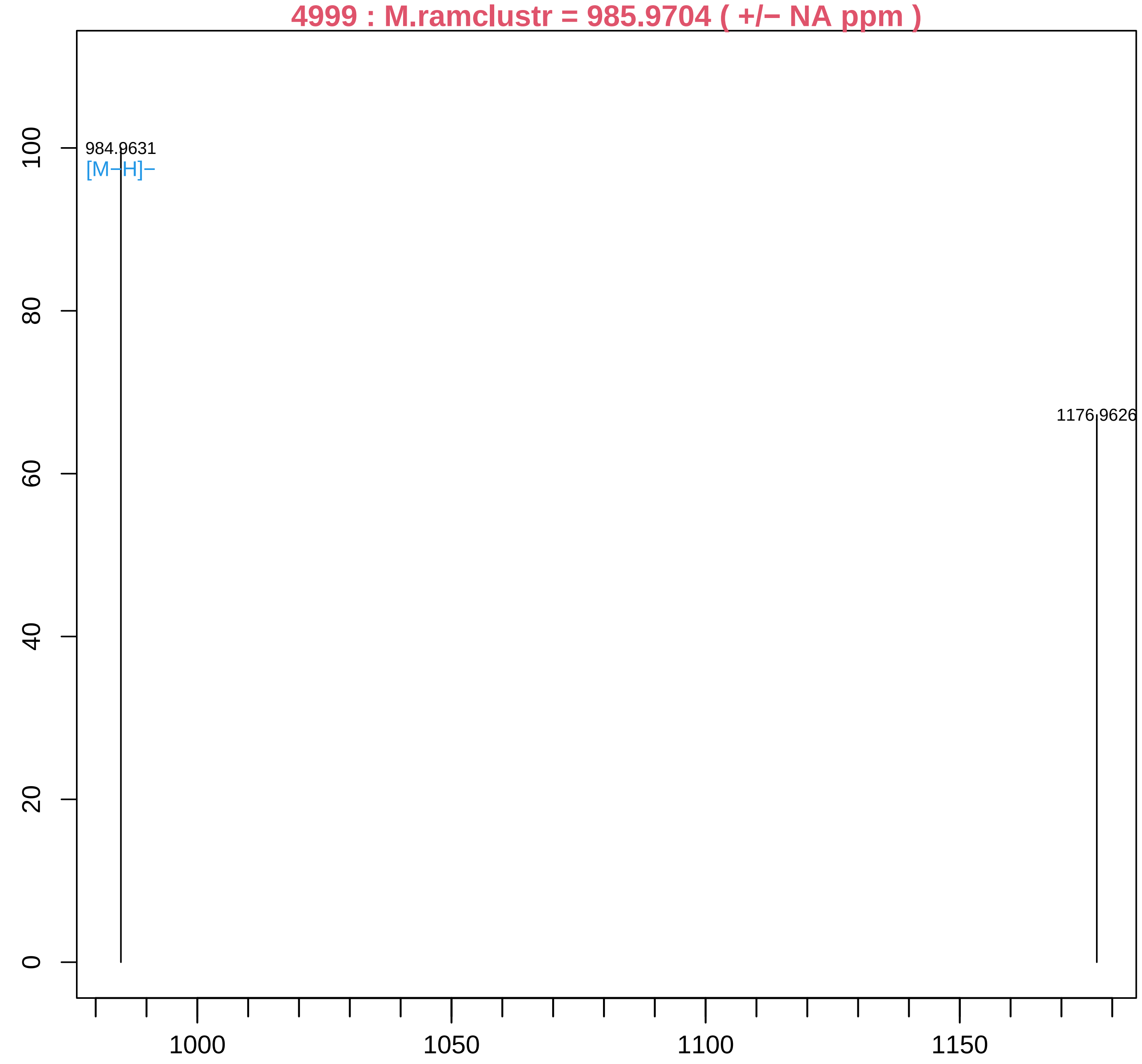
4999 : M.ramclustr = 985.9704 (+/− NA ppm)
984.9631
[M−H]−
1176.9626
0
20
40
60
80
100
1000
1050
1100
1150

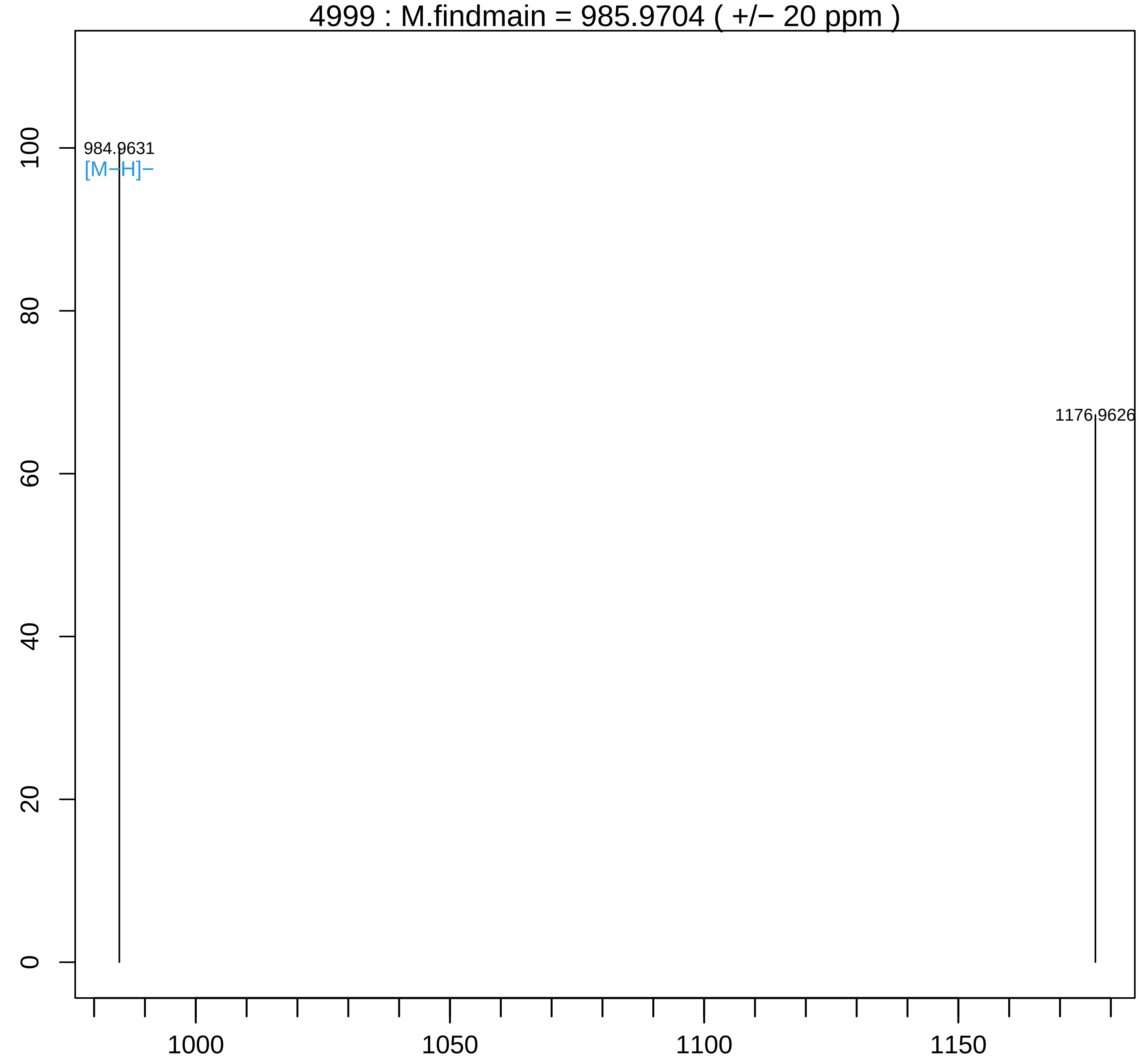
4999 : M.findmain = 985.9704 (+/− 20 ppm)
984.9631
[M−H]−
1176.9626
0
20
40
60
80
100
1000
1050
1100
1150

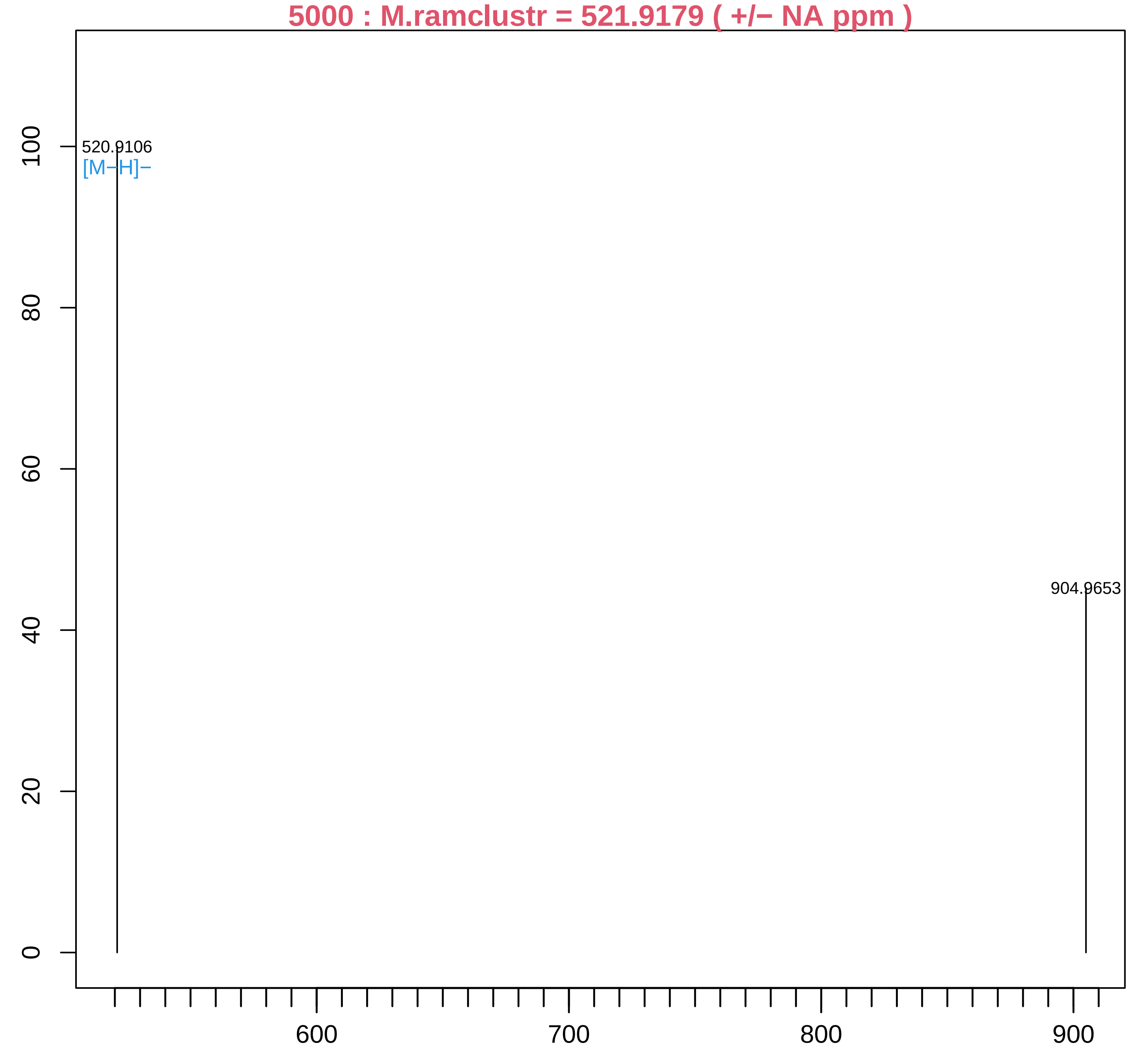
5000 : M.ramclustr = 521.9179 (+/− NA ppm)
520.9106
[M−H]−
904.9653
0
20
40
60
80
100
600
700
800
900

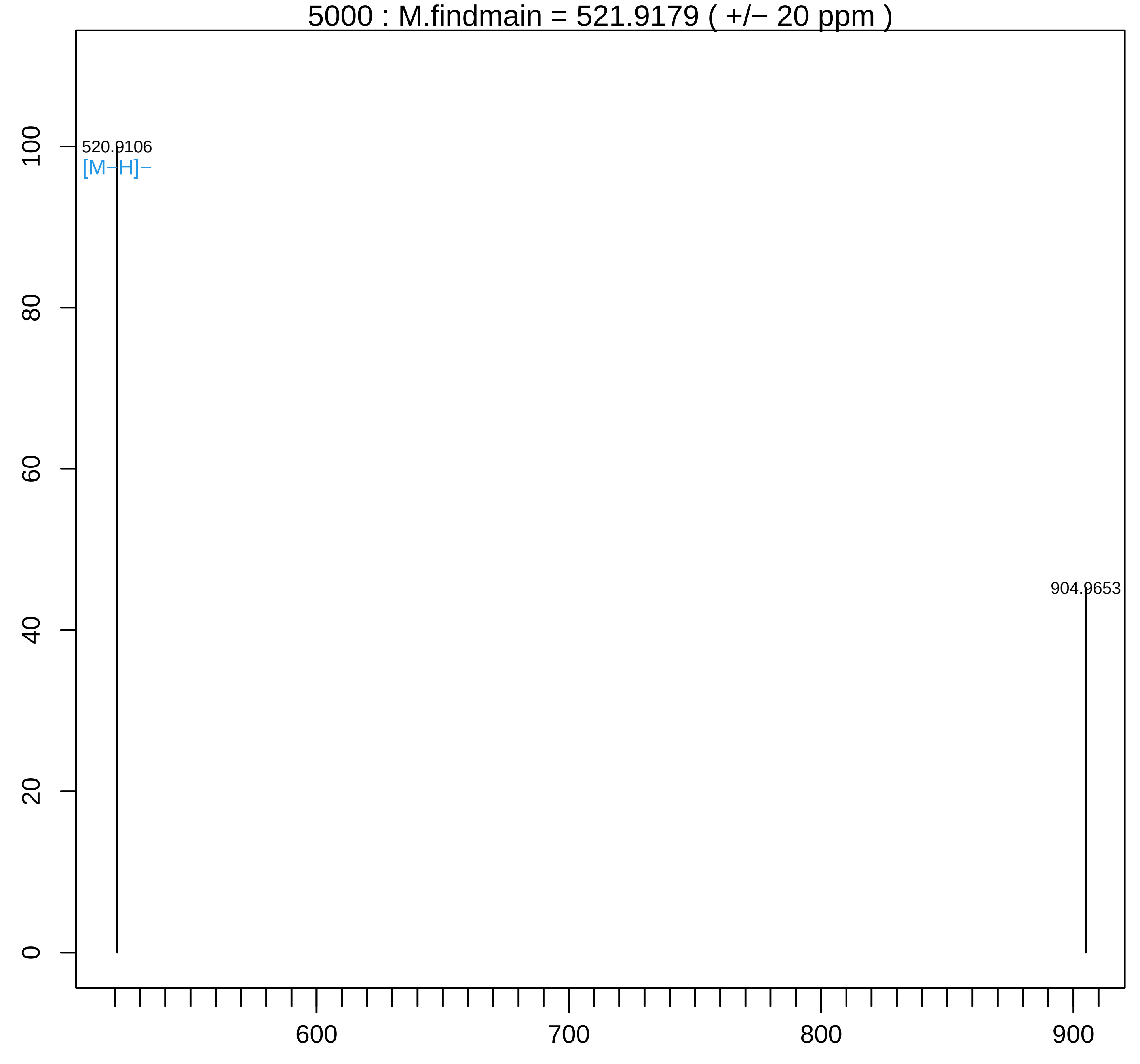
5000 : M.findmain = 521.9179 (+/− 20 ppm)
520.9106
[M−H]−
904.9653
0
20
40
60
80
100
600
700
800
900

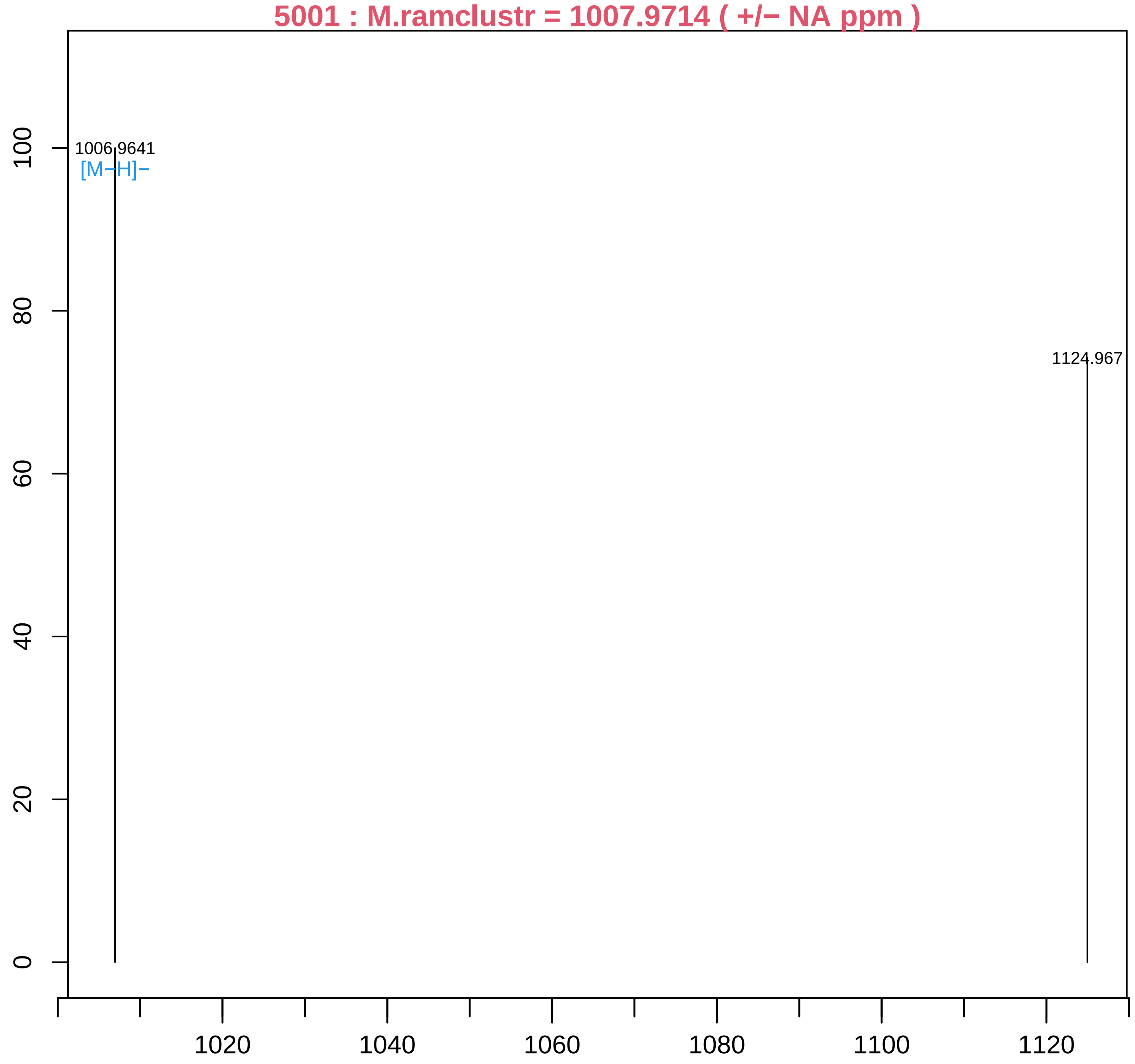
5001 : M.ramclustr = 1007.9714 (+/− NA ppm)
1006.9641
[M−H]−
1124.967
0
20
40
60
80
100
1020
1040
1060
1080
1100
1120

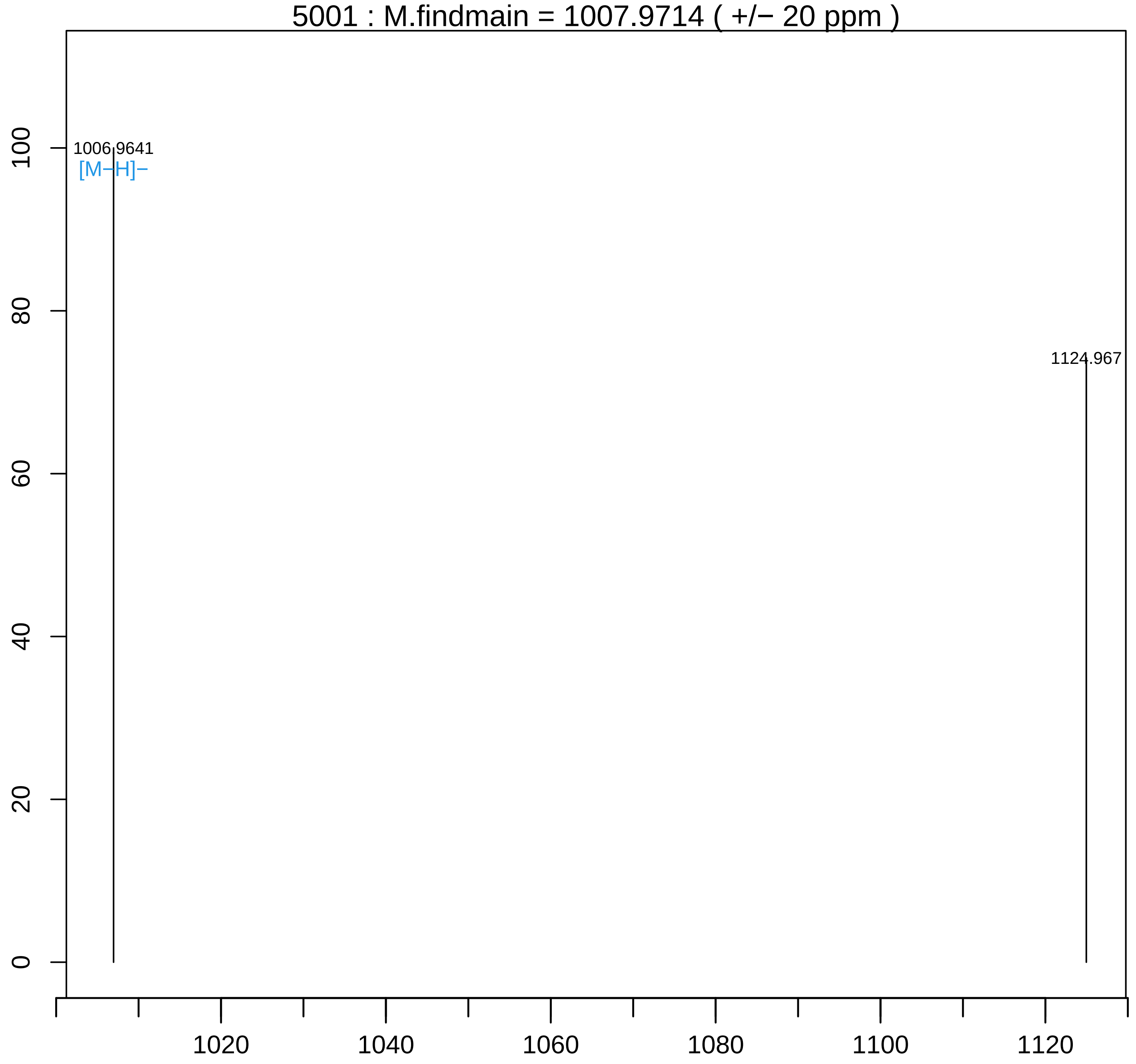
5001 : M.findmain = 1007.9714 (+/− 20 ppm)
1006.9641
[M−H]−
1124.967
0
20
40
60
80
100
1020
1040
1060
1080
1100
1120

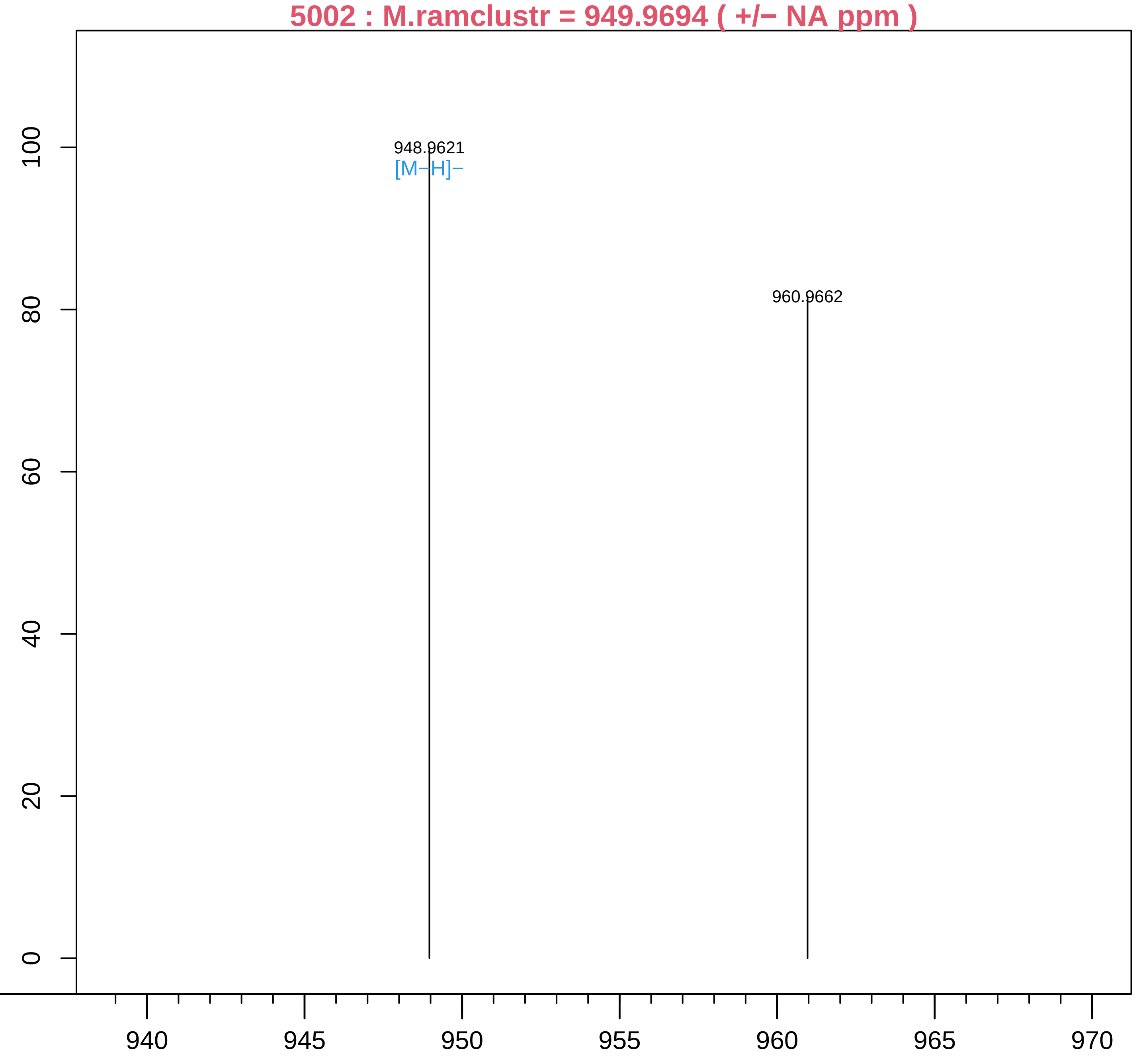
5002 : M.ramclustr = 949.9694 (+/− NA ppm)
948.9621
[M−H]−
960.9662
0
20
40
60
80
100
940
945
950
955
960
965
970

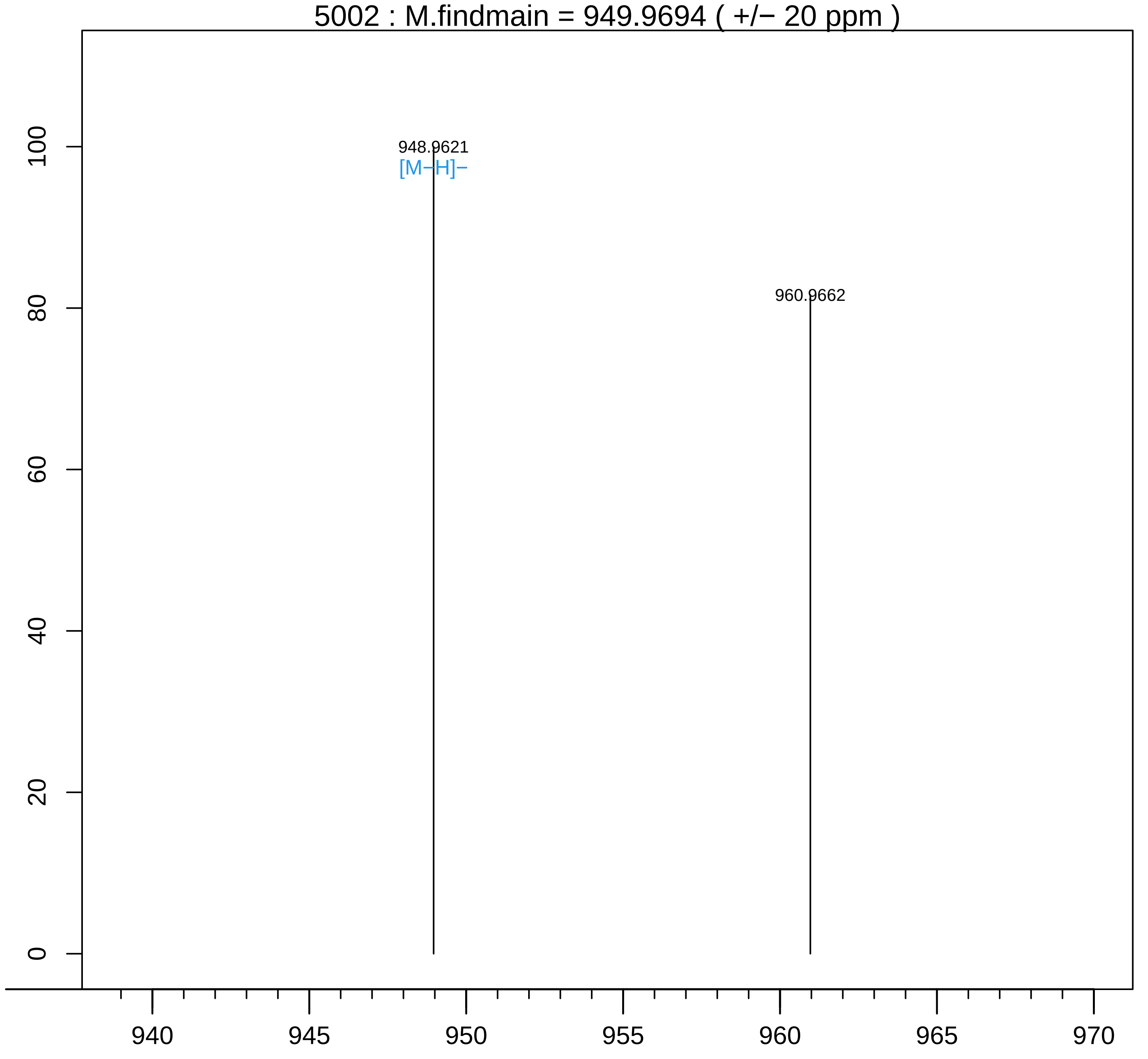
5002 : M.findmain = 949.9694 (+/− 20 ppm)
948.9621
[M−H]−
960.9662
0
20
40
60
80
100
940
945
950
955
960
965
970

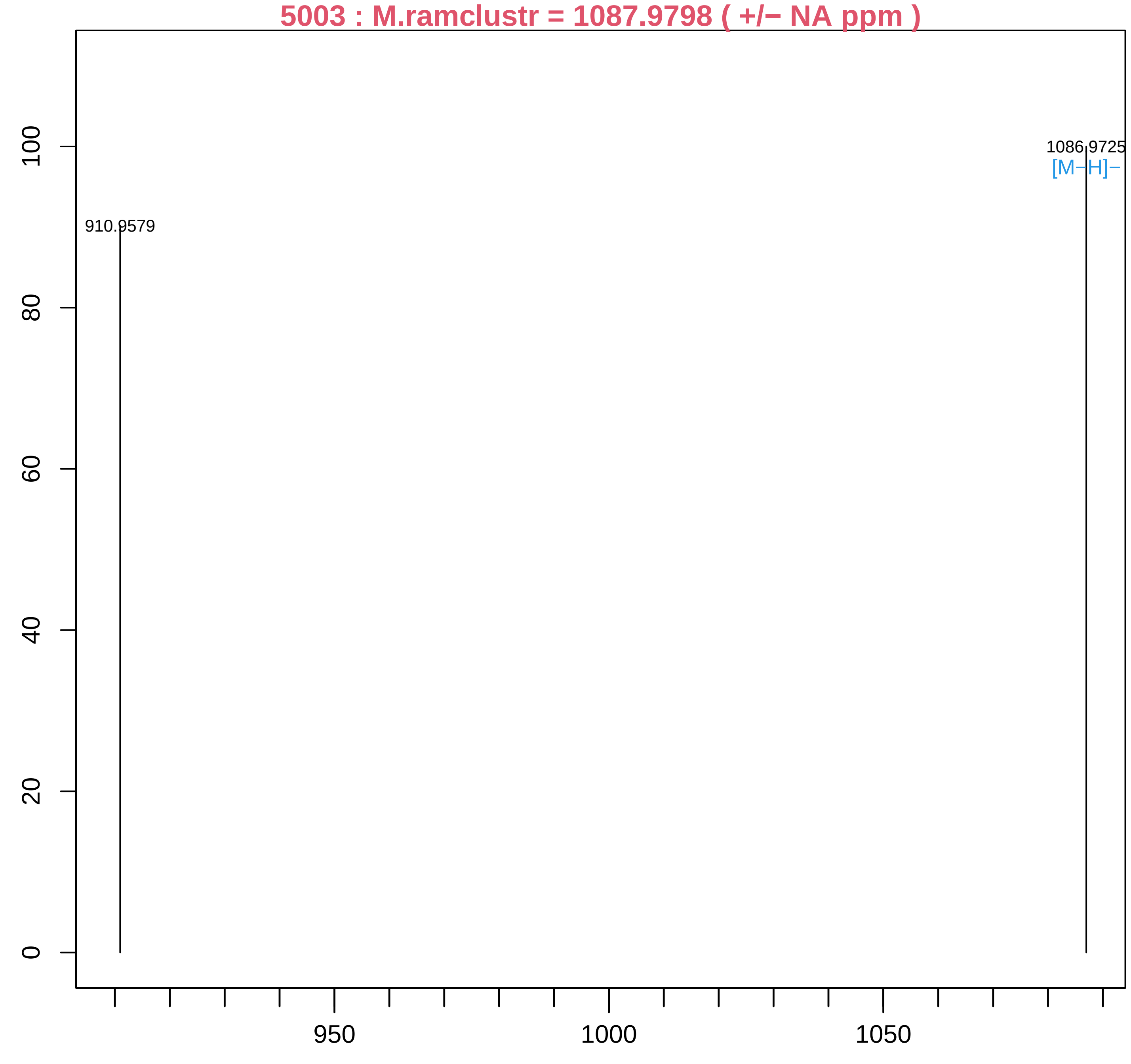
5003 : M.ramclustr = 1087.9798 (+/− NA ppm)
1086.9725
[M−H]−
910.9579
0
20
40
60
80
100
950
1000
1050

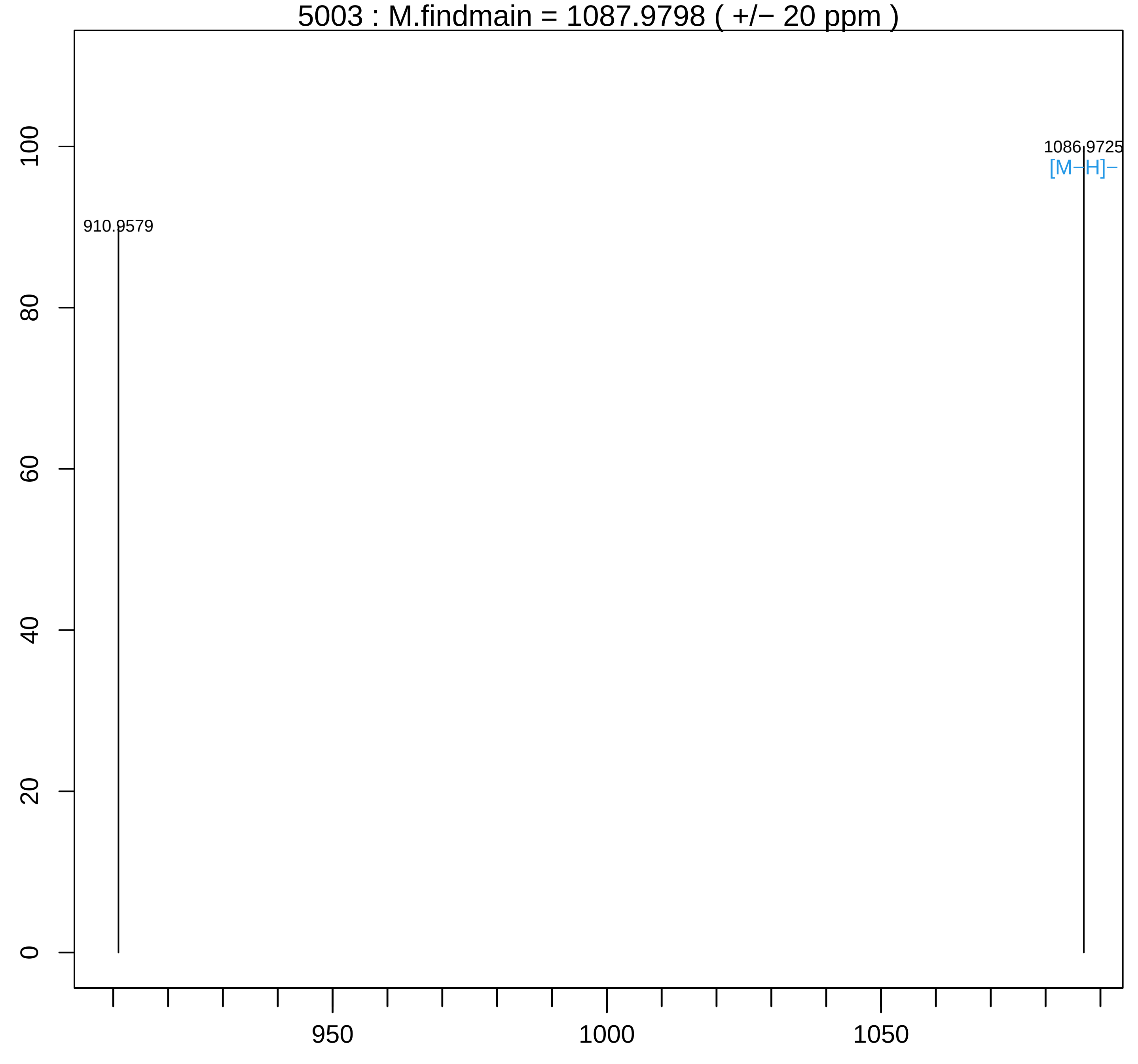
5003 : M.findmain = 1087.9798 (+/− 20 ppm)
1086.9725
[M−H]−
910.9579
0
20
40
60
80
100
950
1000
1050

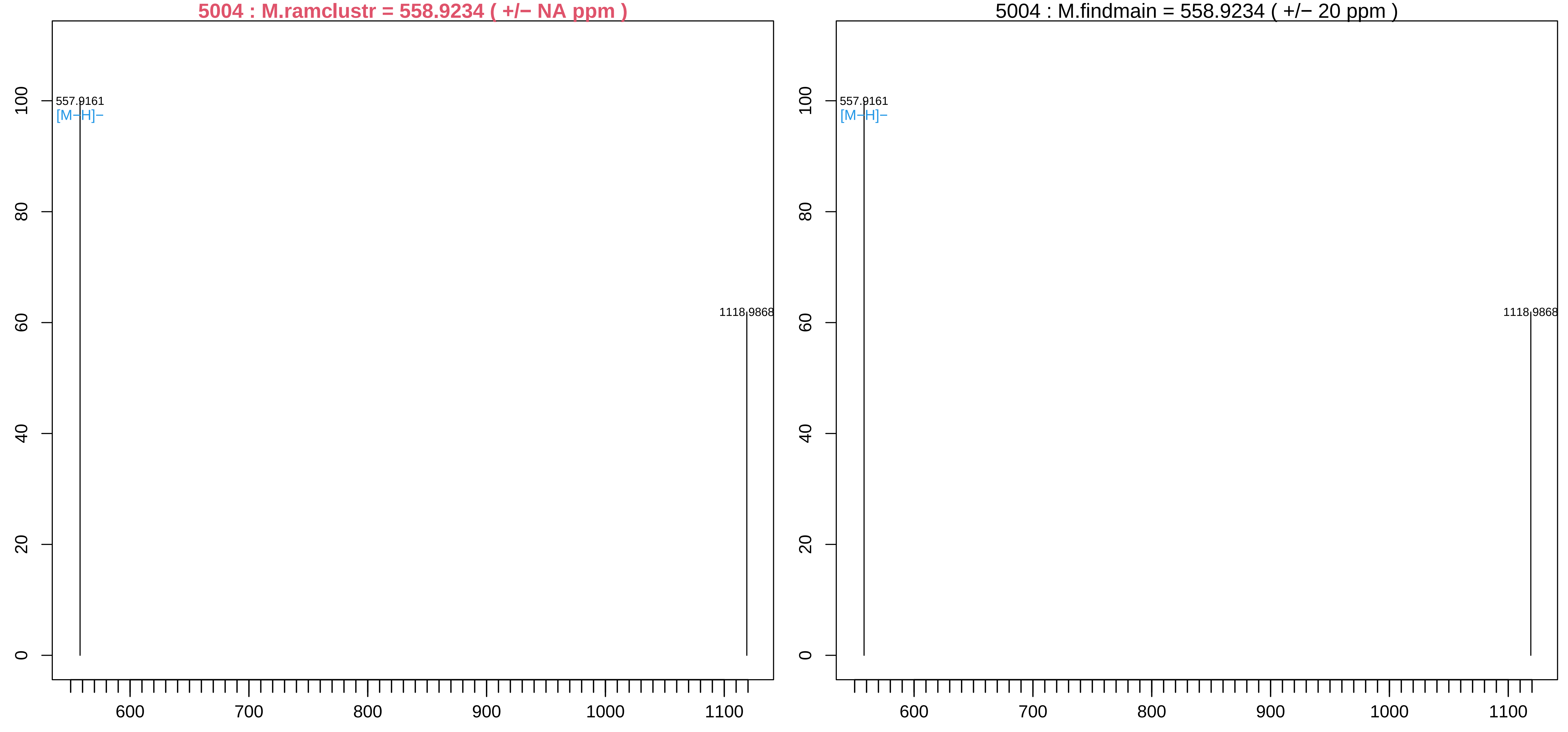

5004 : M.ramclustr = 558.9234 (+/− NA ppm)
557.9161
[M−H]−
1118.9868
0
20
40
60
80
100
600
700
800
900
1000
1100
5004 : M.findmain = 558.9234 (+/− 20 ppm)
557.9161
[M−H]−
1118.9868
0
20
40
60
80
100
600
700
800
900
1000
1100

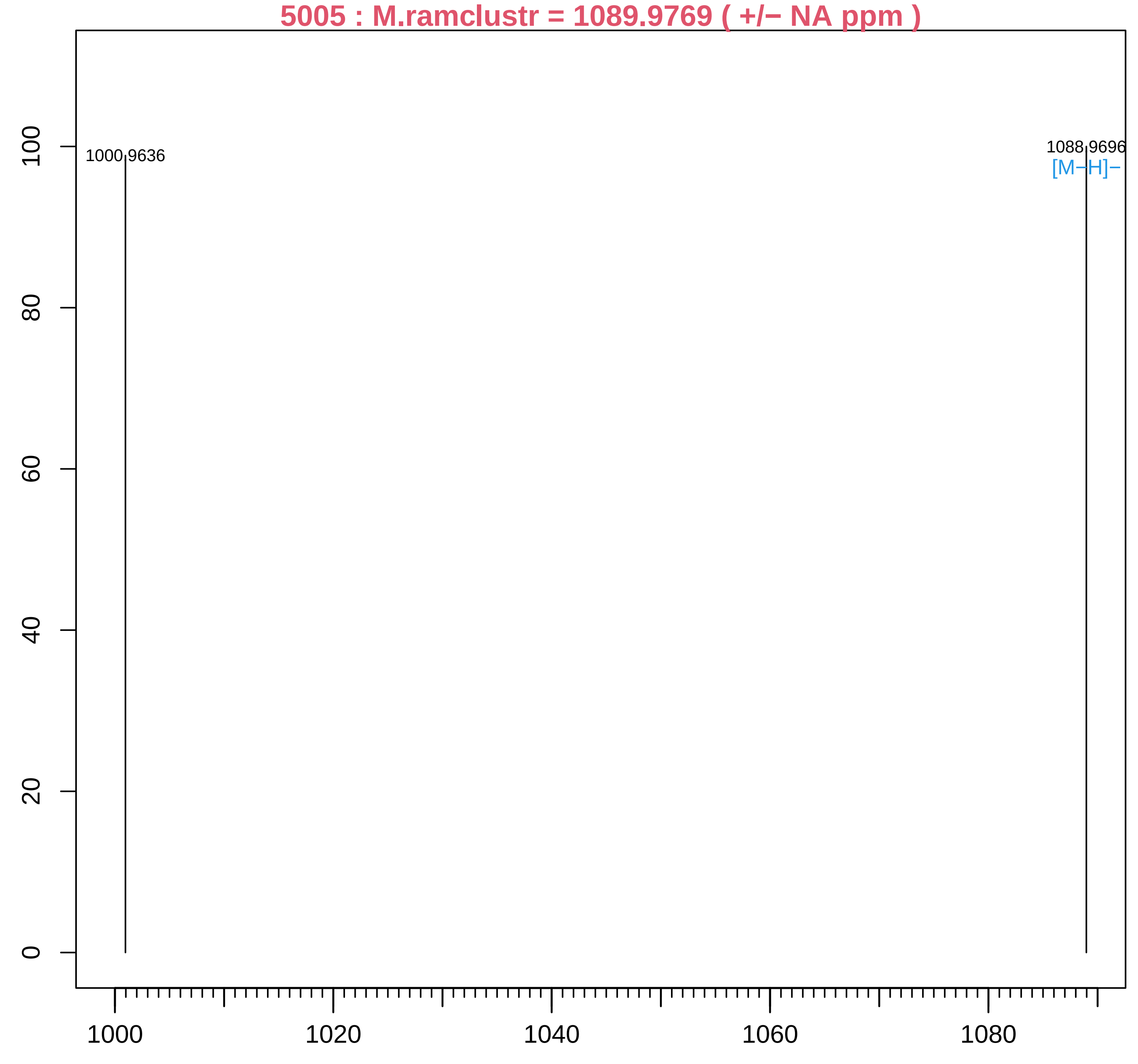
5005 : M.ramclustr = 1089.9769 (+/− NA ppm)
1000.9636
1088.9696
[M−H]−
0
20
40
60
80
100
1000
1020
1040
1060
1080
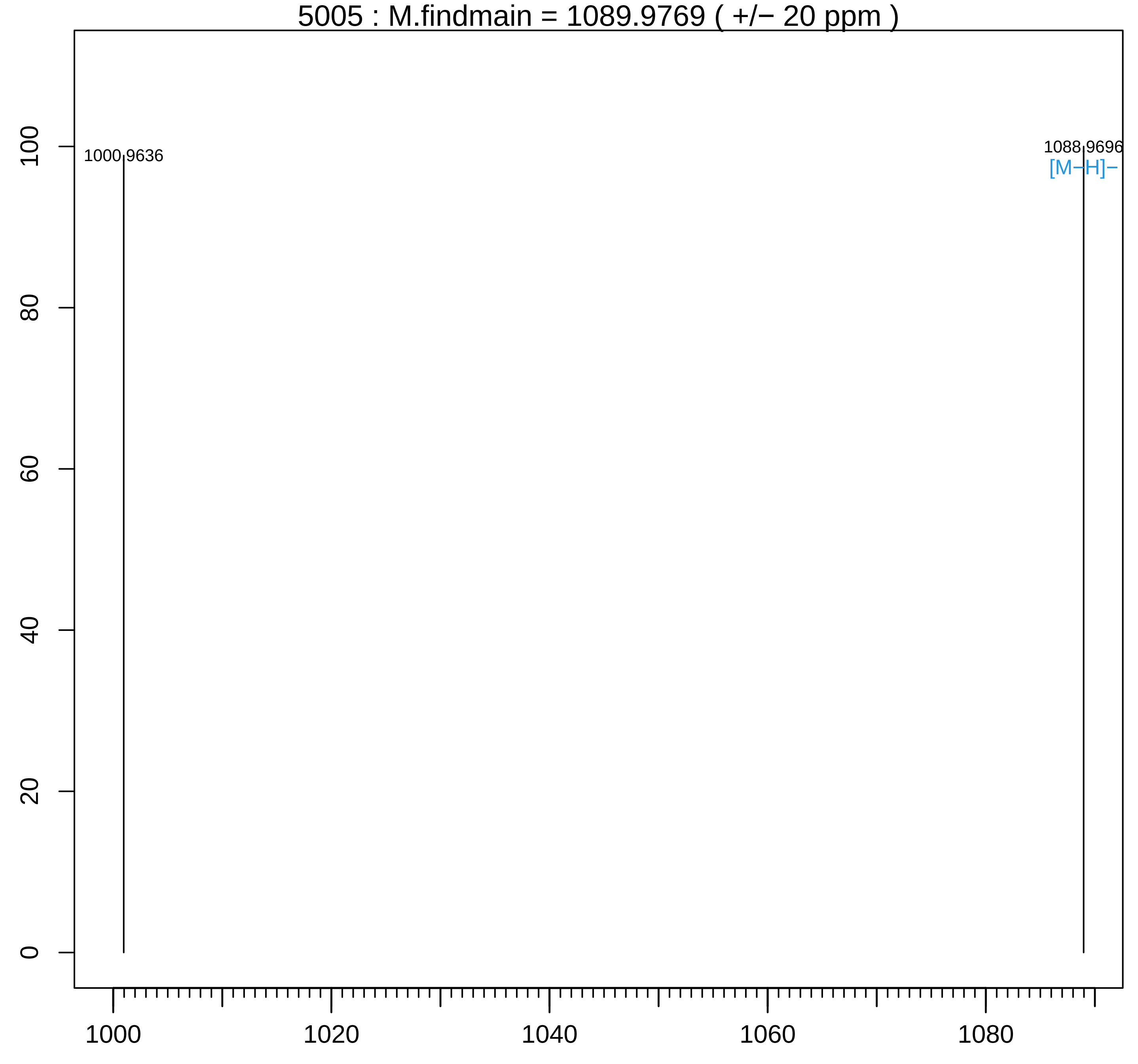
5005 : M.findmain = 1089.9769 (+/− 20 ppm)
1000.9636
1088.9696
[M−H]−
0
20
40
60
80
100
1000
1020
1040
1060
1080

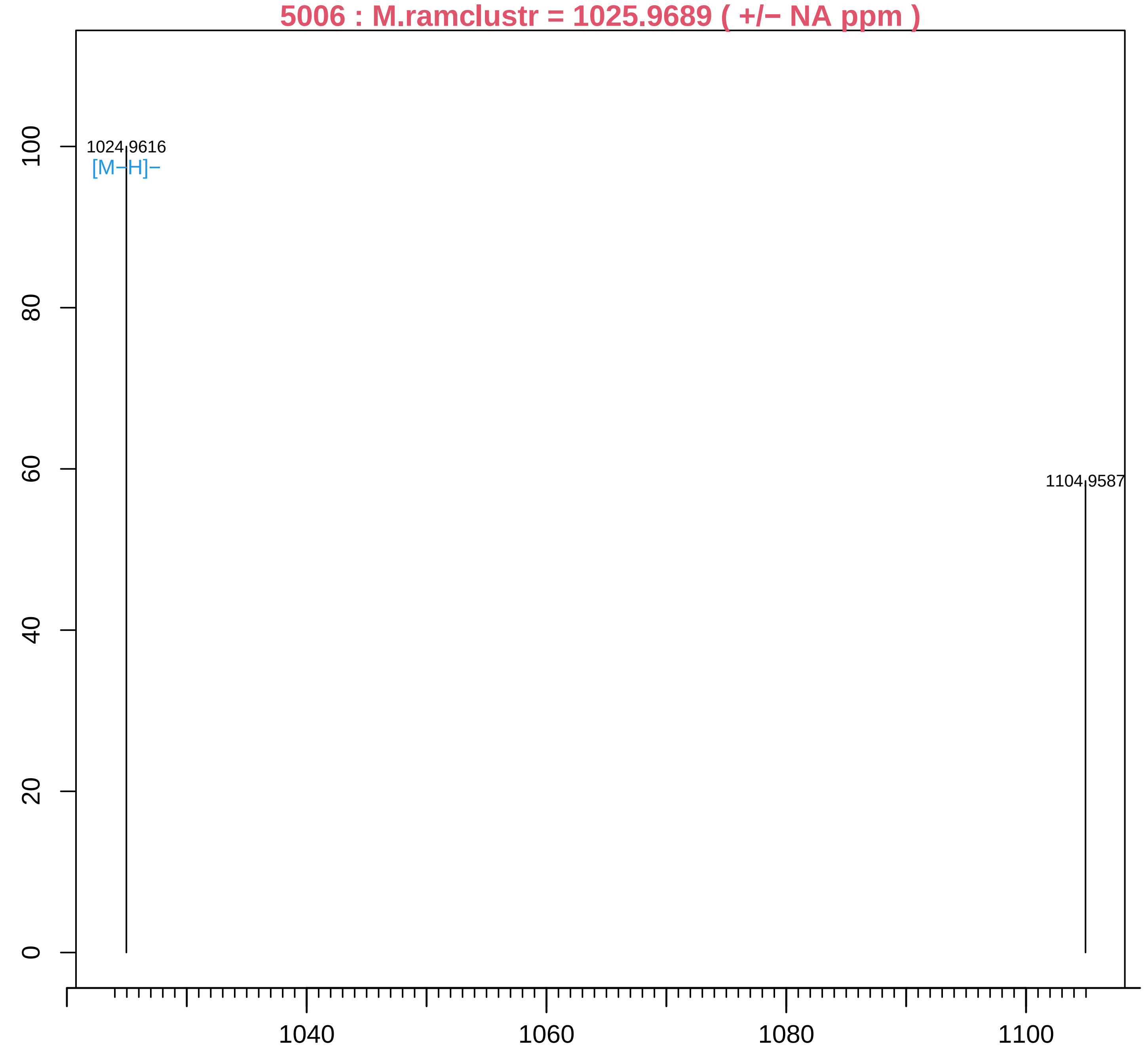
5006 : M.ramclustr = 1025.9689 (+/− NA ppm)
1024.9616
[M−H]−
1104.9587
0
20
40
60
80
100
1040
1060
1080
1100

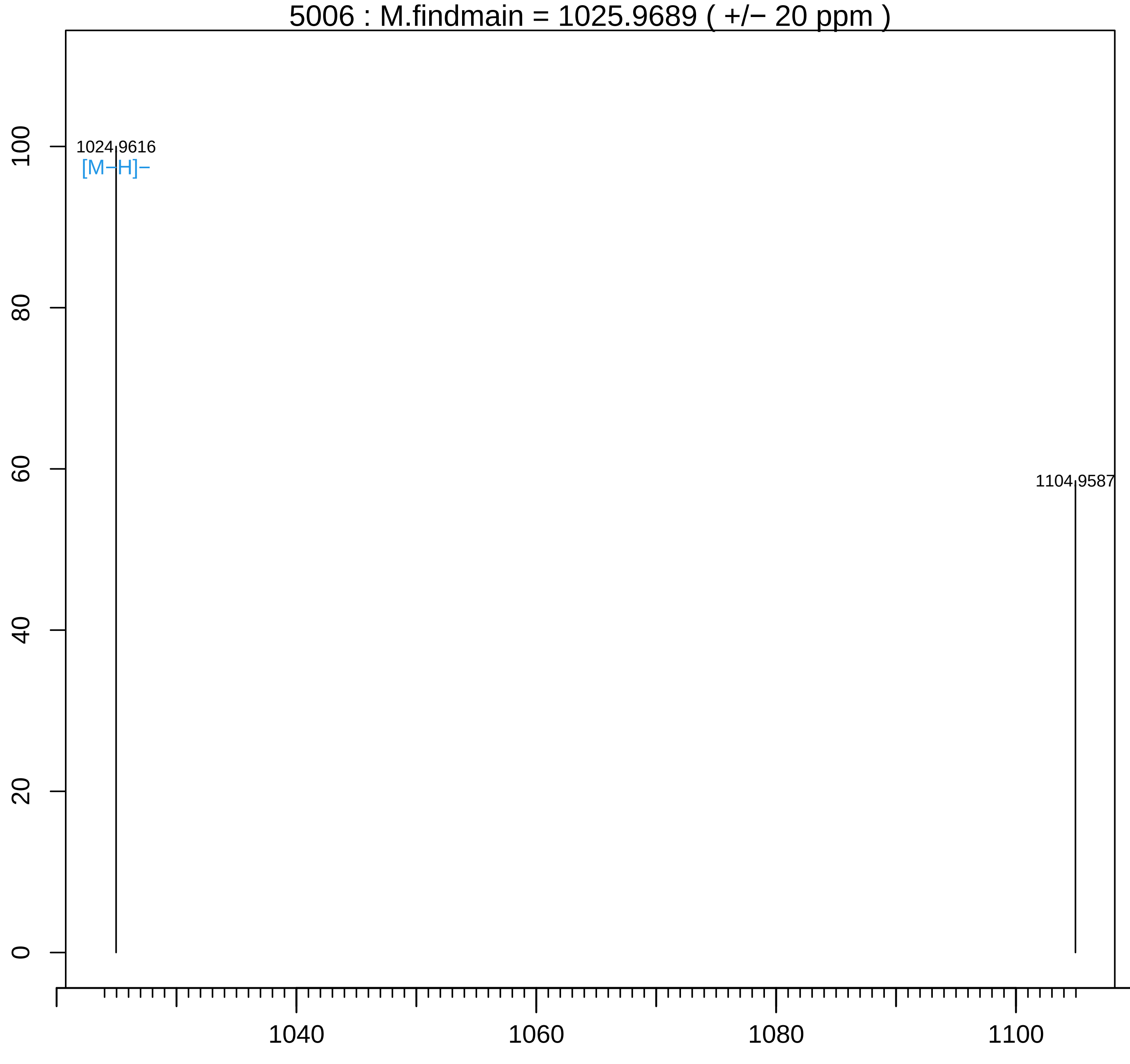
5006 : M.findmain = 1025.9689 (+/− 20 ppm)
1024.9616
[M−H]−
1104.9587
0
20
40
60
80
100
1040
1060
1080
1100

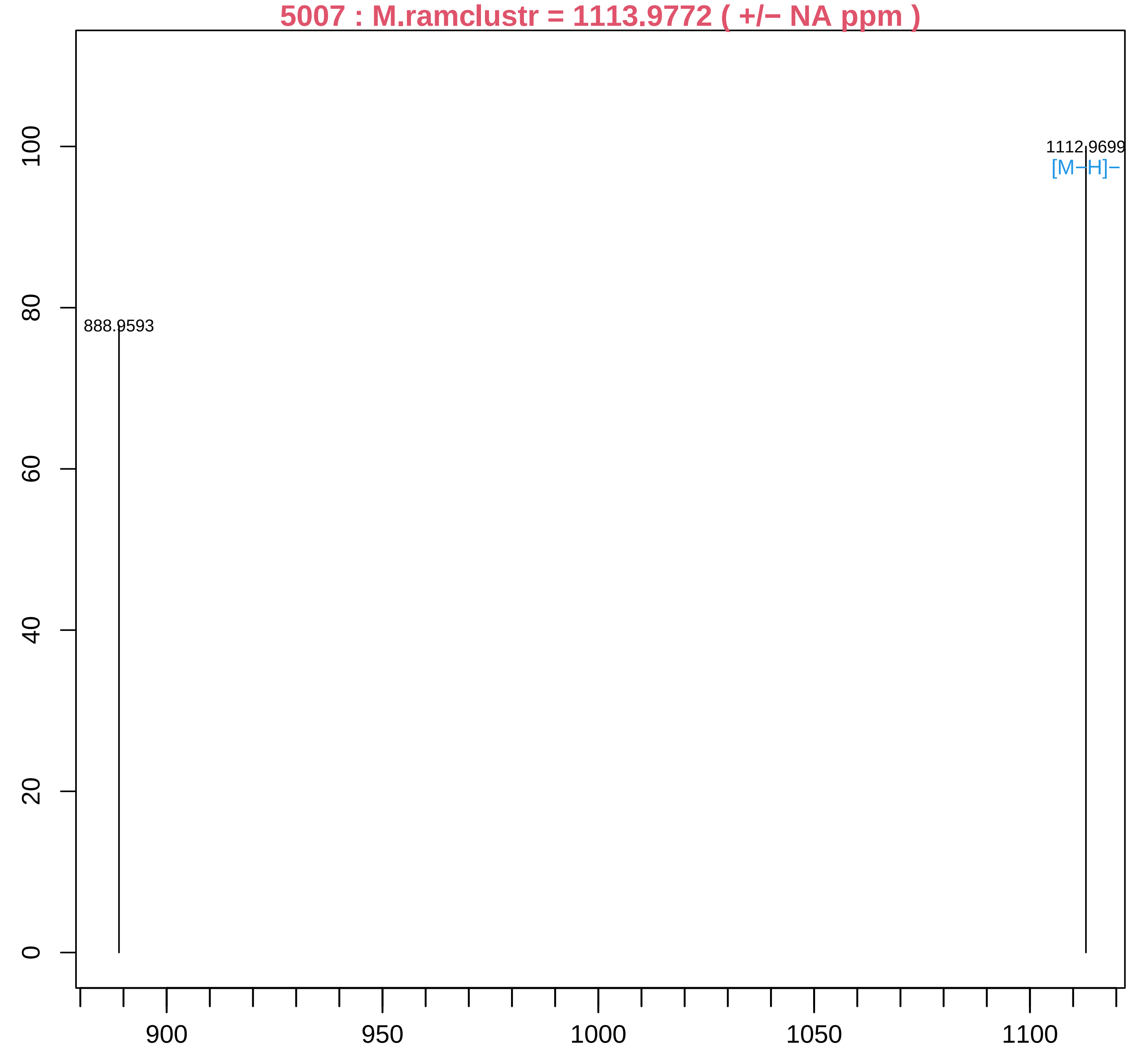
5007 : M.ramclustr = 1113.9772 (+/− NA ppm)
1112.9699
[M−H]−
888.9593
0
20
40
60
80
100
900
950
1000
1050
1100
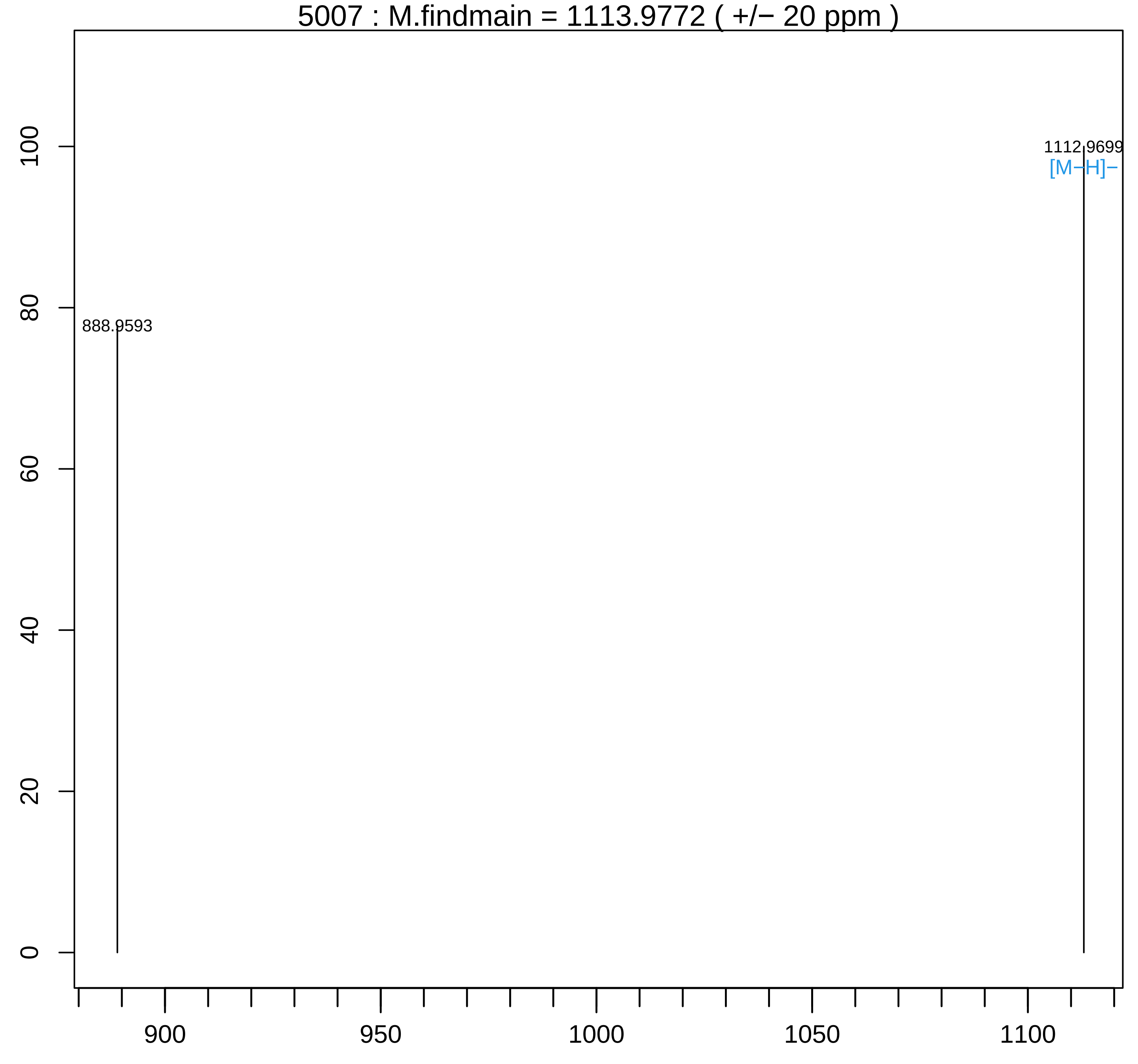
5007 : M.findmain = 1113.9772 (+/− 20 ppm)
1112.9699
[M−H]−
888.9593
0
20
40
60
80
100
900
950
1000
1050
1100

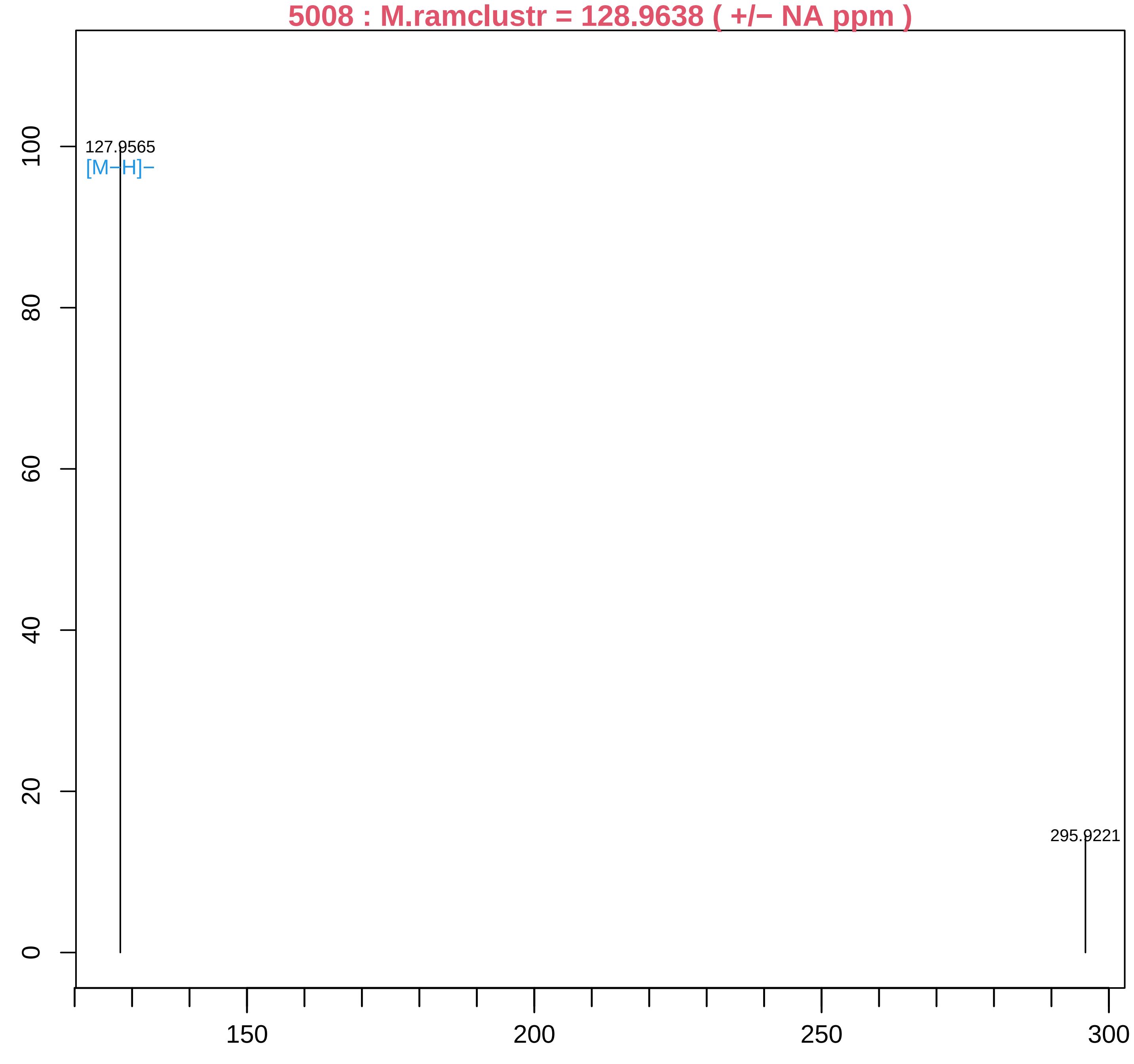
5008 : M.ramclustr = 128.9638 (+/− NA ppm)
127.9565
[M−H]−
295.9221
0
20
40
60
80
100
150
200
250
300

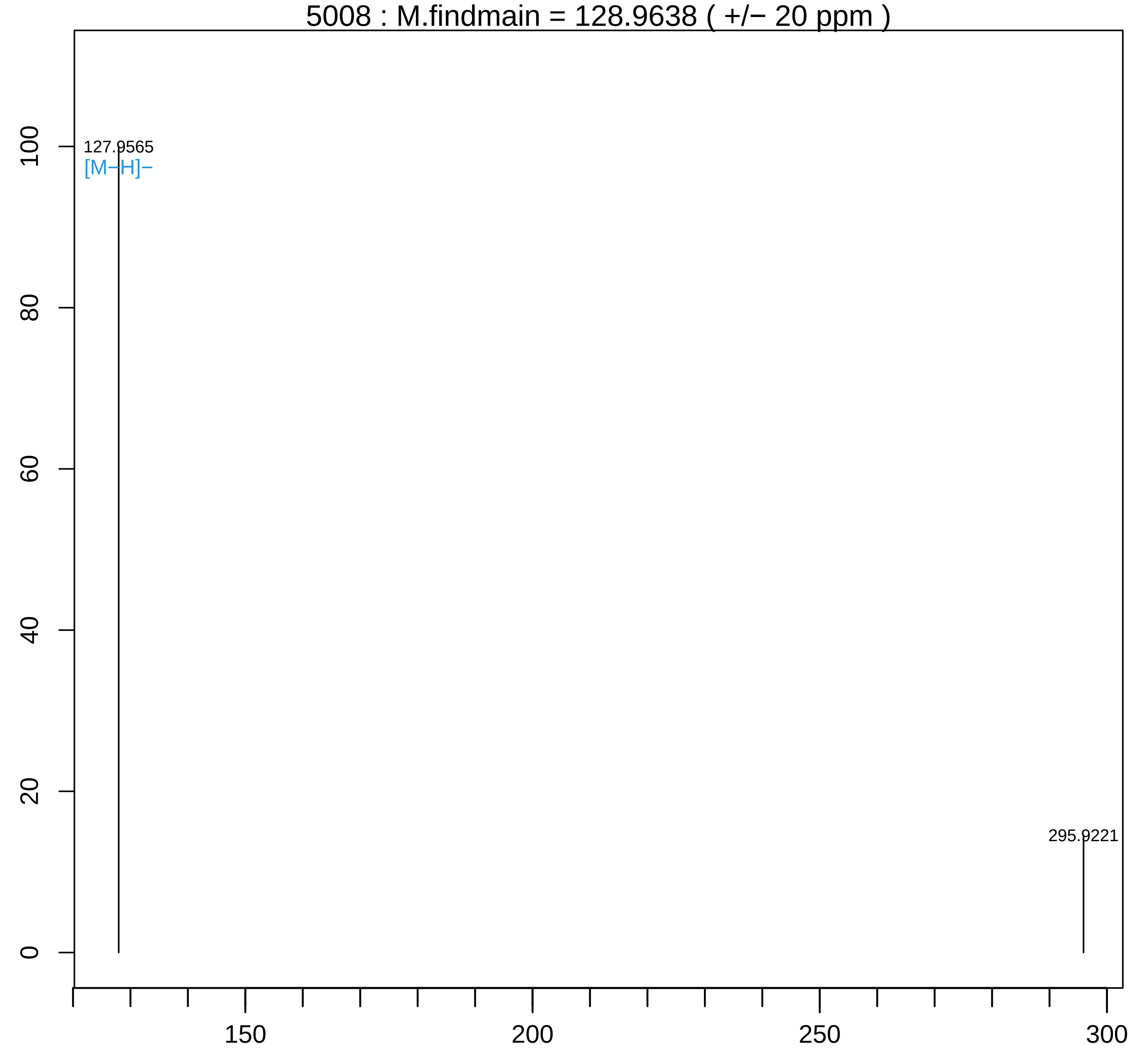
5008 : M.findmain = 128.9638 (+/− 20 ppm)
127.9565
[M−H]−
295.9221
0
20
40
60
80
100
150
200
250
300

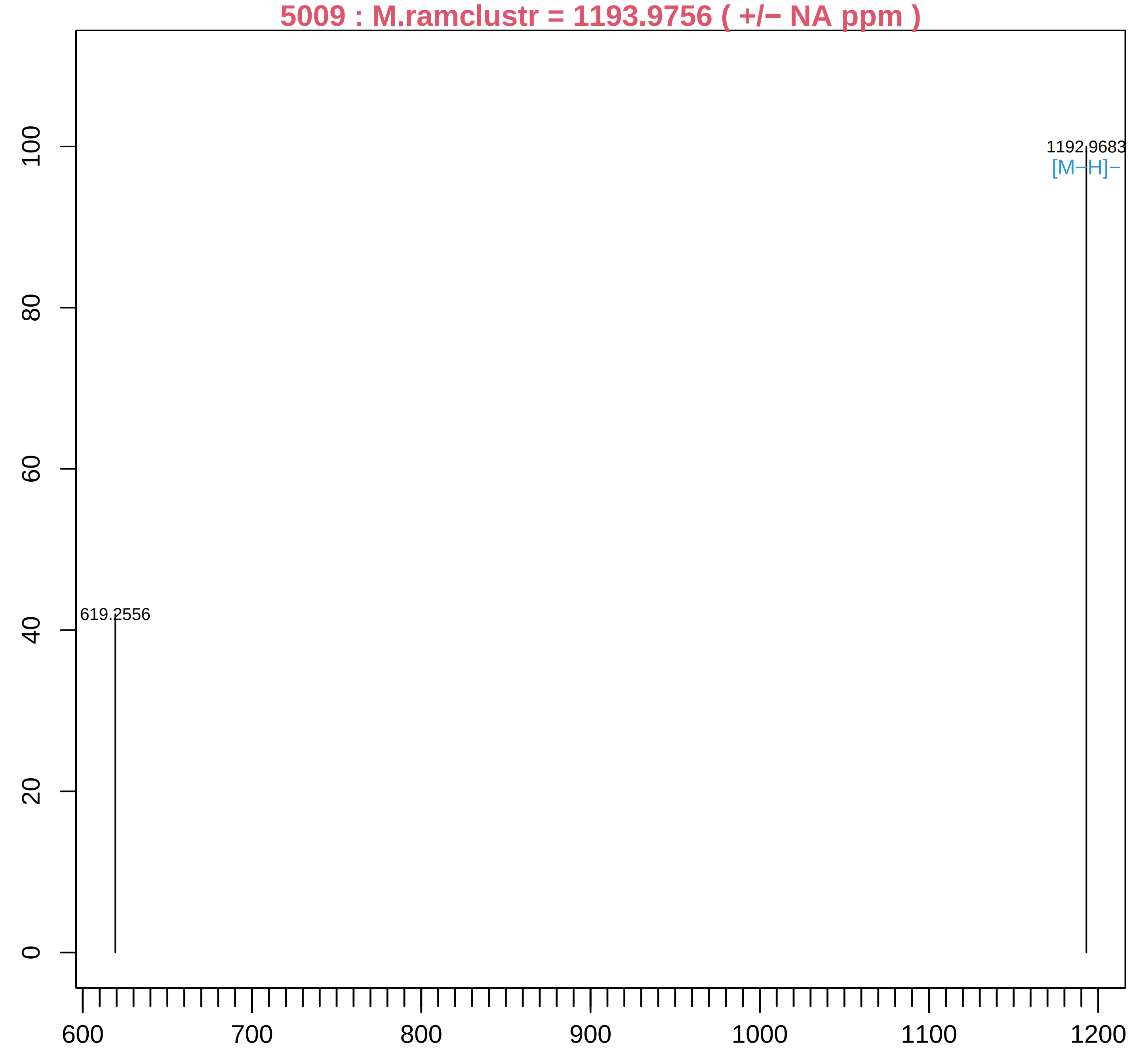
5009 : M.ramclustr = 1193.9756 (+/− NA ppm)
1192.9683
[M−H]−
619.2556
0
20
40
60
80
100
600
700
800
900
1000
1100
1200

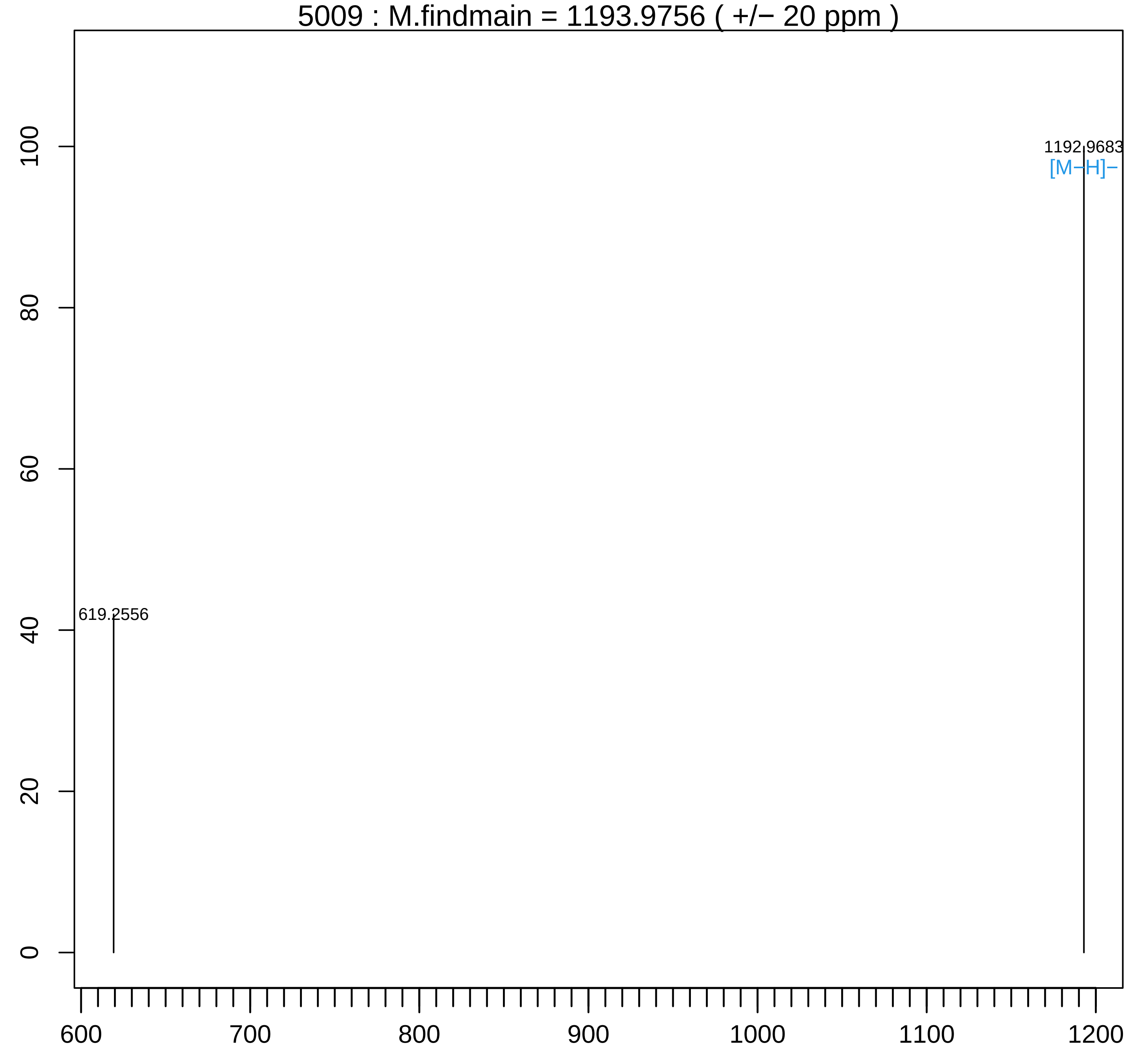
5009 : M.findmain = 1193.9756 (+/− 20 ppm)
1192.9683
[M−H]−
619.2556
0
20
40
60
80
100
600
700
800
900
1000
1100
1200

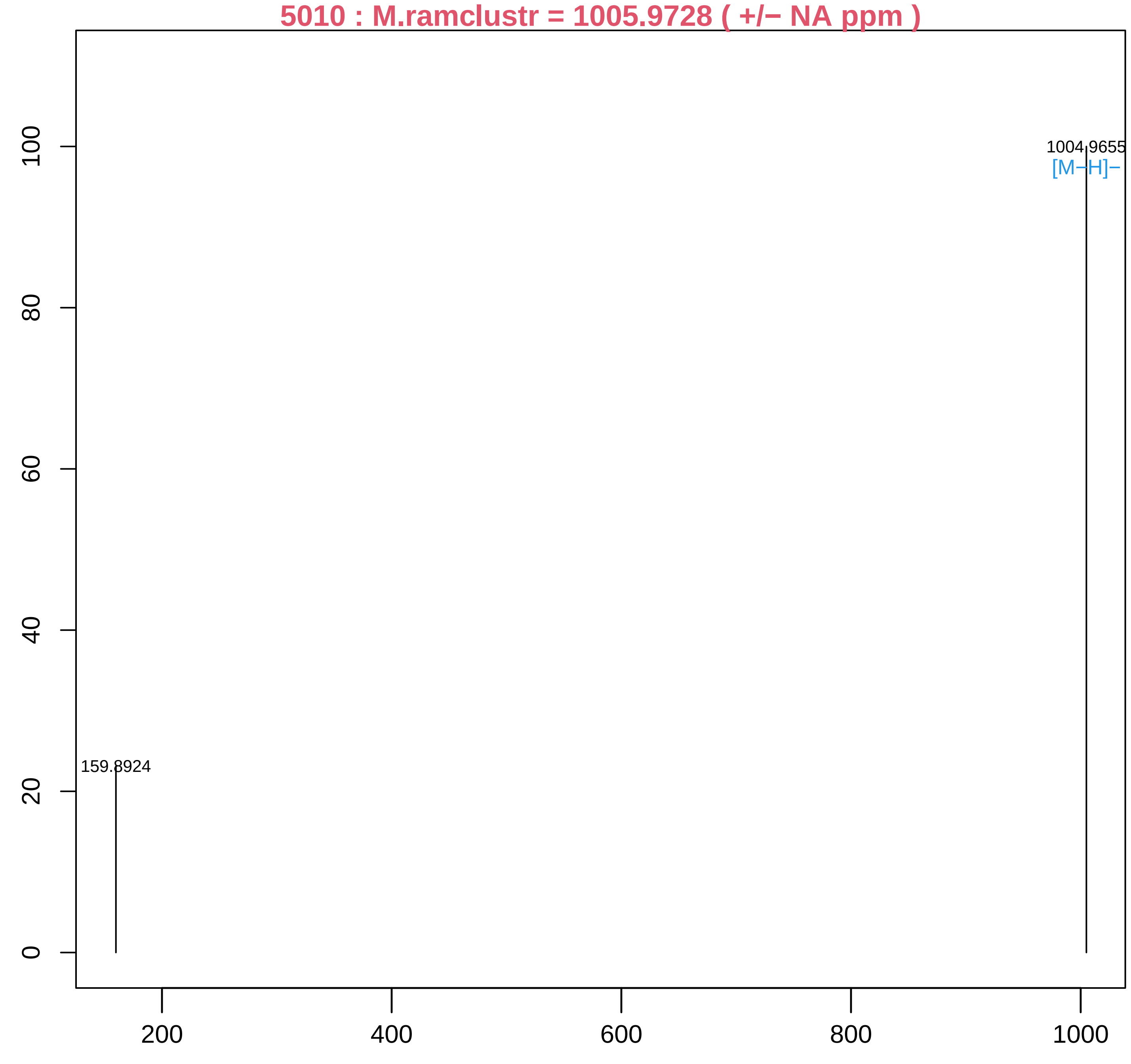

5010 : M.ramclustr = 1005.9728 (+/− NA ppm)
1004.9655
[M−H]−
159.8924
0
20
40
60
80
100
200
400
600
800
1000

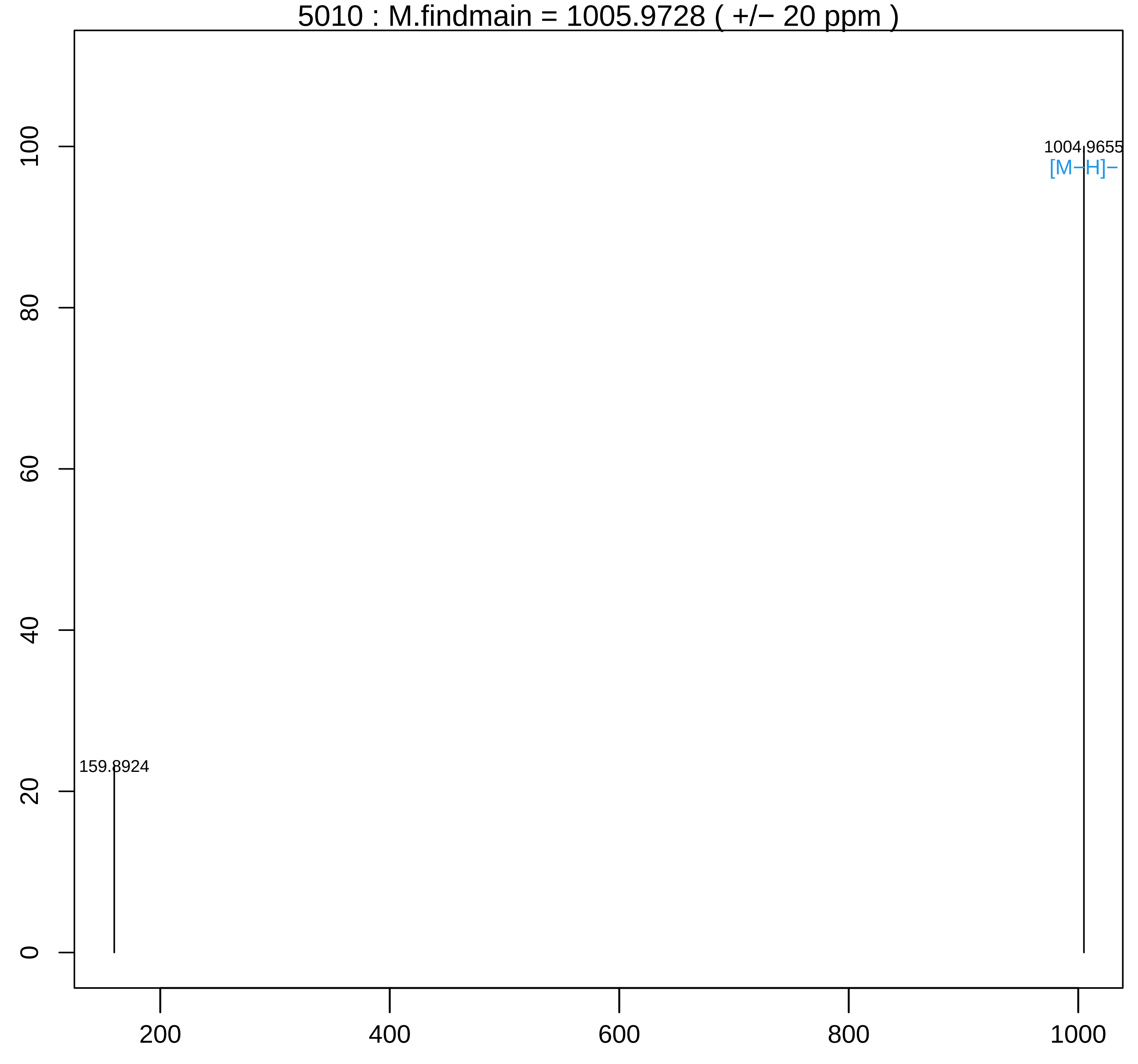

5010 : M.findmain = 1005.9728 (+/− 20 ppm)
1004.9655
[M−H]−
159.8924
0
20
40
60
80
100
200
400
600
800
1000

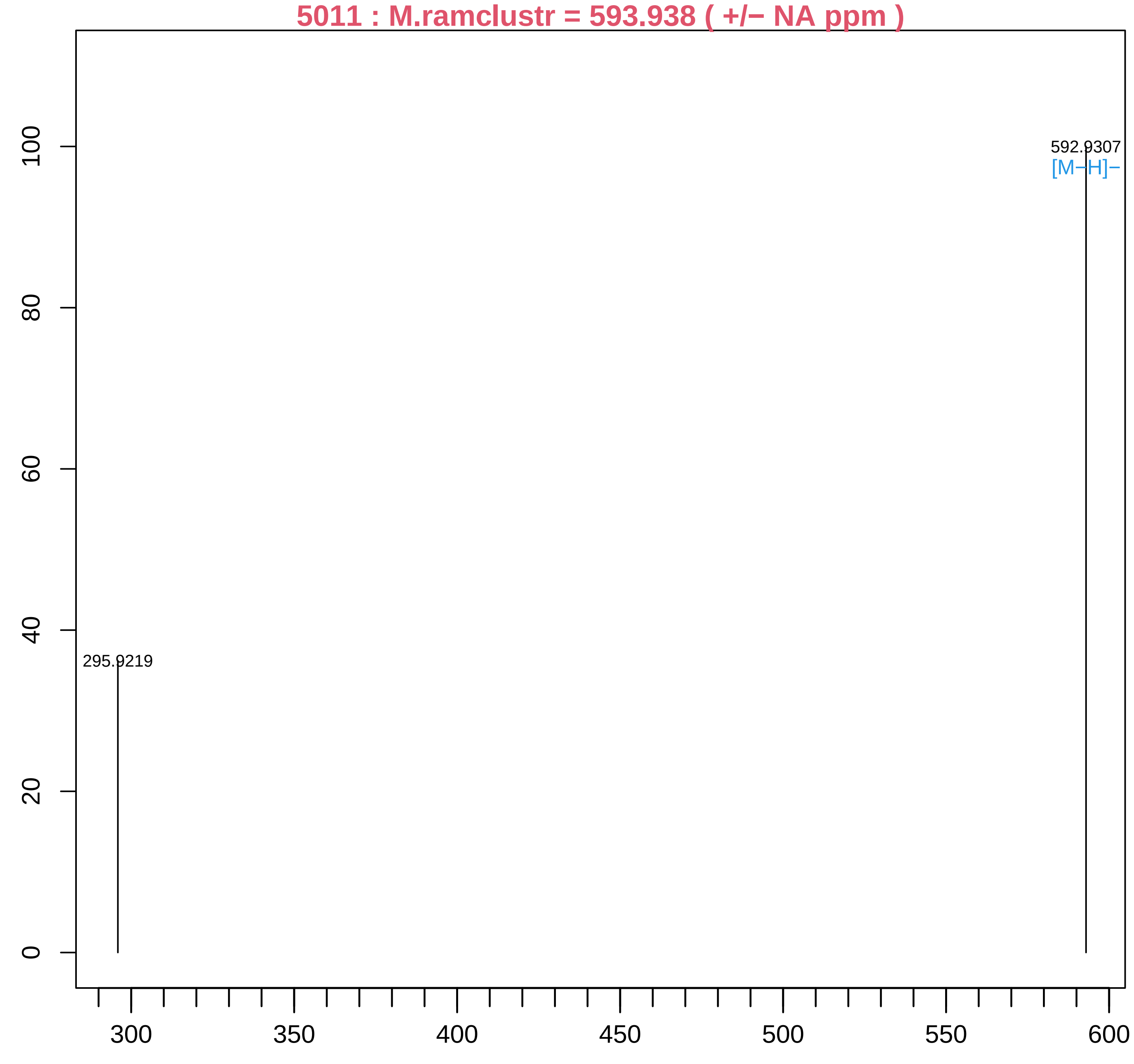
5011 : M.ramclustr = 593.938 (+/− NA ppm)
592.9307
[M−H]−
295.9219
0
20
40
60
80
100
300
350
400
450
500
550
600

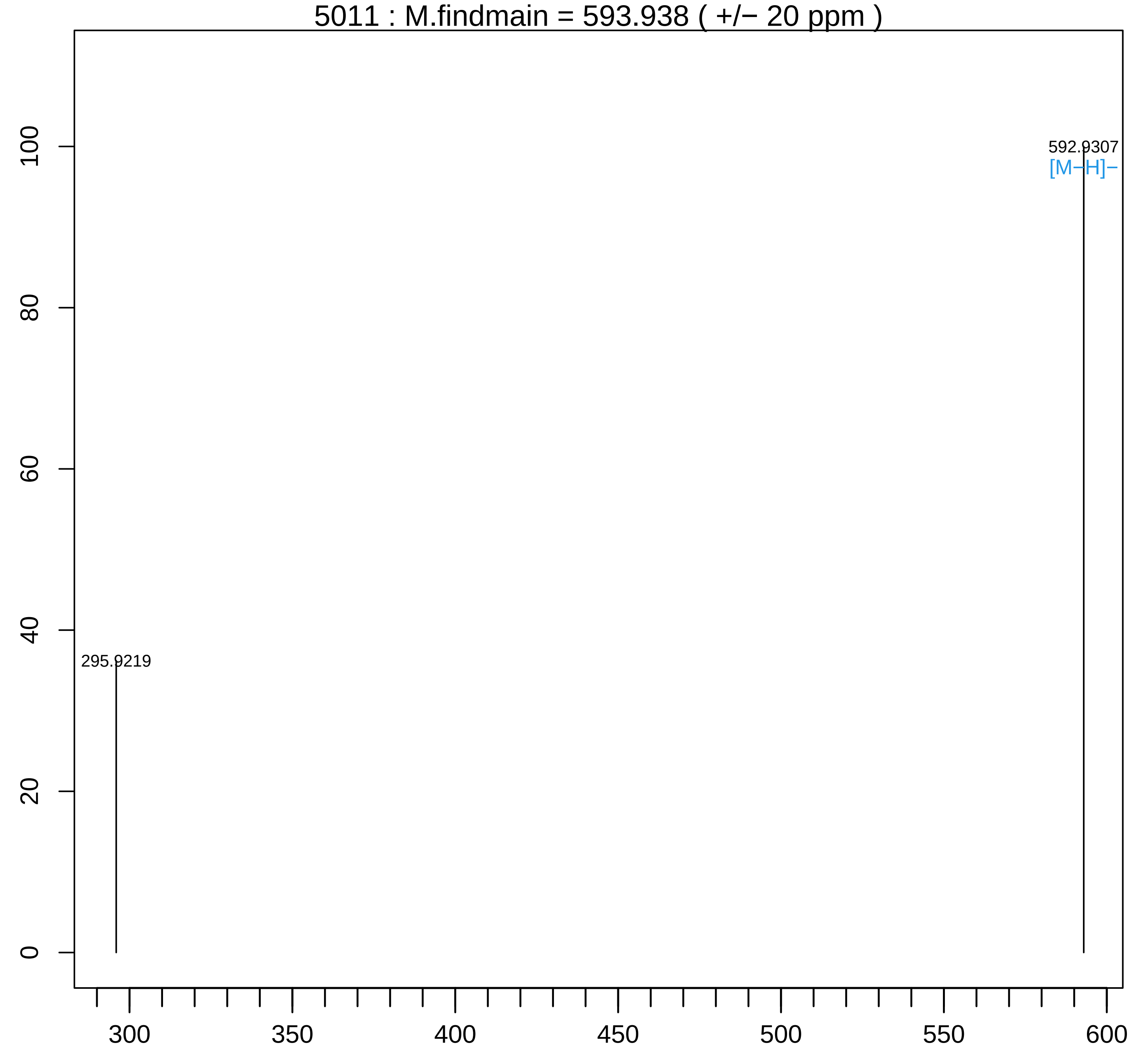
5011 : M.findmain = 593.938 (+/− 20 ppm)
592.9307
[M−H]−
295.9219
0
20
40
60
80
100
300
350
400
450
500
550
600

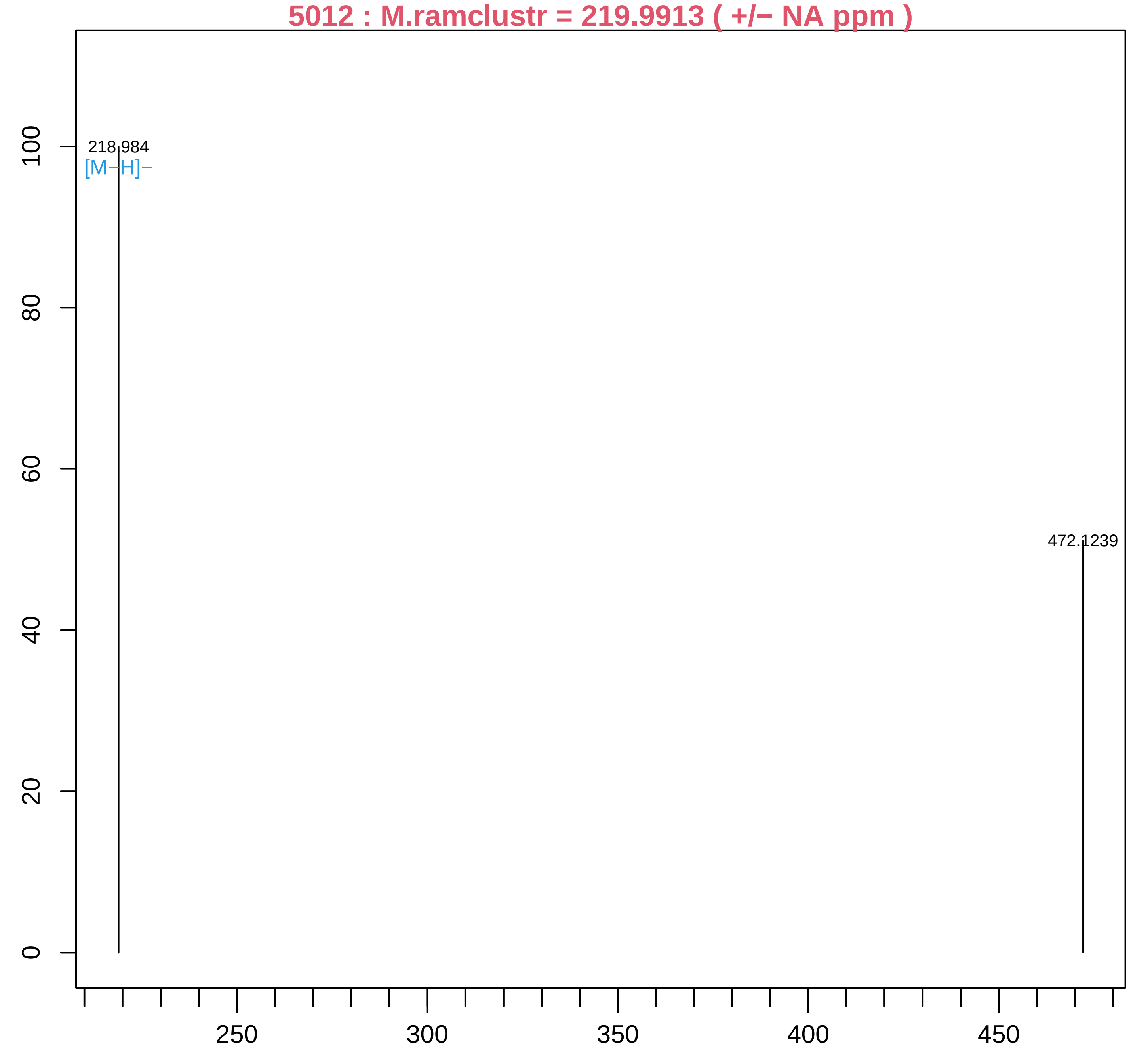
5012 : M.ramclustr = 219.9913 (+/− NA ppm)
218.984
[M−H]−
472.1239
0
20
40
60
80
100
250
300
350
400
450

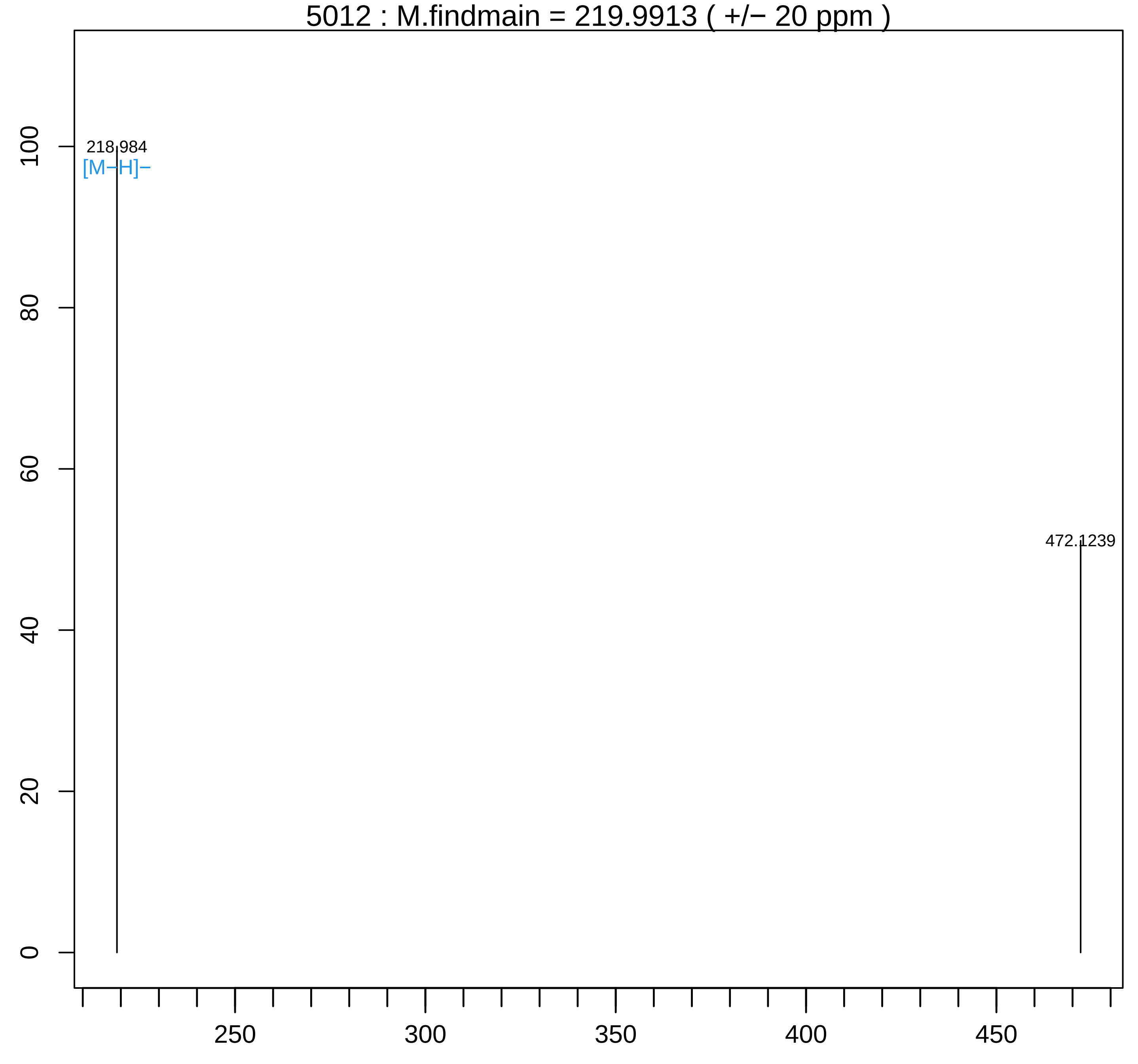
5012 : M.findmain = 219.9913 (+/− 20 ppm)
218.984
[M−H]−
472.1239
0
20
40
60
80
100
250
300
350
400
450

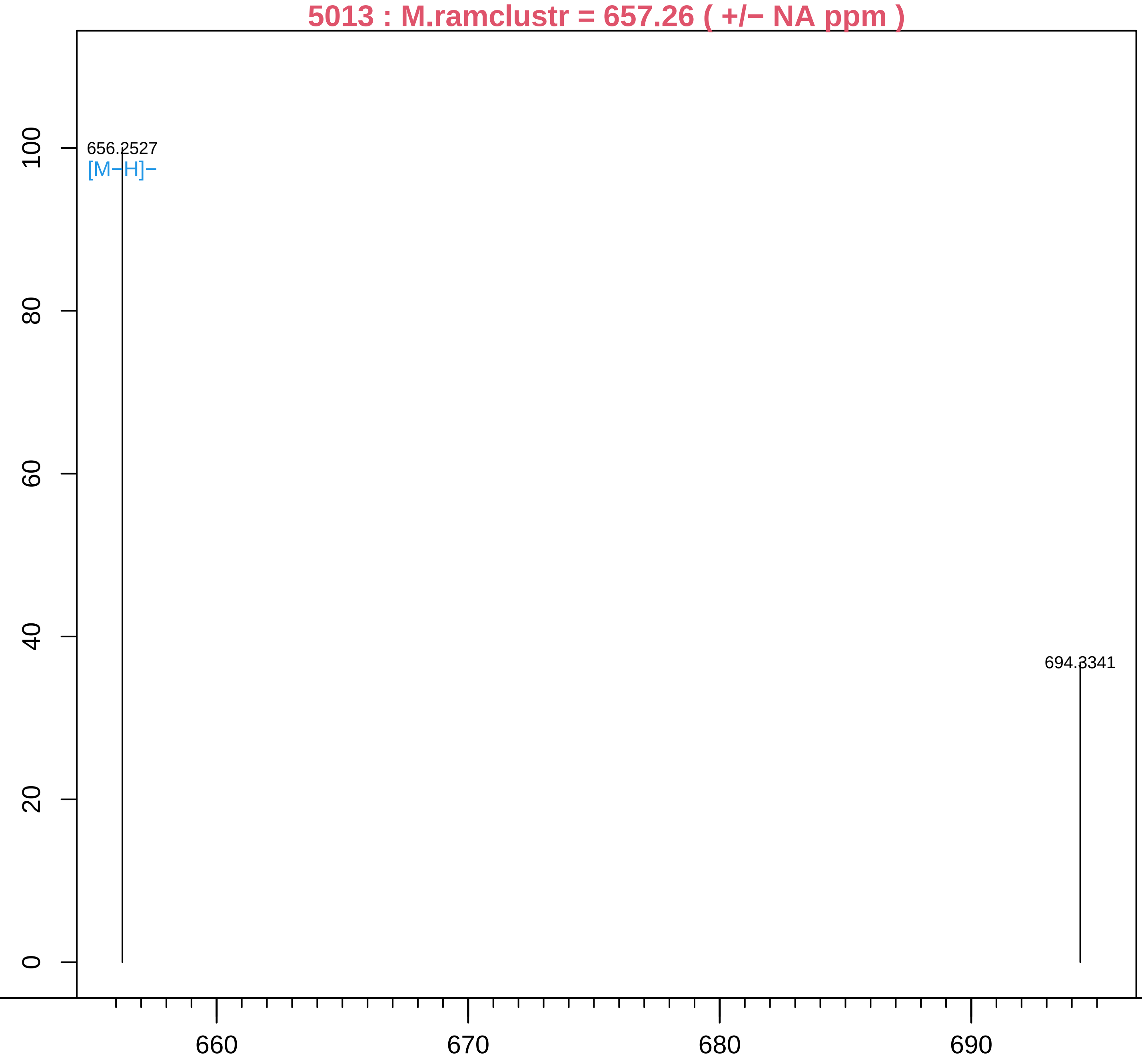
5013 : M.ramclustr = 657.26 (+/− NA ppm)
656.2527
[M−H]−
694.3341
0
20
40
60
80
100
660
670
680
690

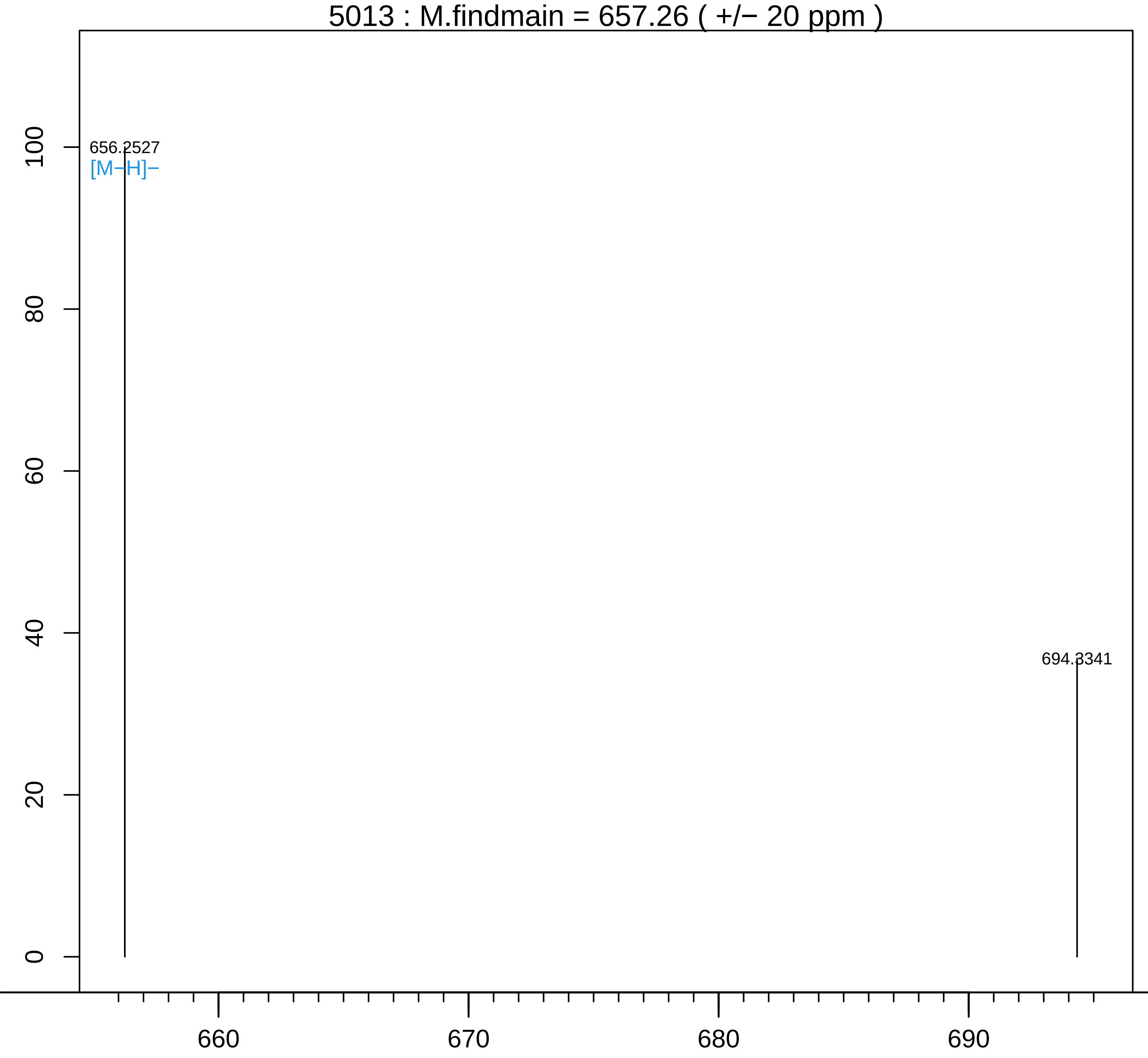
5013 : M.findmain = 657.26 (+/− 20 ppm)
656.2527
[M−H]−
694.3341
0
20
40
60
80
100
660
670
680
690

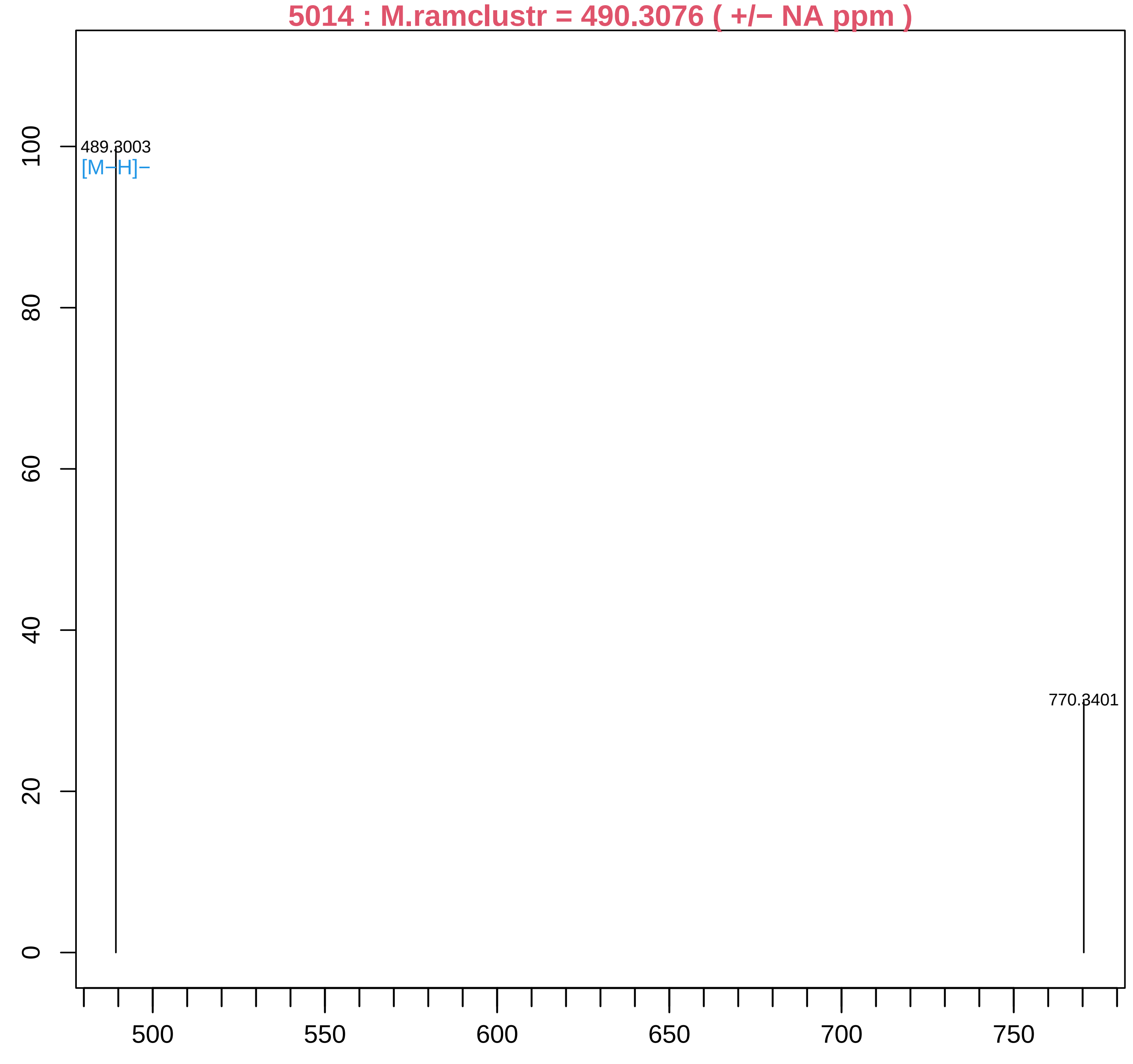
5014 : M.ramclustr = 490.3076 (+/− NA ppm)
489.3003
[M−H]−
770.3401
0
20
40
60
80
100
500
550
600
650
700
750

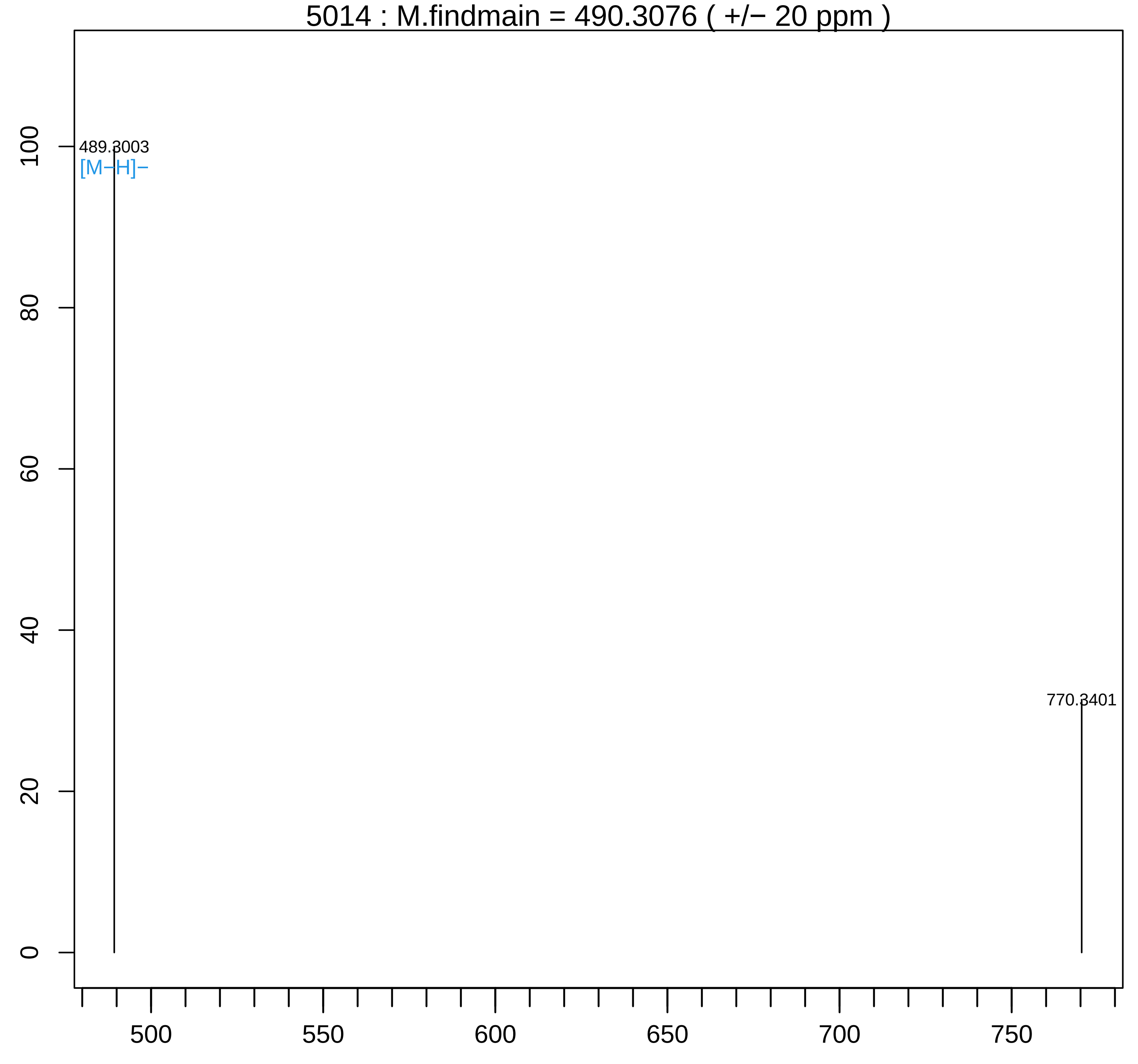
5014 : M.findmain = 490.3076 (+/− 20 ppm)
489.3003
[M−H]−
770.3401
0
20
40
60
80
100
500
550
600
650
700
750

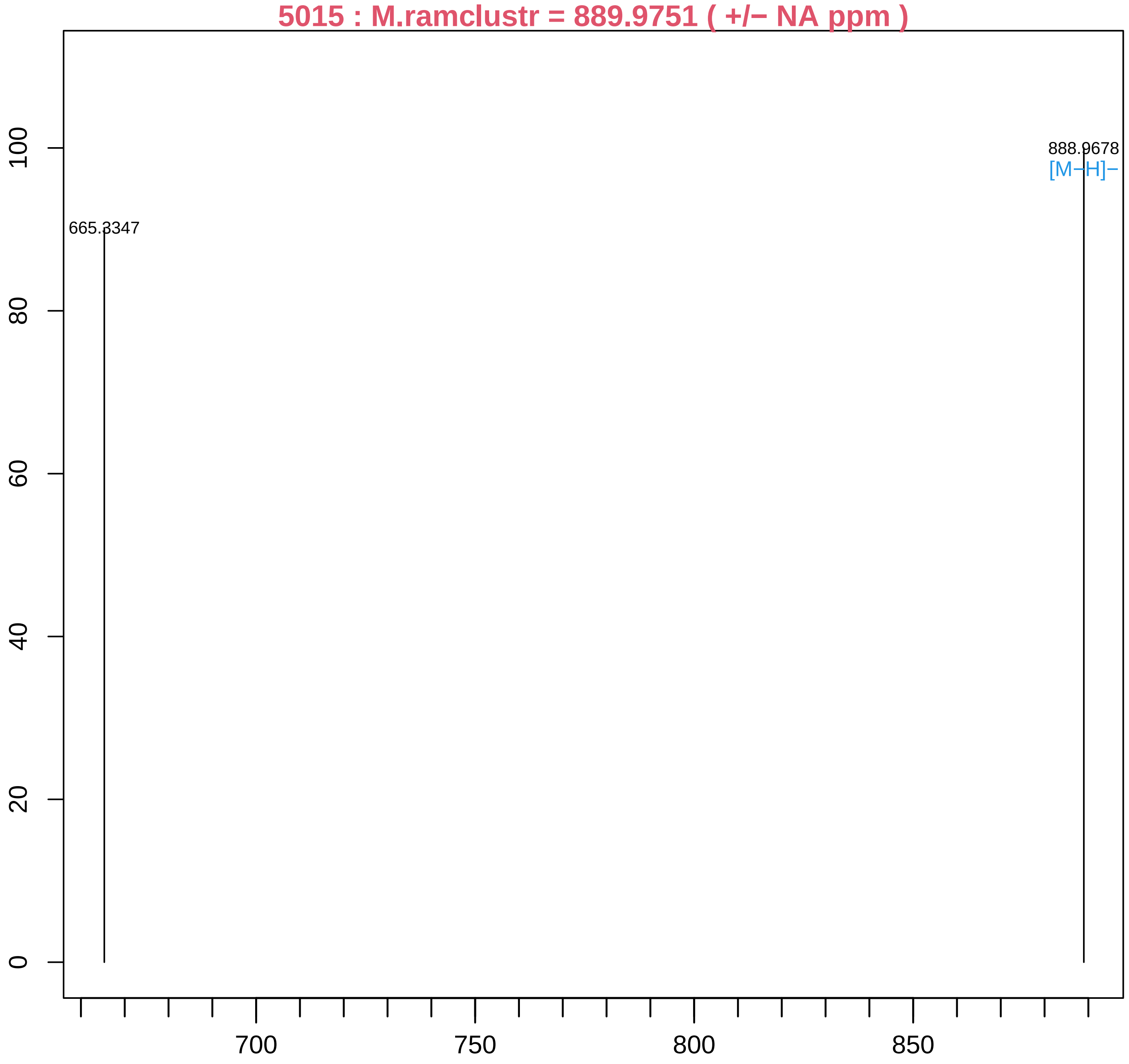
5015 : M.ramclustr = 889.9751 (+/− NA ppm)
888.9678
[M−H]−
665.3347
0
20
40
60
80
100
700
750
800
850

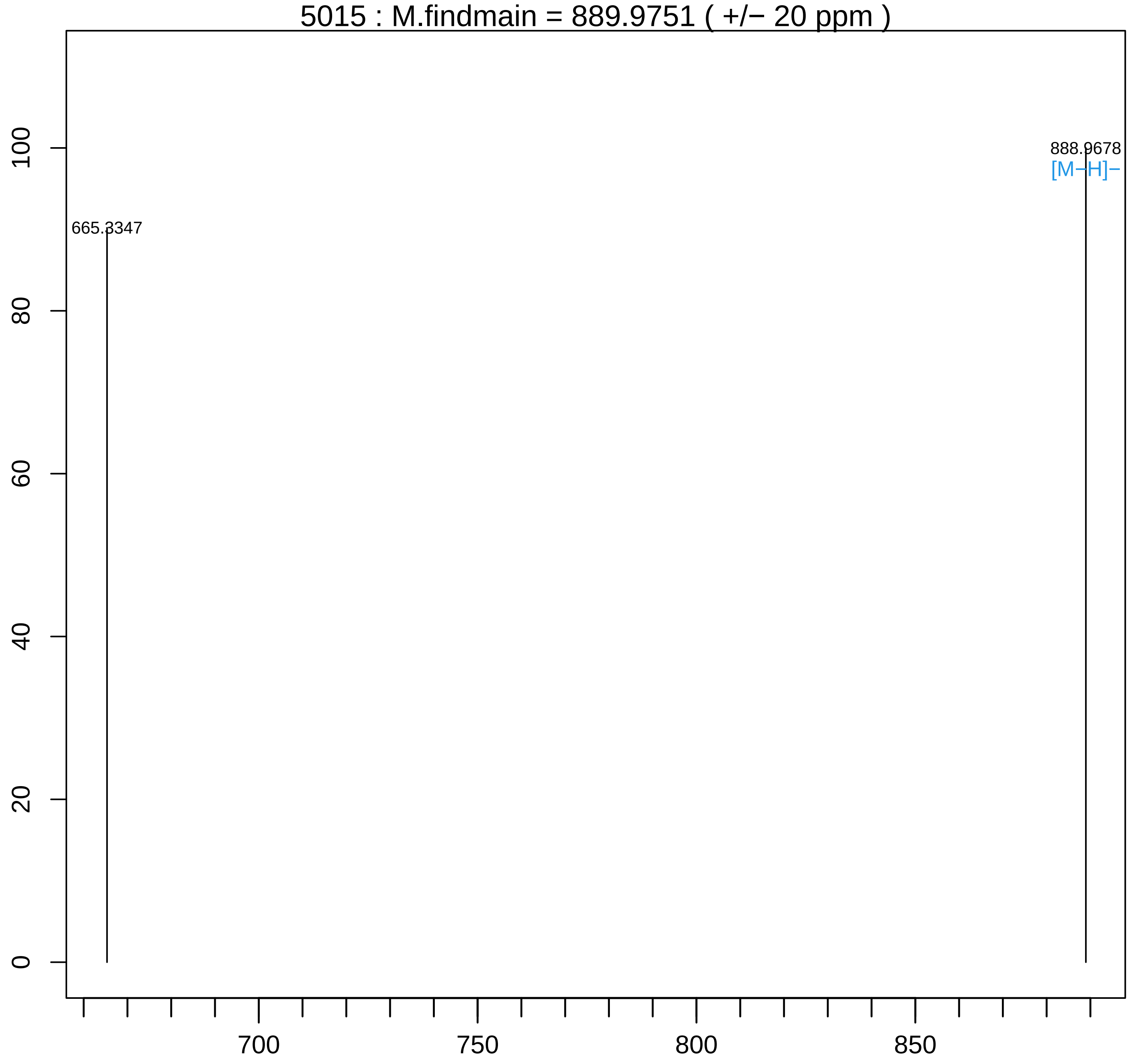
5015 : M.findmain = 889.9751 (+/− 20 ppm)
888.9678
[M−H]−
665.3347
0
20
40
60
80
100
700
750
800
850

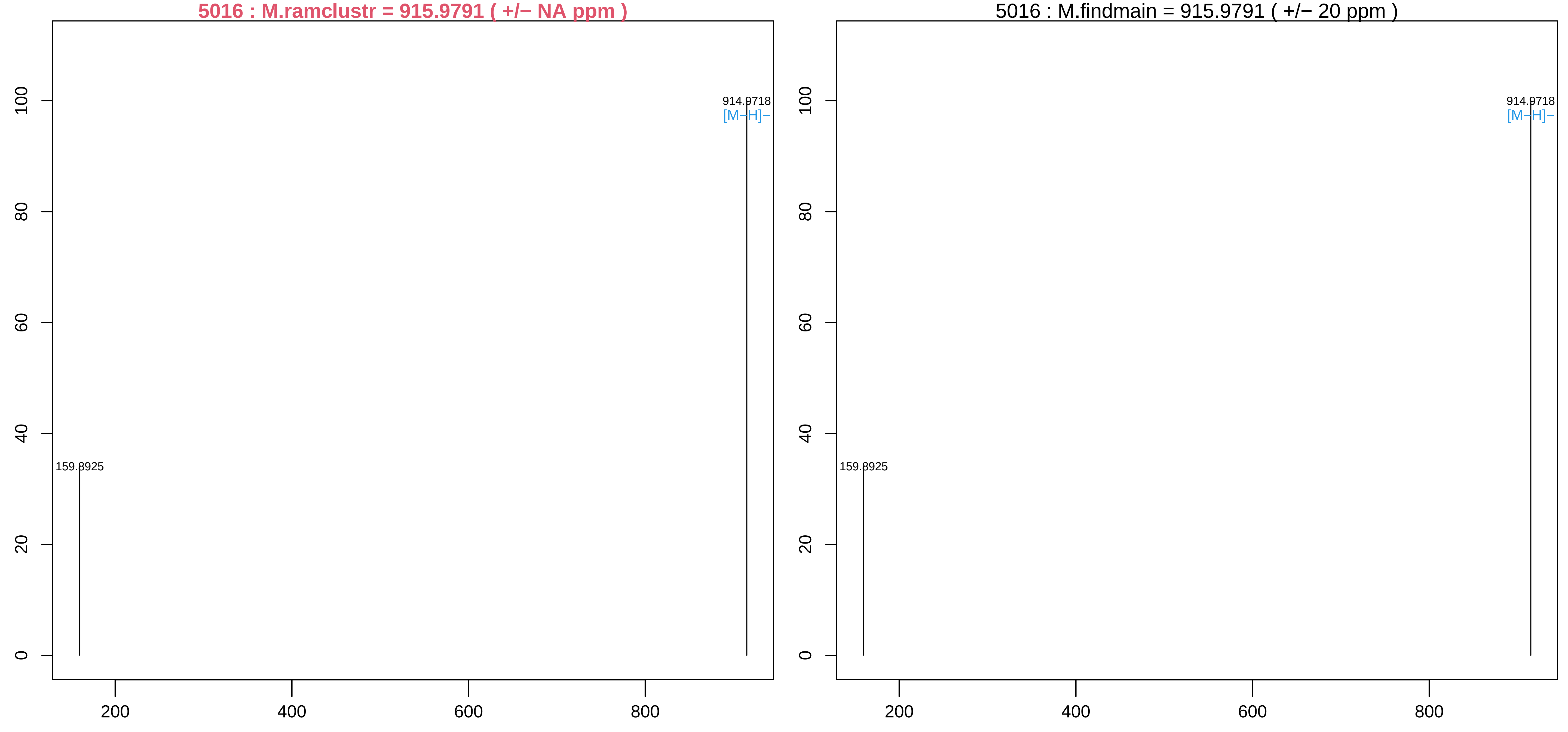
5016 : M.ramclustr = 915.9791 (+/− NA ppm)
914.9718
[M−H]−
159.8925
0
20
40
60
80
100
200
400
600
800
5016 : M.findmain = 915.9791 (+/− 20 ppm)
914.9718
[M−H]−
159.8925
0
20
40
60
80
100
200
400
600
800

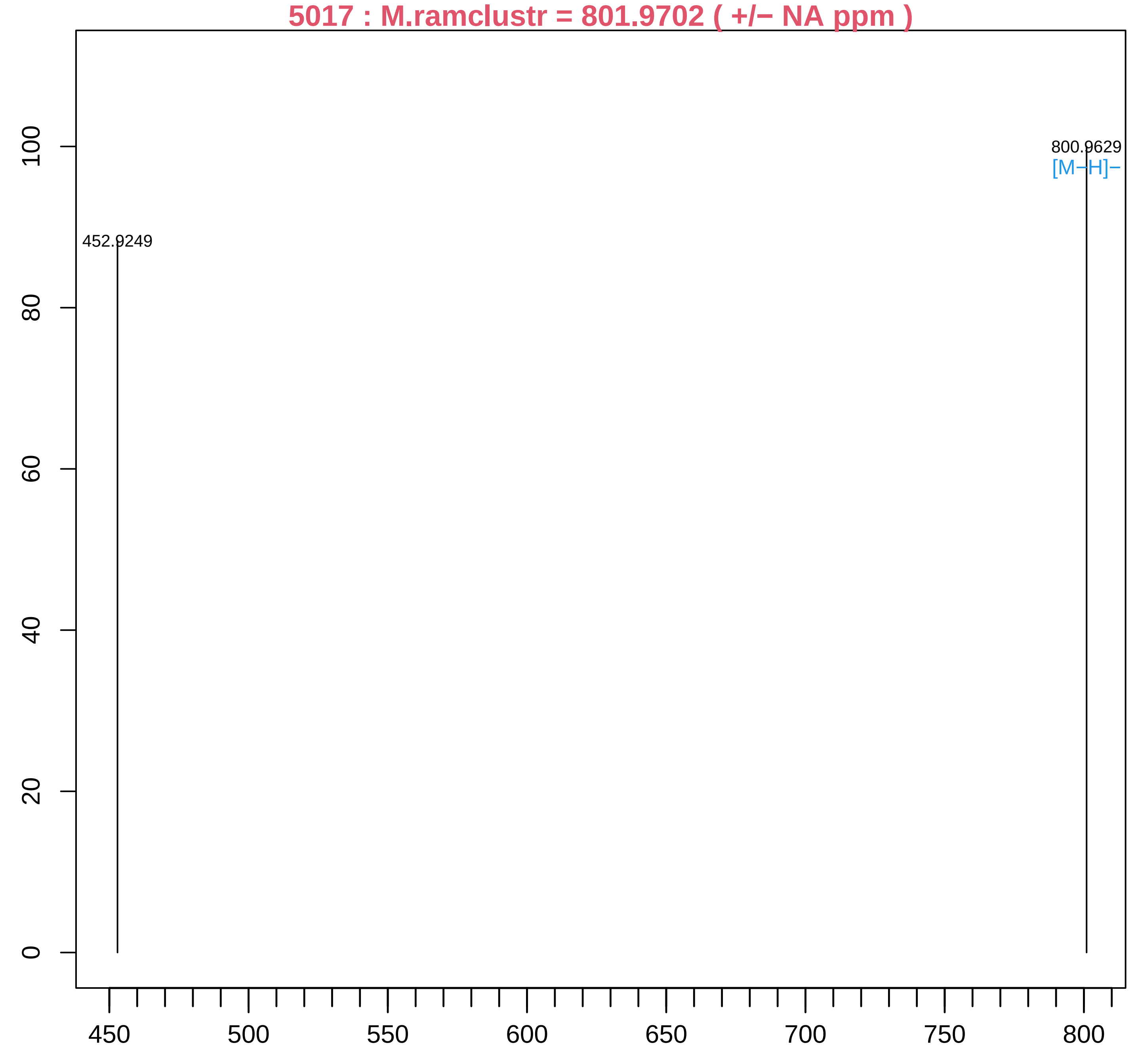
5017 : M.ramclustr = 801.9702 (+/− NA ppm)
800.9629
[M−H]−
452.9249
0
20
40
60
80
100
450
500
550
600
650
700
750
800

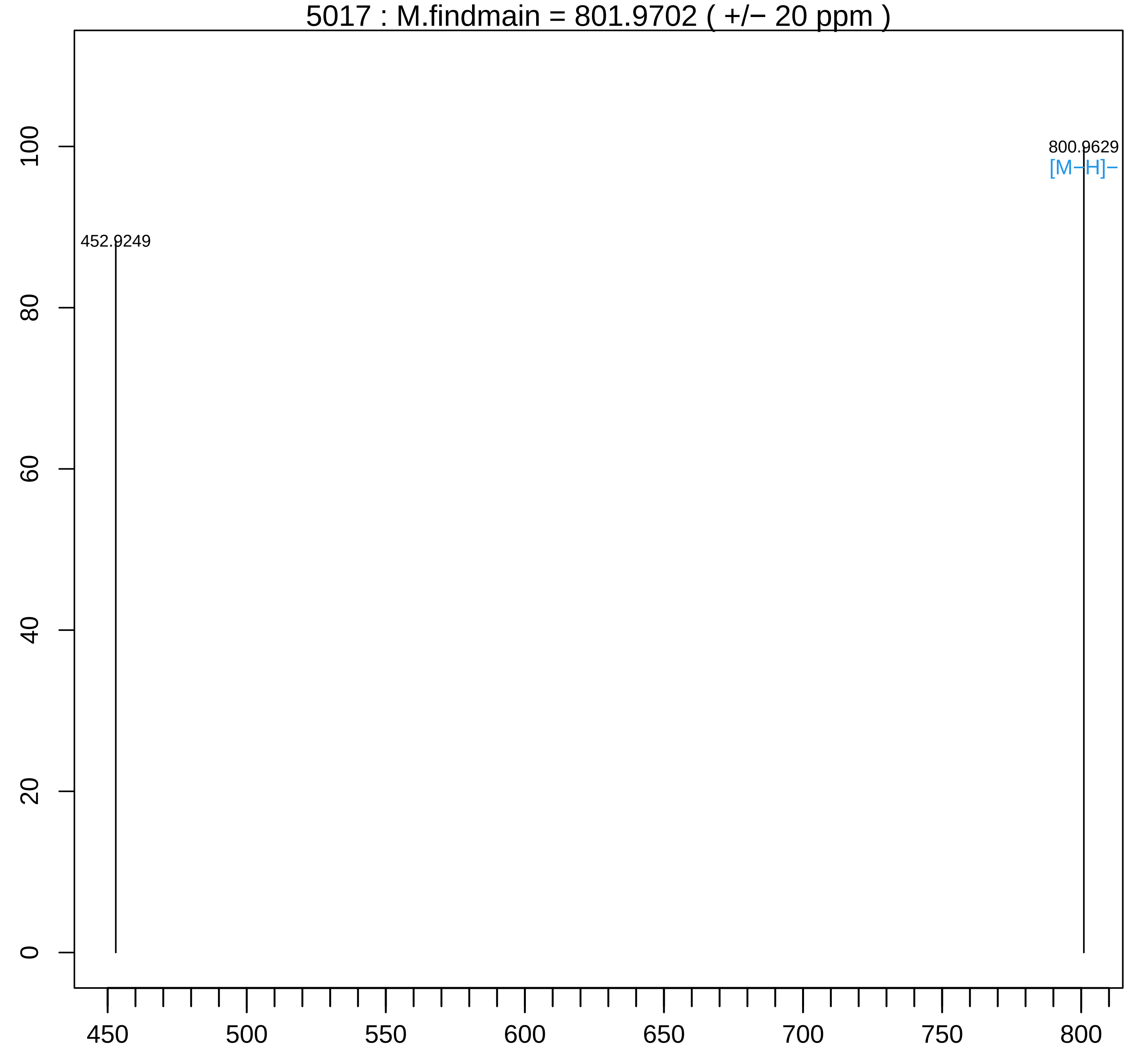
5017 : M.findmain = 801.9702 (+/− 20 ppm)
800.9629
[M−H]−
452.9249
0
20
40
60
80
100
450
500
550
600
650
700
750
800

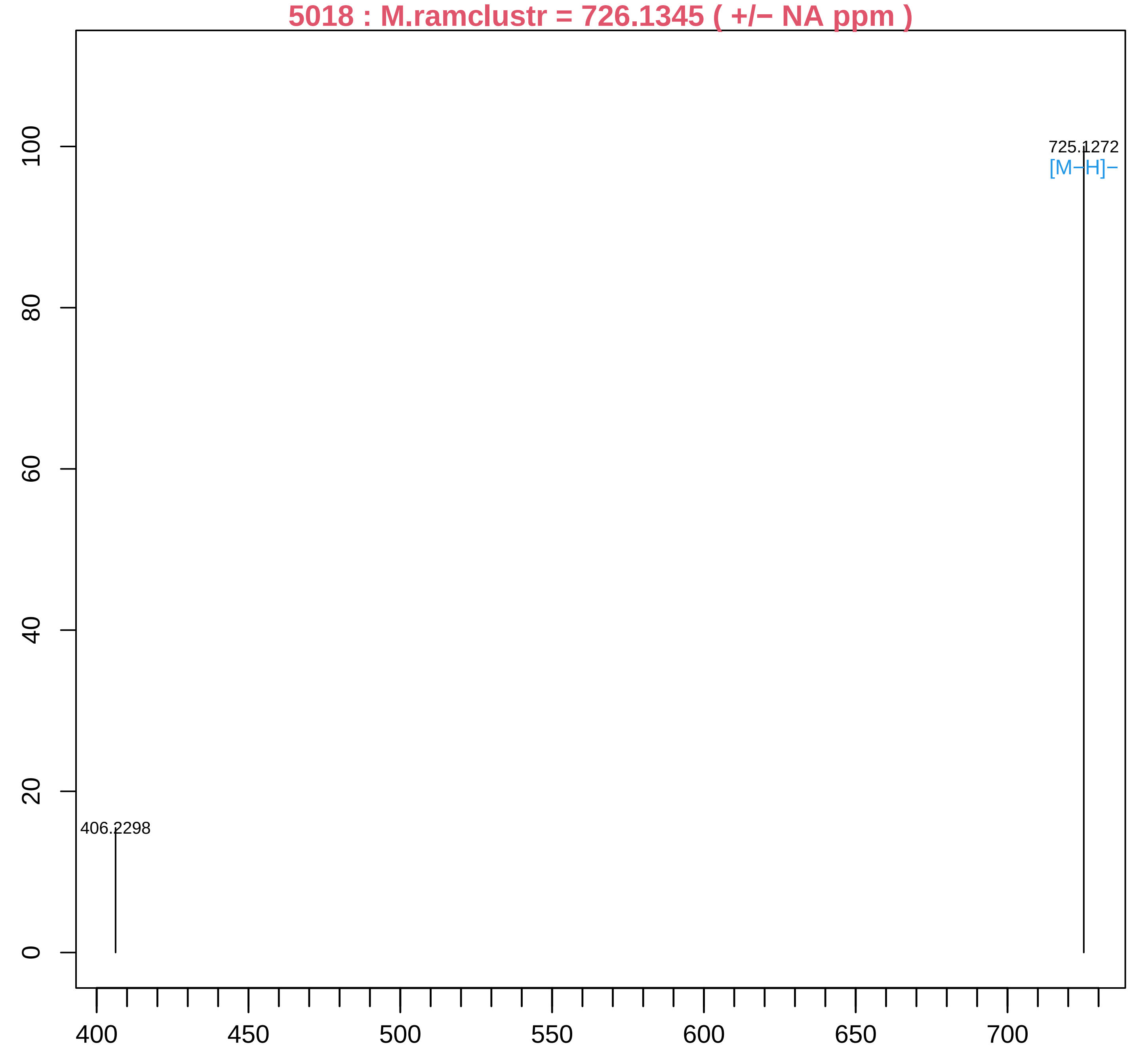

5018 : M.ramclustr = 726.1345 (+/− NA ppm)
725.1272
[M−H]−
406.2298

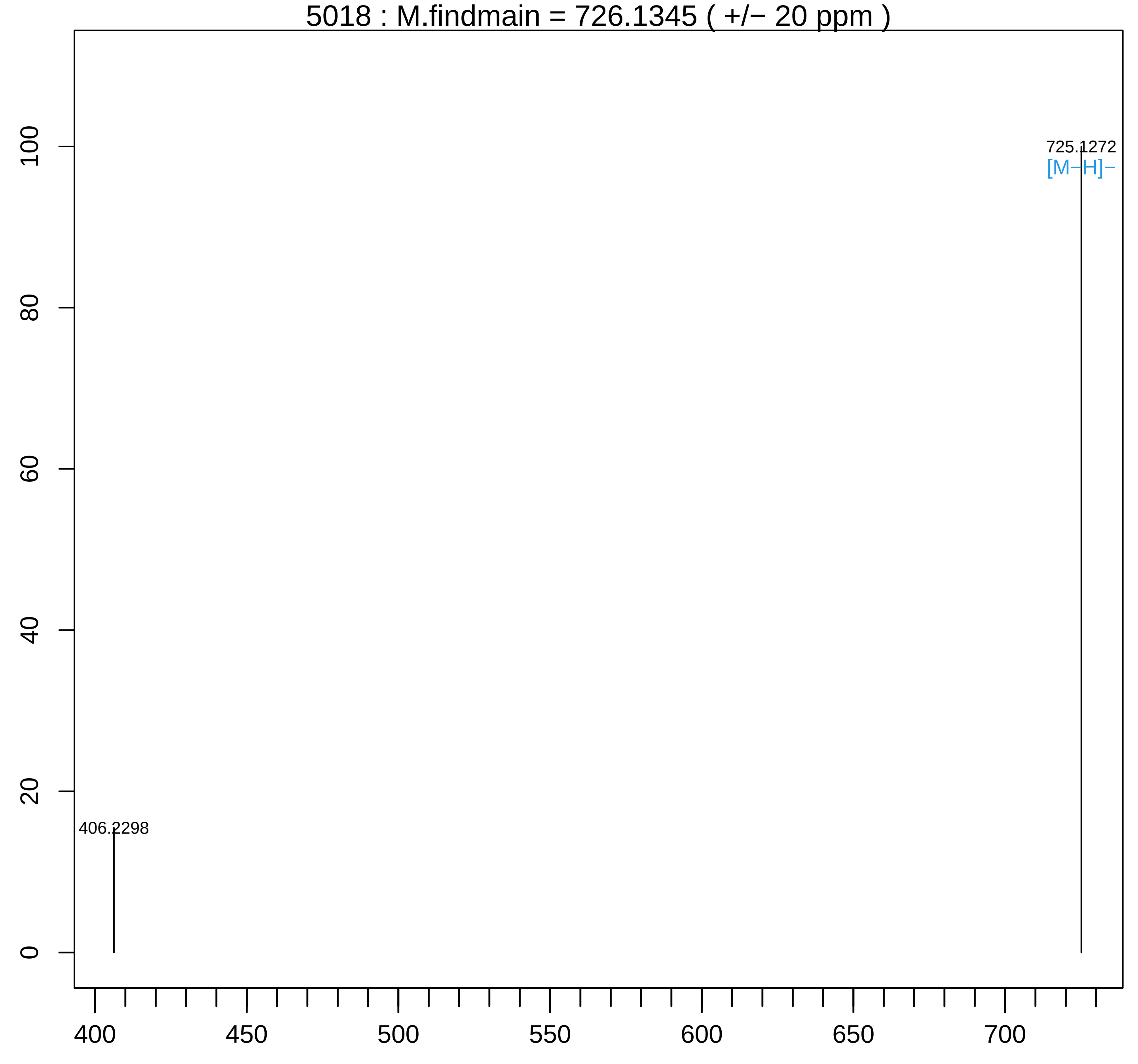

5018 : M.findmain = 726.1345 (+/− 20 ppm)
725.1272
[M−H]−
406.2298

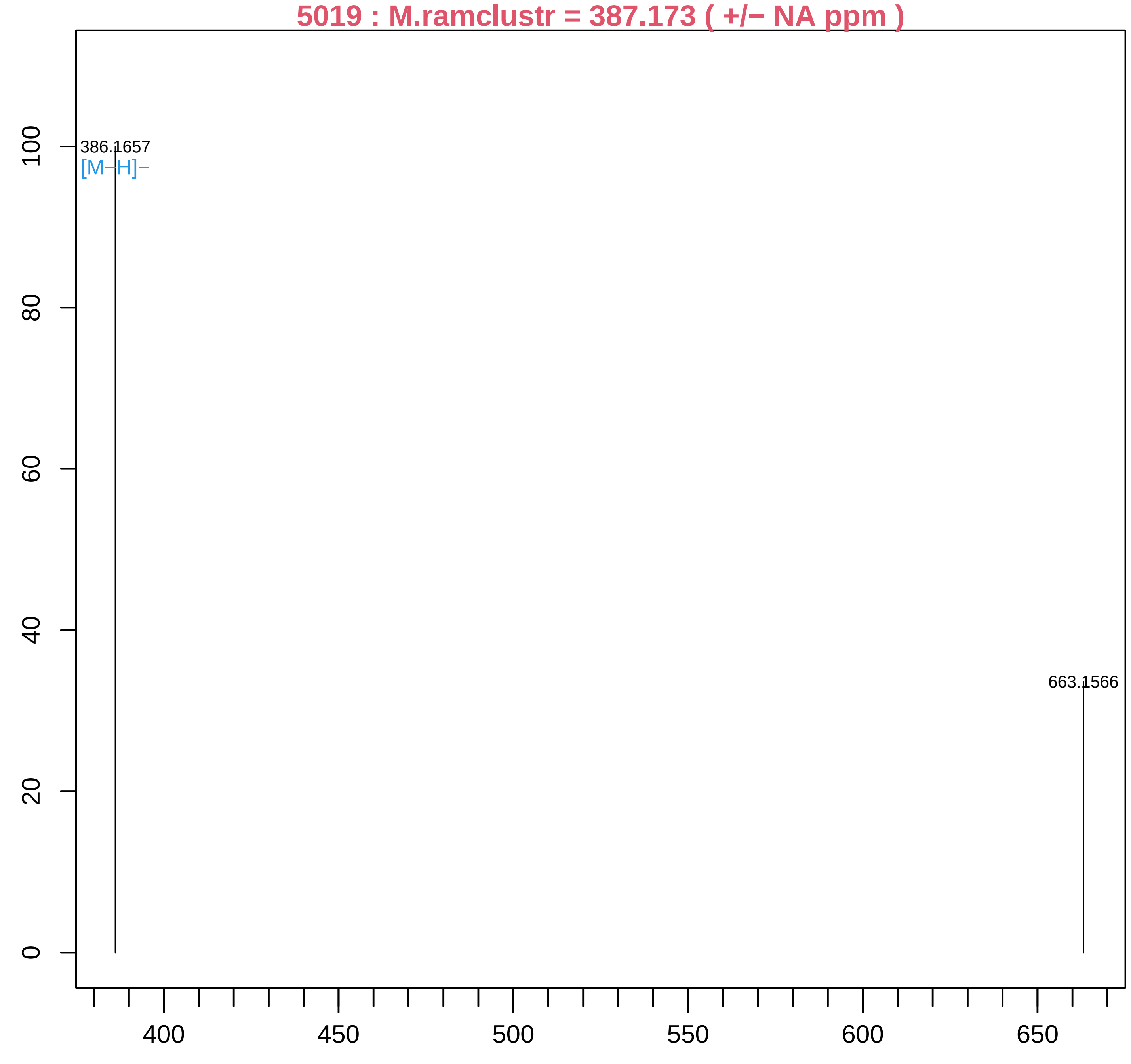

5019 : M.ramclustr = 387.173 (+/− NA ppm)
386.1657
[M−H]−
663.1566
0
20
40
60
80
100
400
450
500
550
600
650

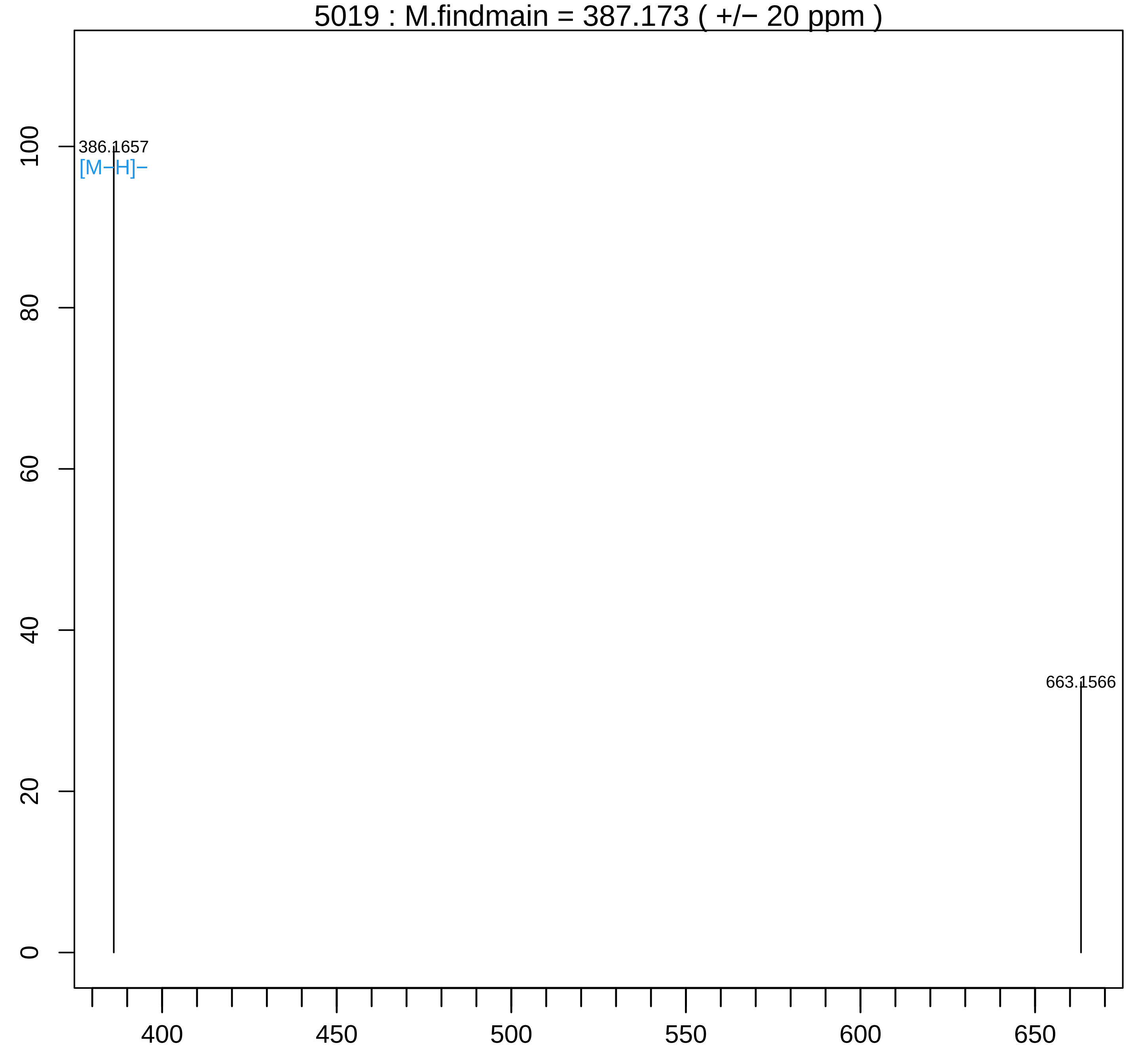

5019 : M.findmain = 387.173 (+/− 20 ppm)
386.1657
[M−H]−
663.1566
0
20
40
60
80
100
400
450
500
550
600
650

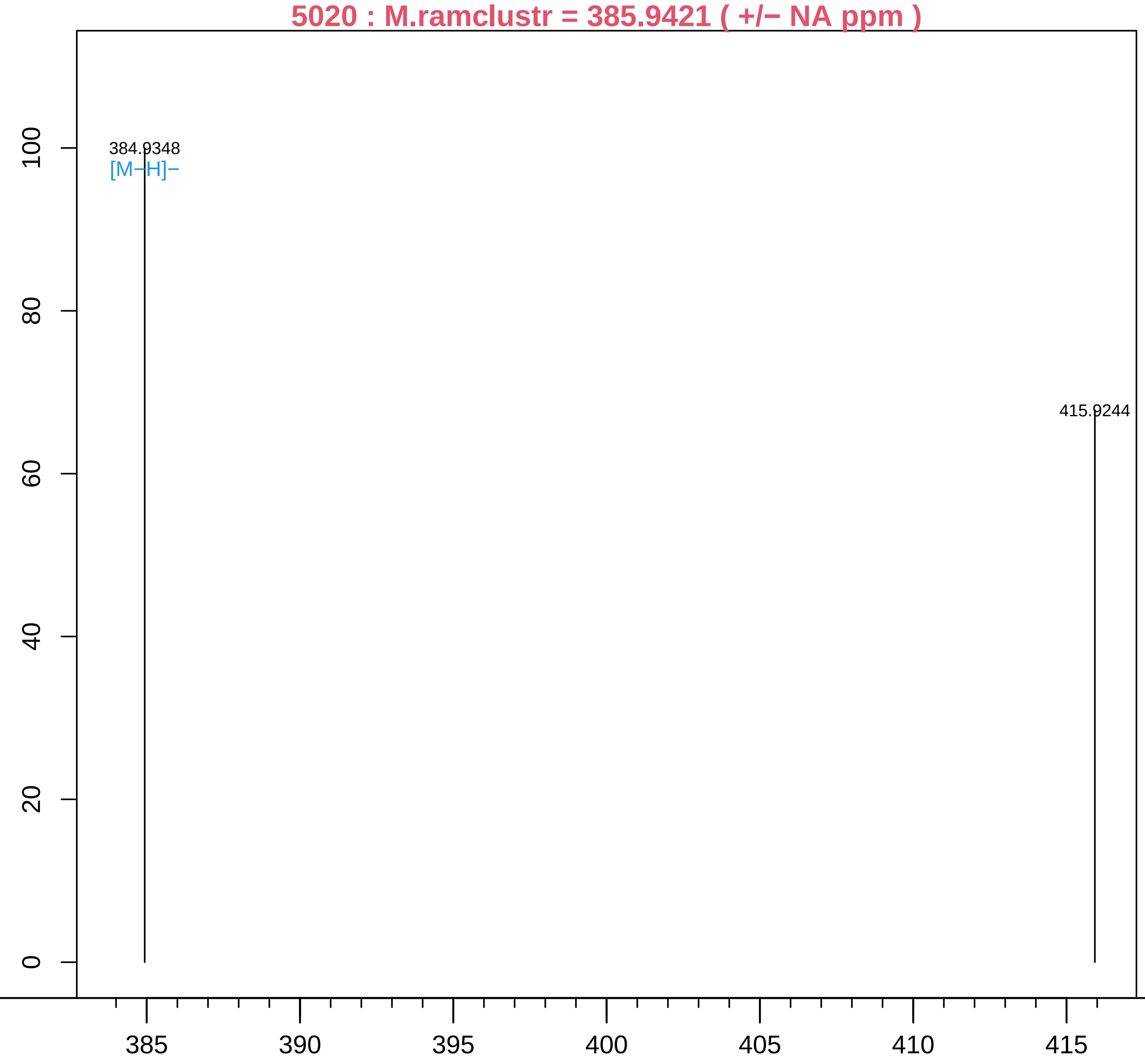

5020 : M.ramclustr = 385.9421 (+/− NA ppm)
384.9348
[M−H]−
415.9244
0
20
40
60
80
100
385
390
395
400
405
410
415

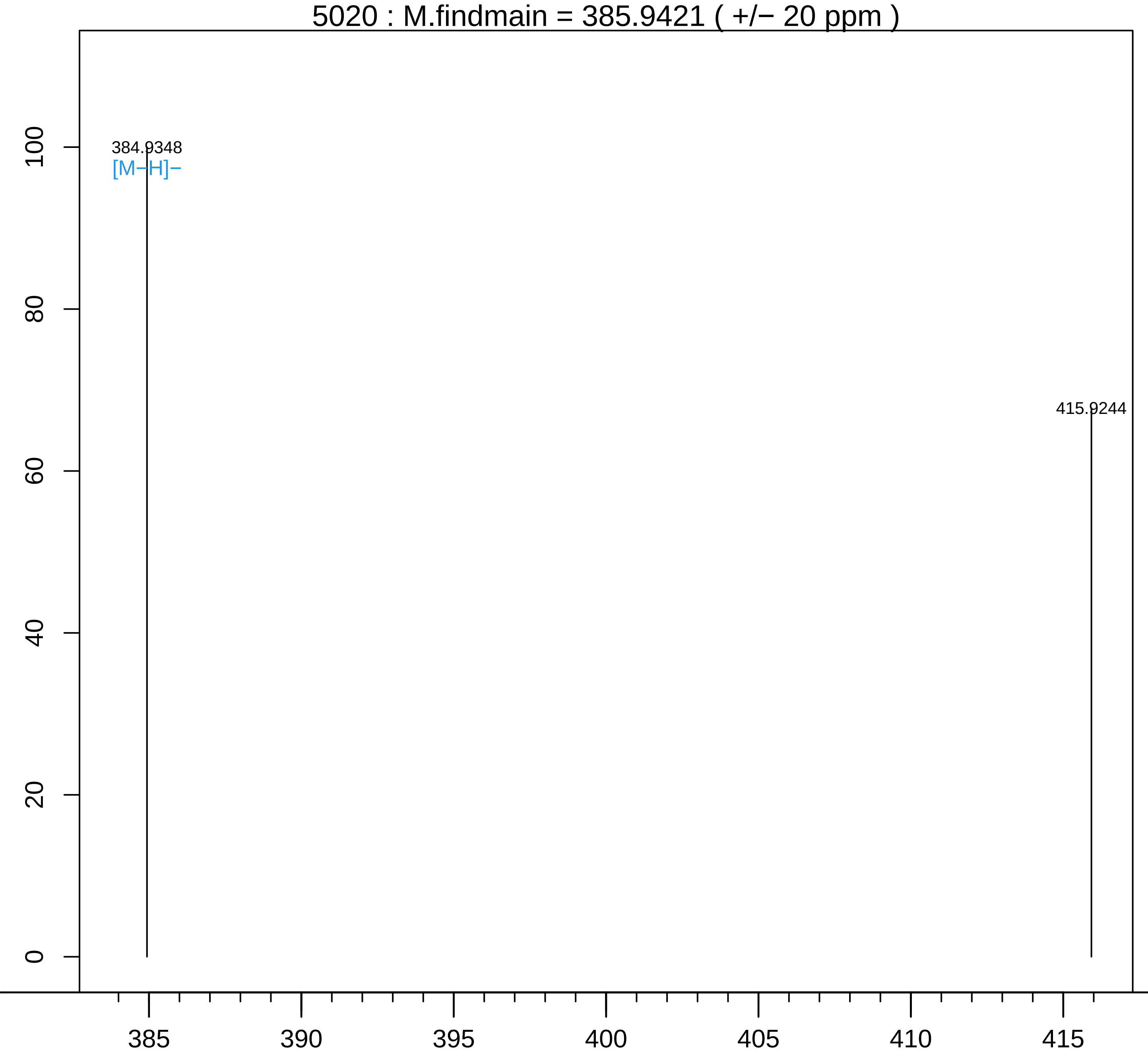

5020 : M.findmain = 385.9421 (+/− 20 ppm)
384.9348
[M−H]−
415.9244
0
20
40
60
80
100
385
390
395
400
405
410
415

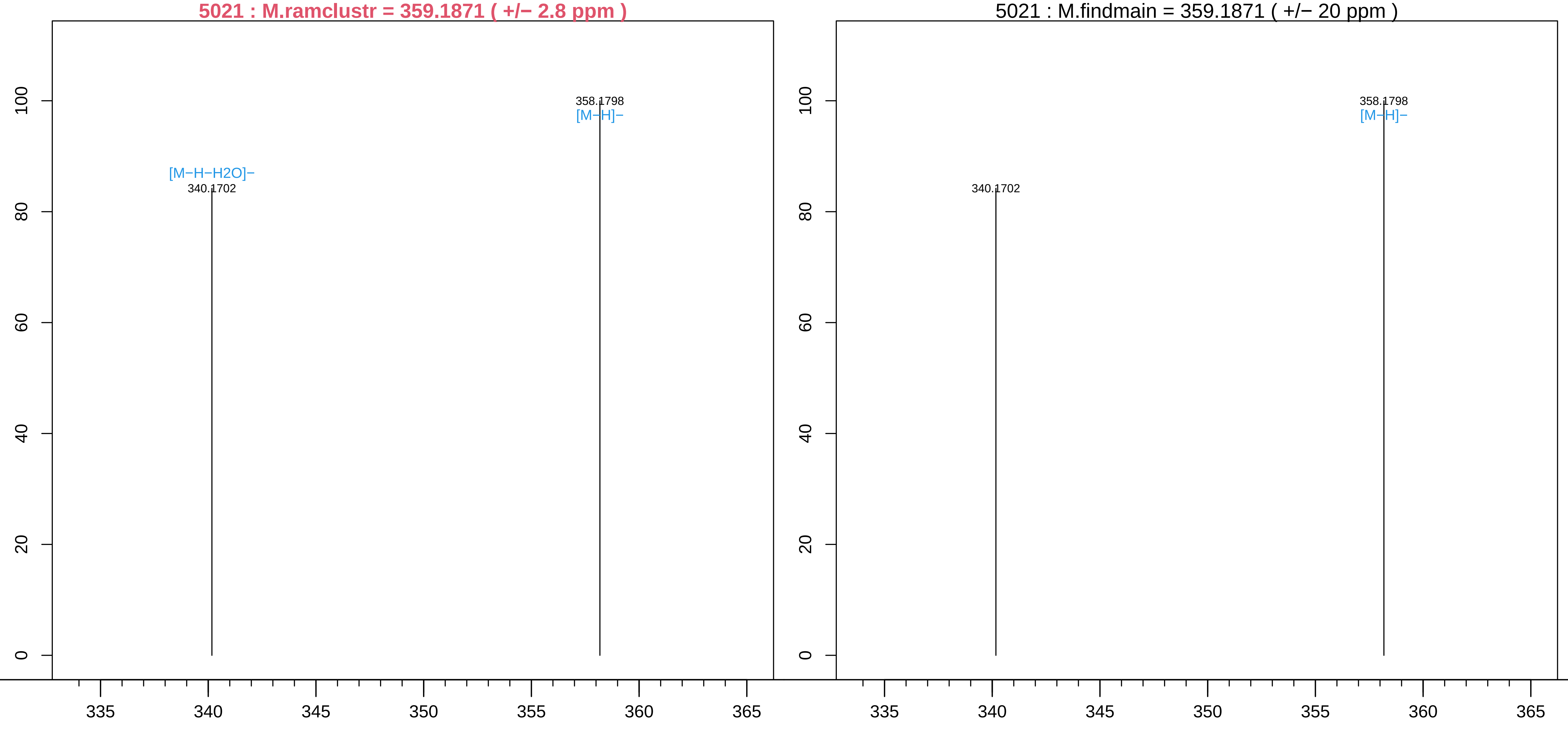
5021 : M.ramclustr = 359.1871 (+/− 2.8 ppm)
5021 : M.findmain = 359.1871 (+/− 20 ppm)
358.1798
[M−H]−
[M−H−H2O]−
340.1702
358.1798
[M−H]−
340.1702
0
20
40
60
80
100
335
340
345
350
355
360
365

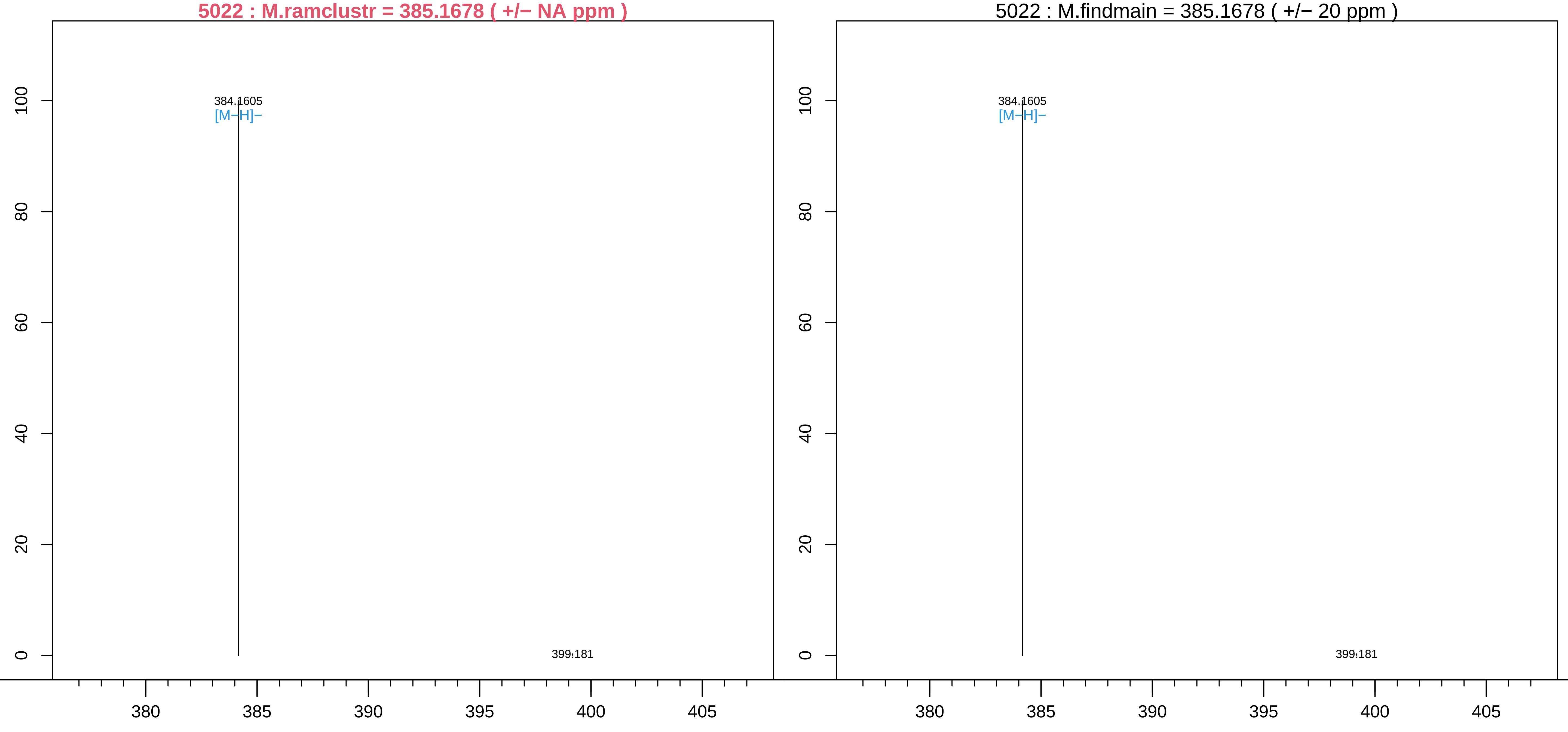

5022 : M.ramclustr = 385.1678 (+/− NA ppm)
384.1605
[M−H]−
399.181
5022 : M.findmain = 385.1678 (+/− 20 ppm)
384.1605
[M−H]−
399.181

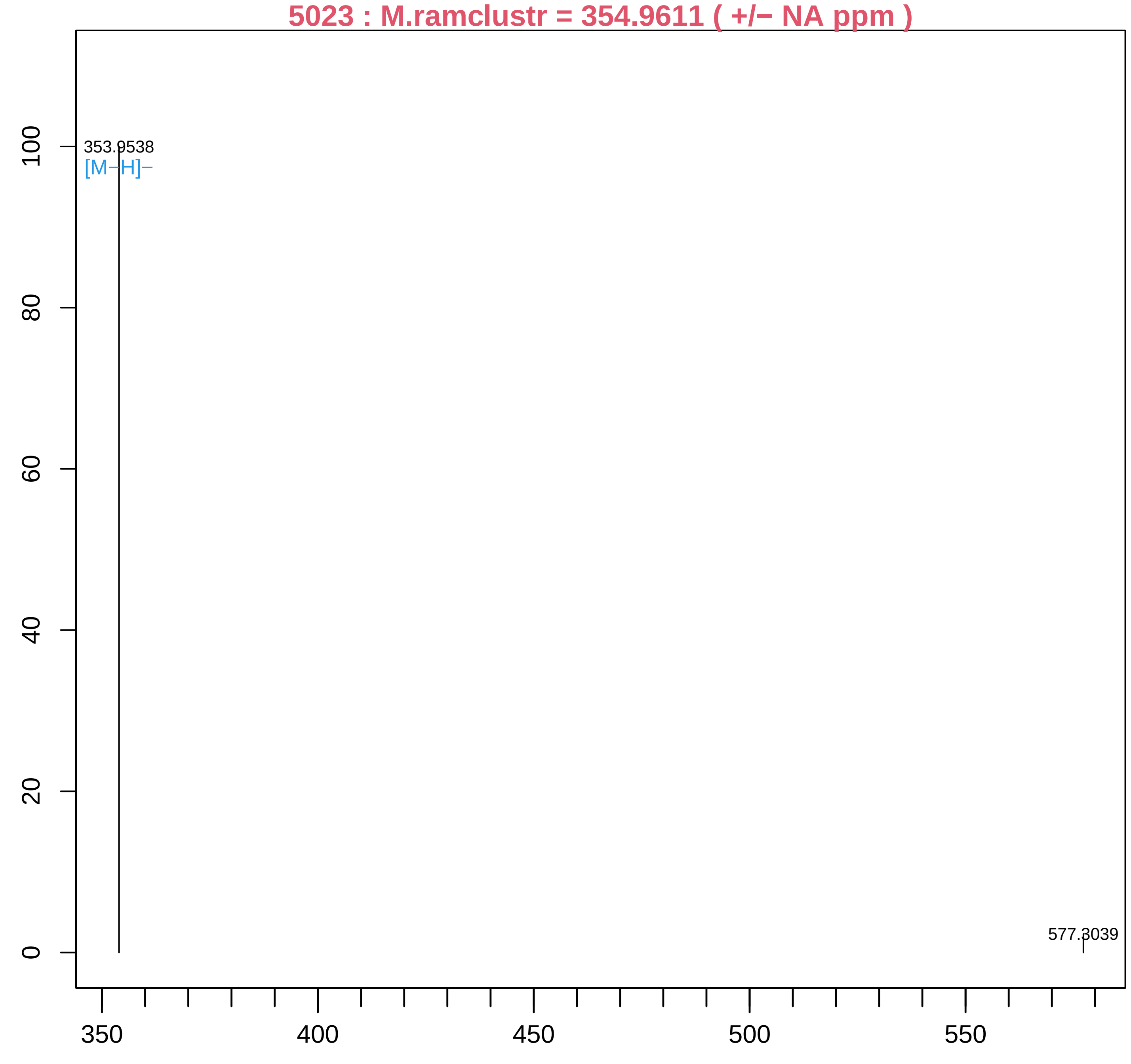
5023 : M.ramclustr = 354.9611 (+/− NA ppm)
353.9538
[M−H]−
577.3039
0
20
40
60
80
100
350
400
450
500
550

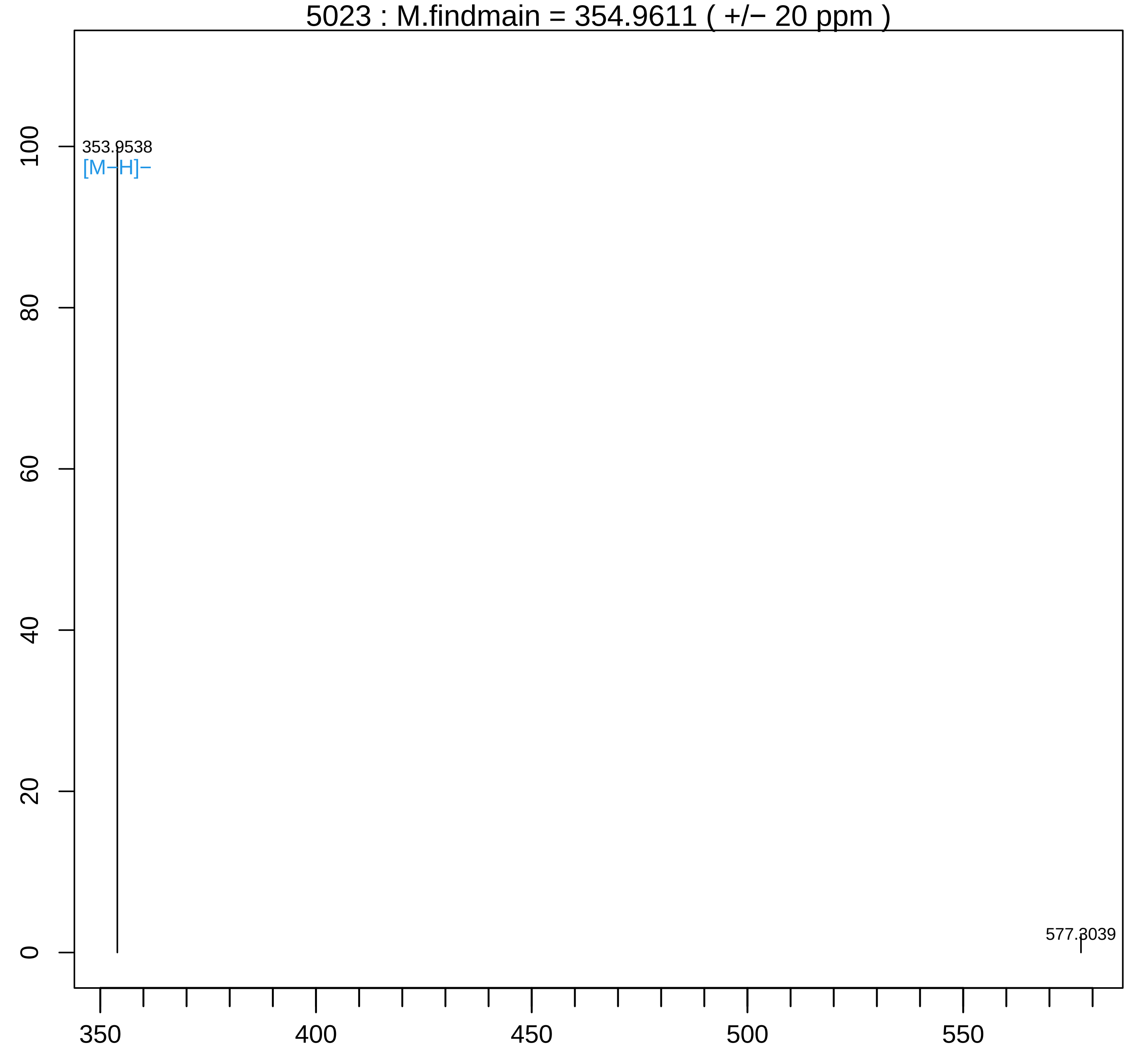
5023 : M.findmain = 354.9611 (+/− 20 ppm)
353.9538
[M−H]−
577.3039
0
20
40
60
80
100
350
400
450
500
550

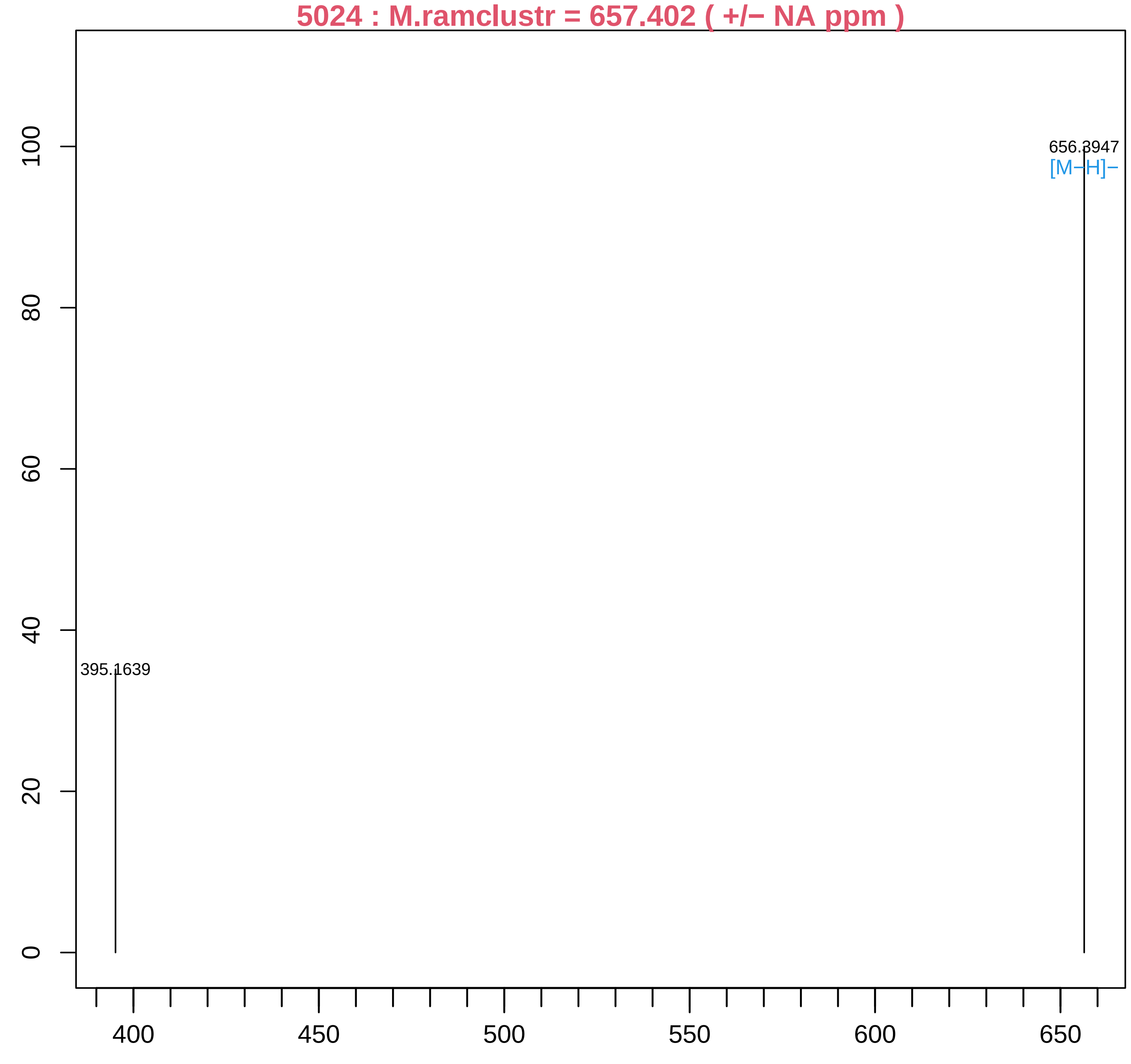
5024 : M.ramclustr = 657.402 (+/− NA ppm)
656.3947
[M−H]−
395.1639
0
20
40
60
80
100
400
450
500
550
600
650

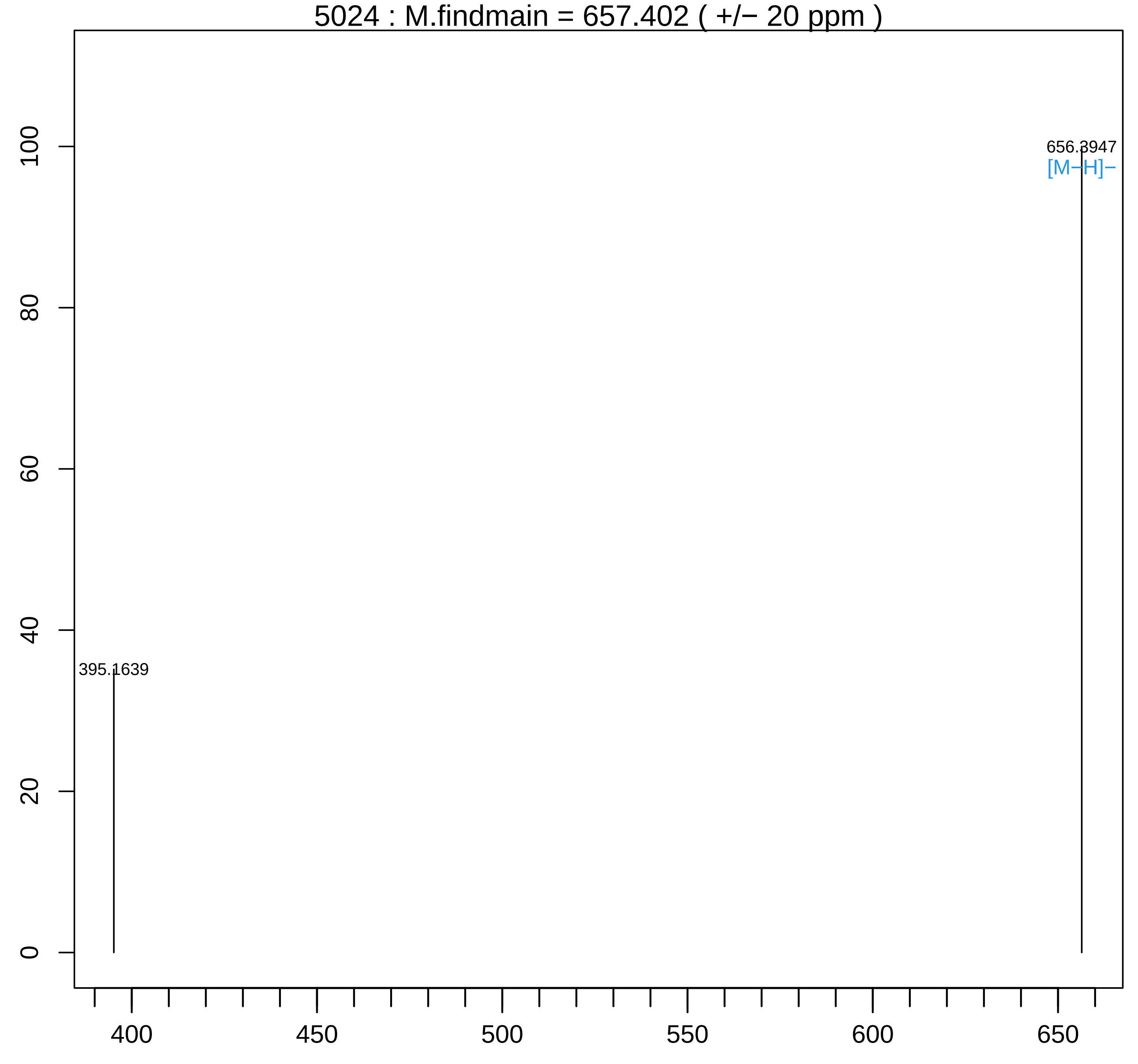
5024 : M.findmain = 657.402 (+/− 20 ppm)
656.3947
[M−H]−
395.1639
0
20
40
60
80
100
400
450
500
550
600
650

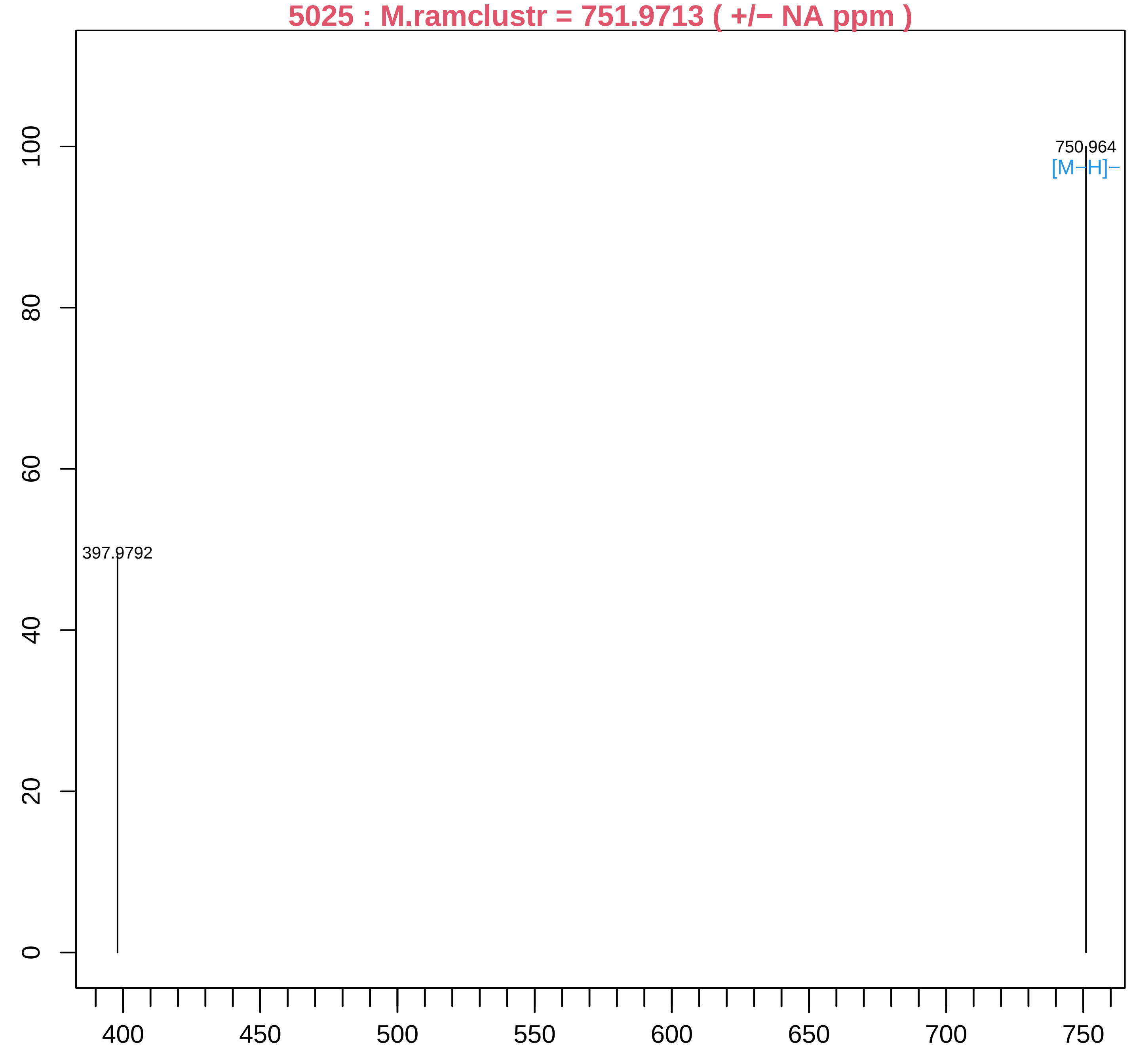

5025 : M.ramclustr = 751.9713 (+/− NA ppm)
750.964
[M−H]−
397.9792
0
20
40
60
80
100
400
450
500
550
600
650
700
750

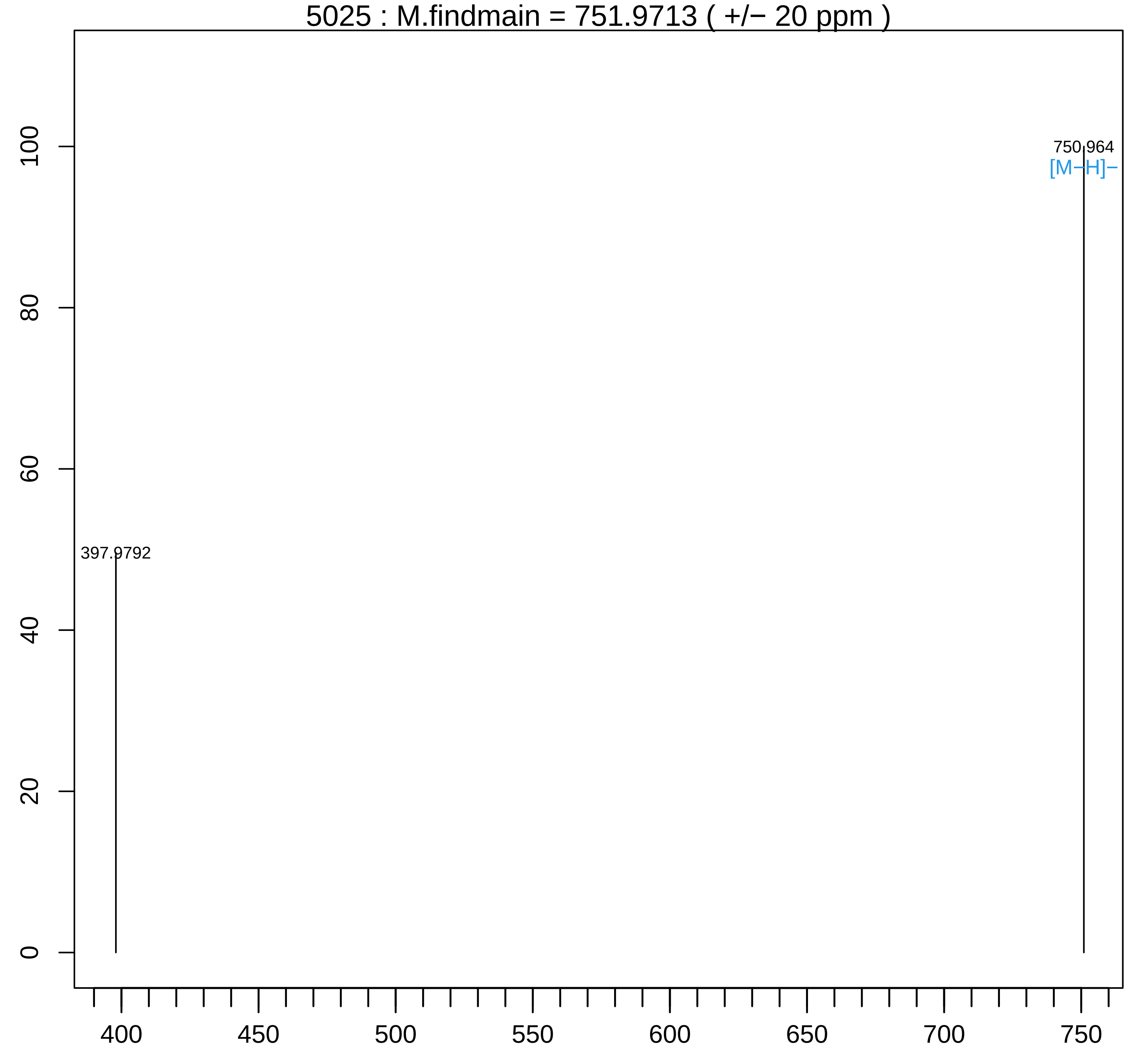

5025 : M.findmain = 751.9713 (+/− 20 ppm)
750.964
[M−H]−
397.9792
0
20
40
60
80
100
400
450
500
550
600
650
700
750

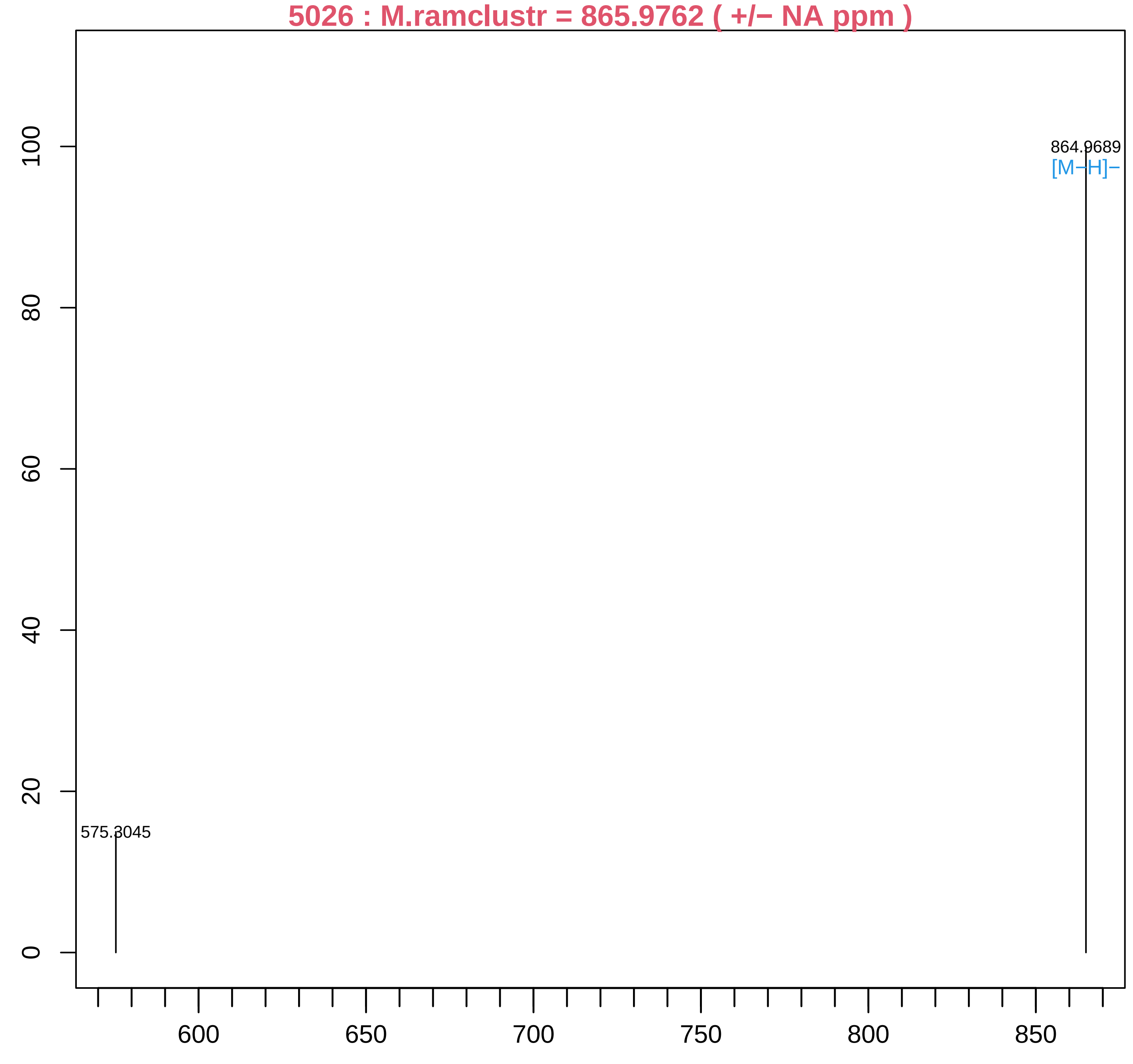
5026 : M.ramclustr = 865.9762 (+/− NA ppm)
864.9689
[M−H]−
575.3045
0
20
40
60
80
100
600
650
700
750
800
850

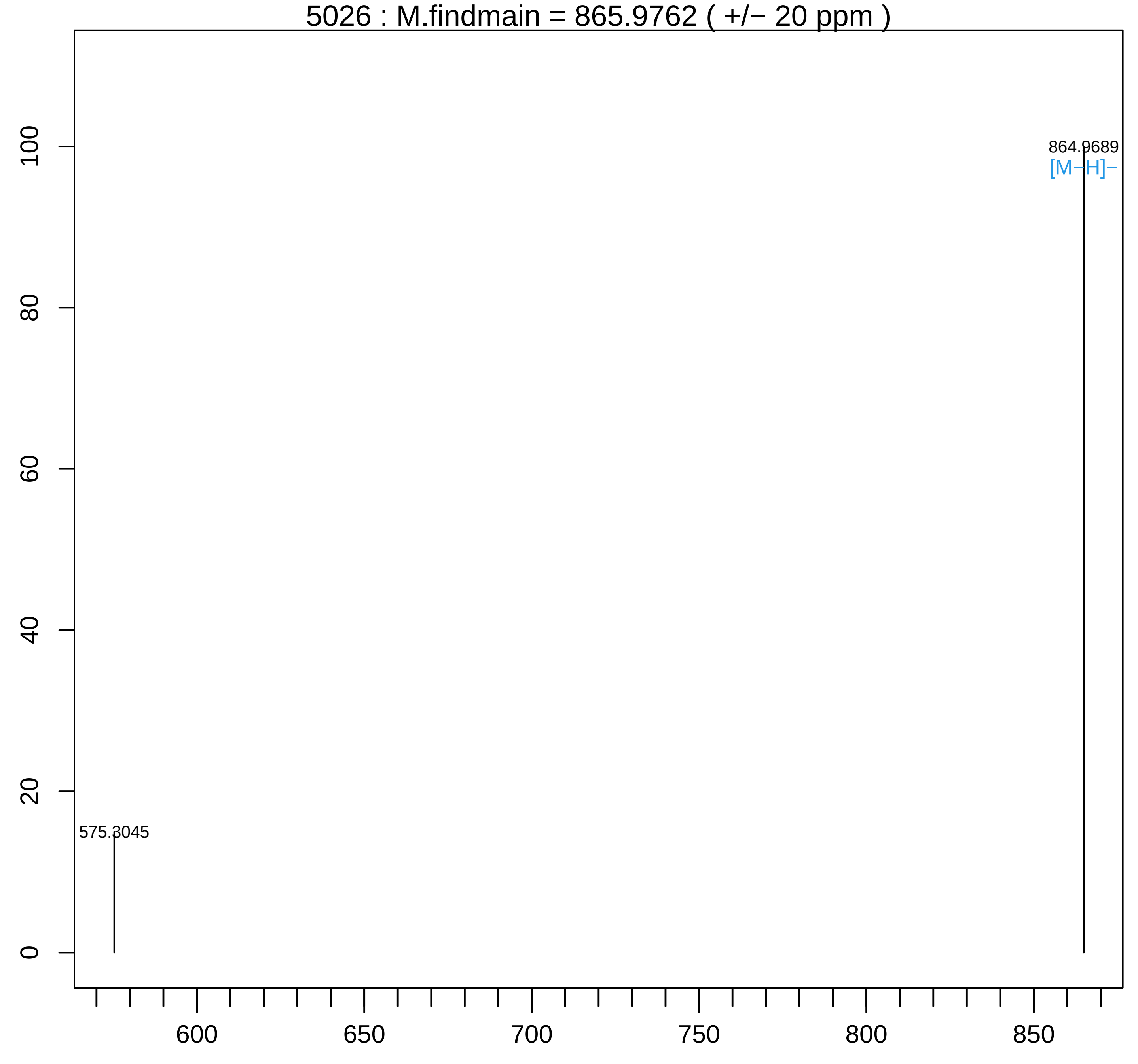
5026 : M.findmain = 865.9762 (+/− 20 ppm)
864.9689
[M−H]−
575.3045
0
20
40
60
80
100
600
650
700
750
800
850

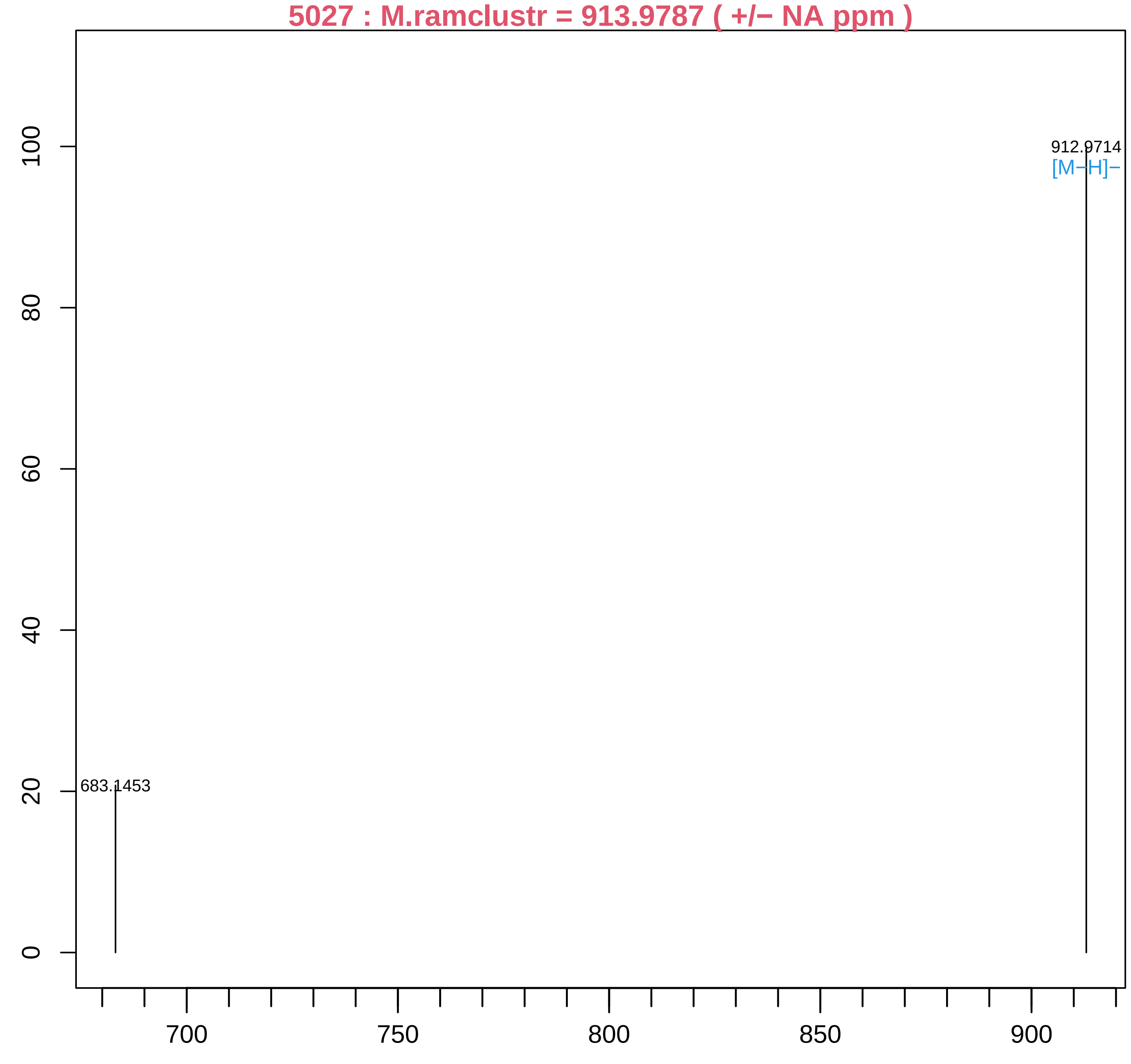
5027 : M.ramclustr = 913.9787 (+/− NA ppm)
912.9714
[M−H]−
683.1453
0
20
40
60
80
100
700
750
800
850
900

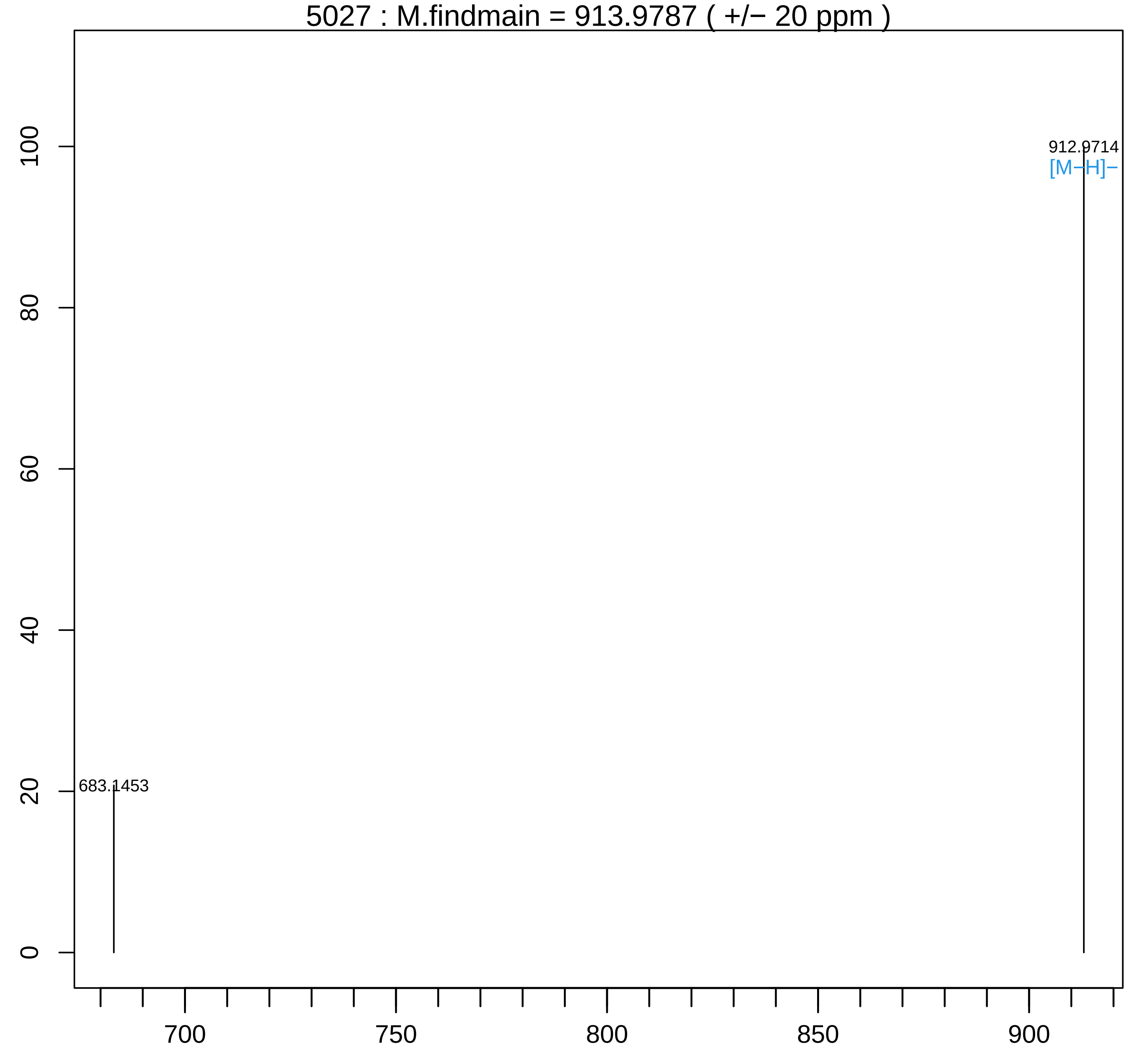
5027 : M.findmain = 913.9787 (+/− 20 ppm)
912.9714
[M−H]−
683.1453
0
20
40
60
80
100
700
750
800
850
900

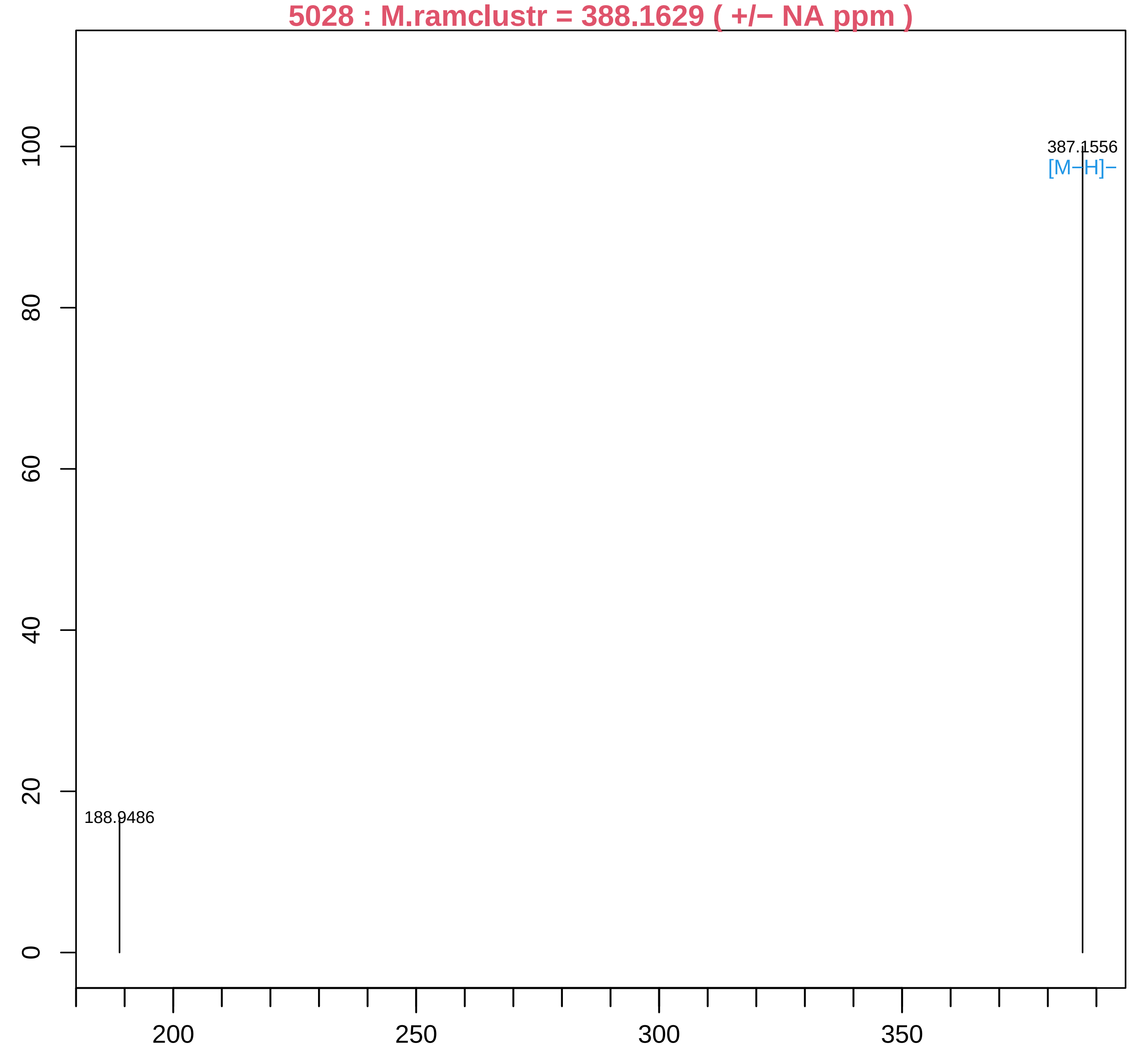
5028 : M.ramclustr = 388.1629 (+/− NA ppm)
387.1556
[M−H]−
188.9486
0
20
40
60
80
100
200
250
300
350

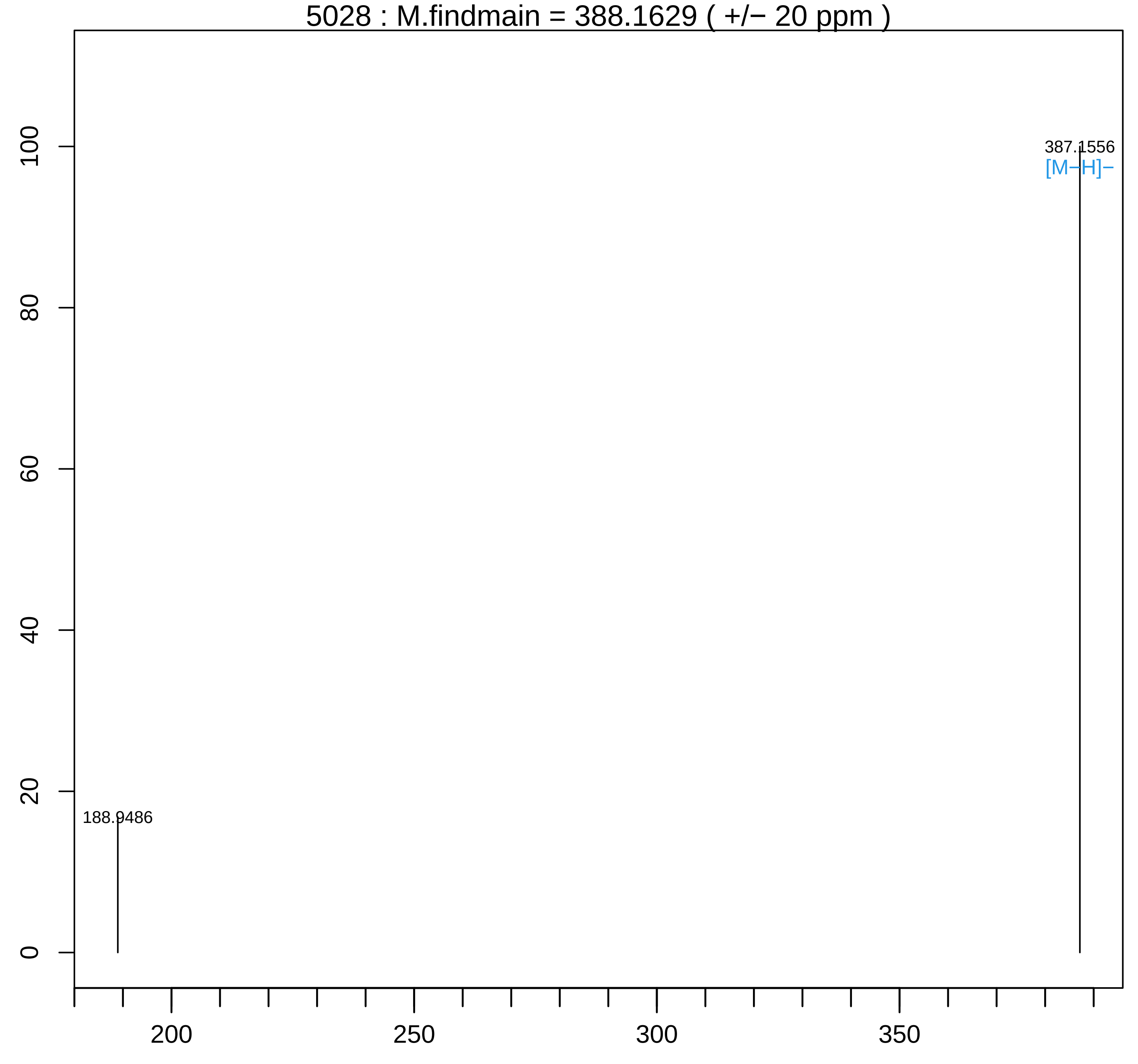
5028 : M.findmain = 388.1629 (+/− 20 ppm)
387.1556
[M−H]−
188.9486
0
20
40
60
80
100
200
250
300
350

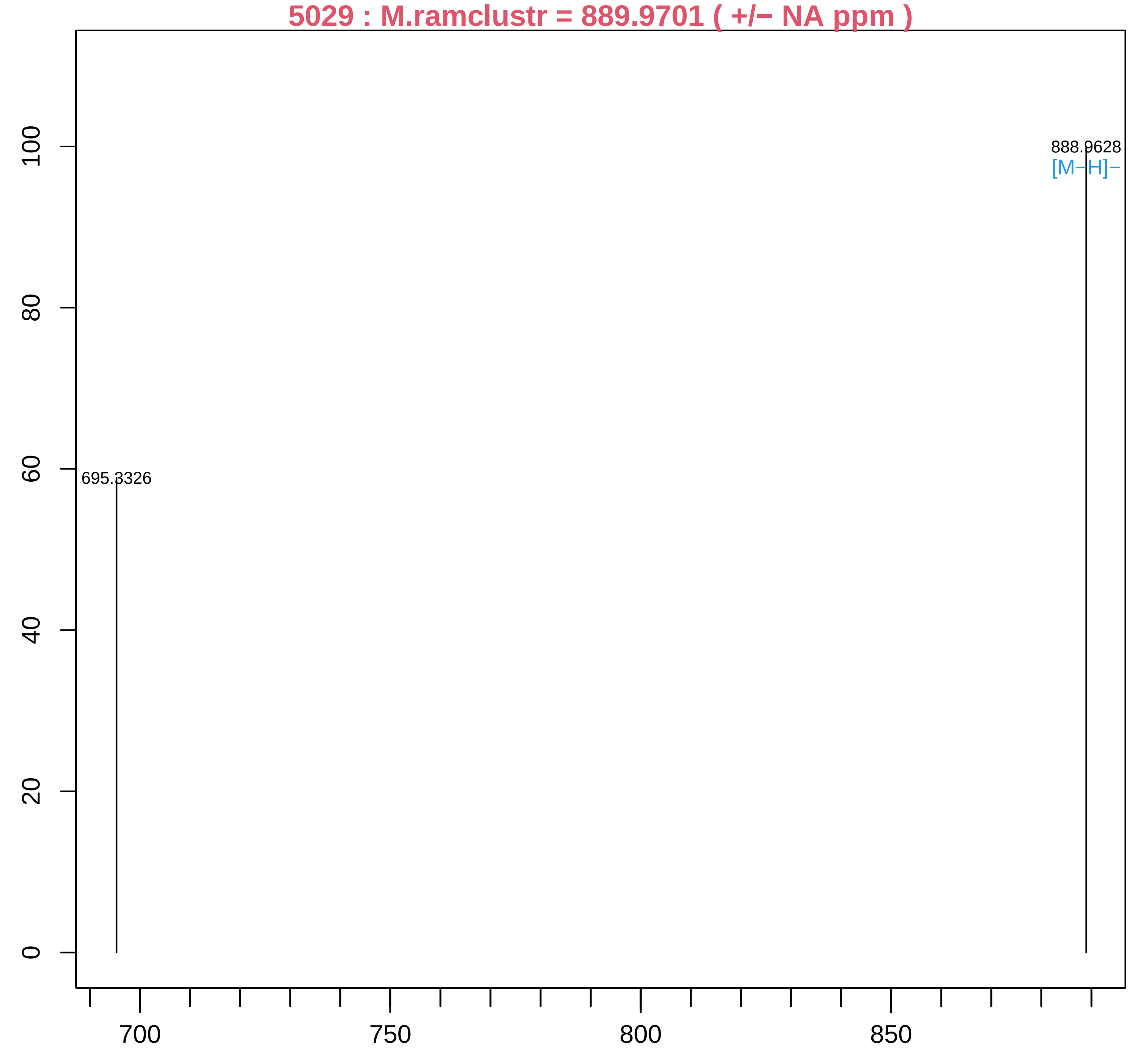

5029 : M.ramclustr = 889.9701 (+/− NA ppm)
888.9628
[M−H]−
695.3326
0
20
40
60
80
100
700
750
800
850

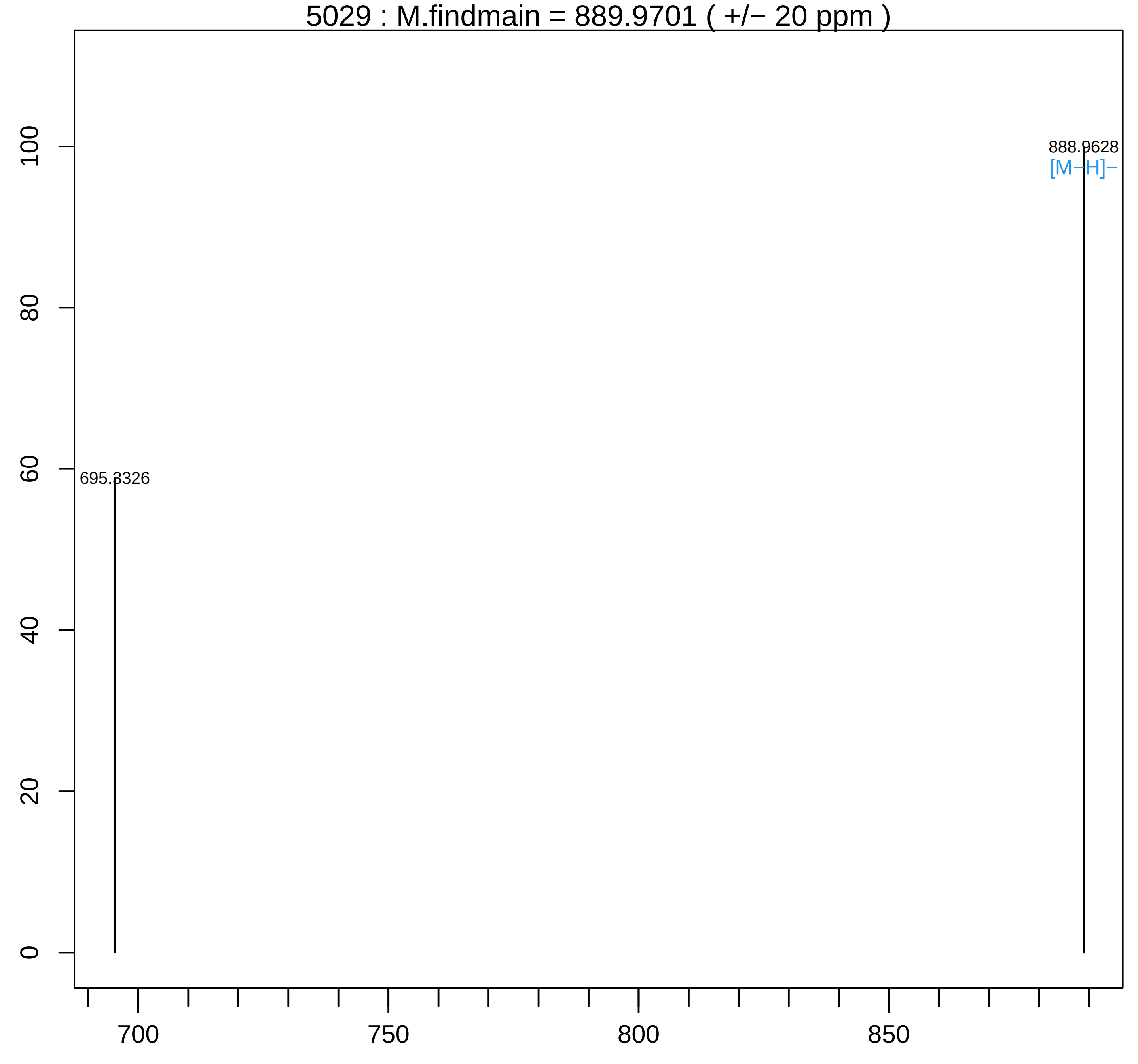

5029 : M.findmain = 889.9701 (+/− 20 ppm)
888.9628
[M−H]−
695.3326
0
20
40
60
80
100
700
750
800
850

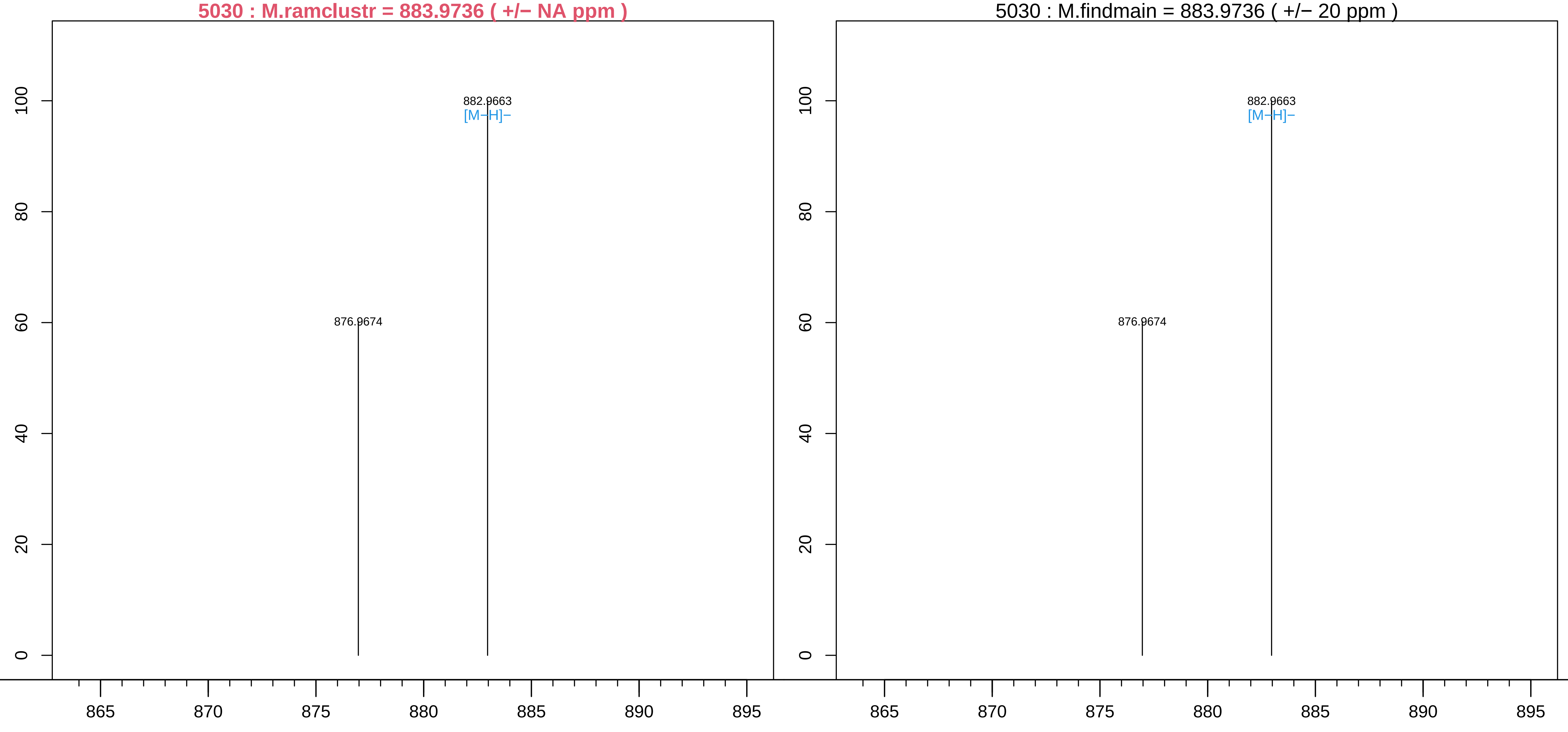
5030 : M.ramclustr = 883.9736 (+/− NA ppm)
882.9663
[M−H]−
876.9674
0
20
40
60
80
100
865
870
875
880
885
890
895
5030 : M.findmain = 883.9736 (+/− 20 ppm)
882.9663
[M−H]−
876.9674
0
20
40
60
80
100
865
870
875
880
885
890
895

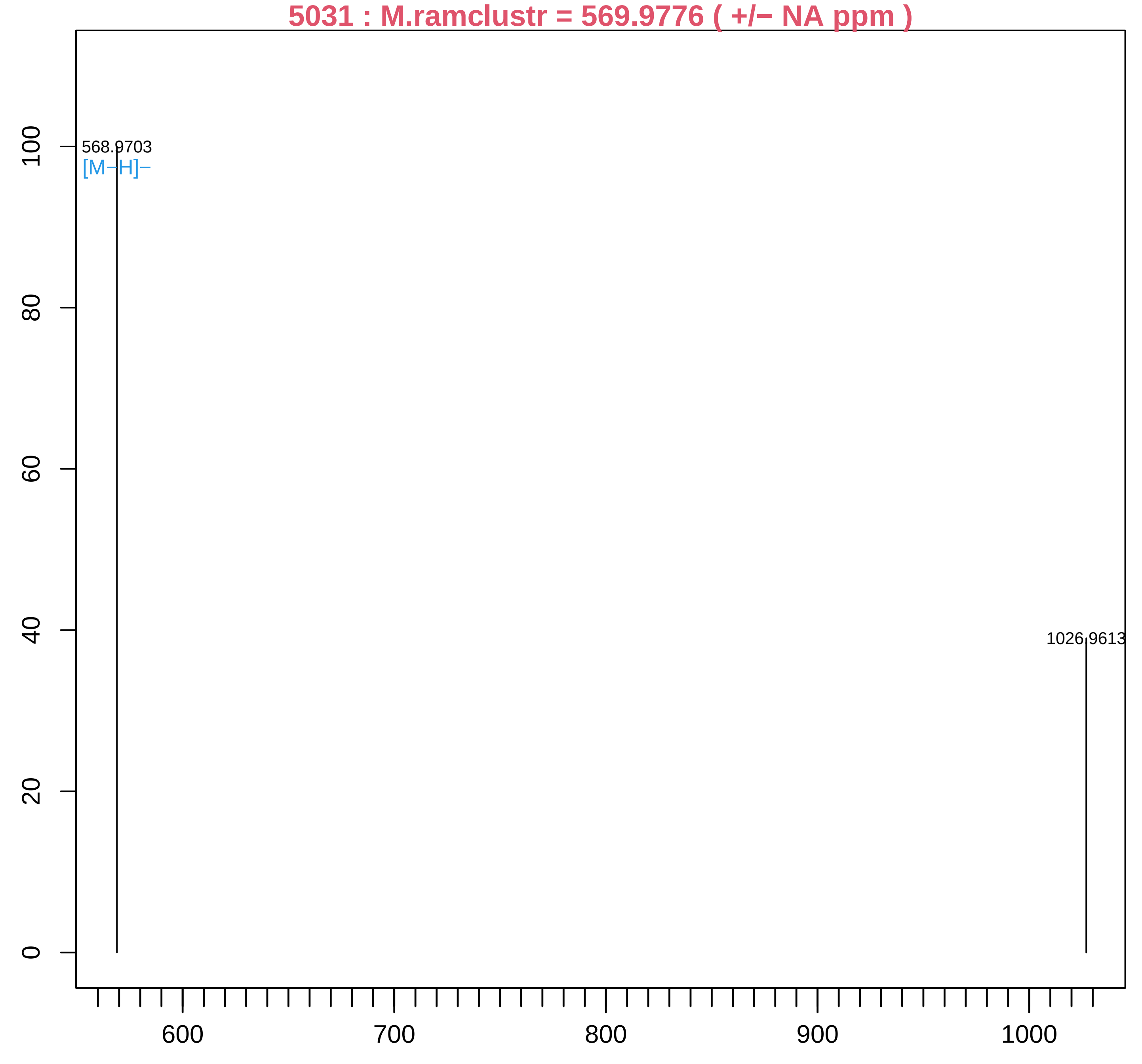
5031 : M.ramclustr = 569.9776 (+/− NA ppm)
568.9703
[M−H]−
1026.9613
0
20
40
60
80
100
600
700
800
900
1000

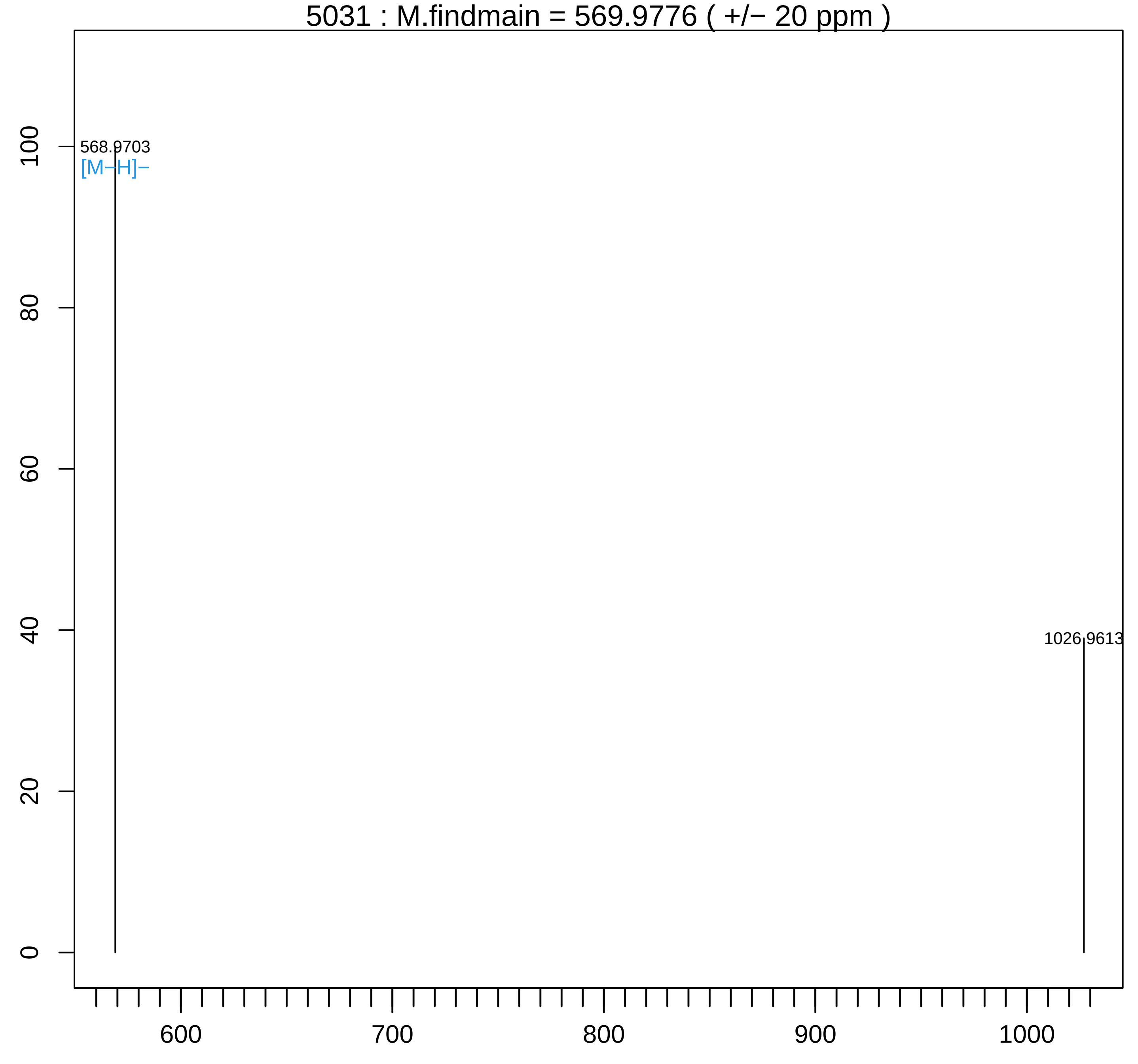
5031 : M.findmain = 569.9776 (+/− 20 ppm)
568.9703
[M−H]−
1026.9613
0
20
40
60
80
100
600
700
800
900
1000

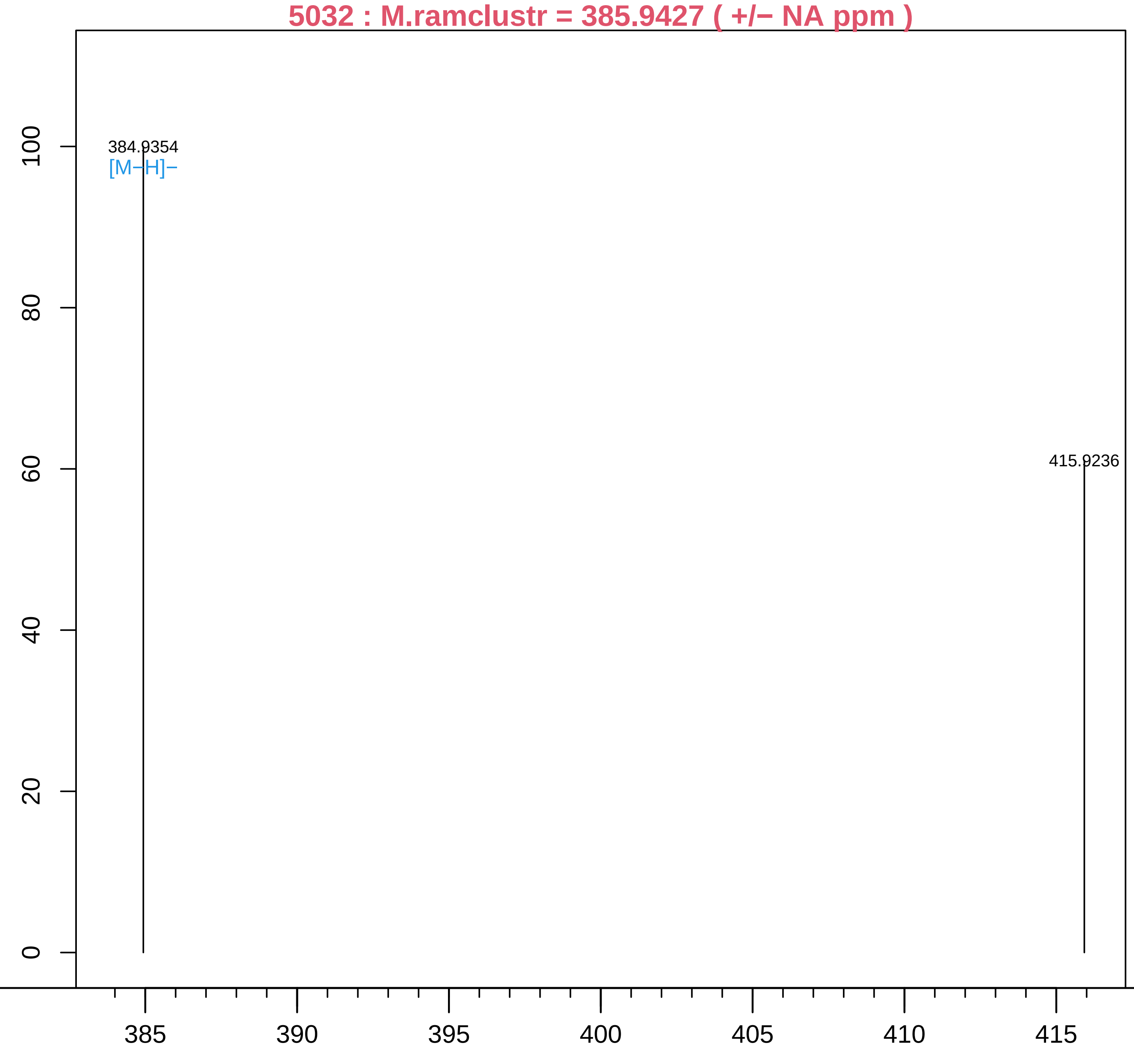
5032 : M.ramclustr = 385.9427 (+/− NA ppm)
384.9354
[M−H]−
415.9236
0
20
40
60
80
100
385
390
395
400
405
410
415

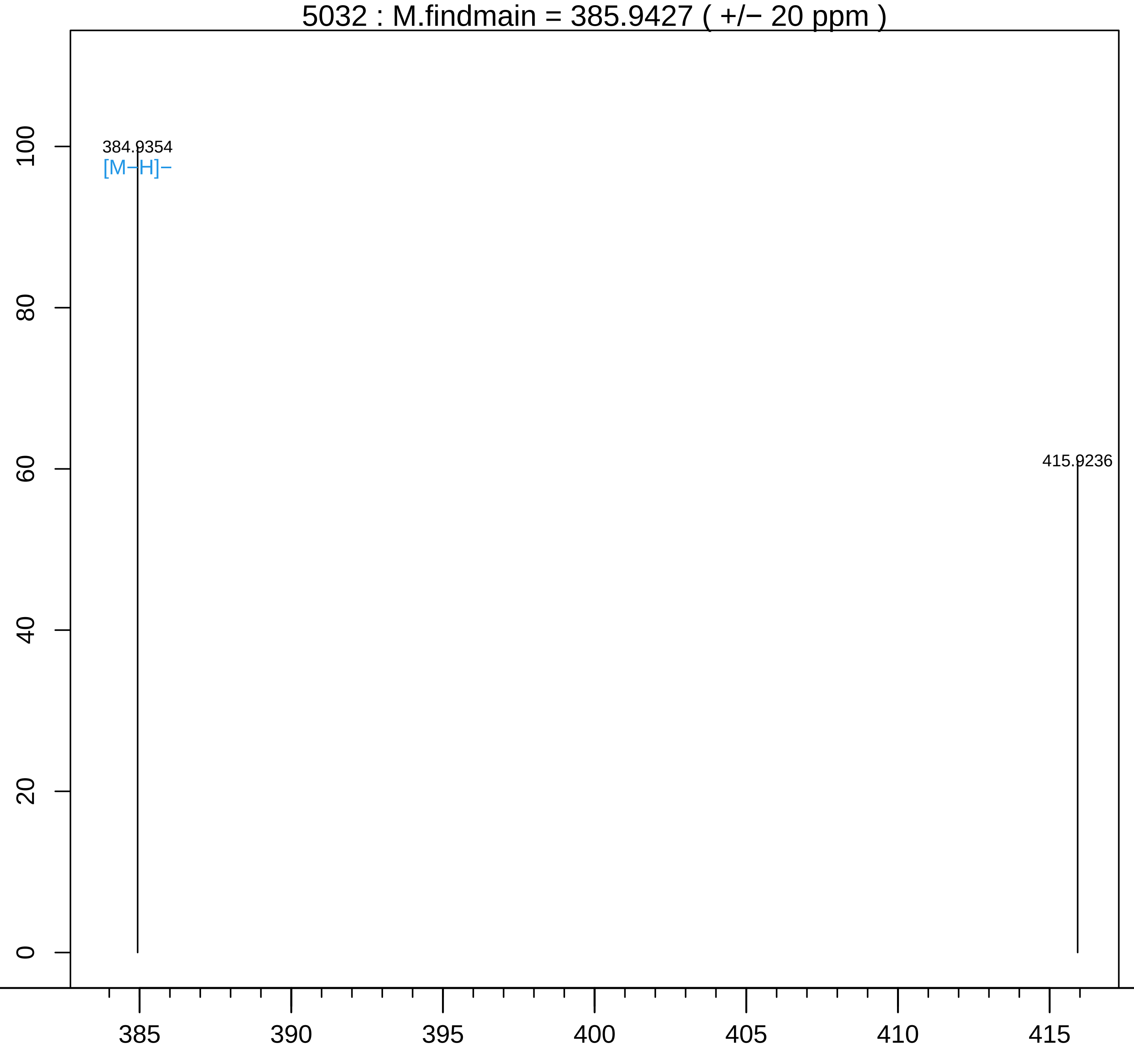
5032 : M.findmain = 385.9427 (+/− 20 ppm)
384.9354
[M−H]−
415.9236
0
20
40
60
80
100
385
390
395
400
405
410
415

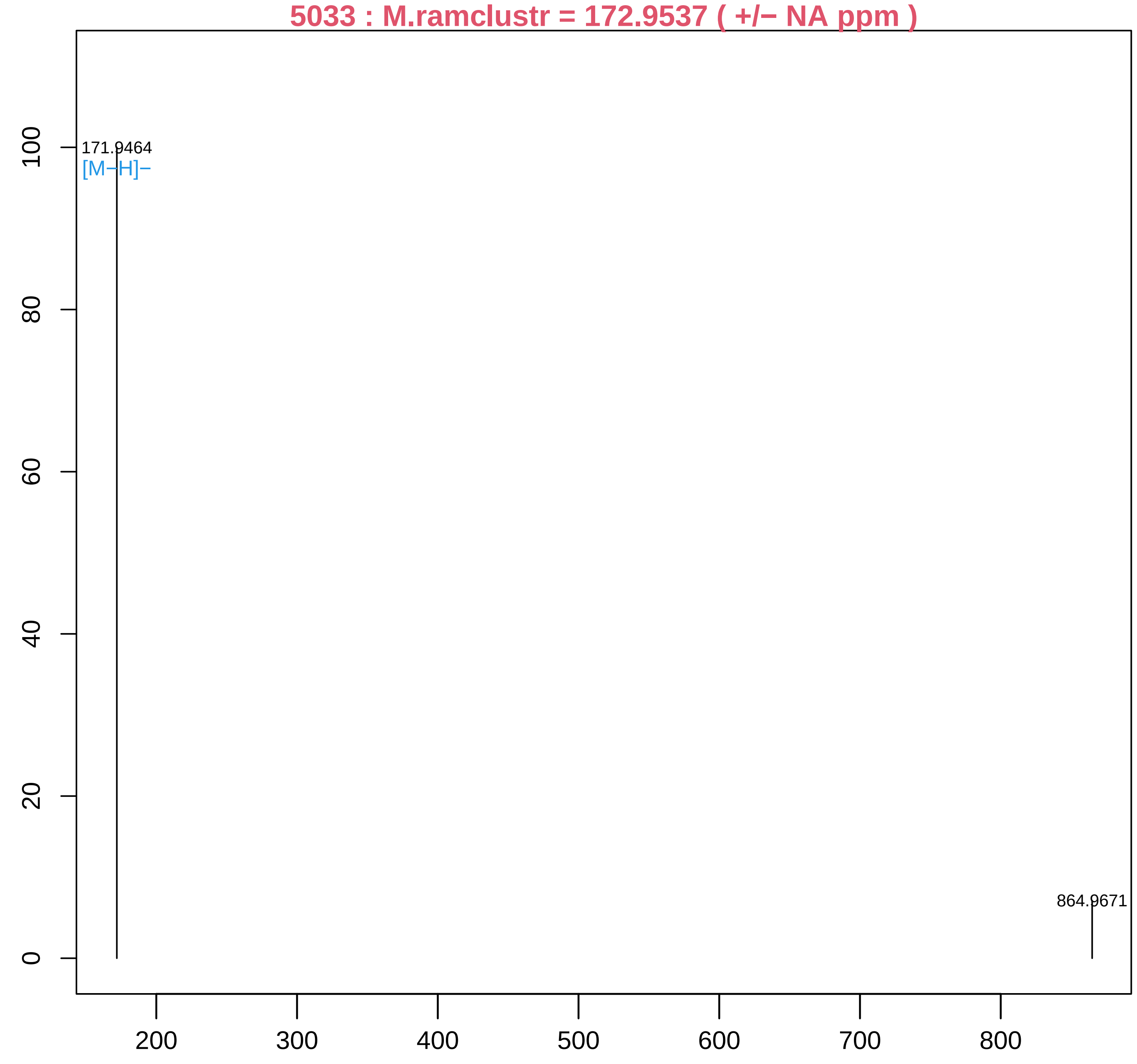
5033 : M.ramclustr = 172.9537 (+/− NA ppm)
171.9464
[M−H]−
864.9671
0
20
40
60
80
100
200
300
400
500
600
700
800

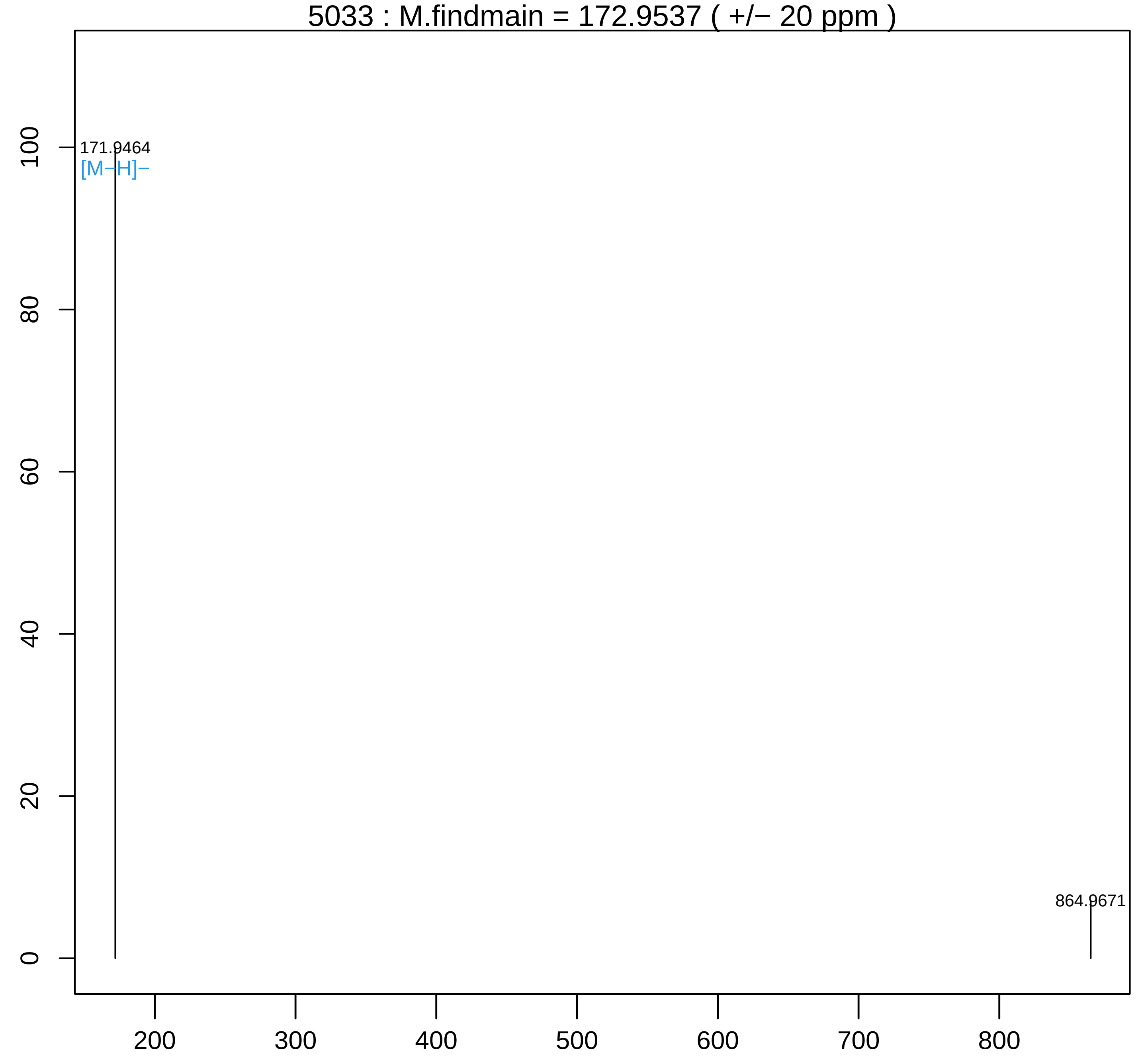
5033 : M.findmain = 172.9537 (+/− 20 ppm)
171.9464
[M−H]−
864.9671
0
20
40
60
80
100
200
300
400
500
600
700
800

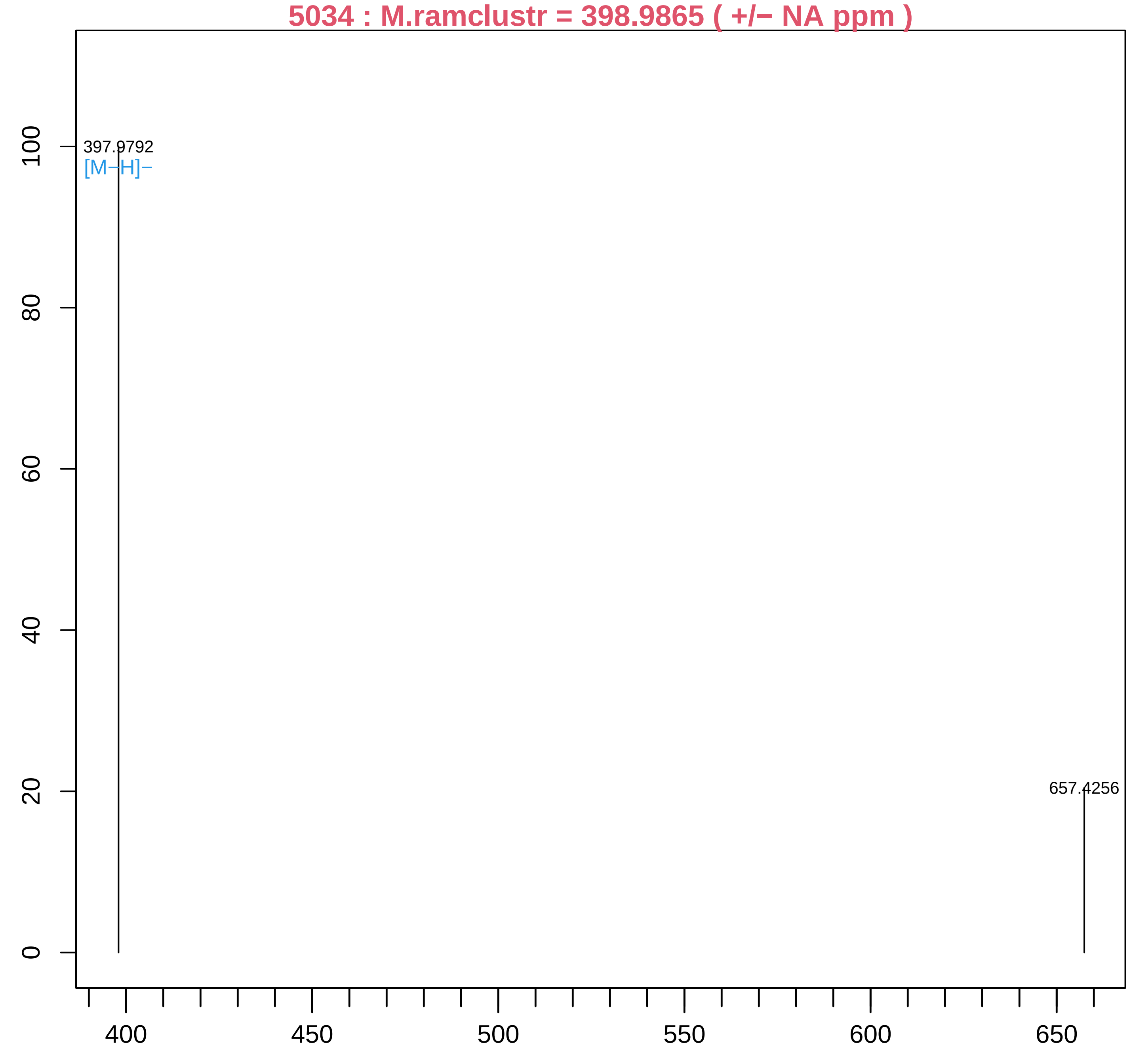
5034 : M.ramclustr = 398.9865 (+/− NA ppm)
397.9792
[M−H]−
657.4256
0
20
40
60
80
100
400
450
500
550
600
650
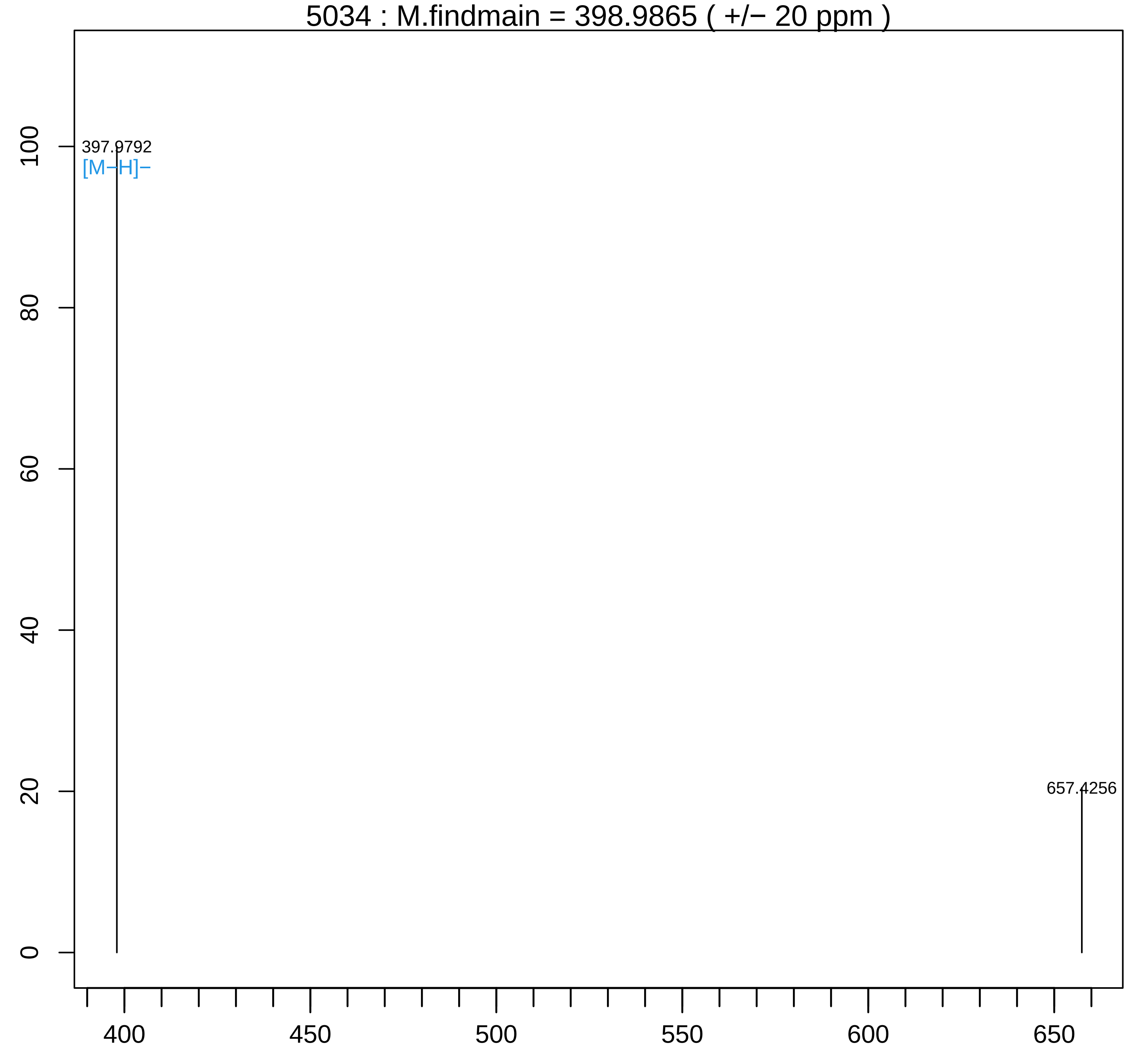
5034 : M.findmain = 398.9865 (+/− 20 ppm)
397.9792
[M−H]−
657.4256
0
20
40
60
80
100
400
450
500
550
600
650

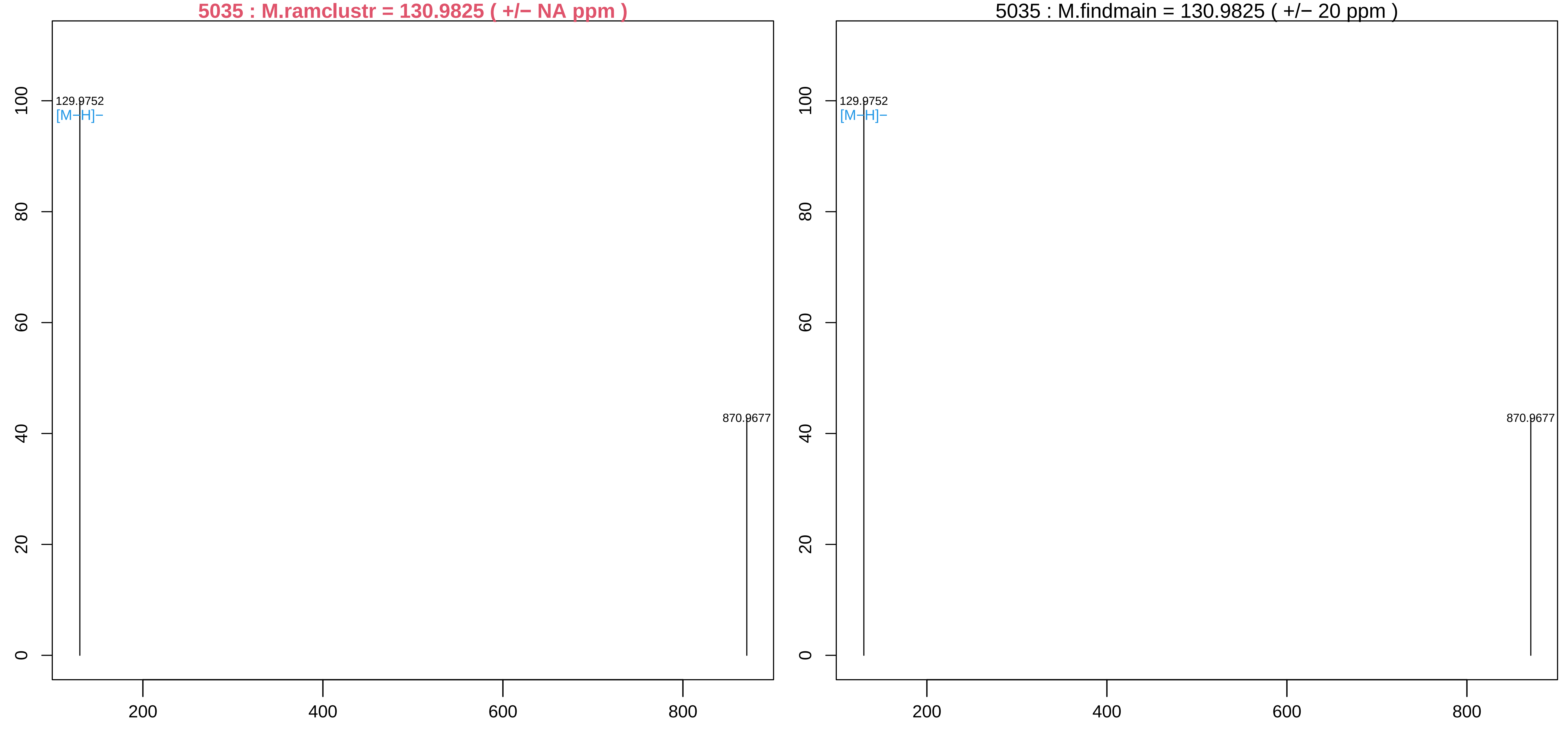
5035 : M.ramclustr = 130.9825 (+/− NA ppm)
129.9752
[M−H]−
870.9677
5035 : M.findmain = 130.9825 (+/− 20 ppm)
129.9752
[M−H]−
870.9677
0
20
40
60
80
100
200
400
600
800

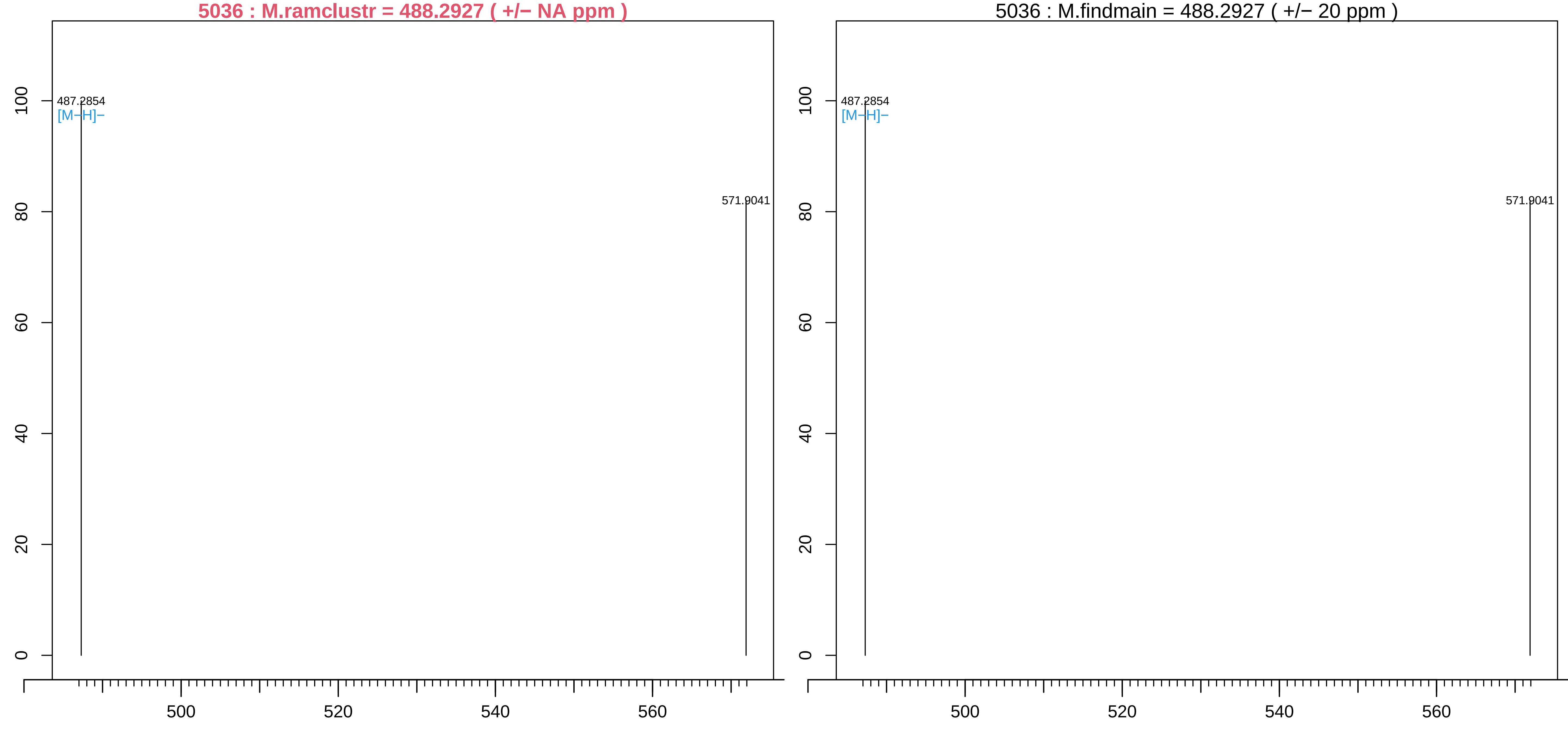
5036 : M.ramclustr = 488.2927 (+/− NA ppm)
487.2854
[M−H]−
571.9041
5036 : M.findmain = 488.2927 (+/− 20 ppm)
487.2854
[M−H]−
571.9041
0
20
40
60
80
100
500
520
540
560

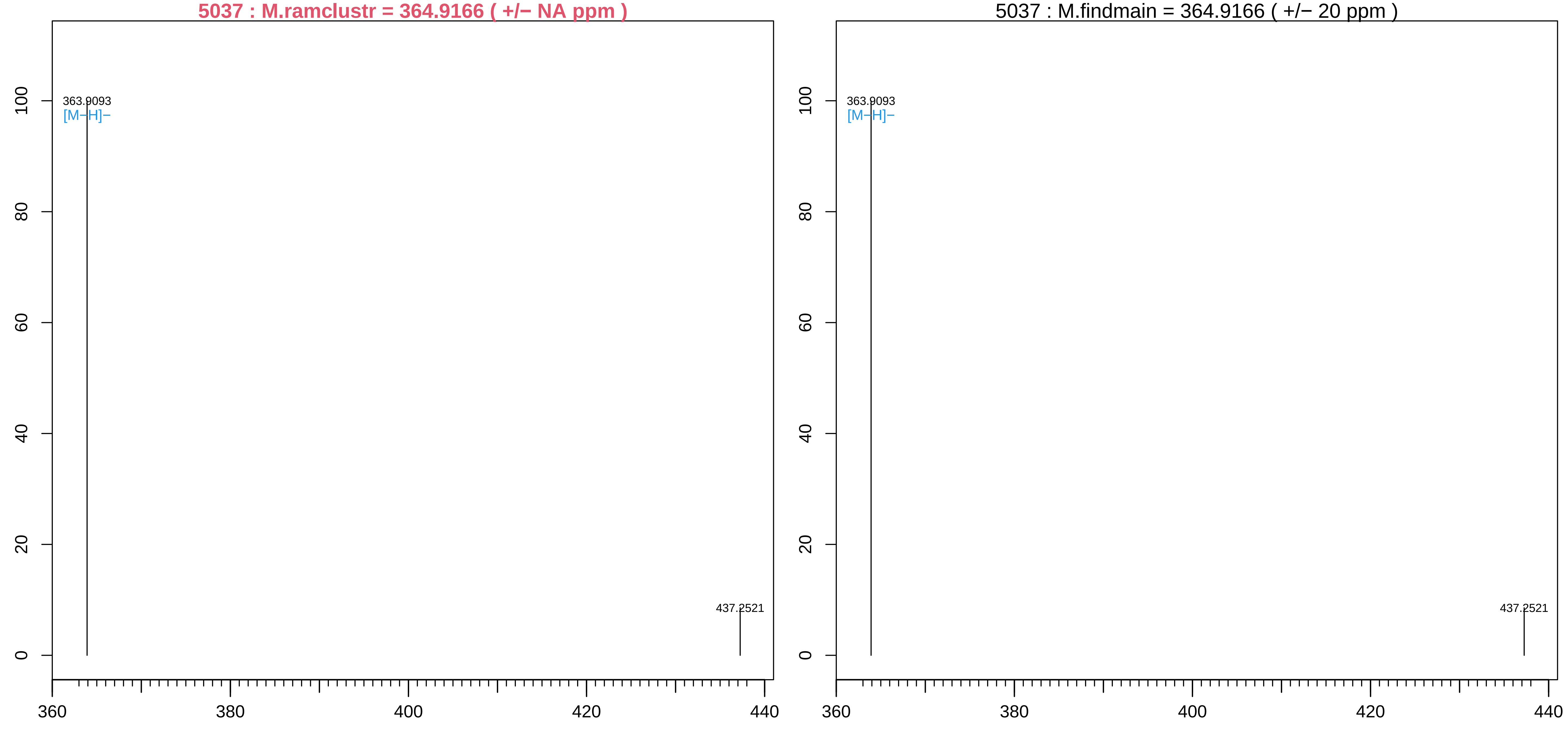

5037 : M.ramclustr = 364.9166 (+/− NA ppm)
363.9093
[M−H]−
437.2521
5037 : M.findmain = 364.9166 (+/− 20 ppm)
363.9093
[M−H]−
437.2521
0
20
40
60
80
100
360
380
400
420
440

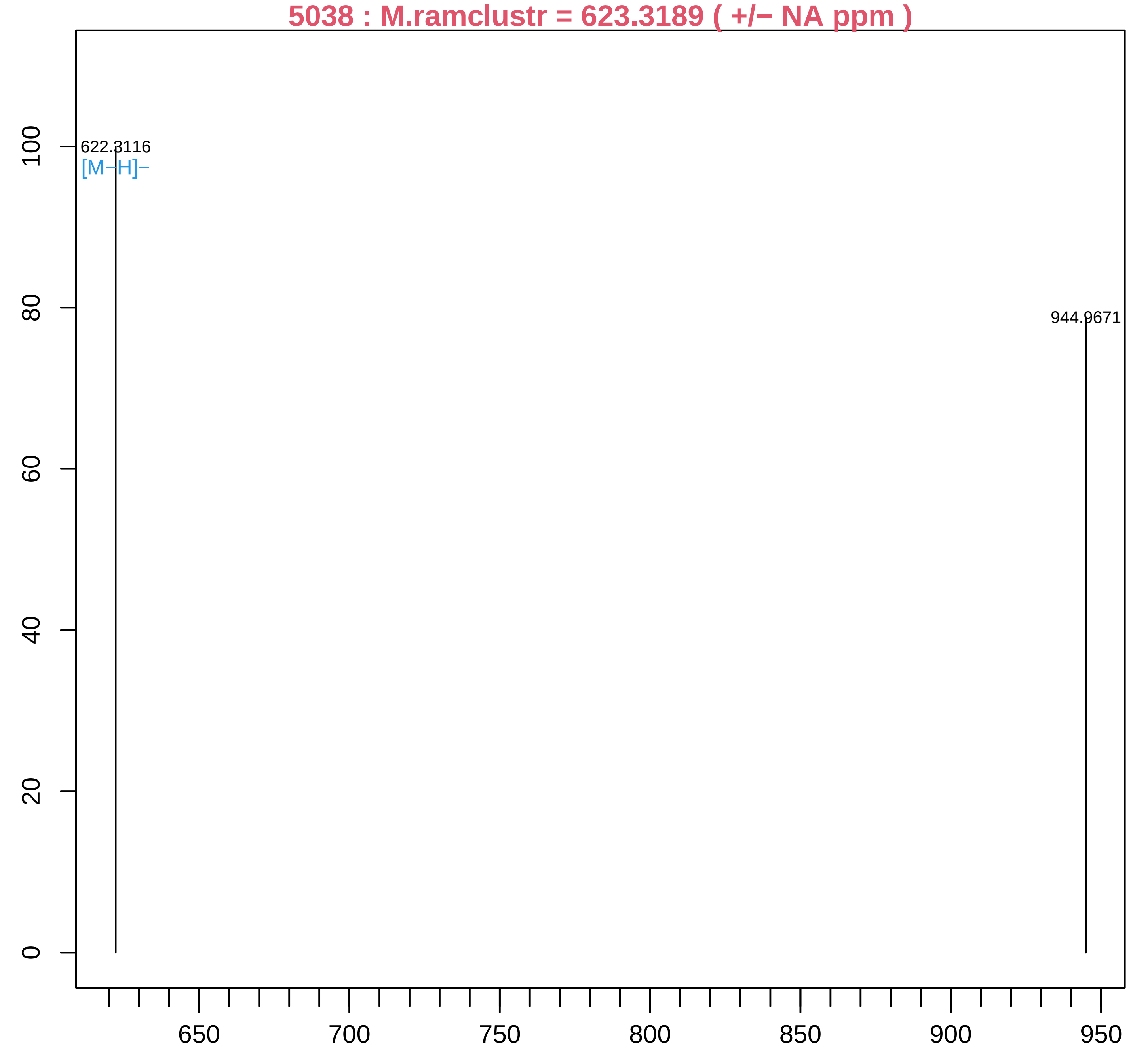
5038 : M.ramclustr = 623.3189 (+/− NA ppm)
622.3116
[M−H]−
944.9671
0
20
40
60
80
100
650
700
750
800
850
900
950

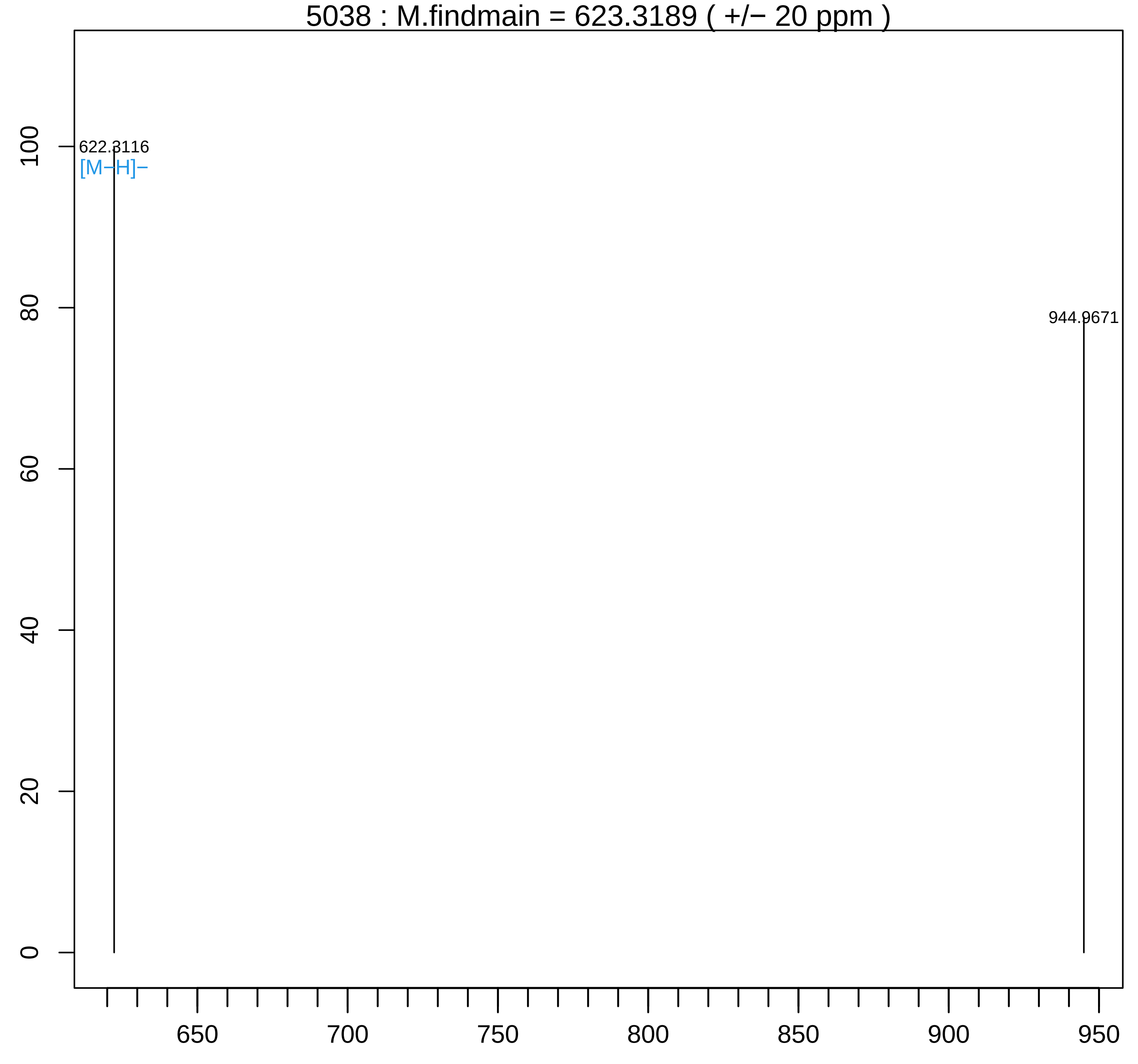
5038 : M.findmain = 623.3189 (+/− 20 ppm)
622.3116
[M−H]−
944.9671
0
20
40
60
80
100
650
700
750
800
850
900
950

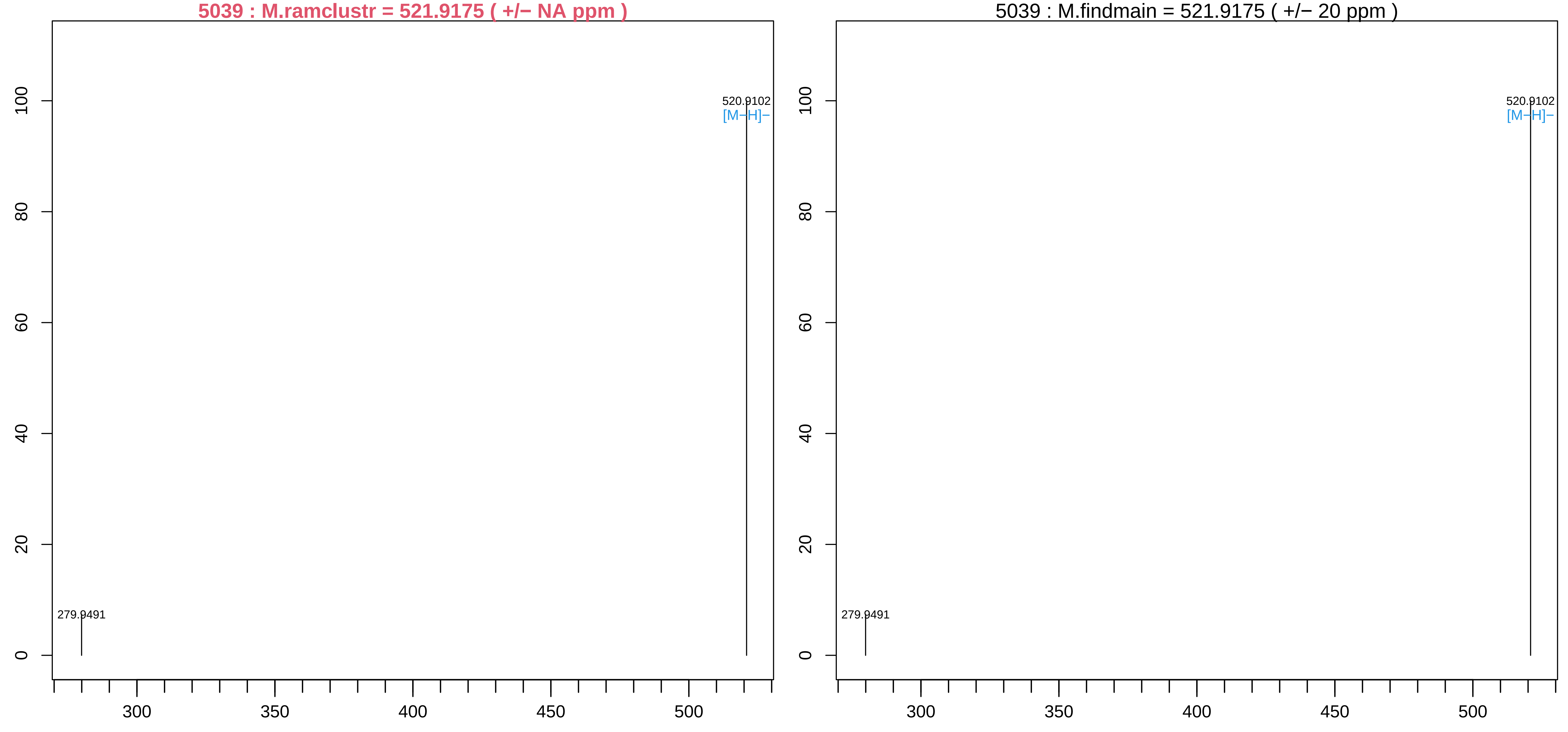
5039 : M.ramclustr = 521.9175 (+/− NA ppm)
520.9102
[M−H]−
279.9491
0
20
40
60
80
100
300
350
400
450
500
5039 : M.findmain = 521.9175 (+/− 20 ppm)
520.9102
[M−H]−
279.9491
0
20
40
60
80
100
300
350
400
450
500

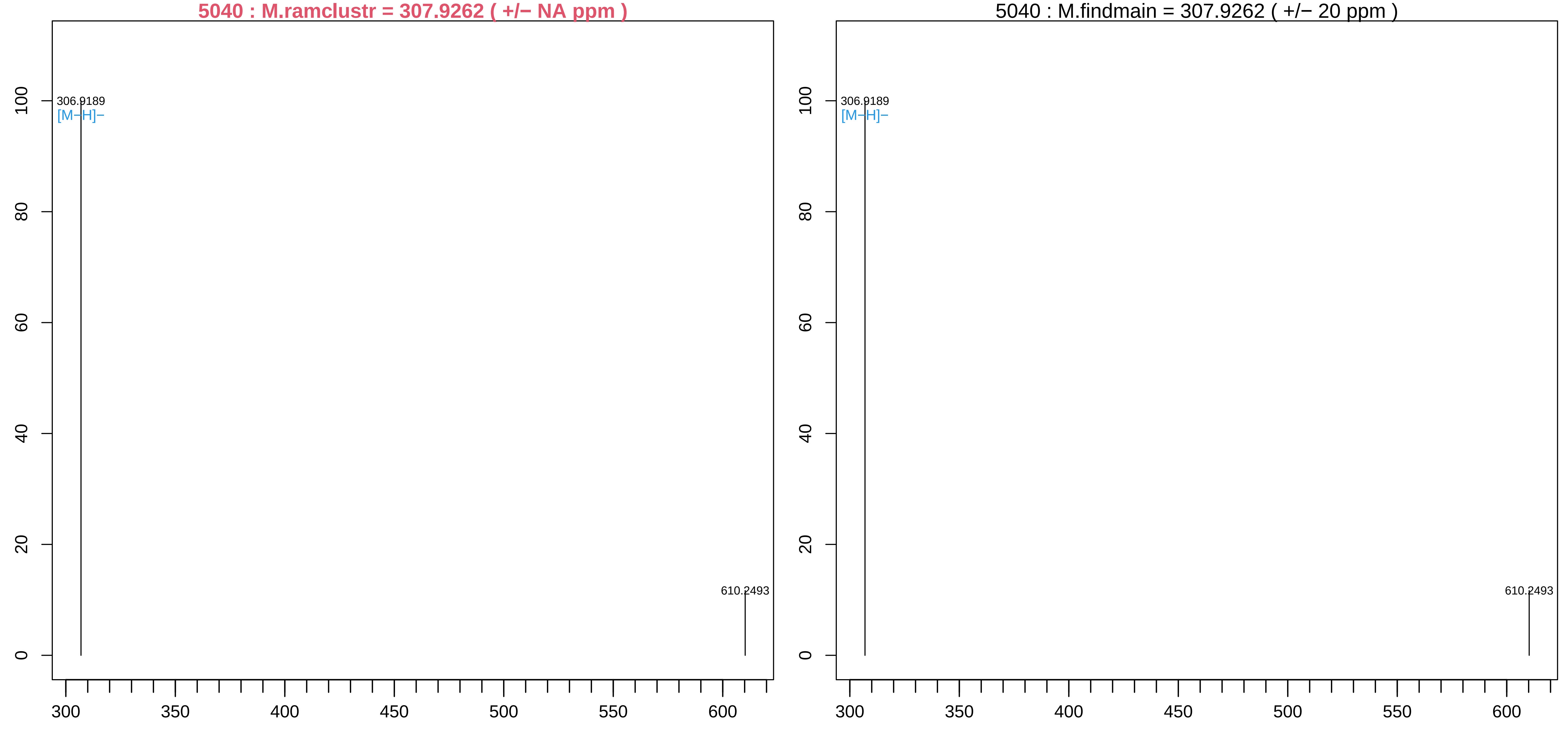
5040 : M.ramclustr = 307.9262 (+/− NA ppm)
306.9189
[M−H]−
610.2493
5040 : M.findmain = 307.9262 (+/− 20 ppm)
306.9189
[M−H]−
610.2493
0
20
40
60
80
100
300
350
400
450
500
550
600

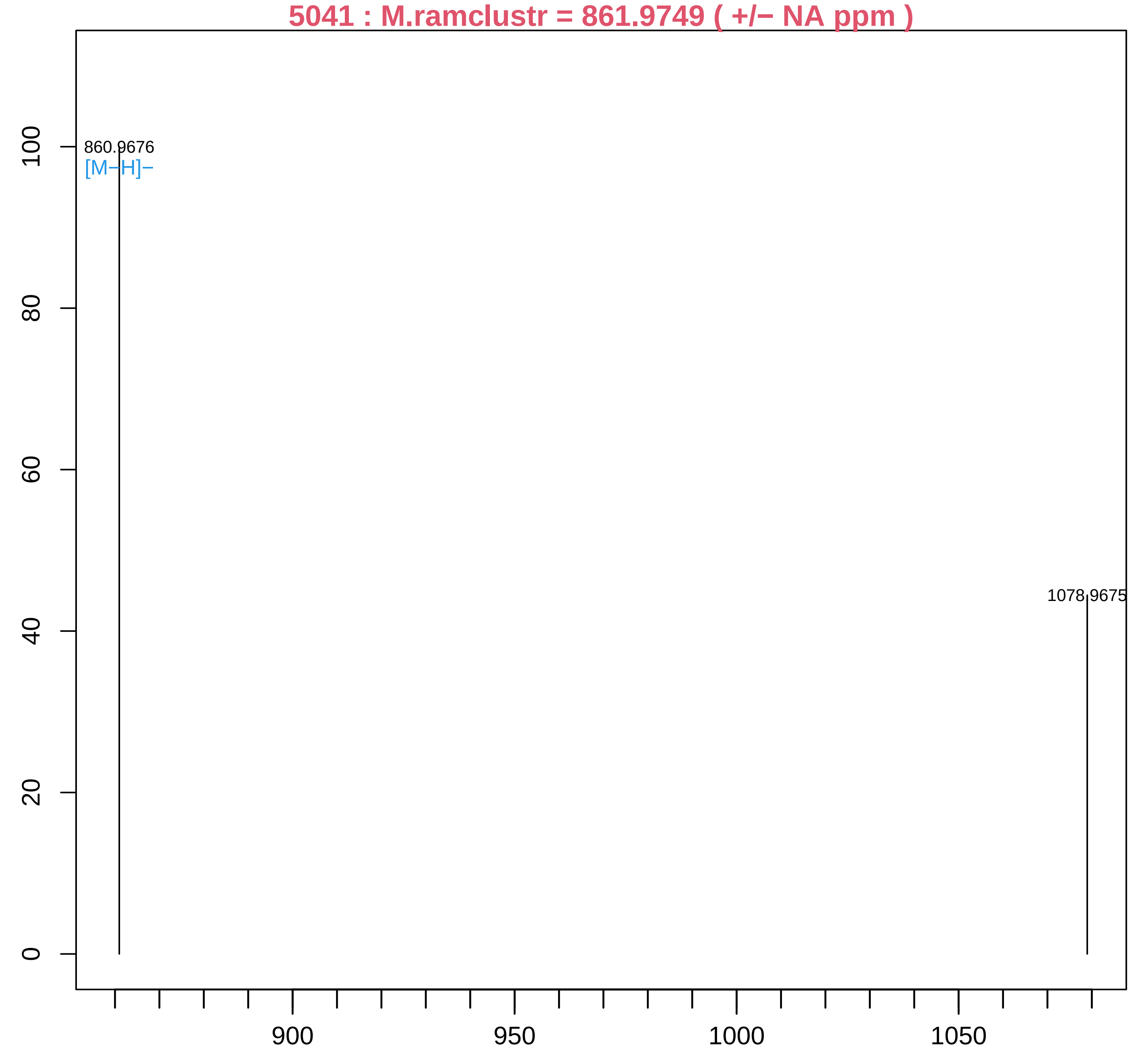

5041 : M.ramclustr = 861.9749 (+/− NA ppm)
860.9676
[M−H]−
1078.9675
0
20
40
60
80
100
900
950
1000
1050

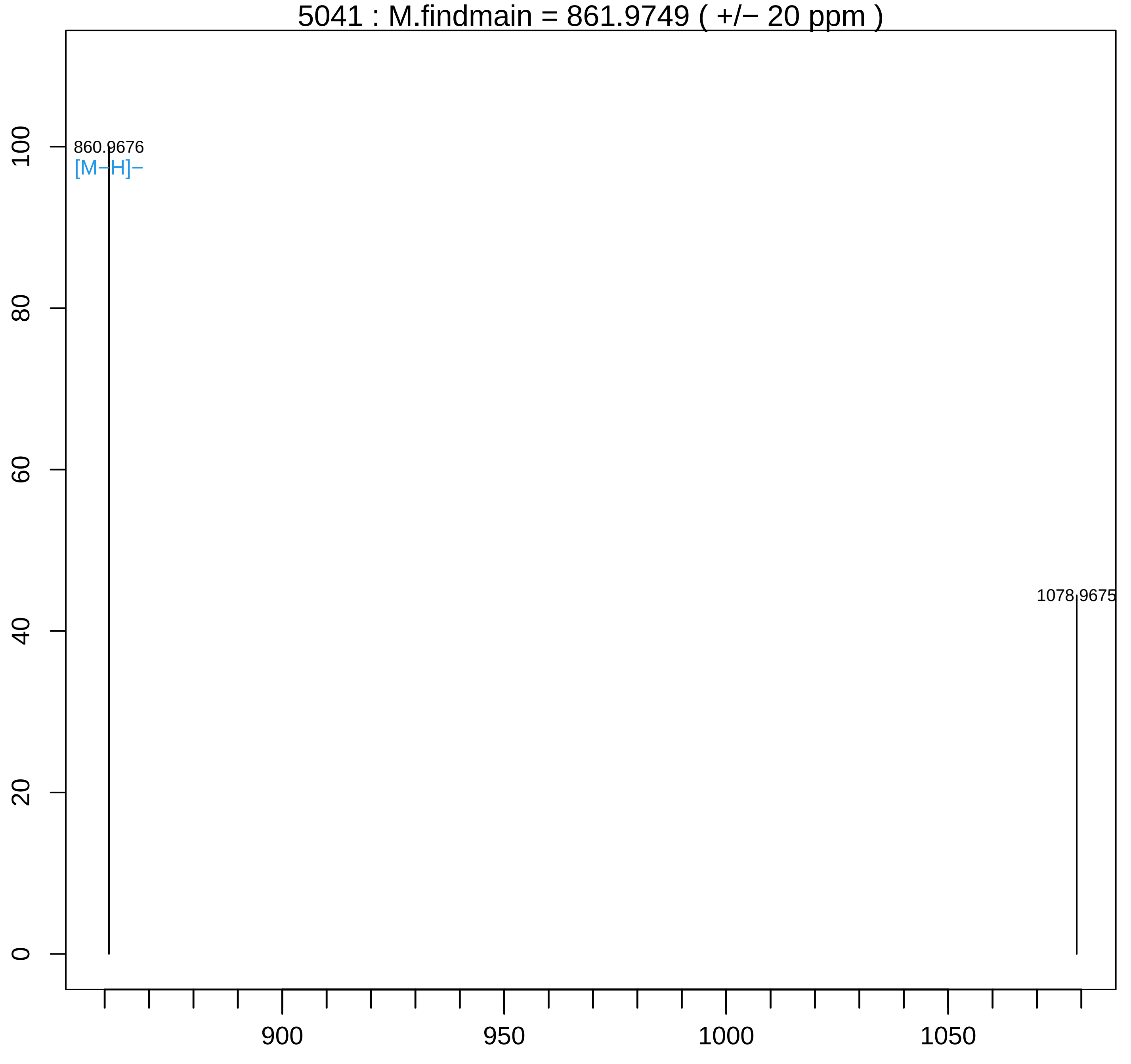

5041 : M.findmain = 861.9749 (+/− 20 ppm)
860.9676
[M−H]−
1078.9675
0
20
40
60
80
100
900
950
1000
1050

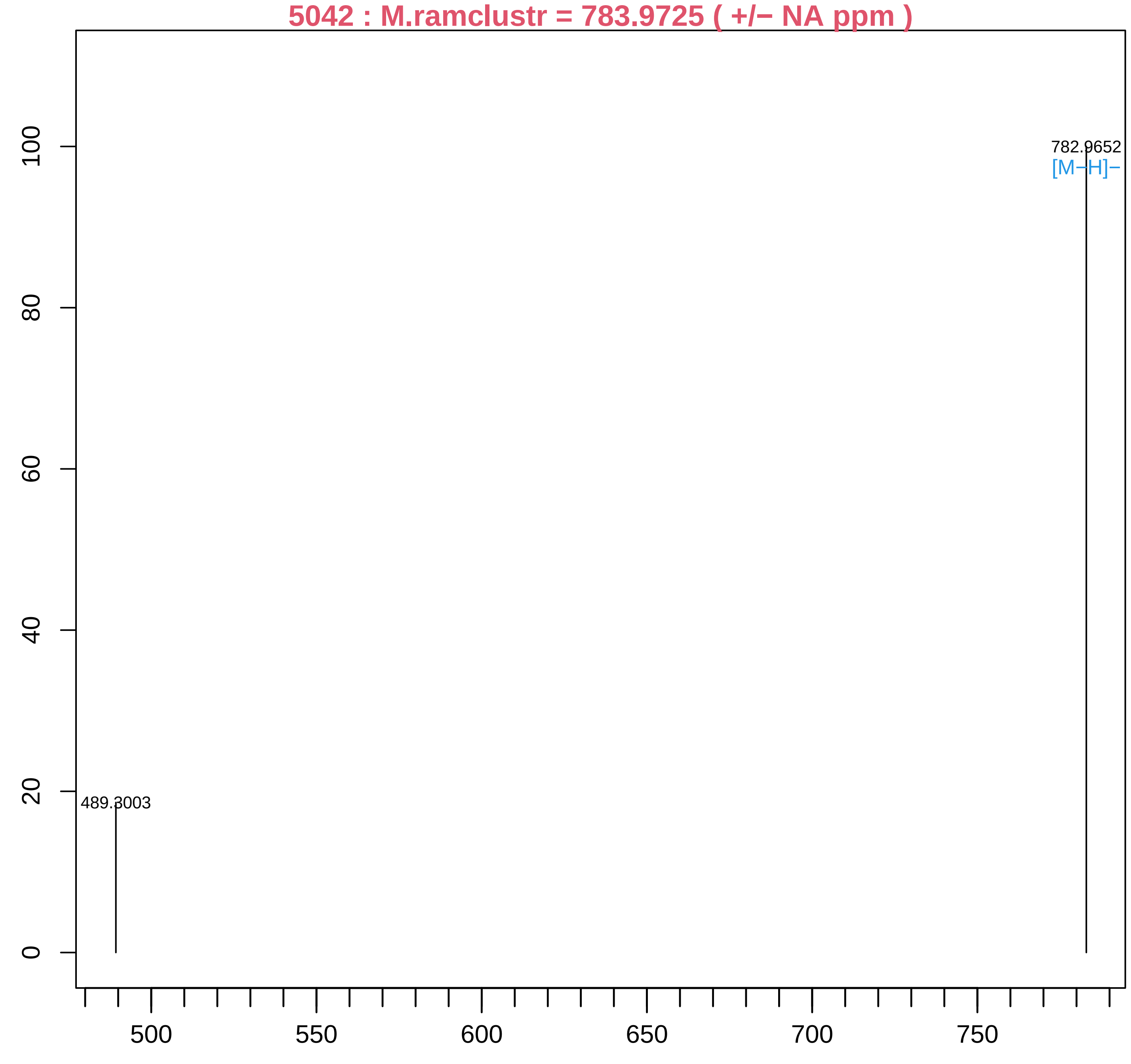
5042 : M.ramclustr = 783.9725 (+/− NA ppm)
782.9652
[M−H]−
489.3003
0
20
40
60
80
100
500
550
600
650
700
750

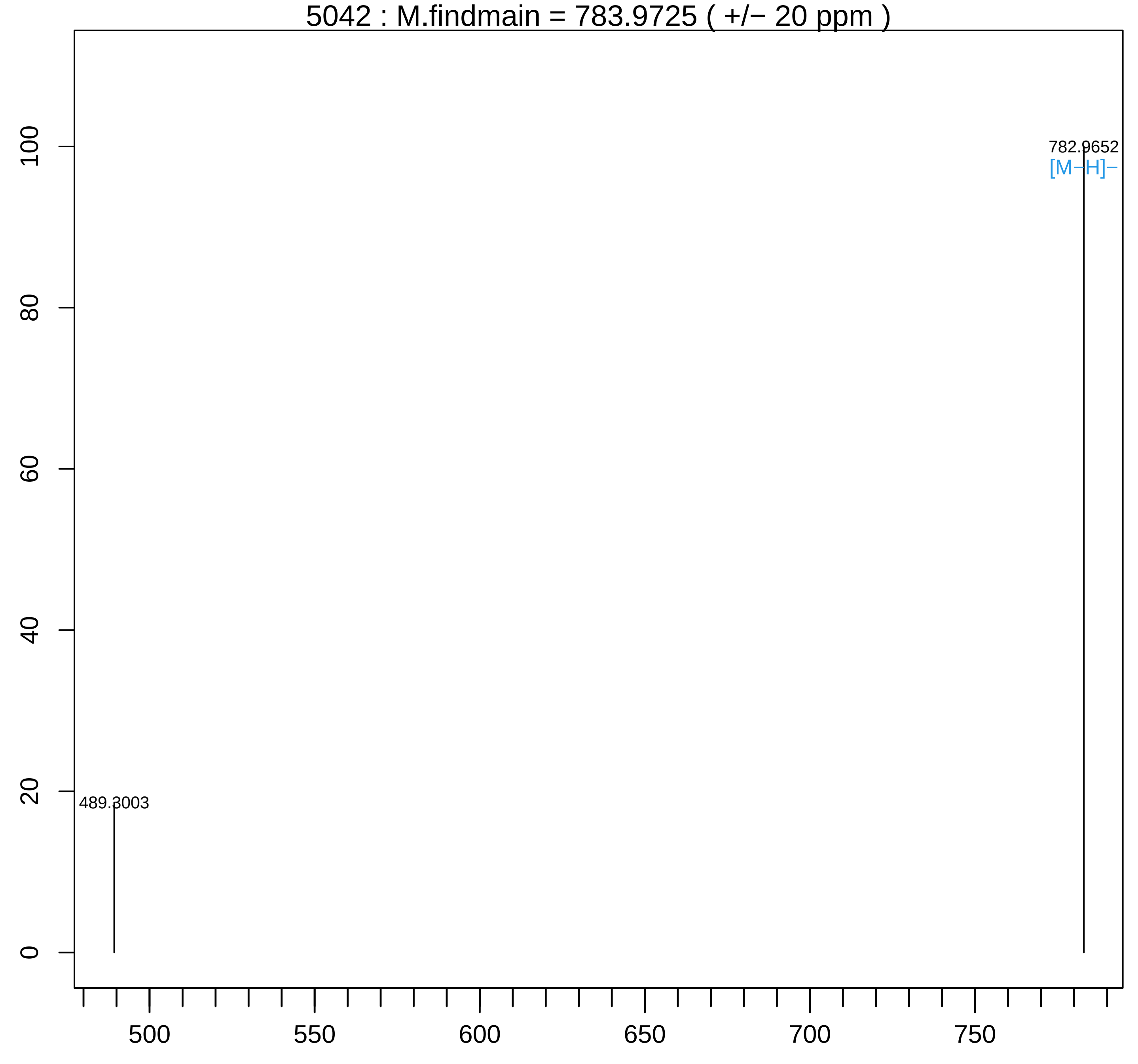
5042 : M.findmain = 783.9725 (+/− 20 ppm)
782.9652
[M−H]−
489.3003
0
20
40
60
80
100
500
550
600
650
700
750

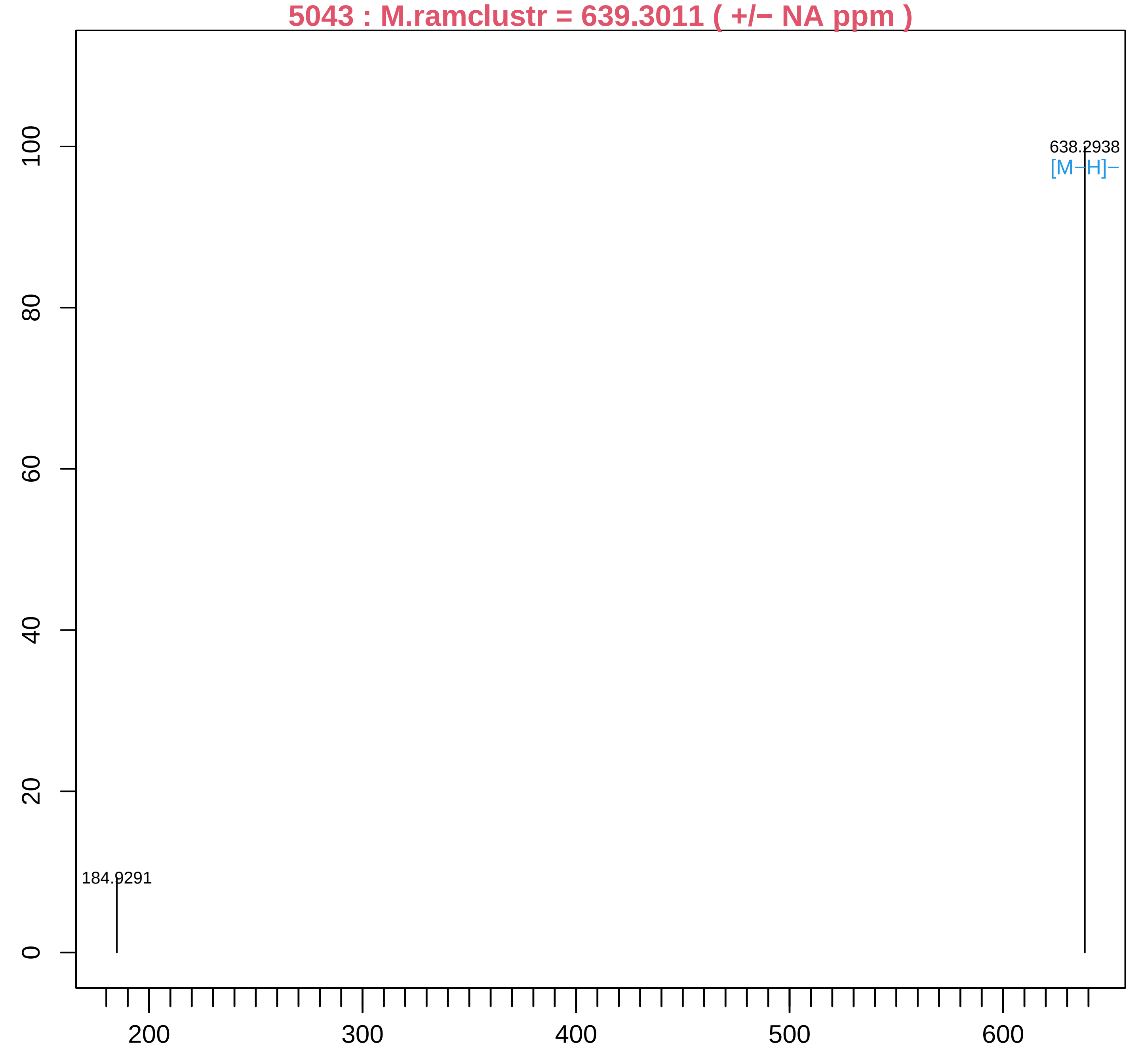
5043 : M.ramclustr = 639.3011 (+/− NA ppm)
638.2938
[M−H]−
184.9291
0
20
40
60
80
100
200
300
400
500
600

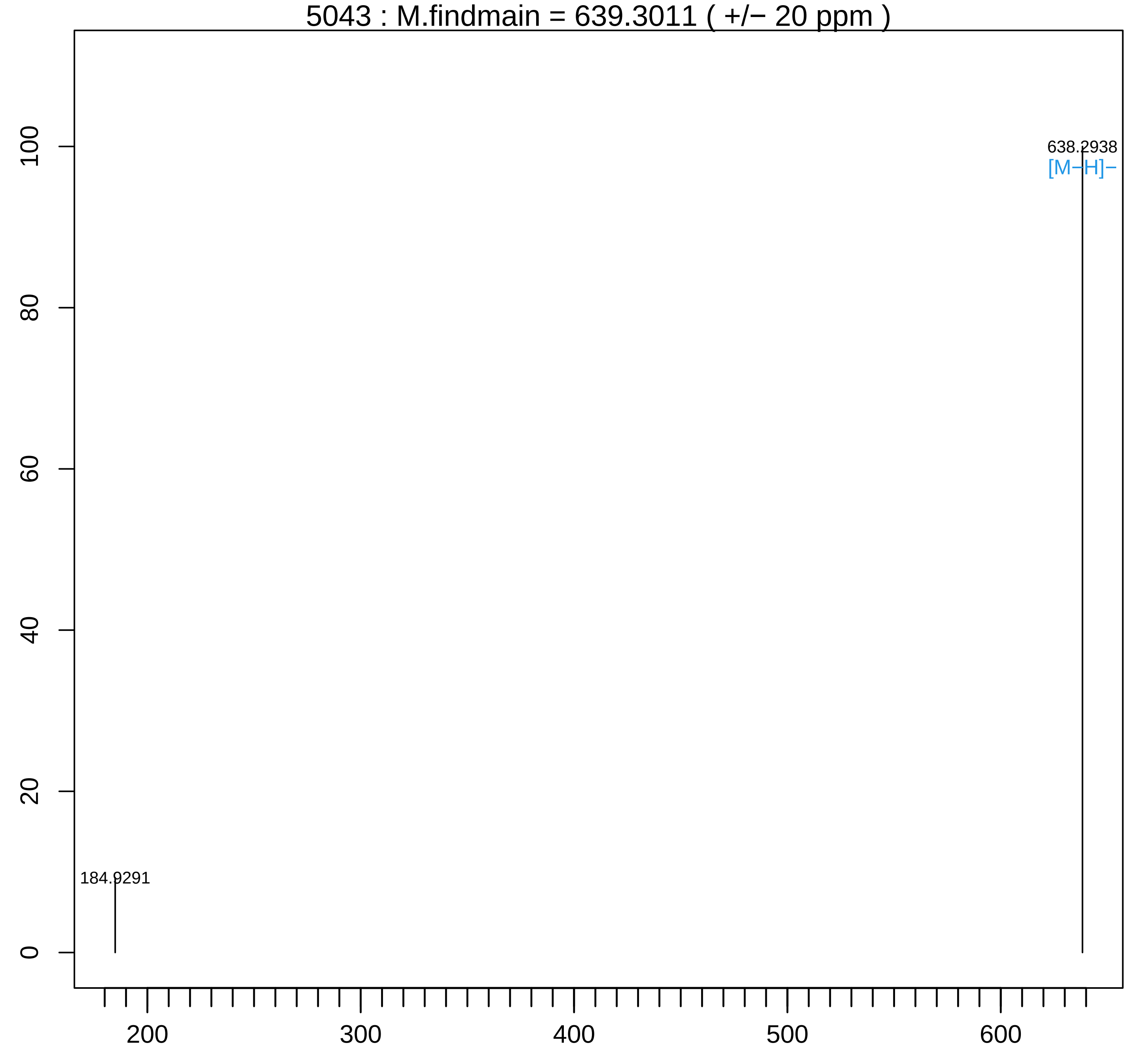
5043 : M.findmain = 639.3011 (+/− 20 ppm)
638.2938
[M−H]−
184.9291
0
20
40
60
80
100
200
300
400
500
600

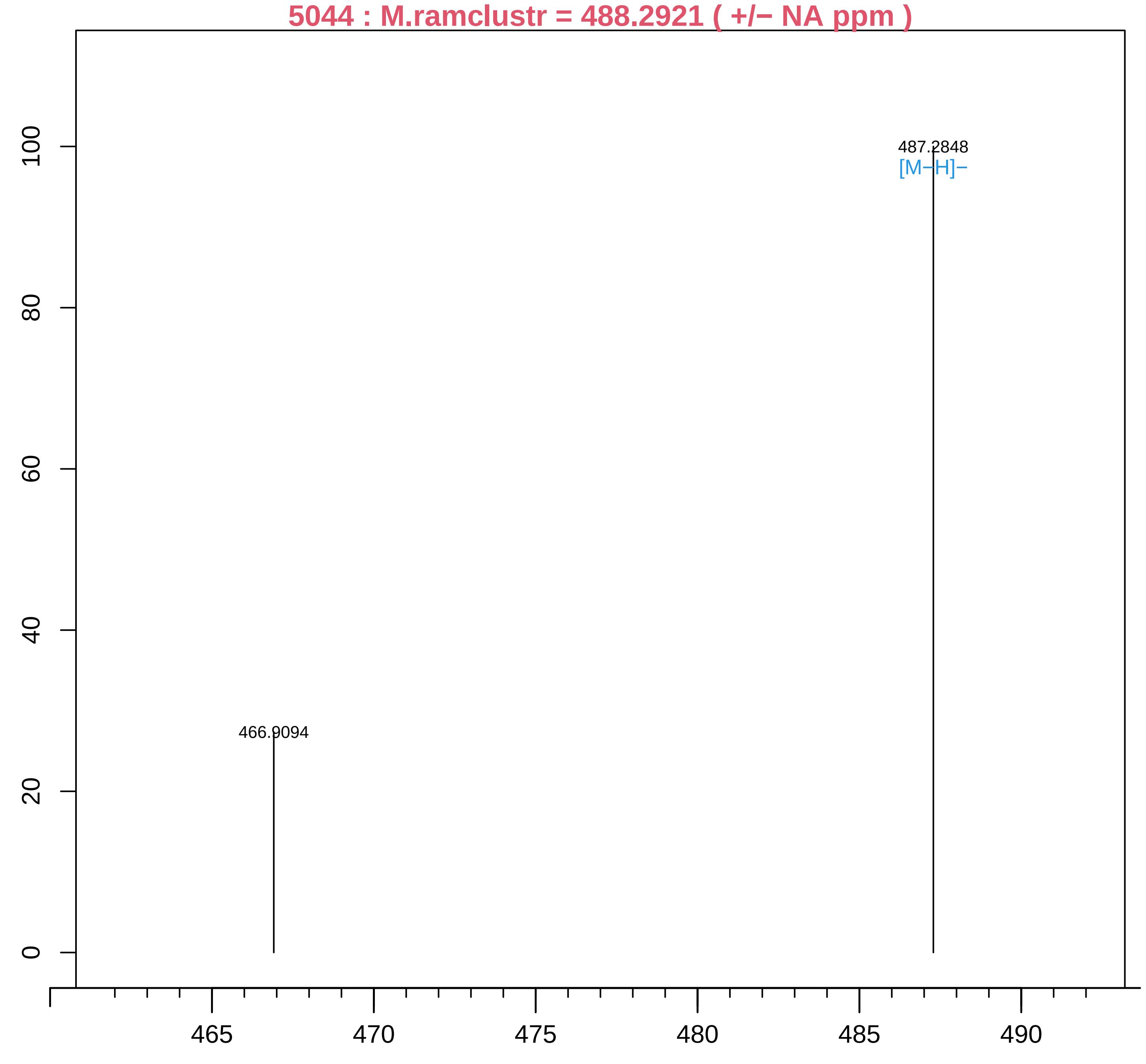
5044 : M.ramclustr = 488.2921 (+/− NA ppm)
487.2848
[M−H]−
466.9094
0
20
40
60
80
100
465
470
475
480
485
490

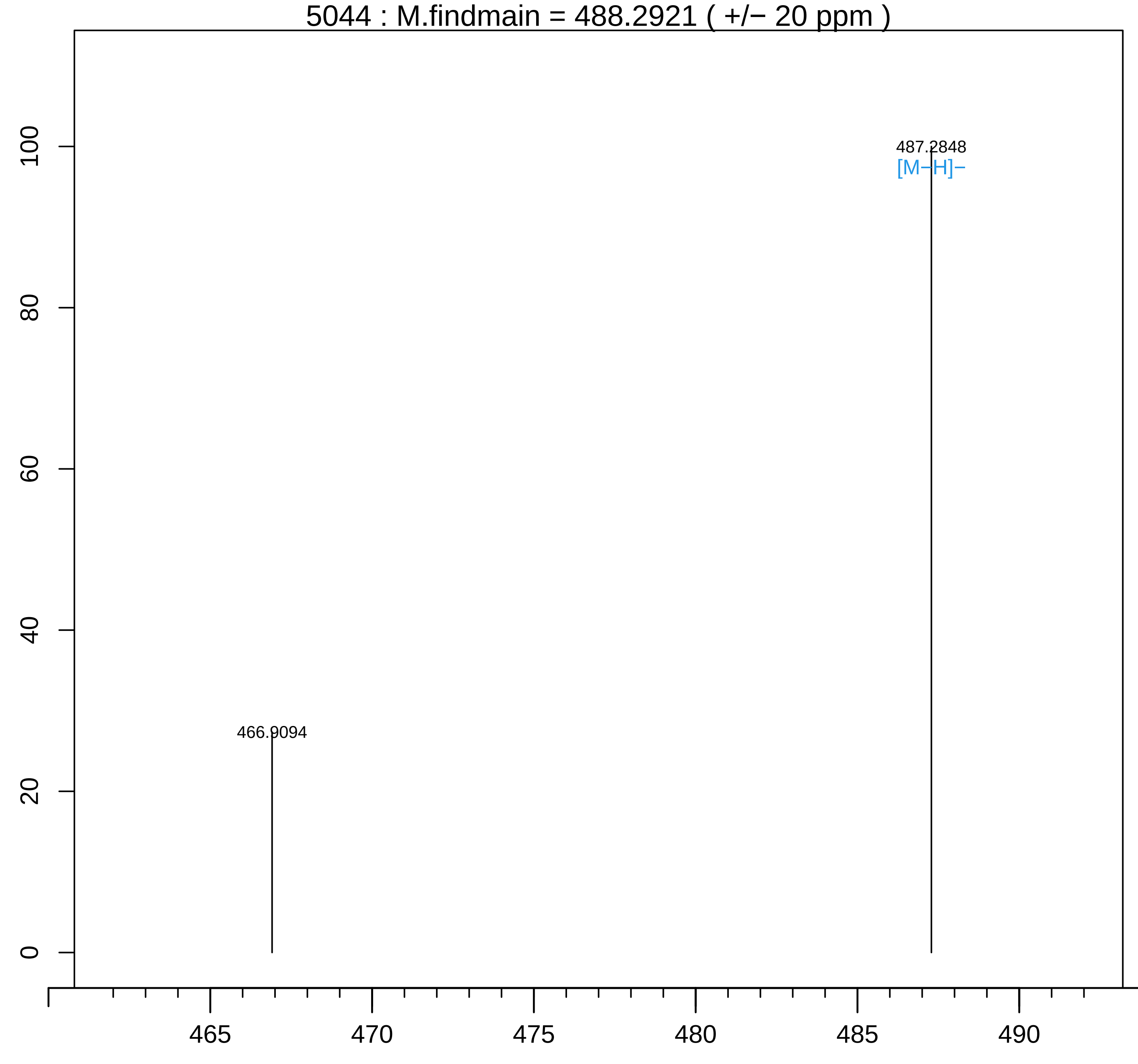
5044 : M.findmain = 488.2921 (+/− 20 ppm)
487.2848
[M−H]−
466.9094
0
20
40
60
80
100
465
470
475
480
485
490

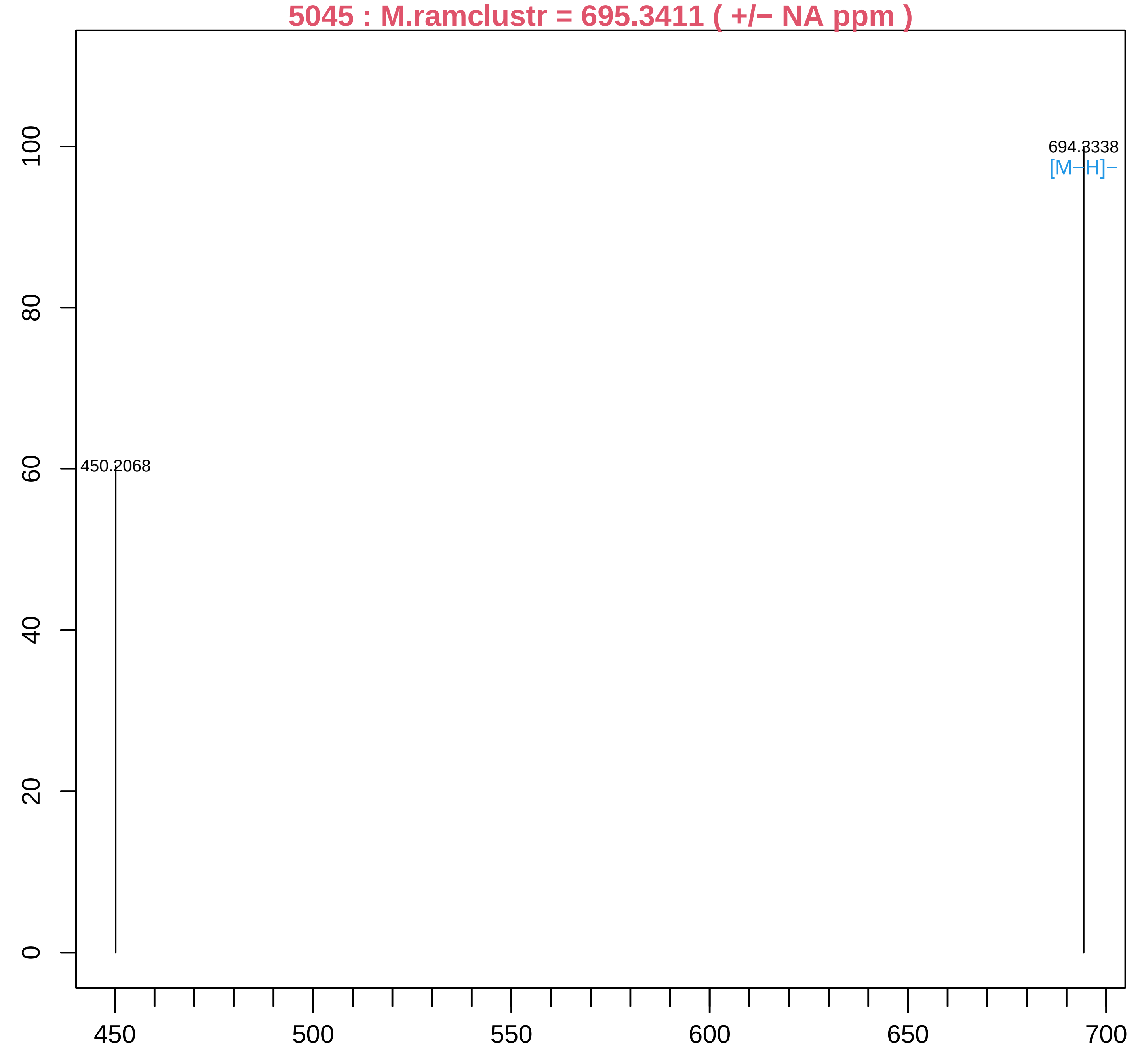

5045 : M.ramclustr = 695.3411 (+/− NA ppm)
694.3338
[M−H]−
450.2068
0
20
40
60
80
100
450
500
550
600
650
700

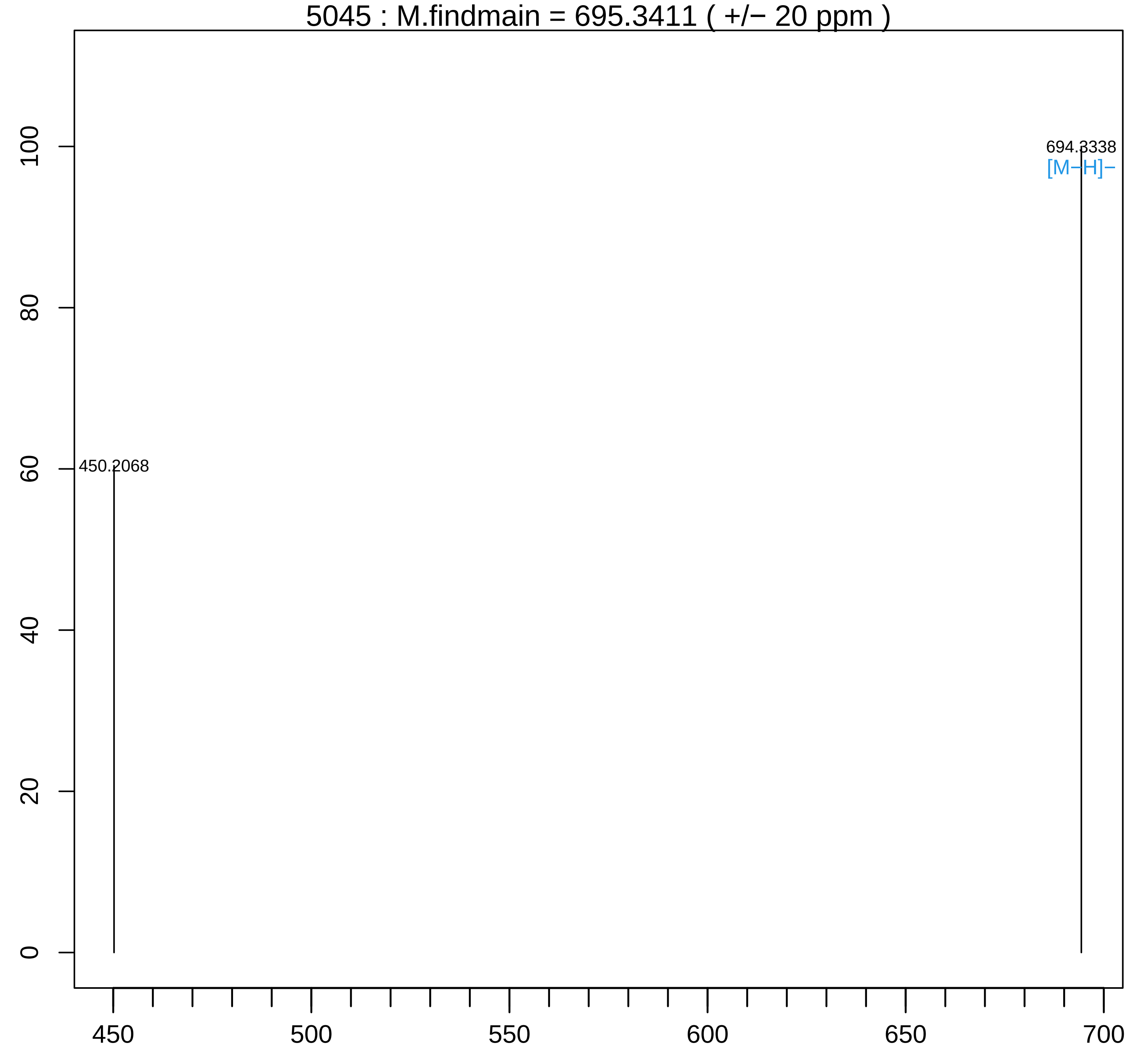

5045 : M.findmain = 695.3411 (+/− 20 ppm)
694.3338
[M−H]−
450.2068
0
20
40
60
80
100
450
500
550
600
650
700

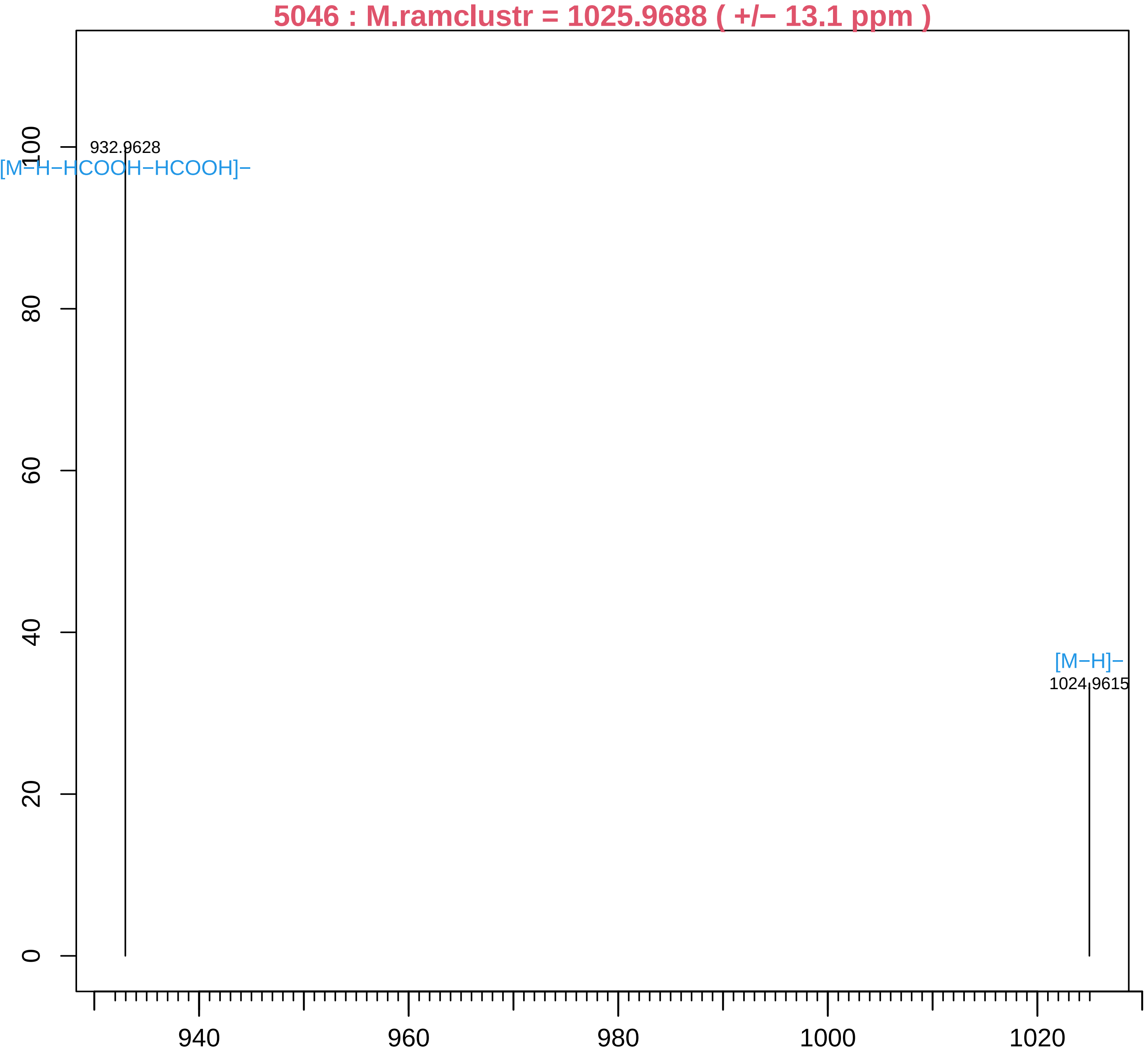
5046 : M.ramclustr = 1025.9688 (+/− 13.1 ppm)
932.9628
[M−H−HCOOH−HCOOH]−
[M−H]−
1024.9615
100
80
60
40
20
0
940
960
980
1000
1020

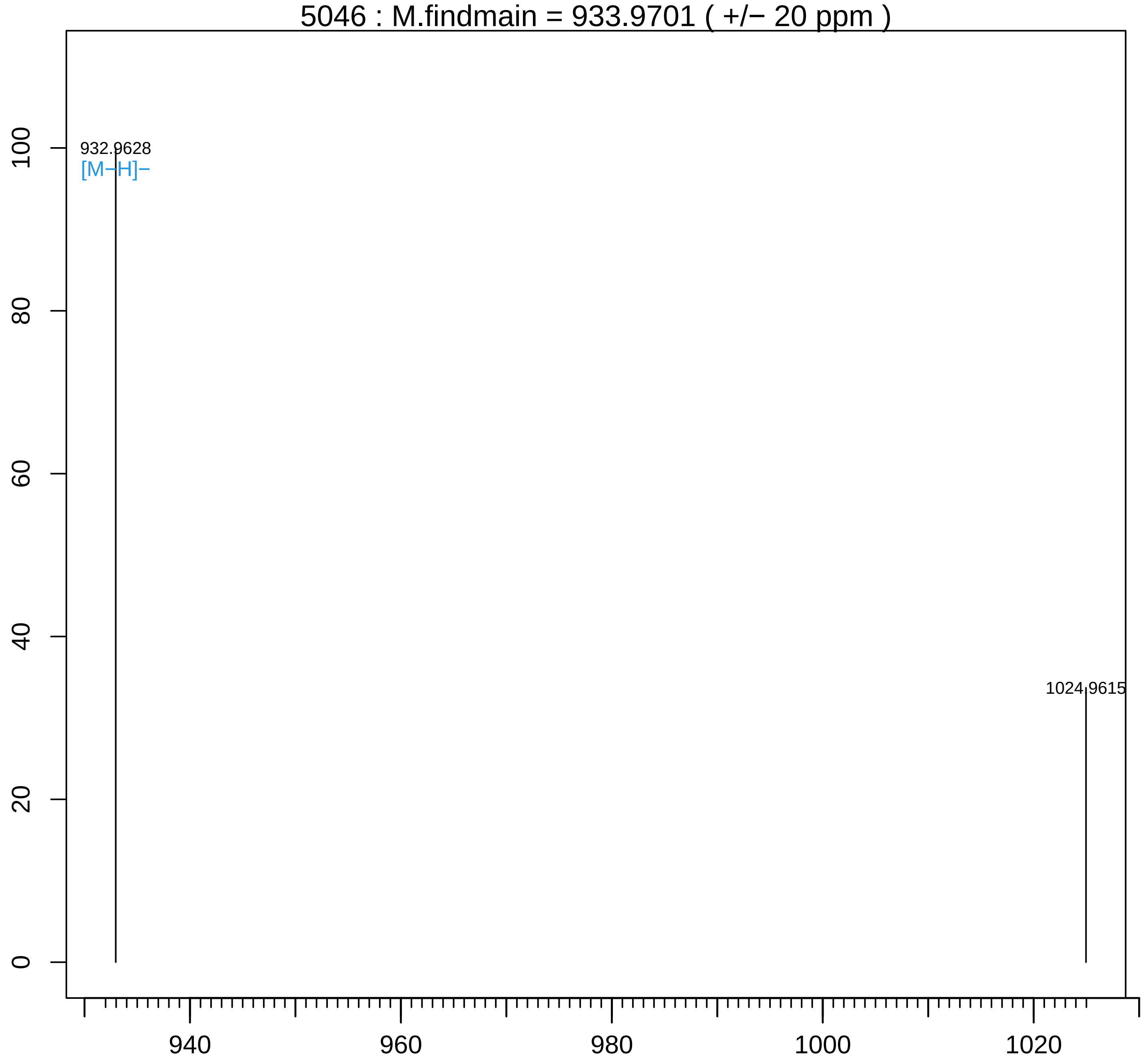
5046 : M.findmain = 933.9701 (+/− 20 ppm)
932.9628
[M−H]−
1024.9615
100
80
60
40
20
0
940
960
980
1000
1020

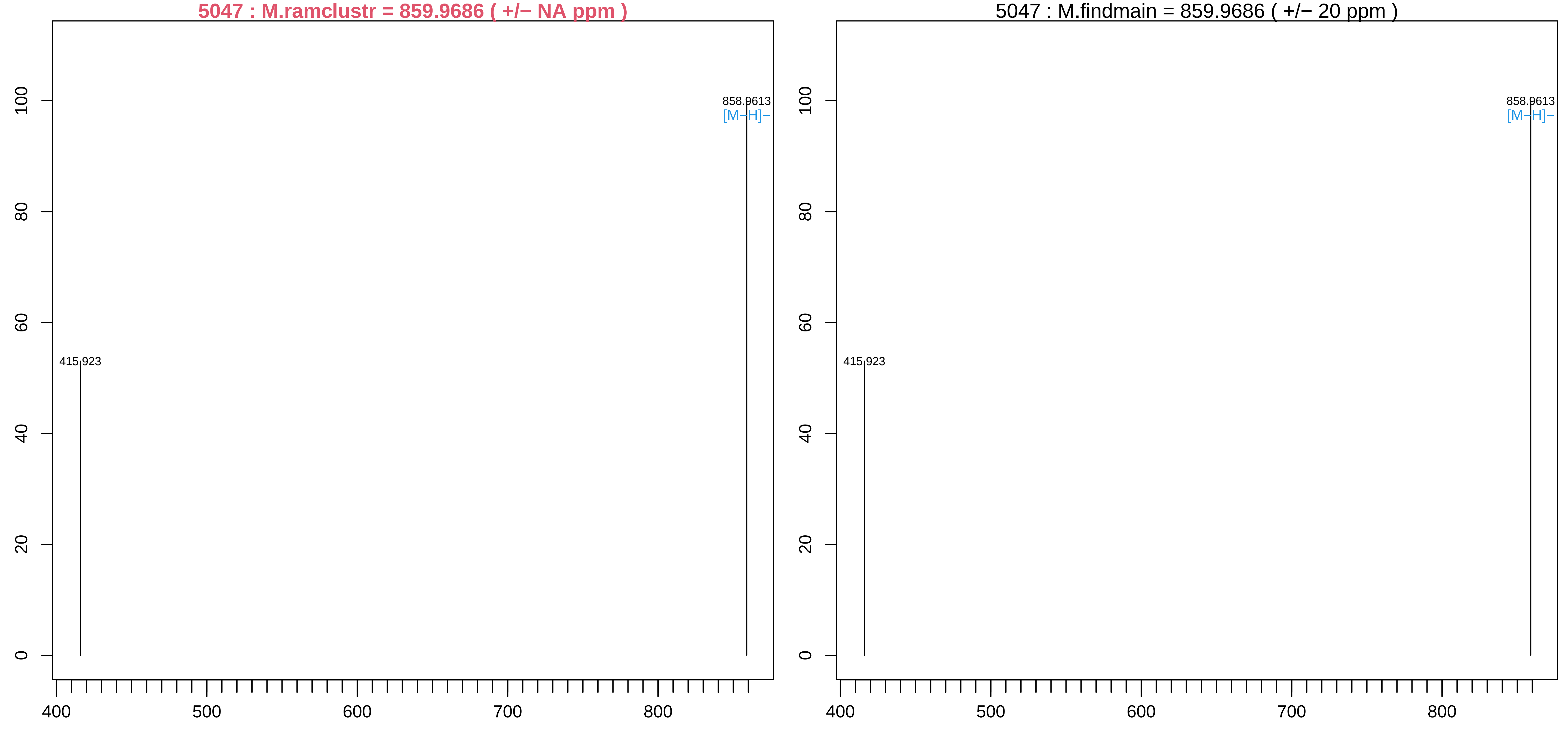

5047 : M.ramclustr = 859.9686 (+/− NA ppm)
858.9613
[M−H]−
415.923
0
20
40
60
80
100
400
500
600
700
800
5047 : M.findmain = 859.9686 (+/− 20 ppm)
858.9613
[M−H]−
415.923
0
20
40
60
80
100
400
500
600
700
800

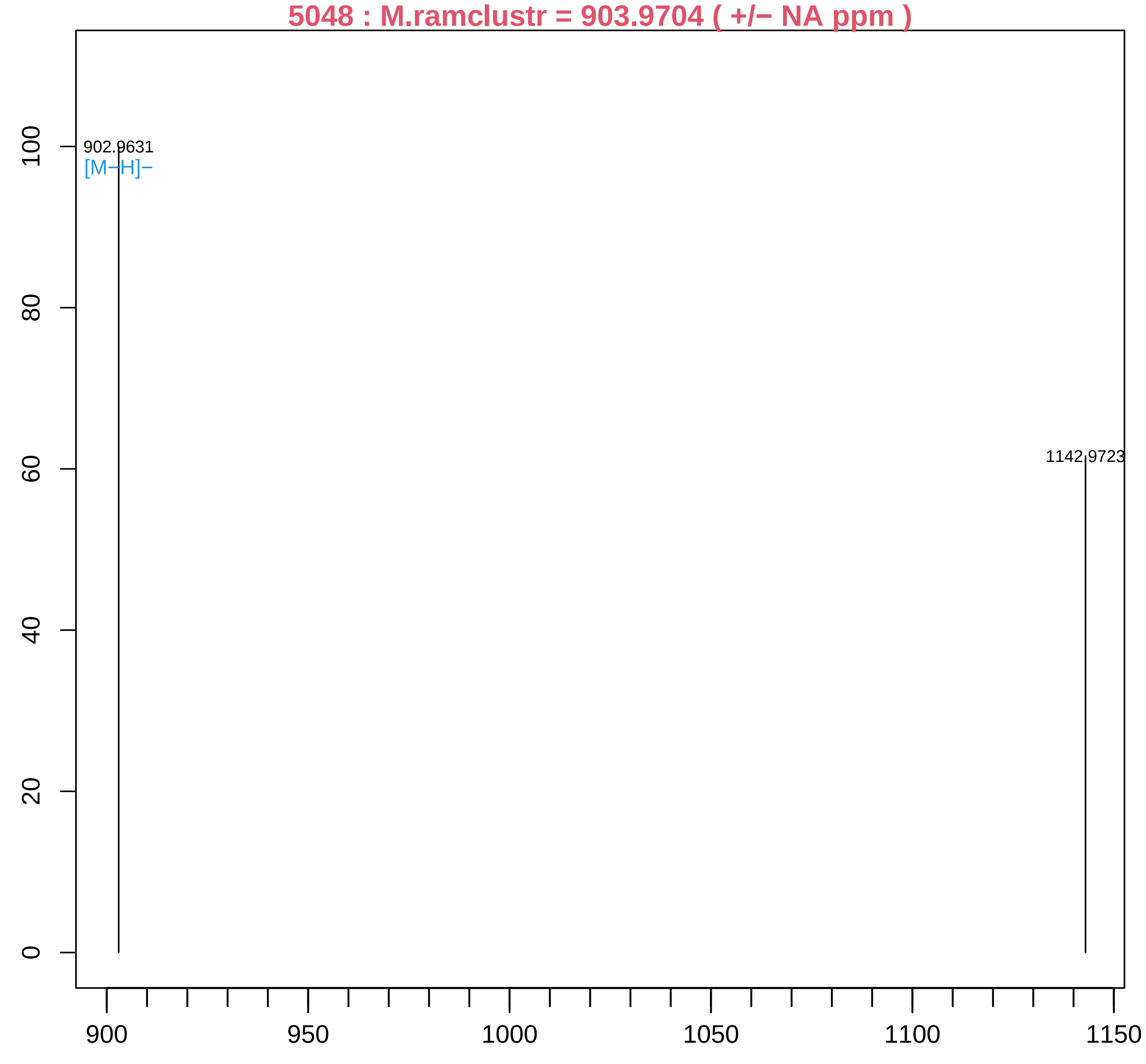
5048 : M.ramclustr = 903.9704 (+/− NA ppm)
902.9631
[M−H]−
1142.9723
0
20
40
60
80
100
900
950
1000
1050
1100
1150

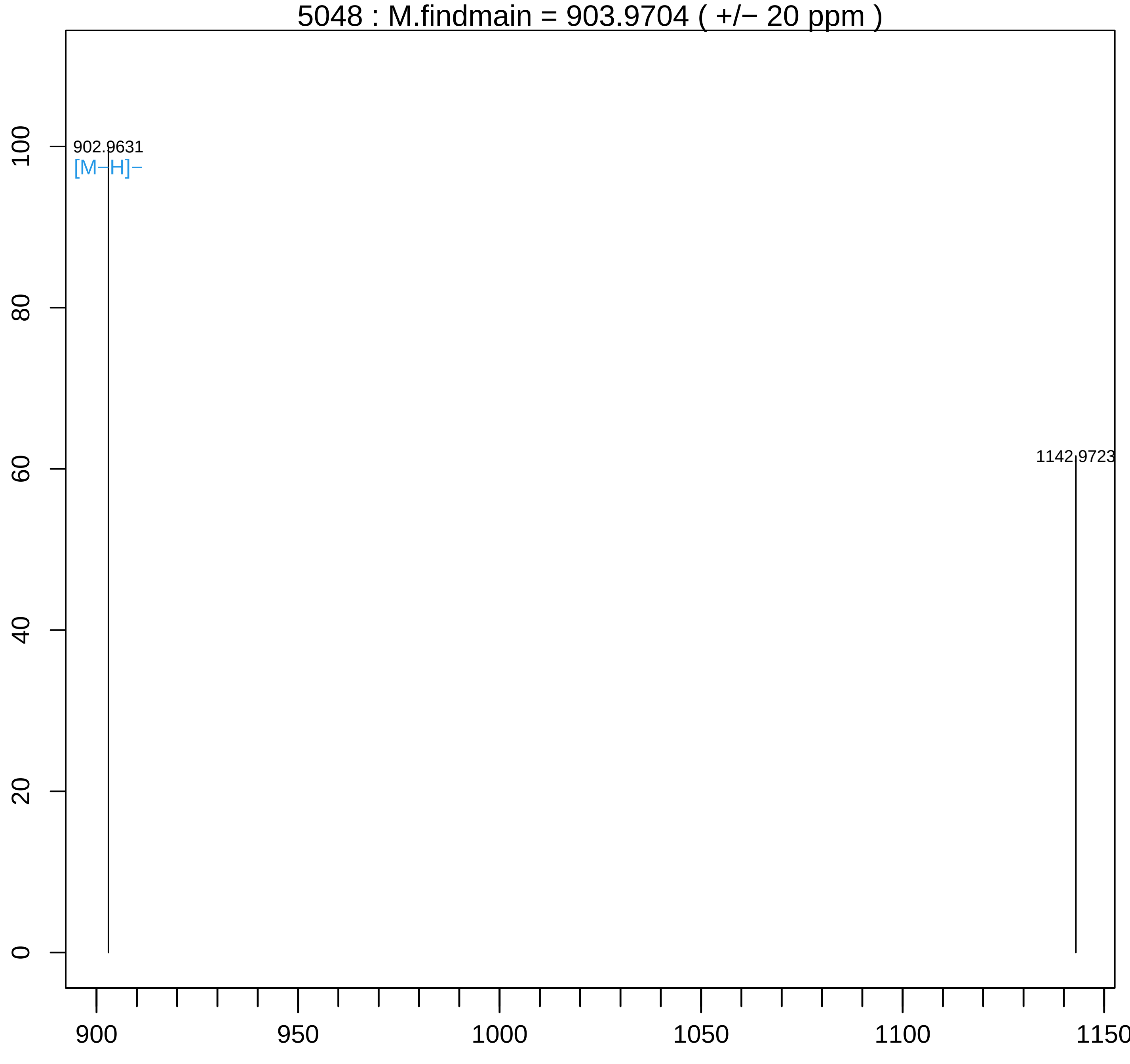
5048 : M.findmain = 903.9704 (+/− 20 ppm)
902.9631
[M−H]−
1142.9723
0
20
40
60
80
100
900
950
1000
1050
1100
1150

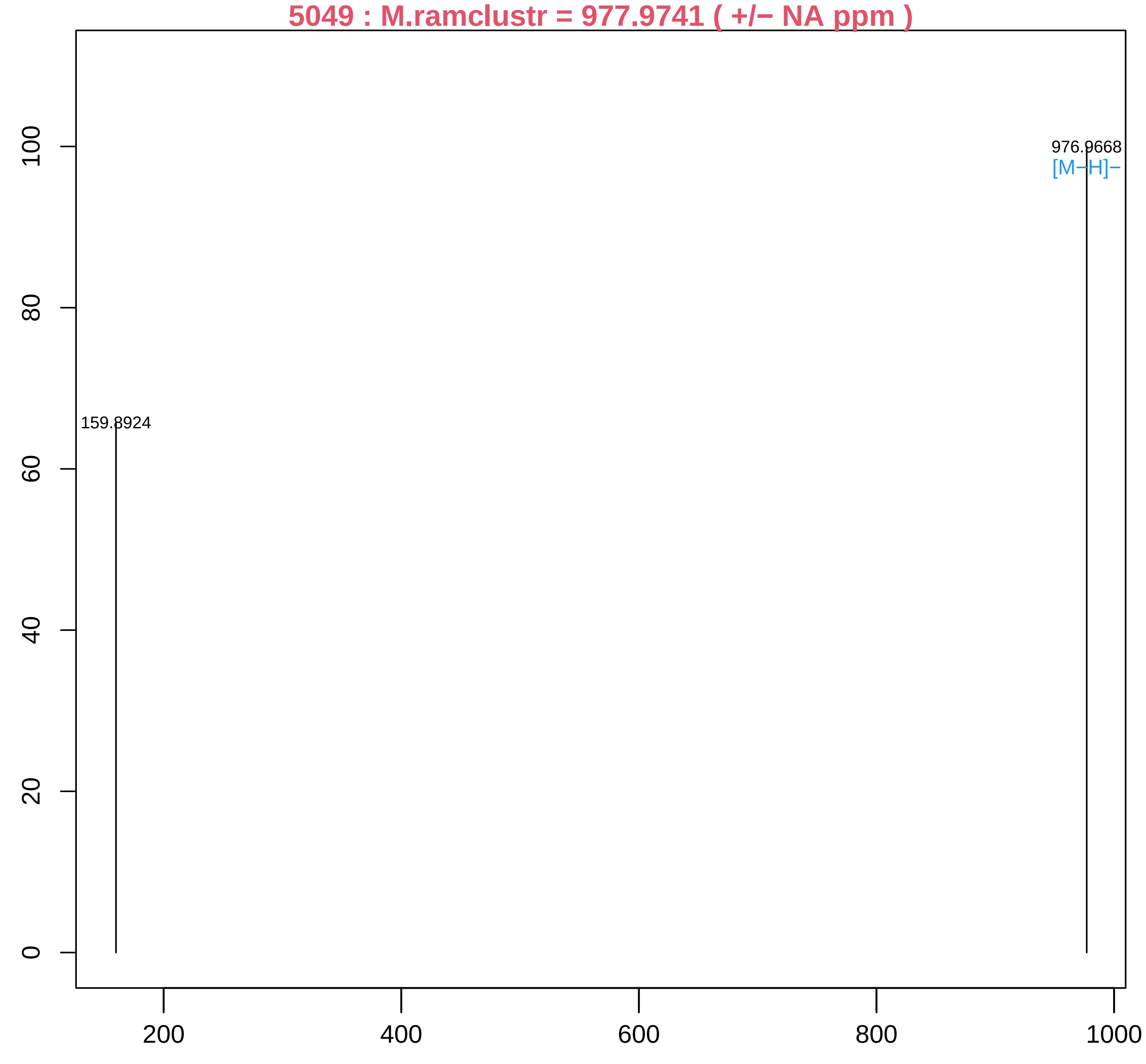

5049 : M.ramclustr = 977.9741 (+/− NA ppm)
976.9668
[M−H]−
159.8924
0
20
40
60
80
100
200
400
600
800
1000

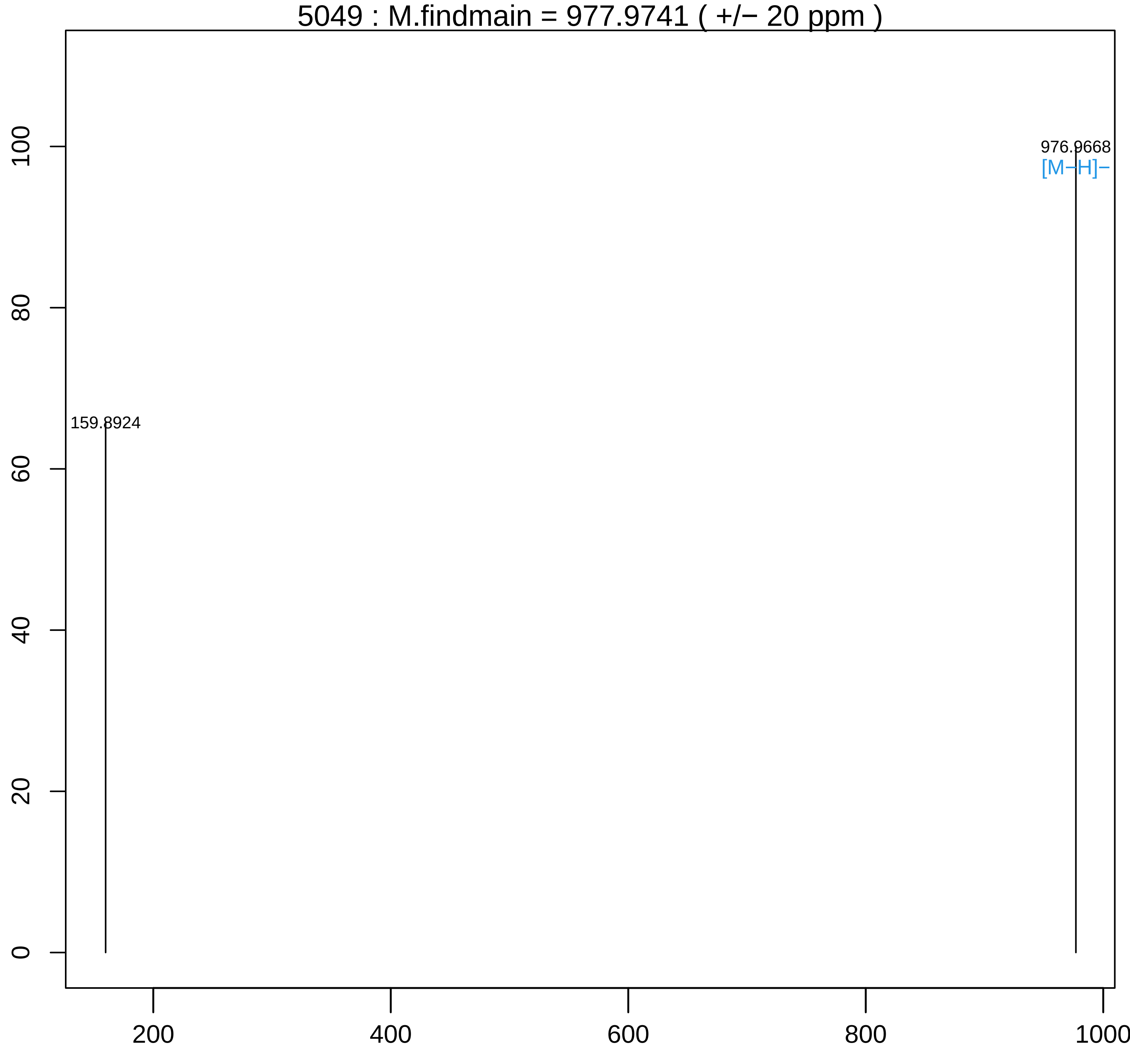

5049 : M.findmain = 977.9741 (+/− 20 ppm)
976.9668
[M−H]−
159.8924
0
20
40
60
80
100
200
400
600
800
1000

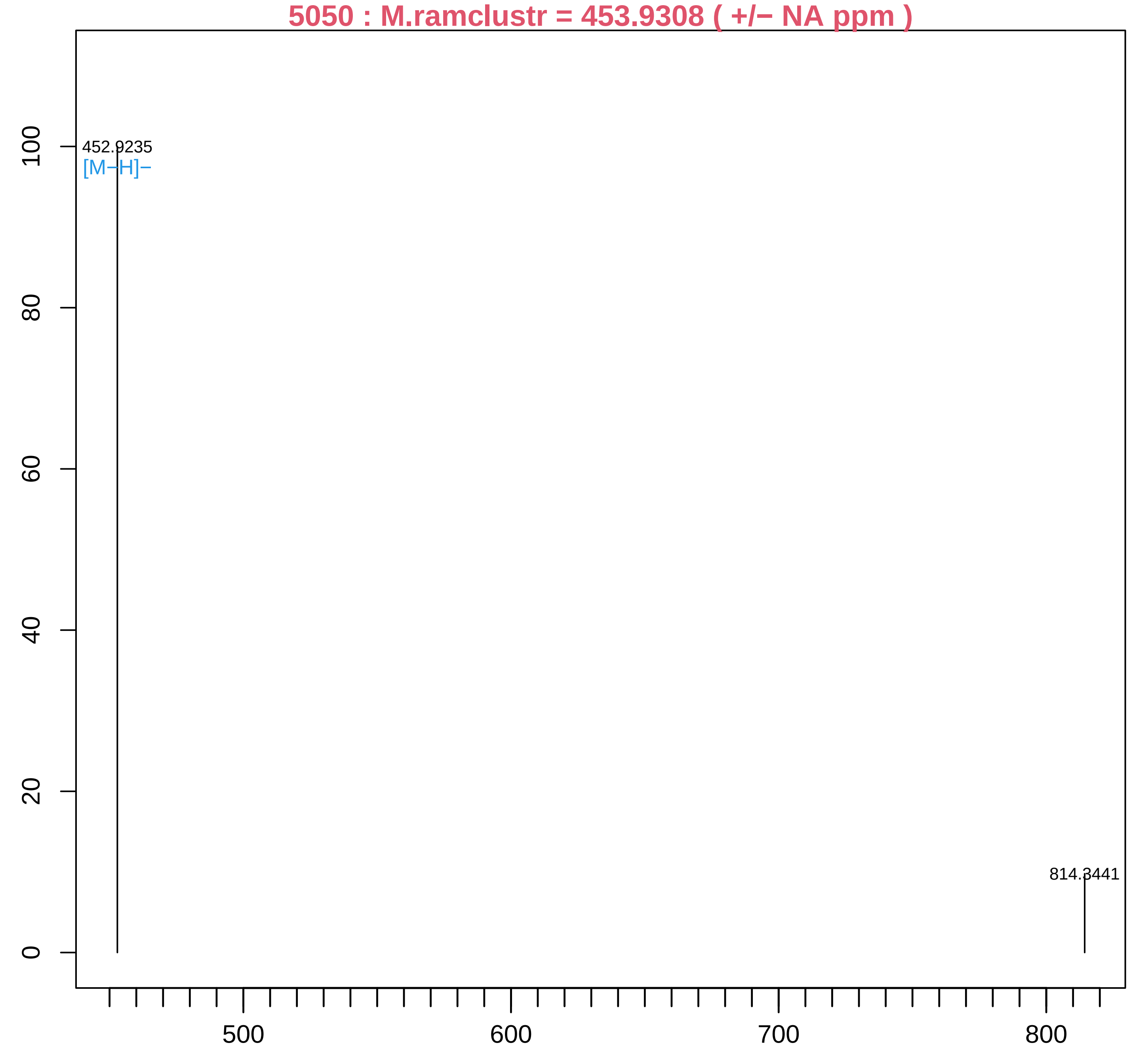
5050 : M.ramclustr = 453.9308 (+/− NA ppm)
452.9235
[M−H]−
814.3441
0
20
40
60
80
100
500
600
700
800

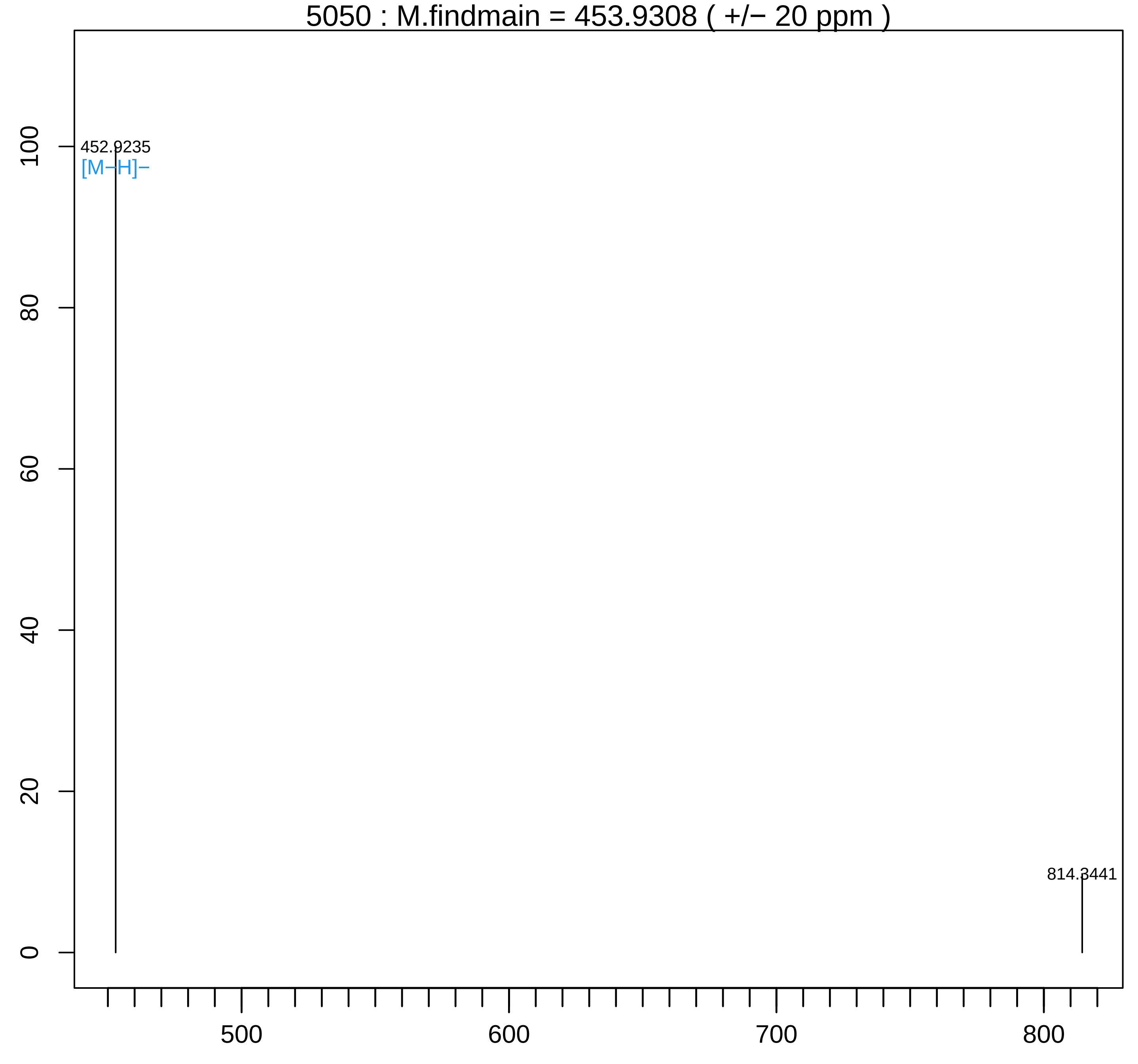
5050 : M.findmain = 453.9308 (+/− 20 ppm)
452.9235
[M−H]−
814.3441
0
20
40
60
80
100
500
600
700
800

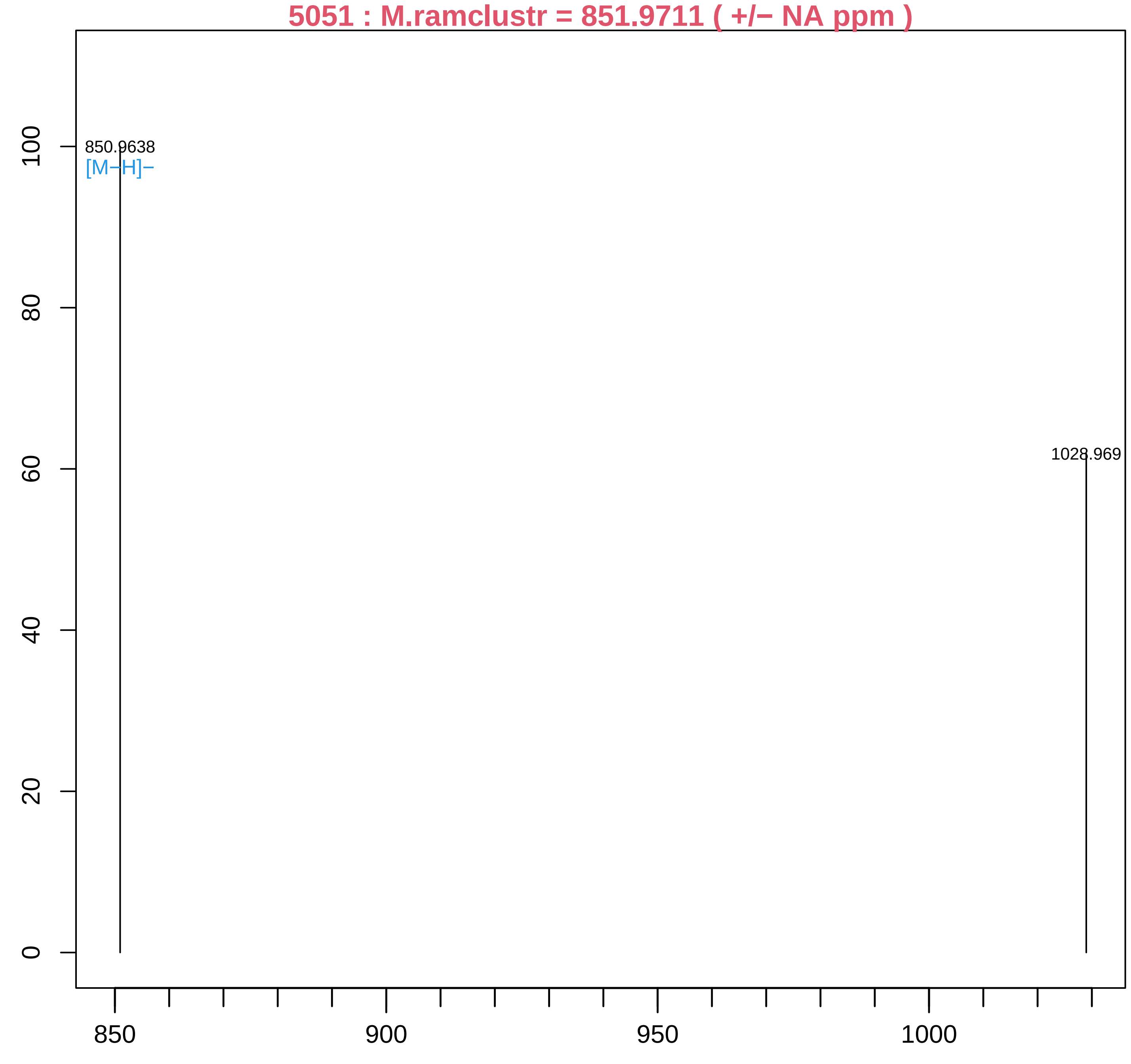
5051 : M.ramclustr = 851.9711 (+/− NA ppm)
850.9638
[M−H]−
1028.969
0
20
40
60
80
100
850
900
950
1000

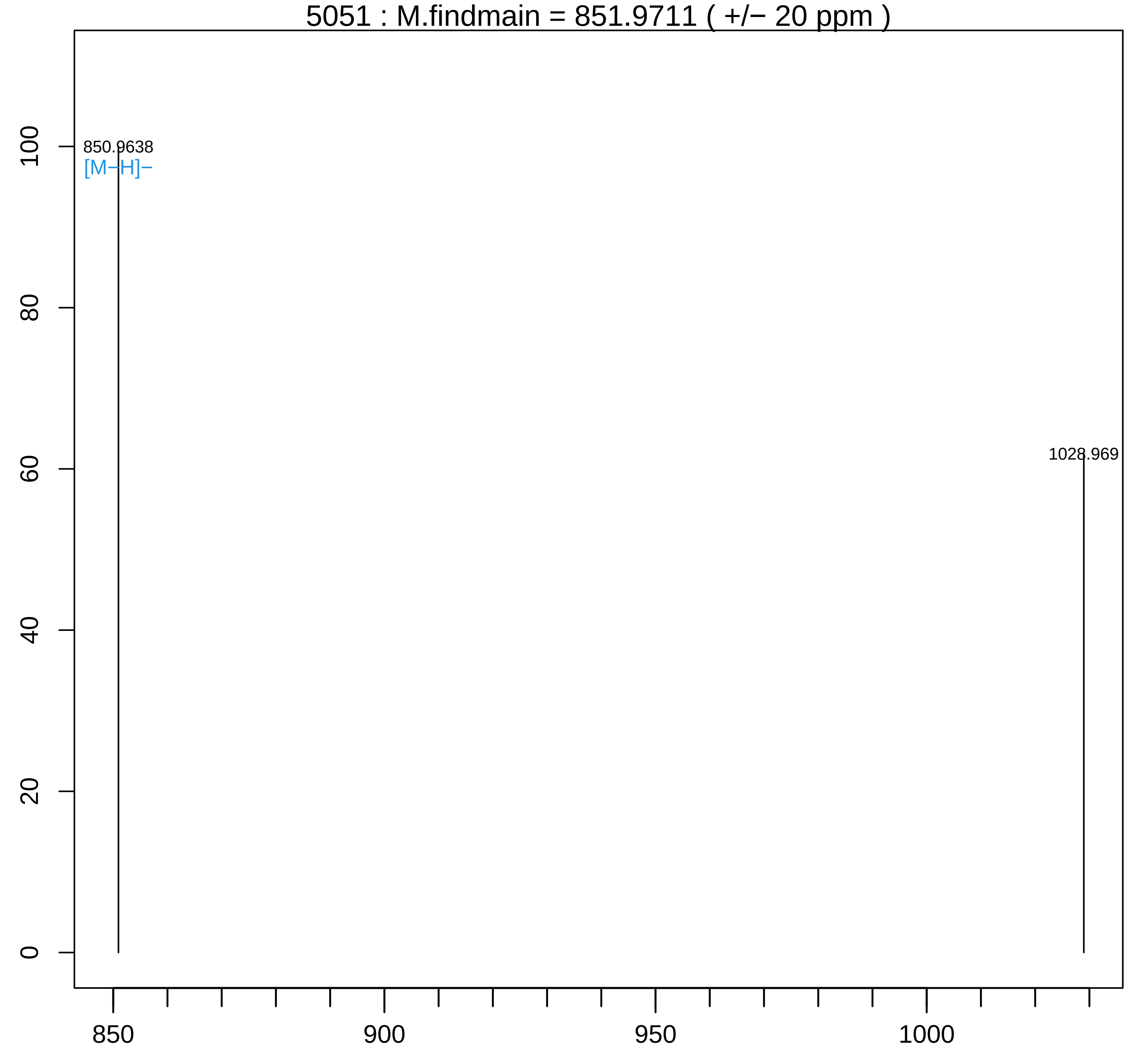
5051 : M.findmain = 851.9711 (+/− 20 ppm)
850.9638
[M−H]−
1028.969
0
20
40
60
80
100
850
900
950
1000

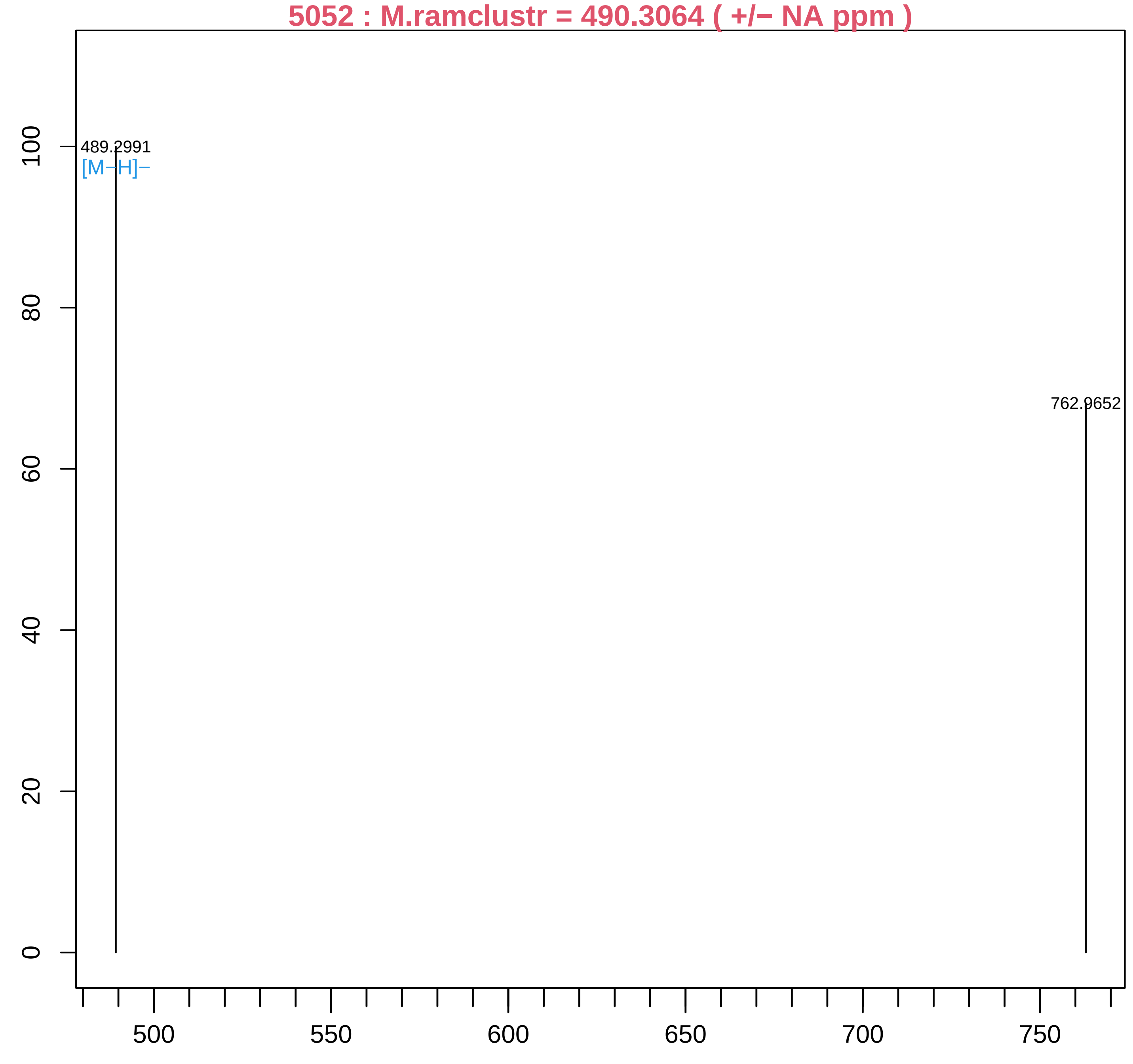

5052 : M.ramclustr = 490.3064 (+/− NA ppm)
489.2991
[M−H]−
762.9652
0
20
40
60
80
100
500
550
600
650
700
750

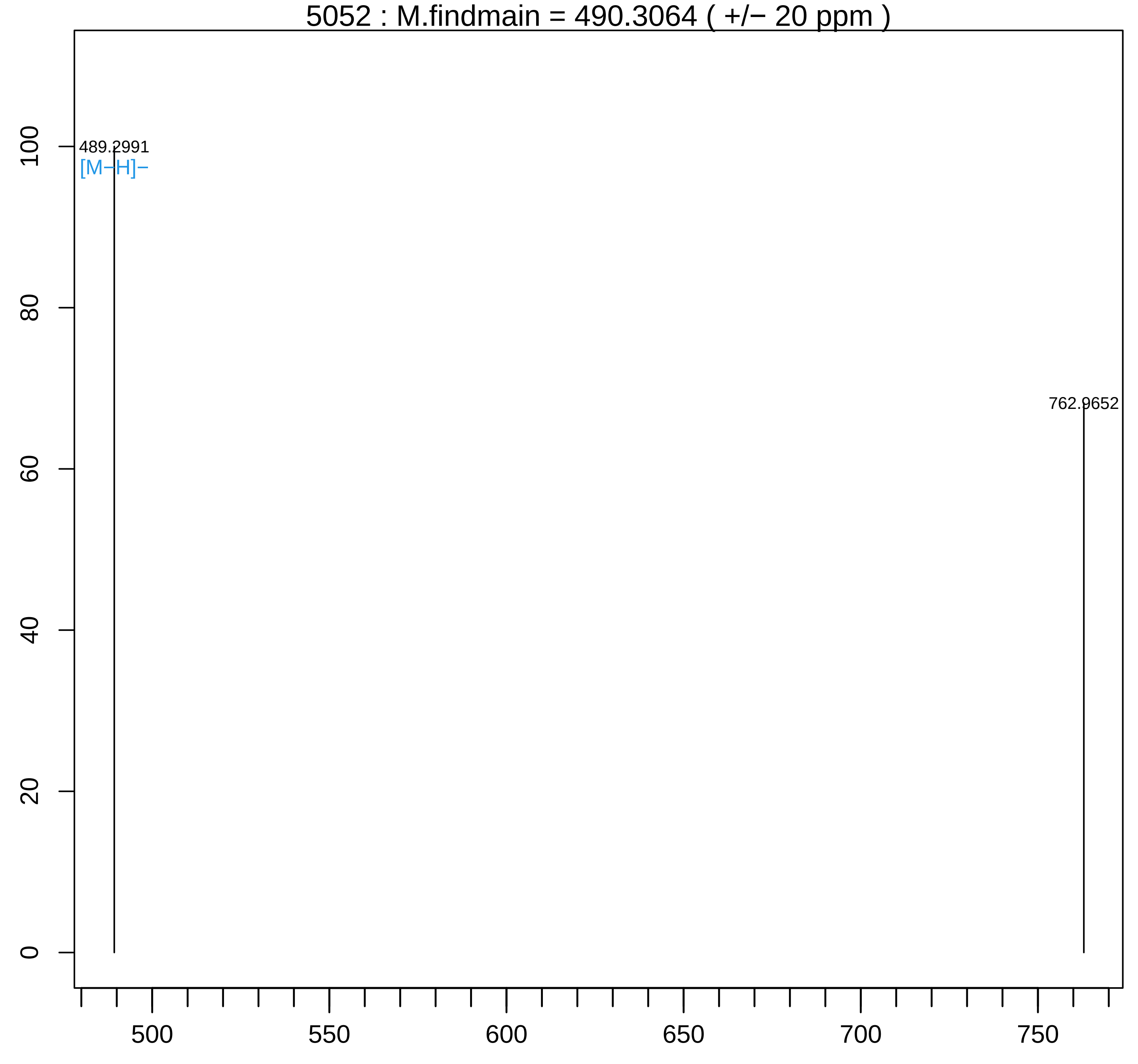

5052 : M.findmain = 490.3064 (+/− 20 ppm)
489.2991
[M−H]−
762.9652
0
20
40
60
80
100
500
550
600
650
700
750

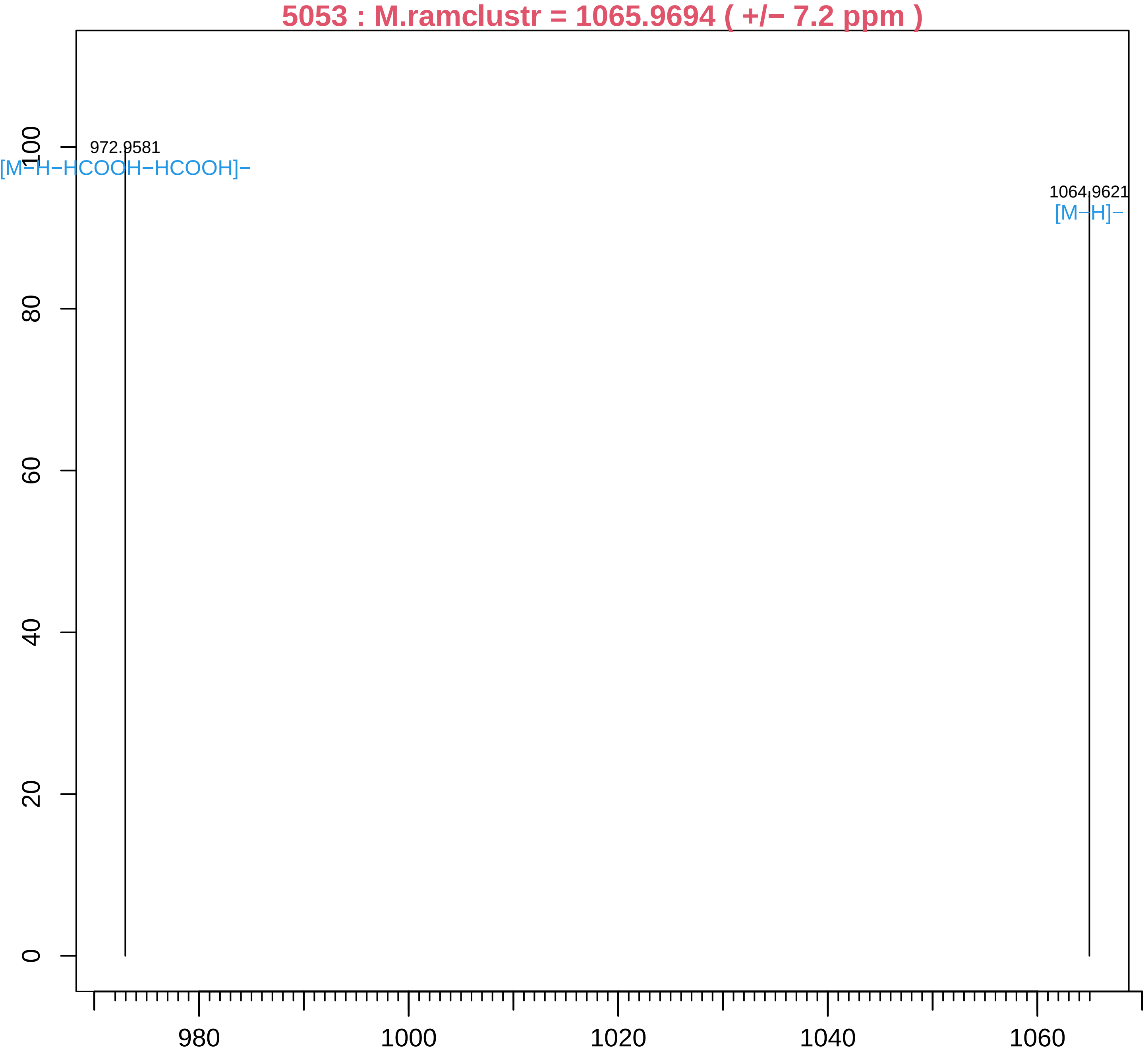
5053 : M.ramclustr = 1065.9694 (+/− 7.2 ppm)
972.9581
[M−H−HCOOH−HCOOH]−
1064.9621
[M−H]−
0
20
40
60
80
100
980
1000
1020
1040
1060

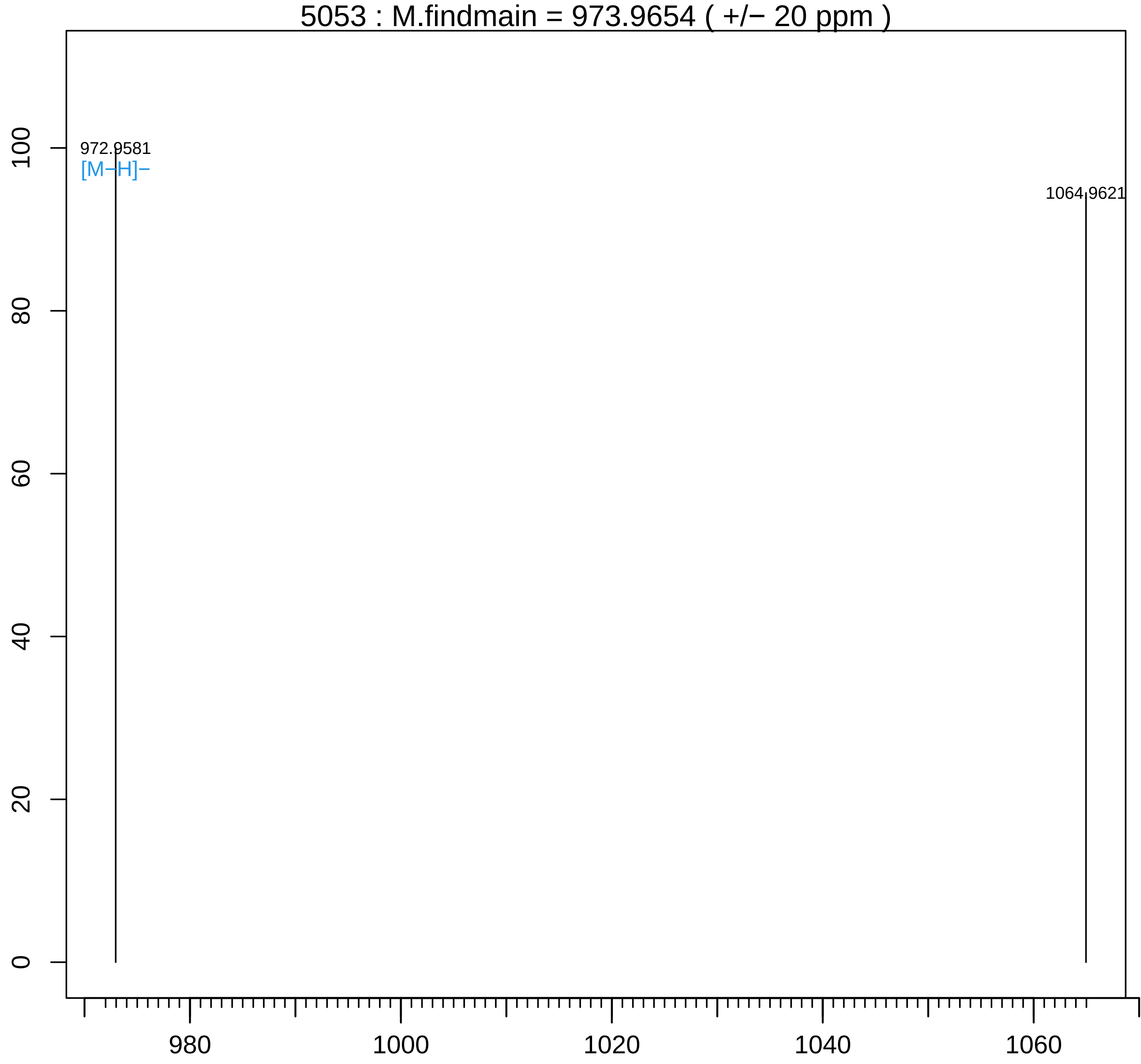
5053 : M.findmain = 973.9654 (+/− 20 ppm)
972.9581
[M−H]−
1064.9621
0
20
40
60
80
100
980
1000
1020
1040
1060

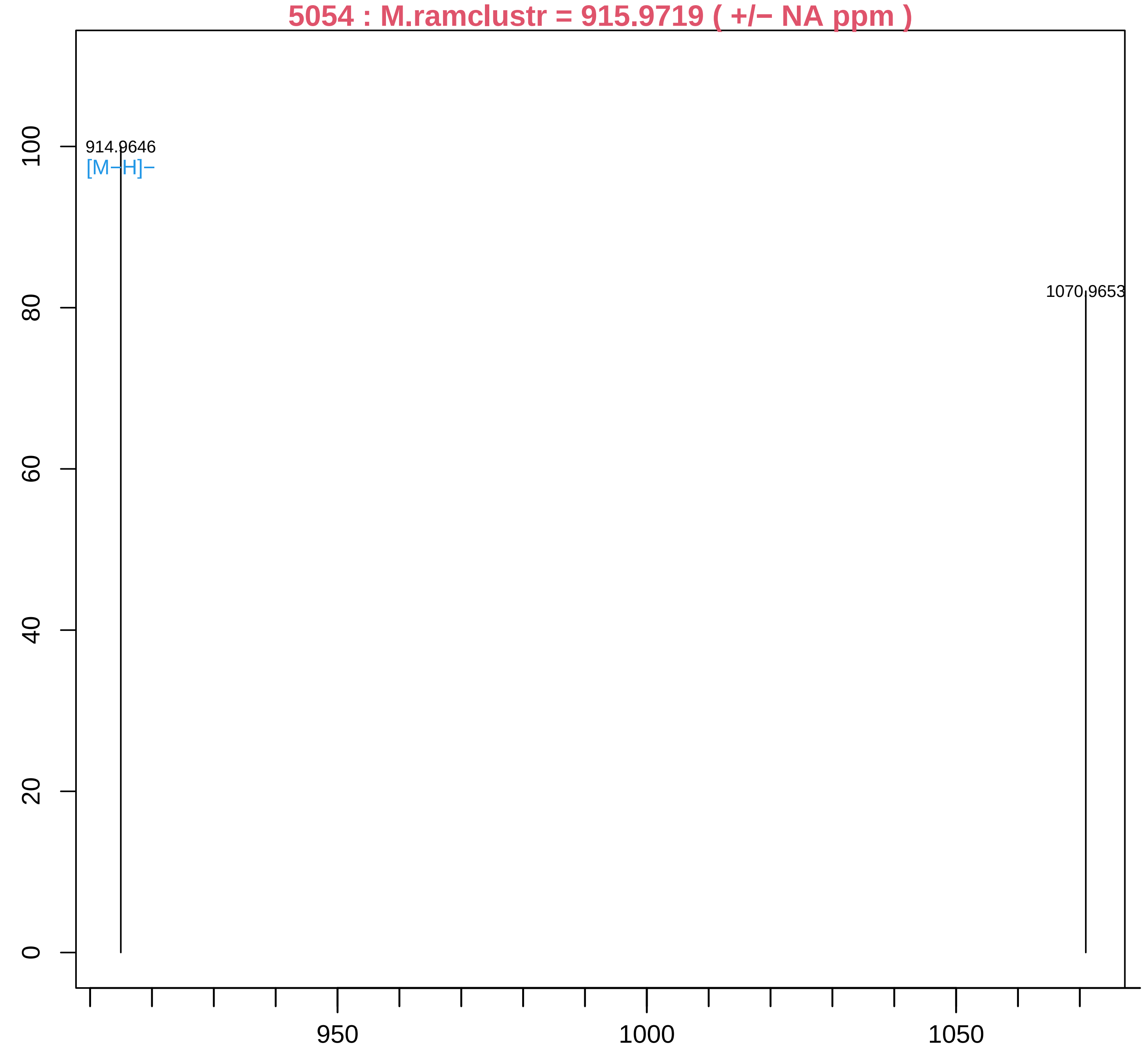
5054 : M.ramclustr = 915.9719 (+/− NA ppm)
914.9646
[M−H]−
1070.9653
0
20
40
60
80
100
950
1000
1050

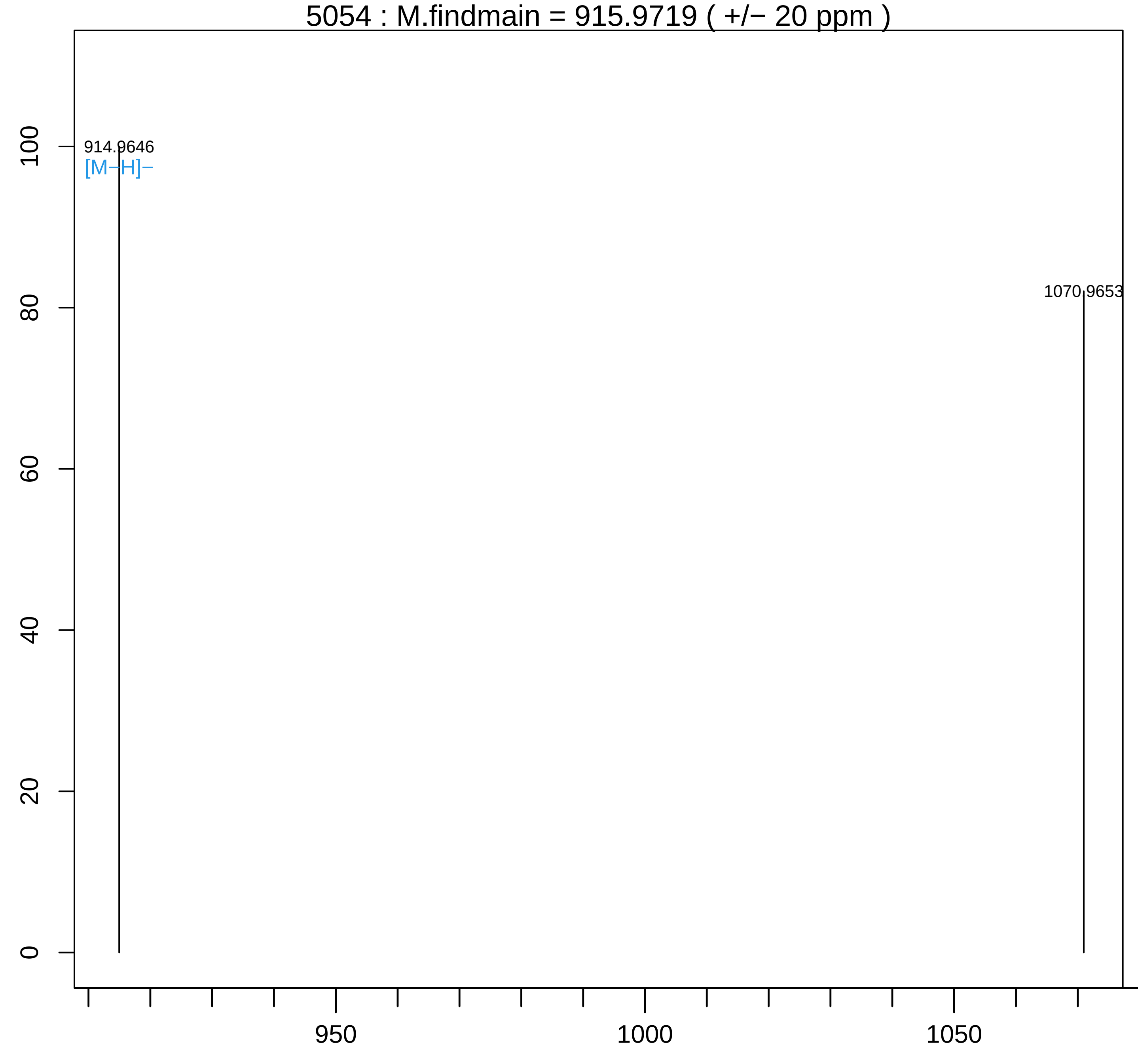
5054 : M.findmain = 915.9719 (+/− 20 ppm)
914.9646
[M−H]−
1070.9653
0
20
40
60
80
100
950
1000
1050

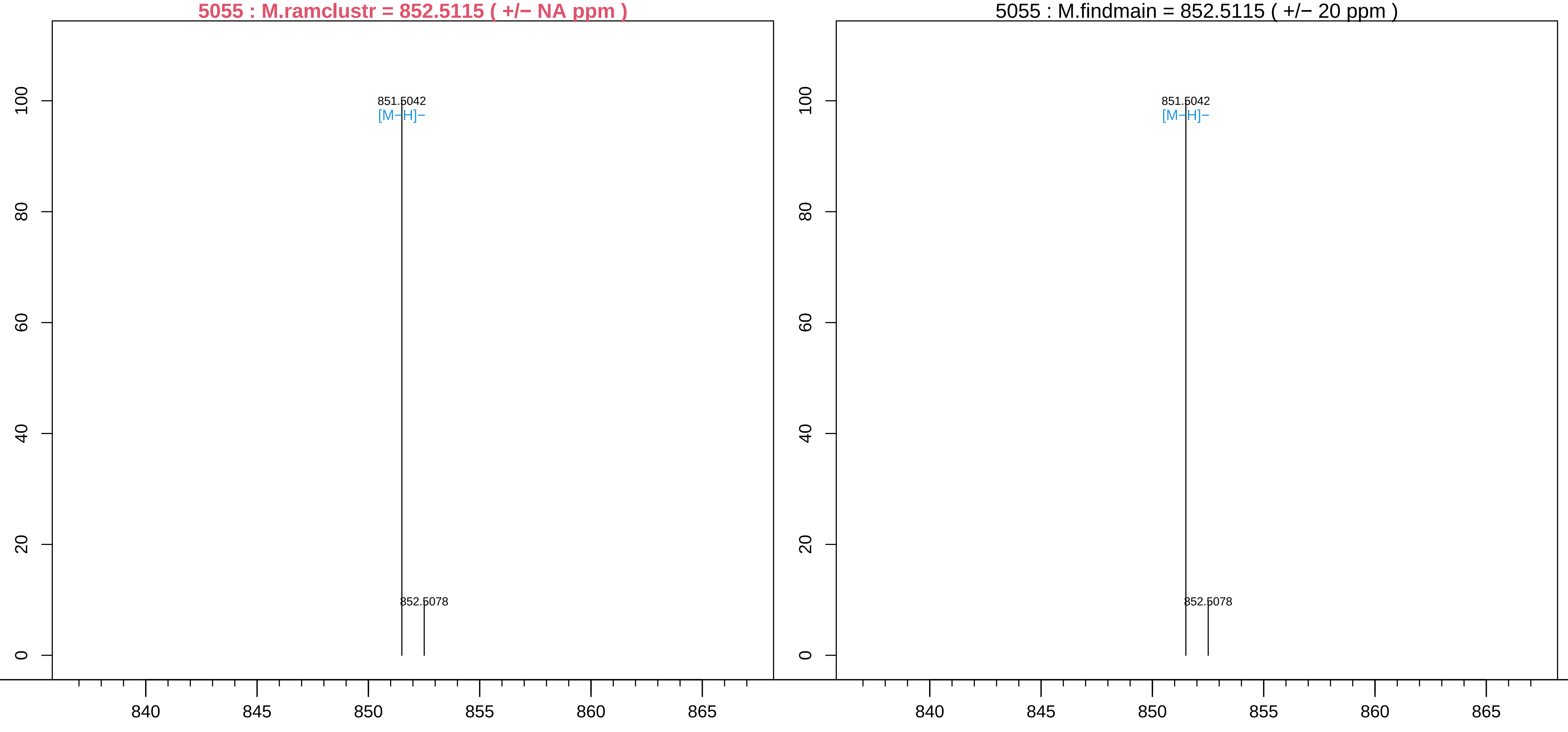
5055 : M.ramclustr = 852.5115 (+/− NA ppm)
851.5042
[M−H]−
852.5078
5055 : M.findmain = 852.5115 (+/− 20 ppm)
851.5042
[M−H]−
852.5078
0
20
40
60
80
100
840
845
850
855
860
865

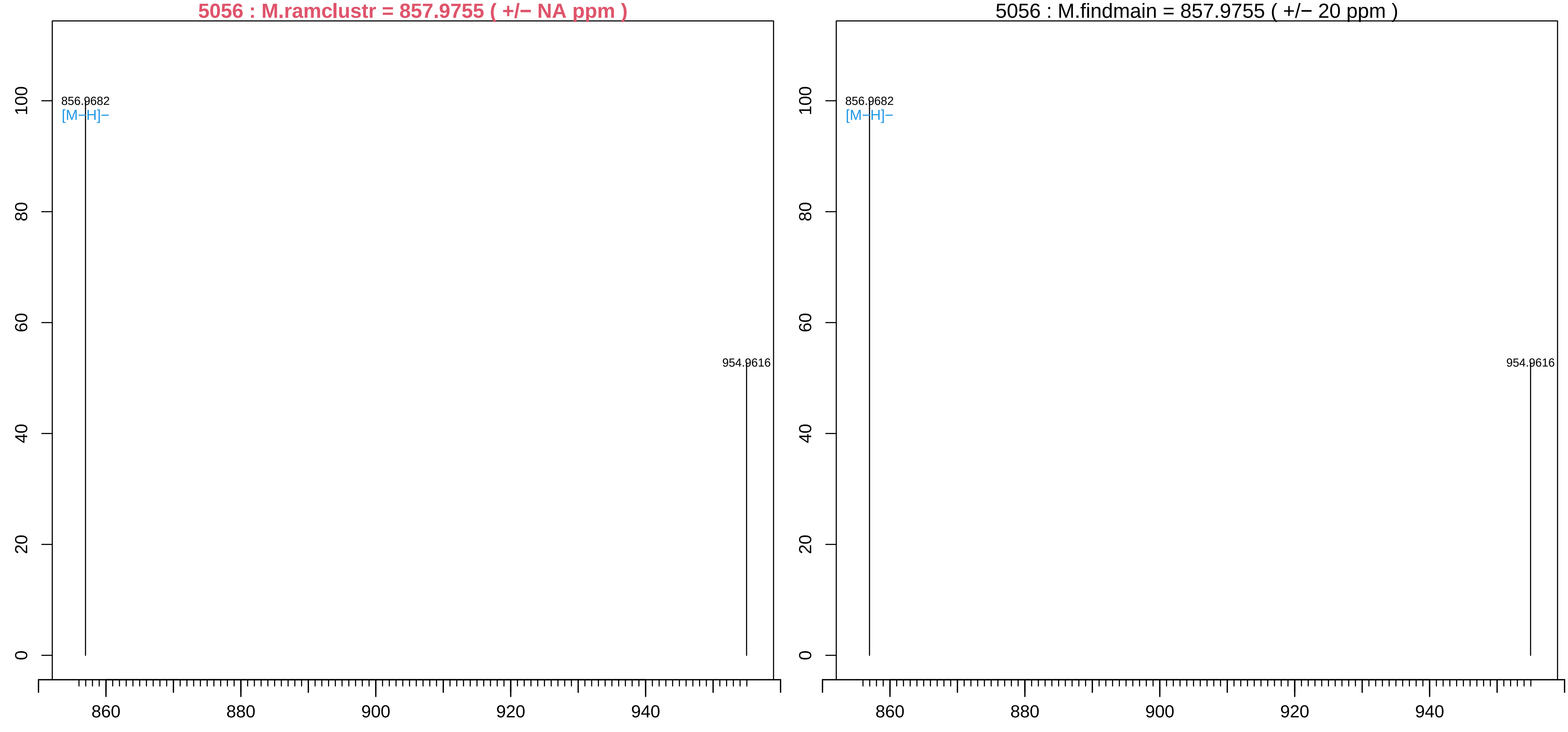
5056 : M.ramclustr = 857.9755 (+/− NA ppm)
856.9682
[M−H]−
954.9616
5056 : M.findmain = 857.9755 (+/− 20 ppm)
856.9682
[M−H]−
954.9616
0
20
40
60
80
100
860
880
900
920
940

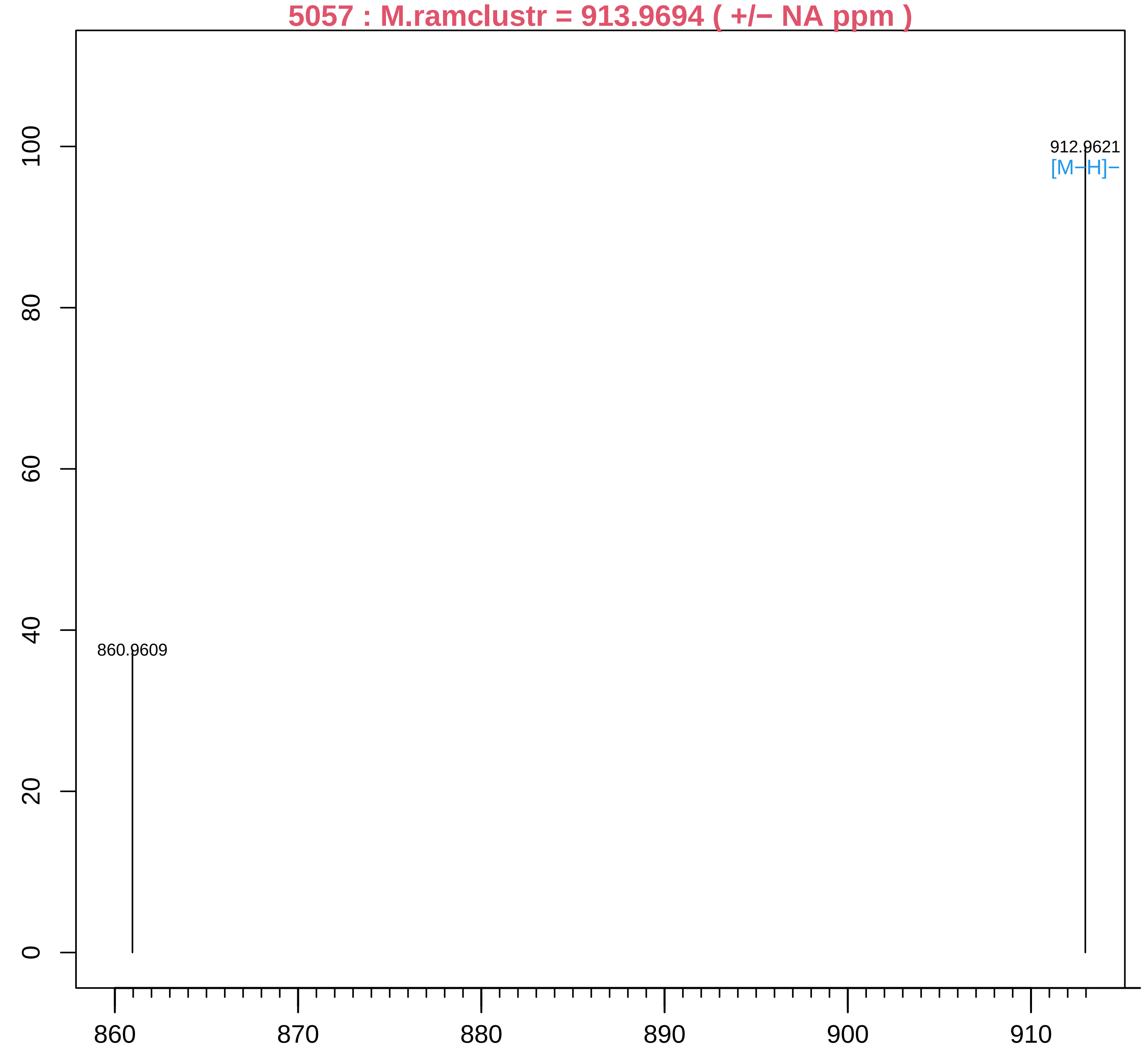
5057 : M.ramclustr = 913.9694 (+/− NA ppm)
912.9621
[M−H]−
860.9609
0
20
40
60
80
100
860
870
880
890
900
910

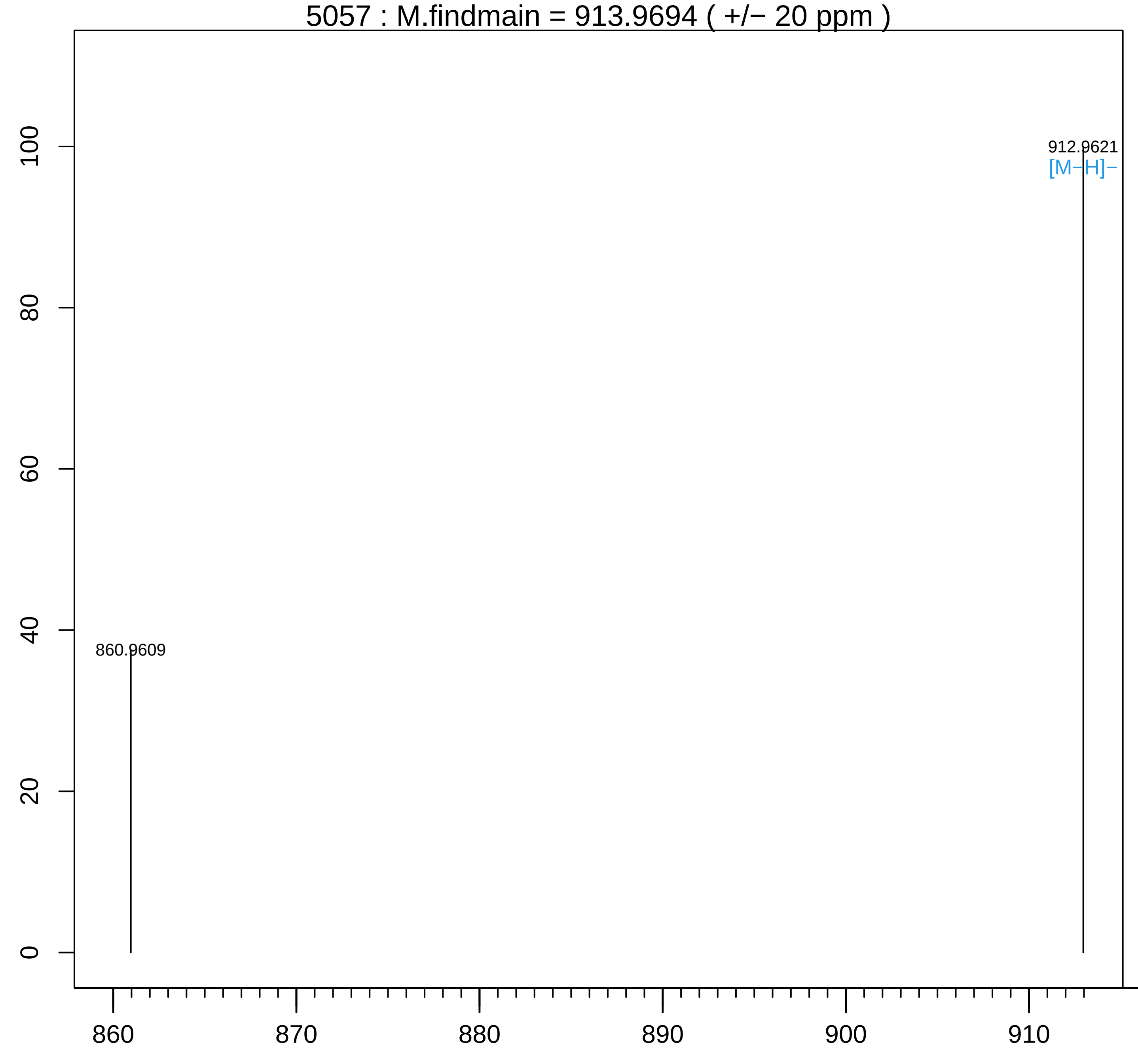
5057 : M.findmain = 913.9694 (+/− 20 ppm)
912.9621
[M−H]−
860.9609
0
20
40
60
80
100
860
870
880
890
900
910

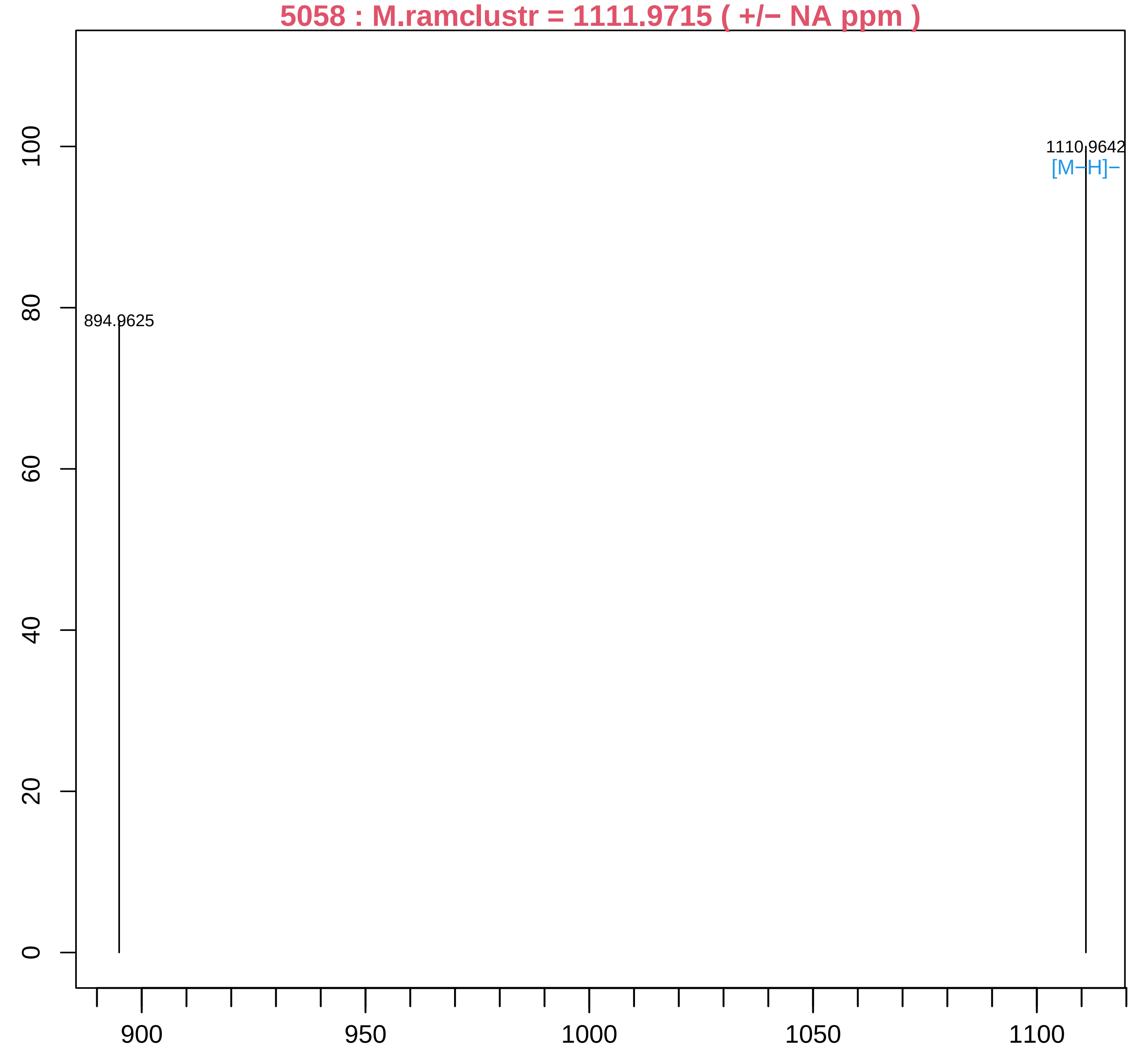
5058 : M.ramclustr = 1111.9715 (+/− NA ppm)
1110.9642
[M−H]−
894.9625
0
20
40
60
80
100
900
950
1000
1050
1100

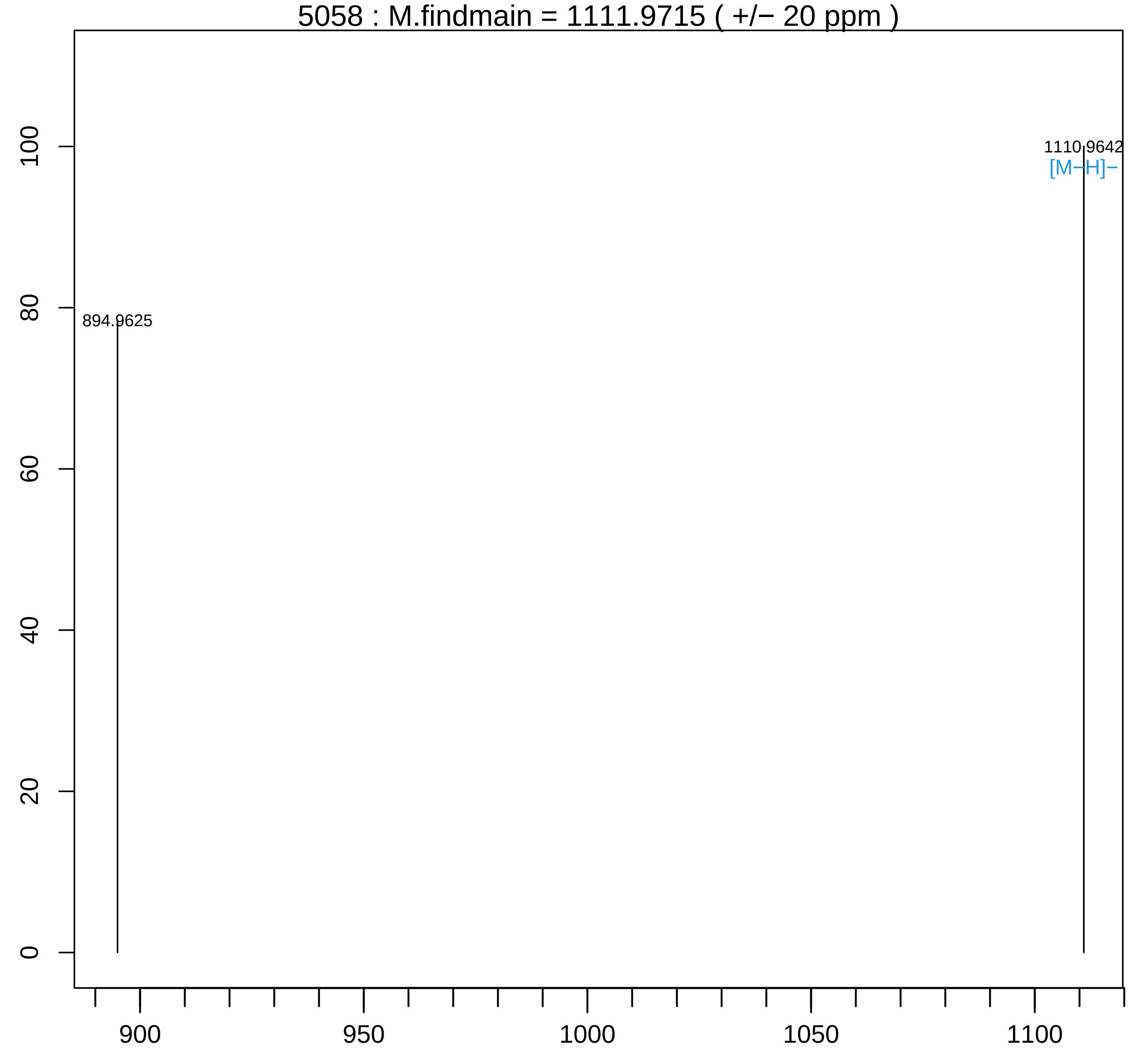
5058 : M.findmain = 1111.9715 (+/− 20 ppm)
1110.9642
[M−H]−
894.9625
0
20
40
60
80
100
900
950
1000
1050
1100

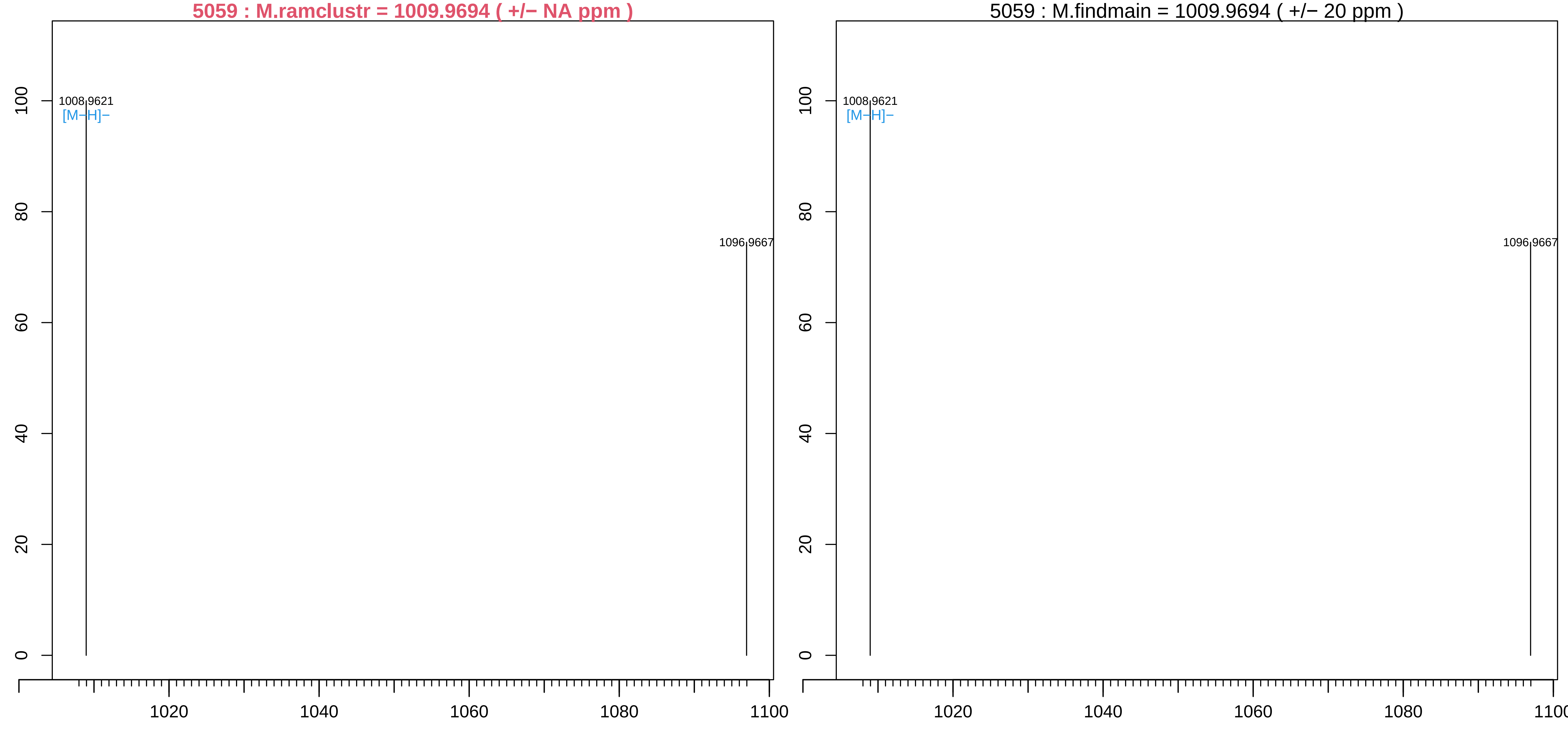
5059 : M.ramclustr = 1009.9694 (+/− NA ppm)
1008.9621
[M−H]−
1096.9667
5059 : M.findmain = 1009.9694 (+/− 20 ppm)
1008.9621
[M−H]−
1096.9667
0
20
40
60
80
100
1020
1040
1060
1080
1100

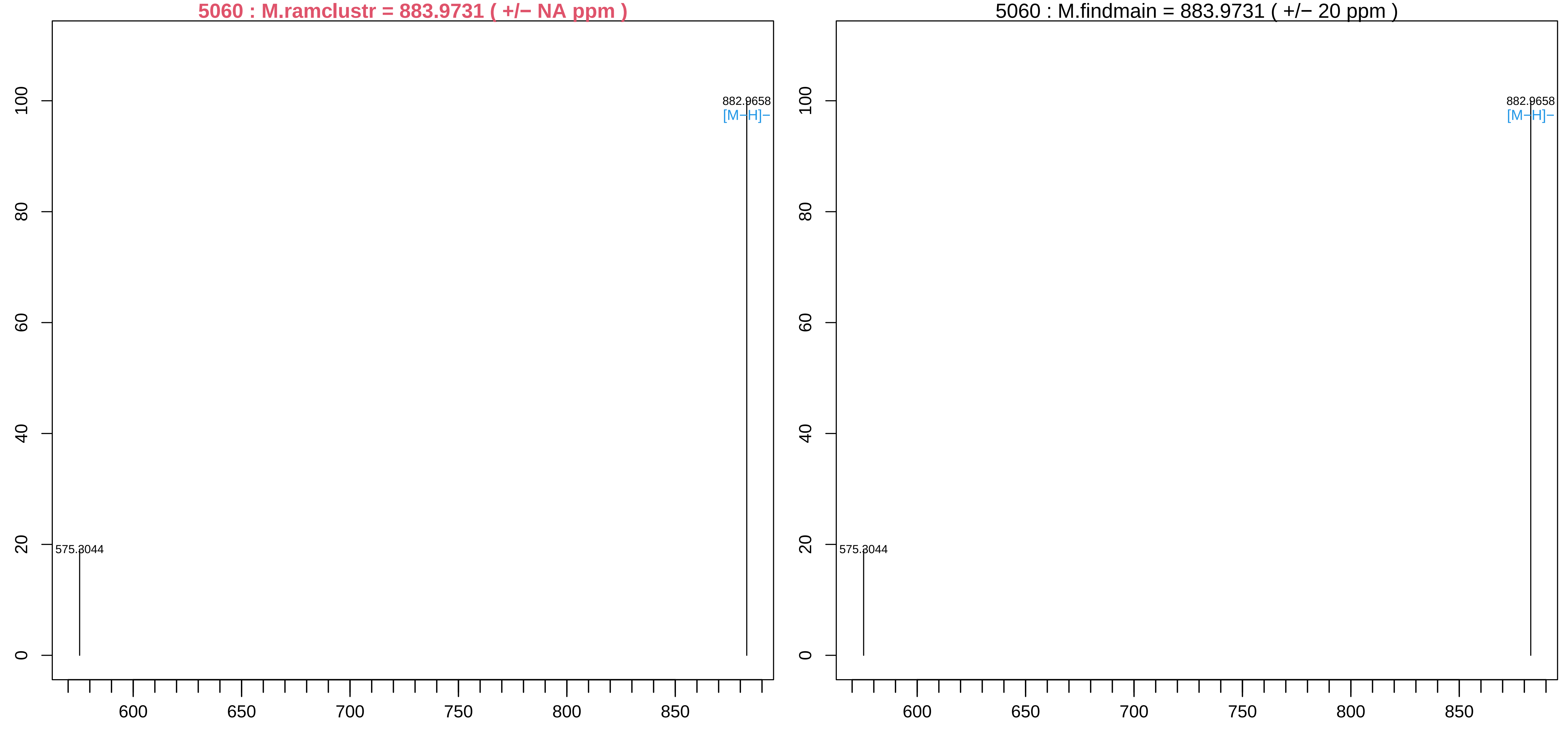
5060 : M.ramclustr = 883.9731 (+/− NA ppm)
5060 : M.findmain = 883.9731 (+/− 20 ppm)
882.9658
[M−H]−
575.3044
0
20
40
60
80
100
600
650
700
750
800
850

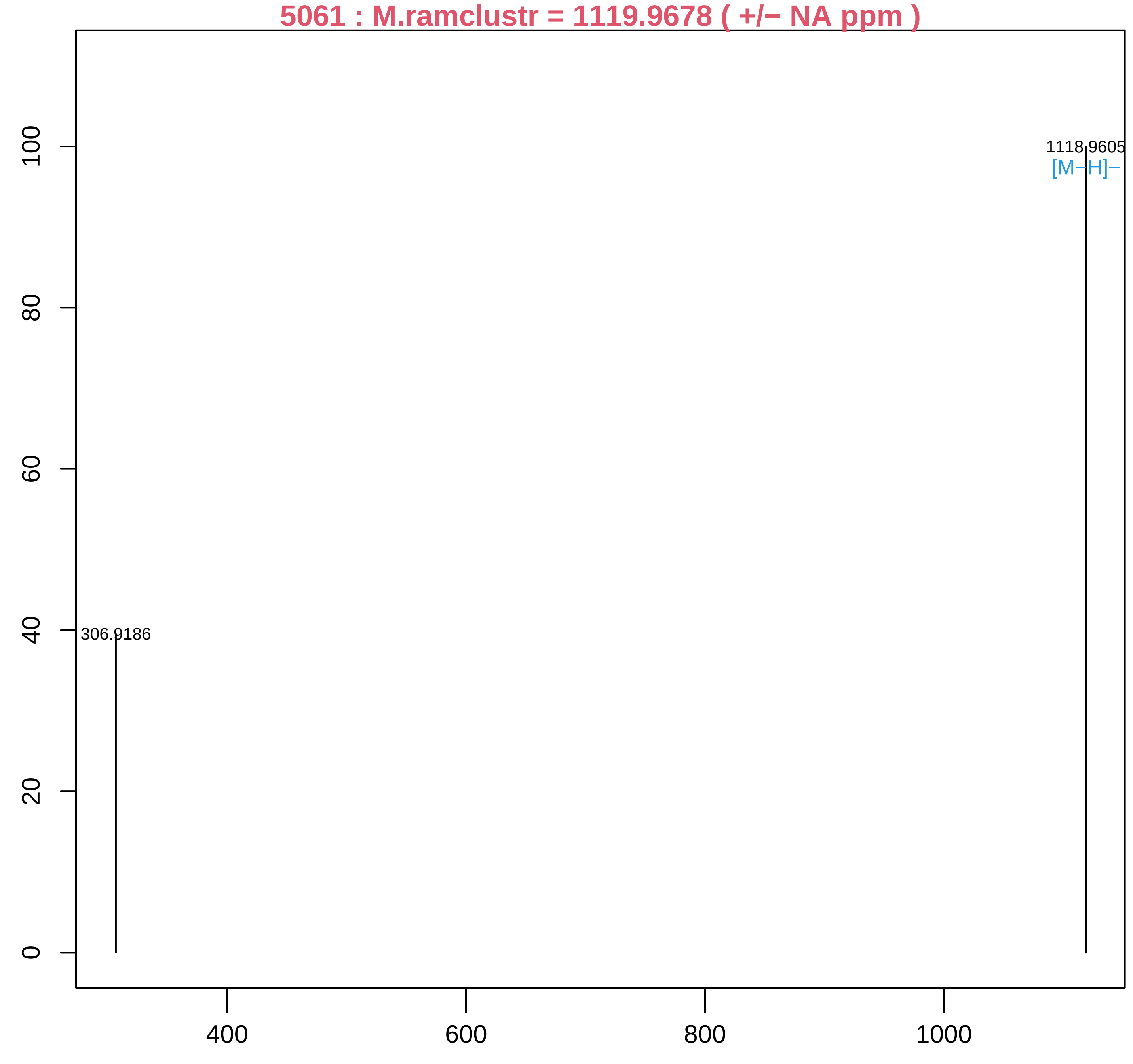

5061 : M.ramclustr = 1119.9678 (+/− NA ppm)
1118.9605
[M−H]−
306.9186
0
20
40
60
80
100
400
600
800
1000

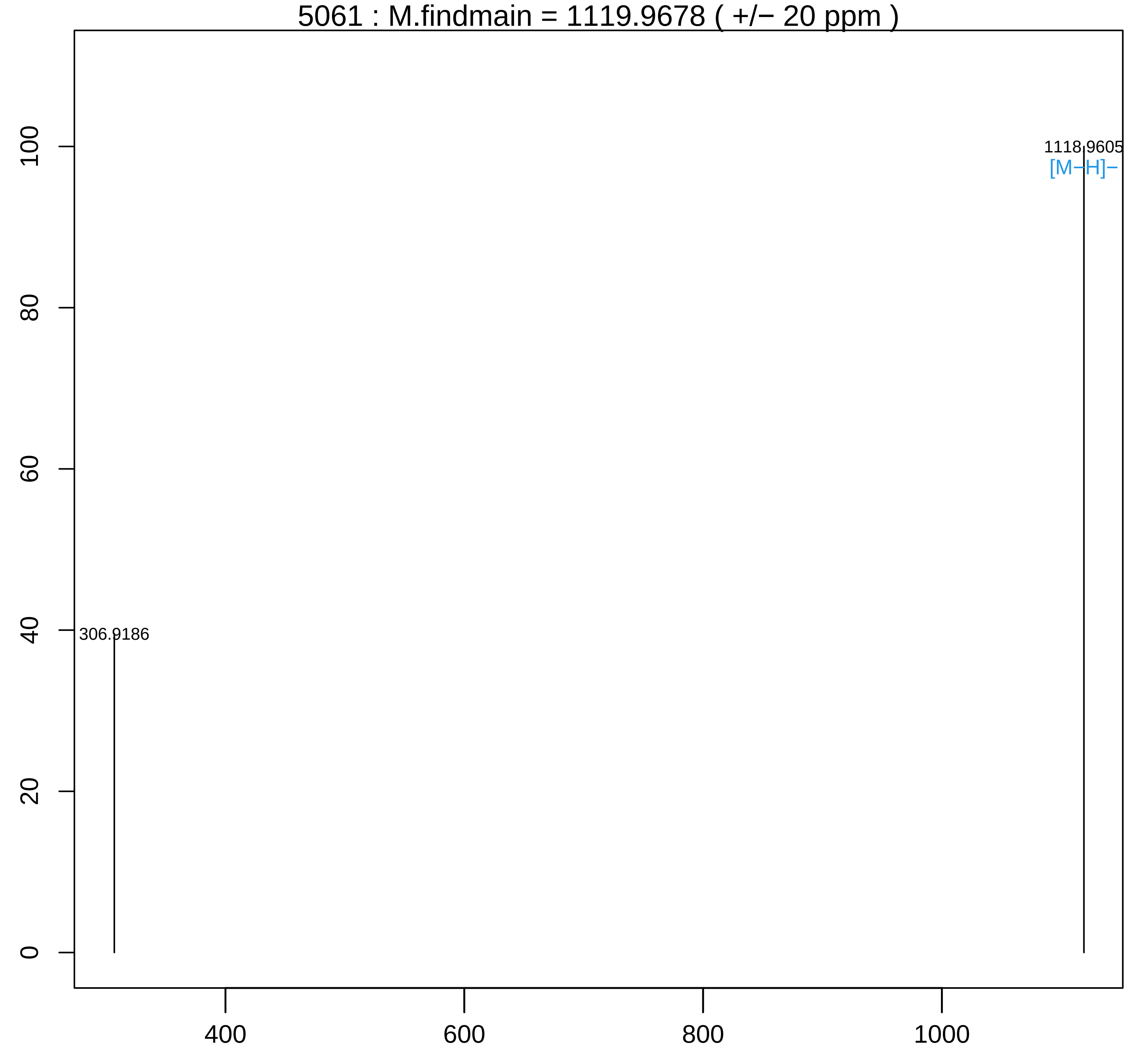

5061 : M.findmain = 1119.9678 (+/− 20 ppm)
1118.9605
[M−H]−
306.9186
0
20
40
60
80
100
400
600
800
1000

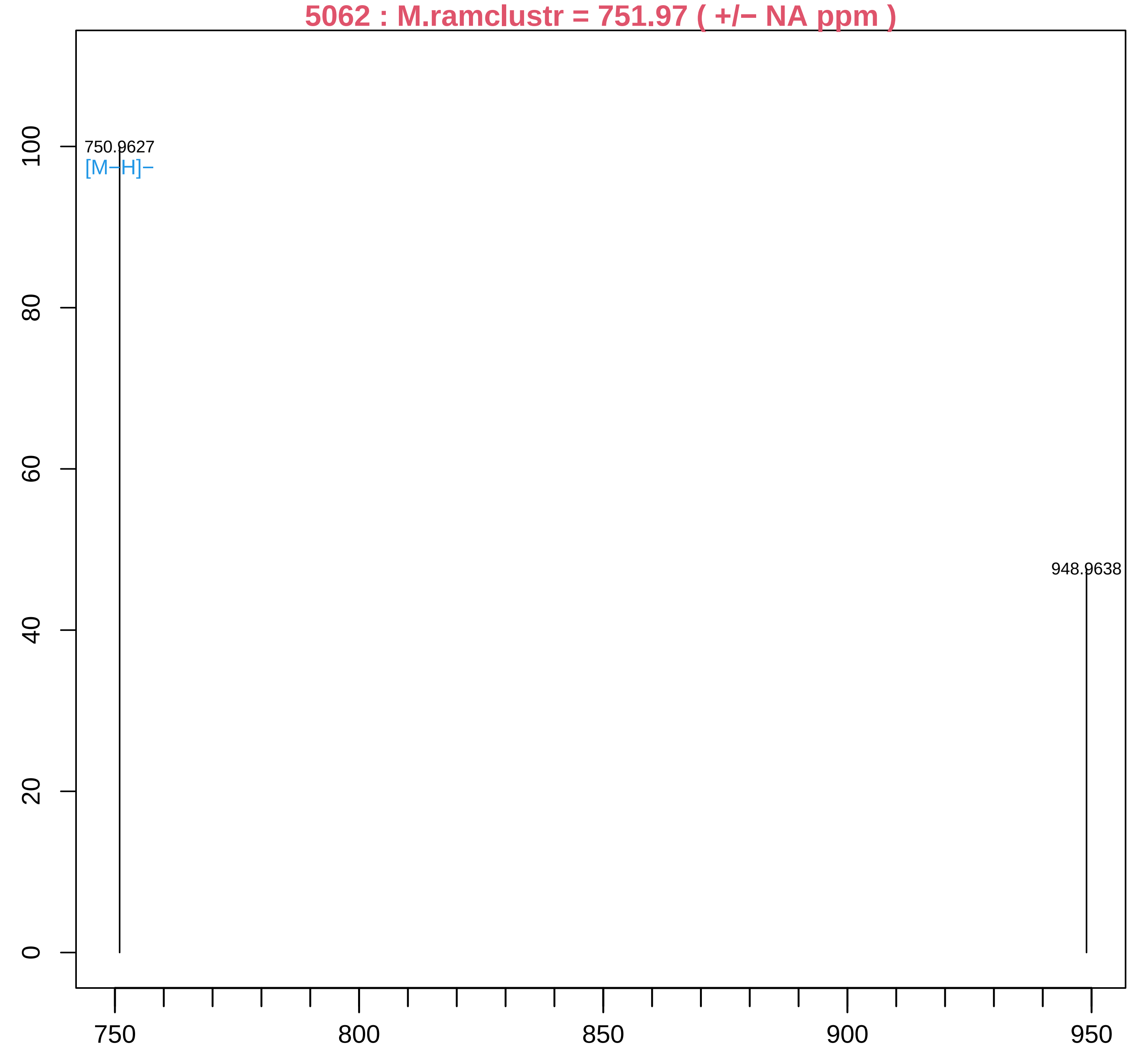
5062 : M.ramclustr = 751.97 (+/− NA ppm)
750.9627
[M−H]−
948.9638
0
20
40
60
80
100
750
800
850
900
950

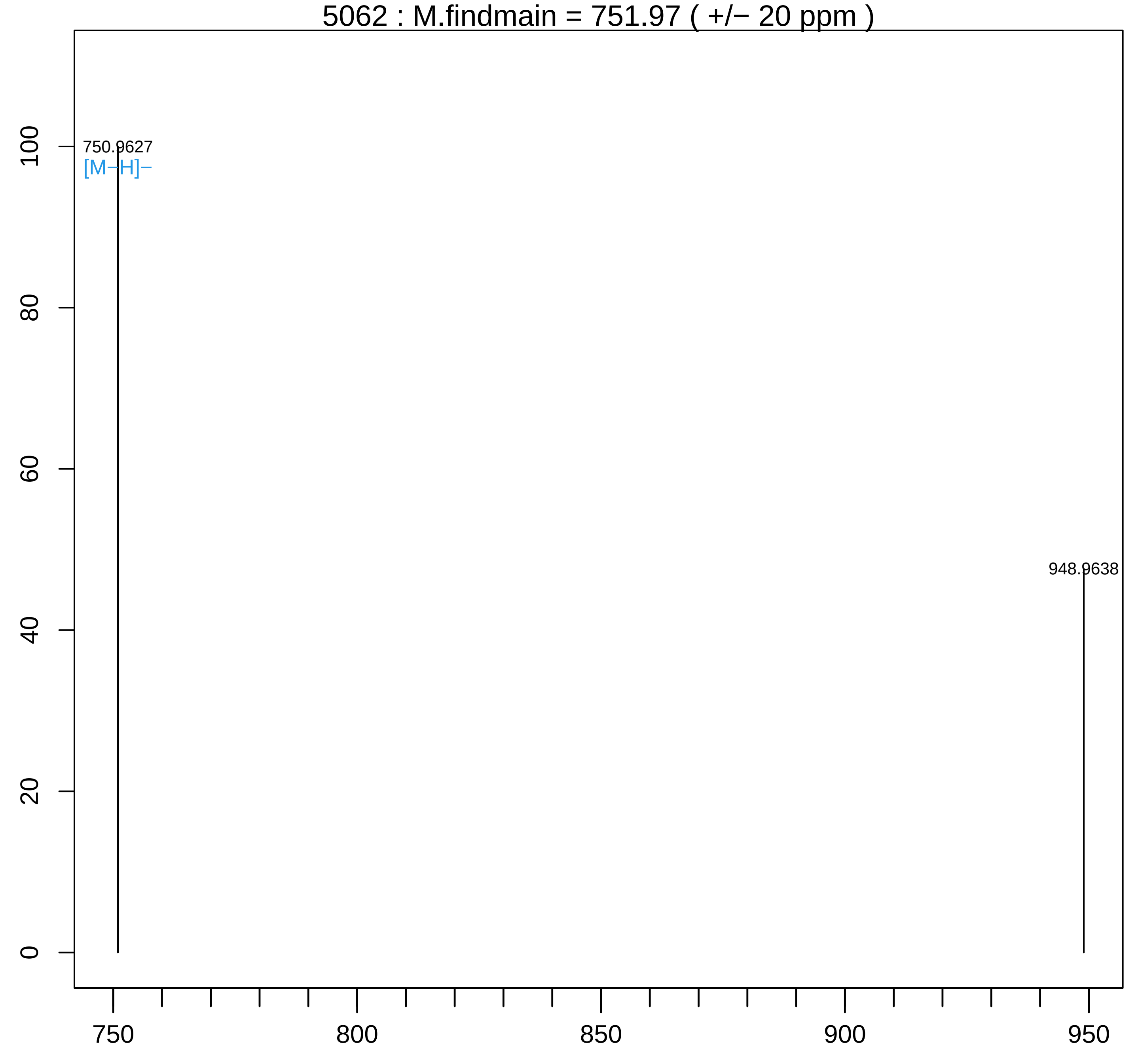
5062 : M.findmain = 751.97 (+/− 20 ppm)
750.9627
[M−H]−
948.9638
0
20
40
60
80
100
750
800
850
900
950

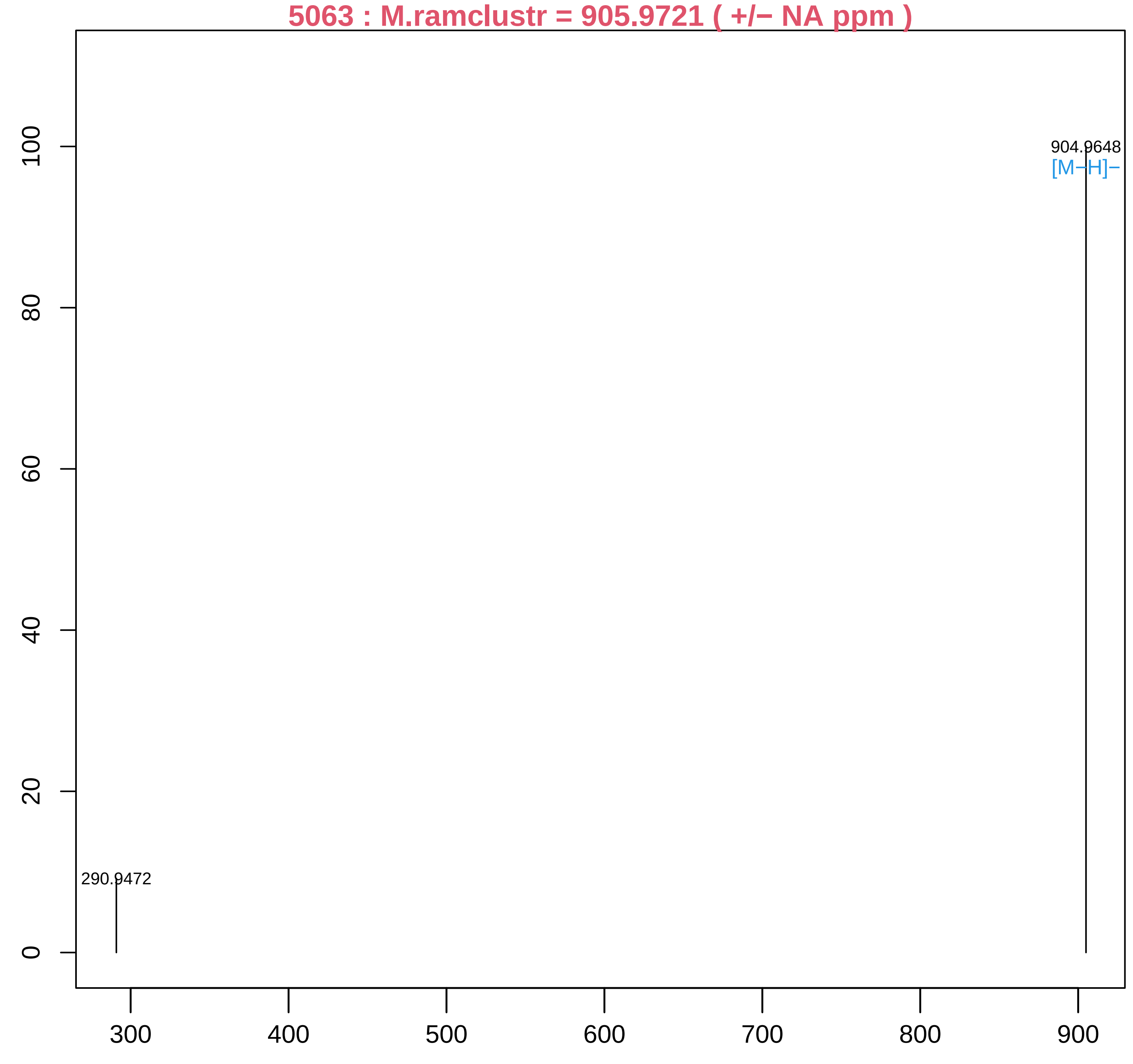
5063 : M.ramclustr = 905.9721 (+/− NA ppm)
904.9648
[M−H]−
290.9472
0
20
40
60
80
100
300
400
500
600
700
800
900

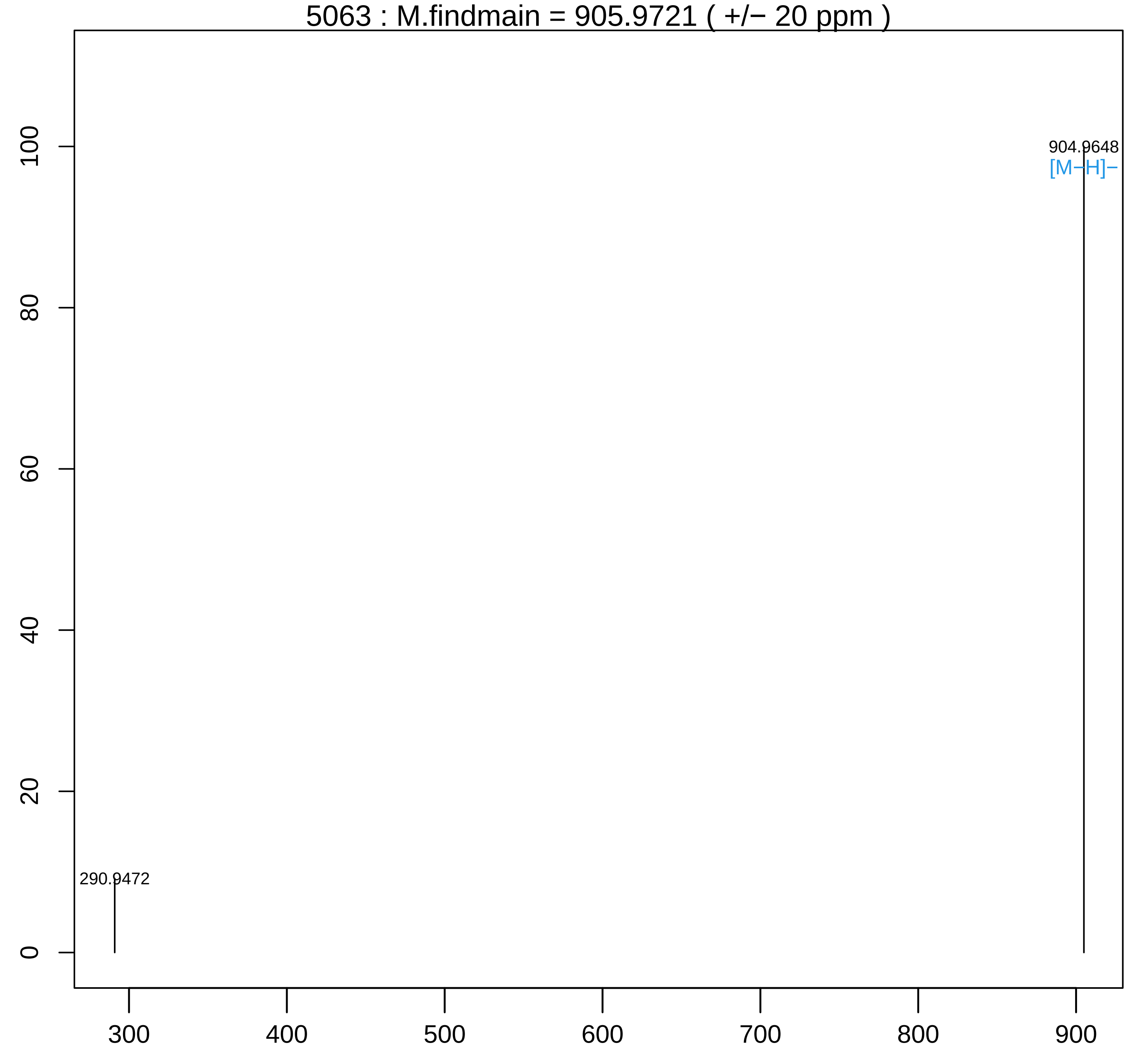
5063 : M.findmain = 905.9721 (+/− 20 ppm)
904.9648
[M−H]−
290.9472
0
20
40
60
80
100
300
400
500
600
700
800
900

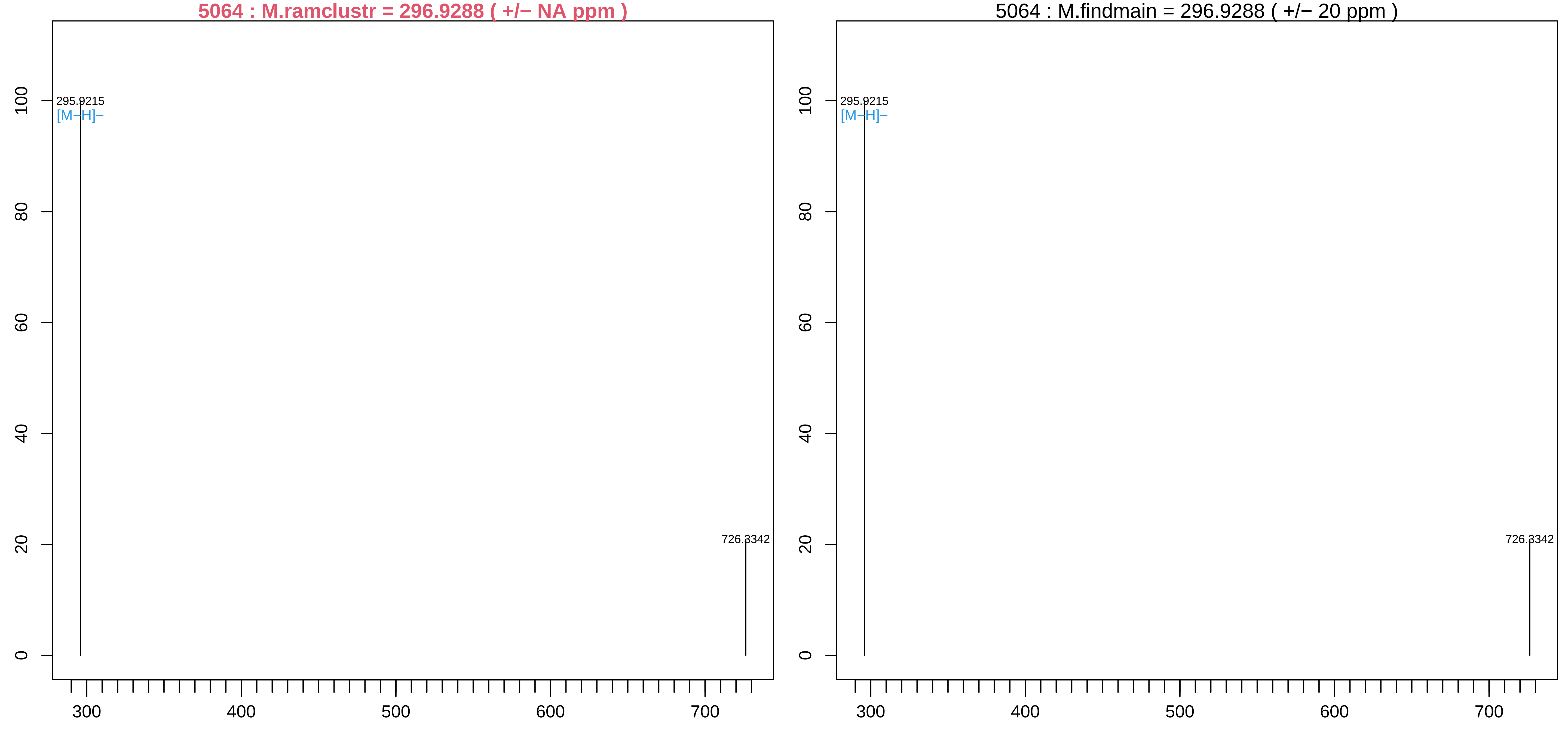
5064 : M.ramclustr = 296.9288 (+/− NA ppm)
295.9215
[M−H]−
726.3342
5064 : M.findmain = 296.9288 (+/− 20 ppm)
295.9215
[M−H]−
726.3342
0
20
40
60
80
100
300
400
500
600
700

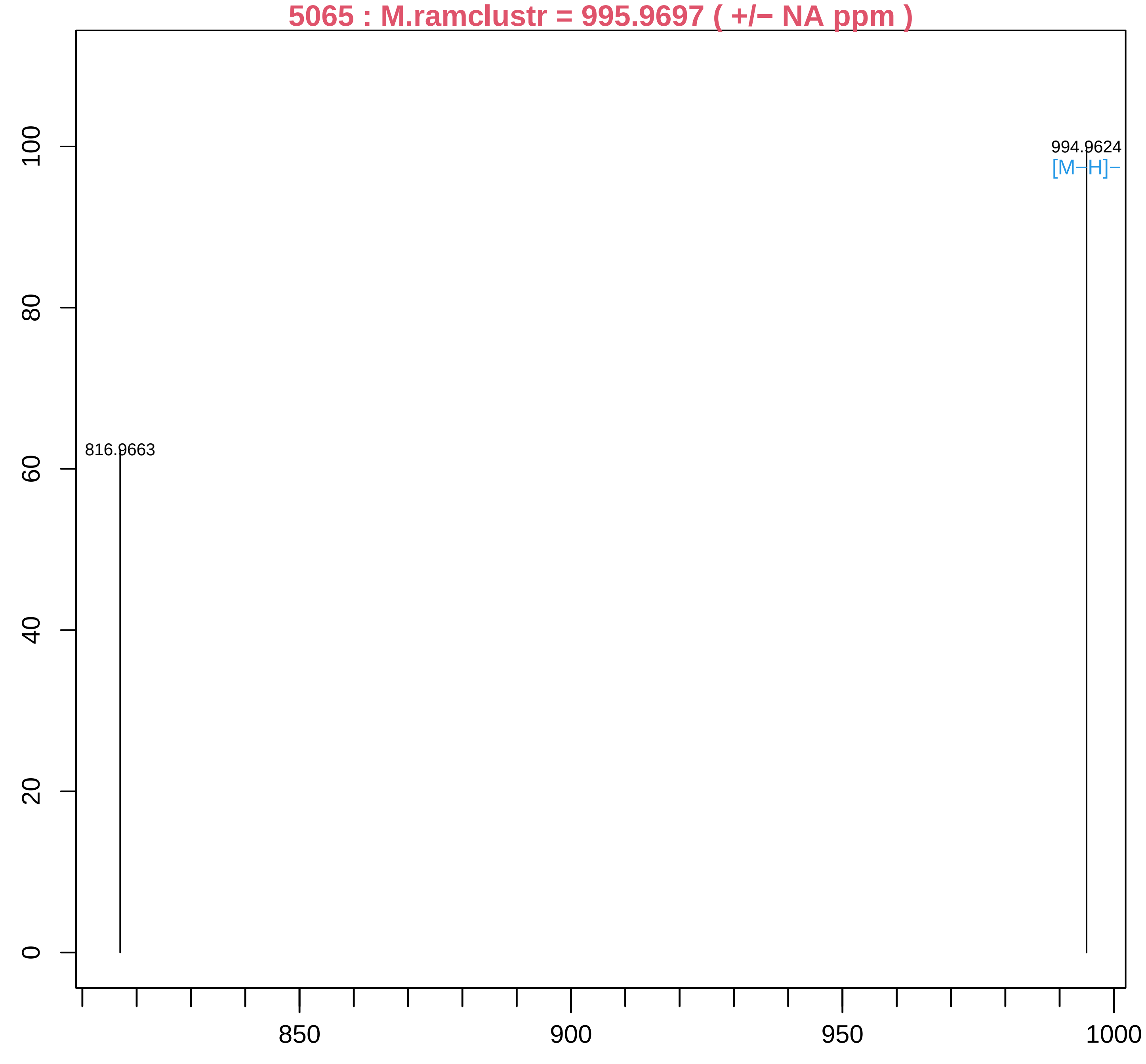

5065 : M.ramclustr = 995.9697 (+/− NA ppm)
994.9624
[M−H]−
816.9663
0
20
40
60
80
100
850
900
950
1000

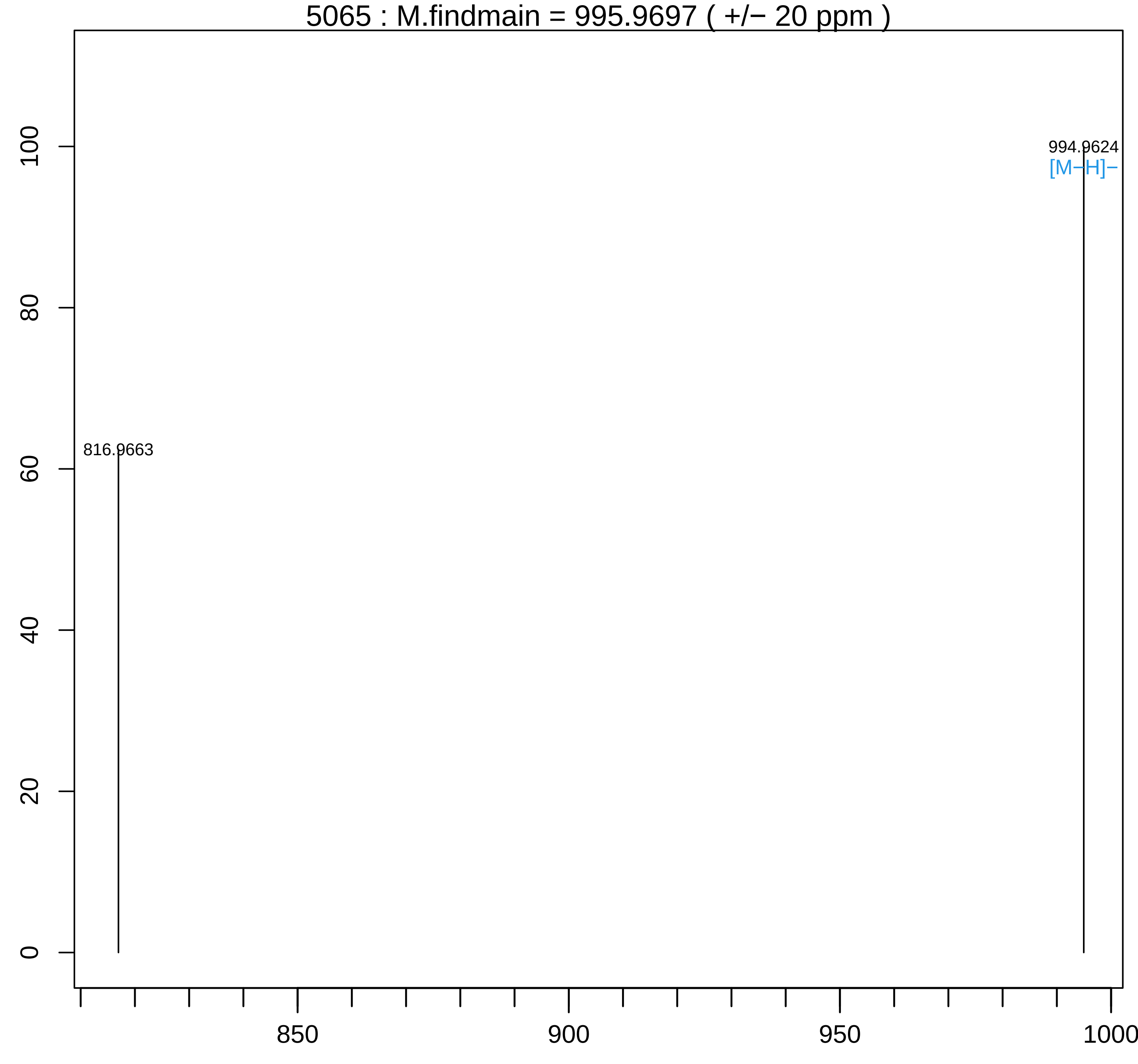

5065 : M.findmain = 995.9697 (+/− 20 ppm)
994.9624
[M−H]−
816.9663
0
20
40
60
80
100
850
900
950
1000

5066 : M.ramclustr = 398.9865 (+/− NA ppm)

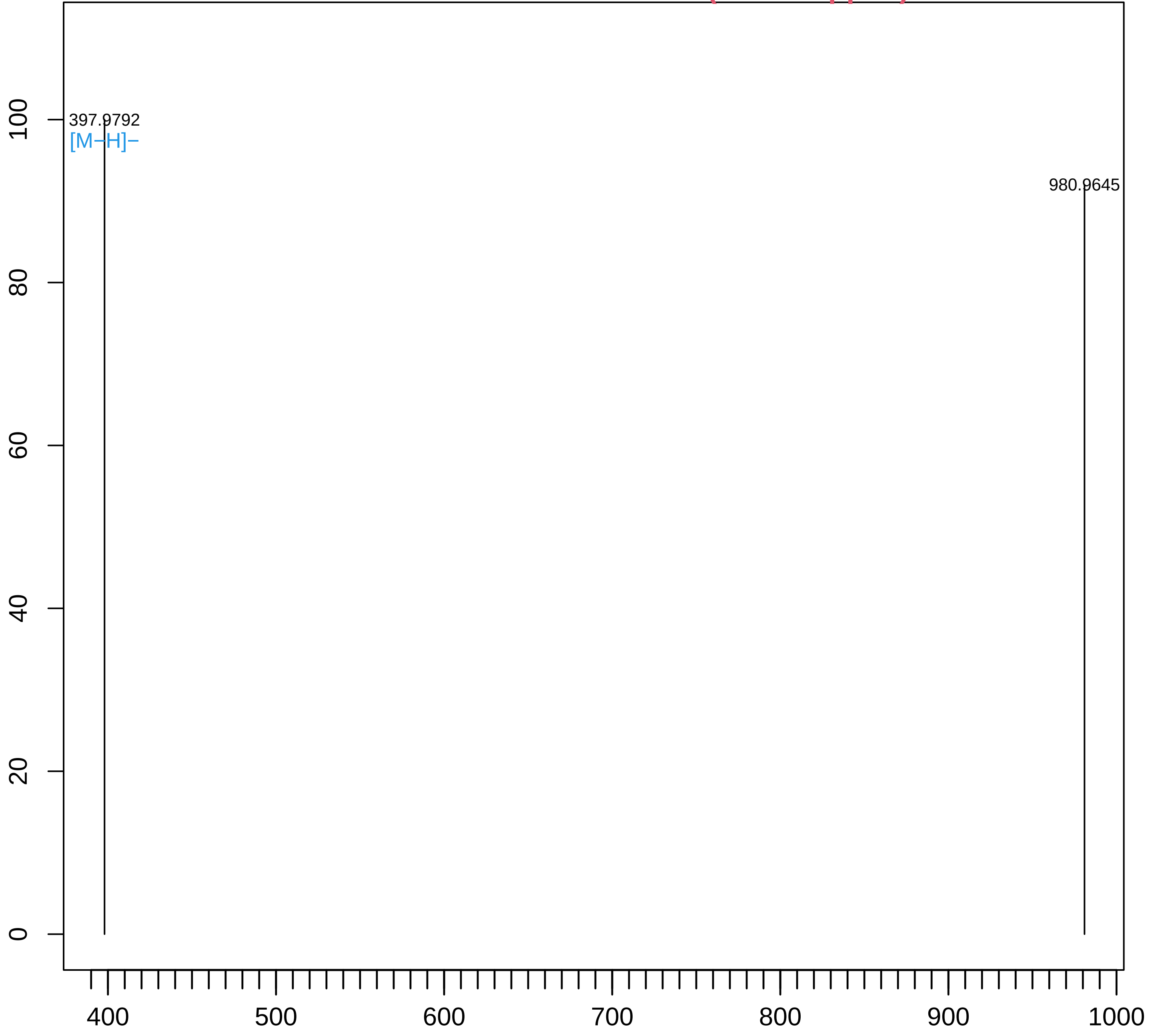

5066 : M.findmain = 398.9865 (+/− 20 ppm)

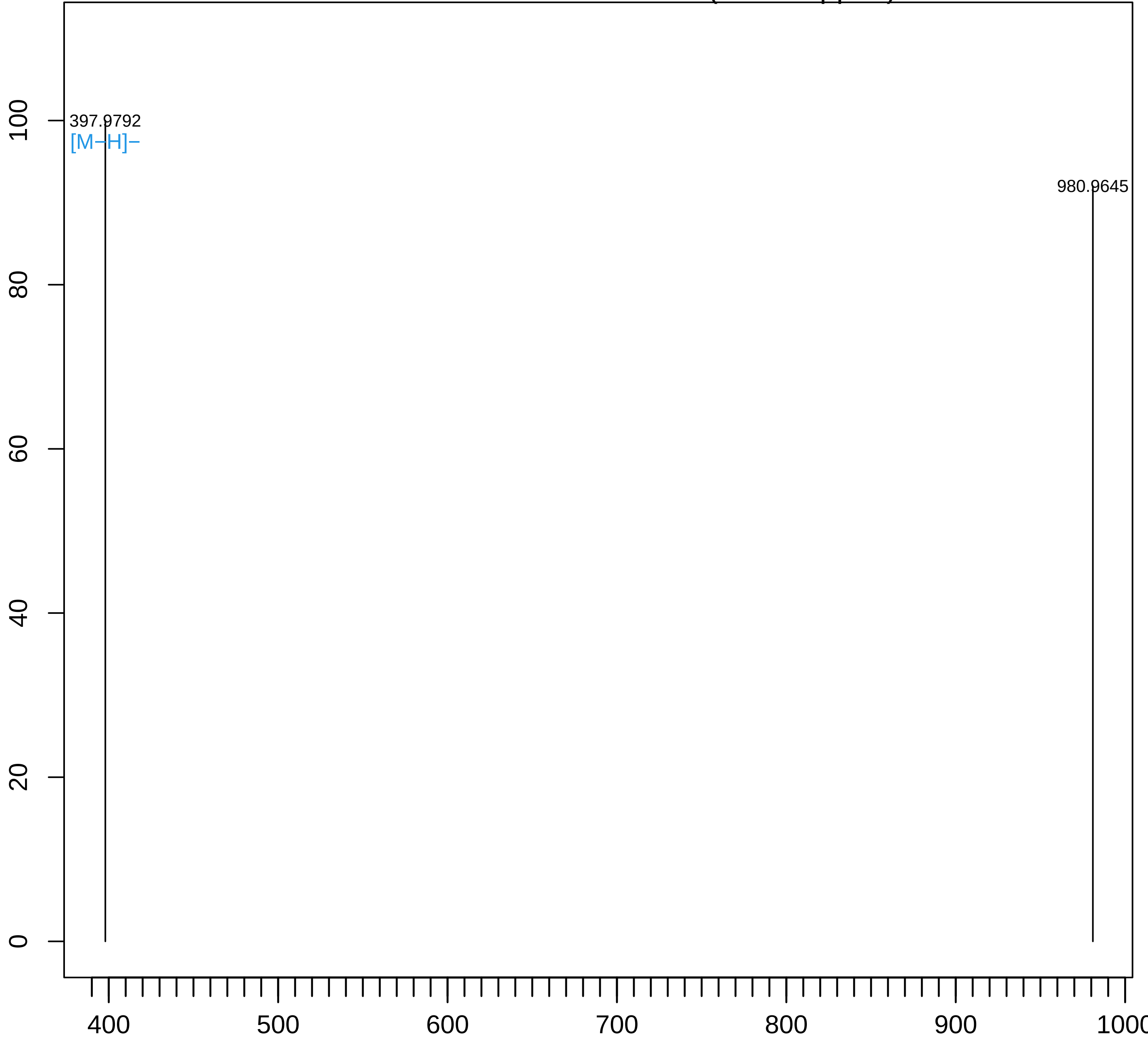

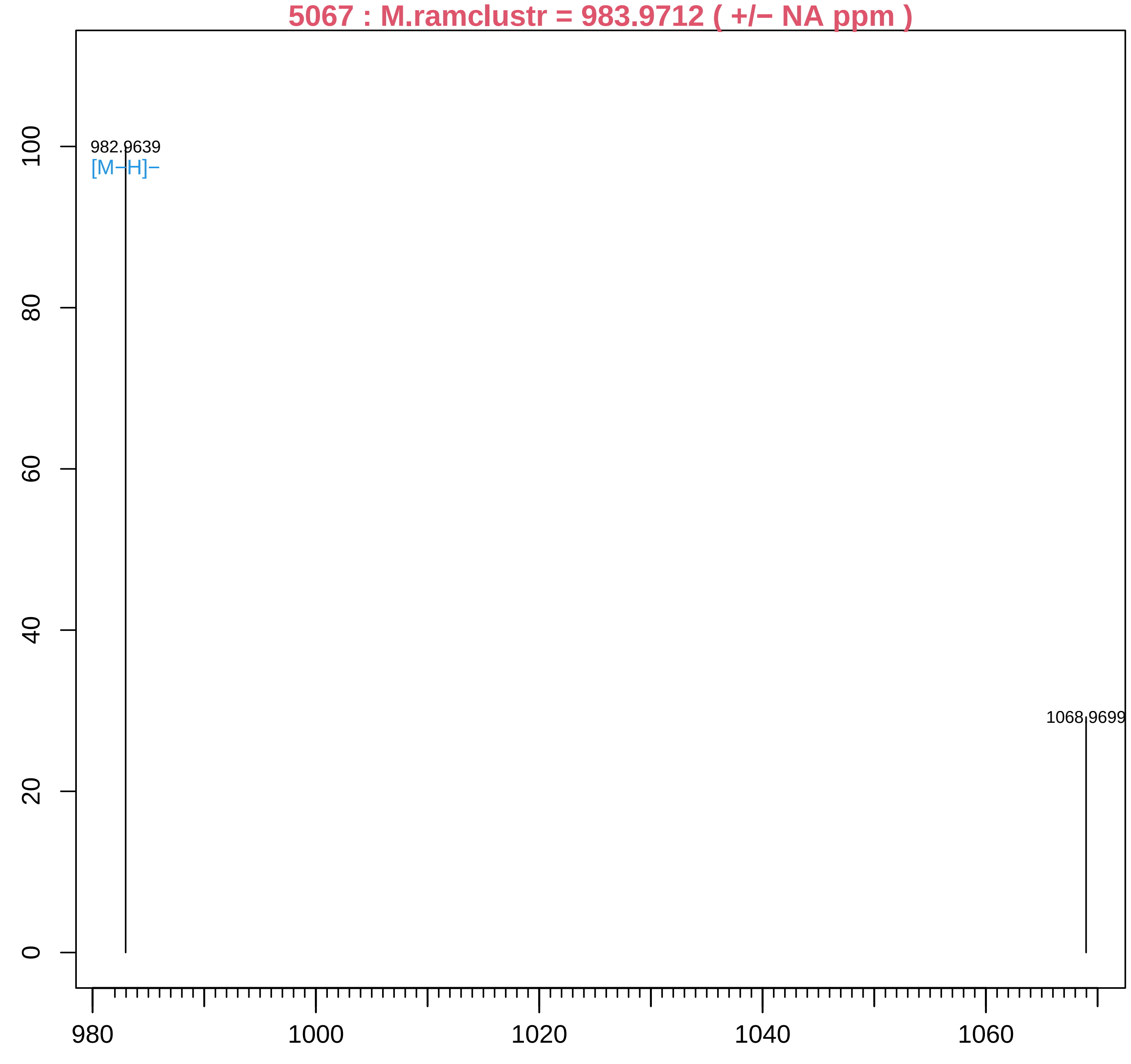
5067 : M.ramclustr = 983.9712 (+/− NA ppm)
982.9639
[M−H]−
1068.9699
0
20
40
60
80
100
980
1000
1020
1040
1060

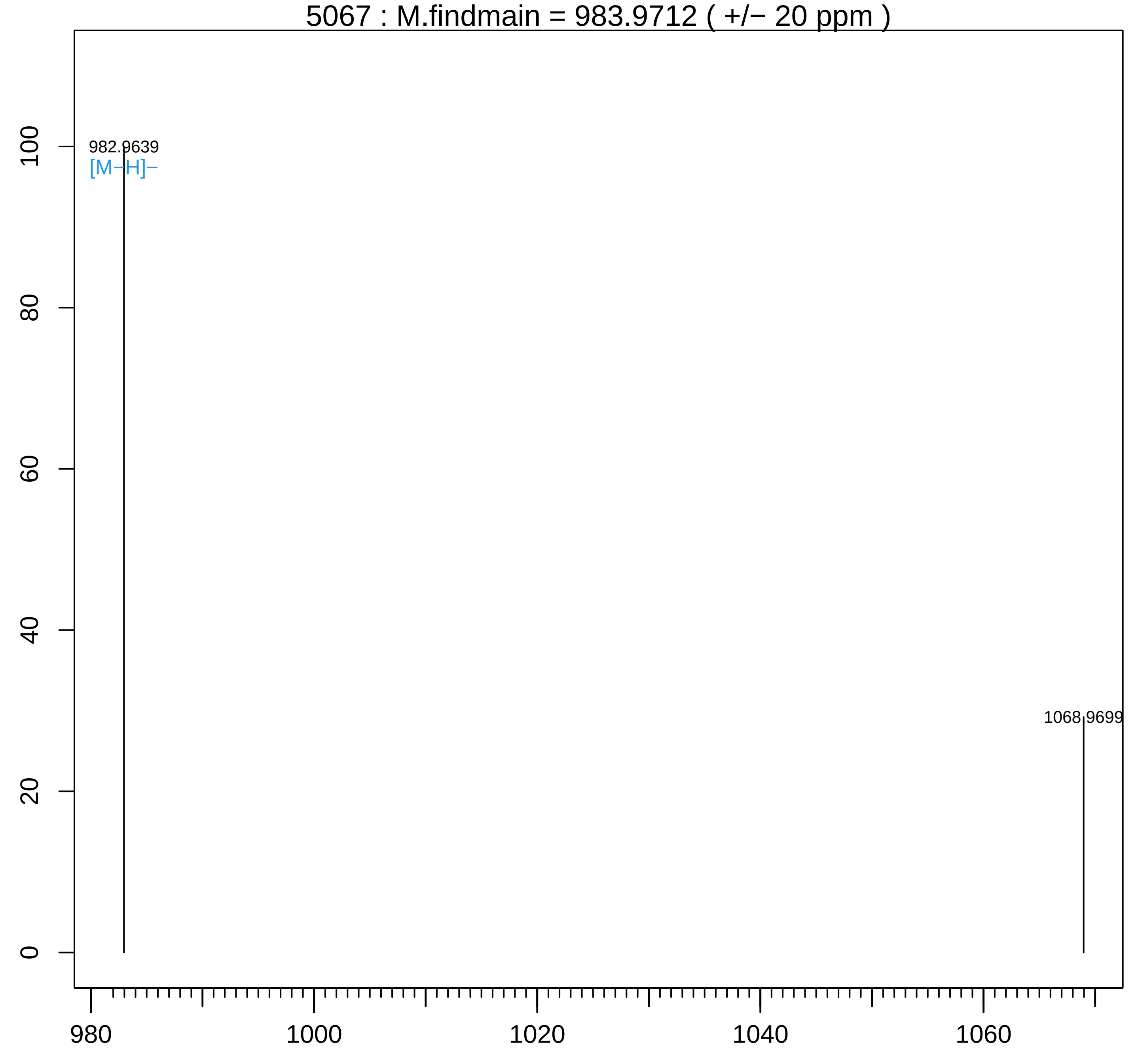
5067 : M.findmain = 983.9712 (+/− 20 ppm)
982.9639
[M−H]−
1068.9699
0
20
40
60
80
100
980
1000
1020
1040
1060

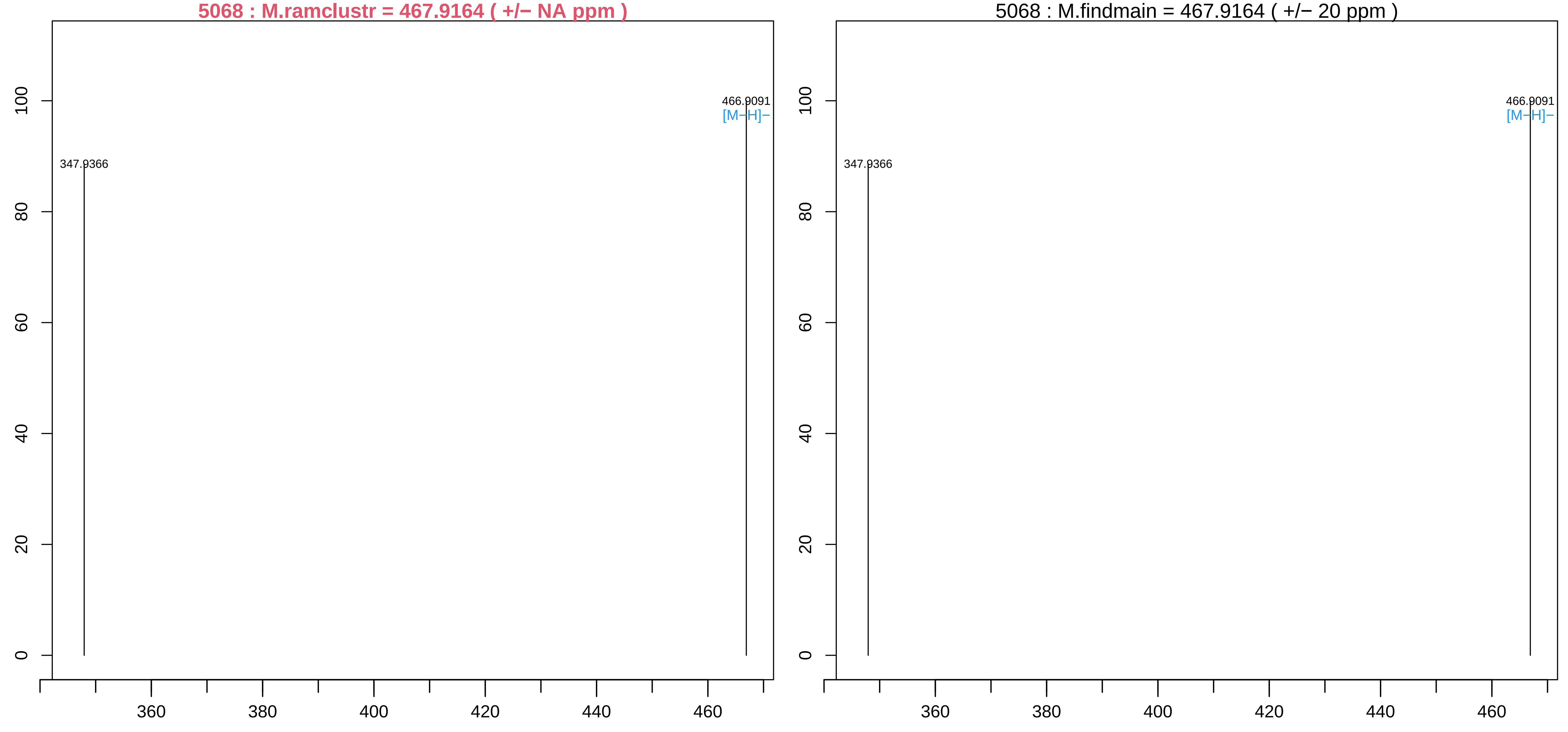

5068 : M.ramclustr = 467.9164 (+/− NA ppm)
5068 : M.findmain = 467.9164 (+/− 20 ppm)
347.9366
466.9091
[M−H]−
347.9366
466.9091
[M−H]−
0
20
40
60
80
100
360
380
400
420
440
460

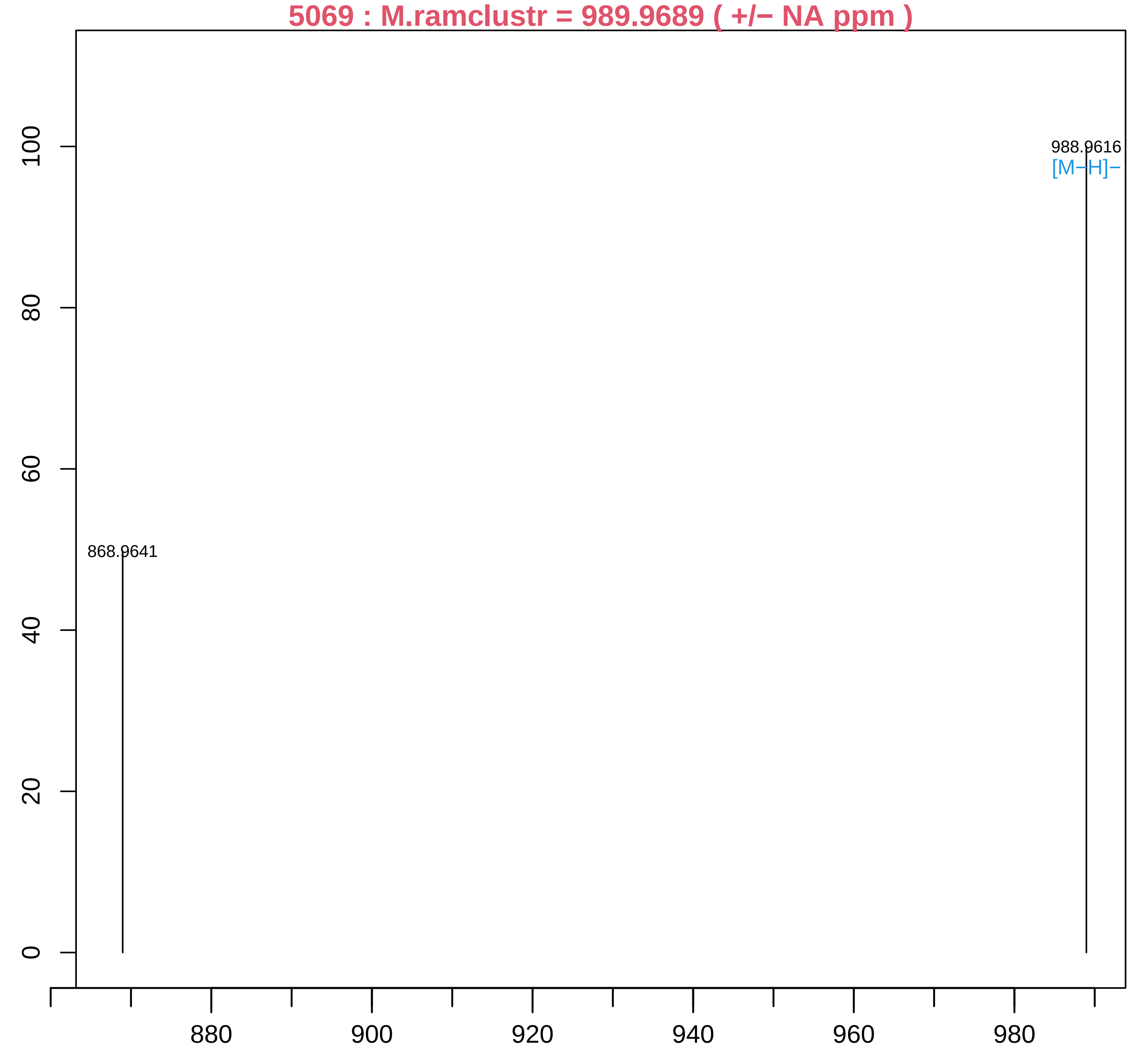
5069 : M.ramclustr = 989.9689 (+/− NA ppm)
988.9616
[M−H]−
868.9641
0
20
40
60
80
100
880
900
920
940
960
980

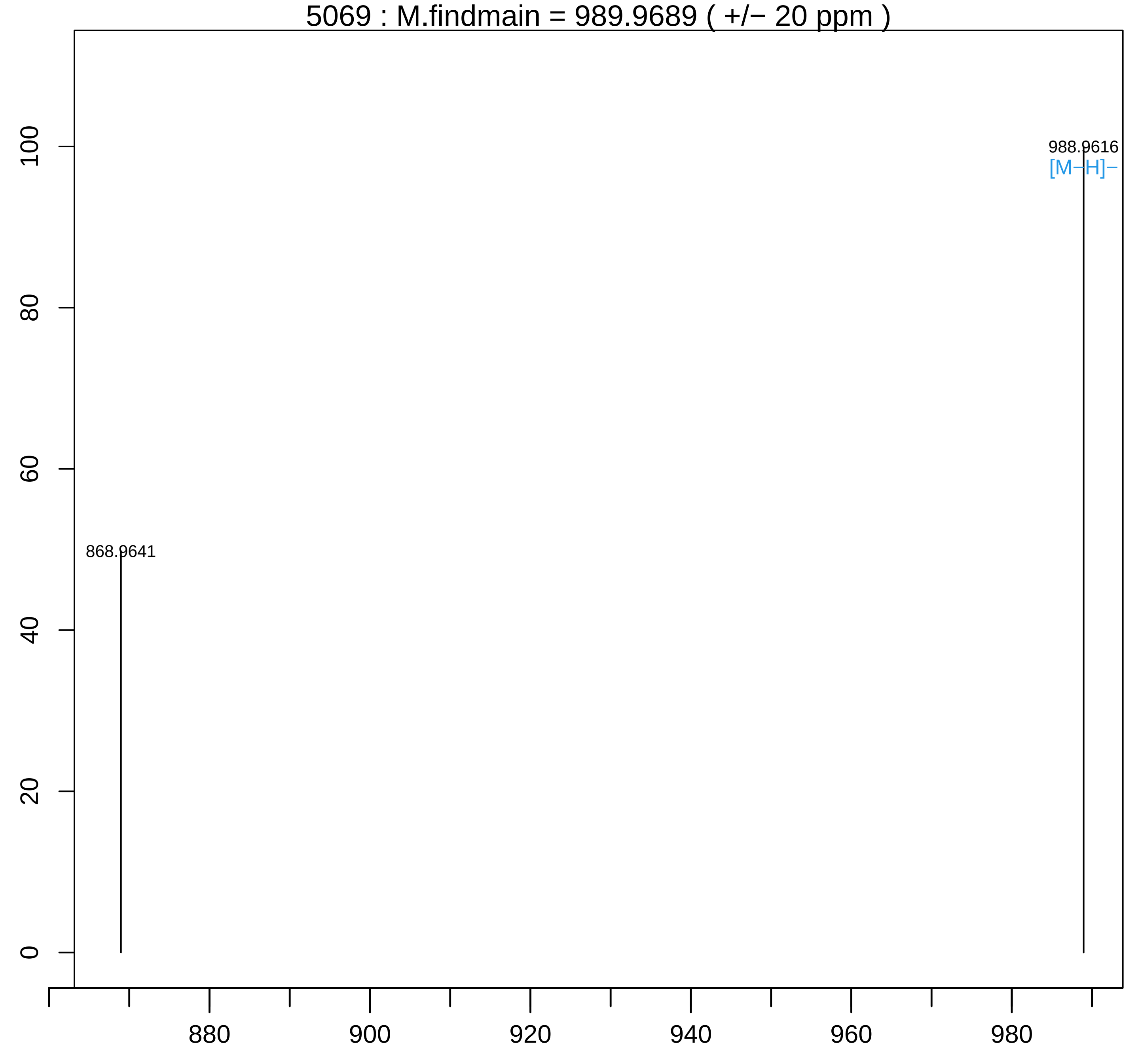
5069 : M.findmain = 989.9689 (+/− 20 ppm)
988.9616
[M−H]−
868.9641
0
20
40
60
80
100
880
900
920
940
960
980

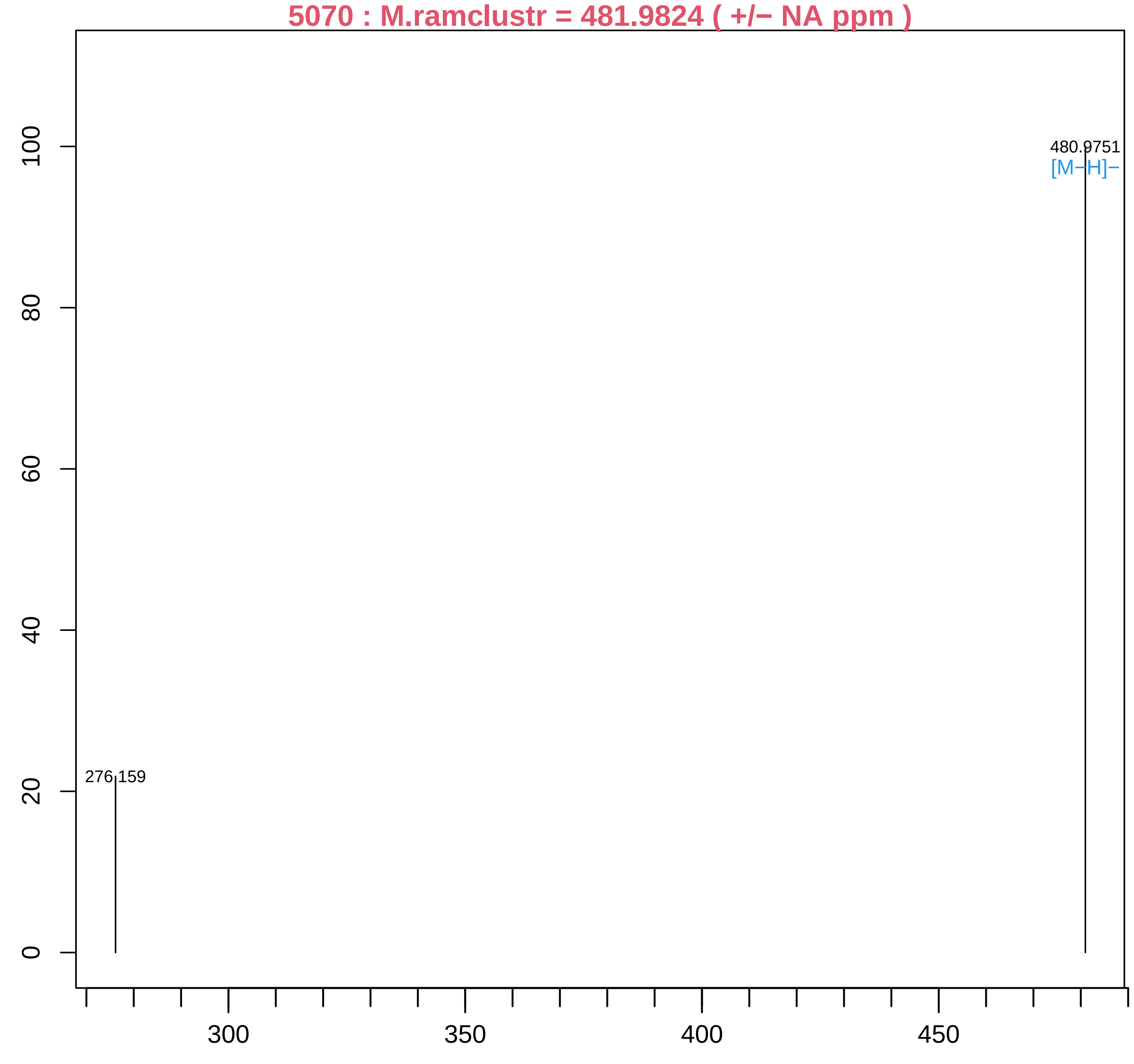
5070 : M.ramclustr = 481.9824 (+/− NA ppm)
480.9751
[M−H]−
276.159
0
20
40
60
80
100
300
350
400
450

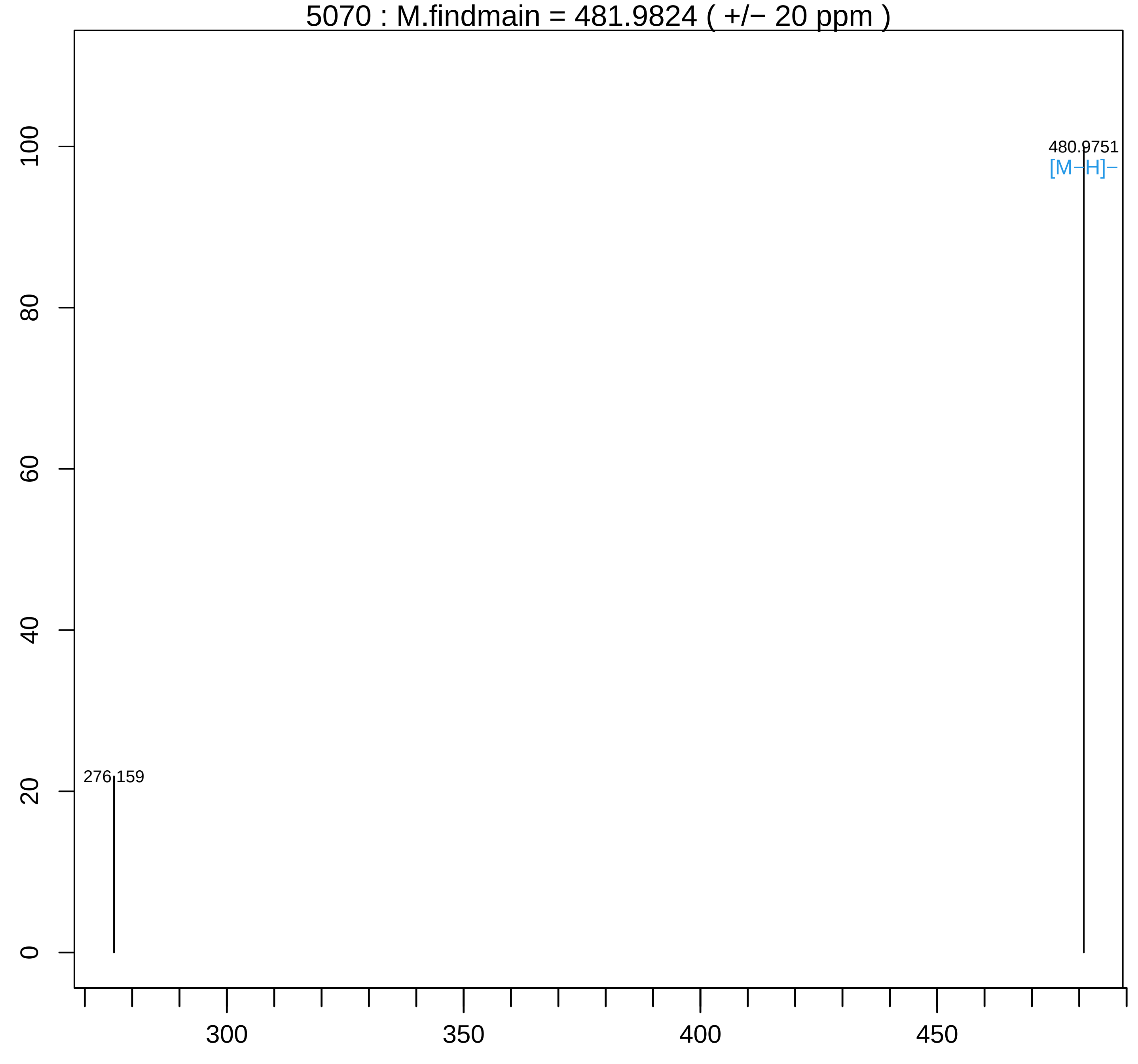
5070 : M.findmain = 481.9824 (+/− 20 ppm)
480.9751
[M−H]−
276.159
0
20
40
60
80
100
300
350
400
450

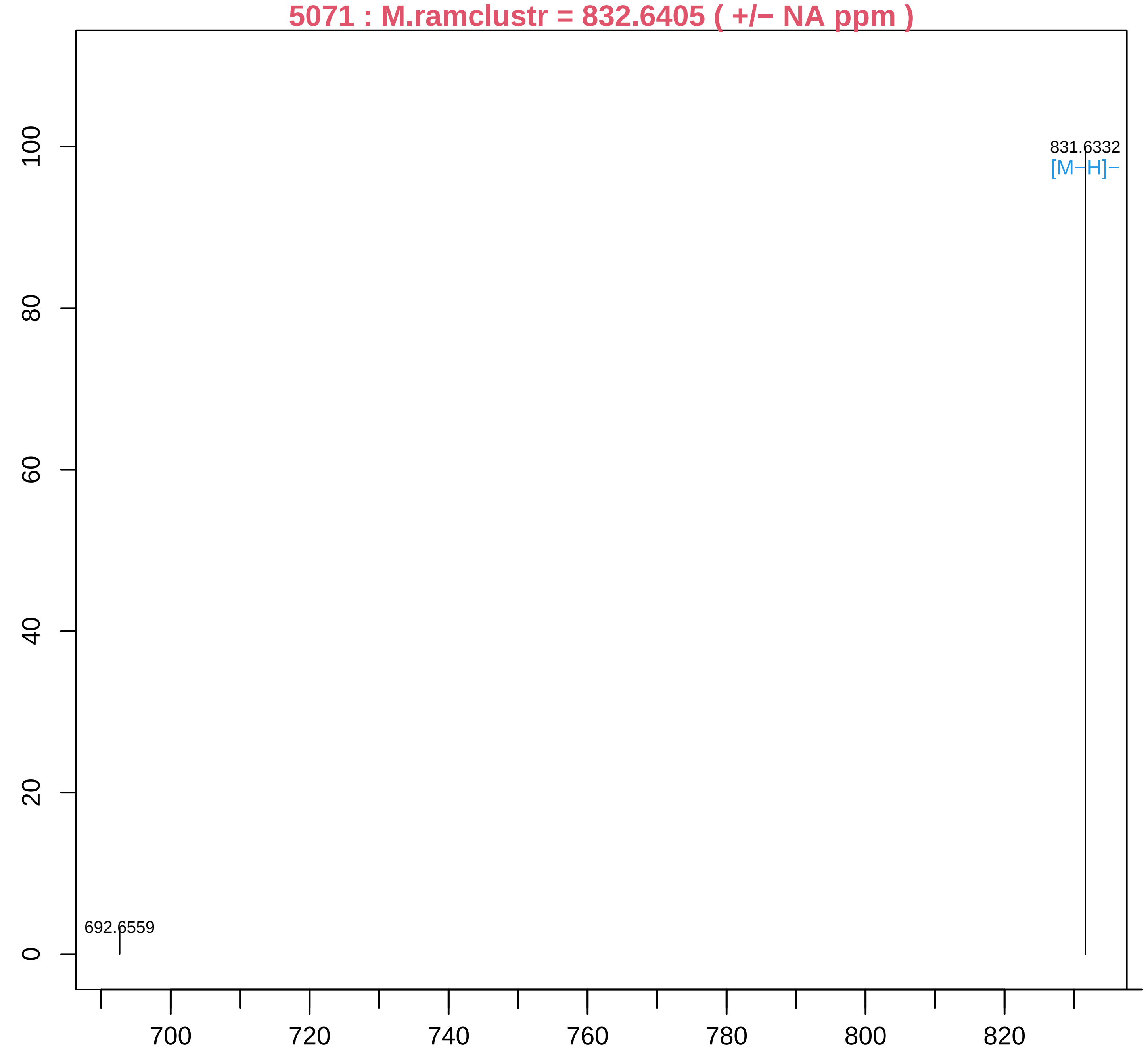
5071 : M.ramclustr = 832.6405 (+/− NA ppm)
831.6332
[M−H]−
692.6559
0
20
40
60
80
100
700
720
740
760
780
800
820

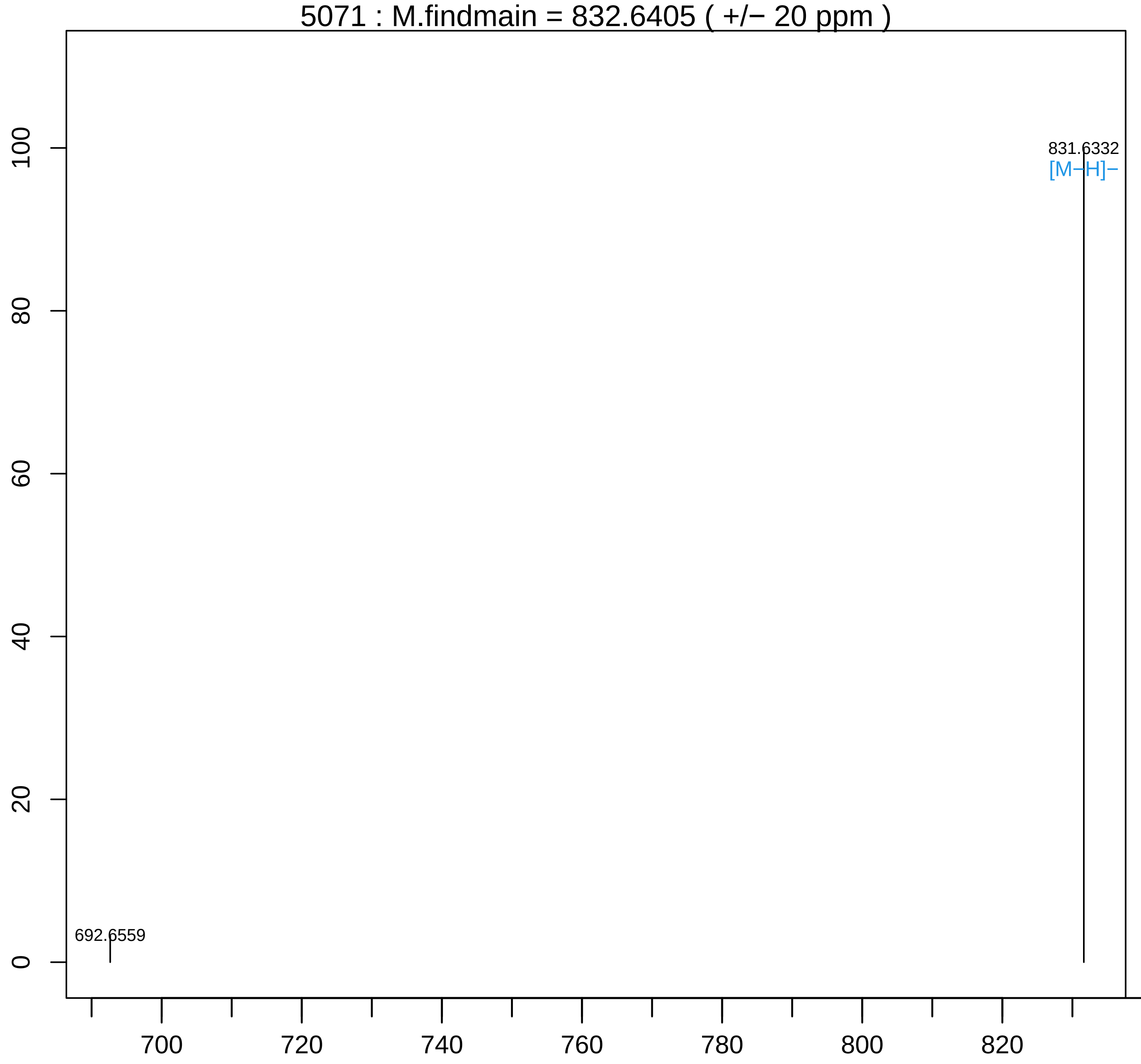
5071 : M.findmain = 832.6405 (+/− 20 ppm)
831.6332
[M−H]−
692.6559
0
20
40
60
80
100
700
720
740
760
780
800
820

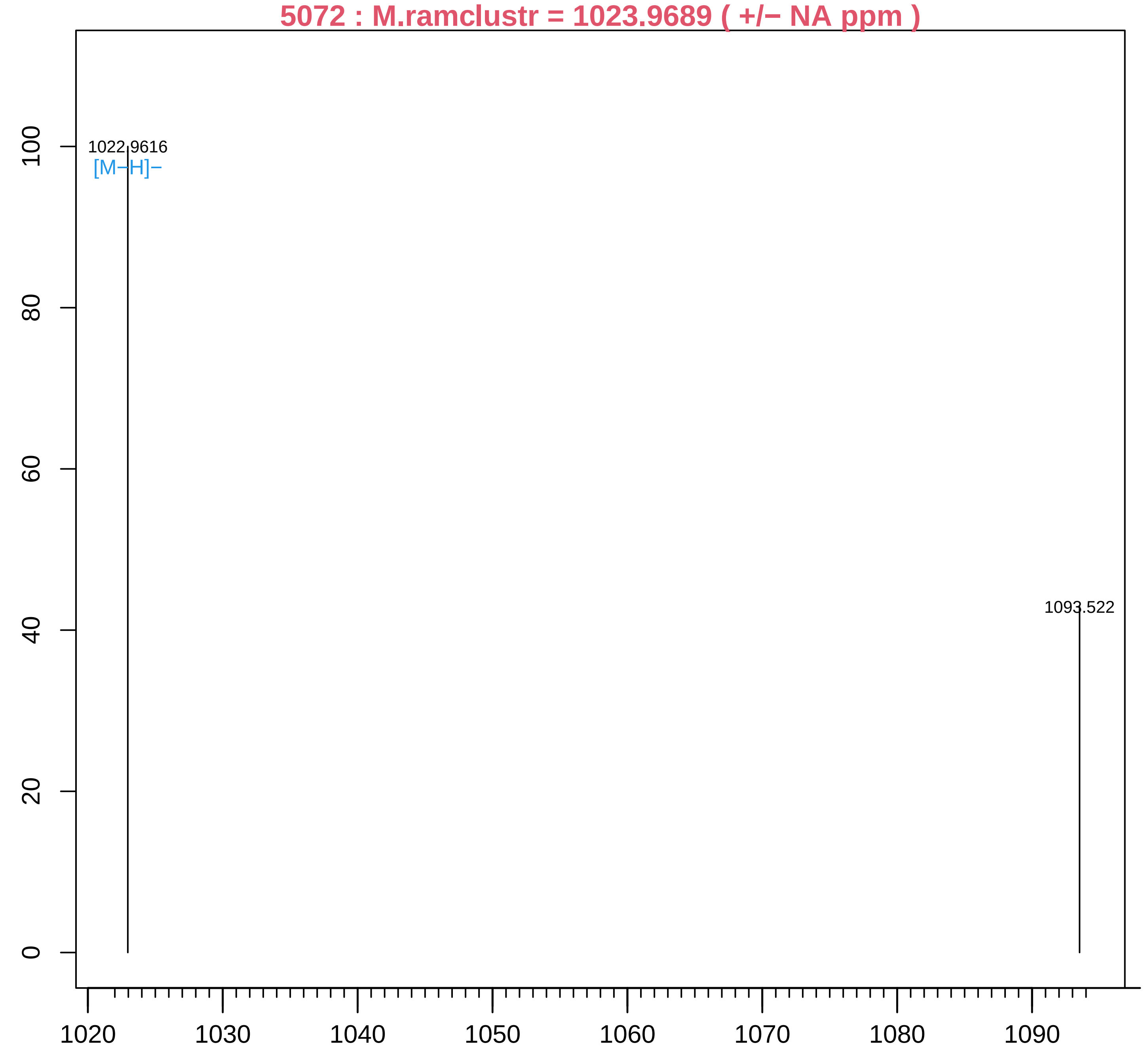

5072 : M.ramclustr = 1023.9689 (+/− NA ppm)
1022.9616
[M−H]−
1093.522
0
20
40
60
80
100
1020
1030
1040
1050
1060
1070
1080
1090

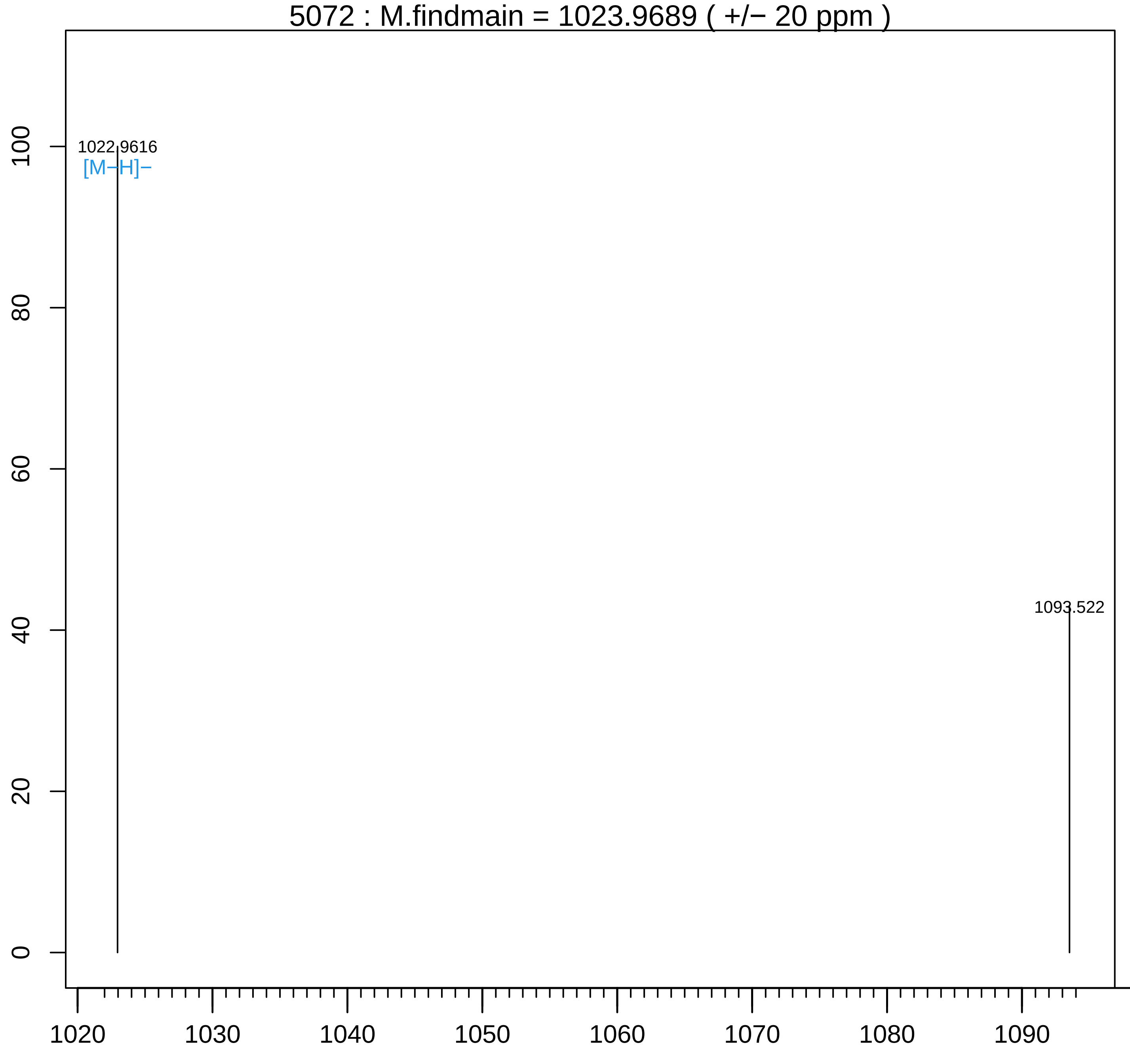

5072 : M.findmain = 1023.9689 (+/− 20 ppm)
1022.9616
[M−H]−
1093.522
0
20
40
60
80
100
1020
1030
1040
1050
1060
1070
1080
1090

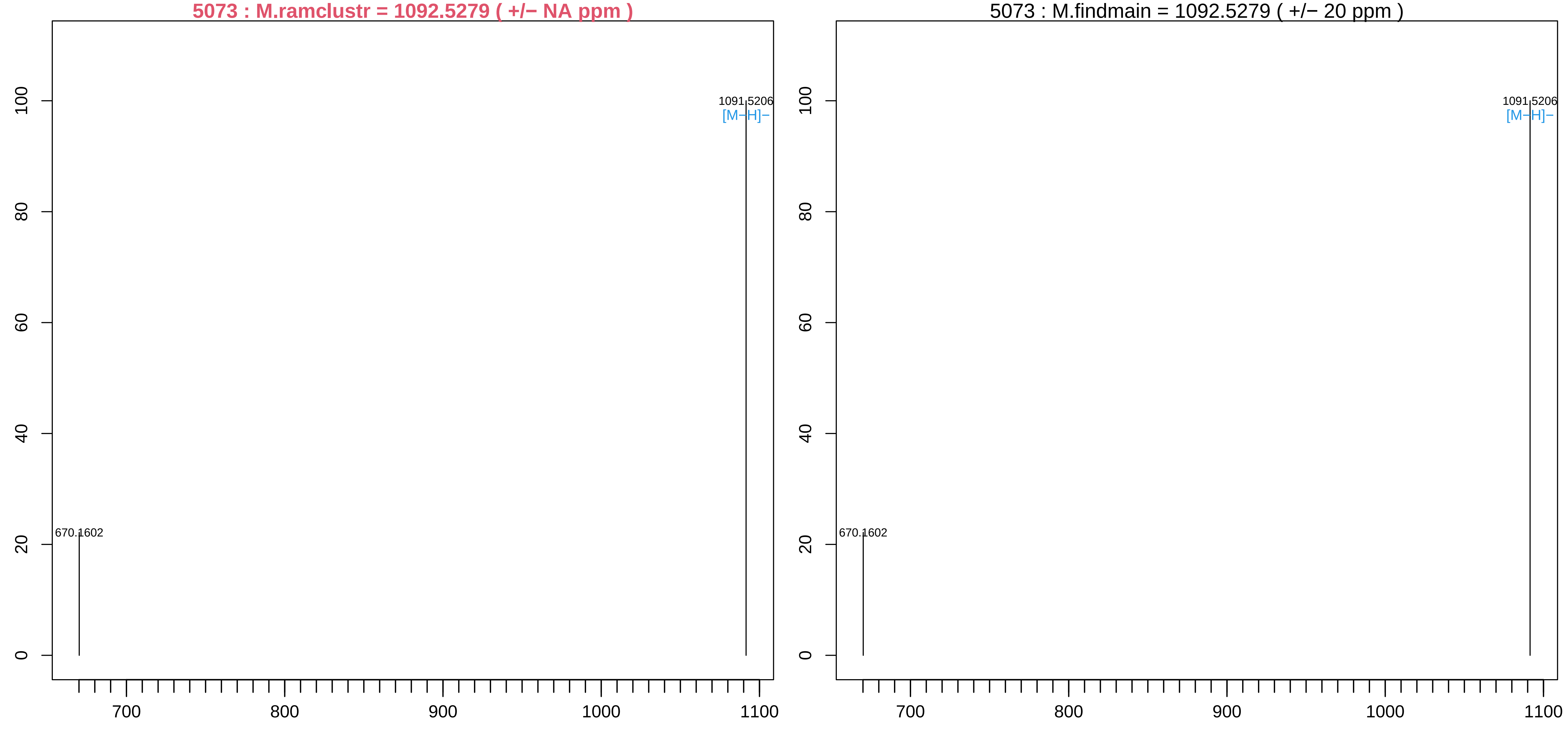
5073 : M.ramclustr = 1092.5279 (+/− NA ppm)
1091.5206
[M−H]−
670.1602
0
20
40
60
80
100
700
800
900
1000
1100
5073 : M.findmain = 1092.5279 (+/− 20 ppm)
1091.5206
[M−H]−
670.1602
0
20
40
60
80
100
700
800
900
1000
1100

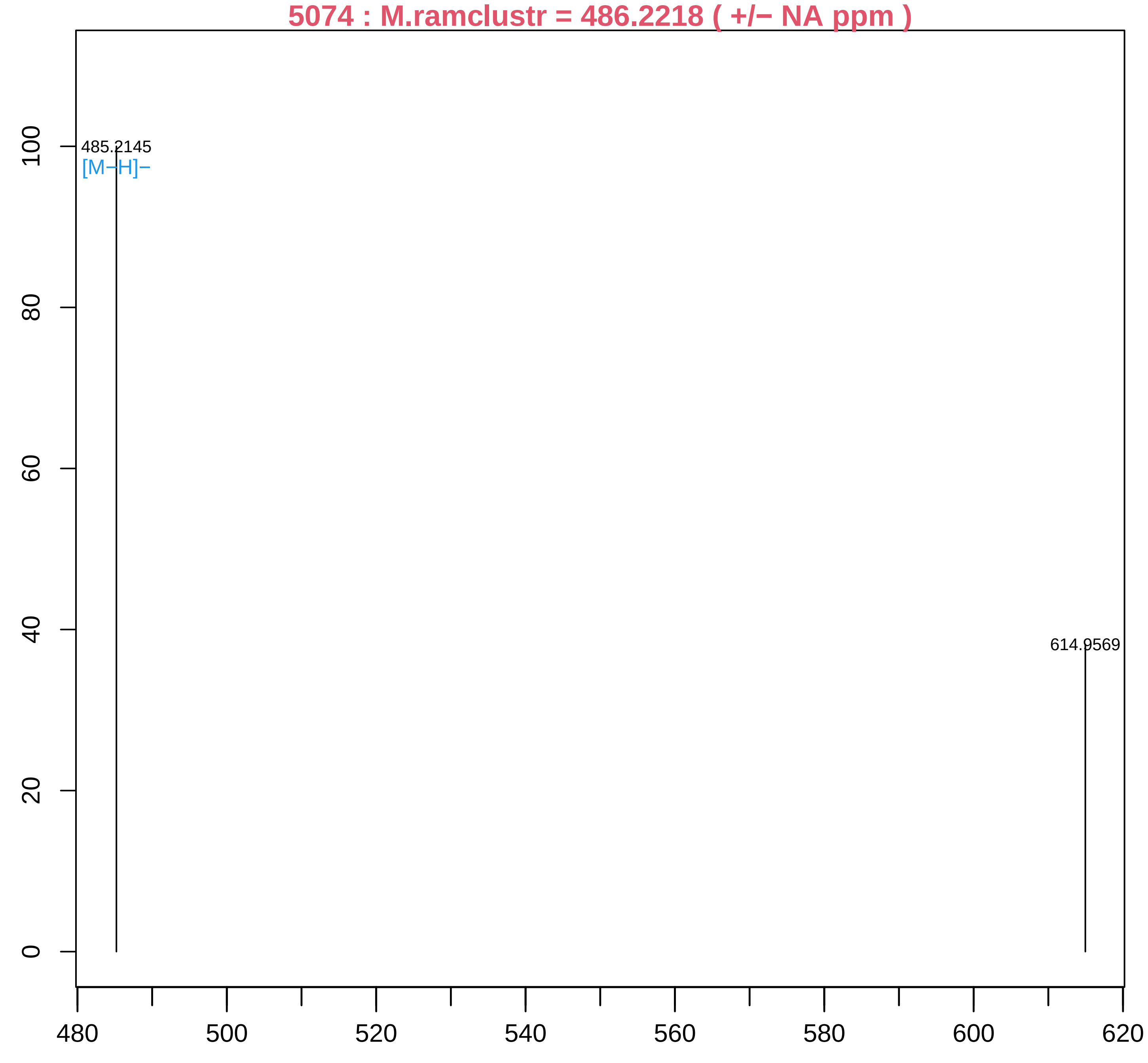
5074 : M.ramclustr = 486.2218 (+/− NA ppm)
485.2145
[M−H]−
614.9569
0
20
40
60
80
100
480
500
520
540
560
580
600
620

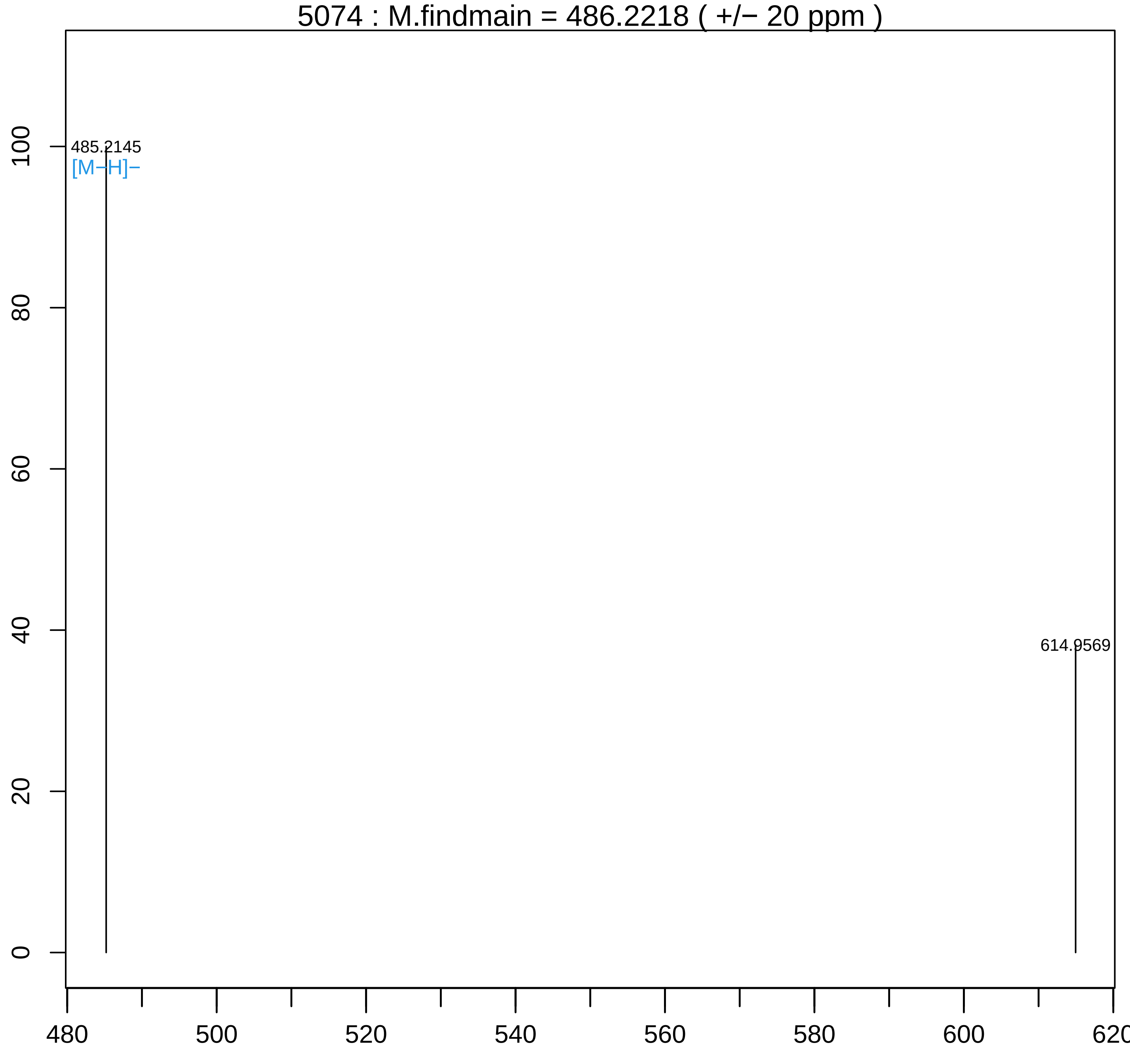
5074 : M.findmain = 486.2218 (+/− 20 ppm)
485.2145
[M−H]−
614.9569
0
20
40
60
80
100
480
500
520
540
560
580
600
620

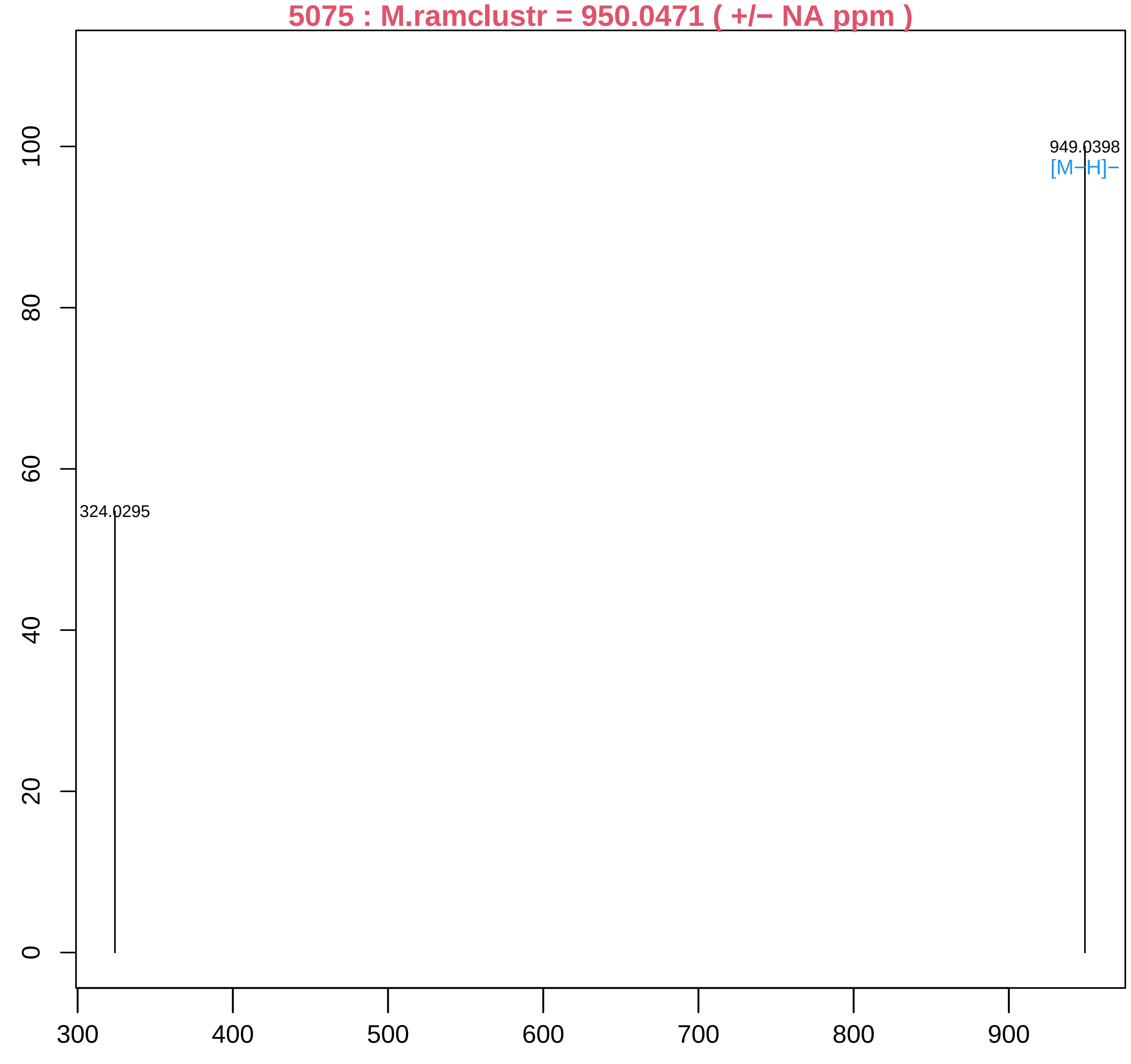

5075 : M.ramclustr = 950.0471 (+/− NA ppm)
949.0398
[M−H]−
324.0295
0
20
40
60
80
100
300
400
500
600
700
800
900

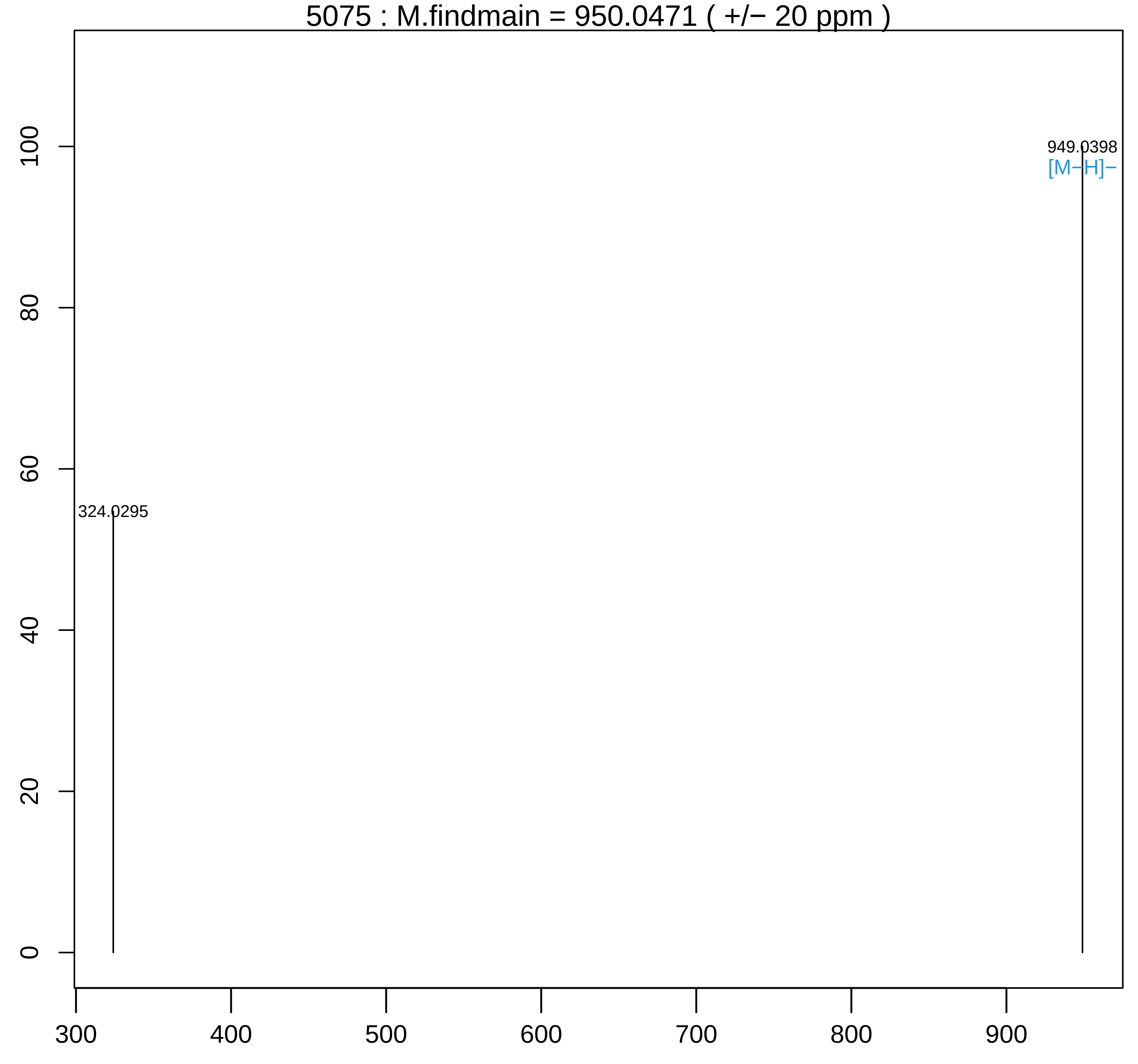

5075 : M.findmain = 950.0471 (+/− 20 ppm)
949.0398
[M−H]−
324.0295
0
20
40
60
80
100
300
400
500
600
700
800
900

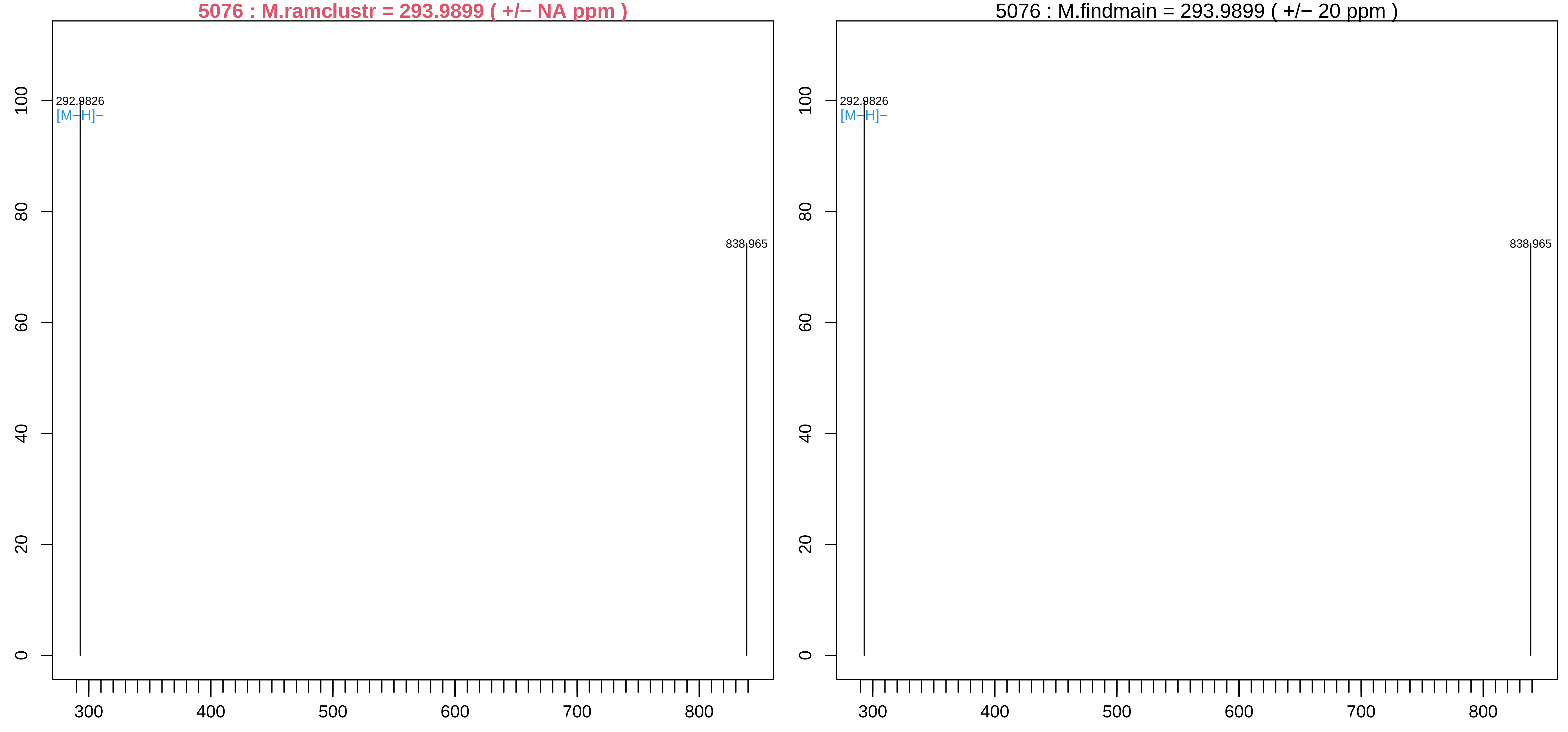
5076 : M.ramclustr = 293.9899 (+/− NA ppm)
292.9826
[M−H]−
838.965
0
20
40
60
80
100
300
400
500
600
700
800
5076 : M.findmain = 293.9899 (+/− 20 ppm)
292.9826
[M−H]−
838.965
0
20
40
60
80
100
300
400
500
600
700
800

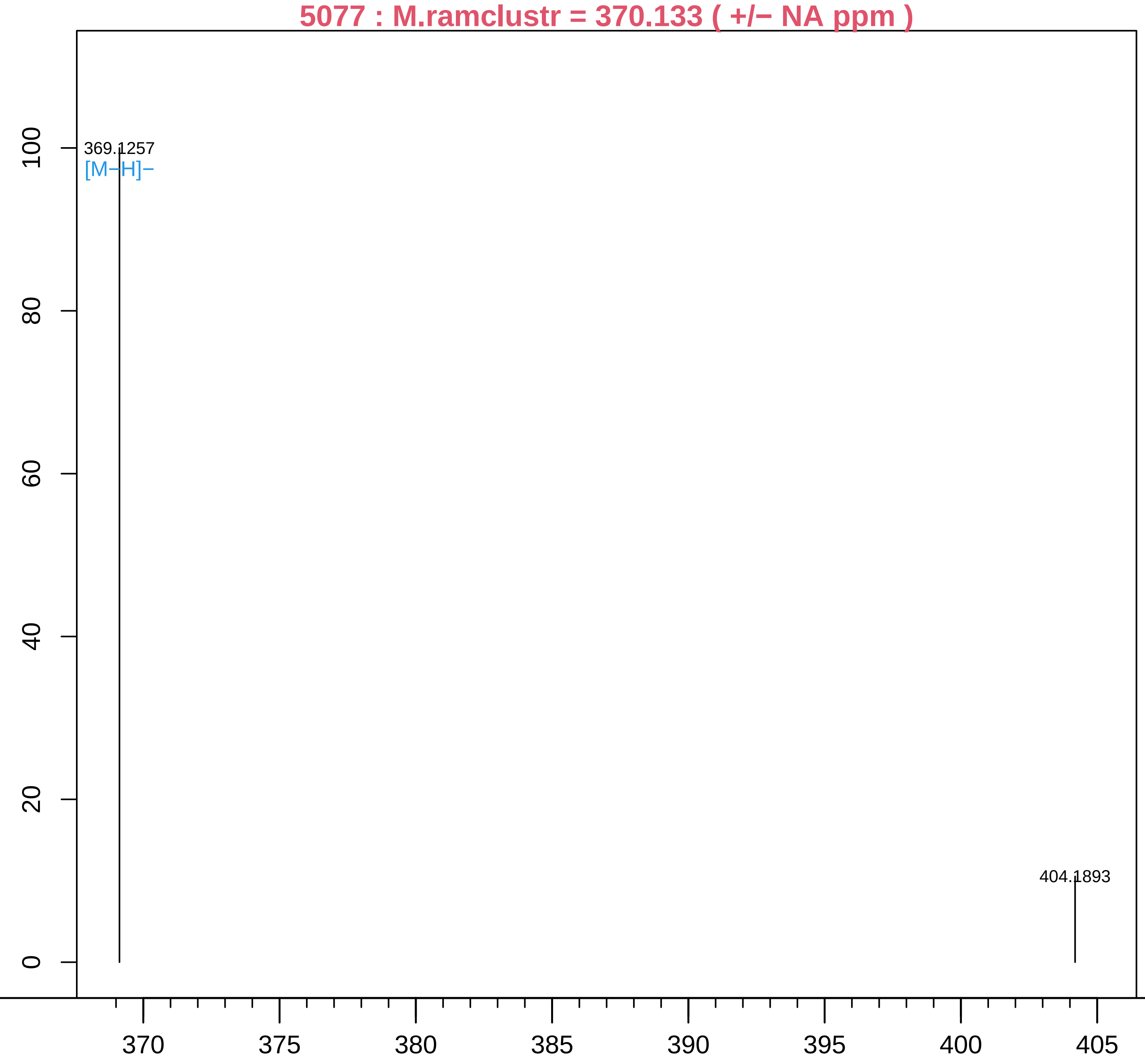

5077 : M.ramclustr = 370.133 (+/− NA ppm)
369.1257
[M−H]−
404.1893
0
20
40
60
80
100
370
375
380
385
390
395
400
405

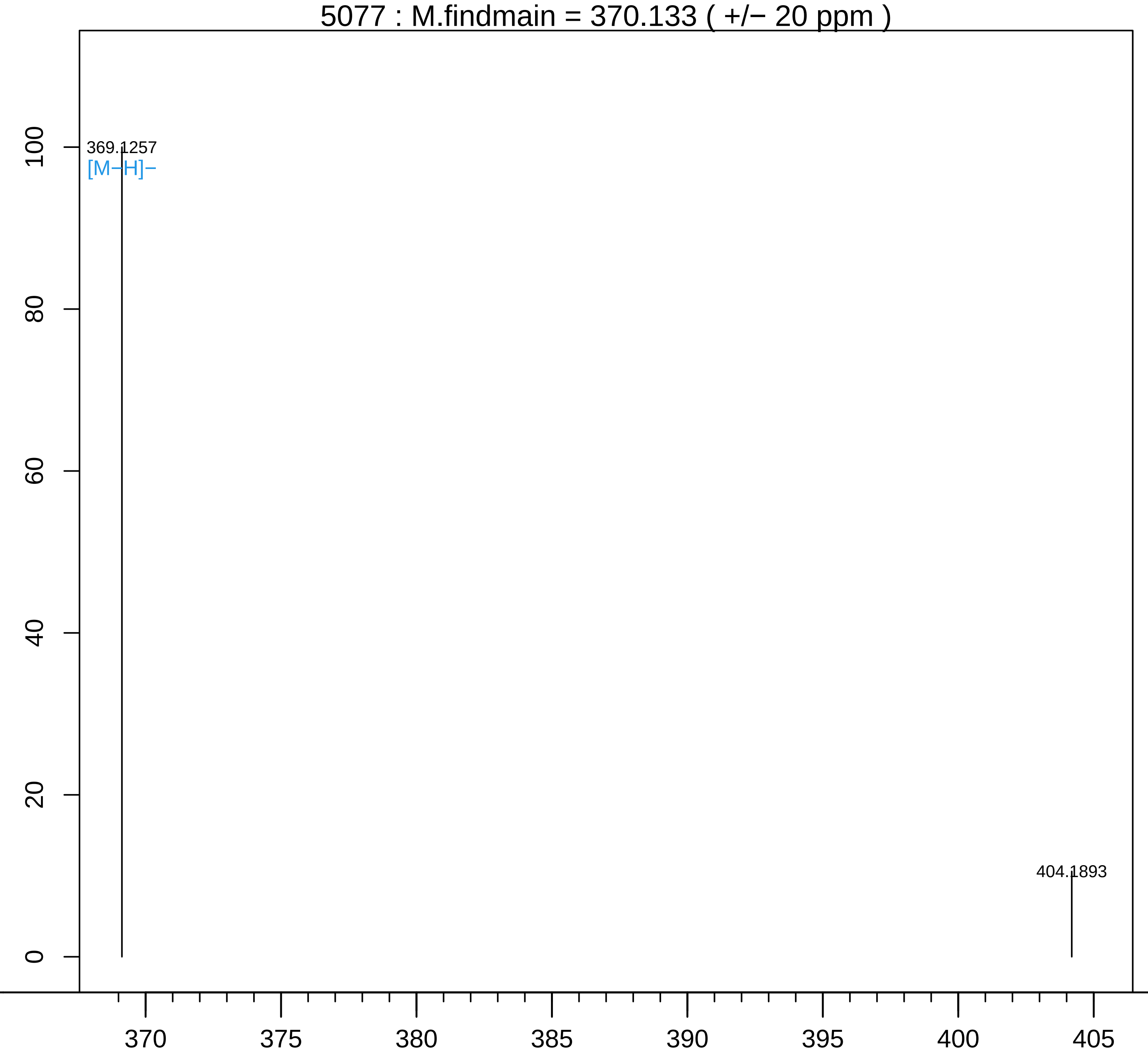

5077 : M.findmain = 370.133 (+/− 20 ppm)
369.1257
[M−H]−
404.1893
0
20
40
60
80
100
370
375
380
385
390
395
400
405

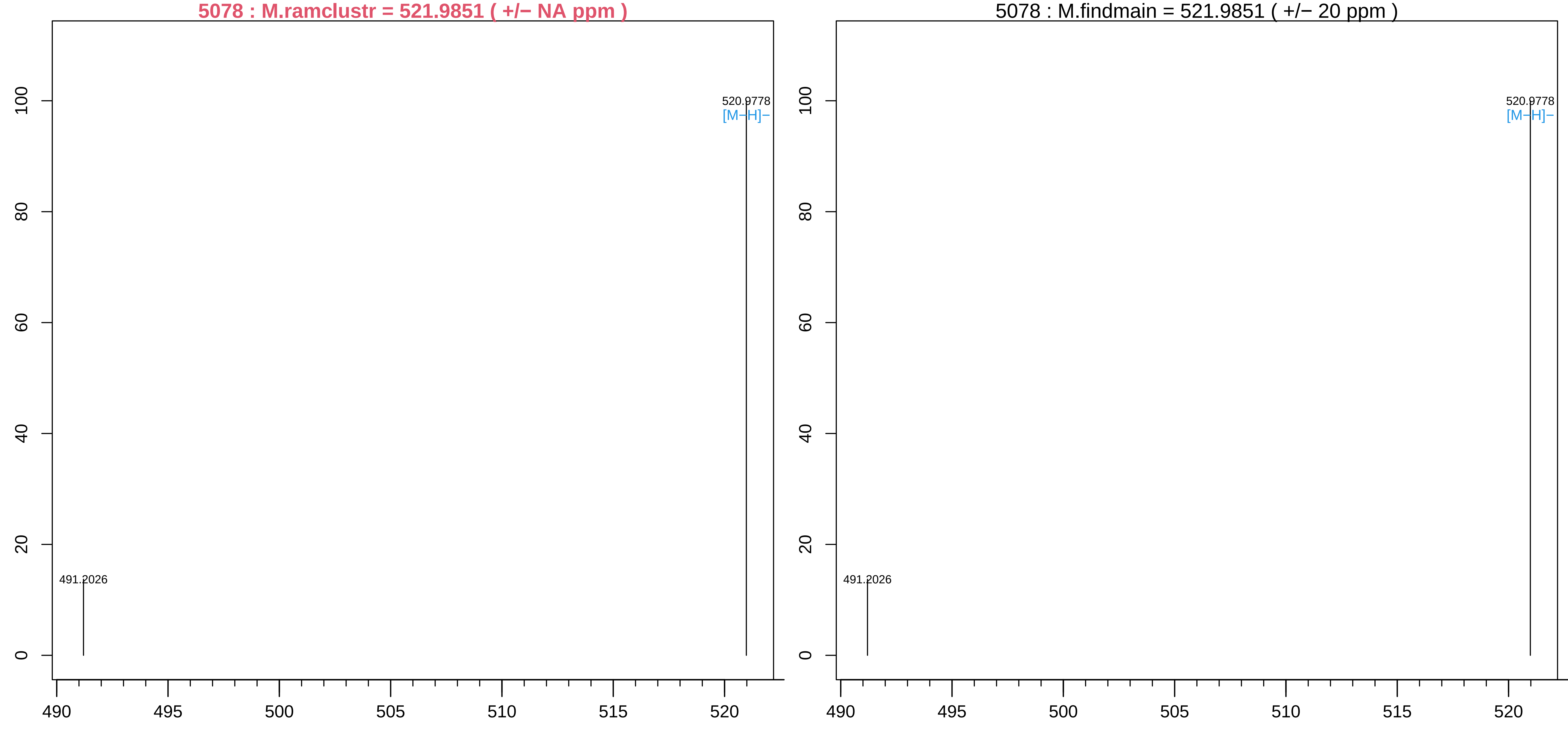
5078 : M.ramclustr = 521.9851 (+/− NA ppm)
520.9778
[M−H]−
491.2026
5078 : M.findmain = 521.9851 (+/− 20 ppm)
520.9778
[M−H]−
491.2026
0
20
40
60
80
100
490
495
500
505
510
515
520

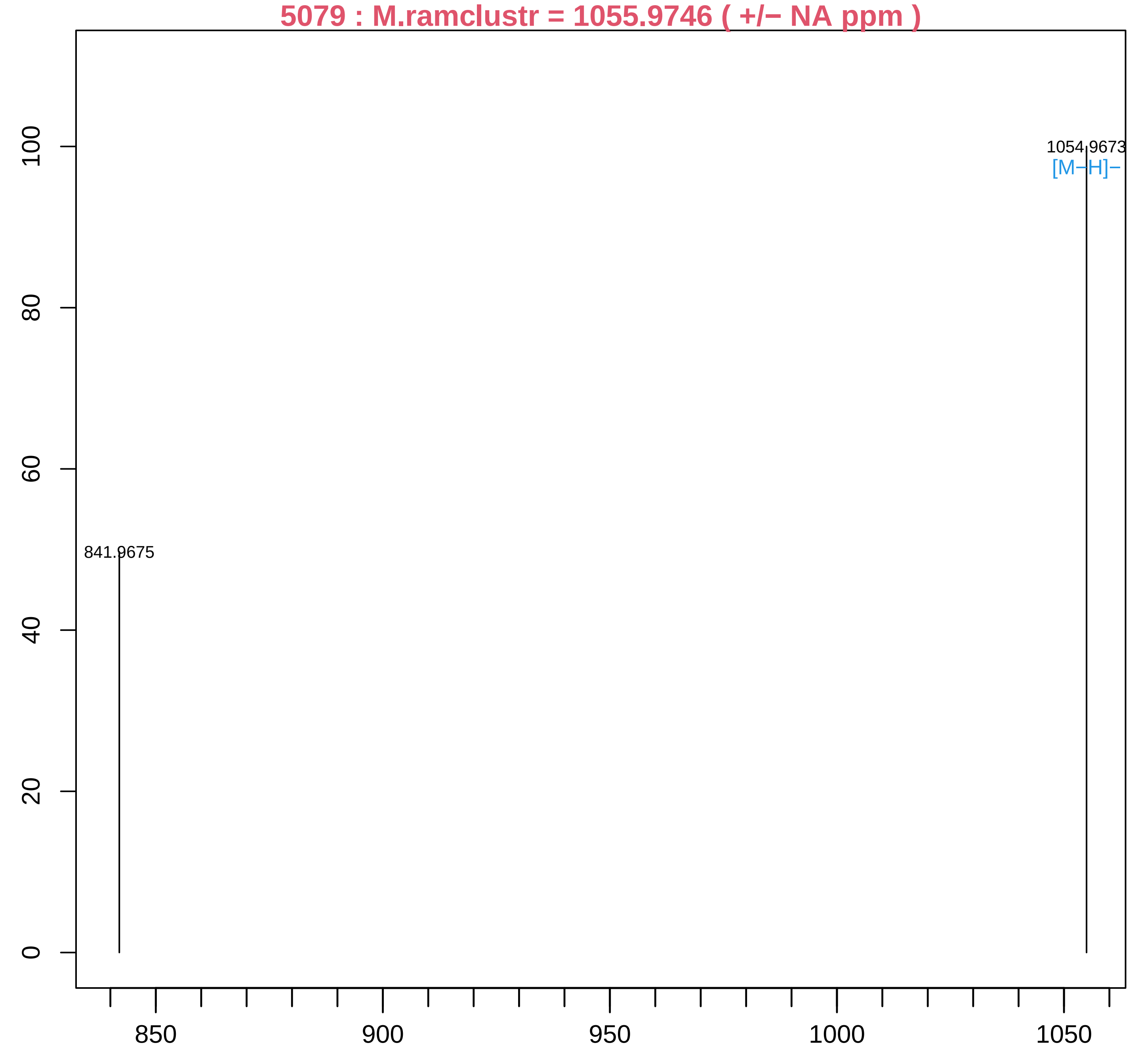
5079 : M.ramclustr = 1055.9746 (+/− NA ppm)
1054.9673
[M−H]−
841.9675
0
20
40
60
80
100
850
900
950
1000
1050

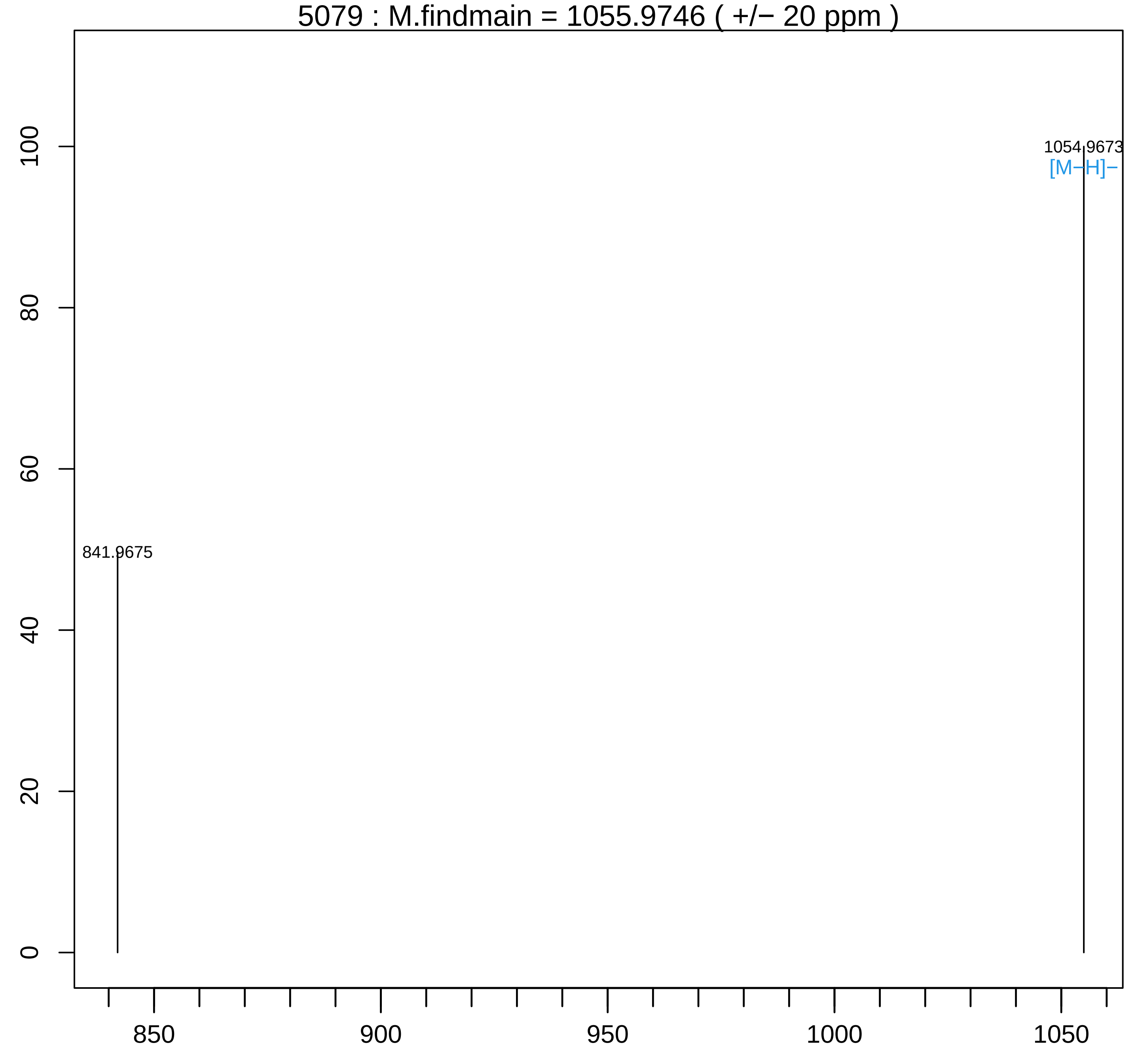
5079 : M.findmain = 1055.9746 (+/− 20 ppm)
1054.9673
[M−H]−
841.9675
0
20
40
60
80
100
850
900
950
1000
1050

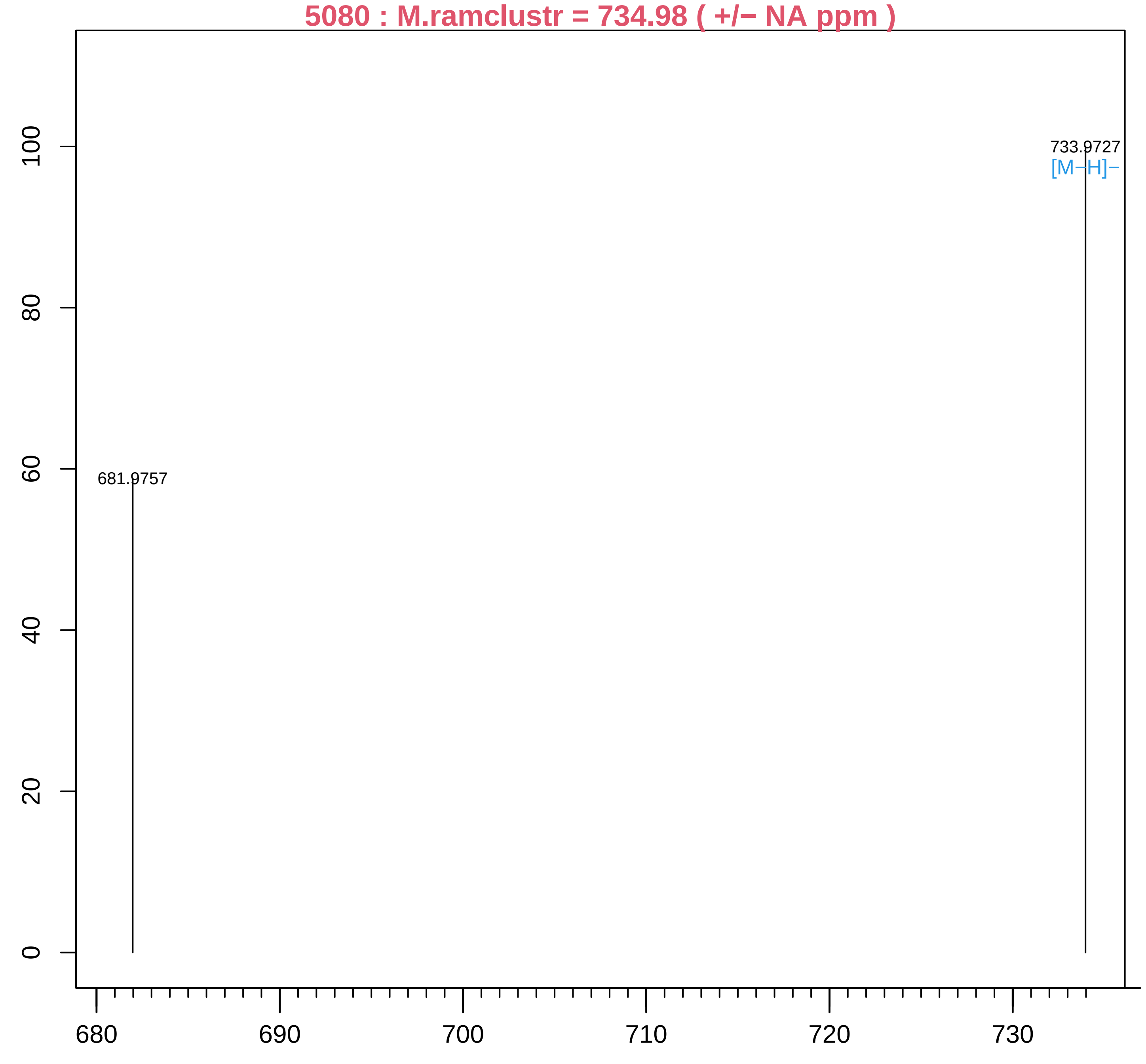
5080 : M.ramclustr = 734.98 (+/− NA ppm)
733.9727
[M−H]−
681.9757
0
20
40
60
80
100
680
690
700
710
720
730

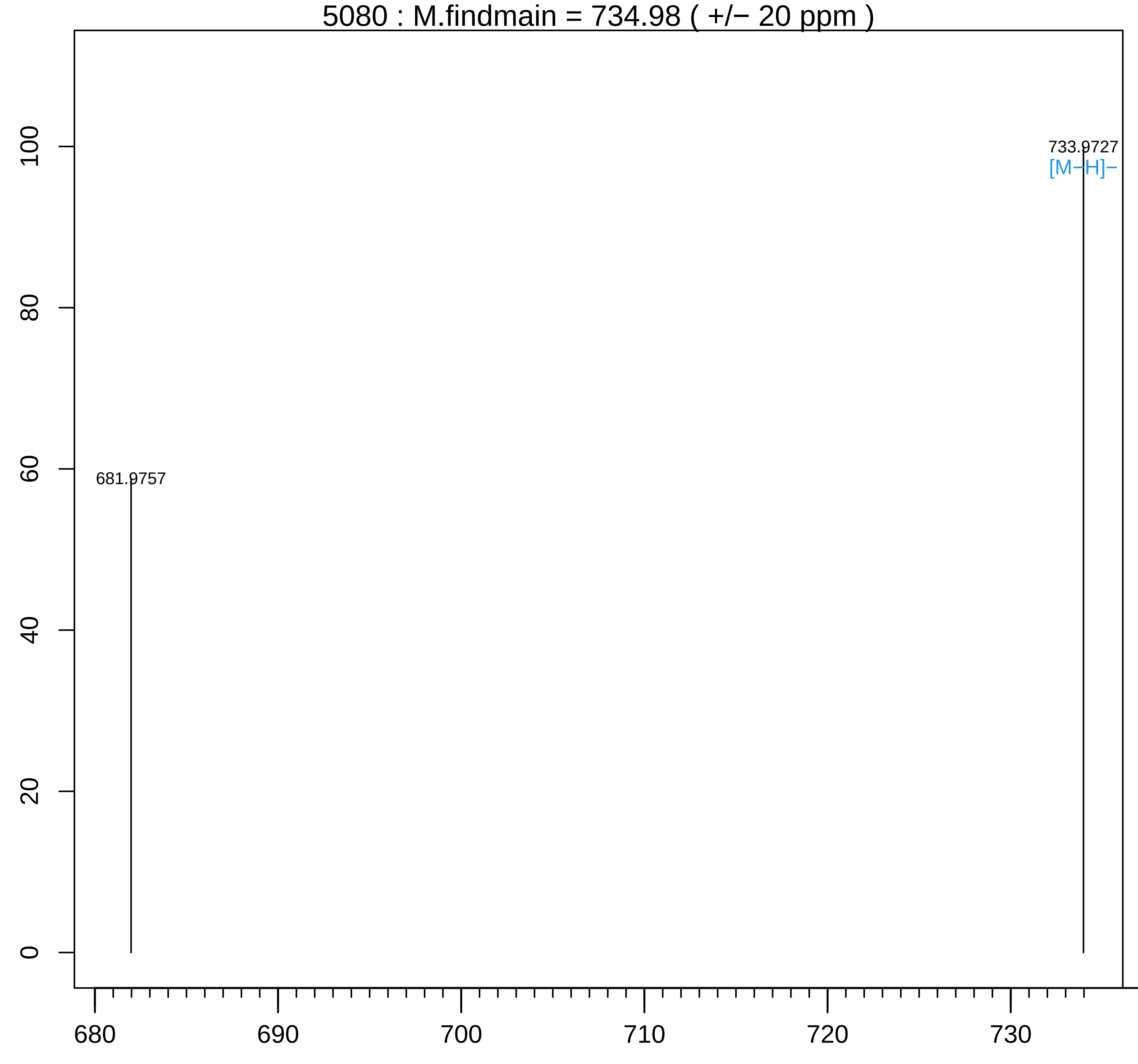
5080 : M.findmain = 734.98 (+/− 20 ppm)
733.9727
[M−H]−
681.9757
0
20
40
60
80
100
680
690
700
710
720
730

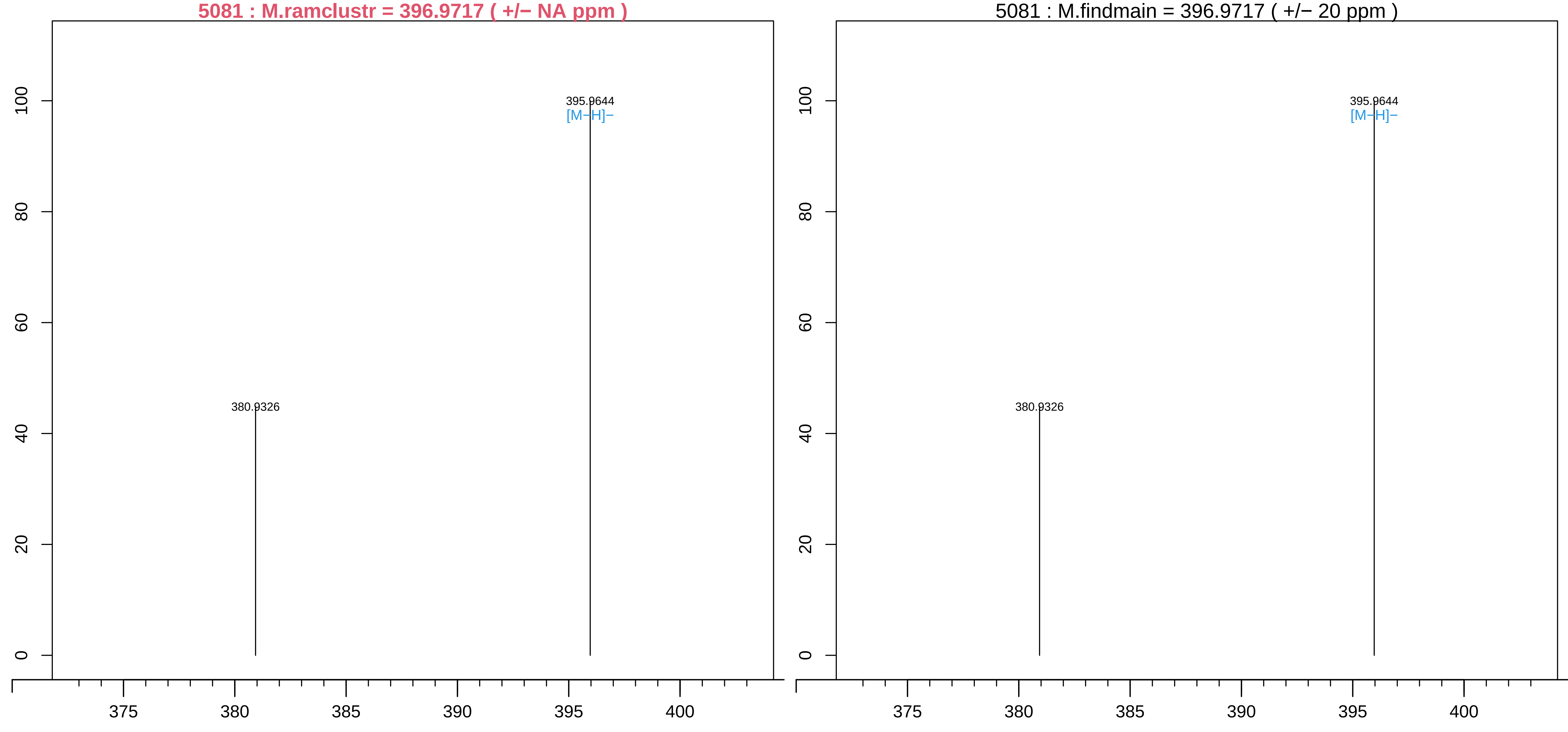

5081 : M.ramclustr = 396.9717 (+/− NA ppm)
395.9644
[M−H]−
380.9326
5081 : M.findmain = 396.9717 (+/− 20 ppm)
395.9644
[M−H]−
380.9326
0
20
40
60
80
100
375
380
385
390
395
400

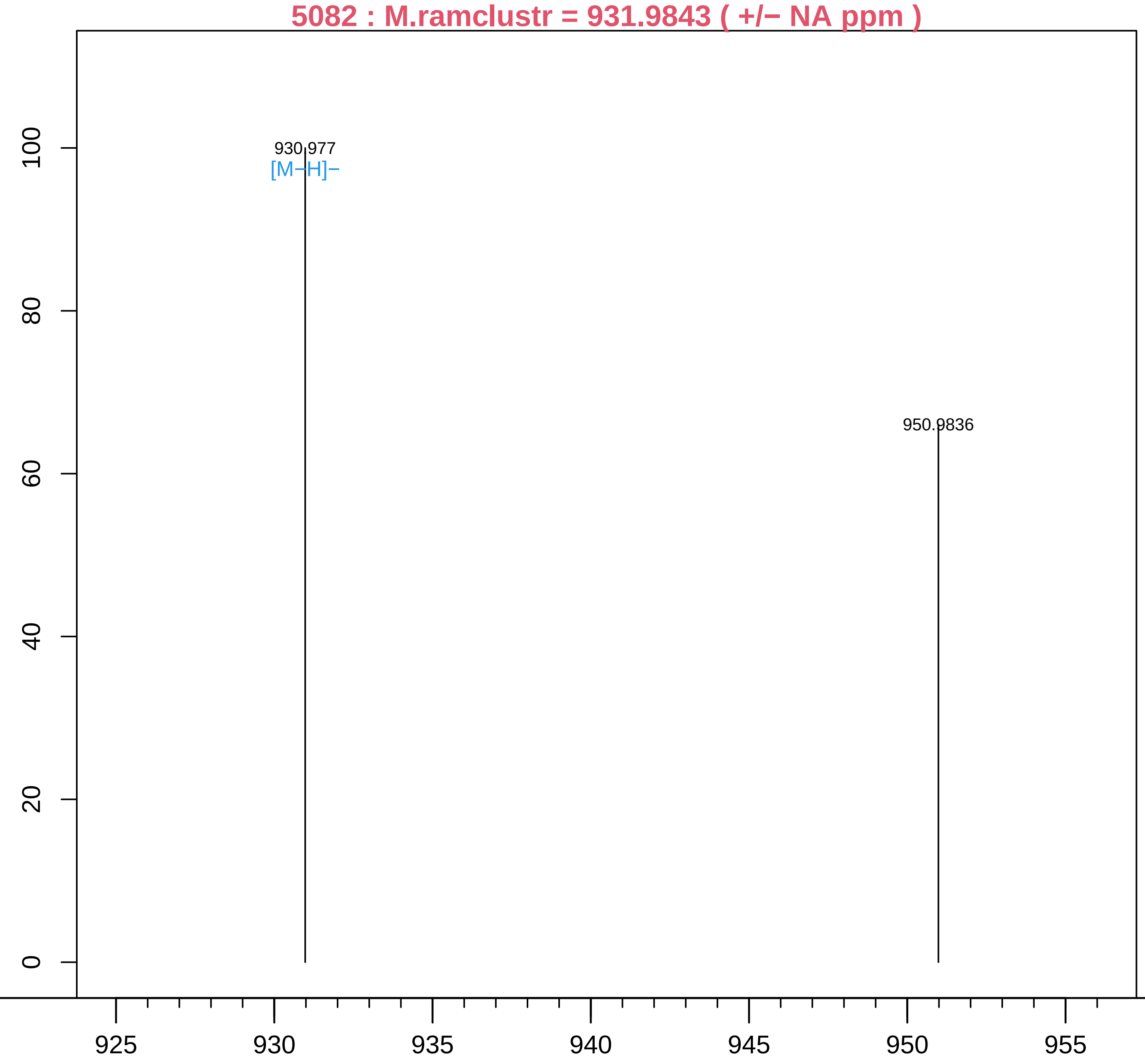
5082 : M.ramclustr = 931.9843 (+/− NA ppm)
930.977
[M−H]−
950.9836
0
20
40
60
80
100
925
930
935
940
945
950
955

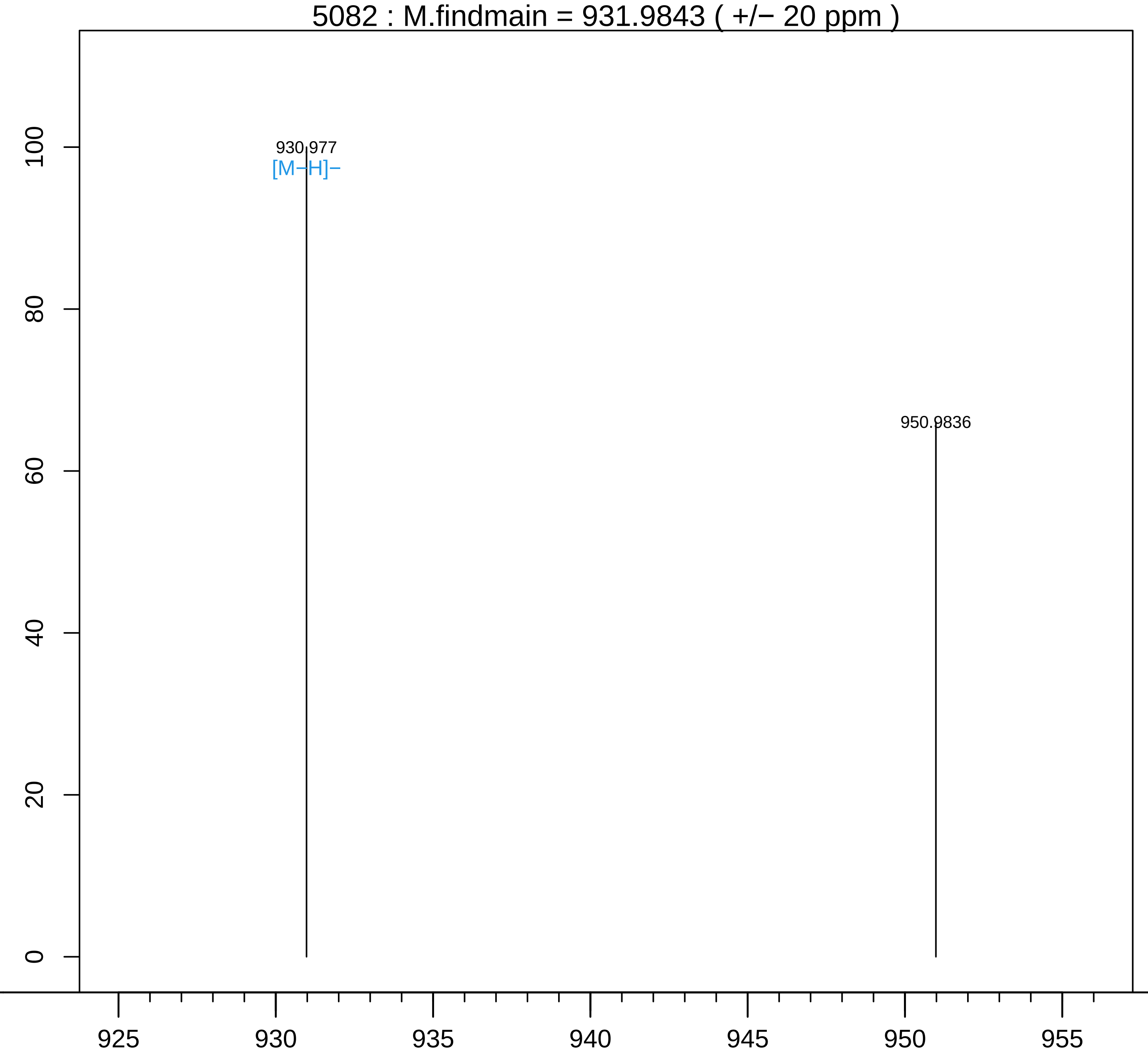
5082 : M.findmain = 931.9843 (+/− 20 ppm)
930.977
[M−H]−
950.9836
0
20
40
60
80
100
925
930
935
940
945
950
955

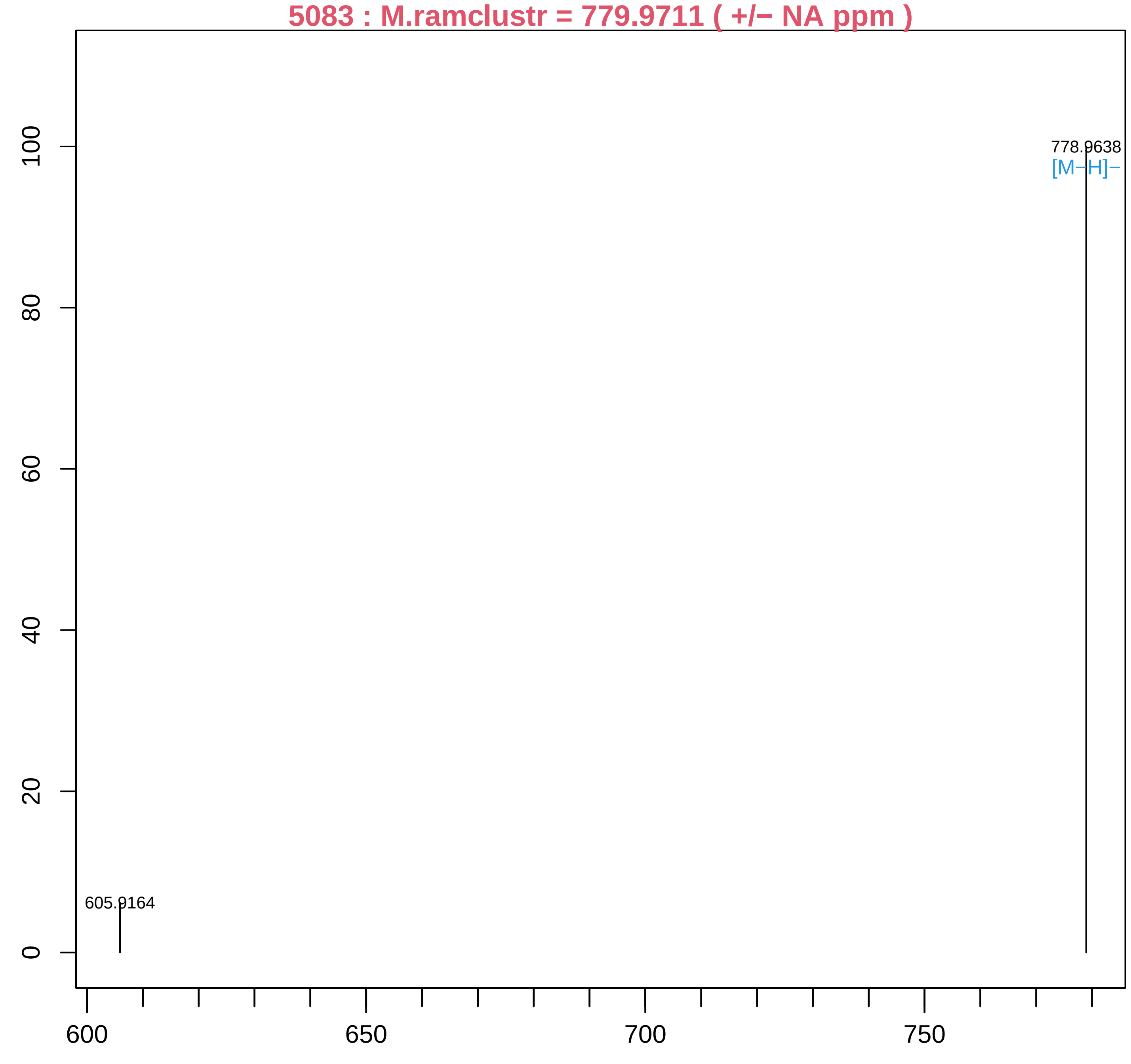

5083 : M.ramclustr = 779.9711 (+/− NA ppm)
778.9638
[M−H]−
605.9164
0
20
40
60
80
100
600
650
700
750

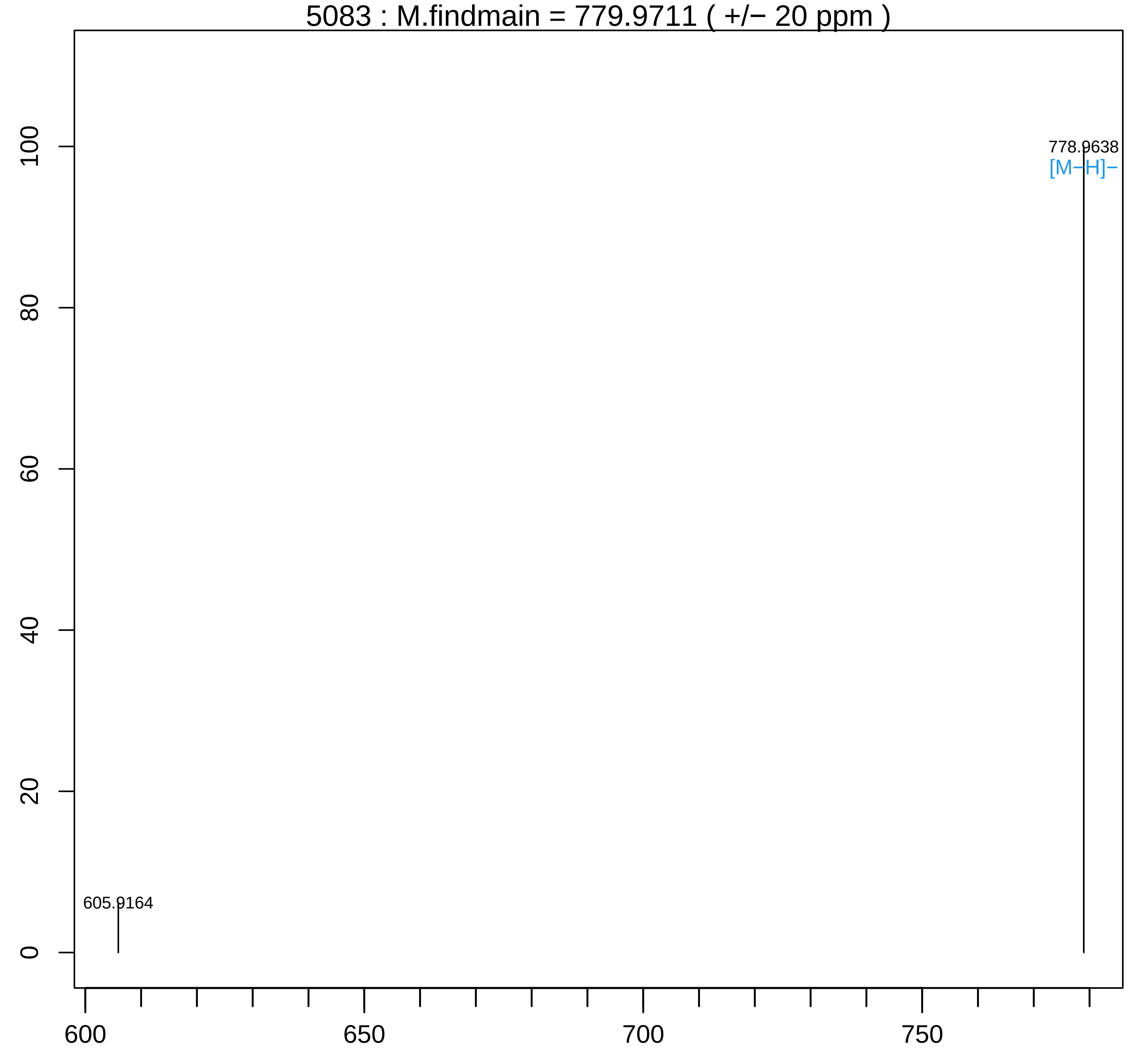

5083 : M.findmain = 779.9711 (+/− 20 ppm)
778.9638
[M−H]−
605.9164
0
20
40
60
80
100
600
650
700
750

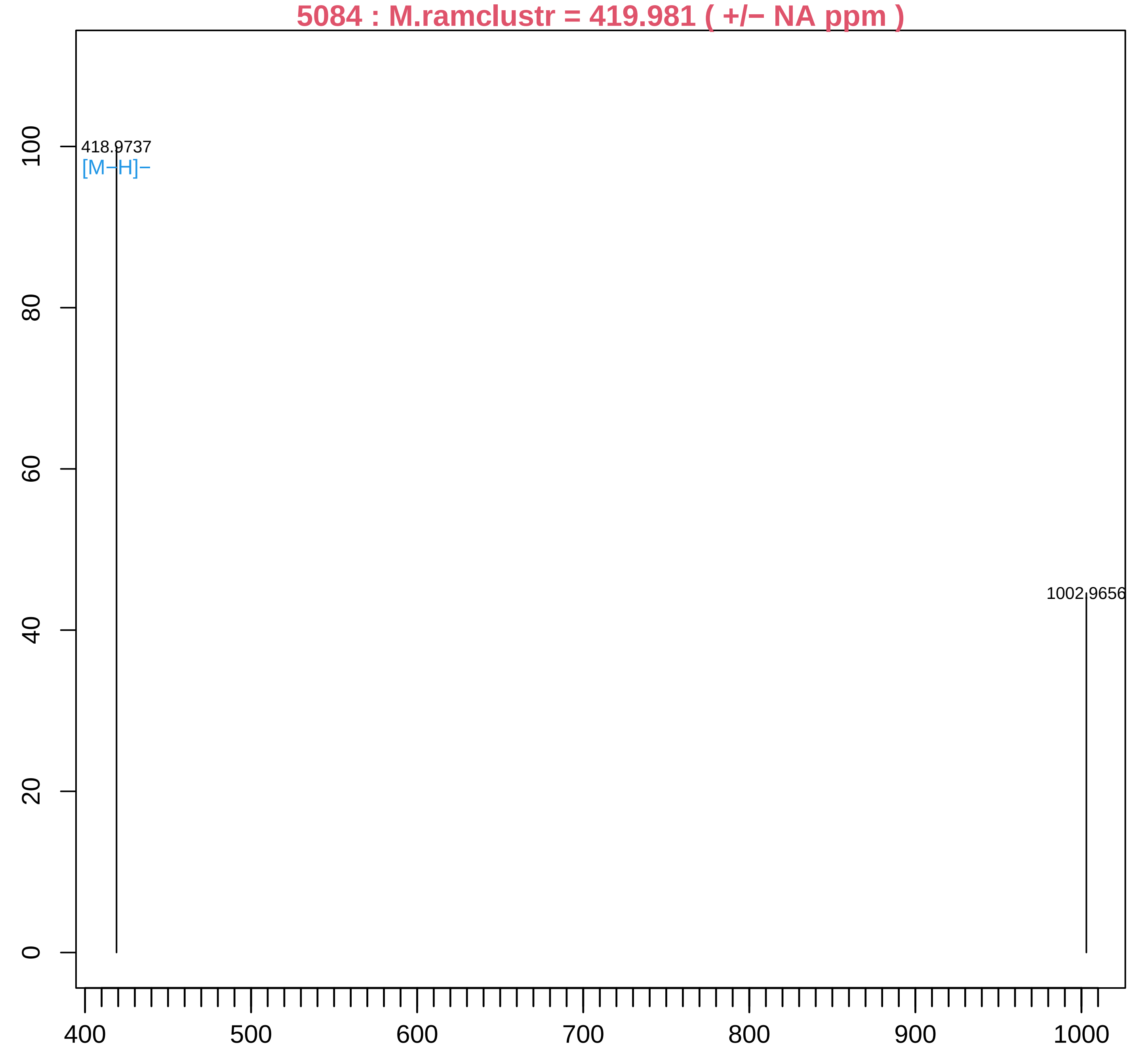
5084 : M.ramclustr = 419.981 (+/− NA ppm)
418.9737
[M−H]−
1002.9656
0
20
40
60
80
100
400
500
600
700
800
900
1000

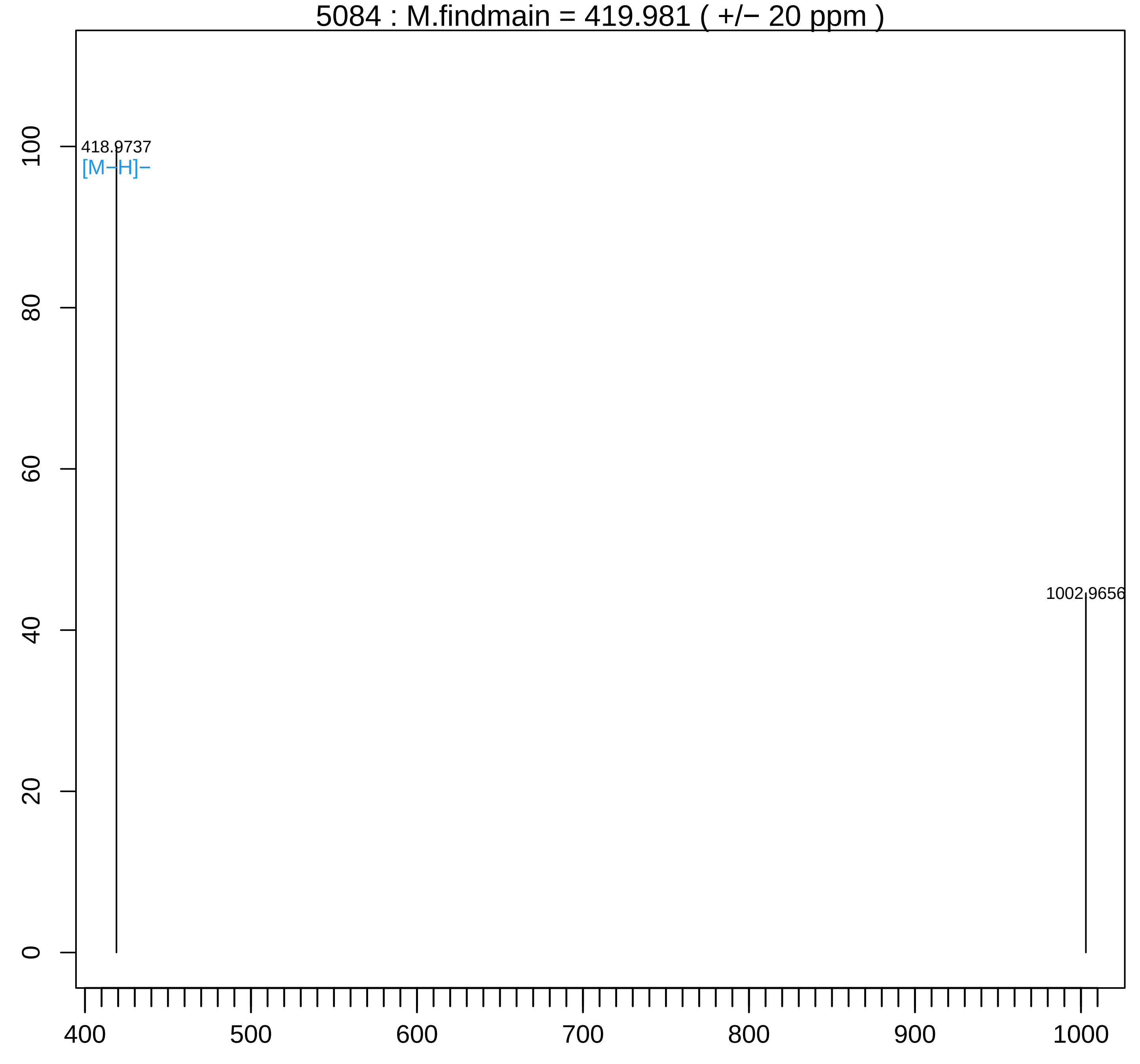
5084 : M.findmain = 419.981 (+/− 20 ppm)
418.9737
[M−H]−
1002.9656
0
20
40
60
80
100
400
500
600
700
800
900
1000

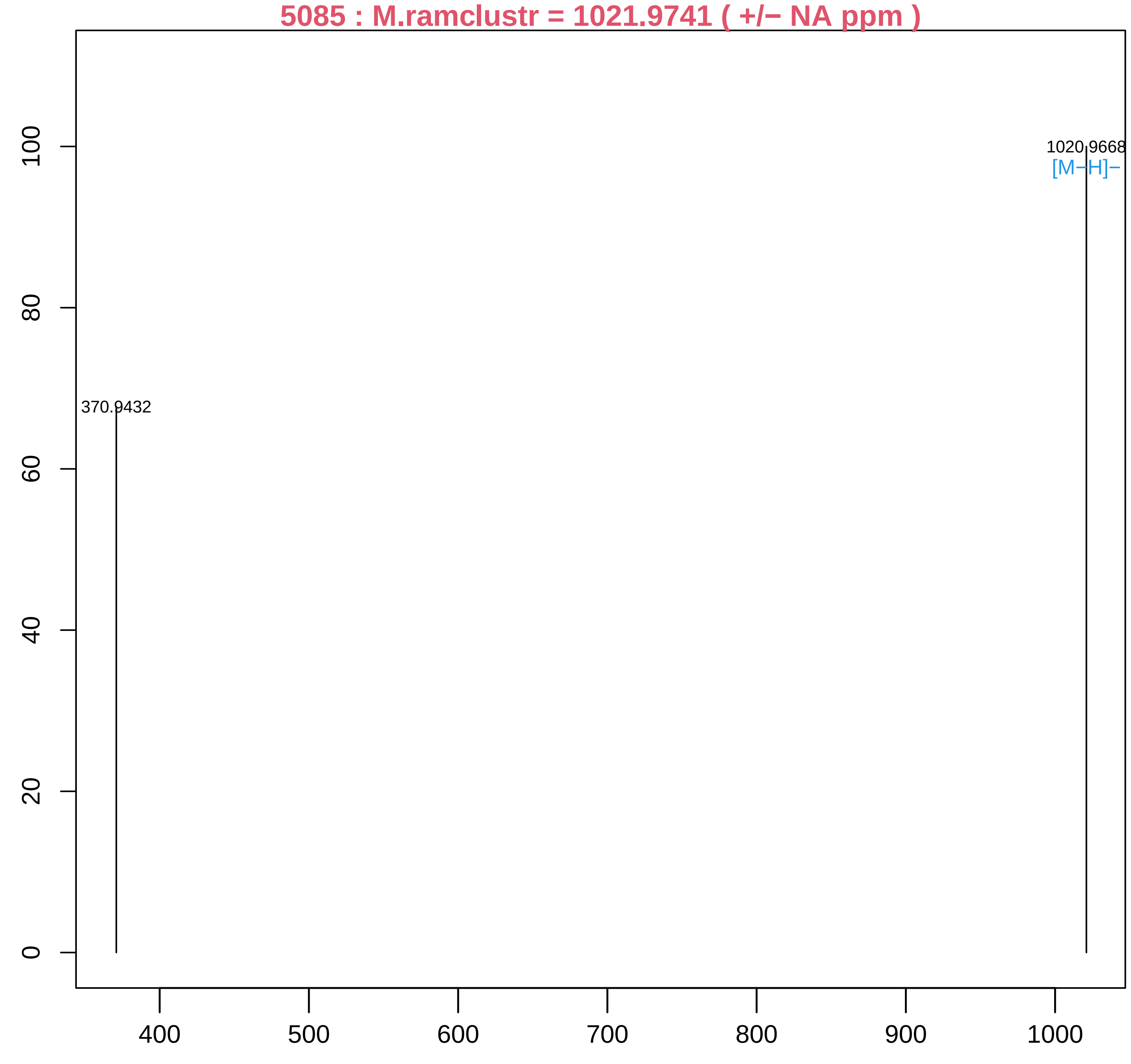

5085 : M.ramclustr = 1021.9741 (+/− NA ppm)
1020.9668
[M−H]−
370.9432
0
20
40
60
80
100
400
500
600
700
800
900
1000

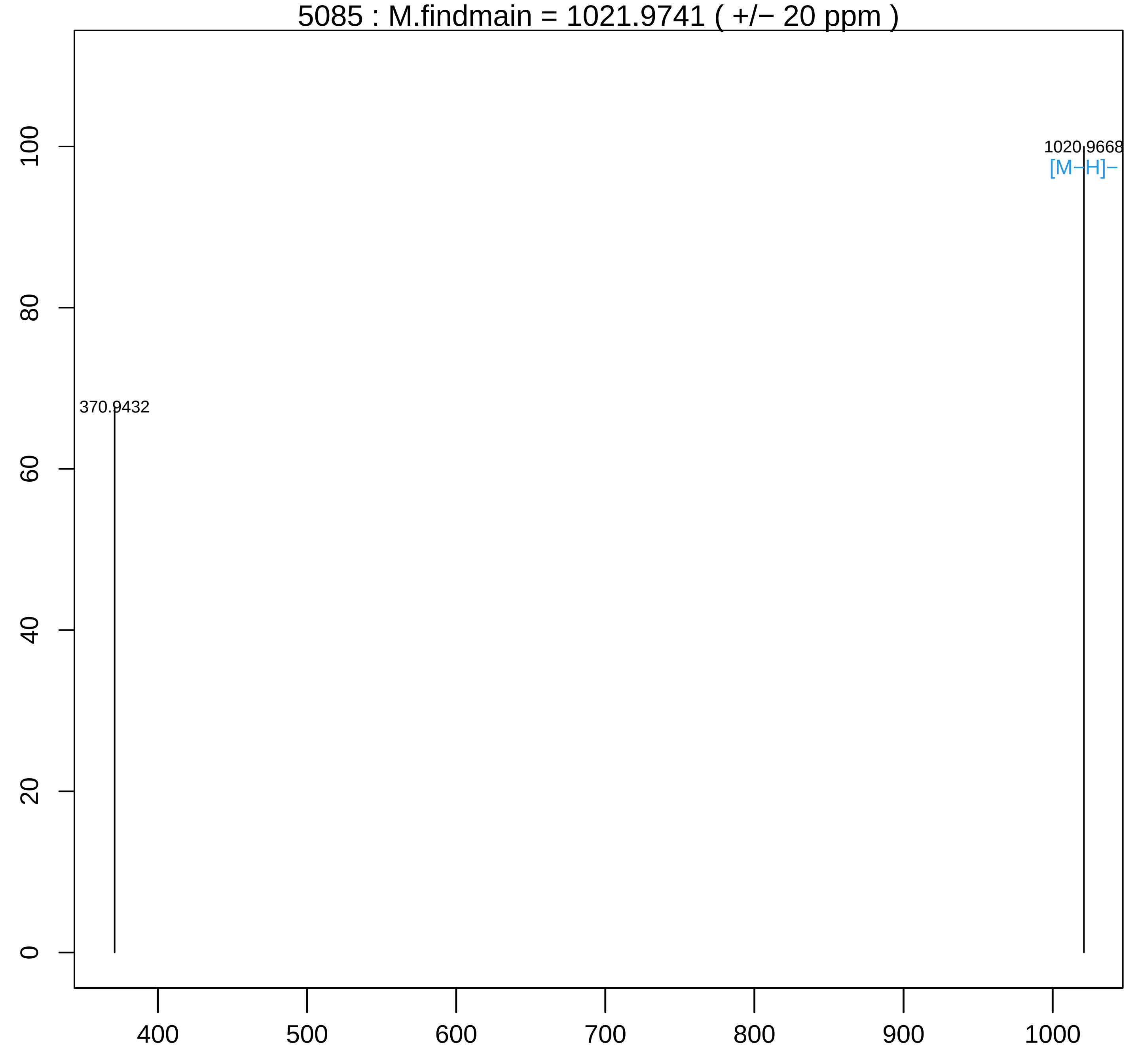

5085 : M.findmain = 1021.9741 (+/− 20 ppm)
1020.9668
[M−H]−
370.9432
0
20
40
60
80
100
400
500
600
700
800
900
1000

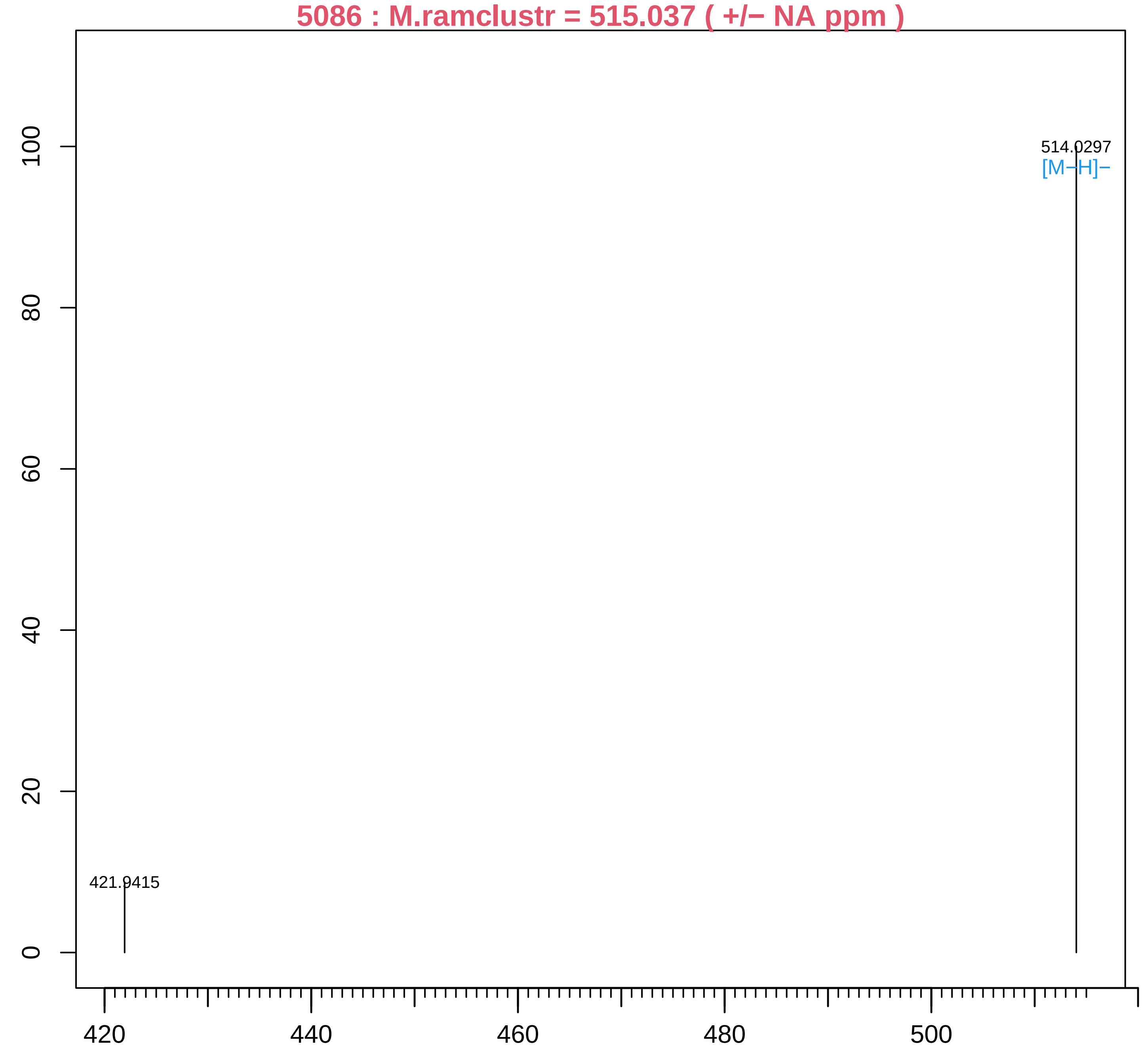
5086 : M.ramclustr = 515.037 (+/− NA ppm)
514.0297
[M−H]−
421.9415
0
20
40
60
80
100
420
440
460
480
500

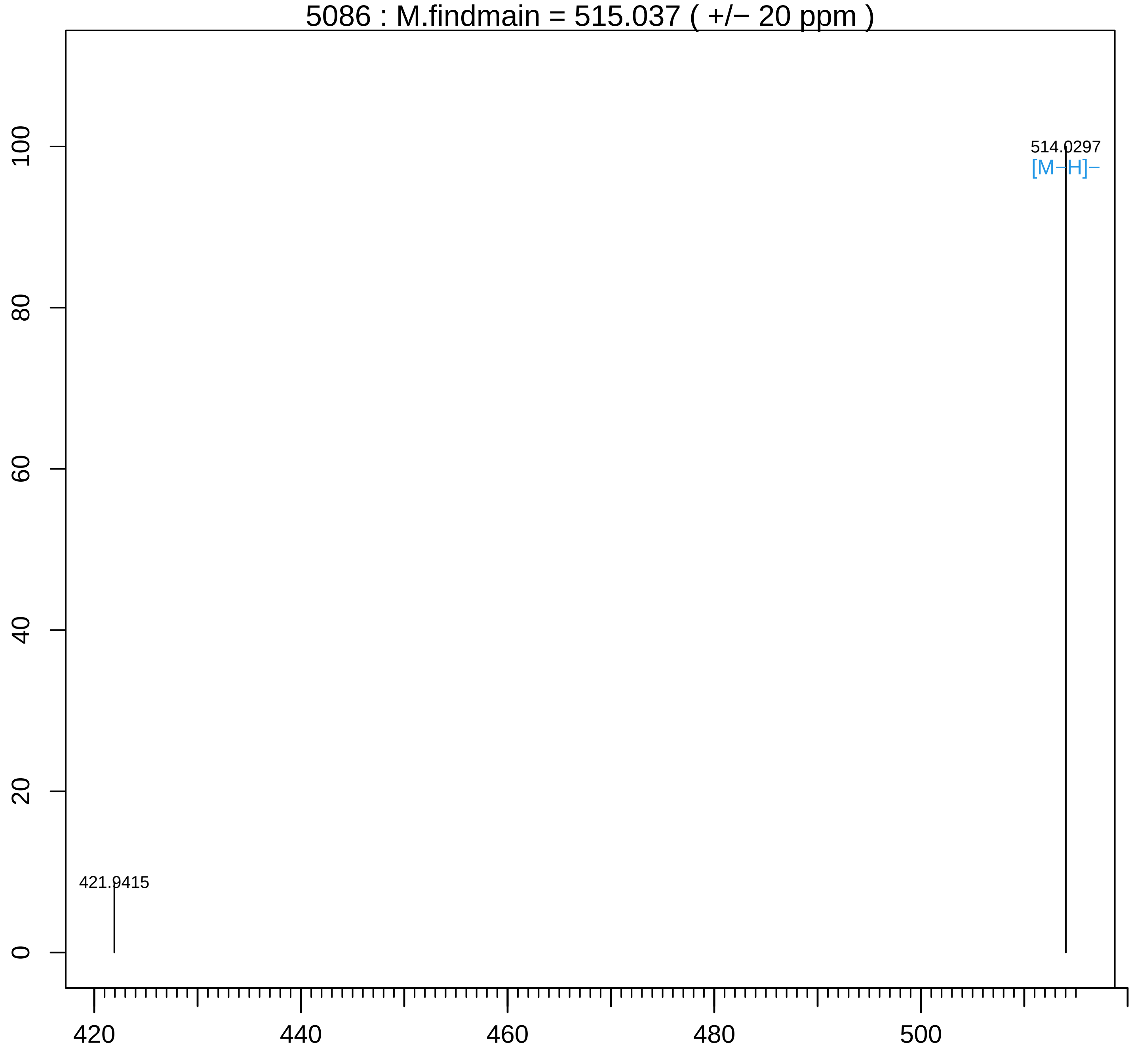
5086 : M.findmain = 515.037 (+/− 20 ppm)
514.0297
[M−H]−
421.9415
0
20
40
60
80
100
420
440
460
480
500

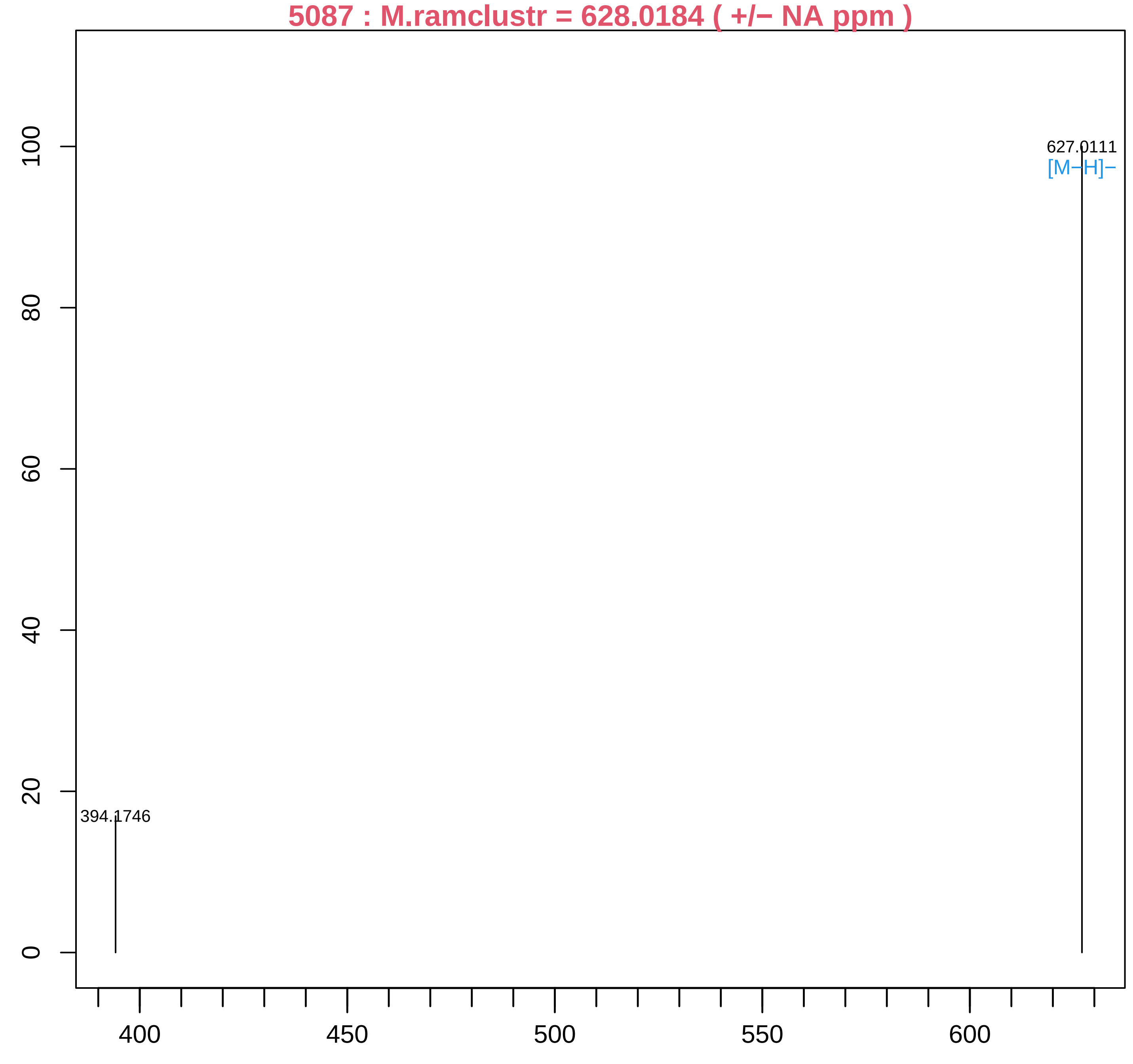
5087 : M.ramclustr = 628.0184 (+/− NA ppm)
627.0111
[M−H]−
394.1746
0
20
40
60
80
100
400
450
500
550
600

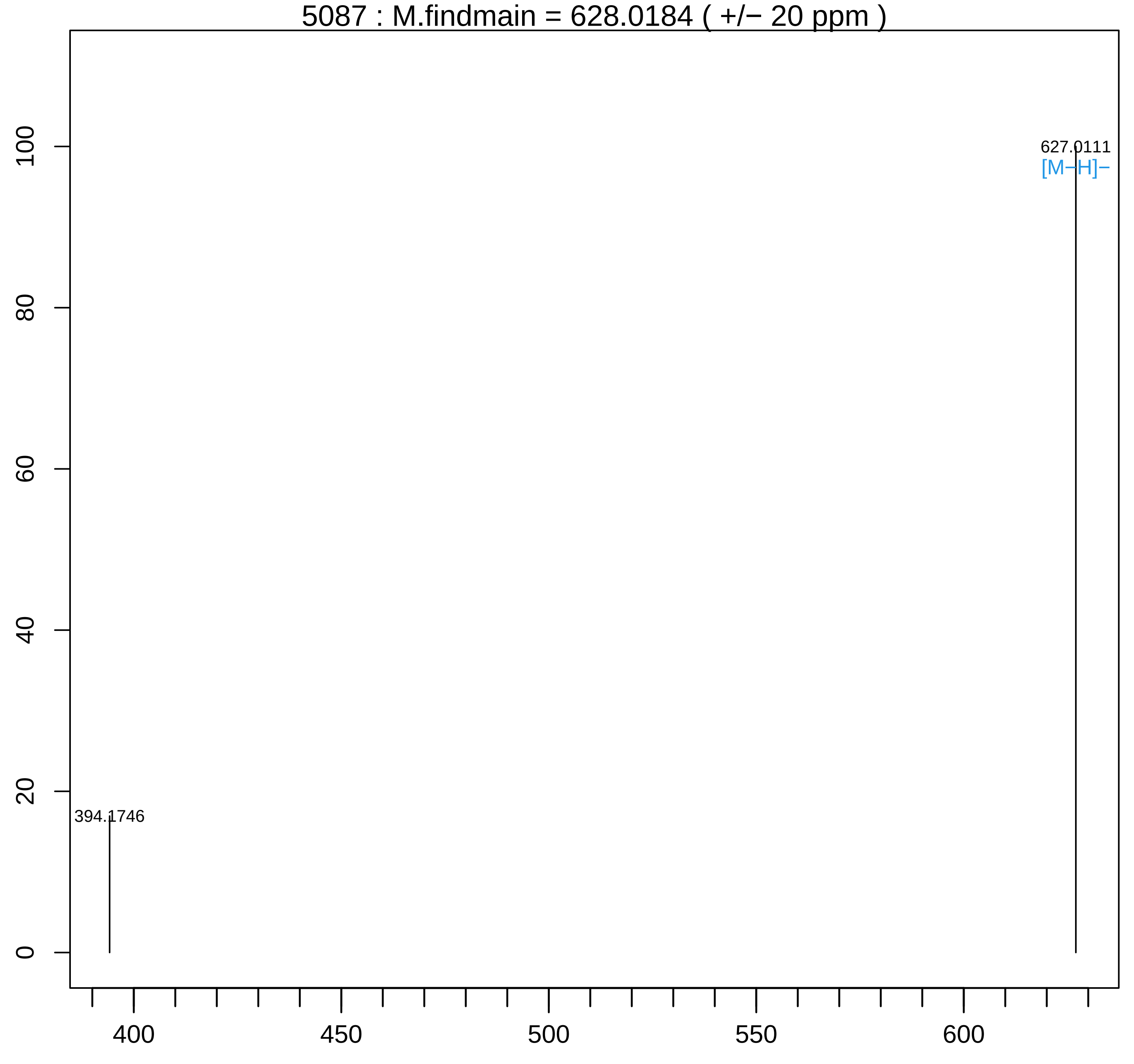
5087 : M.findmain = 628.0184 (+/− 20 ppm)
627.0111
[M−H]−
394.1746
0
20
40
60
80
100
400
450
500
550
600

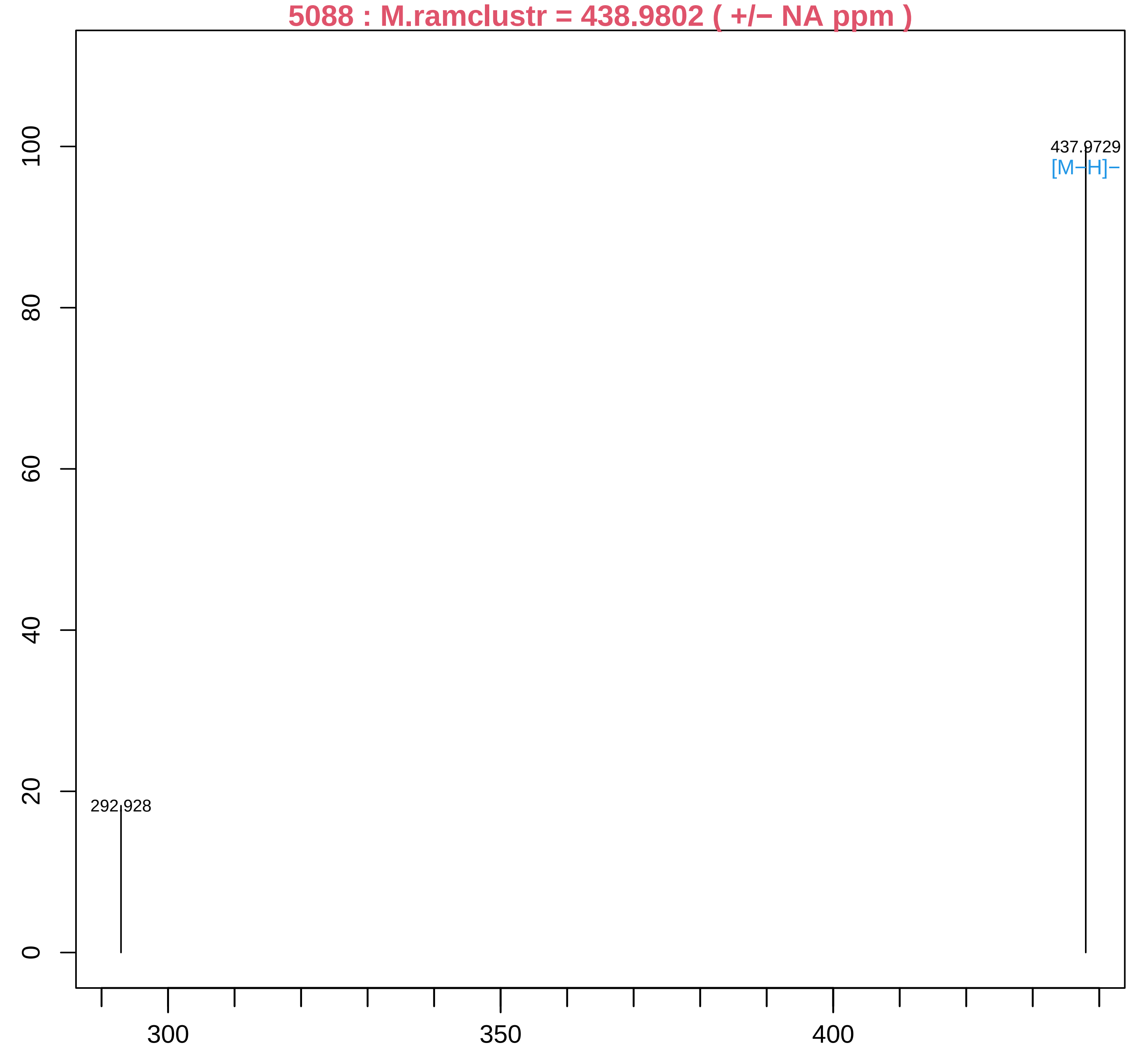
5088 : M.ramclustr = 438.9802 (+/− NA ppm)
437.9729
[M−H]−
292.928
0
20
40
60
80
100
300
350
400

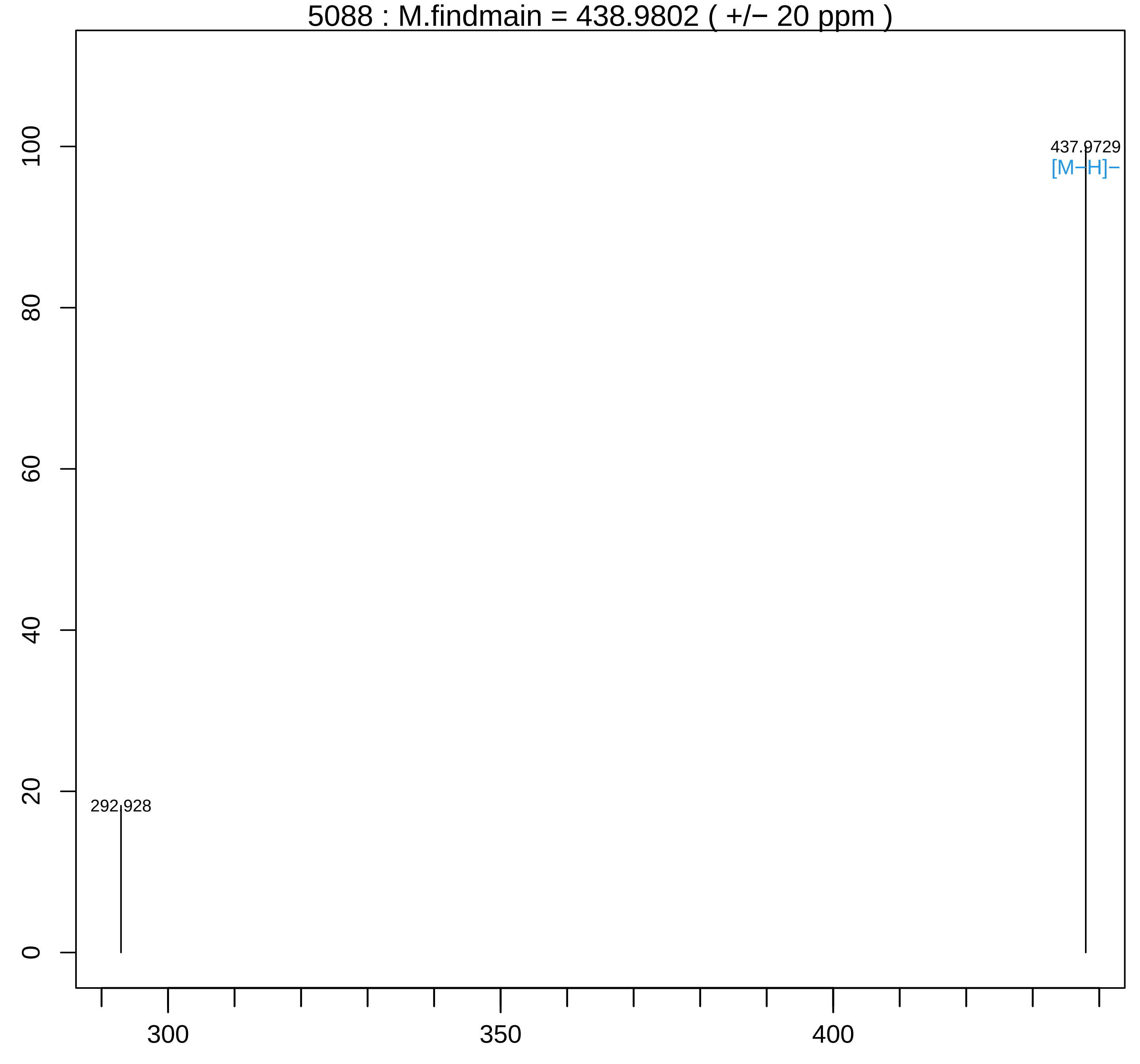
5088 : M.findmain = 438.9802 (+/− 20 ppm)
437.9729
[M−H]−
292.928
0
20
40
60
80
100
300
350
400

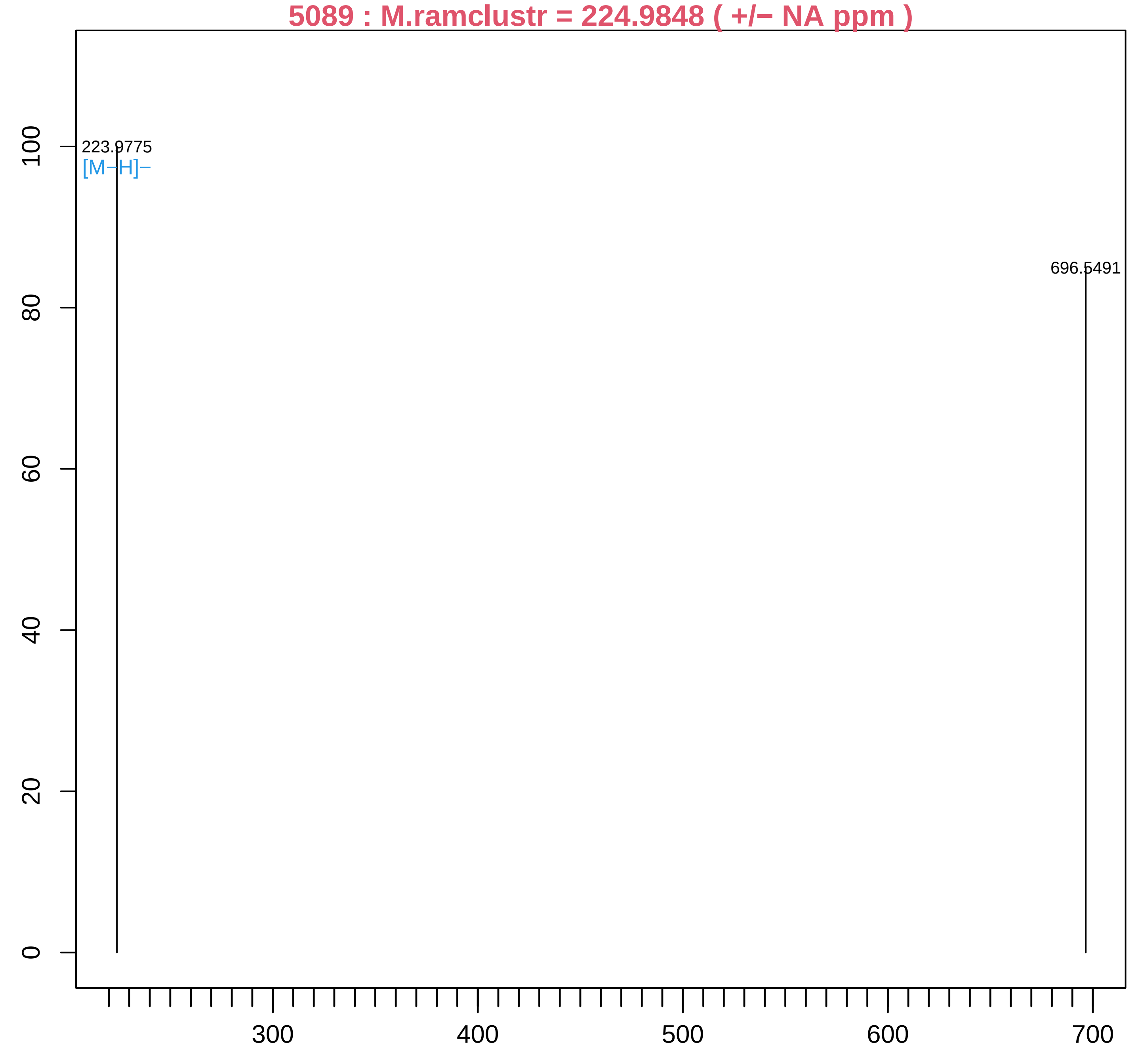
5089 : M.ramclustr = 224.9848 (+/− NA ppm)
223.9775
[M−H]−
696.5491
0
20
40
60
80
100
300
400
500
600
700

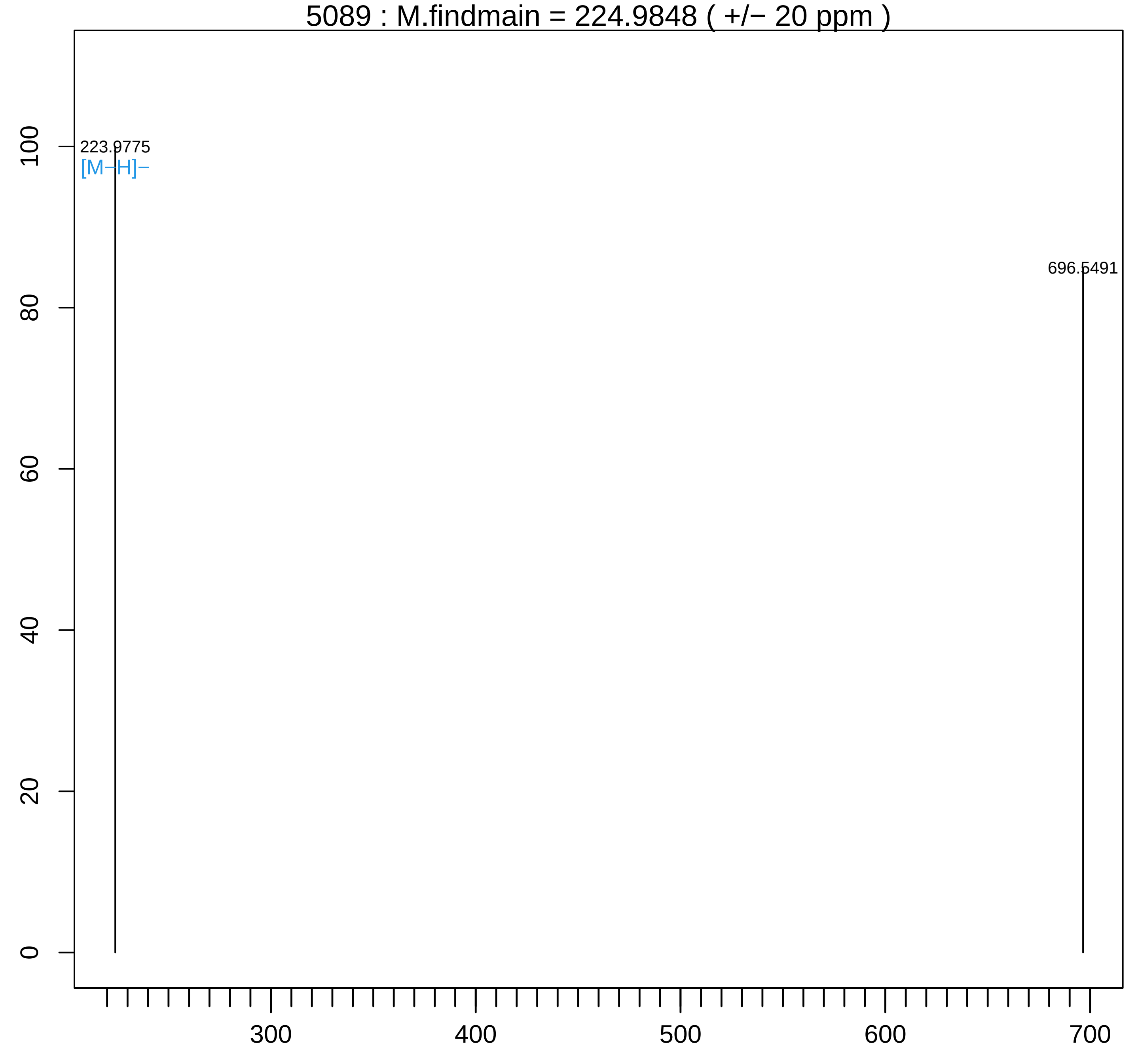
5089 : M.findmain = 224.9848 (+/− 20 ppm)
223.9775
[M−H]−
696.5491
0
20
40
60
80
100
300
400
500
600
700

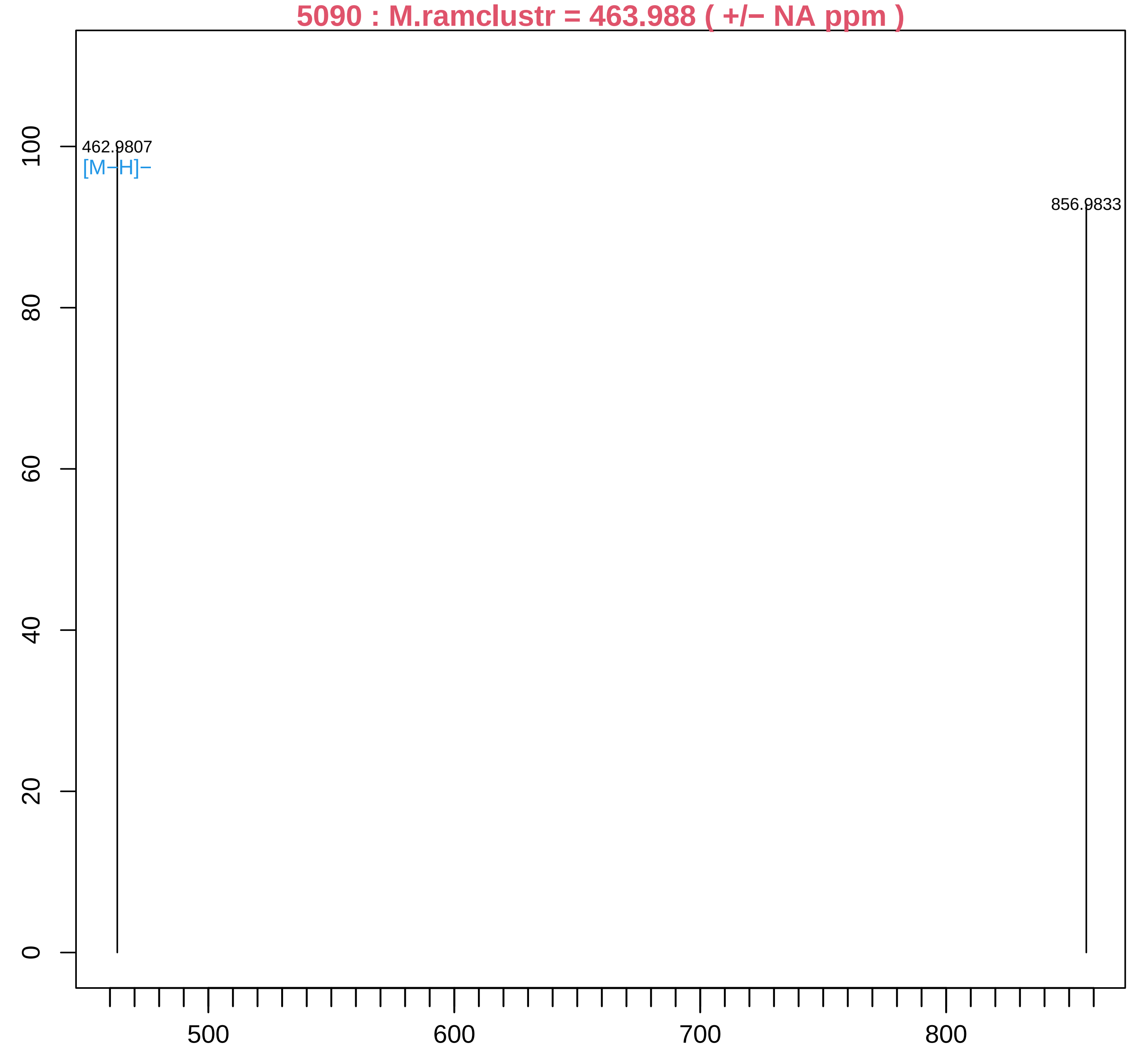
5090 : M.ramclustr = 463.988 (+/− NA ppm)
462.9807
[M−H]−
856.9833
0
20
40
60
80
100
500
600
700
800

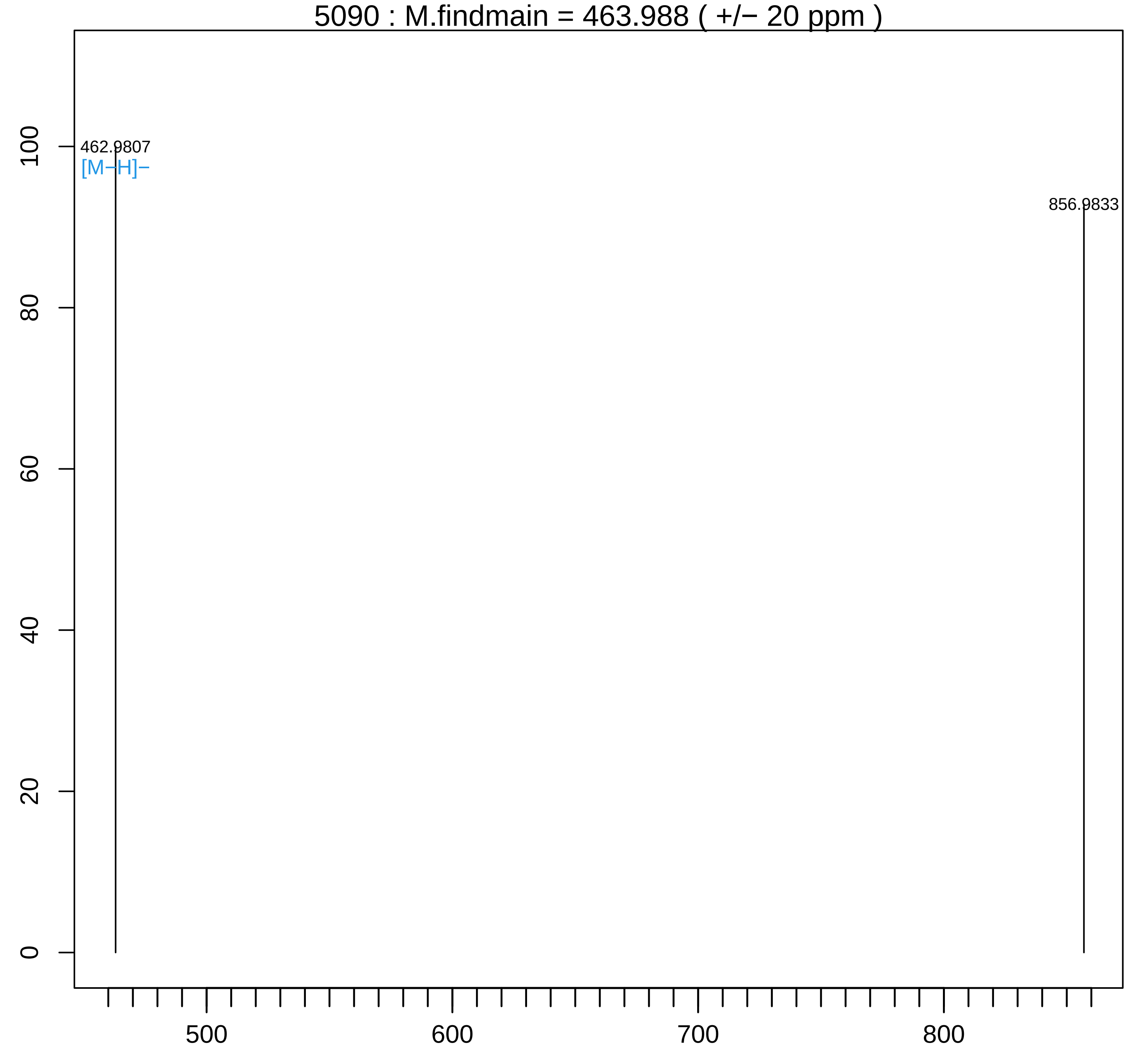
5090 : M.findmain = 463.988 (+/− 20 ppm)
462.9807
[M−H]−
856.9833
0
20
40
60
80
100
500
600
700
800

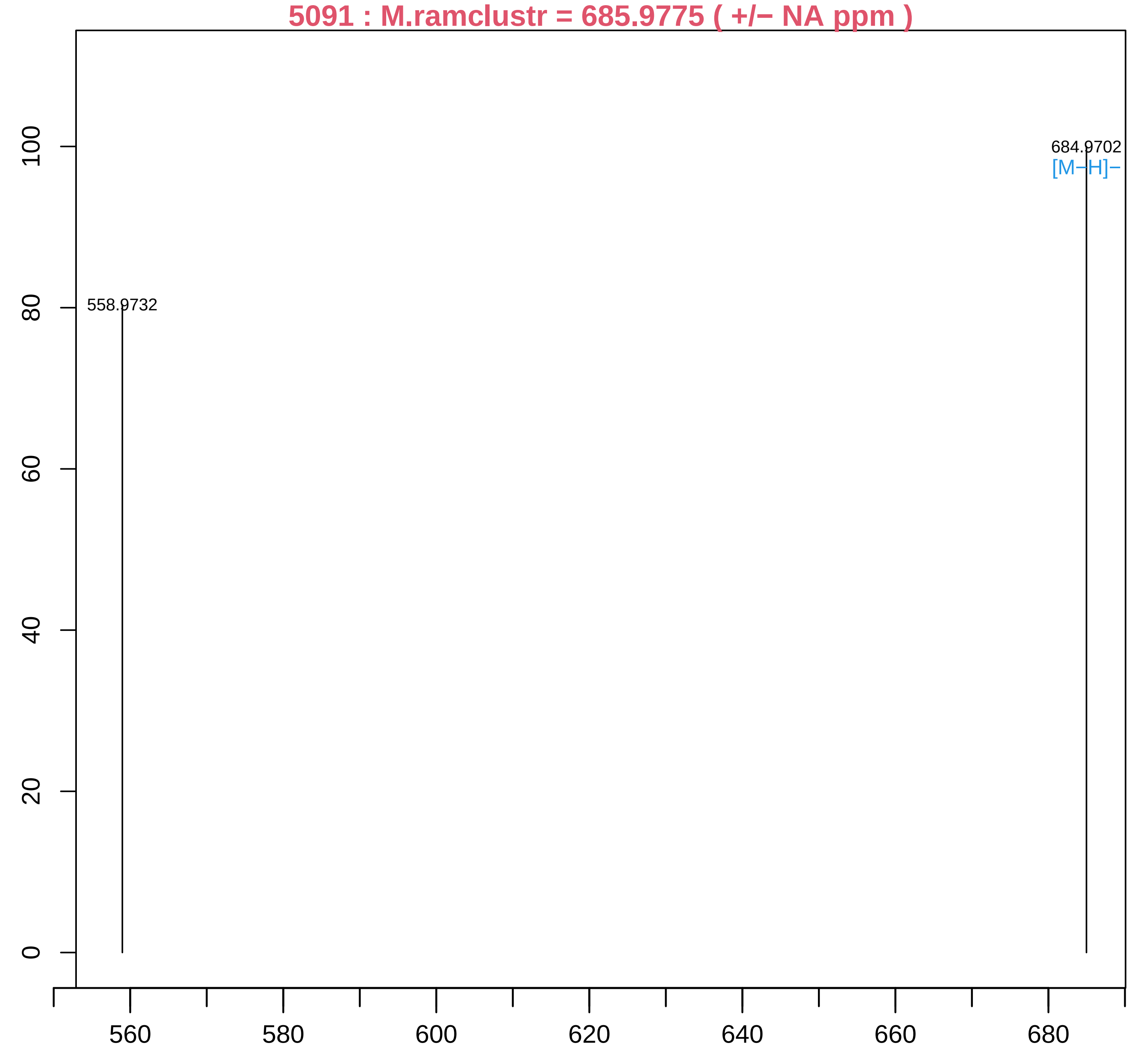
5091 : M.ramclustr = 685.9775 (+/− NA ppm)
684.9702
[M−H]−
558.9732
0
20
40
60
80
100
560
580
600
620
640
660
680

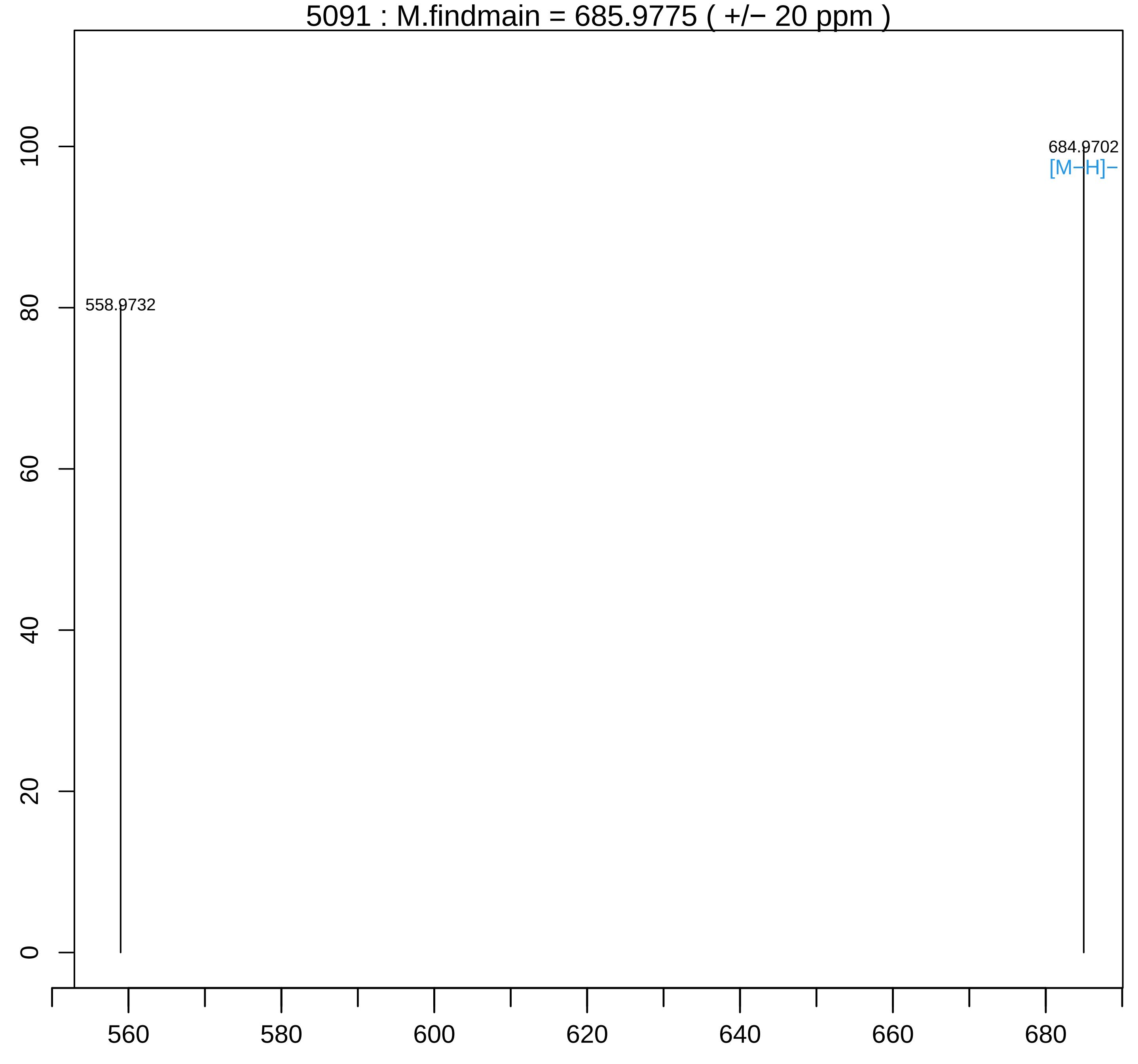
5091 : M.findmain = 685.9775 (+/− 20 ppm)
684.9702
[M−H]−
558.9732
0
20
40
60
80
100
560
580
600
620
640
660
680

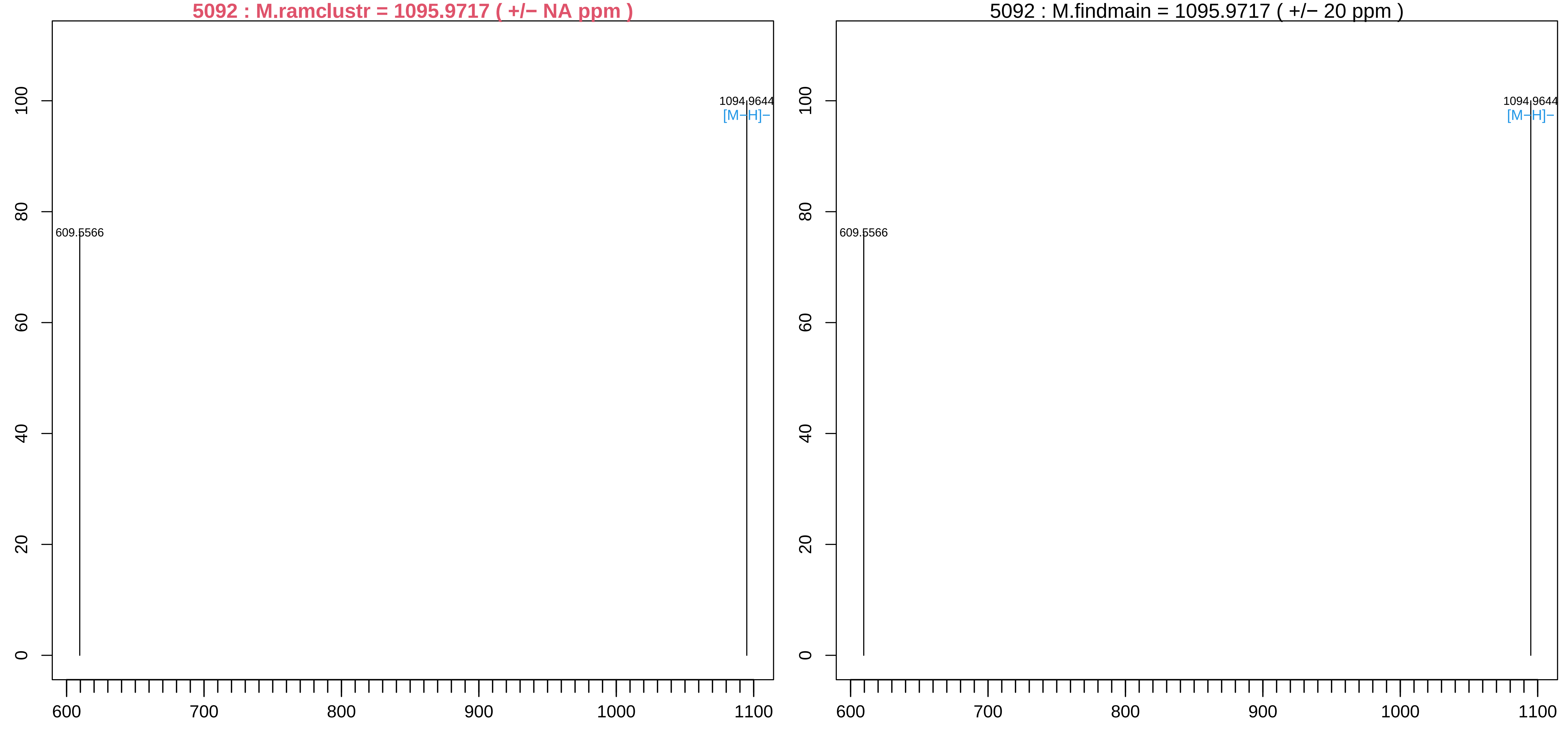
5092 : M.ramclustr = 1095.9717 (+/− NA ppm)
5092 : M.findmain = 1095.9717 (+/− 20 ppm)
1094.9644
[M−H]−
609.5566
0
20
40
60
80
100
600
700
800
900
1000
1100

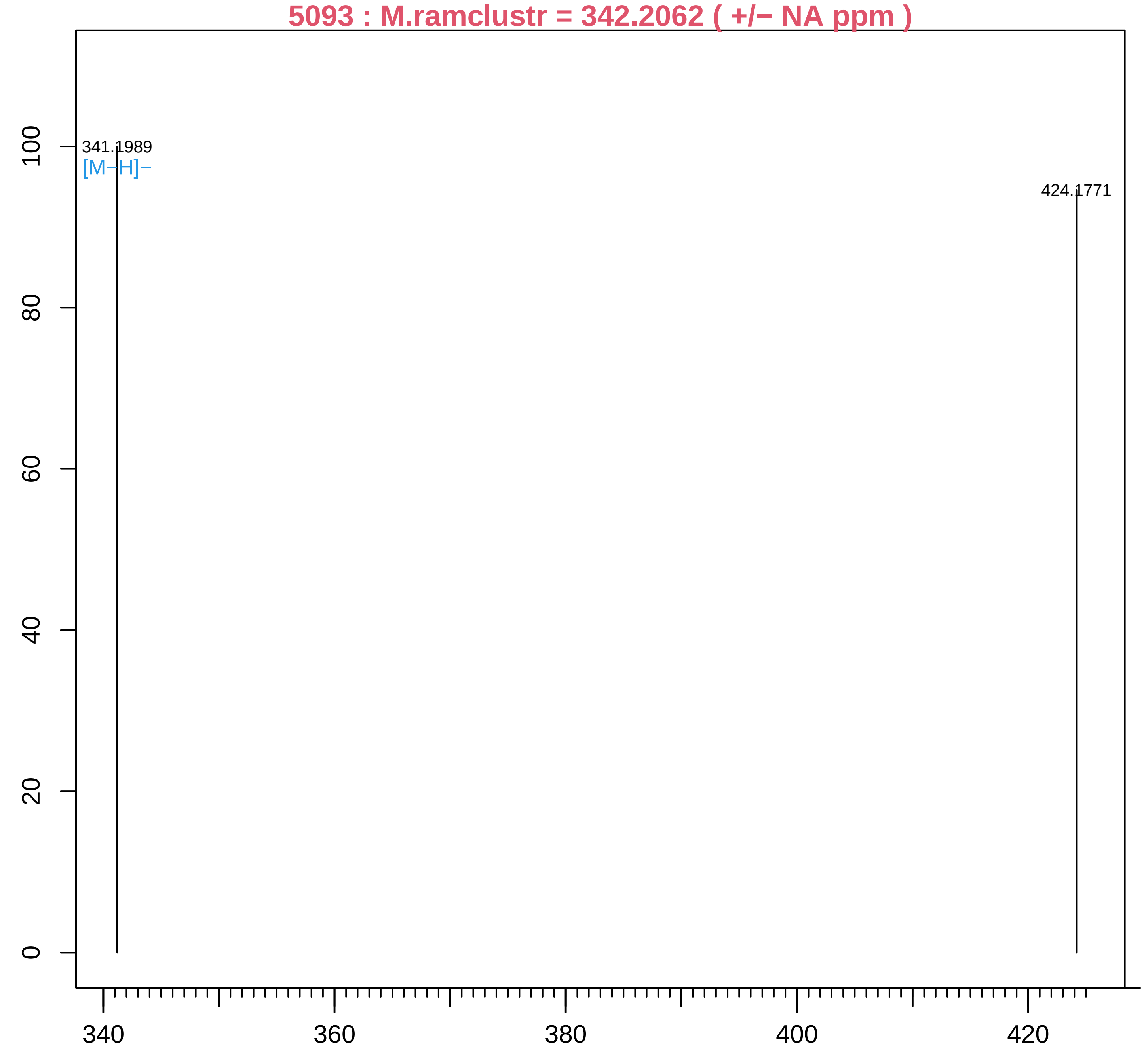
5093 : M.ramclustr = 342.2062 (+/− NA ppm)
341.1989
[M−H]−
424.1771
0
20
40
60
80
100
340
360
380
400
420

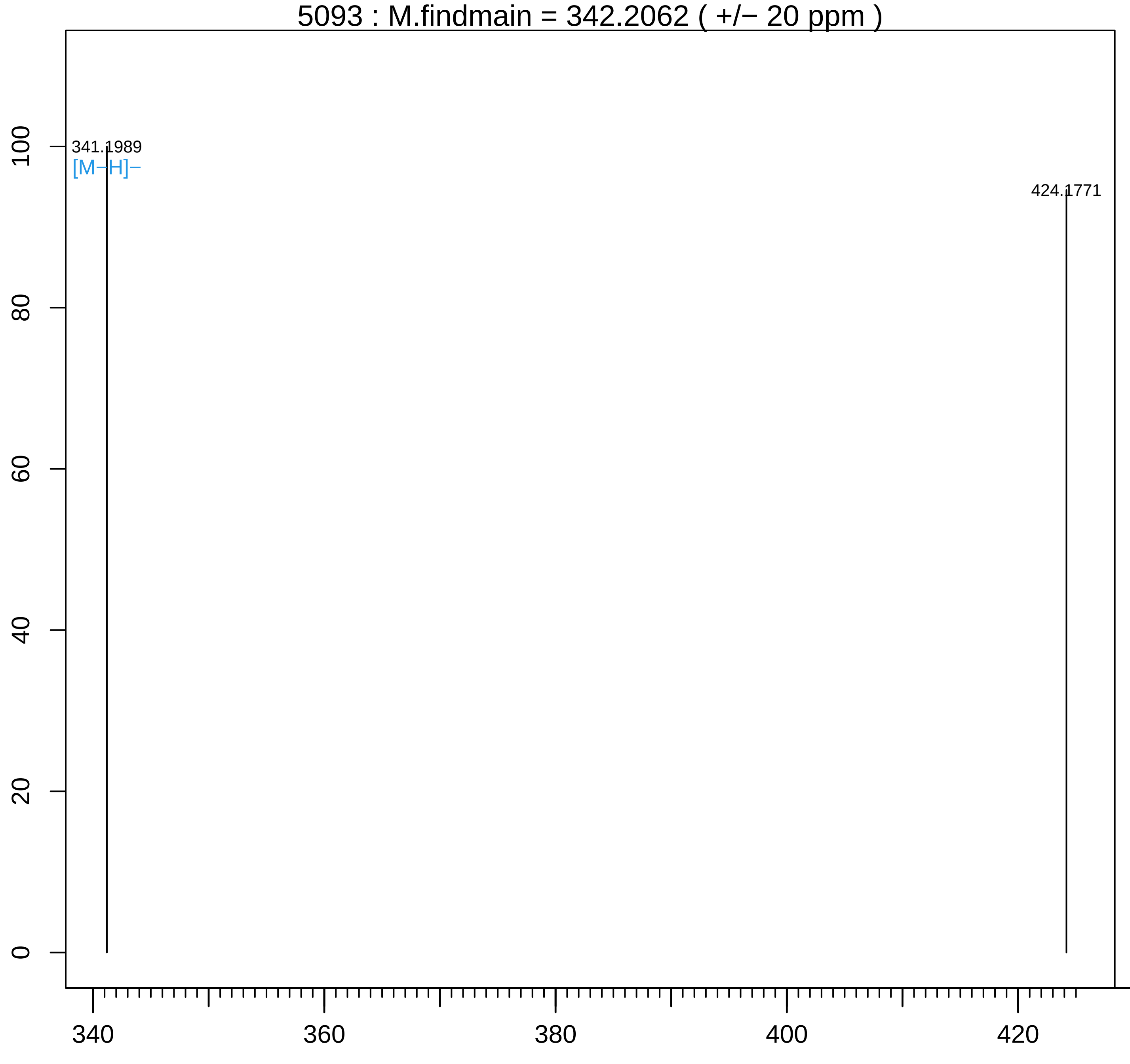
5093 : M.findmain = 342.2062 (+/− 20 ppm)
341.1989
[M−H]−
424.1771
0
20
40
60
80
100
340
360
380
400
420

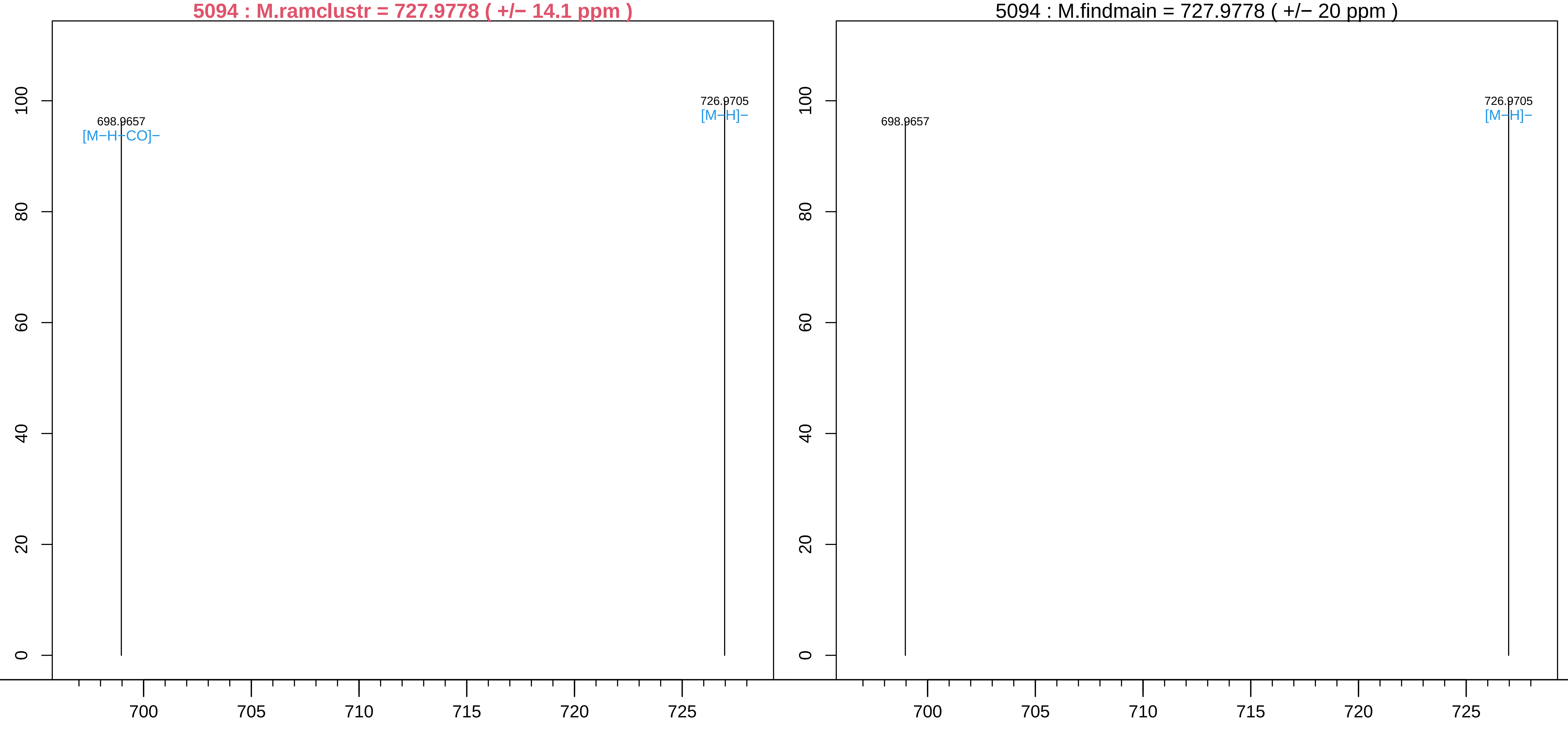
5094 : M.ramclustr = 727.9778 (+/− 14.1 ppm)
698.9657
[M−H−CO]−
726.9705
[M−H]−
5094 : M.findmain = 727.9778 (+/− 20 ppm)
698.9657
726.9705
[M−H]−
0
20
40
60
80
100
700
705
710
715
720
725

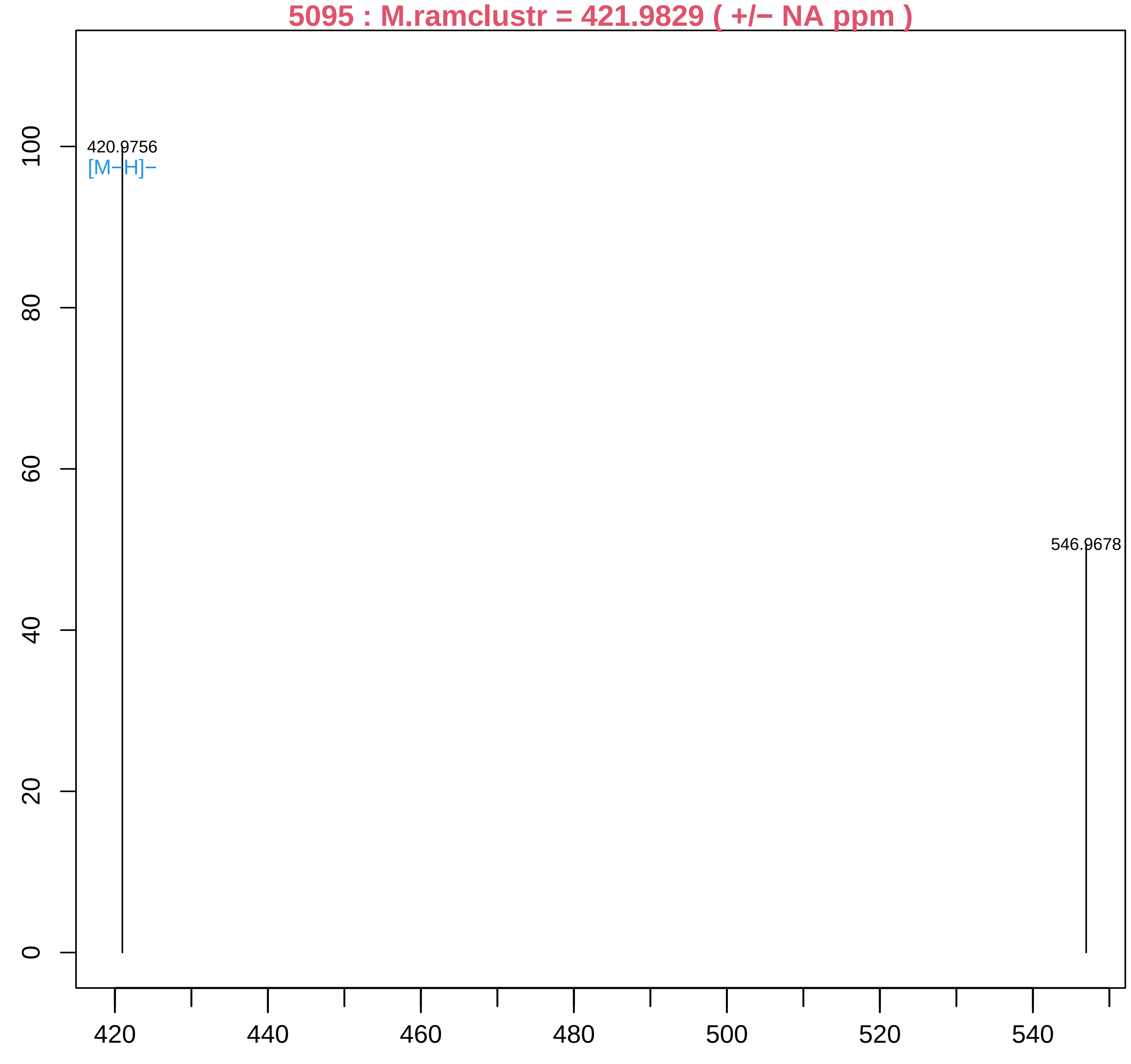
5095 : M.ramclustr = 421.9829 (+/− NA ppm)
420.9756
[M−H]−
546.9678
0
20
40
60
80
100
420
440
460
480
500
520
540

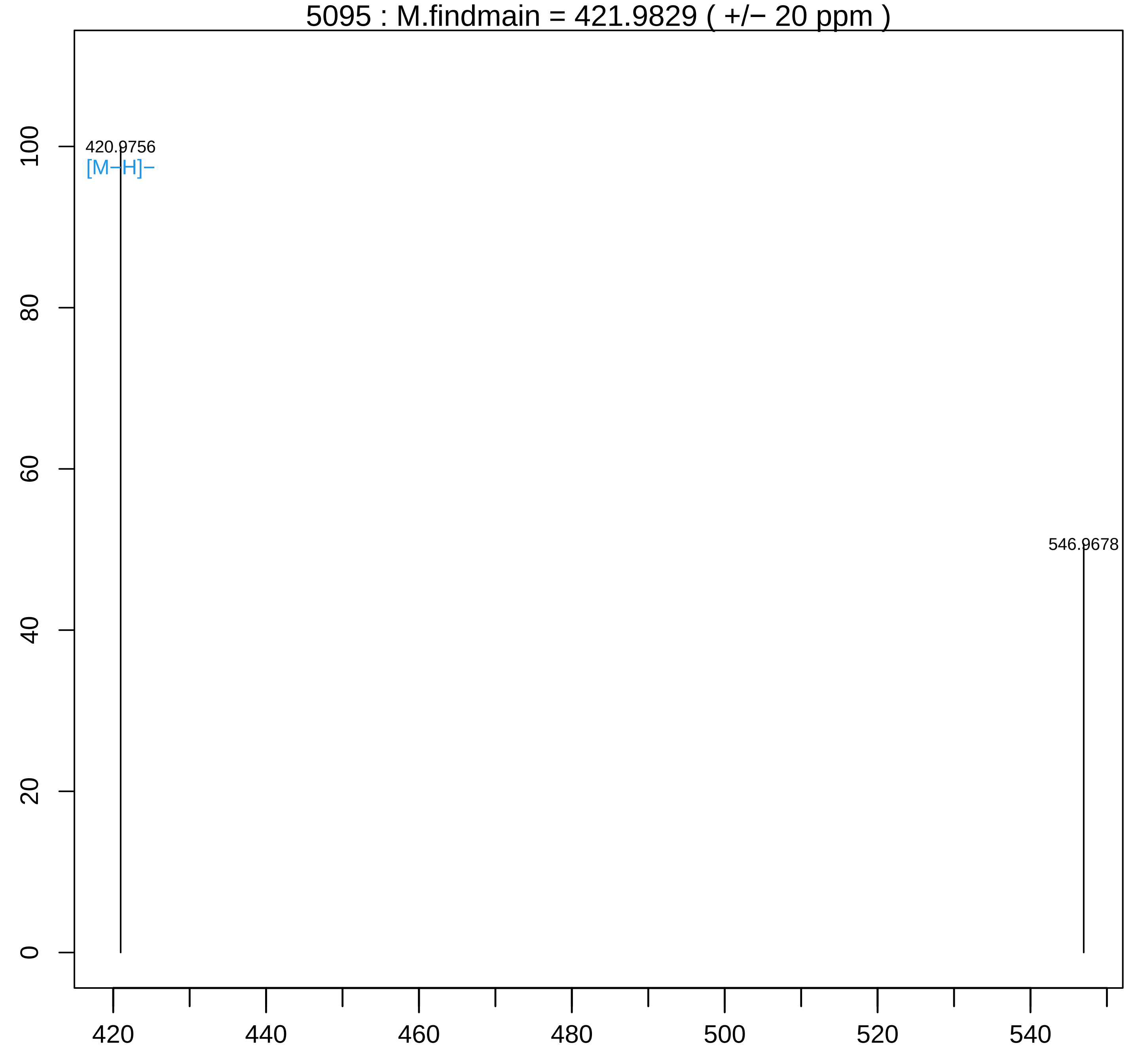
5095 : M.findmain = 421.9829 (+/− 20 ppm)
420.9756
[M−H]−
546.9678
0
20
40
60
80
100
420
440
460
480
500
520
540

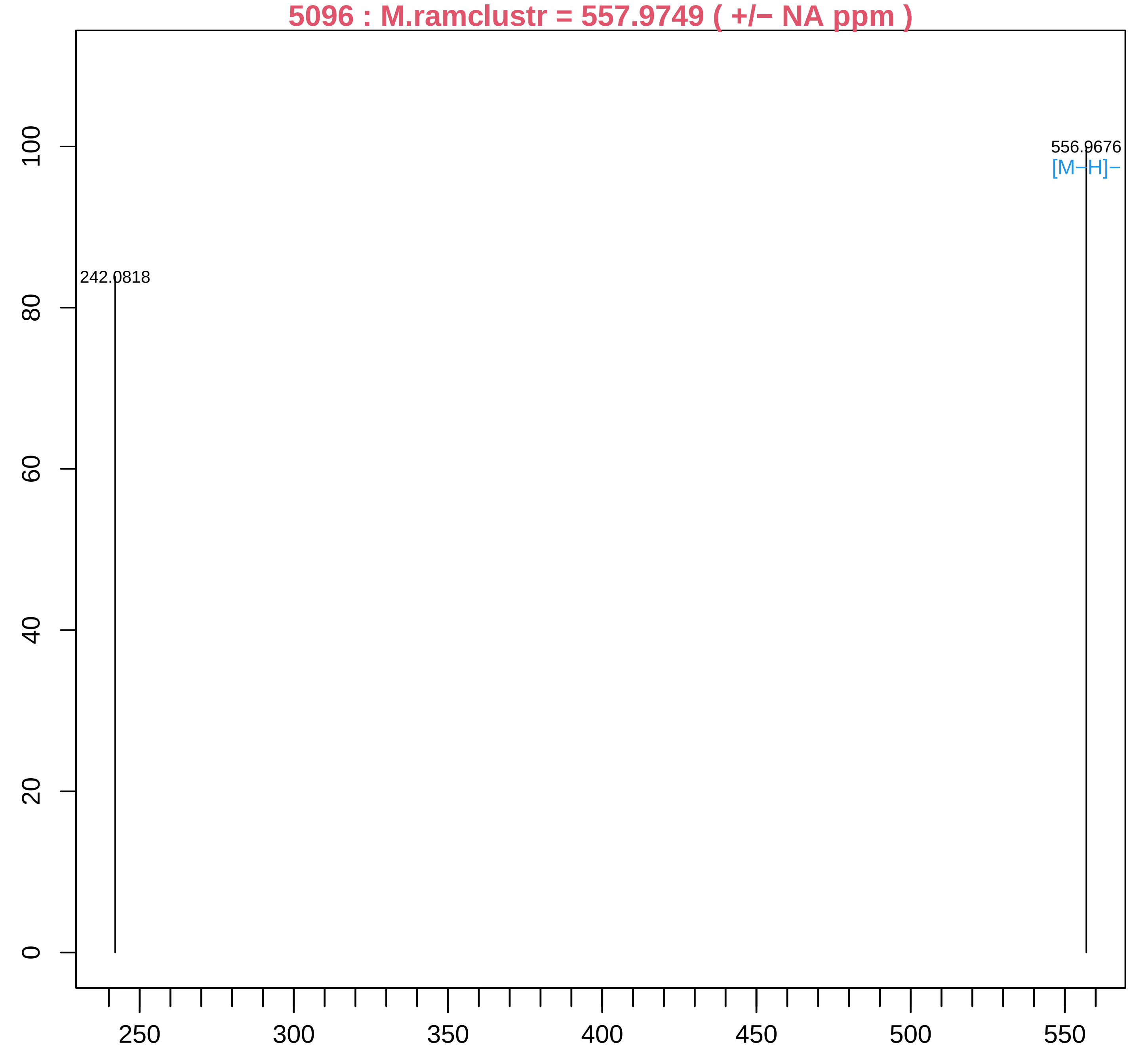
5096 : M.ramclustr = 557.9749 (+/− NA ppm)
556.9676
[M−H]−
242.0818
0
20
40
60
80
100
250
300
350
400
450
500
550

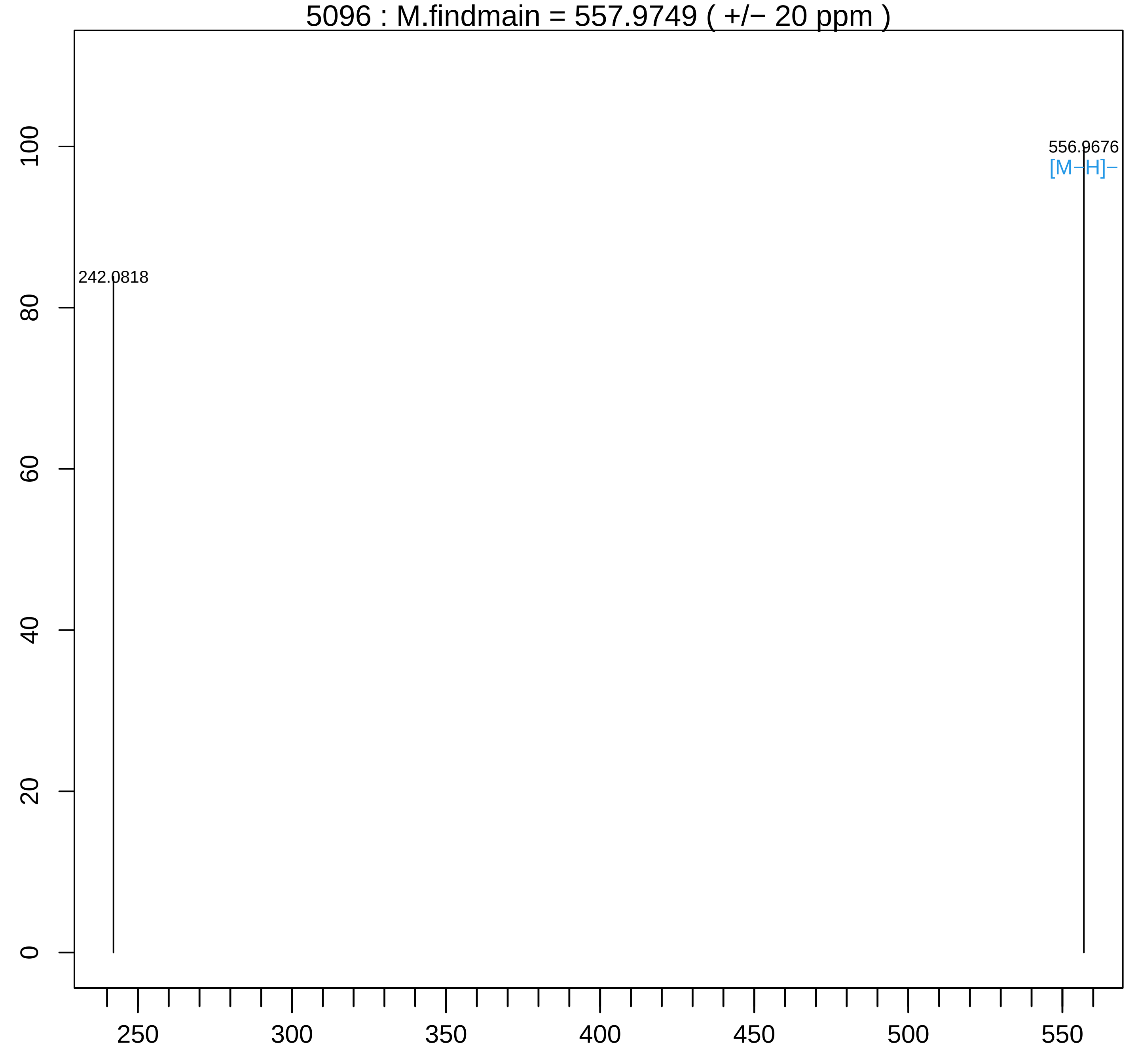
5096 : M.findmain = 557.9749 (+/− 20 ppm)
556.9676
[M−H]−
242.0818
0
20
40
60
80
100
250
300
350
400
450
500
550

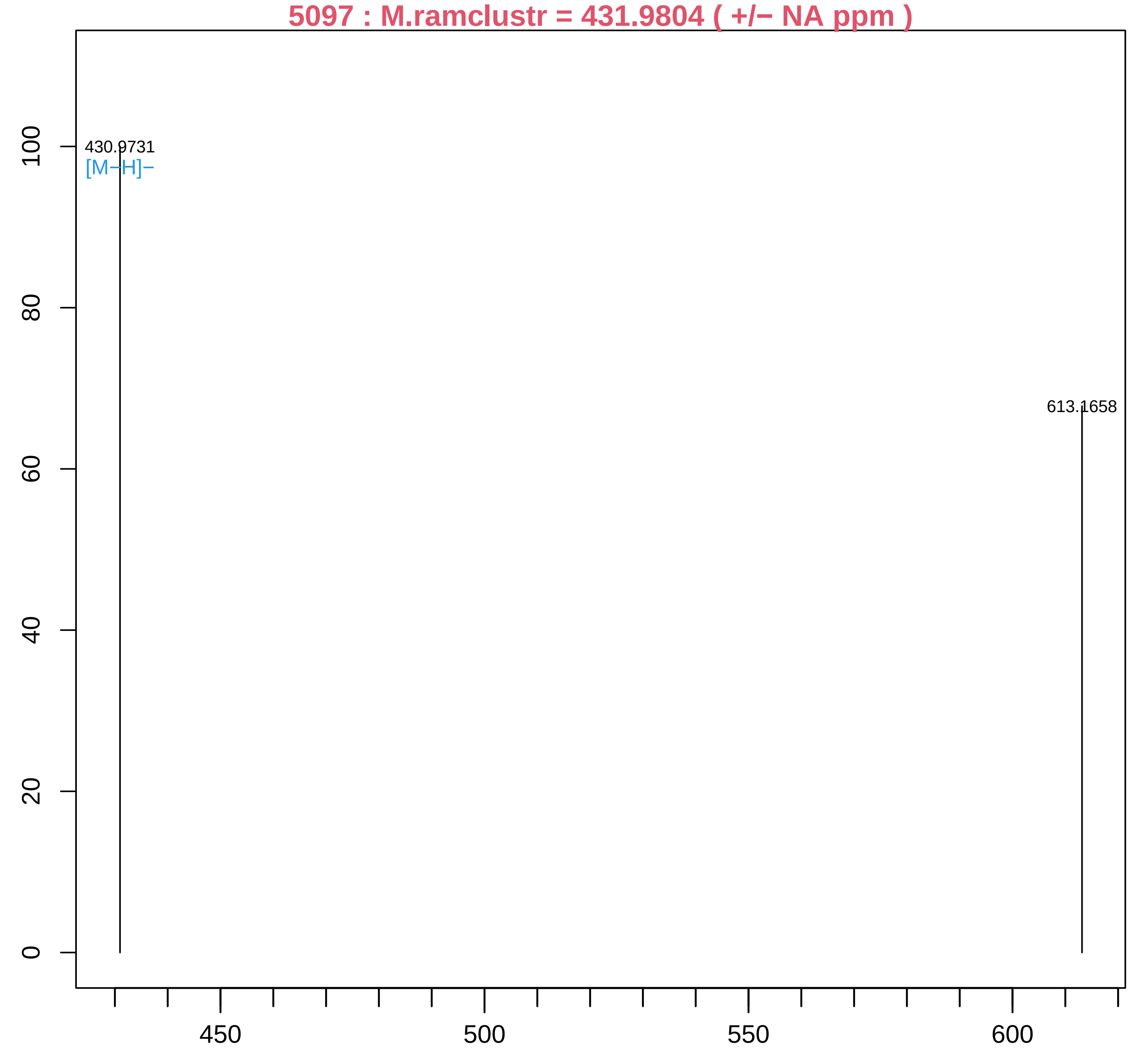

5097 : M.ramclustr = 431.9804 (+/− NA ppm)
430.9731
[M−H]−
613.1658
0
20
40
60
80
100
450
500
550
600

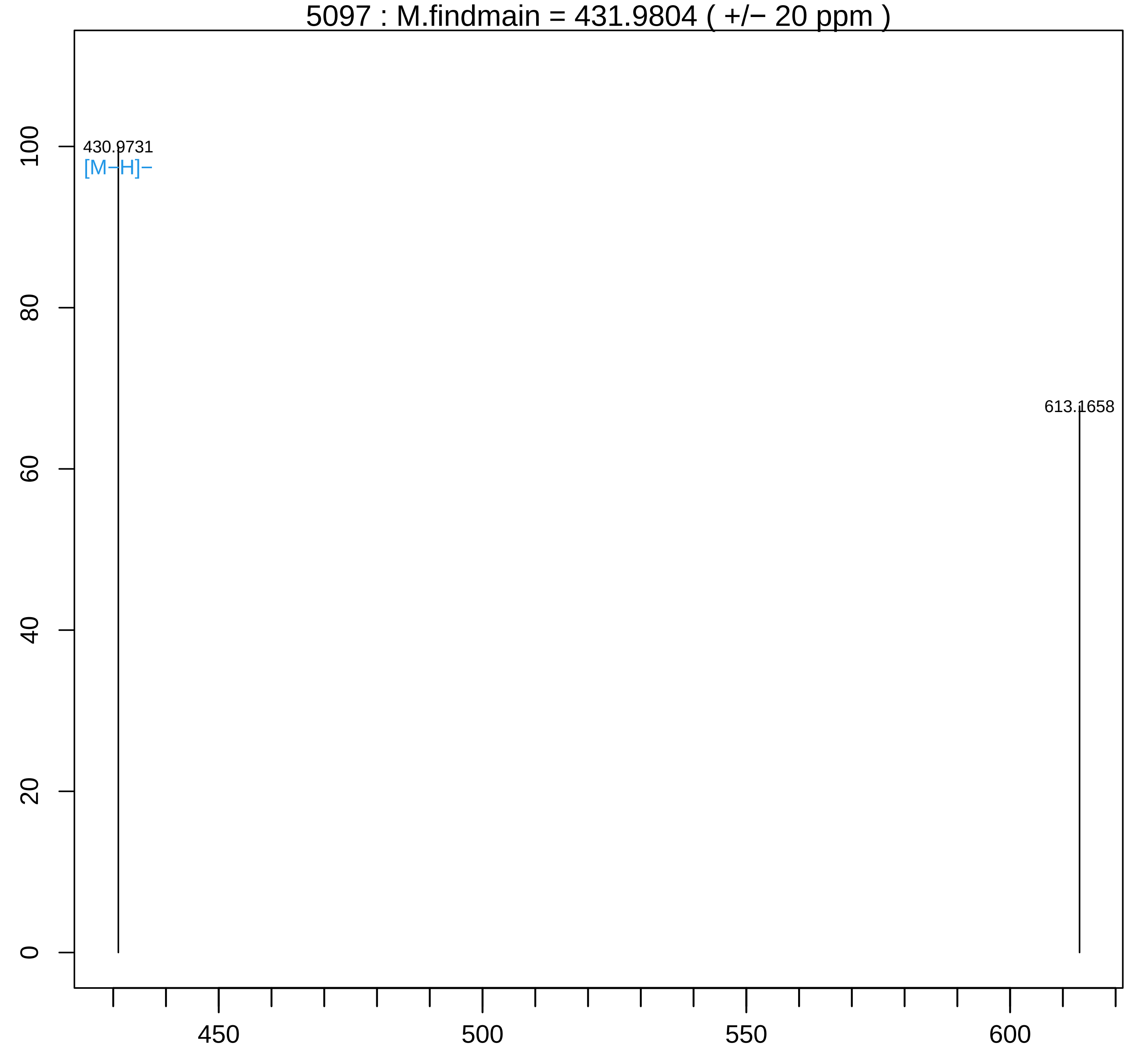

5097 : M.findmain = 431.9804 (+/− 20 ppm)
430.9731
[M−H]−
613.1658
0
20
40
60
80
100
450
500
550
600

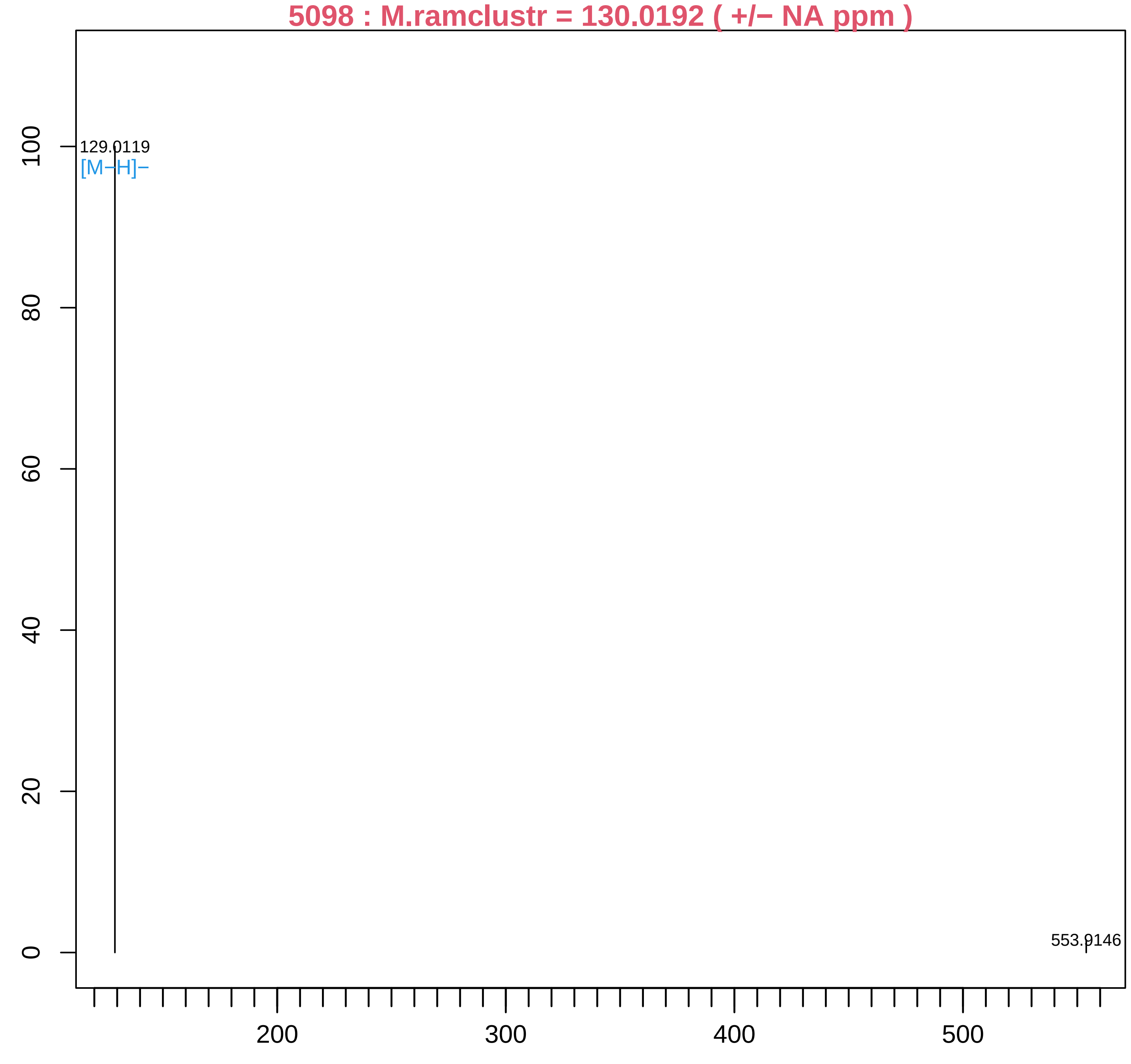
5098 : M.ramclustr = 130.0192 (+/− NA ppm)
129.0119
[M−H]−
553.9146
0
20
40
60
80
100
200
300
400
500

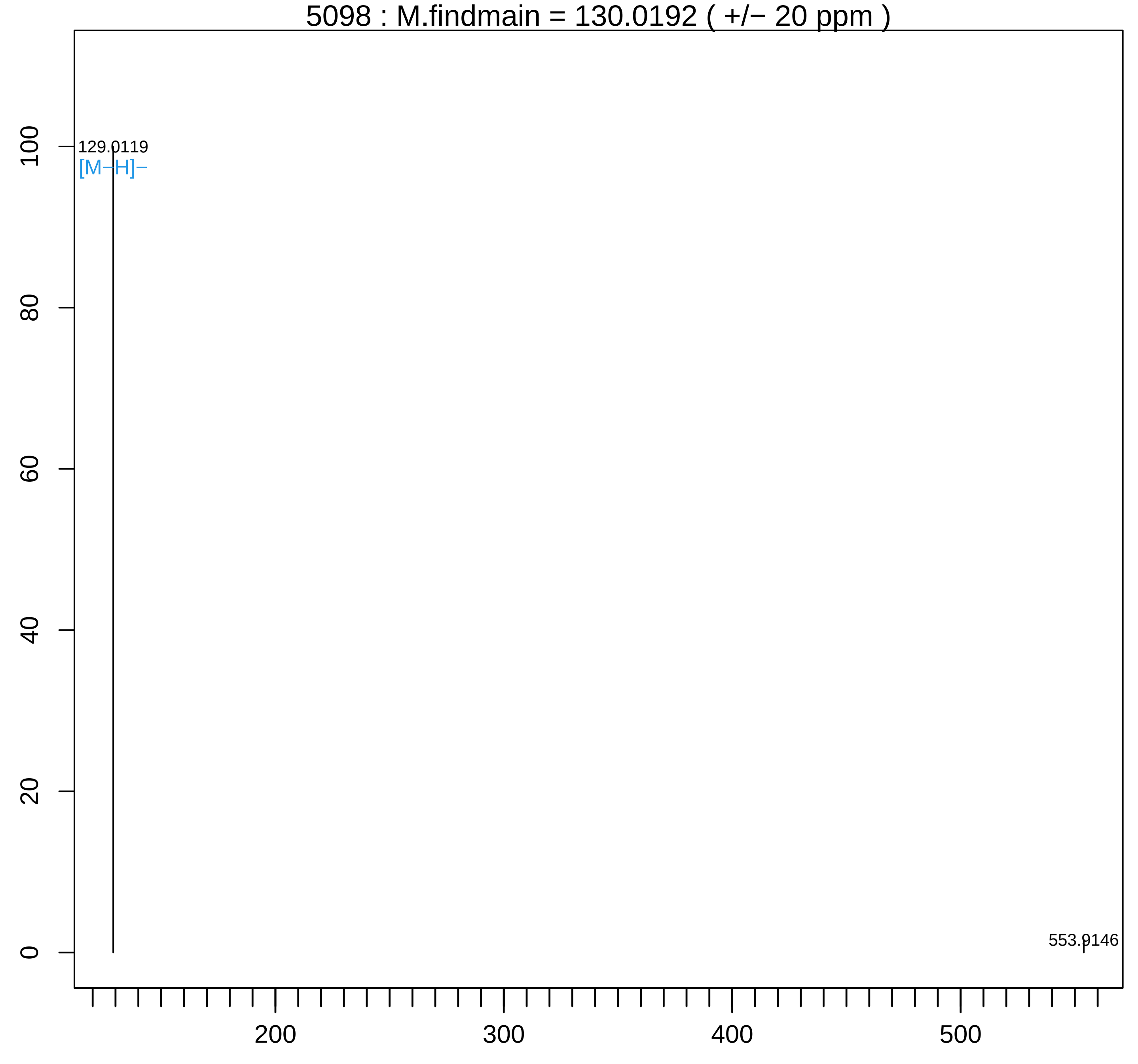
5098 : M.findmain = 130.0192 (+/− 20 ppm)
129.0119
[M−H]−
553.9146
0
20
40
60
80
100
200
300
400
500

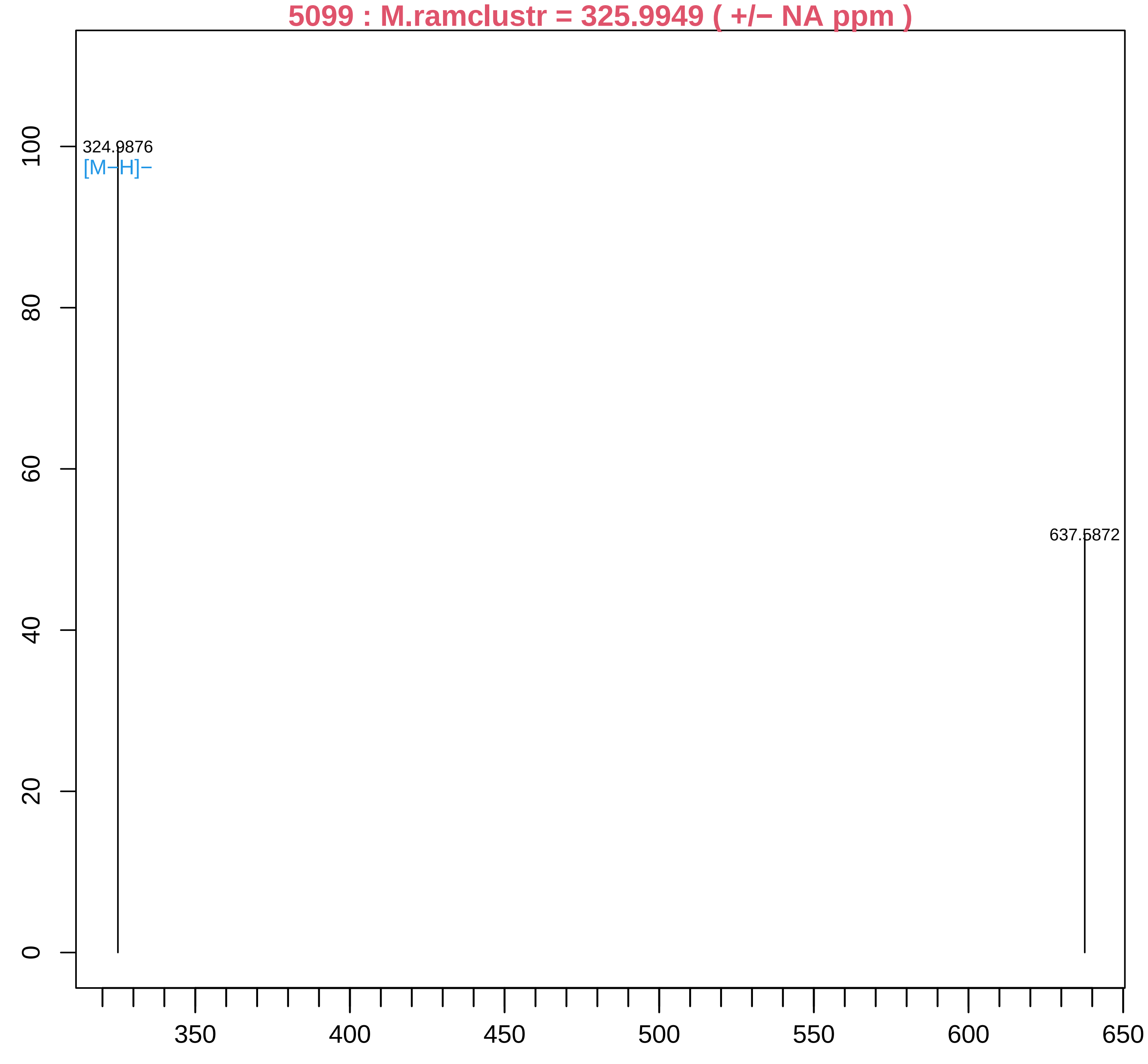
5099 : M.ramclustr = 325.9949 (+/− NA ppm)
324.9876
[M−H]−
637.5872
0
20
40
60
80
100
350
400
450
500
550
600
650

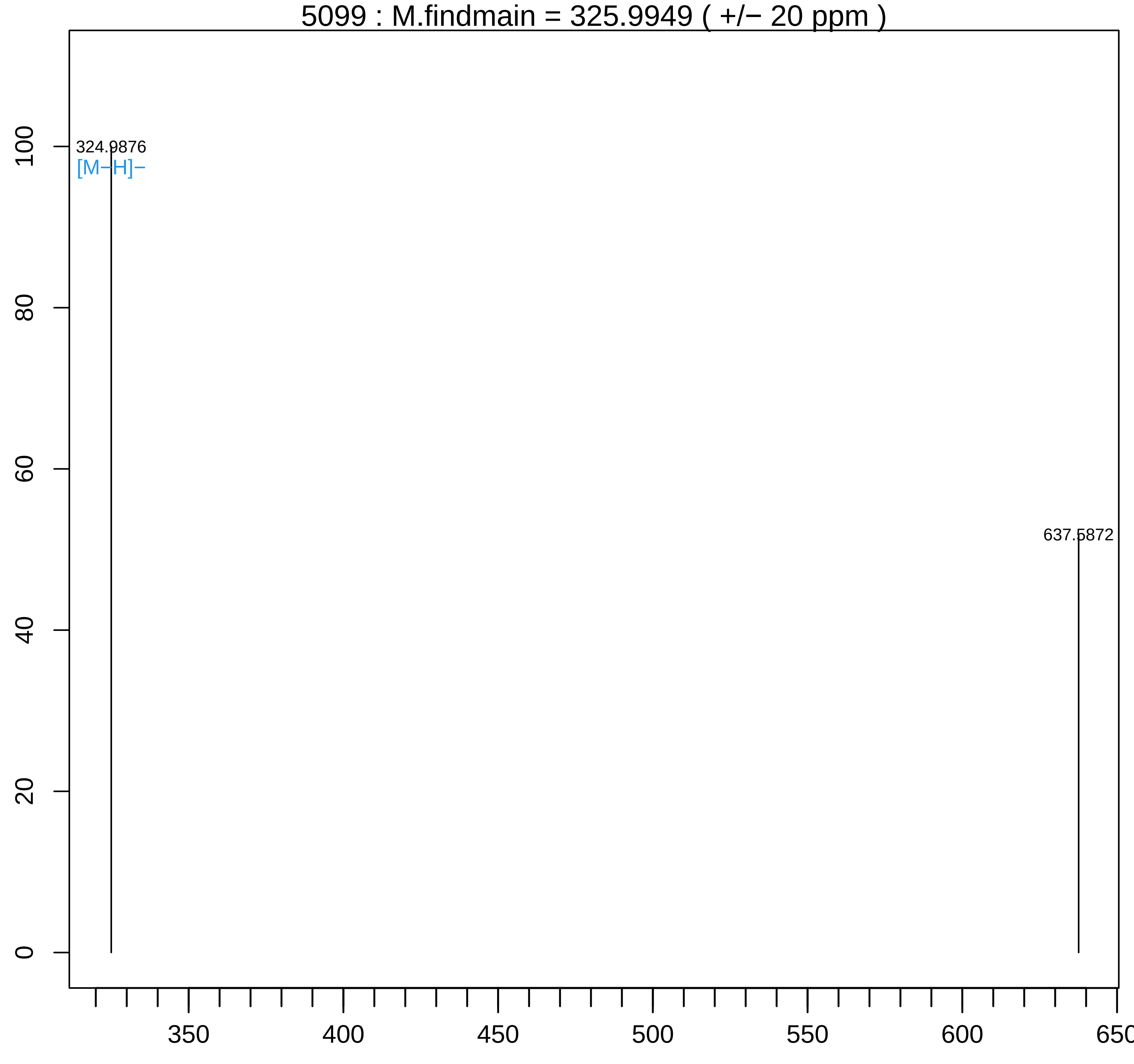
5099 : M.findmain = 325.9949 (+/− 20 ppm)
324.9876
[M−H]−
637.5872
0
20
40
60
80
100
350
400
450
500
550
600
650

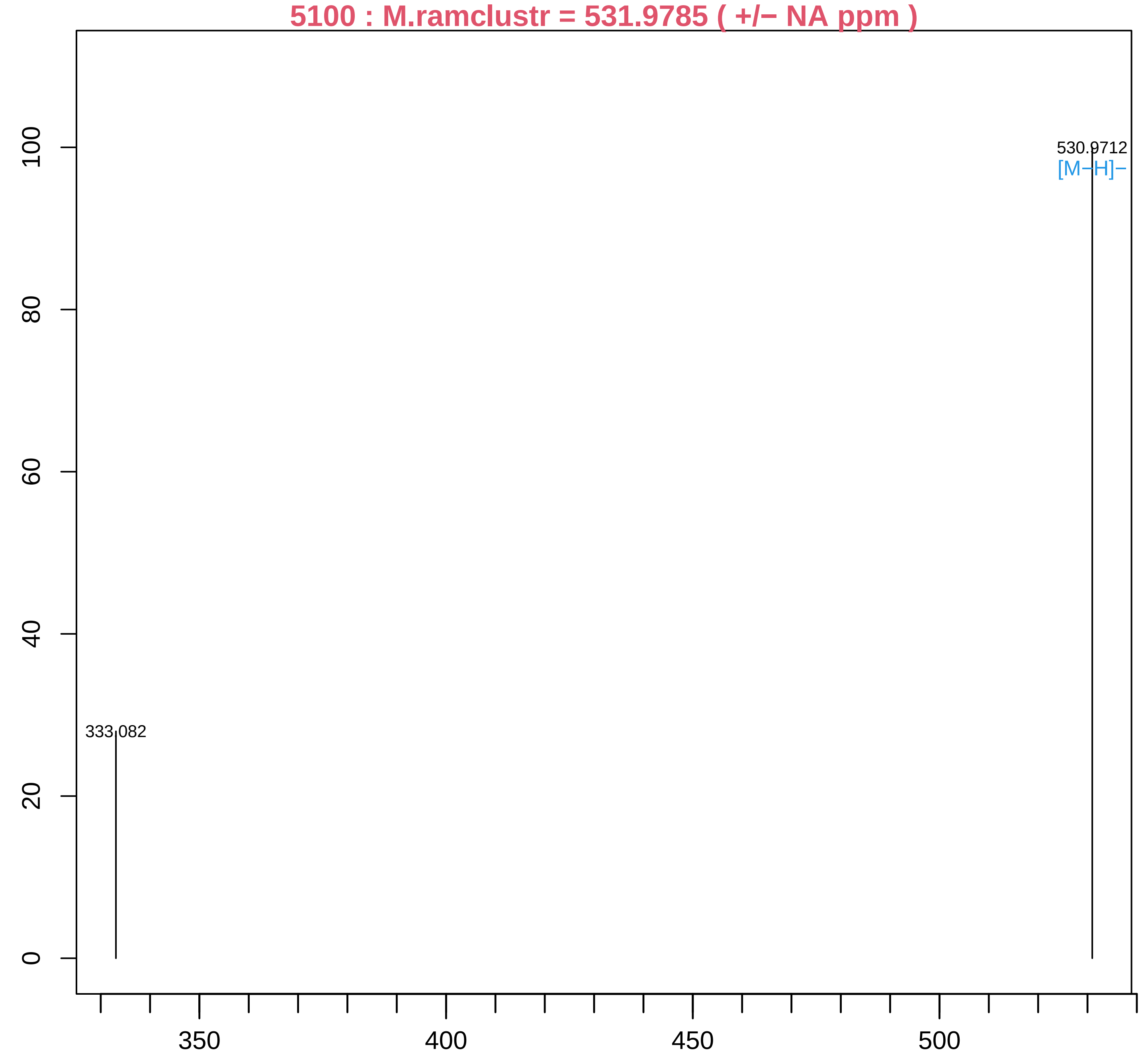

5100 : M.ramclustr = 531.9785 (+/− NA ppm)
530.9712
[M−H]−
333.082
0
20
40
60
80
100
350
400
450
500

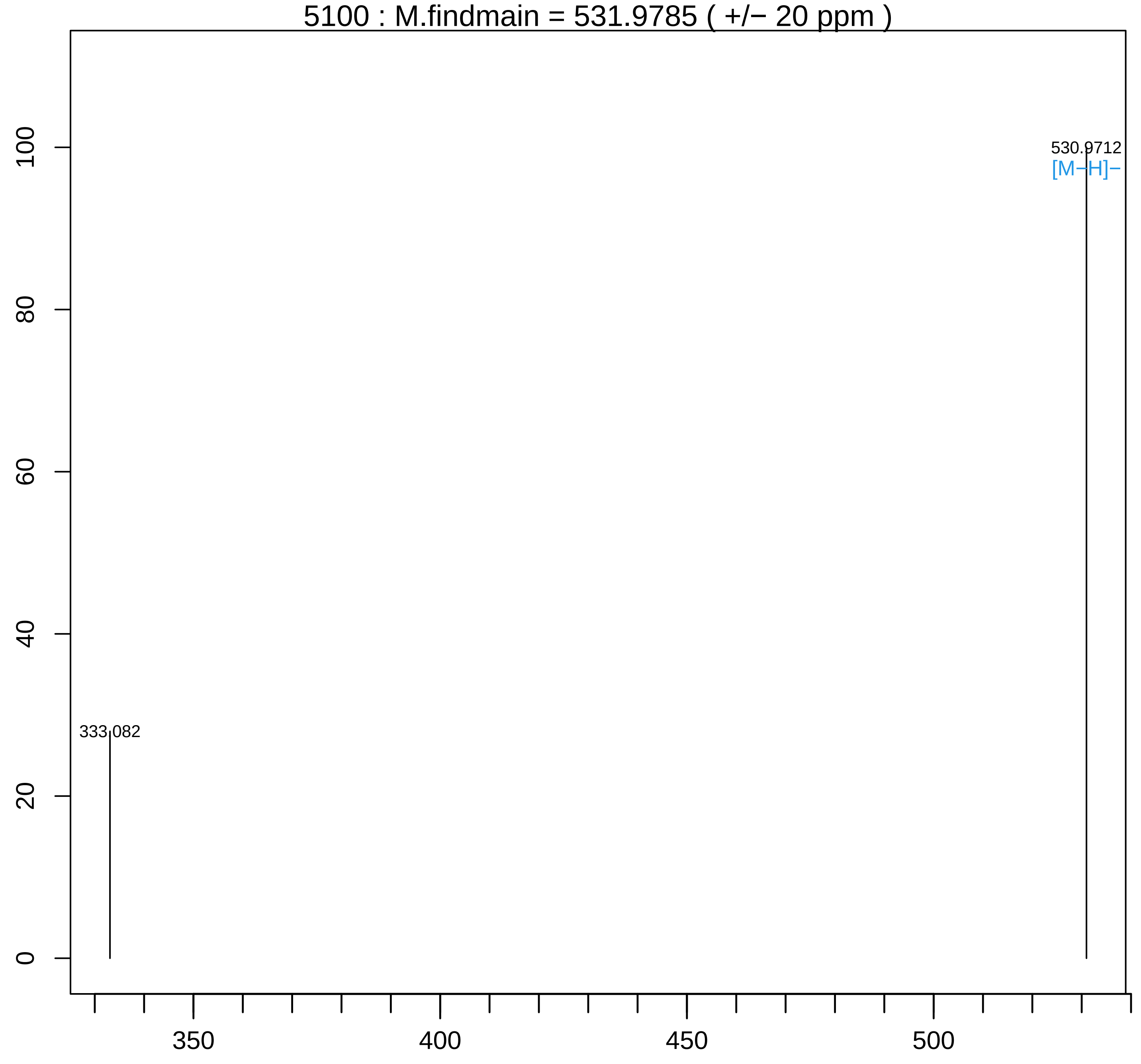

5100 : M.findmain = 531.9785 (+/− 20 ppm)
530.9712
[M−H]−
333.082
0
20
40
60
80
100
350
400
450
500

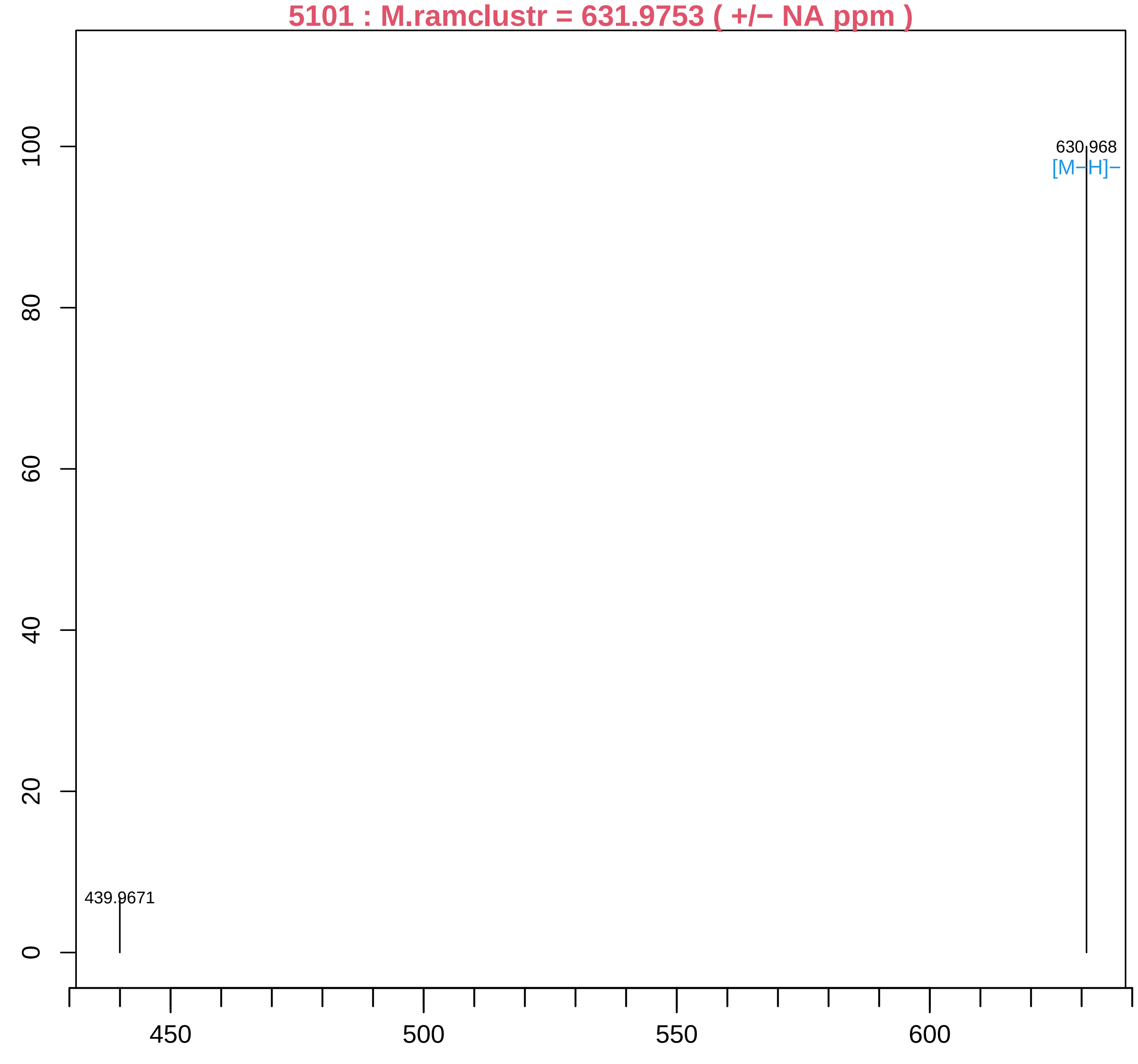
5101 : M.ramclustr = 631.9753 (+/− NA ppm)
630.968
[M−H]−
439.9671
0
20
40
60
80
100
450
500
550
600
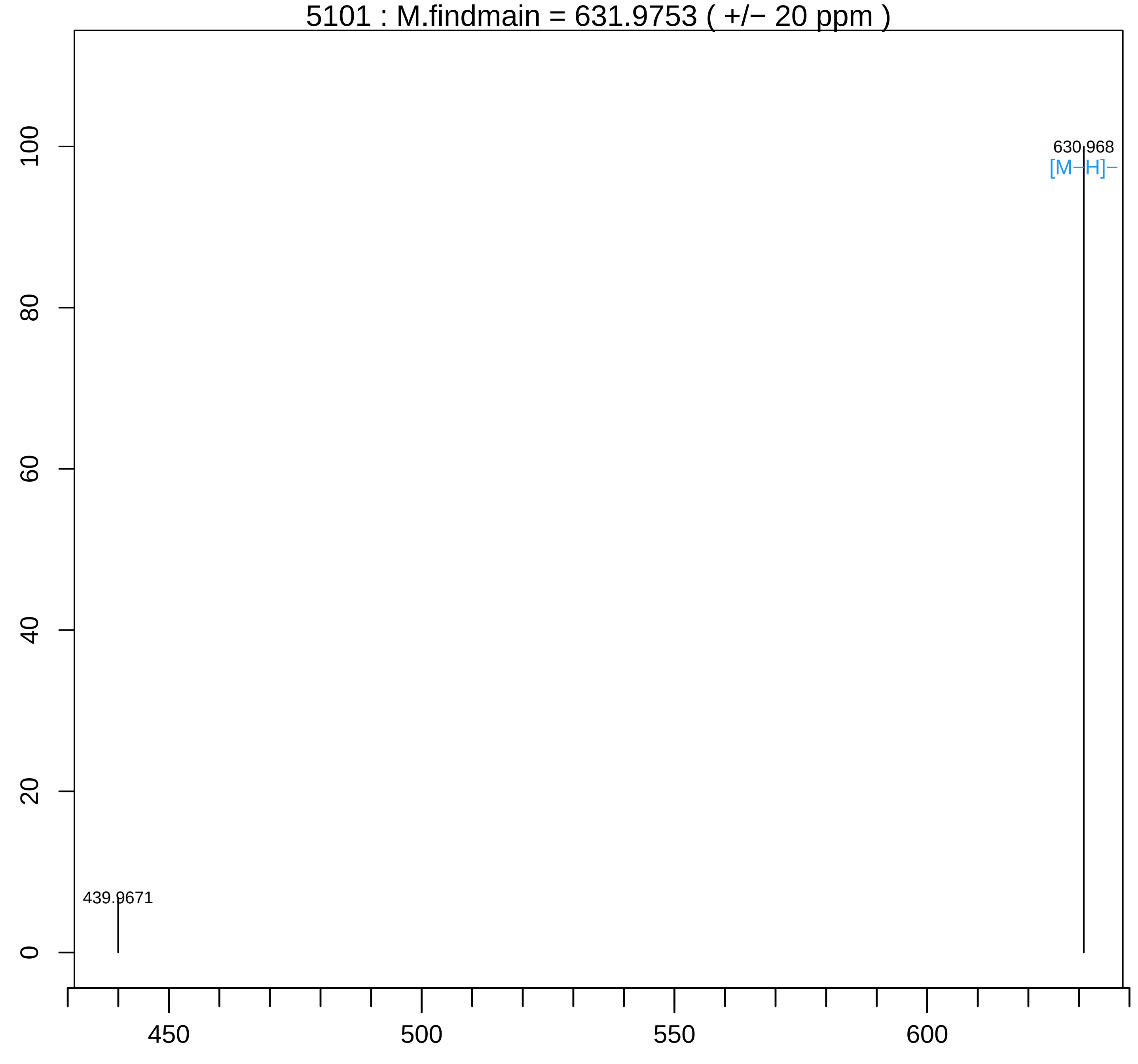
5101 : M.findmain = 631.9753 (+/− 20 ppm)
630.968
[M−H]−
439.9671
0
20
40
60
80
100
450
500
550
600

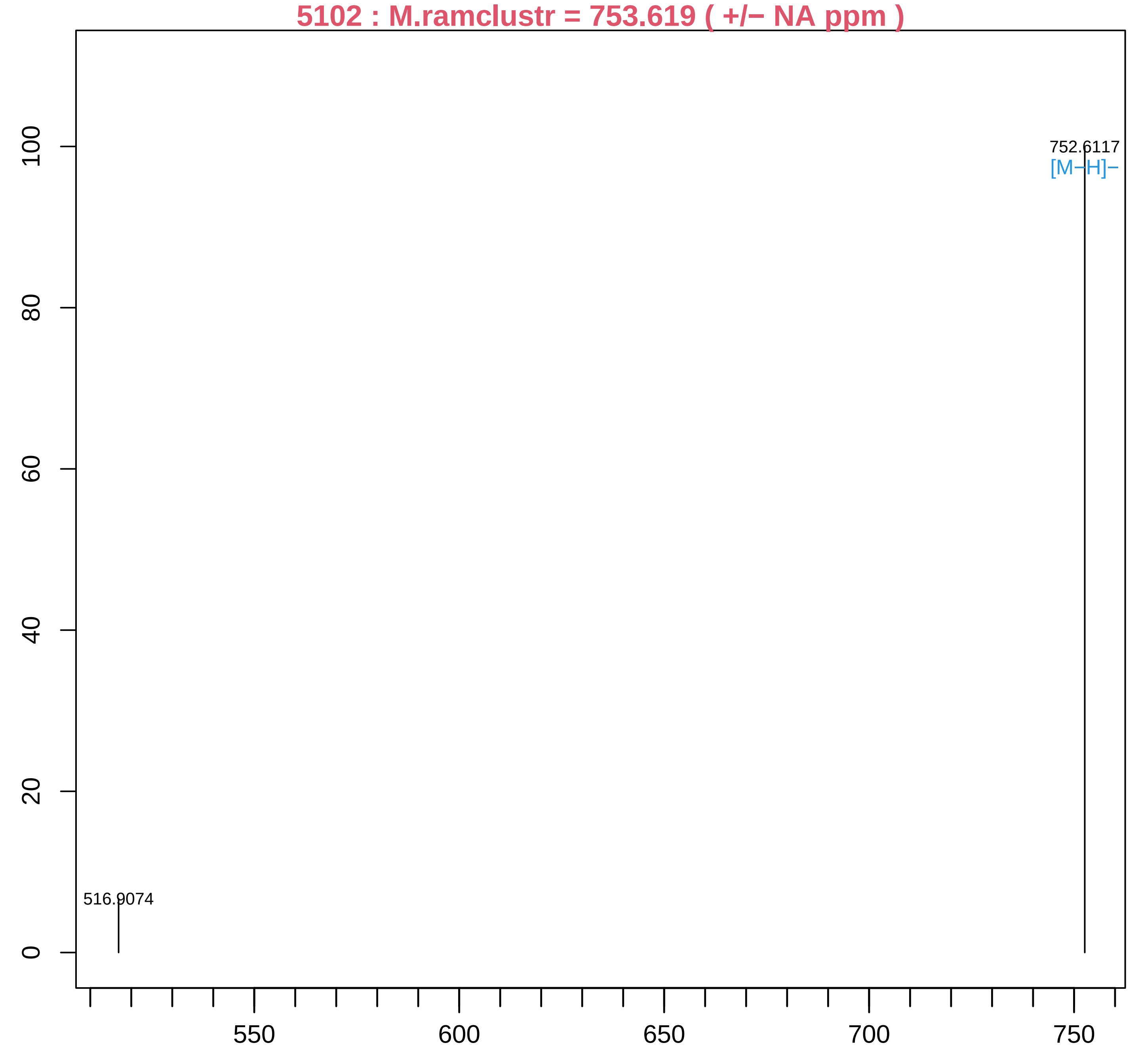
5102 : M.ramclustr = 753.619 (+/− NA ppm)
752.6117
[M−H]−
516.9074
0
20
40
60
80
100
550
600
650
700
750

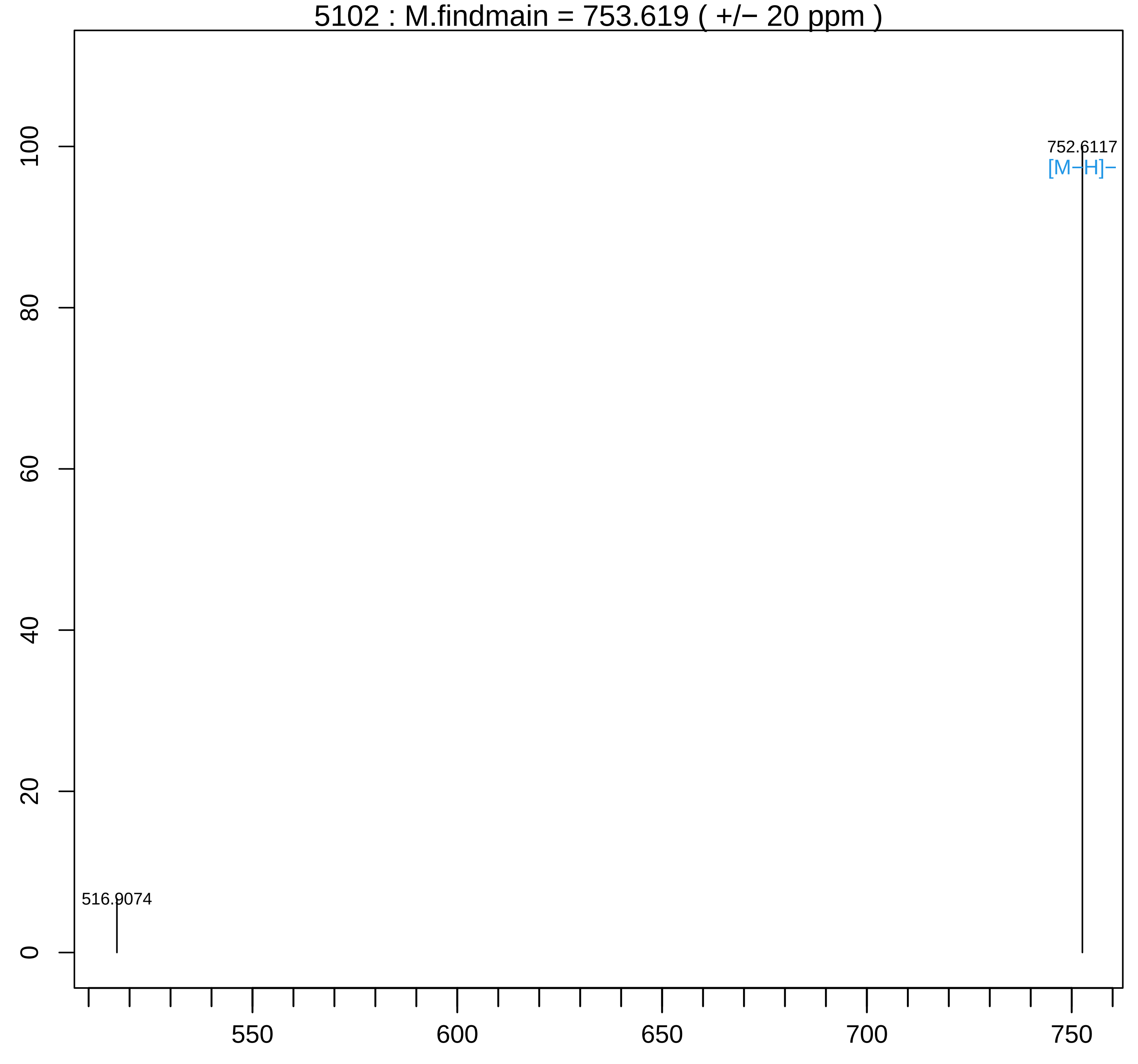
5102 : M.findmain = 753.619 (+/− 20 ppm)
752.6117
[M−H]−
516.9074
0
20
40
60
80
100
550
600
650
700
750

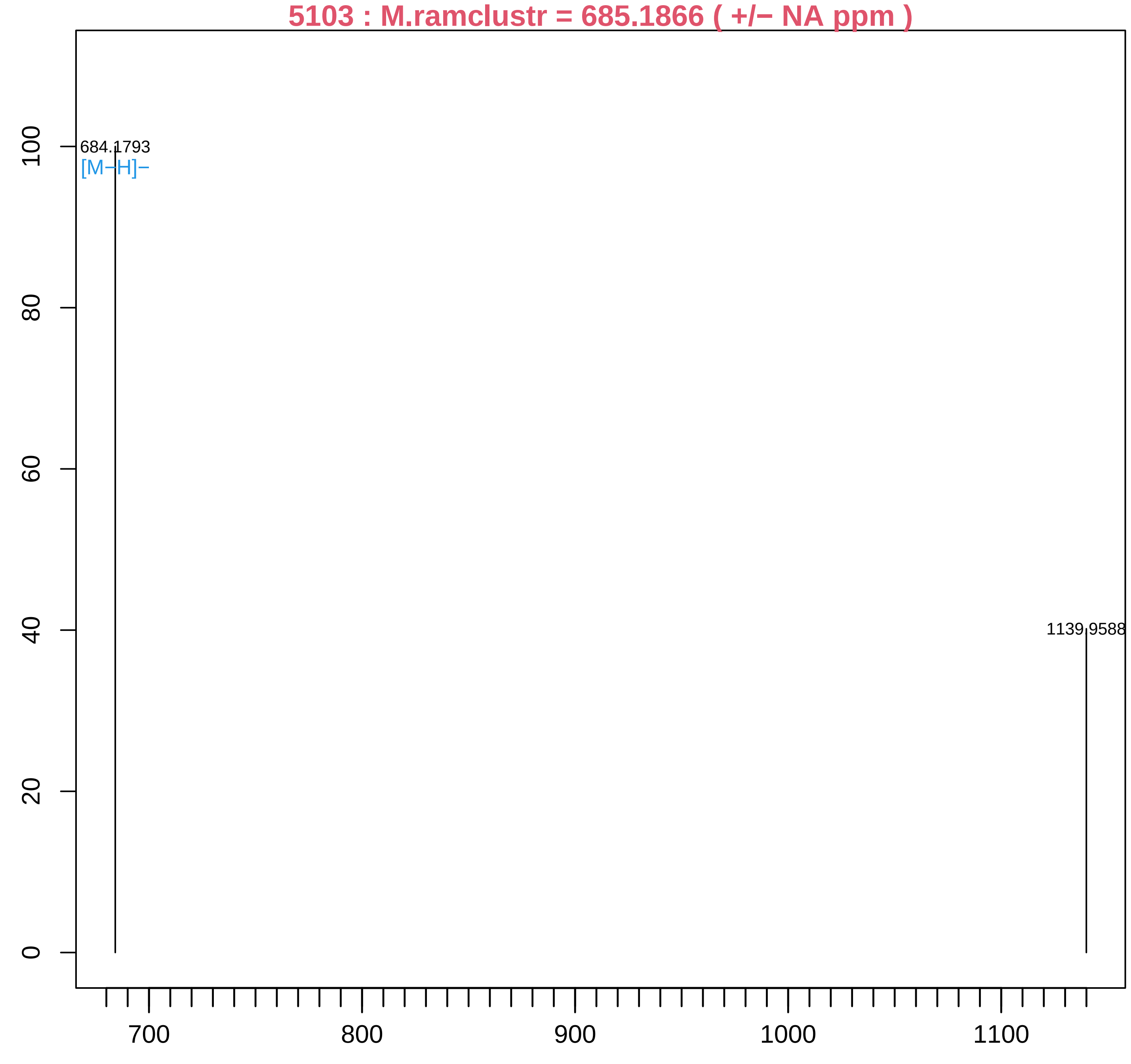
5103 : M.ramclustr = 685.1866 (+/− NA ppm)
684.1793
[M−H]−
1139.9588
0
20
40
60
80
100
700
800
900
1000
1100

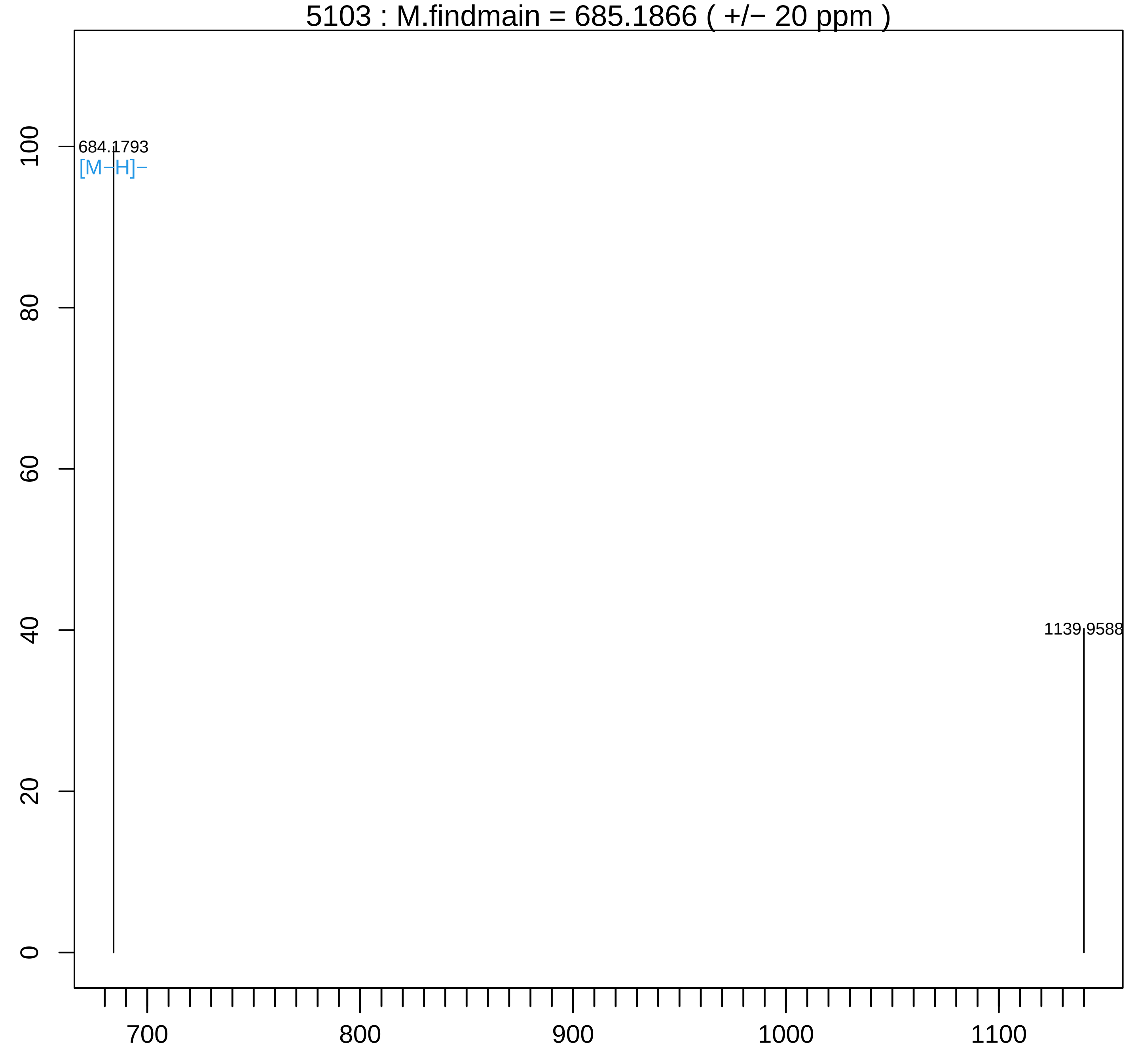
5103 : M.findmain = 685.1866 (+/− 20 ppm)
684.1793
[M−H]−
1139.9588
0
20
40
60
80
100
700
800
900
1000
1100

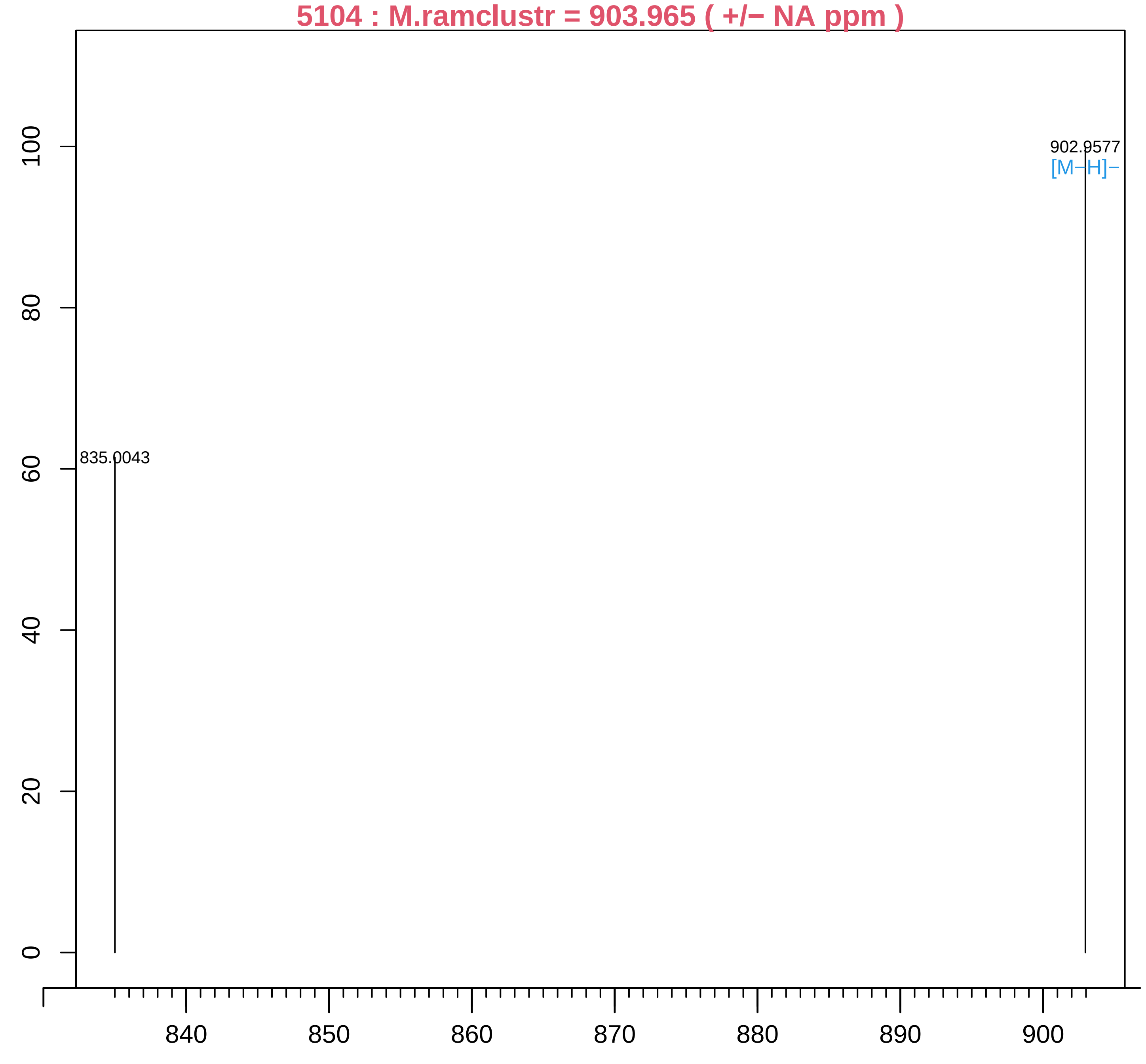
5104 : M.ramclustr = 903.965 (+/− NA ppm)
902.9577
[M−H]−
835.0043
0
20
40
60
80
100
840
850
860
870
880
890
900

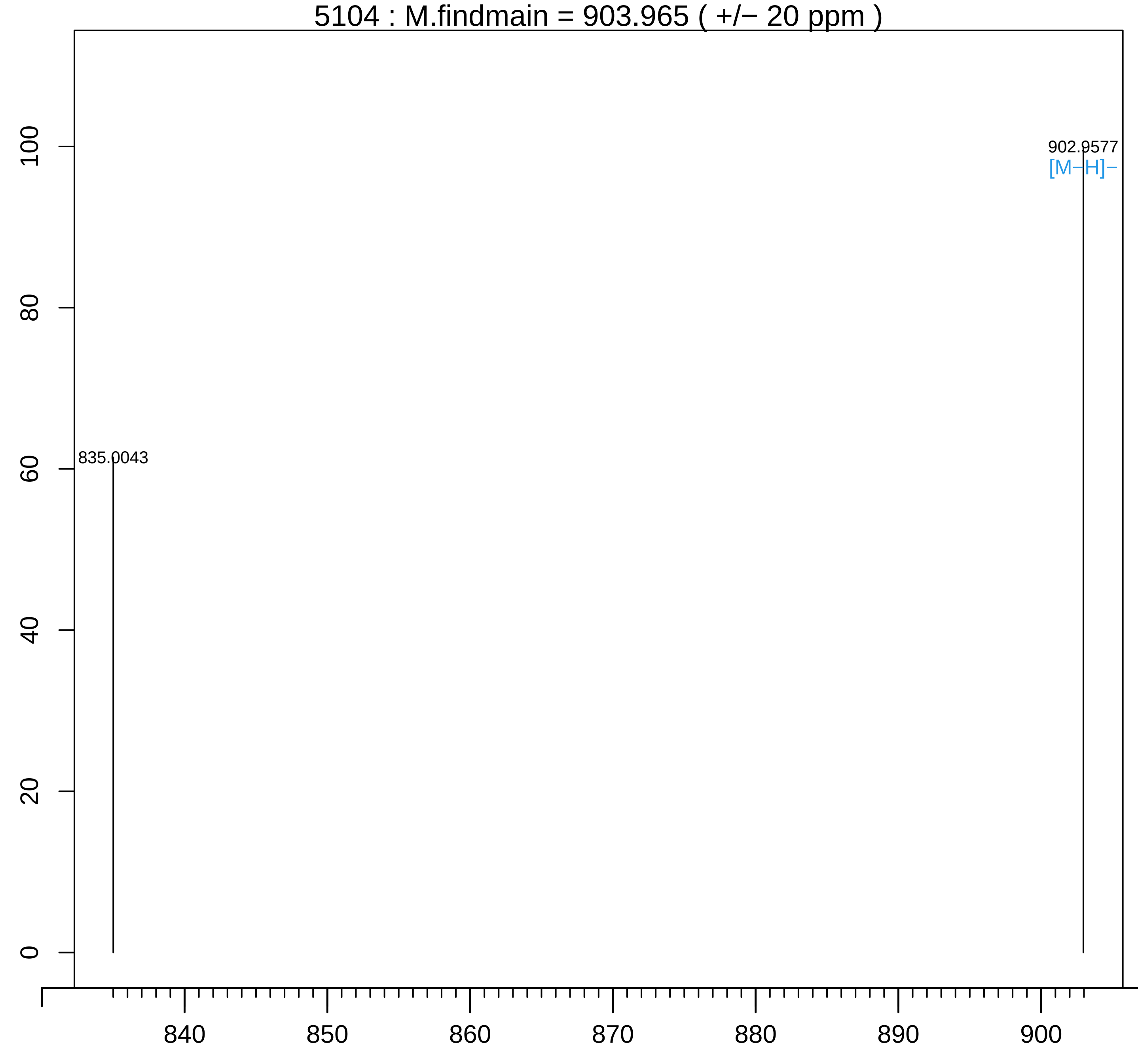
5104 : M.findmain = 903.965 (+/− 20 ppm)
902.9577
[M−H]−
835.0043
0
20
40
60
80
100
840
850
860
870
880
890
900

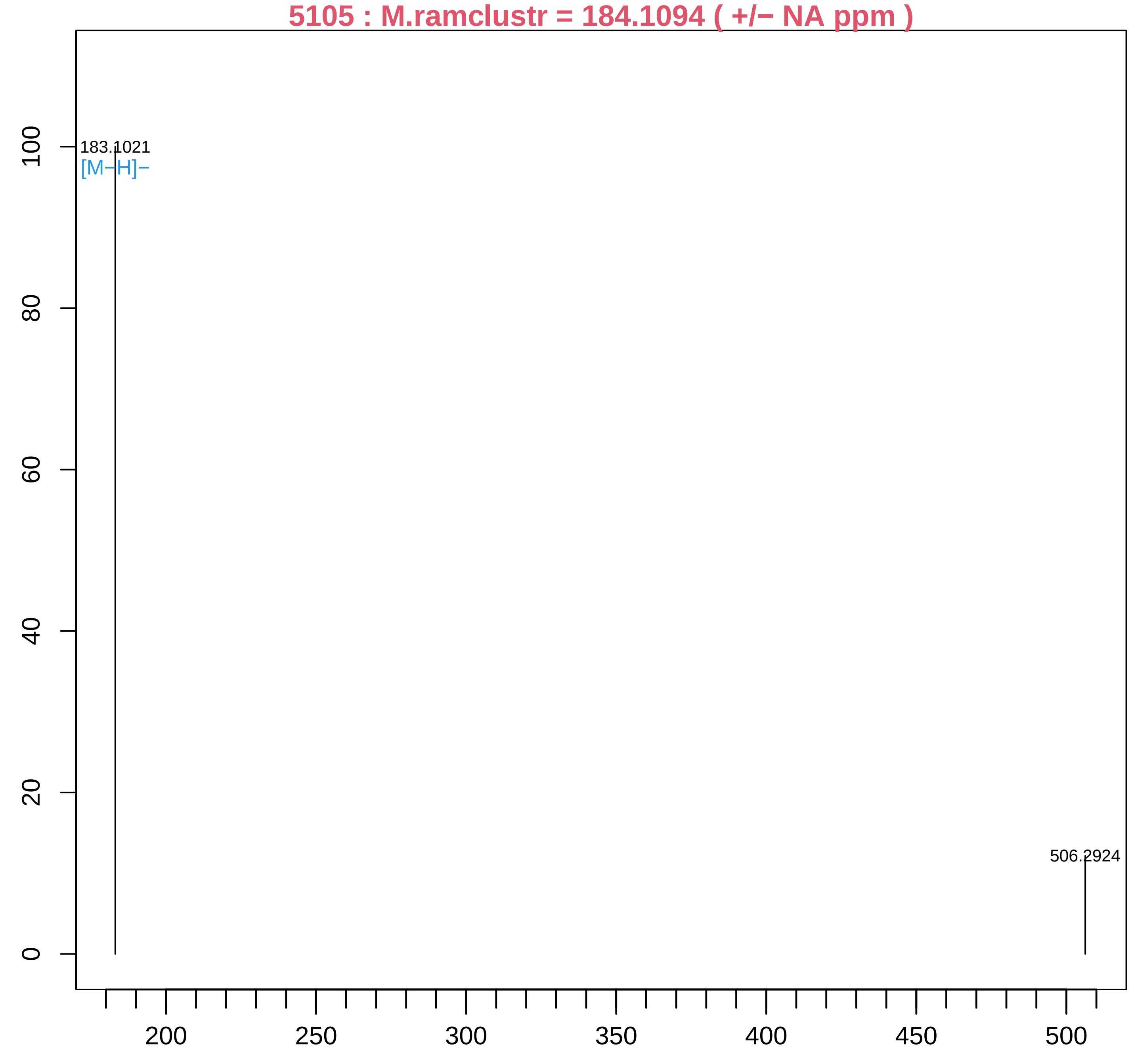

5105 : M.ramclustr = 184.1094 (+/− NA ppm)
183.1021
[M−H]−
506.2924
0
20
40
60
80
100
200
250
300
350
400
450
500

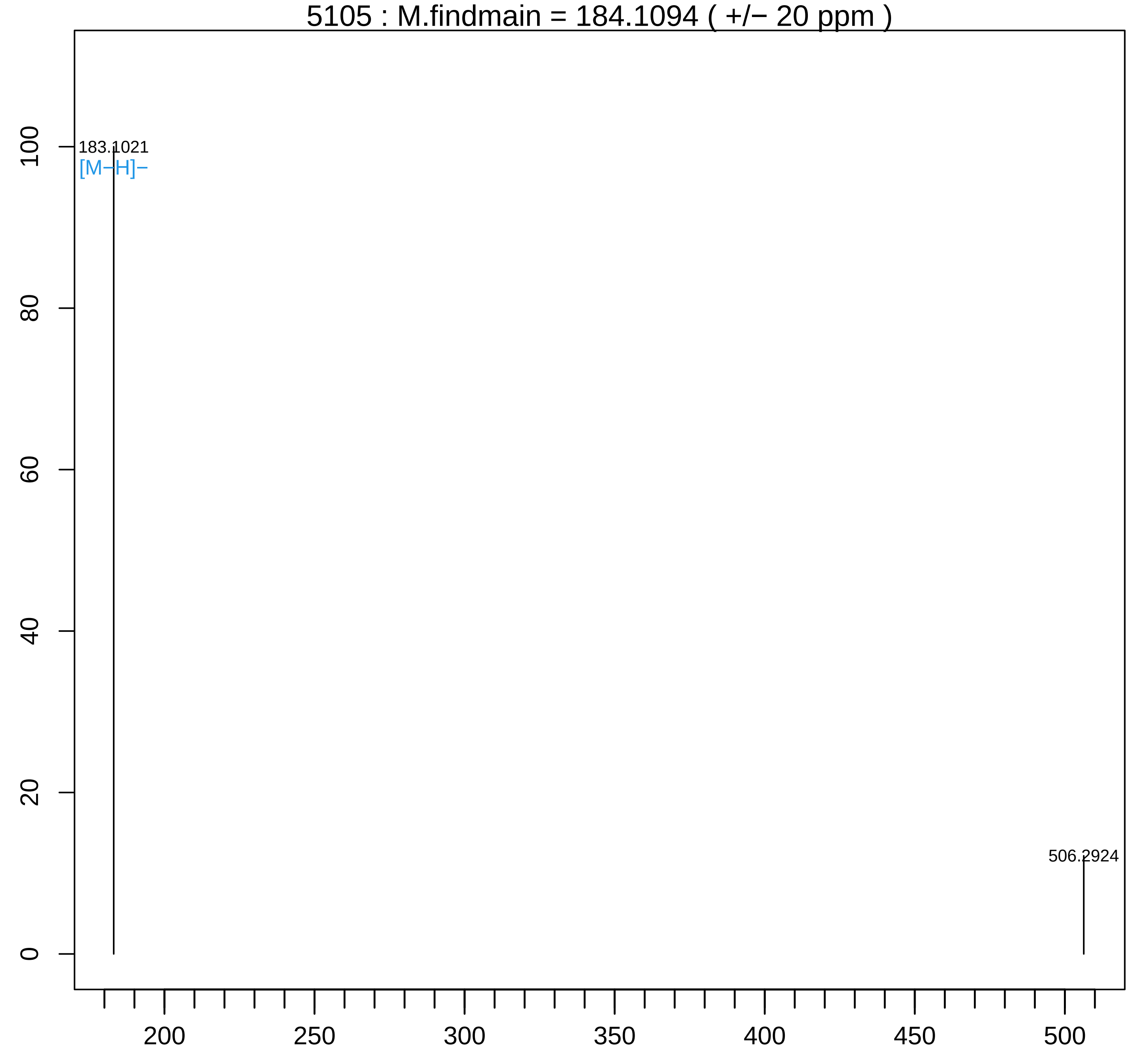

5105 : M.findmain = 184.1094 (+/− 20 ppm)
183.1021
[M−H]−
506.2924
0
20
40
60
80
100
200
250
300
350
400
450
500

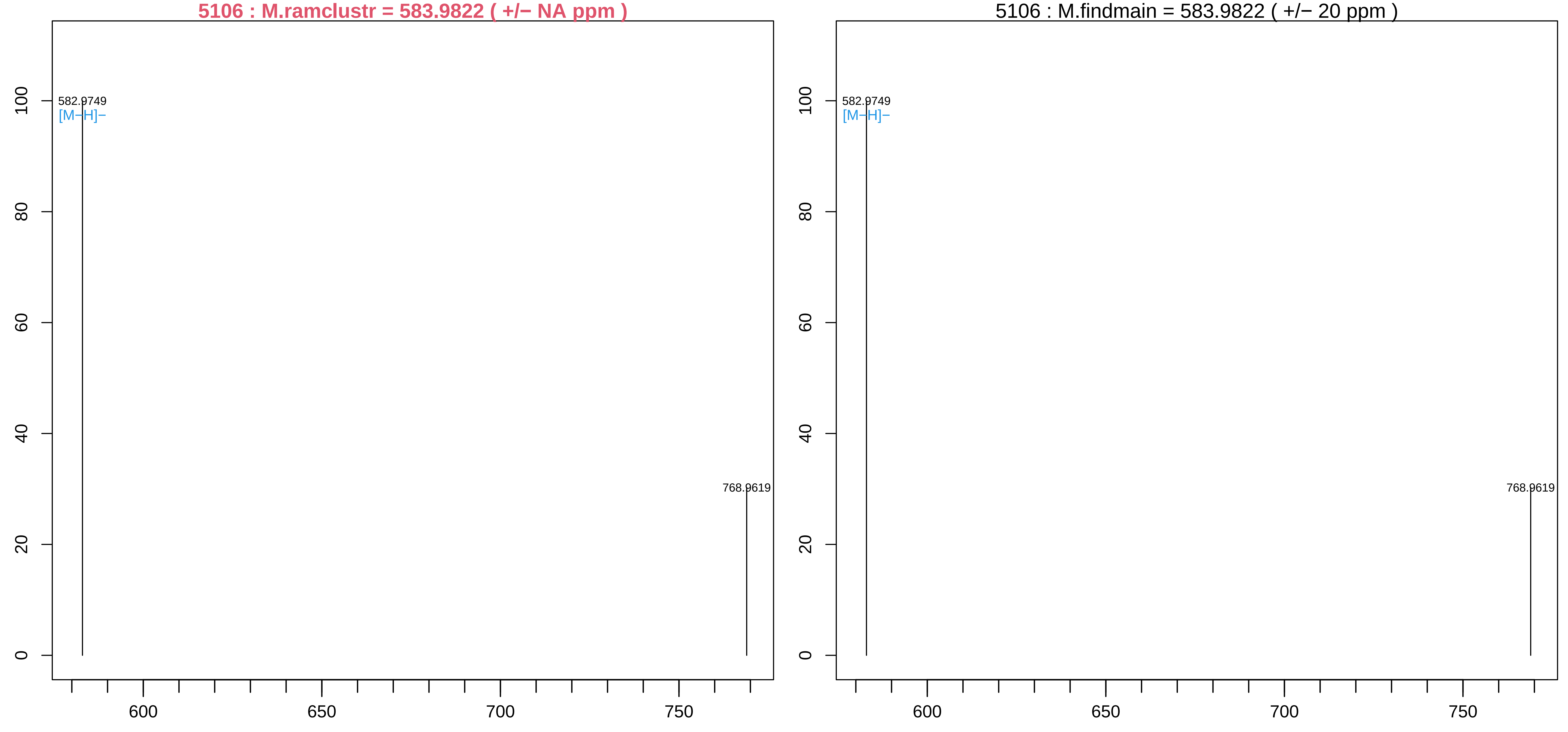
5106 : M.ramclustr = 583.9822 (+/− NA ppm)
582.9749
[M−H]−
768.9619
5106 : M.findmain = 583.9822 (+/− 20 ppm)
582.9749
[M−H]−
768.9619
0
20
40
60
80
100
600
650
700
750

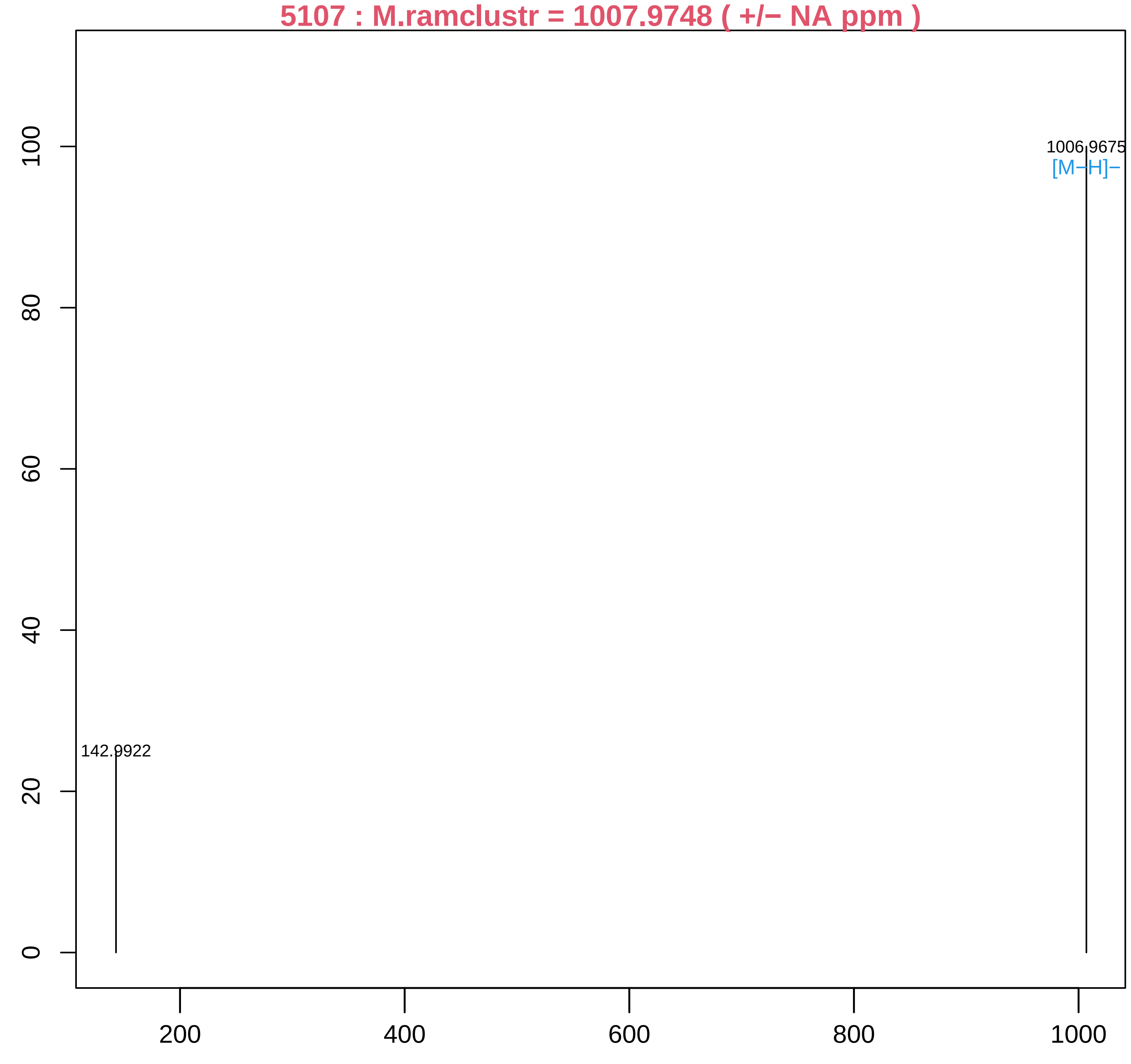
5107 : M.ramclustr = 1007.9748 (+/− NA ppm)
1006.9675
[M−H]−
142.9922
0
20
40
60
80
100
200
400
600
800
1000

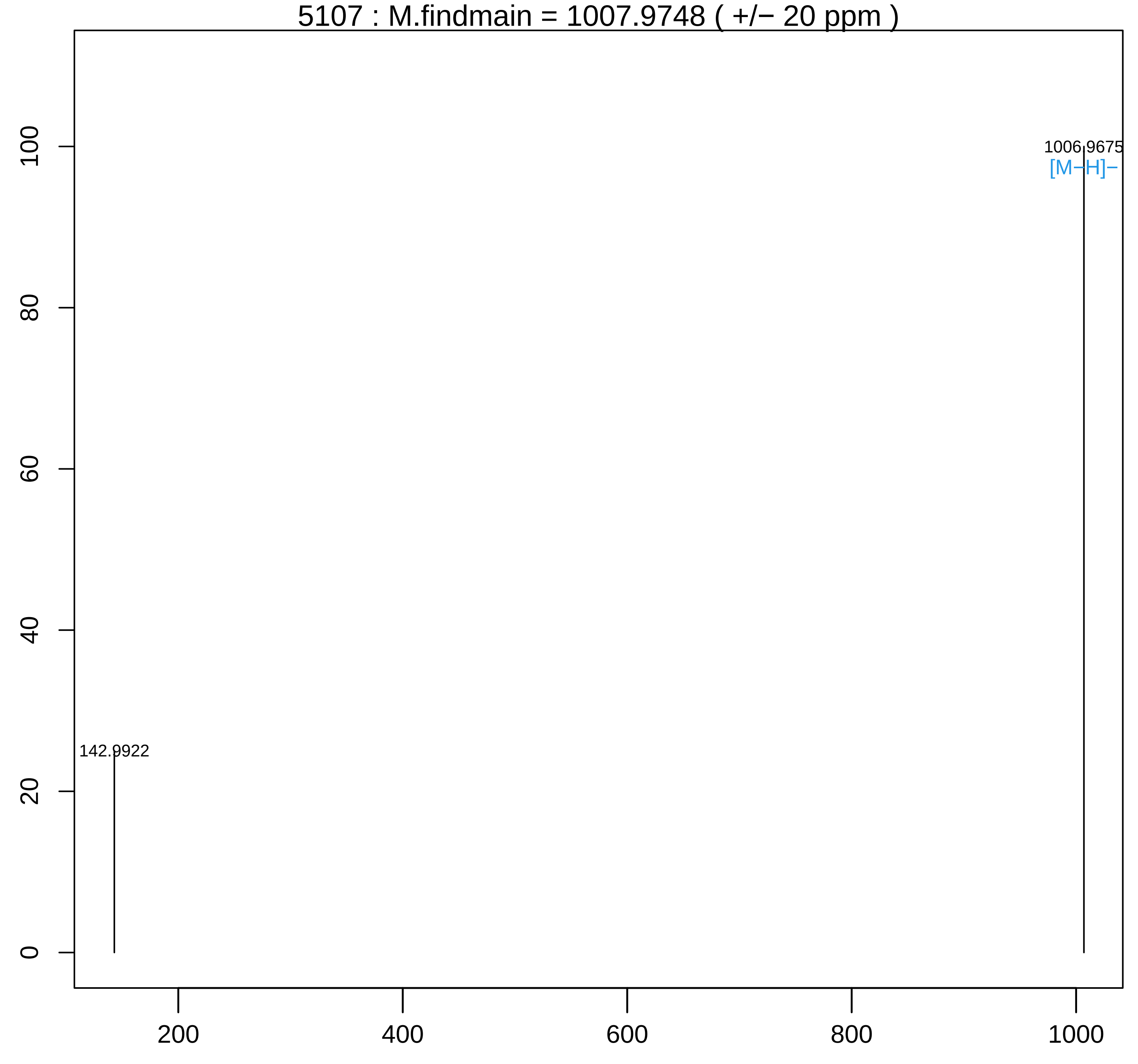
5107 : M.findmain = 1007.9748 (+/− 20 ppm)
1006.9675
[M−H]−
142.9922
0
20
40
60
80
100
200
400
600
800
1000

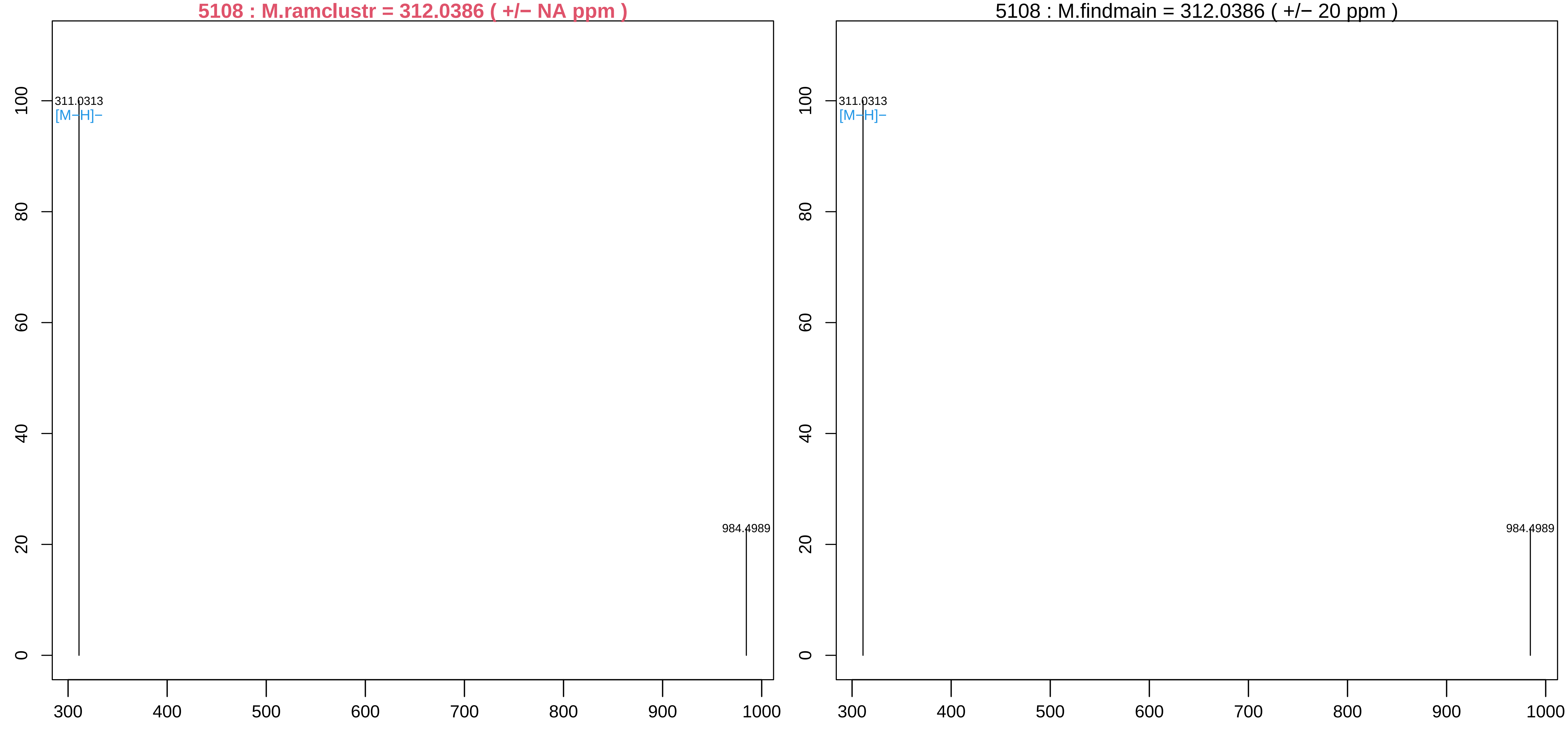
5108 : M.ramclustr = 312.0386 (+/− NA ppm)
311.0313
[M−H]−
984.4989
5108 : M.findmain = 312.0386 (+/− 20 ppm)
311.0313
[M−H]−
984.4989

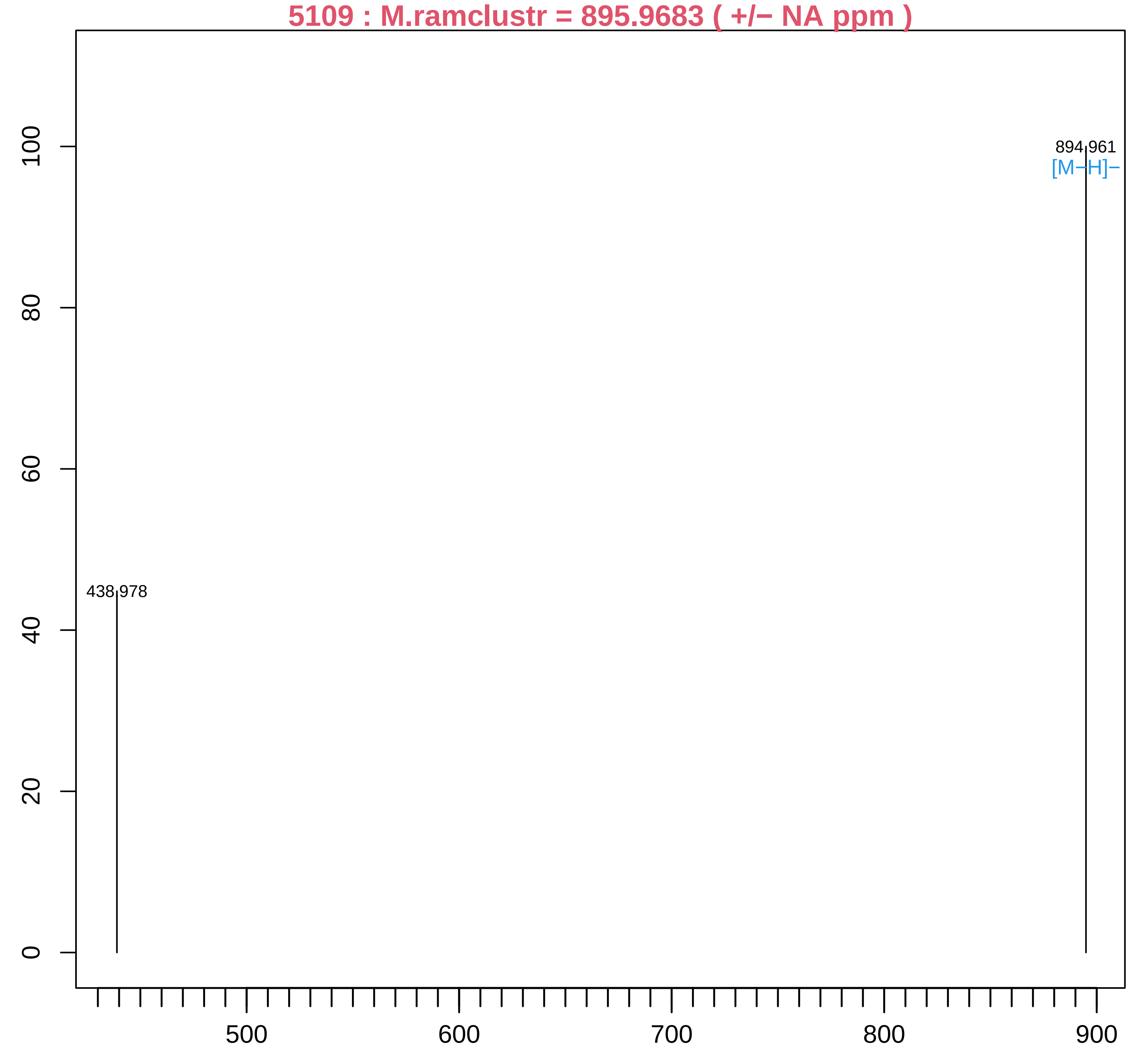
5109 : M.ramclustr = 895.9683 (+/− NA ppm)
894.961
[M−H]−
438.978
0
20
40
60
80
100
500
600
700
800
900

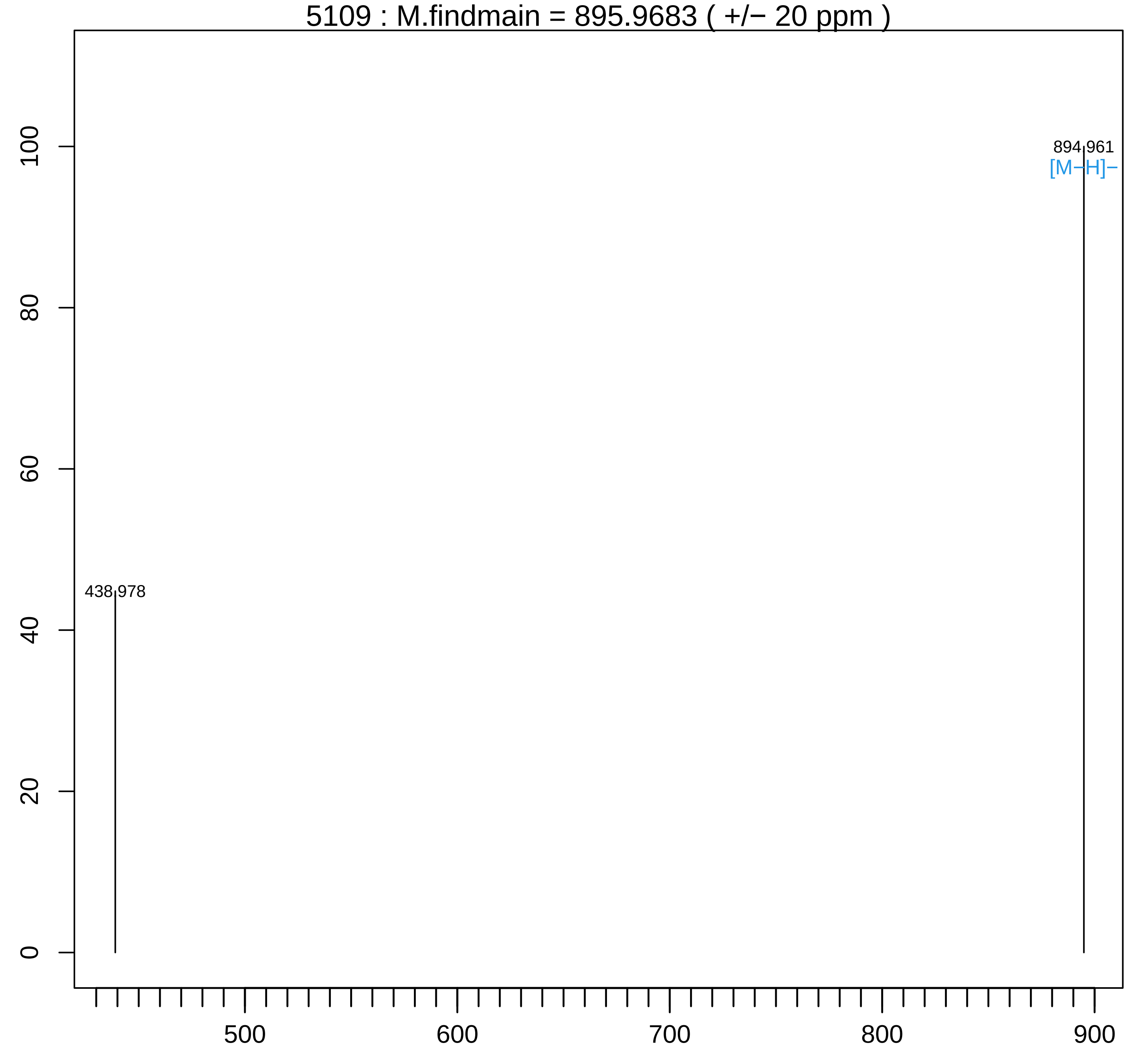
5109 : M.findmain = 895.9683 (+/− 20 ppm)
894.961
[M−H]−
438.978
0
20
40
60
80
100
500
600
700
800
900

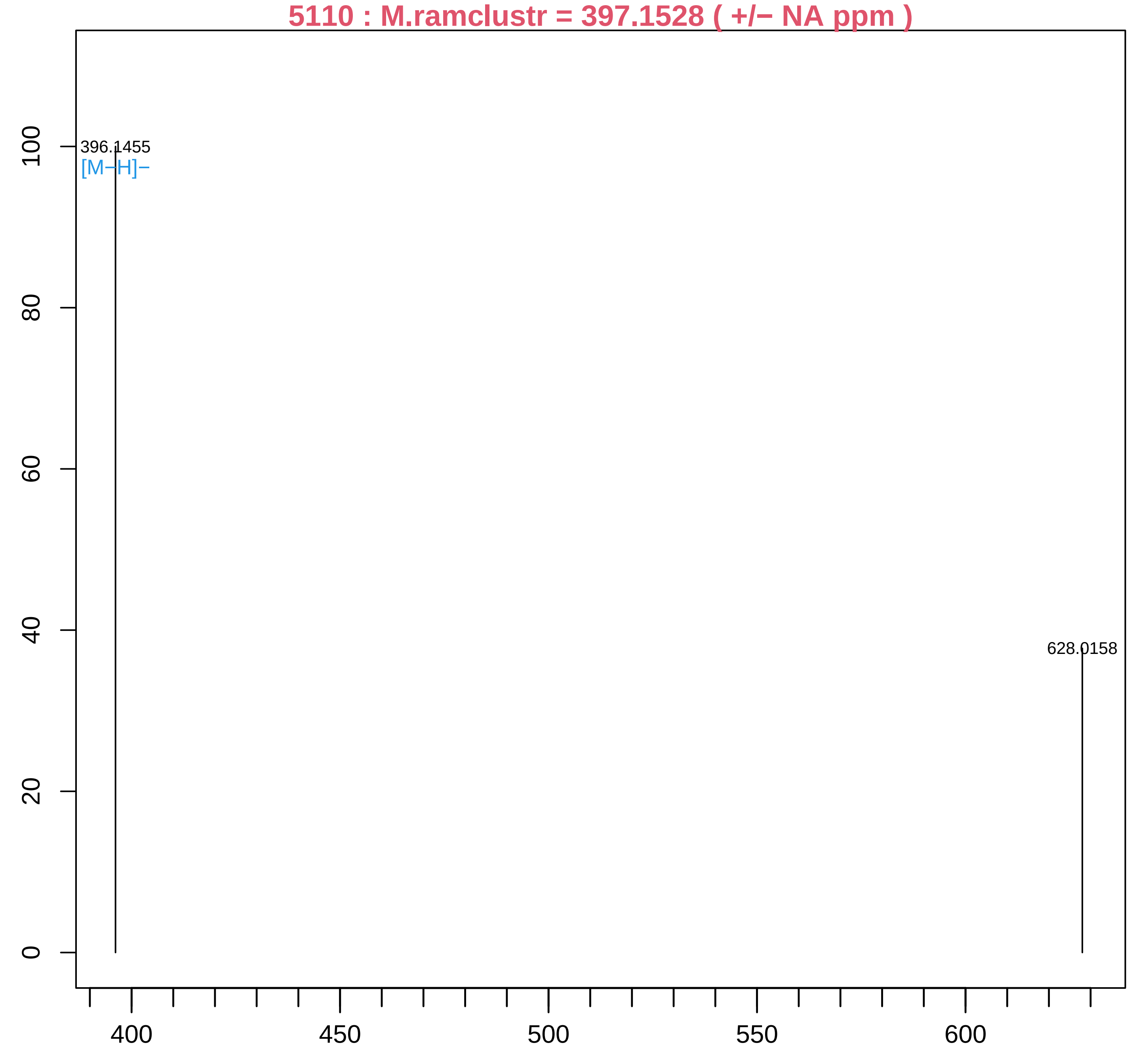
5110 : M.ramclustr = 397.1528 (+/− NA ppm)
396.1455
[M−H]−
628.0158
0
20
40
60
80
100
400
450
500
550
600

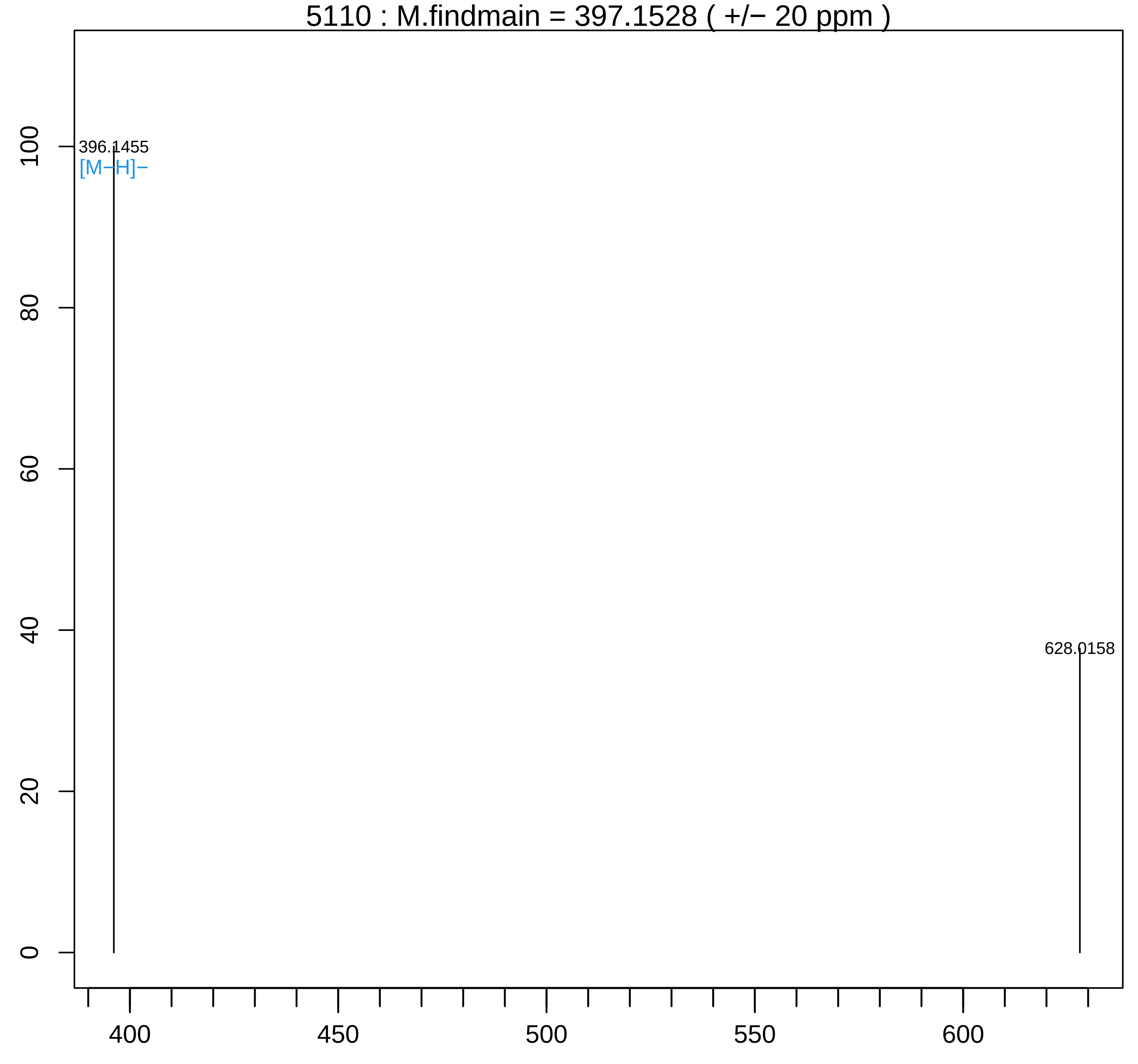
5110 : M.findmain = 397.1528 (+/− 20 ppm)
396.1455
[M−H]−
628.0158
0
20
40
60
80
100
400
450
500
550
600

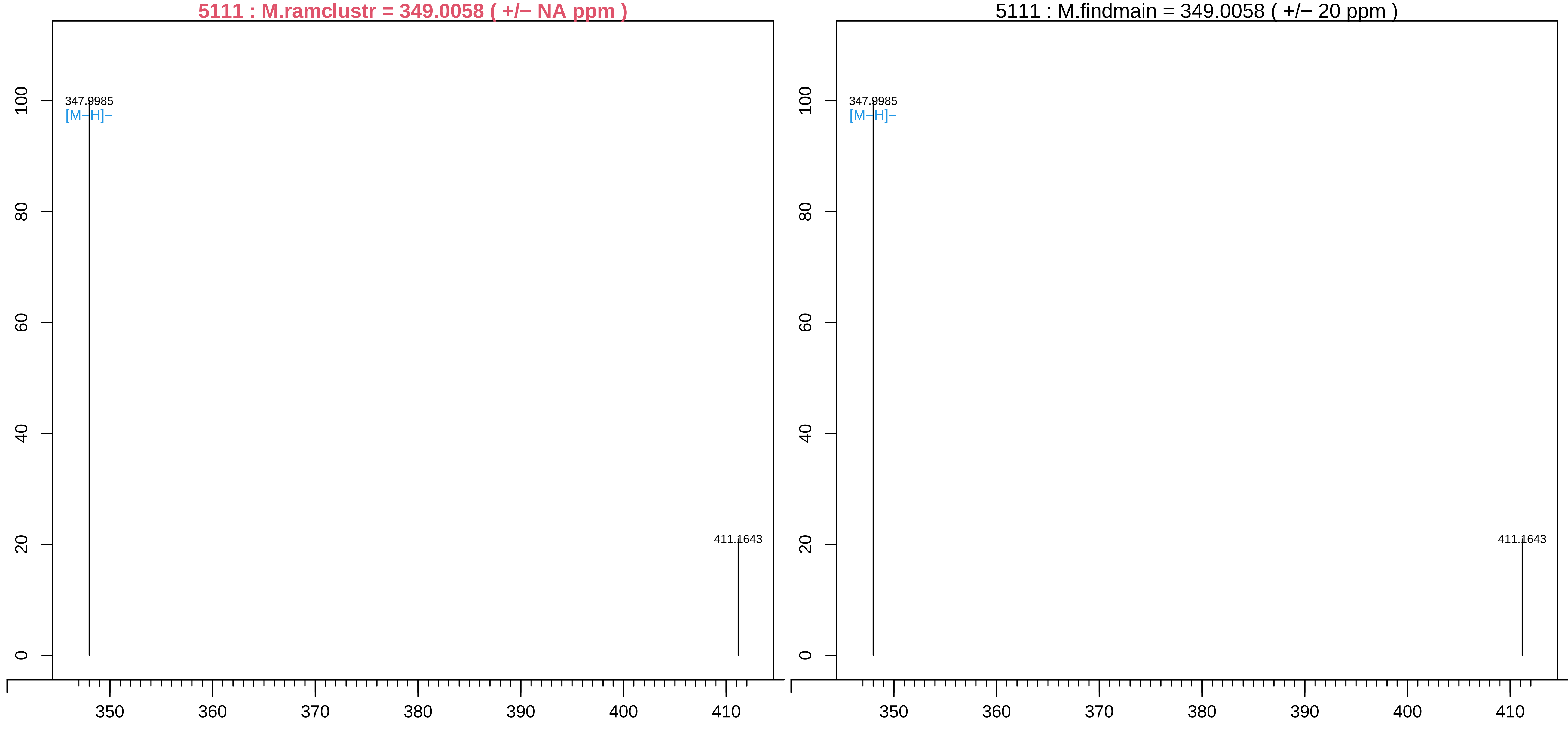
5111 : M.ramclustr = 349.0058 (+/− NA ppm)
347.9985
[M−H]−
411.1643
5111 : M.findmain = 349.0058 (+/− 20 ppm)
347.9985
[M−H]−
411.1643
0
20
40
60
80
100
350
360
370
380
390
400
410

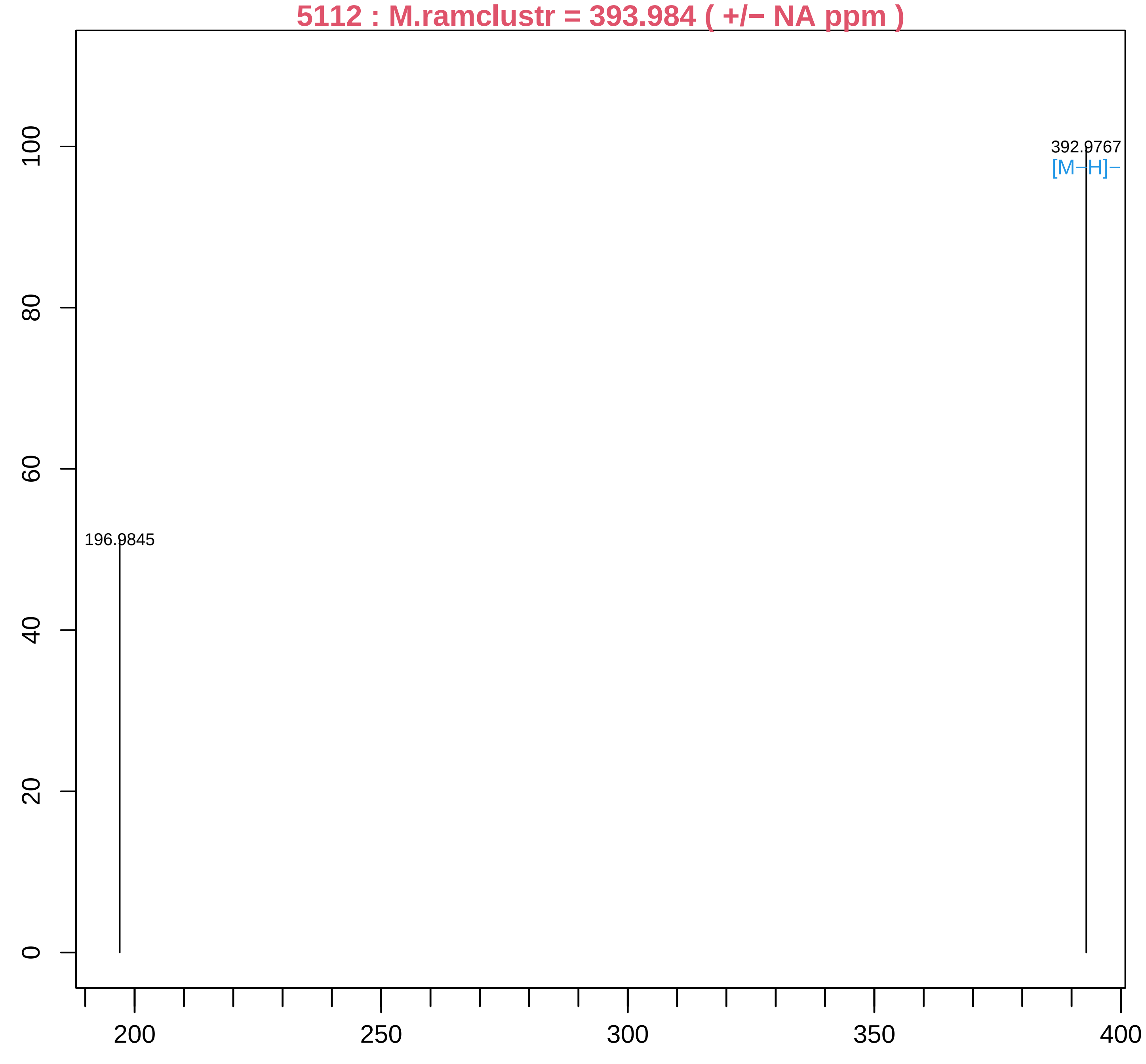
5112 : M.ramclustr = 393.984 (+/− NA ppm)
392.9767
[M−H]−
196.9845
0
20
40
60
80
100
200
250
300
350
400

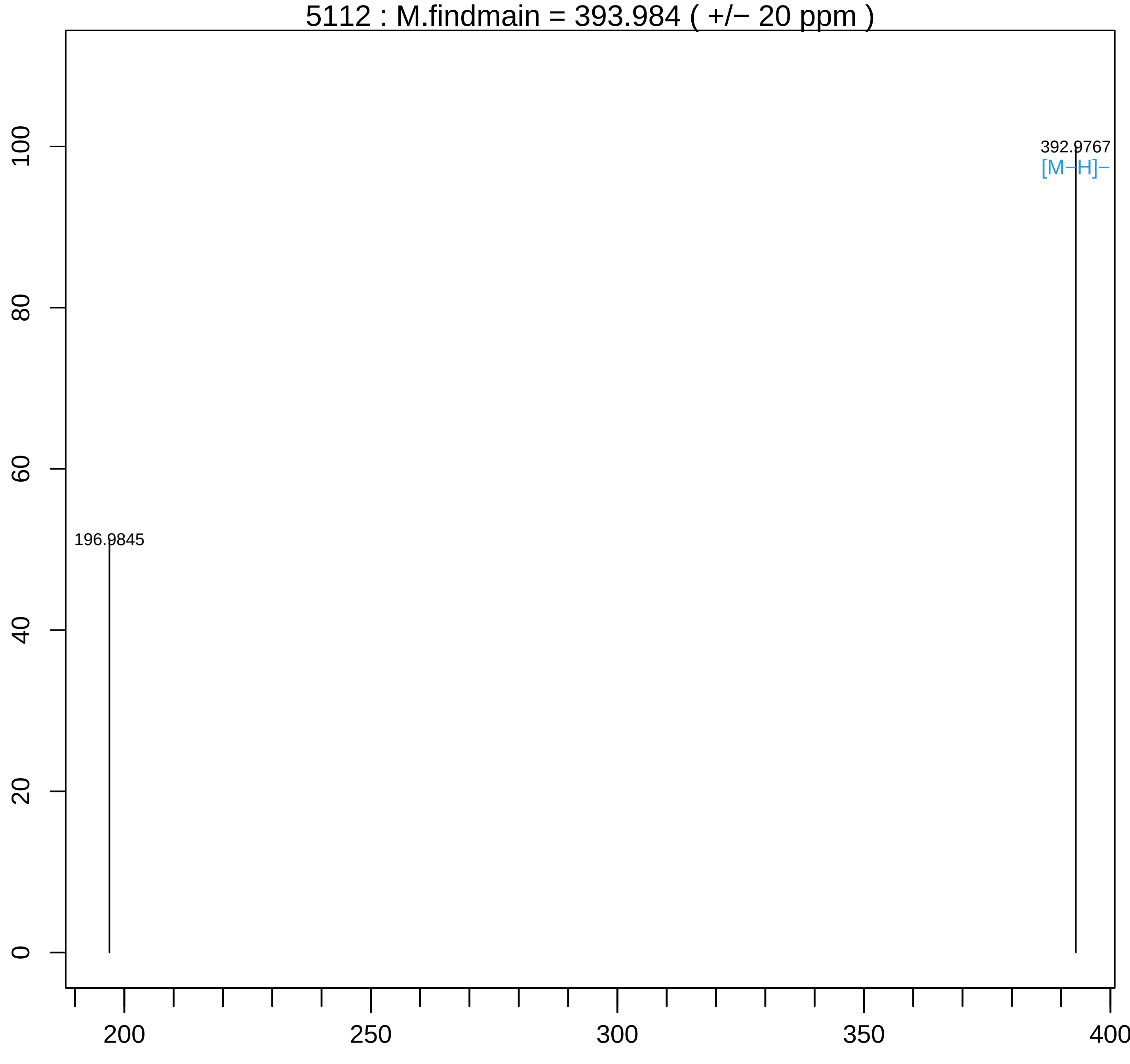
5112 : M.findmain = 393.984 (+/− 20 ppm)
392.9767
[M−H]−
196.9845
0
20
40
60
80
100
200
250
300
350
400

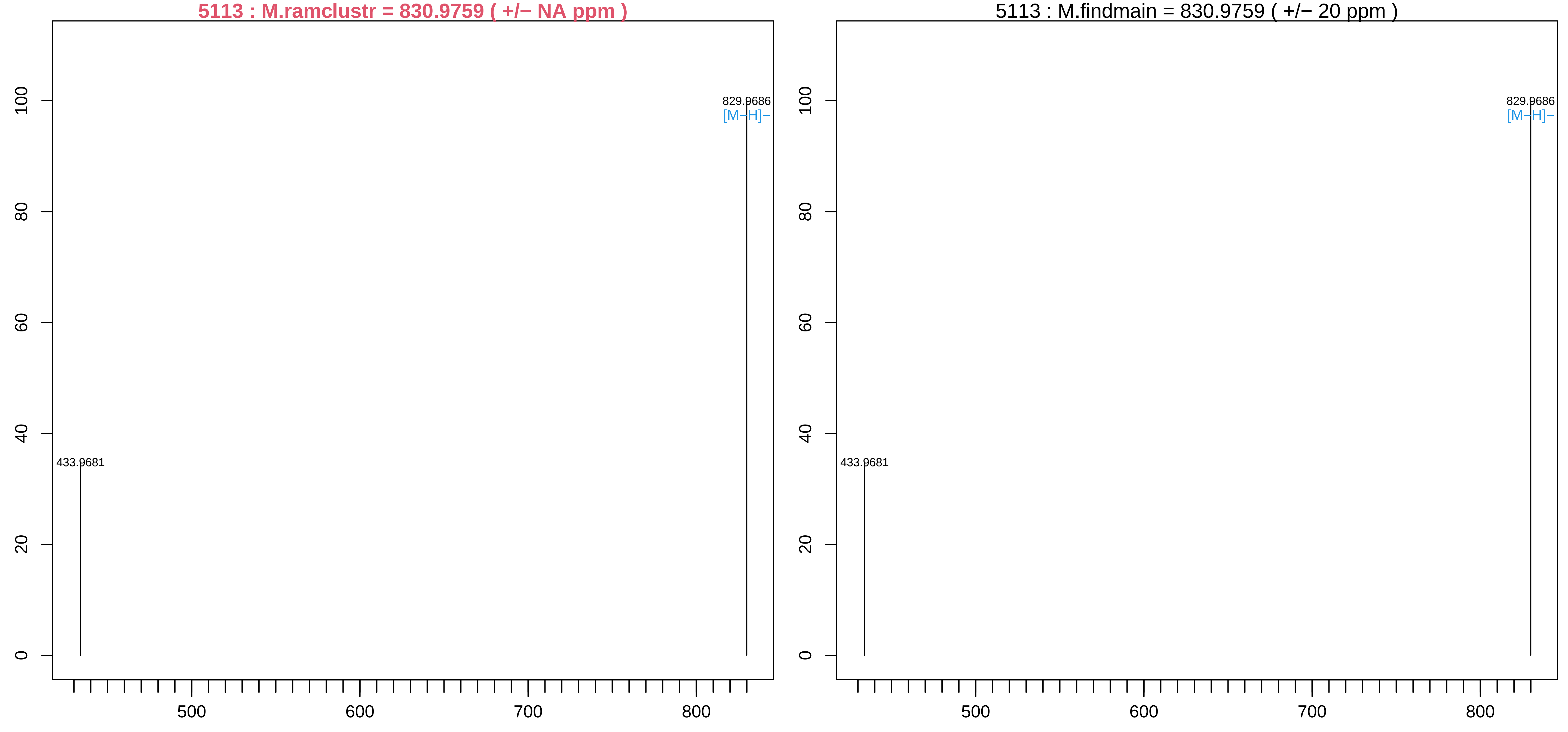
5113 : M.ramclustr = 830.9759 (+/− NA ppm)
829.9686
[M−H]−
433.9681
100
80
60
40
20
0
500
600
700
800
5113 : M.findmain = 830.9759 (+/− 20 ppm)
829.9686
[M−H]−
433.9681
100
80
60
40
20
0
500
600
700
800

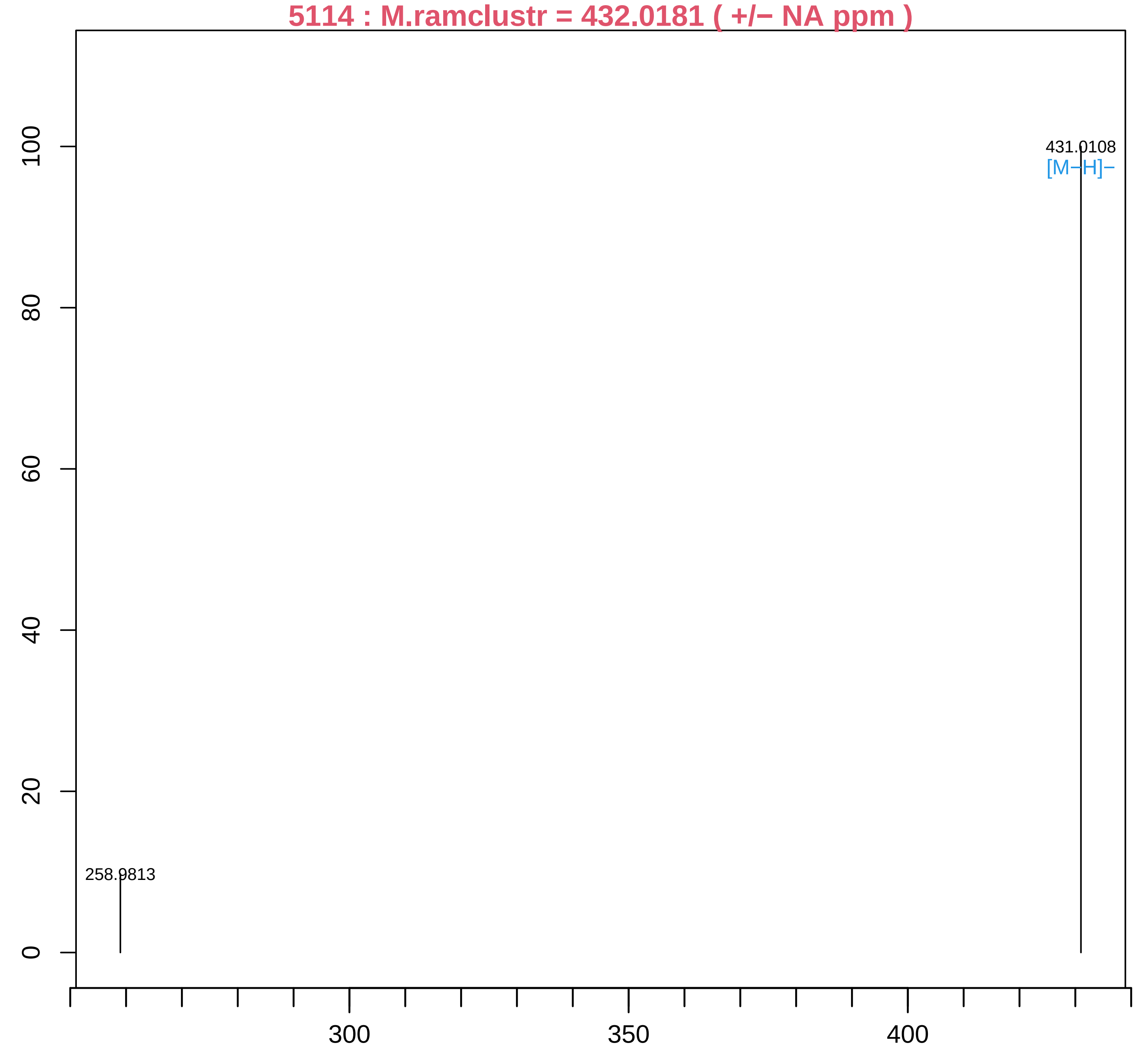
5114 : M.ramclustr = 432.0181 (+/− NA ppm)
431.0108
[M−H]−
258.9813
0
20
40
60
80
100
300
350
400

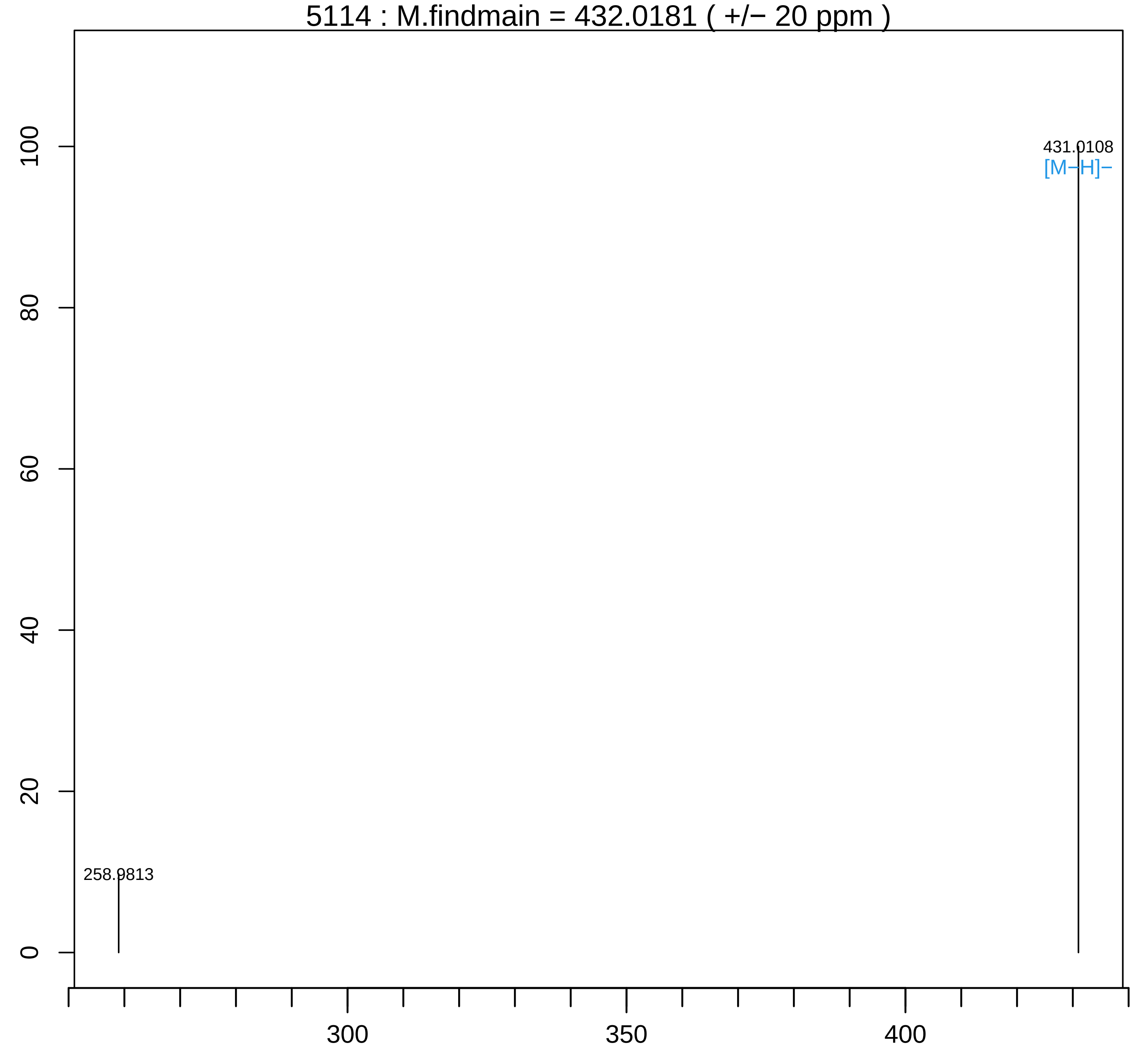
5114 : M.findmain = 432.0181 (+/− 20 ppm)
431.0108
[M−H]−
258.9813
0
20
40
60
80
100
300
350
400

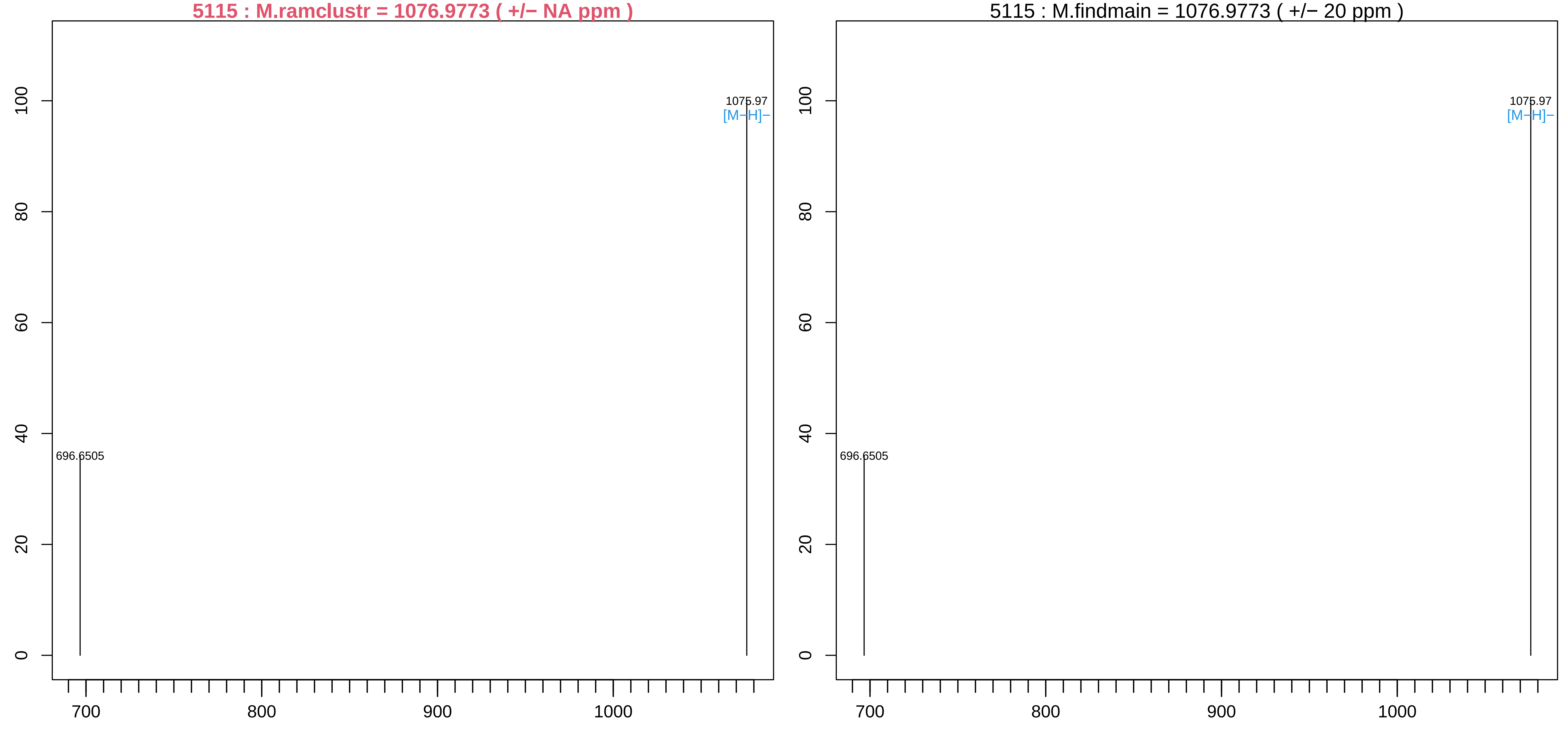
5115 : M.ramclustr = 1076.9773 (+/− NA ppm)
5115 : M.findmain = 1076.9773 (+/− 20 ppm)
1076.97
[M−H]−
696.6505
0
20
40
60
80
100
700
800
900
1000

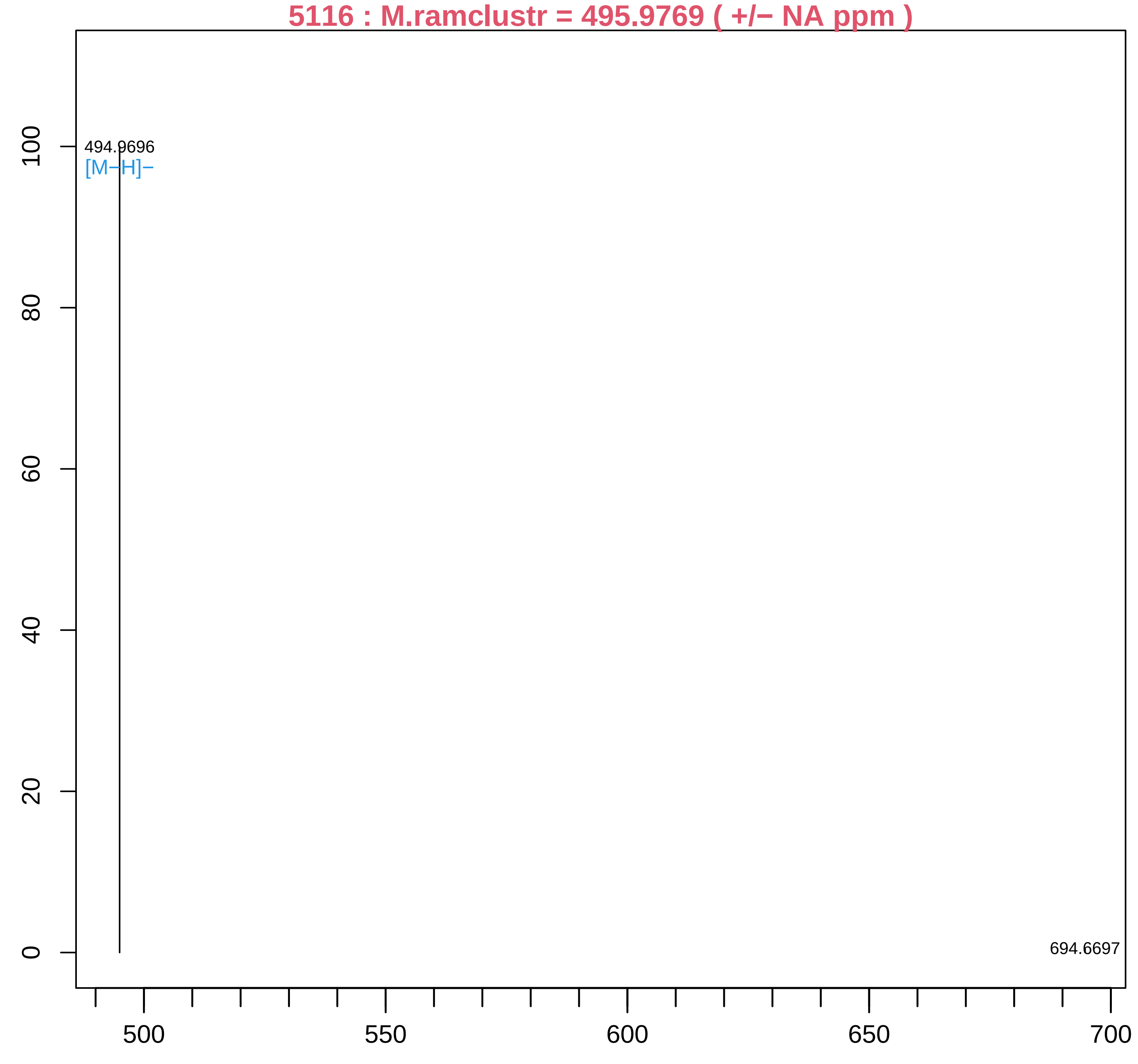
5116 : M.ramclustr = 495.9769 (+/− NA ppm)
494.9696
[M−H]−
694.6697
0
20
40
60
80
100
500
550
600
650
700

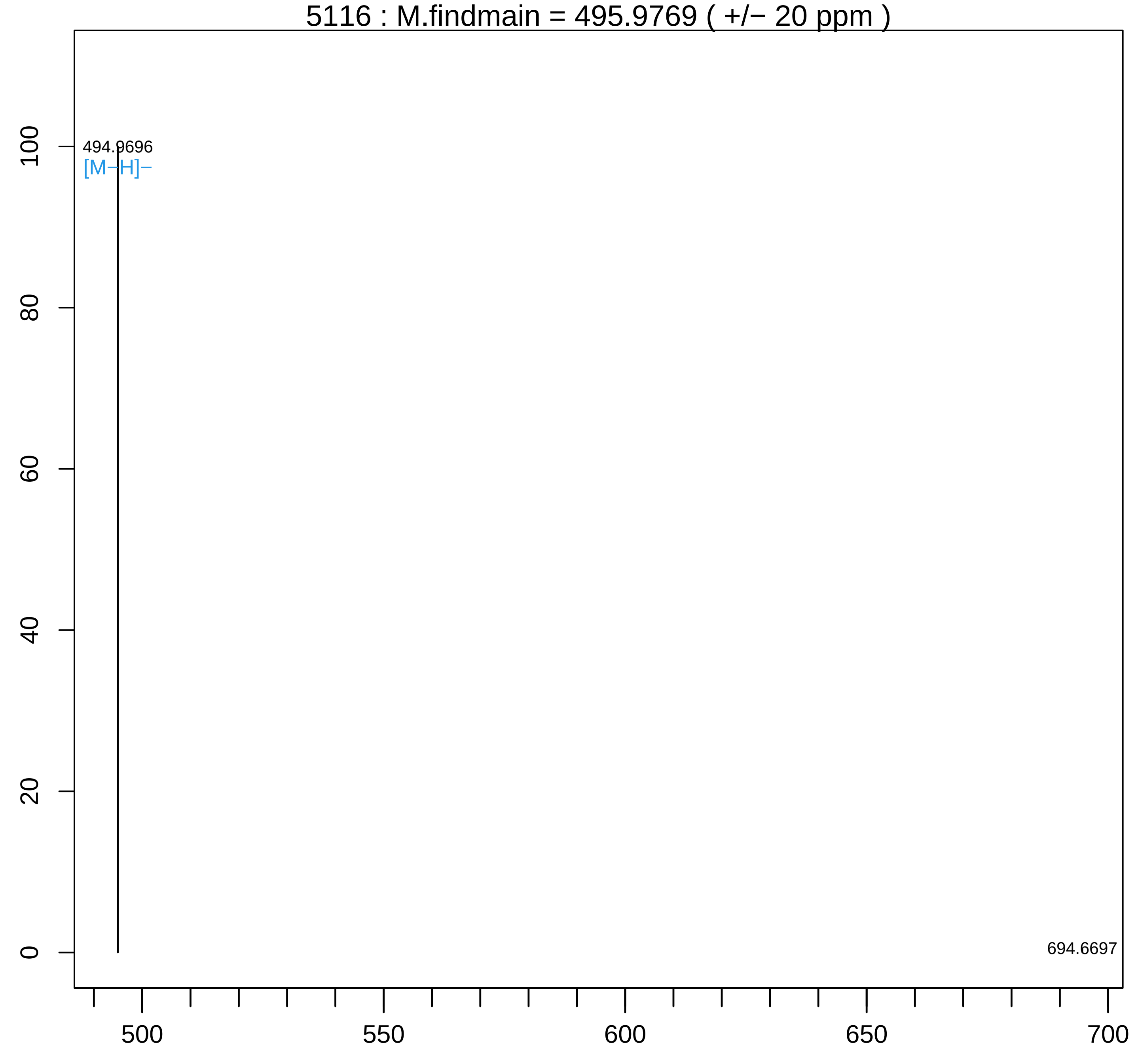
5116 : M.findmain = 495.9769 (+/− 20 ppm)
494.9696
[M−H]−
694.6697
0
20
40
60
80
100
500
550
600
650
700

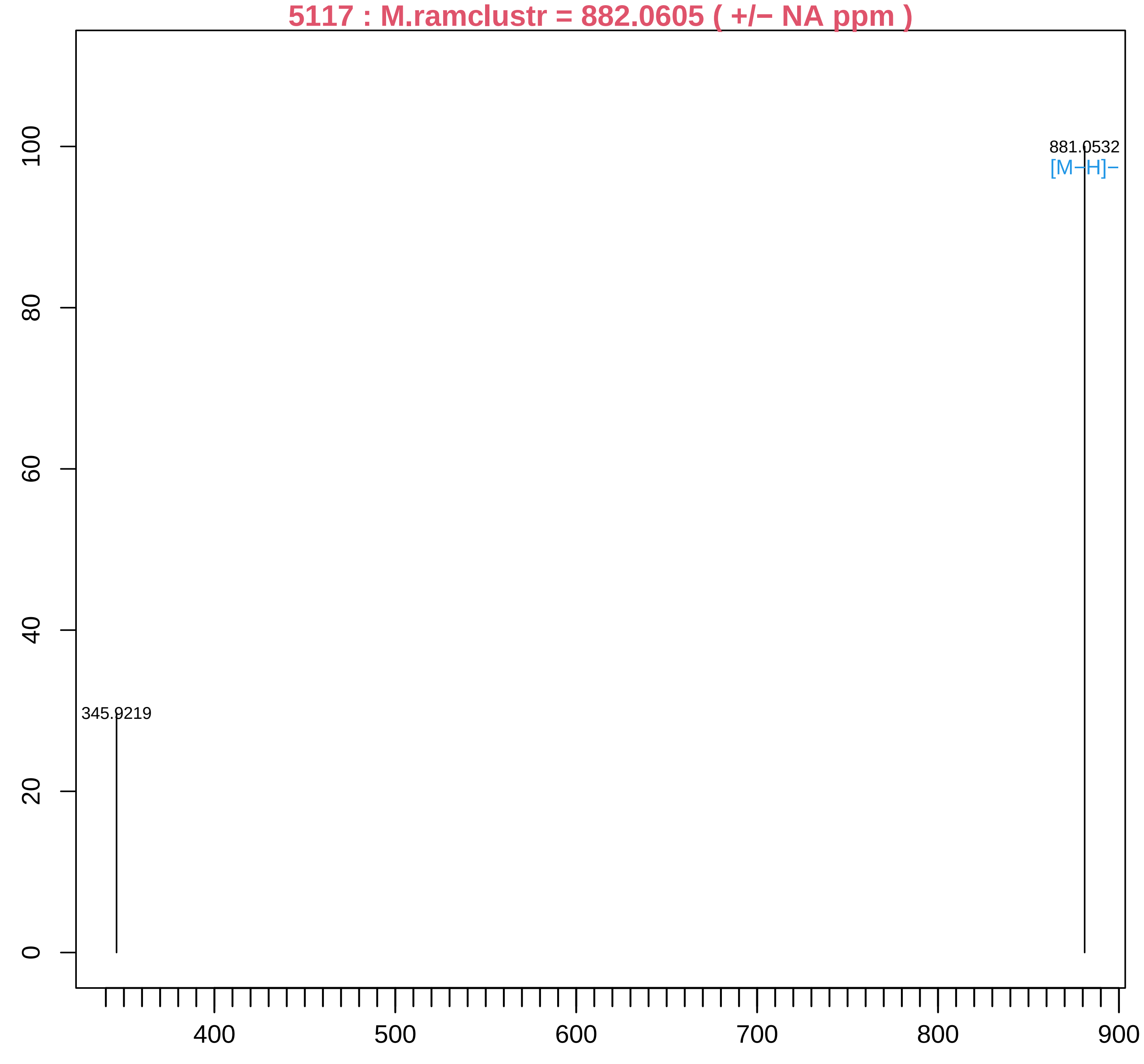
5117 : M.ramclustr = 882.0605 (+/− NA ppm)
881.0532
[M−H]−
345.9219
0
20
40
60
80
100
400
500
600
700
800
900

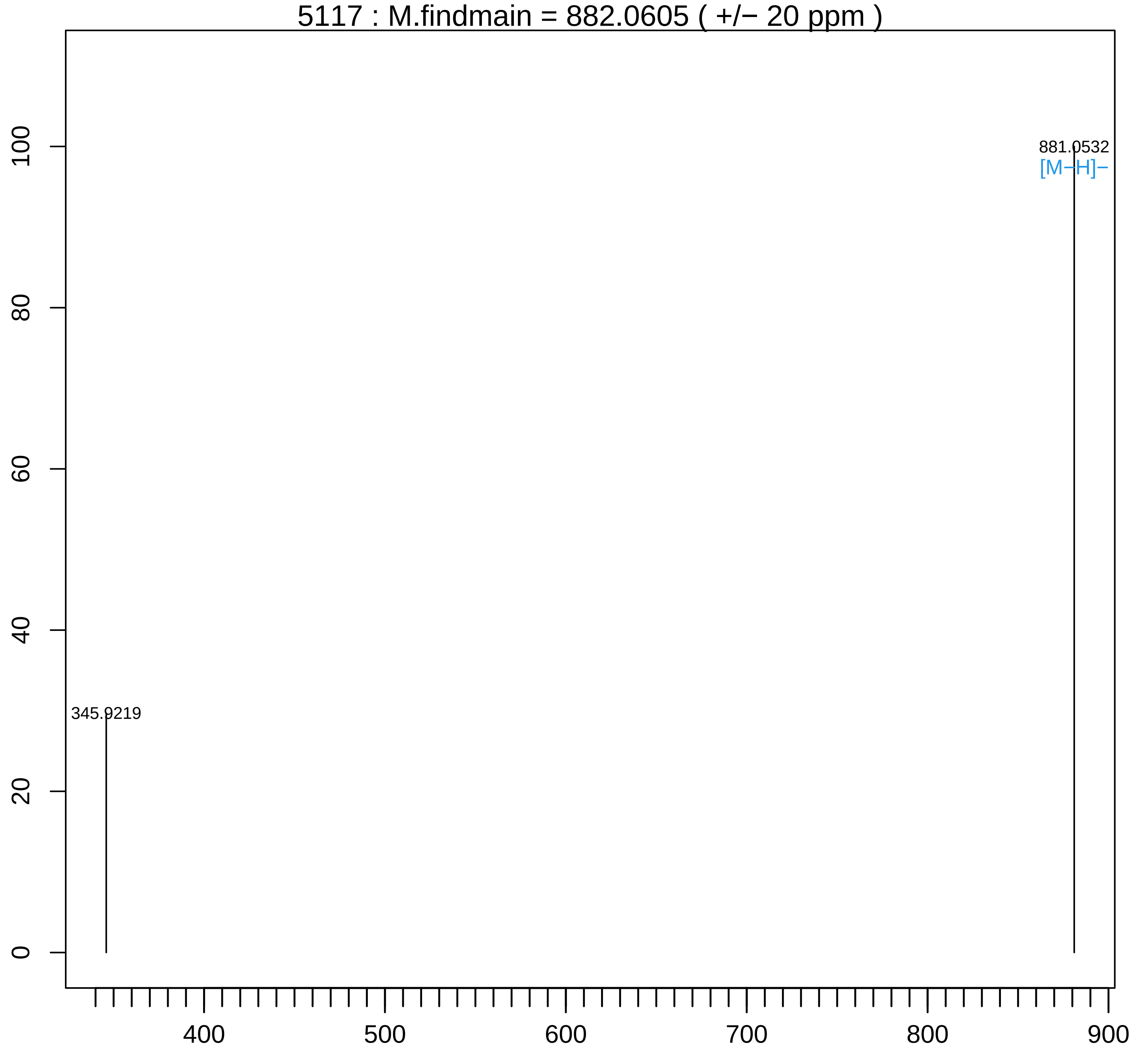
5117 : M.findmain = 882.0605 (+/− 20 ppm)
881.0532
[M−H]−
345.9219
0
20
40
60
80
100
400
500
600
700
800
900

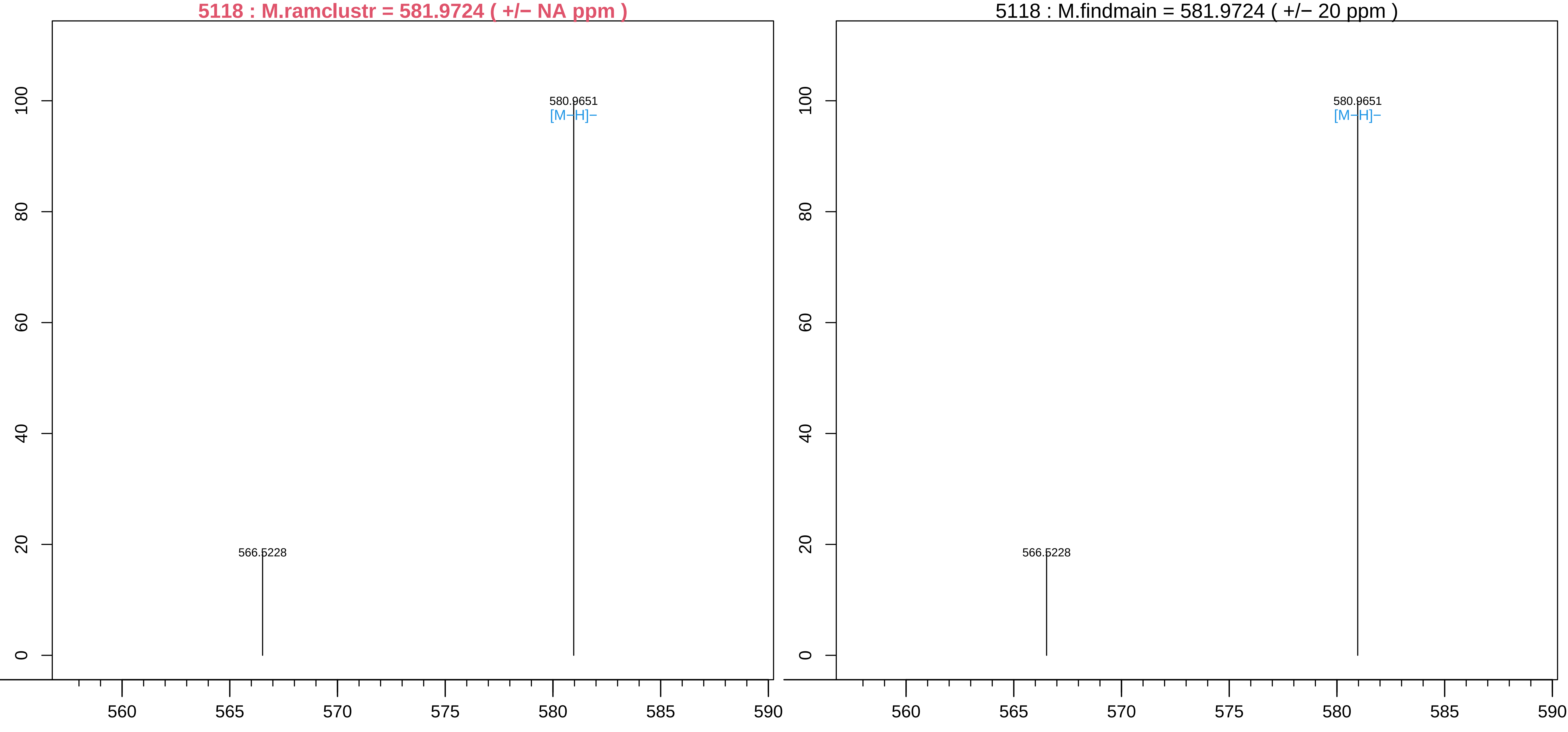
5118 : M.ramclustr = 581.9724 (+/− NA ppm)
580.9651
[M−H]−
566.5228
0
20
40
60
80
100
560
565
570
575
580
585
590
5118 : M.findmain = 581.9724 (+/− 20 ppm)
580.9651
[M−H]−
566.5228
0
20
40
60
80
100
560
565
570
575
580
585
590

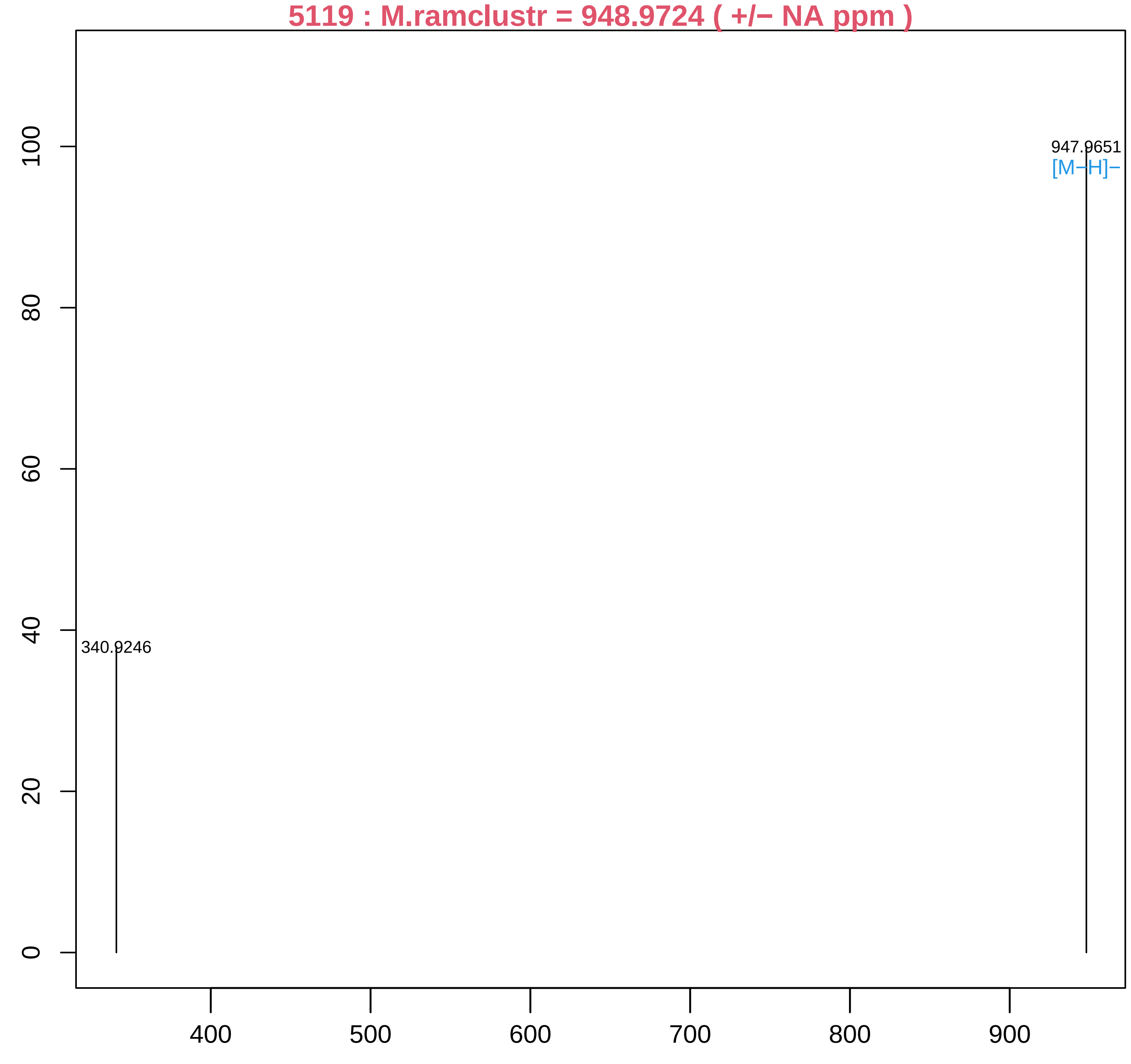
5119 : M.ramclustr = 948.9724 (+/− NA ppm)
947.9651
[M−H]−
340.9246
0
20
40
60
80
100
400
500
600
700
800
900

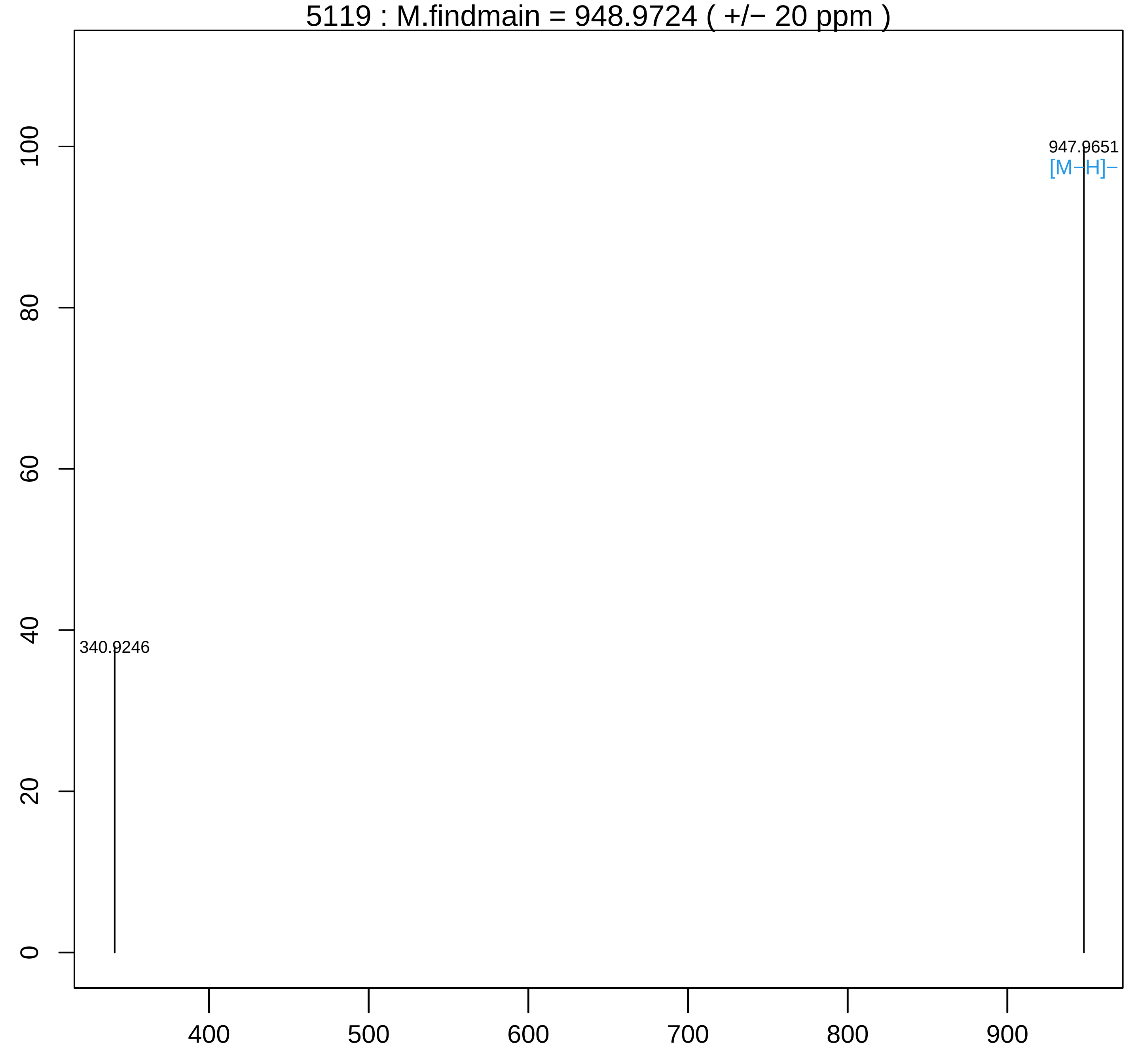
5119 : M.findmain = 948.9724 (+/− 20 ppm)
947.9651
[M−H]−
340.9246
0
20
40
60
80
100
400
500
600
700
800
900

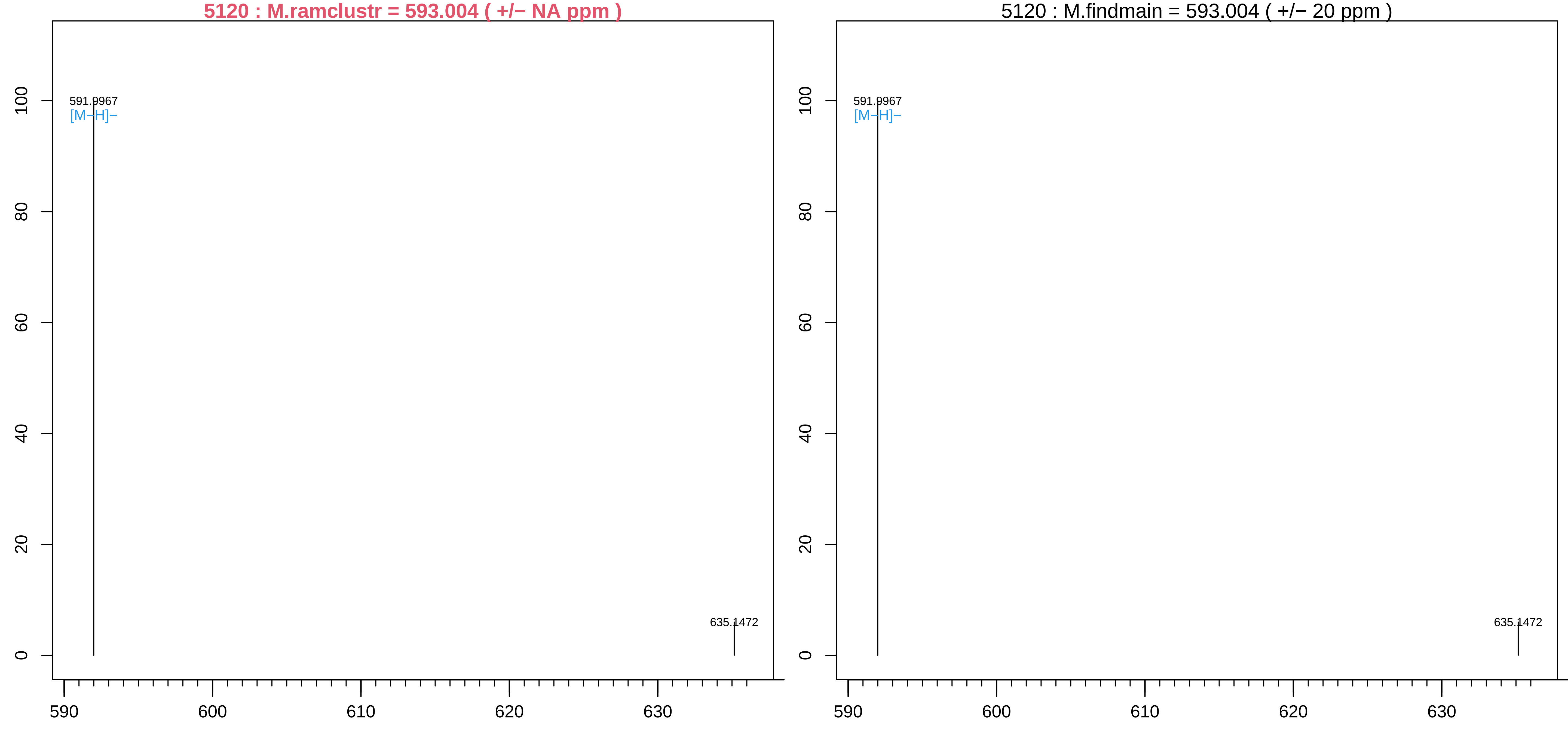
5120 : M.ramclustr = 593.004 (+/− NA ppm)
591.9967
[M−H]−
635.1472
5120 : M.findmain = 593.004 (+/− 20 ppm)
591.9967
[M−H]−
635.1472
0
20
40
60
80
100
590
600
610
620
630

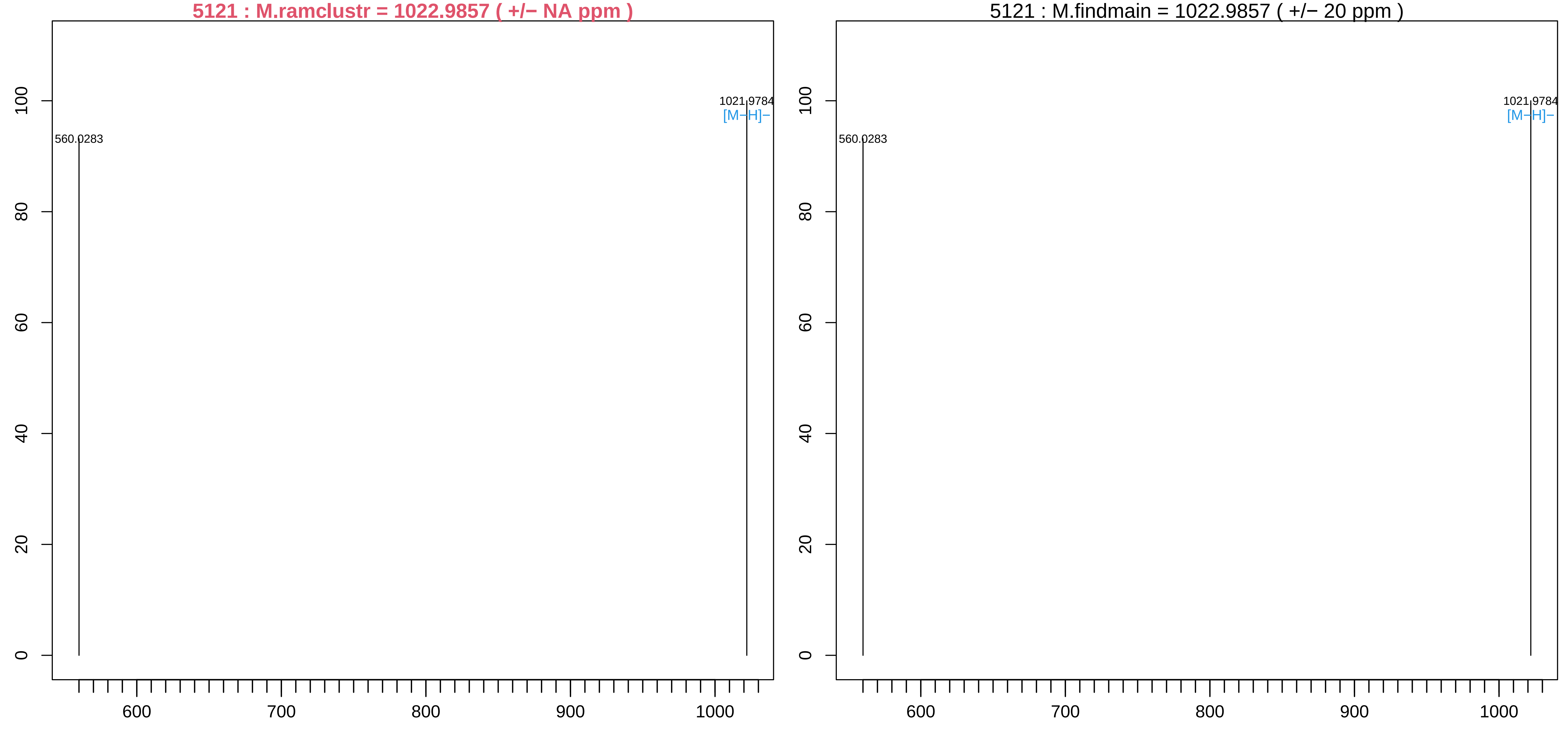
5121 : M.ramclustr = 1022.9857 (+/− NA ppm)
5121 : M.findmain = 1022.9857 (+/− 20 ppm)
1021.9784
[M−H]−
560.0283
1021.9784
[M−H]−
560.0283
0
20
40
60
80
100
600
700
800
900
1000

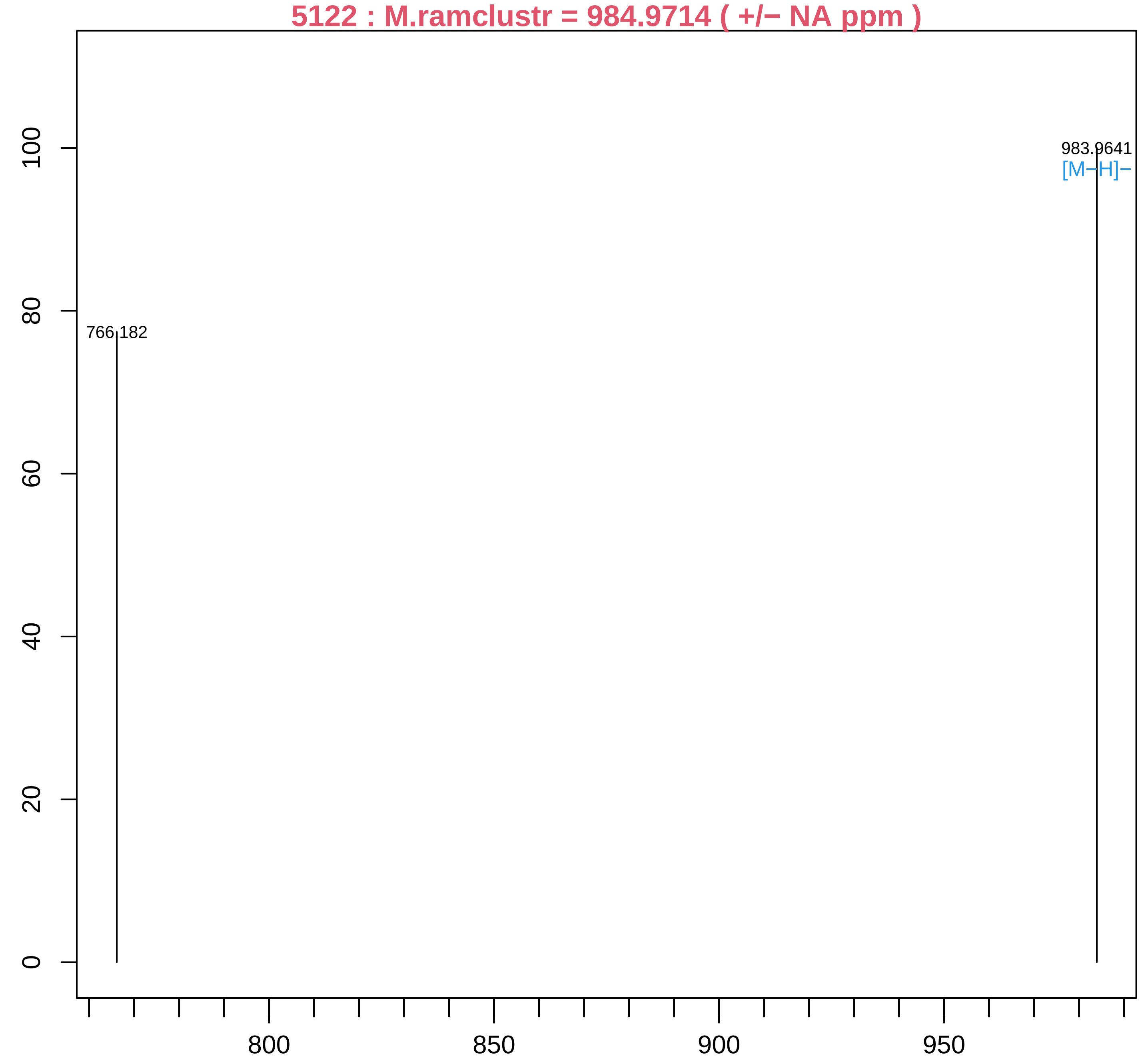
5122 : M.ramclustr = 984.9714 (+/− NA ppm)
983.9641
[M−H]−
766.182
0
20
40
60
80
100
800
850
900
950

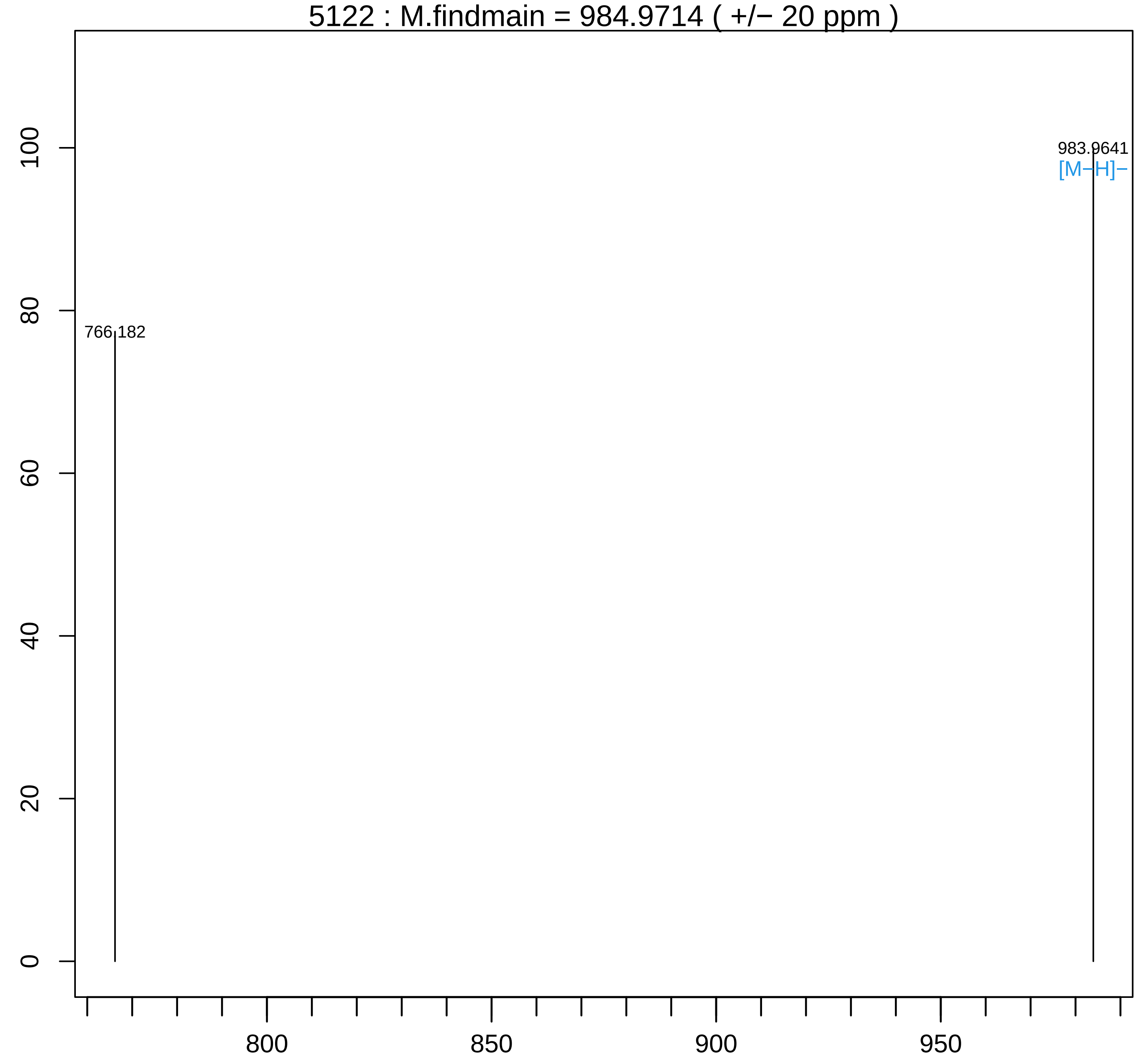
5122 : M.findmain = 984.9714 (+/− 20 ppm)
983.9641
[M−H]−
766.182
0
20
40
60
80
100
800
850
900
950

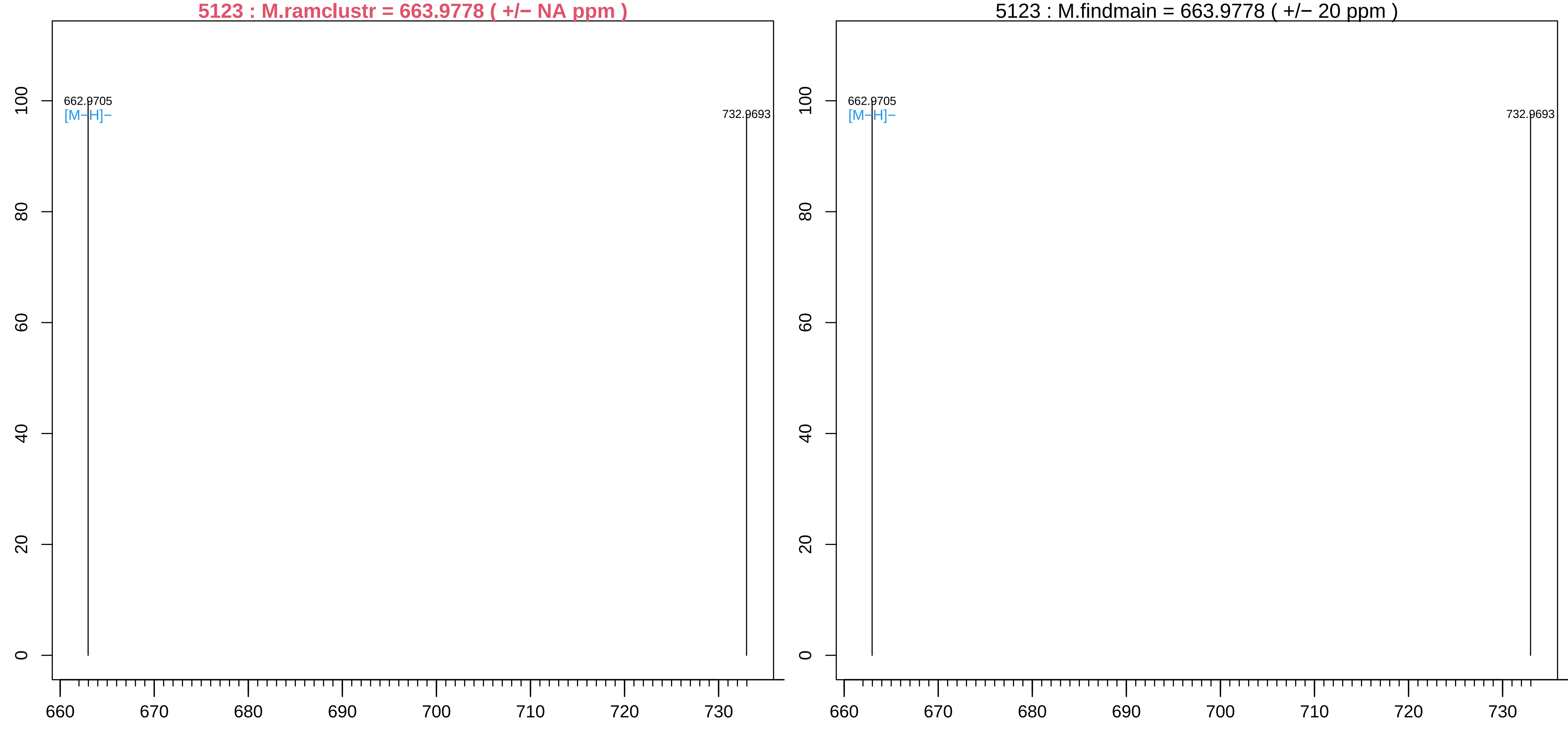
5123 : M.ramclustr = 663.9778 (+/− NA ppm)
662.9705
[M−H]−
732.9693
5123 : M.findmain = 663.9778 (+/− 20 ppm)
662.9705
[M−H]−
732.9693
0
20
40
60
80
100
660
670
680
690
700
710
720
730

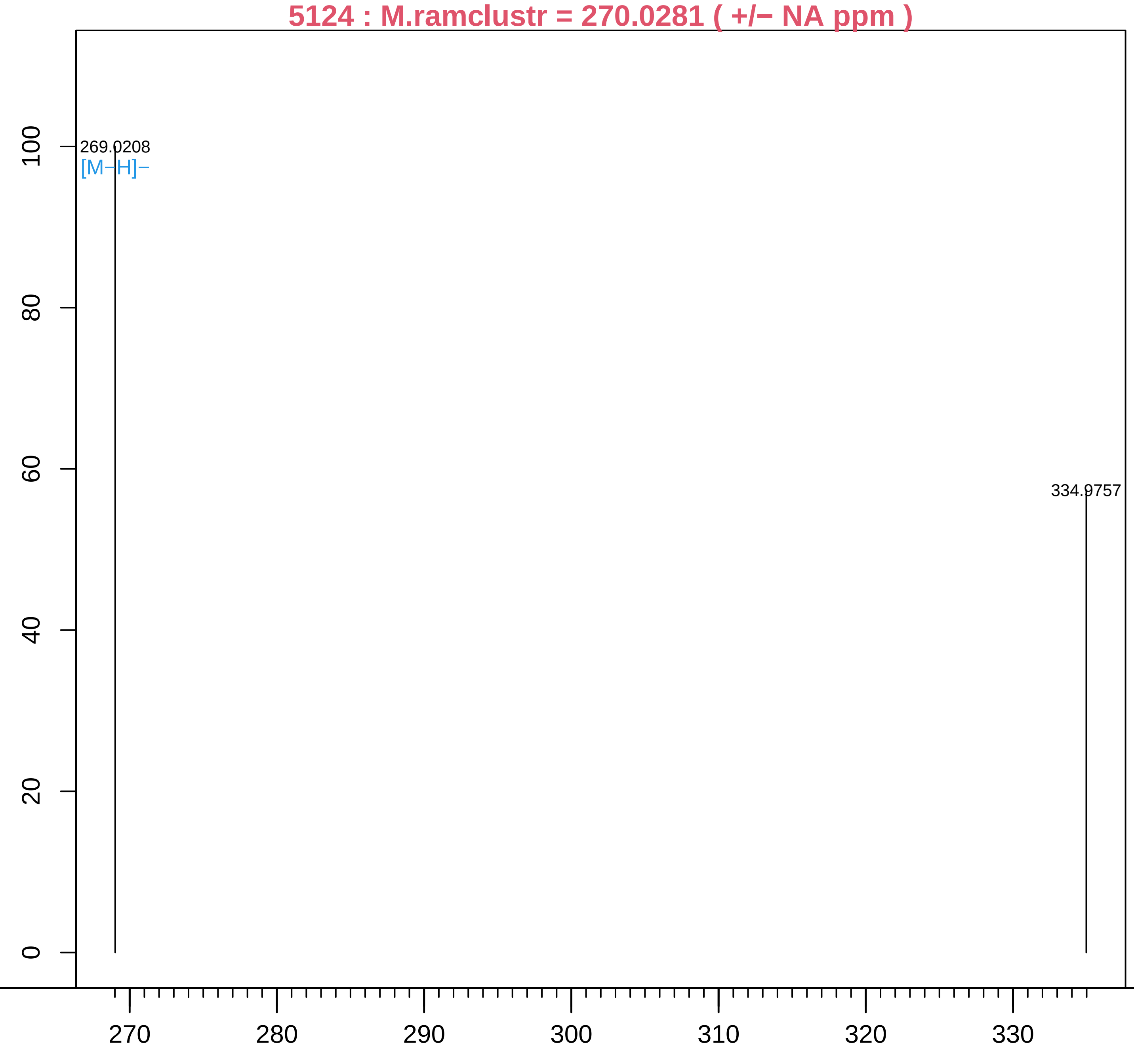

5124 : M.ramclustr = 270.0281 (+/− NA ppm)
269.0208
[M−H]−
334.9757
0
20
40
60
80
100
270
280
290
300
310
320
330

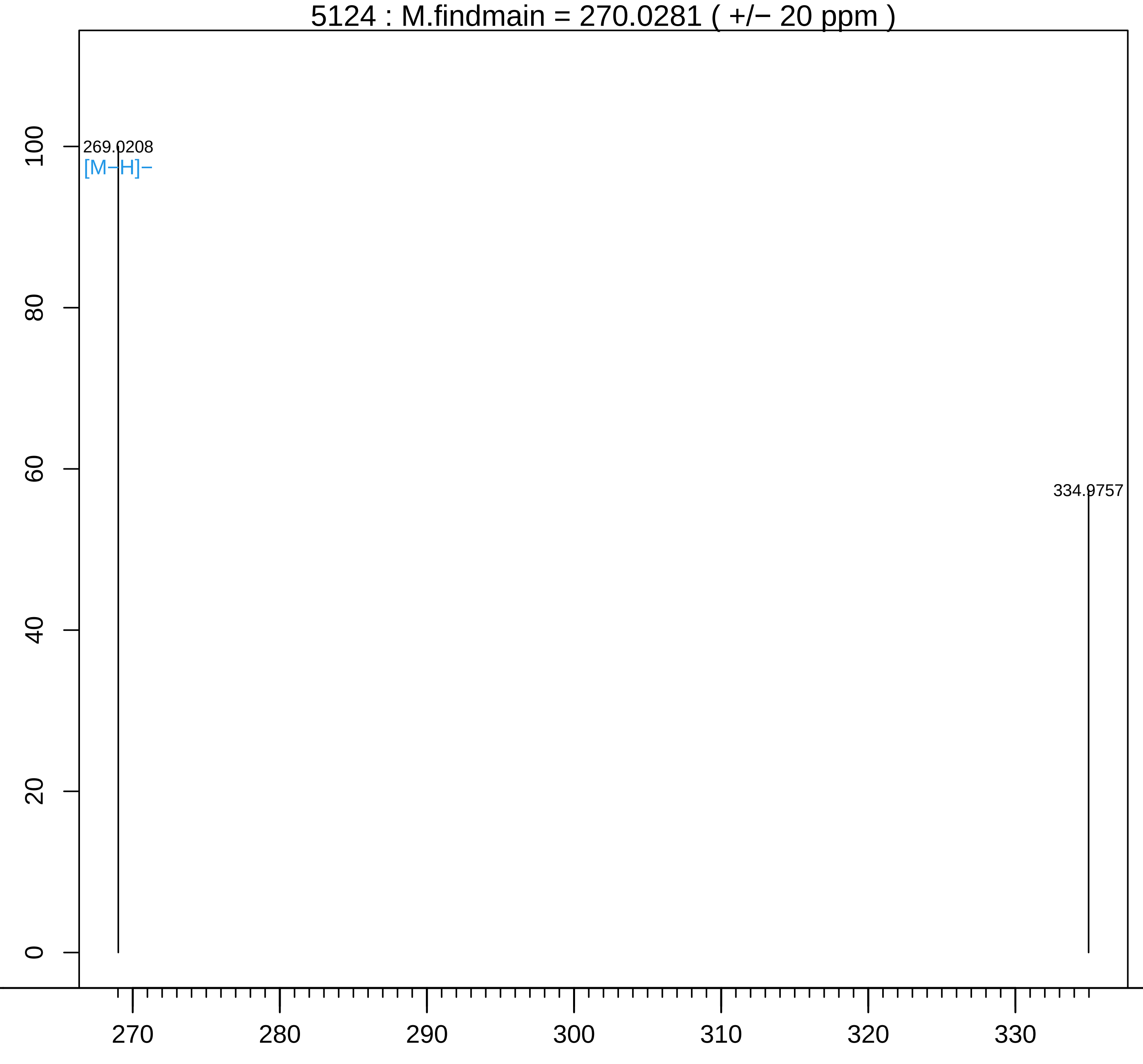

5124 : M.findmain = 270.0281 (+/− 20 ppm)
269.0208
[M−H]−
334.9757
0
20
40
60
80
100
270
280
290
300
310
320
330

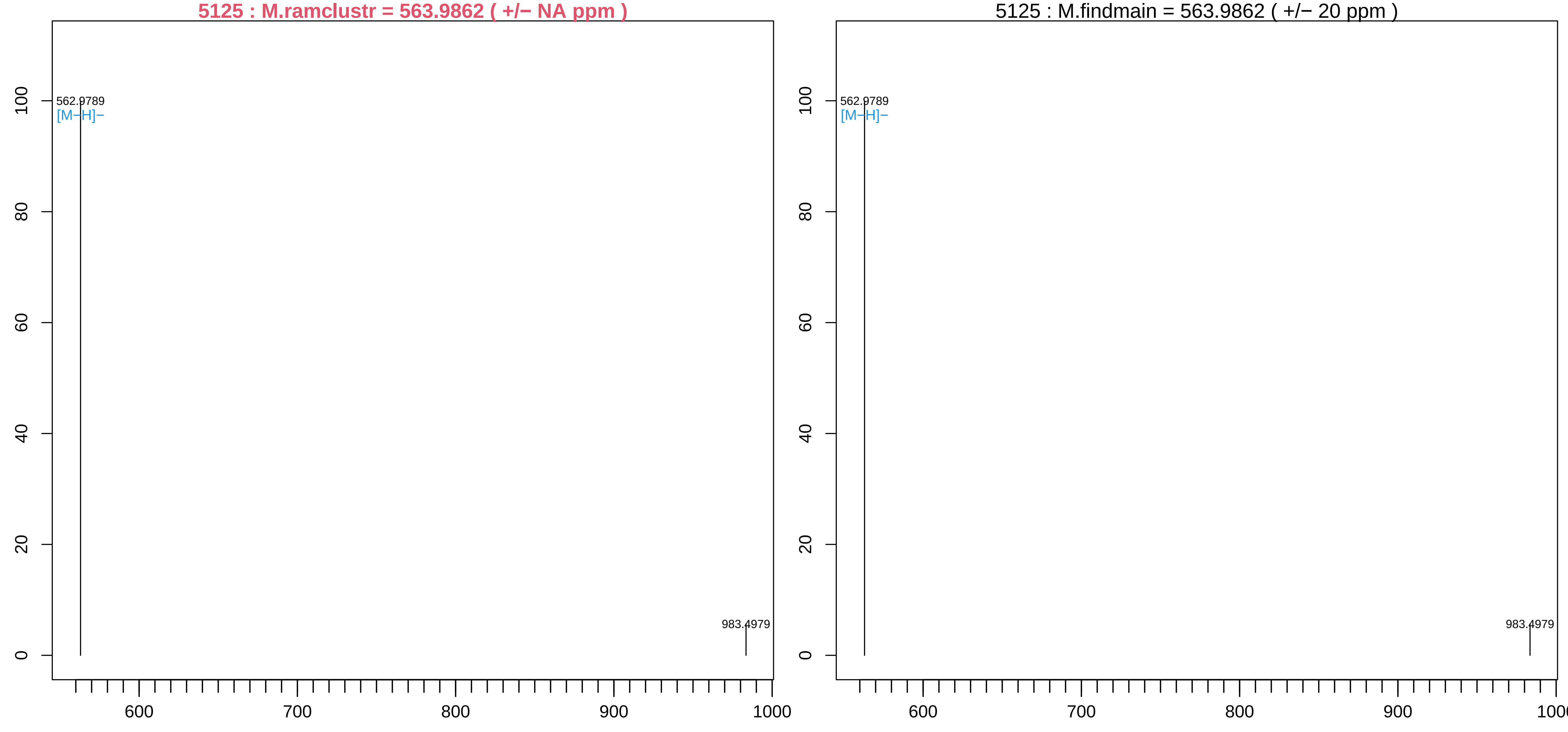

5125 : M.ramclustr = 563.9862 (+/− NA ppm)
562.9789
[M−H]−
983.4979
5125 : M.findmain = 563.9862 (+/− 20 ppm)
562.9789
[M−H]−
983.4979
0
20
40
60
80
100
600
700
800
900
1000

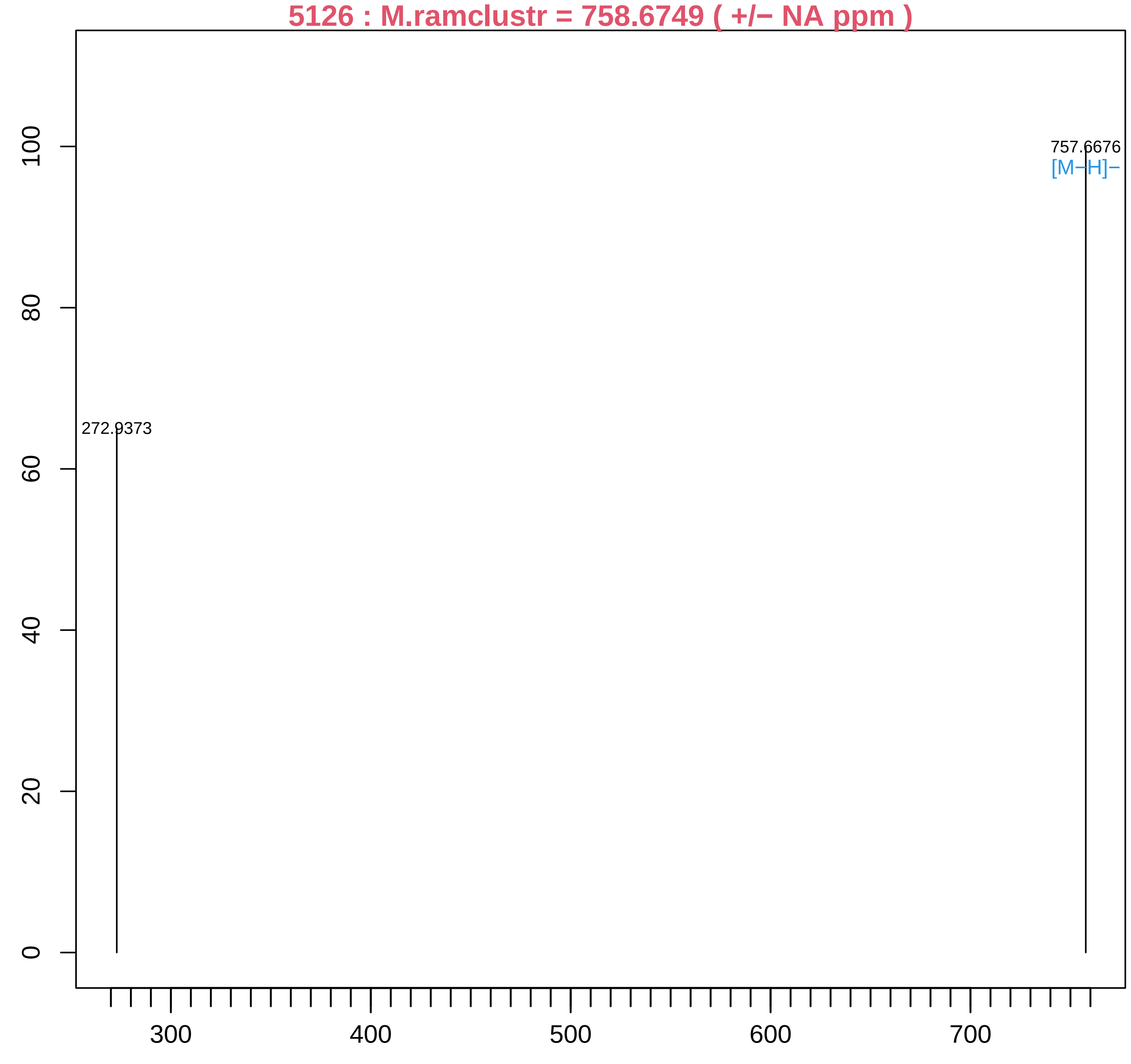
5126 : M.ramclustr = 758.6749 (+/− NA ppm)
272.9373
757.6676
[M−H]−
0
20
40
60
80
100
300
400
500
600
700

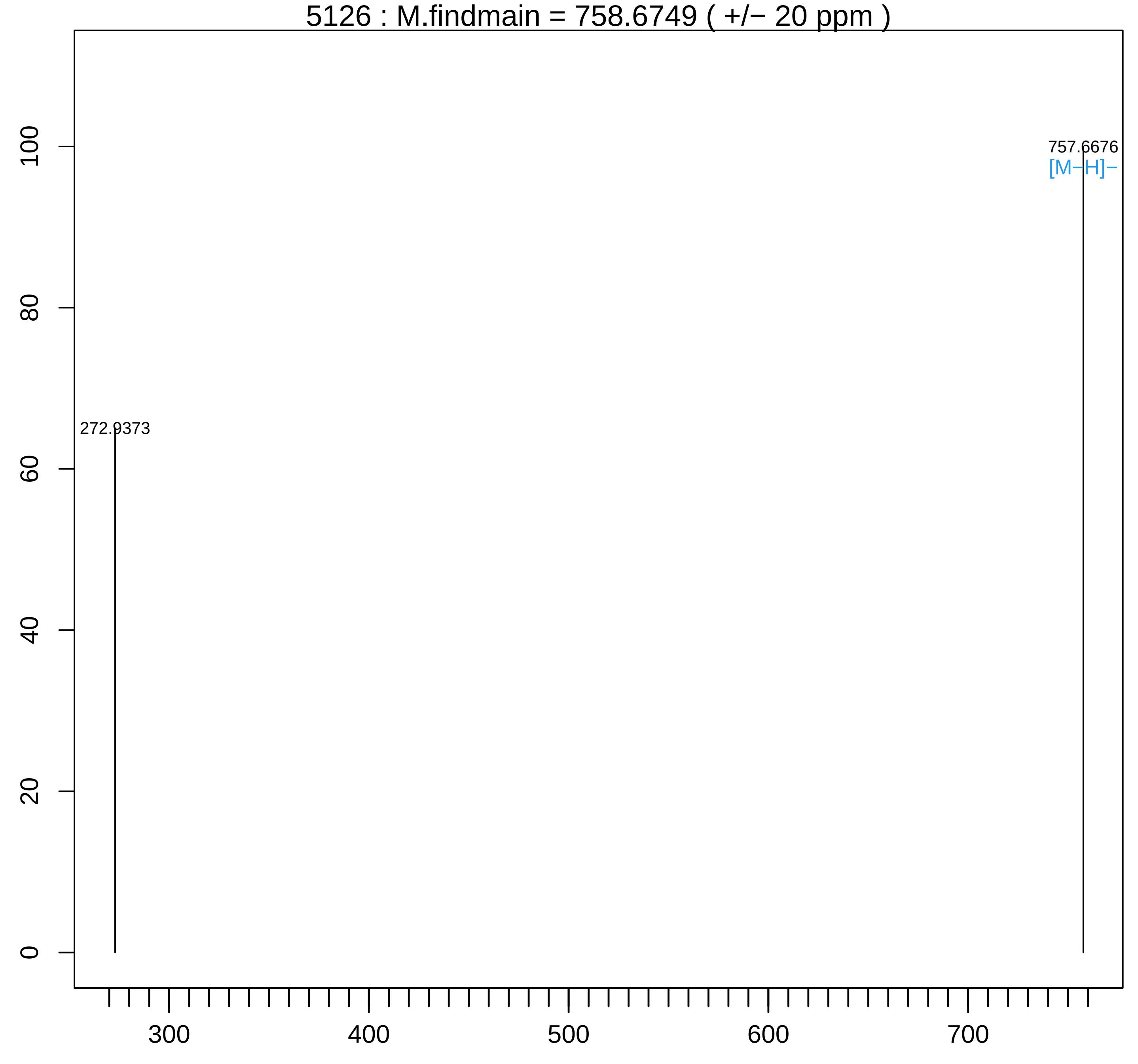
5126 : M.findmain = 758.6749 (+/− 20 ppm)
272.9373
757.6676
[M−H]−
0
20
40
60
80
100
300
400
500
600
700

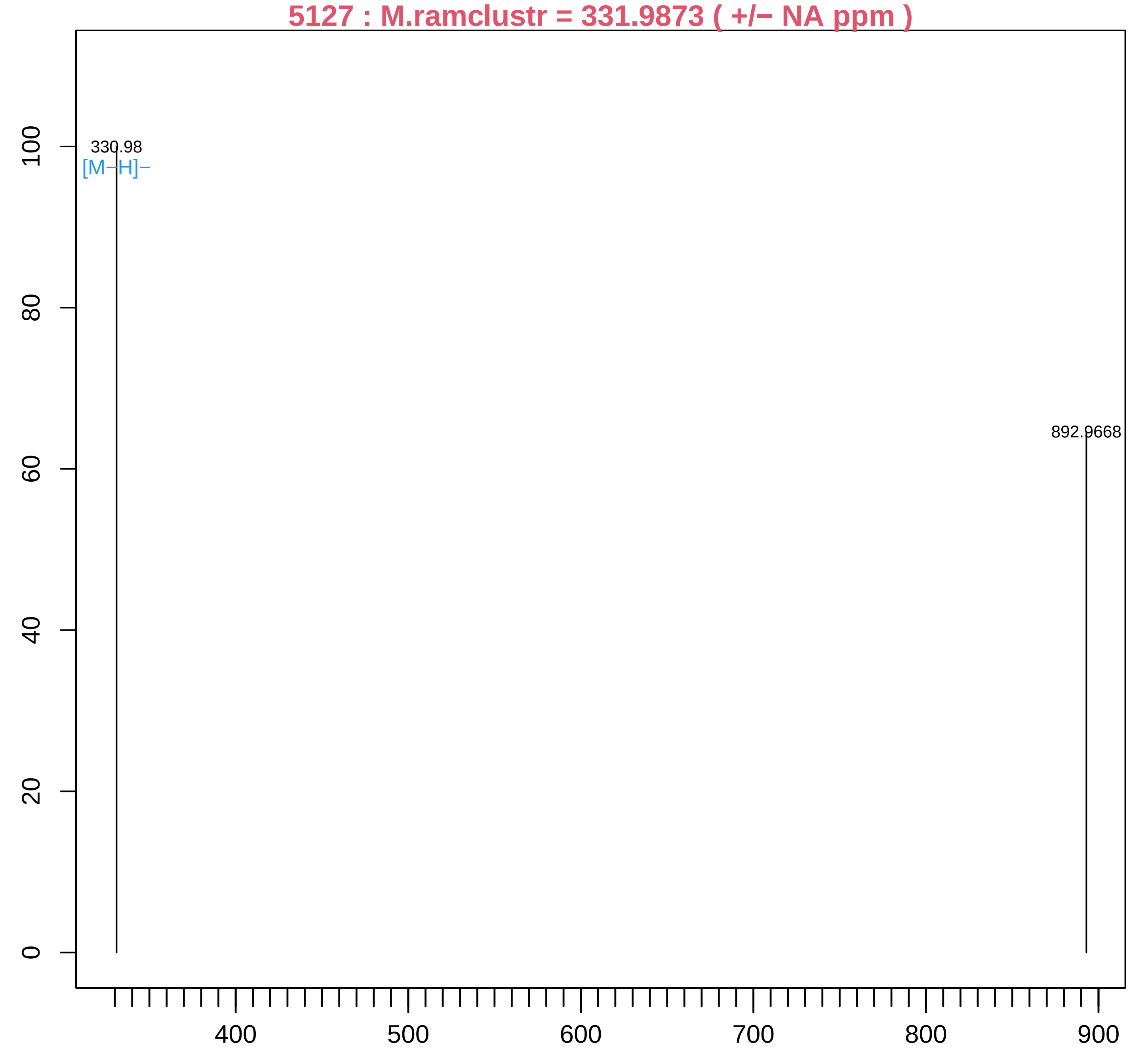
5127 : M.ramclustr = 331.9873 (+/− NA ppm)
330.98
[M−H]−
892.9668
0
20
40
60
80
100
400
500
600
700
800
900

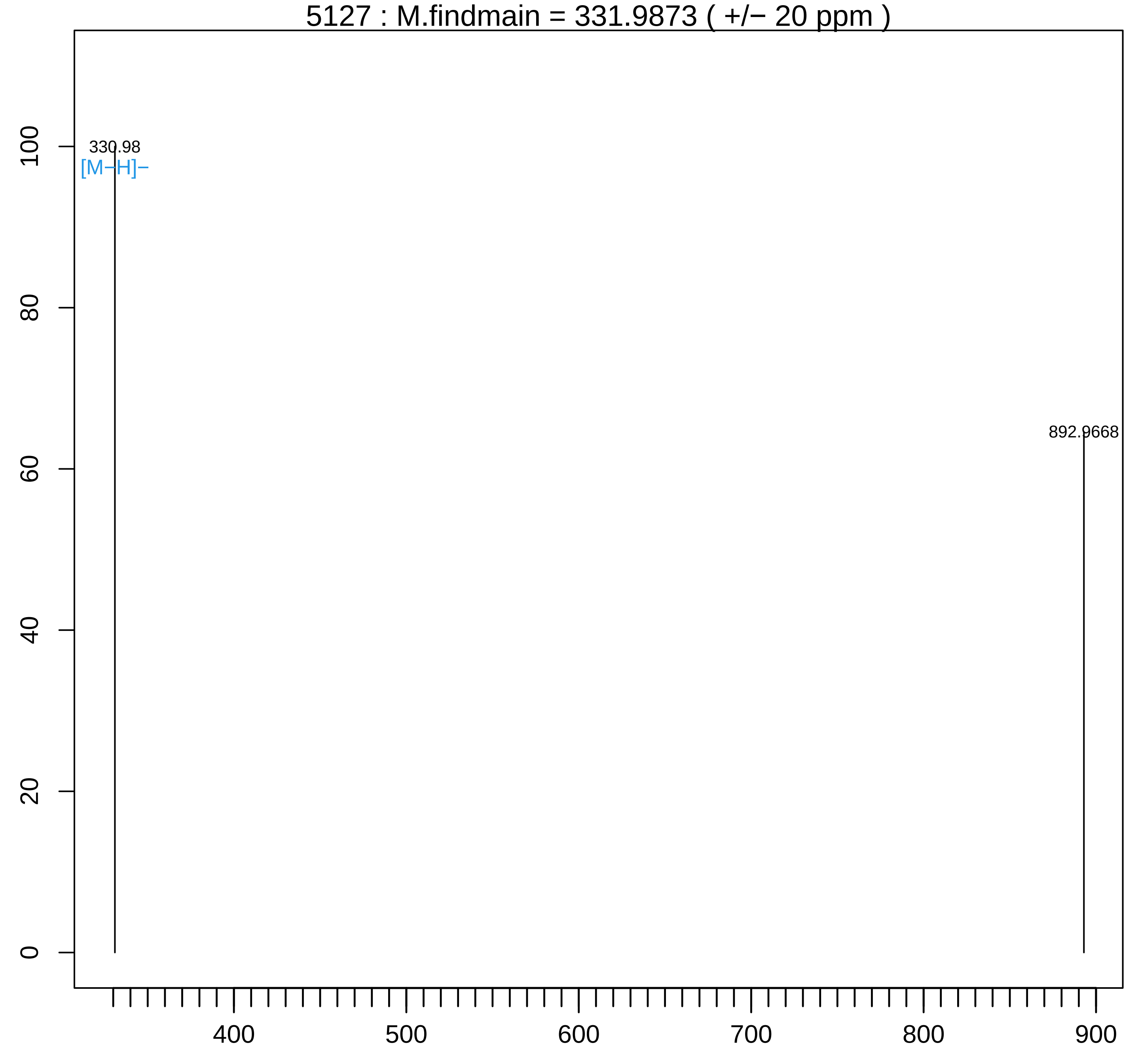
5127 : M.findmain = 331.9873 (+/− 20 ppm)
330.98
[M−H]−
892.9668
0
20
40
60
80
100
400
500
600
700
800
900

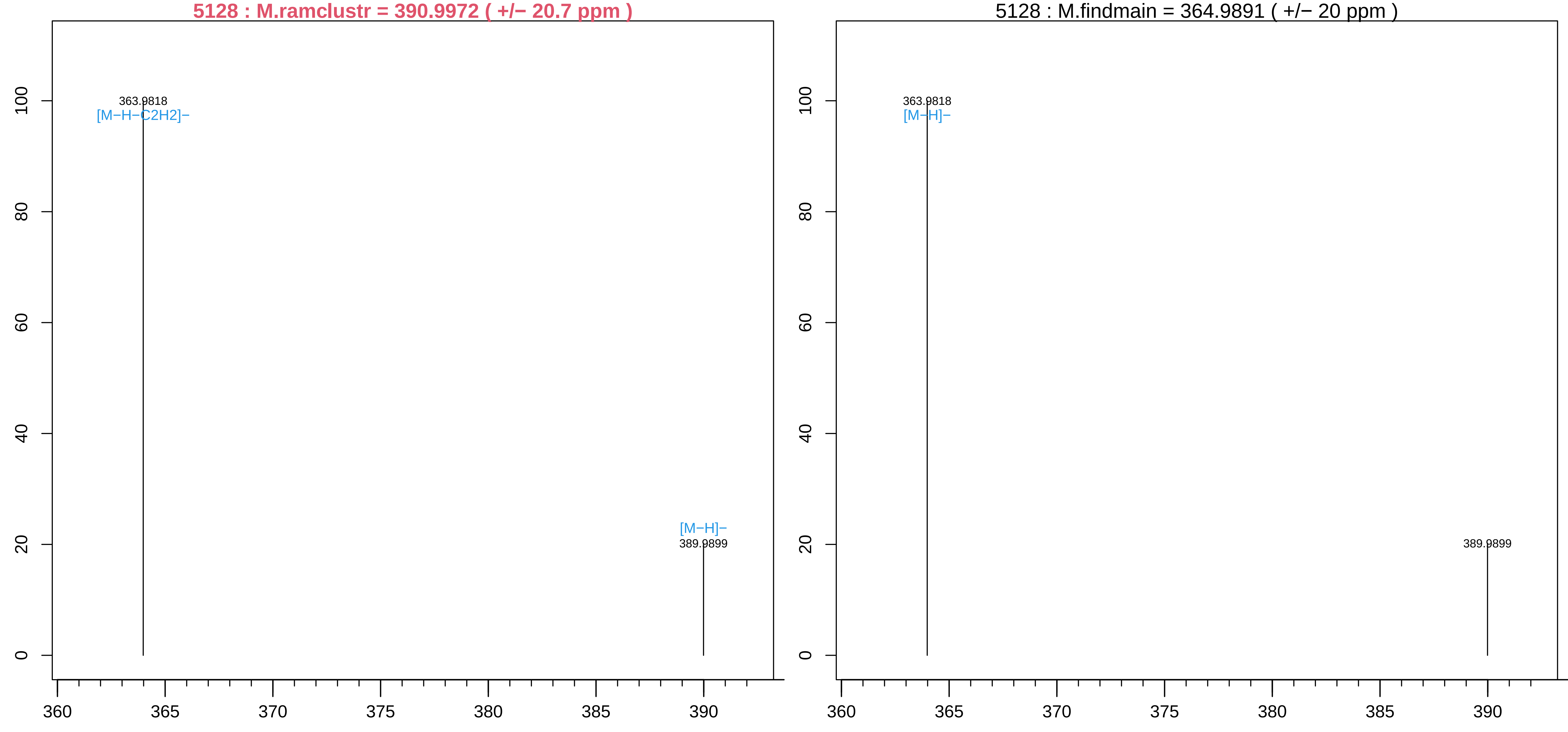
5128 : M.ramclustr = 390.9972 (+/− 20.7 ppm)
363.9818
[M−H−C2H2]−
[M−H]−
389.9899
5128 : M.findmain = 364.9891 (+/− 20 ppm)
363.9818
[M−H]−
389.9899
0
20
40
60
80
100
360
365
370
375
380
385
390

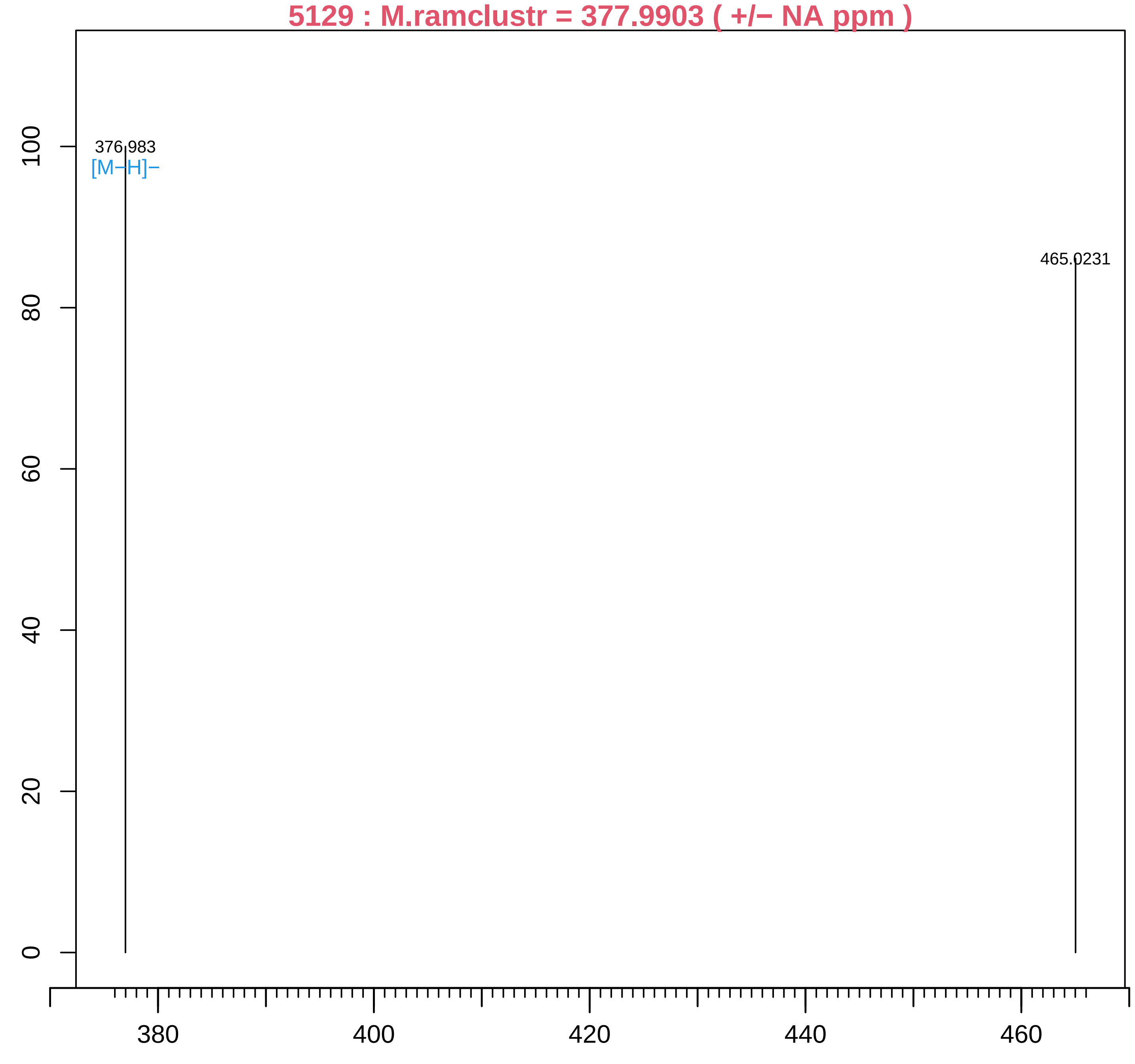
5129 : M.ramclustr = 377.9903 (+/− NA ppm)
376.983
[M−H]−
465.0231
0
20
40
60
80
100
380
400
420
440
460

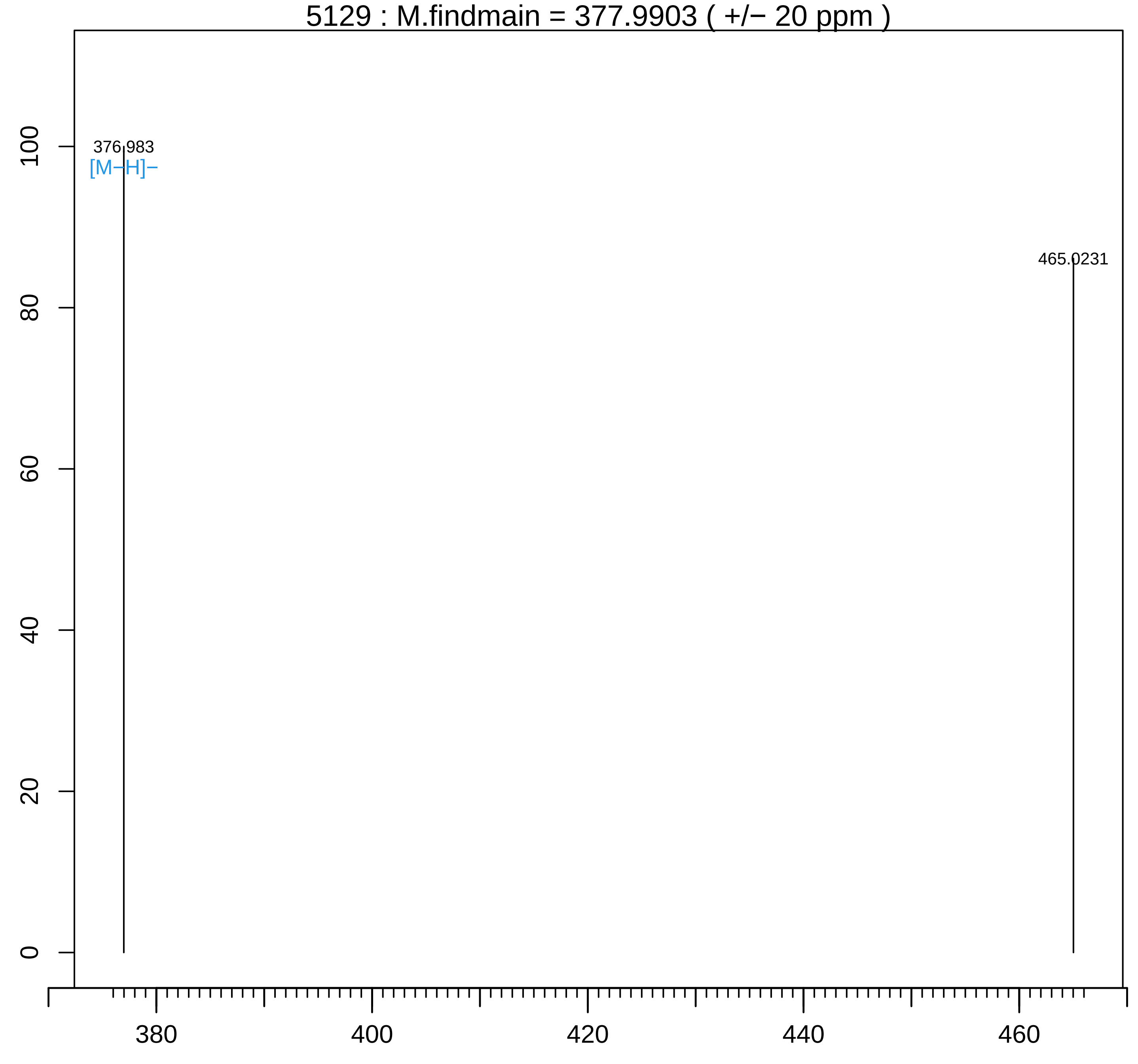
5129 : M.findmain = 377.9903 (+/− 20 ppm)
376.983
[M−H]−
465.0231
0
20
40
60
80
100
380
400
420
440
460

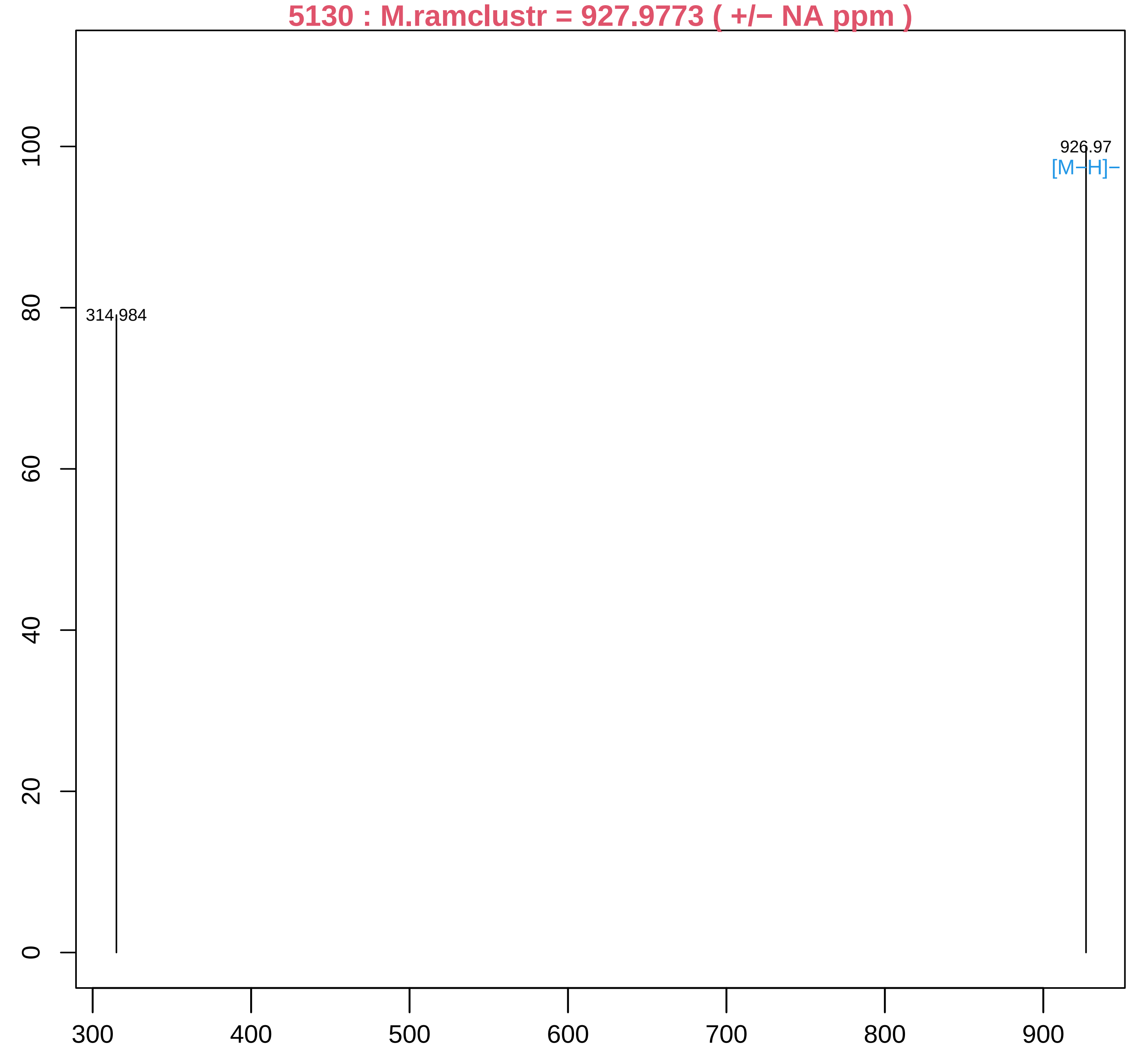
5130 : M.ramclustr = 927.9773 (+/− NA ppm)
926.97
[M−H]−
314.984
0
20
40
60
80
100
300
400
500
600
700
800
900

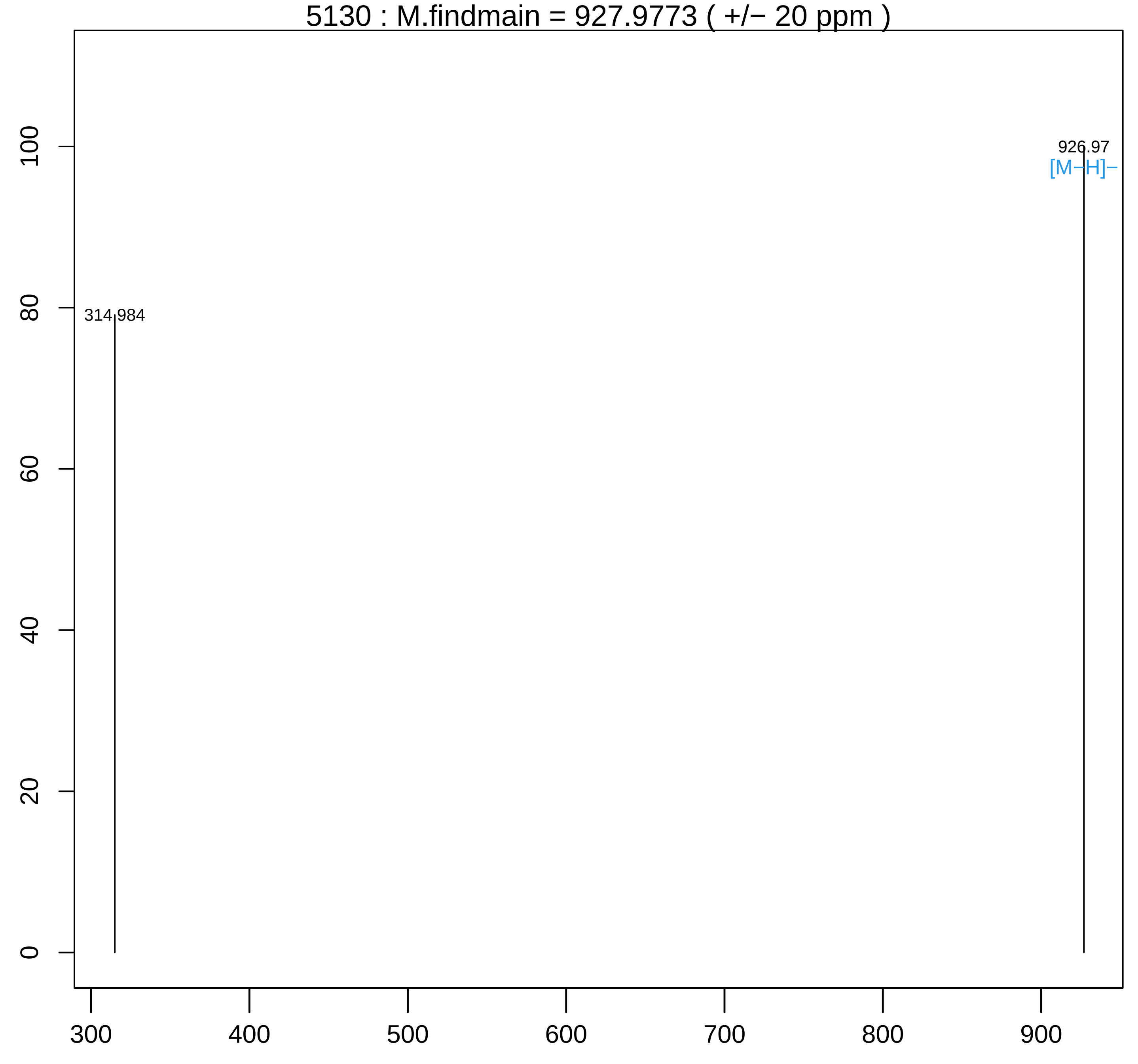
5130 : M.findmain = 927.9773 (+/− 20 ppm)
926.97
[M−H]−
314.984
0
20
40
60
80
100
300
400
500
600
700
800
900

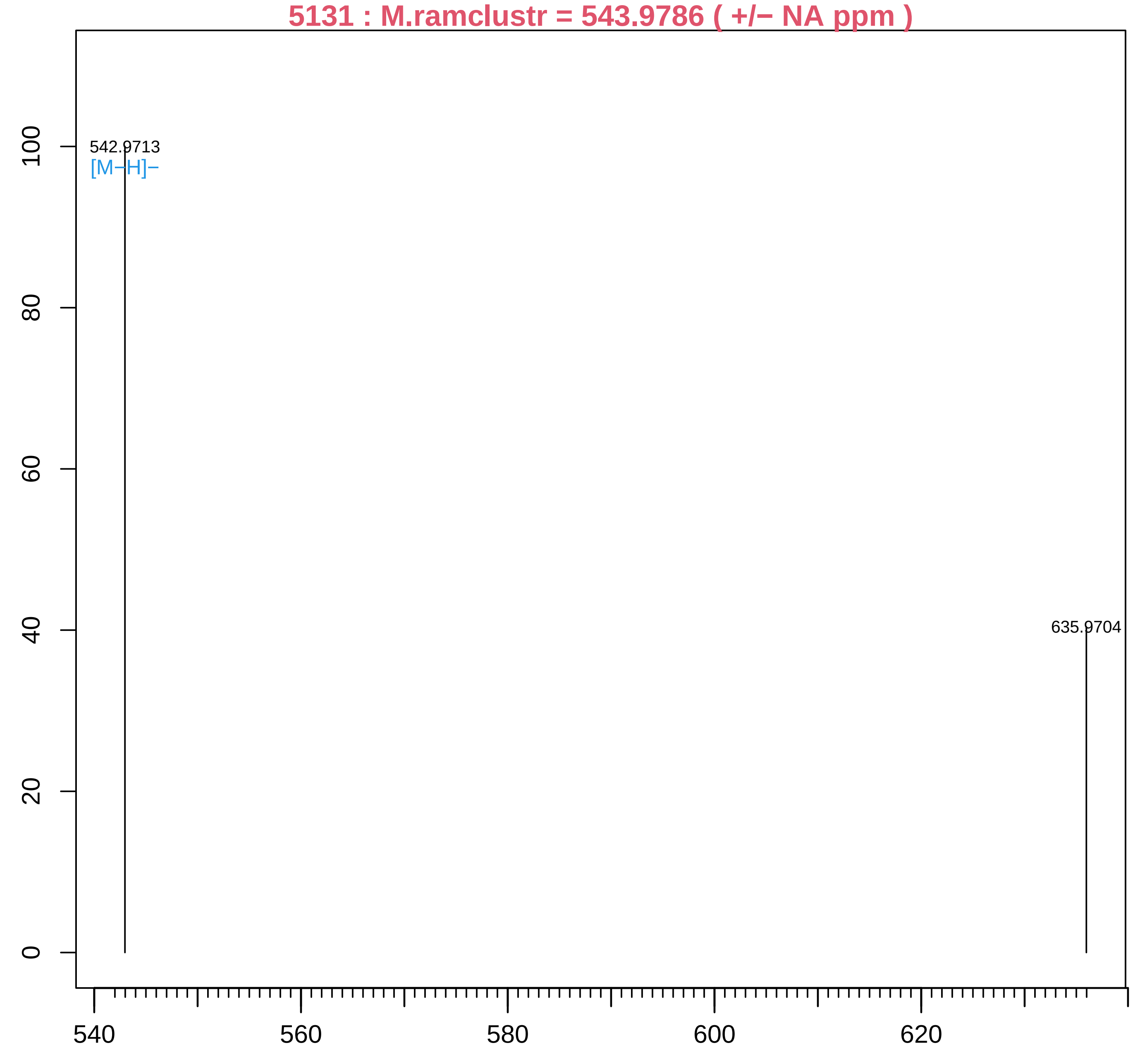

5131 : M.ramclustr = 543.9786 (+/− NA ppm)
542.9713
[M−H]−
635.9704
0
20
40
60
80
100
540
560
580
600
620

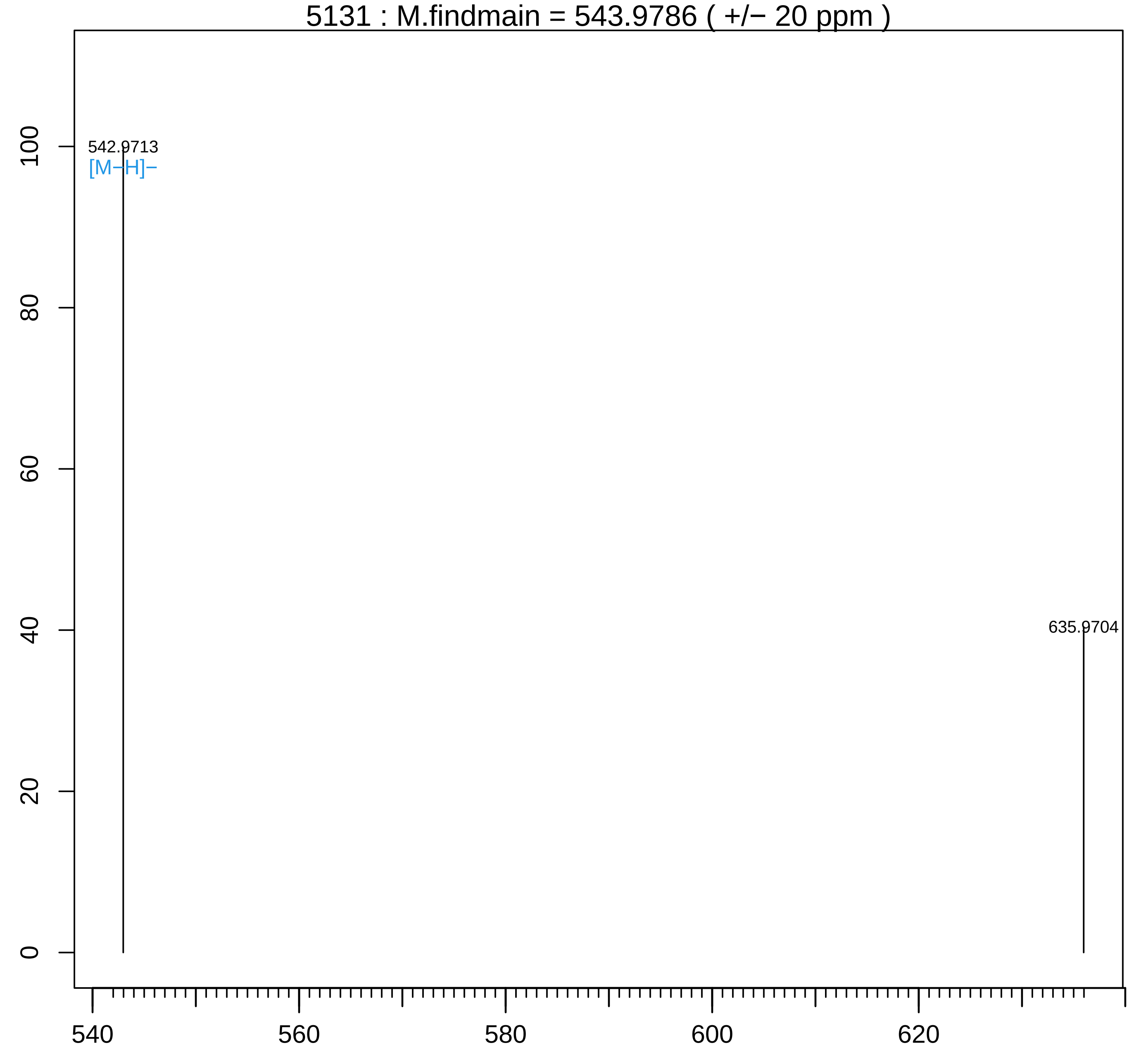

5131 : M.findmain = 543.9786 (+/− 20 ppm)
542.9713
[M−H]−
635.9704
0
20
40
60
80
100
540
560
580
600
620

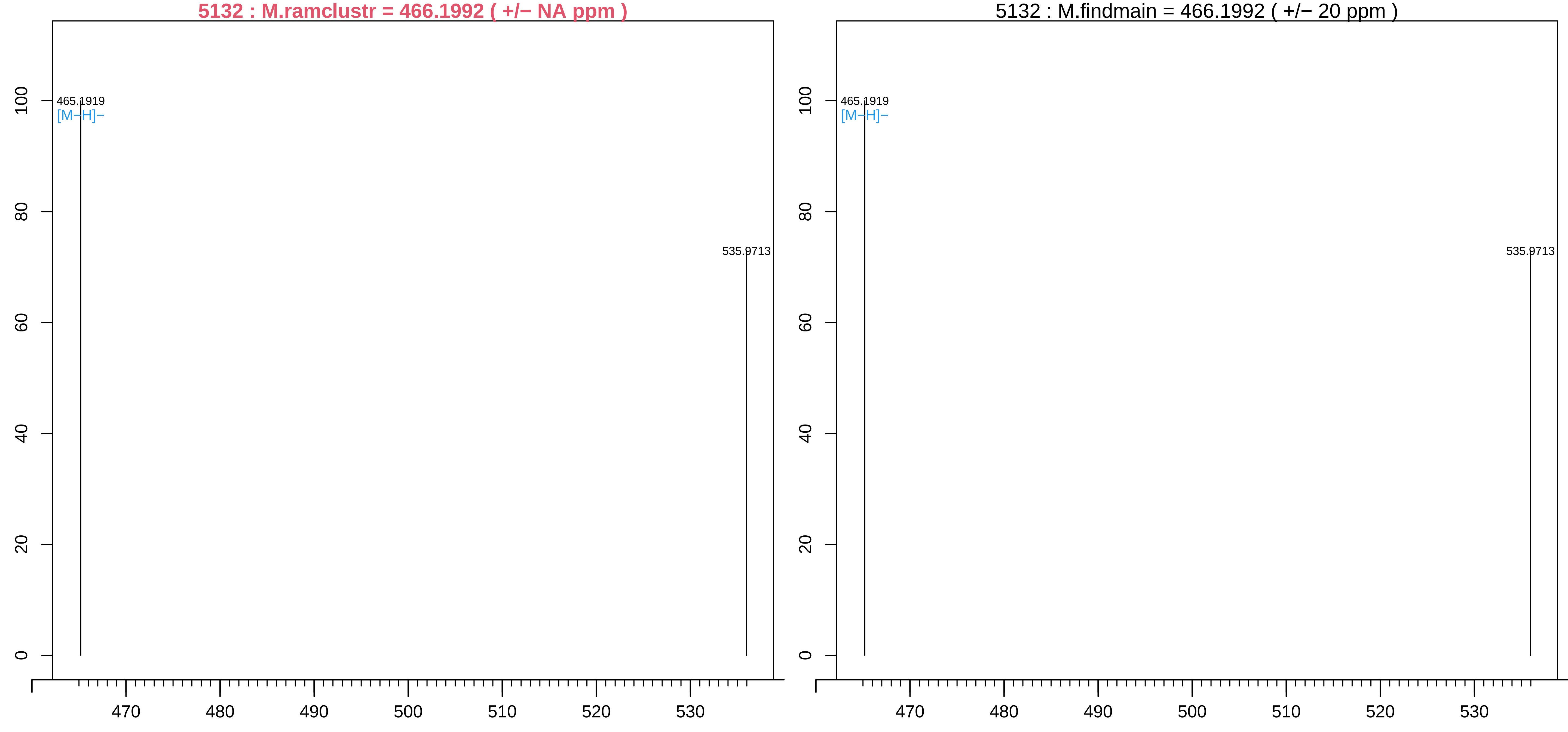

5132 : M.ramclustr = 466.1992 (+/− NA ppm)
465.1919
[M−H]−
535.9713
5132 : M.findmain = 466.1992 (+/− 20 ppm)
465.1919
[M−H]−
535.9713

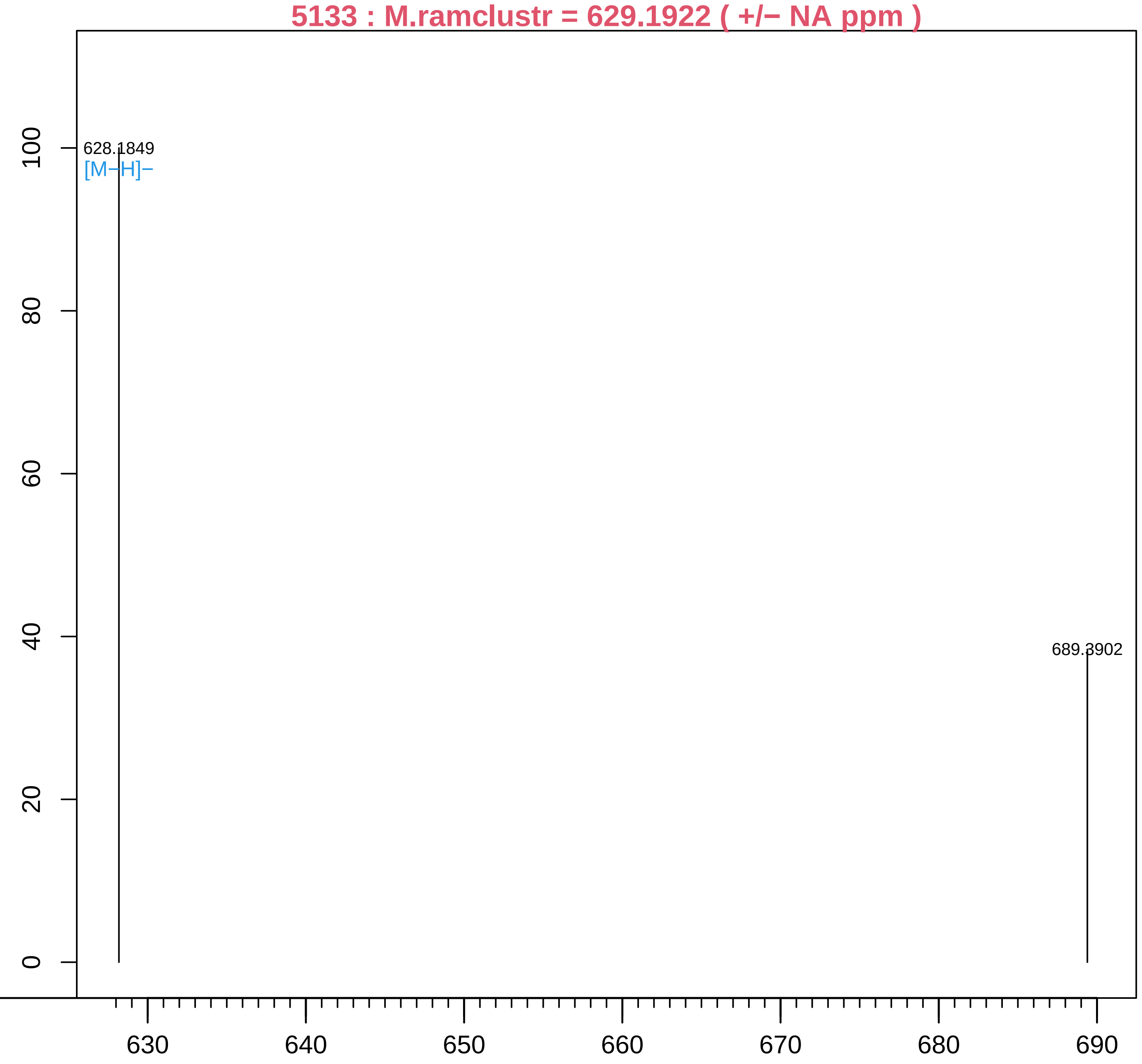

5133 : M.ramclustr = 629.1922 (+/− NA ppm)
628.1849
[M−H]−
689.3902
0
20
40
60
80
100
630
640
650
660
670
680
690

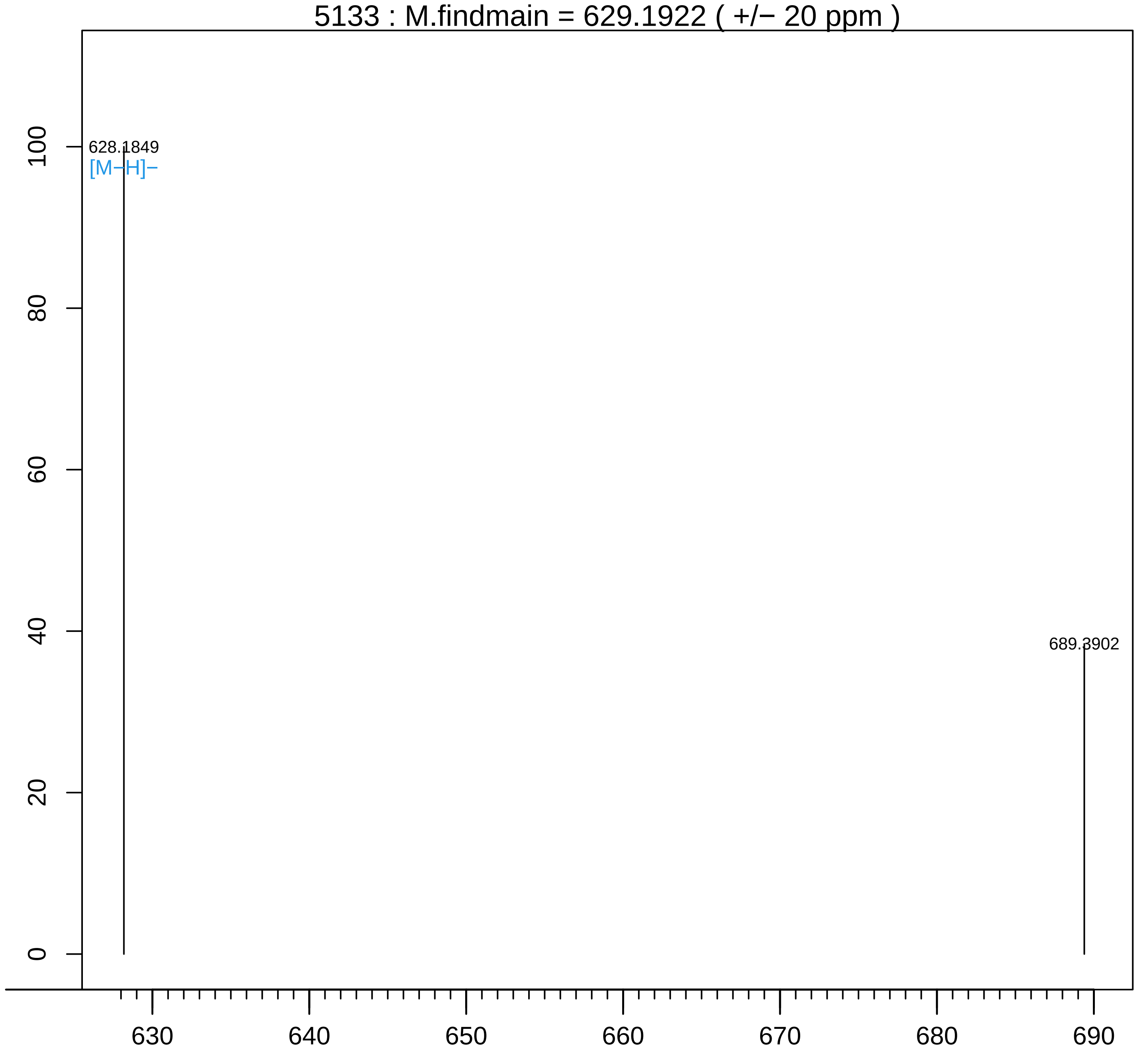

5133 : M.findmain = 629.1922 (+/− 20 ppm)
628.1849
[M−H]−
689.3902
0
20
40
60
80
100
630
640
650
660
670
680
690

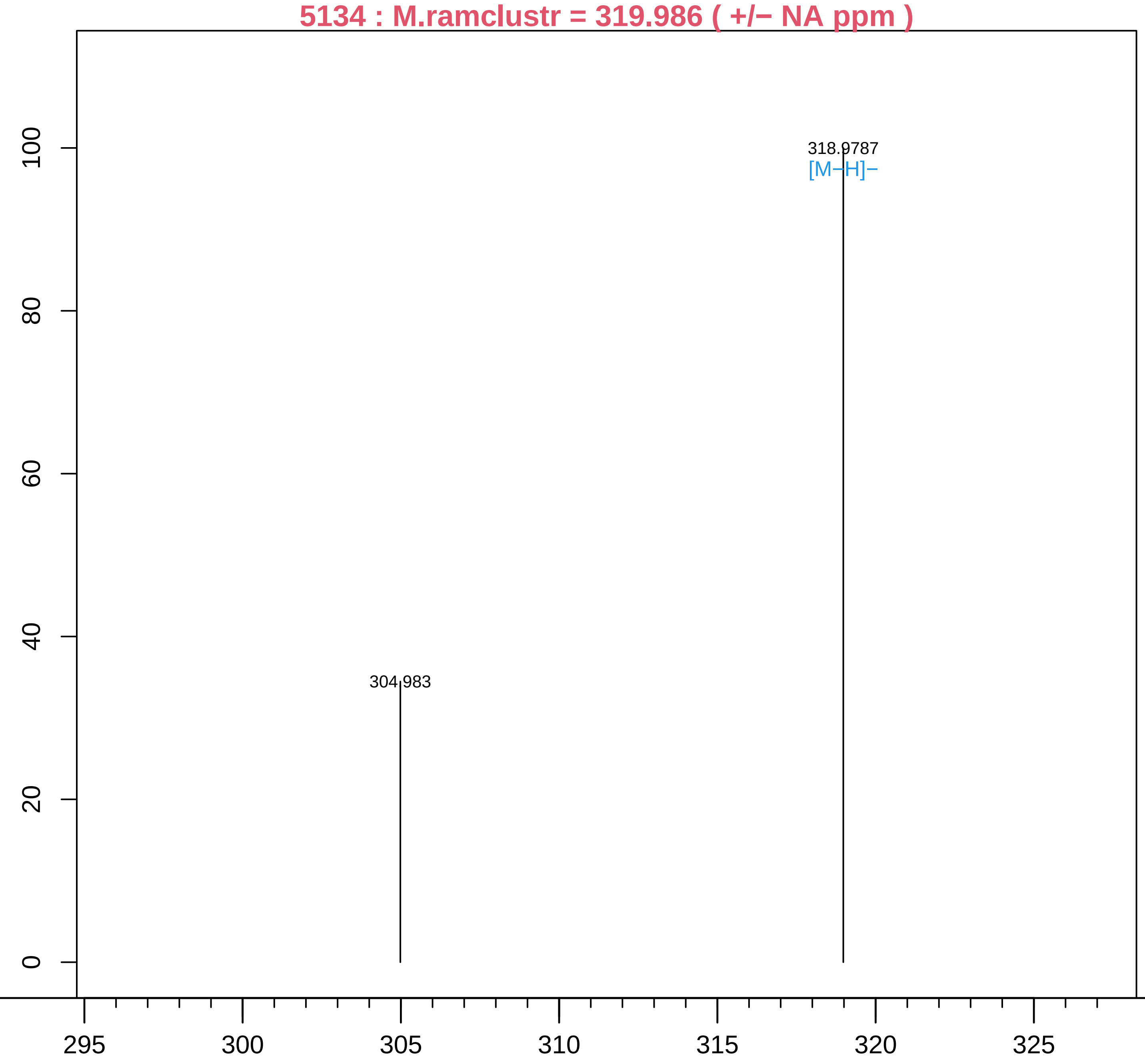
5134 : M.ramclustr = 319.986 (+/− NA ppm)
318.9787
[M−H]−
304.983
0
20
40
60
80
100
295
300
305
310
315
320
325

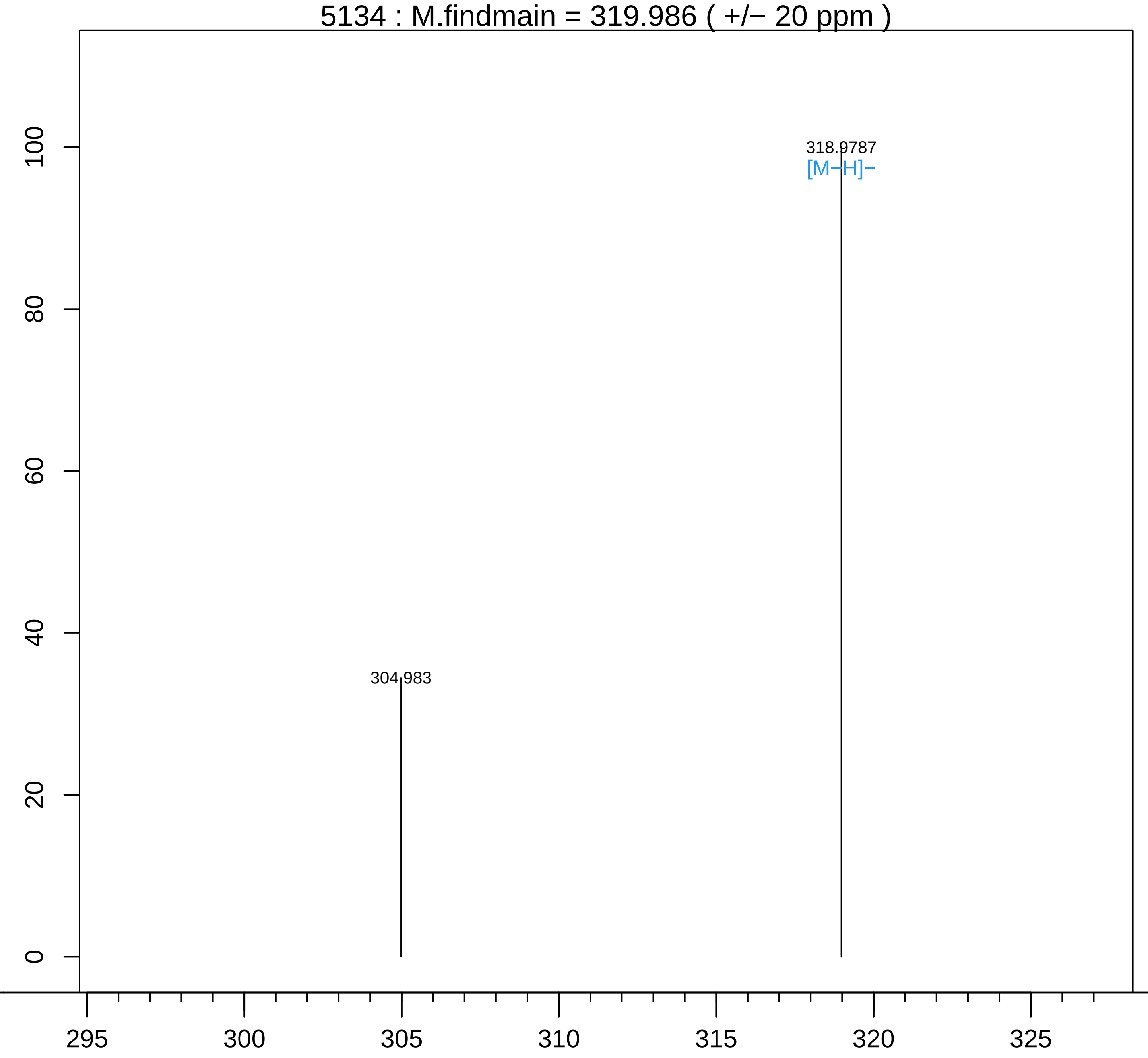
5134 : M.findmain = 319.986 (+/− 20 ppm)
318.9787
[M−H]−
304.983
0
20
40
60
80
100
295
300
305
310
315
320
325

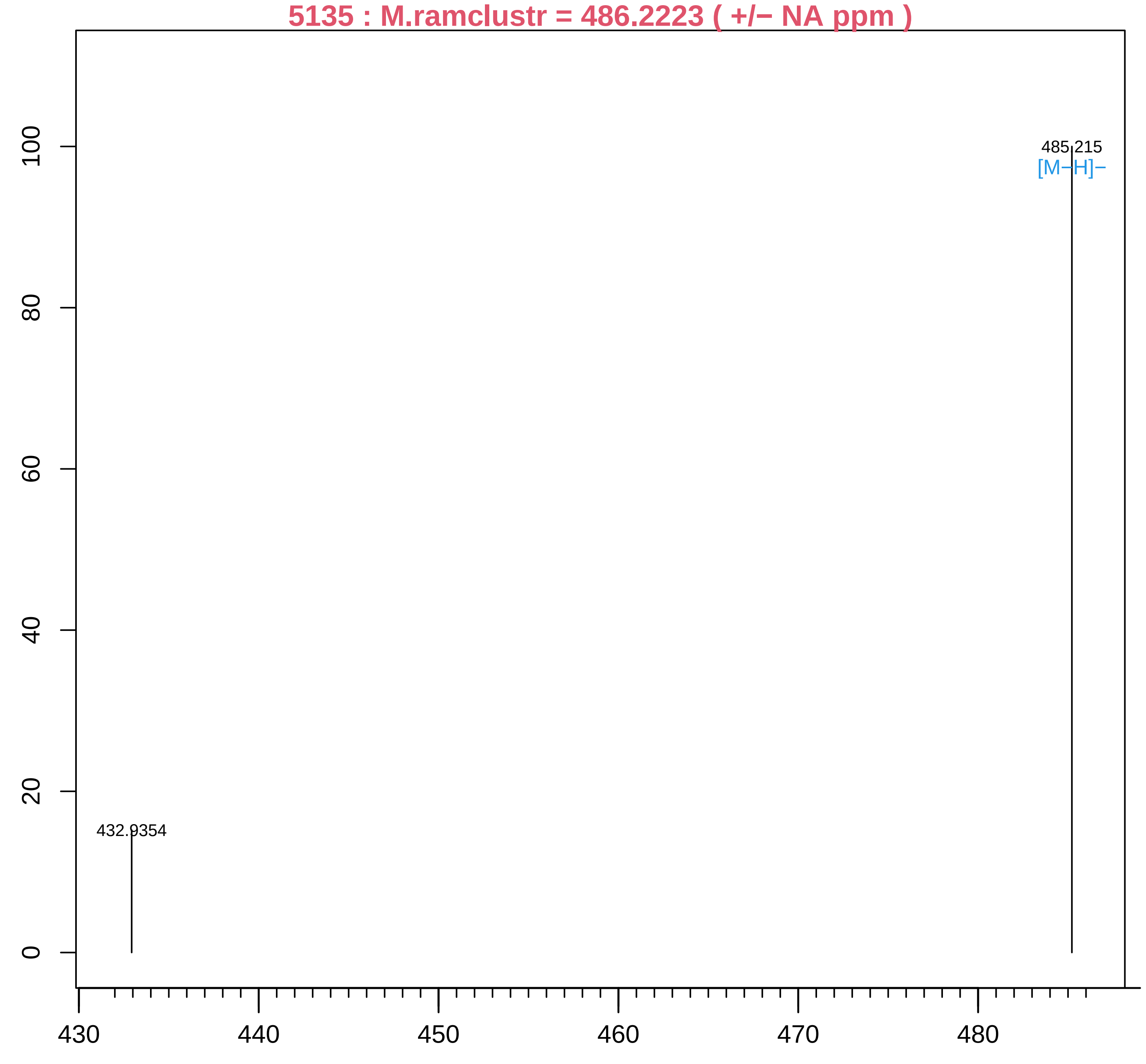

5135 : M.ramclustr = 486.2223 (+/− NA ppm)
485.215
[M−H]−
432.9354
0
20
40
60
80
100
430
440
450
460
470
480

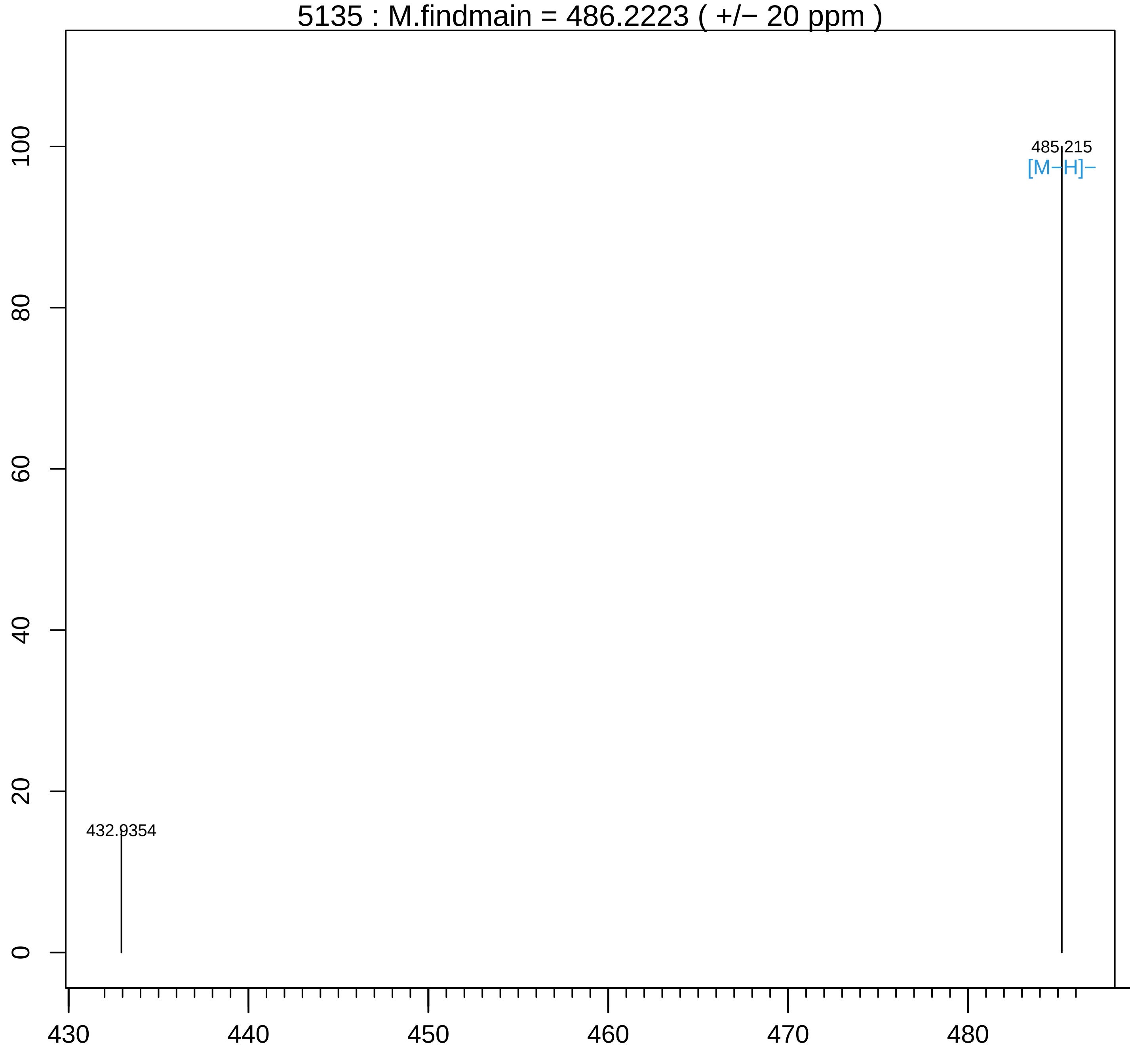

5135 : M.findmain = 486.2223 (+/− 20 ppm)
485.215
[M−H]−
432.9354
0
20
40
60
80
100
430
440
450
460
470
480

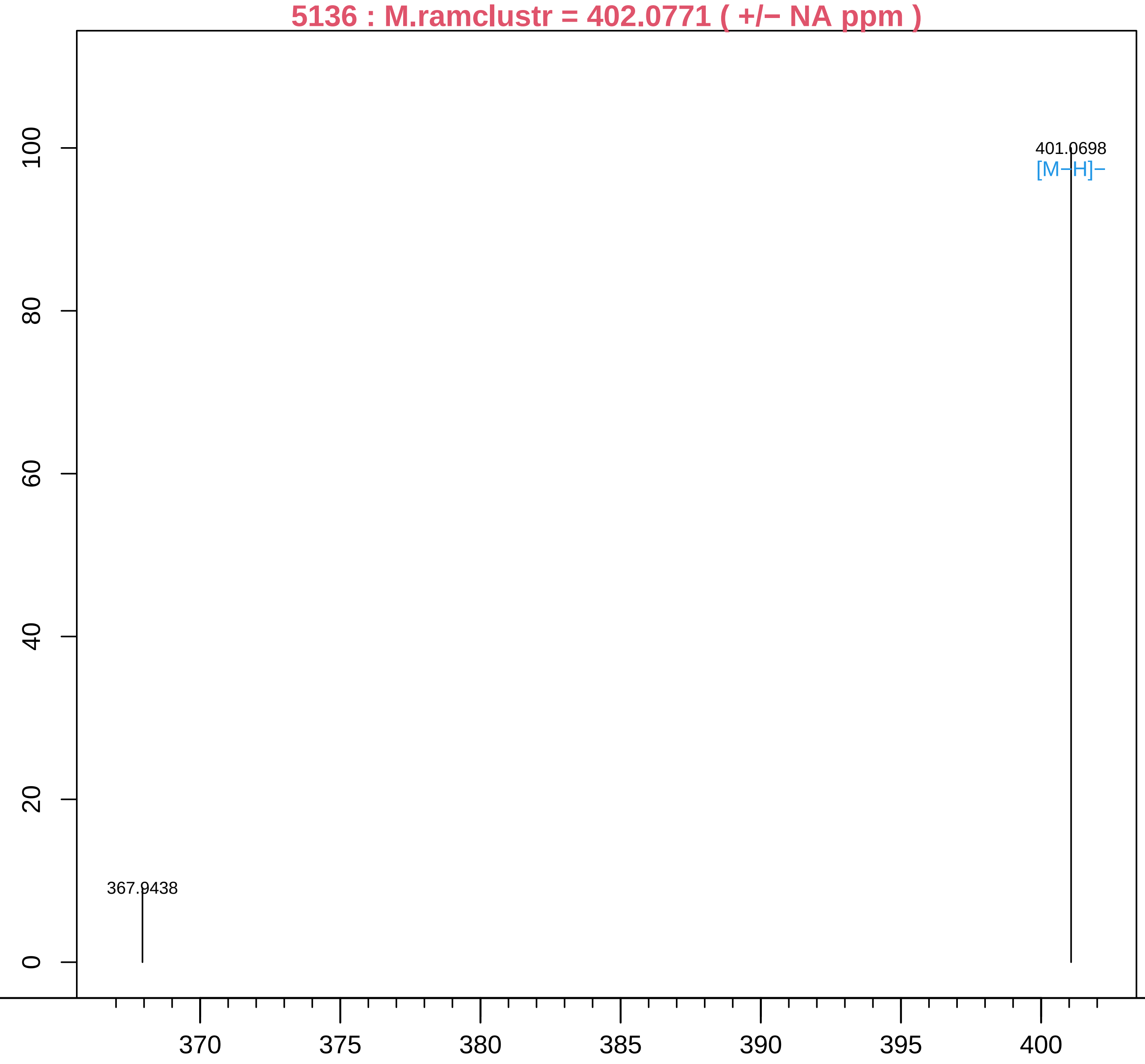

5136 : M.ramclustr = 402.0771 (+/− NA ppm)
401.0698
[M−H]−
367.9438
0
20
40
60
80
100
370
375
380
385
390
395
400

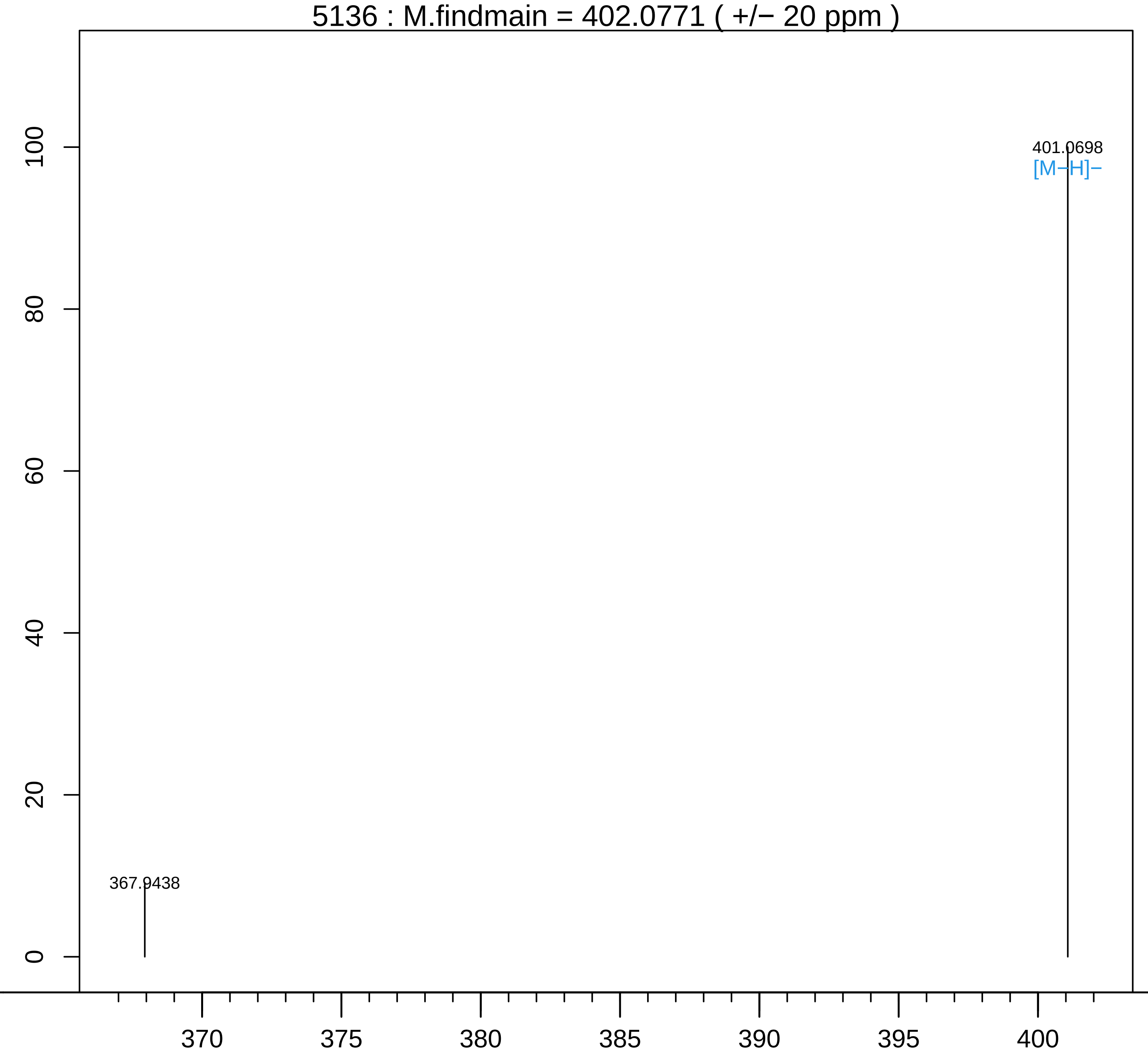

5136 : M.findmain = 402.0771 (+/− 20 ppm)
401.0698
[M−H]−
367.9438
0
20
40
60
80
100
370
375
380
385
390
395
400

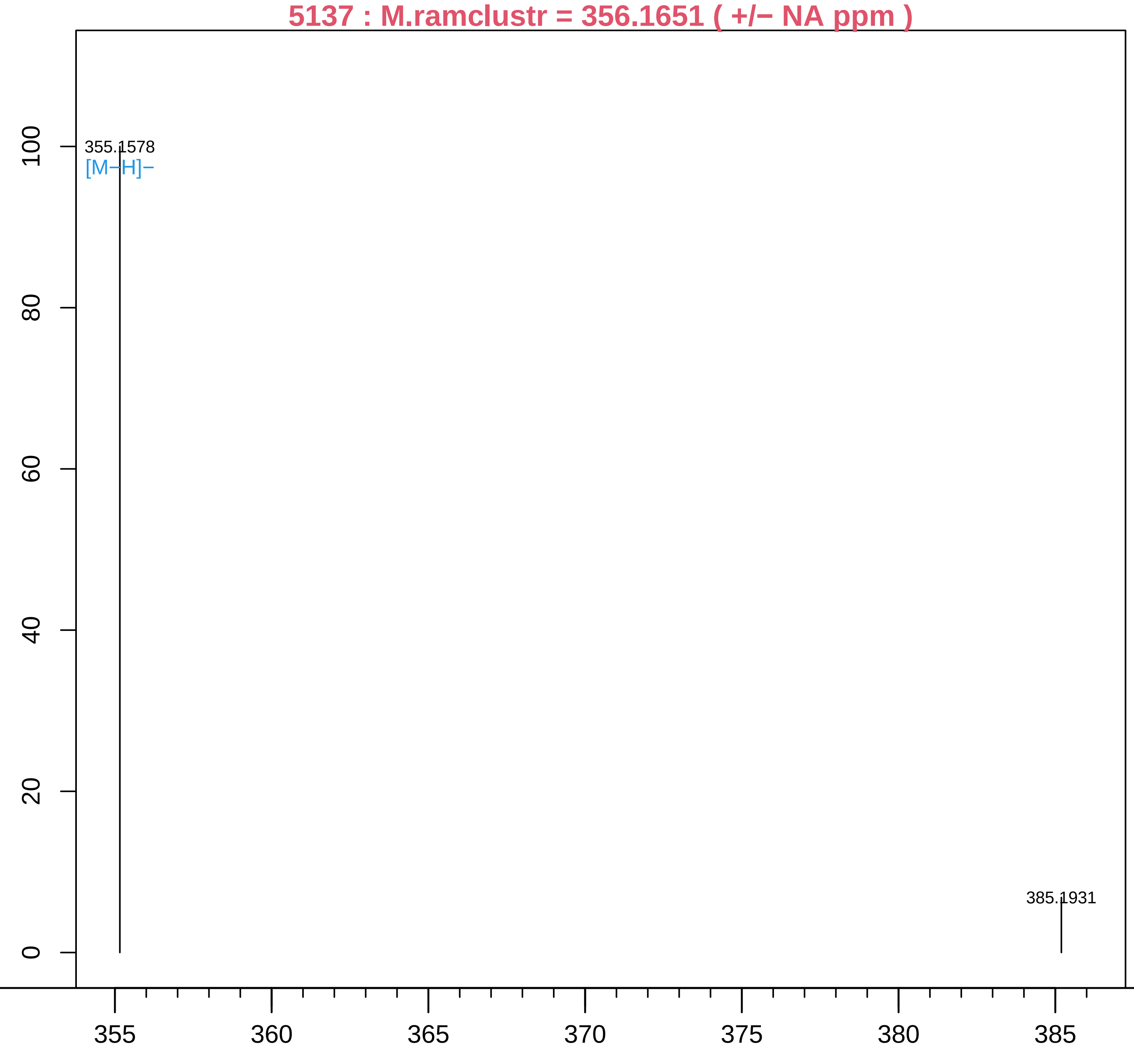
5137 : M.ramclustr = 356.1651 (+/− NA ppm)
355.1578
[M−H]−
385.1931
0
20
40
60
80
100
355
360
365
370
375
380
385

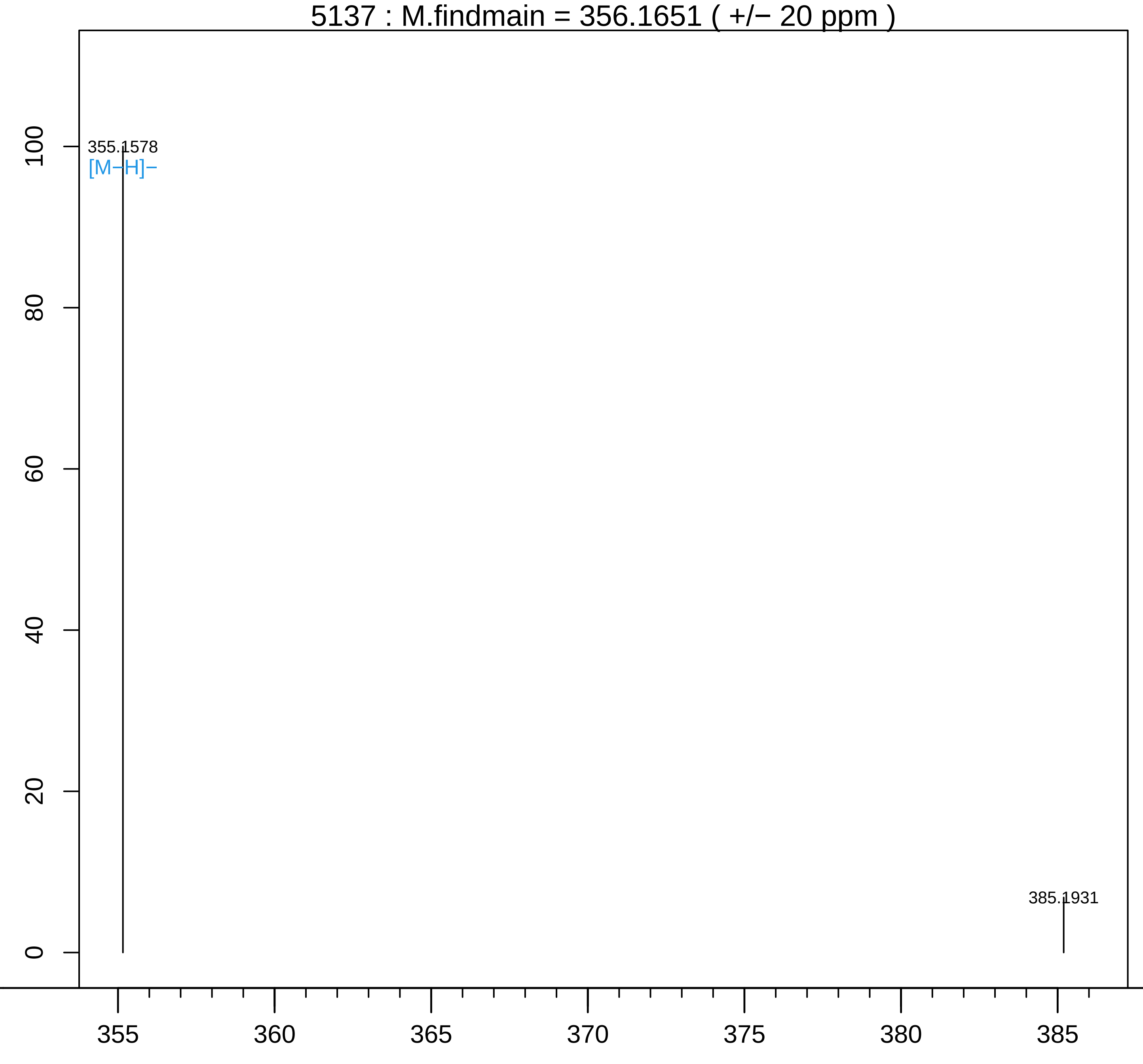
5137 : M.findmain = 356.1651 (+/− 20 ppm)
355.1578
[M−H]−
385.1931
0
20
40
60
80
100
355
360
365
370
375
380
385

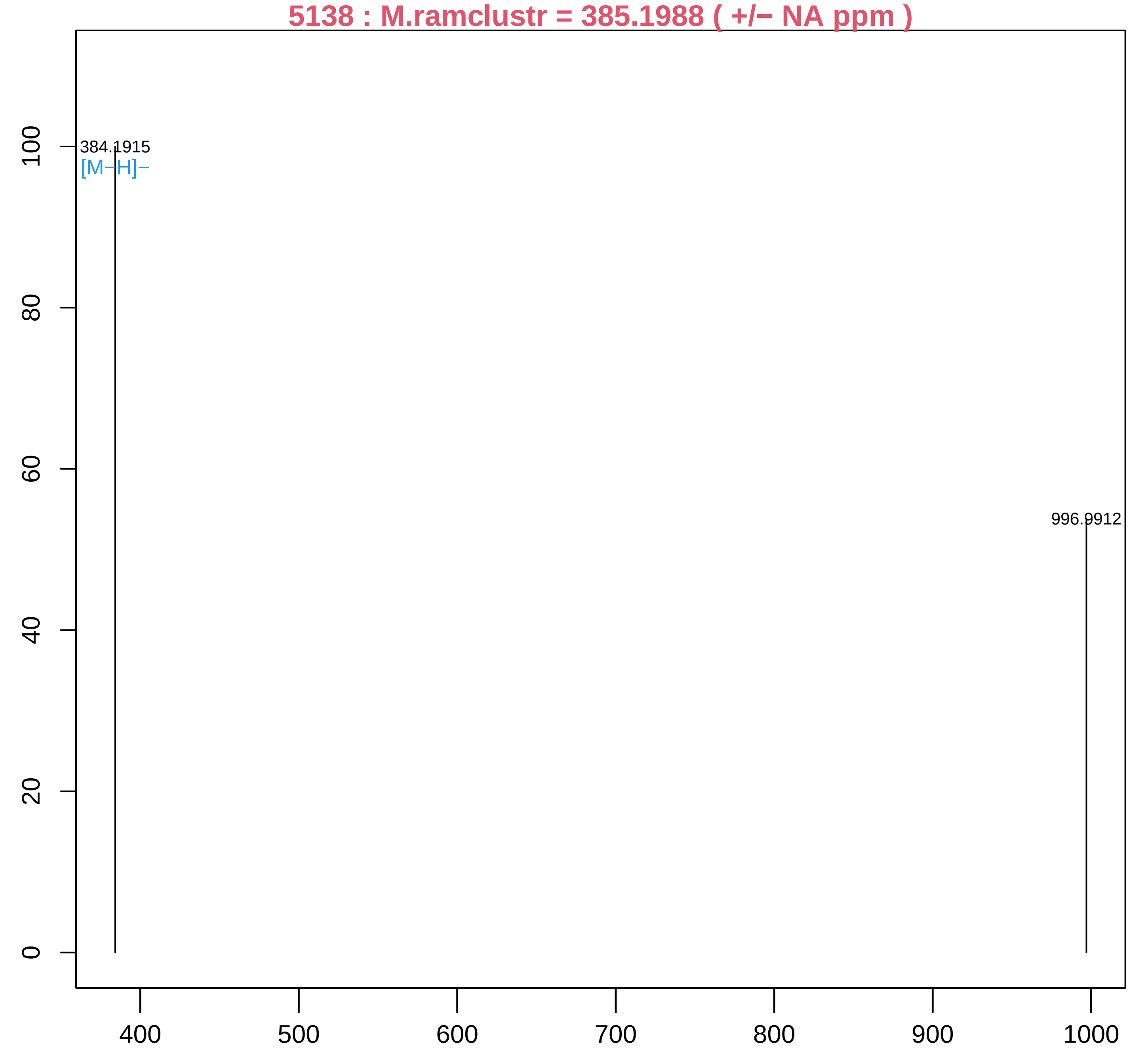
5138 : M.ramclustr = 385.1988 (+/− NA ppm)
384.1915
[M−H]−
996.9912
0
20
40
60
80
100
400
500
600
700
800
900
1000

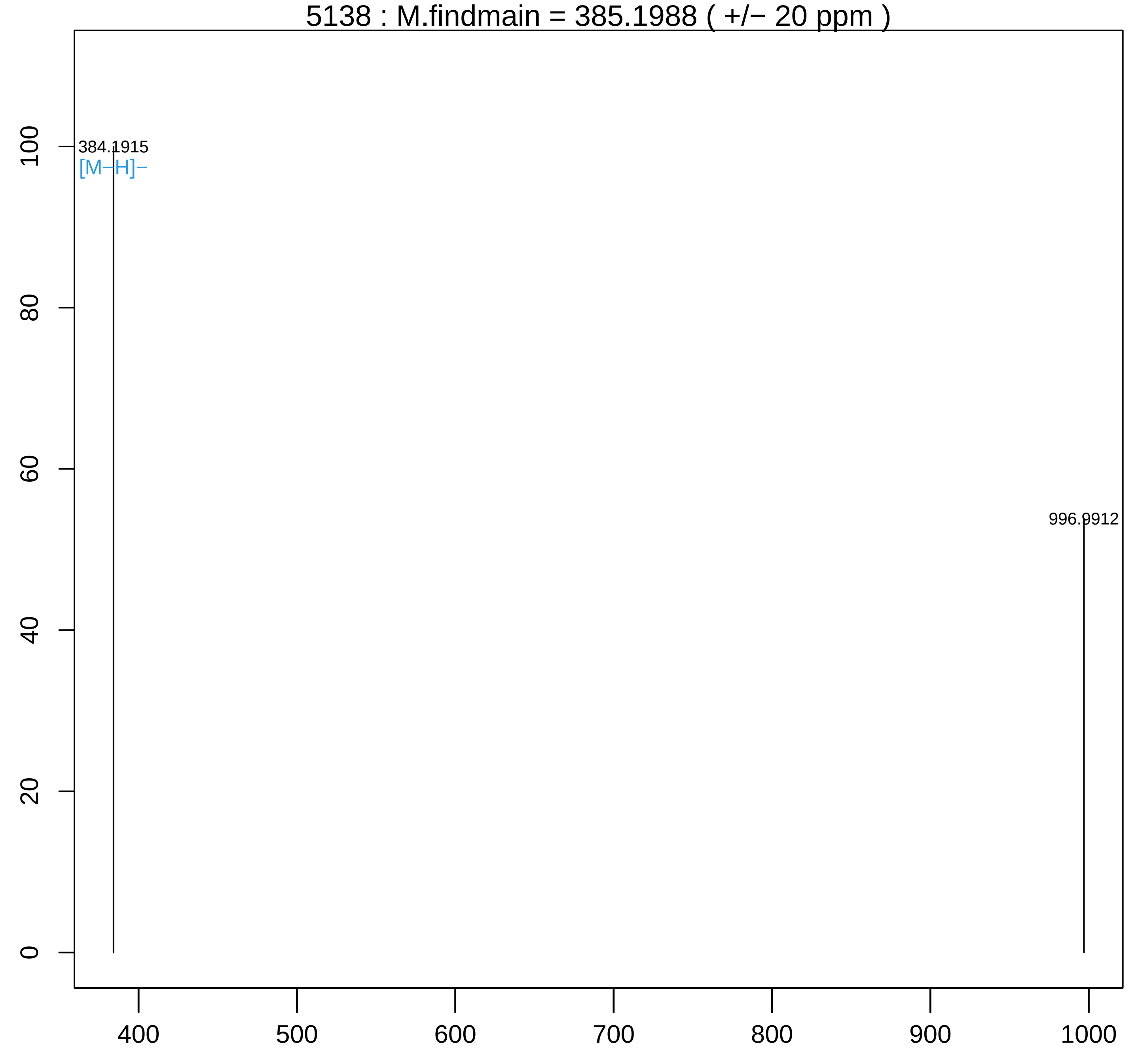
5138 : M.findmain = 385.1988 (+/− 20 ppm)
384.1915
[M−H]−
996.9912
0
20
40
60
80
100
400
500
600
700
800
900
1000

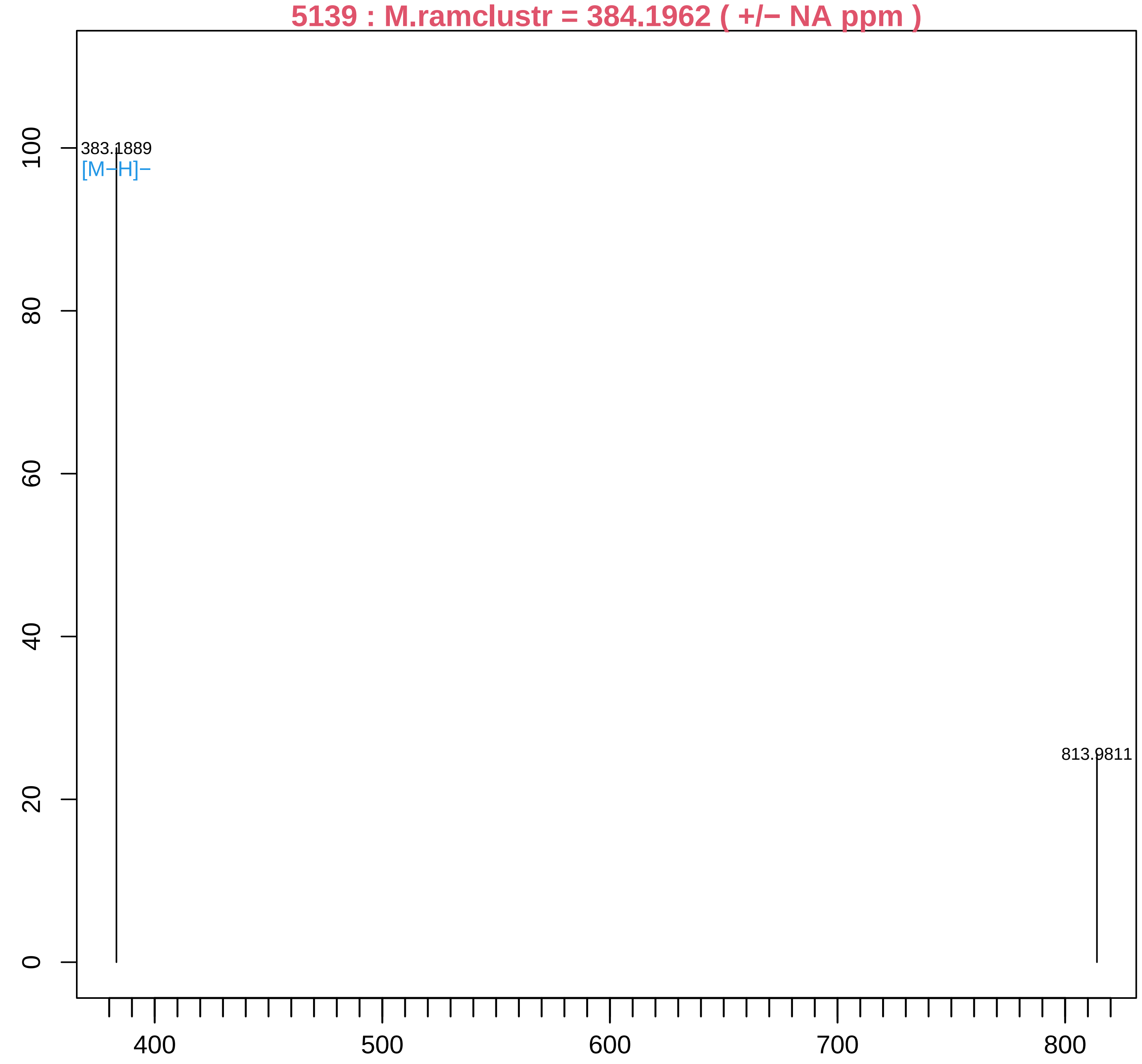
5139 : M.ramclustr = 384.1962 (+/− NA ppm)
383.1889
[M−H]−
813.9811
0
20
40
60
80
100
400
500
600
700
800

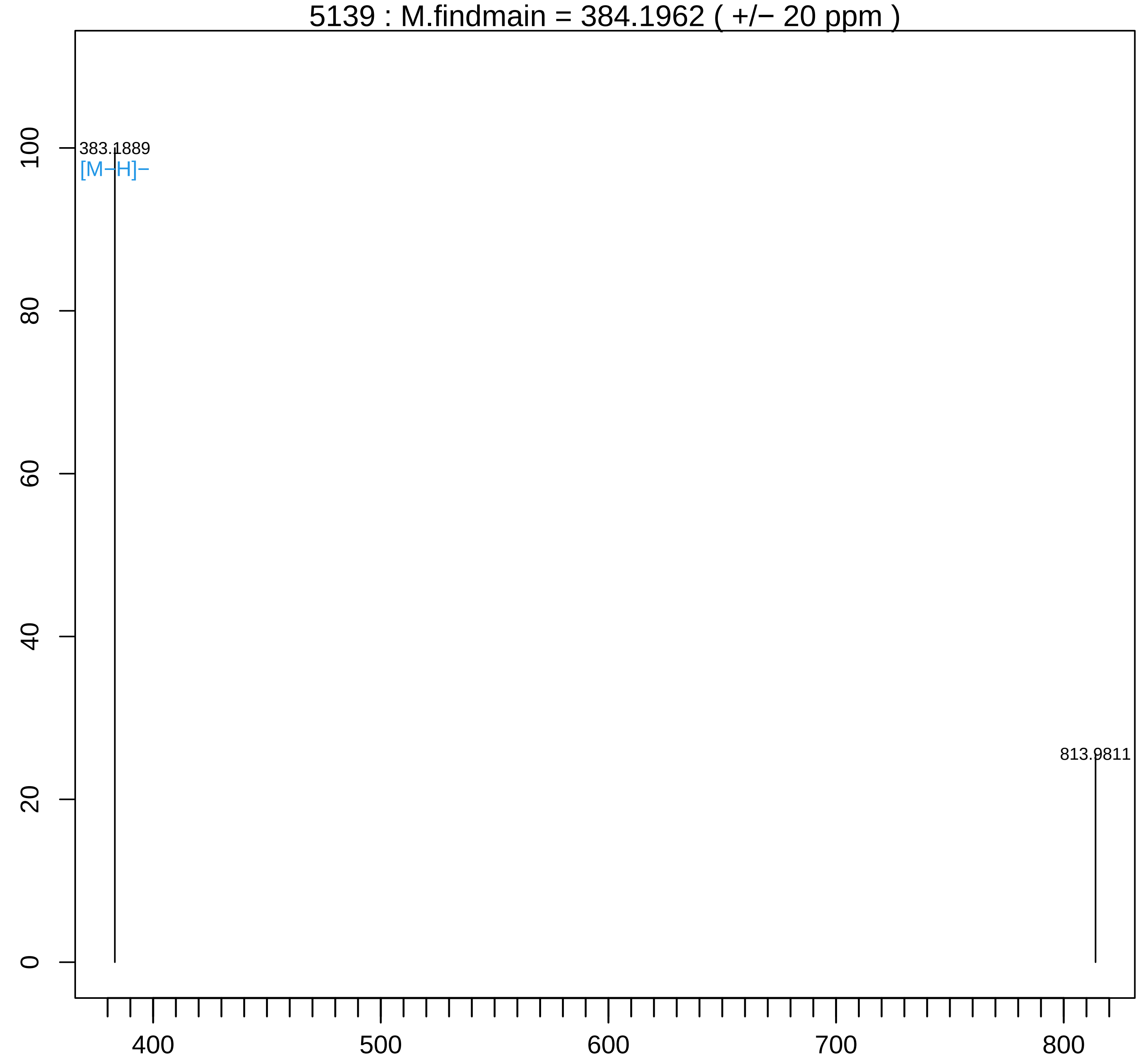
5139 : M.findmain = 384.1962 (+/− 20 ppm)
383.1889
[M−H]−
813.9811
0
20
40
60
80
100
400
500
600
700
800

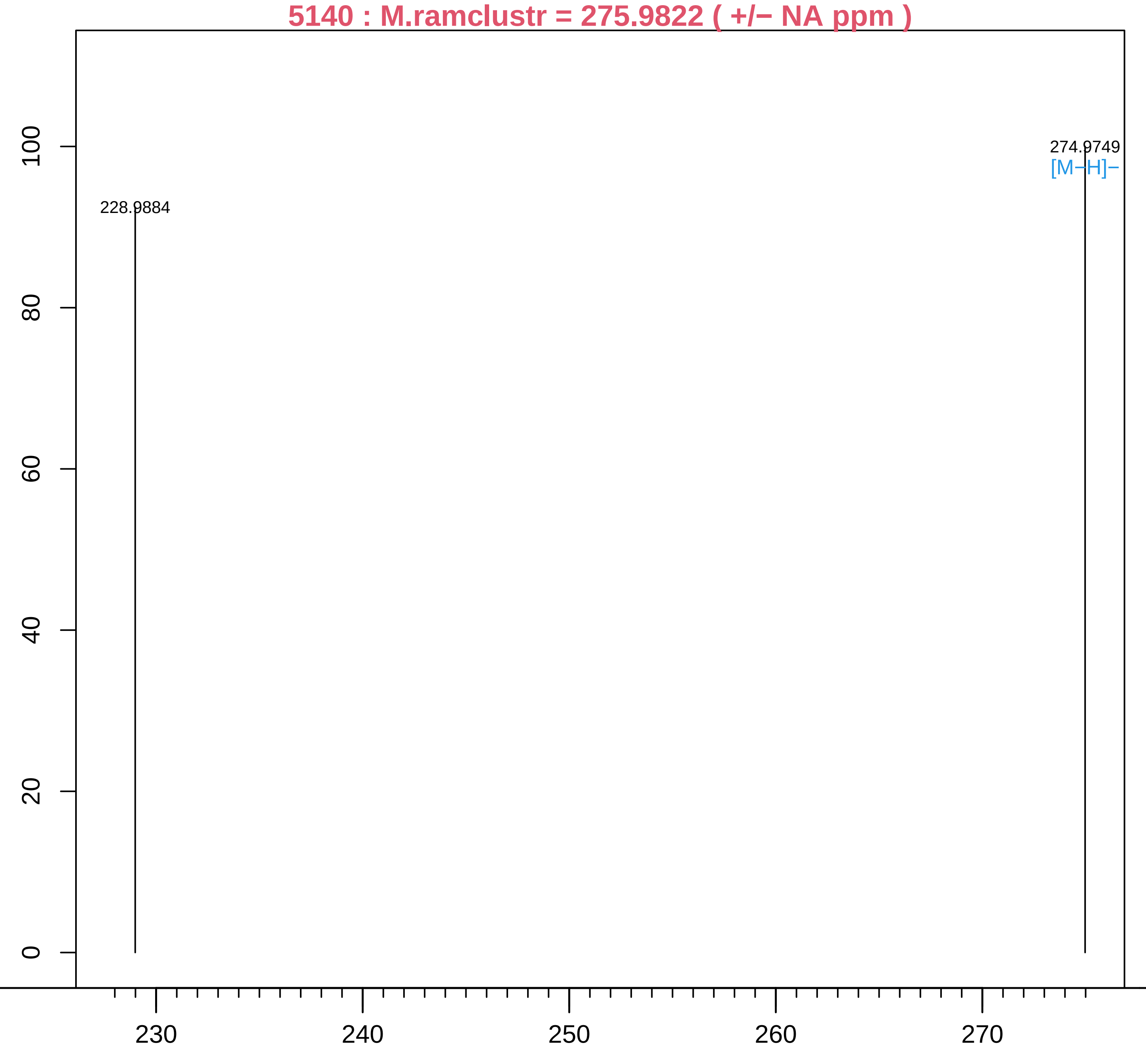
5140 : M.ramclustr = 275.9822 (+/− NA ppm)
228.9884
274.9749
[M−H]−
0
20
40
60
80
100
230
240
250
260
270

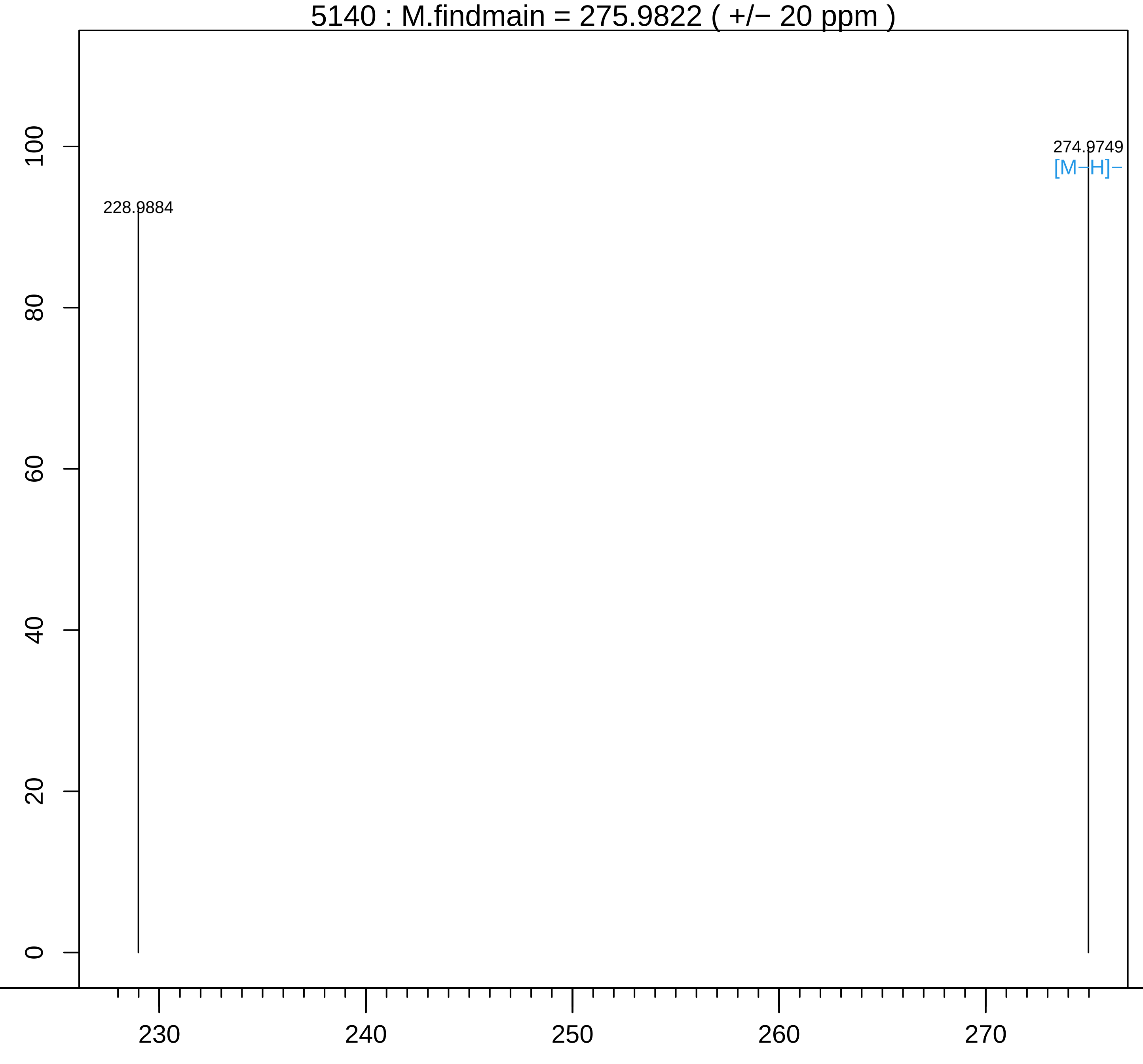
5140 : M.findmain = 275.9822 (+/− 20 ppm)
228.9884
274.9749
[M−H]−
0
20
40
60
80
100
230
240
250
260
270

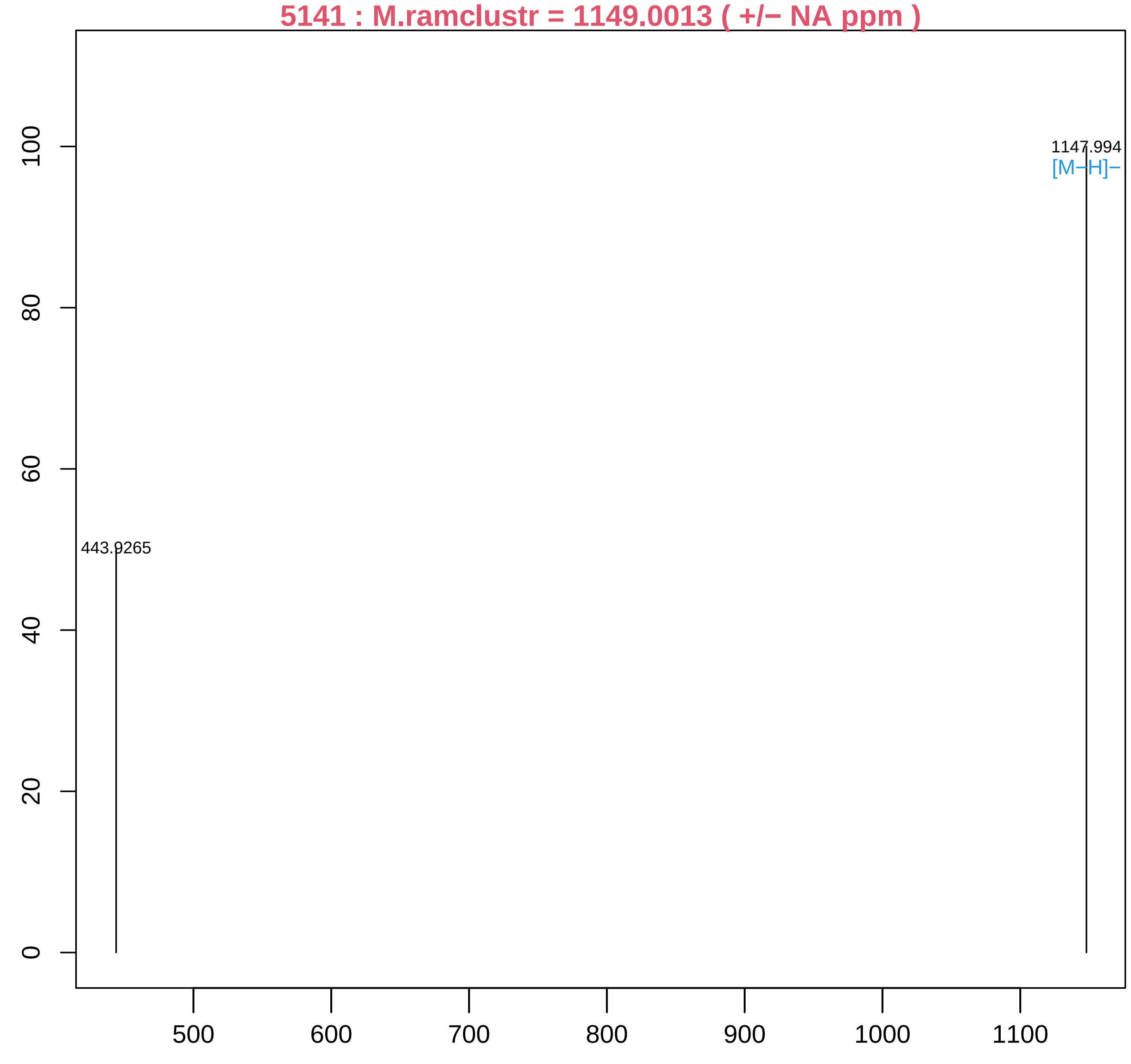

5141 : M.ramclustr = 1149.0013 (+/− NA ppm)
1147.994
[M−H]−
443.9265
0
20
40
60
80
100
500
600
700
800
900
1000
1100

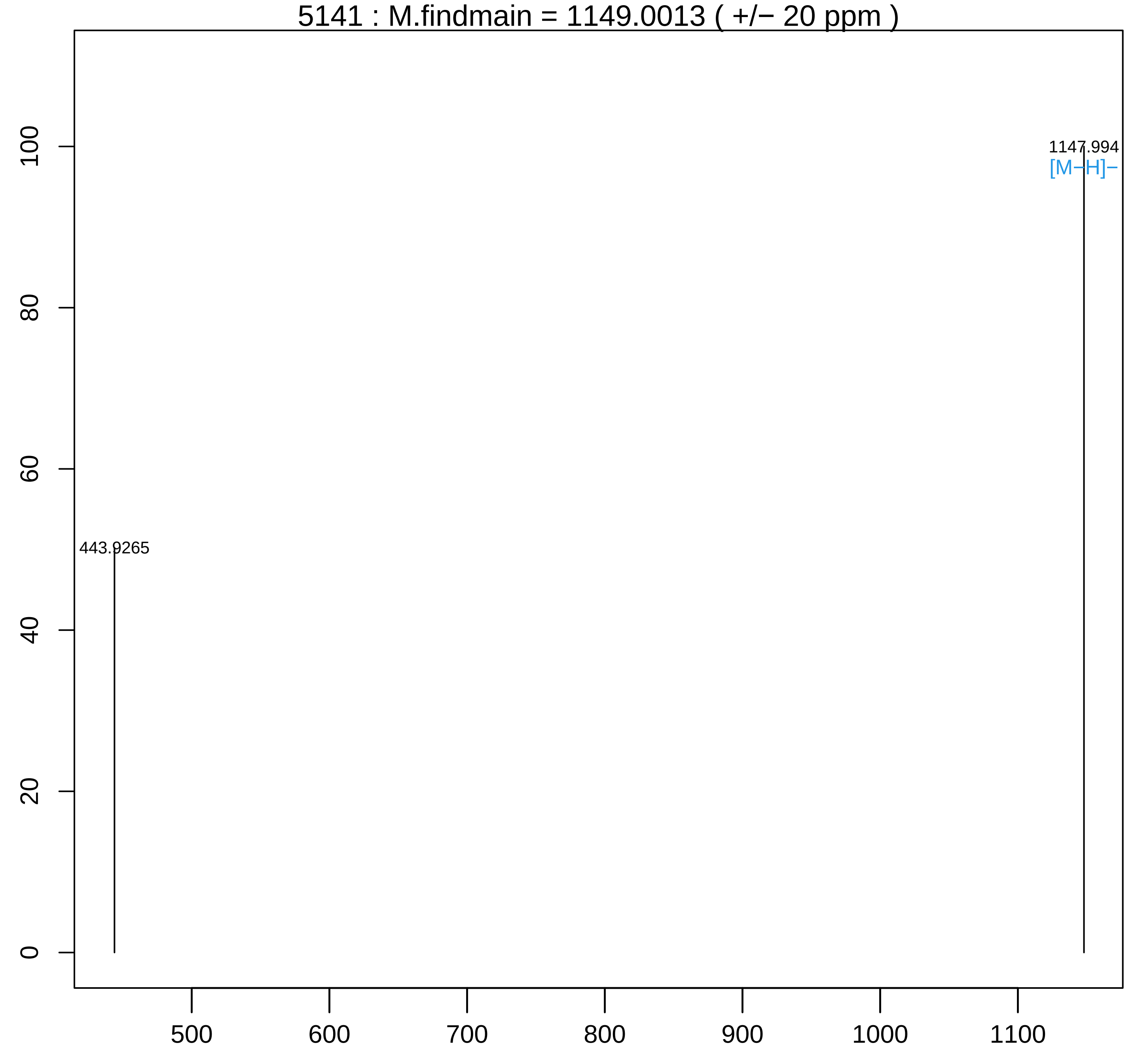

5141 : M.findmain = 1149.0013 (+/− 20 ppm)
1147.994
[M−H]−
443.9265
0
20
40
60
80
100
500
600
700
800
900
1000
1100

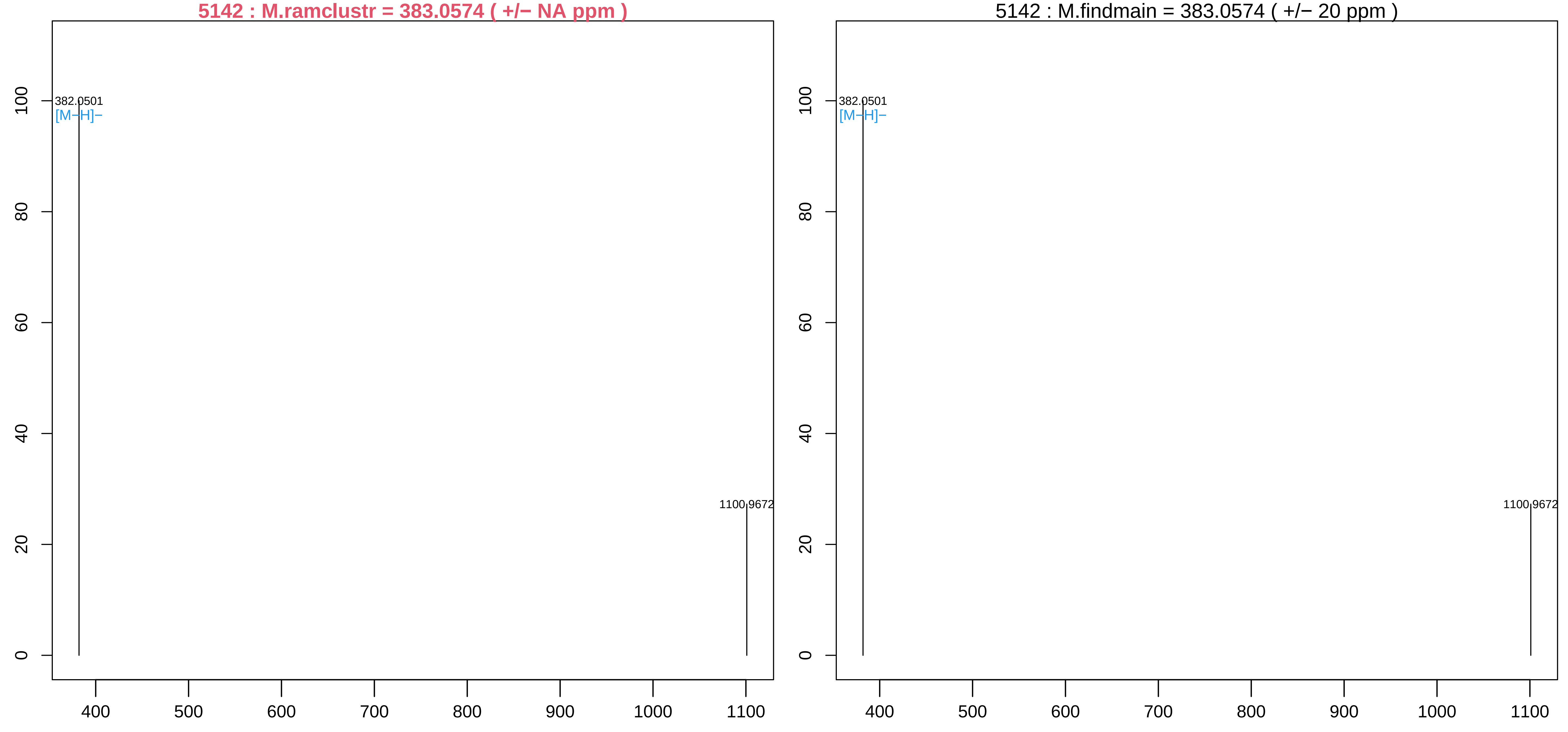
5142 : M.ramclustr = 383.0574 (+/− NA ppm)
382.0501
[M−H]−
1100.9672
5142 : M.findmain = 383.0574 (+/− 20 ppm)
382.0501
[M−H]−
1100.9672

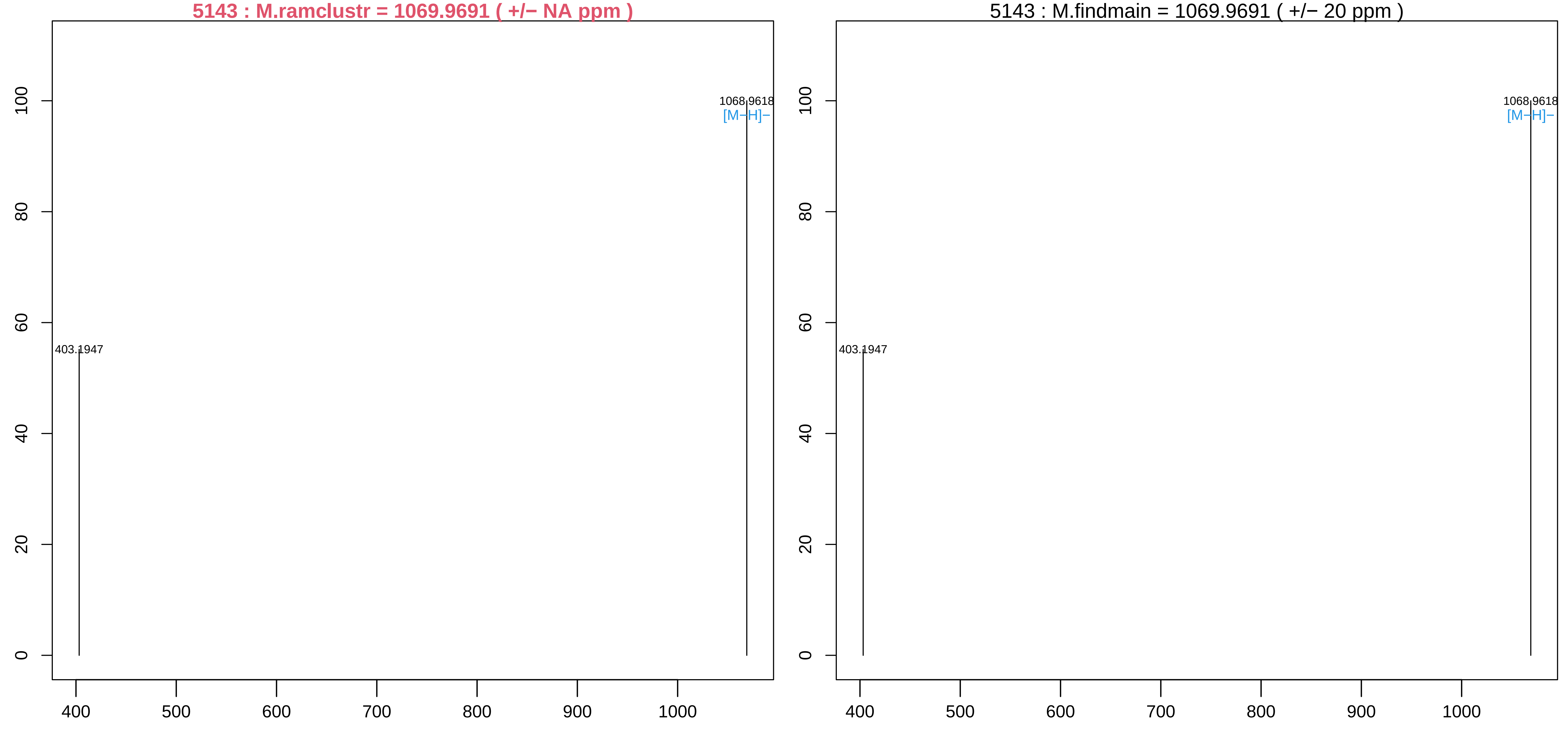
5143 : M.ramclustr = 1069.9691 (+/− NA ppm)
5143 : M.findmain = 1069.9691 (+/− 20 ppm)
1068.9618
[M−H]−
403.1947
0
20
40
60
80
100
400
500
600
700
800
900
1000

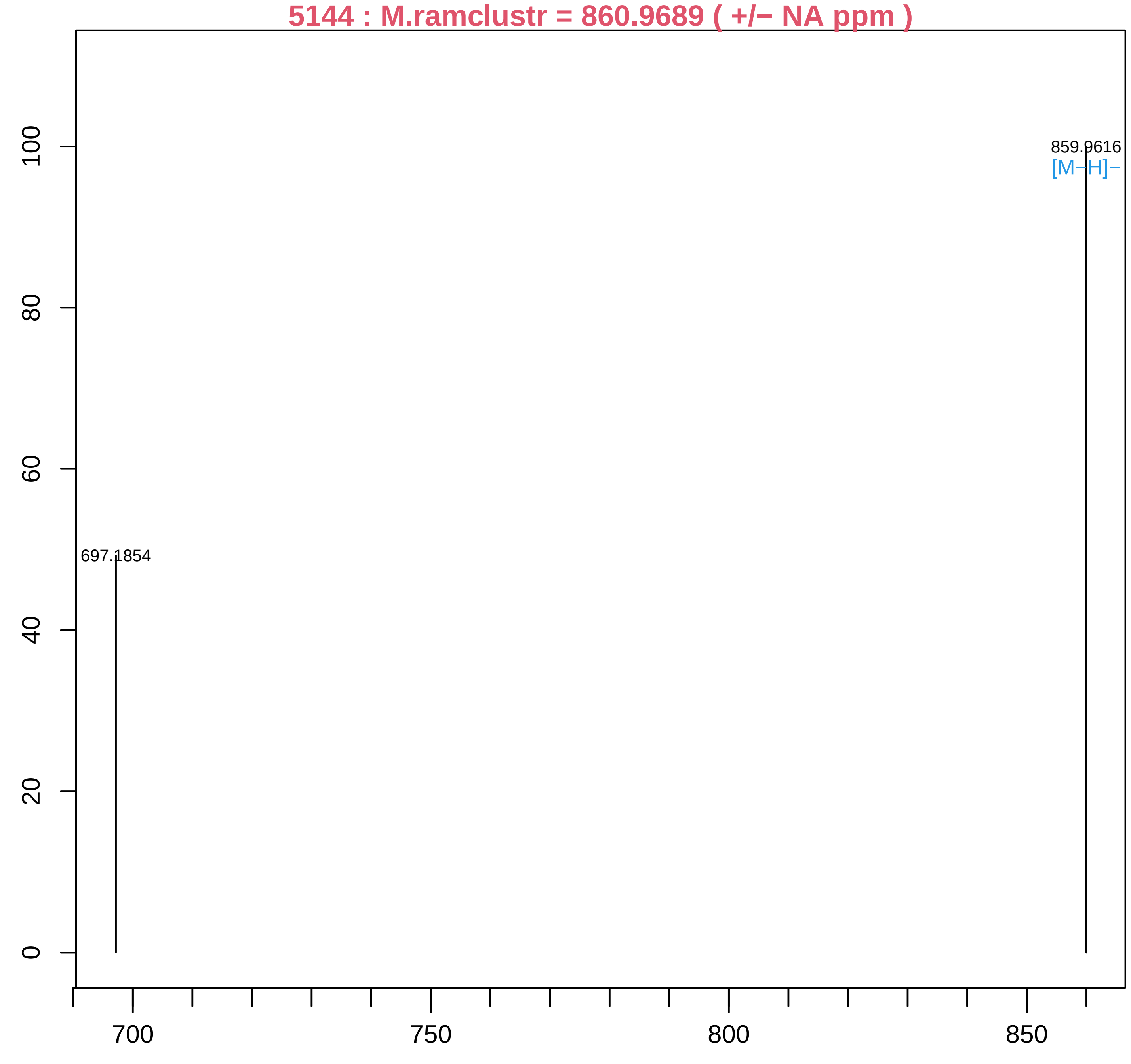
5144 : M.ramclustr = 860.9689 (+/− NA ppm)
859.9616
[M−H]−
697.1854
0
20
40
60
80
100
700
750
800
850

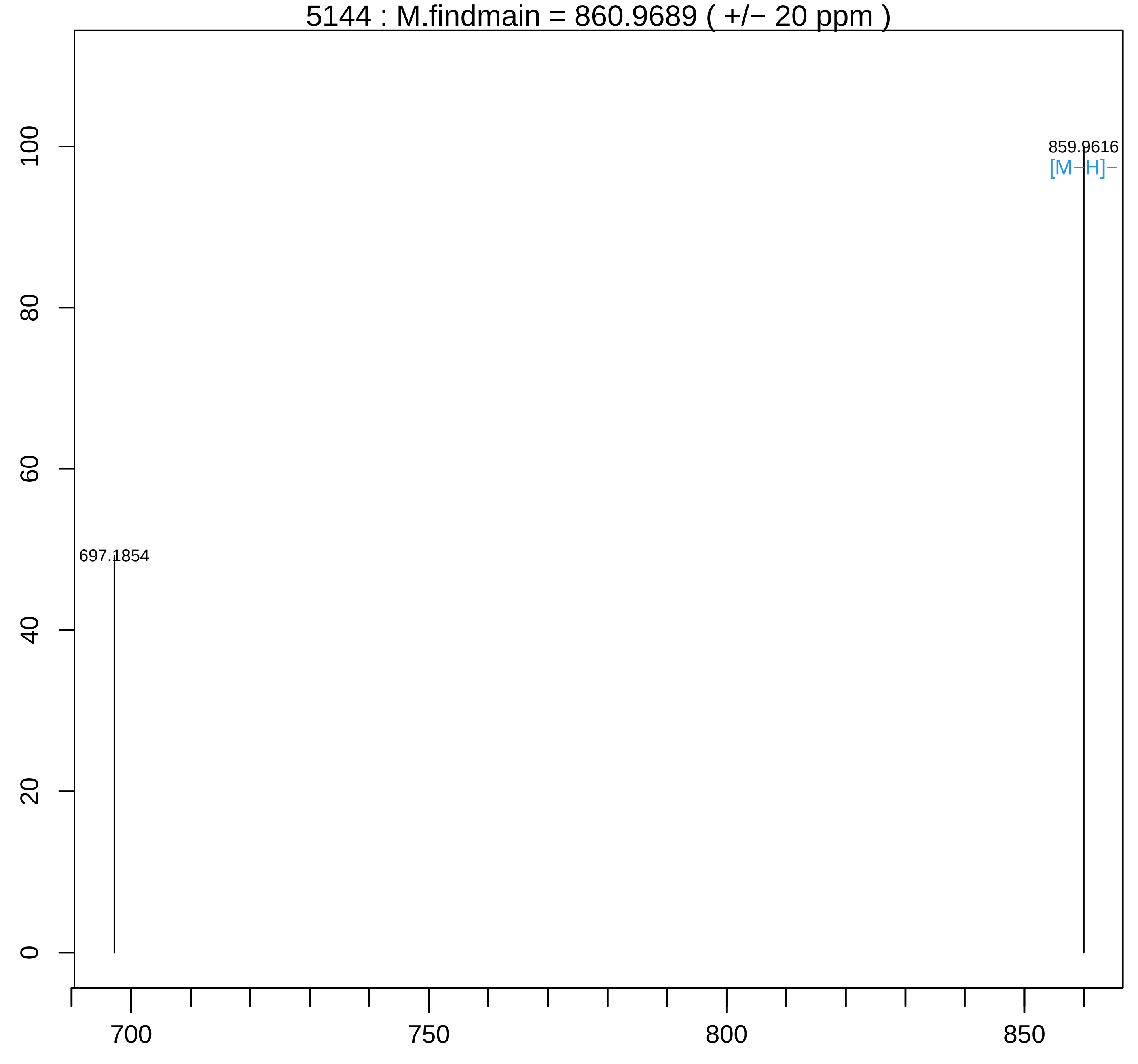
5144 : M.findmain = 860.9689 (+/− 20 ppm)
859.9616
[M−H]−
697.1854
0
20
40
60
80
100
700
750
800
850

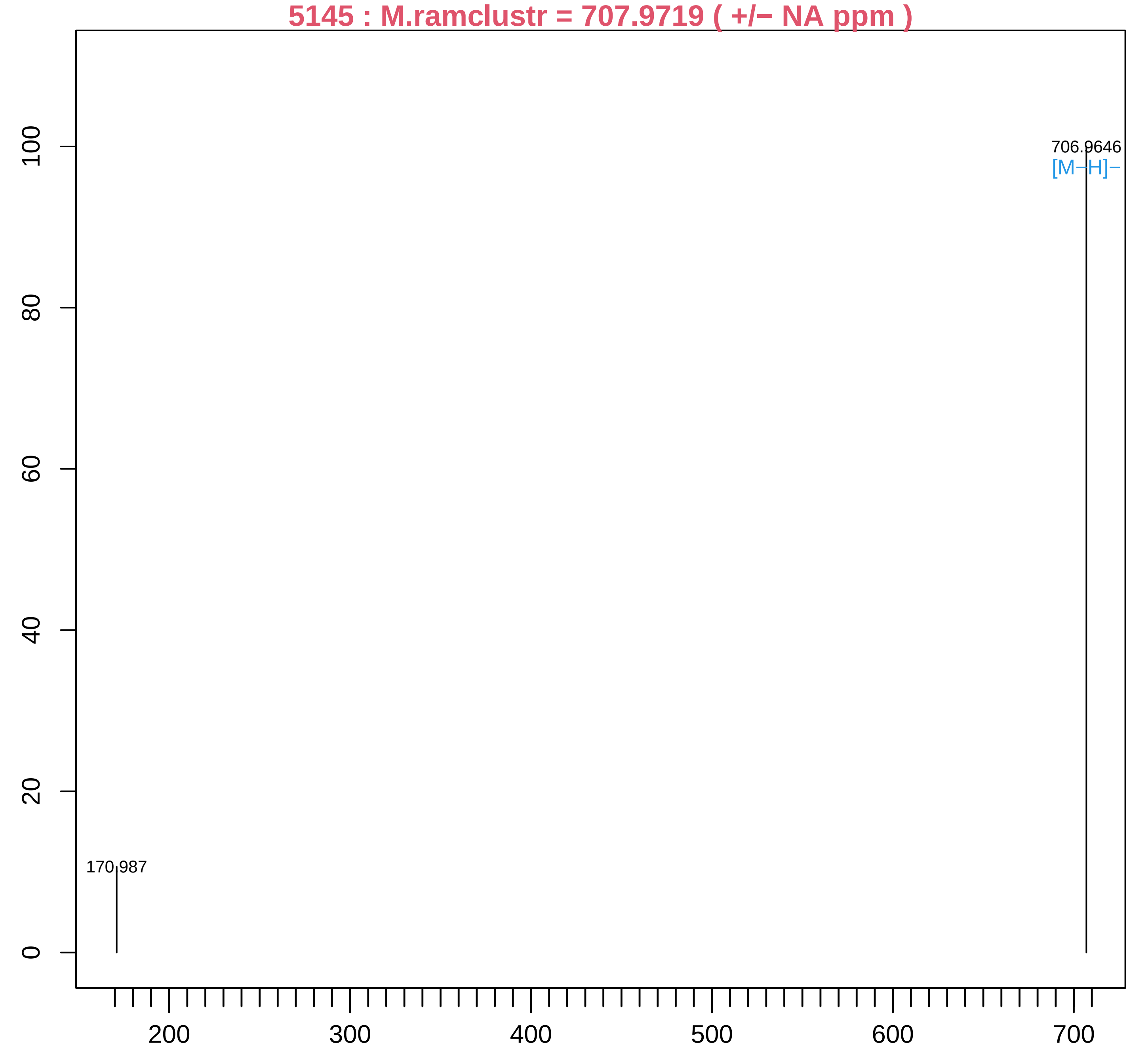

5145 : M.ramclustr = 707.9719 (+/− NA ppm)
706.9646
[M−H]−
170.987
0
20
40
60
80
100
200
300
400
500
600
700

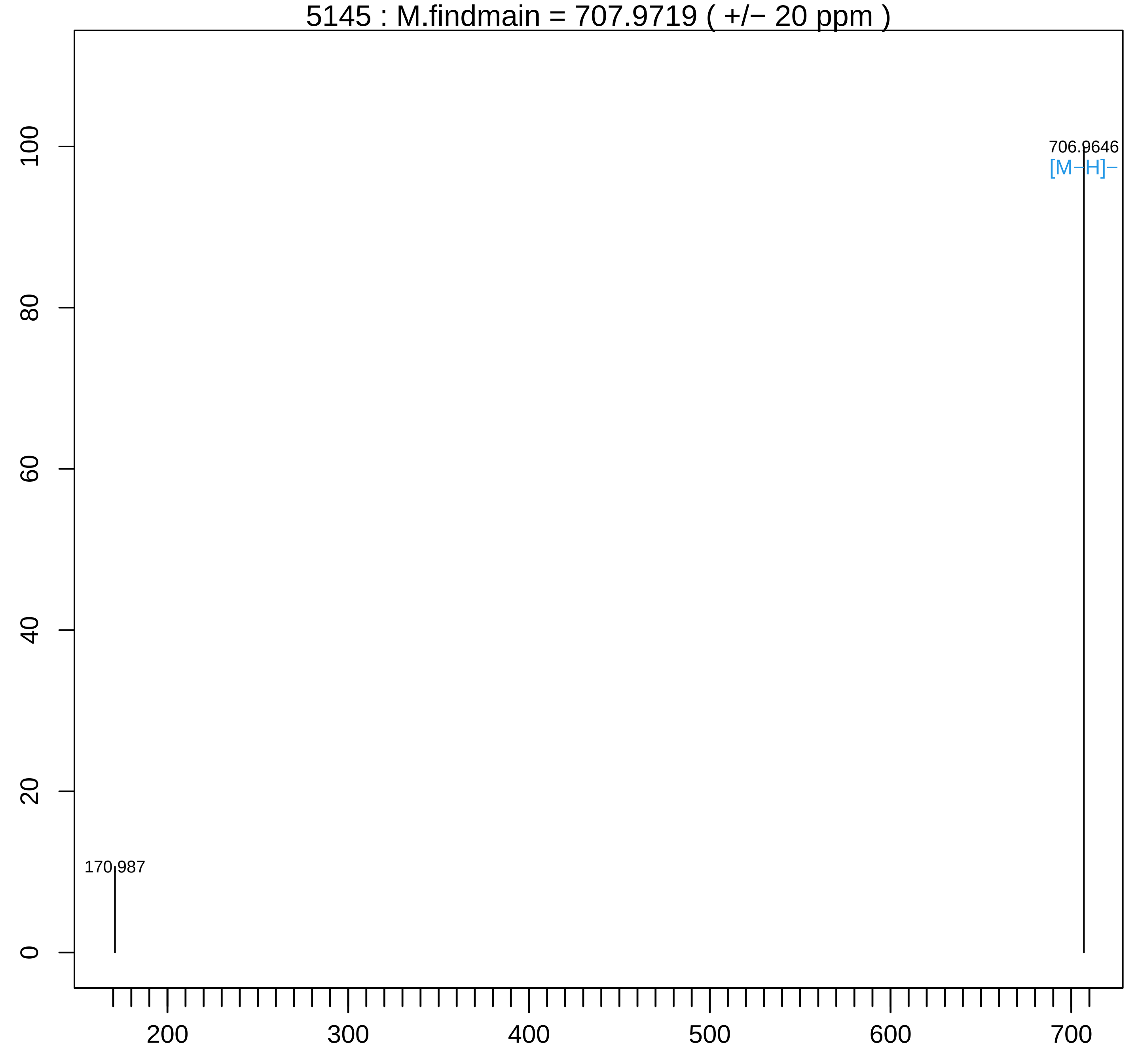

5145 : M.findmain = 707.9719 (+/− 20 ppm)
706.9646
[M−H]−
170.987
0
20
40
60
80
100
200
300
400
500
600
700

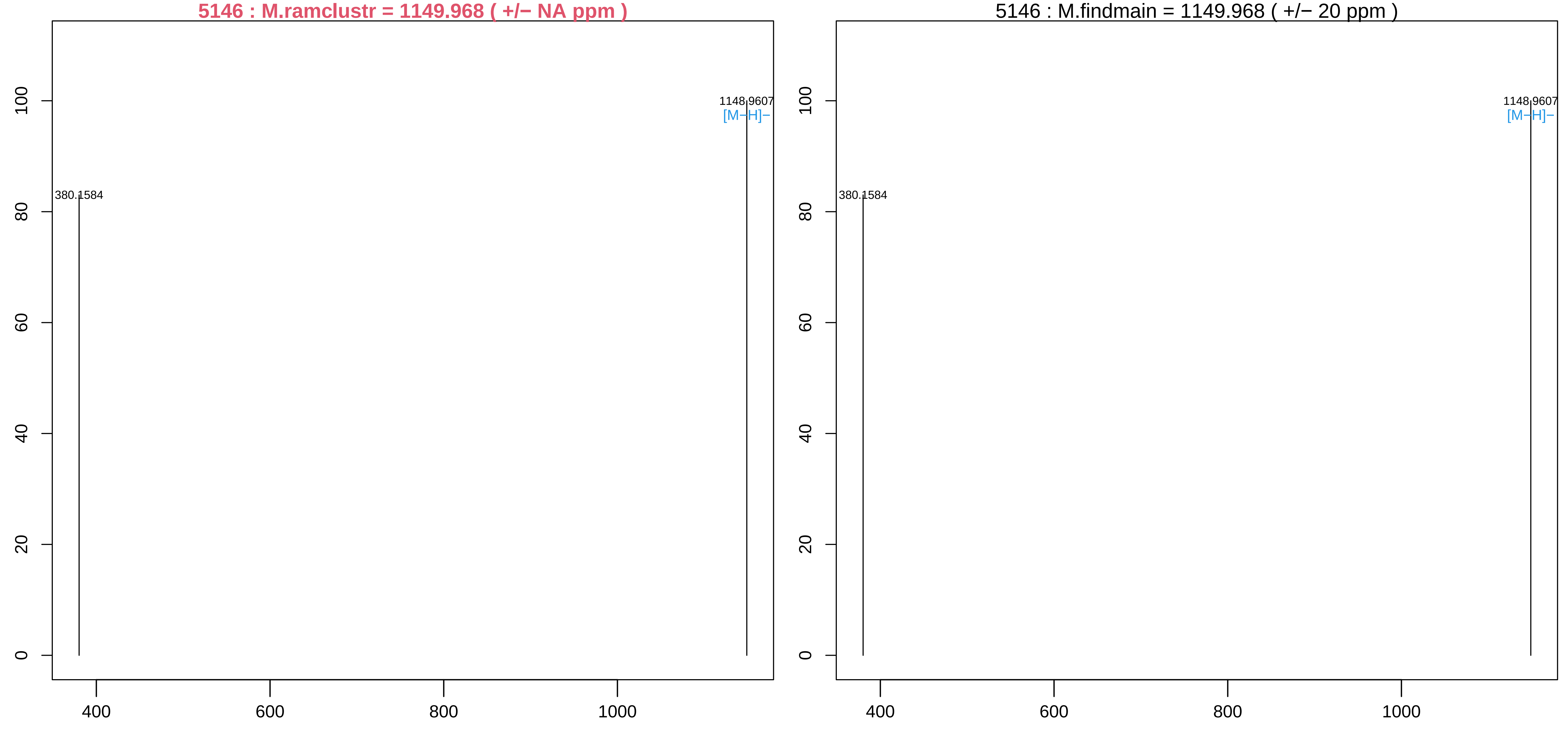
5146 : M.ramclustr = 1149.968 (+/− NA ppm)
1148.9607
[M−H]−
380.1584
0
20
40
60
80
100
400
600
800
1000
5146 : M.findmain = 1149.968 (+/− 20 ppm)
1148.9607
[M−H]−
380.1584
0
20
40
60
80
100
400
600
800
1000

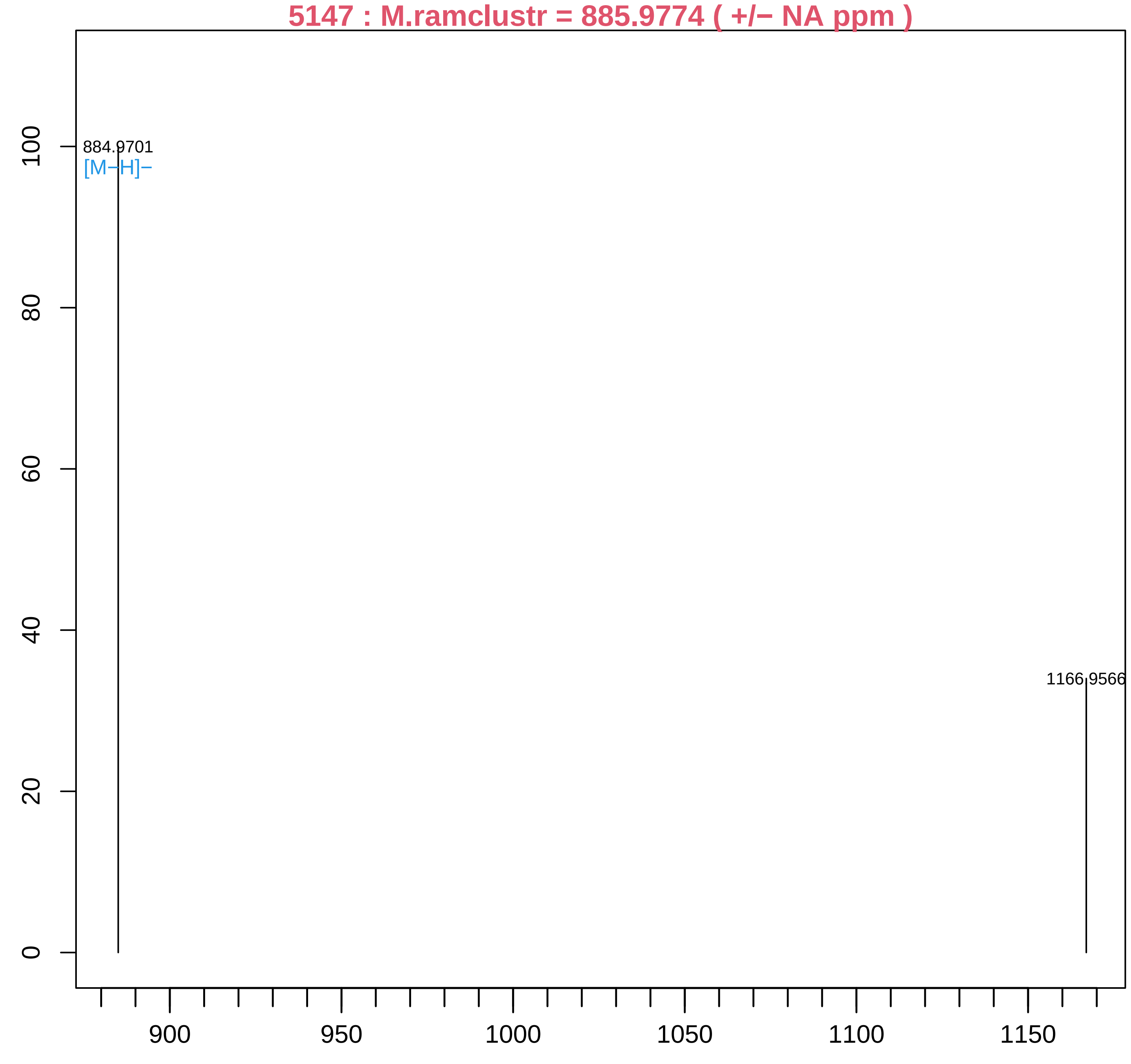
5147 : M.ramclustr = 885.9774 (+/− NA ppm)
884.9701
[M−H]−
1166.9566
0
20
40
60
80
100
900
950
1000
1050
1100
1150

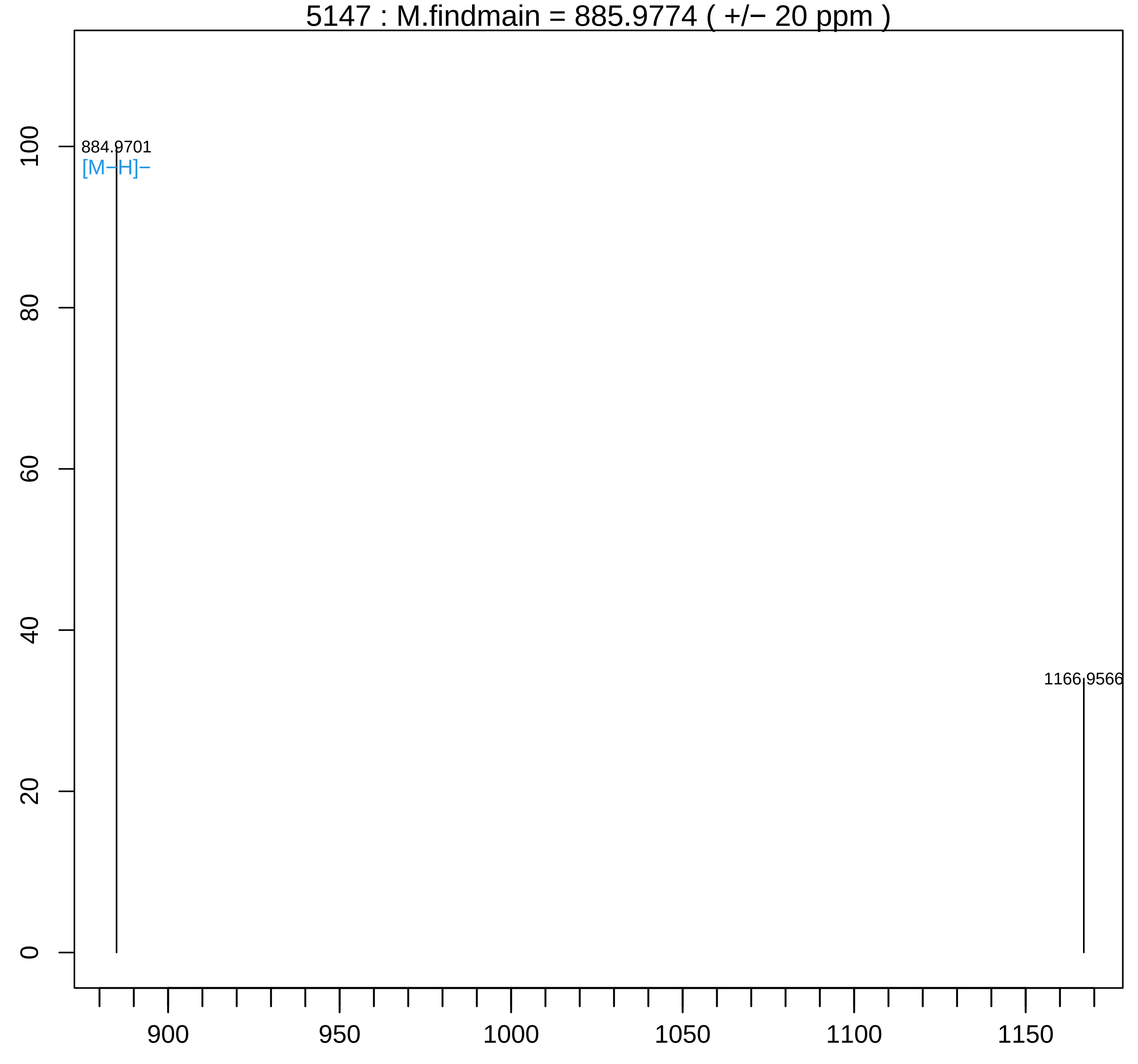
5147 : M.findmain = 885.9774 (+/− 20 ppm)
884.9701
[M−H]−
1166.9566
0
20
40
60
80
100
900
950
1000
1050
1100
1150

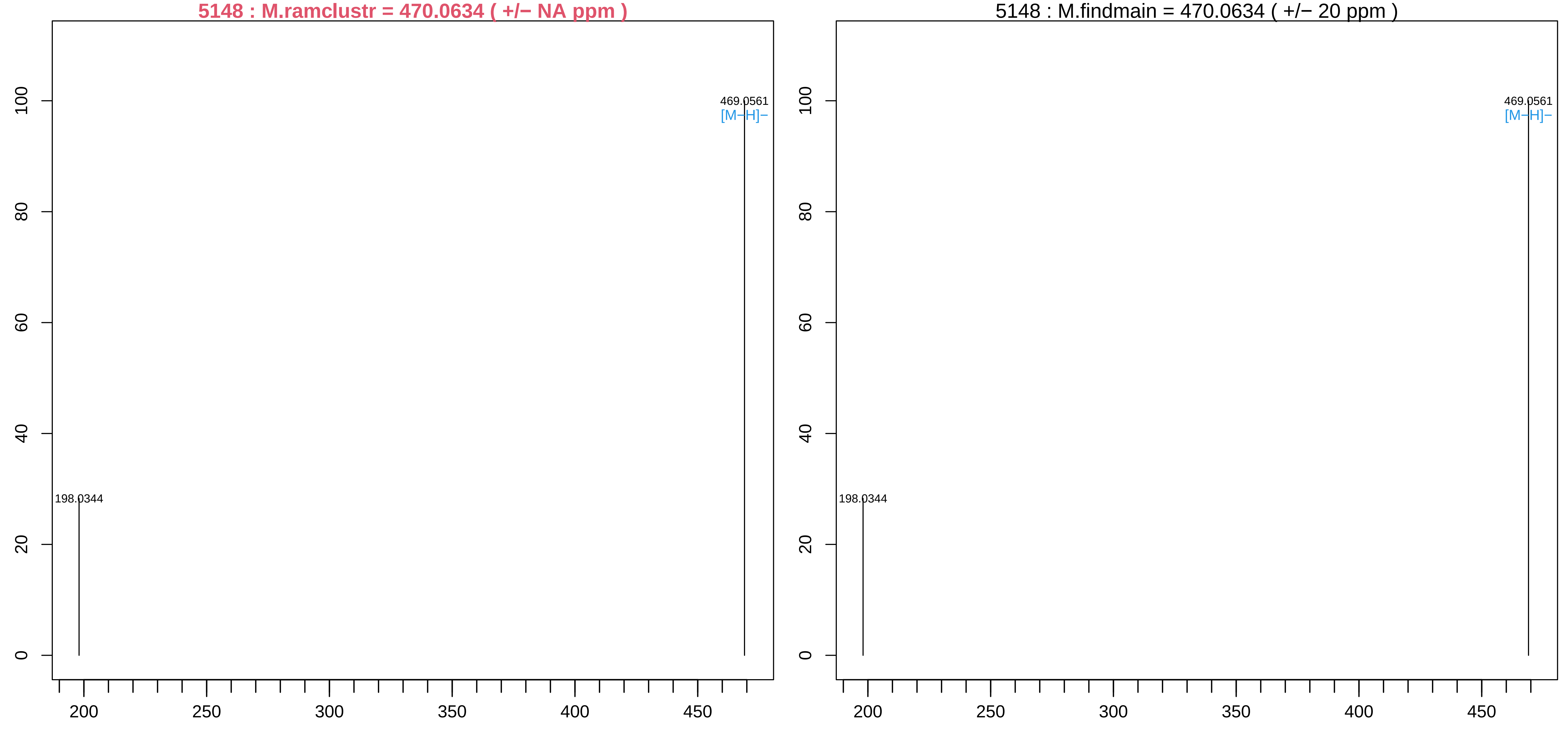

5148 : M.ramclustr = 470.0634 (+/− NA ppm)
469.0561
[M−H]−
198.0344
0
20
40
60
80
100
200
250
300
350
400
450
5148 : M.findmain = 470.0634 (+/− 20 ppm)
469.0561
[M−H]−
198.0344
0
20
40
60
80
100
200
250
300
350
400
450

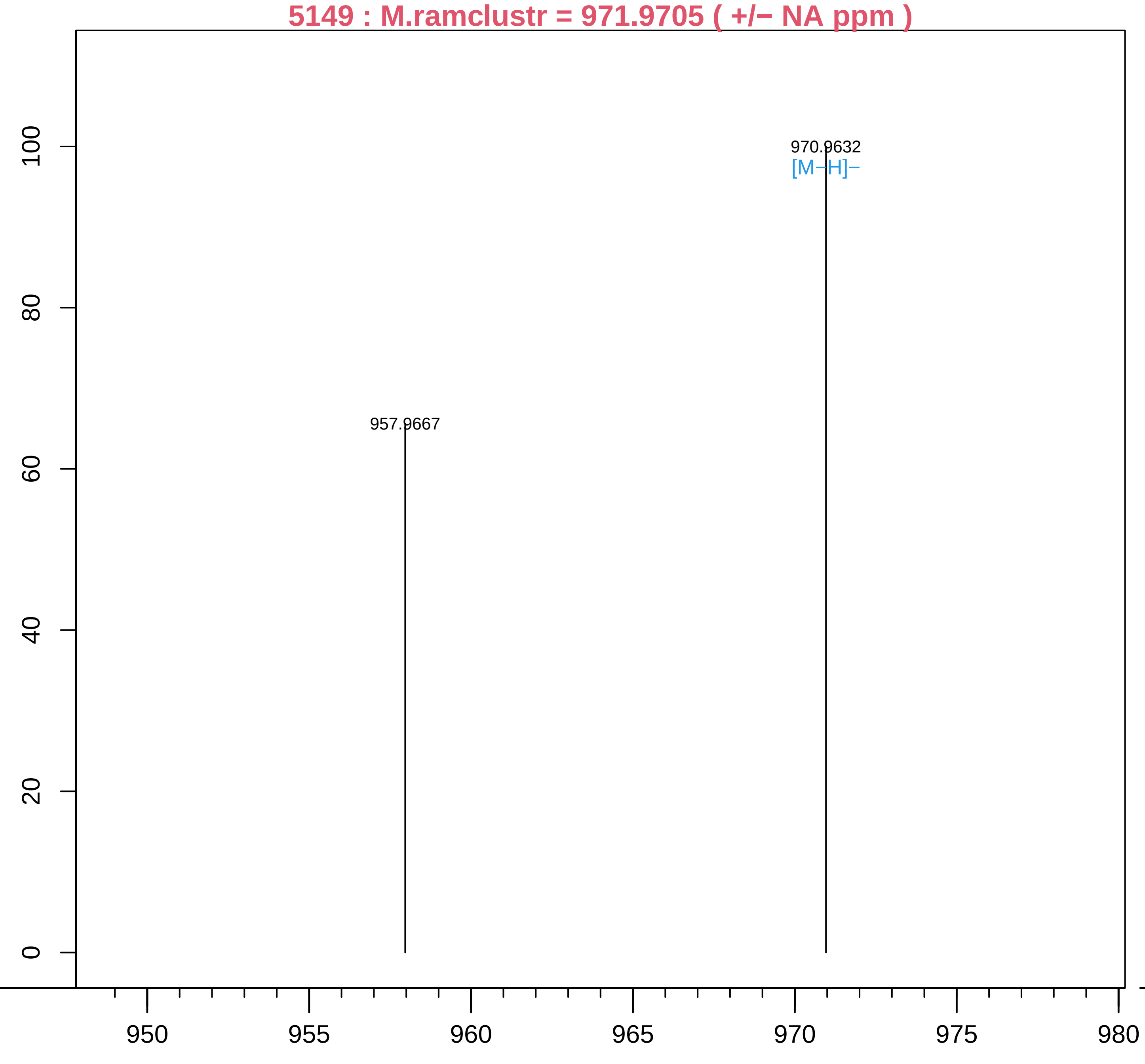
5149 : M.ramclustr = 971.9705 (+/− NA ppm)
970.9632
[M−H]−
957.9667
0
20
40
60
80
100
950
955
960
965
970
975
980

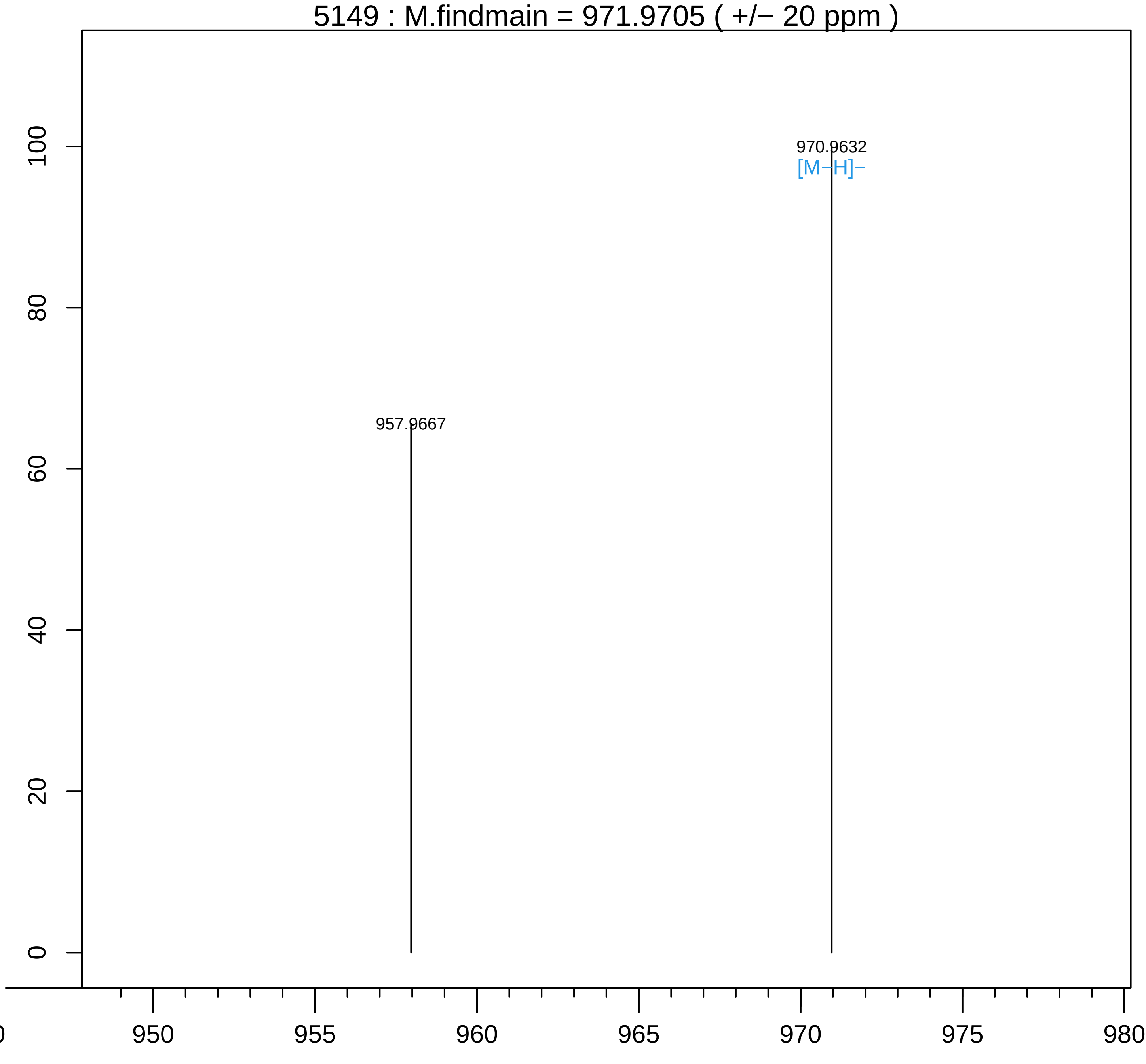
5149 : M.findmain = 971.9705 (+/− 20 ppm)
970.9632
[M−H]−
957.9667
0
20
40
60
80
100
950
955
960
965
970
975
980

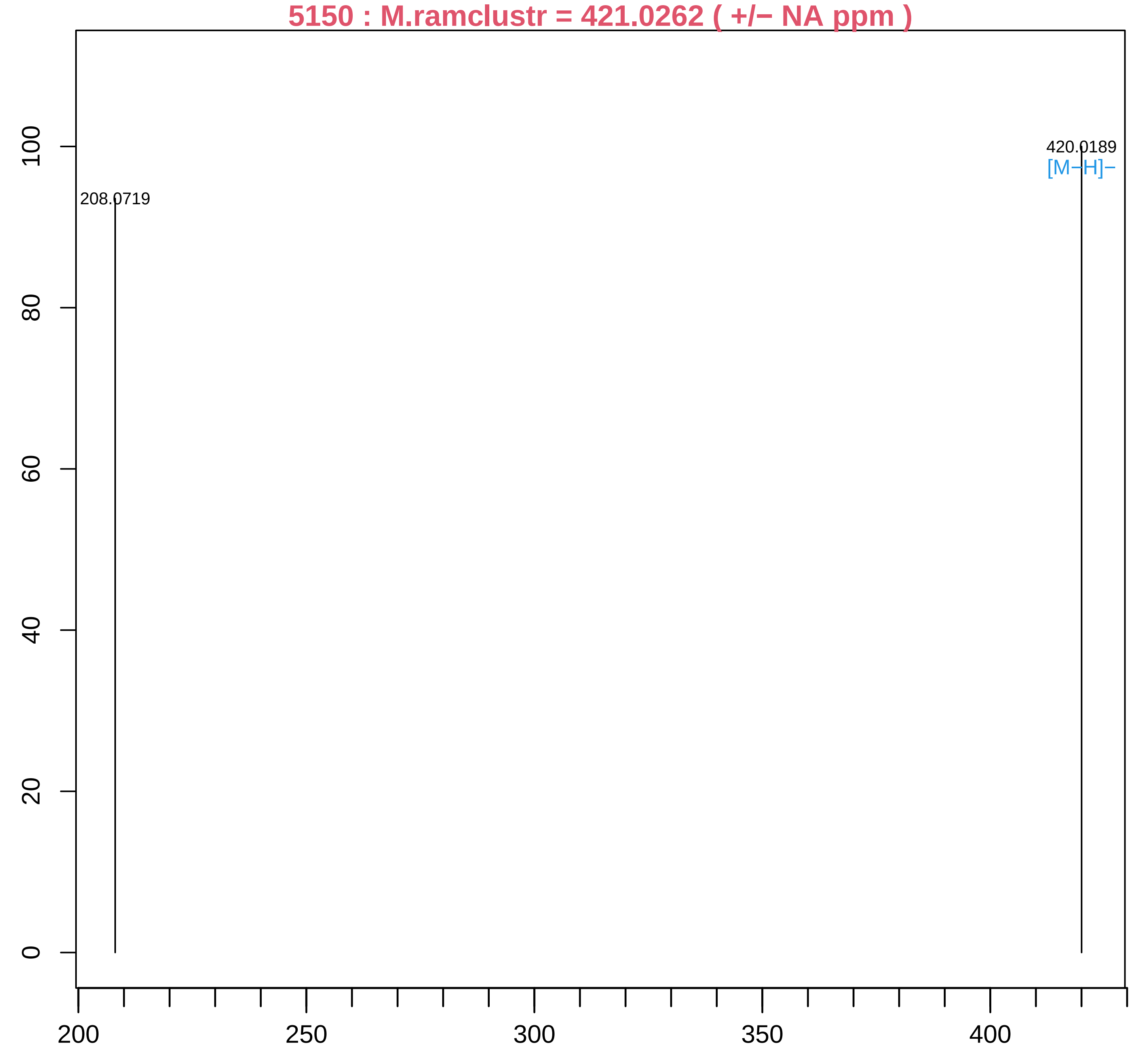
5150 : M.ramclustr = 421.0262 (+/− NA ppm)
208.0719
420.0189
[M−H]−
0
20
40
60
80
100
200
250
300
350
400

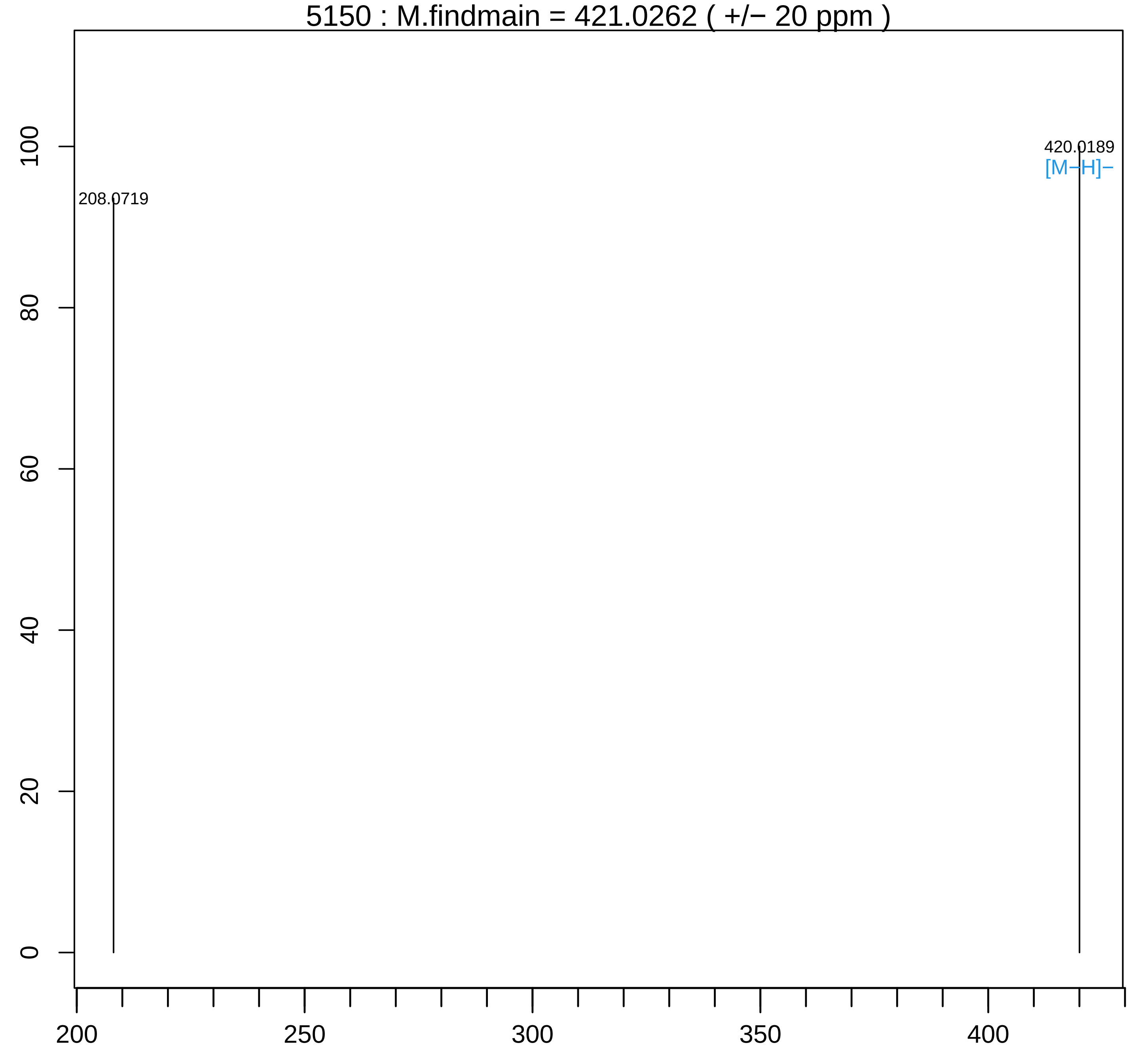
5150 : M.findmain = 421.0262 (+/− 20 ppm)
208.0719
420.0189
[M−H]−
0
20
40
60
80
100
200
250
300
350
400

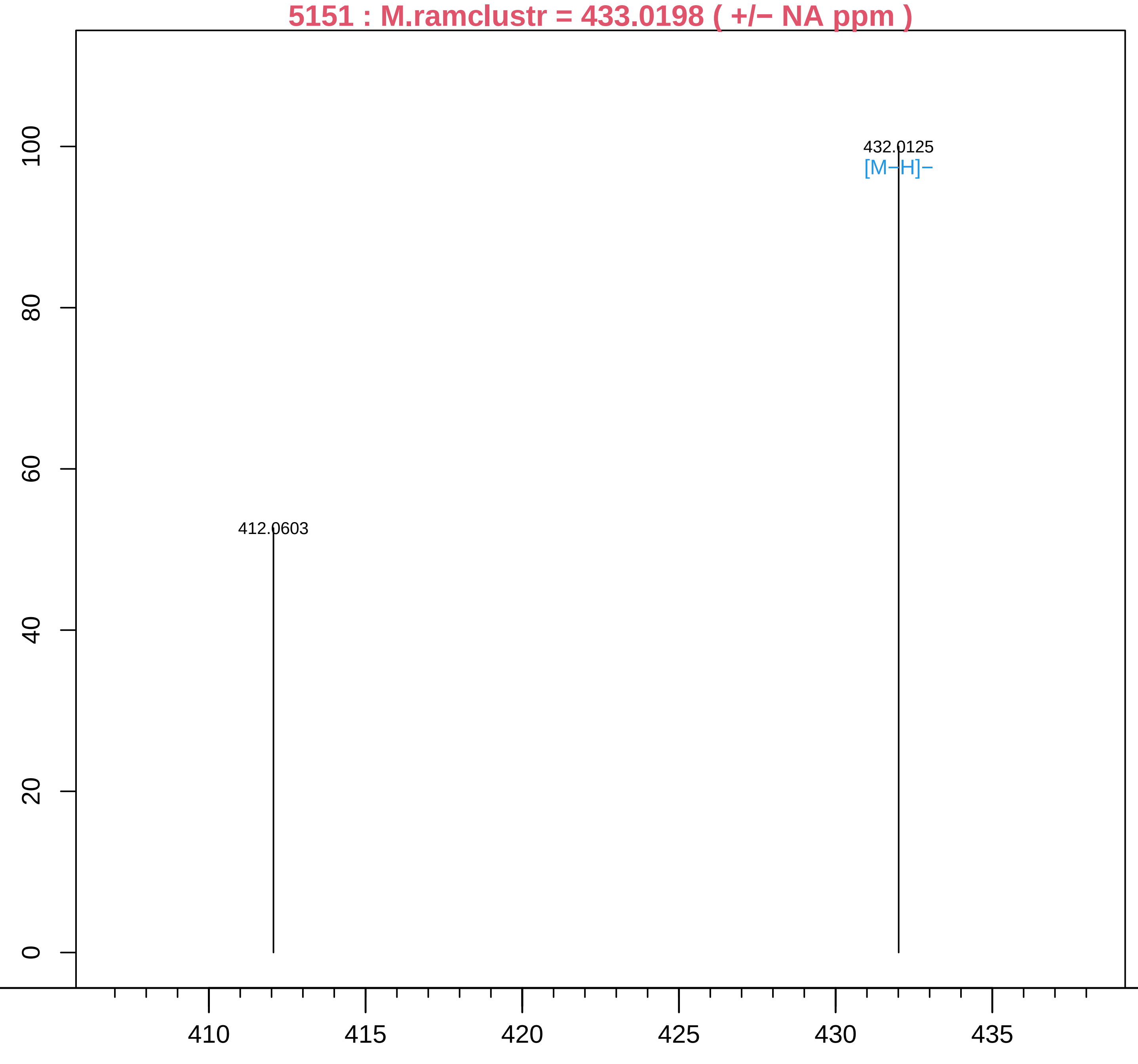
5151 : M.ramclustr = 433.0198 (+/− NA ppm)
432.0125
[M−H]−
412.0603
0
20
40
60
80
100
410
415
420
425
430
435

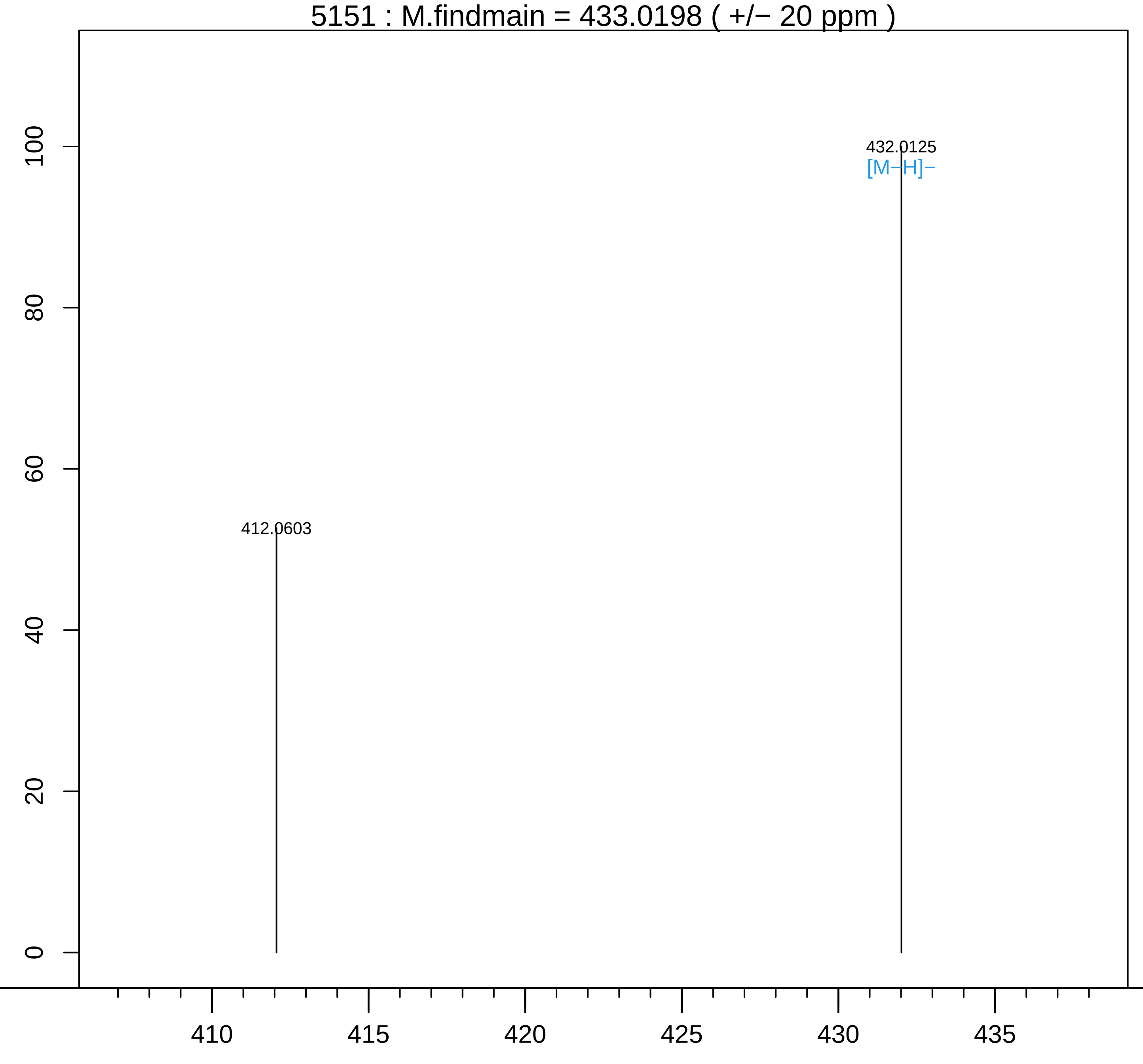
5151 : M.findmain = 433.0198 (+/− 20 ppm)
432.0125
[M−H]−
412.0603
0
20
40
60
80
100
410
415
420
425
430
435

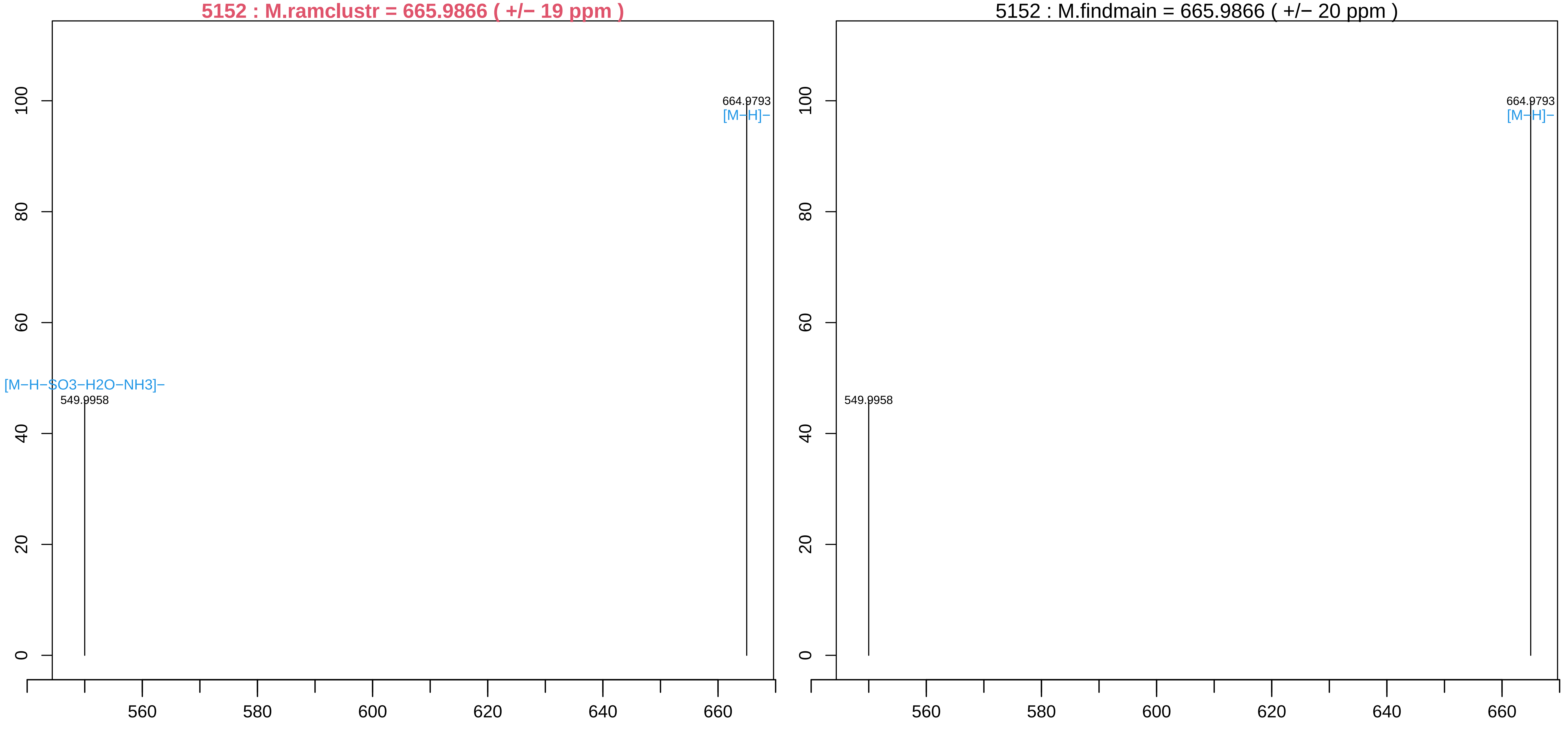
5152 : M.ramclustr = 665.9866 (+/− 19 ppm)
664.9793
[M−H]−
[M−H−SO3−H2O−NH3]−
549.9958
5152 : M.findmain = 665.9866 (+/− 20 ppm)
664.9793
[M−H]−
549.9958
0
20
40
60
80
100
560
580
600
620
640
660

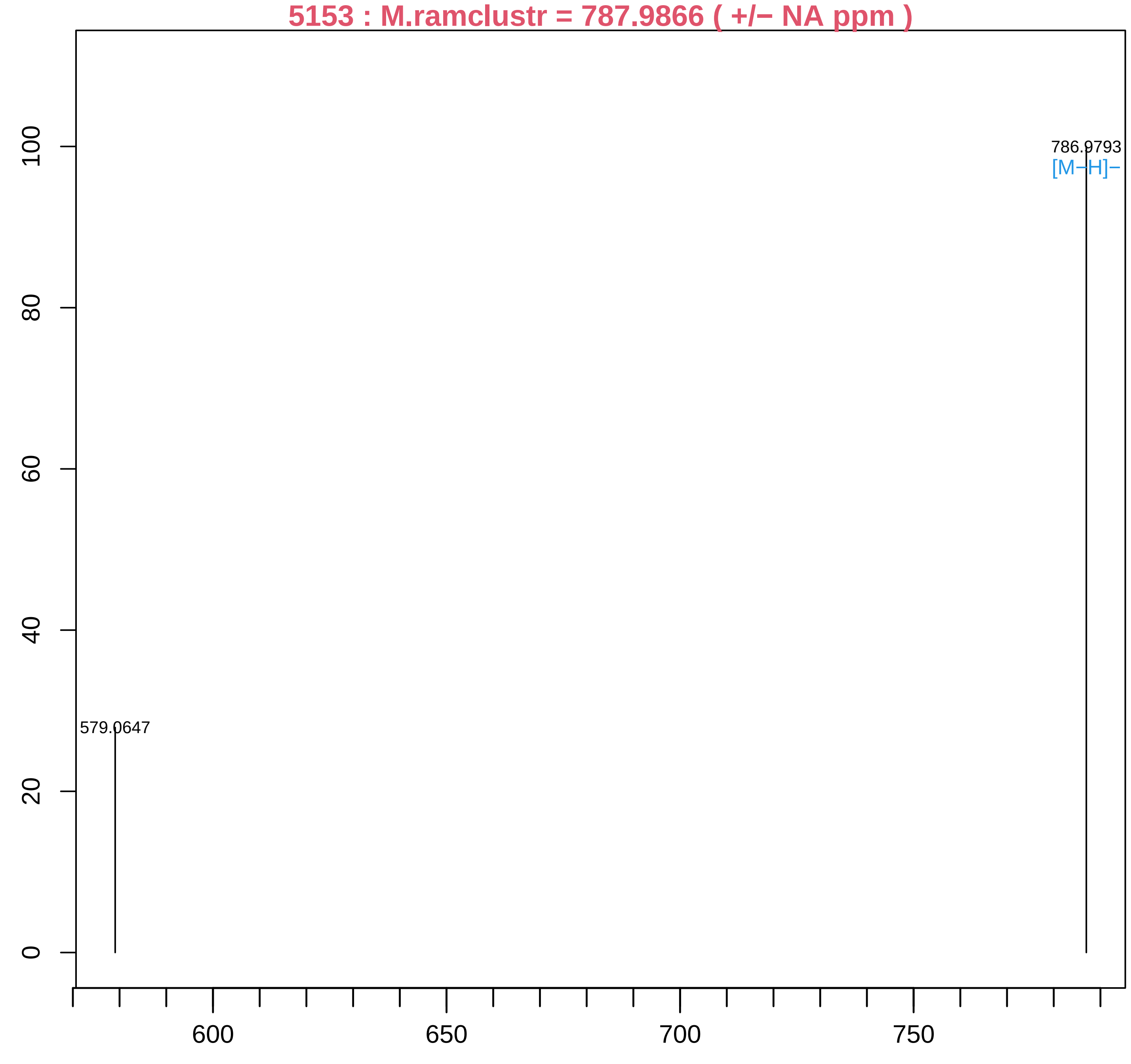
5153 : M.ramclustr = 787.9866 (+/− NA ppm)
786.9793
[M−H]−
579.0647
0
20
40
60
80
100
600
650
700
750

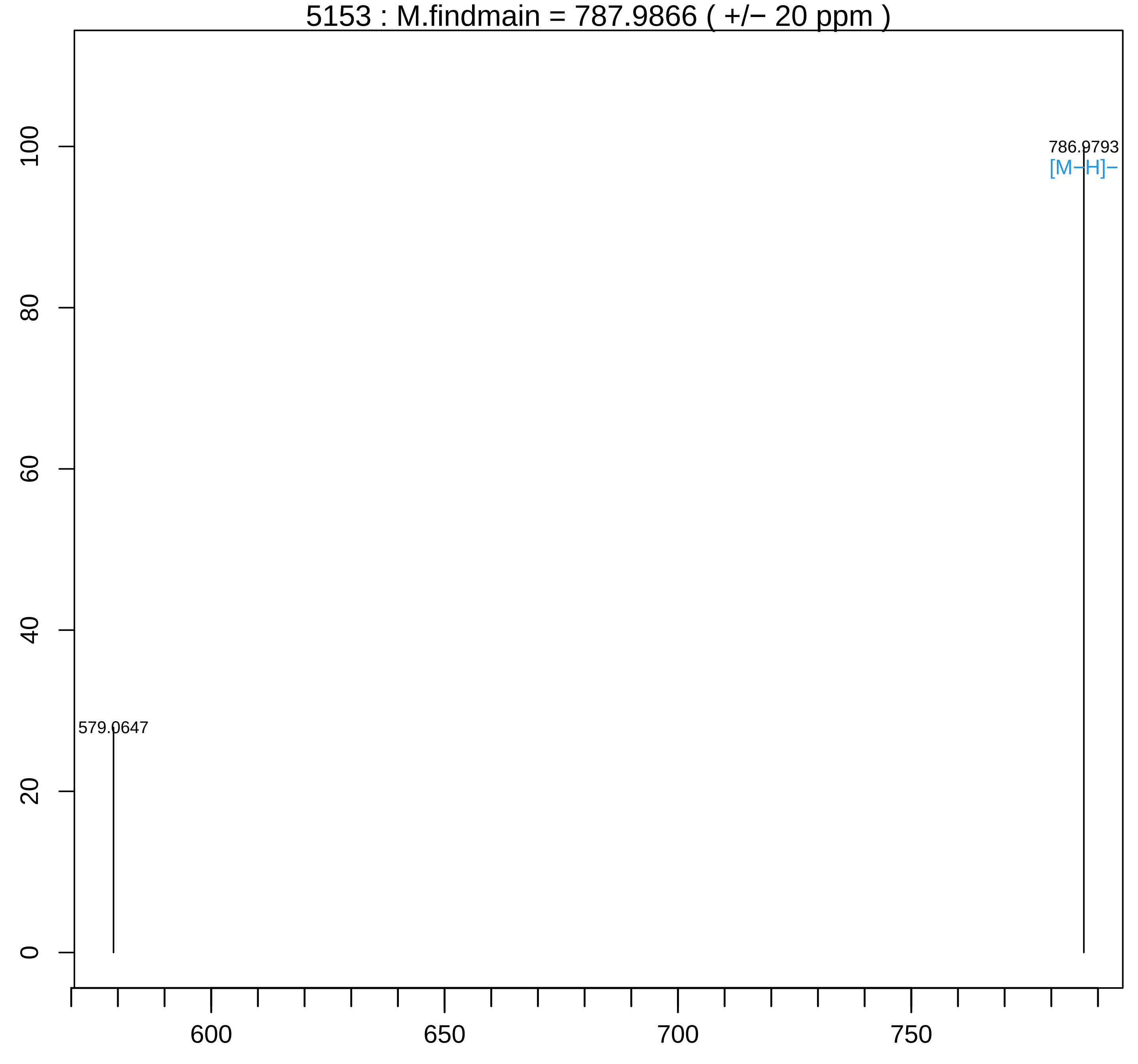
5153 : M.findmain = 787.9866 (+/− 20 ppm)
786.9793
[M−H]−
579.0647
0
20
40
60
80
100
600
650
700
750

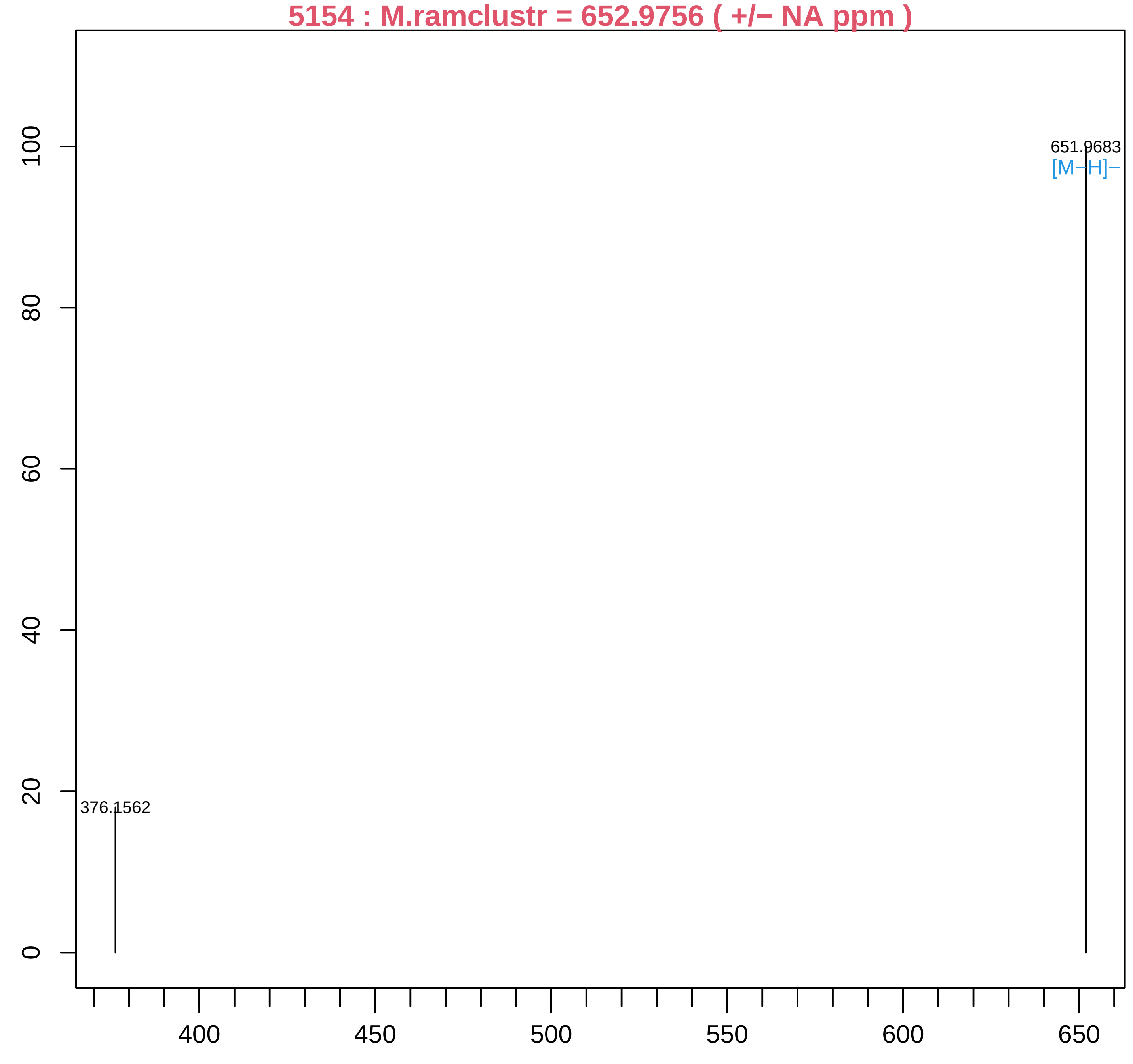
5154 : M.ramclustr = 652.9756 (+/− NA ppm)
651.9683
[M−H]−
376.1562
0
20
40
60
80
100
400
450
500
550
600
650

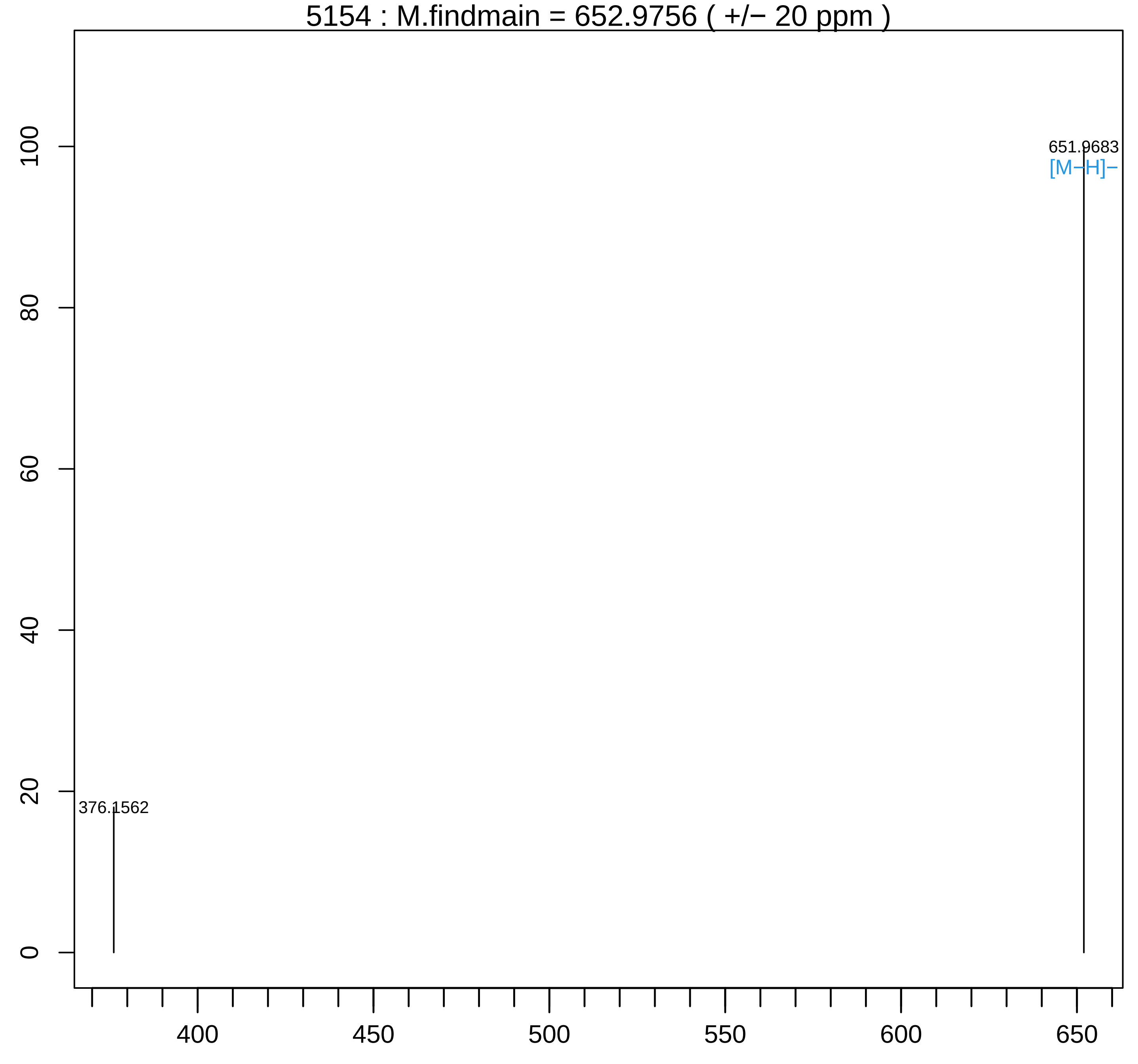
5154 : M.findmain = 652.9756 (+/− 20 ppm)
651.9683
[M−H]−
376.1562
0
20
40
60
80
100
400
450
500
550
600
650

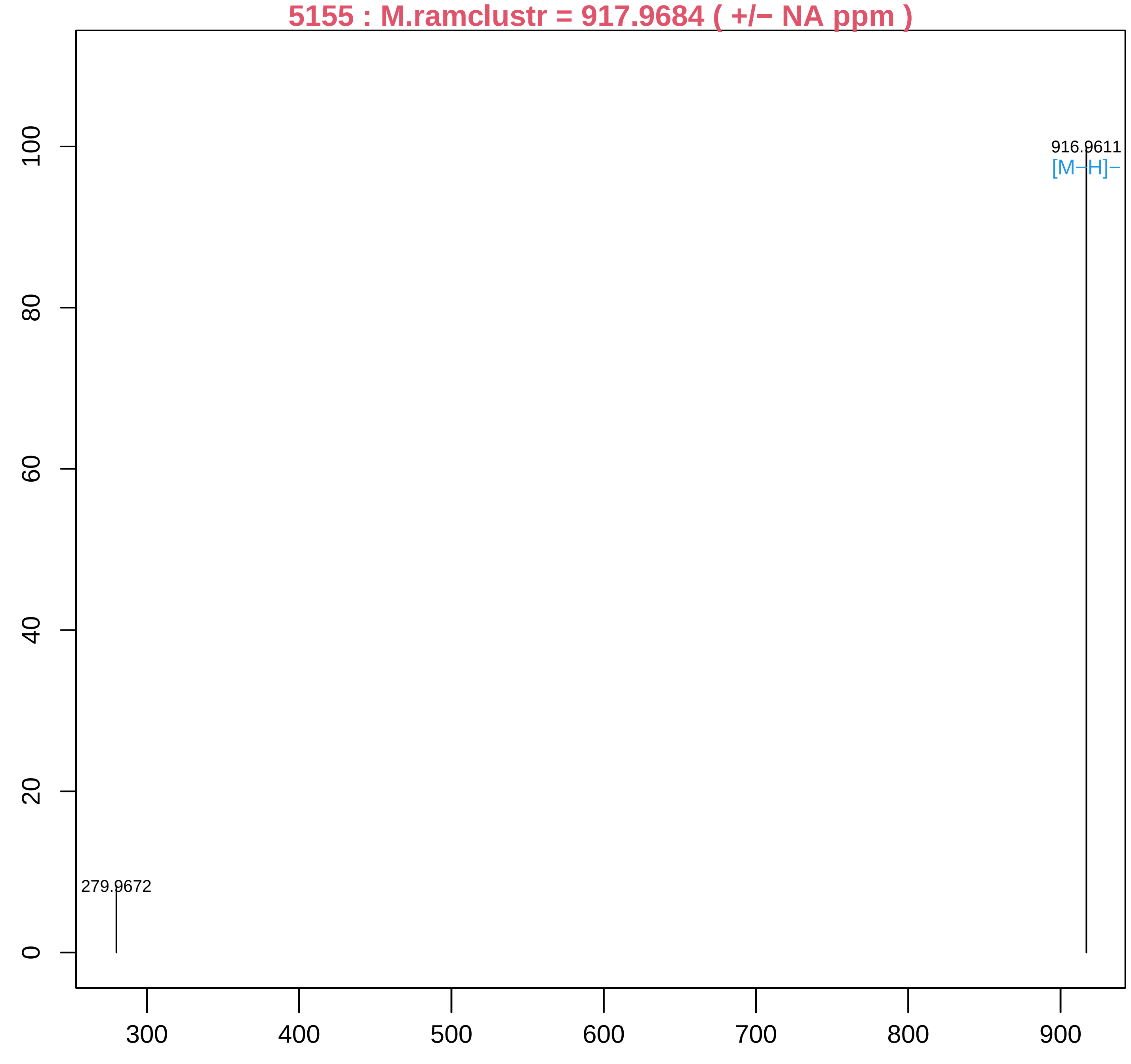
5155 : M.ramclustr = 917.9684 (+/− NA ppm)
916.9611
[M−H]−
279.9672
0
20
40
60
80
100
300
400
500
600
700
800
900

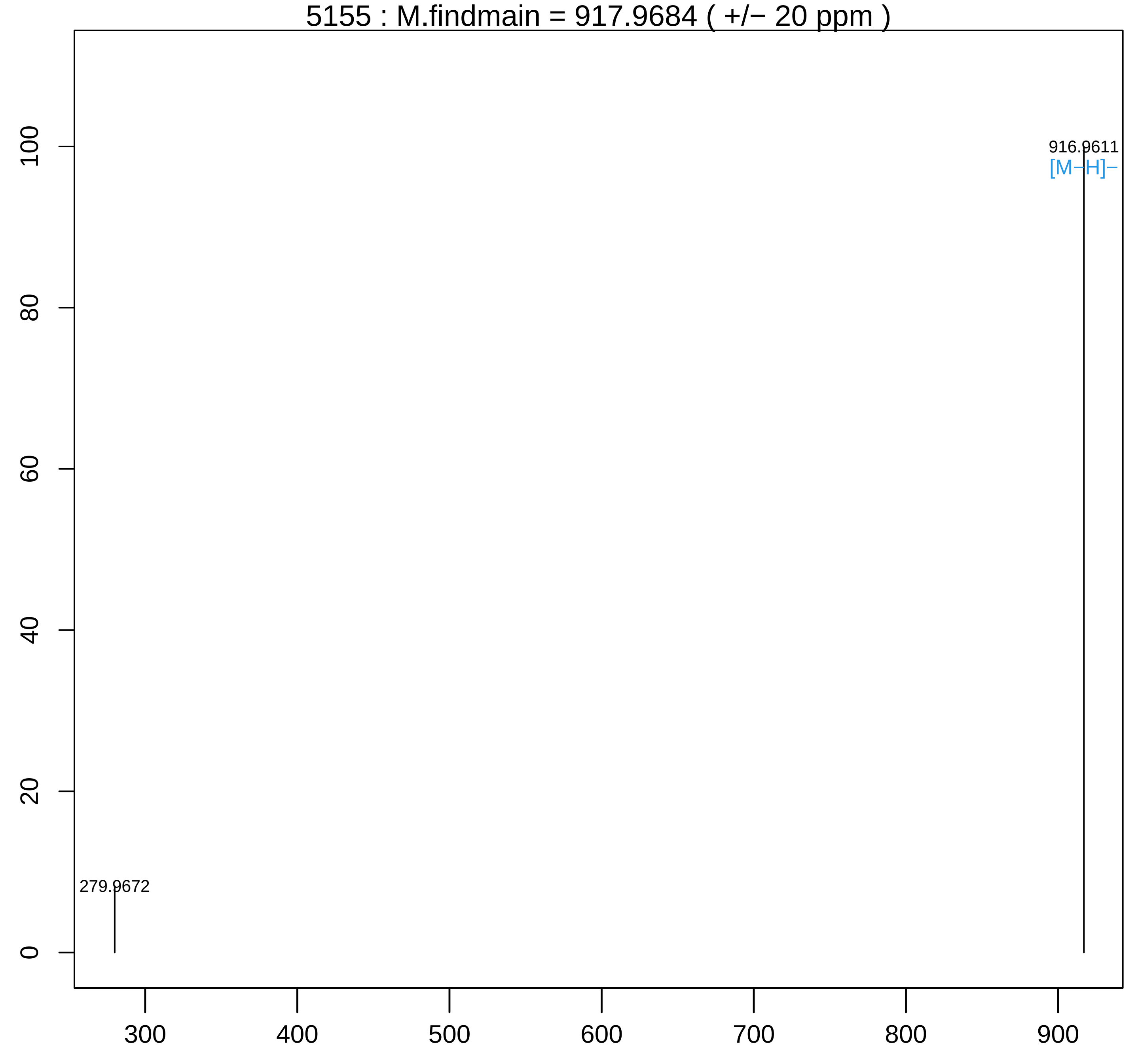
5155 : M.findmain = 917.9684 (+/− 20 ppm)
916.9611
[M−H]−
279.9672
0
20
40
60
80
100
300
400
500
600
700
800
900

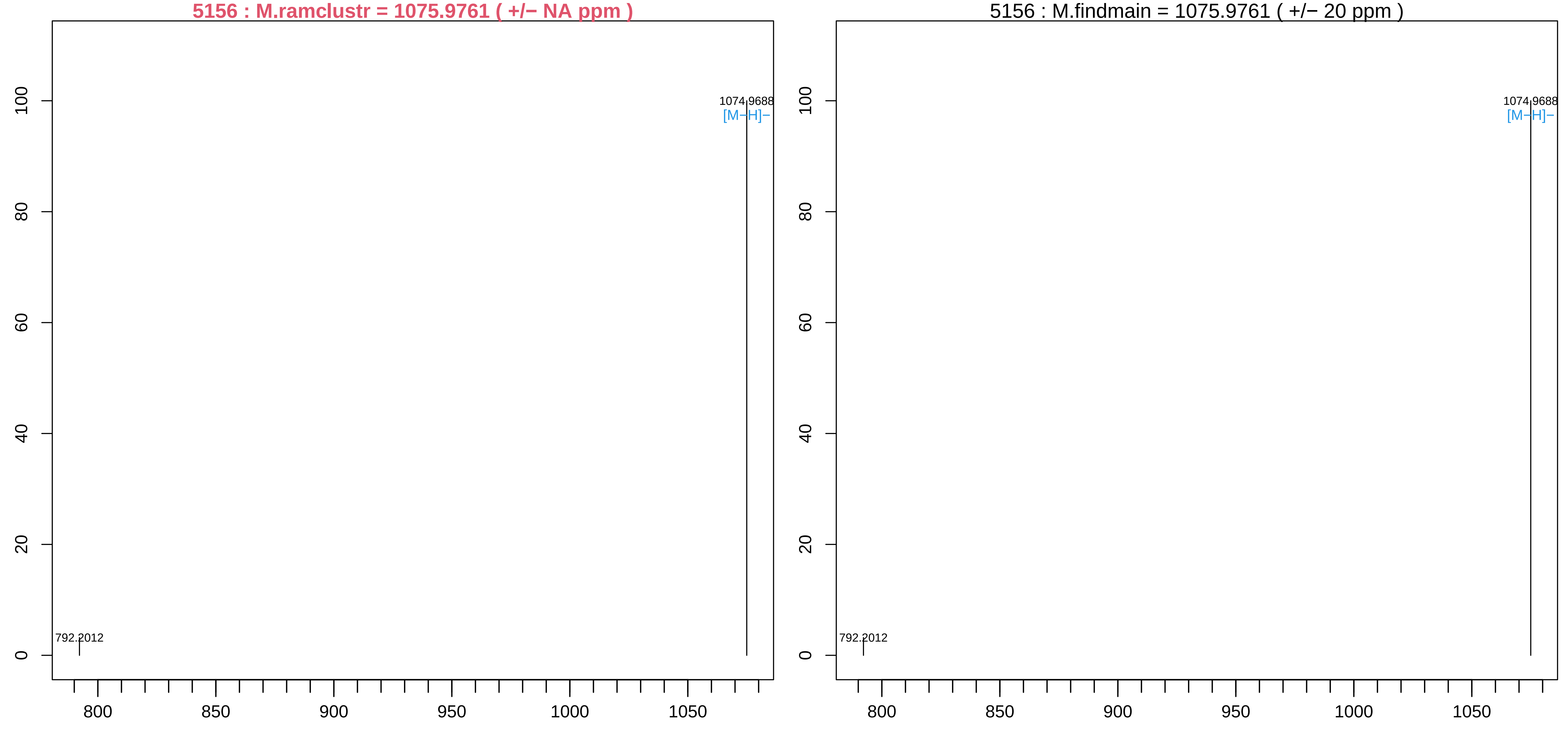

5156 : M.ramclustr = 1075.9761 (+/− NA ppm)
5156 : M.findmain = 1075.9761 (+/− 20 ppm)
1074.9688
[M−H]−
792.2012
0
20
40
60
80
100
800
850
900
950
1000
1050

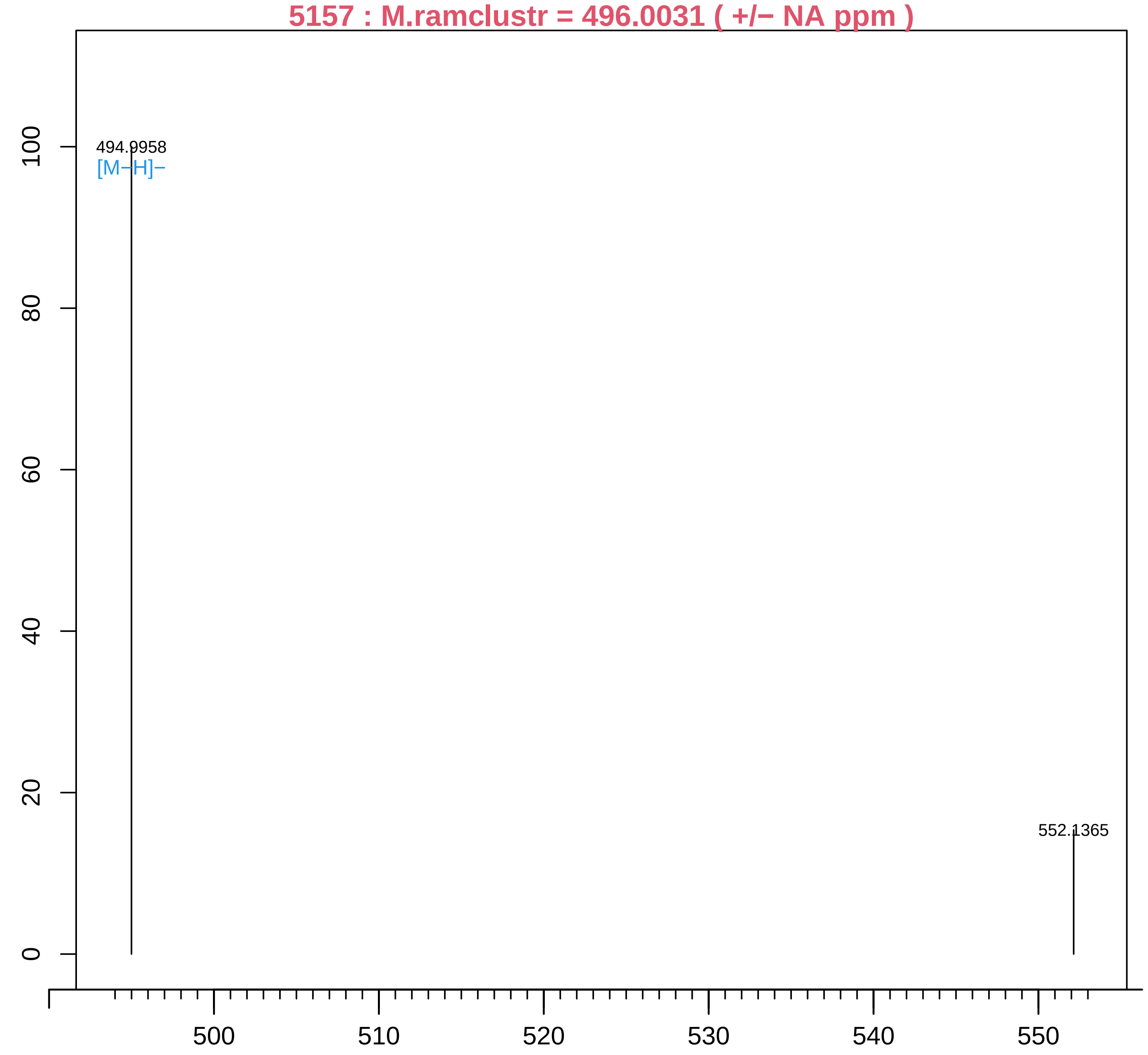
5157 : M.ramclustr = 496.0031 (+/− NA ppm)
494.9958
[M−H]−
552.1365
0
20
40
60
80
100
500
510
520
530
540
550

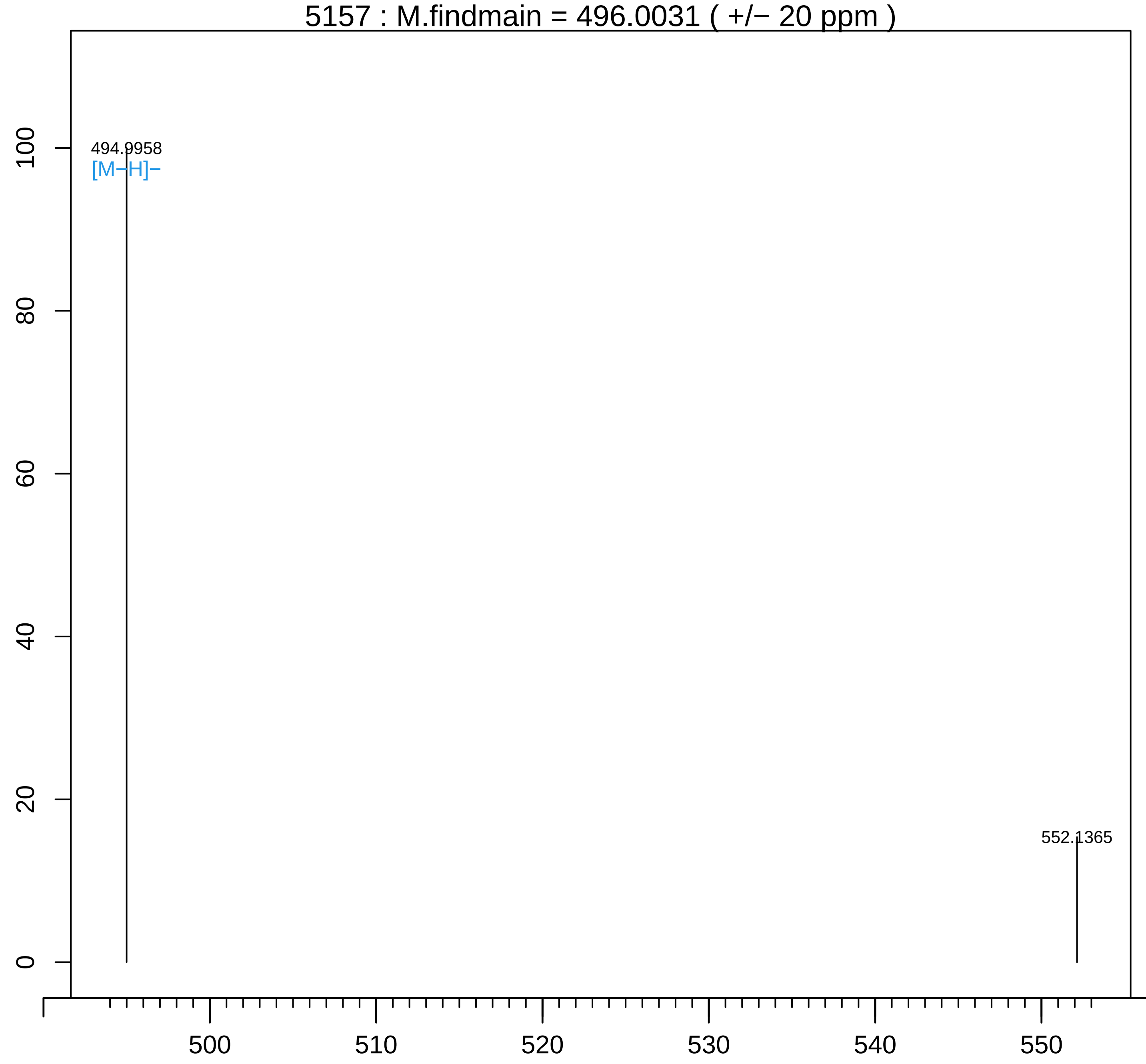
5157 : M.findmain = 496.0031 (+/− 20 ppm)
494.9958
[M−H]−
552.1365
0
20
40
60
80
100
500
510
520
530
540
550

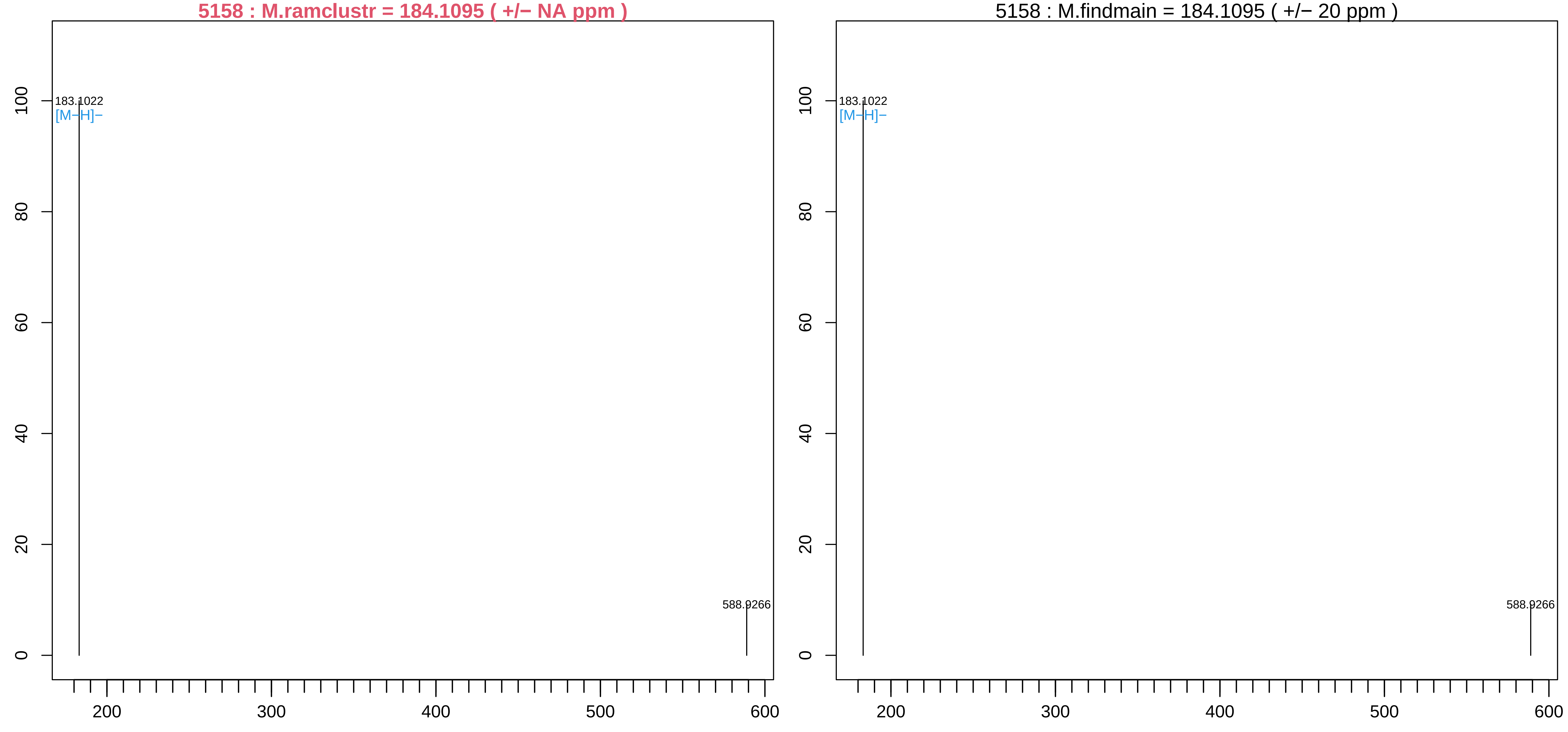
5158 : M.ramclustr = 184.1095 (+/− NA ppm)
183.1022
[M−H]−
588.9266
0
20
40
60
80
100
200
300
400
500
600
5158 : M.findmain = 184.1095 (+/− 20 ppm)
183.1022
[M−H]−
588.9266
0
20
40
60
80
100
200
300
400
500
600

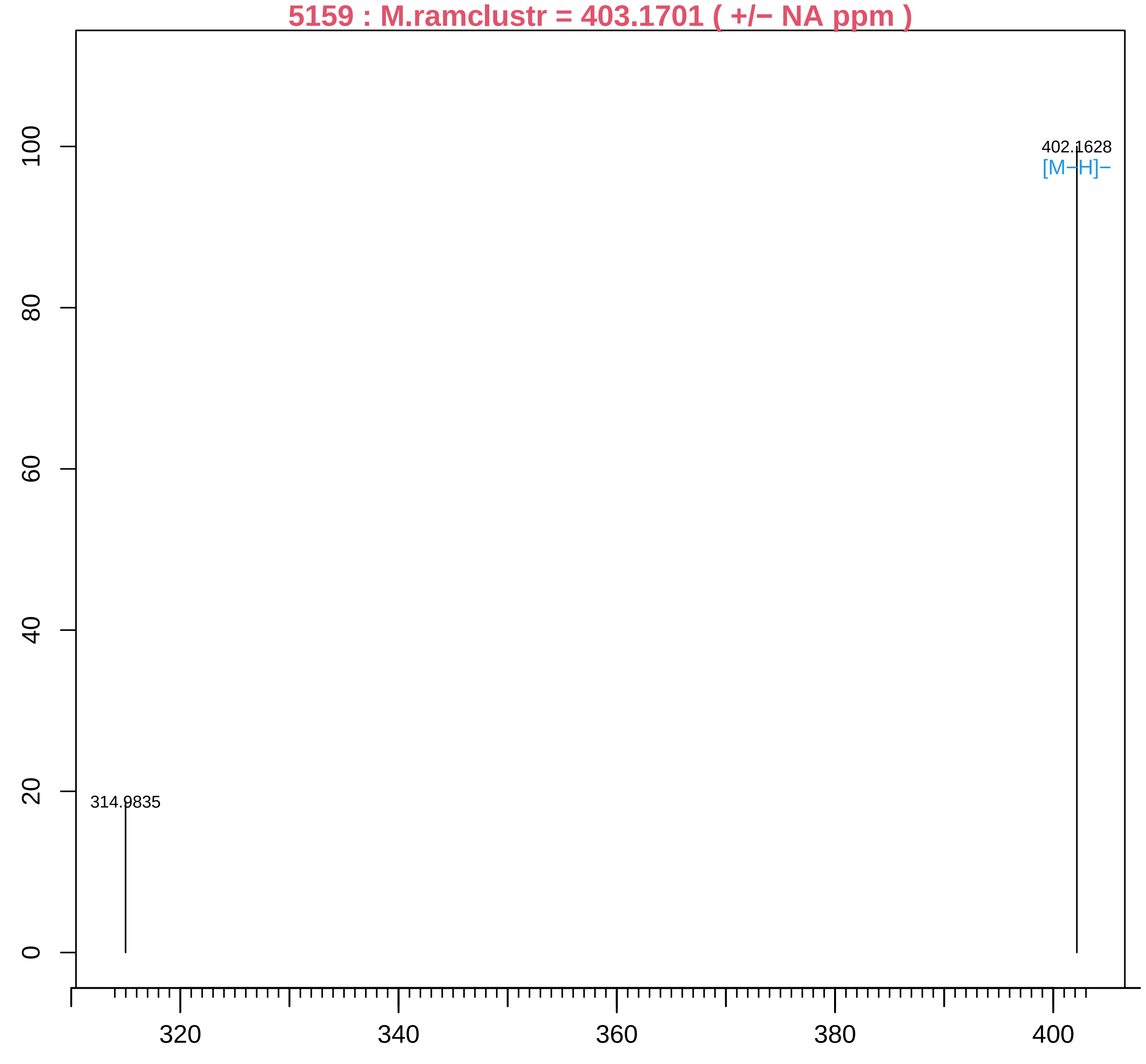
5159 : M.ramclustr = 403.1701 (+/− NA ppm)
402.1628
[M−H]−
314.9835
0
20
40
60
80
100
320
340
360
380
400

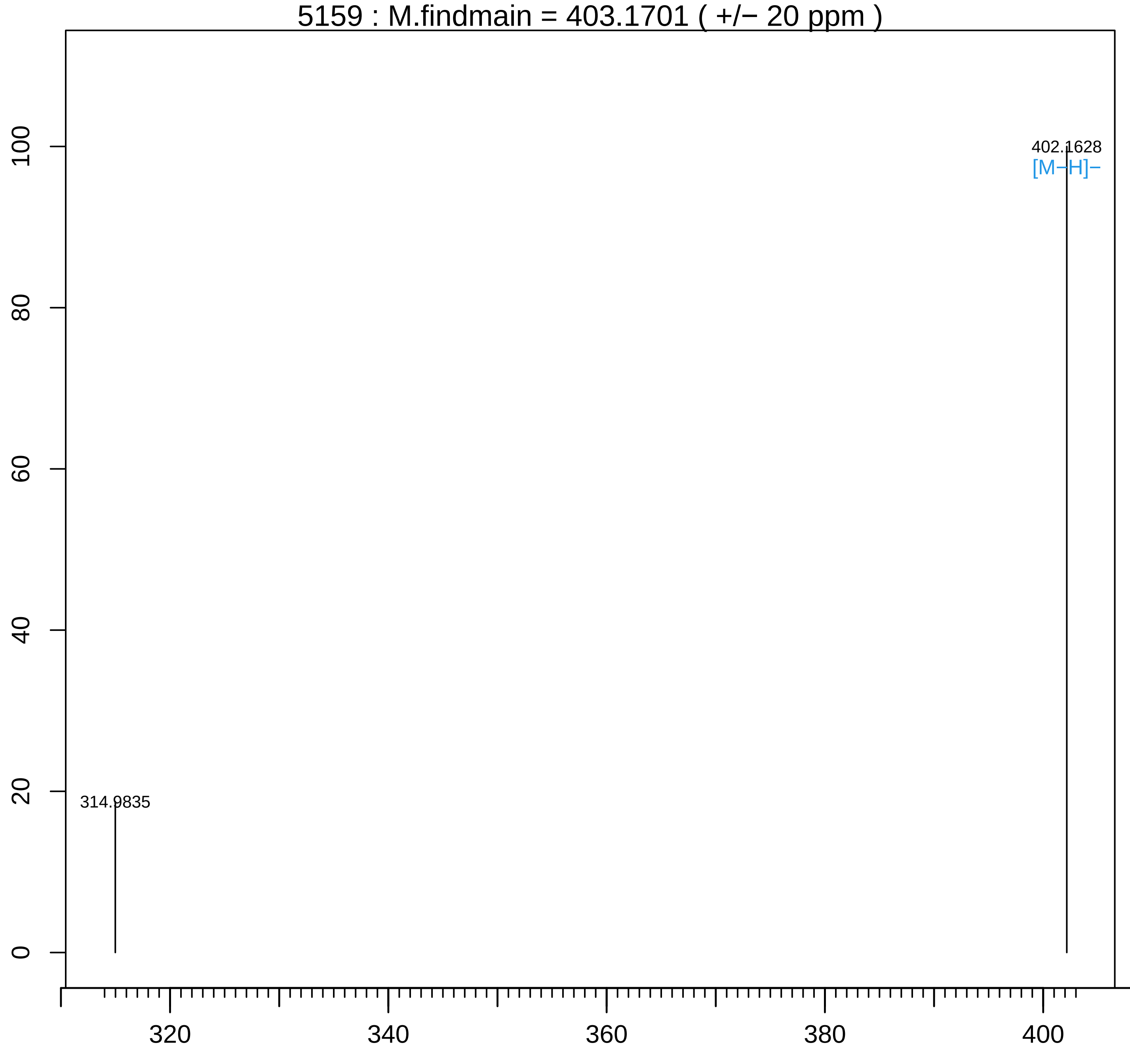
5159 : M.findmain = 403.1701 (+/− 20 ppm)
402.1628
[M−H]−
314.9835
0
20
40
60
80
100
320
340
360
380
400

5160 : M.ramclustr = 591.9767 (+/− NA ppm)

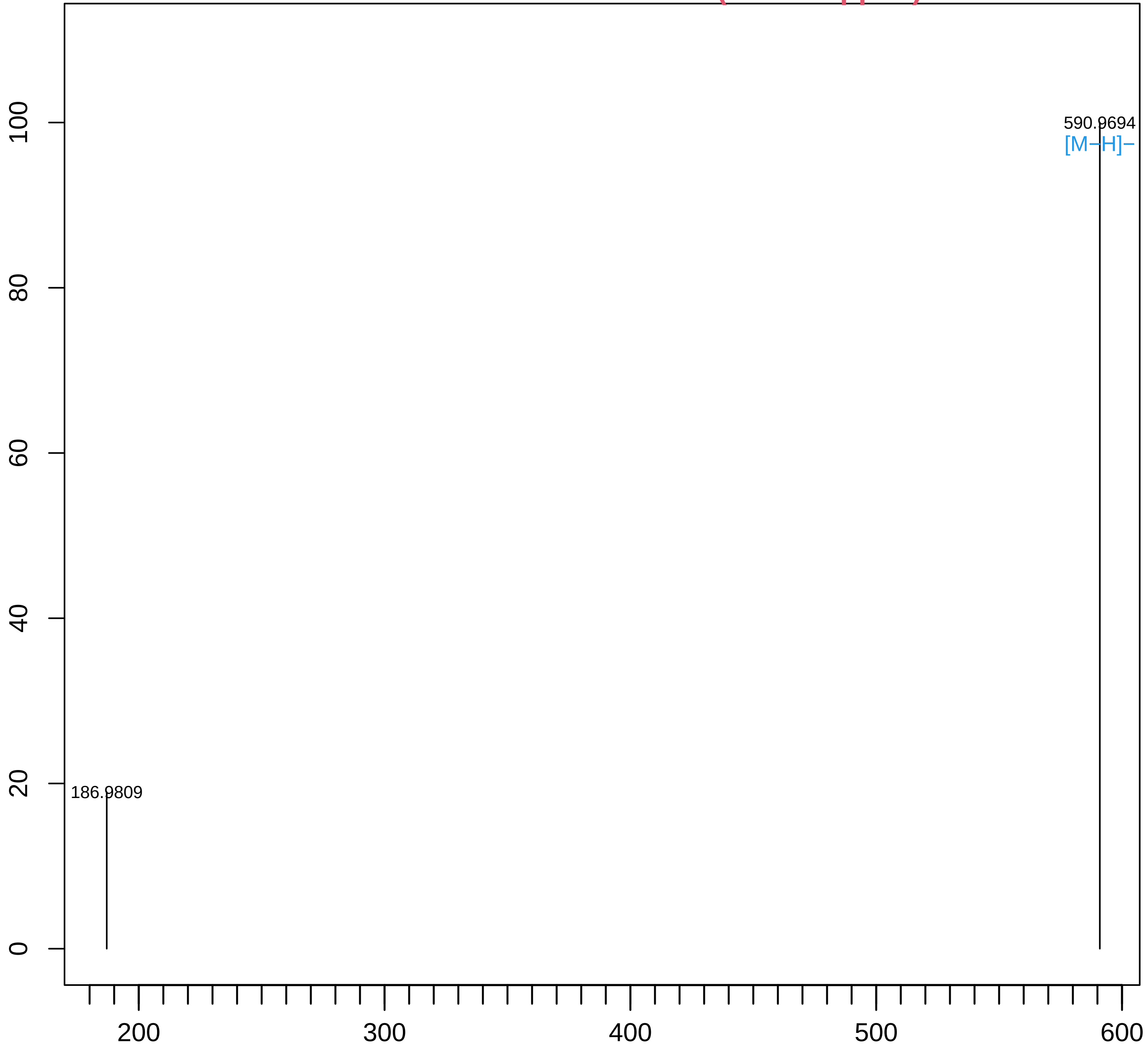

5160 : M.findmain = 591.9767 (+/− 20 ppm)

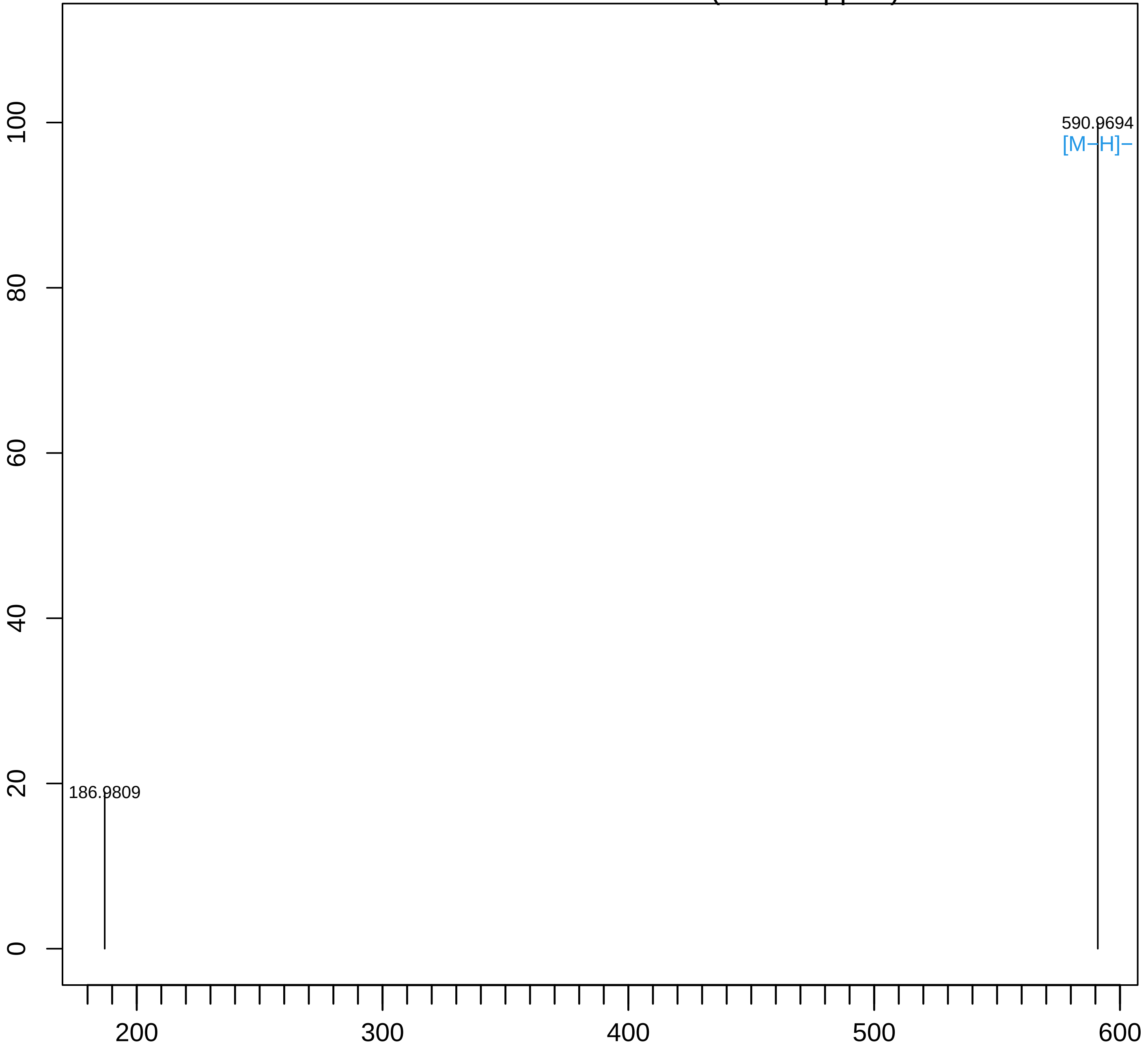

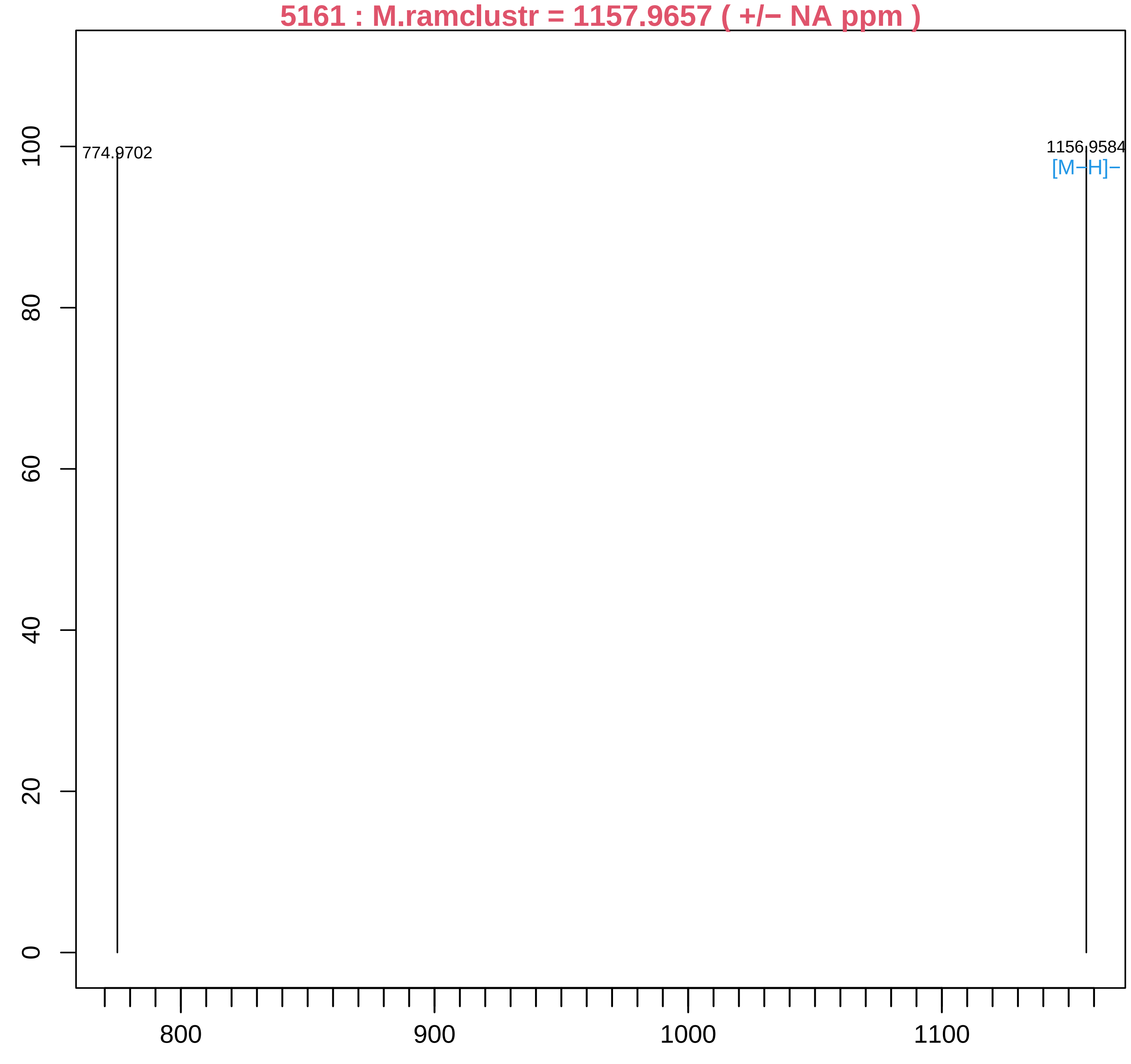
5161 : M.ramclustr = 1157.9657 (+/− NA ppm)
774.9702
1156.9584
[M−H]−
0
20
40
60
80
100
800
900
1000
1100

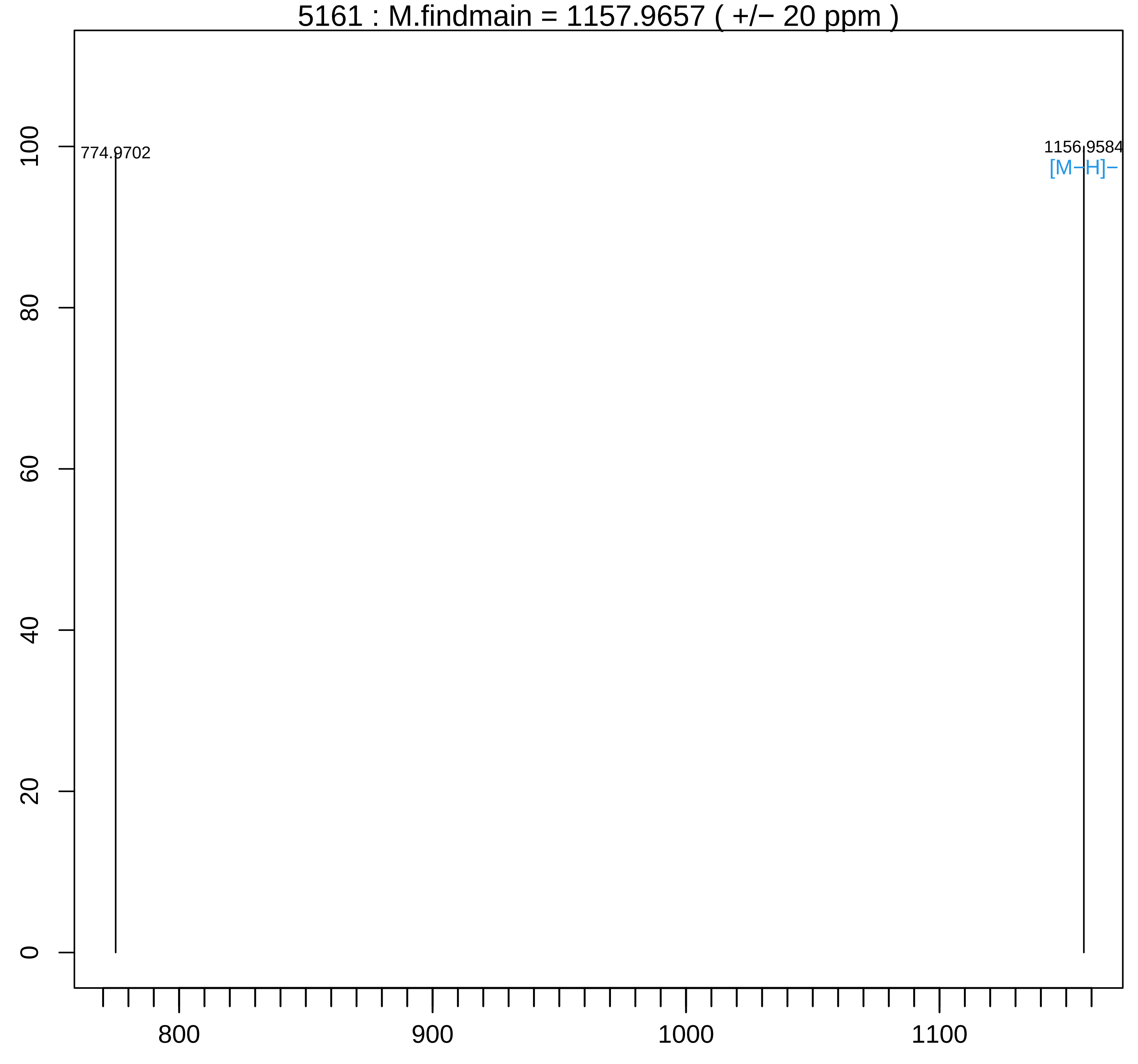
5161 : M.findmain = 1157.9657 (+/− 20 ppm)
774.9702
1156.9584
[M−H]−
0
20
40
60
80
100
800
900
1000
1100

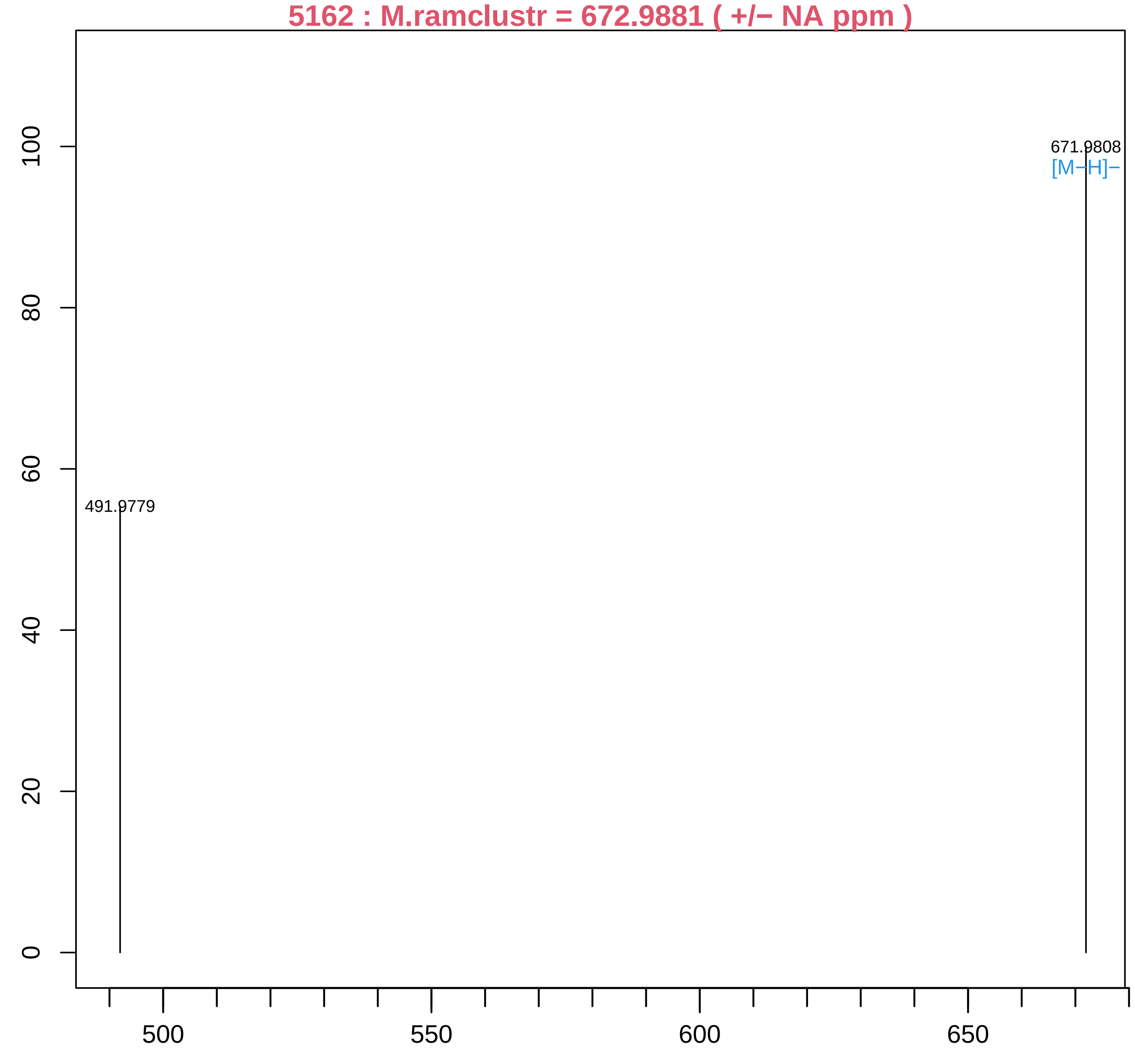
5162 : M.ramclustr = 672.9881 (+/− NA ppm)
671.9808
[M−H]−
491.9779
0
20
40
60
80
100
500
550
600
650

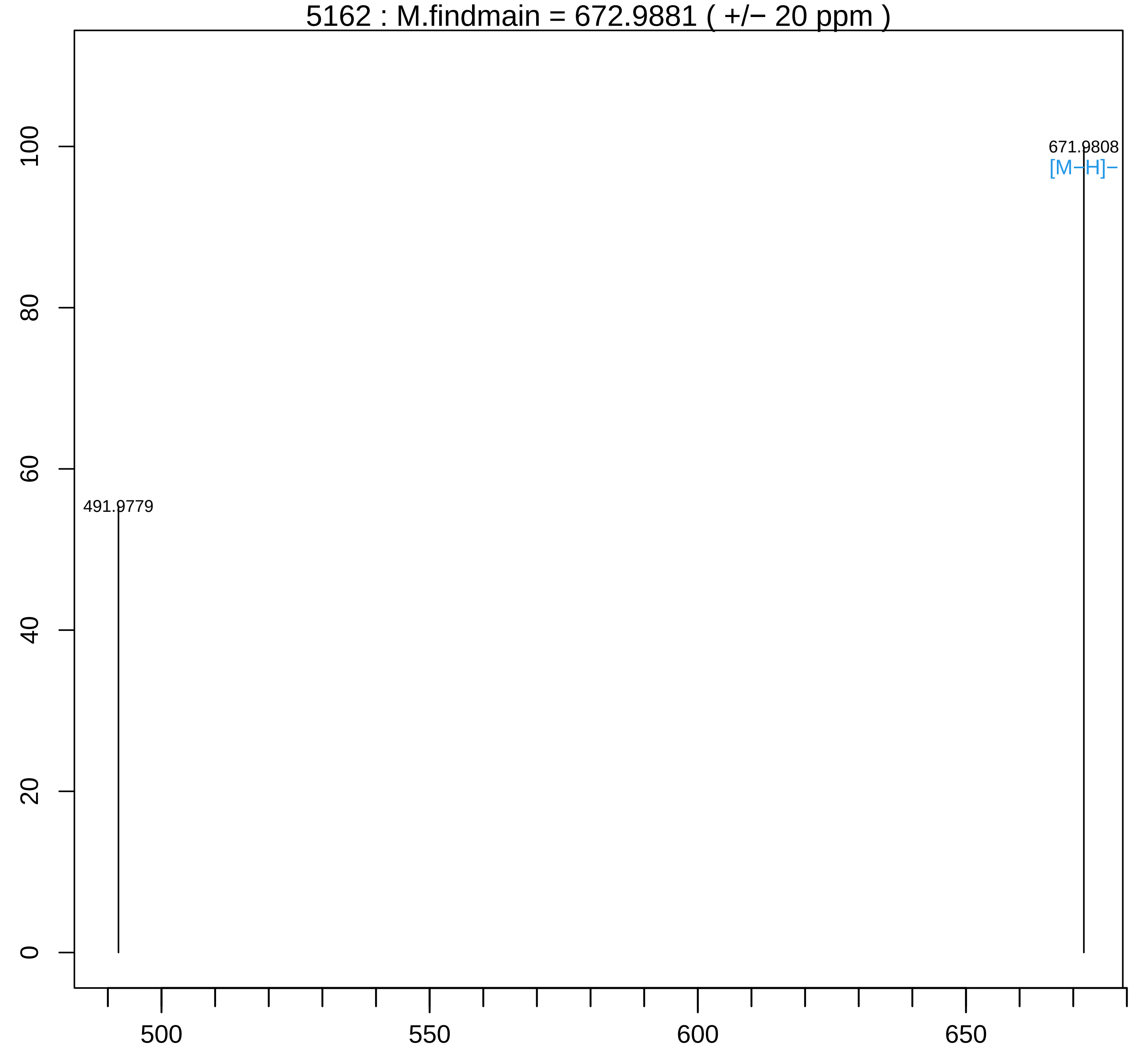
5162 : M.findmain = 672.9881 (+/− 20 ppm)
671.9808
[M−H]−
491.9779
0
20
40
60
80
100
500
550
600
650

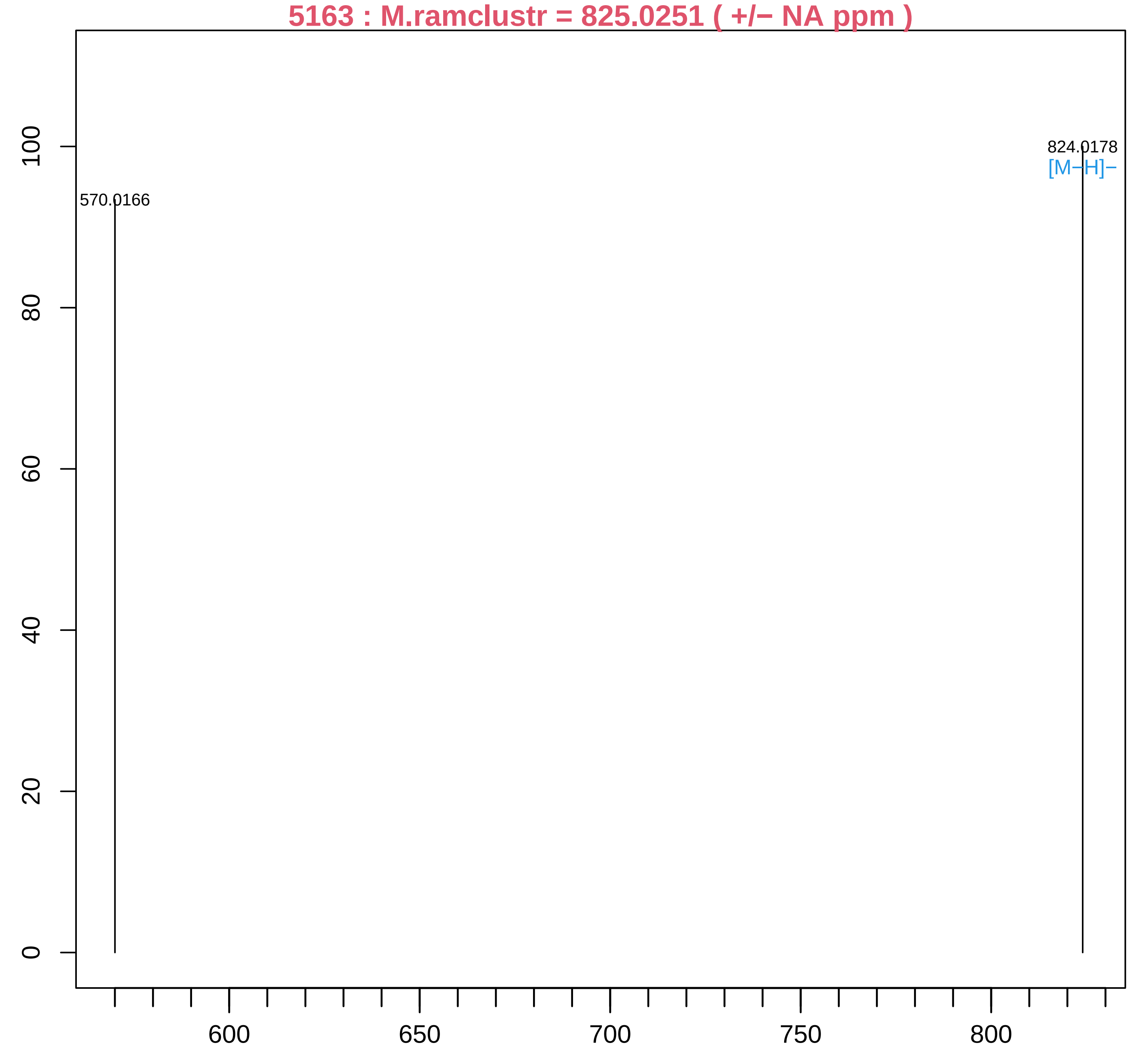
5163 : M.ramclustr = 825.0251 (+/− NA ppm)
824.0178
[M−H]−
570.0166
0
20
40
60
80
100
600
650
700
750
800

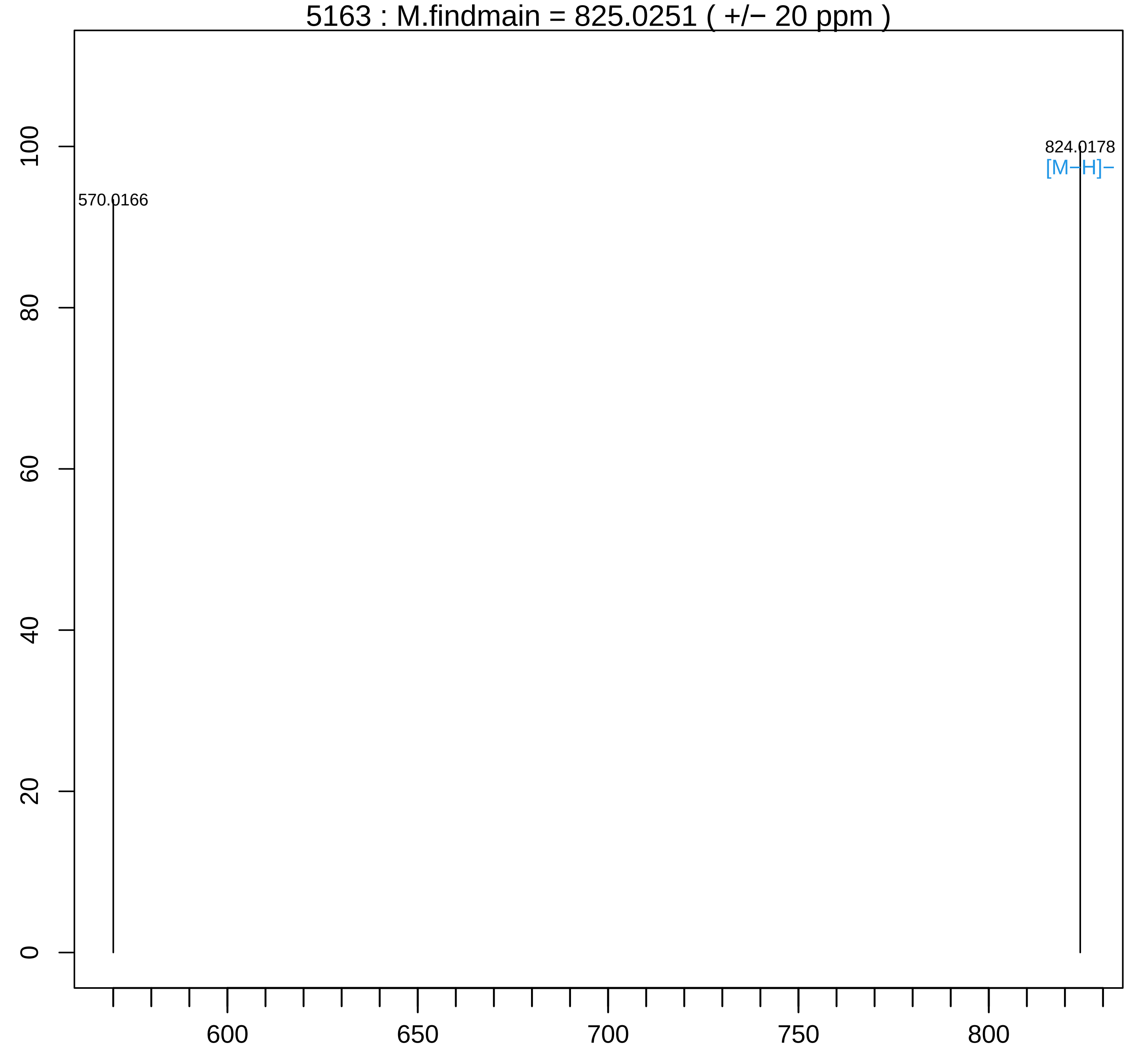
5163 : M.findmain = 825.0251 (+/− 20 ppm)
824.0178
[M−H]−
570.0166
0
20
40
60
80
100
600
650
700
750
800

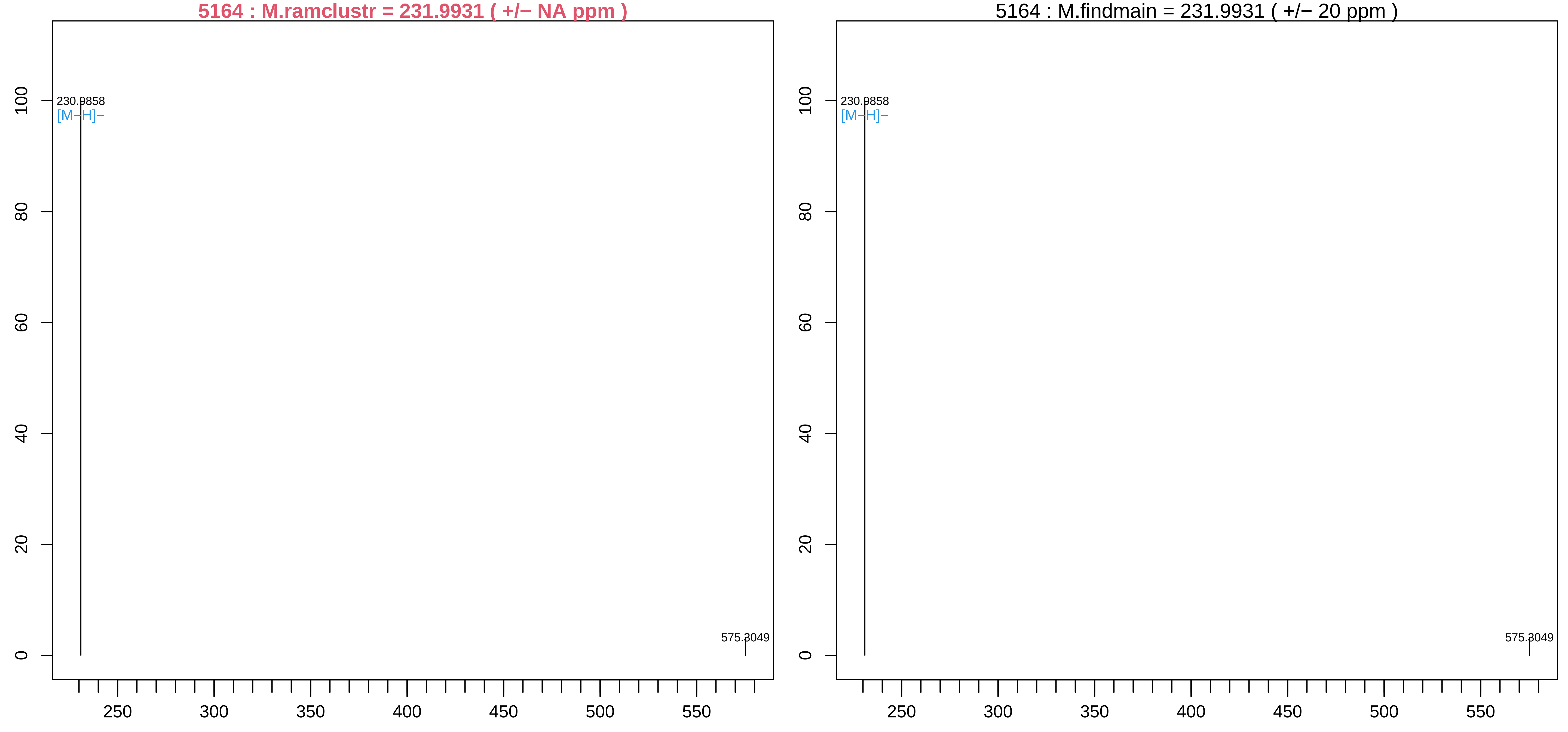

5164 : M.ramclustr = 231.9931 (+/− NA ppm)
230.9858
[M−H]−
575.3049
5164 : M.findmain = 231.9931 (+/− 20 ppm)
230.9858
[M−H]−
575.3049
0
20
40
60
80
100
250
300
350
400
450
500
550

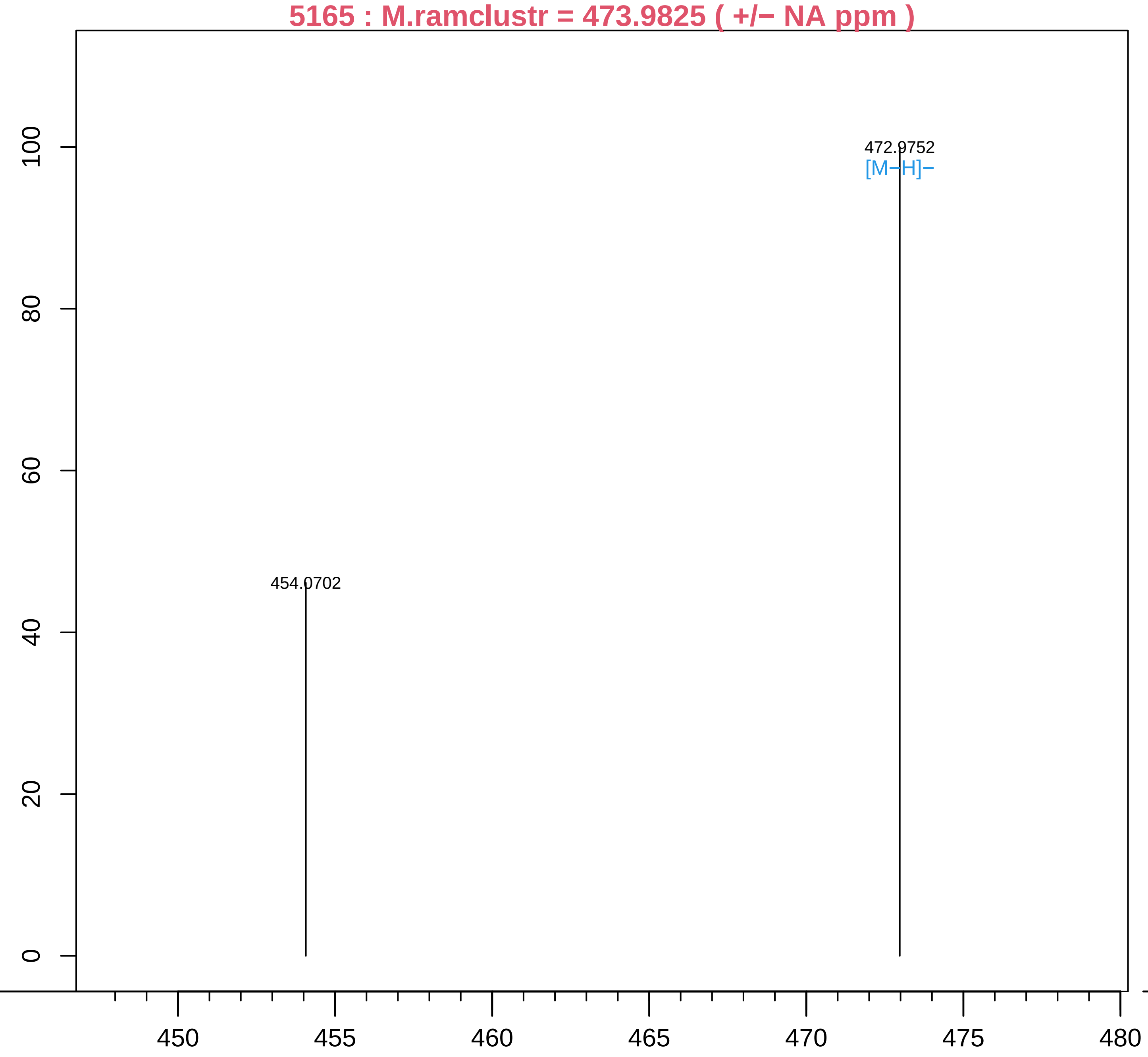

5165 : M.ramclustr = 473.9825 (+/− NA ppm)
472.9752
[M−H]−
454.0702
0
20
40
60
80
100
450
455
460
465
470
475
480

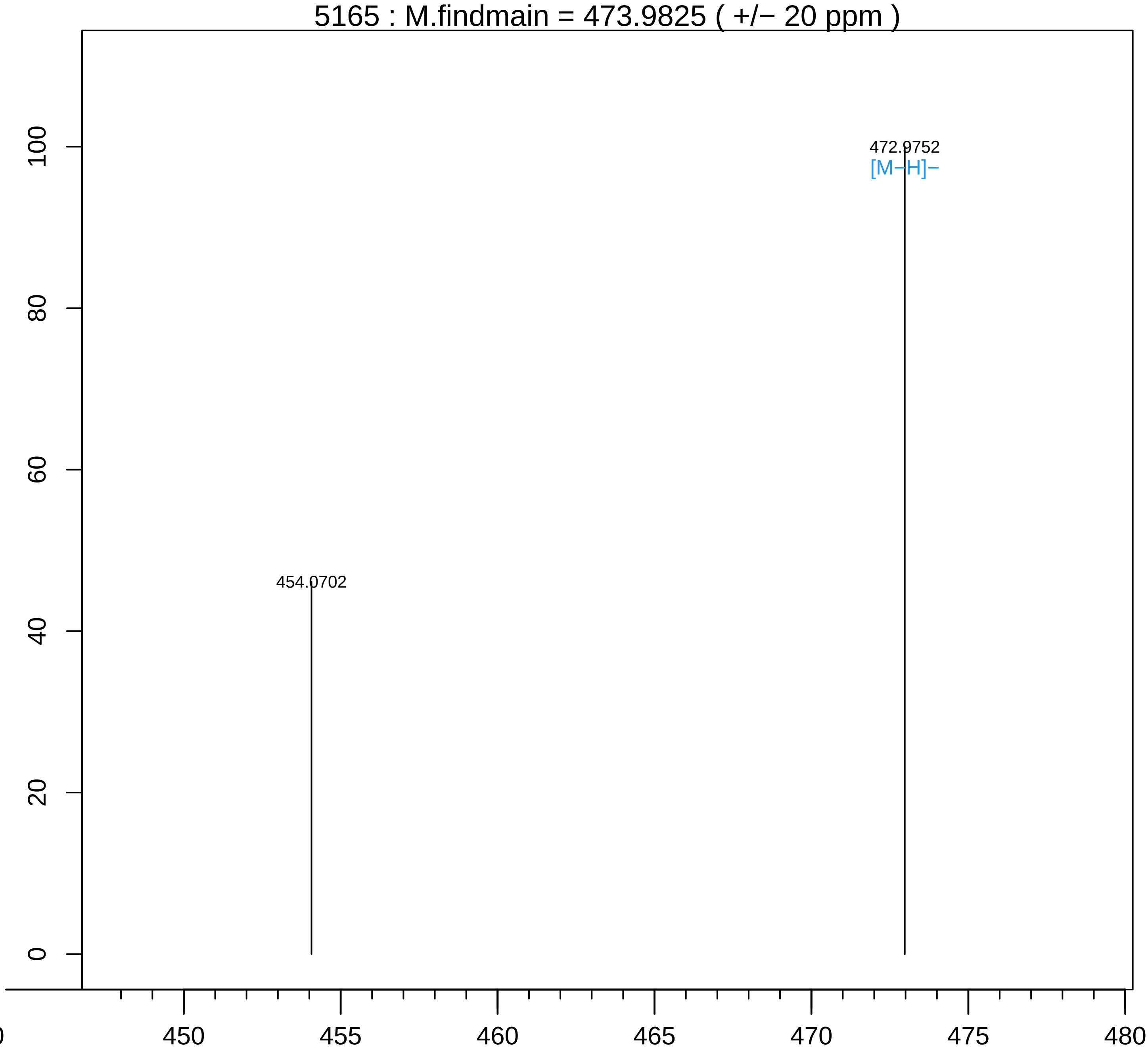

5165 : M.findmain = 473.9825 (+/− 20 ppm)
472.9752
[M−H]−
454.0702
0
20
40
60
80
100
450
455
460
465
470
475
480

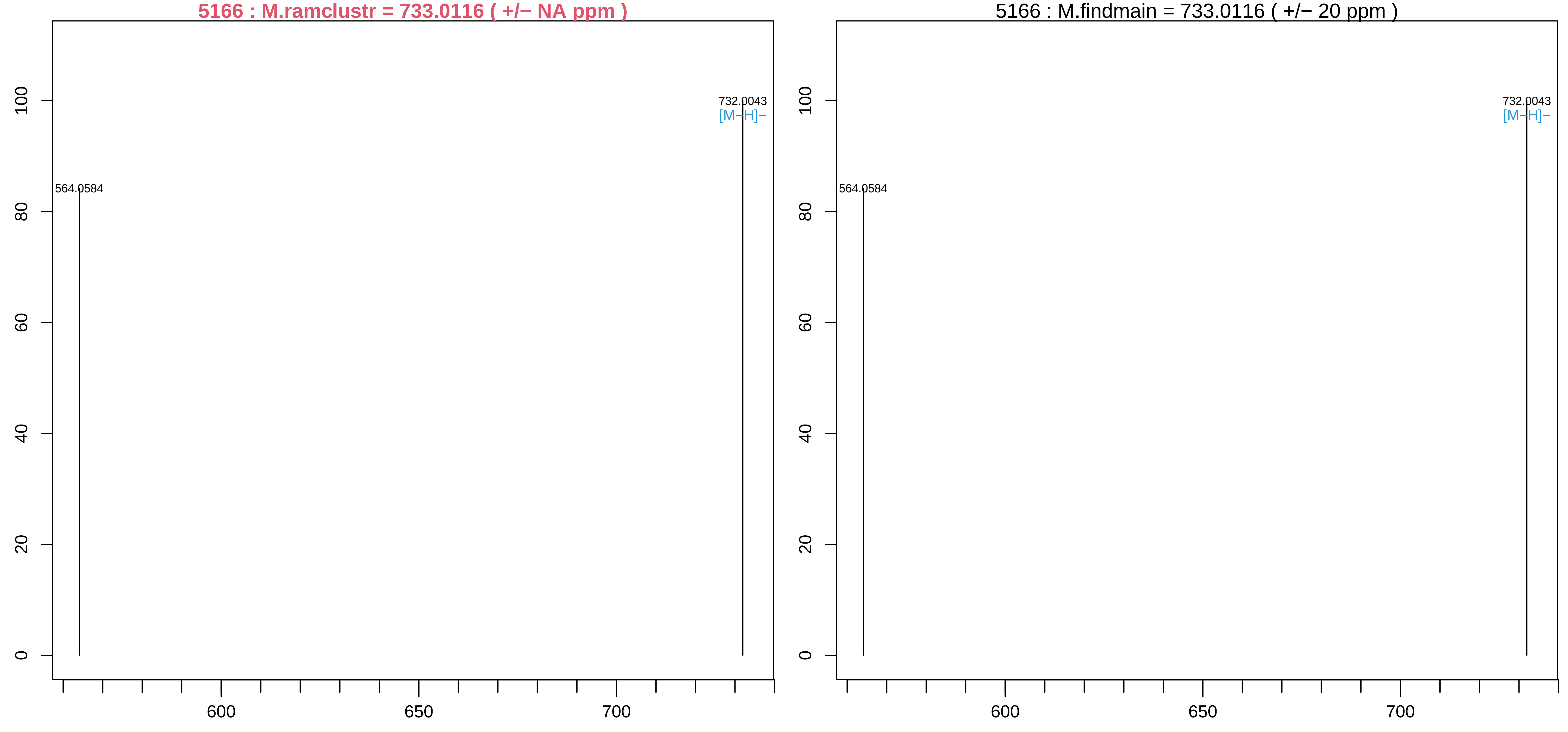
5166 : M.ramclustr = 733.0116 (+/− NA ppm)
564.0584
732.0043
[M−H]−
0
20
40
60
80
100
600
650
700
5166 : M.findmain = 733.0116 (+/− 20 ppm)
564.0584
732.0043
[M−H]−
0
20
40
60
80
100
600
650
700

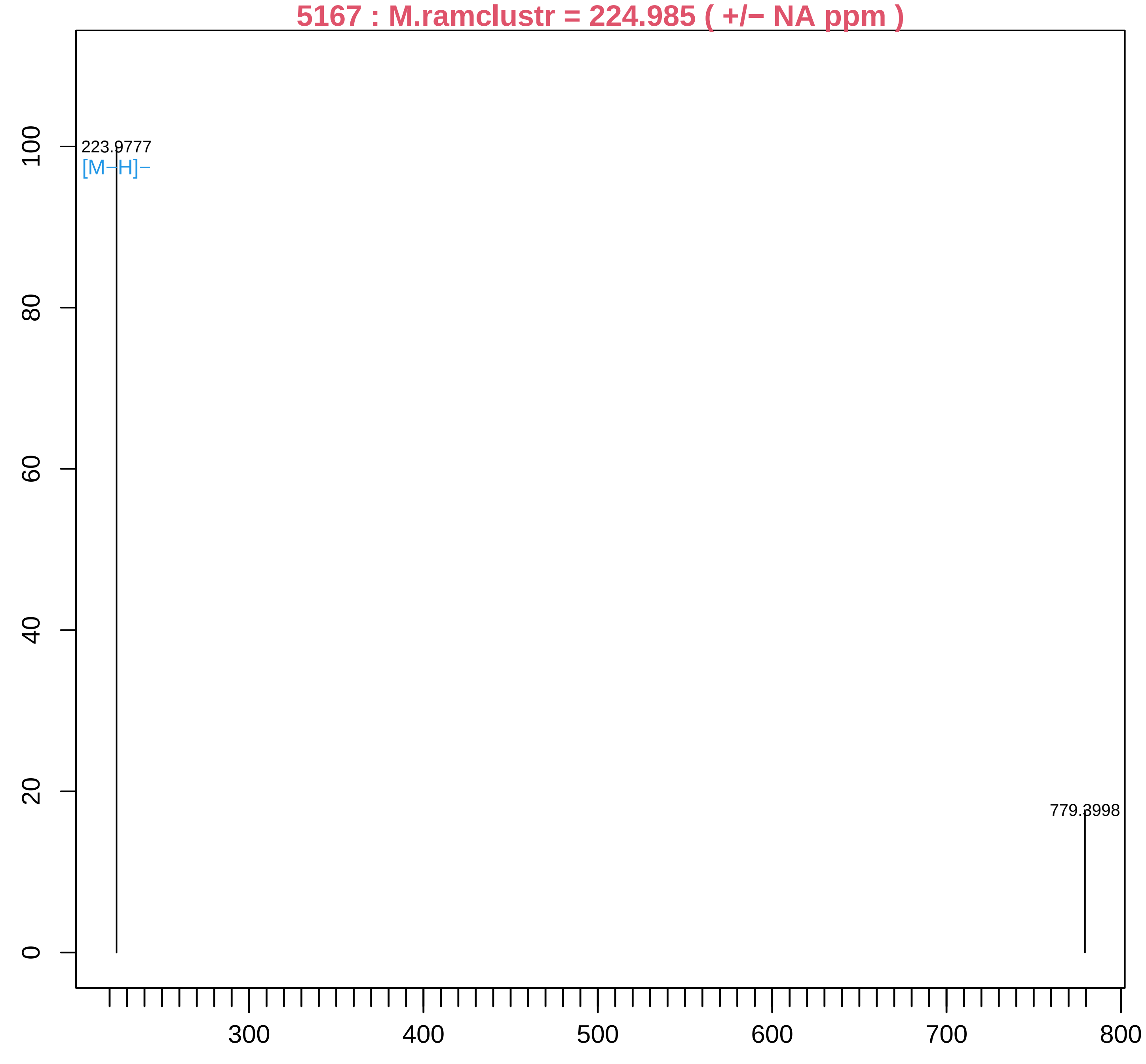
5167 : M.ramclustr = 224.985 (+/− NA ppm)
223.9777
[M−H]−
779.3998
0
20
40
60
80
100
300
400
500
600
700
800

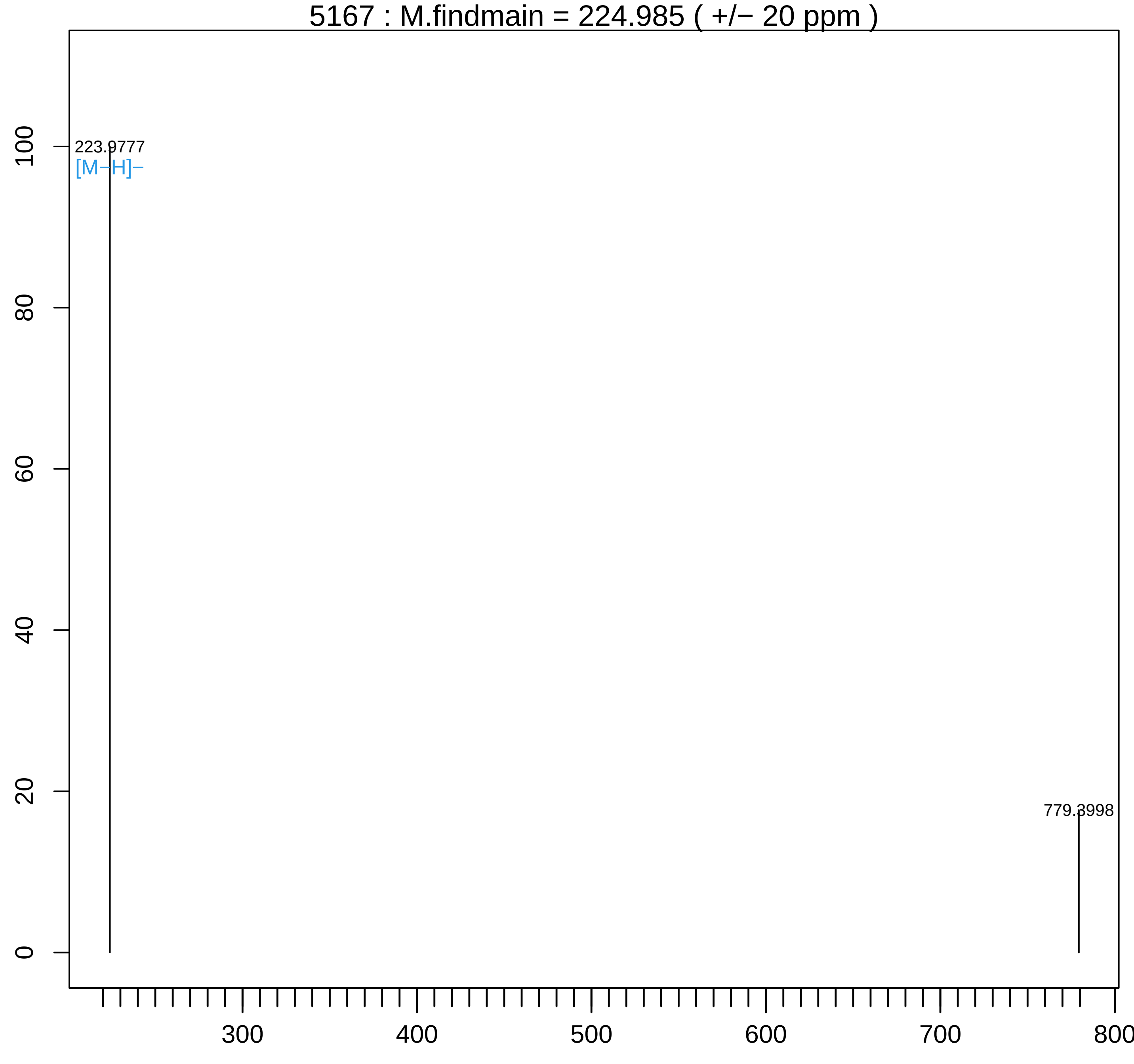
5167 : M.findmain = 224.985 (+/− 20 ppm)
223.9777
[M−H]−
779.3998
0
20
40
60
80
100
300
400
500
600
700
800

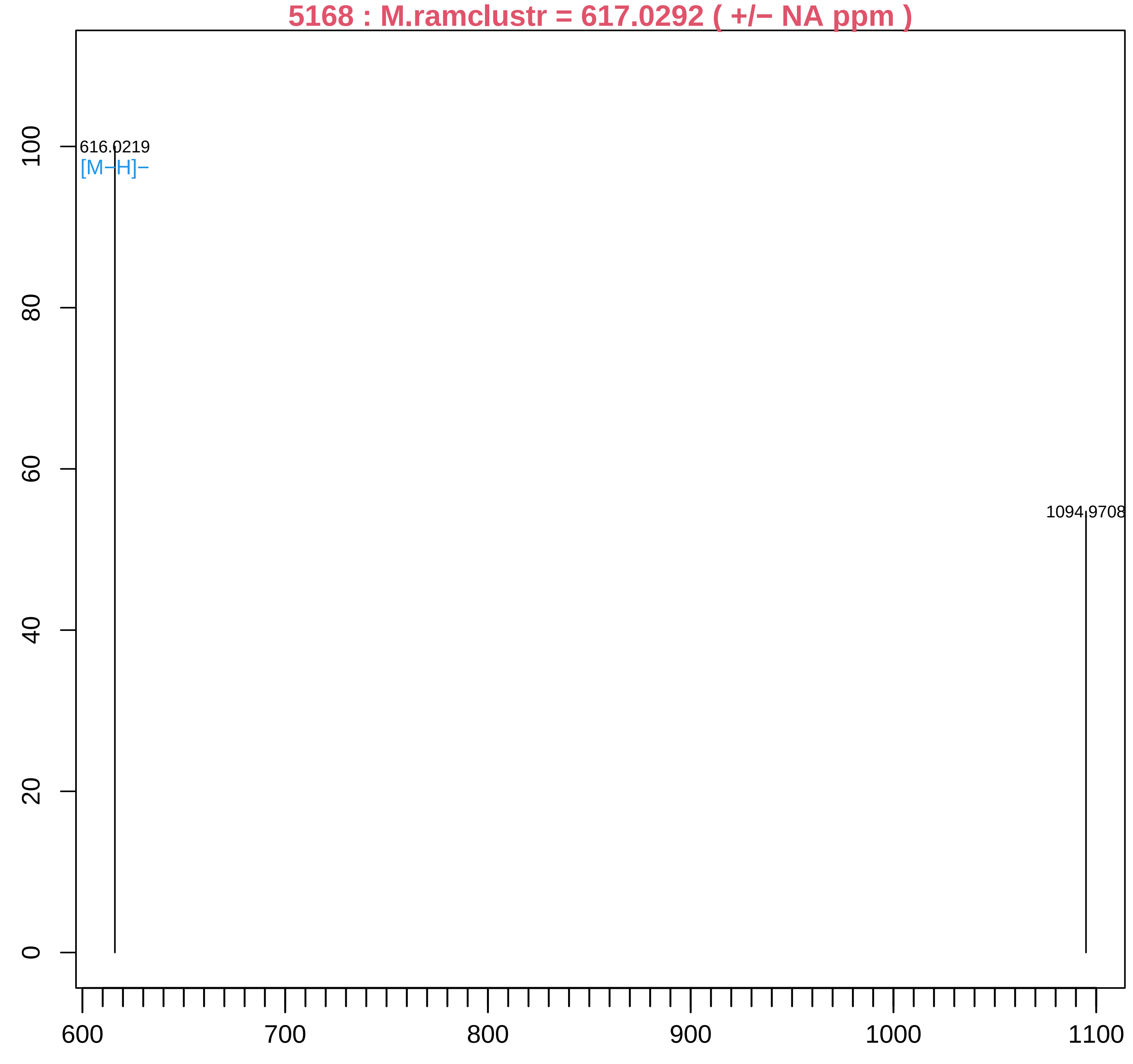

5168 : M.ramclustr = 617.0292 (+/− NA ppm)
616.0219
[M−H]−
1094.9708
0
20
40
60
80
100
600
700
800
900
1000
1100

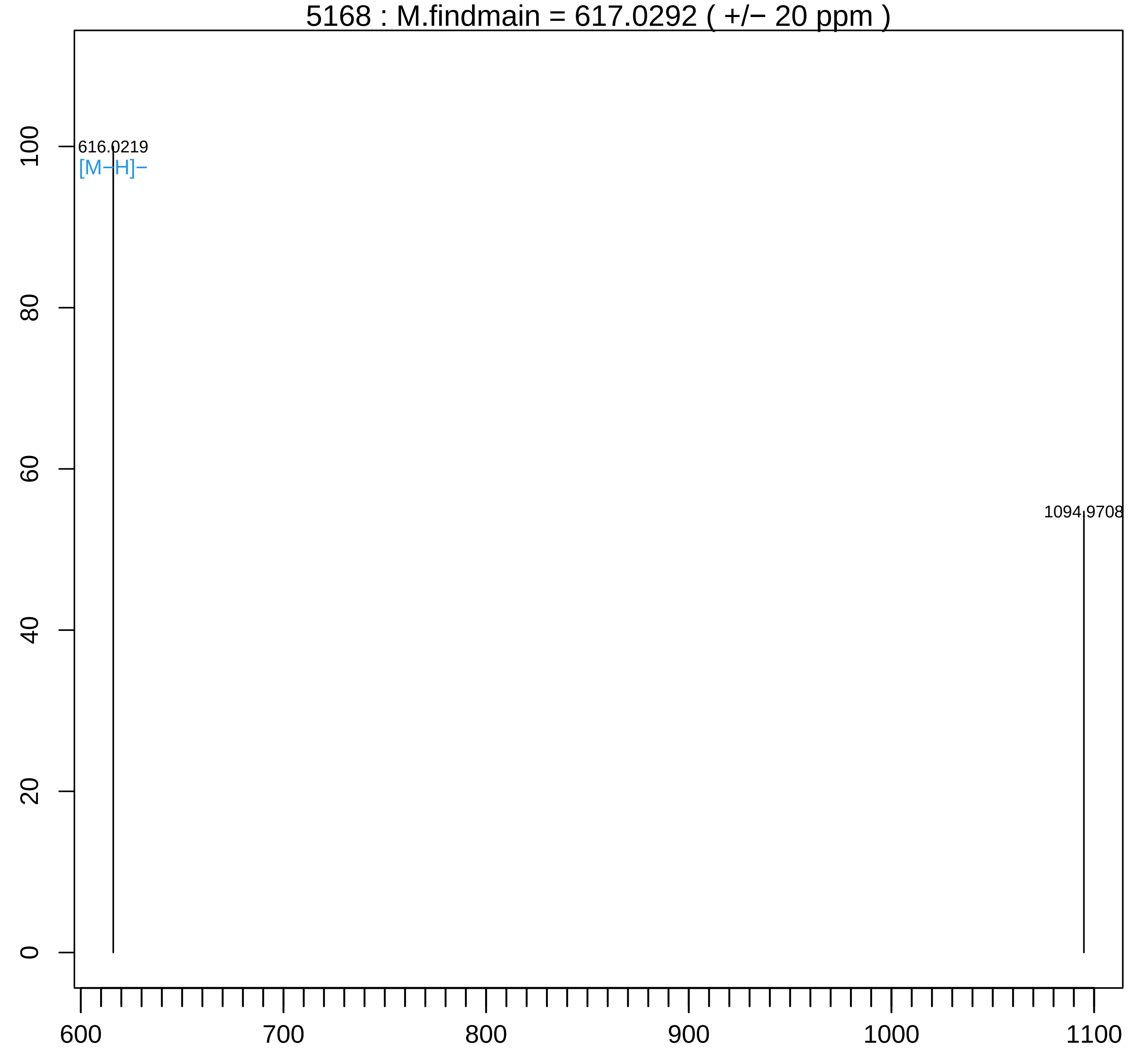

5168 : M.findmain = 617.0292 (+/− 20 ppm)
616.0219
[M−H]−
1094.9708
0
20
40
60
80
100
600
700
800
900
1000
1100

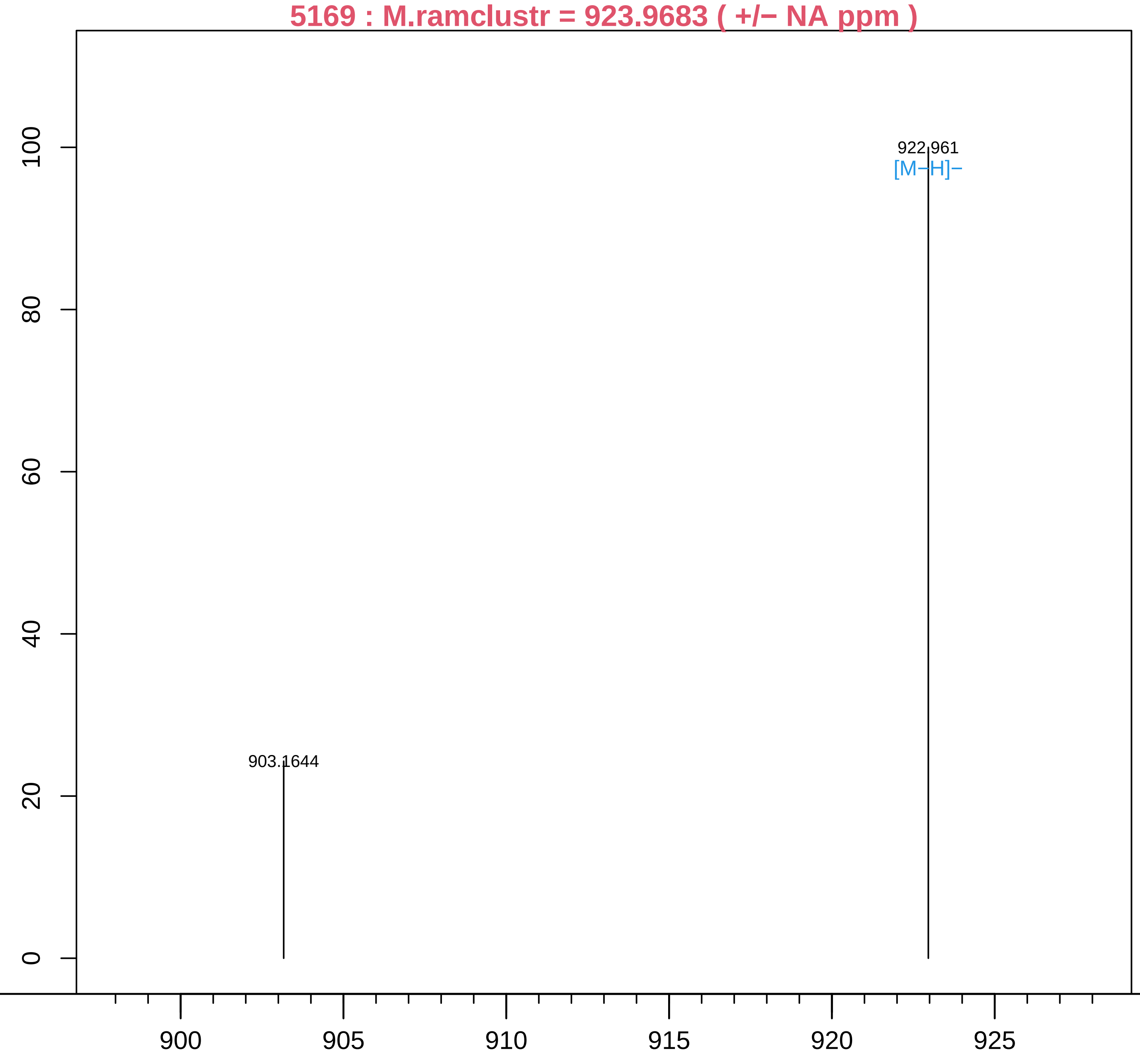
5169 : M.ramclustr = 923.9683 (+/− NA ppm)
922.961
[M−H]−
903.1644
0
20
40
60
80
100
900
905
910
915
920
925

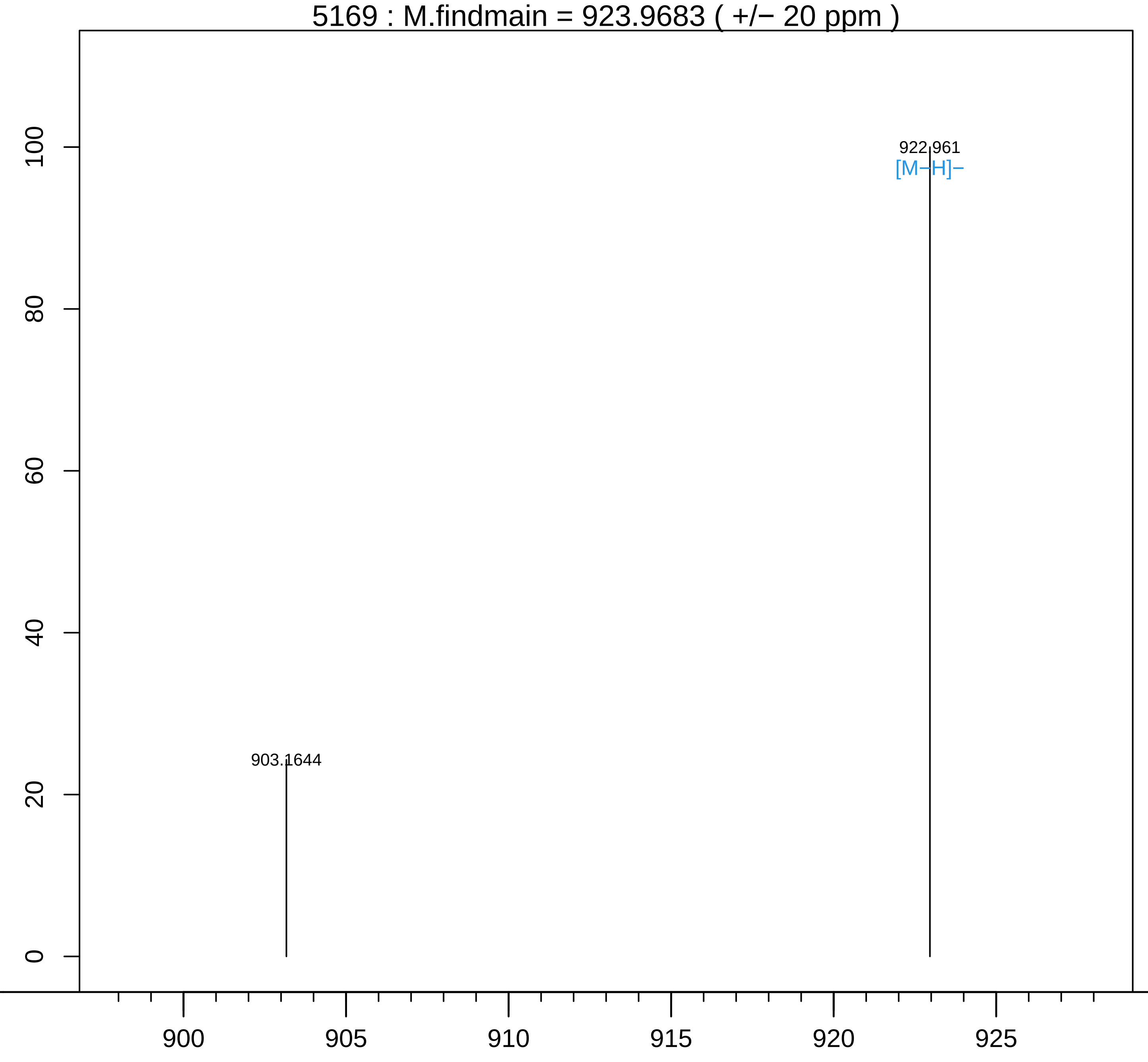
5169 : M.findmain = 923.9683 (+/− 20 ppm)
922.961
[M−H]−
903.1644
0
20
40
60
80
100
900
905
910
915
920
925

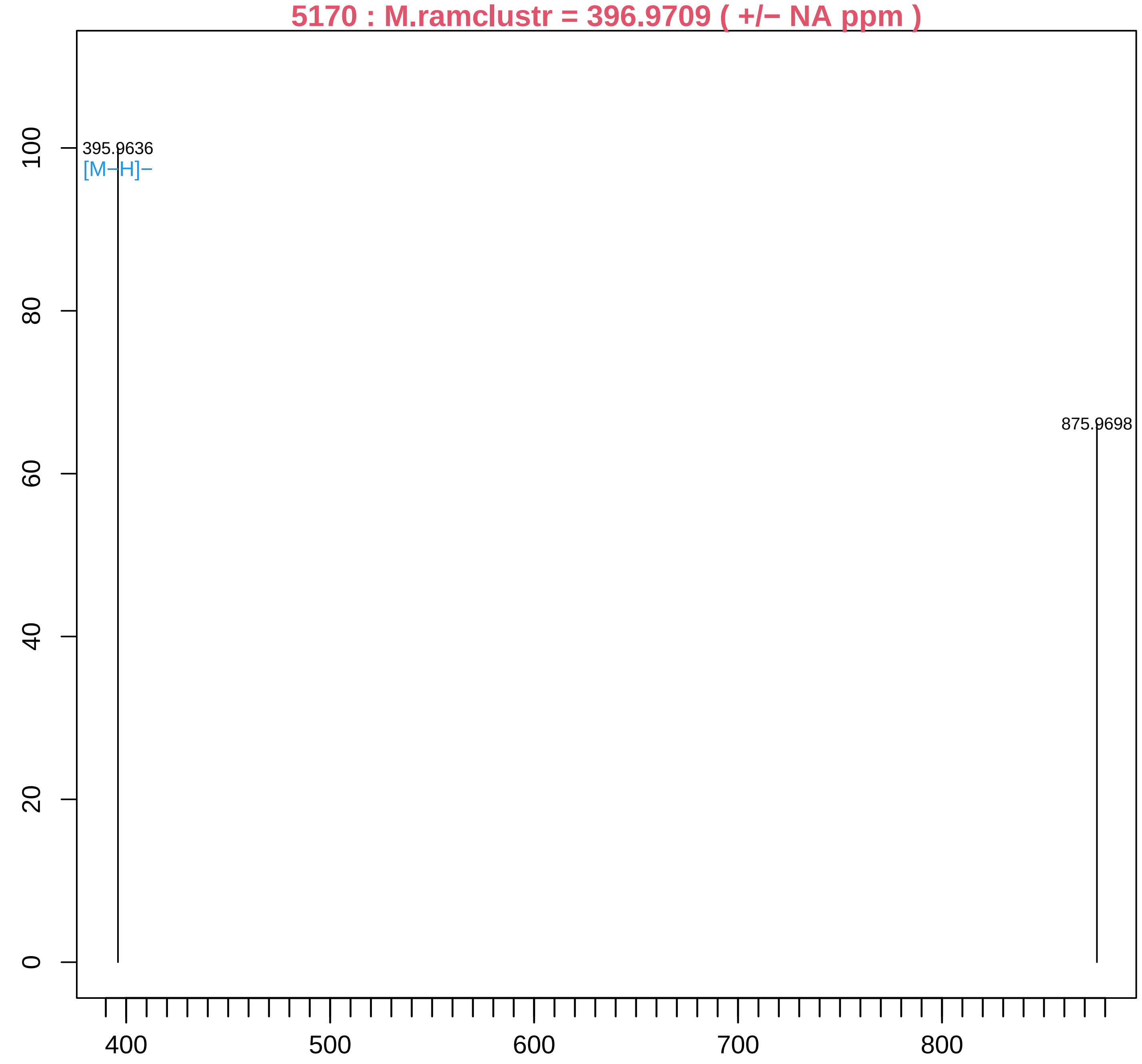
5170 : M.ramclustr = 396.9709 (+/− NA ppm)
395.9636
[M−H]−
875.9698
0
20
40
60
80
100
400
500
600
700
800

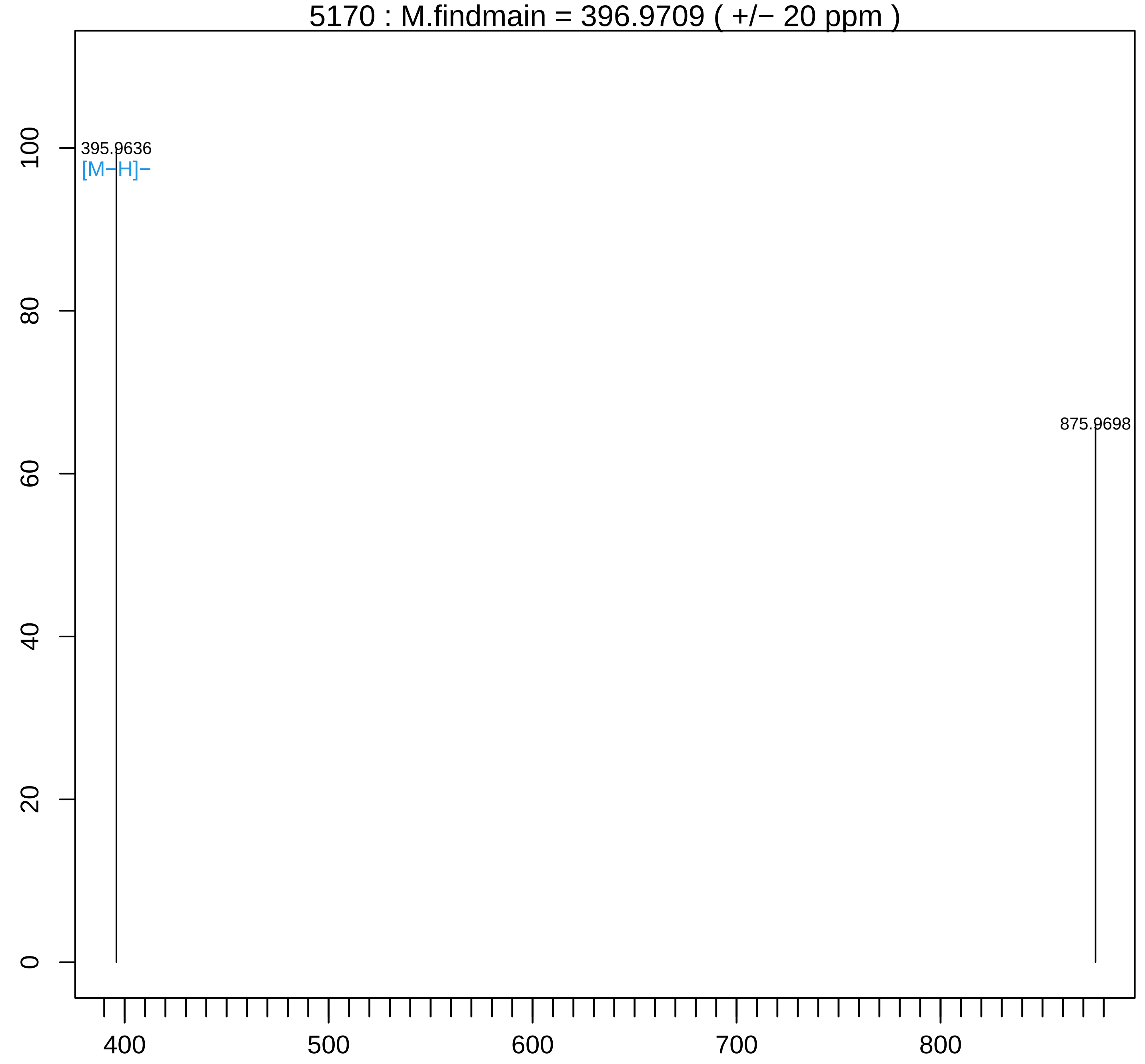
5170 : M.findmain = 396.9709 (+/− 20 ppm)
395.9636
[M−H]−
875.9698
0
20
40
60
80
100
400
500
600
700
800

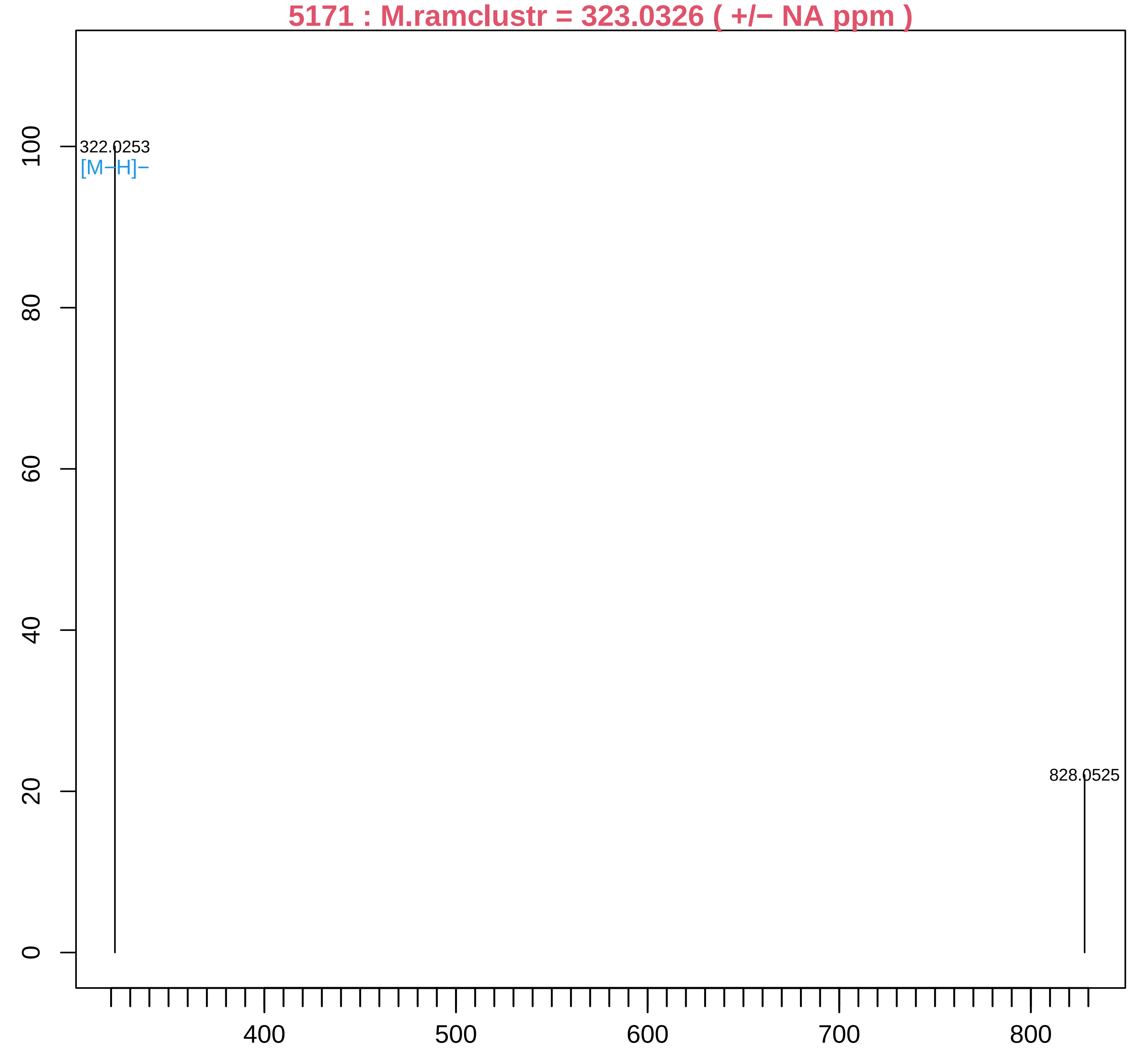
5171 : M.ramclustr = 323.0326 (+/− NA ppm)
322.0253
[M−H]−
828.0525
0
20
40
60
80
100
400
500
600
700
800

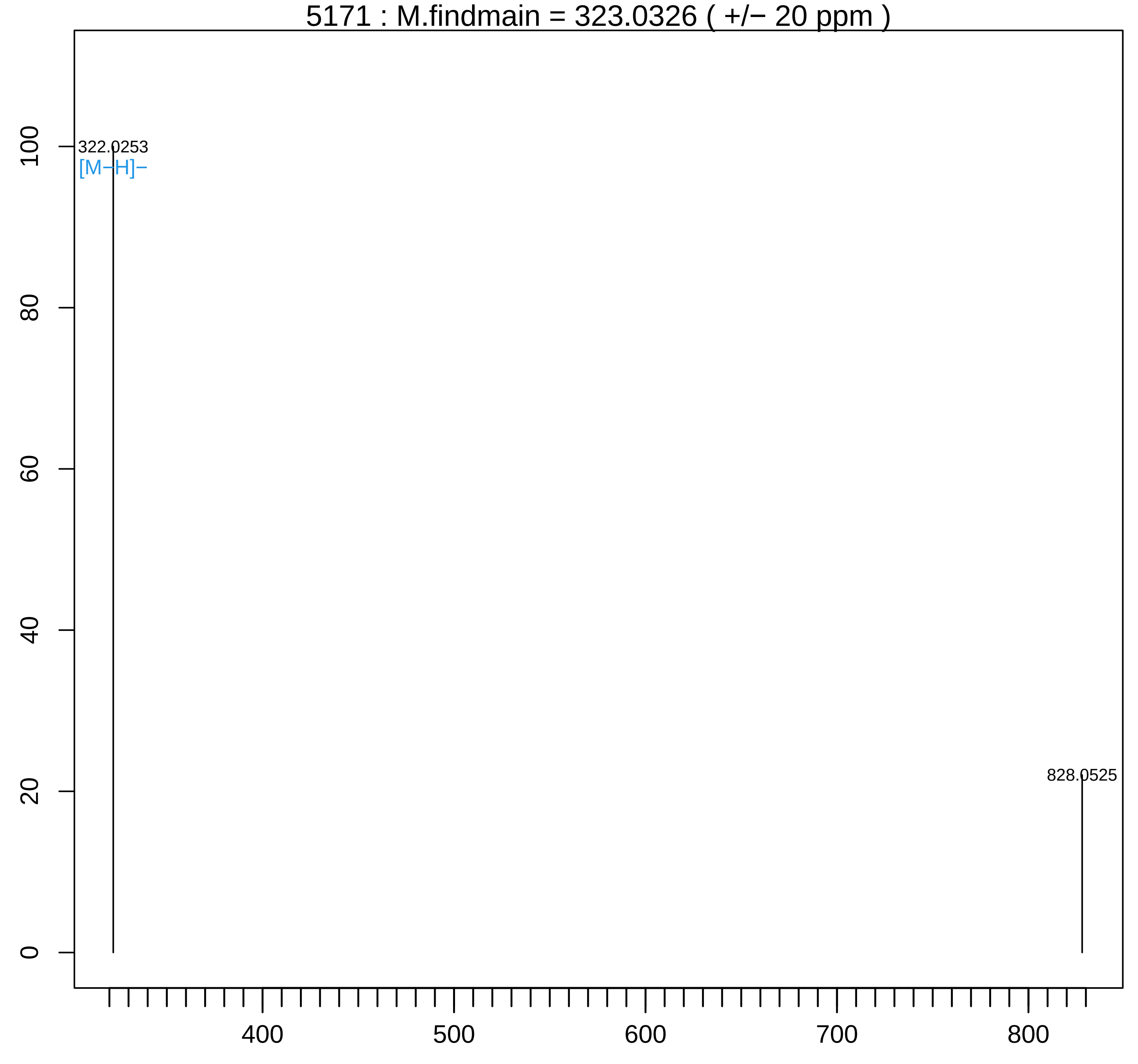
5171 : M.findmain = 323.0326 (+/− 20 ppm)
322.0253
[M−H]−
828.0525
0
20
40
60
80
100
400
500
600
700
800

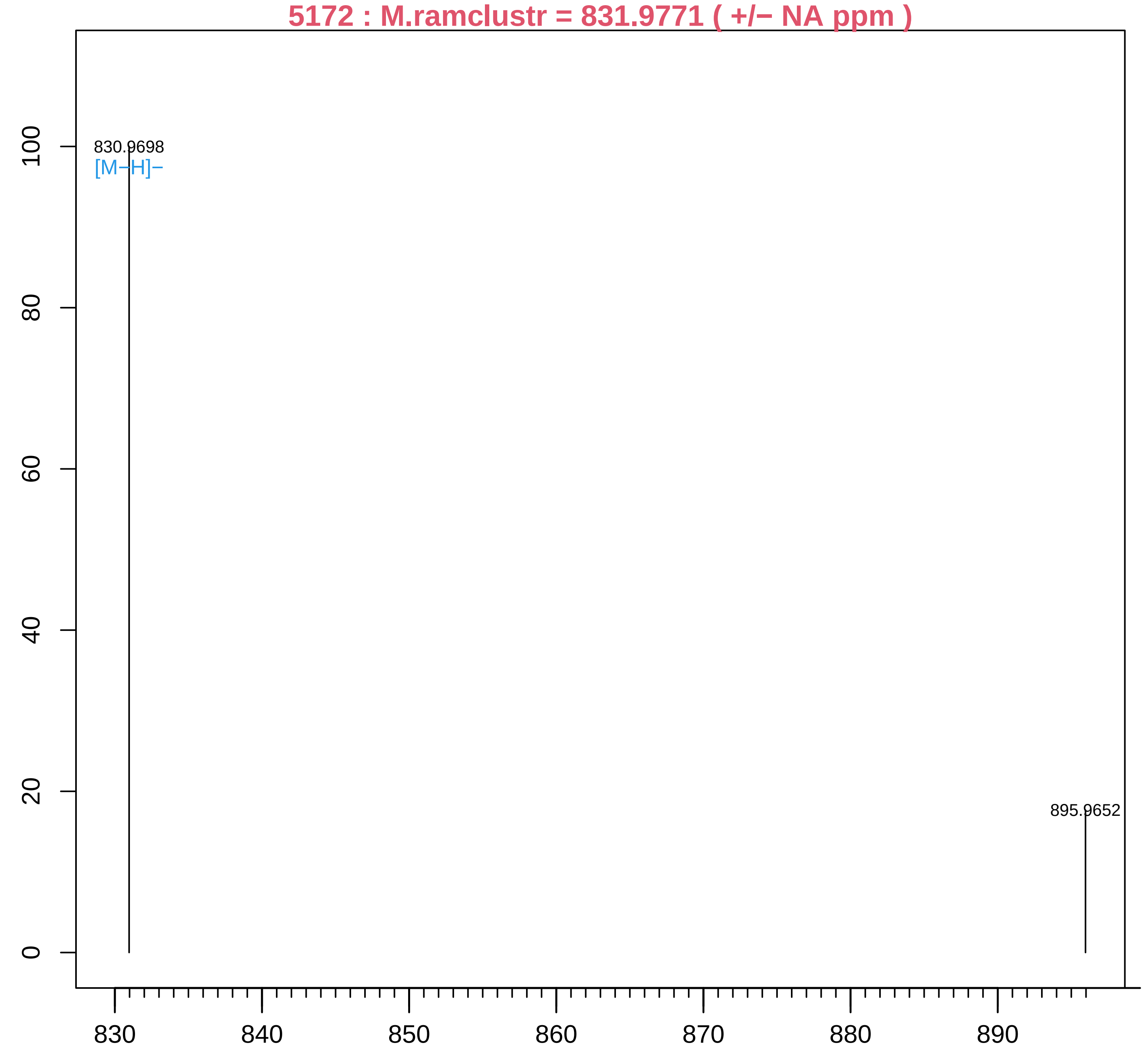
5172 : M.ramclustr = 831.9771 (+/− NA ppm)
830.9698
[M−H]−
895.9652
0
20
40
60
80
100
830
840
850
860
870
880
890

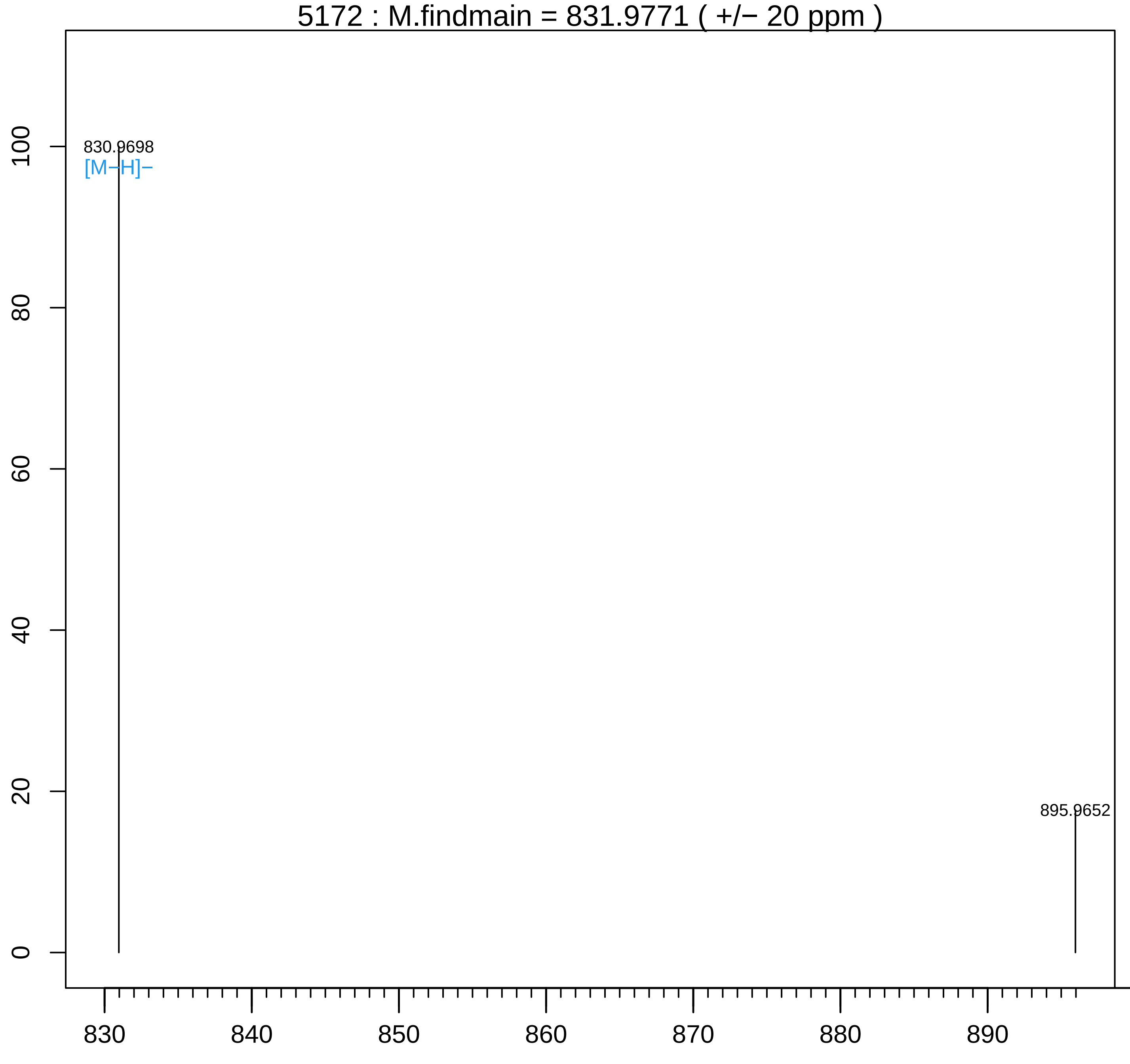
5172 : M.findmain = 831.9771 (+/− 20 ppm)
830.9698
[M−H]−
895.9652
0
20
40
60
80
100
830
840
850
860
870
880
890

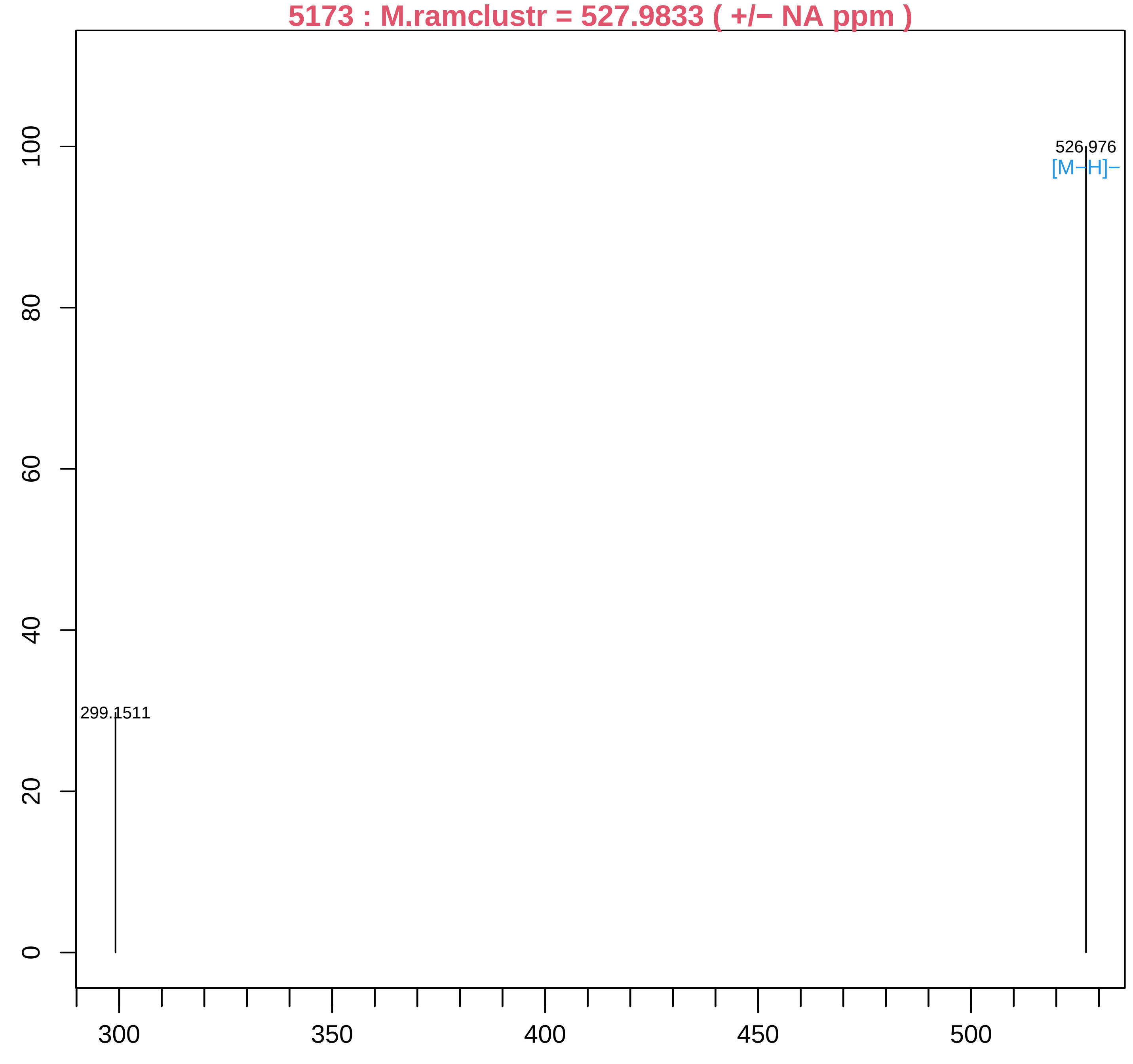
5173 : M.ramclustr = 527.9833 (+/− NA ppm)
526.976
[M−H]−
299.1511
0
20
40
60
80
100
300
350
400
450
500

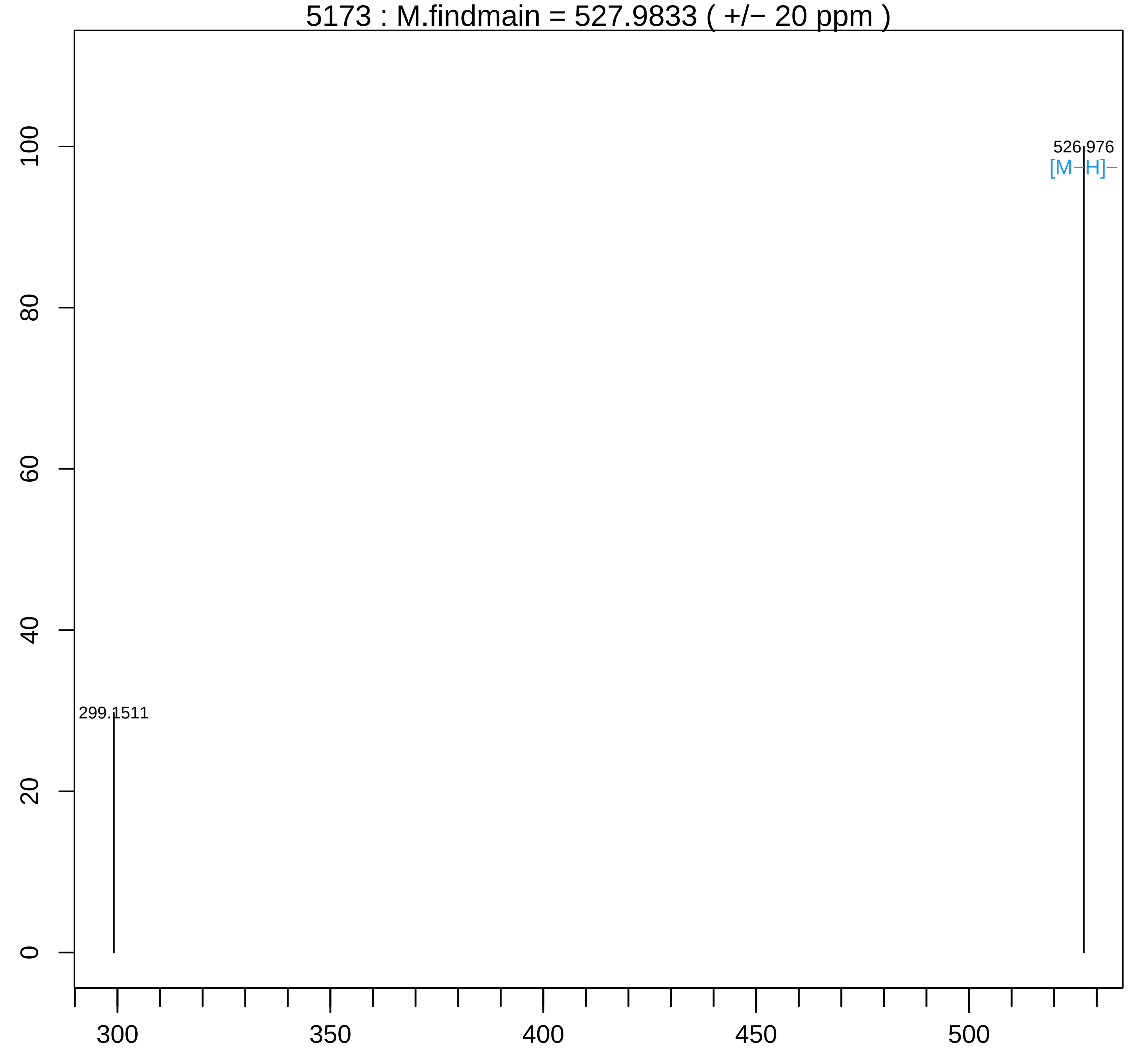
5173 : M.findmain = 527.9833 (+/− 20 ppm)
526.976
[M−H]−
299.1511
0
20
40
60
80
100
300
350
400
450
500

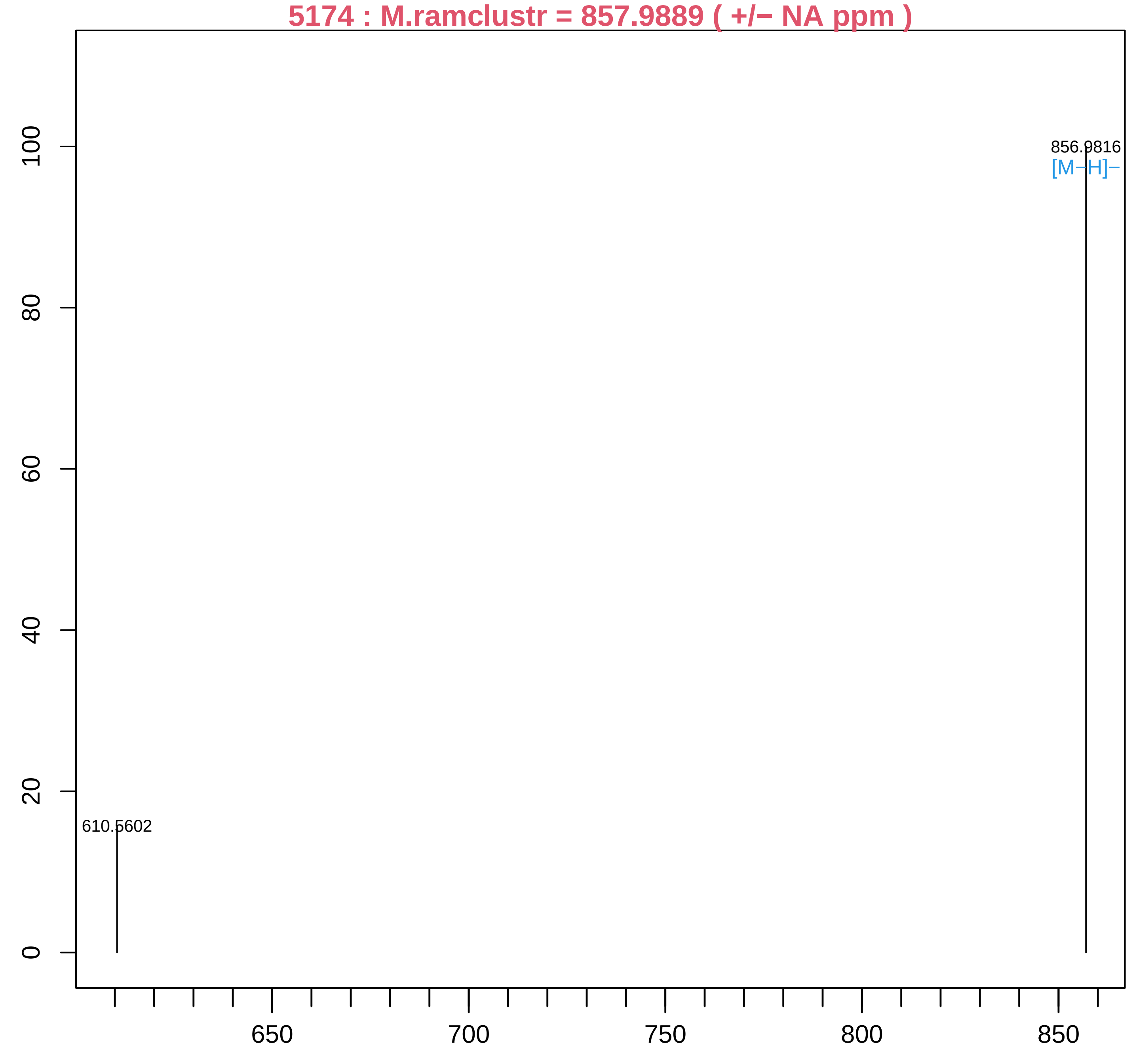
5174 : M.ramclustr = 857.9889 (+/− NA ppm)
856.9816
[M−H]−
610.5602
0
20
40
60
80
100
650
700
750
800
850

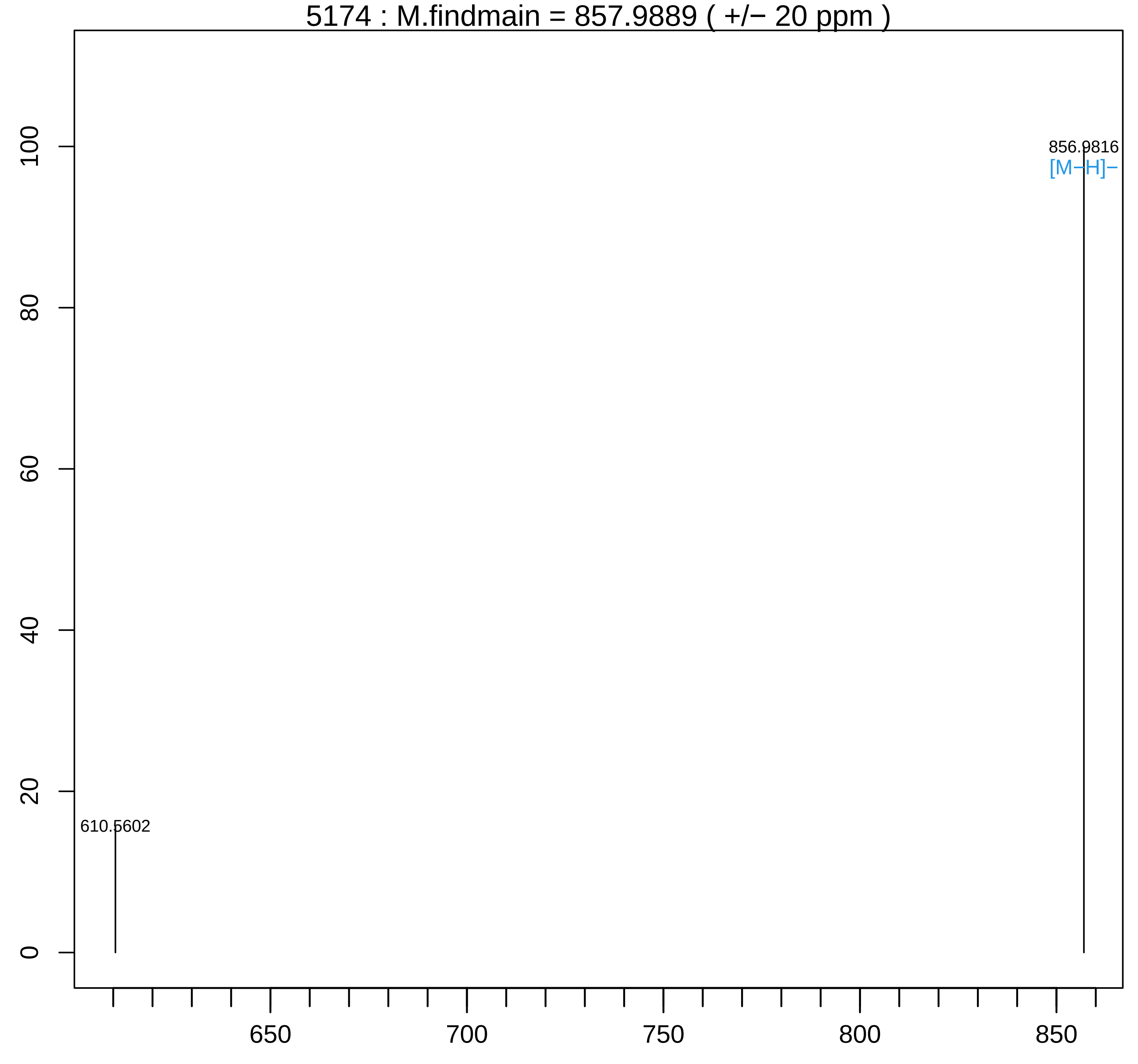
5174 : M.findmain = 857.9889 (+/− 20 ppm)
856.9816
[M−H]−
610.5602
0
20
40
60
80
100
650
700
750
800
850

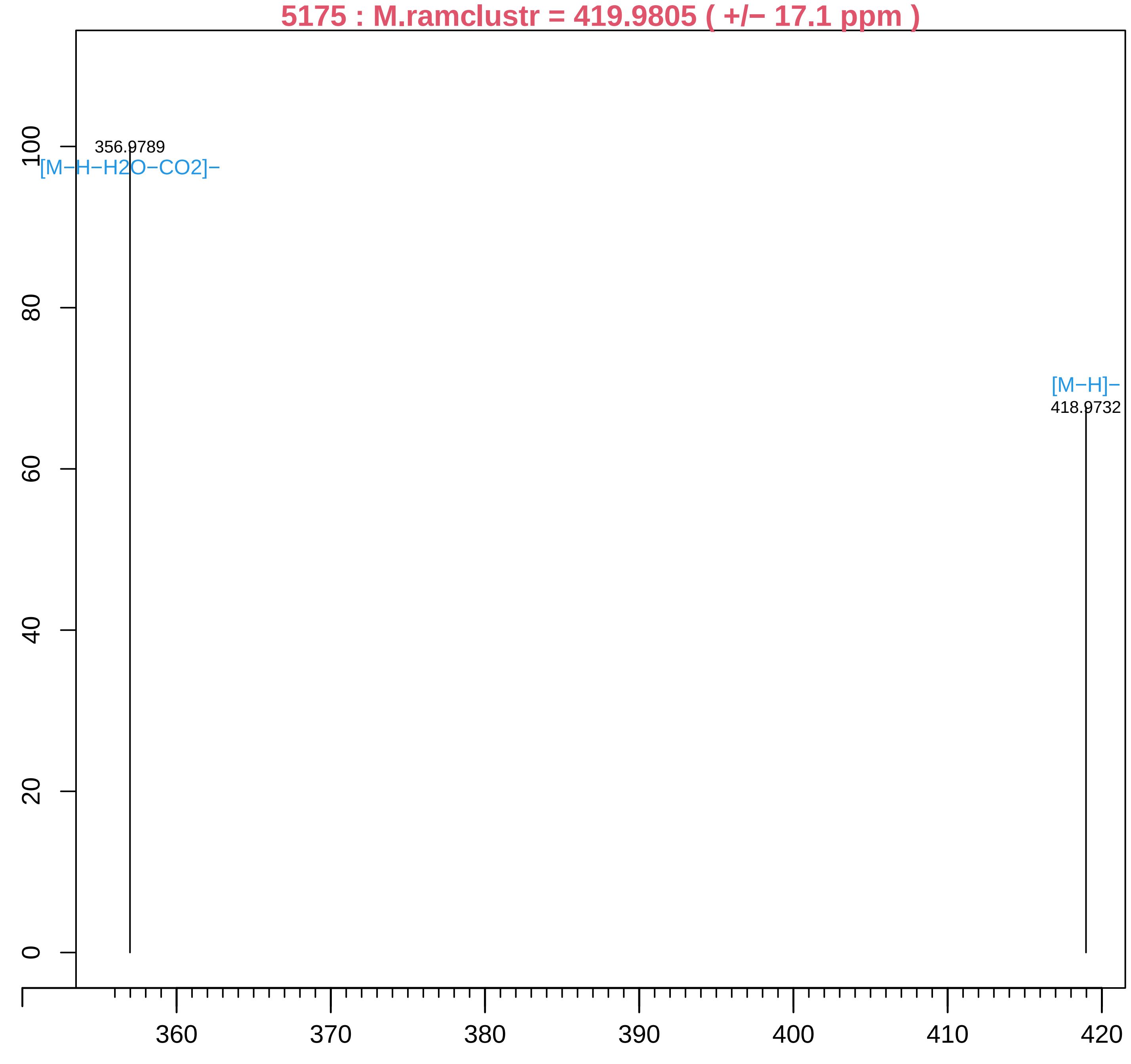

5175 : M.ramclustr = 419.9805 (+/− 17.1 ppm)
356.9789
[M−H−H2O−CO2]−
[M−H]−
418.9732
0
20
40
60
80
100
360
370
380
390
400
410
420

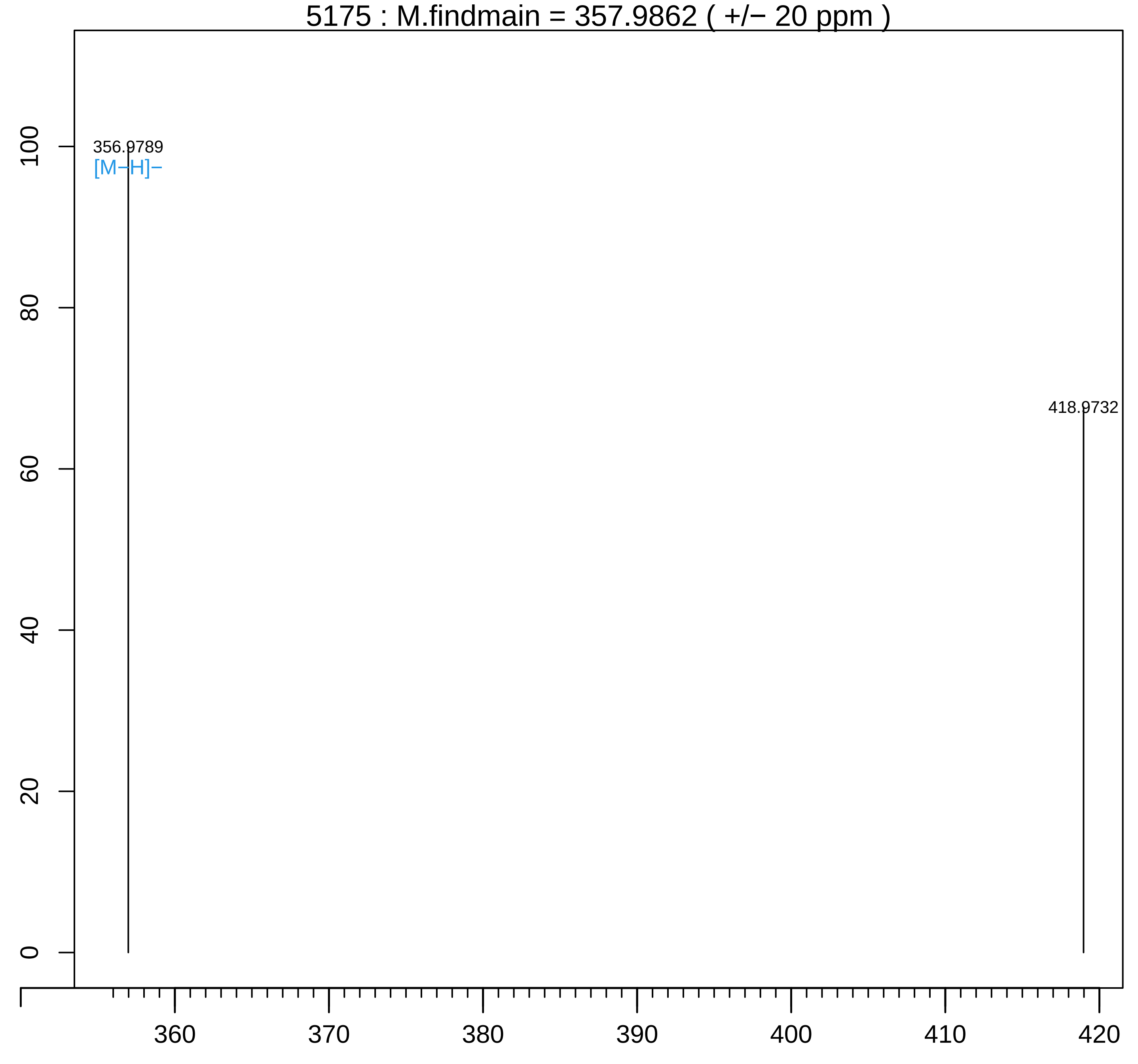

5175 : M.findmain = 357.9862 (+/− 20 ppm)
356.9789
[M−H]−
418.9732
0
20
40
60
80
100
360
370
380
390
400
410
420

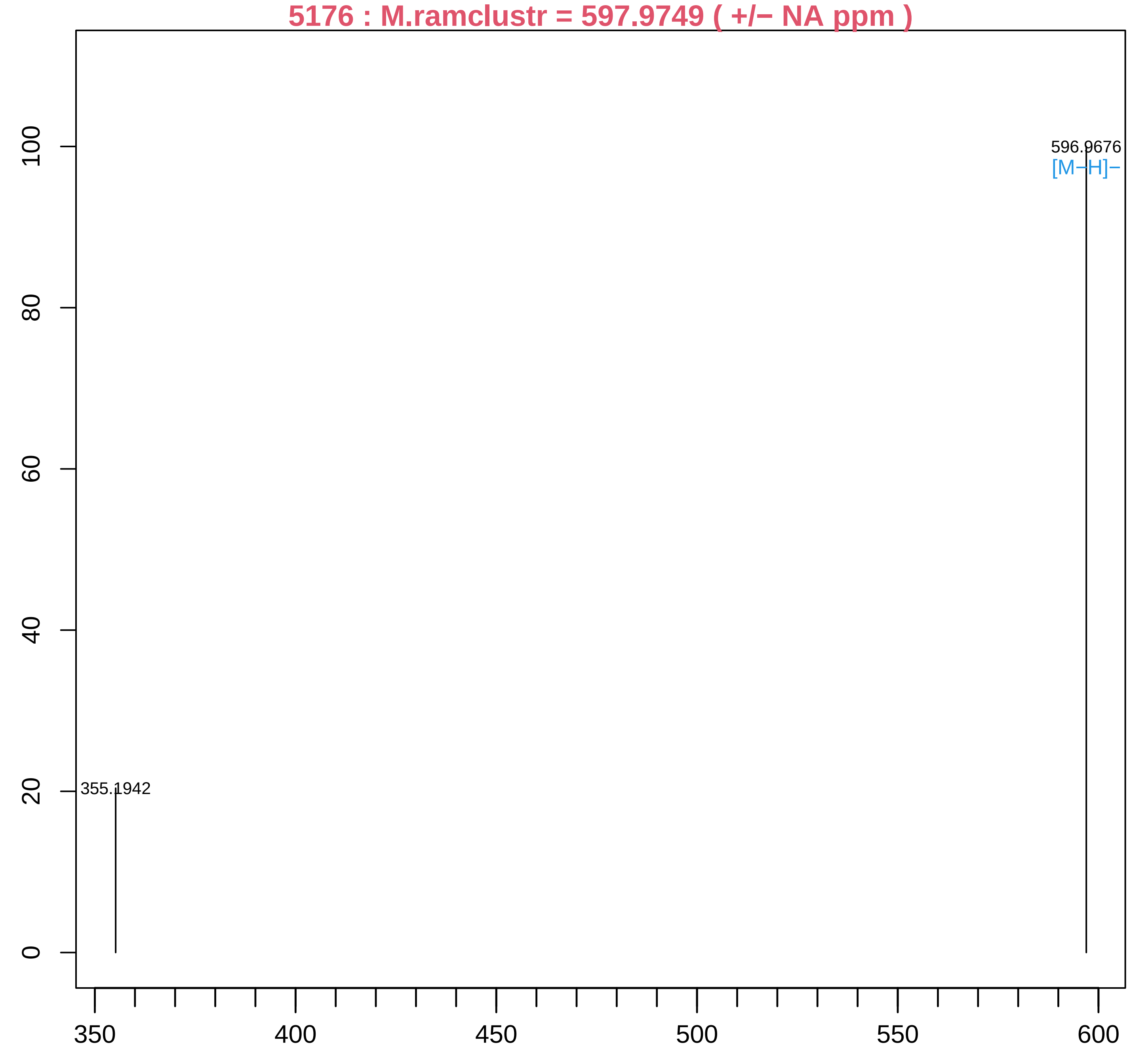
5176 : M.ramclustr = 597.9749 (+/− NA ppm)
596.9676
[M−H]−
355.1942
0
20
40
60
80
100
350
400
450
500
550
600

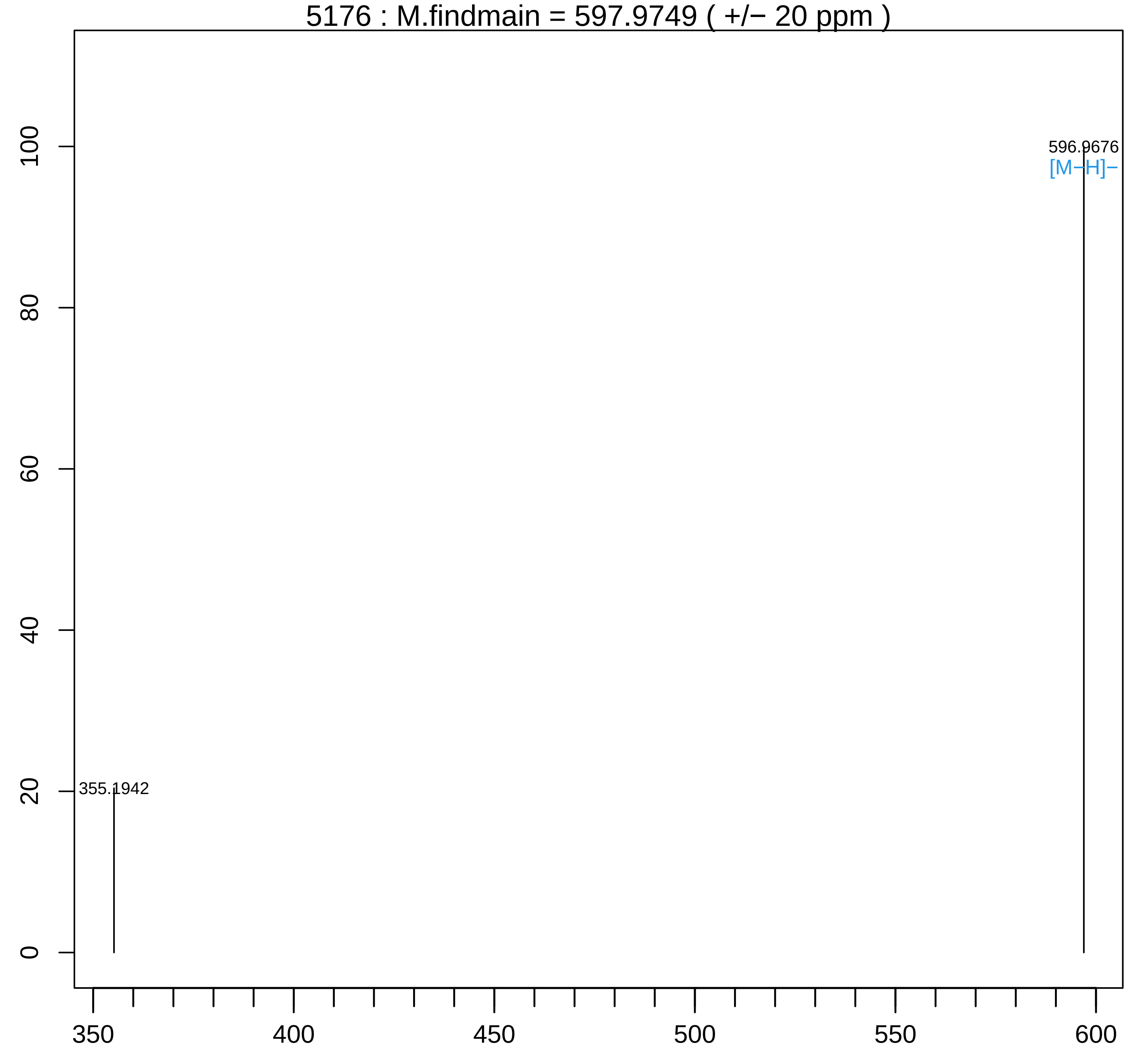
5176 : M.findmain = 597.9749 (+/− 20 ppm)
596.9676
[M−H]−
355.1942
0
20
40
60
80
100
350
400
450
500
550
600

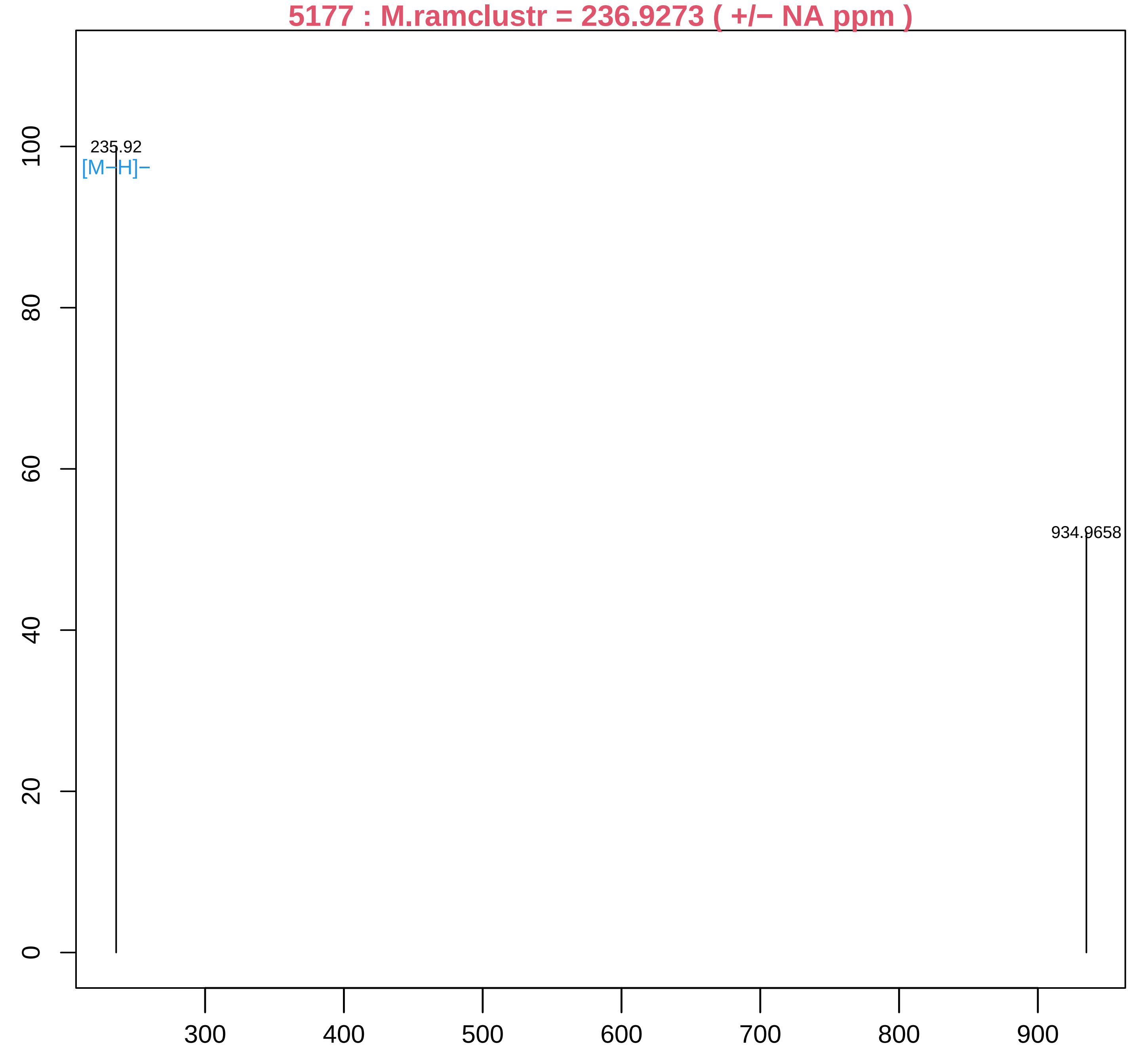
5177 : M.ramclustr = 236.9273 (+/− NA ppm)
235.92
[M−H]−
934.9658
0
20
40
60
80
100
300
400
500
600
700
800
900

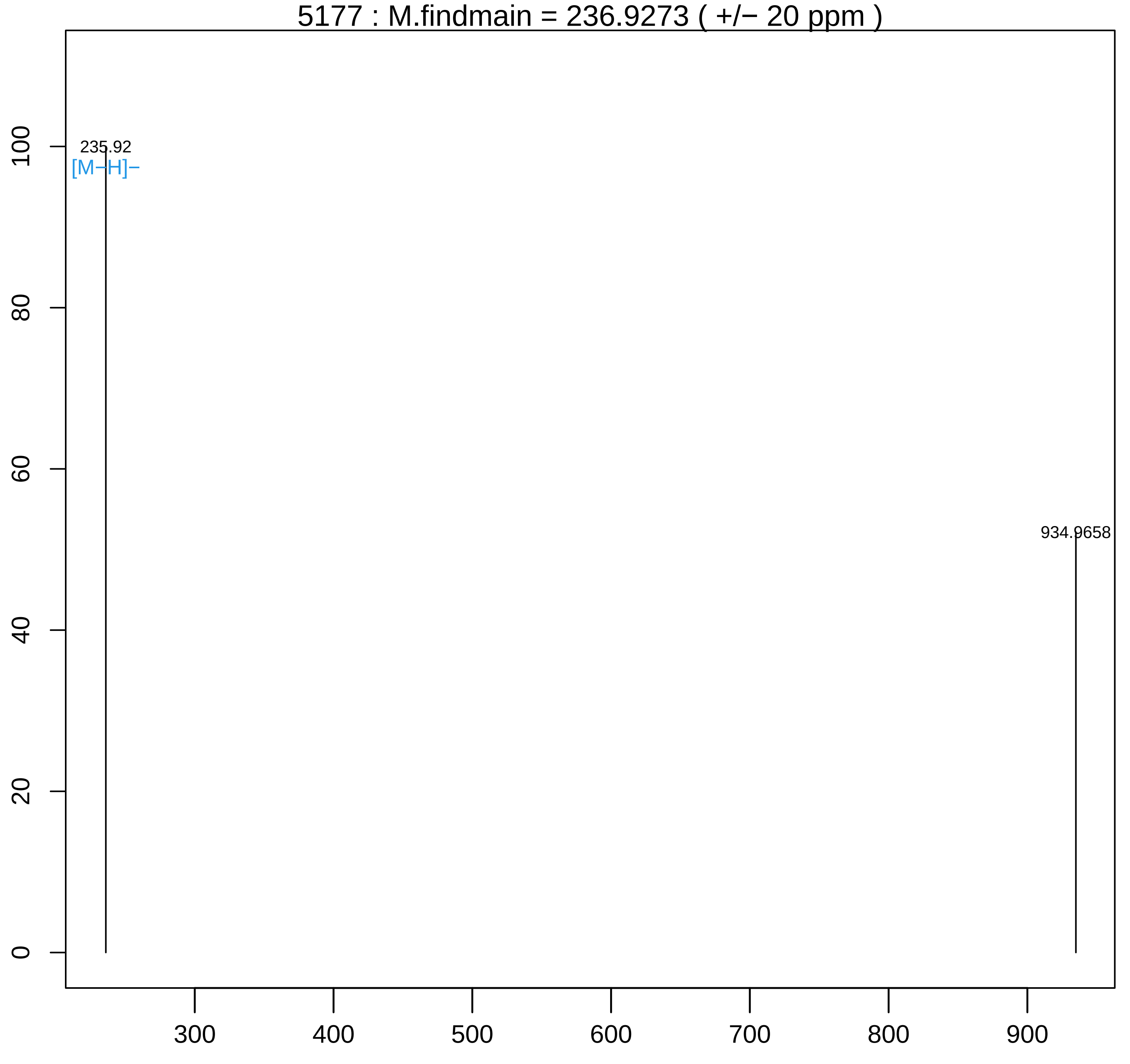
5177 : M.findmain = 236.9273 (+/− 20 ppm)
235.92
[M−H]−
934.9658
0
20
40
60
80
100
300
400
500
600
700
800
900

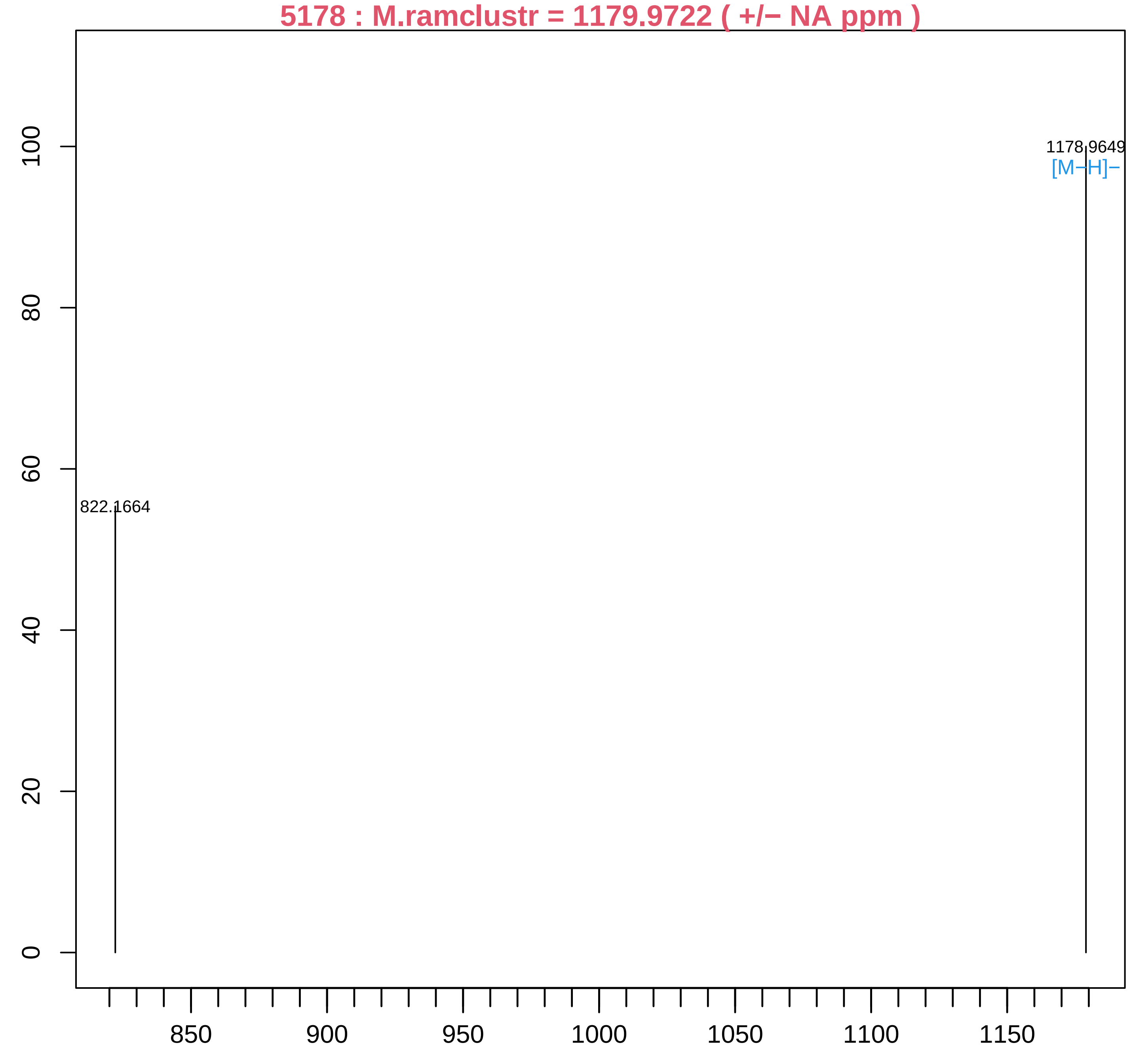

5178 : M.ramclustr = 1179.9722 (+/− NA ppm)
1178.9649
[M−H]−
822.1664
0
20
40
60
80
100
850
900
950
1000
1050
1100
1150

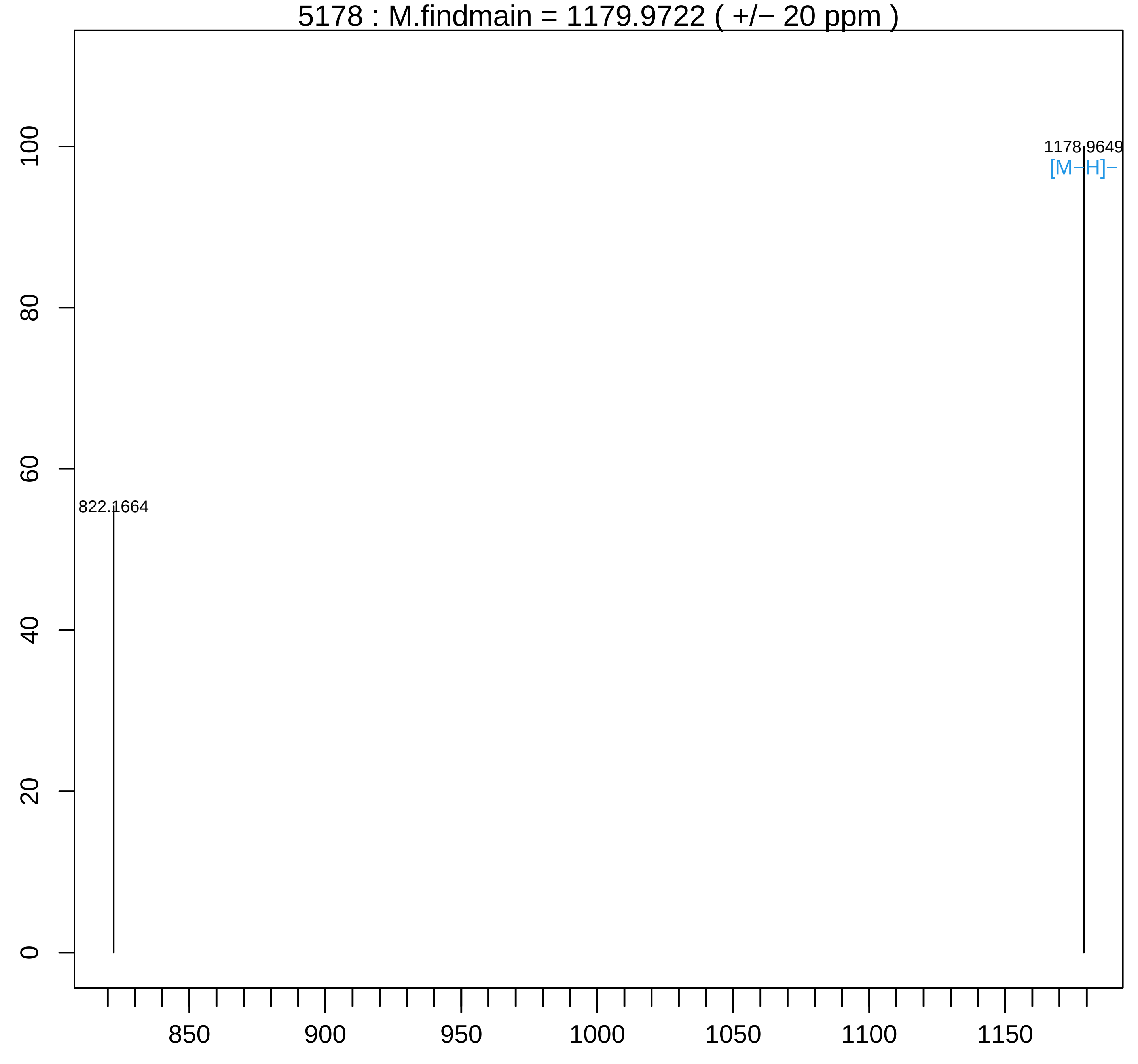

5178 : M.findmain = 1179.9722 (+/− 20 ppm)
1178.9649
[M−H]−
822.1664
0
20
40
60
80
100
850
900
950
1000
1050
1100
1150

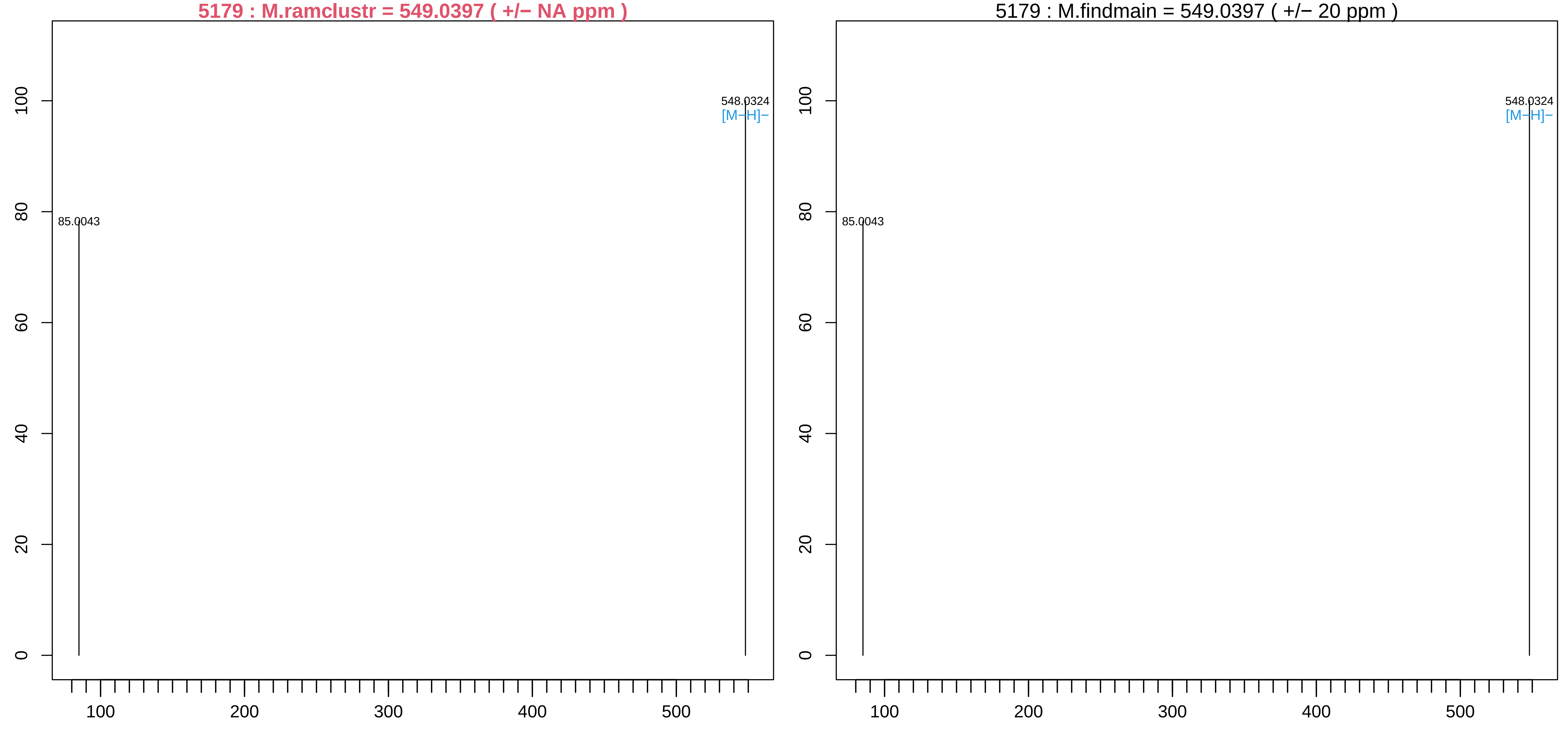
5179 : M.ramclustr = 549.0397 (+/− NA ppm)
548.0324
[M−H]−
85.0043
5179 : M.findmain = 549.0397 (+/− 20 ppm)
548.0324
[M−H]−
85.0043

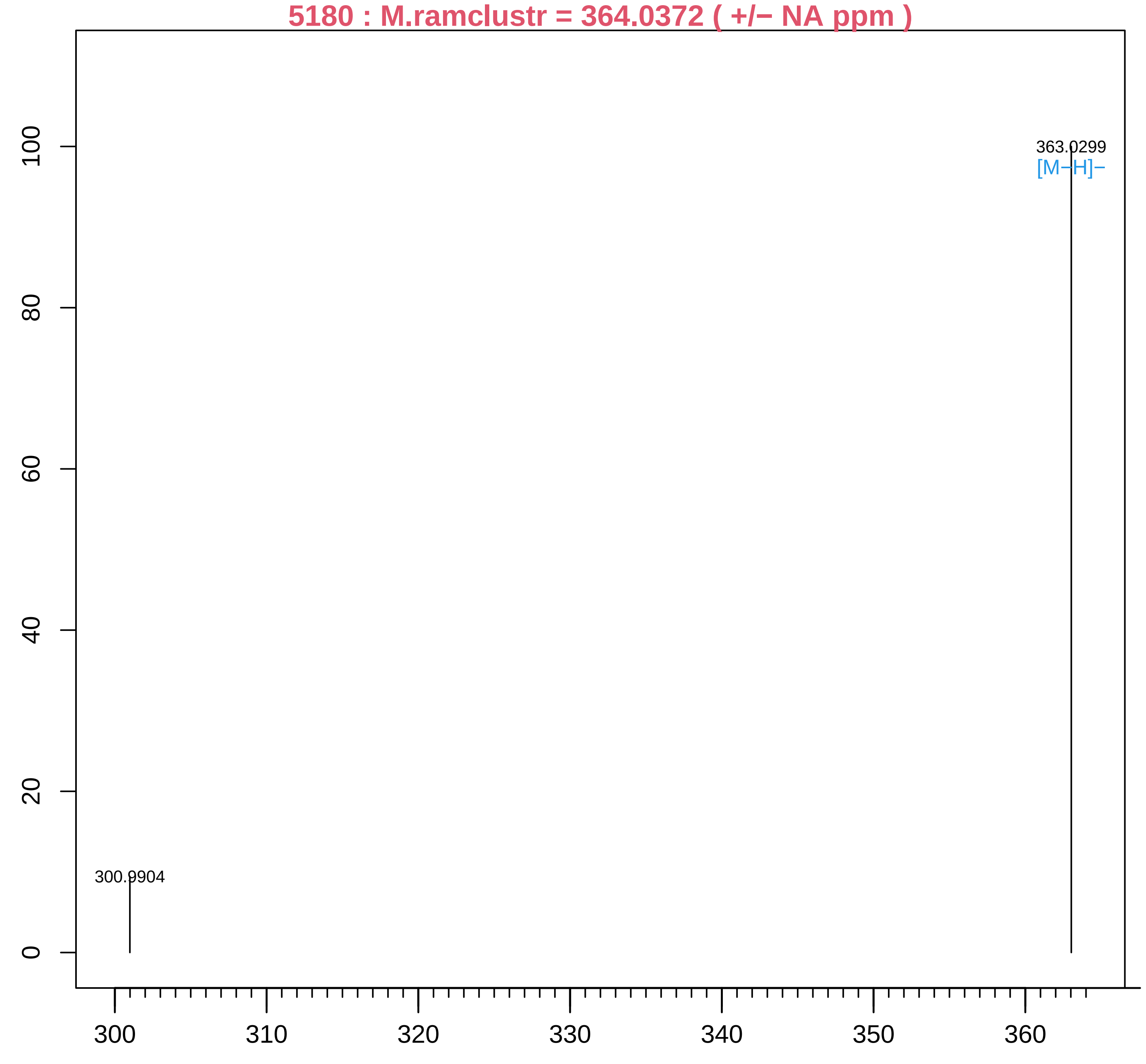
5180 : M.ramclustr = 364.0372 (+/− NA ppm)
363.0299
[M−H]−
300.9904
0
20
40
60
80
100
300
310
320
330
340
350
360

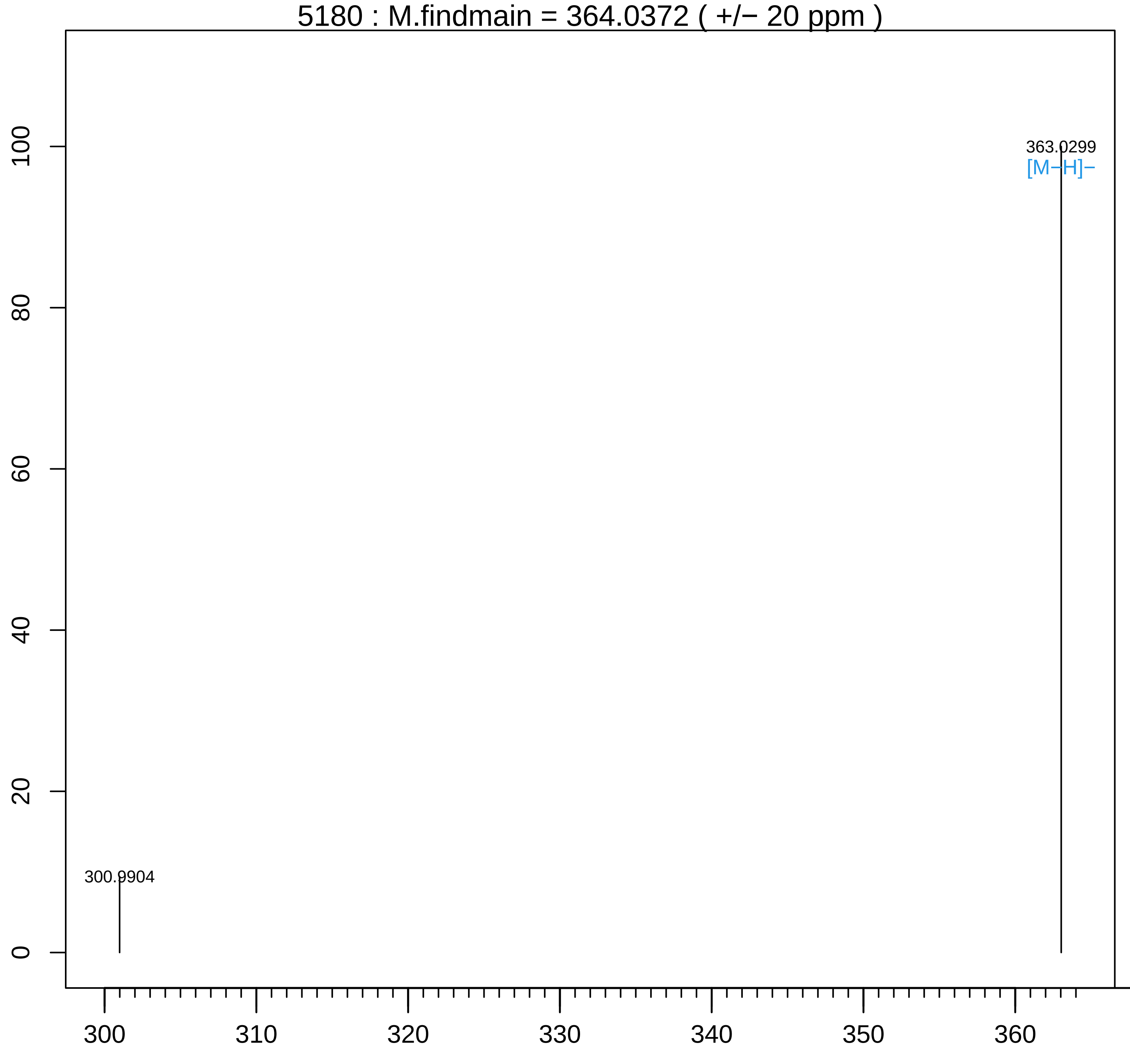
5180 : M.findmain = 364.0372 (+/− 20 ppm)
363.0299
[M−H]−
300.9904
0
20
40
60
80
100
300
310
320
330
340
350
360

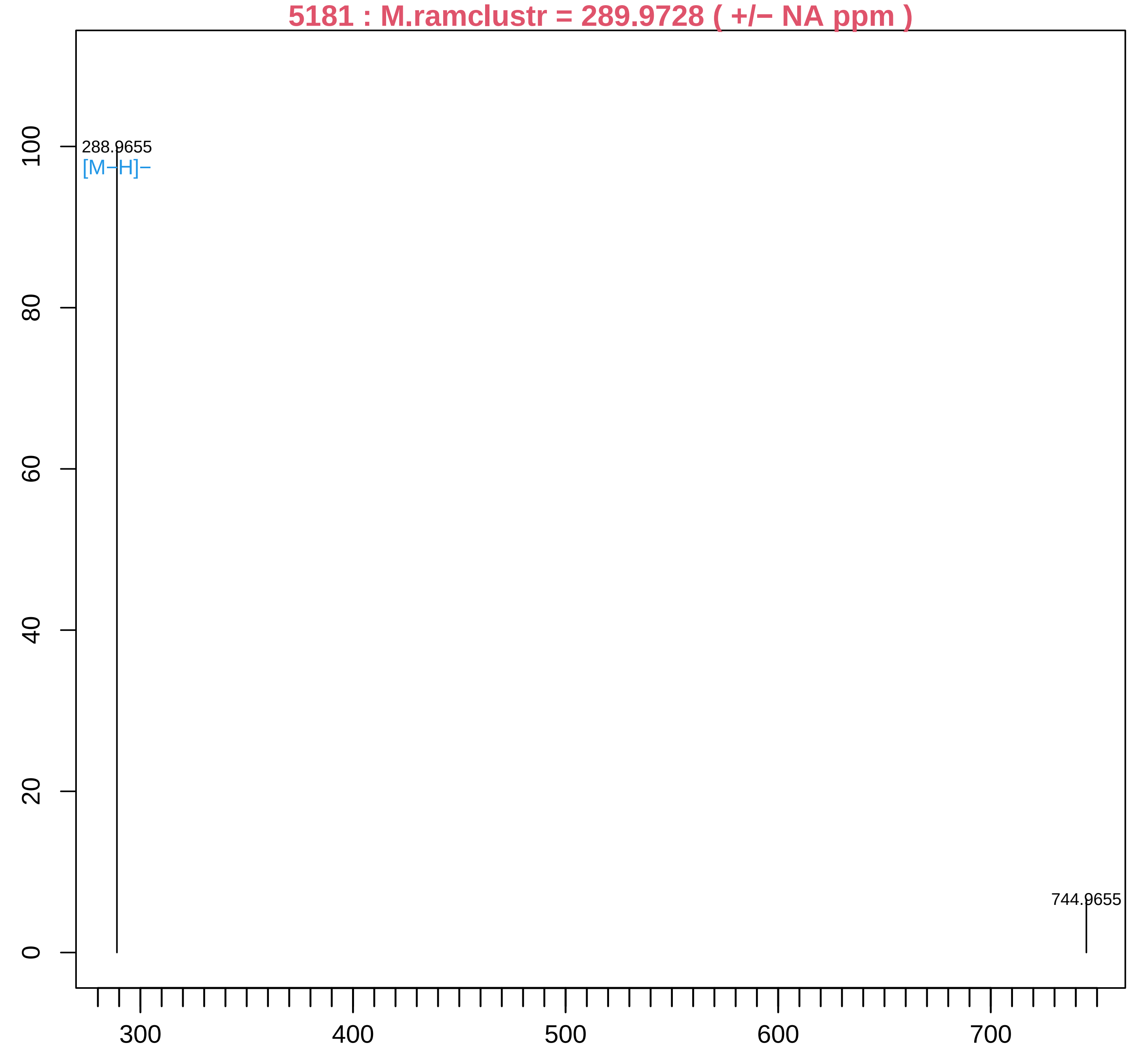

5181 : M.ramclustr = 289.9728 (+/− NA ppm)
288.9655
[M−H]−
744.9655
0
20
40
60
80
100
300
400
500
600
700

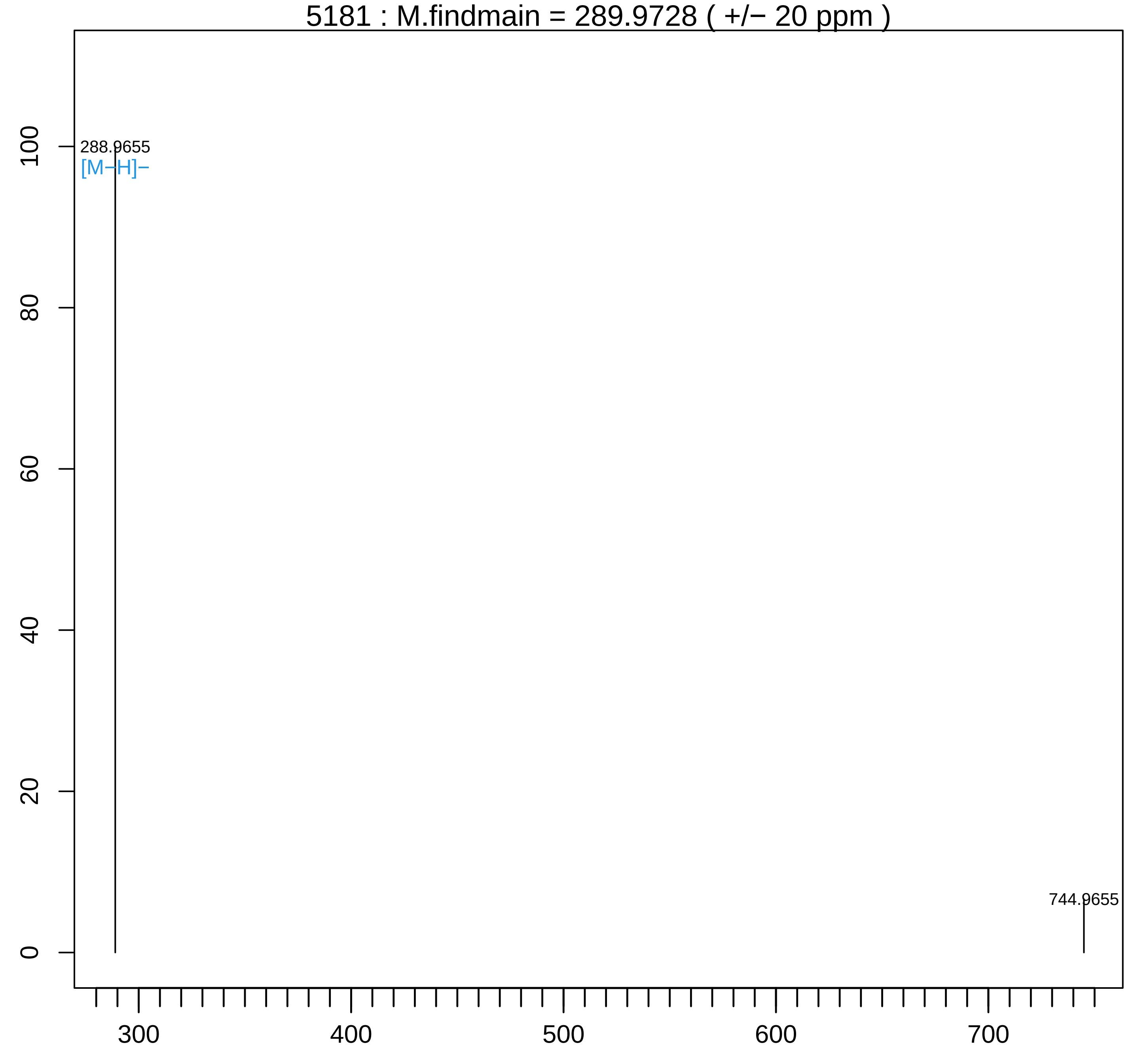

5181 : M.findmain = 289.9728 (+/− 20 ppm)
288.9655
[M−H]−
744.9655
0
20
40
60
80
100
300
400
500
600
700

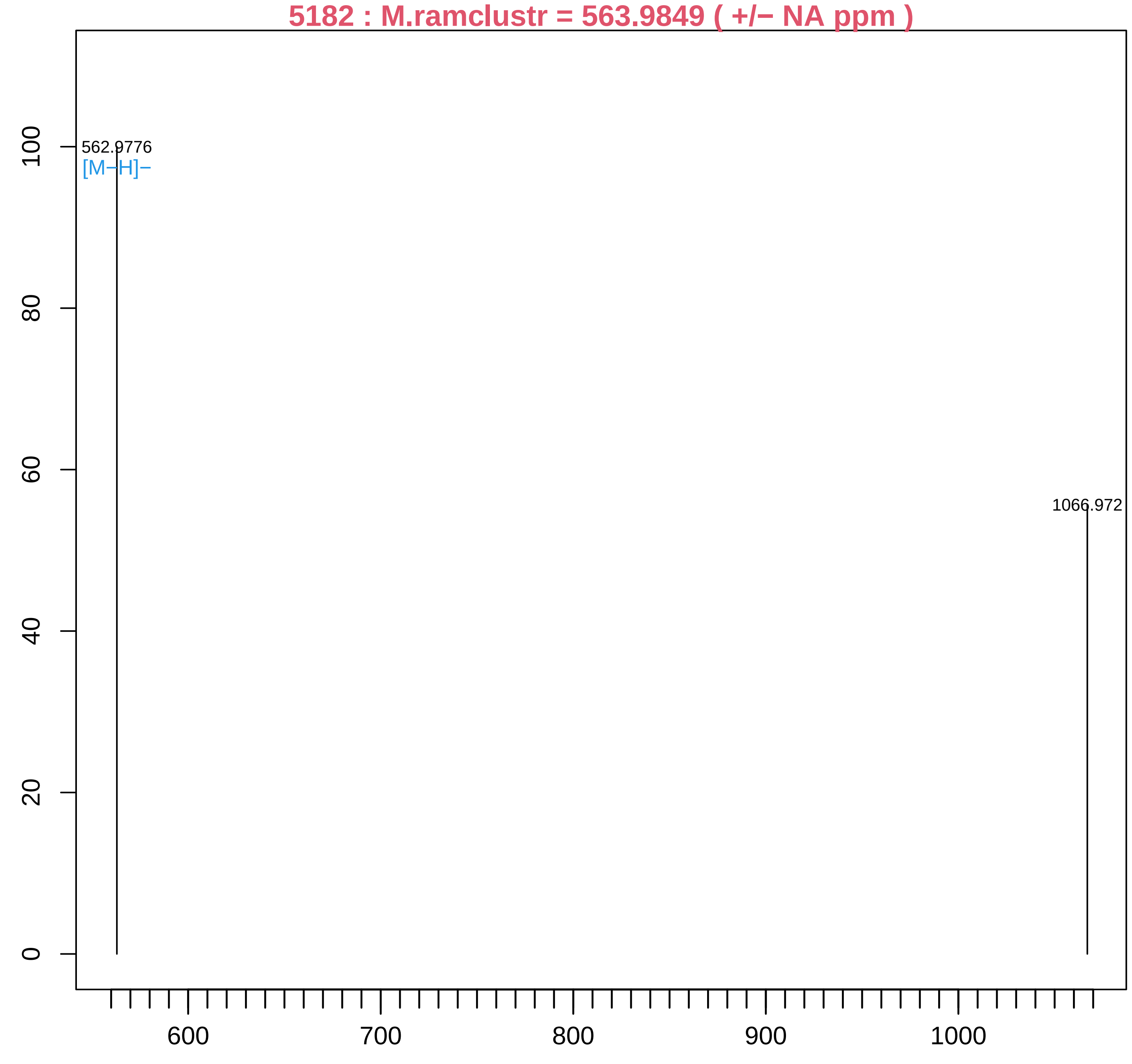
5182 : M.ramclustr = 563.9849 (+/− NA ppm)
562.9776
[M−H]−
1066.972
0
20
40
60
80
100
600
700
800
900
1000

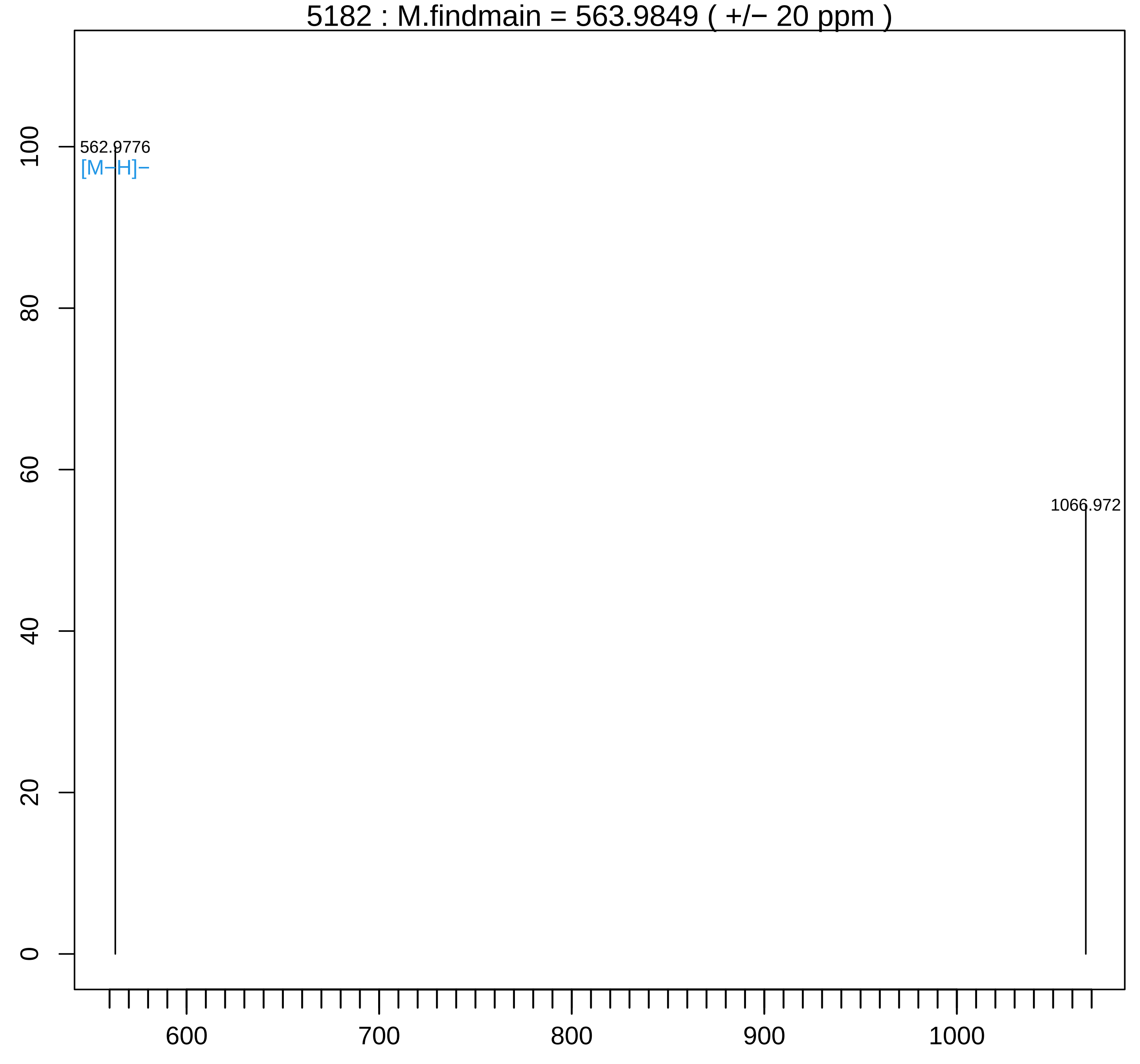
5182 : M.findmain = 563.9849 (+/− 20 ppm)
562.9776
[M−H]−
1066.972
0
20
40
60
80
100
600
700
800
900
1000

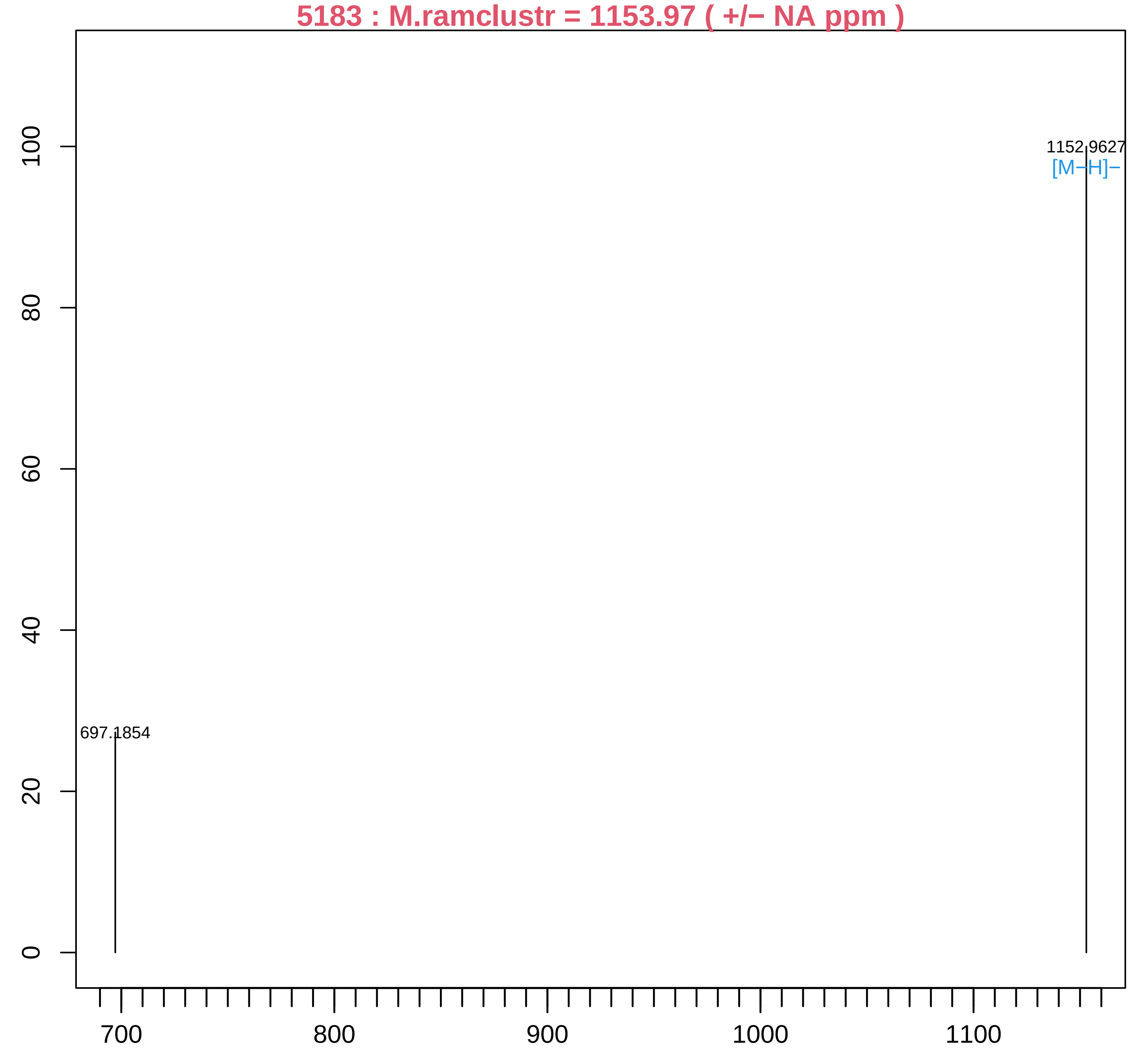
5183 : M.ramclustr = 1153.97 (+/− NA ppm)
1152.9627
[M−H]−
697.1854
0
20
40
60
80
100
700
800
900
1000
1100

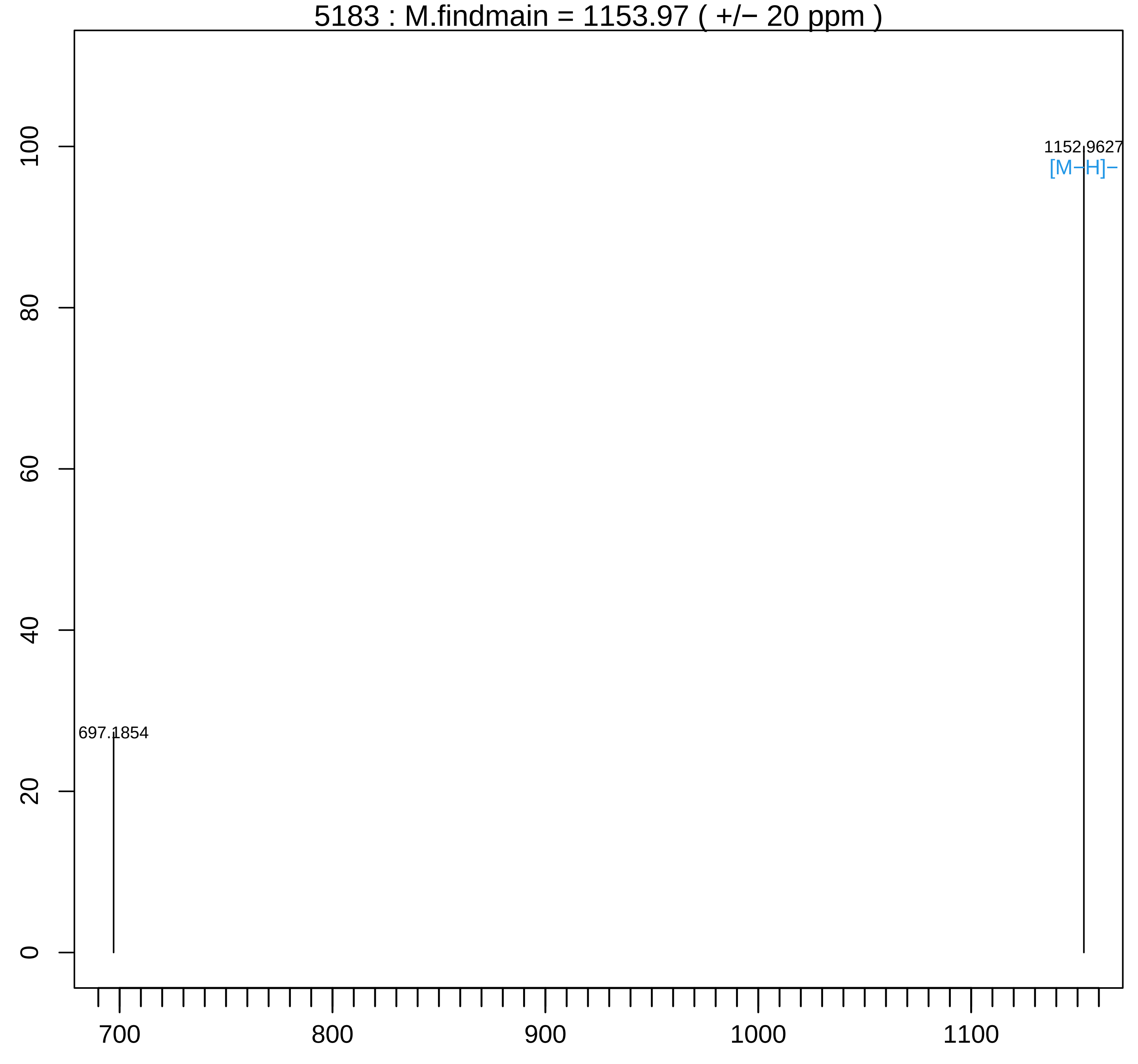
5183 : M.findmain = 1153.97 (+/− 20 ppm)
1152.9627
[M−H]−
697.1854
0
20
40
60
80
100
700
800
900
1000
1100

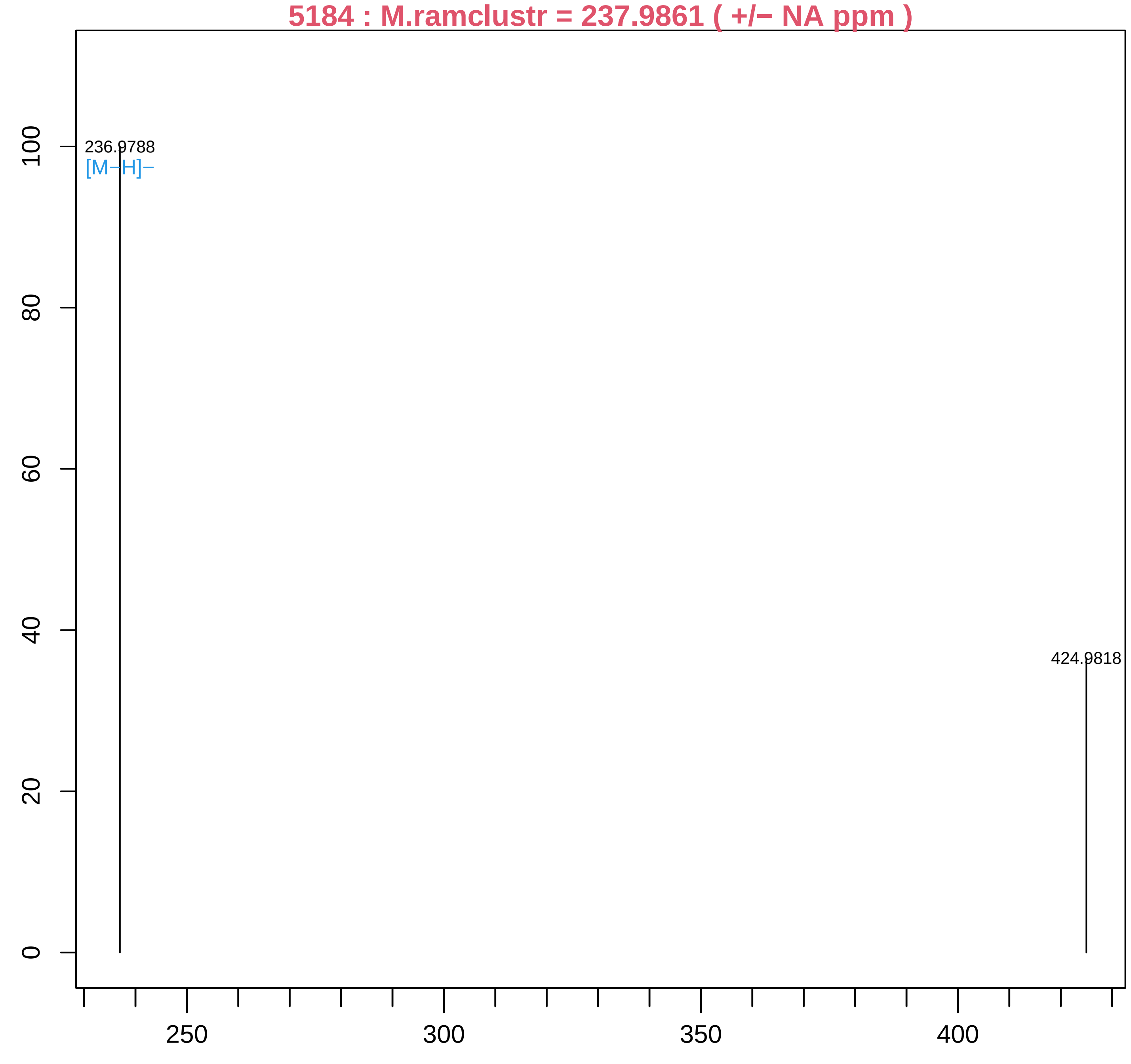

5184 : M.ramclustr = 237.9861 (+/− NA ppm)
236.9788
[M−H]−
424.9818
0
20
40
60
80
100
250
300
350
400

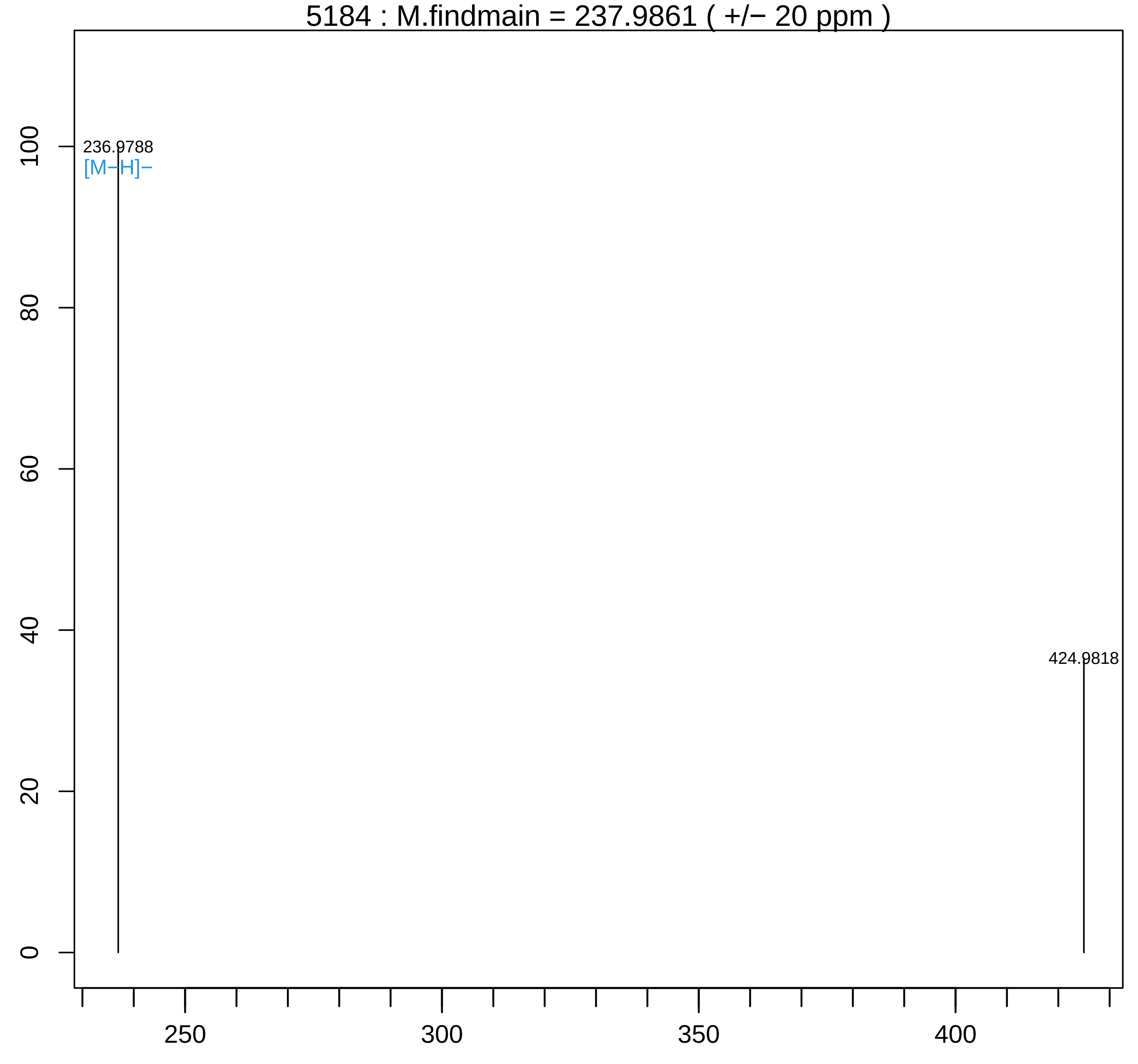

5184 : M.findmain = 237.9861 (+/− 20 ppm)
236.9788
[M−H]−
424.9818
0
20
40
60
80
100
250
300
350
400

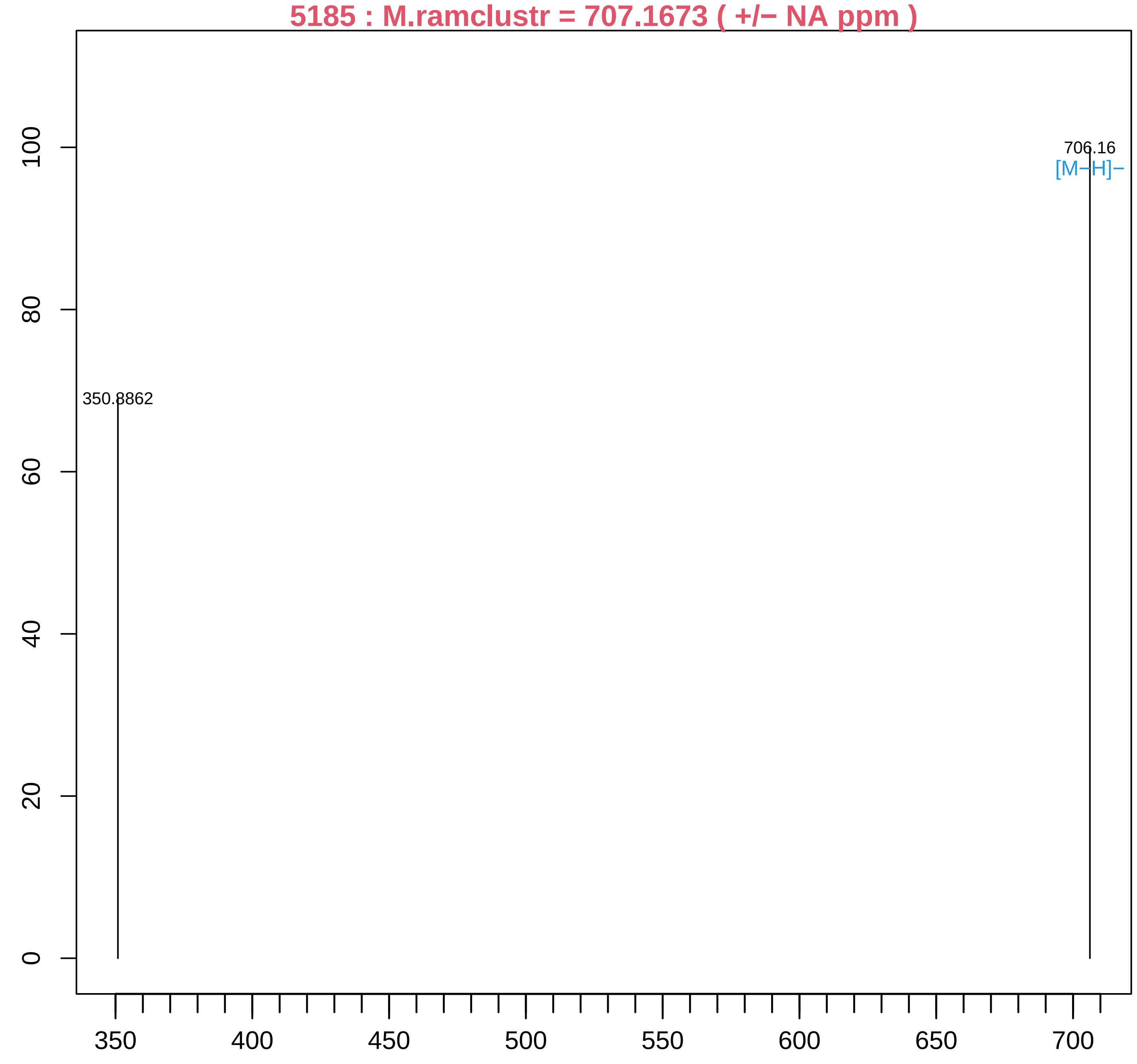
5185 : M.ramclustr = 707.1673 (+/− NA ppm)
706.16
[M−H]−
350.8862
0
20
40
60
80
100
350
400
450
500
550
600
650
700

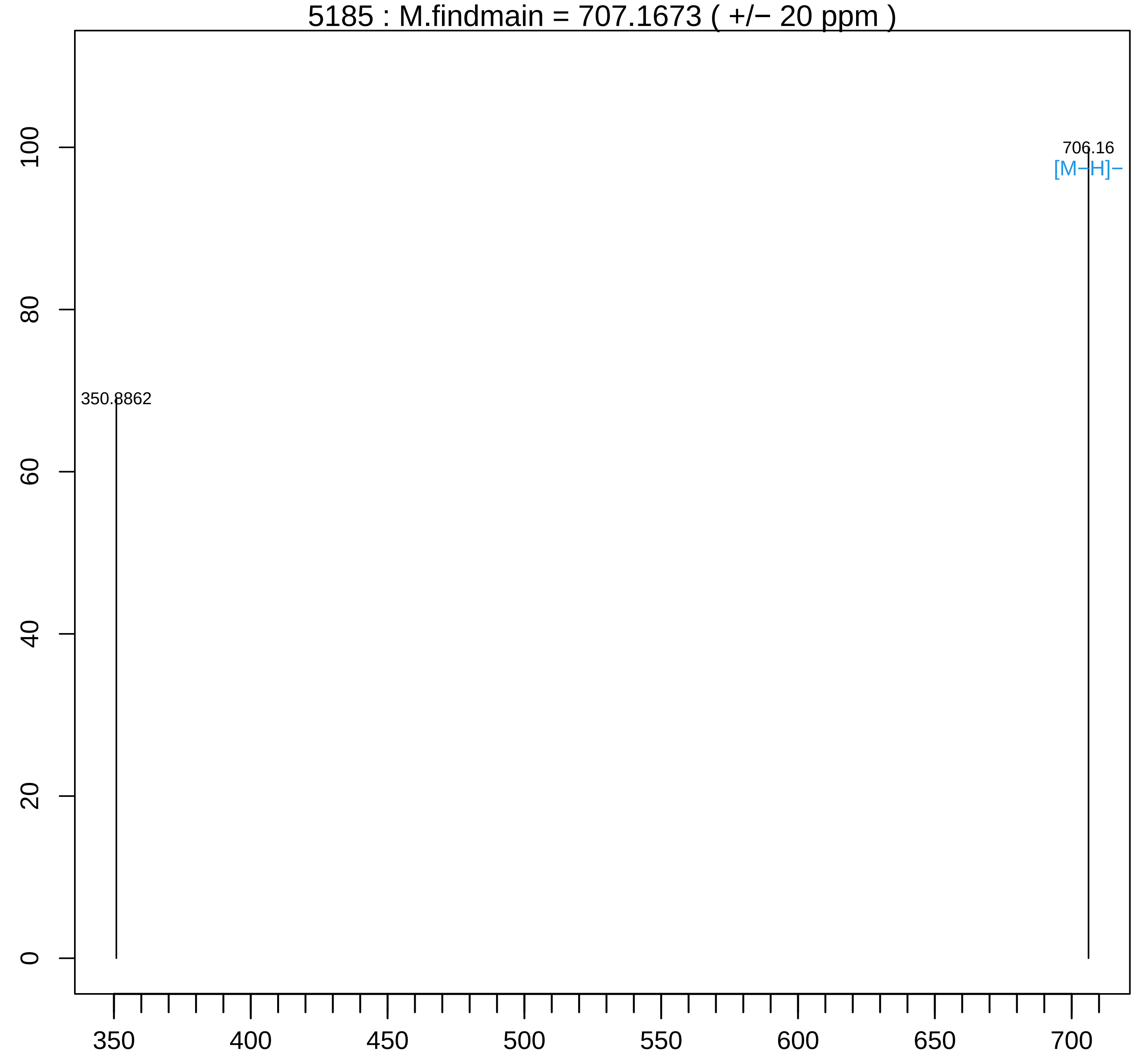
5185 : M.findmain = 707.1673 (+/− 20 ppm)
706.16
[M−H]−
350.8862
0
20
40
60
80
100
350
400
450
500
550
600
650
700

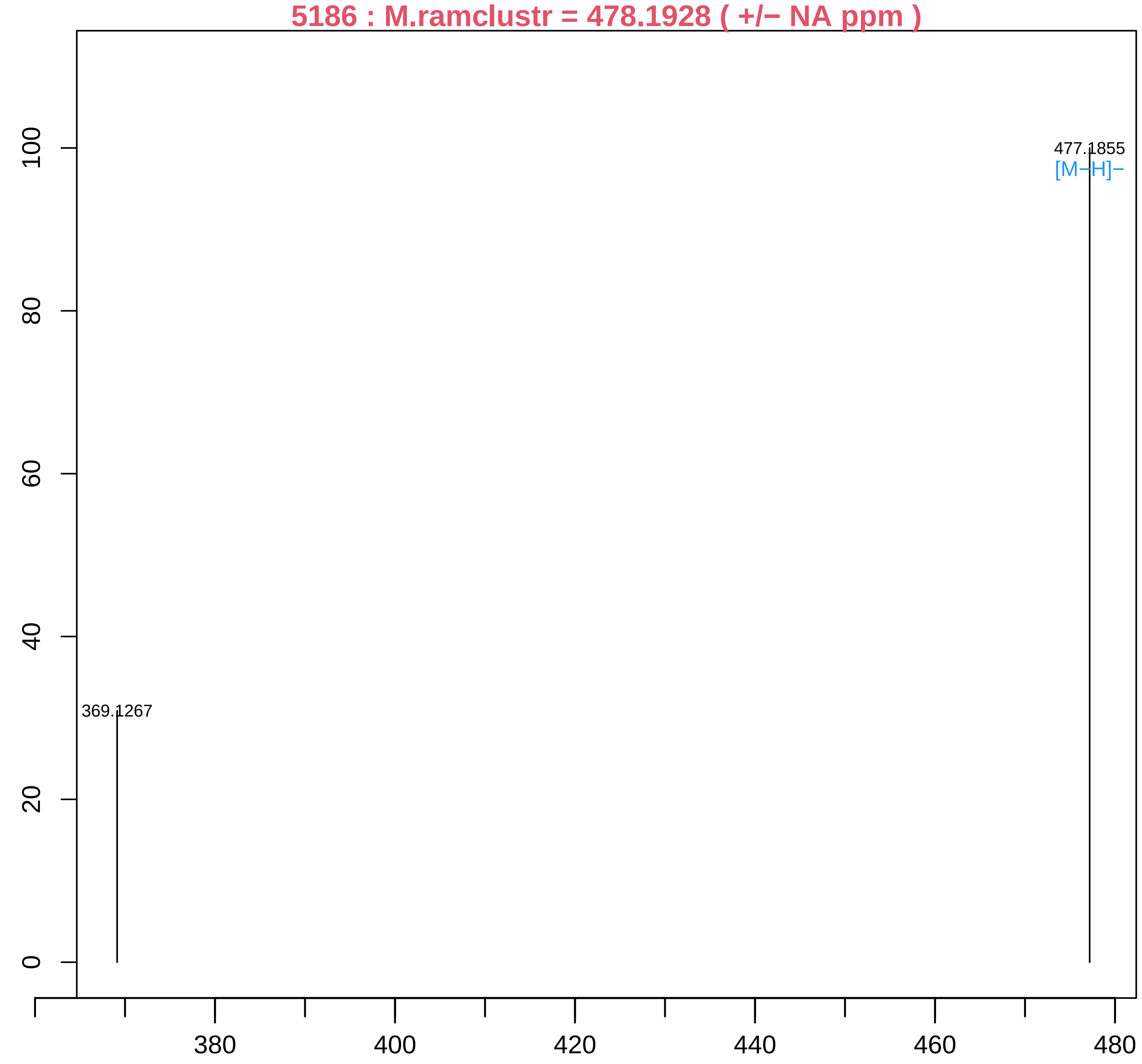
5186 : M.ramclustr = 478.1928 (+/− NA ppm)
477.1855
[M−H]−
369.1267
0
20
40
60
80
100
380
400
420
440
460
480

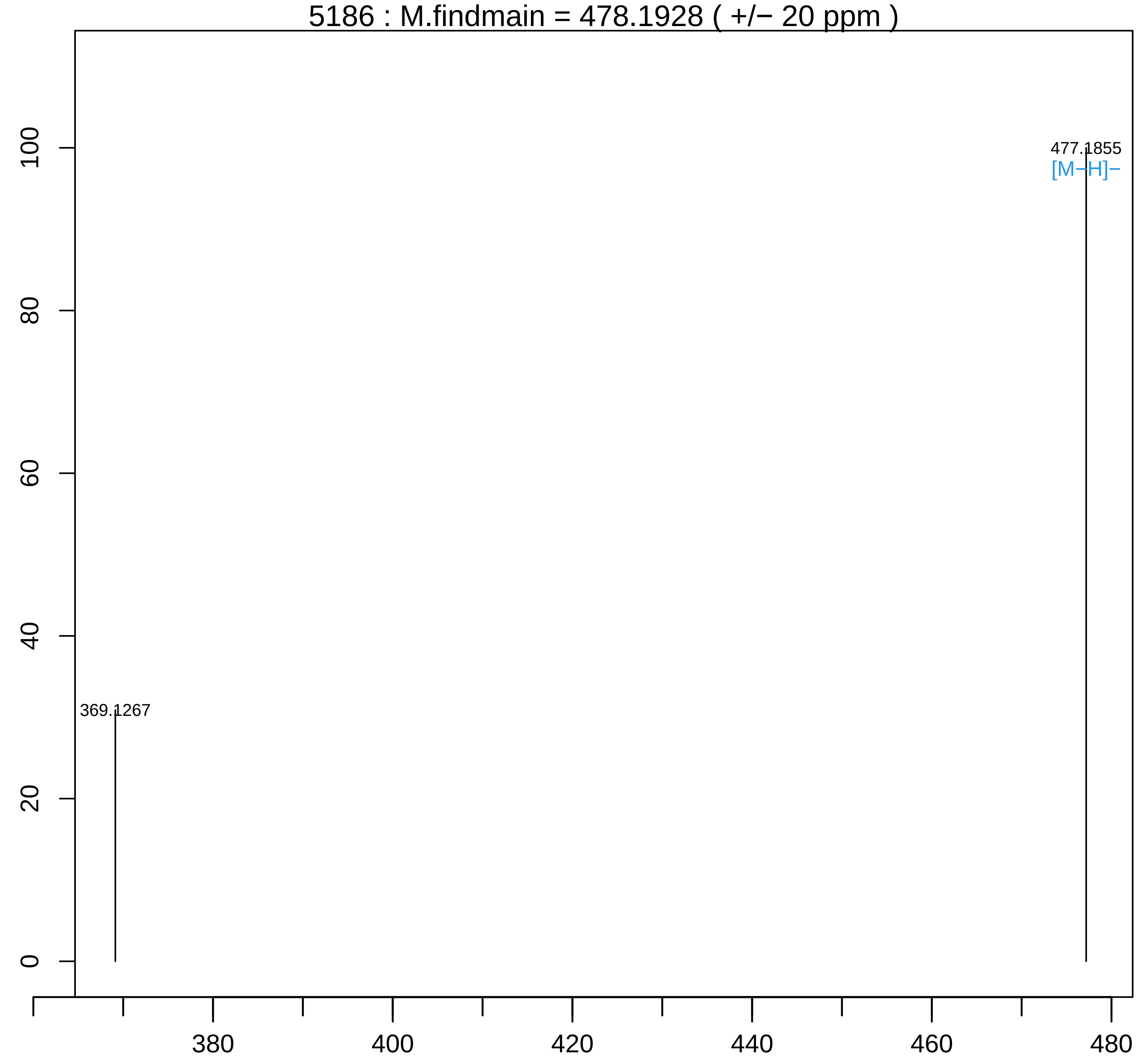
5186 : M.findmain = 478.1928 (+/− 20 ppm)
477.1855
[M−H]−
369.1267
0
20
40
60
80
100
380
400
420
440
460
480

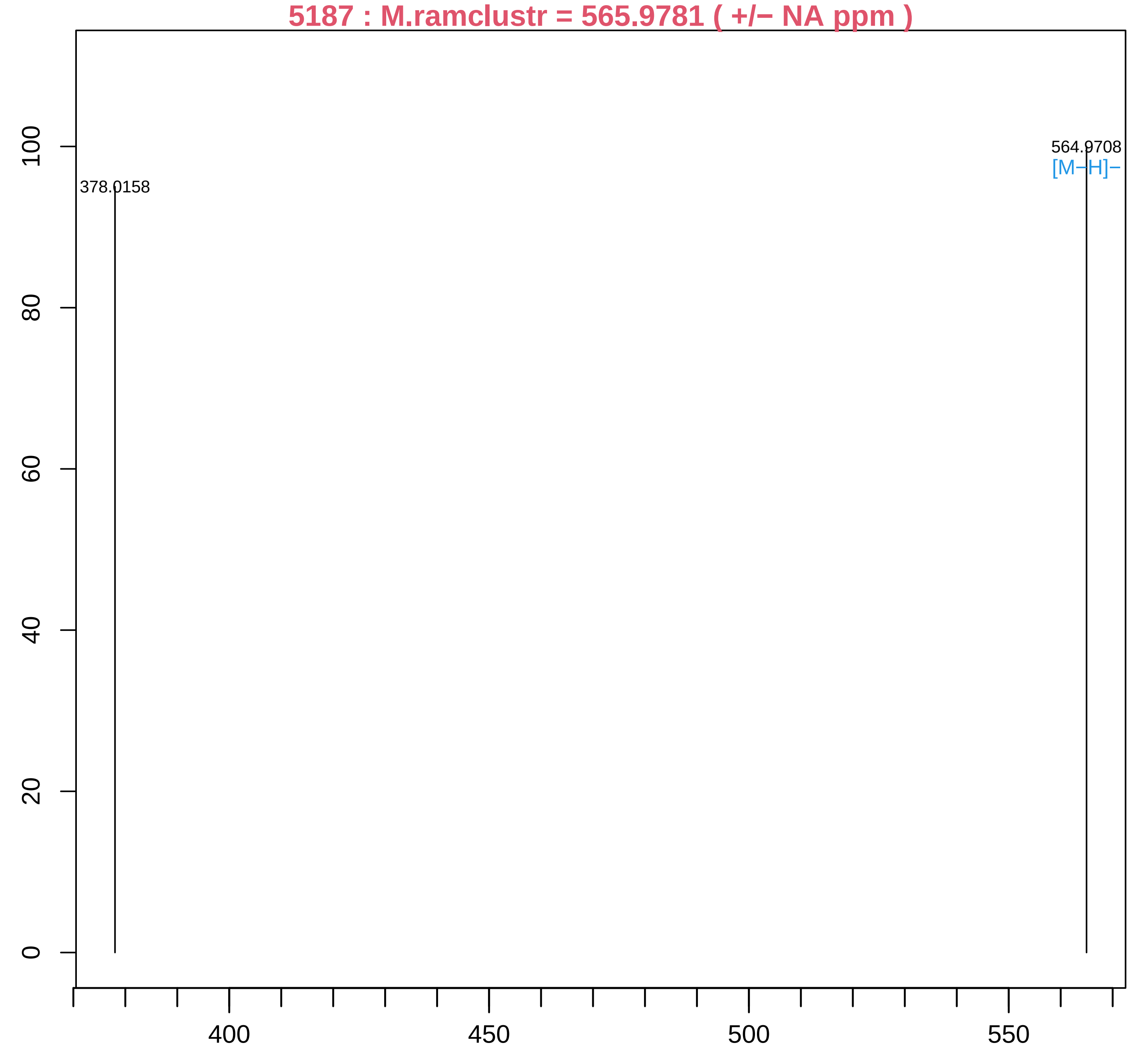
5187 : M.ramclustr = 565.9781 (+/− NA ppm)
378.0158
564.9708
[M−H]−
0
20
40
60
80
100
400
450
500
550

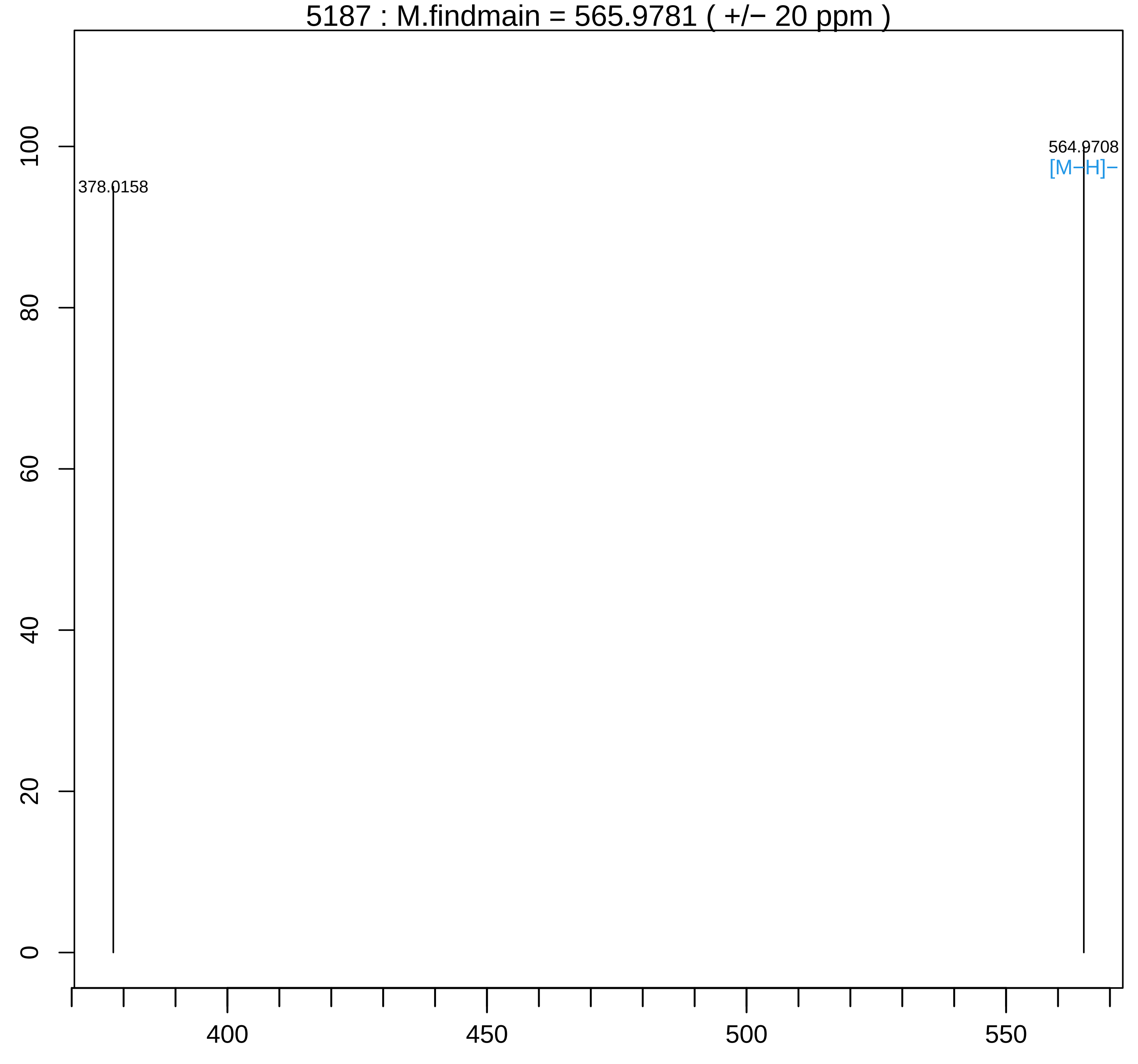
5187 : M.findmain = 565.9781 (+/− 20 ppm)
378.0158
564.9708
[M−H]−
0
20
40
60
80
100
400
450
500
550

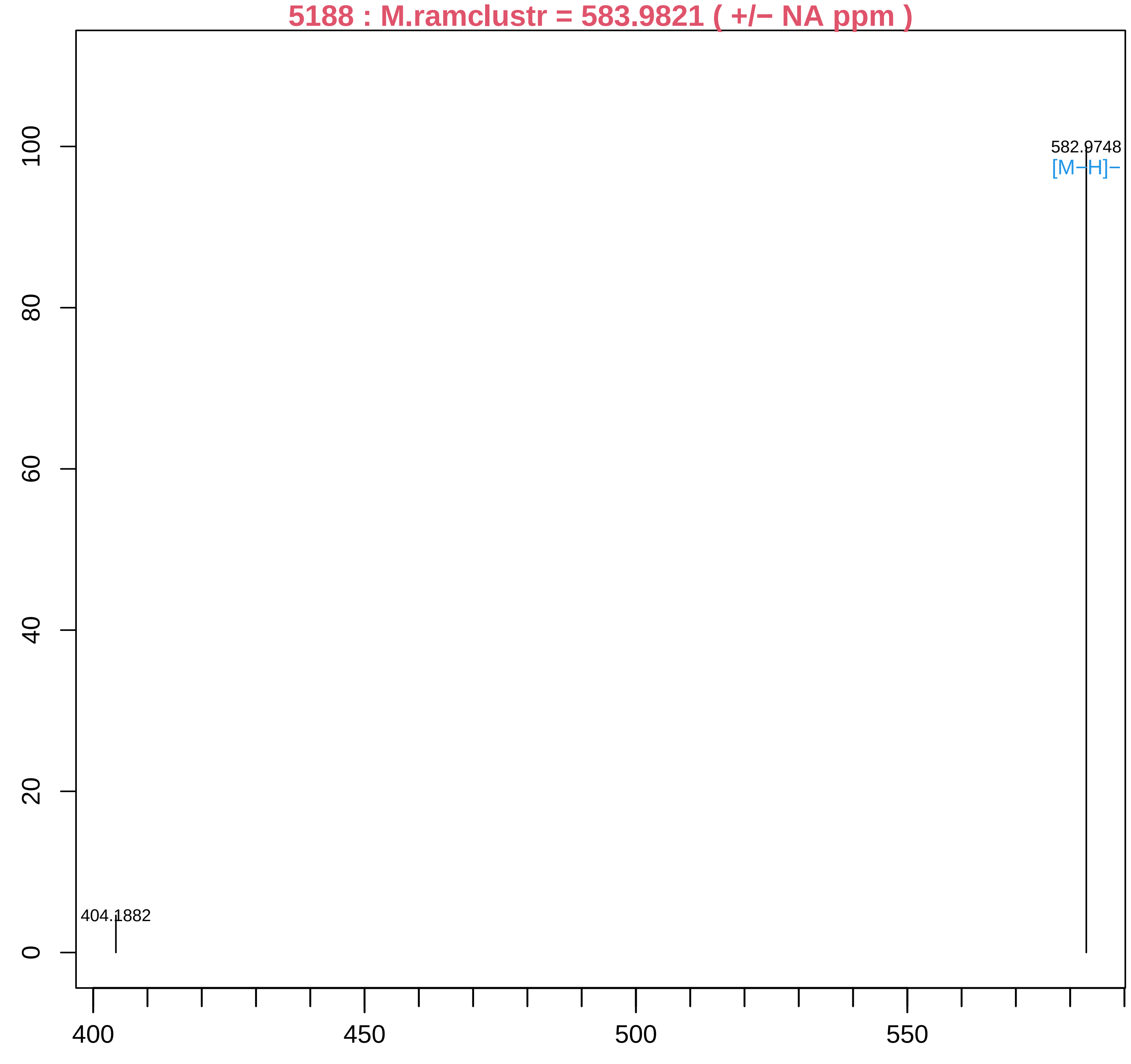

5188 : M.ramclustr = 583.9821 (+/− NA ppm)
582.9748
[M−H]−
404.1882
0
20
40
60
80
100
400
450
500
550

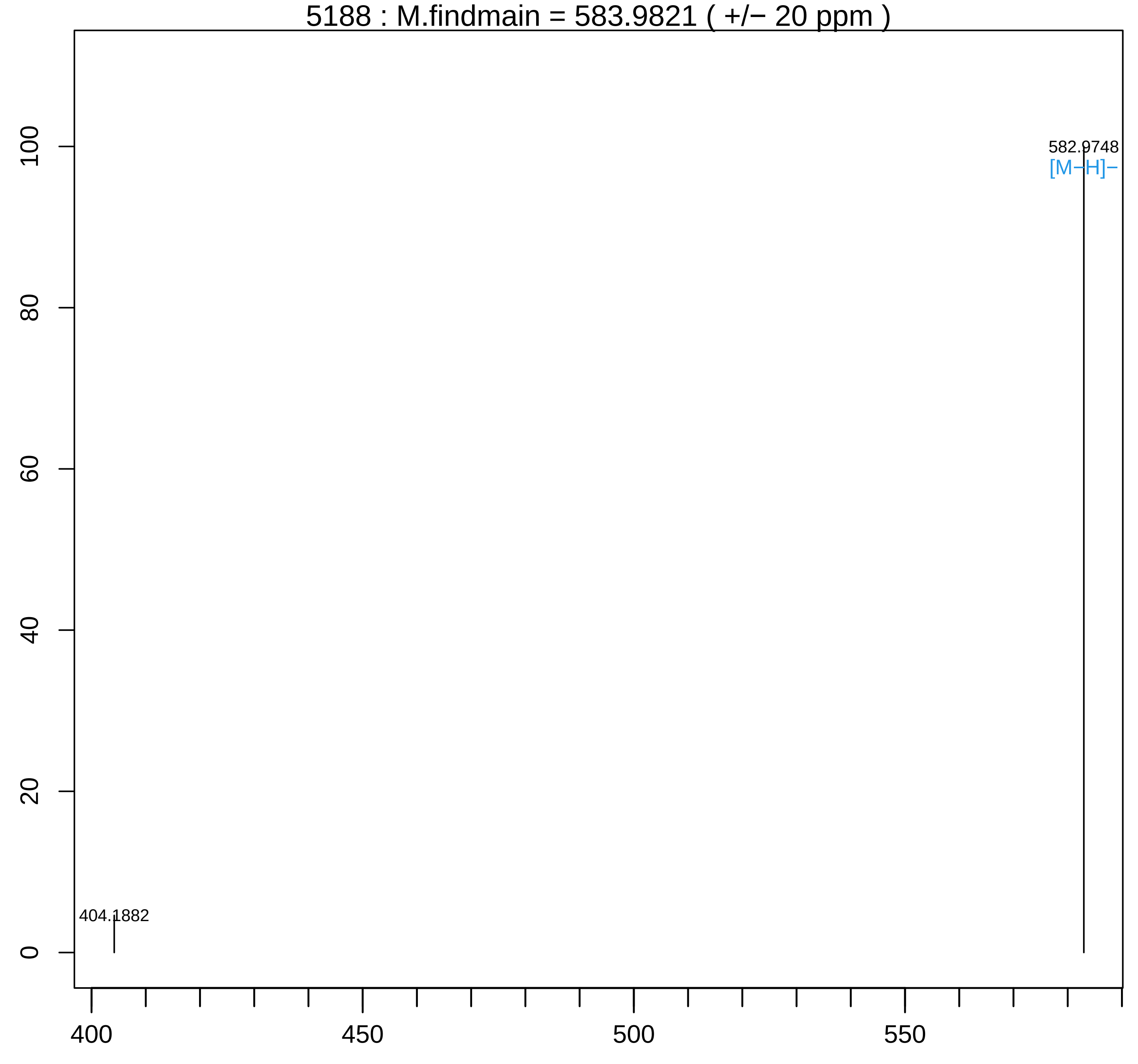

5188 : M.findmain = 583.9821 (+/− 20 ppm)
582.9748
[M−H]−
404.1882
0
20
40
60
80
100
400
450
500
550

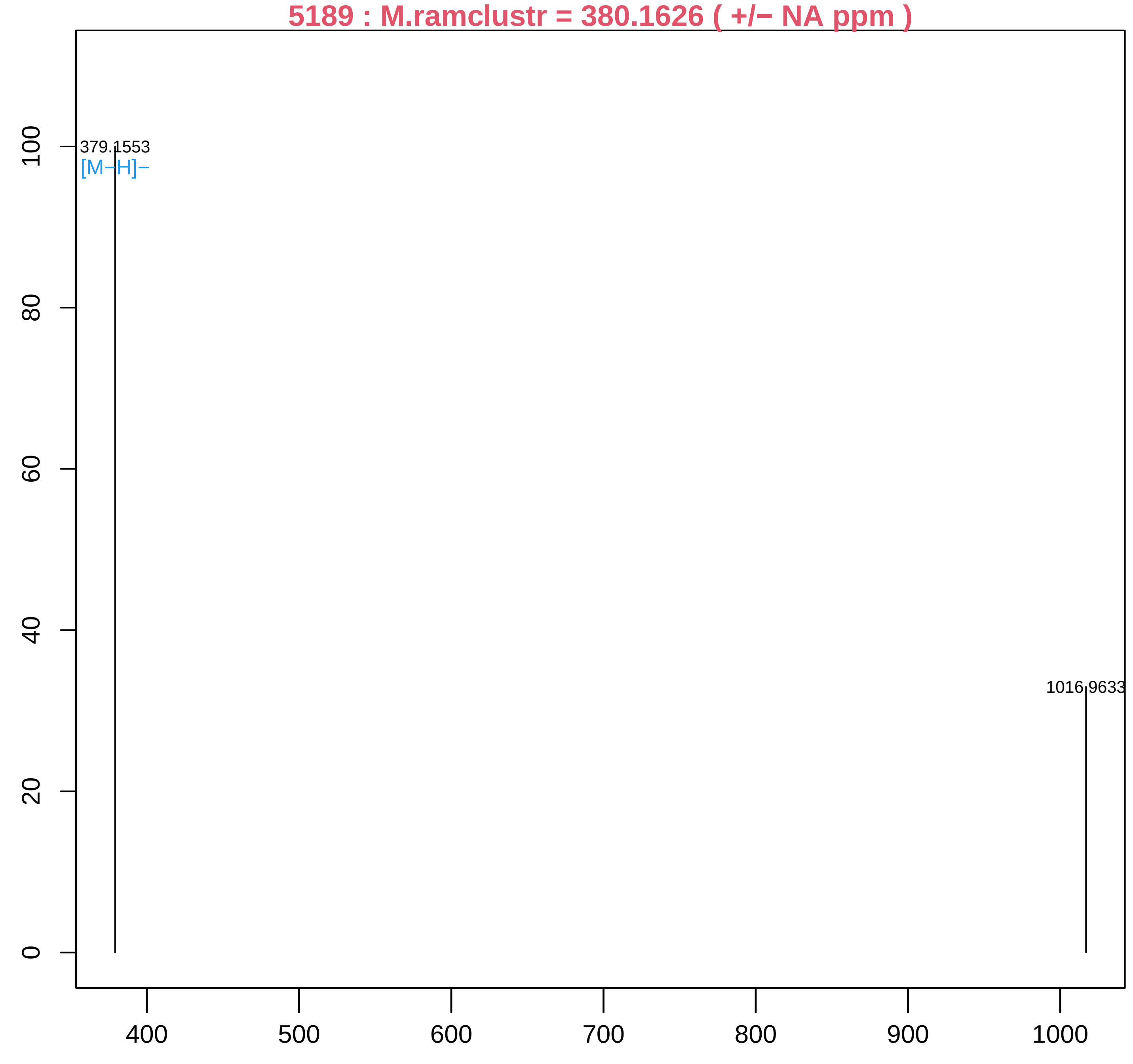

5189 : M.ramclustr = 380.1626 (+/− NA ppm)
379.1553
[M−H]−
1016.9633
0
20
40
60
80
100
400
500
600
700
800
900
1000

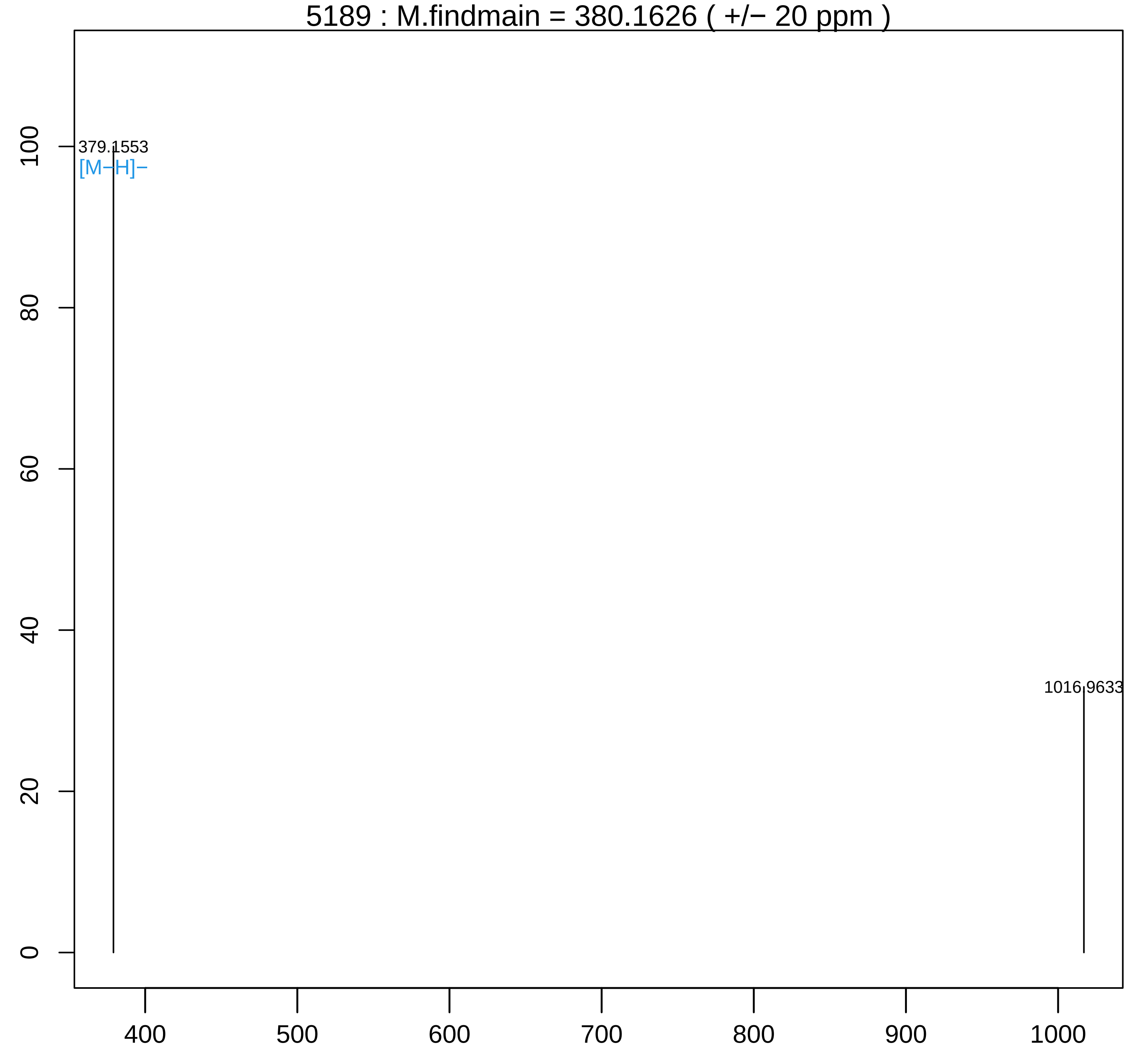

5189 : M.findmain = 380.1626 (+/− 20 ppm)
379.1553
[M−H]−
1016.9633
0
20
40
60
80
100
400
500
600
700
800
900
1000

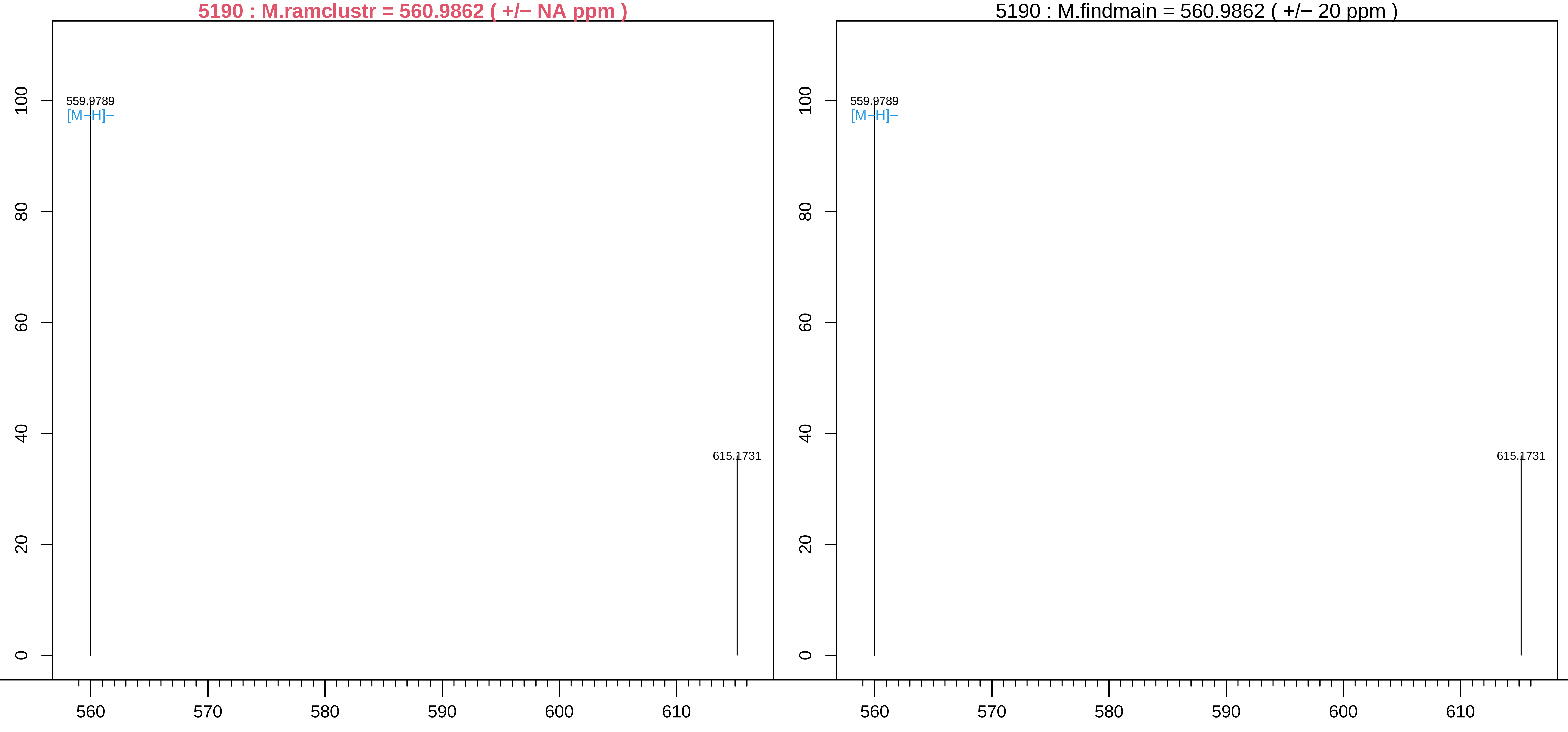
5190 : M.ramclustr = 560.9862 (+/− NA ppm)
559.9789
[M−H]−
615.1731
5190 : M.findmain = 560.9862 (+/− 20 ppm)
559.9789
[M−H]−
615.1731
0
20
40
60
80
100
560
570
580
590
600
610

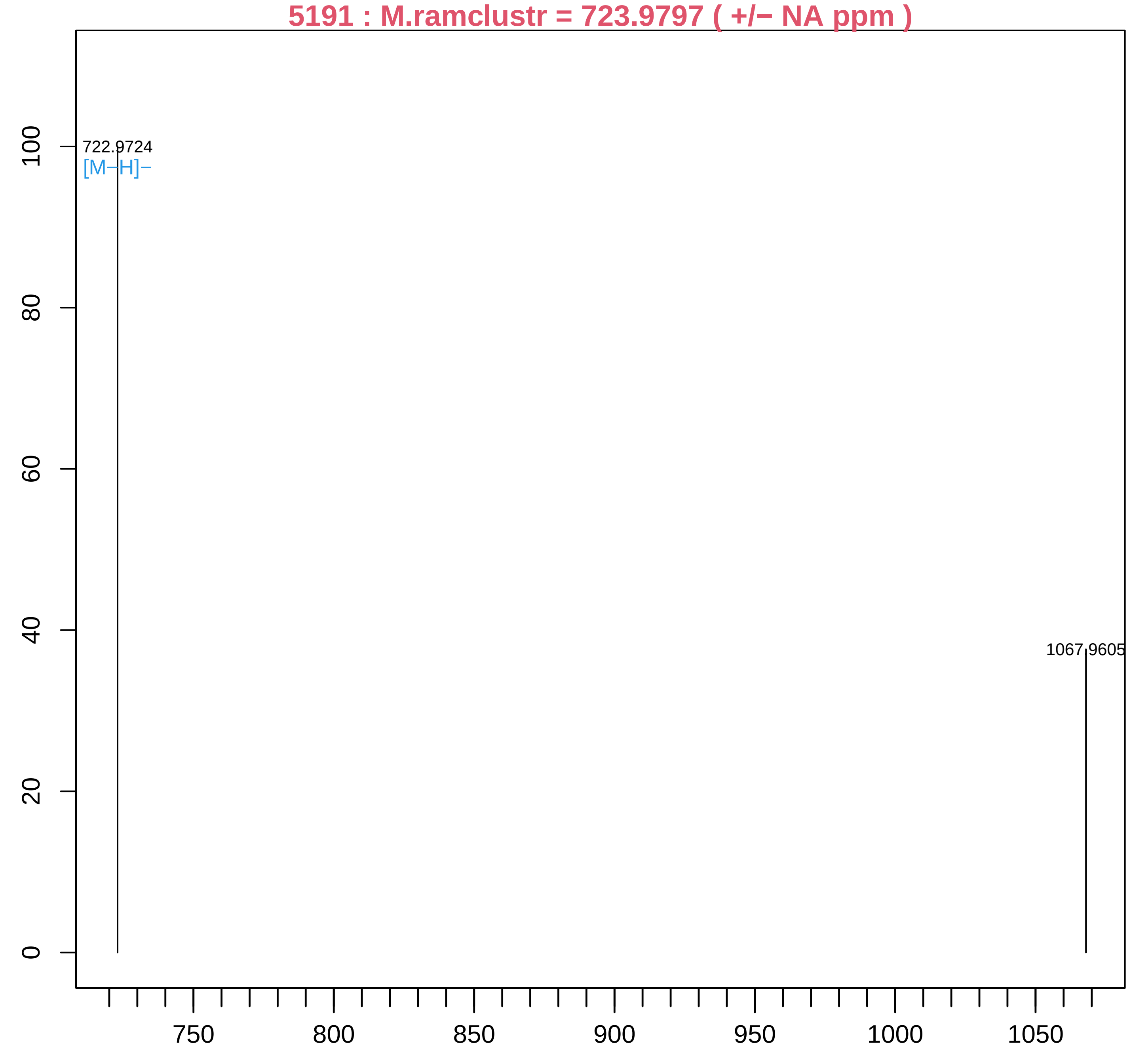
5191 : M.ramclustr = 723.9797 (+/− NA ppm)
722.9724
[M−H]−
1067.9605
0
20
40
60
80
100
750
800
850
900
950
1000
1050

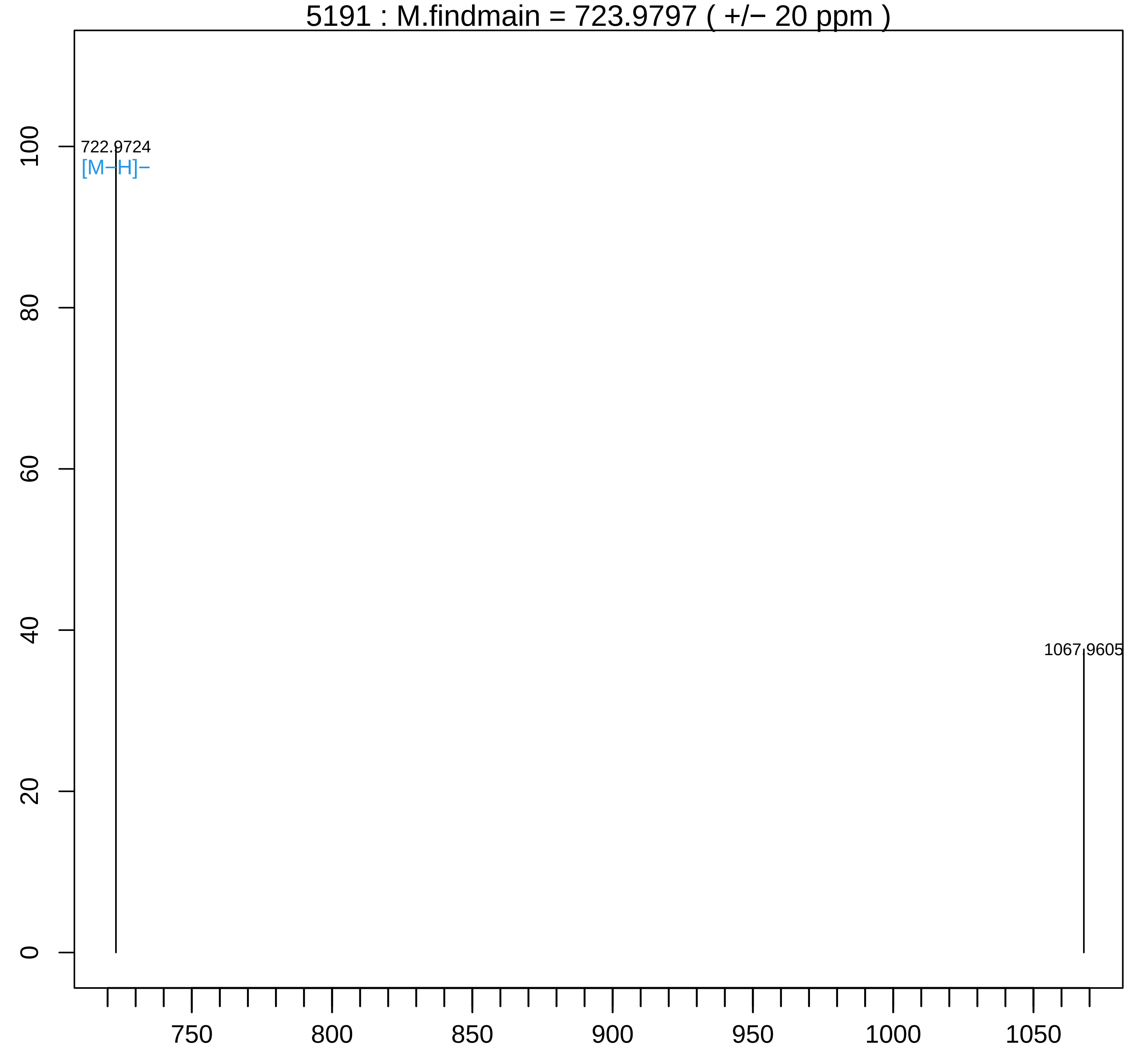
5191 : M.findmain = 723.9797 (+/− 20 ppm)
722.9724
[M−H]−
1067.9605
0
20
40
60
80
100
750
800
850
900
950
1000
1050

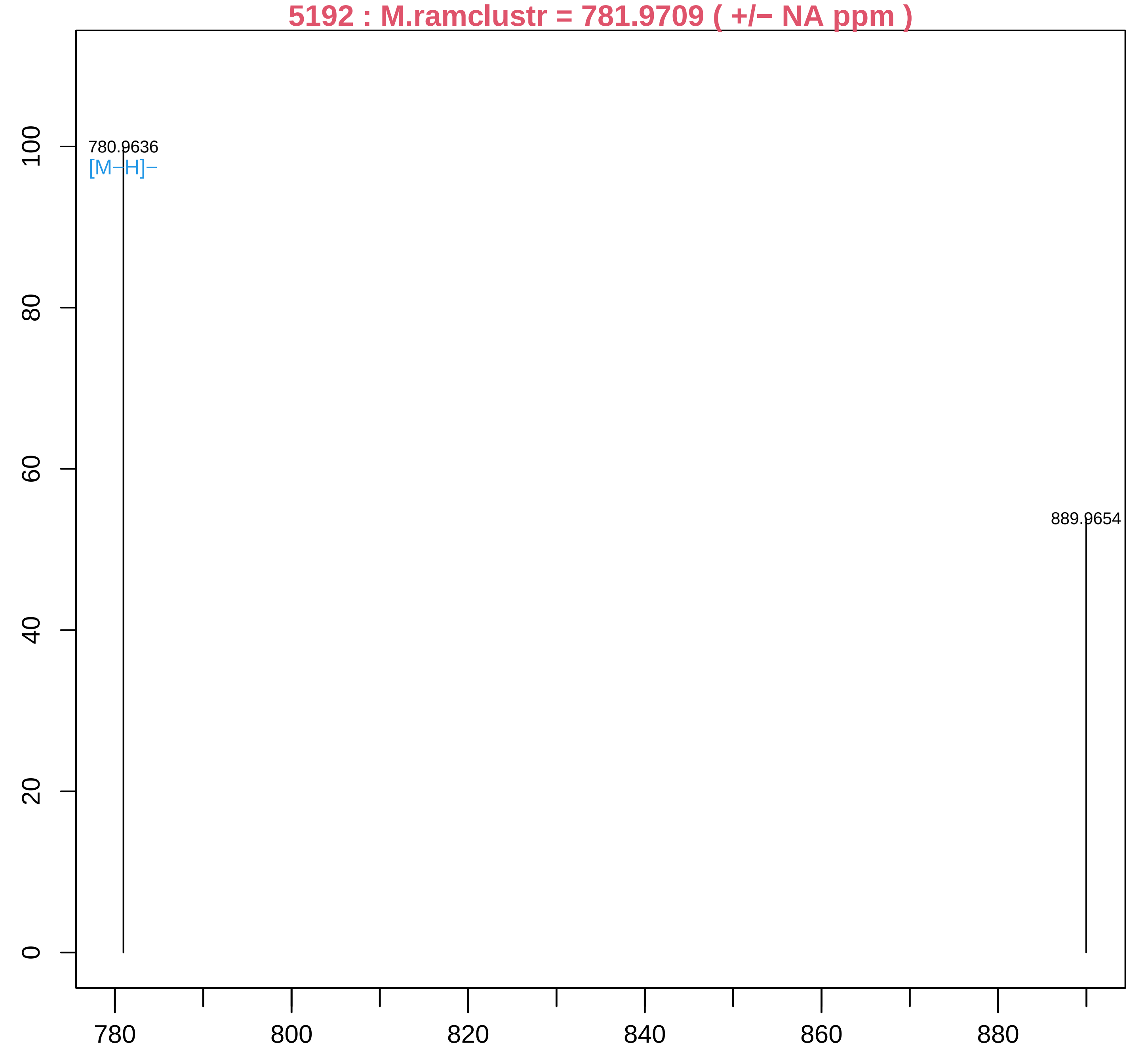

5192 : M.ramclustr = 781.9709 (+/− NA ppm)
780.9636
[M−H]−
889.9654
0
20
40
60
80
100
780
800
820
840
860
880

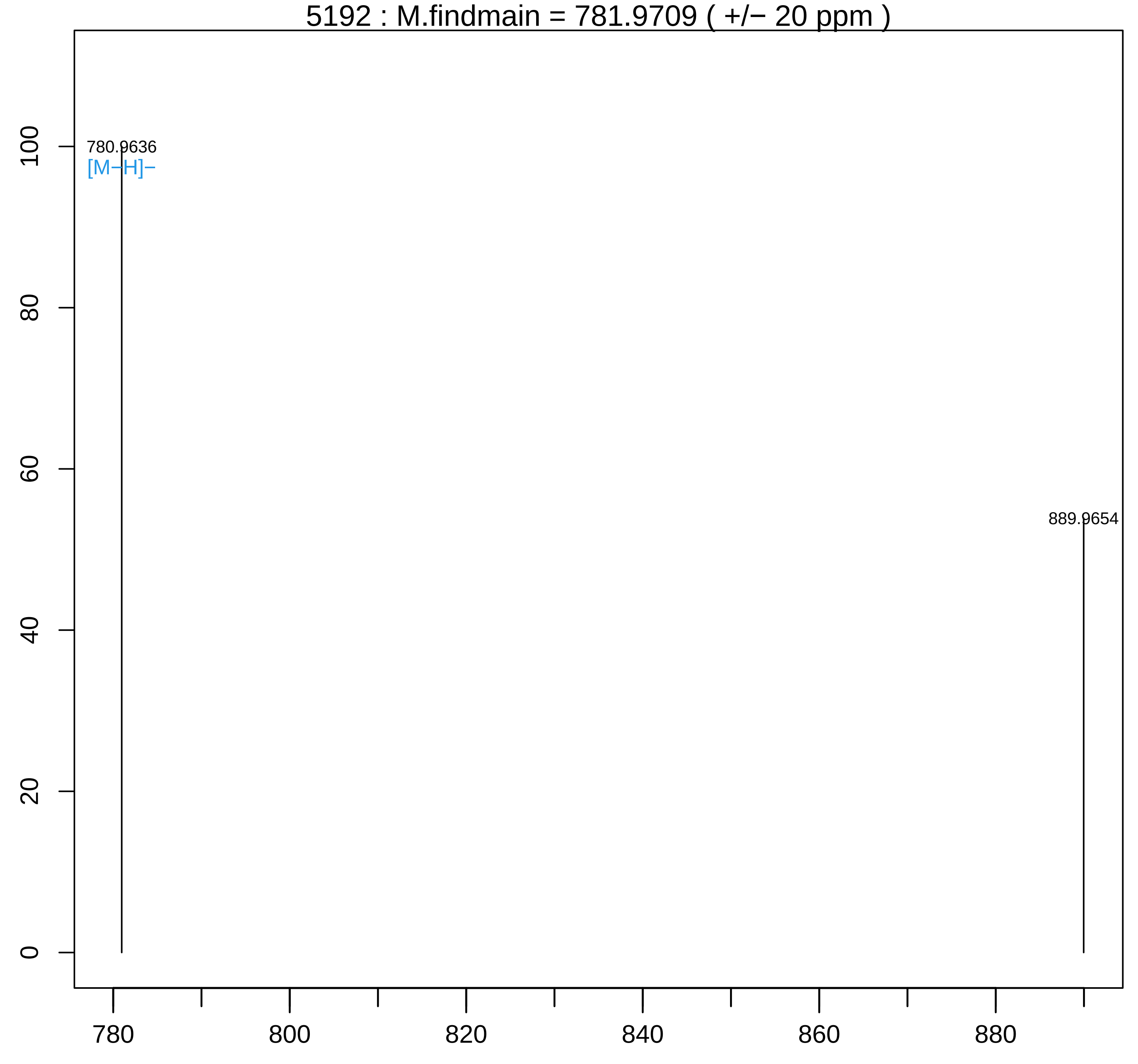

5192 : M.findmain = 781.9709 (+/− 20 ppm)
780.9636
[M−H]−
889.9654
0
20
40
60
80
100
780
800
820
840
860
880

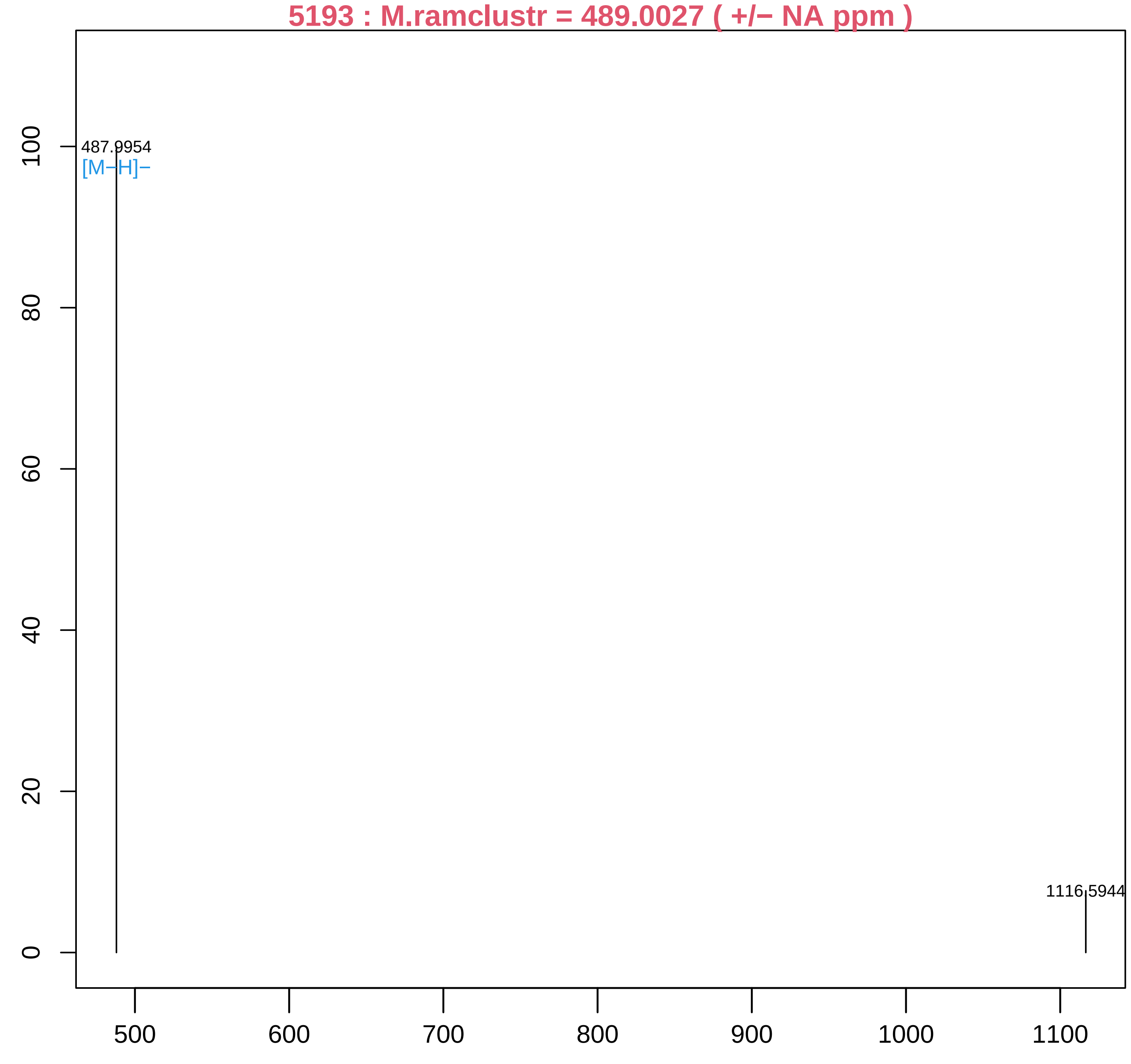
5193 : M.ramclustr = 489.0027 (+/− NA ppm)
487.9954
[M−H]−
1116.5944
0
20
40
60
80
100
500
600
700
800
900
1000
1100

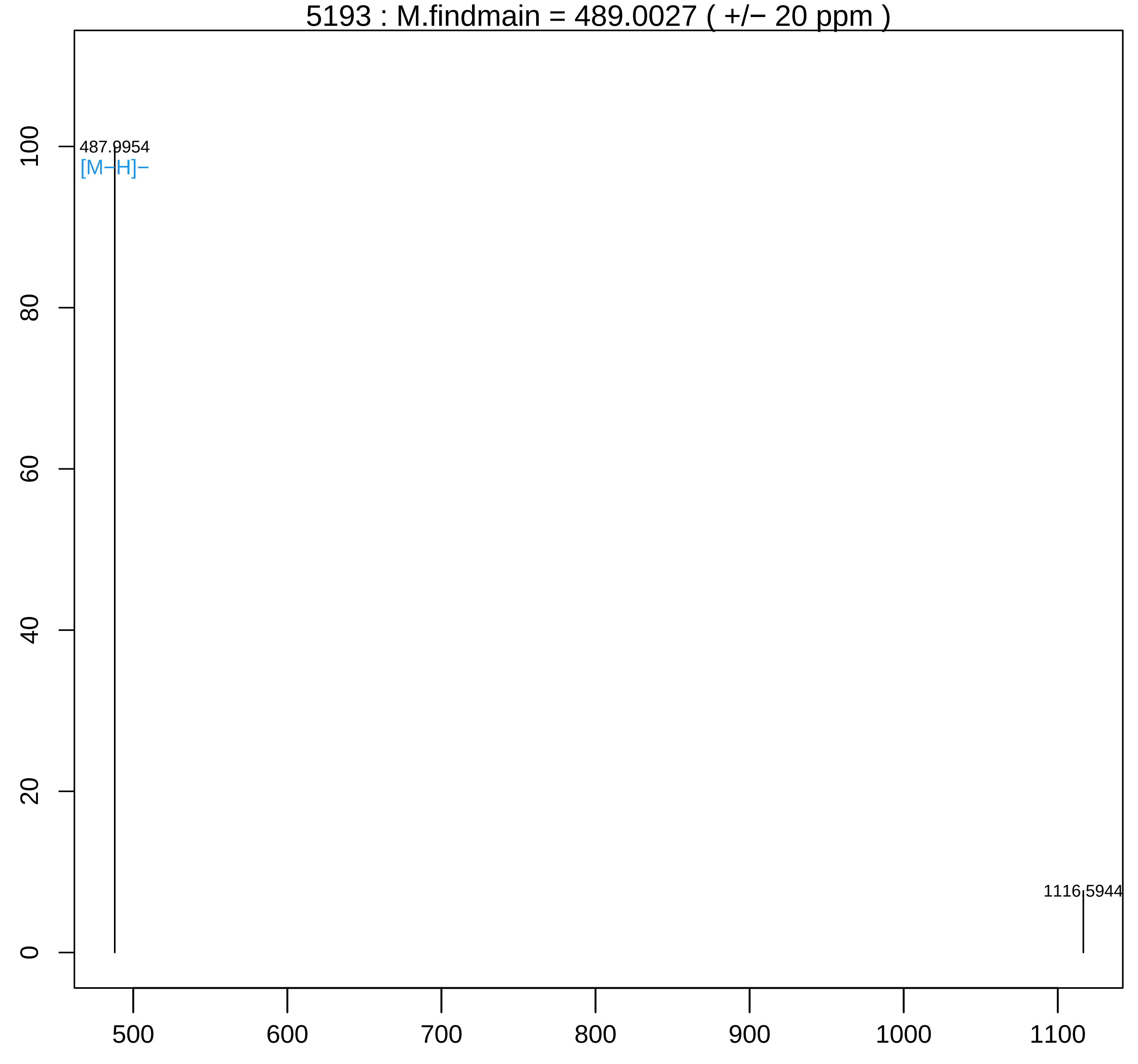
5193 : M.findmain = 489.0027 (+/− 20 ppm)
487.9954
[M−H]−
1116.5944
0
20
40
60
80
100
500
600
700
800
900
1000
1100

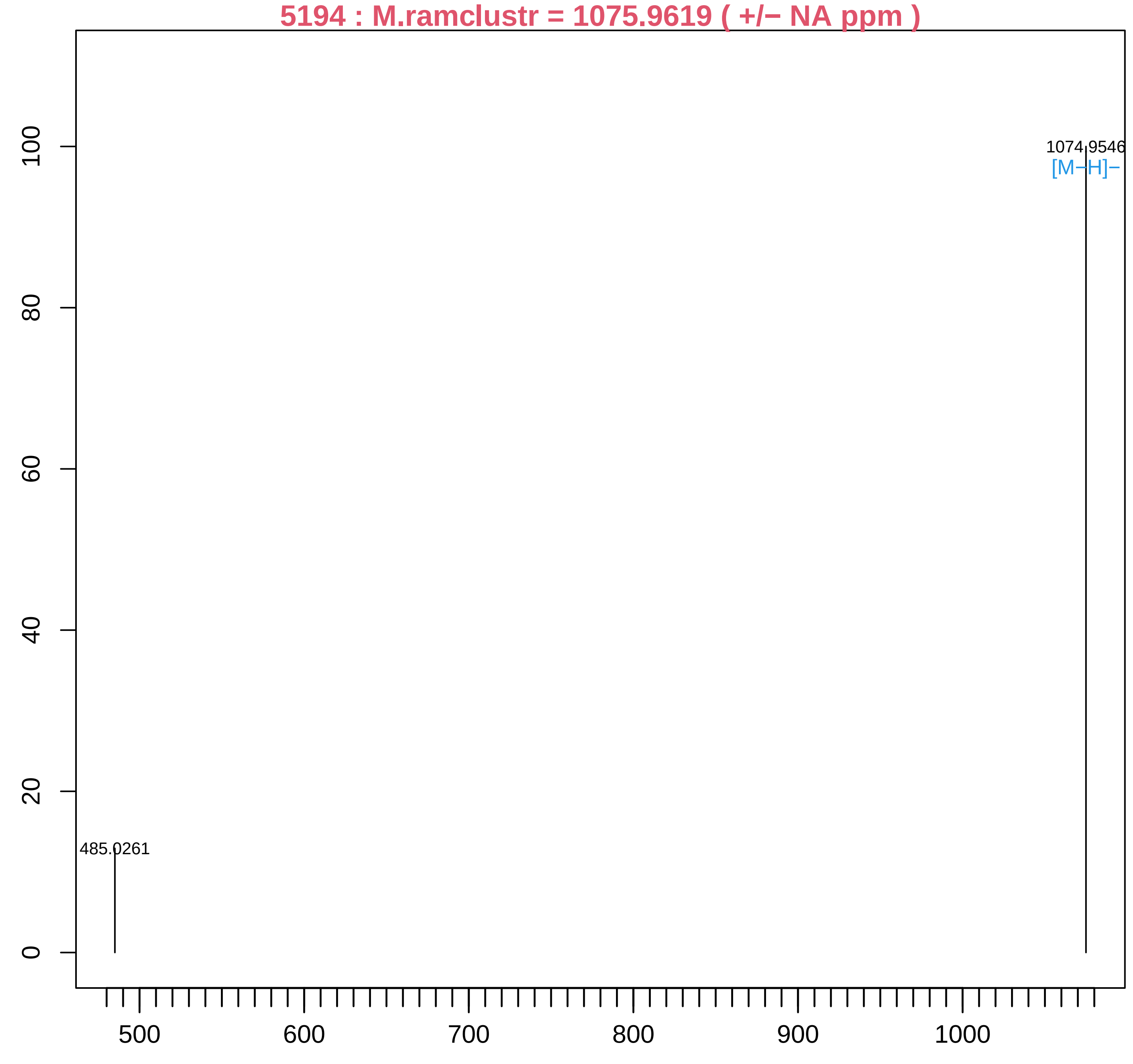
5194 : M.ramclustr = 1075.9619 (+/− NA ppm)
1074.9546
[M−H]−
485.0261
0
20
40
60
80
100
500
600
700
800
900
1000
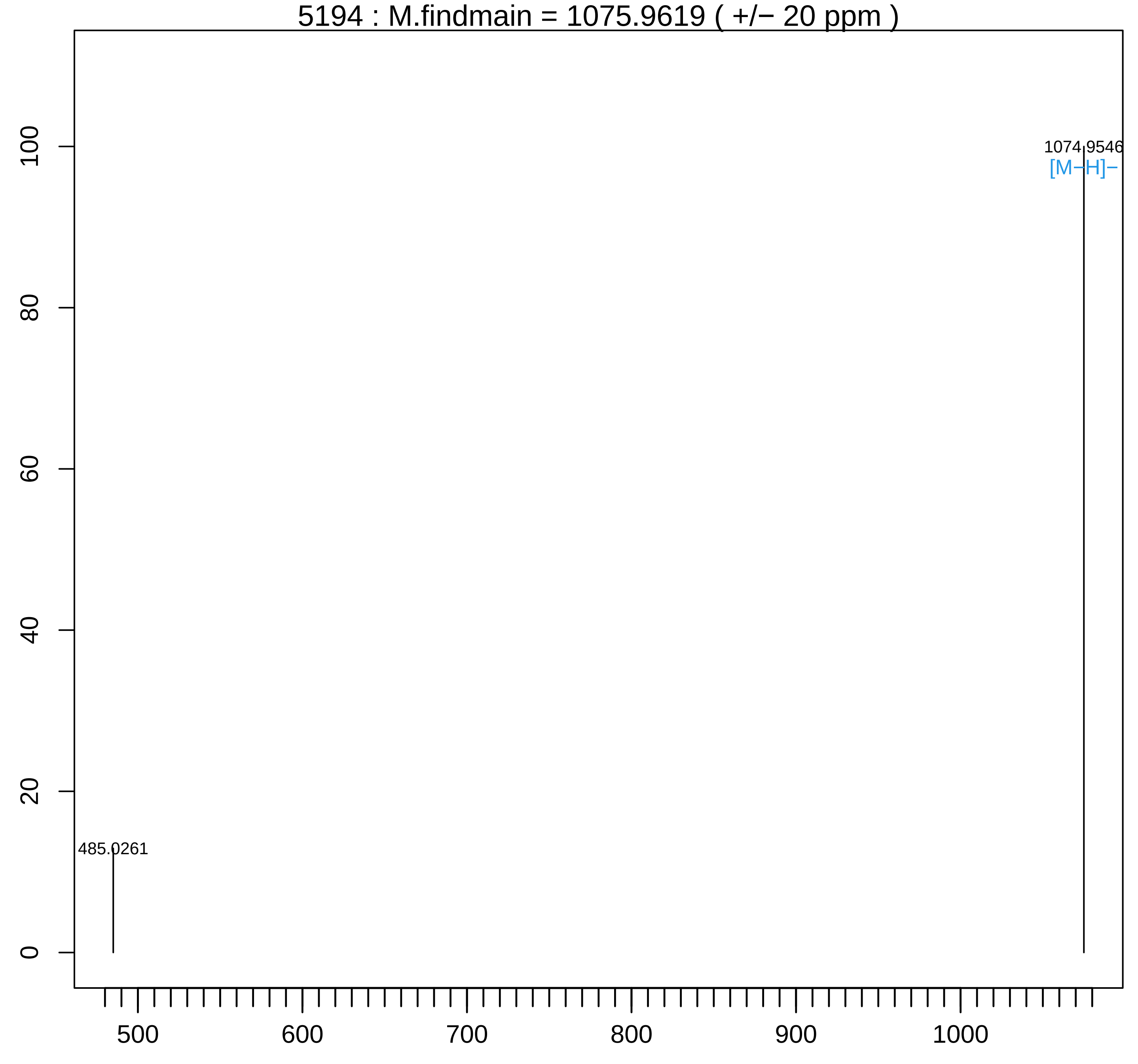
5194 : M.findmain = 1075.9619 (+/− 20 ppm)
1074.9546
[M−H]−
485.0261
0
20
40
60
80
100
500
600
700
800
900
1000

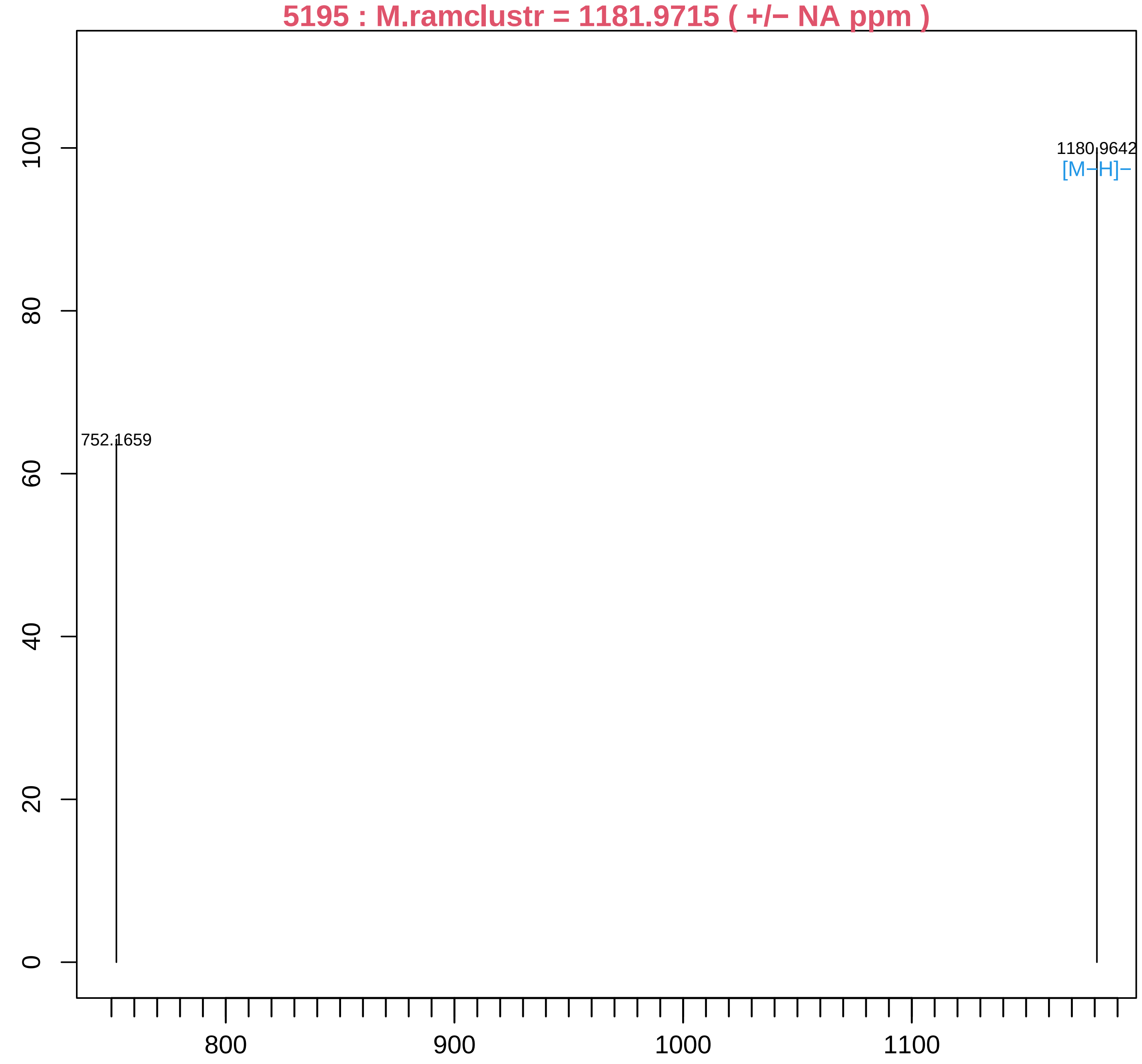
5195 : M.ramclustr = 1181.9715 (+/− NA ppm)
1180.9642
[M−H]−
752.1659
0
20
40
60
80
100
800
900
1000
1100

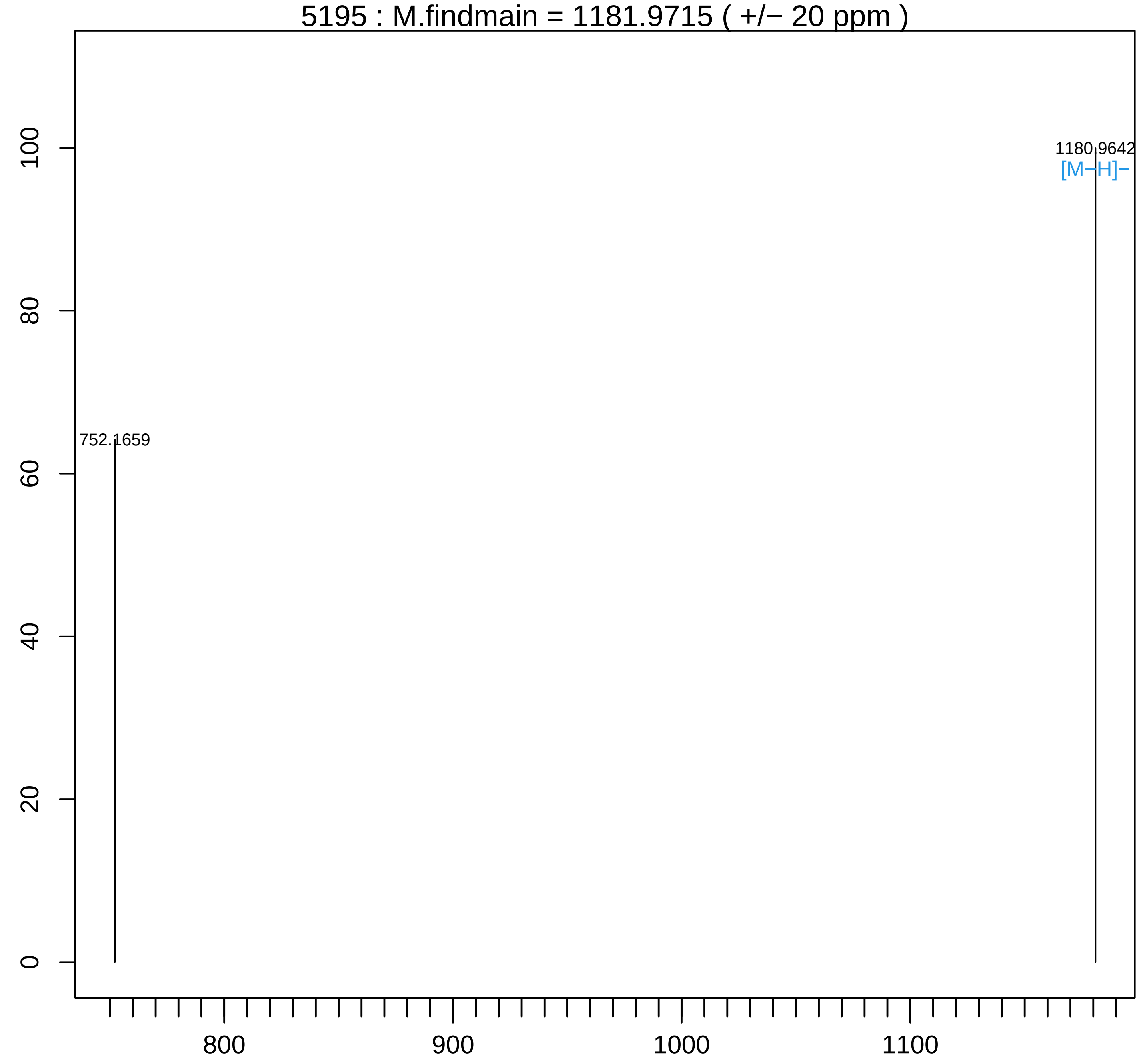
5195 : M.findmain = 1181.9715 (+/− 20 ppm)
1180.9642
[M−H]−
752.1659
0
20
40
60
80
100
800
900
1000
1100

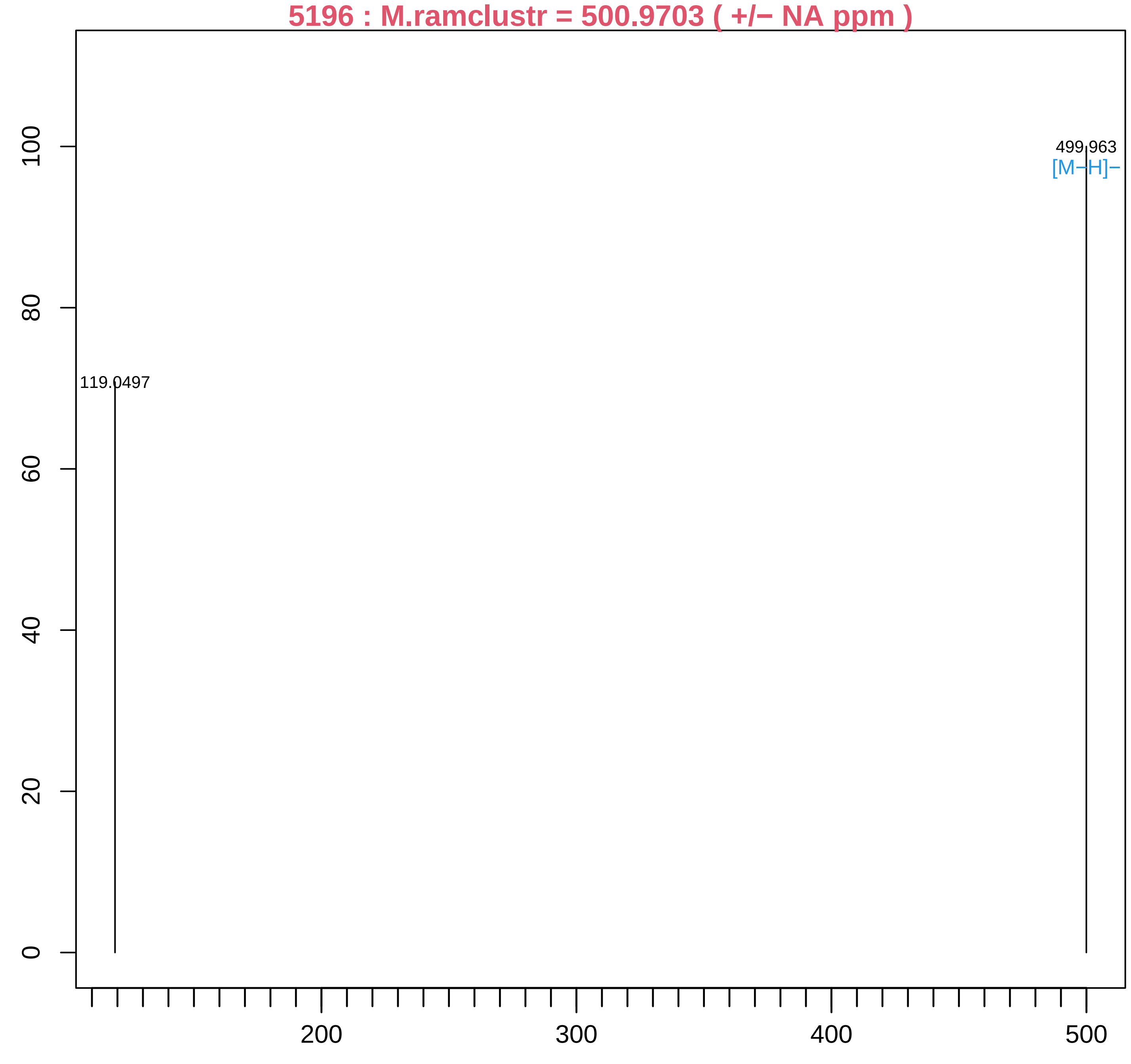
5196 : M.ramclustr = 500.9703 (+/− NA ppm)
100
80
60
40
20
0
119.0497
499.963
[M−H]−
200
300
400
500

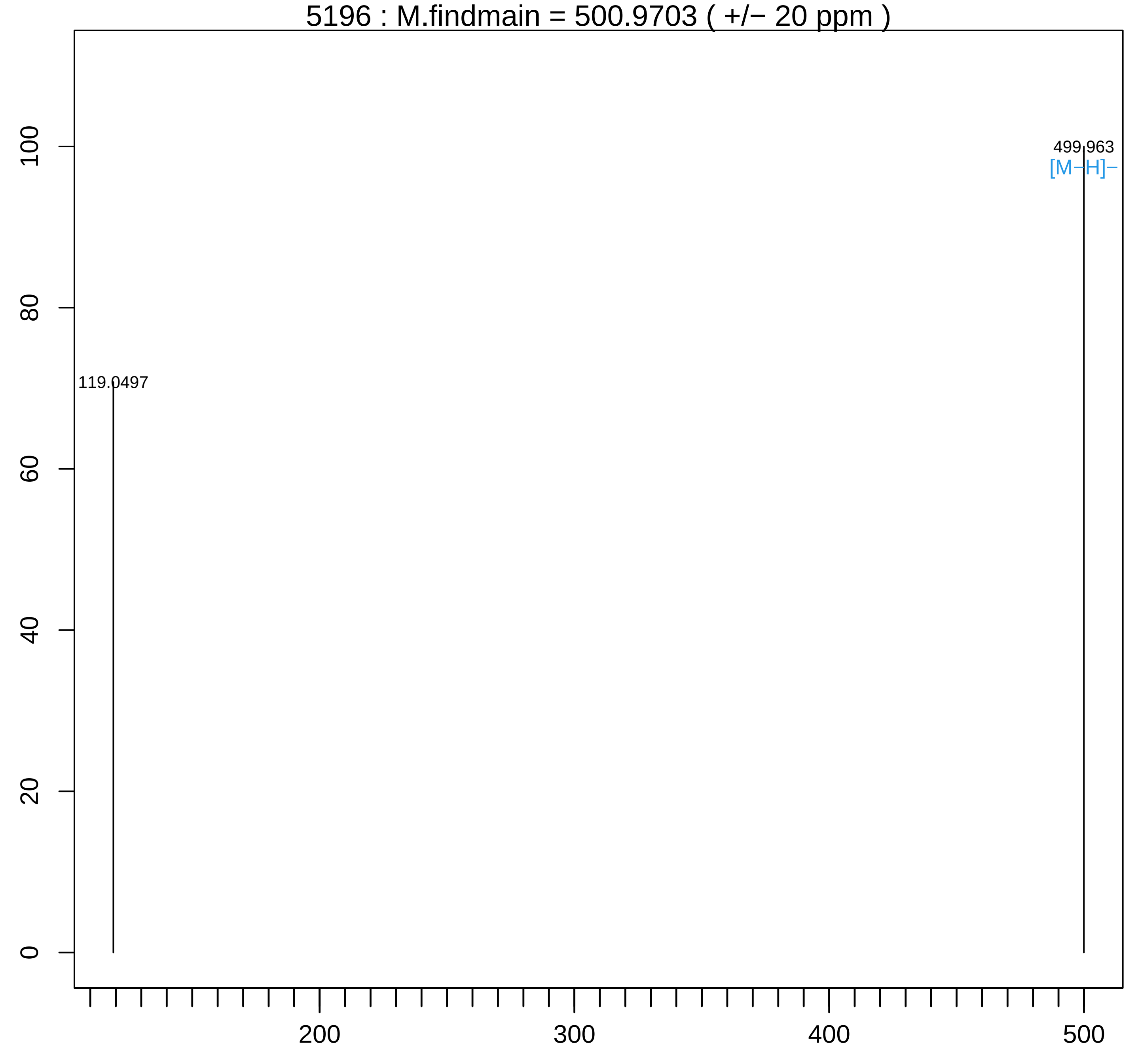
5196 : M.findmain = 500.9703 (+/− 20 ppm)
100
80
60
40
20
0
119.0497
499.963
[M−H]−
200
300
400
500

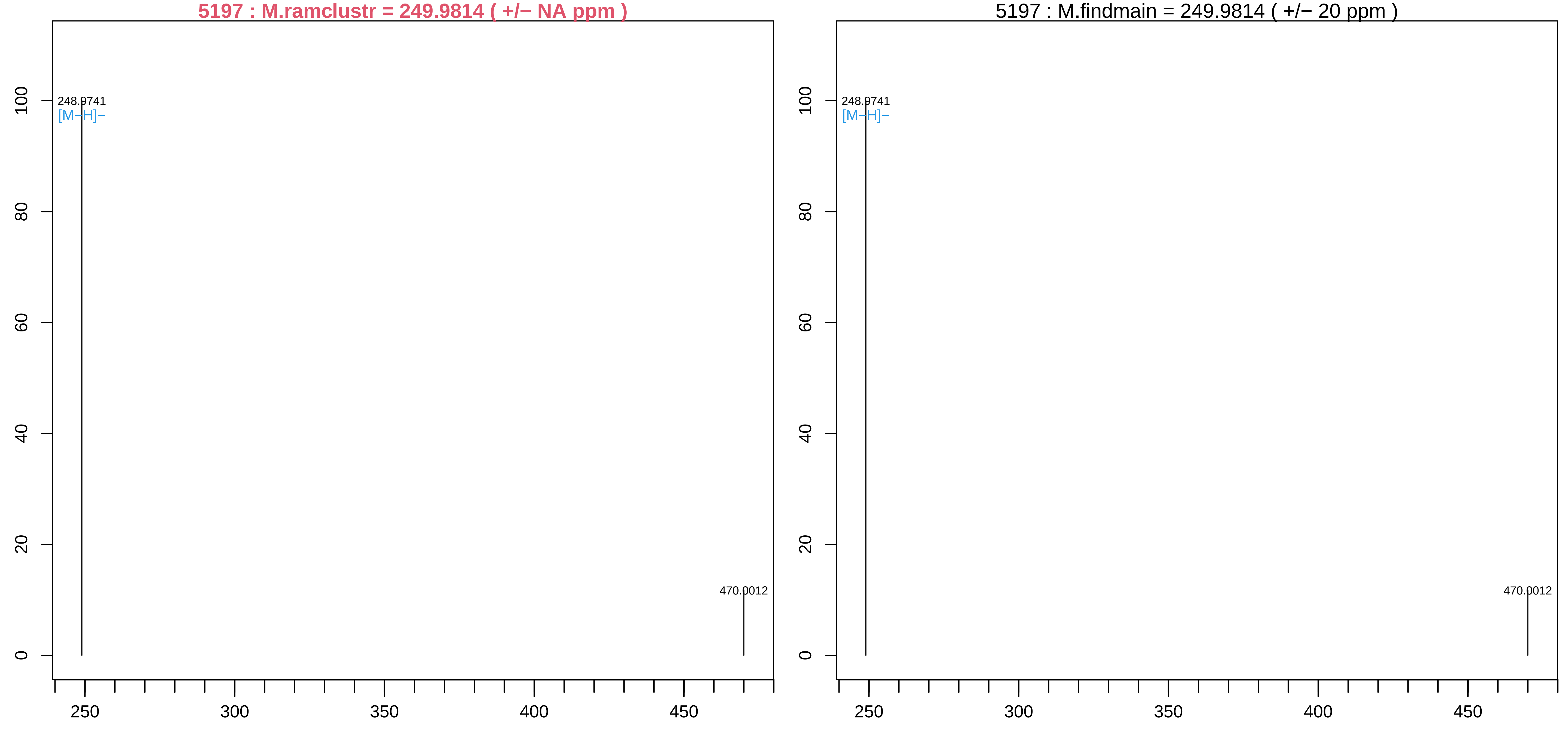
5197 : M.ramclustr = 249.9814 (+/− NA ppm)
248.9741
[M−H]−
470.0012
0
20
40
60
80
100
250
300
350
400
450
5197 : M.findmain = 249.9814 (+/− 20 ppm)
248.9741
[M−H]−
470.0012
0
20
40
60
80
100
250
300
350
400
450

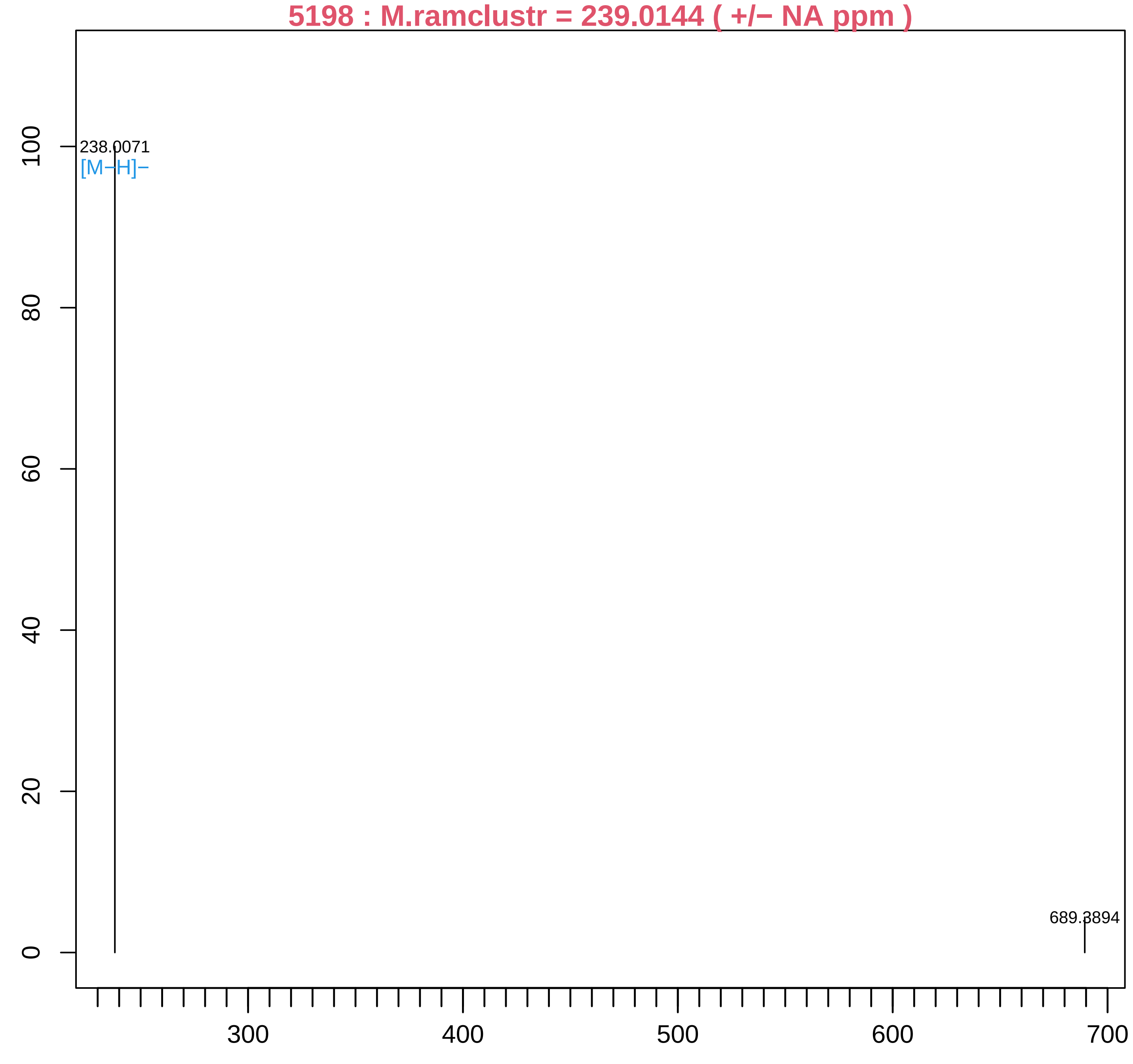
5198 : M.ramclustr = 239.0144 (+/− NA ppm)
238.0071
[M−H]−
689.3894
0
20
40
60
80
100
300
400
500
600
700

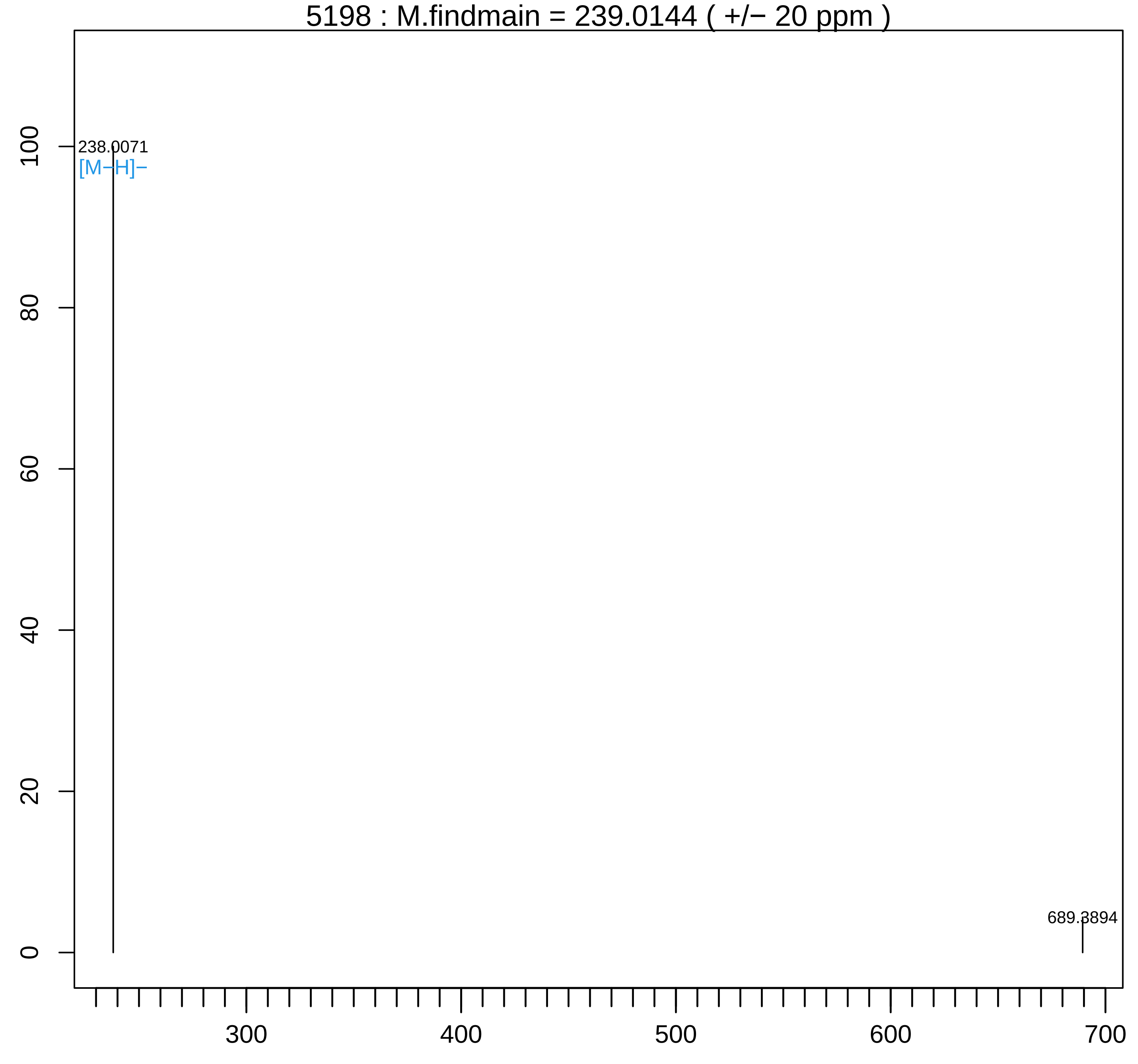
5198 : M.findmain = 239.0144 (+/− 20 ppm)
238.0071
[M−H]−
689.3894
0
20
40
60
80
100
300
400
500
600
700

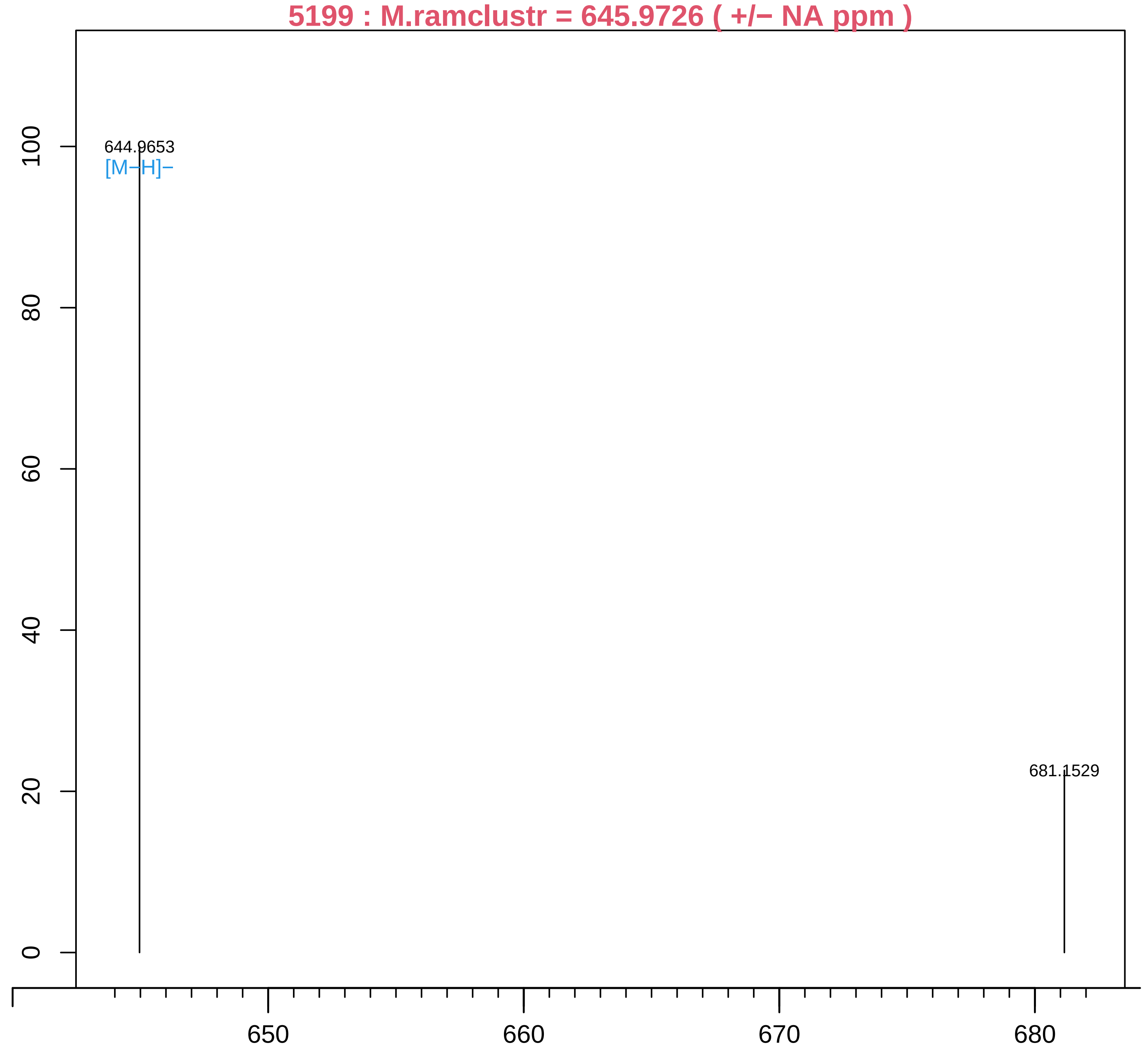
5199 : M.ramclustr = 645.9726 (+/− NA ppm)
644.9653
[M−H]−
681.1529
0
20
40
60
80
100
650
660
670
680

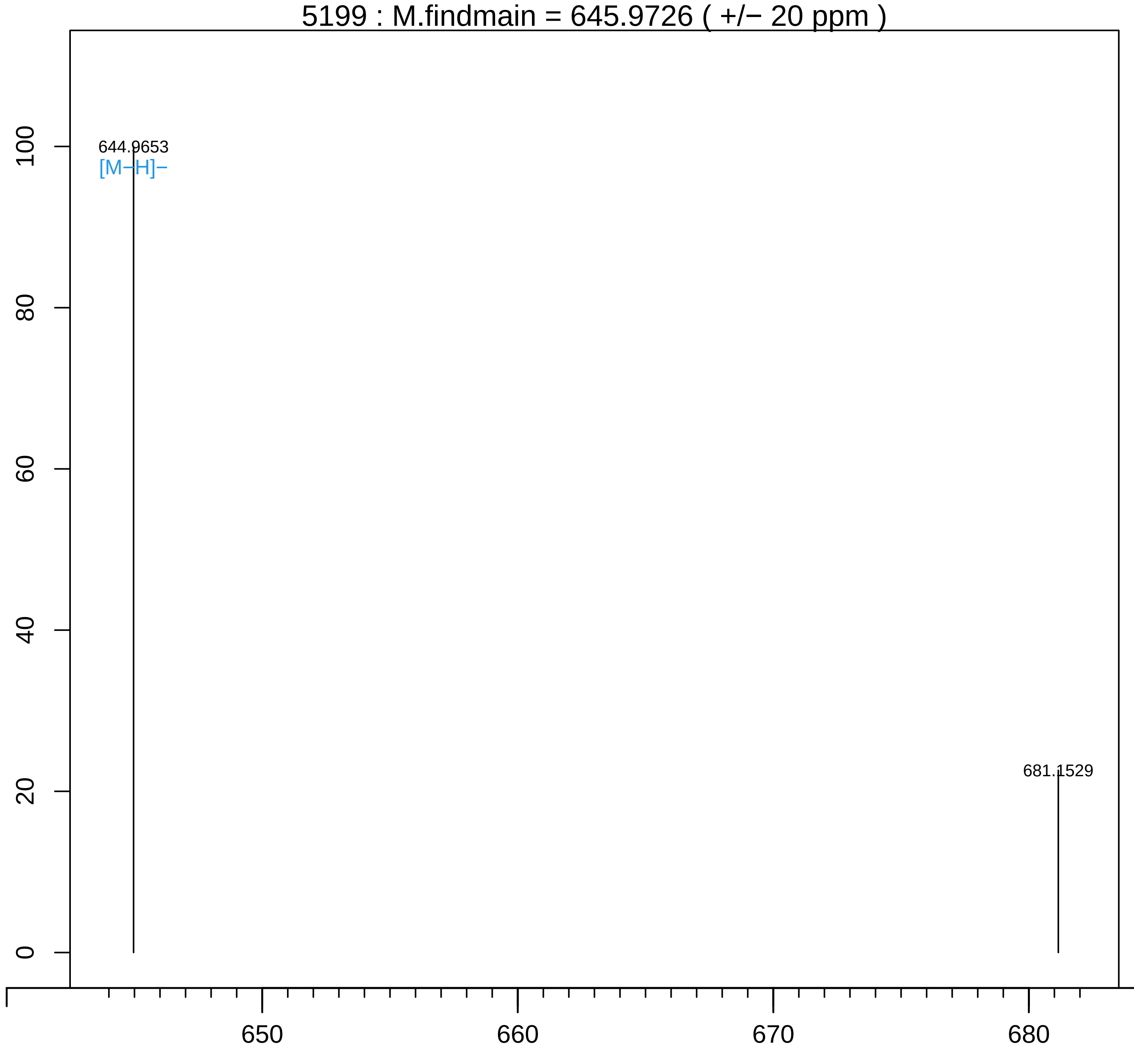
5199 : M.findmain = 645.9726 (+/− 20 ppm)
644.9653
[M−H]−
681.1529
0
20
40
60
80
100
650
660
670
680

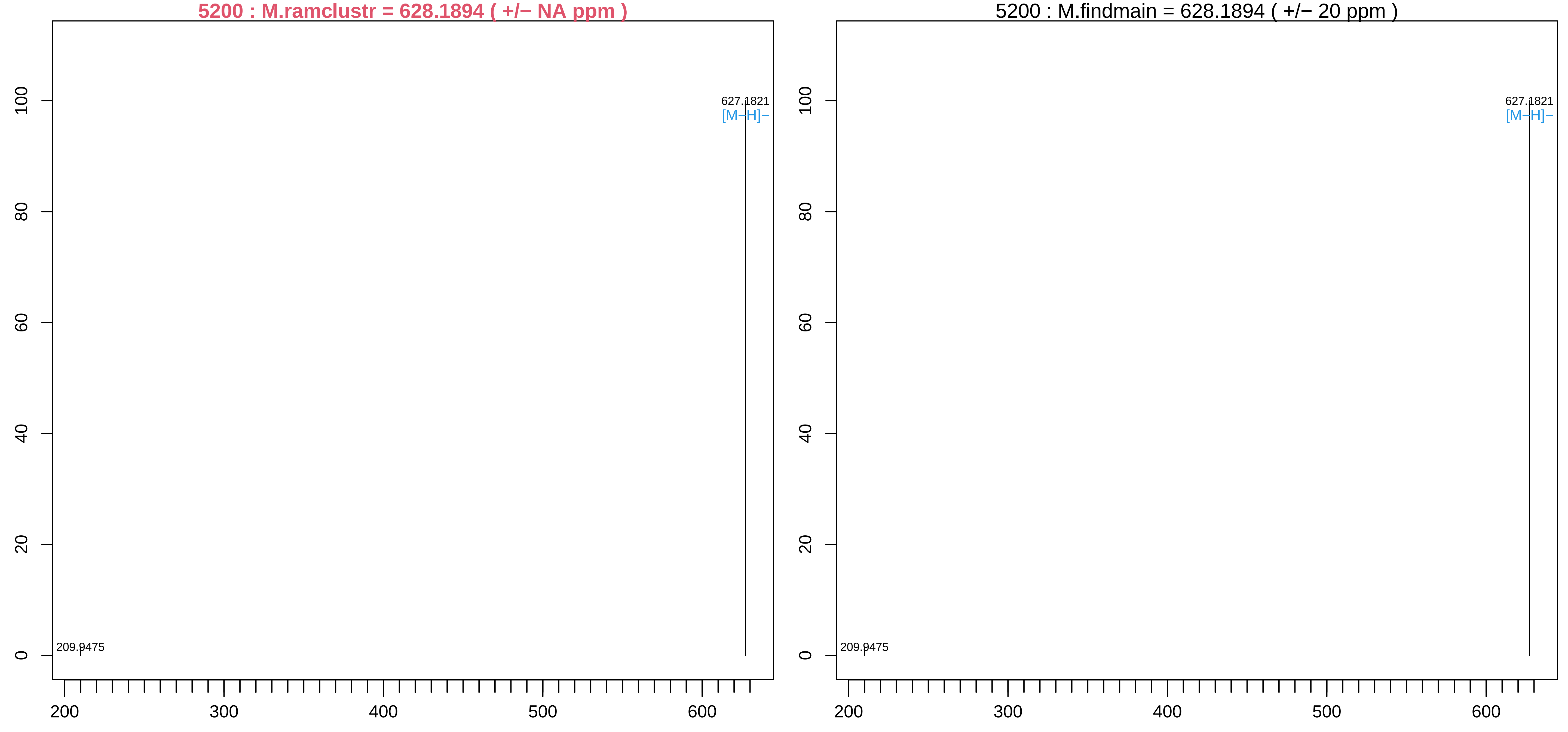
5200 : M.ramclustr = 628.1894 (+/− NA ppm)
627.1821
[M−H]−
209.9475
5200 : M.findmain = 628.1894 (+/− 20 ppm)
627.1821
[M−H]−
209.9475
0
20
40
60
80
100
200
300
400
500
600

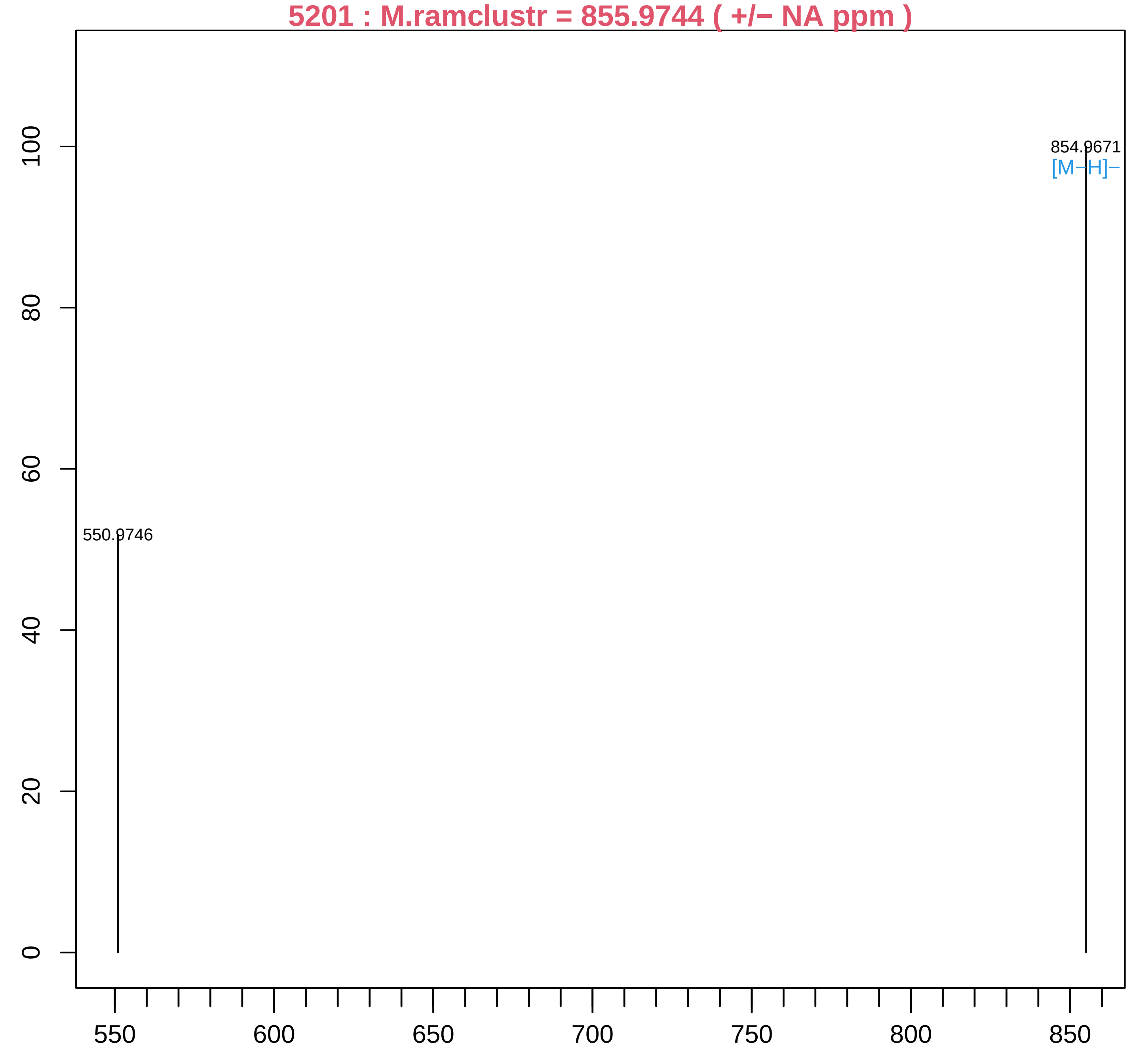
5201 : M.ramclustr = 855.9744 (+/− NA ppm)
854.9671
[M−H]−
550.9746
0
20
40
60
80
100
550
600
650
700
750
800
850

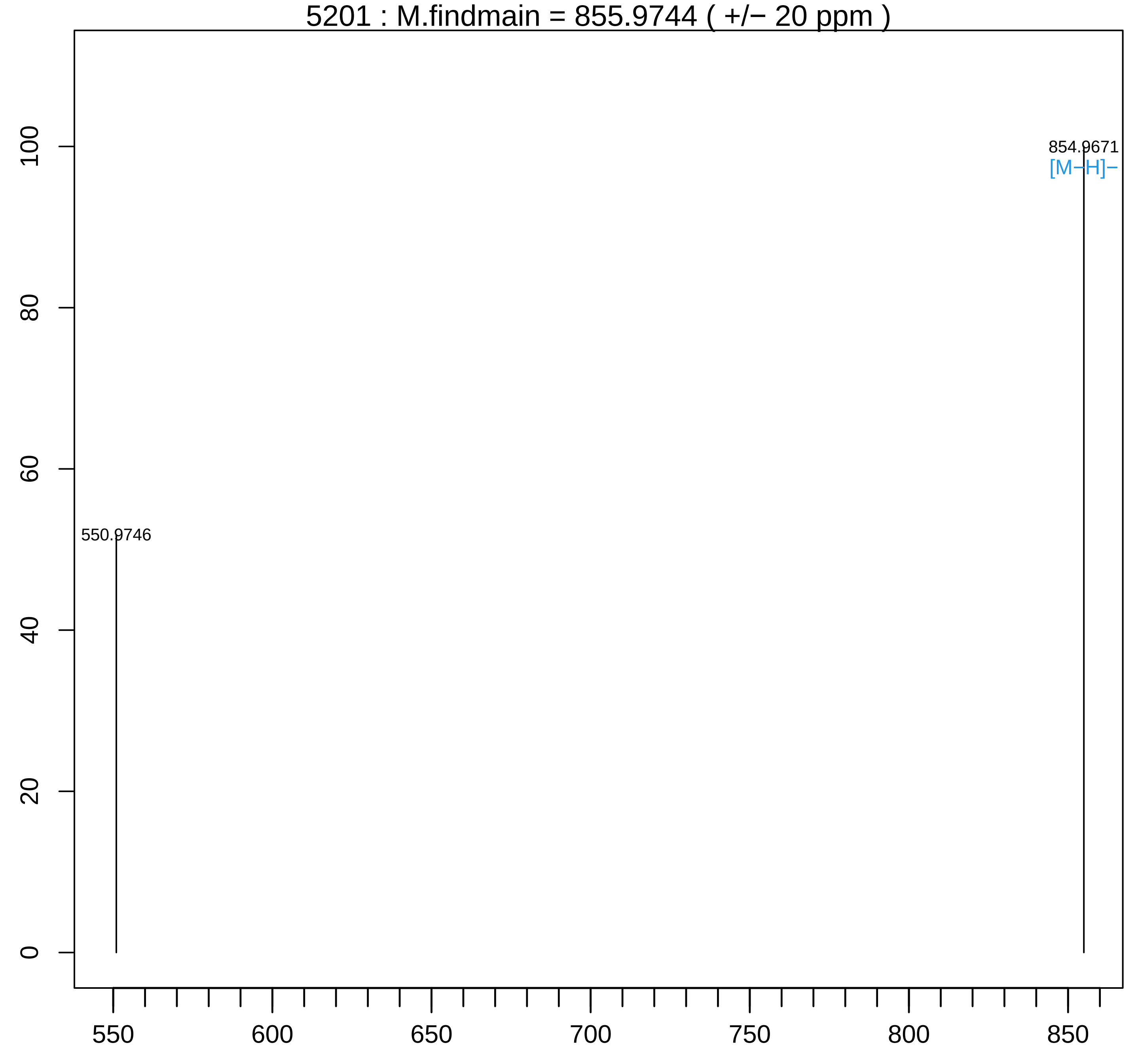
5201 : M.findmain = 855.9744 (+/− 20 ppm)
854.9671
[M−H]−
550.9746
0
20
40
60
80
100
550
600
650
700
750
800
850

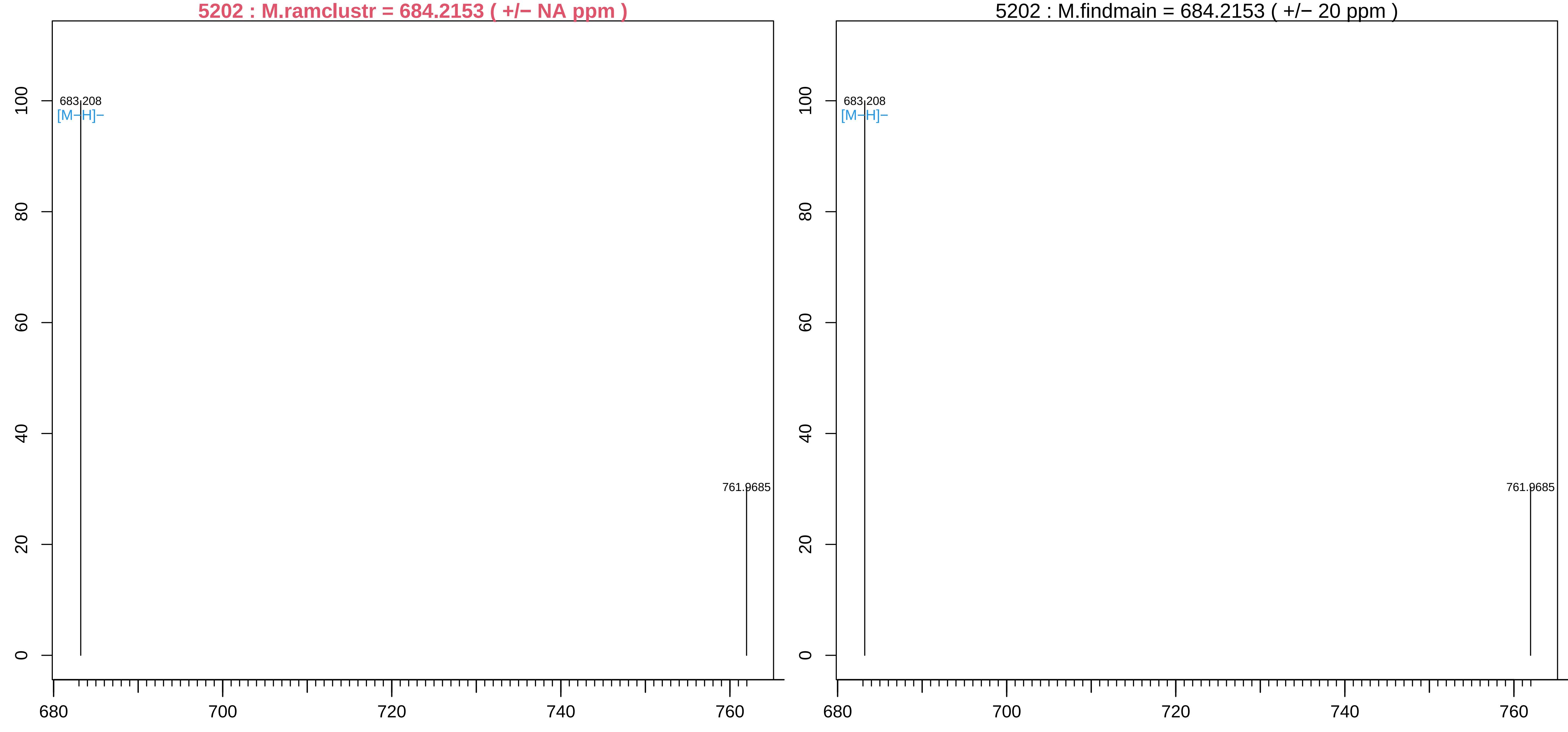

5202 : M.ramclustr = 684.2153 (+/− NA ppm)
683.208
[M−H]−
761.9685
5202 : M.findmain = 684.2153 (+/− 20 ppm)
683.208
[M−H]−
761.9685
0
20
40
60
80
100
680
700
720
740
760

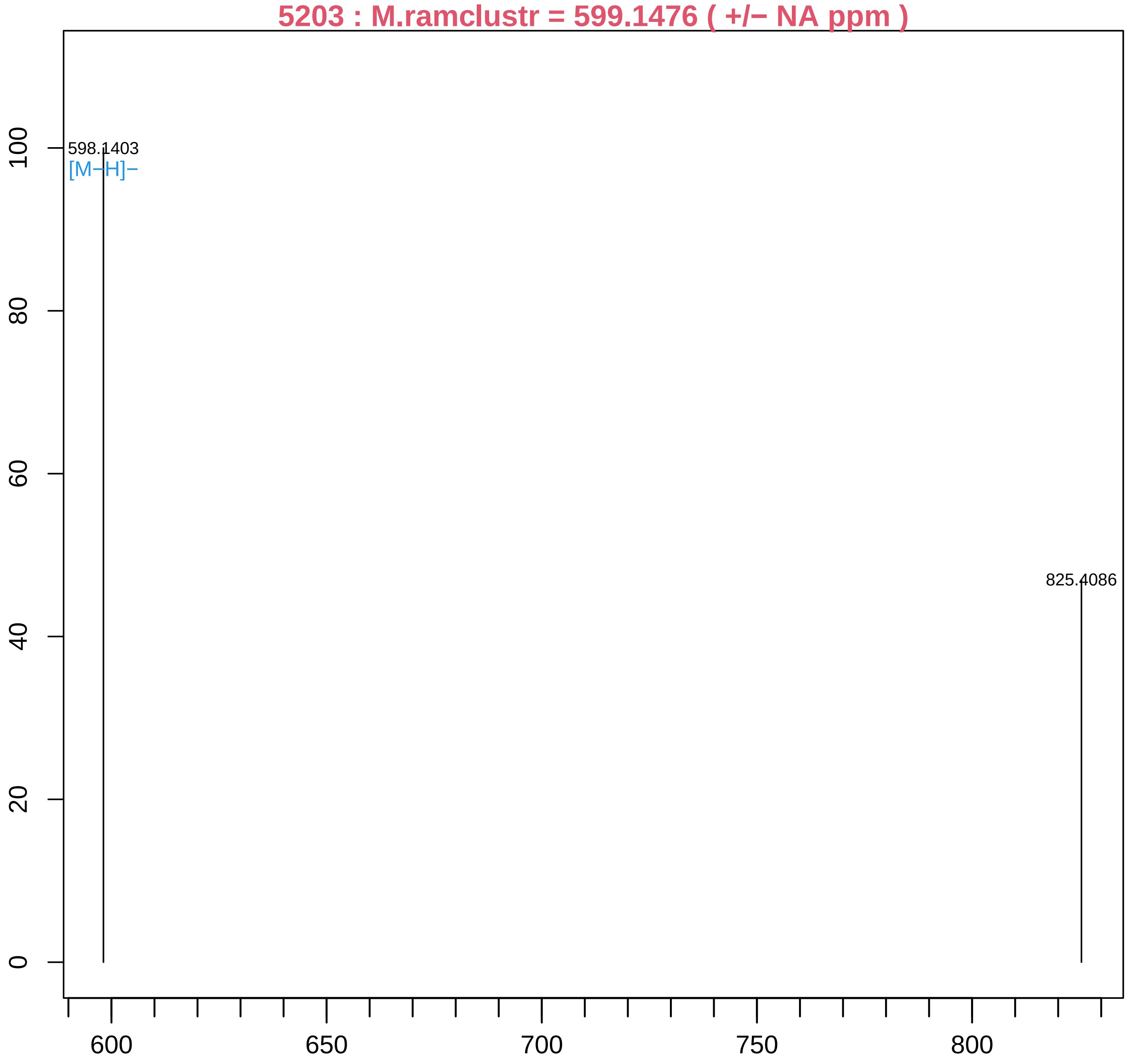
5203 : M.ramclustr = 599.1476 (+/− NA ppm)
598.1403
[M−H]−
825.4086
0
20
40
60
80
100
600
650
700
750
800

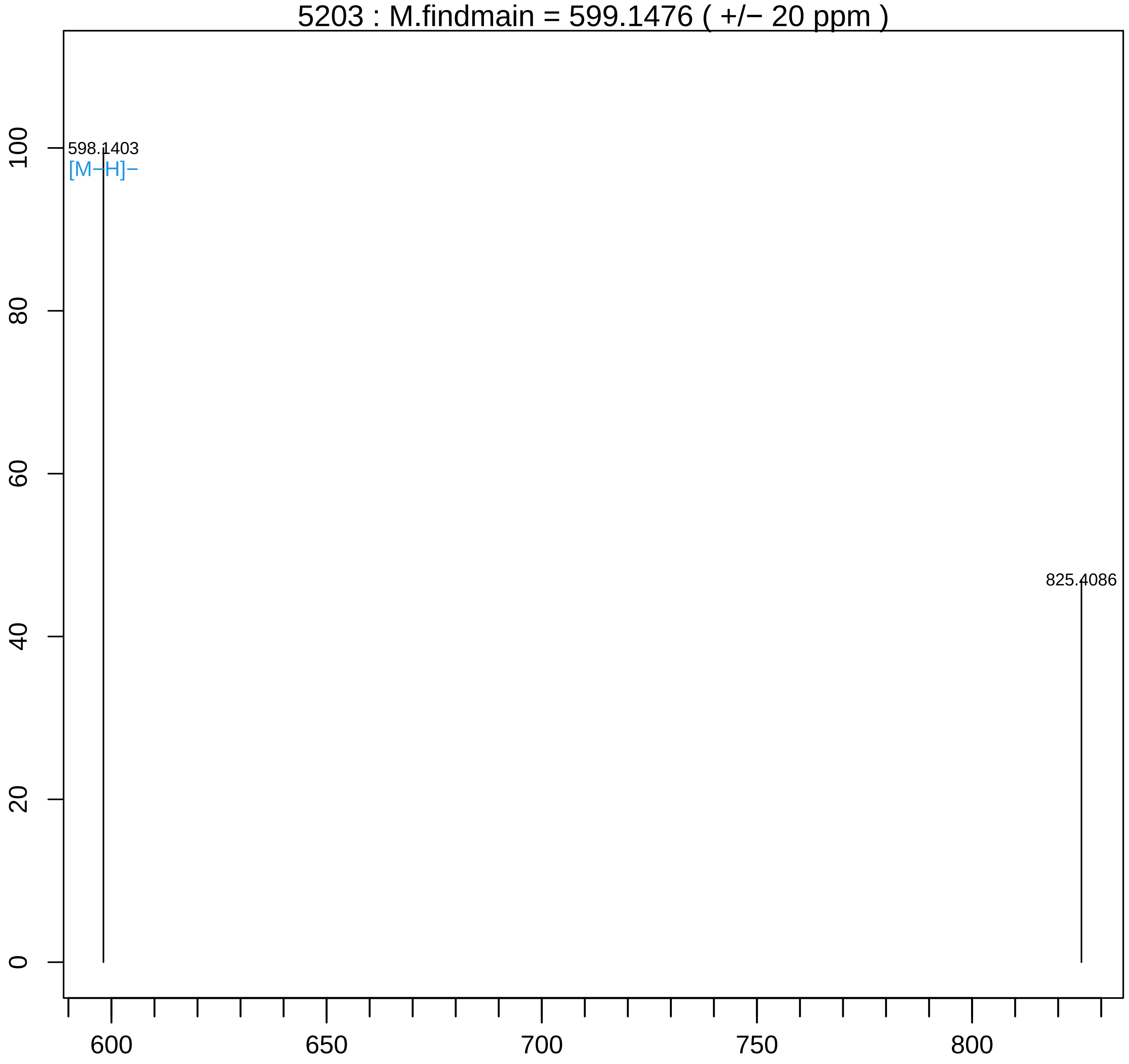
5203 : M.findmain = 599.1476 (+/− 20 ppm)
598.1403
[M−H]−
825.4086
0
20
40
60
80
100
600
650
700
750
800

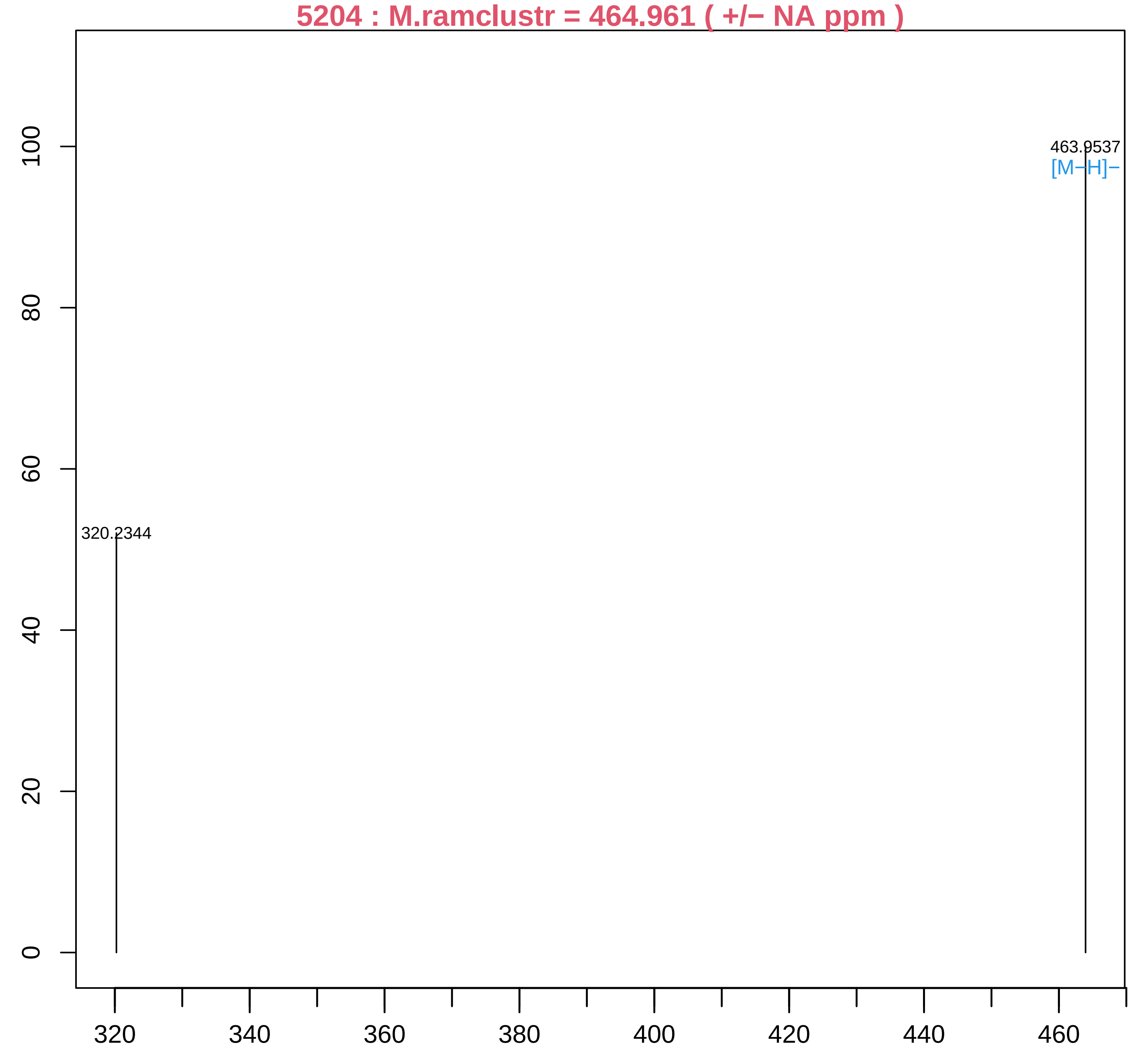

5204 : M.ramclustr = 464.961 (+/− NA ppm)
463.9537
[M−H]−
320.2344

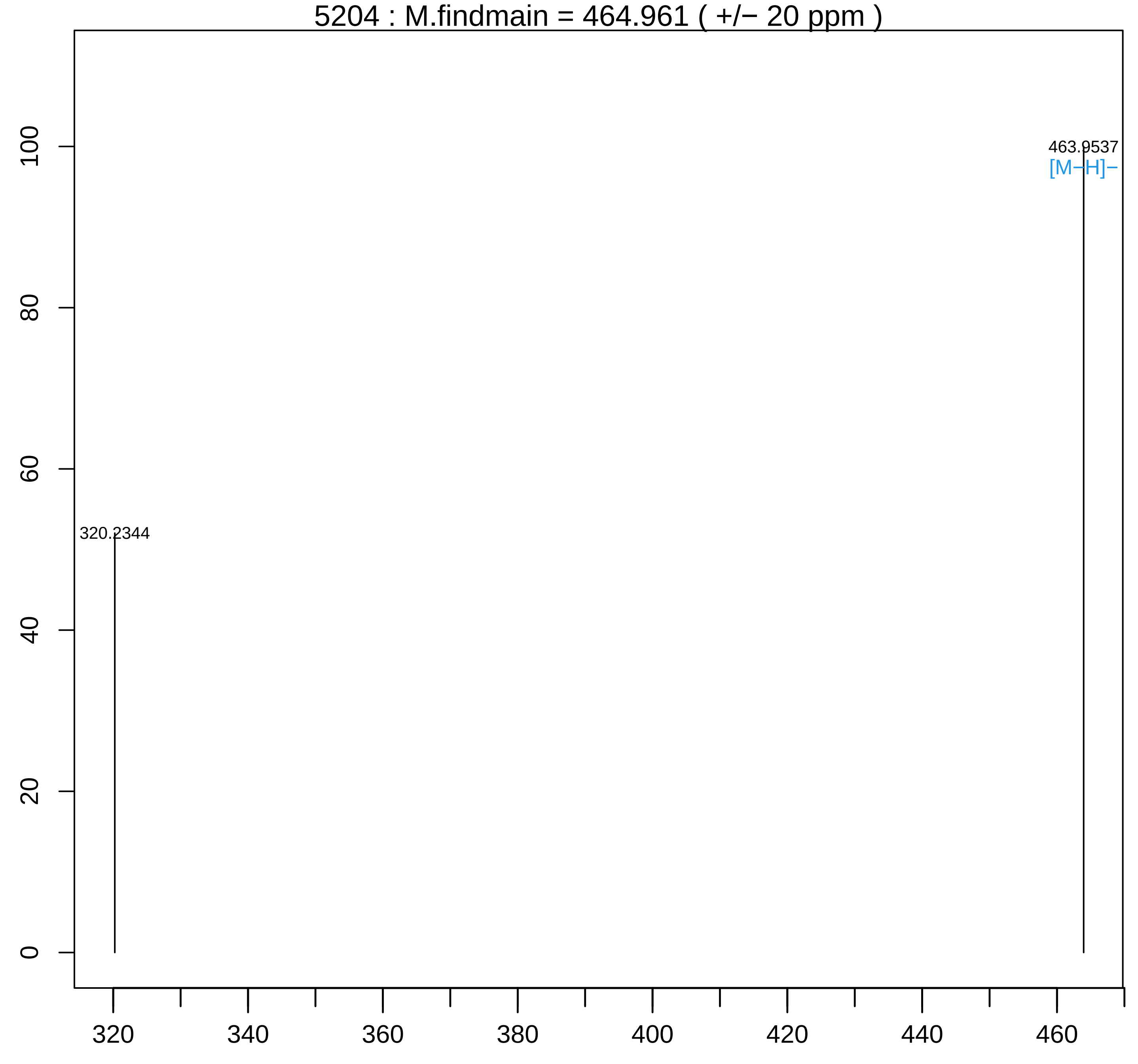

5204 : M.findmain = 464.961 (+/− 20 ppm)
463.9537
[M−H]−
320.2344

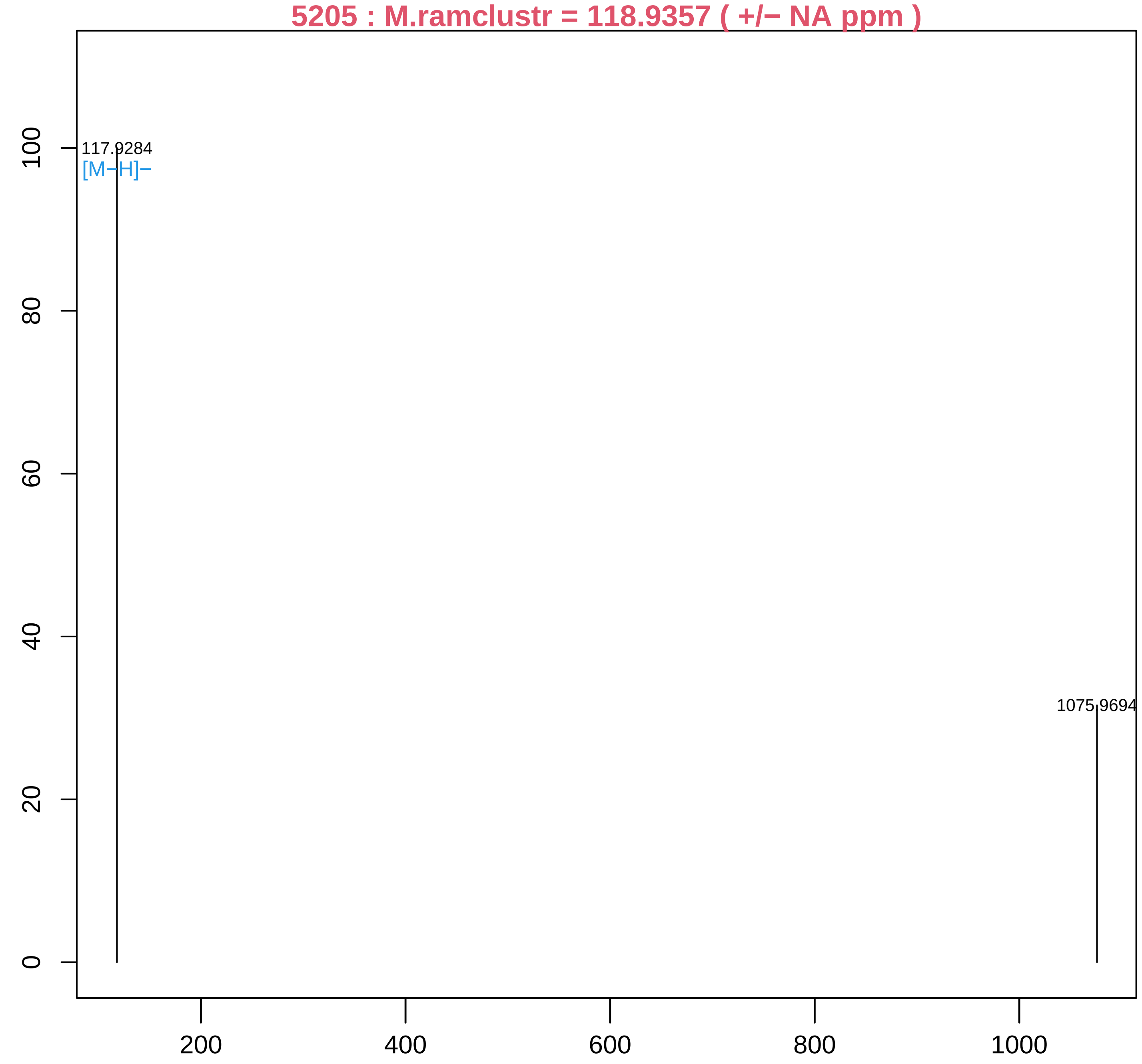

5205 : M.ramclustr = 118.9357 (+/− NA ppm)
117.9284
[M−H]−
1075.9694
0
20
40
60
80
100
200
400
600
800
1000

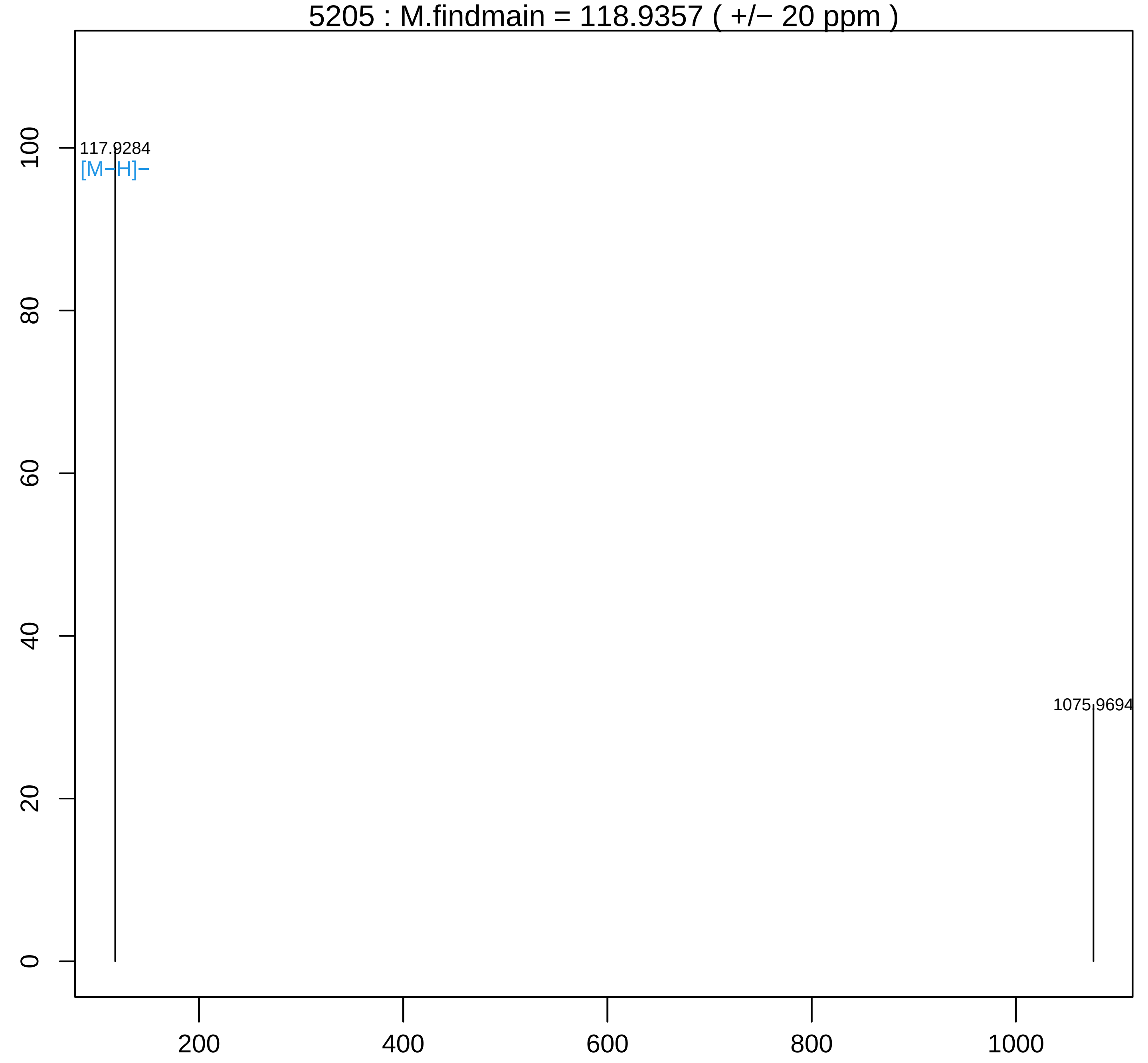

5205 : M.findmain = 118.9357 (+/− 20 ppm)
117.9284
[M−H]−
1075.9694
0
20
40
60
80
100
200
400
600
800
1000

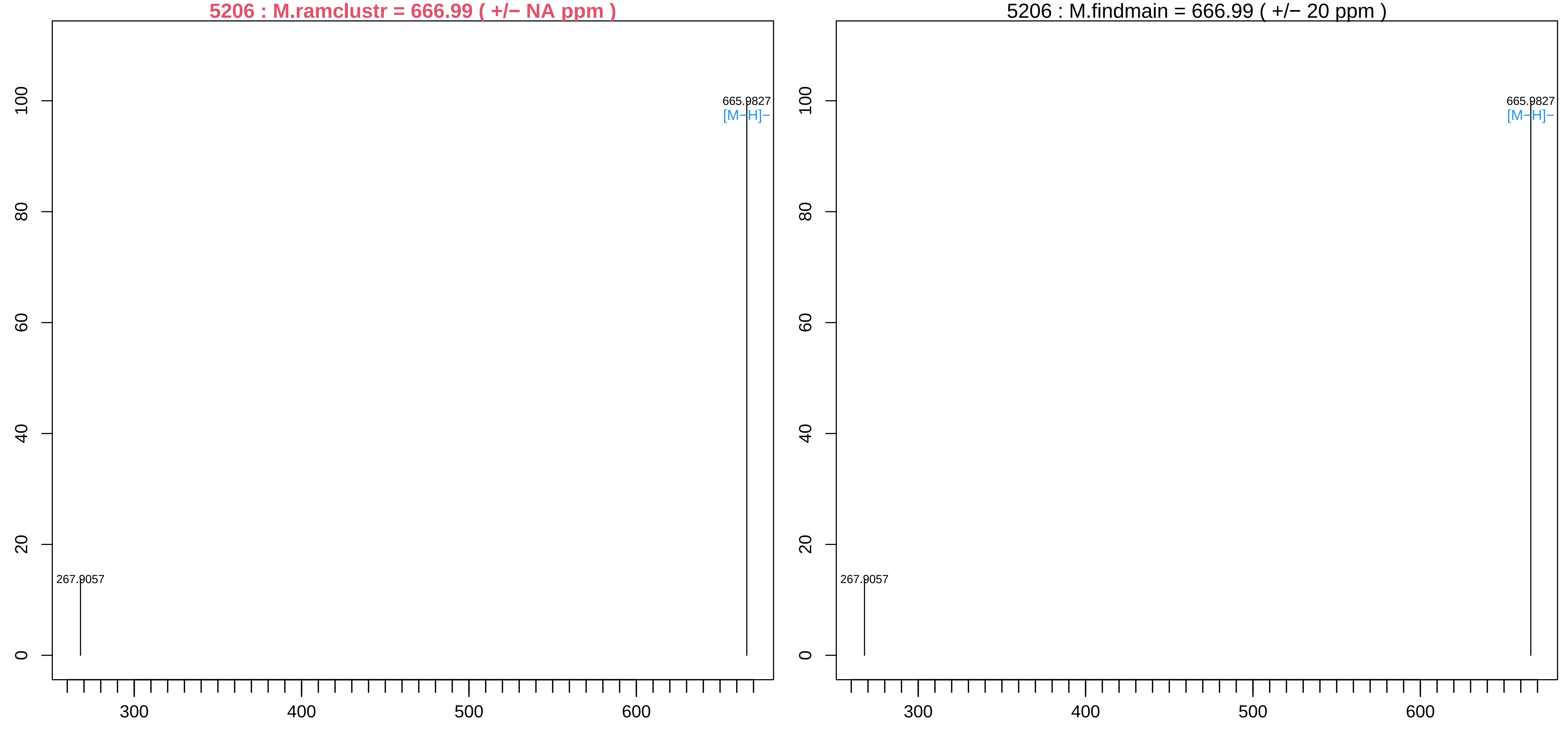

5206 : M.ramclustr = 666.99 (+/− NA ppm)
665.9827
[M−H]−
267.9057
0
20
40
60
80
100
300
400
500
600
5206 : M.findmain = 666.99 (+/− 20 ppm)
665.9827
[M−H]−
267.9057
0
20
40
60
80
100
300
400
500
600

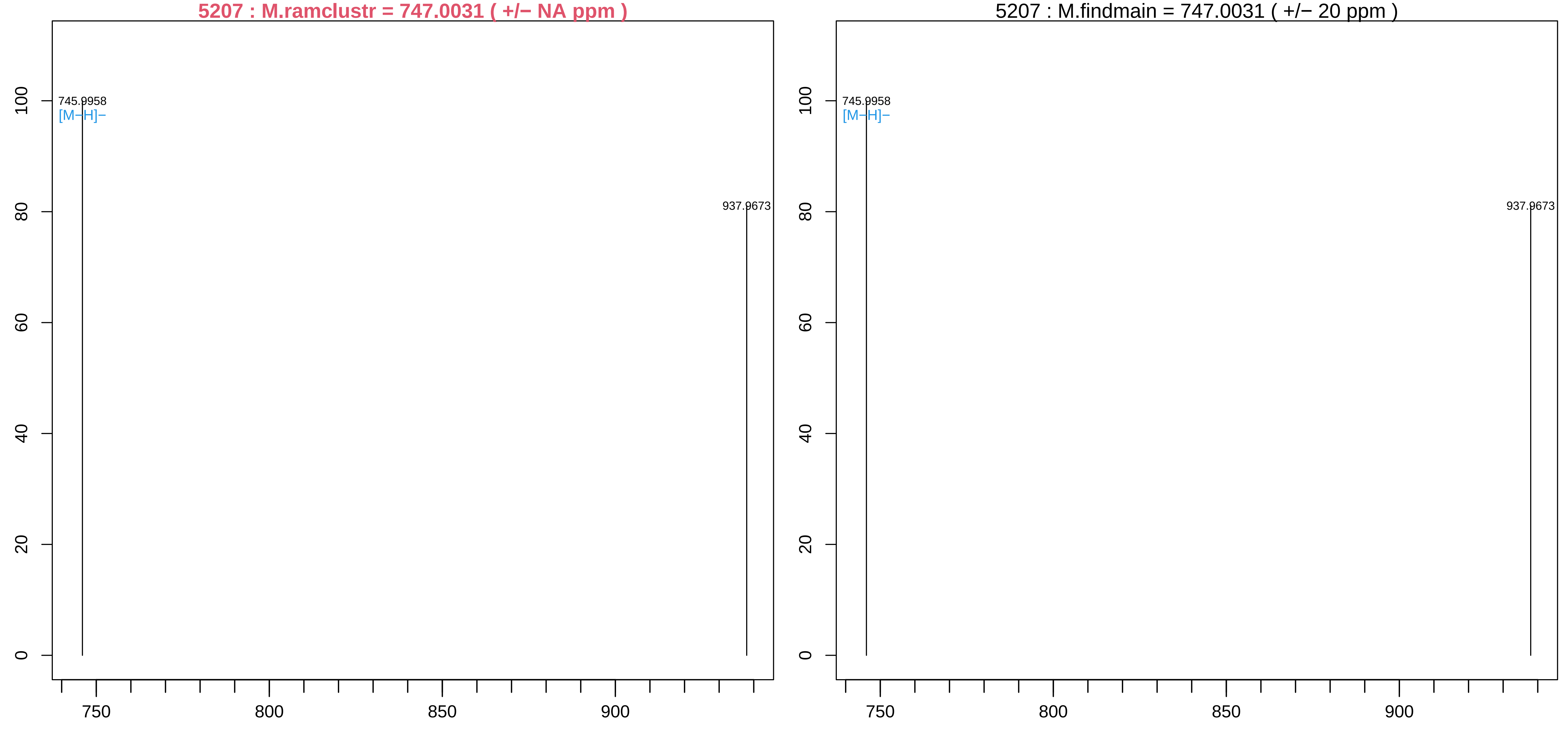

5207 : M.ramclustr = 747.0031 (+/− NA ppm)
745.9958
[M−H]−
937.9673
5207 : M.findmain = 747.0031 (+/− 20 ppm)
745.9958
[M−H]−
937.9673
0
20
40
60
80
100
750
800
850
900

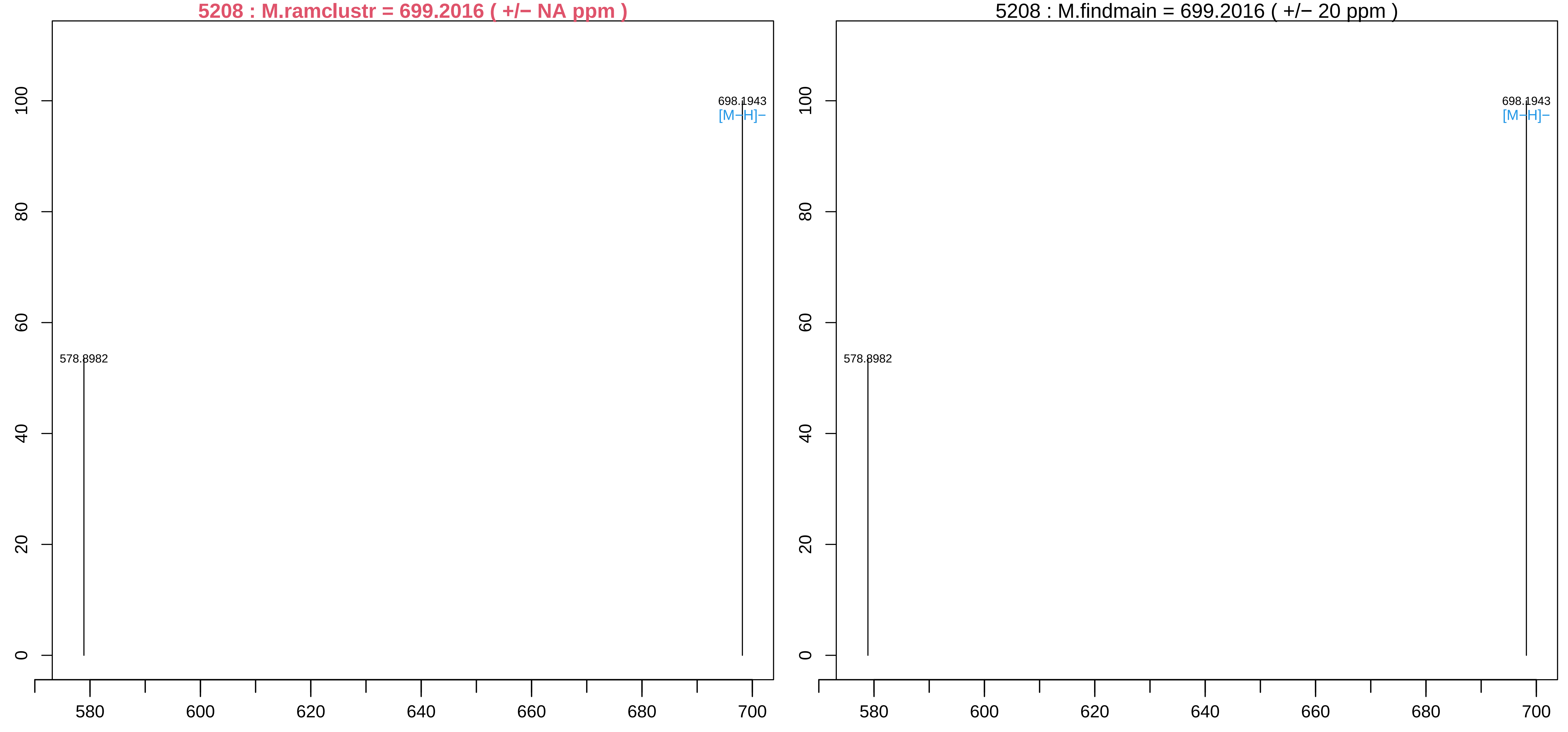
5208 : M.ramclustr = 699.2016 (+/− NA ppm)
5208 : M.findmain = 699.2016 (+/− 20 ppm)
698.1943
[M−H]−
578.8982
0
20
40
60
80
100
580
600
620
640
660
680
700

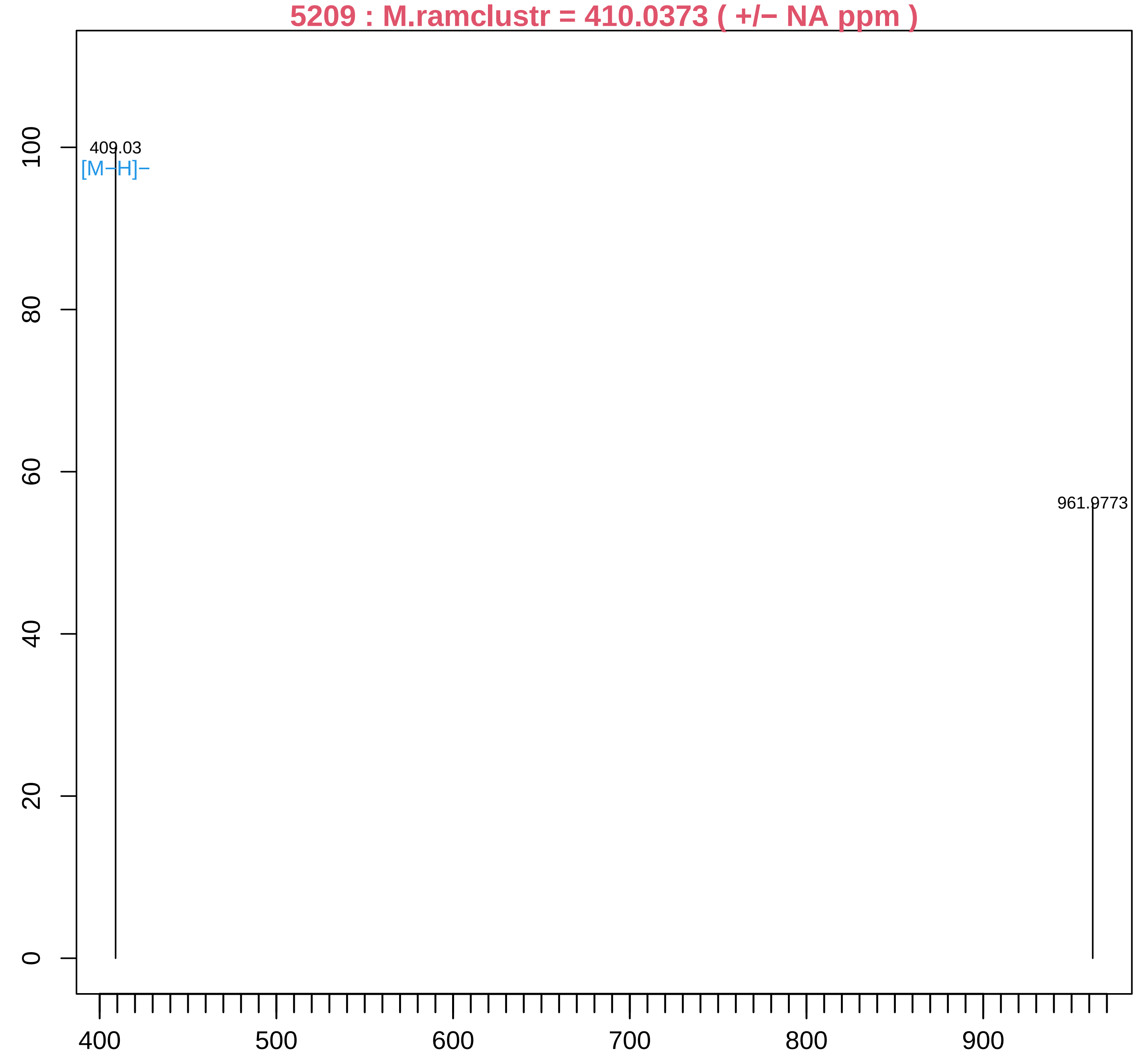
5209 : M.ramclustr = 410.0373 (+/− NA ppm)
409.03
[M−H]−
961.9773
0
20
40
60
80
100
400
500
600
700
800
900

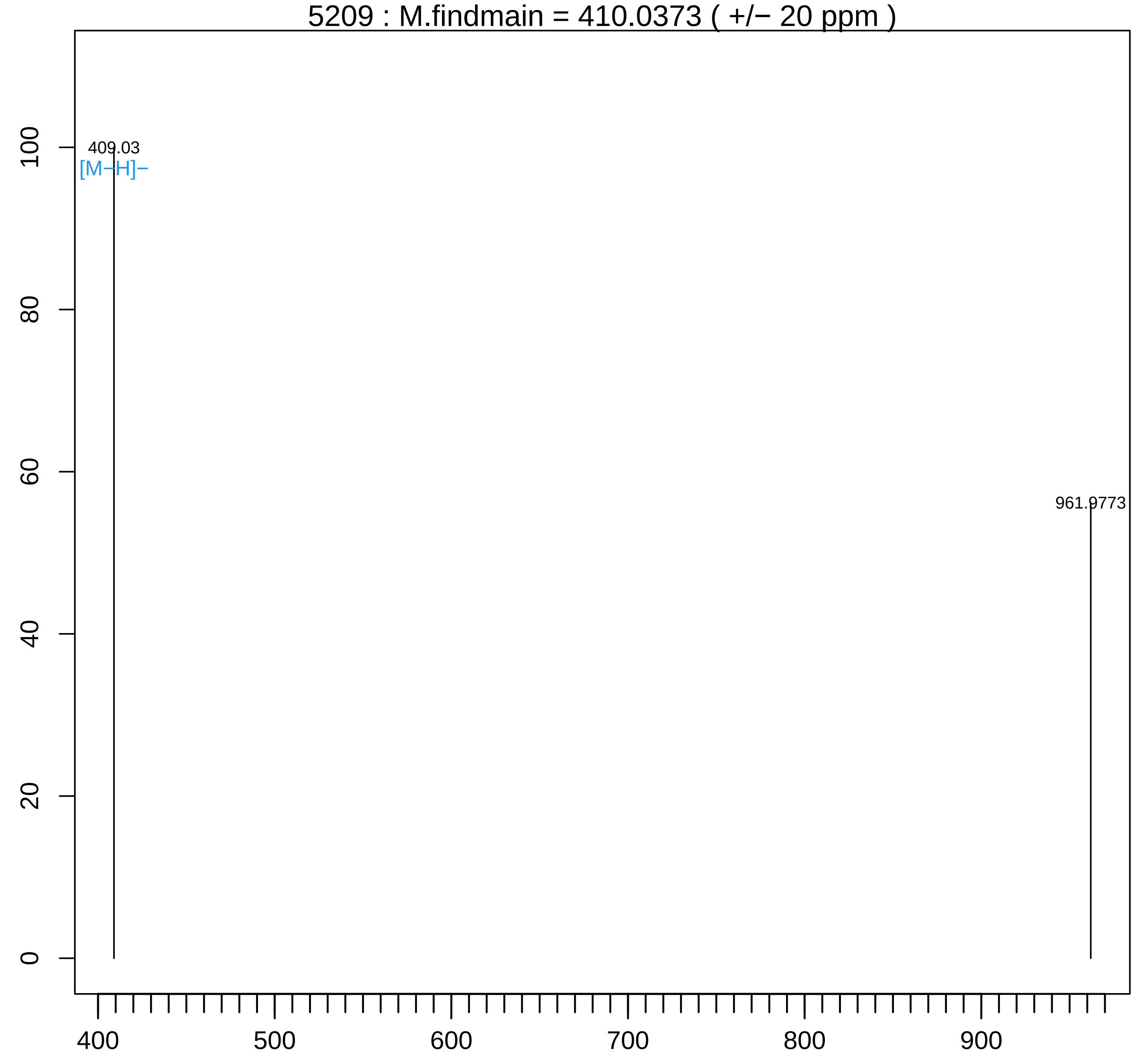
5209 : M.findmain = 410.0373 (+/− 20 ppm)
409.03
[M−H]−
961.9773
0
20
40
60
80
100
400
500
600
700
800
900

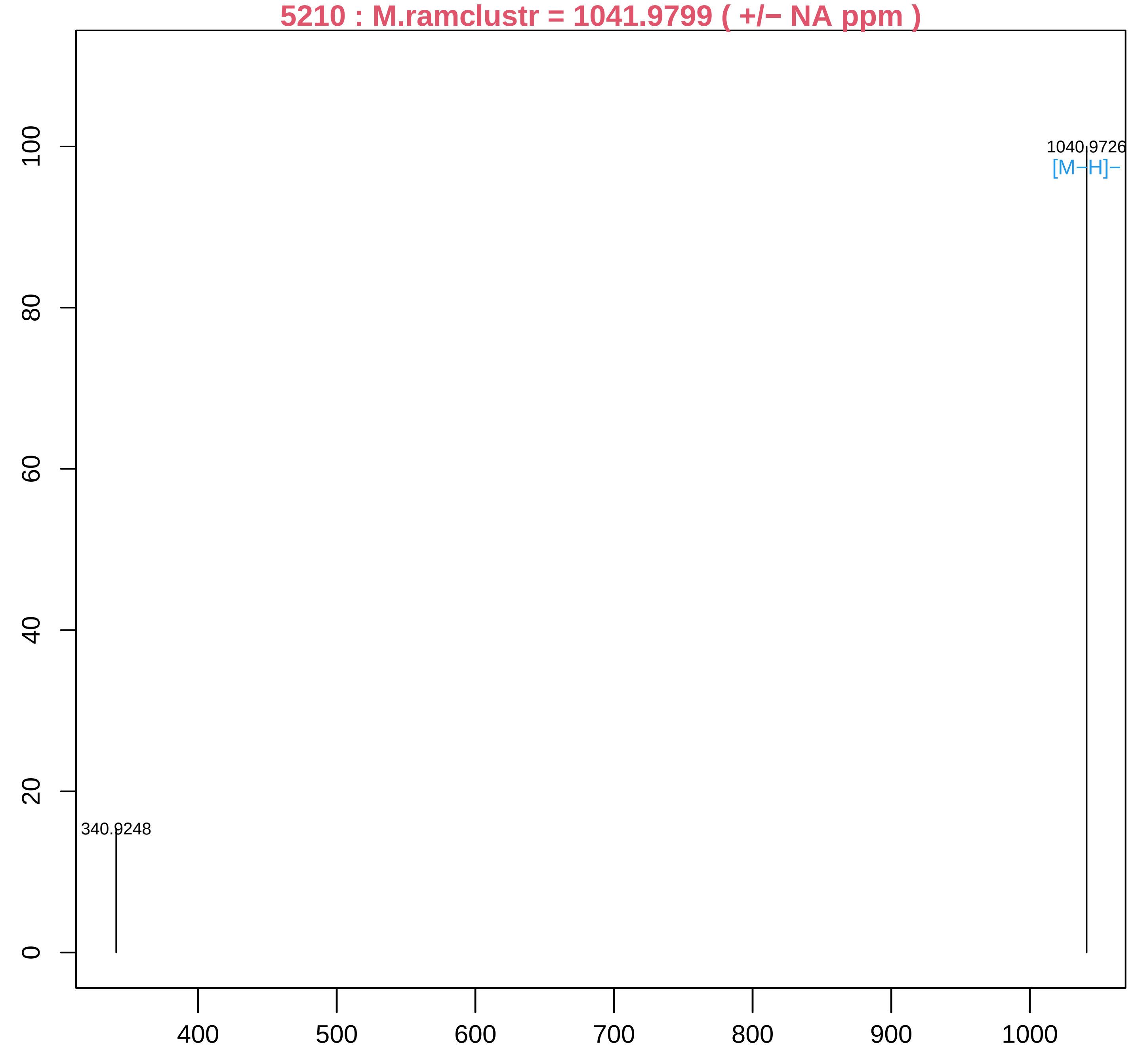
5210 : M.ramclustr = 1041.9799 (+/− NA ppm)
1040.9726
[M−H]−
340.9248
0
20
40
60
80
100
400
500
600
700
800
900
1000

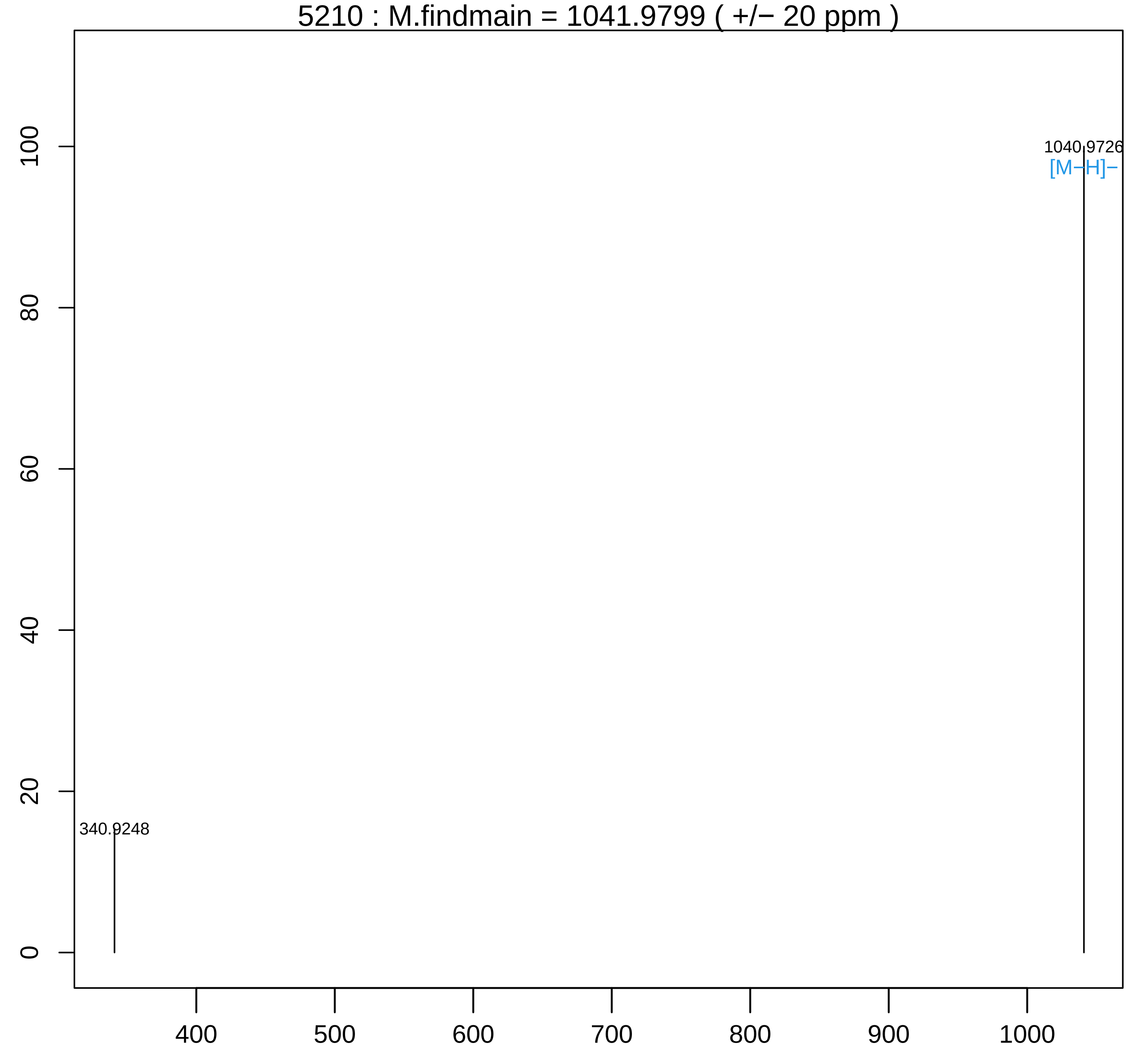
5210 : M.findmain = 1041.9799 (+/− 20 ppm)
1040.9726
[M−H]−
340.9248
0
20
40
60
80
100
400
500
600
700
800
900
1000

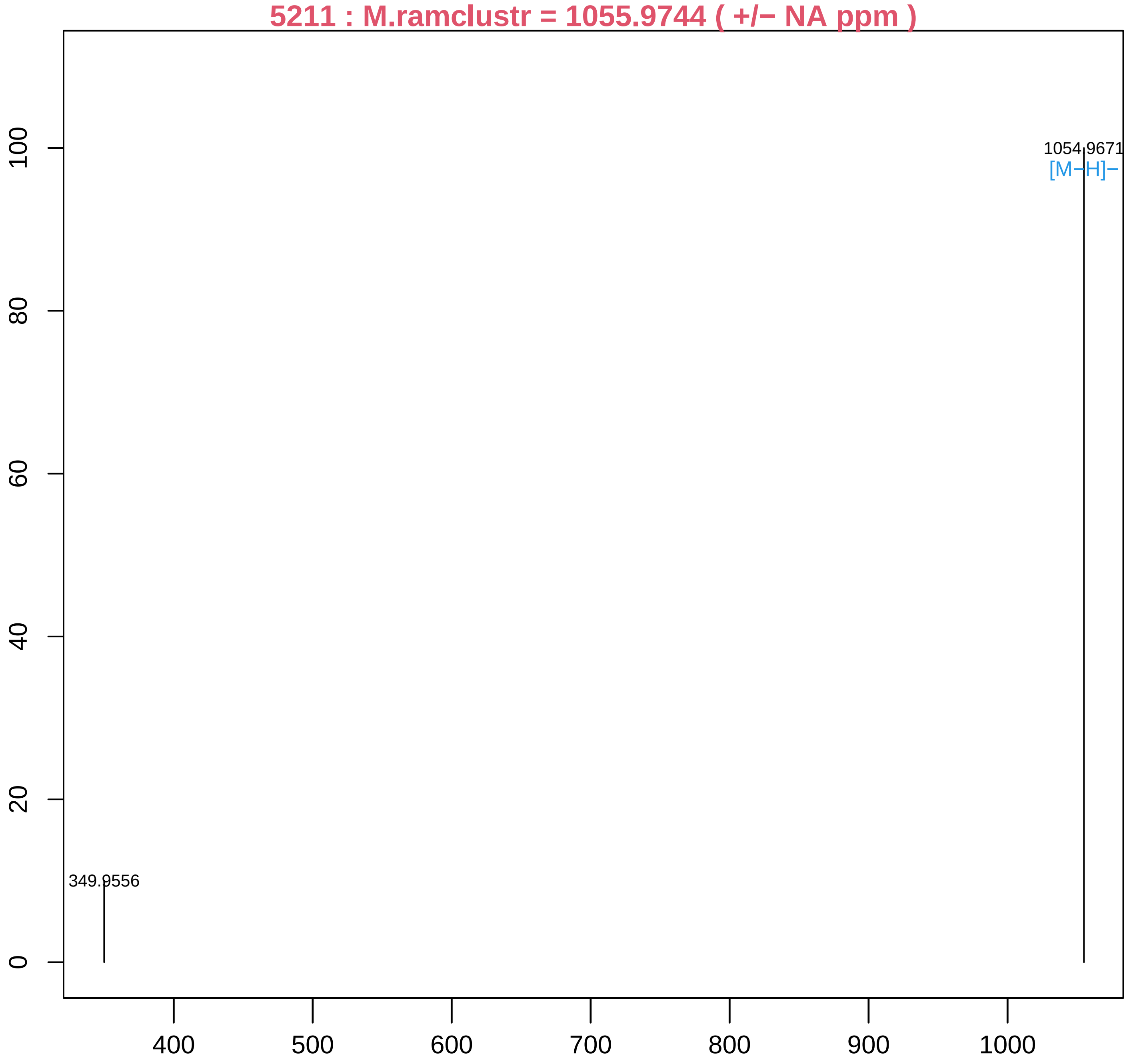
5211 : M.ramclustr = 1055.9744 (+/− NA ppm)
1054.9671
[M−H]−
349.9556
0
20
40
60
80
100
400
500
600
700
800
900
1000

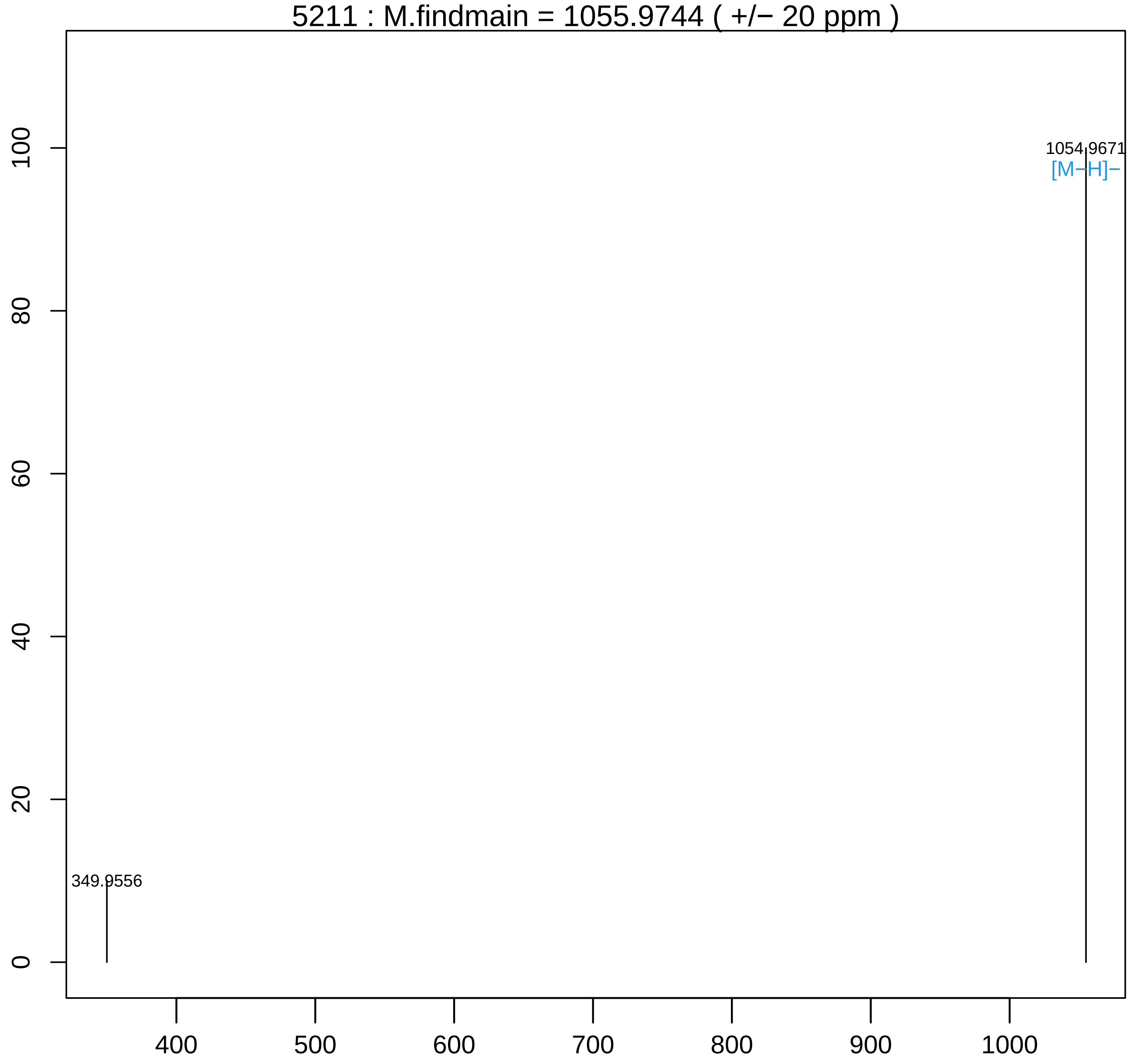
5211 : M.findmain = 1055.9744 (+/− 20 ppm)
1054.9671
[M−H]−
349.9556
0
20
40
60
80
100
400
500
600
700
800
900
1000

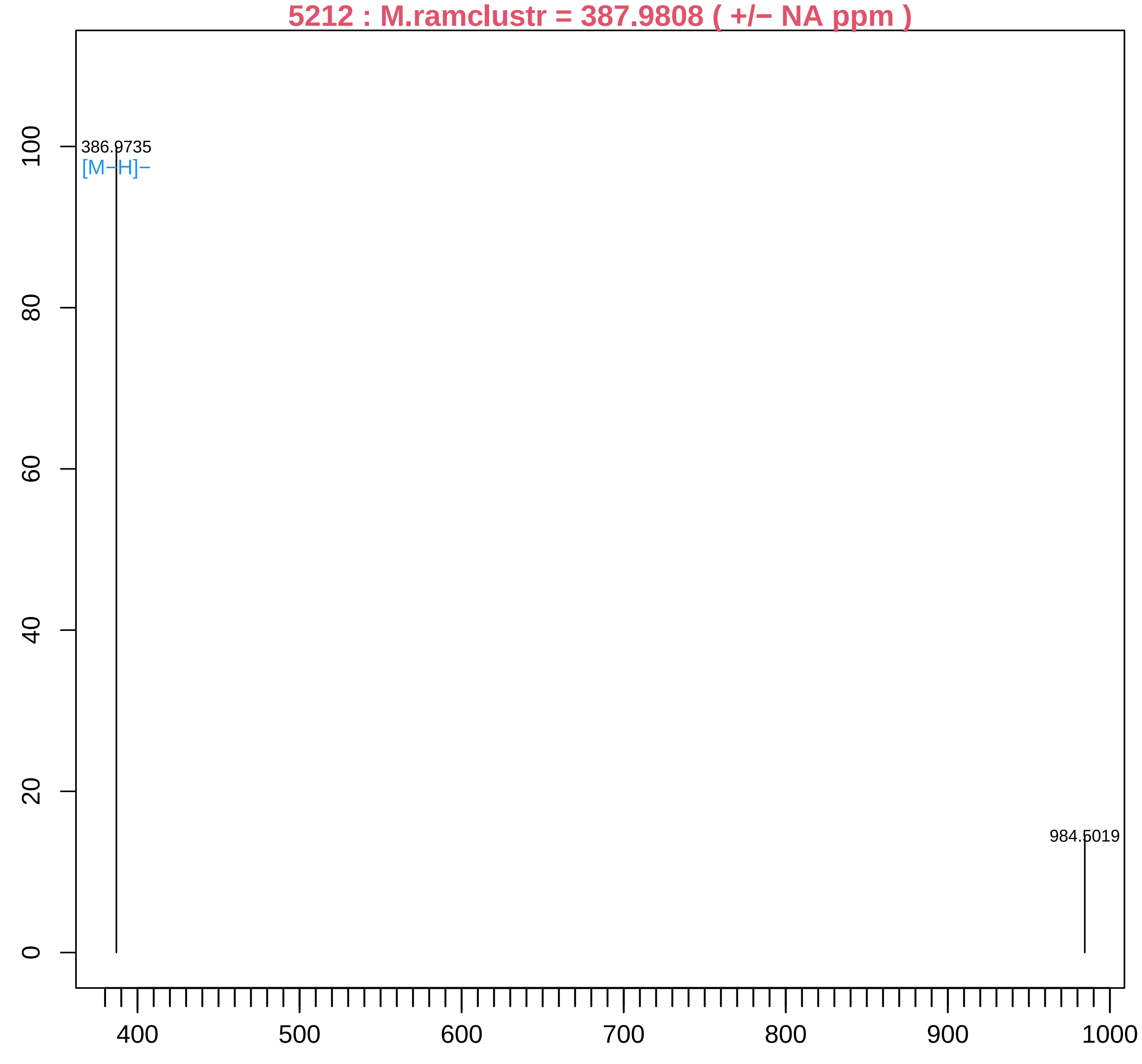
5212 : M.ramclustr = 387.9808 (+/− NA ppm)
386.9735
[M−H]−
984.5019
0
20
40
60
80
100
400
500
600
700
800
900
1000

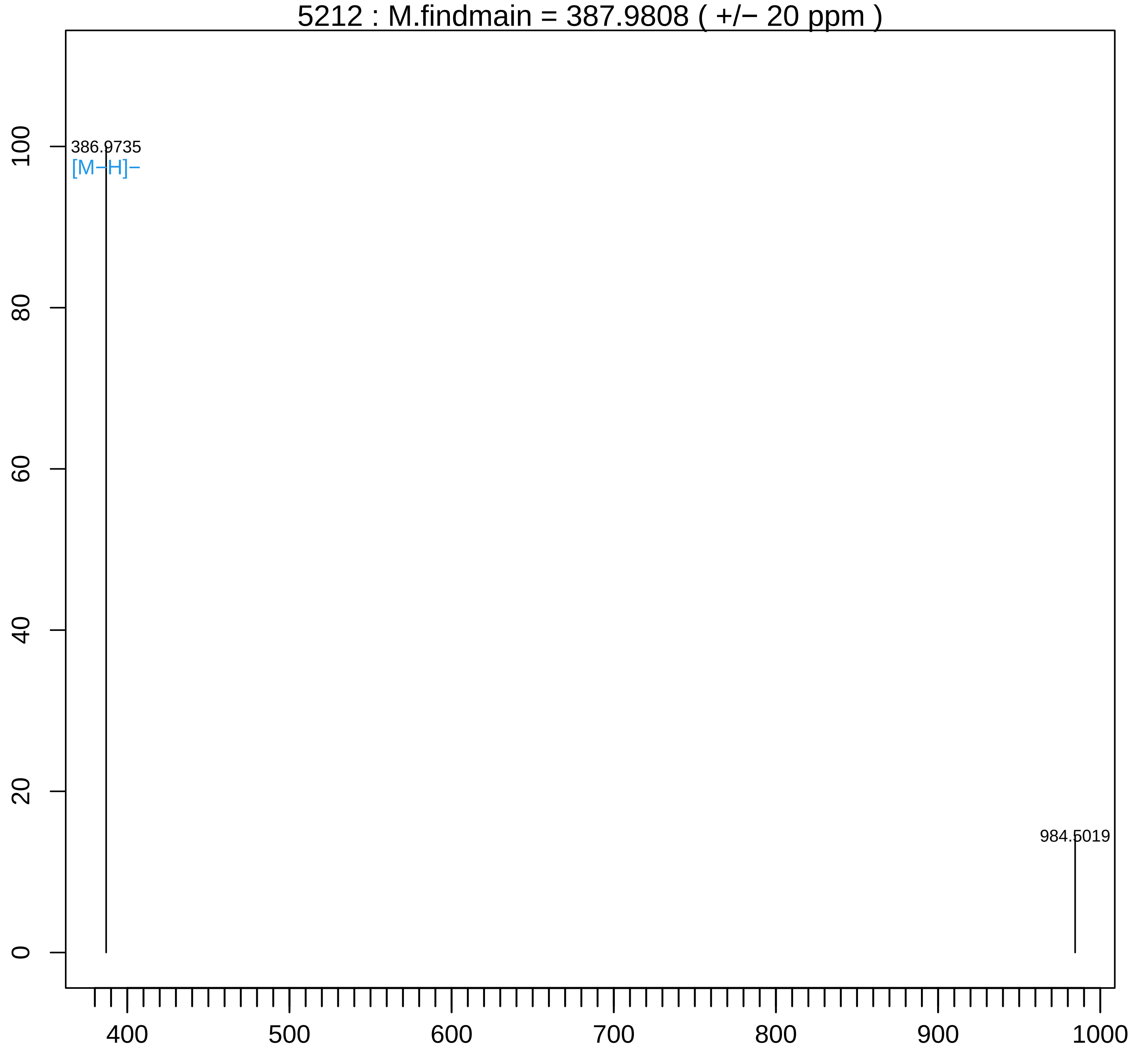
5212 : M.findmain = 387.9808 (+/− 20 ppm)
386.9735
[M−H]−
984.5019
0
20
40
60
80
100
400
500
600
700
800
900
1000

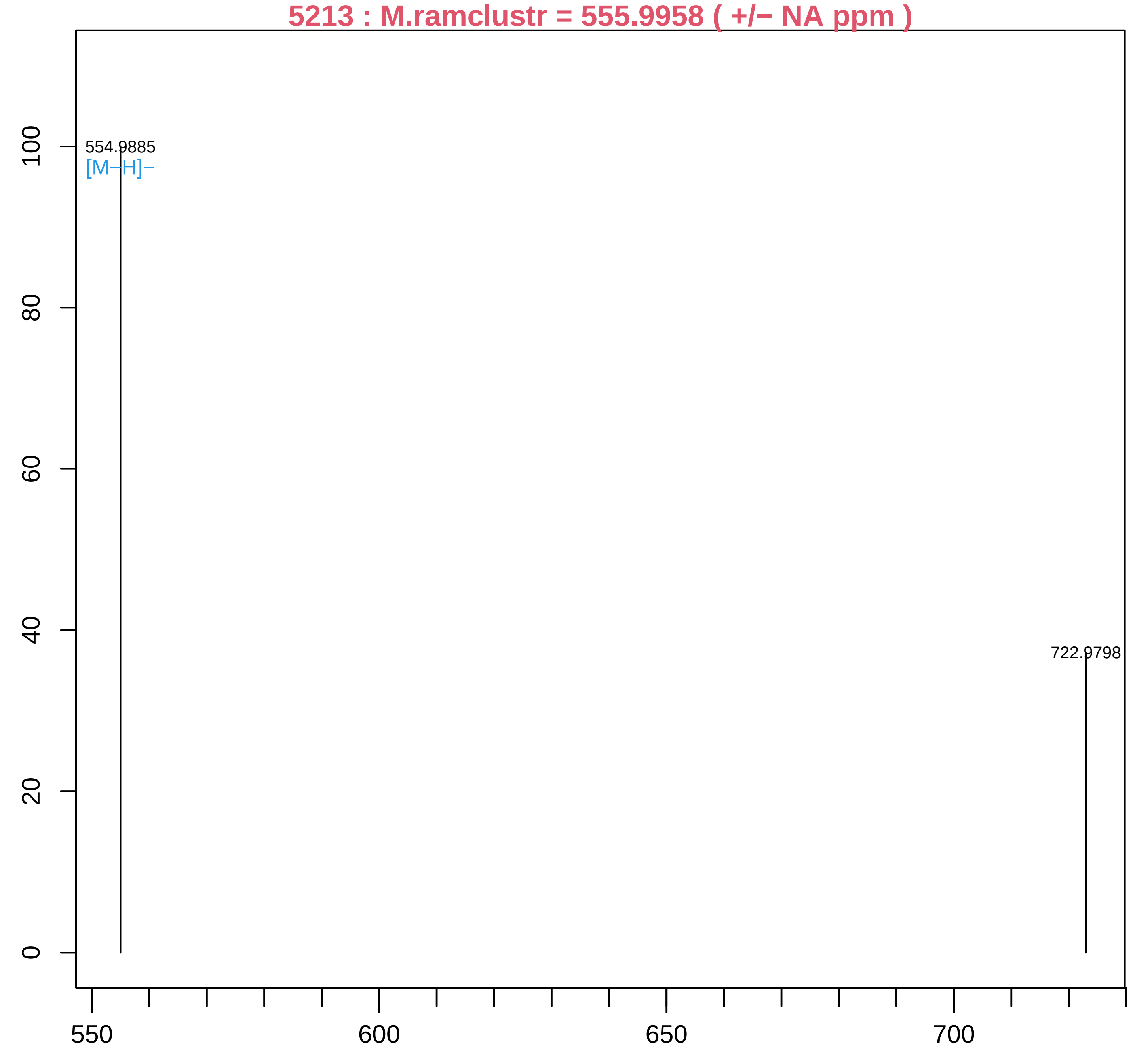
5213 : M.ramclustr = 555.9958 (+/− NA ppm)
554.9885
[M−H]−
722.9798
0
20
40
60
80
100
550
600
650
700

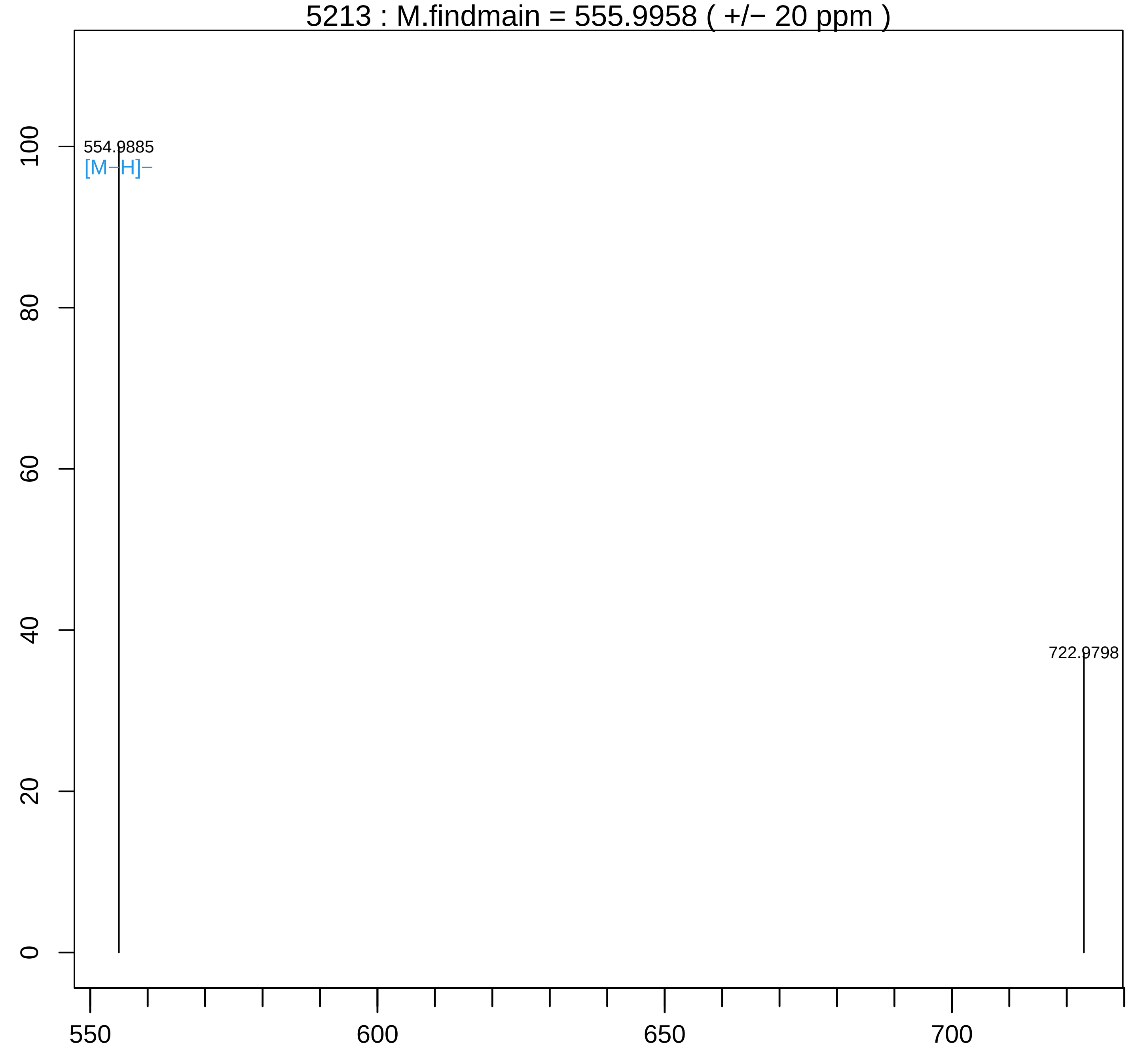
5213 : M.findmain = 555.9958 (+/− 20 ppm)
554.9885
[M−H]−
722.9798
0
20
40
60
80
100
550
600
650
700

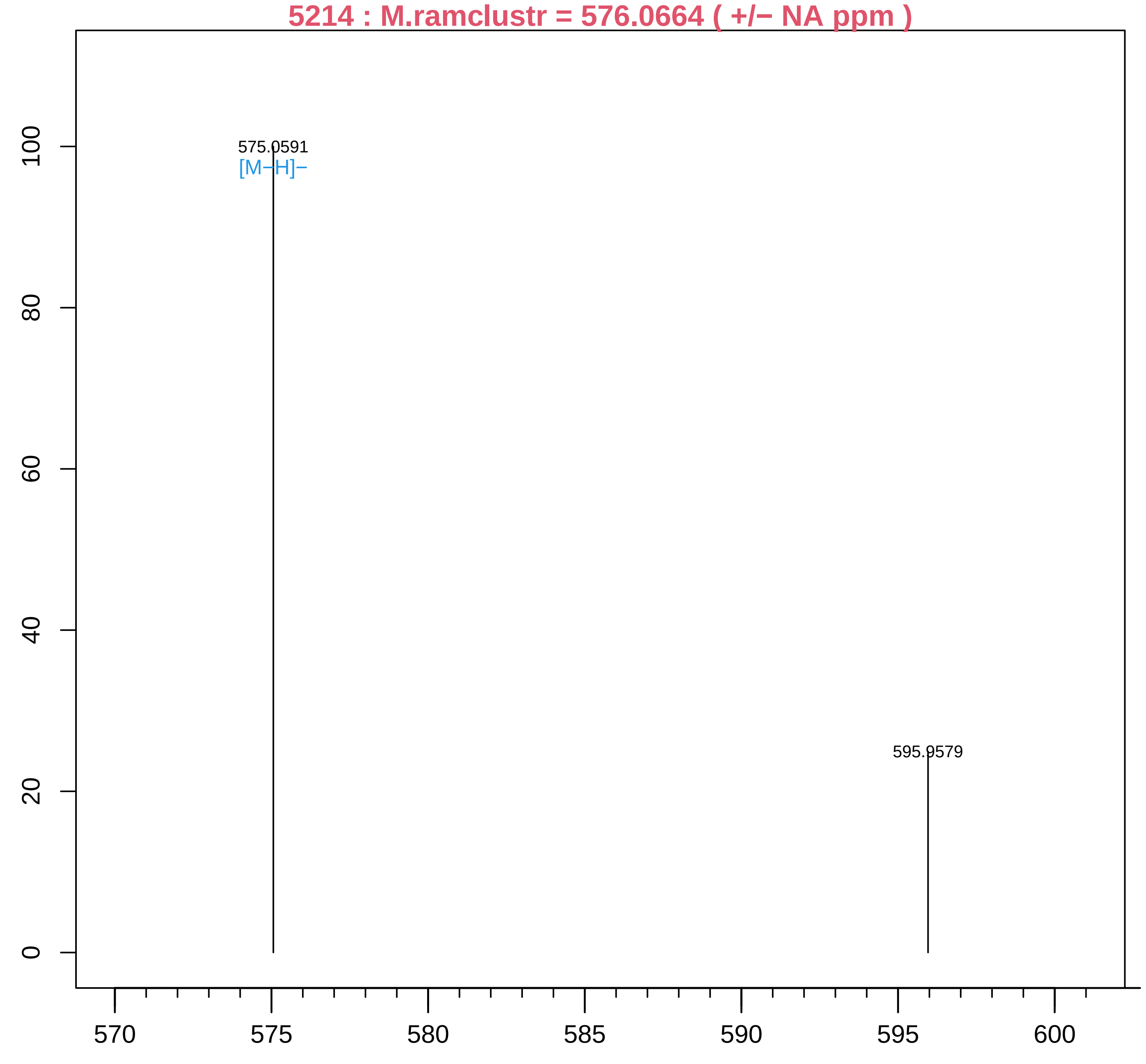
5214 : M.ramclustr = 576.0664 (+/− NA ppm)
575.0591
[M−H]−
595.9579
0
20
40
60
80
100
570
575
580
585
590
595
600

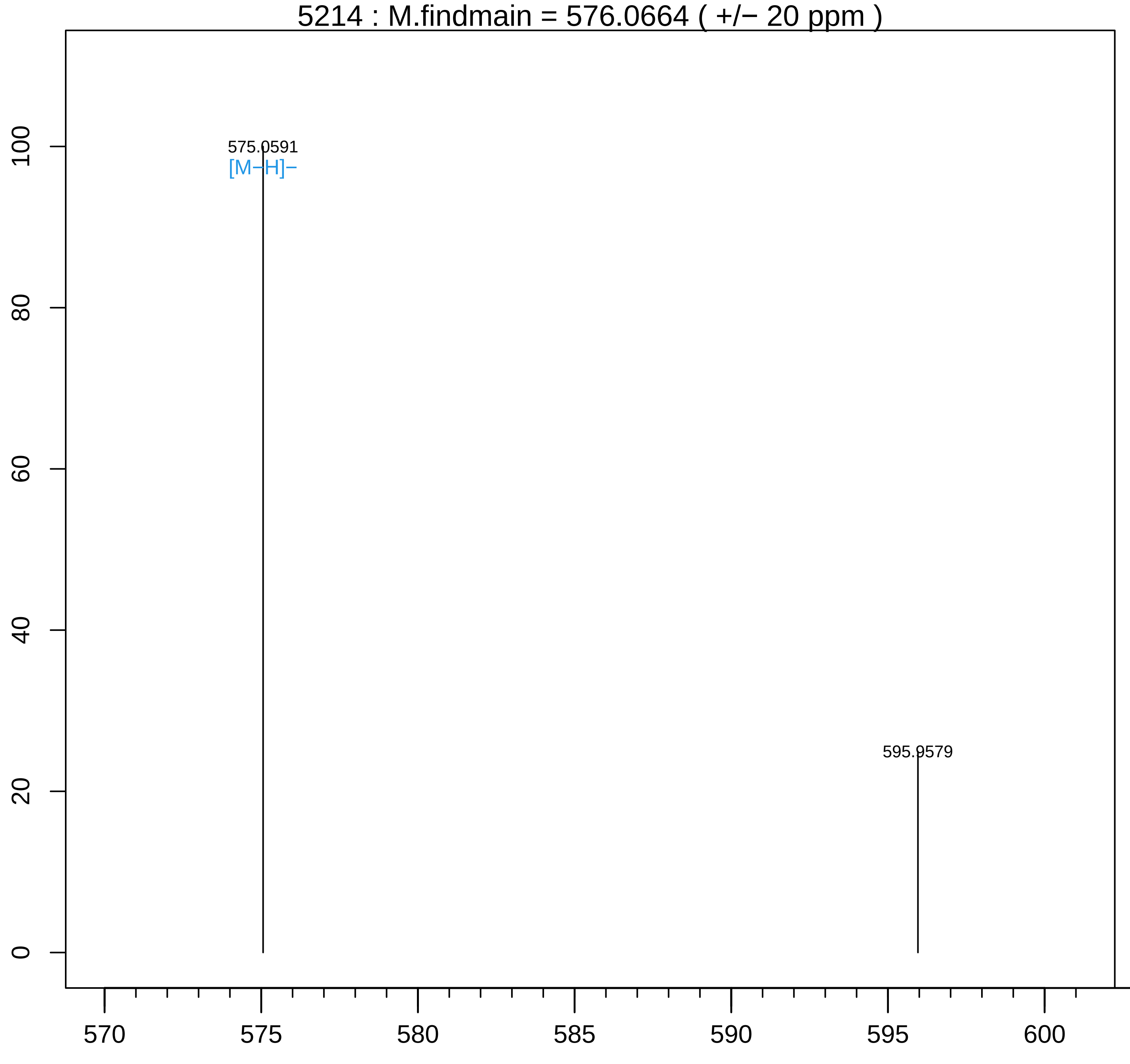
5214 : M.findmain = 576.0664 (+/− 20 ppm)
575.0591
[M−H]−
595.9579
0
20
40
60
80
100
570
575
580
585
590
595
600

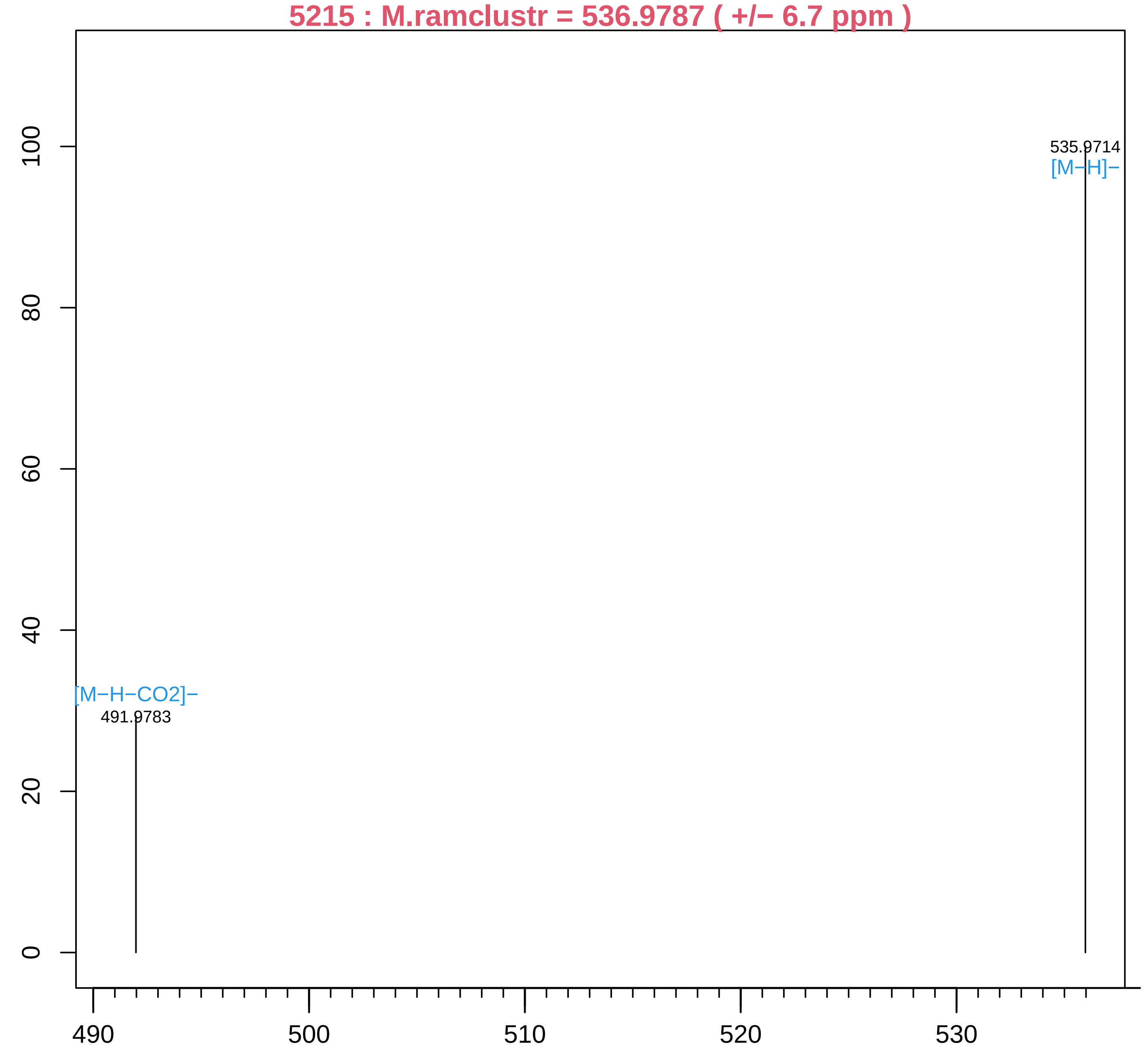
5215 : M.ramclustr = 536.9787 (+/− 6.7 ppm)
535.9714
[M−H]−
[M−H−CO2]−
491.9783
0
20
40
60
80
100
490
500
510
520
530

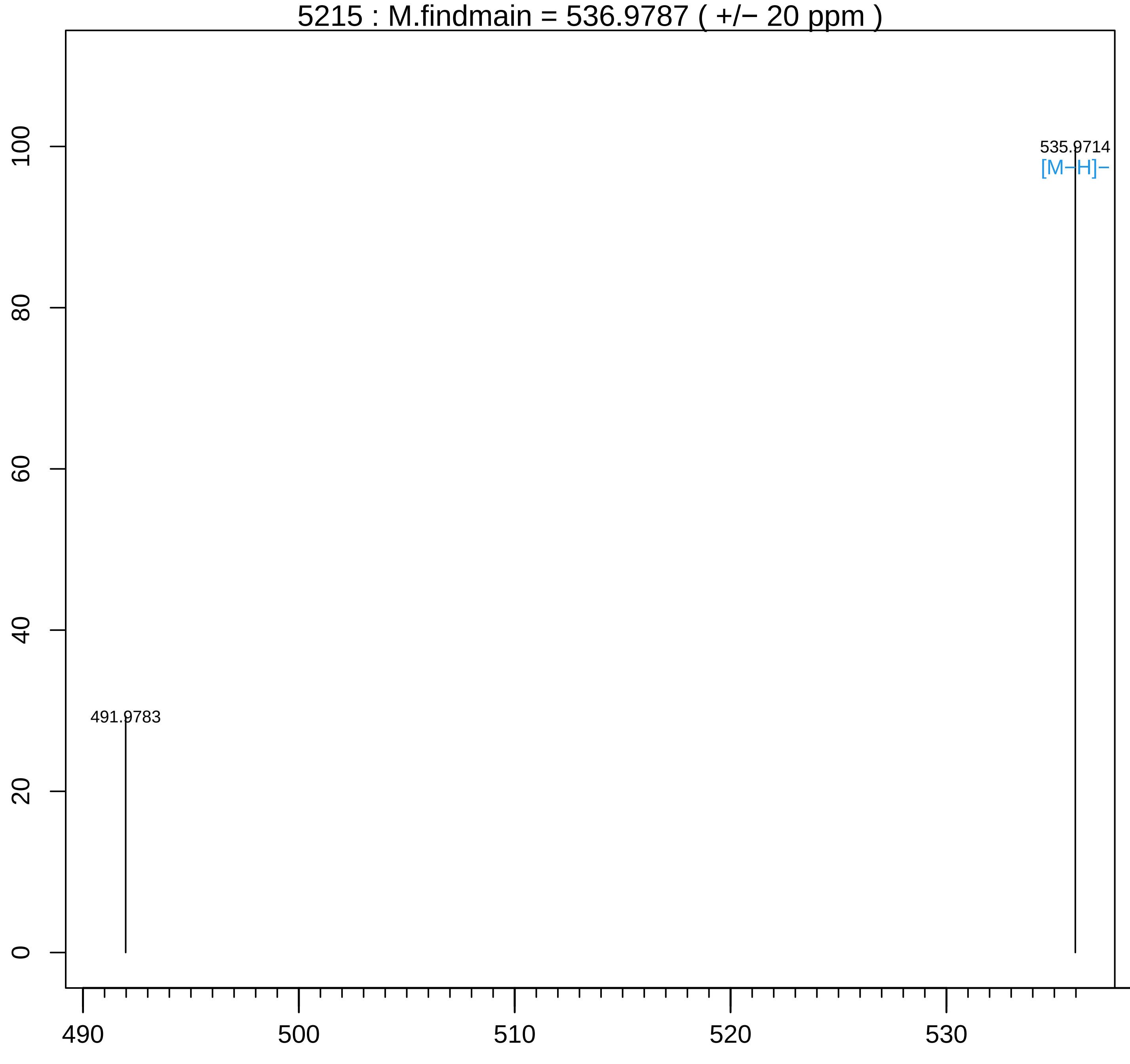
5215 : M.findmain = 536.9787 (+/− 20 ppm)
535.9714
[M−H]−
491.9783
0
20
40
60
80
100
490
500
510
520
530

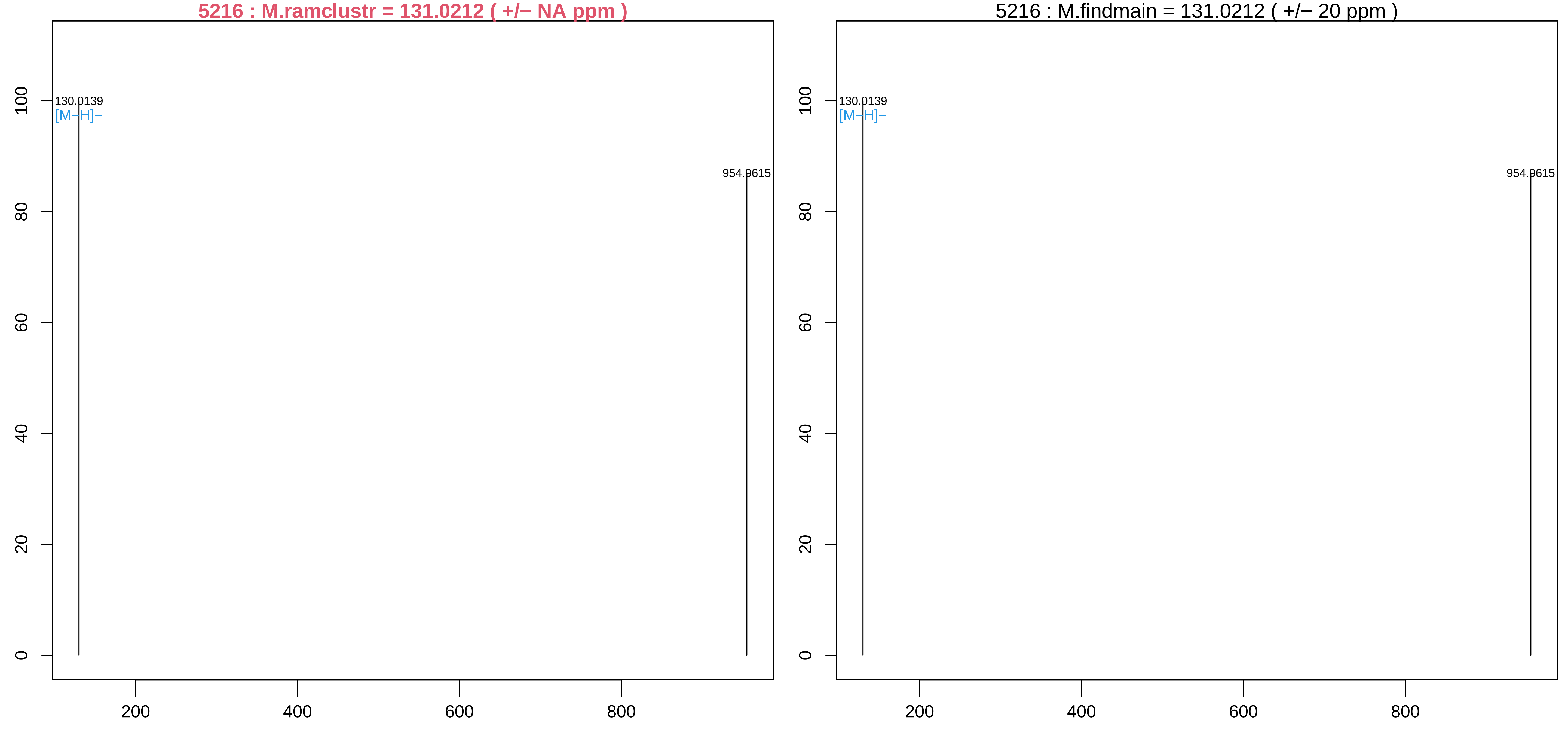

5216 : M.ramclustr = 131.0212 (+/− NA ppm)
130.0139
[M−H]−
954.9615
0
20
40
60
80
100
200
400
600
800
5216 : M.findmain = 131.0212 (+/− 20 ppm)
130.0139
[M−H]−
954.9615
0
20
40
60
80
100
200
400
600
800

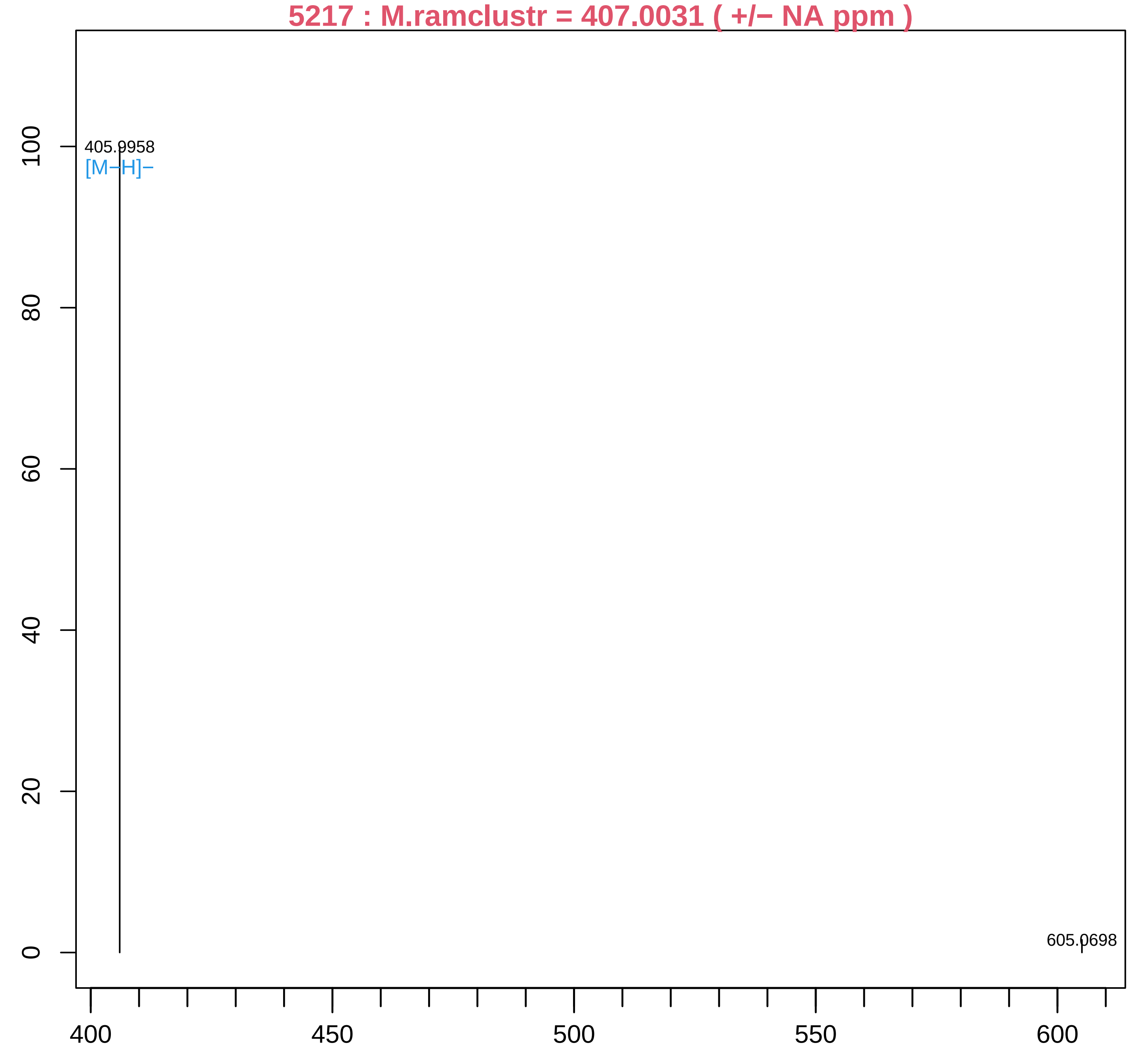

5217 : M.ramclustr = 407.0031 (+/− NA ppm)
405.9958
[M−H]−
605.0698
0
20
40
60
80
100
400
450
500
550
600

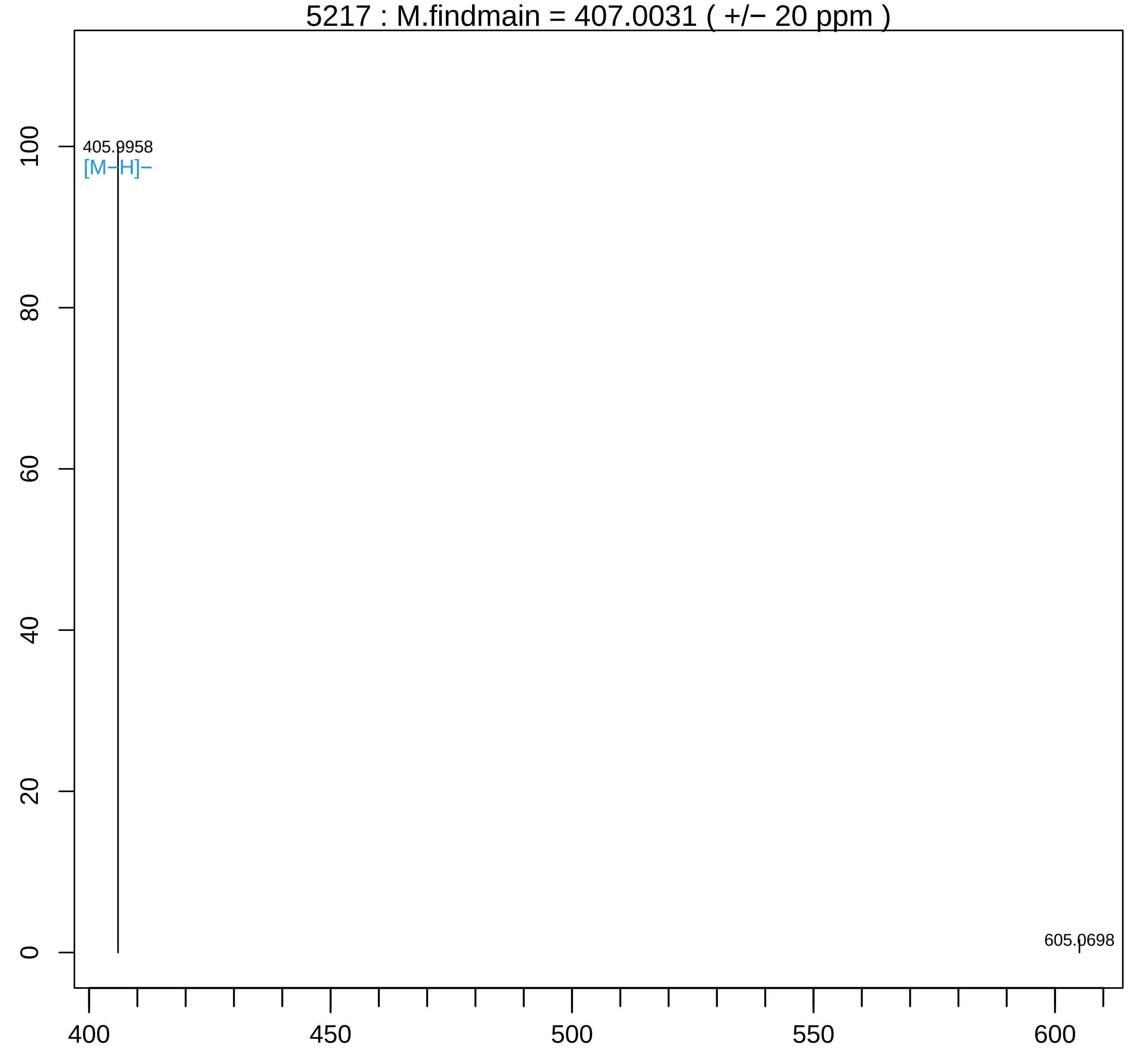

5217 : M.findmain = 407.0031 (+/− 20 ppm)
405.9958
[M−H]−
605.0698
0
20
40
60
80
100
400
450
500
550
600

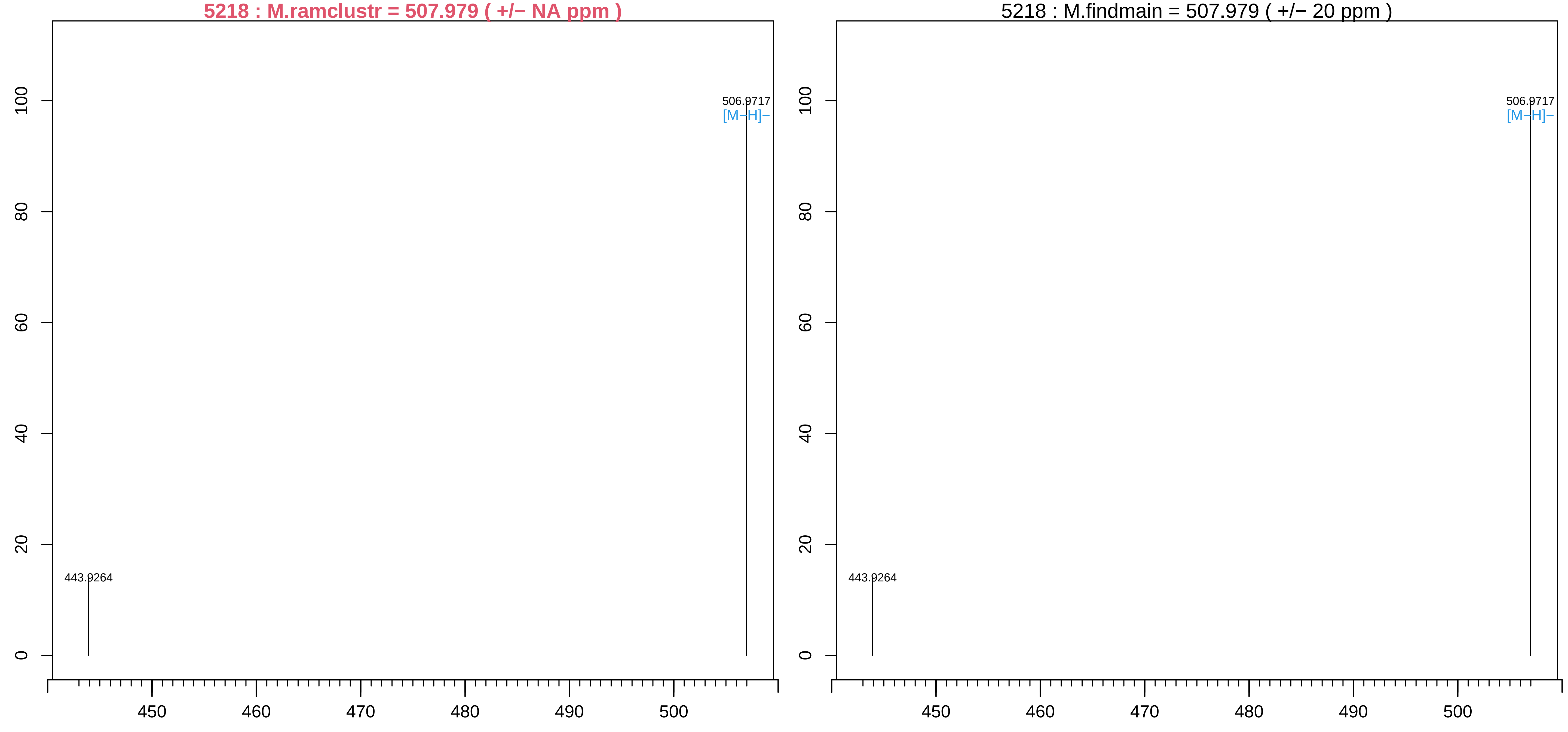
5218 : M.ramclustr = 507.979 (+/− NA ppm)
5218 : M.findmain = 507.979 (+/− 20 ppm)
506.9717
[M−H]−
443.9264
506.9717
[M−H]−
443.9264
0
20
40
60
80
100
450
460
470
480
490
500

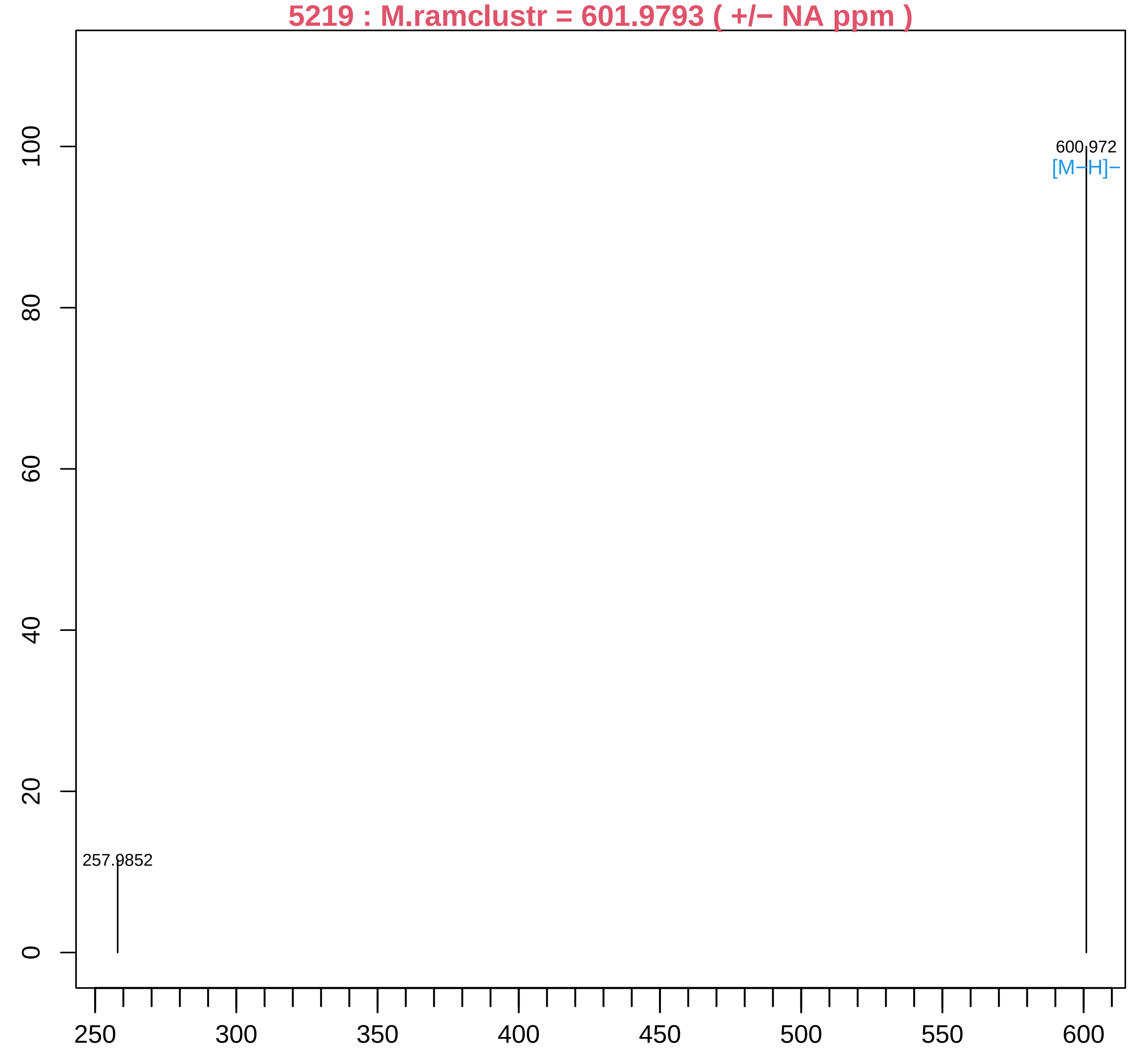

5219 : M.ramclustr = 601.9793 (+/− NA ppm)
600.972
[M−H]−
257.9852
0
20
40
60
80
100
250
300
350
400
450
500
550
600

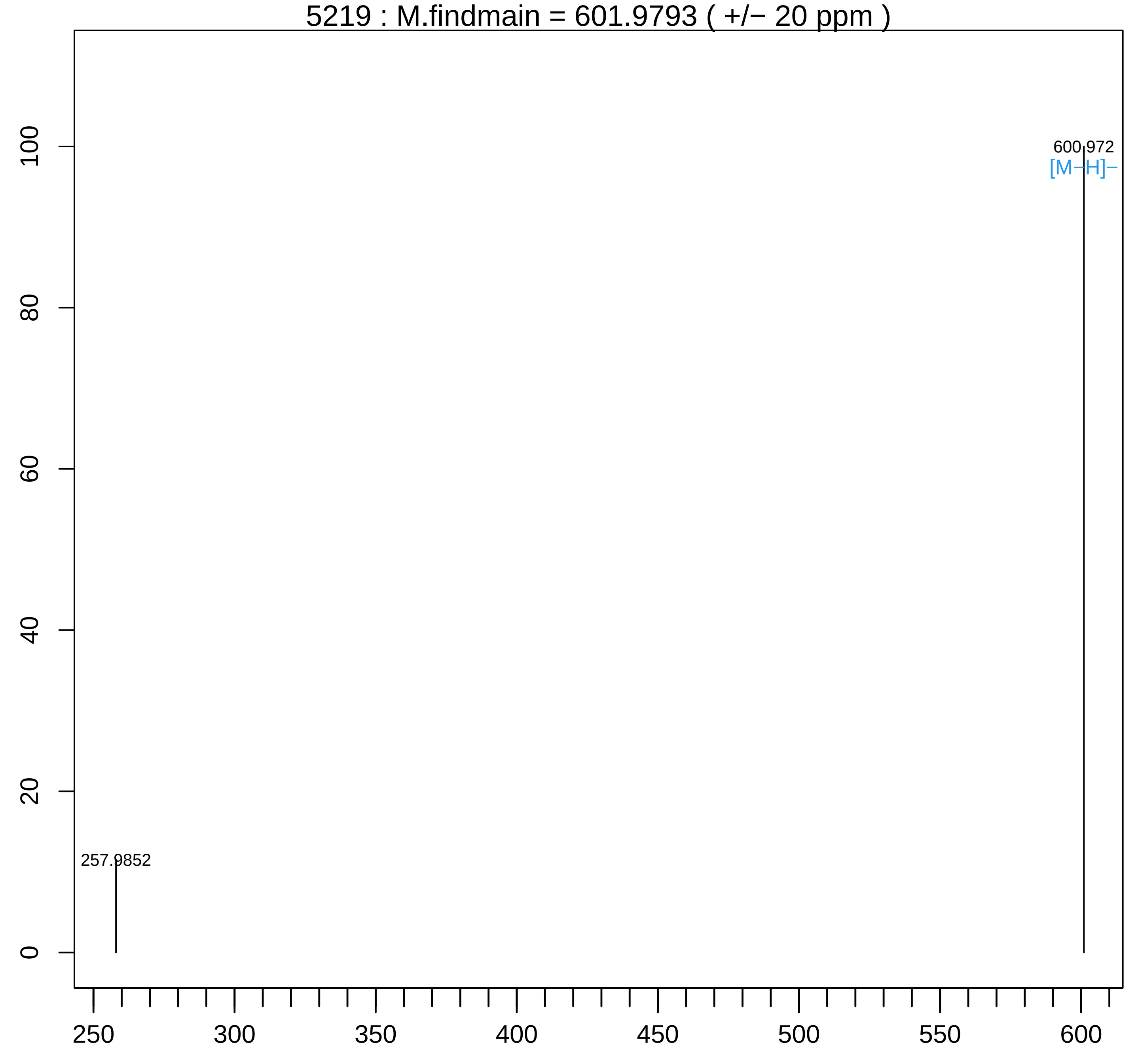

5219 : M.findmain = 601.9793 (+/− 20 ppm)
600.972
[M−H]−
257.9852
0
20
40
60
80
100
250
300
350
400
450
500
550
600

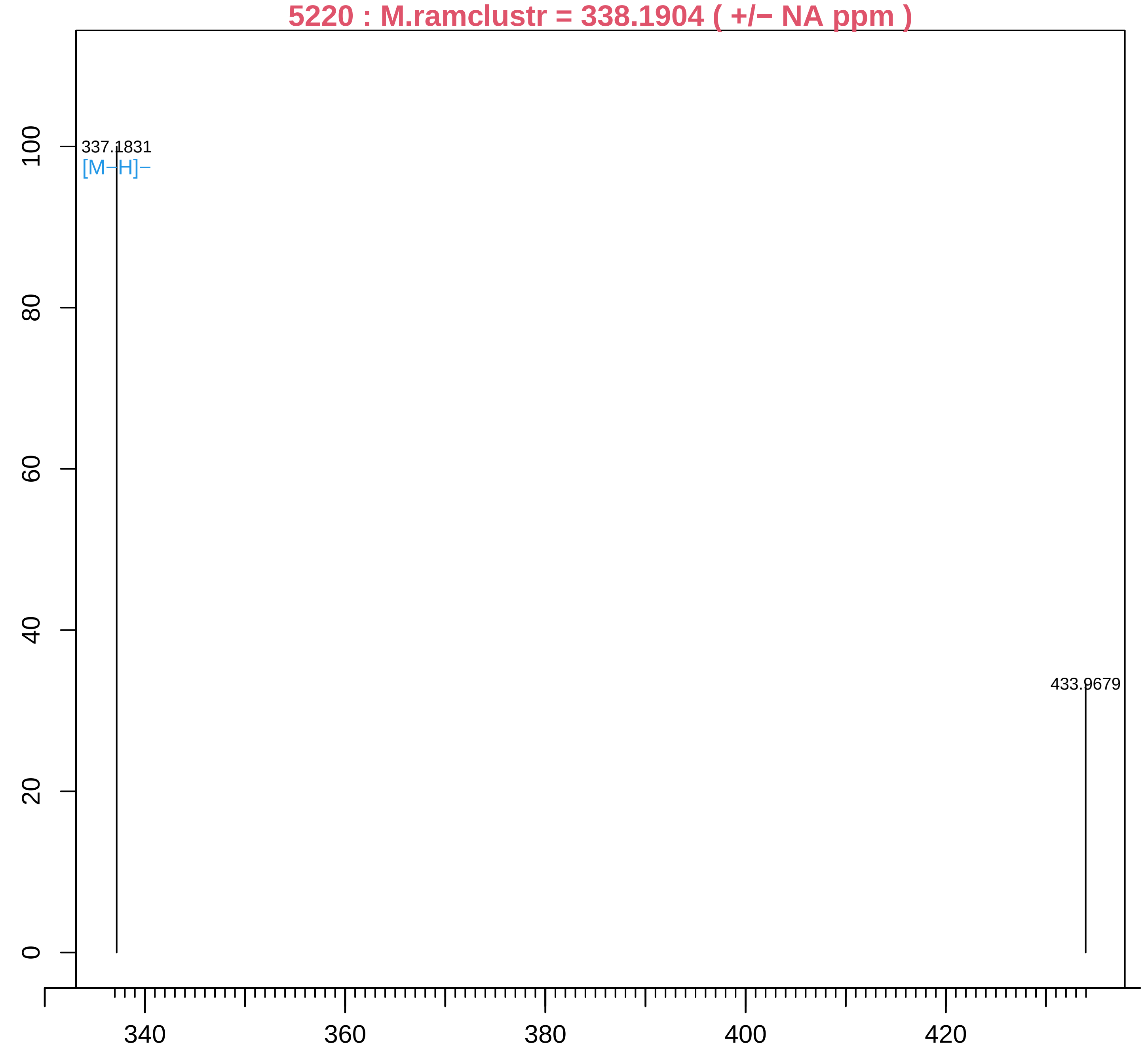
5220 : M.ramclustr = 338.1904 (+/− NA ppm)
337.1831
[M−H]−
433.9679
0
20
40
60
80
100
340
360
380
400
420

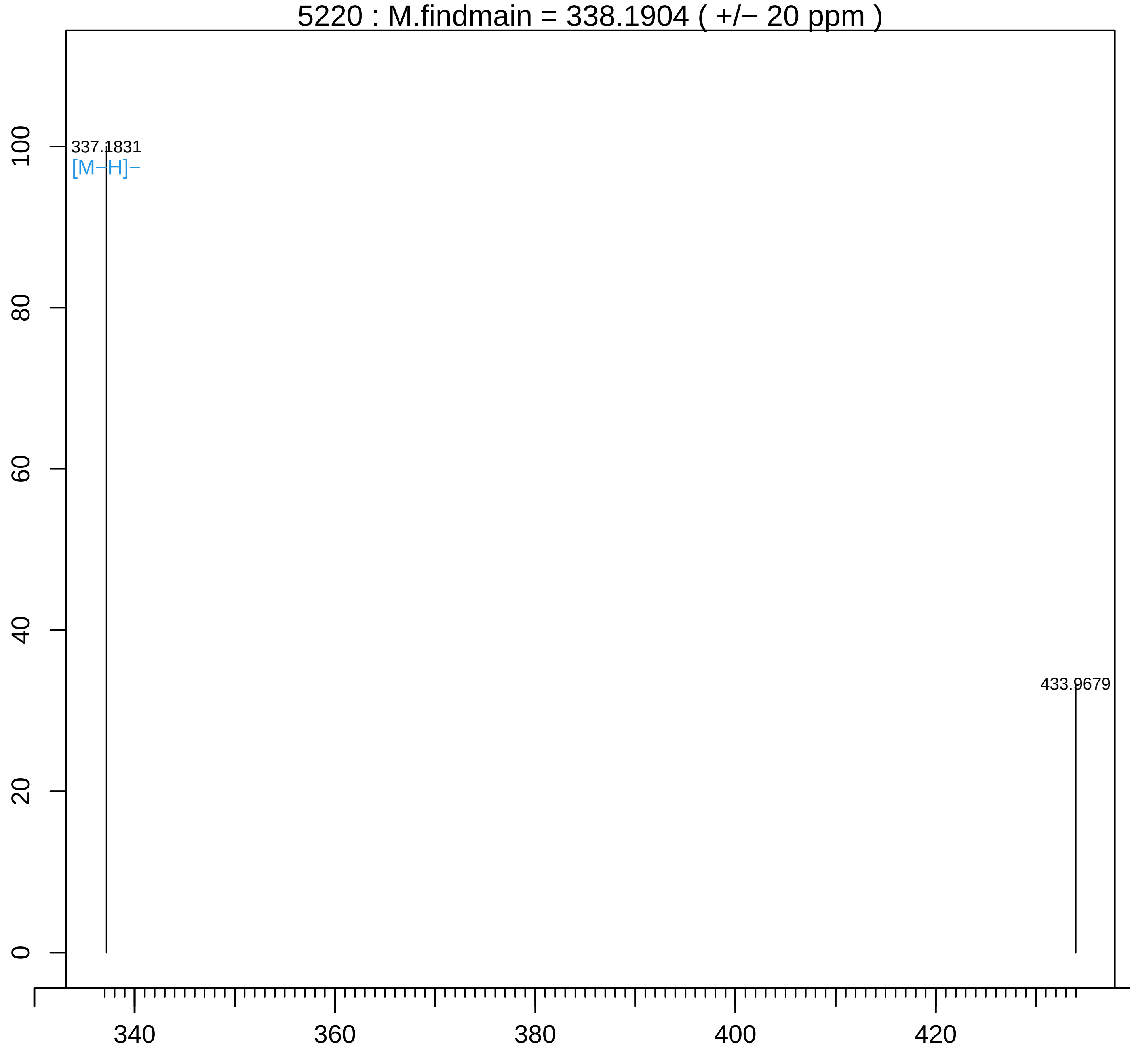
5220 : M.findmain = 338.1904 (+/− 20 ppm)
337.1831
[M−H]−
433.9679
0
20
40
60
80
100
340
360
380
400
420

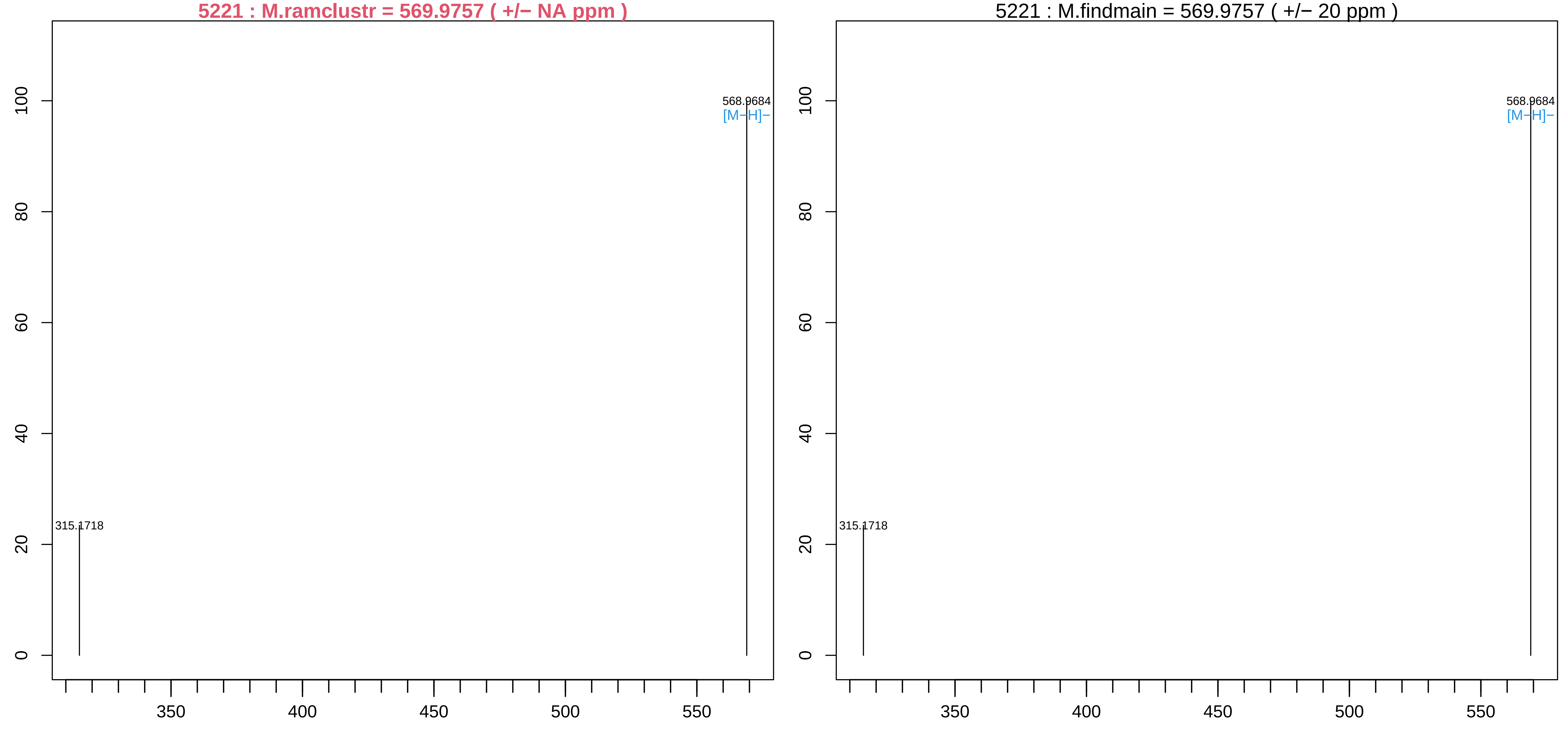

5221 : M.ramclustr = 569.9757 (+/− NA ppm)
568.9684
[M−H]−
315.1718
5221 : M.findmain = 569.9757 (+/− 20 ppm)
568.9684
[M−H]−
315.1718
0
20
40
60
80
100
350
400
450
500
550

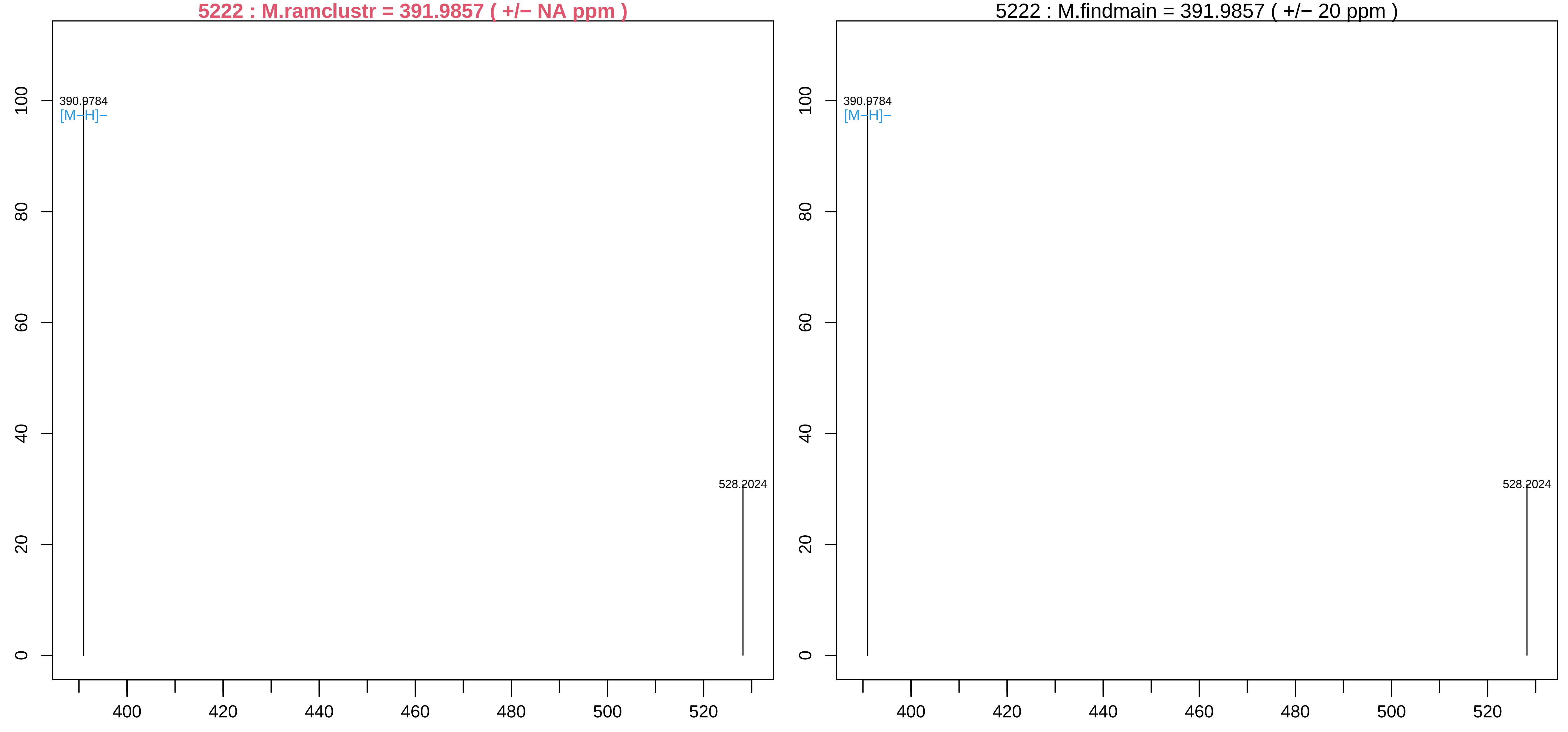

5222 : M.ramclustr = 391.9857 (+/− NA ppm)
390.9784
[M−H]−
528.2024
5222 : M.findmain = 391.9857 (+/− 20 ppm)
390.9784
[M−H]−
528.2024

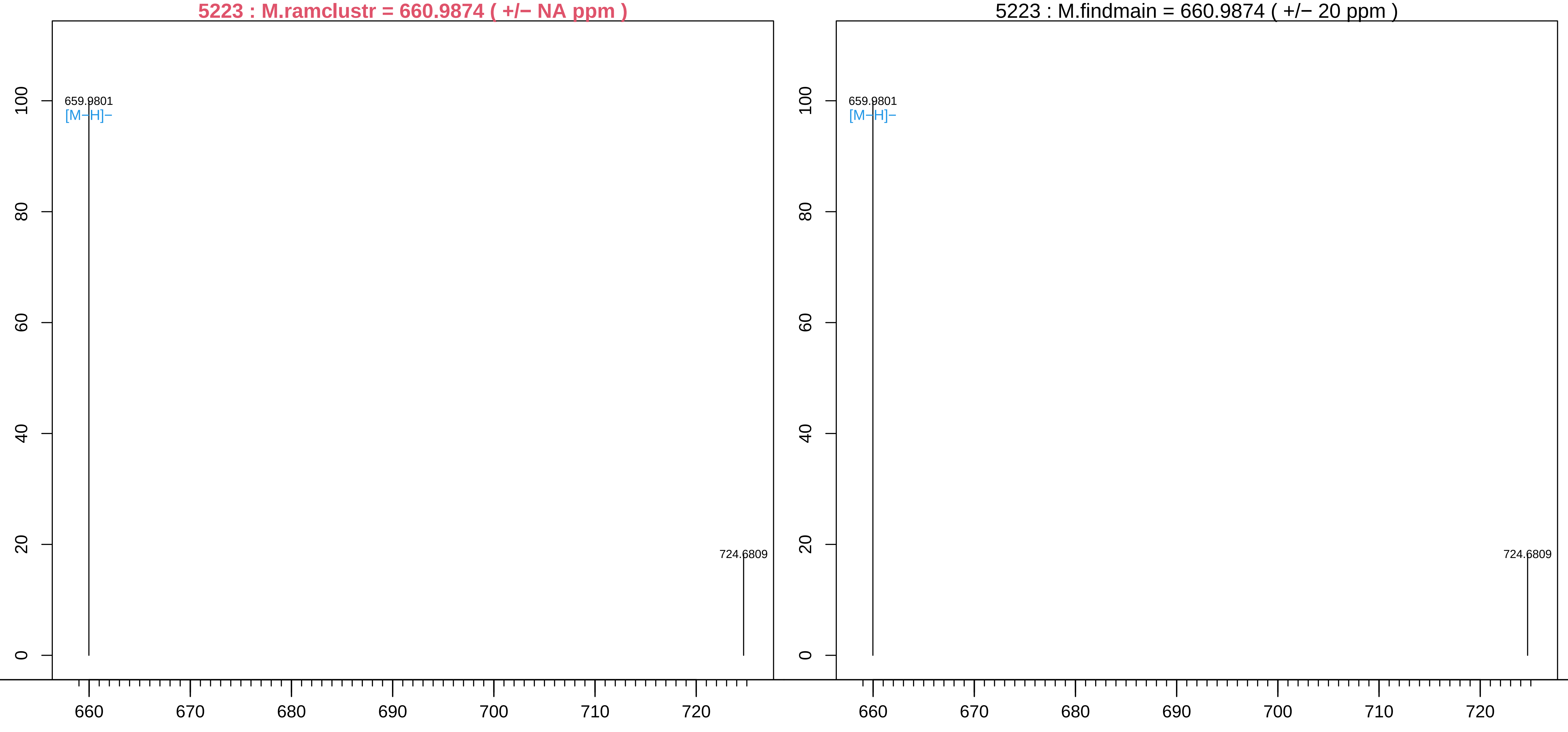
5223 : M.ramclustr = 660.9874 (+/− NA ppm)
659.9801
[M−H]−
724.6809
5223 : M.findmain = 660.9874 (+/− 20 ppm)
659.9801
[M−H]−
724.6809
0
20
40
60
80
100
660
670
680
690
700
710
720

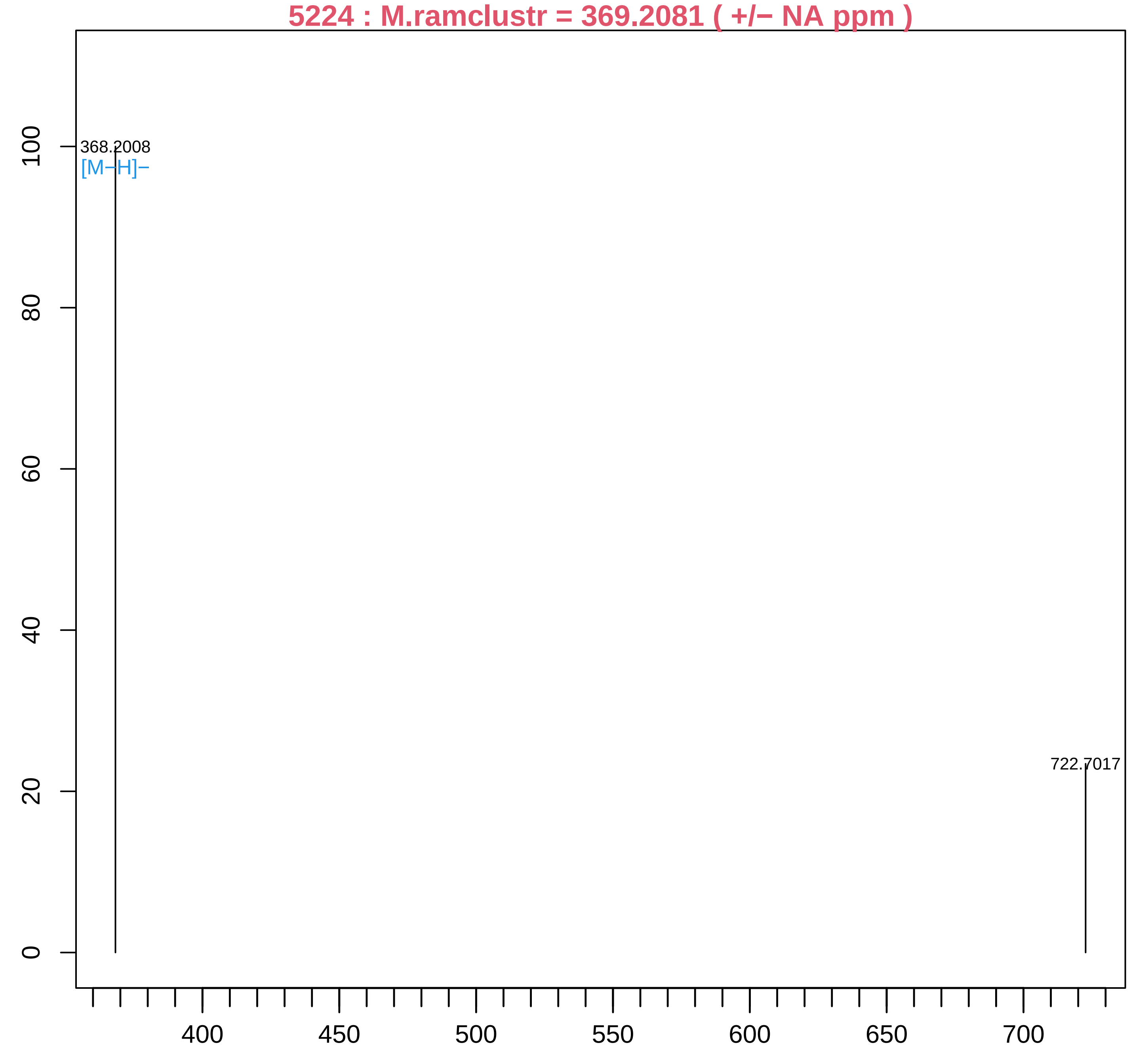
5224 : M.ramclustr = 369.2081 (+/− NA ppm)
368.2008
[M−H]−
722.7017
0
20
40
60
80
100
400
450
500
550
600
650
700

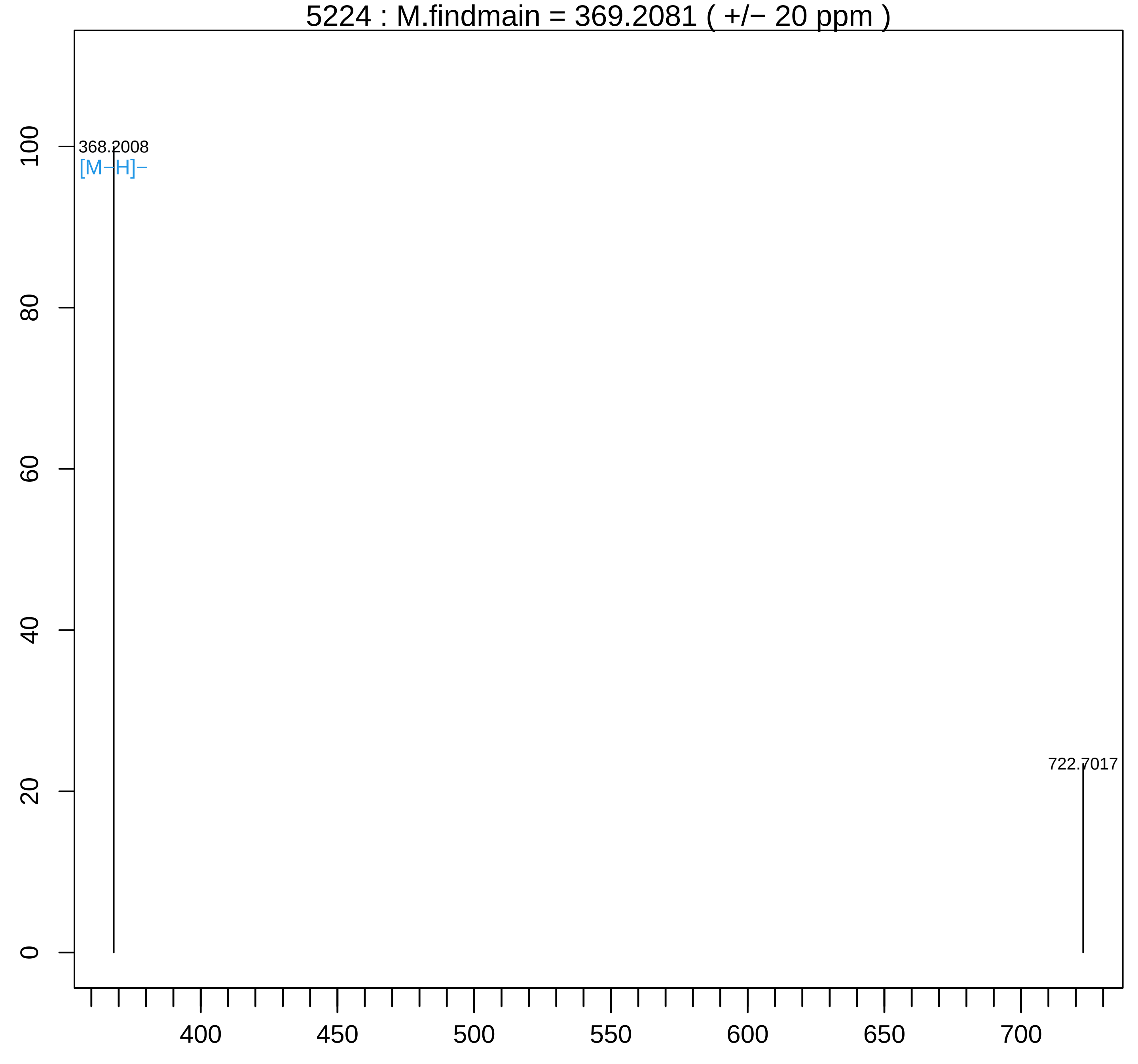
5224 : M.findmain = 369.2081 (+/− 20 ppm)
368.2008
[M−H]−
722.7017
0
20
40
60
80
100
400
450
500
550
600
650
700

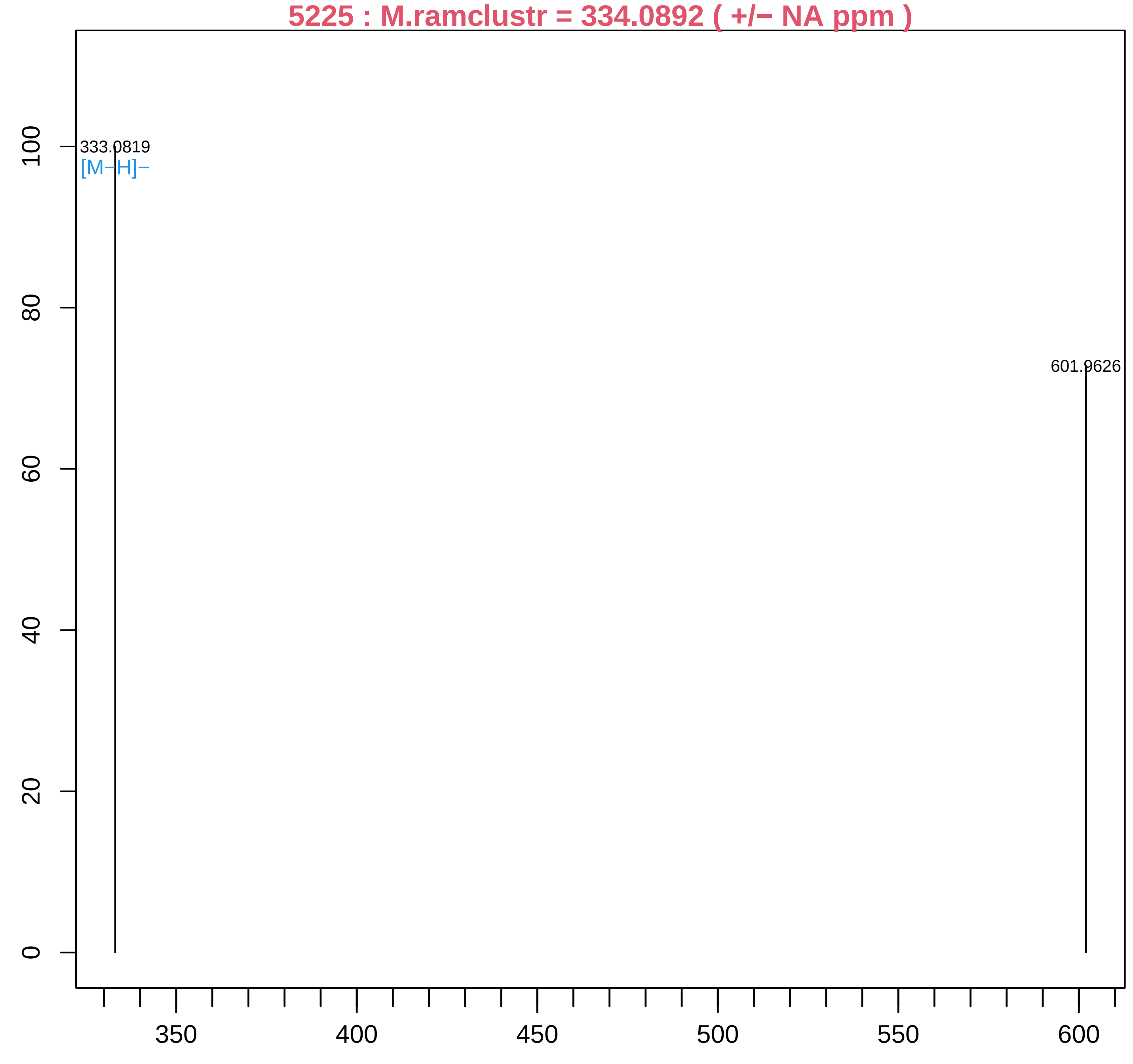
5225 : M.ramclustr = 334.0892 (+/− NA ppm)
333.0819
[M−H]−
601.9626
0
20
40
60
80
100
350
400
450
500
550
600

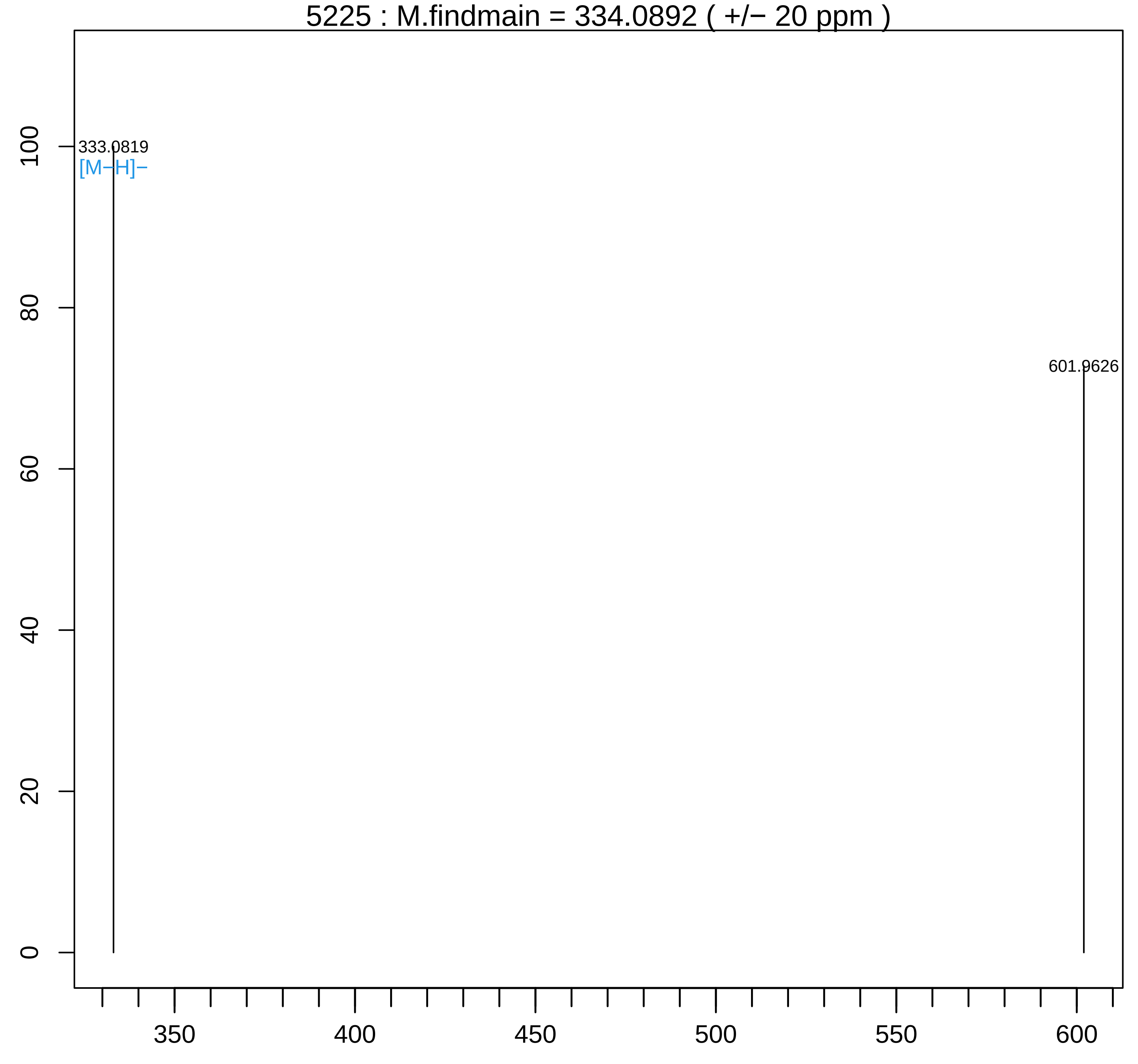
5225 : M.findmain = 334.0892 (+/− 20 ppm)
333.0819
[M−H]−
601.9626
0
20
40
60
80
100
350
400
450
500
550
600

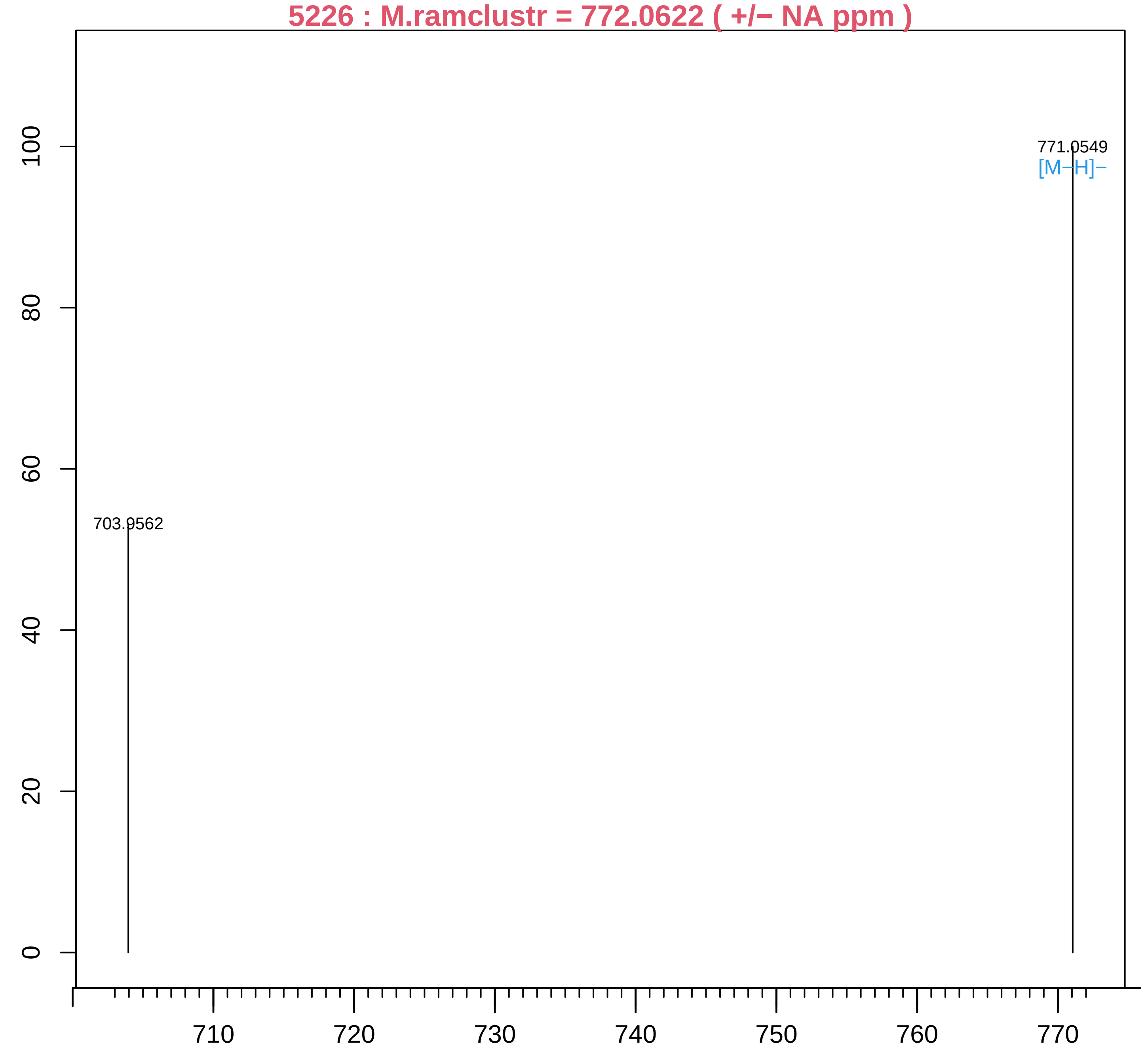
5226 : M.ramclustr = 772.0622 (+/− NA ppm)
771.0549
[M−H]−
703.9562
0
20
40
60
80
100
710
720
730
740
750
760
770

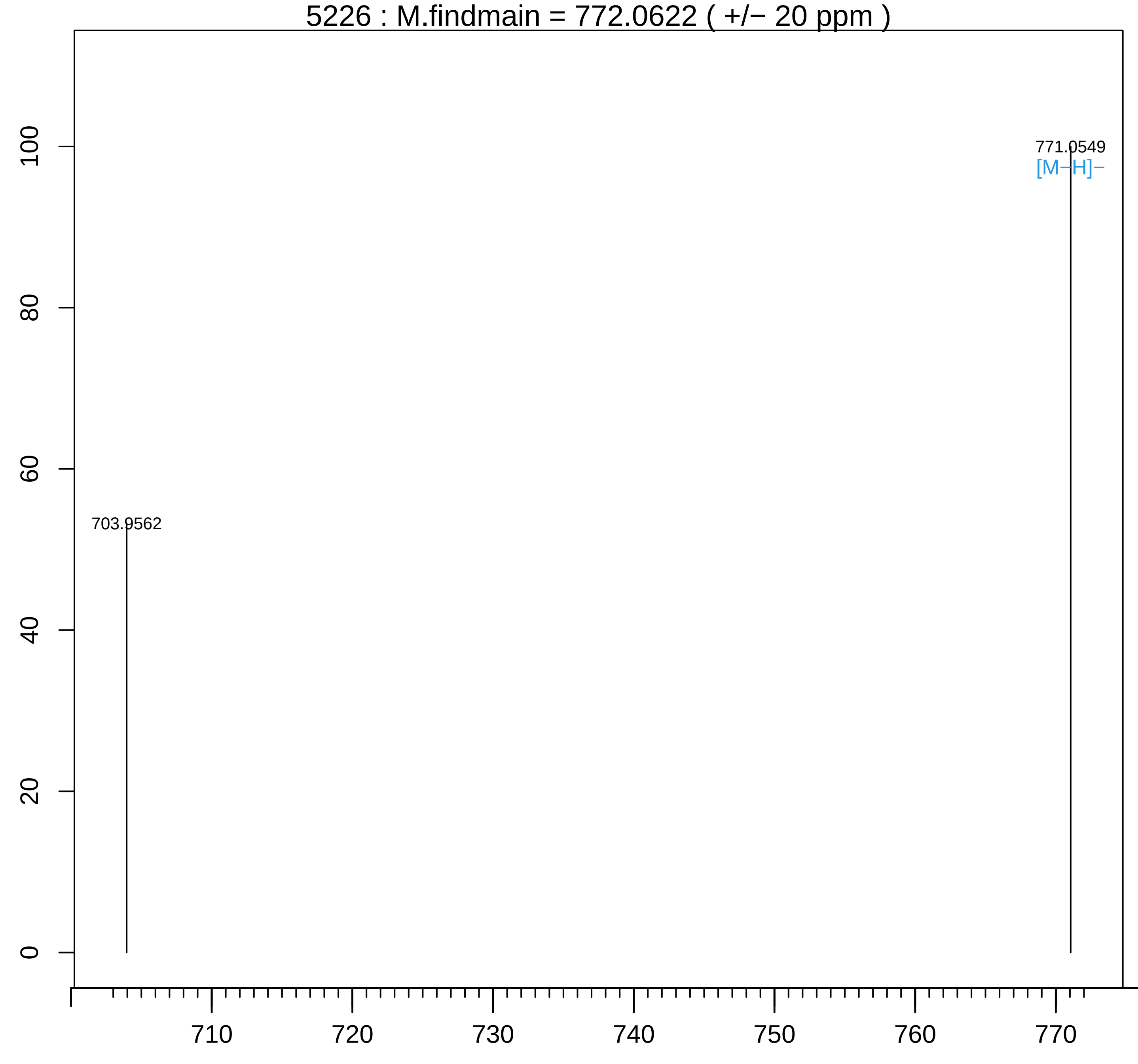
5226 : M.findmain = 772.0622 (+/− 20 ppm)
771.0549
[M−H]−
703.9562
0
20
40
60
80
100
710
720
730
740
750
760
770

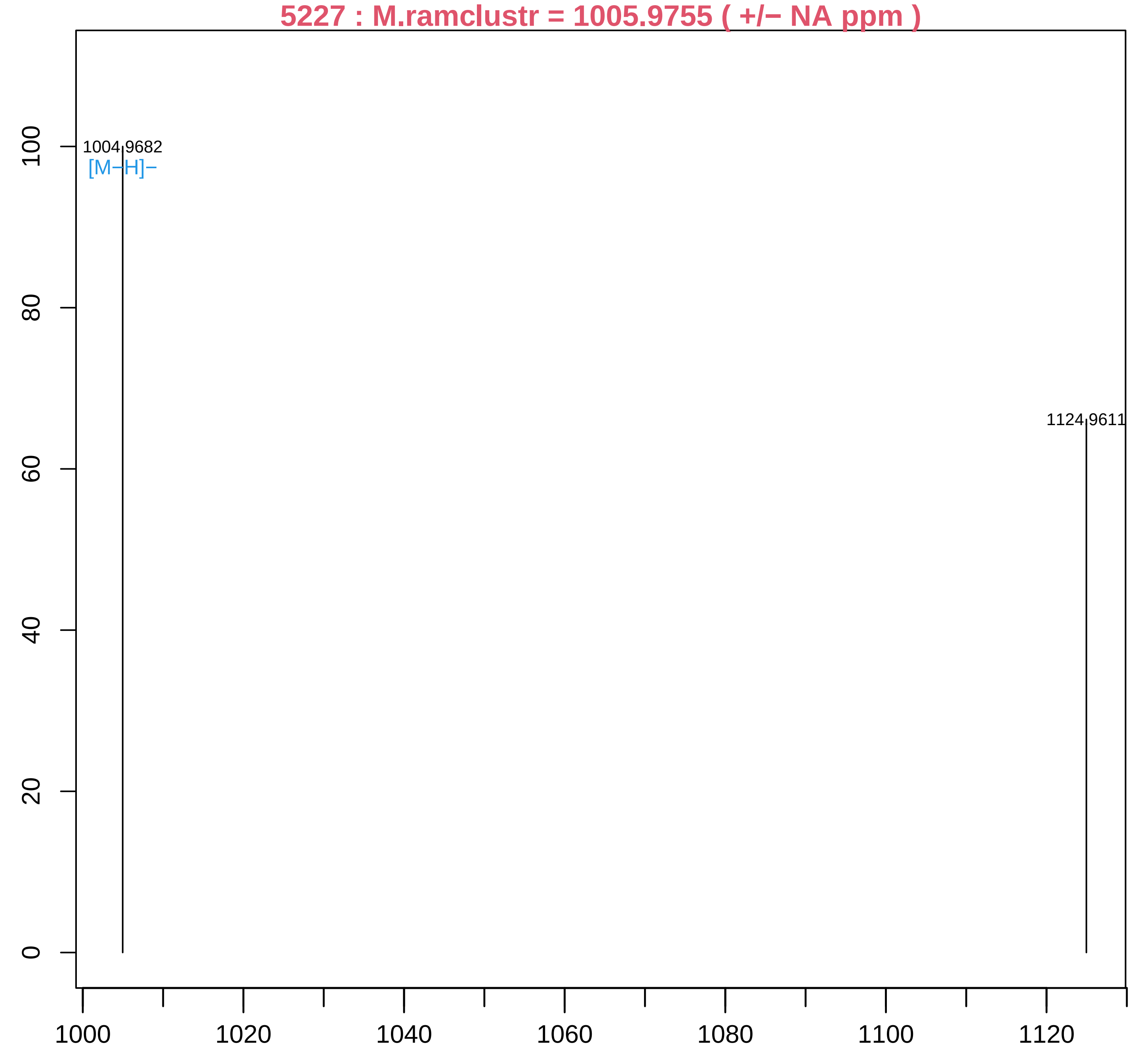
5227 : M.ramclustr = 1005.9755 (+/− NA ppm)
1004.9682
[M−H]−
1124.9611
0
20
40
60
80
100
1000
1020
1040
1060
1080
1100
1120

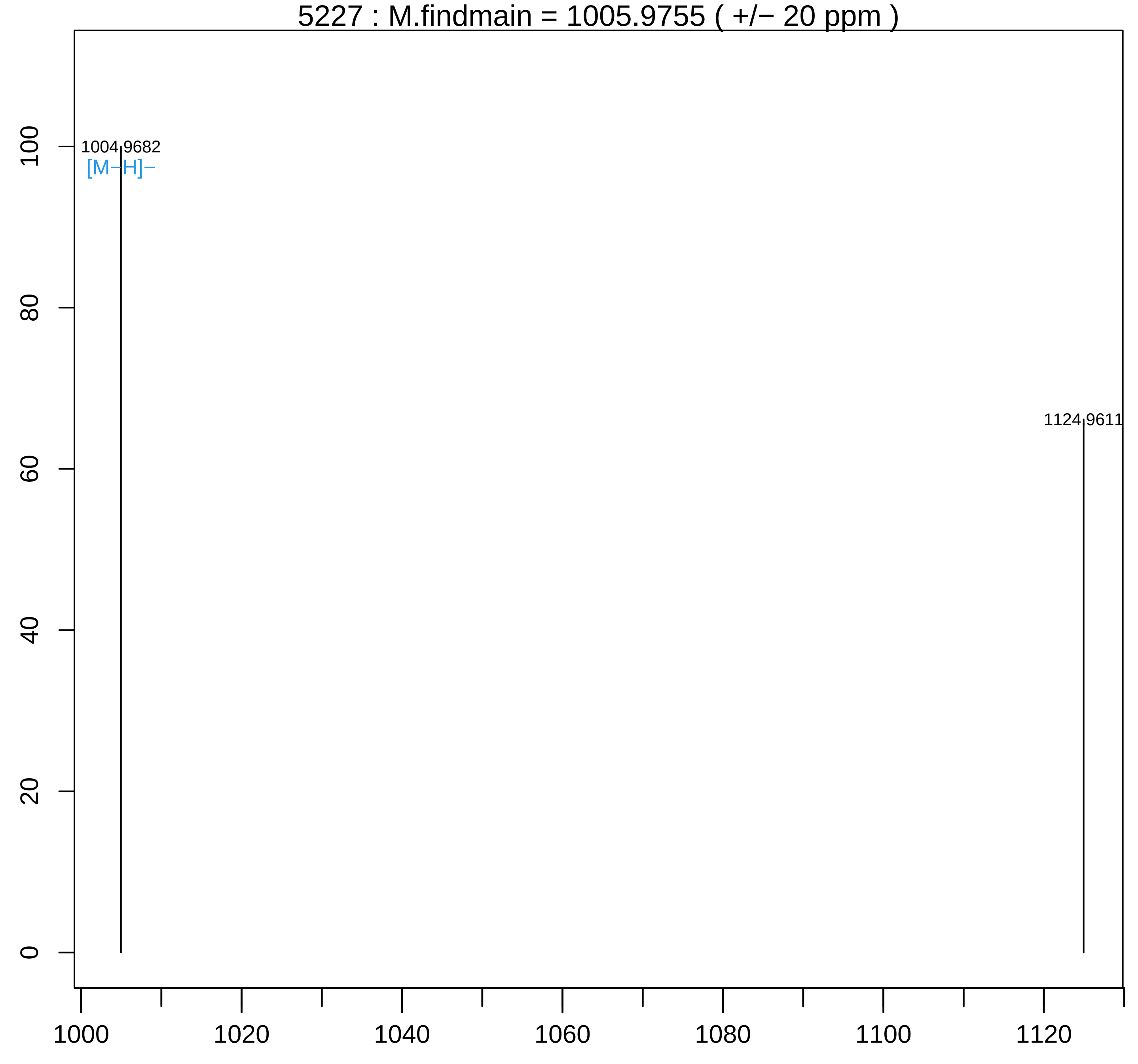
5227 : M.findmain = 1005.9755 (+/− 20 ppm)
1004.9682
[M−H]−
1124.9611
0
20
40
60
80
100
1000
1020
1040
1060
1080
1100
1120

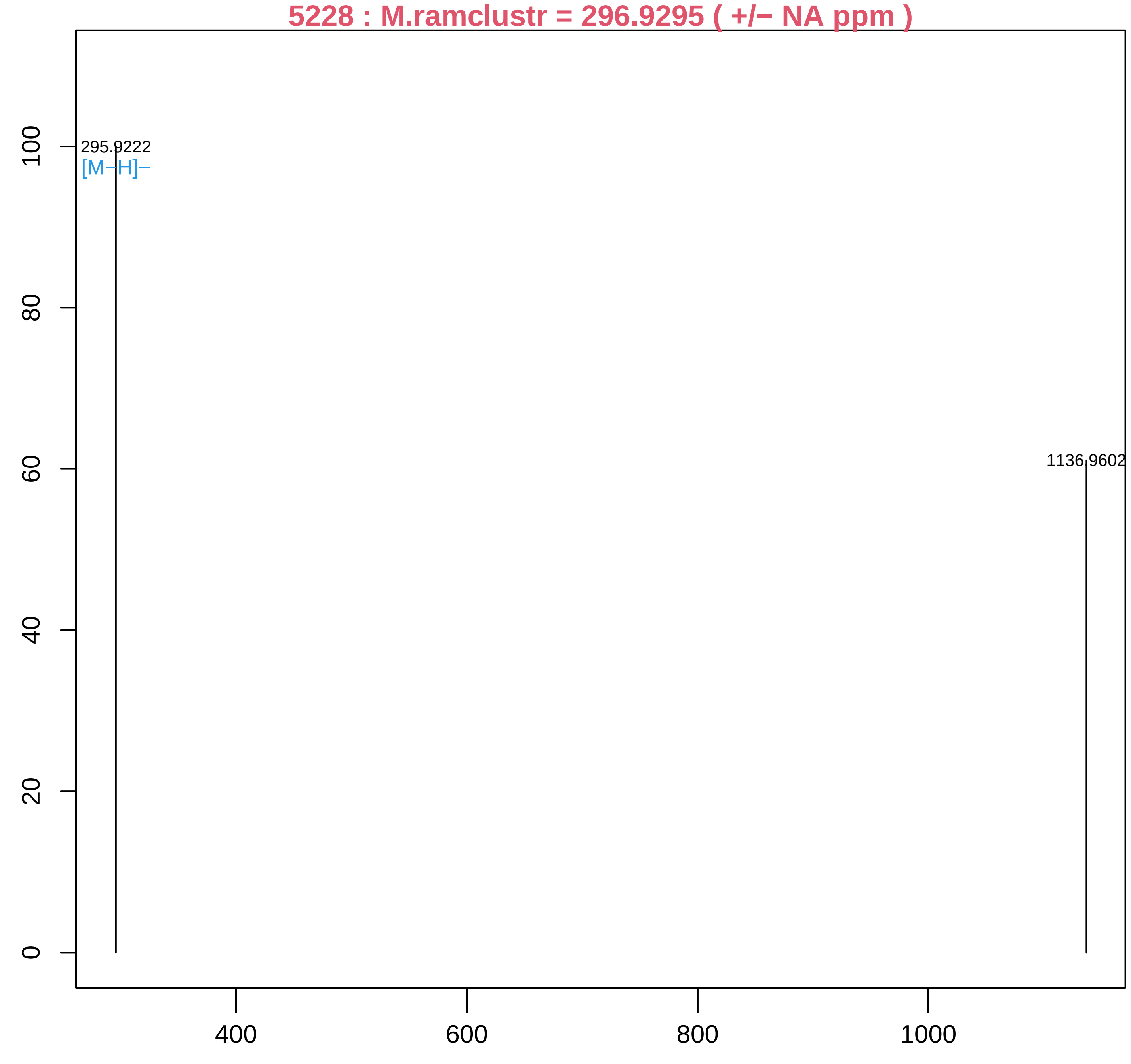
5228 : M.ramclustr = 296.9295 (+/− NA ppm)
295.9222
[M−H]−
1136.9602
0
20
40
60
80
100
400
600
800
1000

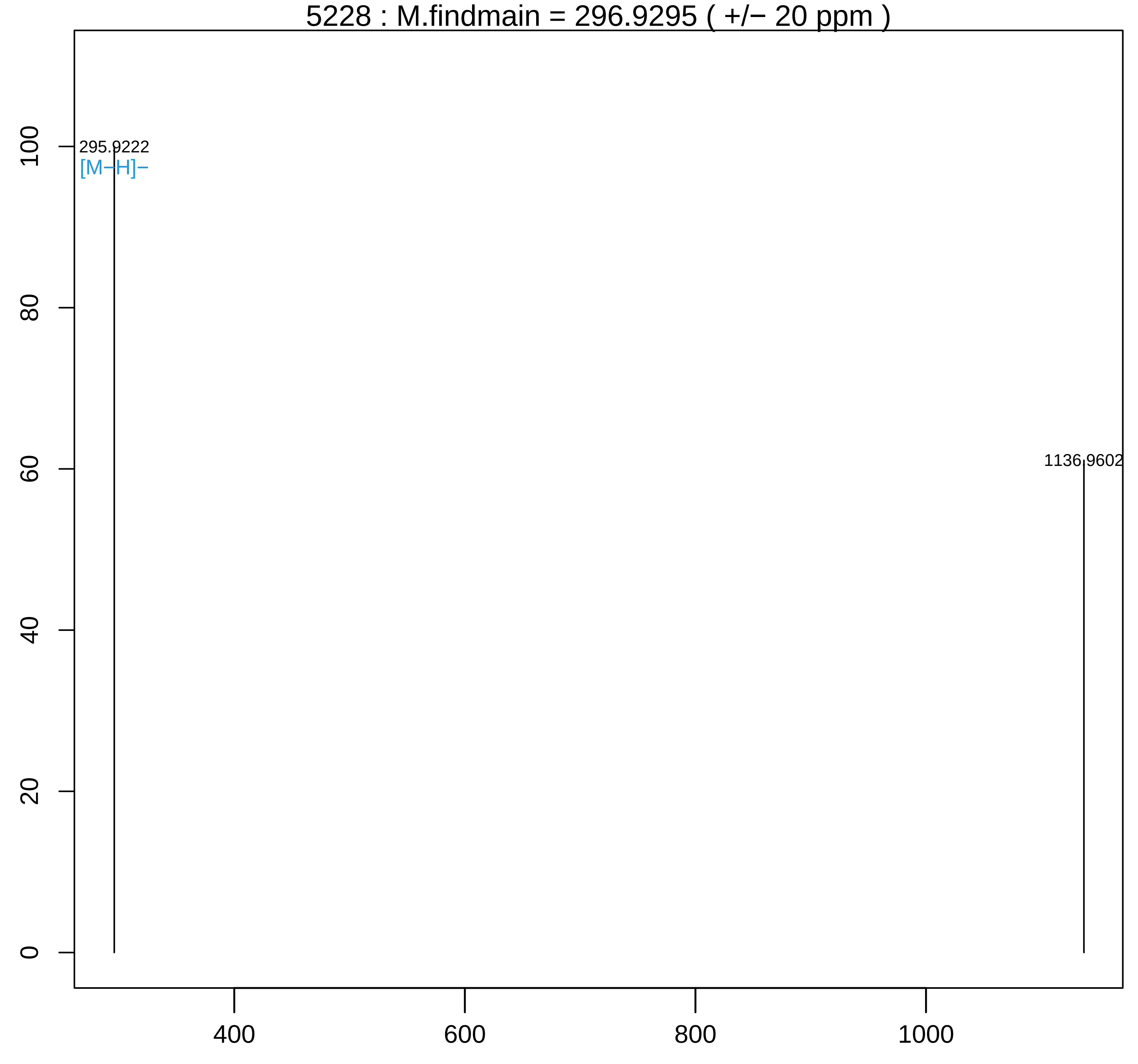
5228 : M.findmain = 296.9295 (+/− 20 ppm)
295.9222
[M−H]−
1136.9602
0
20
40
60
80
100
400
600
800
1000

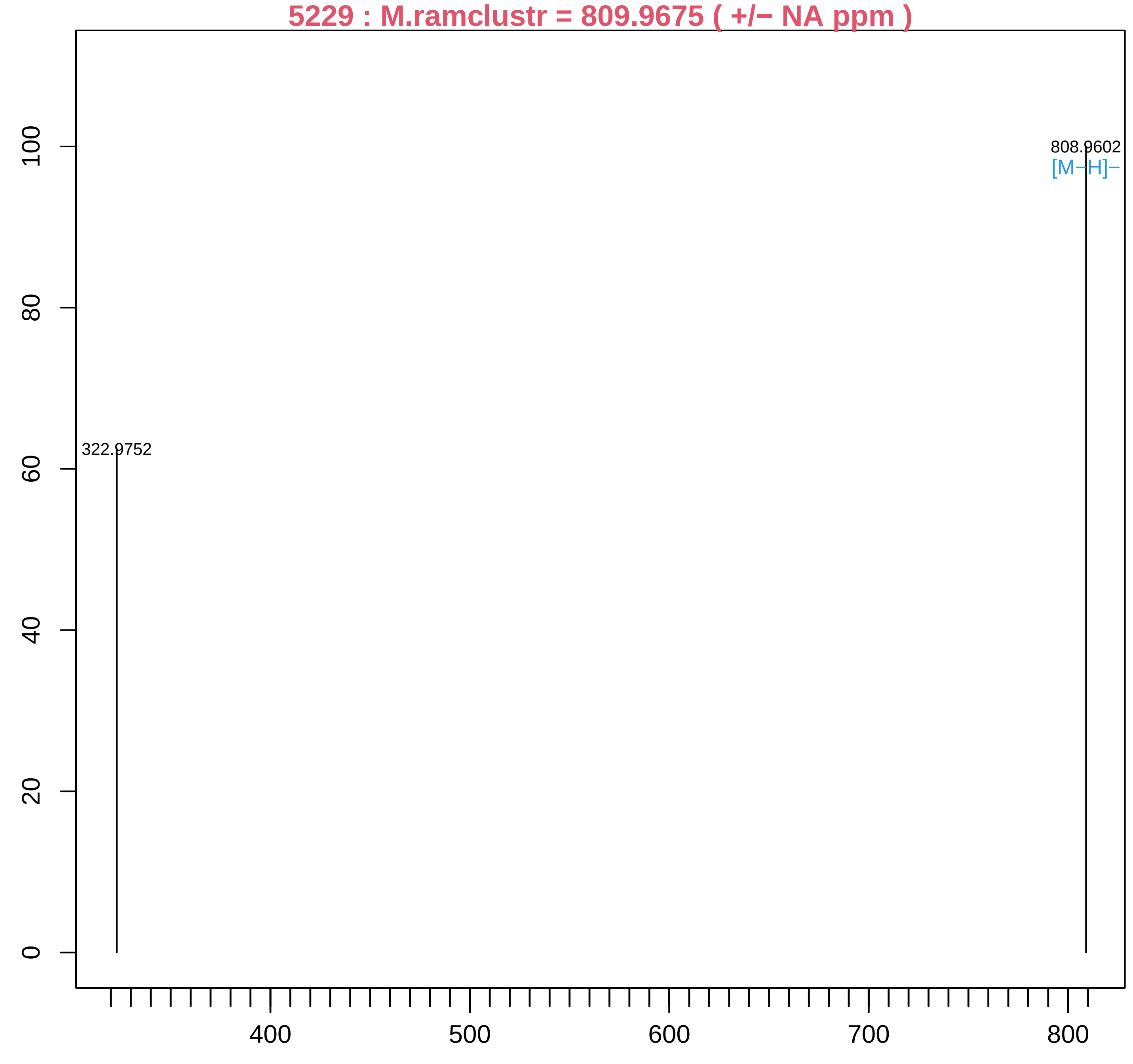
5229 : M.ramclustr = 809.9675 (+/− NA ppm)
808.9602
[M−H]−
322.9752
0
20
40
60
80
100
400
500
600
700
800

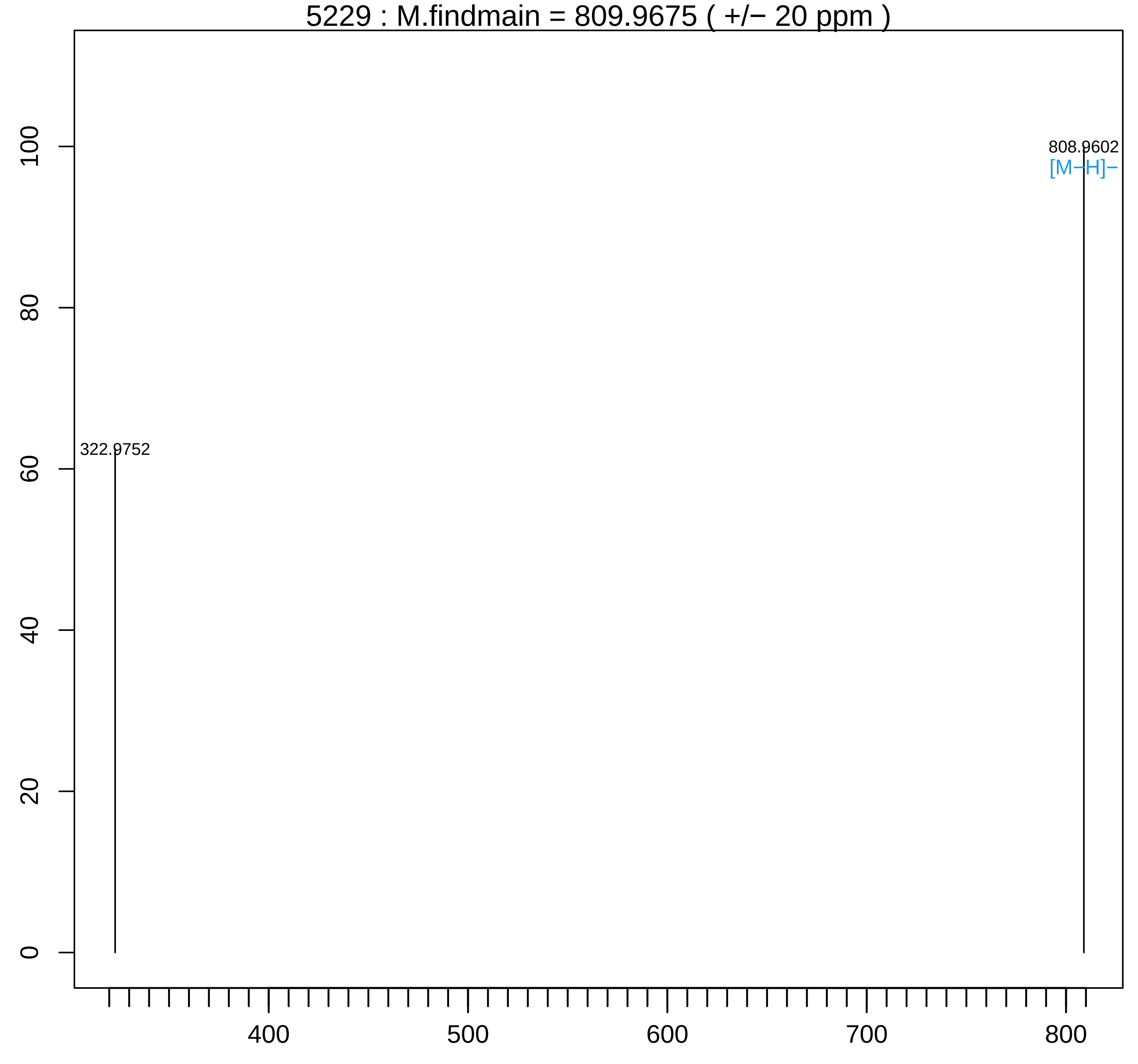
5229 : M.findmain = 809.9675 (+/− 20 ppm)
808.9602
[M−H]−
322.9752
0
20
40
60
80
100
400
500
600
700
800

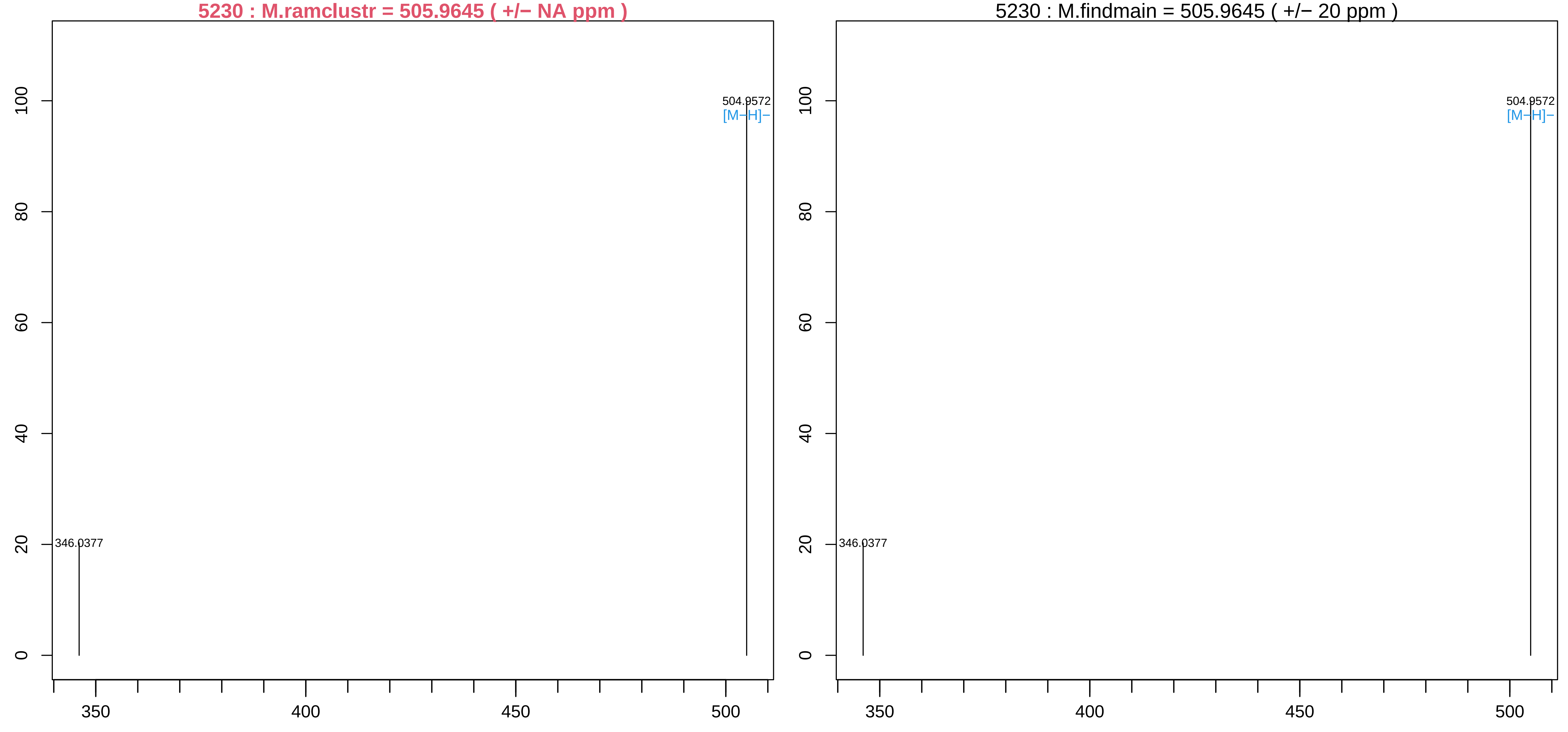
5230 : M.ramclustr = 505.9645 (+/− NA ppm)
504.9572
[M−H]−
346.0377
0
20
40
60
80
100
350
400
450
500
5230 : M.findmain = 505.9645 (+/− 20 ppm)
504.9572
[M−H]−
346.0377
0
20
40
60
80
100
350
400
450
500

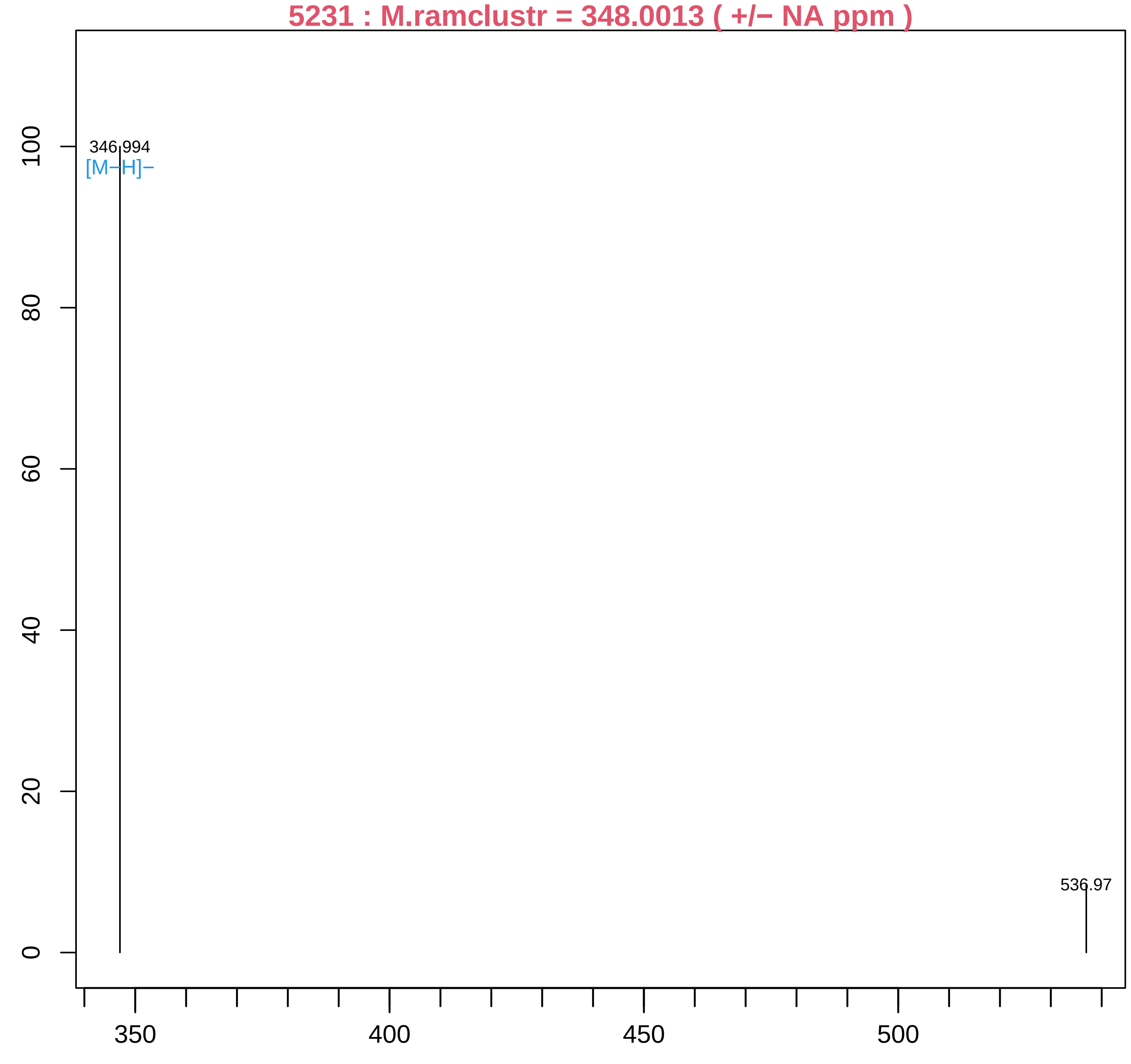
5231 : M.ramclustr = 348.0013 (+/− NA ppm)
346.994
[M−H]−
536.97
0
20
40
60
80
100
350
400
450
500

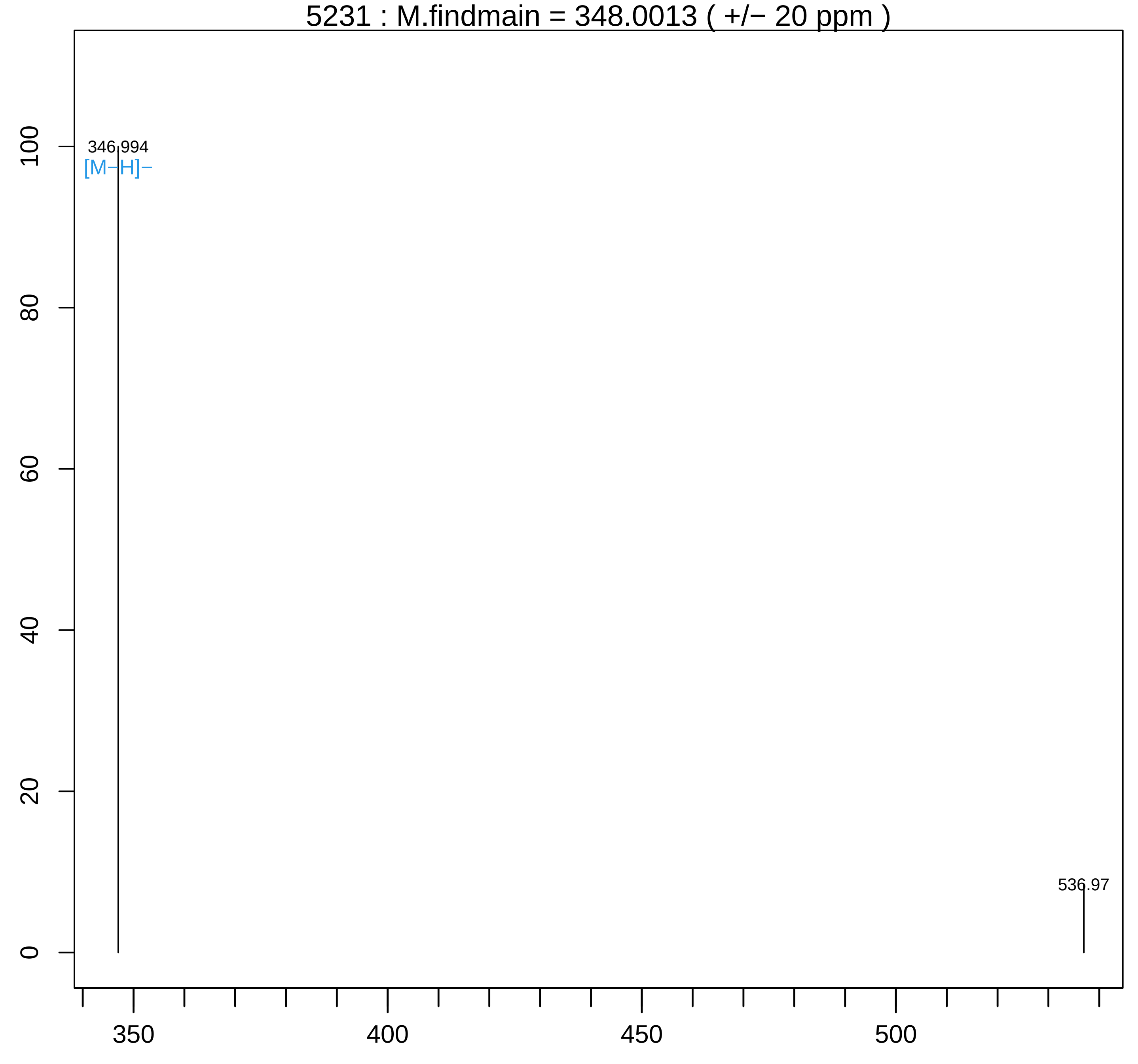
5231 : M.findmain = 348.0013 (+/− 20 ppm)
346.994
[M−H]−
536.97
0
20
40
60
80
100
350
400
450
500

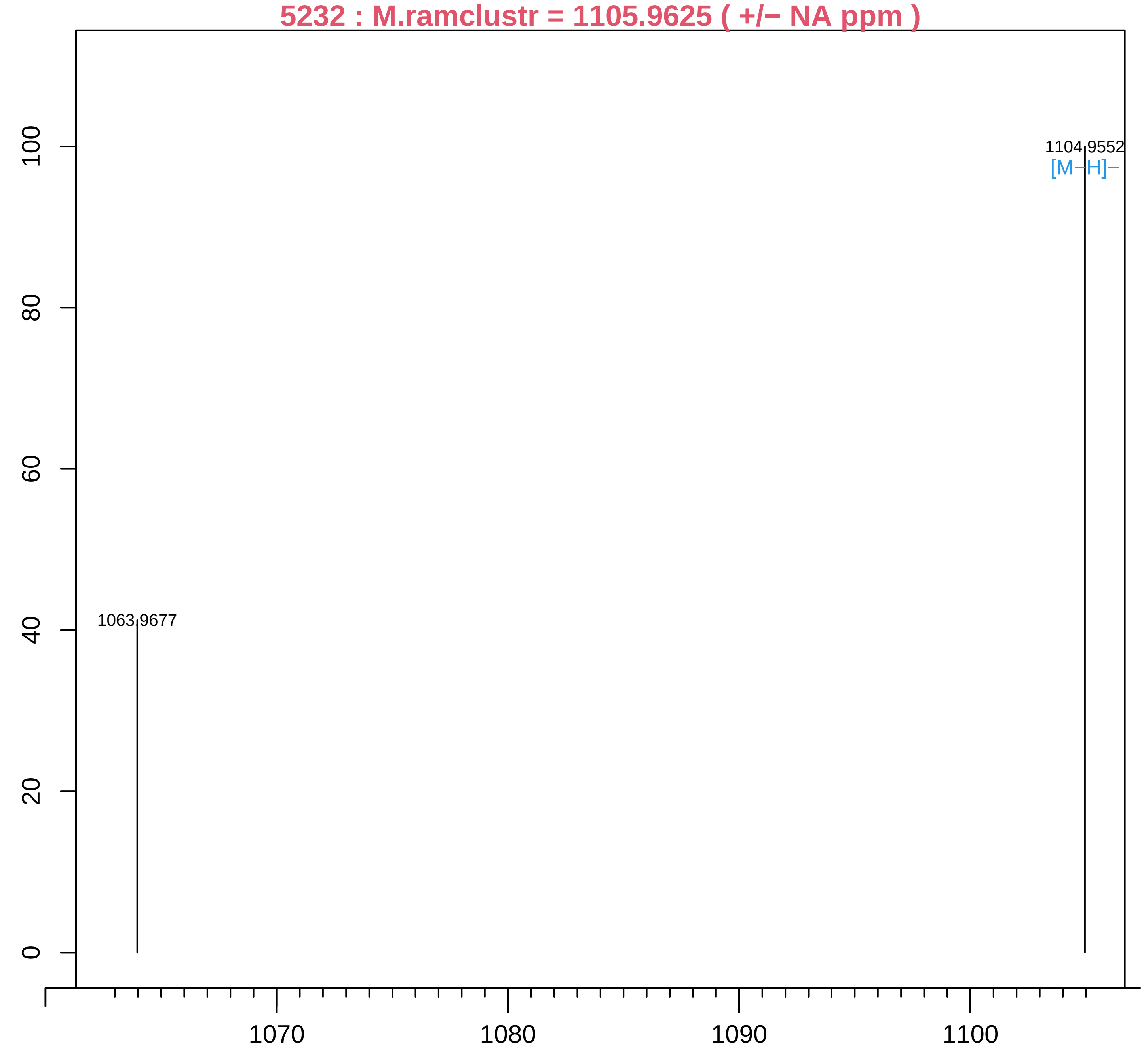
5232 : M.ramclustr = 1105.9625 (+/− NA ppm)
1104.9552
[M−H]−
1063.9677
0
20
40
60
80
100
1070
1080
1090
1100

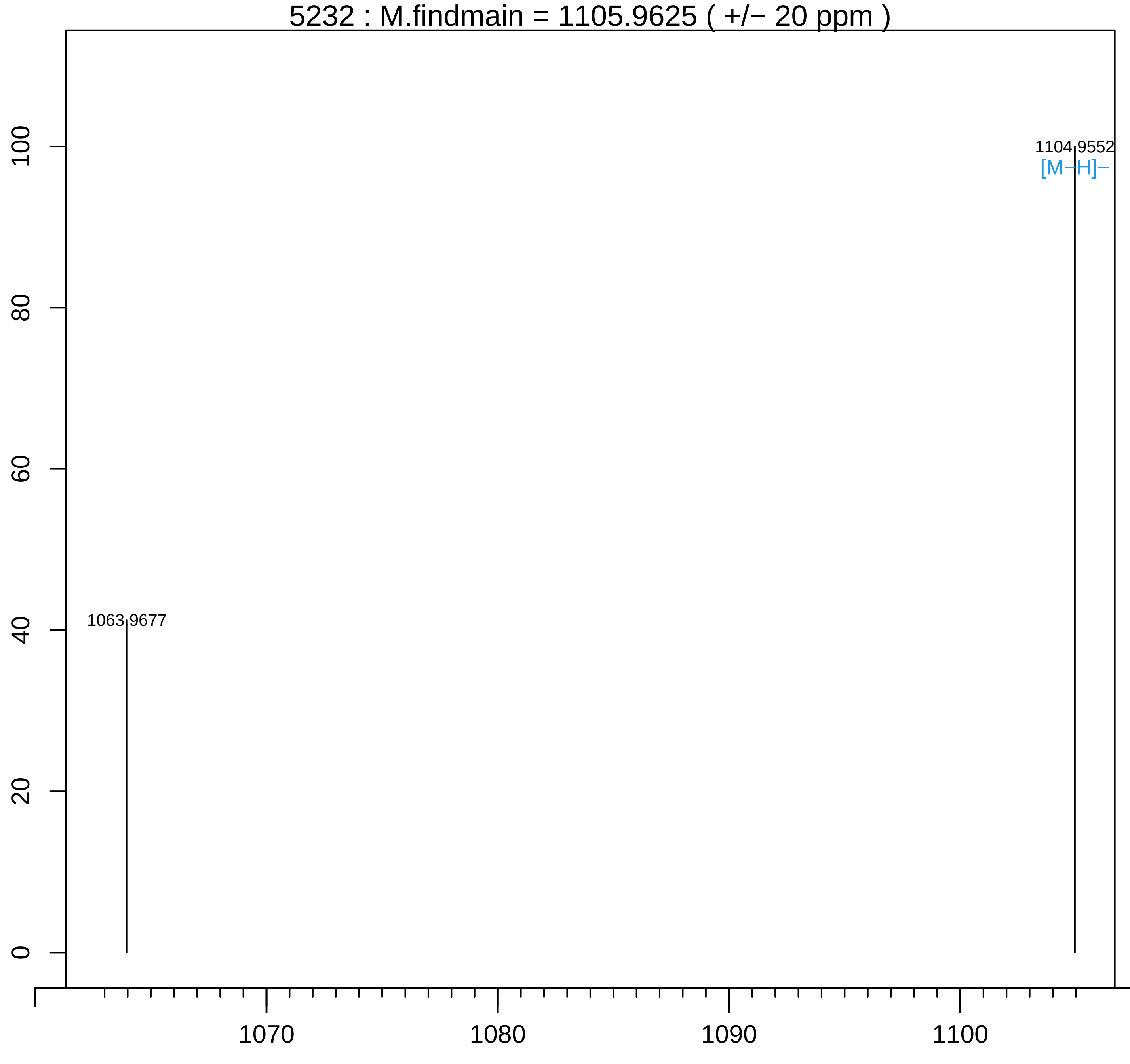
5232 : M.findmain = 1105.9625 (+/− 20 ppm)
1104.9552
[M−H]−
1063.9677
0
20
40
60
80
100
1070
1080
1090
1100

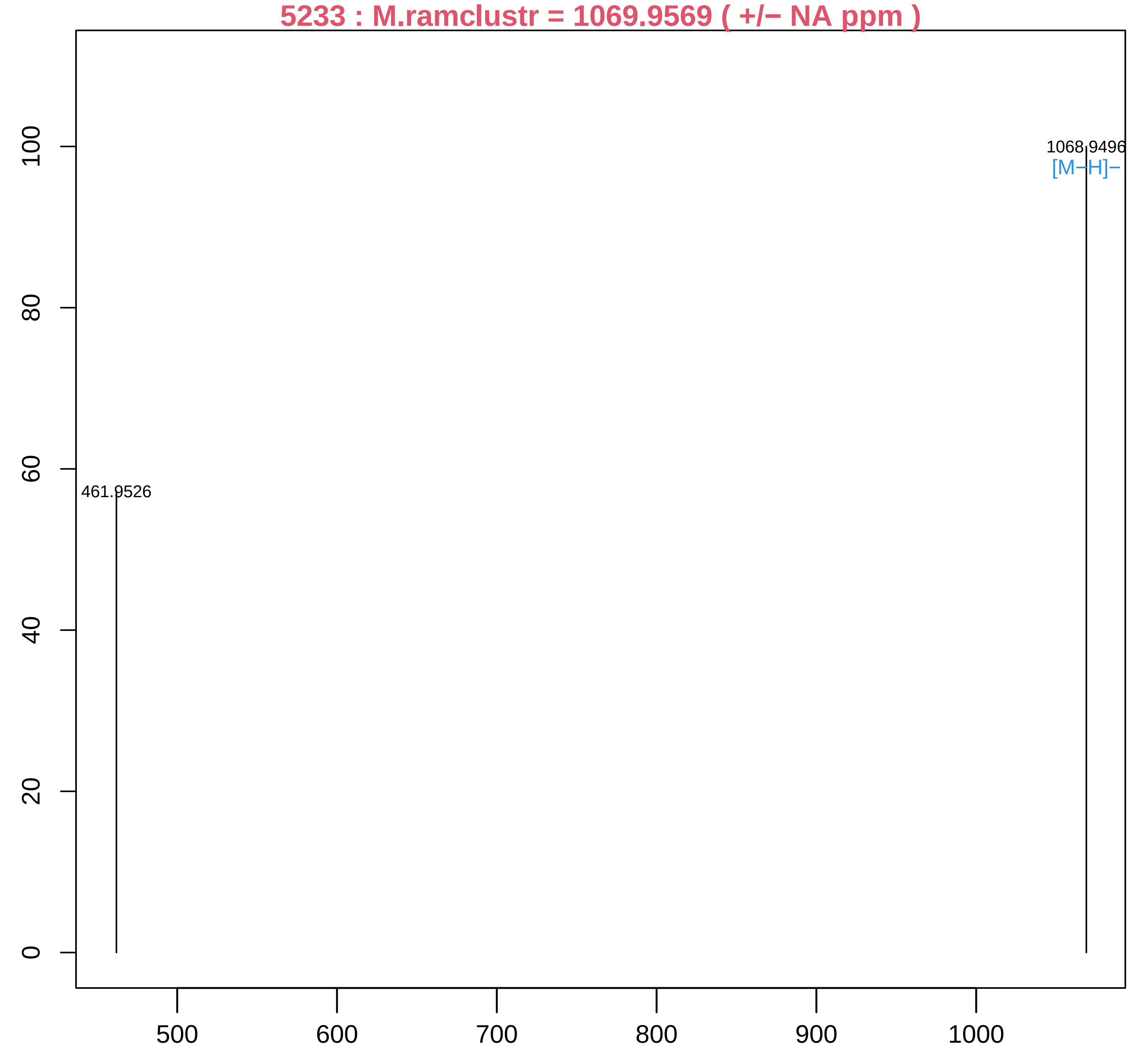
5233 : M.ramclustr = 1069.9569 (+/− NA ppm)
1068.9496
[M−H]−
461.9526
0
20
40
60
80
100
500
600
700
800
900
1000

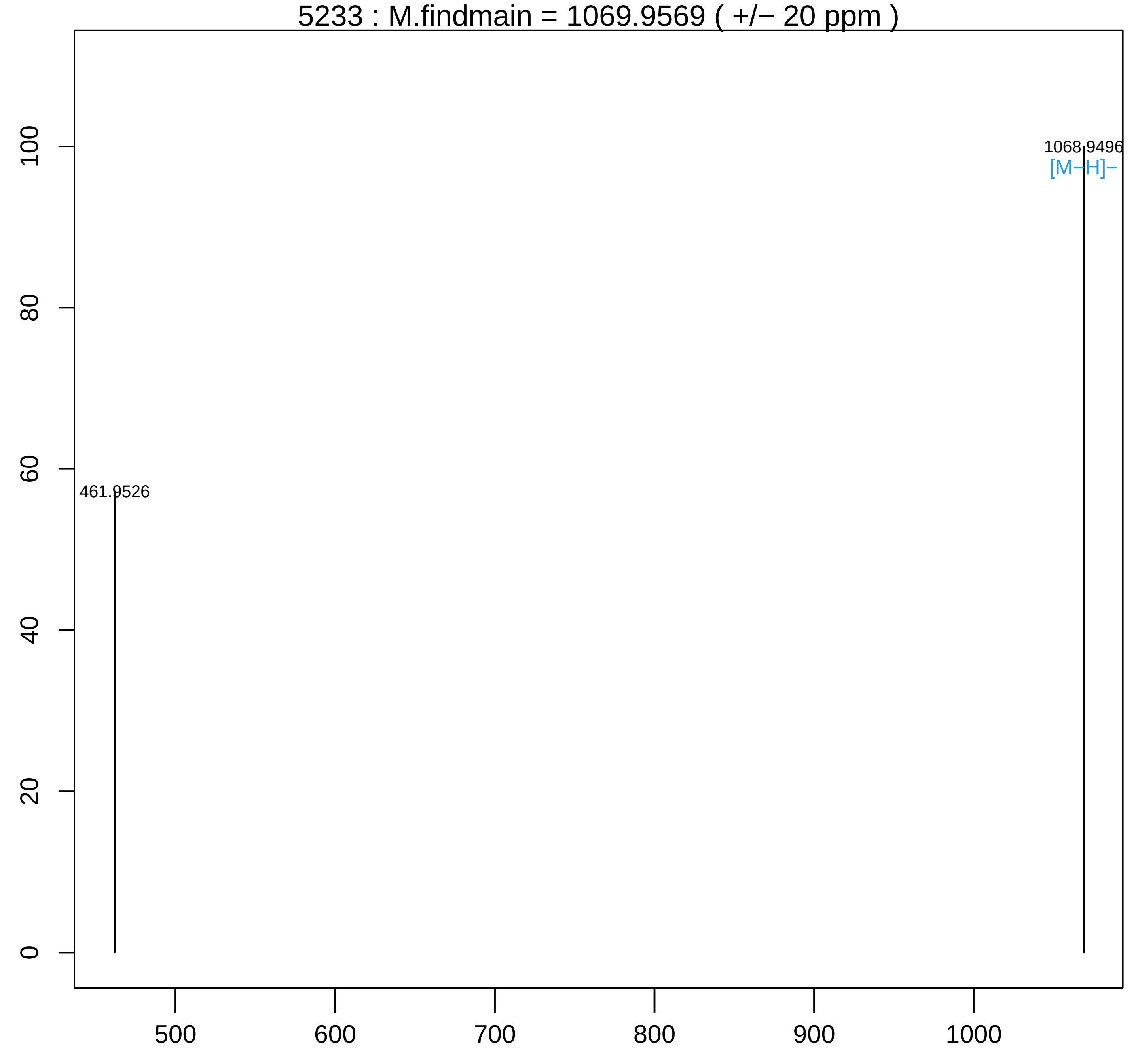
5233 : M.findmain = 1069.9569 (+/− 20 ppm)
1068.9496
[M−H]−
461.9526
0
20
40
60
80
100
500
600
700
800
900
1000

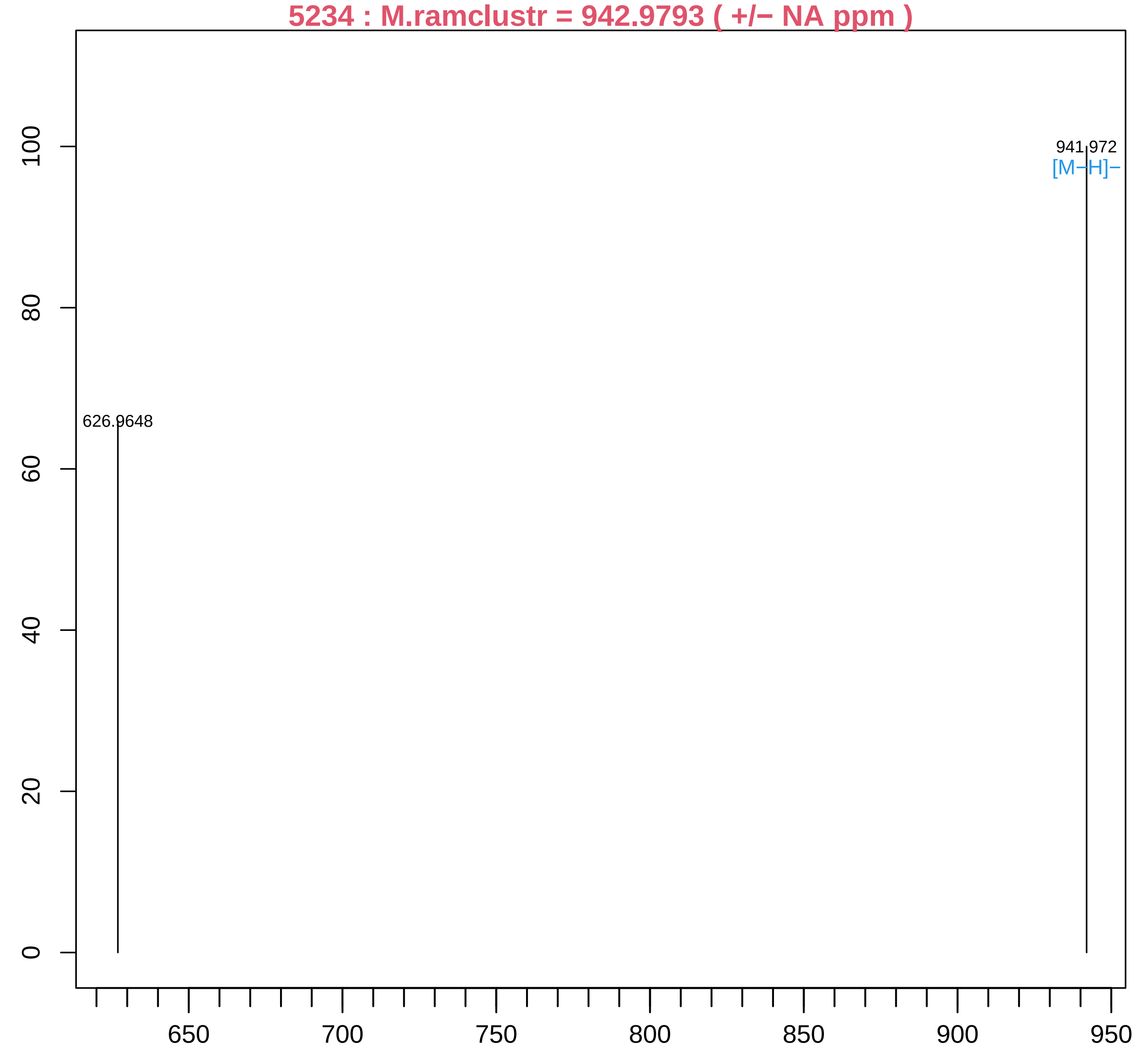
5234 : M.ramclustr = 942.9793 (+/− NA ppm)
941.972
[M−H]−
626.9648
0
20
40
60
80
100
650
700
750
800
850
900
950

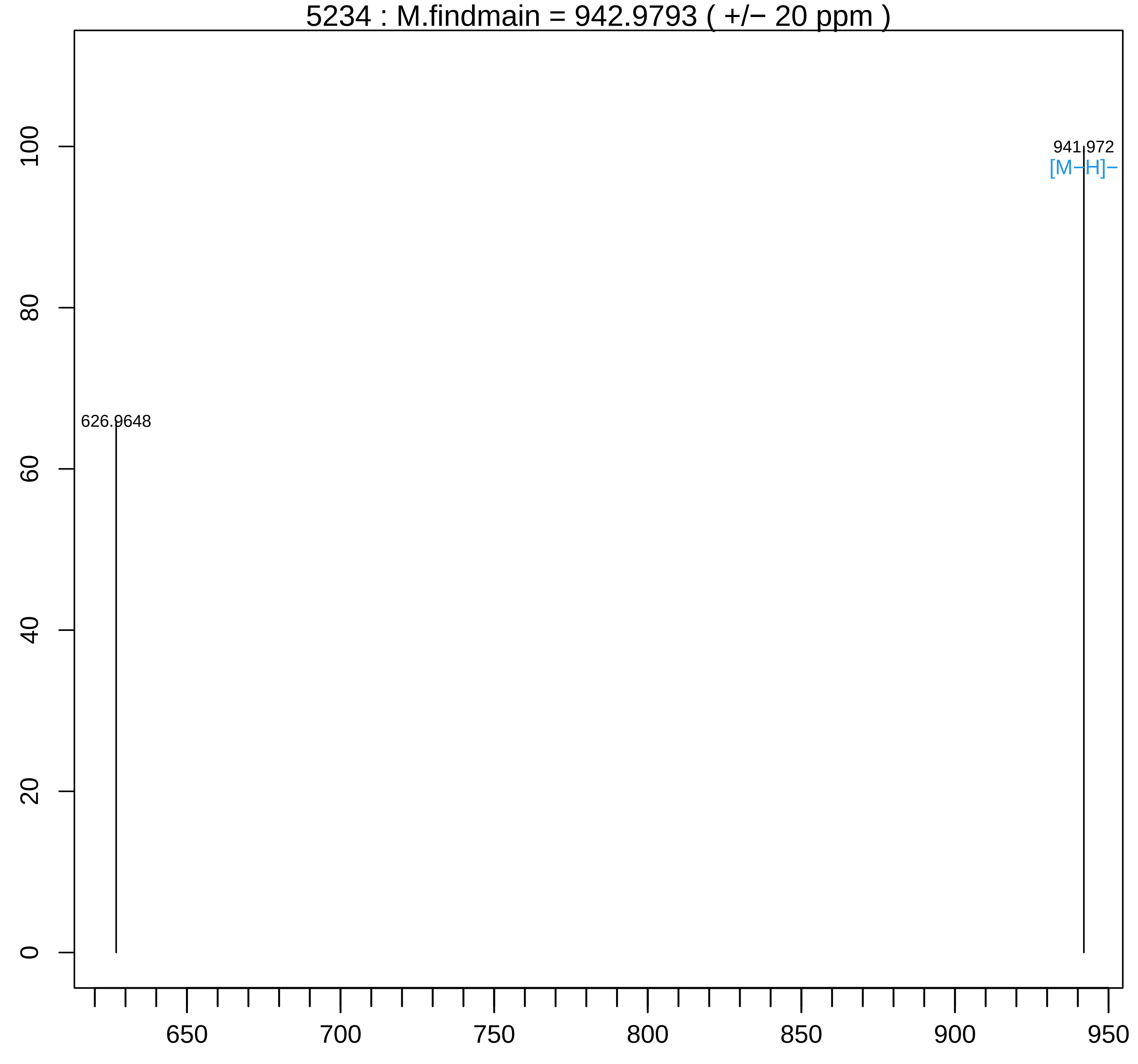
5234 : M.findmain = 942.9793 (+/− 20 ppm)
941.972
[M−H]−
626.9648
0
20
40
60
80
100
650
700
750
800
850
900
950

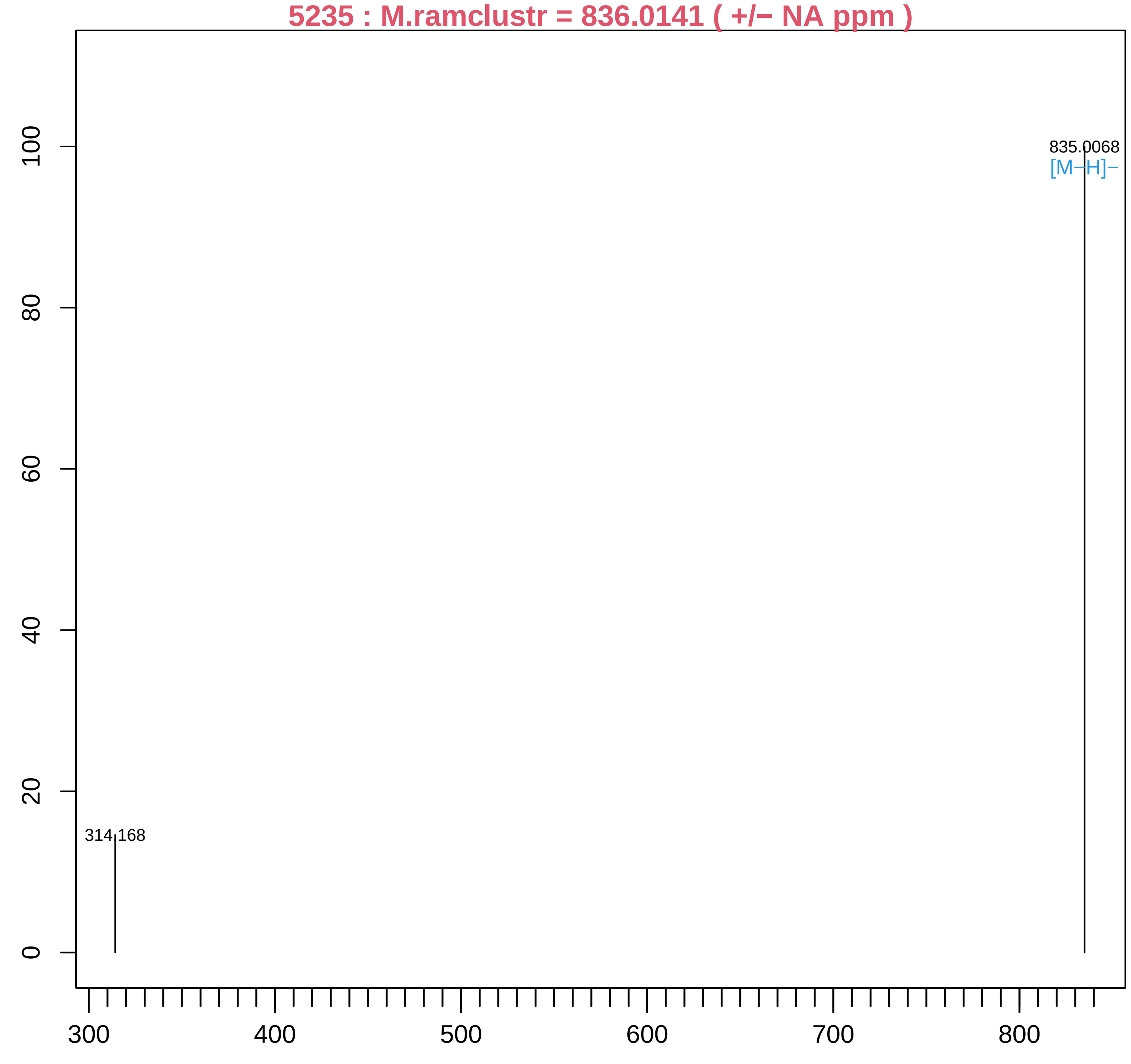
5235 : M.ramclustr = 836.0141 (+/− NA ppm)
835.0068
[M−H]−
314.168
0
20
40
60
80
100
300
400
500
600
700
800

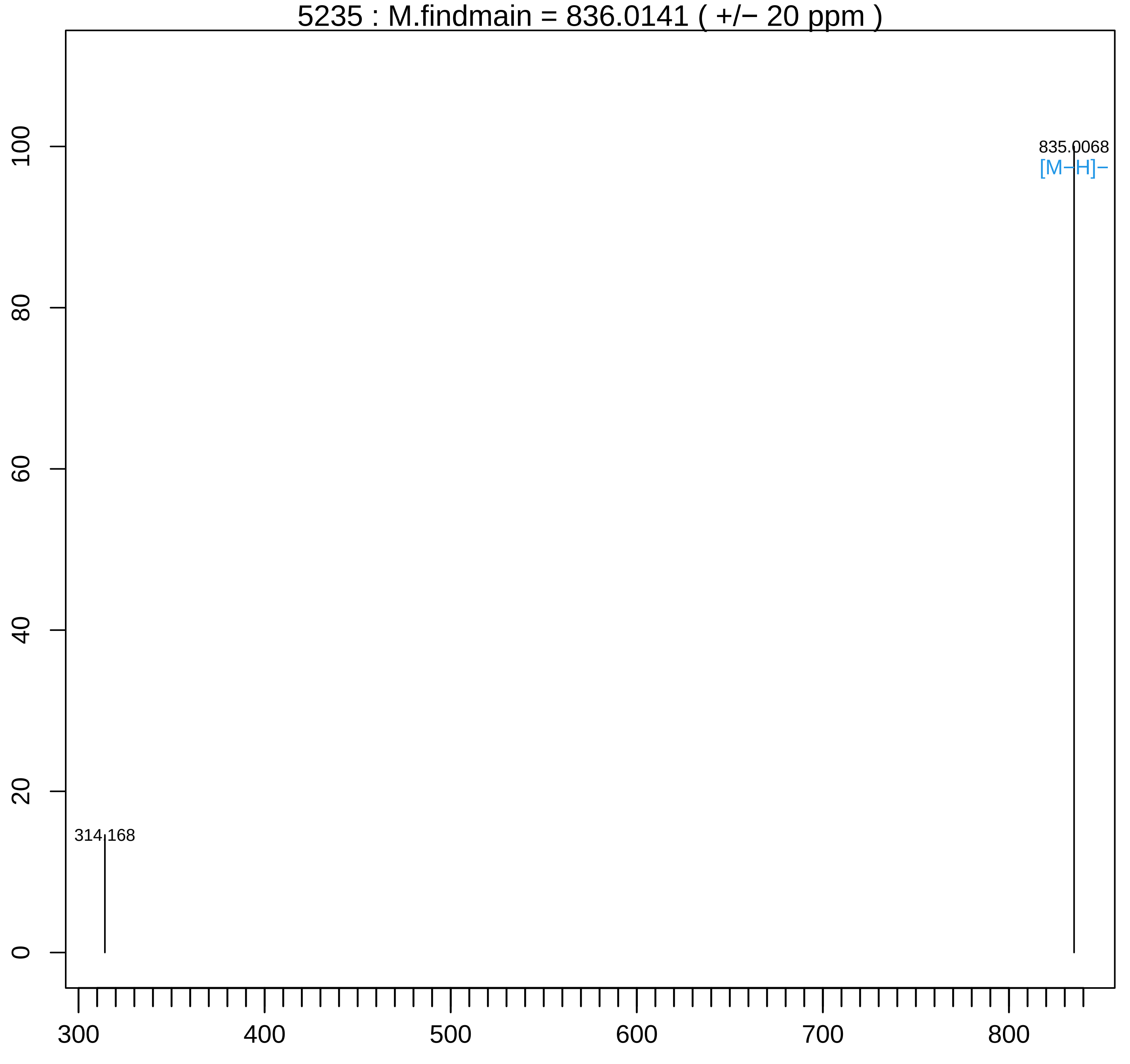
5235 : M.findmain = 836.0141 (+/− 20 ppm)
835.0068
[M−H]−
314.168
0
20
40
60
80
100
300
400
500
600
700
800

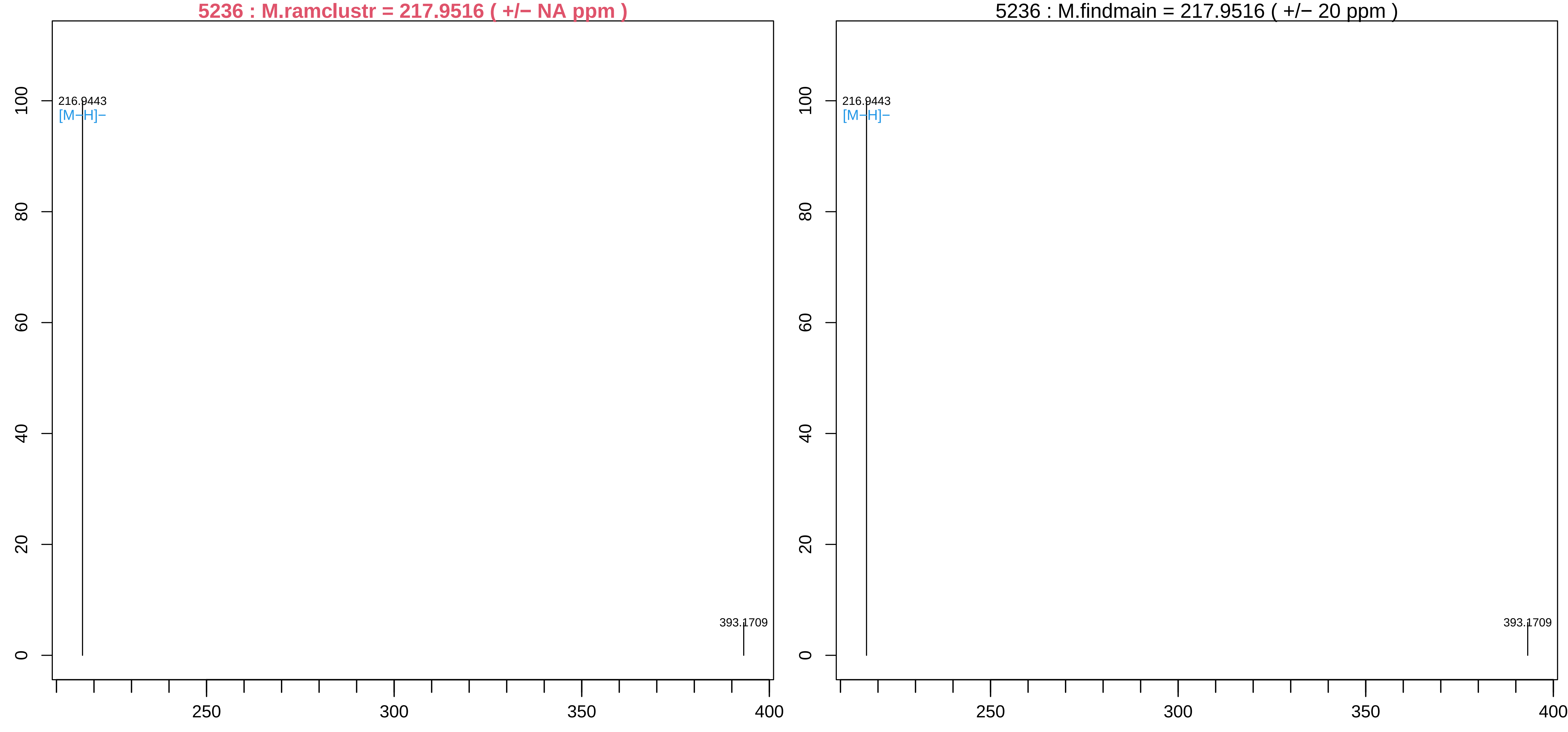

5236 : M.ramclustr = 217.9516 (+/− NA ppm)
216.9443
[M−H]−
393.1709
0
20
40
60
80
100
250
300
350
400
5236 : M.findmain = 217.9516 (+/− 20 ppm)
216.9443
[M−H]−
393.1709
0
20
40
60
80
100
250
300
350
400

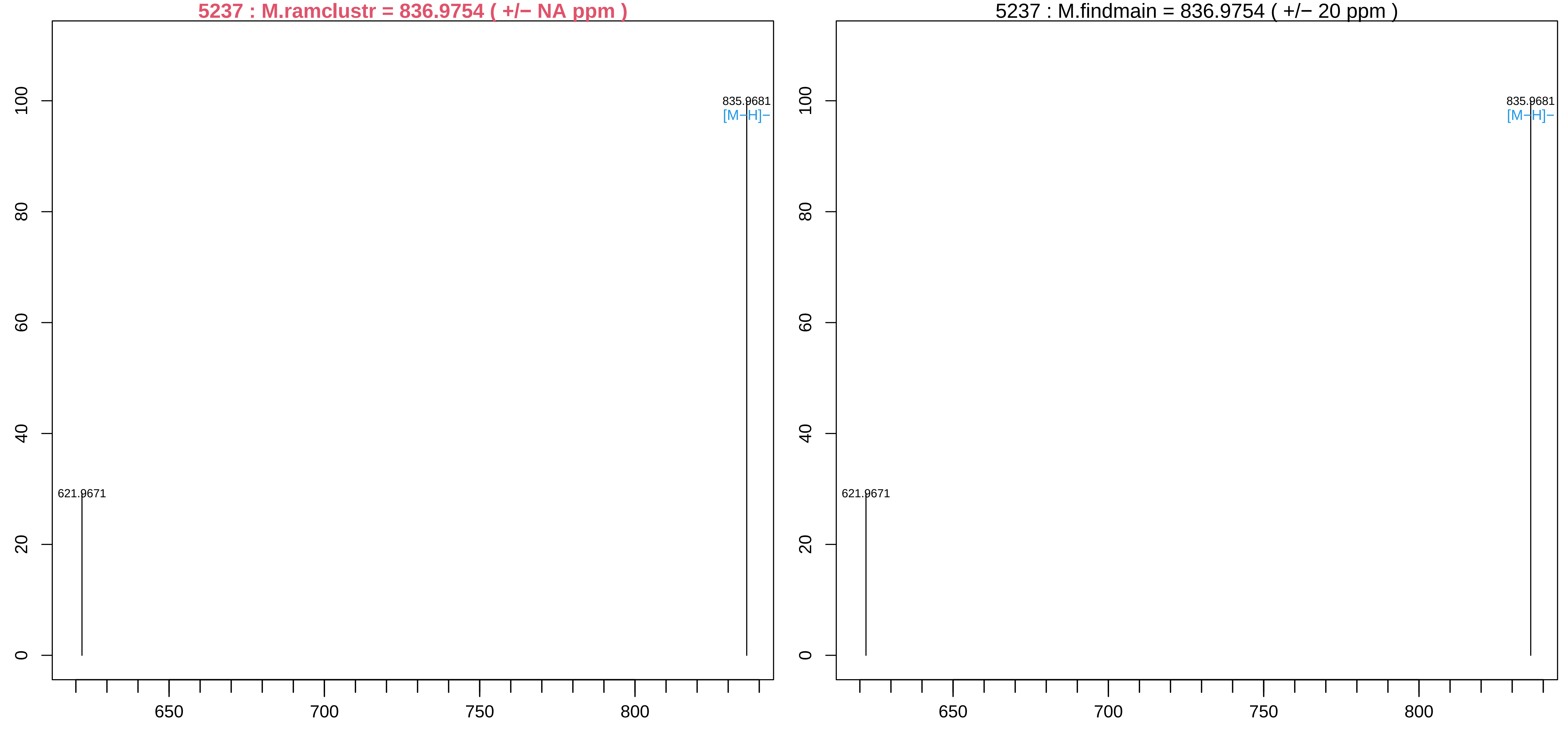
5237 : M.ramclustr = 836.9754 (+/− NA ppm)
835.9681
[M−H]−
621.9671
0
20
40
60
80
100
650
700
750
800
5237 : M.findmain = 836.9754 (+/− 20 ppm)
835.9681
[M−H]−
621.9671
0
20
40
60
80
100
650
700
750
800

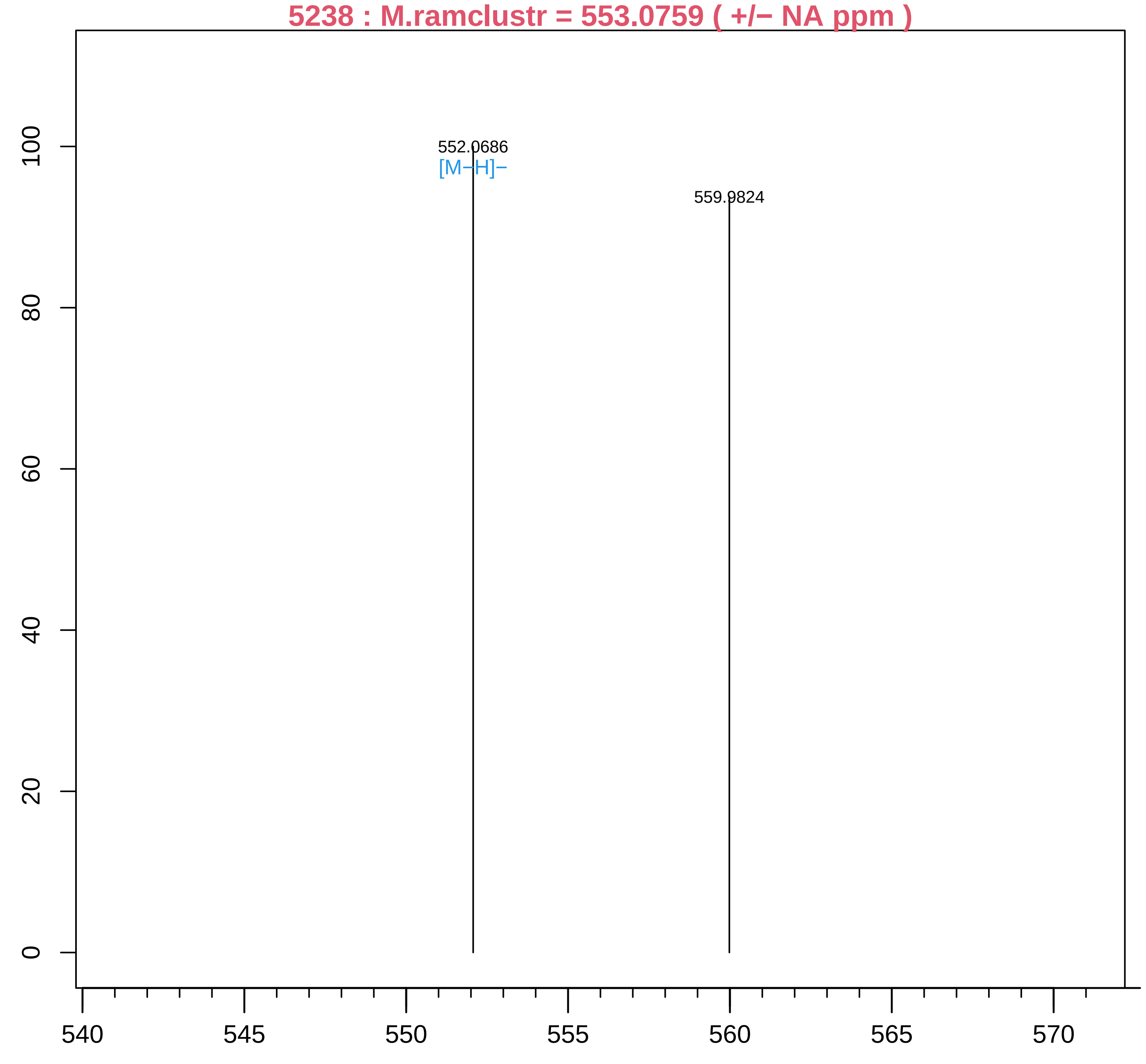
5238 : M.ramclustr = 553.0759 (+/− NA ppm)
552.0686
[M−H]−
559.9824
0
20
40
60
80
100
540
545
550
555
560
565
570

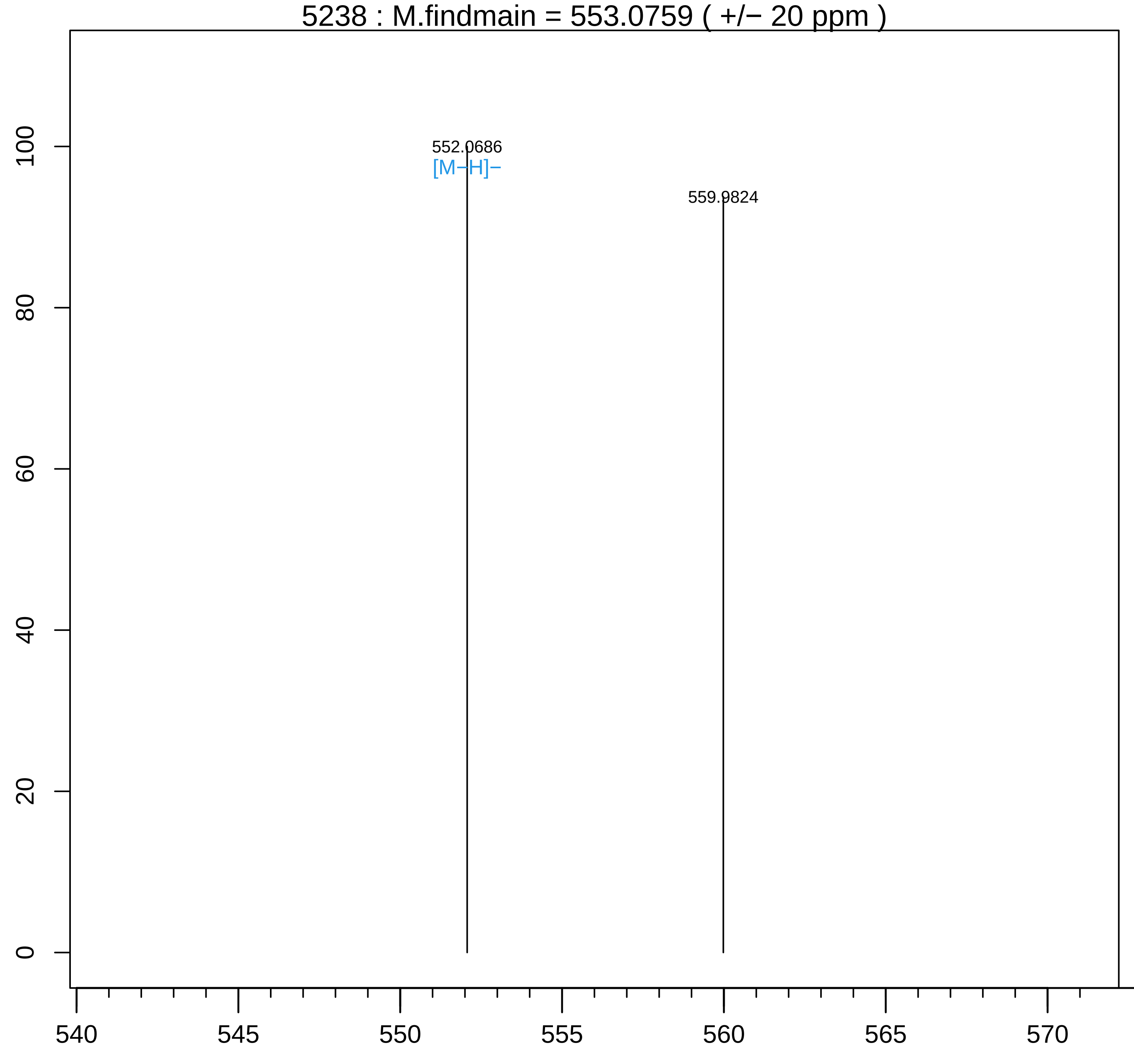
5238 : M.findmain = 553.0759 (+/− 20 ppm)
552.0686
[M−H]−
559.9824
0
20
40
60
80
100
540
545
550
555
560
565
570

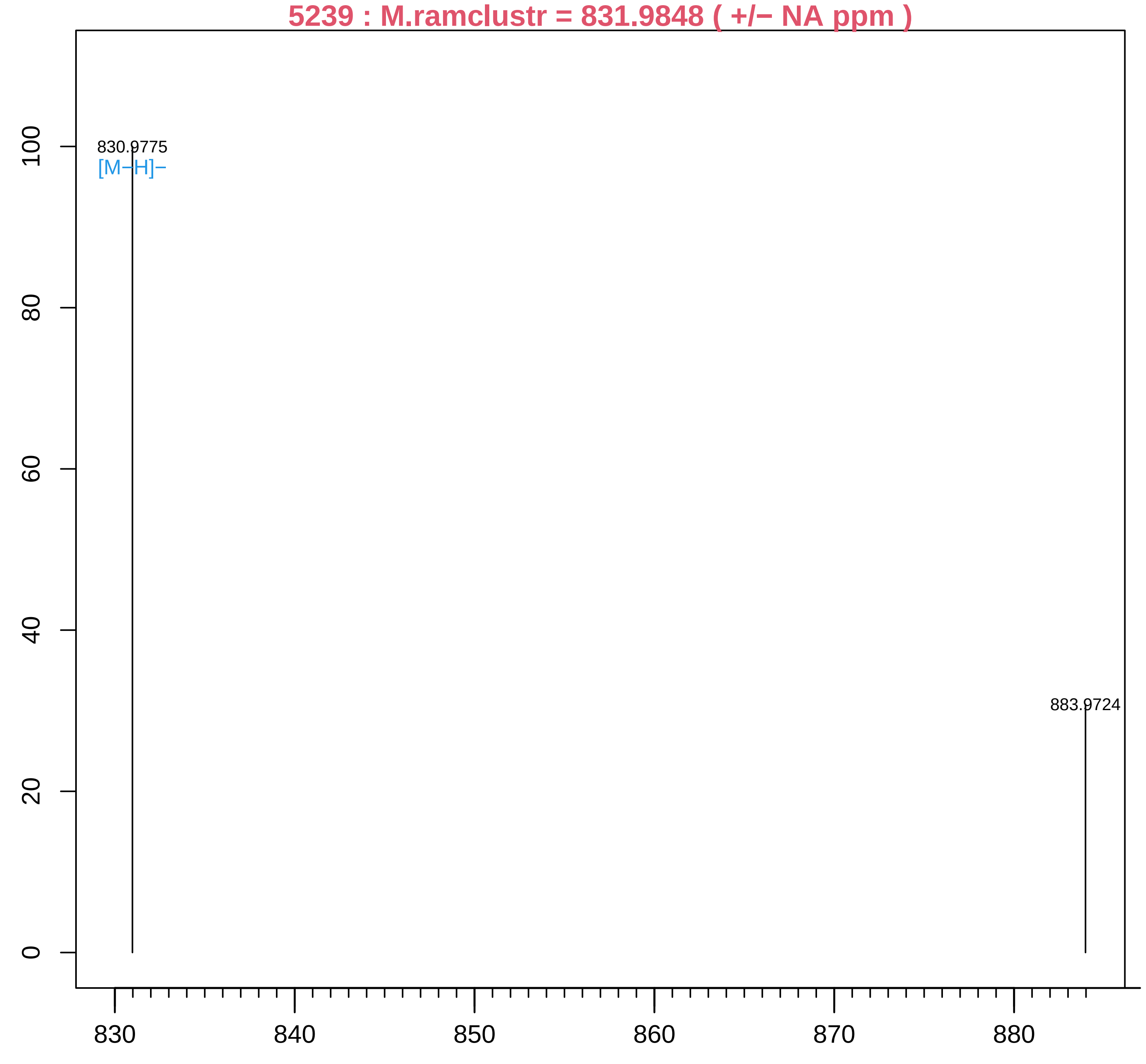
5239 : M.ramclustr = 831.9848 (+/− NA ppm)
830.9775
[M−H]−
883.9724
0
20
40
60
80
100
830
840
850
860
870
880

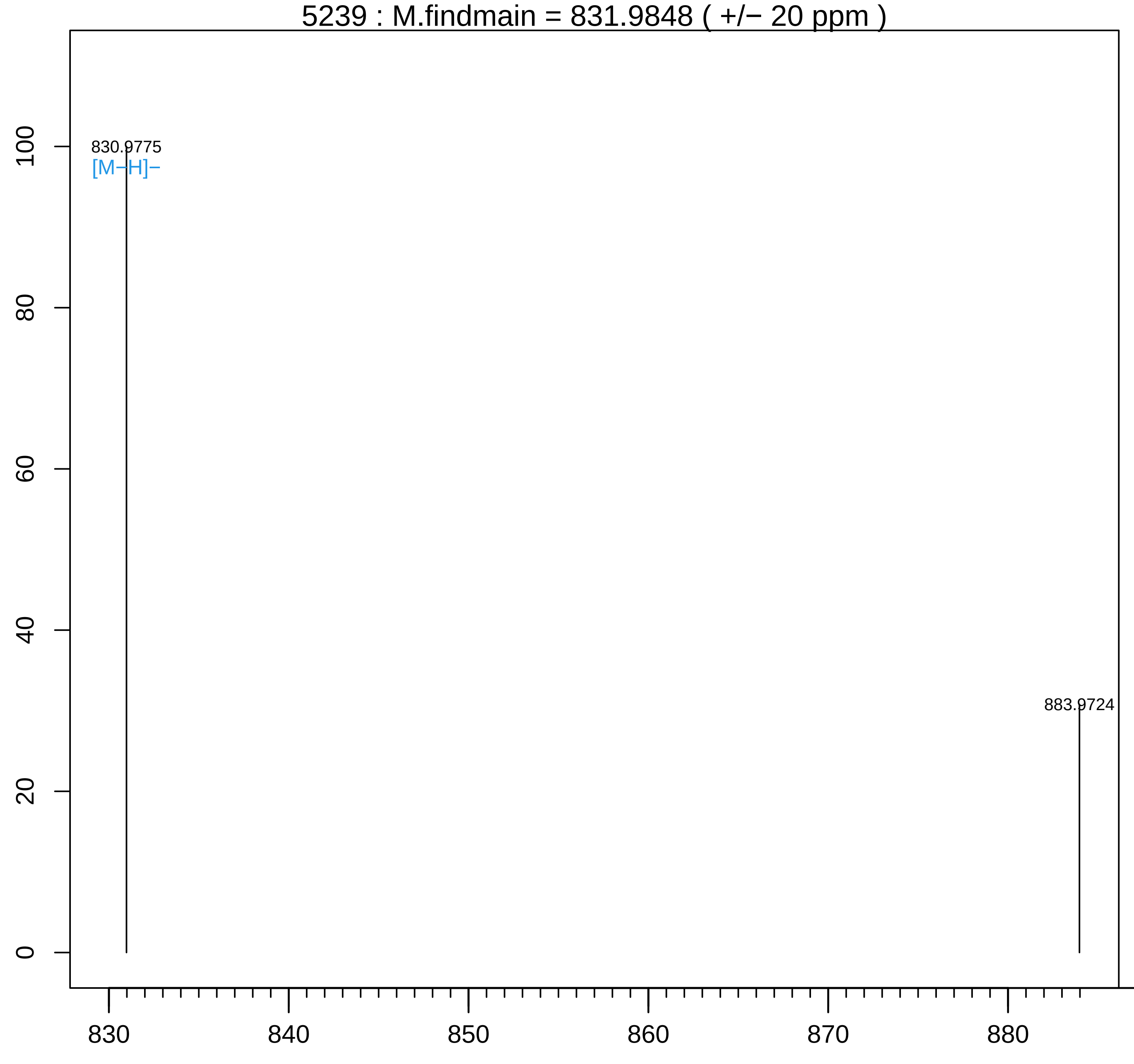
5239 : M.findmain = 831.9848 (+/− 20 ppm)
830.9775
[M−H]−
883.9724
0
20
40
60
80
100
830
840
850
860
870
880

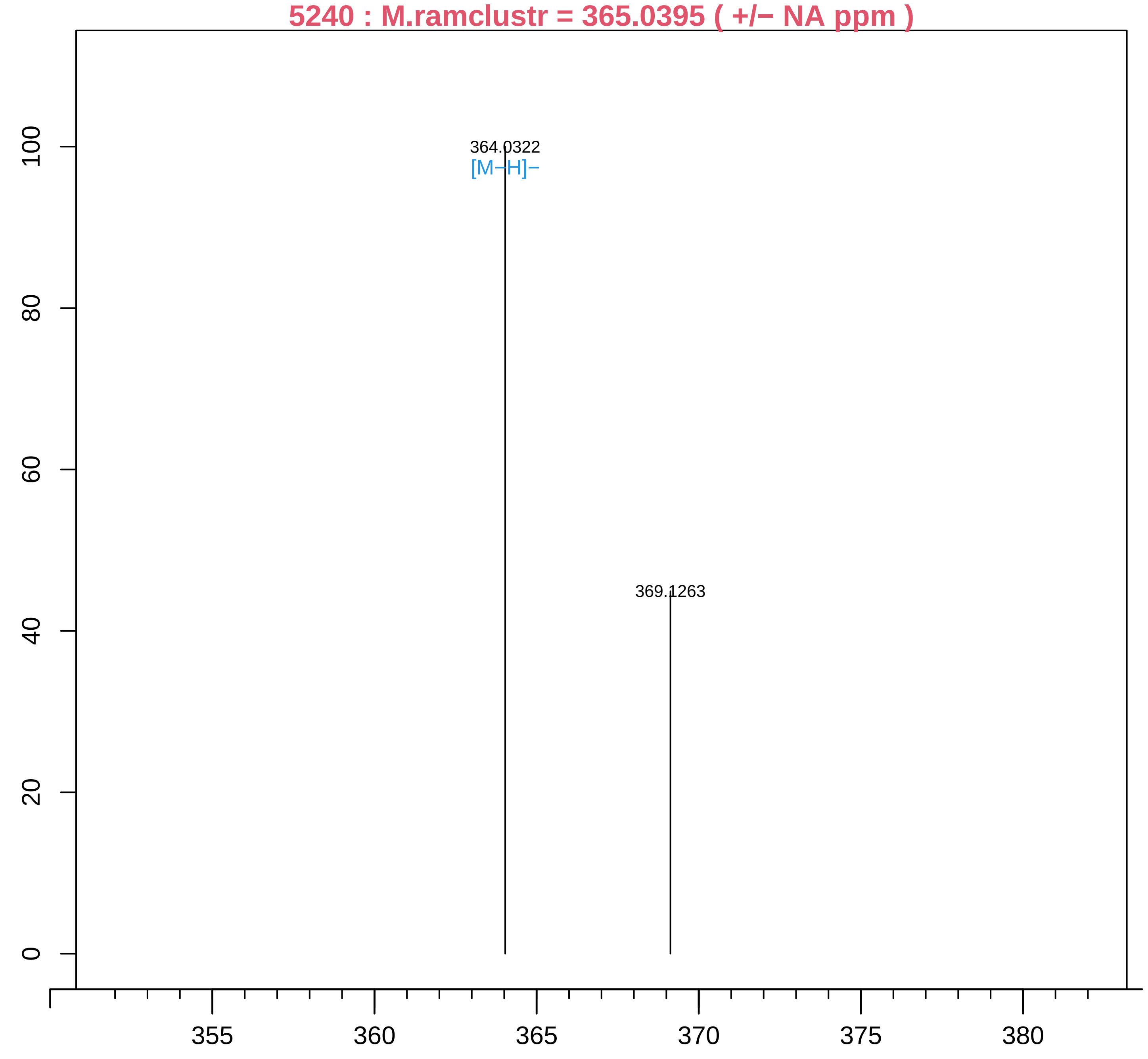

5240 : M.ramclustr = 365.0395 (+/− NA ppm)
364.0322
[M−H]−
369.1263
0
20
40
60
80
100
355
360
365
370
375
380

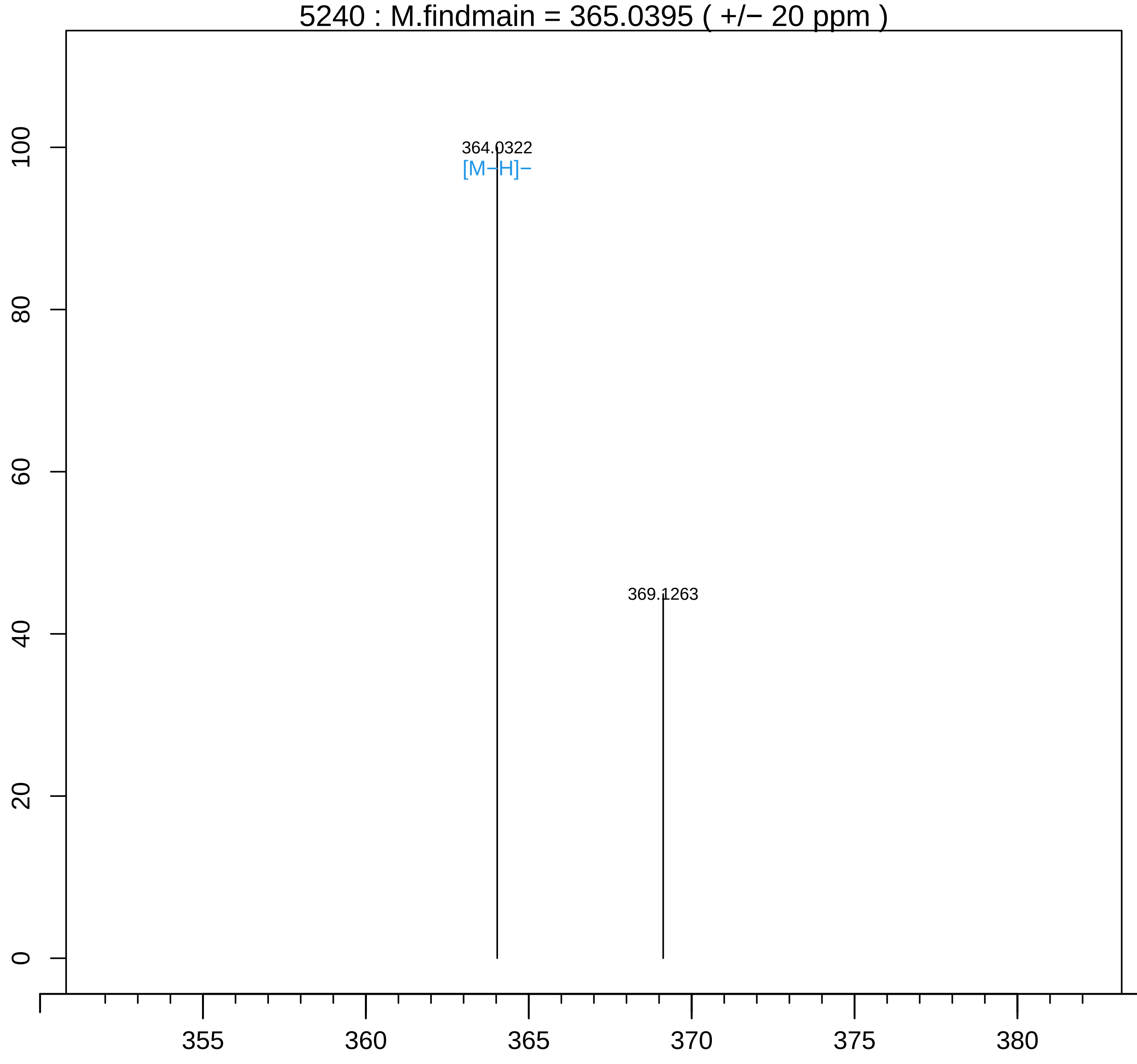

5240 : M.findmain = 365.0395 (+/− 20 ppm)
364.0322
[M−H]−
369.1263
0
20
40
60
80
100
355
360
365
370
375
380

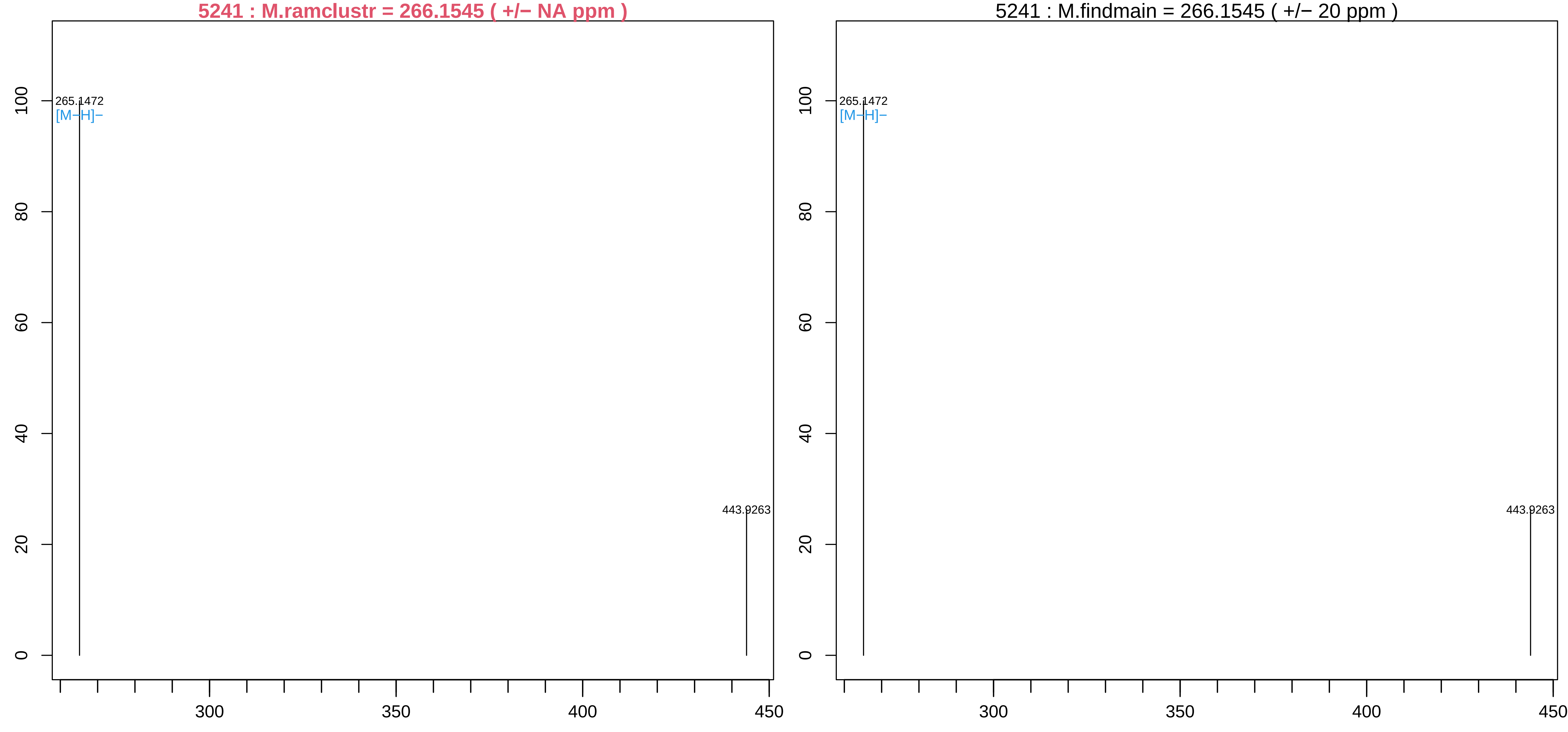

5241 : M.ramclustr = 266.1545 (+/− NA ppm)
265.1472
[M−H]−
443.9263
5241 : M.findmain = 266.1545 (+/− 20 ppm)
265.1472
[M−H]−
443.9263
0
20
40
60
80
100
300
350
400
450

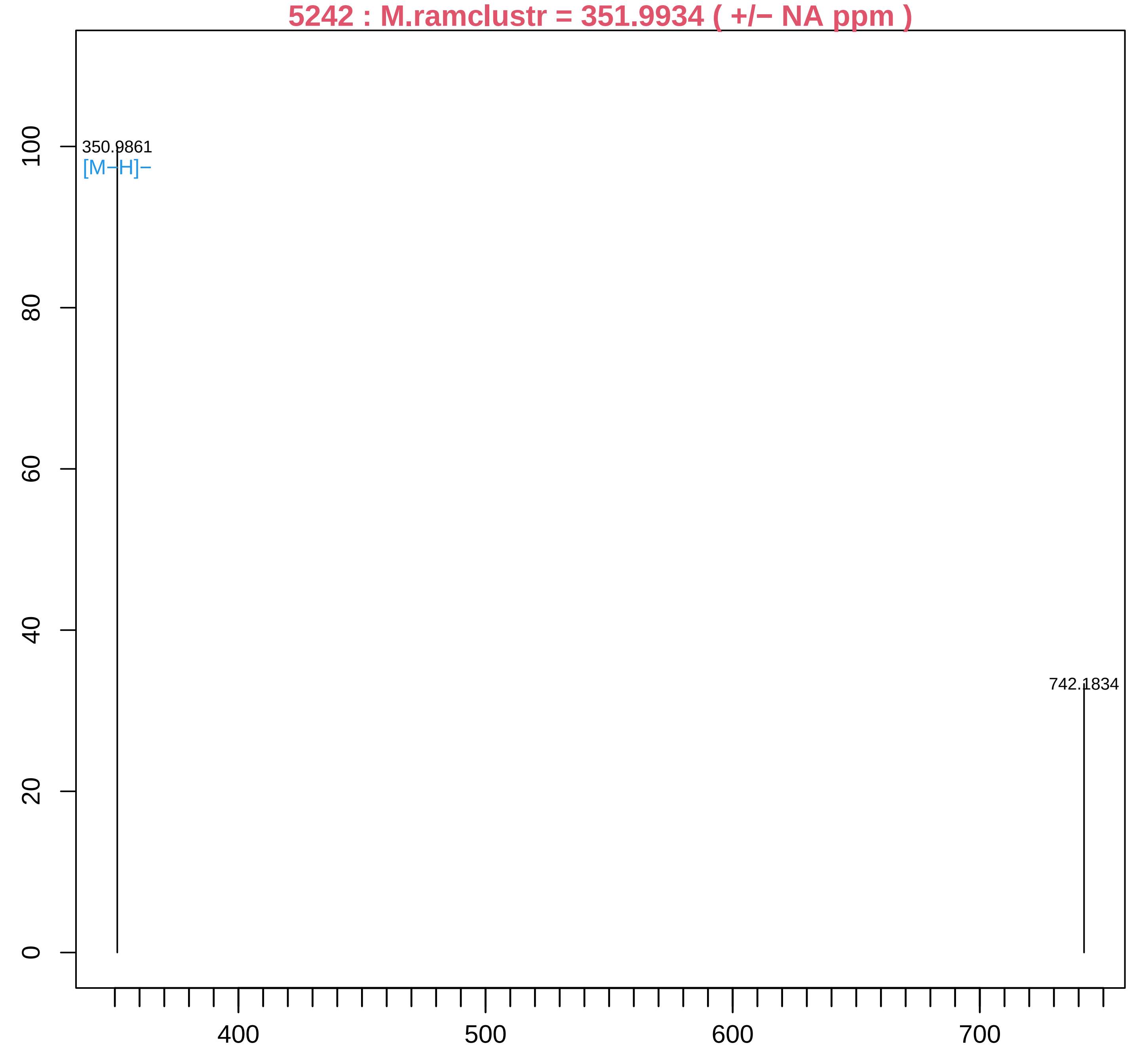

5242 : M.ramclustr = 351.9934 (+/− NA ppm)
350.9861
[M−H]−
742.1834
0
20
40
60
80
100
400
500
600
700

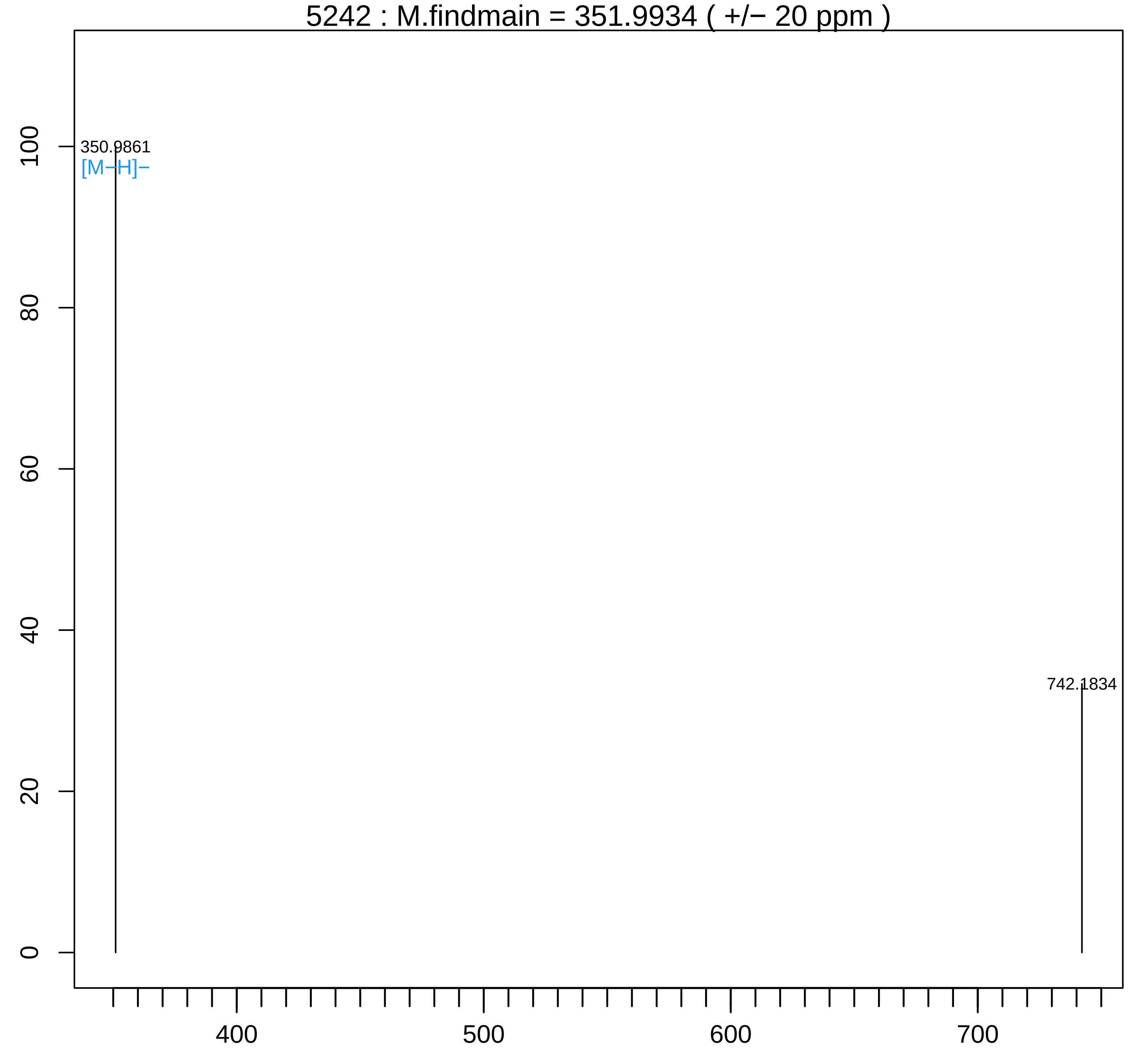

5242 : M.findmain = 351.9934 (+/− 20 ppm)
350.9861
[M−H]−
742.1834
0
20
40
60
80
100
400
500
600
700

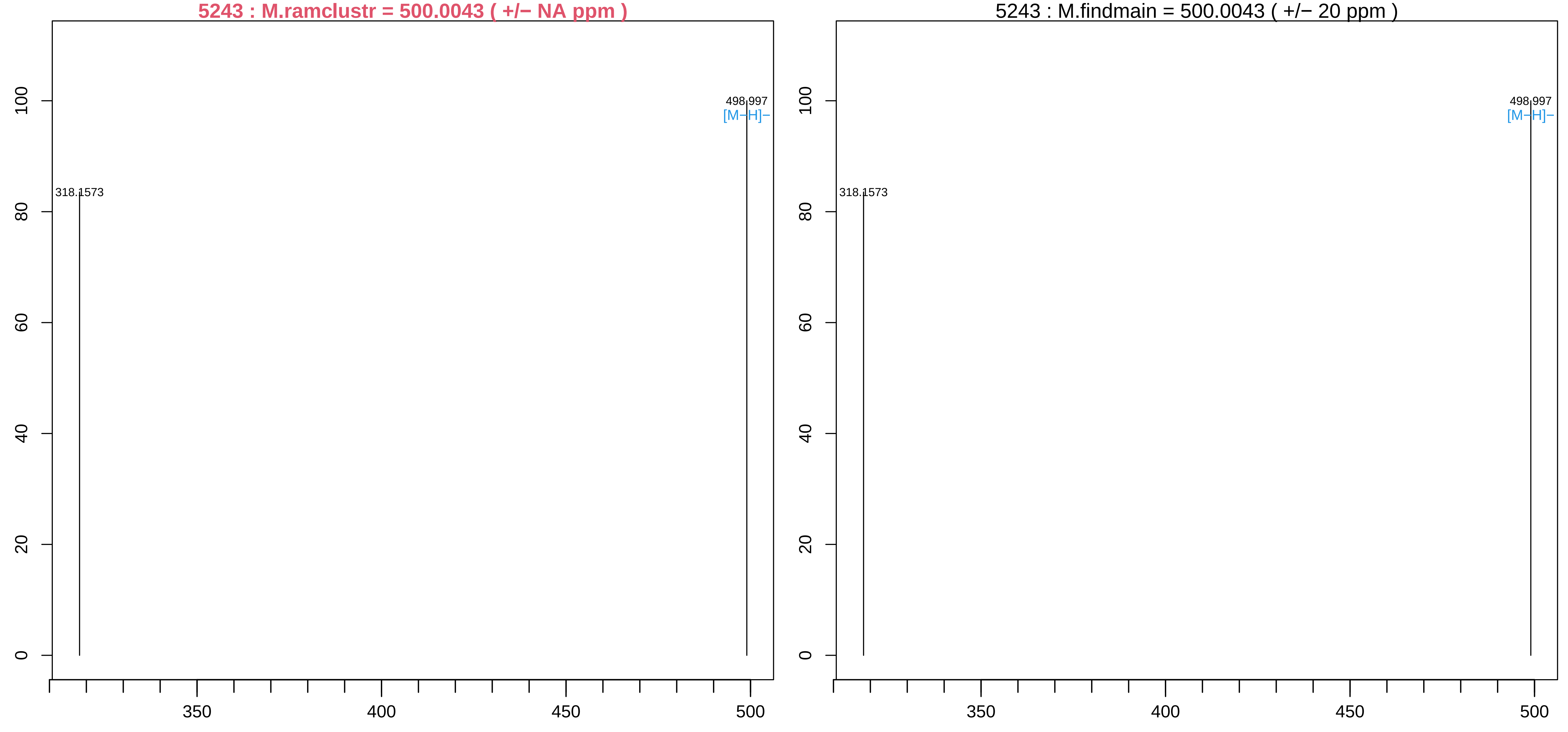

5243 : M.ramclustr = 500.0043 (+/− NA ppm)
318.1573
498.997
[M−H]−
0
20
40
60
80
100
350
400
450
500
5243 : M.findmain = 500.0043 (+/− 20 ppm)
318.1573
498.997
[M−H]−
0
20
40
60
80
100
350
400
450
500

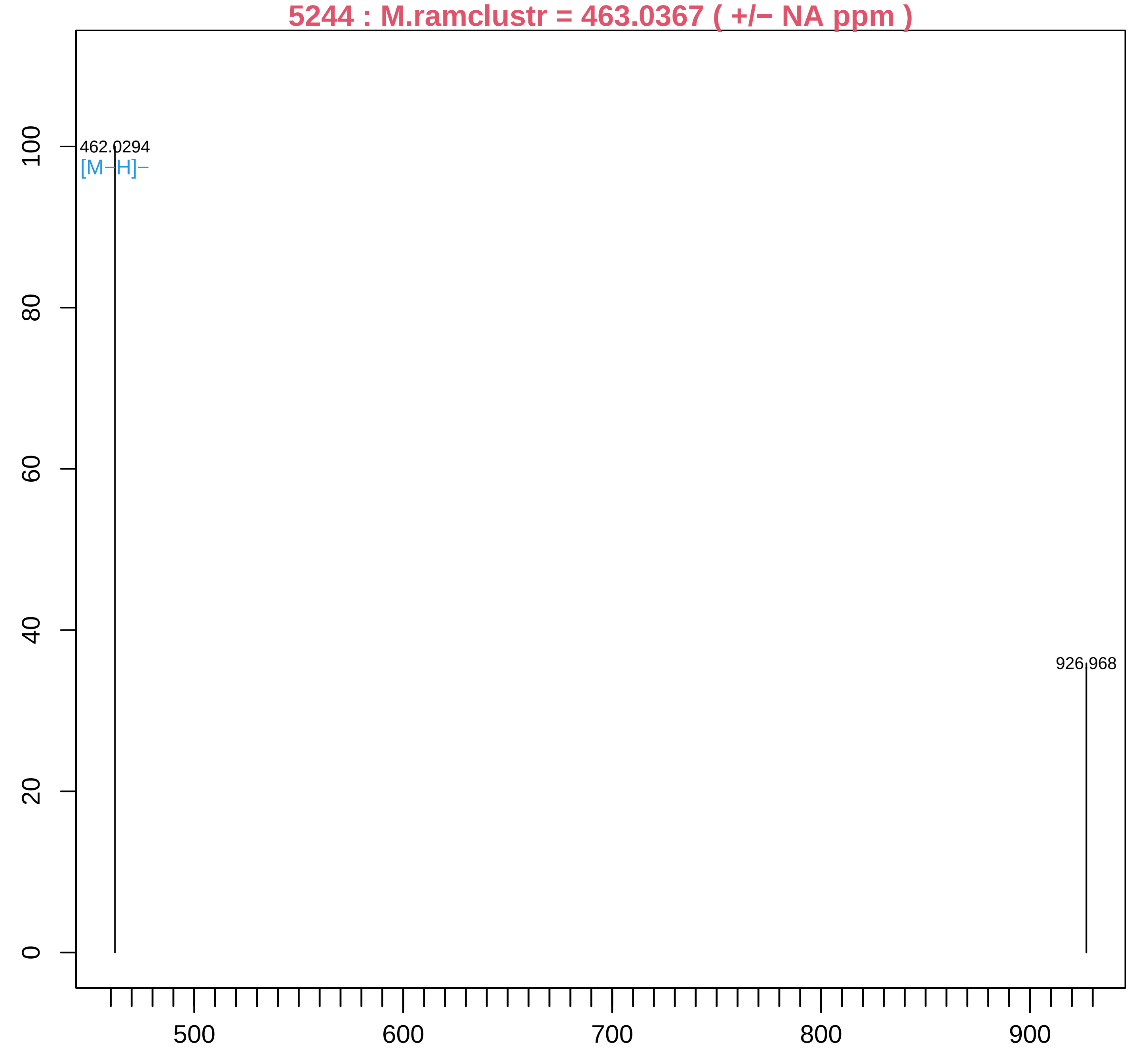
5244 : M.ramclustr = 463.0367 (+/− NA ppm)
462.0294
[M−H]−
926.968
0
20
40
60
80
100
500
600
700
800
900

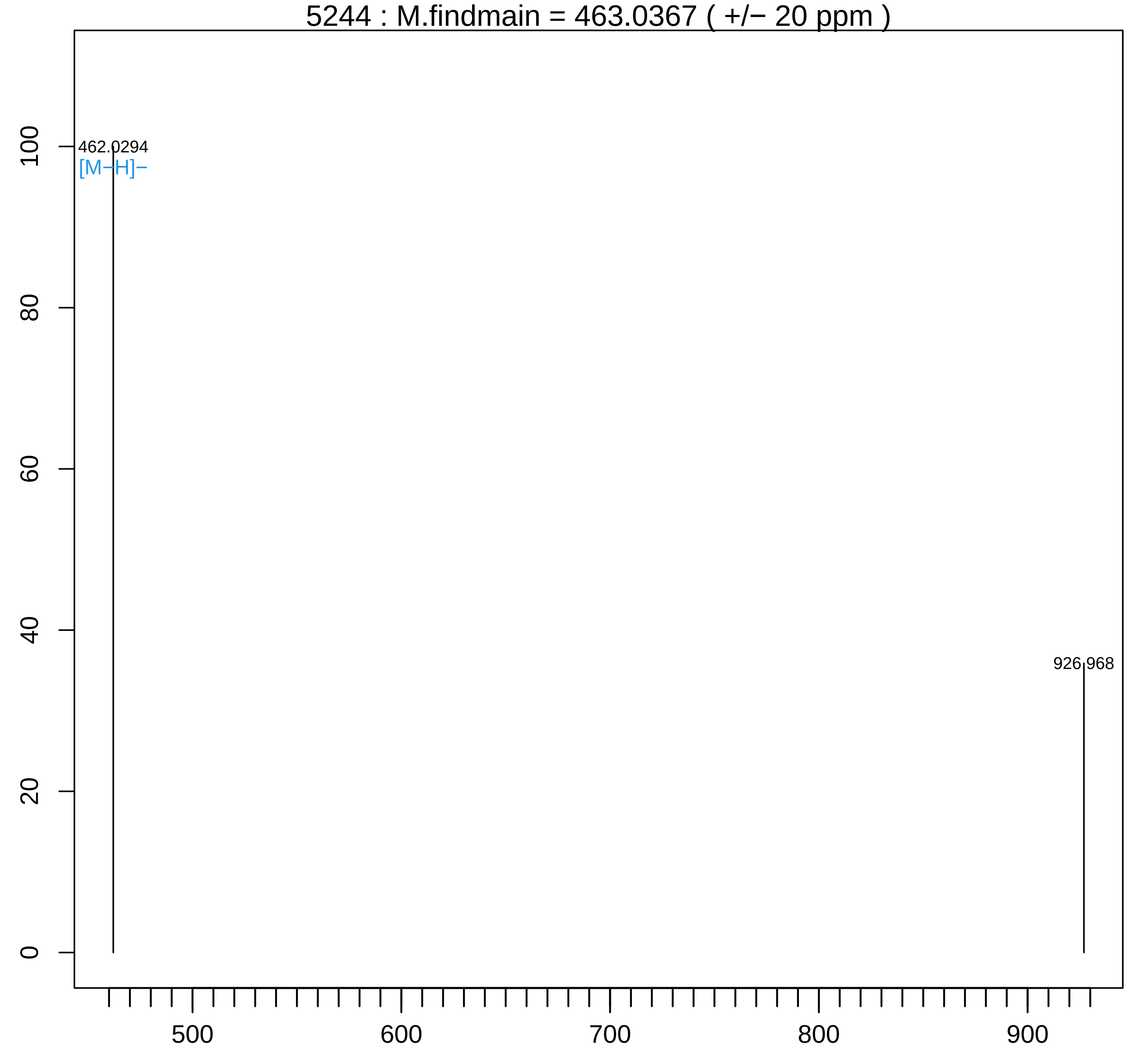
5244 : M.findmain = 463.0367 (+/− 20 ppm)
462.0294
[M−H]−
926.968
0
20
40
60
80
100
500
600
700
800
900

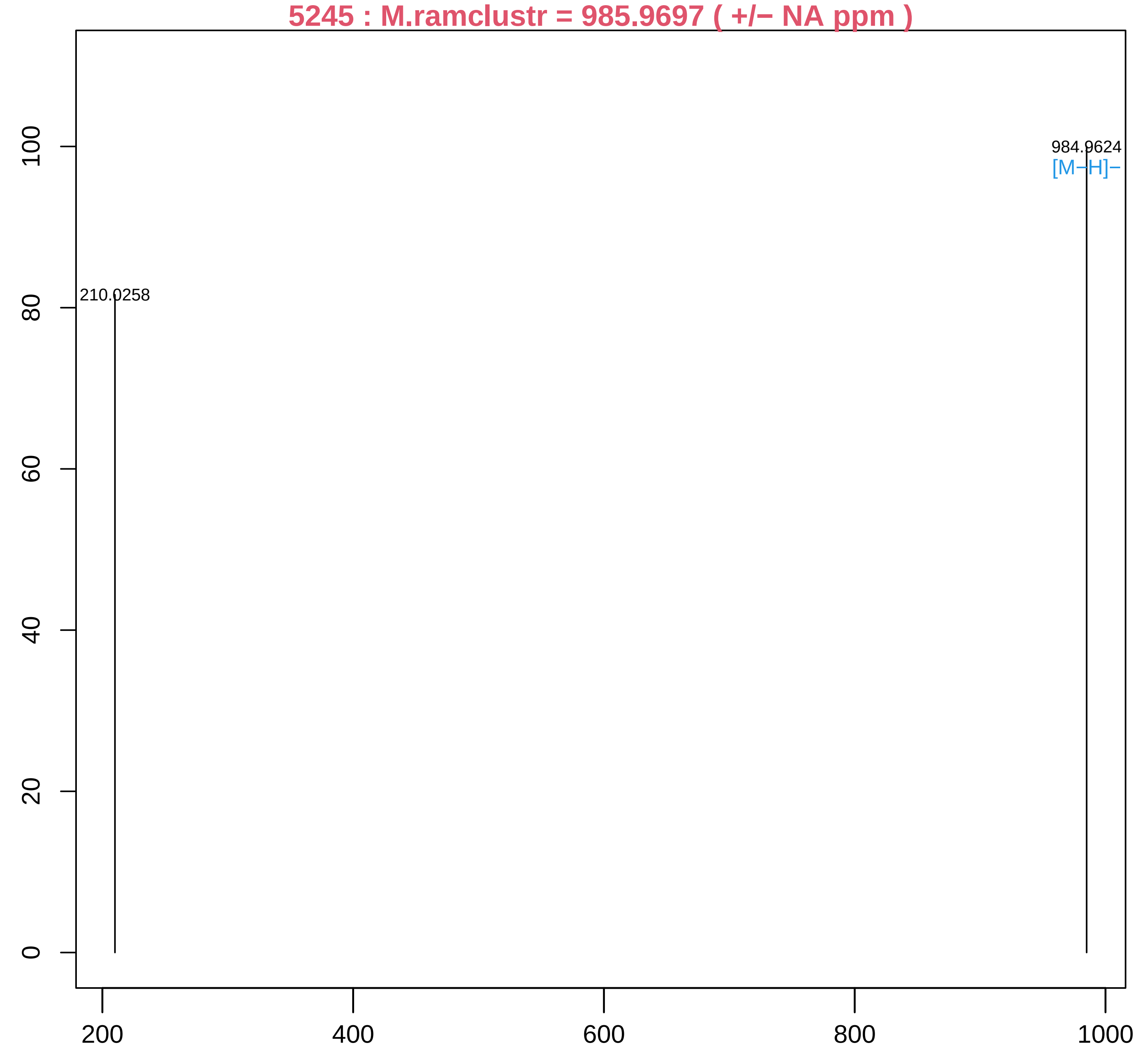
5245 : M.ramclustr = 985.9697 (+/− NA ppm)
984.9624
[M−H]−
210.0258
0
20
40
60
80
100
200
400
600
800
1000

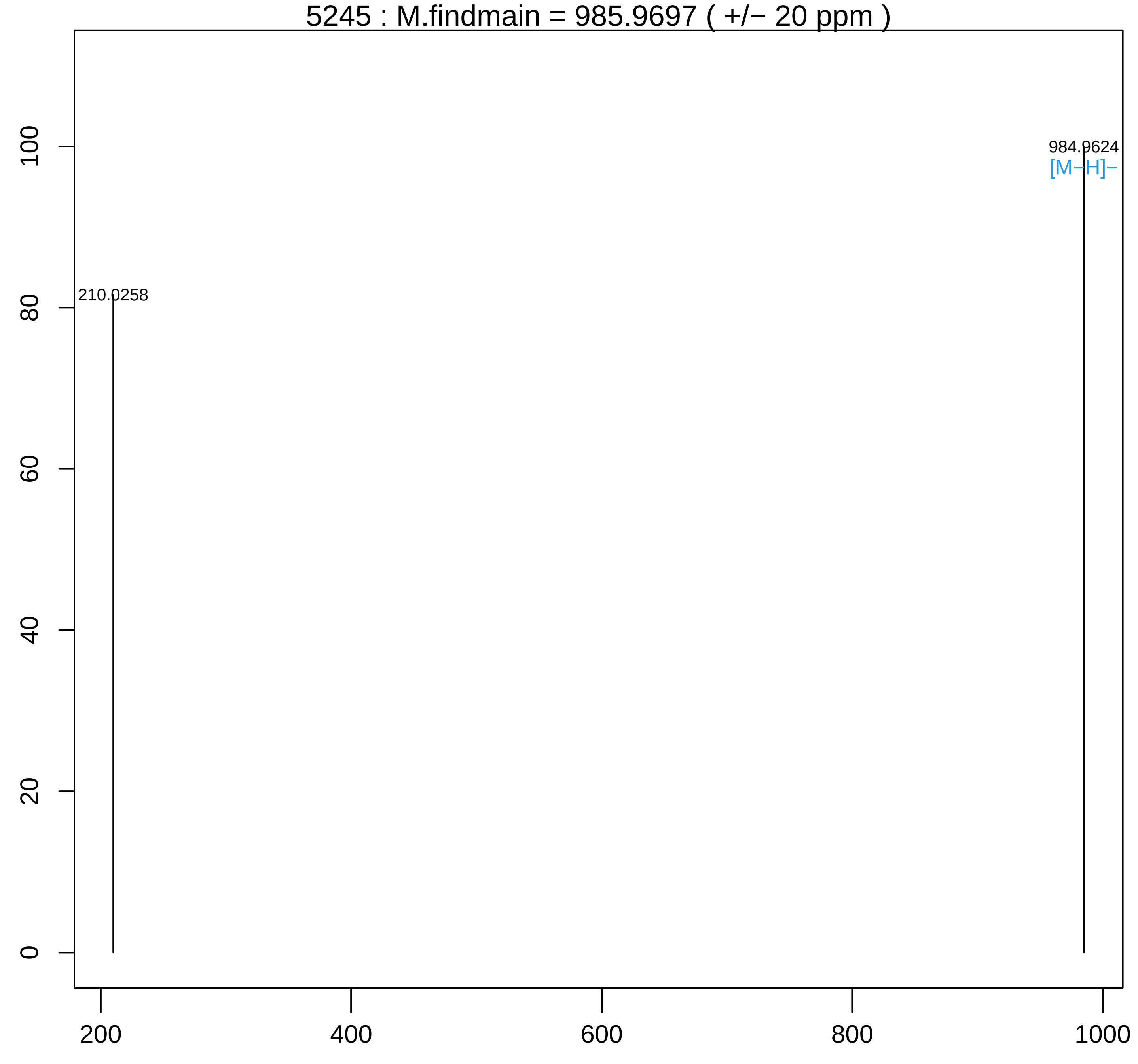
5245 : M.findmain = 985.9697 (+/− 20 ppm)
984.9624
[M−H]−
210.0258
0
20
40
60
80
100
200
400
600
800
1000

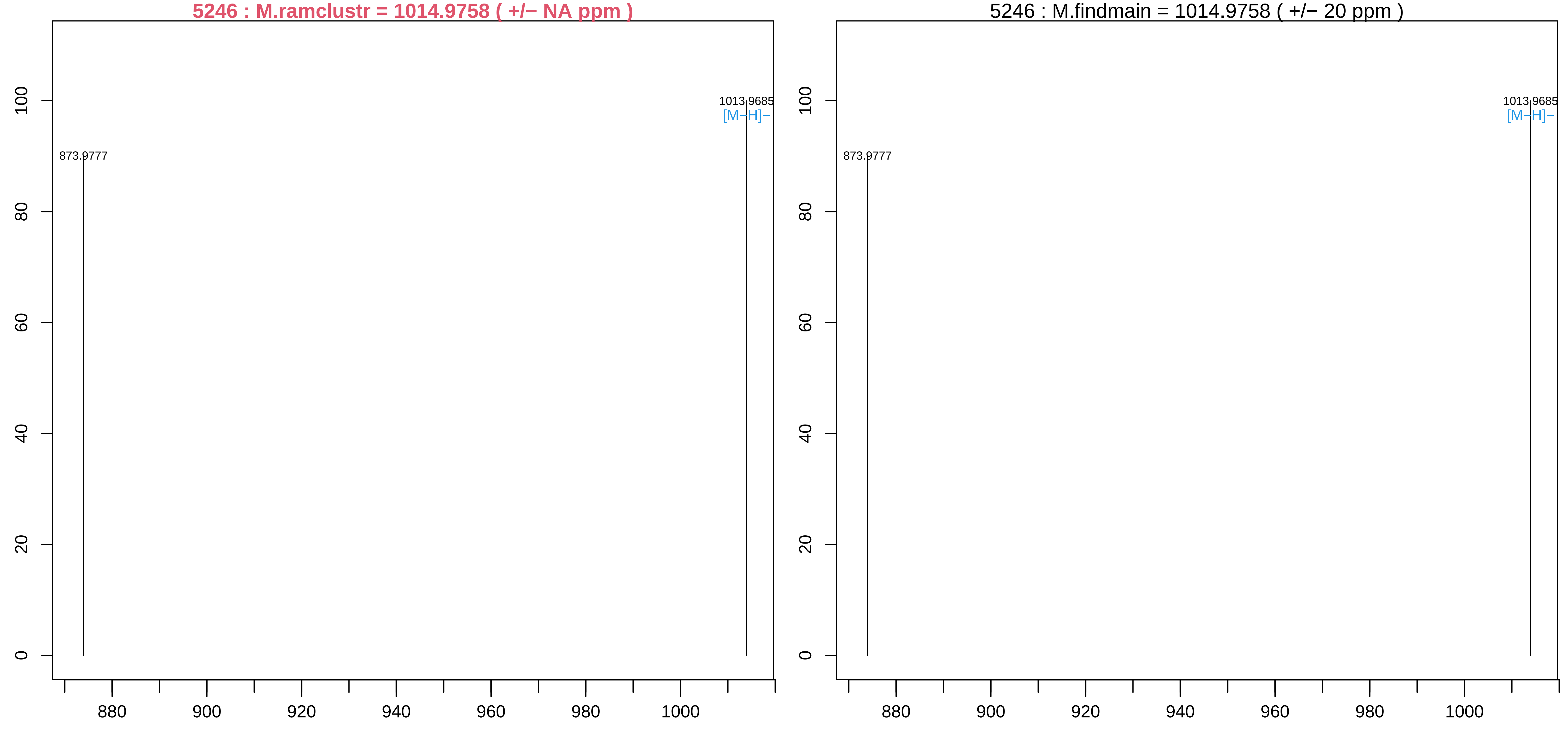
5246 : M.ramclustr = 1014.9758 (+/− NA ppm)
873.9777
1013.9685
[M−H]−
0
20
40
60
80
100
880
900
920
940
960
980
1000
5246 : M.findmain = 1014.9758 (+/− 20 ppm)
873.9777
1013.9685
[M−H]−
0
20
40
60
80
100
880
900
920
940
960
980
1000

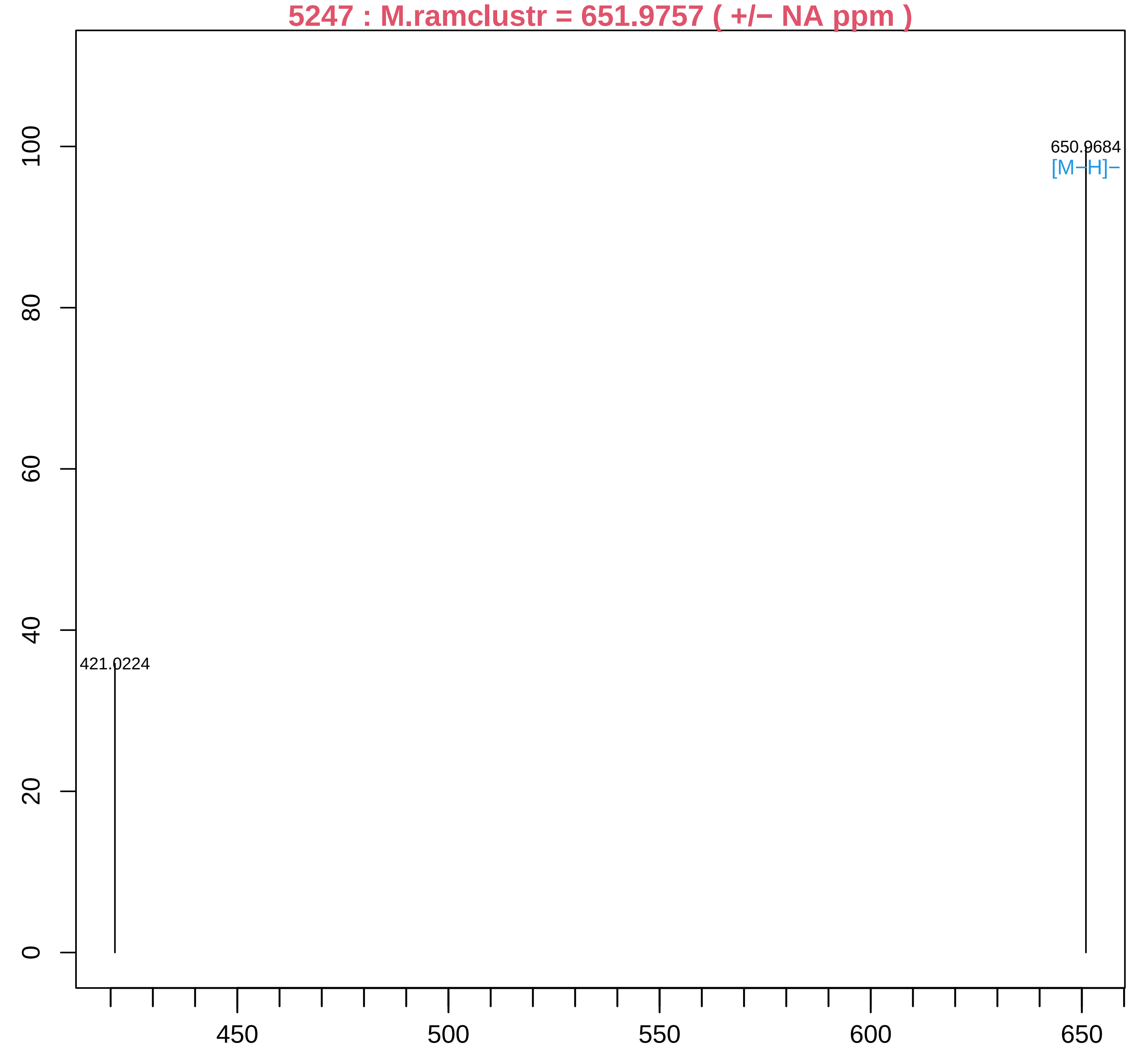
5247 : M.ramclustr = 651.9757 (+/− NA ppm)
650.9684
[M−H]−
421.0224
0
20
40
60
80
100
450
500
550
600
650

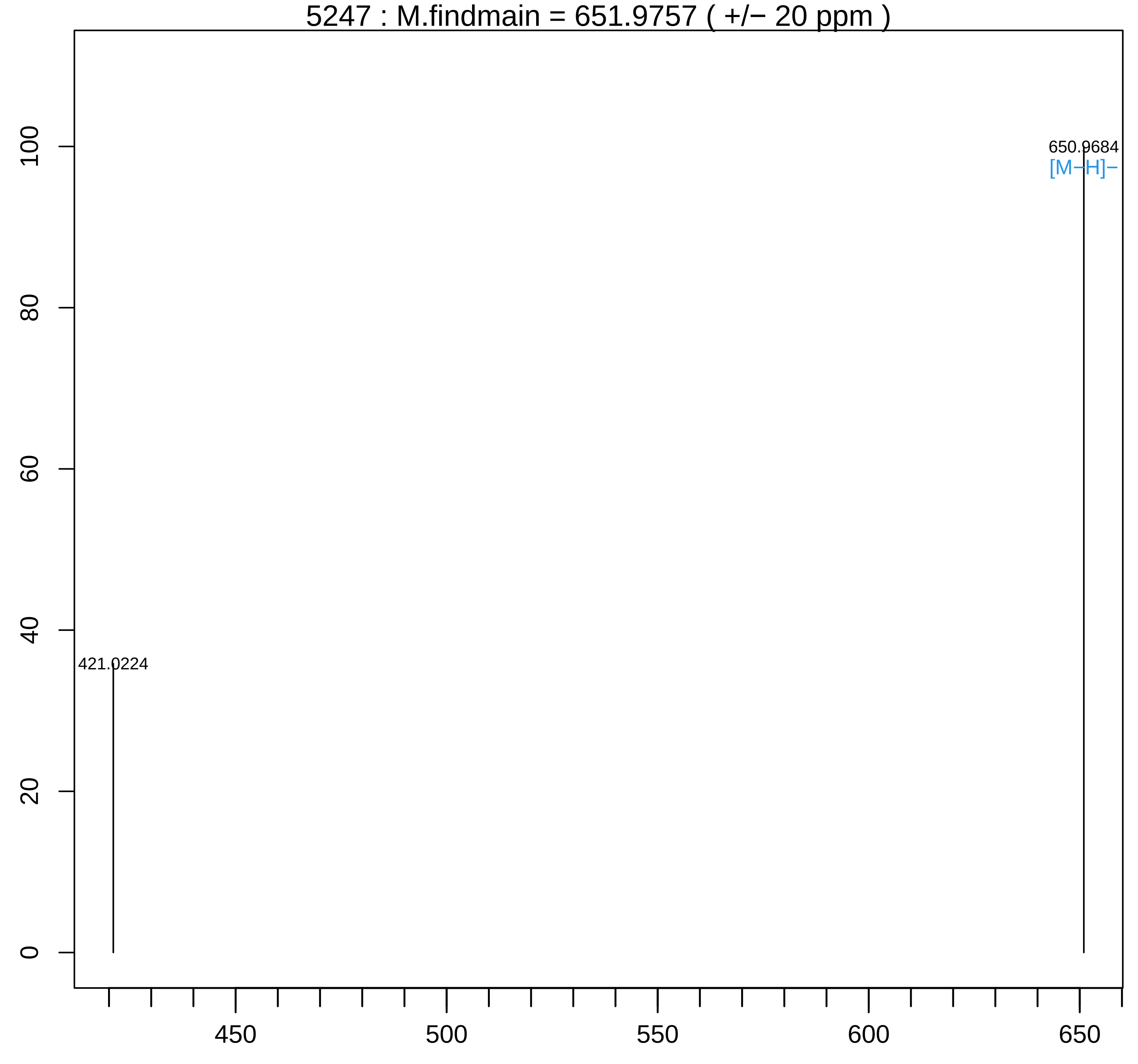
5247 : M.findmain = 651.9757 (+/− 20 ppm)
650.9684
[M−H]−
421.0224
0
20
40
60
80
100
450
500
550
600
650

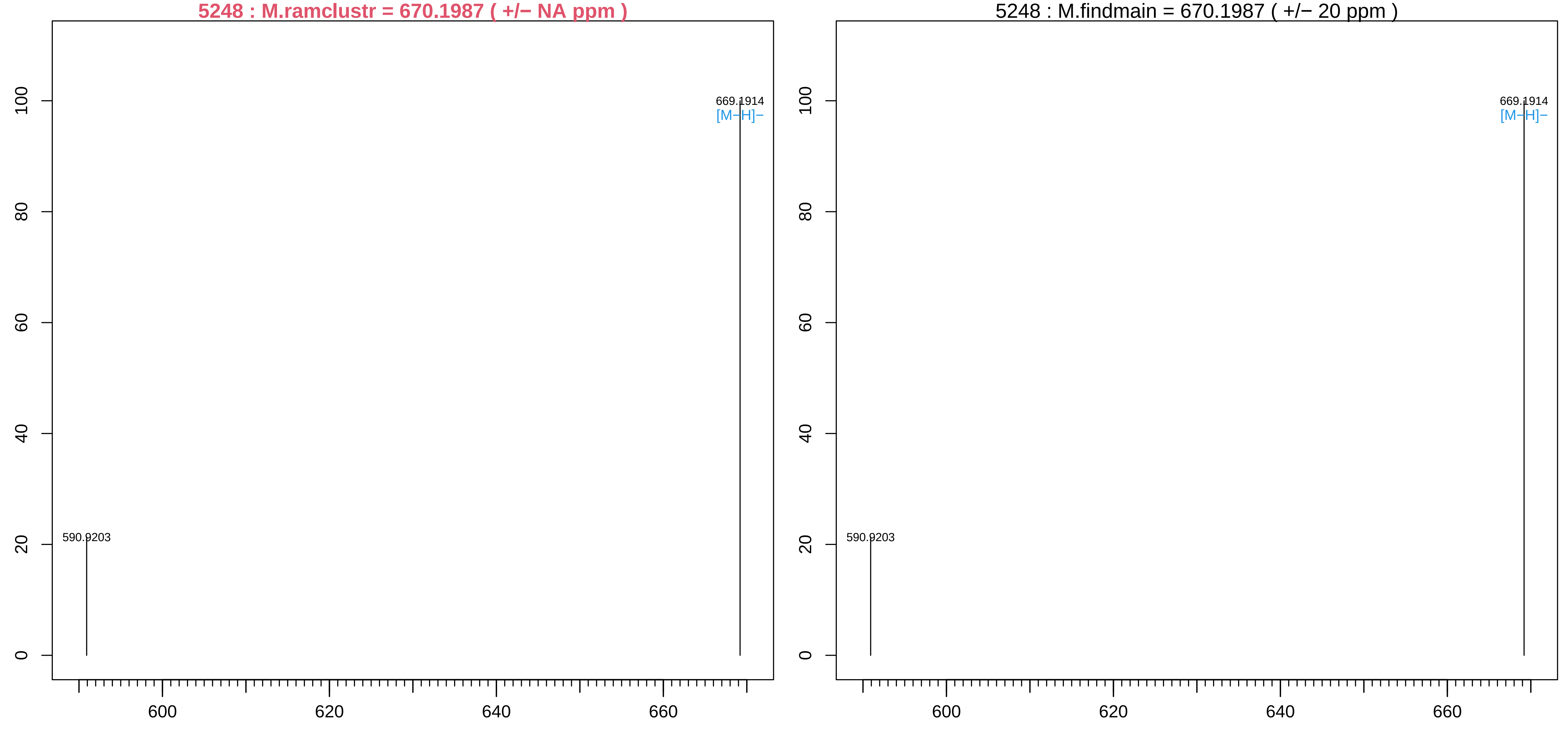
5248 : M.ramclustr = 670.1987 (+/− NA ppm)
669.1914
[M−H]−
590.9203
0
20
40
60
80
100
600
620
640
660
5248 : M.findmain = 670.1987 (+/− 20 ppm)
669.1914
[M−H]−
590.9203
0
20
40
60
80
100
600
620
640
660

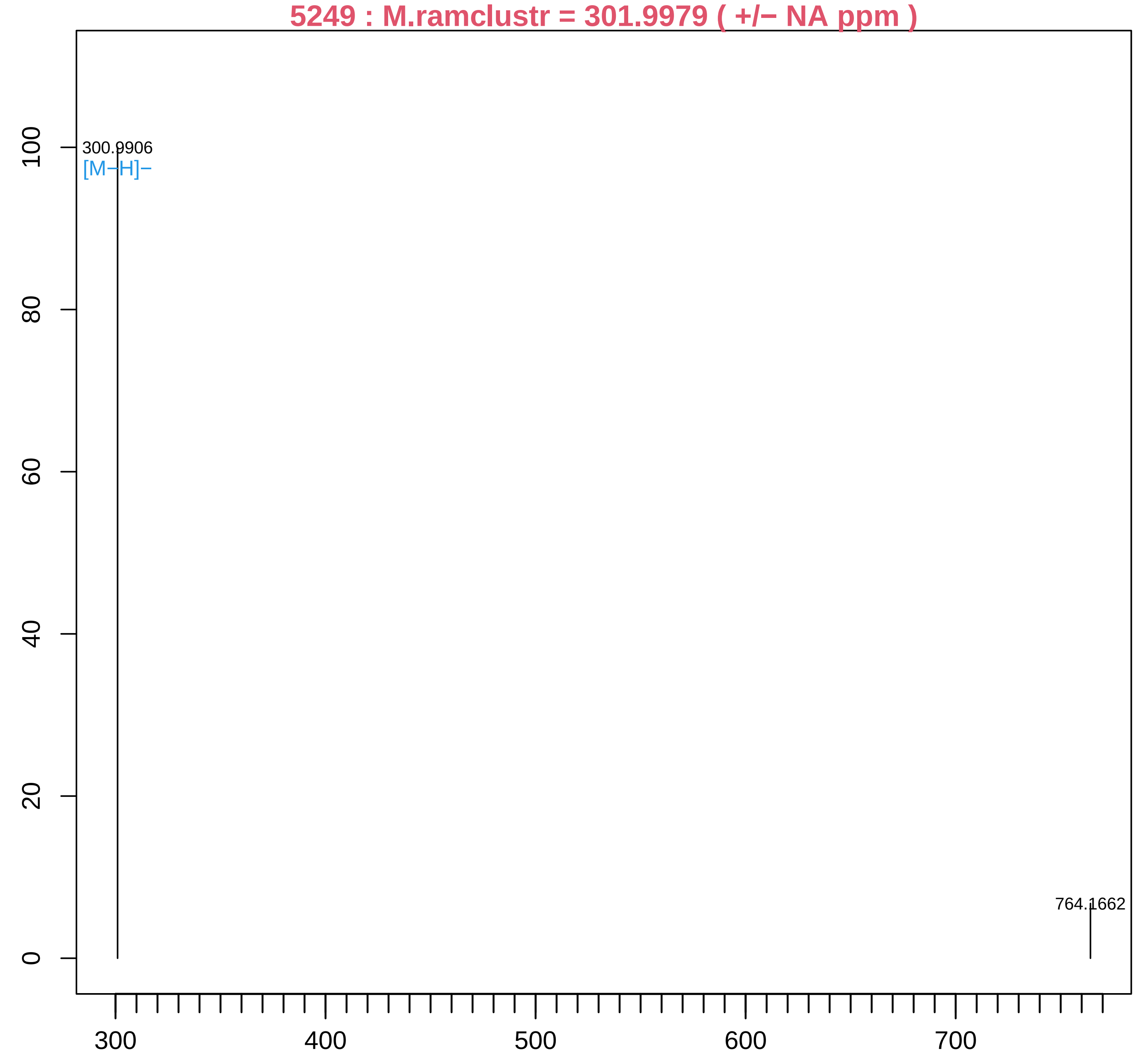

5249 : M.ramclustr = 301.9979 (+/− NA ppm)
300.9906
[M−H]−
764.1662
0
20
40
60
80
100
300
400
500
600
700

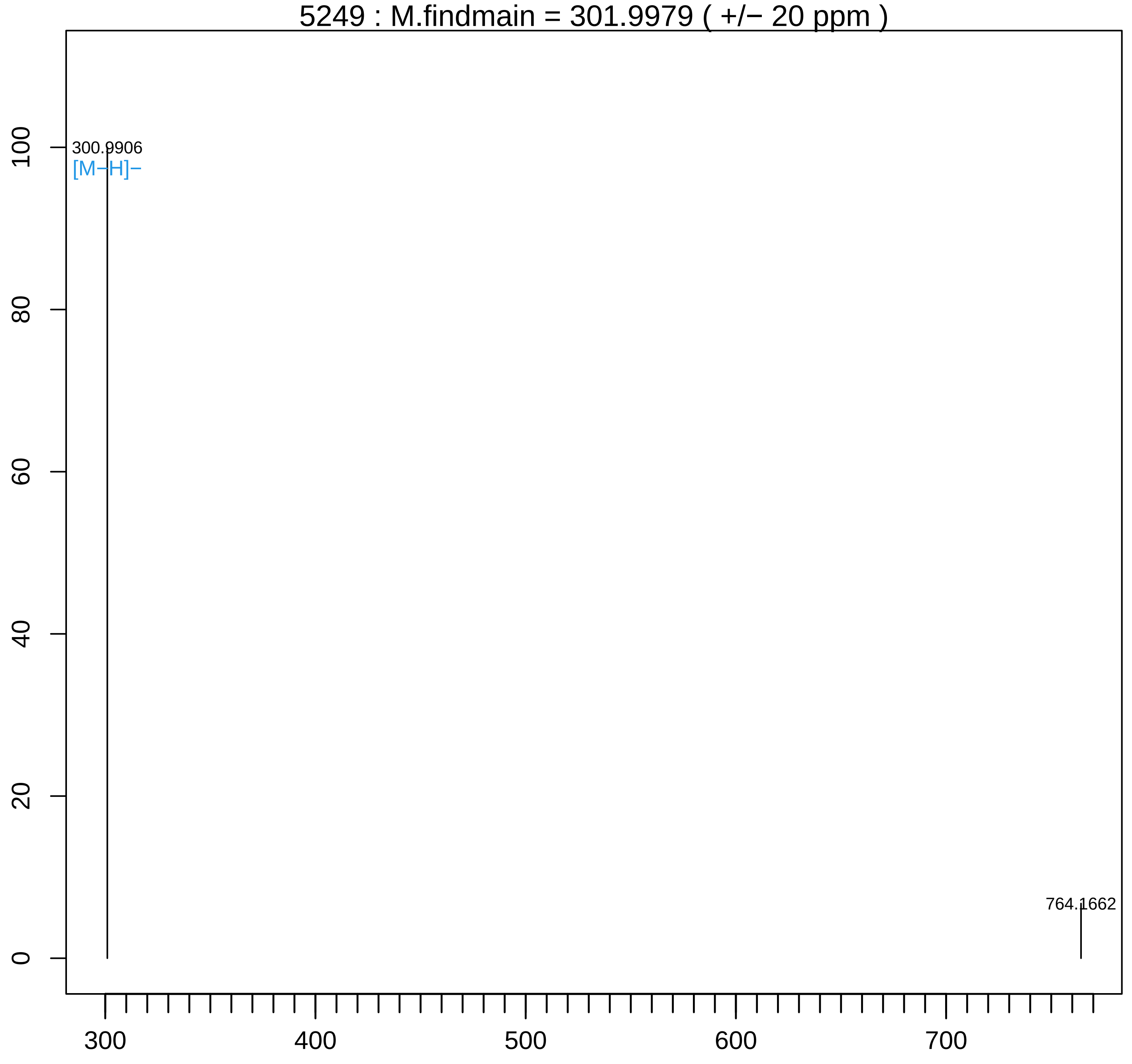

5249 : M.findmain = 301.9979 (+/− 20 ppm)
300.9906
[M−H]−
764.1662
0
20
40
60
80
100
300
400
500
600
700

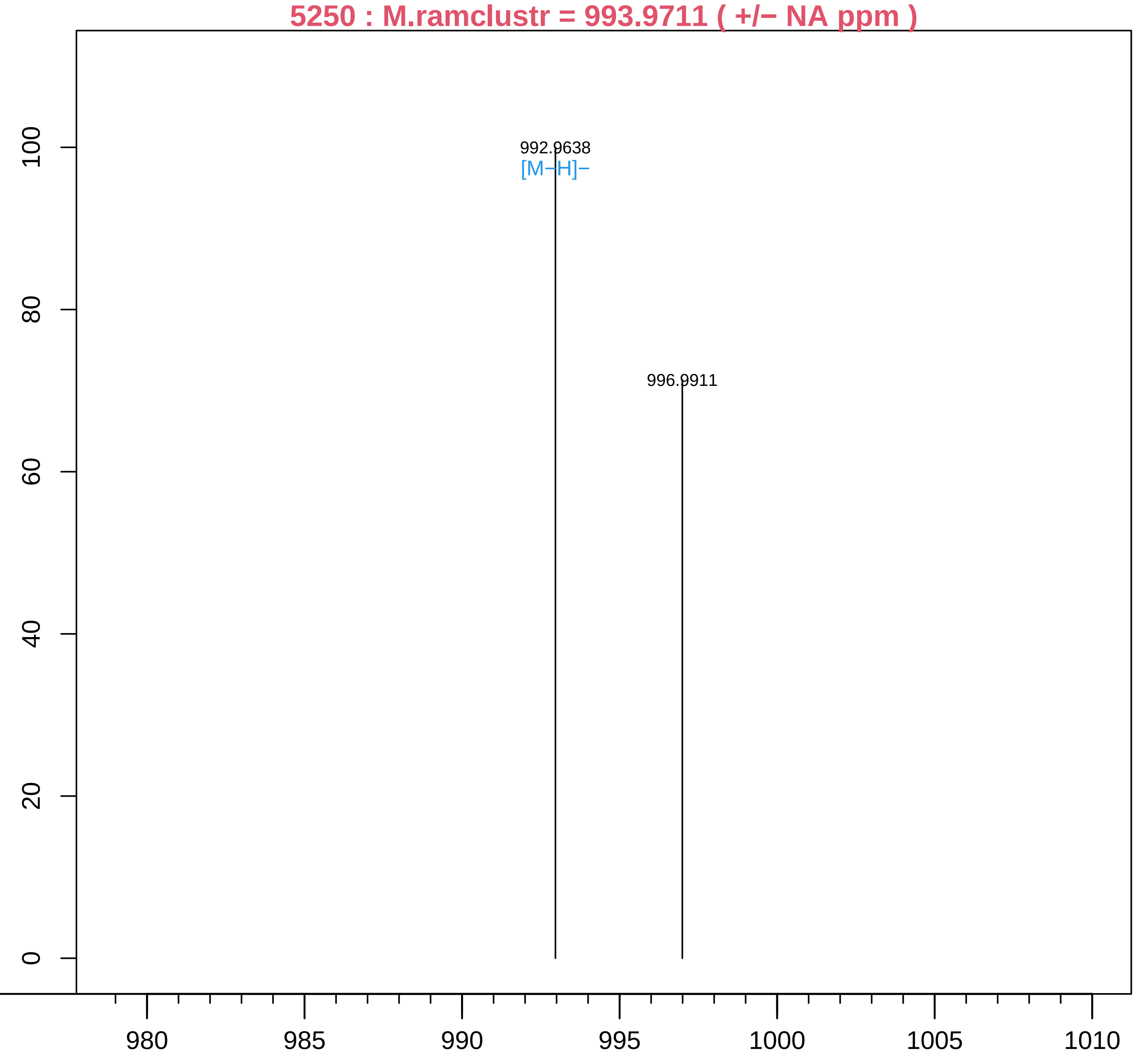
5250 : M.ramclustr = 993.9711 (+/− NA ppm)
992.9638
[M−H]−
996.9911
0
20
40
60
80
100
980
985
990
995
1000
1005
1010

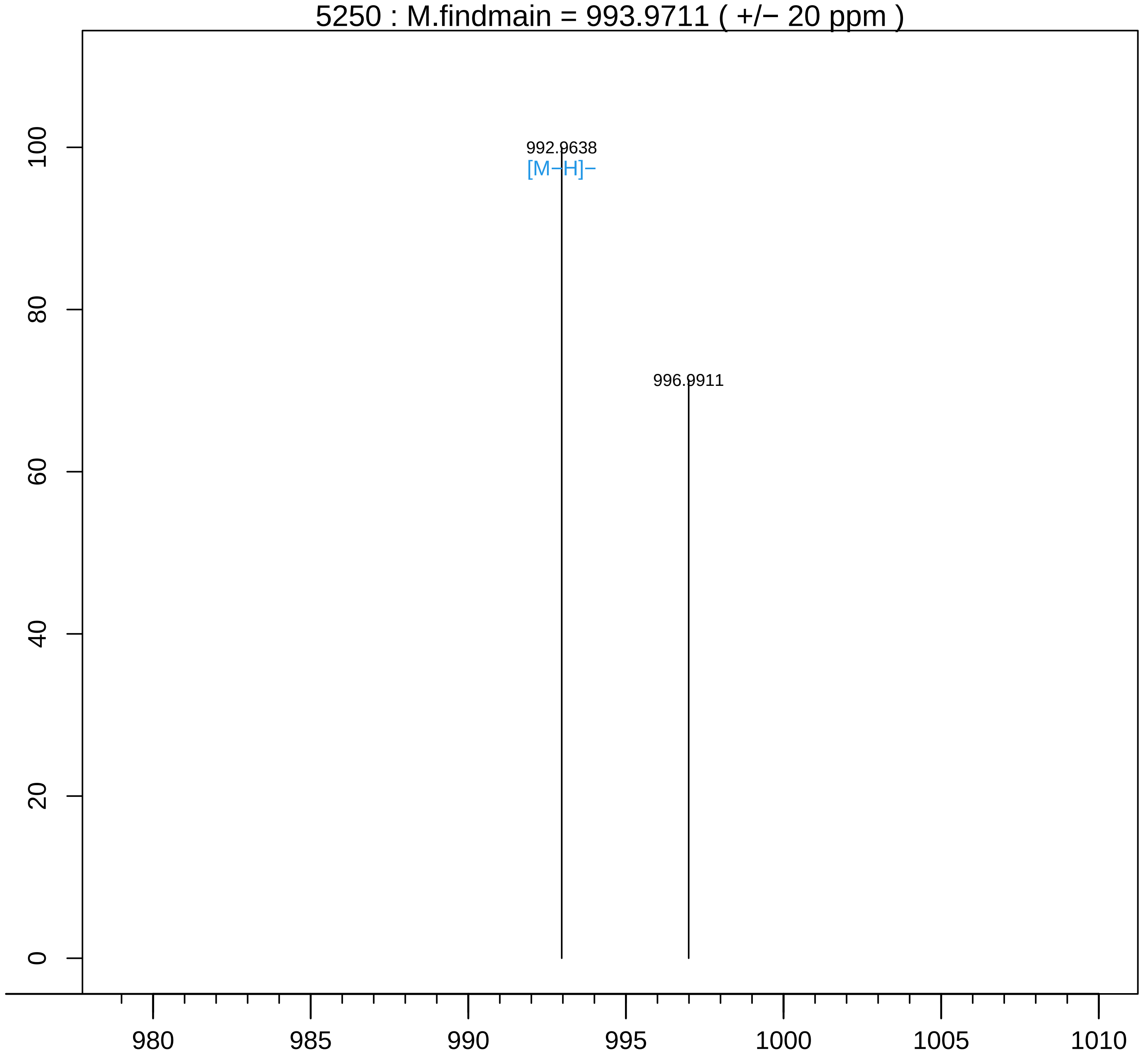
5250 : M.findmain = 993.9711 (+/− 20 ppm)
992.9638
[M−H]−
996.9911
0
20
40
60
80
100
980
985
990
995
1000
1005
1010

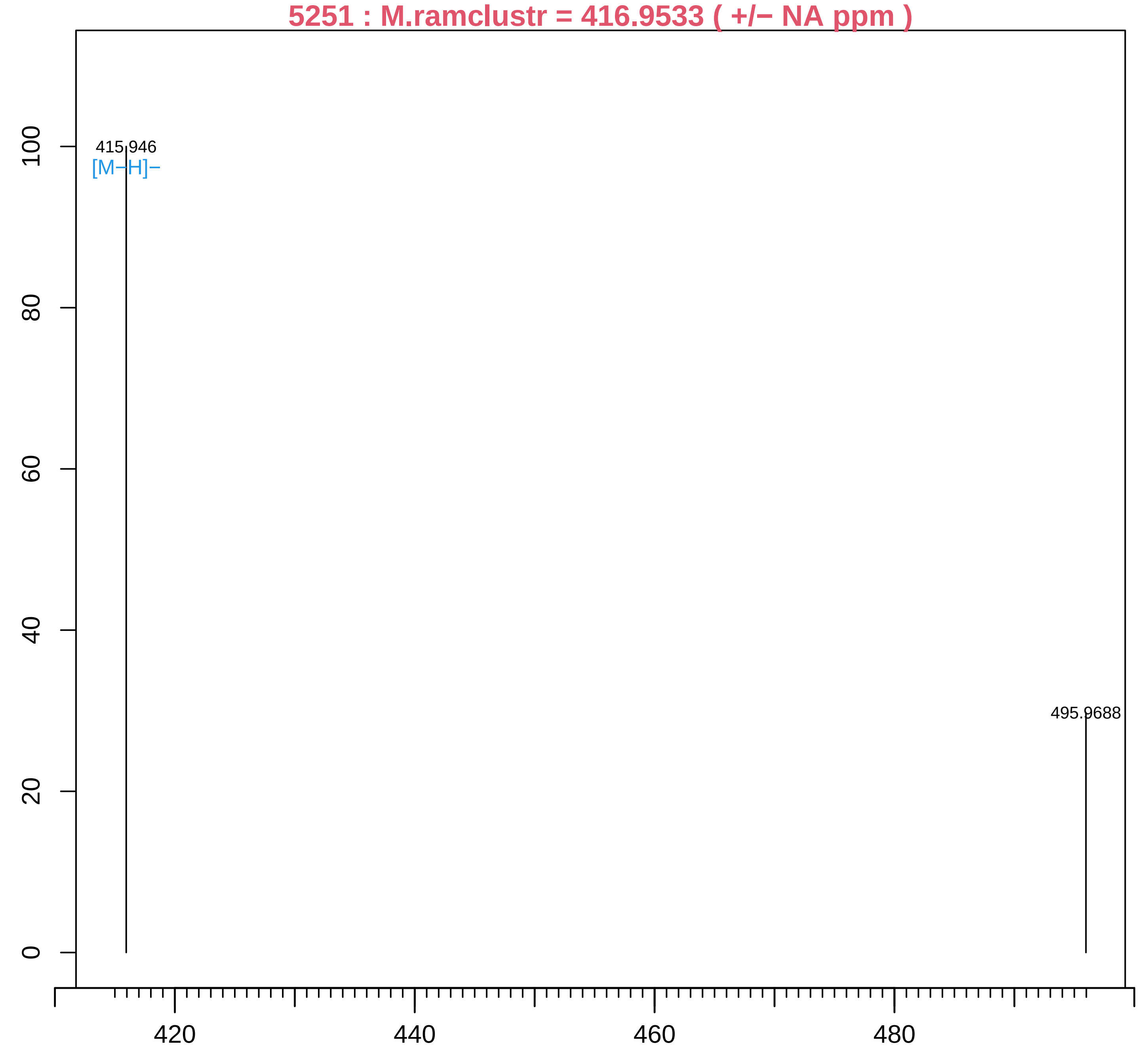

5251 : M.ramclustr = 416.9533 (+/− NA ppm)
415.946
[M−H]−
495.9688

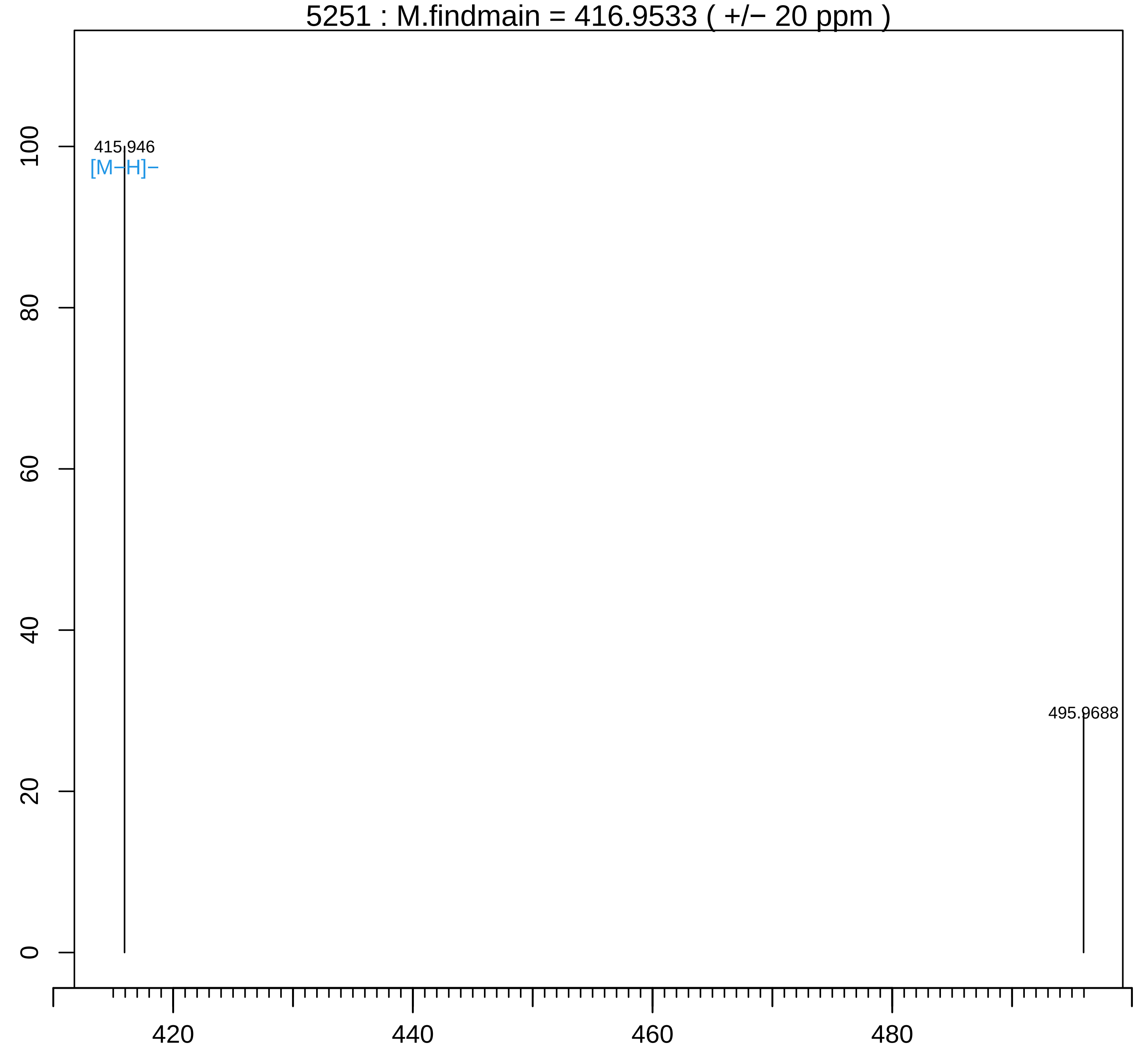

5251 : M.findmain = 416.9533 (+/− 20 ppm)
415.946
[M−H]−
495.9688

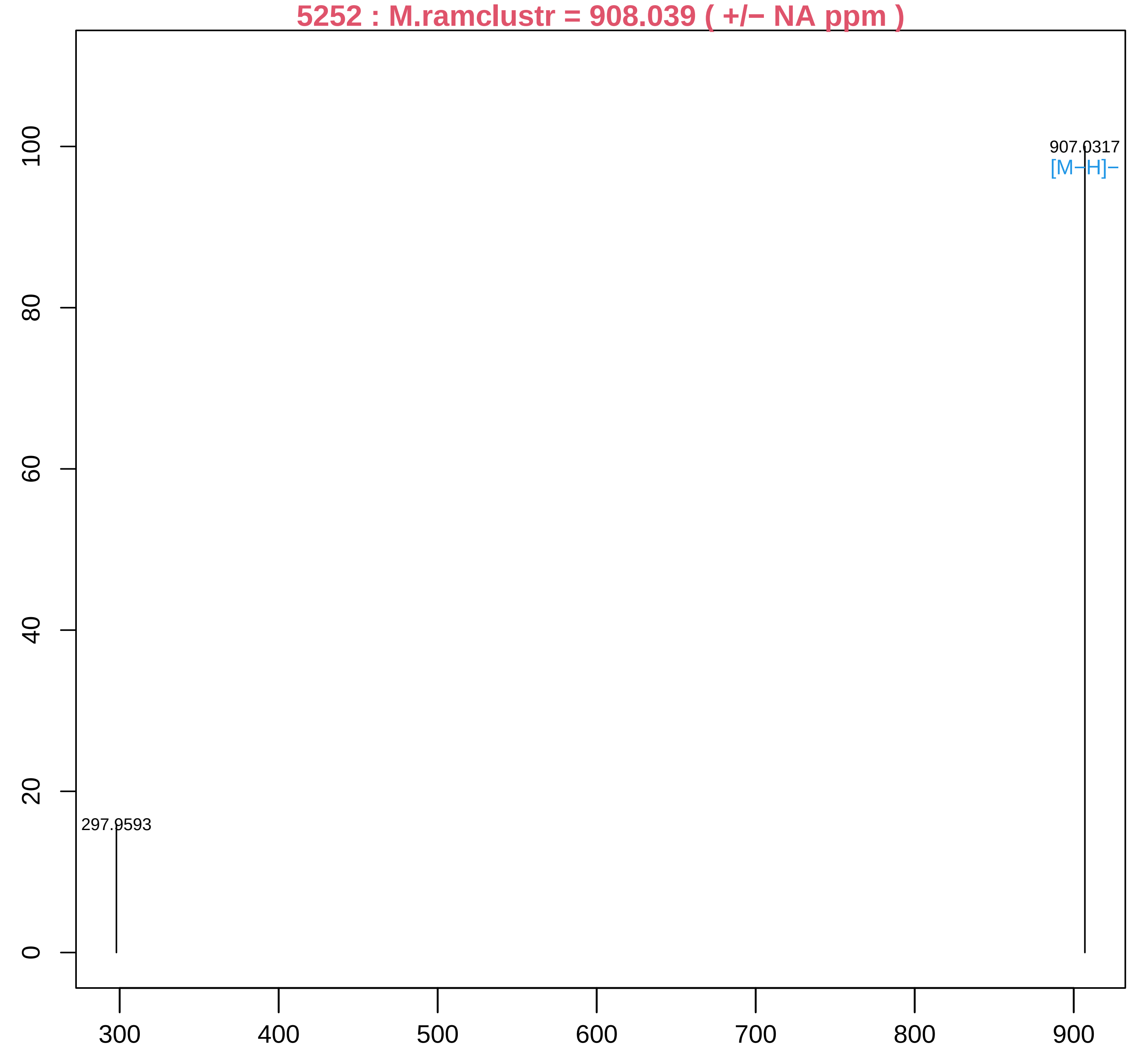

5252 : M.ramclustr = 908.039 (+/− NA ppm)
907.0317
[M−H]−
297.9593
0
20
40
60
80
100
300
400
500
600
700
800
900

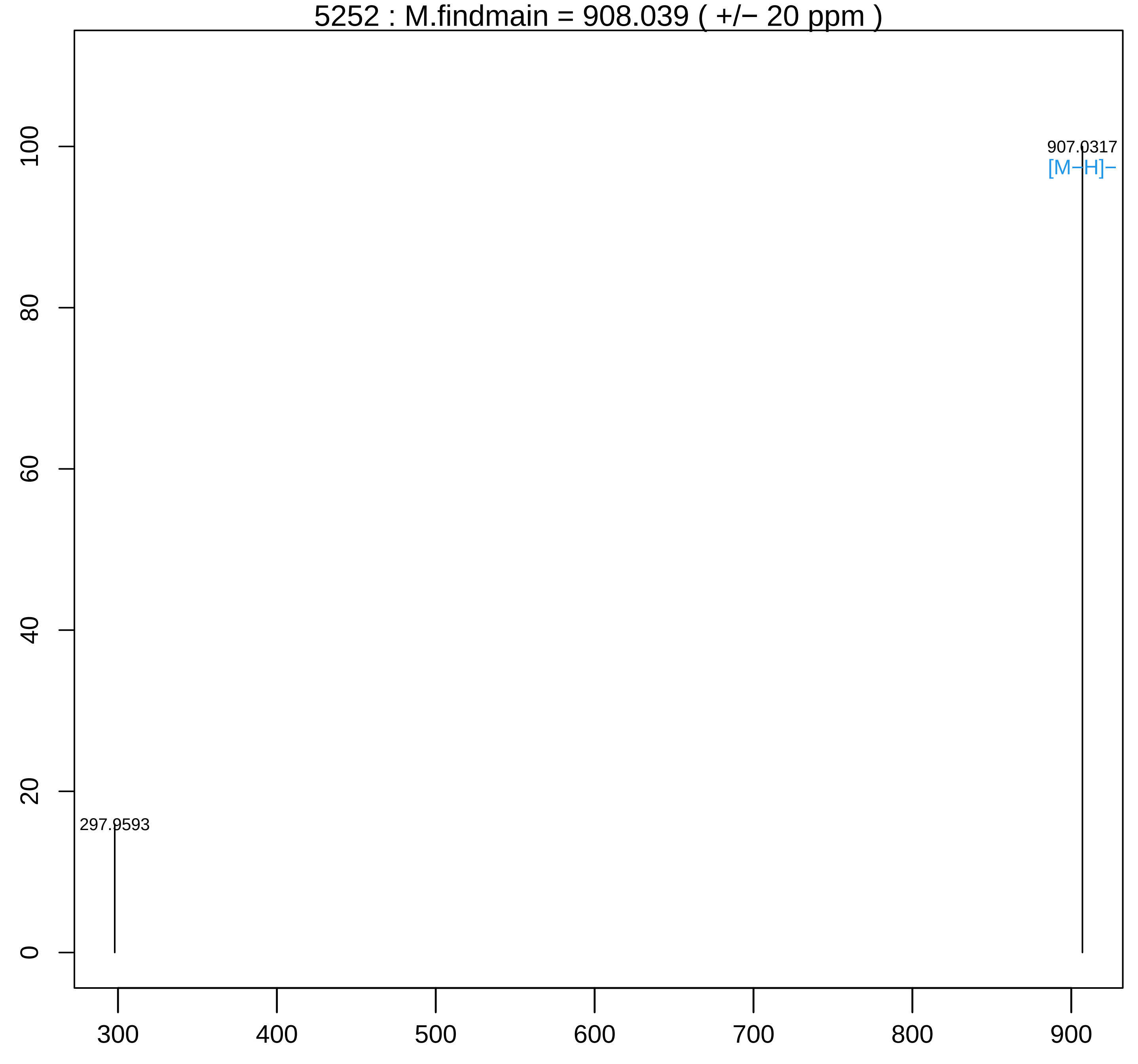

5252 : M.findmain = 908.039 (+/− 20 ppm)
907.0317
[M−H]−
297.9593
0
20
40
60
80
100
300
400
500
600
700
800
900

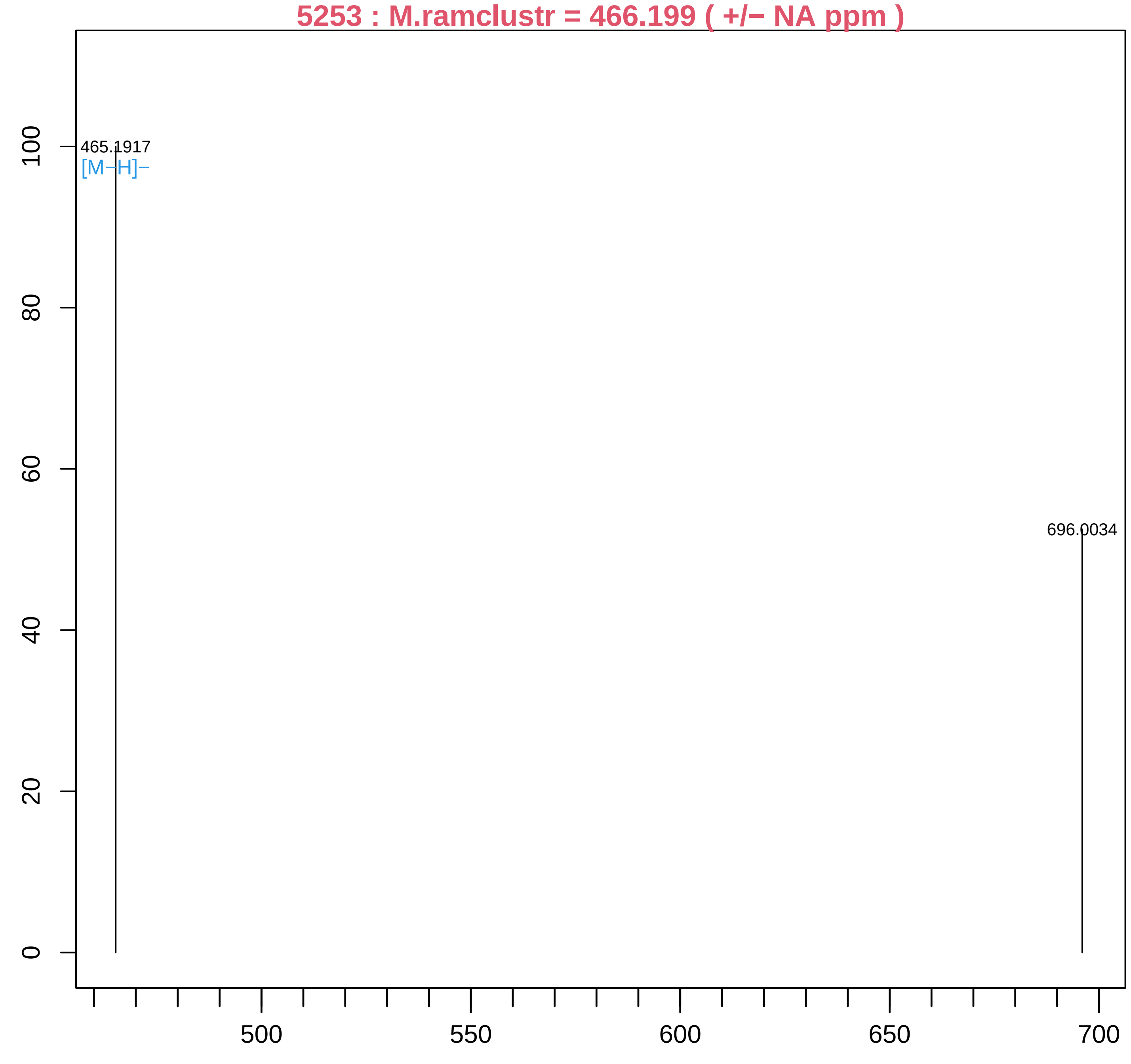
5253 : M.ramclustr = 466.199 (+/− NA ppm)
465.1917
[M−H]−
696.0034
0
20
40
60
80
100
500
550
600
650
700
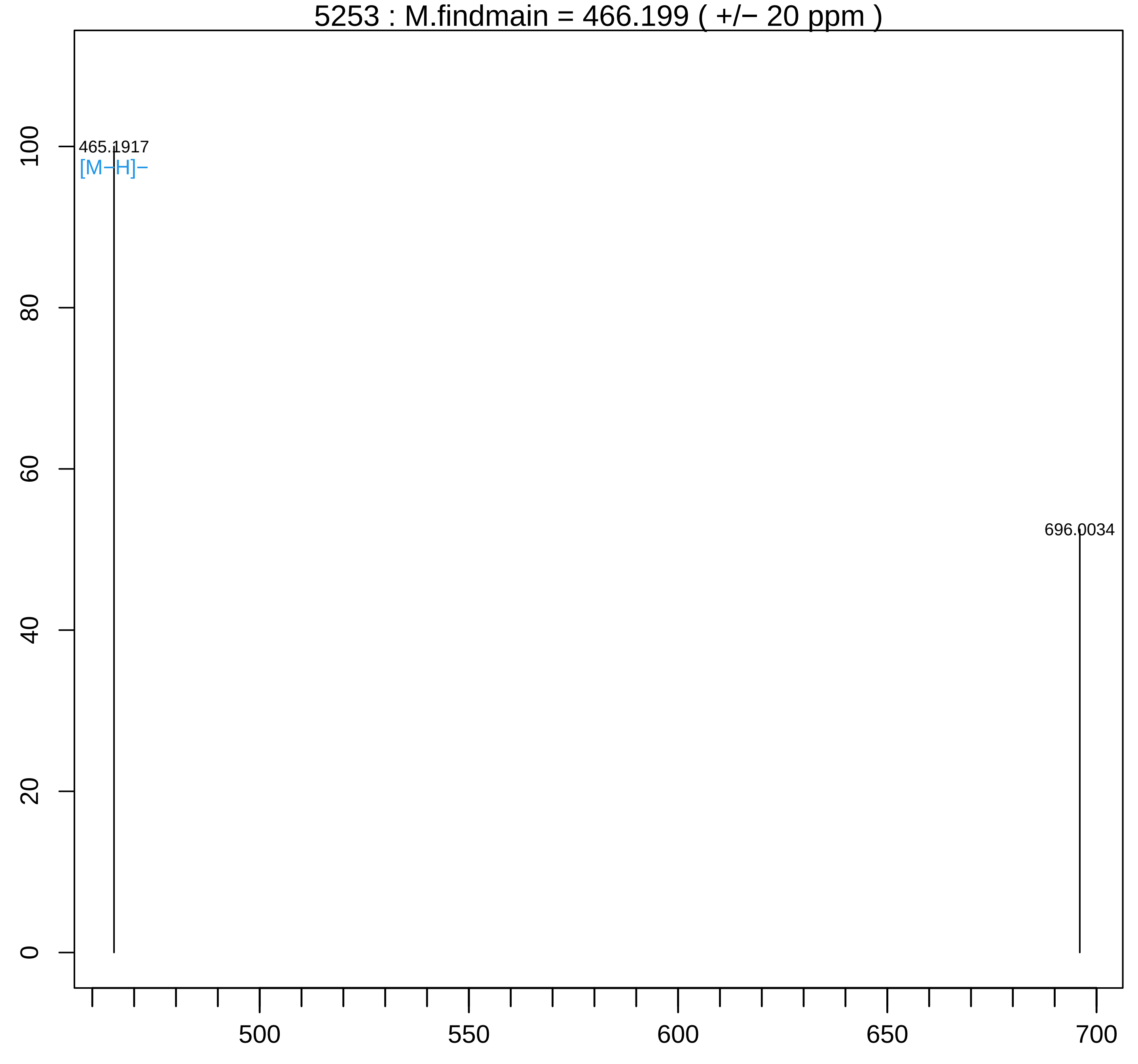
5253 : M.findmain = 466.199 (+/− 20 ppm)
465.1917
[M−H]−
696.0034
0
20
40
60
80
100
500
550
600
650
700

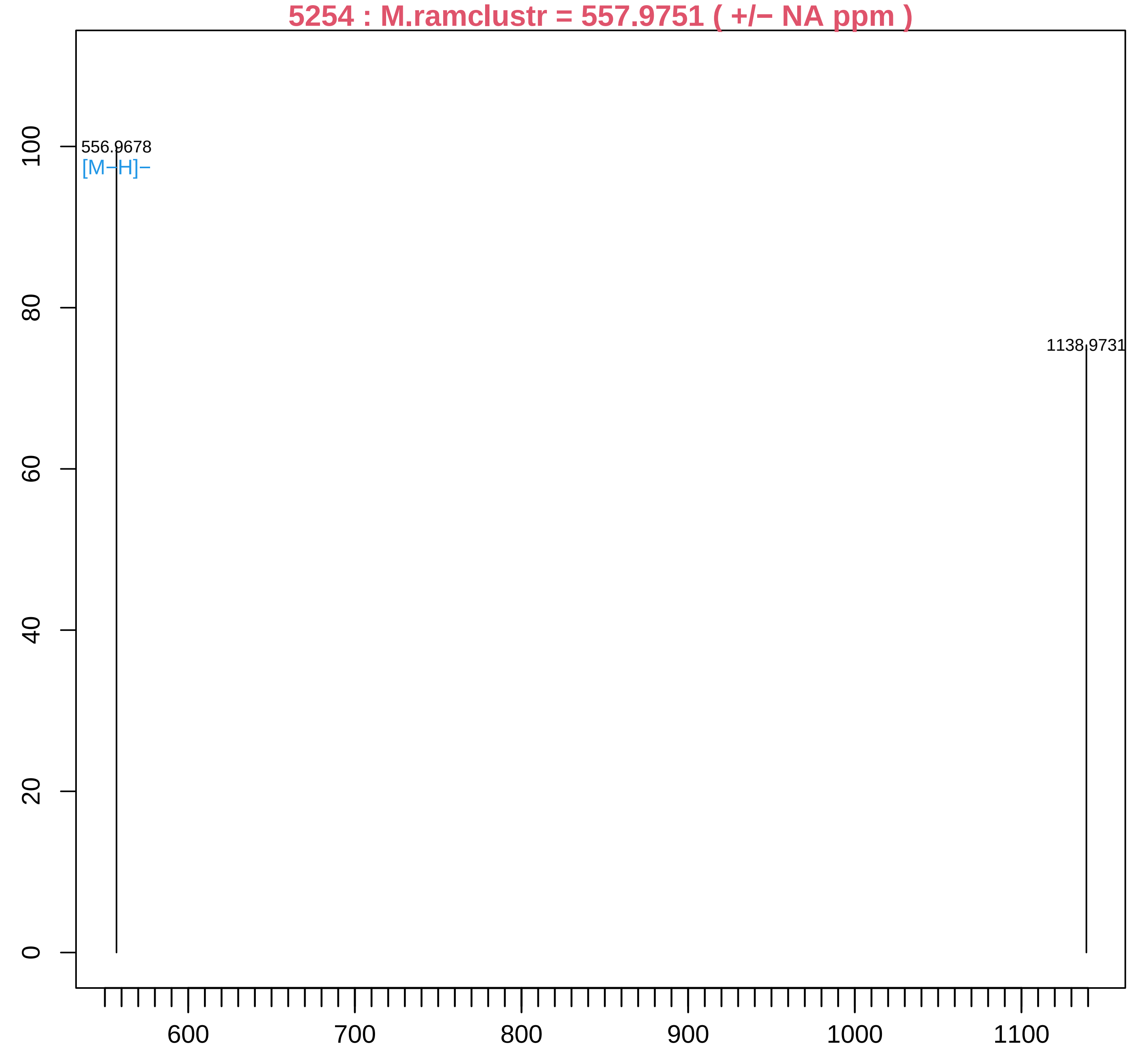

5254 : M.ramclustr = 557.9751 (+/− NA ppm)
556.9678
[M−H]−
1138.9731
0
20
40
60
80
100
600
700
800
900
1000
1100

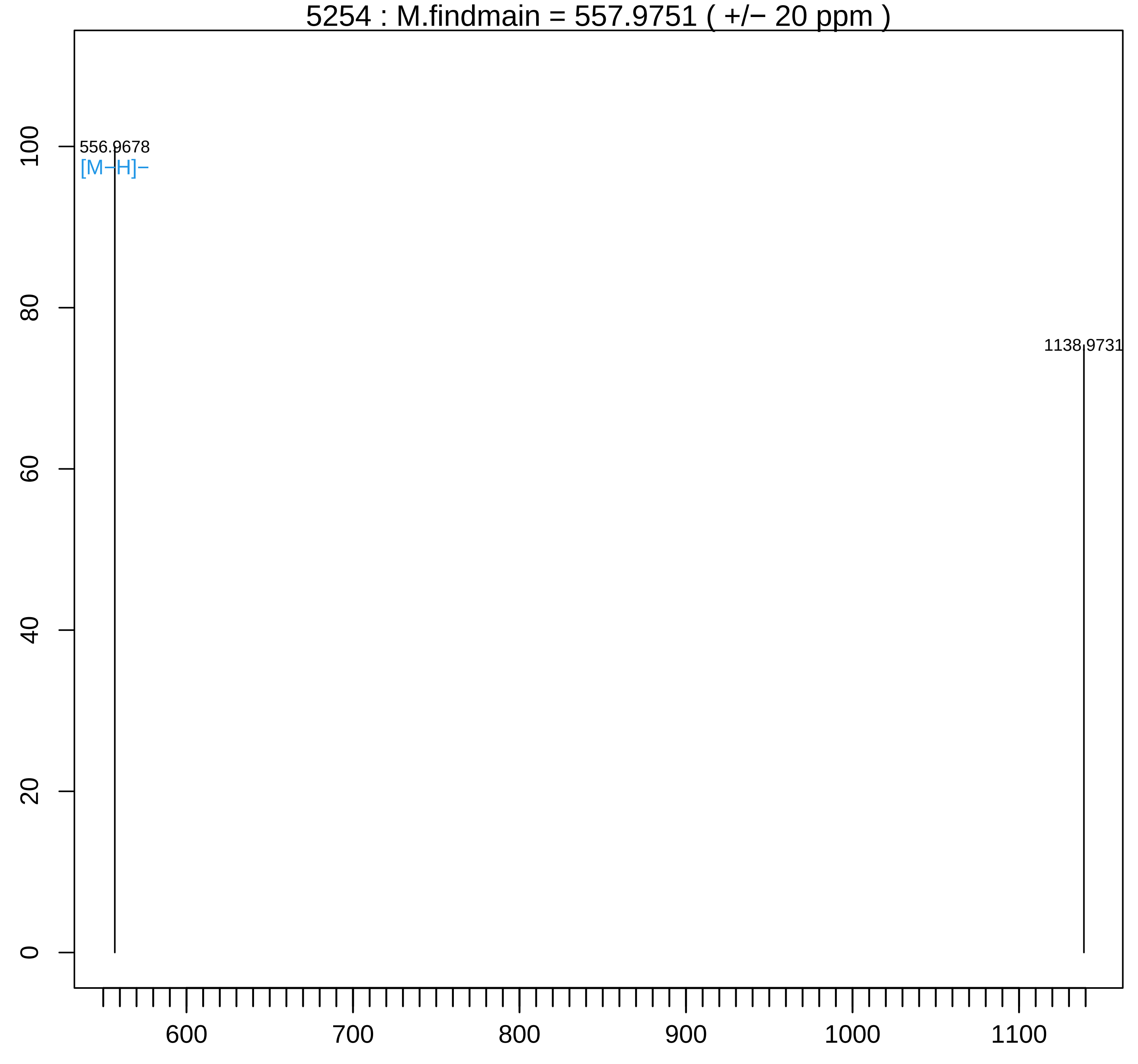

5254 : M.findmain = 557.9751 (+/− 20 ppm)
556.9678
[M−H]−
1138.9731
0
20
40
60
80
100
600
700
800
900
1000
1100

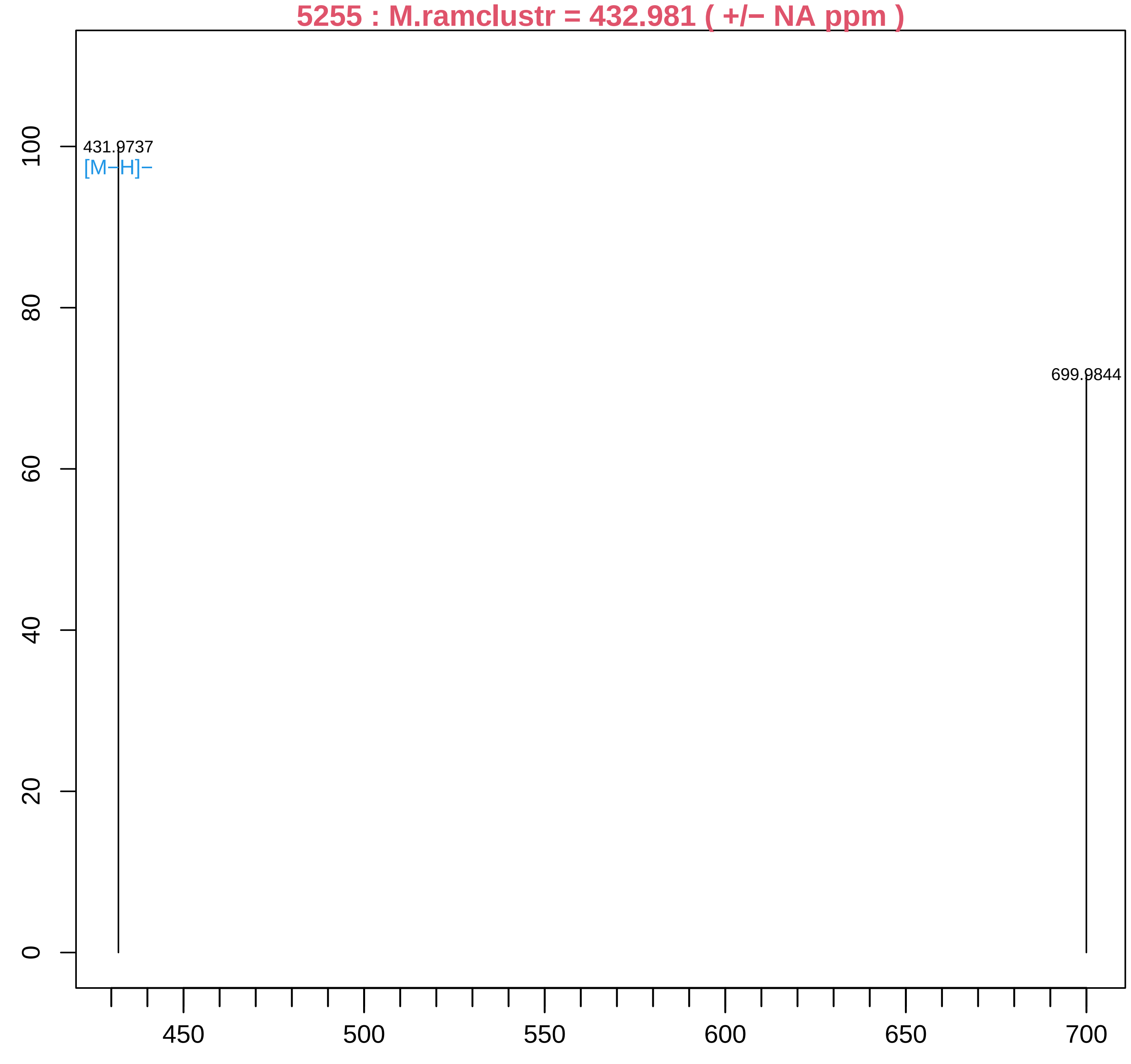
5255 : M.ramclustr = 432.981 (+/− NA ppm)
431.9737
[M−H]−
699.9844
0
20
40
60
80
100
450
500
550
600
650
700

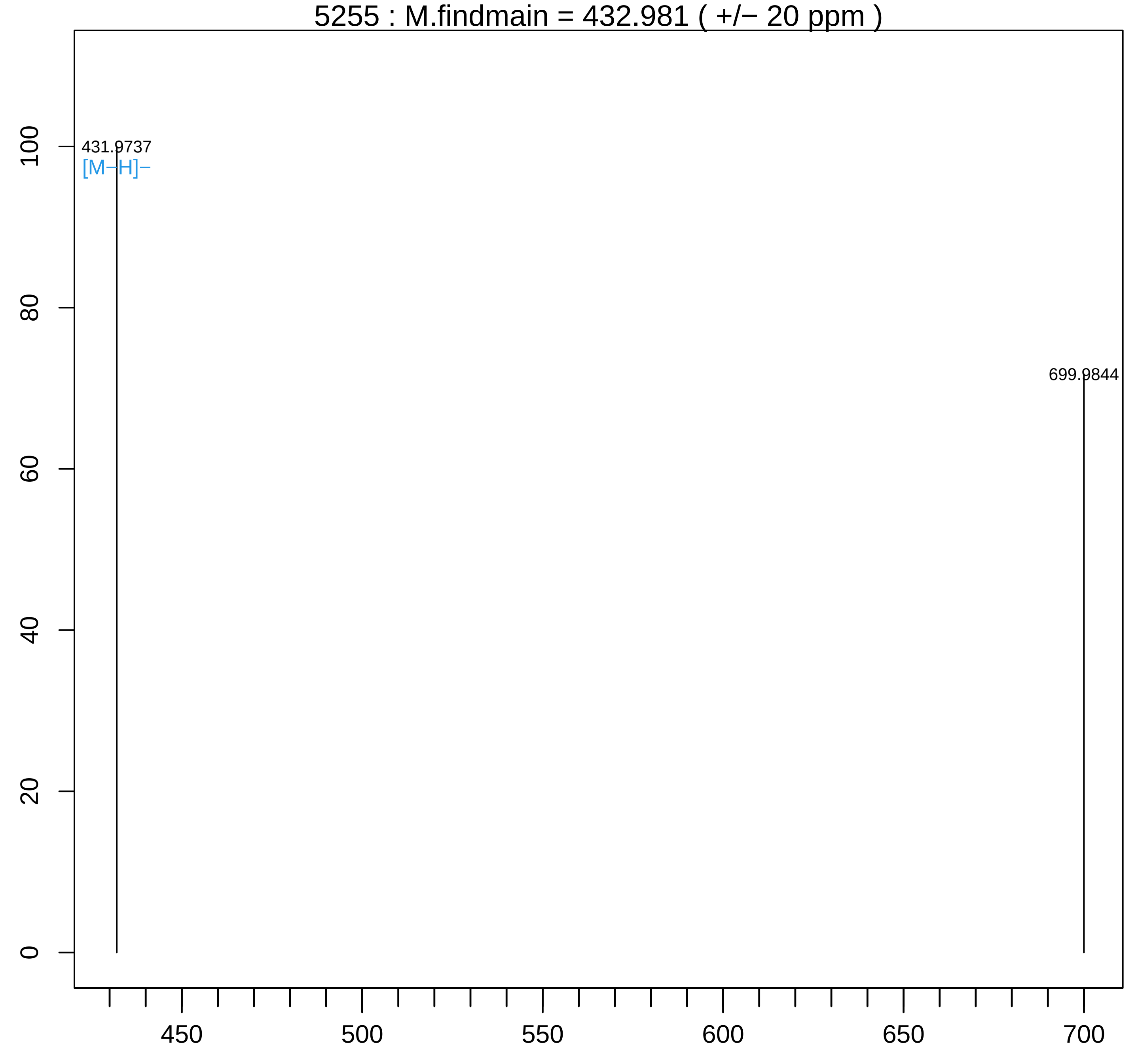
5255 : M.findmain = 432.981 (+/− 20 ppm)
431.9737
[M−H]−
699.9844
0
20
40
60
80
100
450
500
550
600
650
700

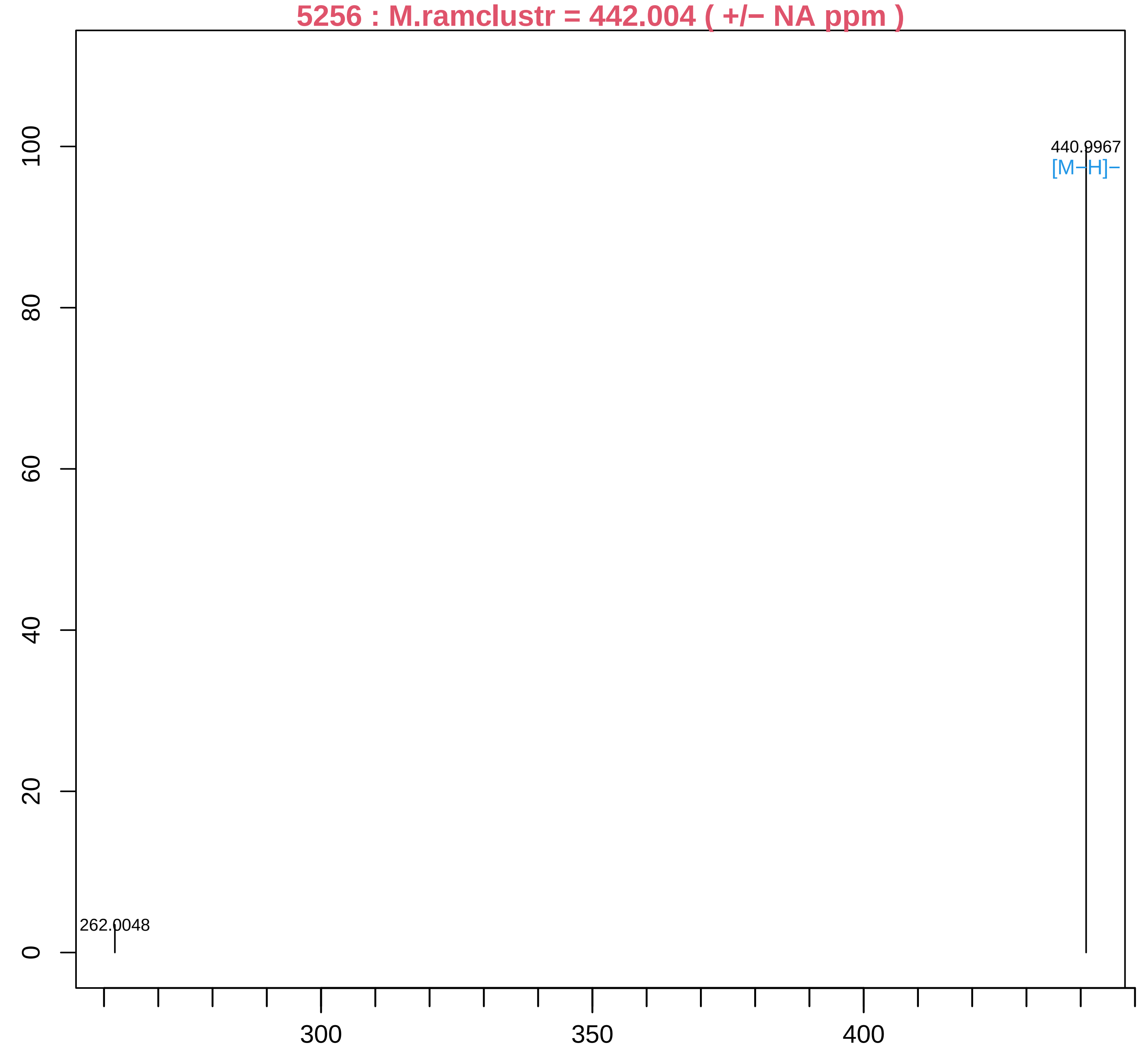
5256 : M.ramclustr = 442.004 (+/− NA ppm)
440.9967
[M−H]−
262.0048
0
20
40
60
80
100
300
350
400

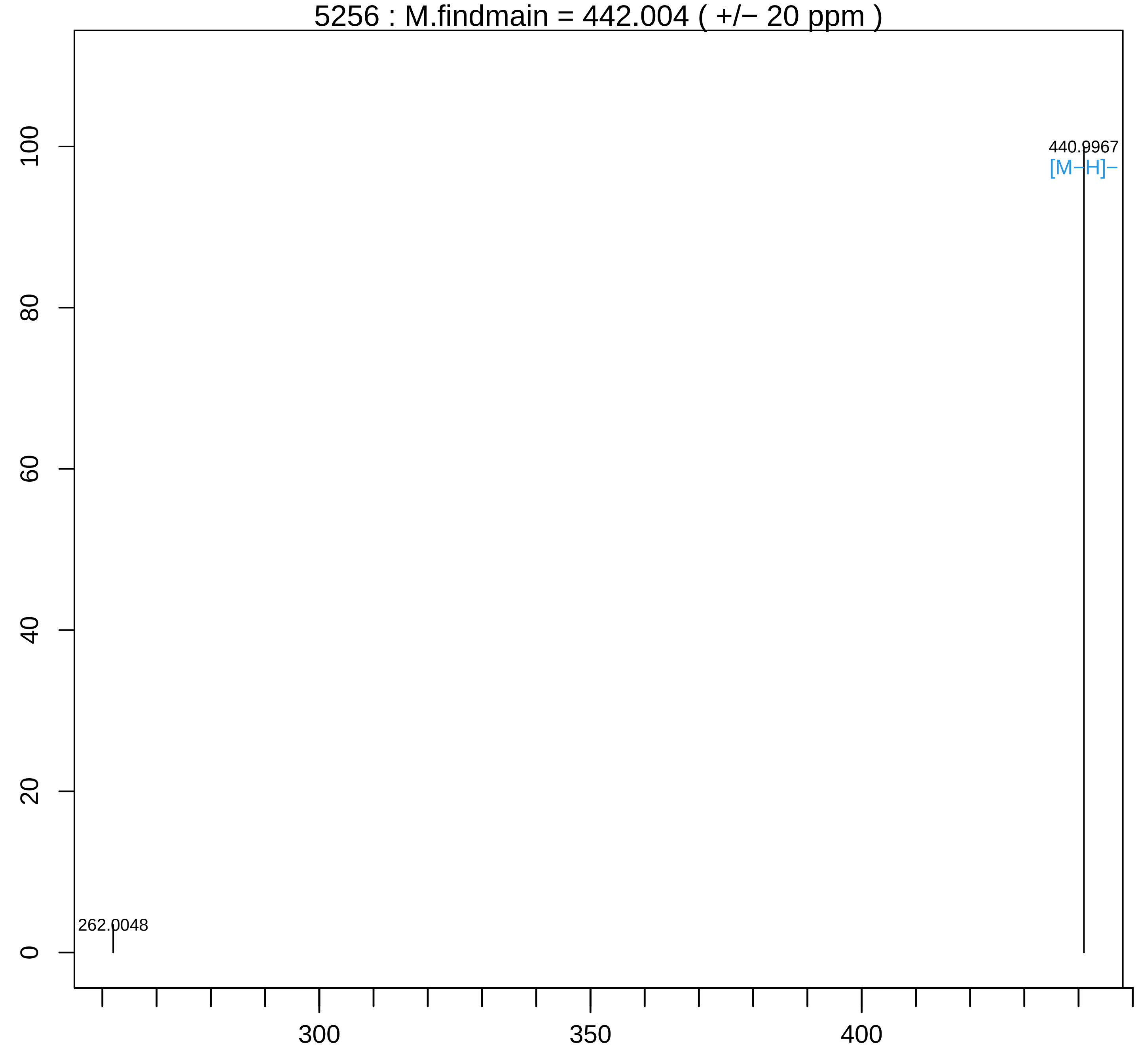
5256 : M.findmain = 442.004 (+/− 20 ppm)
440.9967
[M−H]−
262.0048
0
20
40
60
80
100
300
350
400

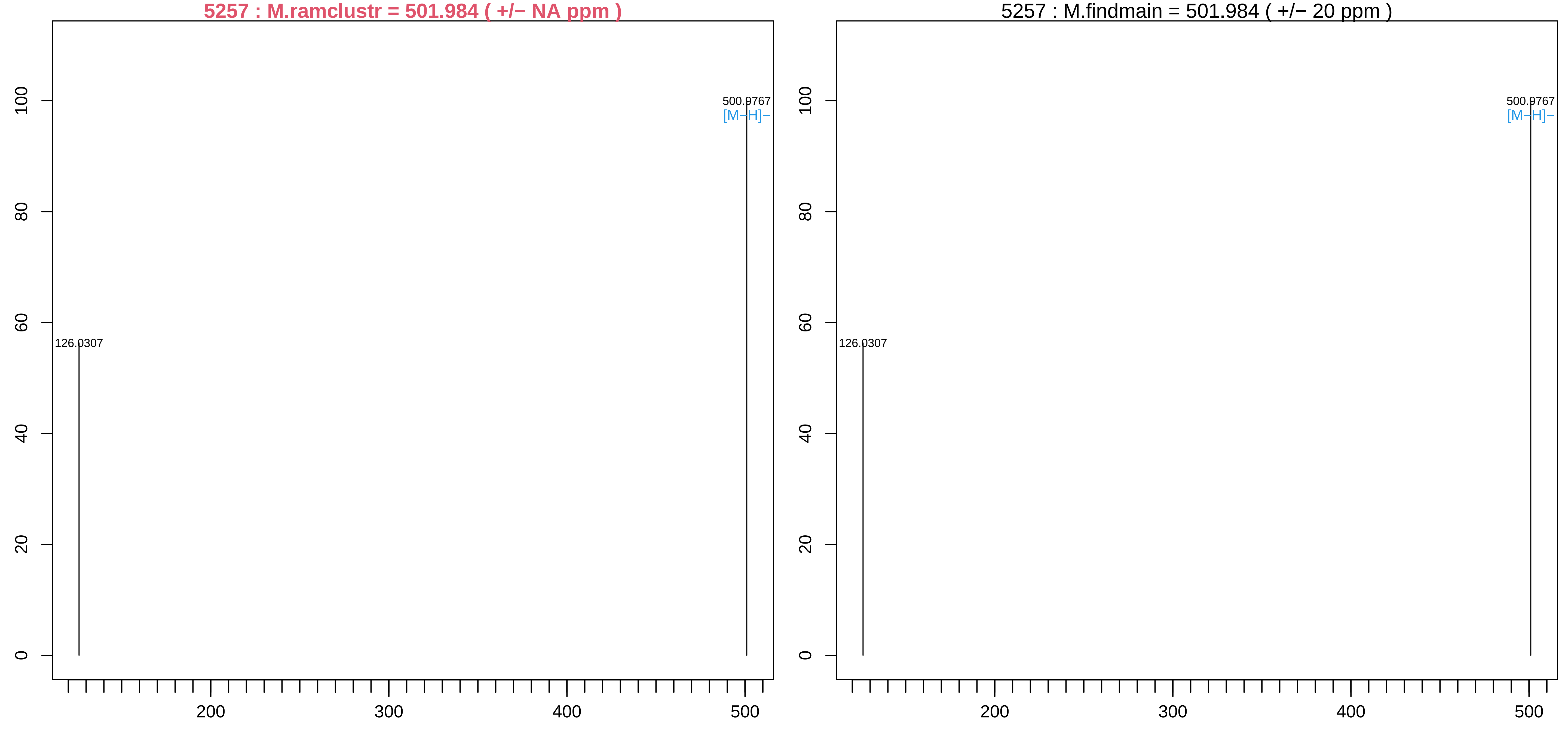
5257 : M.ramclustr = 501.984 (+/− NA ppm)
5257 : M.findmain = 501.984 (+/− 20 ppm)
500.9767
[M−H]−
126.0307
0
20
40
60
80
100
200
300
400
500

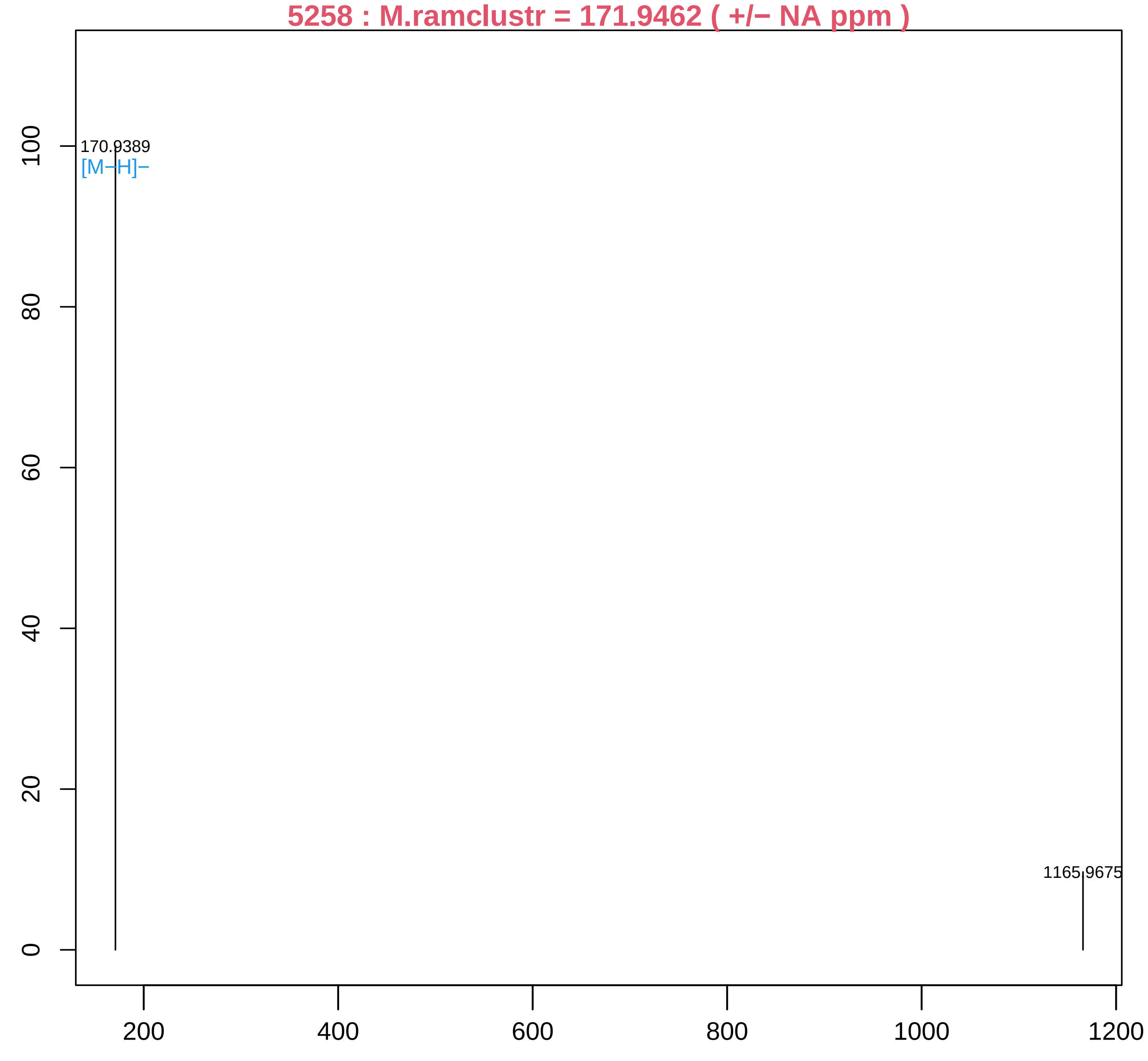
5258 : M.ramclustr = 171.9462 (+/− NA ppm)
170.9389
[M−H]−
1165.9675
0
20
40
60
80
100
200
400
600
800
1000
1200

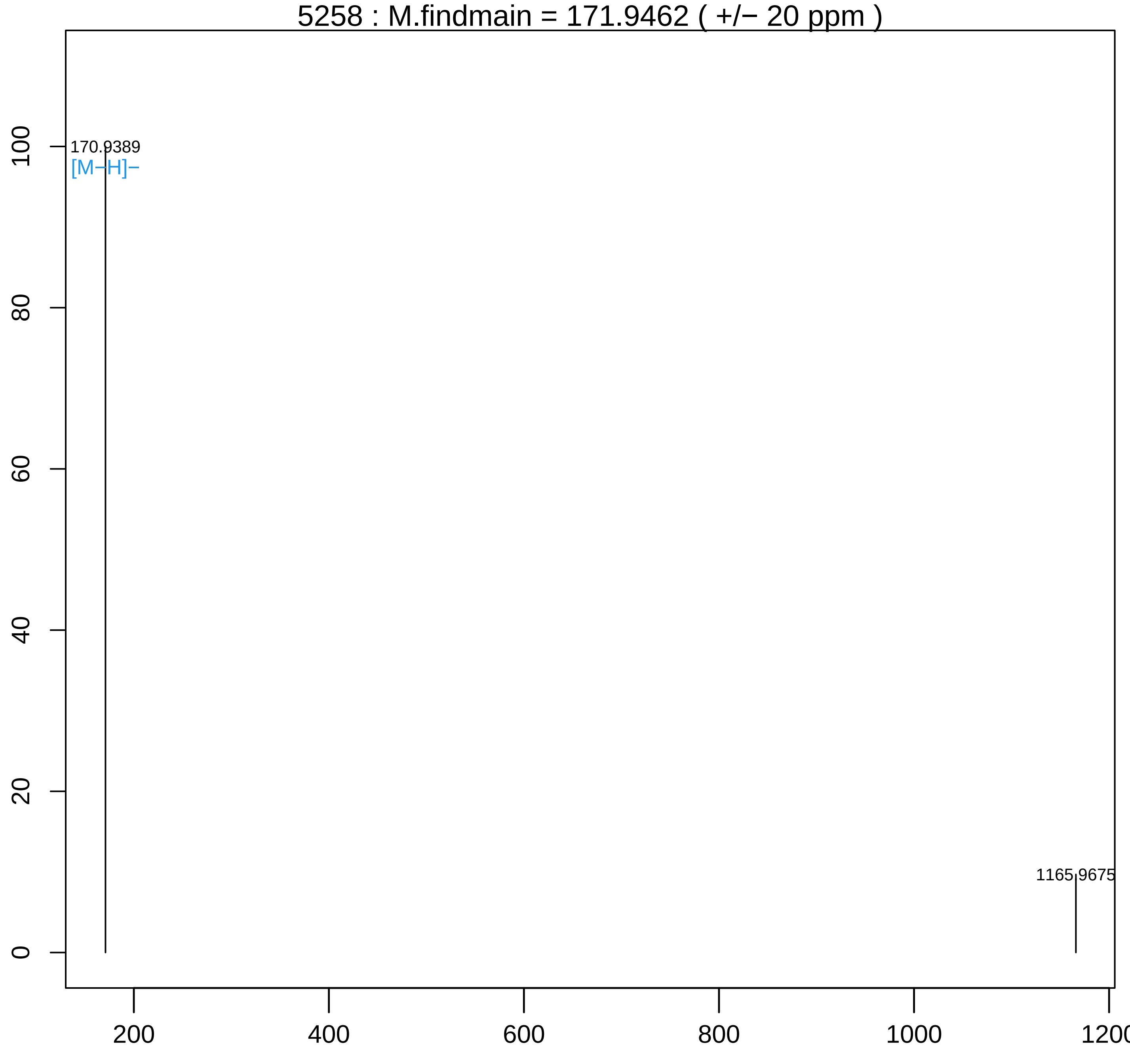
5258 : M.findmain = 171.9462 (+/− 20 ppm)
170.9389
[M−H]−
1165.9675
0
20
40
60
80
100
200
400
600
800
1000

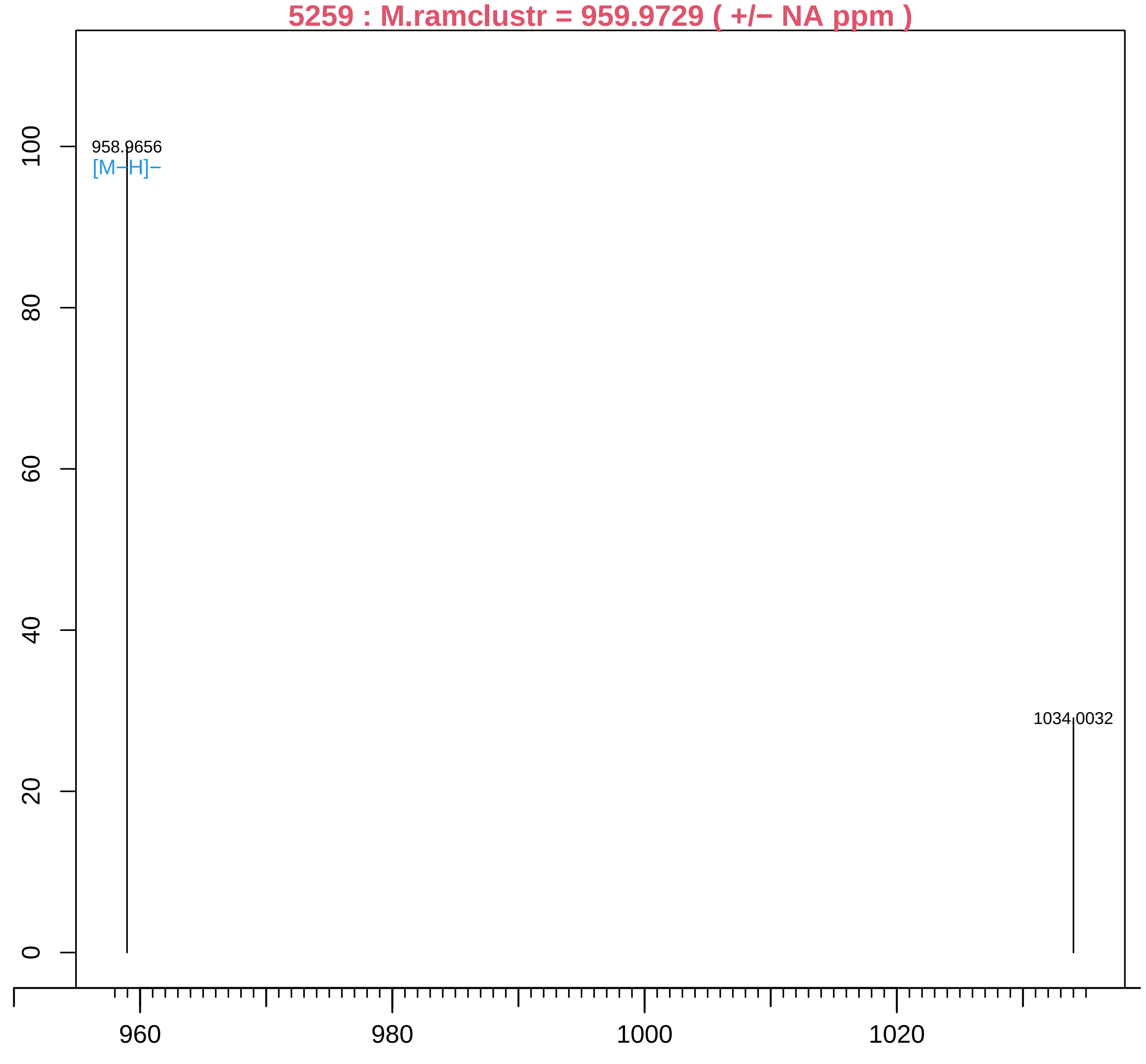
5259 : M.ramclustr = 959.9729 (+/− NA ppm)
958.9656
[M−H]−
1034.0032
0
20
40
60
80
100
960
980
1000
1020

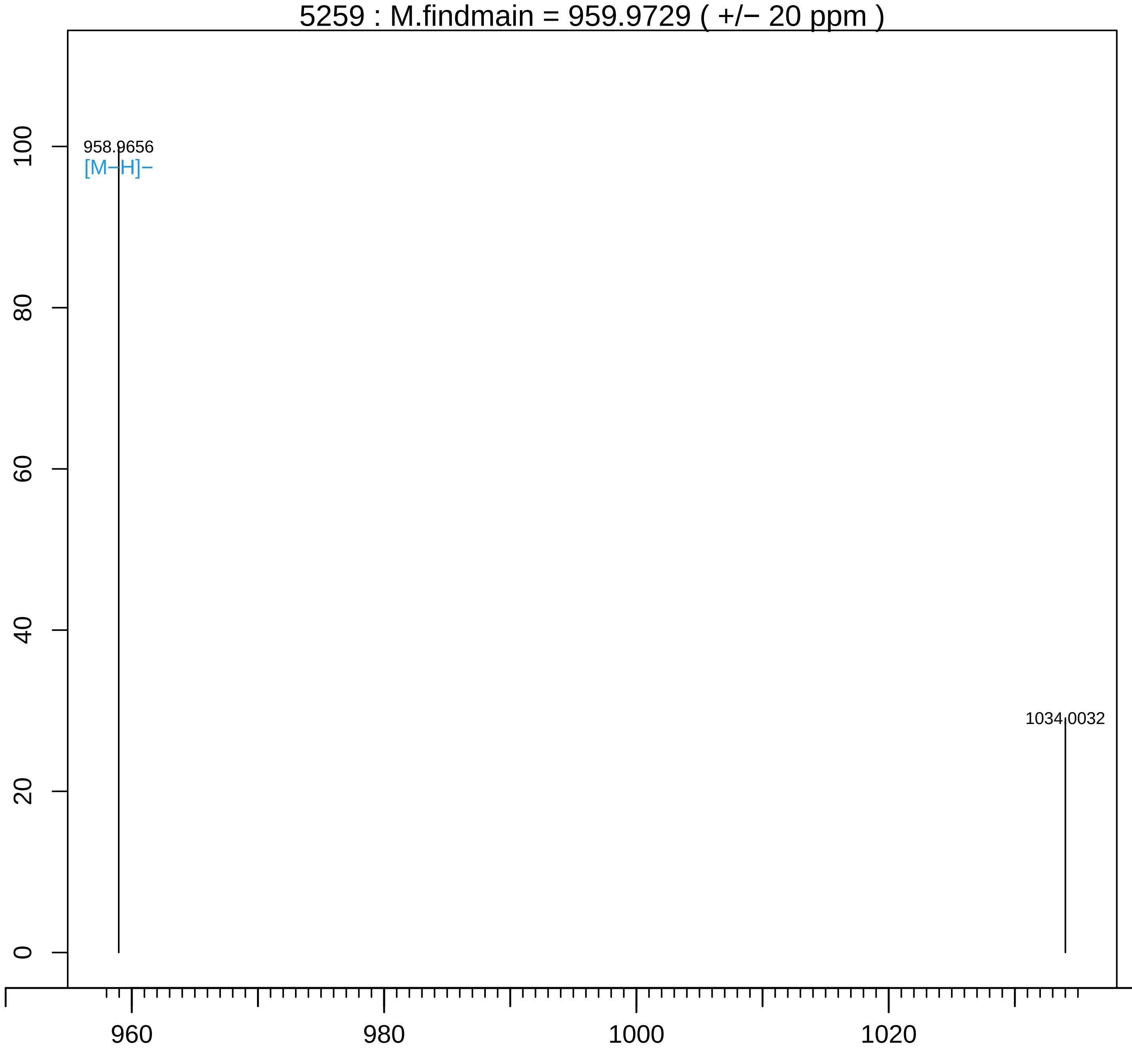
5259 : M.findmain = 959.9729 (+/− 20 ppm)
958.9656
[M−H]−
1034.0032
0
20
40
60
80
100
960
980
1000
1020

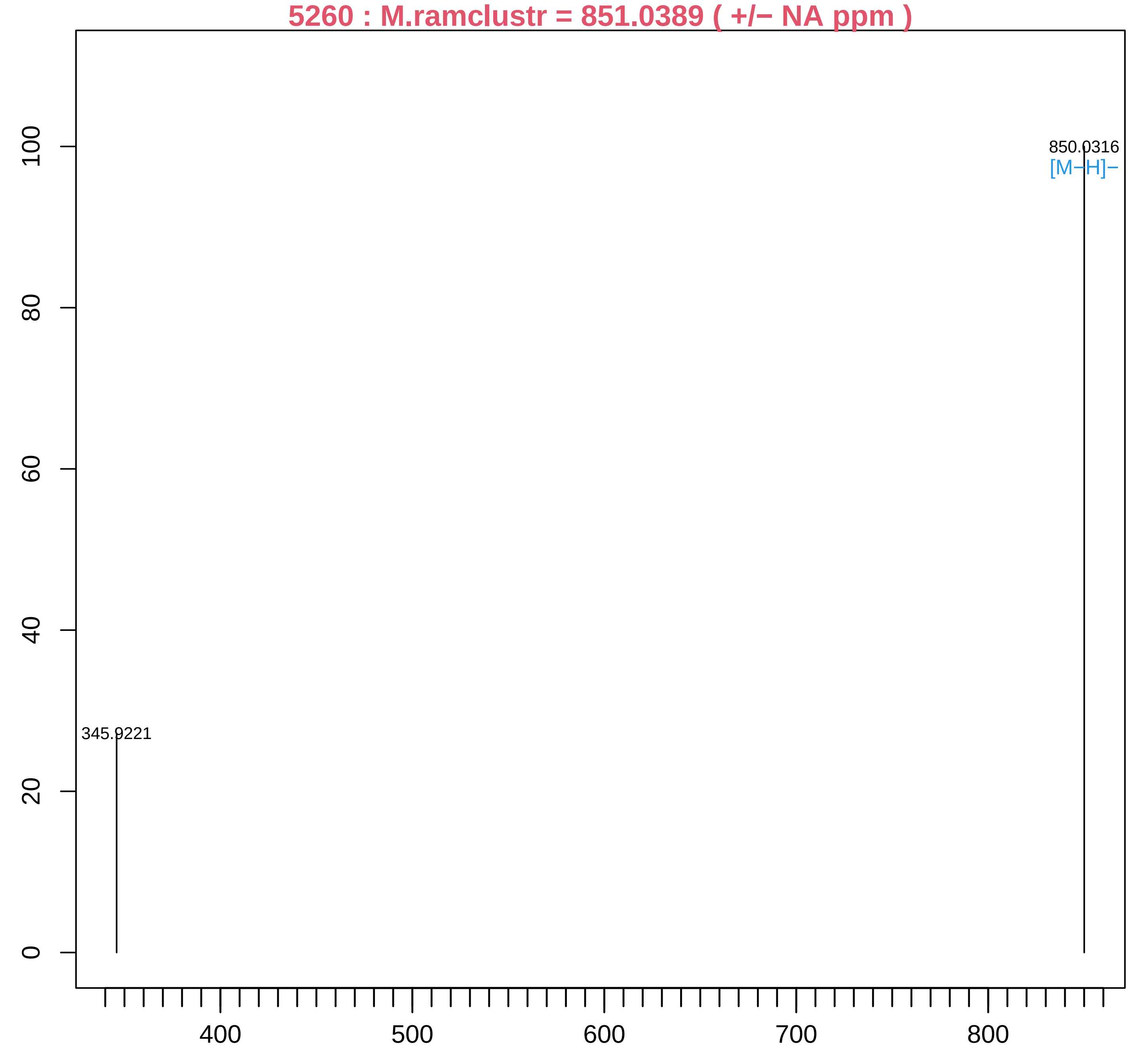

5260 : M.ramclustr = 851.0389 (+/− NA ppm)
850.0316
[M−H]−
345.9221
0
20
40
60
80
100
400
500
600
700
800

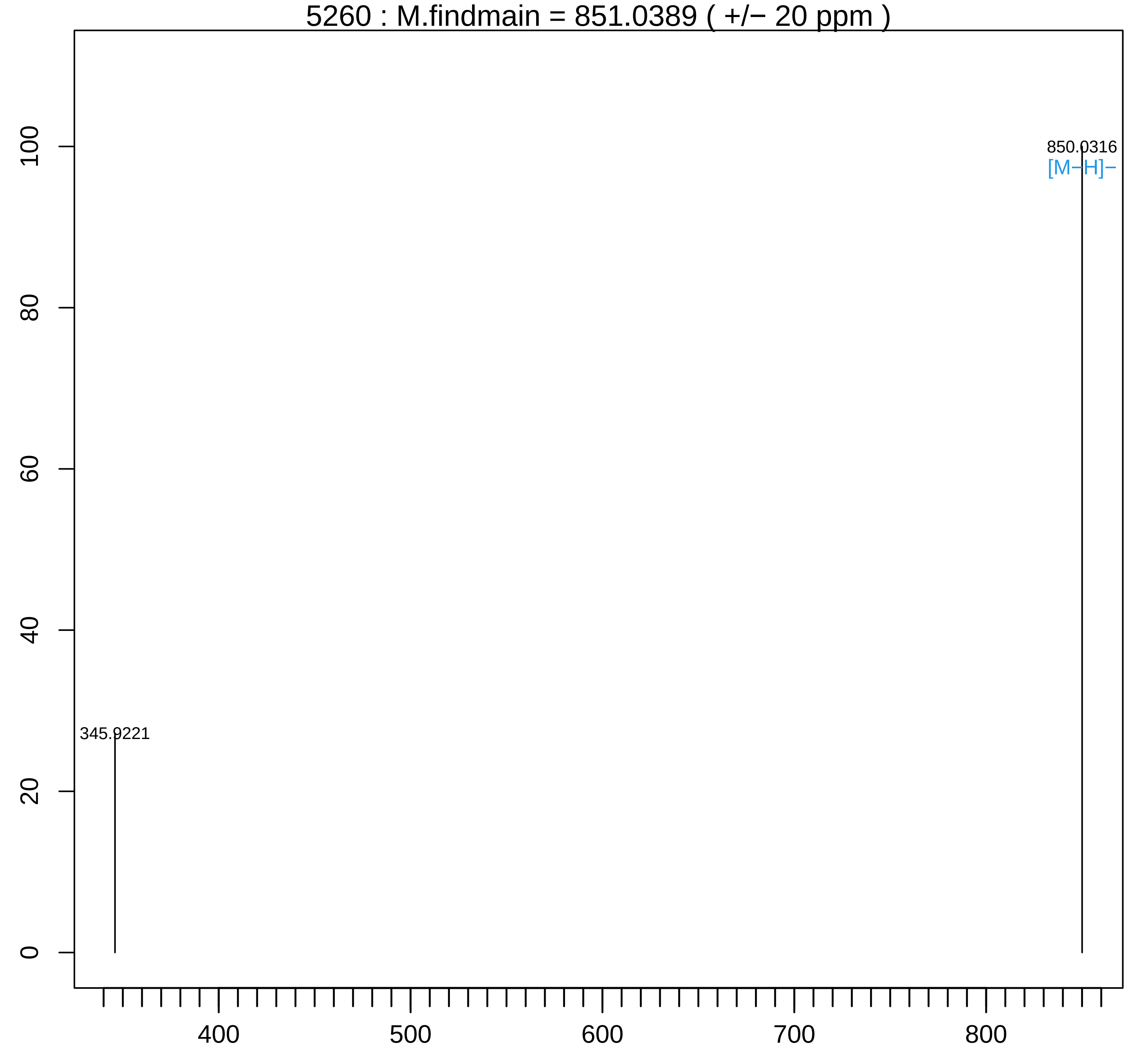

5260 : M.findmain = 851.0389 (+/− 20 ppm)
850.0316
[M−H]−
345.9221
0
20
40
60
80
100
400
500
600
700
800

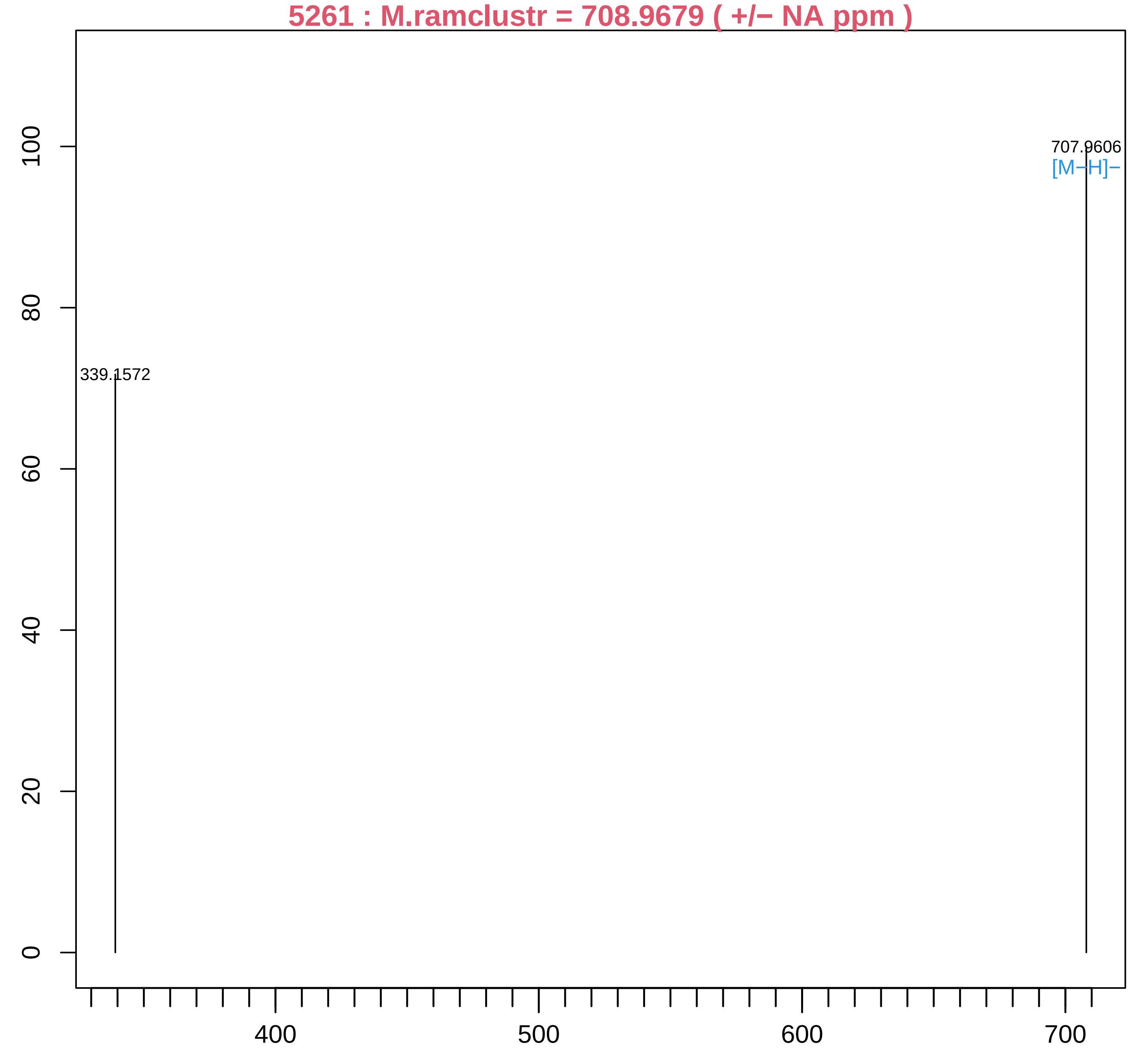
5261 : M.ramclustr = 708.9679 (+/− NA ppm)
707.9606
[M−H]−
339.1572
0
20
40
60
80
100
400
500
600
700

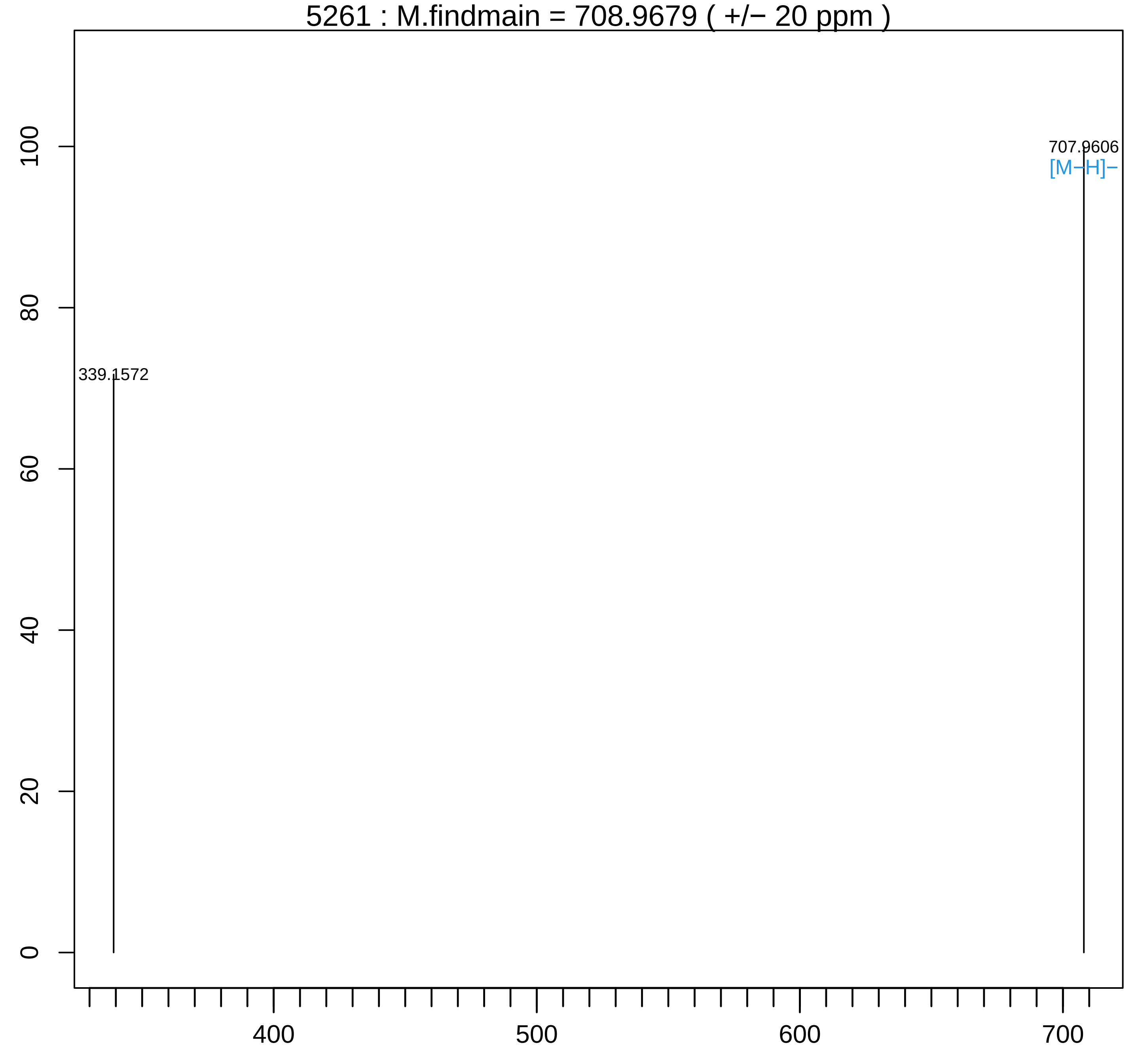
5261 : M.findmain = 708.9679 (+/− 20 ppm)
707.9606
[M−H]−
339.1572
0
20
40
60
80
100
400
500
600
700

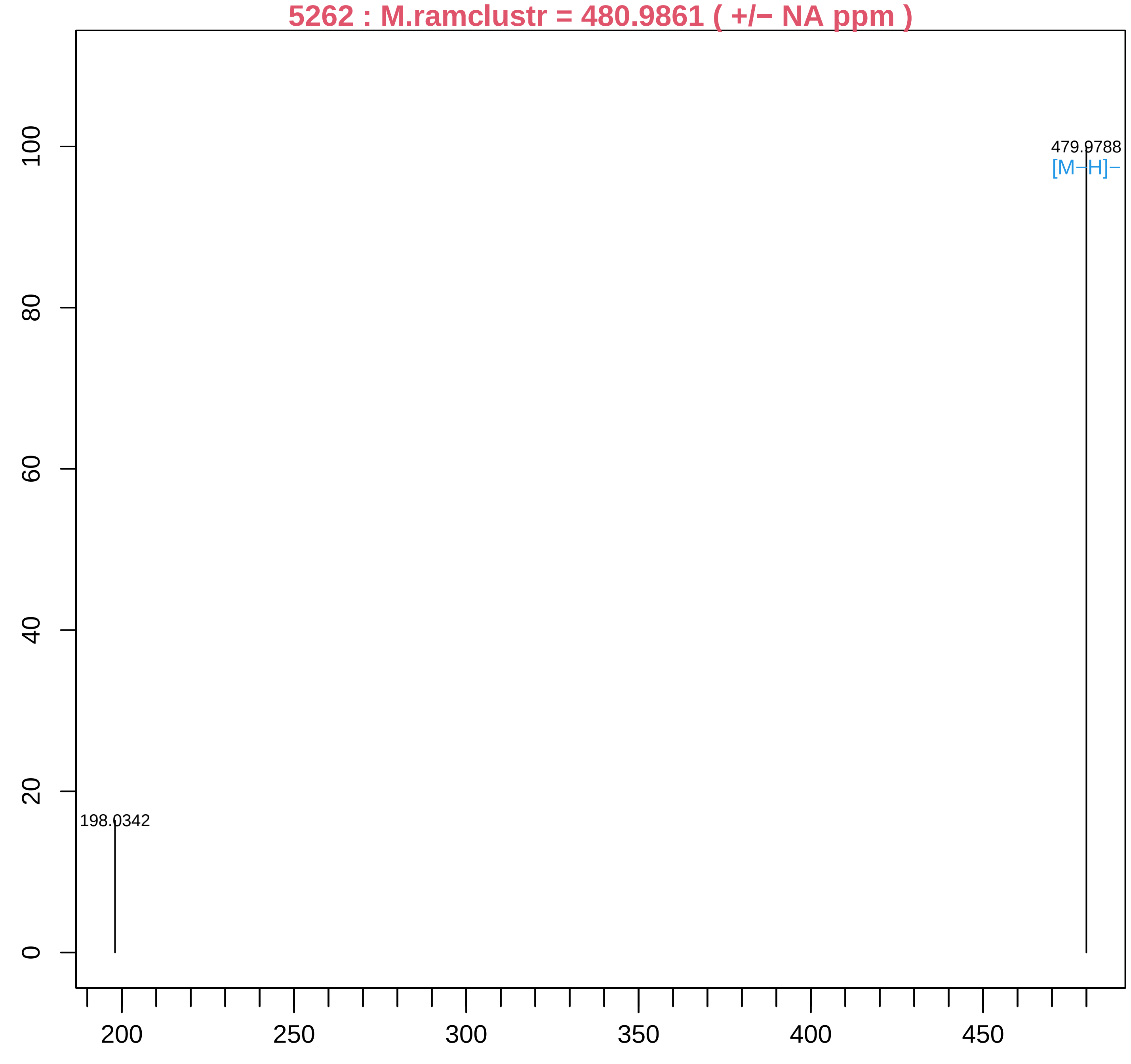

5262 : M.ramclustr = 480.9861 (+/− NA ppm)
479.9788
[M−H]−
198.0342
0
20
40
60
80
100
200
250
300
350
400
450

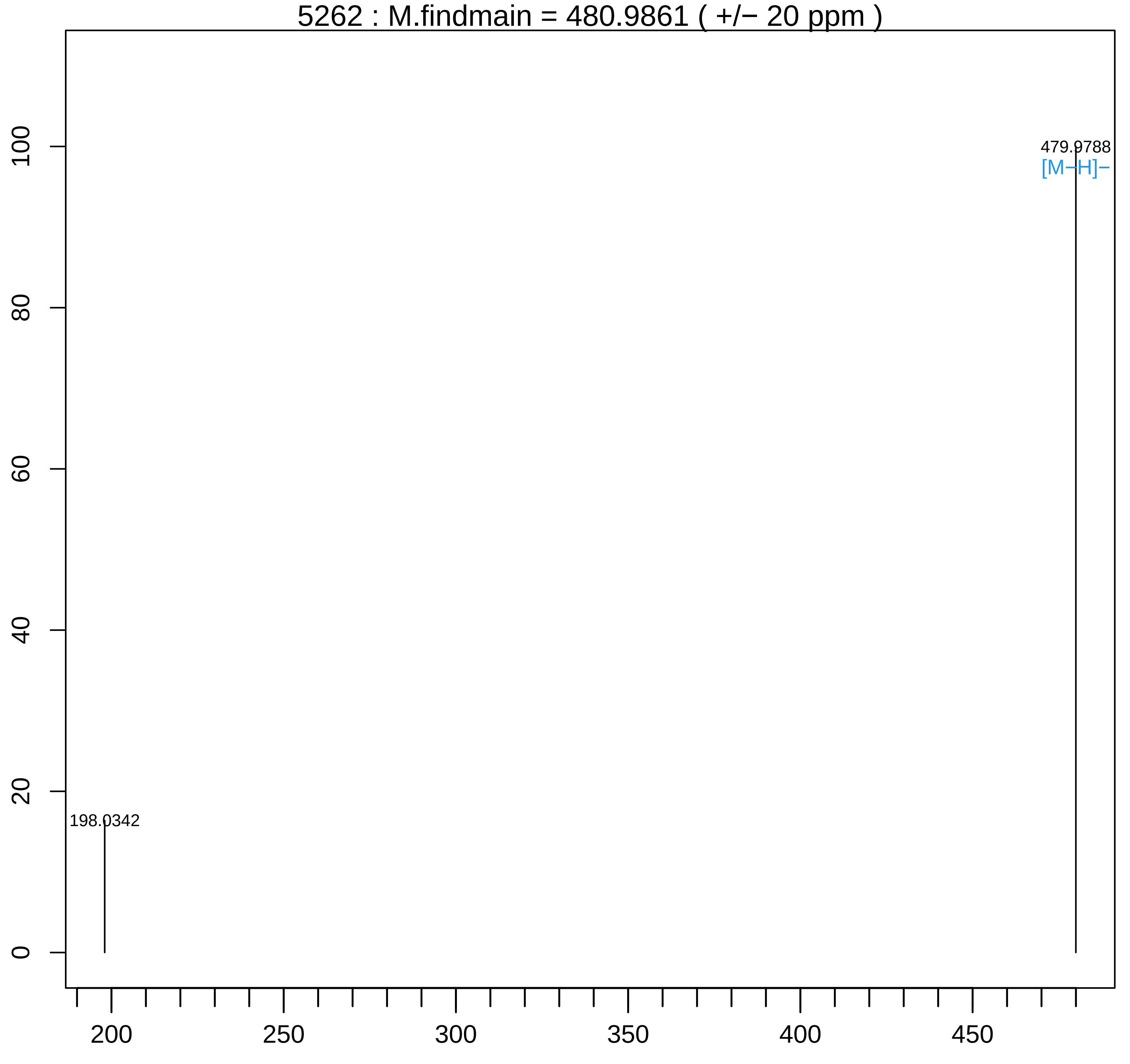

5262 : M.findmain = 480.9861 (+/− 20 ppm)
479.9788
[M−H]−
198.0342
0
20
40
60
80
100
200
250
300
350
400
450

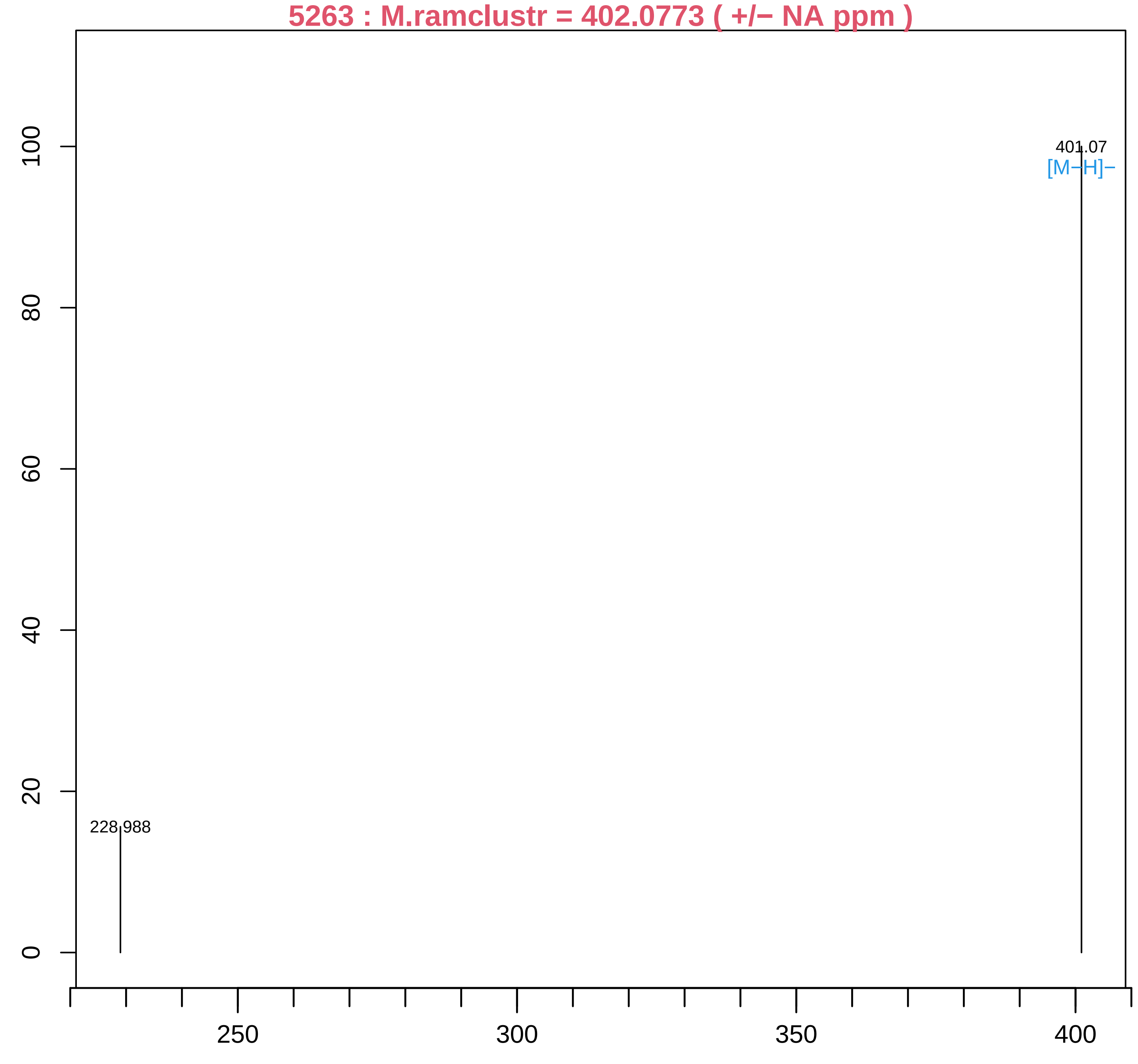
5263 : M.ramclustr = 402.0773 (+/− NA ppm)
401.07
[M−H]−
228.988
0
20
40
60
80
100
250
300
350
400

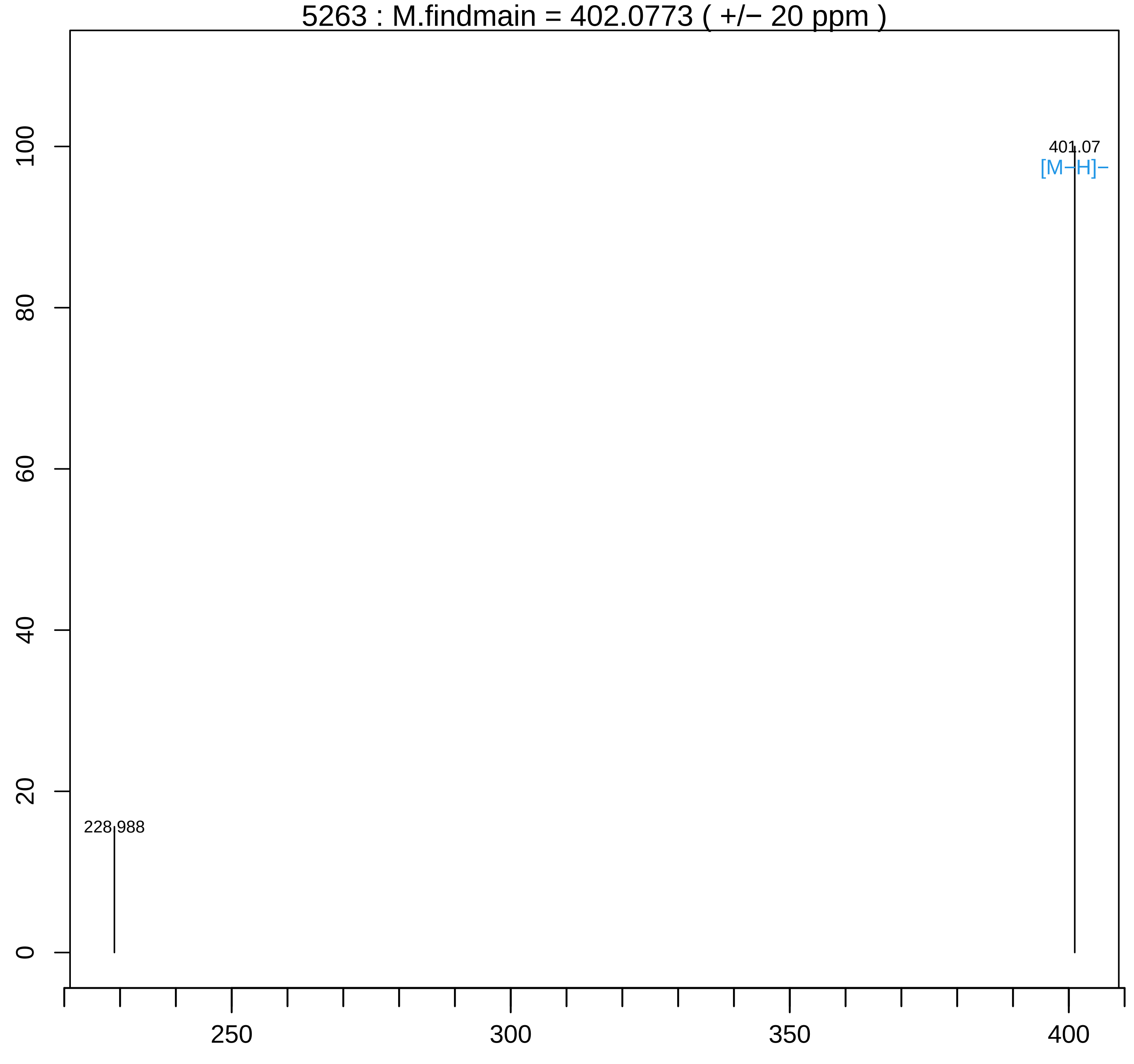
5263 : M.findmain = 402.0773 (+/− 20 ppm)
401.07
[M−H]−
228.988
0
20
40
60
80
100
250
300
350
400

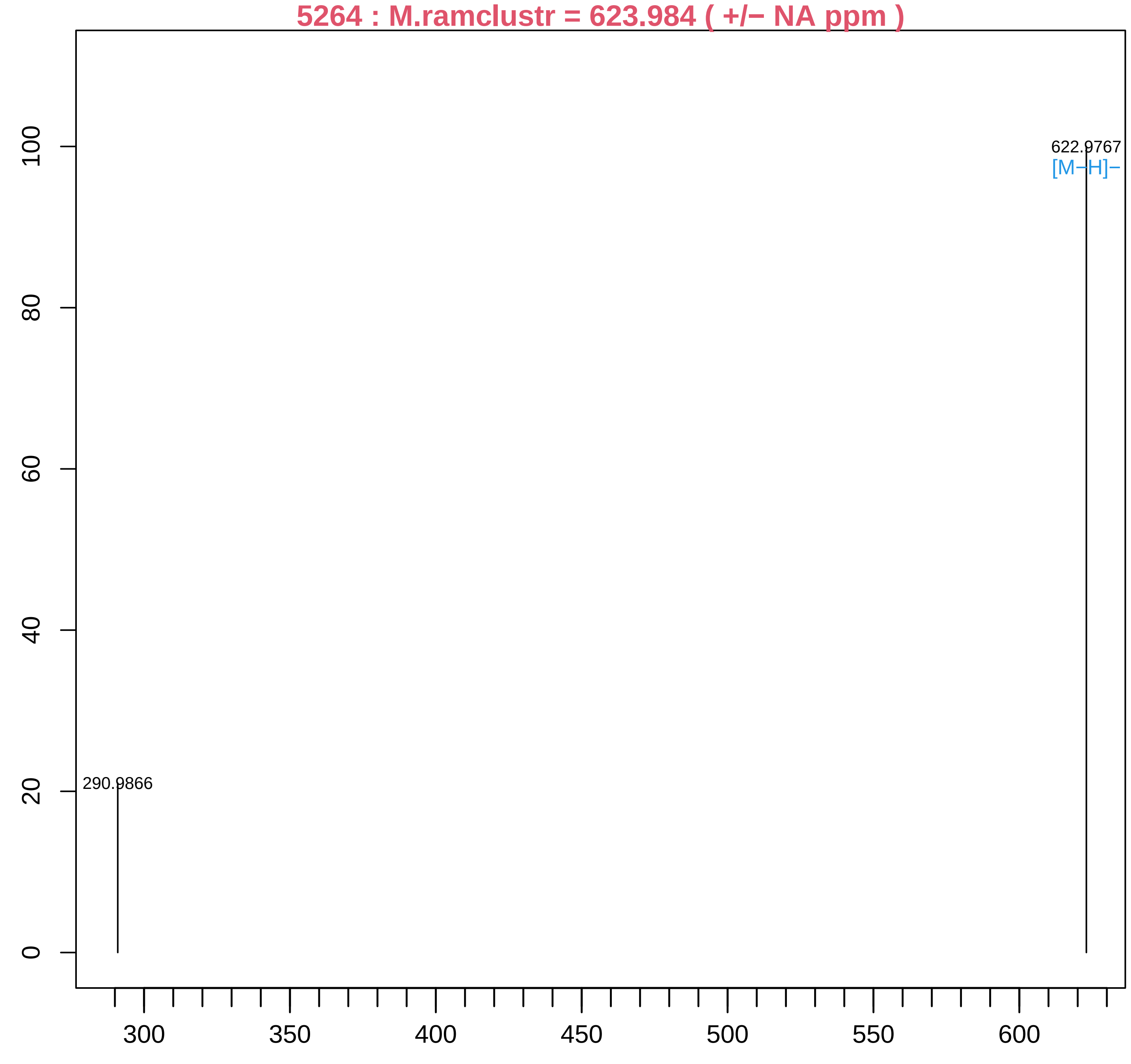
5264 : M.ramclustr = 623.984 (+/− NA ppm)
622.9767
[M−H]−
290.9866
0
20
40
60
80
100
300
350
400
450
500
550
600

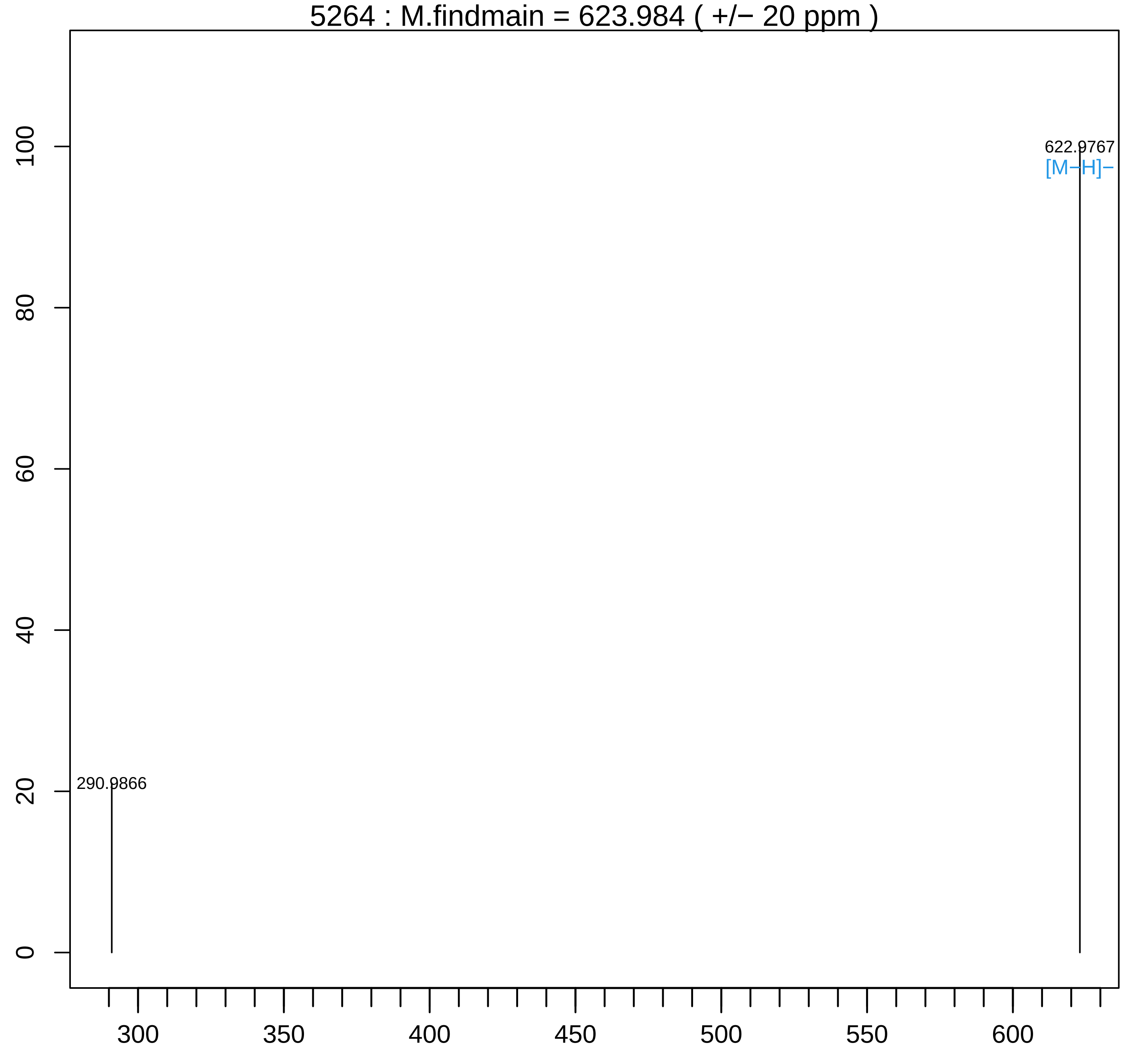
5264 : M.findmain = 623.984 (+/− 20 ppm)
622.9767
[M−H]−
290.9866
0
20
40
60
80
100
300
350
400
450
500
550
600

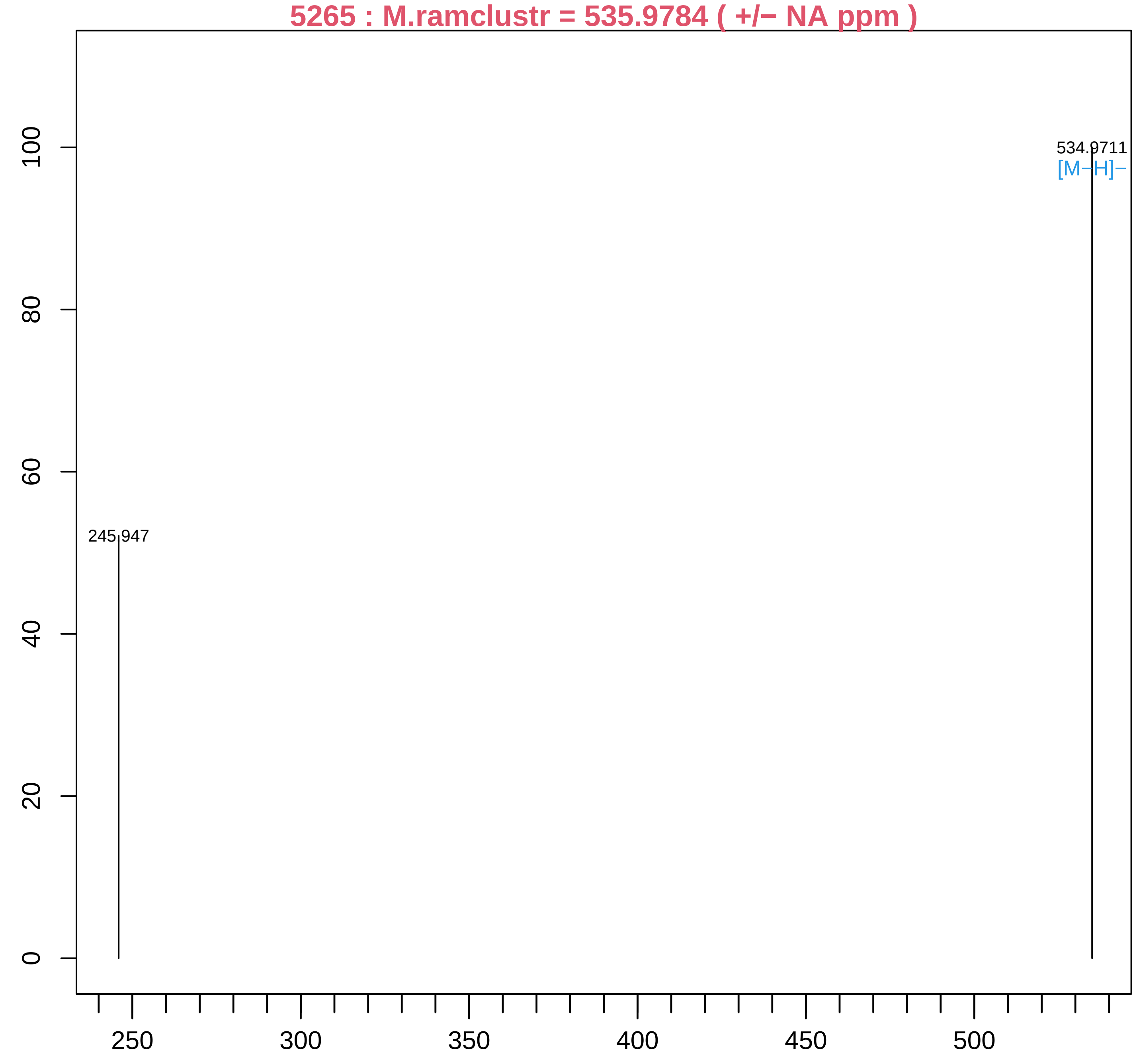

5265 : M.ramclustr = 535.9784 (+/− NA ppm)
534.9711
[M−H]−
245.947
0
20
40
60
80
100
250
300
350
400
450
500

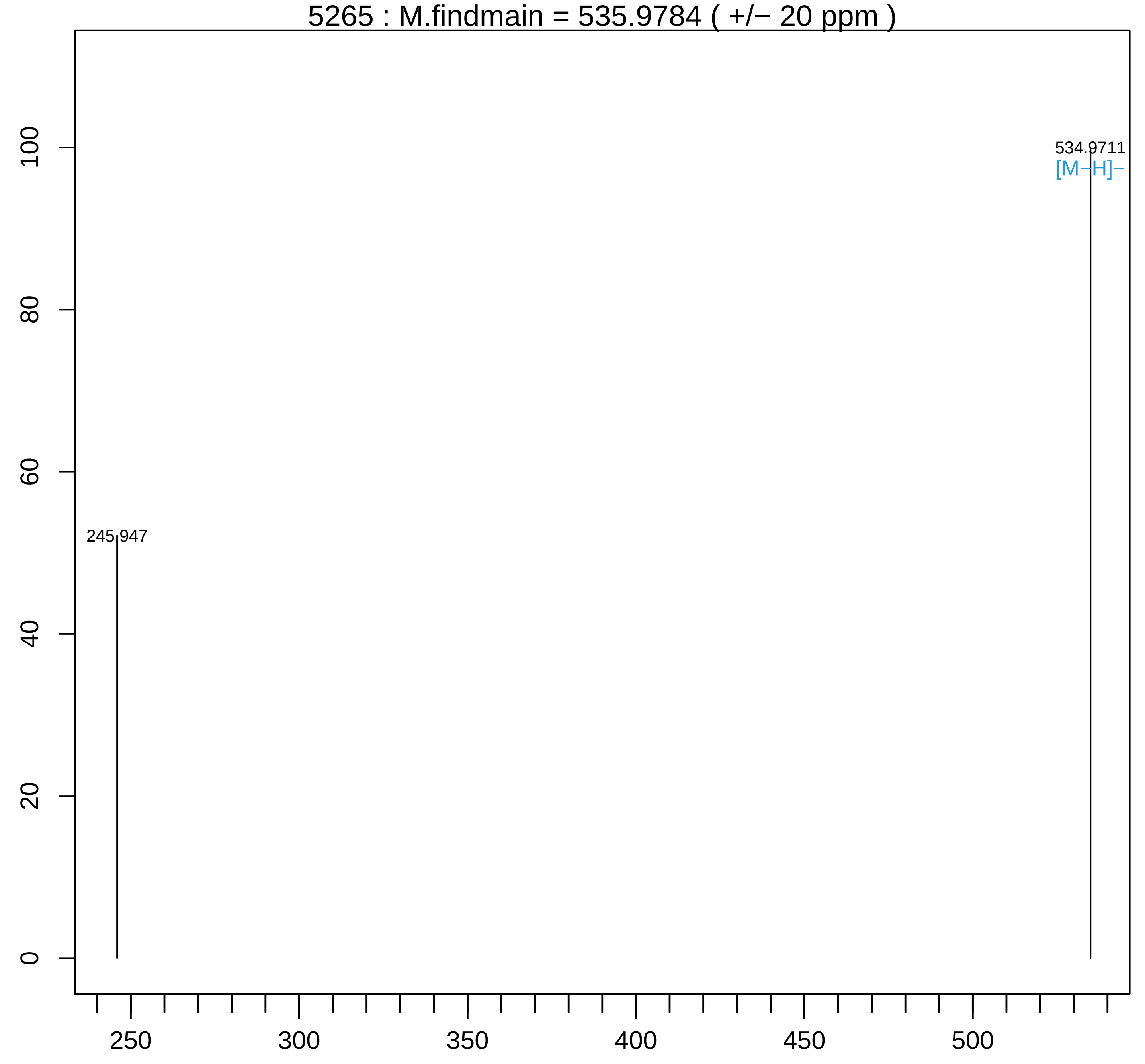

5265 : M.findmain = 535.9784 (+/− 20 ppm)
534.9711
[M−H]−
245.947
0
20
40
60
80
100
250
300
350
400
450
500

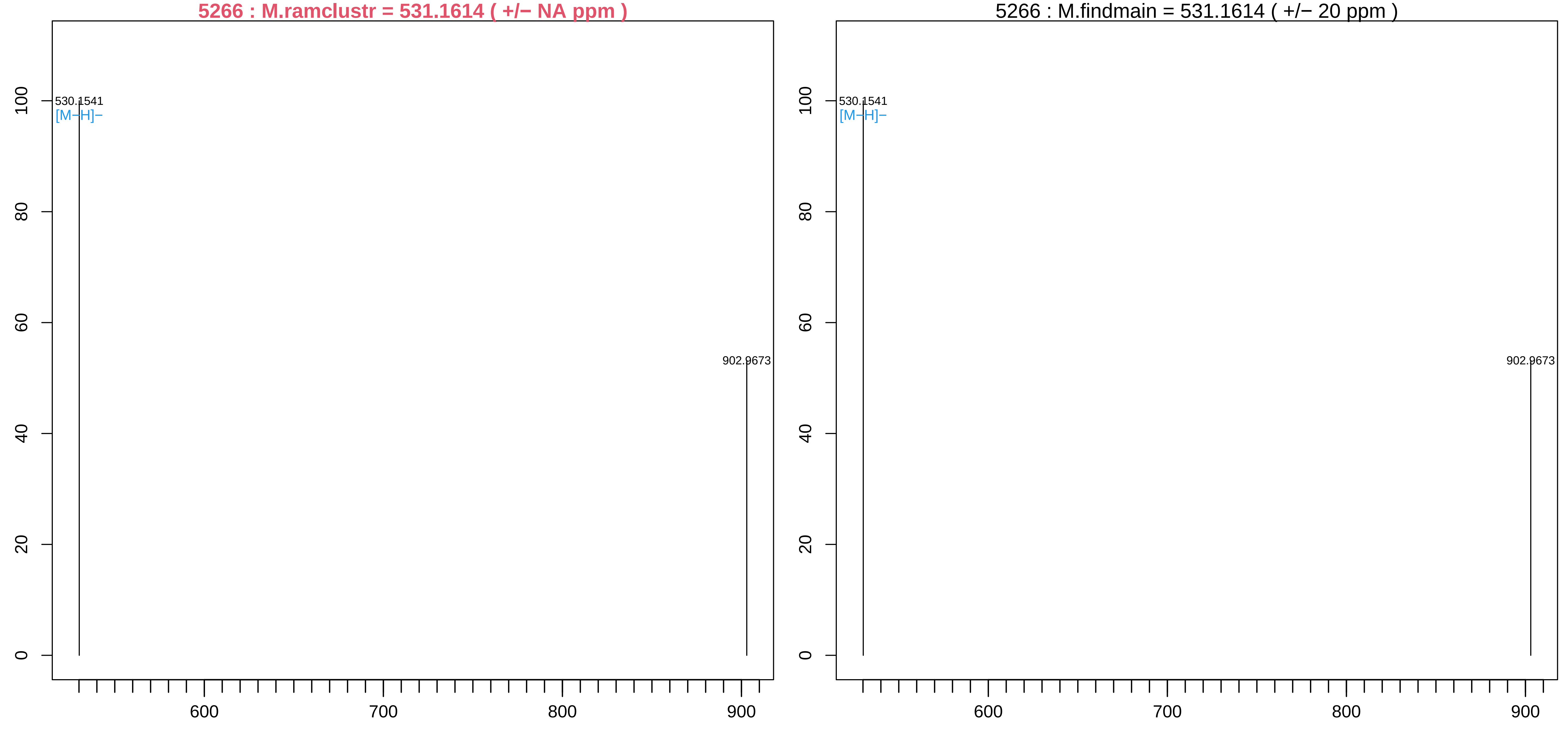
5266 : M.ramclustr = 531.1614 (+/− NA ppm)
530.1541
[M−H]−
902.9673
0
20
40
60
80
100
600
700
800
900
5266 : M.findmain = 531.1614 (+/− 20 ppm)
530.1541
[M−H]−
902.9673
0
20
40
60
80
100
600
700
800
900

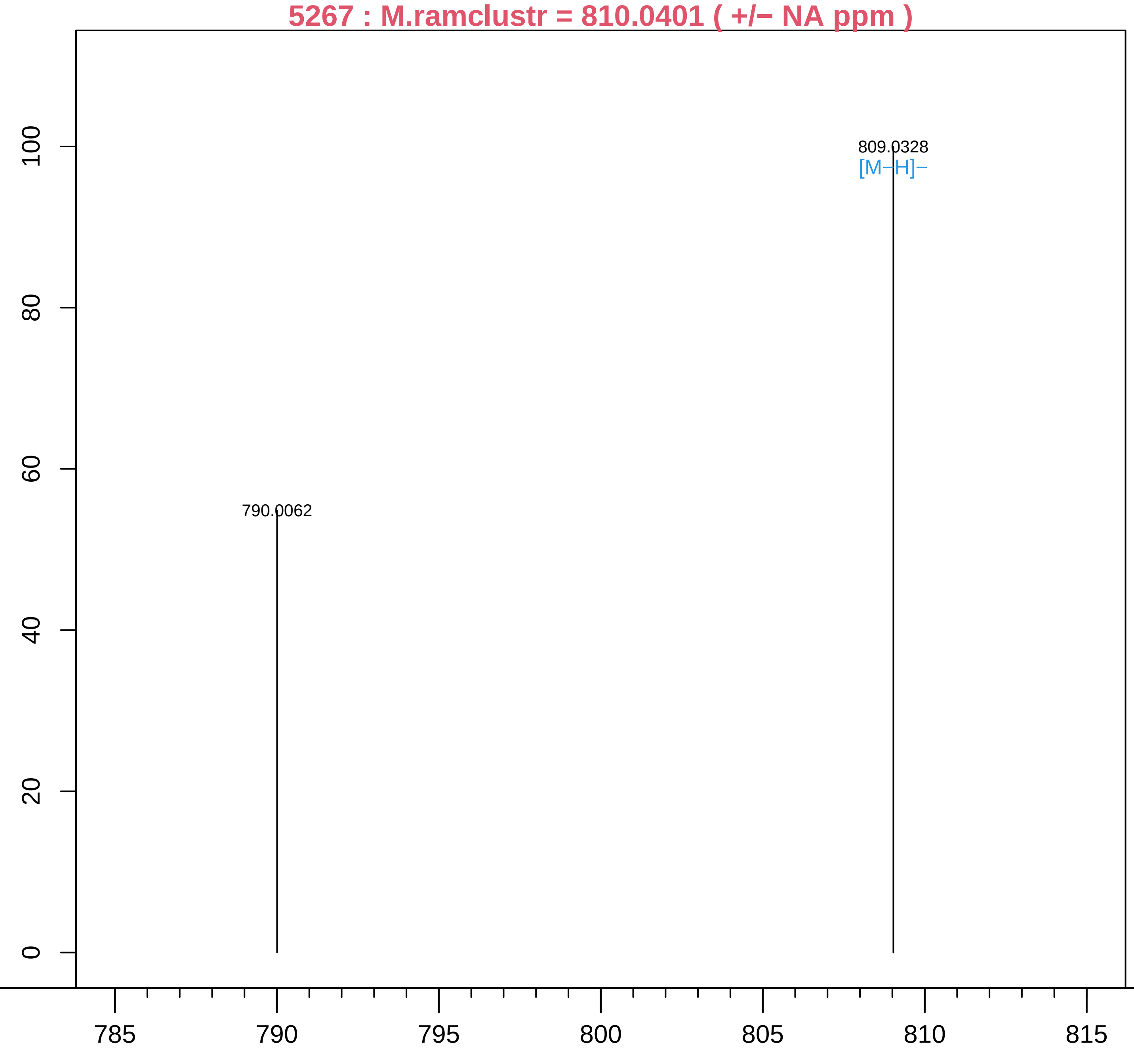
5267 : M.ramclustr = 810.0401 (+/− NA ppm)
809.0328
[M−H]−
790.0062
0
20
40
60
80
100
785
790
795
800
805
810
815

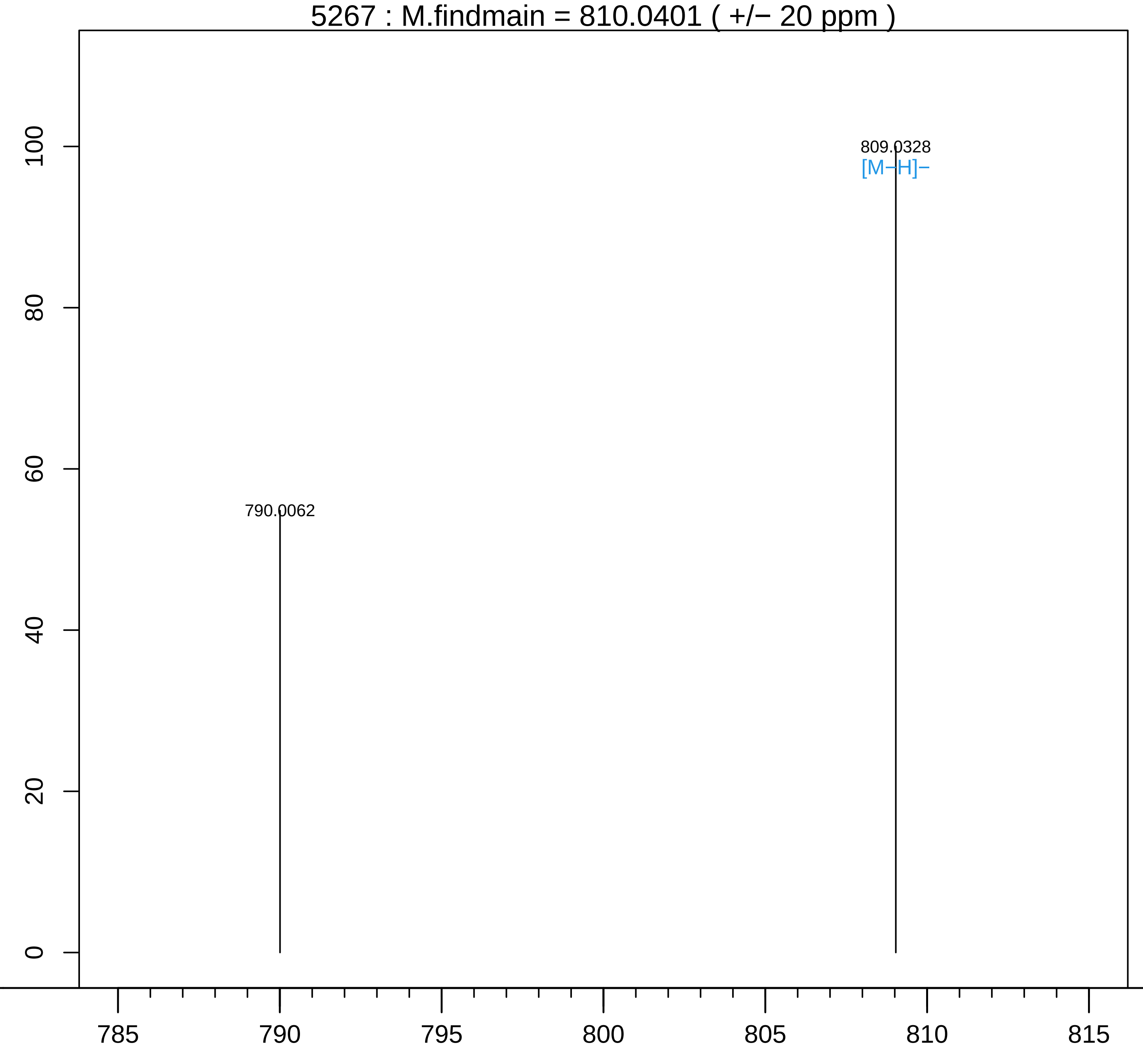
5267 : M.findmain = 810.0401 (+/− 20 ppm)
809.0328
[M−H]−
790.0062
0
20
40
60
80
100
785
790
795
800
805
810
815

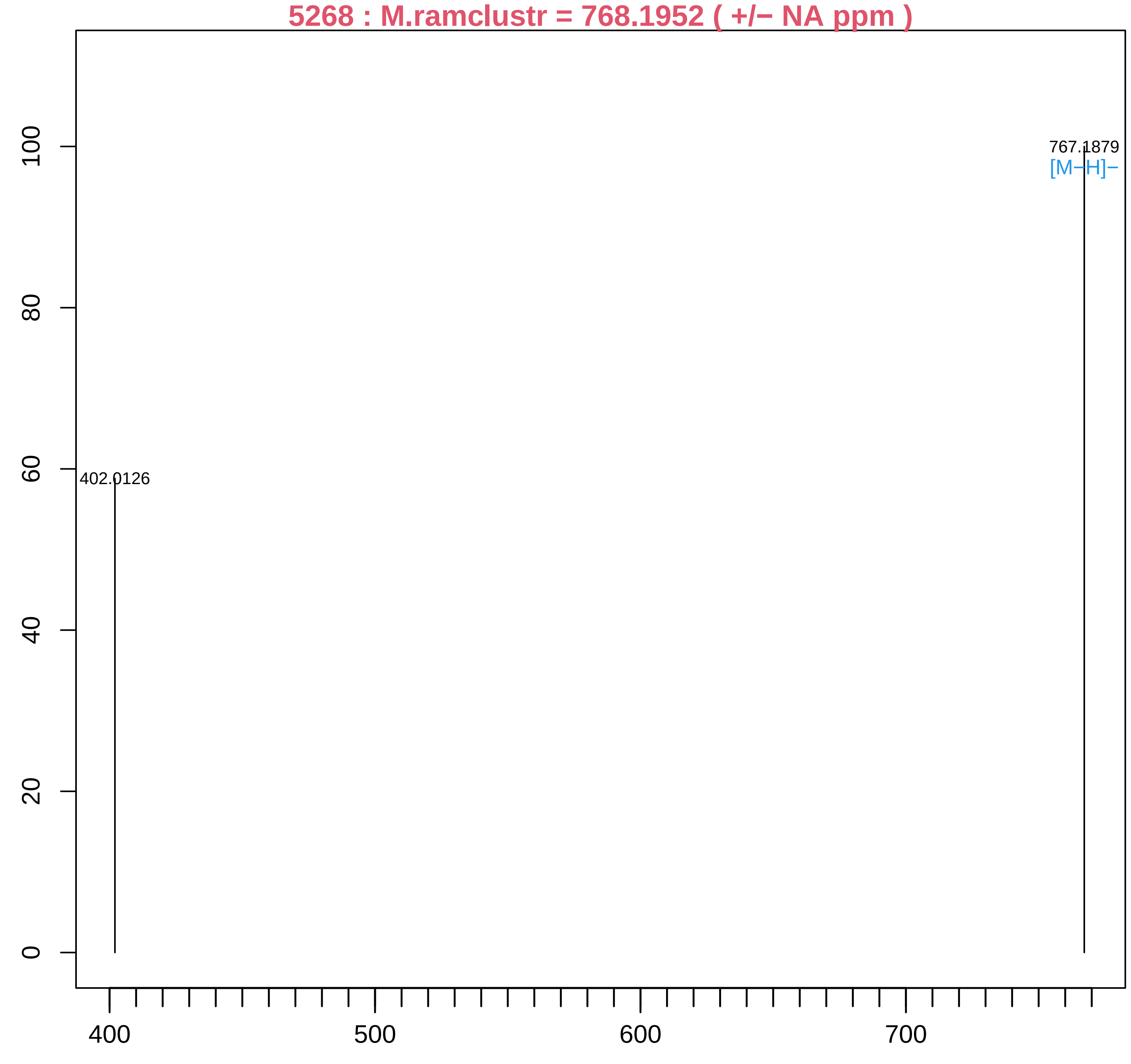
5268 : M.ramclustr = 768.1952 (+/− NA ppm)
767.1879
[M−H]−
402.0126
0
20
40
60
80
100
400
500
600
700

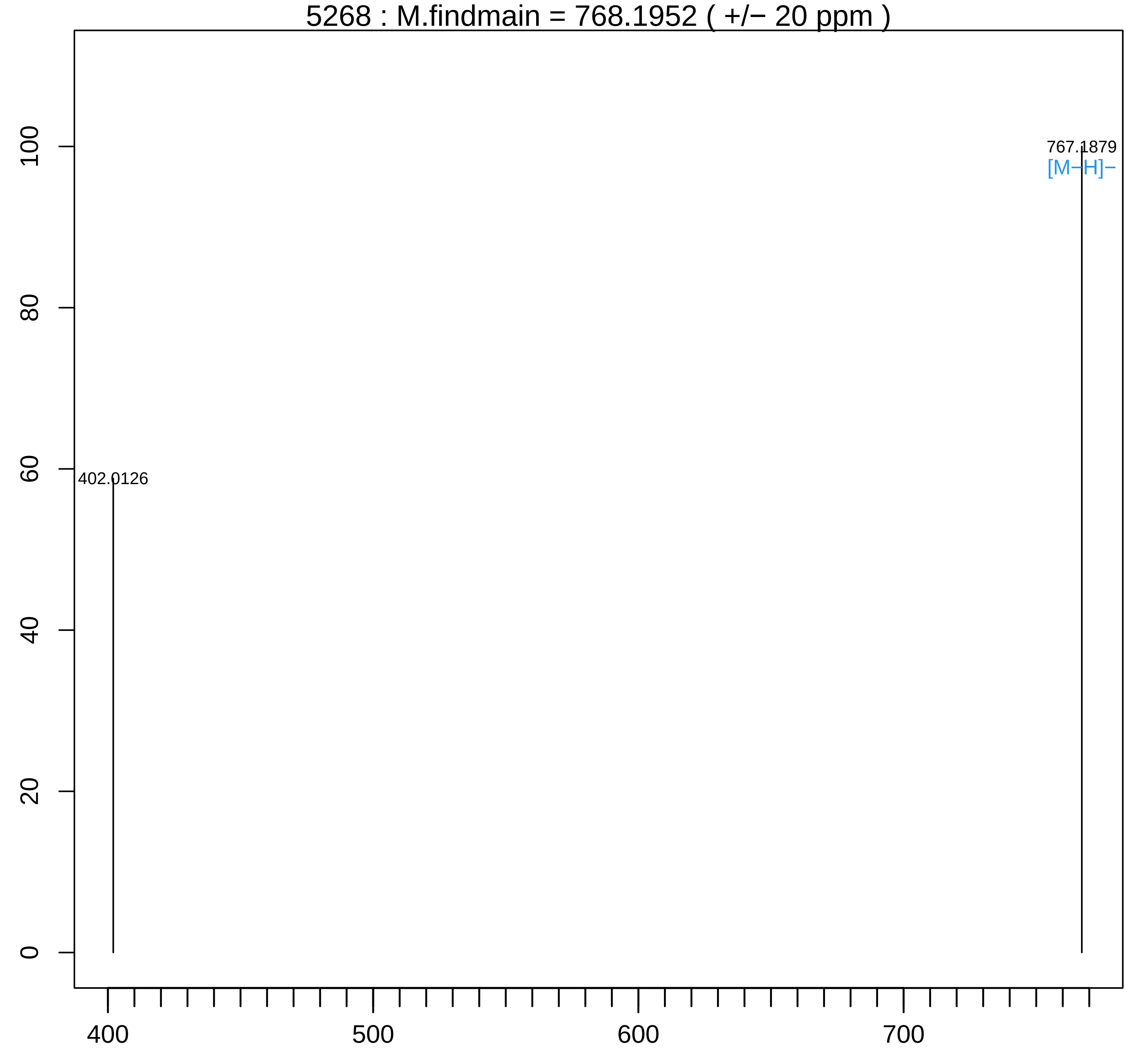
5268 : M.findmain = 768.1952 (+/− 20 ppm)
767.1879
[M−H]−
402.0126
0
20
40
60
80
100
400
500
600
700

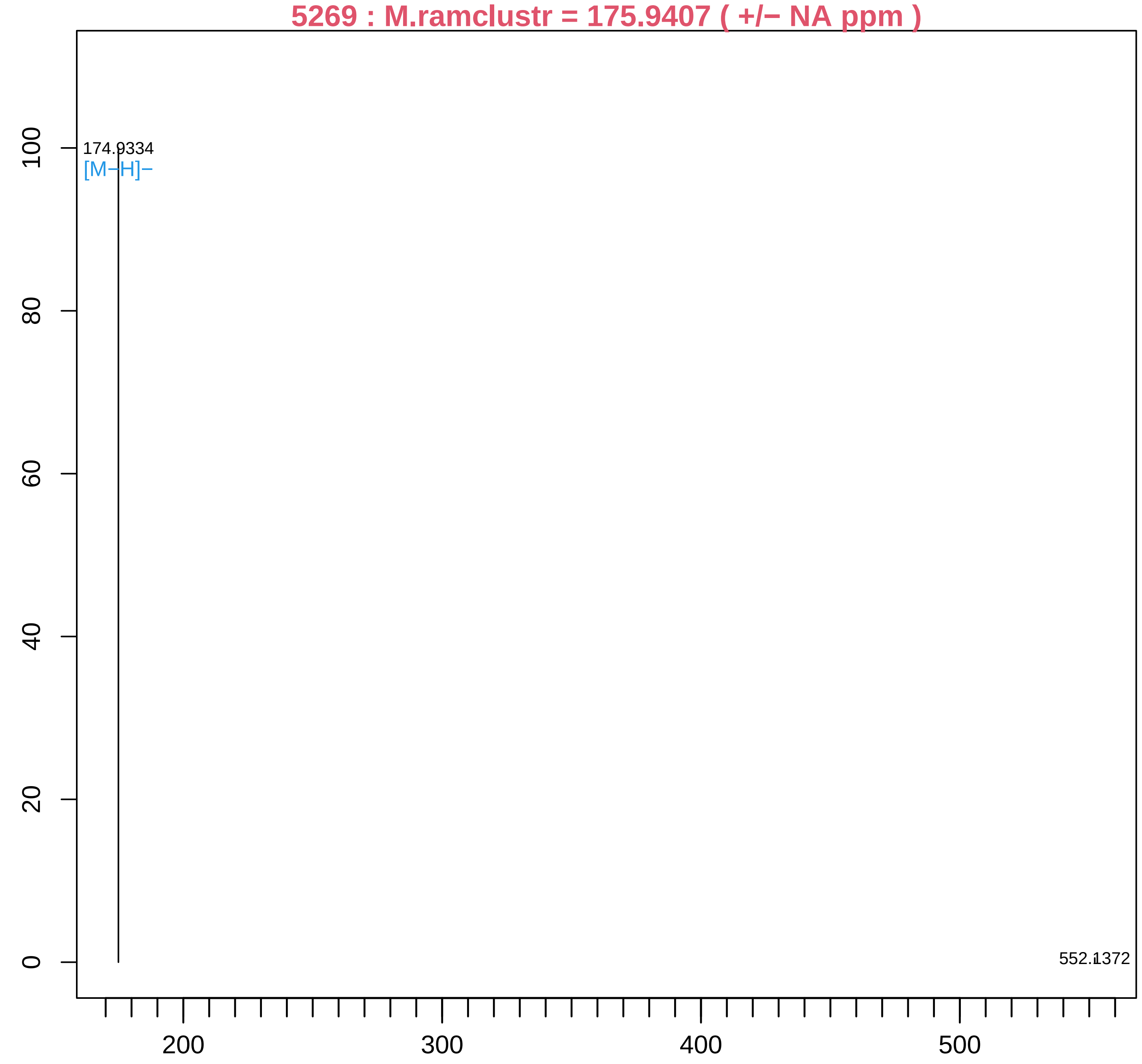
5269 : M.ramclustr = 175.9407 (+/− NA ppm)
174.9334
[M−H]−
552.1372
0
20
40
60
80
100
200
300
400
500

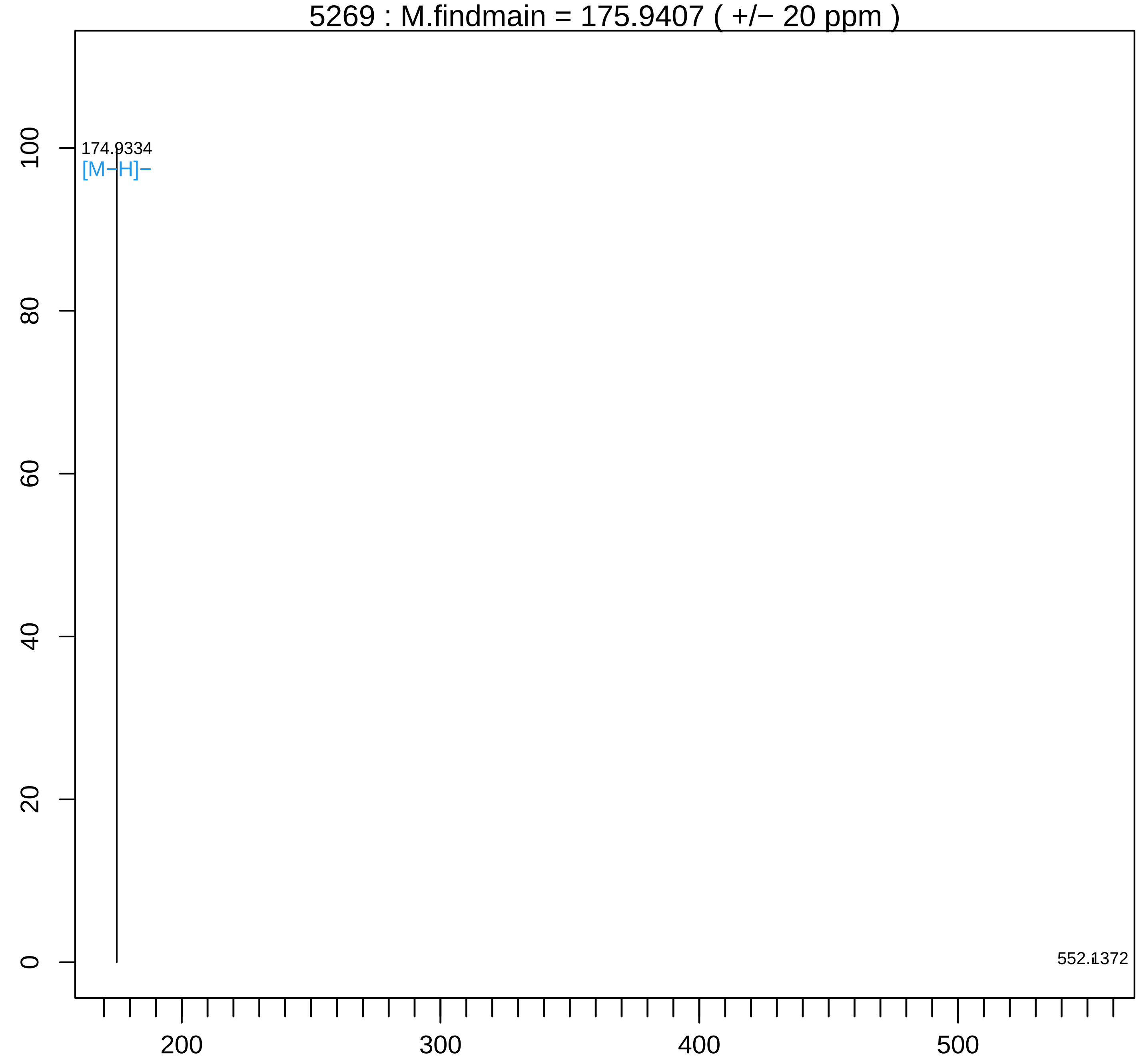
5269 : M.findmain = 175.9407 (+/− 20 ppm)
174.9334
[M−H]−
552.1372
0
20
40
60
80
100
200
300
400
500

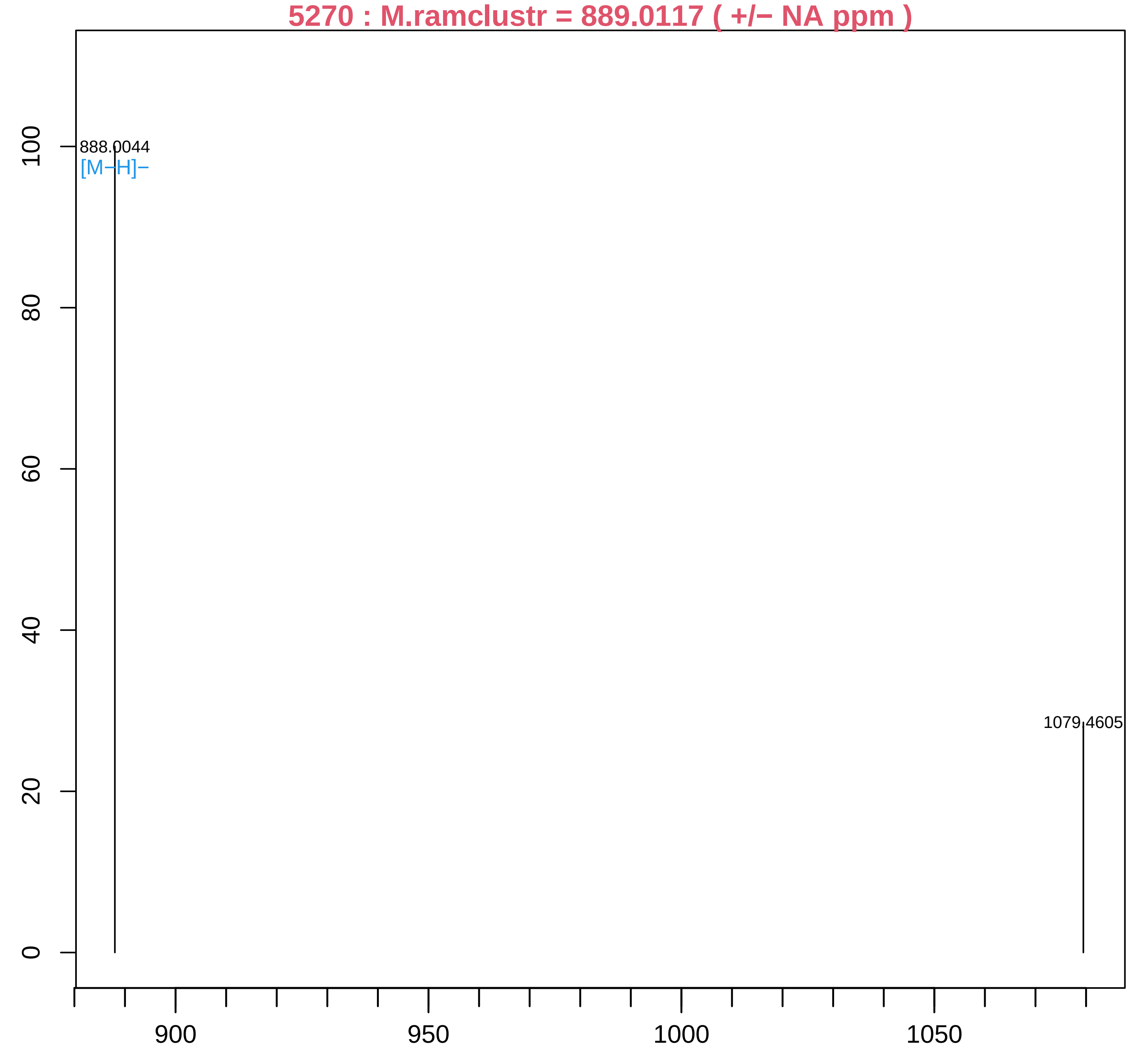

5270 : M.ramclustr = 889.0117 (+/− NA ppm)
888.0044
[M−H]−
1079.4605
0
20
40
60
80
100
900
950
1000
1050

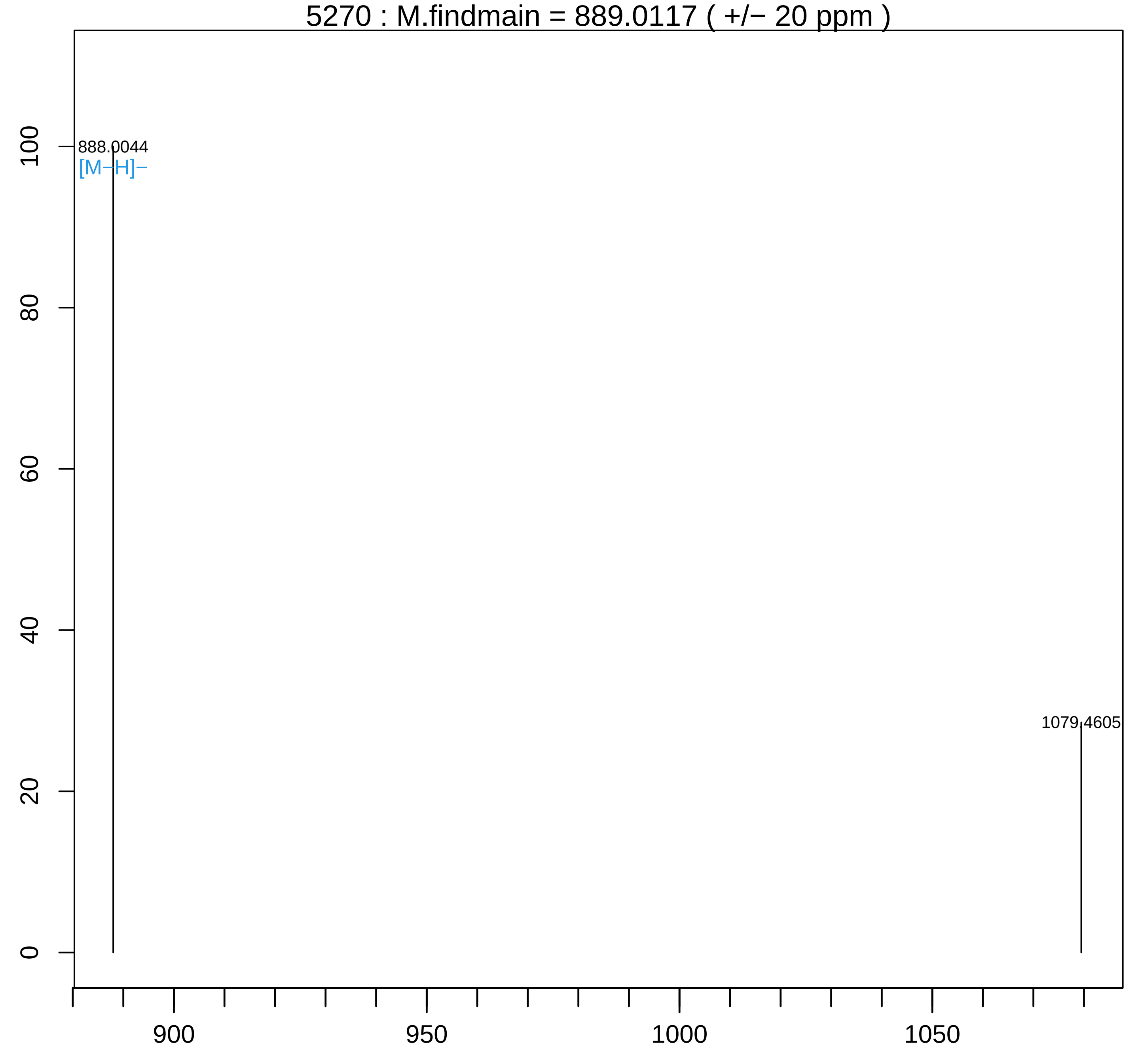

5270 : M.findmain = 889.0117 (+/− 20 ppm)
888.0044
[M−H]−
1079.4605
0
20
40
60
80
100
900
950
1000
1050

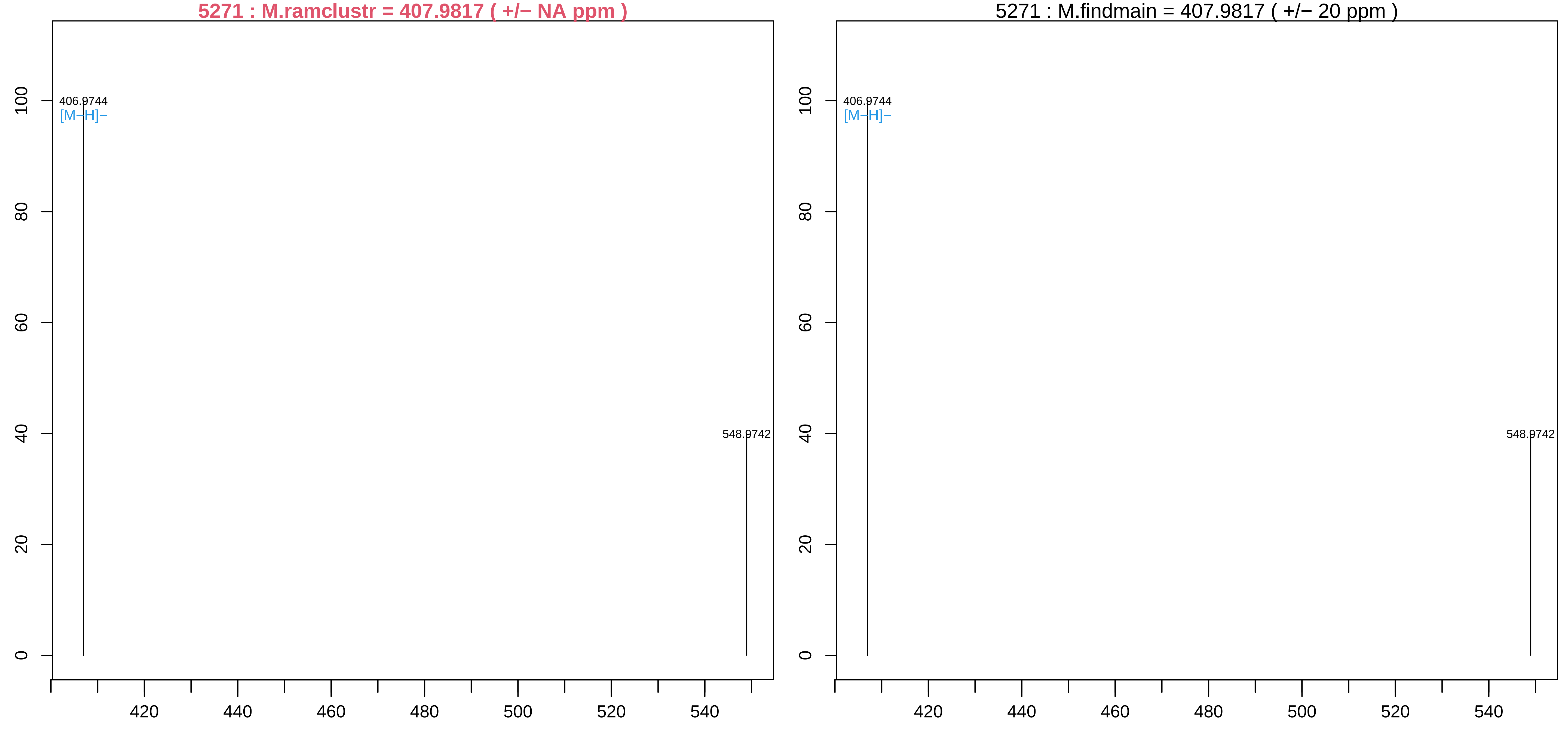
5271 : M.ramclustr = 407.9817 (+/− NA ppm)
406.9744
[M−H]−
548.9742
0
20
40
60
80
100
420
440
460
480
500
520
540
5271 : M.findmain = 407.9817 (+/− 20 ppm)
406.9744
[M−H]−
548.9742
0
20
40
60
80
100
420
440
460
480
500
520
540

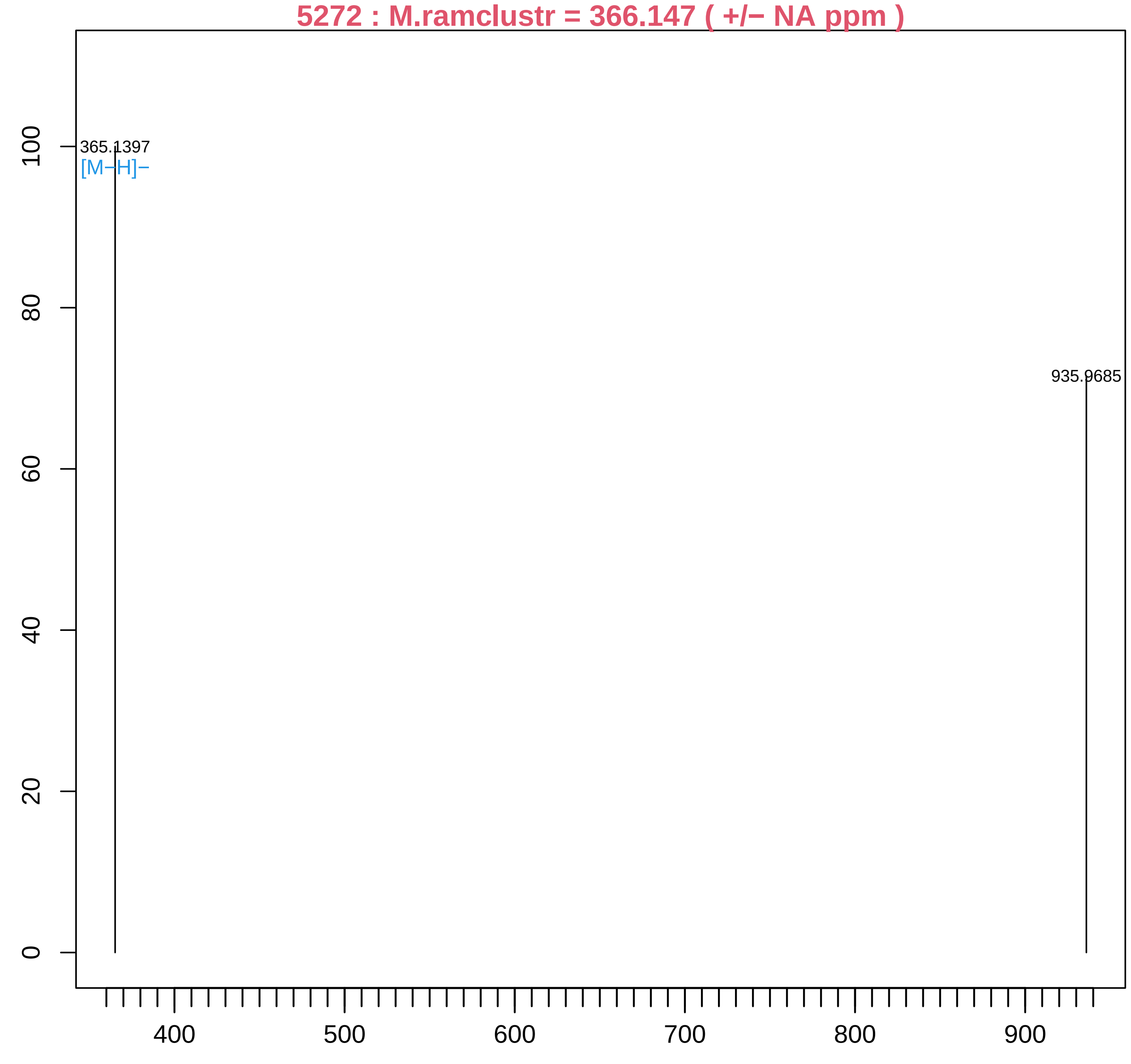
5272 : M.ramclustr = 366.147 (+/− NA ppm)
365.1397
[M−H]−
935.9685
0
20
40
60
80
100
400
500
600
700
800
900

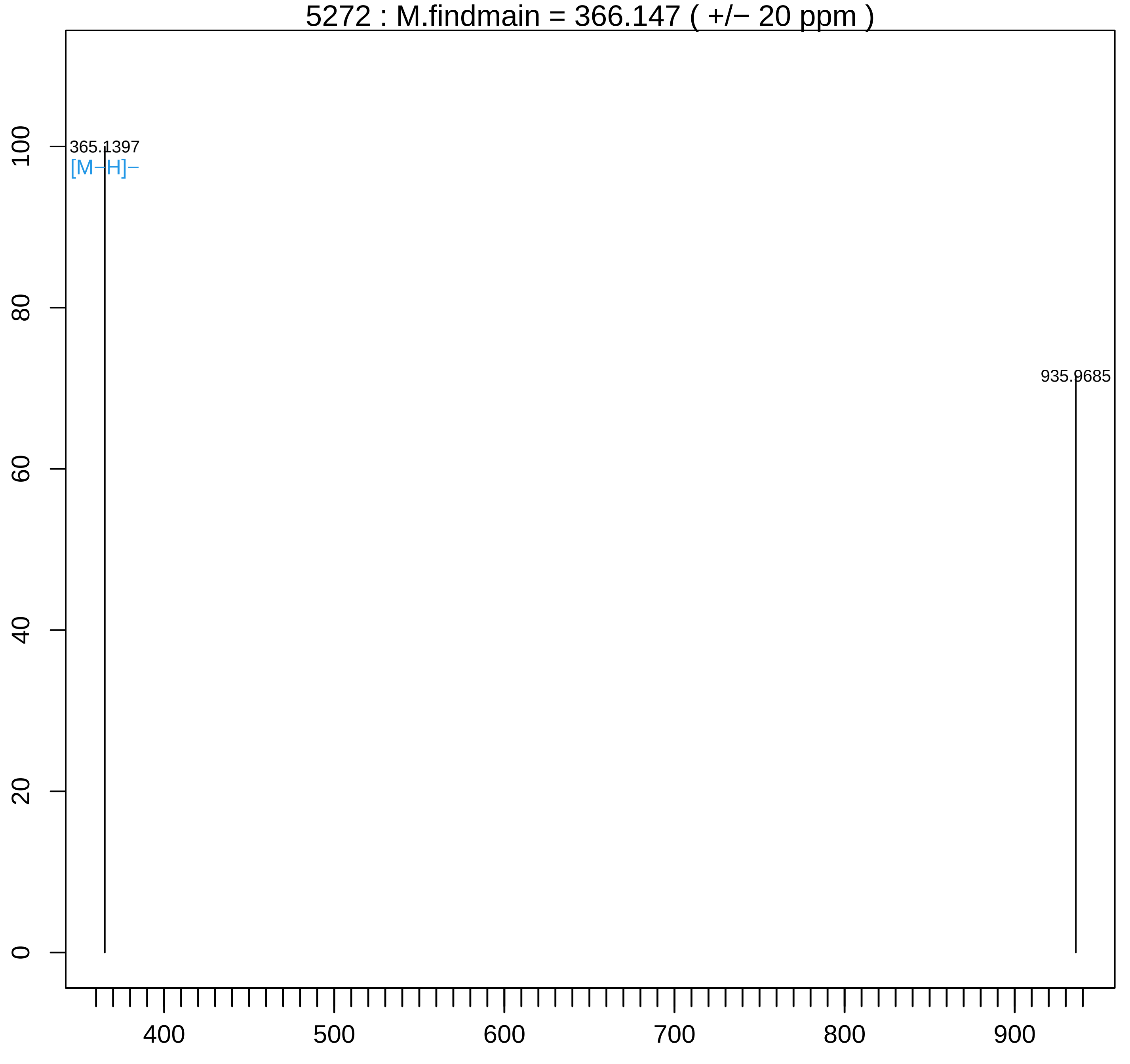
5272 : M.findmain = 366.147 (+/− 20 ppm)
365.1397
[M−H]−
935.9685
0
20
40
60
80
100
400
500
600
700
800
900

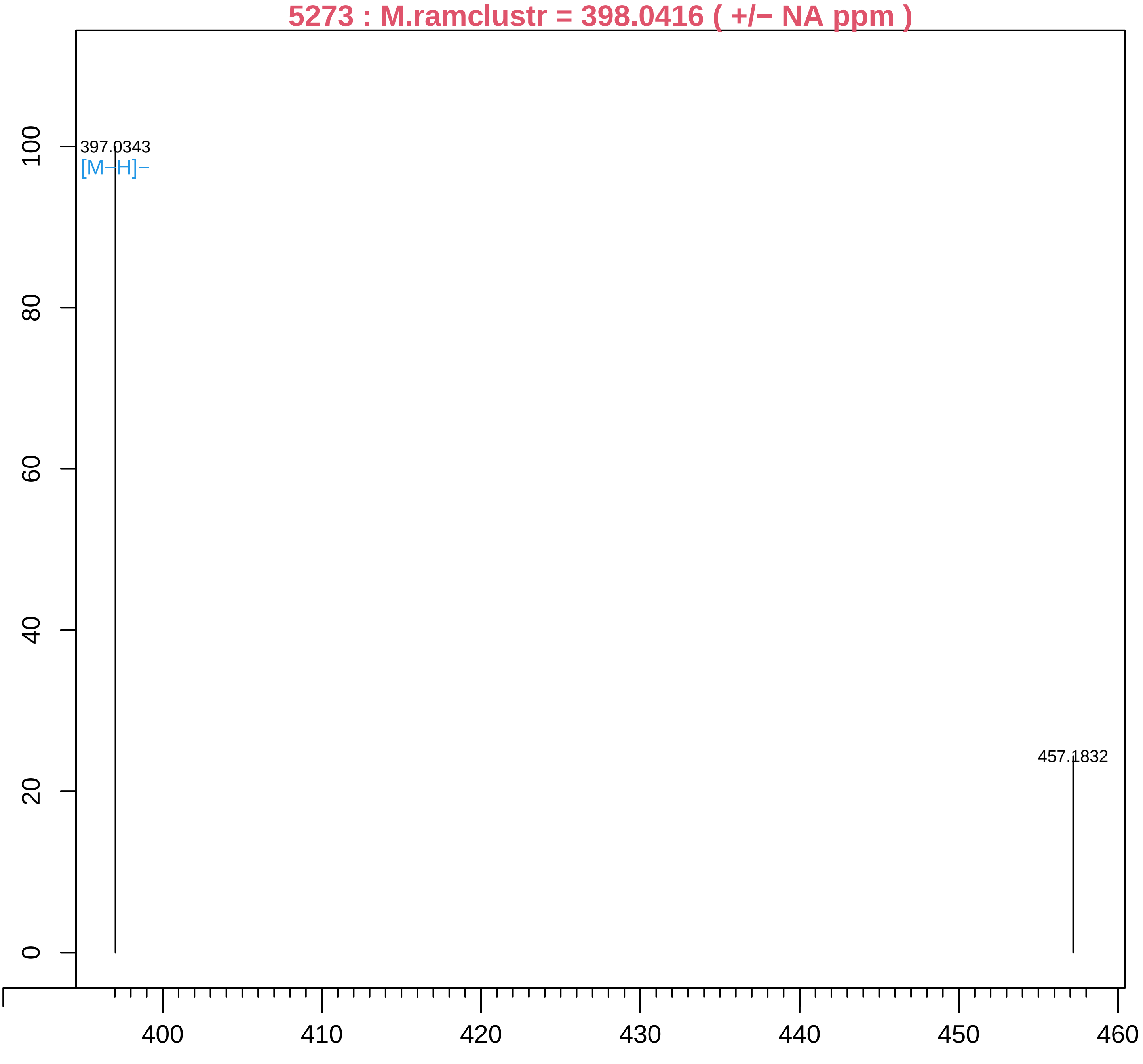
5273 : M.ramclustr = 398.0416 (+/− NA ppm)
397.0343
[M−H]−
457.1832
0
20
40
60
80
100
400
410
420
430
440
450
460

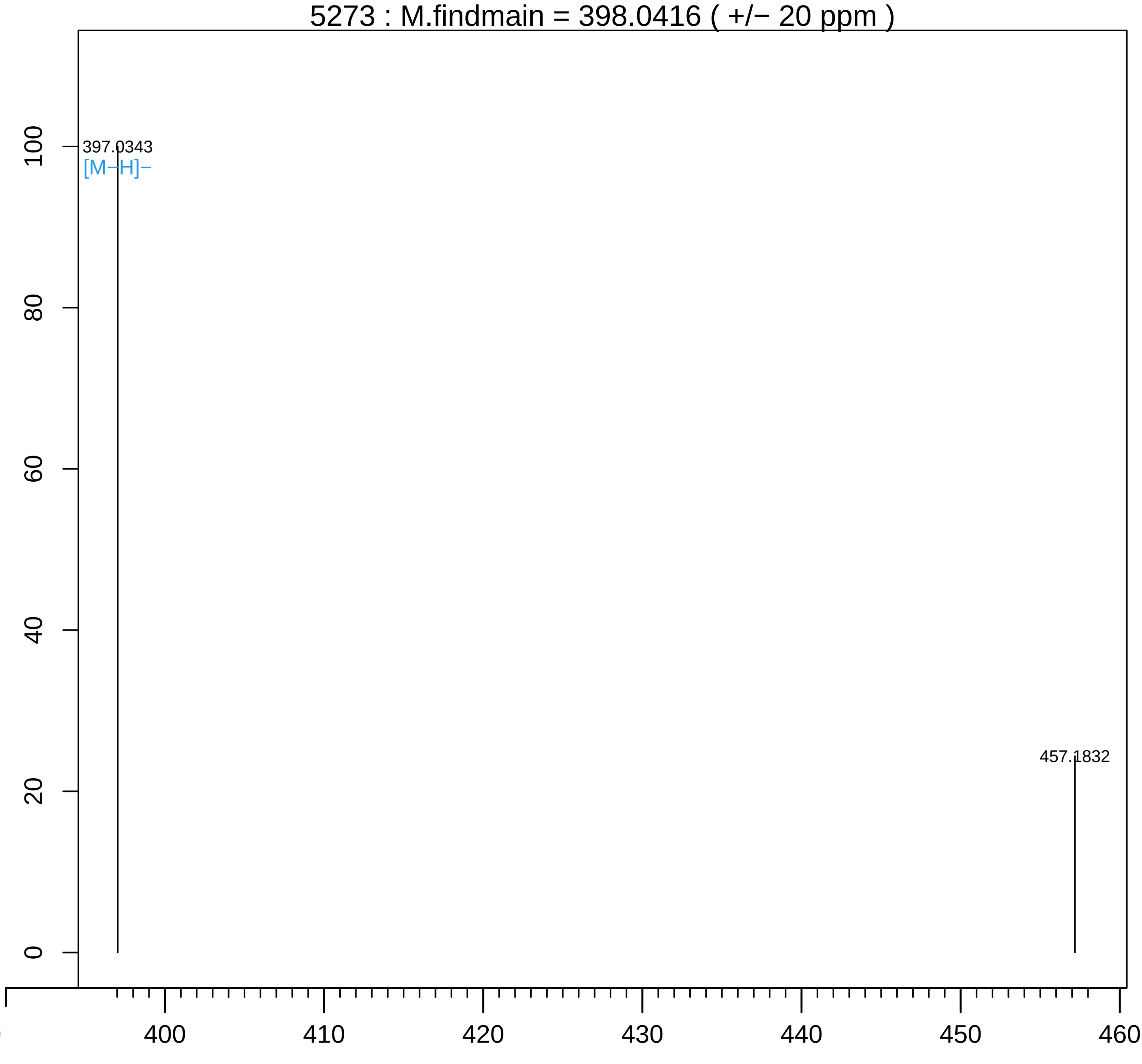
5273 : M.findmain = 398.0416 (+/− 20 ppm)
397.0343
[M−H]−
457.1832
0
20
40
60
80
100
400
410
420
430
440
450
460

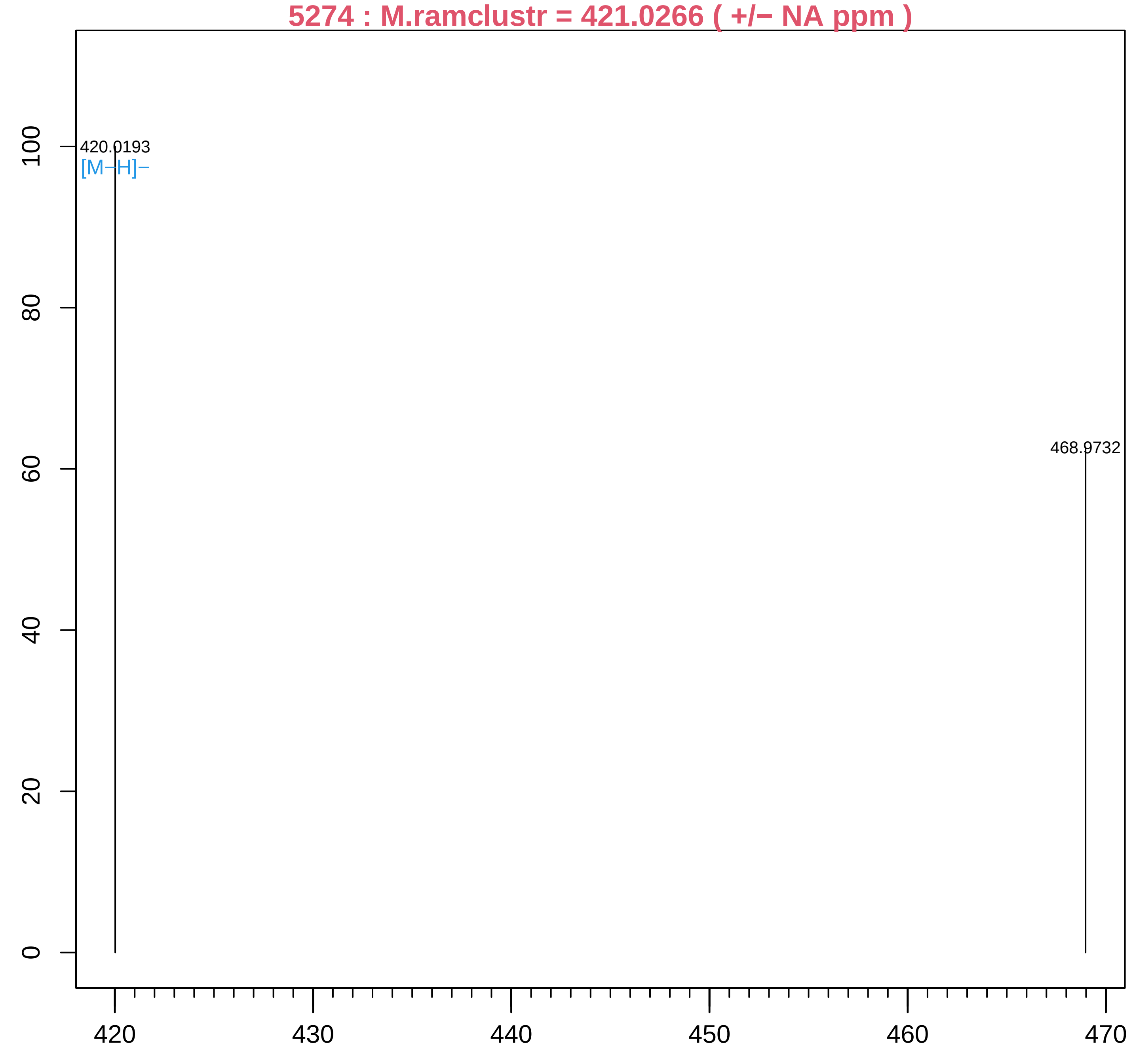

5274 : M.ramclustr = 421.0266 (+/− NA ppm)
420.0193
[M−H]−
468.9732
0
20
40
60
80
100
420
430
440
450
460
470

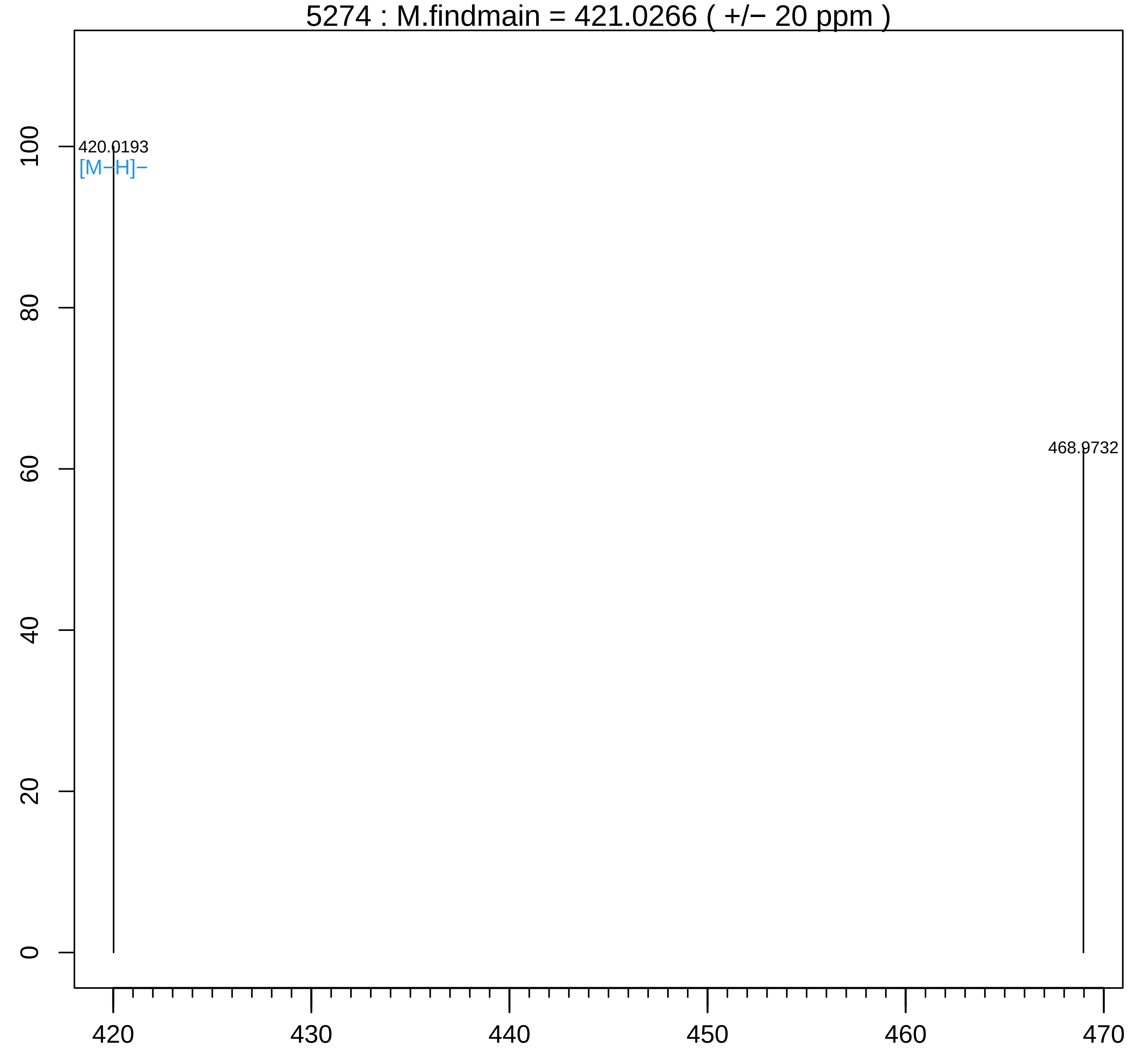

5274 : M.findmain = 421.0266 (+/− 20 ppm)
420.0193
[M−H]−
468.9732
0
20
40
60
80
100
420
430
440
450
460
470

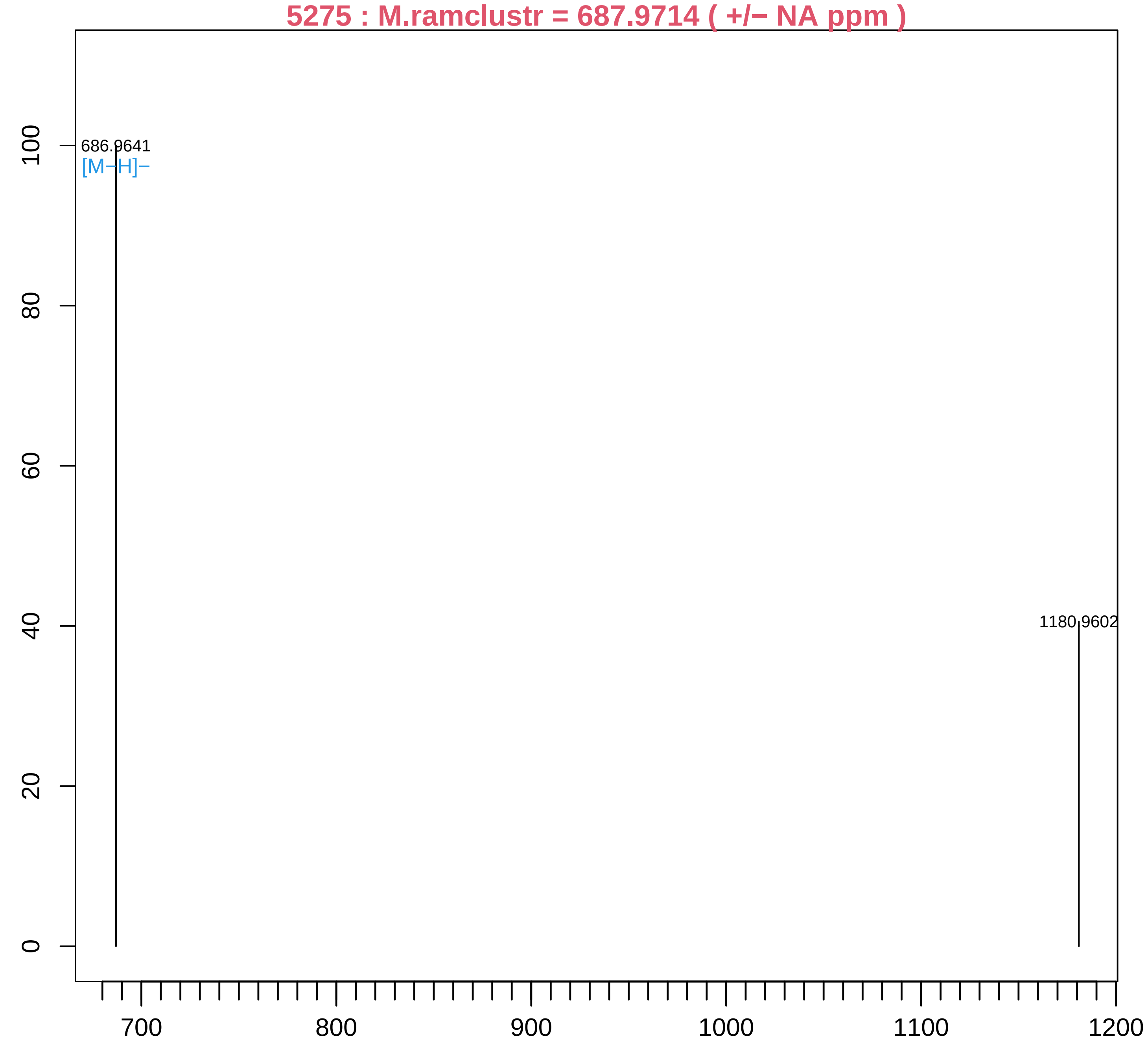
5275 : M.ramclustr = 687.9714 (+/− NA ppm)
686.9641
[M−H]−
1180.9602
0
20
40
60
80
100
700
800
900
1000
1100
1200
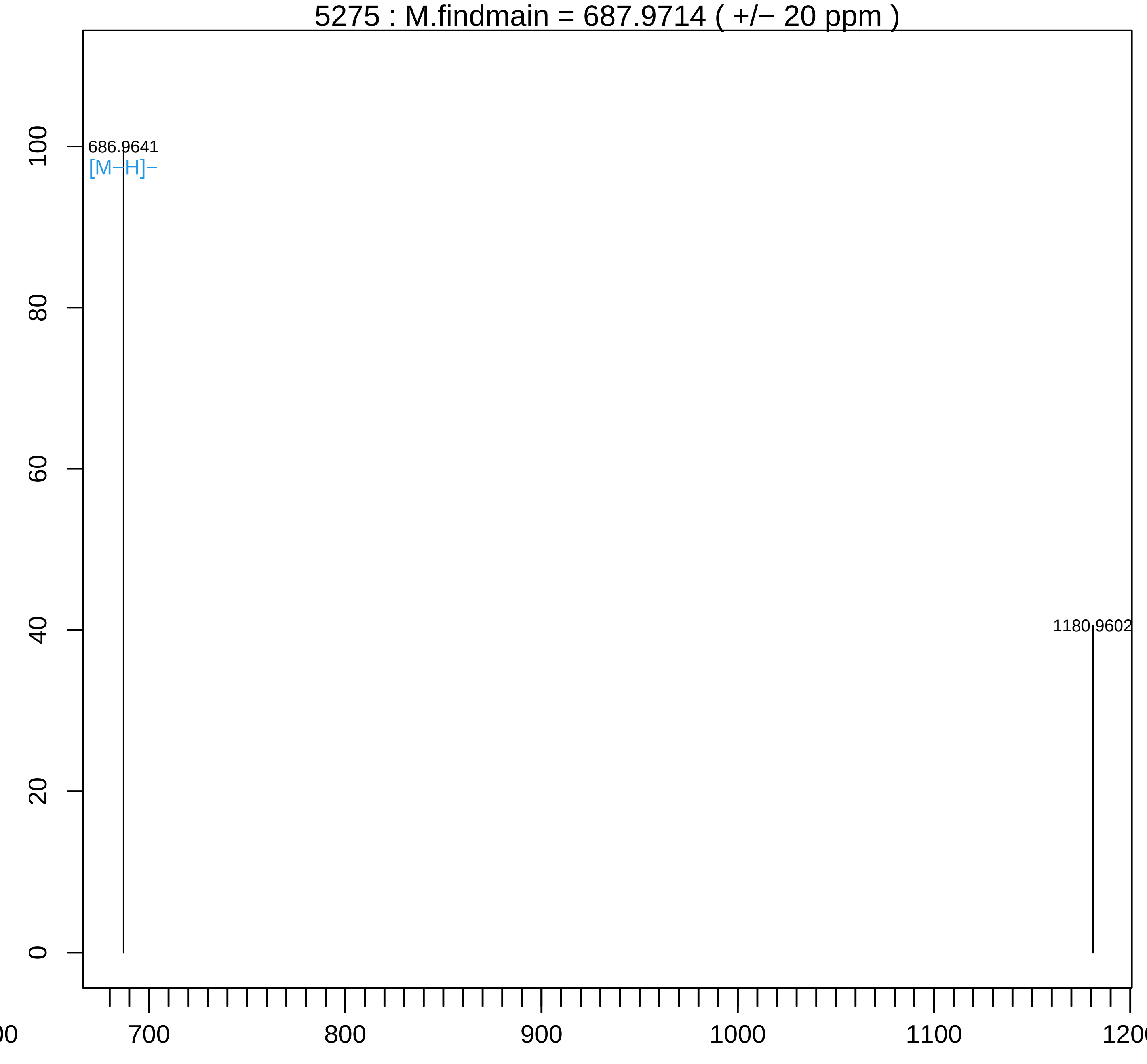
5275 : M.findmain = 687.9714 (+/− 20 ppm)
686.9641
[M−H]−
1180.9602
0
20
40
60
80
100
700
800
900
1000
1100
1200

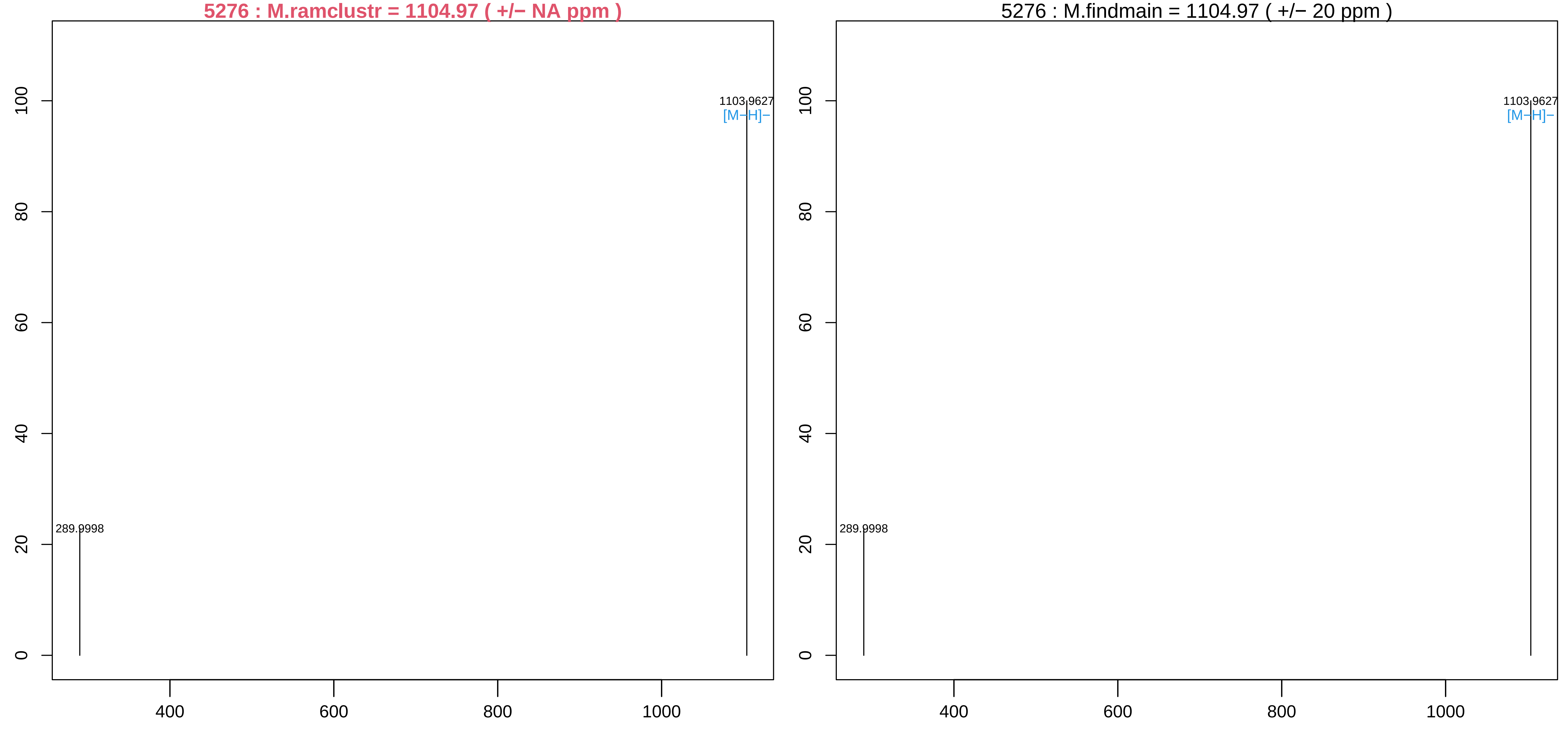
5276 : M.ramclustr = 1104.97 (+/− NA ppm)
1103.9627
[M−H]−
289.9998
0
20
40
60
80
100
400
600
800
1000
5276 : M.findmain = 1104.97 (+/− 20 ppm)
1103.9627
[M−H]−
289.9998
0
20
40
60
80
100
400
600
800
1000

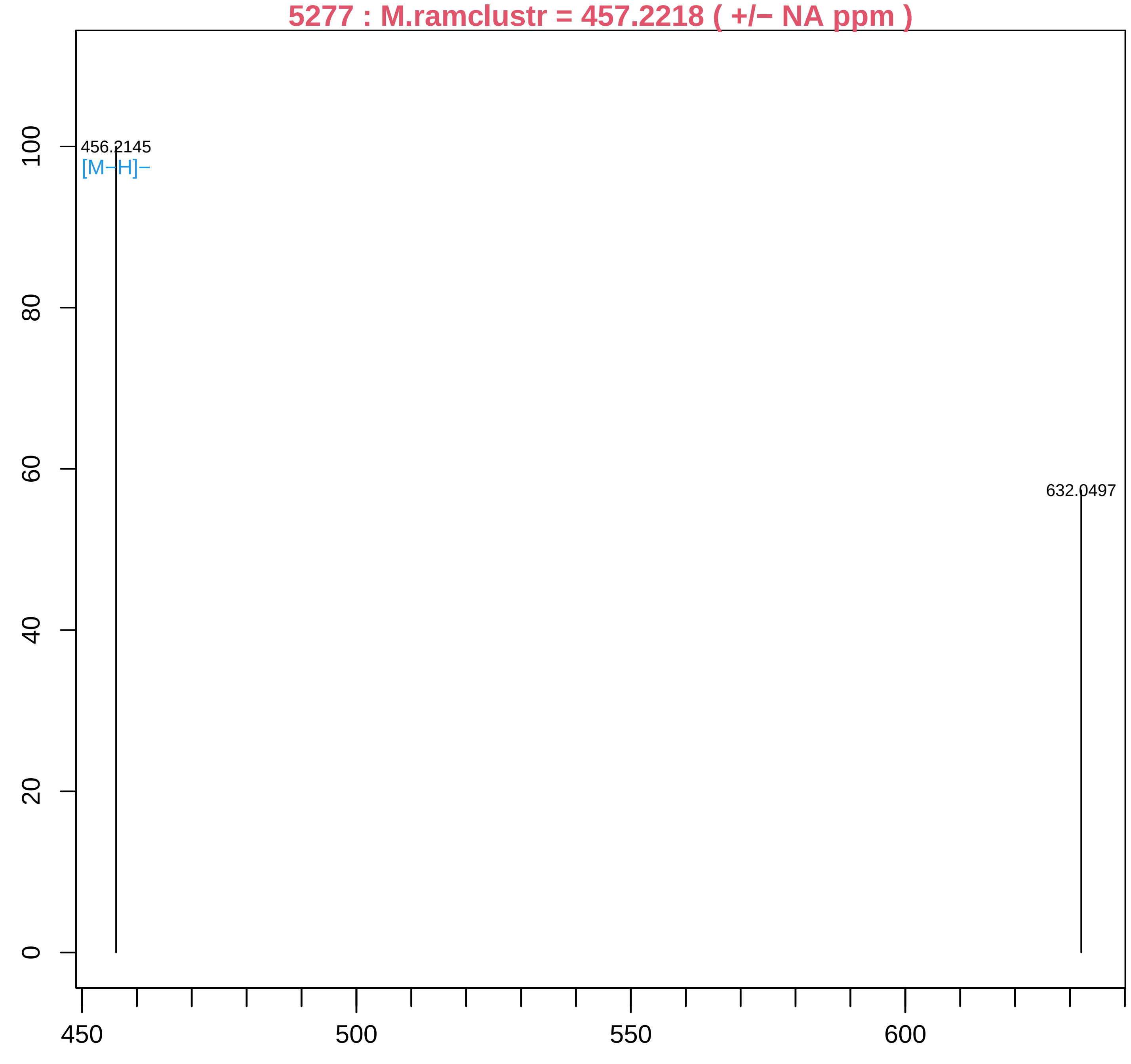

5277 : M.ramclustr = 457.2218 (+/− NA ppm)
456.2145
[M−H]−
632.0497
0
20
40
60
80
100
450
500
550
600

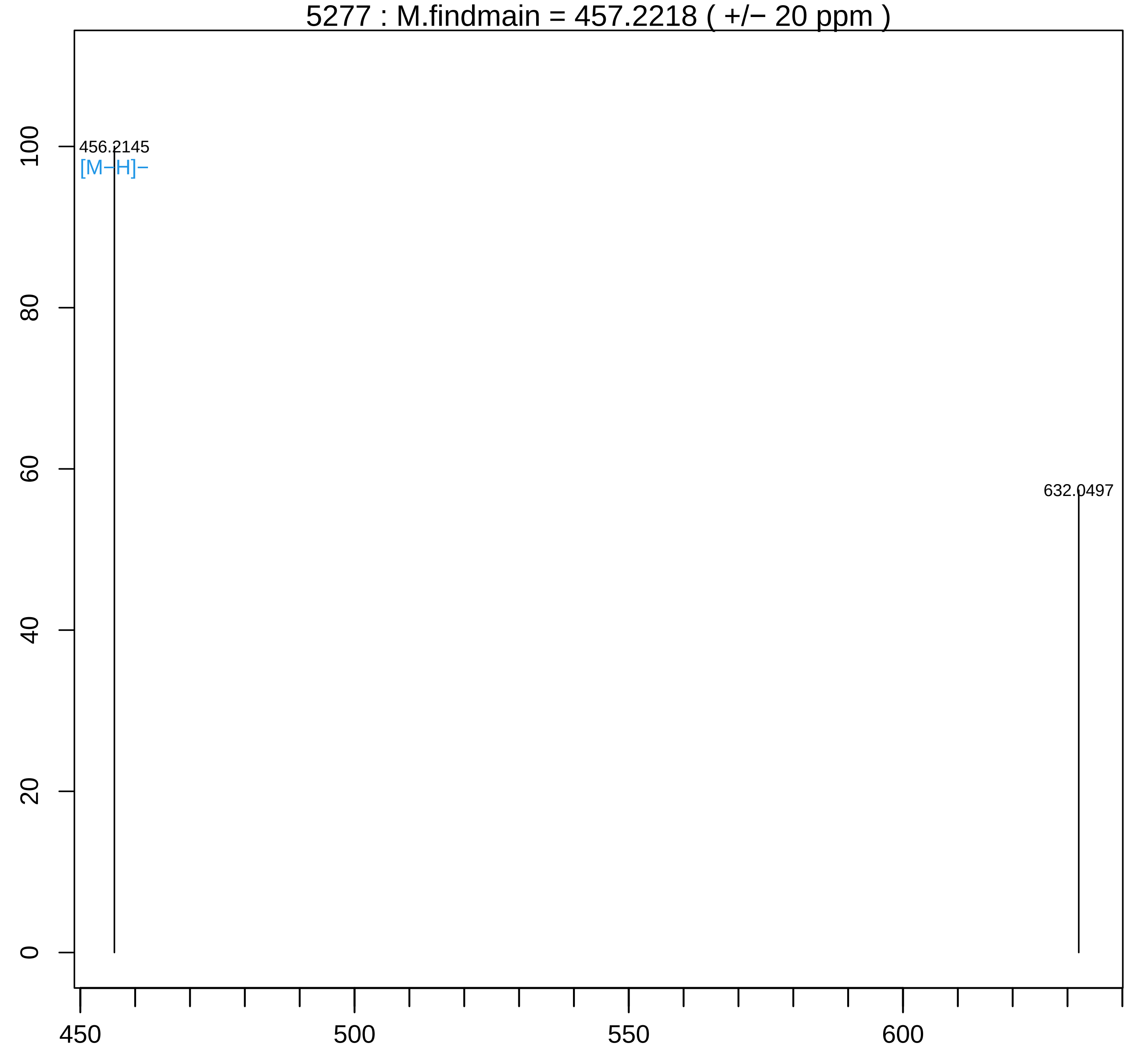

5277 : M.findmain = 457.2218 (+/− 20 ppm)
456.2145
[M−H]−
632.0497
0
20
40
60
80
100
450
500
550
600

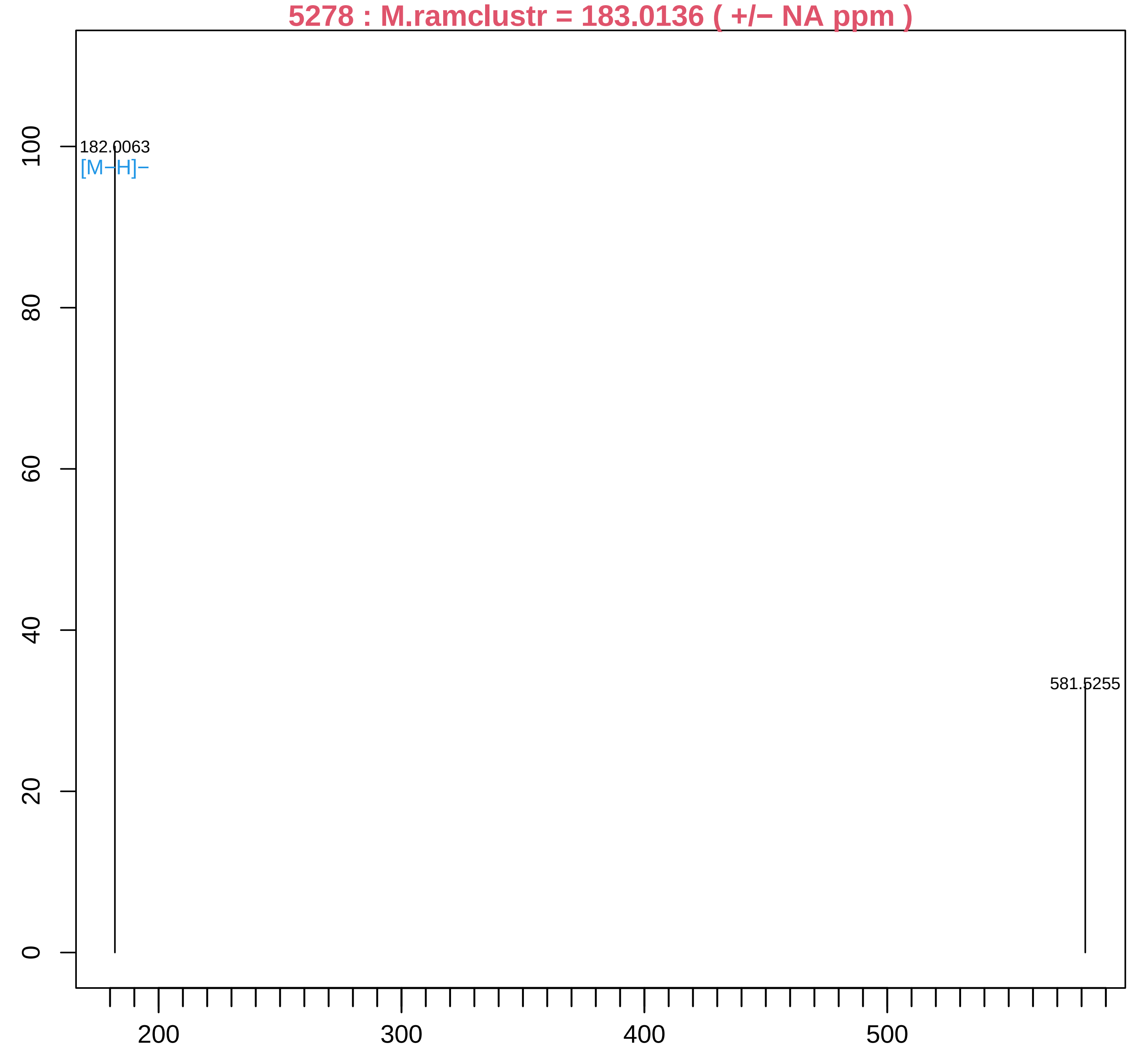

5278 : M.ramclustr = 183.0136 (+/− NA ppm)
182.0063
[M−H]−
581.5255
0
20
40
60
80
100
200
300
400
500

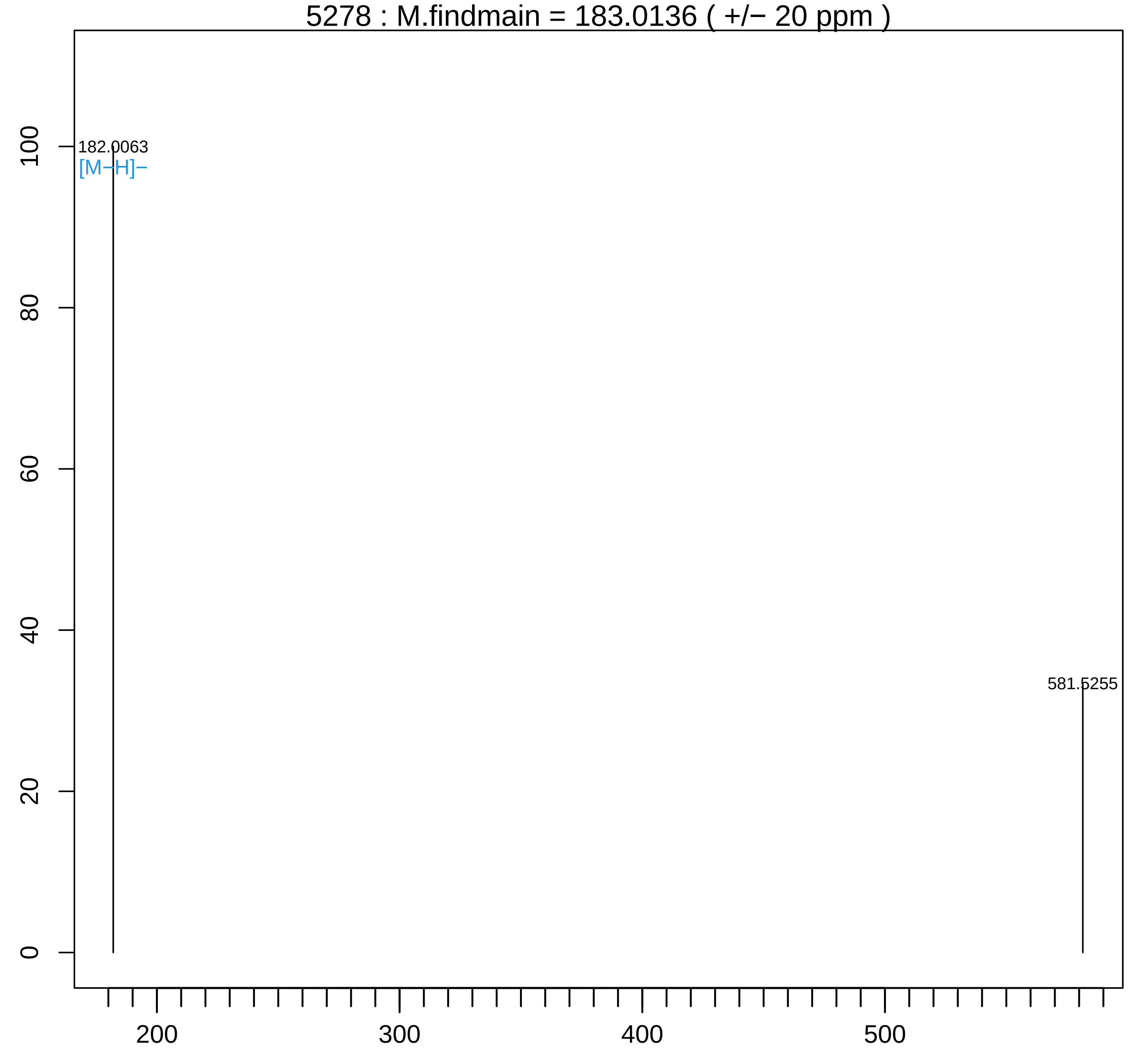

5278 : M.findmain = 183.0136 (+/− 20 ppm)
182.0063
[M−H]−
581.5255
0
20
40
60
80
100
200
300
400
500

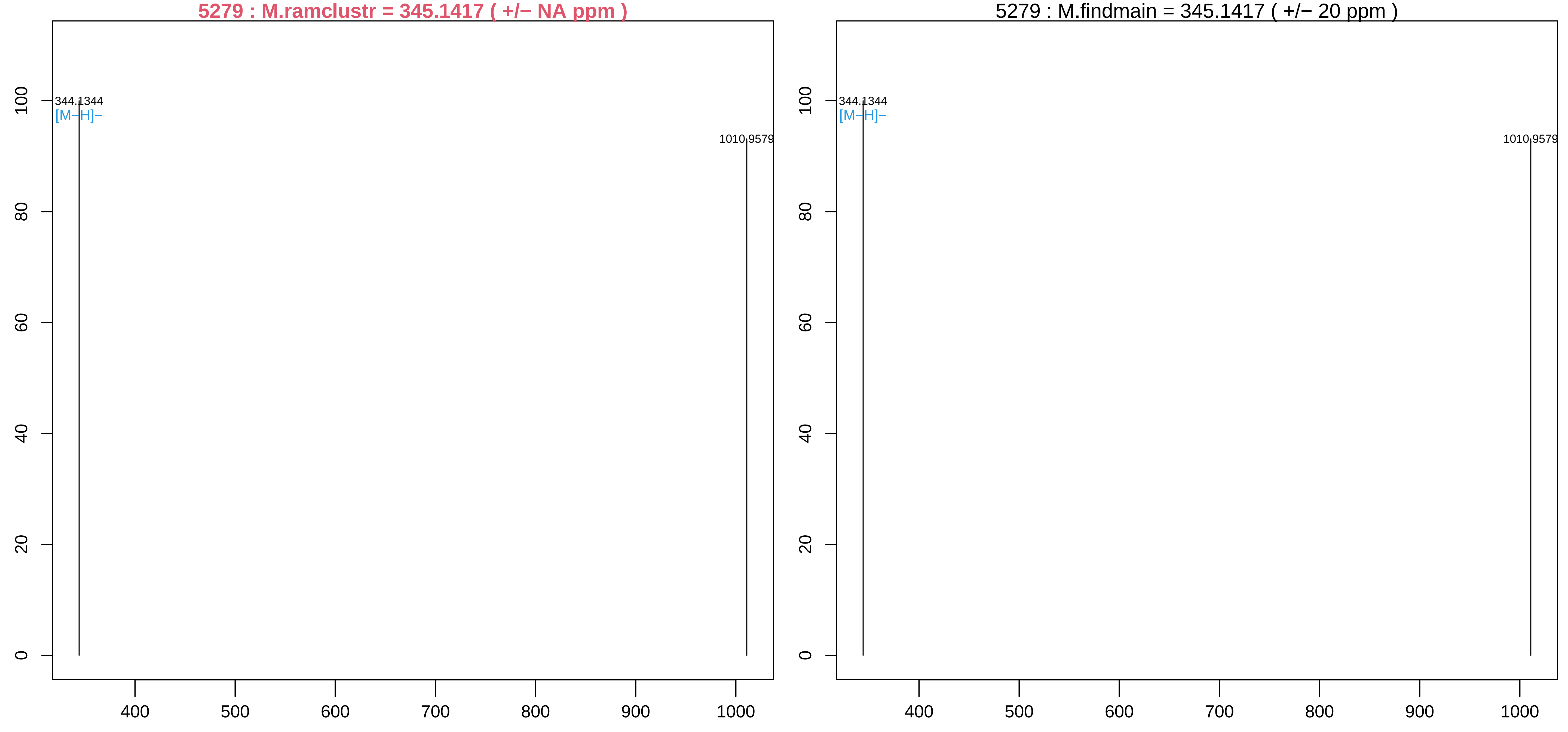
5279 : M.ramclustr = 345.1417 (+/− NA ppm)
344.1344
[M−H]−
1010.9579
5279 : M.findmain = 345.1417 (+/− 20 ppm)
344.1344
[M−H]−
1010.9579

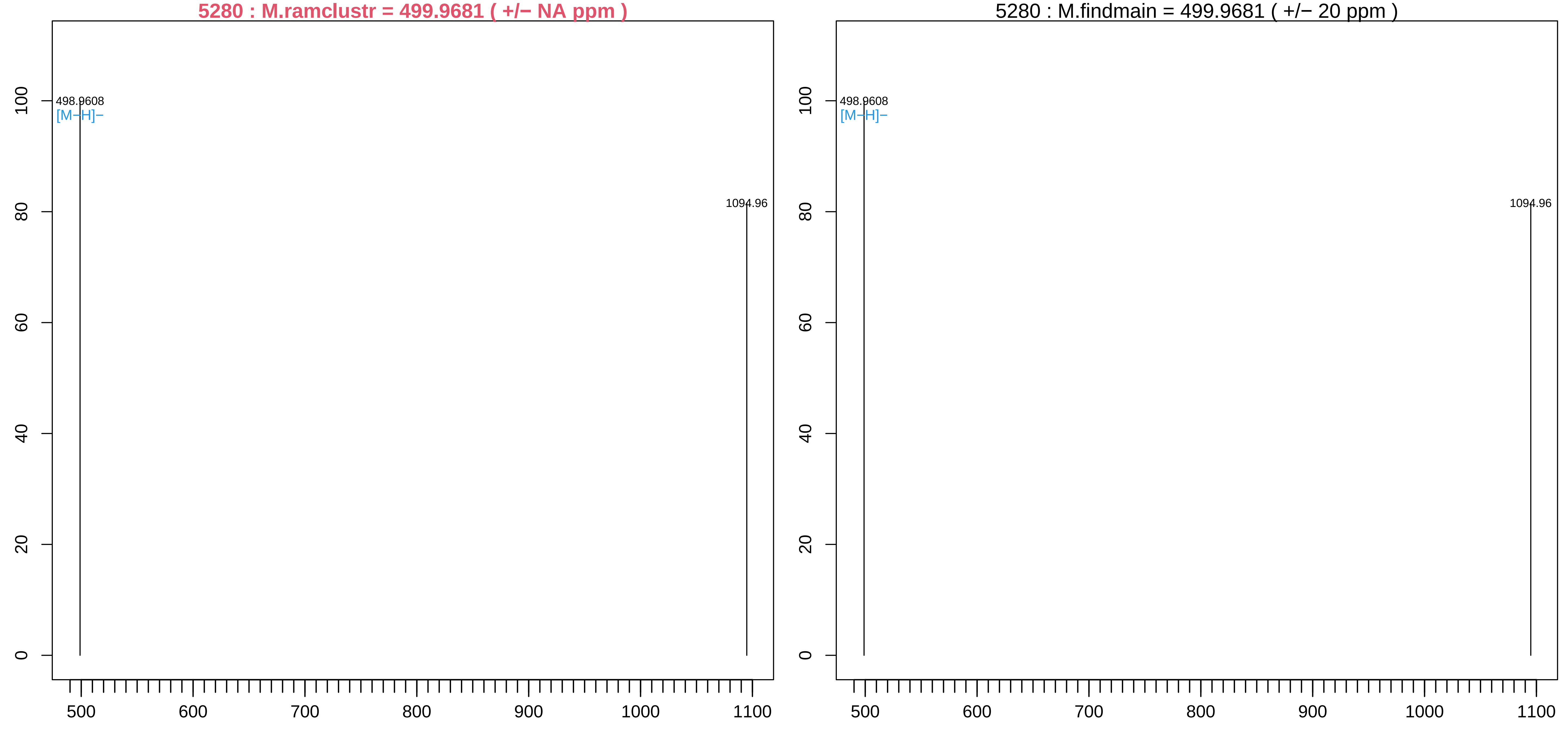

5280 : M.ramclustr = 499.9681 (+/− NA ppm)
498.9608
[M−H]−
1094.96
0
20
40
60
80
100
500
600
700
800
900
1000
1100
5280 : M.findmain = 499.9681 (+/− 20 ppm)
498.9608
[M−H]−
1094.96
0
20
40
60
80
100
500
600
700
800
900
1000
1100

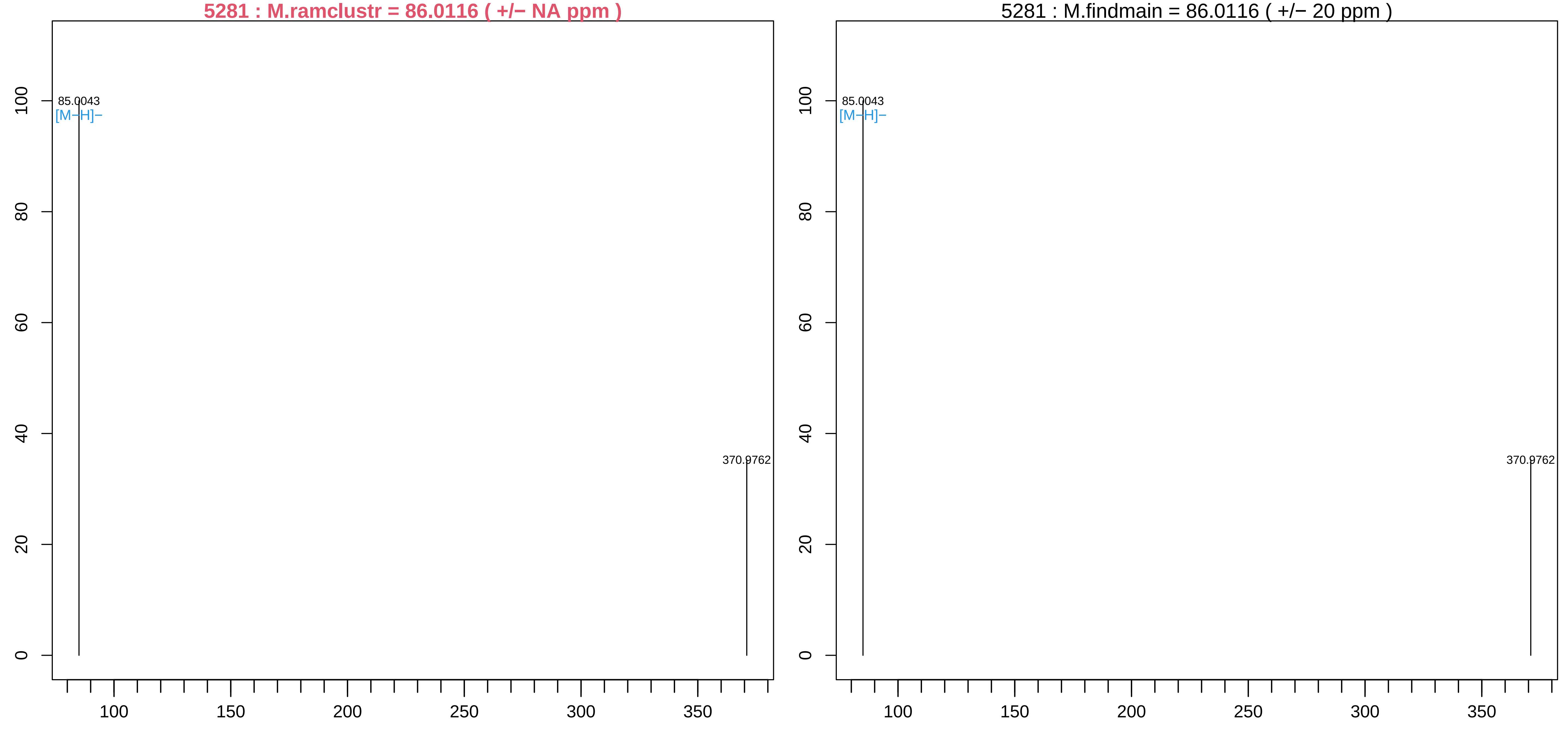

5281 : M.ramclustr = 86.0116 (+/− NA ppm)
85.0043
[M−H]−
370.9762
5281 : M.findmain = 86.0116 (+/− 20 ppm)
85.0043
[M−H]−
370.9762

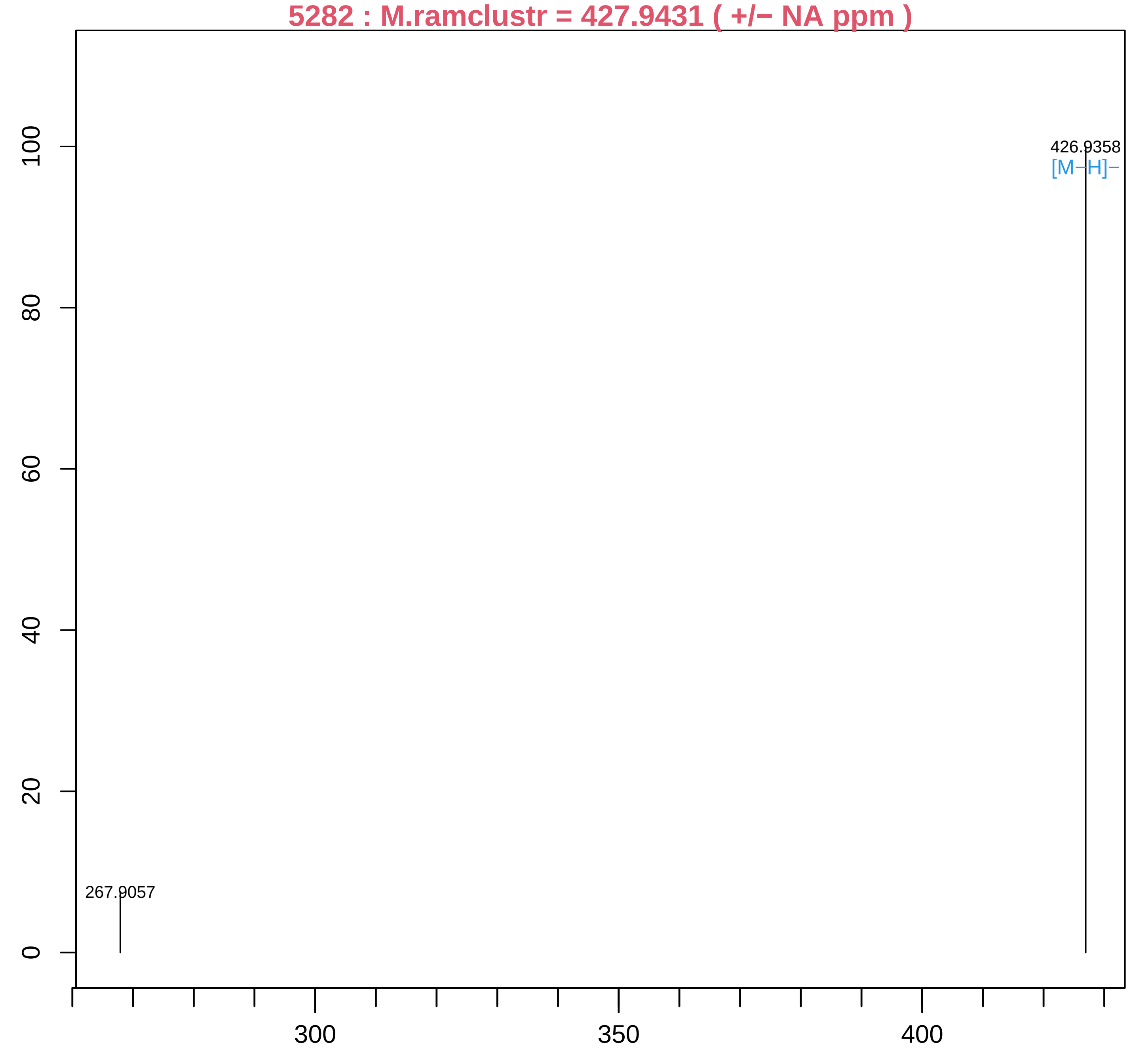

5282 : M.ramclustr = 427.9431 (+/− NA ppm)
426.9358
[M−H]−
267.9057
0
20
40
60
80
100
300
350
400

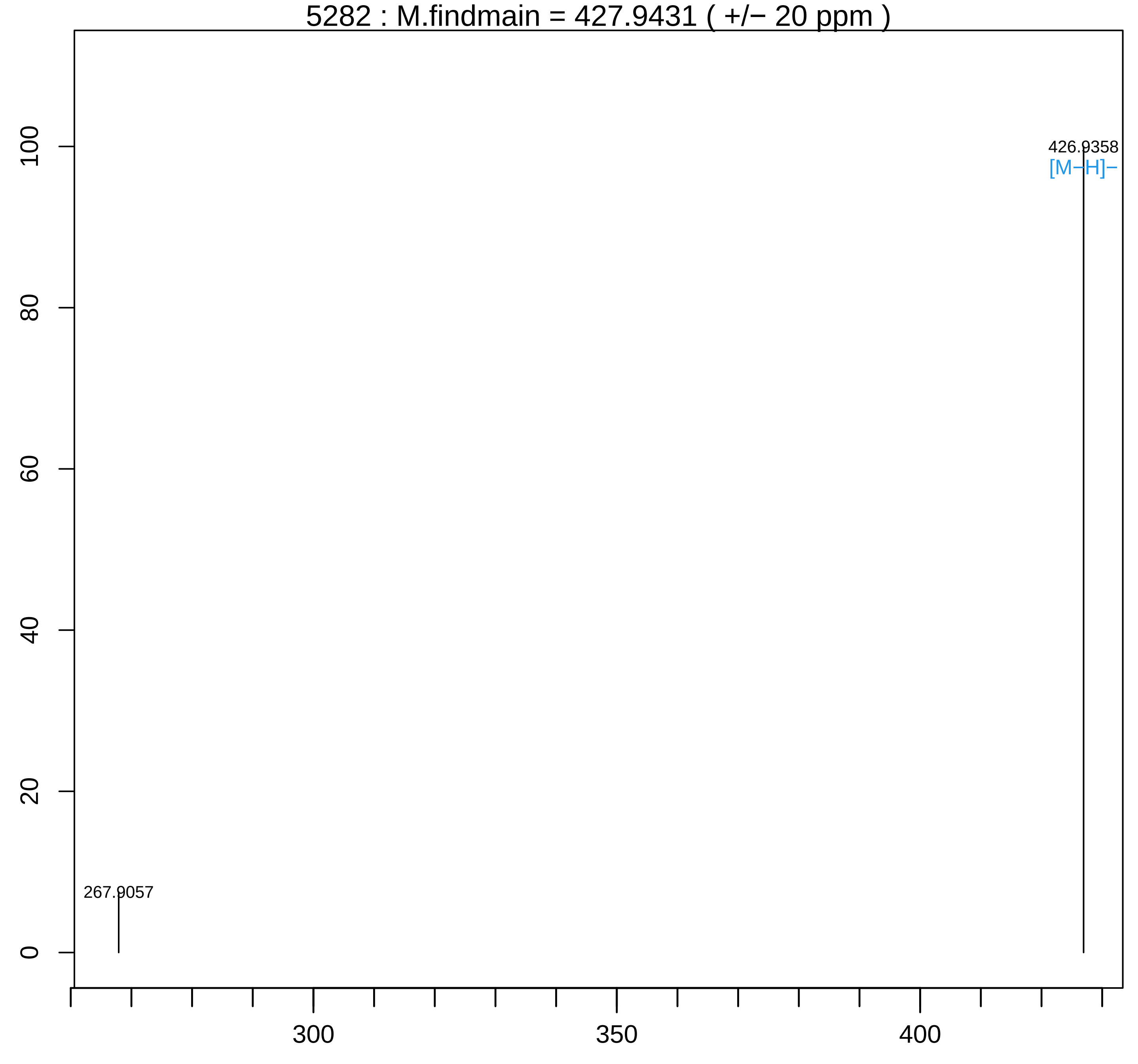

5282 : M.findmain = 427.9431 (+/− 20 ppm)
426.9358
[M−H]−
267.9057
0
20
40
60
80
100
300
350
400

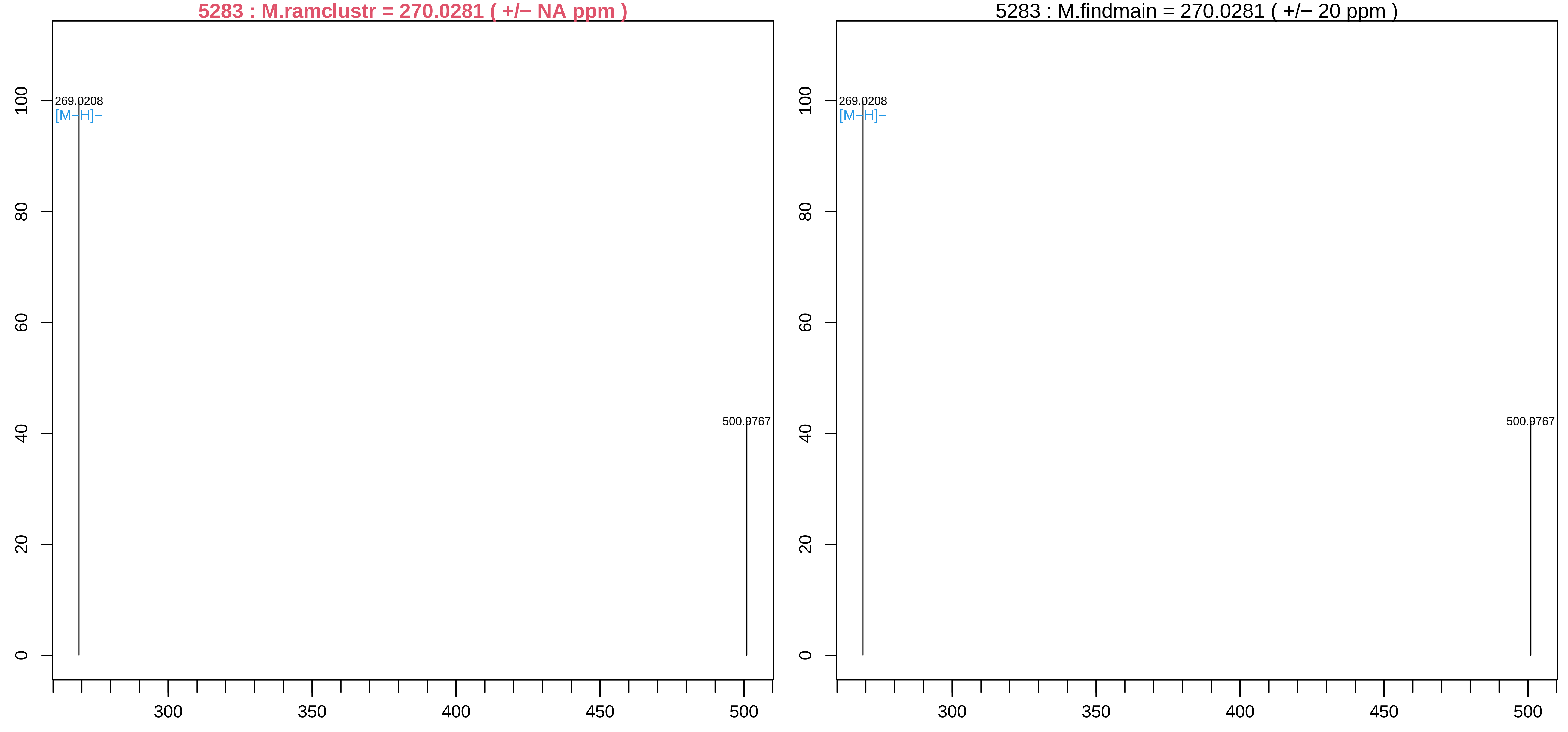
5283 : M.ramclustr = 270.0281 (+/− NA ppm)
269.0208
[M−H]−
500.9767
5283 : M.findmain = 270.0281 (+/− 20 ppm)
269.0208
[M−H]−
500.9767
0
20
40
60
80
100
300
350
400
450
500

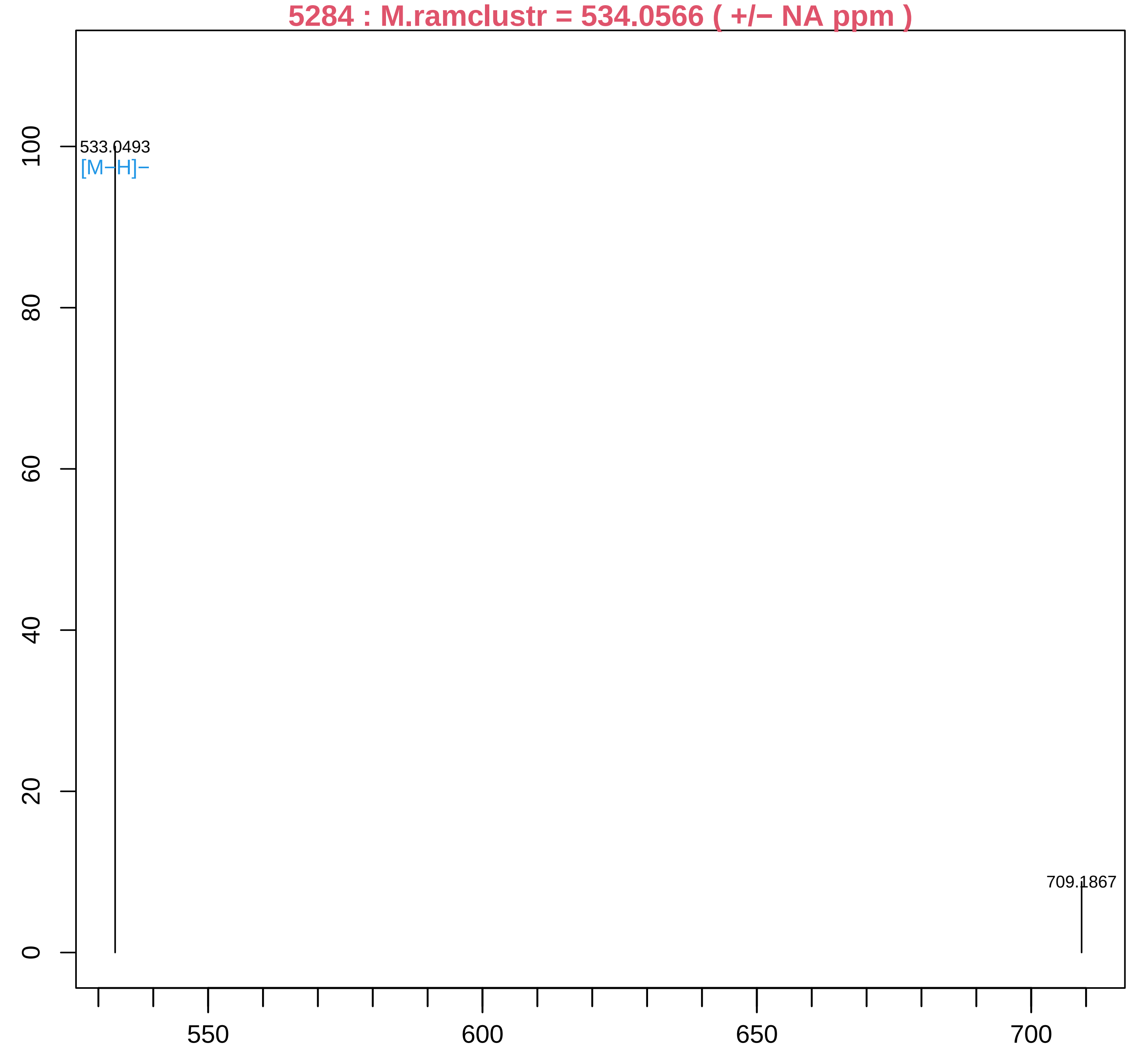

5284 : M.ramclustr = 534.0566 (+/− NA ppm)
533.0493
[M−H]−
709.1867

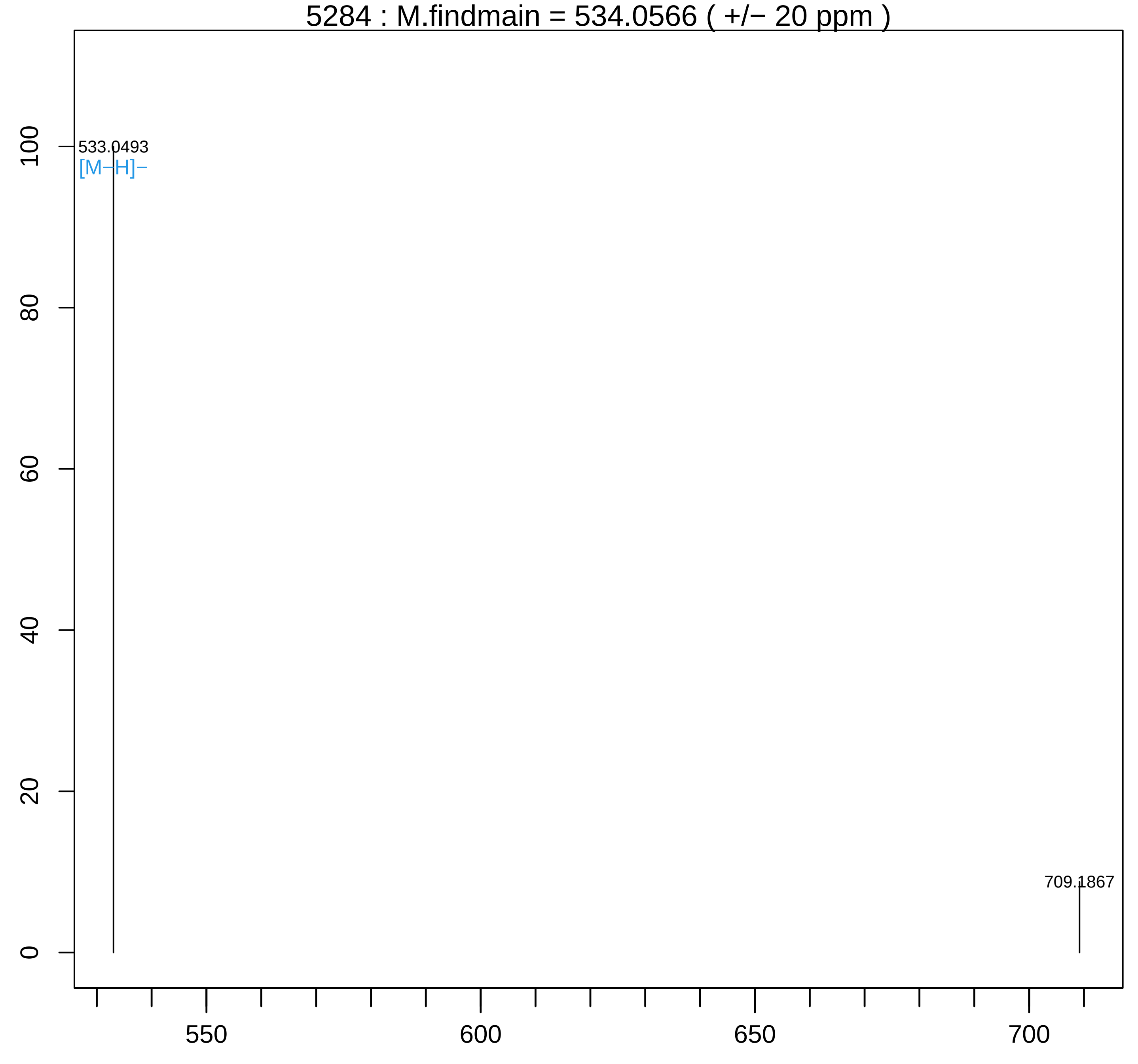

5284 : M.findmain = 534.0566 (+/− 20 ppm)
533.0493
[M−H]−
709.1867

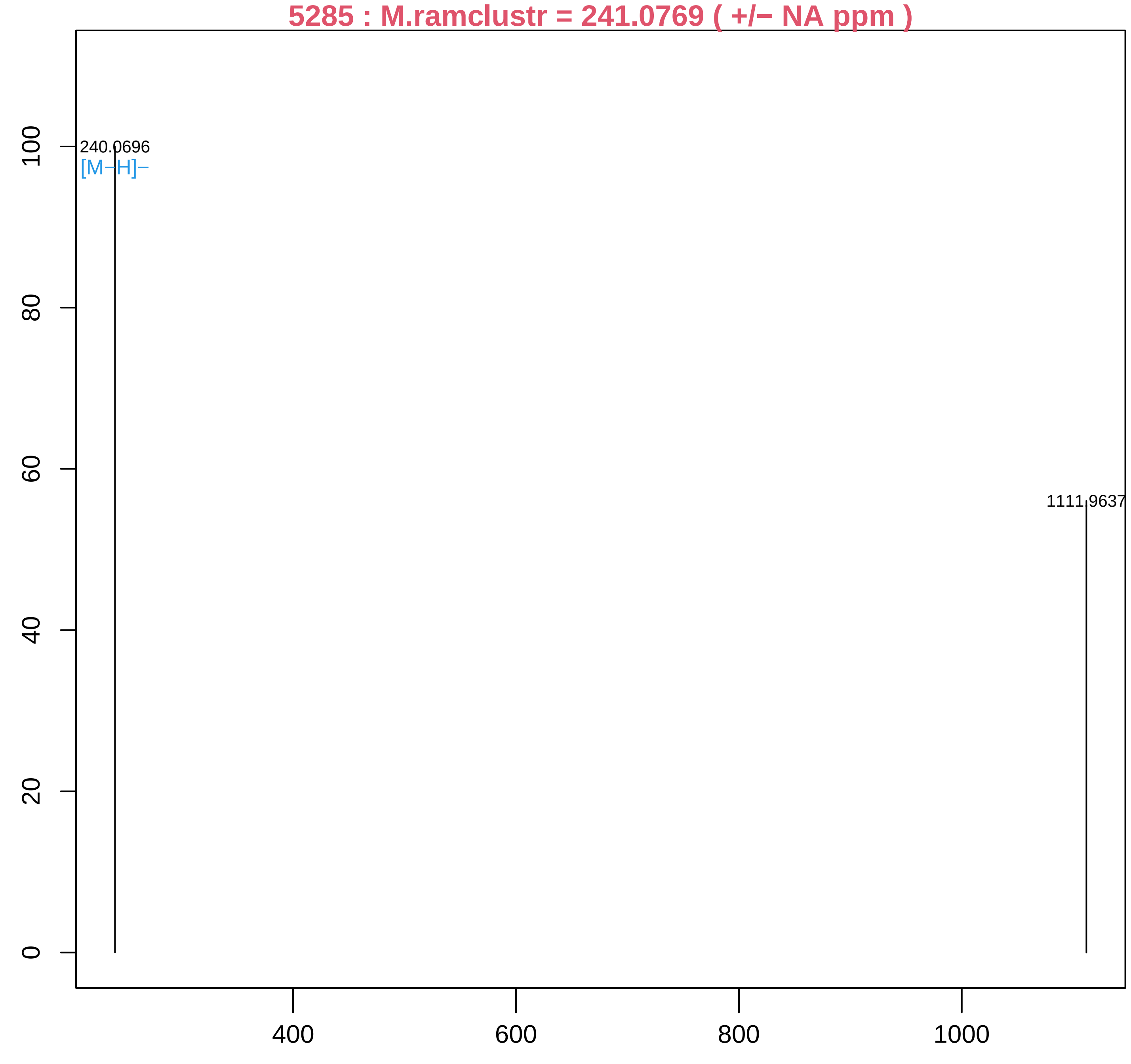
5285 : M.ramclustr = 241.0769 (+/− NA ppm)
240.0696
[M−H]−
1111.9637
0
20
40
60
80
100
400
600
800
1000

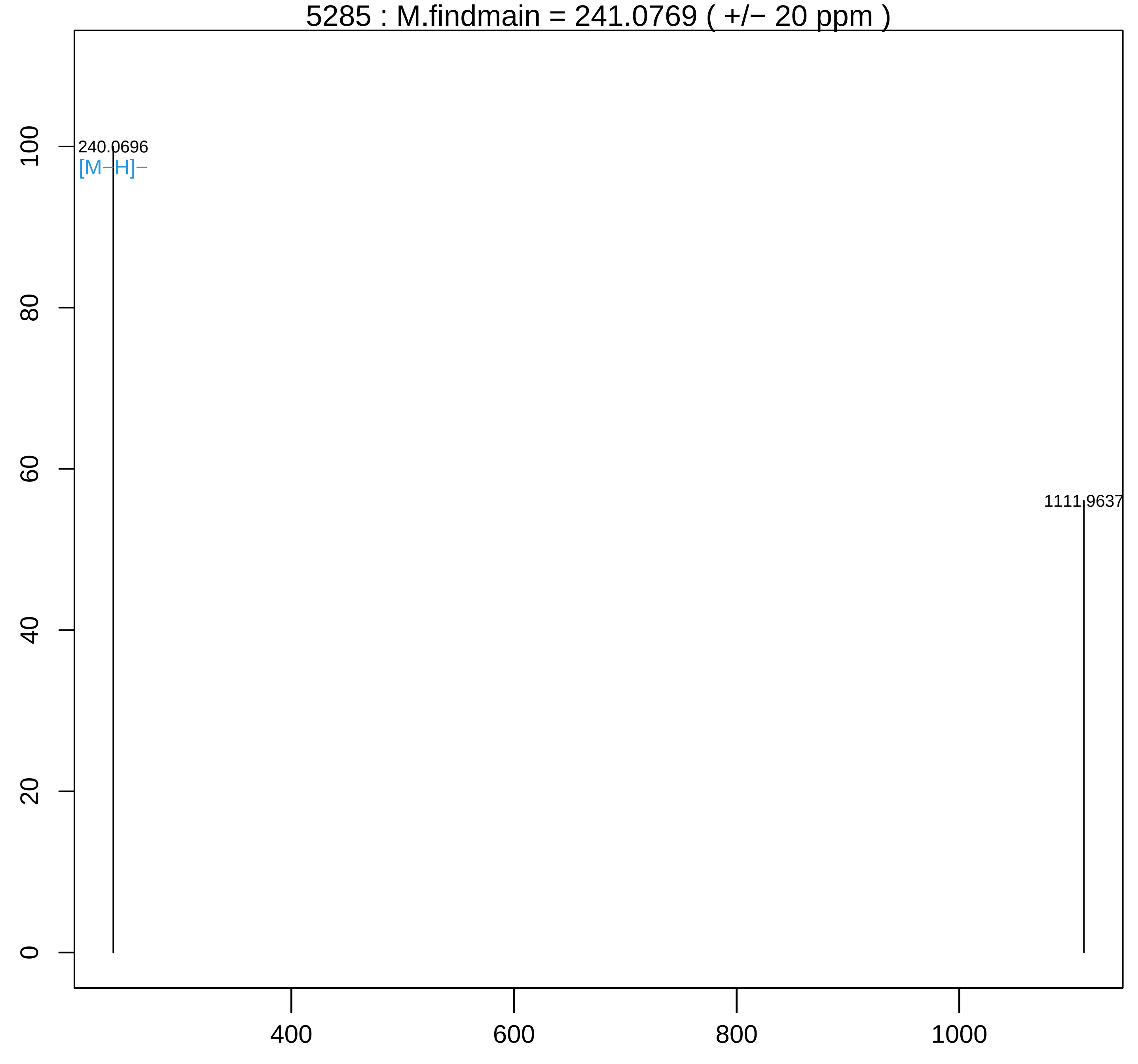
5285 : M.findmain = 241.0769 (+/− 20 ppm)
240.0696
[M−H]−
1111.9637
0
20
40
60
80
100
400
600
800
1000

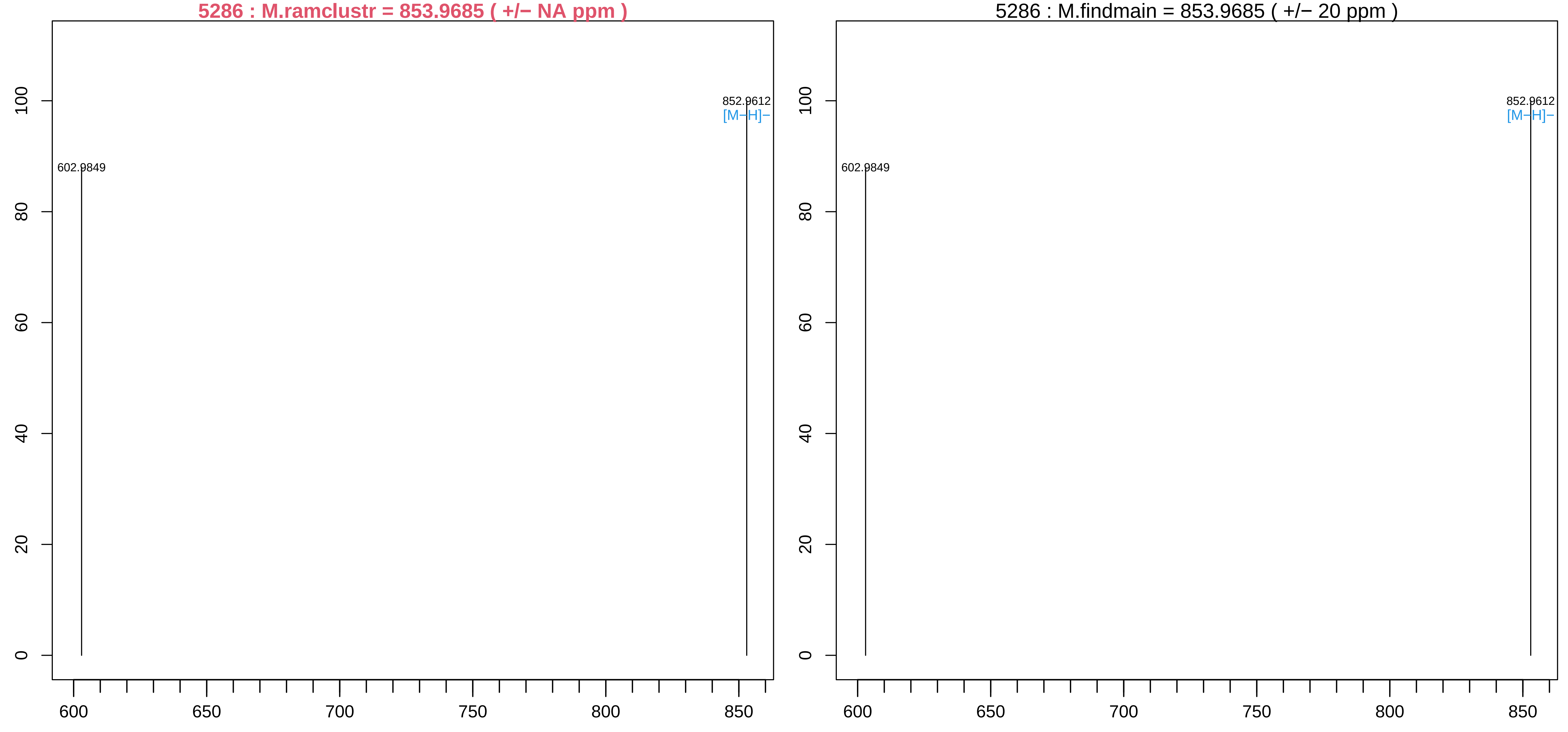
5286 : M.ramclustr = 853.9685 (+/− NA ppm)
852.9612
[M−H]−
602.9849
0
20
40
60
80
100
600
650
700
750
800
850
5286 : M.findmain = 853.9685 (+/− 20 ppm)
852.9612
[M−H]−
602.9849
0
20
40
60
80
100
600
650
700
750
800
850

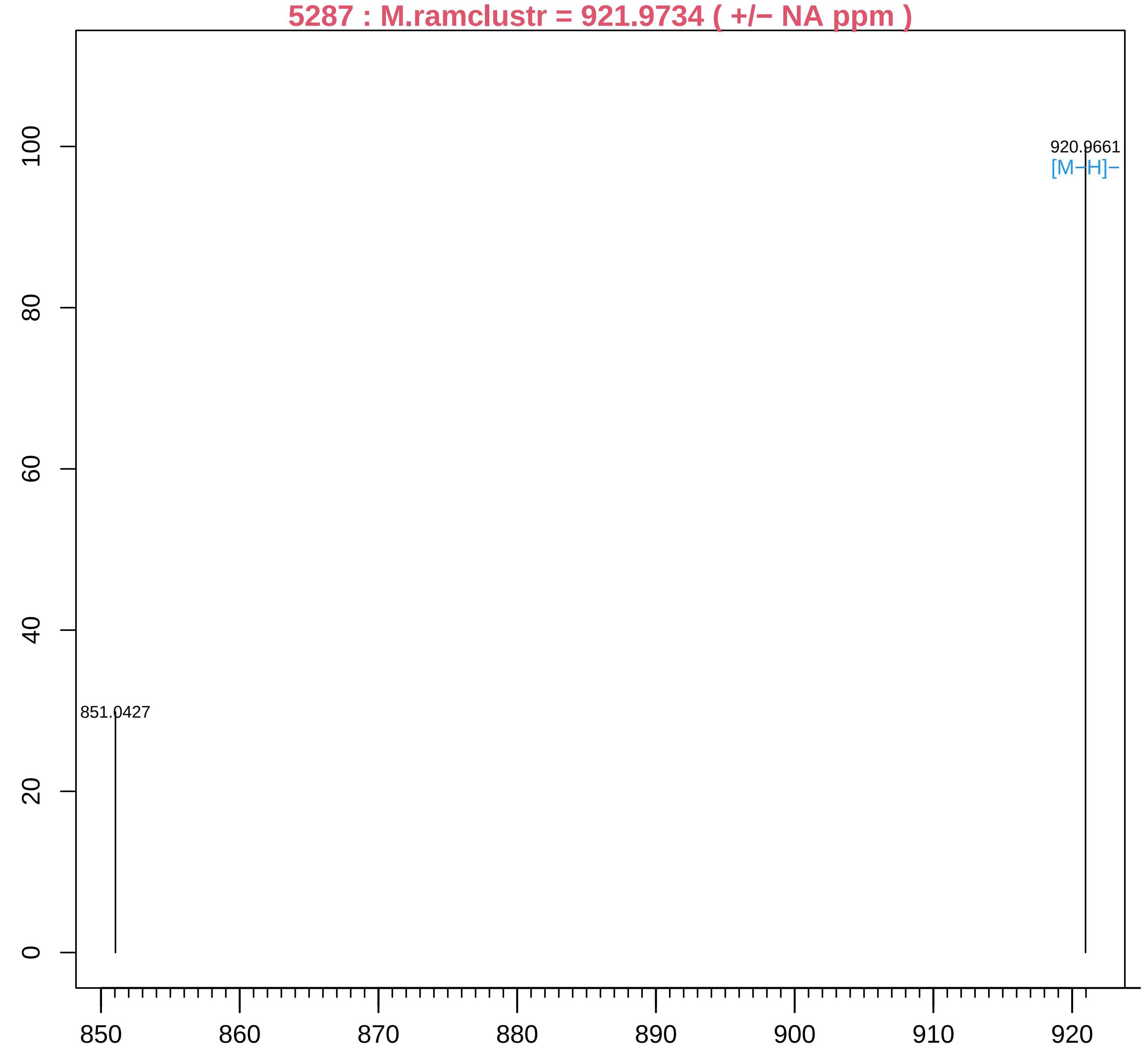
5287 : M.ramclustr = 921.9734 (+/− NA ppm)
920.9661
[M−H]−
851.0427
0
20
40
60
80
100
850
860
870
880
890
900
910
920

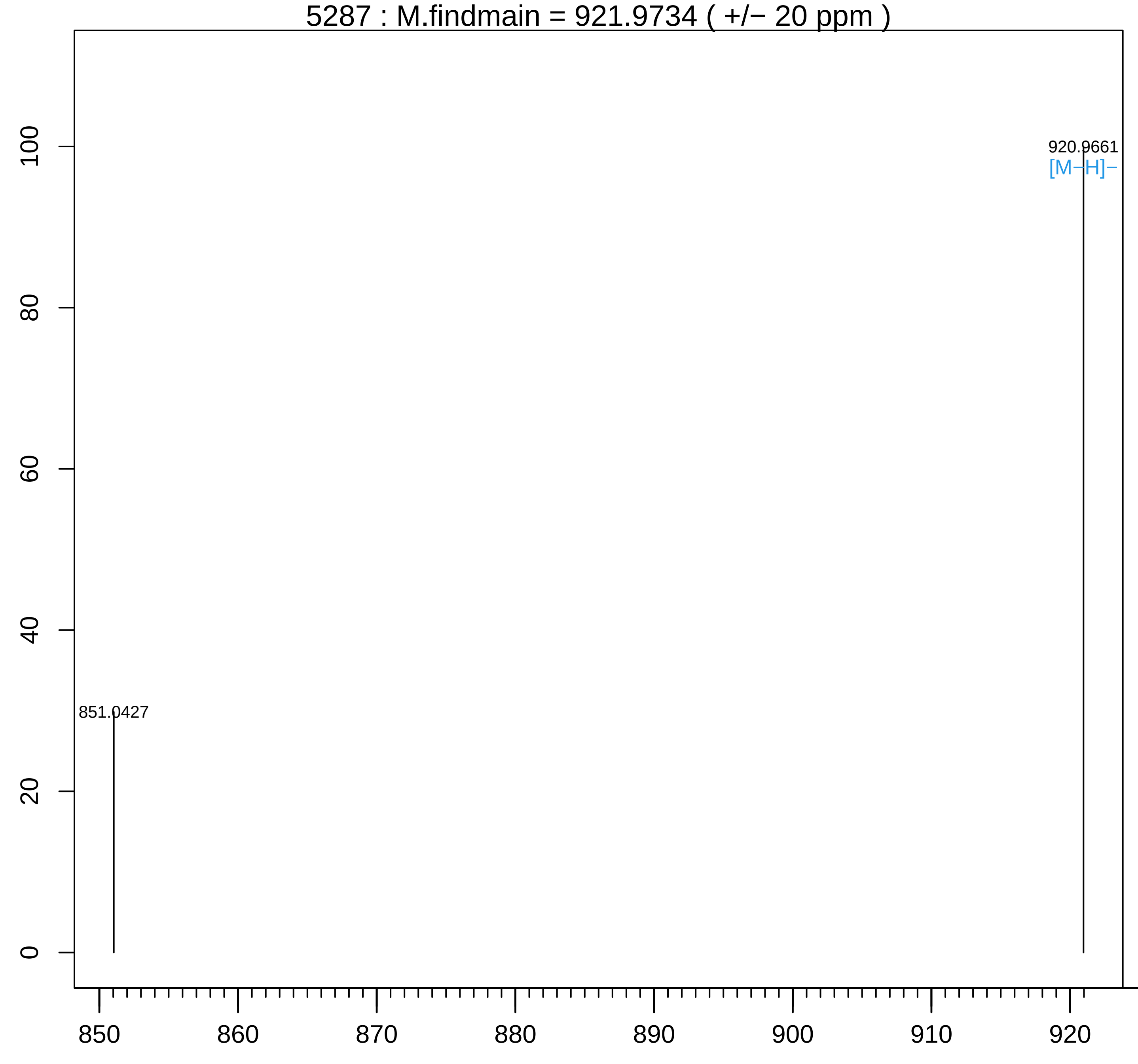
5287 : M.findmain = 921.9734 (+/− 20 ppm)
920.9661
[M−H]−
851.0427
0
20
40
60
80
100
850
860
870
880
890
900
910
920

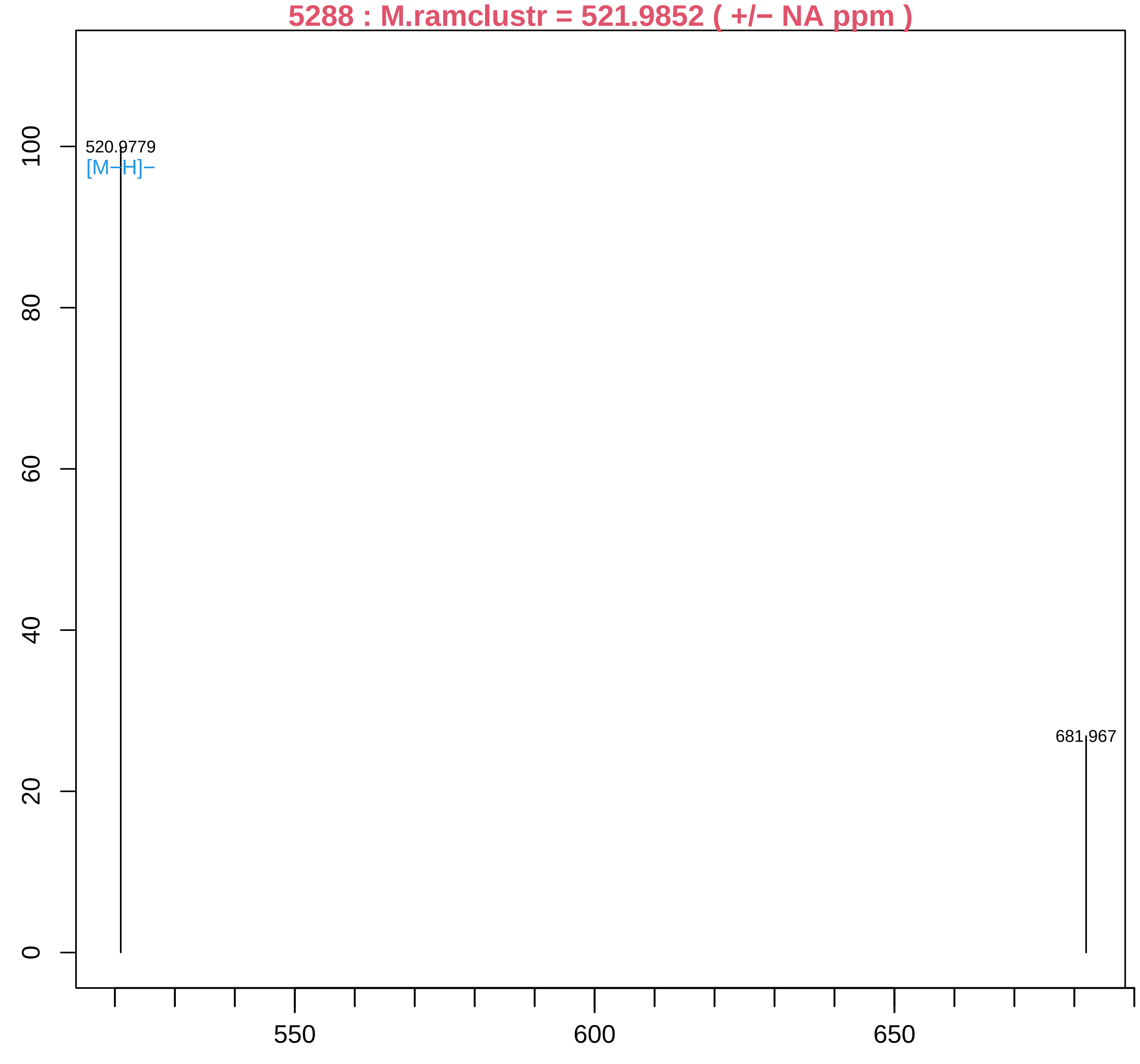
5288 : M.ramclustr = 521.9852 (+/− NA ppm)
520.9779
[M−H]−
681.967
0
20
40
60
80
100
550
600
650

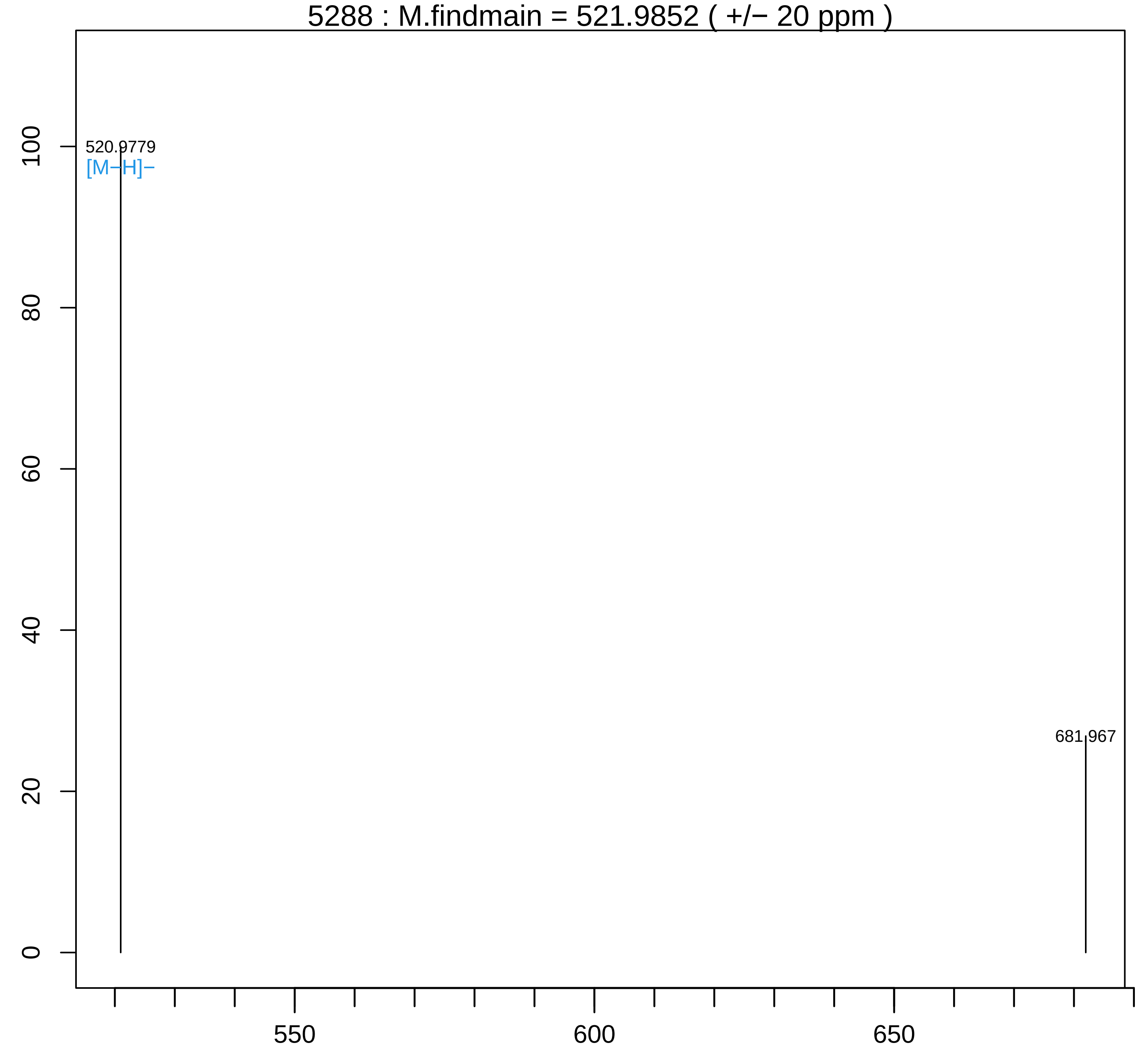
5288 : M.findmain = 521.9852 (+/− 20 ppm)
520.9779
[M−H]−
681.967
0
20
40
60
80
100
550
600
650

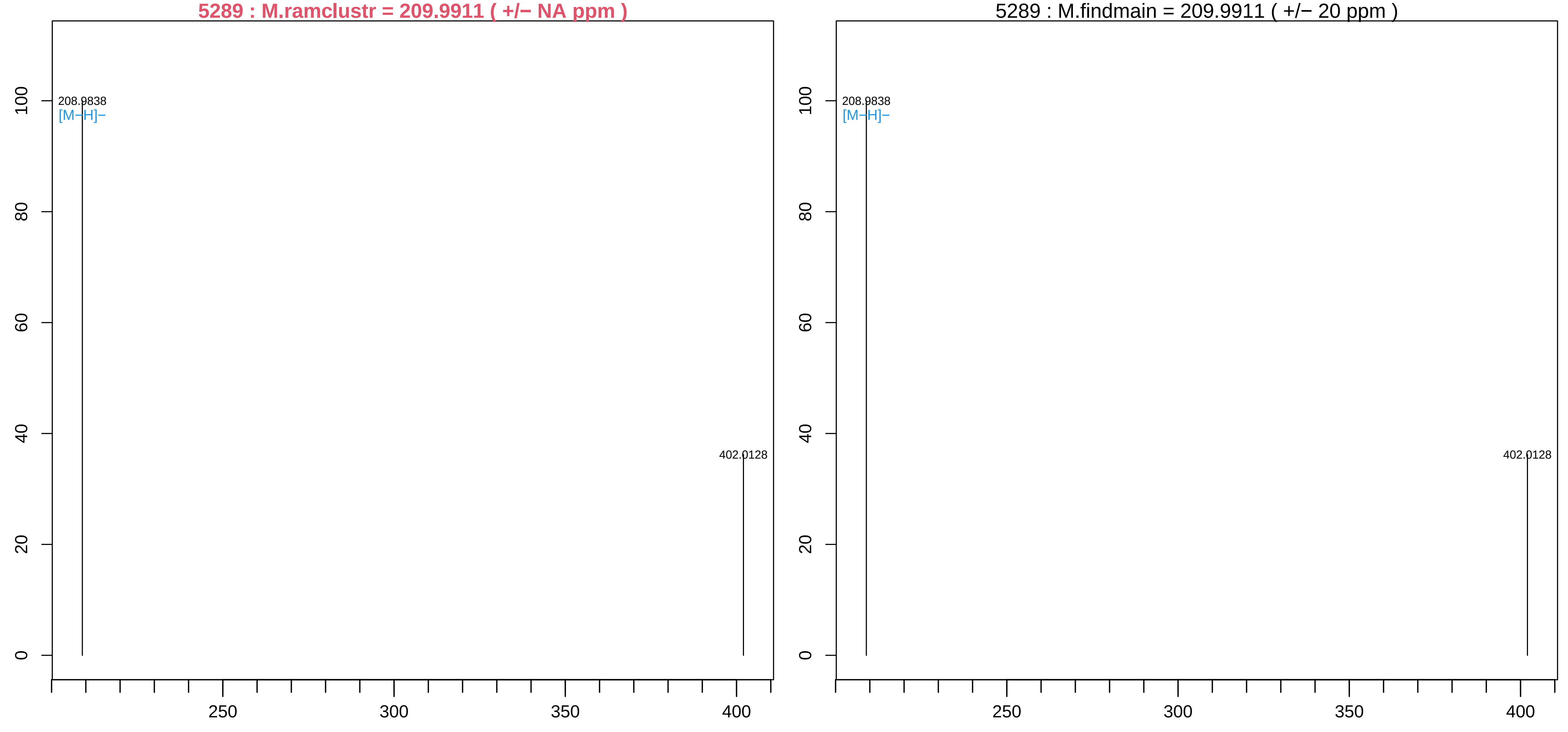
5289 : M.ramclustr = 209.9911 (+/− NA ppm)
208.9838
[M−H]−
402.0128
5289 : M.findmain = 209.9911 (+/− 20 ppm)
208.9838
[M−H]−
402.0128
0
20
40
60
80
100
250
300
350
400

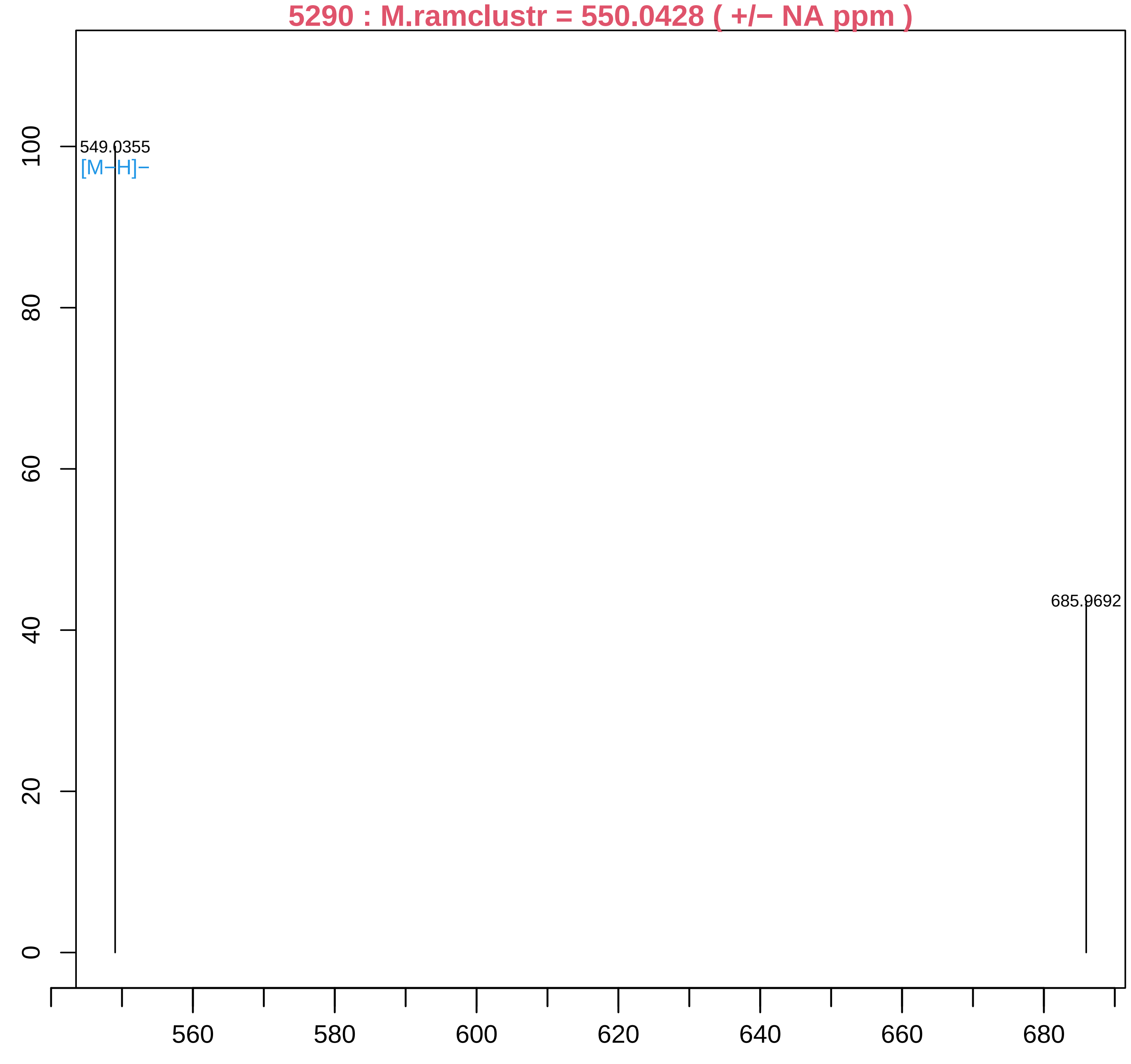
5290 : M.ramclustr = 550.0428 (+/− NA ppm)
549.0355
[M−H]−
685.9692
0
20
40
60
80
100
560
580
600
620
640
660
680

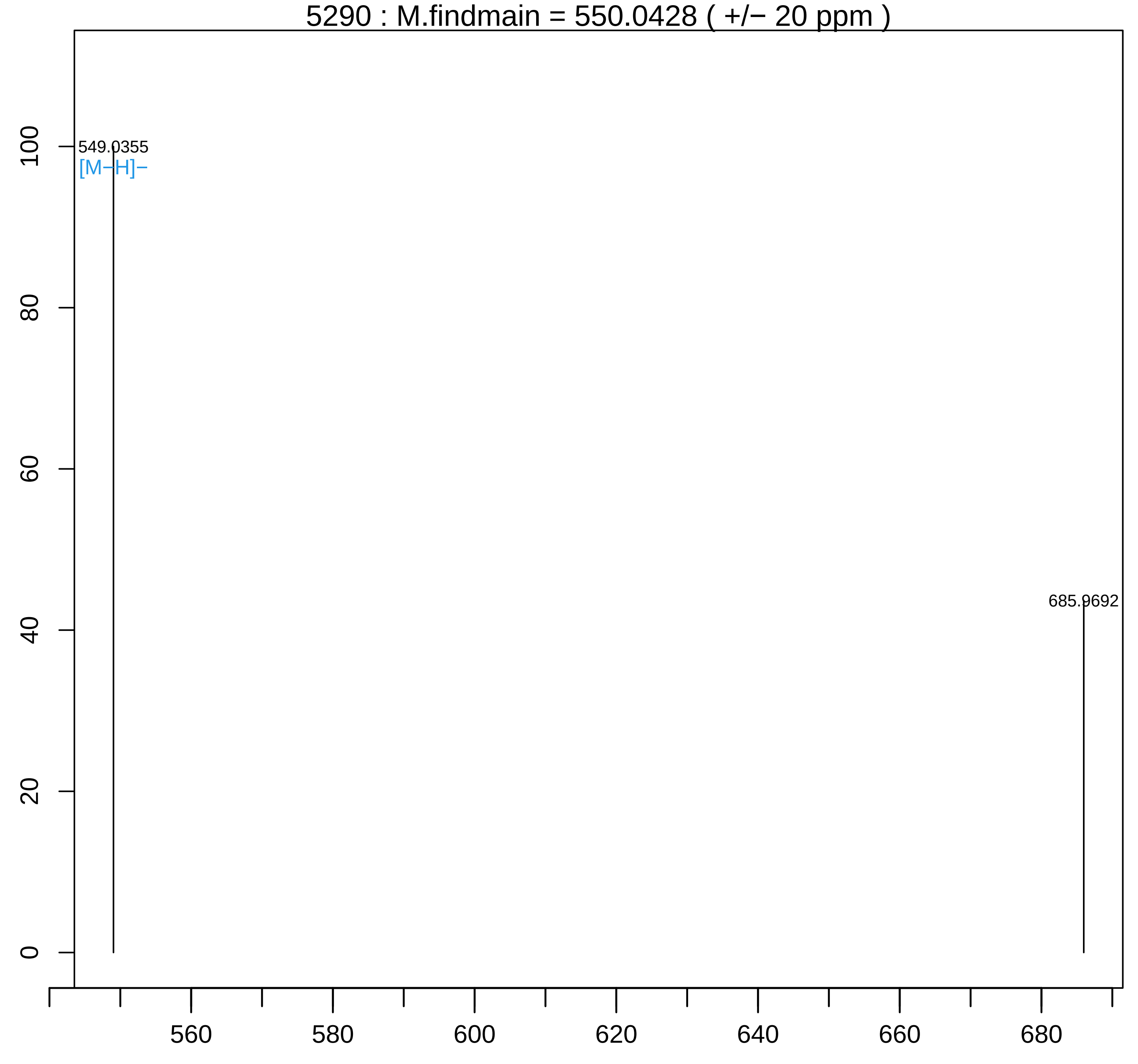
5290 : M.findmain = 550.0428 (+/− 20 ppm)
549.0355
[M−H]−
685.9692
0
20
40
60
80
100
560
580
600
620
640
660
680

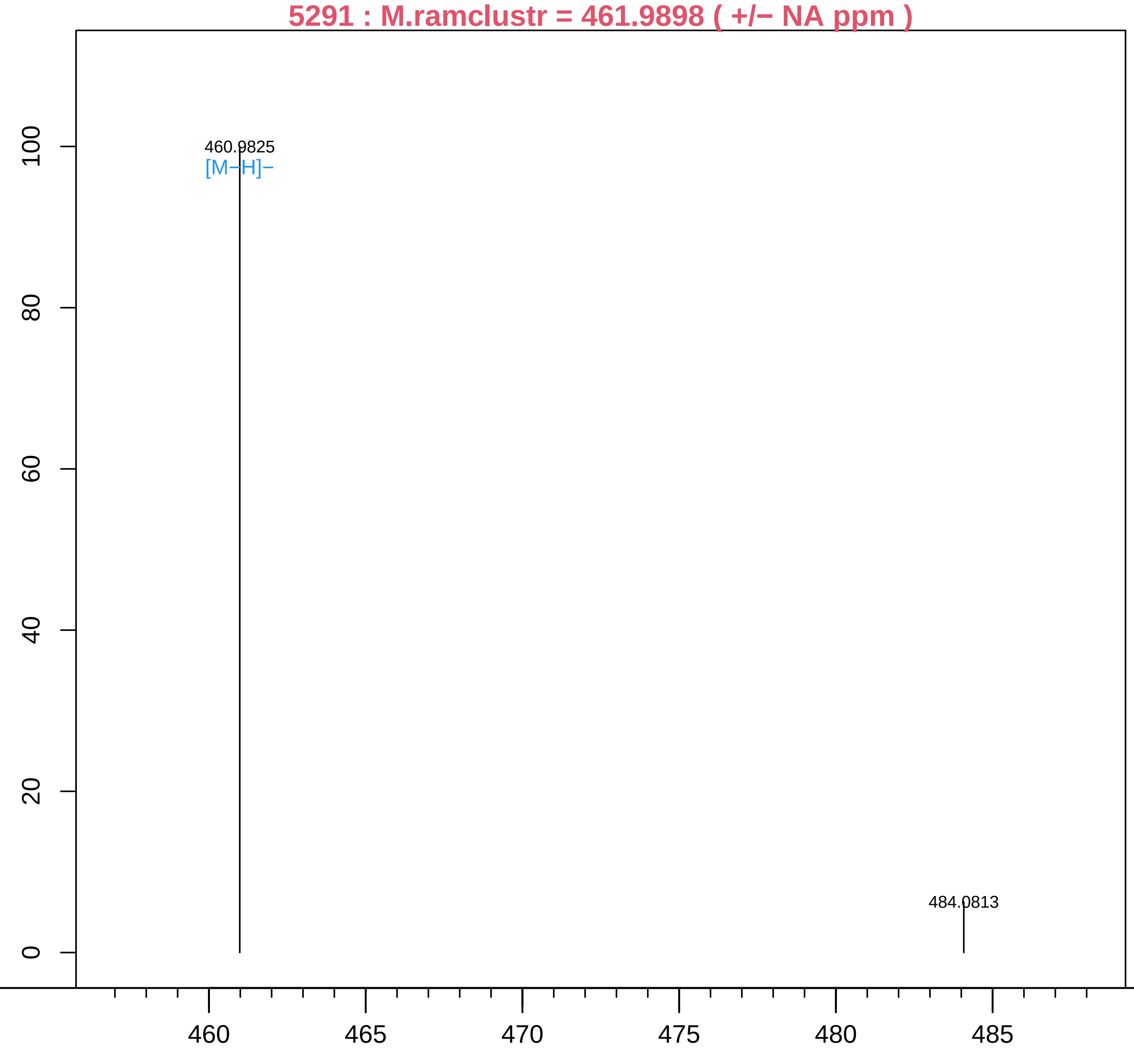

5291 : M.ramclustr = 461.9898 (+/− NA ppm)
460.9825
[M−H]−
484.0813
0
20
40
60
80
100
460
465
470
475
480
485

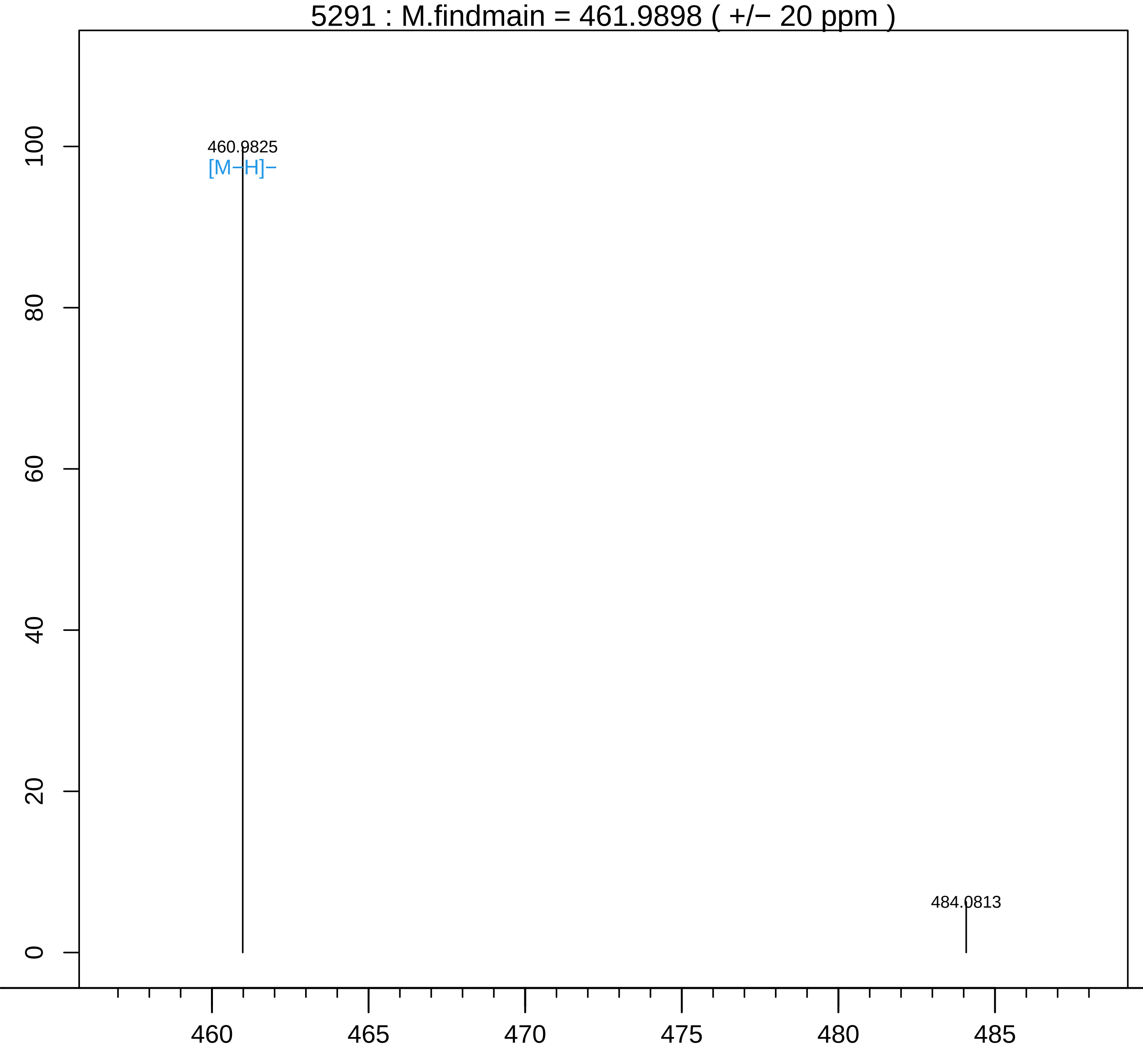

5291 : M.findmain = 461.9898 (+/− 20 ppm)
460.9825
[M−H]−
484.0813
0
20
40
60
80
100
460
465
470
475
480
485

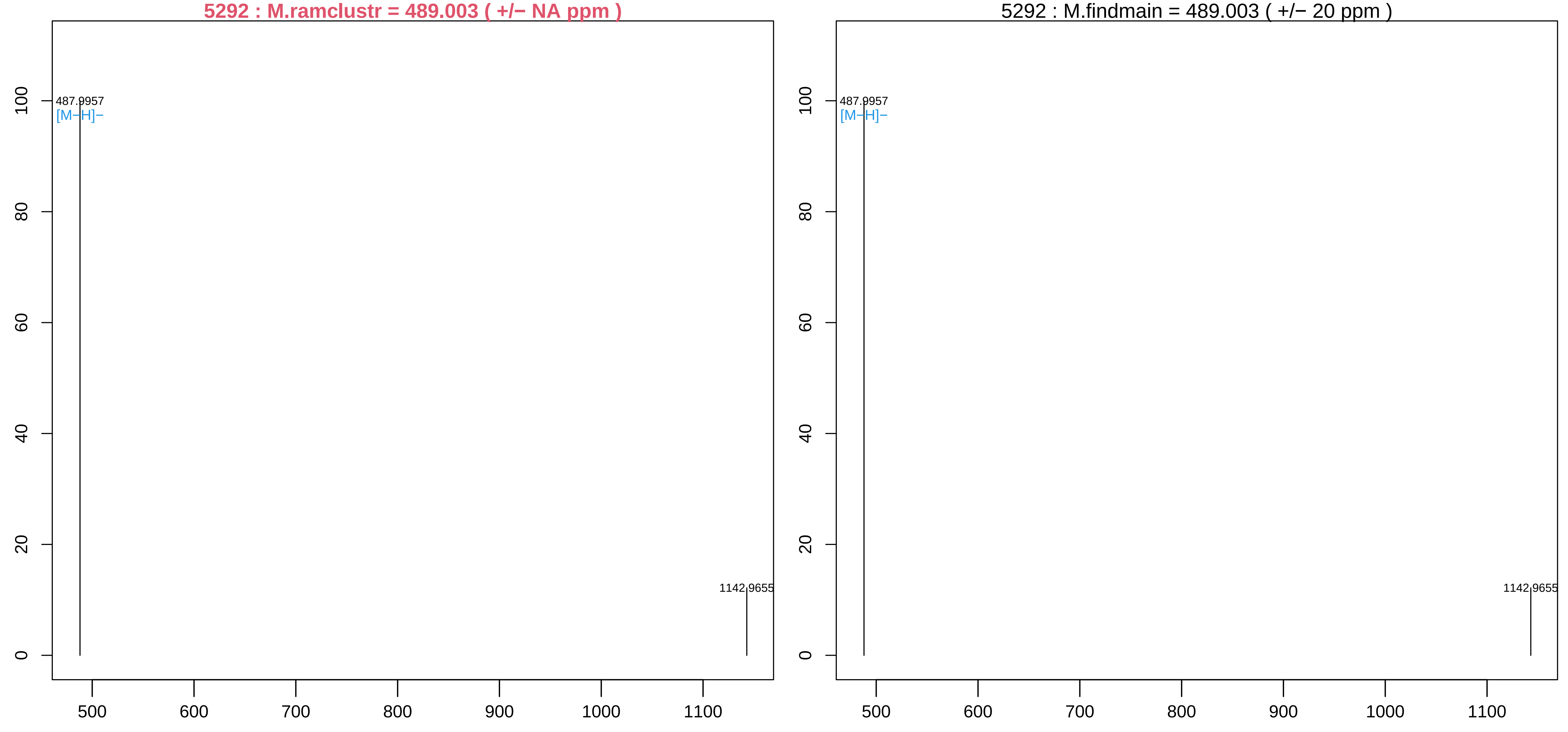

5292 : M.ramclustr = 489.003 (+/− NA ppm)
487.9957
[M−H]−
1142.9655
0
20
40
60
80
100
500
600
700
800
900
1000
1100
5292 : M.findmain = 489.003 (+/− 20 ppm)
487.9957
[M−H]−
1142.9655
0
20
40
60
80
100
500
600
700
800
900
1000
1100

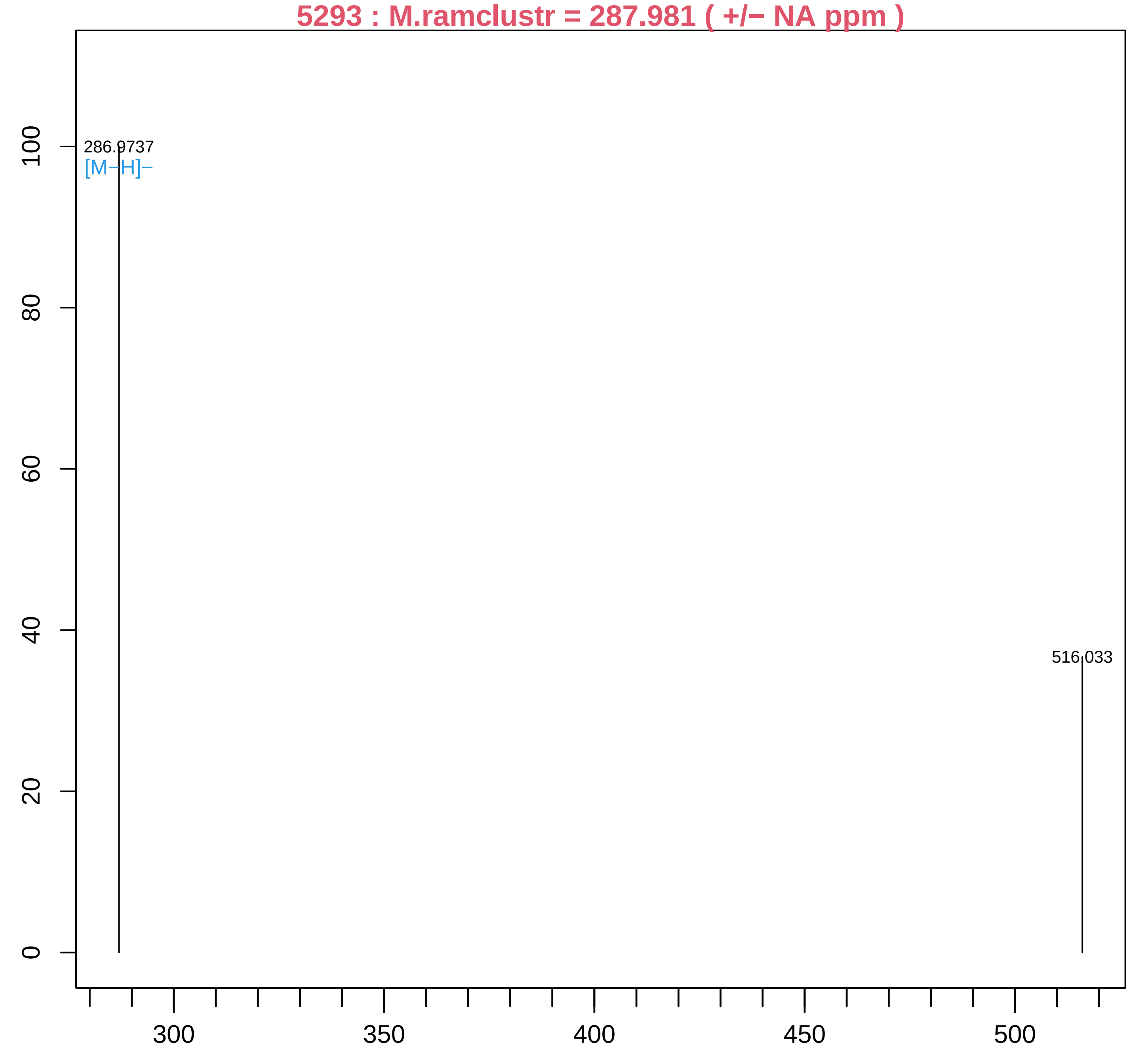
5293 : M.ramclustr = 287.981 (+/− NA ppm)
286.9737
[M−H]−
516.033
0
20
40
60
80
100
300
350
400
450
500

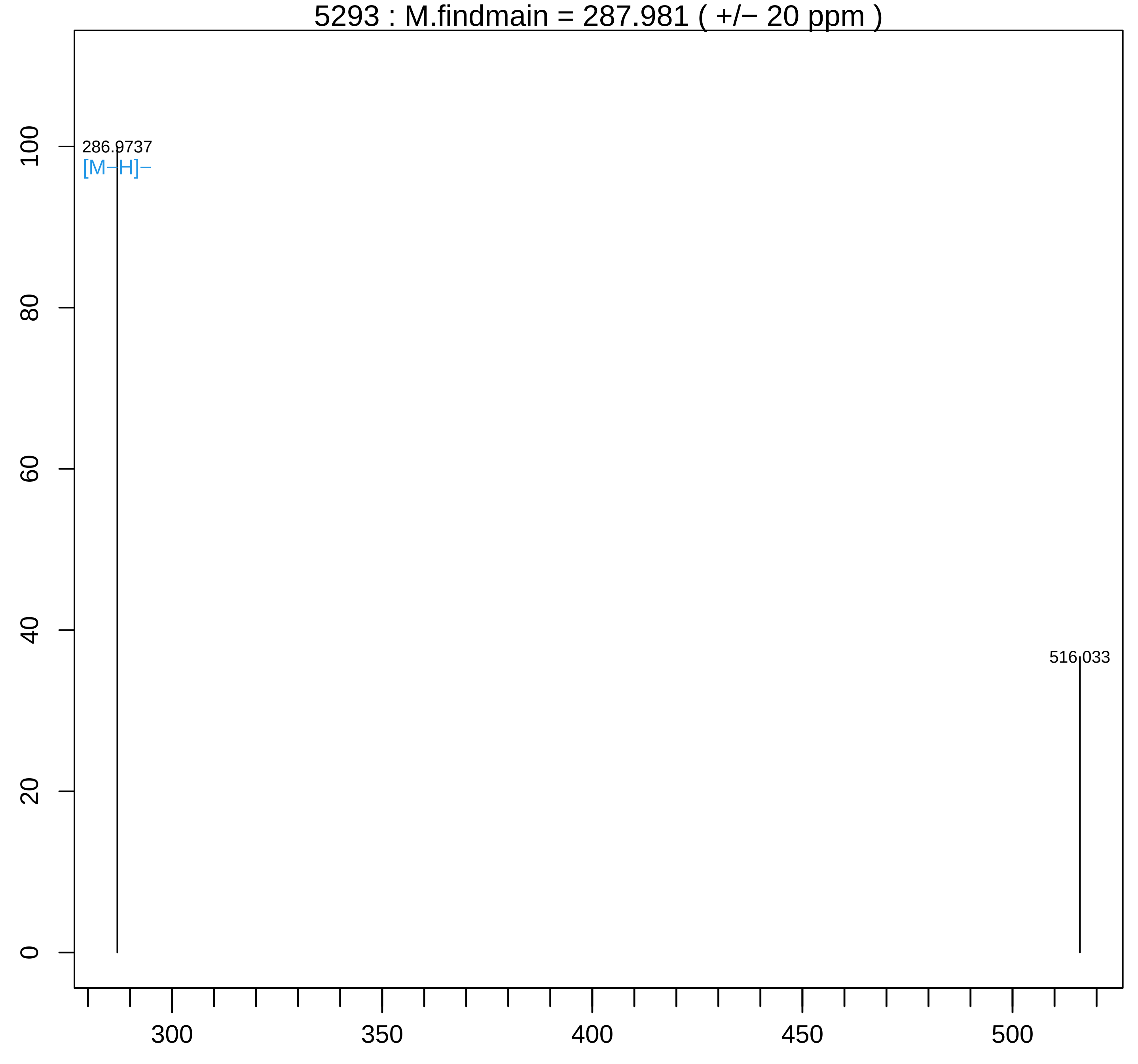
5293 : M.findmain = 287.981 (+/− 20 ppm)
286.9737
[M−H]−
516.033
0
20
40
60
80
100
300
350
400
450
500

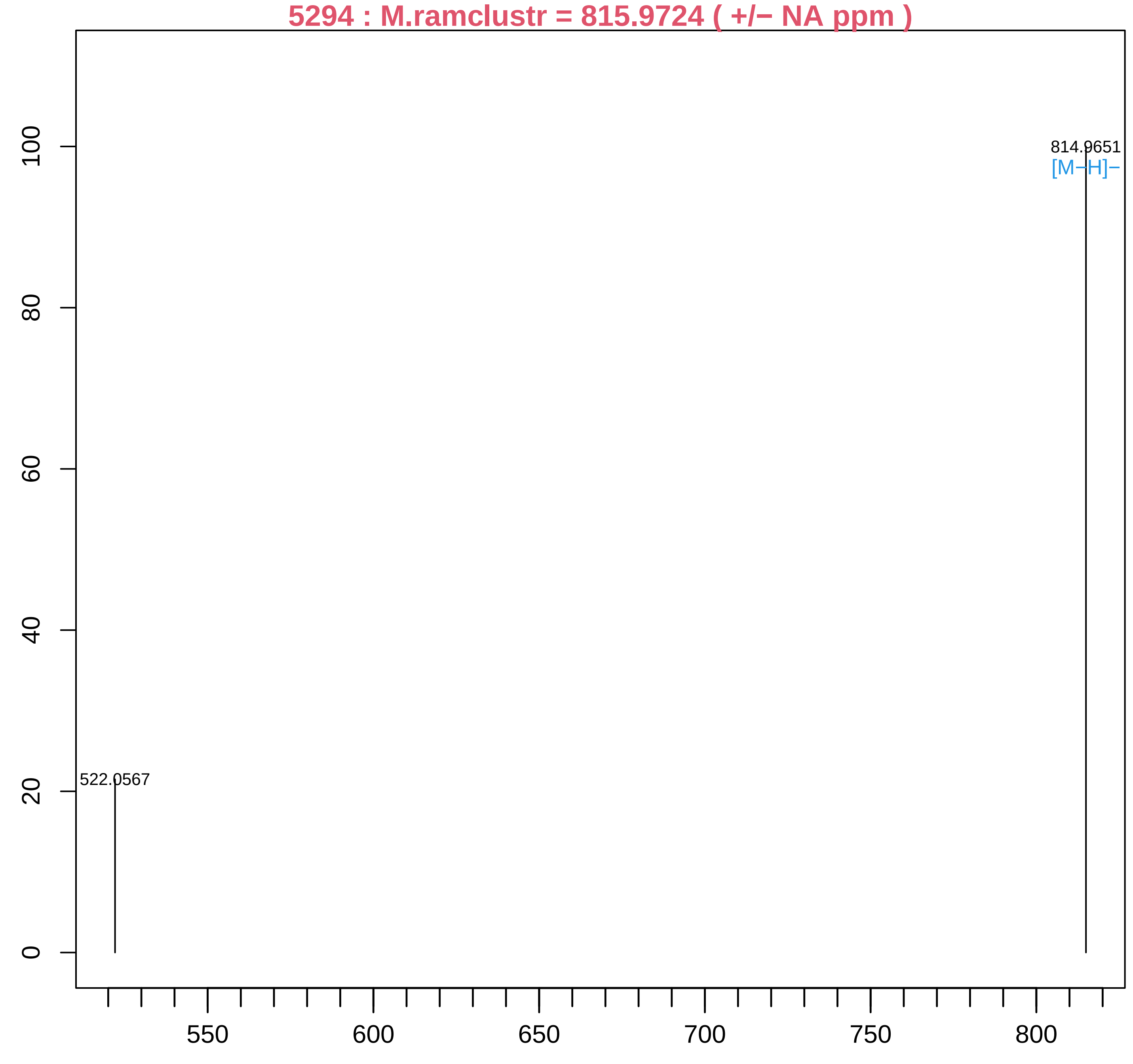
5294 : M.ramclustr = 815.9724 (+/− NA ppm)
814.9651
[M−H]−
522.0567
0
20
40
60
80
100
550
600
650
700
750
800

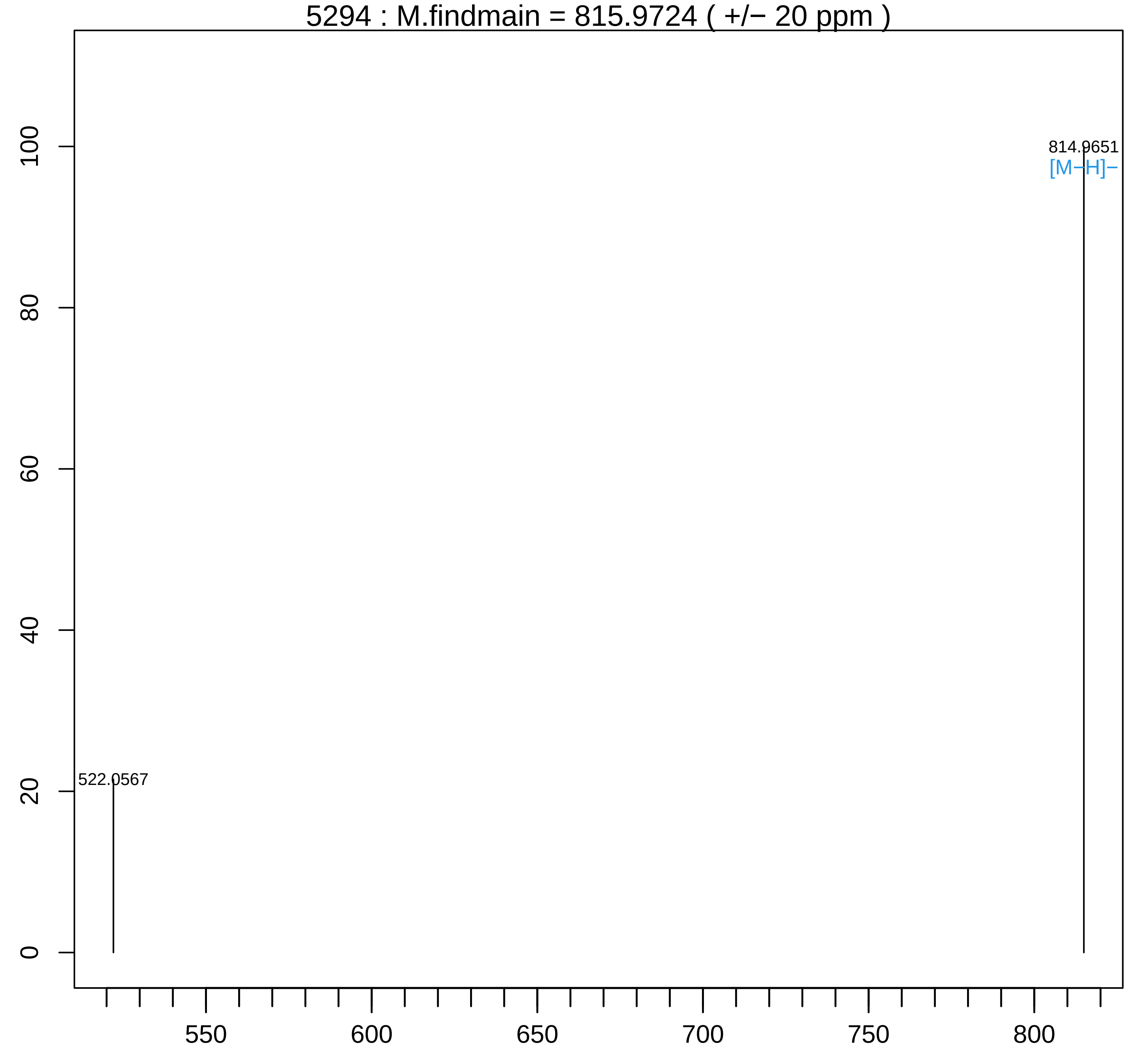
5294 : M.findmain = 815.9724 (+/− 20 ppm)
814.9651
[M−H]−
522.0567
0
20
40
60
80
100
550
600
650
700
750
800

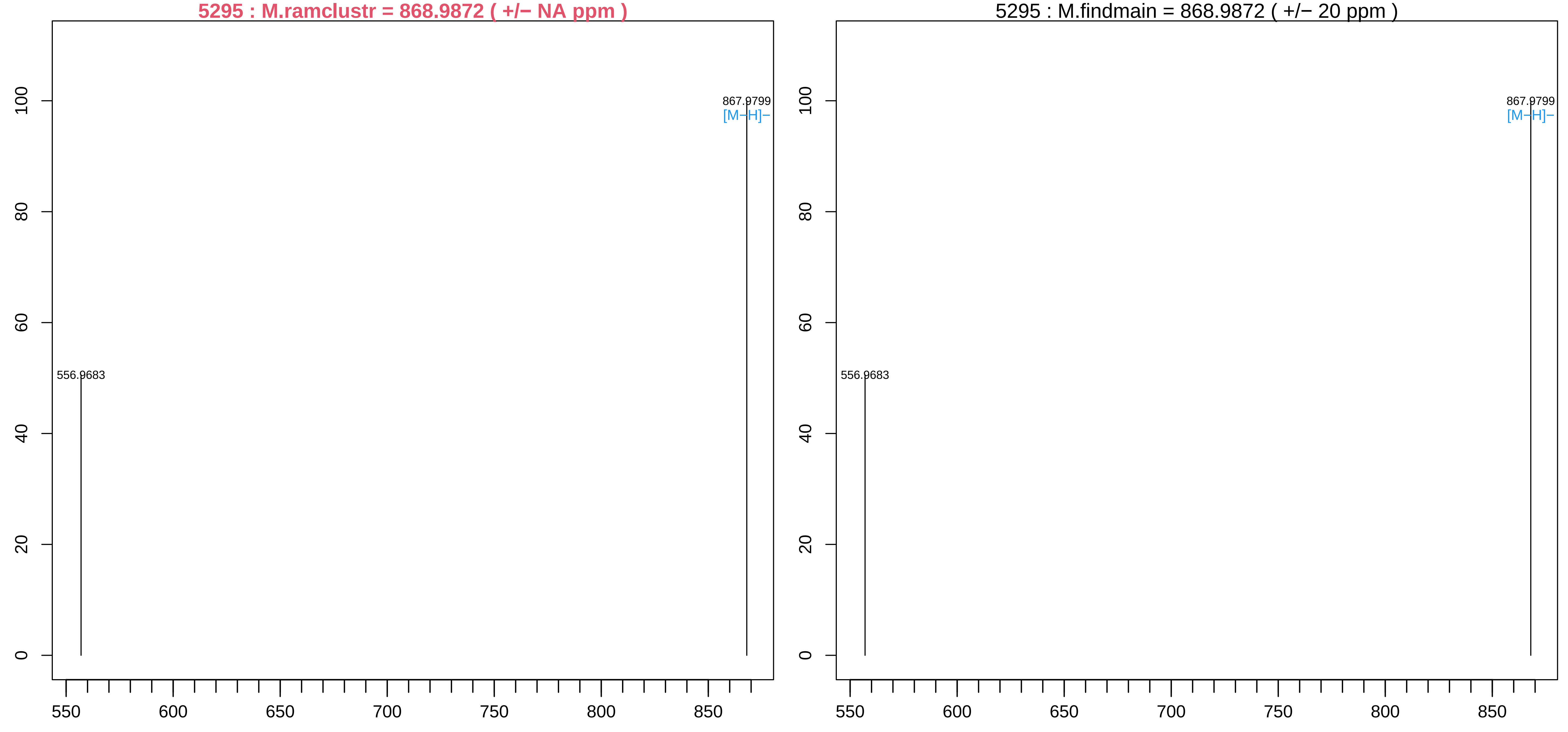

5295 : M.ramclustr = 868.9872 (+/− NA ppm)
867.9799
[M−H]−
556.9683
0
20
40
60
80
100
550
600
650
700
750
800
850
5295 : M.findmain = 868.9872 (+/− 20 ppm)
867.9799
[M−H]−
556.9683
0
20
40
60
80
100
550
600
650
700
750
800
850

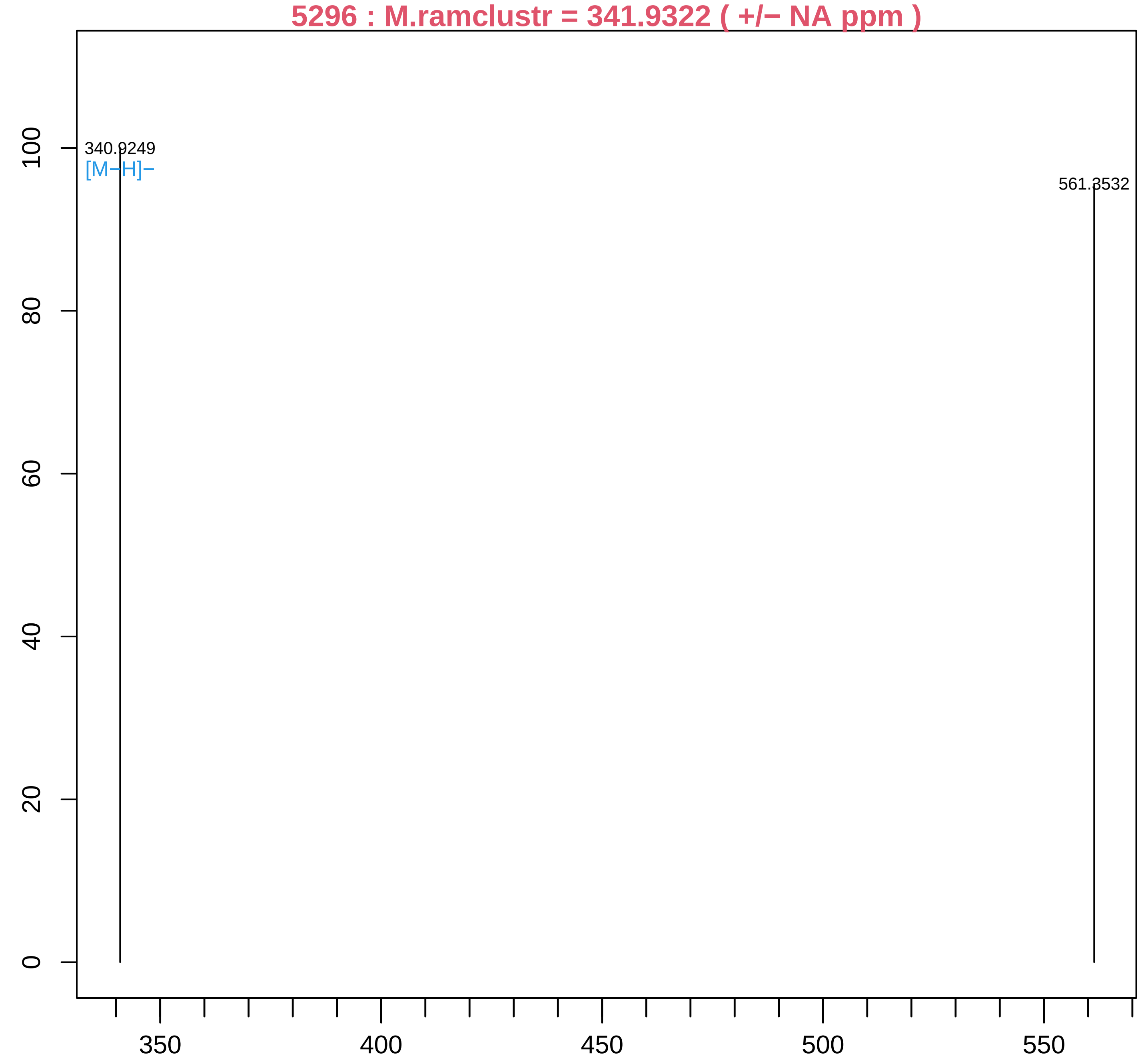
5296 : M.ramclustr = 341.9322 (+/− NA ppm)
340.9249
[M−H]−
561.3532
0
20
40
60
80
100
350
400
450
500
550

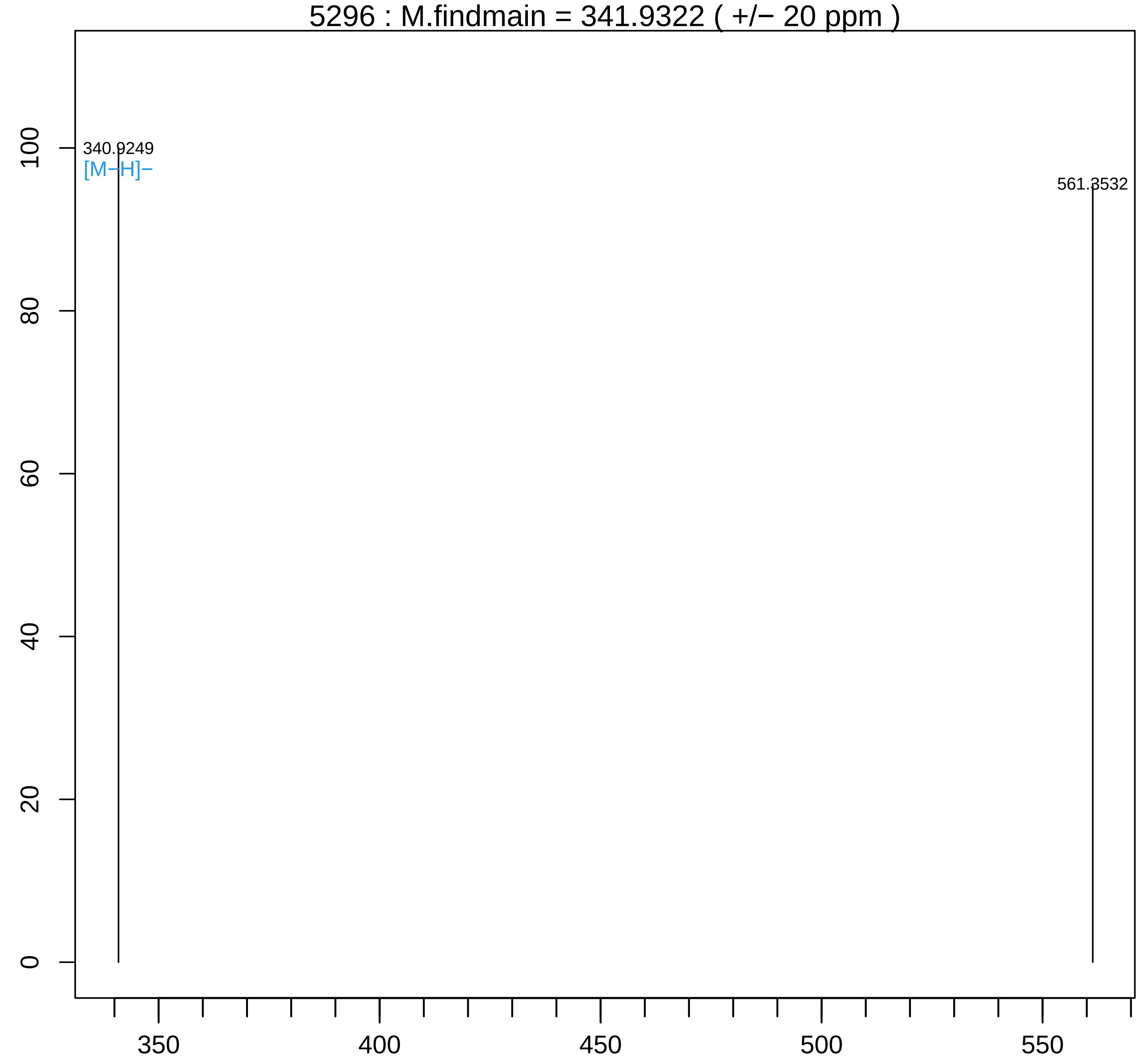
5296 : M.findmain = 341.9322 (+/− 20 ppm)
340.9249
[M−H]−
561.3532
0
20
40
60
80
100
350
400
450
500
550

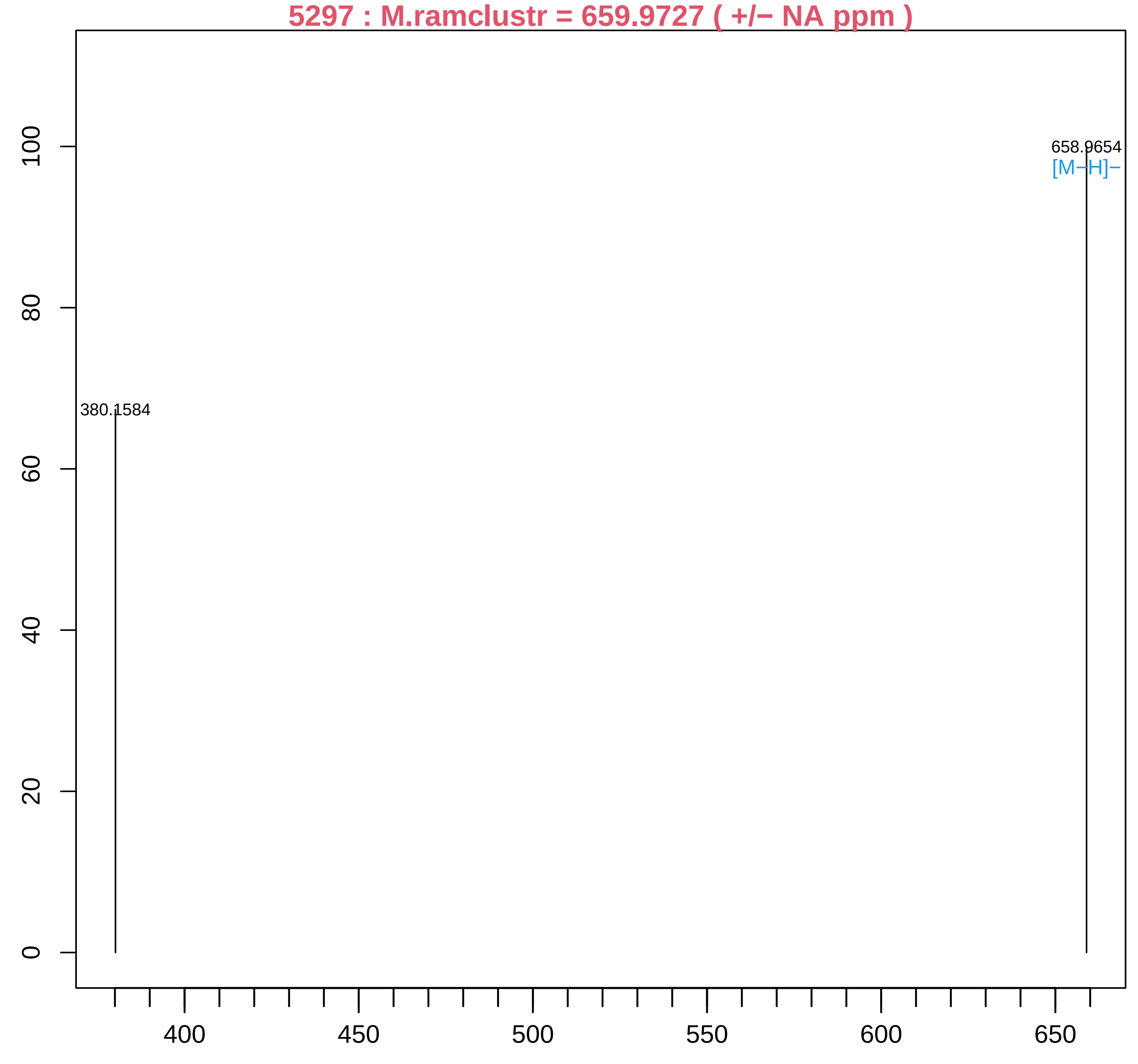

5297 : M.ramclustr = 659.9727 (+/− NA ppm)
658.9654
[M−H]−
380.1584
0
20
40
60
80
100
400
450
500
550
600
650

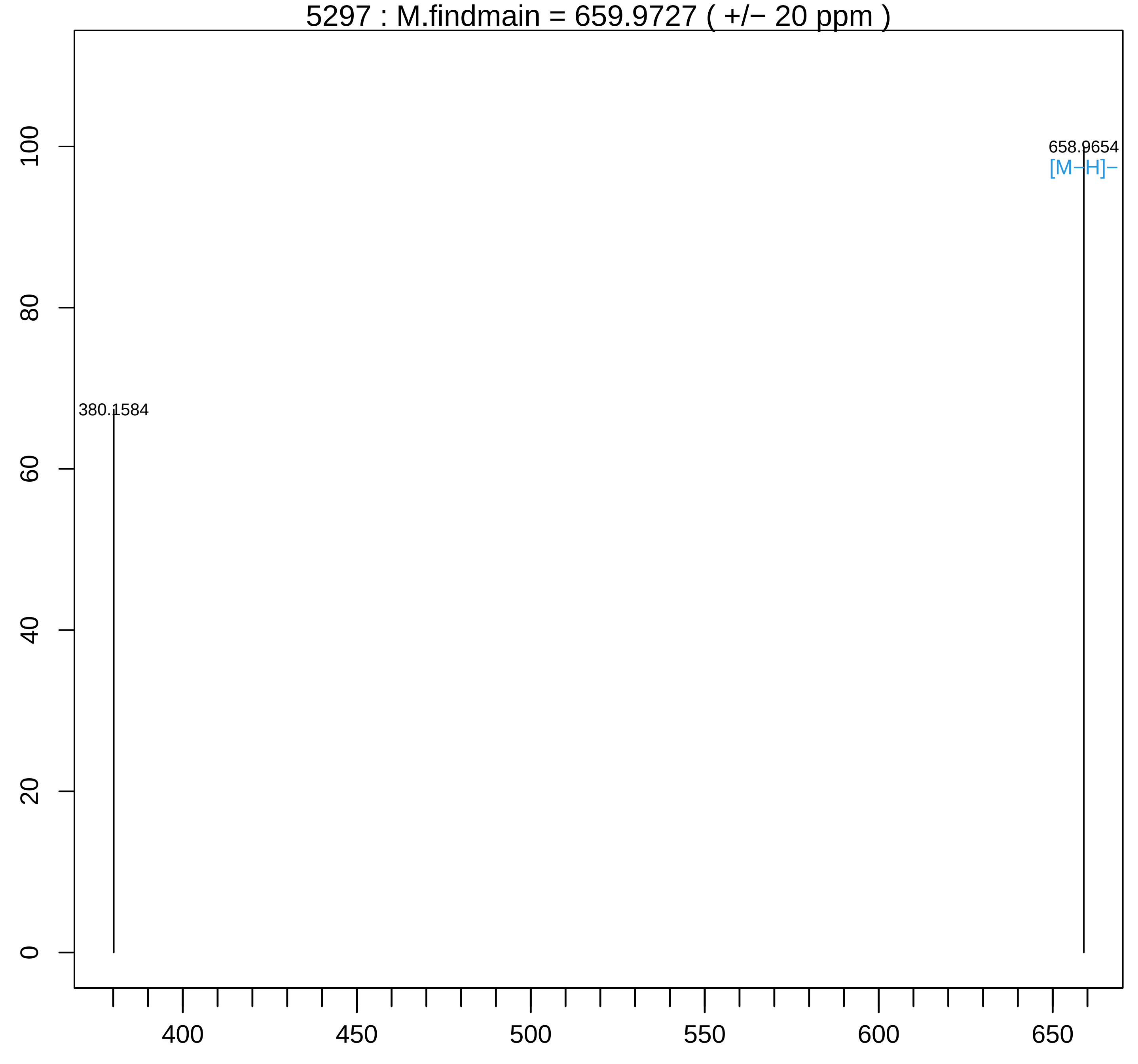

5297 : M.findmain = 659.9727 (+/− 20 ppm)
658.9654
[M−H]−
380.1584
0
20
40
60
80
100
400
450
500
550
600
650

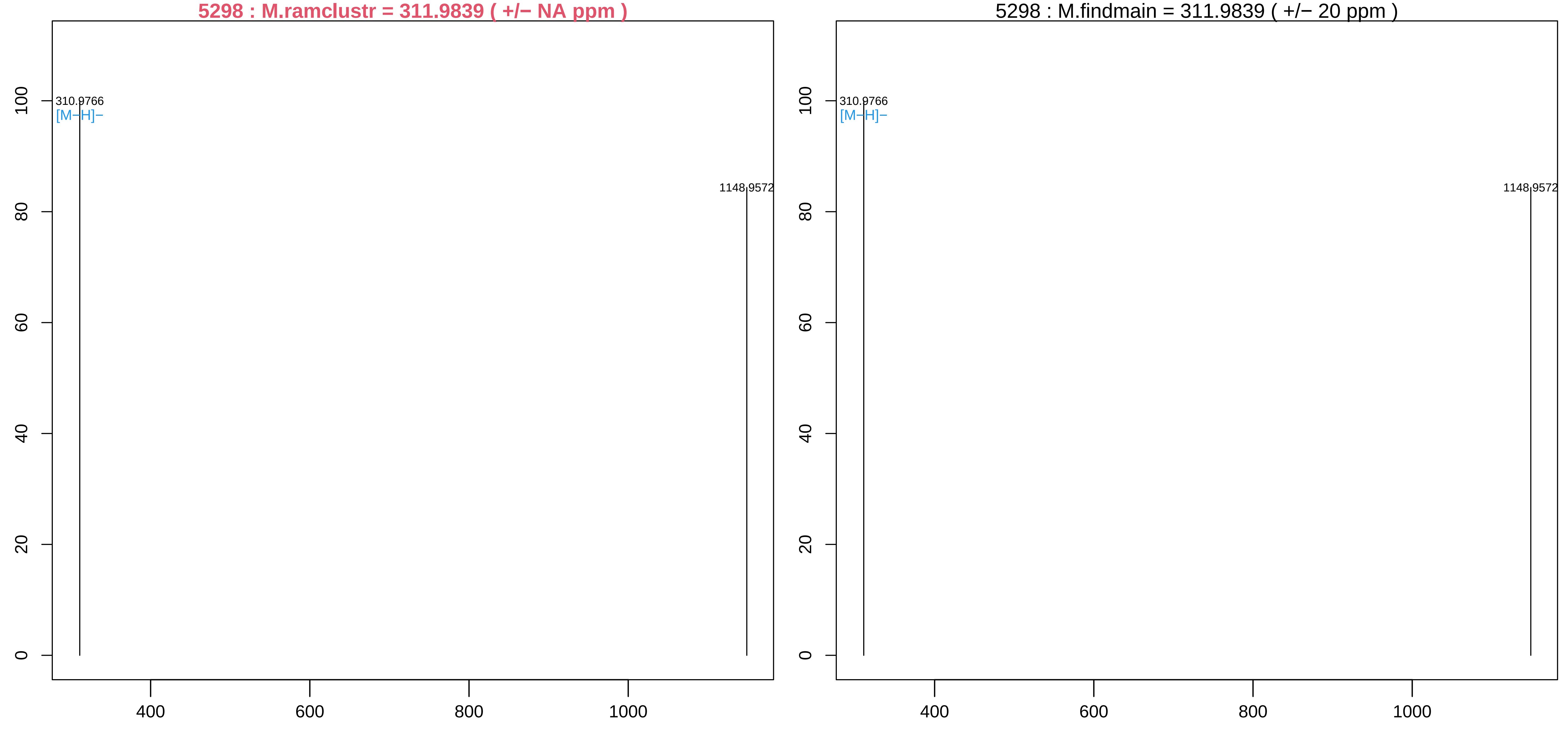
5298 : M.ramclustr = 311.9839 (+/− NA ppm)
310.9766
[M−H]−
1148.9572
0
20
40
60
80
100
400
600
800
1000
5298 : M.findmain = 311.9839 (+/− 20 ppm)
310.9766
[M−H]−
1148.9572
0
20
40
60
80
100
400
600
800
1000

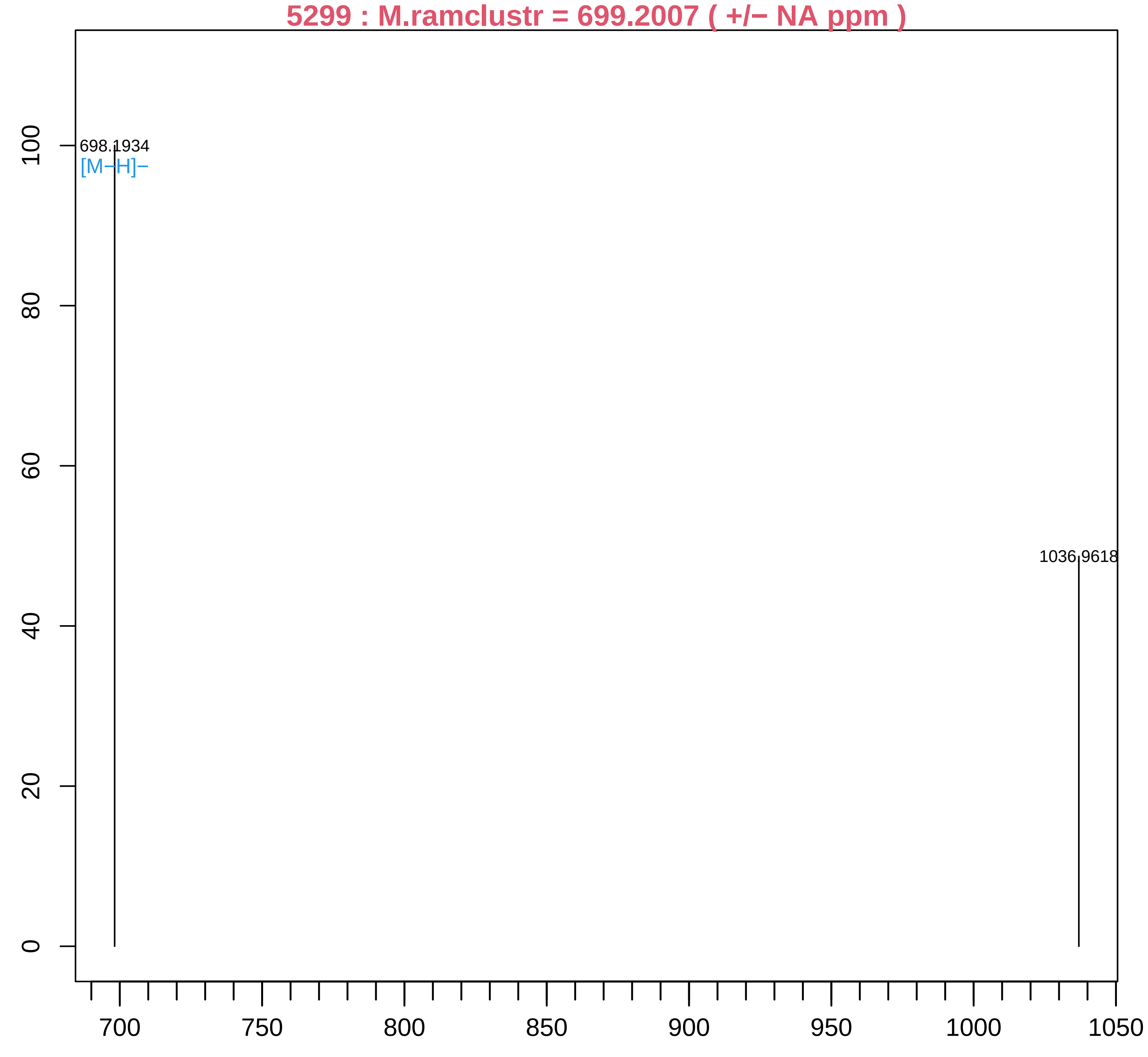
5299 : M.ramclustr = 699.2007 (+/− NA ppm)
698.1934
[M−H]−
1036.9618
0
20
40
60
80
100
700
750
800
850
900
950
1000
1050

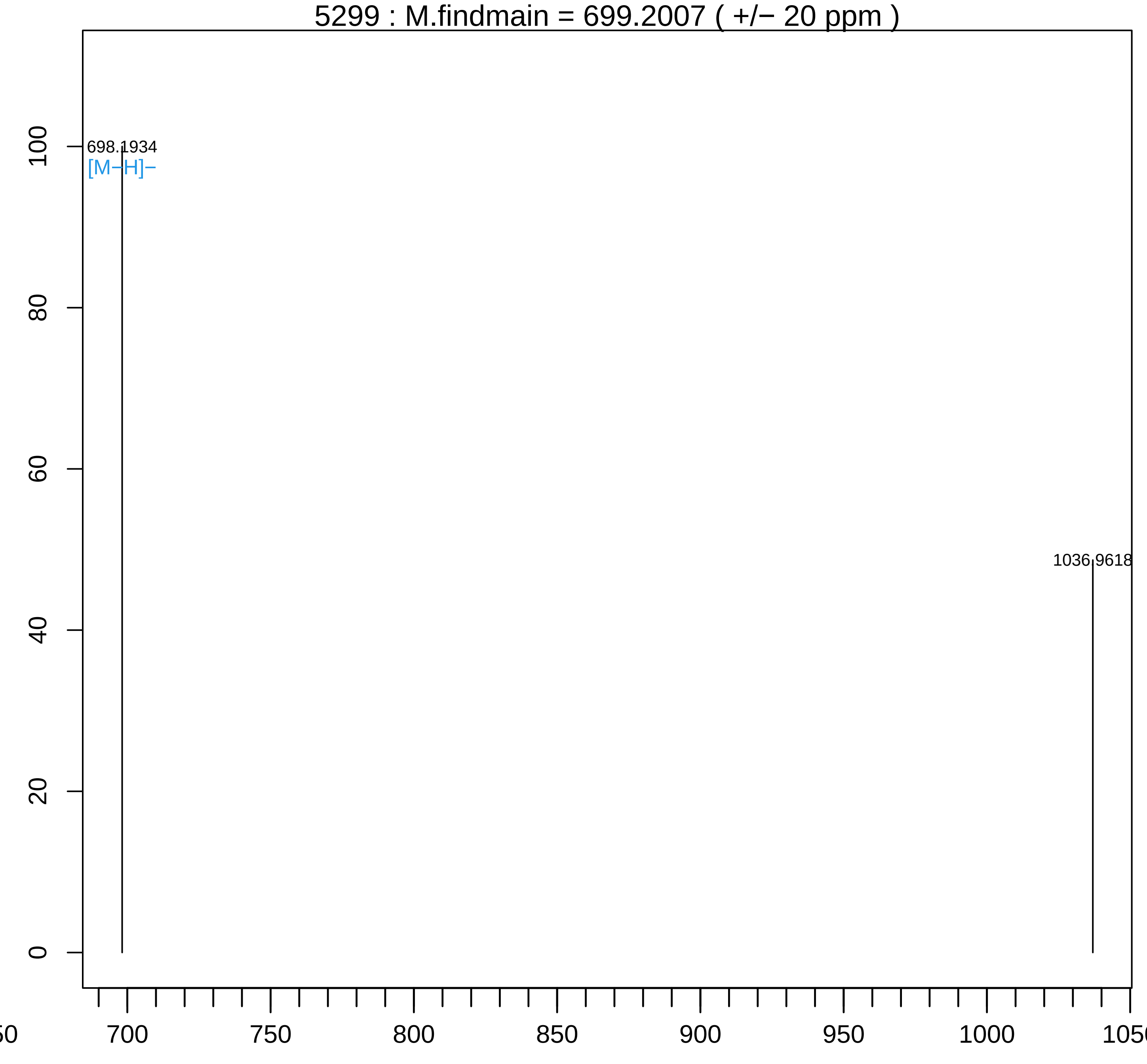
5299 : M.findmain = 699.2007 (+/− 20 ppm)
698.1934
[M−H]−
1036.9618
0
20
40
60
80
100
700
750
800
850
900
950
1000

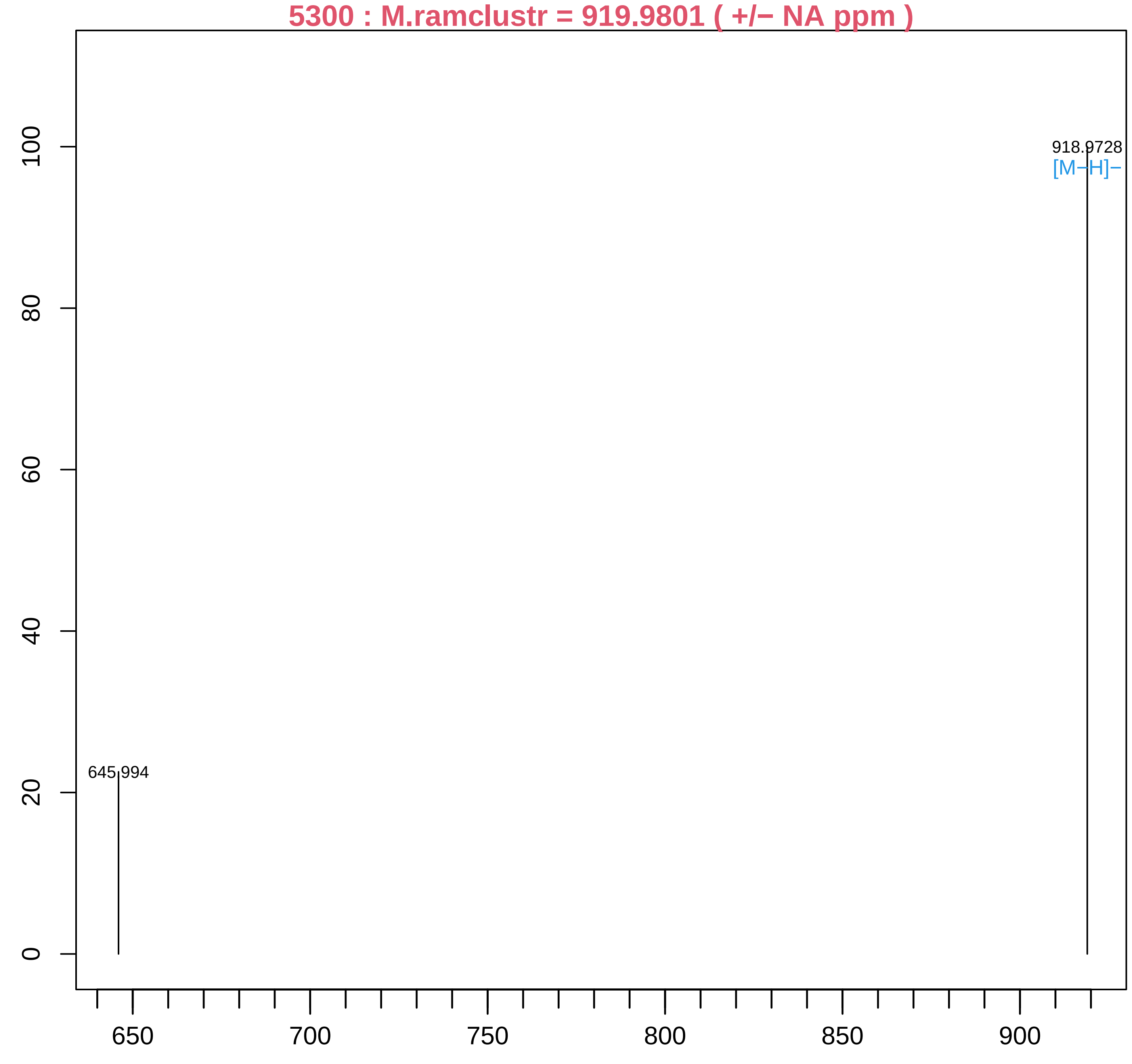

5300 : M.ramclustr = 919.9801 (+/− NA ppm)
918.9728
[M−H]−
645.994
0
20
40
60
80
100
650
700
750
800
850
900

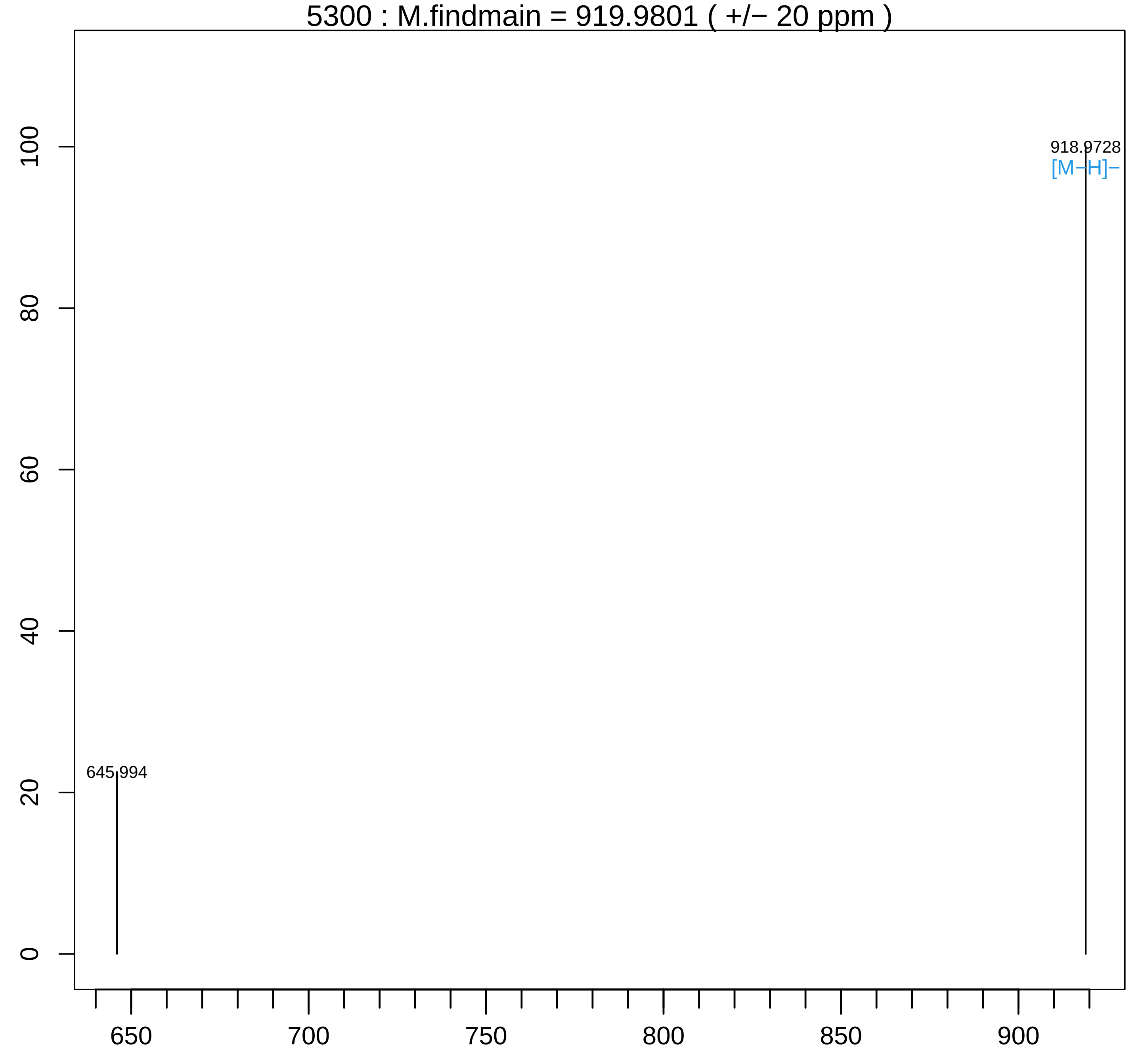

5300 : M.findmain = 919.9801 (+/− 20 ppm)
918.9728
[M−H]−
645.994
0
20
40
60
80
100
650
700
750
800
850
900

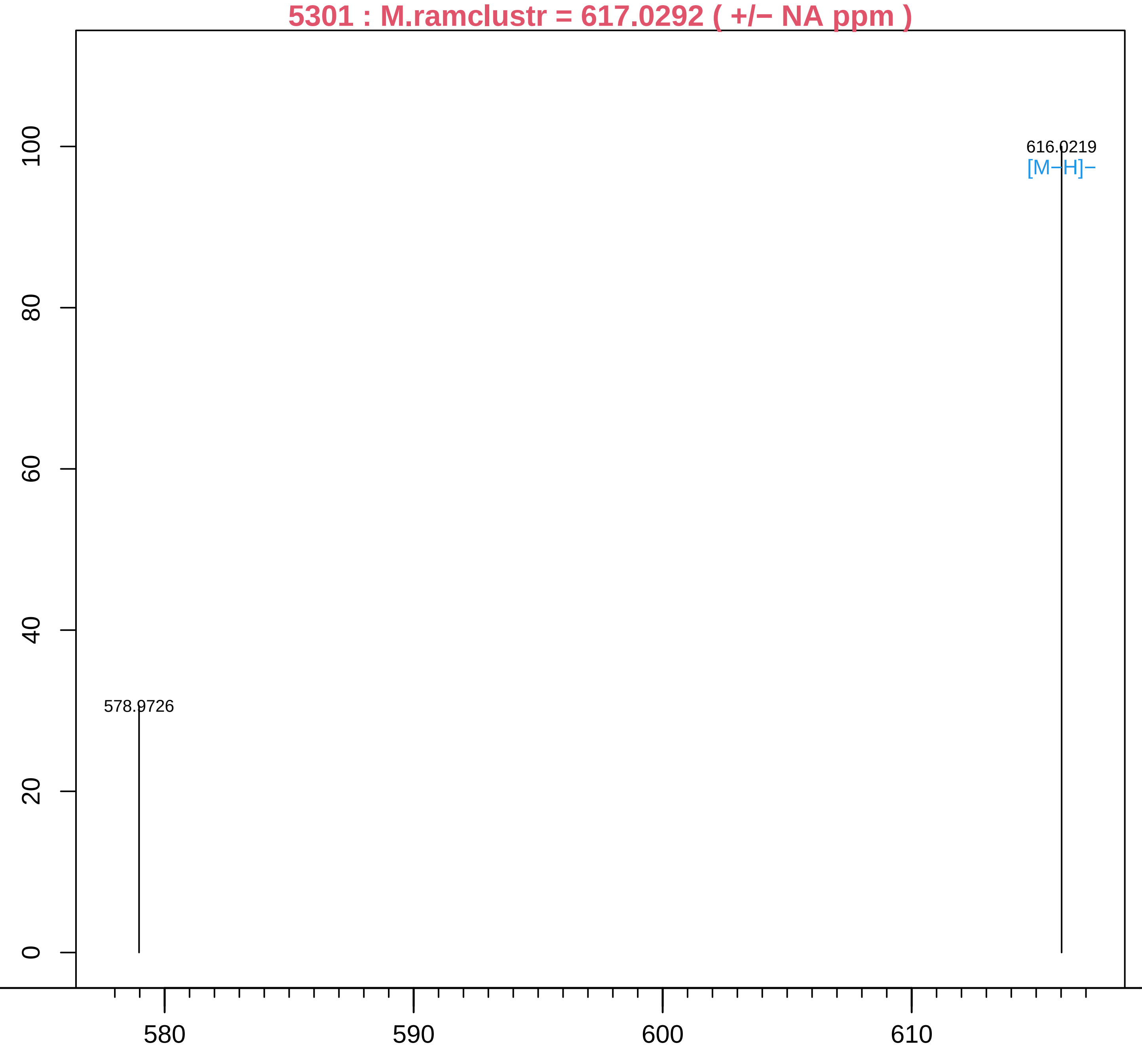
5301 : M.ramclustr = 617.0292 (+/− NA ppm)
616.0219
[M−H]−
578.9726
0
20
40
60
80
100
580
590
600
610

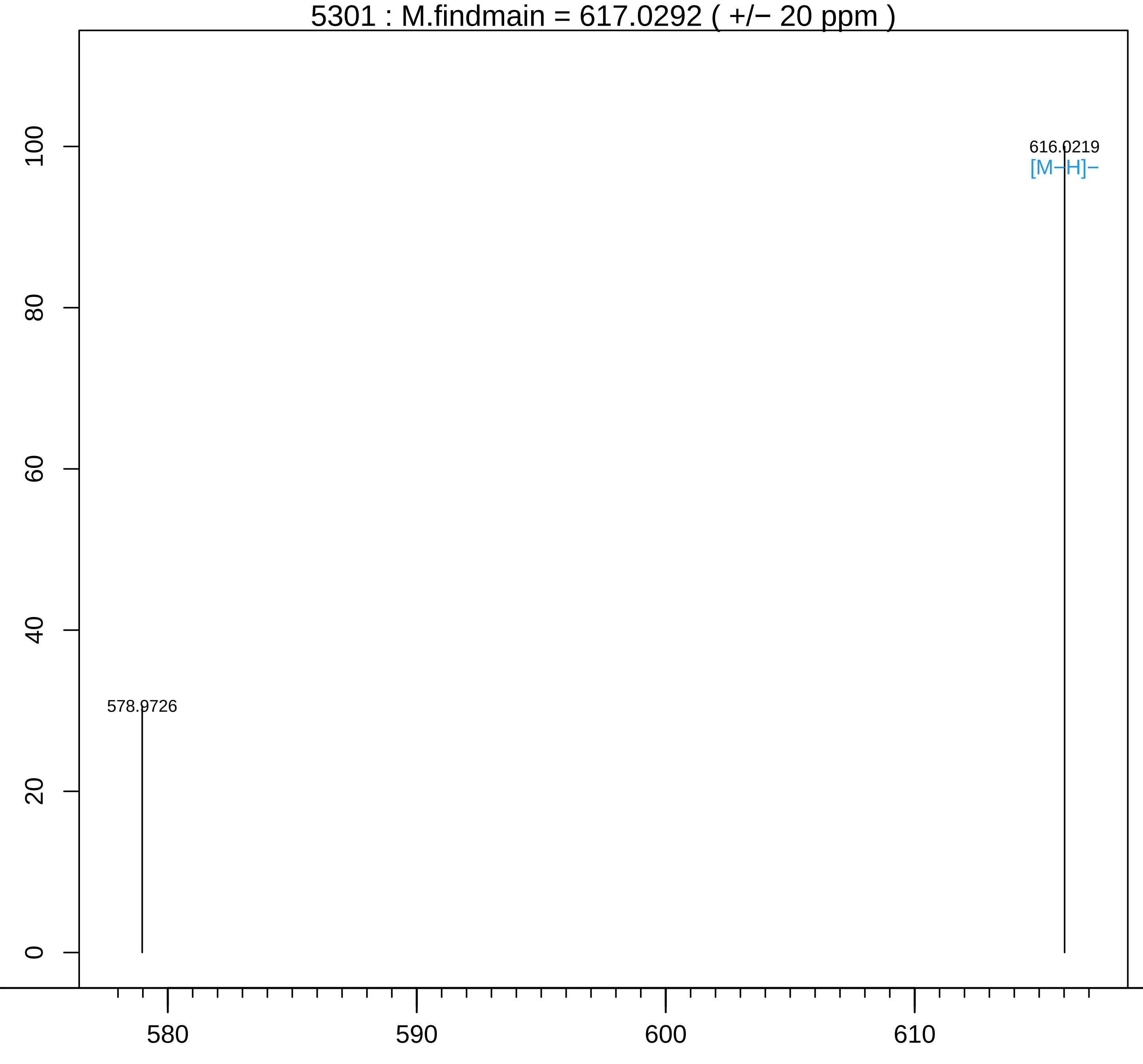
5301 : M.findmain = 617.0292 (+/− 20 ppm)
616.0219
[M−H]−
578.9726
0
20
40
60
80
100
580
590
600
610

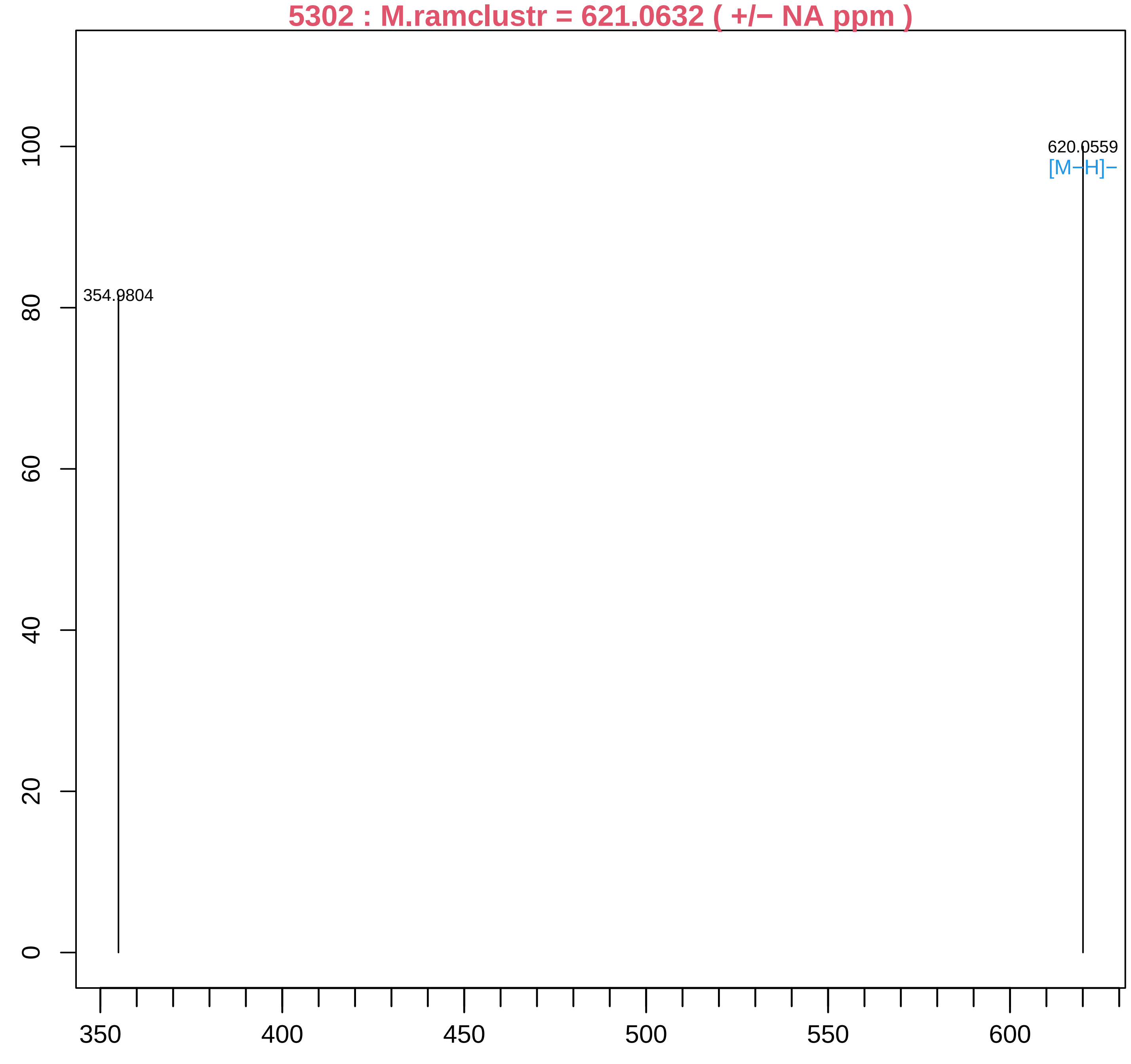
5302 : M.ramclustr = 621.0632 (+/− NA ppm)
620.0559
[M−H]−
354.9804
0
20
40
60
80
100
350
400
450
500
550
600

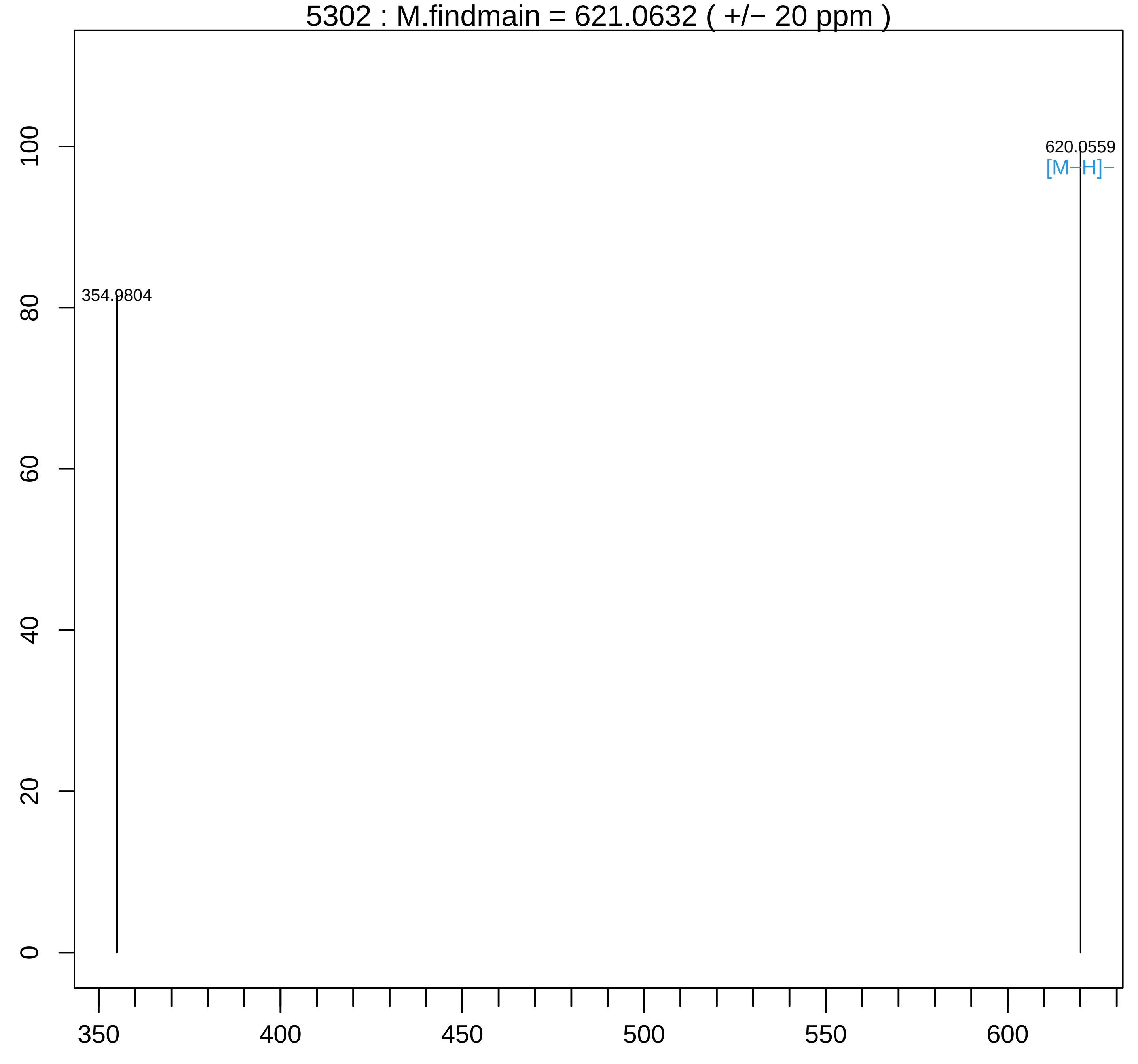
5302 : M.findmain = 621.0632 (+/− 20 ppm)
620.0559
[M−H]−
354.9804
0
20
40
60
80
100
350
400
450
500
550
600

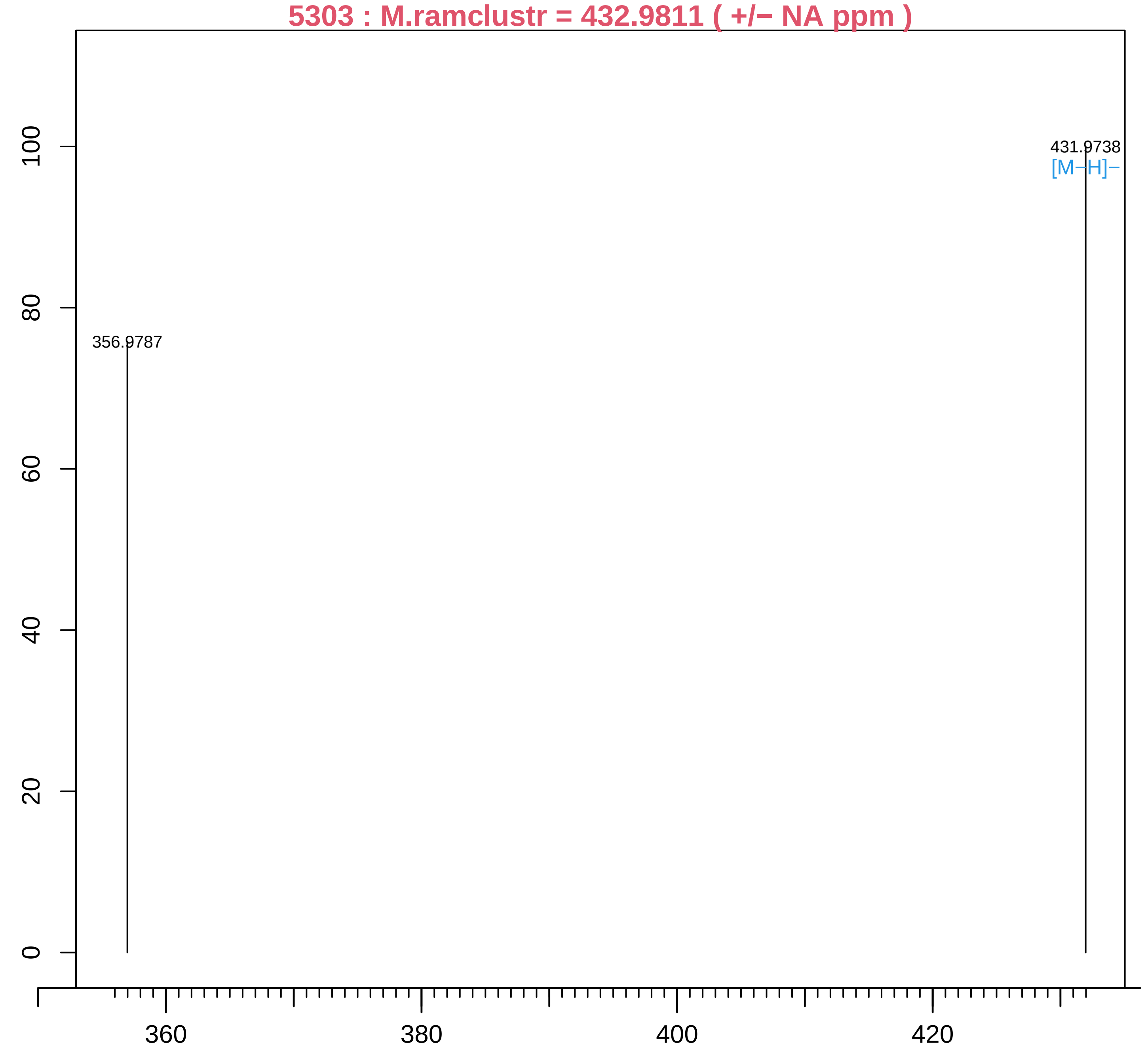
5303 : M.ramclustr = 432.9811 (+/− NA ppm)
431.9738
[M−H]−
356.9787
0
20
40
60
80
100
360
380
400
420

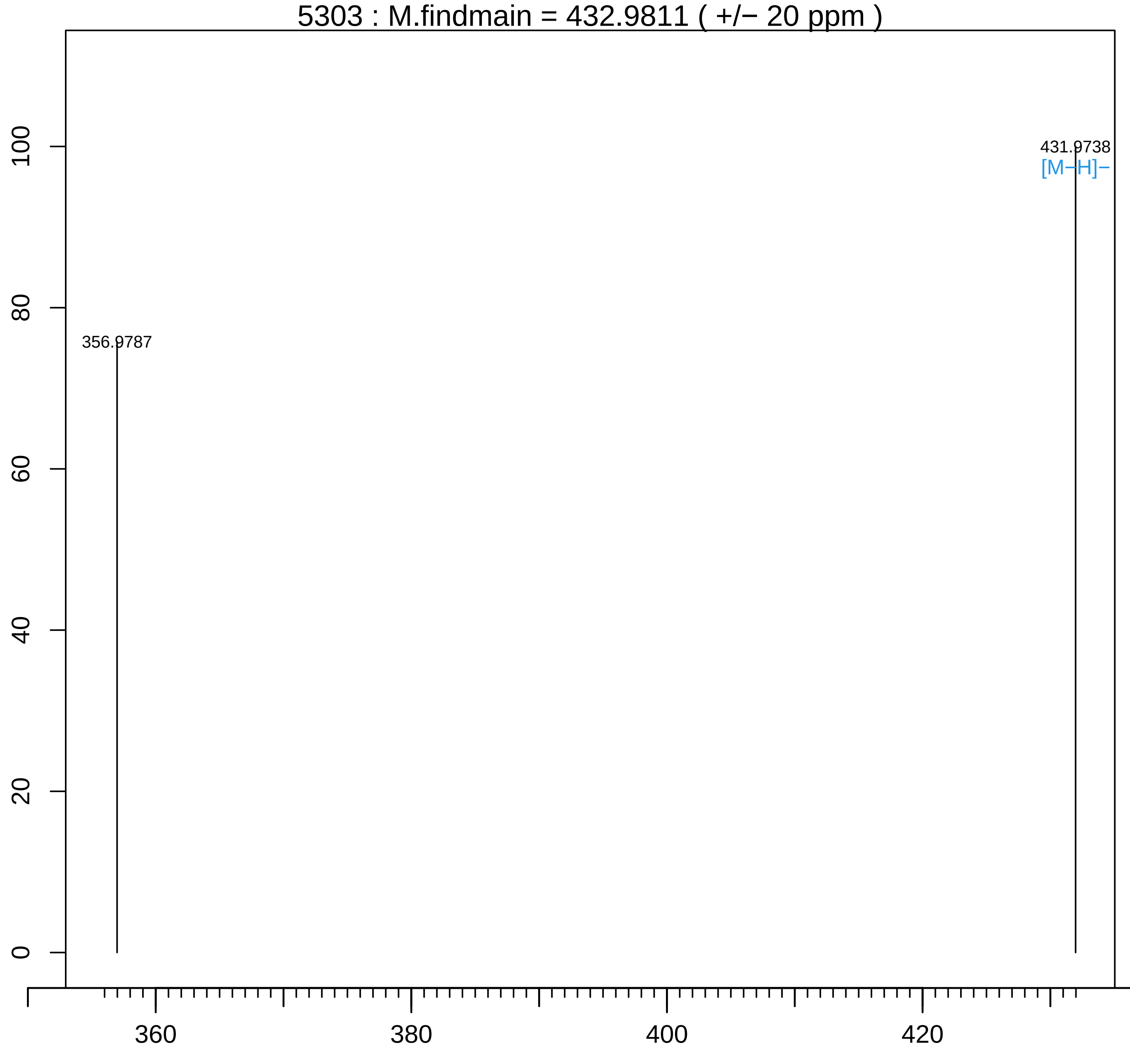
5303 : M.findmain = 432.9811 (+/− 20 ppm)
431.9738
[M−H]−
356.9787
0
20
40
60
80
100
360
380
400
420

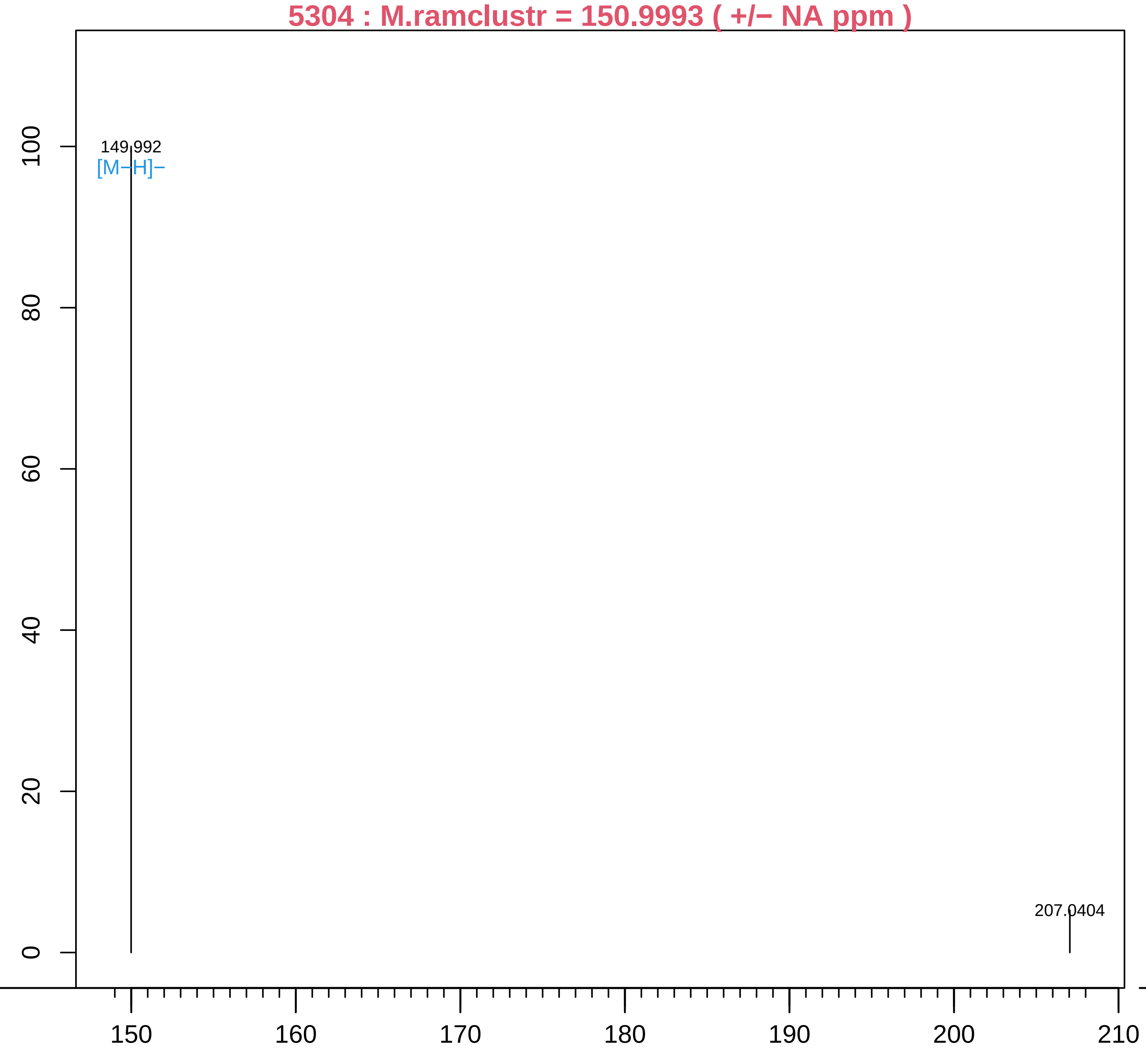
5304 : M.ramclustr = 150.9993 (+/− NA ppm)
149.992
[M−H]−
207.0404
0
20
40
60
80
100
150
160
170
180
190
200
210

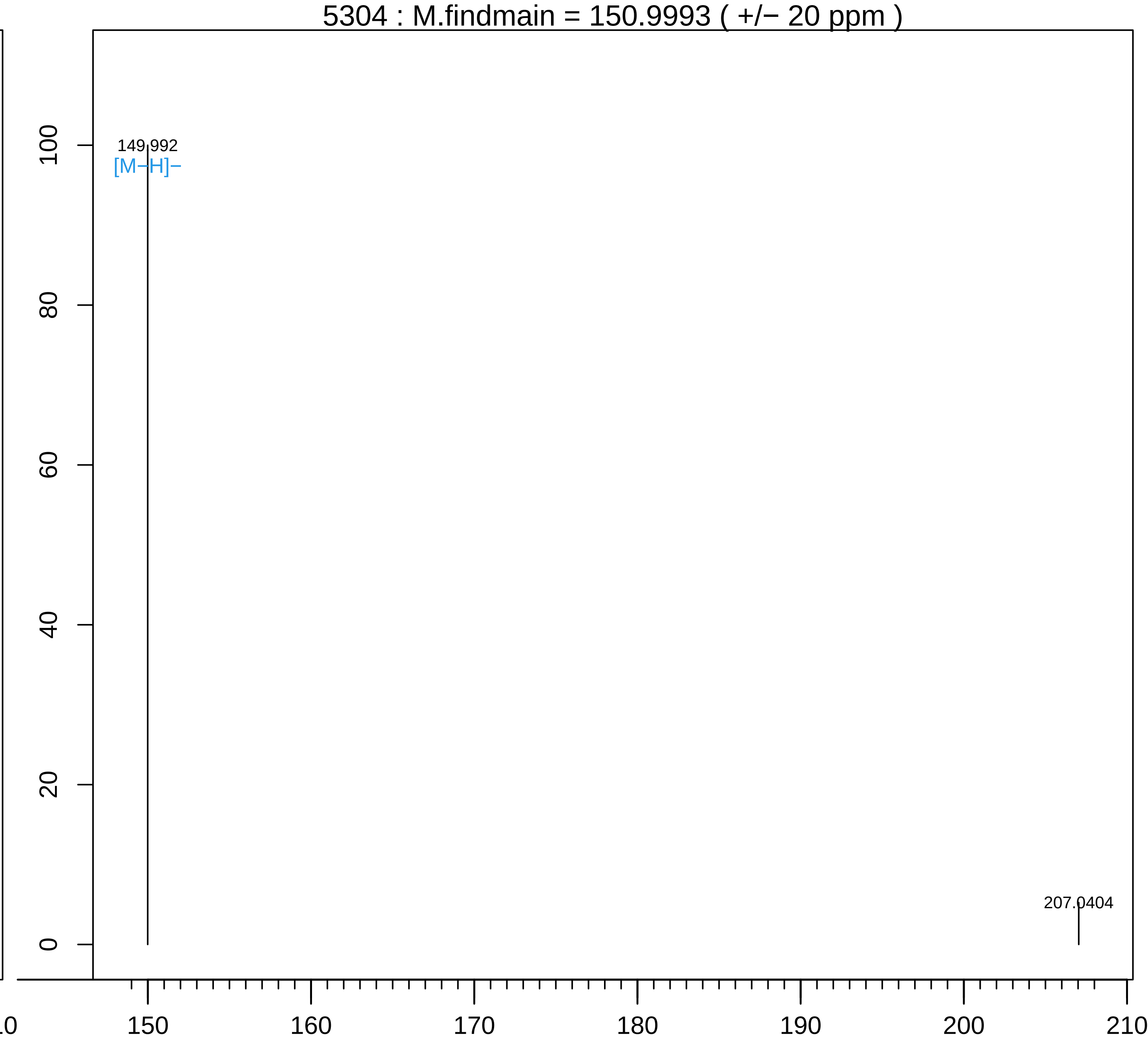
5304 : M.findmain = 150.9993 (+/− 20 ppm)
149.992
[M−H]−
207.0404
0
20
40
60
80
100
150
160
170
180
190
200
210

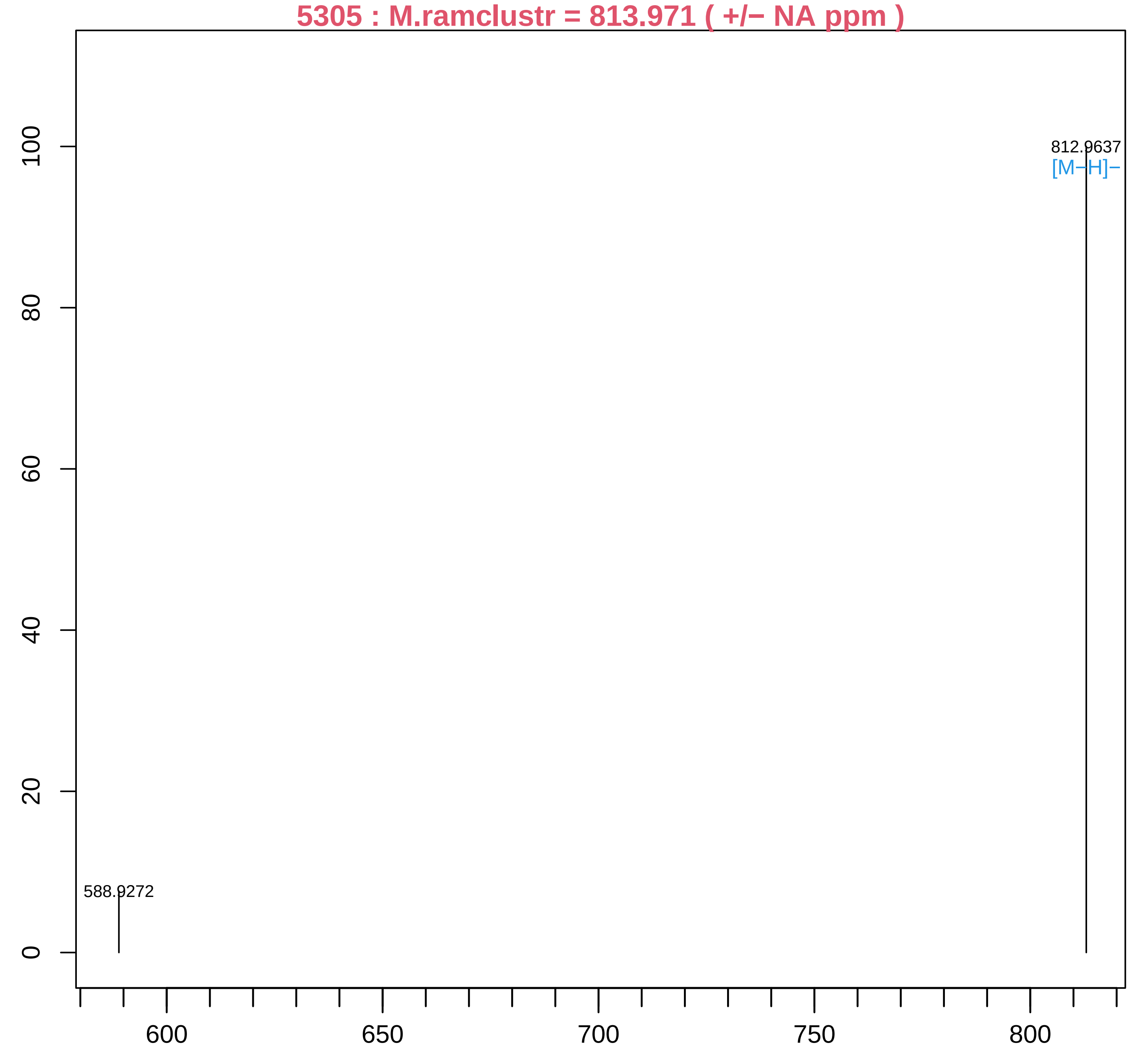
5305 : M.ramclustr = 813.971 (+/− NA ppm)
812.9637
[M−H]−
588.9272
0
20
40
60
80
100
600
650
700
750
800

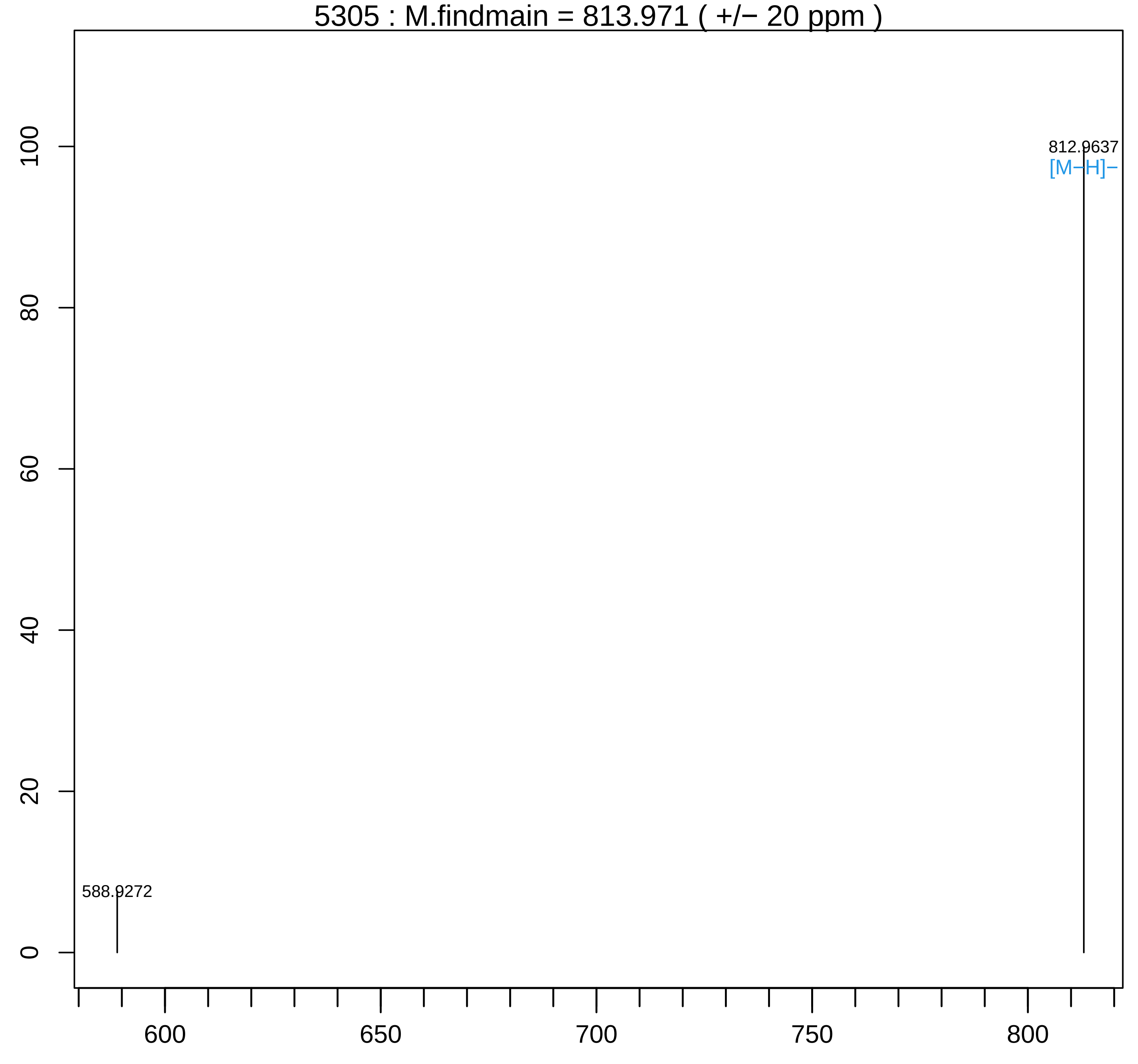
5305 : M.findmain = 813.971 (+/− 20 ppm)
812.9637
[M−H]−
588.9272
0
20
40
60
80
100
600
650
700
750
800

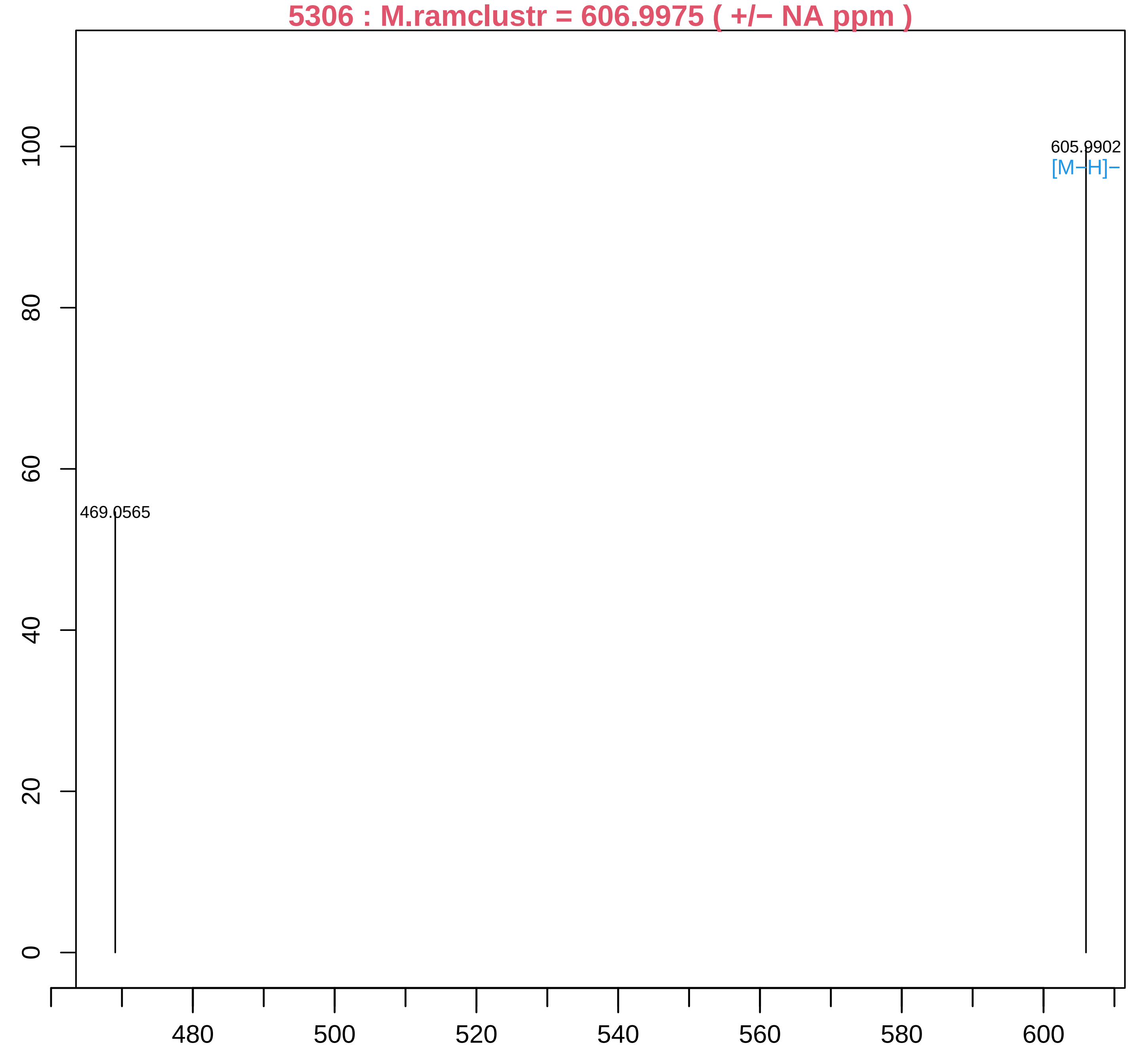
5306 : M.ramclustr = 606.9975 (+/− NA ppm)
605.9902
[M−H]−
469.0565
0
20
40
60
80
100
480
500
520
540
560
580
600

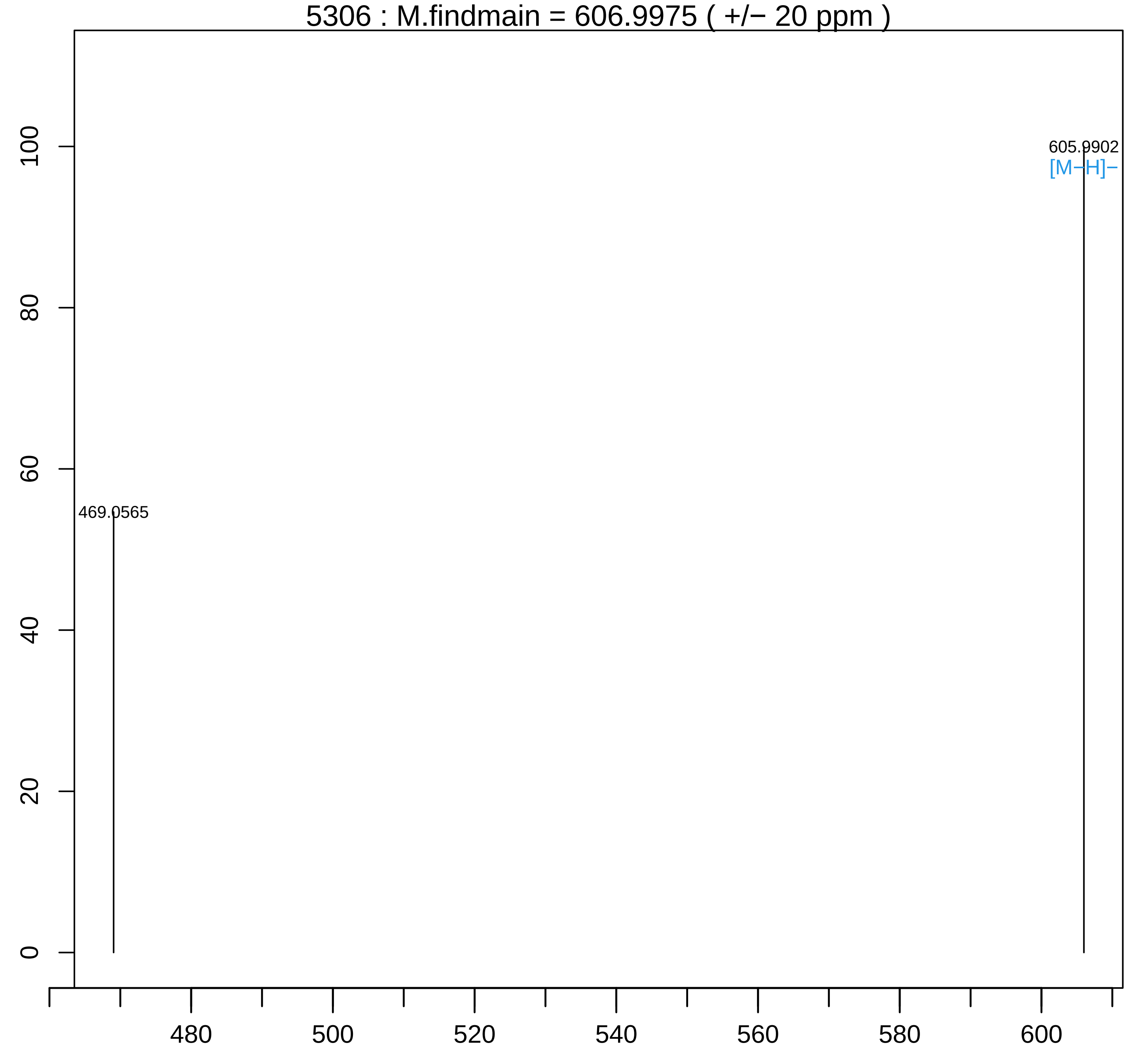
5306 : M.findmain = 606.9975 (+/− 20 ppm)
605.9902
[M−H]−
469.0565
0
20
40
60
80
100
480
500
520
540
560
580
600

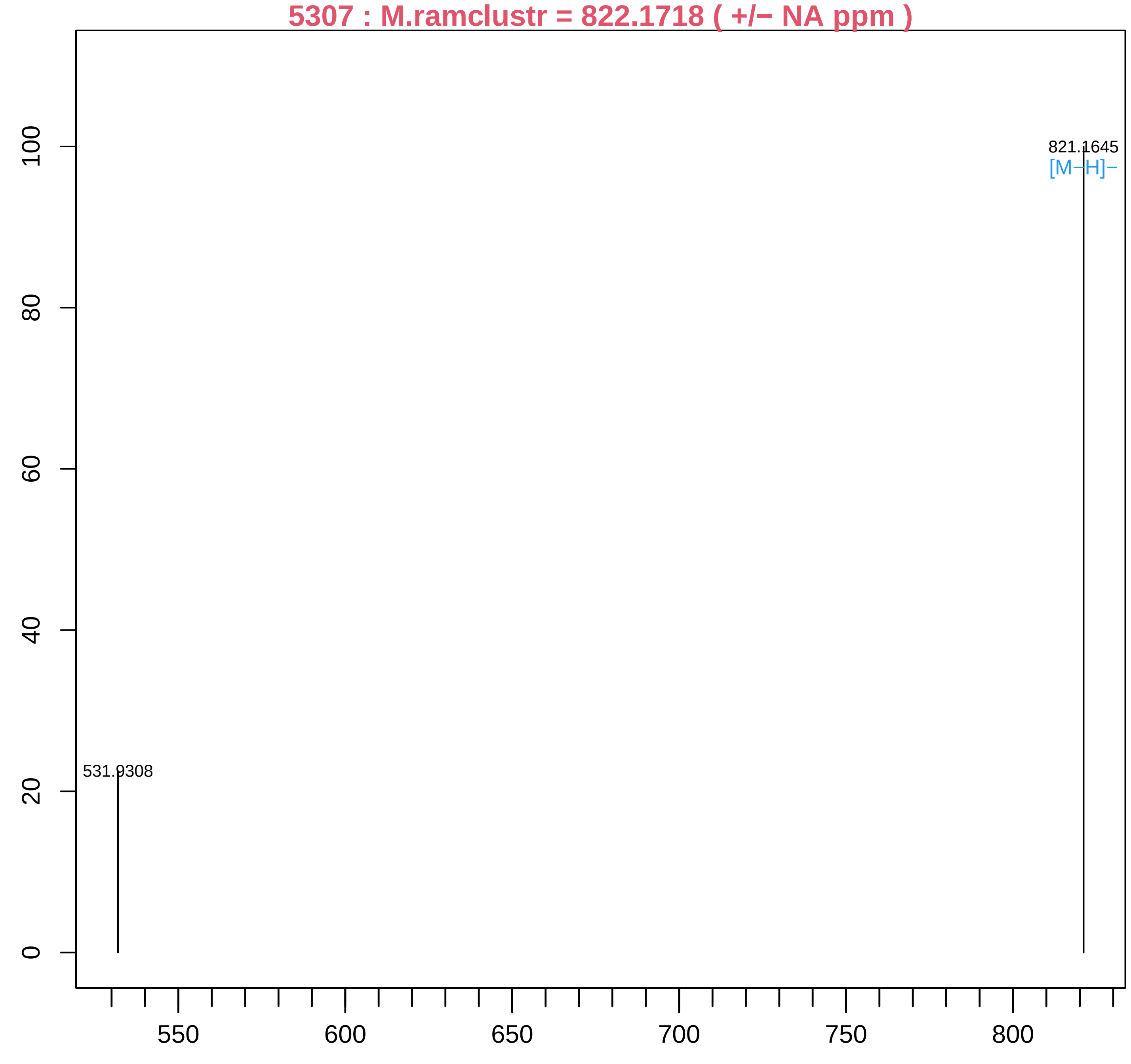
5307 : M.ramclustr = 822.1718 (+/− NA ppm)
821.1645
[M−H]−
531.9308
0
20
40
60
80
100
550
600
650
700
750
800

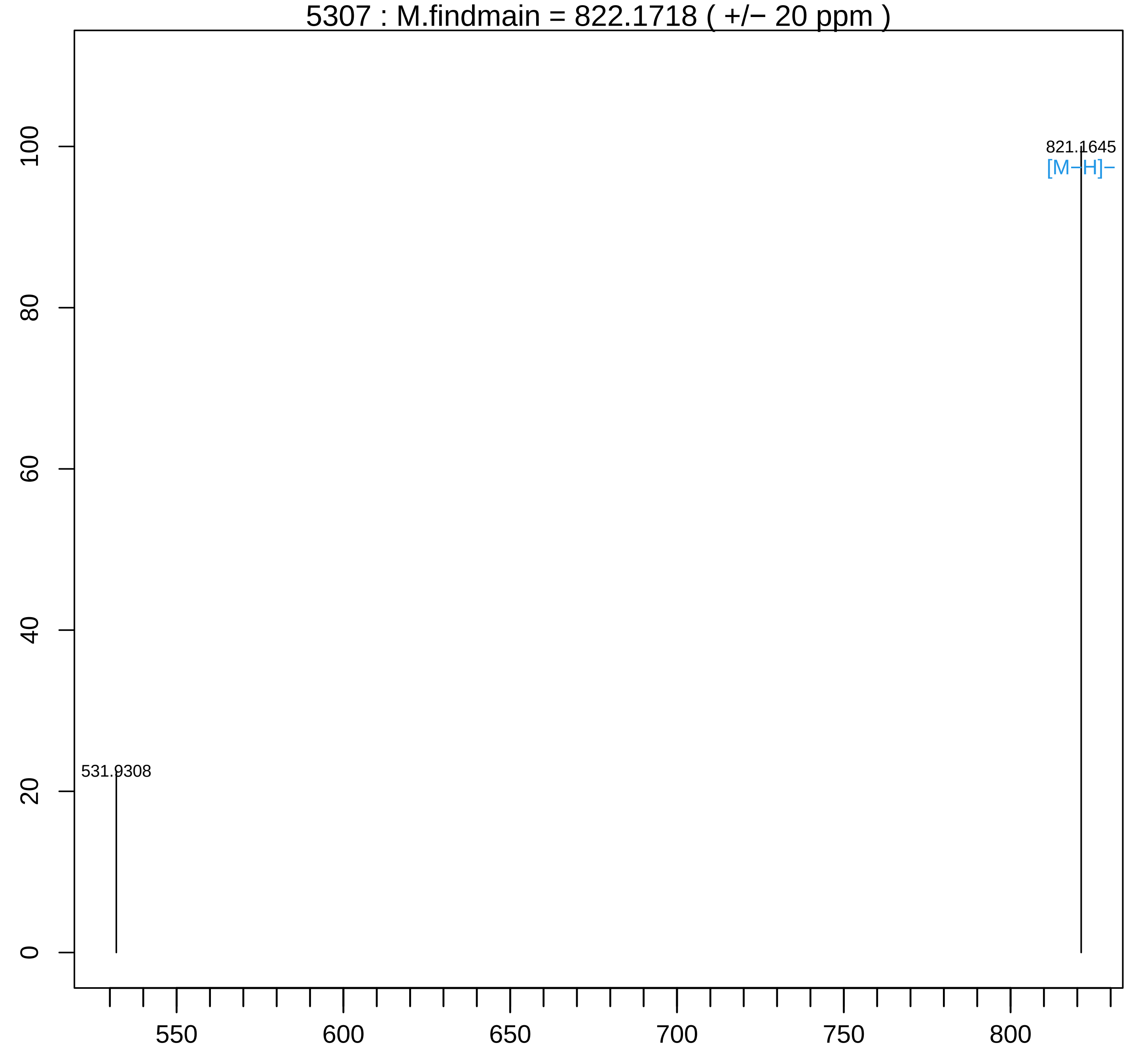
5307 : M.findmain = 822.1718 (+/− 20 ppm)
821.1645
[M−H]−
531.9308
0
20
40
60
80
100
550
600
650
700
750
800

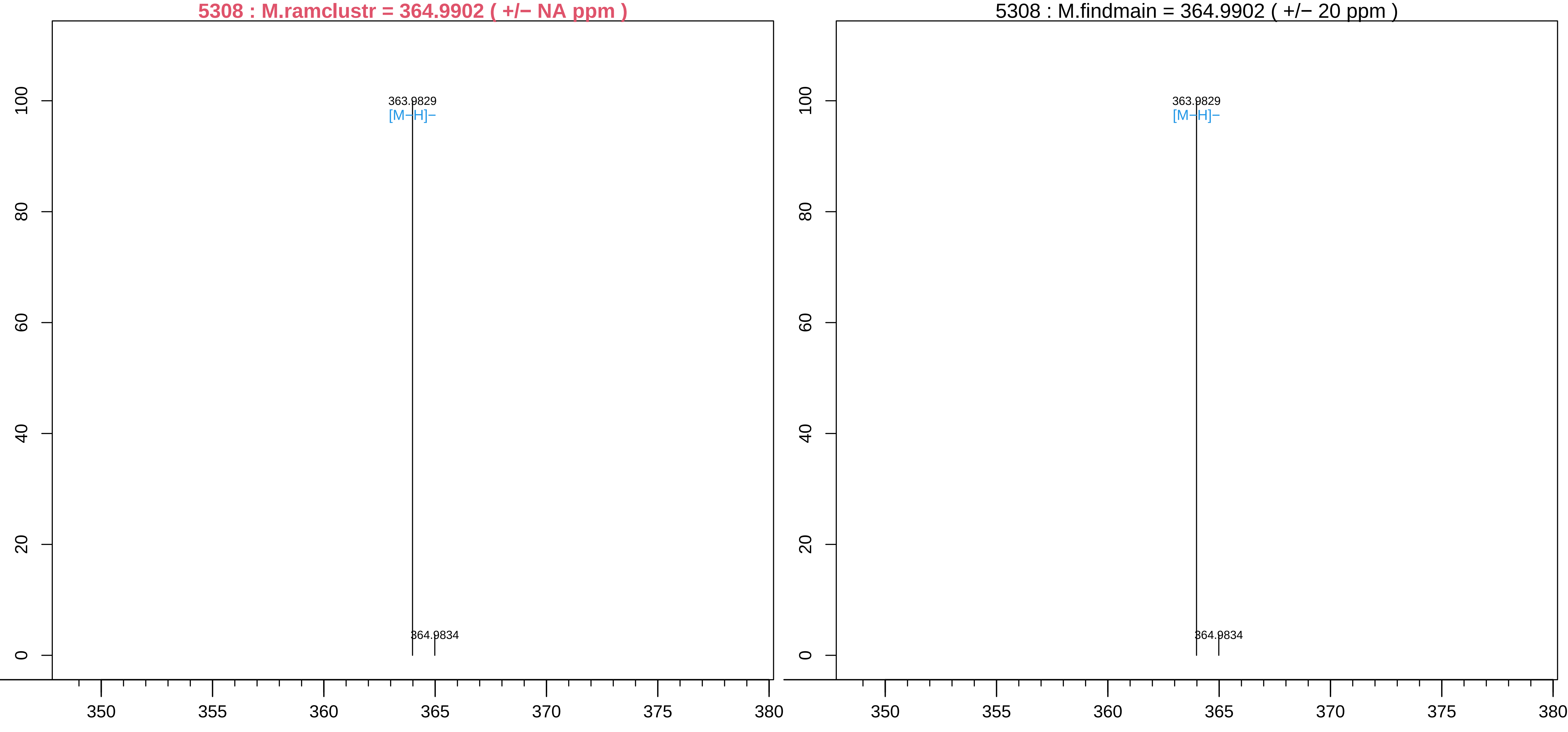
5308 : M.ramclustr = 364.9902 (+/− NA ppm)
363.9829
[M−H]−
364.9834
0
20
40
60
80
100
350
355
360
365
370
375
380
5308 : M.findmain = 364.9902 (+/− 20 ppm)
363.9829
[M−H]−
364.9834
0
20
40
60
80
100
350
355
360
365
370
375
380

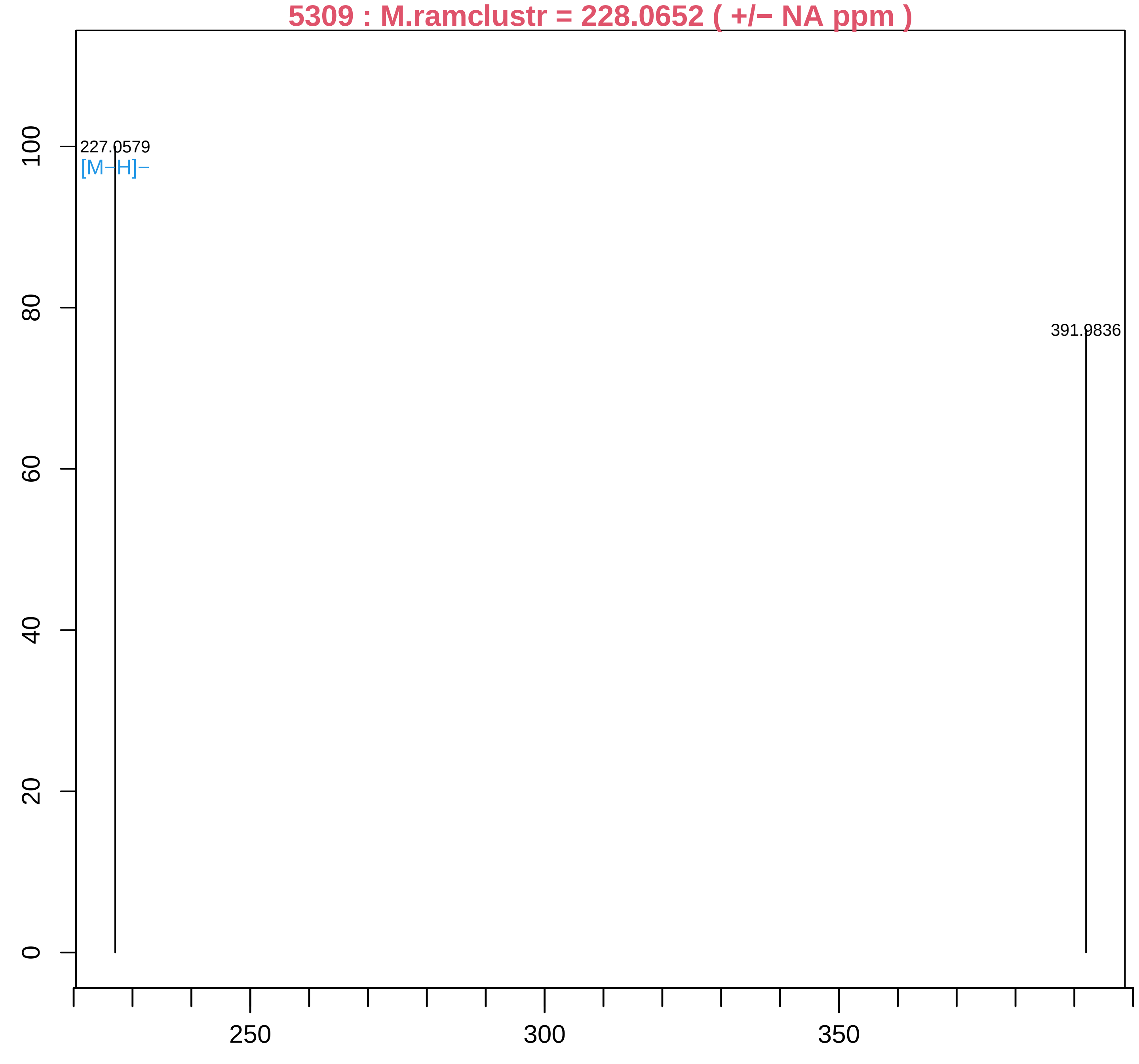

5309 : M.ramclustr = 228.0652 (+/− NA ppm)
227.0579
[M−H]−
391.9836
0
20
40
60
80
100
250
300
350

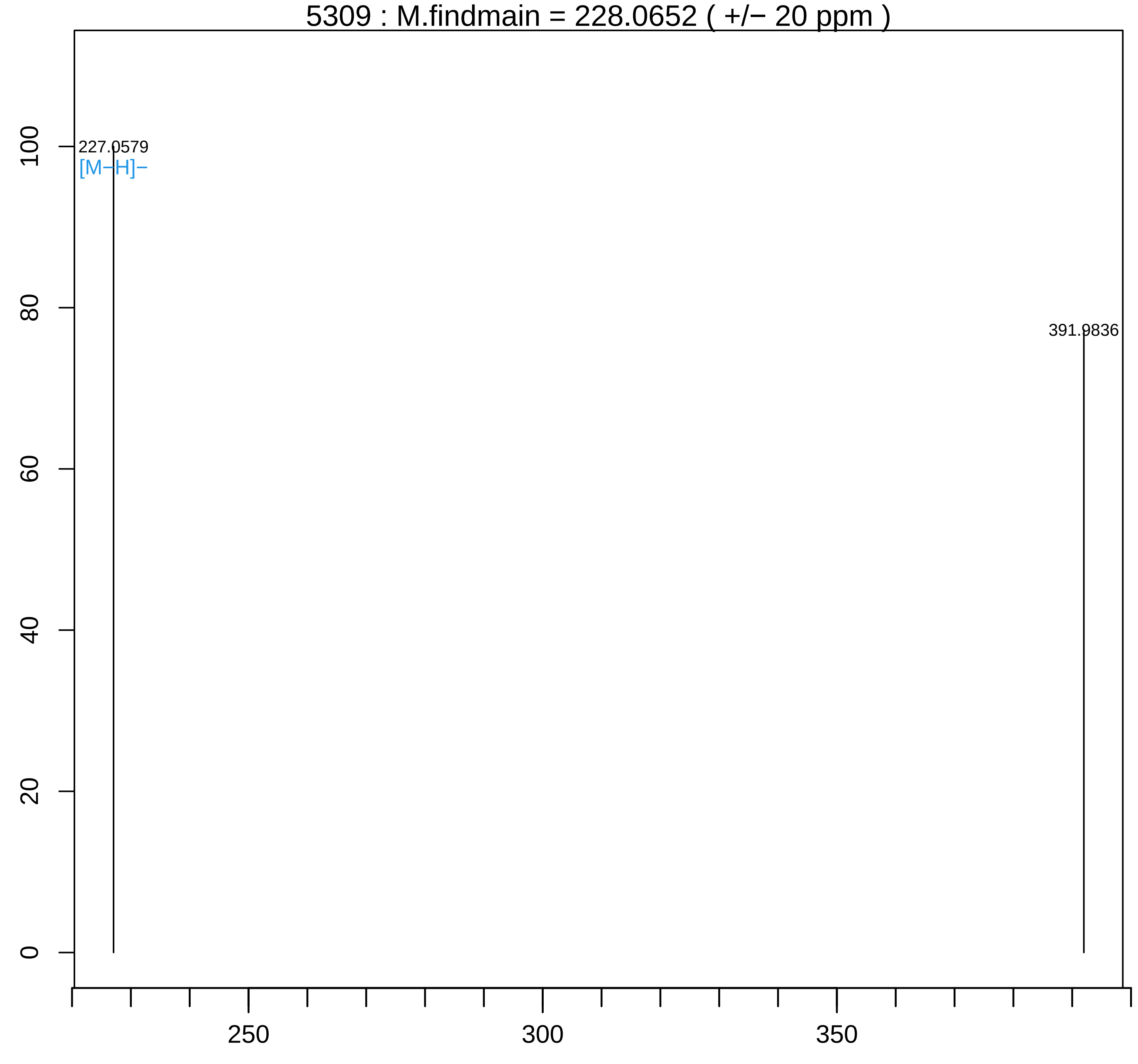

5309 : M.findmain = 228.0652 (+/− 20 ppm)
227.0579
[M−H]−
391.9836
0
20
40
60
80
100
250
300
350

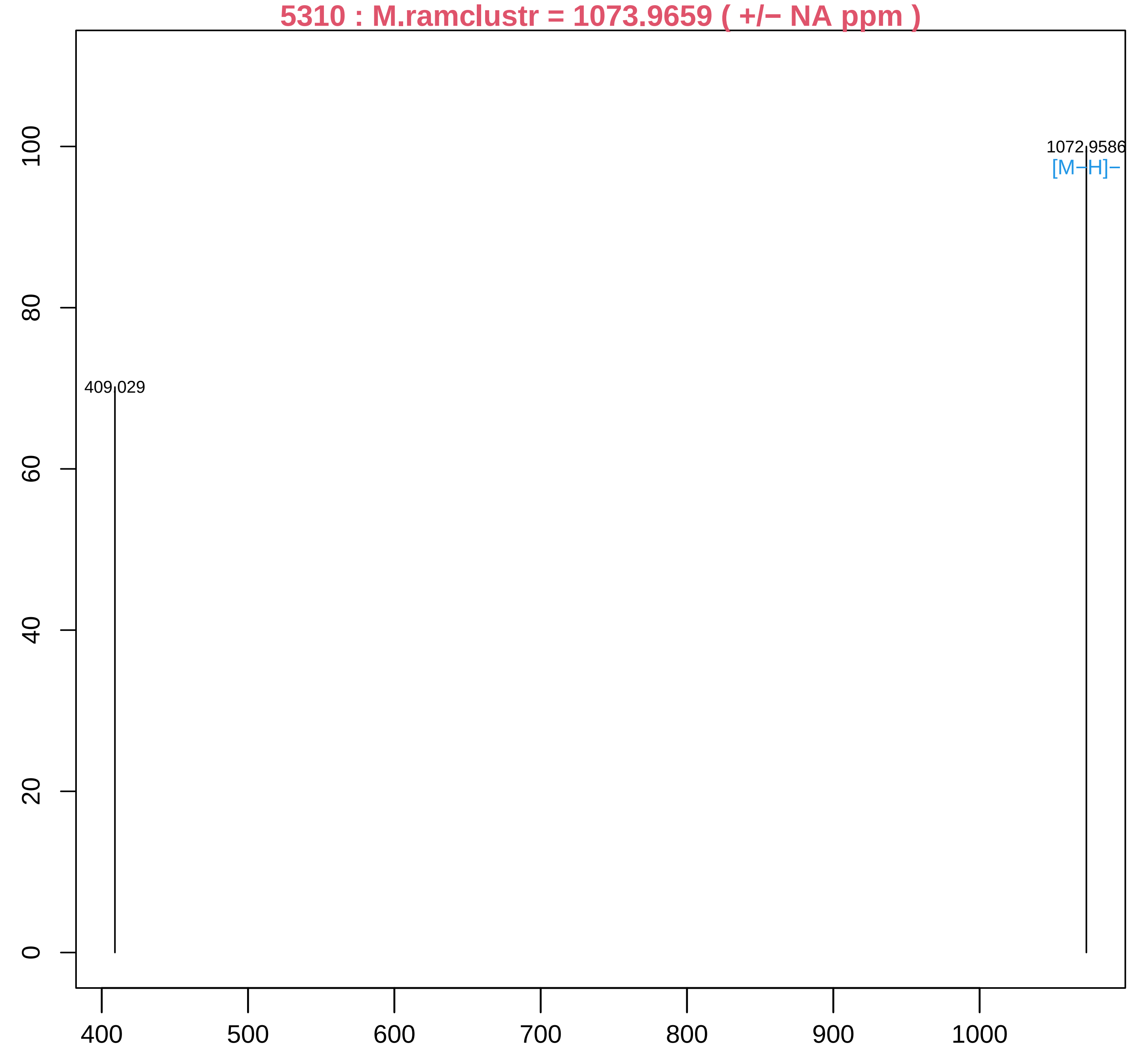
5310 : M.ramclustr = 1073.9659 (+/− NA ppm)
1072.9586
[M−H]−
409.029
0
20
40
60
80
100
400
500
600
700
800
900
1000

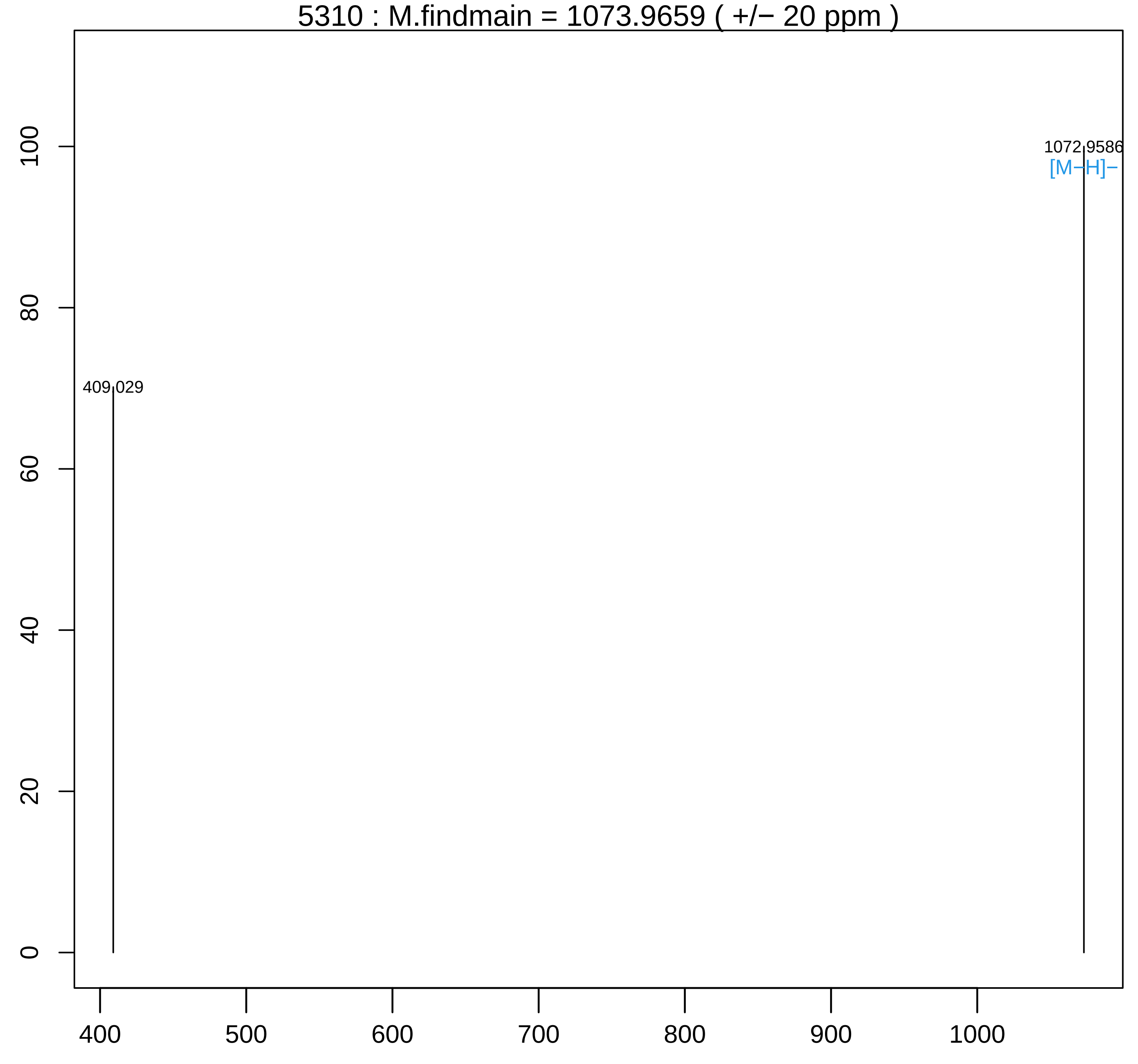
5310 : M.findmain = 1073.9659 (+/− 20 ppm)
1072.9586
[M−H]−
409.029
0
20
40
60
80
100
400
500
600
700
800
900
1000

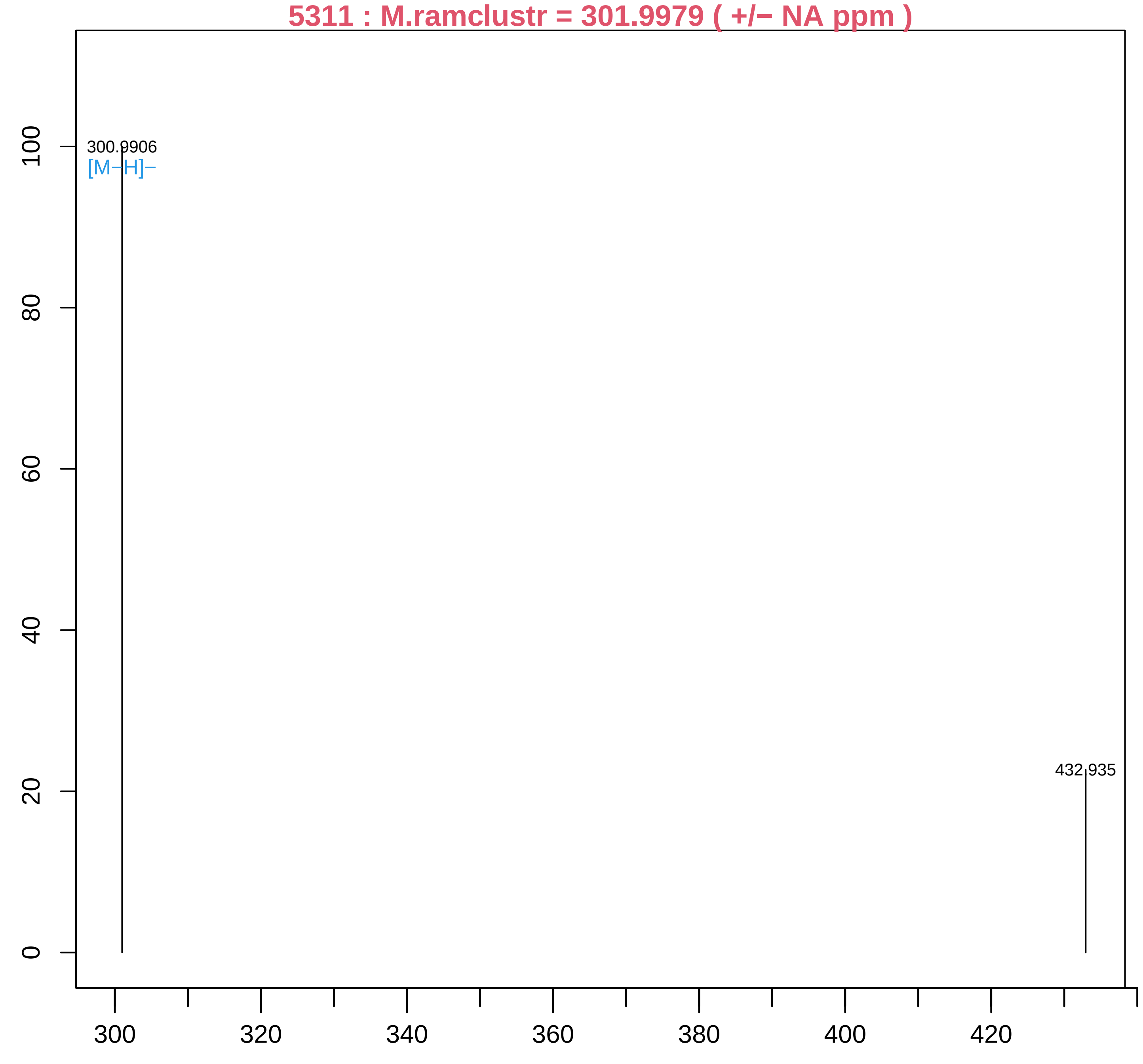

5311 : M.ramclustr = 301.9979 (+/− NA ppm)
300.9906
[M−H]−
432.935
0
20
40
60
80
100
300
320
340
360
380
400
420

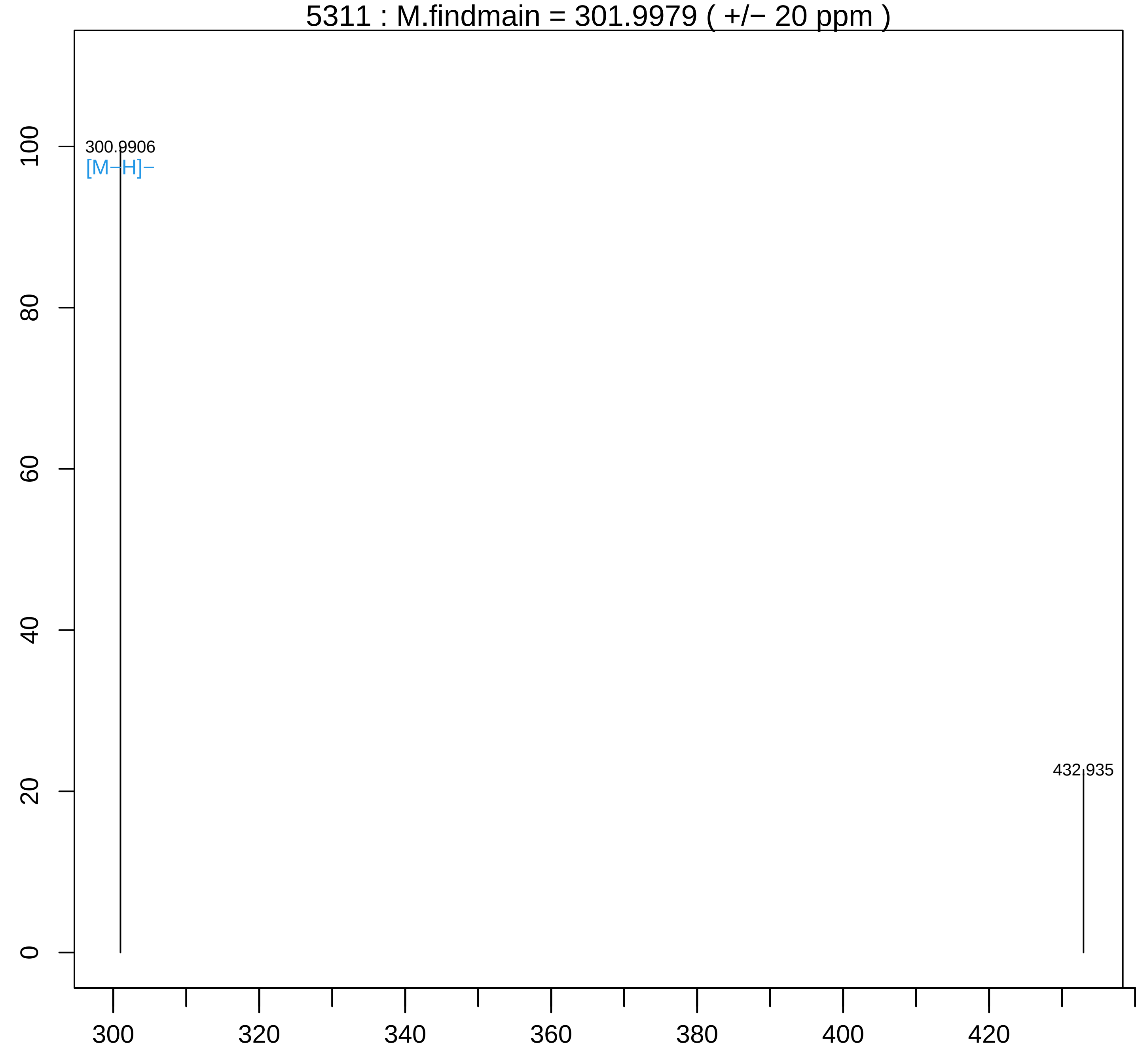

5311 : M.findmain = 301.9979 (+/− 20 ppm)
300.9906
[M−H]−
432.935
0
20
40
60
80
100
300
320
340
360
380
400
420

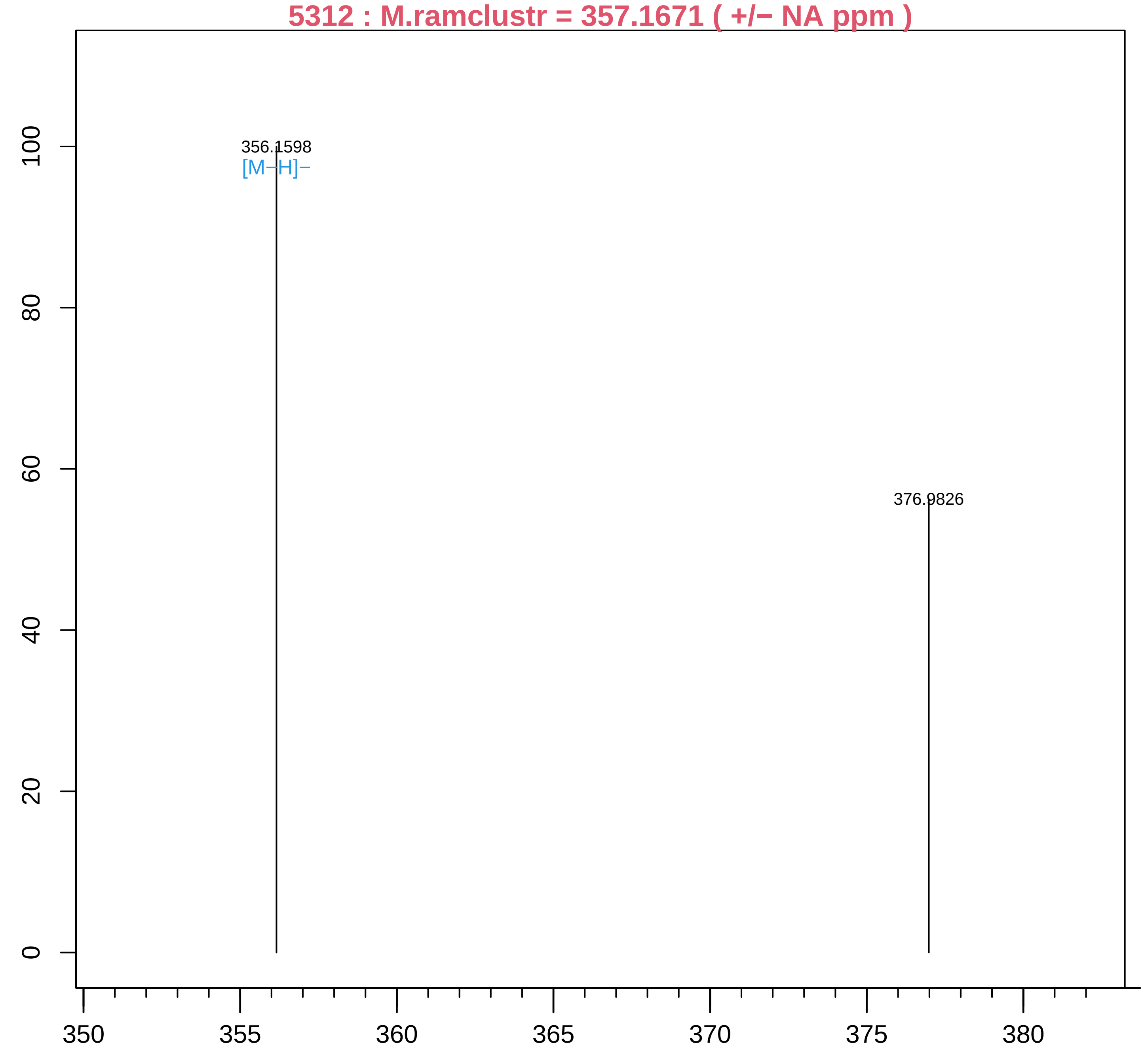
5312 : M.ramclustr = 357.1671 (+/− NA ppm)
356.1598
[M−H]−
376.9826
0
20
40
60
80
100
350
355
360
365
370
375
380

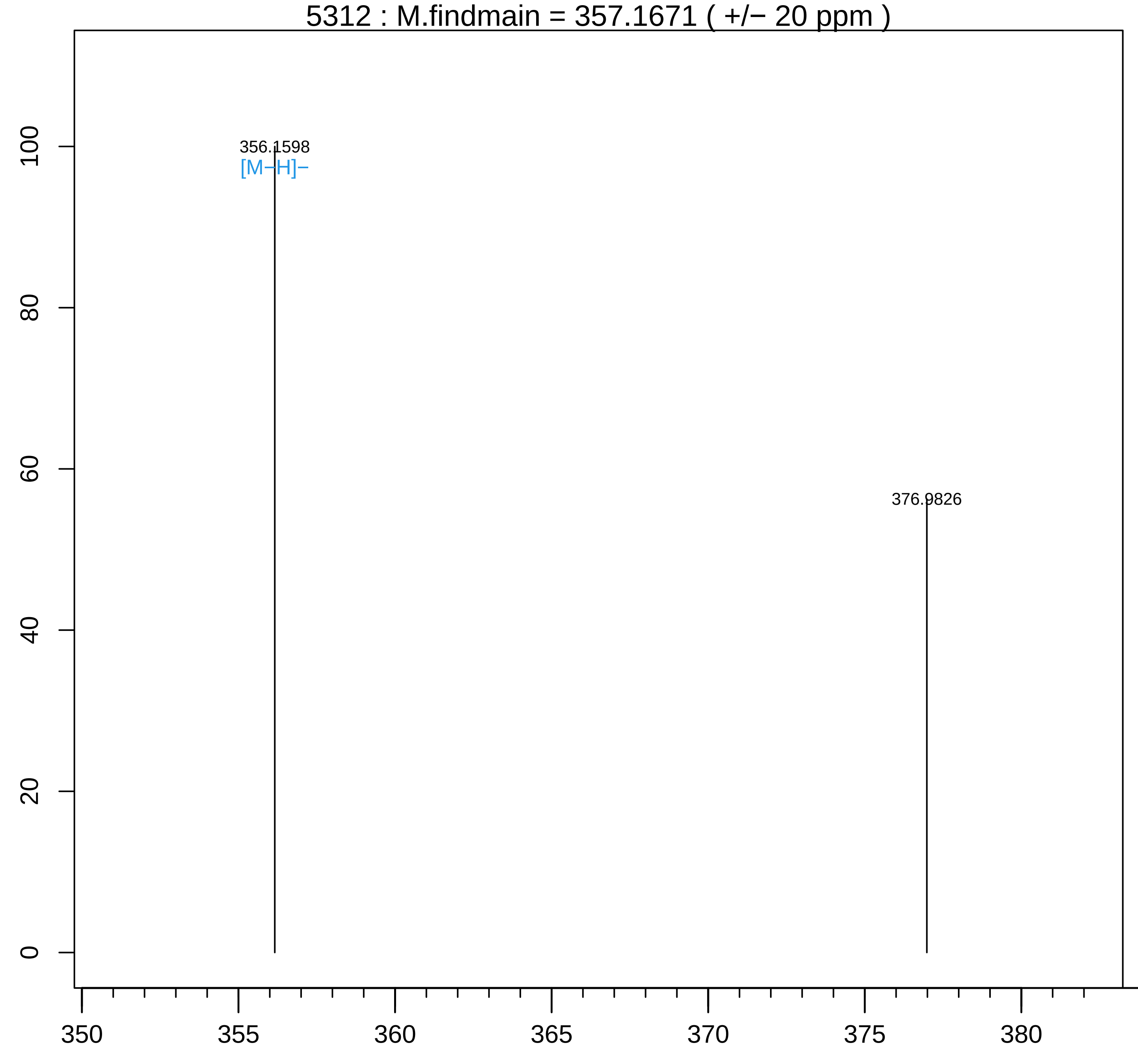
5312 : M.findmain = 357.1671 (+/− 20 ppm)
356.1598
[M−H]−
376.9826
0
20
40
60
80
100
350
355
360
365
370
375
380

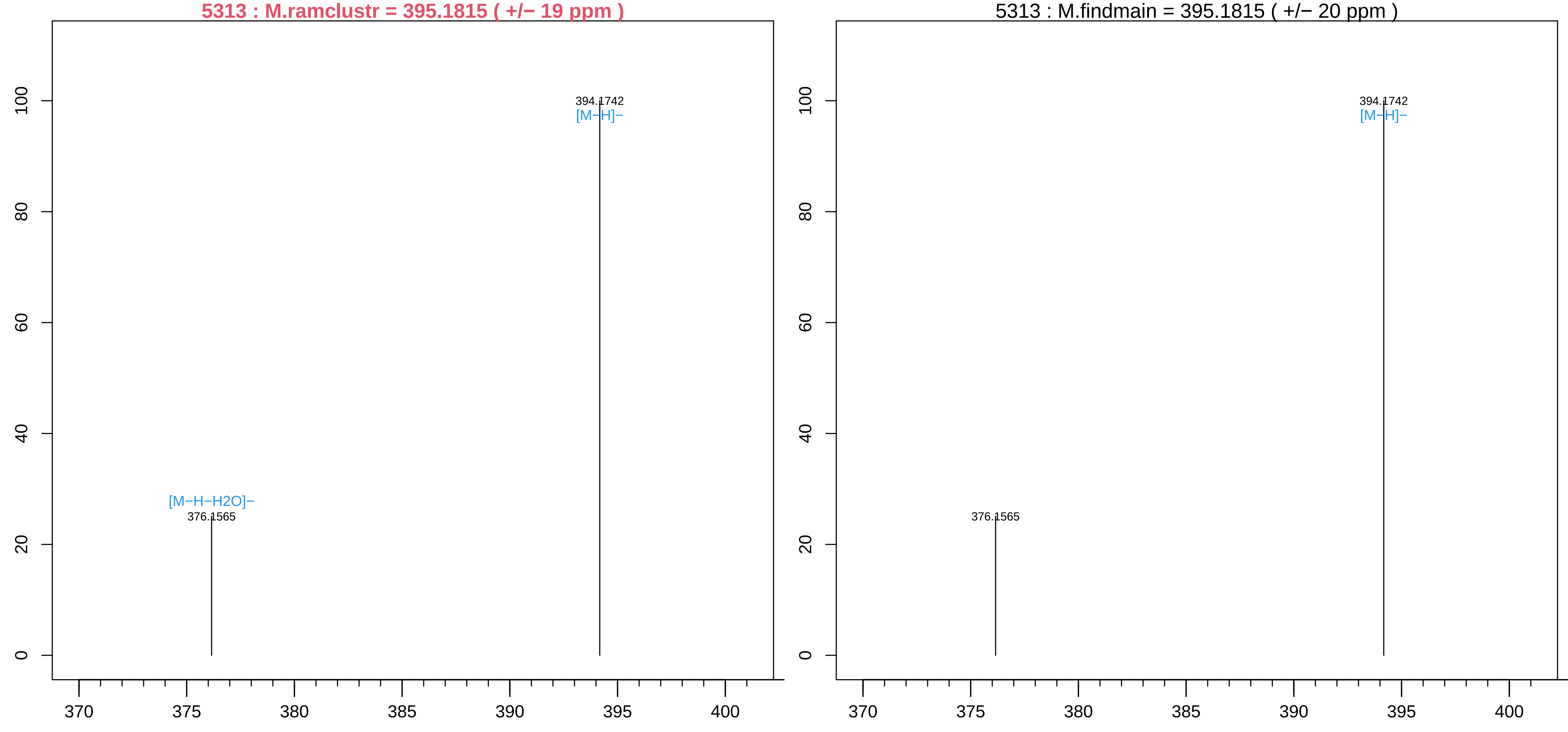
5313 : M.ramclustr = 395.1815 (+/− 19 ppm)
394.1742
[M−H]−
[M−H−H2O]−
376.1565
5313 : M.findmain = 395.1815 (+/− 20 ppm)
394.1742
[M−H]−
376.1565
0
20
40
60
80
100
370
375
380
385
390
395
400

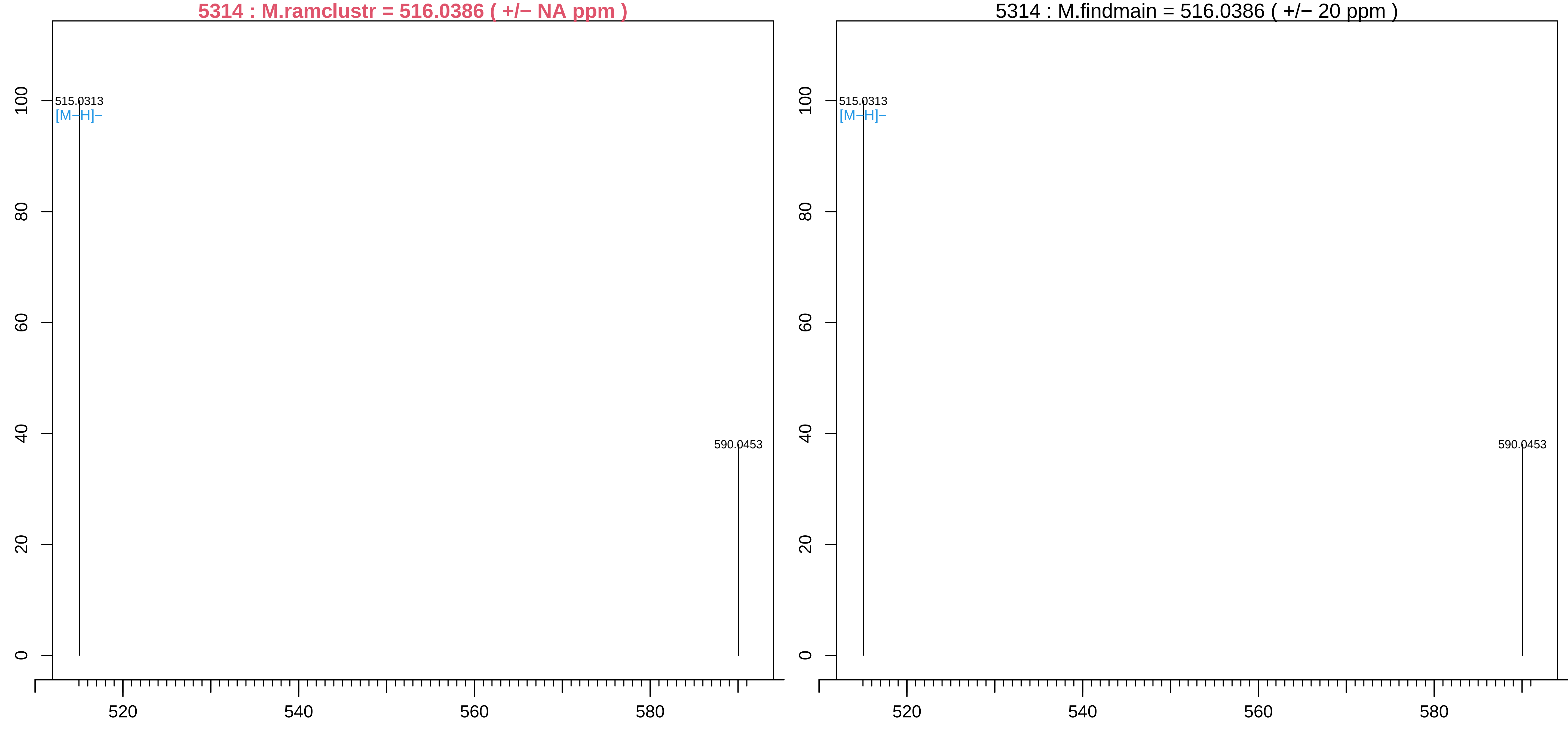

5314 : M.ramclustr = 516.0386 (+/− NA ppm)
515.0313
[M−H]−
590.0453
0
20
40
60
80
100
520
540
560
580
5314 : M.findmain = 516.0386 (+/− 20 ppm)
515.0313
[M−H]−
590.0453
0
20
40
60
80
100
520
540
560
580

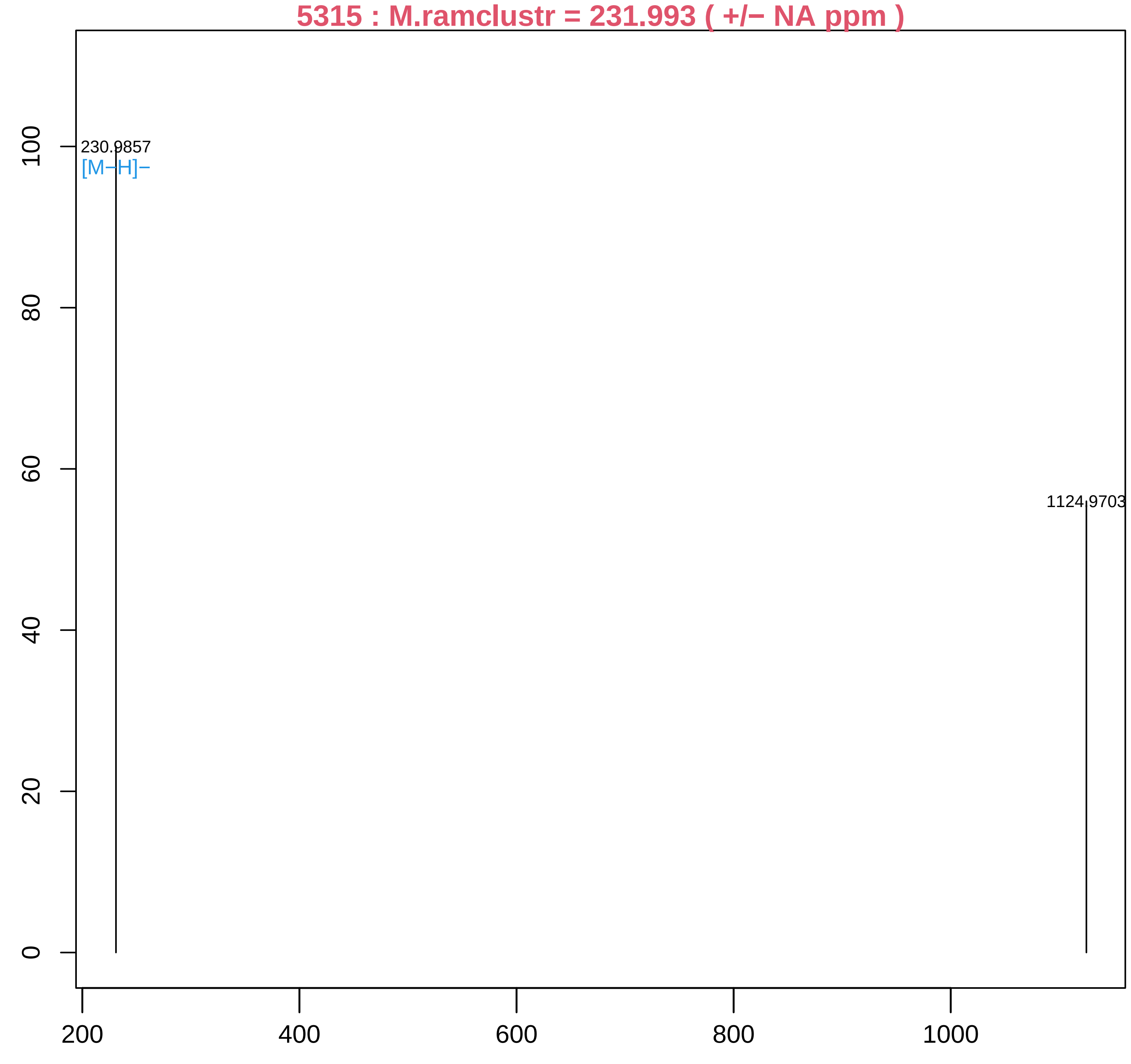

5315 : M.ramclustr = 231.993 (+/− NA ppm)
230.9857
[M−H]−
1124.9703

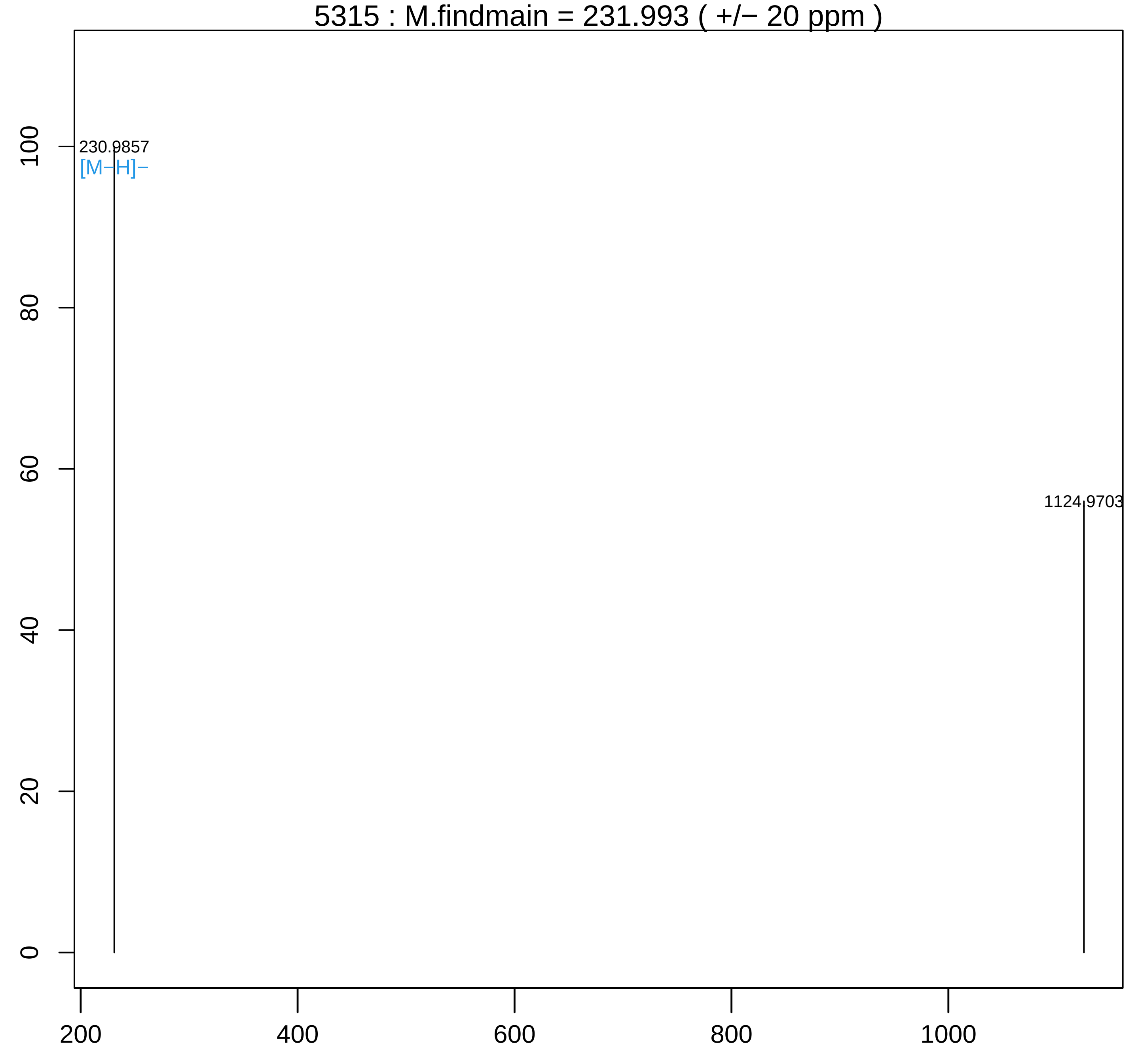

5315 : M.findmain = 231.993 (+/− 20 ppm)
230.9857
[M−H]−
1124.9703

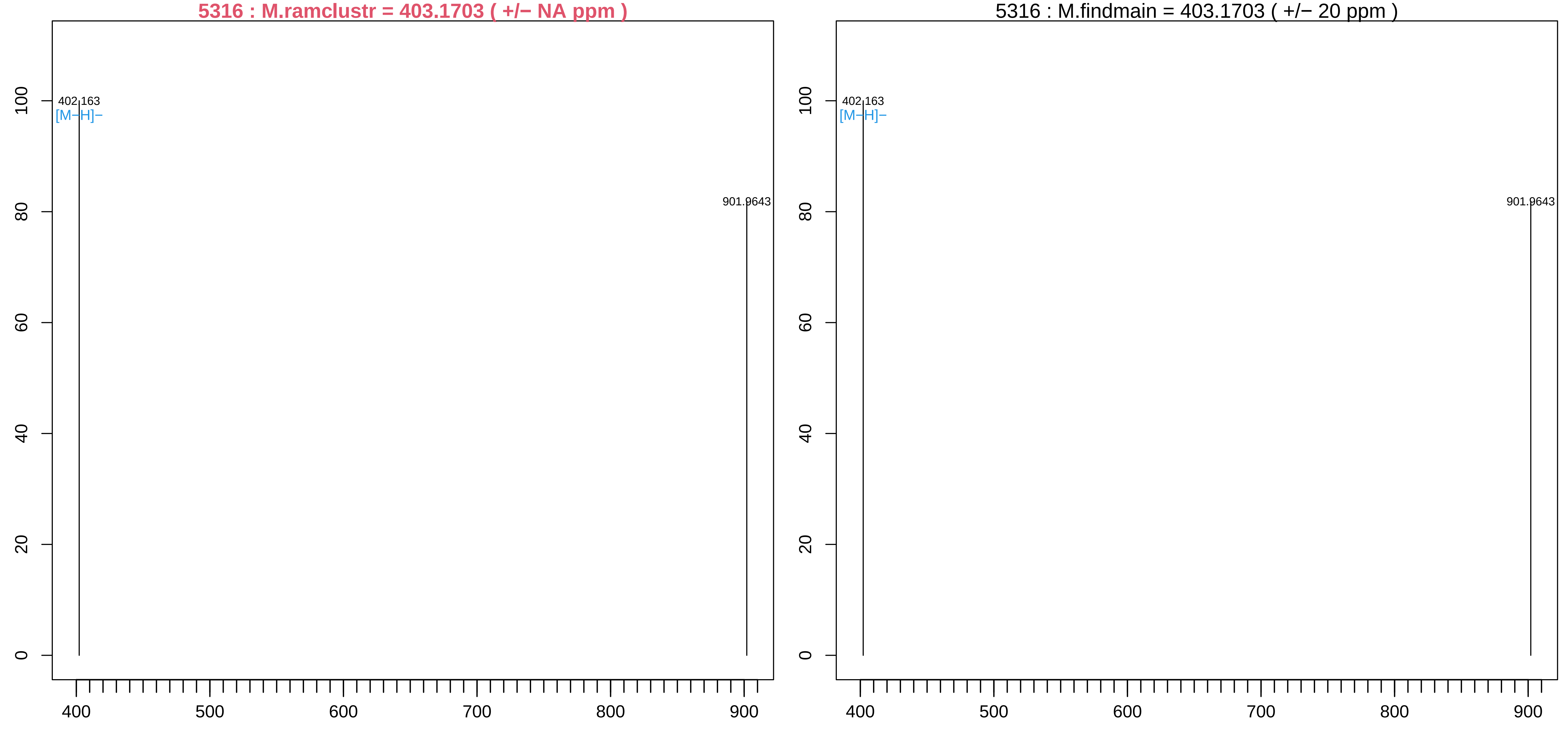

5316 : M.ramclustr = 403.1703 (+/− NA ppm)
402.163
[M−H]−
901.9643
0
20
40
60
80
100
400
500
600
700
800
900
5316 : M.findmain = 403.1703 (+/− 20 ppm)
402.163
[M−H]−
901.9643
0
20
40
60
80
100
400
500
600
700
800
900

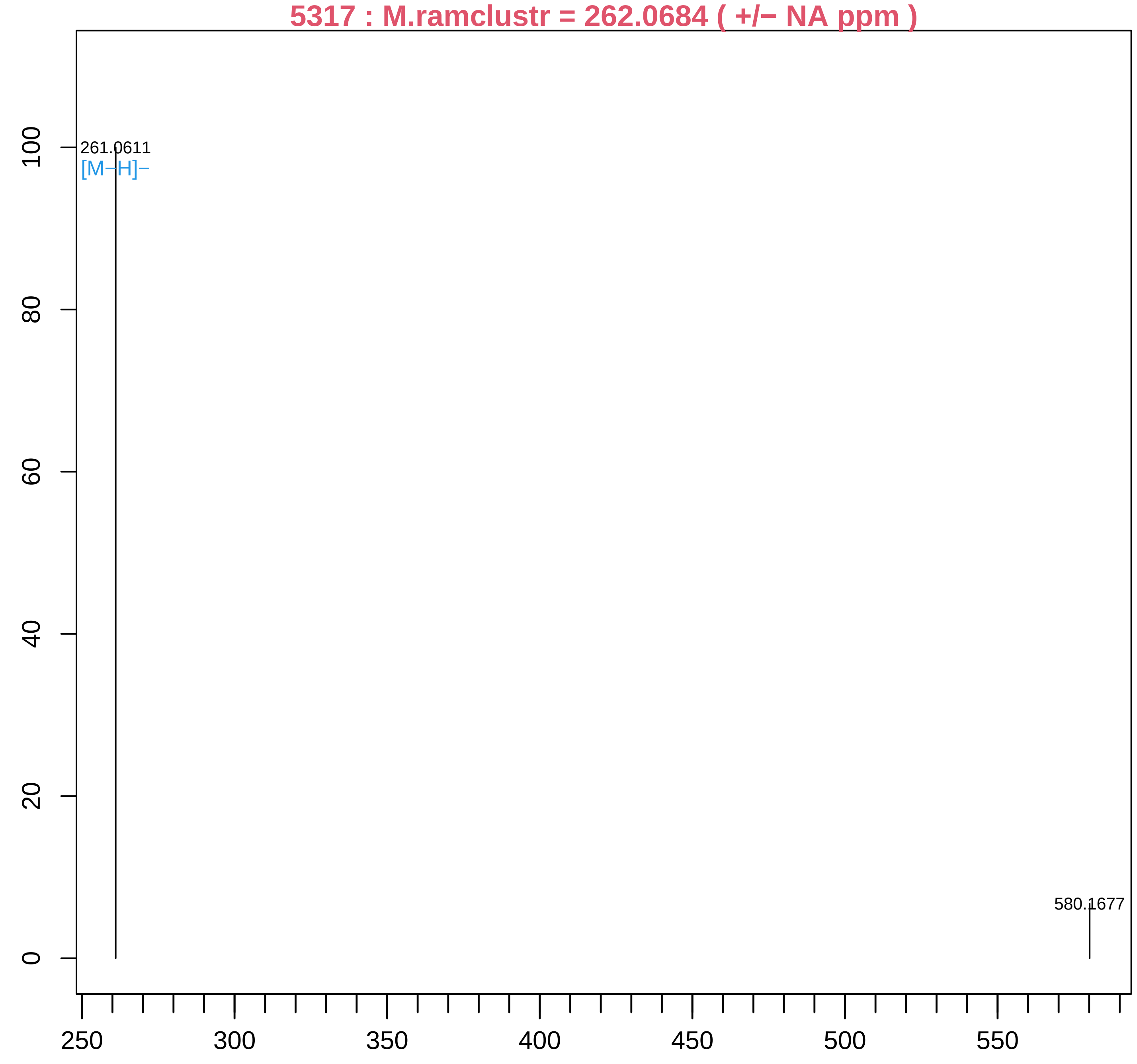

5317 : M.ramclustr = 262.0684 (+/− NA ppm)
261.0611
[M−H]−
580.1677

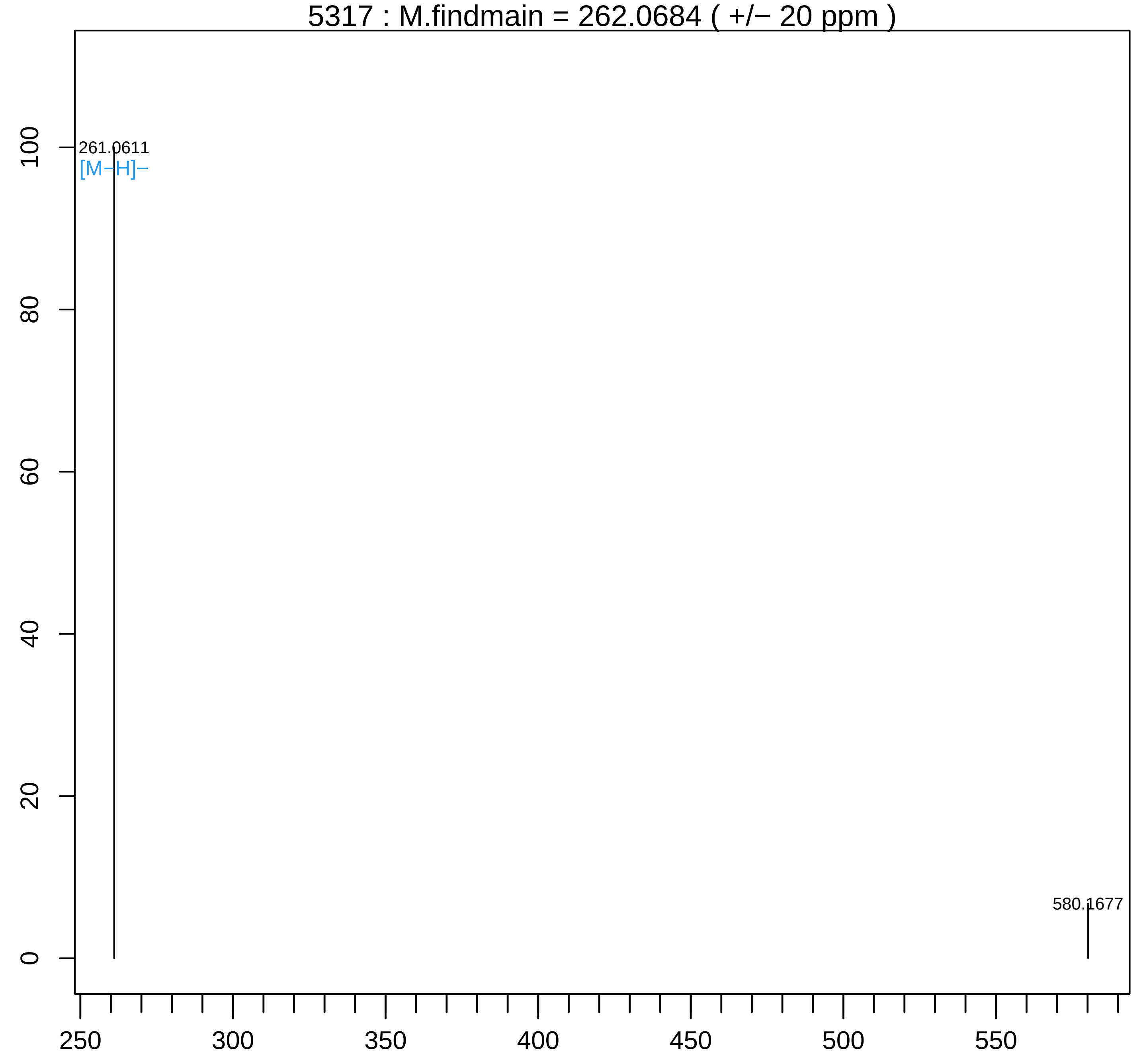

5317 : M.findmain = 262.0684 (+/− 20 ppm)
261.0611
[M−H]−
580.1677

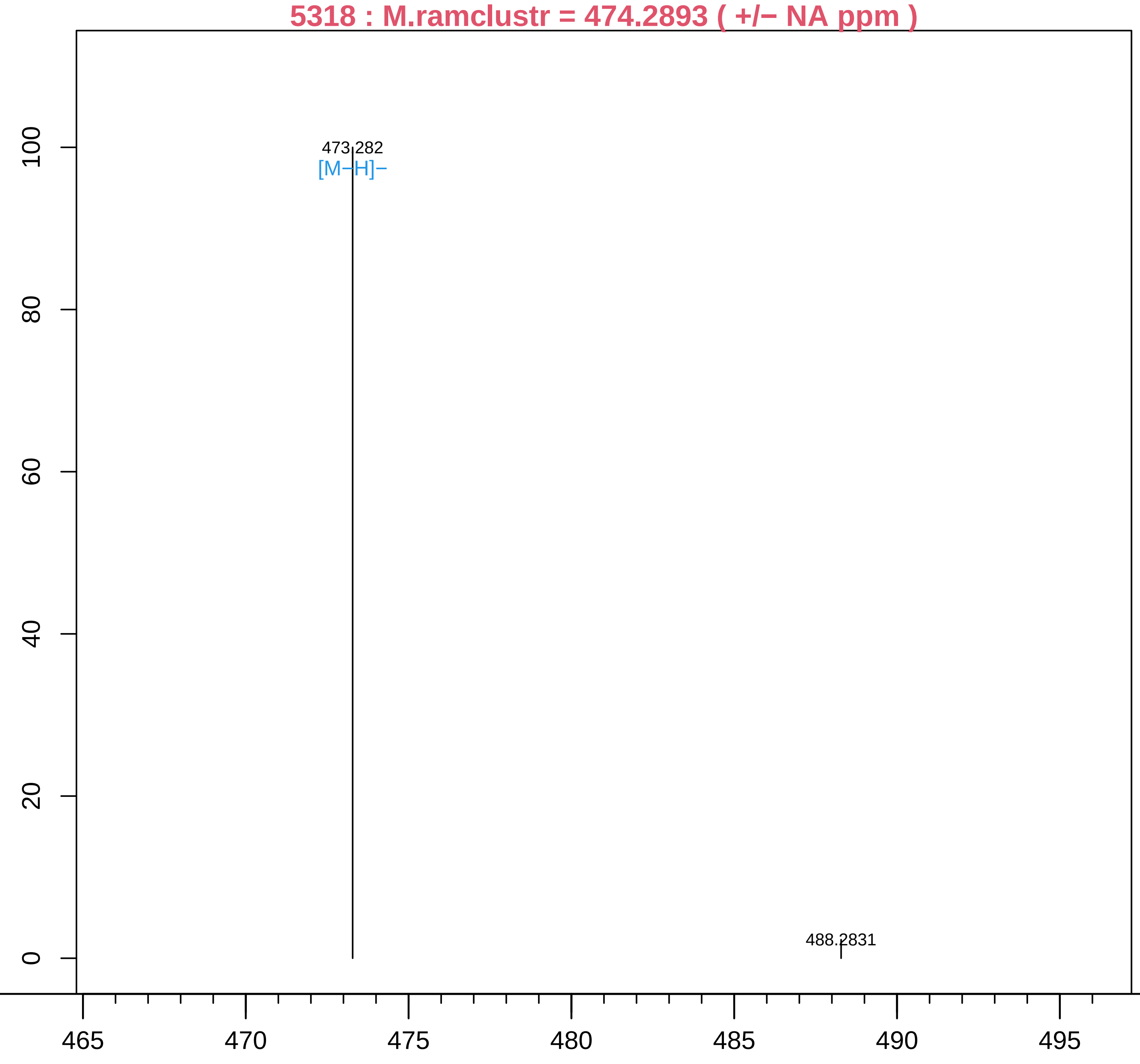
5318 : M.ramclustr = 474.2893 (+/− NA ppm)
473.282
[M−H]−
488.2831
0
20
40
60
80
100
465
470
475
480
485
490
495

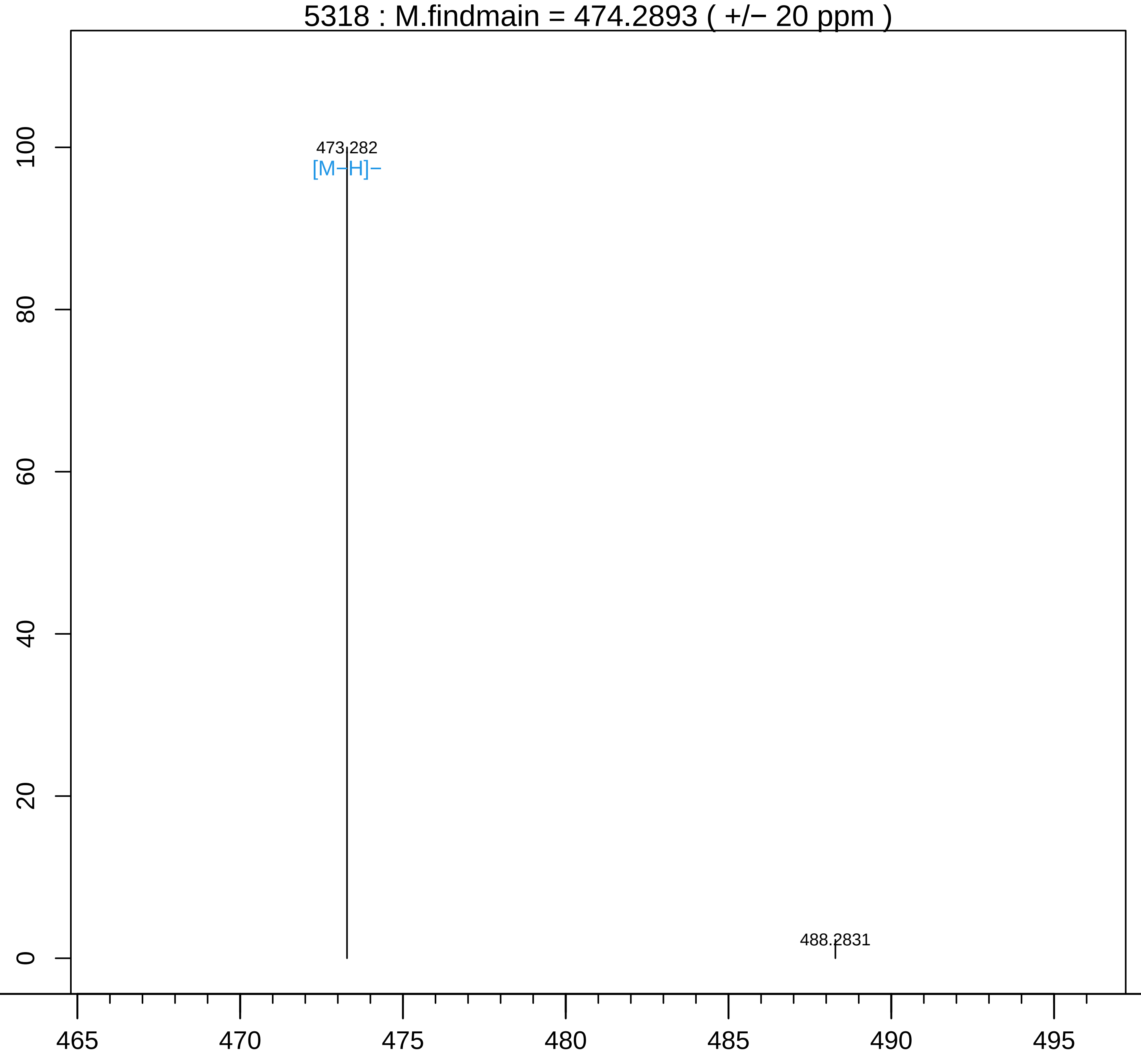
5318 : M.findmain = 474.2893 (+/− 20 ppm)
473.282
[M−H]−
488.2831
0
20
40
60
80
100
465
470
475
480
485
490
495

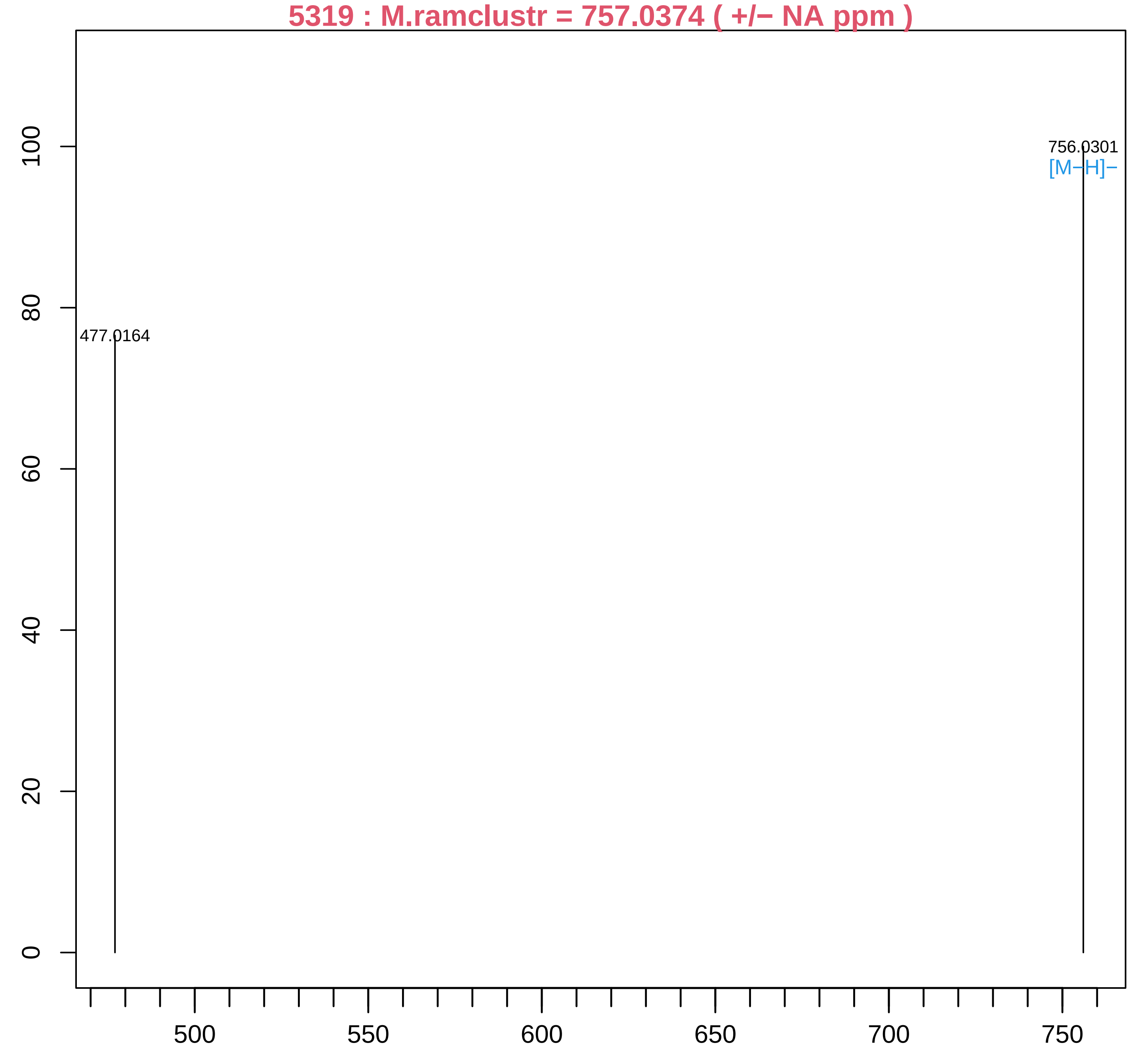
5319 : M.ramclustr = 757.0374 (+/− NA ppm)
756.0301
[M−H]−
477.0164
0
20
40
60
80
100
500
550
600
650
700
750

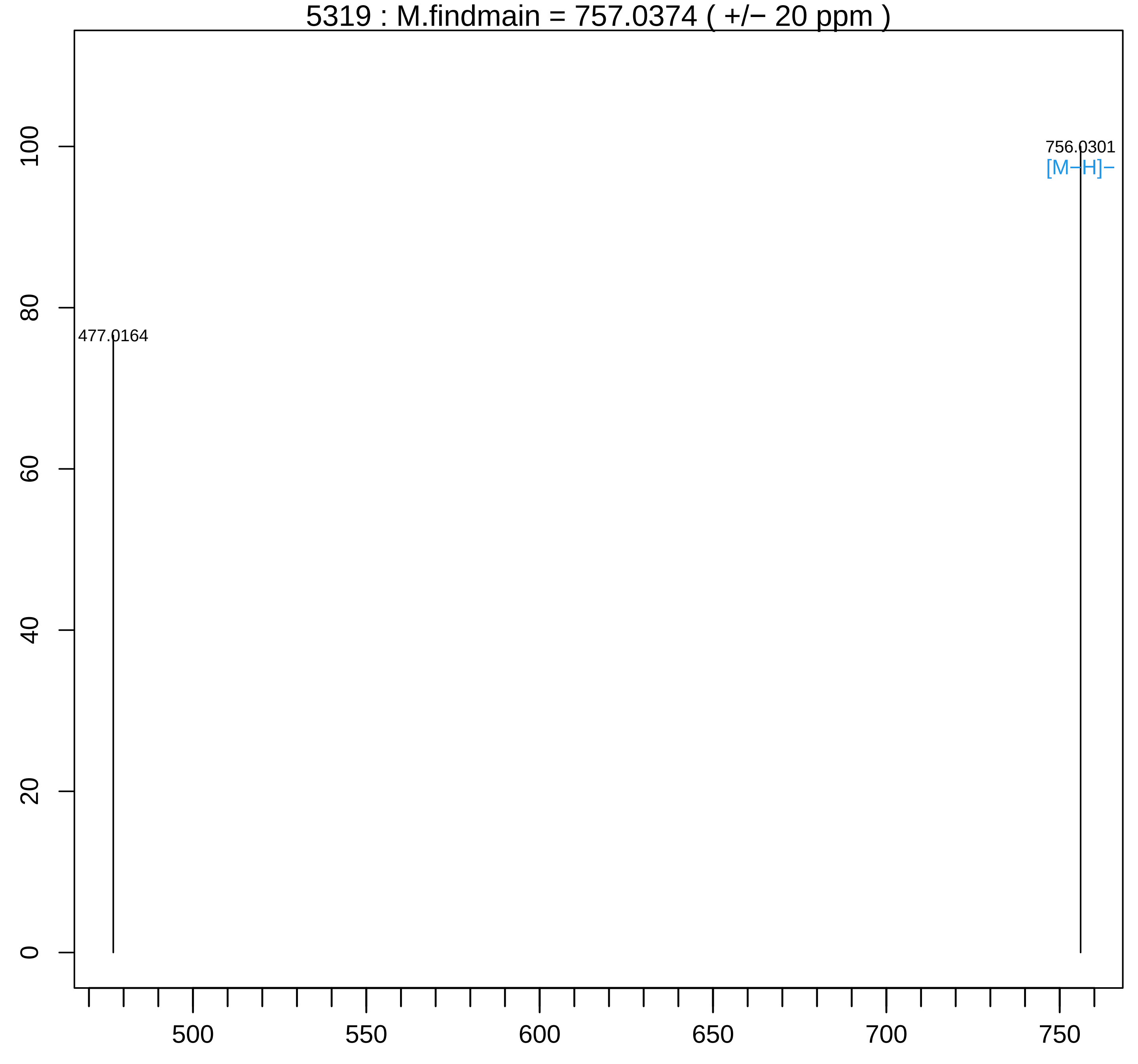
5319 : M.findmain = 757.0374 (+/− 20 ppm)
756.0301
[M−H]−
477.0164
0
20
40
60
80
100
500
550
600
650
700
750

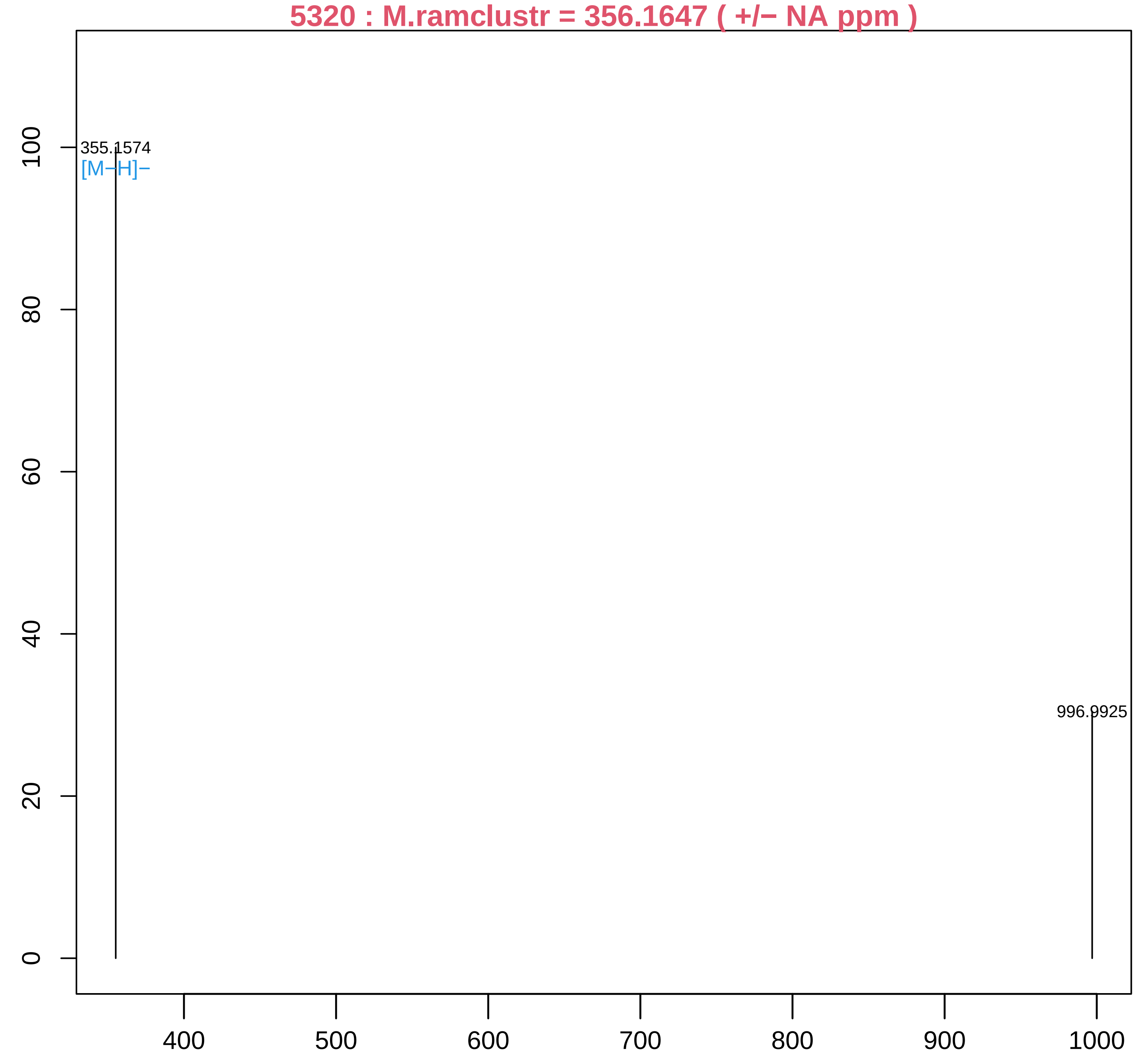

5320 : M.ramclustr = 356.1647 (+/− NA ppm)
355.1574
[M−H]−
996.9925
0
20
40
60
80
100
400
500
600
700
800
900
1000

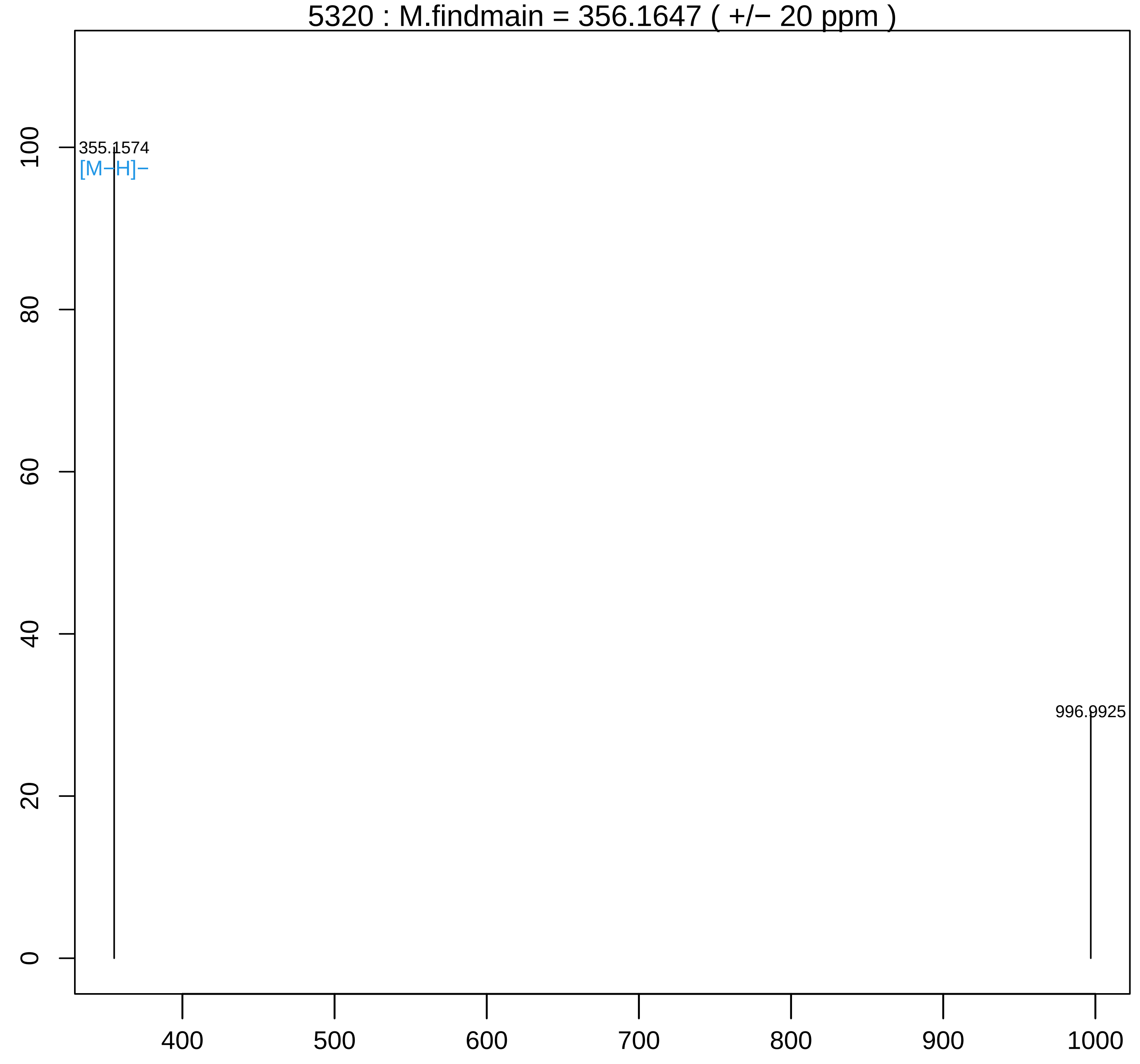

5320 : M.findmain = 356.1647 (+/− 20 ppm)
355.1574
[M−H]−
996.9925
0
20
40
60
80
100
400
500
600
700
800
900
1000

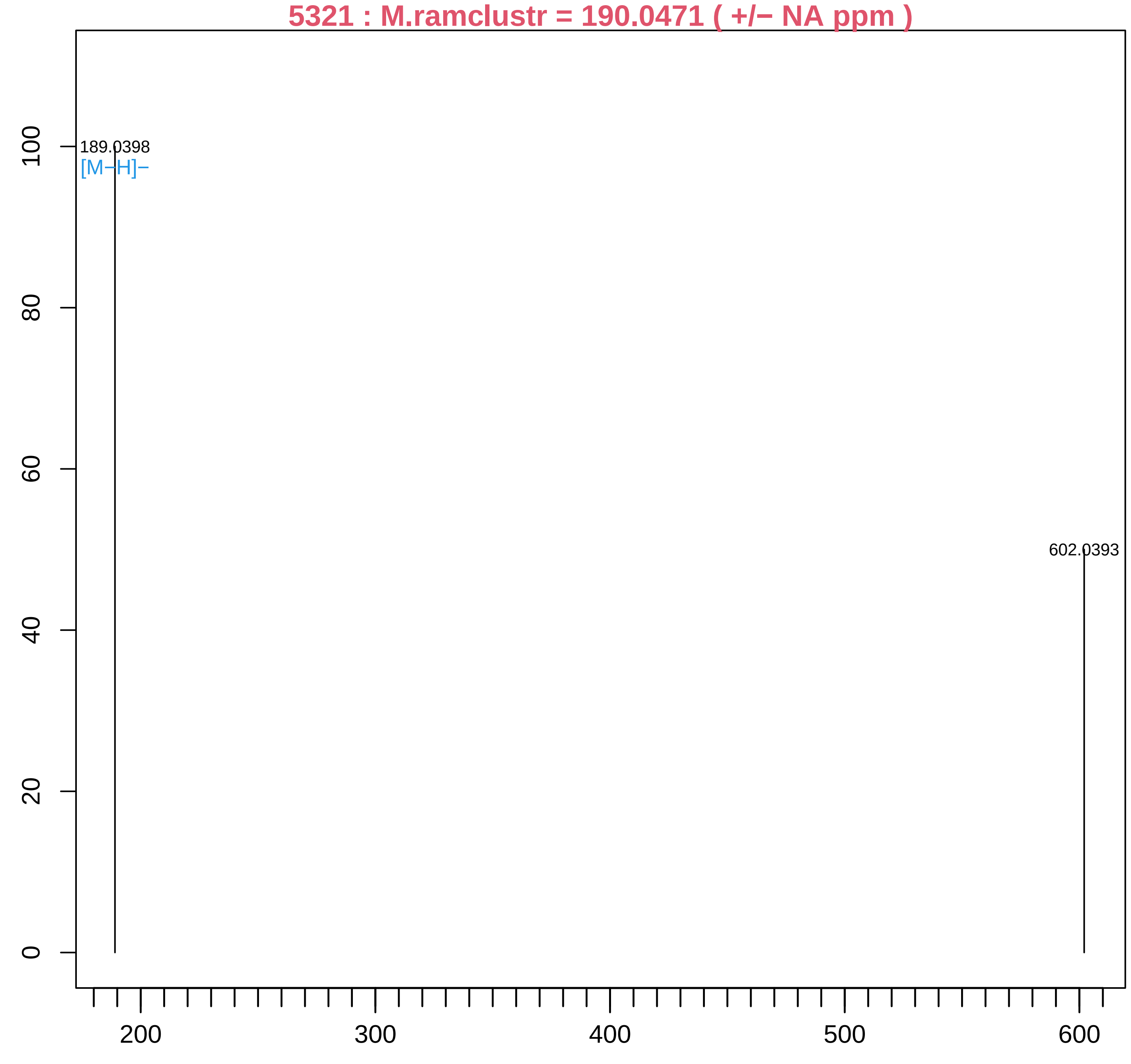
5321 : M.ramclustr = 190.0471 (+/− NA ppm)
189.0398
[M−H]−
602.0393
0
20
40
60
80
100
200
300
400
500
600

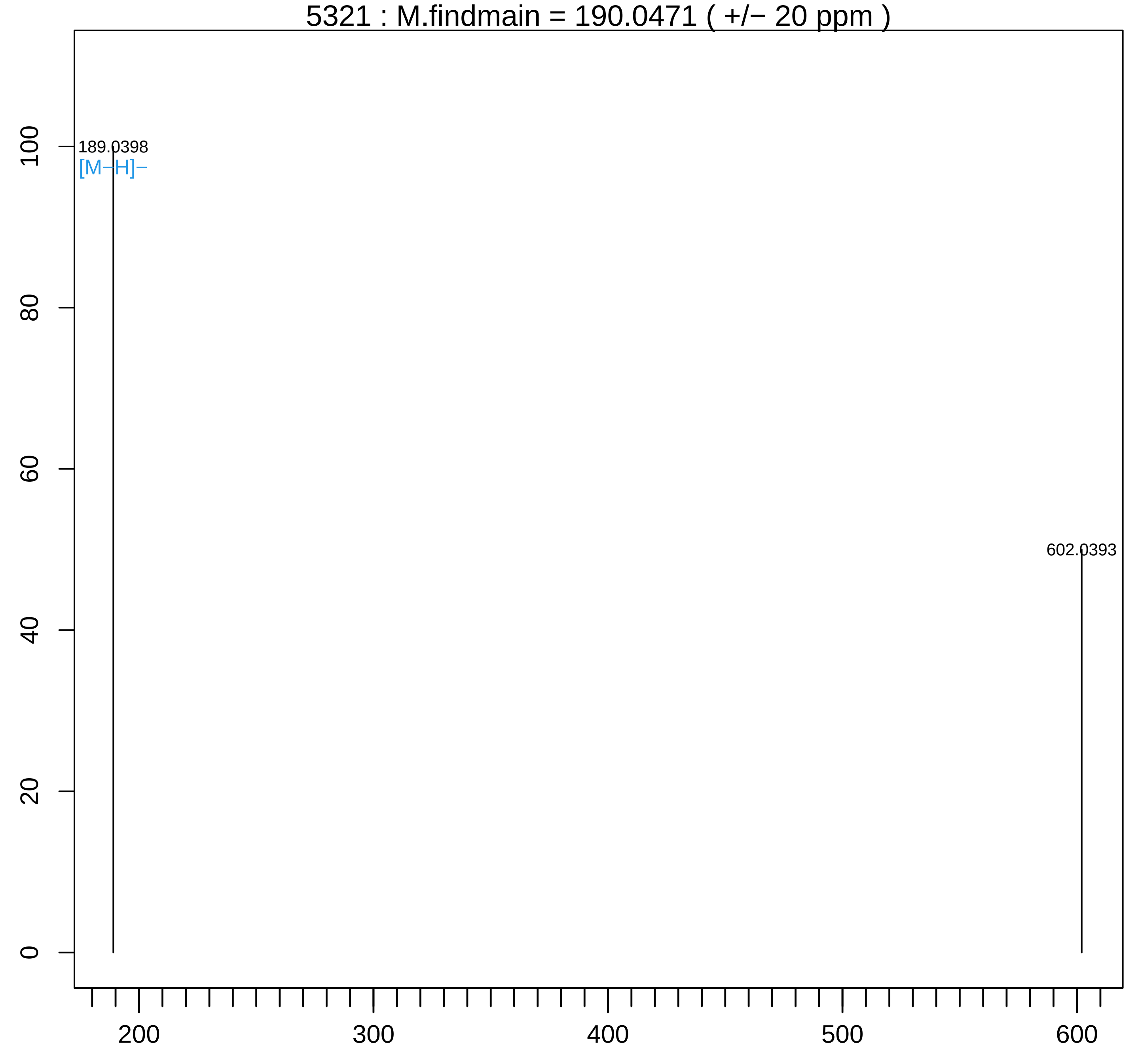
5321 : M.findmain = 190.0471 (+/− 20 ppm)
189.0398
[M−H]−
602.0393
0
20
40
60
80
100
200
300
400
500
600

5322 : M.ramclustr = 563.9867 (+/− NA ppm)

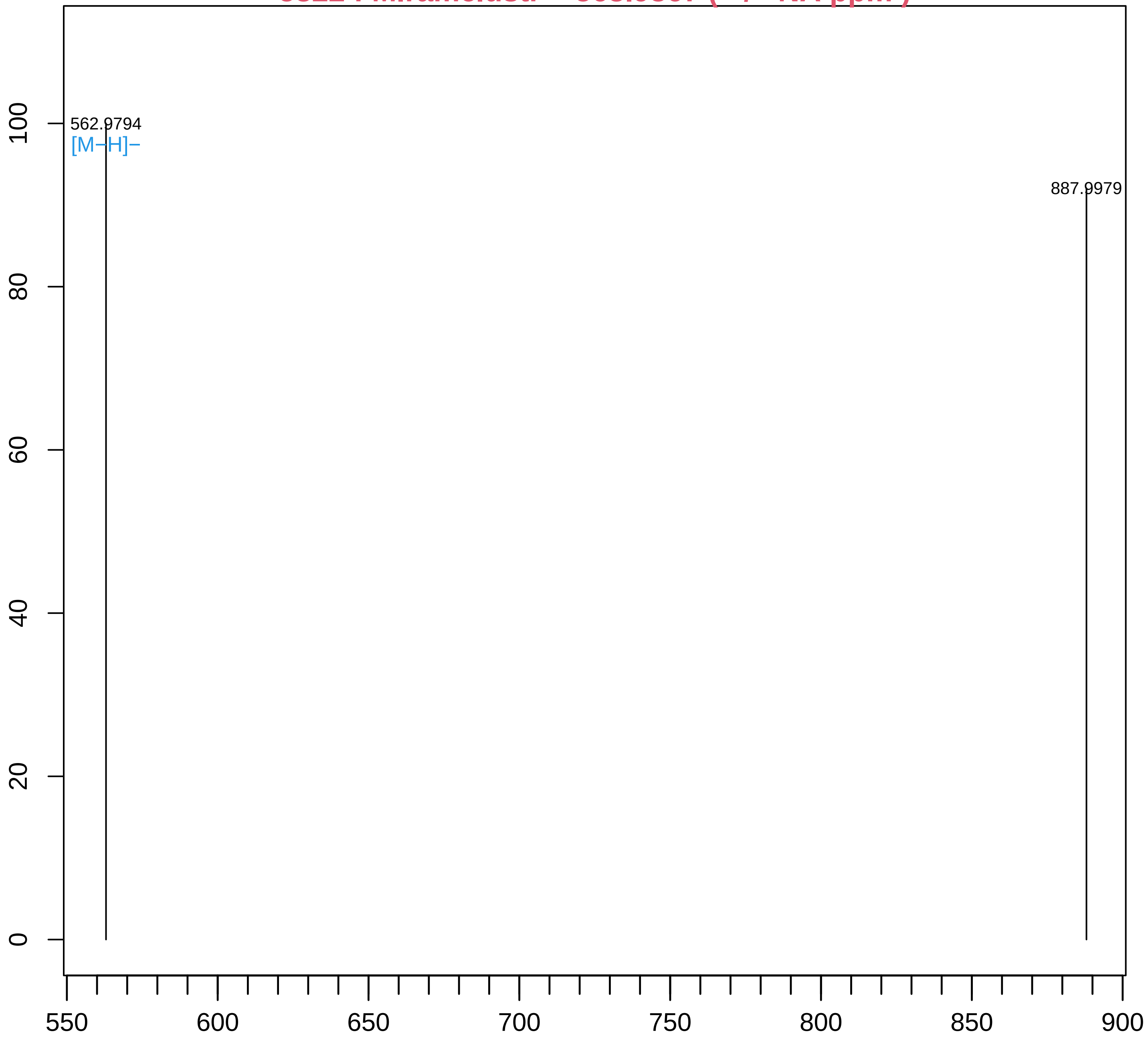

5322 : M.findmain = 563.9867 (+/− 20 ppm)

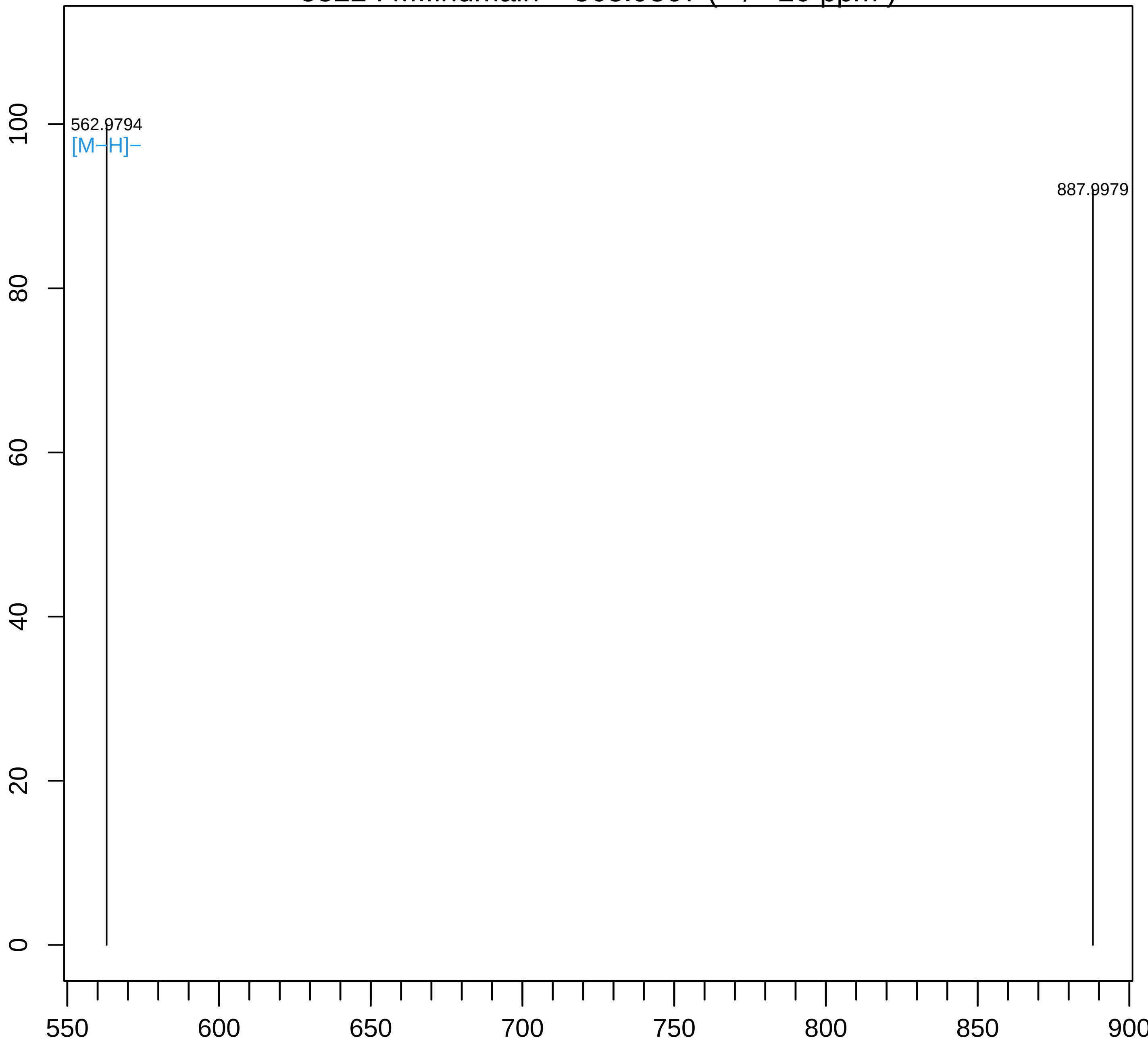

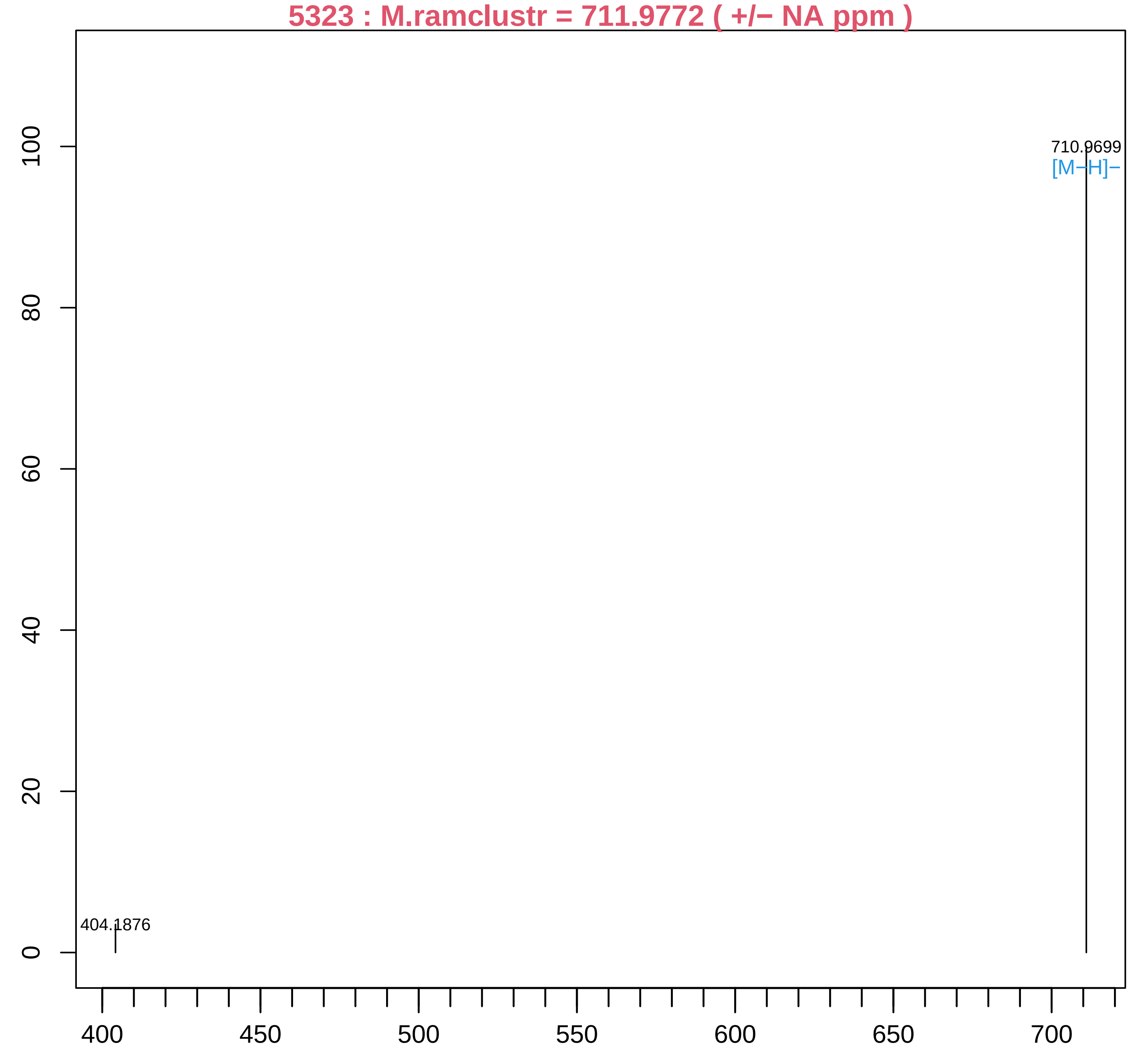
5323 : M.ramclustr = 711.9772 (+/− NA ppm)
710.9699
[M−H]−
404.1876
0
20
40
60
80
100
400
450
500
550
600
650
700

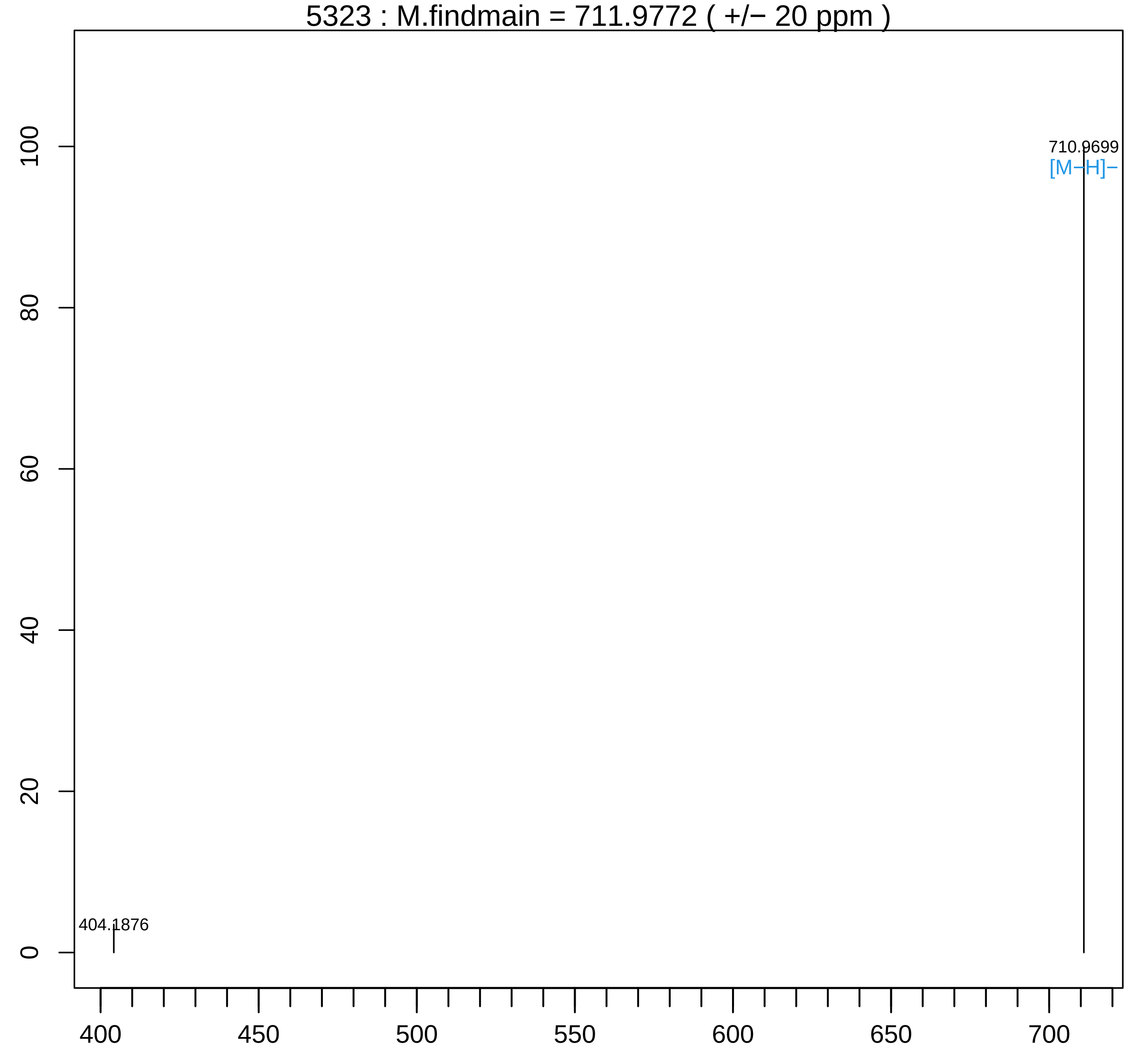
5323 : M.findmain = 711.9772 (+/− 20 ppm)
710.9699
[M−H]−
404.1876
0
20
40
60
80
100
400
450
500
550
600
650
700

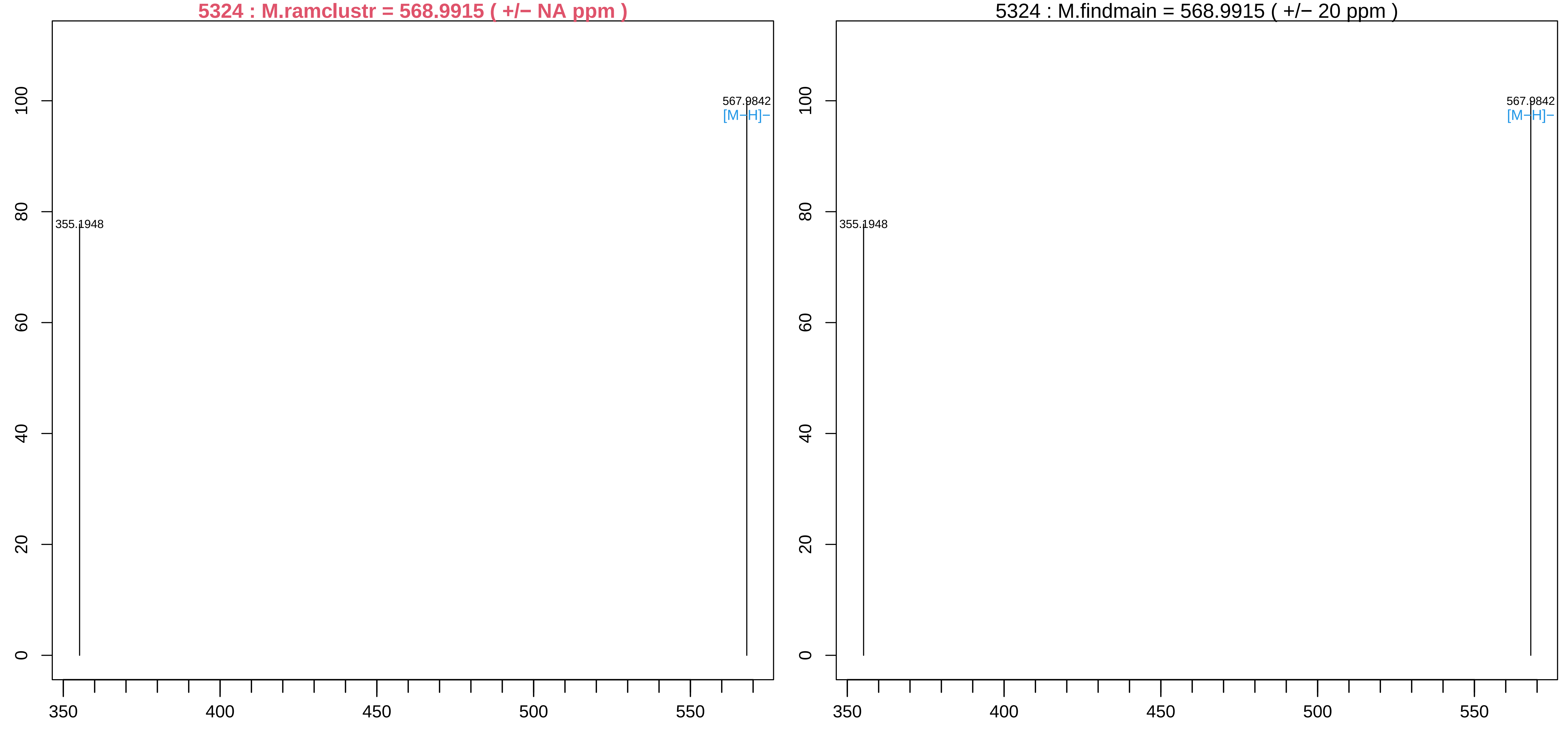

5324 : M.ramclustr = 568.9915 (+/− NA ppm)
567.9842
[M−H]−
355.1948
5324 : M.findmain = 568.9915 (+/− 20 ppm)
567.9842
[M−H]−
355.1948
0
20
40
60
80
100
350
400
450
500
550

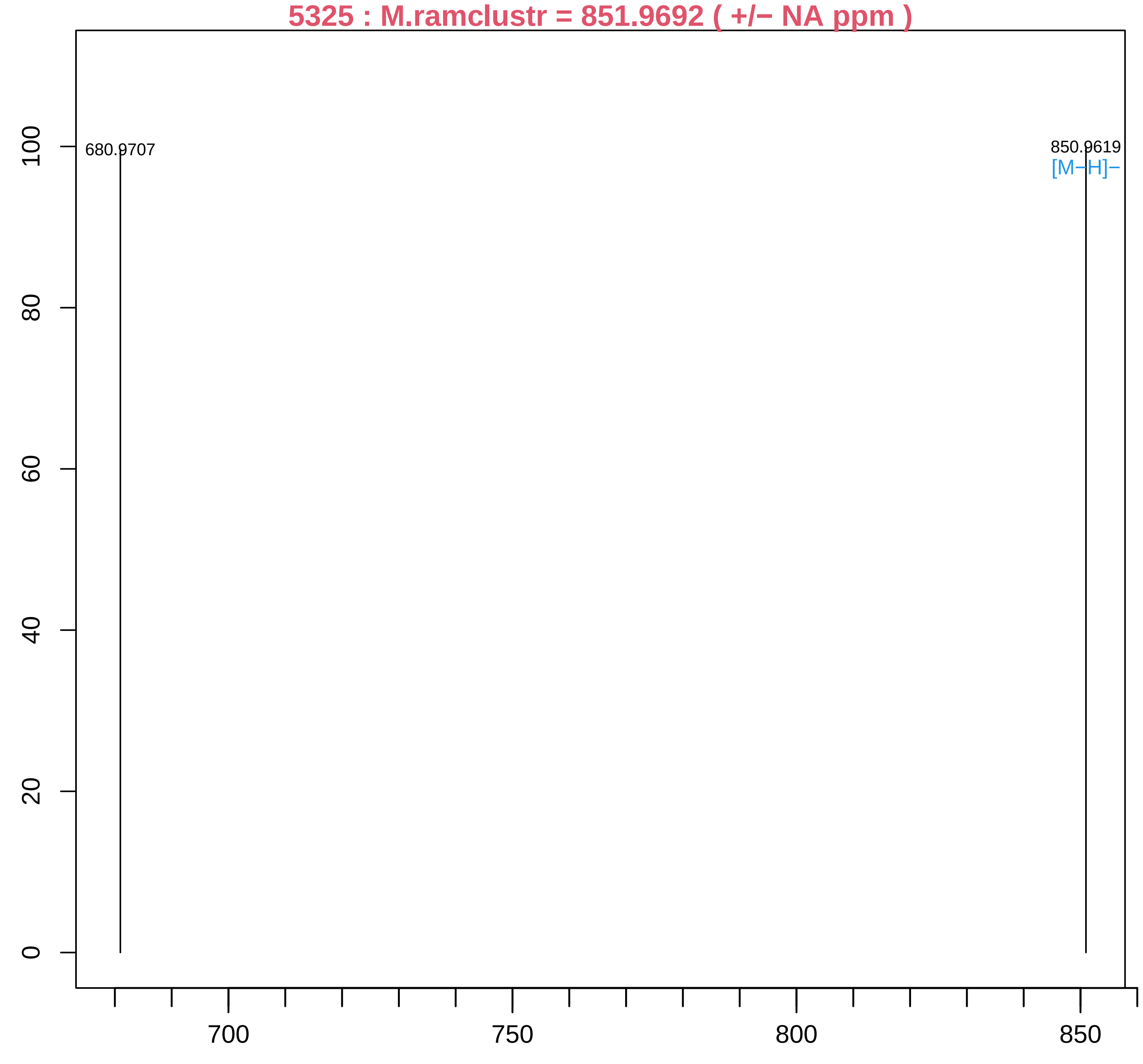
5325 : M.ramclustr = 851.9692 (+/− NA ppm)
680.9707
850.9619
[M−H]−
0
20
40
60
80
100
700
750
800
850

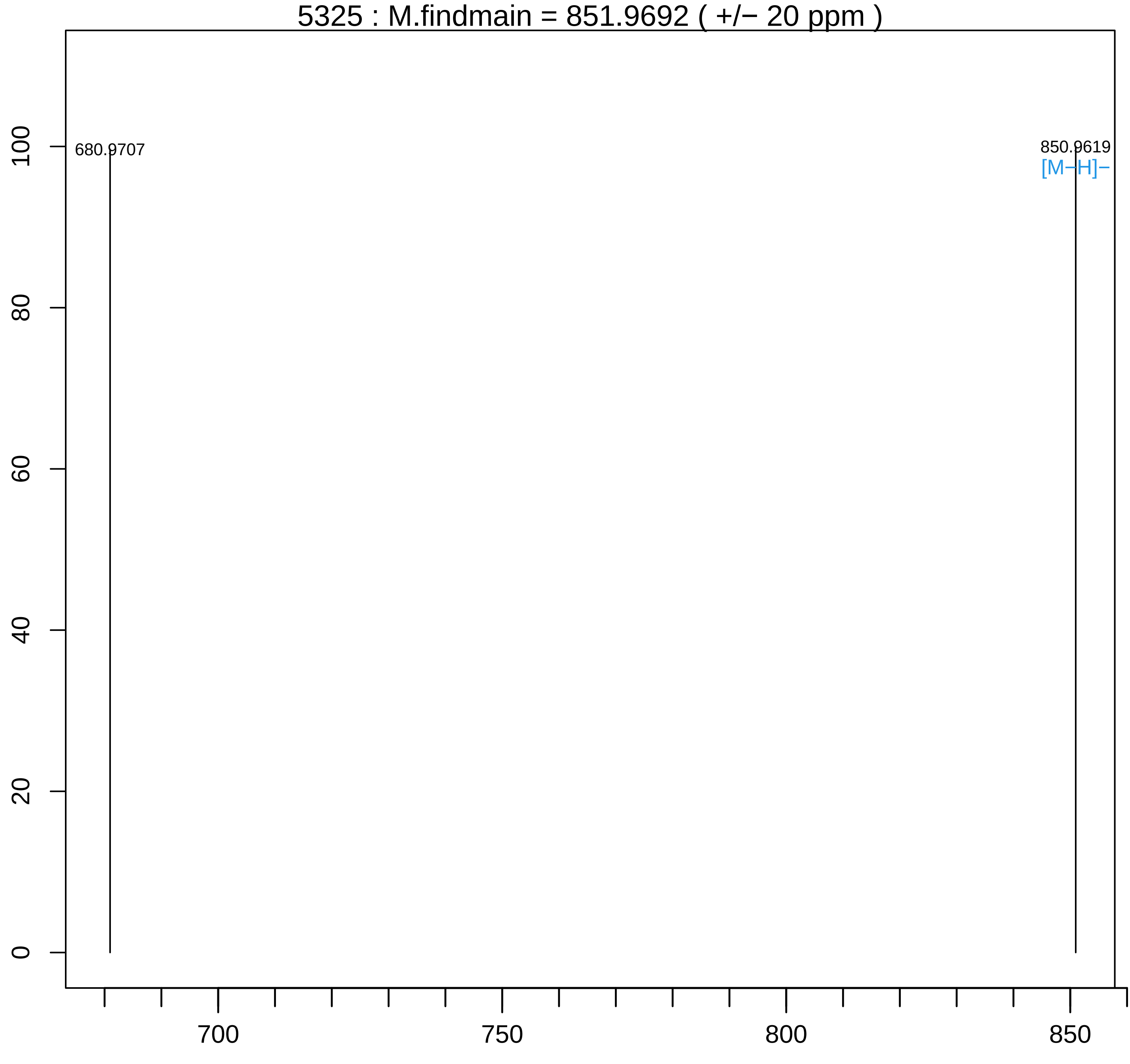
5325 : M.findmain = 851.9692 (+/− 20 ppm)
680.9707
850.9619
[M−H]−
0
20
40
60
80
100
700
750
800
850

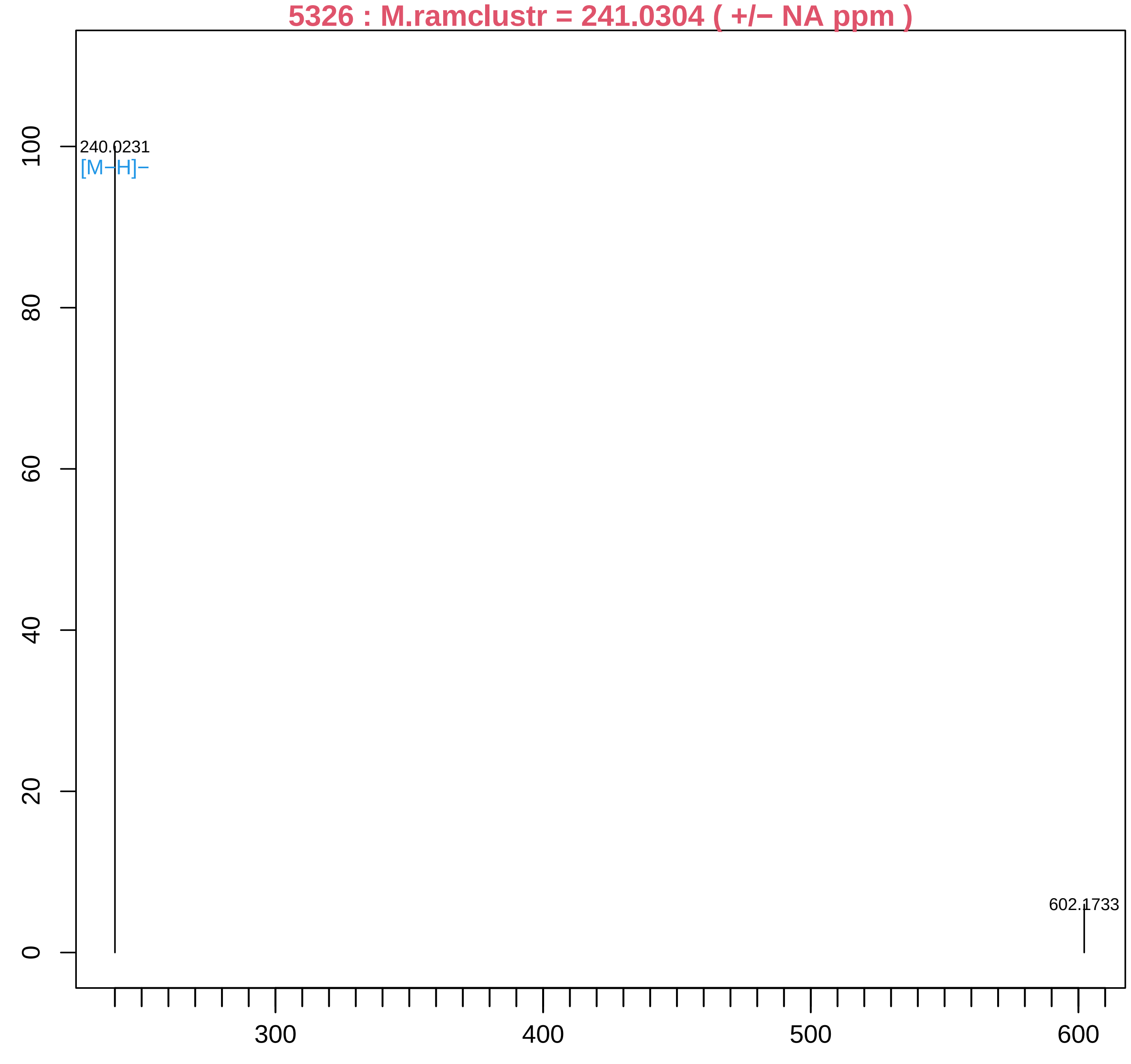
5326 : M.ramclustr = 241.0304 (+/− NA ppm)
240.0231
[M−H]−
602.1733
0
20
40
60
80
100
300
400
500
600

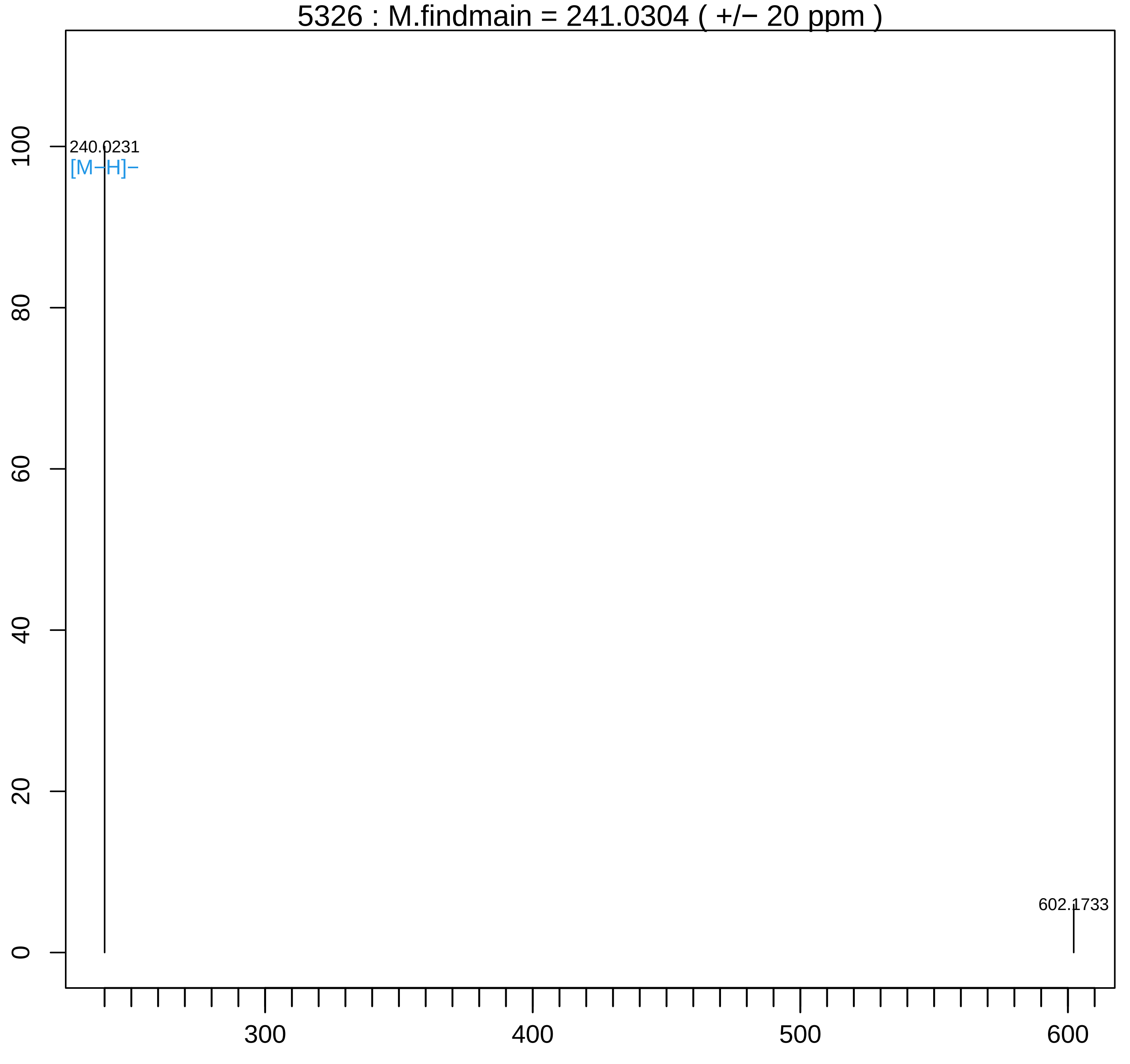
5326 : M.findmain = 241.0304 (+/− 20 ppm)
240.0231
[M−H]−
602.1733
0
20
40
60
80
100
300
400
500
600

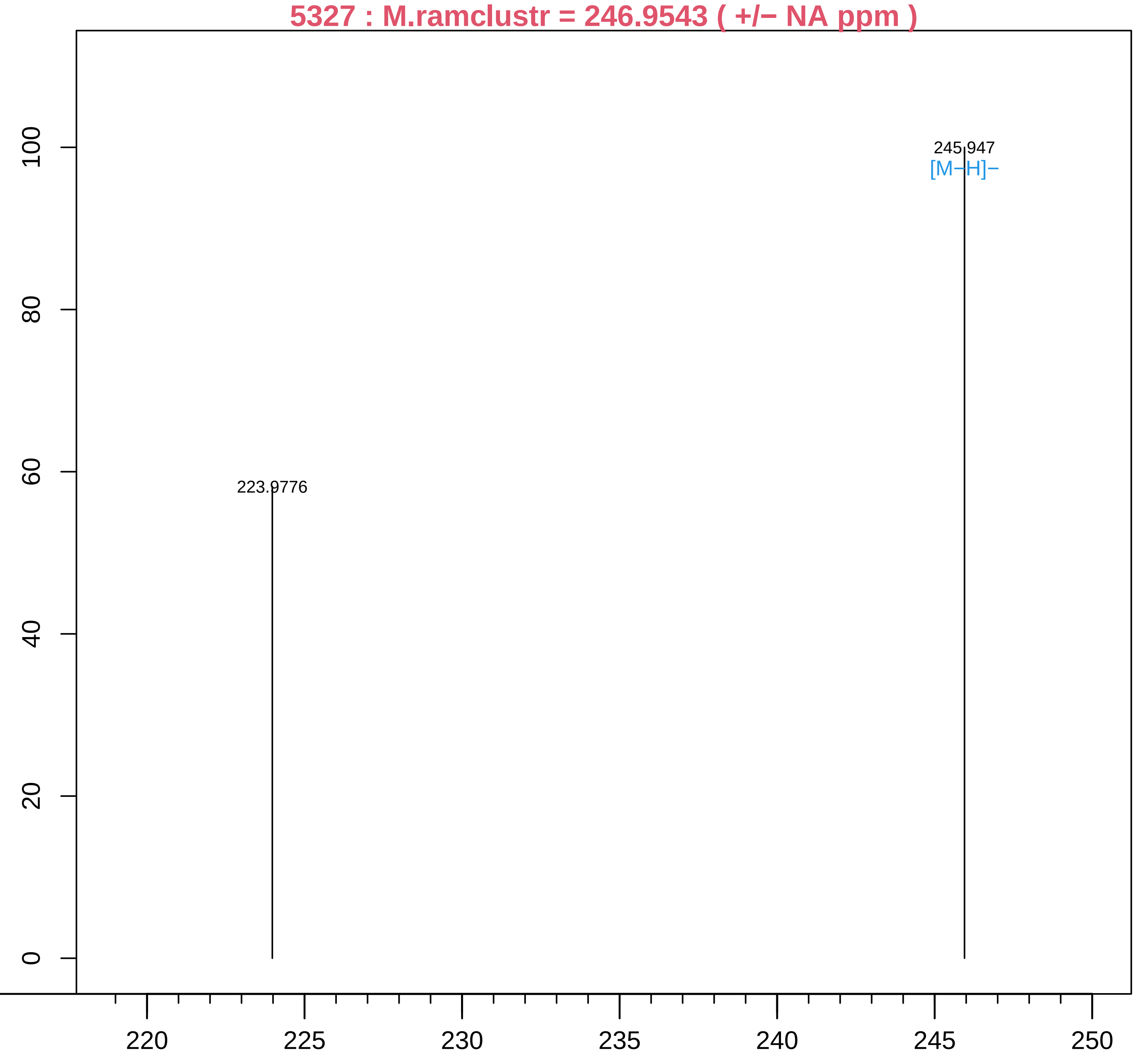
5327 : M.ramclustr = 246.9543 (+/− NA ppm)
245.947
[M−H]−
223.9776
0
20
40
60
80
100
220
225
230
235
240
245
250

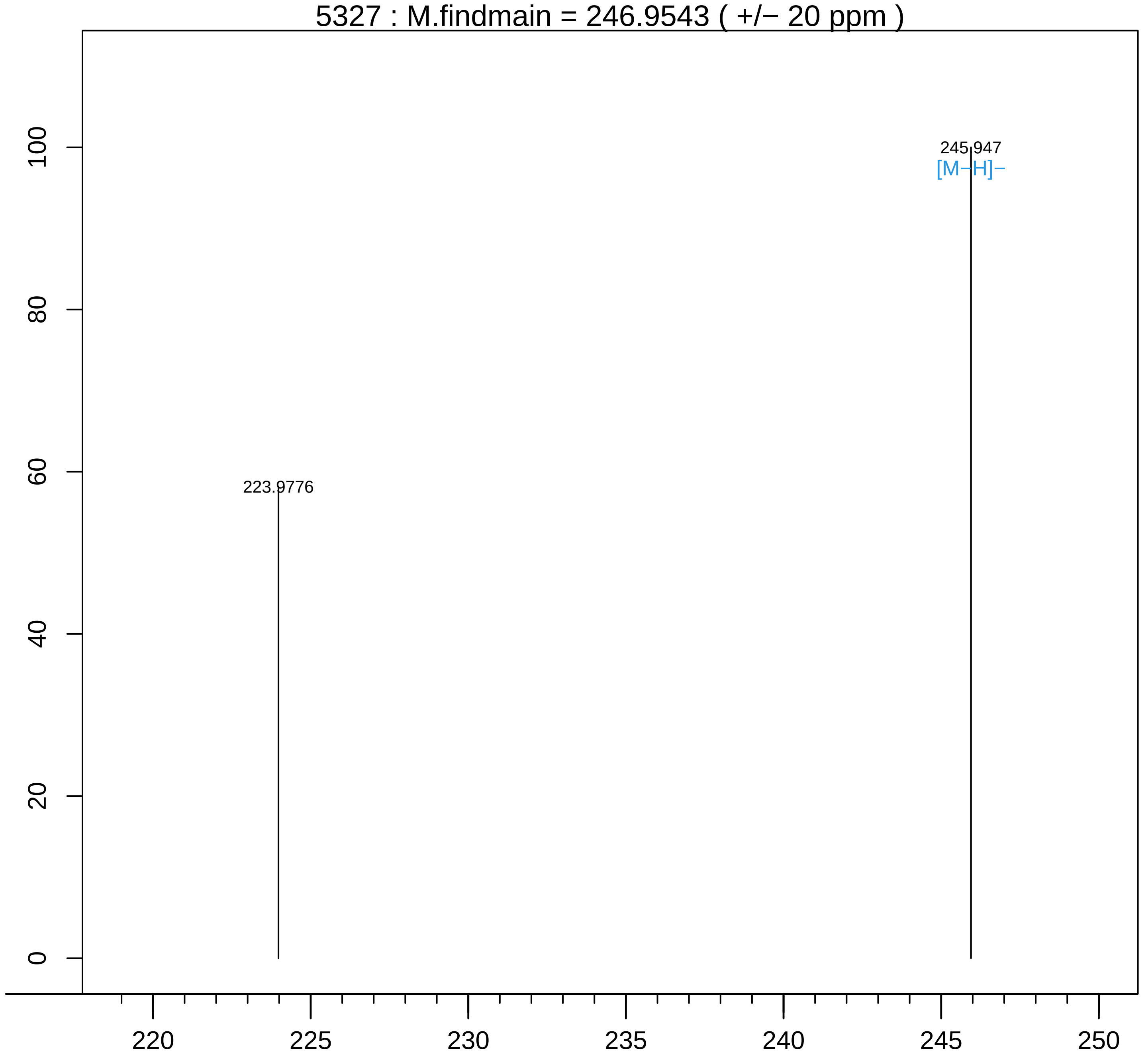
5327 : M.findmain = 246.9543 (+/− 20 ppm)
245.947
[M−H]−
223.9776
0
20
40
60
80
100
220
225
230
235
240
245
250

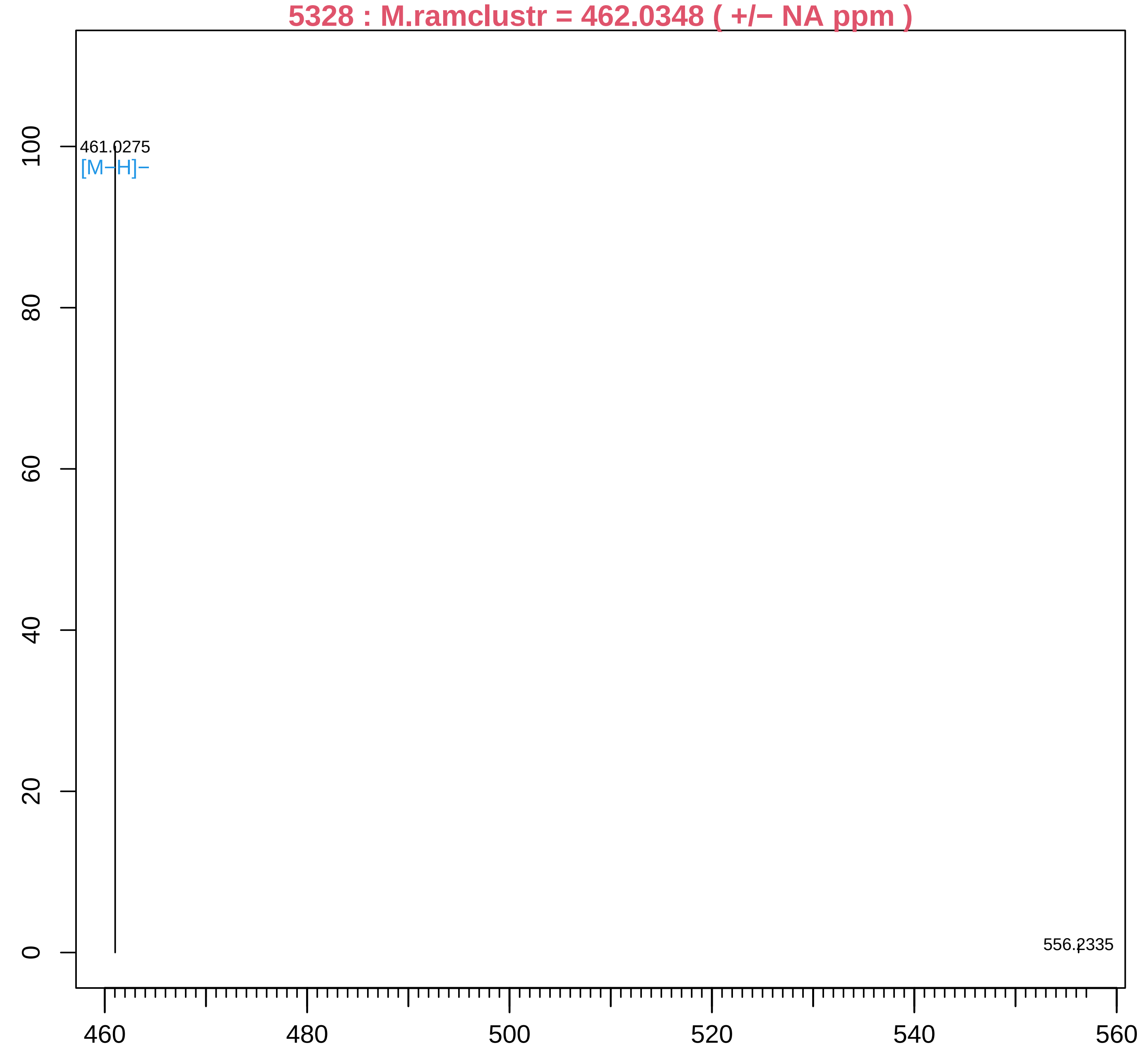
5328 : M.ramclustr = 462.0348 (+/− NA ppm)
461.0275
[M−H]−
556.2335
0
20
40
60
80
100
460
480
500
520
540
560

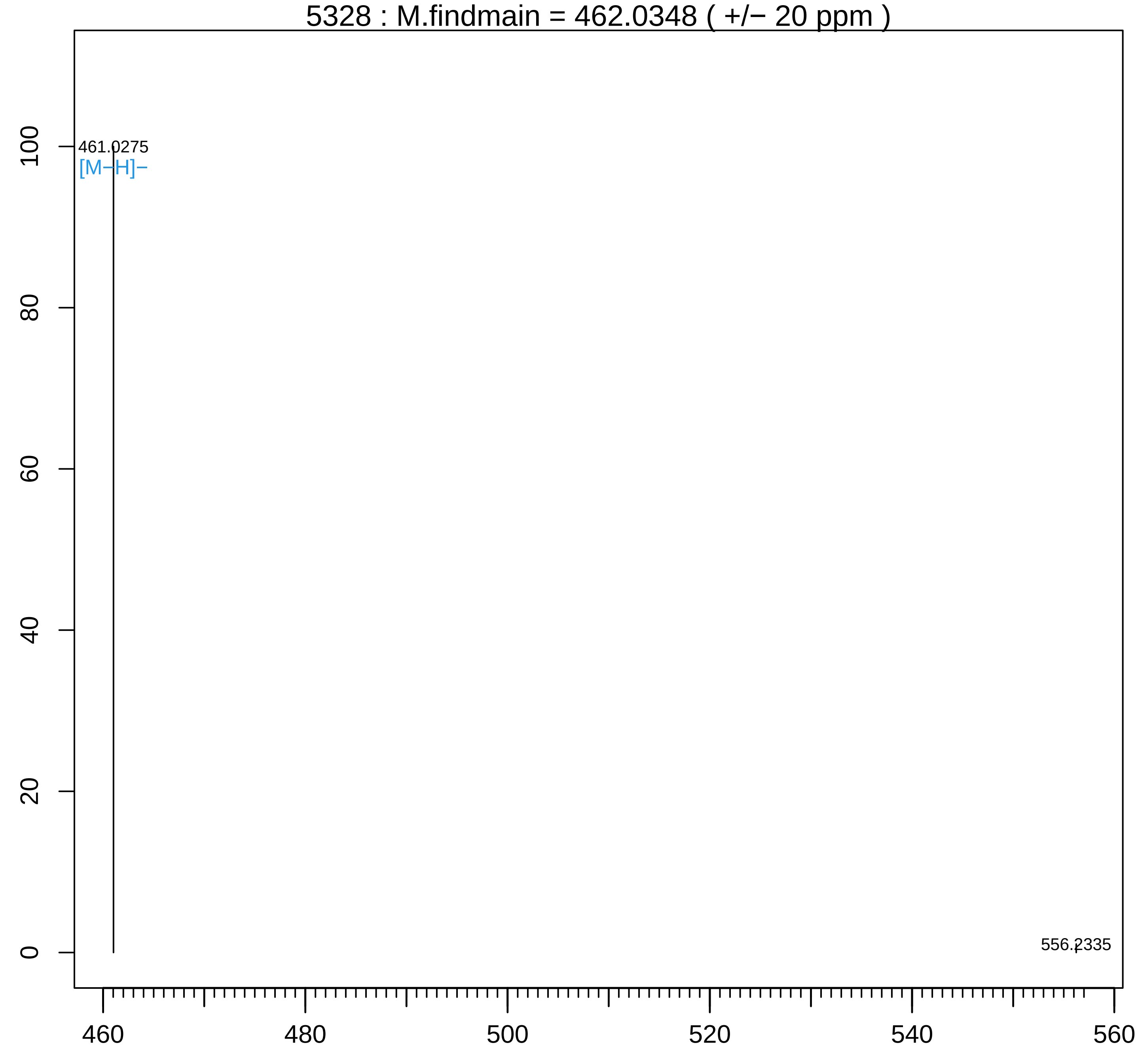
5328 : M.findmain = 462.0348 (+/− 20 ppm)
461.0275
[M−H]−
556.2335
0
20
40
60
80
100
460
480
500
520
540
560

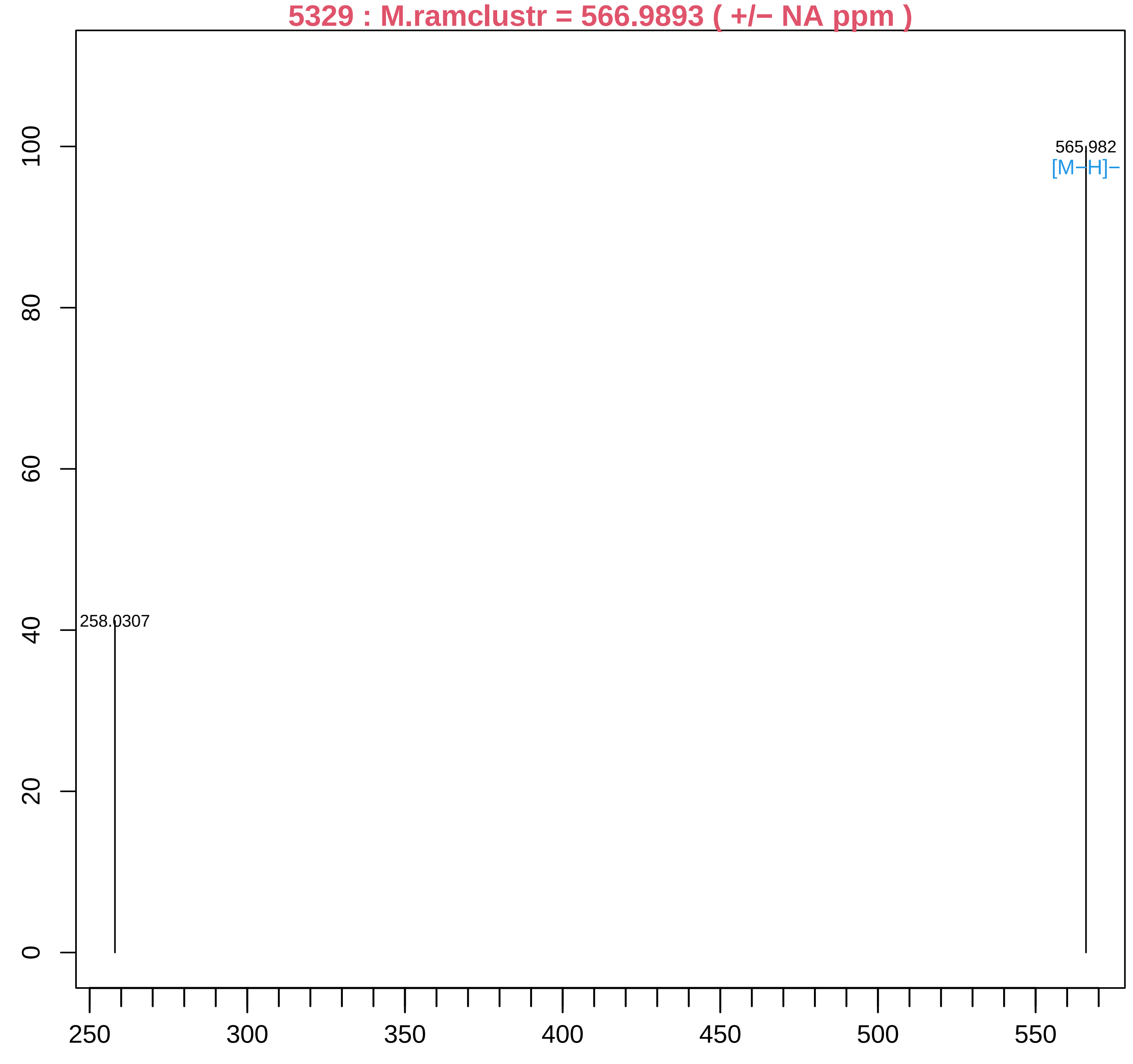
5329 : M.ramclustr = 566.9893 (+/− NA ppm)
565.982
[M−H]−
258.0307
0
20
40
60
80
100
250
300
350
400
450
500
550

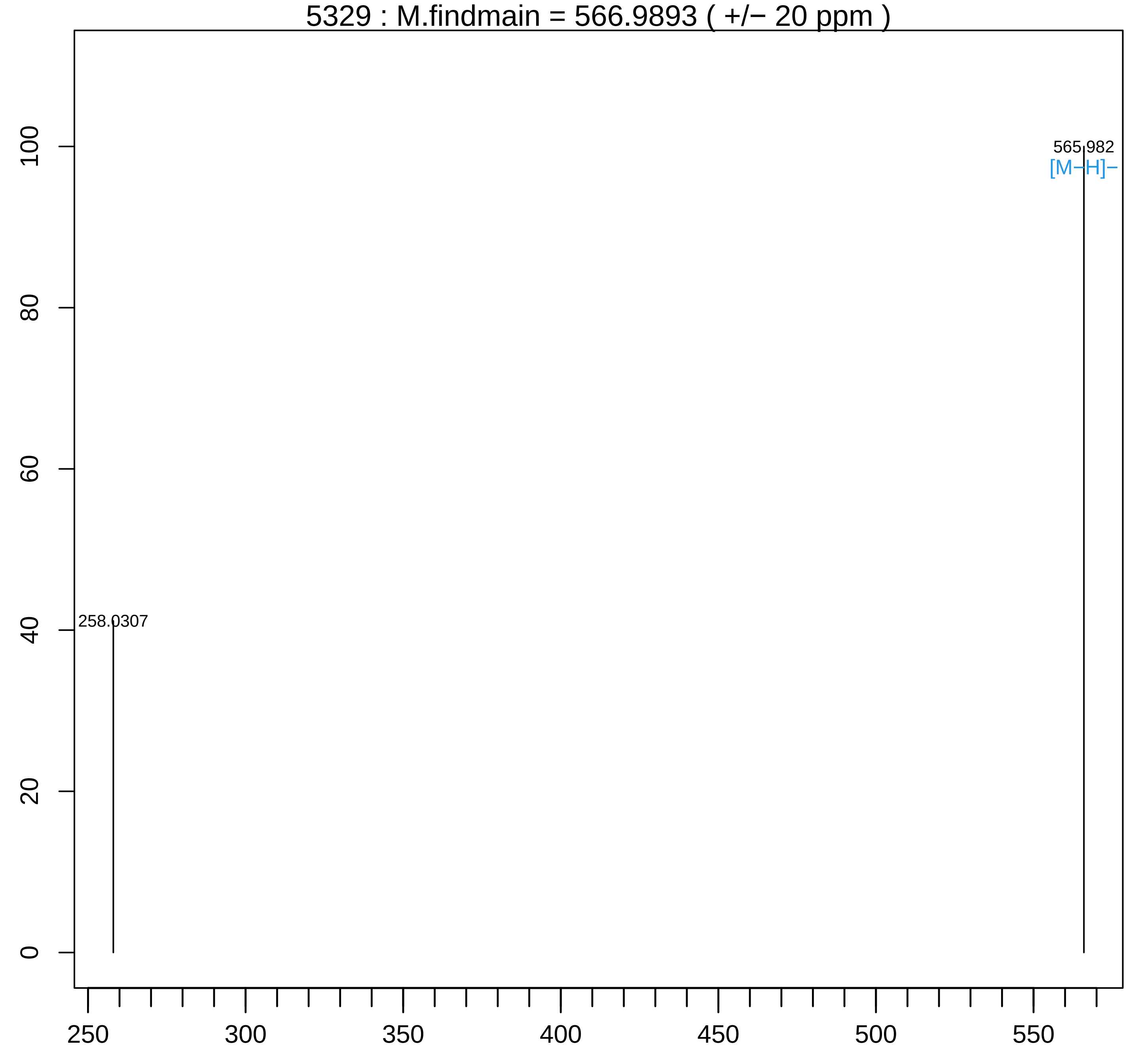
5329 : M.findmain = 566.9893 (+/− 20 ppm)
565.982
[M−H]−
258.0307
0
20
40
60
80
100
250
300
350
400
450
500
550

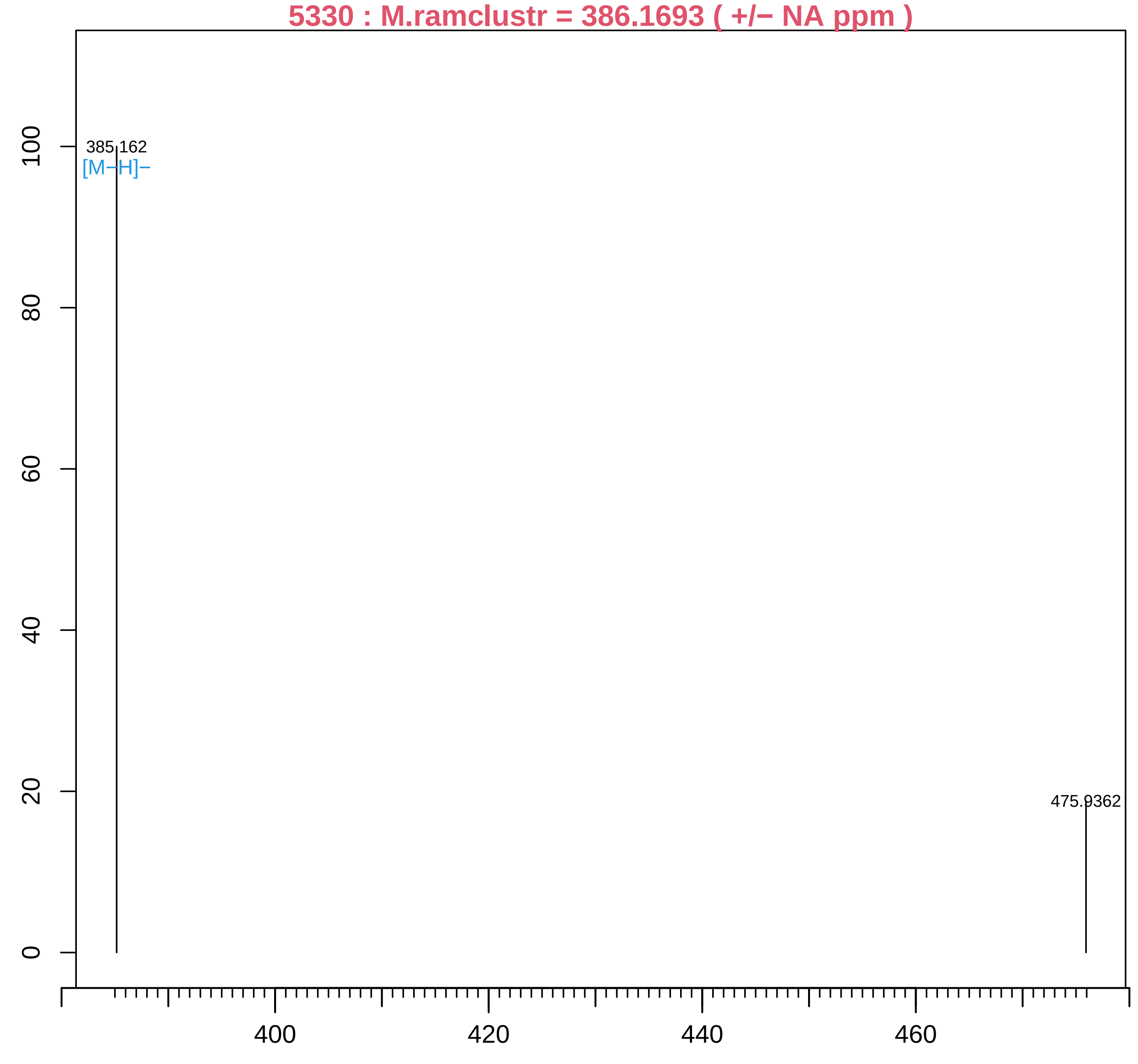
5330 : M.ramclustr = 386.1693 (+/− NA ppm)
385.162
[M−H]−
475.9362
0
20
40
60
80
100
400
420
440
460

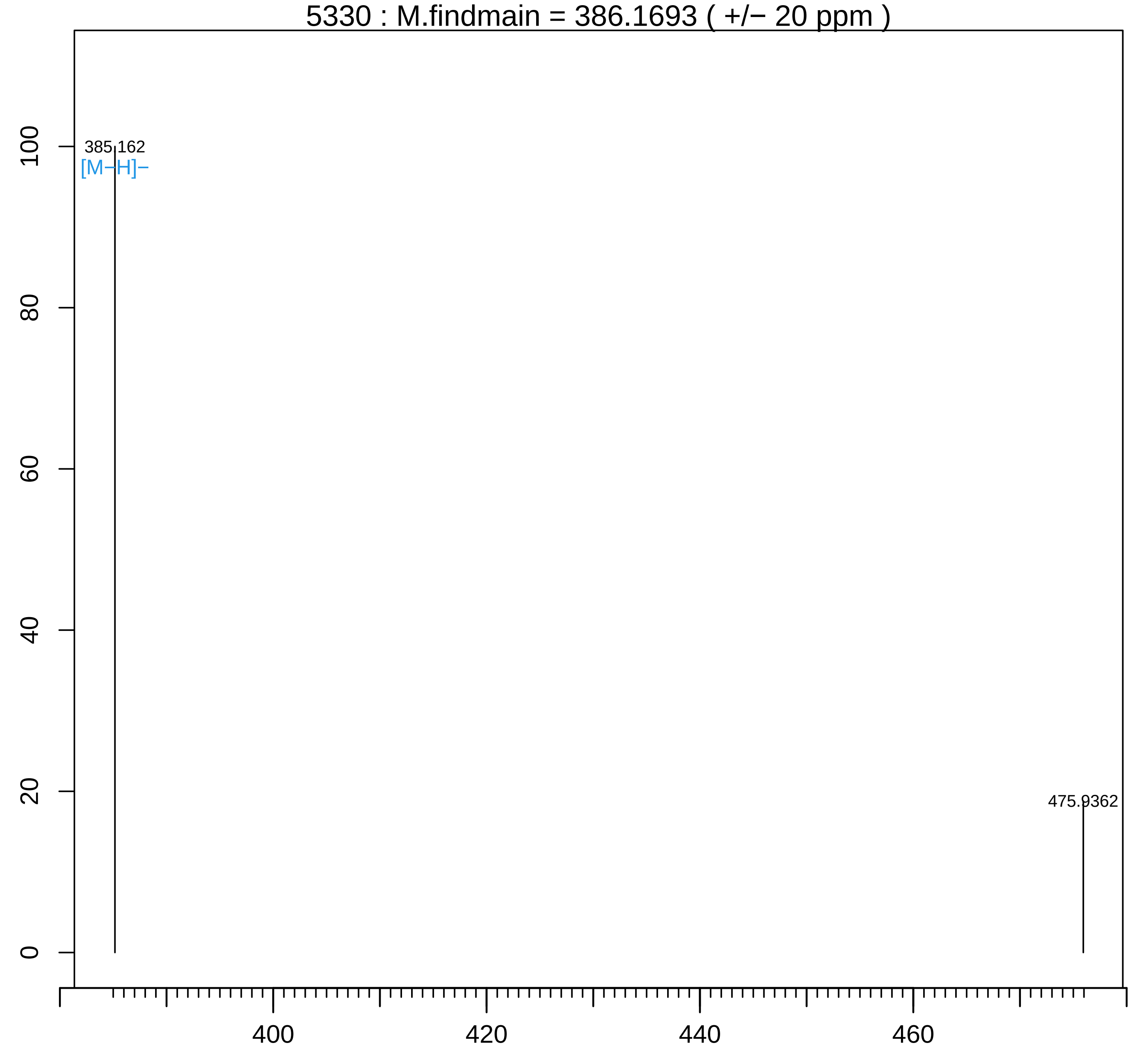
5330 : M.findmain = 386.1693 (+/− 20 ppm)
385.162
[M−H]−
475.9362
0
20
40
60
80
100
400
420
440
460

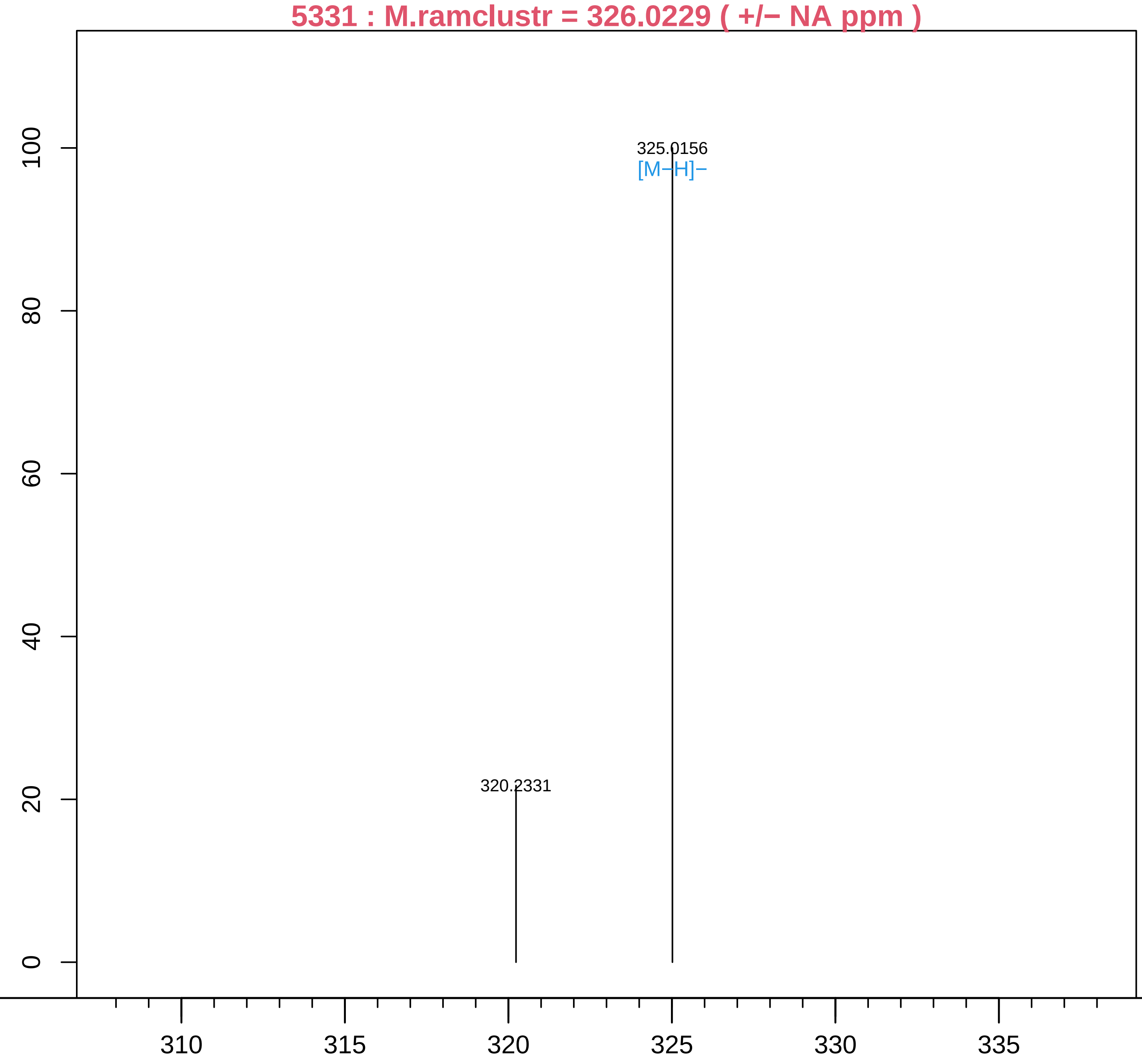
5331 : M.ramclustr = 326.0229 (+/− NA ppm)
325.0156
[M−H]−
320.2331
0
20
40
60
80
100
310
315
320
325
330
335

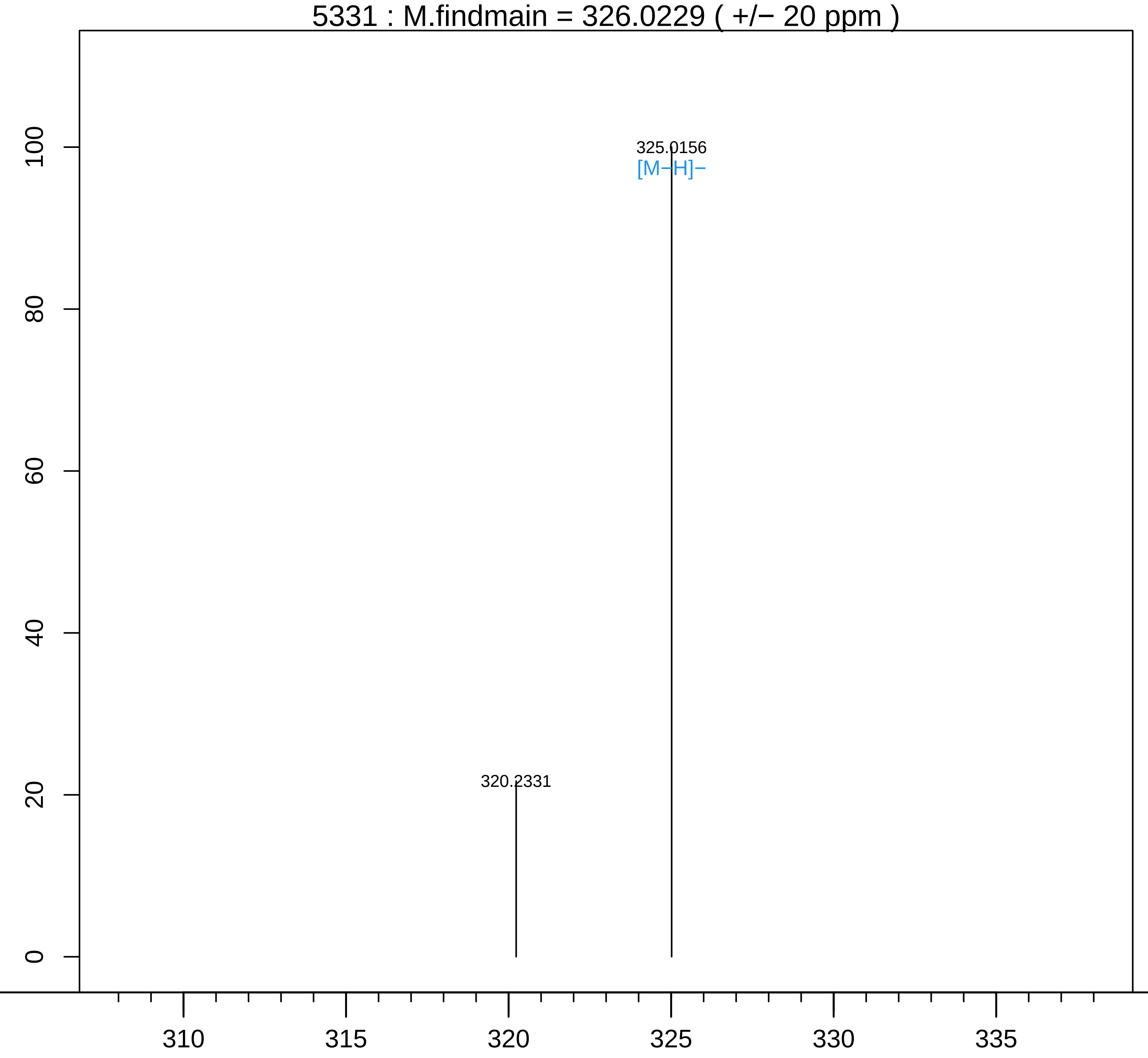
5331 : M.findmain = 326.0229 (+/− 20 ppm)
325.0156
[M−H]−
320.2331
0
20
40
60
80
100
310
315
320
325
330
335

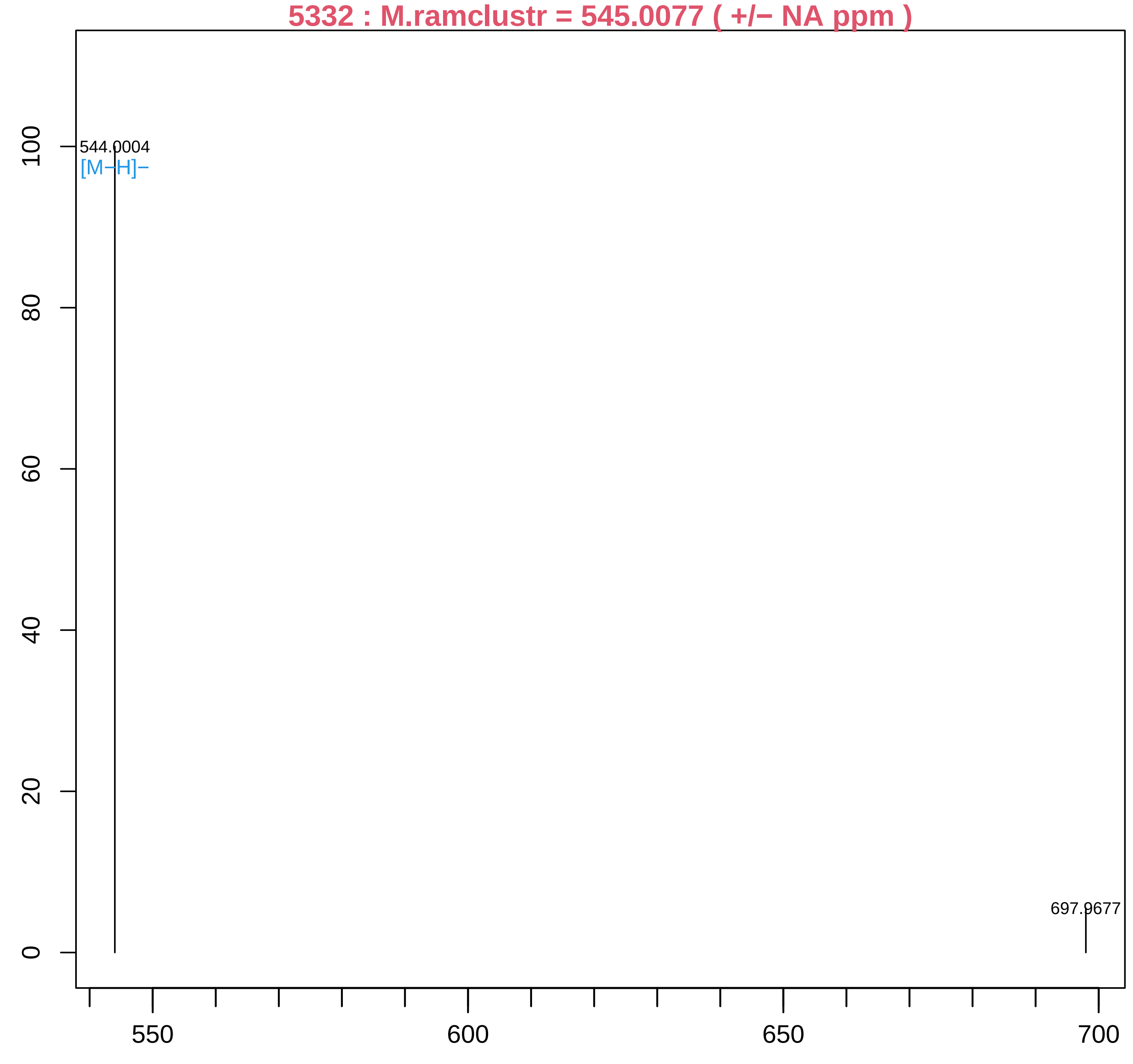
5332 : M.ramclustr = 545.0077 (+/− NA ppm)
544.0004
[M−H]−
697.9677
0
20
40
60
80
100
550
600
650
700

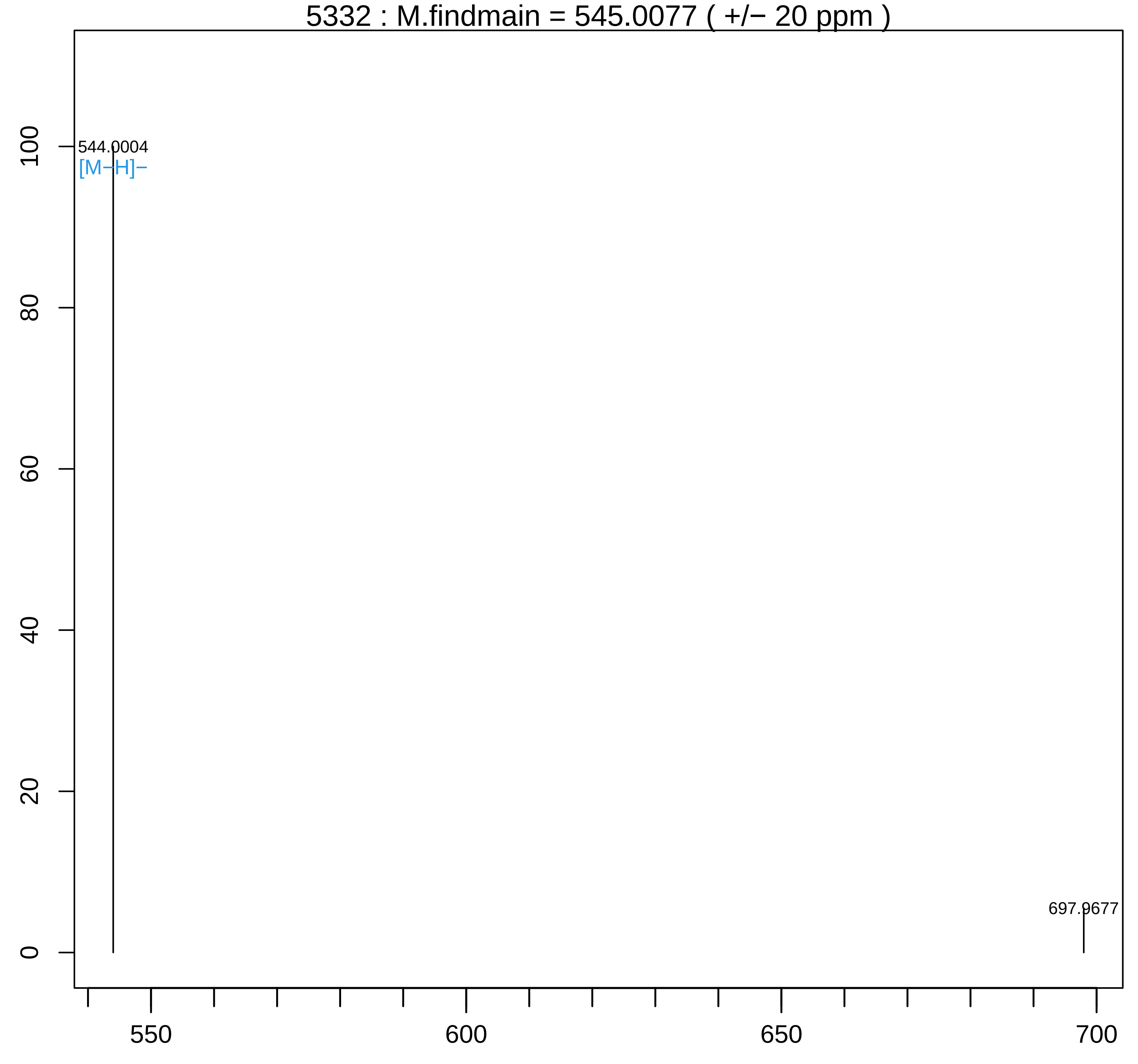
5332 : M.findmain = 545.0077 (+/− 20 ppm)
544.0004
[M−H]−
697.9677
0
20
40
60
80
100
550
600
650
700

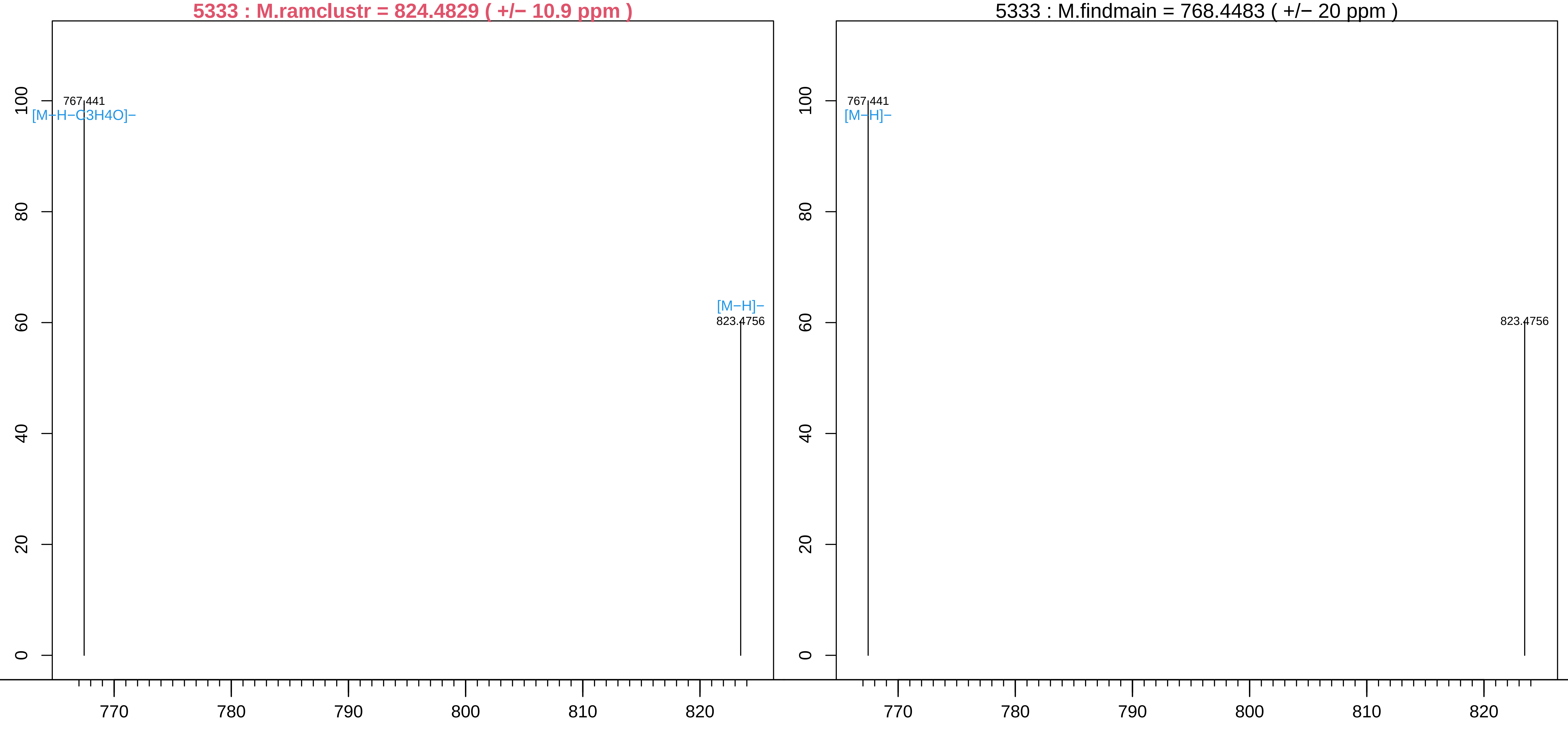
5333 : M.ramclustr = 824.4829 (+/− 10.9 ppm)
767.441
[M−H−C3H4O]−
[M−H]−
823.4756
5333 : M.findmain = 768.4483 (+/− 20 ppm)
767.441
[M−H]−
823.4756
0
20
40
60
80
100
770
780
790
800
810
820

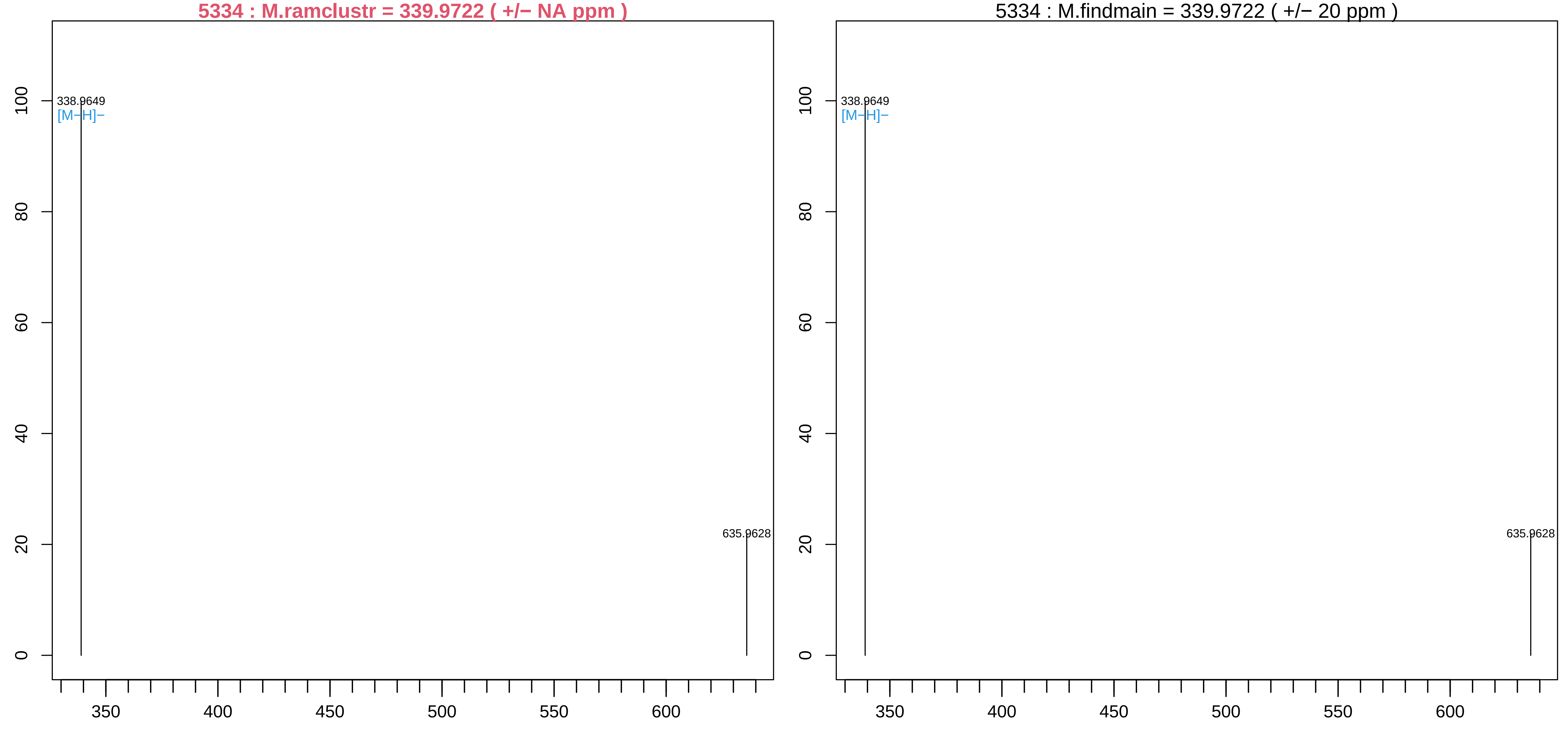
5334 : M.ramclustr = 339.9722 (+/− NA ppm)
338.9649
[M−H]−
635.9628
5334 : M.findmain = 339.9722 (+/− 20 ppm)
338.9649
[M−H]−
635.9628
0
20
40
60
80
100
350
400
450
500
550
600

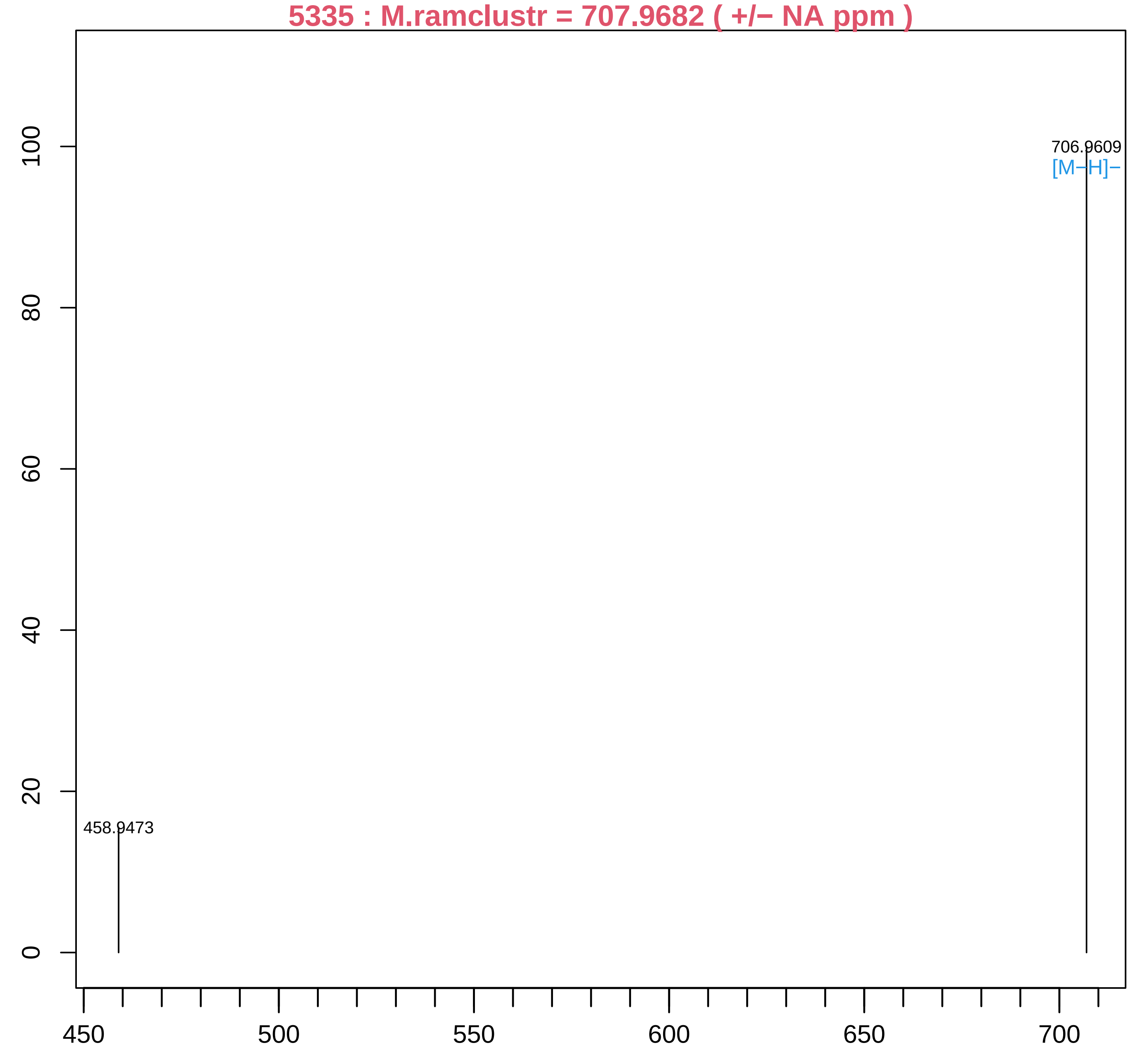
5335 : M.ramclustr = 707.9682 (+/− NA ppm)
706.9609
[M−H]−
458.9473
0
20
40
60
80
100
450
500
550
600
650
700

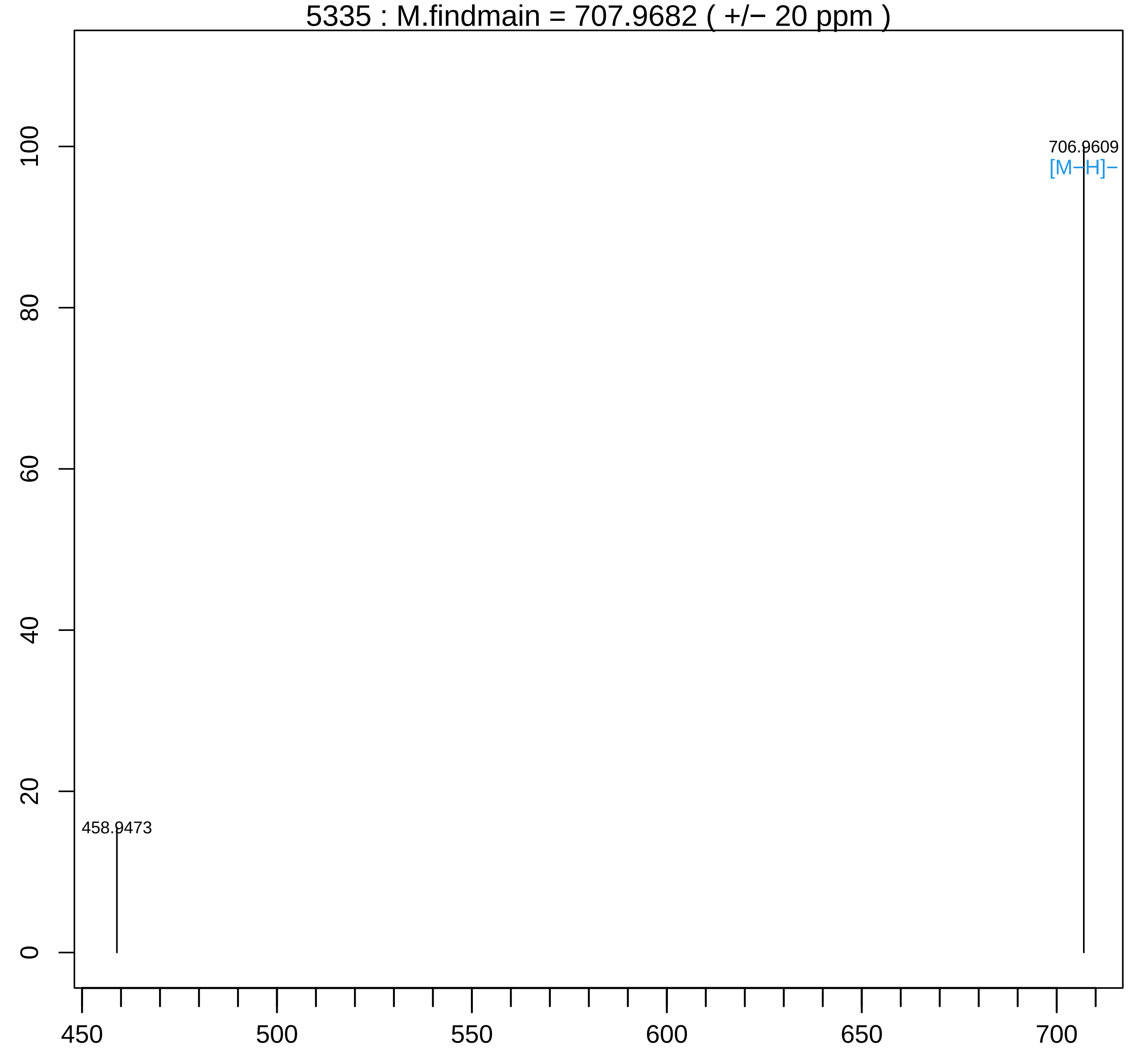
5335 : M.findmain = 707.9682 (+/− 20 ppm)
706.9609
[M−H]−
458.9473
0
20
40
60
80
100
450
500
550
600
650
700

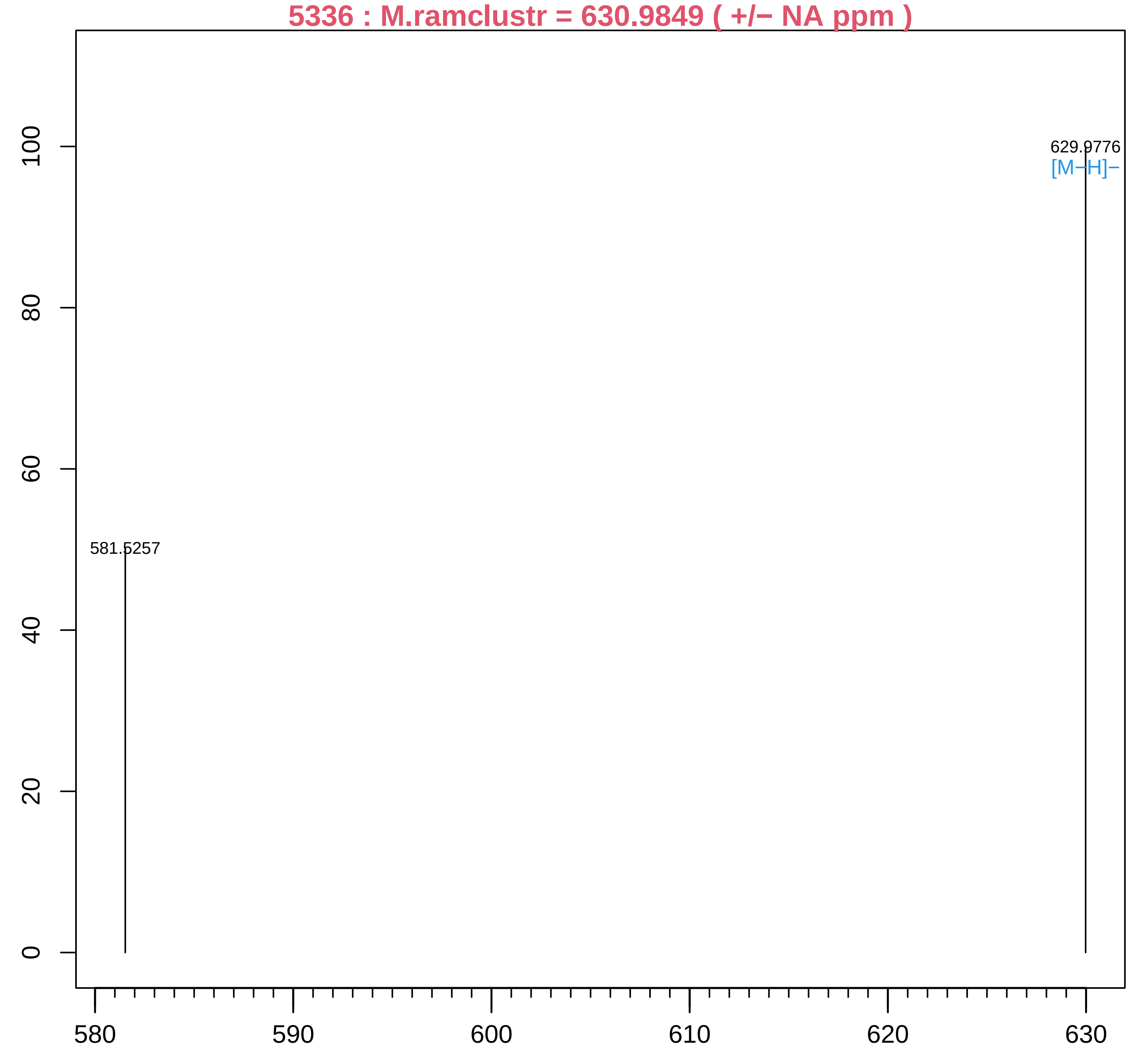

5336 : M.ramclustr = 630.9849 (+/− NA ppm)
629.9776
[M−H]−
581.5257
0
20
40
60
80
100
580
590
600
610
620
630

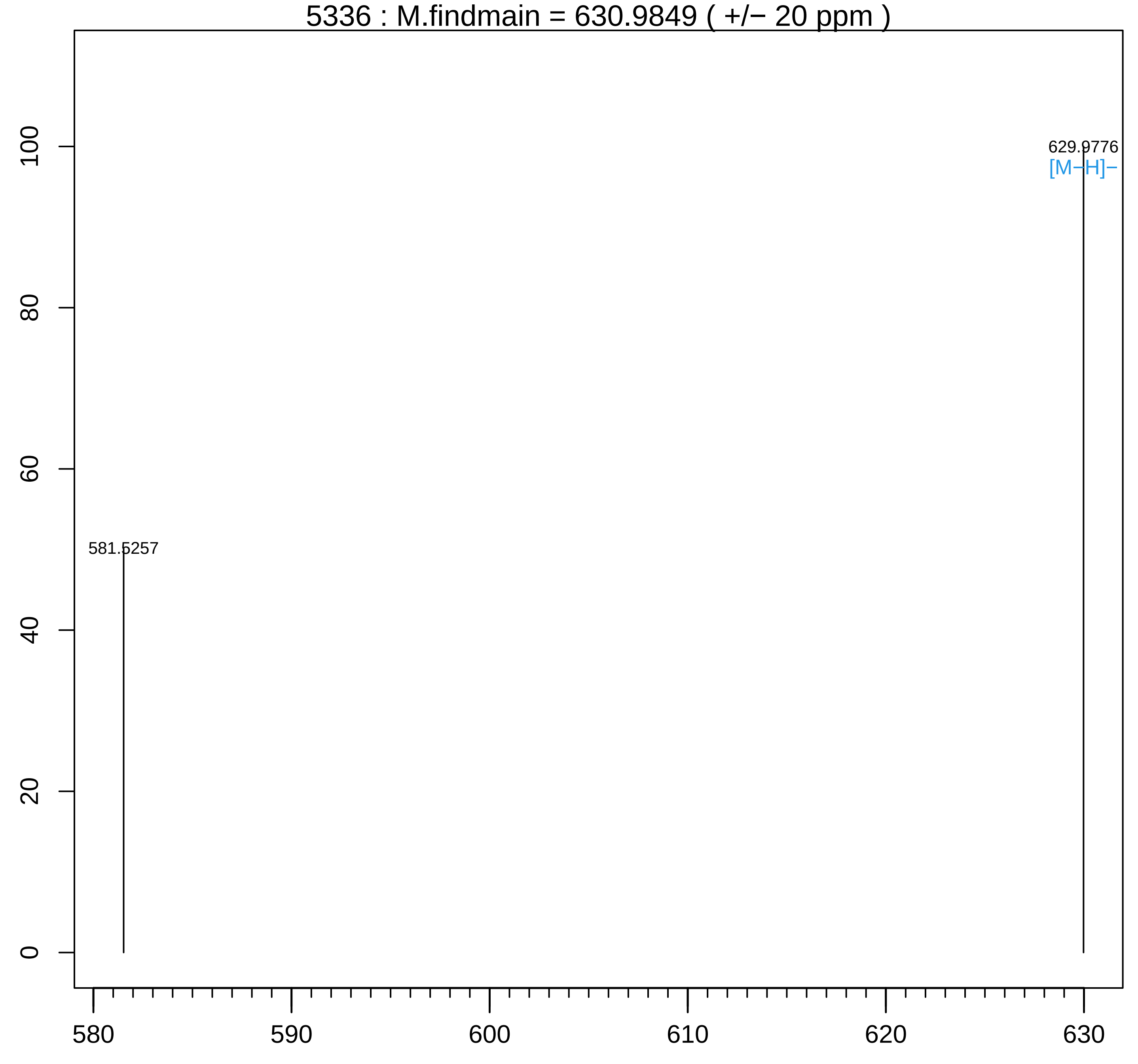

5336 : M.findmain = 630.9849 (+/− 20 ppm)
629.9776
[M−H]−
581.5257
0
20
40
60
80
100
580
590
600
610
620
630

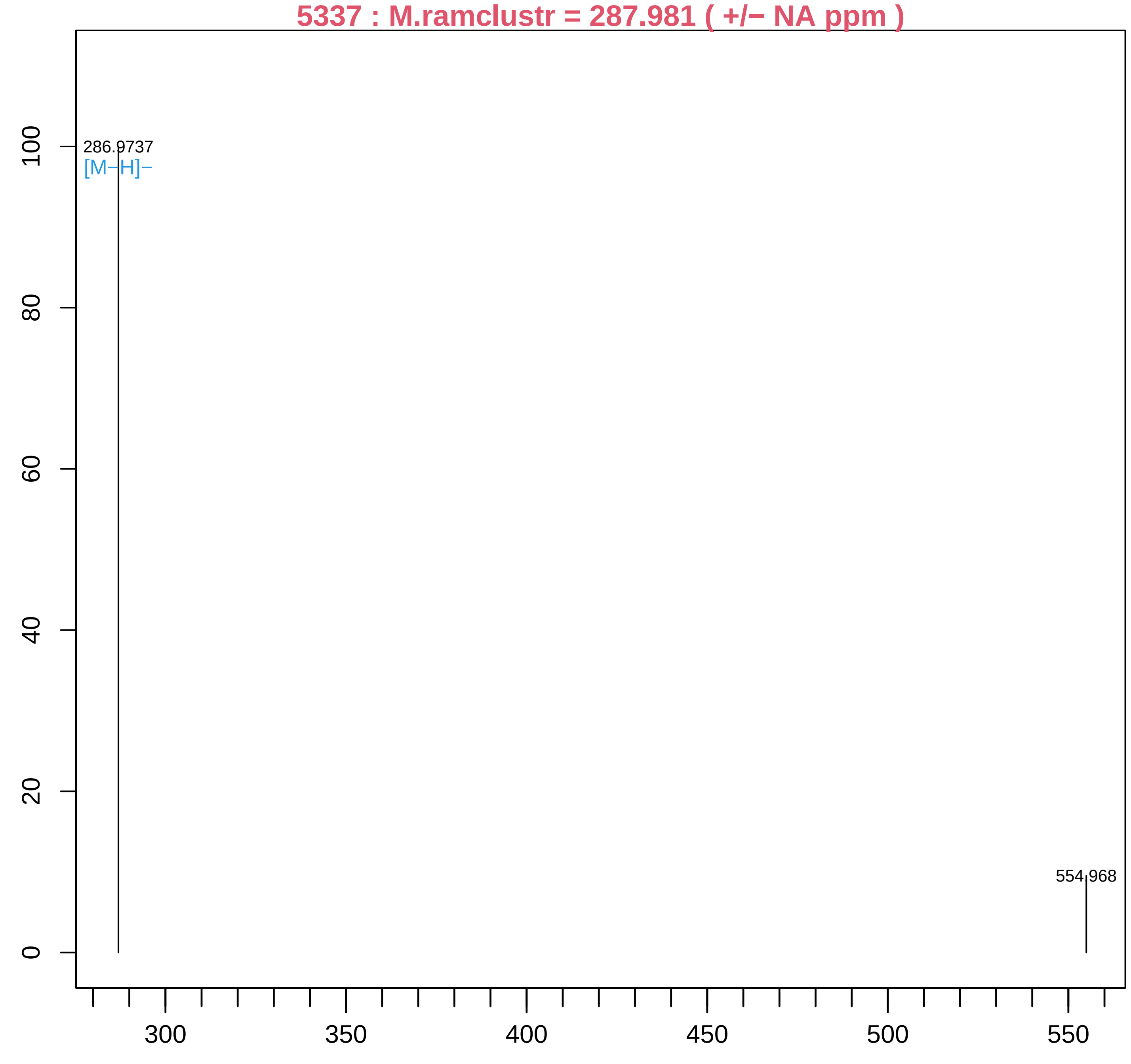

5337 : M.ramclustr = 287.981 (+/− NA ppm)
286.9737
[M−H]−
554.968
0
20
40
60
80
100
300
350
400
450
500
550

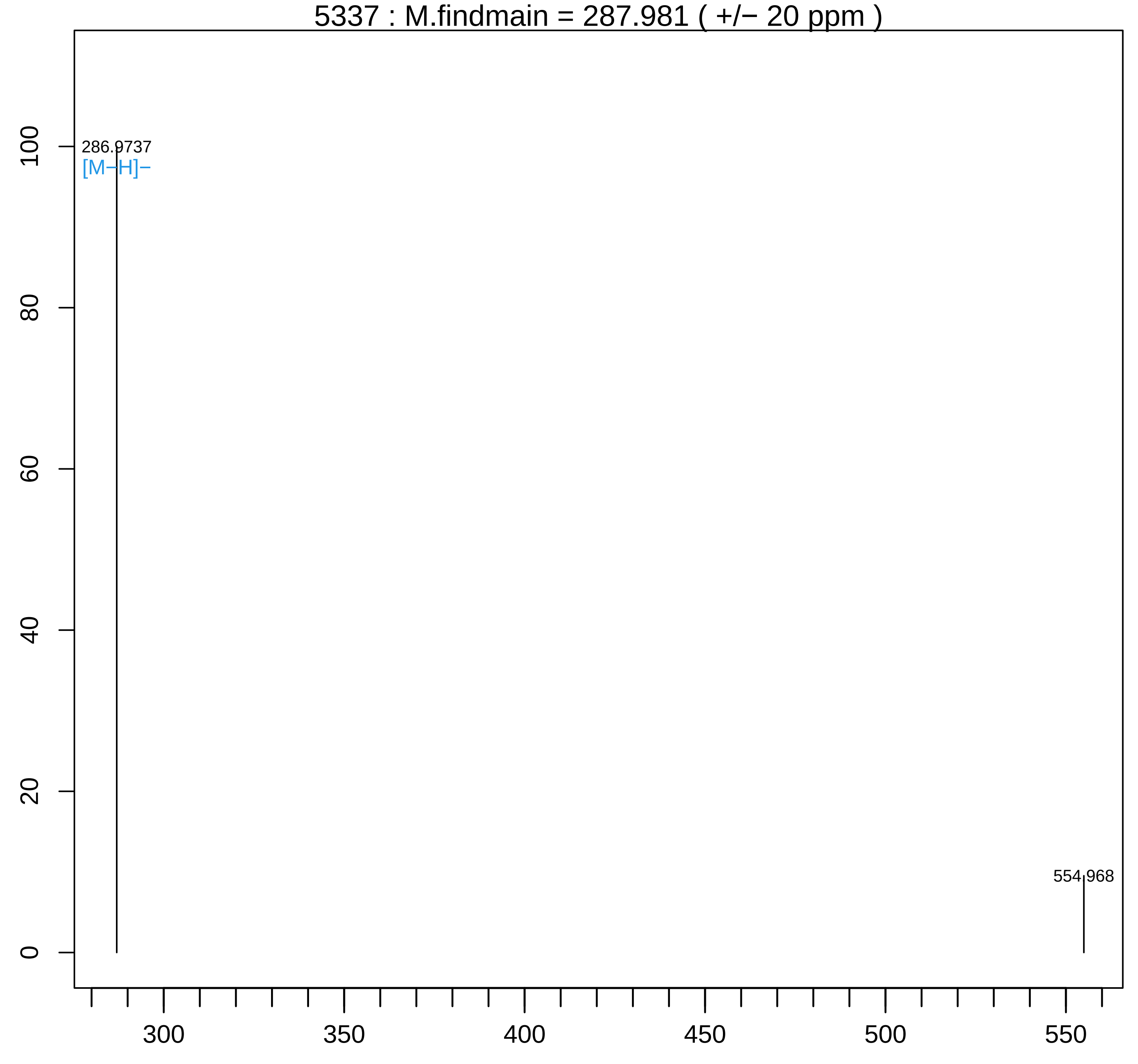

5337 : M.findmain = 287.981 (+/− 20 ppm)
286.9737
[M−H]−
554.968
0
20
40
60
80
100
300
350
400
450
500
550

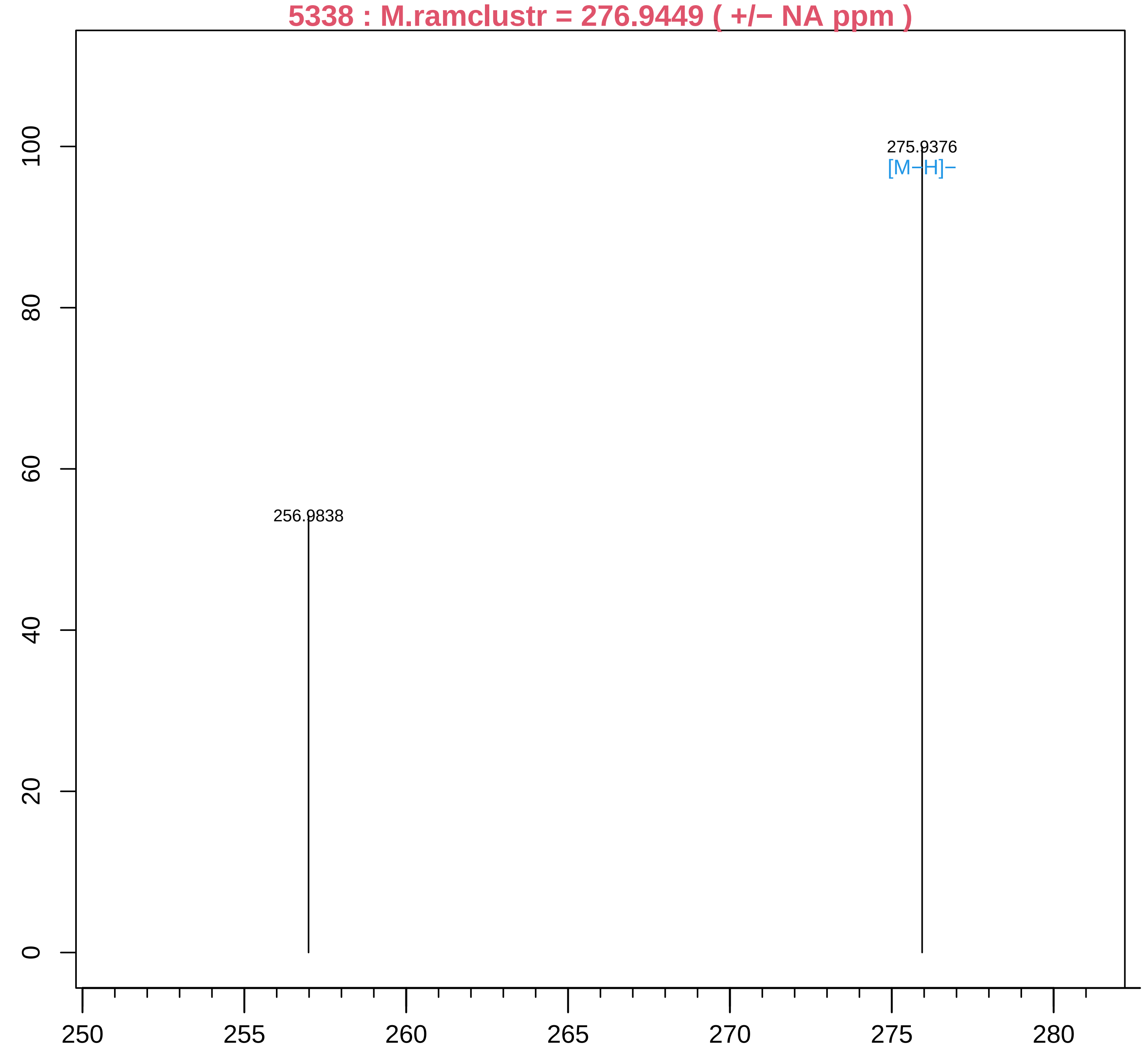
5338 : M.ramclustr = 276.9449 (+/− NA ppm)
275.9376
[M−H]−
256.9838
0
20
40
60
80
100
250
255
260
265
270
275
280

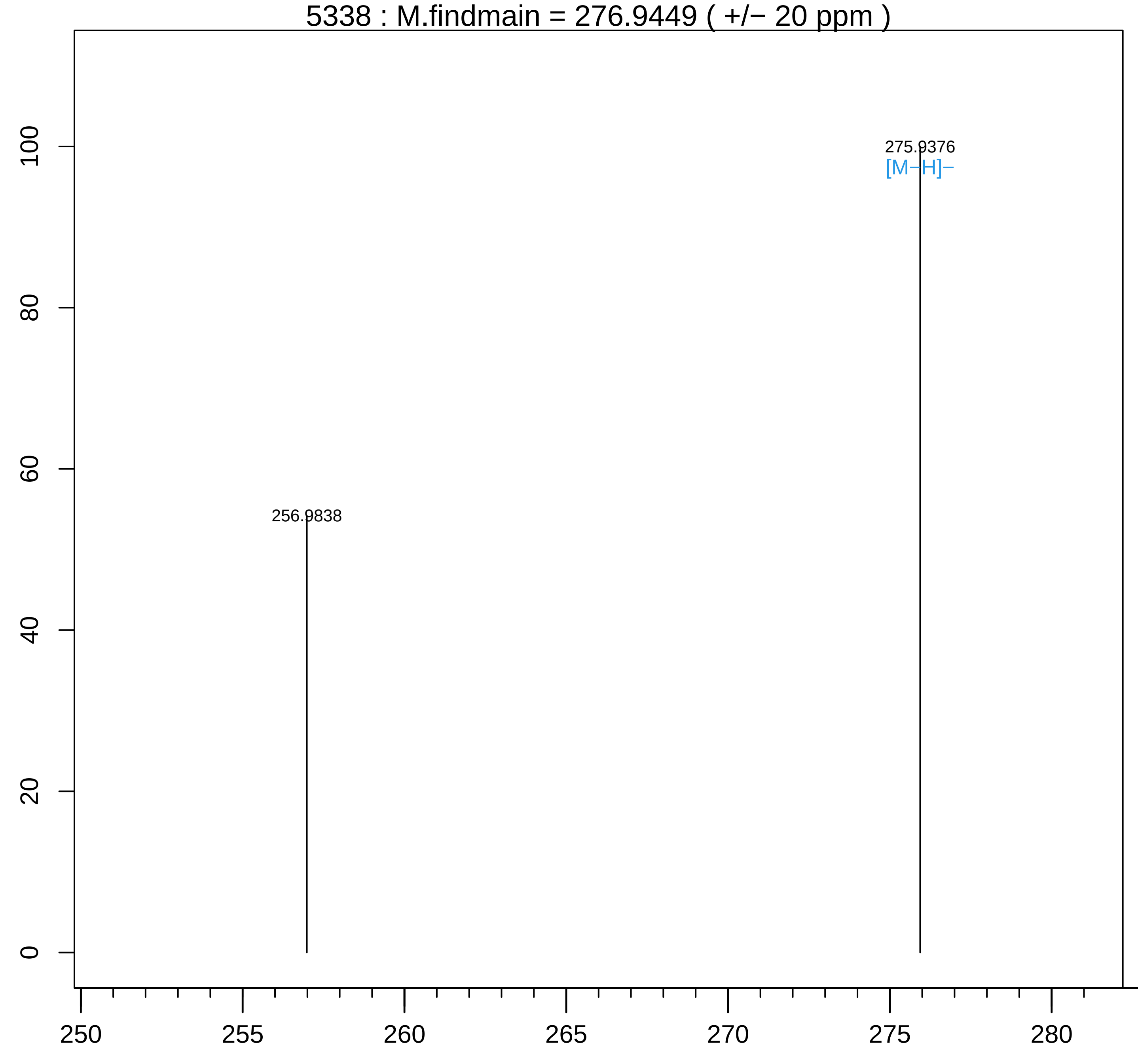
5338 : M.findmain = 276.9449 (+/− 20 ppm)
275.9376
[M−H]−
256.9838
0
20
40
60
80
100
250
255
260
265
270
275
280

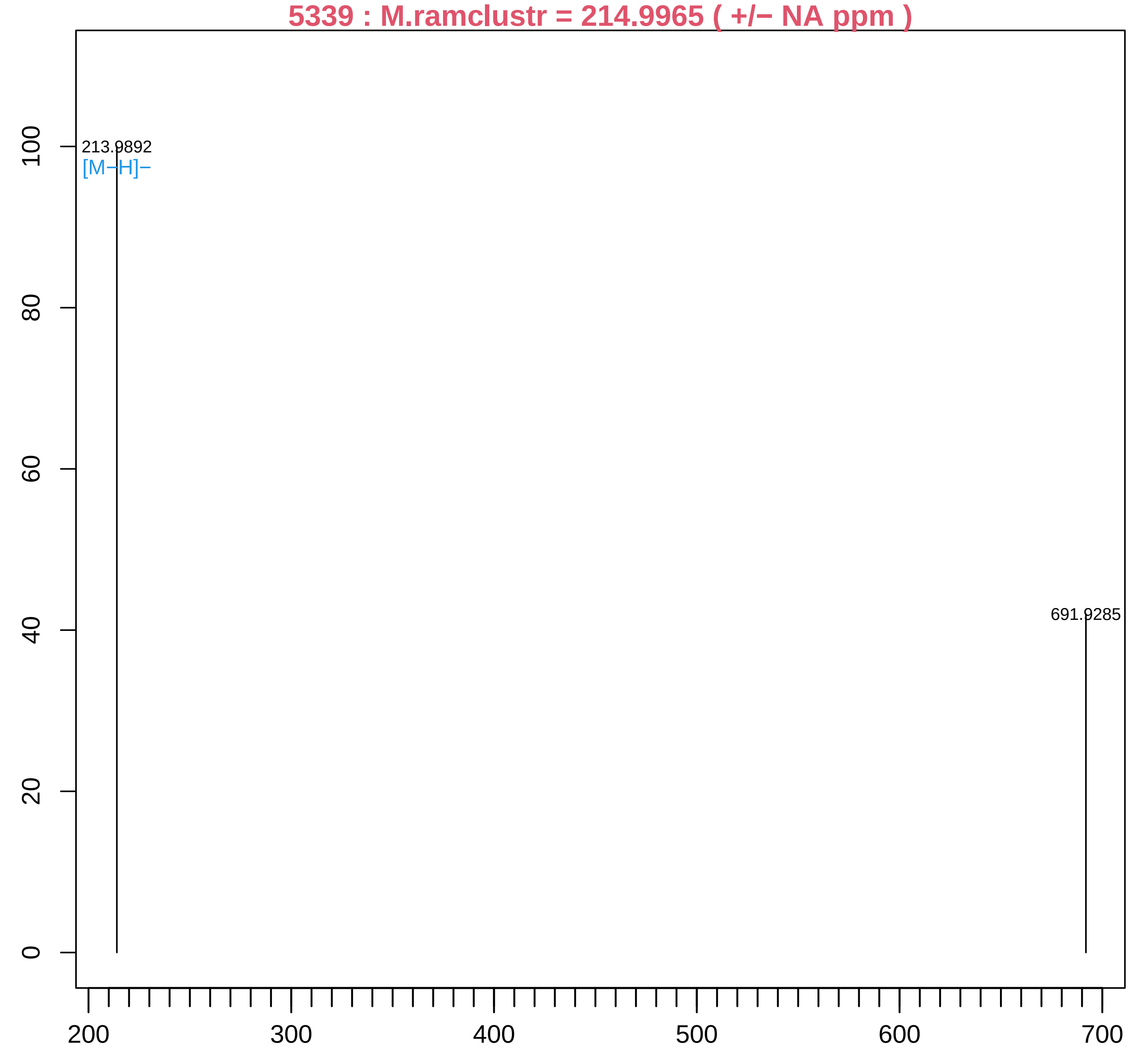
5339 : M.ramclustr = 214.9965 (+/− NA ppm)
213.9892
[M−H]−
691.9285
0
20
40
60
80
100
200
300
400
500
600
700

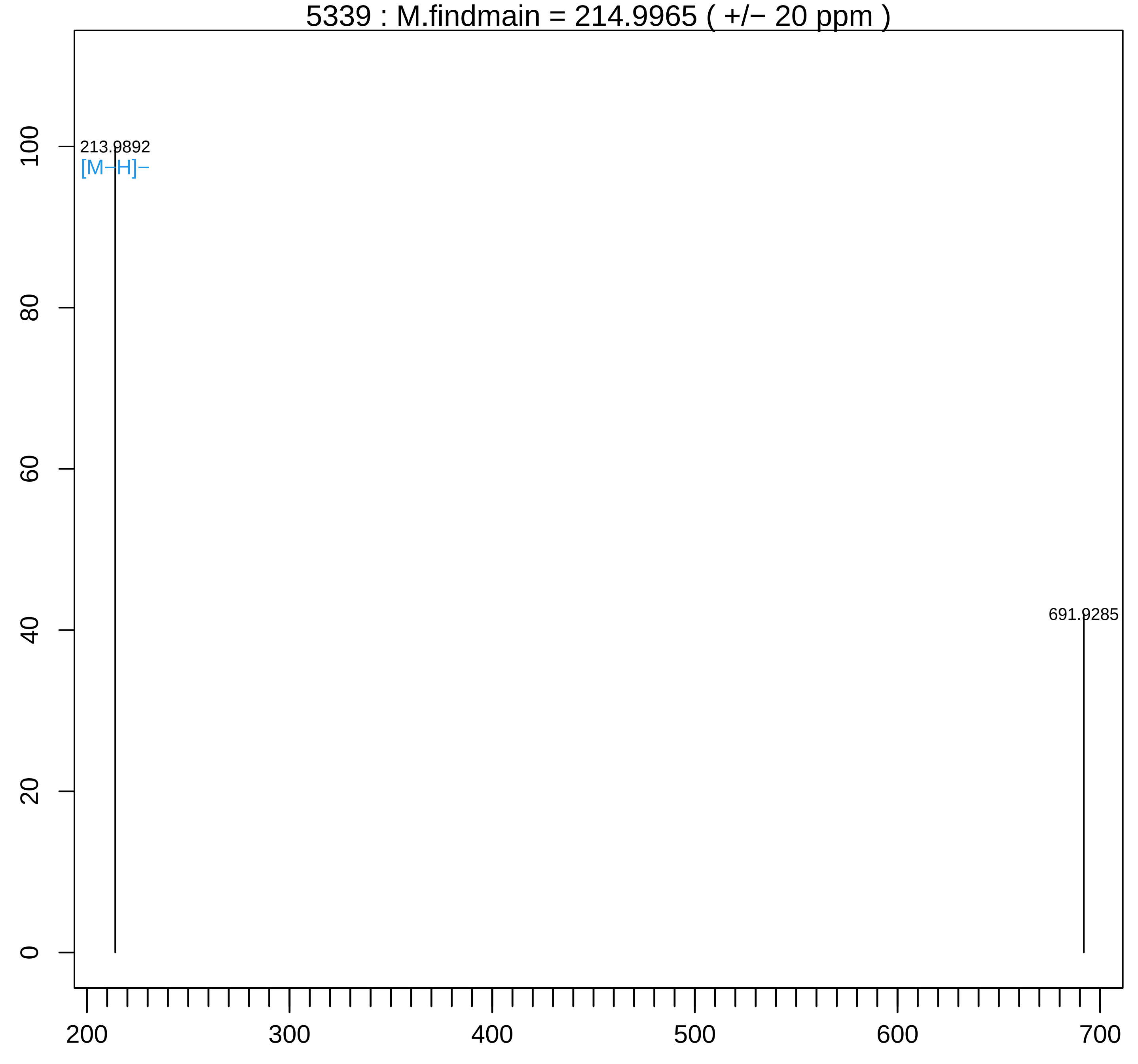
5339 : M.findmain = 214.9965 (+/− 20 ppm)
213.9892
[M−H]−
691.9285
0
20
40
60
80
100
200
300
400
500
600
700

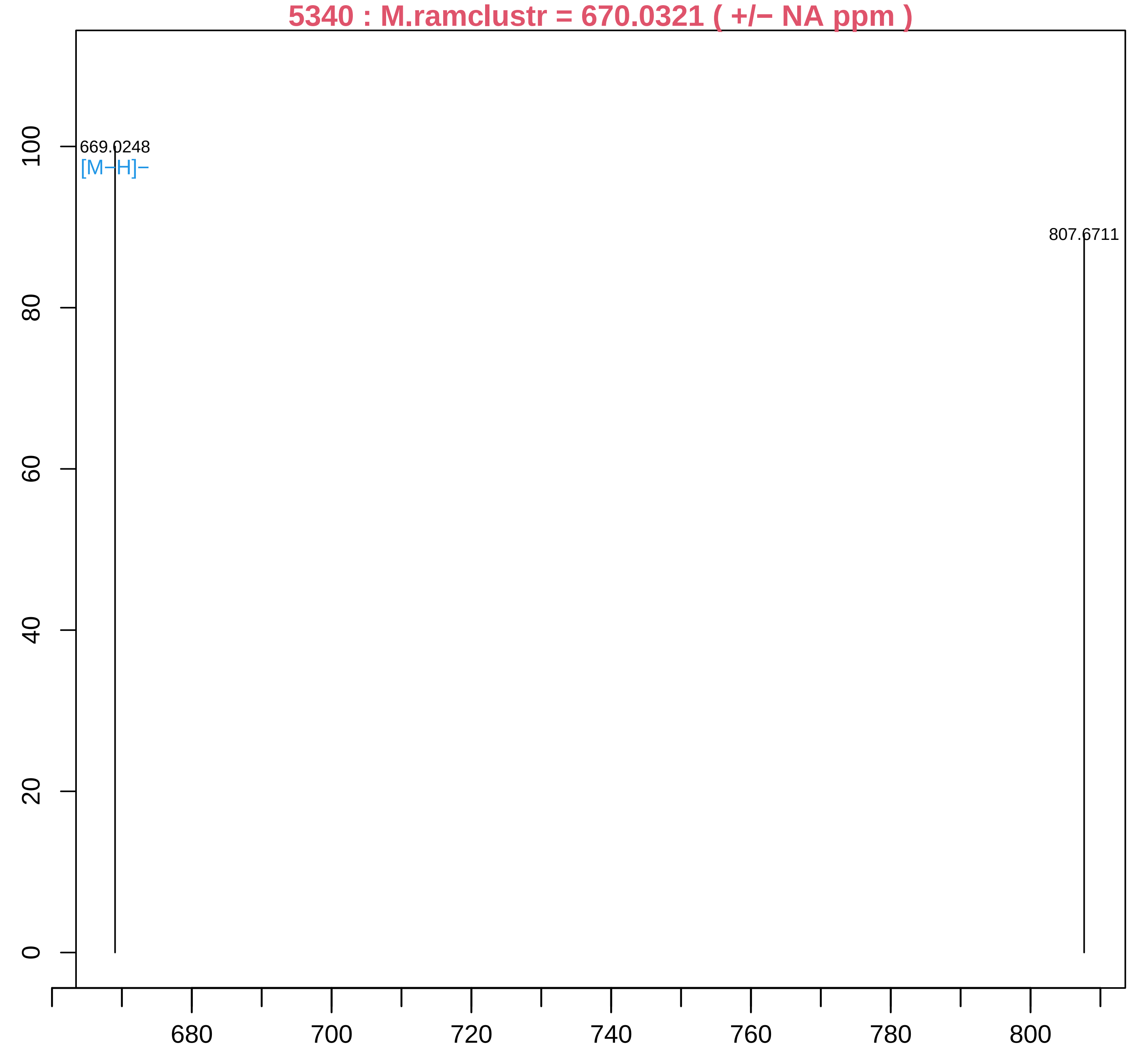
5340 : M.ramclustr = 670.0321 (+/− NA ppm)
669.0248
[M−H]−
807.6711
0
20
40
60
80
100
680
700
720
740
760
780
800

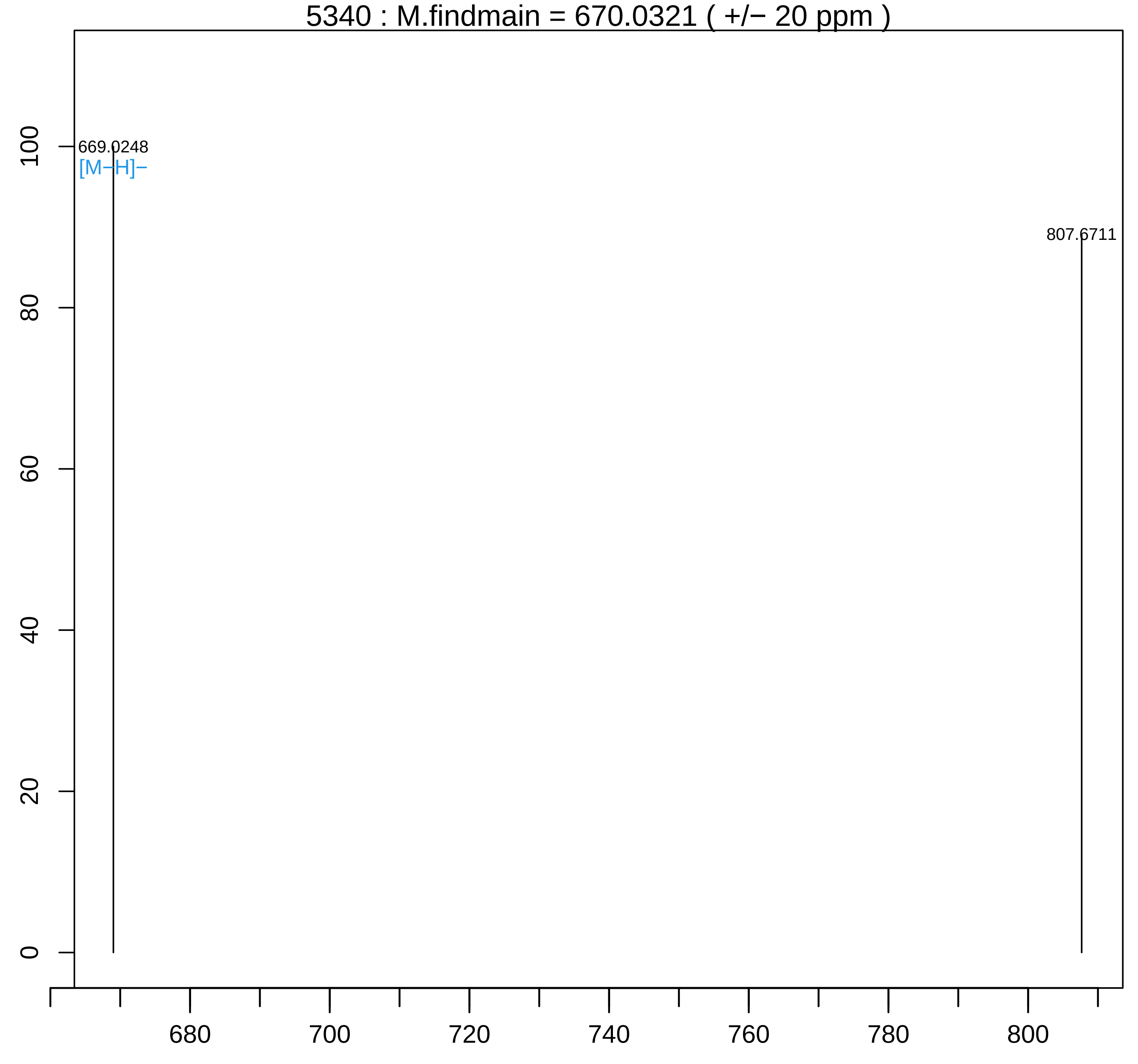
5340 : M.findmain = 670.0321 (+/− 20 ppm)
669.0248
[M−H]−
807.6711
0
20
40
60
80
100
680
700
720
740
760
780
800

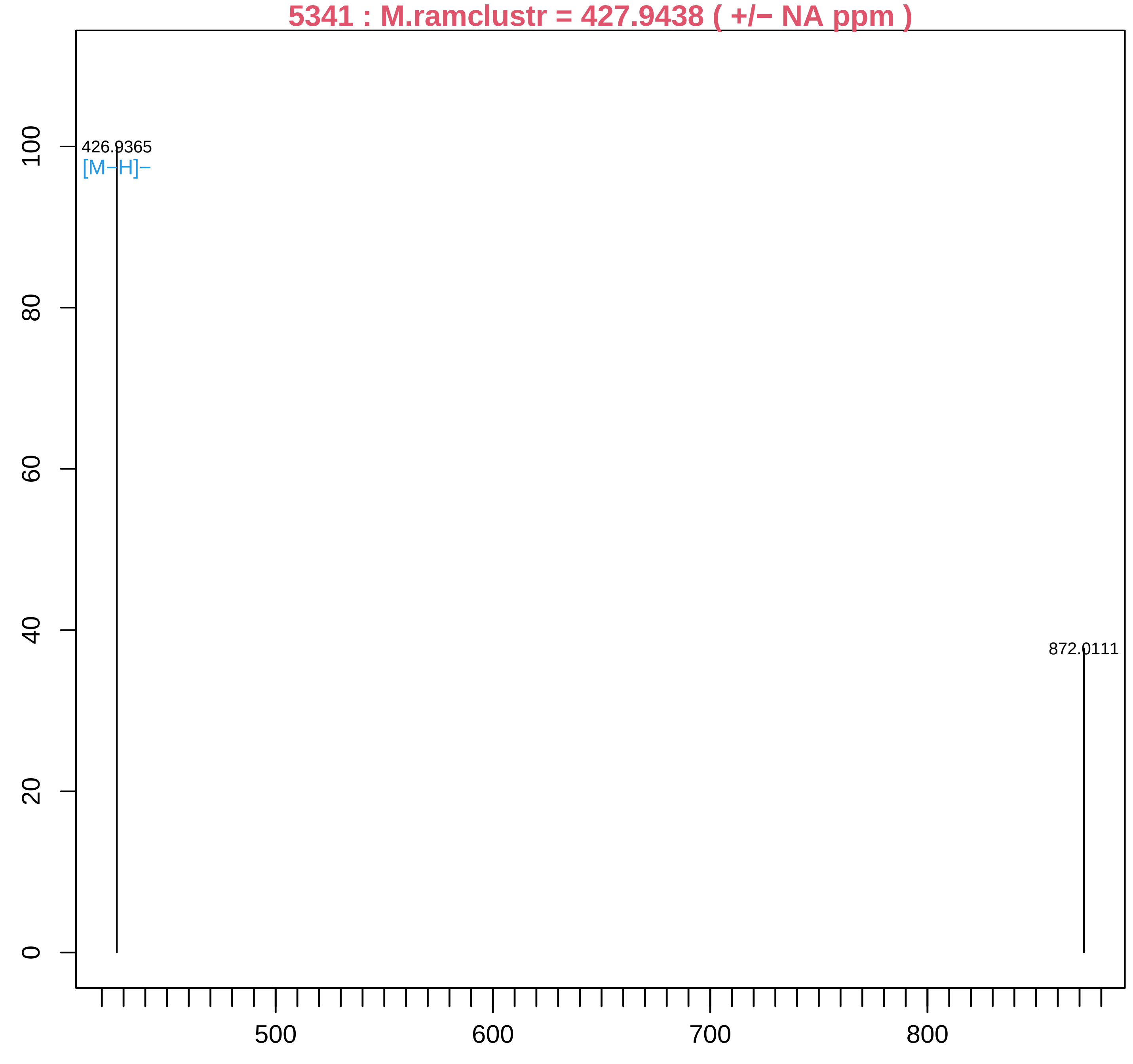
5341 : M.ramclustr = 427.9438 (+/− NA ppm)
426.9365
[M−H]−
872.0111
0
20
40
60
80
100
500
600
700
800

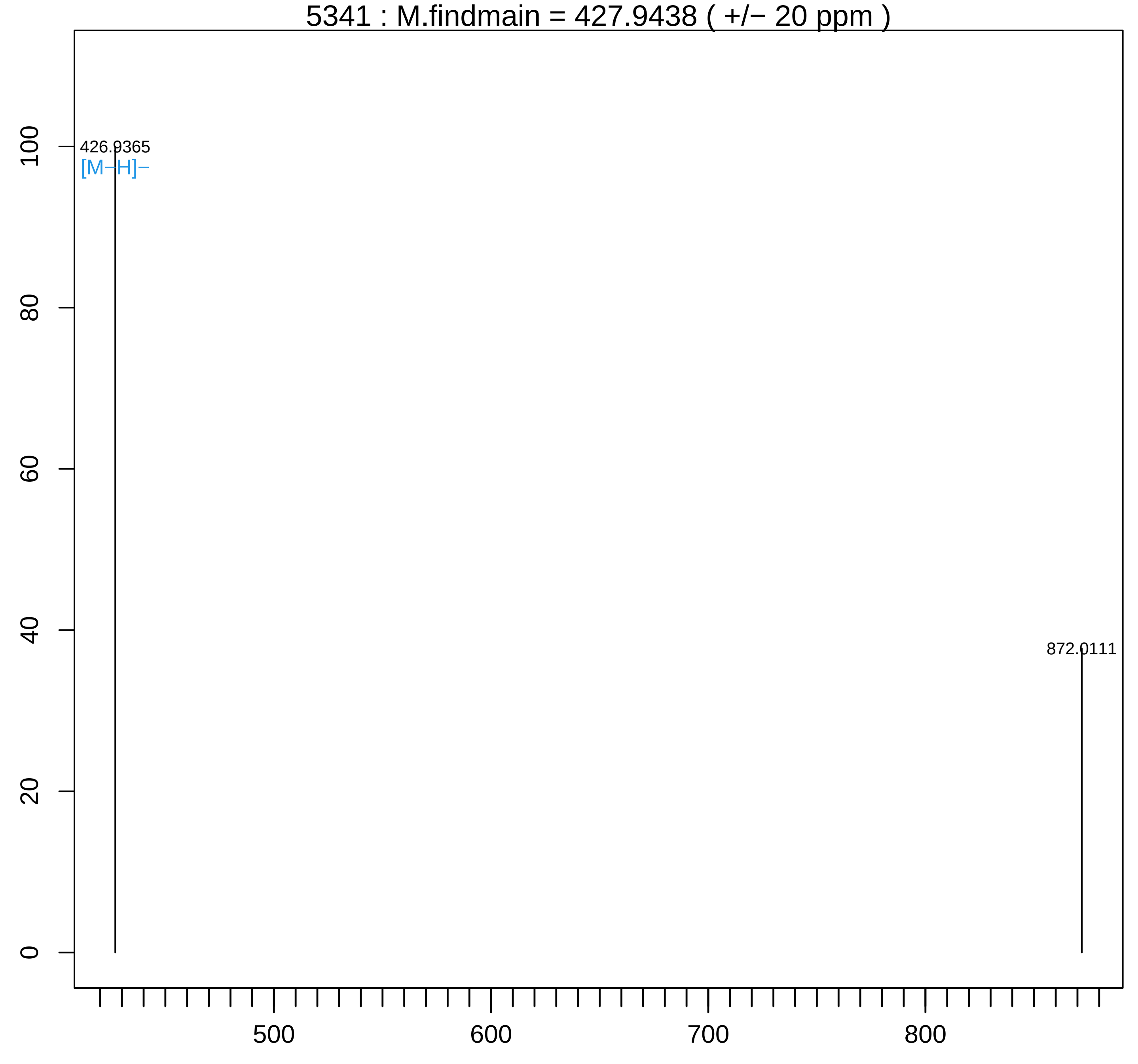
5341 : M.findmain = 427.9438 (+/− 20 ppm)
426.9365
[M−H]−
872.0111
0
20
40
60
80
100
500
600
700
800

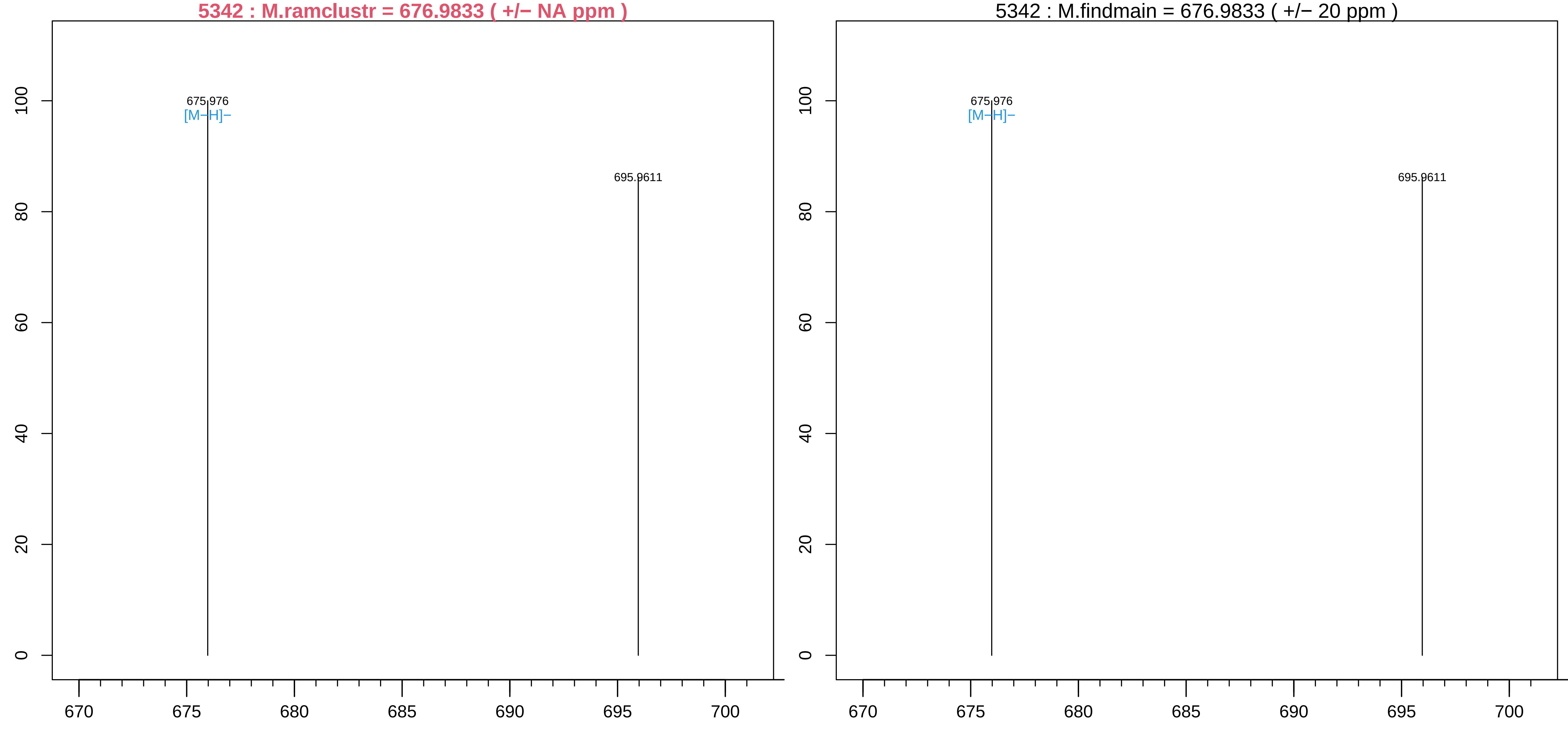
5342 : M.ramclustr = 676.9833 (+/− NA ppm)
675.976
[M−H]−
695.9611
5342 : M.findmain = 676.9833 (+/− 20 ppm)
675.976
[M−H]−
695.9611
0
20
40
60
80
100
670
675
680
685
690
695
700

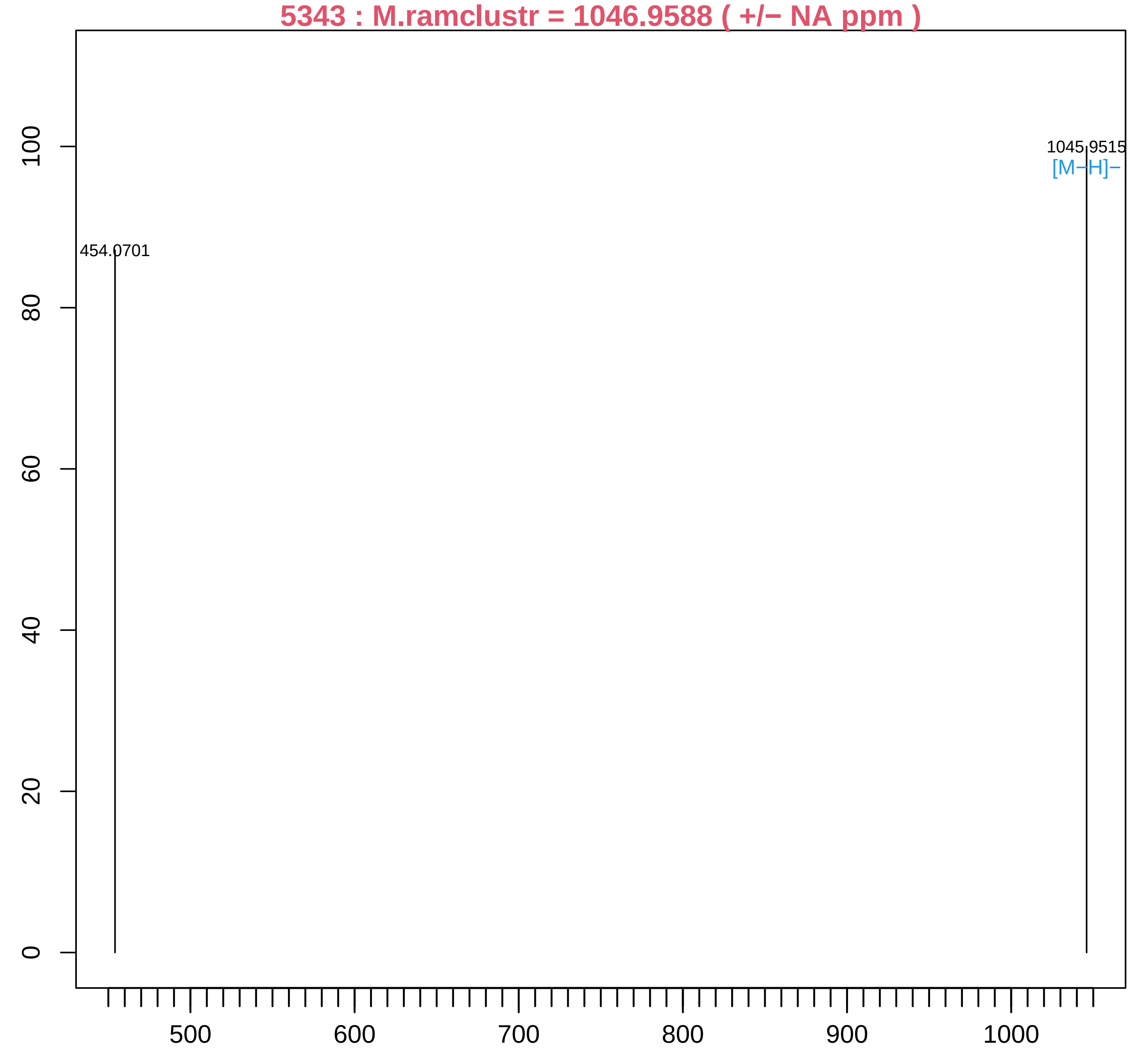

5343 : M.ramclustr = 1046.9588 (+/− NA ppm)
454.0701
1045.9515
[M−H]−
0
20
40
60
80
100
500
600
700
800
900
1000

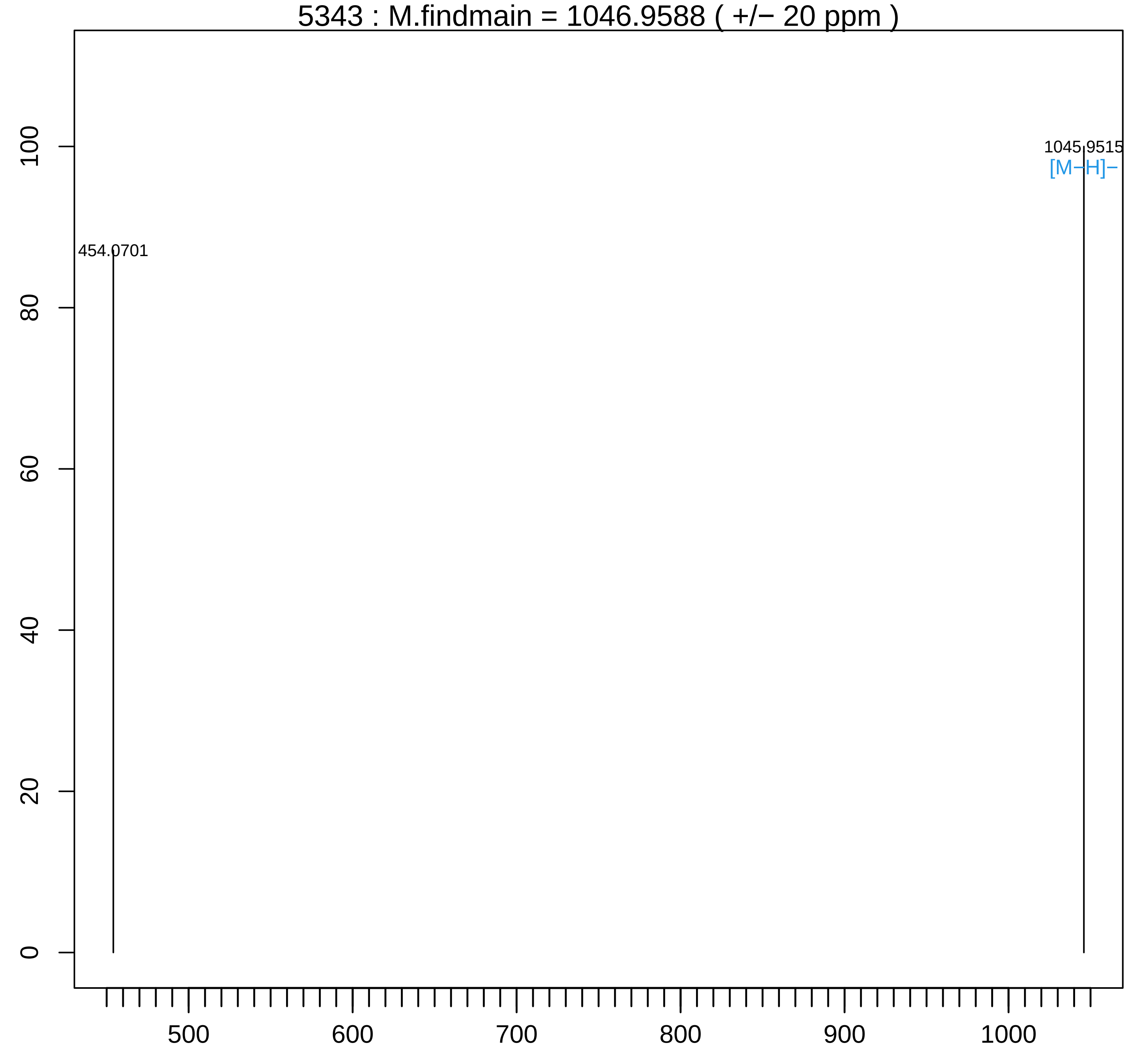

5343 : M.findmain = 1046.9588 (+/− 20 ppm)
454.0701
1045.9515
[M−H]−
0
20
40
60
80
100
500
600
700
800
900
1000

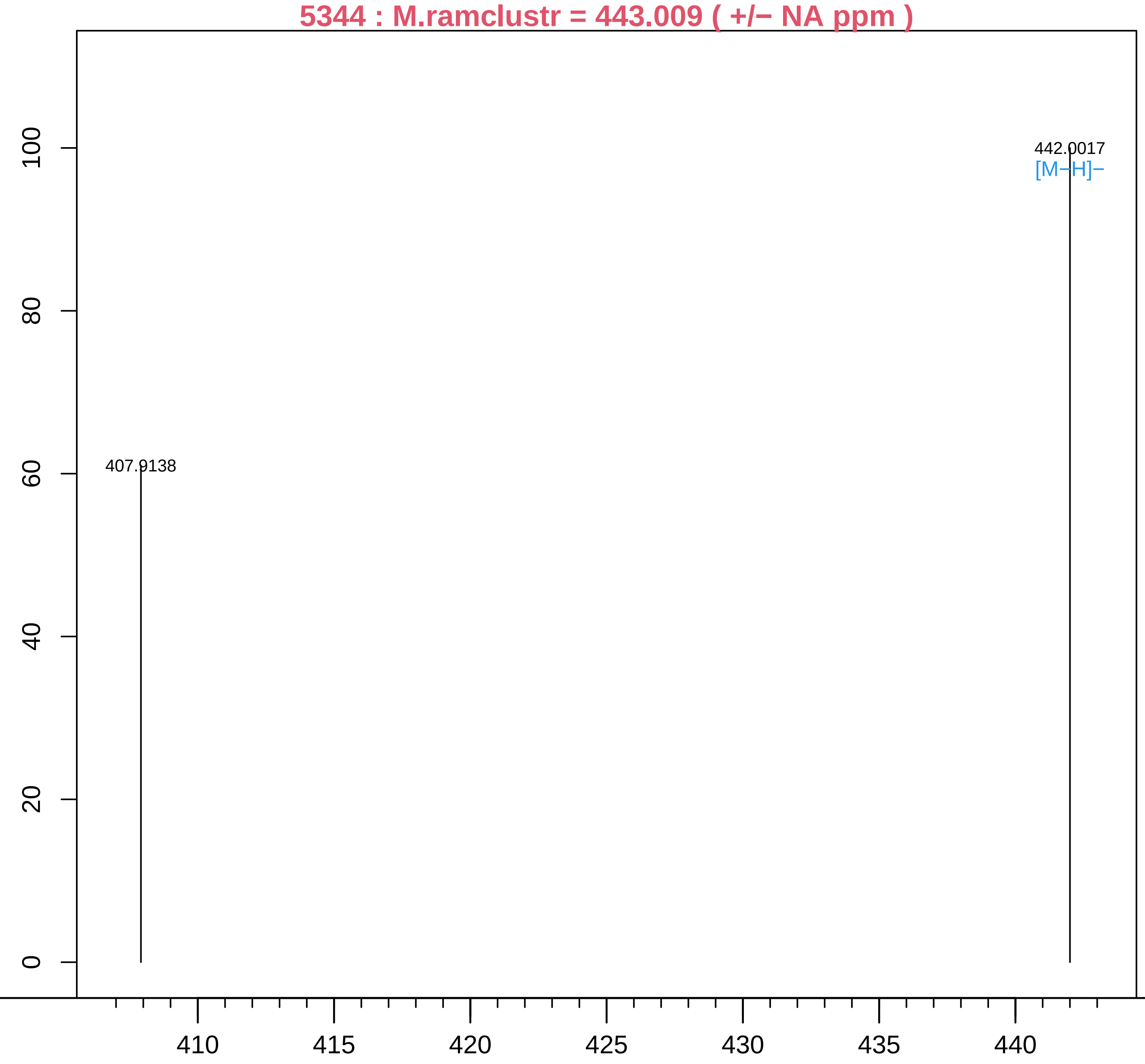
5344 : M.ramclustr = 443.009 (+/− NA ppm)
442.0017
[M−H]−
407.9138
0
20
40
60
80
100
410
415
420
425
430
435
440

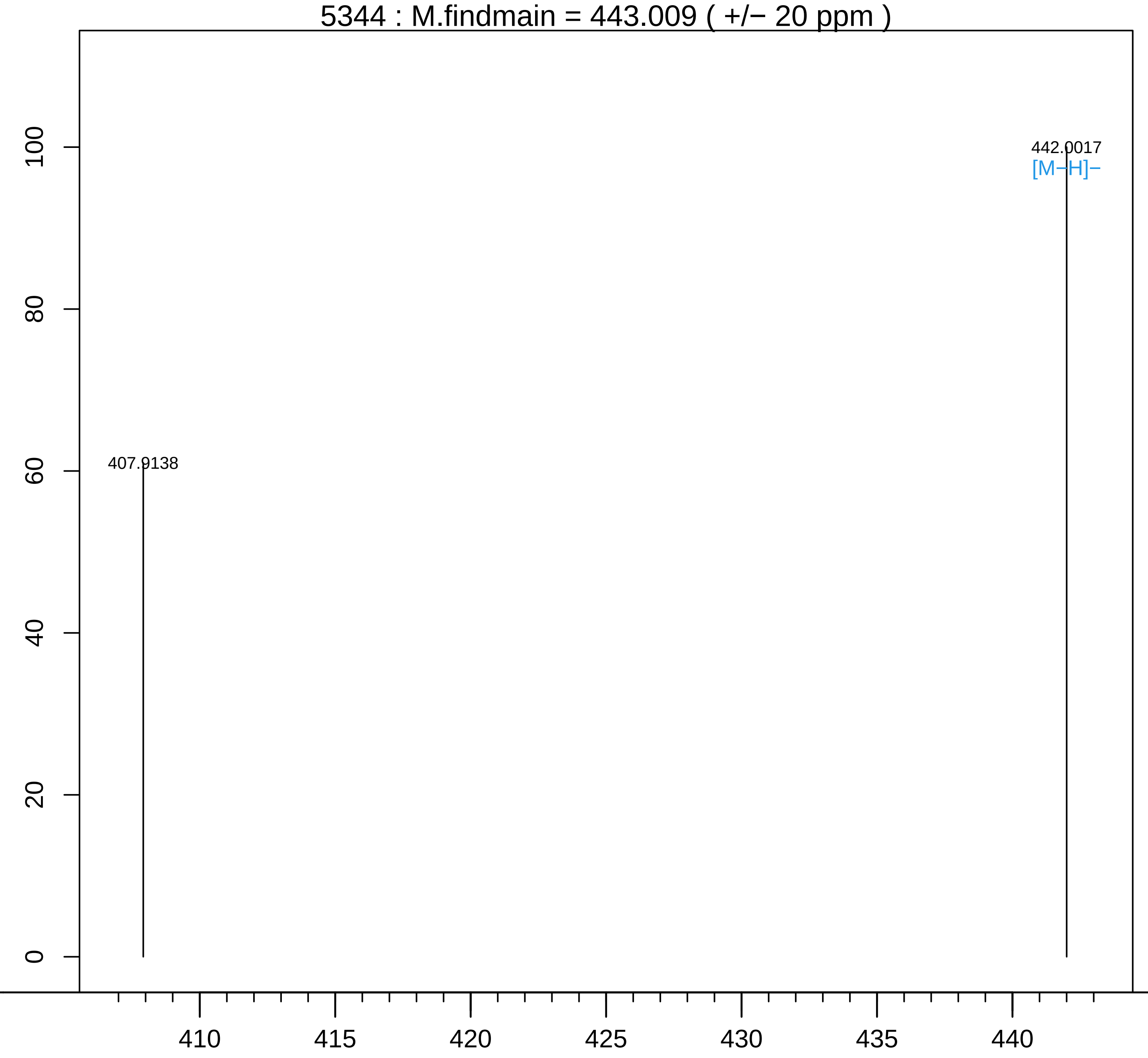
5344 : M.findmain = 443.009 (+/− 20 ppm)
442.0017
[M−H]−
407.9138
0
20
40
60
80
100
410
415
420
425
430
435
440

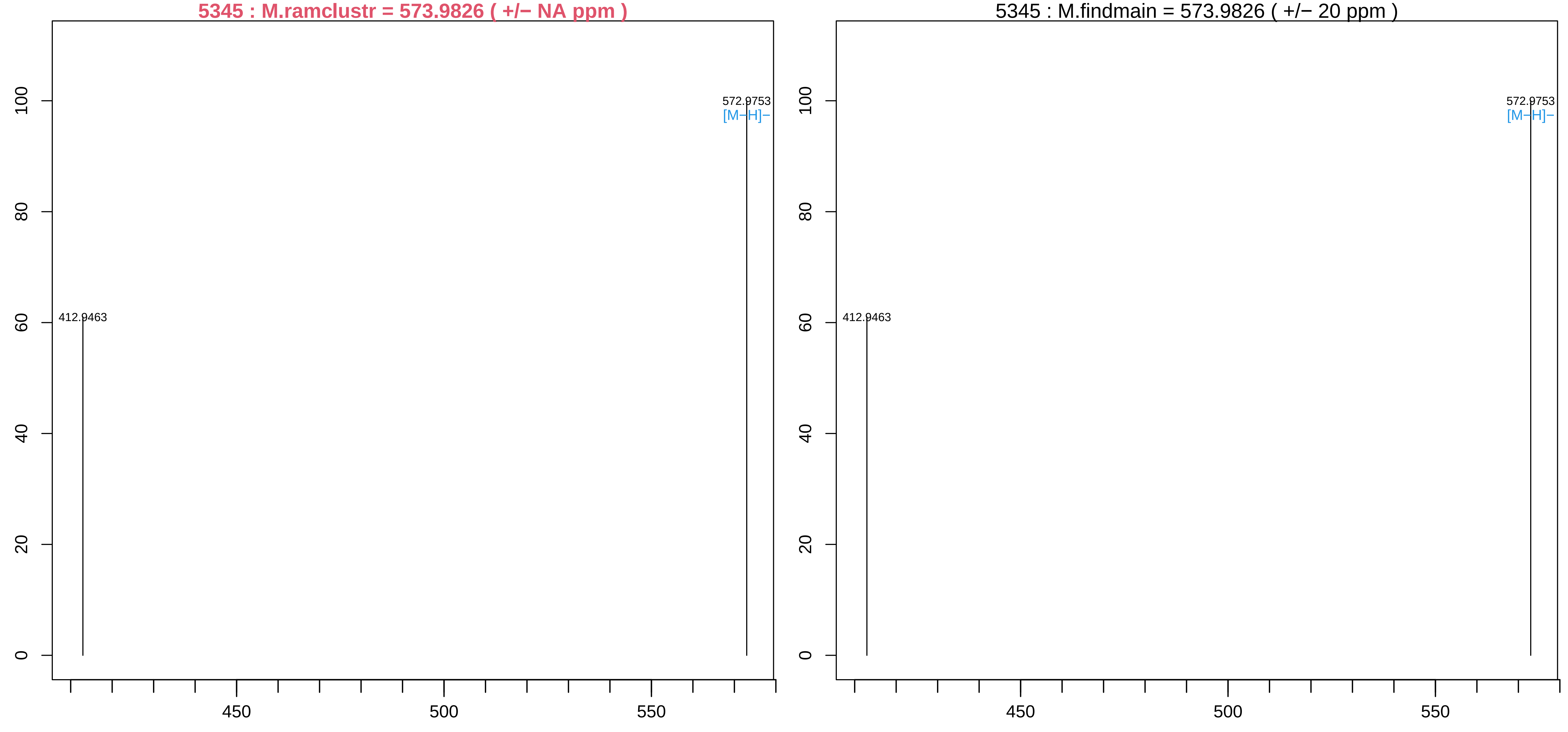
5345 : M.ramclustr = 573.9826 (+/− NA ppm)
572.9753
[M−H]−
412.9463
0
20
40
60
80
100
450
500
550
5345 : M.findmain = 573.9826 (+/− 20 ppm)
572.9753
[M−H]−
412.9463
0
20
40
60
80
100
450
500
550

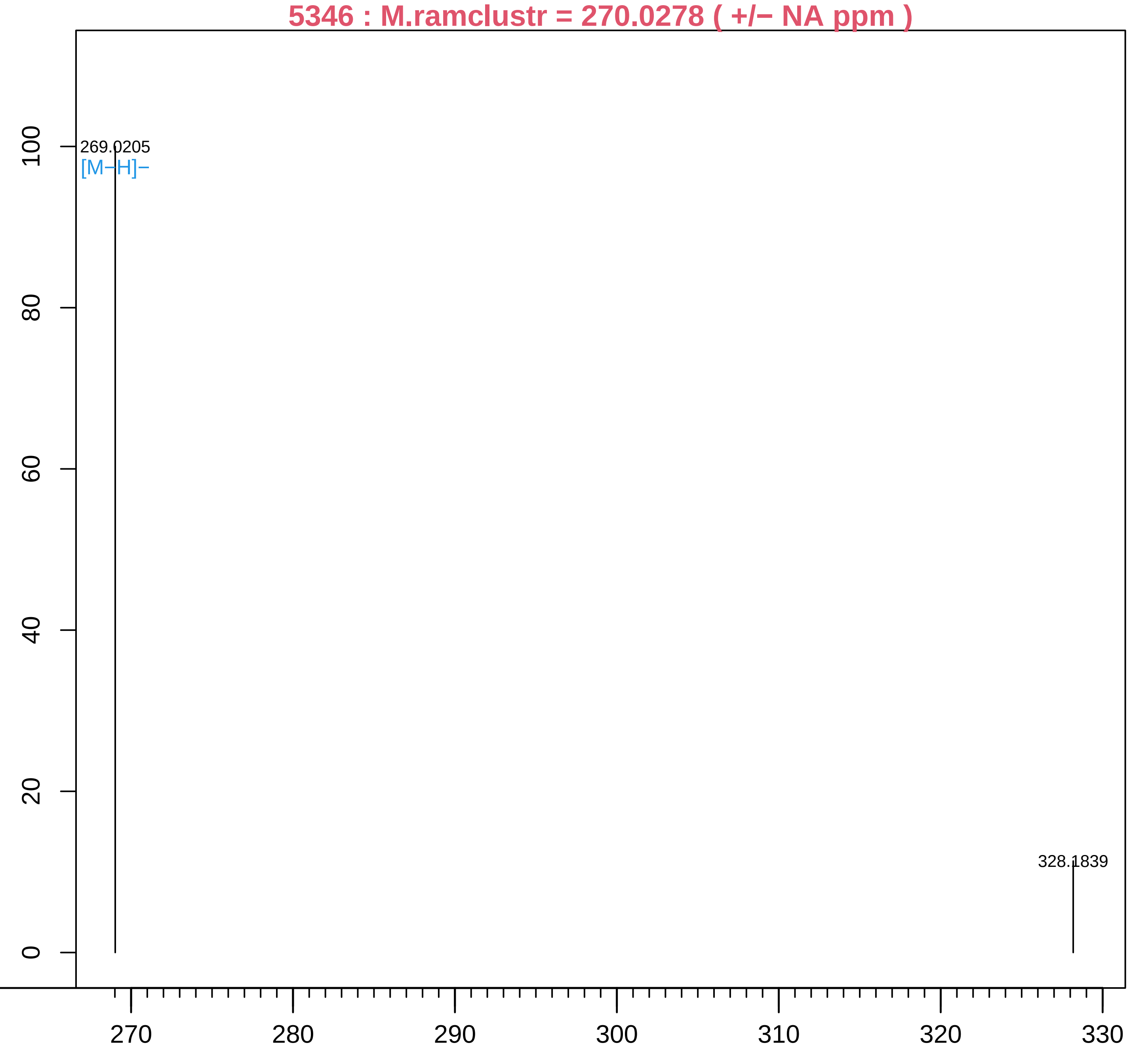
5346 : M.ramclustr = 270.0278 (+/− NA ppm)
269.0205
[M−H]−
328.1839

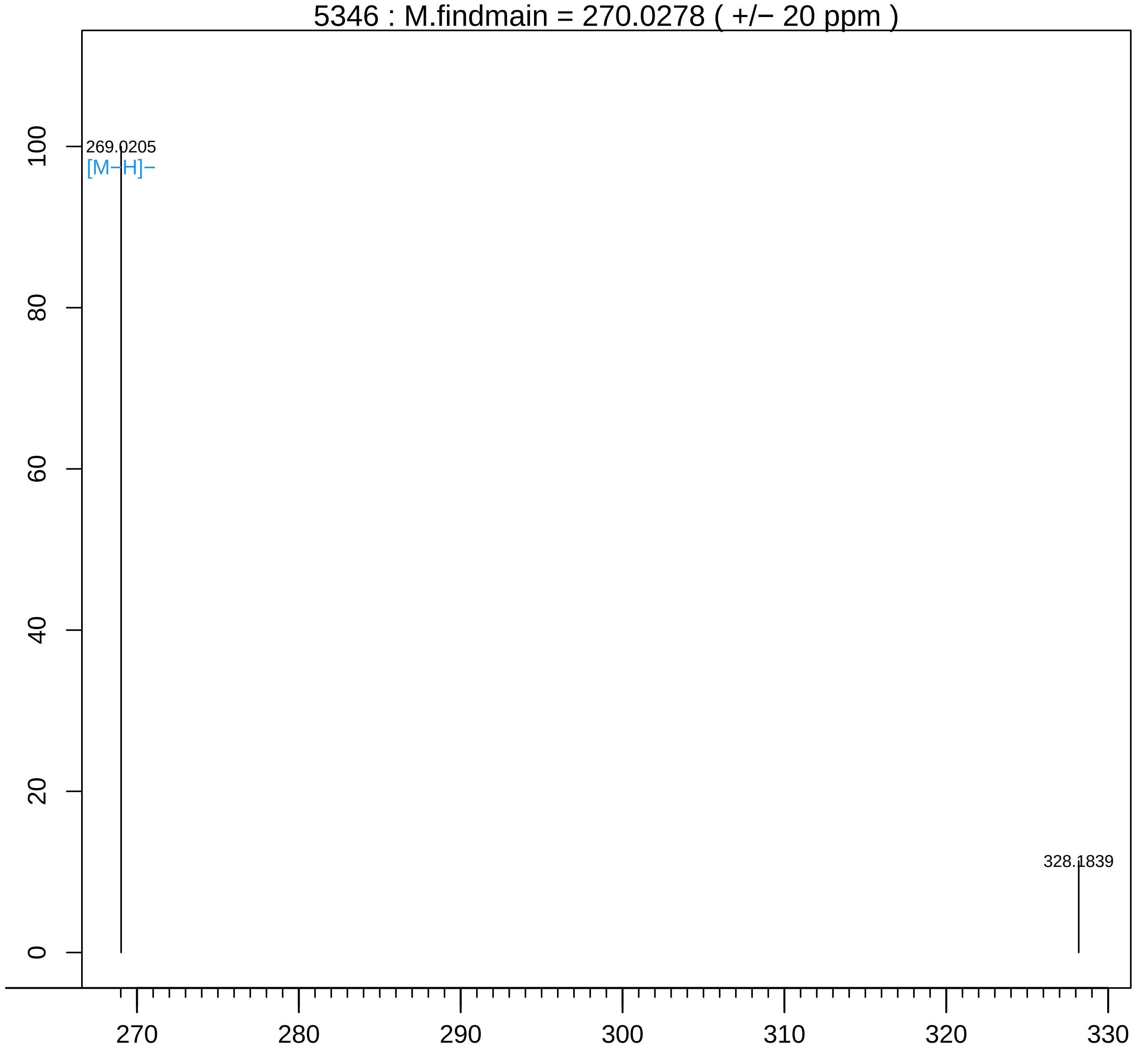
5346 : M.findmain = 270.0278 (+/− 20 ppm)
269.0205
[M−H]−
328.1839

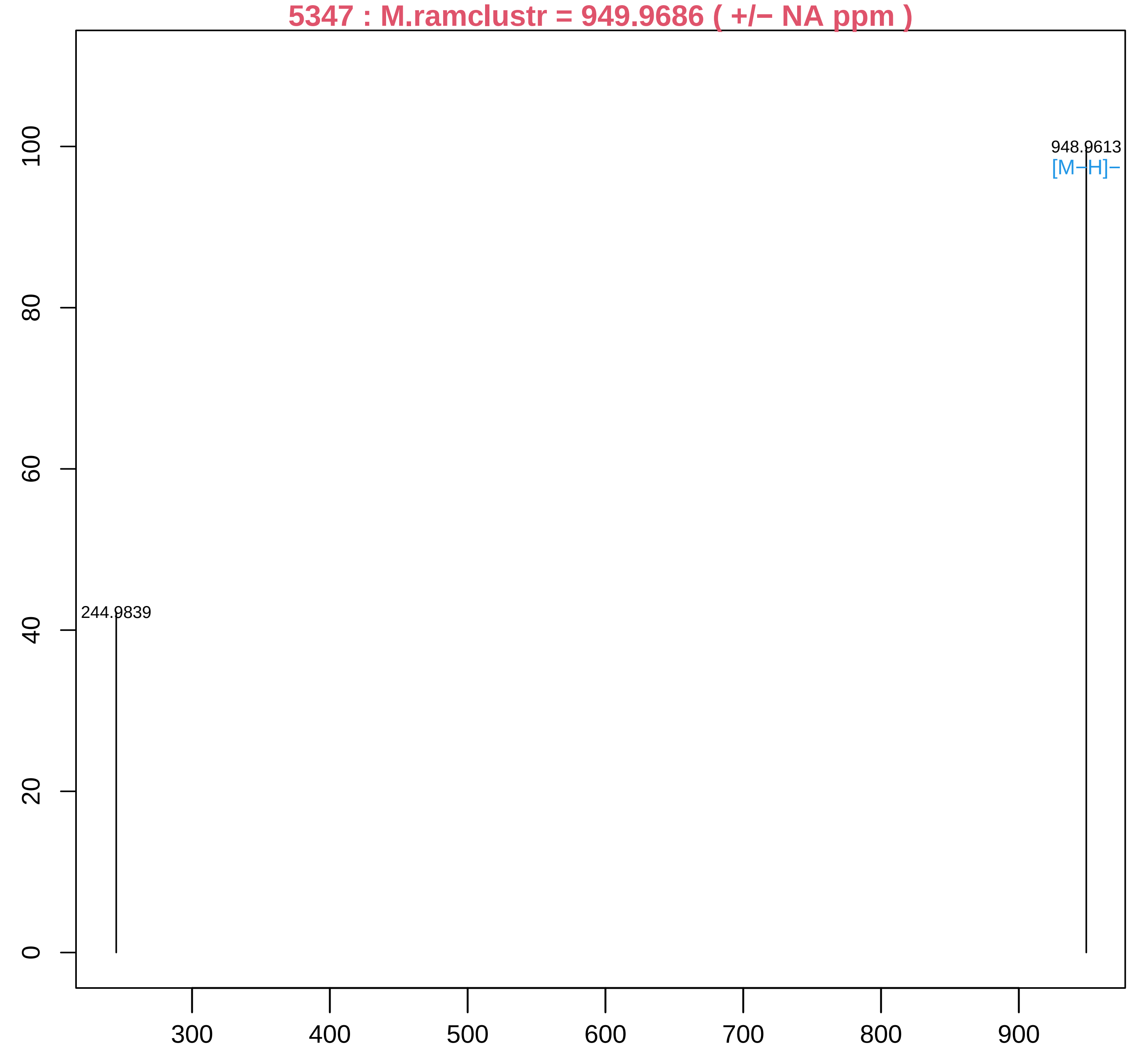
5347 : M.ramclustr = 949.9686 (+/− NA ppm)
948.9613
[M−H]−
244.9839
0
20
40
60
80
100
300
400
500
600
700
800
900

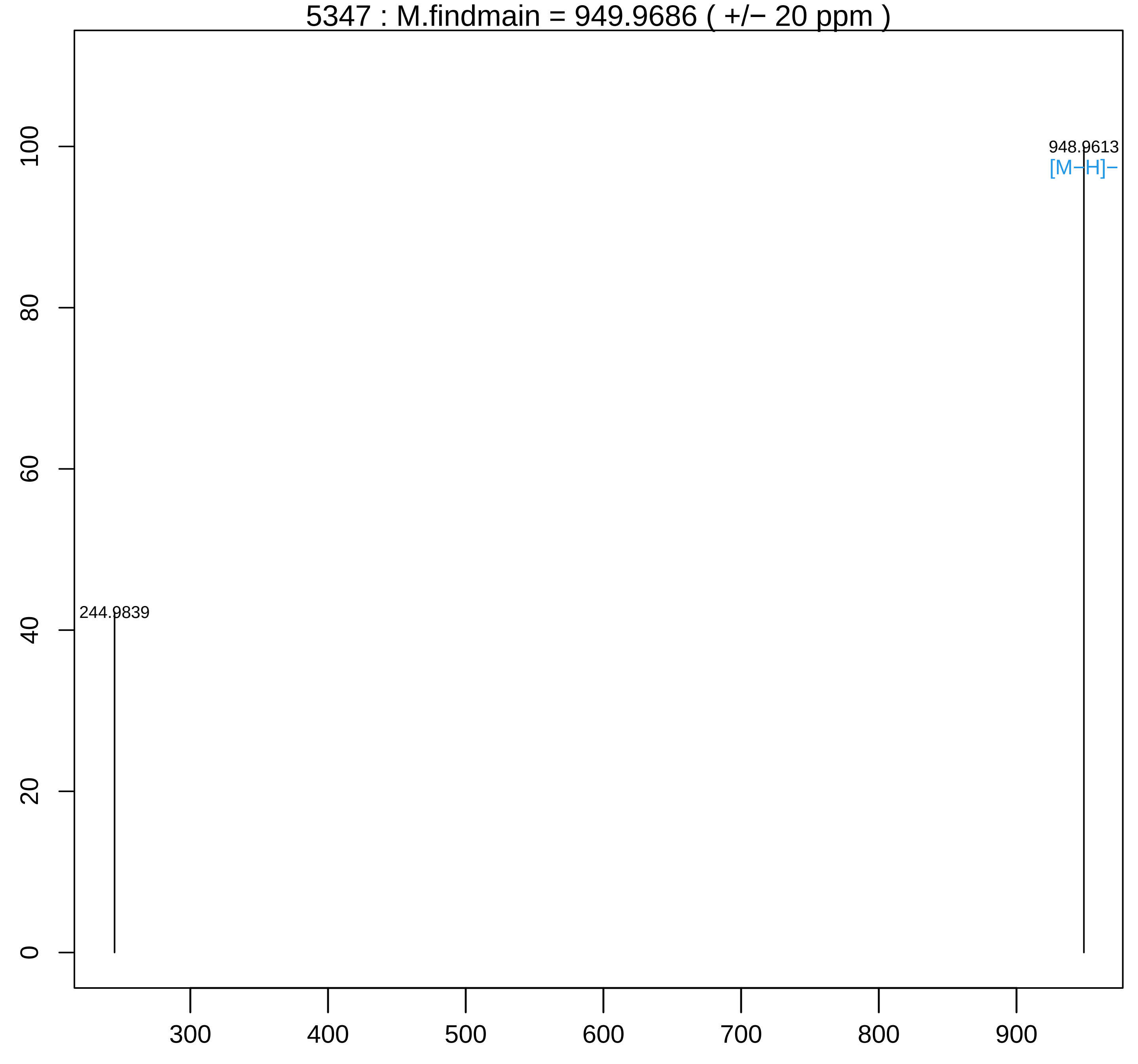
5347 : M.findmain = 949.9686 (+/− 20 ppm)
948.9613
[M−H]−
244.9839
0
20
40
60
80
100
300
400
500
600
700
800
900

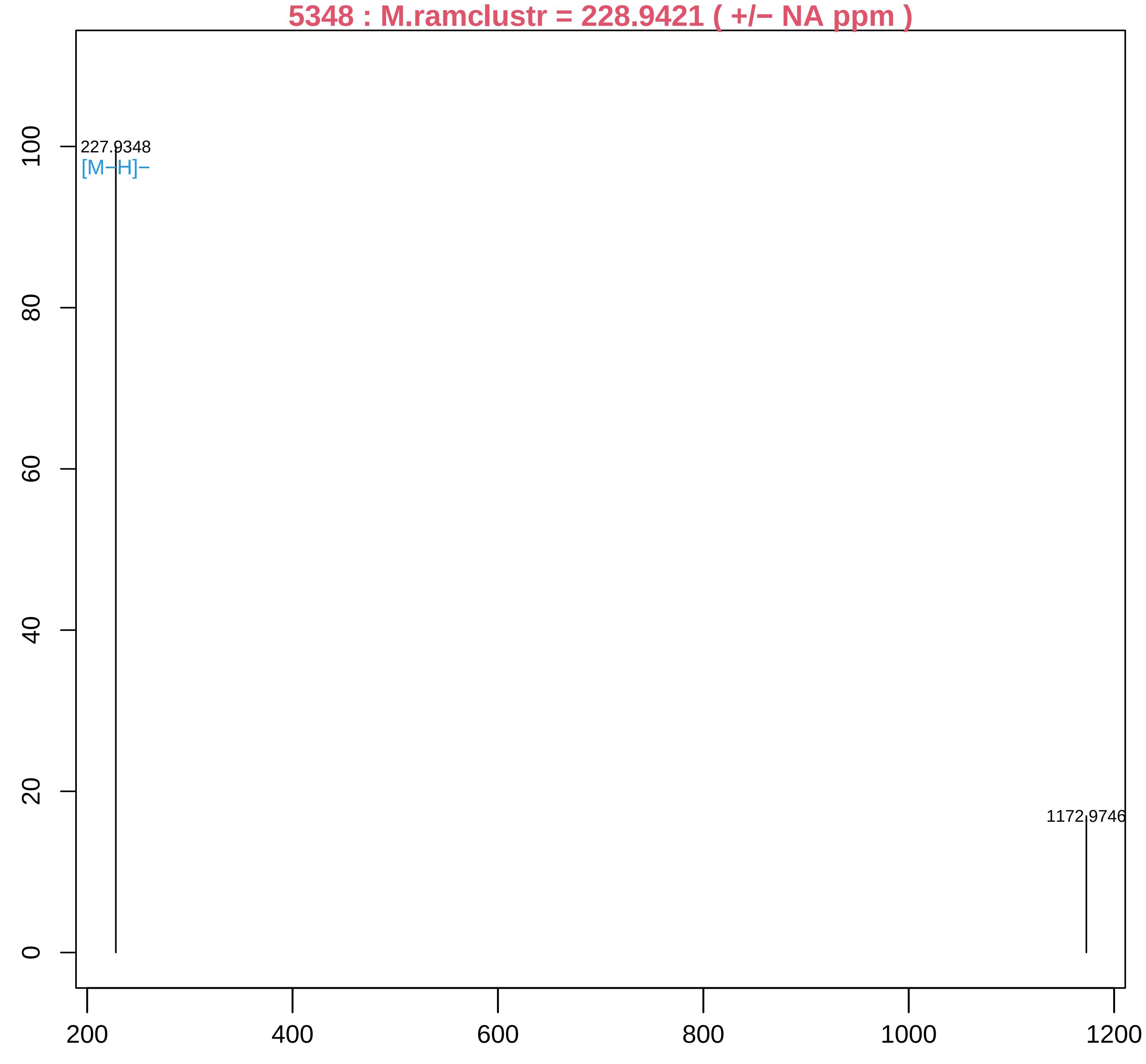
5348 : M.ramclustr = 228.9421 (+/− NA ppm)
227.9348
[M−H]−
1172.9746
0
20
40
60
80
100
200
400
600
800
1000
1200

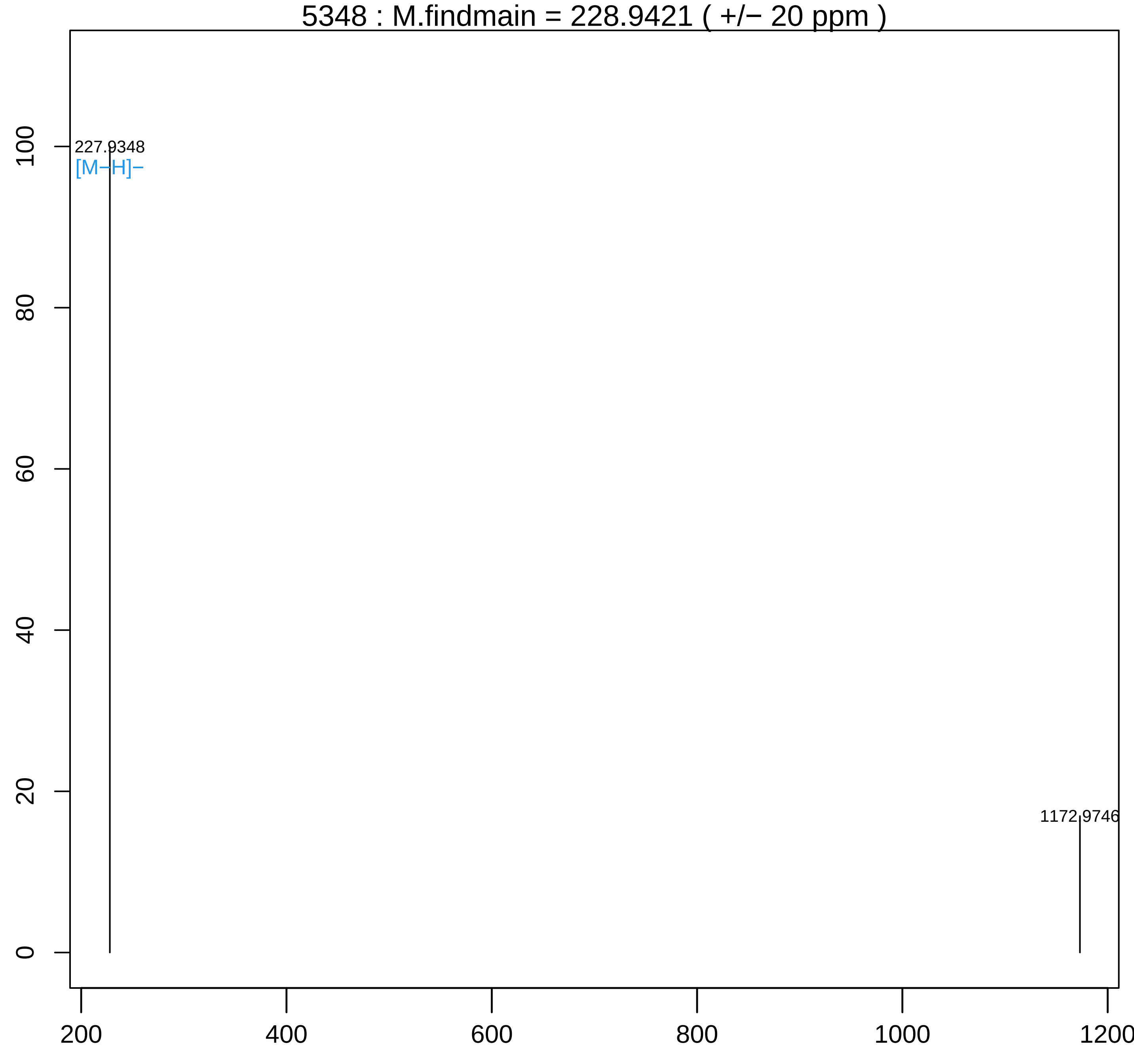
5348 : M.findmain = 228.9421 (+/− 20 ppm)
227.9348
[M−H]−
1172.9746
0
20
40
60
80
100
200
400
600
800
1000
1200

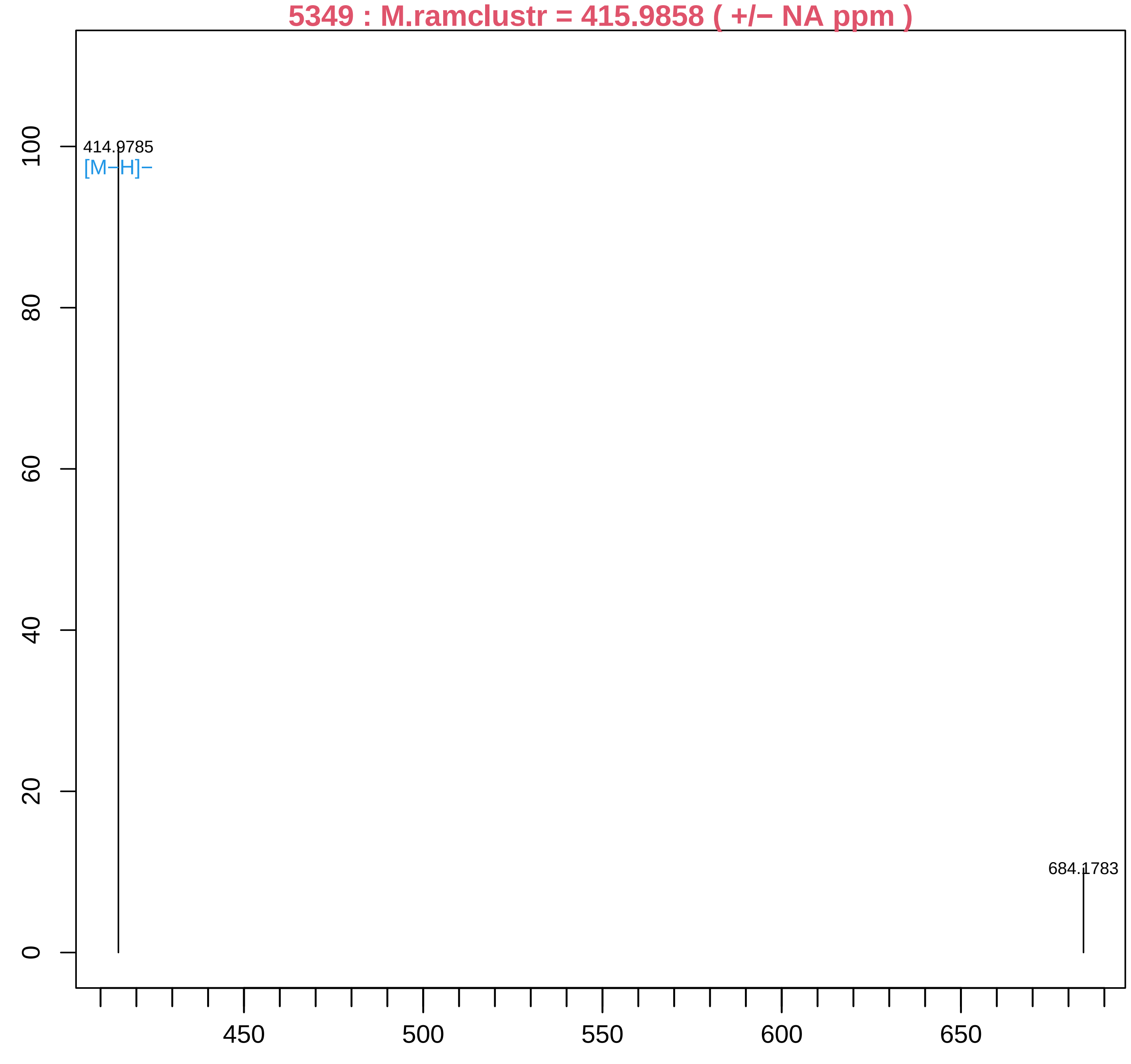
5349 : M.ramclustr = 415.9858 (+/− NA ppm)
414.9785
[M−H]−
684.1783
0
20
40
60
80
100
450
500
550
600
650

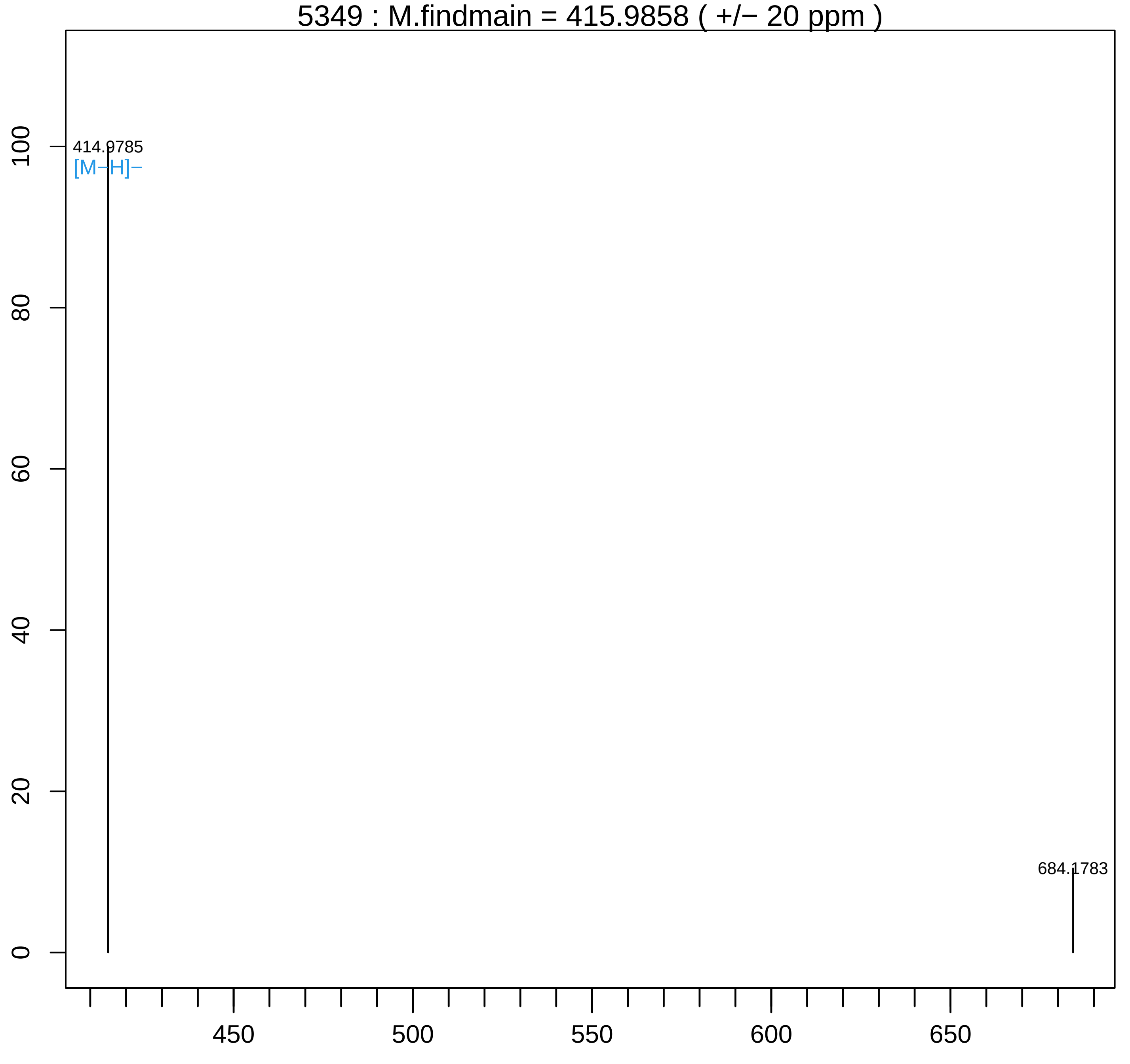
5349 : M.findmain = 415.9858 (+/− 20 ppm)
414.9785
[M−H]−
684.1783
0
20
40
60
80
100
450
500
550
600
650

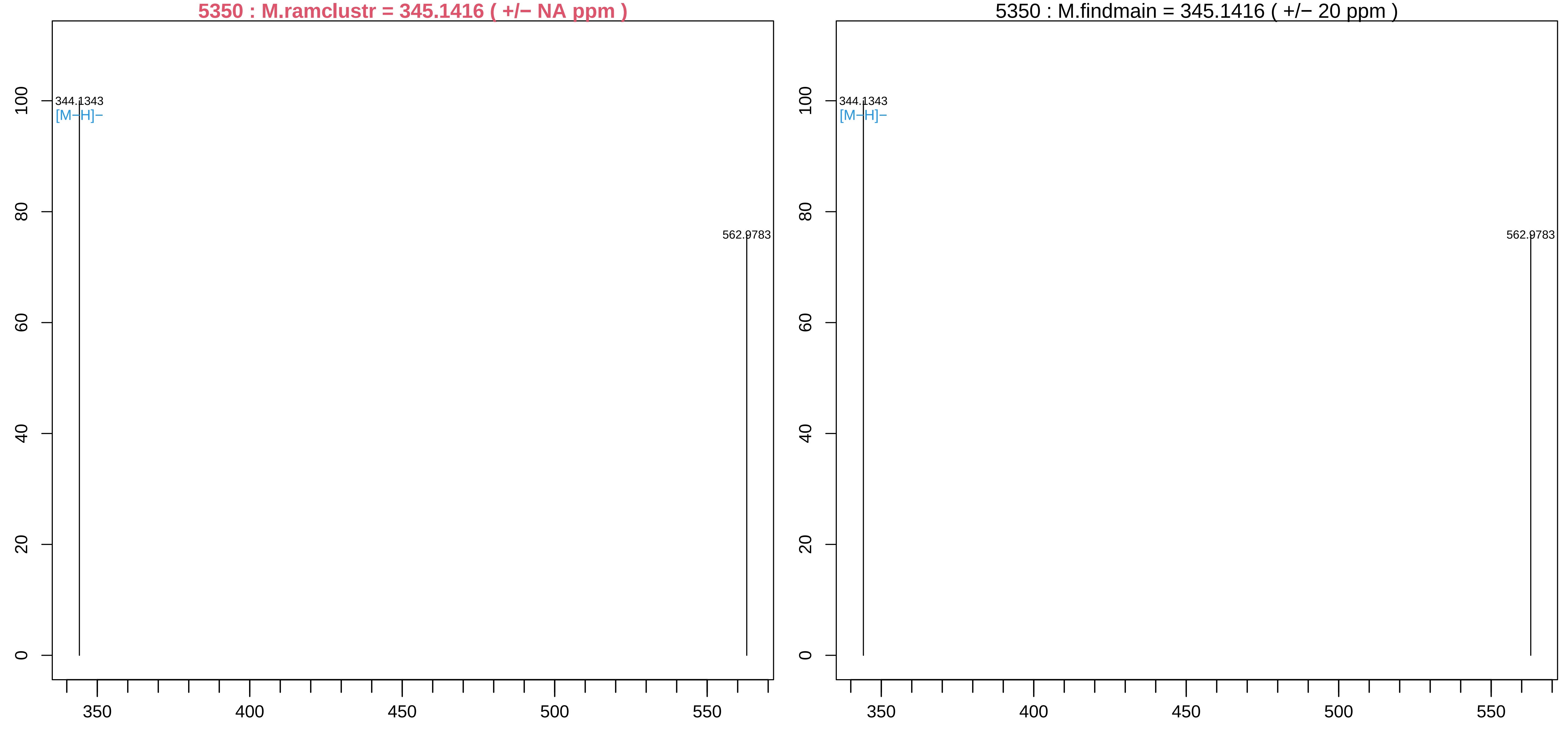
5350 : M.ramclustr = 345.1416 (+/− NA ppm)
344.1343
[M−H]−
562.9783
0
20
40
60
80
100
350
400
450
500
550
5350 : M.findmain = 345.1416 (+/− 20 ppm)
344.1343
[M−H]−
562.9783
0
20
40
60
80
100
350
400
450
500
550

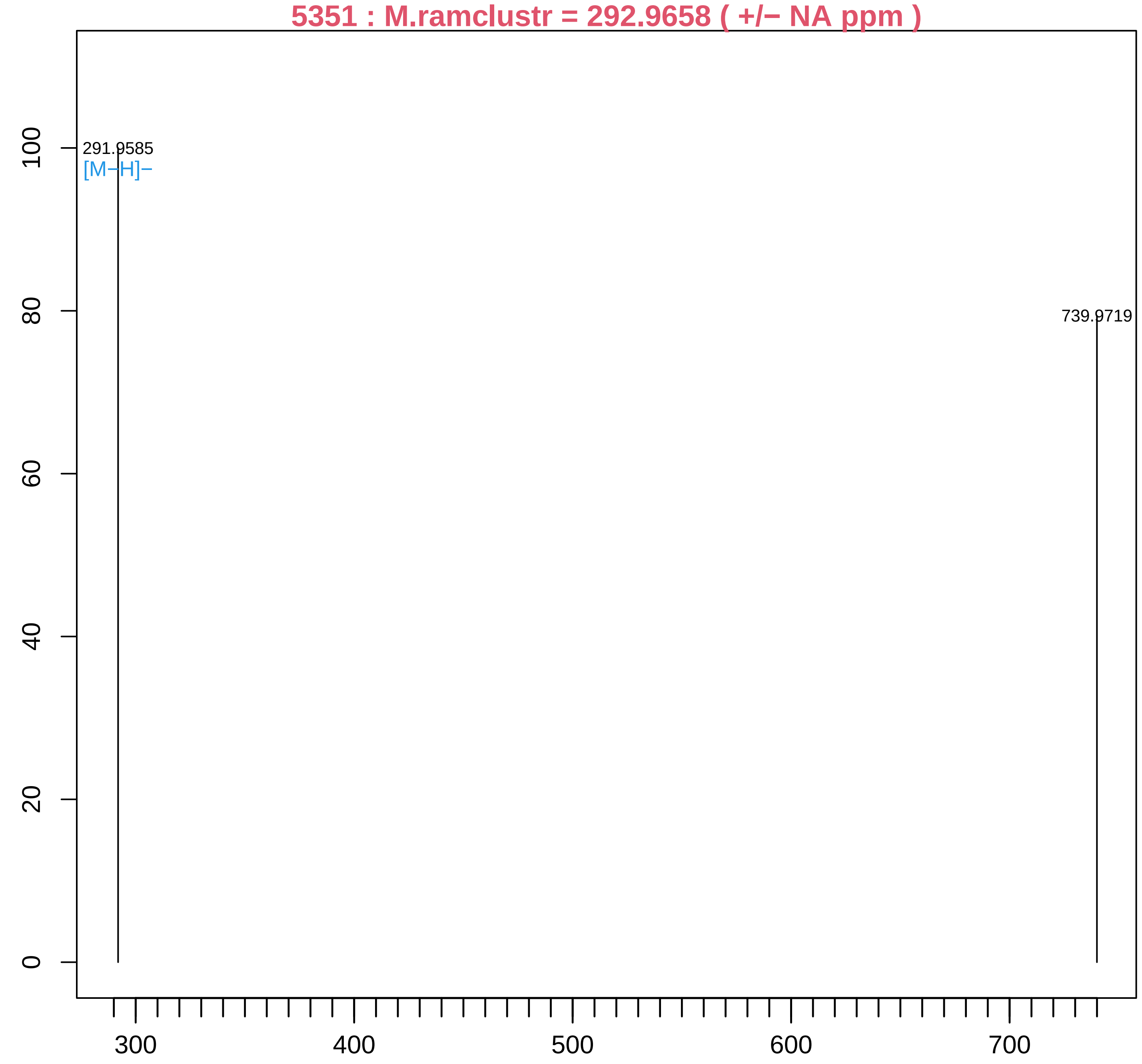
5351 : M.ramclustr = 292.9658 (+/− NA ppm)
291.9585
[M−H]−
739.9719
0
20
40
60
80
100
300
400
500
600
700

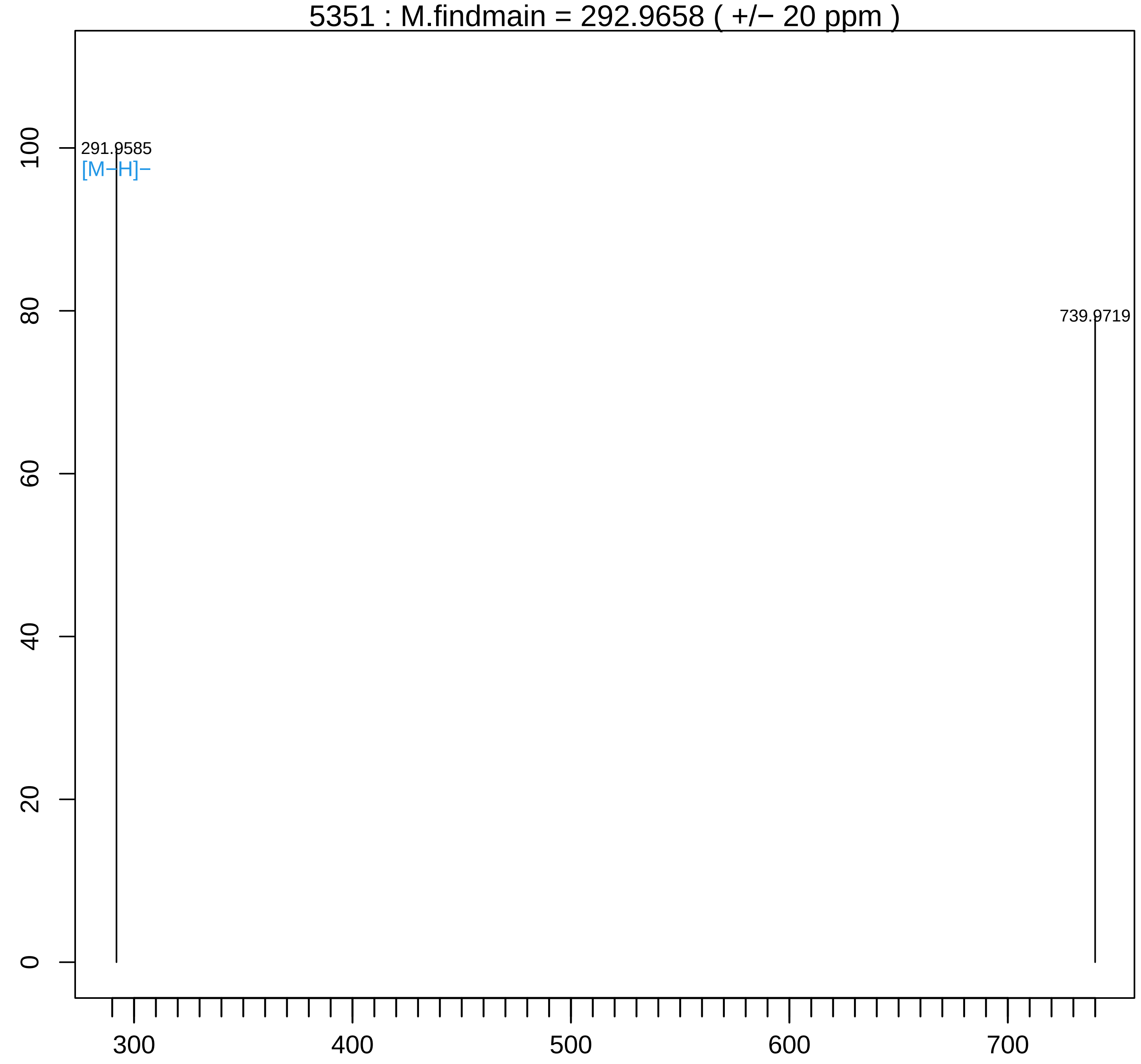
5351 : M.findmain = 292.9658 (+/− 20 ppm)
291.9585
[M−H]−
739.9719
0
20
40
60
80
100
300
400
500
600
700

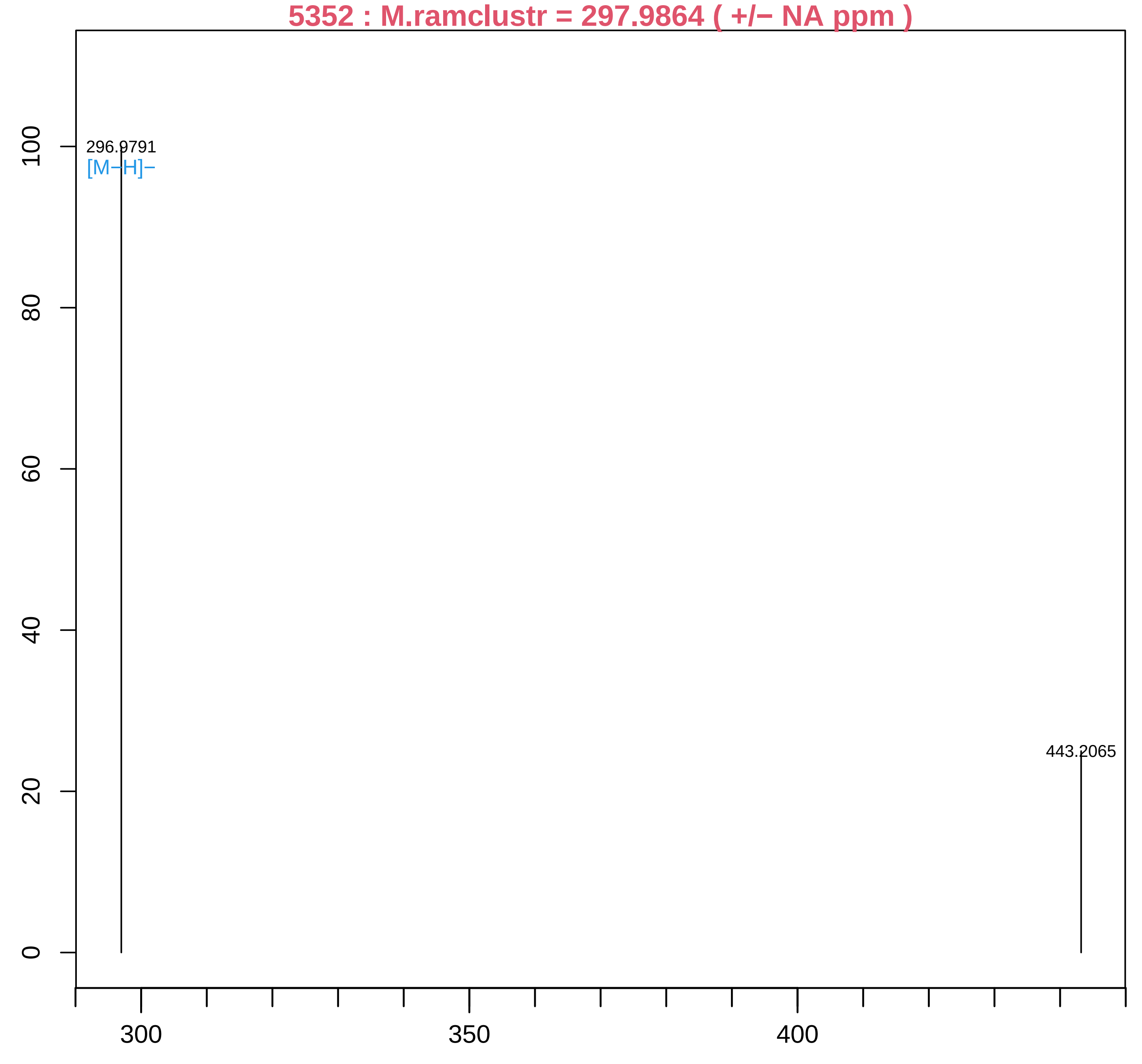
5352 : M.ramclustr = 297.9864 (+/− NA ppm)
296.9791
[M−H]−
443.2065
0
20
40
60
80
100
300
350
400

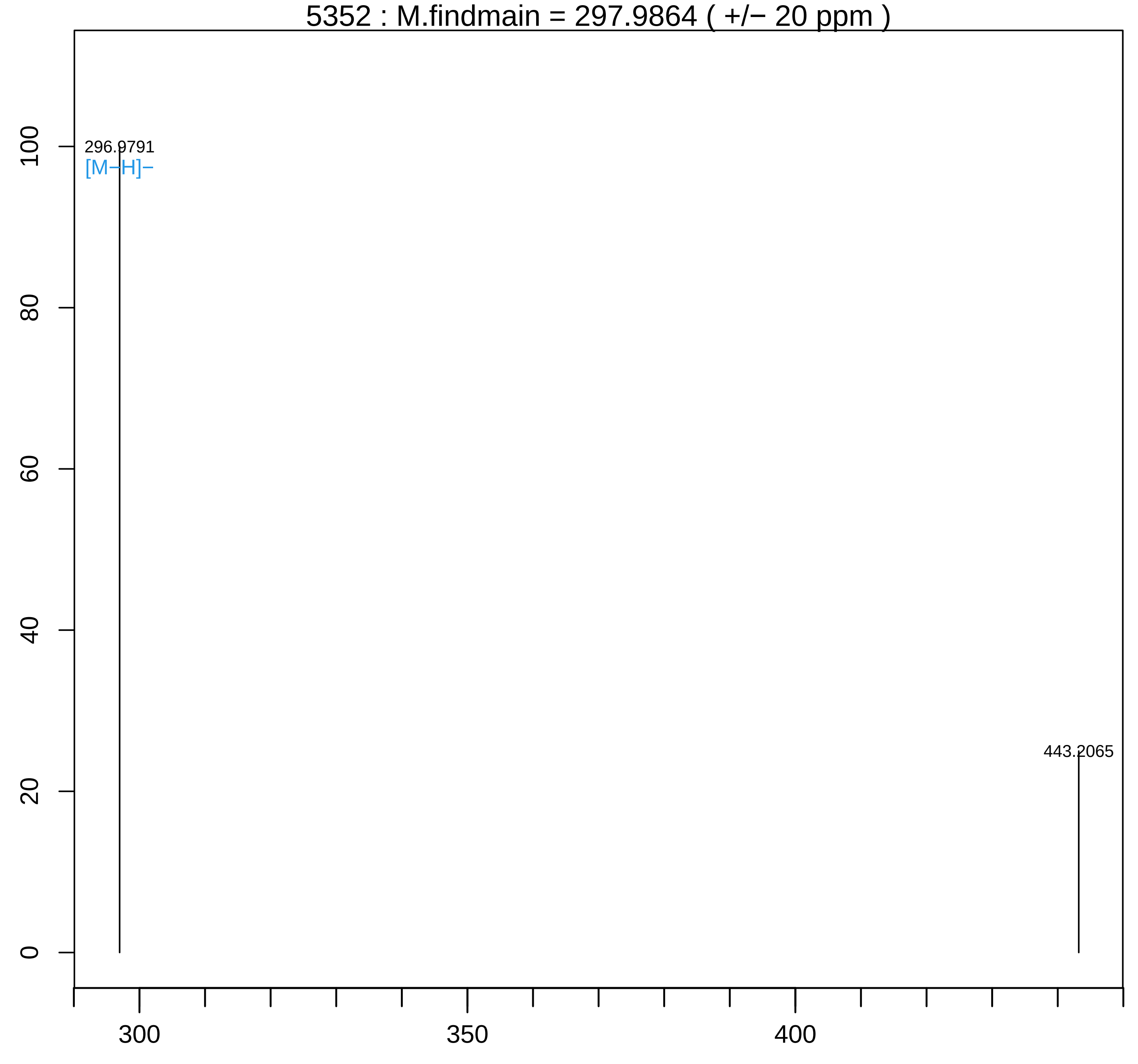
5352 : M.findmain = 297.9864 (+/− 20 ppm)
296.9791
[M−H]−
443.2065
0
20
40
60
80
100
300
350
400

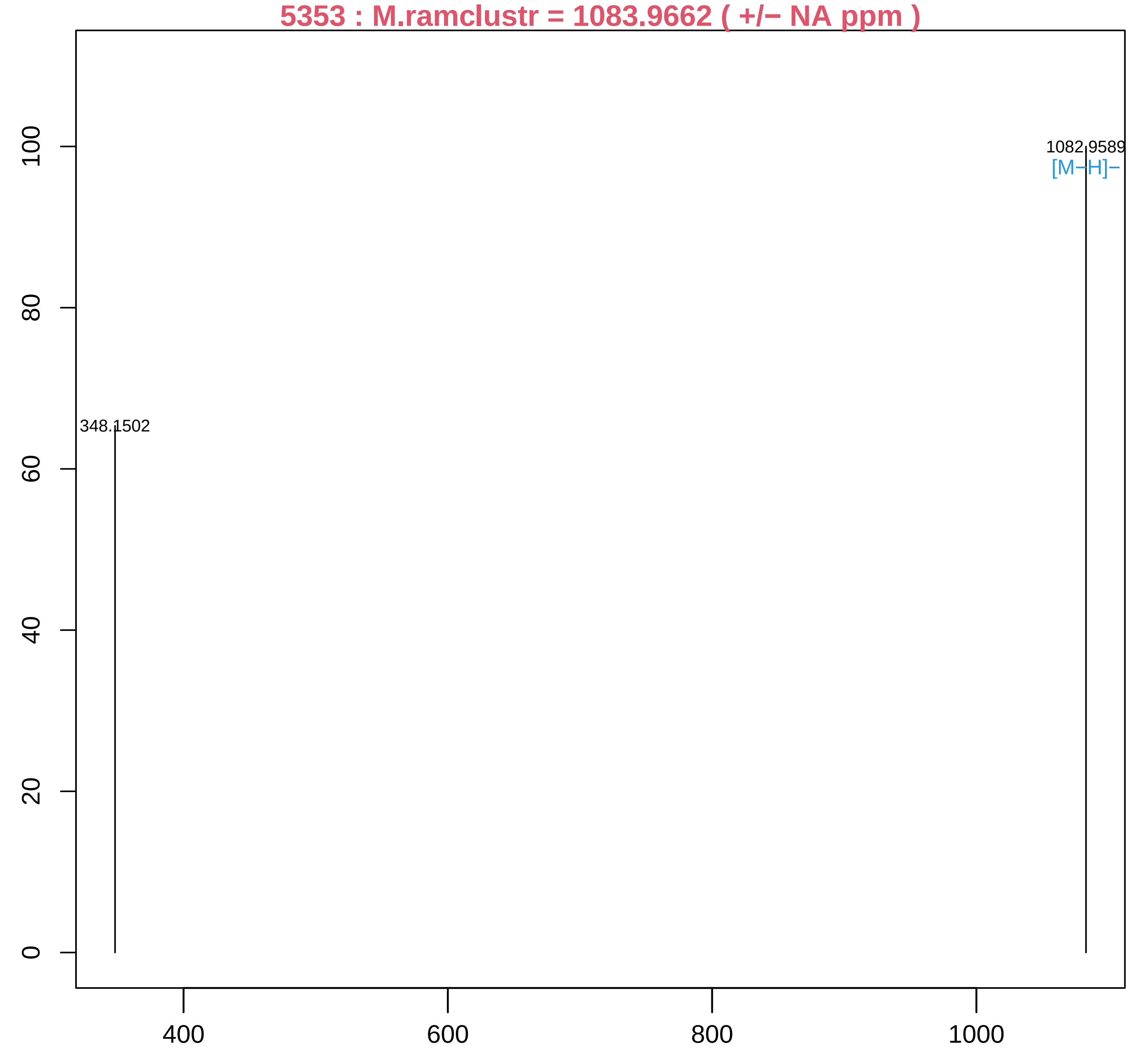

5353 : M.ramclustr = 1083.9662 (+/− NA ppm)
1082.9589
[M−H]−
348.1502
0
20
40
60
80
100
400
600
800
1000

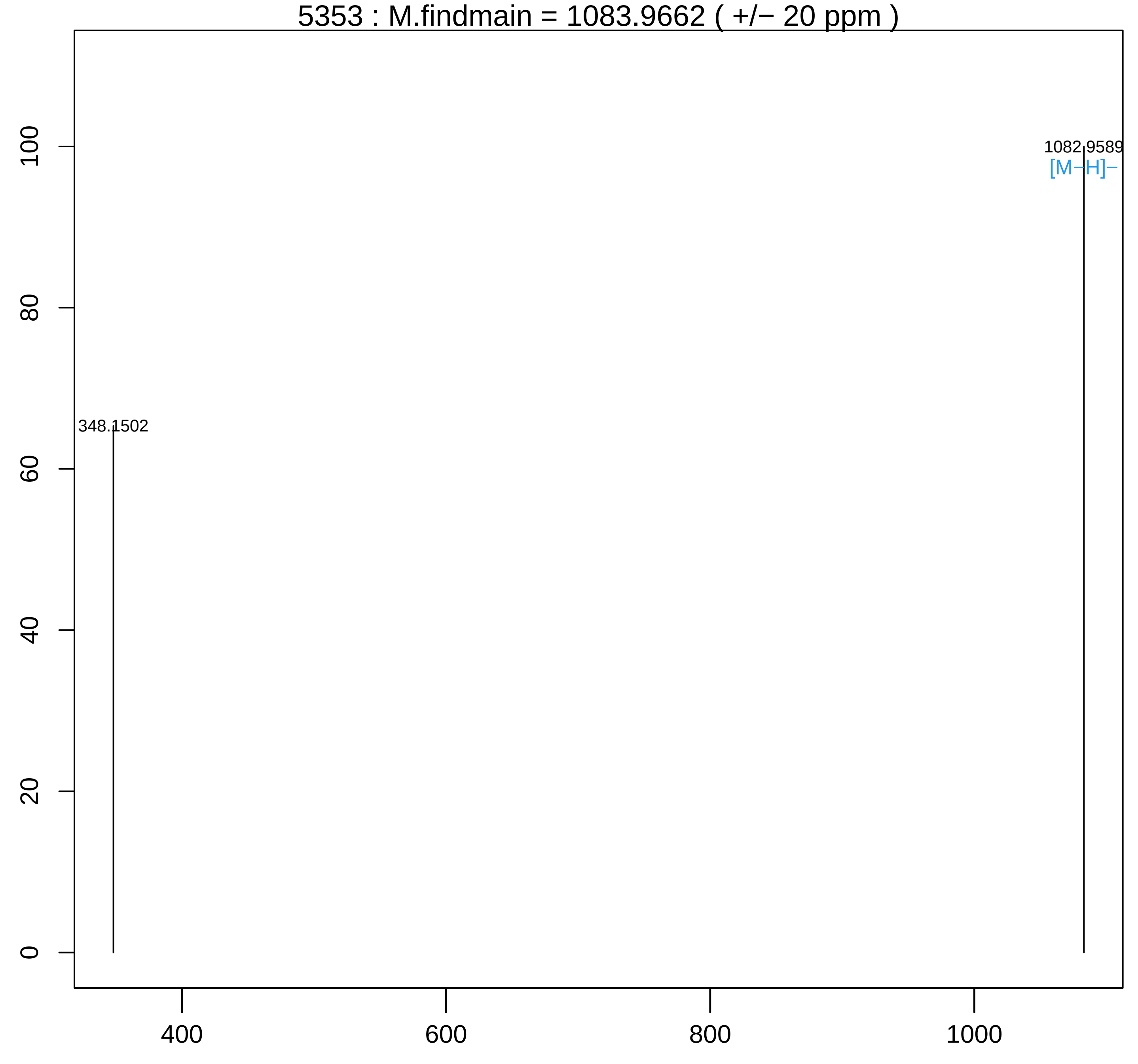

5353 : M.findmain = 1083.9662 (+/− 20 ppm)
1082.9589
[M−H]−
348.1502
0
20
40
60
80
100
400
600
800
1000

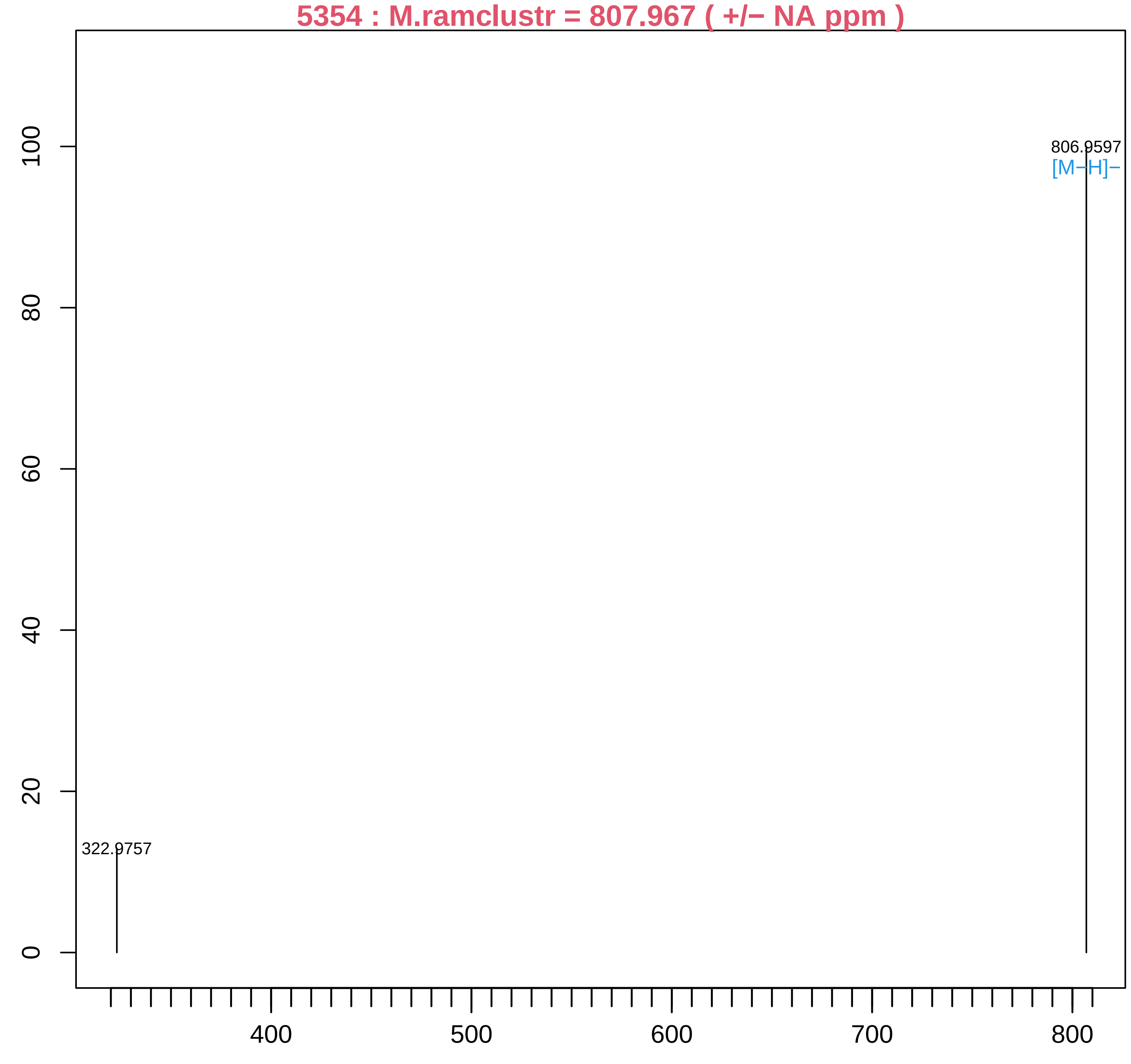
5354 : M.ramclustr = 807.967 (+/− NA ppm)
806.9597
[M−H]−
322.9757
0
20
40
60
80
100
400
500
600
700
800
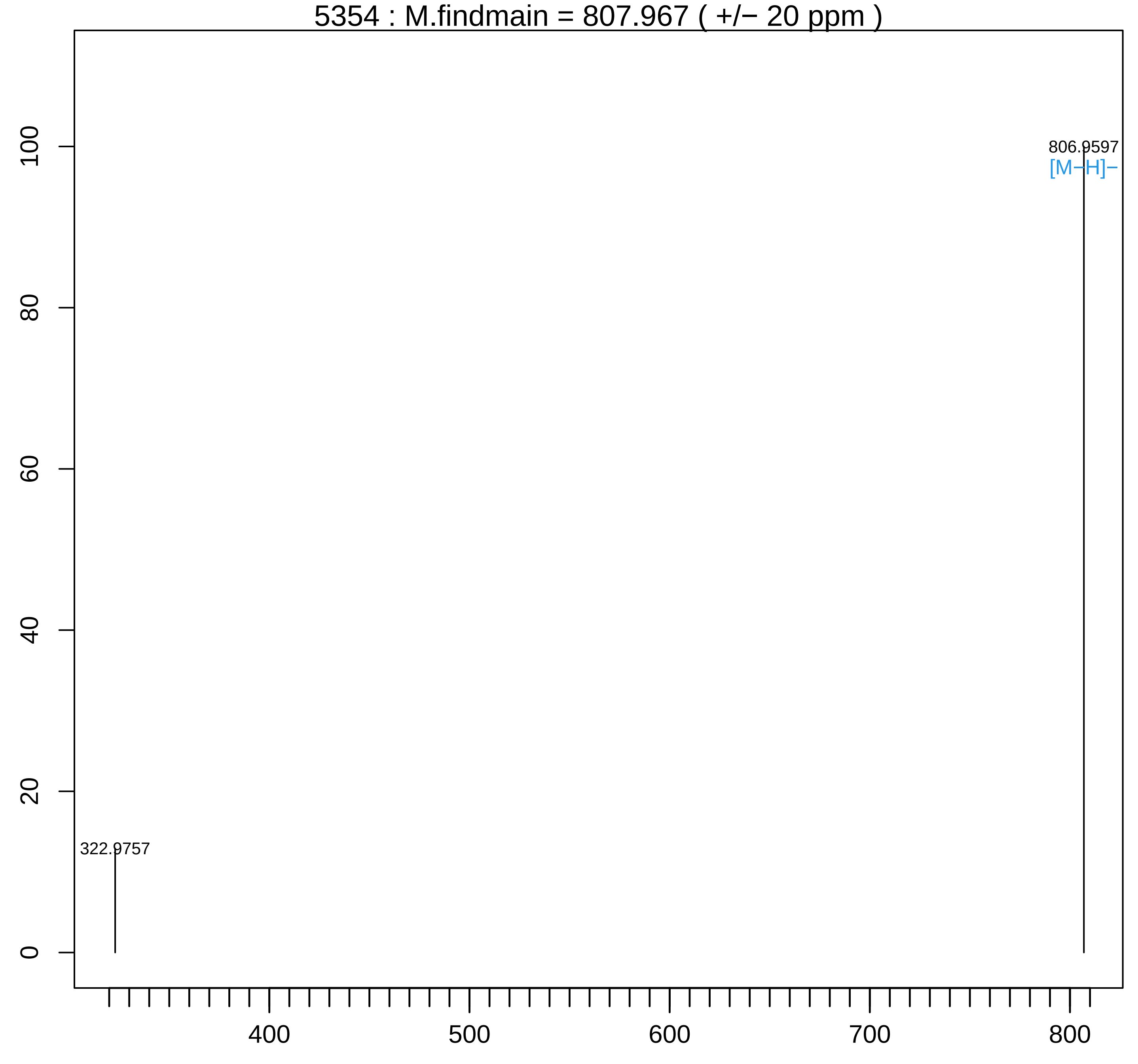
5354 : M.findmain = 807.967 (+/− 20 ppm)
806.9597
[M−H]−
322.9757
0
20
40
60
80
100
400
500
600
700
800

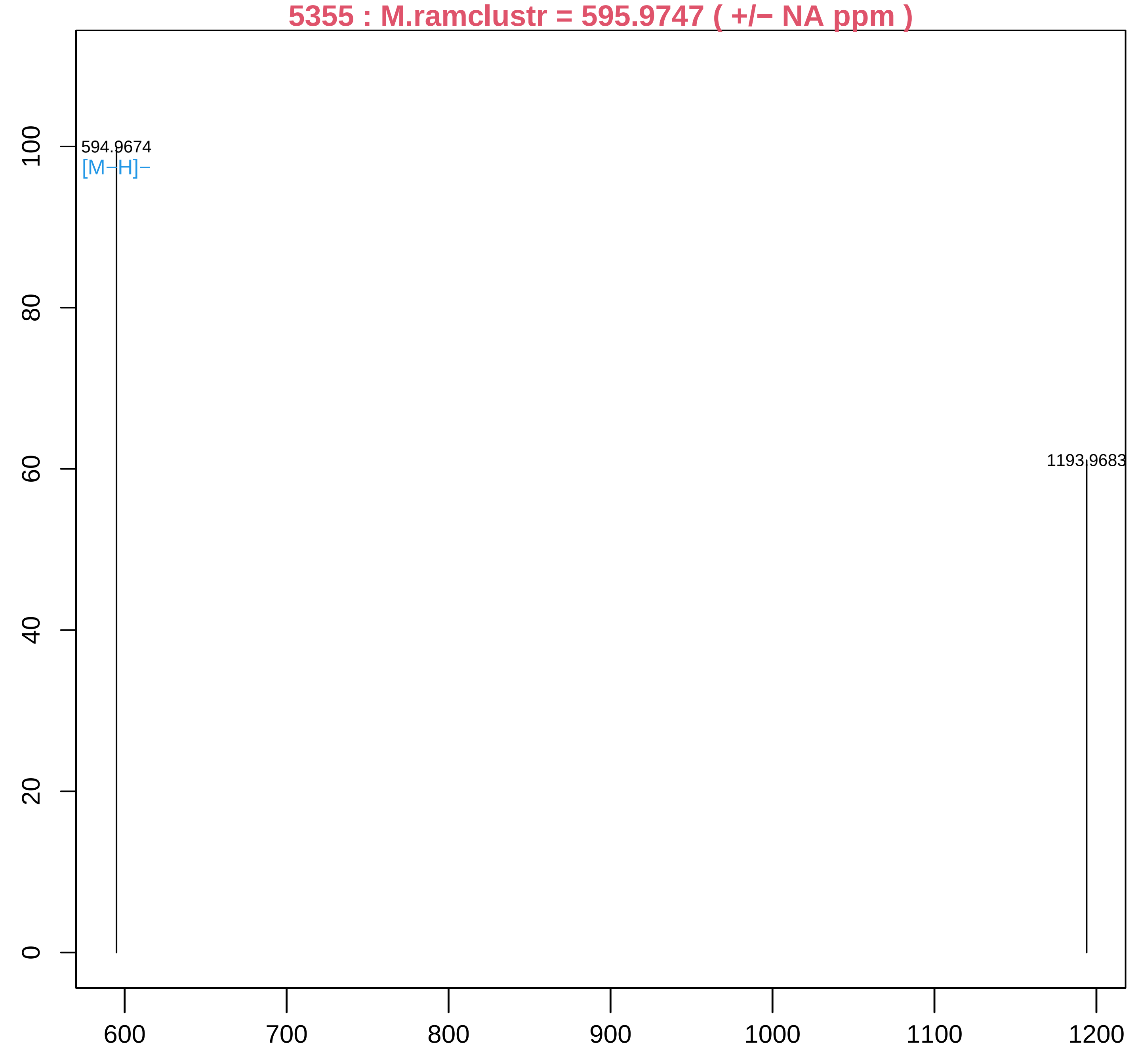
5355 : M.ramclustr = 595.9747 (+/− NA ppm)
594.9674
[M−H]−
1193.9683
0
20
40
60
80
100
600
700
800
900
1000
1100
1200

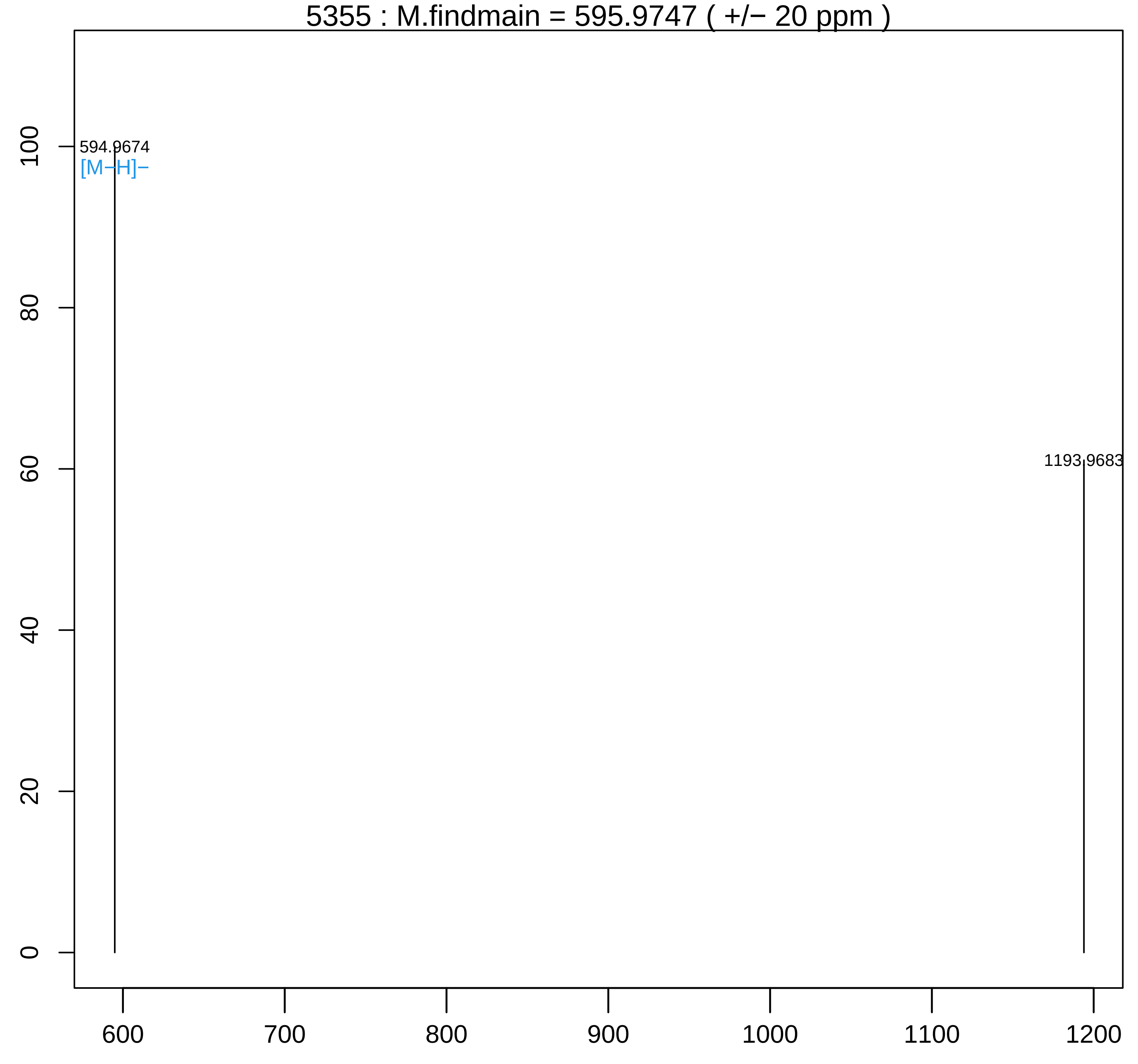
5355 : M.findmain = 595.9747 (+/− 20 ppm)
594.9674
[M−H]−
1193.9683
0
20
40
60
80
100
600
700
800
900
1000
1100
1200

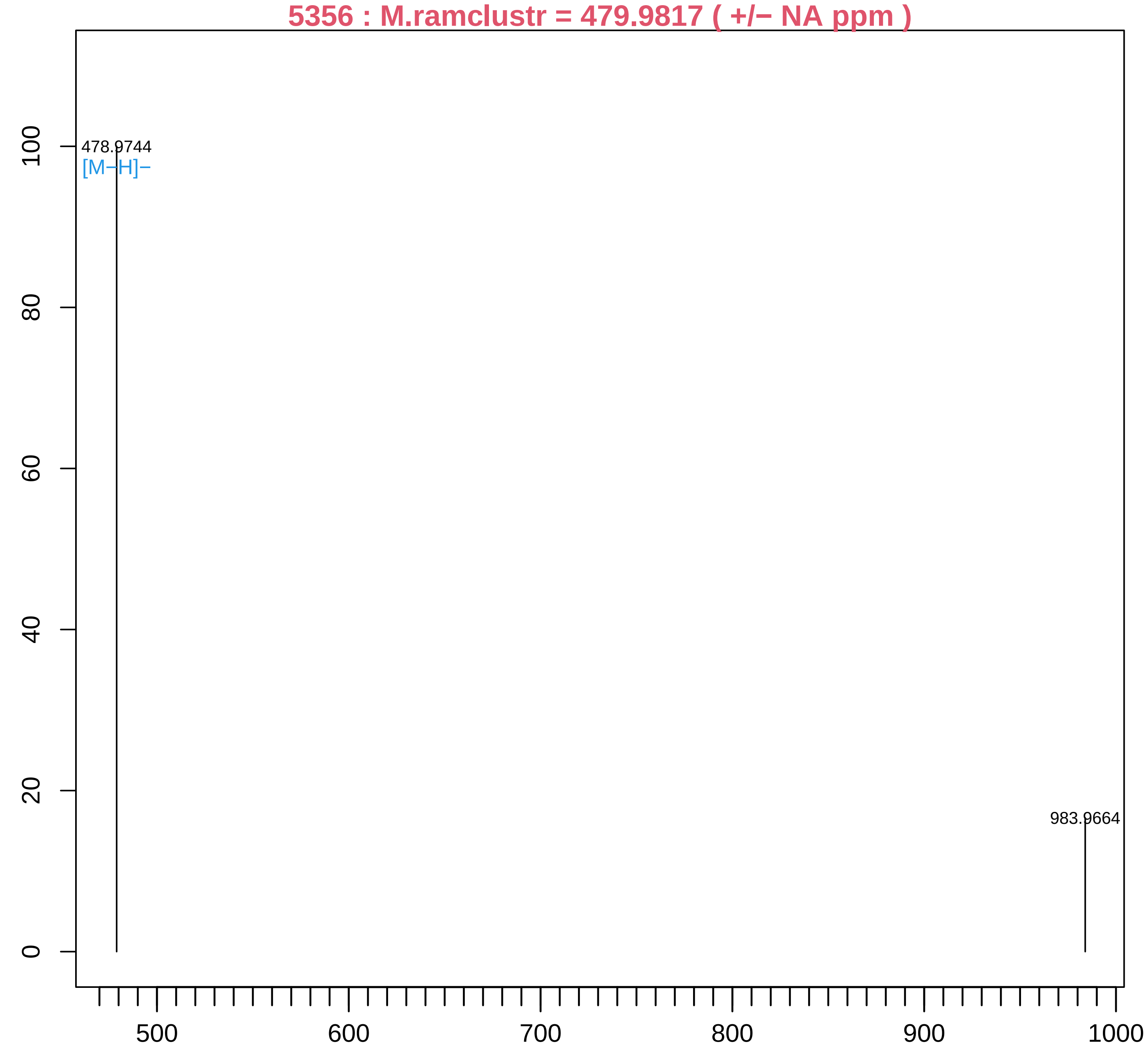

5356 : M.ramclustr = 479.9817 (+/− NA ppm)
478.9744
[M−H]−
983.9664
0
20
40
60
80
100
500
600
700
800
900
1000

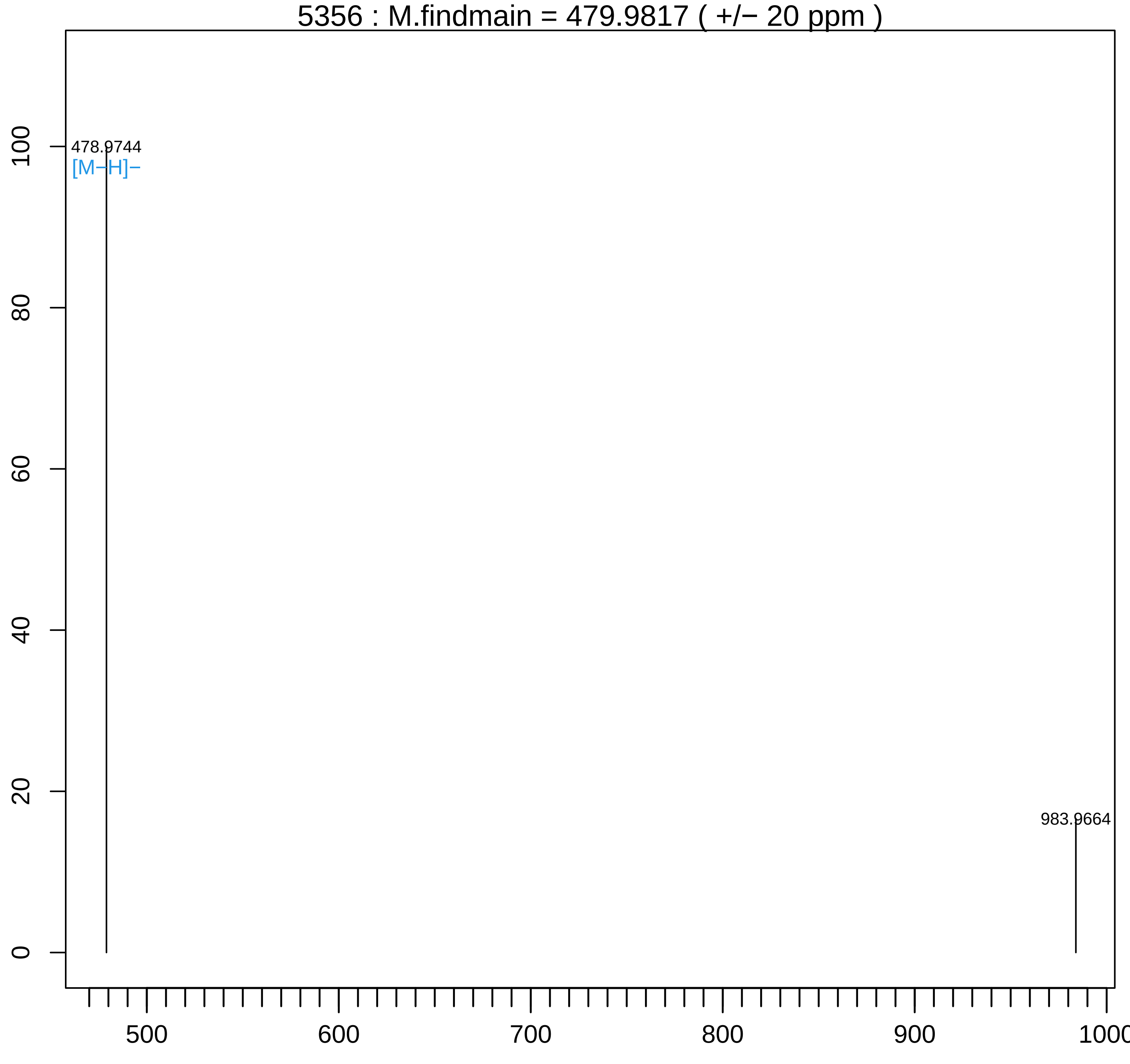

5356 : M.findmain = 479.9817 (+/− 20 ppm)
478.9744
[M−H]−
983.9664
0
20
40
60
80
100
500
600
700
800
900
1000

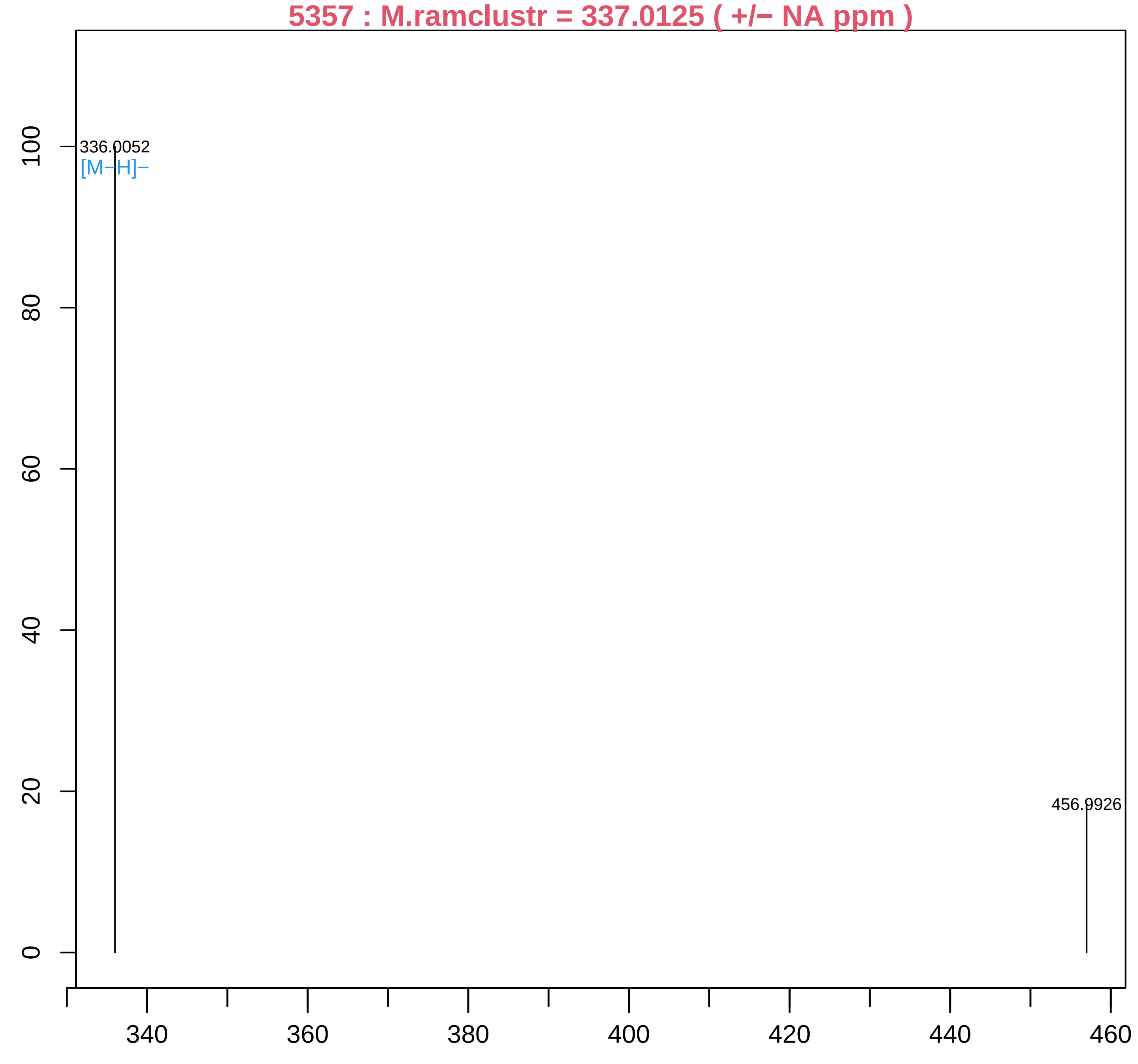

5357 : M.ramclustr = 337.0125 (+/− NA ppm)
336.0052
[M−H]−
456.9926
0
20
40
60
80
100
340
360
380
400
420
440
460

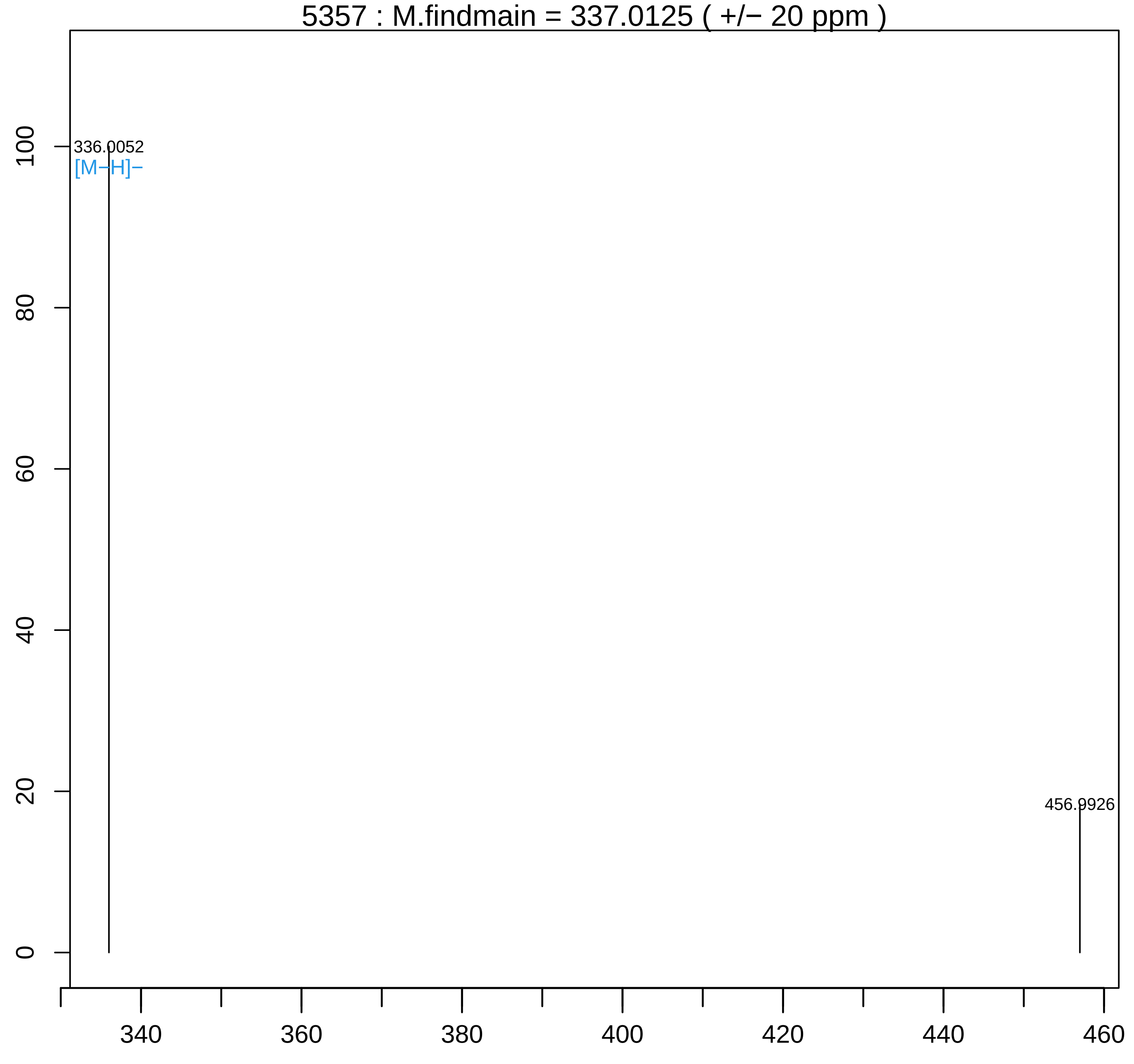

5357 : M.findmain = 337.0125 (+/− 20 ppm)
336.0052
[M−H]−
456.9926
0
20
40
60
80
100
340
360
380
400
420
440
460

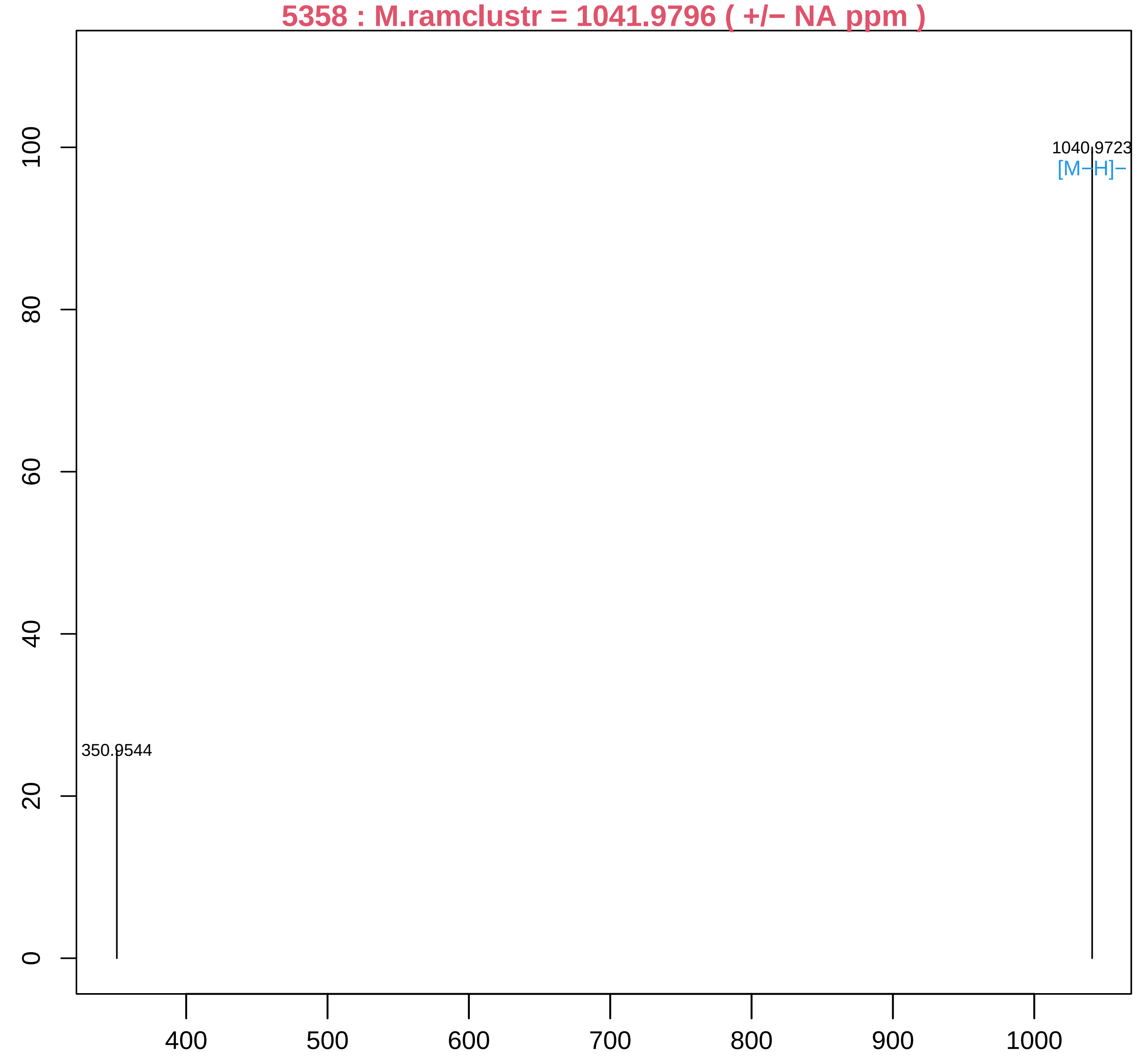
5358 : M.ramclustr = 1041.9796 (+/− NA ppm)
1040.9723
[M−H]−
350.9544
0
20
40
60
80
100
400
500
600
700
800
900
1000

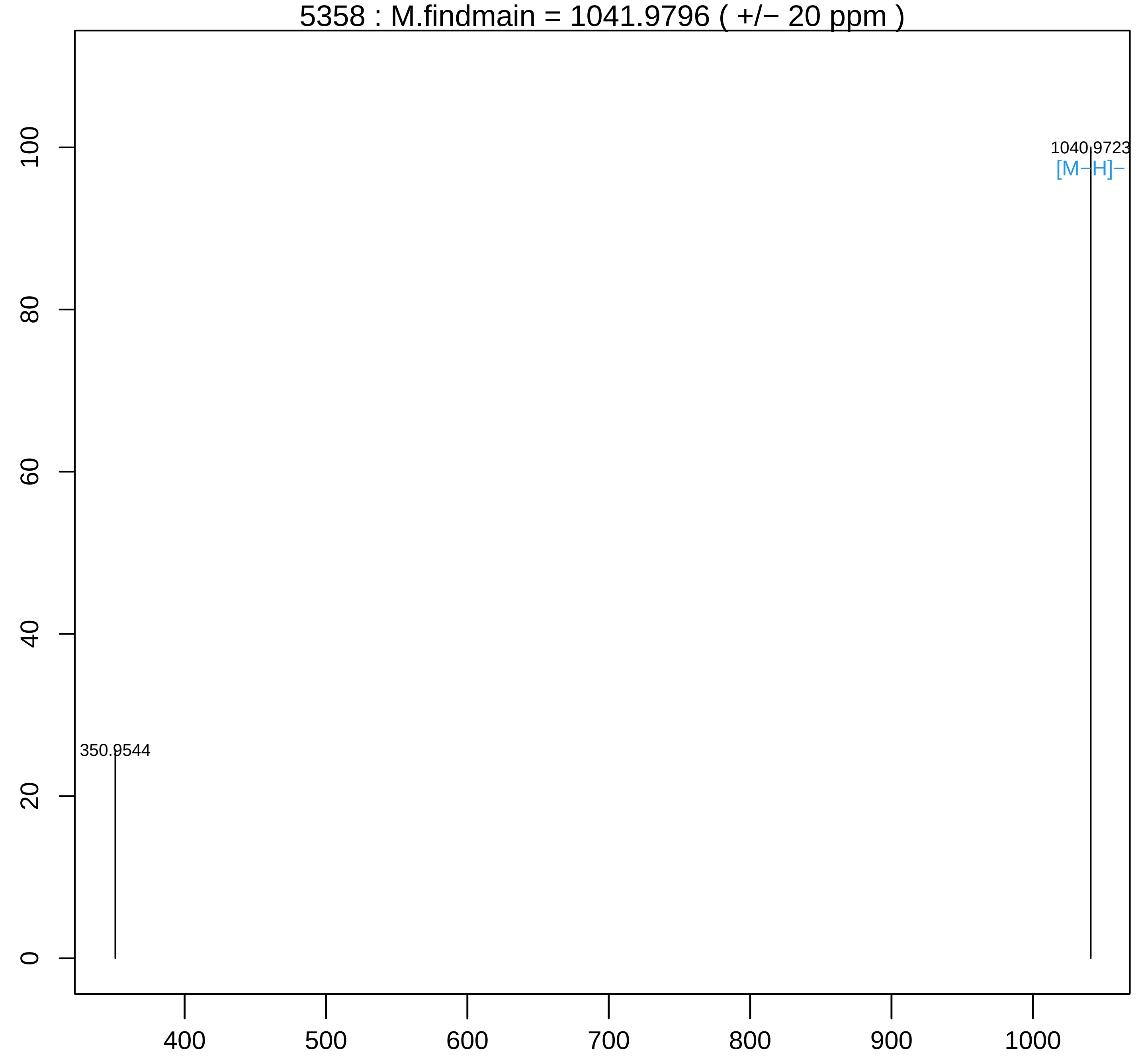
5358 : M.findmain = 1041.9796 (+/− 20 ppm)
1040.9723
[M−H]−
350.9544
0
20
40
60
80
100
400
500
600
700
800
900
1000

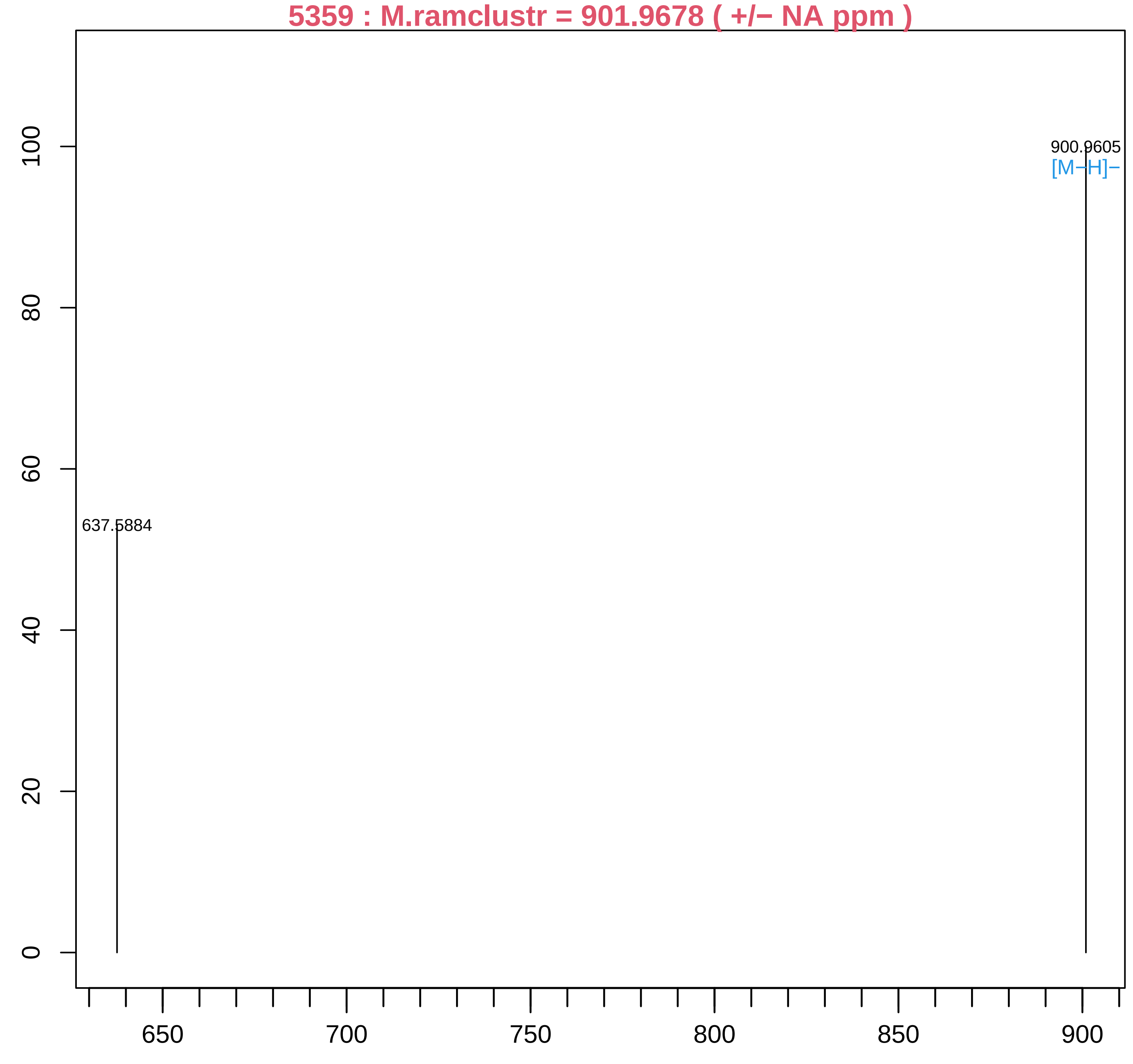
5359 : M.ramclustr = 901.9678 (+/− NA ppm)
900.9605
[M−H]−
637.5884
0
20
40
60
80
100
650
700
750
800
850
900

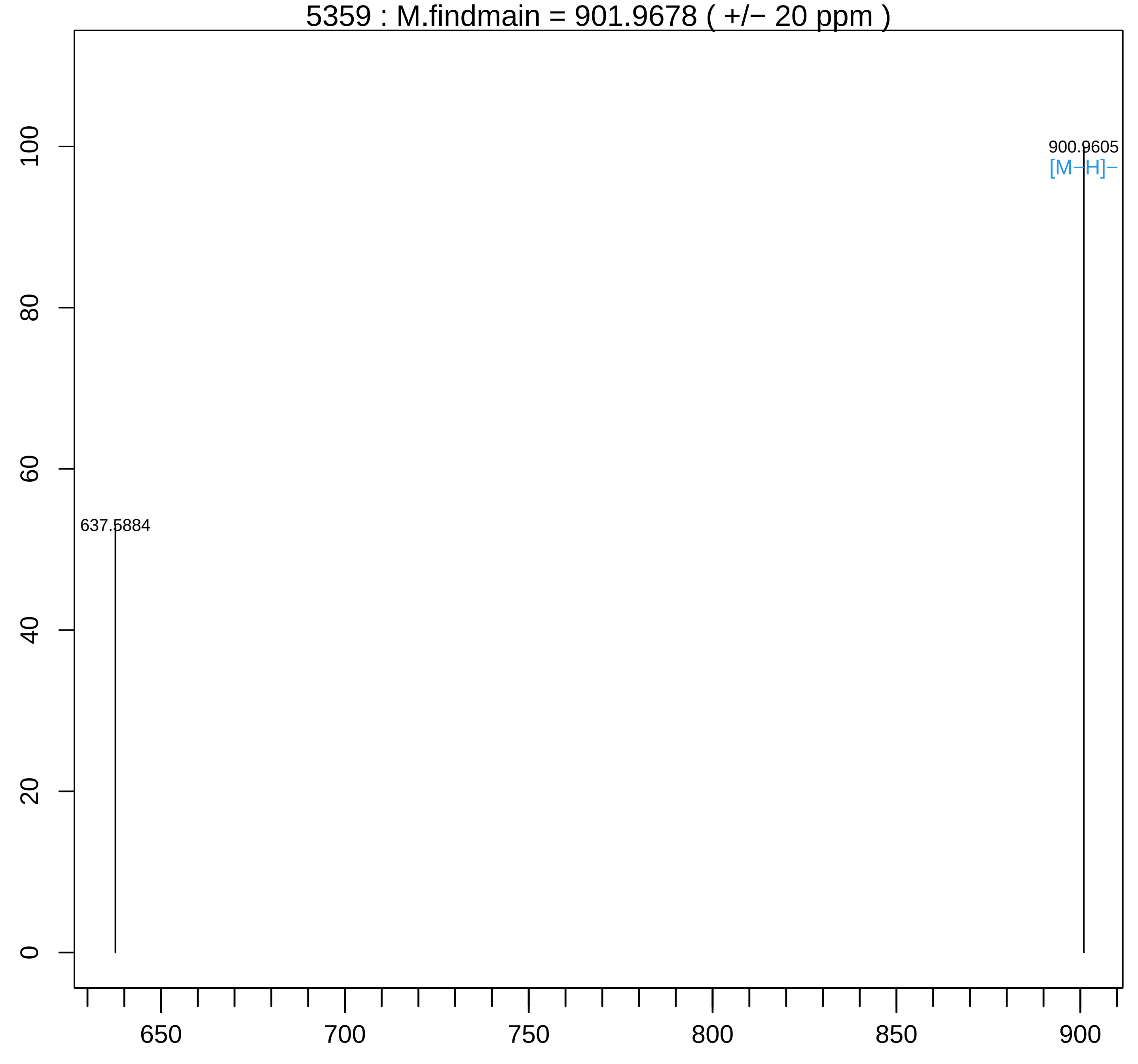
5359 : M.findmain = 901.9678 (+/− 20 ppm)
900.9605
[M−H]−
637.5884
0
20
40
60
80
100
650
700
750
800
850
900

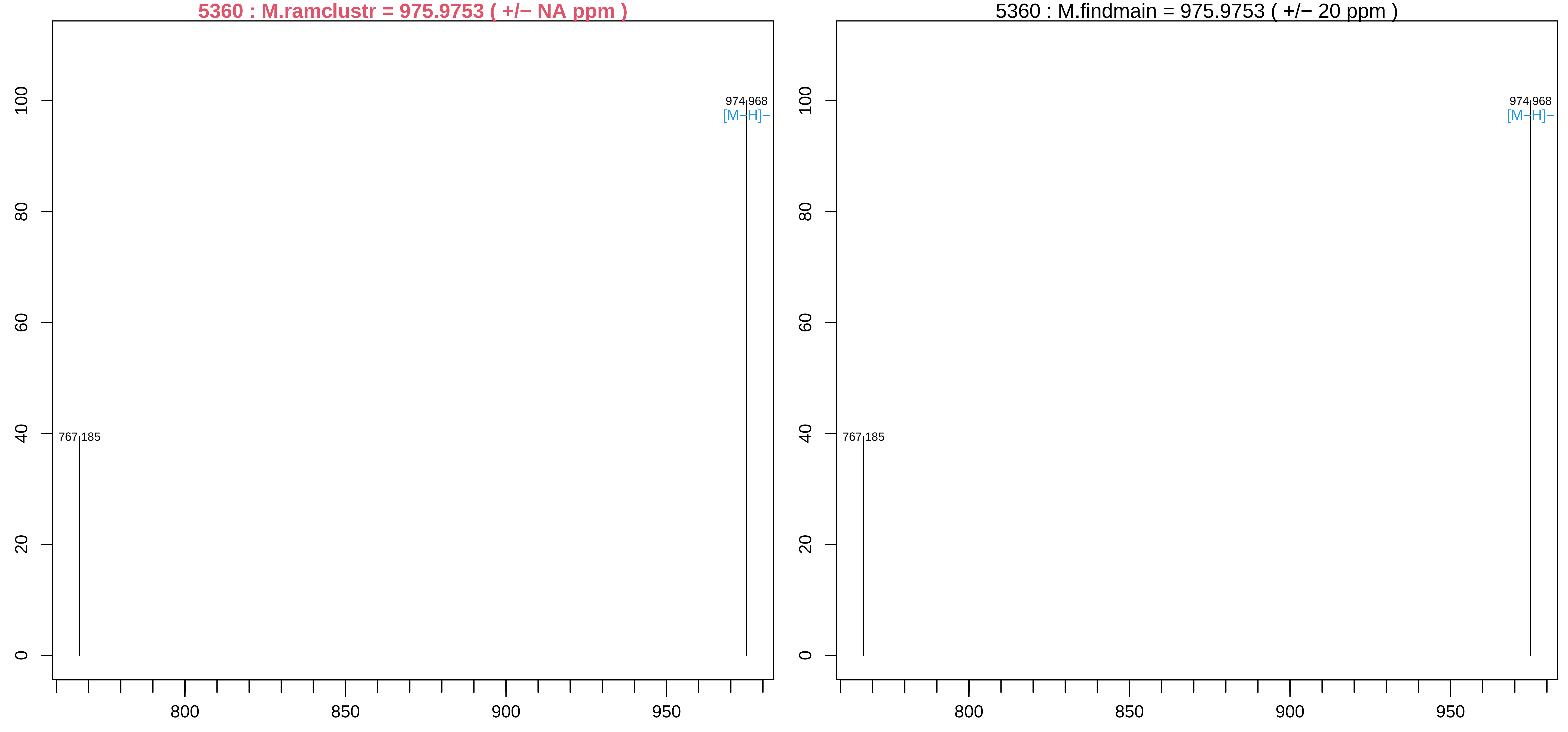
5360 : M.ramclustr = 975.9753 (+/− NA ppm)
5360 : M.findmain = 975.9753 (+/− 20 ppm)
974.968
[M−H]−
767.185
0
20
40
60
80
100
800
850
900
950

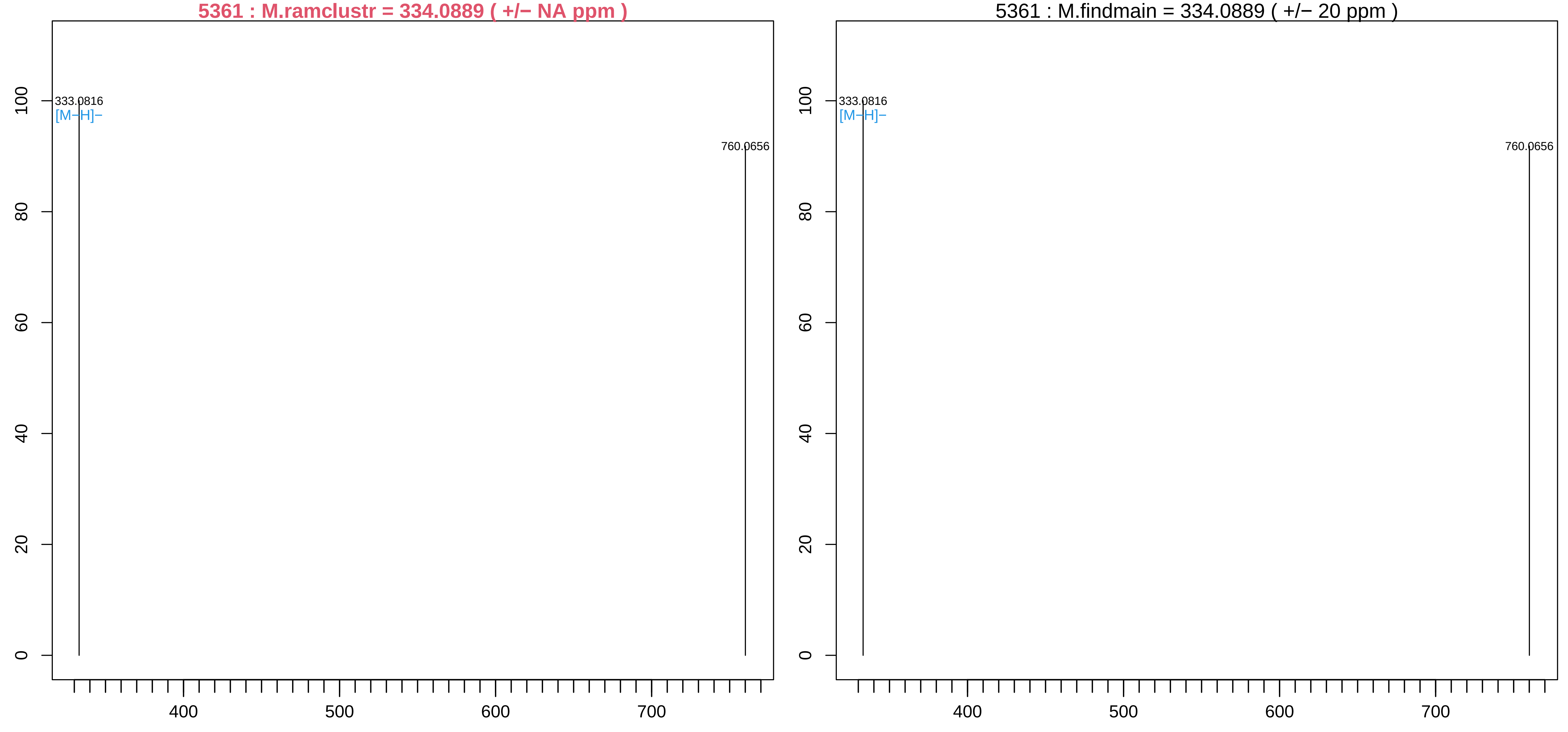
5361 : M.ramclustr = 334.0889 (+/− NA ppm)
5361 : M.findmain = 334.0889 (+/− 20 ppm)
333.0816
[M−H]−
760.0656
0
20
40
60
80
100
400
500
600
700

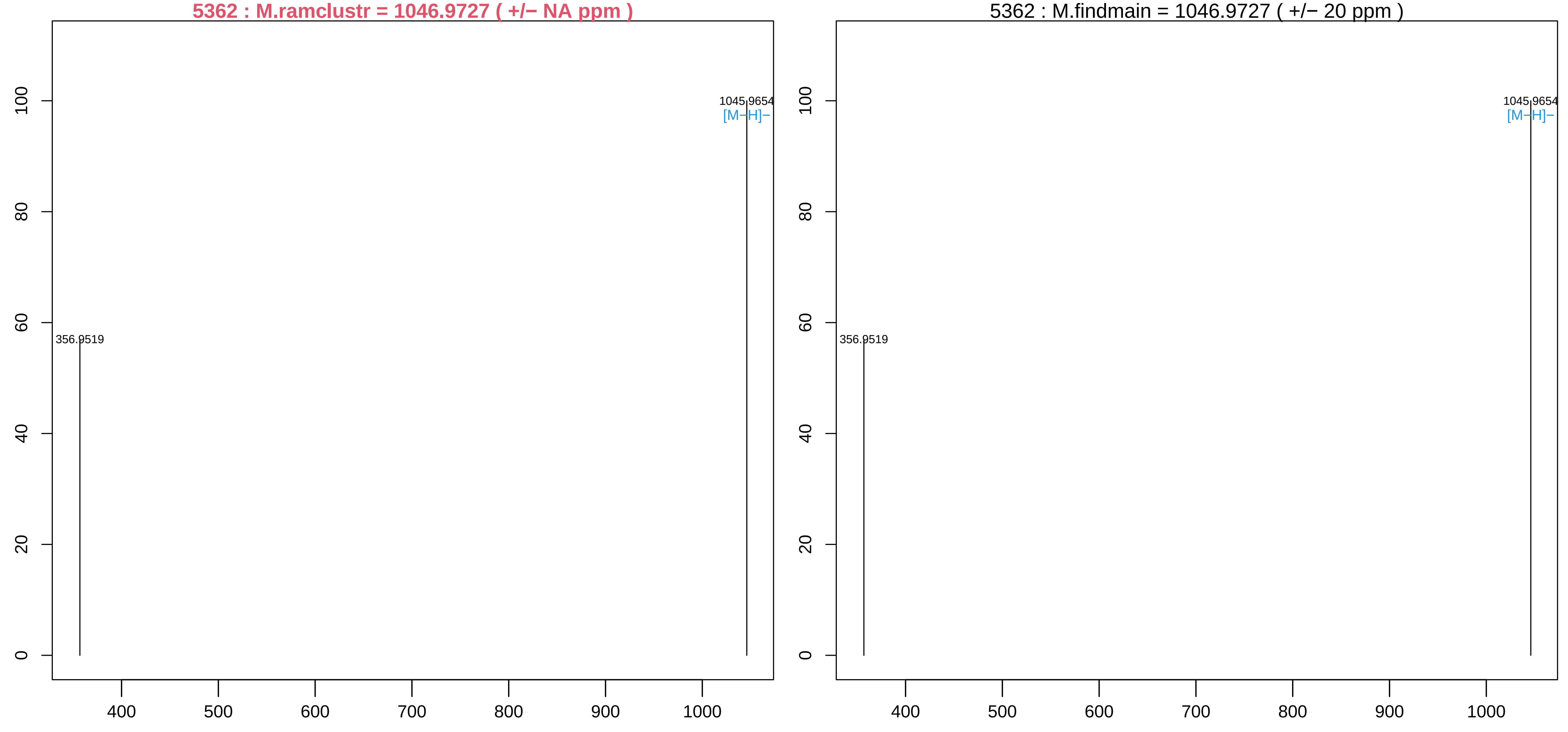
5362 : M.ramclustr = 1046.9727 (+/− NA ppm)
5362 : M.findmain = 1046.9727 (+/− 20 ppm)
1045.9654
[M−H]−
356.9519
0
20
40
60
80
100
400
500
600
700
800
900
1000

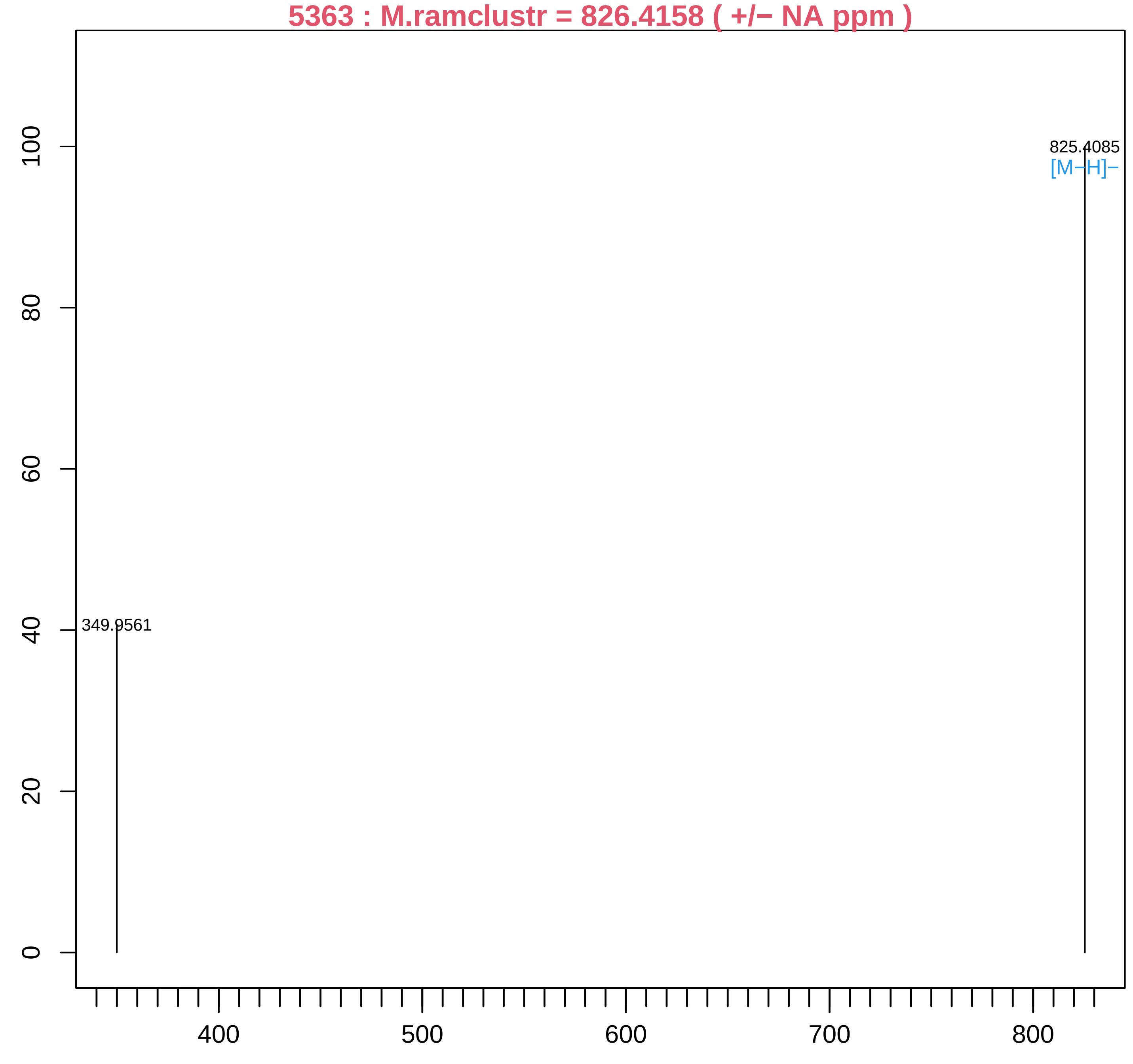
5363 : M.ramclustr = 826.4158 (+/− NA ppm)
825.4085
[M−H]−
349.9561
0
20
40
60
80
100
400
500
600
700
800

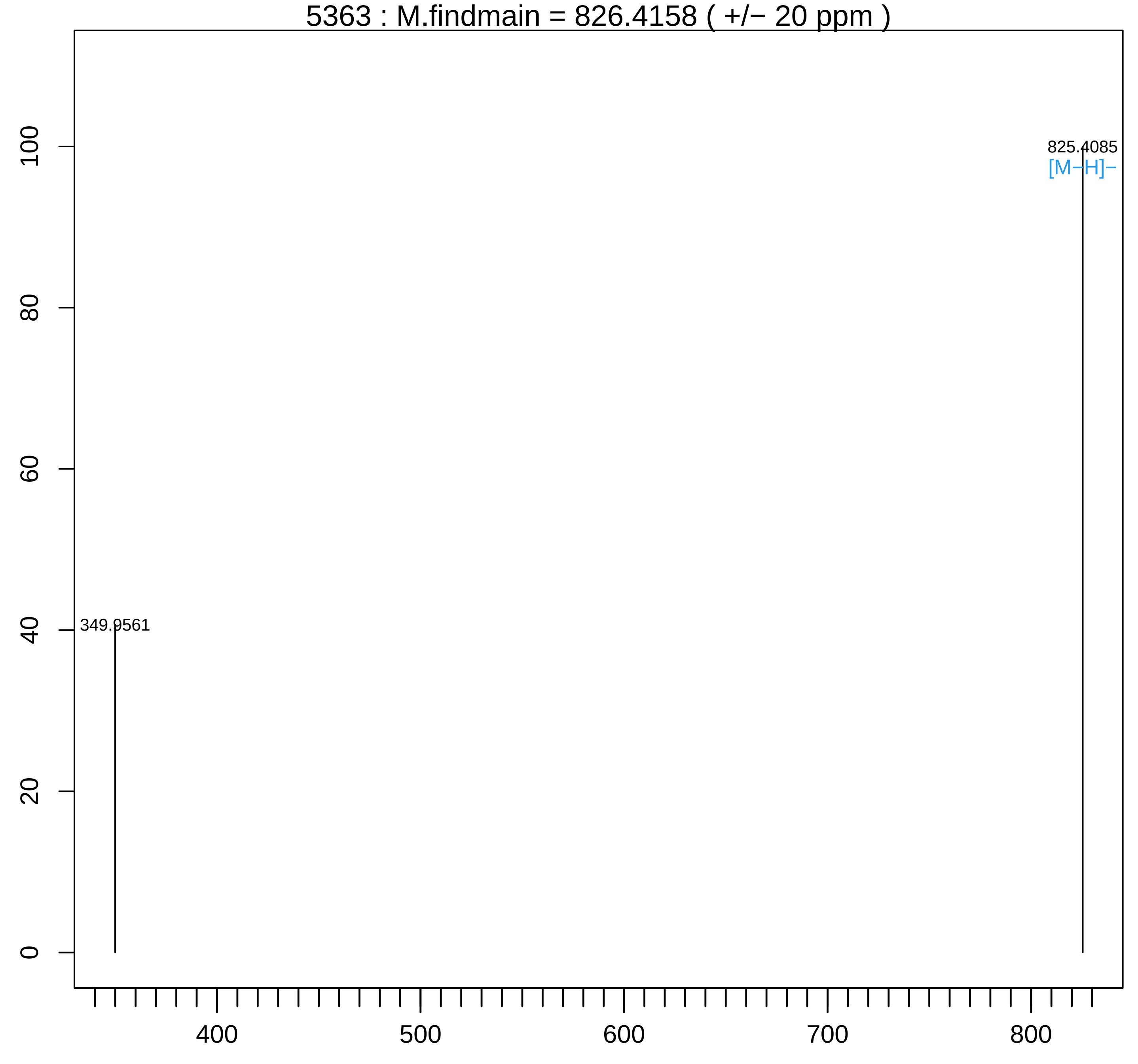
5363 : M.findmain = 826.4158 (+/− 20 ppm)
825.4085
[M−H]−
349.9561
0
20
40
60
80
100
400
500
600
700
800

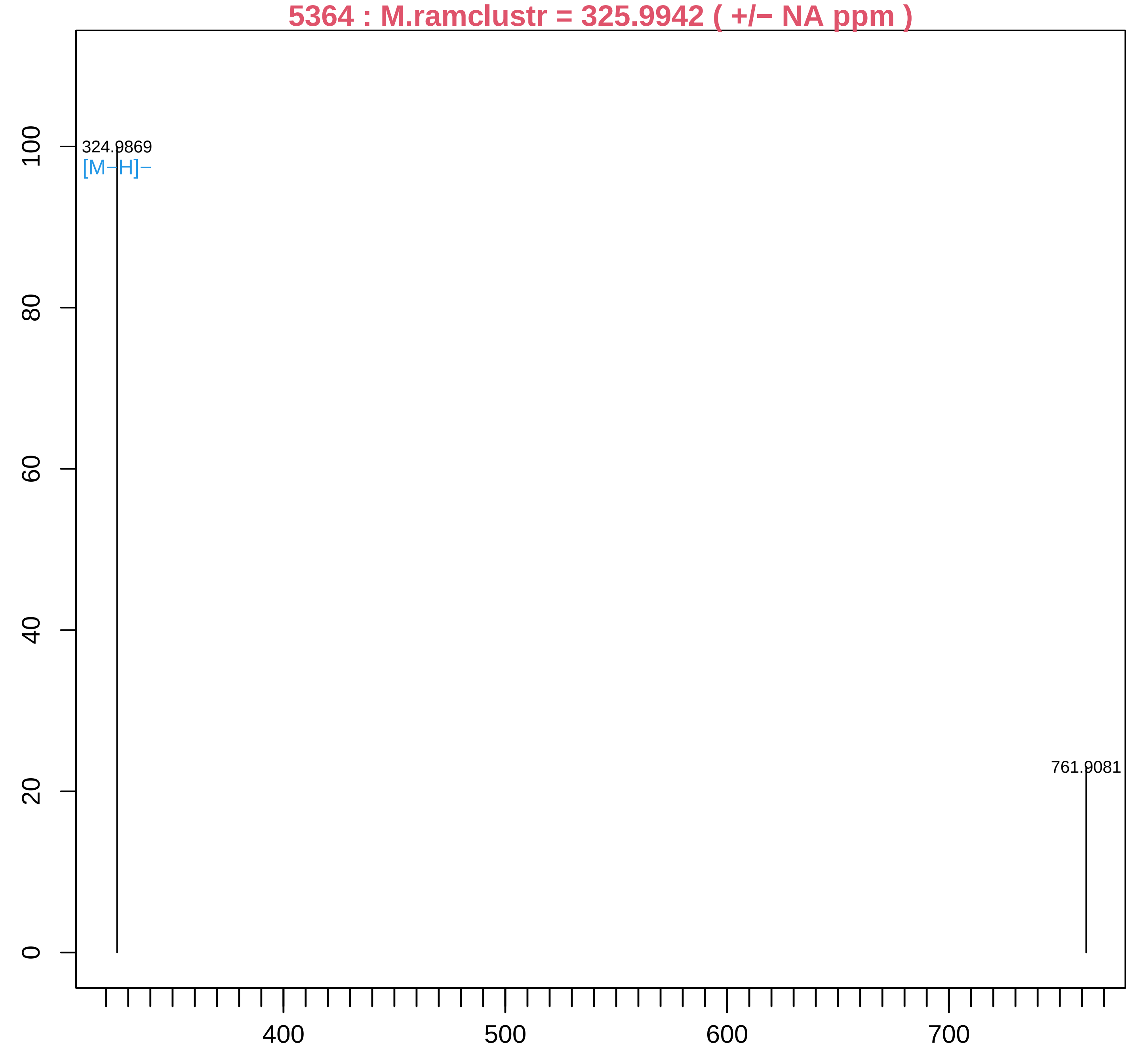

5364 : M.ramclustr = 325.9942 (+/− NA ppm)
324.9869
[M−H]−
761.9081
0
20
40
60
80
100
400
500
600
700

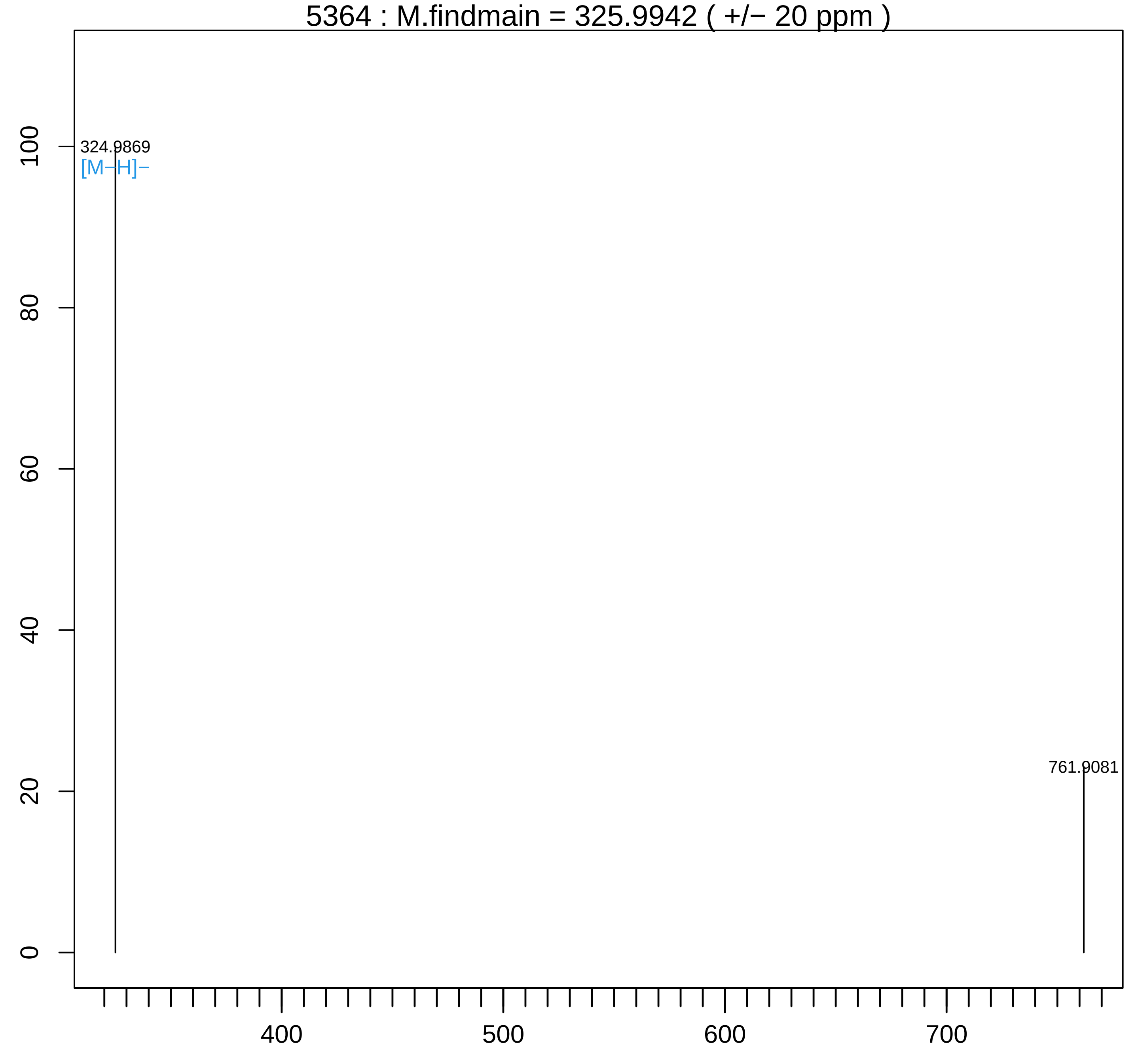

5364 : M.findmain = 325.9942 (+/− 20 ppm)
324.9869
[M−H]−
761.9081
0
20
40
60
80
100
400
500
600
700

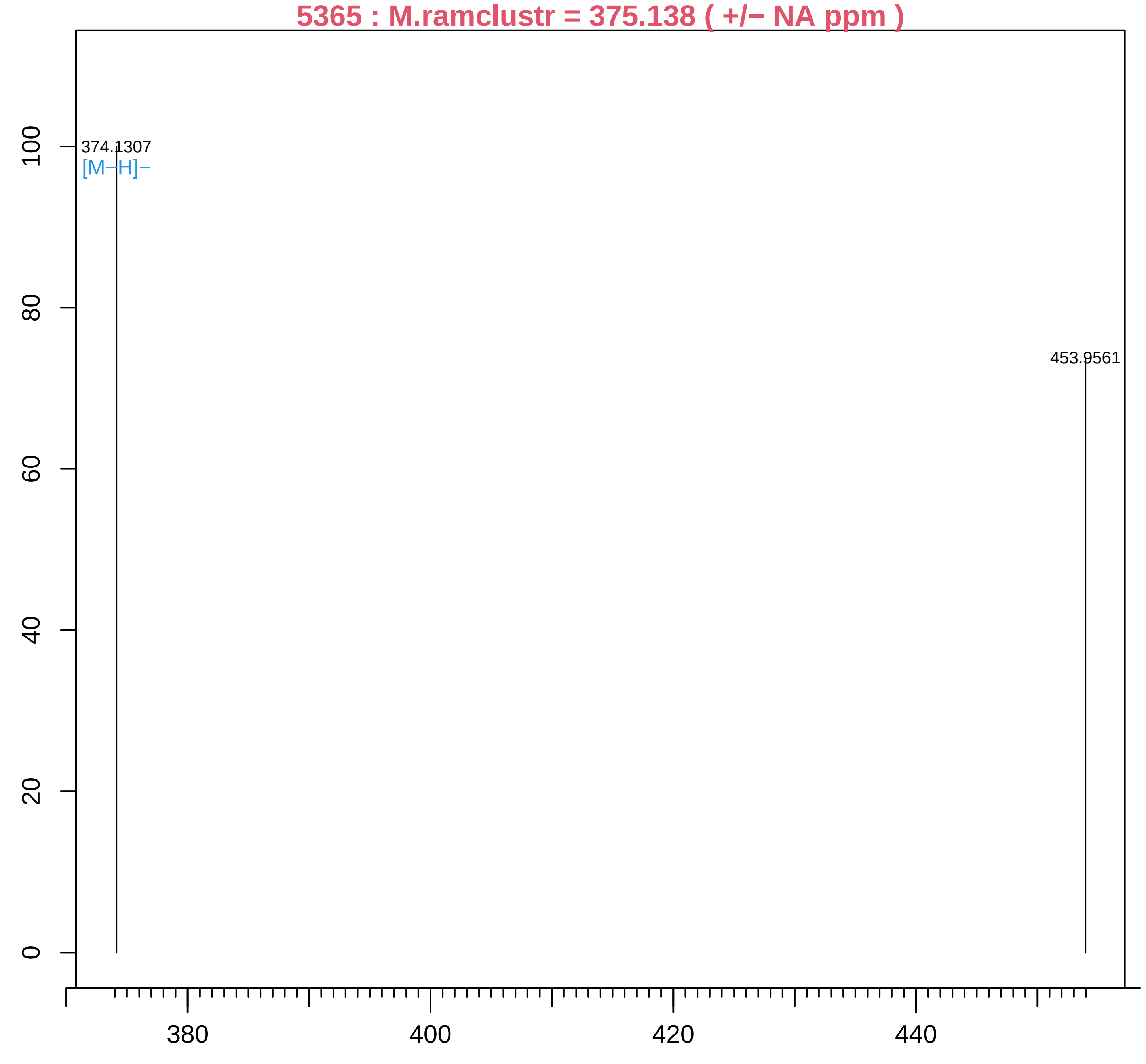

5365 : M.ramclustr = 375.138 (+/− NA ppm)
374.1307
[M−H]−
453.9561
0
20
40
60
80
100
380
400
420
440

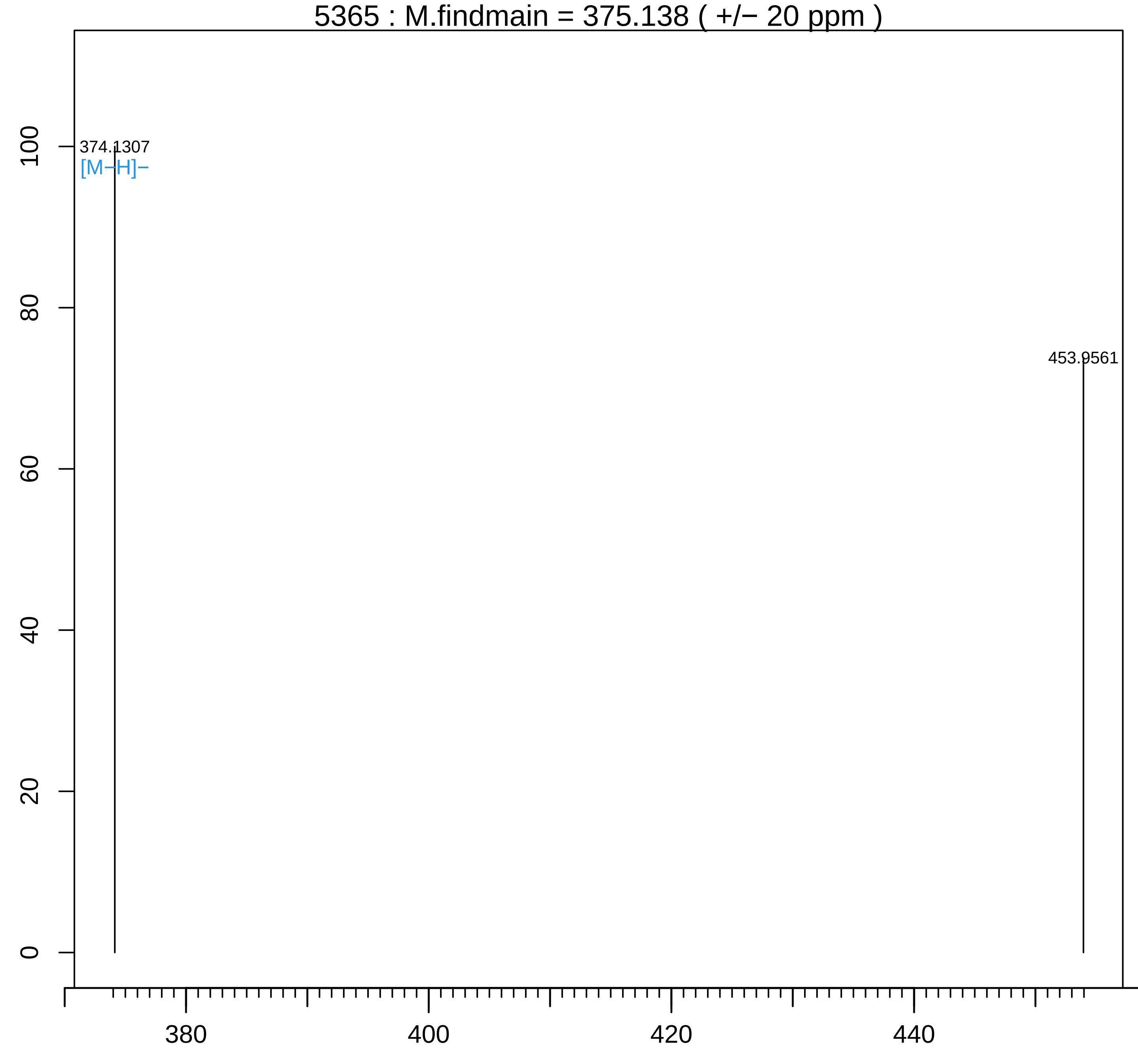

5365 : M.findmain = 375.138 (+/− 20 ppm)
374.1307
[M−H]−
453.9561
0
20
40
60
80
100
380
400
420
440

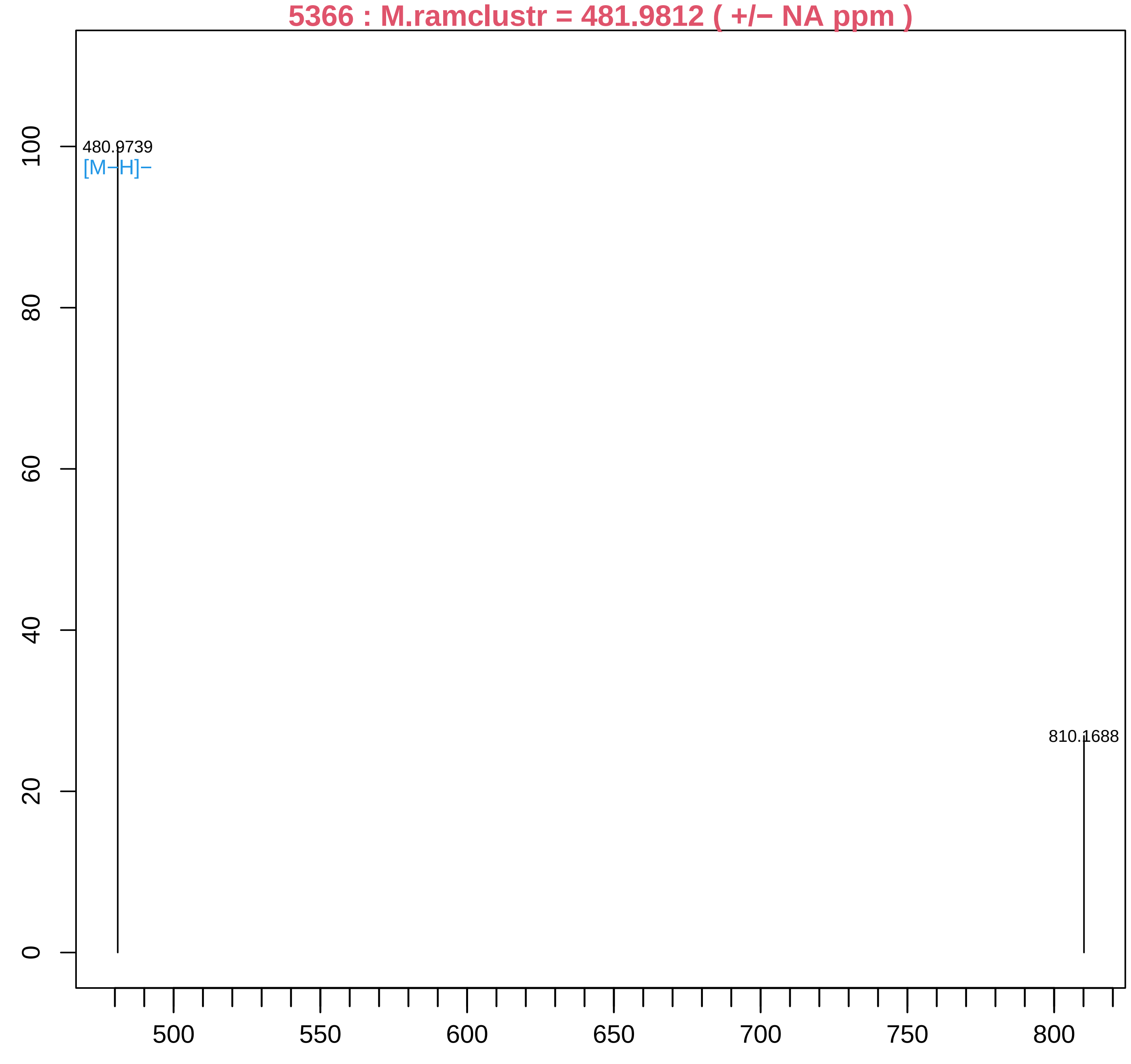
5366 : M.ramclustr = 481.9812 (+/− NA ppm)
480.9739
[M−H]−
810.1688
0
20
40
60
80
100
500
550
600
650
700
750
800

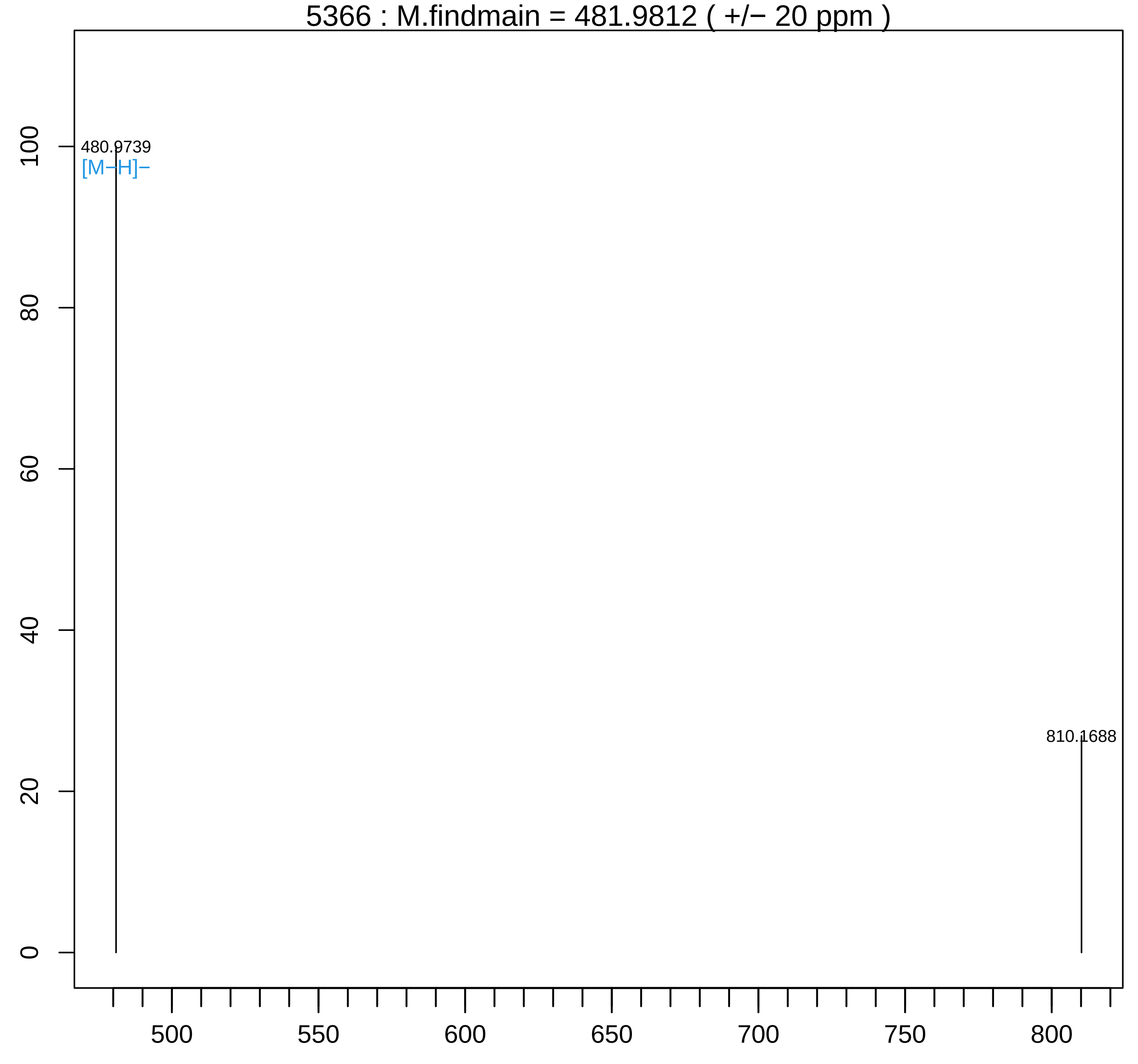
5366 : M.findmain = 481.9812 (+/− 20 ppm)
480.9739
[M−H]−
810.1688
0
20
40
60
80
100
500
550
600
650
700
750
800

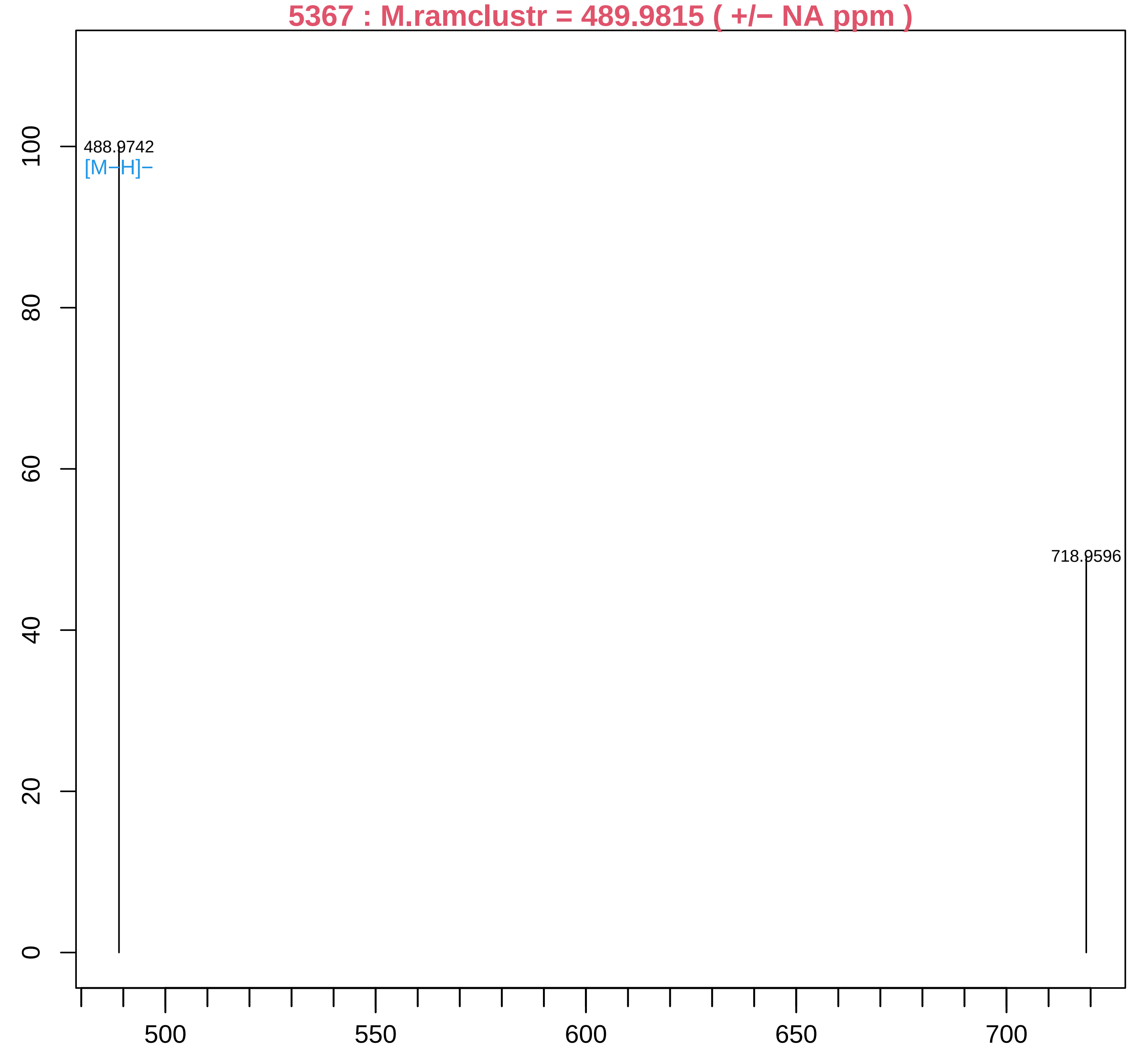

5367 : M.ramclustr = 489.9815 (+/− NA ppm)
488.9742
[M−H]−
718.9596
0
20
40
60
80
100
500
550
600
650
700

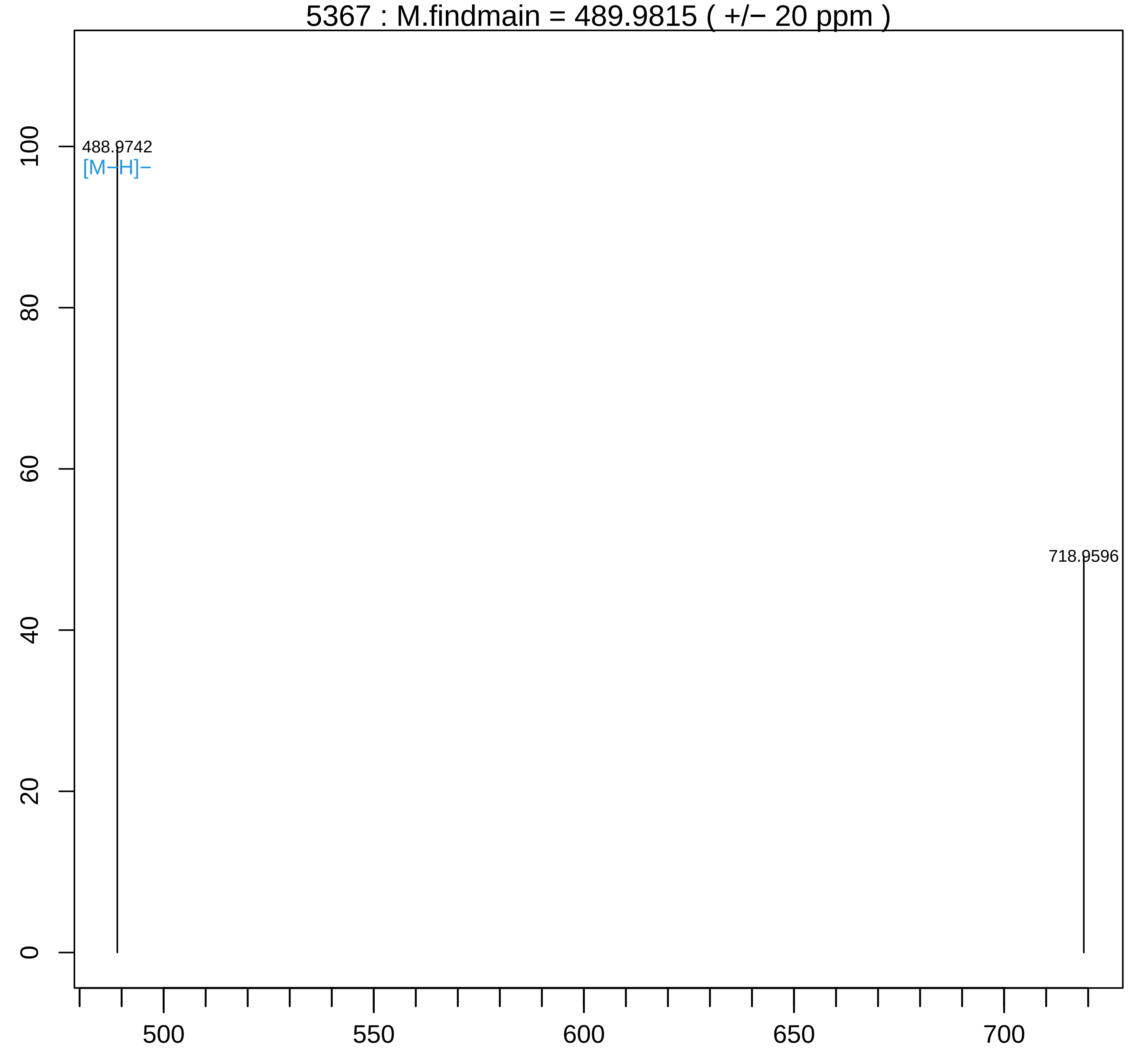

5367 : M.findmain = 489.9815 (+/− 20 ppm)
488.9742
[M−H]−
718.9596
0
20
40
60
80
100
500
550
600
650
700

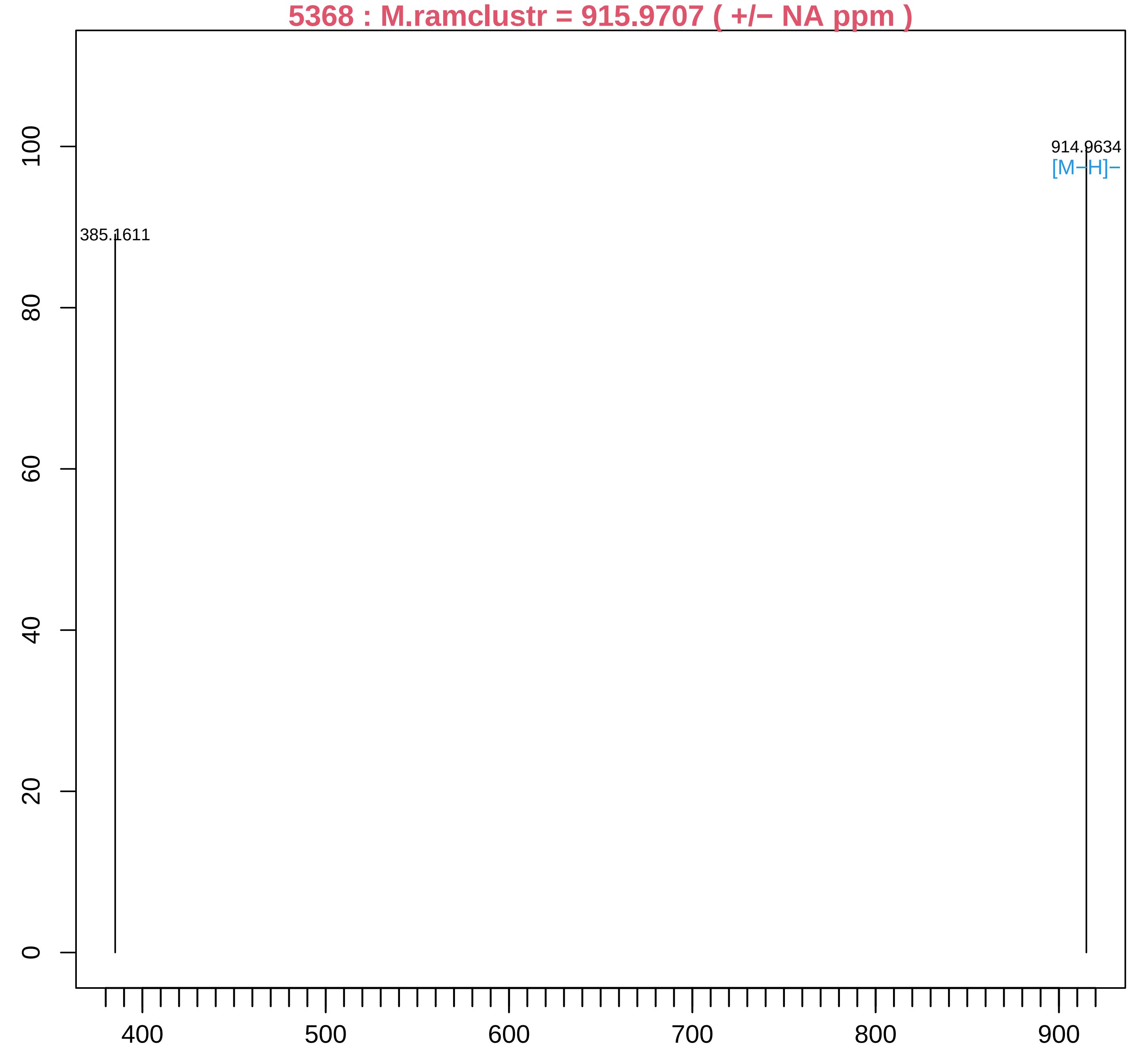
5368 : M.ramclustr = 915.9707 (+/− NA ppm)
914.9634
[M−H]−
385.1611
0
20
40
60
80
100
400
500
600
700
800
900

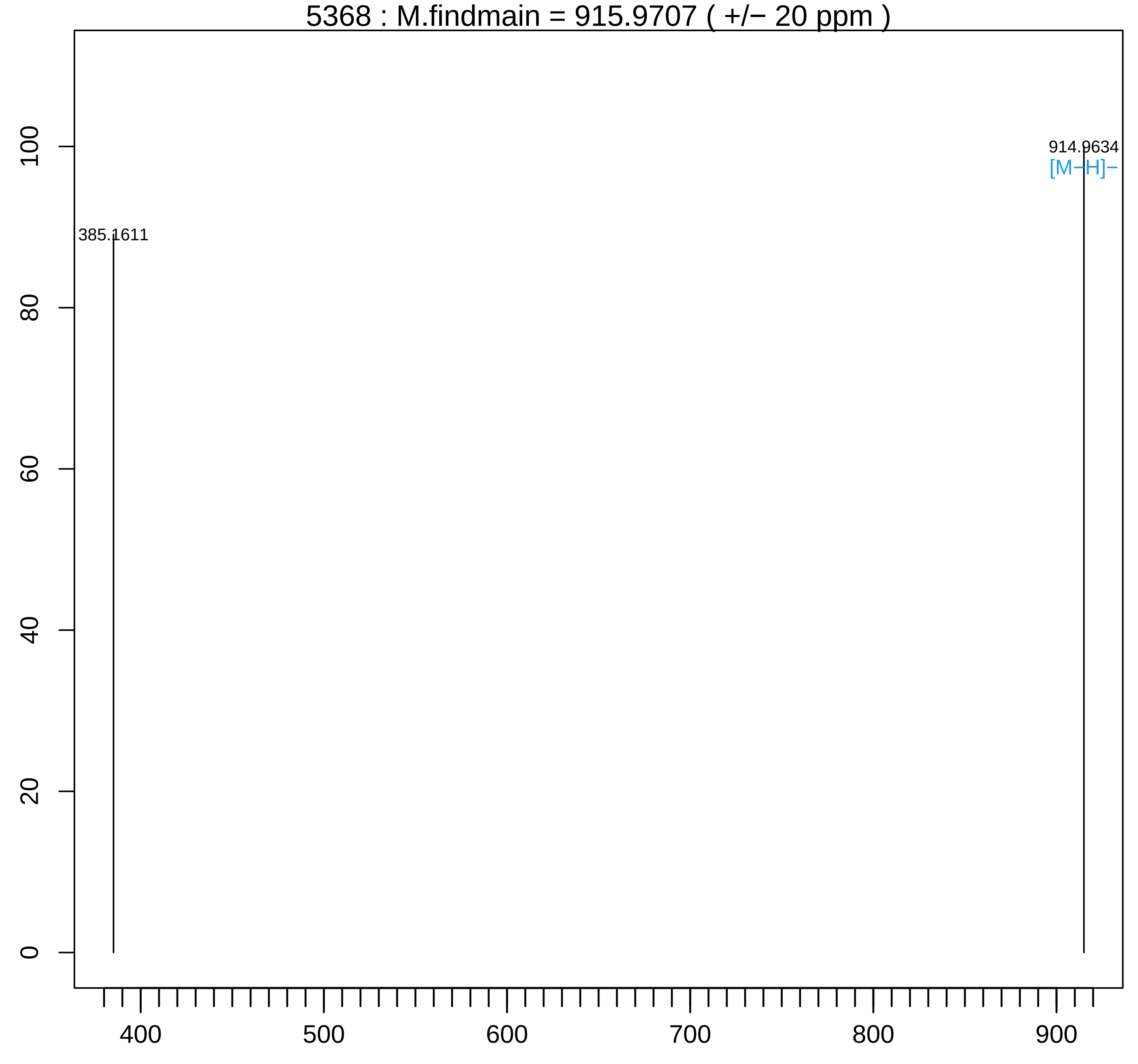
5368 : M.findmain = 915.9707 (+/− 20 ppm)
914.9634
[M−H]−
385.1611
0
20
40
60
80
100
400
500
600
700
800
900

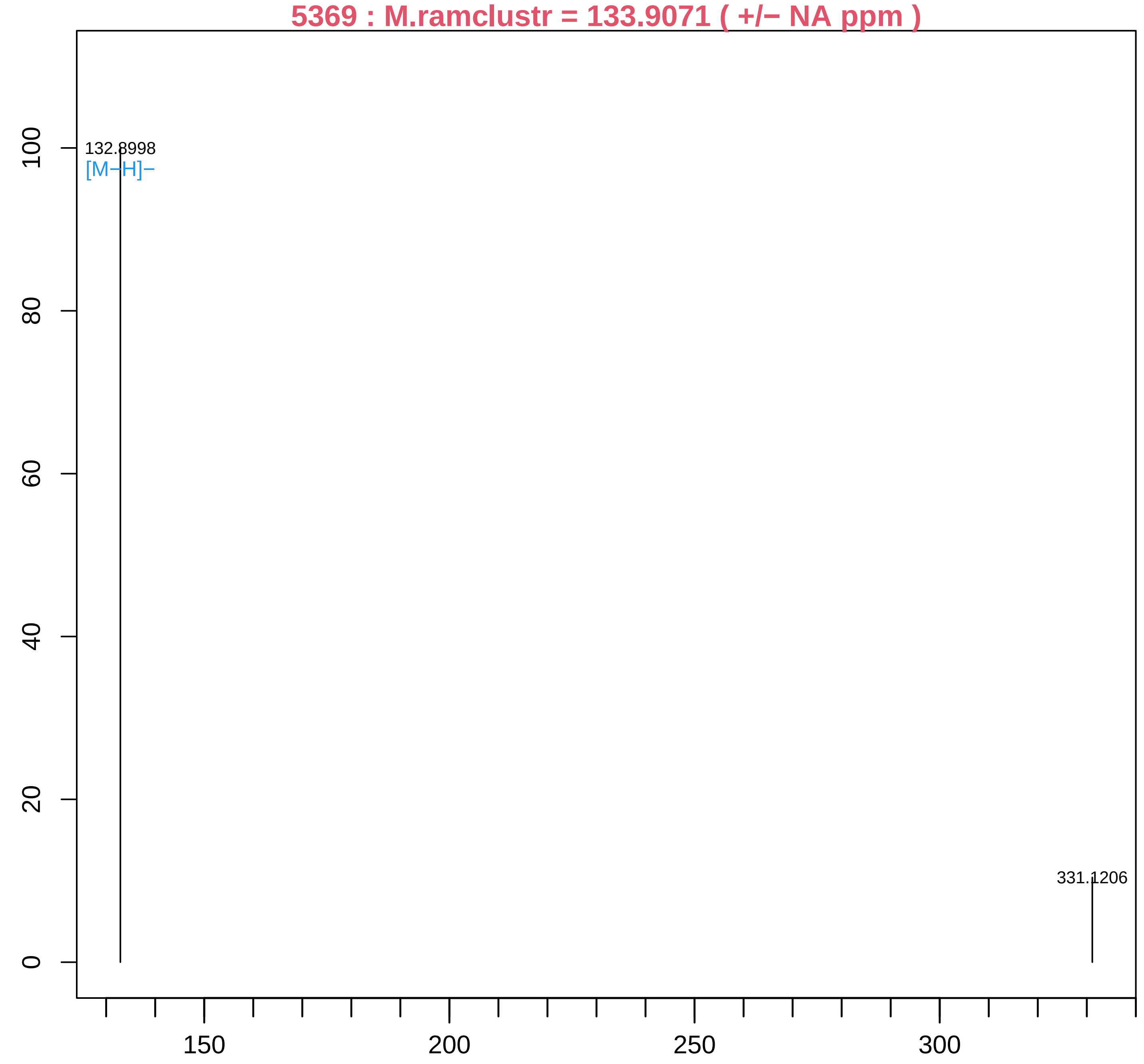
5369 : M.ramclustr = 133.9071 (+/− NA ppm)
132.8998
[M−H]−
331.1206
0
20
40
60
80
100
150
200
250
300

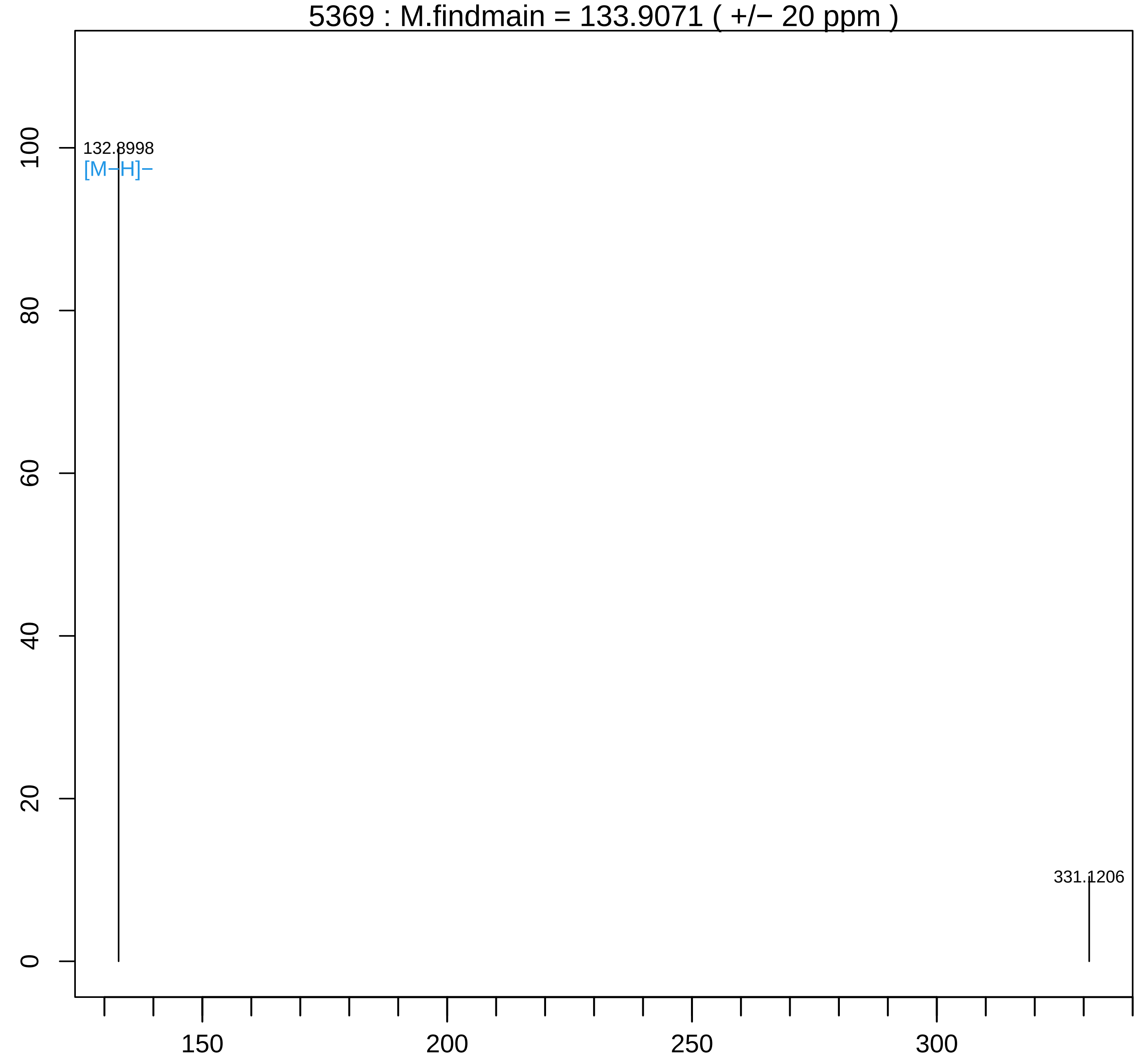
5369 : M.findmain = 133.9071 (+/− 20 ppm)
132.8998
[M−H]−
331.1206
0
20
40
60
80
100
150
200
250
300

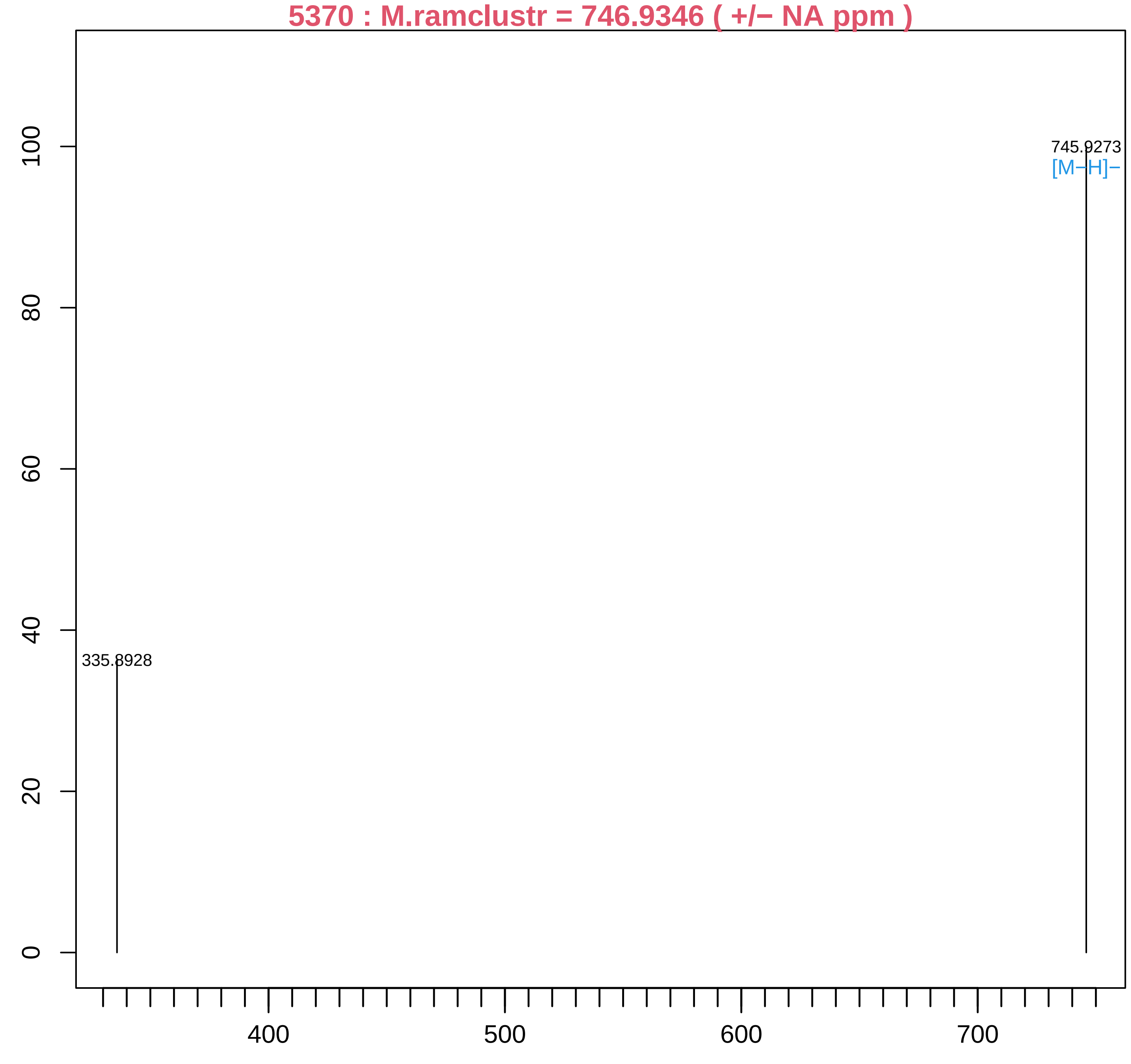
5370 : M.ramclustr = 746.9346 (+/− NA ppm)
745.9273
[M−H]−
335.8928
0
20
40
60
80
100
400
500
600
700

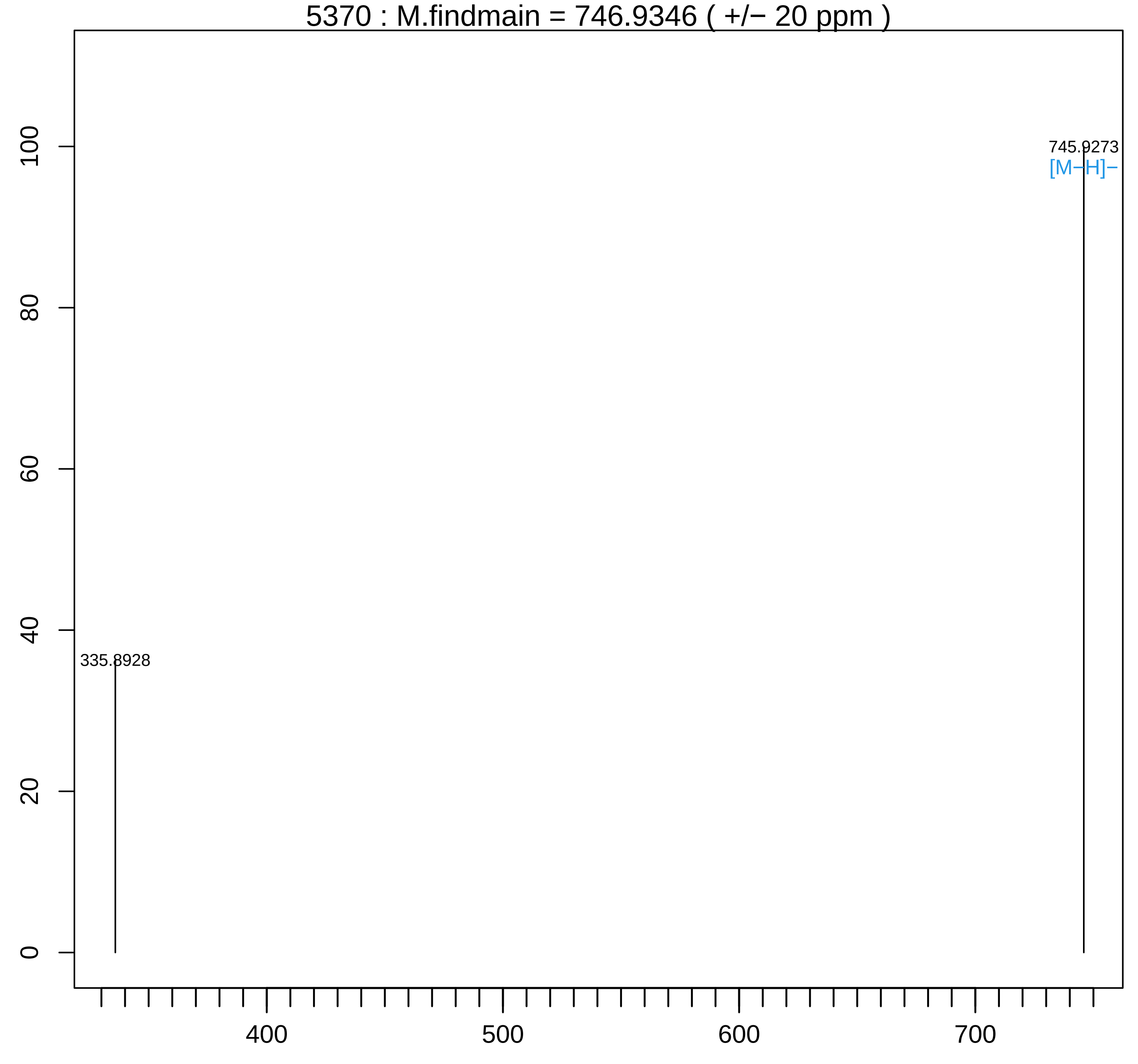
5370 : M.findmain = 746.9346 (+/− 20 ppm)
745.9273
[M−H]−
335.8928
0
20
40
60
80
100
400
500
600
700

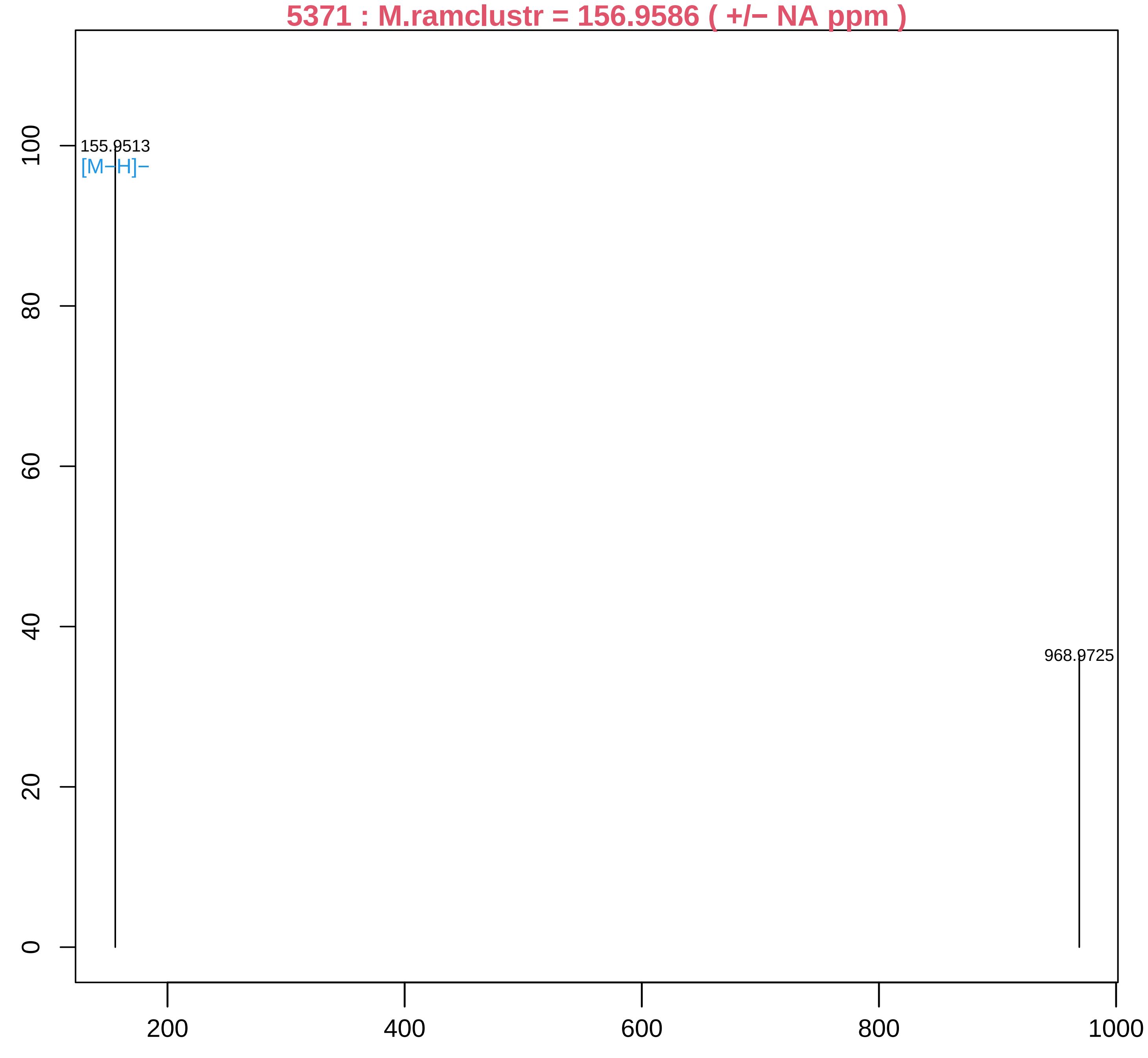
5371 : M.ramclustr = 156.9586 (+/− NA ppm)
155.9513
[M−H]−
968.9725
0
20
40
60
80
100
200
400
600
800
1000

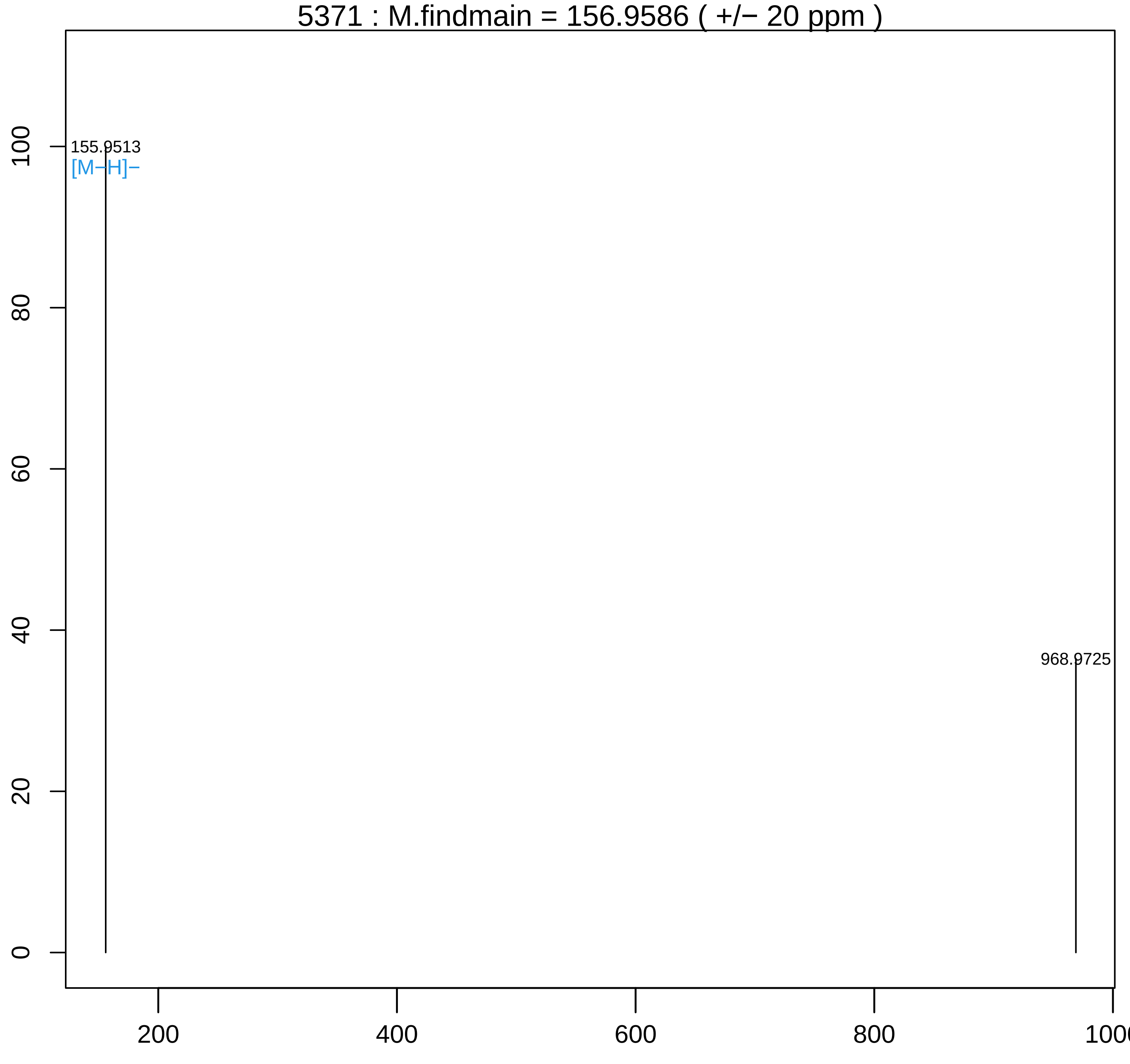
5371 : M.findmain = 156.9586 (+/− 20 ppm)
155.9513
[M−H]−
968.9725
0
20
40
60
80
100
200
400
600
800

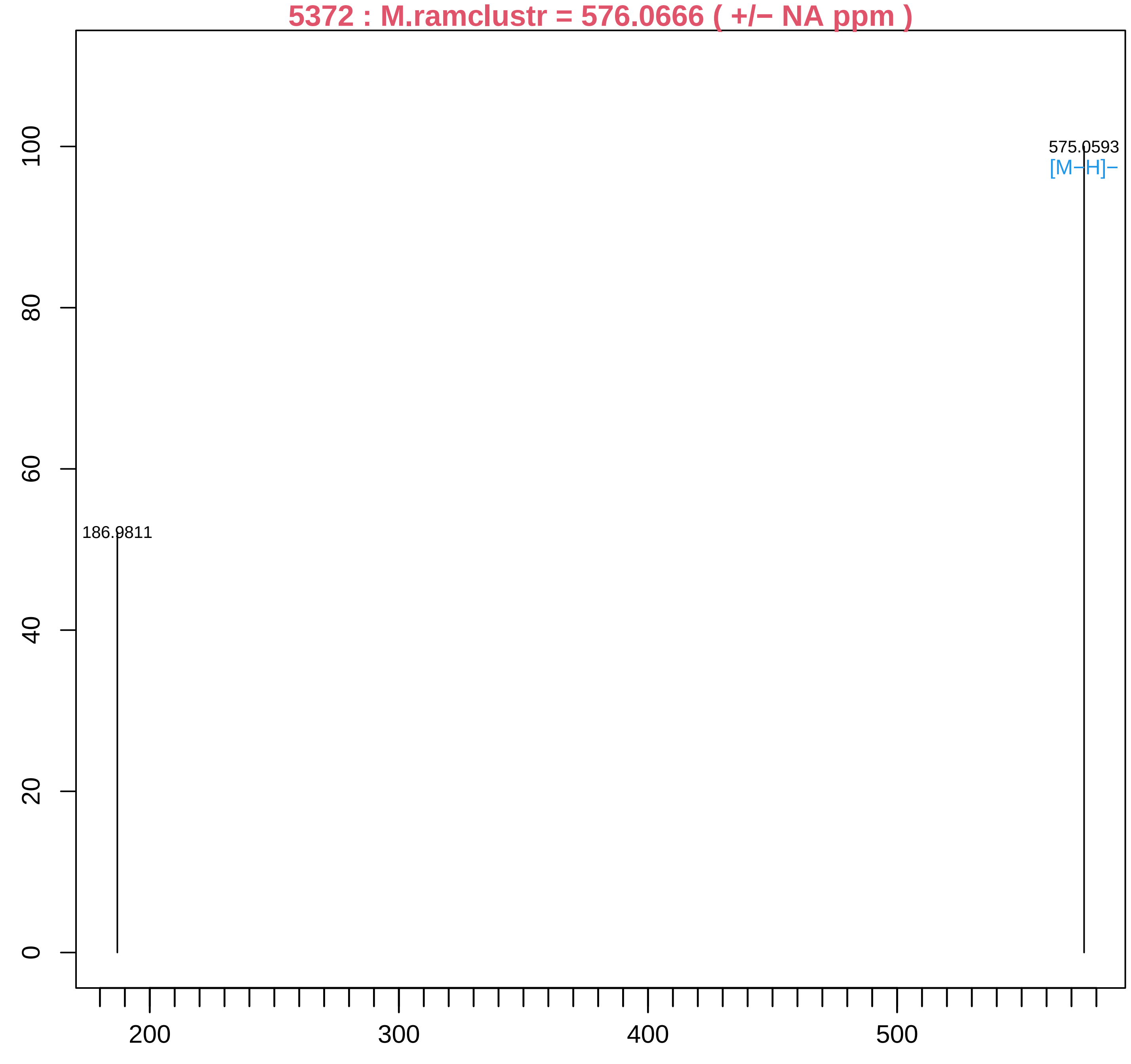
5372 : M.ramclustr = 576.0666 (+/− NA ppm)
575.0593
[M−H]−
186.9811
0
20
40
60
80
100
200
300
400
500

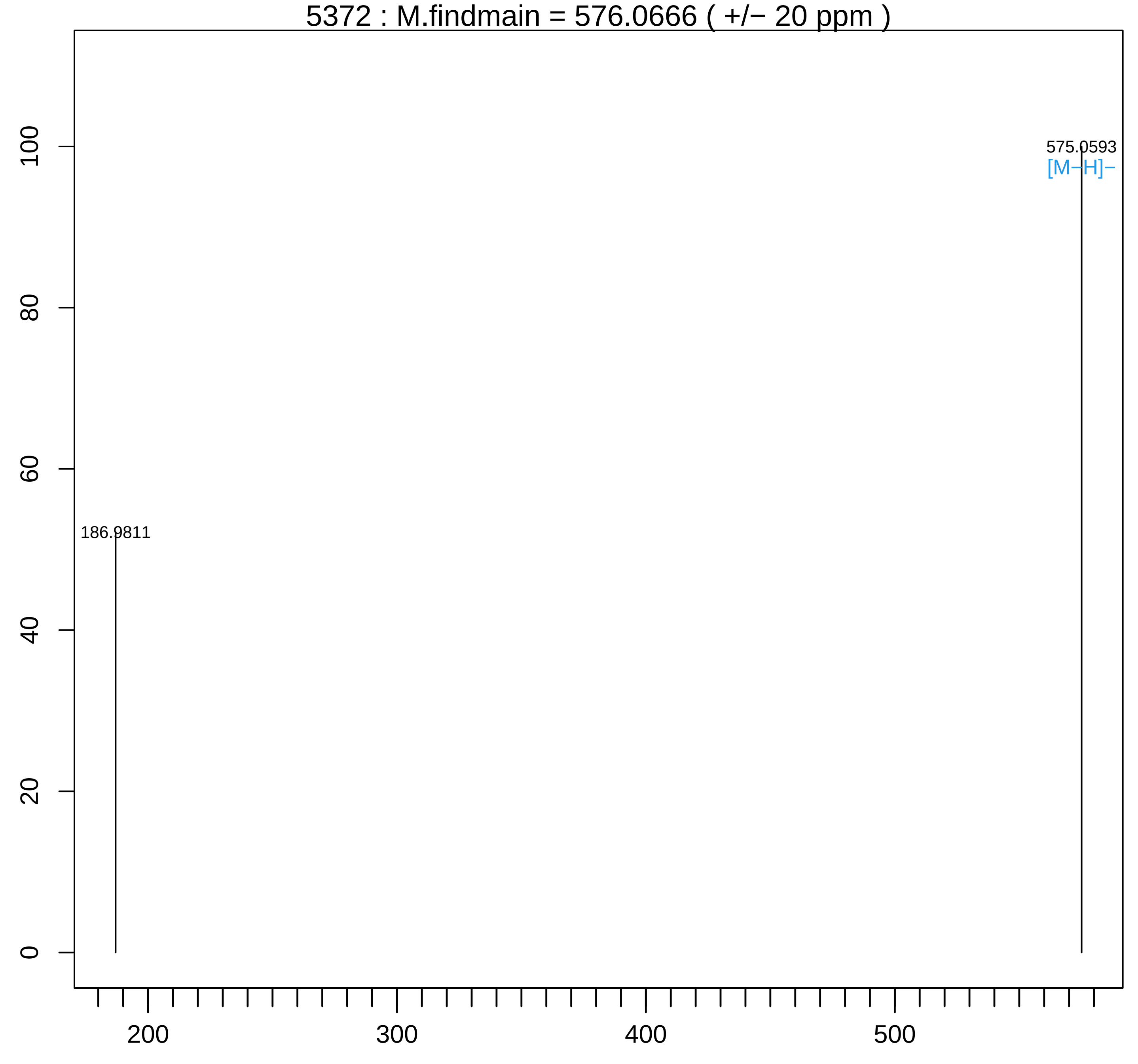
5372 : M.findmain = 576.0666 (+/− 20 ppm)
575.0593
[M−H]−
186.9811
0
20
40
60
80
100
200
300
400
500

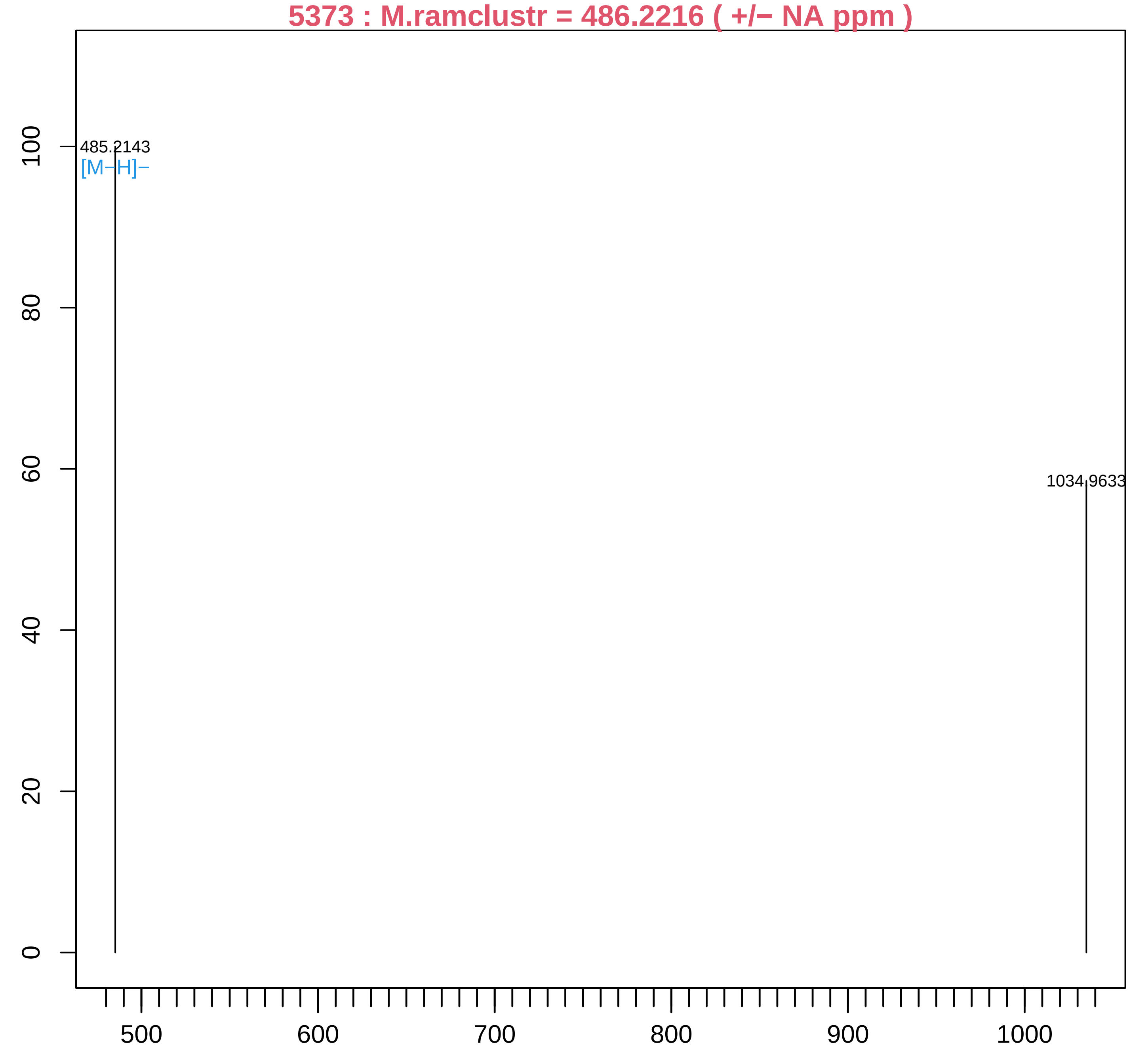

5373 : M.ramclustr = 486.2216 (+/− NA ppm)
485.2143
[M−H]−
1034.9633
0
20
40
60
80
100
500
600
700
800
900
1000

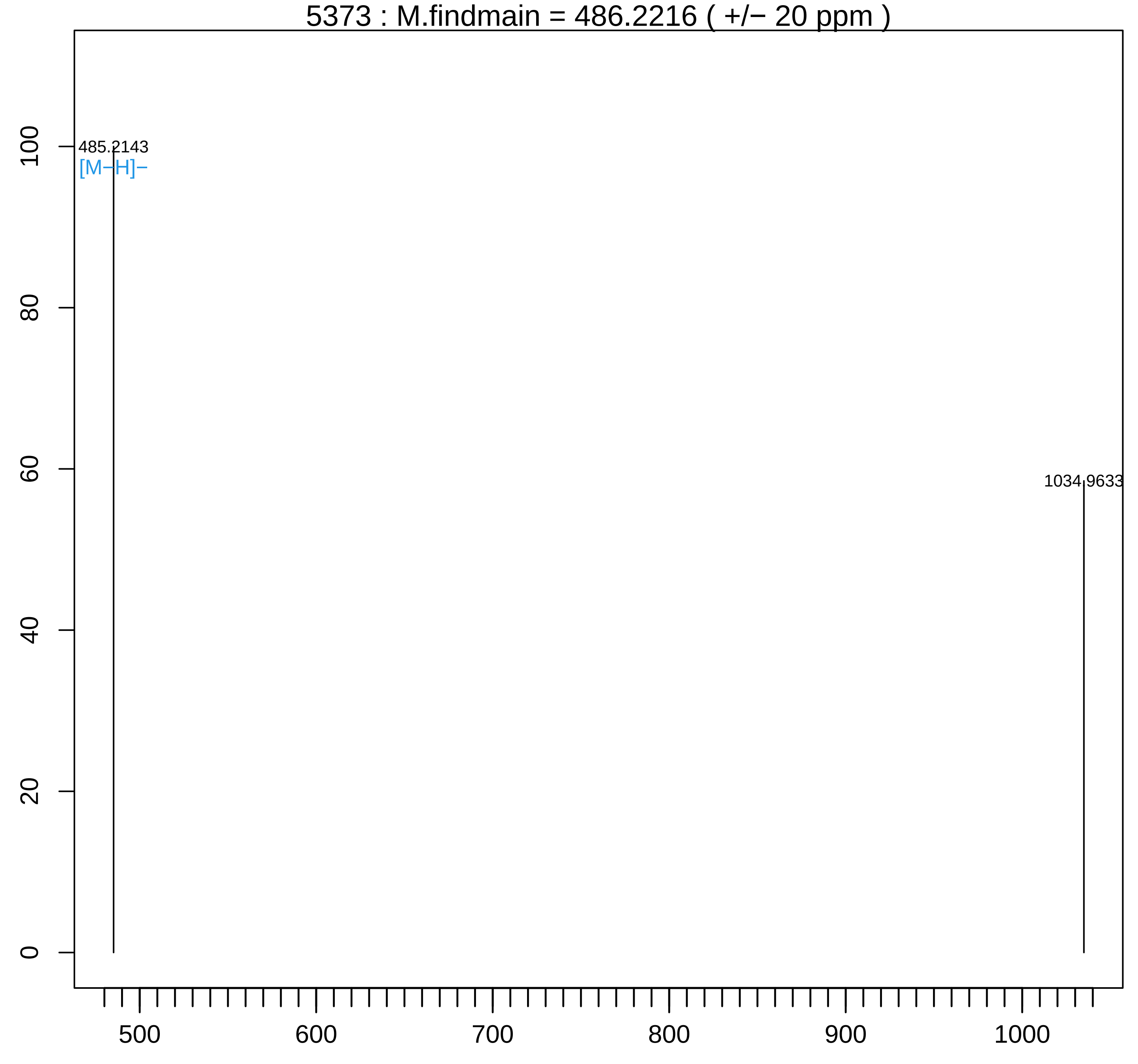

5373 : M.findmain = 486.2216 (+/− 20 ppm)
485.2143
[M−H]−
1034.9633
0
20
40
60
80
100
500
600
700
800
900
1000

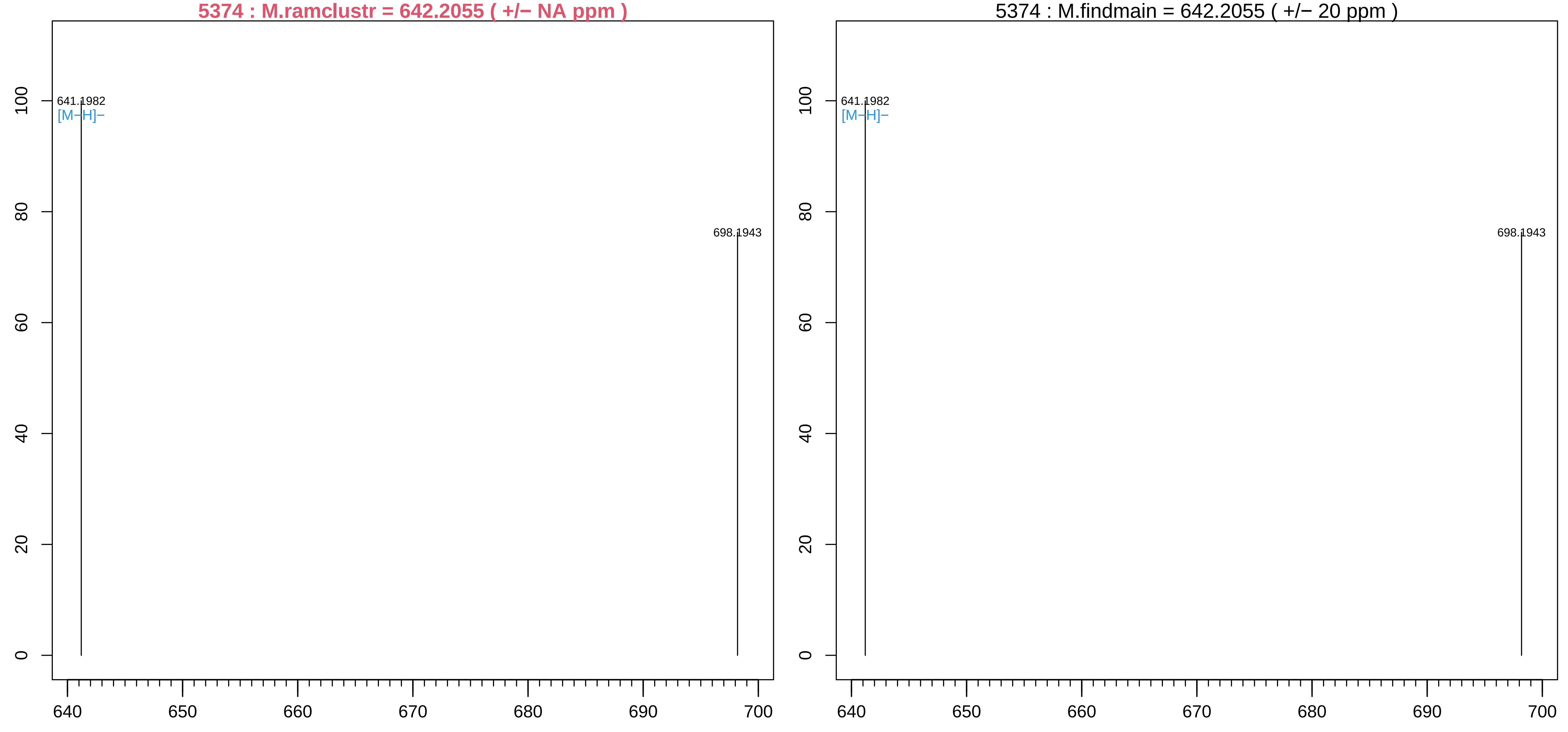

5374 : M.ramclustr = 642.2055 (+/− NA ppm)
641.1982
[M−H]−
698.1943
5374 : M.findmain = 642.2055 (+/− 20 ppm)
641.1982
[M−H]−
698.1943
0
20
40
60
80
100
640
650
660
670
680
690
700

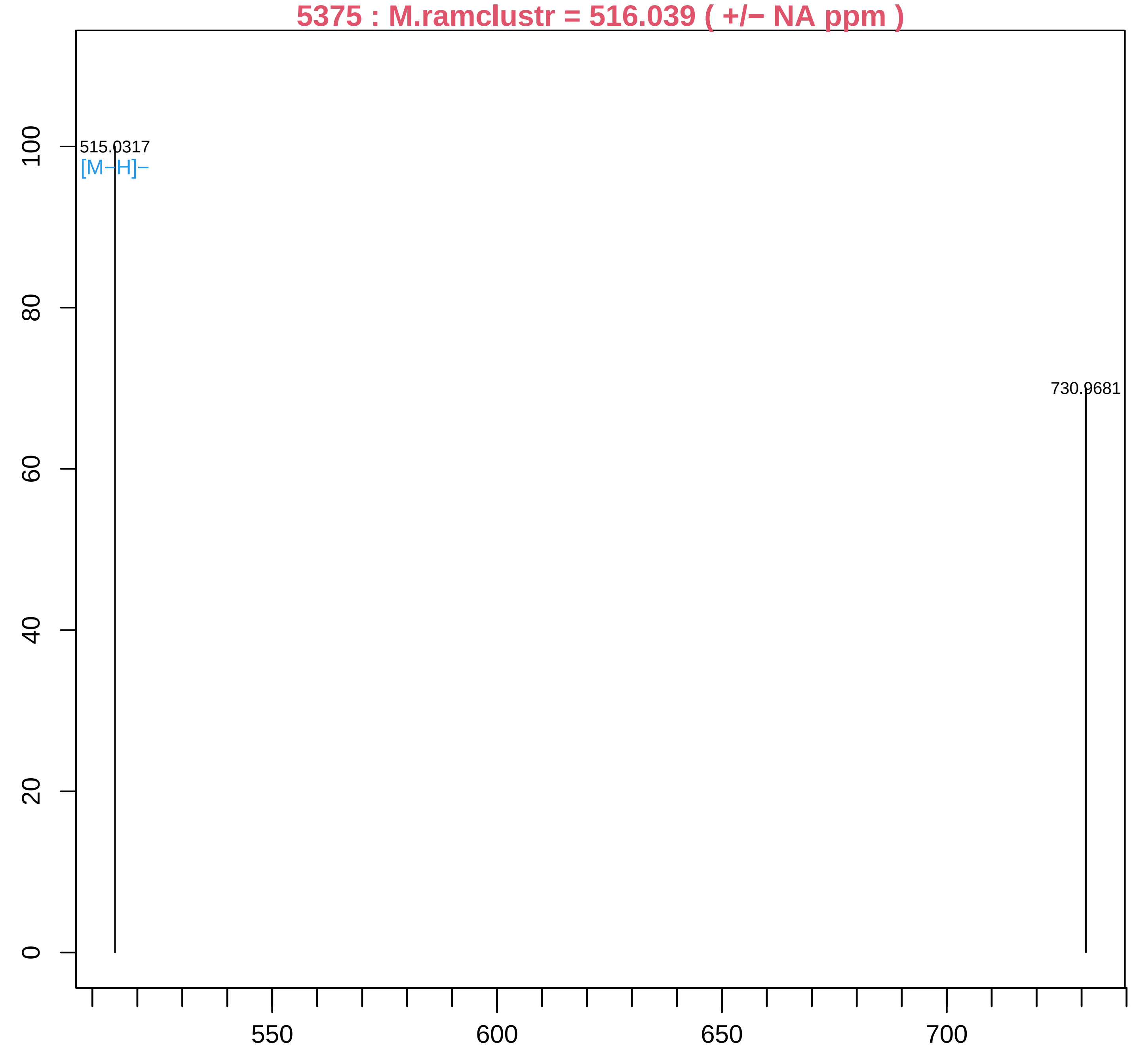
5375 : M.ramclustr = 516.039 (+/− NA ppm)
515.0317
[M−H]−
730.9681
0
20
40
60
80
100
550
600
650
700

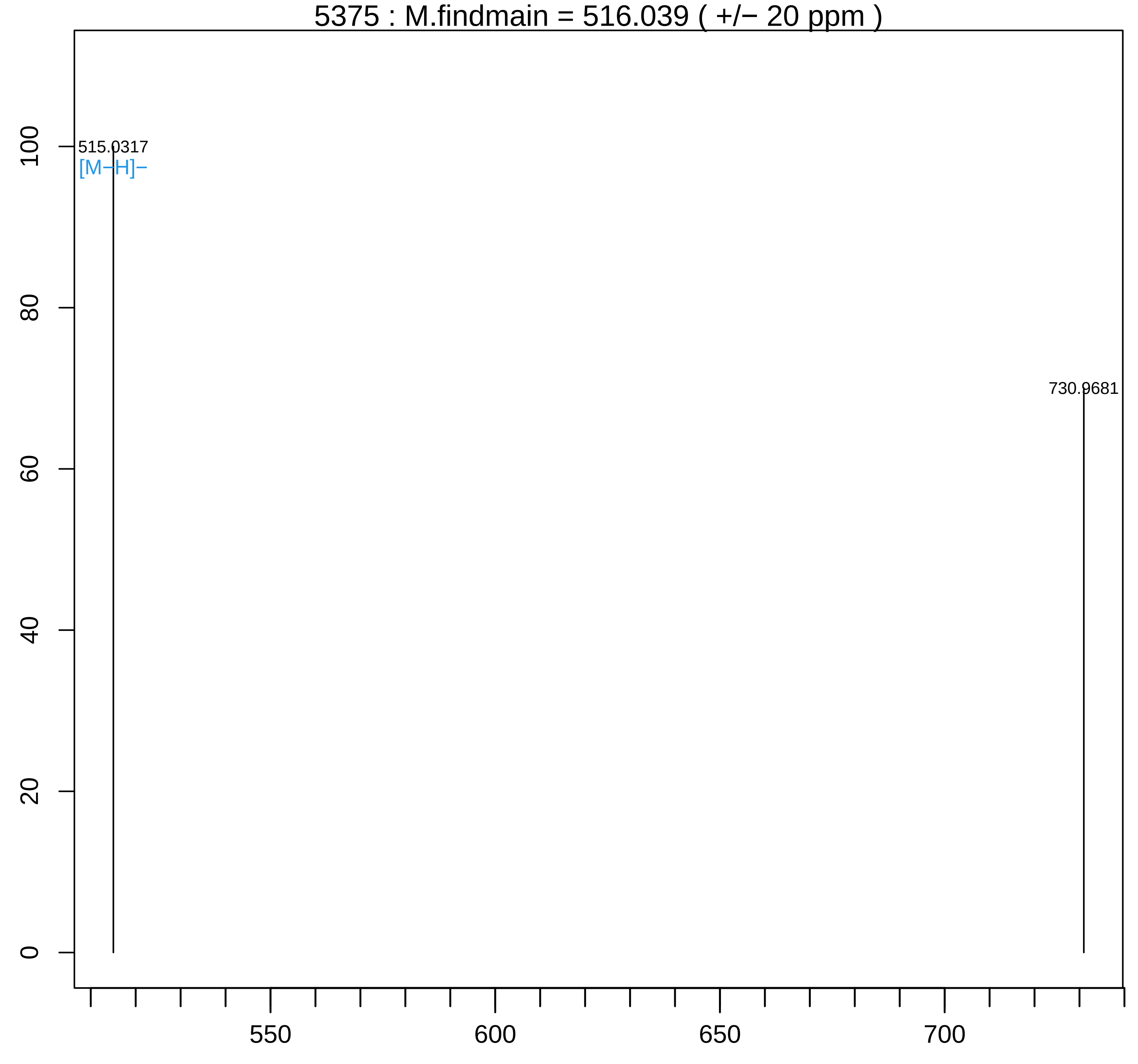
5375 : M.findmain = 516.039 (+/− 20 ppm)
515.0317
[M−H]−
730.9681
0
20
40
60
80
100
550
600
650
700

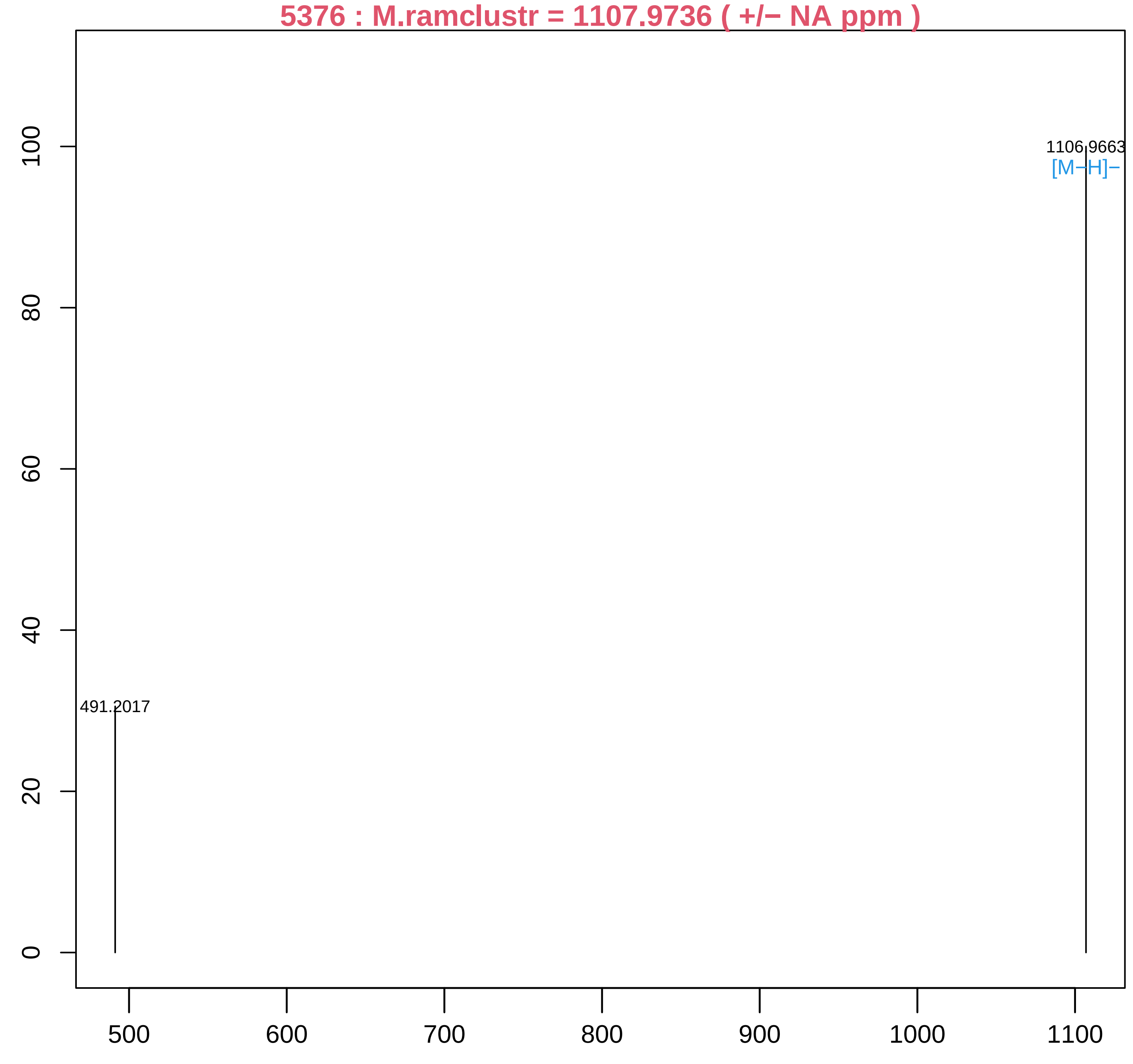
5376 : M.ramclustr = 1107.9736 (+/− NA ppm)
1106.9663
[M−H]−
491.2017
0
20
40
60
80
100
500
600
700
800
900
1000
1100

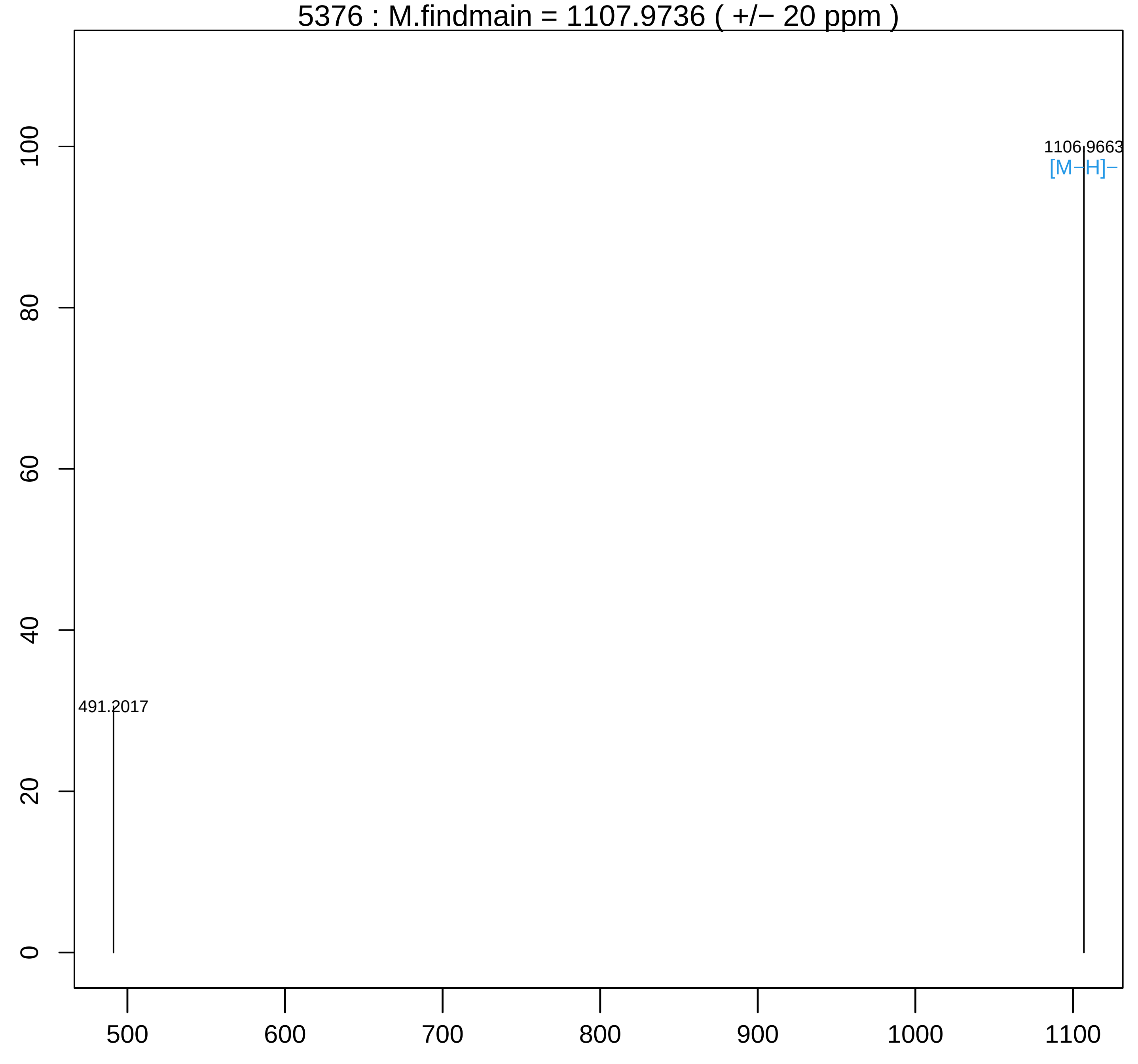
5376 : M.findmain = 1107.9736 (+/− 20 ppm)
1106.9663
[M−H]−
491.2017
0
20
40
60
80
100
500
600
700
800
900
1000
1100

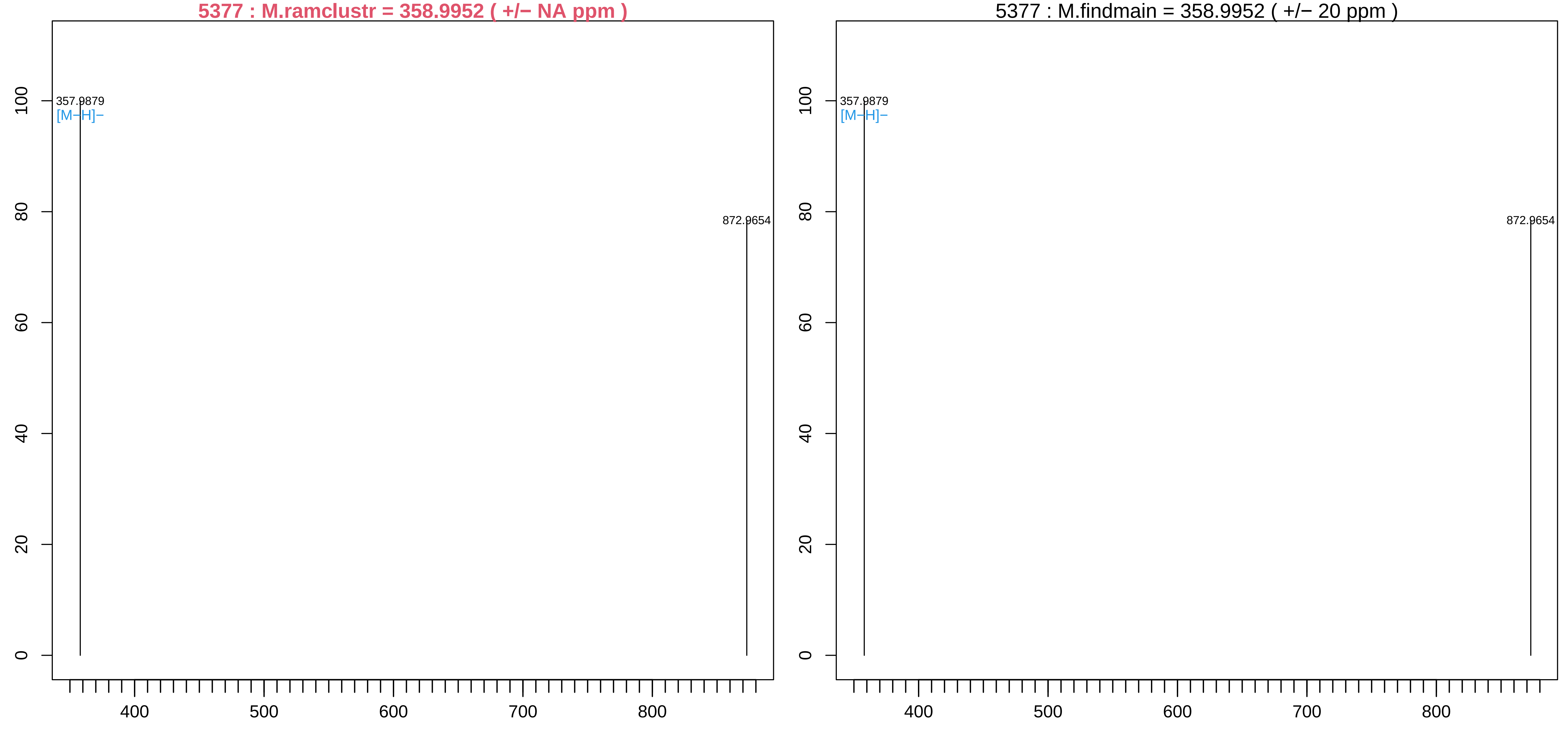
5377 : M.ramclustr = 358.9952 (+/− NA ppm)
357.9879
[M−H]−
872.9654
5377 : M.findmain = 358.9952 (+/− 20 ppm)
357.9879
[M−H]−
872.9654
0
20
40
60
80
100
400
500
600
700
800

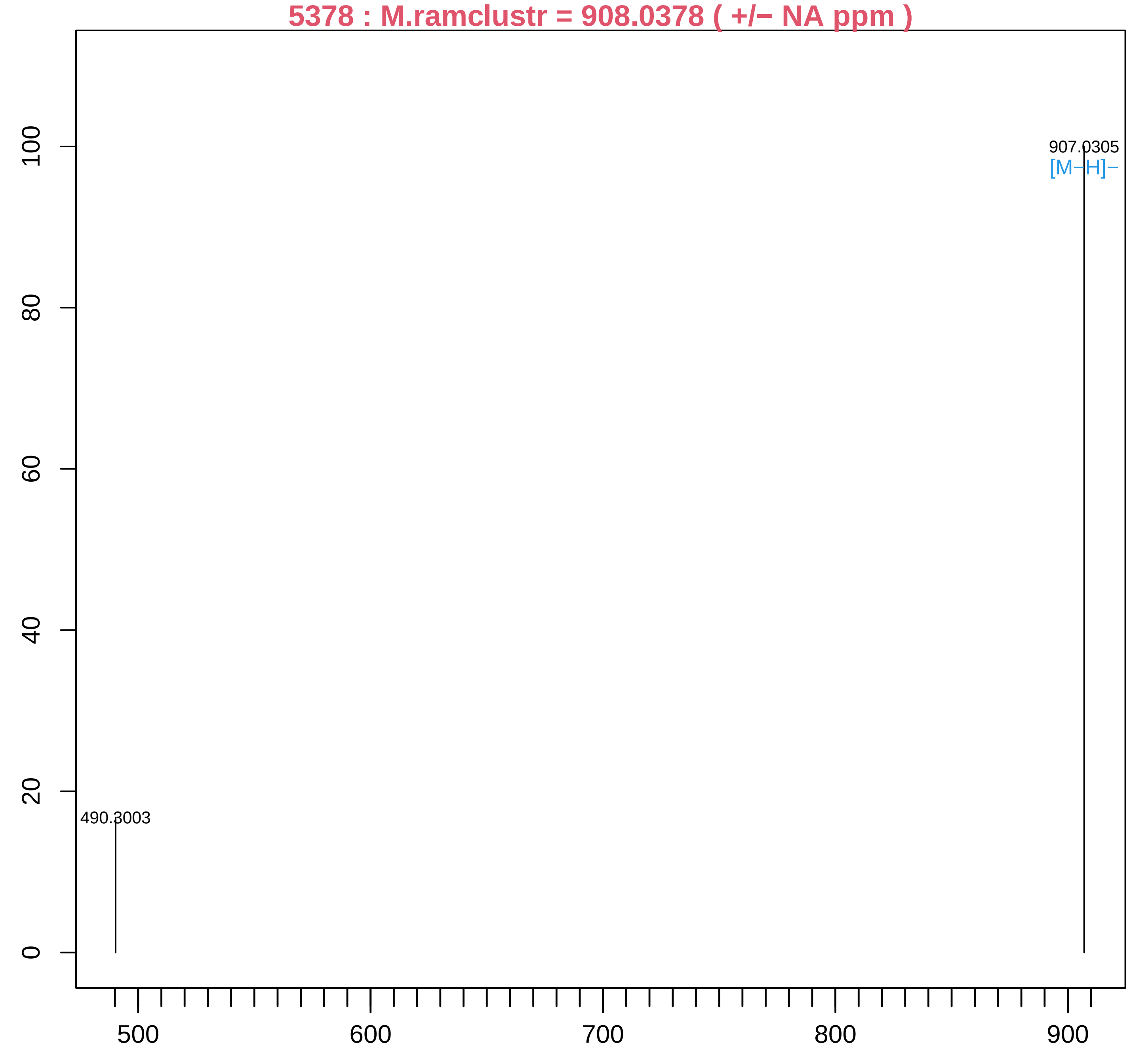
5378 : M.ramclustr = 908.0378 (+/− NA ppm)
907.0305
[M−H]−
490.3003
0
20
40
60
80
100
500
600
700
800
900

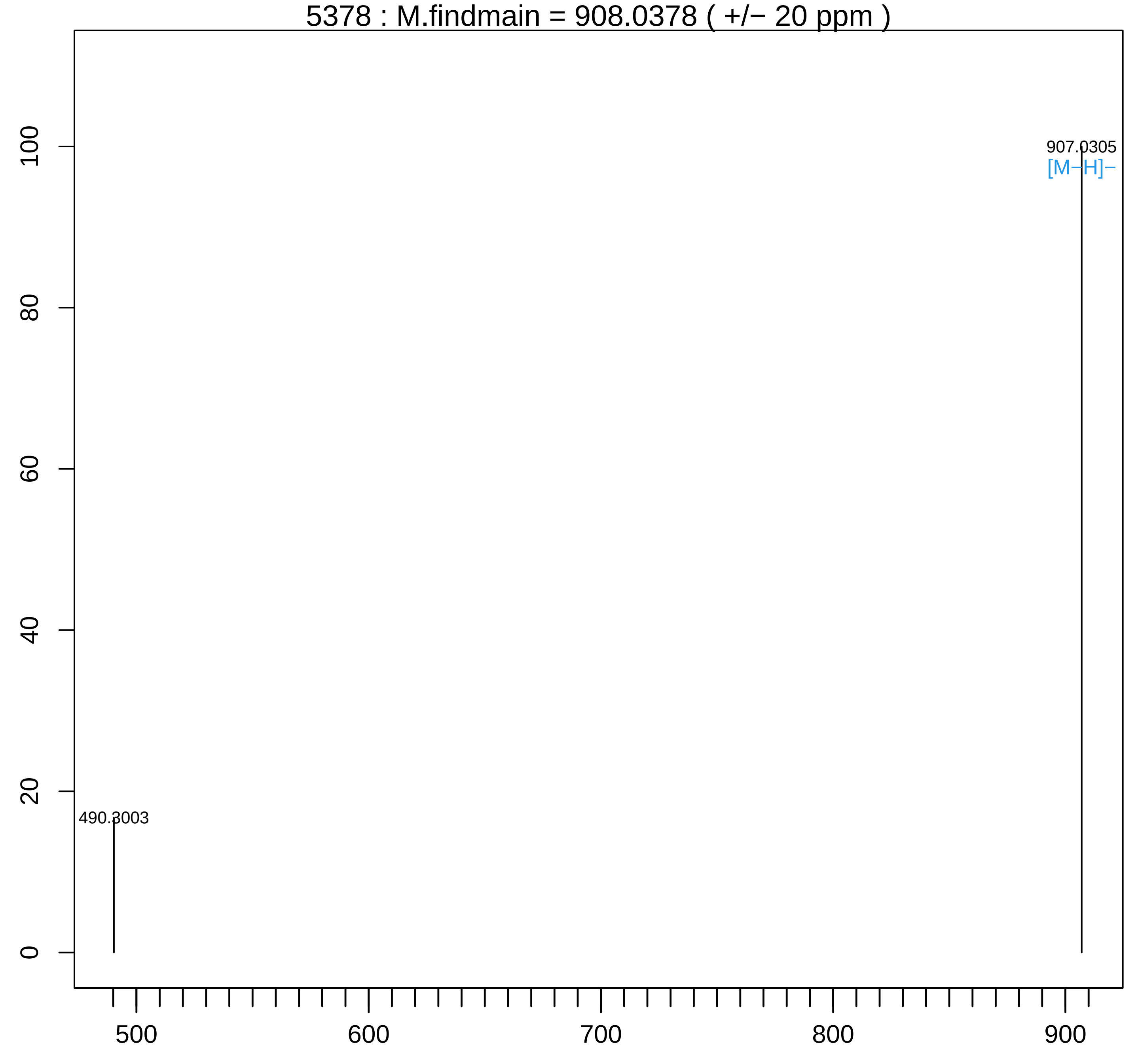
5378 : M.findmain = 908.0378 (+/− 20 ppm)
907.0305
[M−H]−
490.3003
0
20
40
60
80
100
500
600
700
800
900

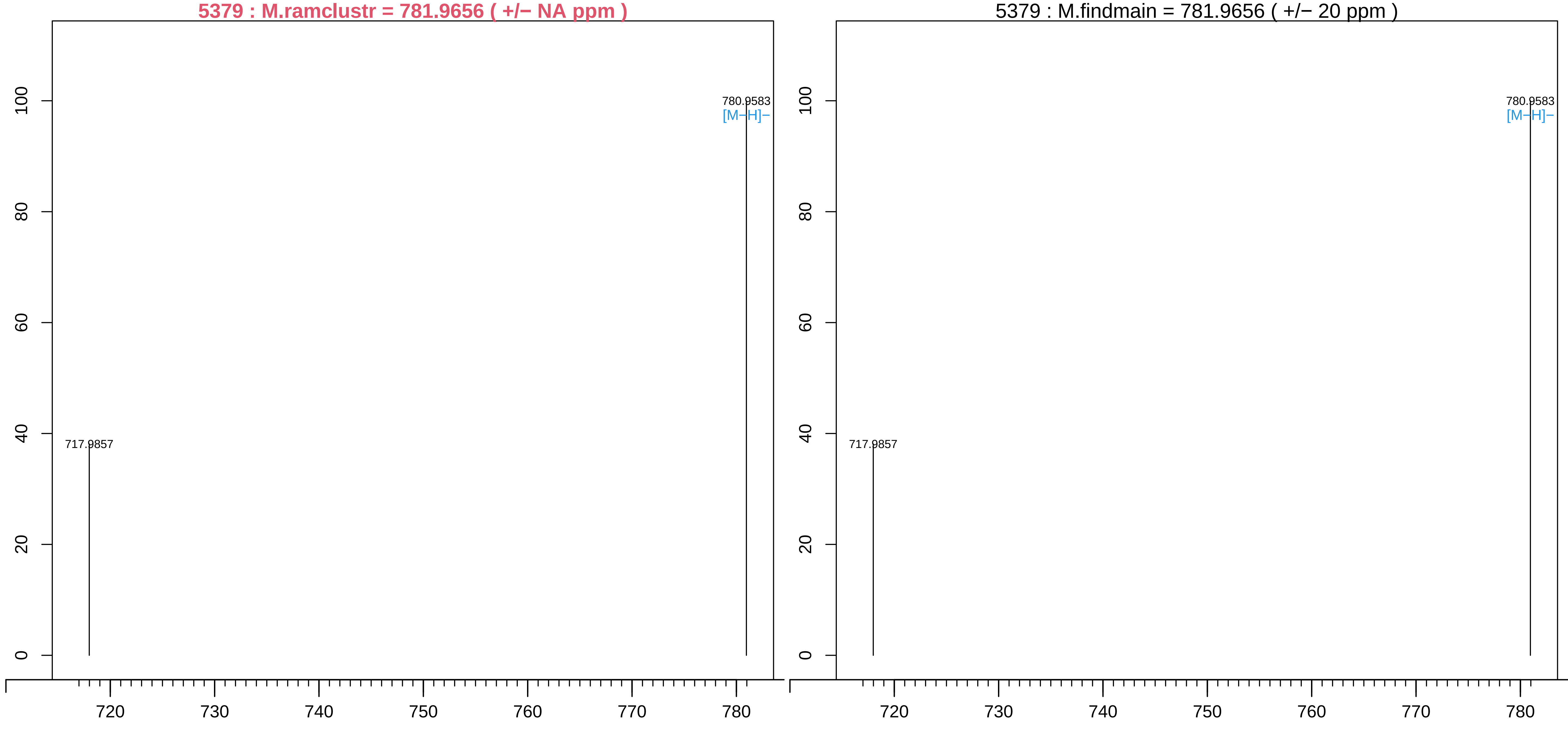
5379 : M.ramclustr = 781.9656 (+/− NA ppm)
780.9583
[M−H]−
717.9857
5379 : M.findmain = 781.9656 (+/− 20 ppm)
780.9583
[M−H]−
717.9857
0
20
40
60
80
100
720
730
740
750
760
770
780

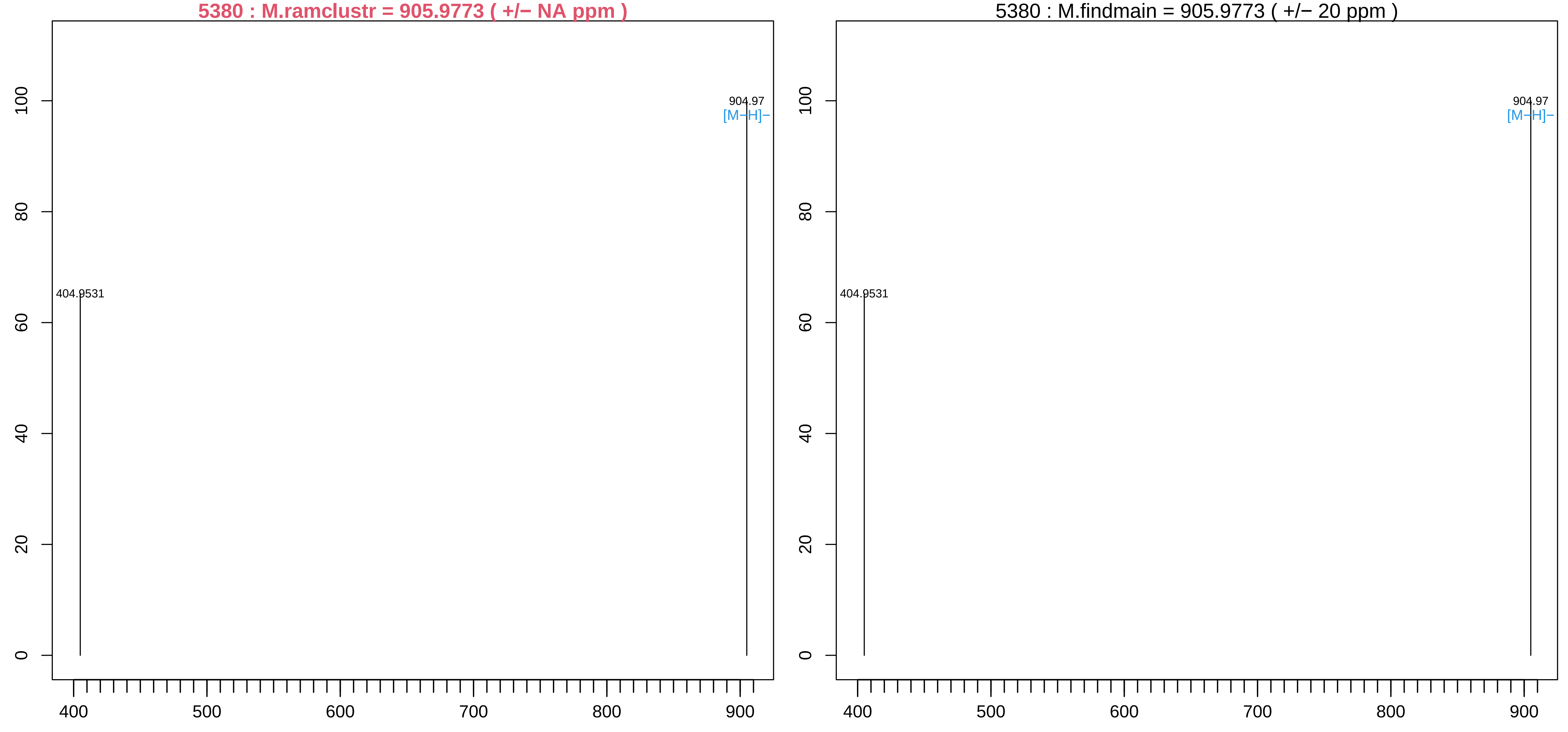
5380 : M.ramclustr = 905.9773 (+/− NA ppm)
5380 : M.findmain = 905.9773 (+/− 20 ppm)
904.97
[M−H]−
404.9531
0
20
40
60
80
100
400
500
600
700
800
900

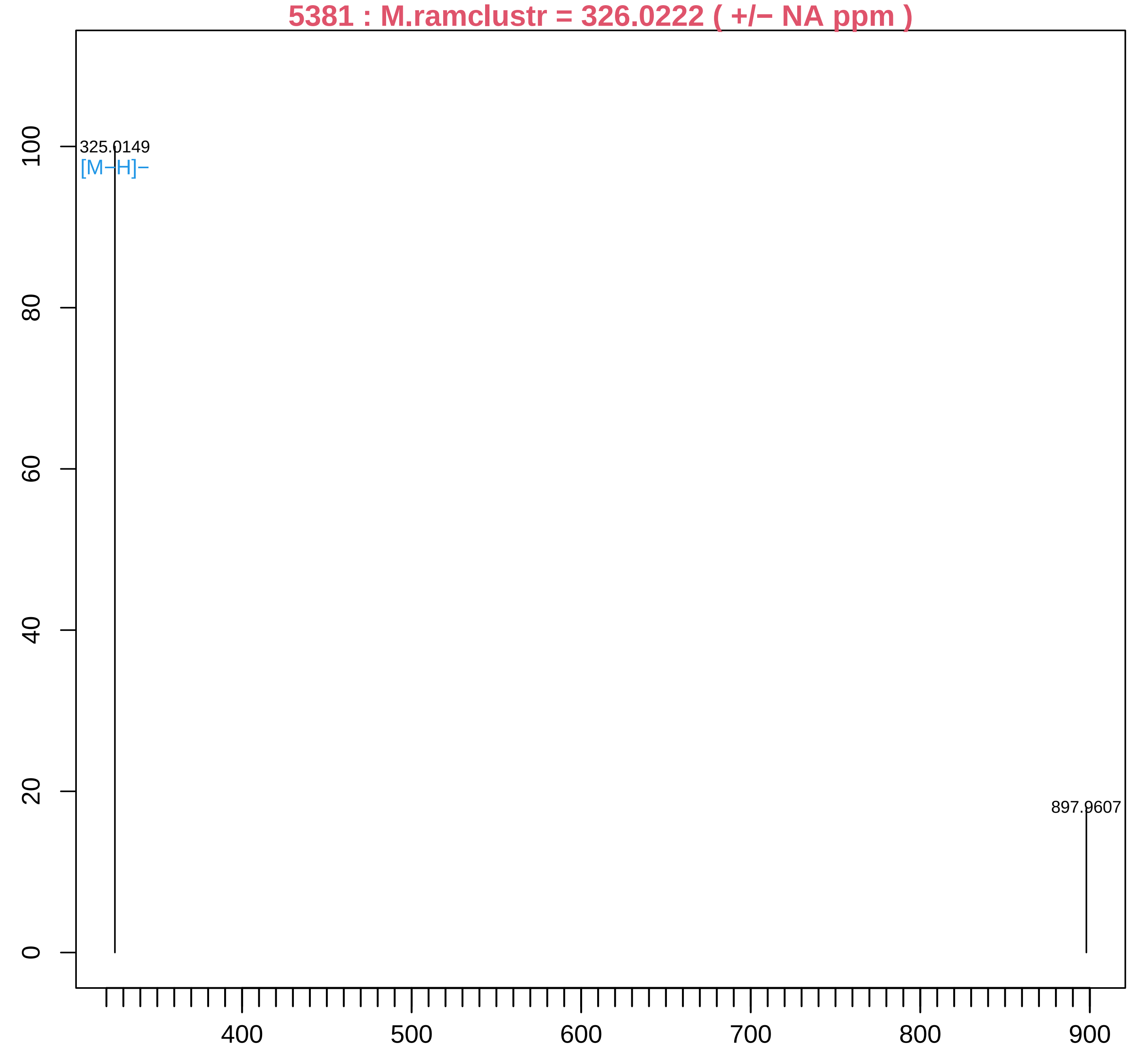
5381 : M.ramclustr = 326.0222 (+/− NA ppm)
325.0149
[M−H]−
897.9607
0
20
40
60
80
100
400
500
600
700
800
900

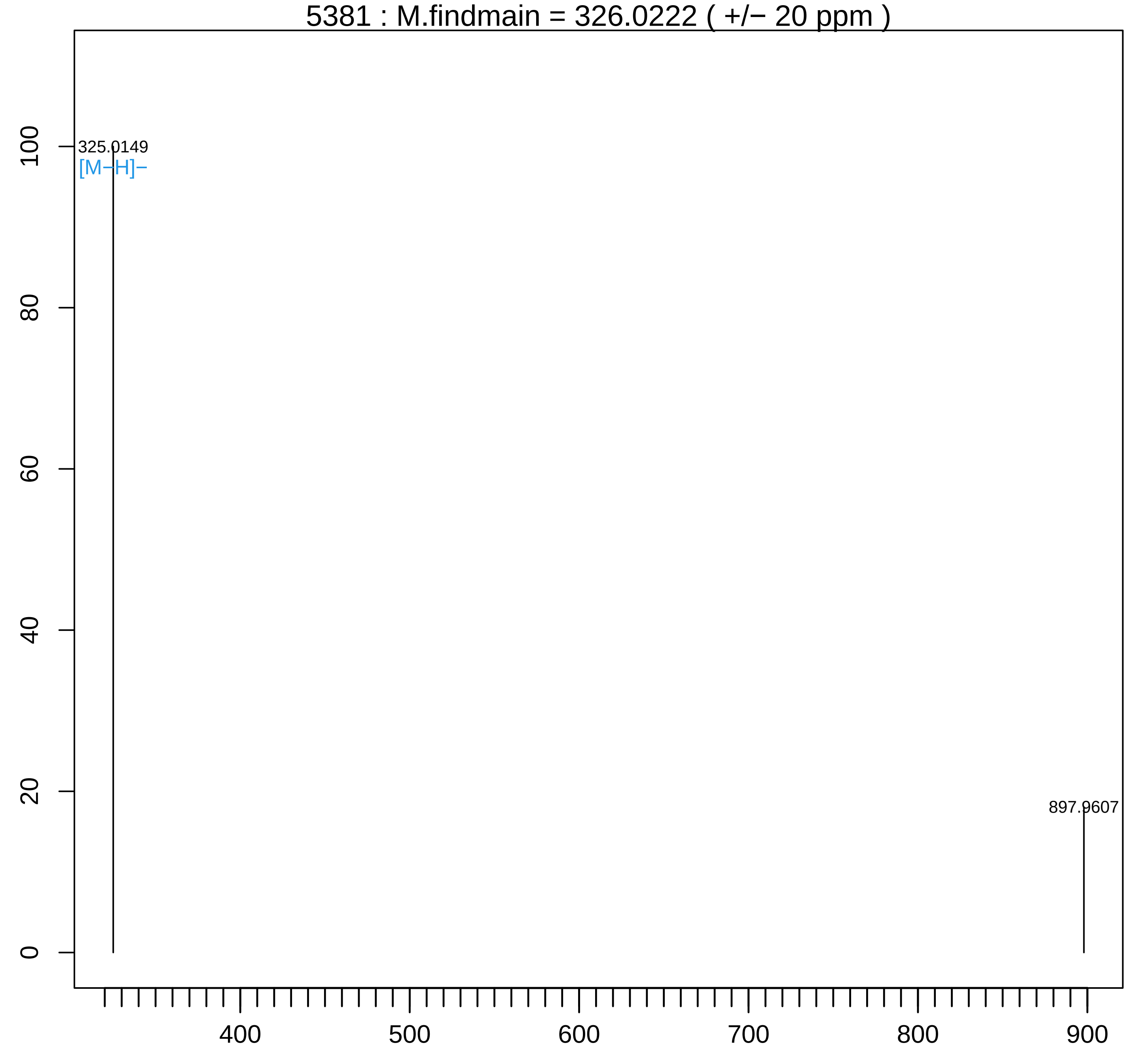
5381 : M.findmain = 326.0222 (+/− 20 ppm)
325.0149
[M−H]−
897.9607
0
20
40
60
80
100
400
500
600
700
800
900

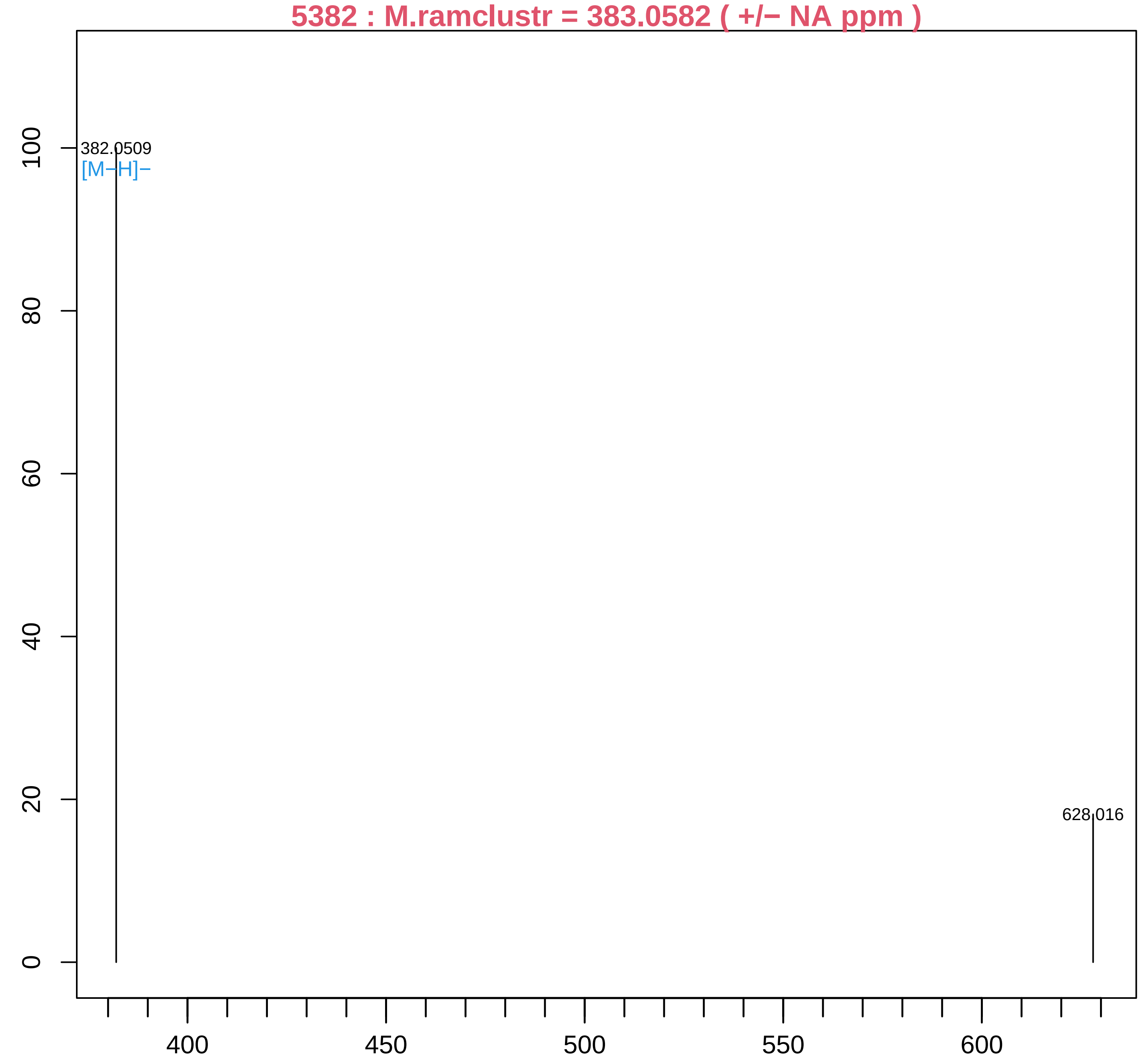
5382 : M.ramclustr = 383.0582 (+/− NA ppm)
382.0509
[M−H]−
628.016
0
20
40
60
80
100
400
450
500
550
600

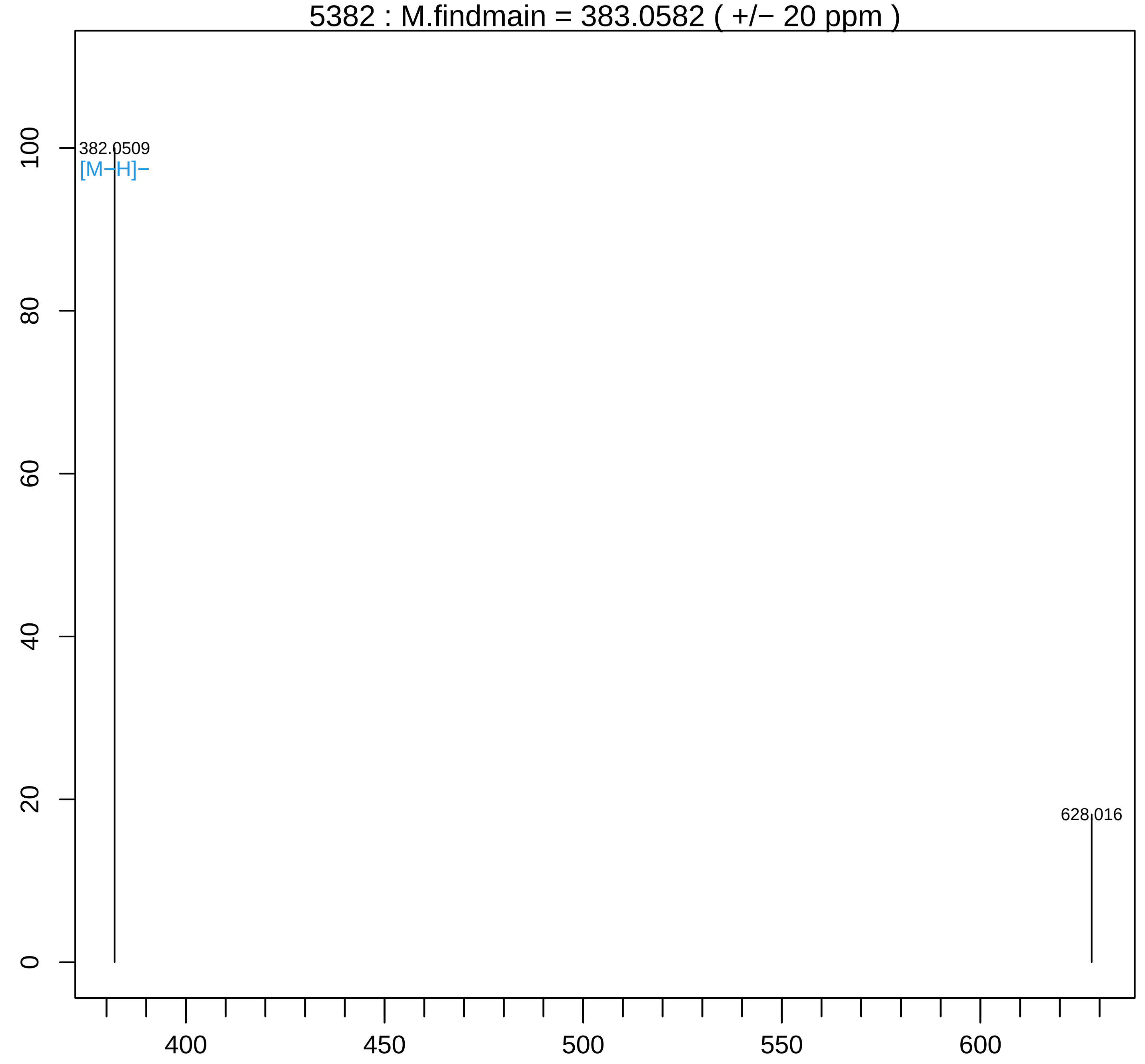
5382 : M.findmain = 383.0582 (+/− 20 ppm)
382.0509
[M−H]−
628.016
0
20
40
60
80
100
400
450
500
550
600

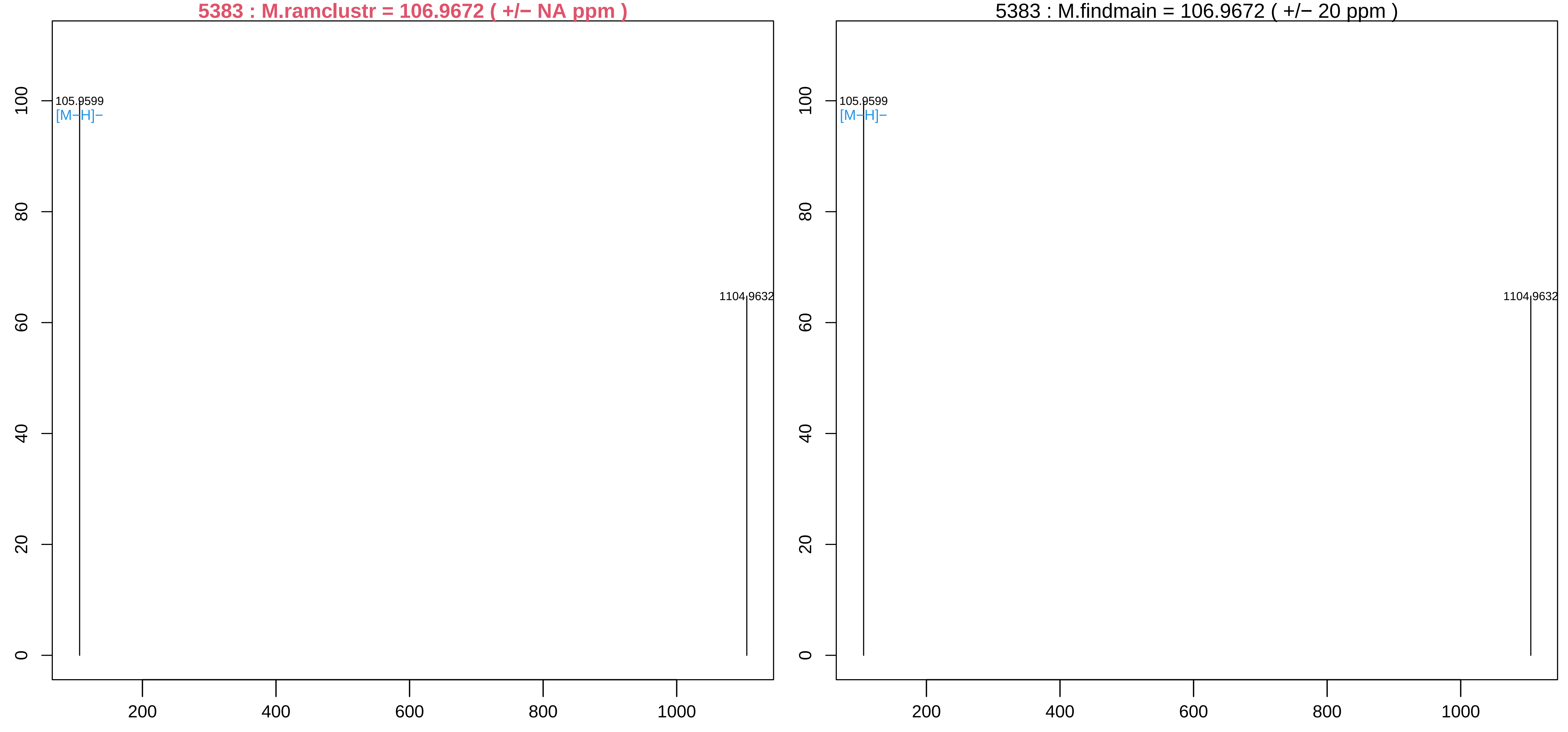
5383 : M.ramclustr = 106.9672 (+/− NA ppm)
105.9599
[M−H]−
1104.9632
0
20
40
60
80
100
200
400
600
800
1000
5383 : M.findmain = 106.9672 (+/− 20 ppm)
105.9599
[M−H]−
1104.9632
0
20
40
60
80
100
200
400
600
800
1000

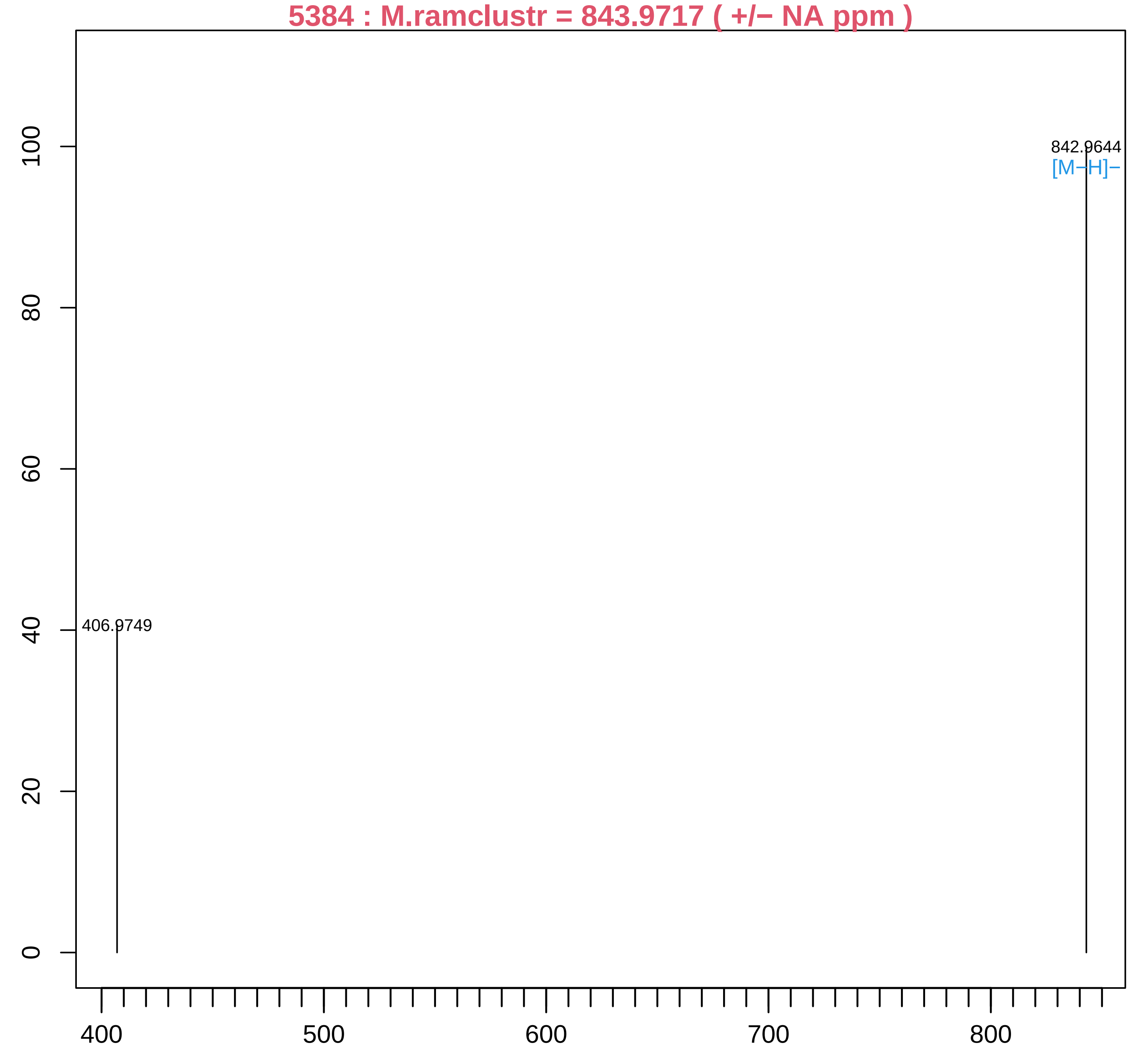
5384 : M.ramclustr = 843.9717 (+/− NA ppm)
842.9644
[M−H]−
406.9749
0
20
40
60
80
100
400
500
600
700
800

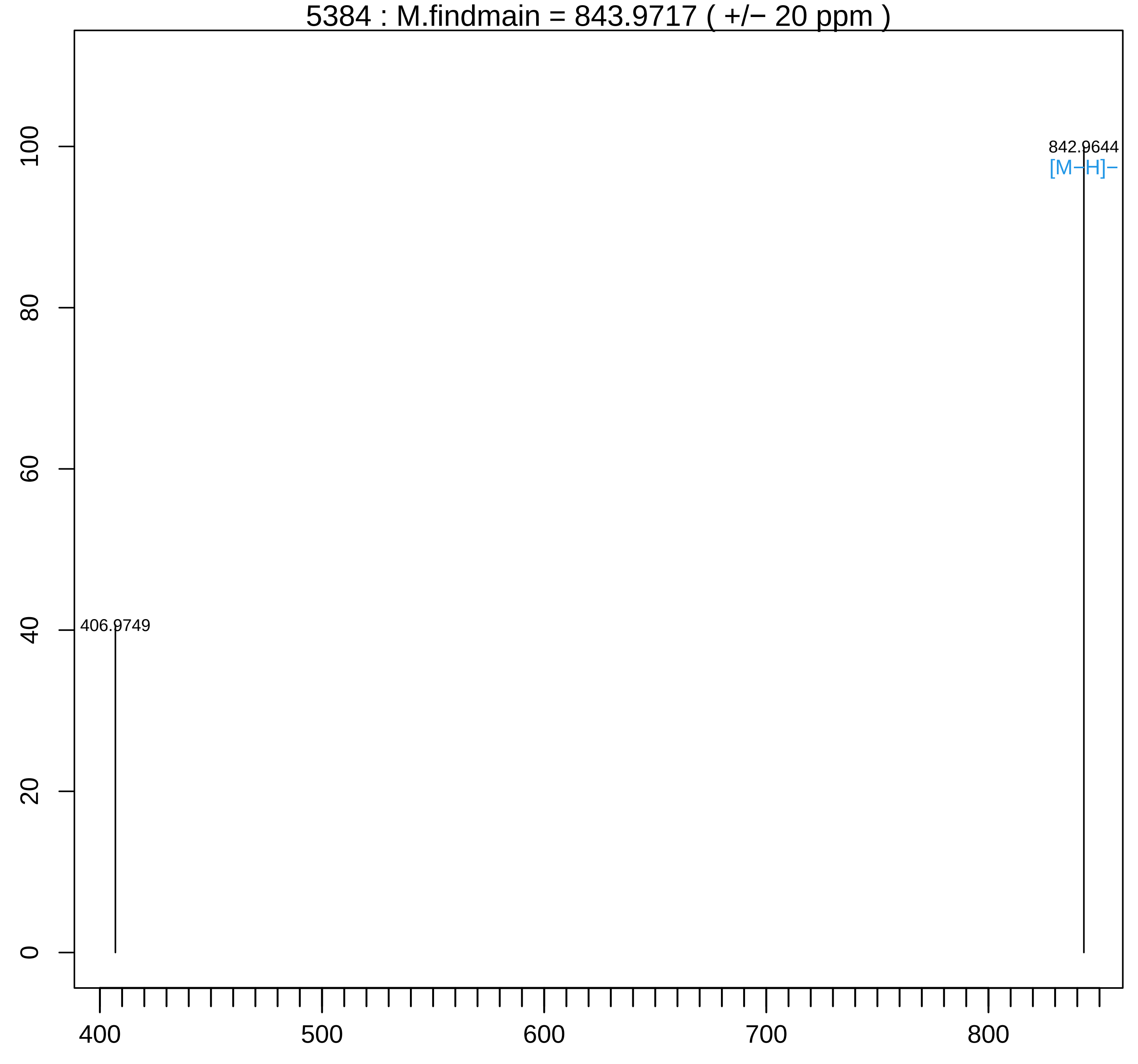
5384 : M.findmain = 843.9717 (+/− 20 ppm)
842.9644
[M−H]−
406.9749
0
20
40
60
80
100
400
500
600
700
800

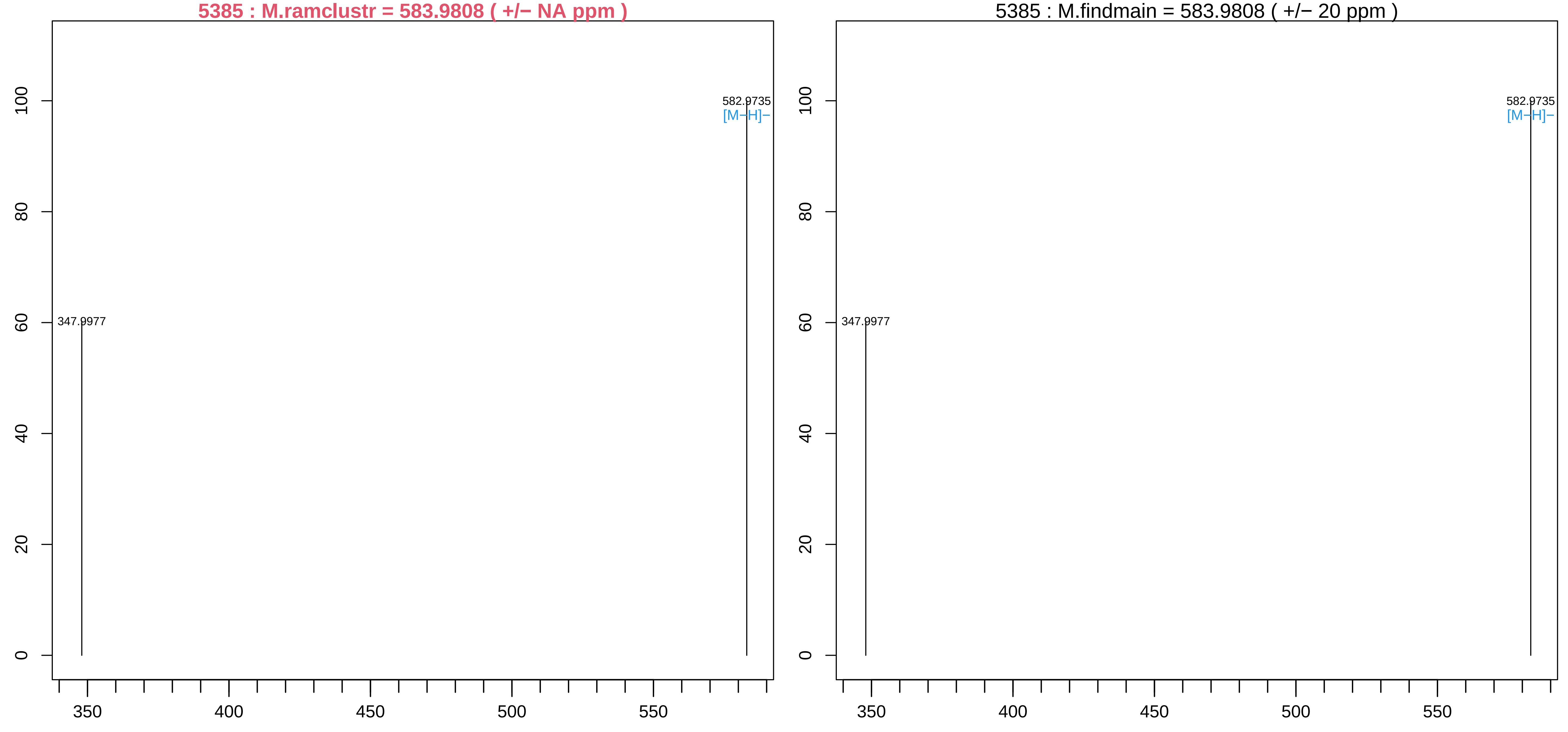

5385 : M.ramclustr = 583.9808 (+/− NA ppm)
5385 : M.findmain = 583.9808 (+/− 20 ppm)
582.9735
[M−H]−
347.9977
582.9735
[M−H]−
347.9977
0
20
40
60
80
100
350
400
450
500
550

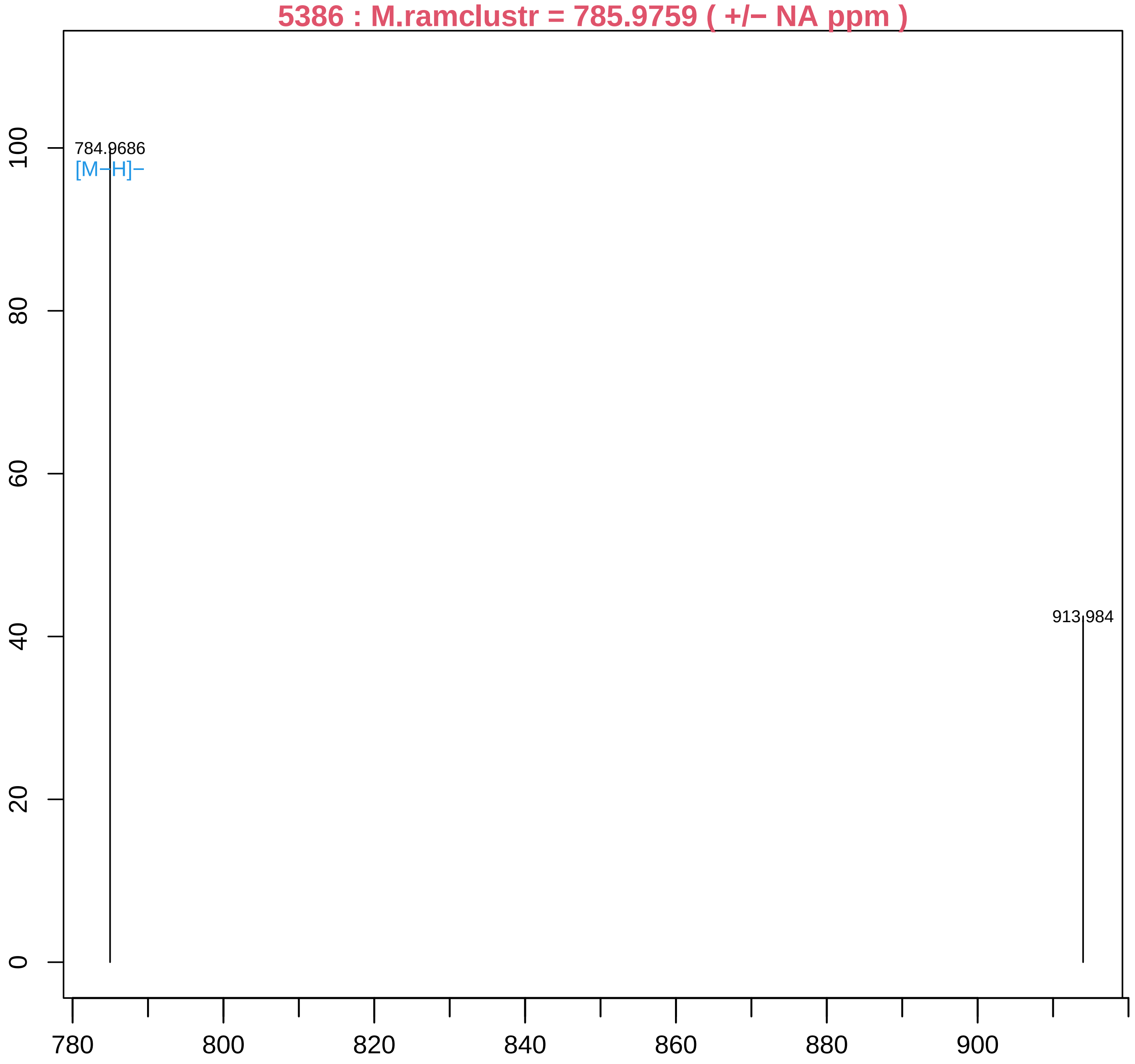
5386 : M.ramclustr = 785.9759 (+/− NA ppm)
784.9686
[M−H]−
913.984
0
20
40
60
80
100
780
800
820
840
860
880
900

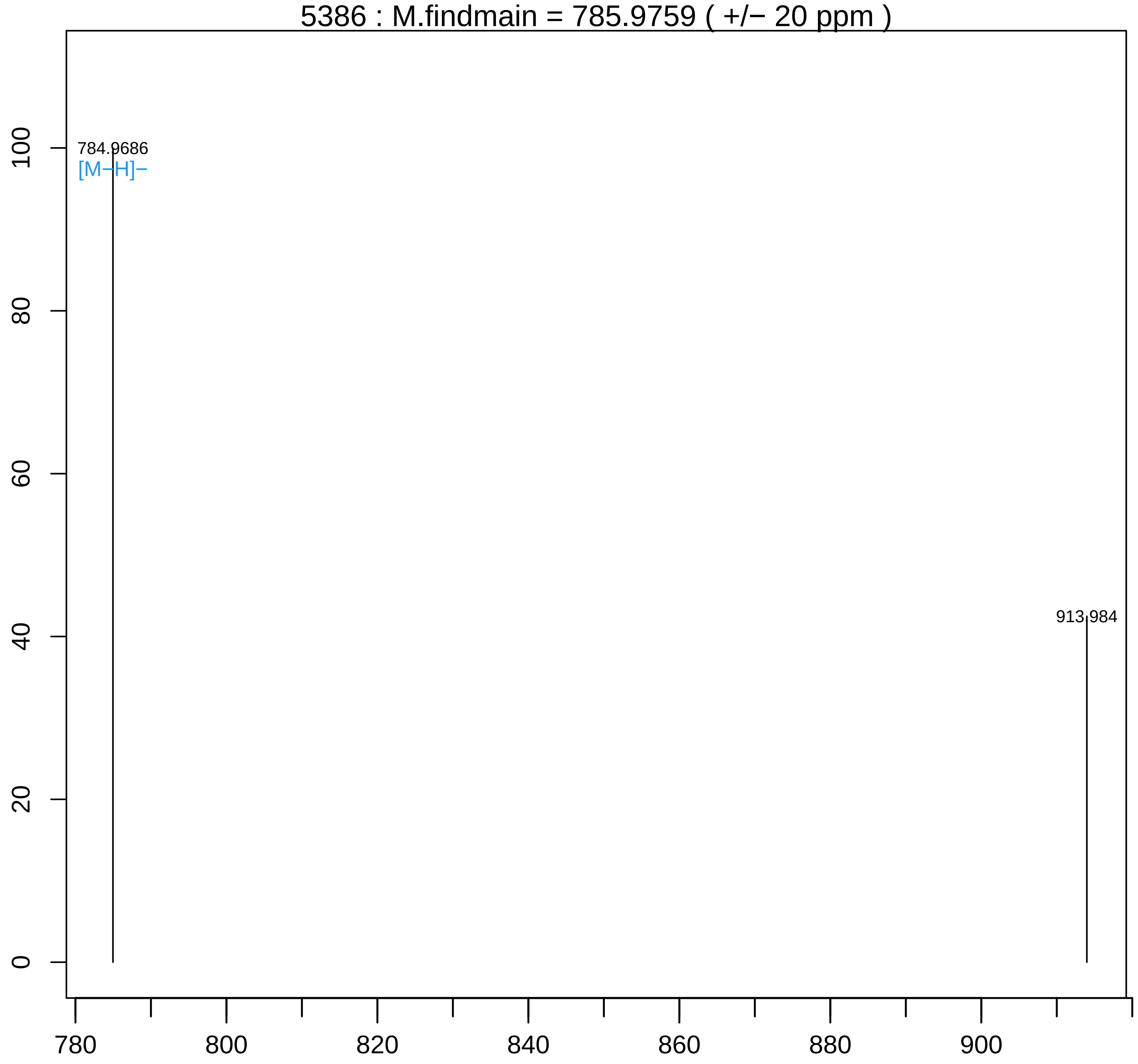
5386 : M.findmain = 785.9759 (+/− 20 ppm)
784.9686
[M−H]−
913.984
0
20
40
60
80
100
780
800
820
840
860
880
900

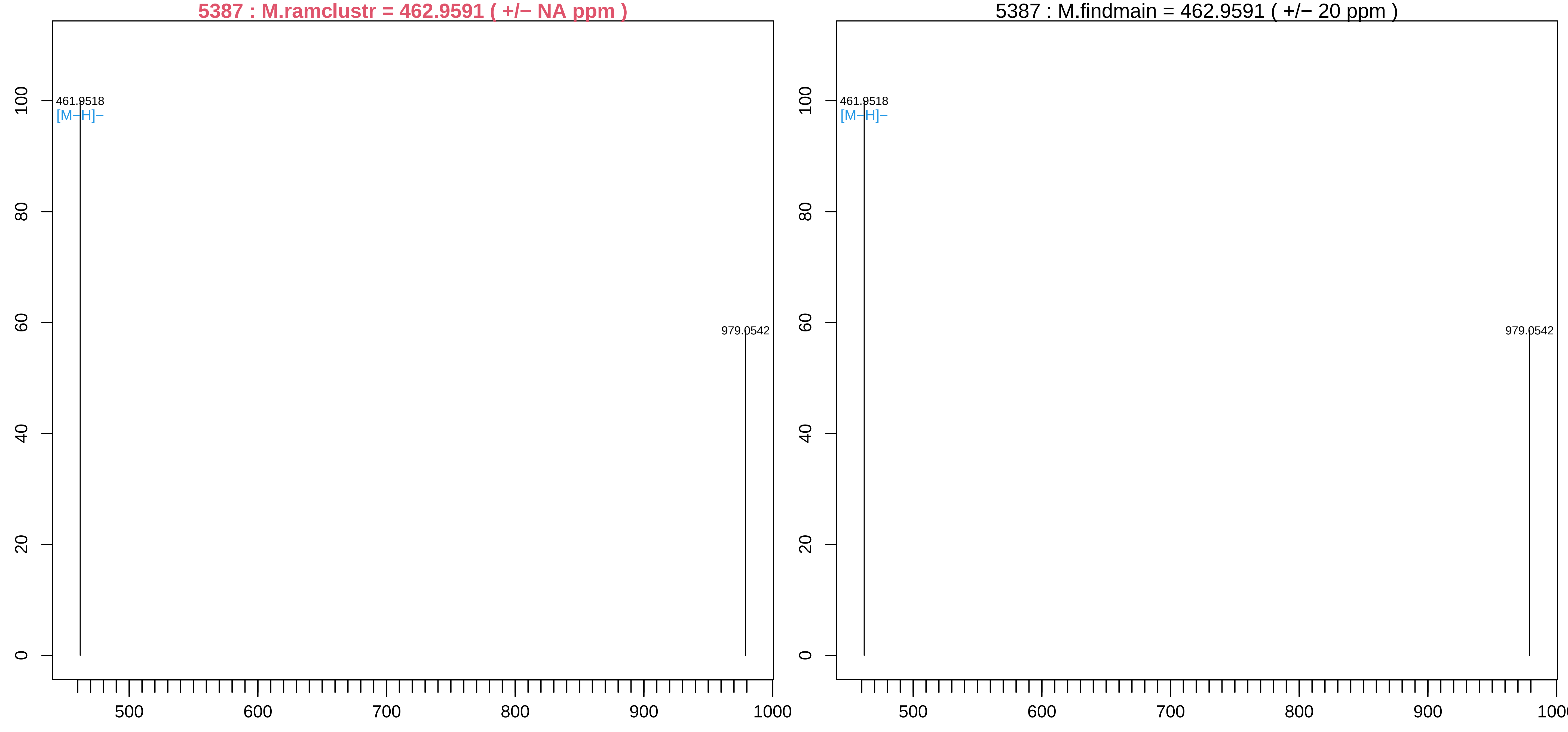
5387 : M.ramclustr = 462.9591 (+/− NA ppm)
461.9518
[M−H]−
979.0542
0
20
40
60
80
100
500
600
700
800
900
1000
5387 : M.findmain = 462.9591 (+/− 20 ppm)
461.9518
[M−H]−
979.0542
0
20
40
60
80
100
500
600
700
800
900

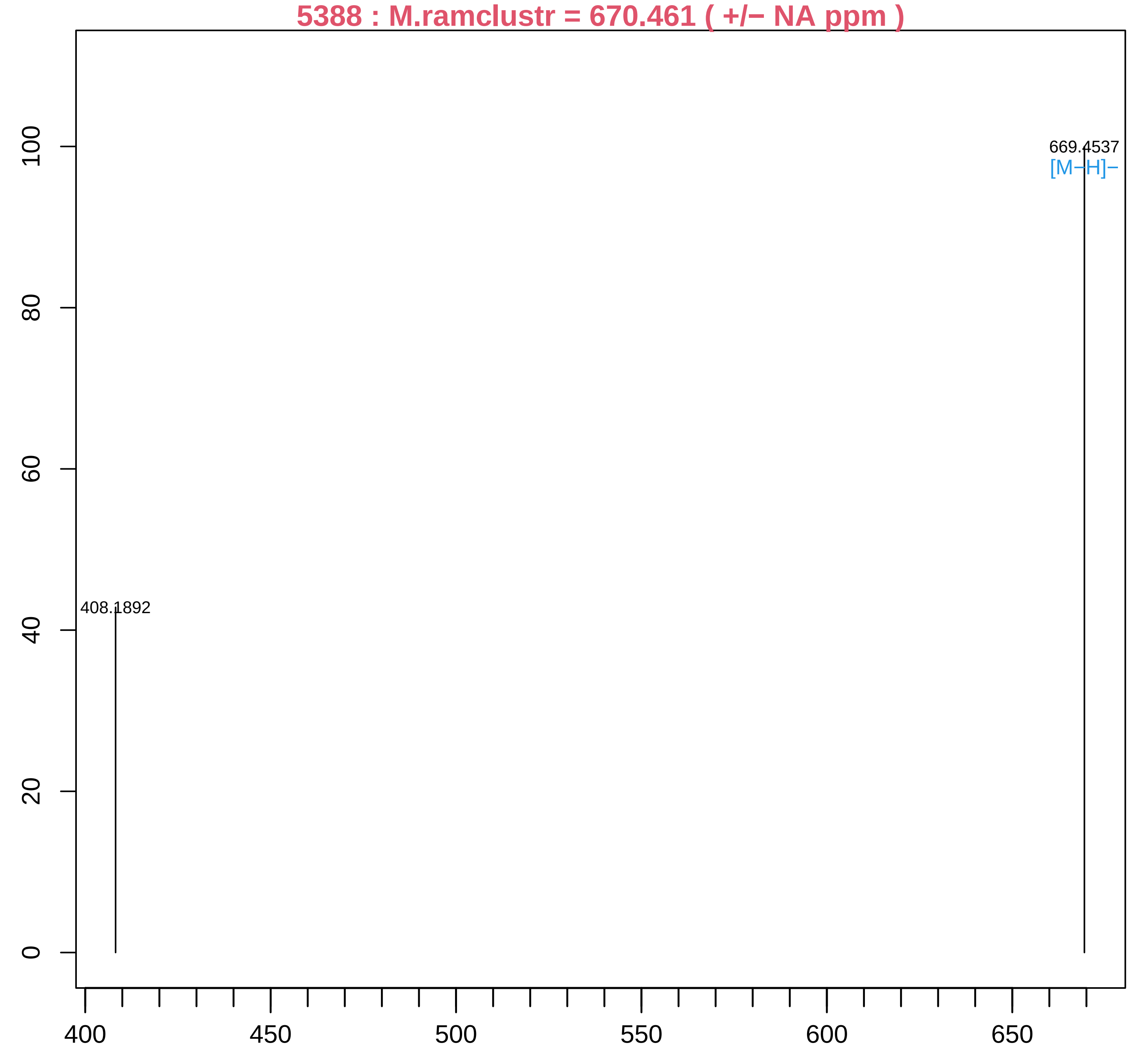
5388 : M.ramclustr = 670.461 (+/− NA ppm)
669.4537
[M−H]−
408.1892
0
20
40
60
80
100
400
450
500
550
600
650

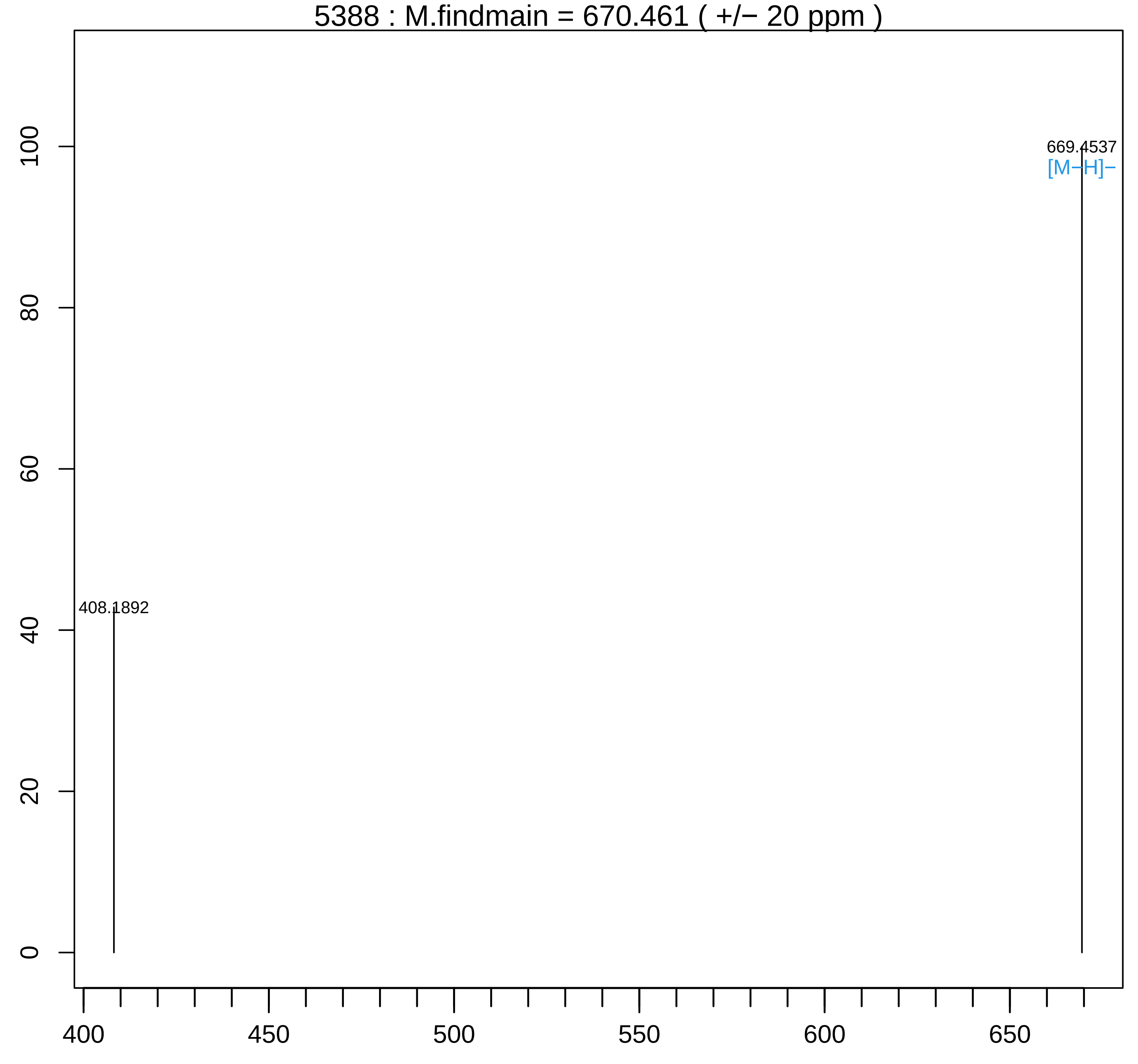
5388 : M.findmain = 670.461 (+/− 20 ppm)
669.4537
[M−H]−
408.1892
0
20
40
60
80
100
400
450
500
550
600
650

5389 : M.ramclustr = 961.9703 (+/− NA ppm)

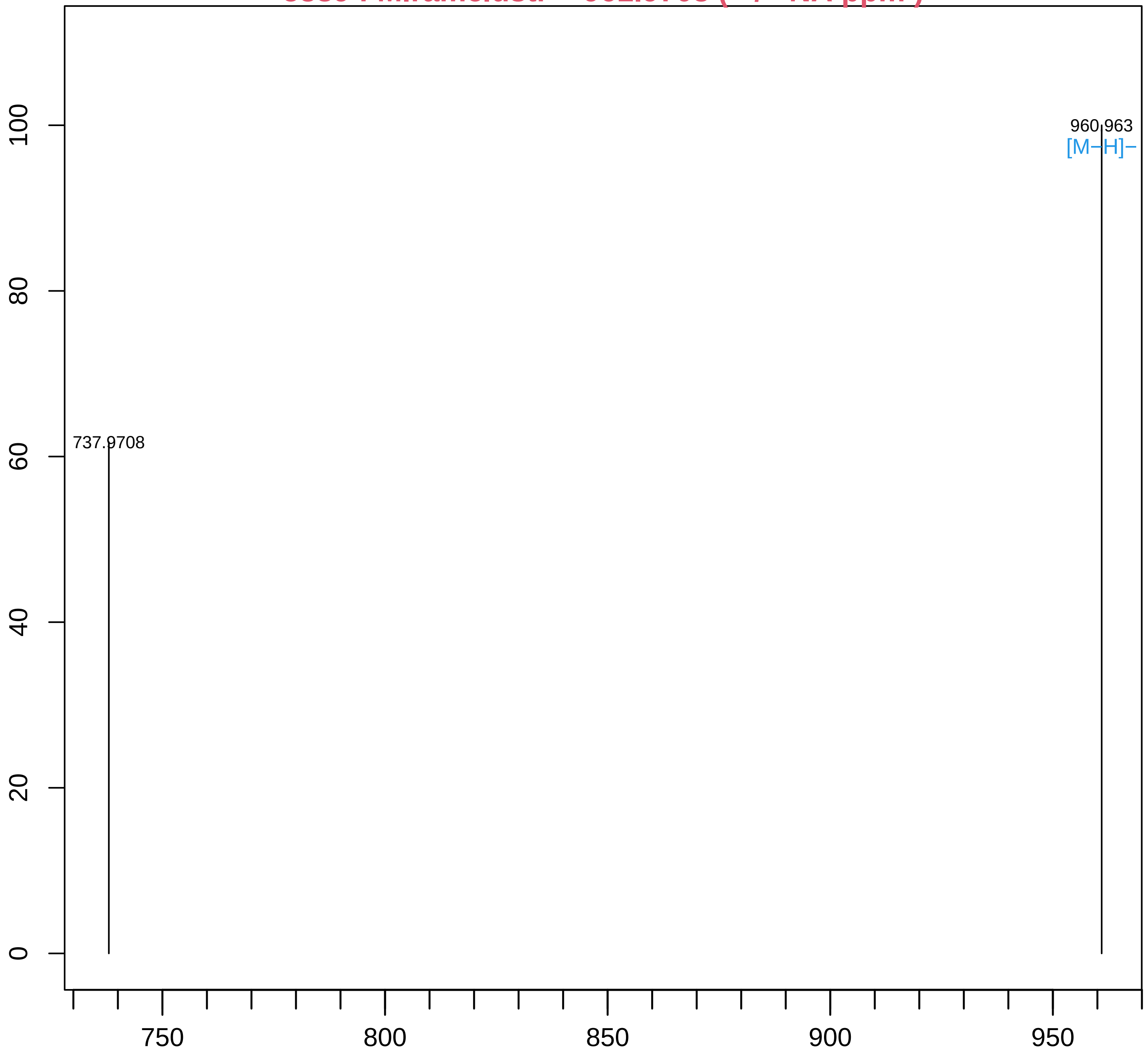

5389 : M.findmain = 961.9703 (+/− 20 ppm)

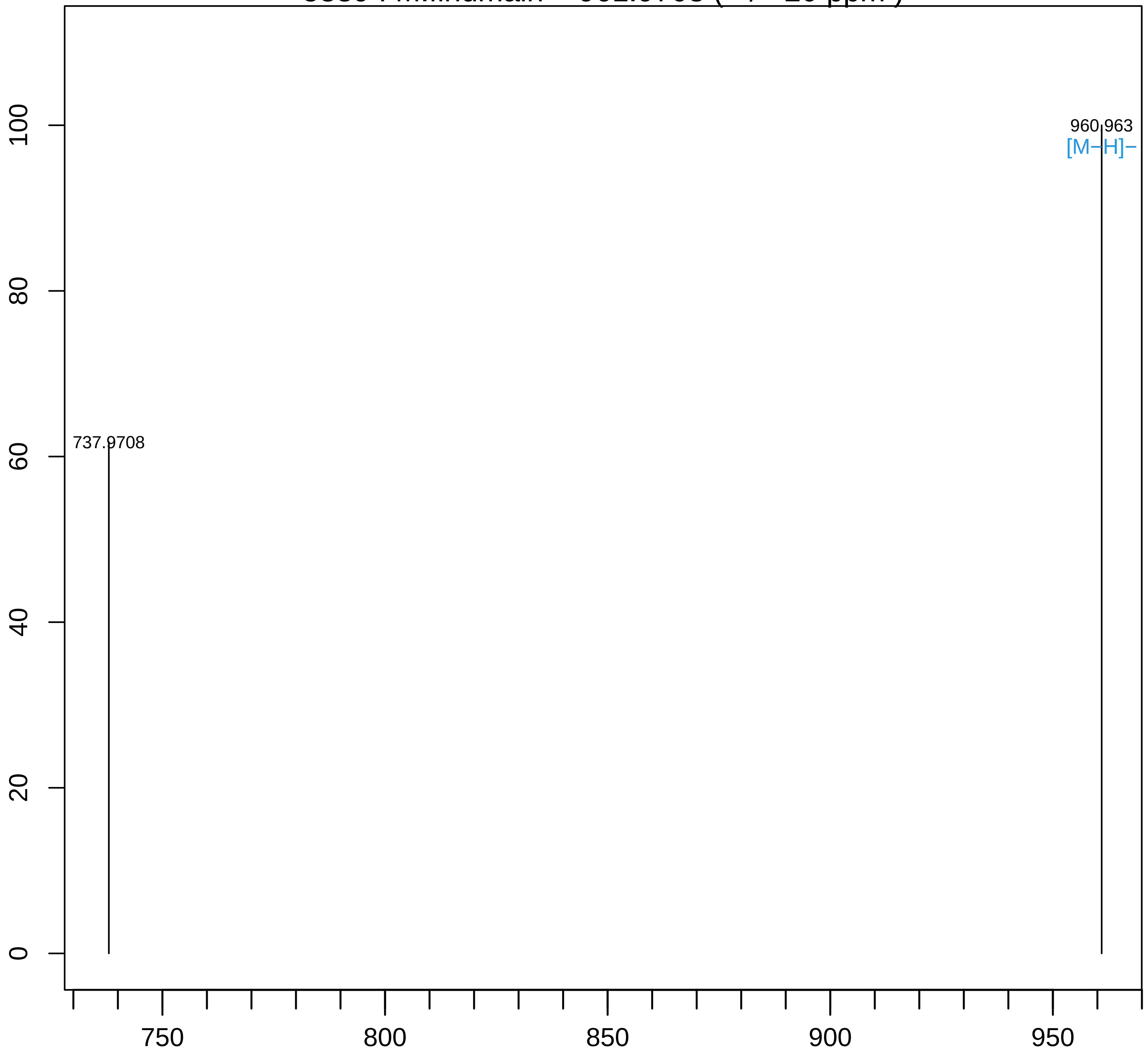

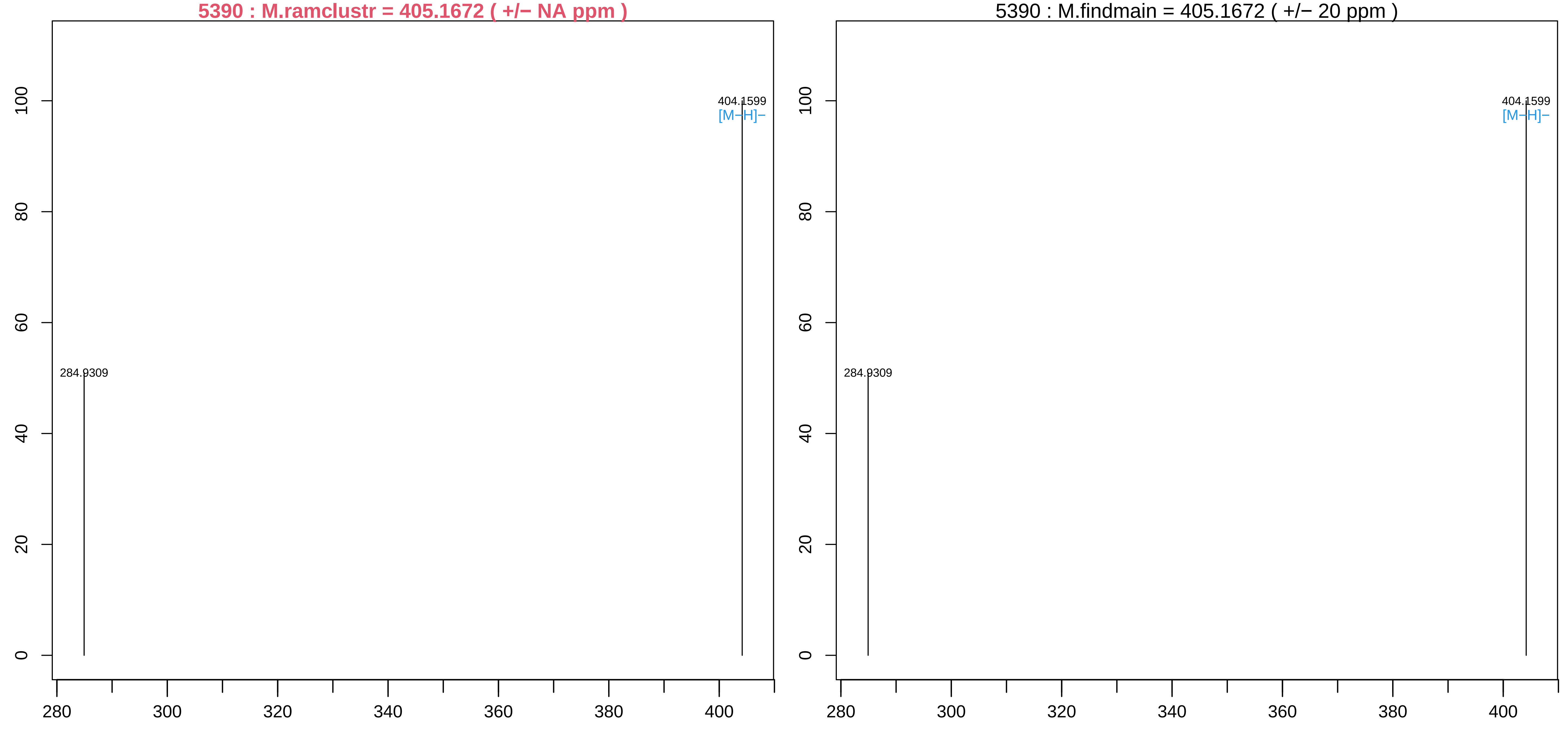
5390 : M.ramclustr = 405.1672 (+/− NA ppm)
404.1599
[M−H]−
284.9309
5390 : M.findmain = 405.1672 (+/− 20 ppm)
404.1599
[M−H]−
284.9309
0
20
40
60
80
100
280
300
320
340
360
380
400

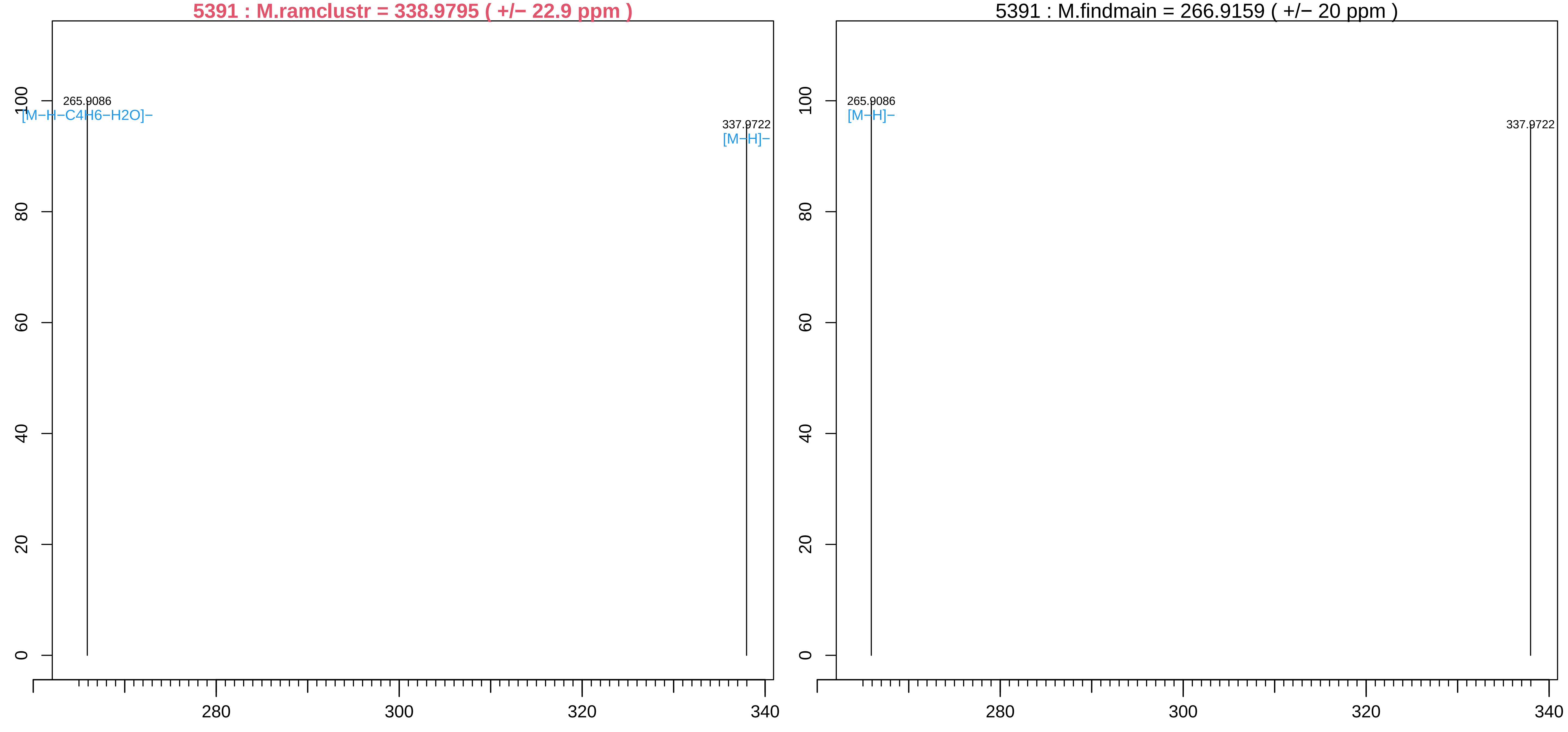

5391 : M.ramclustr = 338.9795 (+/− 22.9 ppm)
265.9086
[M−H−C4H6−H2O]−
337.9722
[M−H]−
0
20
40
60
80
100
280
300
320
340
5391 : M.findmain = 266.9159 (+/− 20 ppm)
265.9086
[M−H]−
337.9722
0
20
40
60
80
100
280
300
320
340

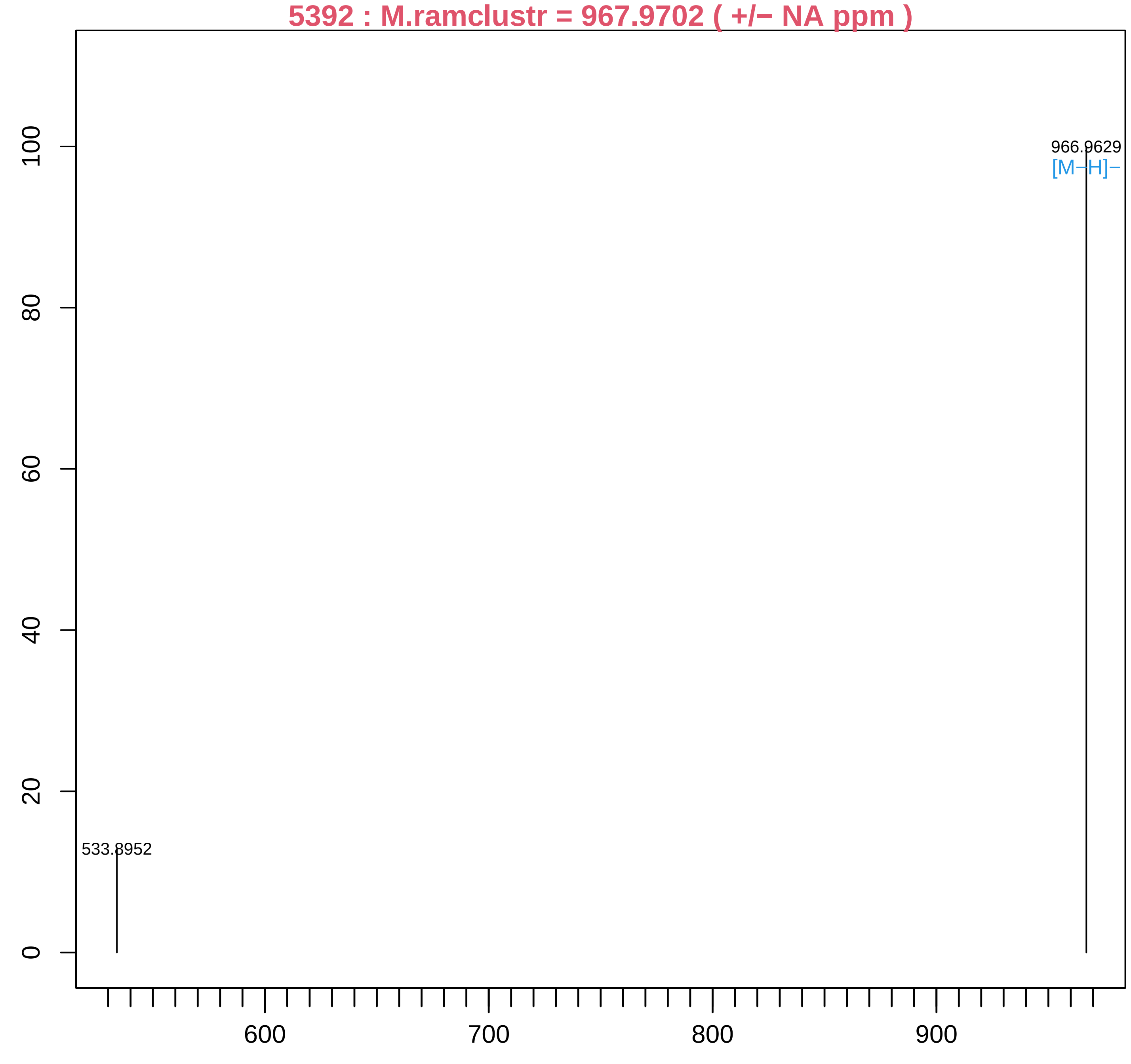
5392 : M.ramclustr = 967.9702 (+/− NA ppm)
966.9629
[M−H]−
533.8952
0
20
40
60
80
100
600
700
800
900

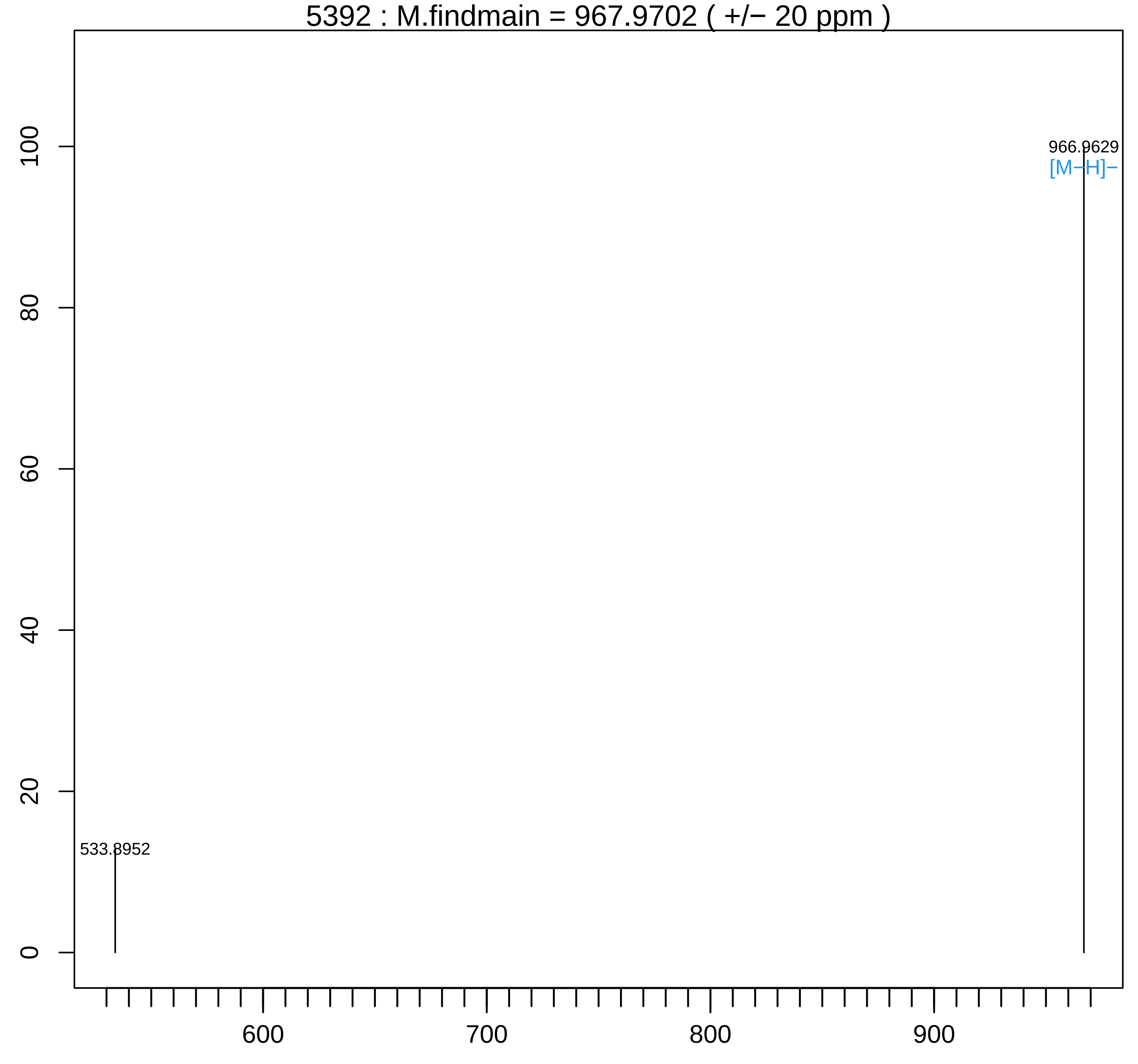
5392 : M.findmain = 967.9702 (+/− 20 ppm)
966.9629
[M−H]−
533.8952
0
20
40
60
80
100
600
700
800
900

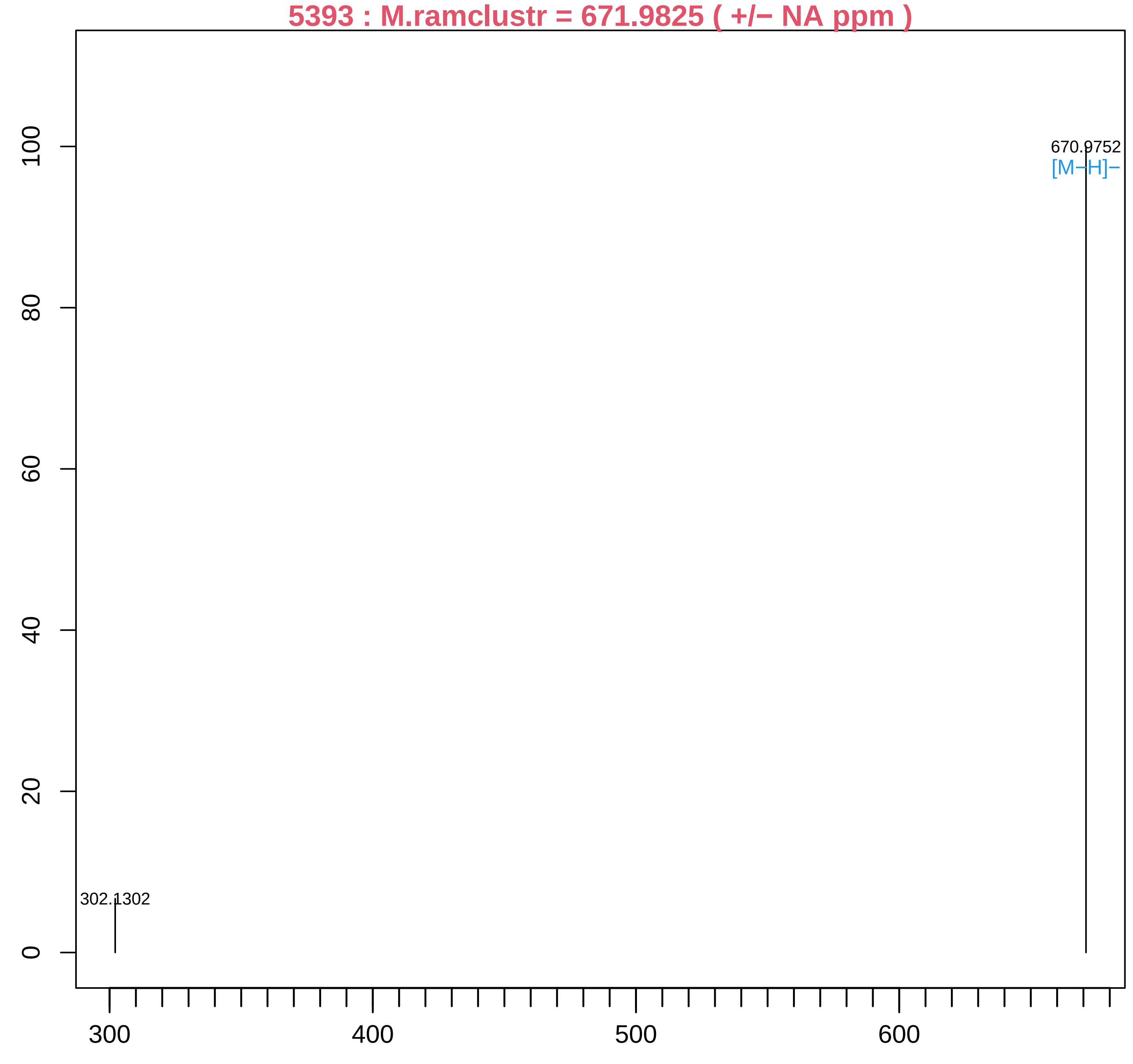

5393 : M.ramclustr = 671.9825 (+/− NA ppm)
670.9752
[M−H]−
302.1302
0
20
40
60
80
100
300
400
500
600

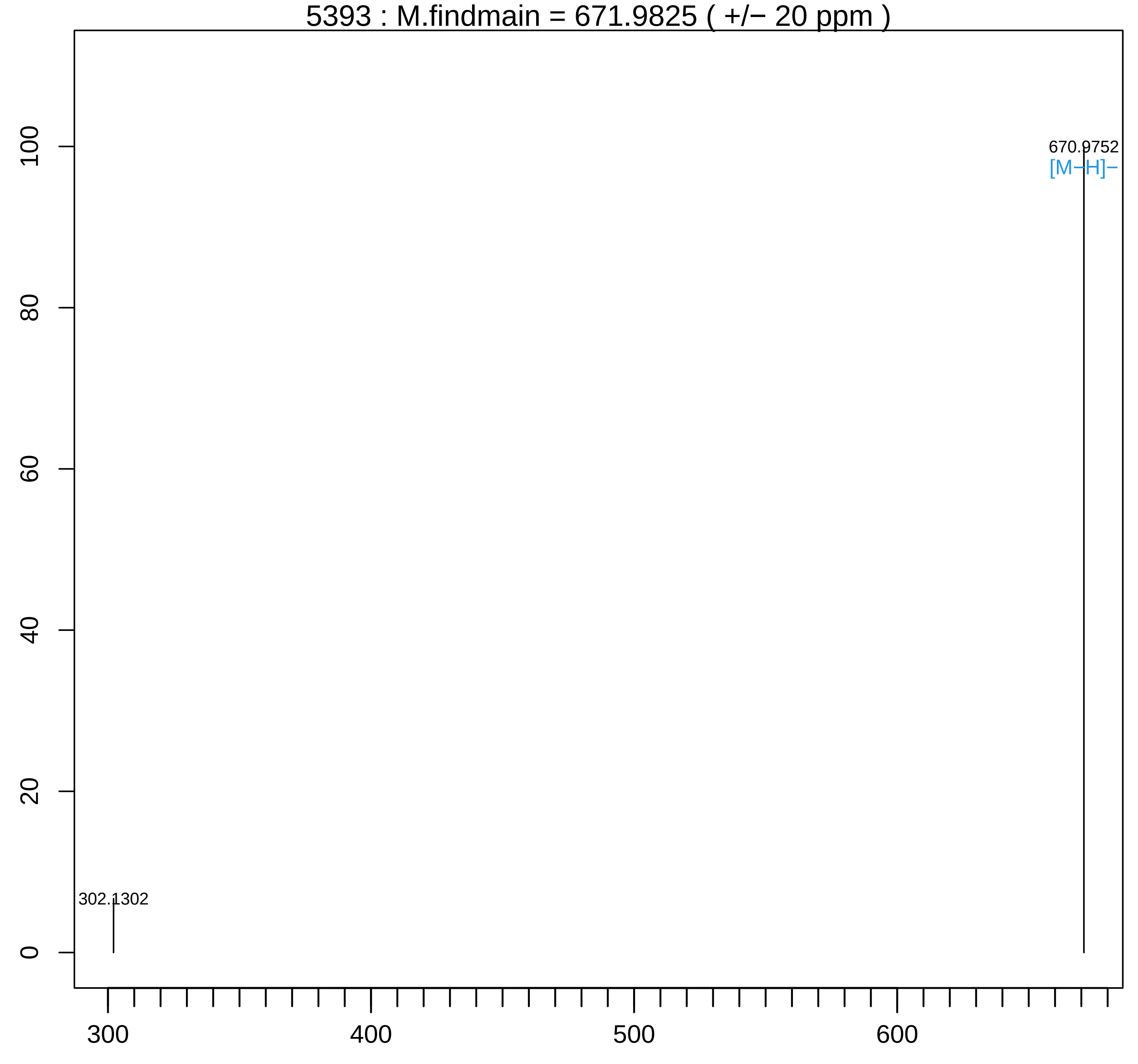

5393 : M.findmain = 671.9825 (+/− 20 ppm)
670.9752
[M−H]−
302.1302
0
20
40
60
80
100
300
400
500
600

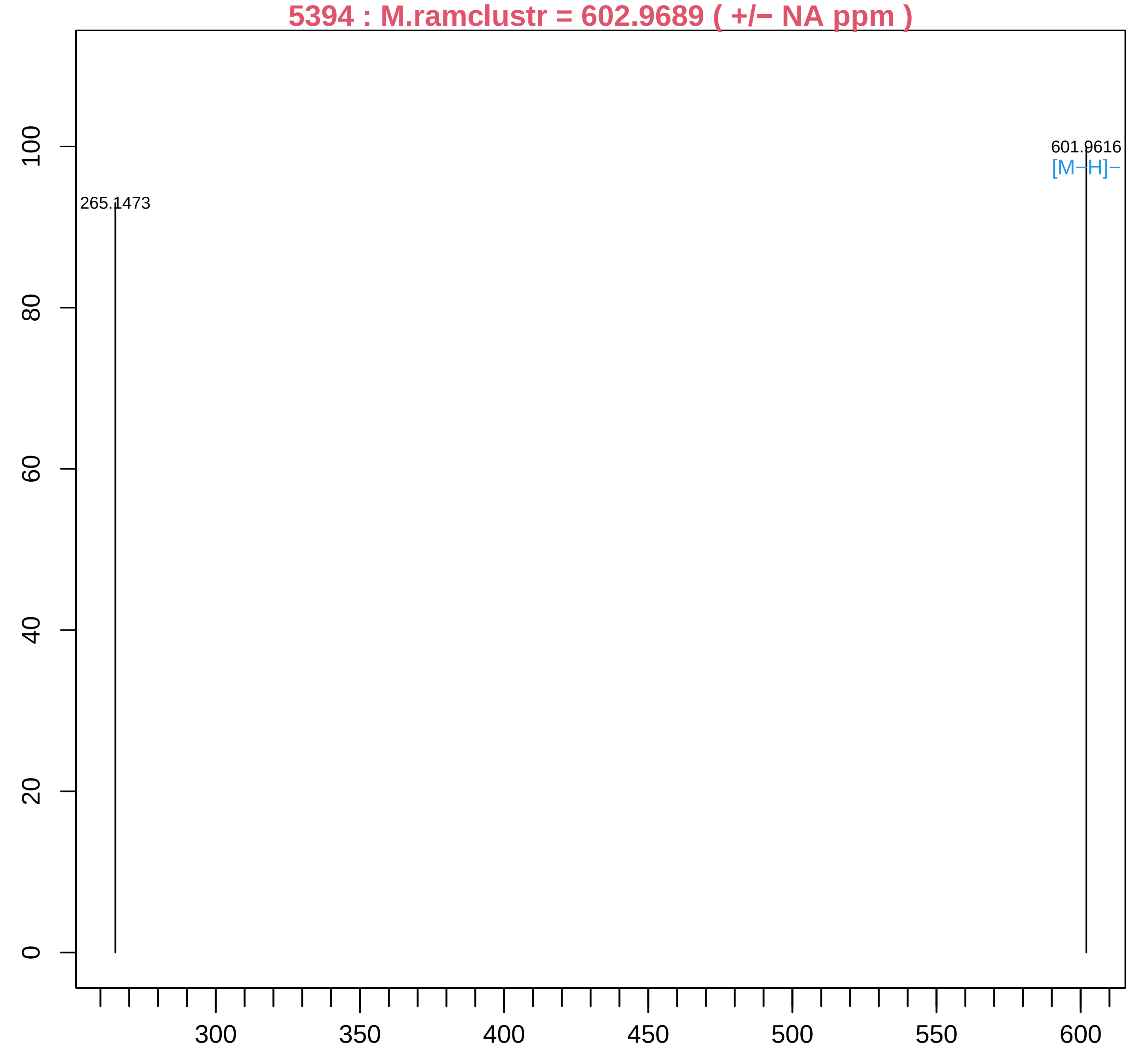
5394 : M.ramclustr = 602.9689 (+/− NA ppm)
601.9616
[M−H]−
265.1473
0
20
40
60
80
100
300
350
400
450
500
550
600

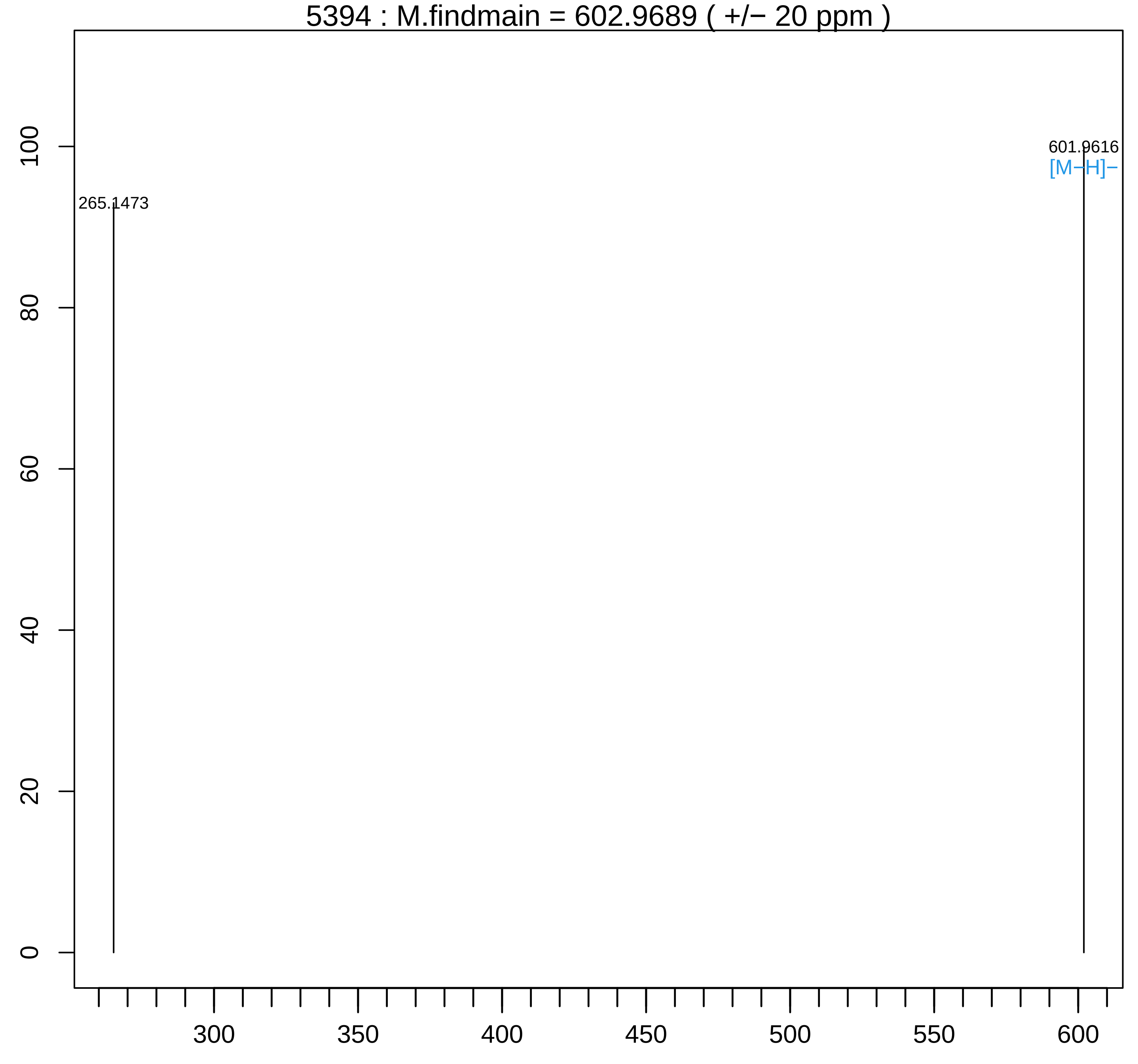
5394 : M.findmain = 602.9689 (+/− 20 ppm)
601.9616
[M−H]−
265.1473
0
20
40
60
80
100
300
350
400
450
500
550
600

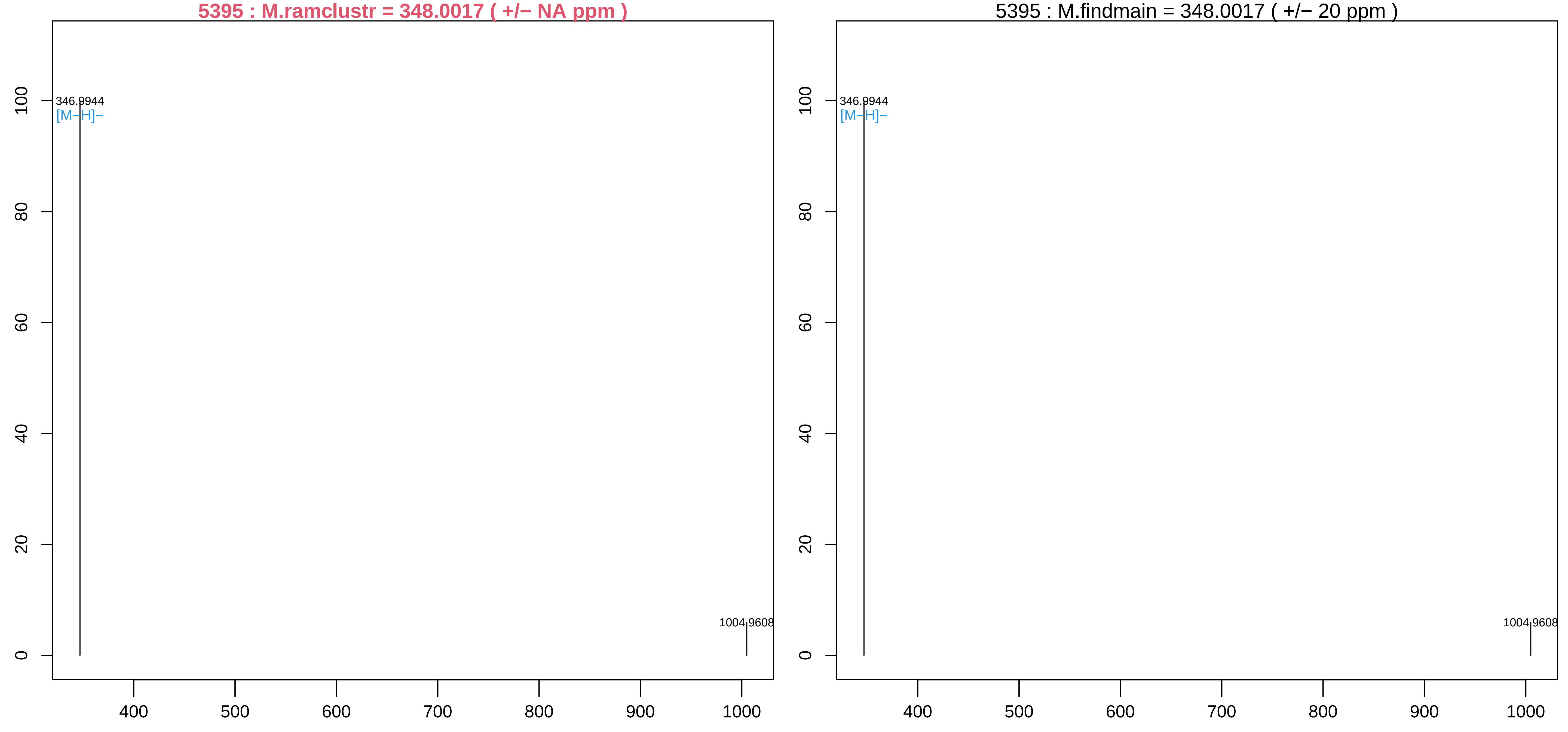
5395 : M.ramclustr = 348.0017 (+/− NA ppm)
346.9944
[M−H]−
1004.9608
5395 : M.findmain = 348.0017 (+/− 20 ppm)
346.9944
[M−H]−
1004.9608
0
20
40
60
80
100
400
500
600
700
800
900
1000

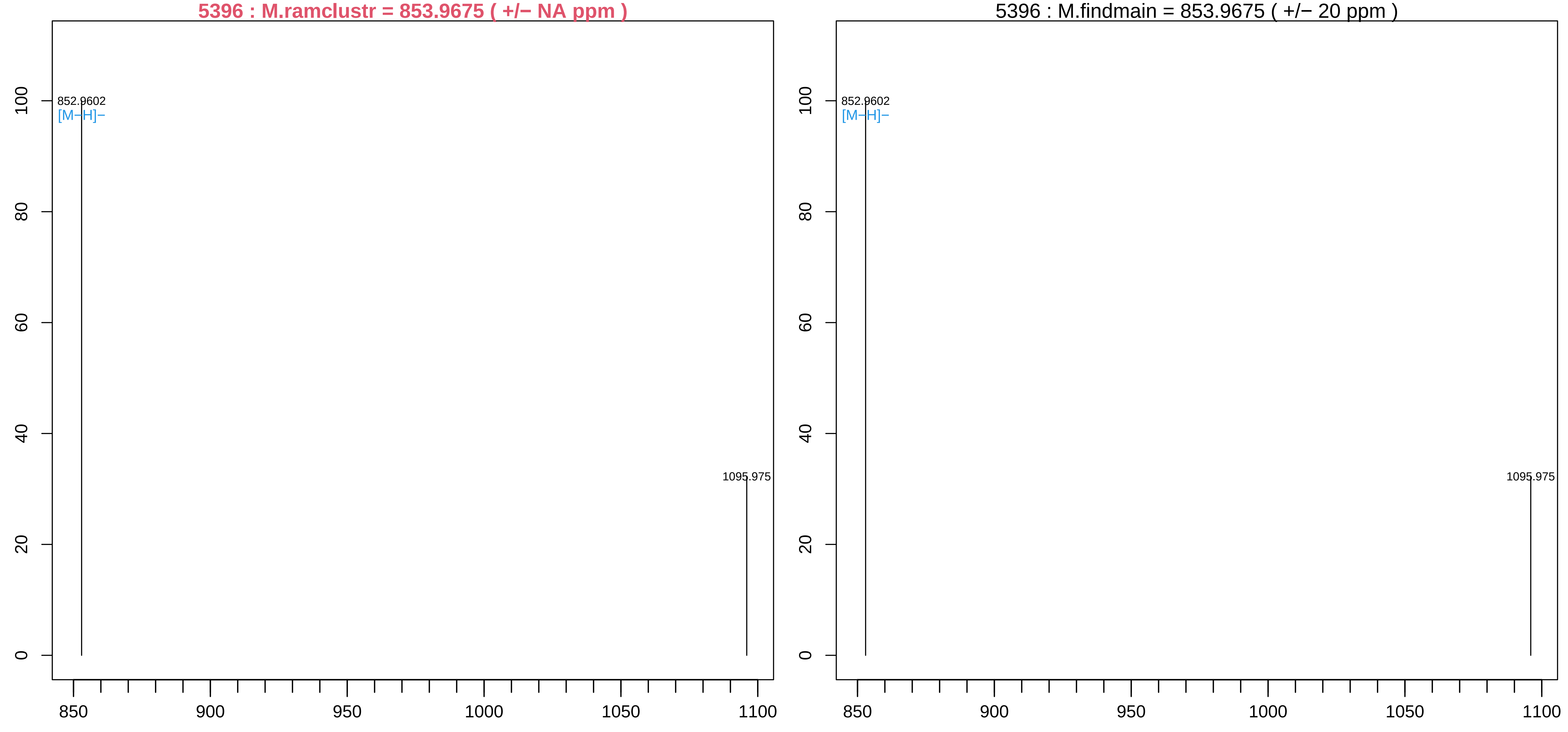

5396 : M.ramclustr = 853.9675 (+/− NA ppm)
852.9602
[M−H]−
1095.975
0
20
40
60
80
100
850
900
950
1000
1050
1100
5396 : M.findmain = 853.9675 (+/− 20 ppm)
852.9602
[M−H]−
1095.975
0
20
40
60
80
100
850
900
950
1000
1050
1100

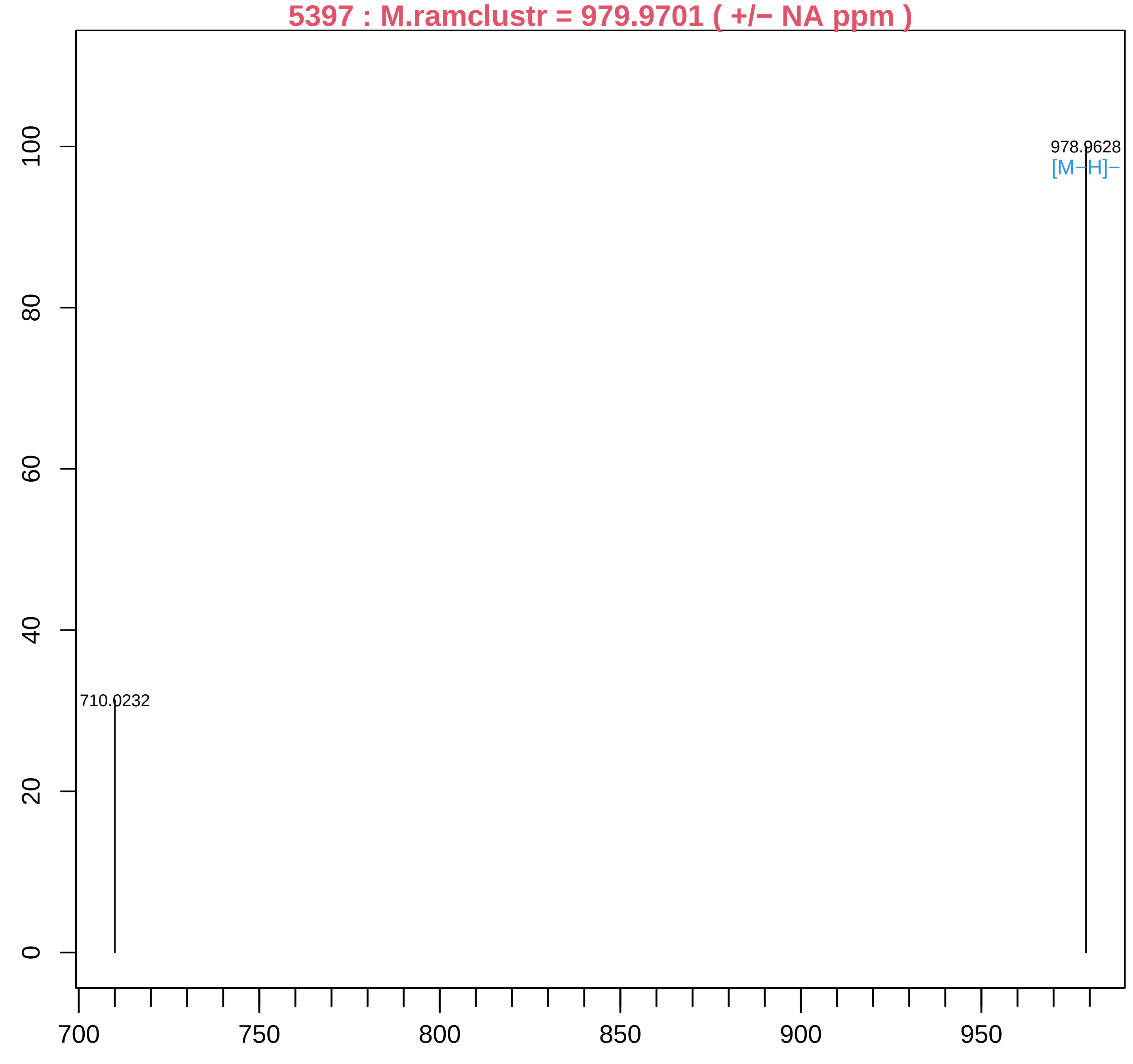
5397 : M.ramclustr = 979.9701 (+/− NA ppm)
978.9628
[M−H]−
710.0232
0
20
40
60
80
100
700
750
800
850
900
950

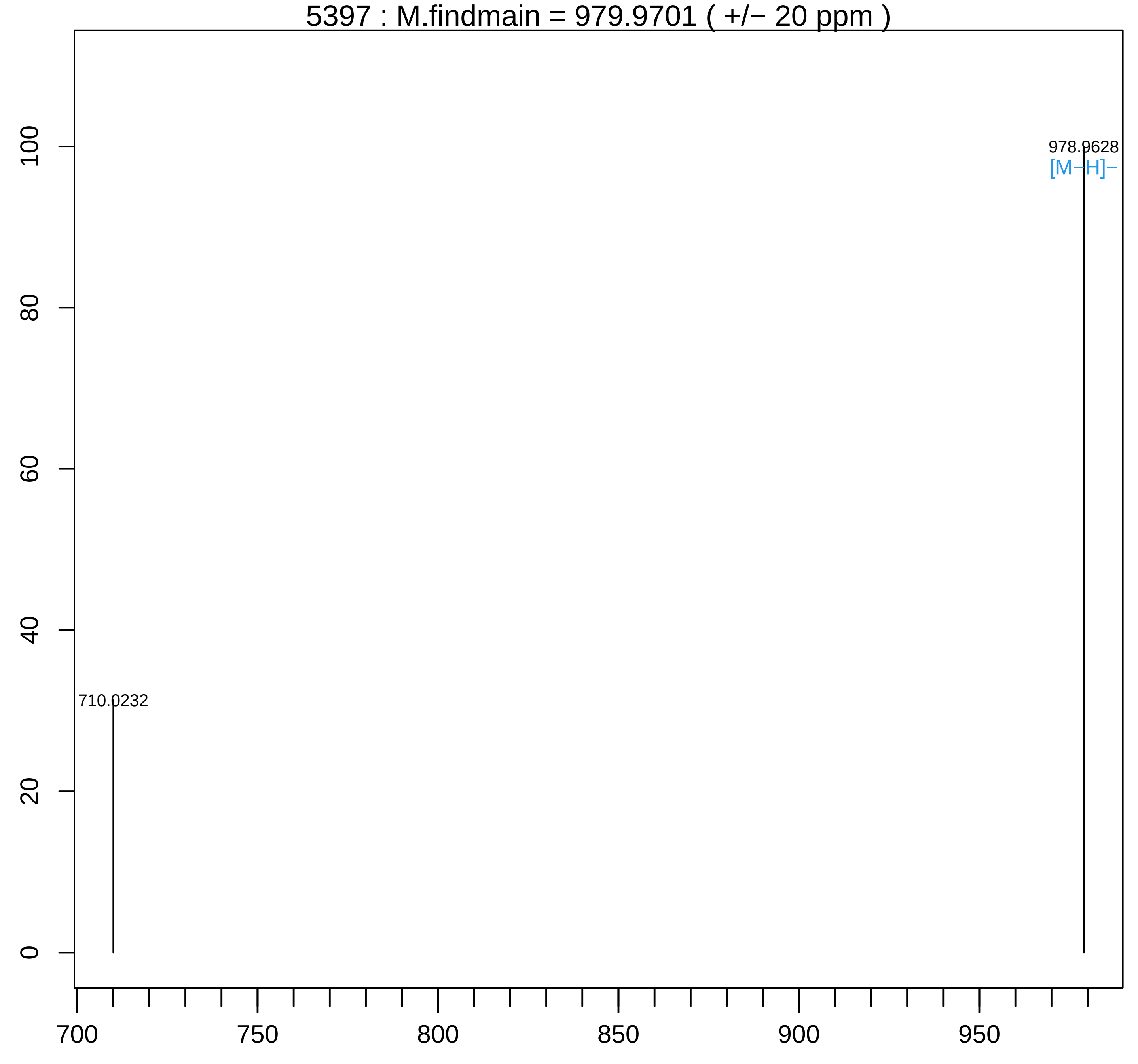
5397 : M.findmain = 979.9701 (+/− 20 ppm)
978.9628
[M−H]−
710.0232
0
20
40
60
80
100
700
750
800
850
900
950

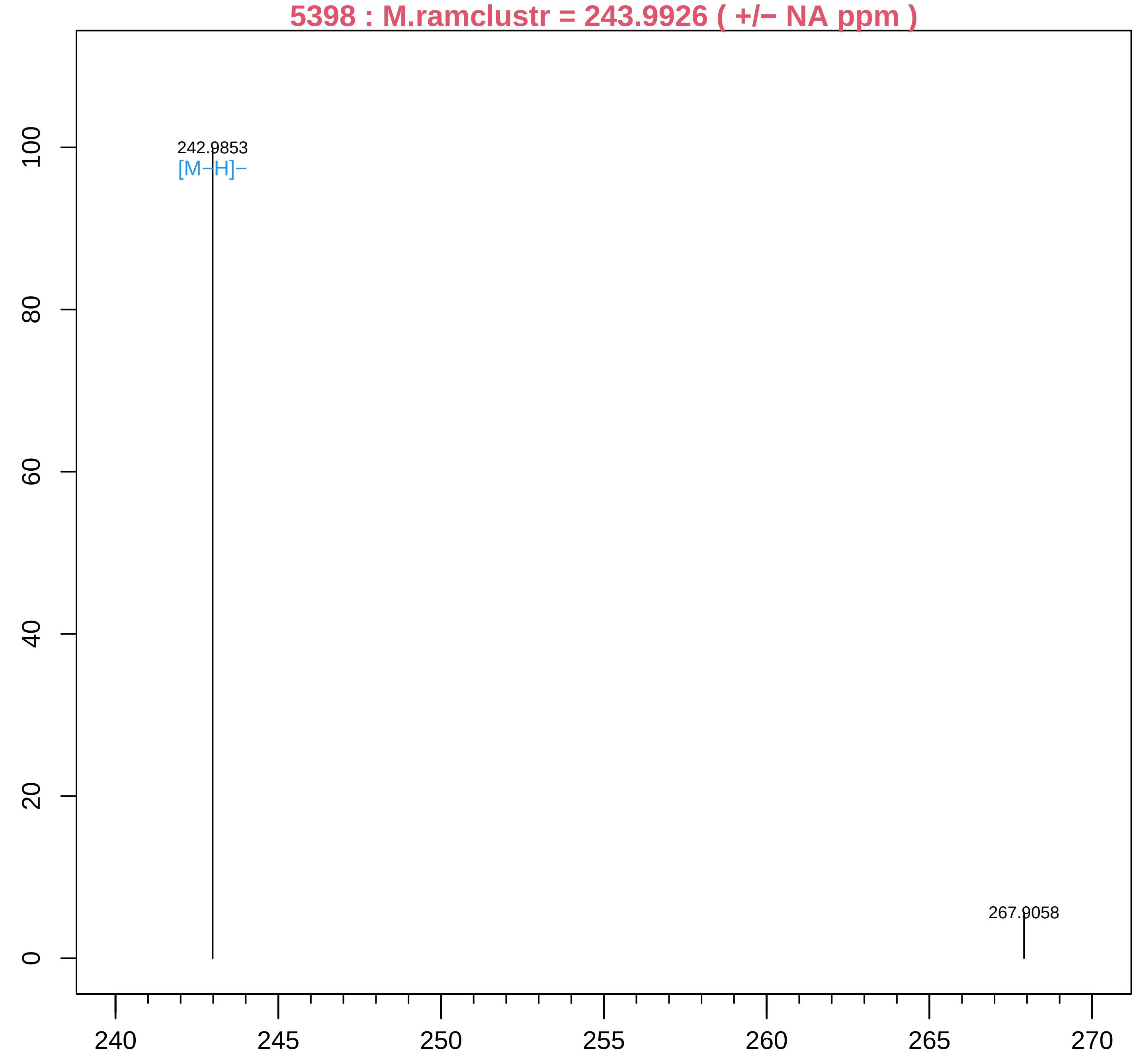
5398 : M.ramclustr = 243.9926 (+/− NA ppm)
242.9853
[M−H]−
267.9058
0
20
40
60
80
100
240
245
250
255
260
265
270

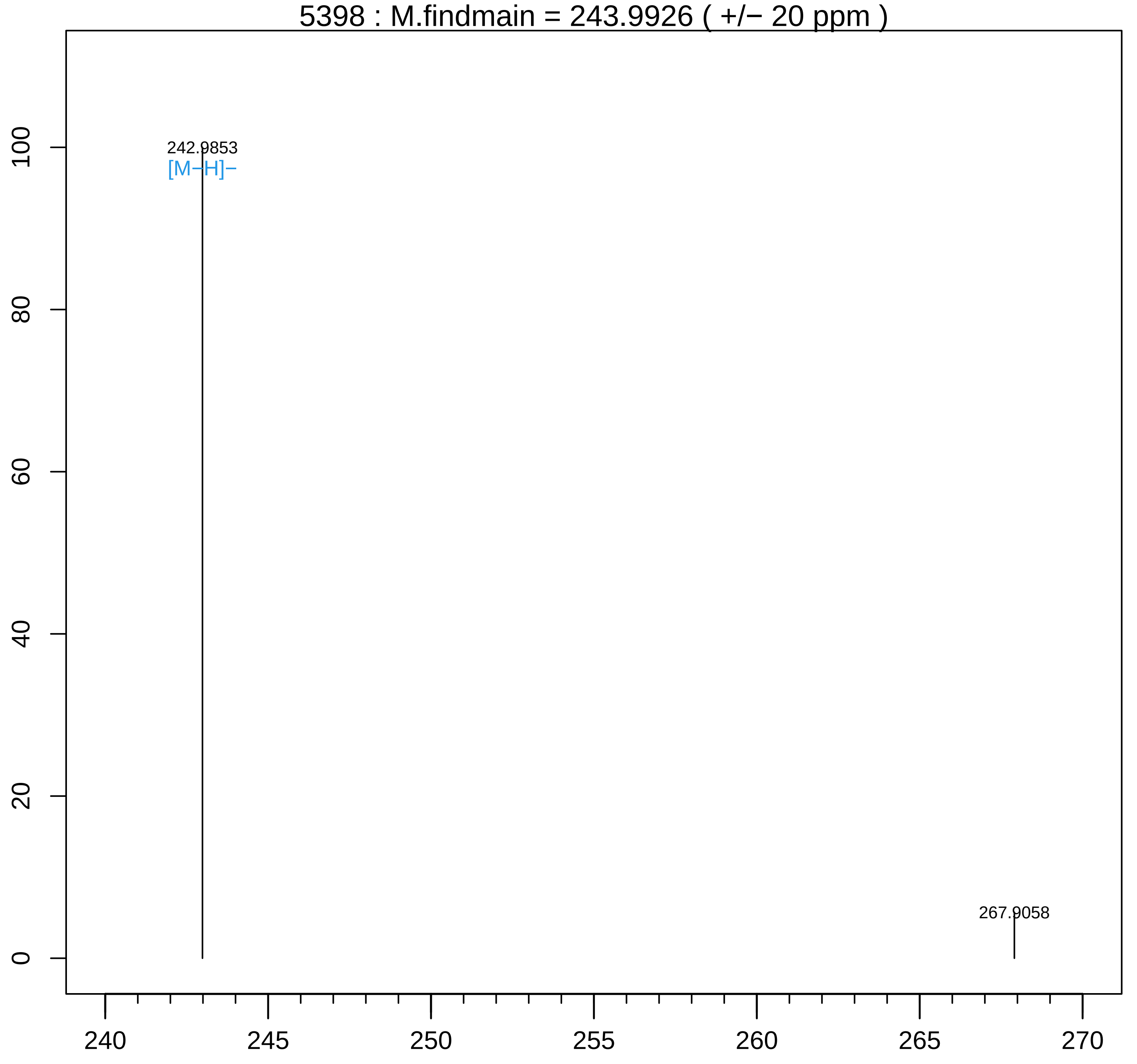
5398 : M.findmain = 243.9926 (+/− 20 ppm)
242.9853
[M−H]−
267.9058
0
20
40
60
80
100
240
245
250
255
260
265
270

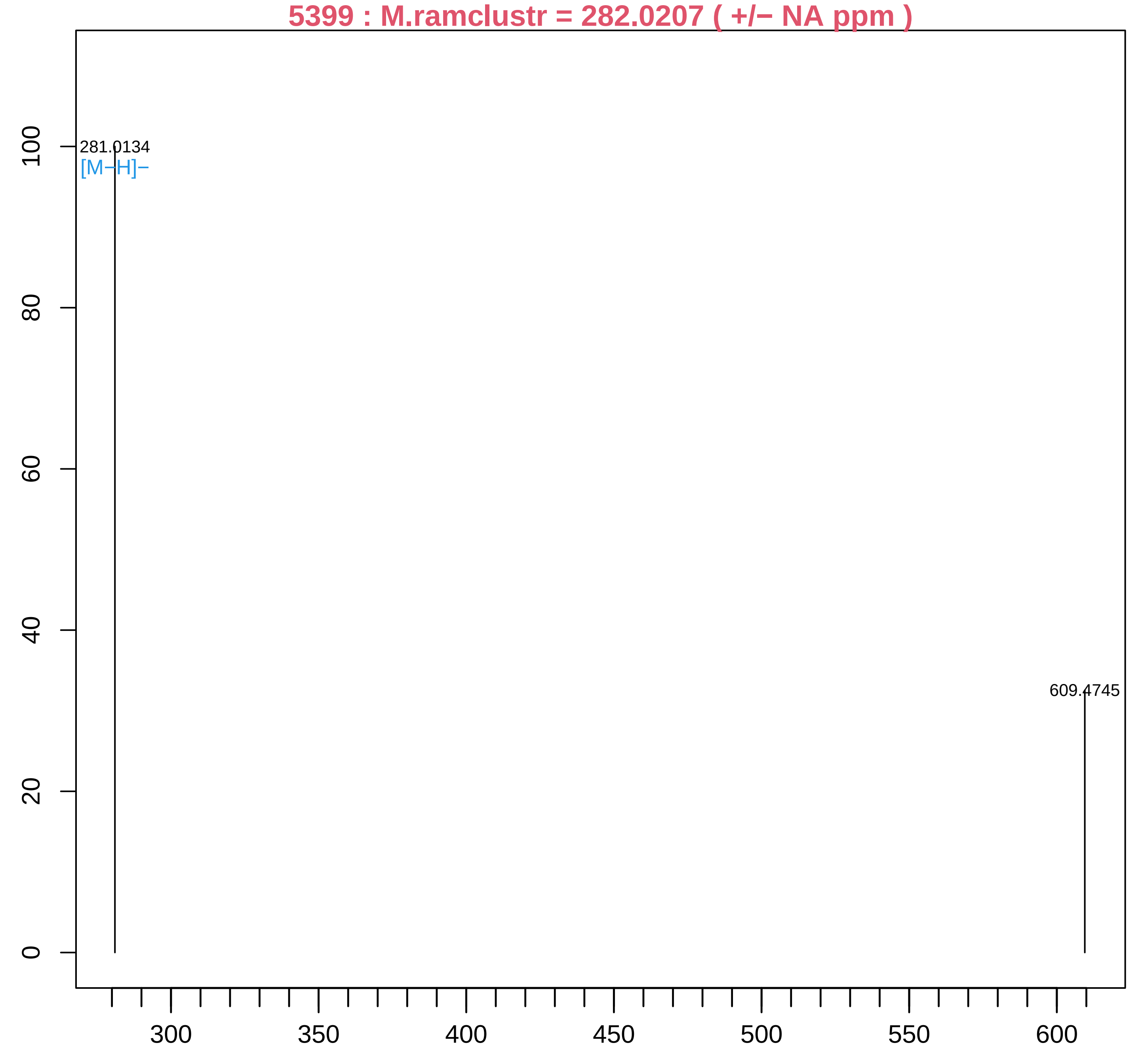
5399 : M.ramclustr = 282.0207 (+/− NA ppm)
281.0134
[M−H]−
609.4745
0
20
40
60
80
100
300
350
400
450
500
550
600

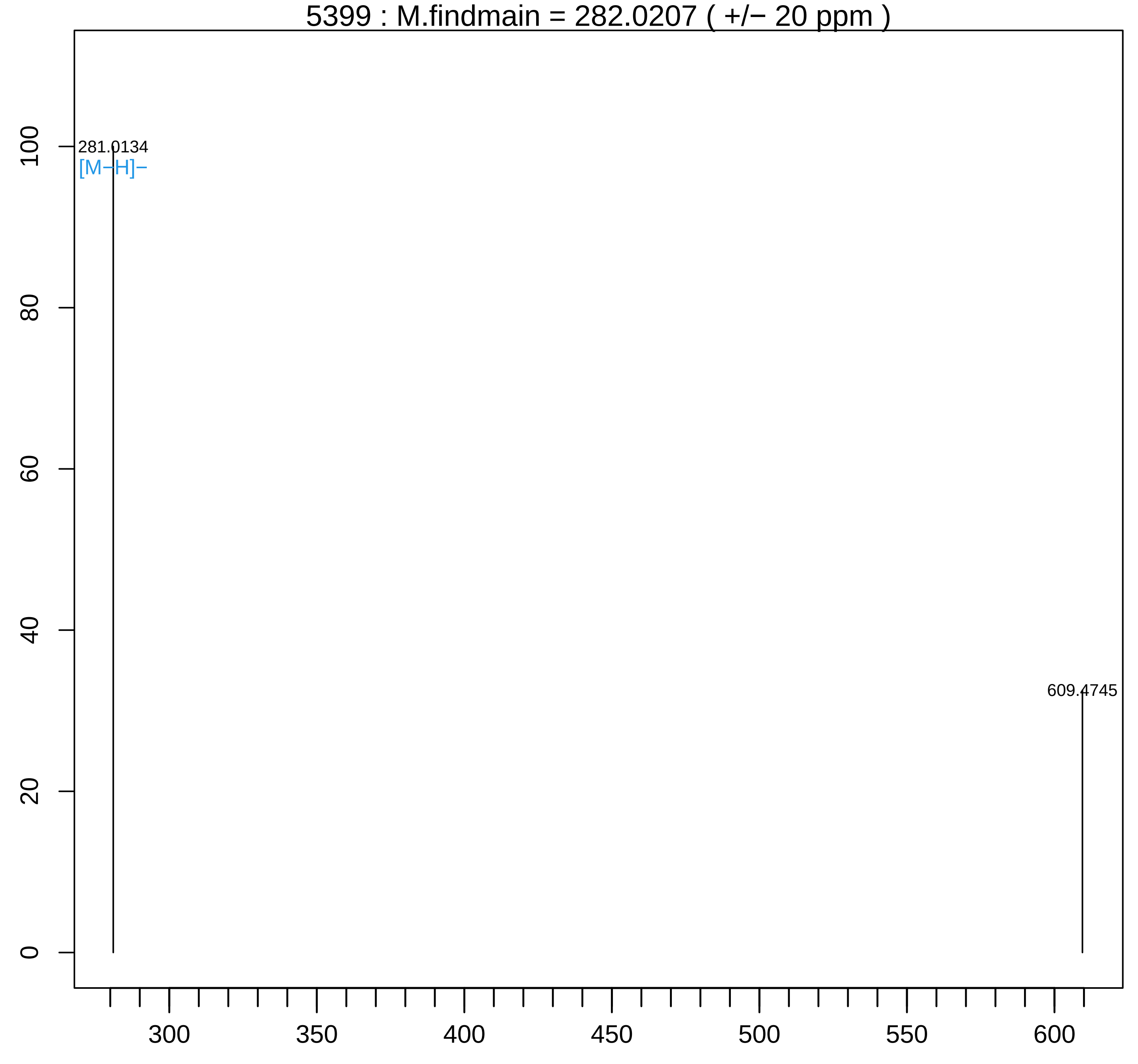
5399 : M.findmain = 282.0207 (+/− 20 ppm)
281.0134
[M−H]−
609.4745
0
20
40
60
80
100
300
350
400
450
500
550
600

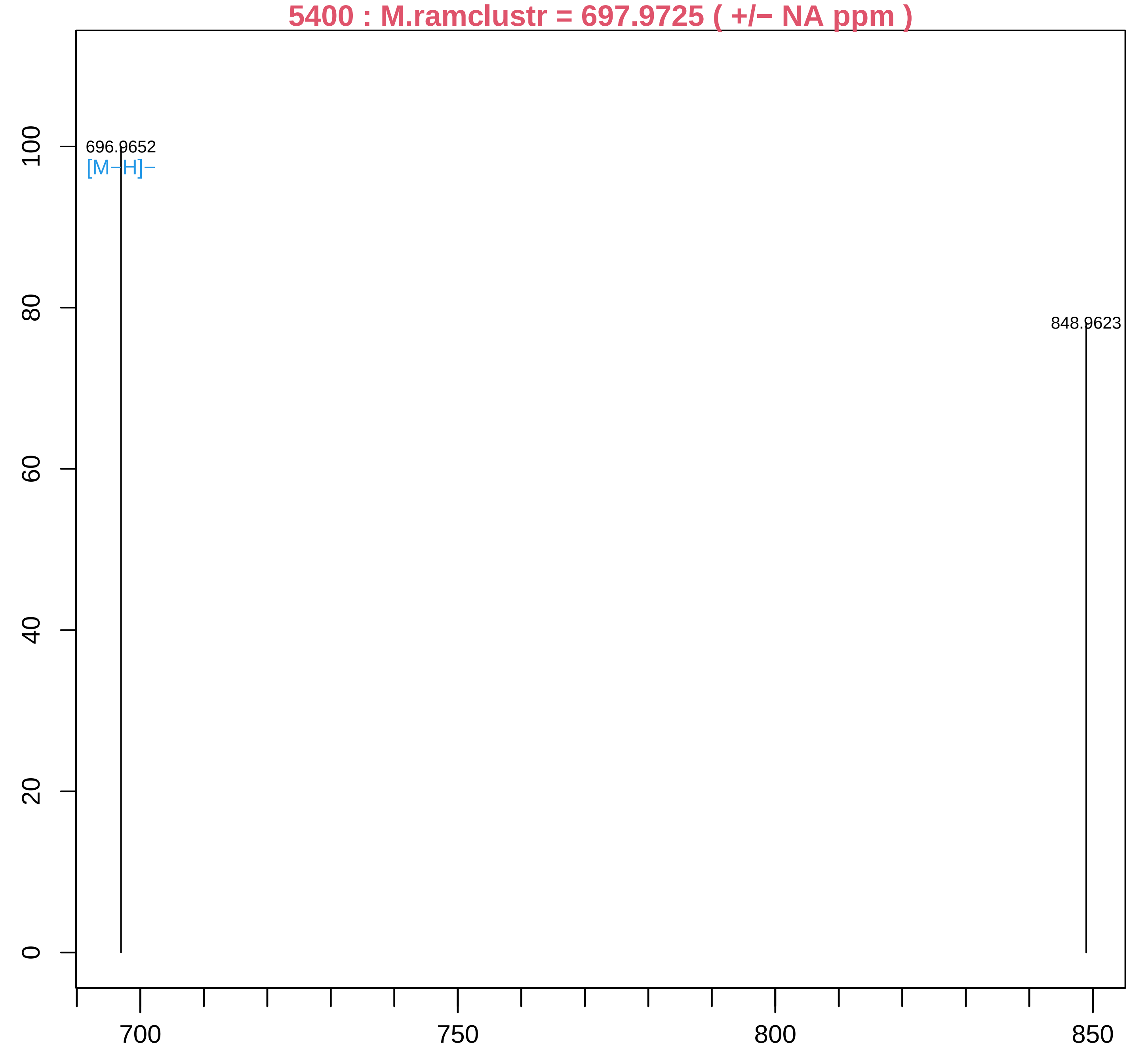
5400 : M.ramclustr = 697.9725 (+/− NA ppm)
696.9652
[M−H]−
848.9623
0
20
40
60
80
100
700
750
800
850

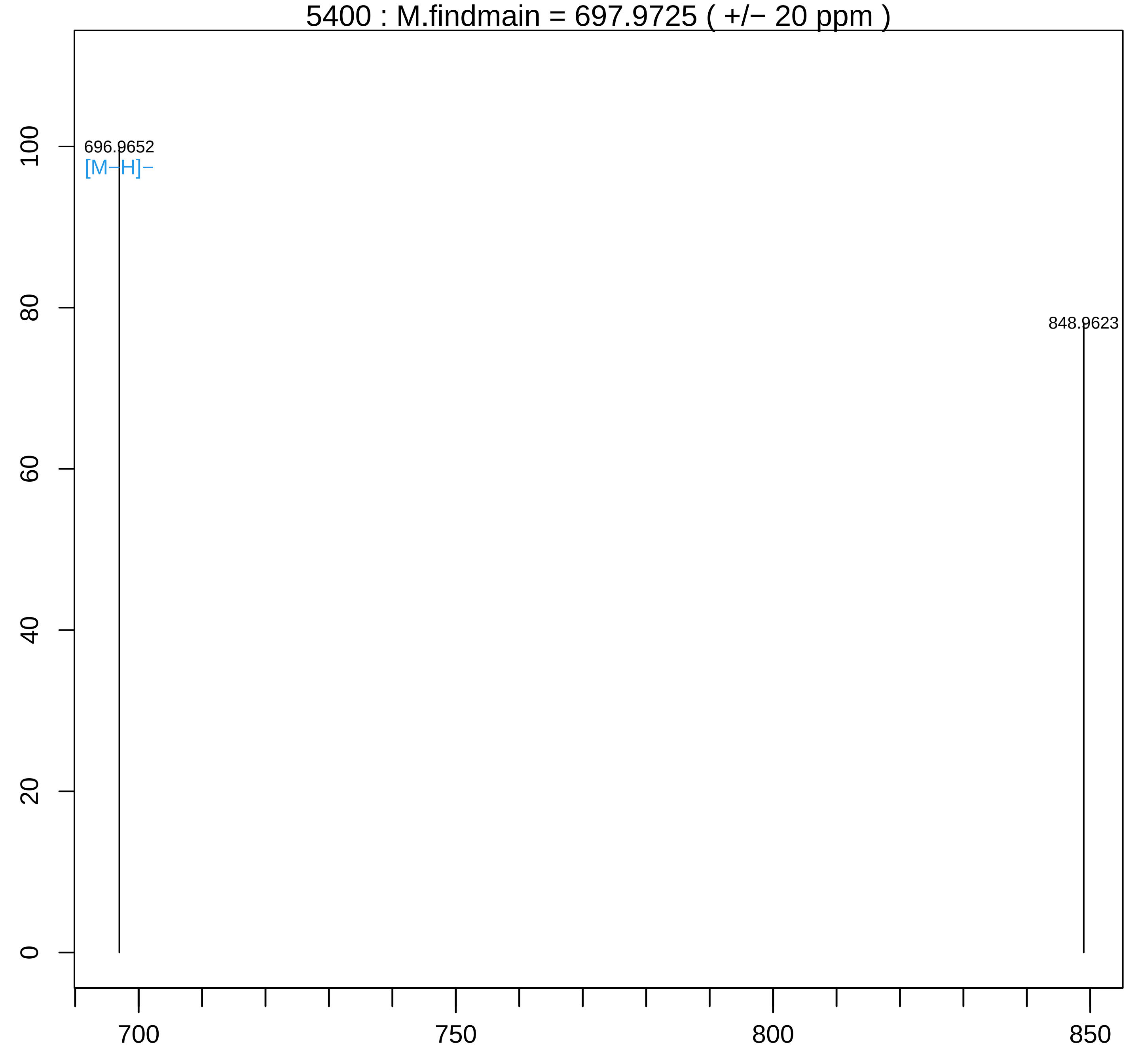
5400 : M.findmain = 697.9725 (+/− 20 ppm)
696.9652
[M−H]−
848.9623
0
20
40
60
80
100
700
750
800
850

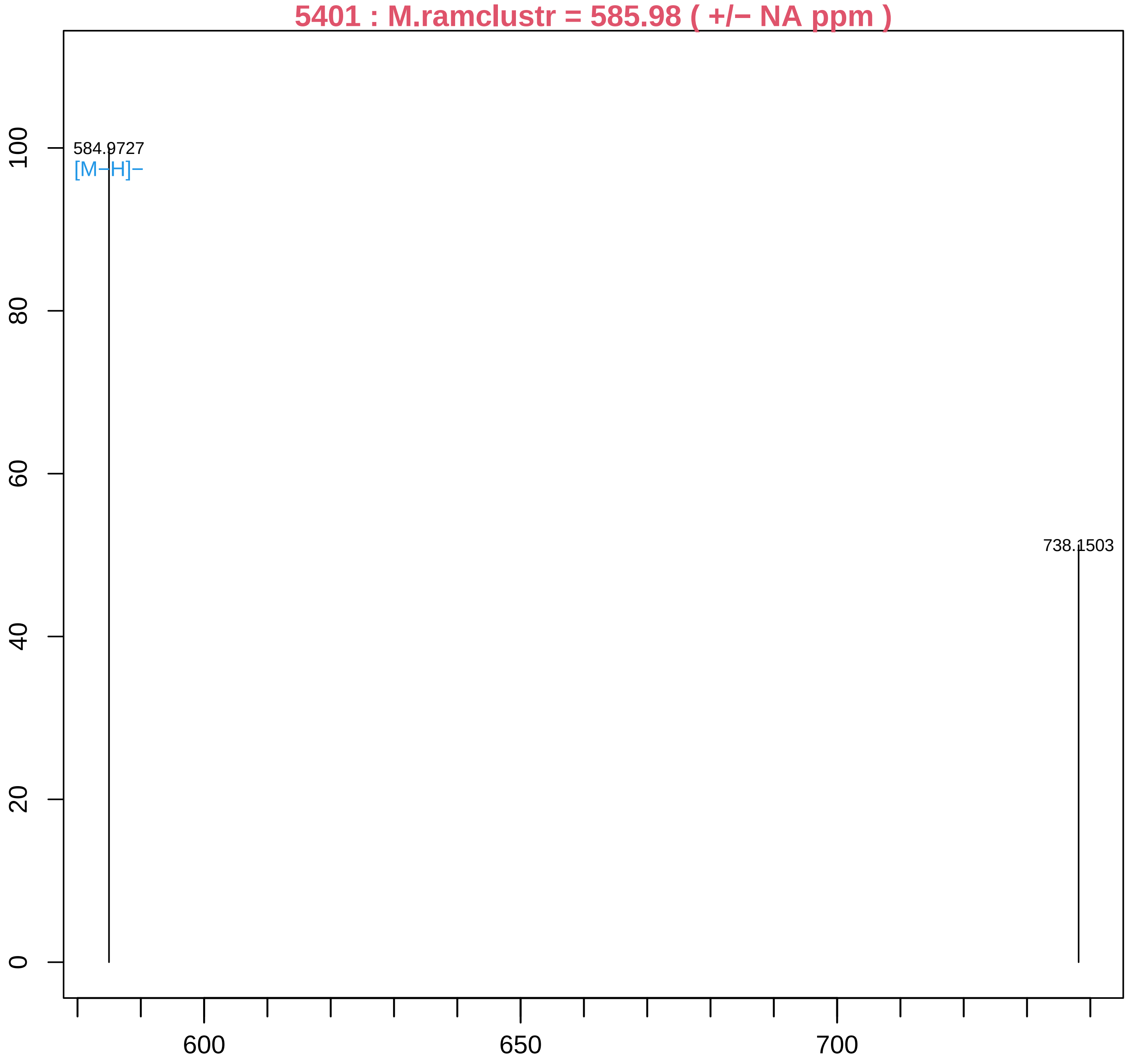
5401 : M.ramclustr = 585.98 (+/− NA ppm)
584.9727
[M−H]−
738.1503
0
20
40
60
80
100
600
650
700

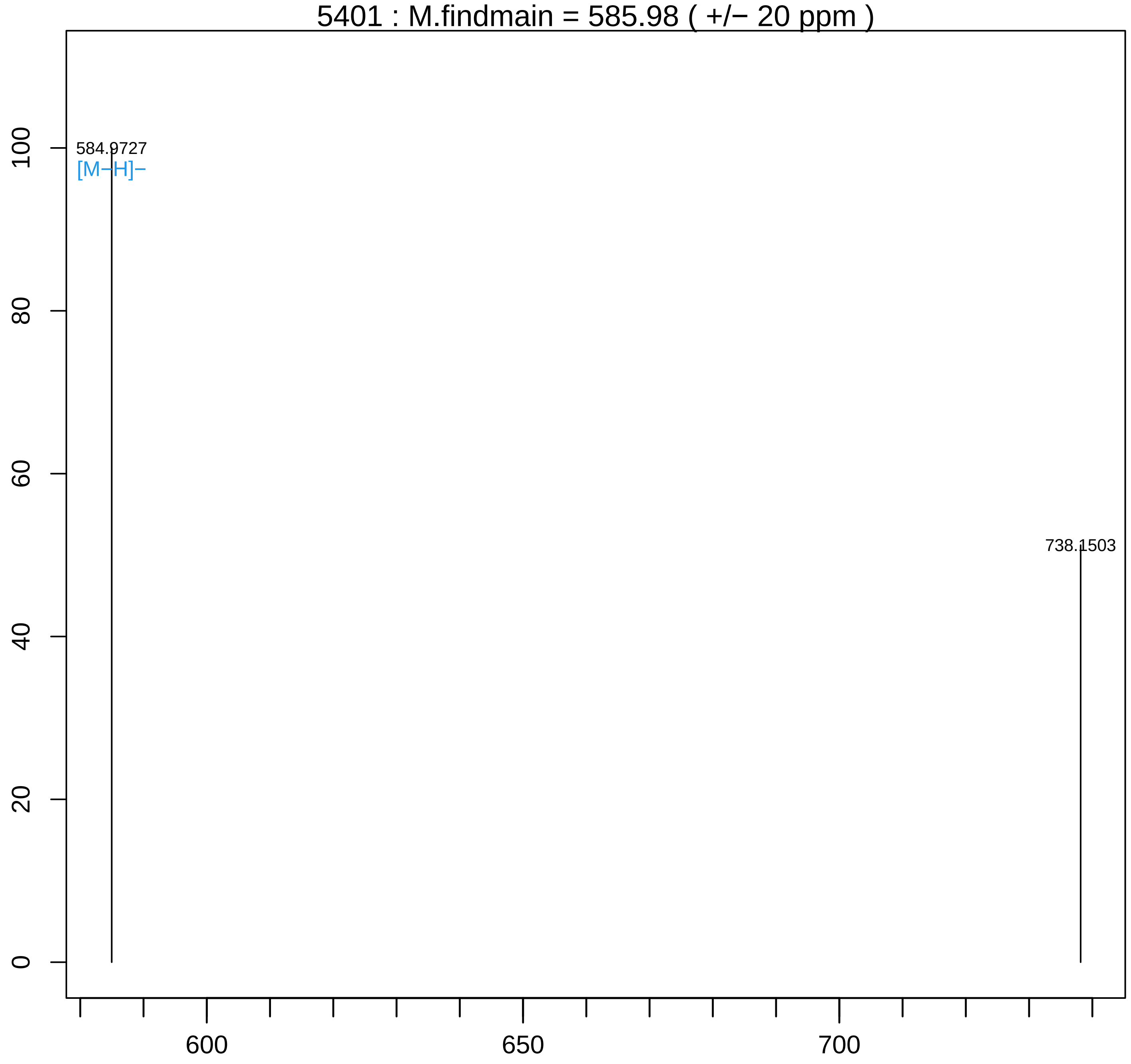
5401 : M.findmain = 585.98 (+/− 20 ppm)
584.9727
[M−H]−
738.1503
0
20
40
60
80
100
600
650
700

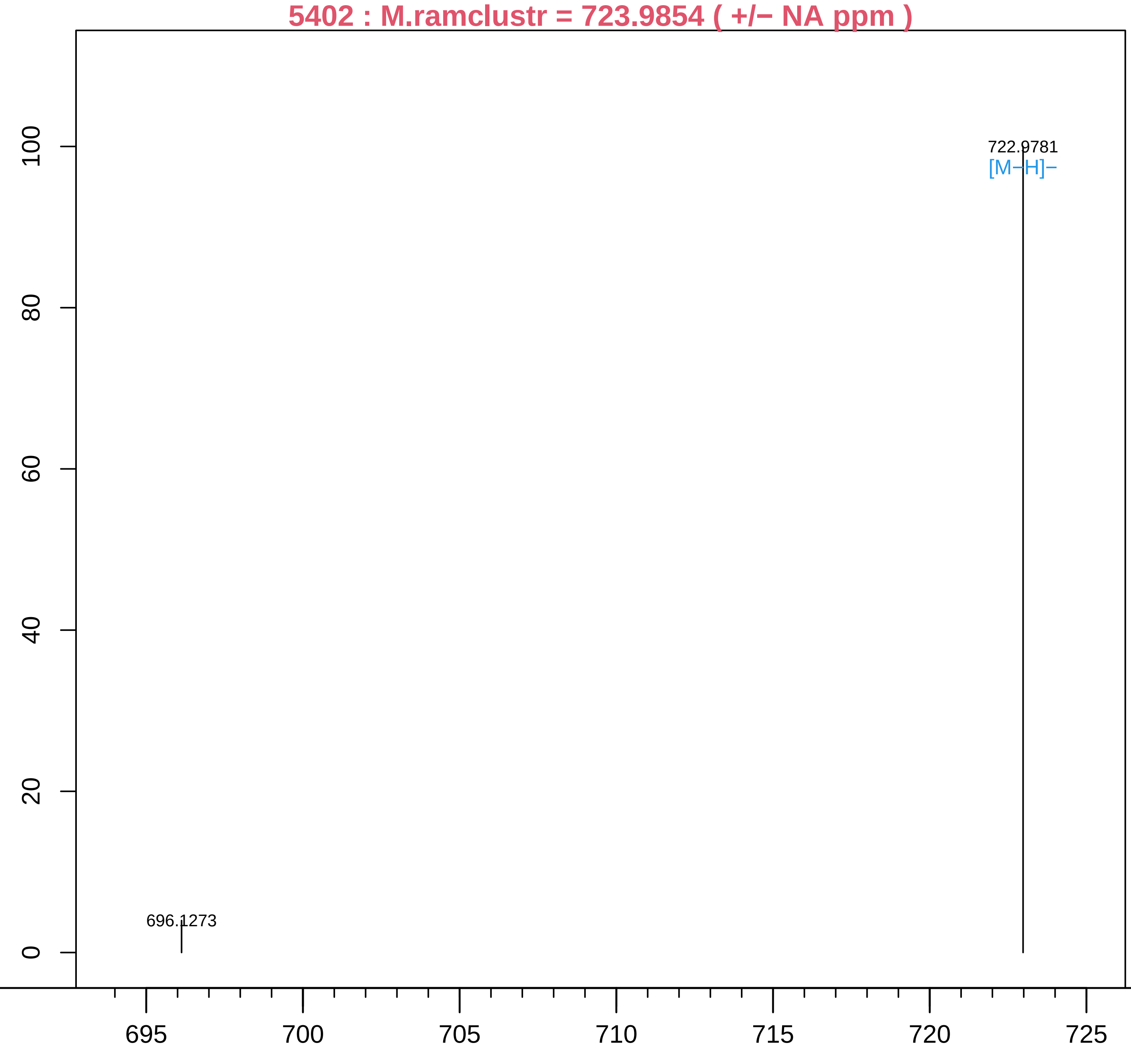
5402 : M.ramclustr = 723.9854 (+/− NA ppm)
722.9781
[M−H]−
696.1273
0
20
40
60
80
100
695
700
705
710
715
720
725

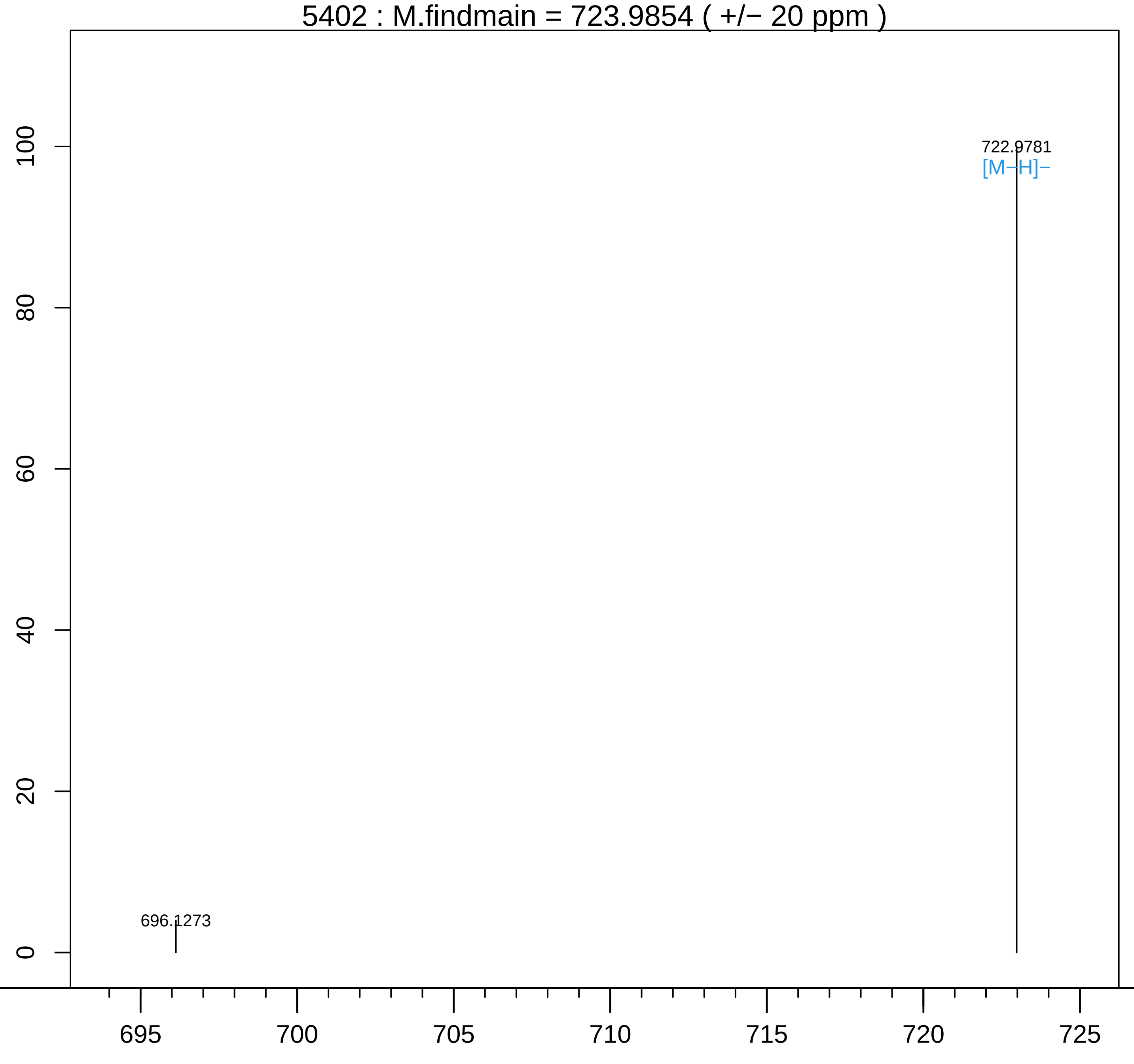
5402 : M.findmain = 723.9854 (+/− 20 ppm)
722.9781
[M−H]−
696.1273
0
20
40
60
80
100
695
700
705
710
715
720
725

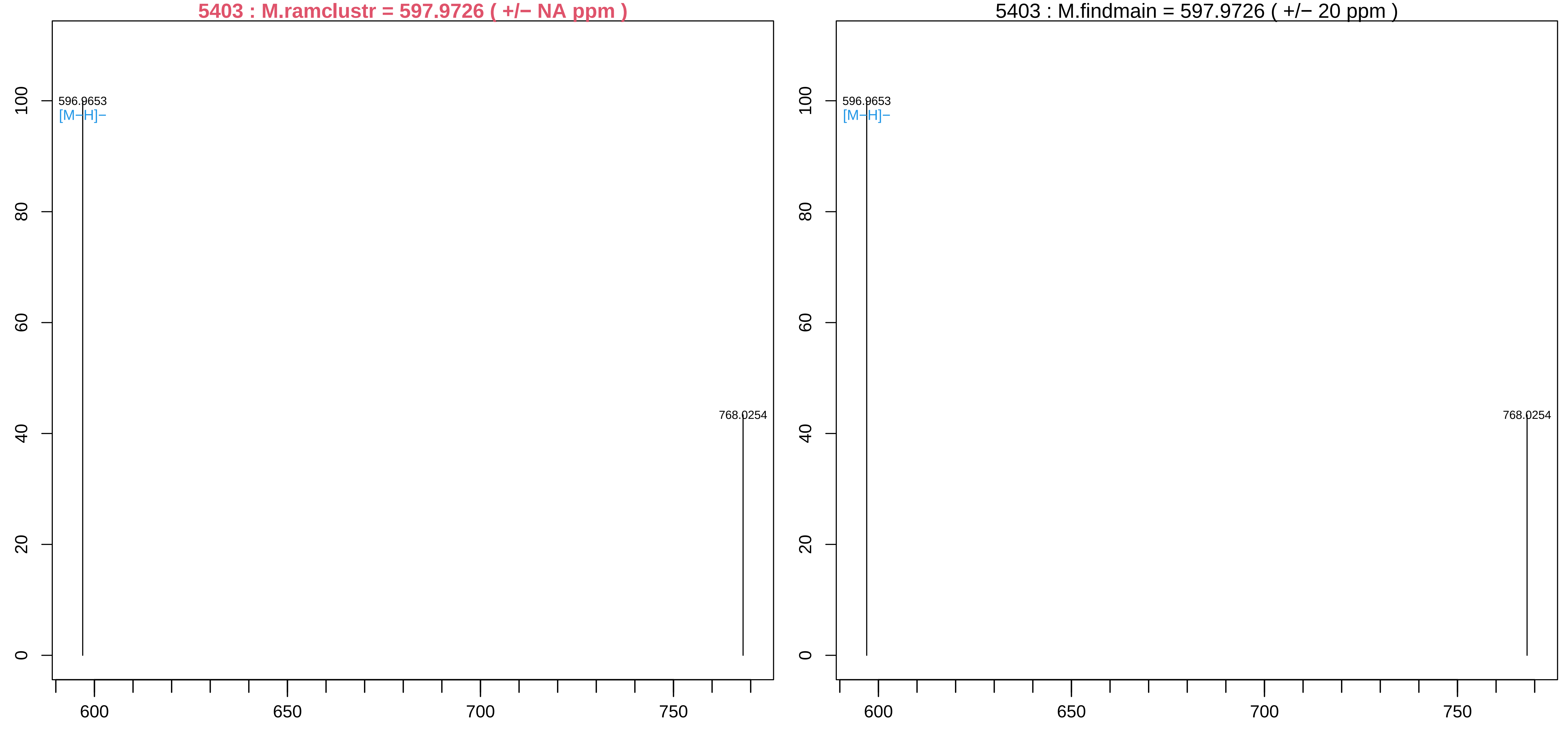
5403 : M.ramclustr = 597.9726 (+/− NA ppm)
596.9653
[M−H]−
768.0254
0
20
40
60
80
100
600
650
700
750
5403 : M.findmain = 597.9726 (+/− 20 ppm)
596.9653
[M−H]−
768.0254
0
20
40
60
80
100
600
650
700
750

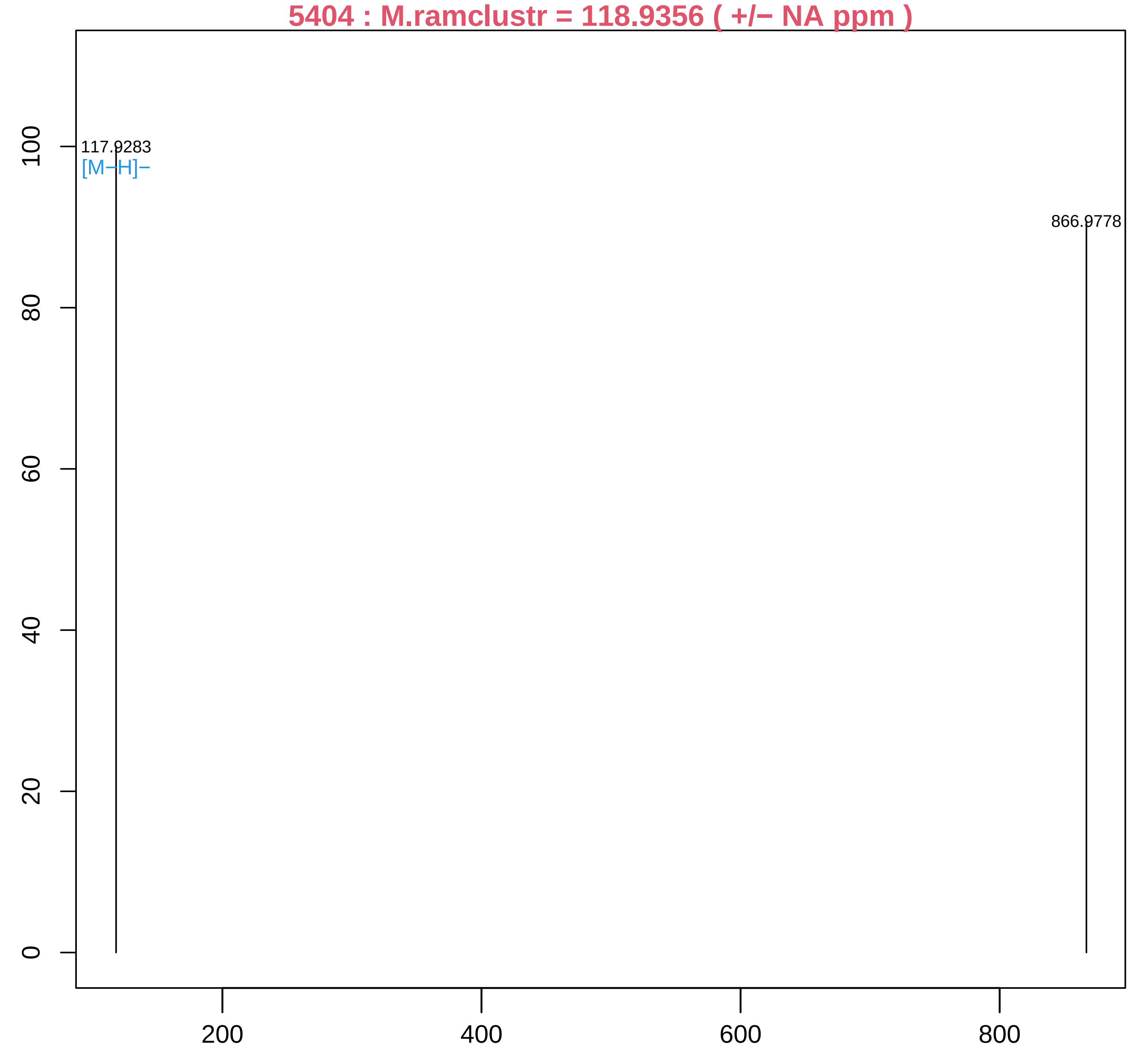

5404 : M.ramclustr = 118.9356 (+/− NA ppm)
117.9283
[M−H]−
866.9778
0
20
40
60
80
100
200
400
600
800

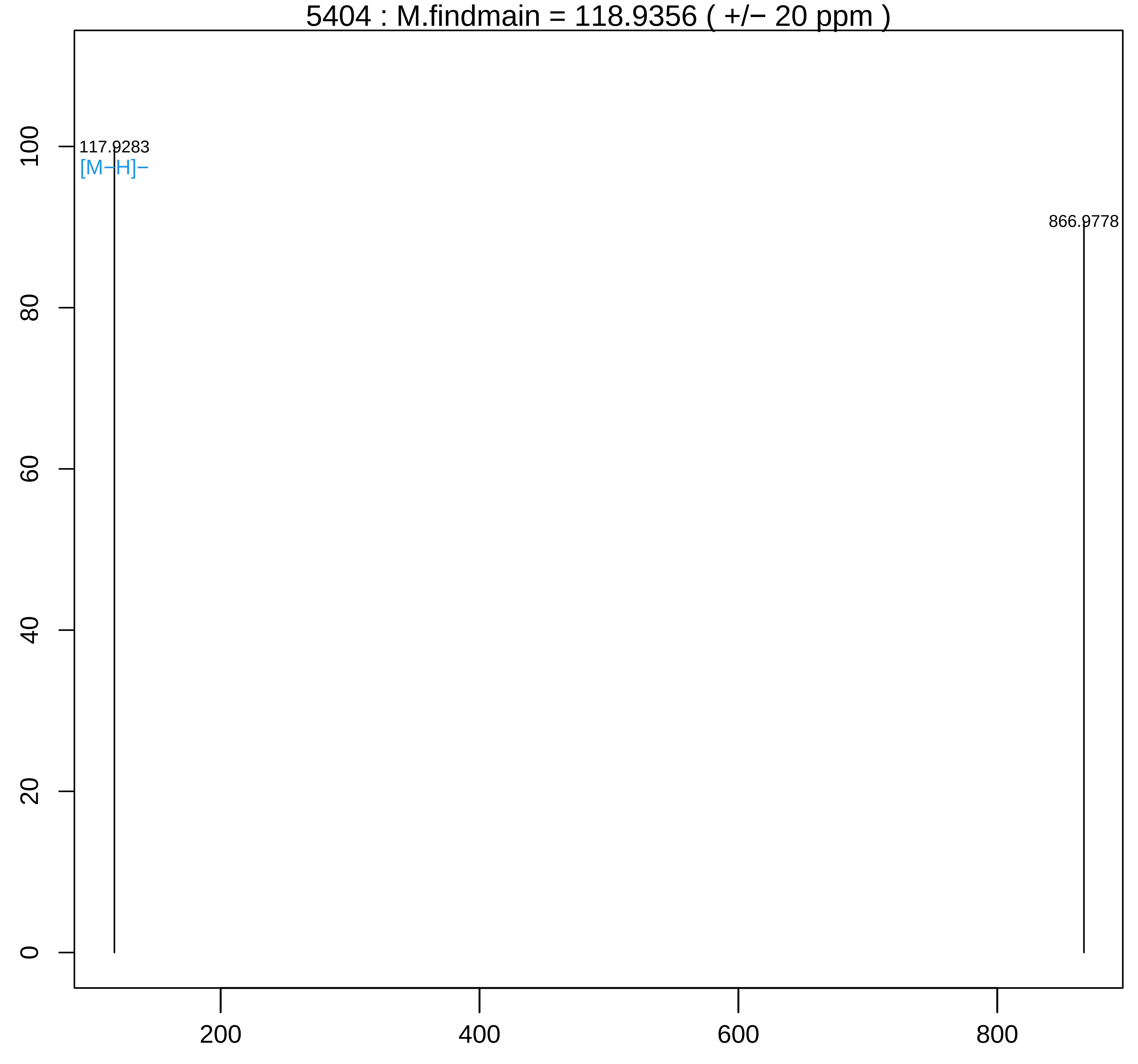

5404 : M.findmain = 118.9356 (+/− 20 ppm)
117.9283
[M−H]−
866.9778
0
20
40
60
80
100
200
400
600
800

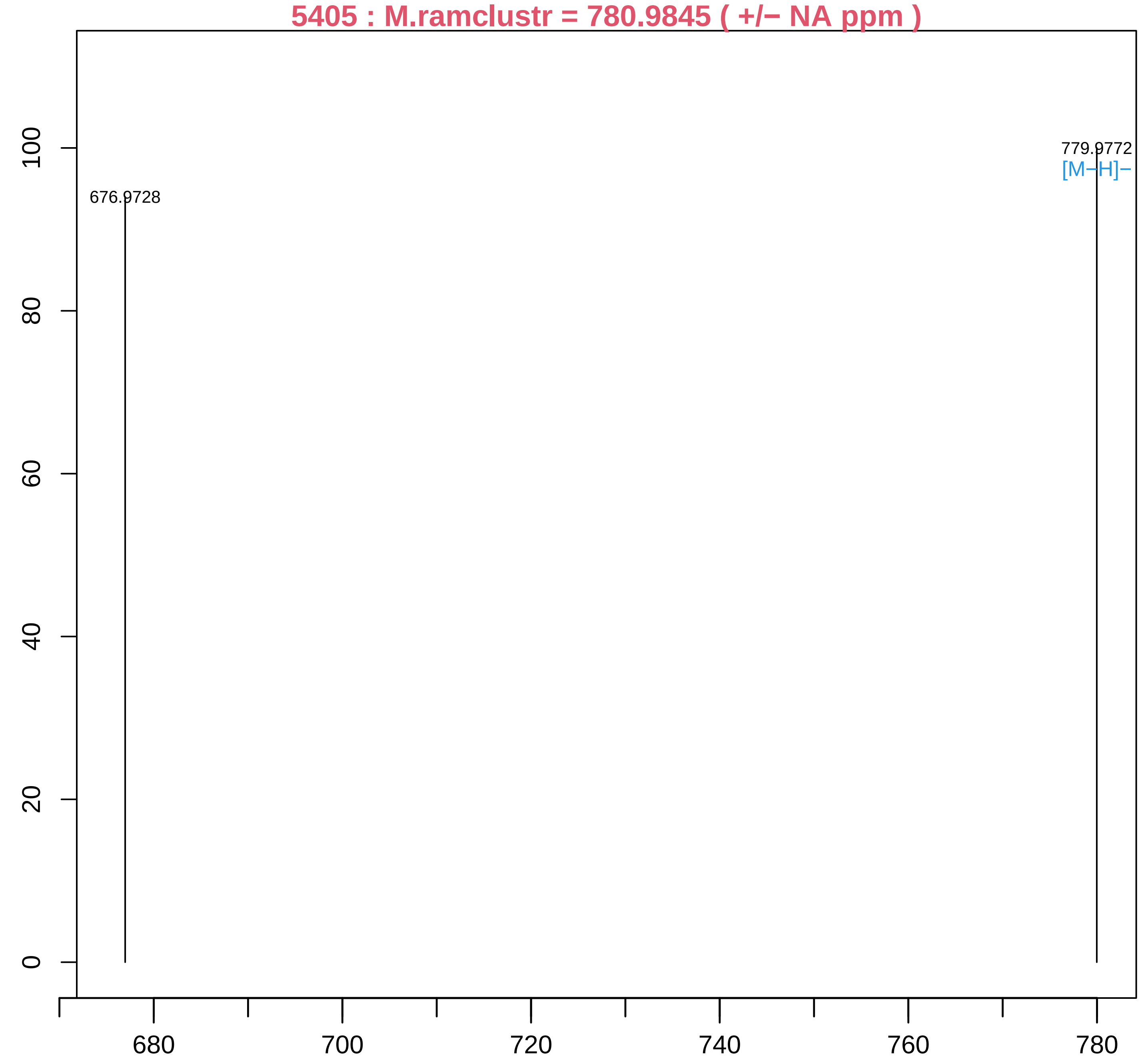
5405 : M.ramclustr = 780.9845 (+/− NA ppm)
779.9772
[M−H]−
676.9728
0
20
40
60
80
100
680
700
720
740
760
780

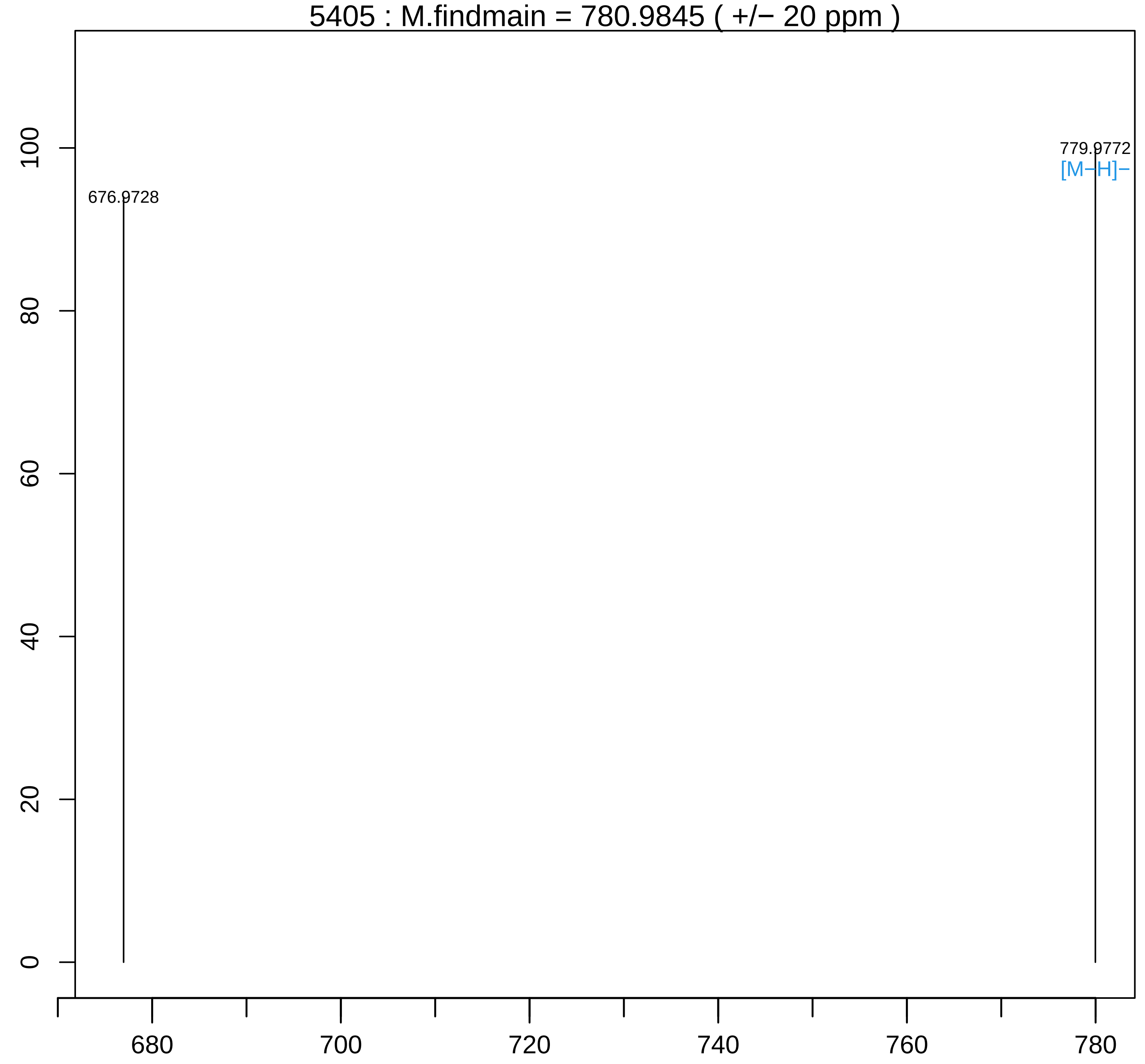
5405 : M.findmain = 780.9845 (+/− 20 ppm)
779.9772
[M−H]−
676.9728
0
20
40
60
80
100
680
700
720
740
760
780

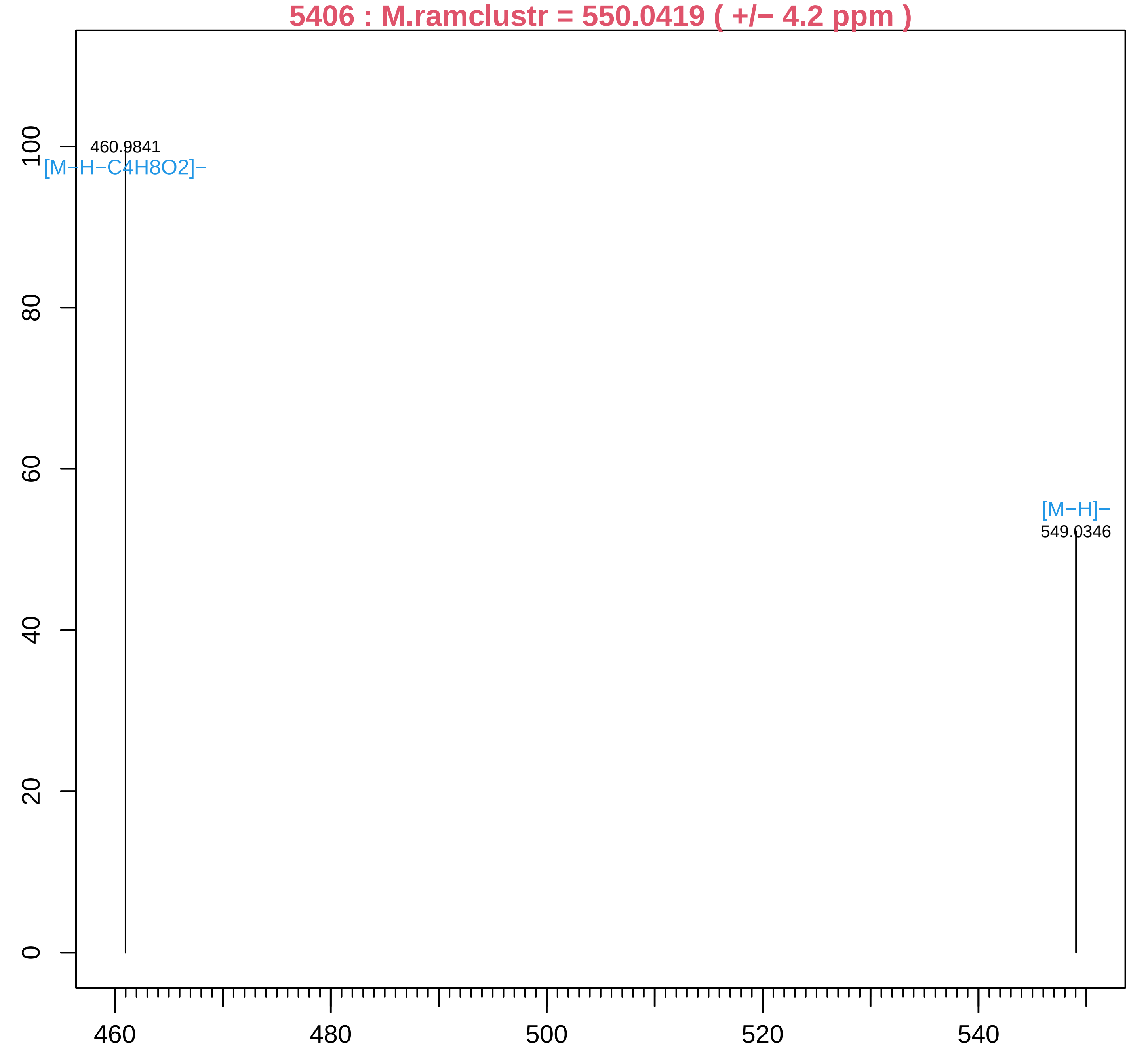
5406 : M.ramclustr = 550.0419 (+/− 4.2 ppm)
460.9841
[M−H−C4H8O2]−
[M−H]−
549.0346
0
20
40
60
80
100
460
480
500
520
540

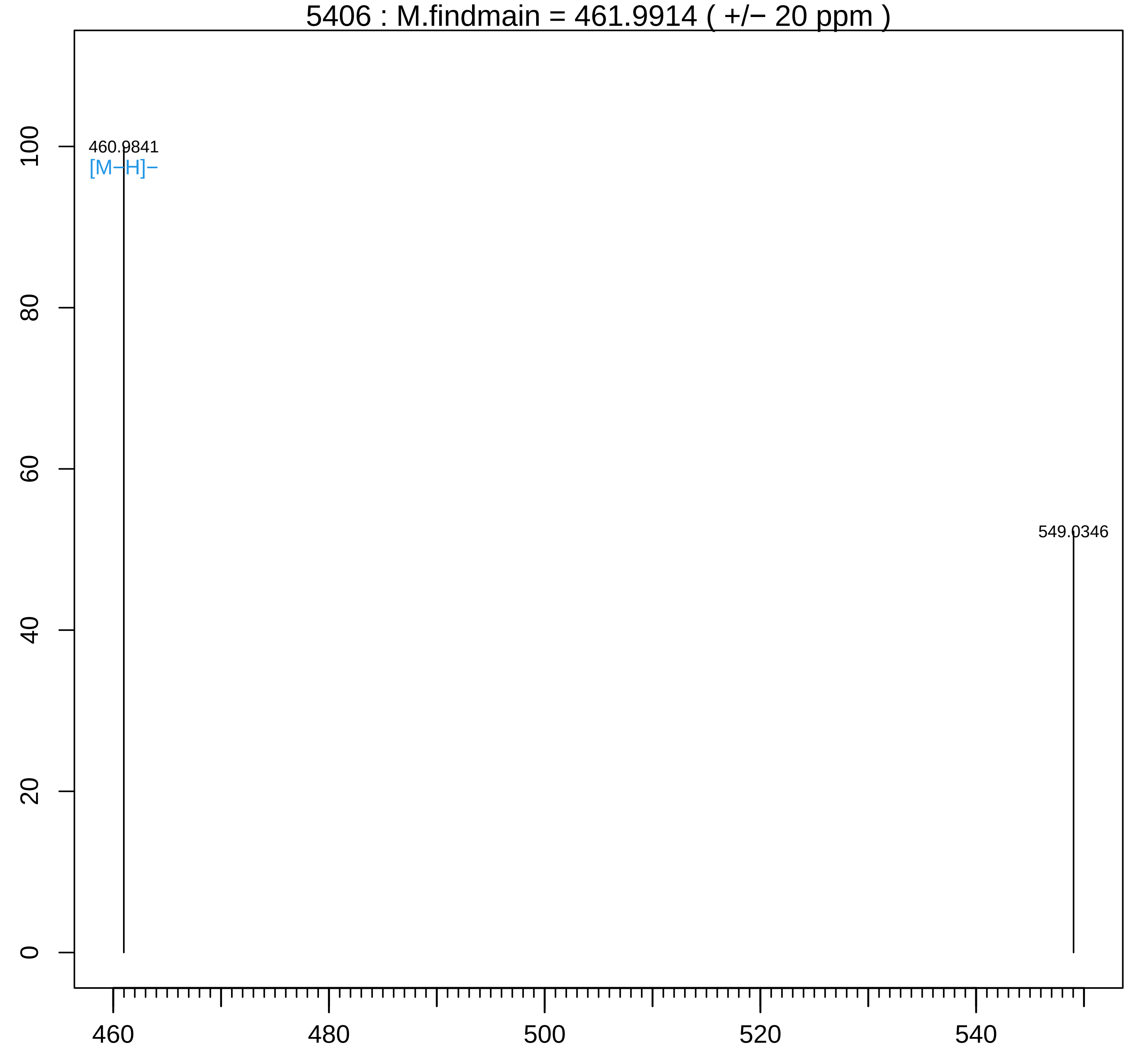
5406 : M.findmain = 461.9914 (+/− 20 ppm)
460.9841
[M−H]−
549.0346
0
20
40
60
80
100
460
480
500
520
540

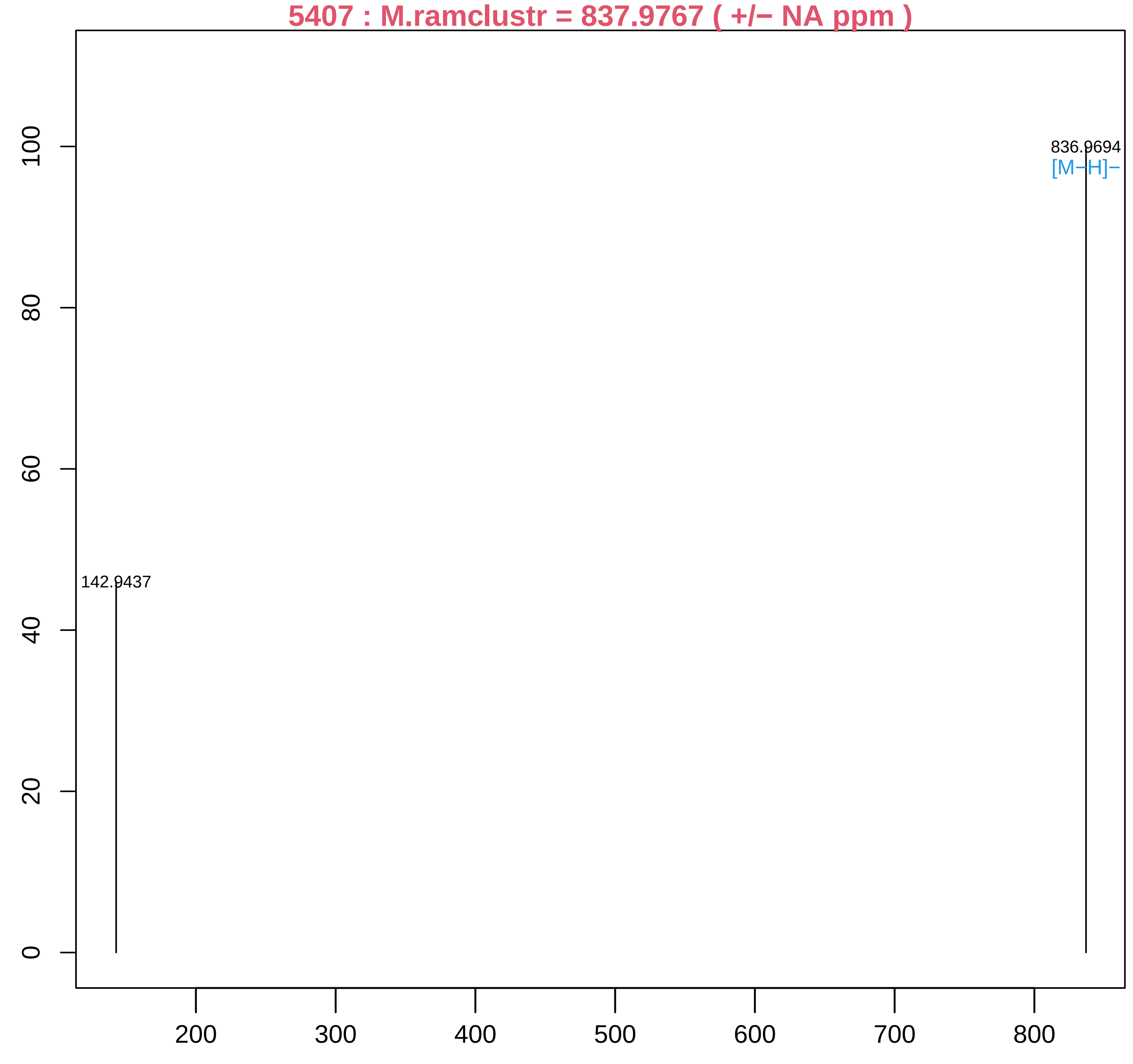
5407 : M.ramclustr = 837.9767 (+/− NA ppm)
836.9694
[M−H]−
142.9437
0
20
40
60
80
100
200
300
400
500
600
700
800

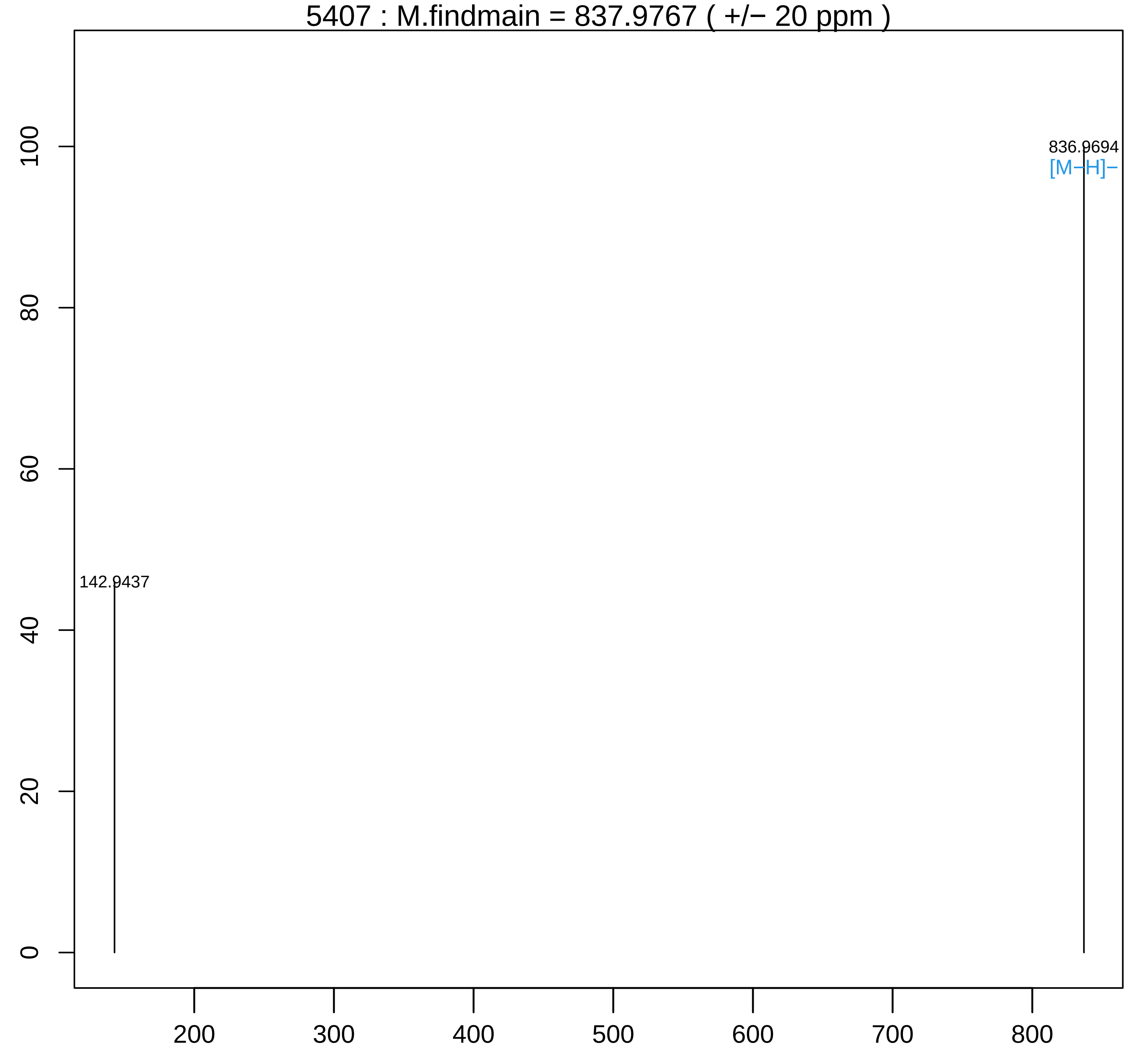
5407 : M.findmain = 837.9767 (+/− 20 ppm)
836.9694
[M−H]−
142.9437
0
20
40
60
80
100
200
300
400
500
600
700
800

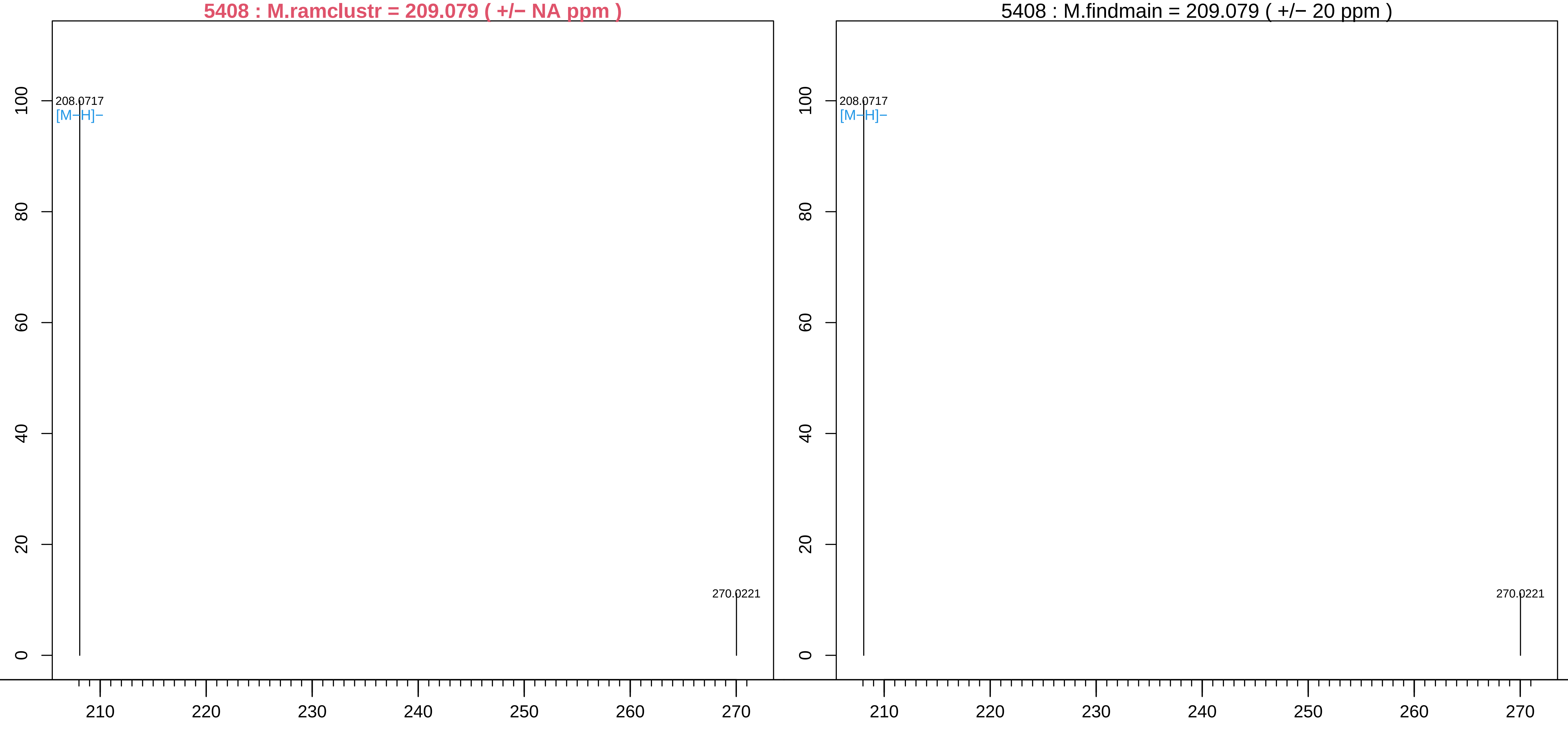
5408 : M.ramclustr = 209.079 (+/− NA ppm)
208.0717
[M−H]−
270.0221
0
20
40
60
80
100
210
220
230
240
250
260
270
5408 : M.findmain = 209.079 (+/− 20 ppm)
208.0717
[M−H]−
270.0221
0
20
40
60
80
100
210
220
230
240
250
260
270

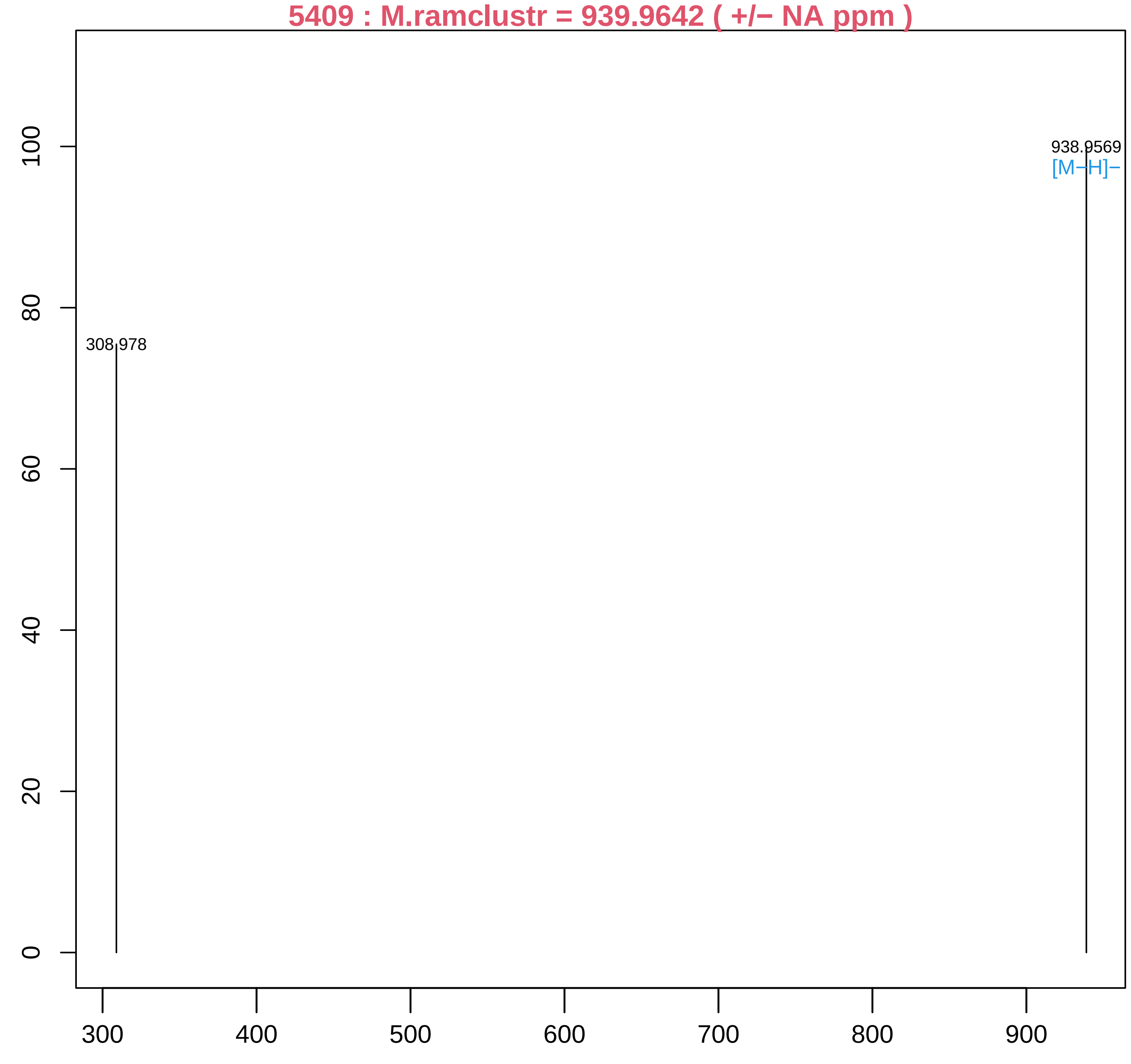
5409 : M.ramclustr = 939.9642 (+/− NA ppm)
938.9569
[M−H]−
308.978
0
20
40
60
80
100
300
400
500
600
700
800
900

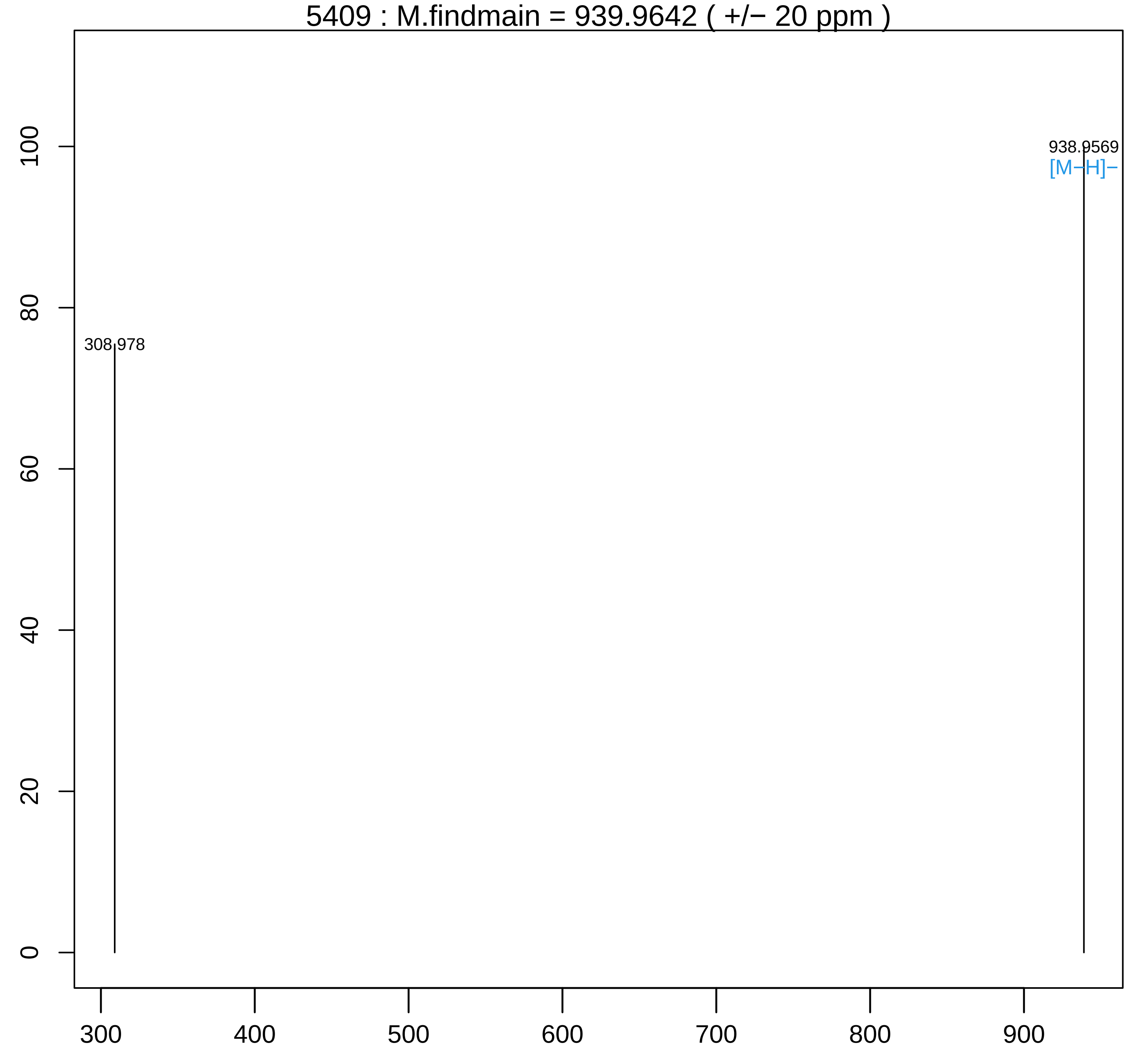
5409 : M.findmain = 939.9642 (+/− 20 ppm)
938.9569
[M−H]−
308.978
0
20
40
60
80
100
300
400
500
600
700
800
900

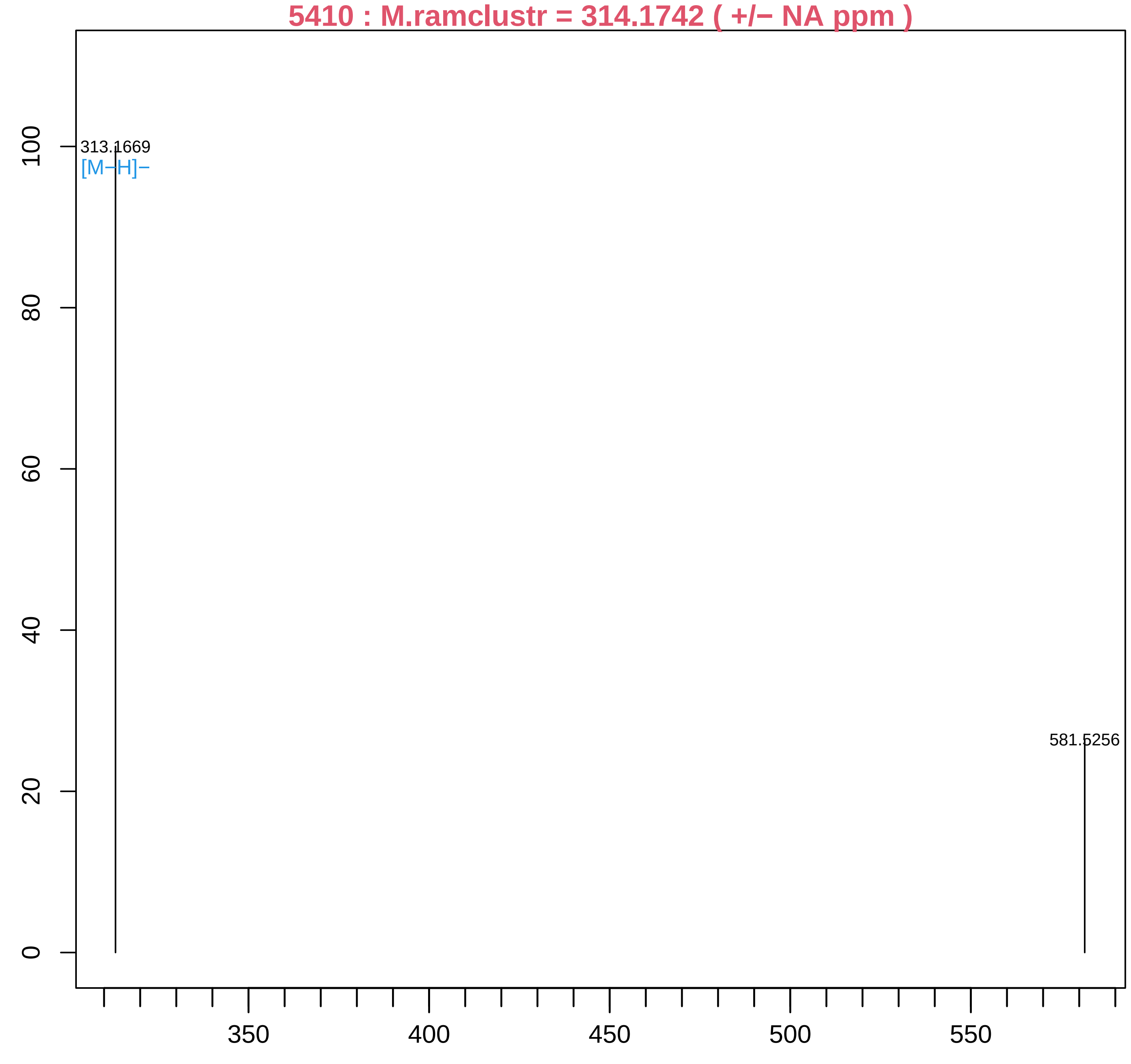

5410 : M.ramclustr = 314.1742 (+/− NA ppm)
313.1669
[M−H]−
581.5256
0
20
40
60
80
100
350
400
450
500
550

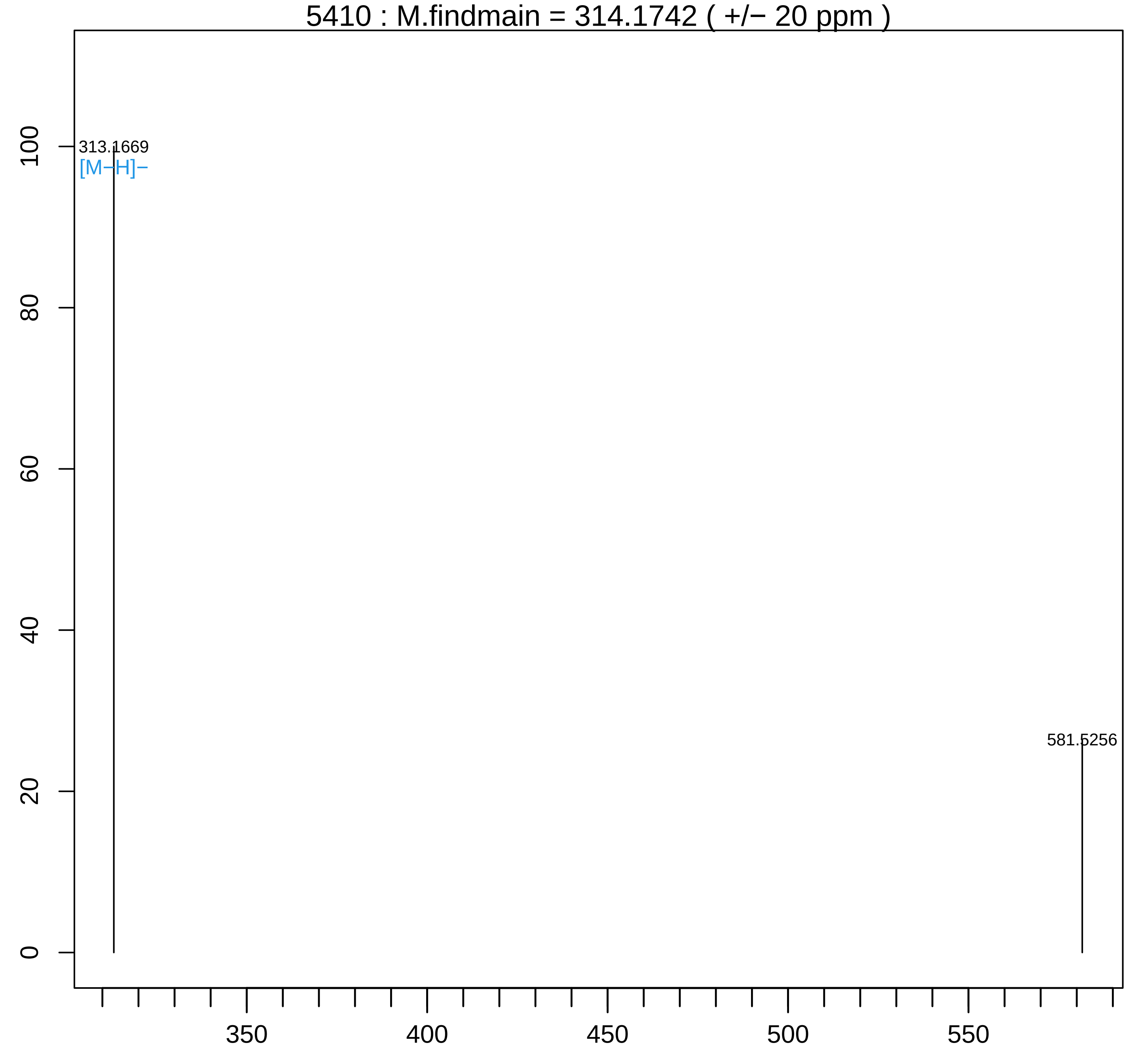

5410 : M.findmain = 314.1742 (+/− 20 ppm)
313.1669
[M−H]−
581.5256
0
20
40
60
80
100
350
400
450
500
550

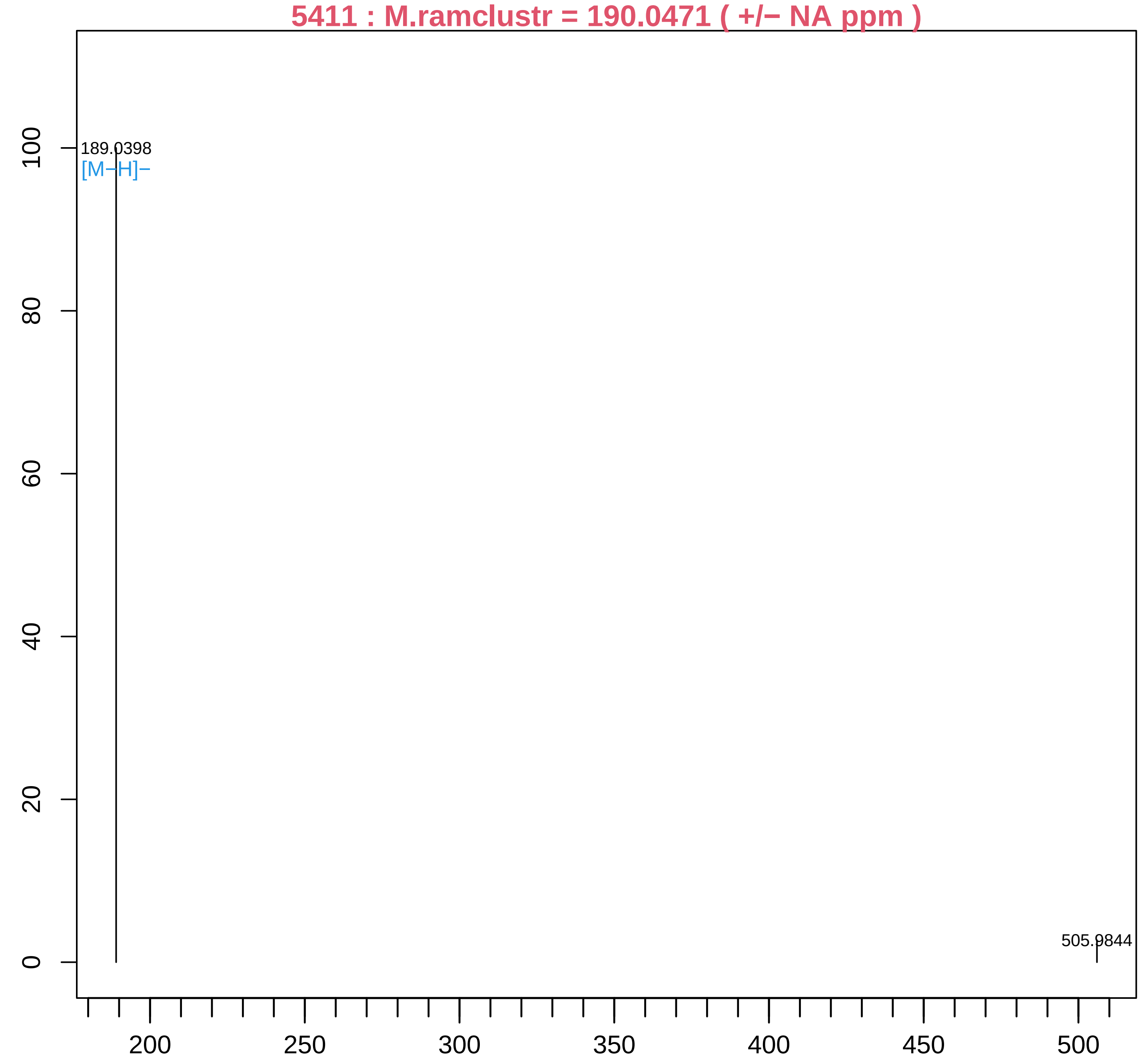
5411 : M.ramclustr = 190.0471 (+/− NA ppm)
189.0398
[M−H]−
505.9844
0
20
40
60
80
100
200
250
300
350
400
450
500

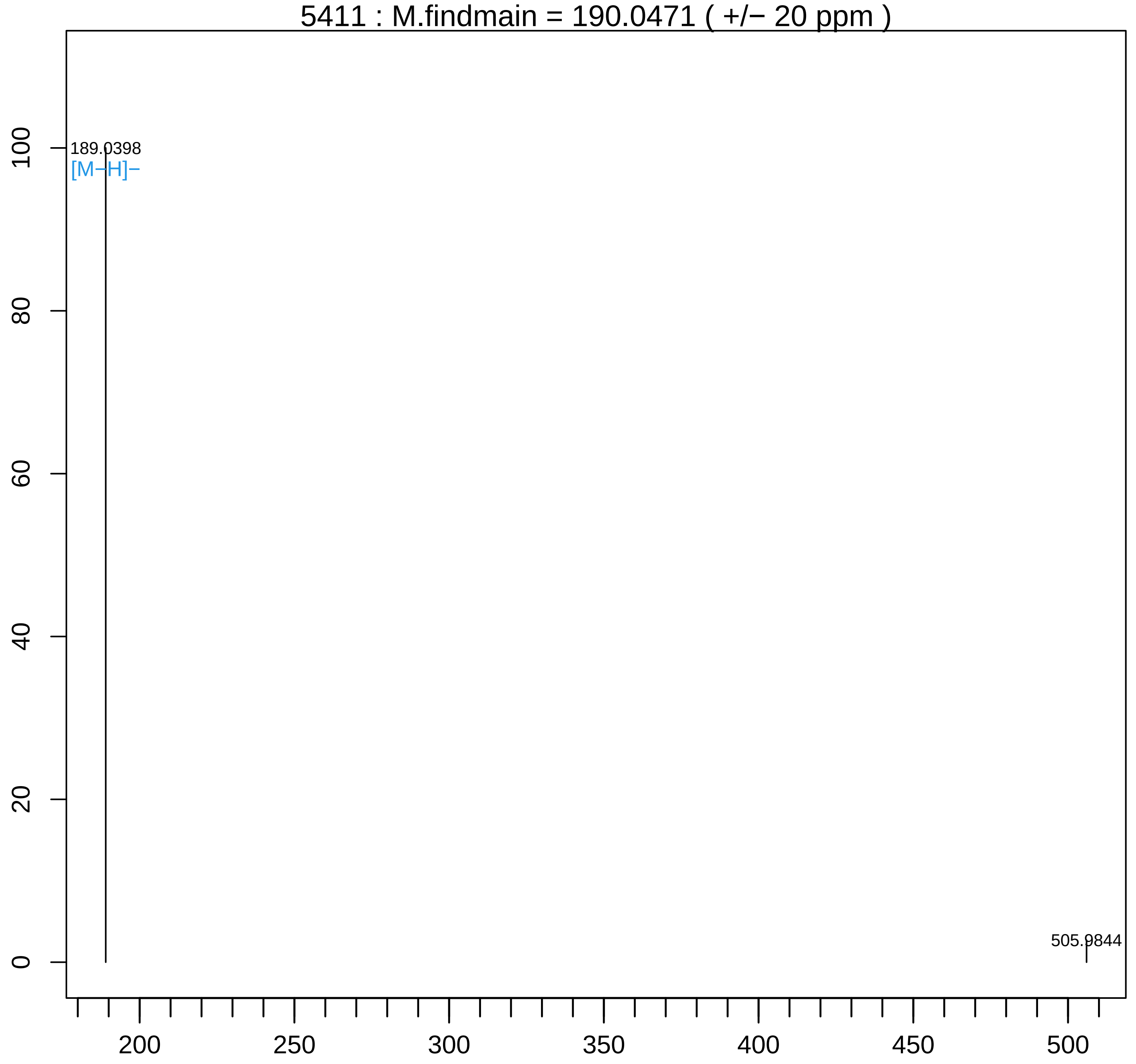
5411 : M.findmain = 190.0471 (+/− 20 ppm)
189.0398
[M−H]−
505.9844
0
20
40
60
80
100
200
250
300
350
400
450
500

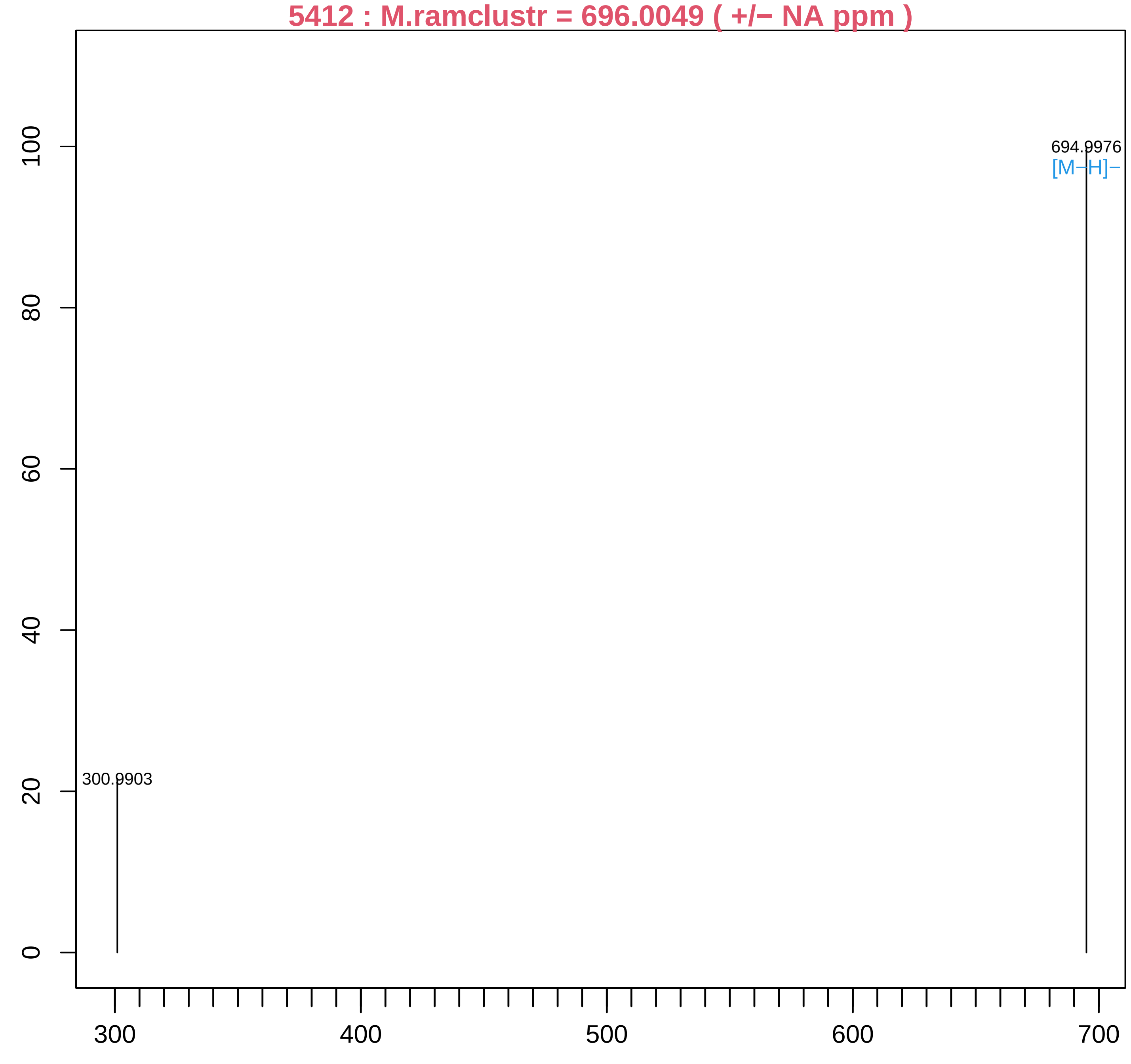
5412 : M.ramclustr = 696.0049 (+/− NA ppm)
694.9976
[M−H]−
300.9903
0
20
40
60
80
100
300
400
500
600
700

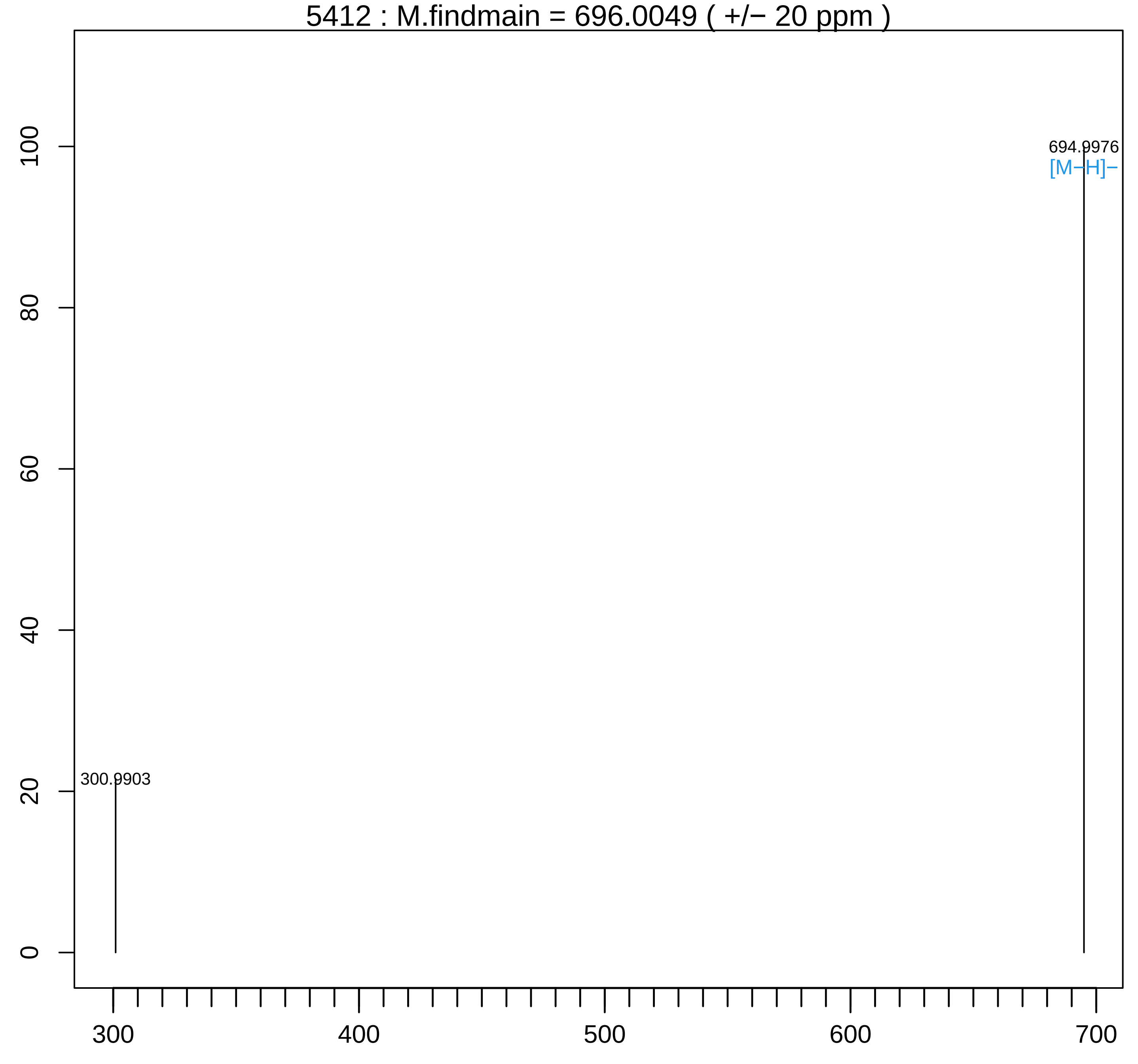
5412 : M.findmain = 696.0049 (+/− 20 ppm)
694.9976
[M−H]−
300.9903
0
20
40
60
80
100
300
400
500
600
700

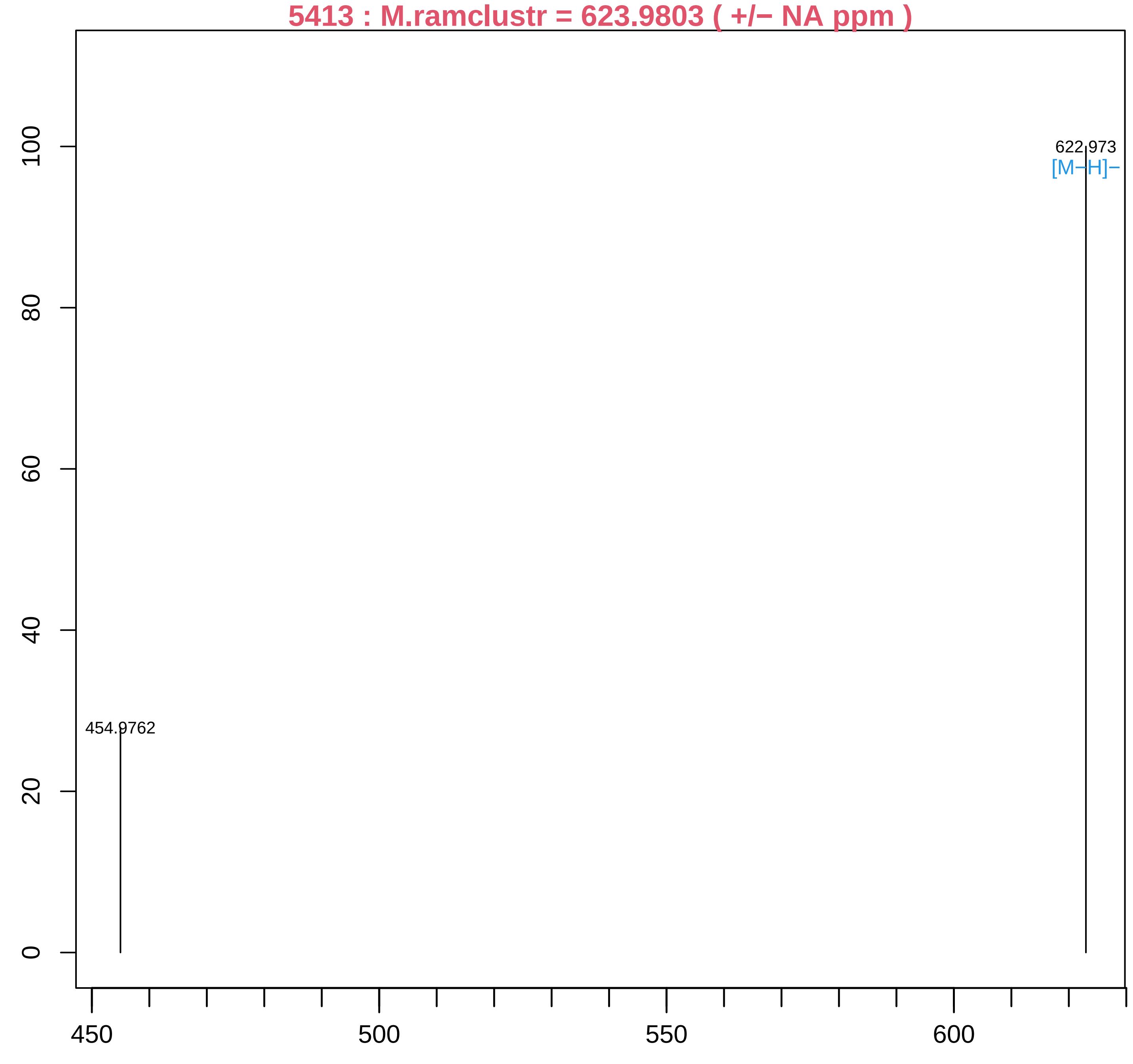

5413 : M.ramclustr = 623.9803 (+/− NA ppm)
0
20
40
60
80
100
450
500
550
600
454.9762
622.973
[M−H]−

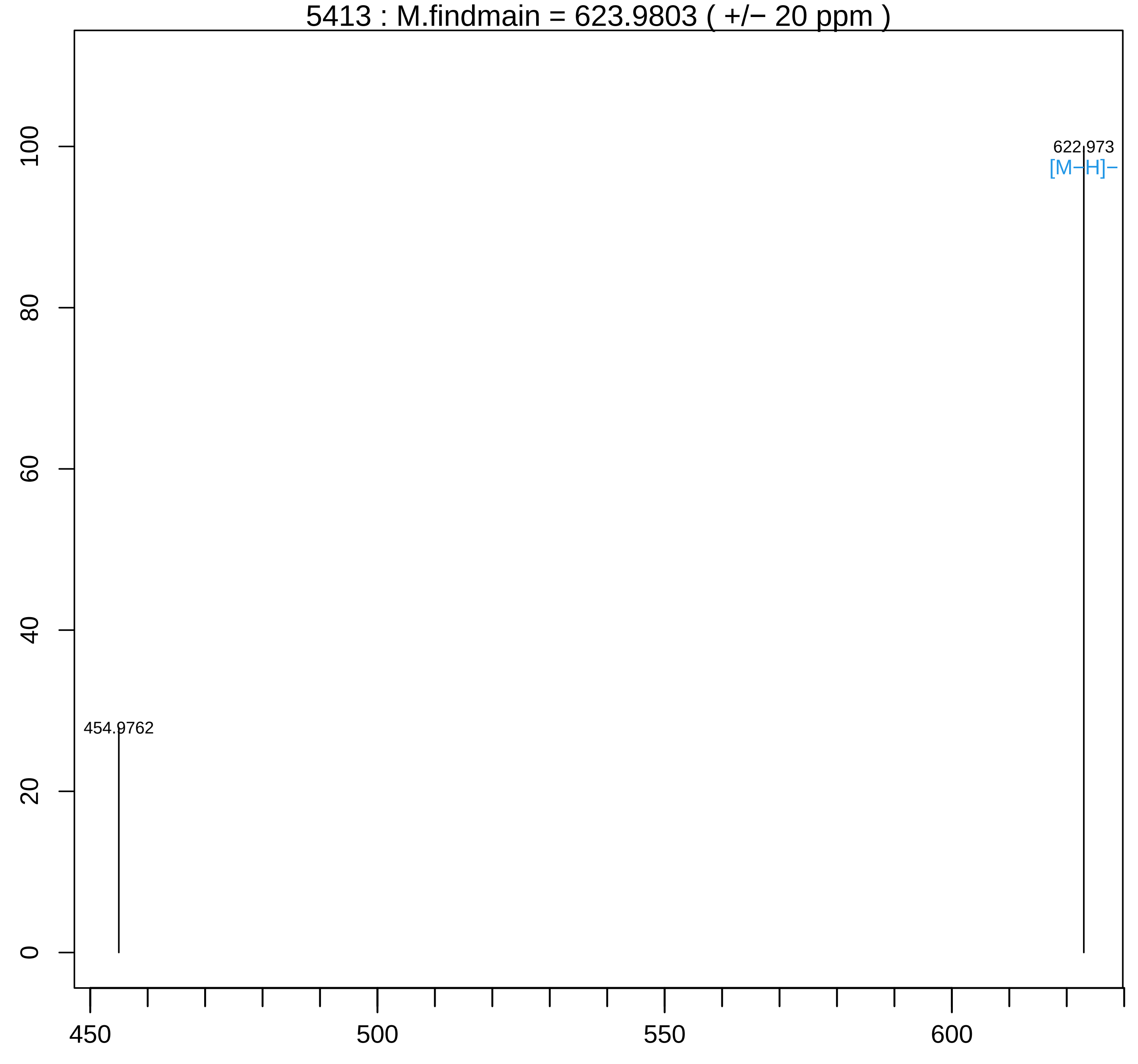

5413 : M.findmain = 623.9803 (+/− 20 ppm)
0
20
40
60
80
100
450
500
550
600
454.9762
622.973
[M−H]−

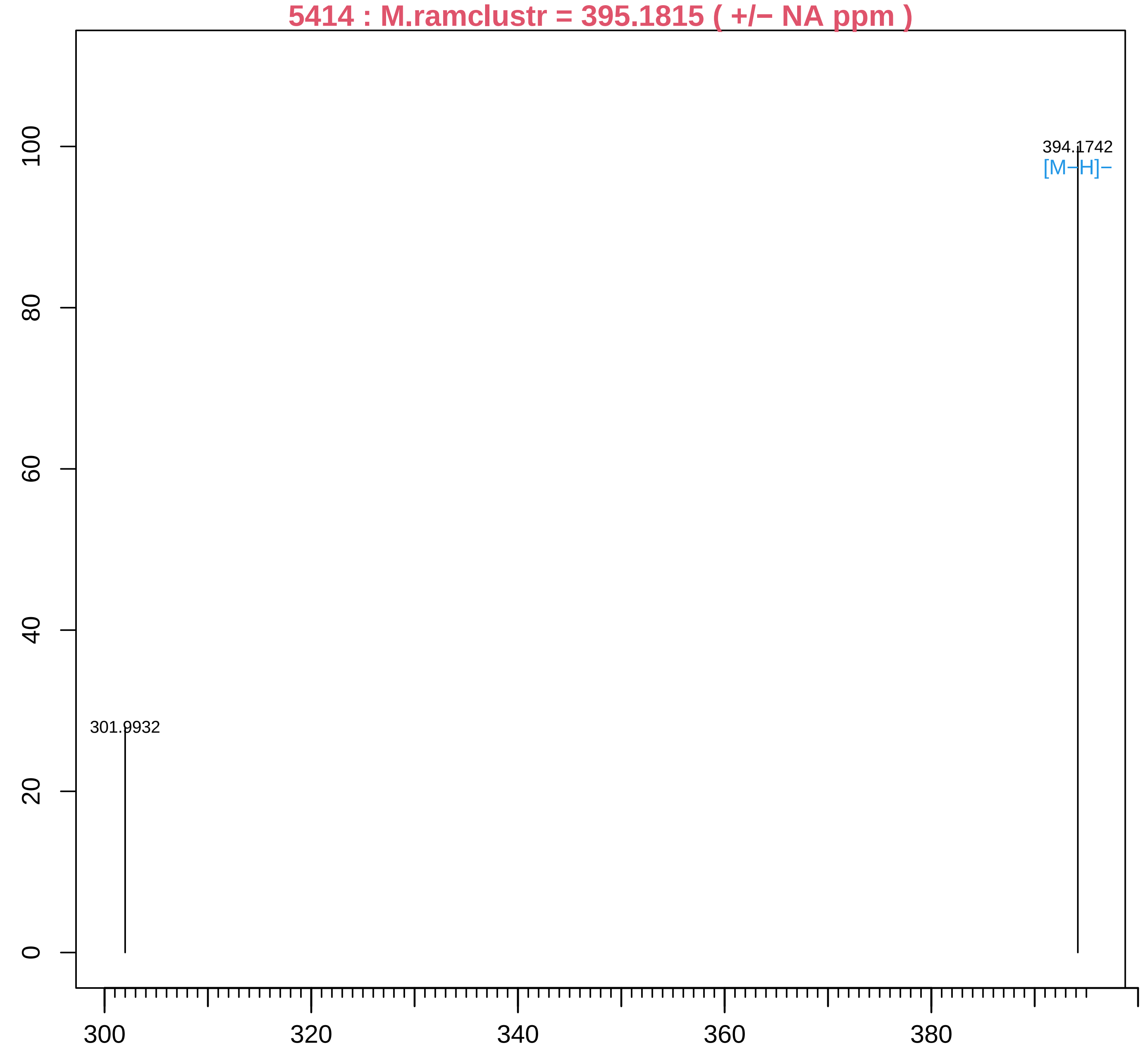

5414 : M.ramclustr = 395.1815 (+/− NA ppm)
394.1742
[M−H]−
301.9932
0
20
40
60
80
100
300
320
340
360
380

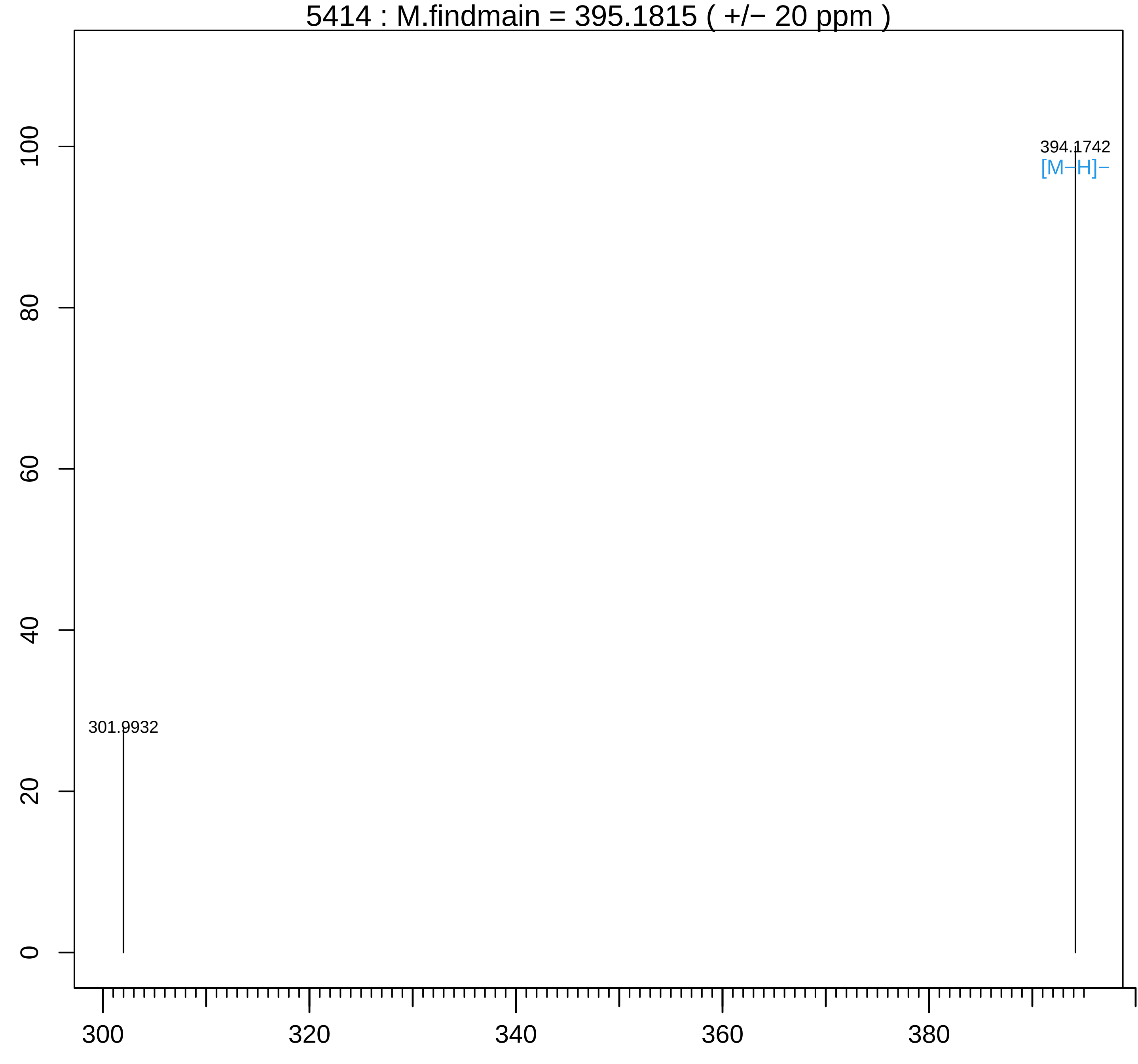

5414 : M.findmain = 395.1815 (+/− 20 ppm)
394.1742
[M−H]−
301.9932
0
20
40
60
80
100
300
320
340
360
380

5415 : M.ramclustr = 561.9938 (+/− NA ppm)

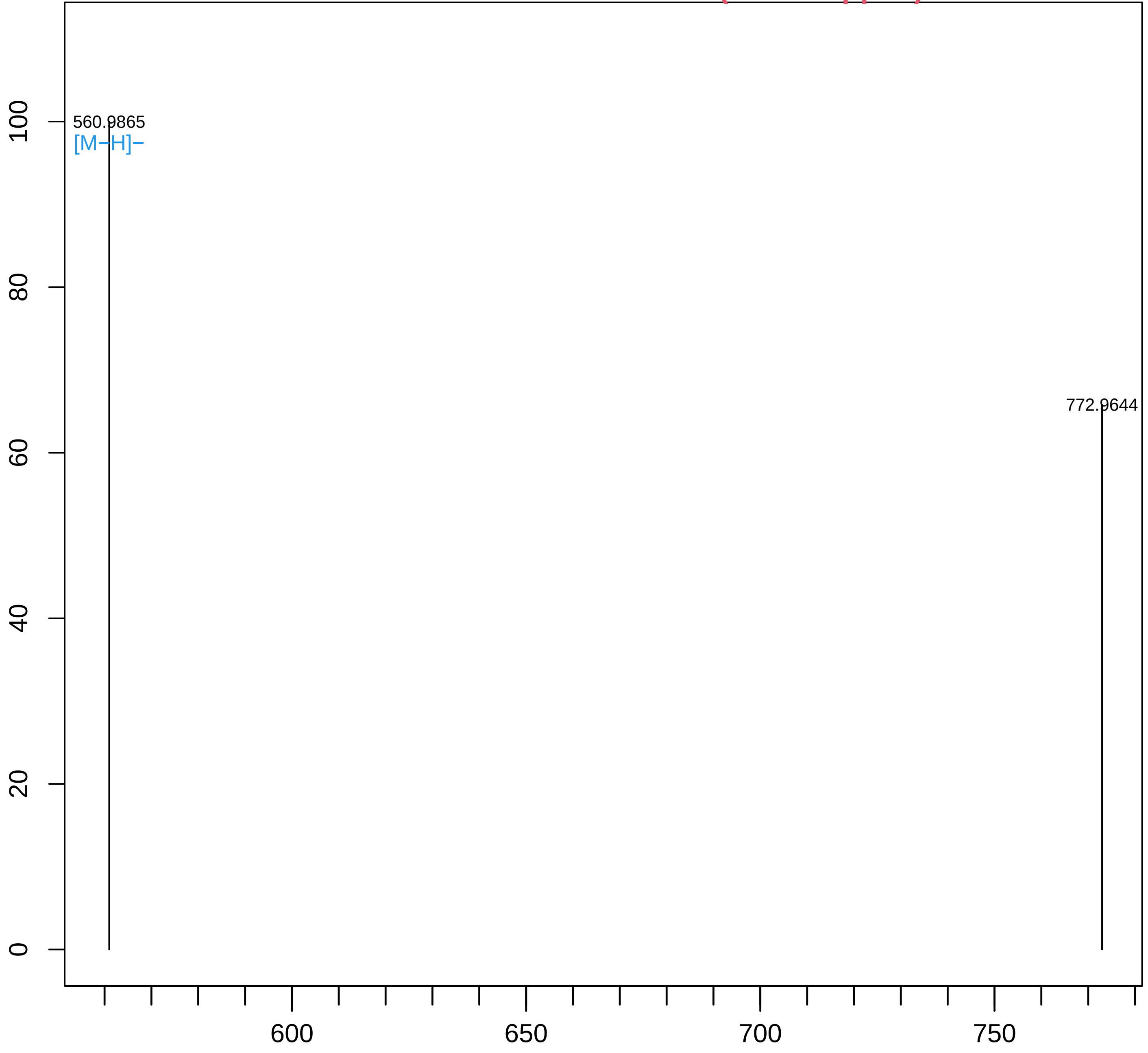

5415 : M.findmain = 561.9938 (+/− 20 ppm)

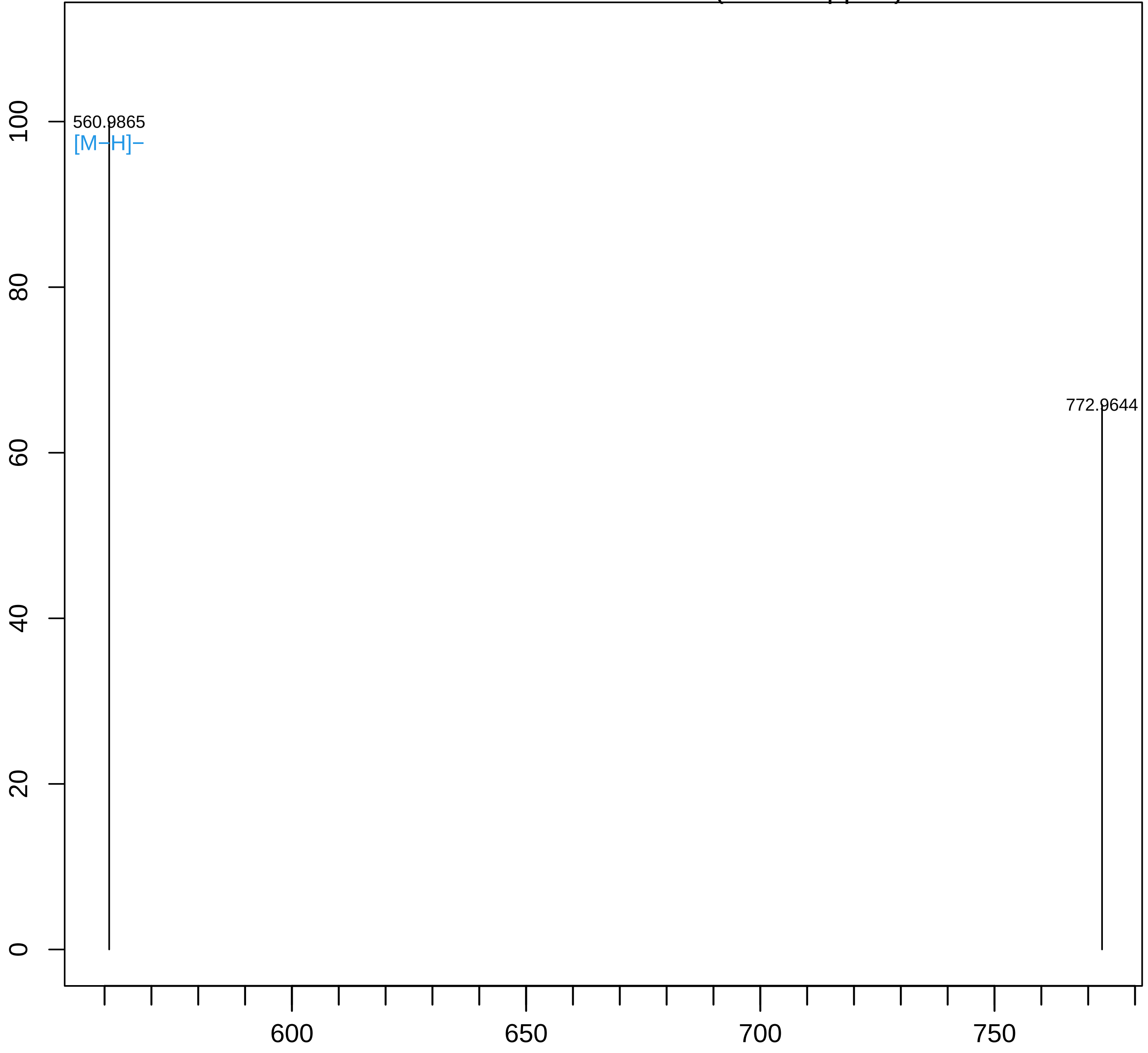

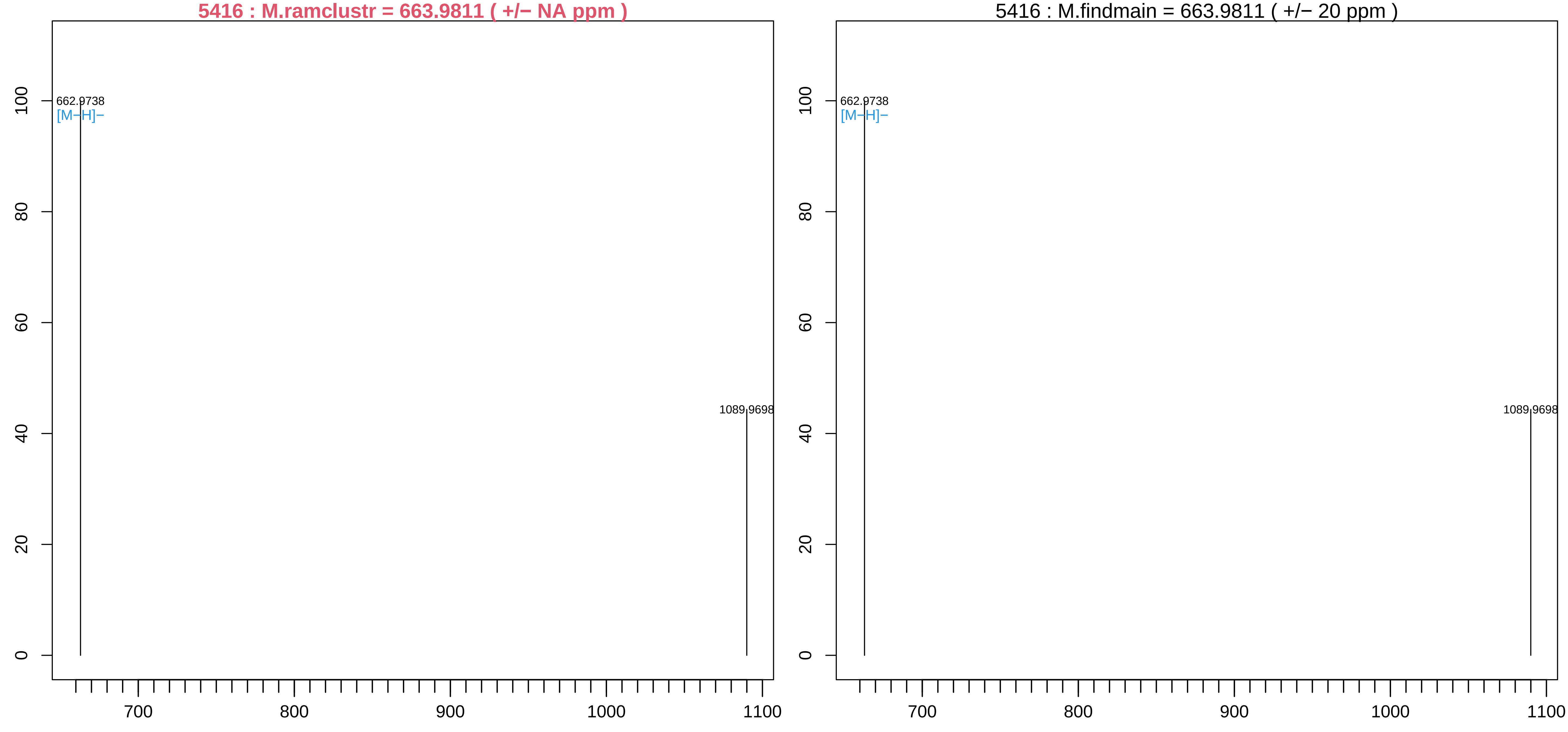
5416 : M.ramclustr = 663.9811 (+/− NA ppm)
662.9738
[M−H]−
1089.9698
5416 : M.findmain = 663.9811 (+/− 20 ppm)
662.9738
[M−H]−
1089.9698
0
20
40
60
80
100
700
800
900
1000
1100

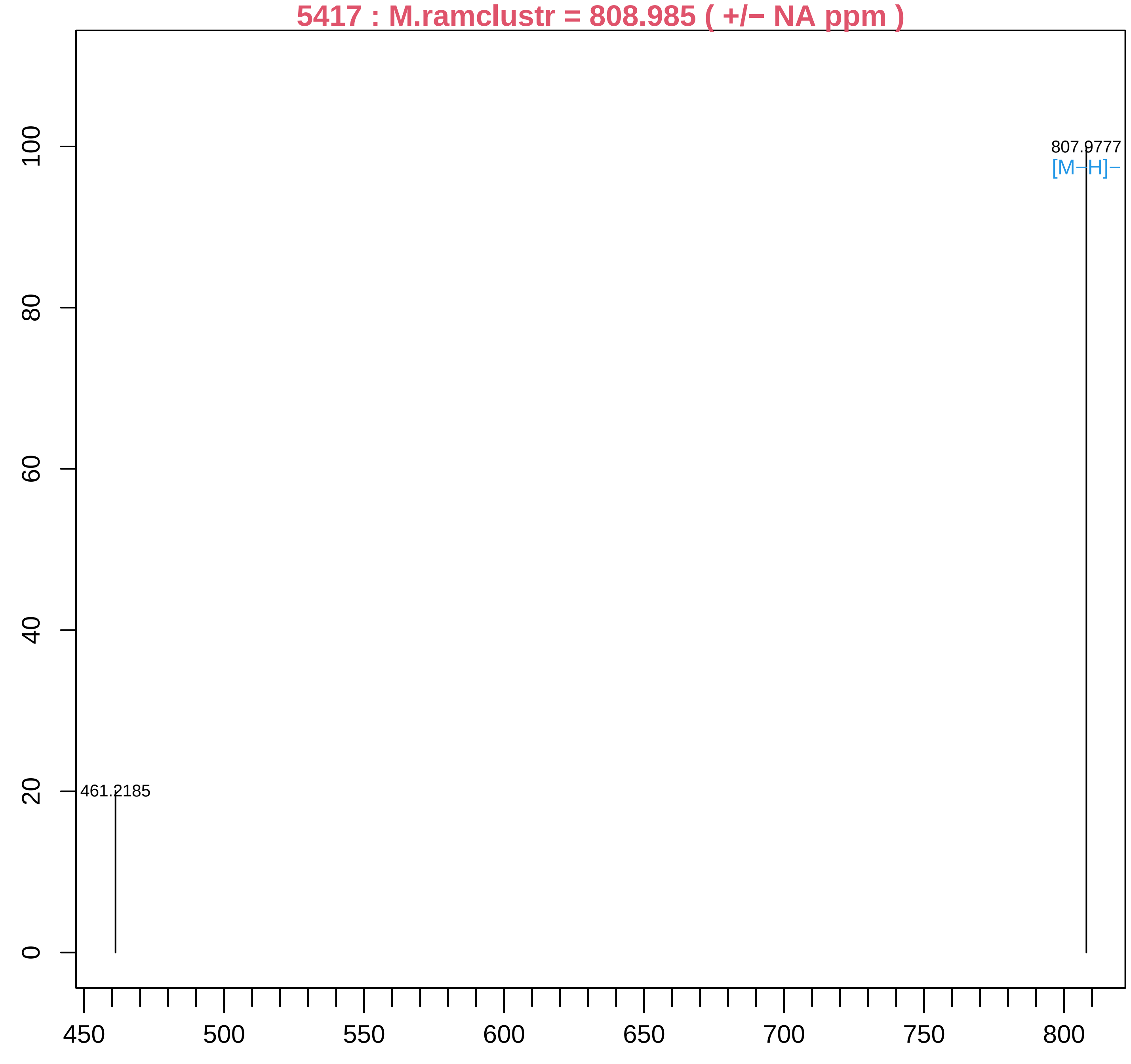
5417 : M.ramclustr = 808.985 (+/− NA ppm)
807.9777
[M−H]−
461.2185
0
20
40
60
80
100
450
500
550
600
650
700
750
800

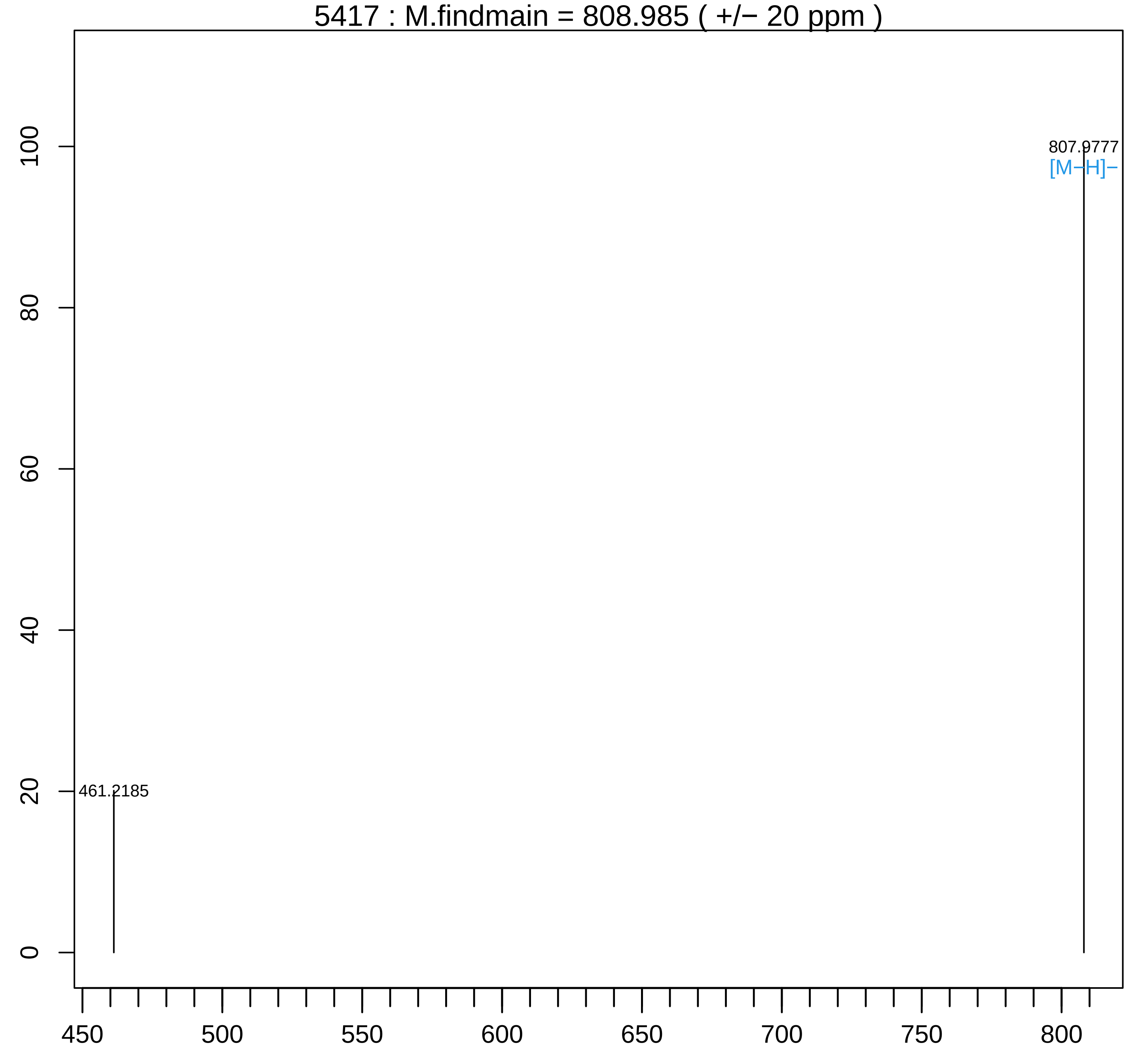
5417 : M.findmain = 808.985 (+/− 20 ppm)
807.9777
[M−H]−
461.2185
0
20
40
60
80
100
450
500
550
600
650
700
750
800

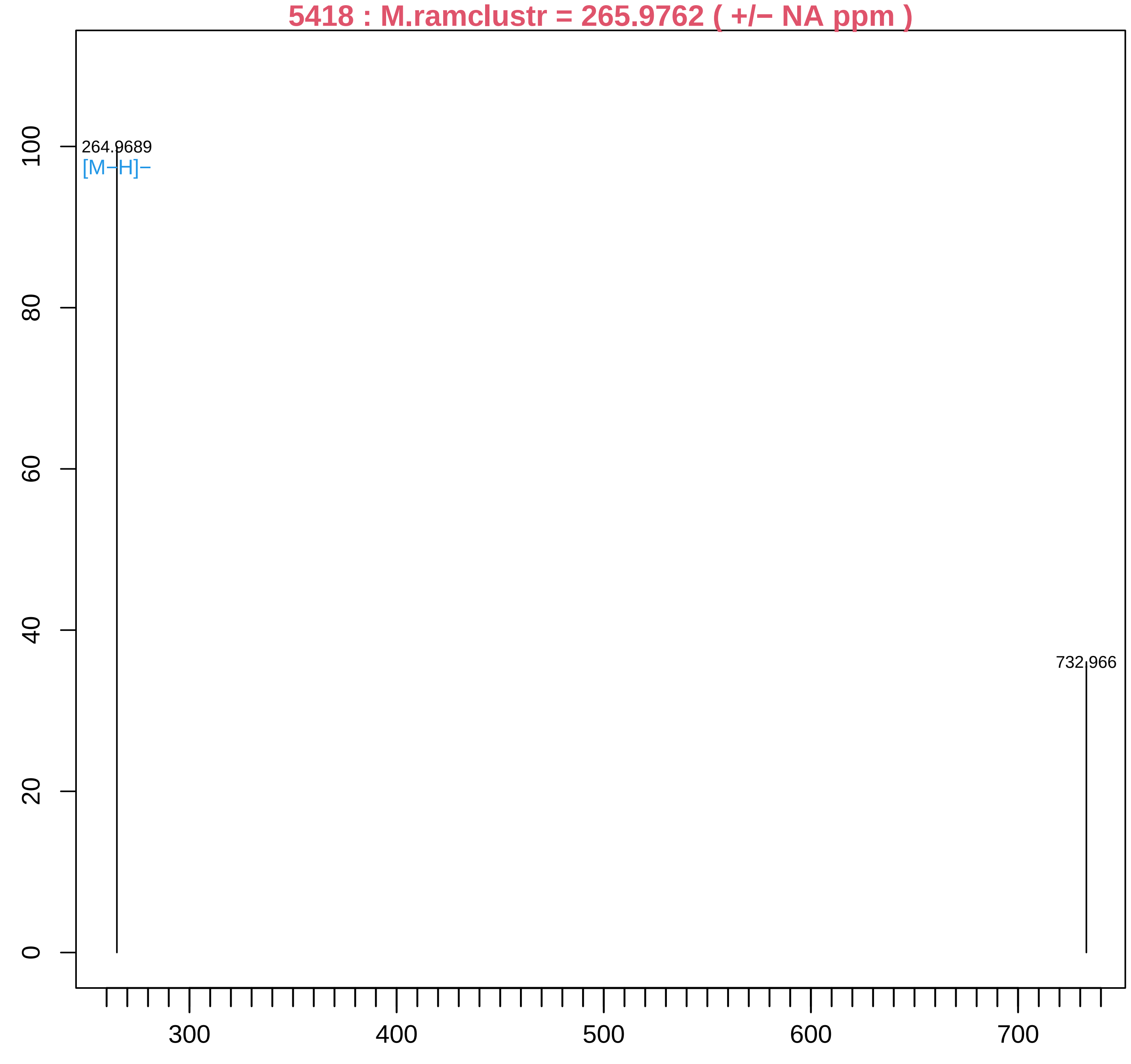
5418 : M.ramclustr = 265.9762 (+/− NA ppm)
264.9689
[M−H]−
732.966
0
20
40
60
80
100
300
400
500
600
700

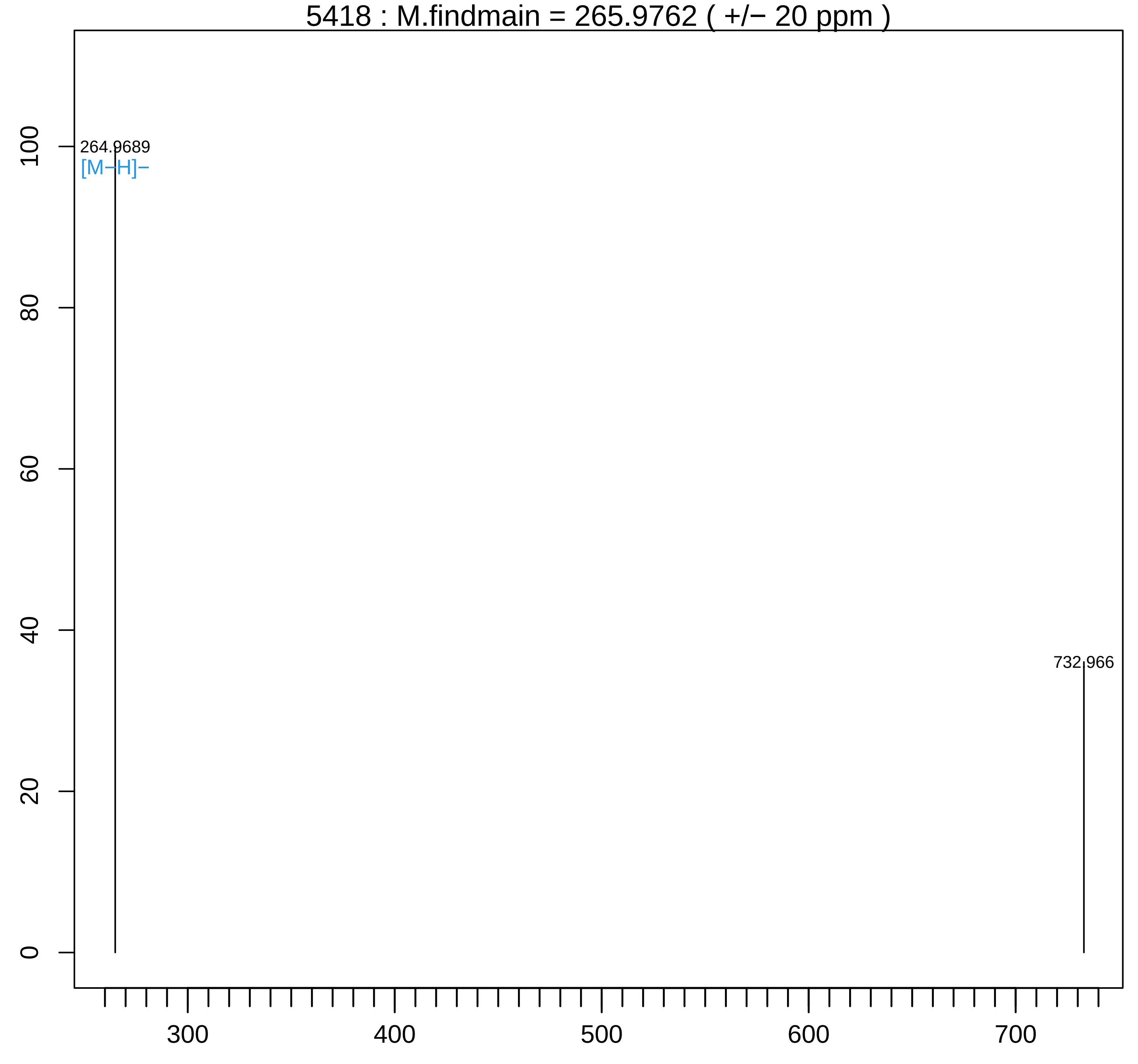
5418 : M.findmain = 265.9762 (+/− 20 ppm)
264.9689
[M−H]−
732.966
0
20
40
60
80
100
300
400
500
600
700

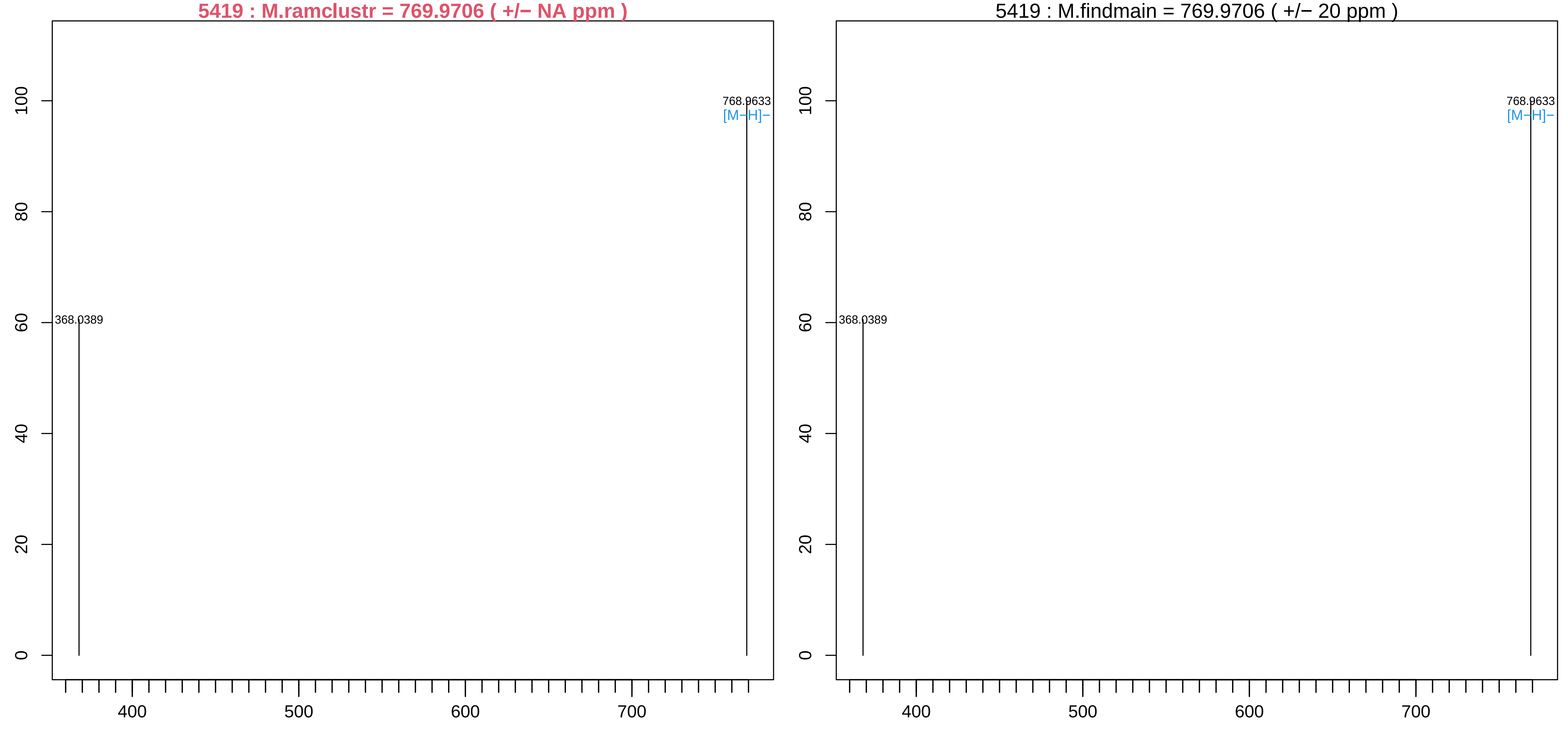

5419 : M.ramclustr = 769.9706 (+/− NA ppm)
768.9633
[M−H]−
368.0389
5419 : M.findmain = 769.9706 (+/− 20 ppm)
768.9633
[M−H]−
368.0389
0
20
40
60
80
100
400
500
600
700

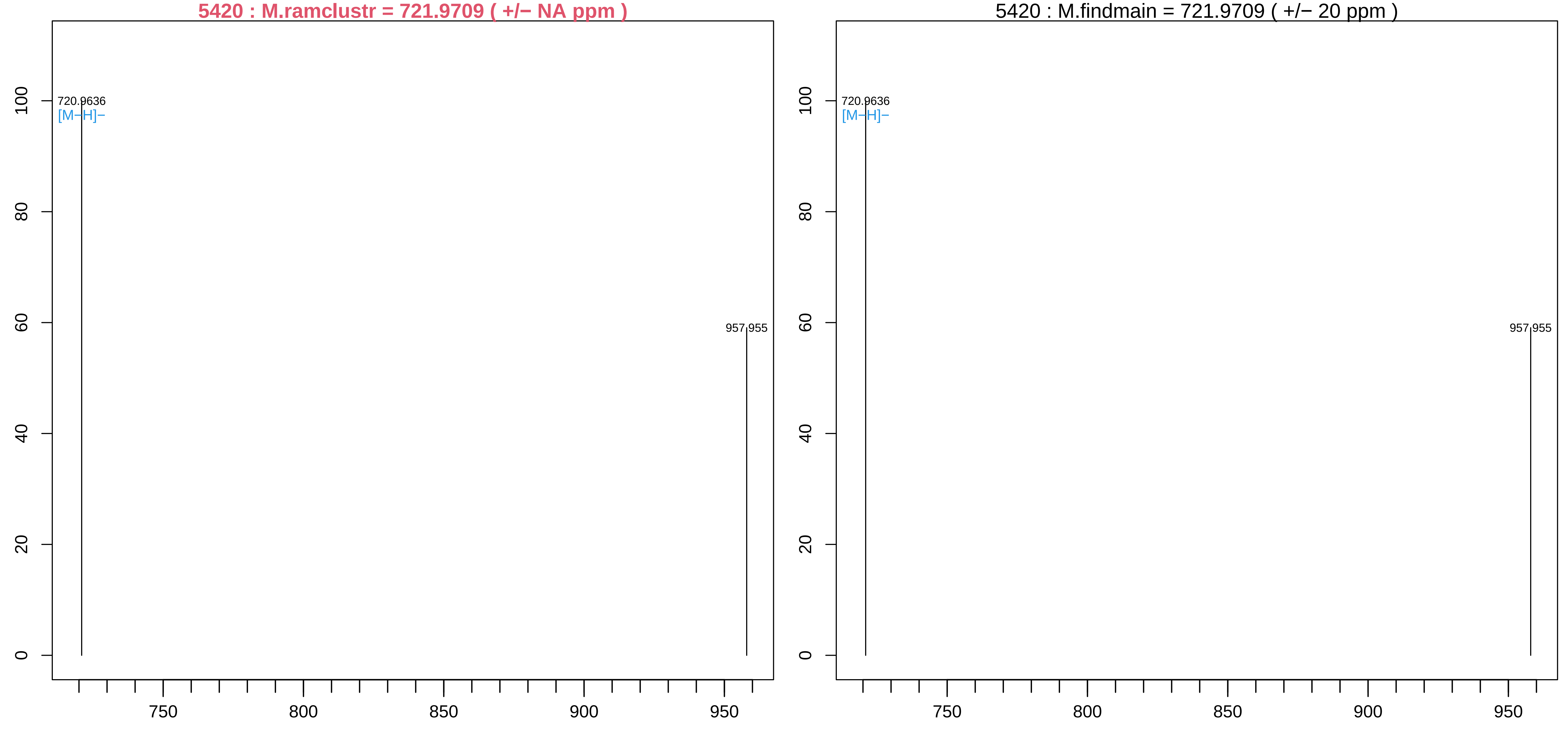
5420 : M.ramclustr = 721.9709 (+/− NA ppm)
720.9636
[M−H]−
957.955
5420 : M.findmain = 721.9709 (+/− 20 ppm)
720.9636
[M−H]−
957.955
0
20
40
60
80
100
750
800
850
900
950

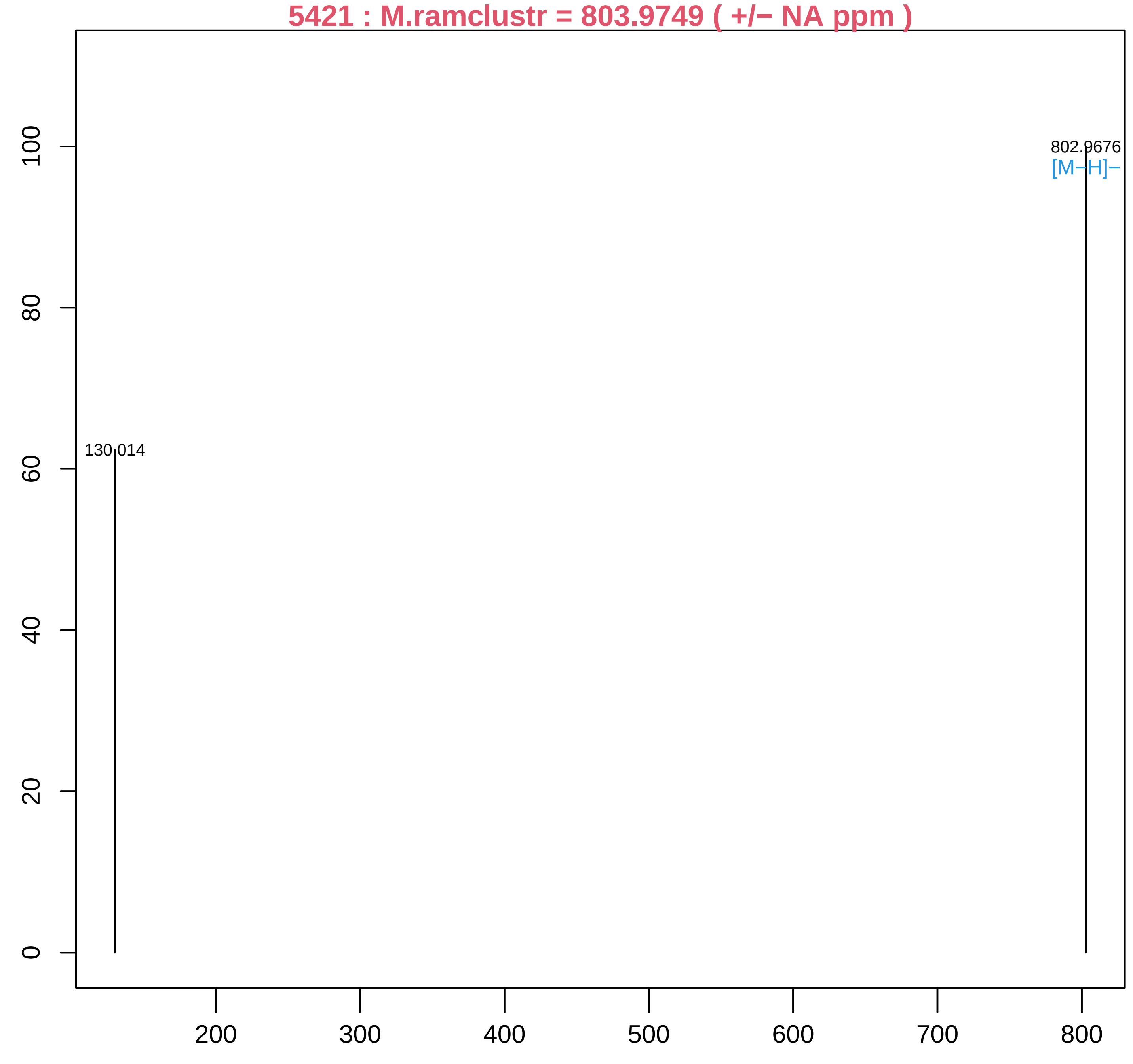

5421 : M.ramclustr = 803.9749 (+/− NA ppm)
802.9676
[M−H]−
130.014
0
20
40
60
80
100
200
300
400
500
600
700
800

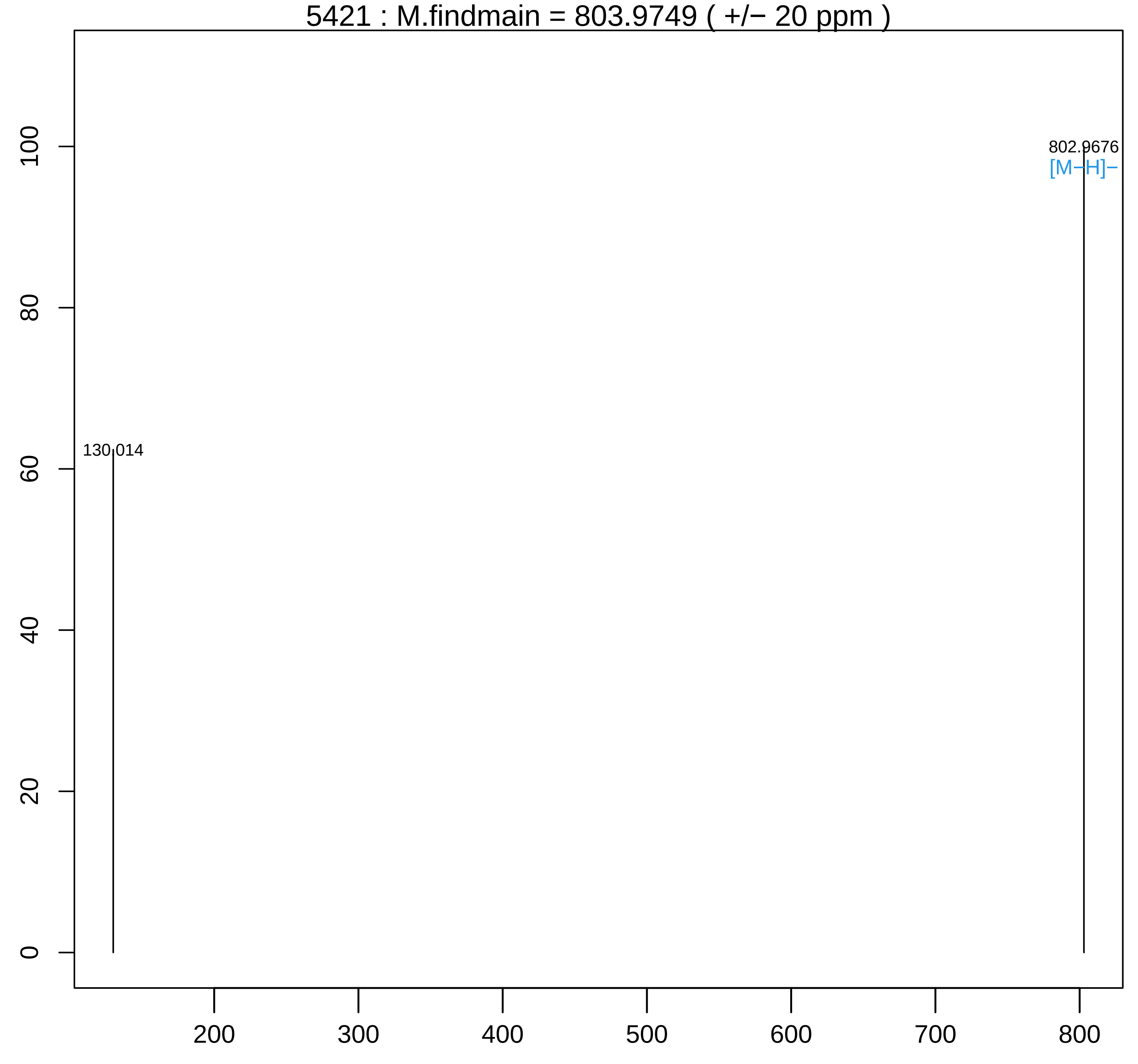

5421 : M.findmain = 803.9749 (+/− 20 ppm)
802.9676
[M−H]−
130.014
0
20
40
60
80
100
200
300
400
500
600
700
800

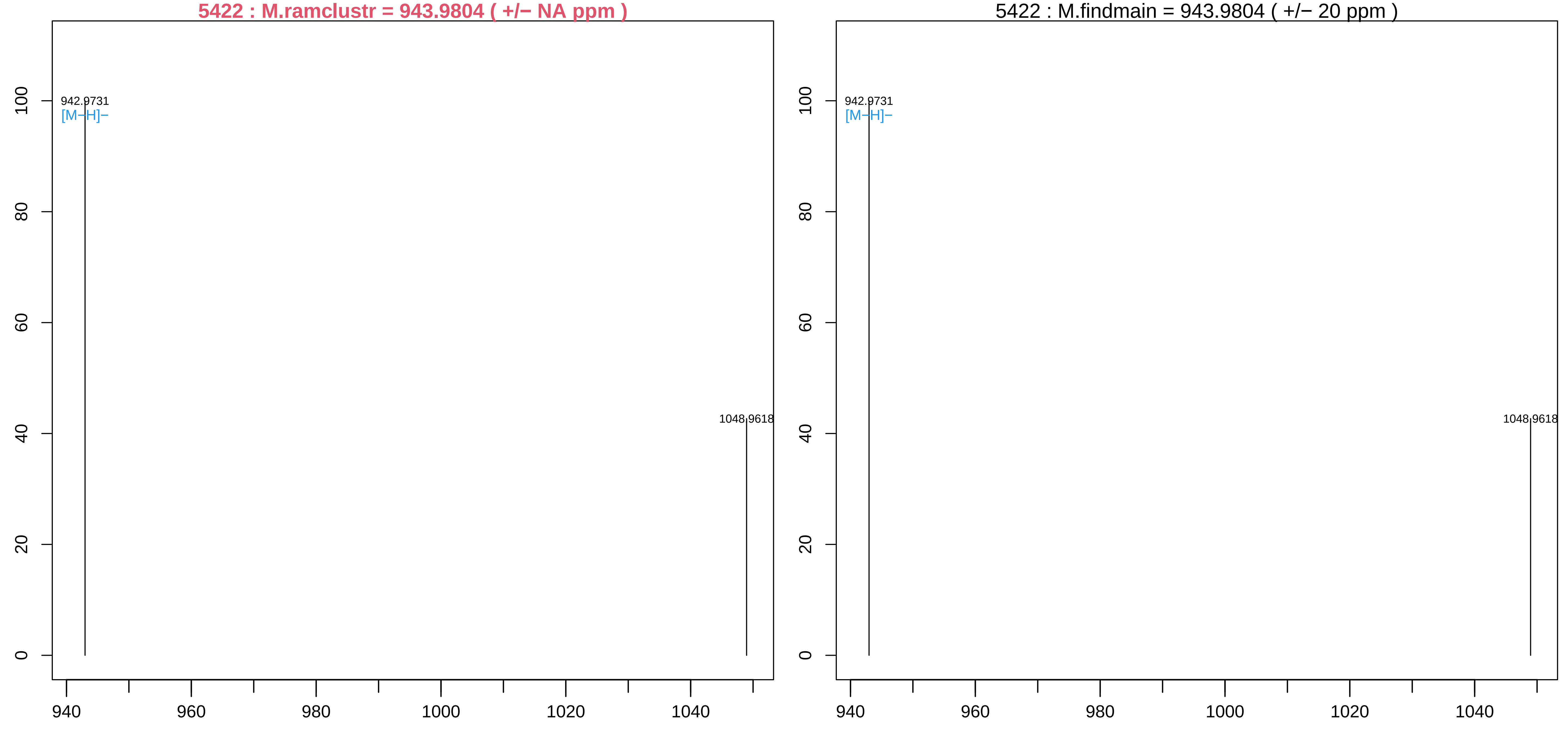
5422 : M.ramclustr = 943.9804 (+/− NA ppm)
942.9731
[M−H]−
1048.9618
5422 : M.findmain = 943.9804 (+/− 20 ppm)
942.9731
[M−H]−
1048.9618
0
20
40
60
80
100
940
960
980
1000
1020
1040

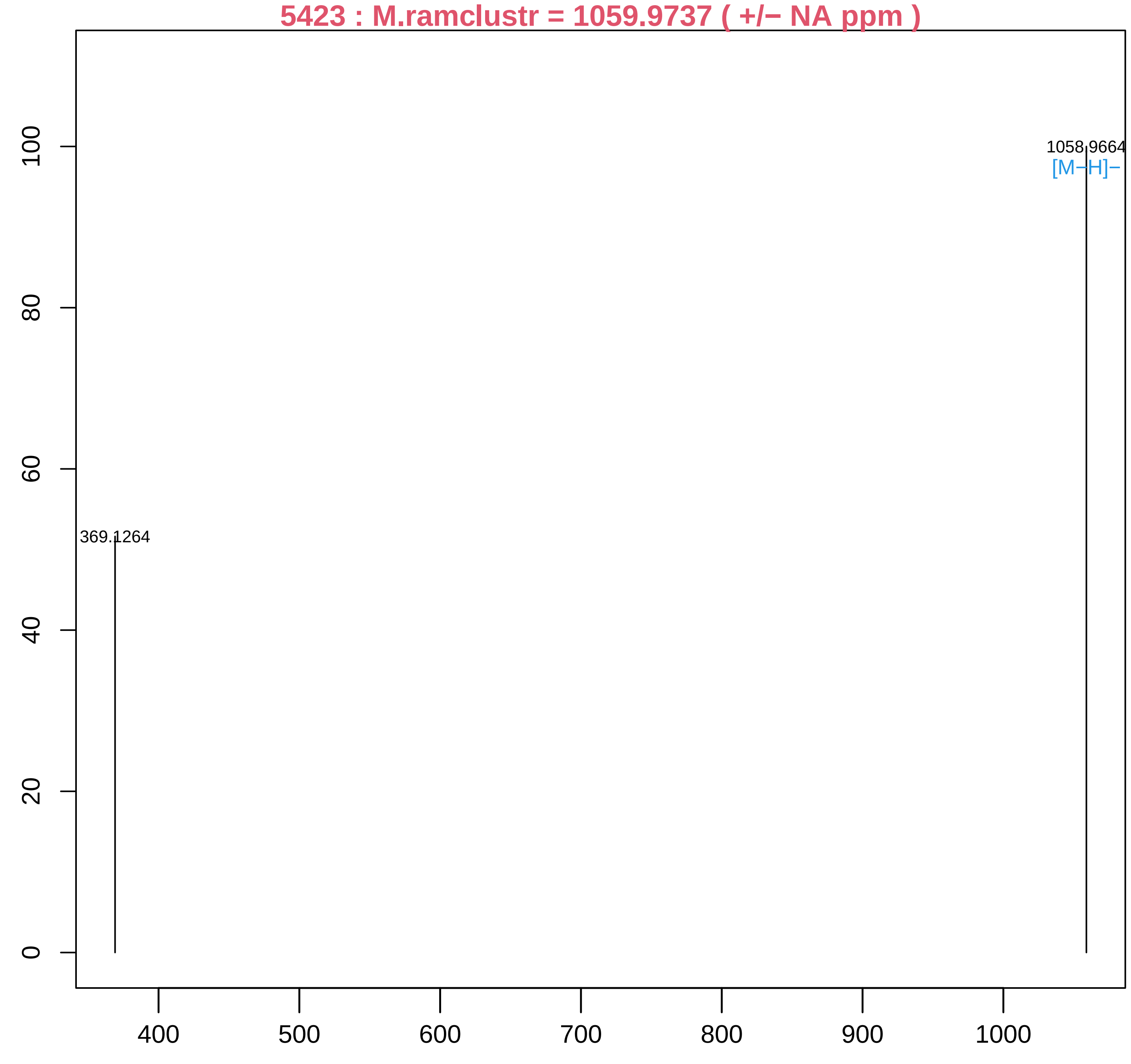
5423 : M.ramclustr = 1059.9737 (+/− NA ppm)
1058.9664
[M−H]−
369.1264
0
20
40
60
80
100
400
500
600
700
800
900
1000

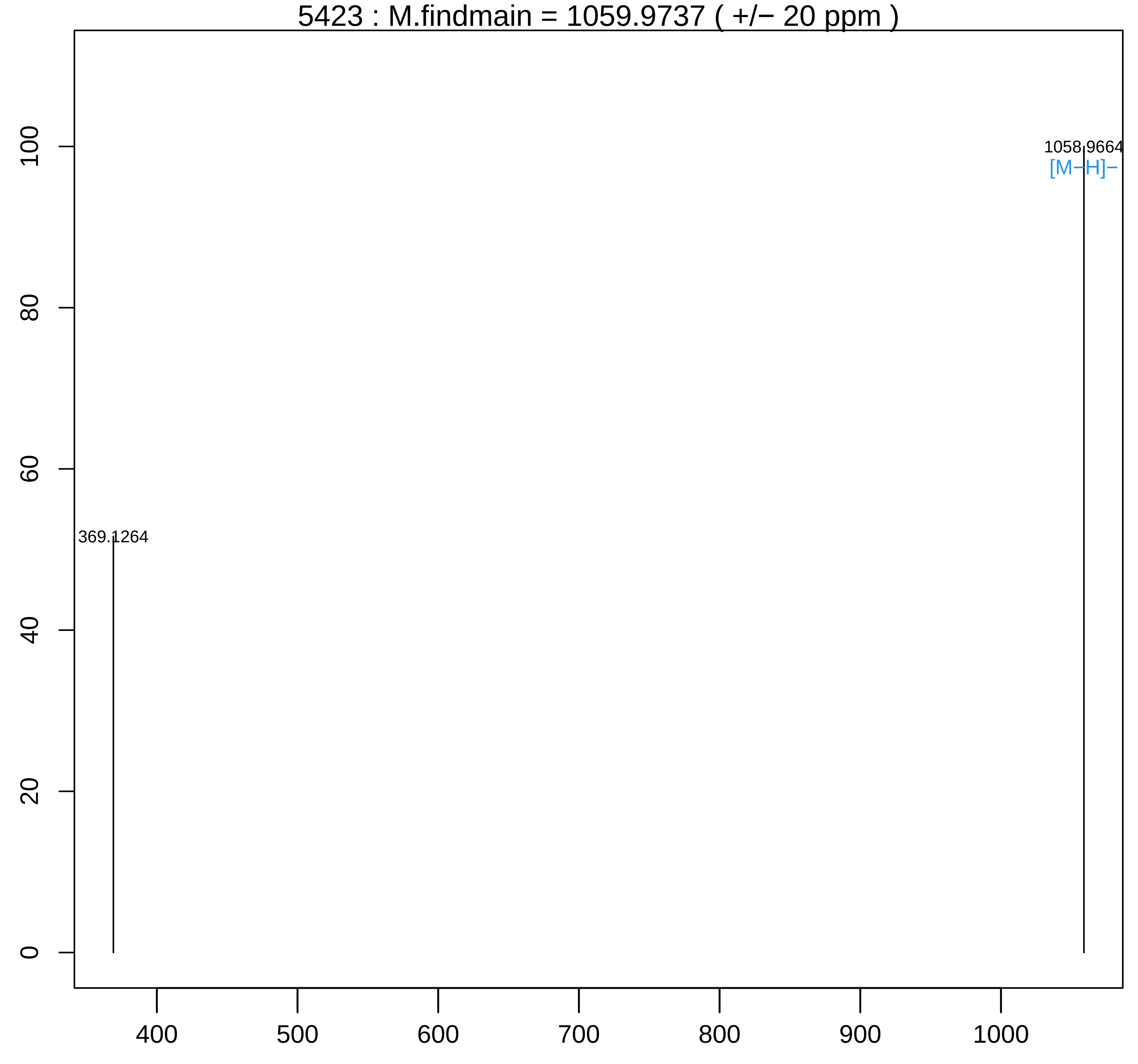
5423 : M.findmain = 1059.9737 (+/− 20 ppm)
1058.9664
[M−H]−
369.1264
0
20
40
60
80
100
400
500
600
700
800
900
1000

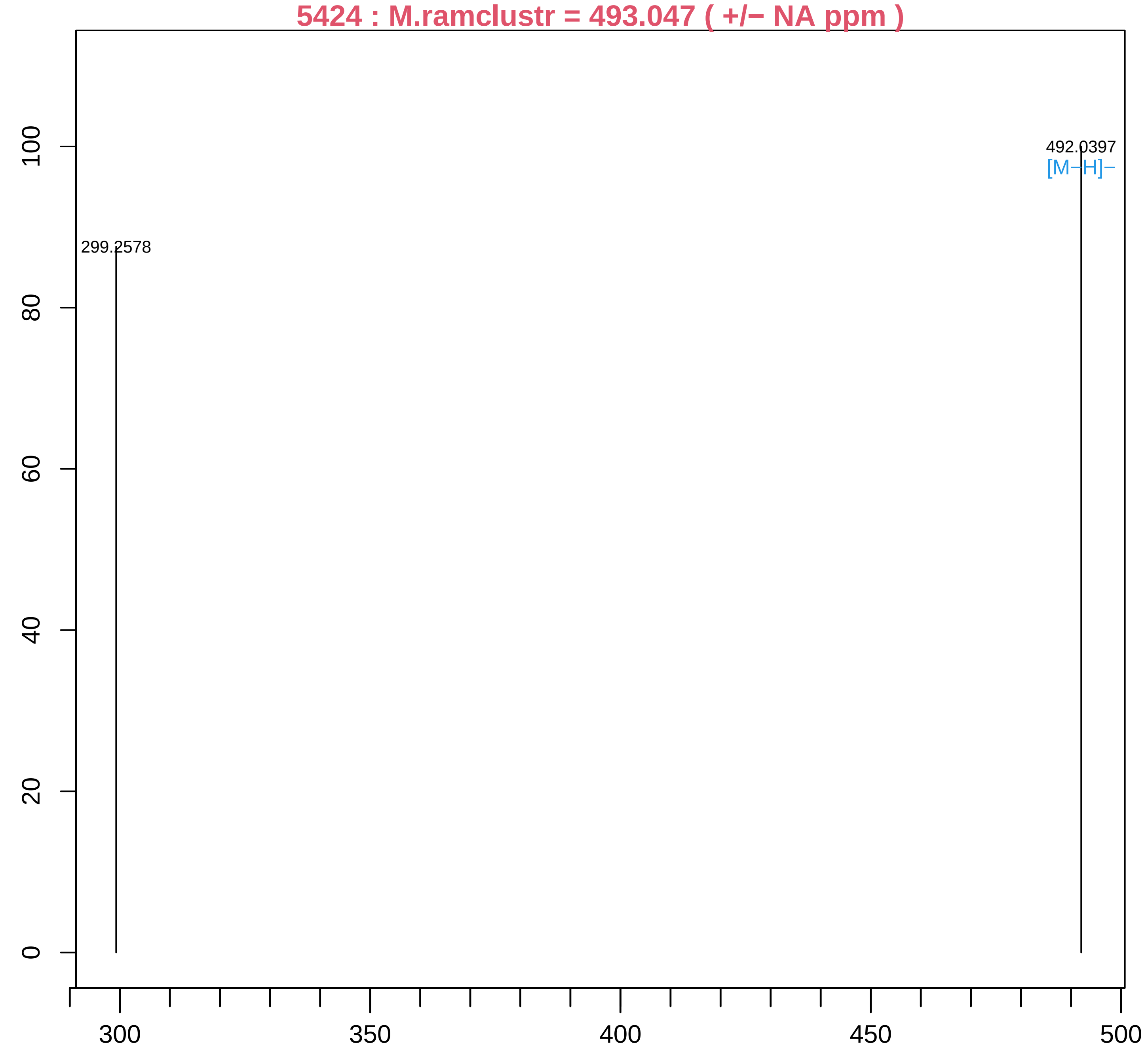
5424 : M.ramclustr = 493.047 (+/− NA ppm)
492.0397
[M−H]−
299.2578
0
20
40
60
80
100
300
350
400
450
500

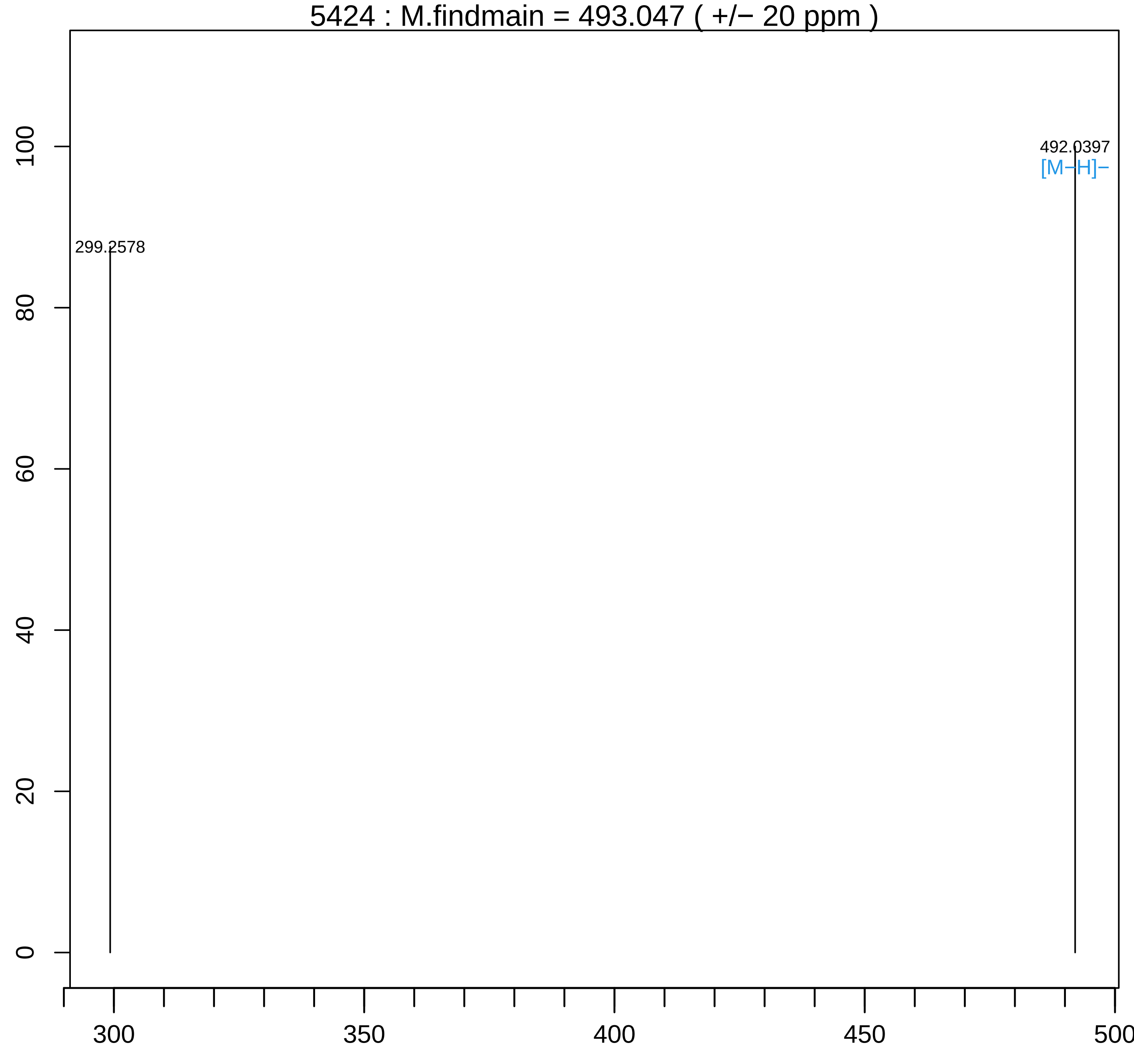
5424 : M.findmain = 493.047 (+/− 20 ppm)
492.0397
[M−H]−
299.2578
0
20
40
60
80
100
300
350
400
450
500

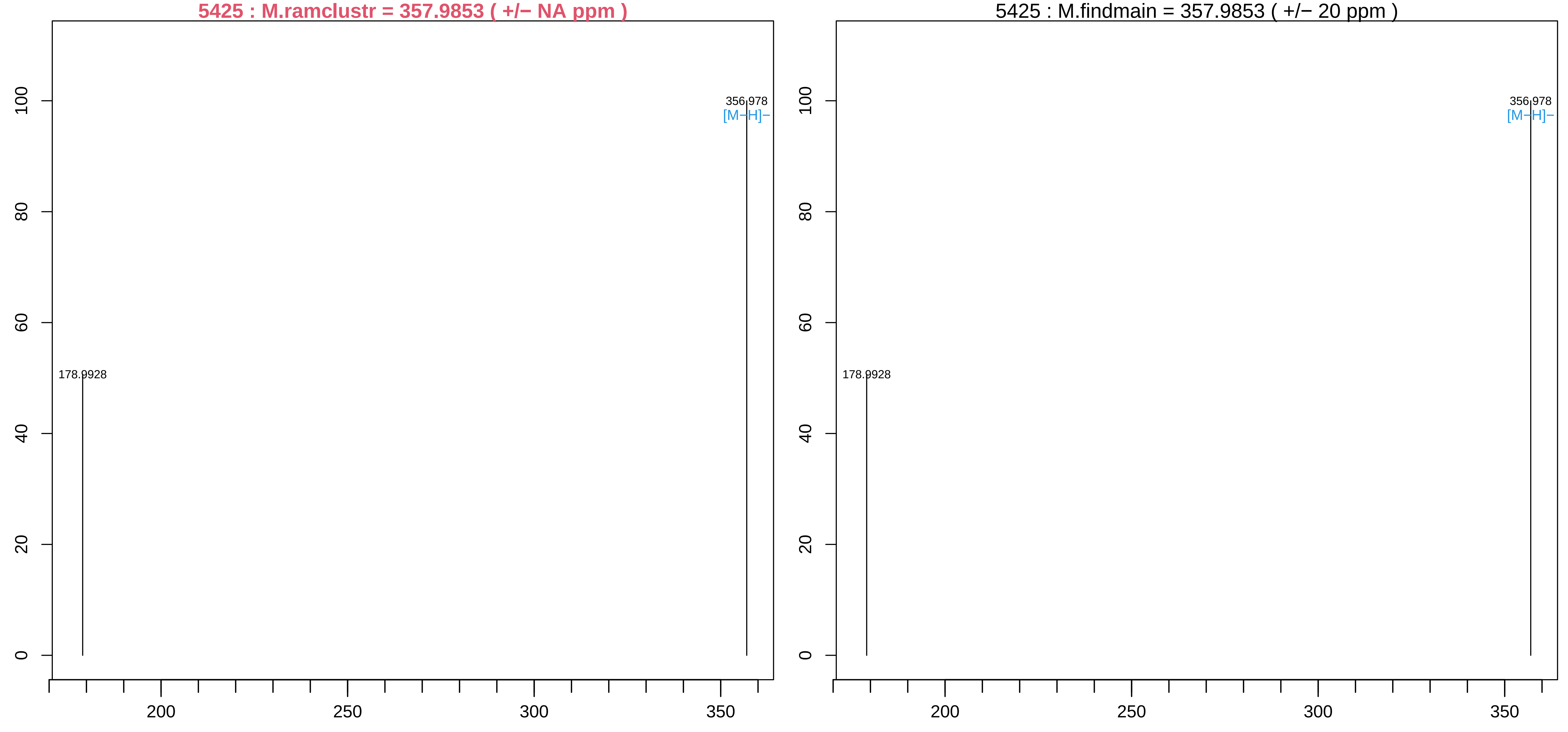
5425 : M.ramclustr = 357.9853 (+/− NA ppm)
5425 : M.findmain = 357.9853 (+/− 20 ppm)
356.978
[M−H]−
178.9928
0
20
40
60
80
100
200
250
300
350

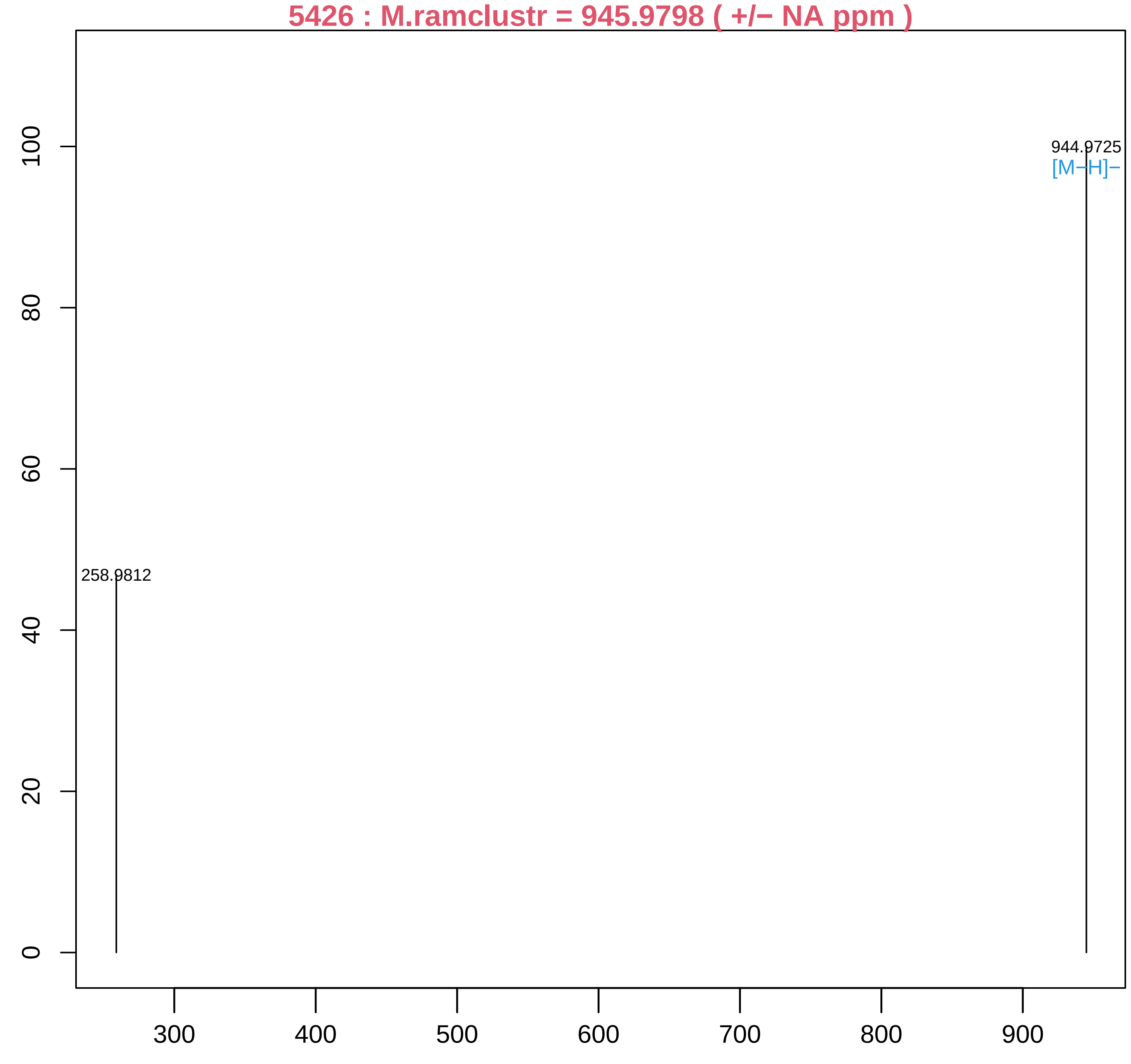
5426 : M.ramclustr = 945.9798 (+/− NA ppm)
944.9725
[M−H]−
258.9812
0
20
40
60
80
100
300
400
500
600
700
800
900

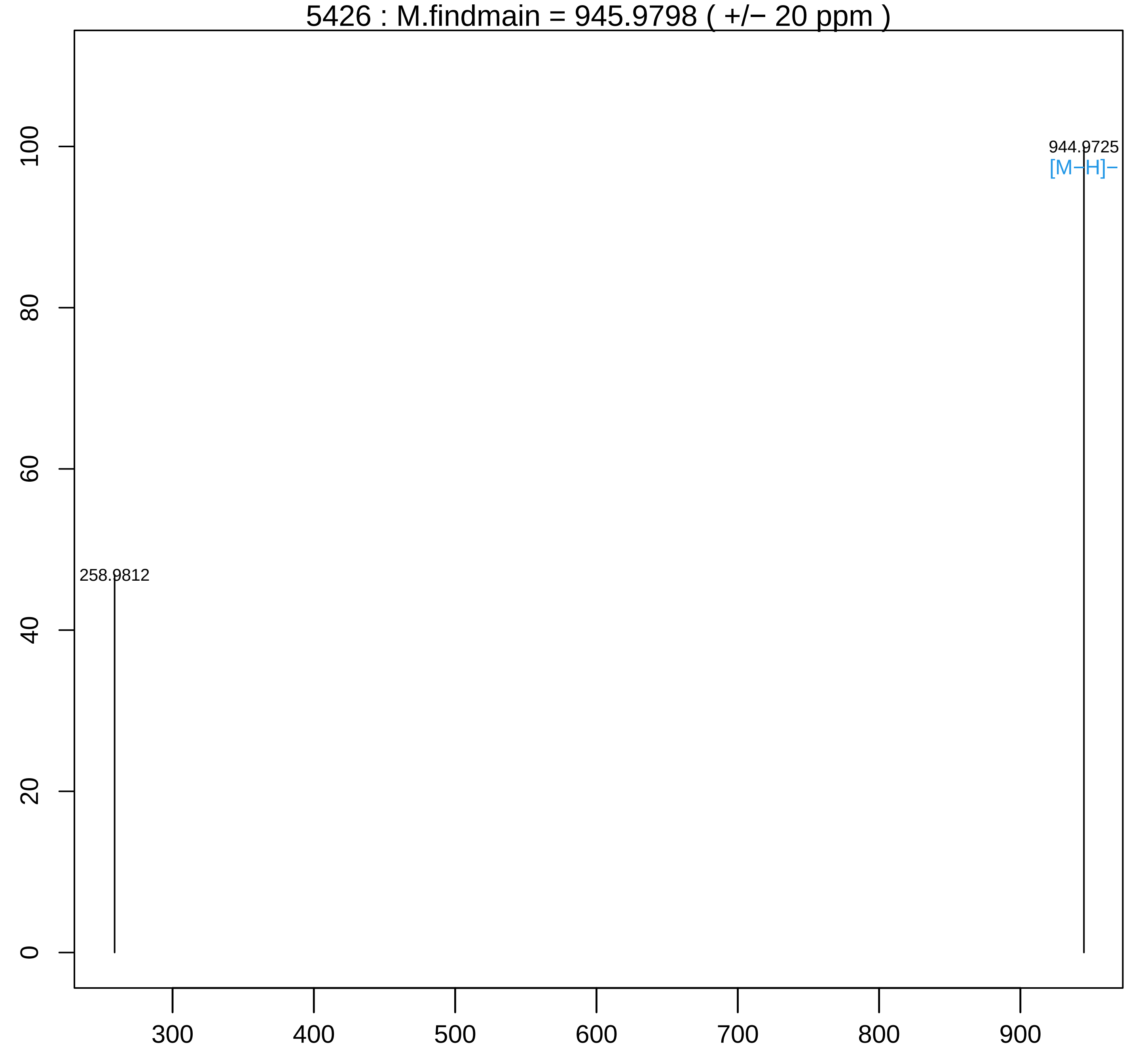
5426 : M.findmain = 945.9798 (+/− 20 ppm)
944.9725
[M−H]−
258.9812
0
20
40
60
80
100
300
400
500
600
700
800
900

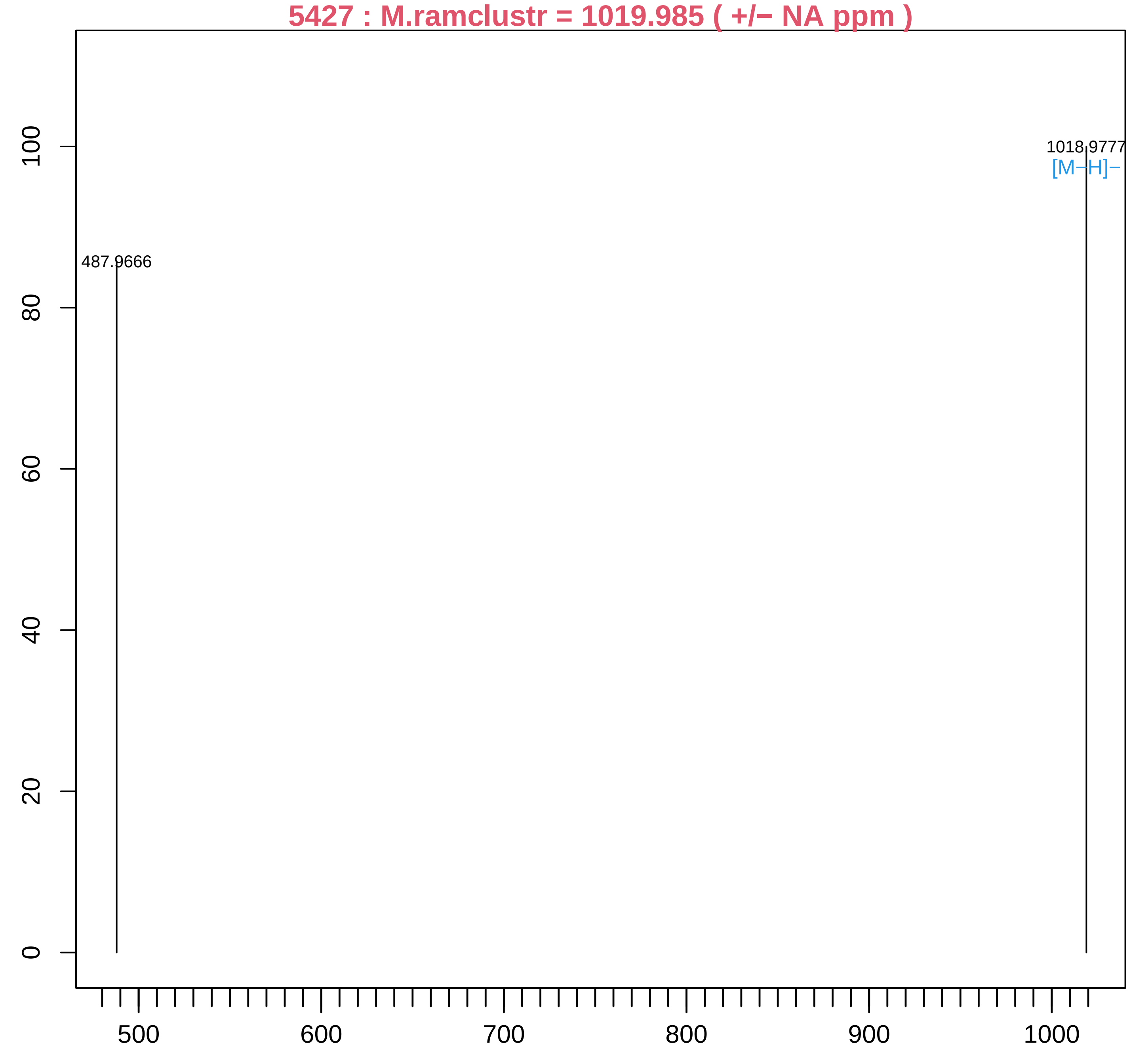
5427 : M.ramclustr = 1019.985 (+/− NA ppm)
1018.9777
[M−H]−
487.9666
0
20
40
60
80
100
500
600
700
800
900
1000

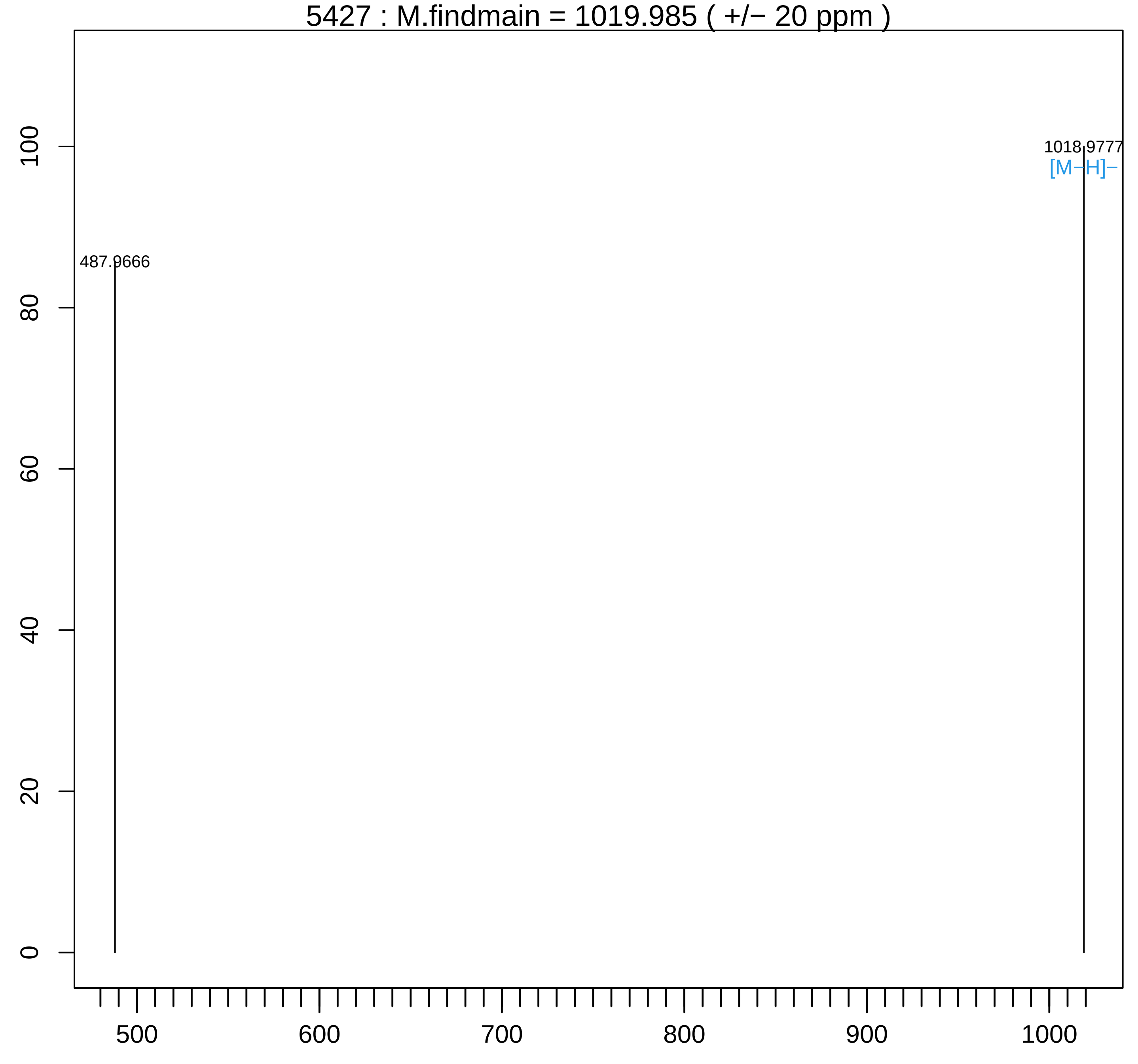
5427 : M.findmain = 1019.985 (+/− 20 ppm)
1018.9777
[M−H]−
487.9666
0
20
40
60
80
100
500
600
700
800
900
1000

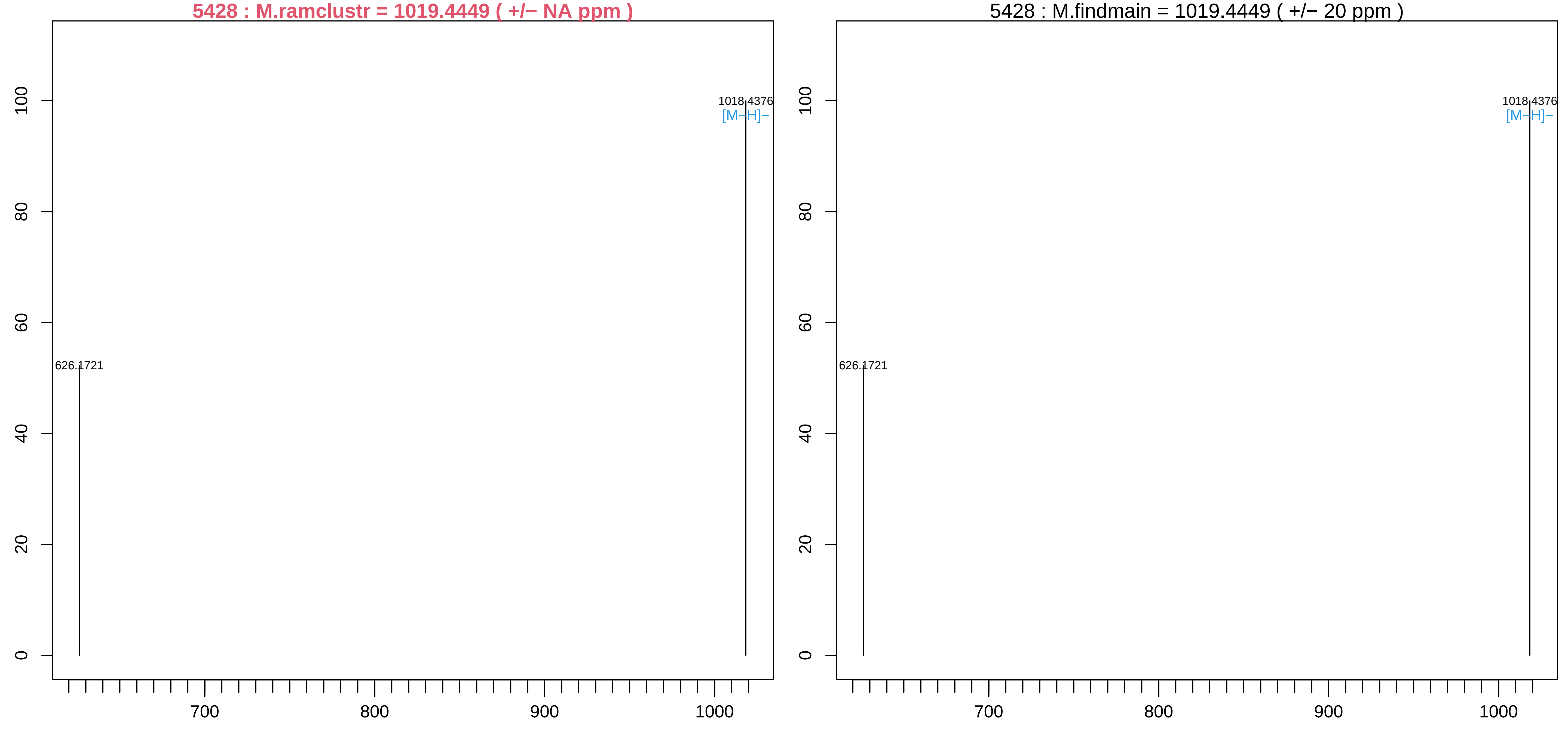
5428 : M.ramclustr = 1019.4449 (+/− NA ppm)
1018.4376
[M−H]−
626.1721
0
20
40
60
80
100
700
800
900
1000
5428 : M.findmain = 1019.4449 (+/− 20 ppm)
1018.4376
[M−H]−
626.1721
0
20
40
60
80
100
700
800
900
1000

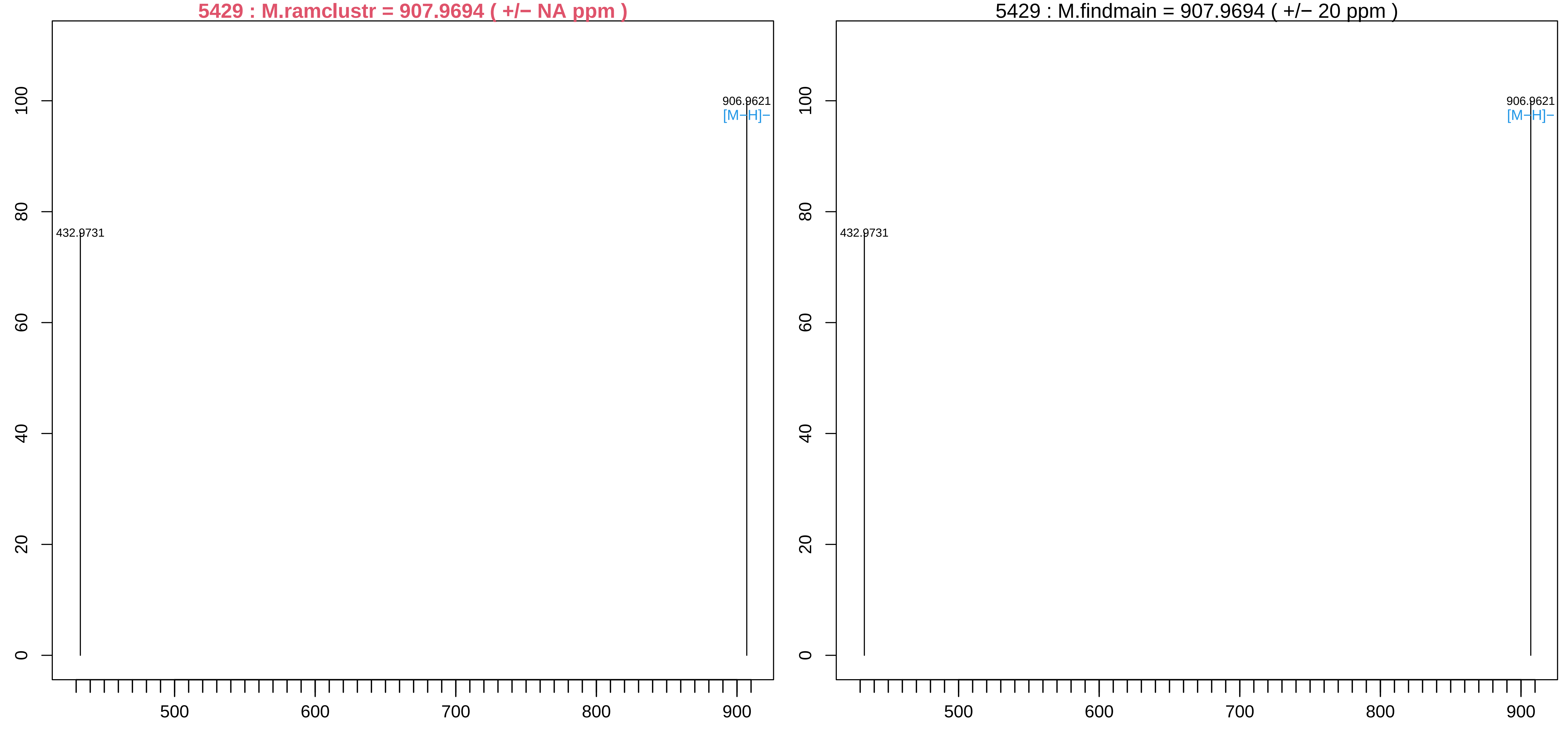

5429 : M.ramclustr = 907.9694 (+/− NA ppm)
5429 : M.findmain = 907.9694 (+/− 20 ppm)
906.9621
[M−H]−
432.9731
906.9621
[M−H]−
432.9731
0
20
40
60
80
100
500
600
700
800
900

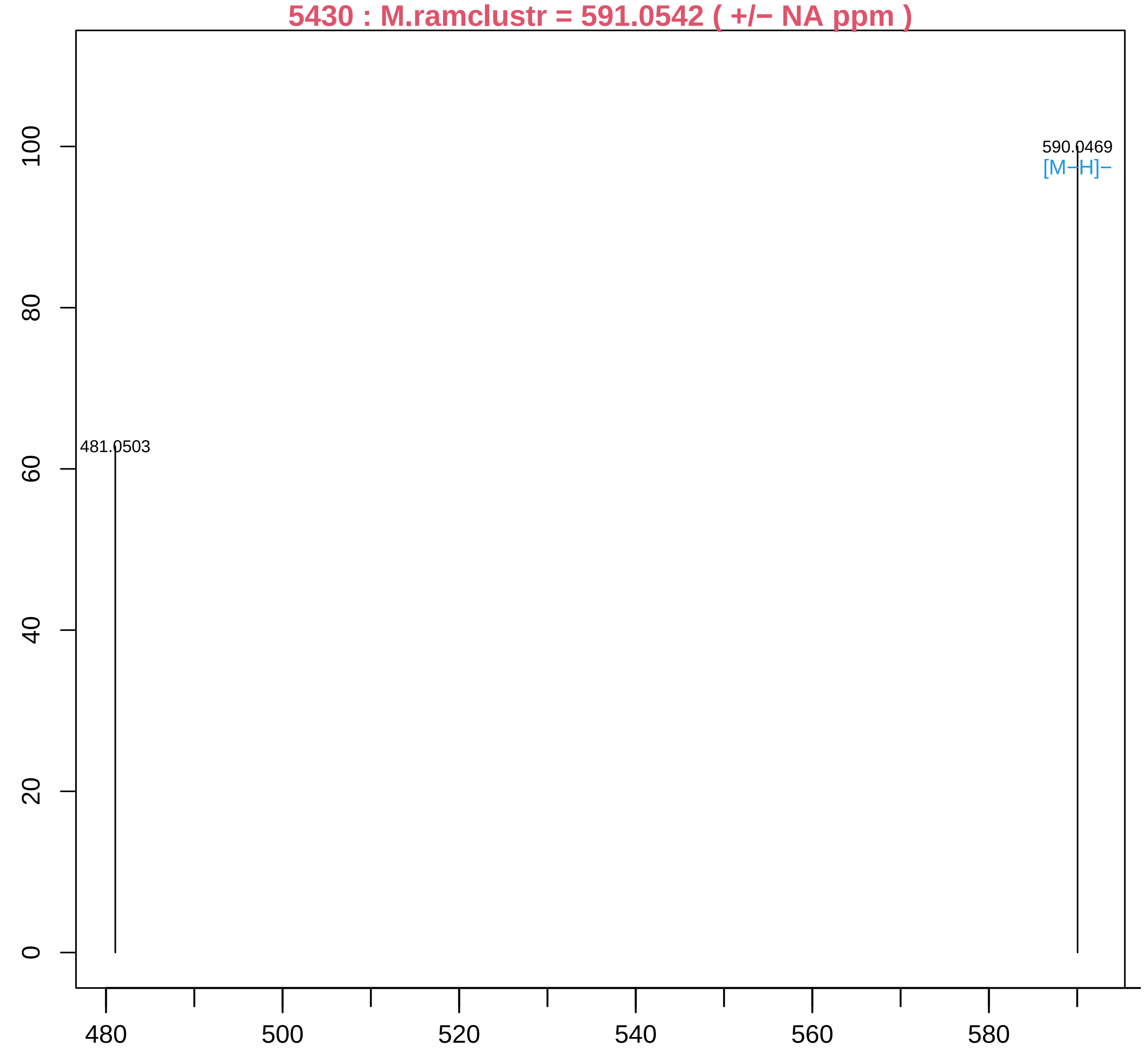
5430 : M.ramclustr = 591.0542 (+/− NA ppm)
590.0469
[M−H]−
481.0503
0
20
40
60
80
100
480
500
520
540
560
580

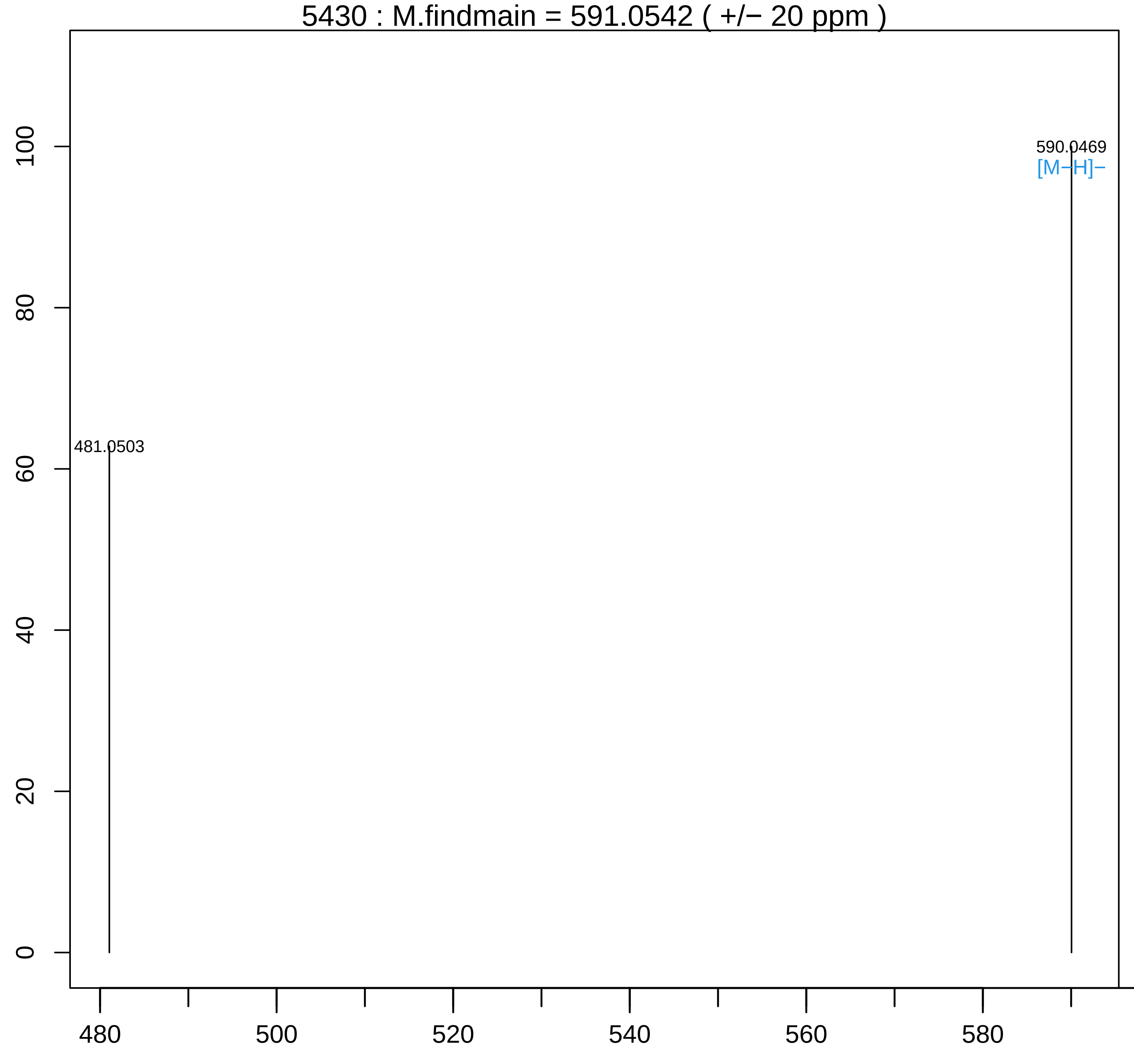
5430 : M.findmain = 591.0542 (+/− 20 ppm)
590.0469
[M−H]−
481.0503
0
20
40
60
80
100
480
500
520
540
560
580

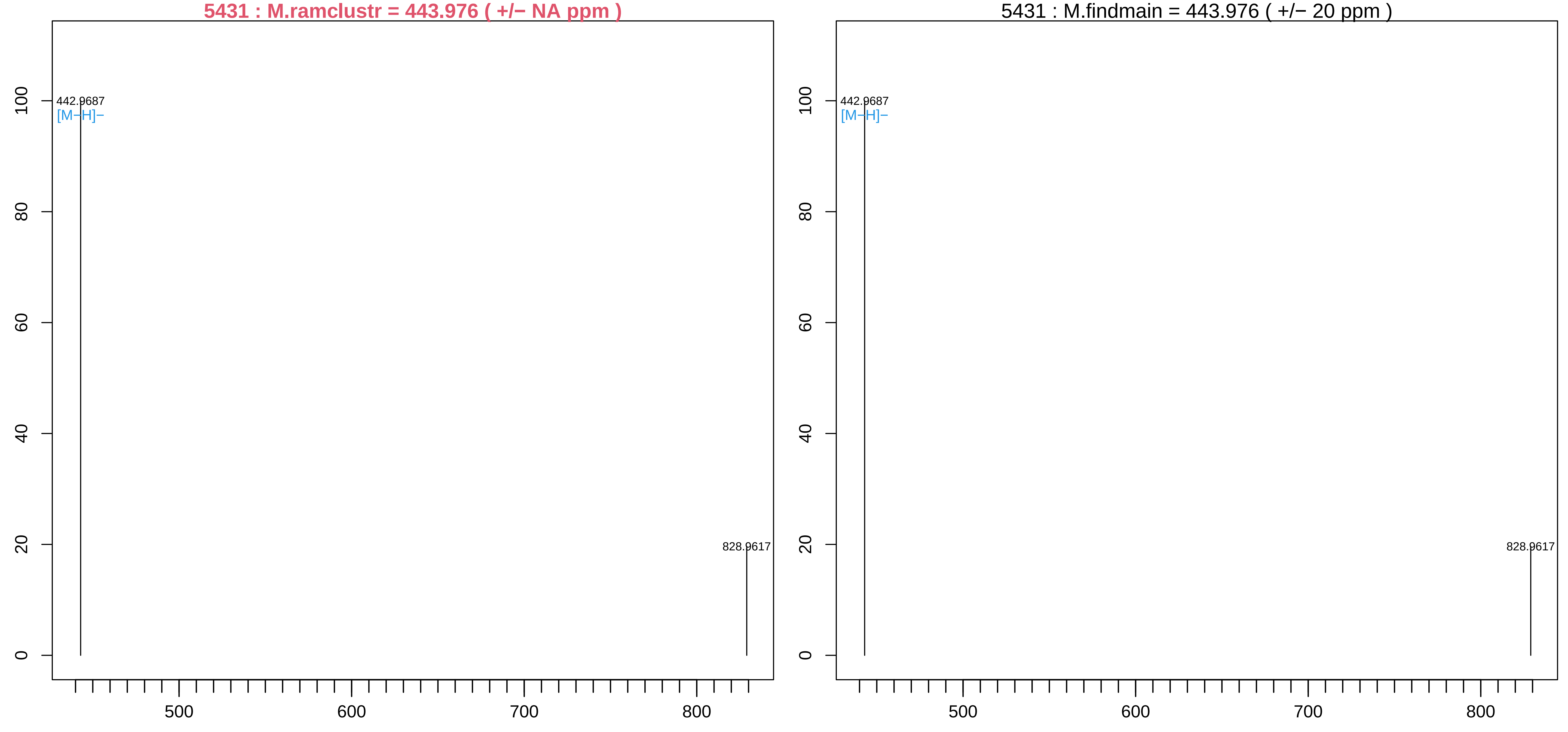
5431 : M.ramclustr = 443.976 (+/− NA ppm)
442.9687
[M−H]−
828.9617
5431 : M.findmain = 443.976 (+/− 20 ppm)
442.9687
[M−H]−
828.9617
0
20
40
60
80
100
500
600
700
800

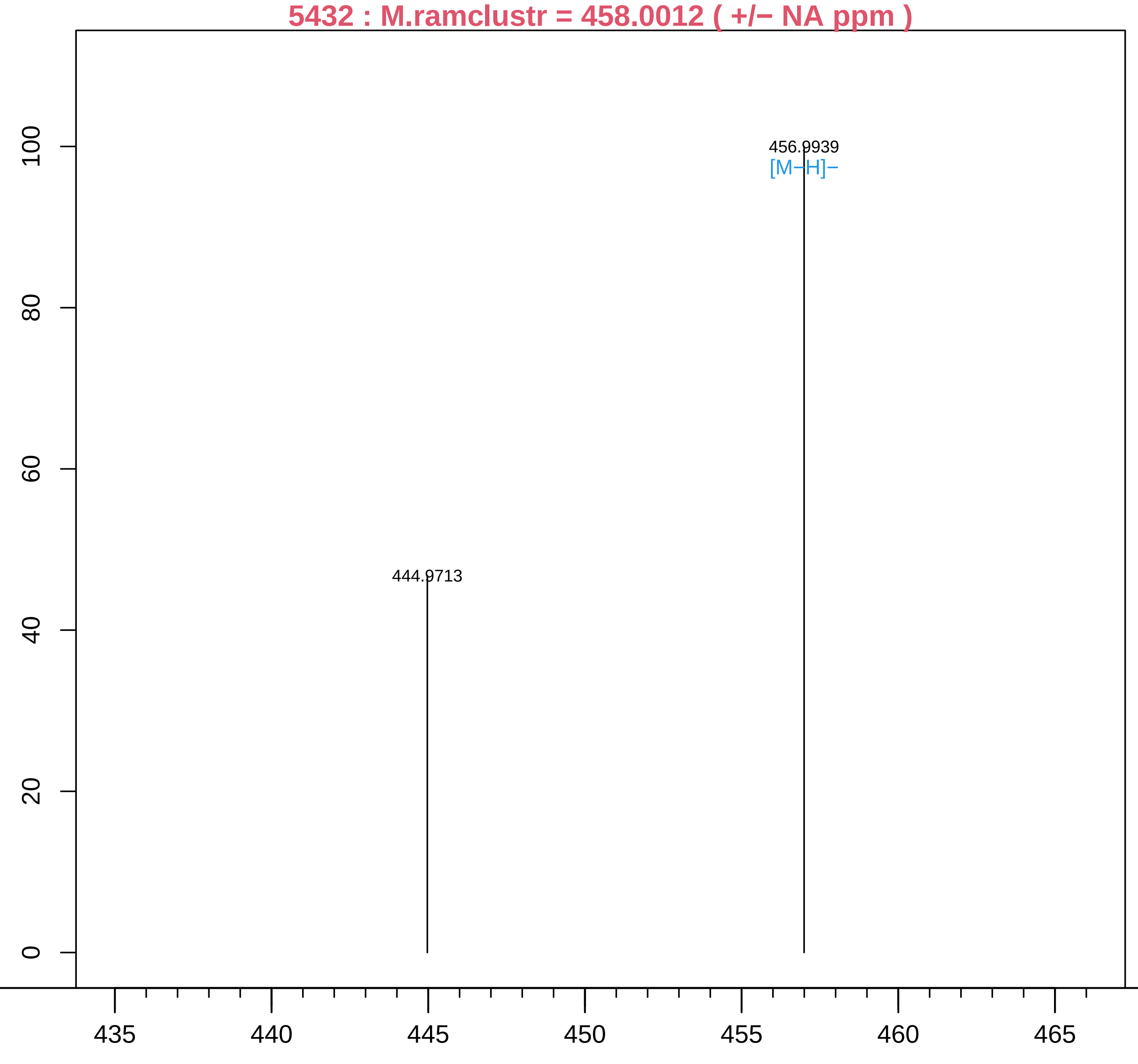
5432 : M.ramclustr = 458.0012 (+/− NA ppm)
456.9939
[M−H]−
444.9713
0
20
40
60
80
100
435
440
445
450
455
460
465

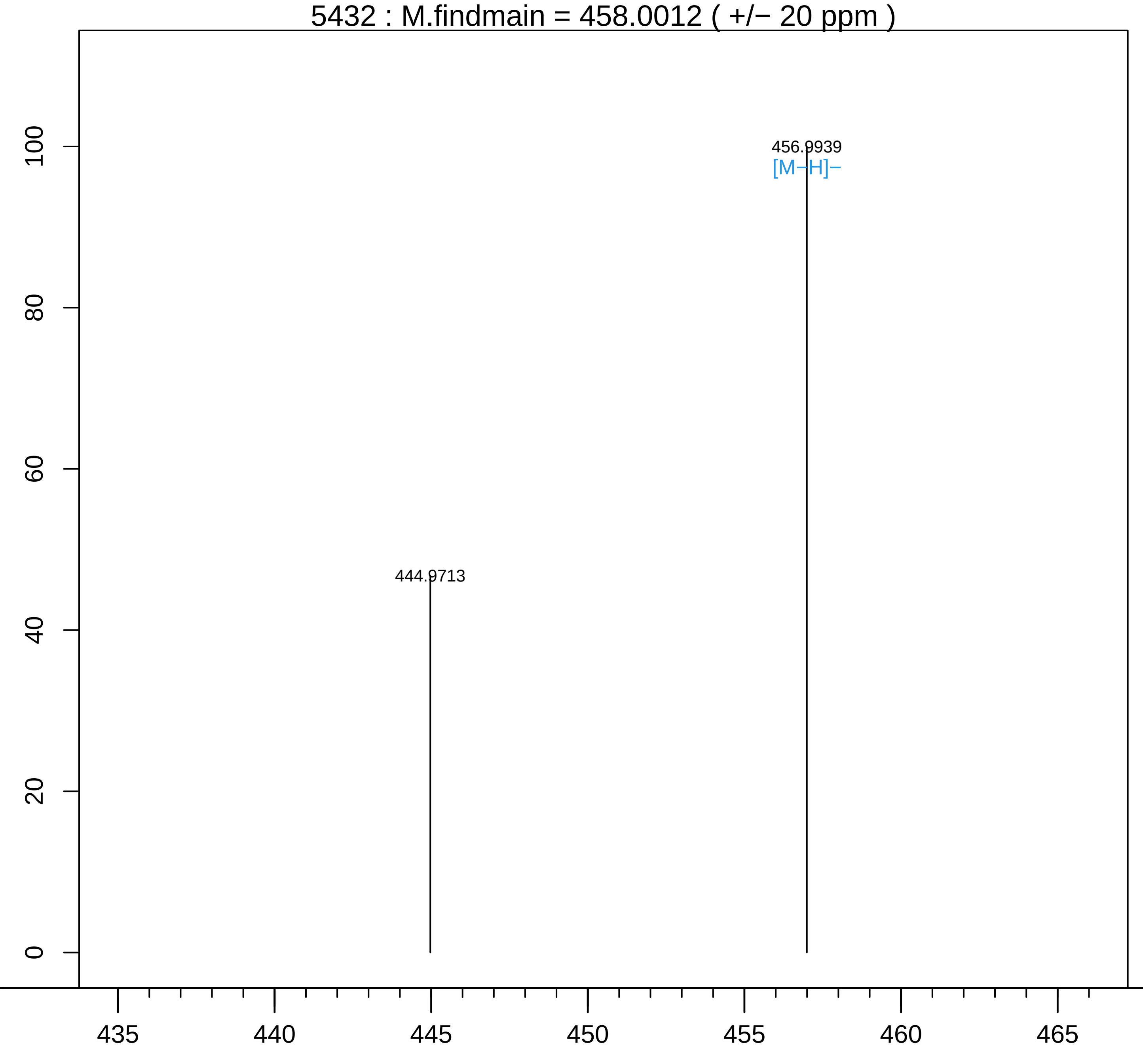
5432 : M.findmain = 458.0012 (+/− 20 ppm)
456.9939
[M−H]−
444.9713
0
20
40
60
80
100
435
440
445
450
455
460
465

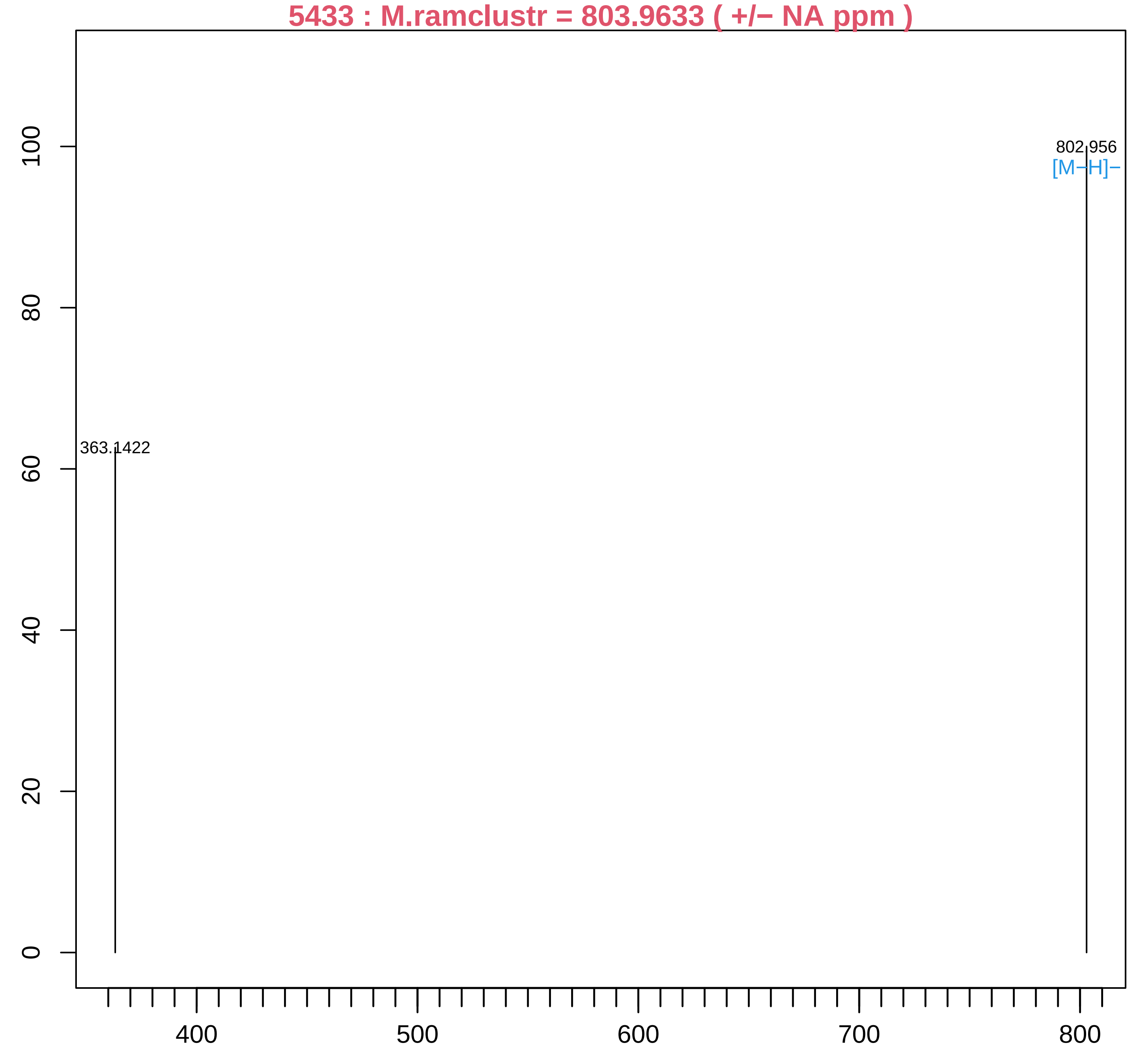
5433 : M.ramclustr = 803.9633 (+/− NA ppm)
802.956
[M−H]−
363.1422
0
20
40
60
80
100
400
500
600
700
800

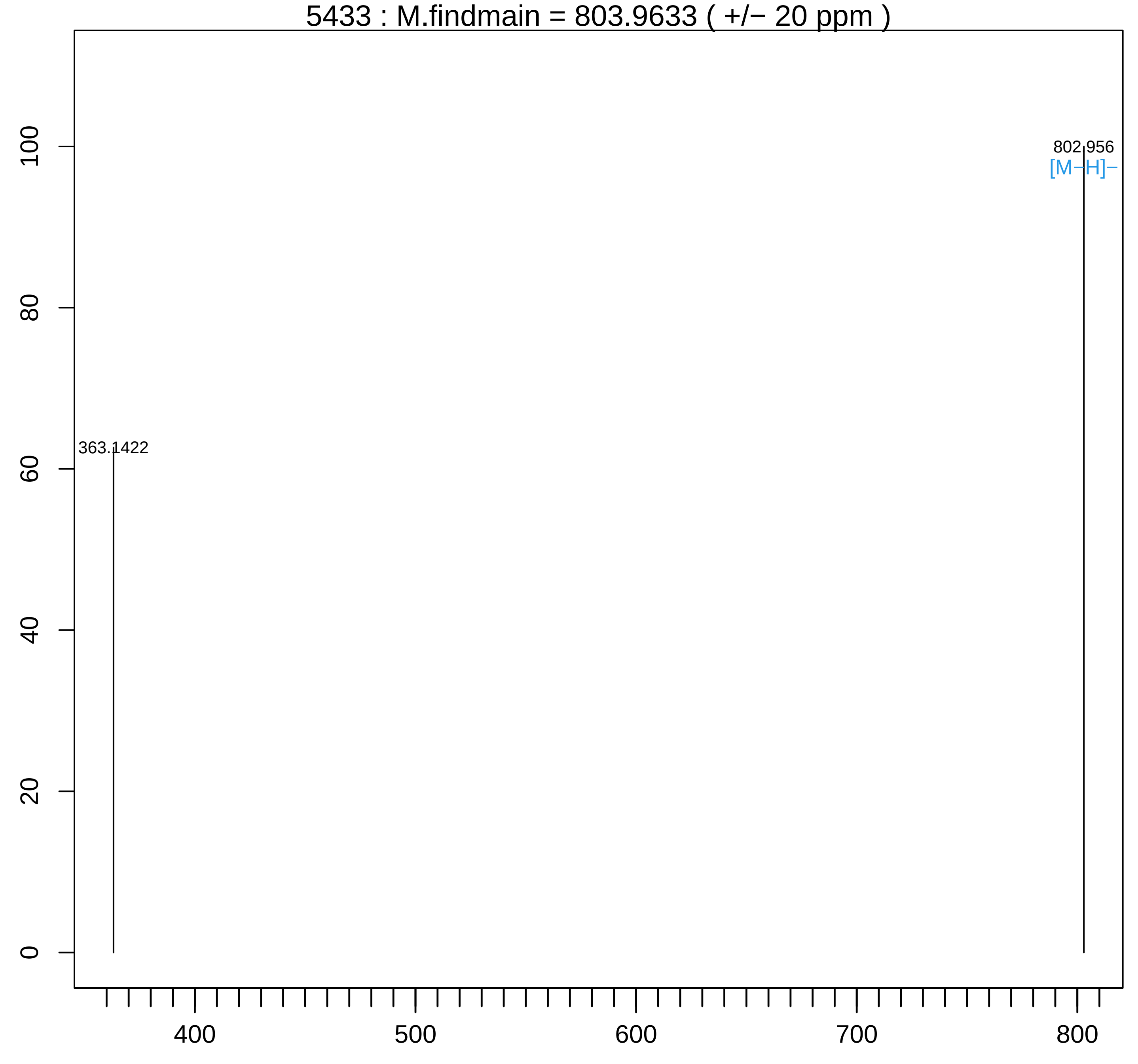
5433 : M.findmain = 803.9633 (+/− 20 ppm)
802.956
[M−H]−
363.1422
0
20
40
60
80
100
400
500
600
700
800

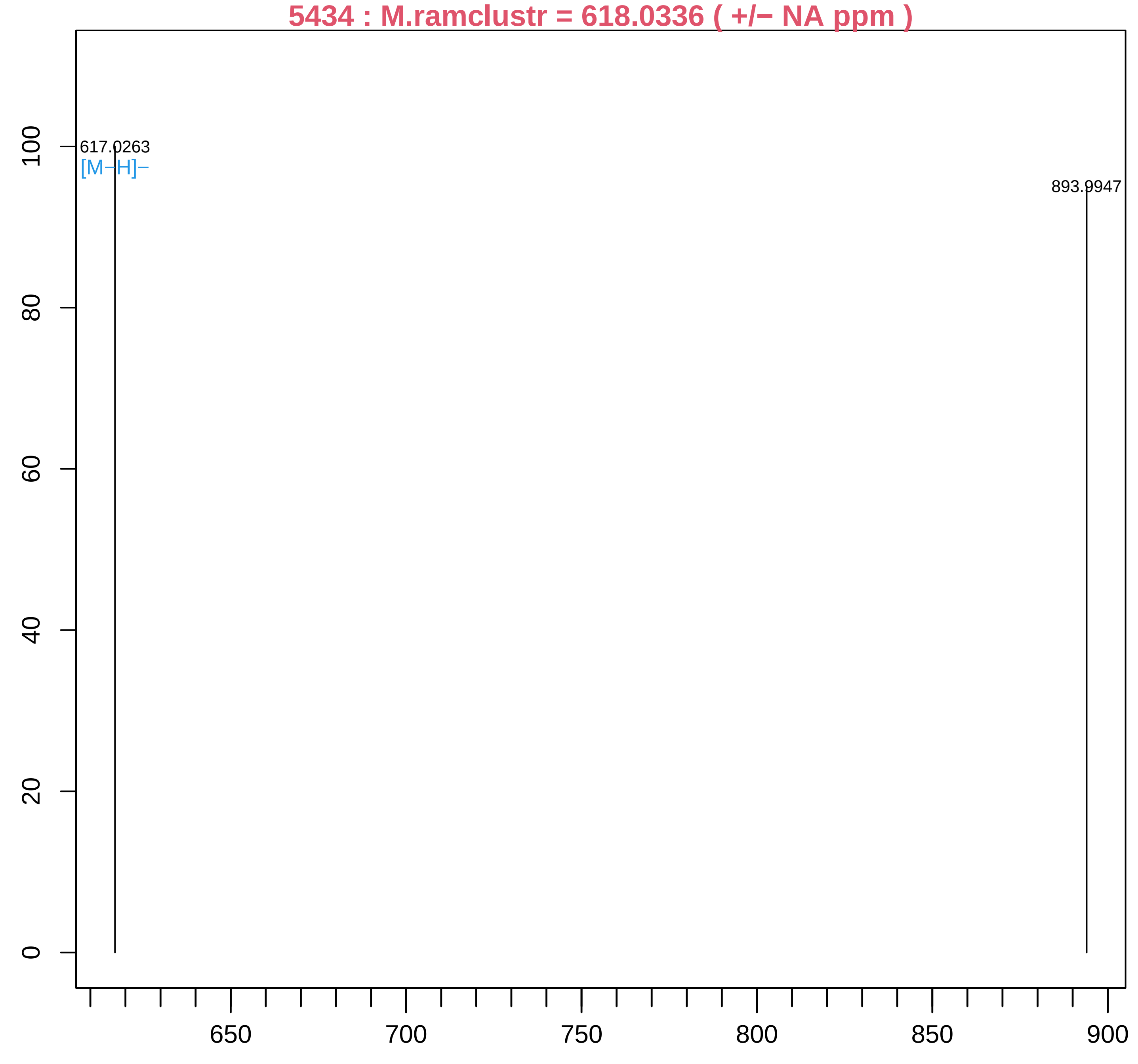
5434 : M.ramclustr = 618.0336 (+/− NA ppm)
617.0263
[M−H]−
893.9947
0
20
40
60
80
100
650
700
750
800
850
900

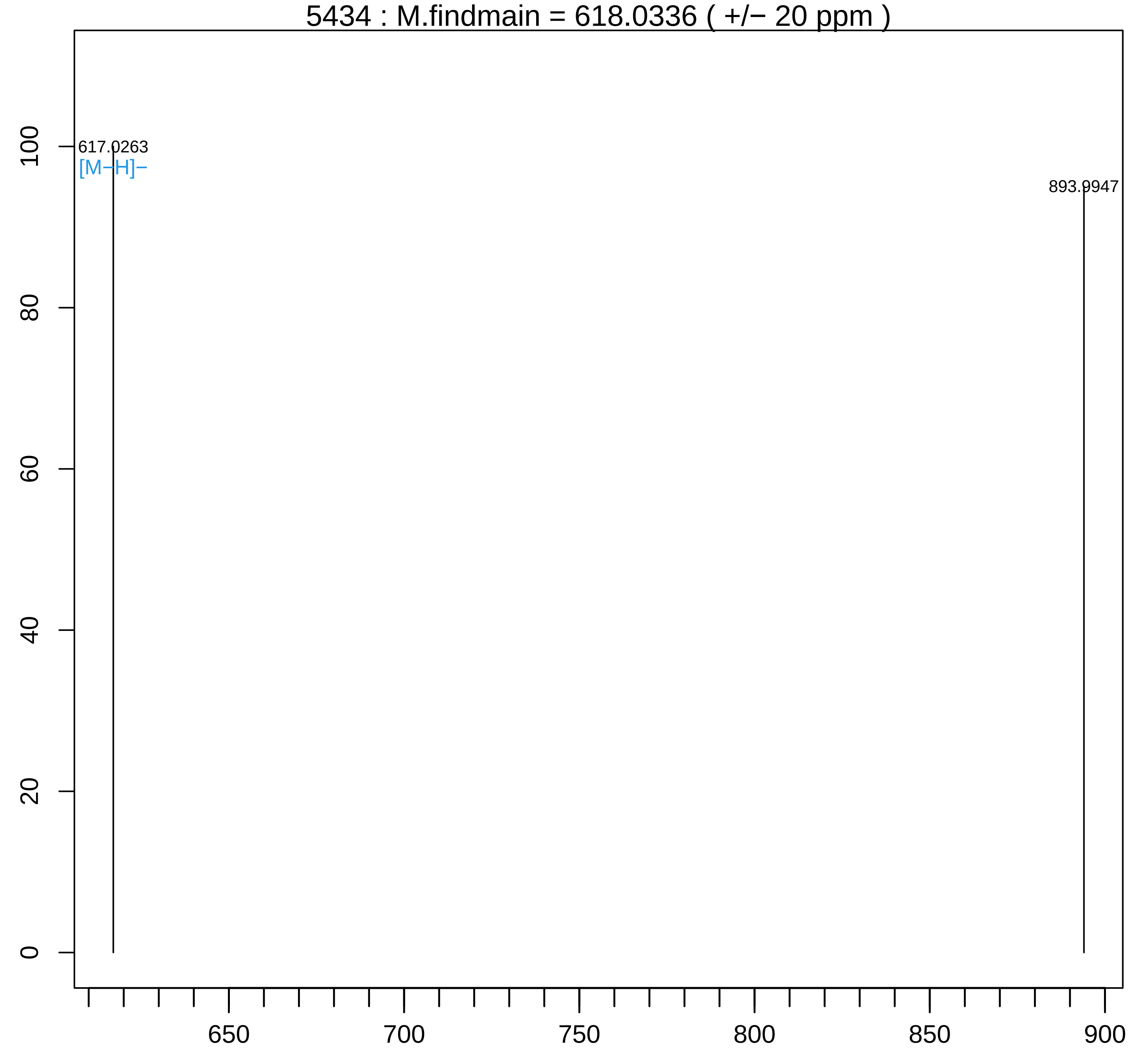
5434 : M.findmain = 618.0336 (+/− 20 ppm)
617.0263
[M−H]−
893.9947
0
20
40
60
80
100
650
700
750
800
850
900

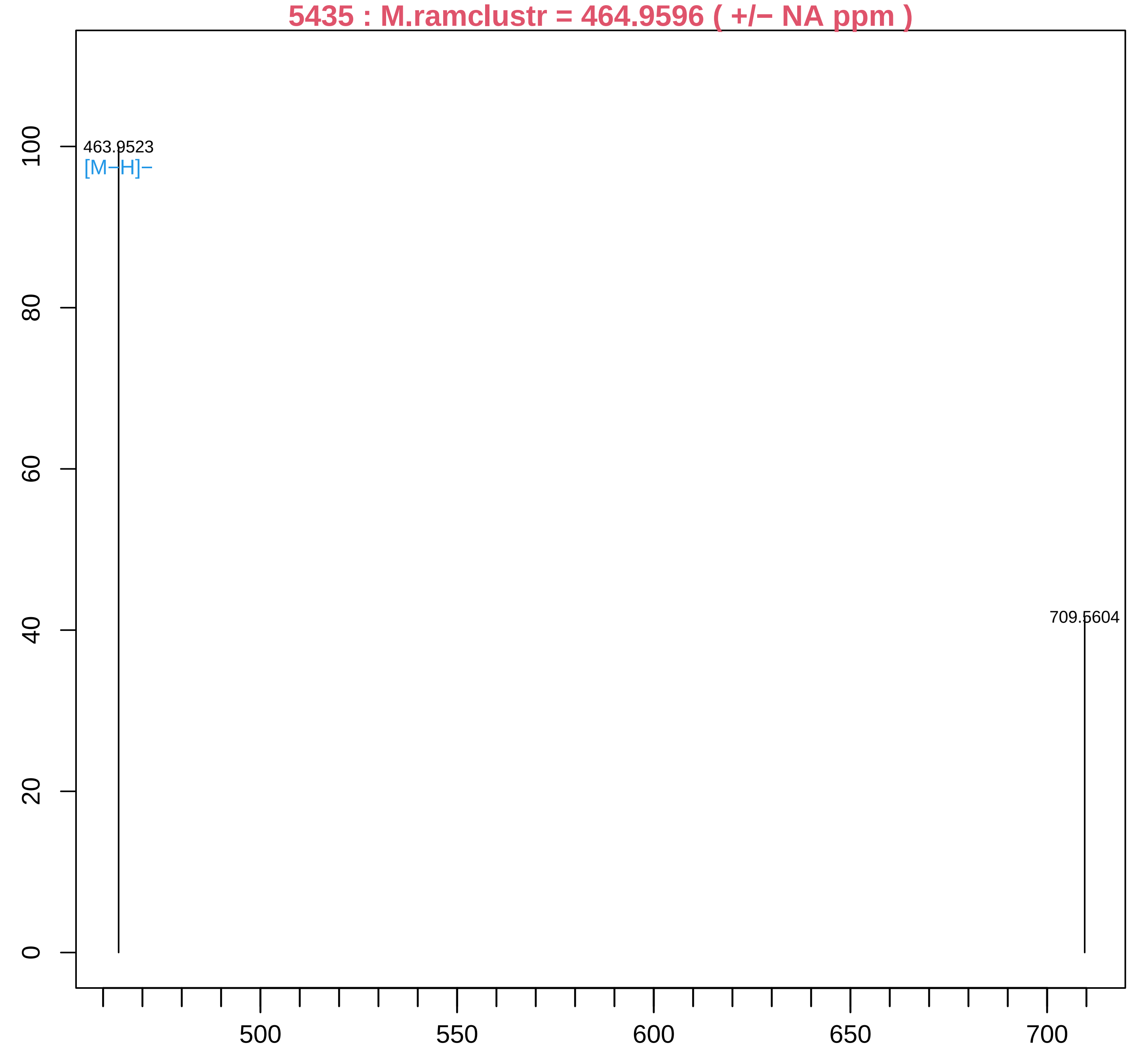
5435 : M.ramclustr = 464.9596 (+/− NA ppm)
463.9523
[M−H]−
709.5604
0
20
40
60
80
100
500
550
600
650
700

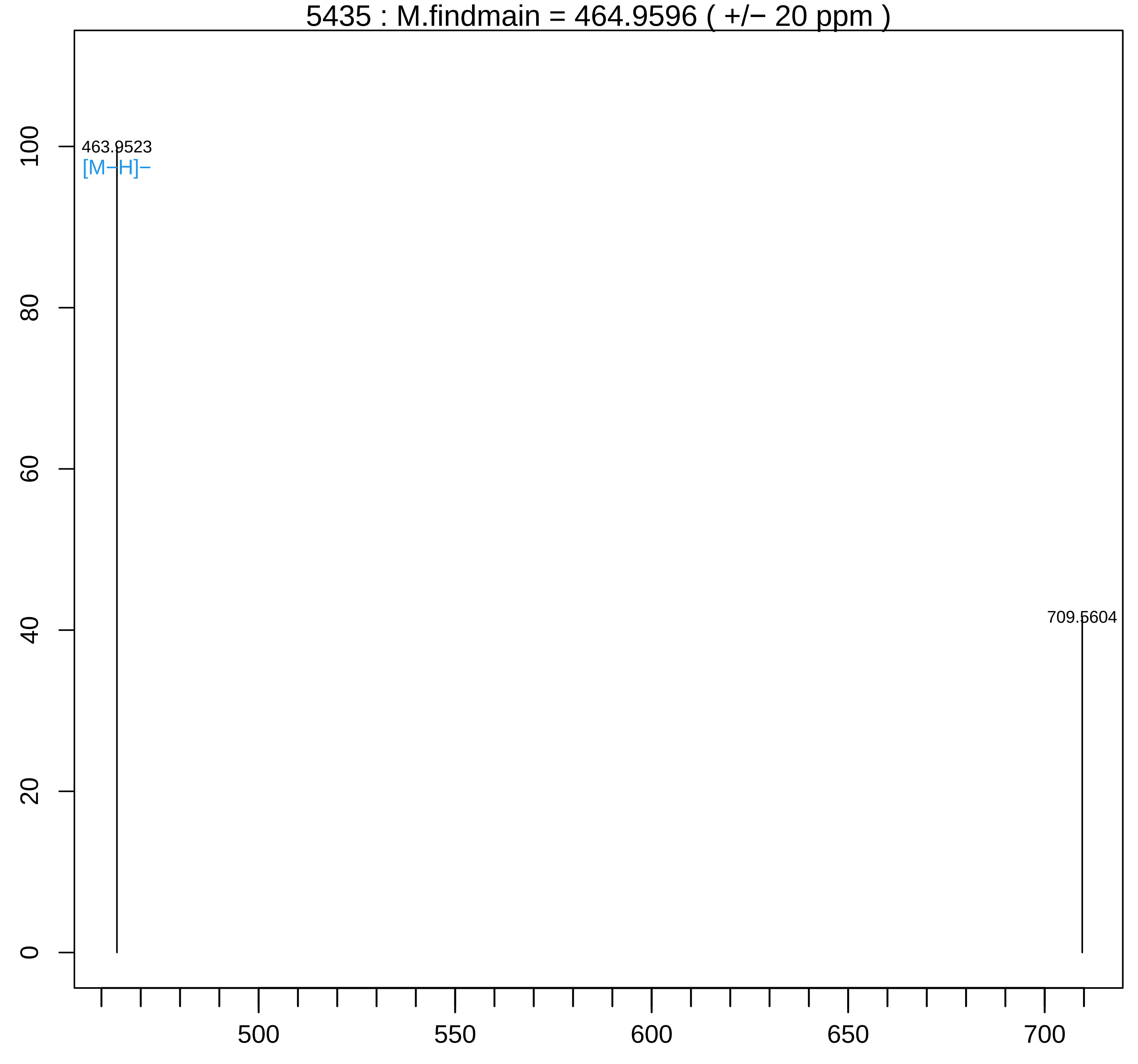
5435 : M.findmain = 464.9596 (+/− 20 ppm)
463.9523
[M−H]−
709.5604
0
20
40
60
80
100
500
550
600
650
700

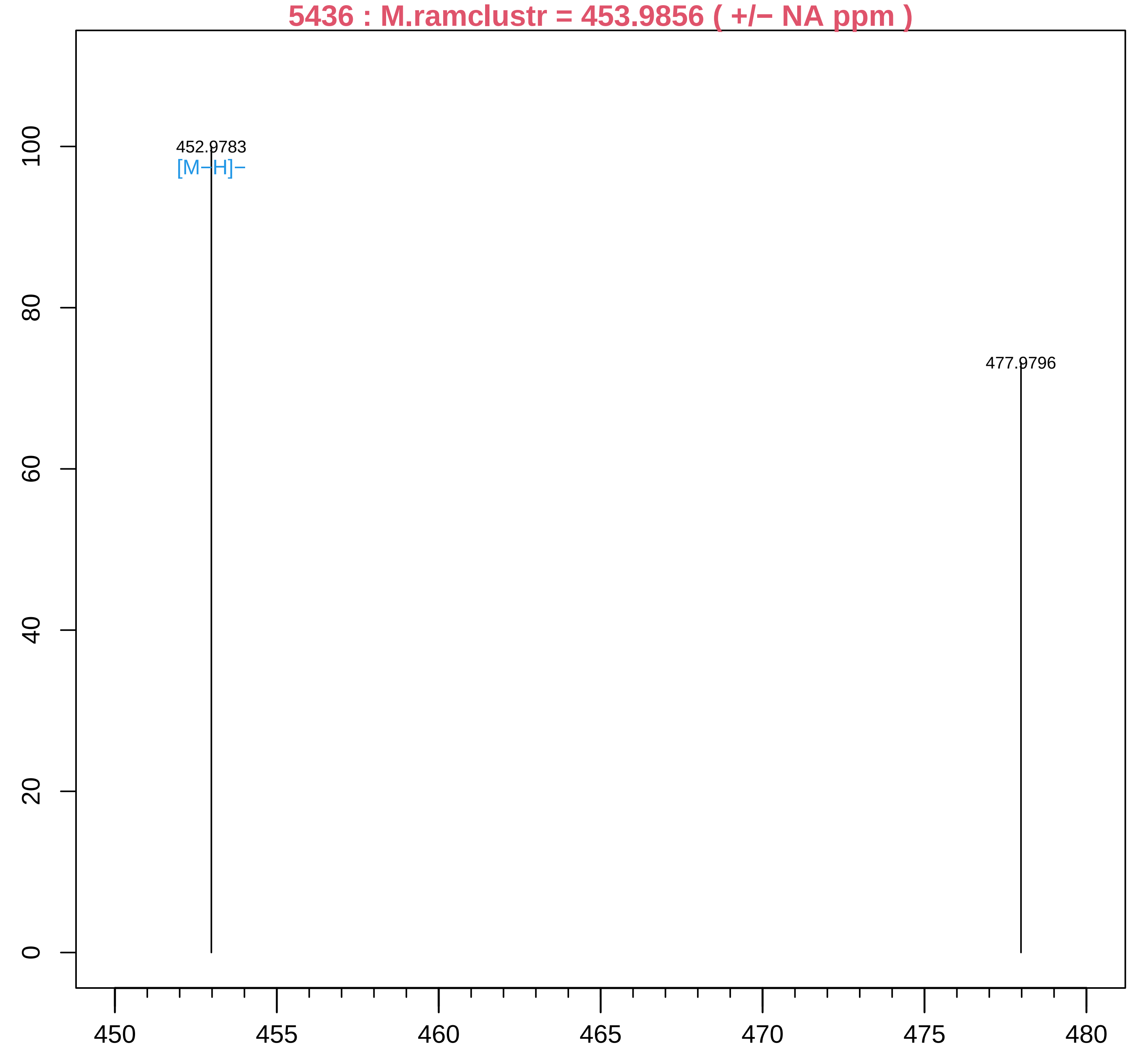
5436 : M.ramclustr = 453.9856 (+/− NA ppm)
452.9783
[M−H]−
477.9796
0
20
40
60
80
100
450
455
460
465
470
475
480

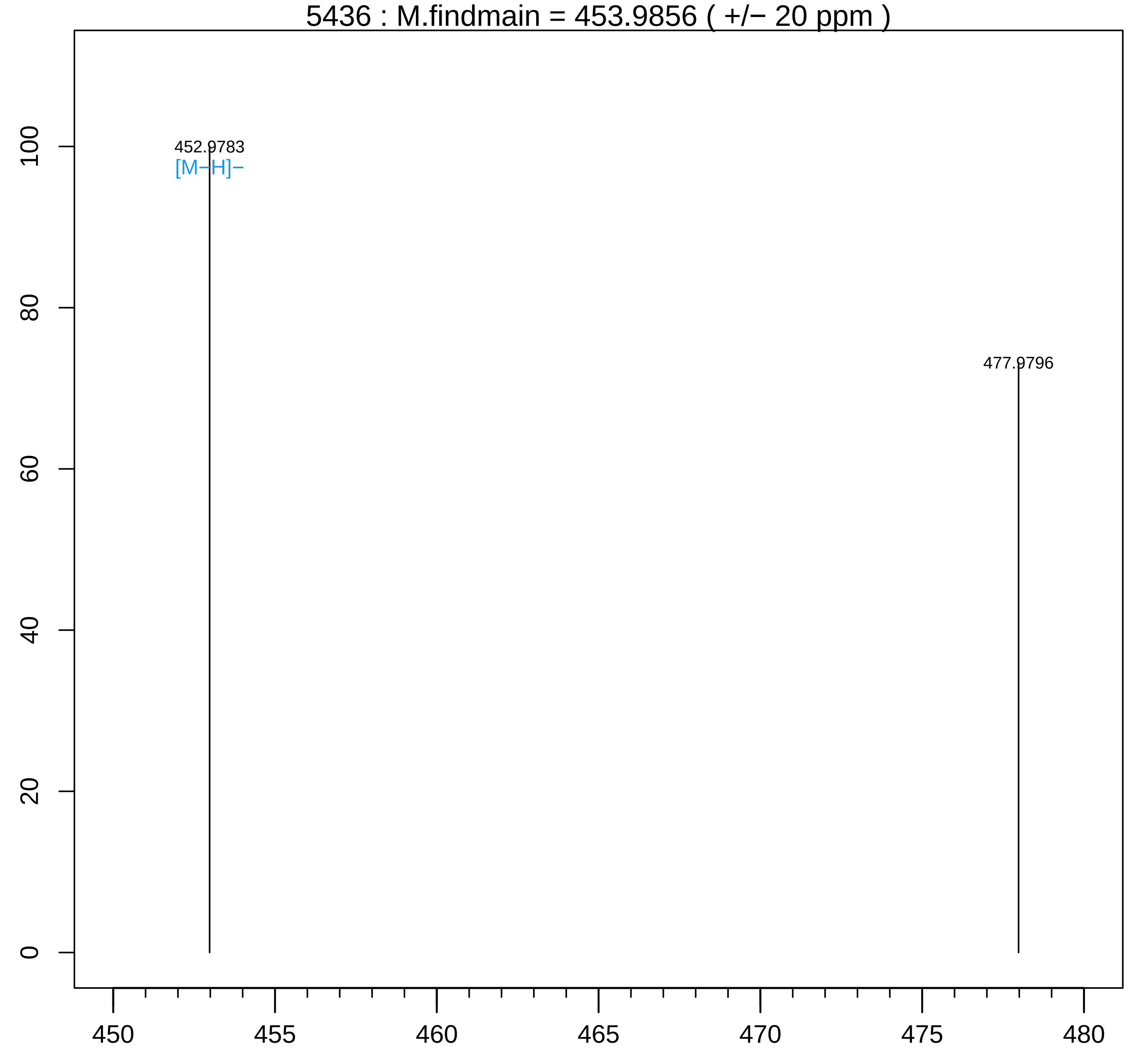
5436 : M.findmain = 453.9856 (+/− 20 ppm)
452.9783
[M−H]−
477.9796
0
20
40
60
80
100
450
455
460
465
470
475
480

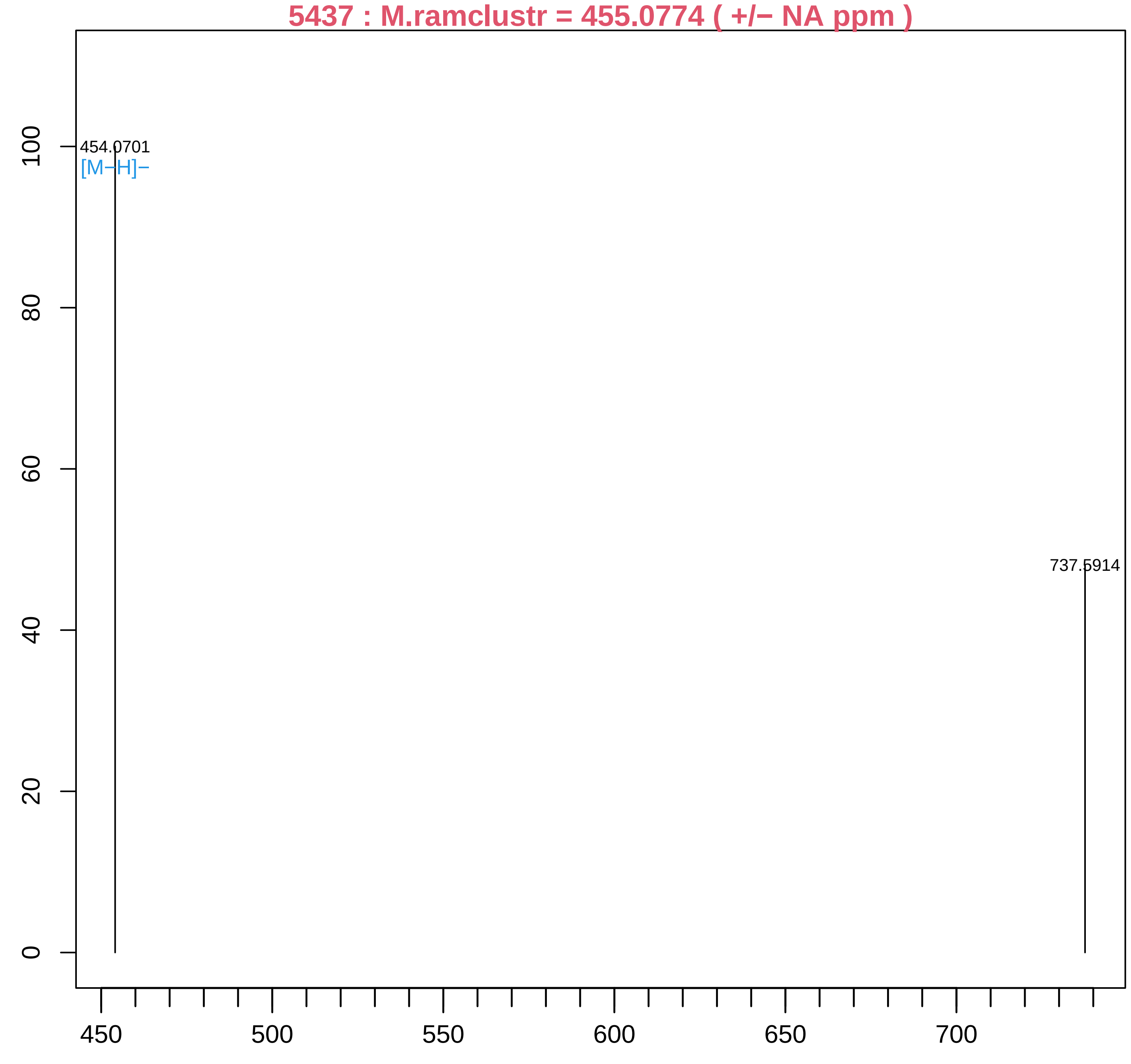
5437 : M.ramclustr = 455.0774 (+/− NA ppm)
454.0701
[M−H]−
737.5914
0
20
40
60
80
100
450
500
550
600
650
700

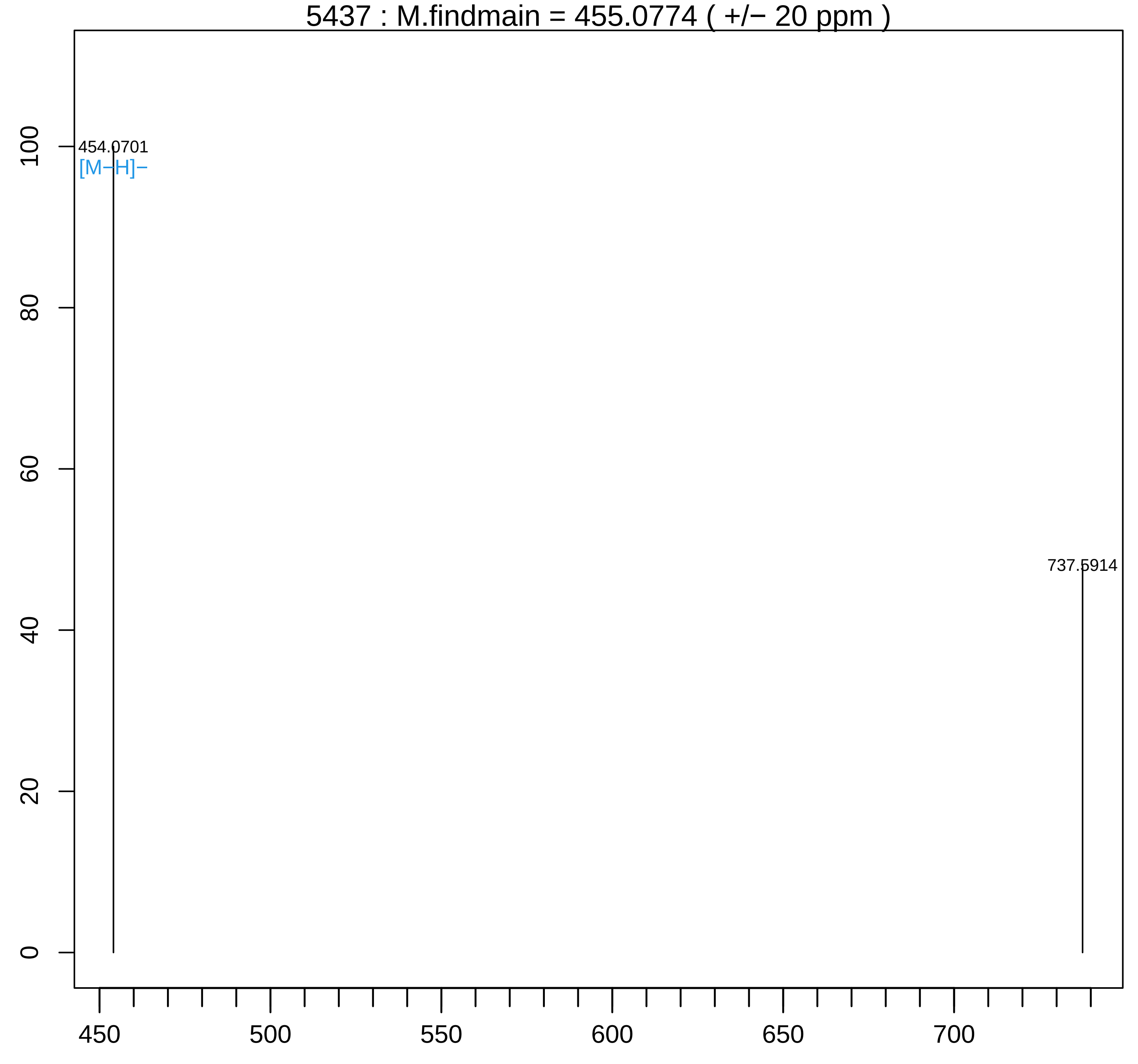
5437 : M.findmain = 455.0774 (+/− 20 ppm)
454.0701
[M−H]−
737.5914
0
20
40
60
80
100
450
500
550
600
650
700

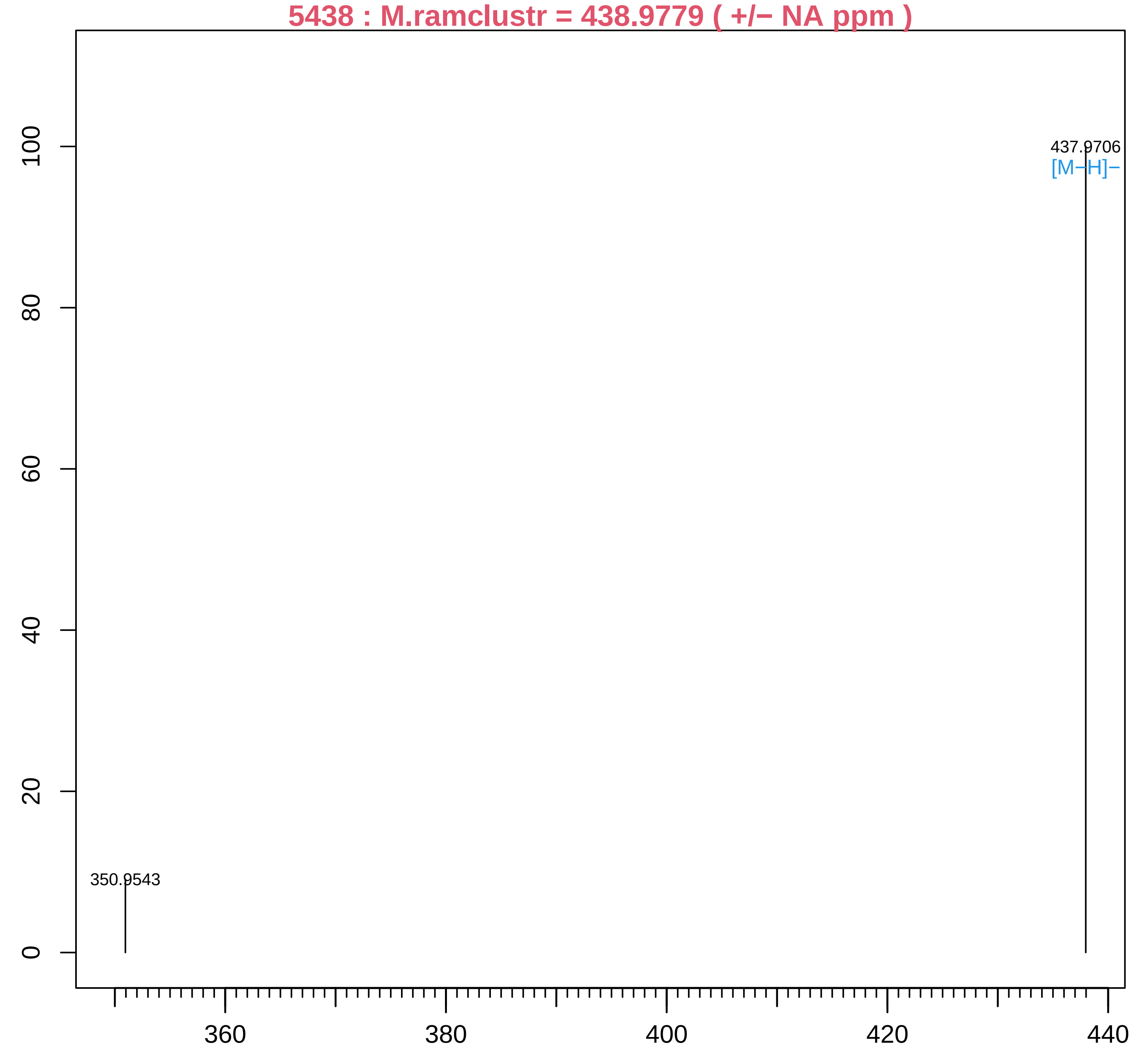
5438 : M.ramclustr = 438.9779 (+/− NA ppm)
437.9706
[M−H]−
350.9543
0
20
40
60
80
100
360
380
400
420
440

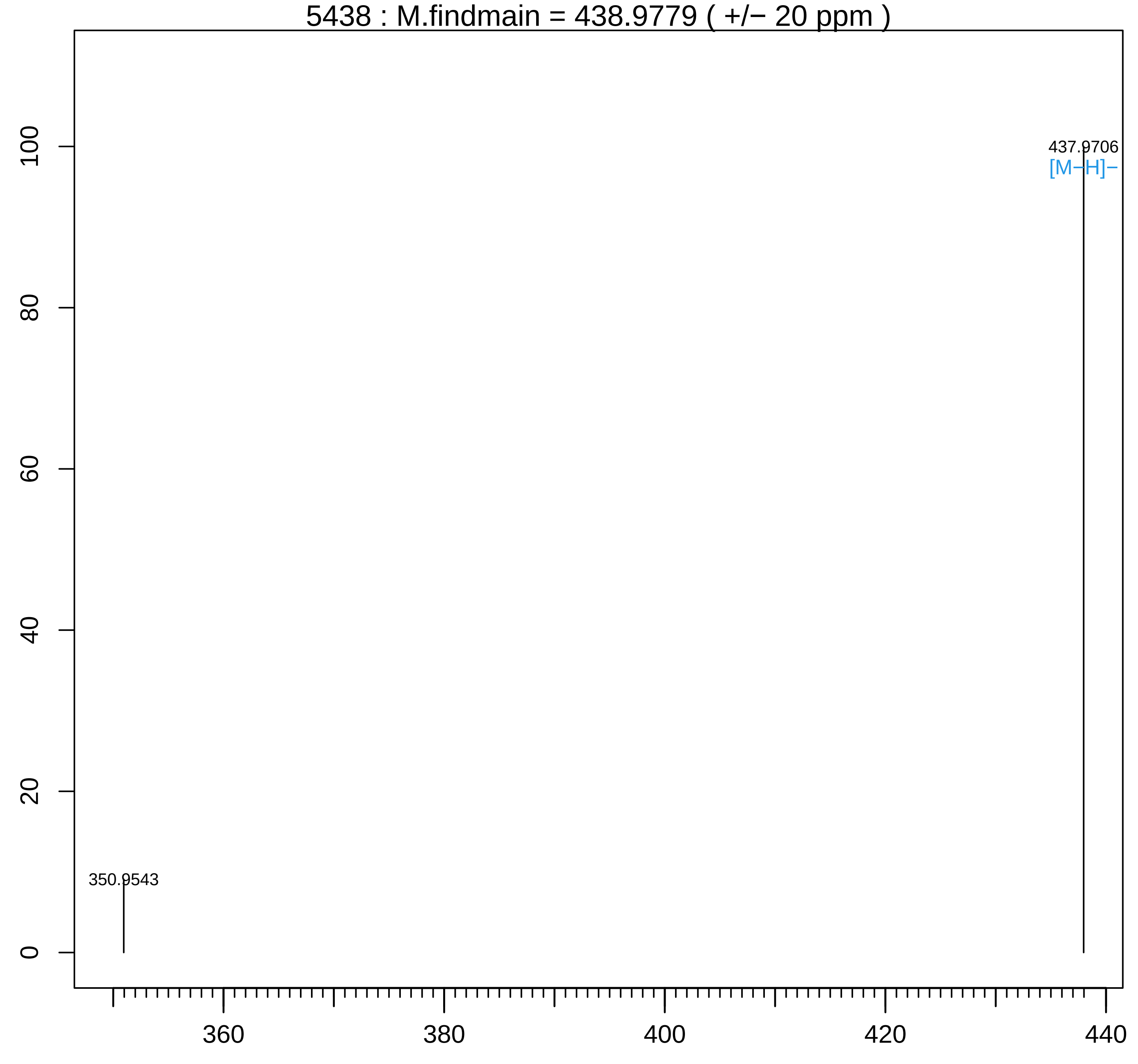
5438 : M.findmain = 438.9779 (+/− 20 ppm)
437.9706
[M−H]−
350.9543
0
20
40
60
80
100
360
380
400
420
440

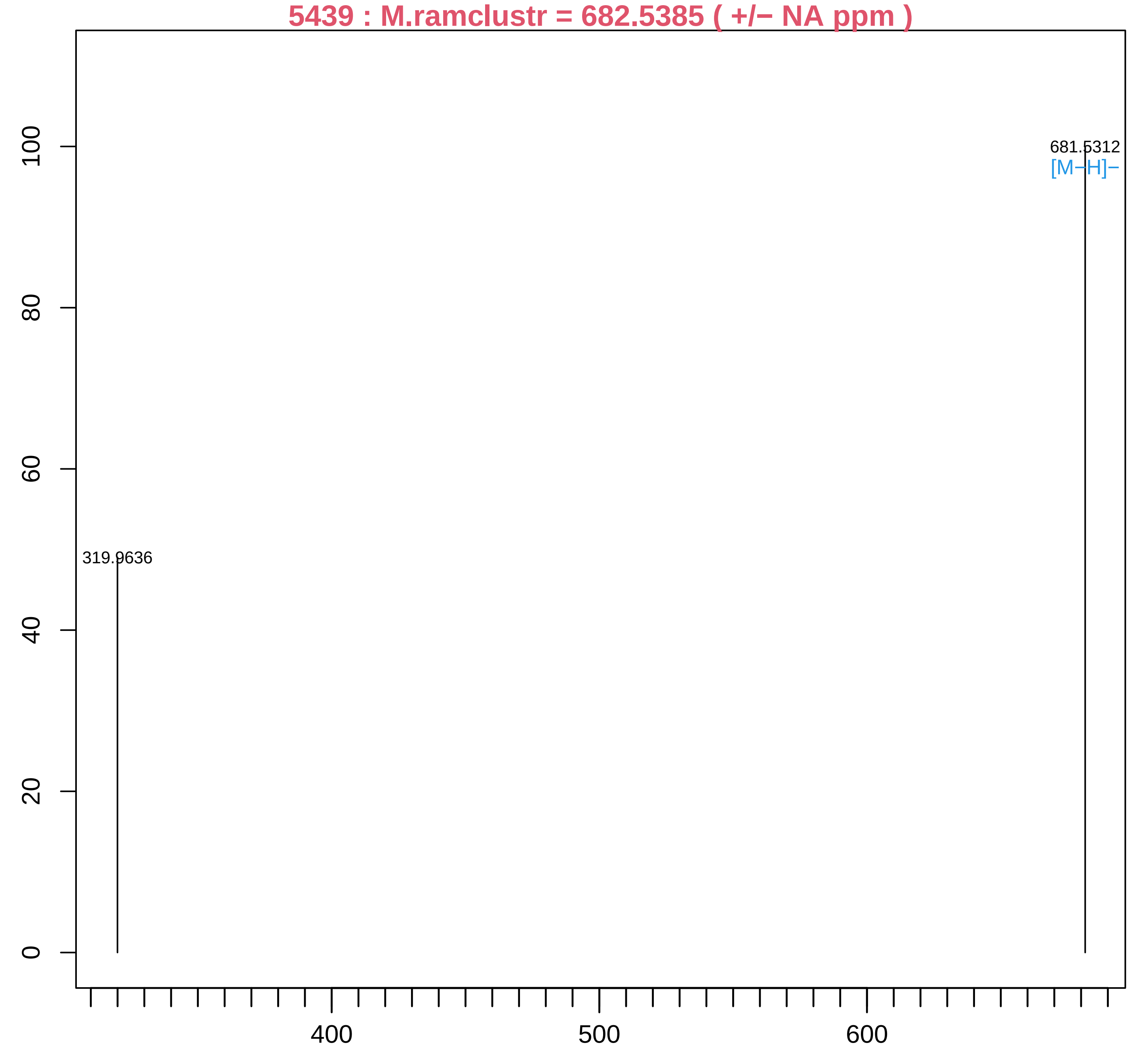
5439 : M.ramclustr = 682.5385 (+/− NA ppm)
681.5312
[M−H]−
319.9636
0
20
40
60
80
100
400
500
600

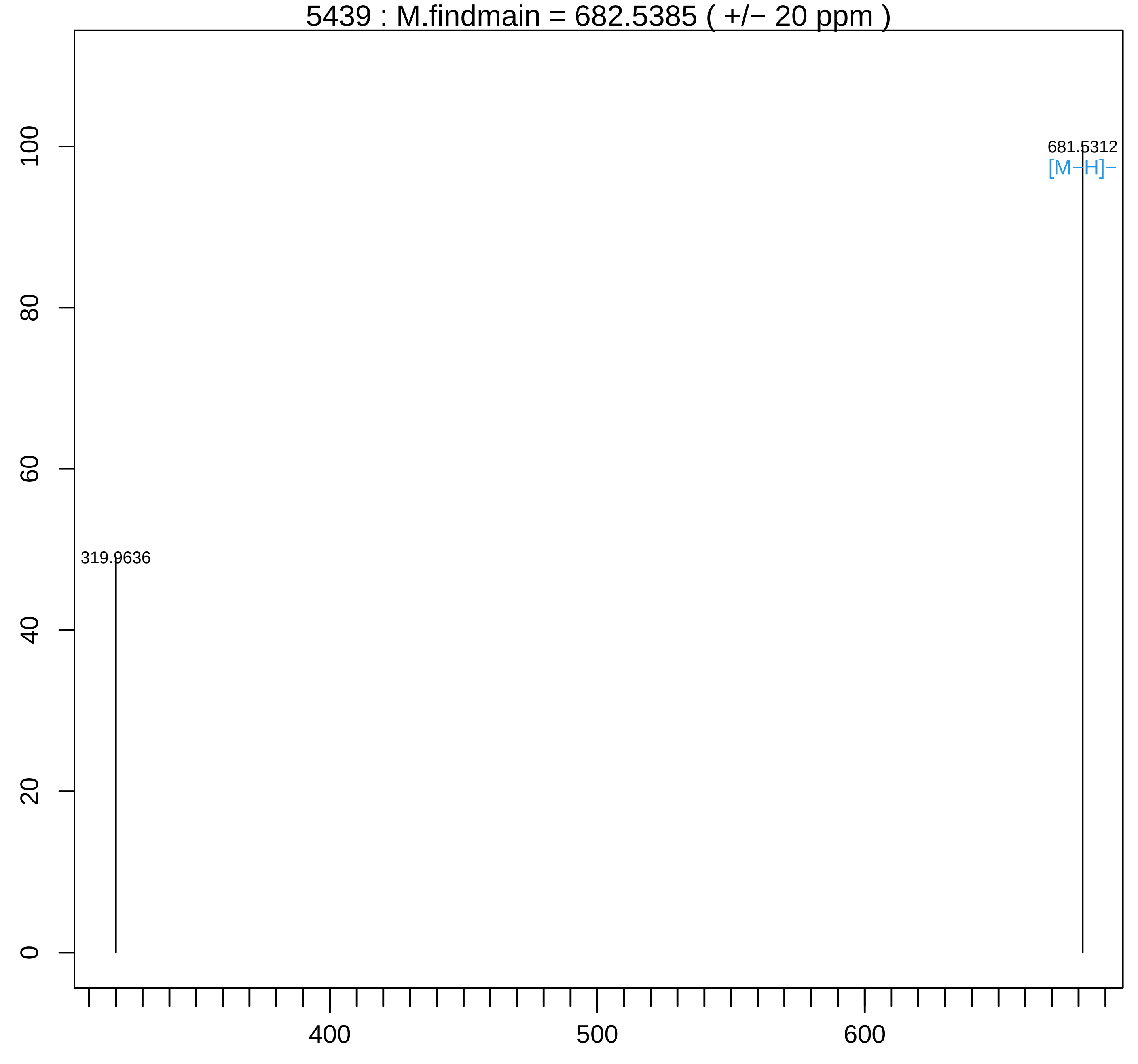
5439 : M.findmain = 682.5385 (+/− 20 ppm)
681.5312
[M−H]−
319.9636
0
20
40
60
80
100
400
500
600

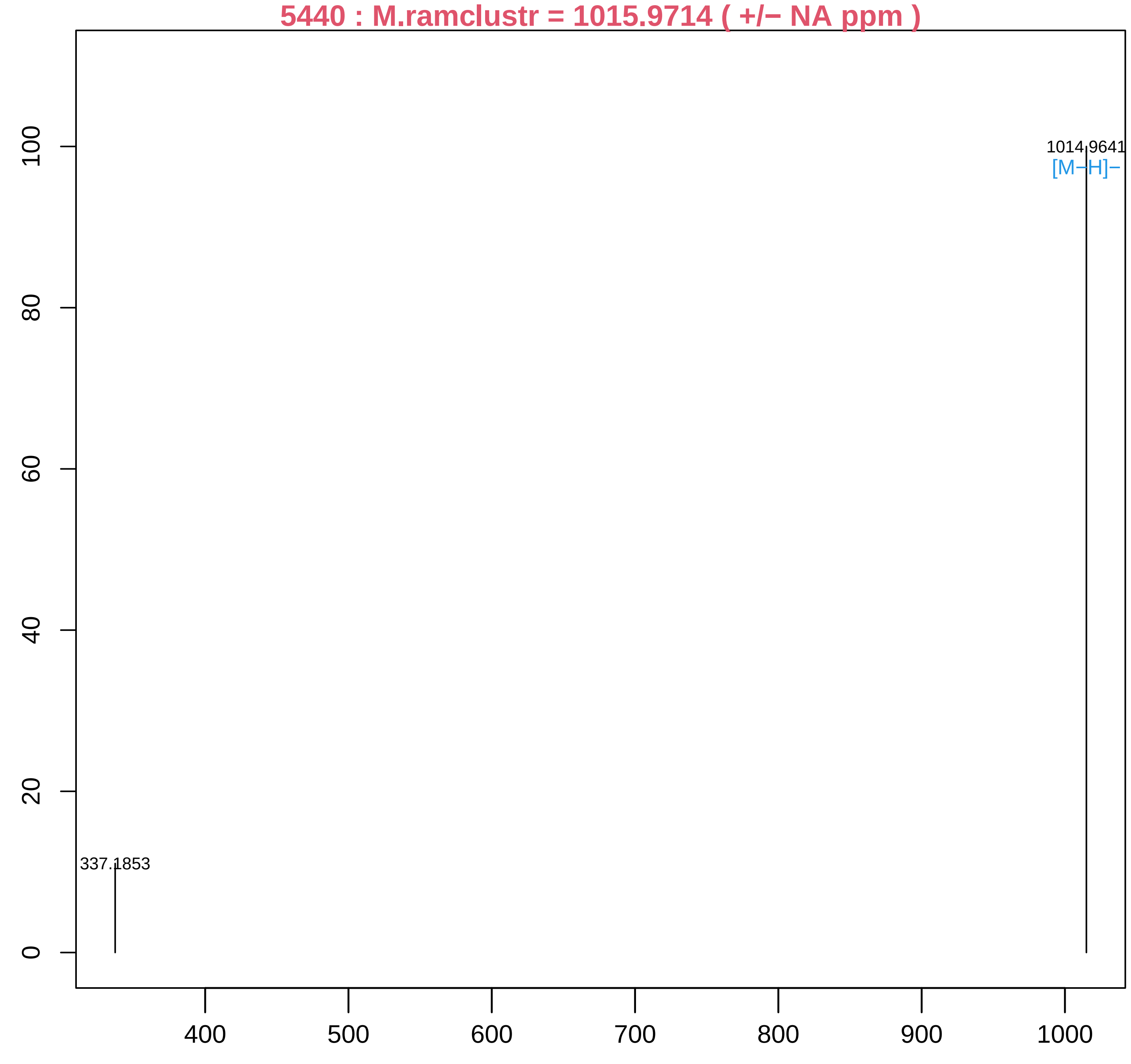
5440 : M.ramclustr = 1015.9714 (+/− NA ppm)
1014.9641
[M−H]−
337.1853
0
20
40
60
80
100
400
500
600
700
800
900
1000

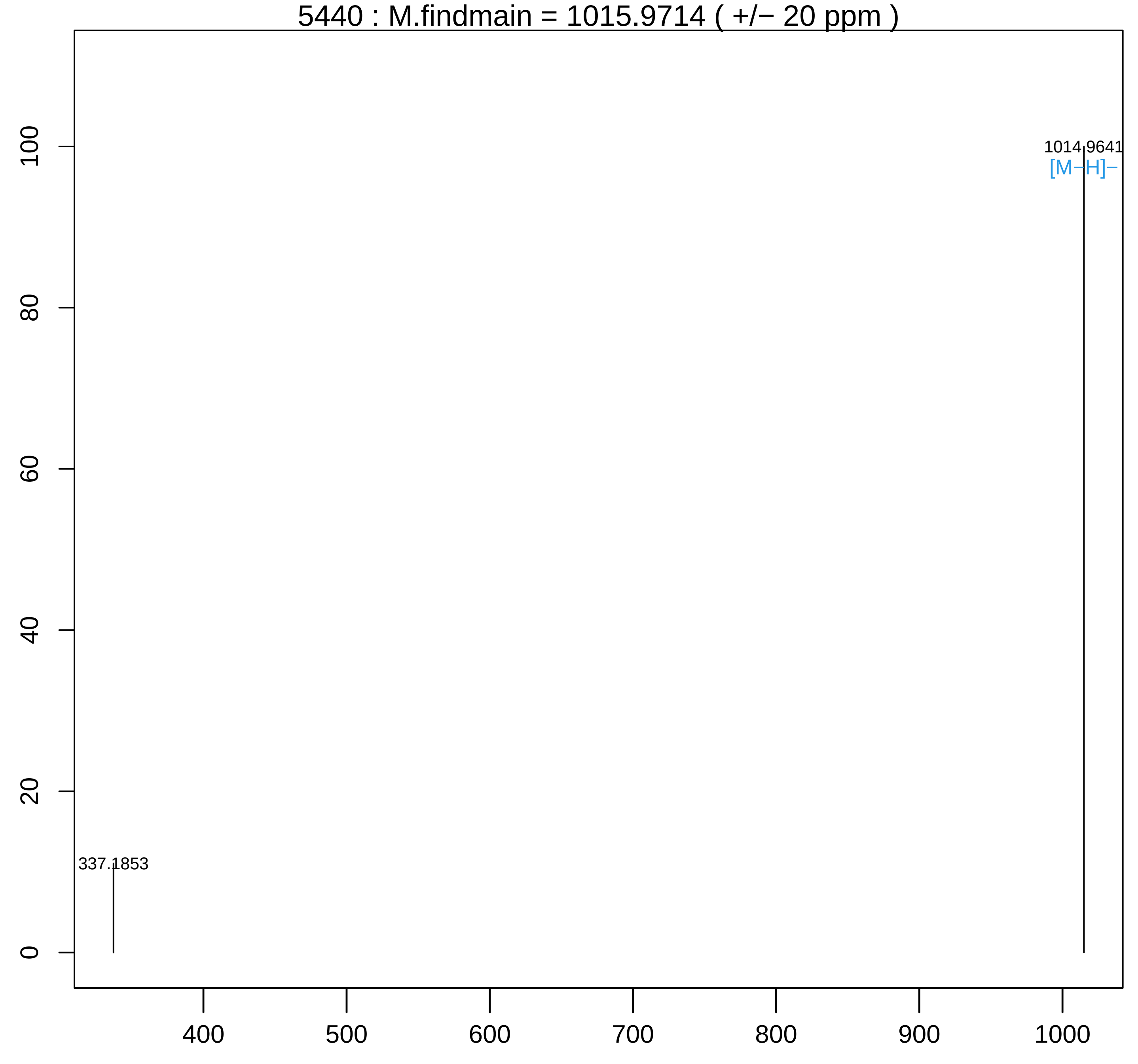
5440 : M.findmain = 1015.9714 (+/− 20 ppm)
1014.9641
[M−H]−
337.1853
0
20
40
60
80
100
400
500
600
700
800
900
1000

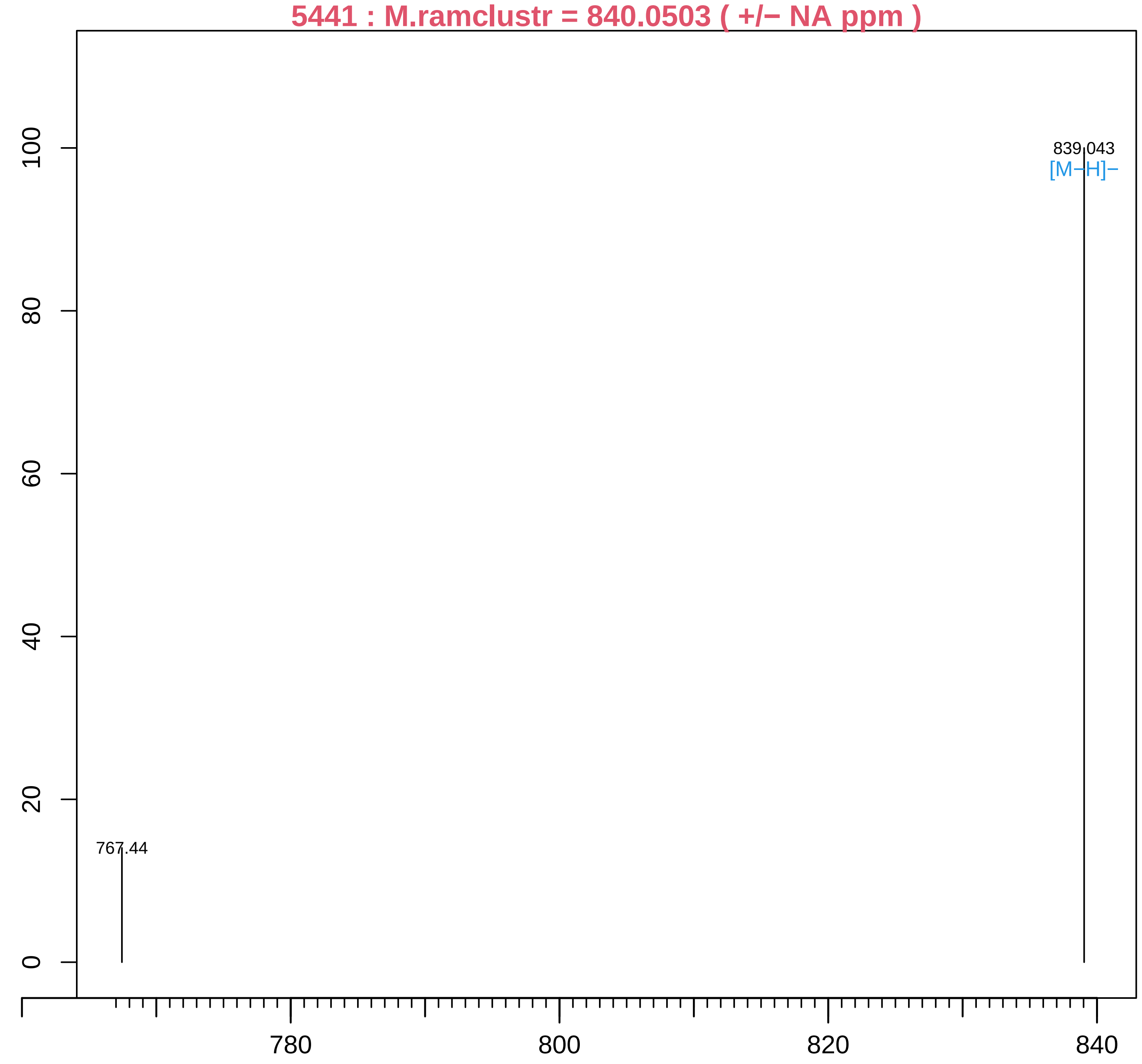
5441 : M.ramclustr = 840.0503 (+/− NA ppm)
839.043
[M−H]−
767.44
0
20
40
60
80
100
780
800
820
840

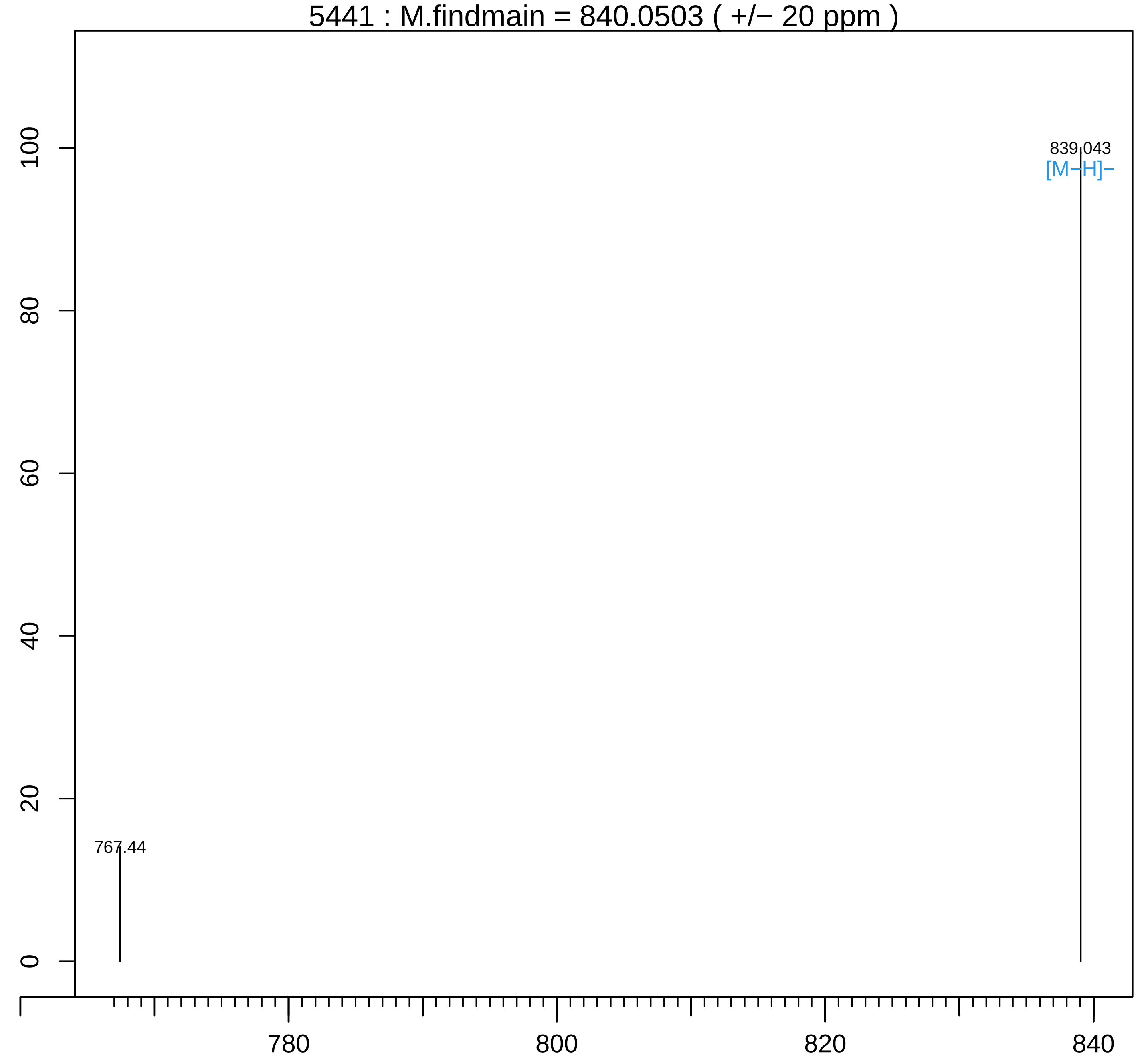
5441 : M.findmain = 840.0503 (+/− 20 ppm)
839.043
[M−H]−
767.44
0
20
40
60
80
100
780
800
820
840

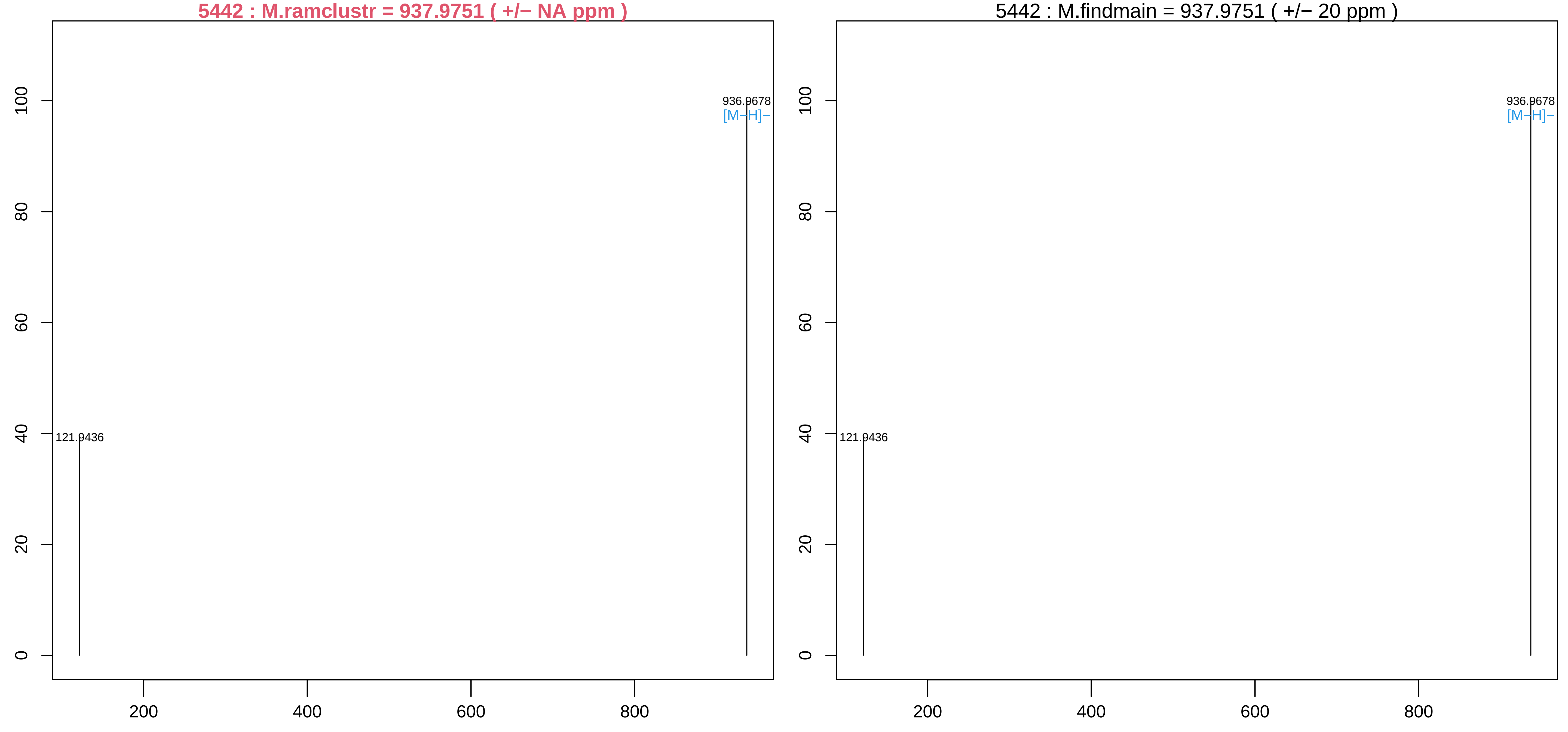

5442 : M.ramclustr = 937.9751 (+/− NA ppm)
936.9678
[M−H]−
121.9436
0
20
40
60
80
100
200
400
600
800
5442 : M.findmain = 937.9751 (+/− 20 ppm)
936.9678
[M−H]−
121.9436
0
20
40
60
80
100
200
400
600
800

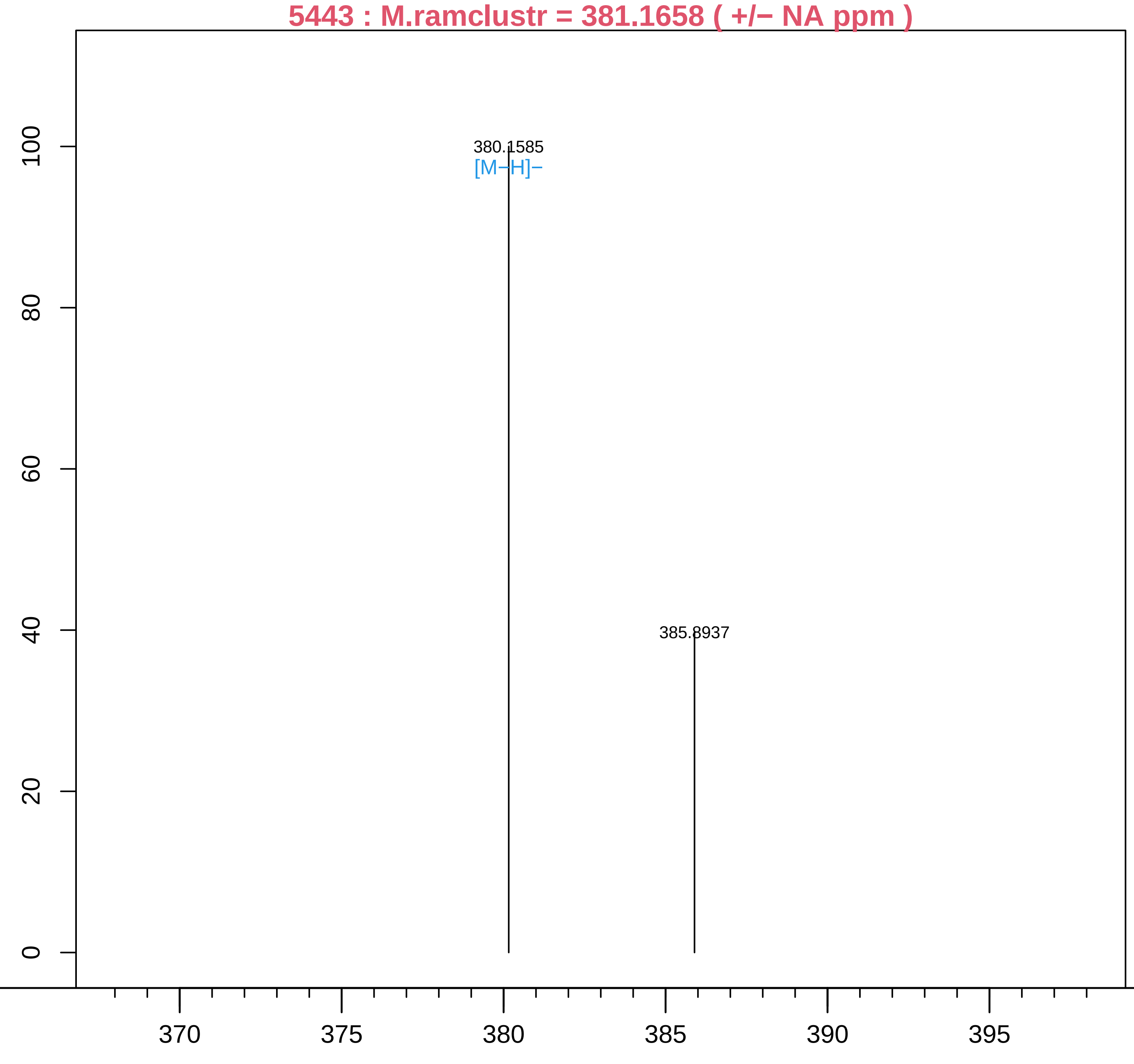

5443 : M.ramclustr = 381.1658 (+/− NA ppm)
380.1585
[M−H]−
385.8937
0
20
40
60
80
100
370
375
380
385
390
395

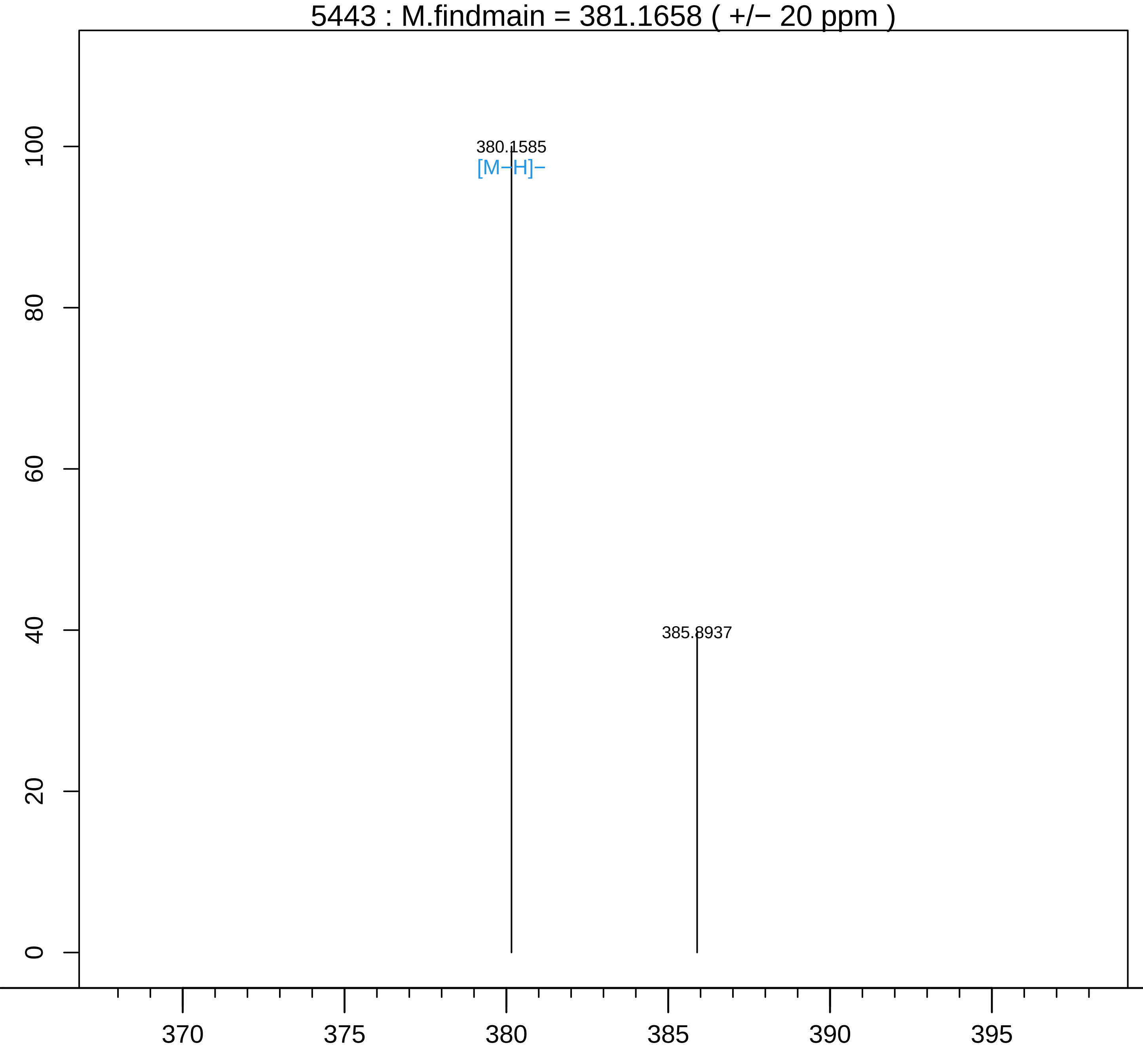

5443 : M.findmain = 381.1658 (+/− 20 ppm)
380.1585
[M−H]−
385.8937
0
20
40
60
80
100
370
375
380
385
390
395

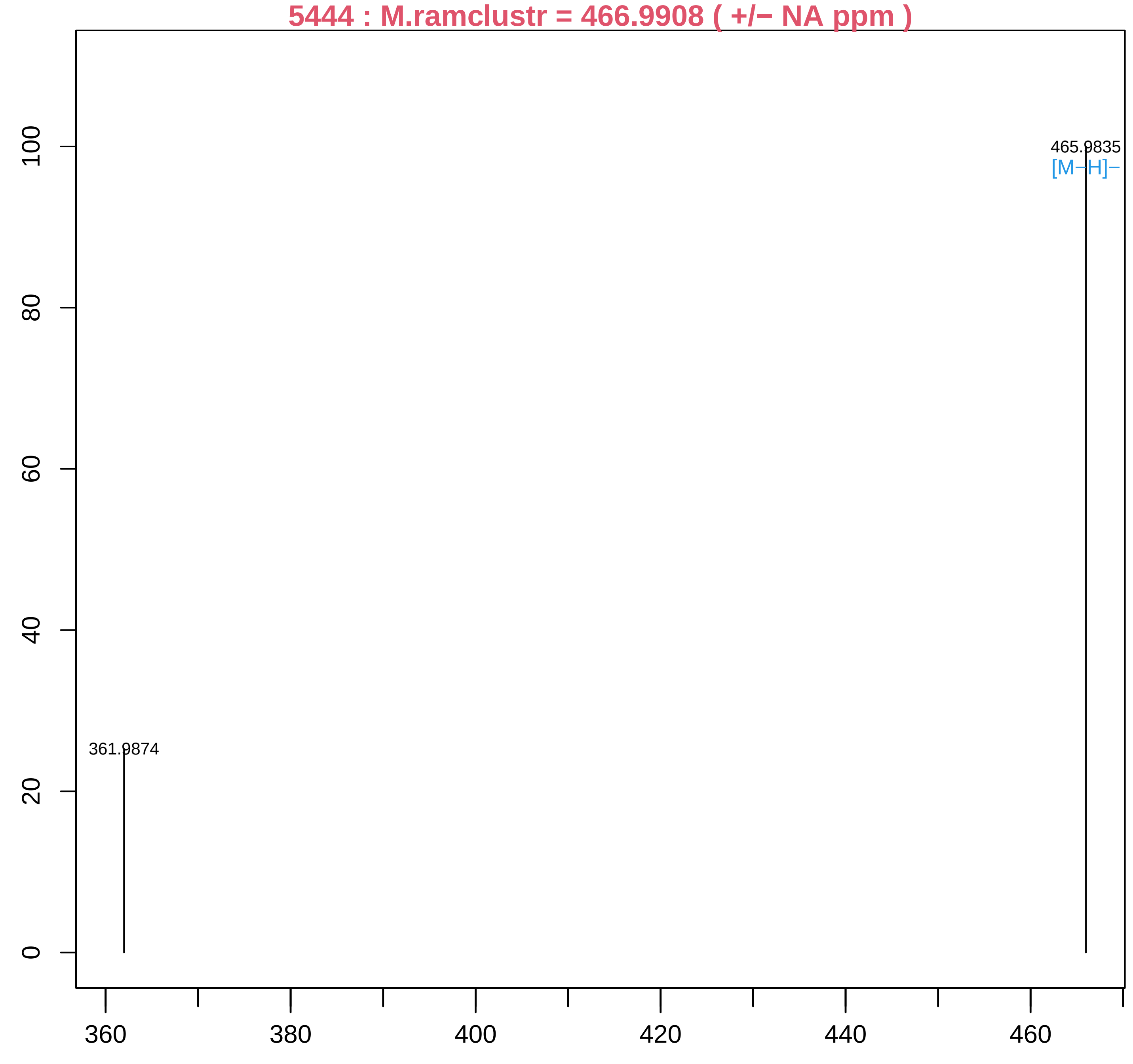
5444 : M.ramclustr = 466.9908 (+/− NA ppm)
465.9835
[M−H]−
361.9874
0
20
40
60
80
100
360
380
400
420
440
460

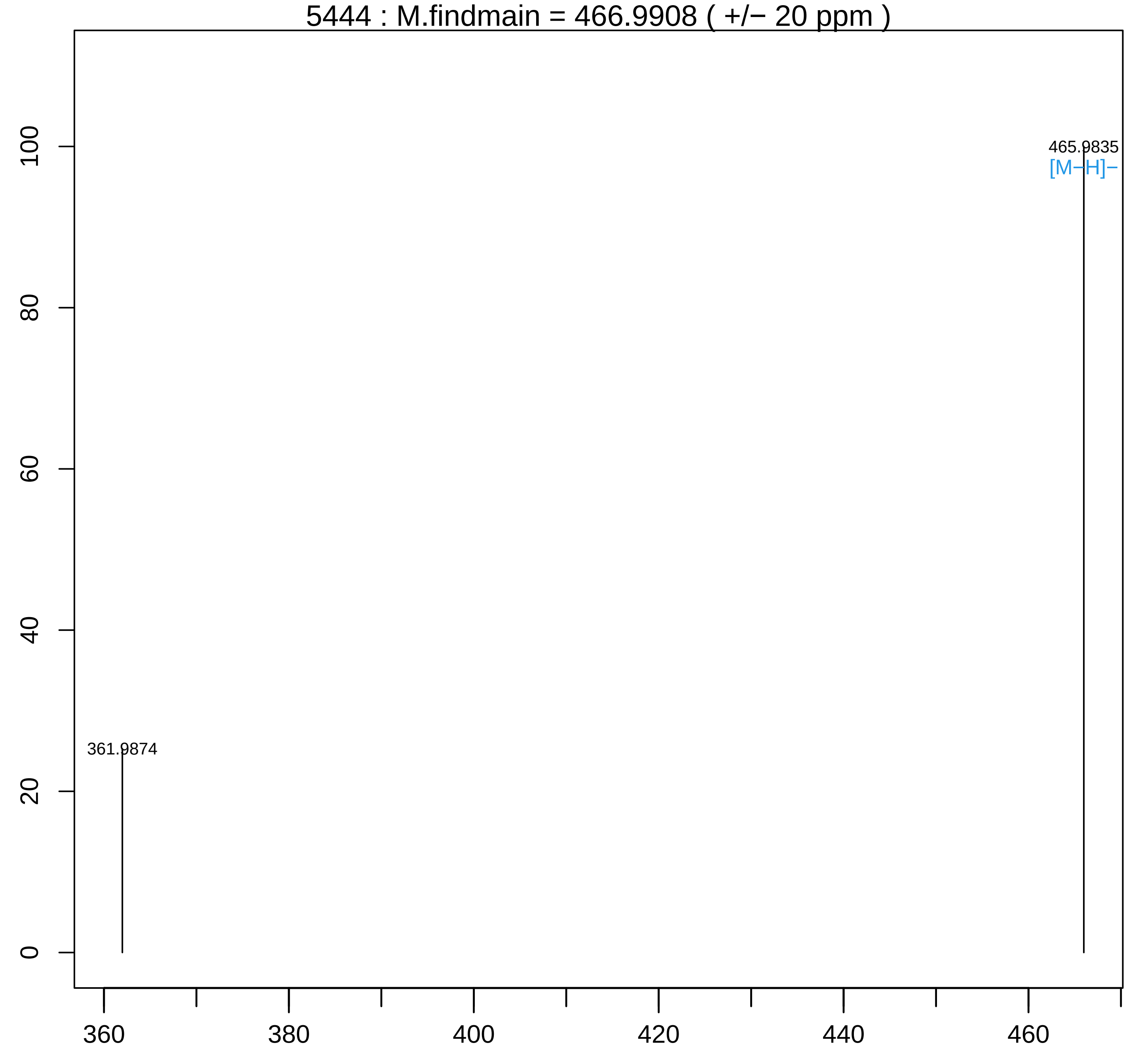
5444 : M.findmain = 466.9908 (+/− 20 ppm)
465.9835
[M−H]−
361.9874
0
20
40
60
80
100
360
380
400
420
440
460

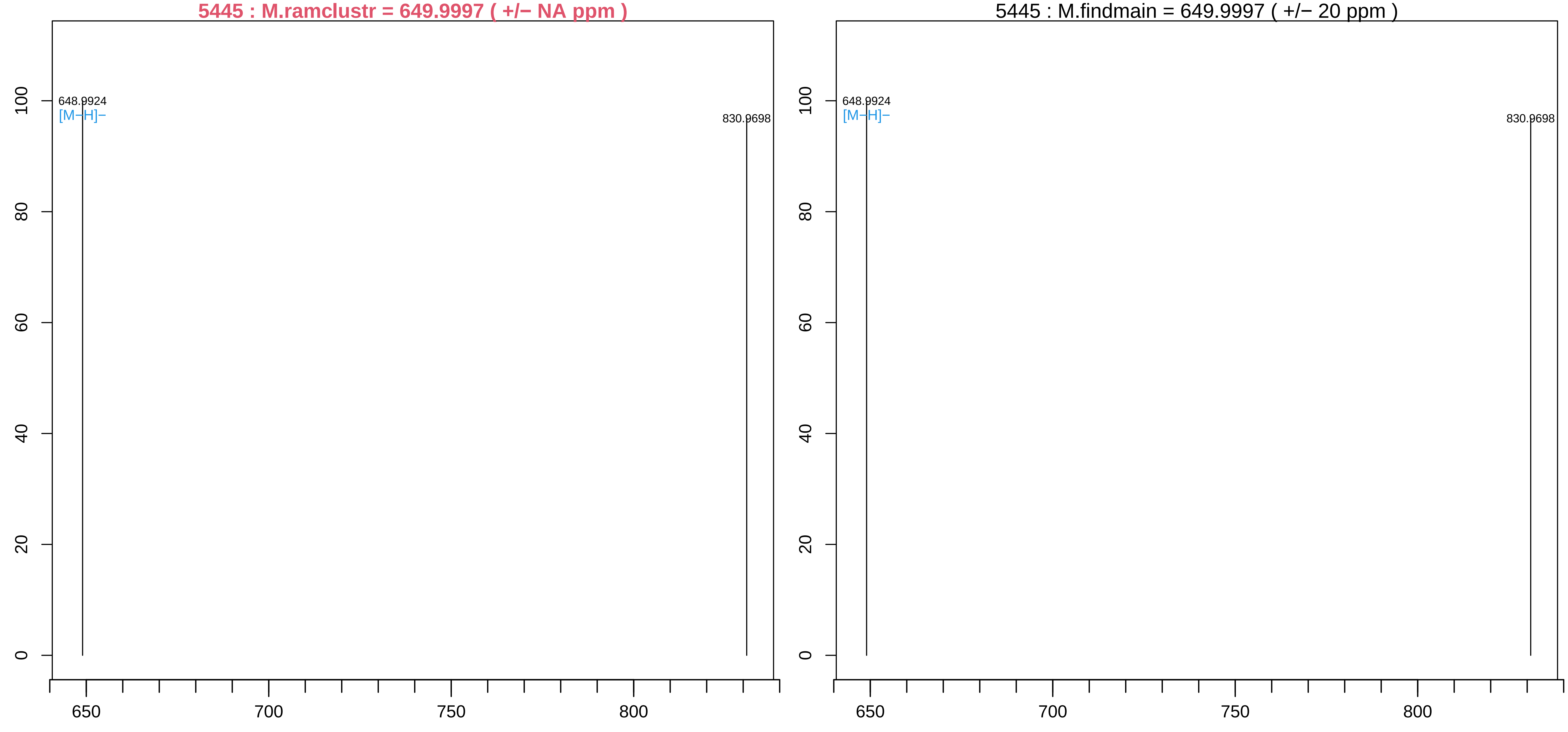

5445 : M.ramclustr = 649.9997 (+/− NA ppm)
648.9924
[M−H]−
830.9698
5445 : M.findmain = 649.9997 (+/− 20 ppm)
648.9924
[M−H]−
830.9698

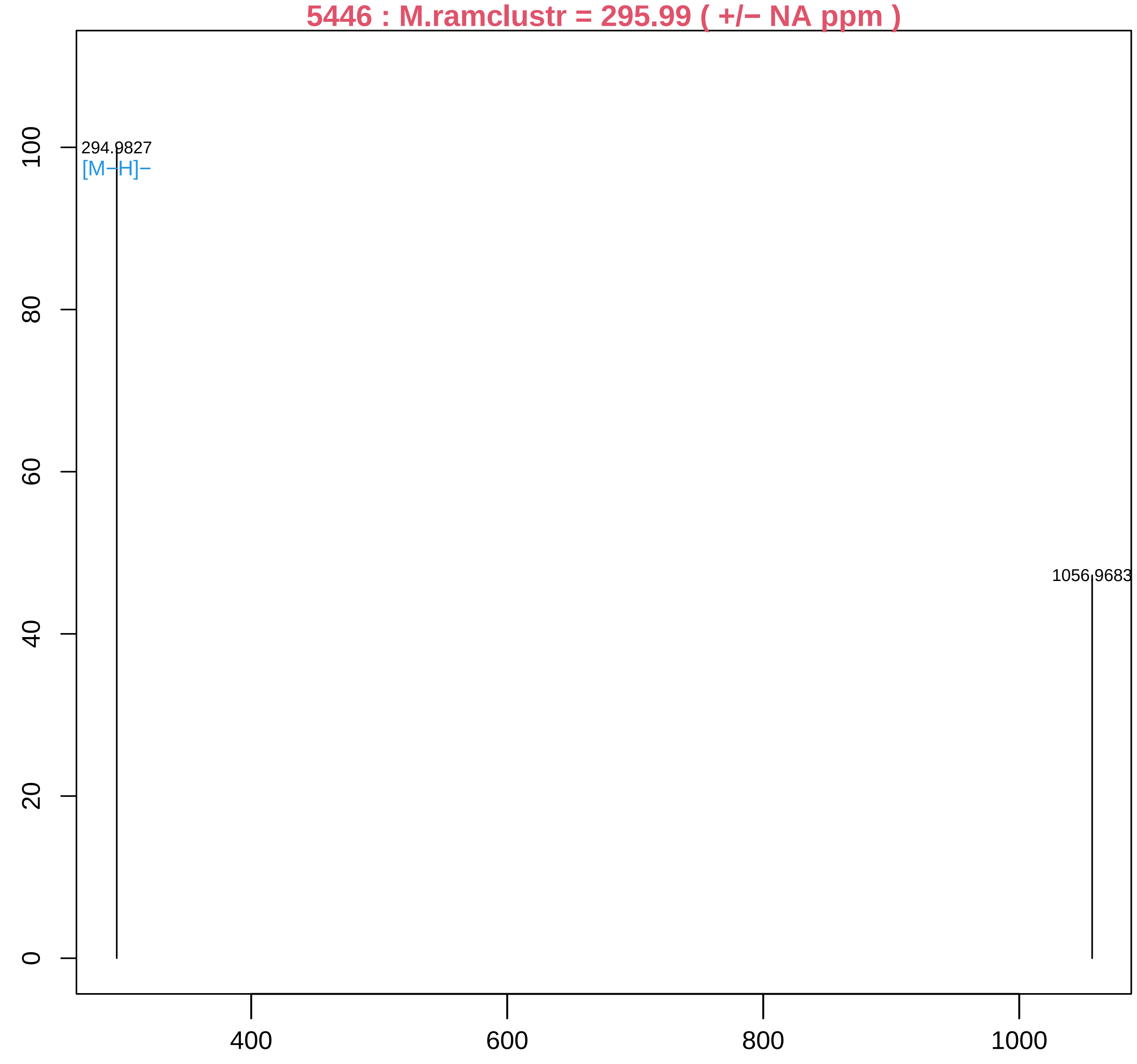
5446 : M.ramclustr = 295.99 (+/− NA ppm)
294.9827
[M−H]−
1056.9683
0
20
40
60
80
100
400
600
800
1000

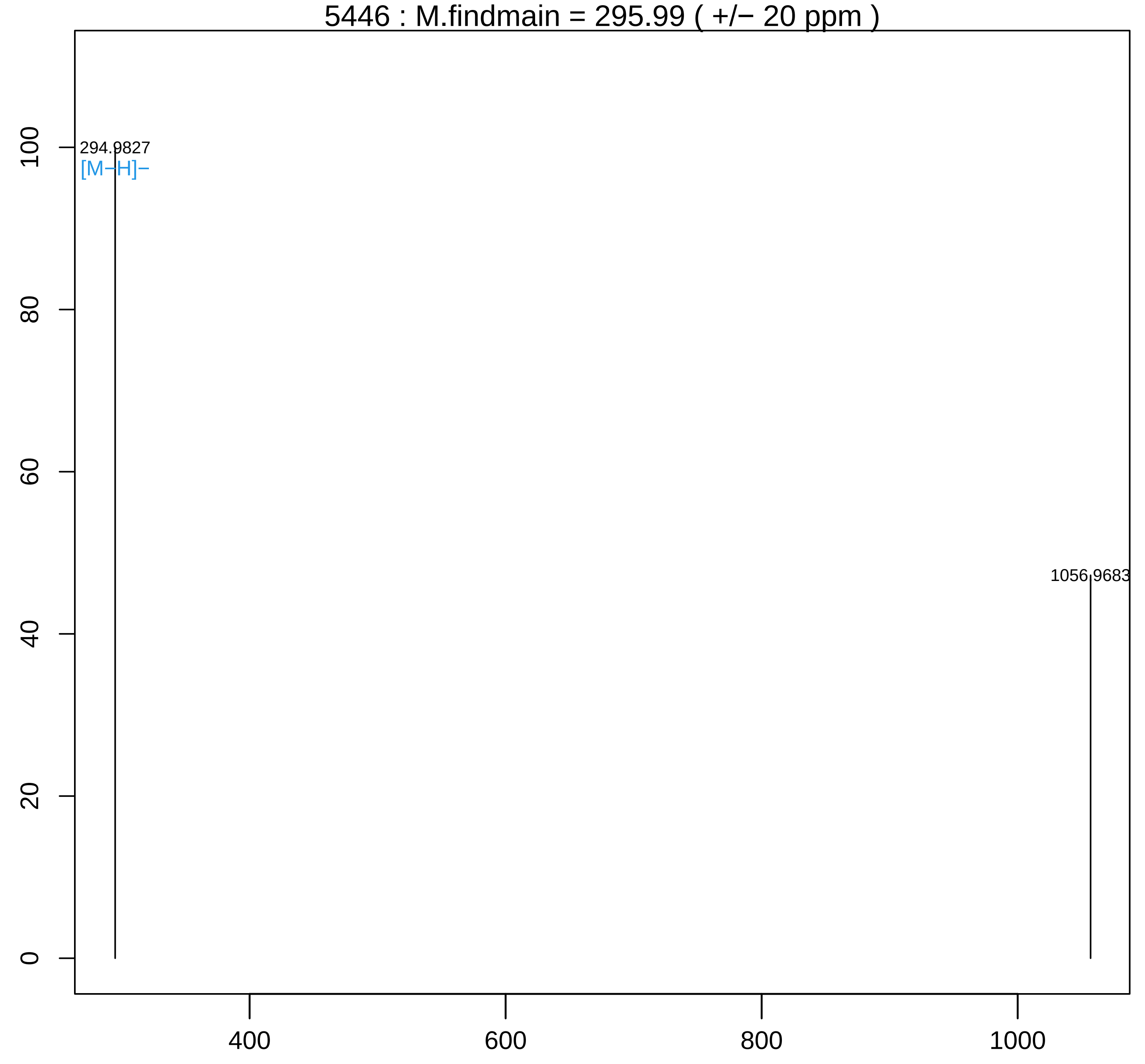
5446 : M.findmain = 295.99 (+/− 20 ppm)
294.9827
[M−H]−
1056.9683
0
20
40
60
80
100
400
600
800
1000

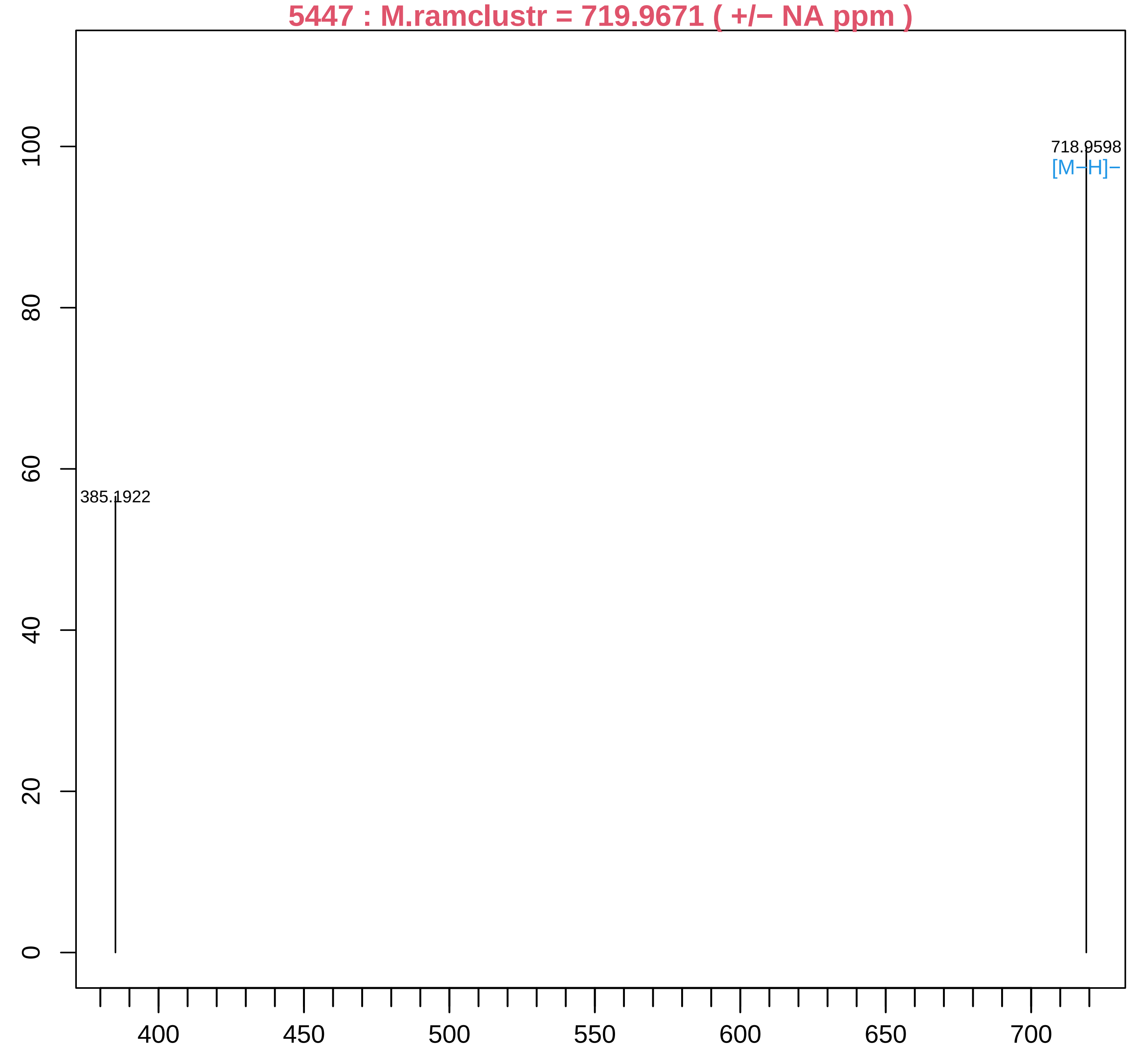
5447 : M.ramclustr = 719.9671 (+/− NA ppm)
718.9598
[M−H]−
385.1922
0
20
40
60
80
100
400
450
500
550
600
650
700

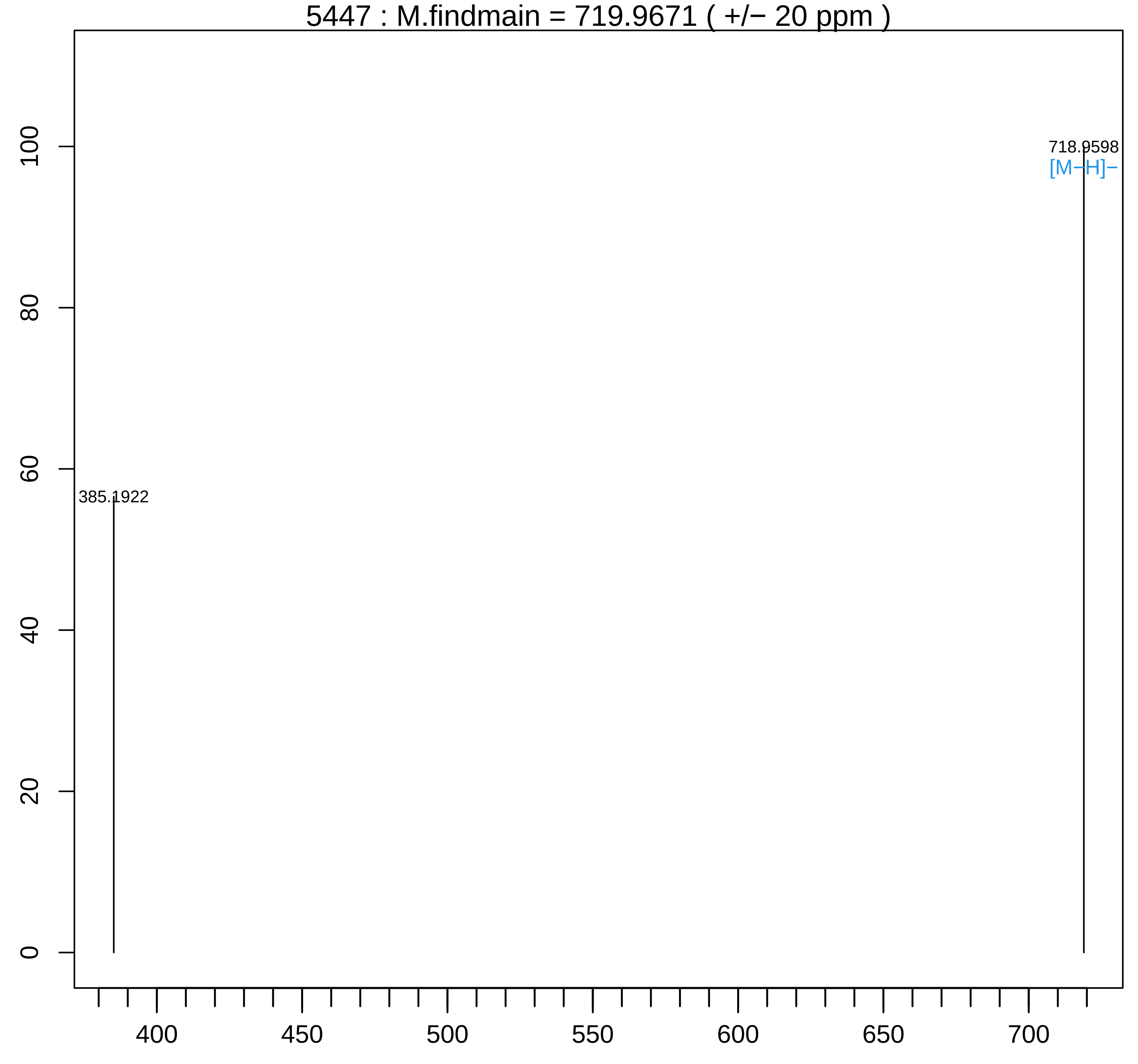
5447 : M.findmain = 719.9671 (+/− 20 ppm)
718.9598
[M−H]−
385.1922
0
20
40
60
80
100
400
450
500
550
600
650
700

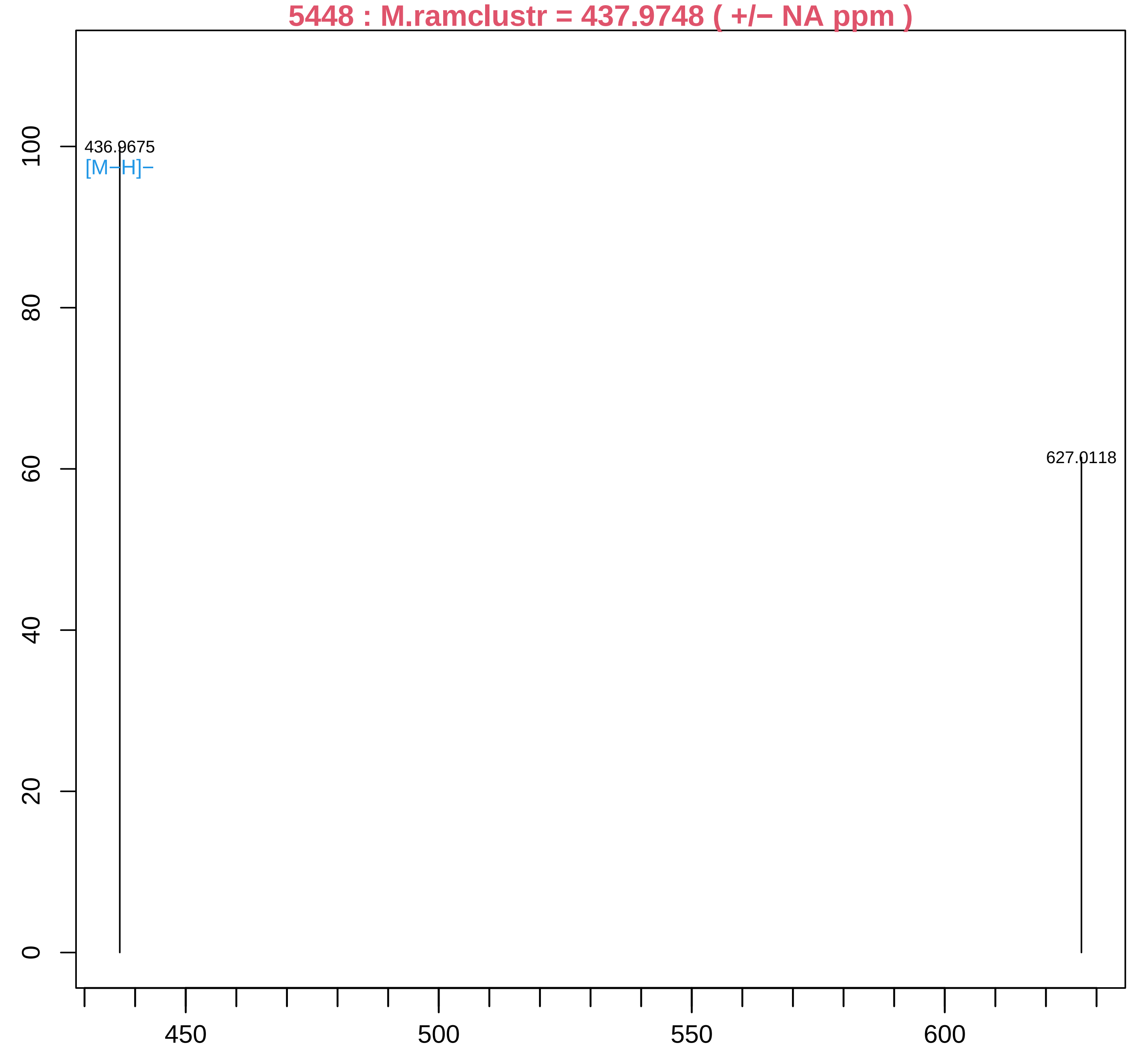
5448 : M.ramclustr = 437.9748 (+/− NA ppm)
436.9675
[M−H]−
627.0118
0
20
40
60
80
100
450
500
550
600

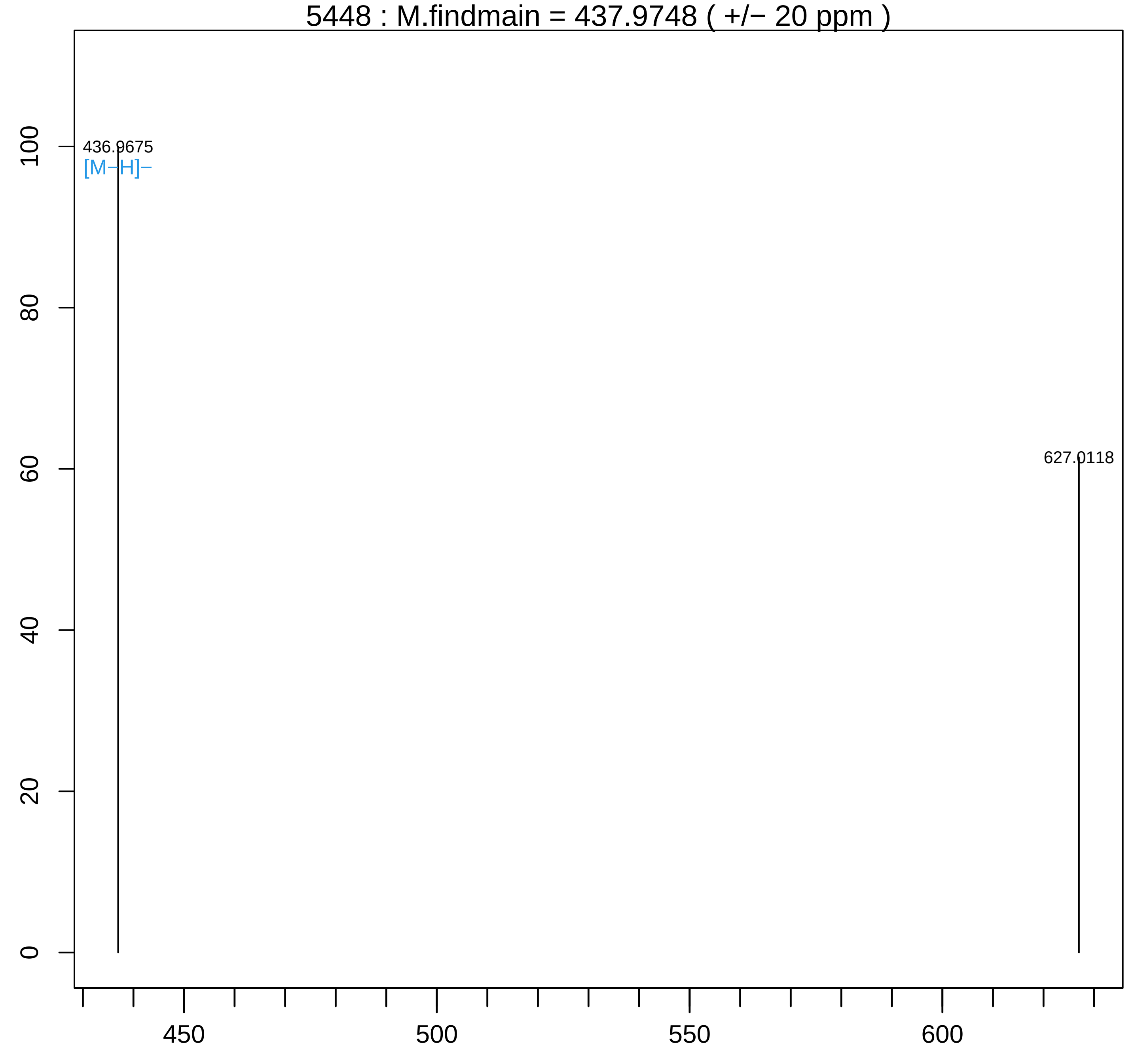
5448 : M.findmain = 437.9748 (+/− 20 ppm)
436.9675
[M−H]−
627.0118
0
20
40
60
80
100
450
500
550
600

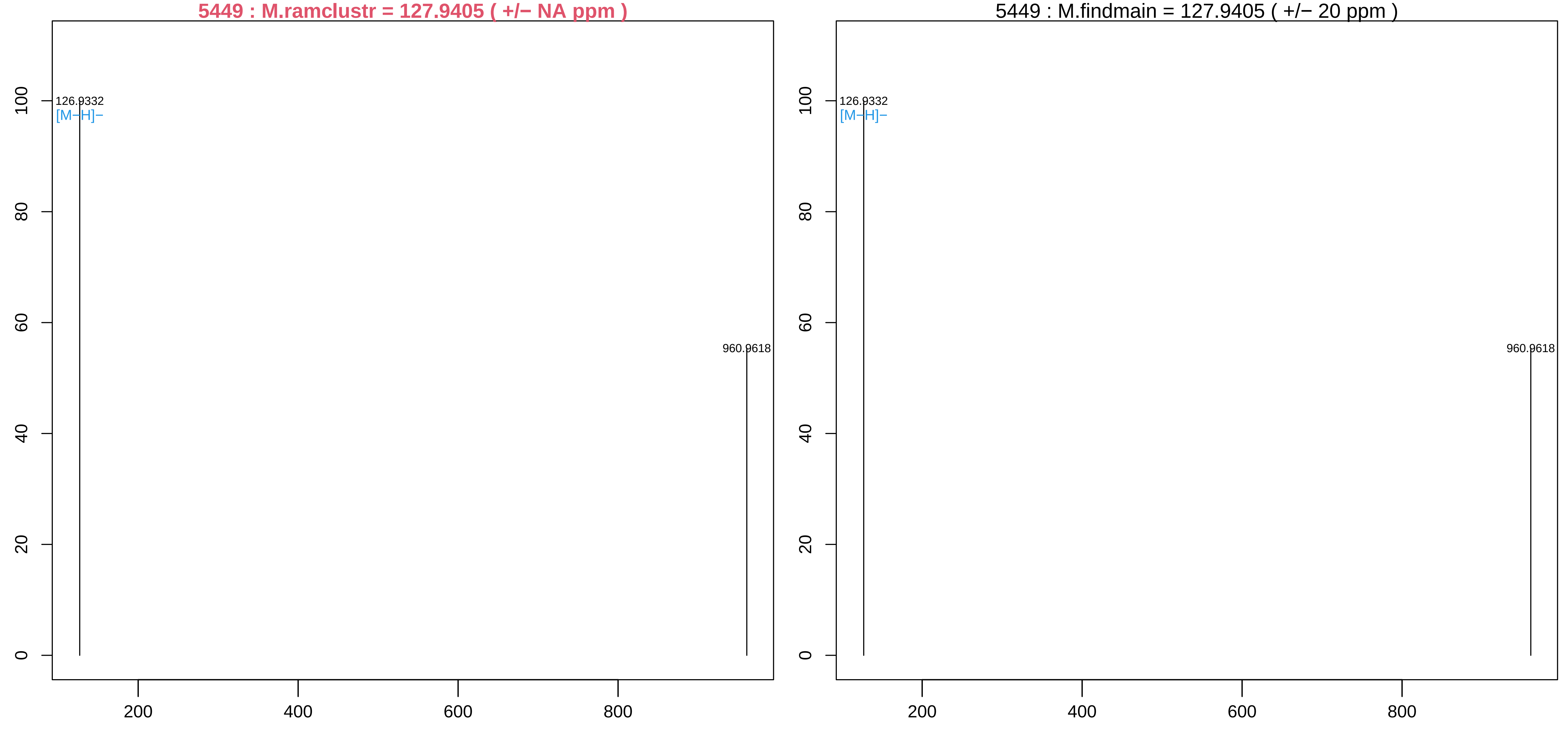
5449 : M.ramclustr = 127.9405 (+/− NA ppm)
126.9332
[M−H]−
960.9618
0
20
40
60
80
100
200
400
600
800
5449 : M.findmain = 127.9405 (+/− 20 ppm)
126.9332
[M−H]−
960.9618
0
20
40
60
80
100
200
400
600
800

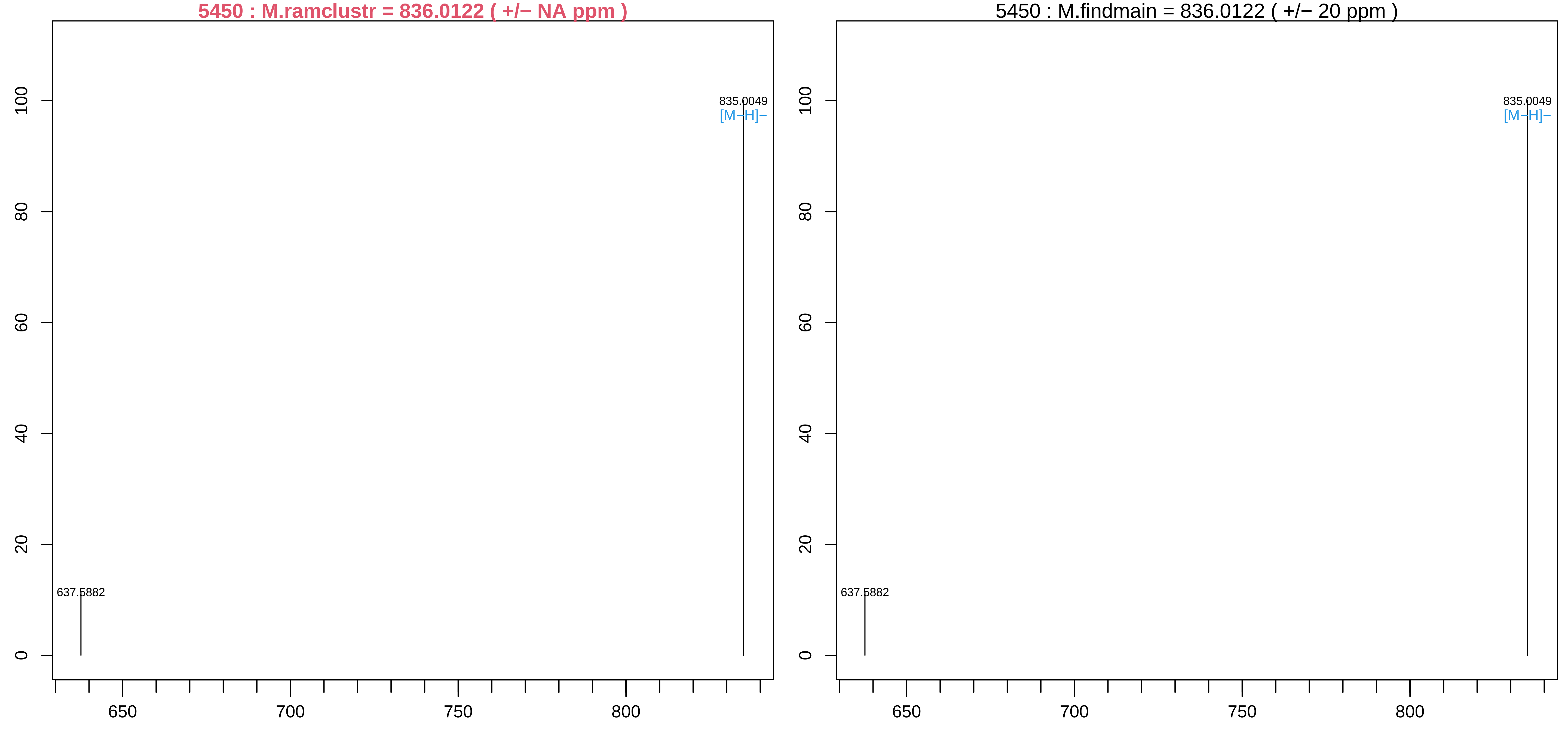
5450 : M.ramclustr = 836.0122 (+/− NA ppm)
835.0049
[M−H]−
637.5882
0
20
40
60
80
100
650
700
750
800
5450 : M.findmain = 836.0122 (+/− 20 ppm)
835.0049
[M−H]−
637.5882
0
20
40
60
80
100
650
700
750
800

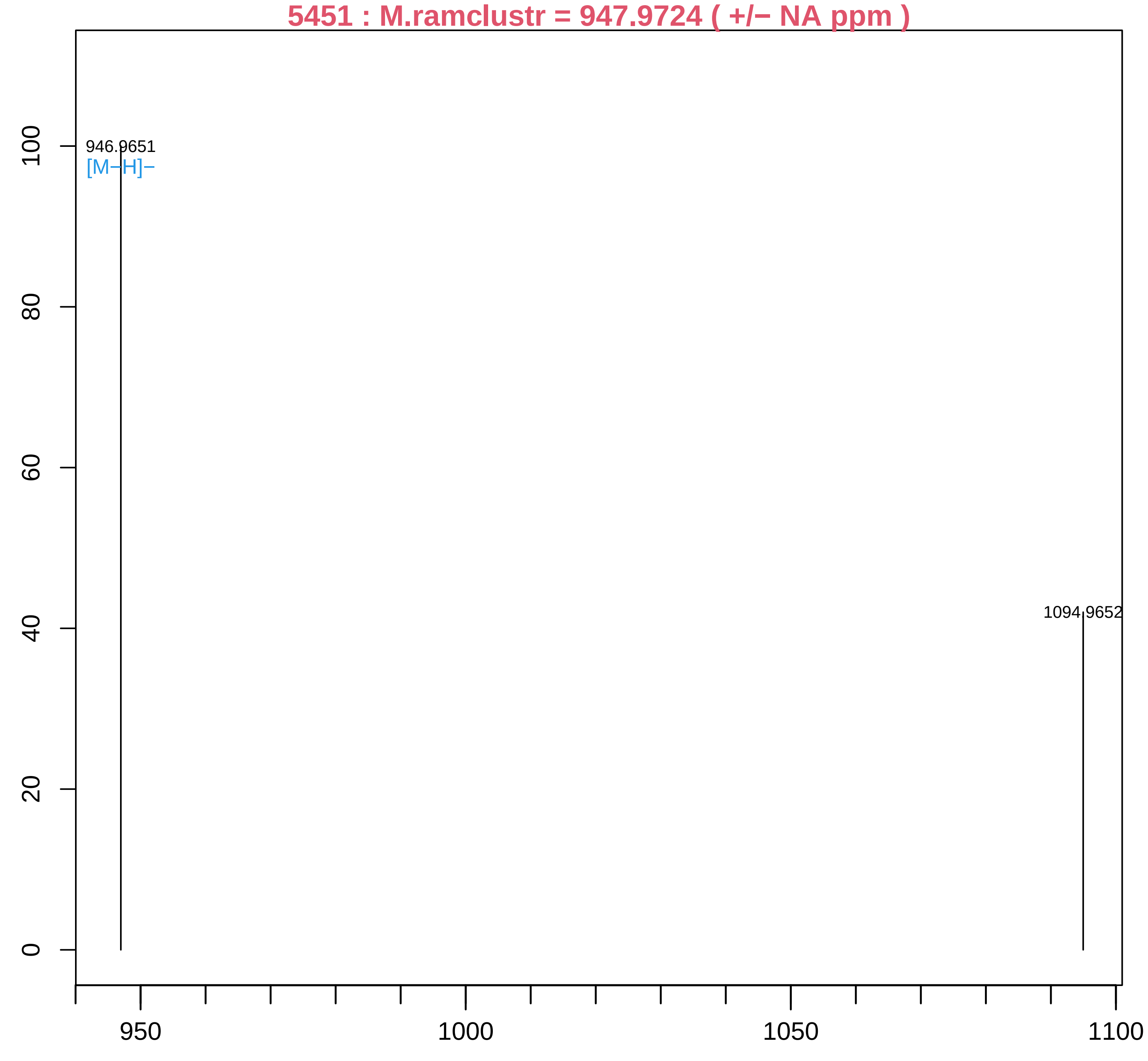
5451 : M.ramclustr = 947.9724 (+/− NA ppm)
946.9651
[M−H]−
1094.9652
0
20
40
60
80
100
950
1000
1050
1100

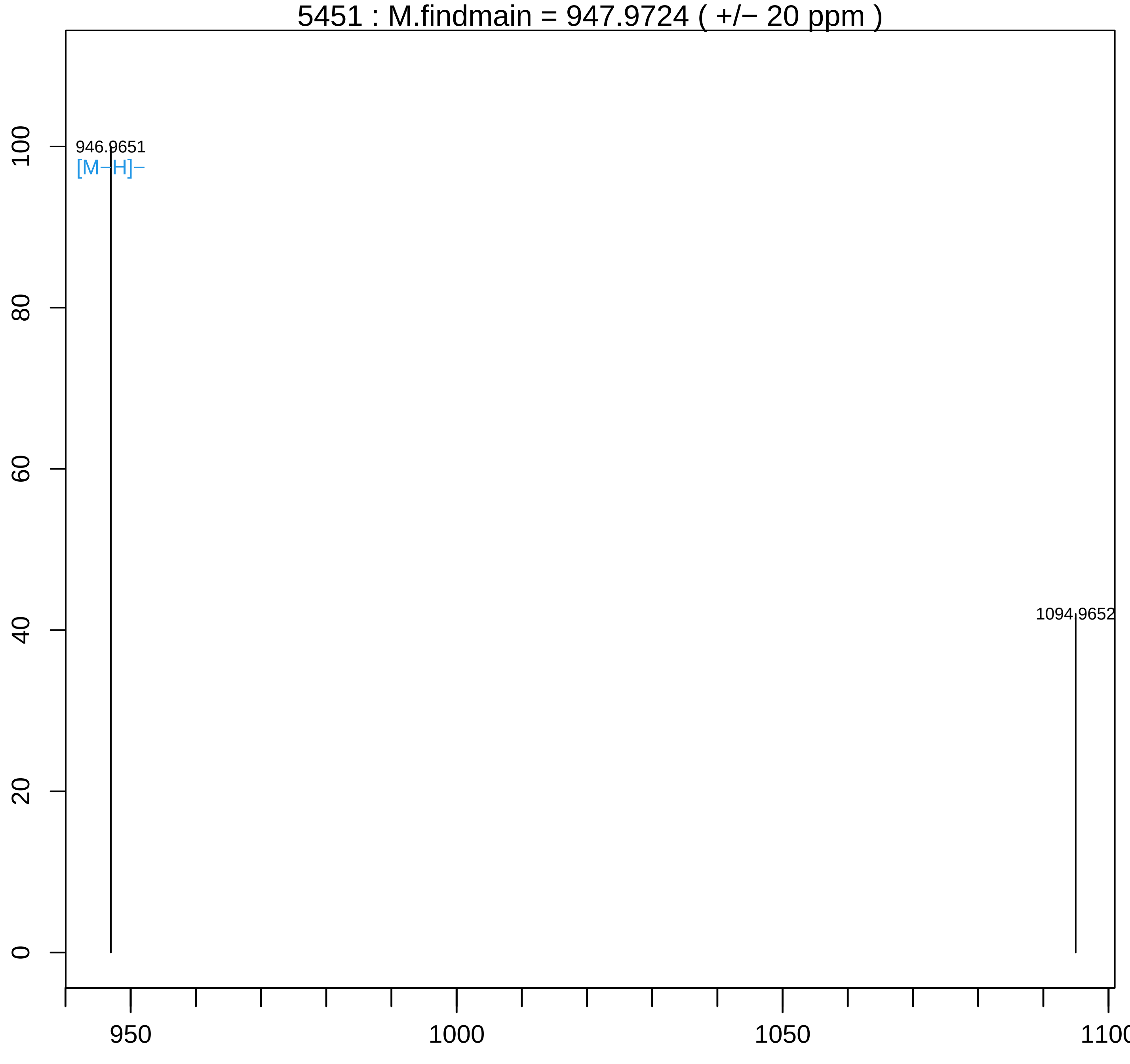
5451 : M.findmain = 947.9724 (+/− 20 ppm)
946.9651
[M−H]−
1094.9652
0
20
40
60
80
100
950
1000
1050

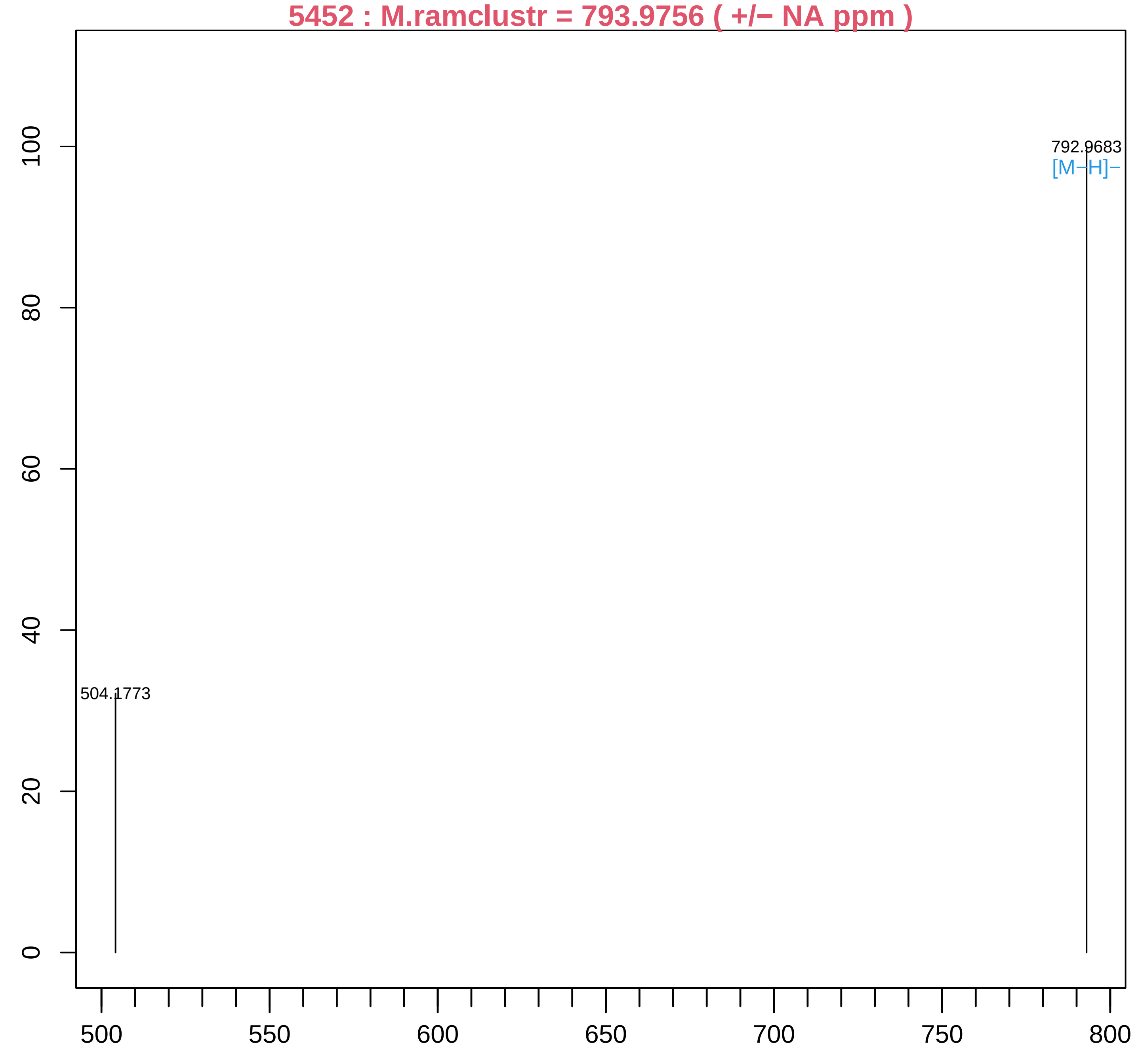

5452 : M.ramclustr = 793.9756 (+/− NA ppm)
792.9683
[M−H]−
504.1773
0
20
40
60
80
100
500
550
600
650
700
750
800

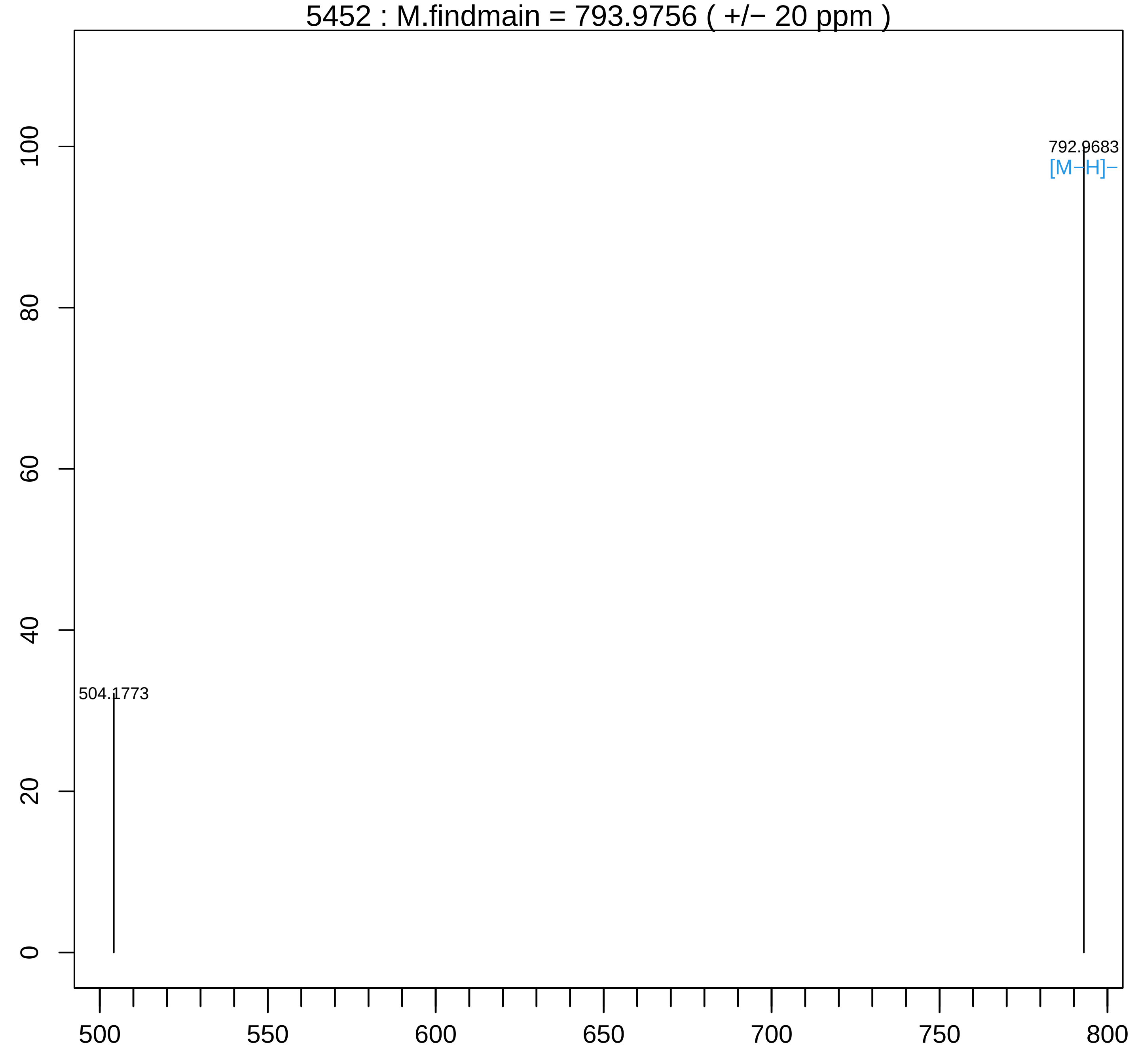

5452 : M.findmain = 793.9756 (+/− 20 ppm)
792.9683
[M−H]−
504.1773
0
20
40
60
80
100
500
550
600
650
700
750
800

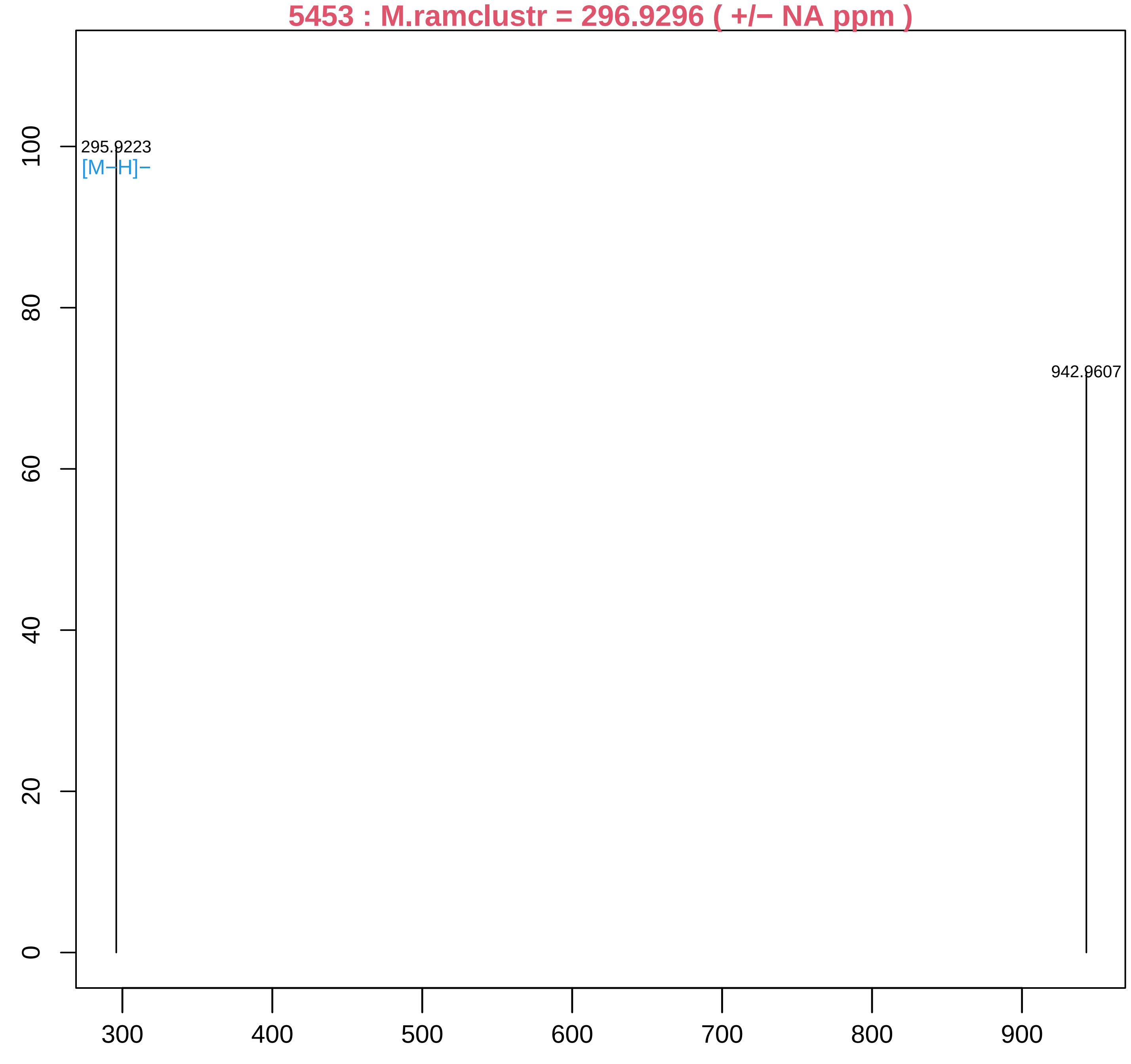
5453 : M.ramclustr = 296.9296 (+/− NA ppm)
295.9223
[M−H]−
942.9607
0
20
40
60
80
100
300
400
500
600
700
800
900

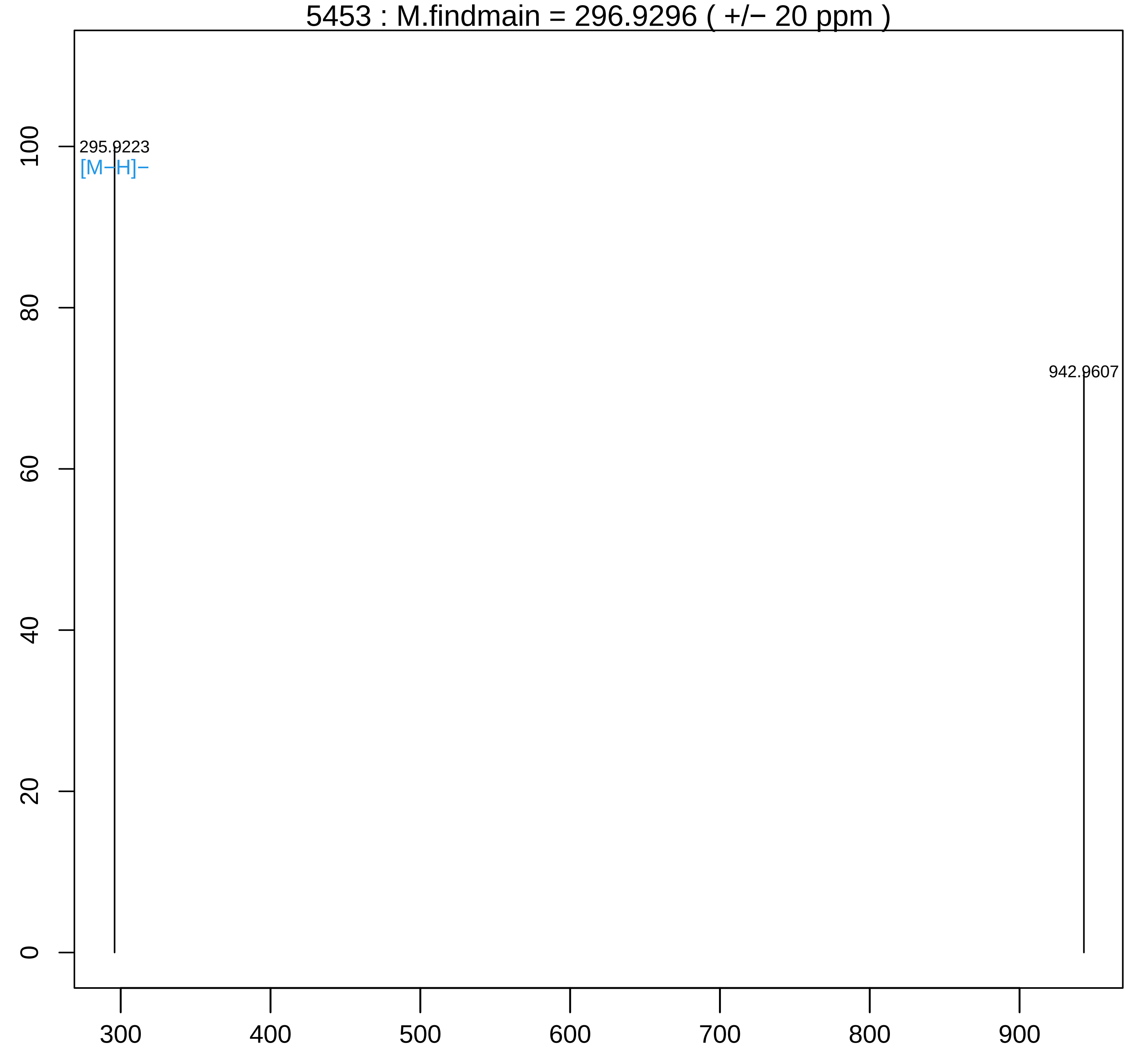
5453 : M.findmain = 296.9296 (+/− 20 ppm)
295.9223
[M−H]−
942.9607
0
20
40
60
80
100
300
400
500
600
700
800
900

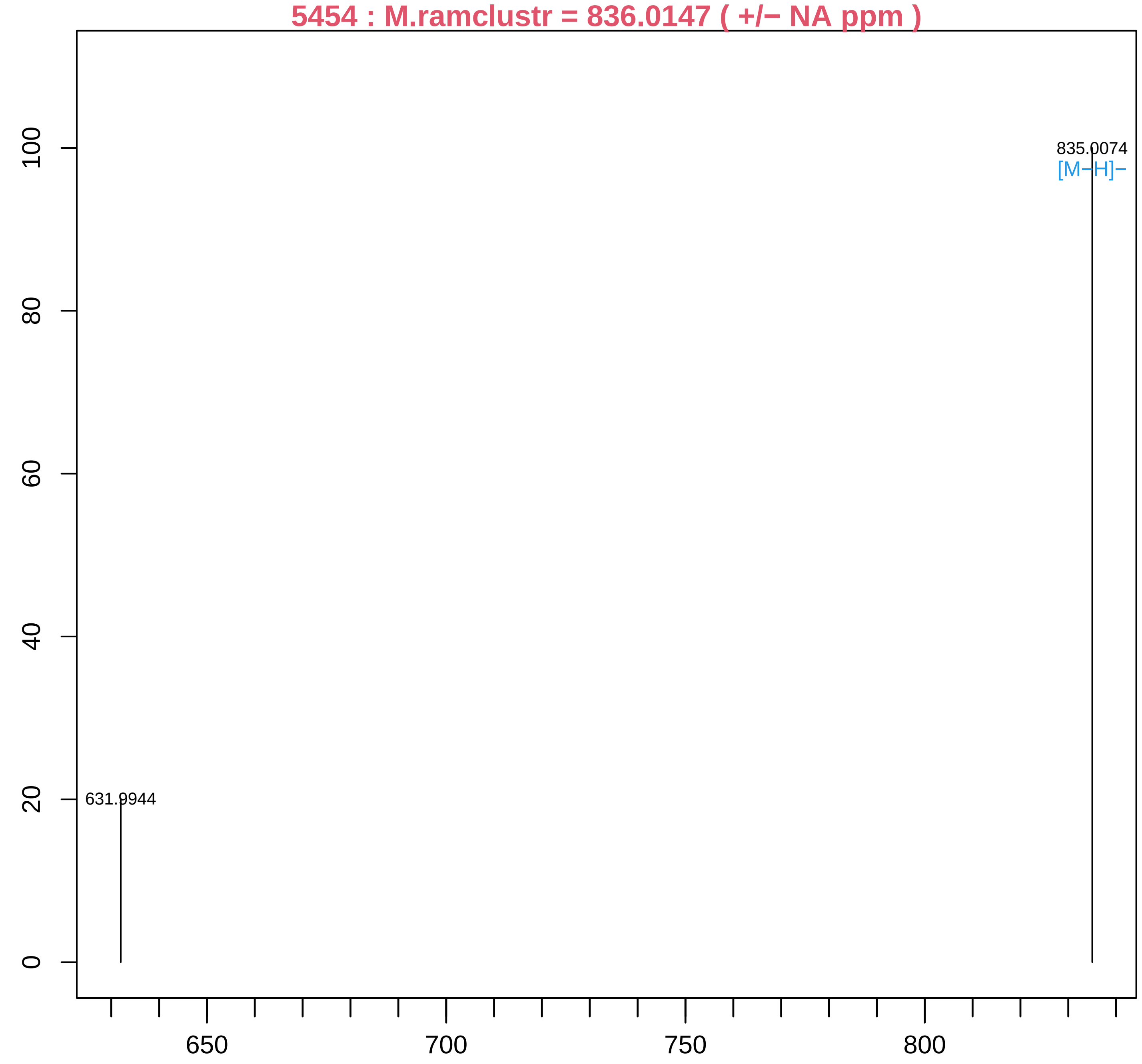

5454 : M.ramclustr = 836.0147 (+/− NA ppm)
835.0074
[M−H]−
631.9944
0
20
40
60
80
100
650
700
750
800

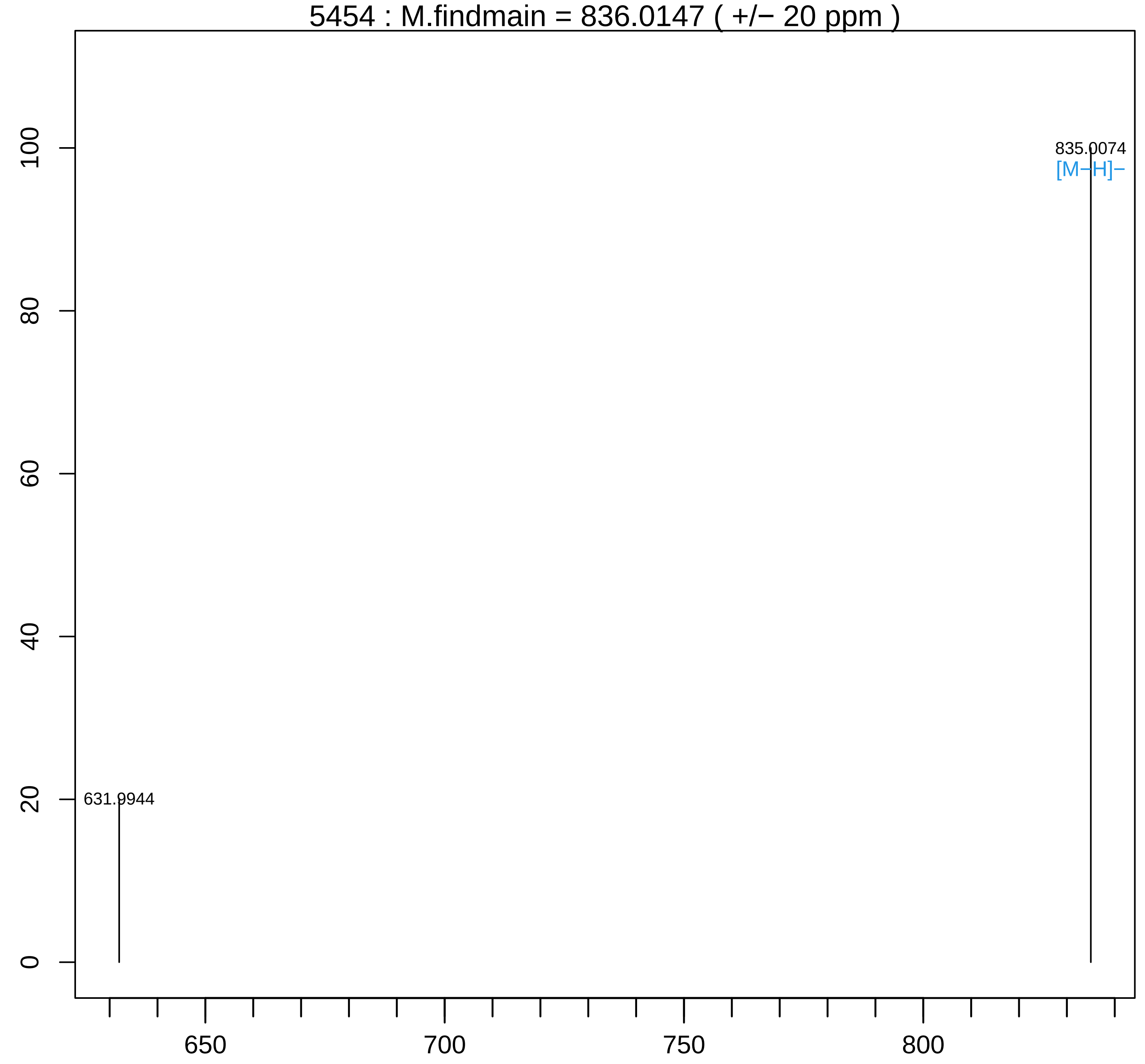

5454 : M.findmain = 836.0147 (+/− 20 ppm)
835.0074
[M−H]−
631.9944
0
20
40
60
80
100
650
700
750
800

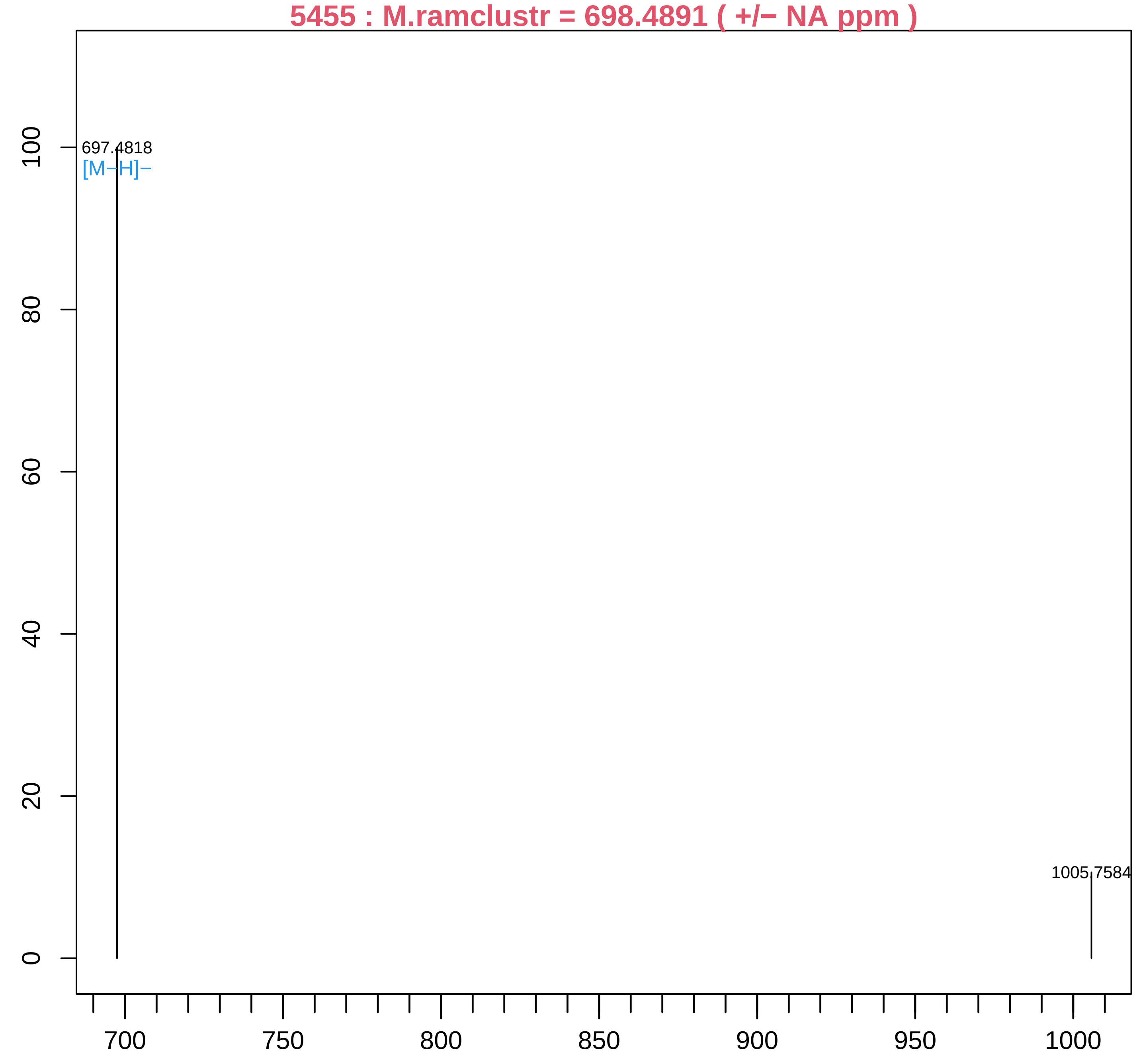
5455 : M.ramclustr = 698.4891 (+/− NA ppm)
697.4818
[M−H]−
1005.7584
0
20
40
60
80
100
700
750
800
850
900
950
1000

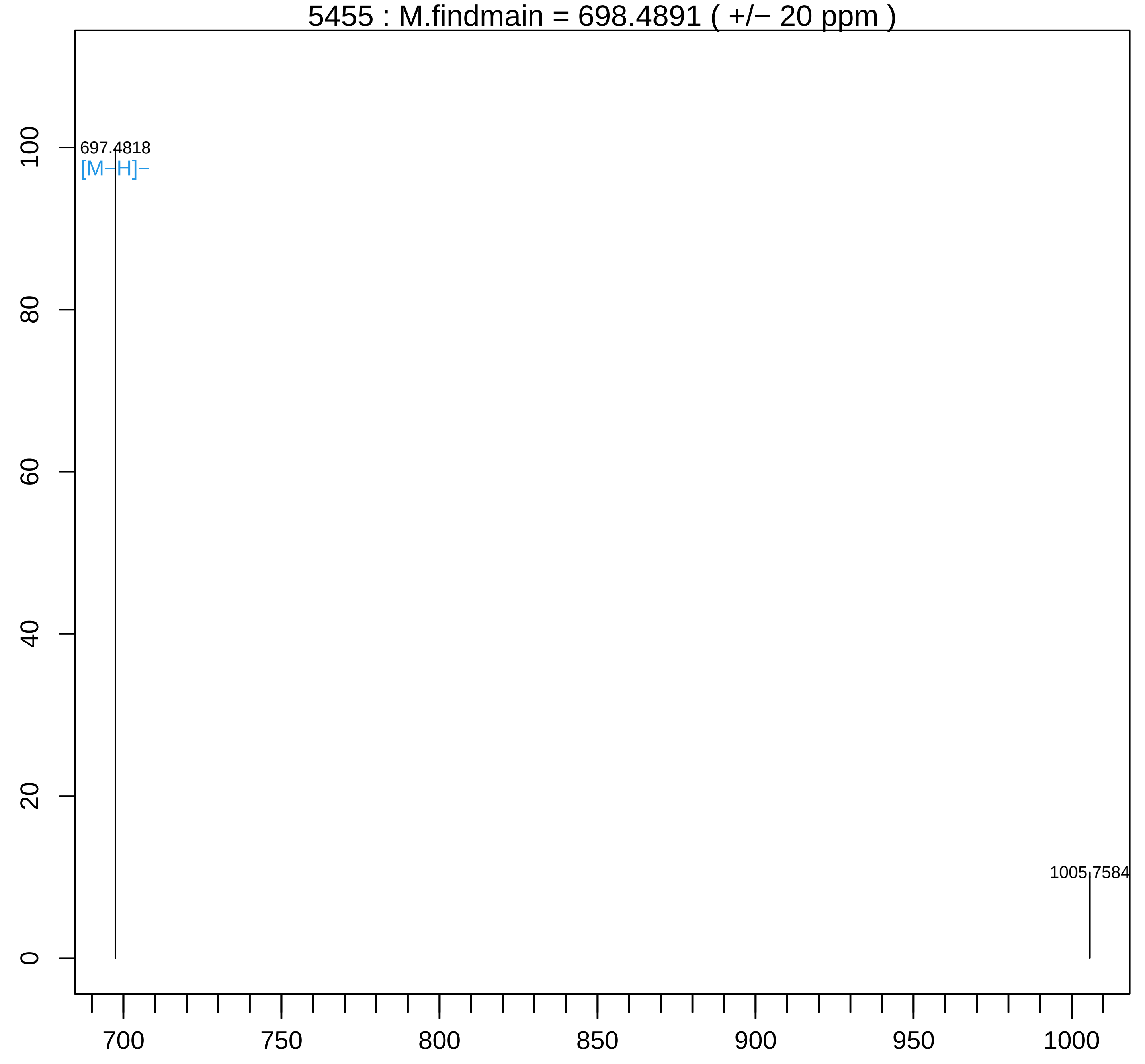
5455 : M.findmain = 698.4891 (+/− 20 ppm)
697.4818
[M−H]−
1005.7584
0
20
40
60
80
100
700
750
800
850
900
950
1000

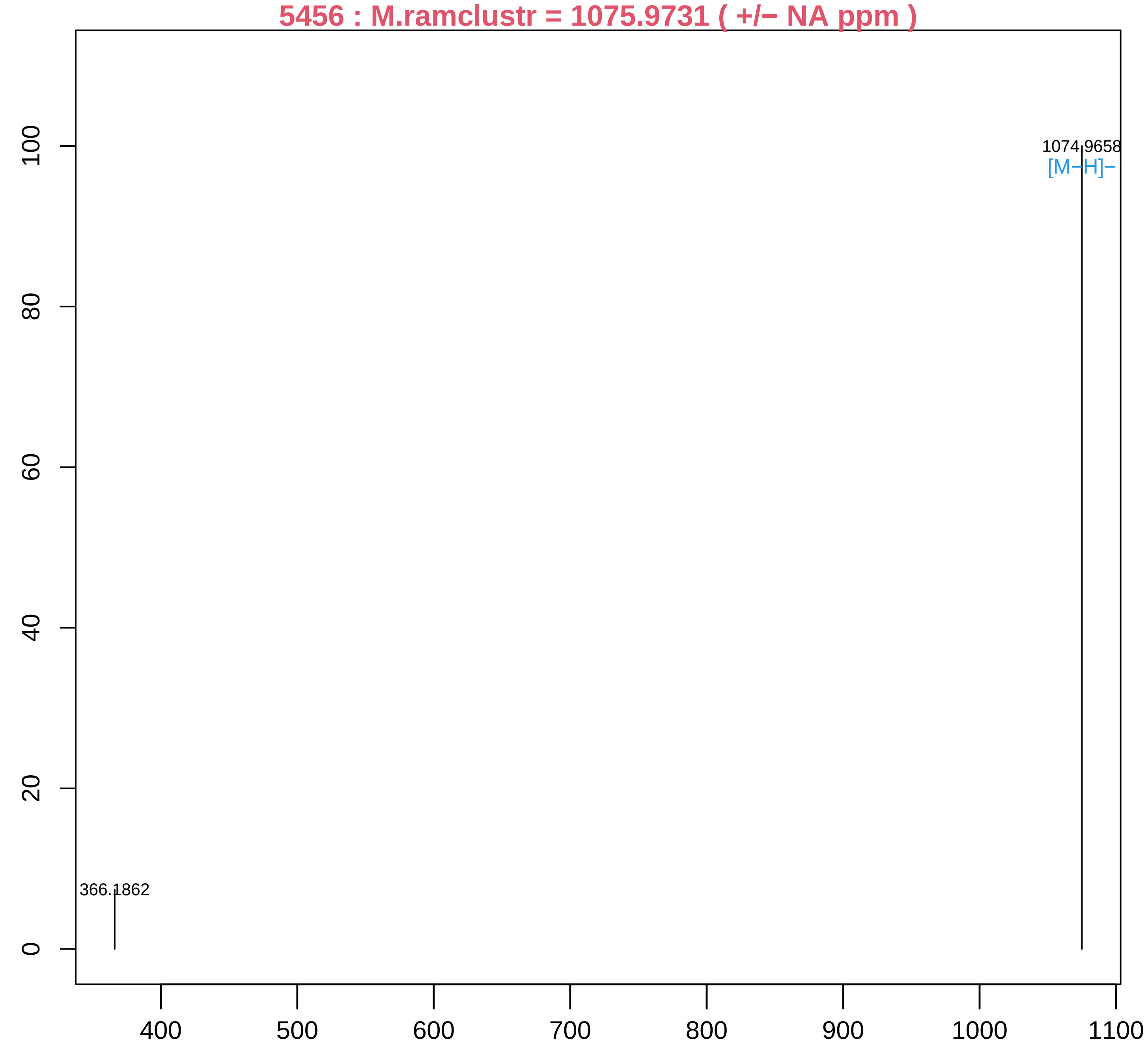
5456 : M.ramclustr = 1075.9731 (+/− NA ppm)
1074.9658
[M−H]−
366.1862
0
20
40
60
80
100
400
500
600
700
800
900
1000
1100

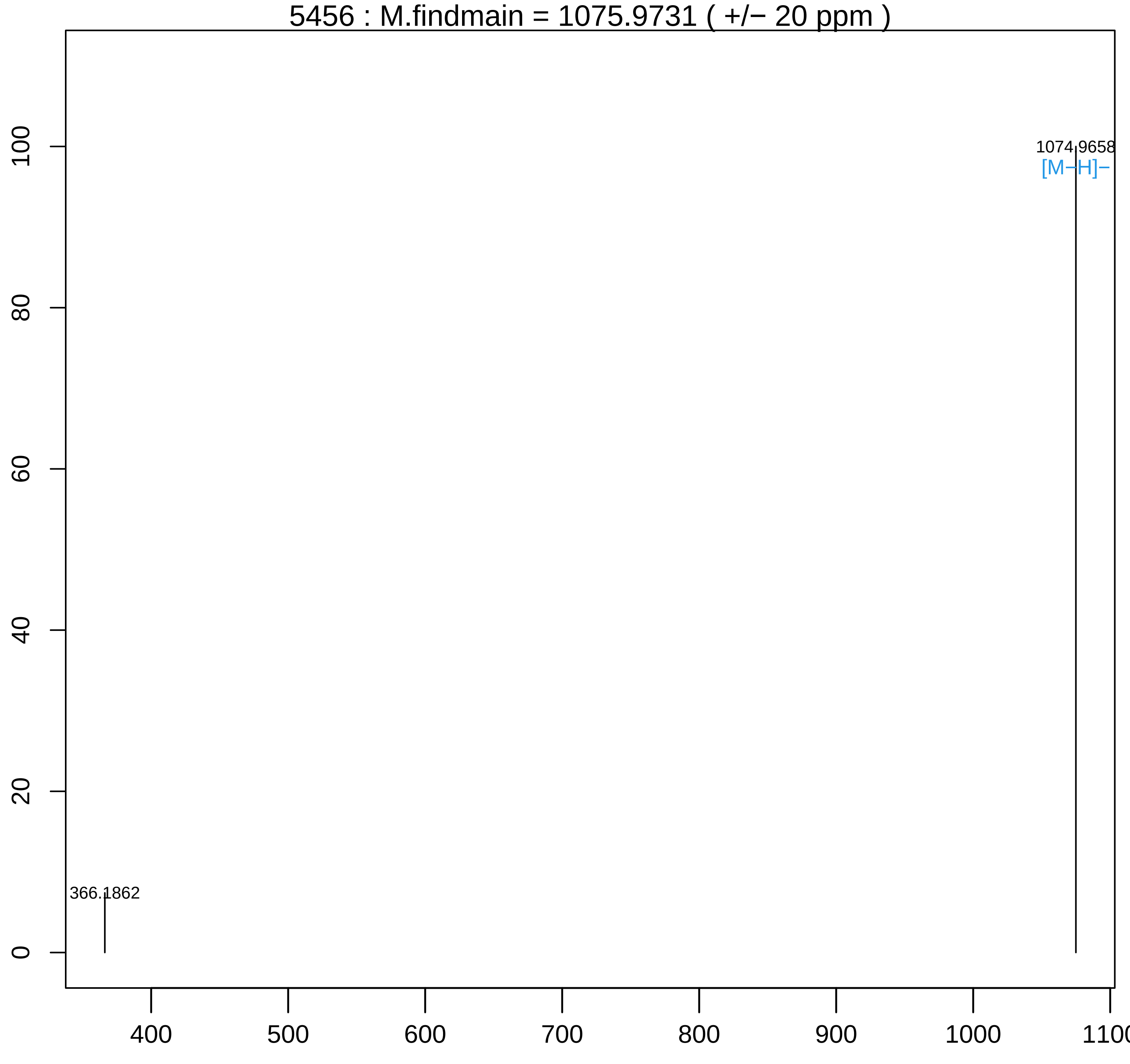
5456 : M.findmain = 1075.9731 (+/− 20 ppm)
1074.9658
[M−H]−
366.1862
0
20
40
60
80
100
400
500
600
700
800
900
1000
1100

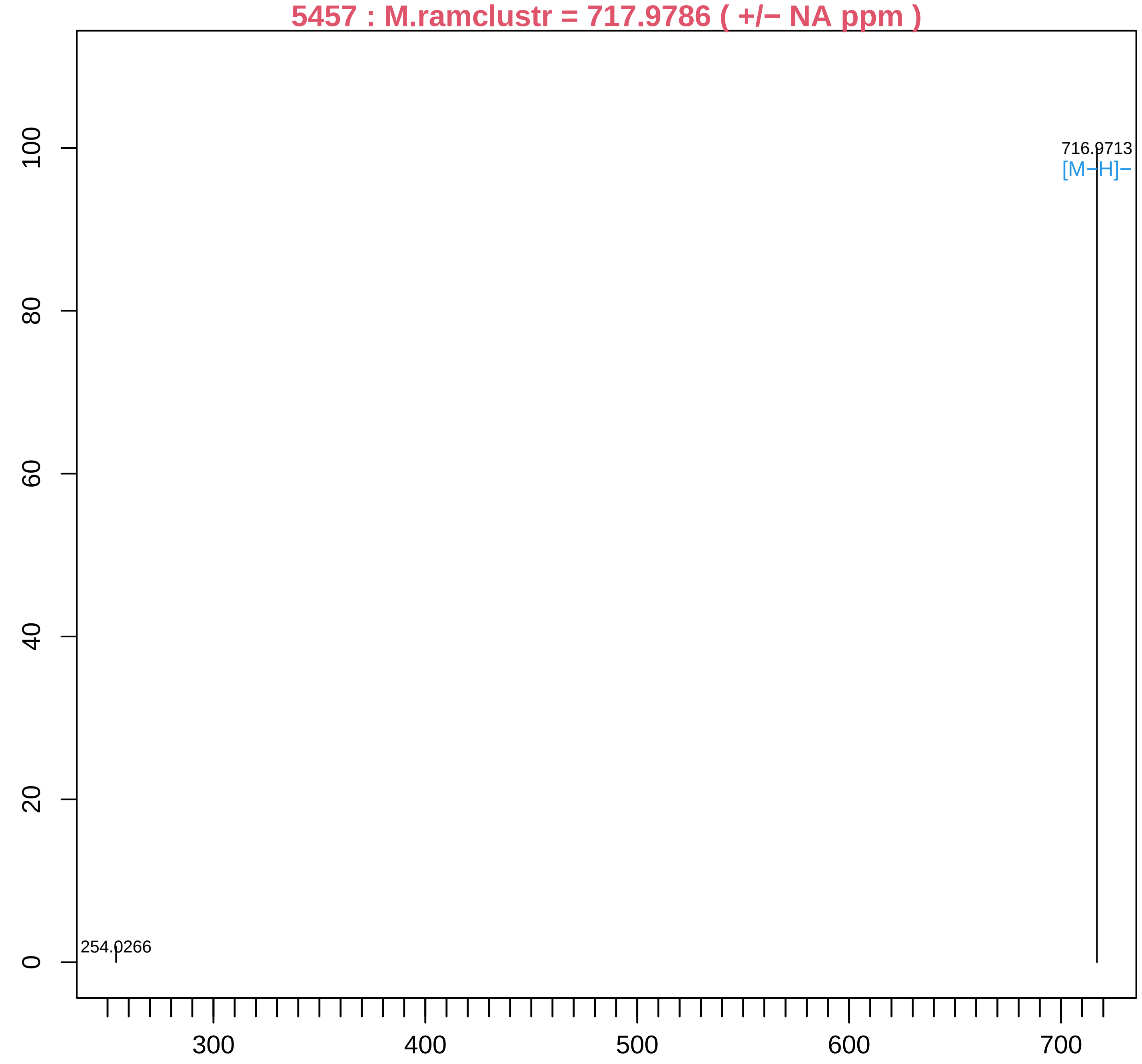
5457 : M.ramclustr = 717.9786 (+/− NA ppm)
716.9713
[M−H]−
254.0266
0
20
40
60
80
100
300
400
500
600
700

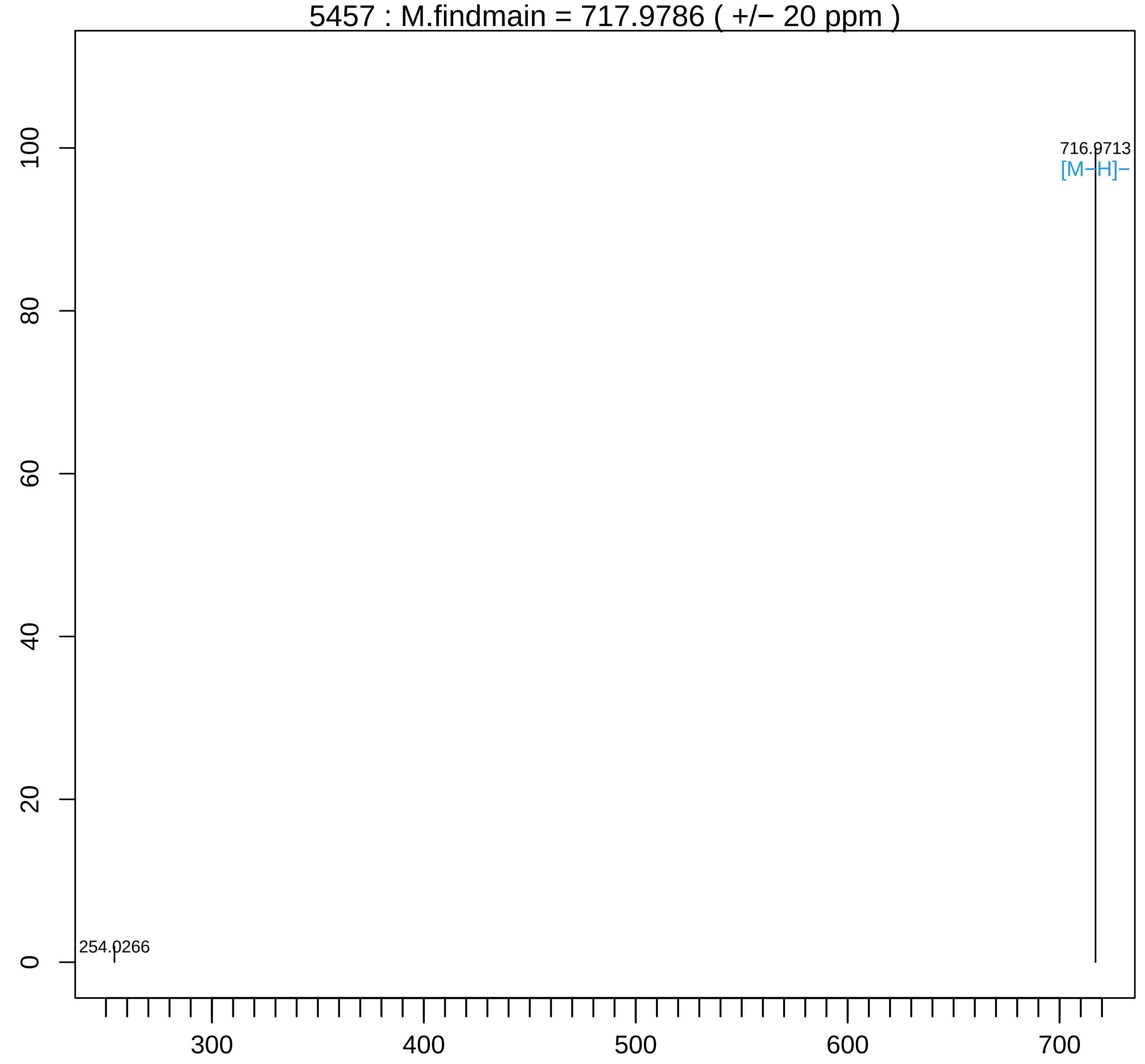
5457 : M.findmain = 717.9786 (+/− 20 ppm)
716.9713
[M−H]−
254.0266
0
20
40
60
80
100
300
400
500
600
700

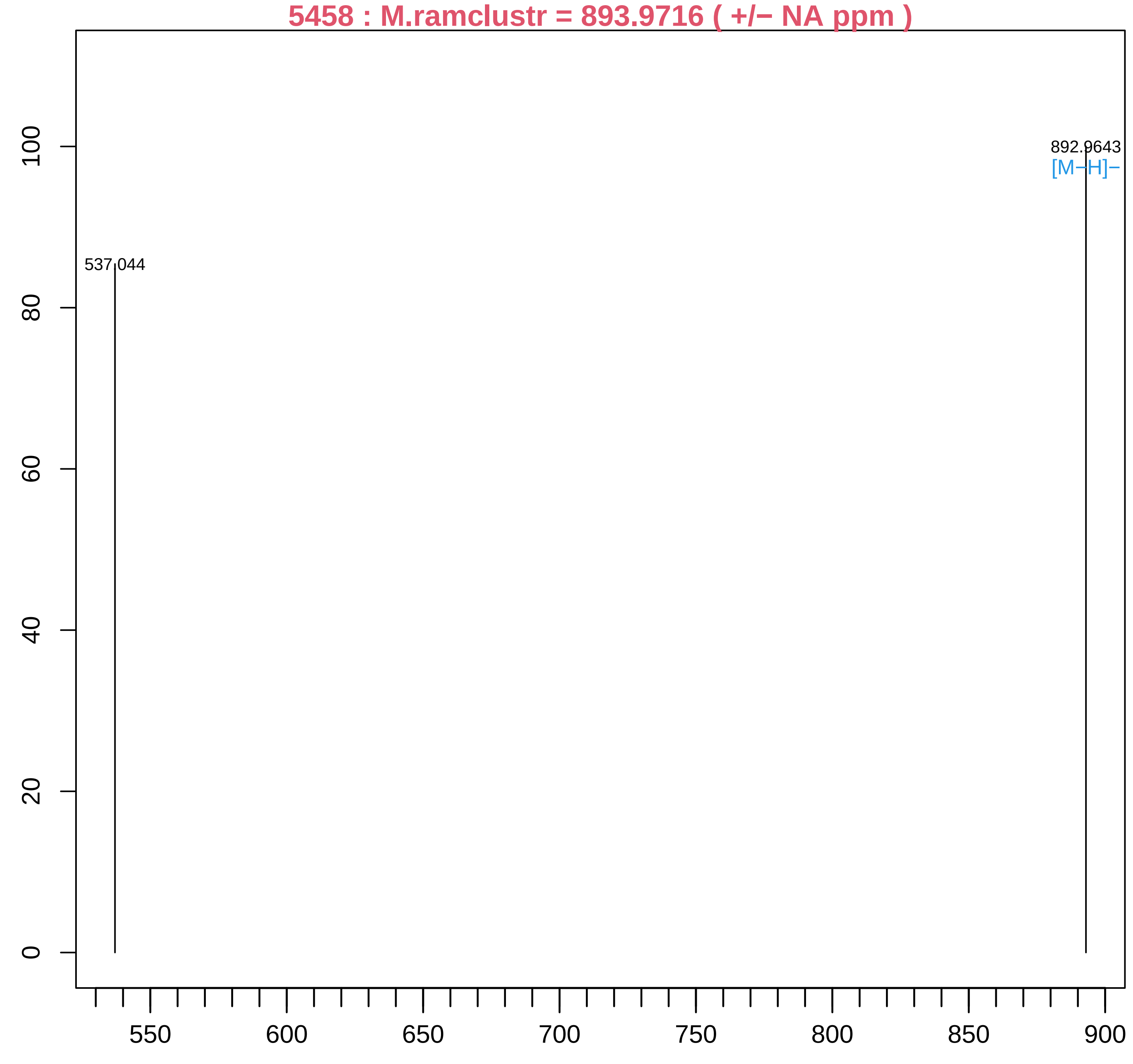
5458 : M.ramclustr = 893.9716 (+/− NA ppm)
892.9643
[M−H]−
537.044
0
20
40
60
80
100
550
600
650
700
750
800
850
900

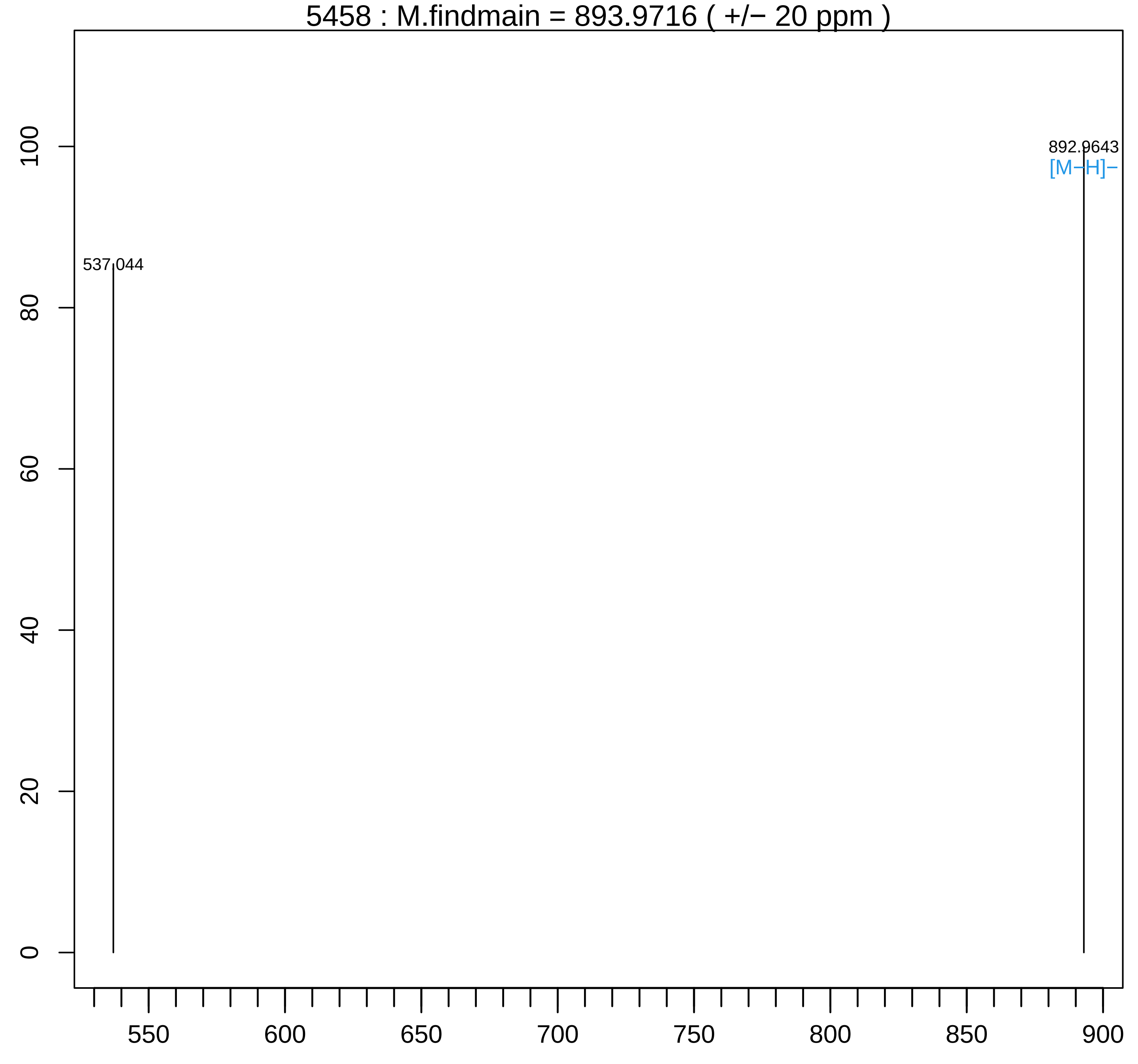
5458 : M.findmain = 893.9716 (+/− 20 ppm)
892.9643
[M−H]−
537.044
0
20
40
60
80
100
550
600
650
700
750
800
850
900

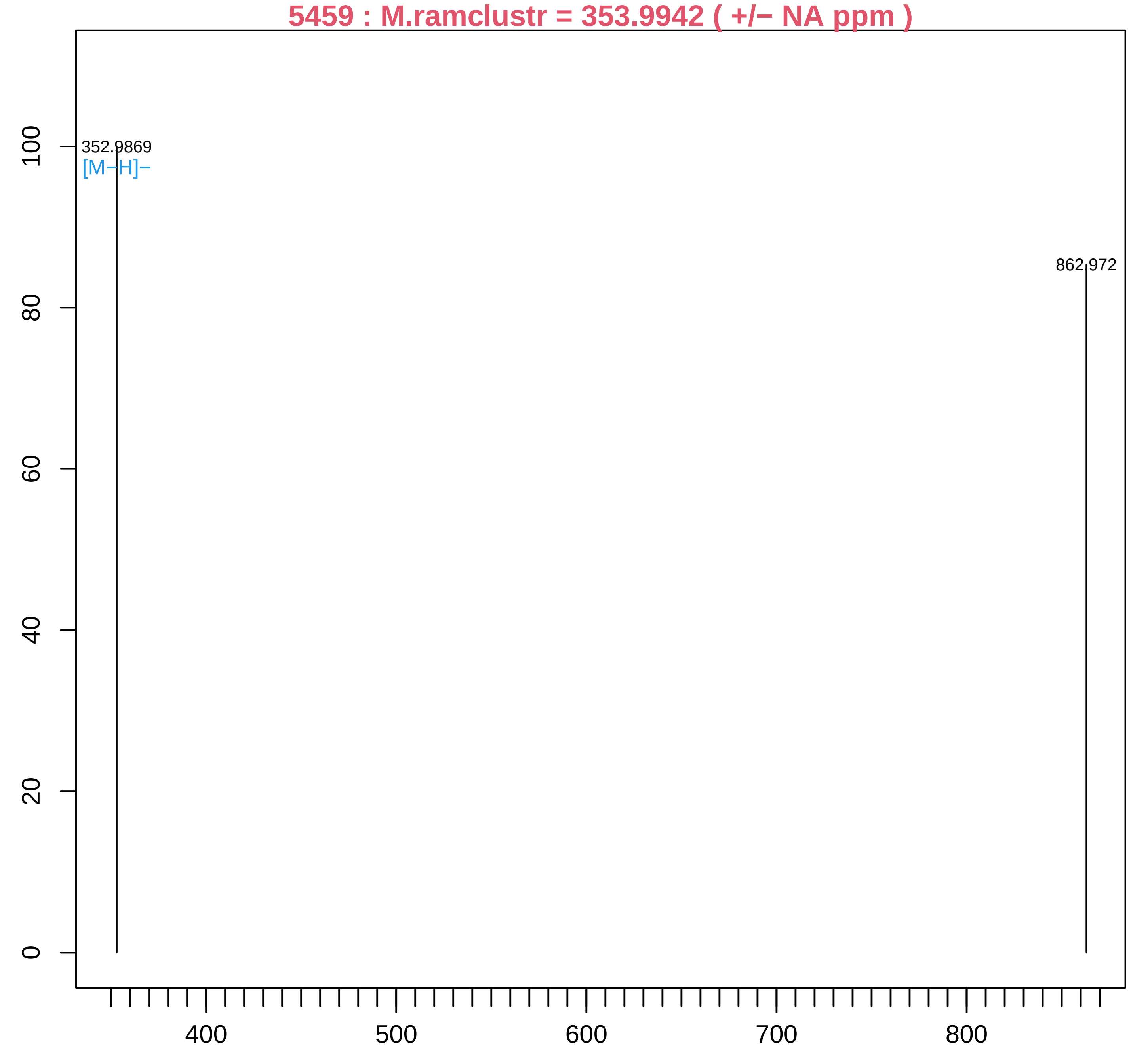

5459 : M.ramclustr = 353.9942 (+/− NA ppm)
352.9869
[M−H]−
862.972
0
20
40
60
80
100
400
500
600
700
800

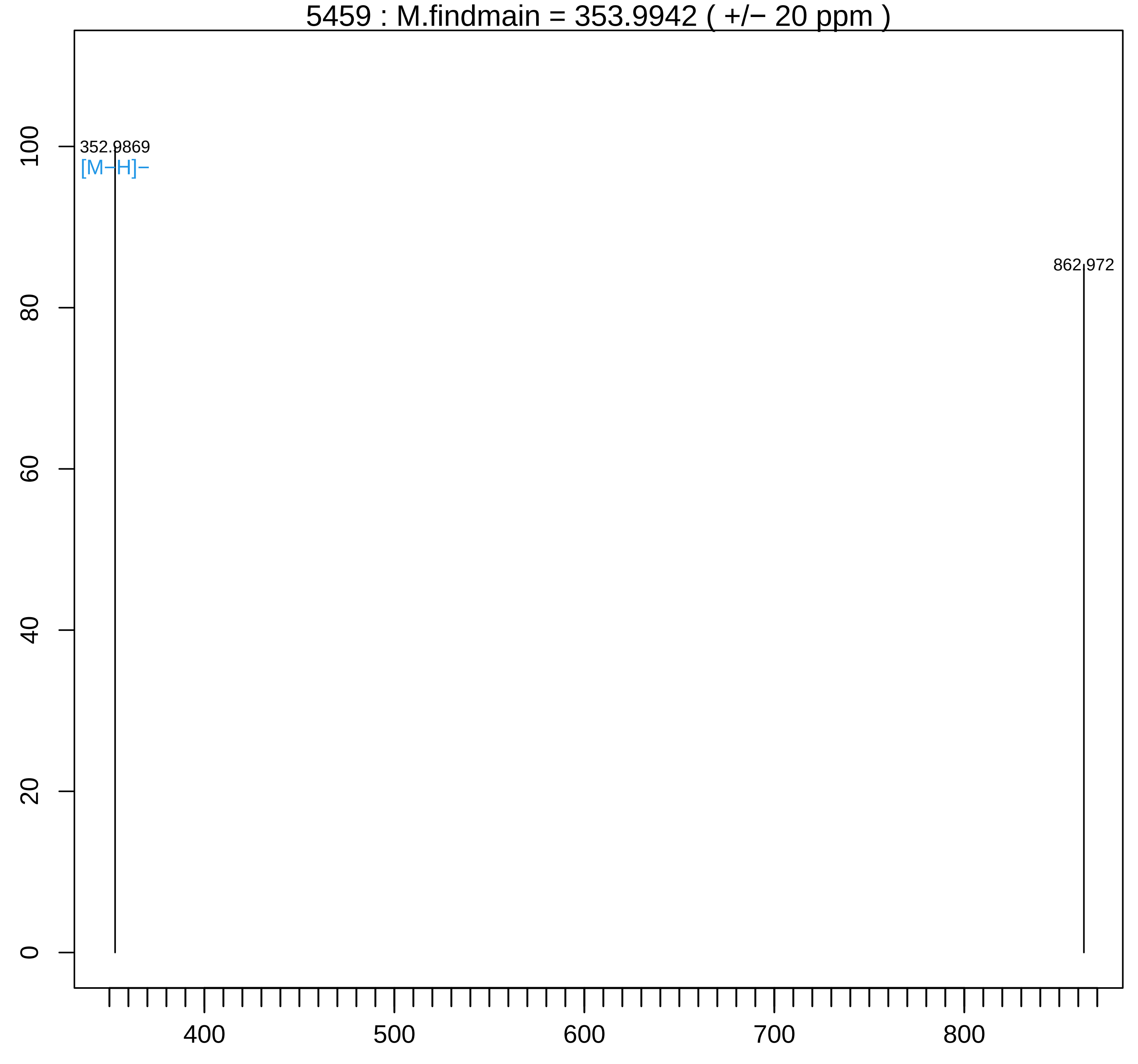

5459 : M.findmain = 353.9942 (+/− 20 ppm)
352.9869
[M−H]−
862.972
0
20
40
60
80
100
400
500
600
700
800

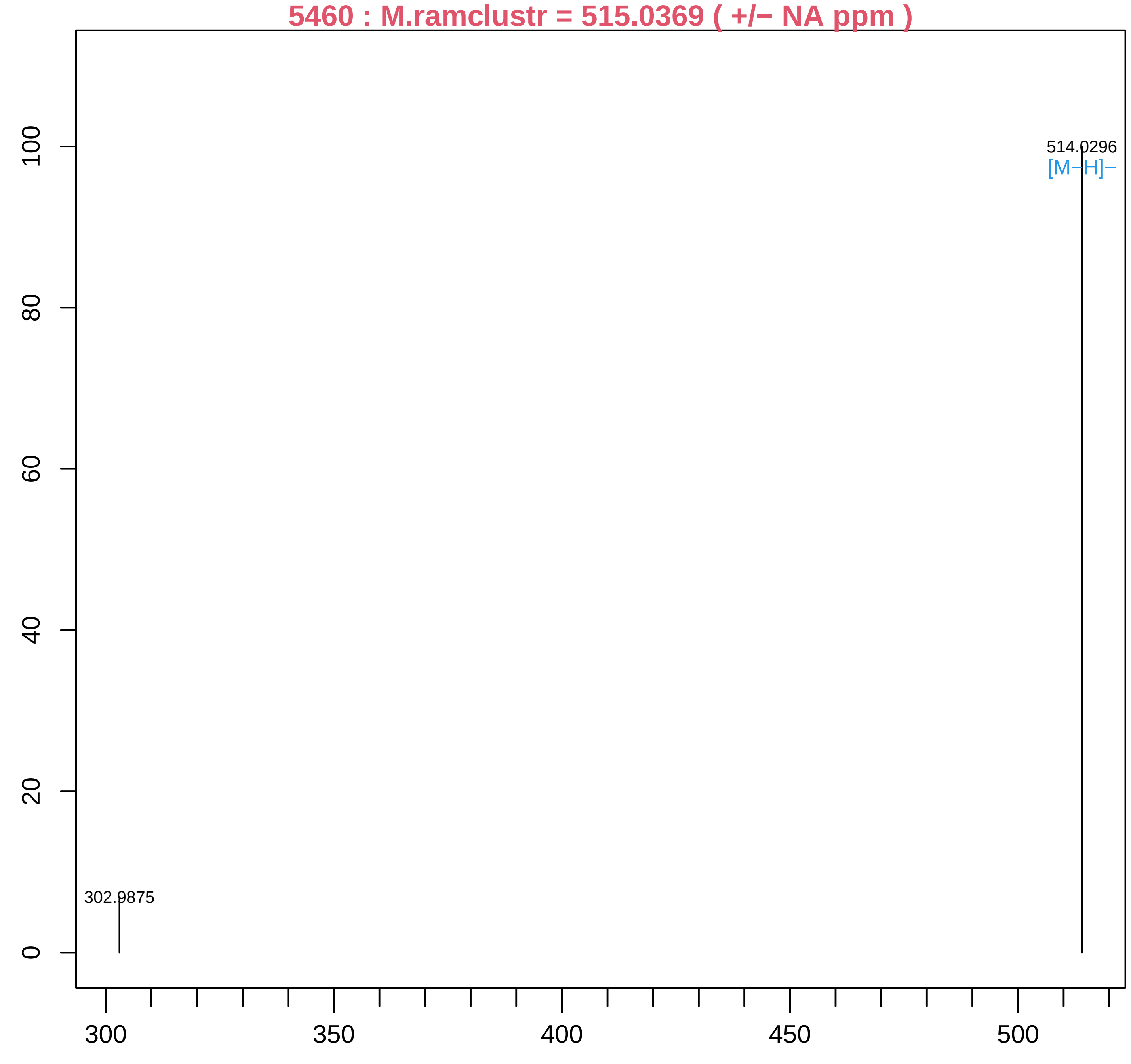

5460 : M.ramclustr = 515.0369 (+/− NA ppm)
514.0296
[M−H]−
302.9875
0
20
40
60
80
100
300
350
400
450
500

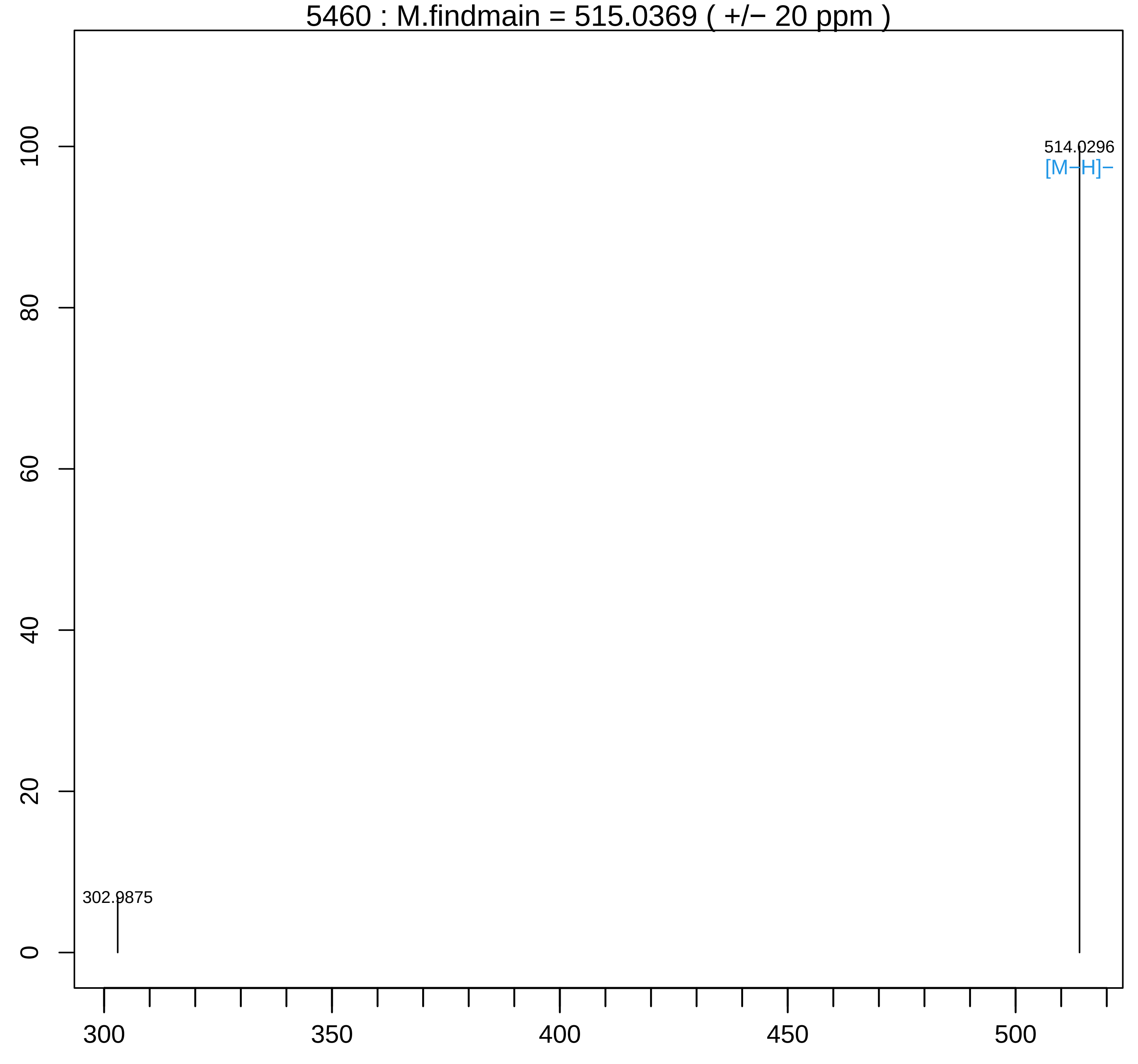

5460 : M.findmain = 515.0369 (+/− 20 ppm)
514.0296
[M−H]−
302.9875
0
20
40
60
80
100
300
350
400
450
500

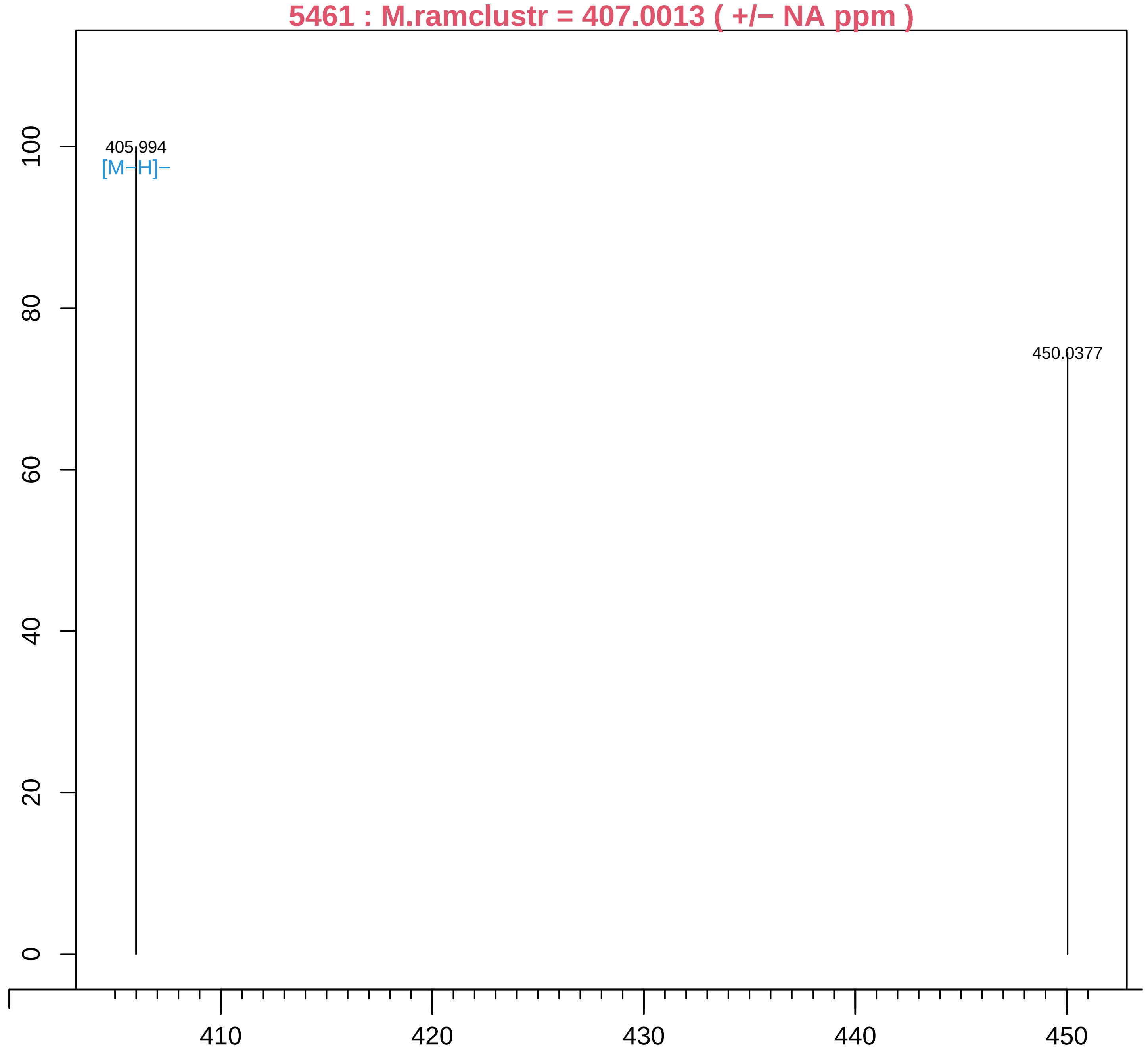

5461 : M.ramclustr = 407.0013 (+/− NA ppm)
405.994
[M−H]−
450.0377
0
20
40
60
80
100
410
420
430
440
450

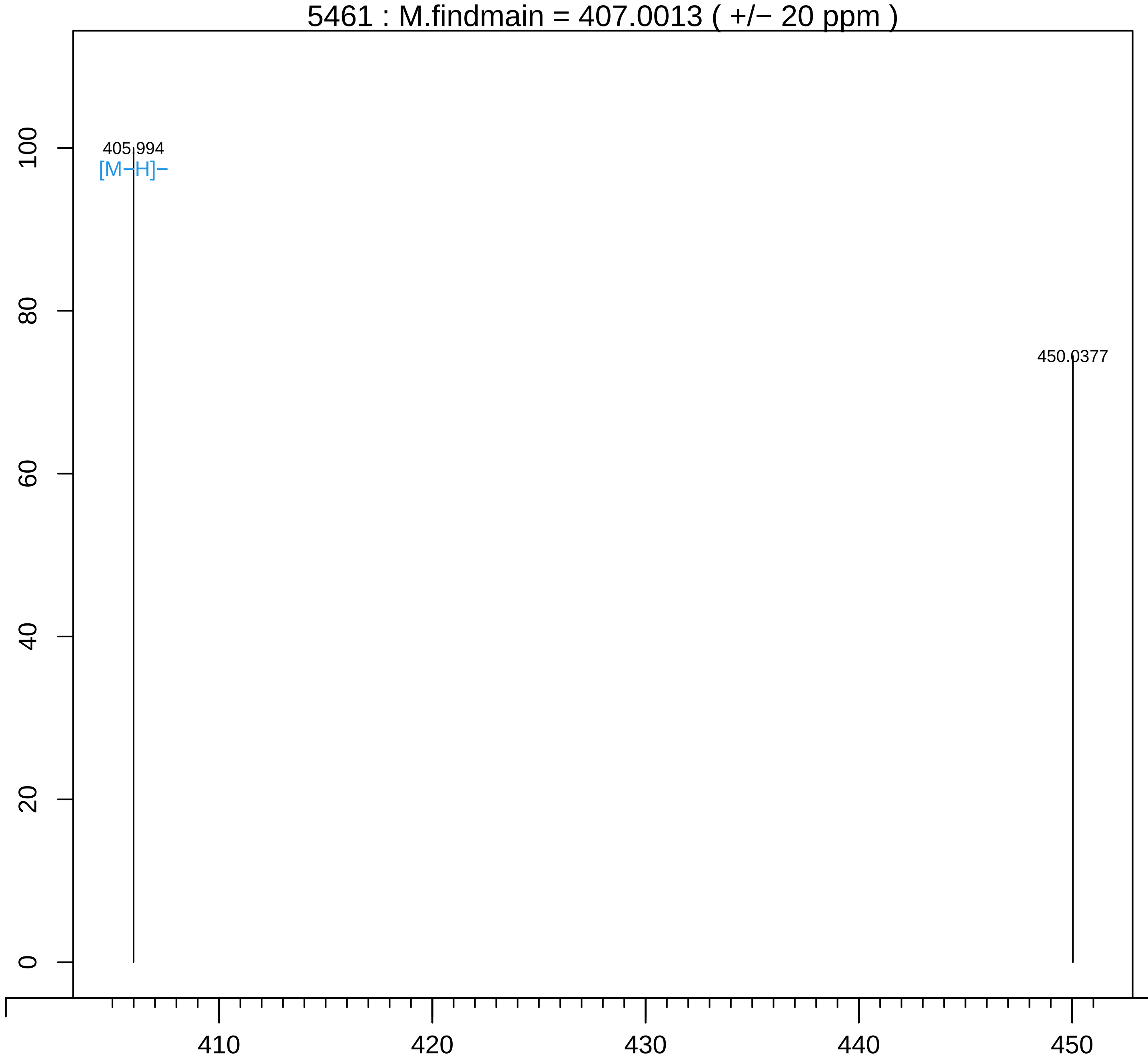

5461 : M.findmain = 407.0013 (+/− 20 ppm)
405.994
[M−H]−
450.0377
0
20
40
60
80
100
410
420
430
440
450

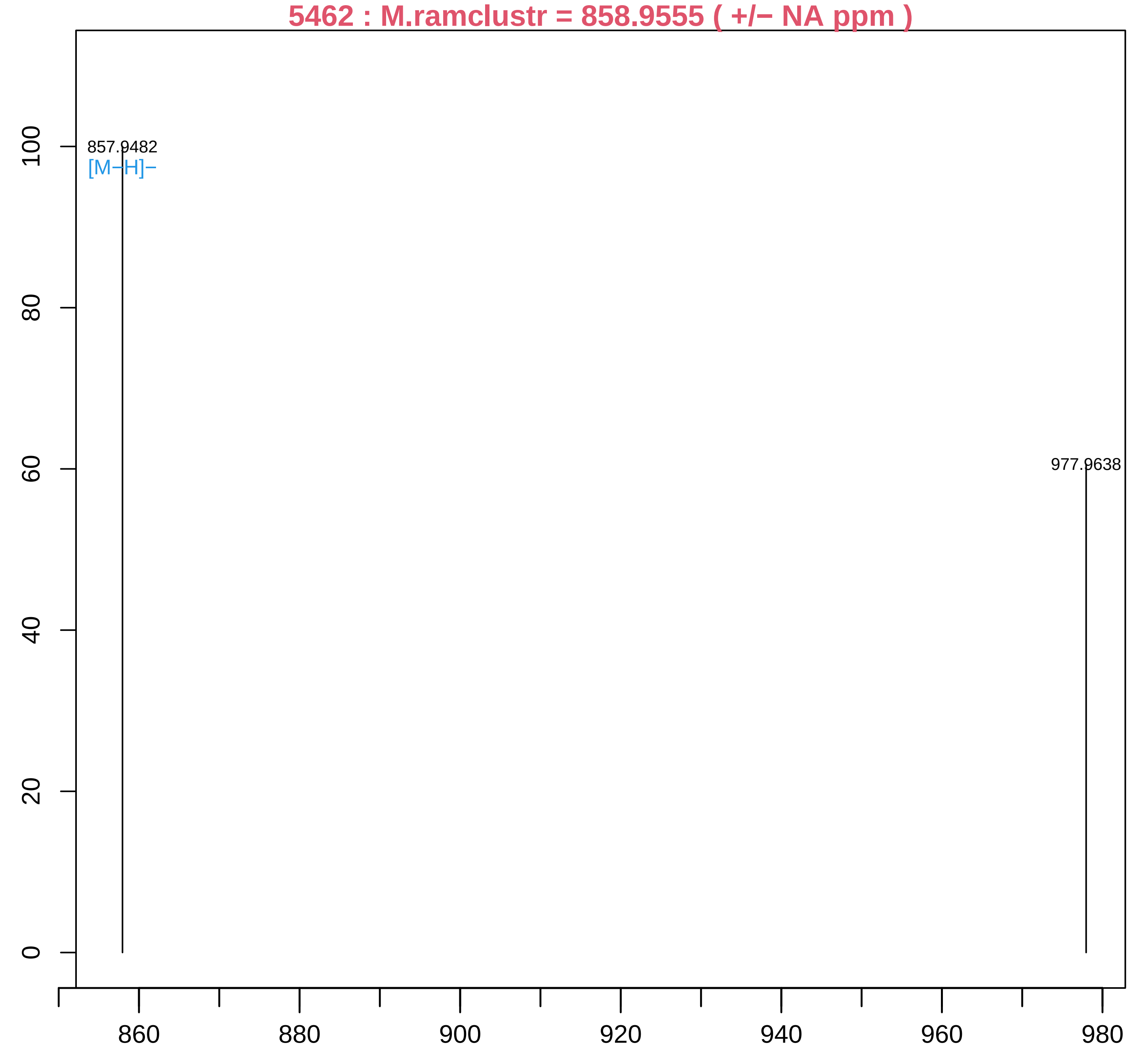
5462 : M.ramclustr = 858.9555 (+/− NA ppm)
857.9482
[M−H]−
977.9638
0
20
40
60
80
100
860
880
900
920
940
960
980

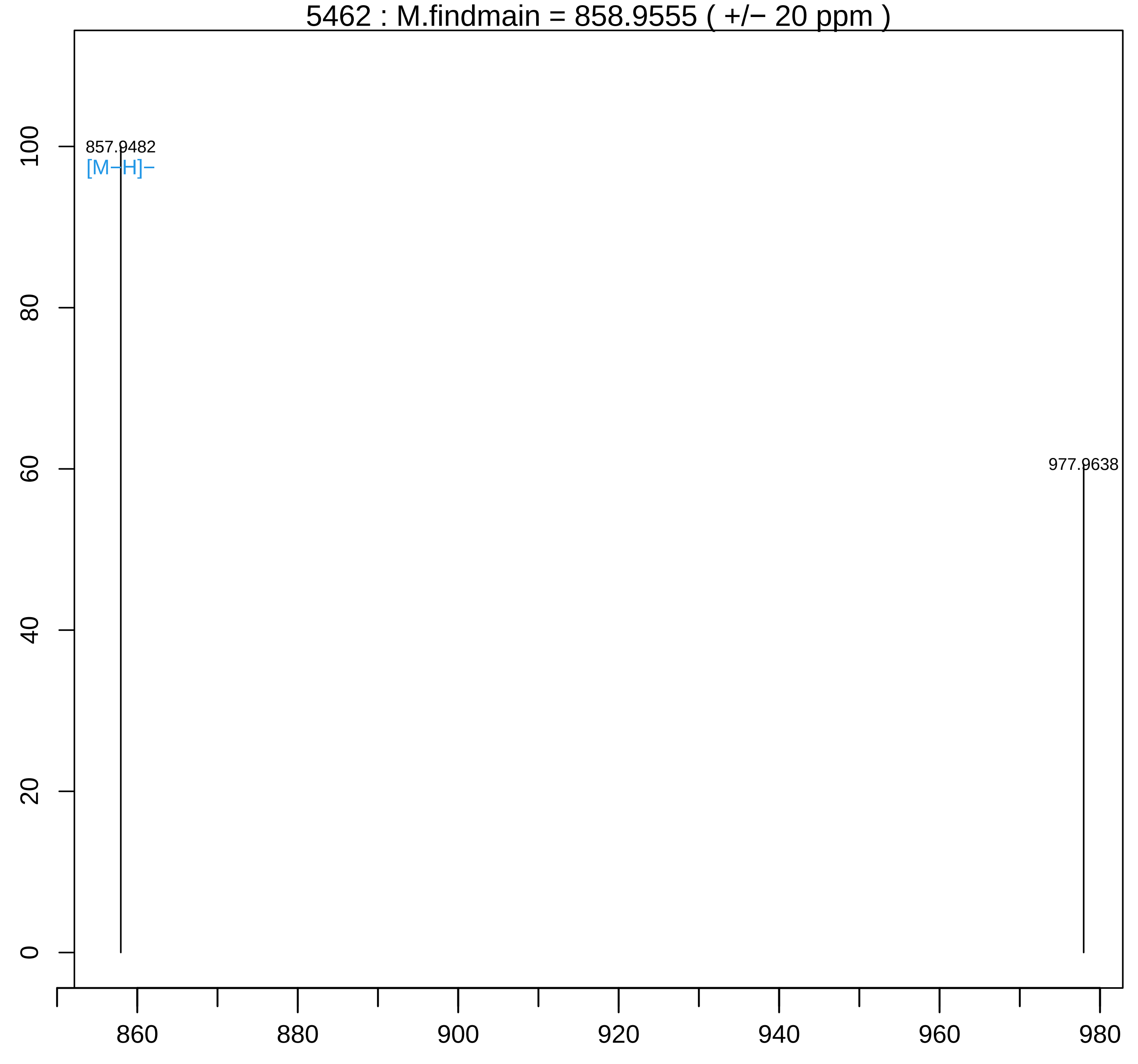
5462 : M.findmain = 858.9555 (+/− 20 ppm)
857.9482
[M−H]−
977.9638
0
20
40
60
80
100
860
880
900
920
940
960
980

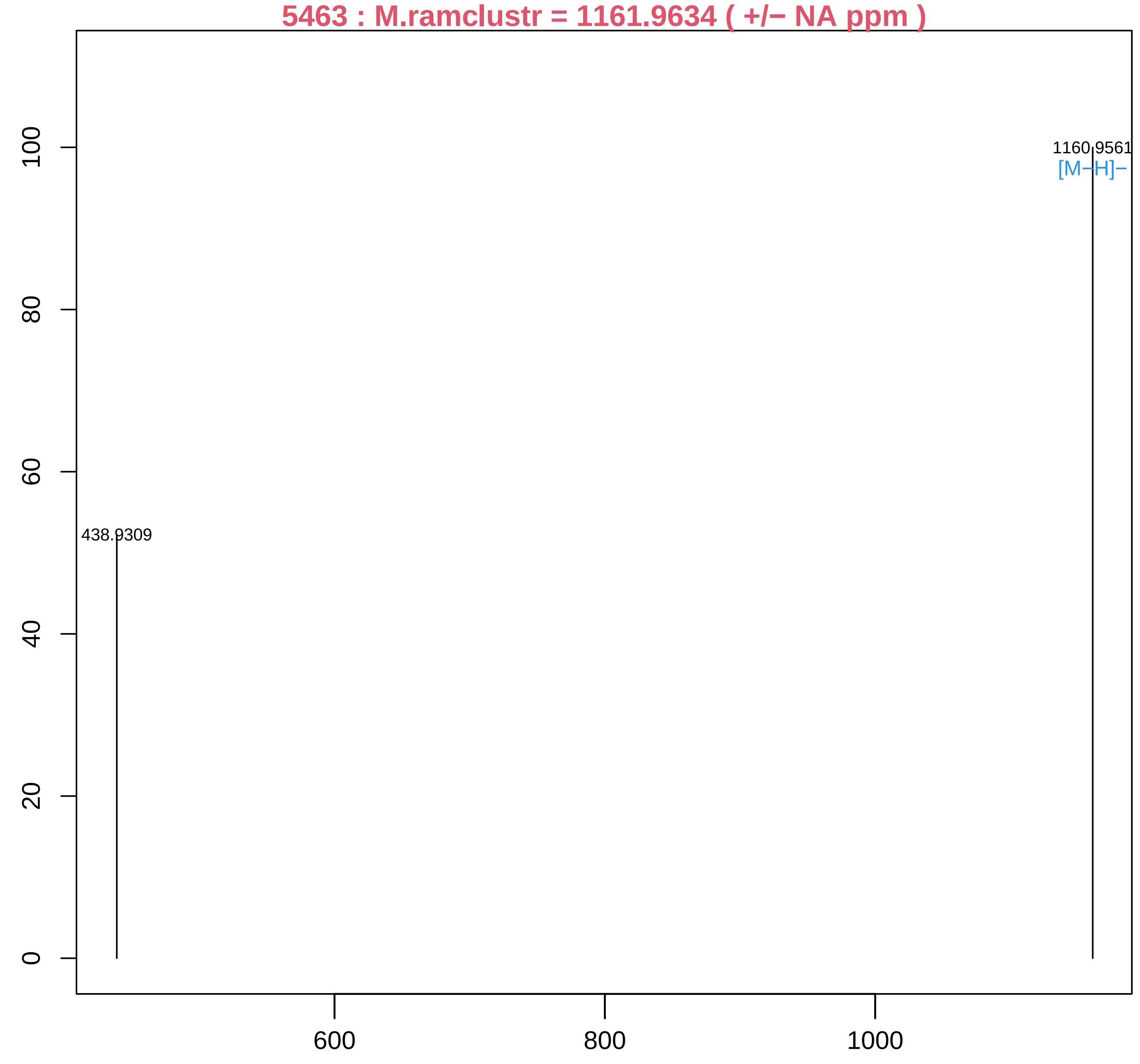

5463 : M.ramclustr = 1161.9634 (+/− NA ppm)
1160.9561
[M−H]−
438.9309
0
20
40
60
80
100
600
800
1000

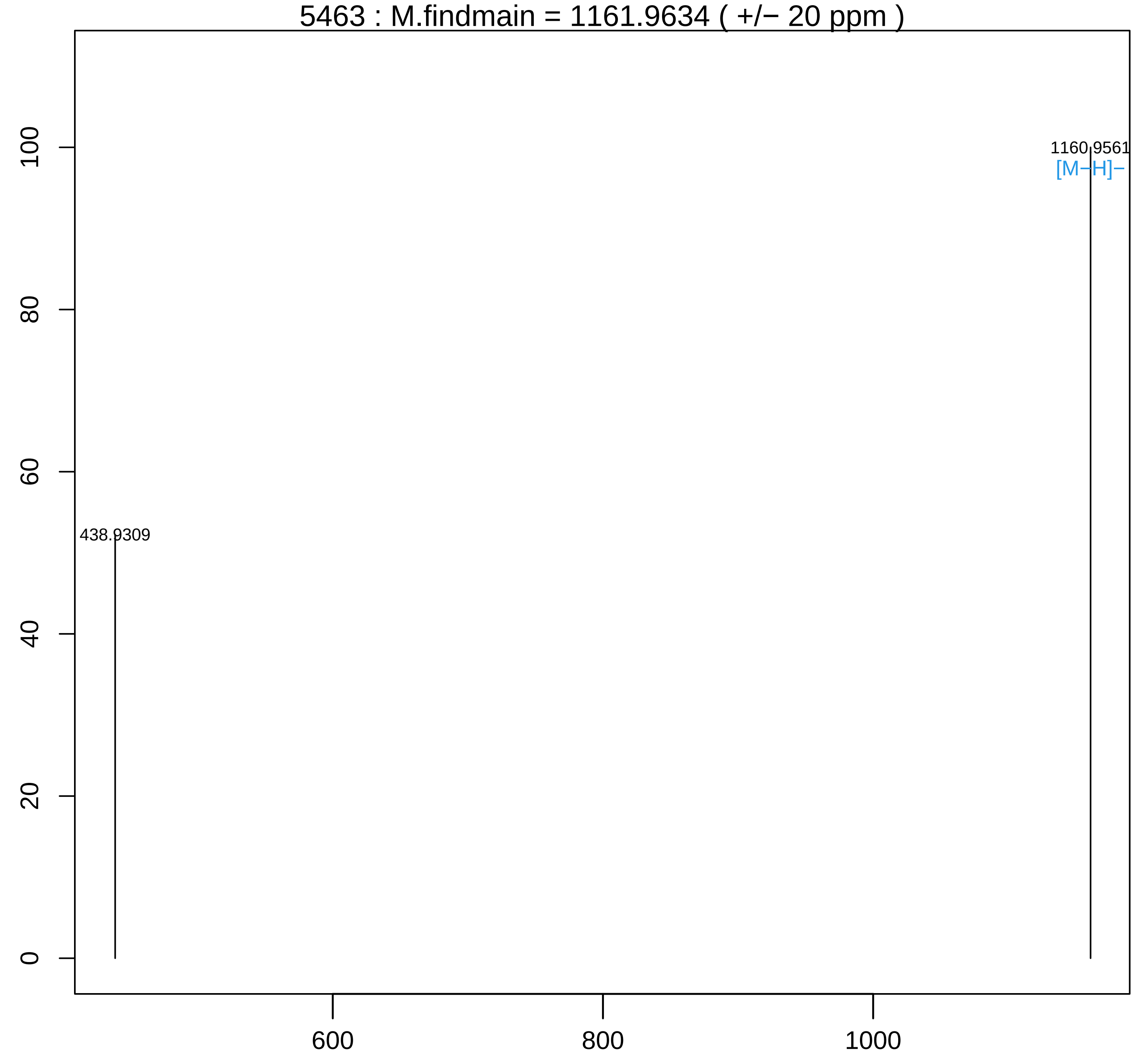

5463 : M.findmain = 1161.9634 (+/− 20 ppm)
1160.9561
[M−H]−
438.9309
0
20
40
60
80
100
600
800
1000

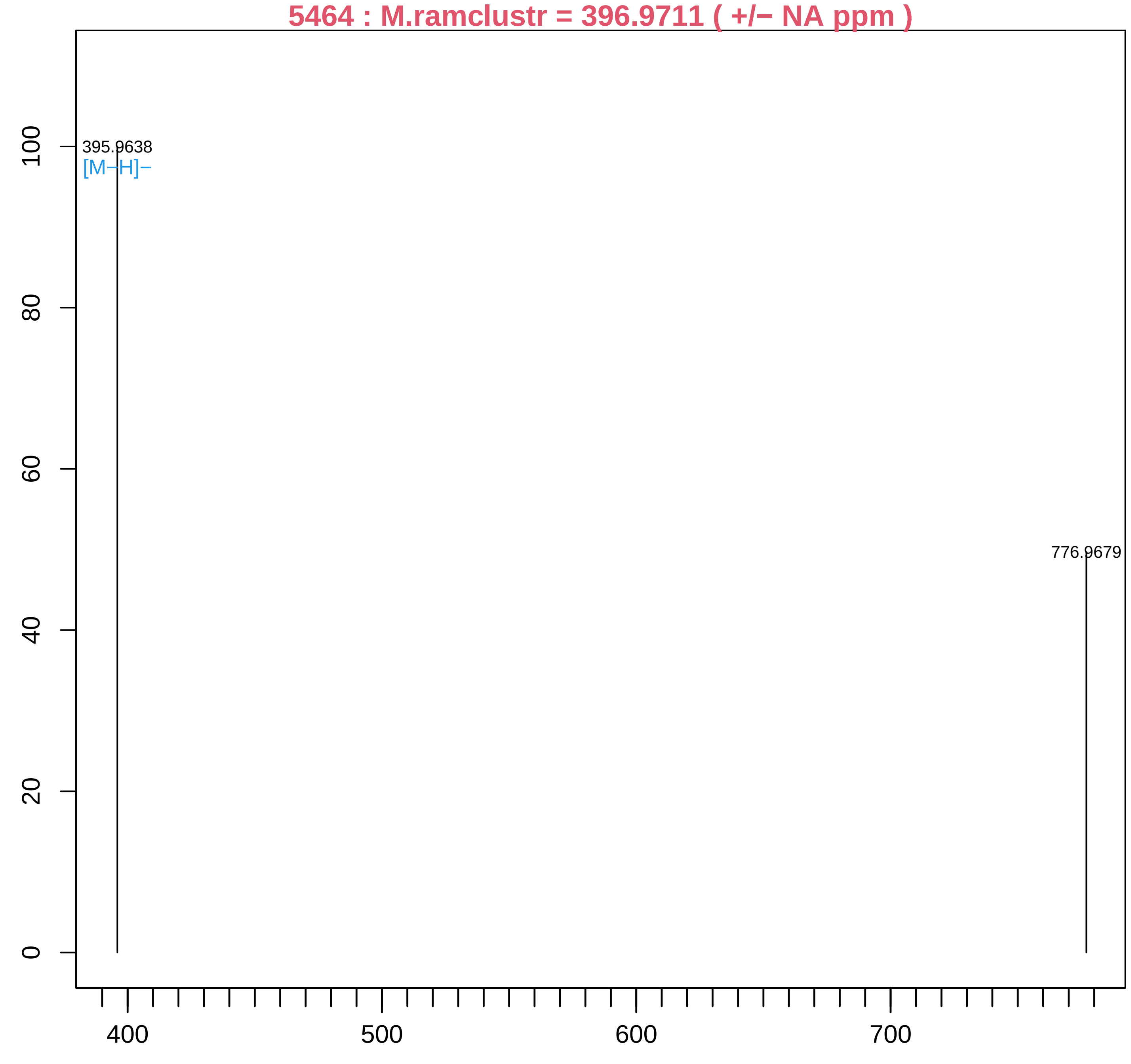

5464 : M.ramclustr = 396.9711 (+/− NA ppm)
395.9638
[M−H]−
776.9679
0
20
40
60
80
100
400
500
600
700

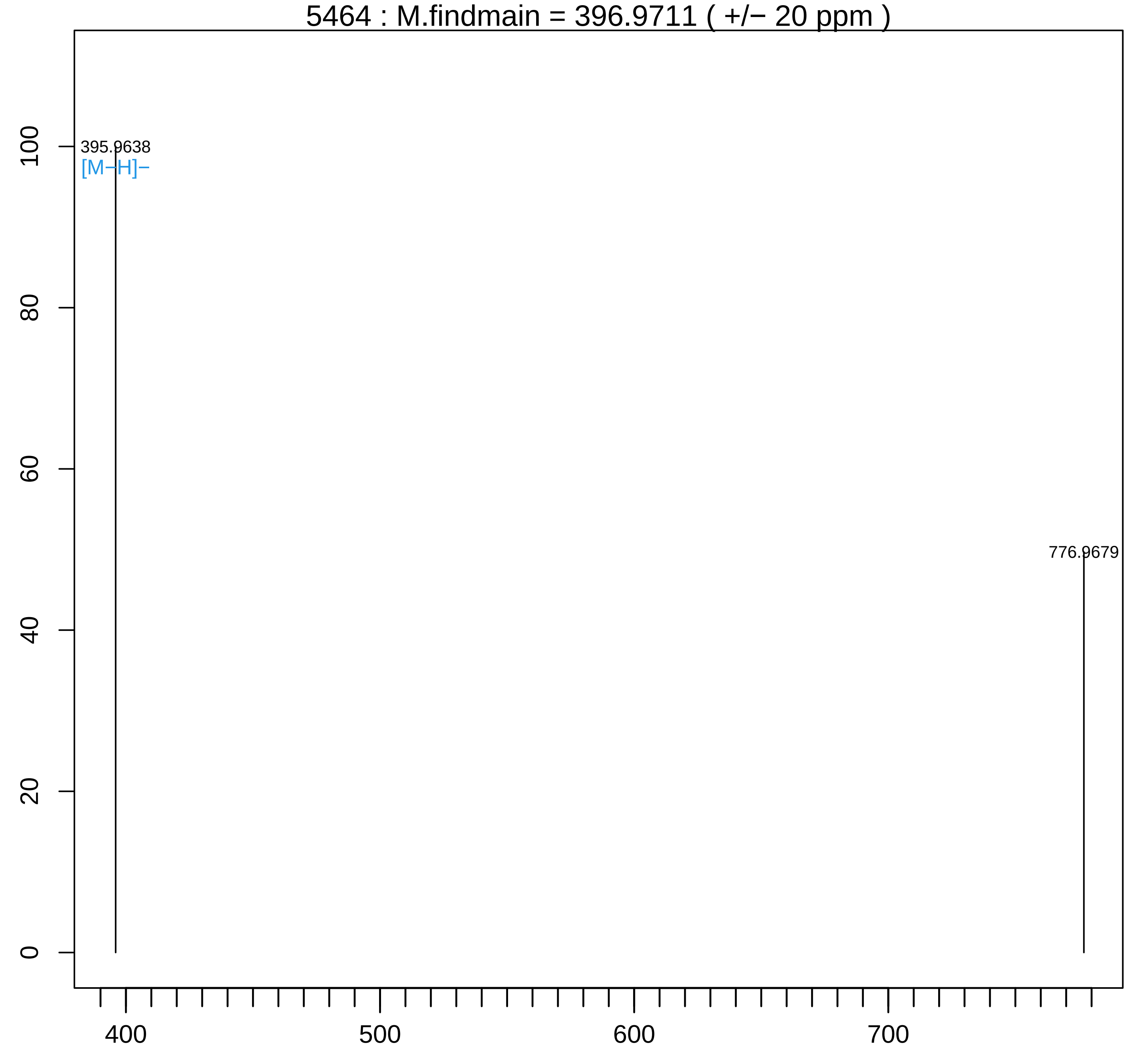

5464 : M.findmain = 396.9711 (+/− 20 ppm)
395.9638
[M−H]−
776.9679
0
20
40
60
80
100
400
500
600
700

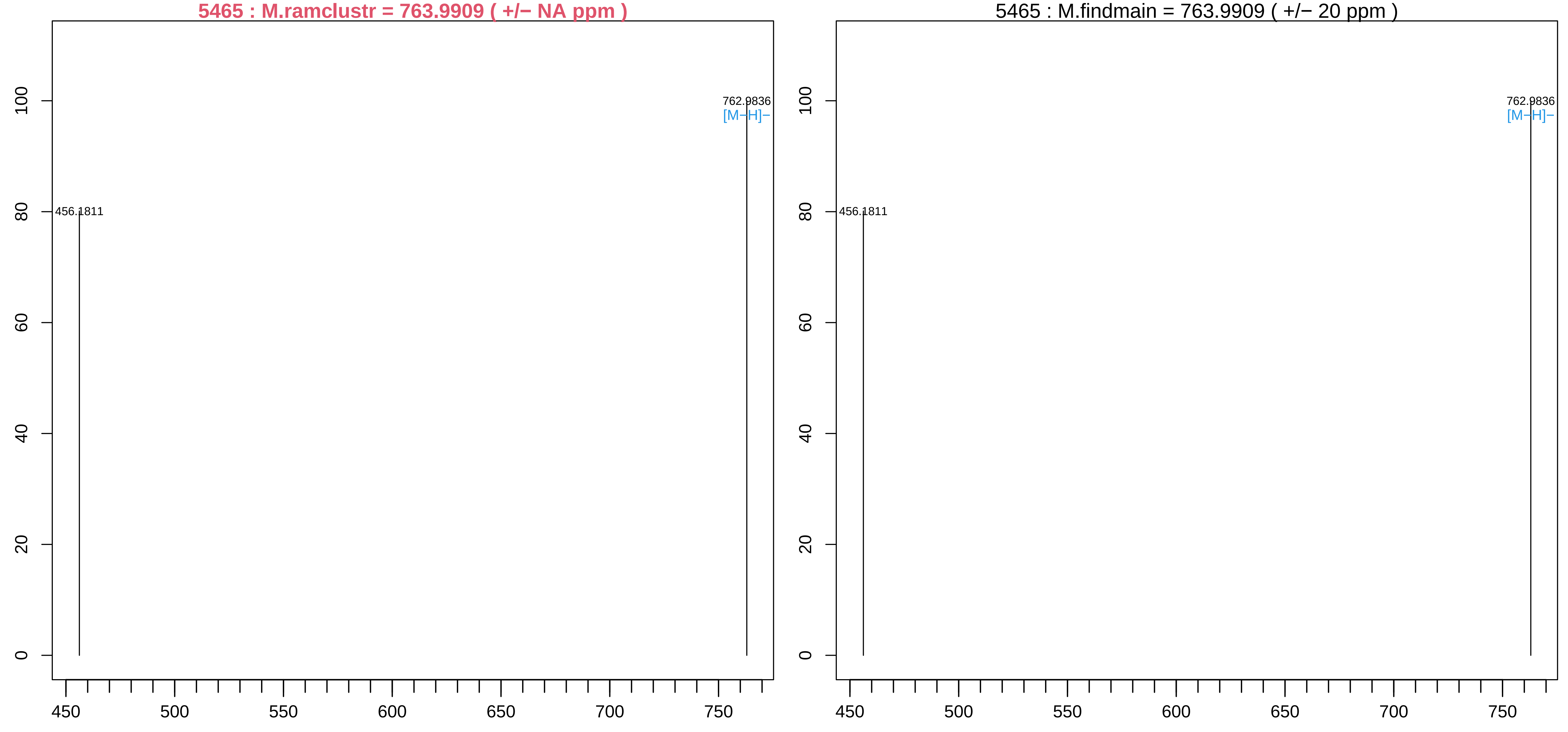
5465 : M.ramclustr = 763.9909 (+/− NA ppm)
762.9836
[M−H]−
456.1811
0
20
40
60
80
100
450
500
550
600
650
700
750
5465 : M.findmain = 763.9909 (+/− 20 ppm)
762.9836
[M−H]−
456.1811
0
20
40
60
80
100
450
500
550
600
650
700
750

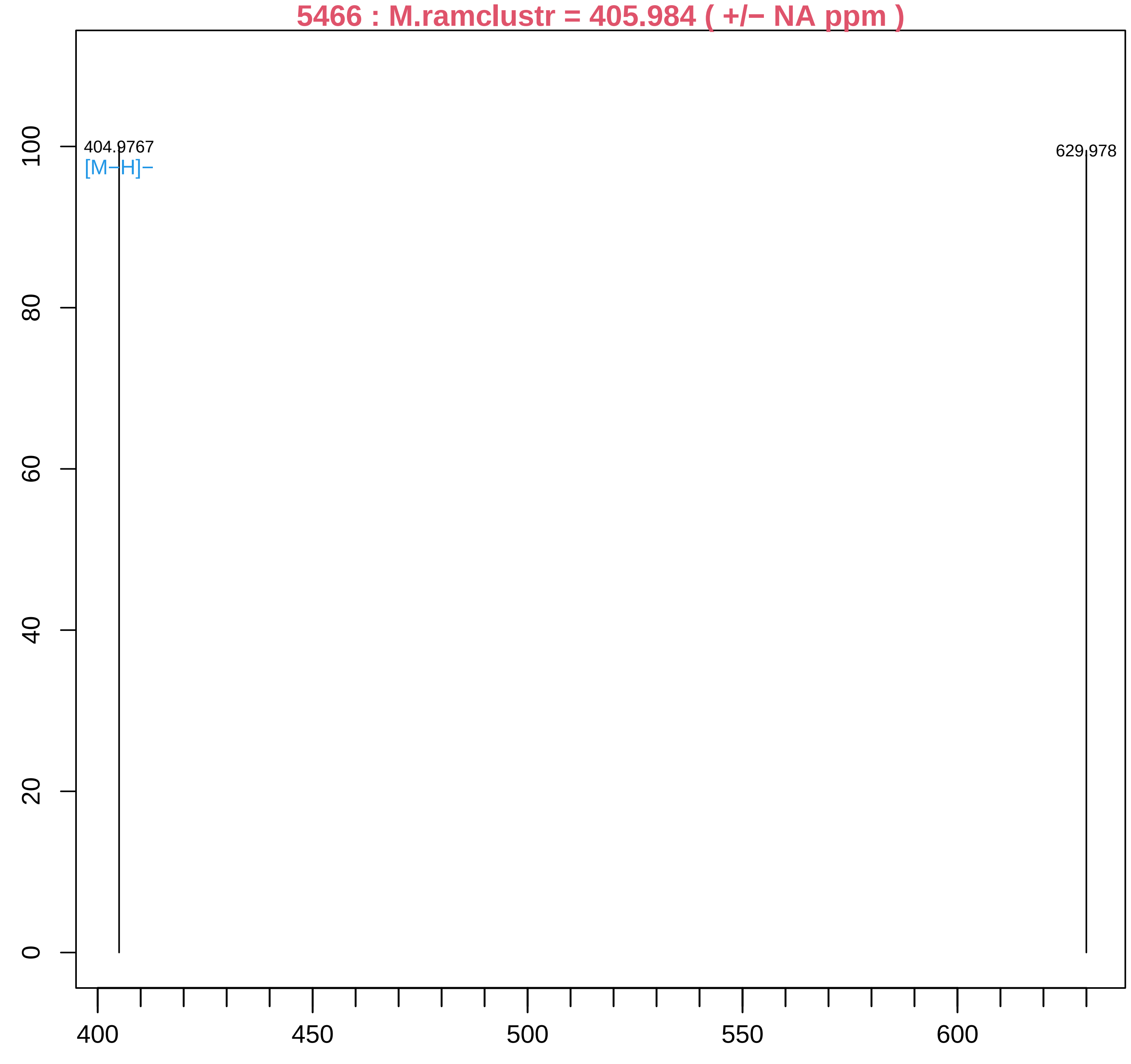
5466 : M.ramclustr = 405.984 (+/− NA ppm)
404.9767
[M−H]−
629.978
0
20
40
60
80
100
400
450
500
550
600

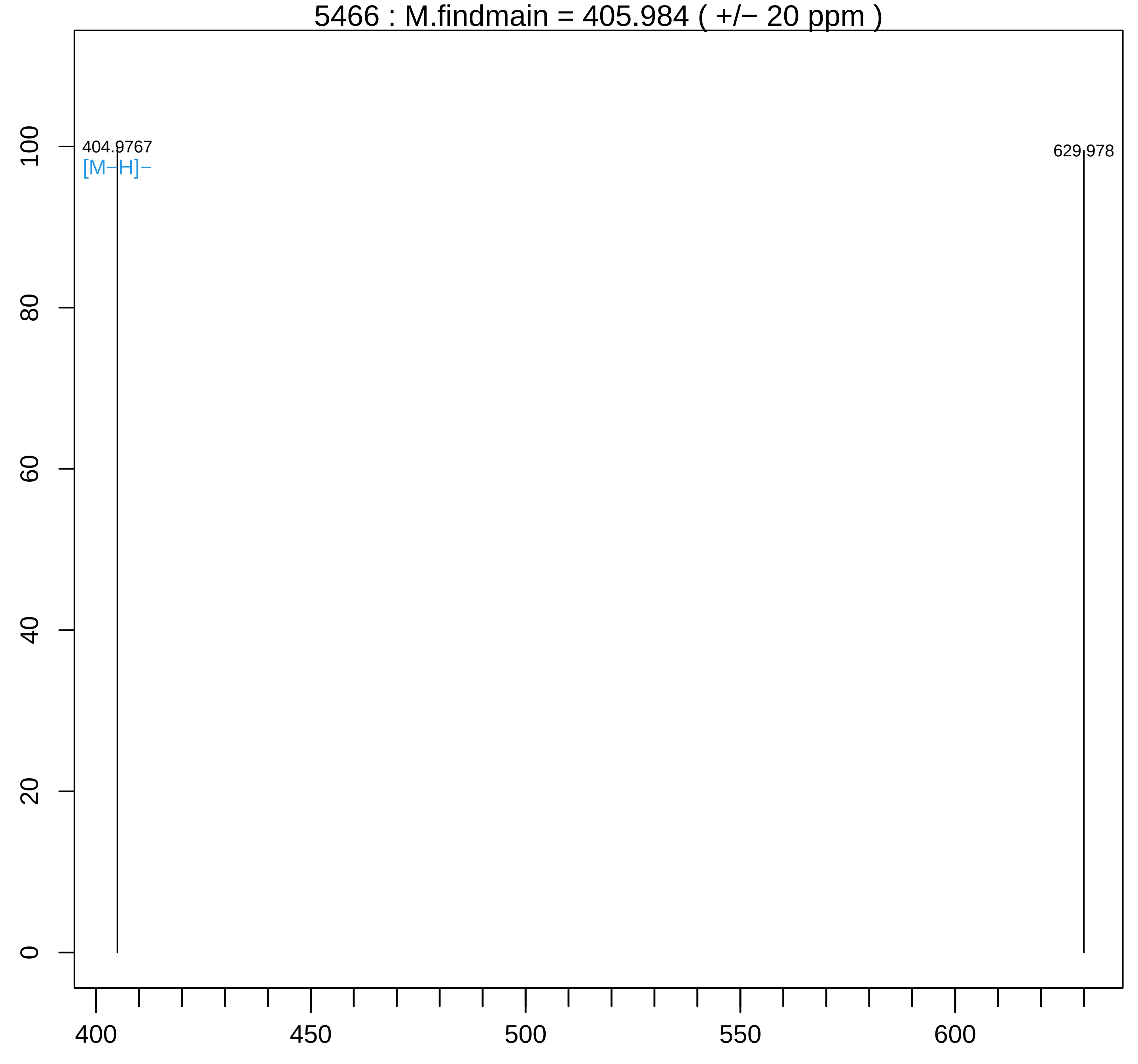
5466 : M.findmain = 405.984 (+/− 20 ppm)
404.9767
[M−H]−
629.978
0
20
40
60
80
100
400
450
500
550
600

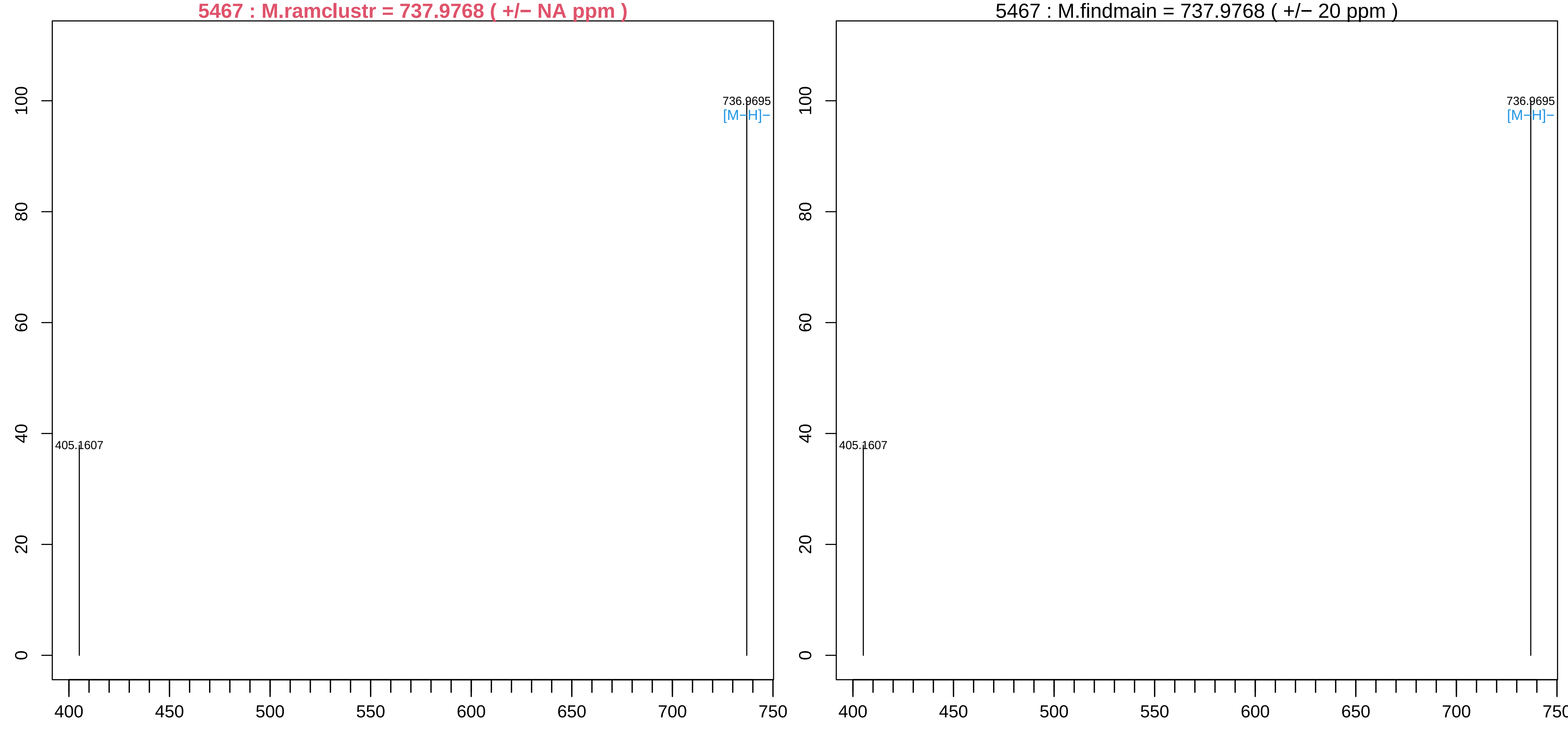

5467 : M.ramclustr = 737.9768 (+/− NA ppm)
405.1607
736.9695
[M−H]−
5467 : M.findmain = 737.9768 (+/− 20 ppm)
405.1607
736.9695
[M−H]−

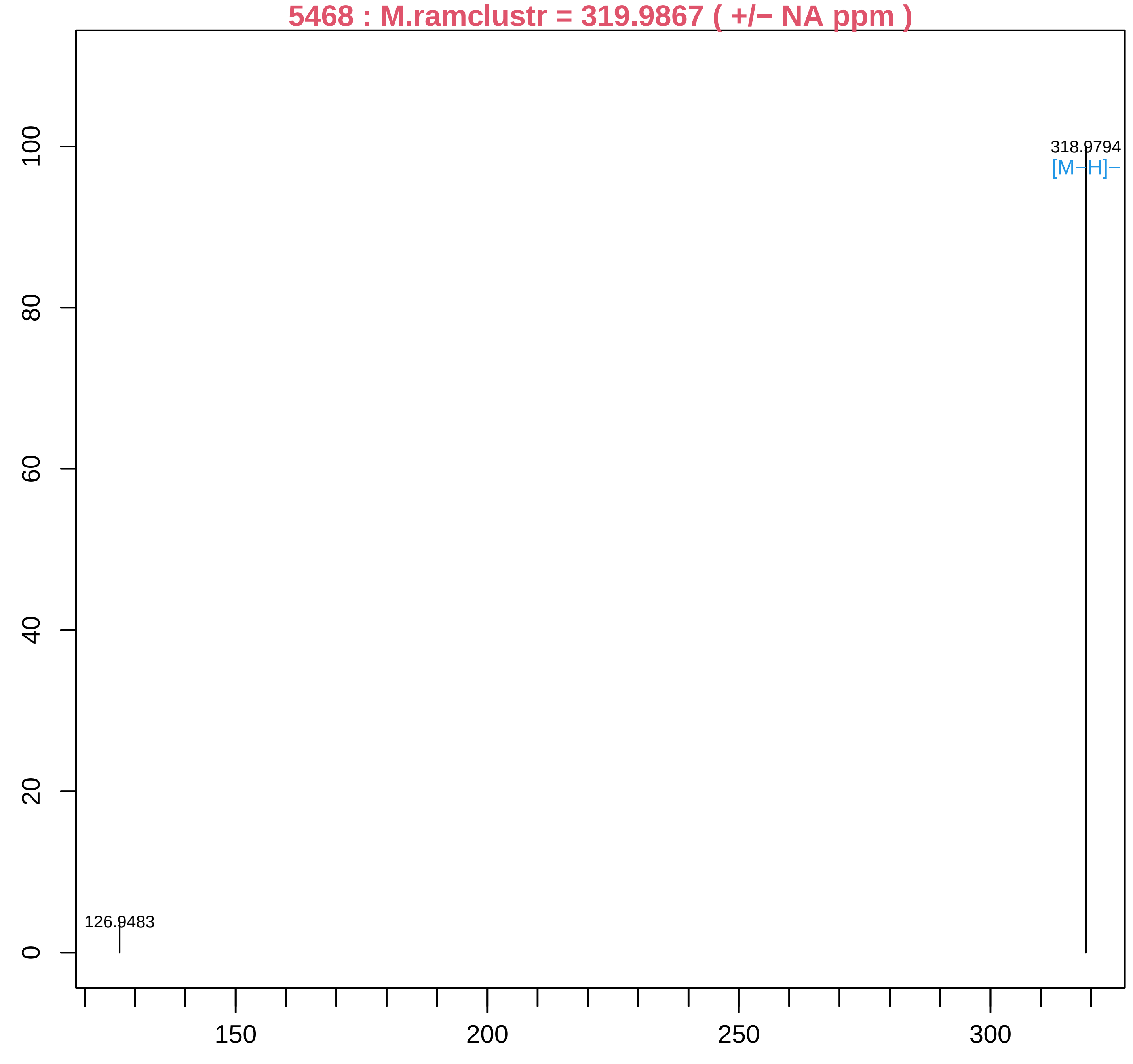
5468 : M.ramclustr = 319.9867 (+/− NA ppm)
318.9794
[M−H]−
126.9483
0
20
40
60
80
100
150
200
250
300

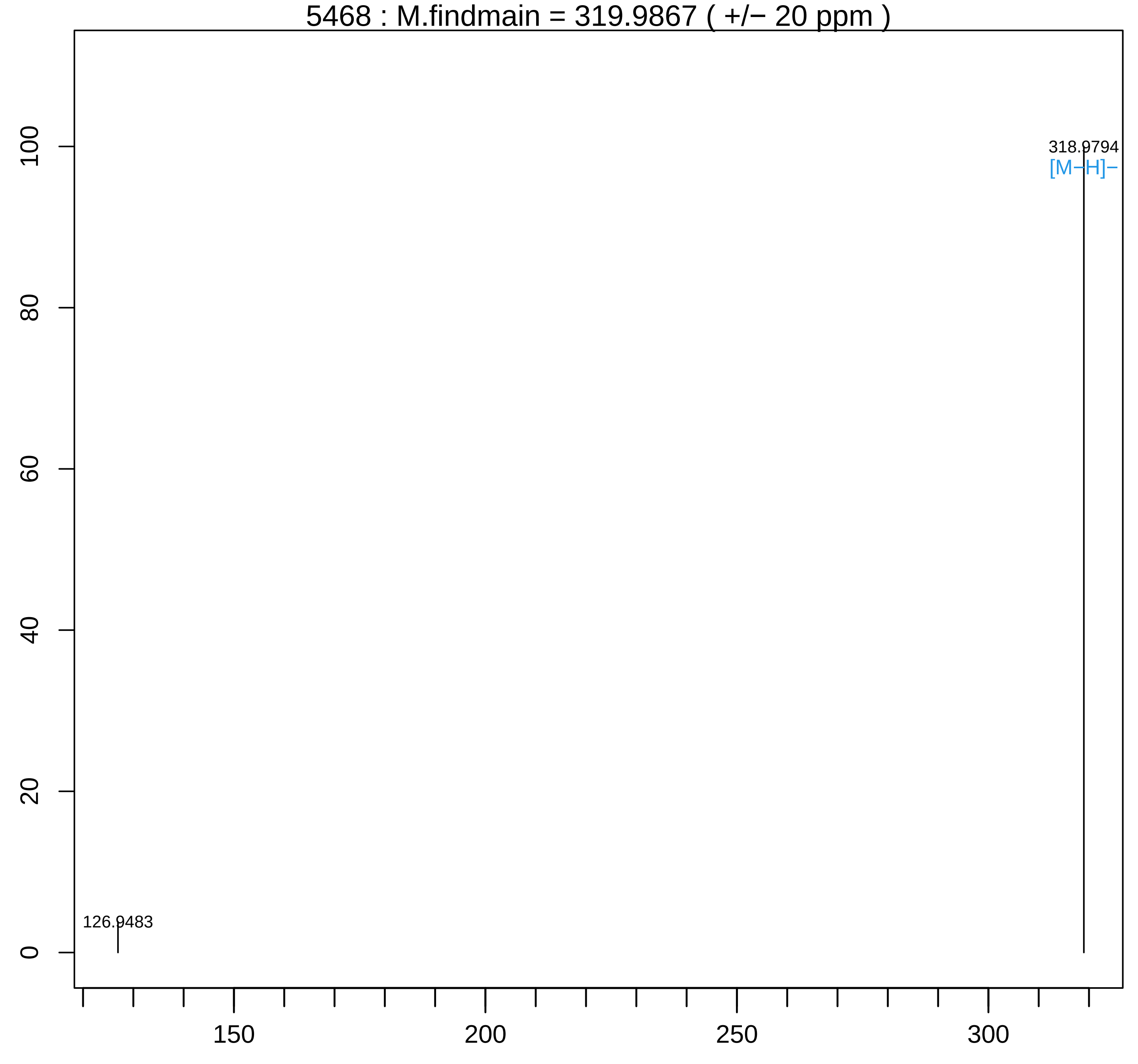
5468 : M.findmain = 319.9867 (+/− 20 ppm)
318.9794
[M−H]−
126.9483
0
20
40
60
80
100
150
200
250
300

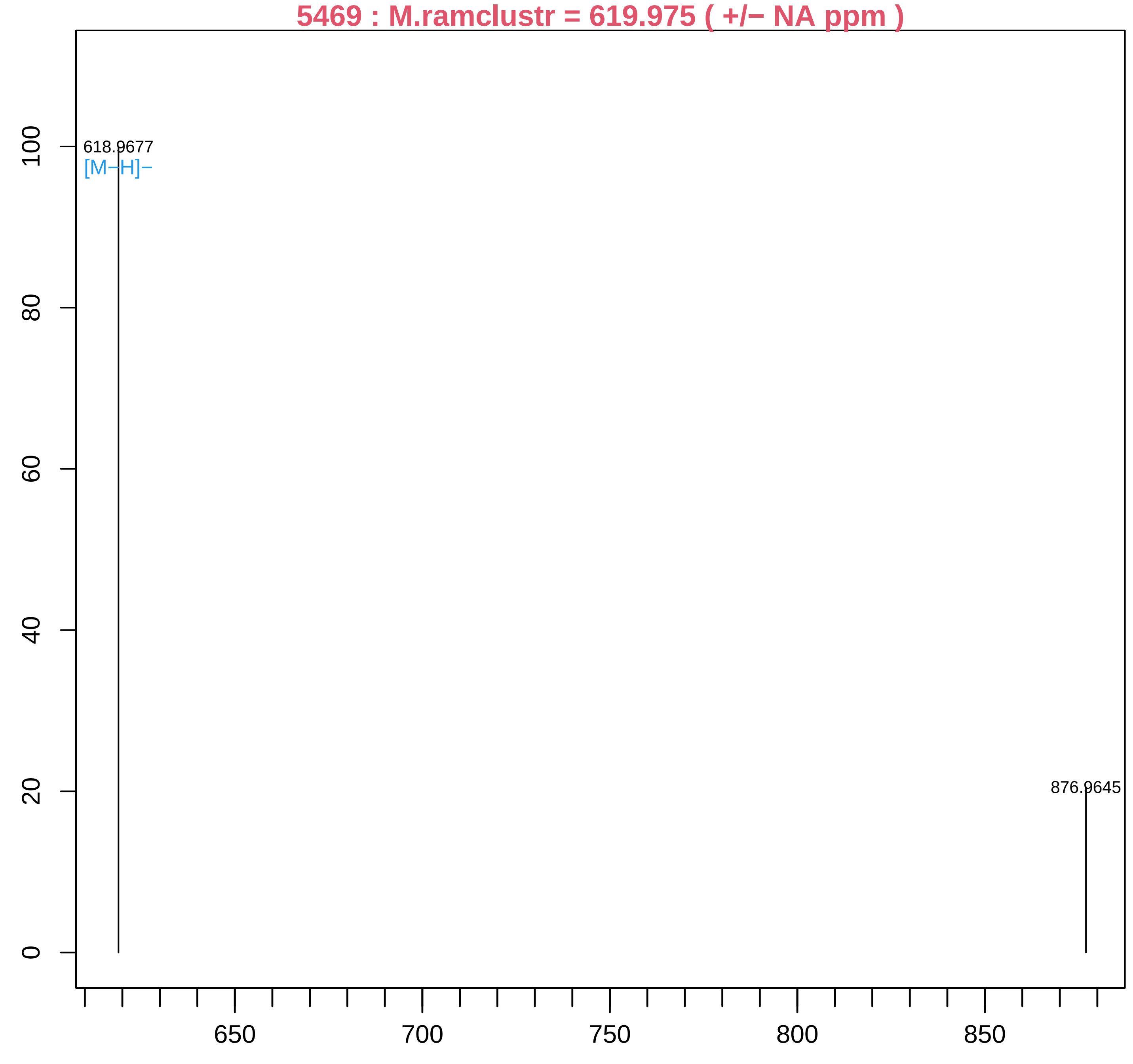
5469 : M.ramclustr = 619.975 (+/− NA ppm)
618.9677
[M−H]−
876.9645
0
20
40
60
80
100
650
700
750
800
850

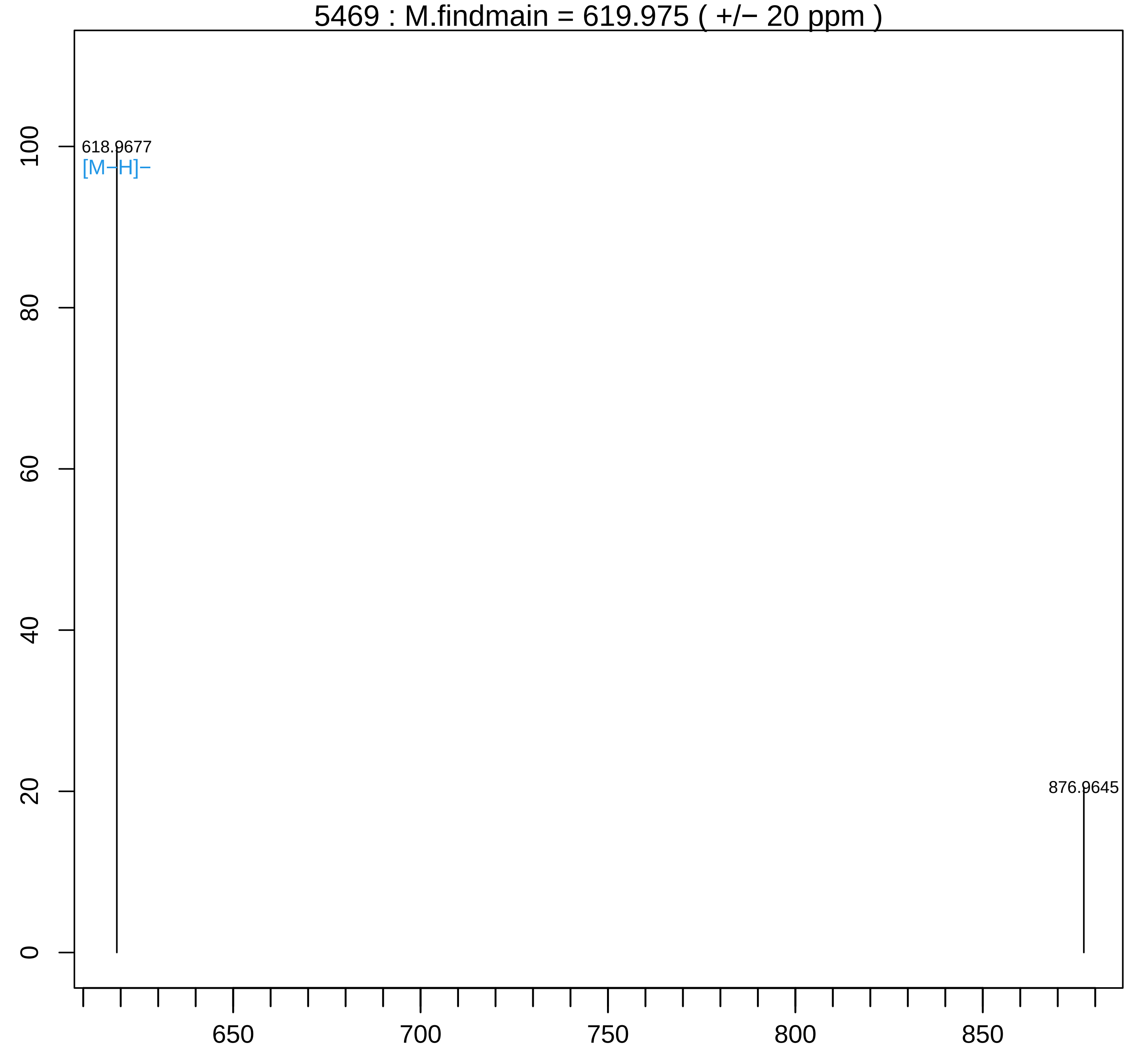
5469 : M.findmain = 619.975 (+/− 20 ppm)
618.9677
[M−H]−
876.9645
0
20
40
60
80
100
650
700
750
800
850

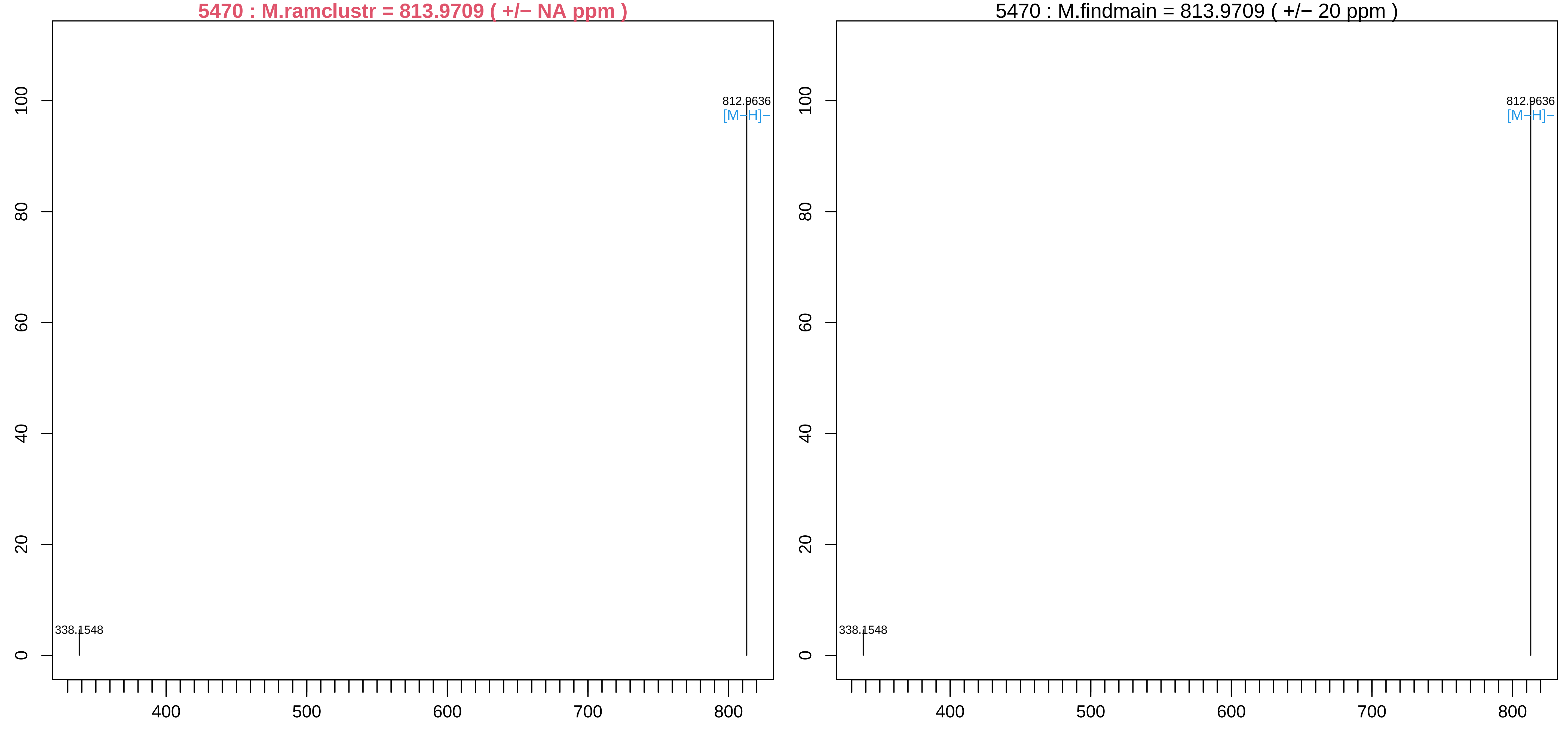

5470 : M.ramclustr = 813.9709 (+/− NA ppm)
812.9636
[M−H]−
338.1548
5470 : M.findmain = 813.9709 (+/− 20 ppm)
812.9636
[M−H]−
338.1548
0
20
40
60
80
100
400
500
600
700
800

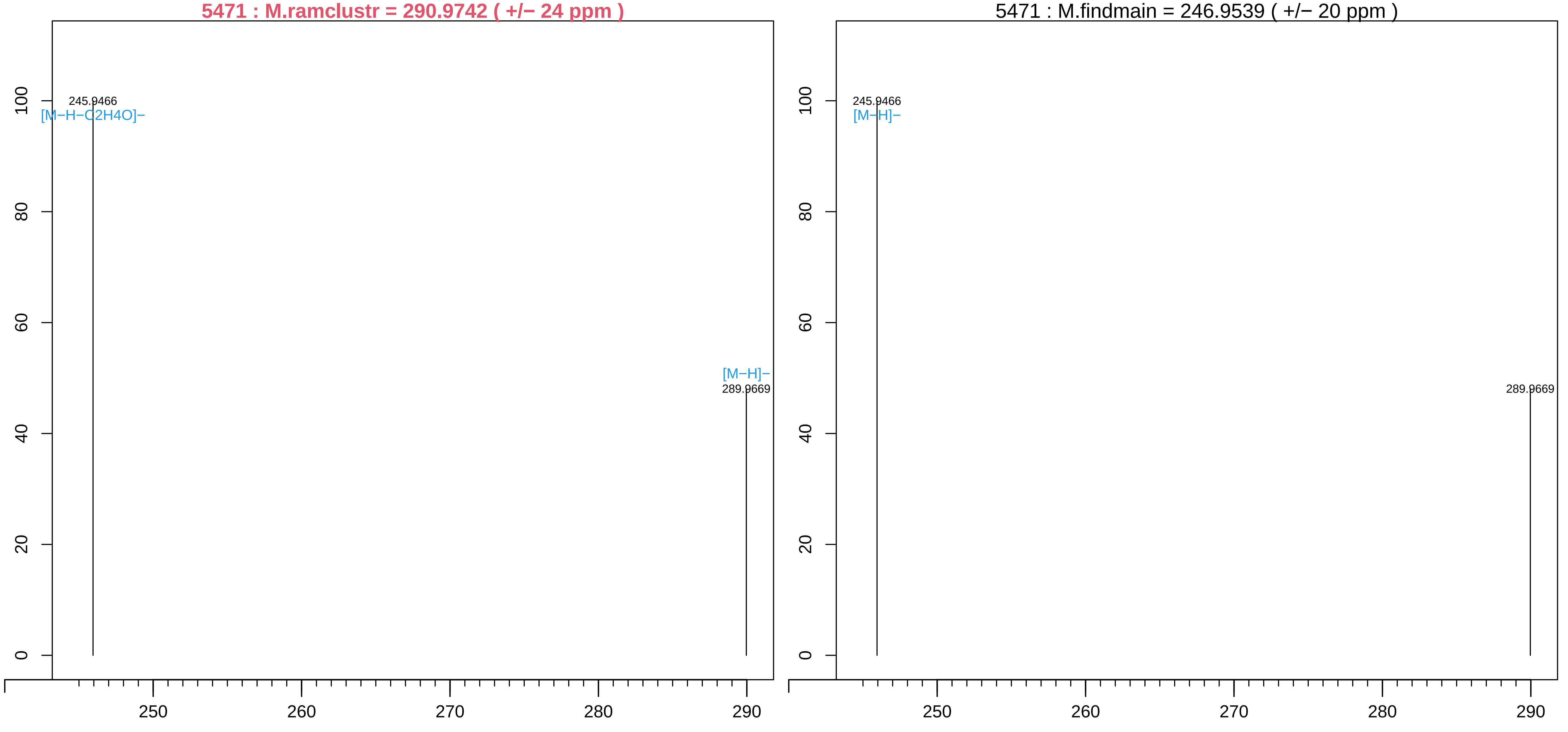
5471 : M.ramclustr = 290.9742 (+/− 24 ppm)
245.9466
[M−H−C2H4O]−
[M−H]−
289.9669
5471 : M.findmain = 246.9539 (+/− 20 ppm)
245.9466
[M−H]−
289.9669
0
20
40
60
80
100
250
260
270
280
290

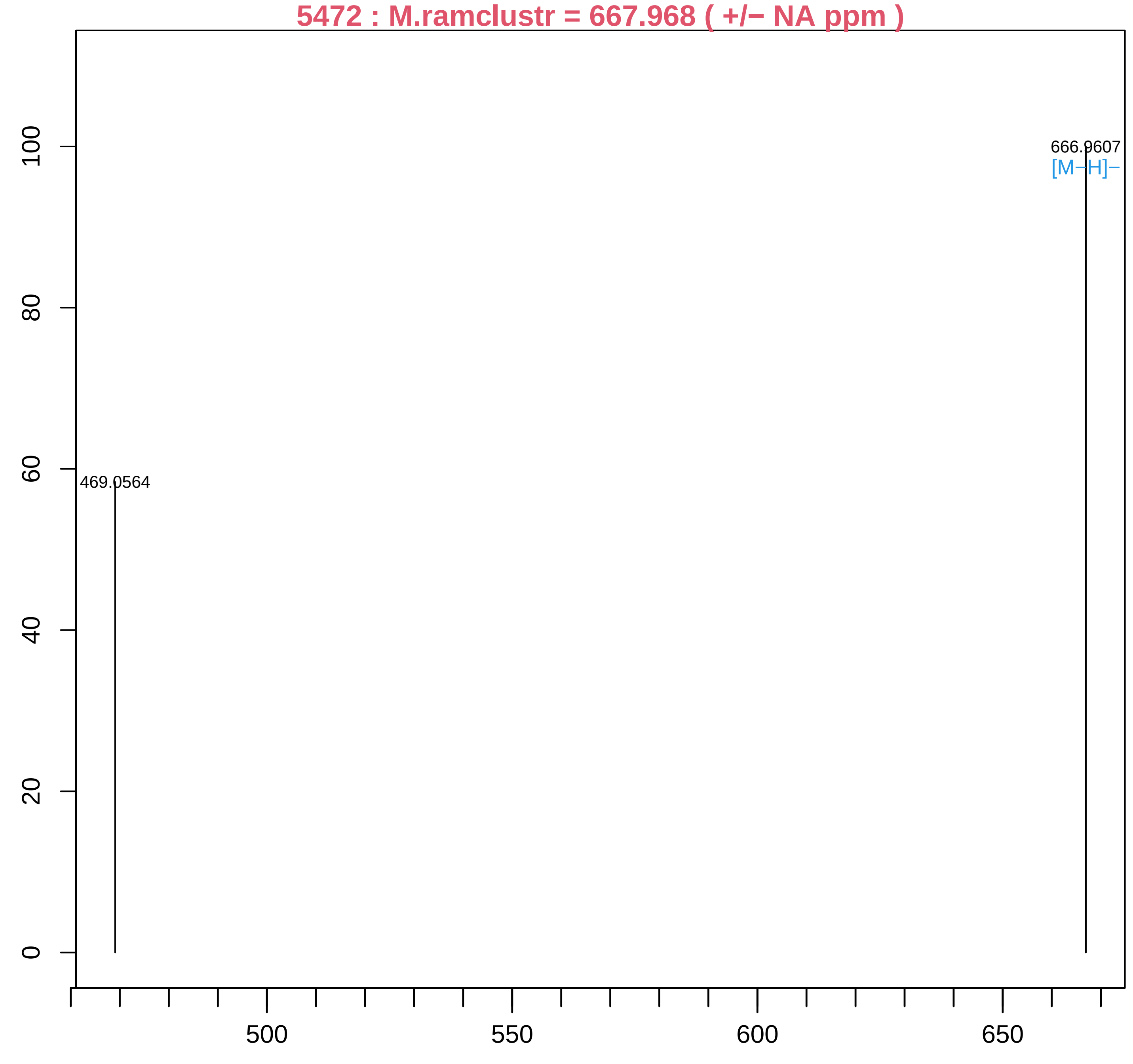

5472 : M.ramclustr = 667.968 (+/− NA ppm)
666.9607
[M−H]−
469.0564
0
20
40
60
80
100
500
550
600
650

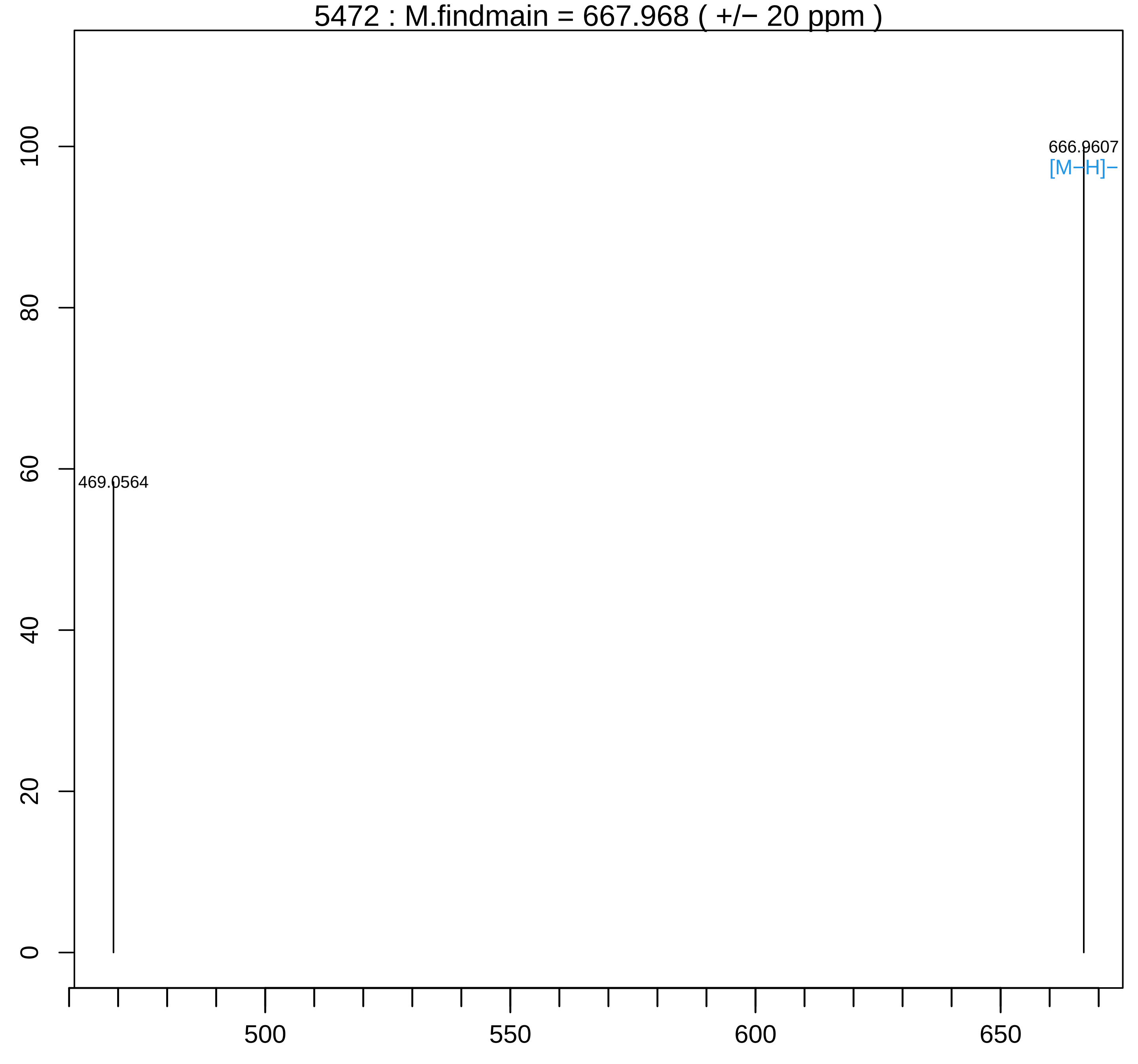

5472 : M.findmain = 667.968 (+/− 20 ppm)
666.9607
[M−H]−
469.0564
0
20
40
60
80
100
500
550
600
650

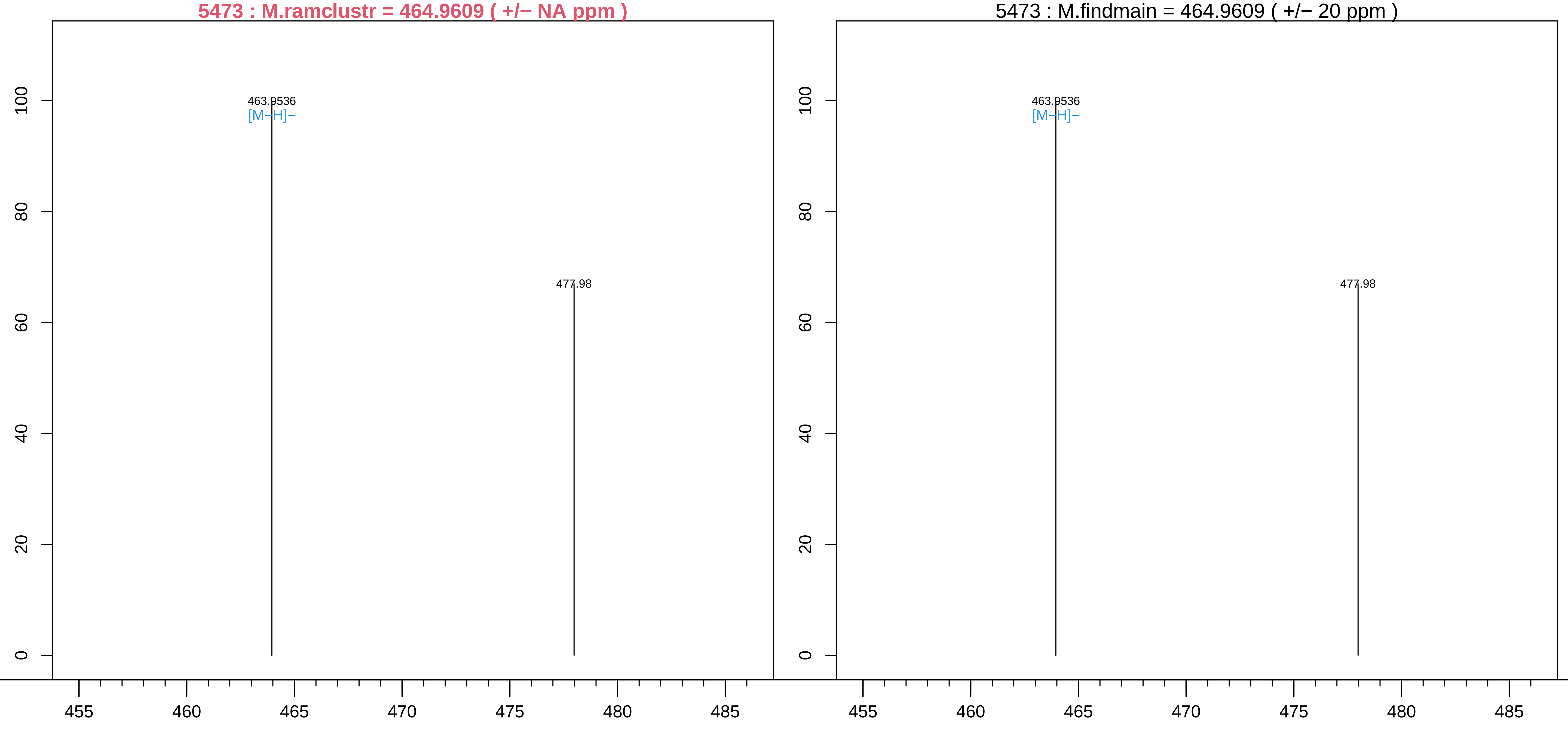
5473 : M.ramclustr = 464.9609 (+/− NA ppm)
463.9536
[M−H]−
477.98
5473 : M.findmain = 464.9609 (+/− 20 ppm)
463.9536
[M−H]−
477.98
0
20
40
60
80
100
455
460
465
470
475
480
485

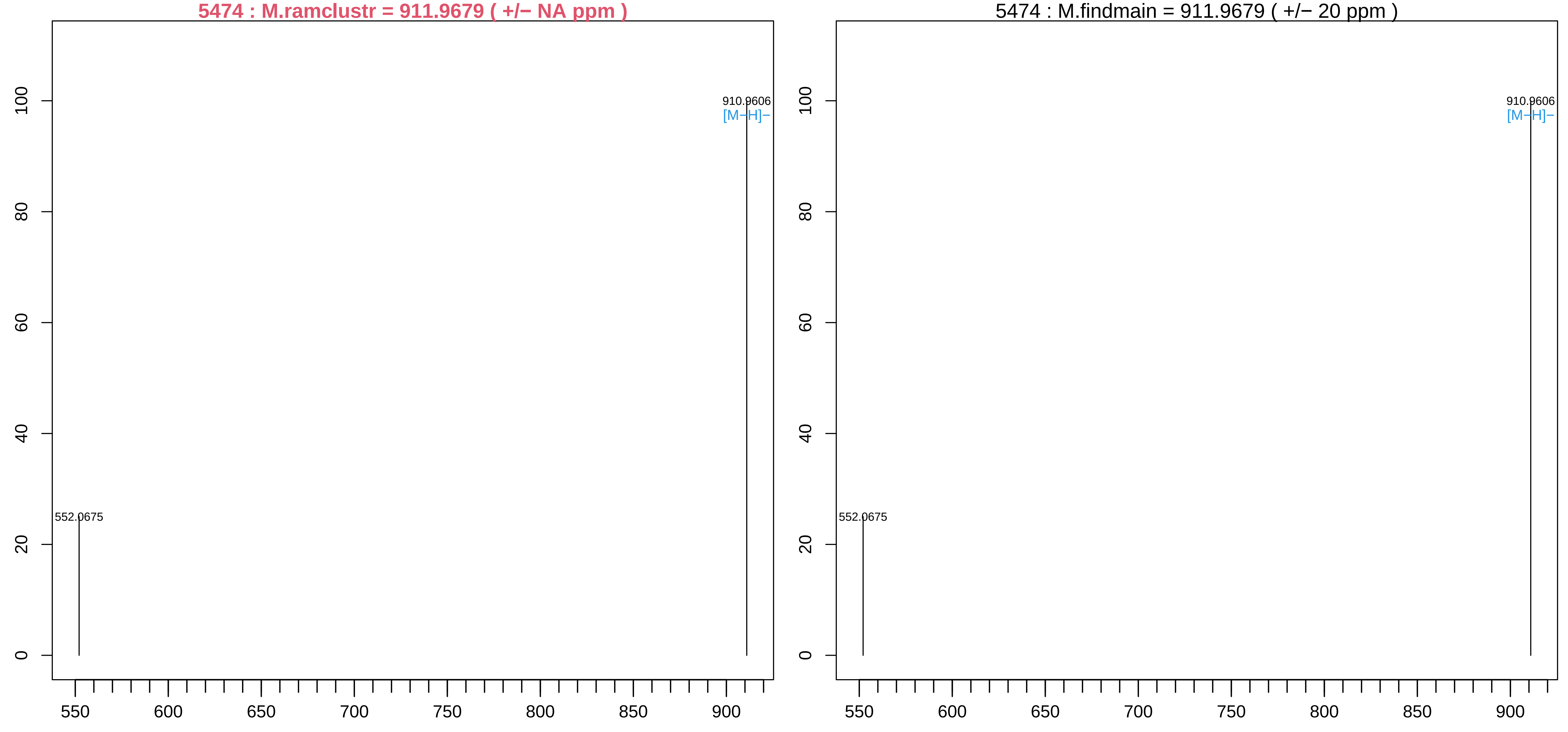
5474 : M.ramclustr = 911.9679 (+/− NA ppm)
910.9606
[M−H]−
552.0675
5474 : M.findmain = 911.9679 (+/− 20 ppm)
910.9606
[M−H]−
552.0675
0
20
40
60
80
100
550
600
650
700
750
800
850
900

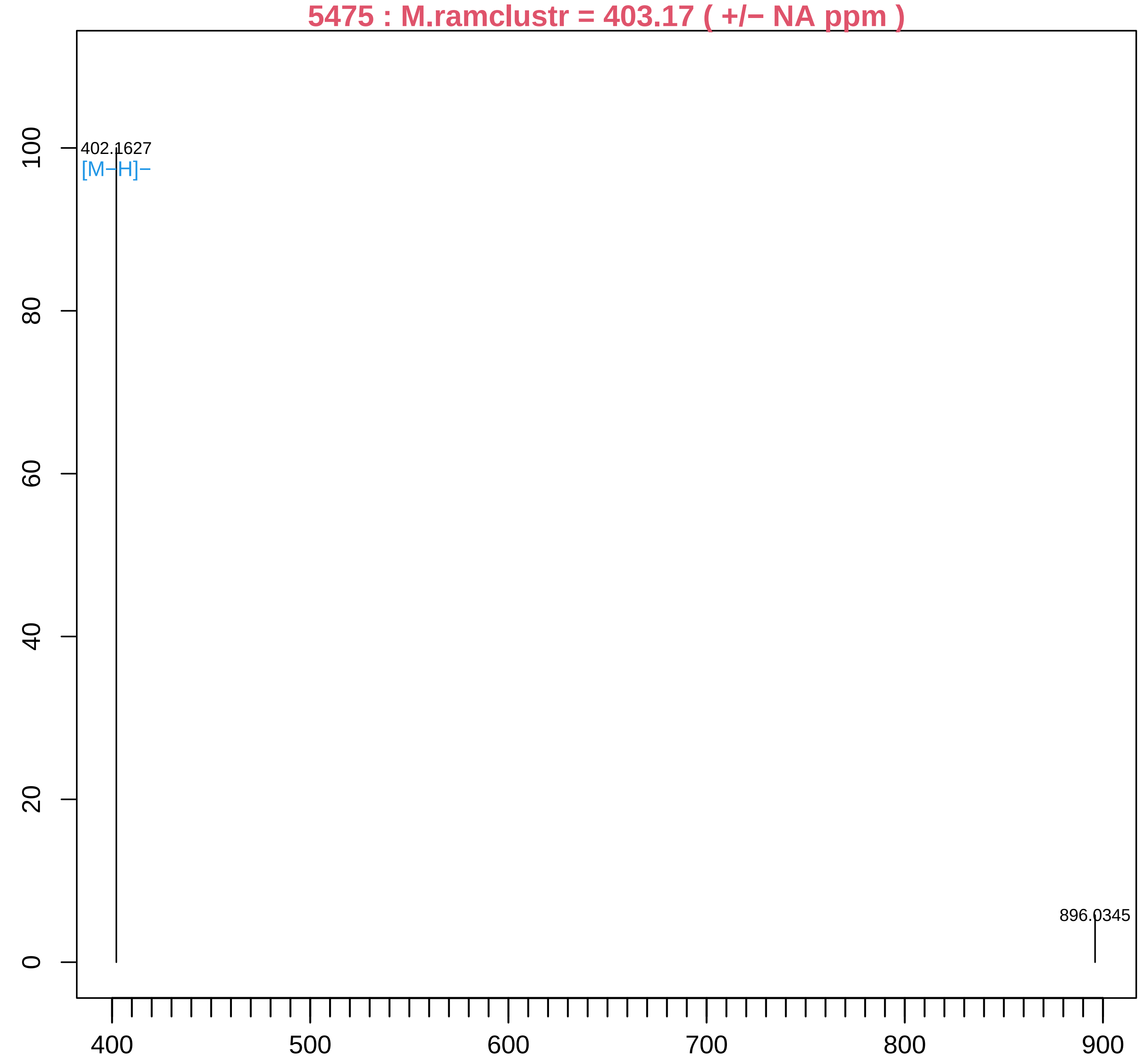

5475 : M.ramclustr = 403.17 (+/− NA ppm)
402.1627
[M−H]−
896.0345
0
20
40
60
80
100
400
500
600
700
800
900

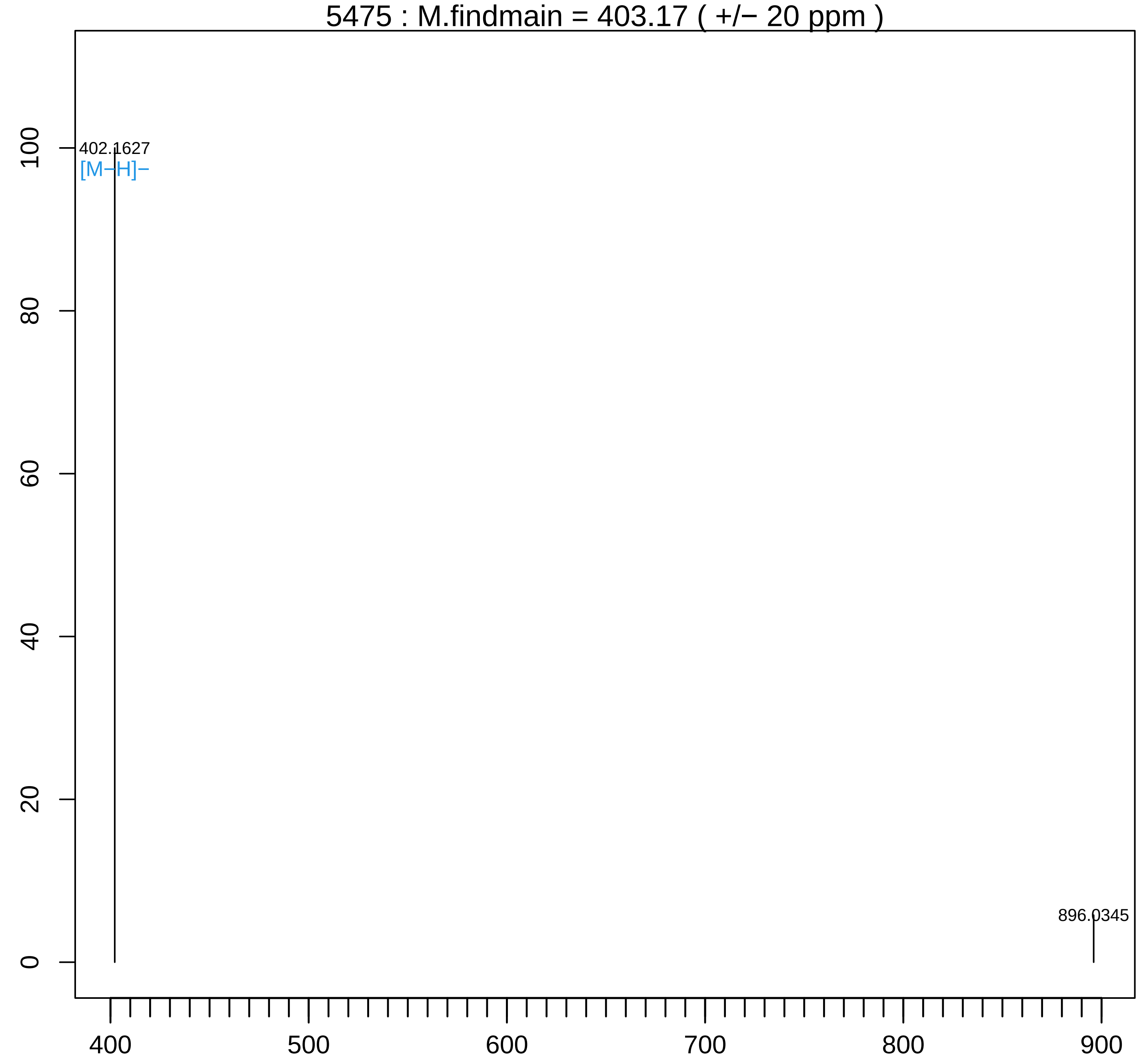

5475 : M.findmain = 403.17 (+/− 20 ppm)
402.1627
[M−H]−
896.0345
0
20
40
60
80
100
400
500
600
700
800
900

5476 : M.ramclustr = 171.9462 (+/− NA ppm)

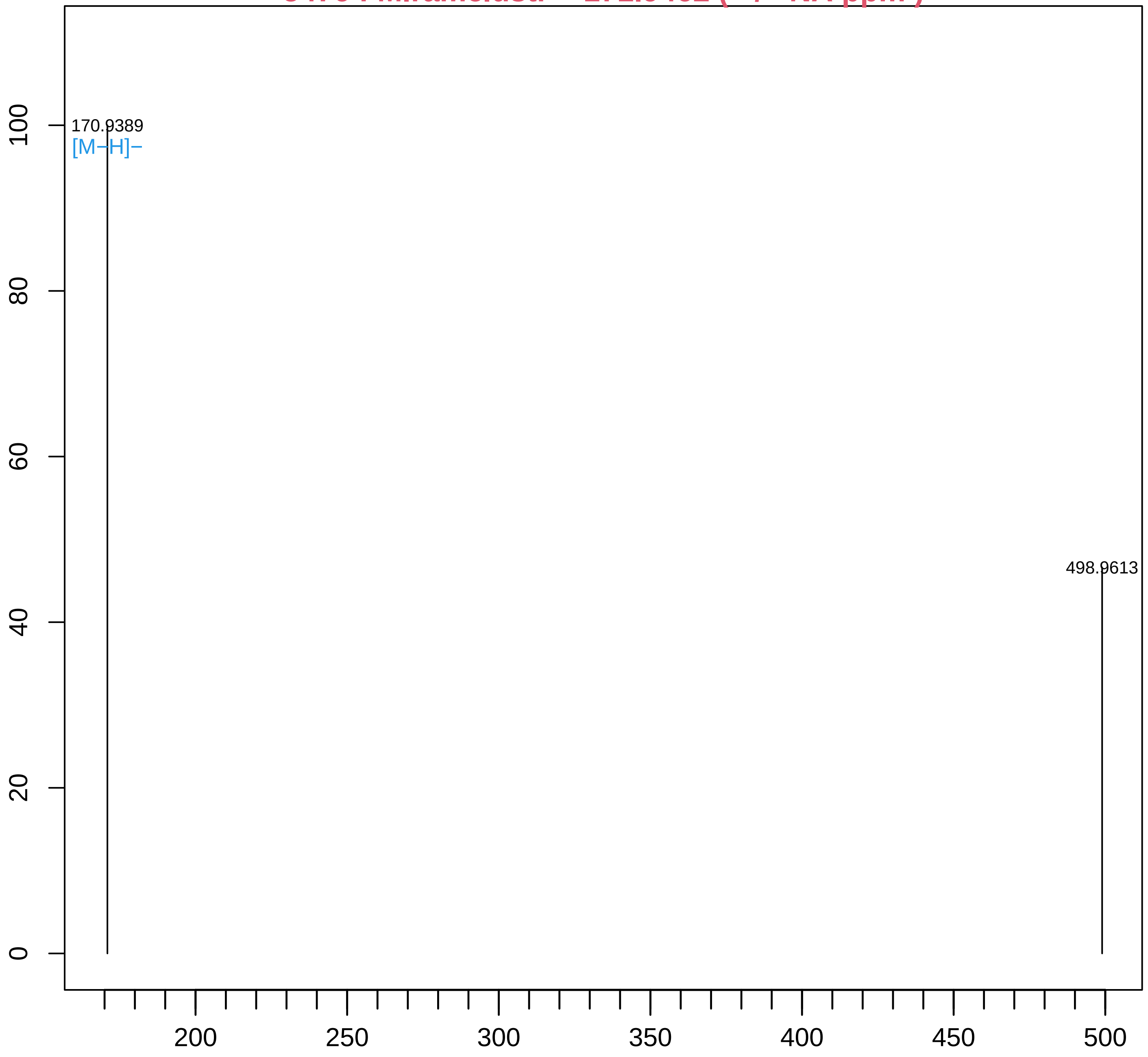

5476 : M.findmain = 171.9462 (+/− 20 ppm)

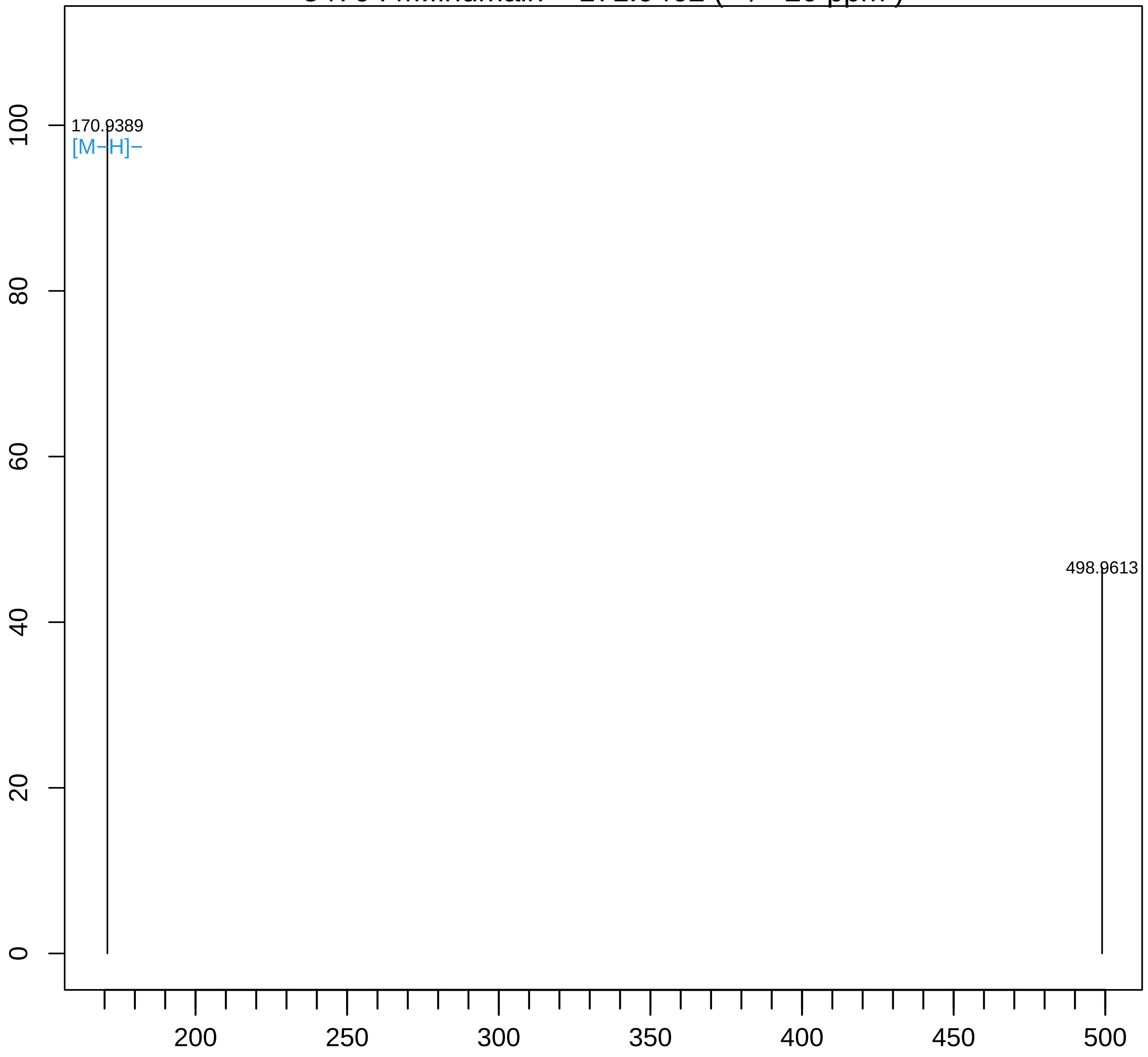

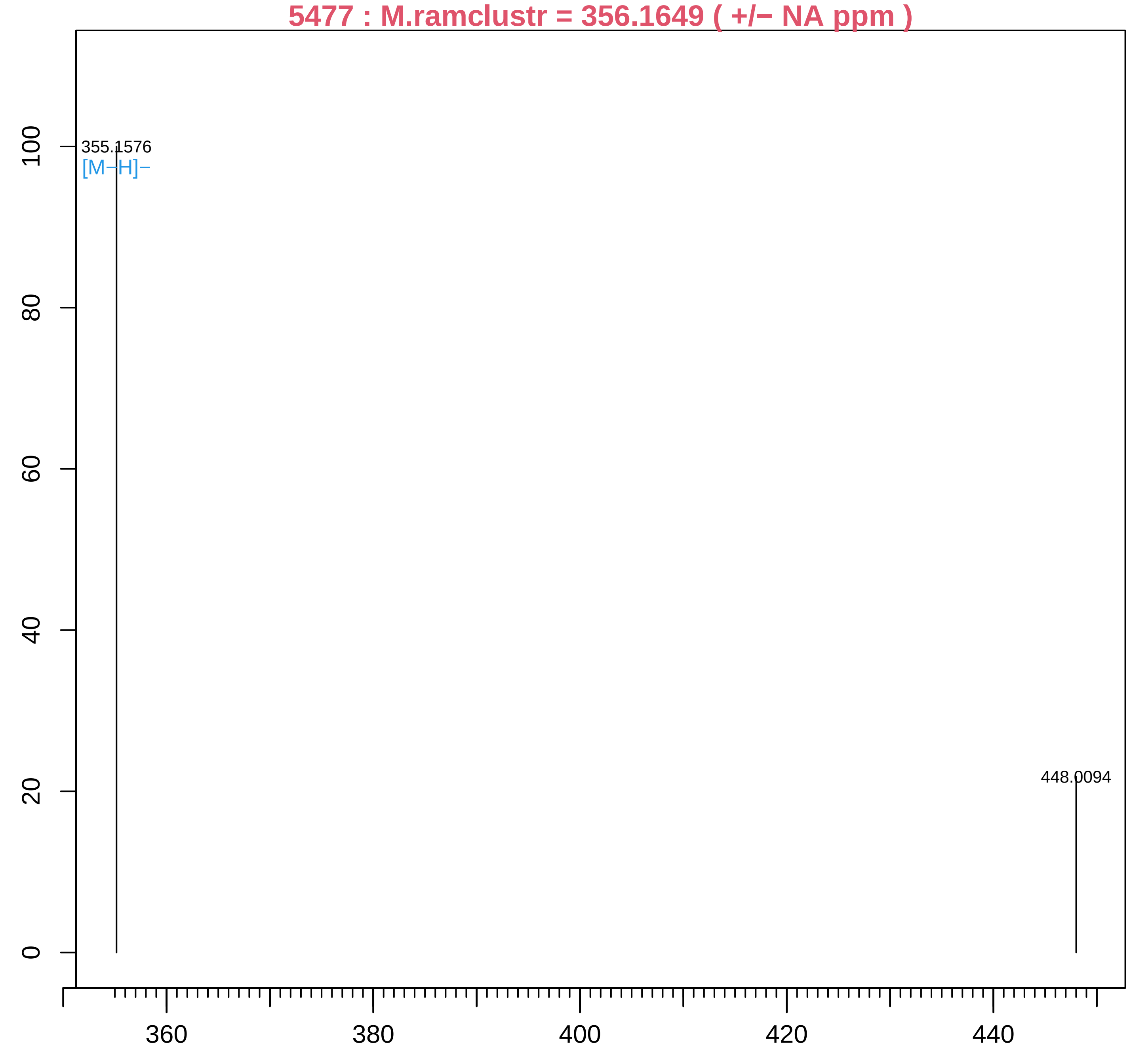
5477 : M.ramclustr = 356.1649 (+/− NA ppm)
355.1576
[M−H]−
448.0094
0
20
40
60
80
100
360
380
400
420
440

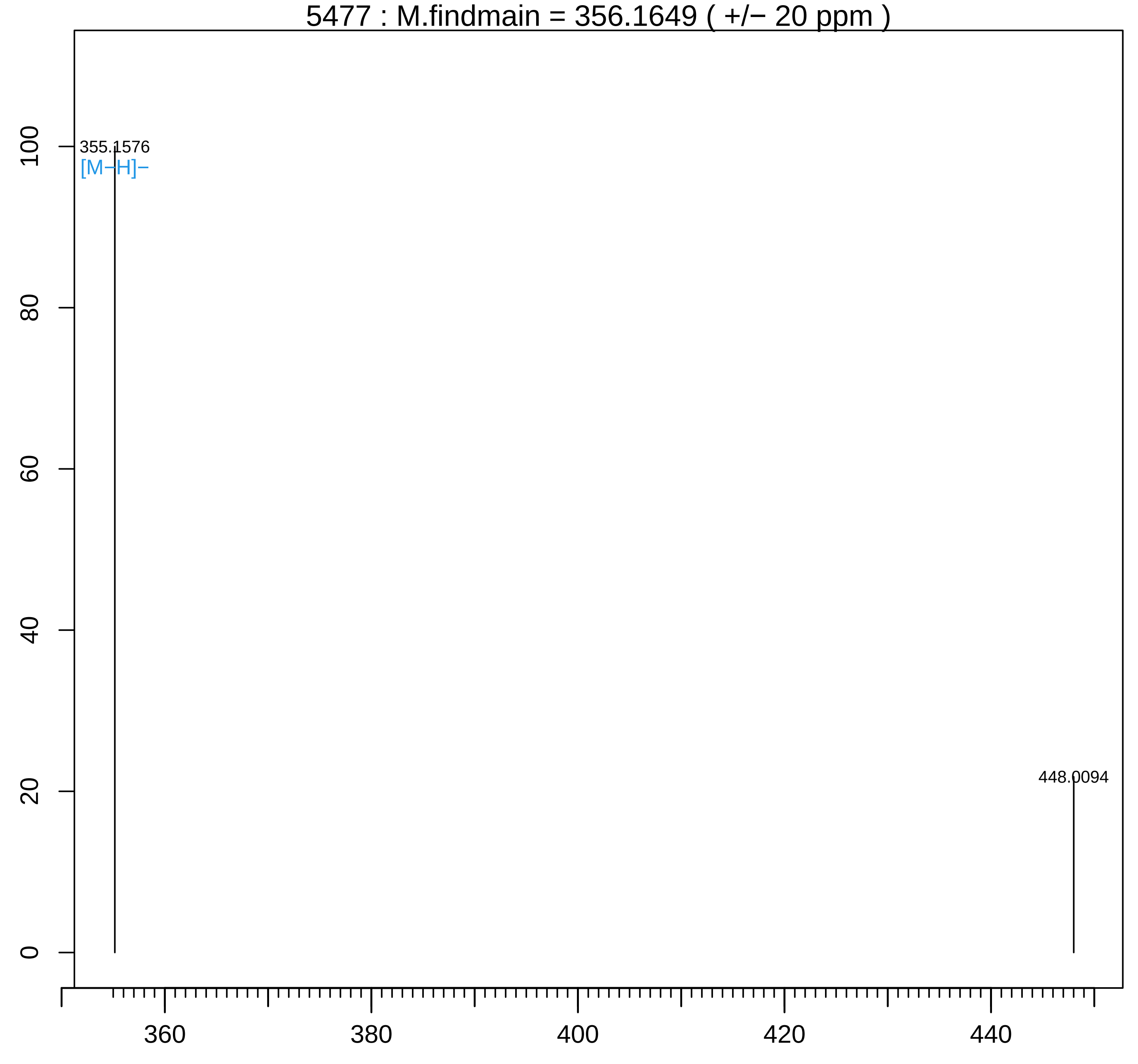
5477 : M.findmain = 356.1649 (+/− 20 ppm)
355.1576
[M−H]−
448.0094
0
20
40
60
80
100
360
380
400
420
440

5478 : M.ramclustr = 591.0516 (+/− NA ppm)

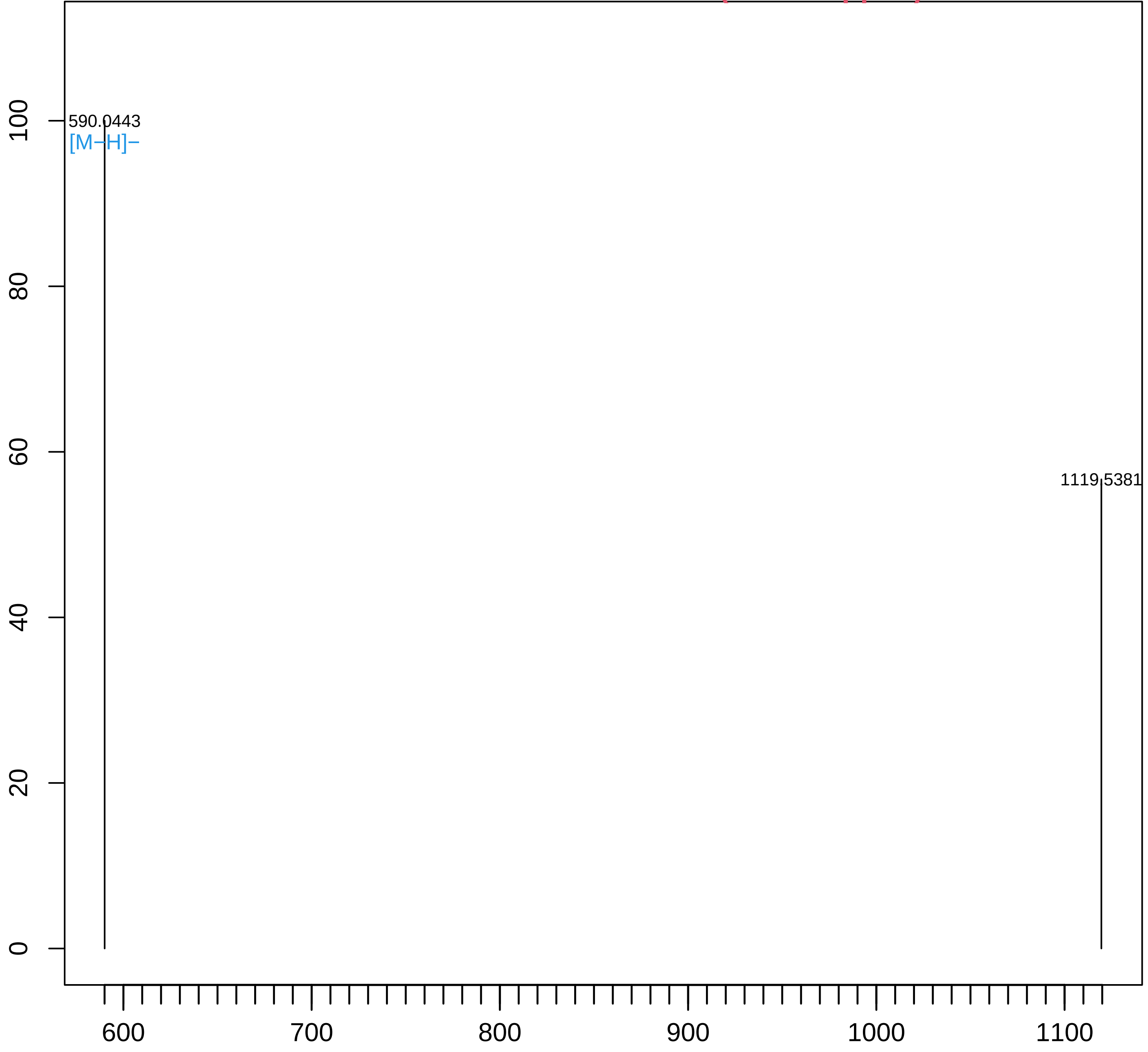

5478 : M.findmain = 591.0516 (+/− 20 ppm)

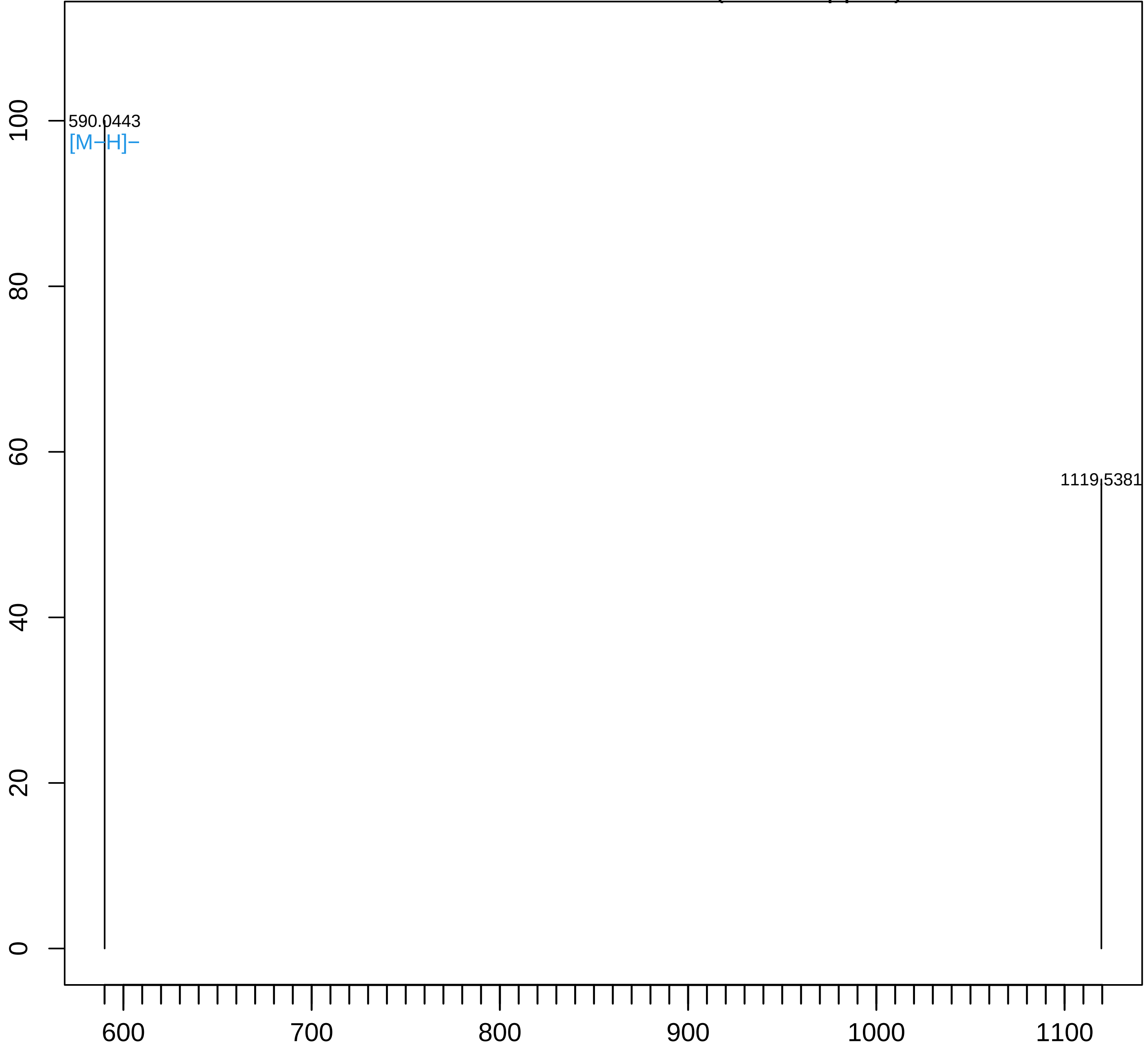

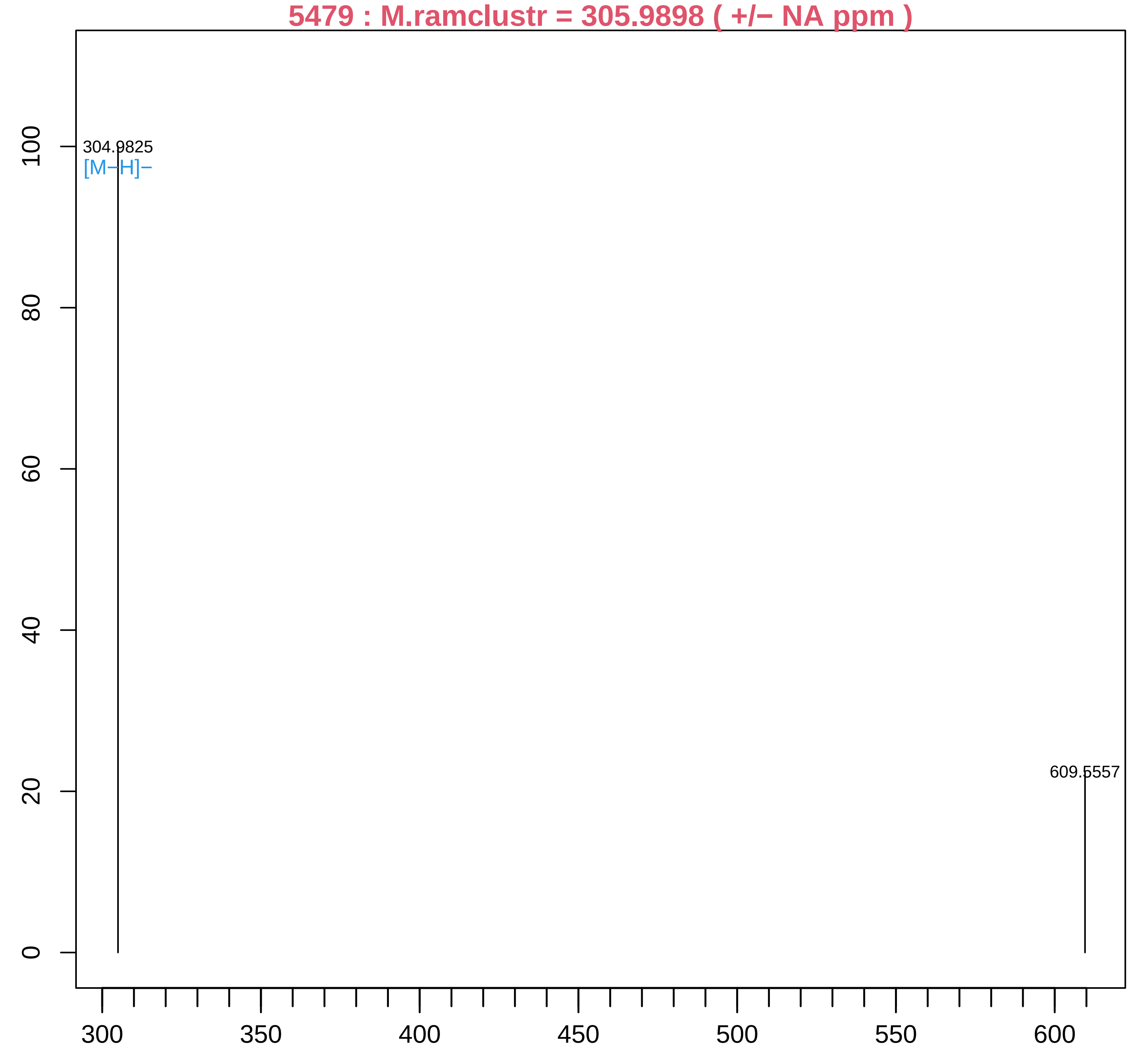

5479 : M.ramclustr = 305.9898 (+/− NA ppm)
304.9825
[M−H]−
609.5557
0
20
40
60
80
100
300
350
400
450
500
550
600

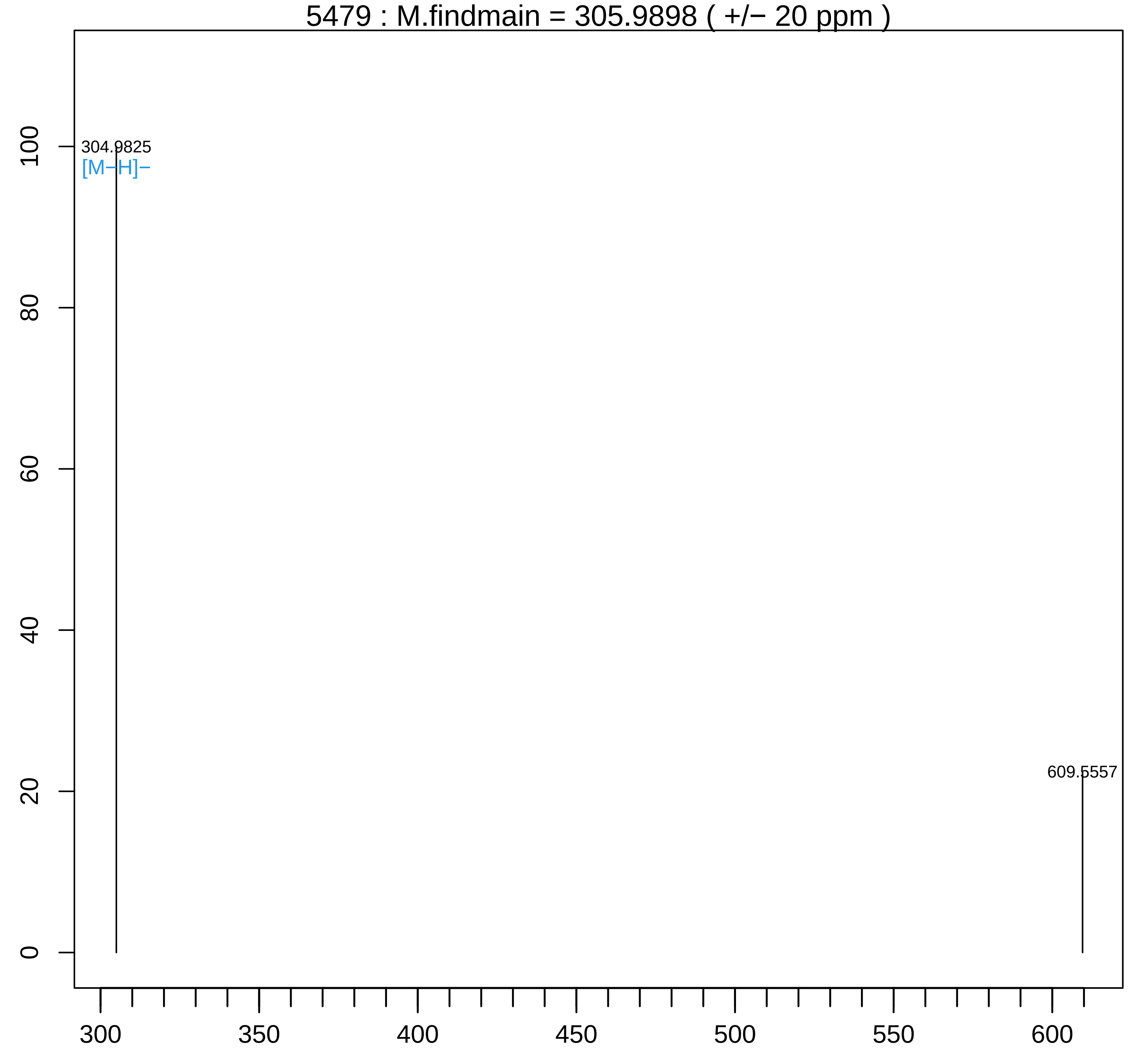

5479 : M.findmain = 305.9898 (+/− 20 ppm)
304.9825
[M−H]−
609.5557
0
20
40
60
80
100
300
350
400
450
500
550
600

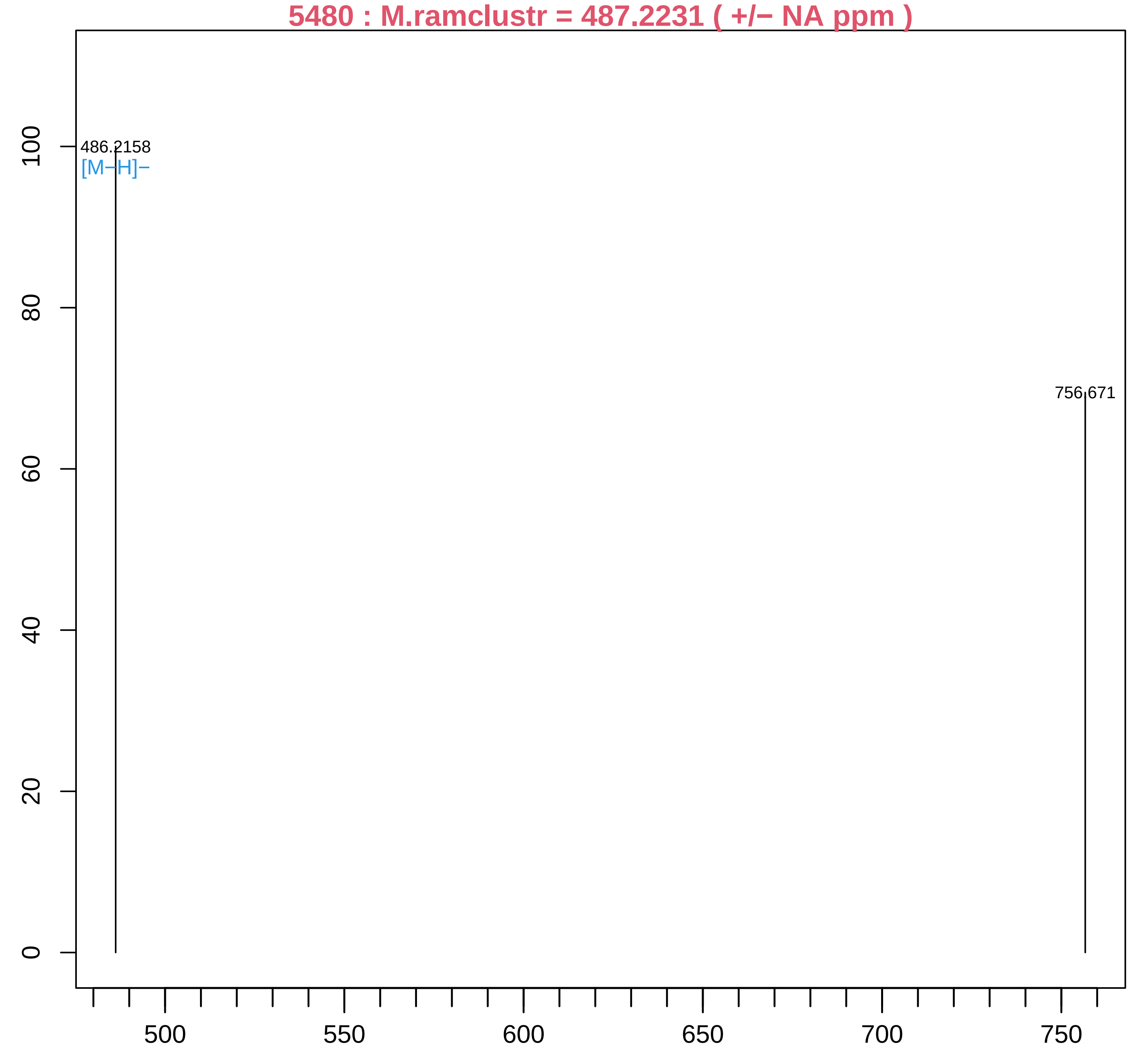
5480 : M.ramclustr = 487.2231 (+/− NA ppm)
486.2158
[M−H]−
756.671
0
20
40
60
80
100
500
550
600
650
700
750

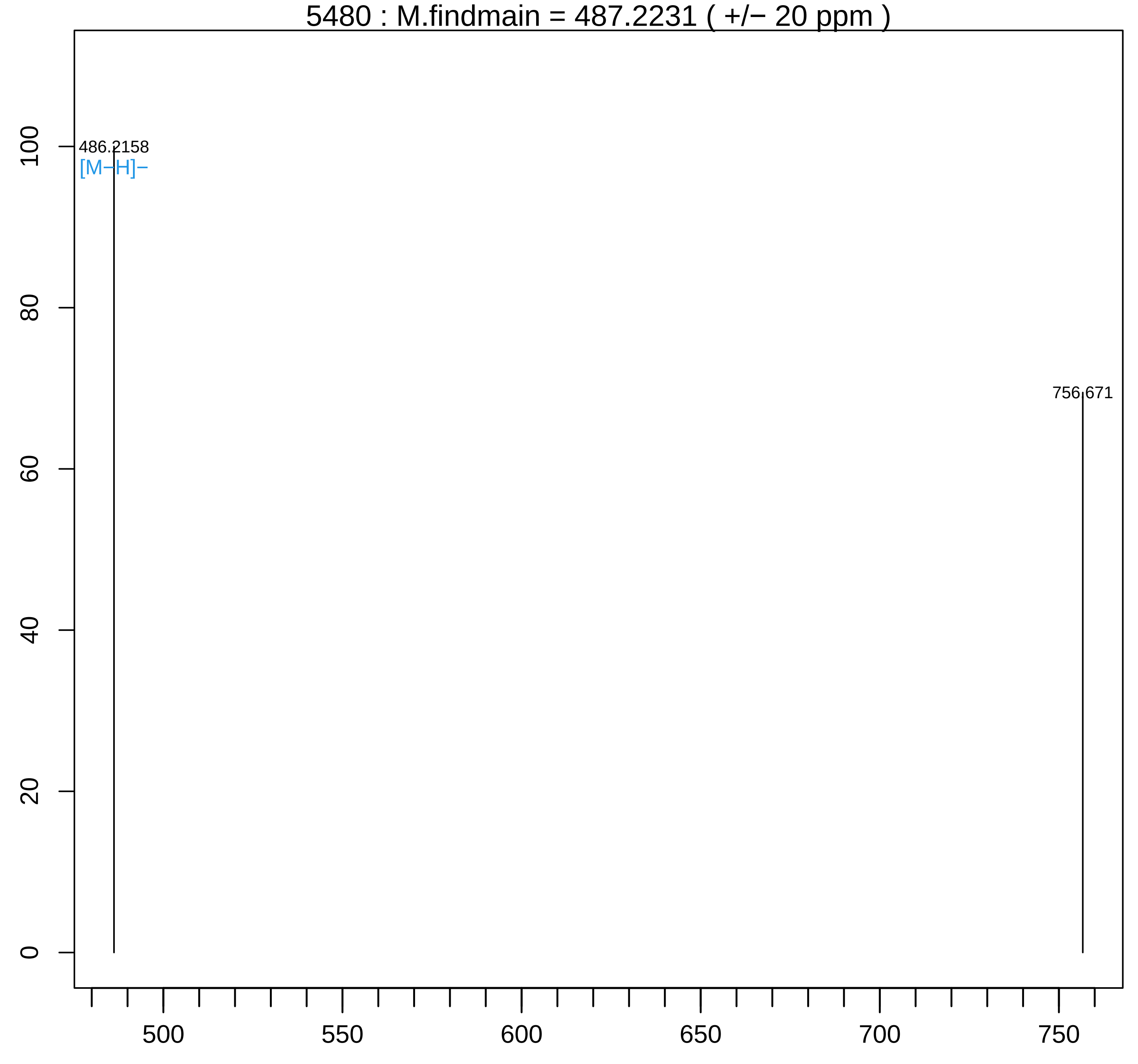
5480 : M.findmain = 487.2231 (+/− 20 ppm)
486.2158
[M−H]−
756.671
0
20
40
60
80
100
500
550
600
650
700
750

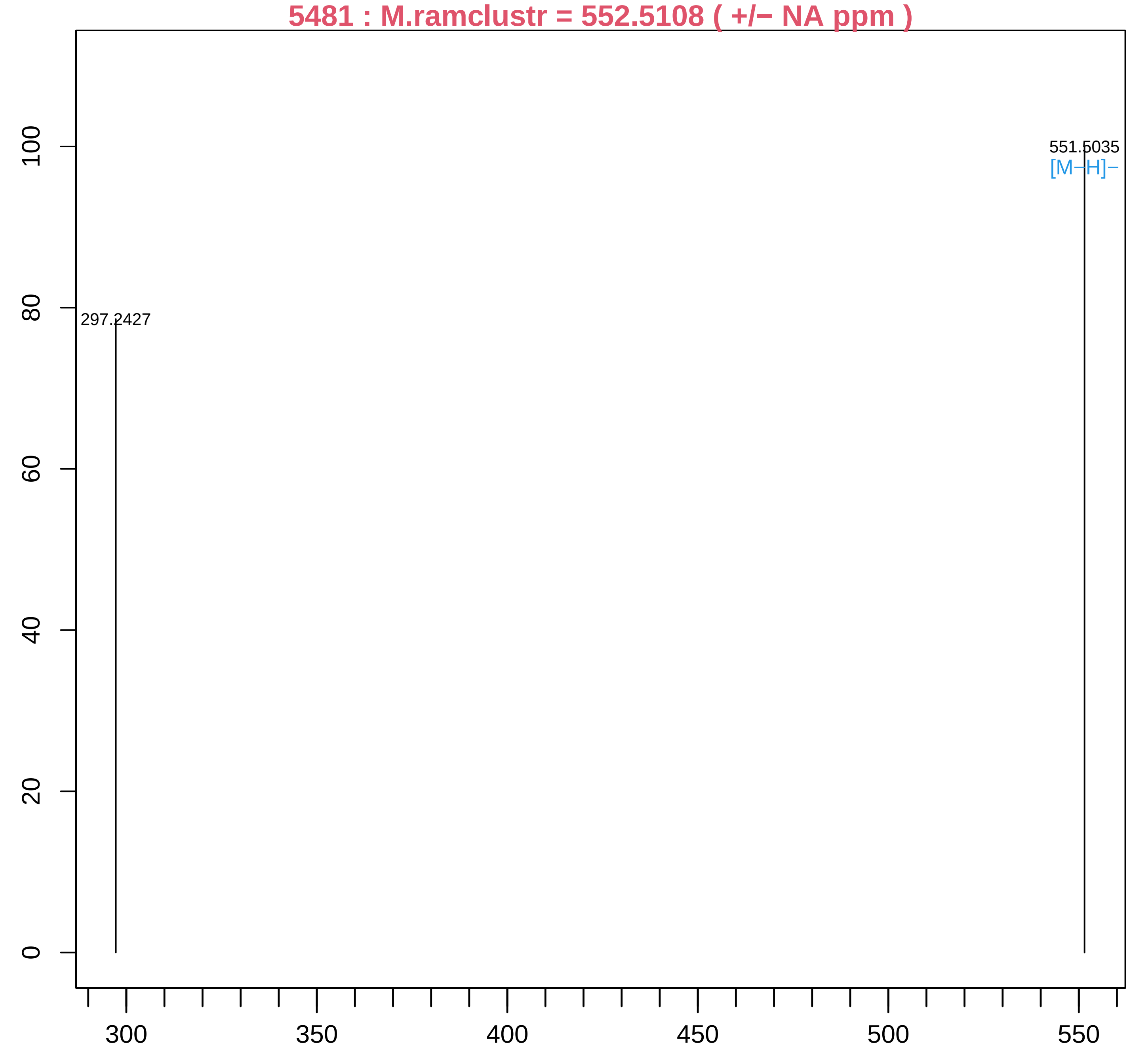

5481 : M.ramclustr = 552.5108 (+/− NA ppm)
551.5035
[M−H]−
297.2427
0
20
40
60
80
100
300
350
400
450
500
550

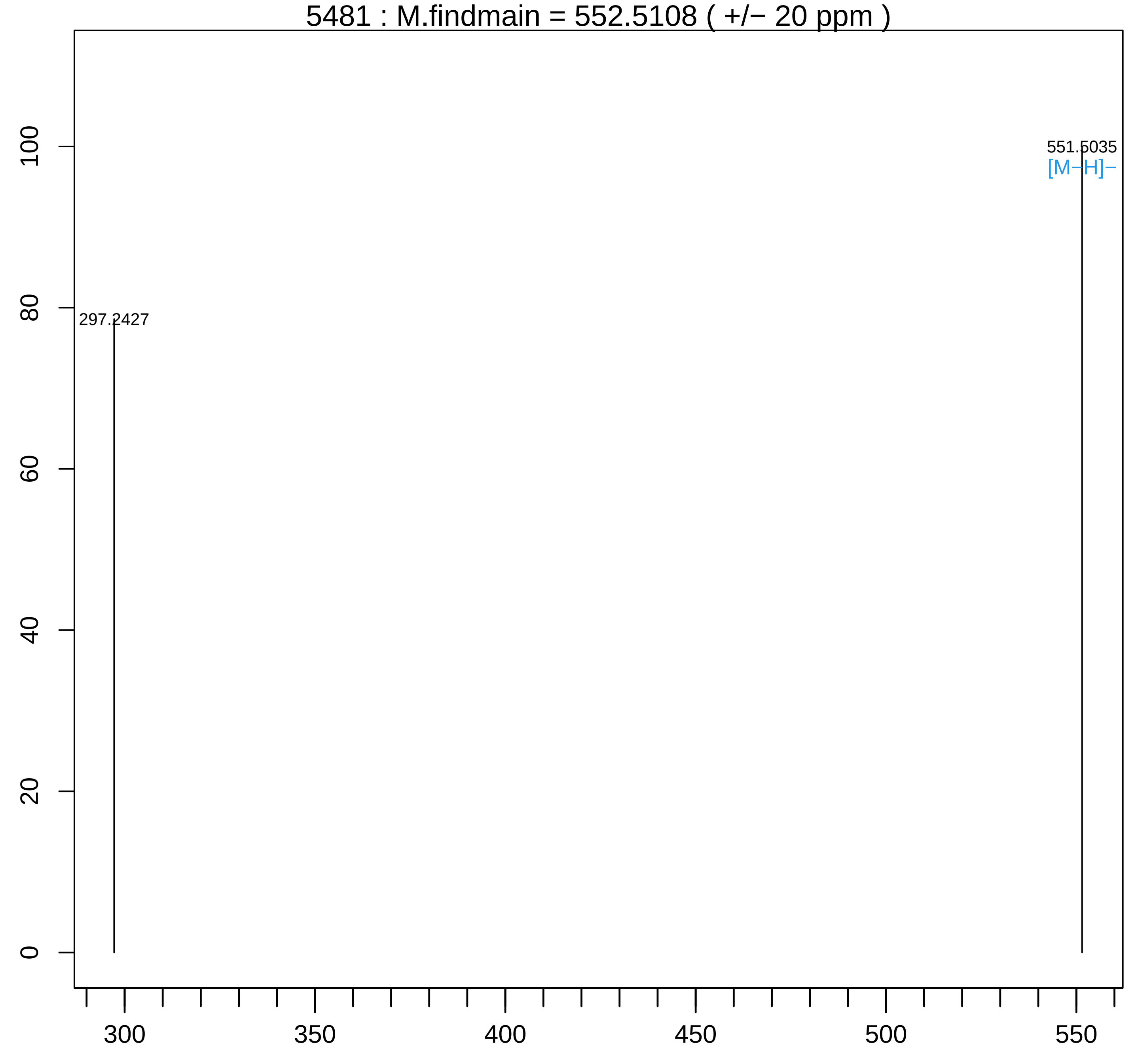

5481 : M.findmain = 552.5108 (+/− 20 ppm)
551.5035
[M−H]−
297.2427
0
20
40
60
80
100
300
350
400
450
500
550

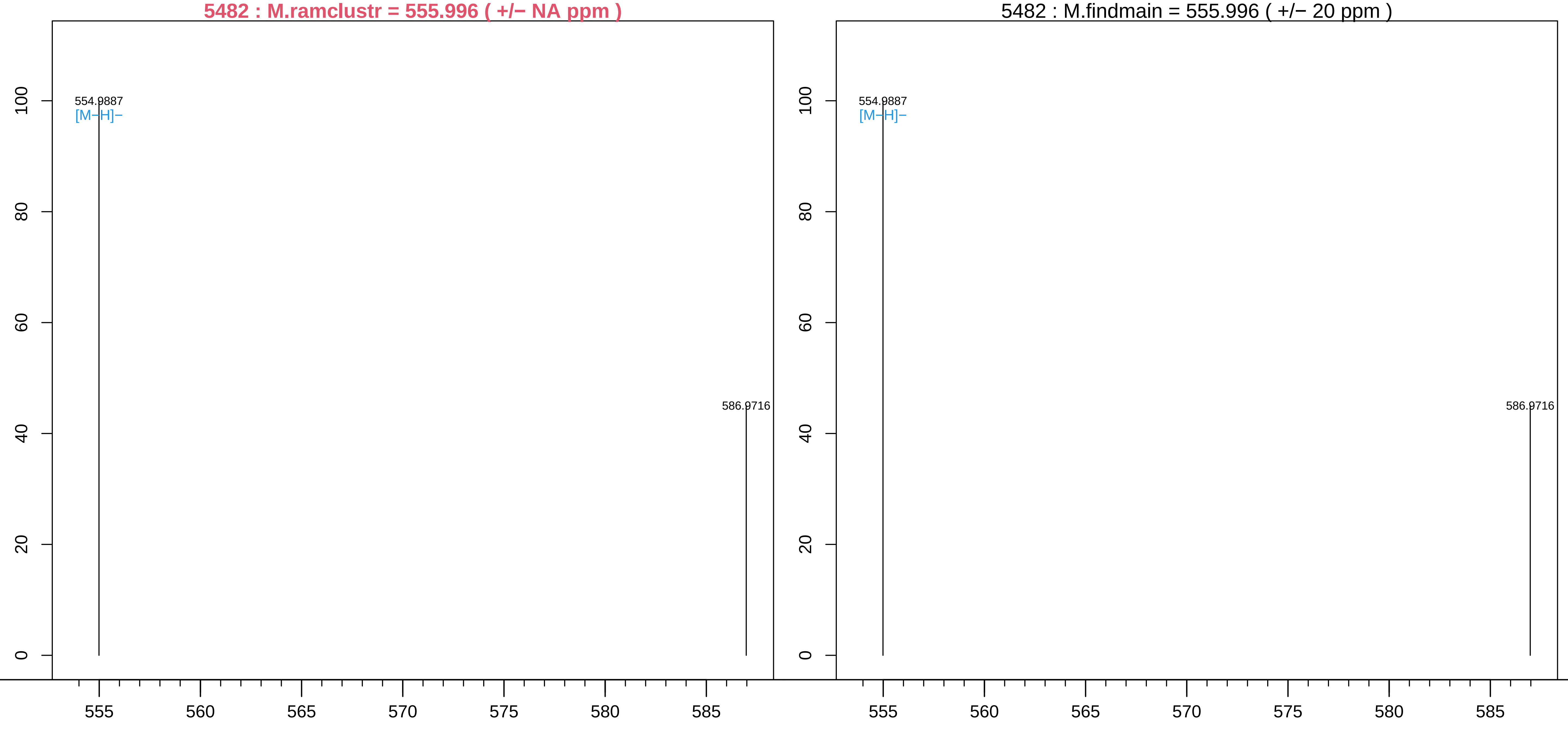
5482 : M.ramclustr = 555.996 (+/− NA ppm)
554.9887
[M−H]−
586.9716
5482 : M.findmain = 555.996 (+/− 20 ppm)
554.9887
[M−H]−
586.9716

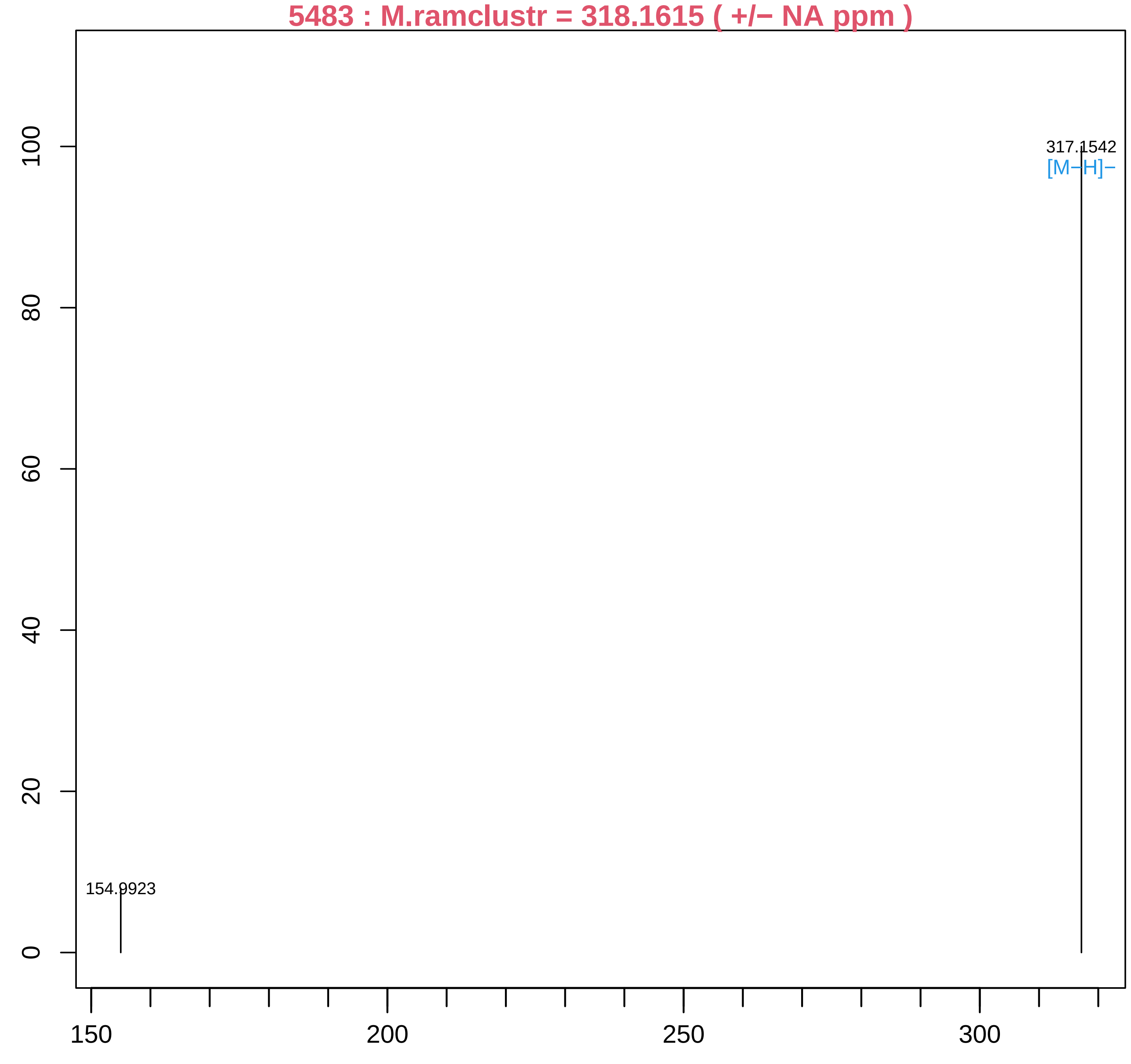
5483 : M.ramclustr = 318.1615 (+/− NA ppm)
317.1542
[M−H]−
154.9923
0
20
40
60
80
100
150
200
250
300

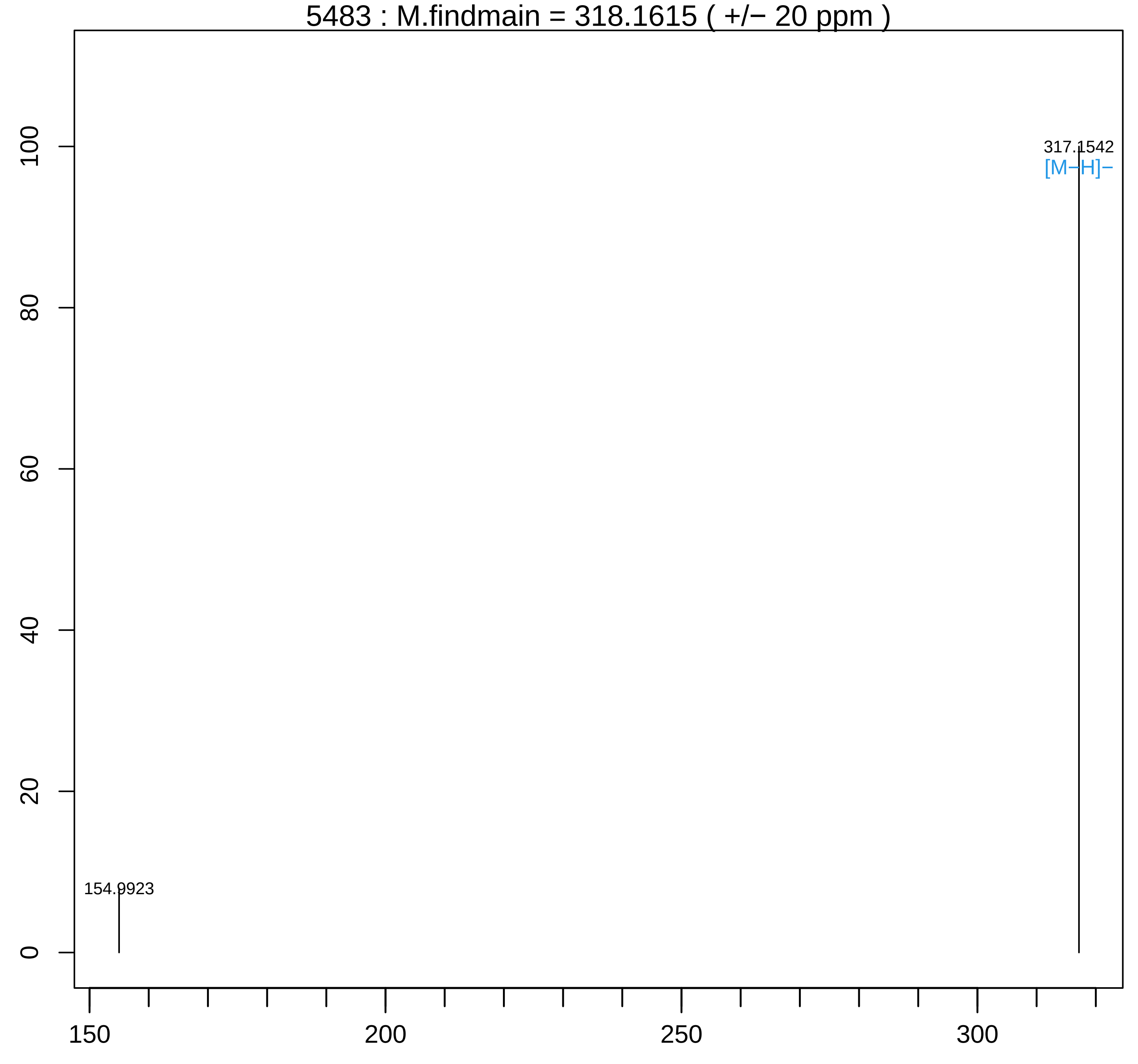
5483 : M.findmain = 318.1615 (+/− 20 ppm)
317.1542
[M−H]−
154.9923
0
20
40
60
80
100
150
200
250
300

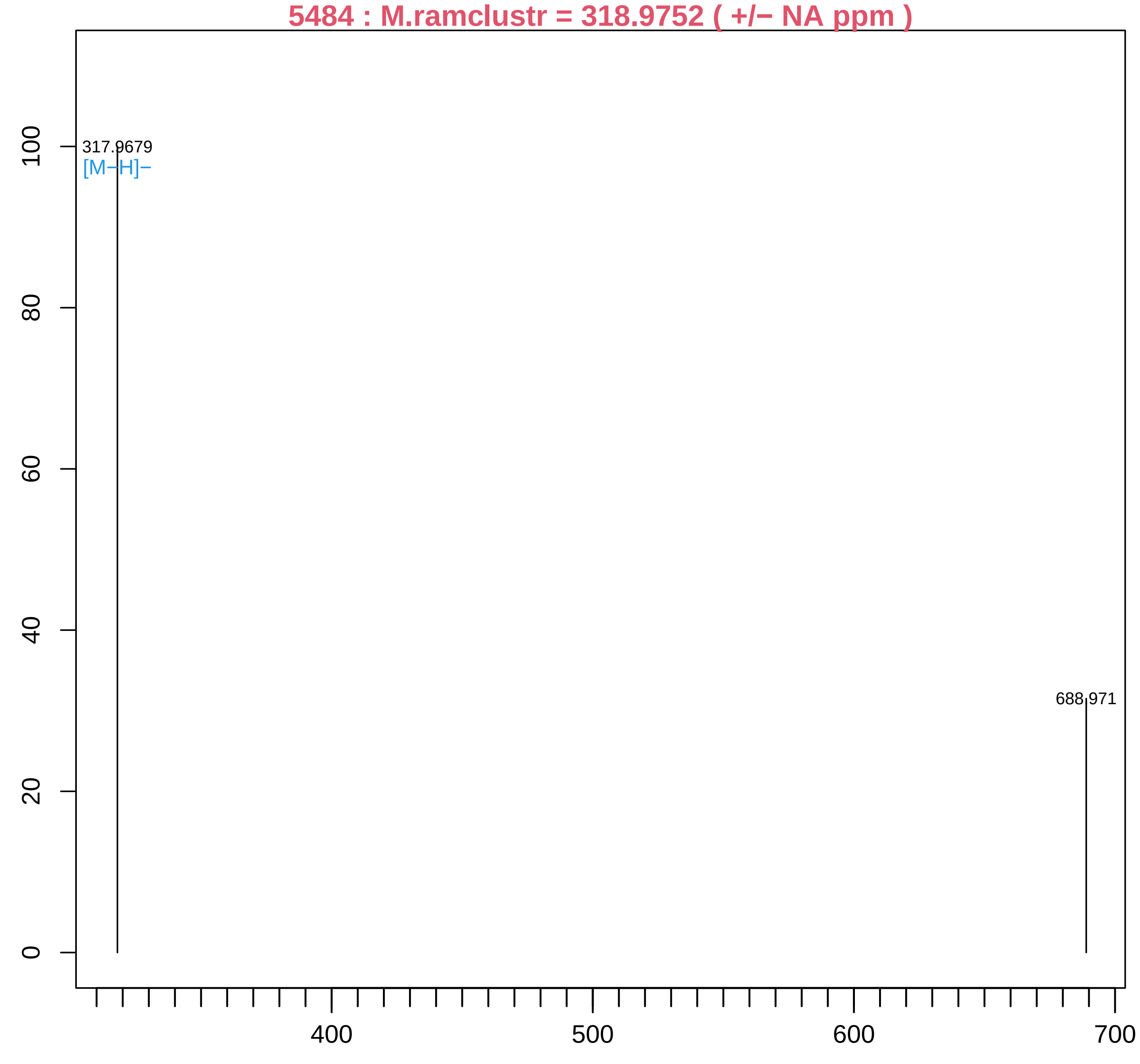
5484 : M.ramclustr = 318.9752 (+/− NA ppm)
317.9679
[M−H]−
688.971
0
20
40
60
80
100
400
500
600
700

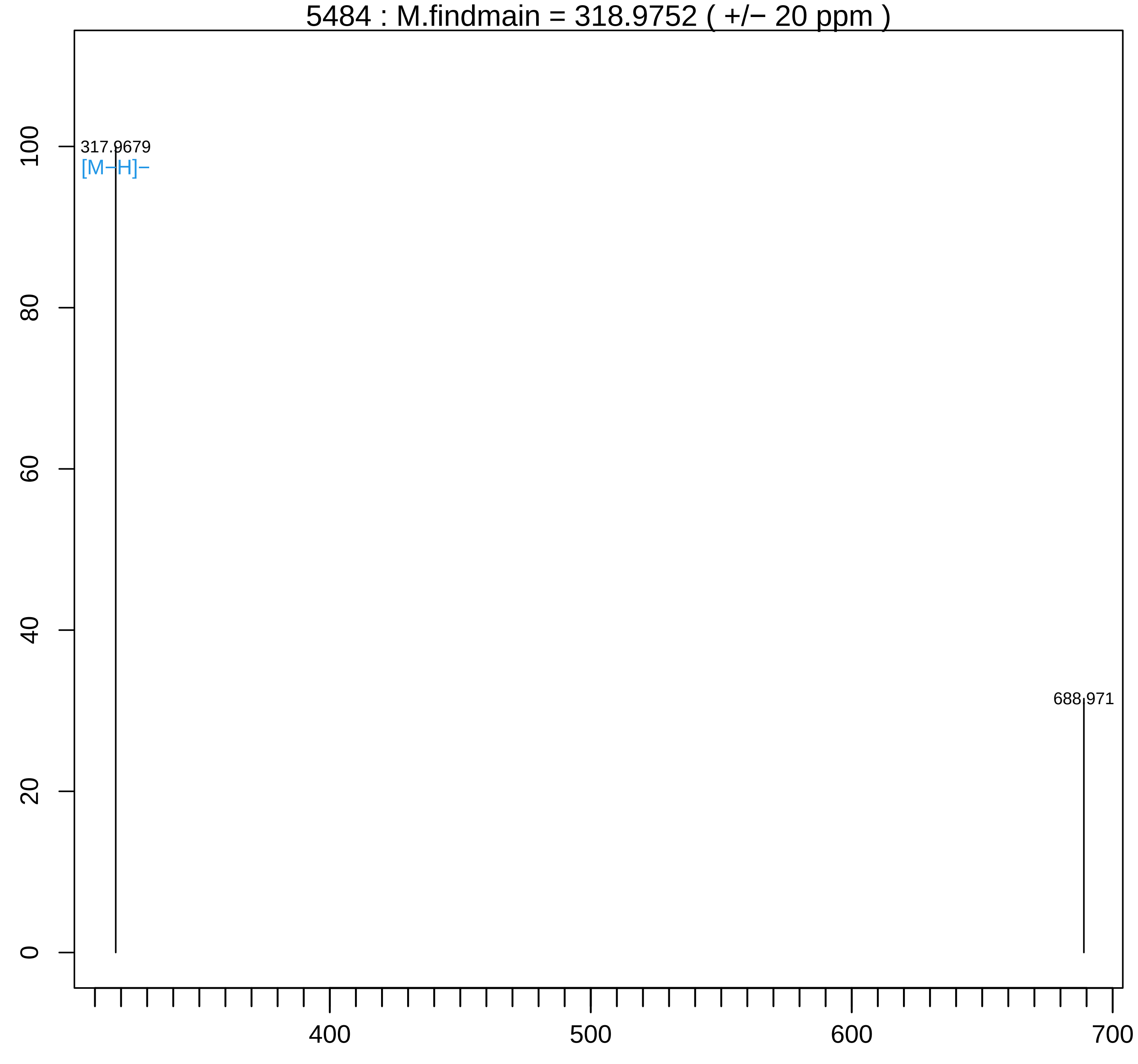
5484 : M.findmain = 318.9752 (+/− 20 ppm)
317.9679
[M−H]−
688.971
0
20
40
60
80
100
400
500
600
700

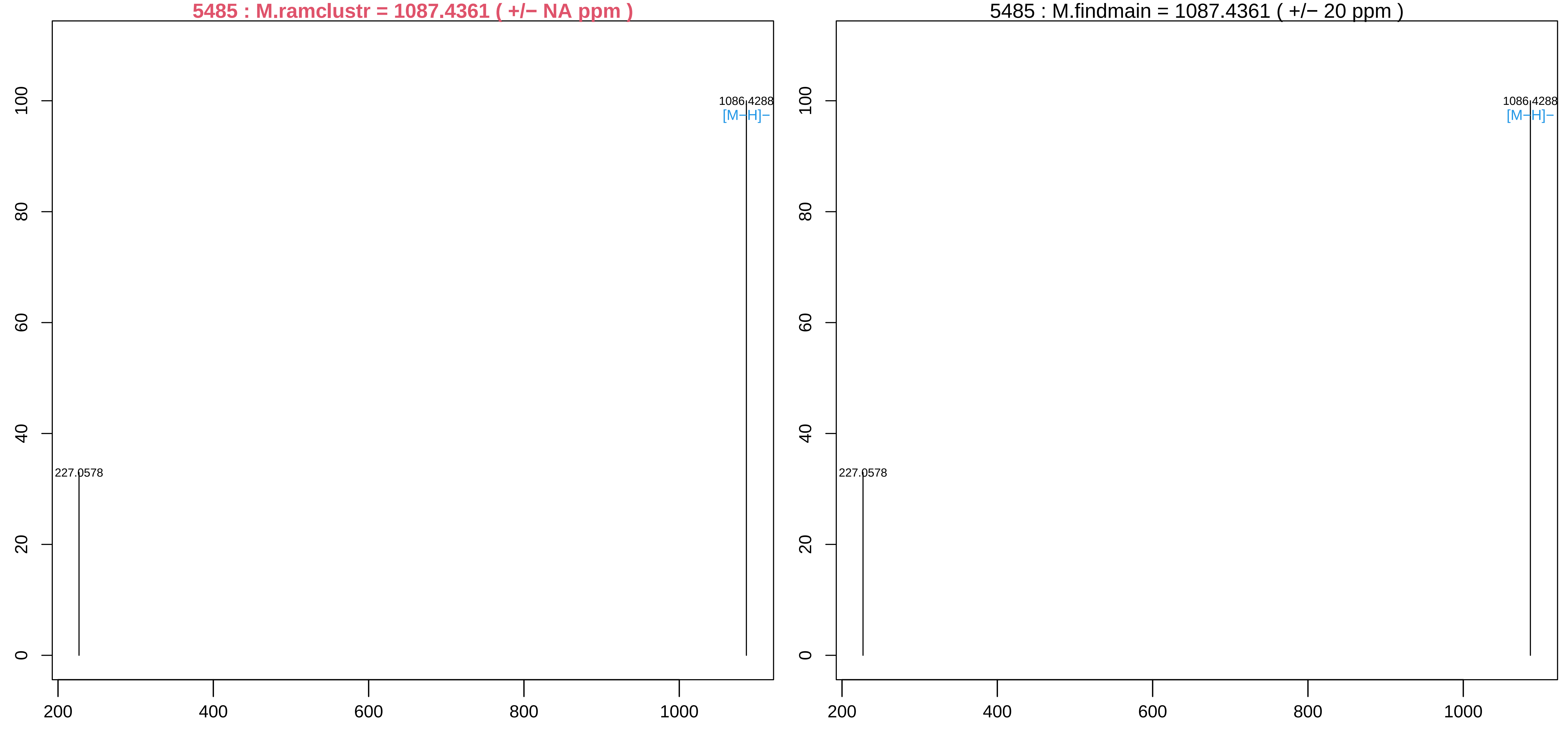
5485 : M.ramclustr = 1087.4361 (+/− NA ppm)
1086.4288
[M−H]−
227.0578
5485 : M.findmain = 1087.4361 (+/− 20 ppm)
1086.4288
[M−H]−
227.0578

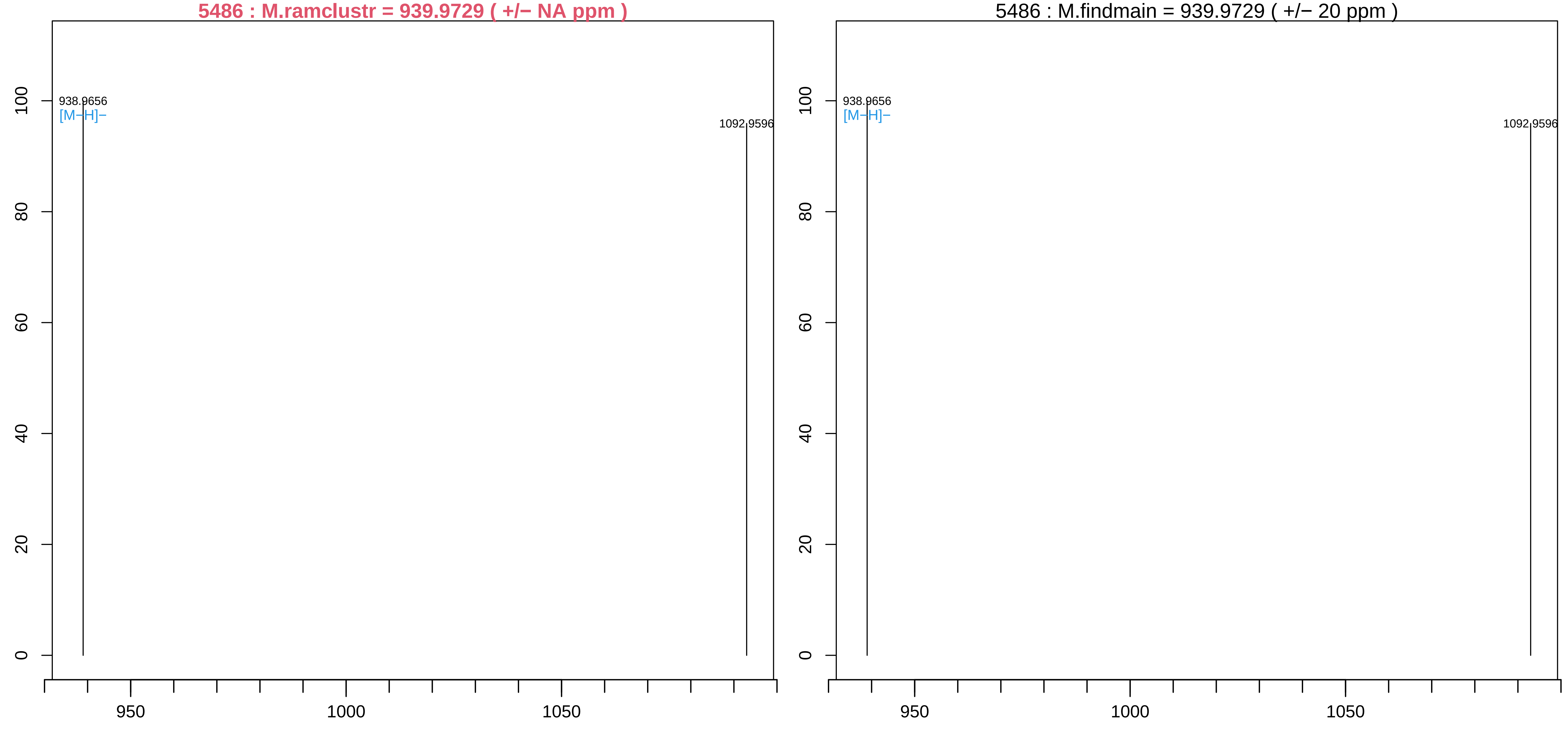
5486 : M.ramclustr = 939.9729 (+/− NA ppm)
938.9656
[M−H]−
1092.9596
5486 : M.findmain = 939.9729 (+/− 20 ppm)
938.9656
[M−H]−
1092.9596
0
20
40
60
80
100
950
1000
1050

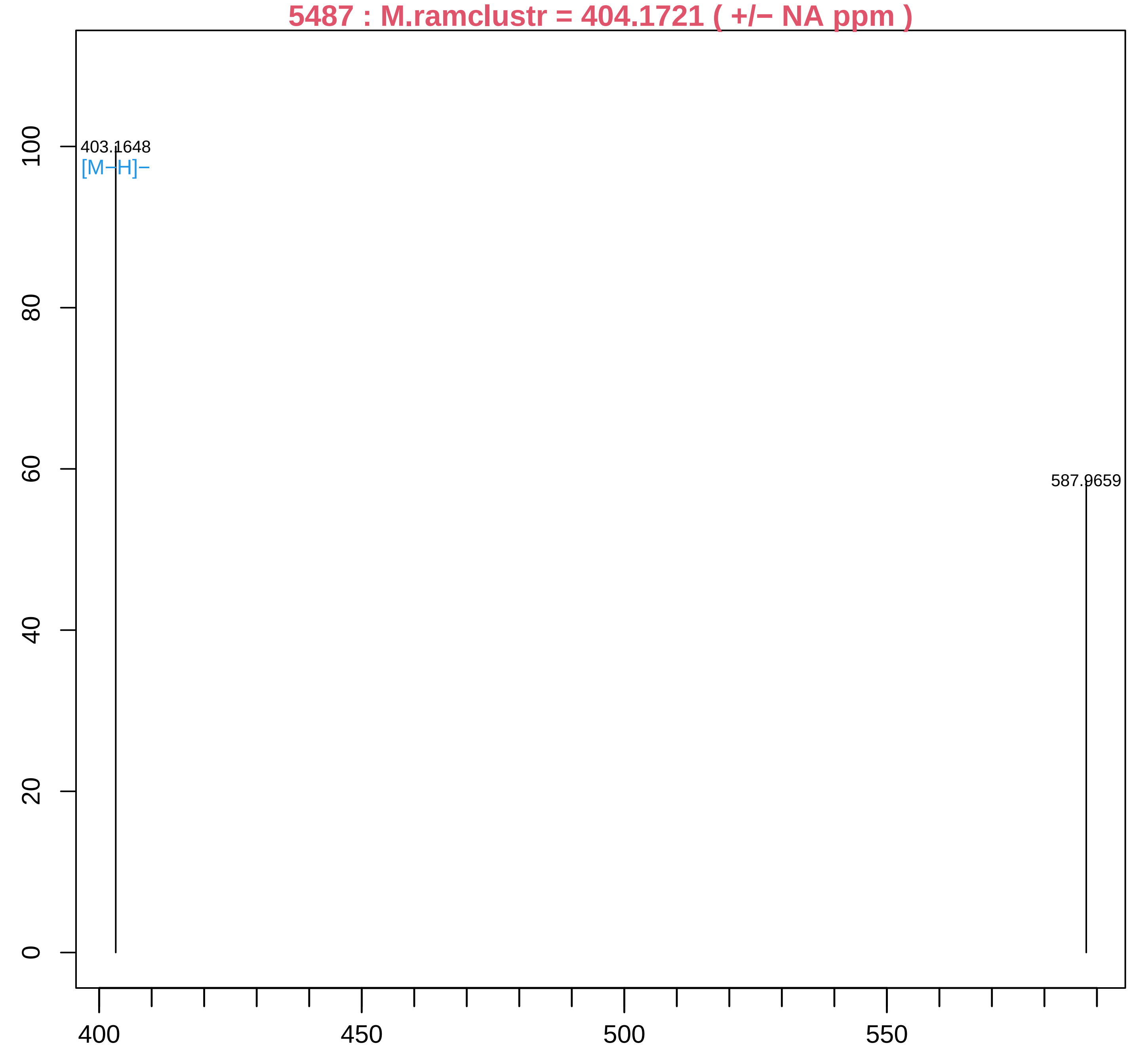
5487 : M.ramclustr = 404.1721 (+/− NA ppm)
403.1648
[M−H]−
587.9659
0
20
40
60
80
100
400
450
500
550

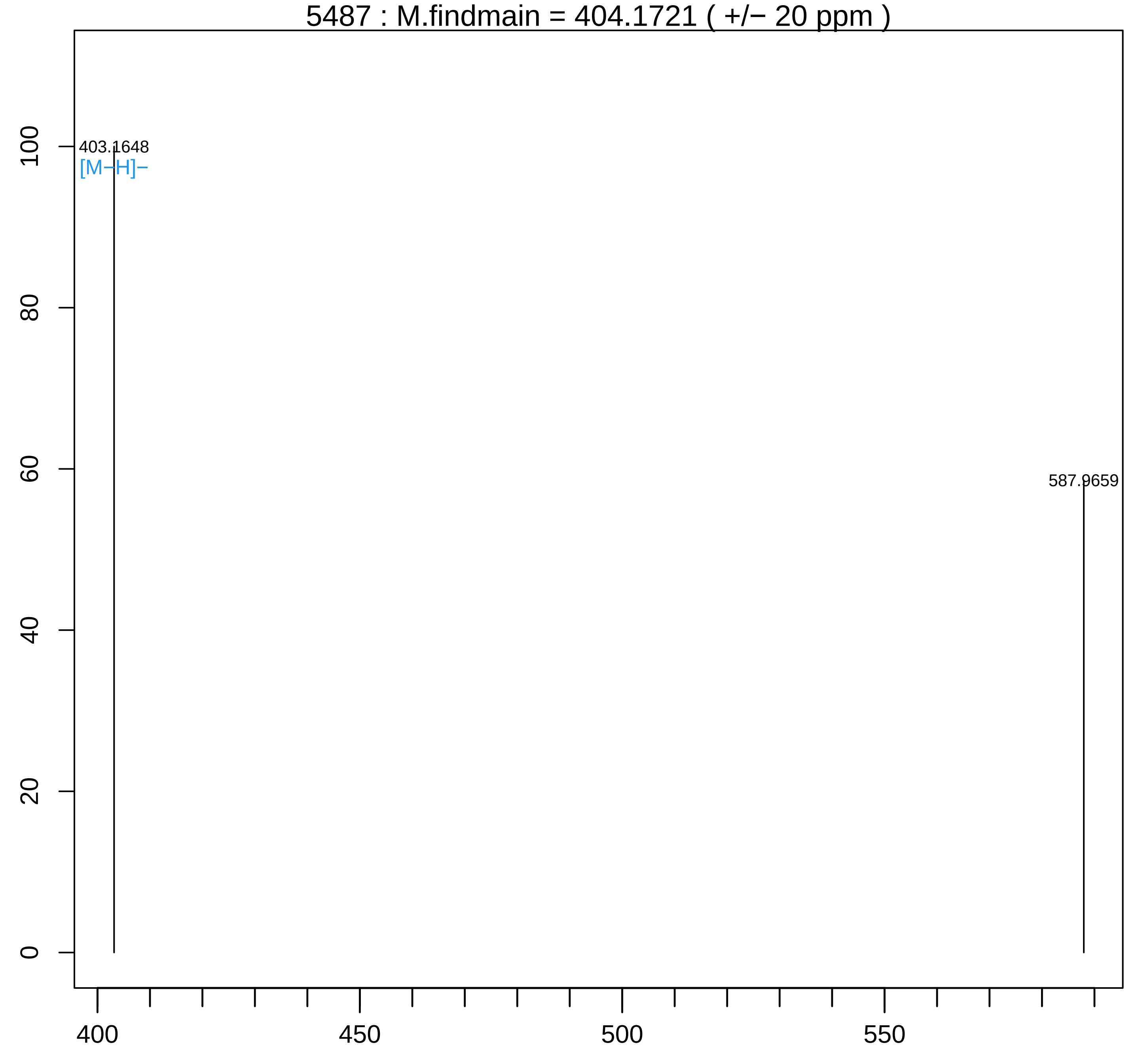
5487 : M.findmain = 404.1721 (+/− 20 ppm)
403.1648
[M−H]−
587.9659
0
20
40
60
80
100
400
450
500
550

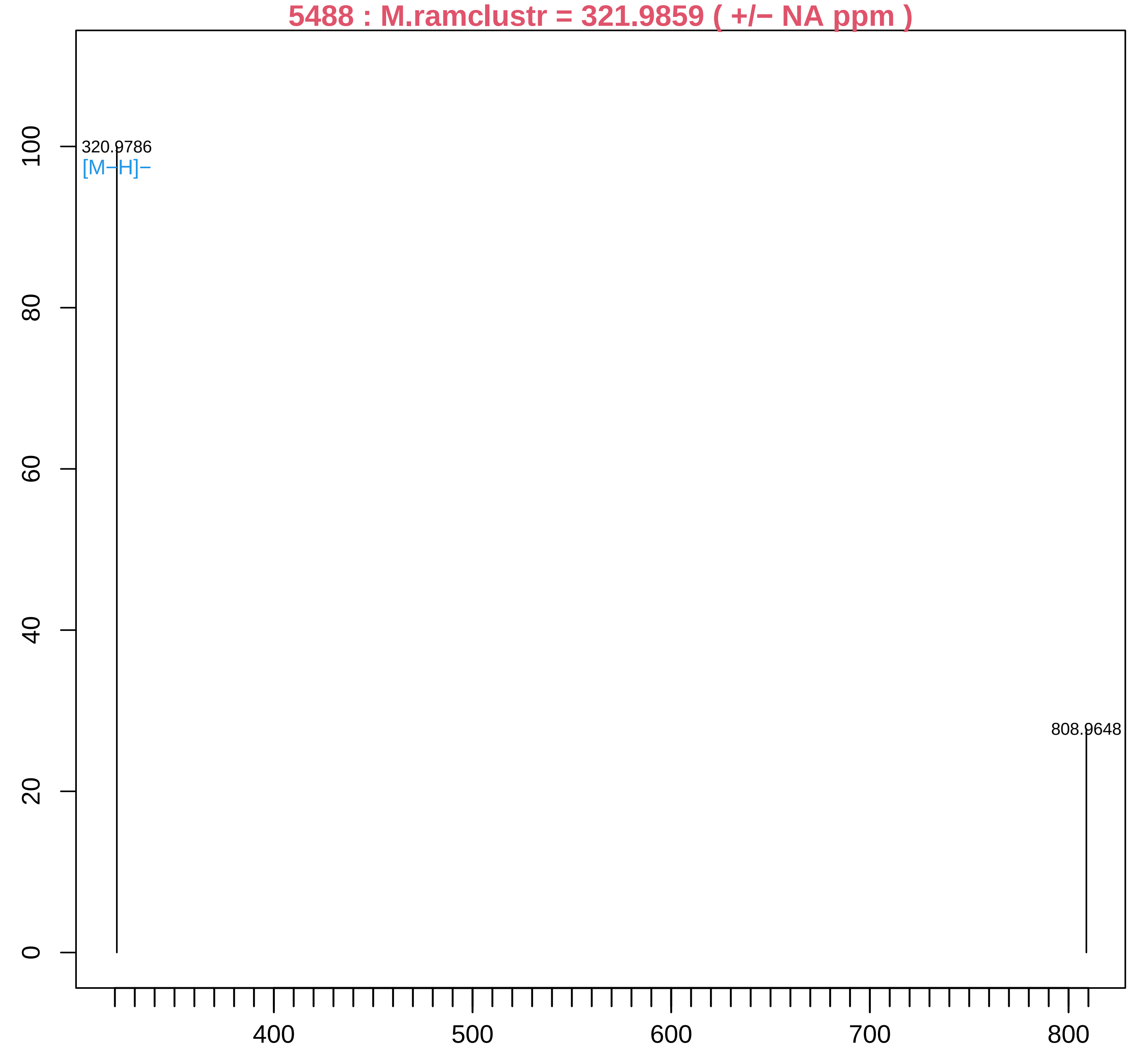
5488 : M.ramclustr = 321.9859 (+/− NA ppm)
320.9786
[M−H]−
808.9648
0
20
40
60
80
100
400
500
600
700
800

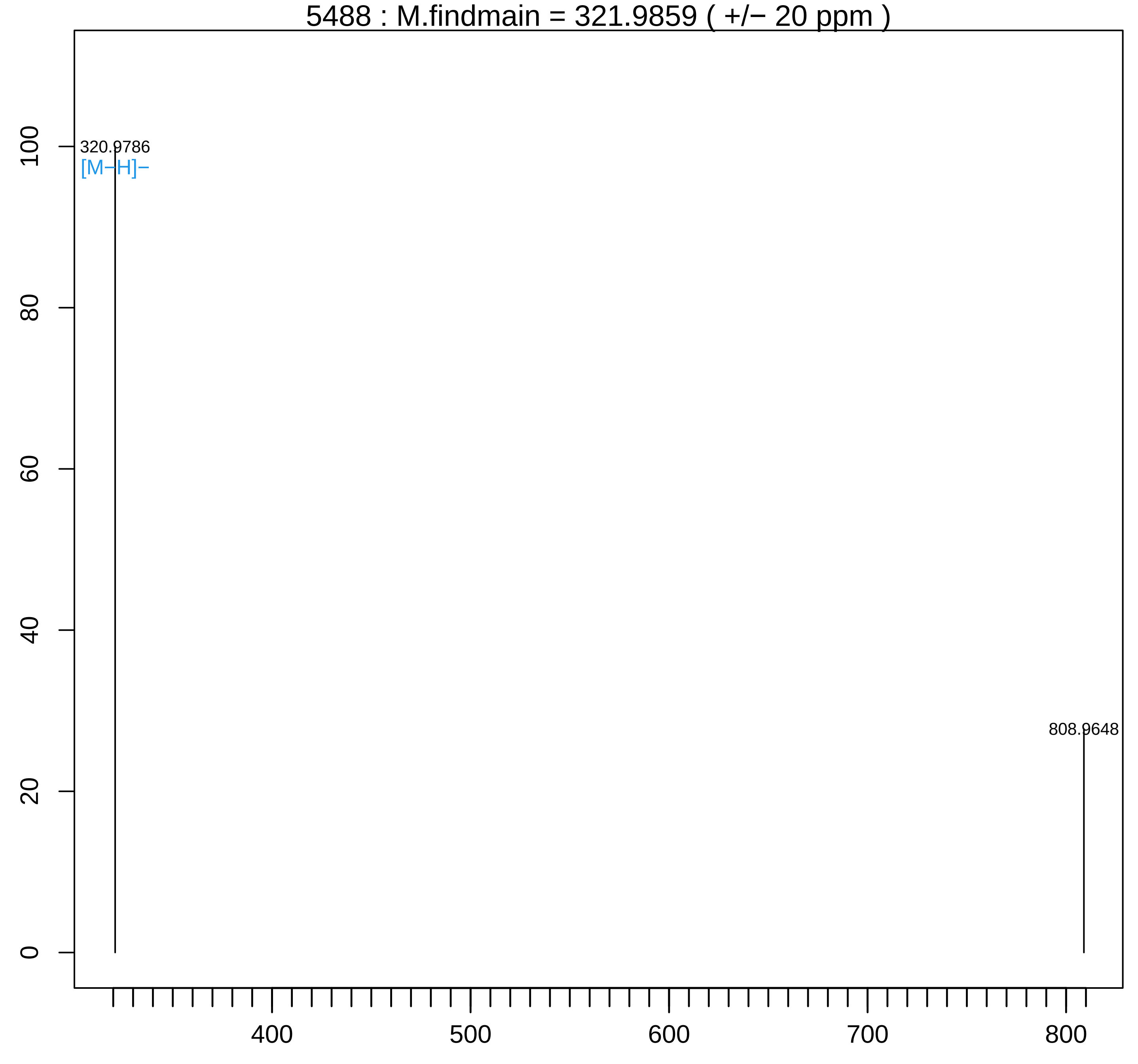
5488 : M.findmain = 321.9859 (+/− 20 ppm)
320.9786
[M−H]−
808.9648
0
20
40
60
80
100
400
500
600
700
800

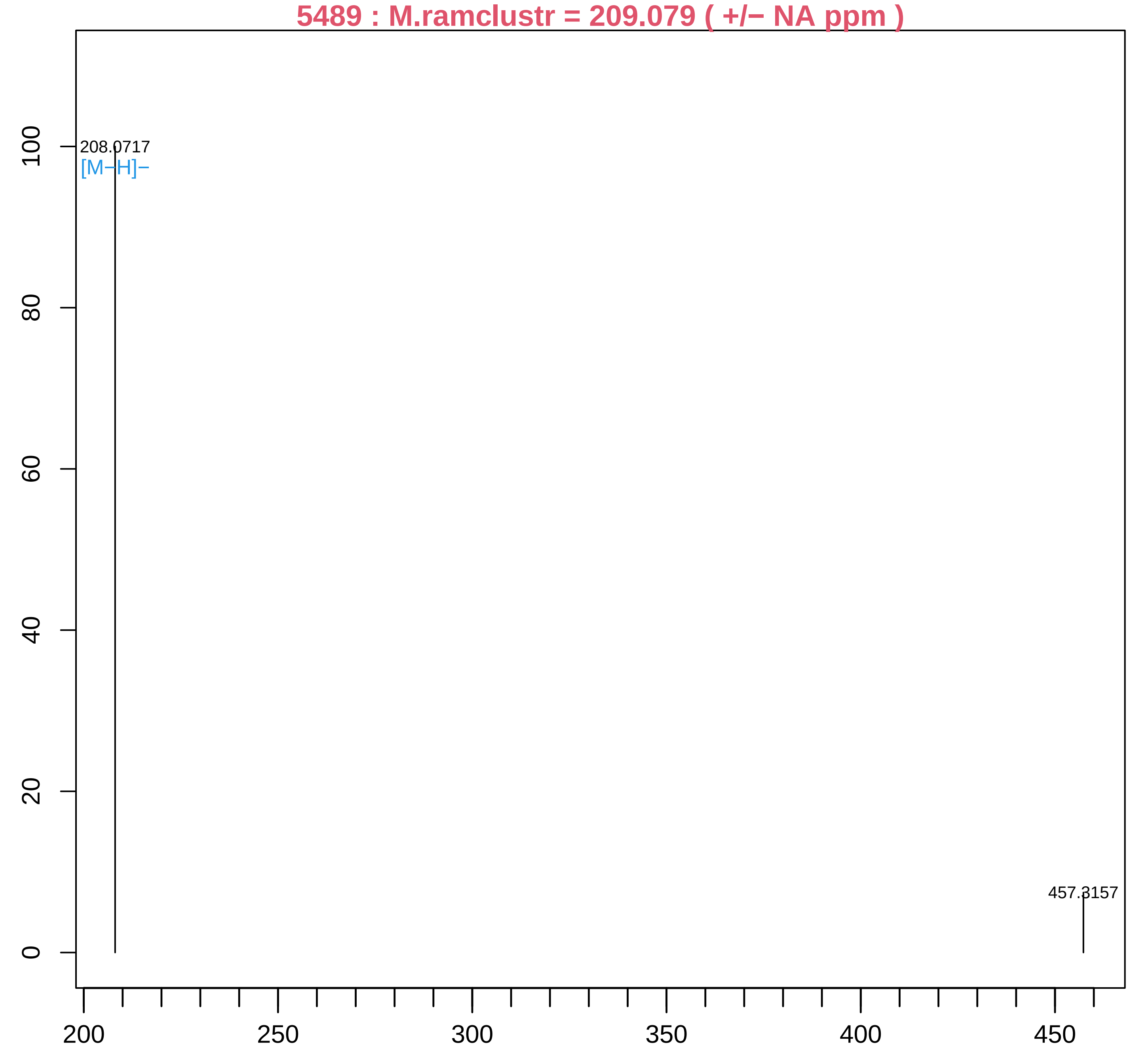
5489 : M.ramclustr = 209.079 (+/− NA ppm)
208.0717
[M−H]−
457.3157
100
80
60
40
20
0
200
250
300
350
400
450

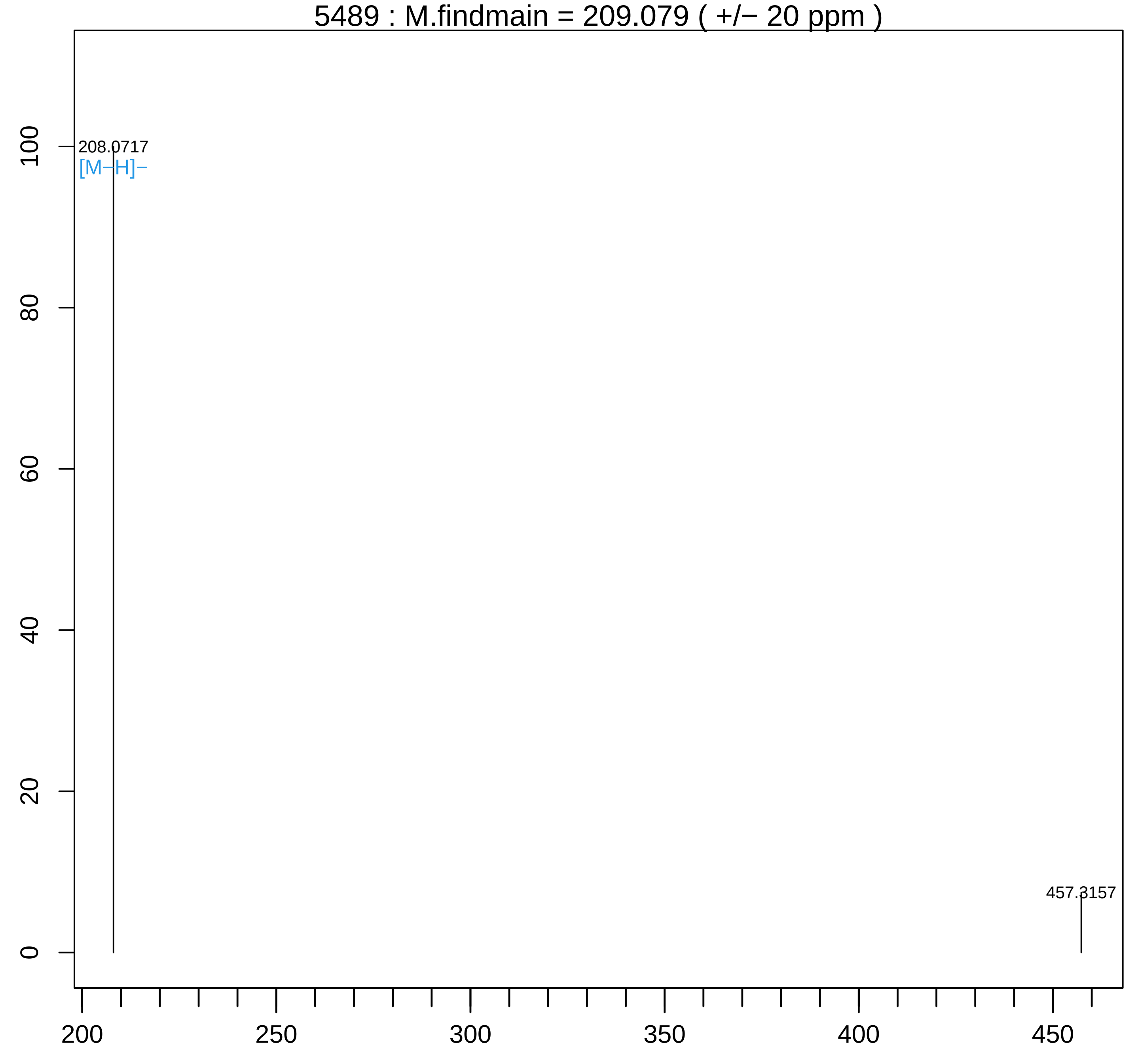
5489 : M.findmain = 209.079 (+/− 20 ppm)
208.0717
[M−H]−
457.3157
100
80
60
40
20
0
200
250
300
350
400
450

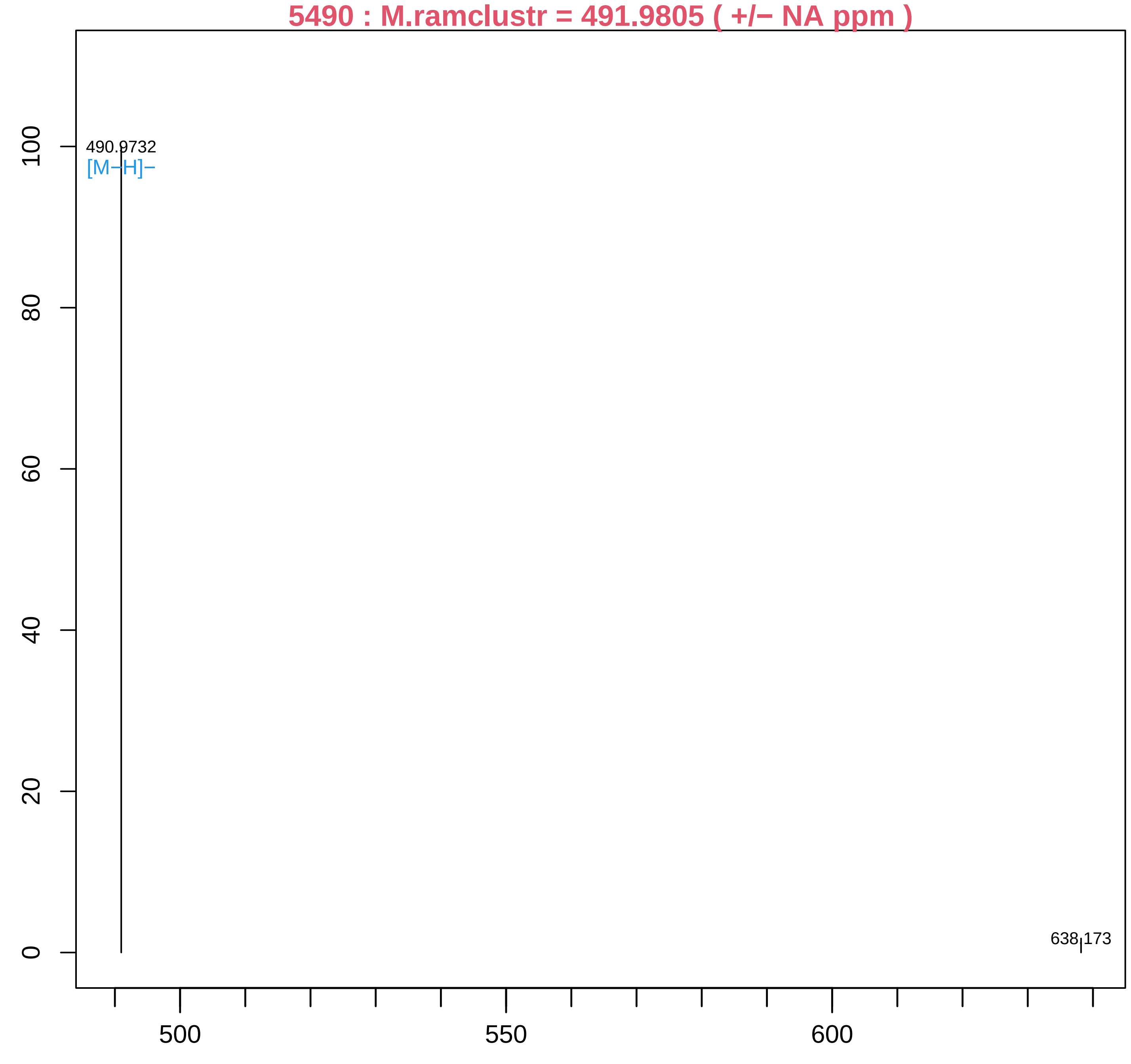
5490 : M.ramclustr = 491.9805 (+/− NA ppm)
490.9732
[M−H]−
638.173
0
20
40
60
80
100
500
550
600

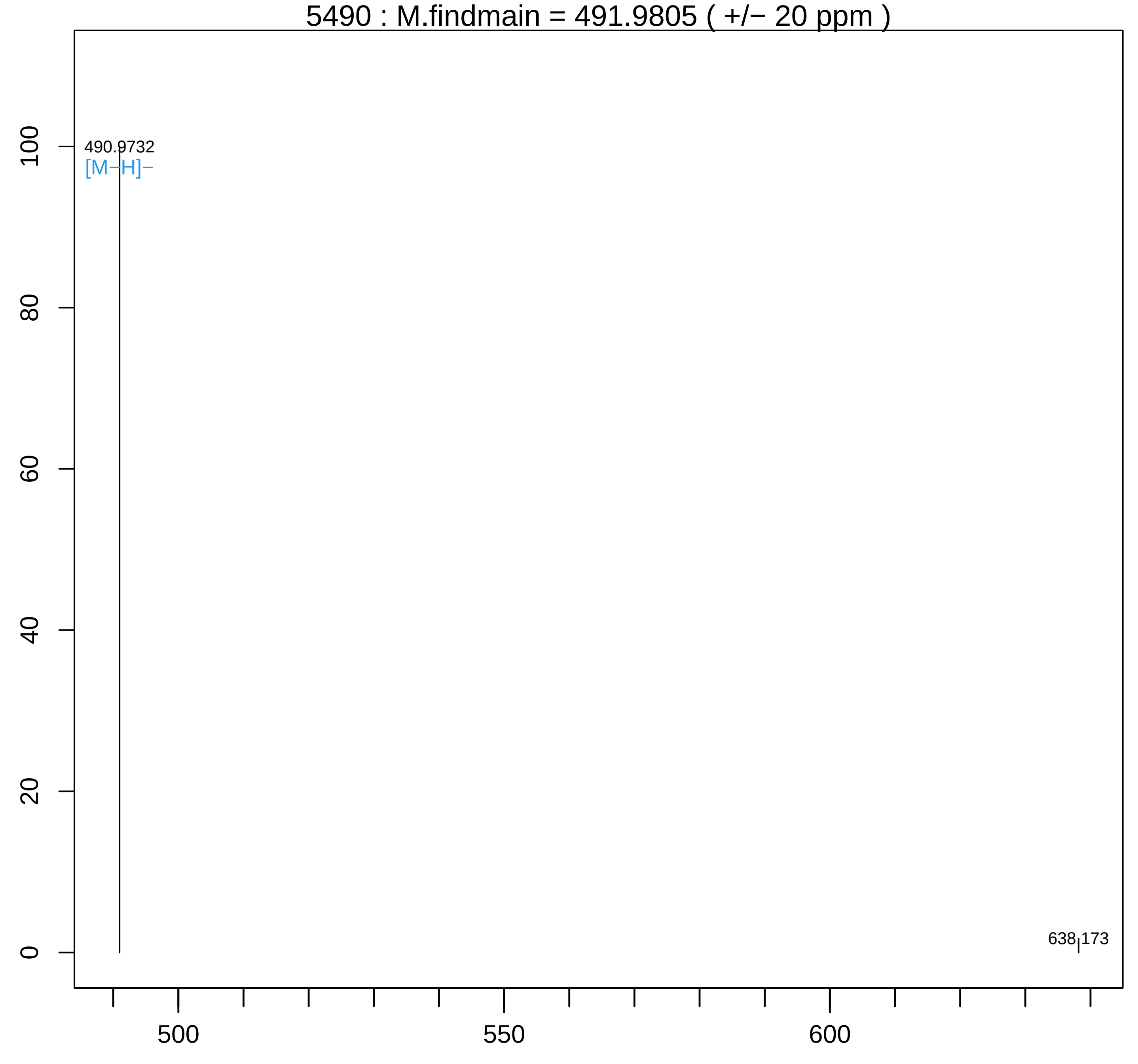
5490 : M.findmain = 491.9805 (+/− 20 ppm)
490.9732
[M−H]−
638.173
0
20
40
60
80
100
500
550
600

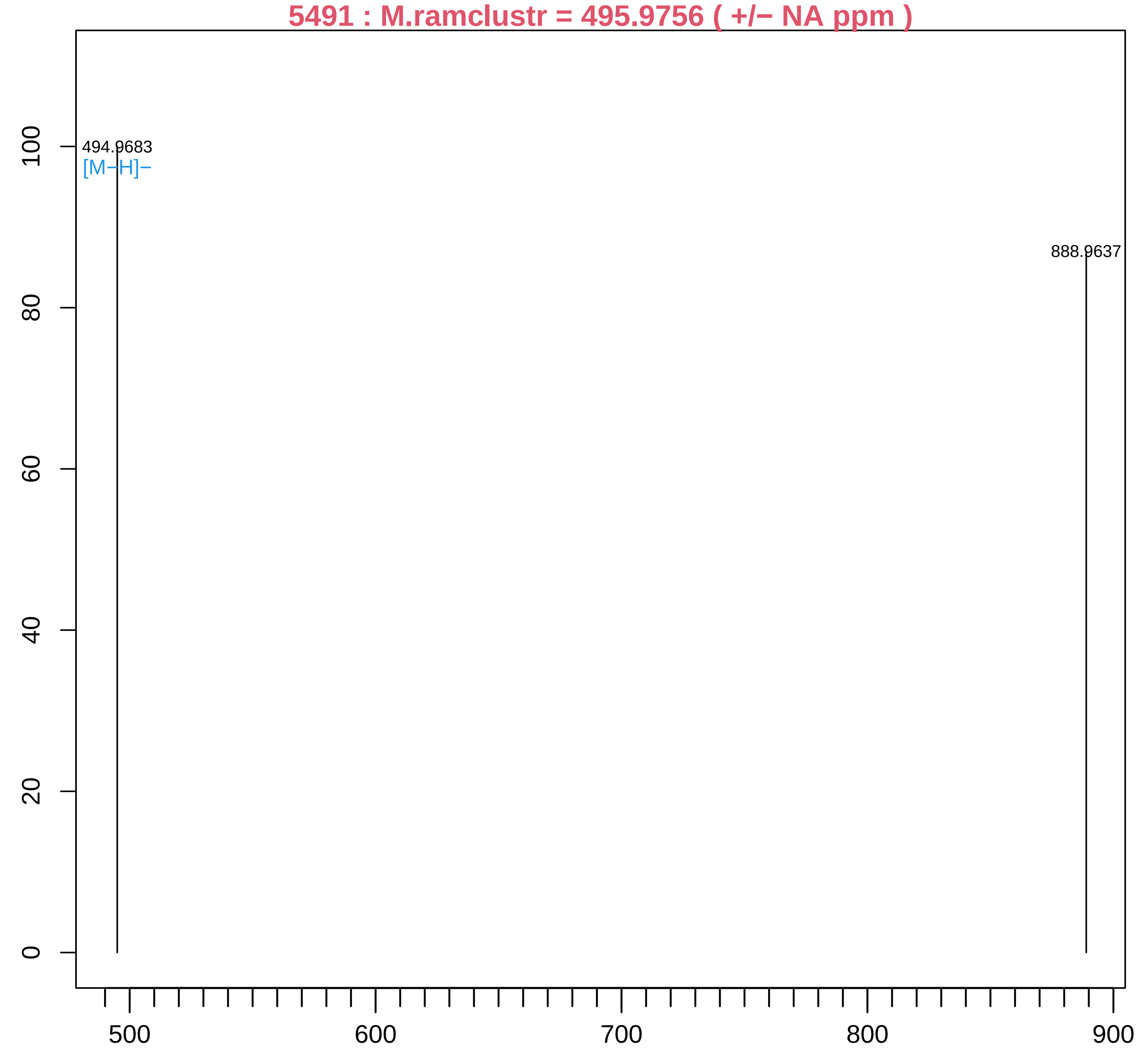

5491 : M.ramclustr = 495.9756 (+/− NA ppm)
494.9683
[M−H]−
888.9637
0
20
40
60
80
100
500
600
700
800
900

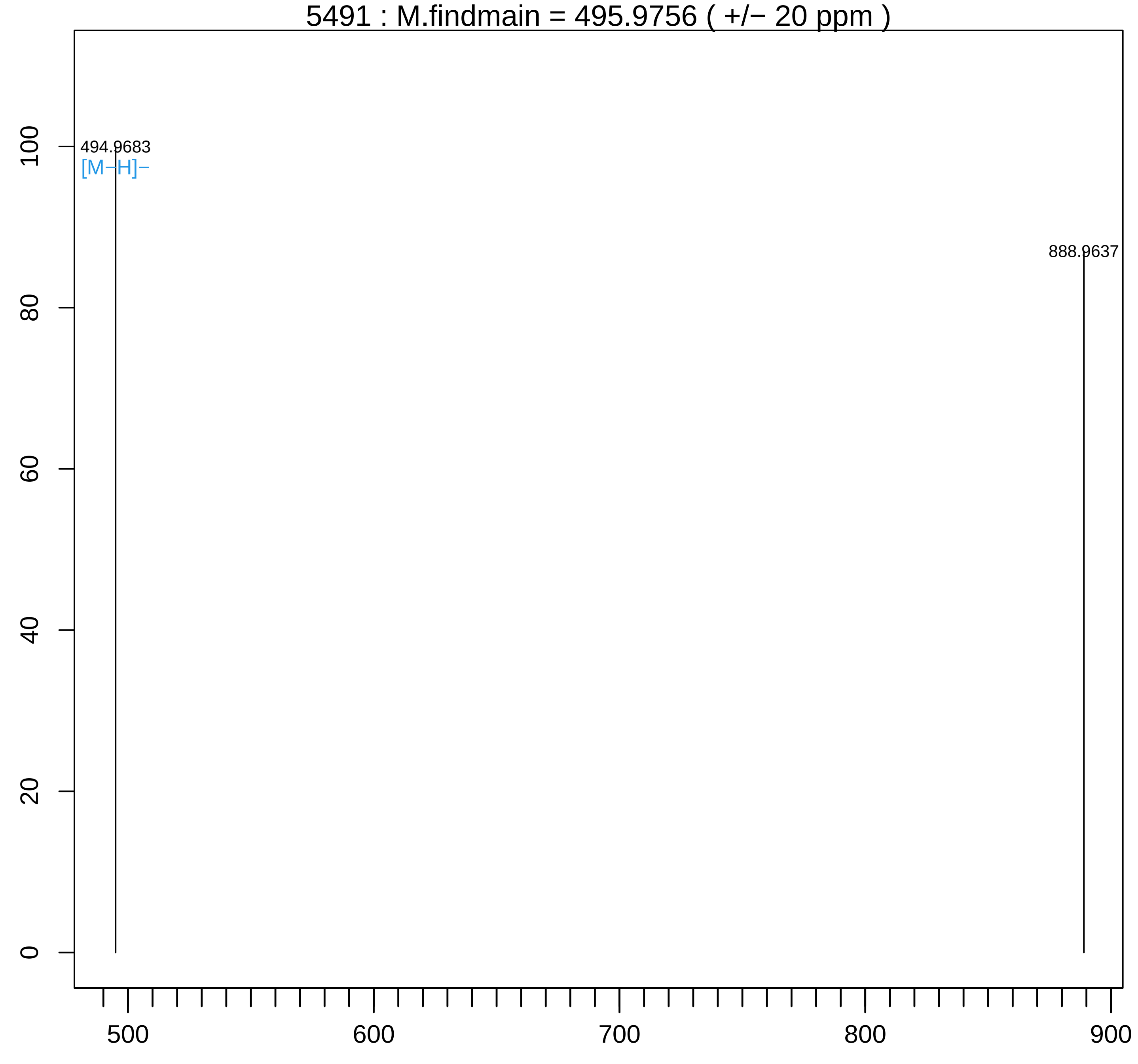

5491 : M.findmain = 495.9756 (+/− 20 ppm)
494.9683
[M−H]−
888.9637
0
20
40
60
80
100
500
600
700
800
900

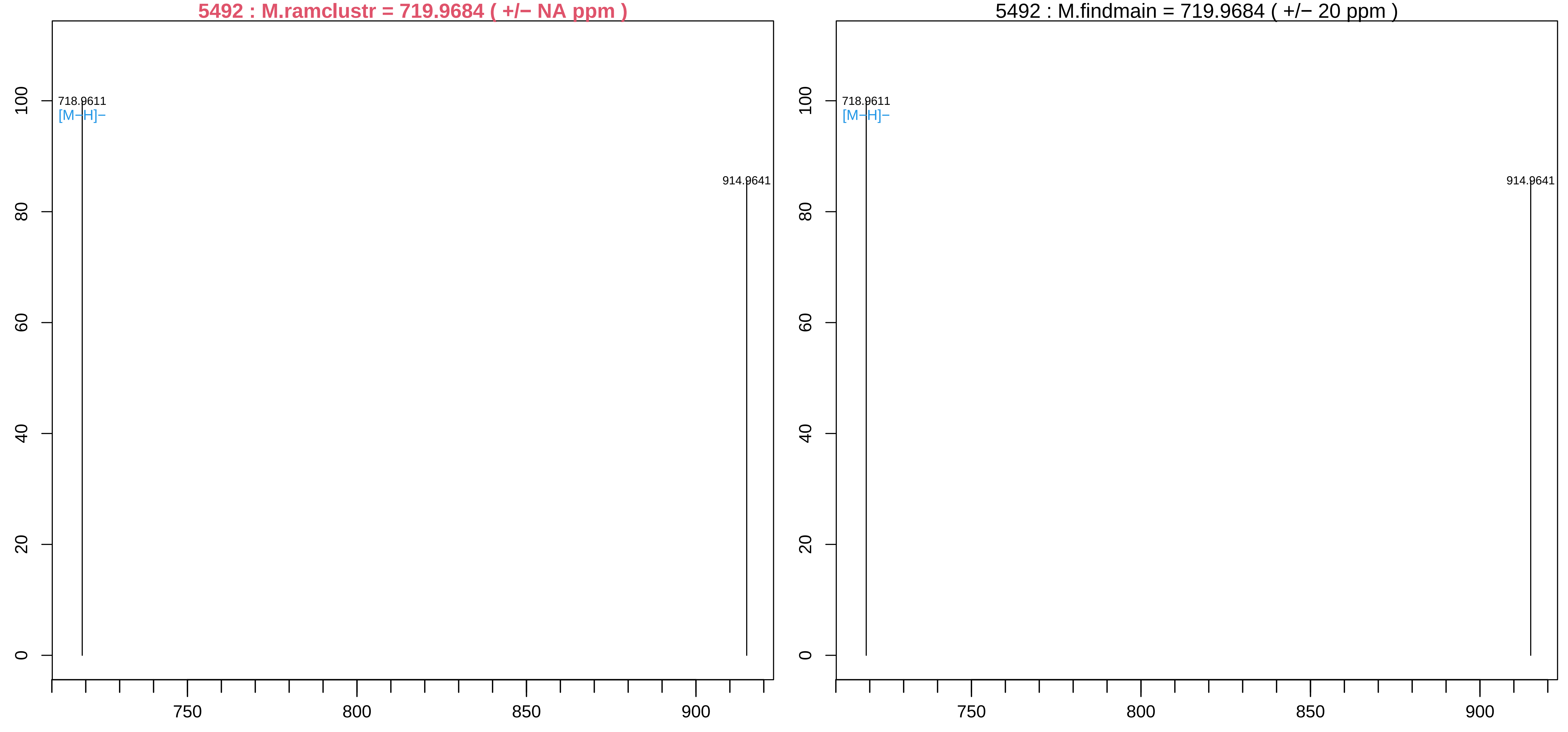
5492 : M.ramclustr = 719.9684 (+/− NA ppm)
718.9611
[M−H]−
914.9641
0
20
40
60
80
100
750
800
850
900
5492 : M.findmain = 719.9684 (+/− 20 ppm)
718.9611
[M−H]−
914.9641
0
20
40
60
80
100
750
800
850
900

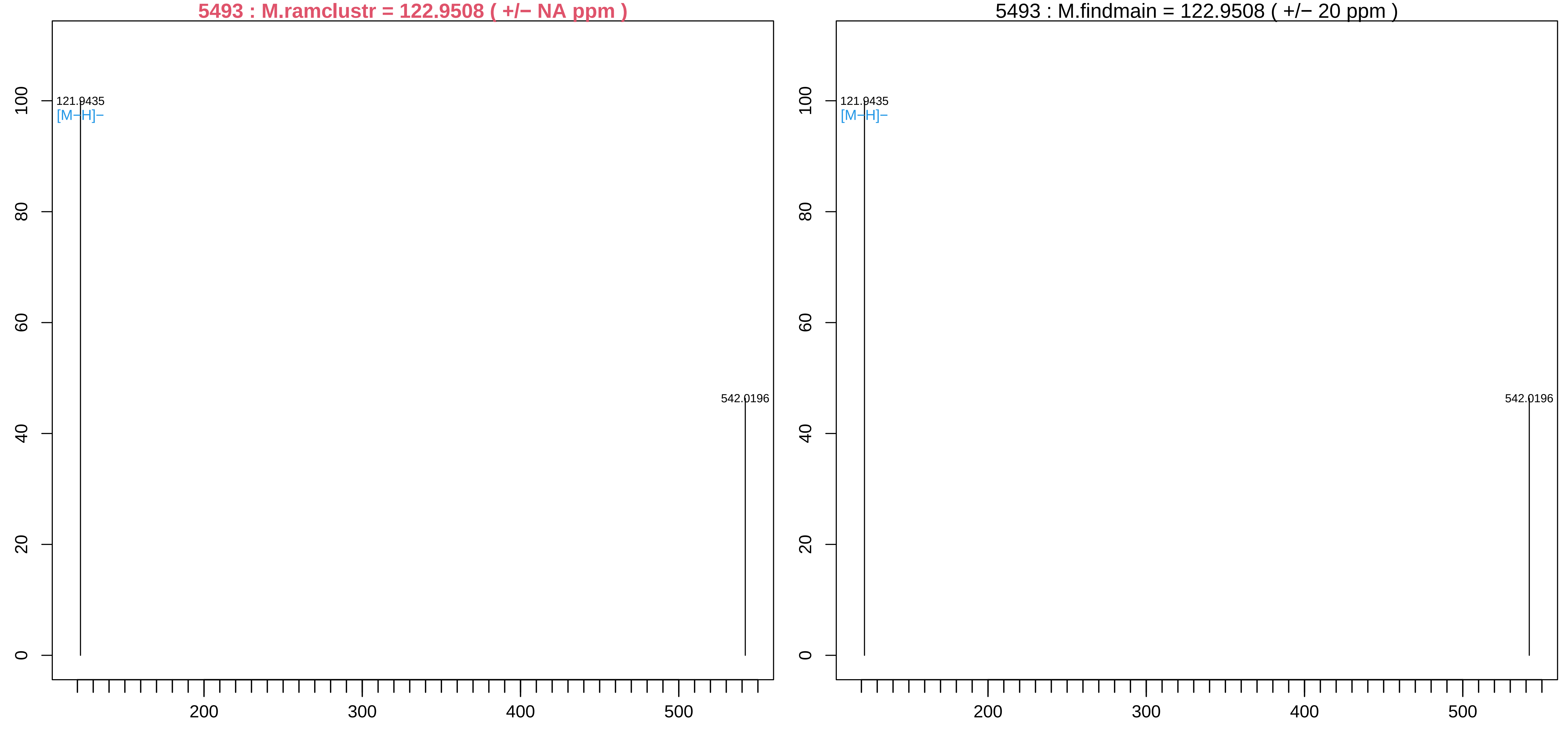
5493 : M.ramclustr = 122.9508 (+/− NA ppm)
121.9435
[M−H]−
542.0196
5493 : M.findmain = 122.9508 (+/− 20 ppm)
121.9435
[M−H]−
542.0196
0
20
40
60
80
100
200
300
400
500

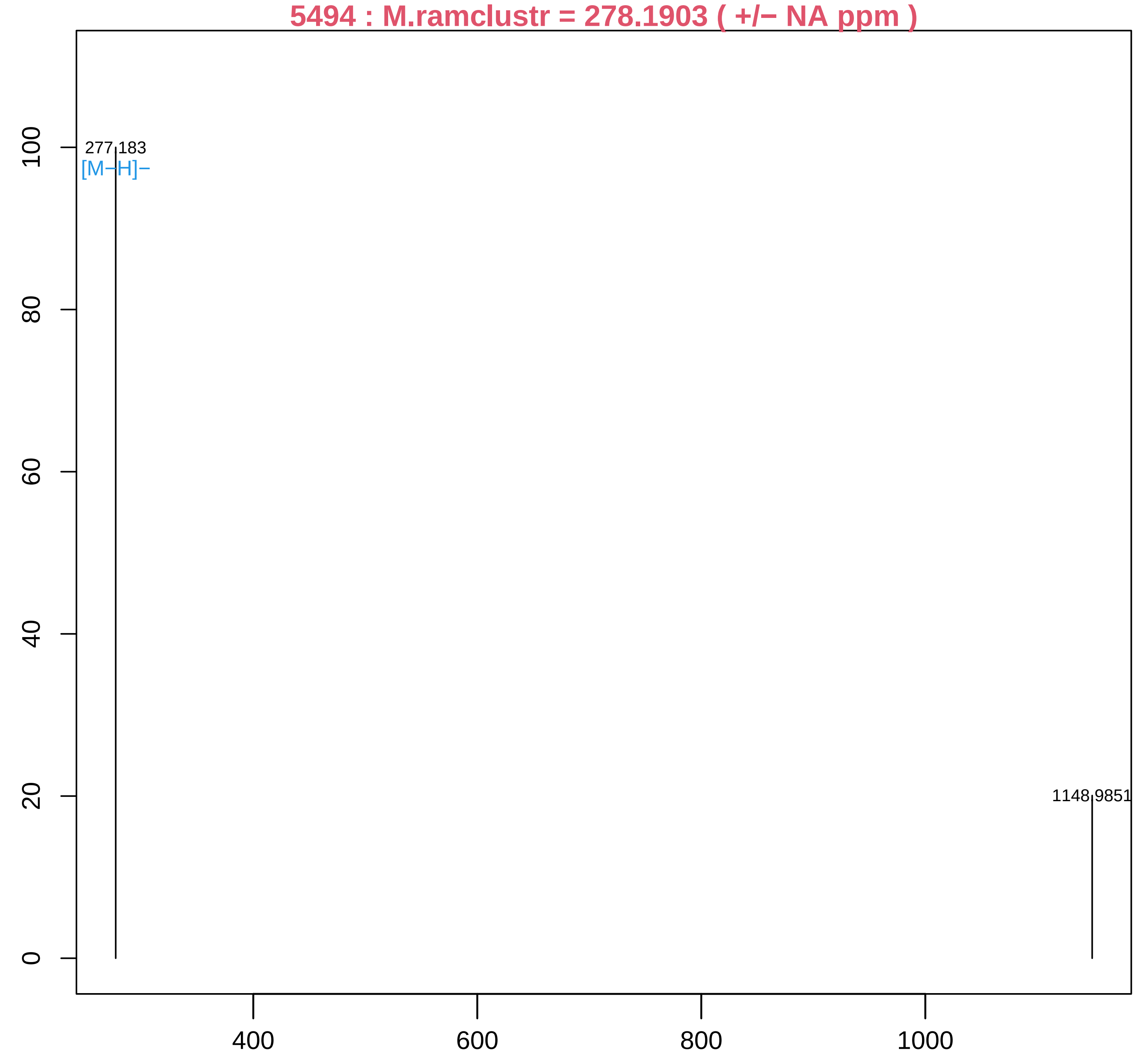
5494 : M.ramclustr = 278.1903 (+/− NA ppm)
277.183
[M−H]−
1148.9851
0
20
40
60
80
100
400
600
800
1000

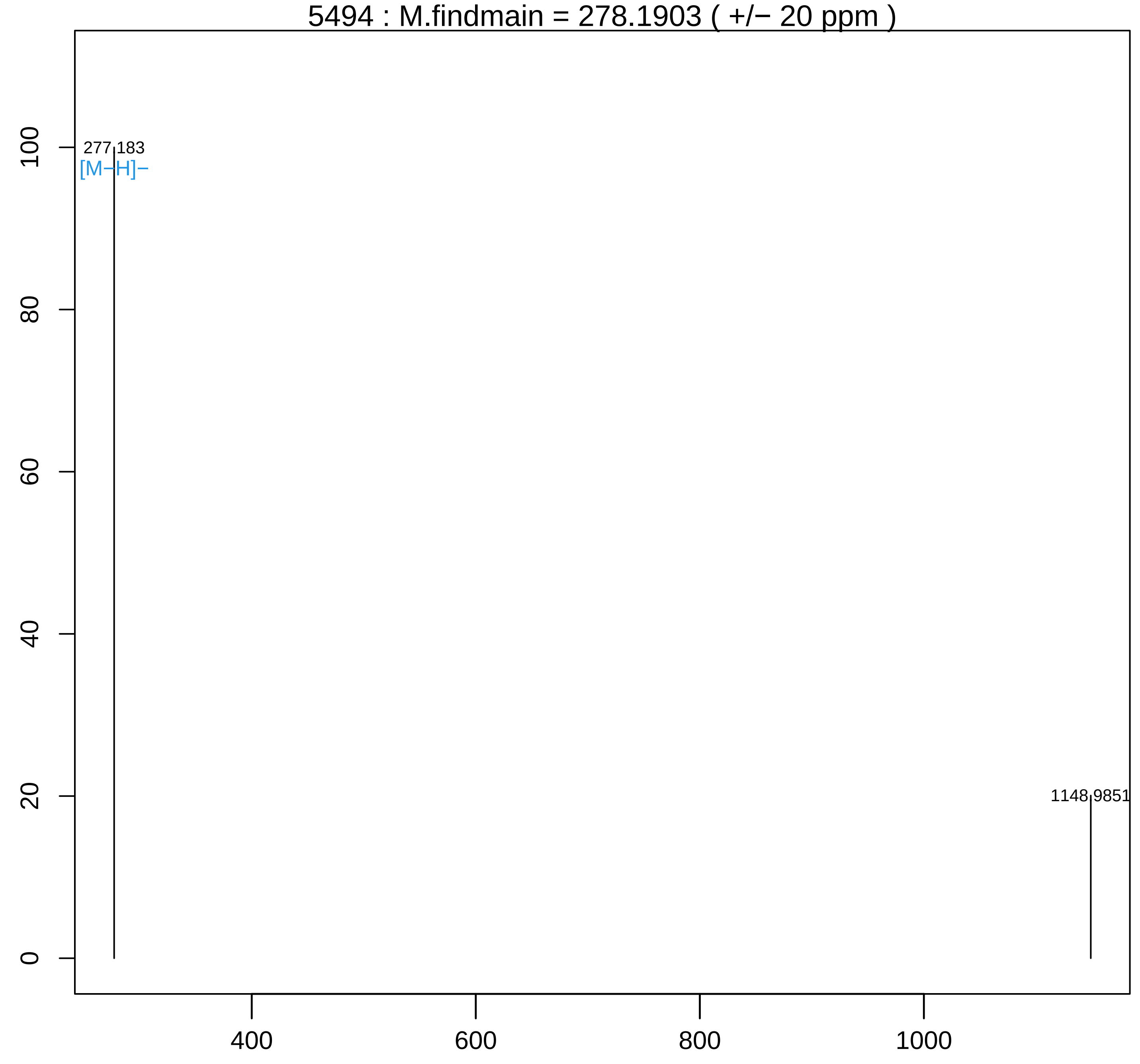
5494 : M.findmain = 278.1903 (+/− 20 ppm)
277.183
[M−H]−
1148.9851
0
20
40
60
80
100
400
600
800
1000

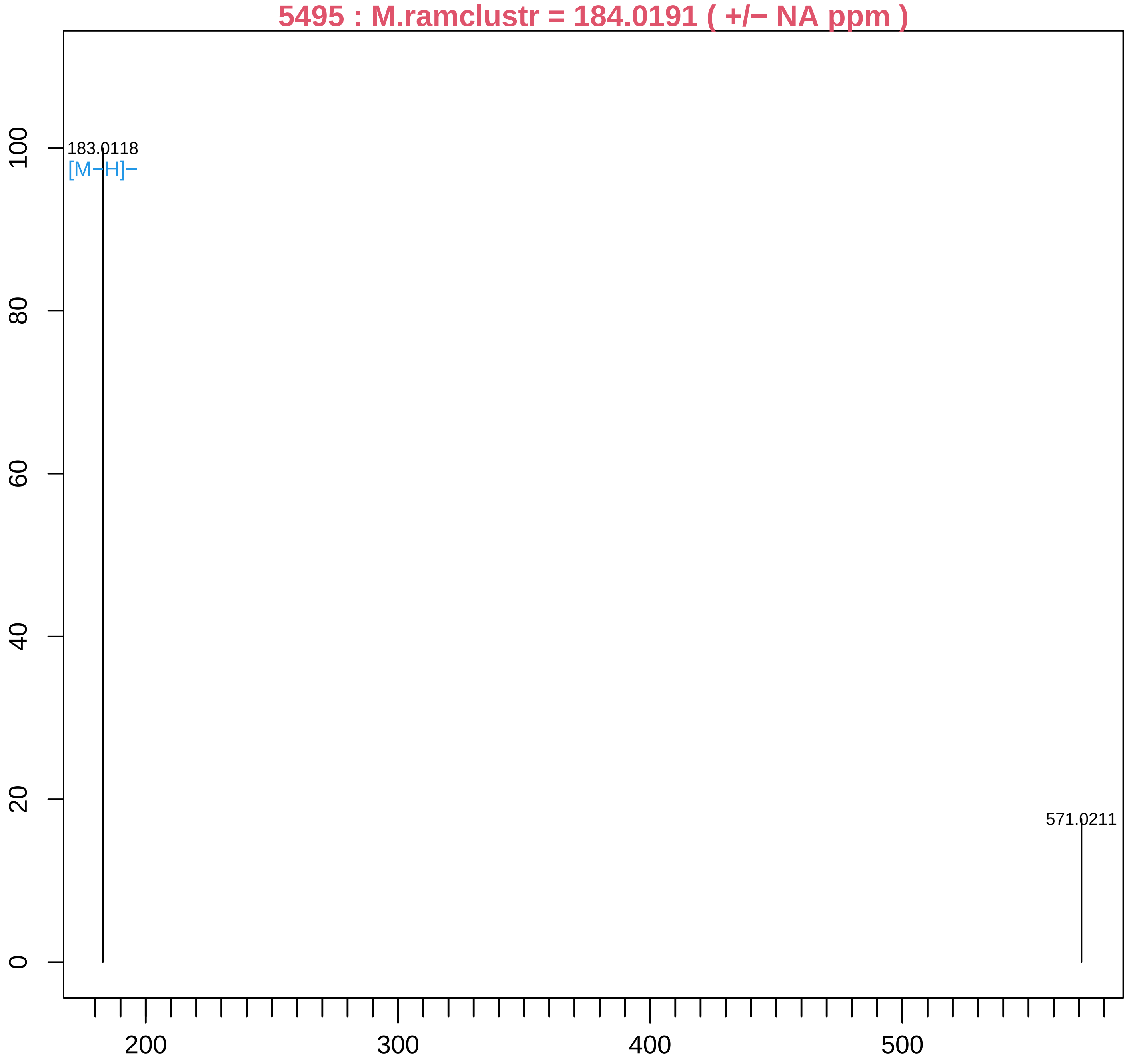
5495 : M.ramclustr = 184.0191 (+/− NA ppm)
183.0118
[M−H]−
571.0211
0
20
40
60
80
100
200
300
400
500

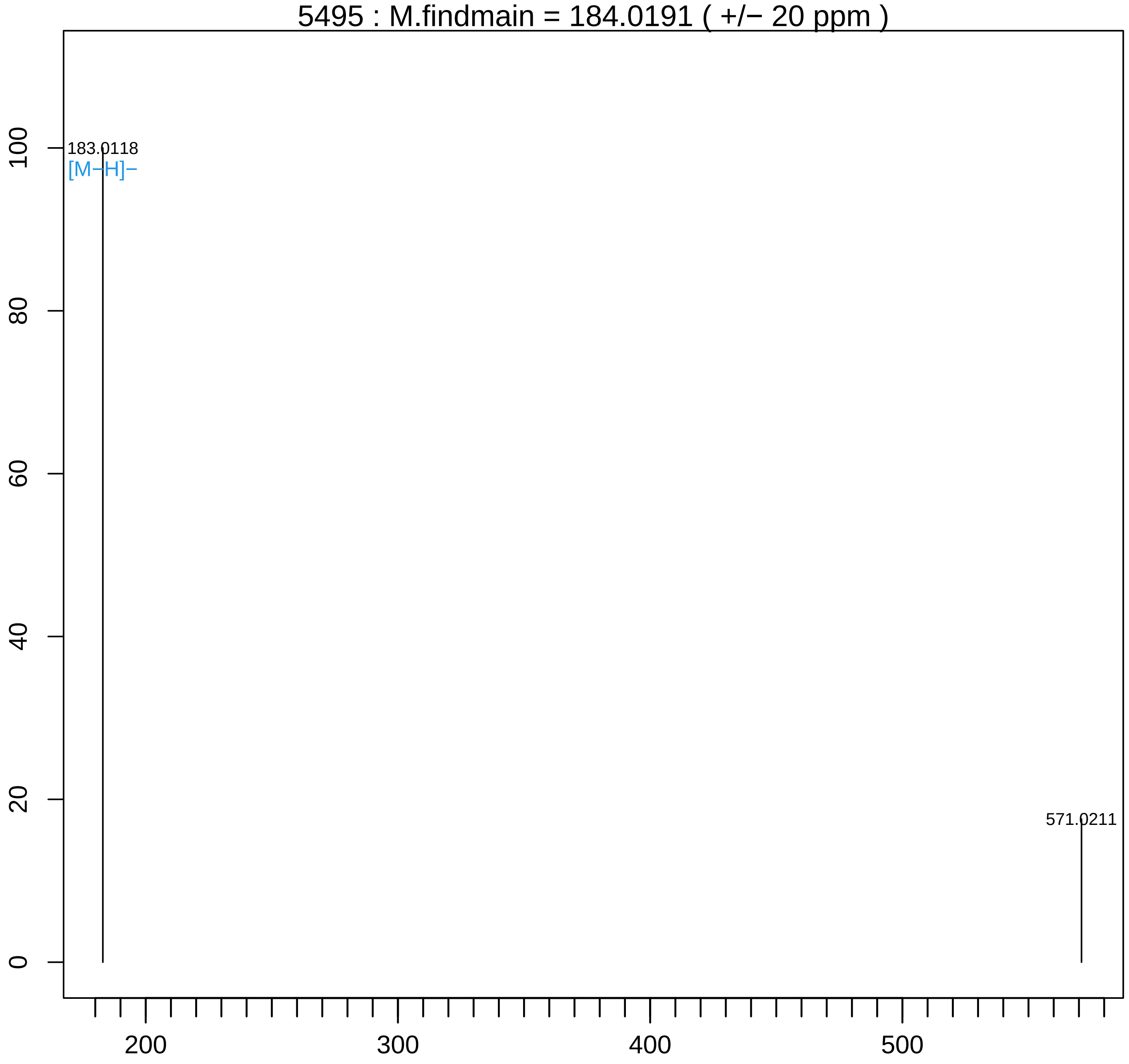
5495 : M.findmain = 184.0191 (+/− 20 ppm)
183.0118
[M−H]−
571.0211
0
20
40
60
80
100
200
300
400
500

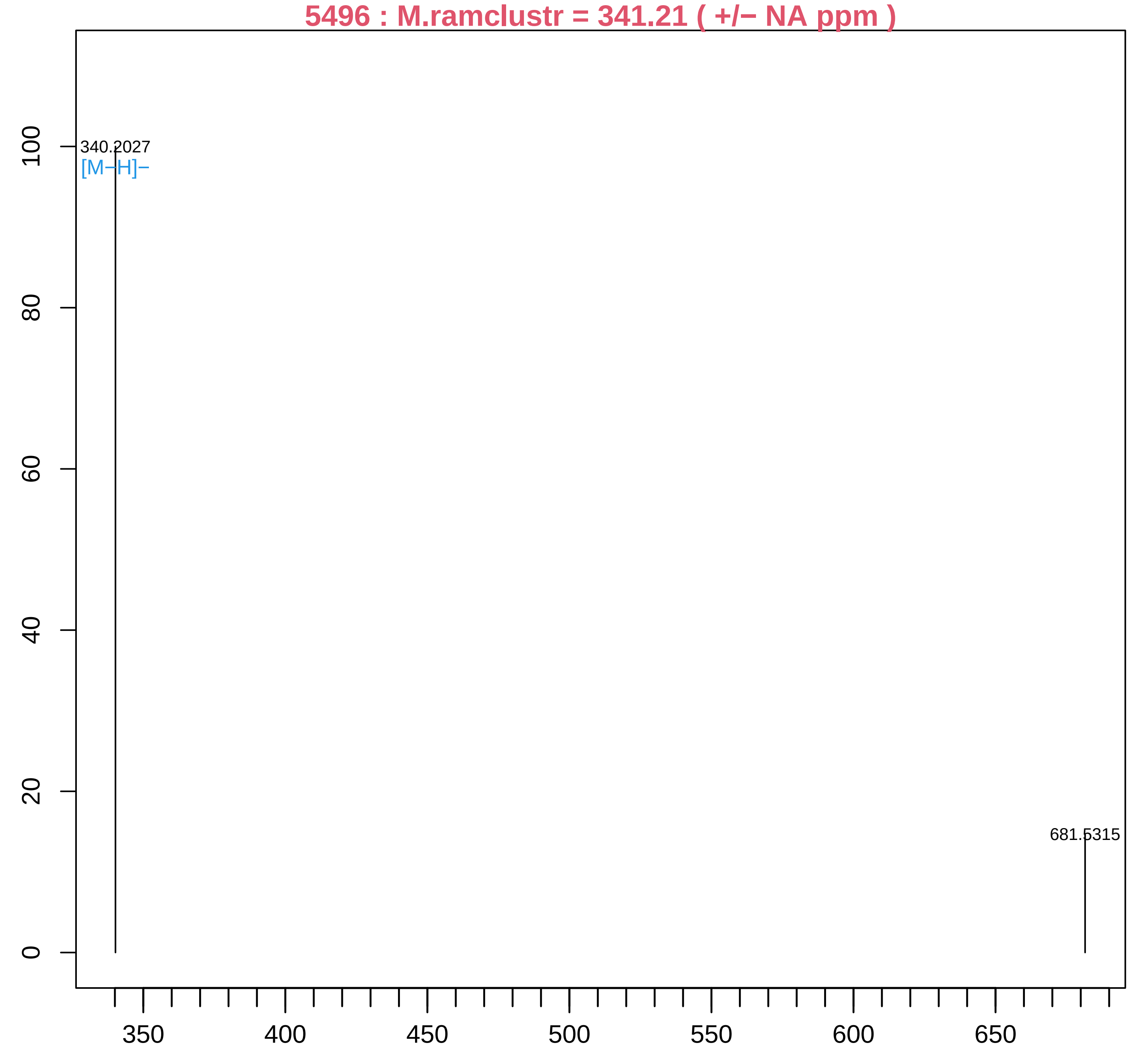

5496 : M.ramclustr = 341.21 (+/− NA ppm)
340.2027
[M−H]−
681.5315
0
20
40
60
80
100
350
400
450
500
550
600
650

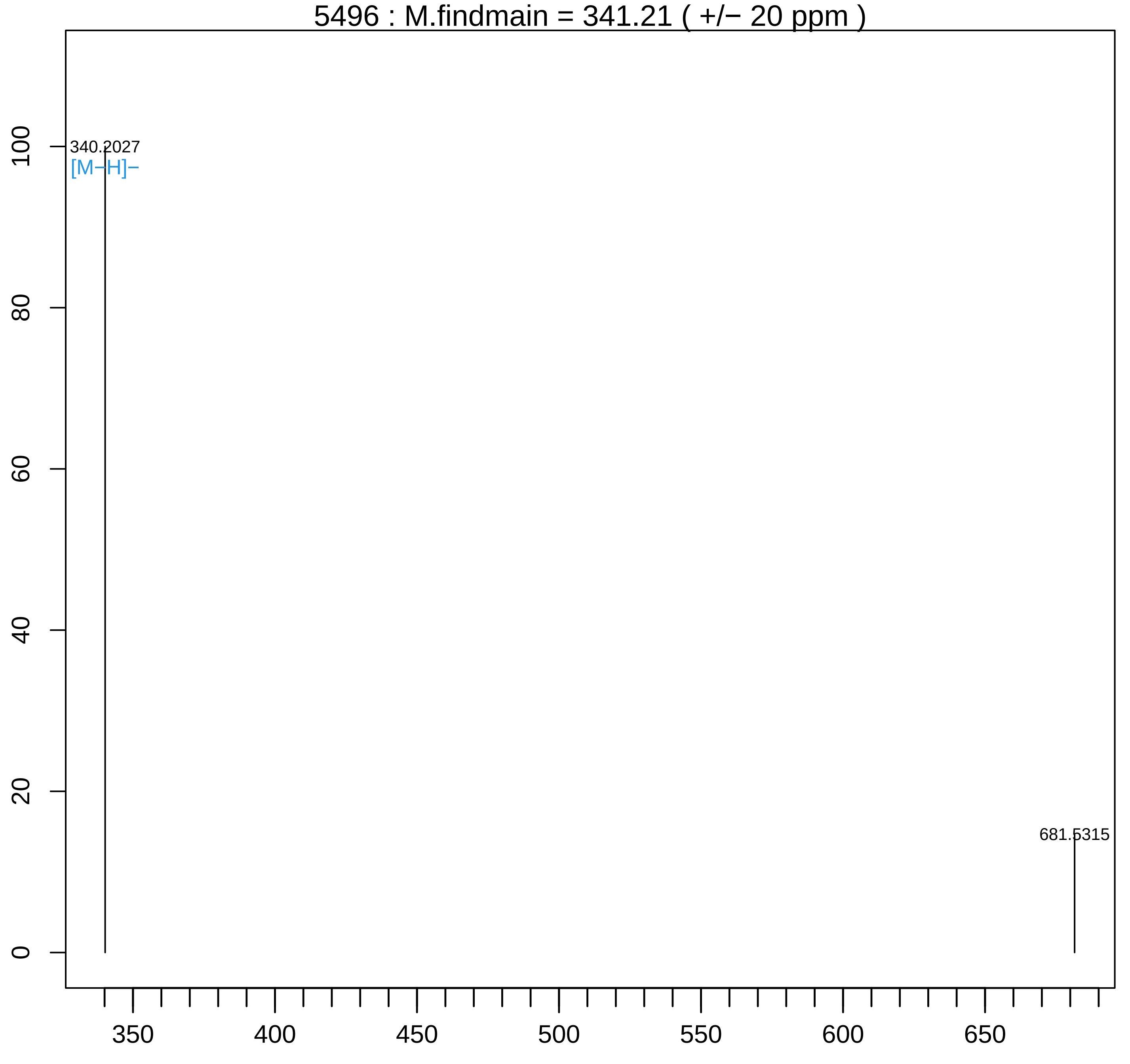

5496 : M.findmain = 341.21 (+/− 20 ppm)
340.2027
[M−H]−
681.5315
0
20
40
60
80
100
350
400
450
500
550
600
650

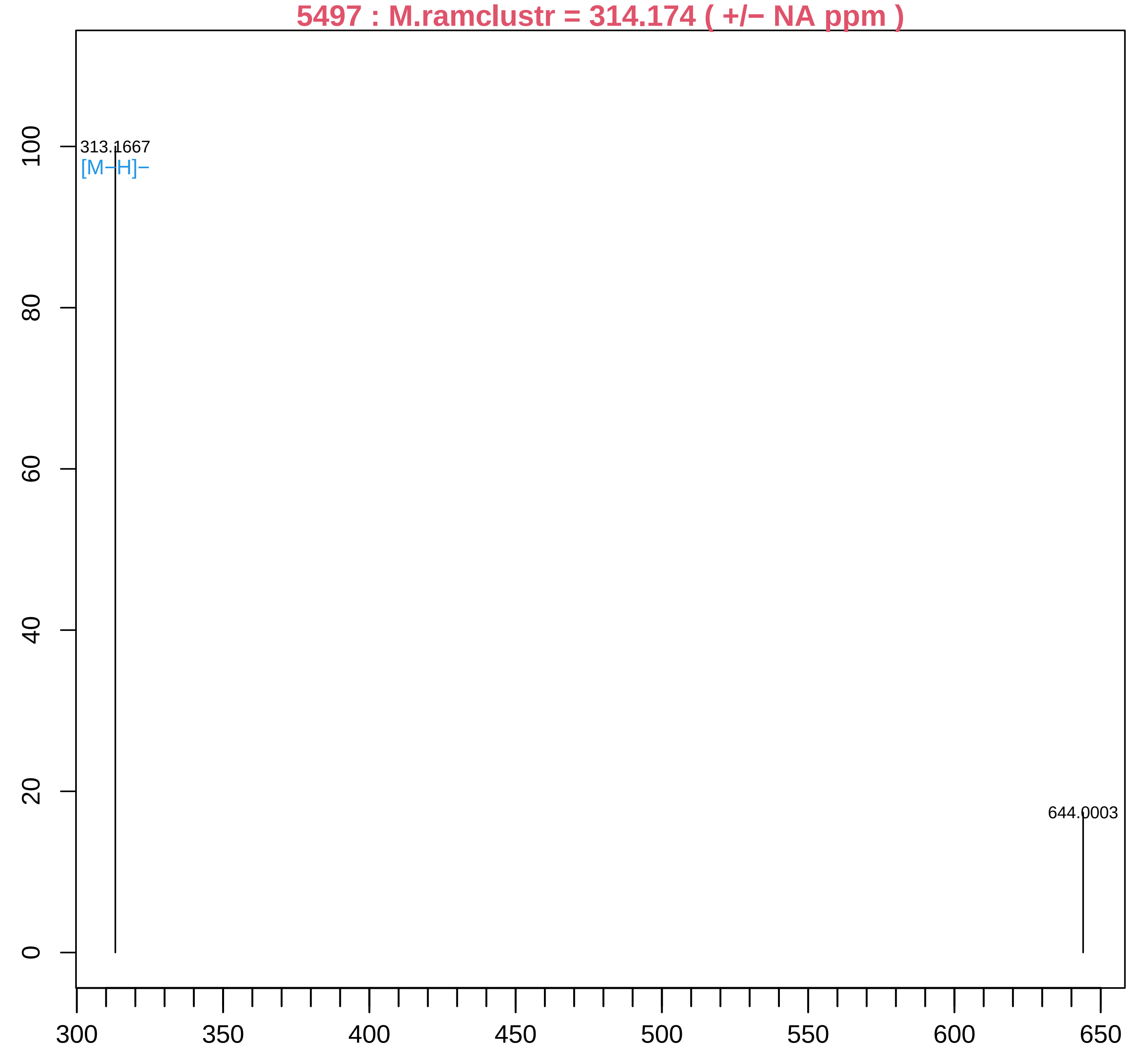
5497 : M.ramclustr = 314.174 (+/− NA ppm)
313.1667
[M−H]−
644.0003
0
20
40
60
80
100
300
350
400
450
500
550
600
650

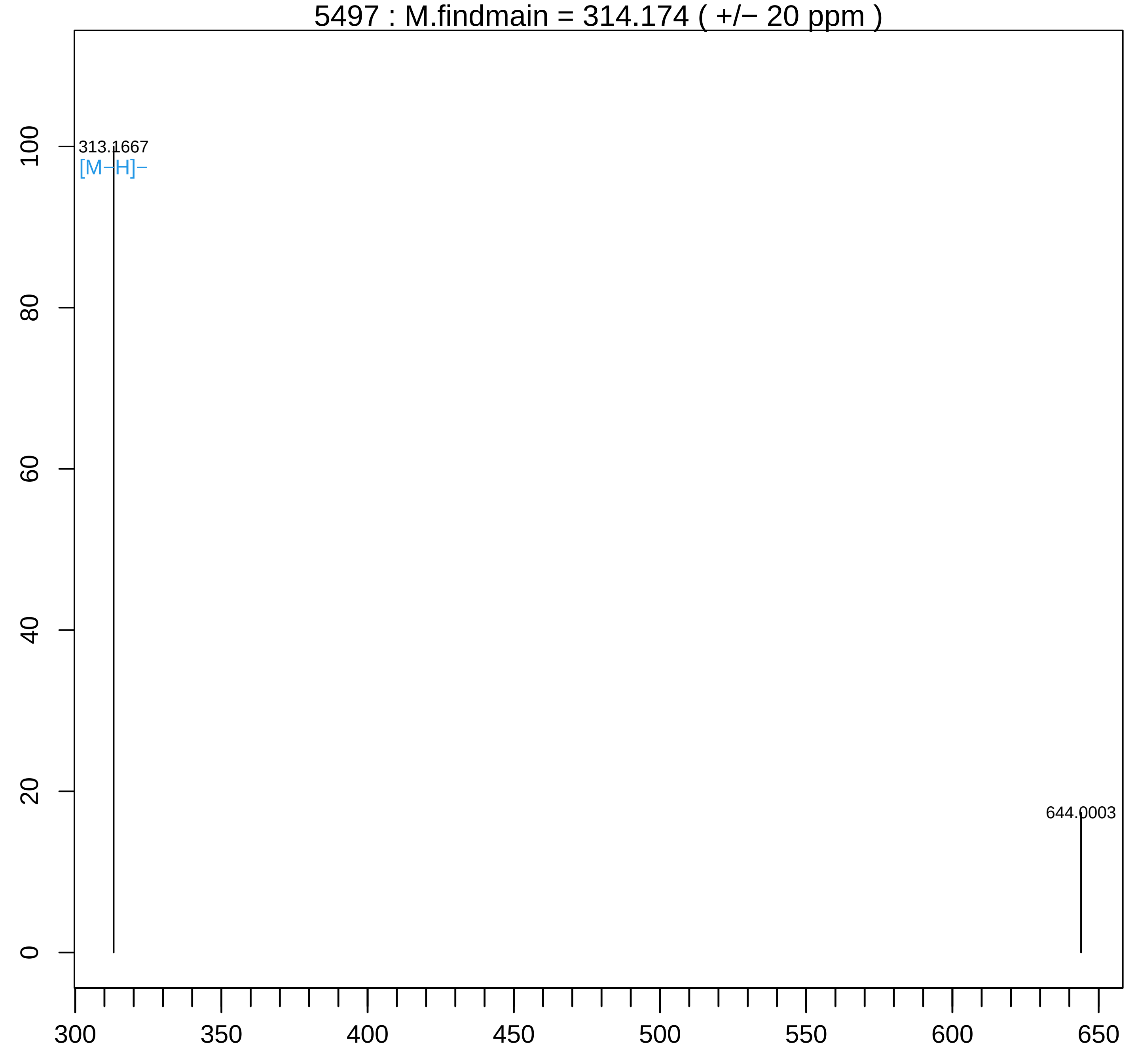
5497 : M.findmain = 314.174 (+/− 20 ppm)
313.1667
[M−H]−
644.0003
0
20
40
60
80
100
300
350
400
450
500
550
600
650

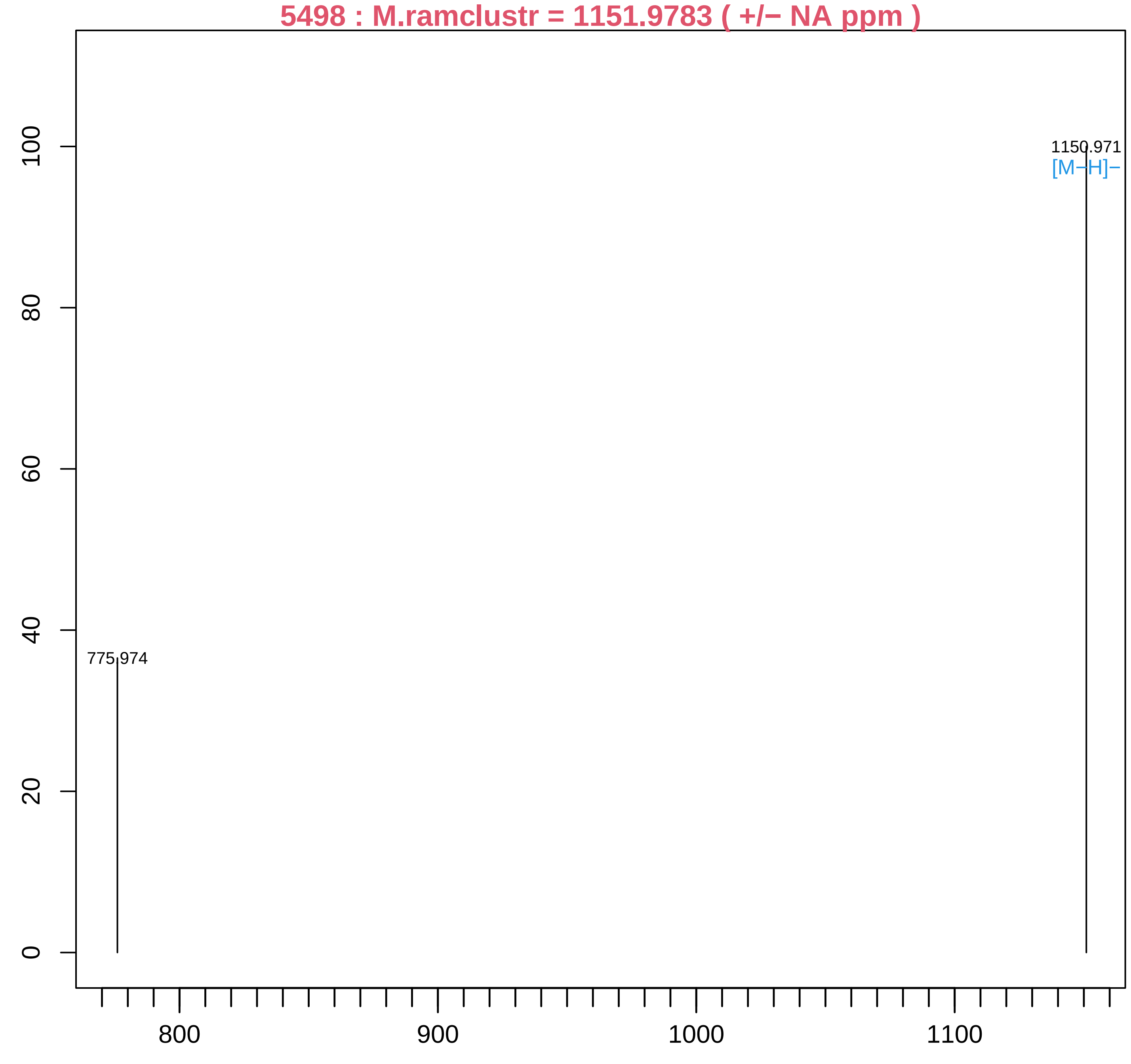
5498 : M.ramclustr = 1151.9783 (+/− NA ppm)
1150.971
[M−H]−
775.974
0
20
40
60
80
100
800
900
1000
1100

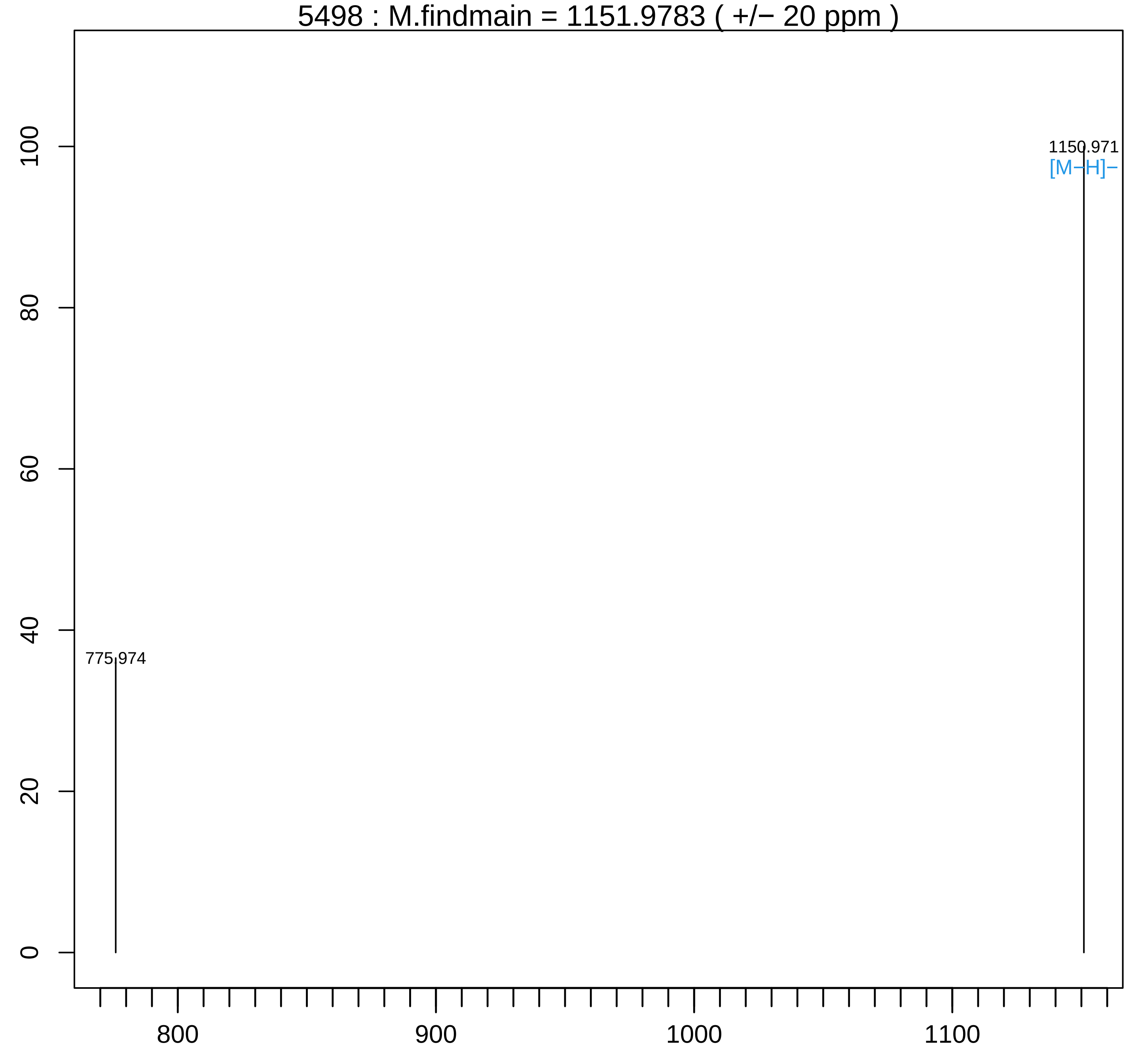
5498 : M.findmain = 1151.9783 (+/− 20 ppm)
1150.971
[M−H]−
775.974
0
20
40
60
80
100
800
900
1000
1100

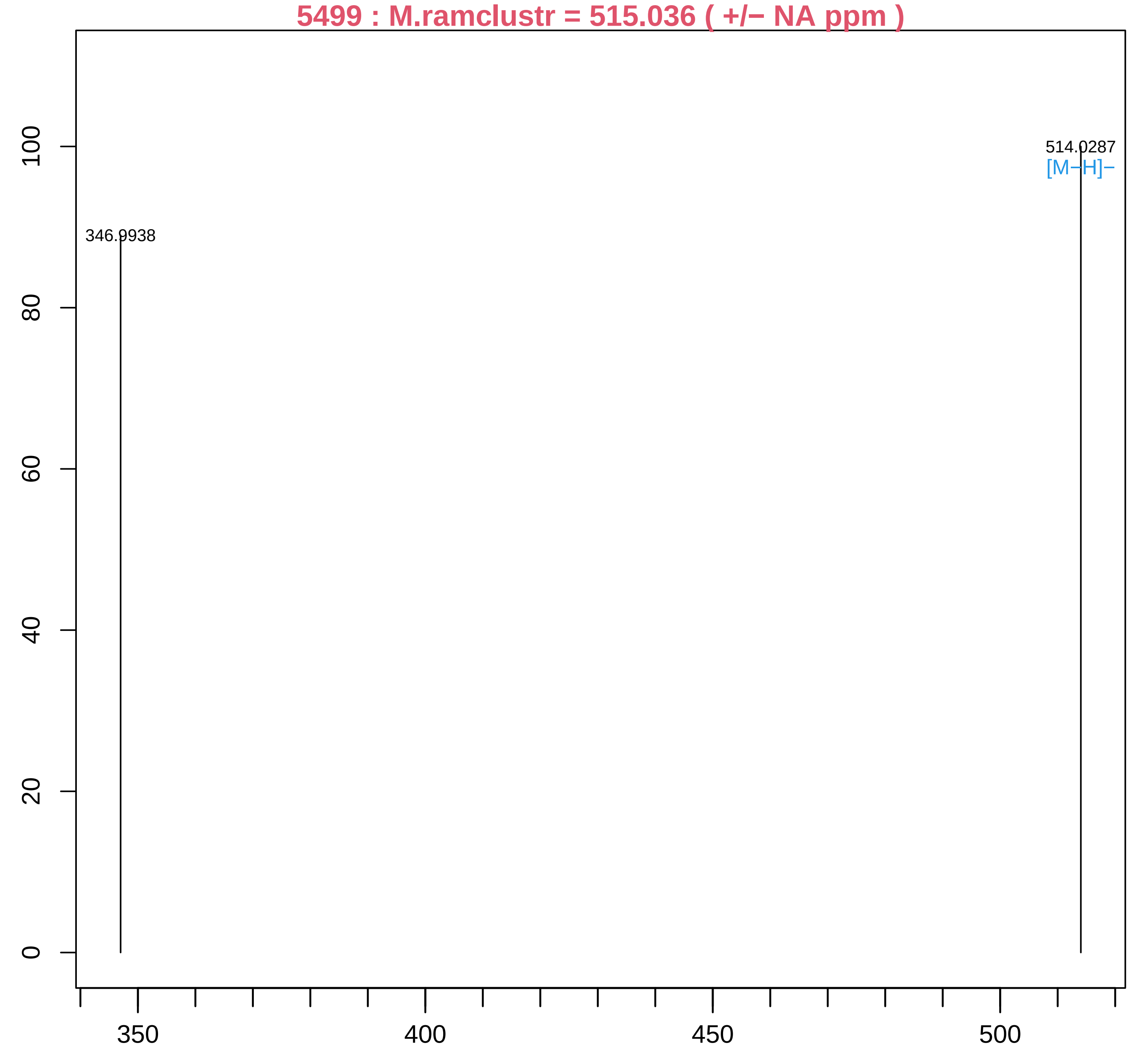
5499 : M.ramclustr = 515.036 (+/− NA ppm)
514.0287
[M−H]−
346.9938
0
20
40
60
80
100
350
400
450
500
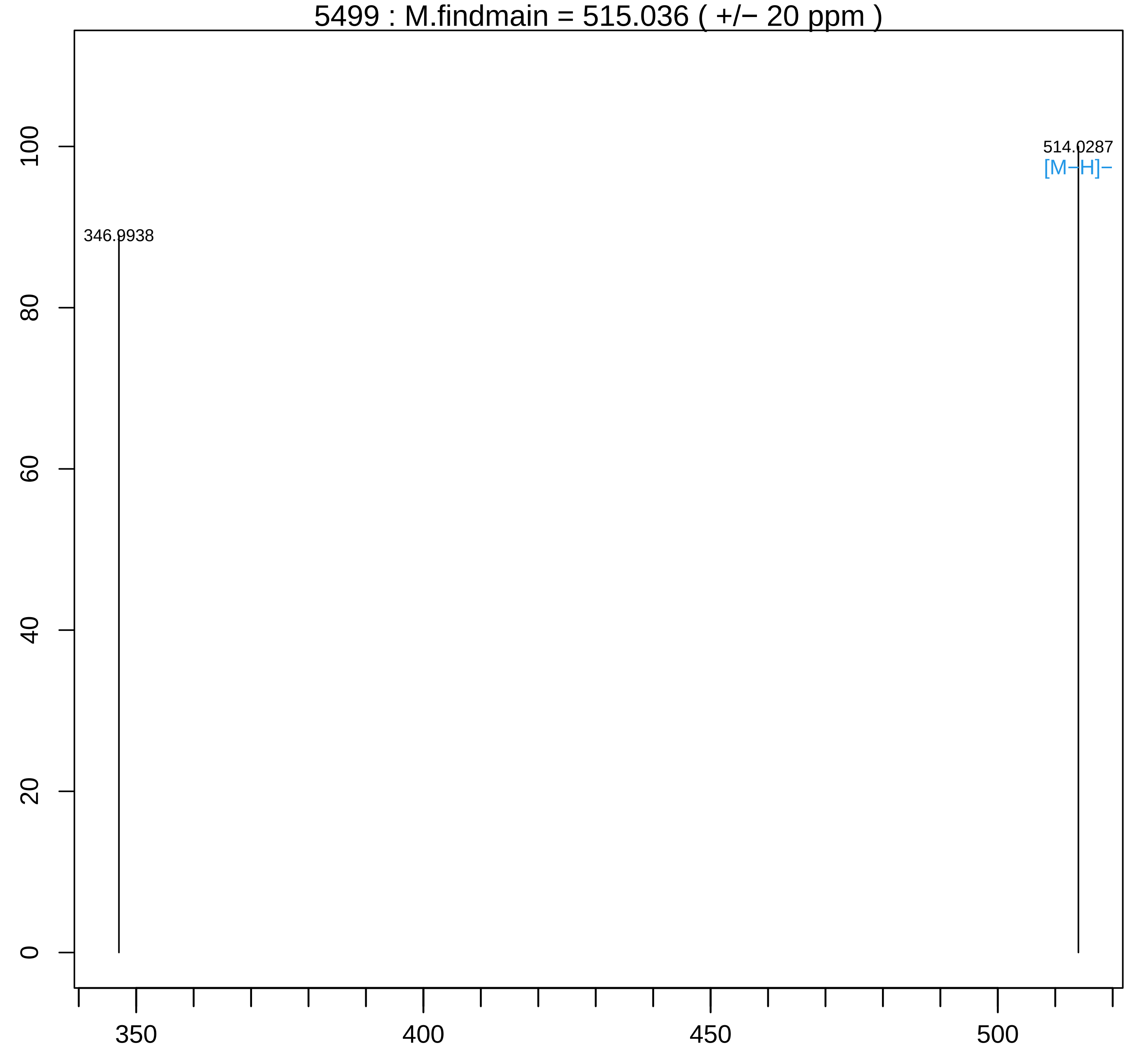
5499 : M.findmain = 515.036 (+/− 20 ppm)
514.0287
[M−H]−
346.9938
0
20
40
60
80
100
350
400
450
500

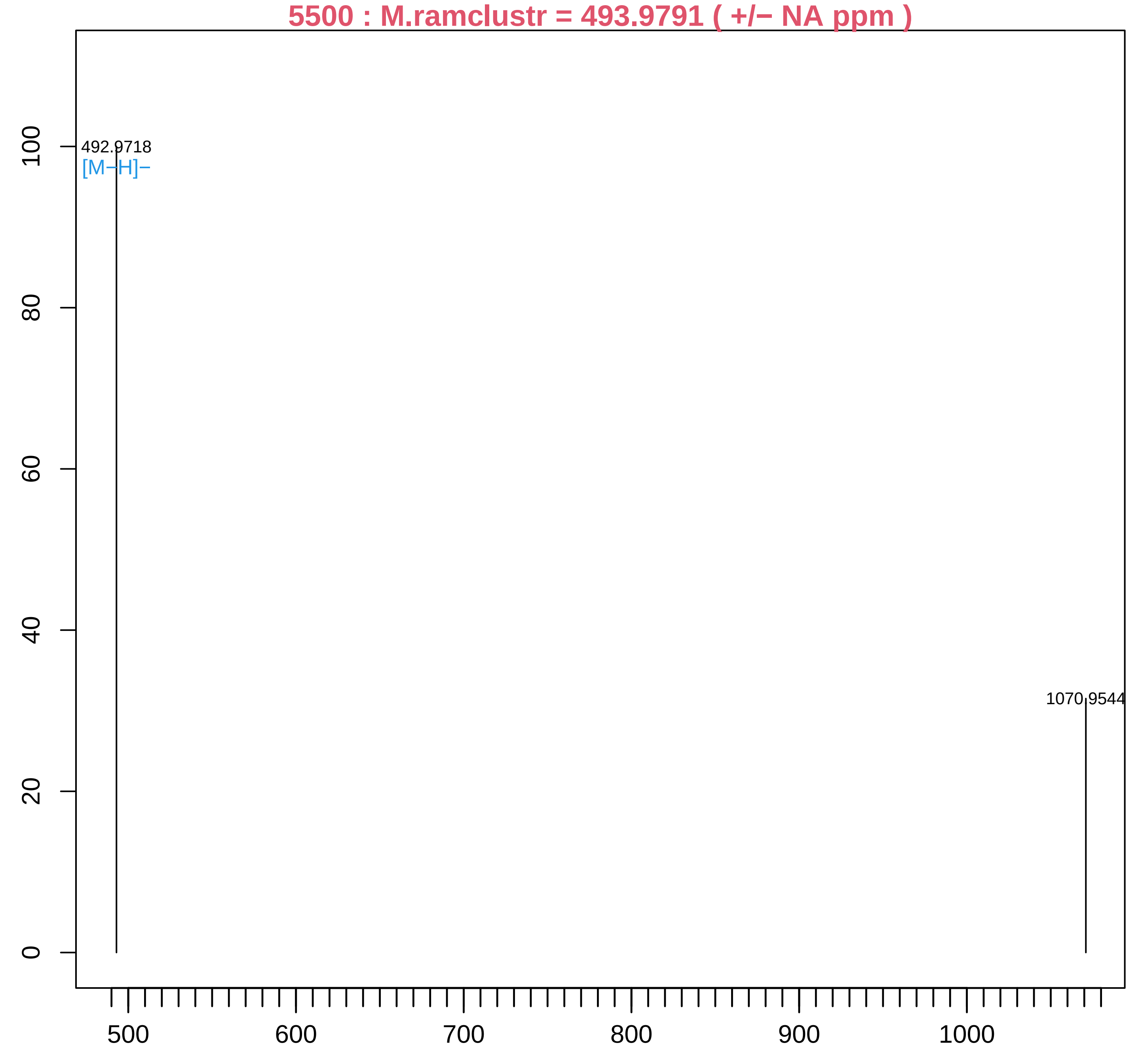
5500 : M.ramclustr = 493.9791 (+/− NA ppm)
492.9718
[M−H]−
1070.9544
0
20
40
60
80
100
500
600
700
800
900
1000

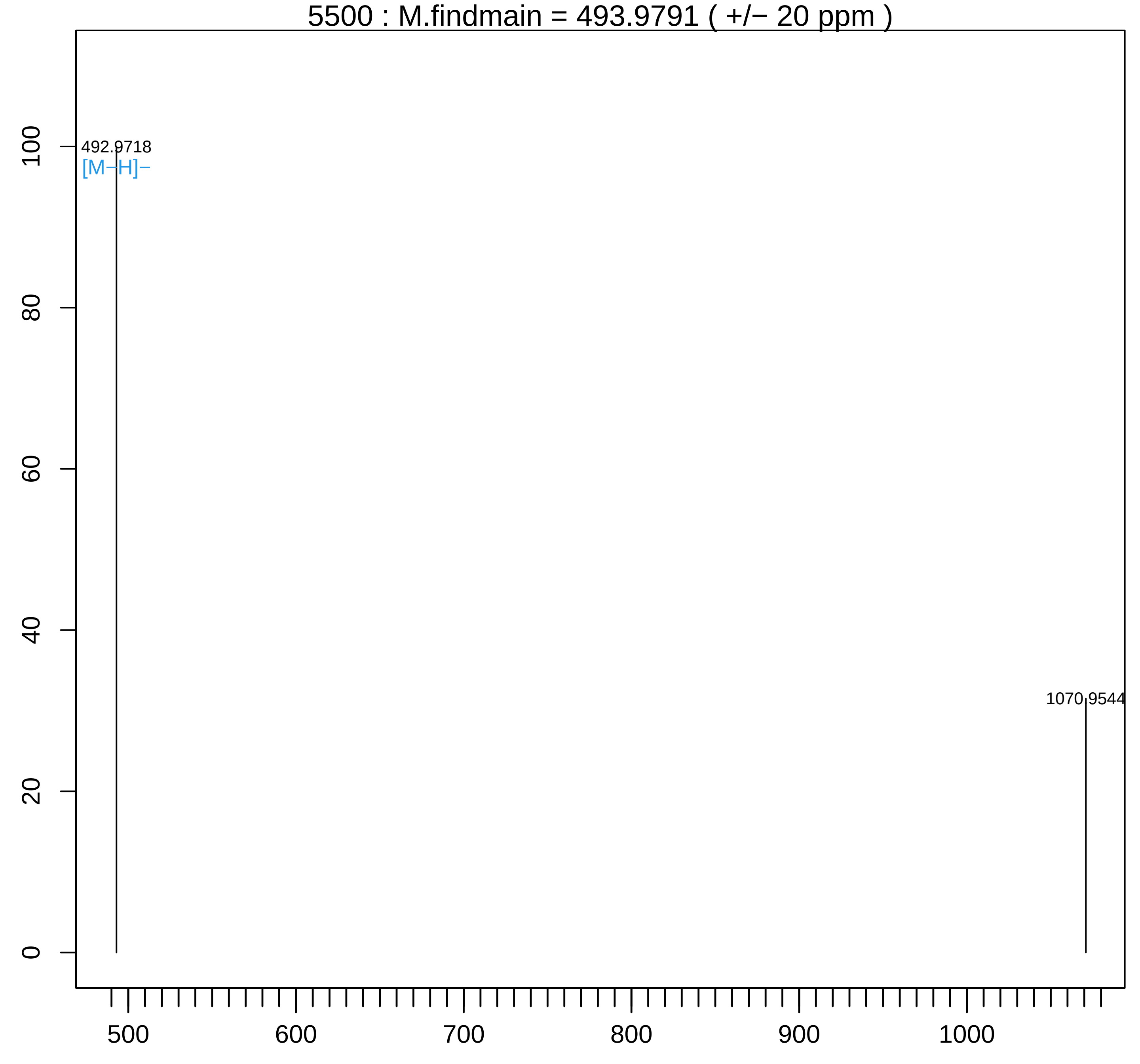
5500 : M.findmain = 493.9791 (+/− 20 ppm)
492.9718
[M−H]−
1070.9544
0
20
40
60
80
100
500
600
700
800
900
1000

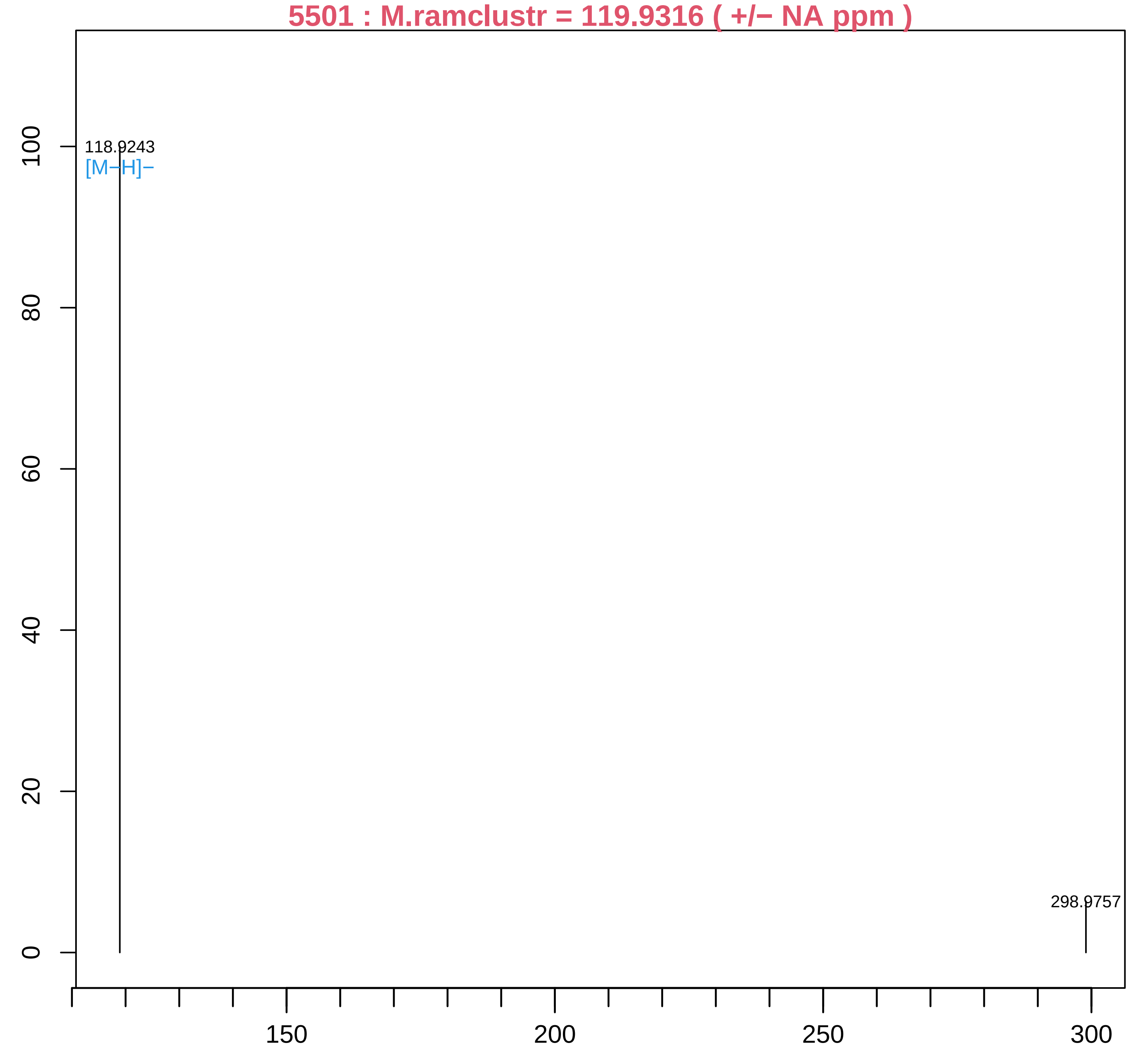
5501 : M.ramclustr = 119.9316 (+/− NA ppm)
118.9243
[M−H]−
298.9757
0
20
40
60
80
100
150
200
250
300

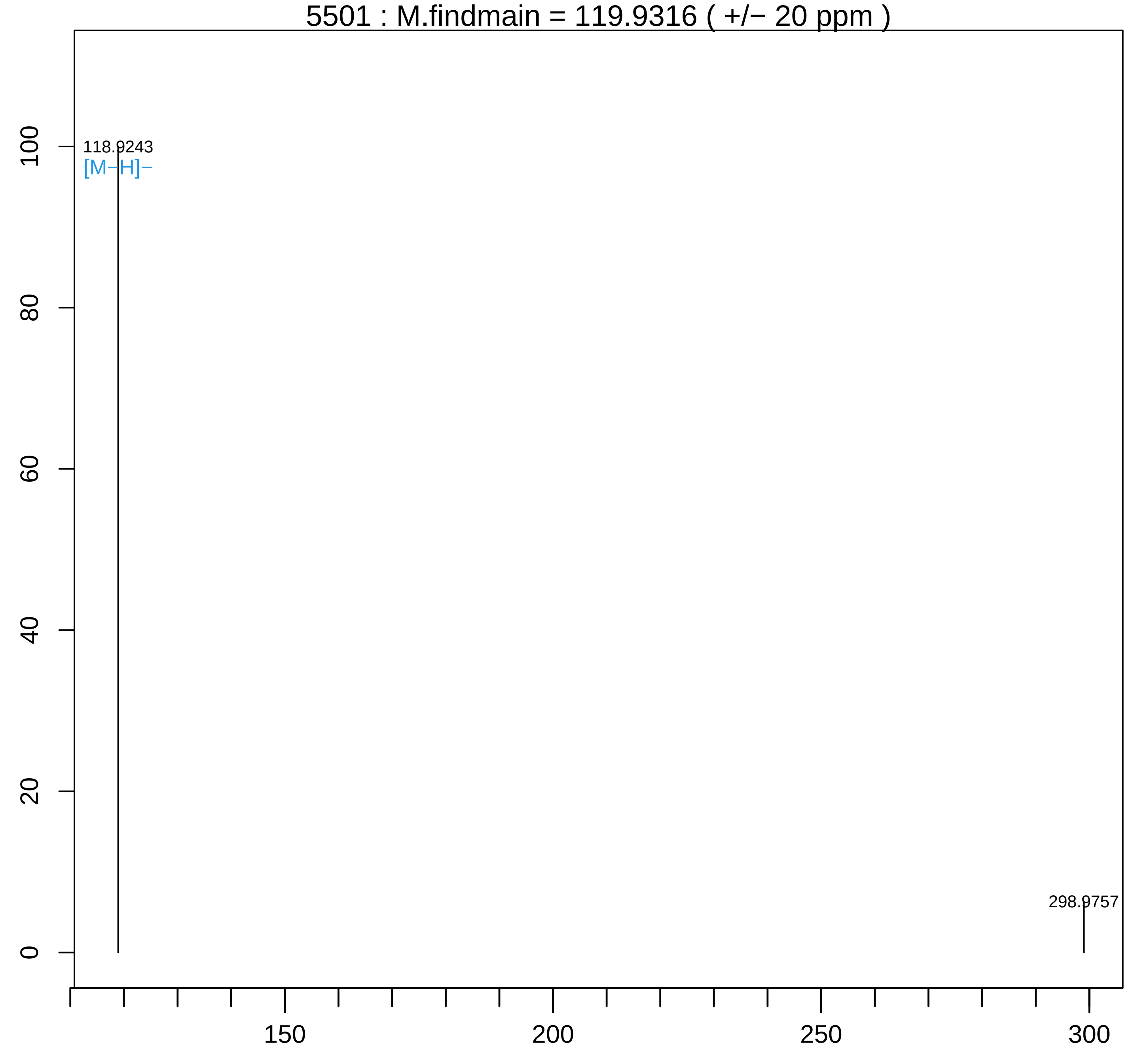
5501 : M.findmain = 119.9316 (+/− 20 ppm)
118.9243
[M−H]−
298.9757
0
20
40
60
80
100
150
200
250
300

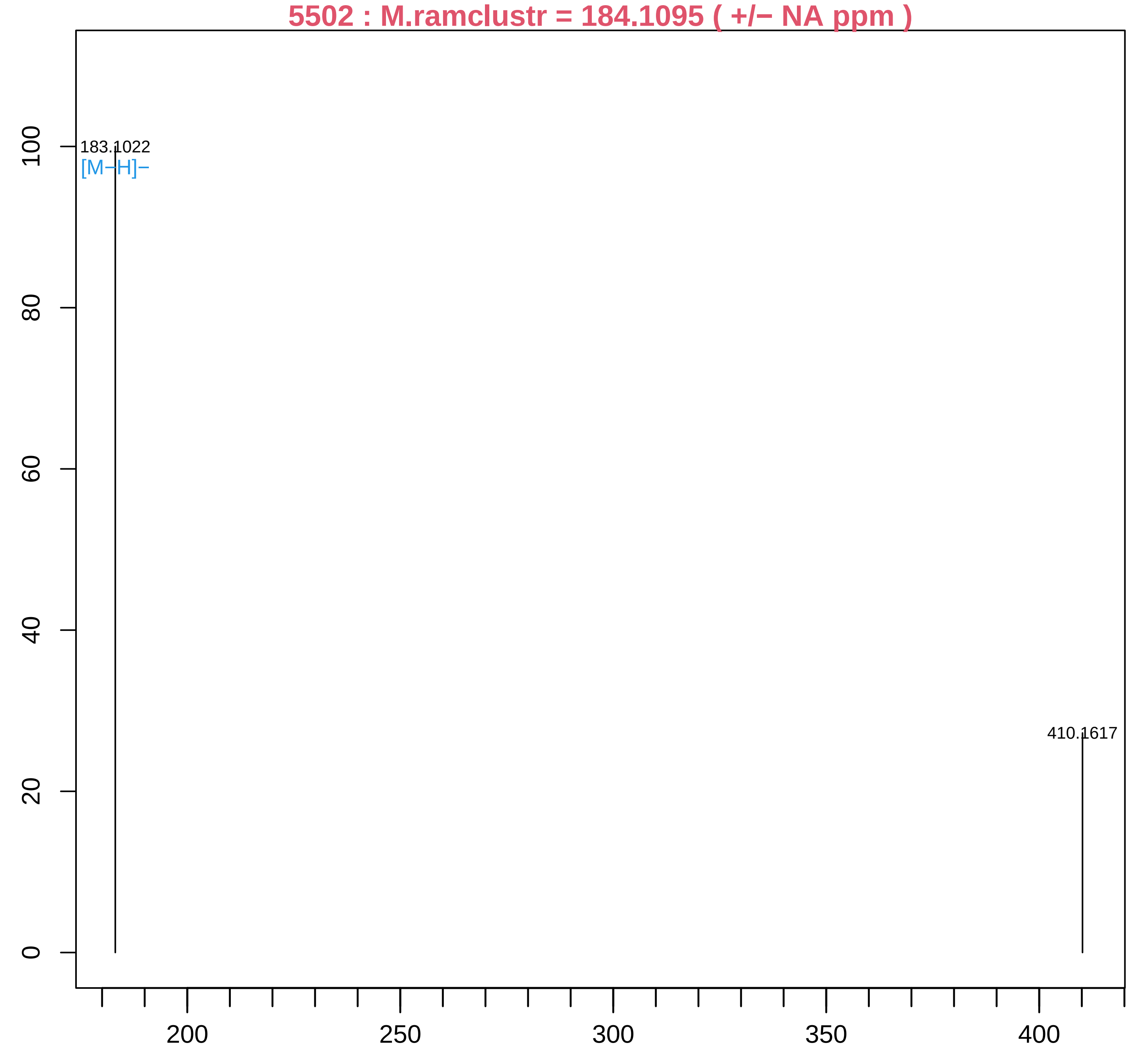

5502 : M.ramclustr = 184.1095 (+/− NA ppm)
183.1022
[M−H]−
410.1617
0
20
40
60
80
100
200
250
300
350
400

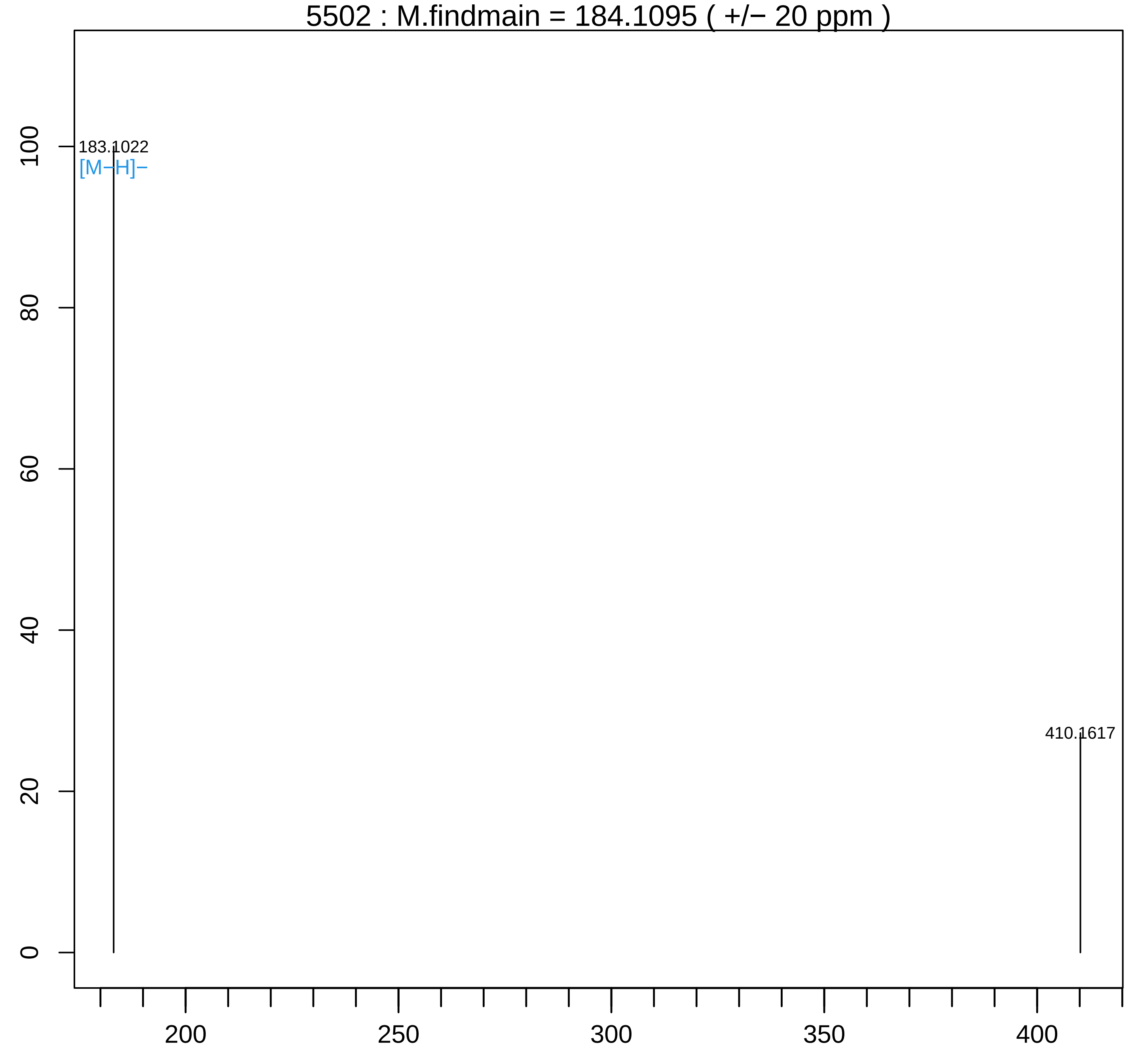

5502 : M.findmain = 184.1095 (+/− 20 ppm)
183.1022
[M−H]−
410.1617
0
20
40
60
80
100
200
250
300
350
400

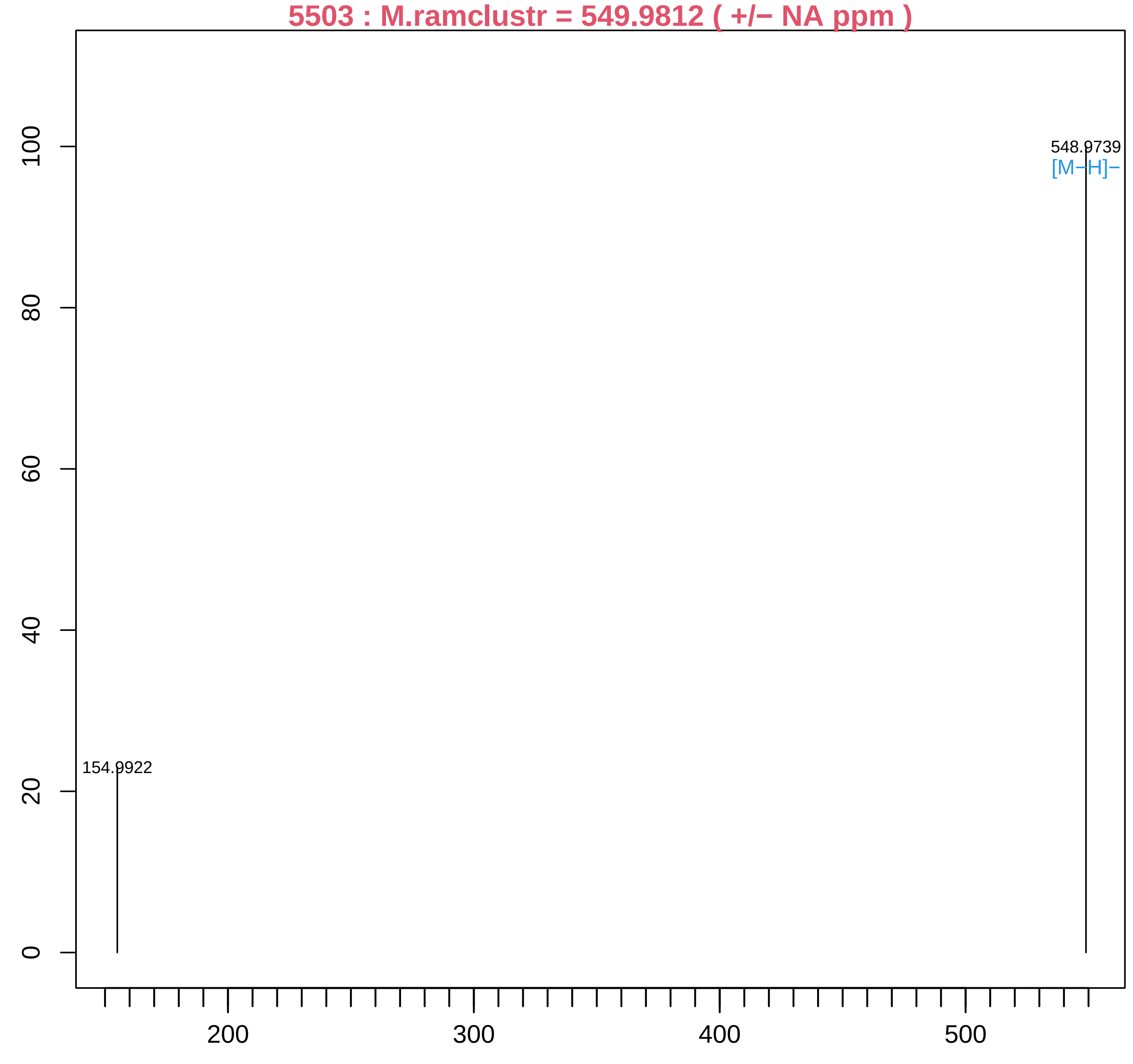
5503 : M.ramclustr = 549.9812 (+/− NA ppm)
548.9739
[M−H]−
154.9922
0
20
40
60
80
100
200
300
400
500

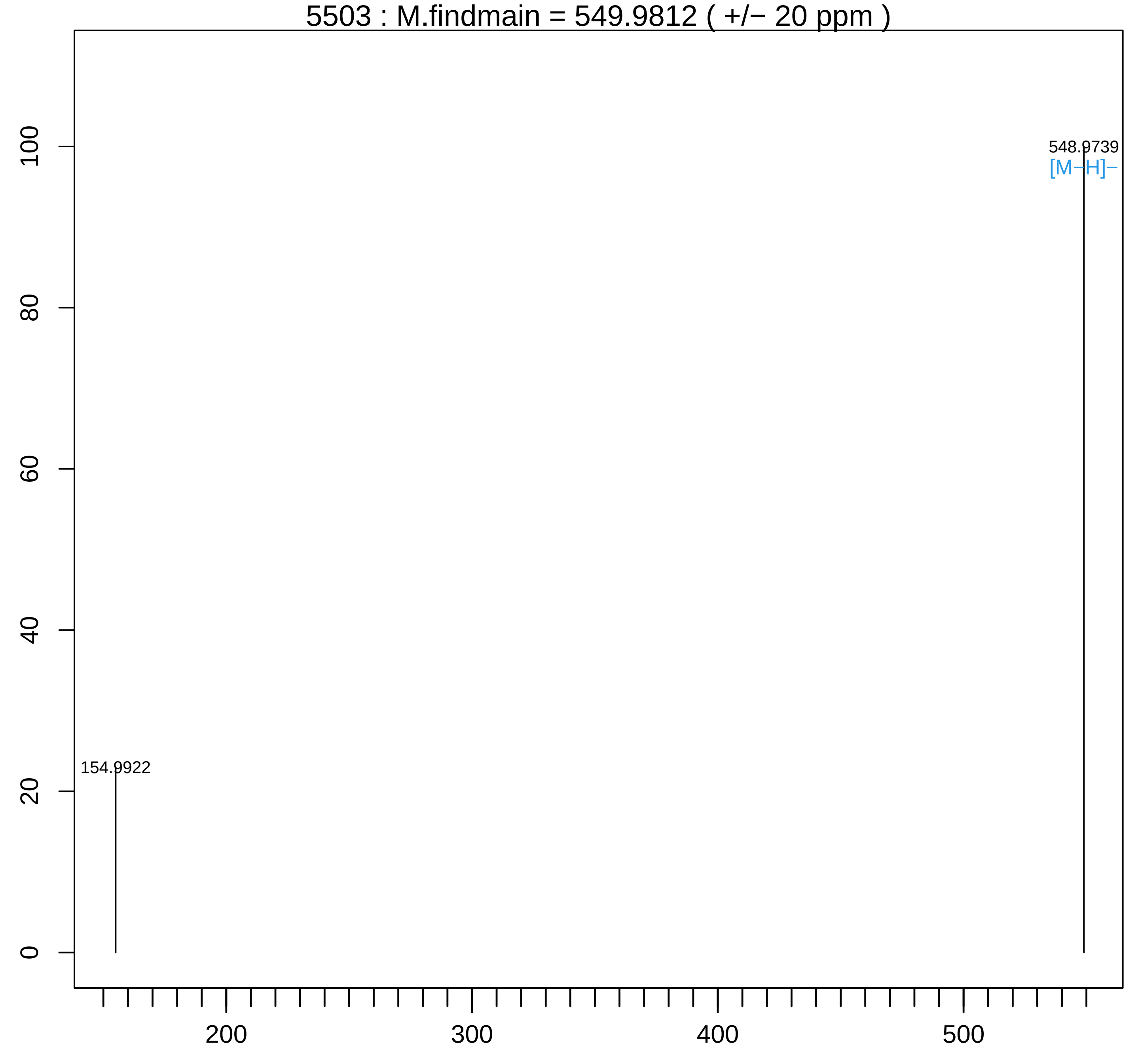
5503 : M.findmain = 549.9812 (+/− 20 ppm)
548.9739
[M−H]−
154.9922
0
20
40
60
80
100
200
300
400
500

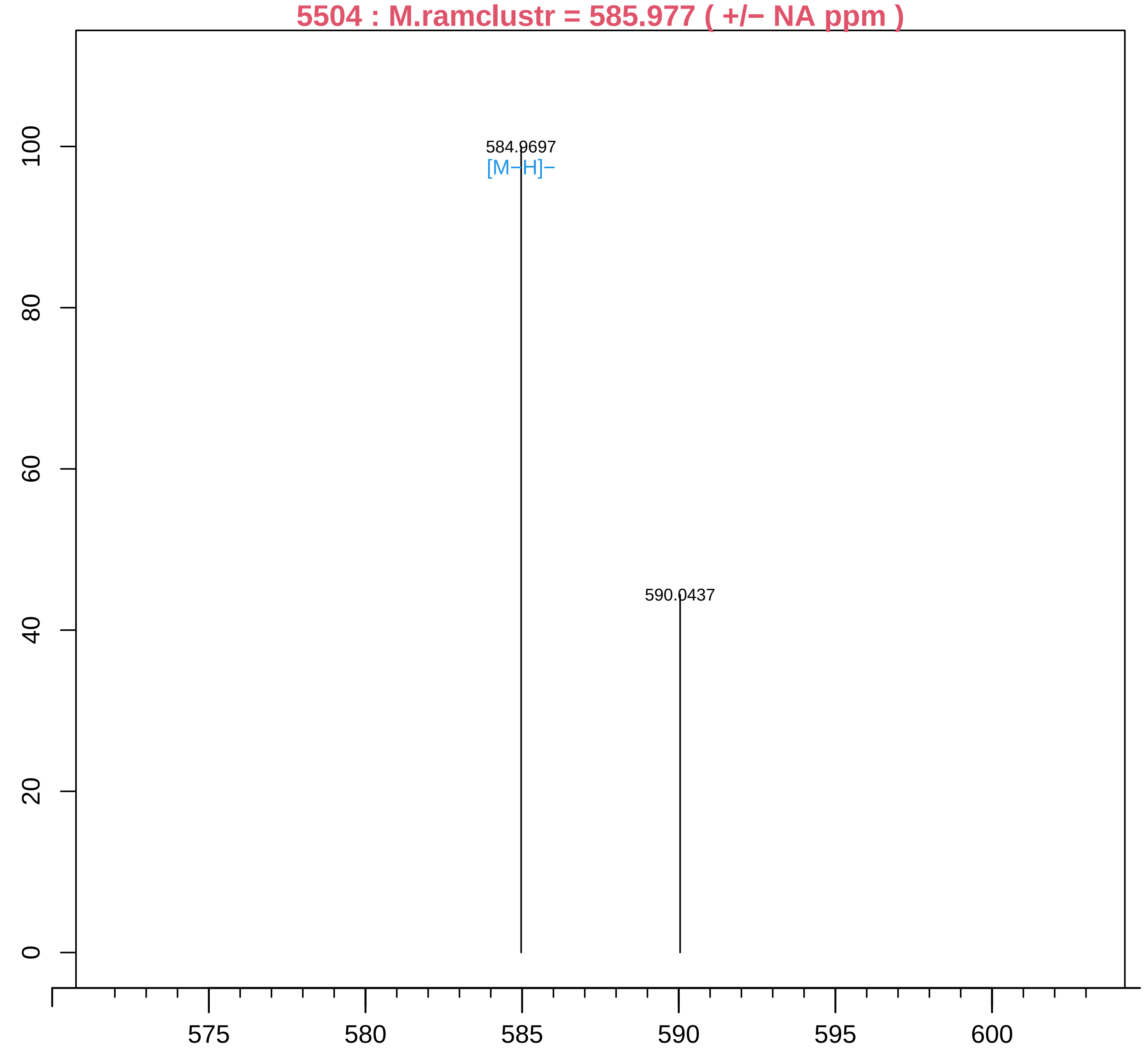
5504 : M.ramclustr = 585.977 (+/− NA ppm)
584.9697
[M−H]−
590.0437
0
20
40
60
80
100
575
580
585
590
595
600

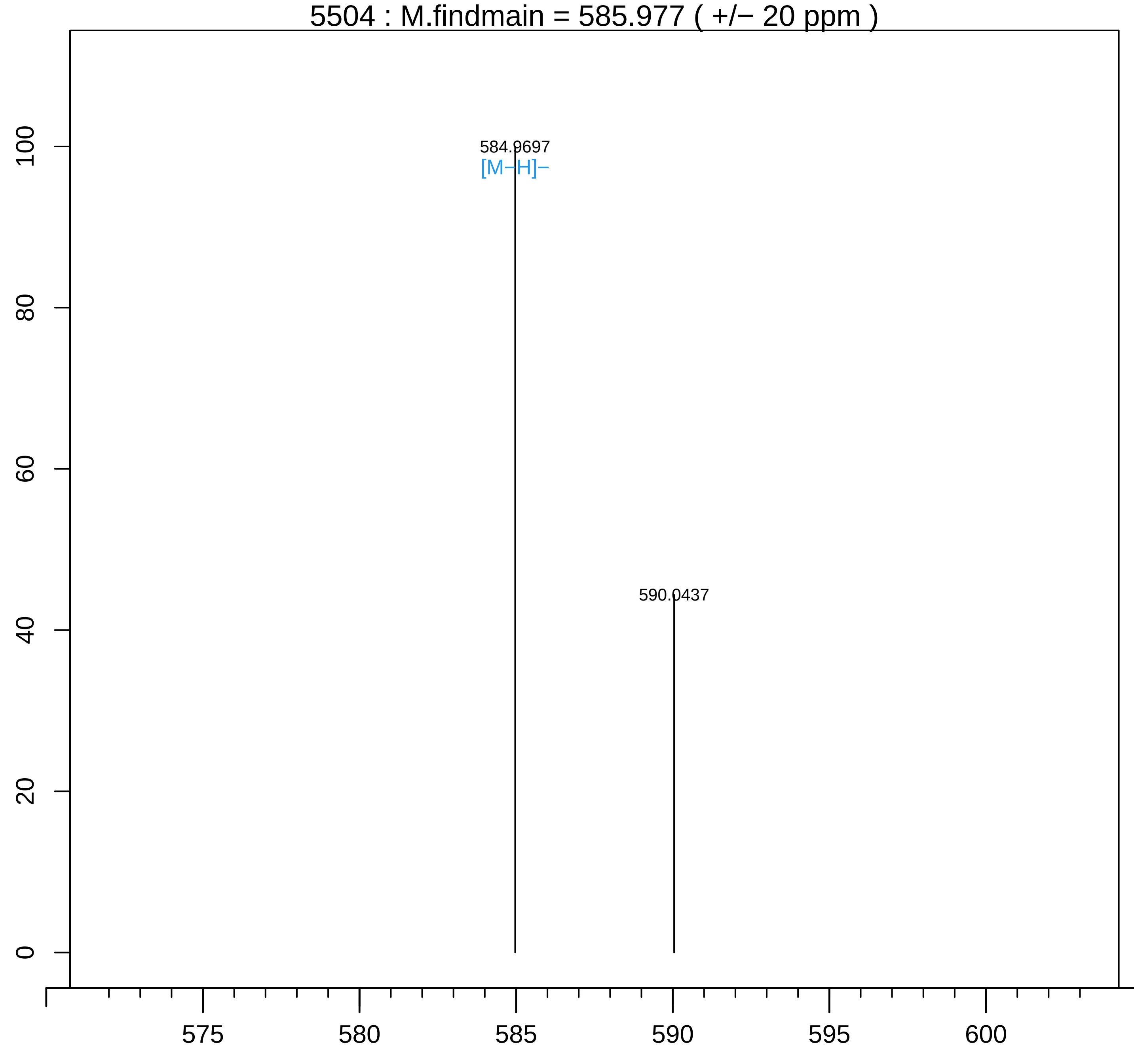
5504 : M.findmain = 585.977 (+/− 20 ppm)
584.9697
[M−H]−
590.0437
0
20
40
60
80
100
575
580
585
590
595
600

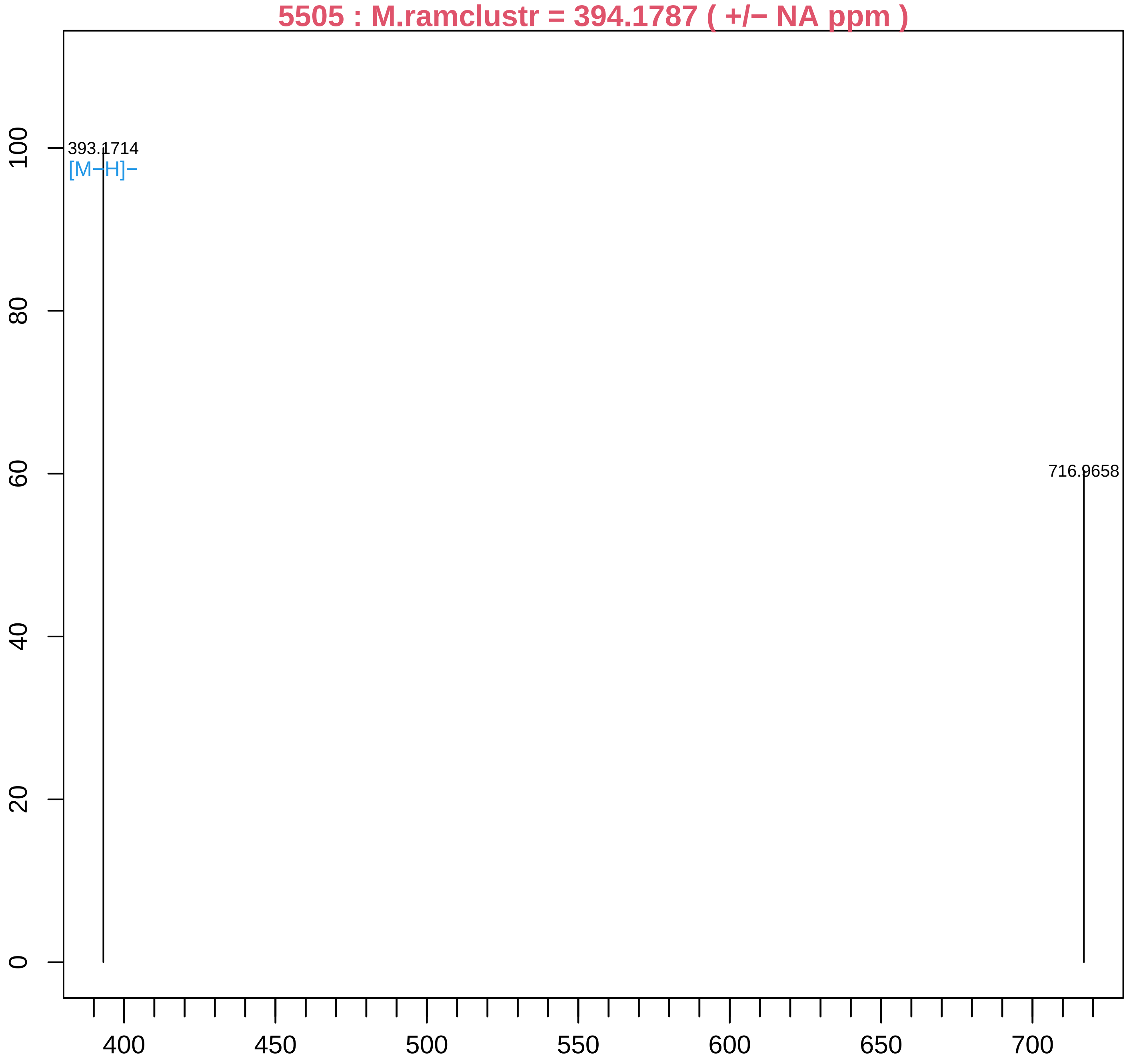
5505 : M.ramclustr = 394.1787 (+/− NA ppm)
393.1714
[M−H]−
716.9658
0
20
40
60
80
100
400
450
500
550
600
650
700

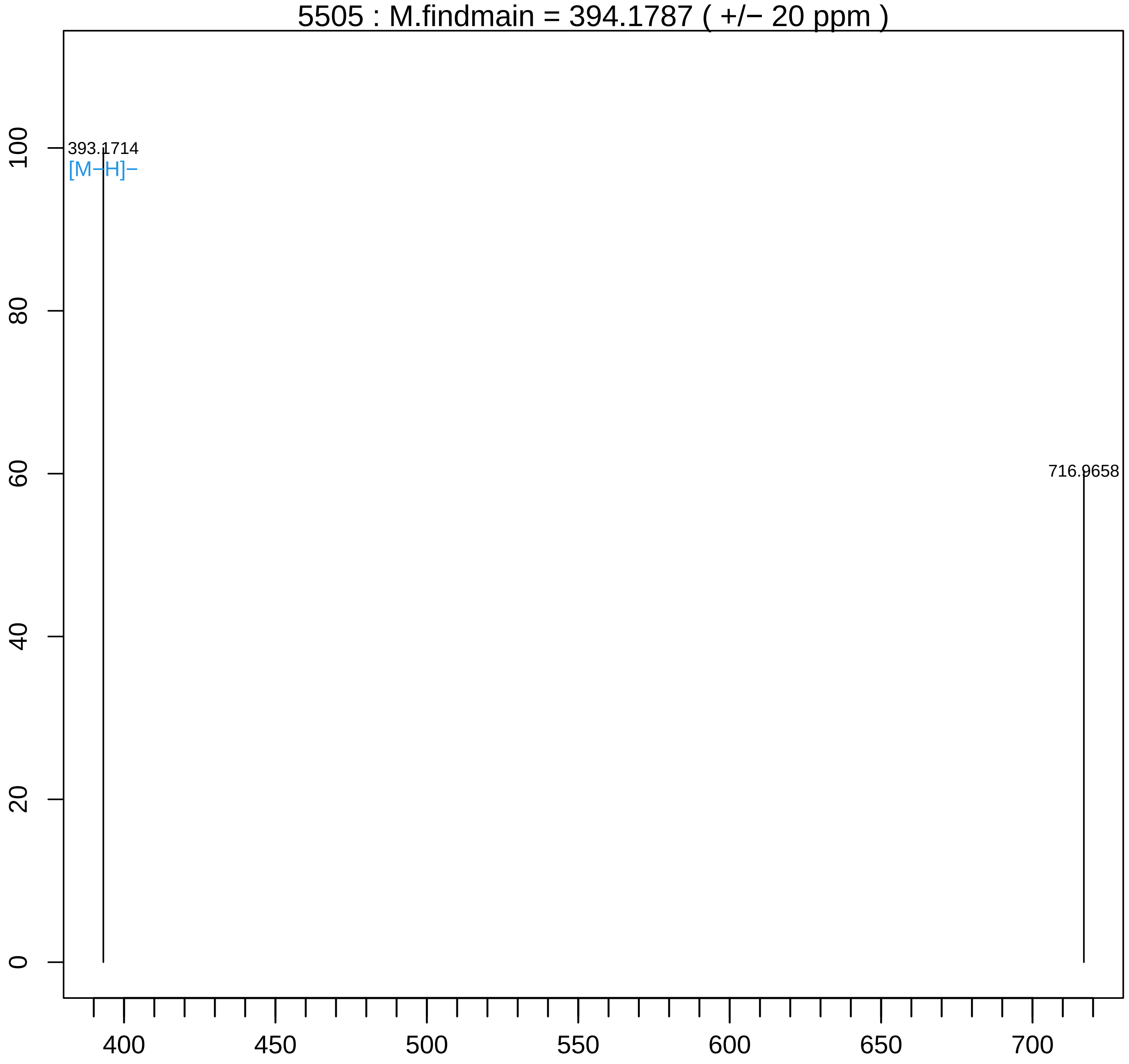
5505 : M.findmain = 394.1787 (+/− 20 ppm)
393.1714
[M−H]−
716.9658
0
20
40
60
80
100
400
450
500
550
600
650
700

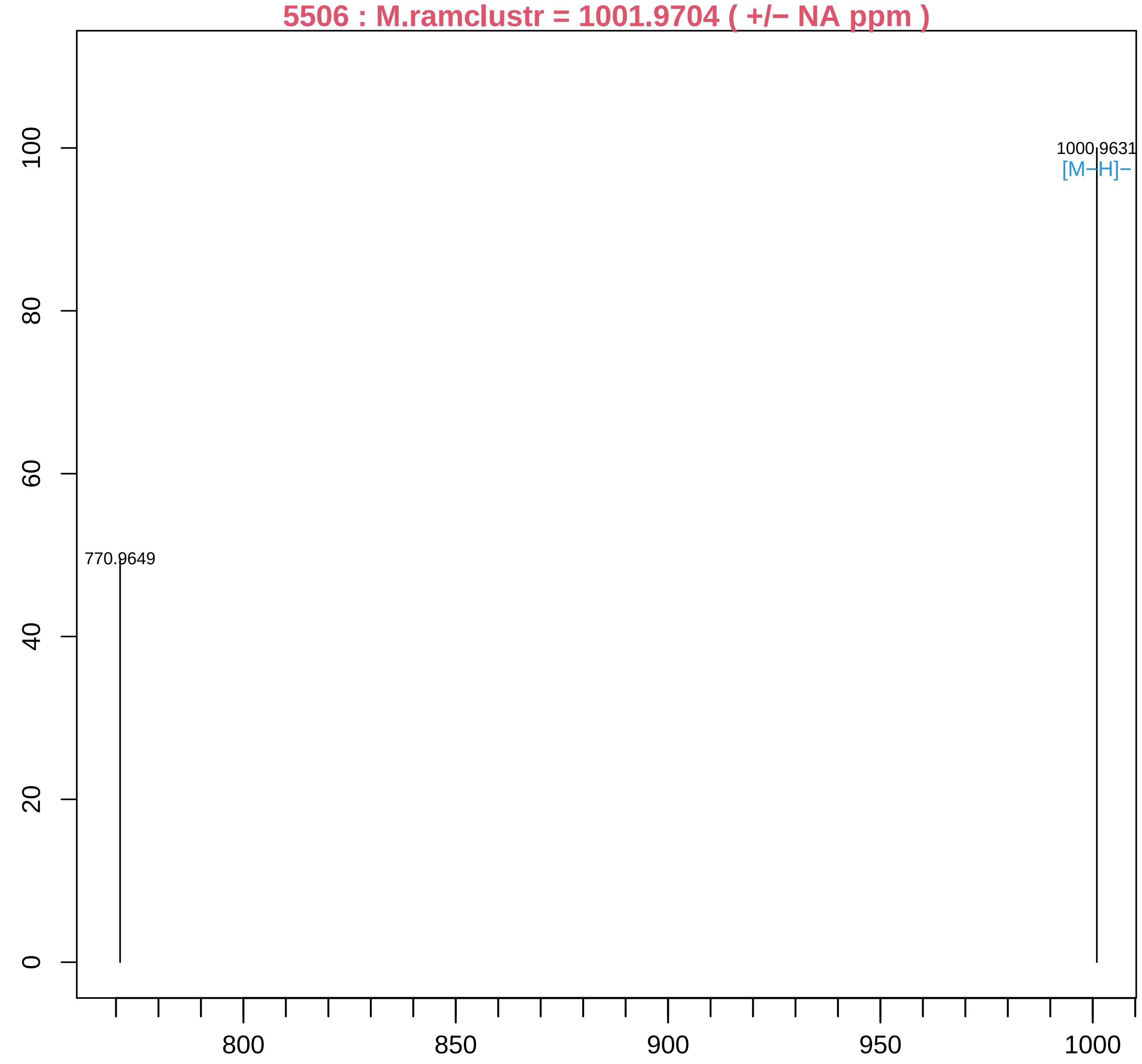
5506 : M.ramclustr = 1001.9704 (+/− NA ppm)
1000.9631
[M−H]−
770.9649
0
20
40
60
80
100
800
850
900
950
1000

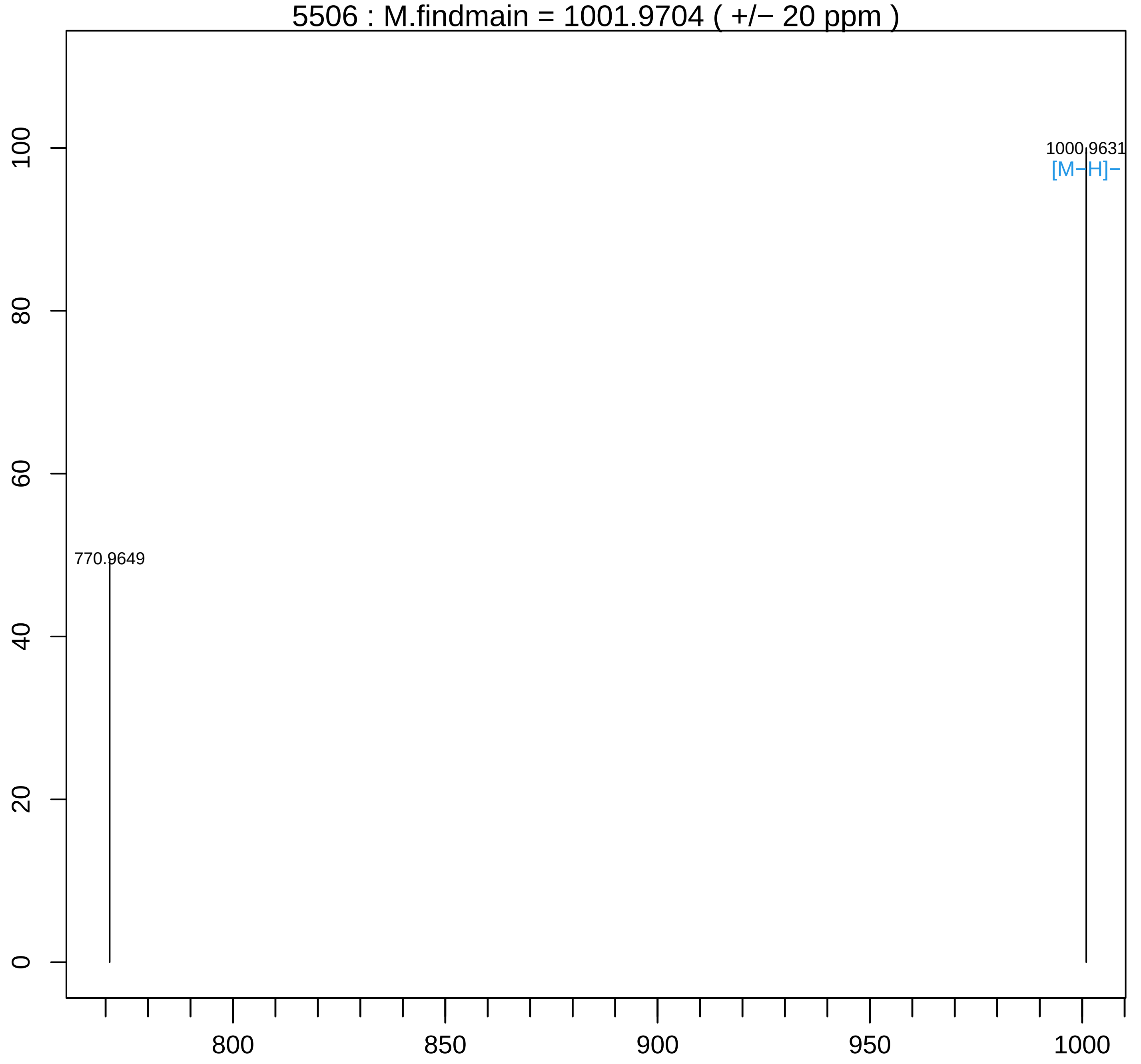
5506 : M.findmain = 1001.9704 (+/− 20 ppm)
1000.9631
[M−H]−
770.9649
0
20
40
60
80
100
800
850
900
950
1000

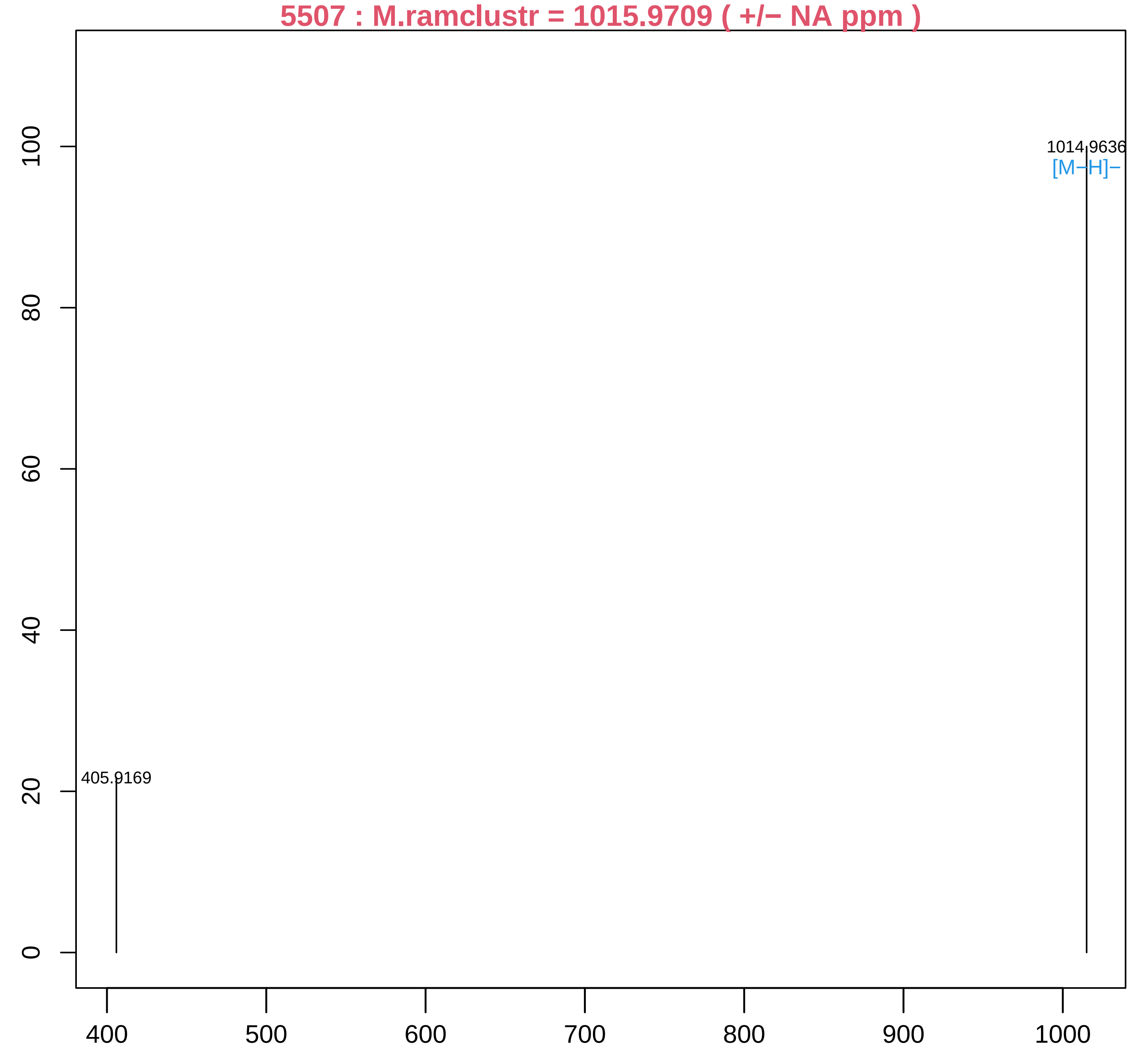
5507 : M.ramclustr = 1015.9709 (+/− NA ppm)
1014.9636
[M−H]−
405.9169
0
20
40
60
80
100
400
500
600
700
800
900
1000

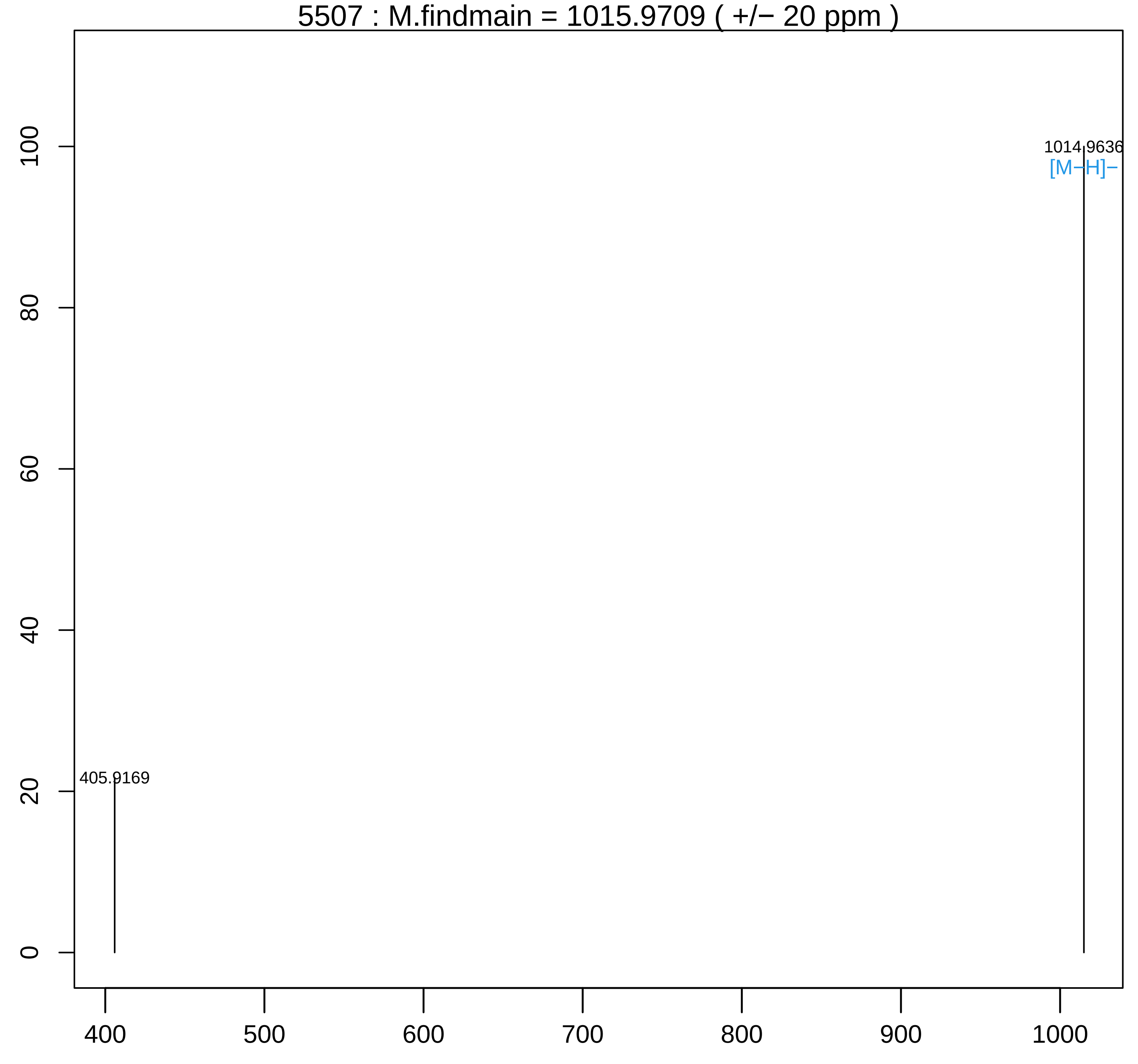
5507 : M.findmain = 1015.9709 (+/− 20 ppm)
1014.9636
[M−H]−
405.9169
0
20
40
60
80
100
400
500
600
700
800
900
1000

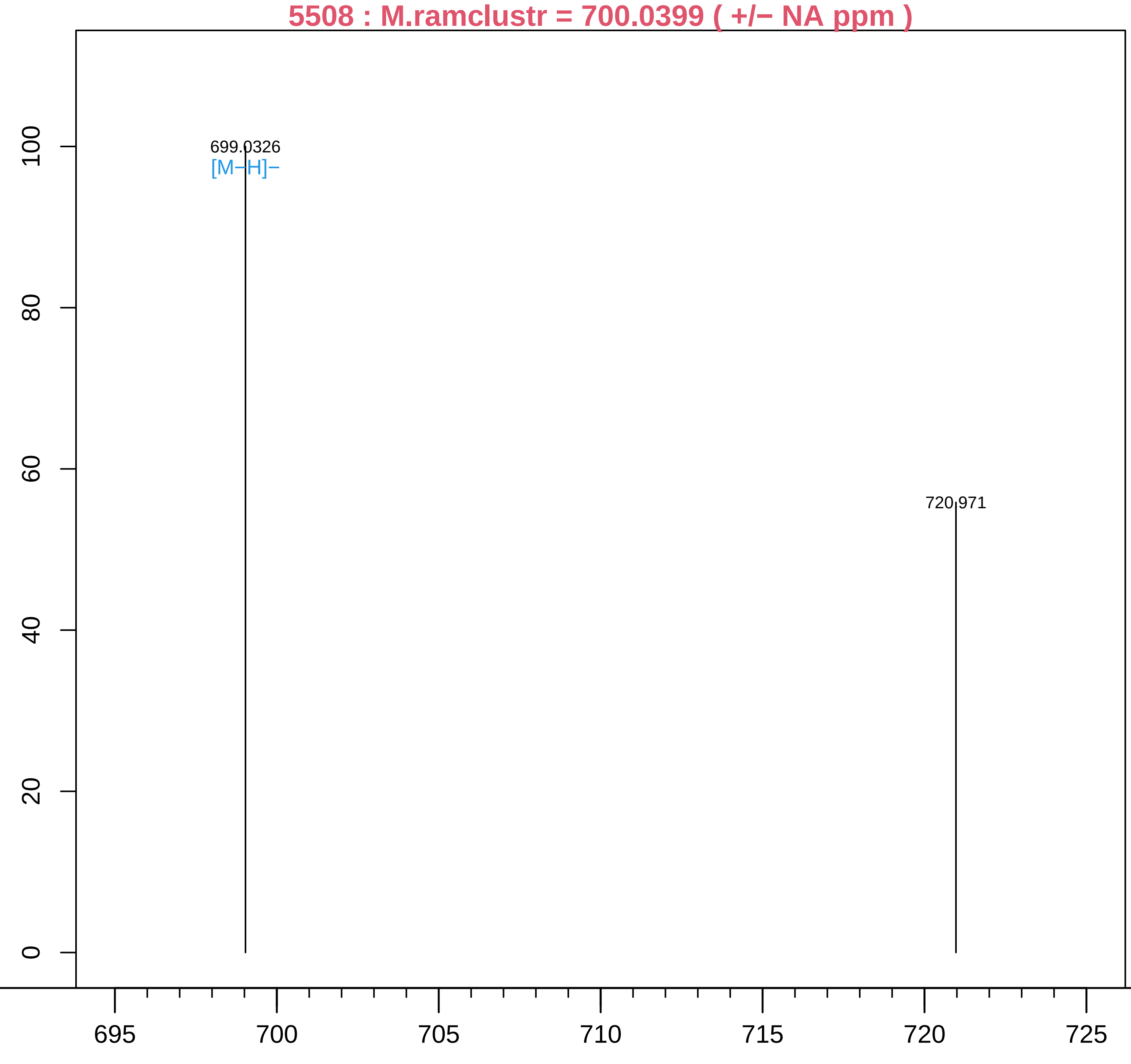
5508 : M.ramclustr = 700.0399 (+/− NA ppm)
699.0326
[M−H]−
720.971
0
20
40
60
80
100
695
700
705
710
715
720
725

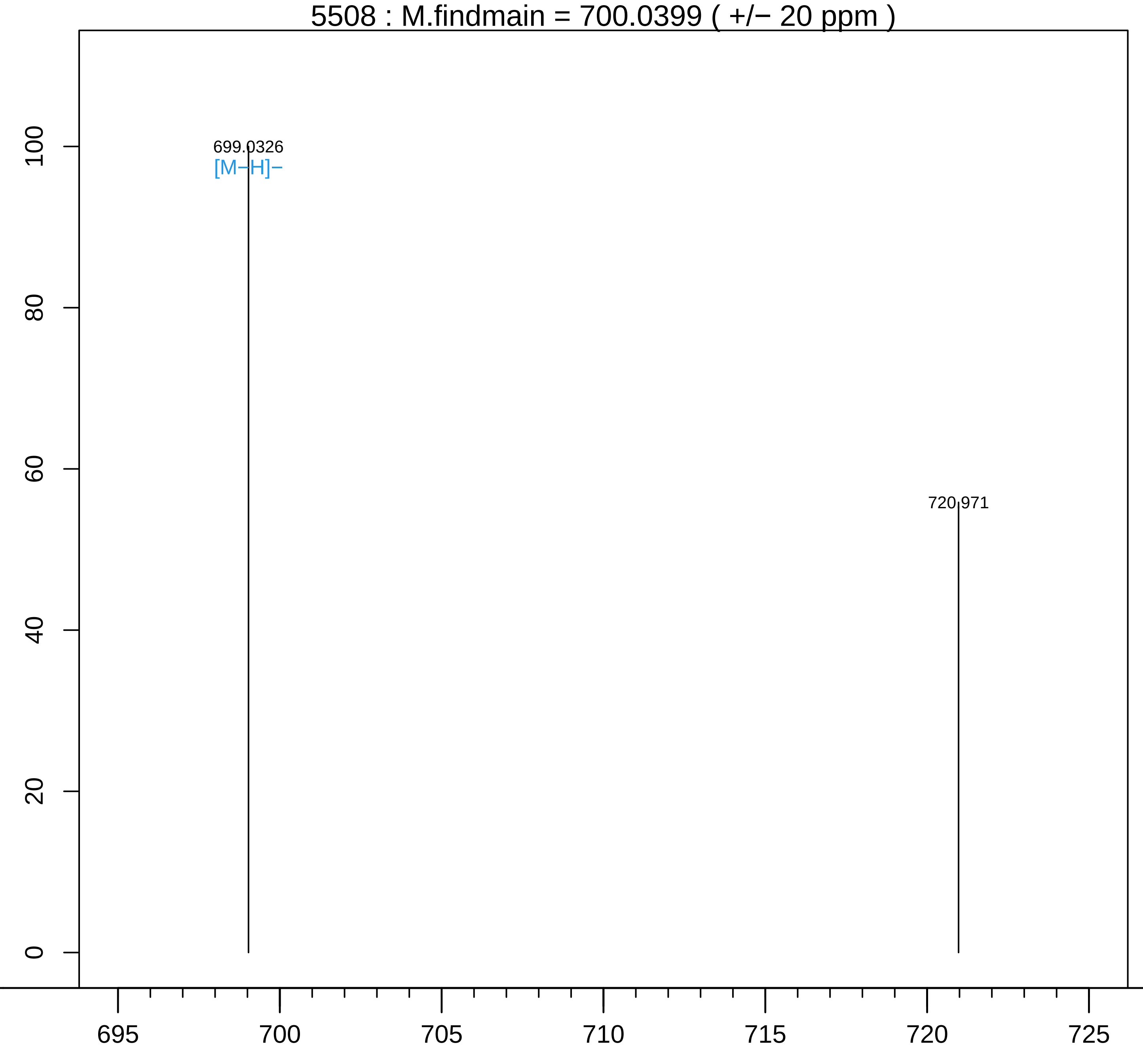
5508 : M.findmain = 700.0399 (+/− 20 ppm)
699.0326
[M−H]−
720.971
0
20
40
60
80
100
695
700
705
710
715
720
725

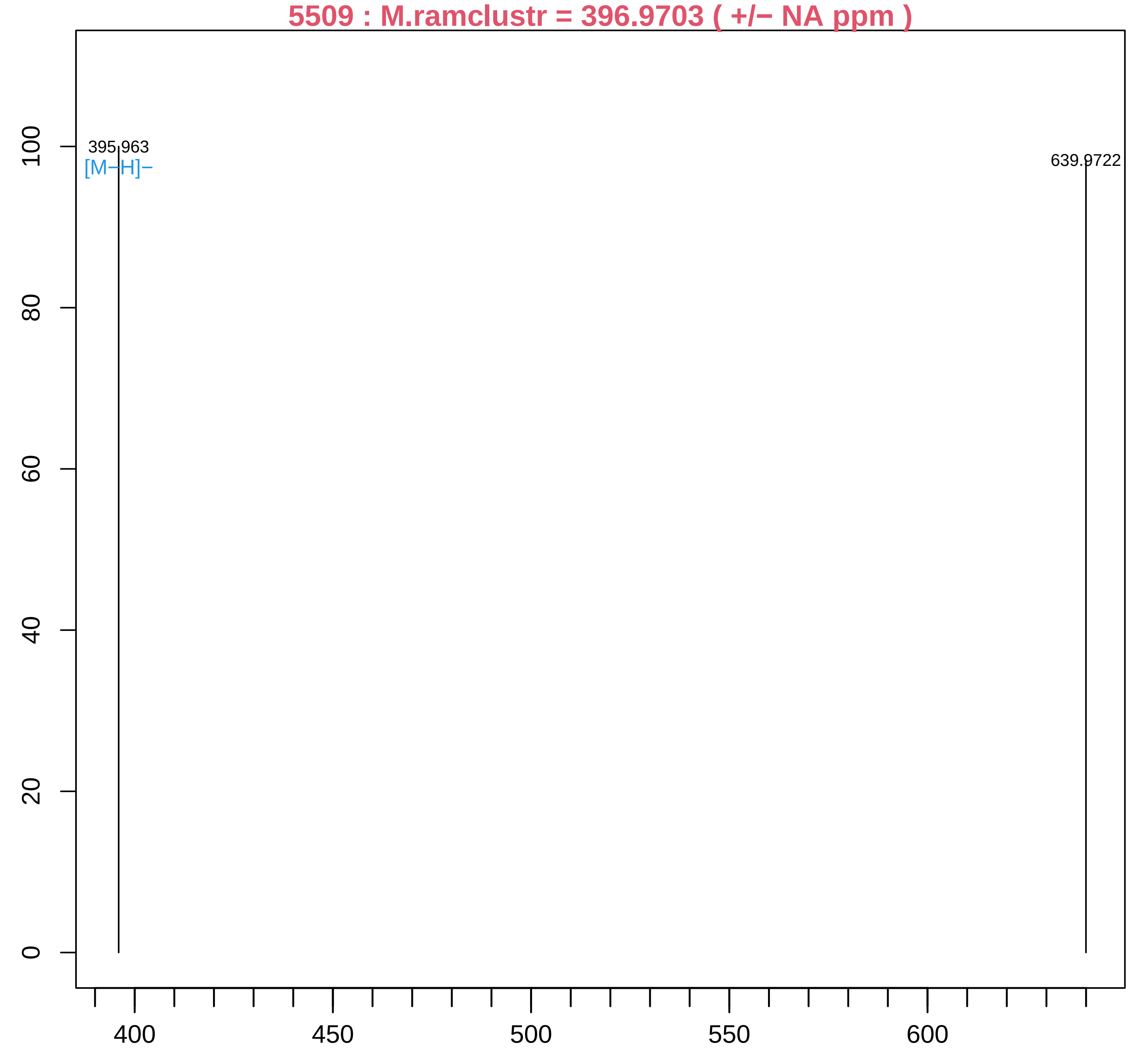

5509 : M.ramclustr = 396.9703 (+/− NA ppm)
395.963
[M−H]−
639.9722
0
20
40
60
80
100
400
450
500
550
600

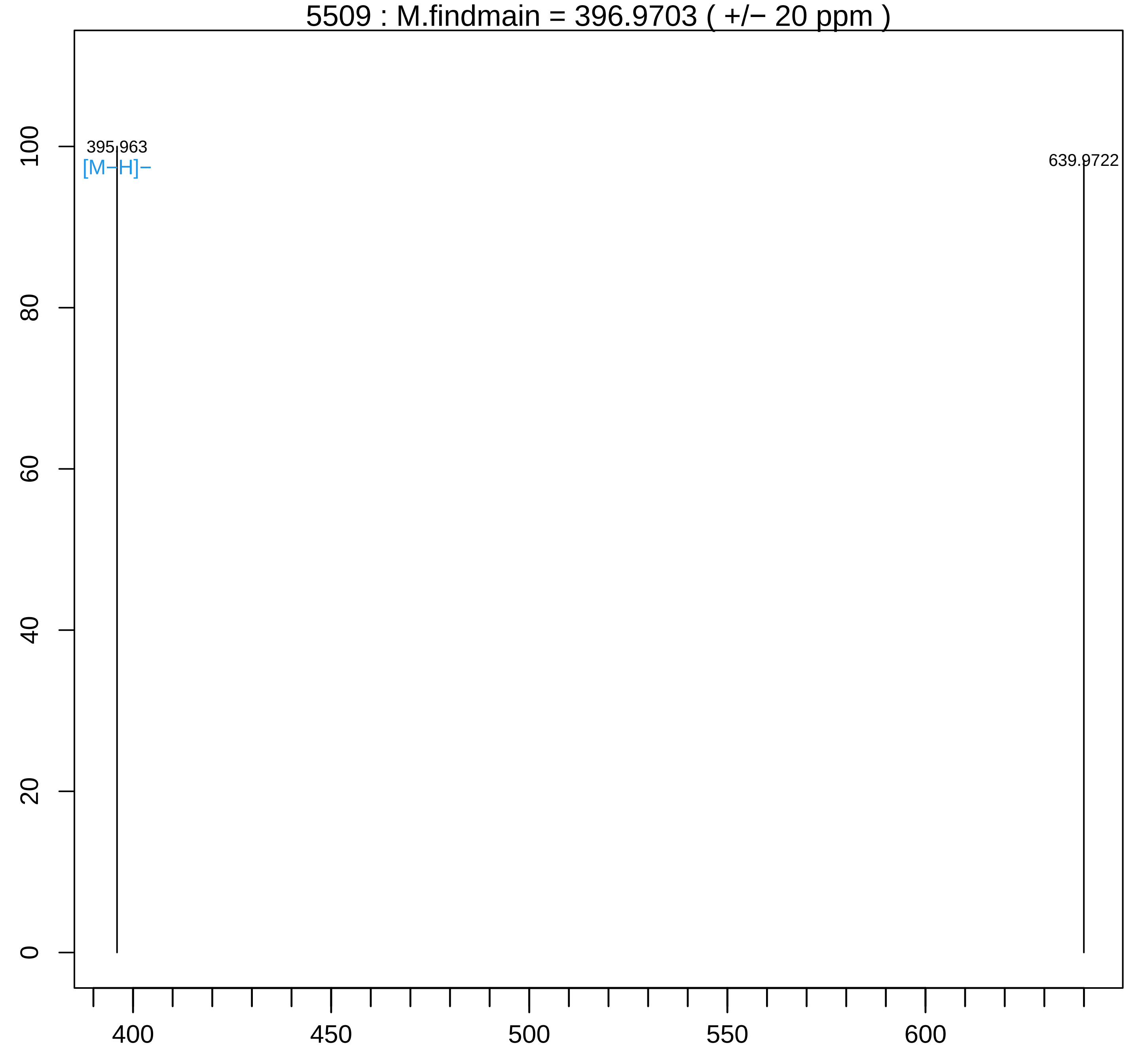

5509 : M.findmain = 396.9703 (+/− 20 ppm)
395.963
[M−H]−
639.9722
0
20
40
60
80
100
400
450
500
550
600

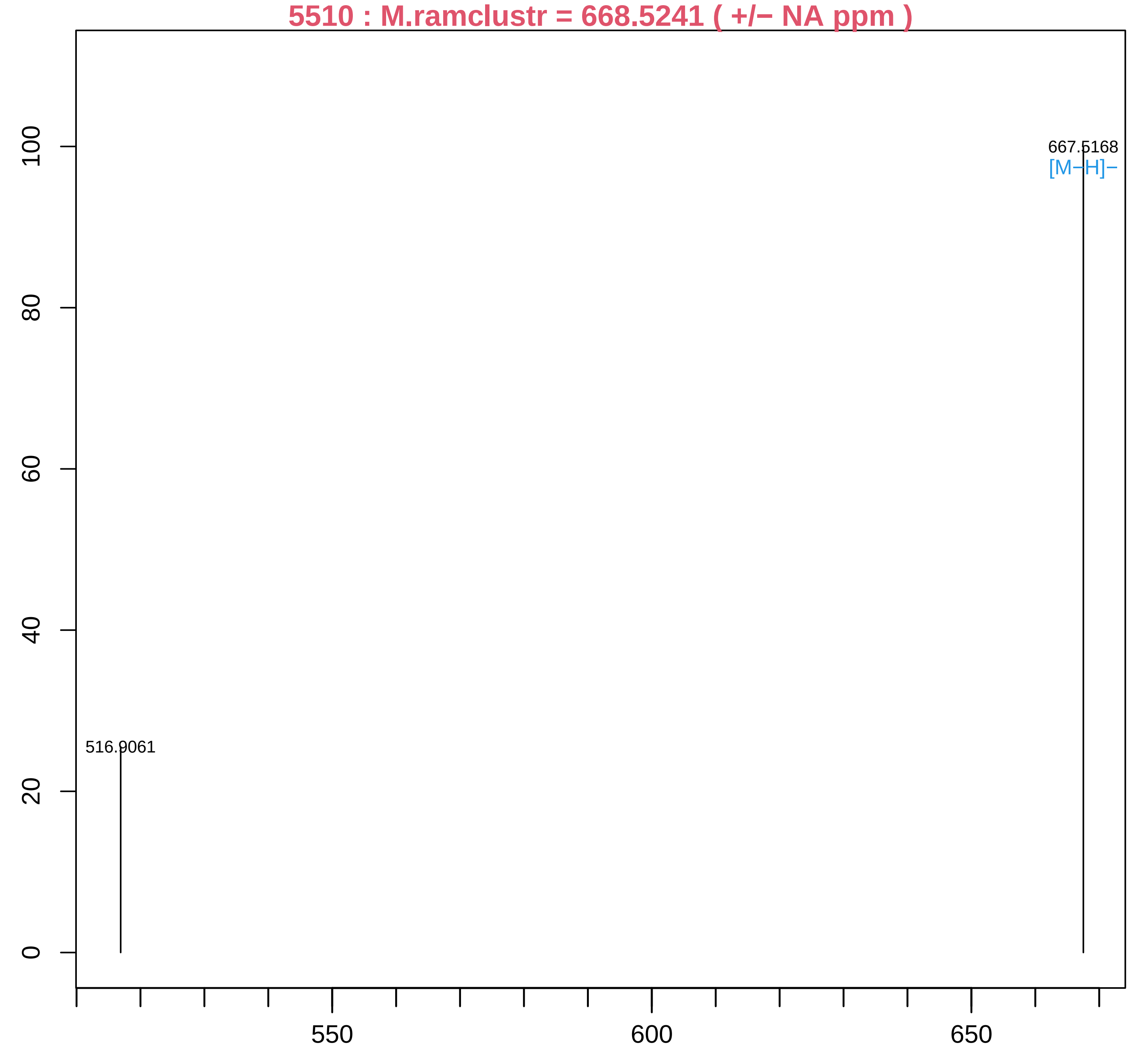
5510 : M.ramclustr = 668.5241 (+/− NA ppm)
667.5168
[M−H]−
516.9061
0
20
40
60
80
100
550
600
650

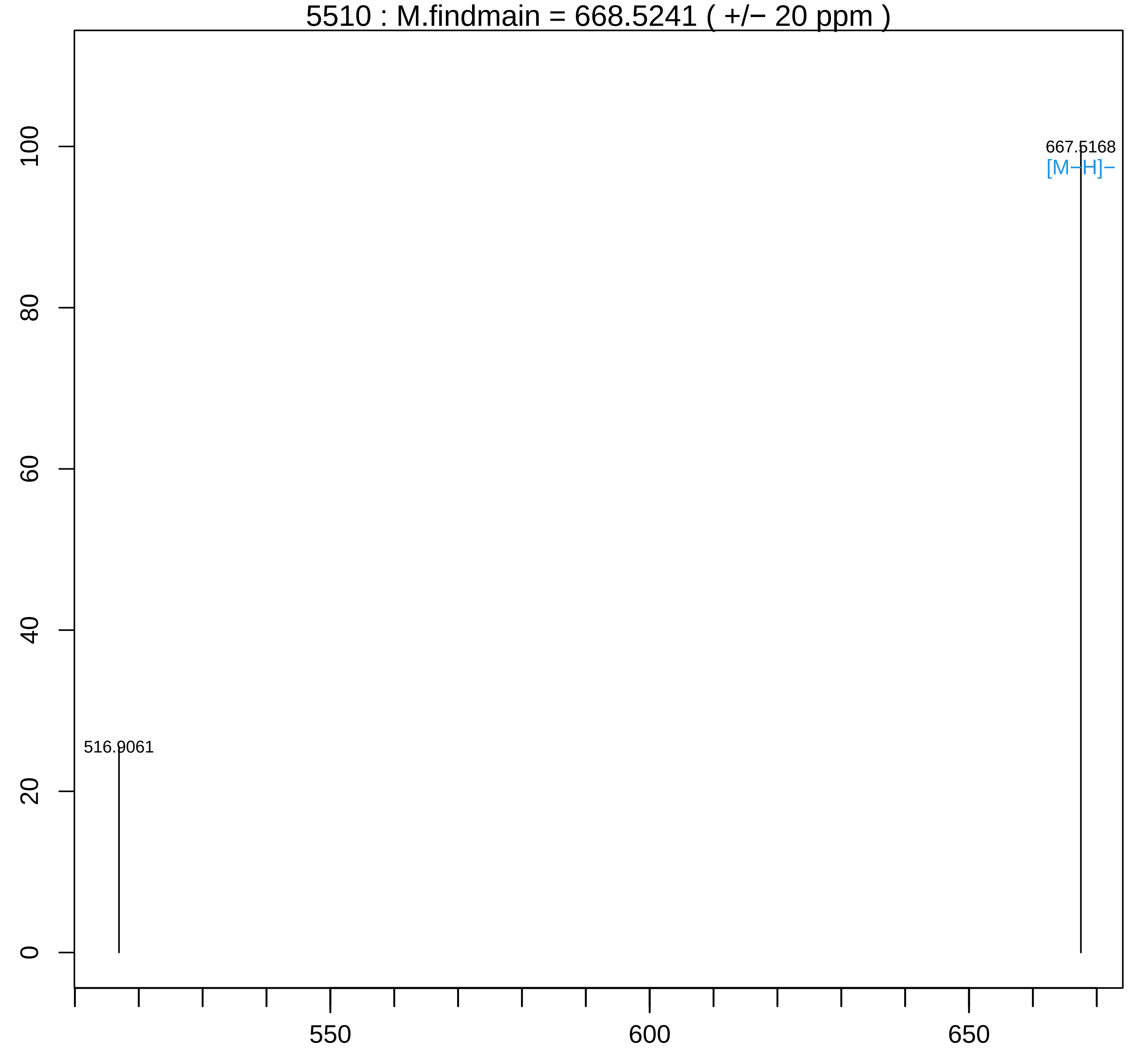
5510 : M.findmain = 668.5241 (+/− 20 ppm)
667.5168
[M−H]−
516.9061
0
20
40
60
80
100
550
600
650

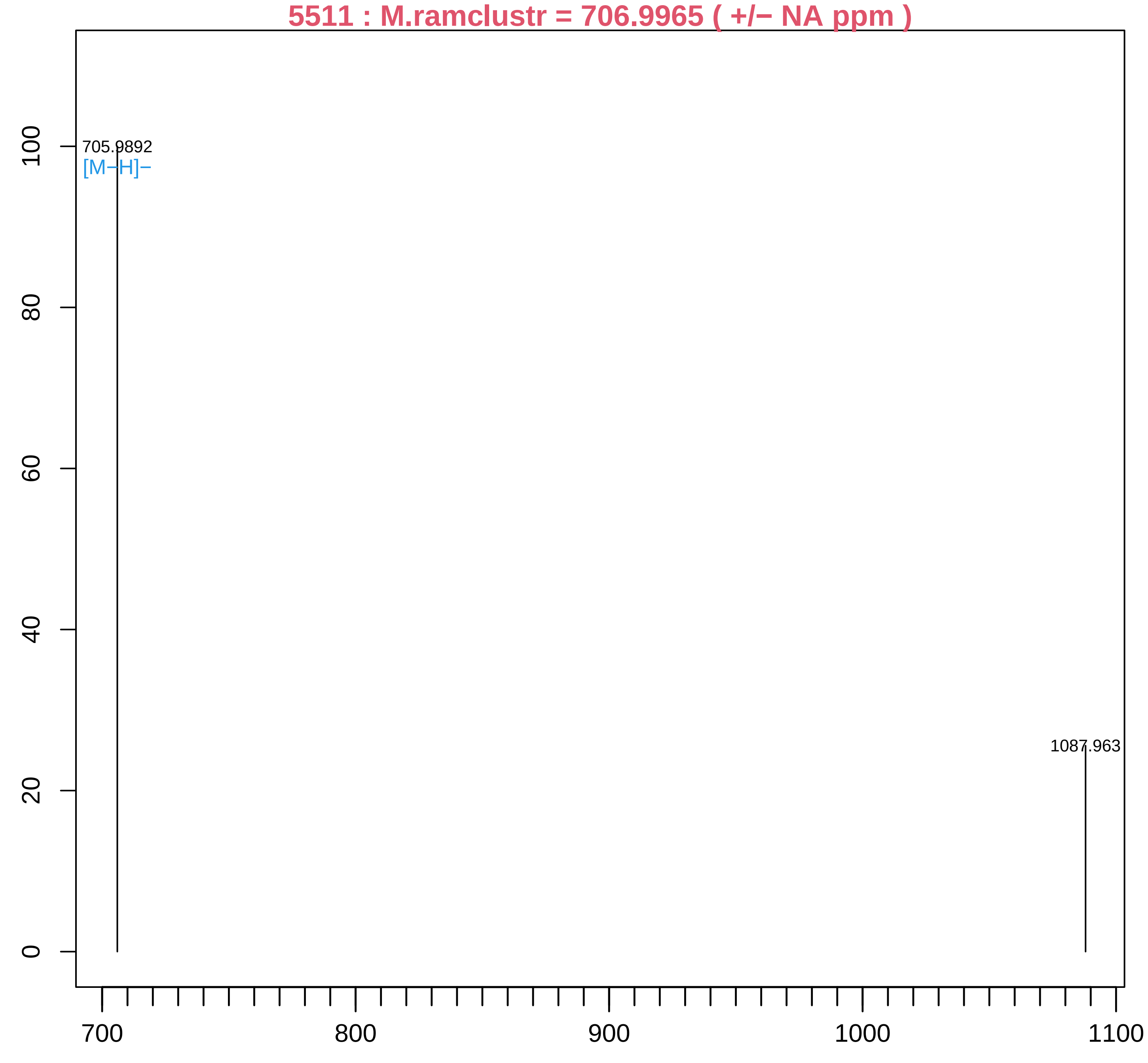
5511 : M.ramclustr = 706.9965 (+/− NA ppm)
705.9892
[M−H]−
1087.963
0
20
40
60
80
100
700
800
900
1000
1100

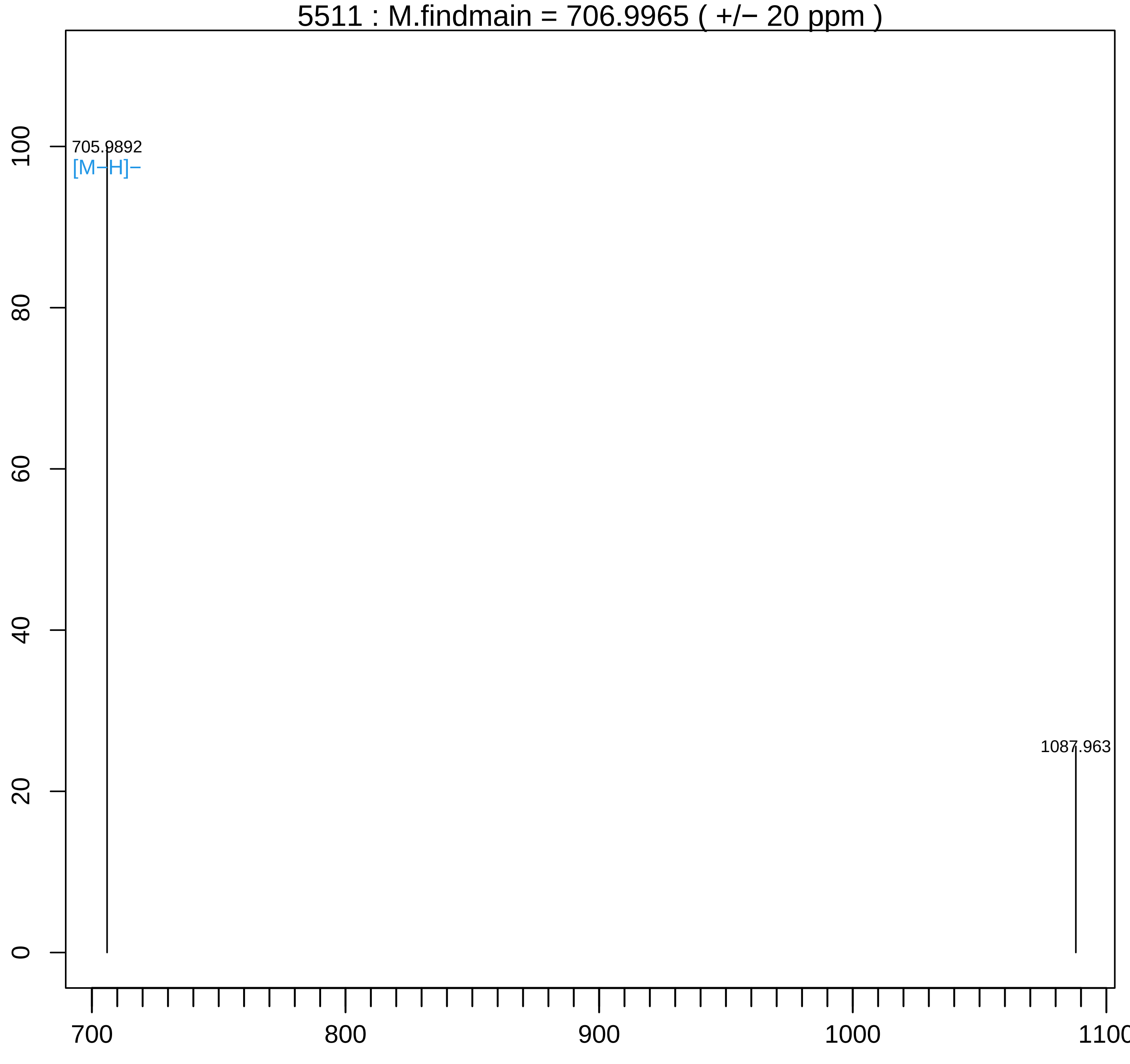
5511 : M.findmain = 706.9965 (+/− 20 ppm)
705.9892
[M−H]−
1087.963
0
20
40
60
80
100
700
800
900
1000
1100

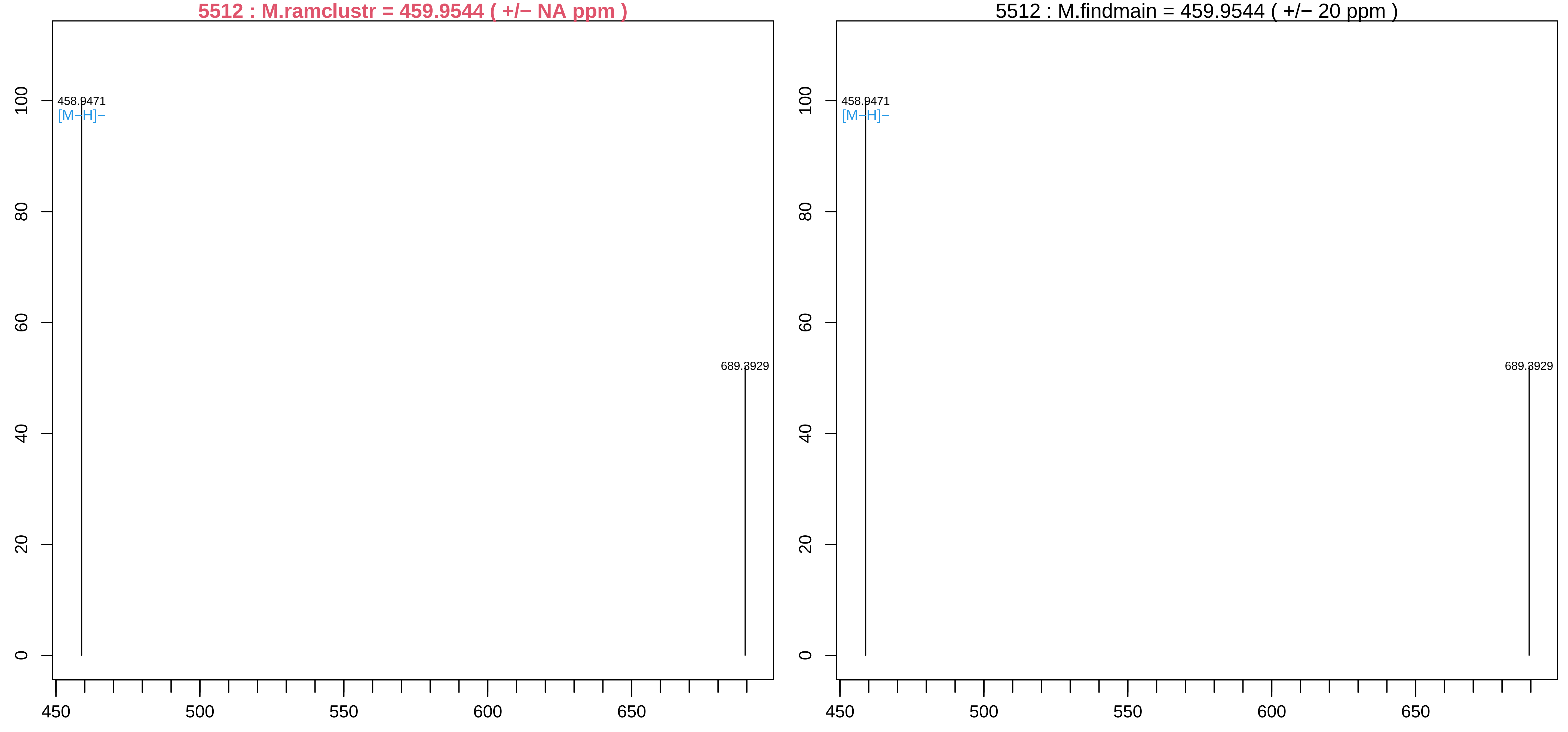

5512 : M.ramclustr = 459.9544 (+/− NA ppm)
458.9471
[M−H]−
689.3929
0
20
40
60
80
100
450
500
550
600
650
5512 : M.findmain = 459.9544 (+/− 20 ppm)
458.9471
[M−H]−
689.3929
0
20
40
60
80
100
450
500
550
600
650

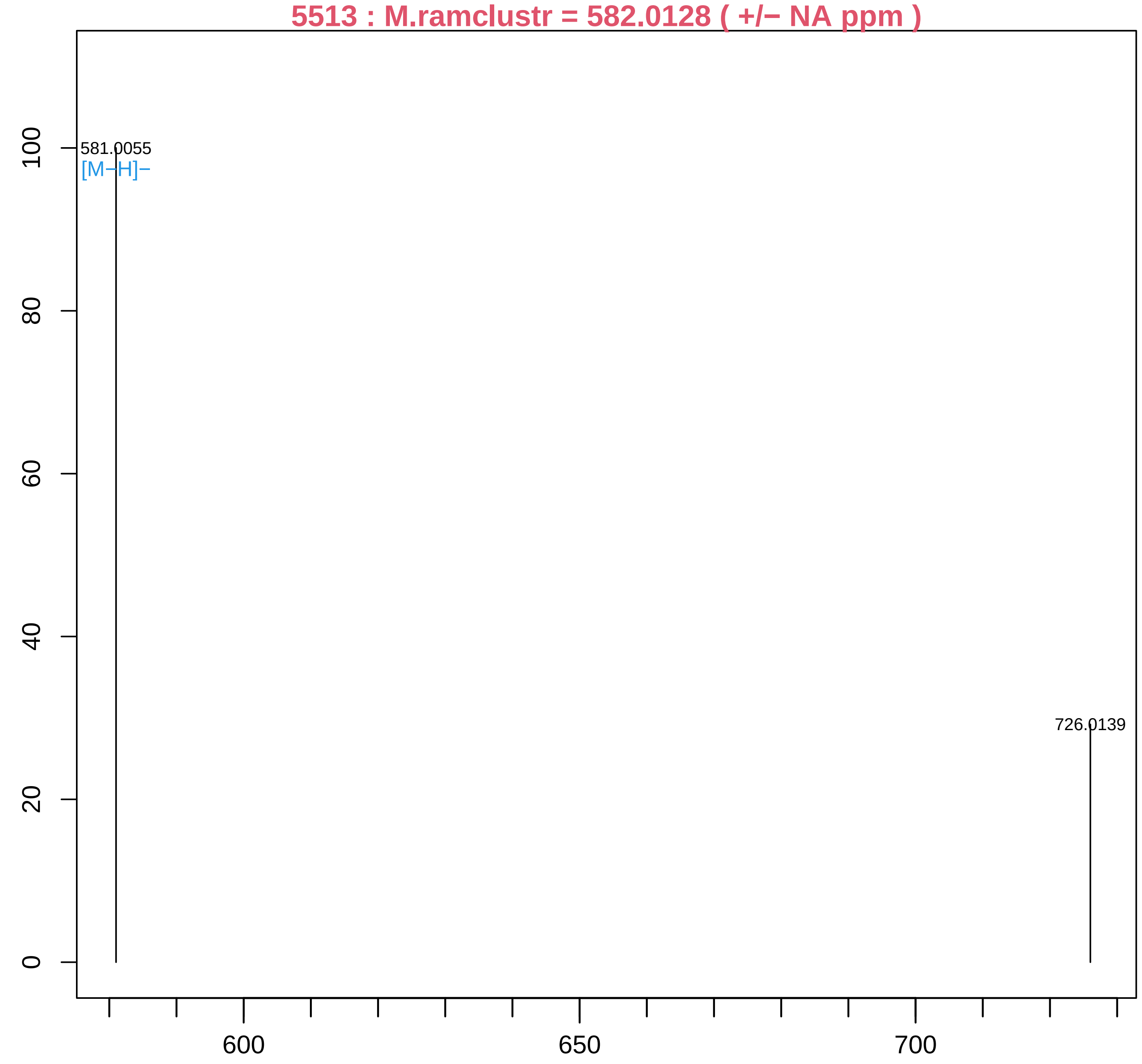
5513 : M.ramclustr = 582.0128 (+/− NA ppm)
581.0055
[M−H]−
726.0139
0
20
40
60
80
100
600
650
700

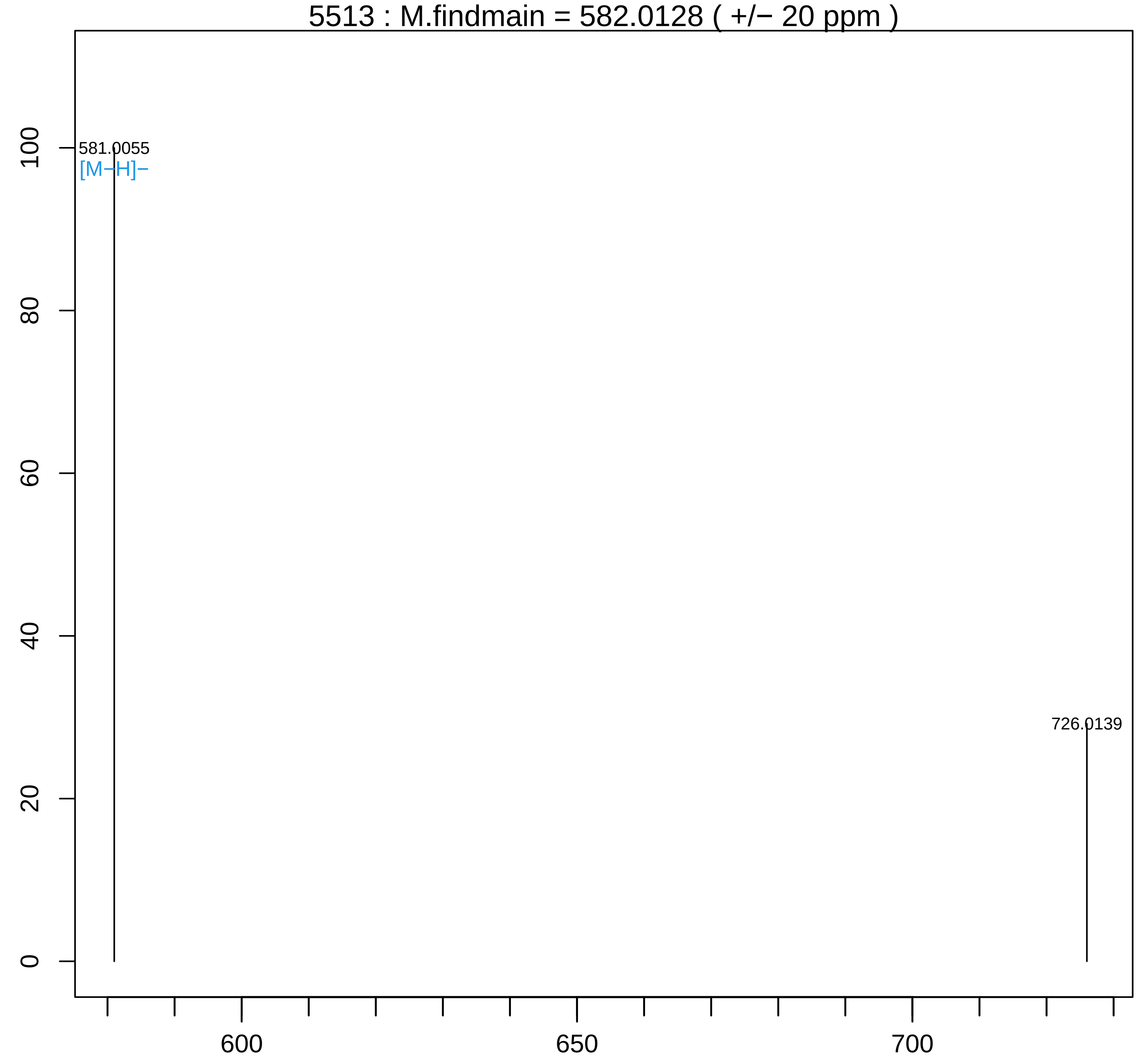
5513 : M.findmain = 582.0128 (+/− 20 ppm)
581.0055
[M−H]−
726.0139
0
20
40
60
80
100
600
650
700

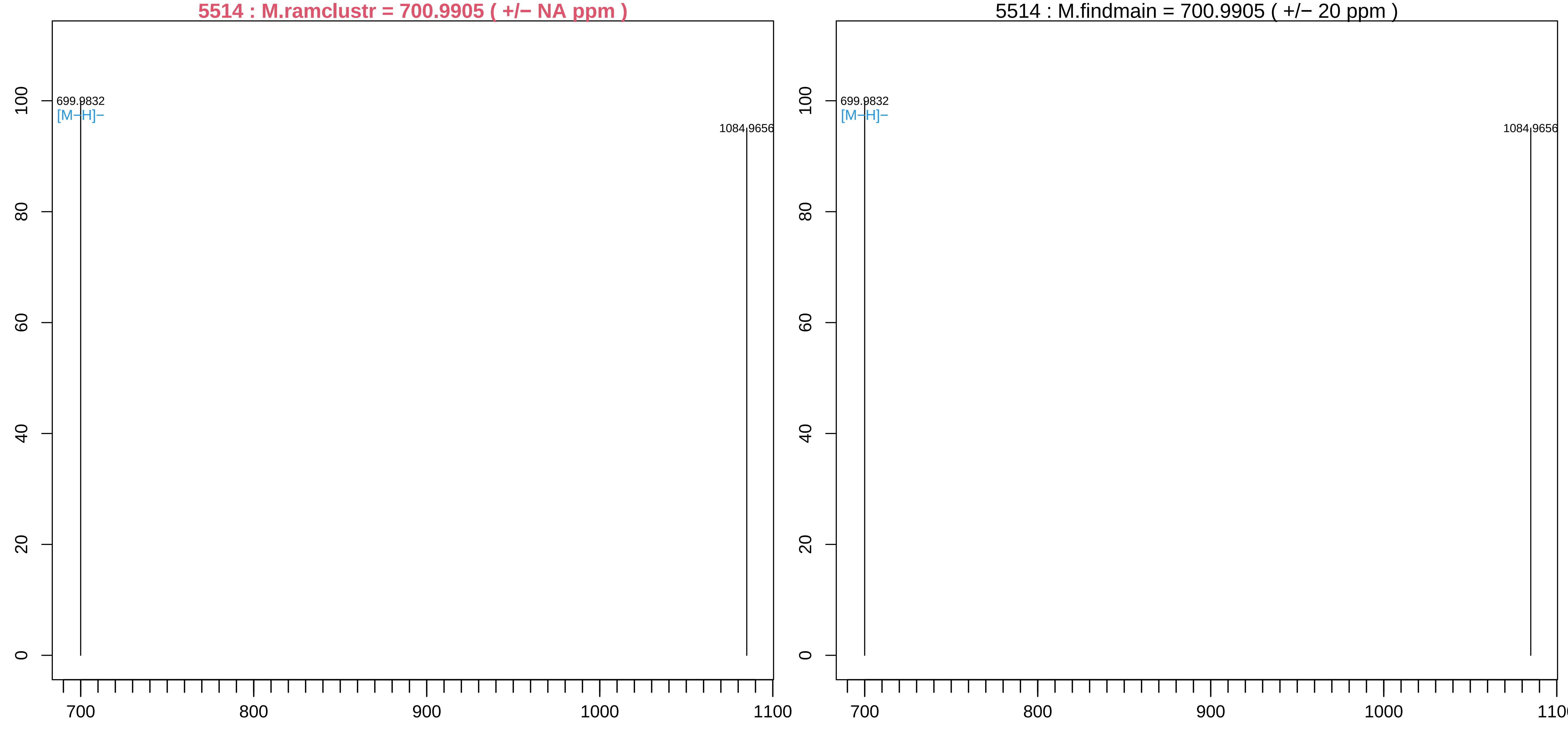
5514 : M.ramclustr = 700.9905 (+/− NA ppm)
699.9832
[M−H]−
1084.9656
0
20
40
60
80
100
700
800
900
1000
1100
5514 : M.findmain = 700.9905 (+/− 20 ppm)
699.9832
[M−H]−
1084.9656
0
20
40
60
80
100
700
800
900
1000
1100

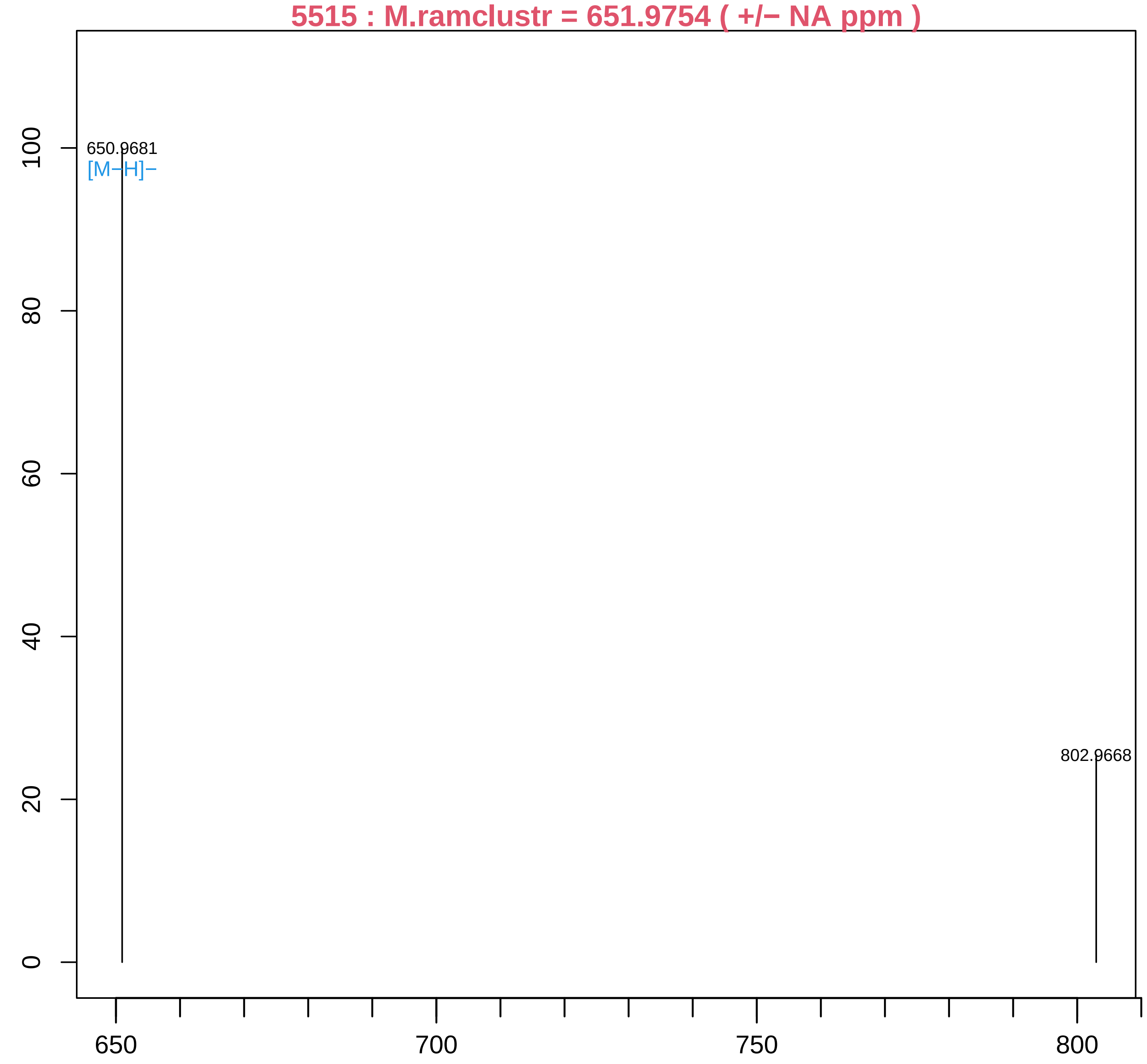

5515 : M.ramclustr = 651.9754 (+/− NA ppm)
650.9681
[M−H]−
802.9668
0
20
40
60
80
100
650
700
750
800

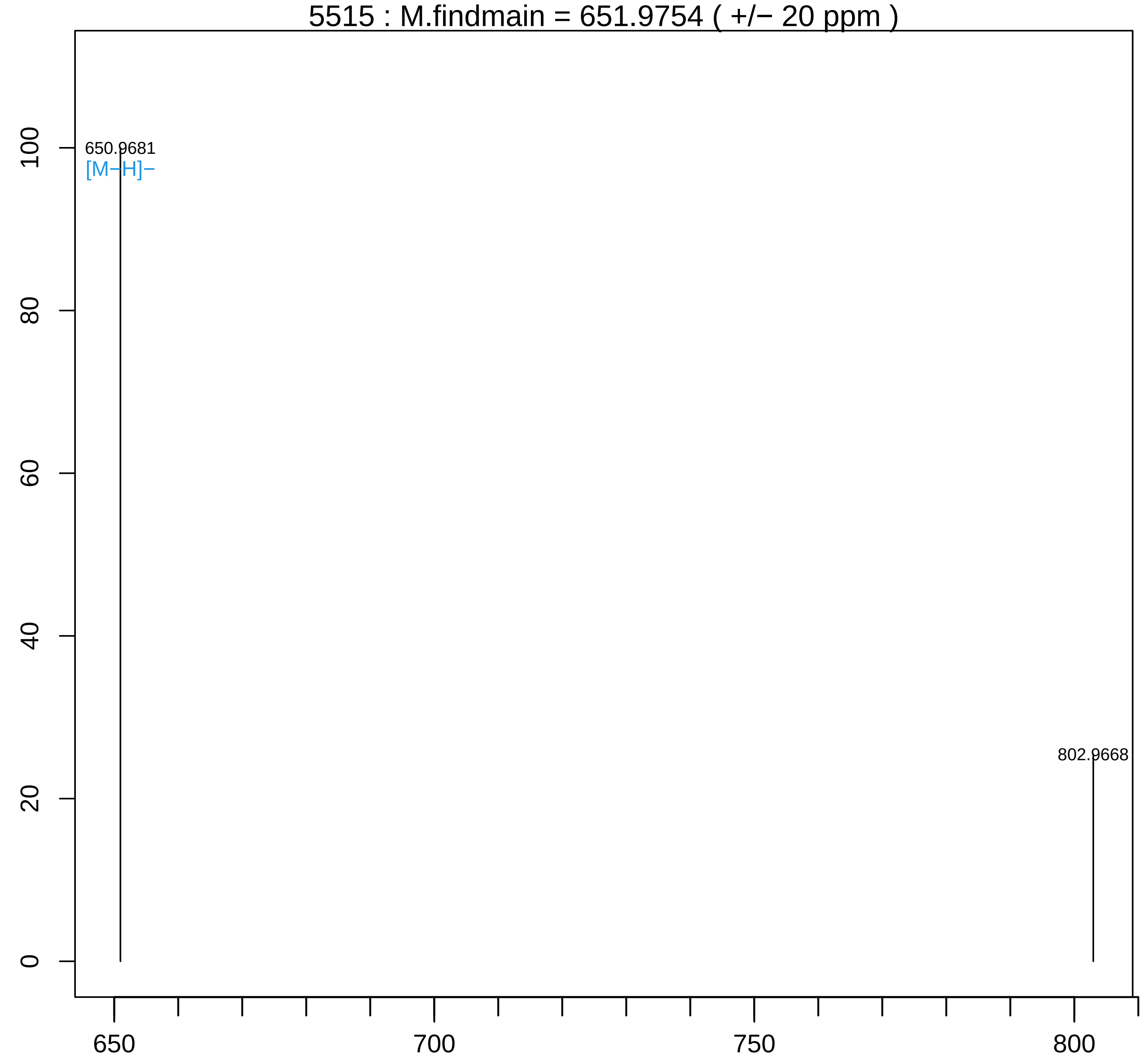

5515 : M.findmain = 651.9754 (+/− 20 ppm)
650.9681
[M−H]−
802.9668
0
20
40
60
80
100
650
700
750
800

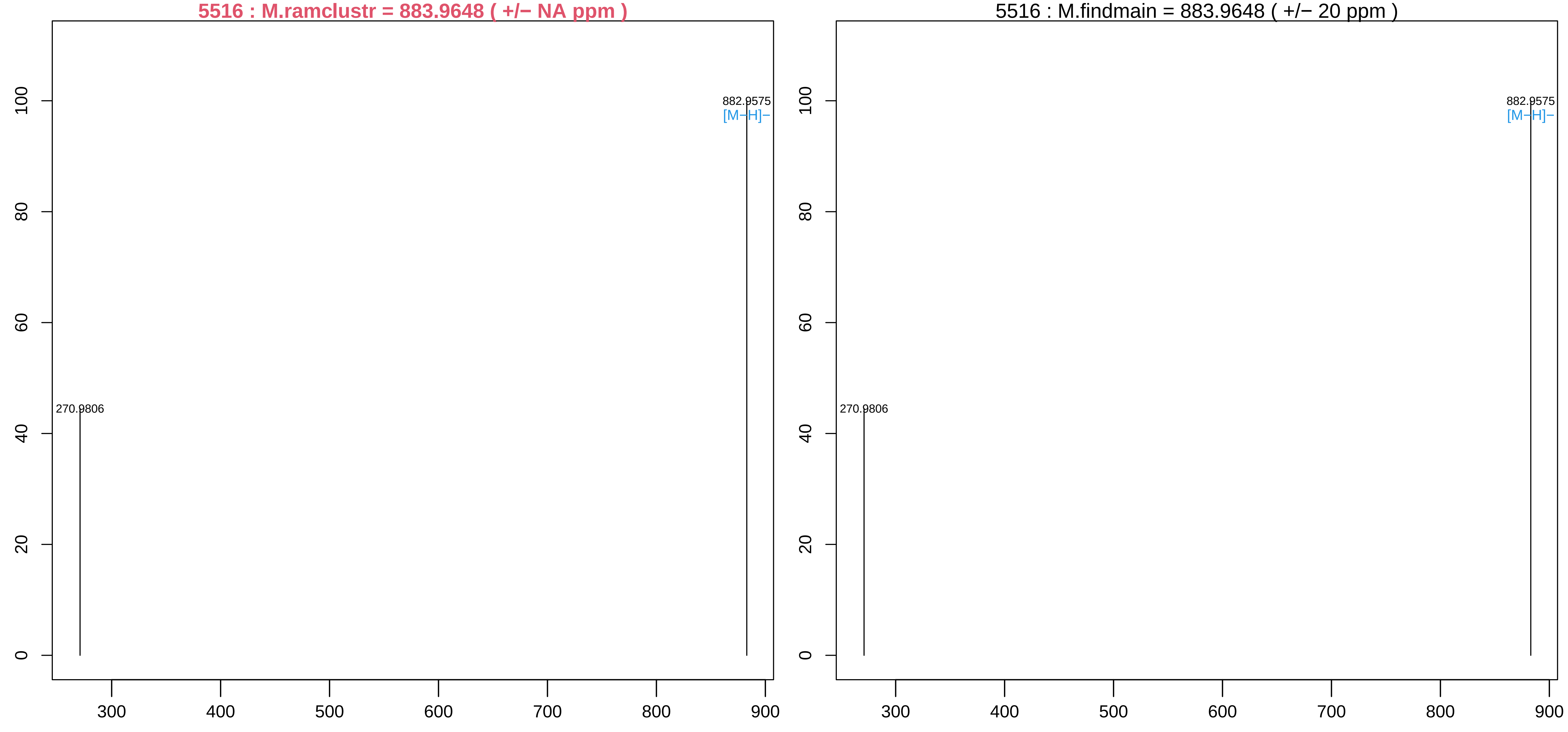
5516 : M.ramclustr = 883.9648 (+/− NA ppm)
882.9575
[M−H]−
270.9806
0
20
40
60
80
100
300
400
500
600
700
800
900
5516 : M.findmain = 883.9648 (+/− 20 ppm)
882.9575
[M−H]−
270.9806
0
20
40
60
80
100
300
400
500
600
700
800
900

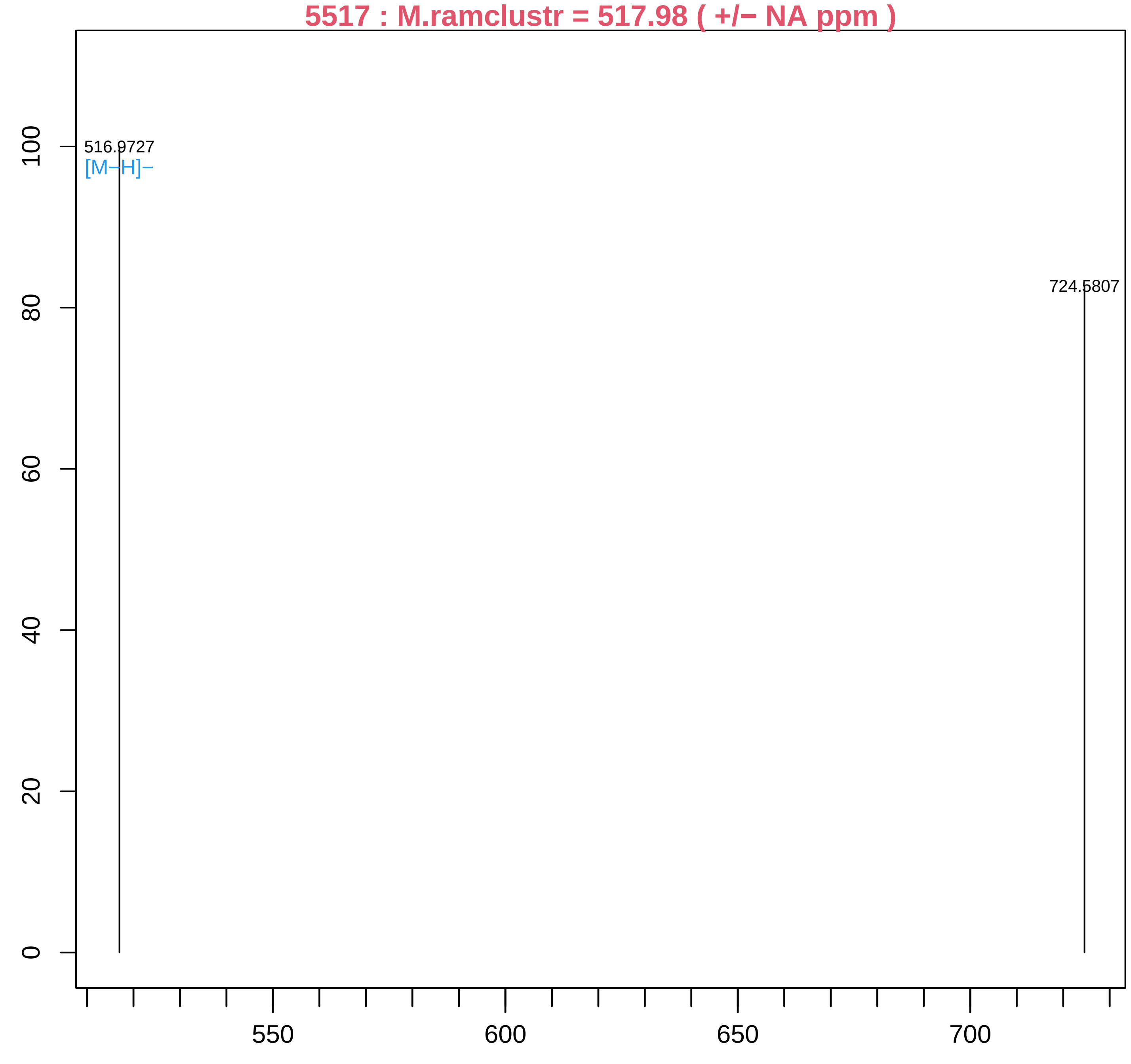
5517 : M.ramclustr = 517.98 (+/− NA ppm)
516.9727
[M−H]−
724.5807
0
20
40
60
80
100
550
600
650
700

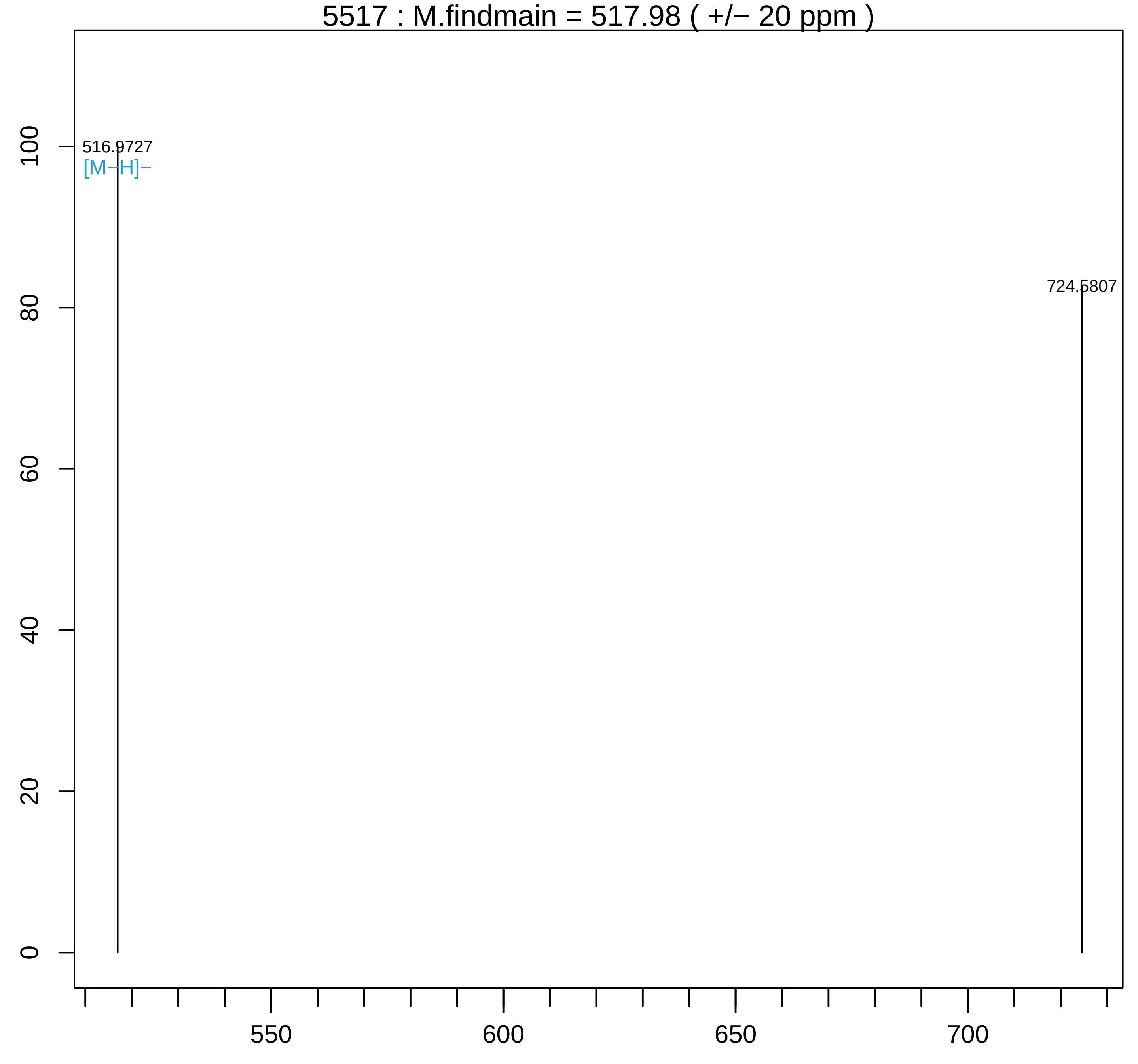
5517 : M.findmain = 517.98 (+/− 20 ppm)
516.9727
[M−H]−
724.5807
0
20
40
60
80
100
550
600
650
700

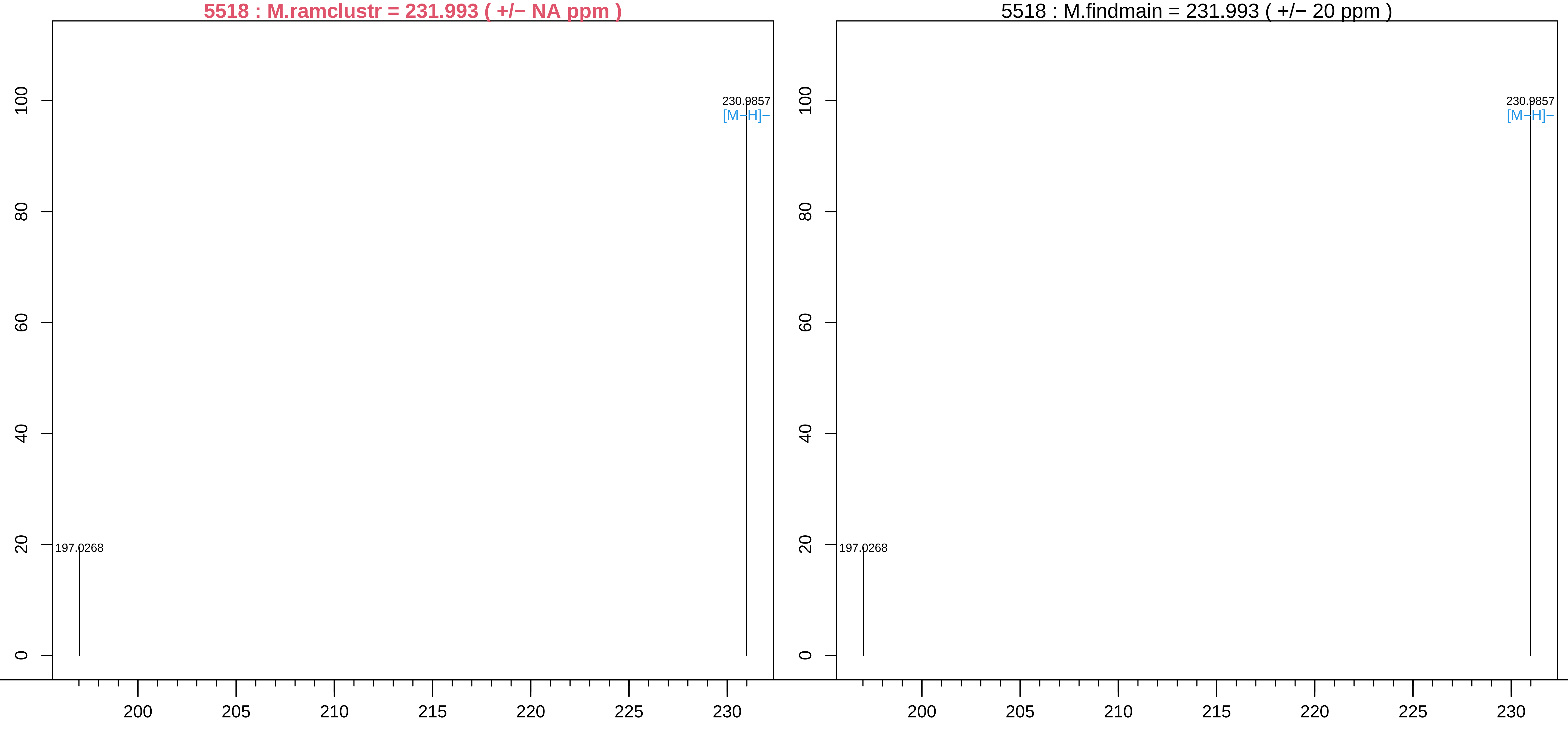

5518 : M.ramclustr = 231.993 (+/− NA ppm)
5518 : M.findmain = 231.993 (+/− 20 ppm)
230.9857
[M−H]−
197.0268
230.9857
[M−H]−
197.0268

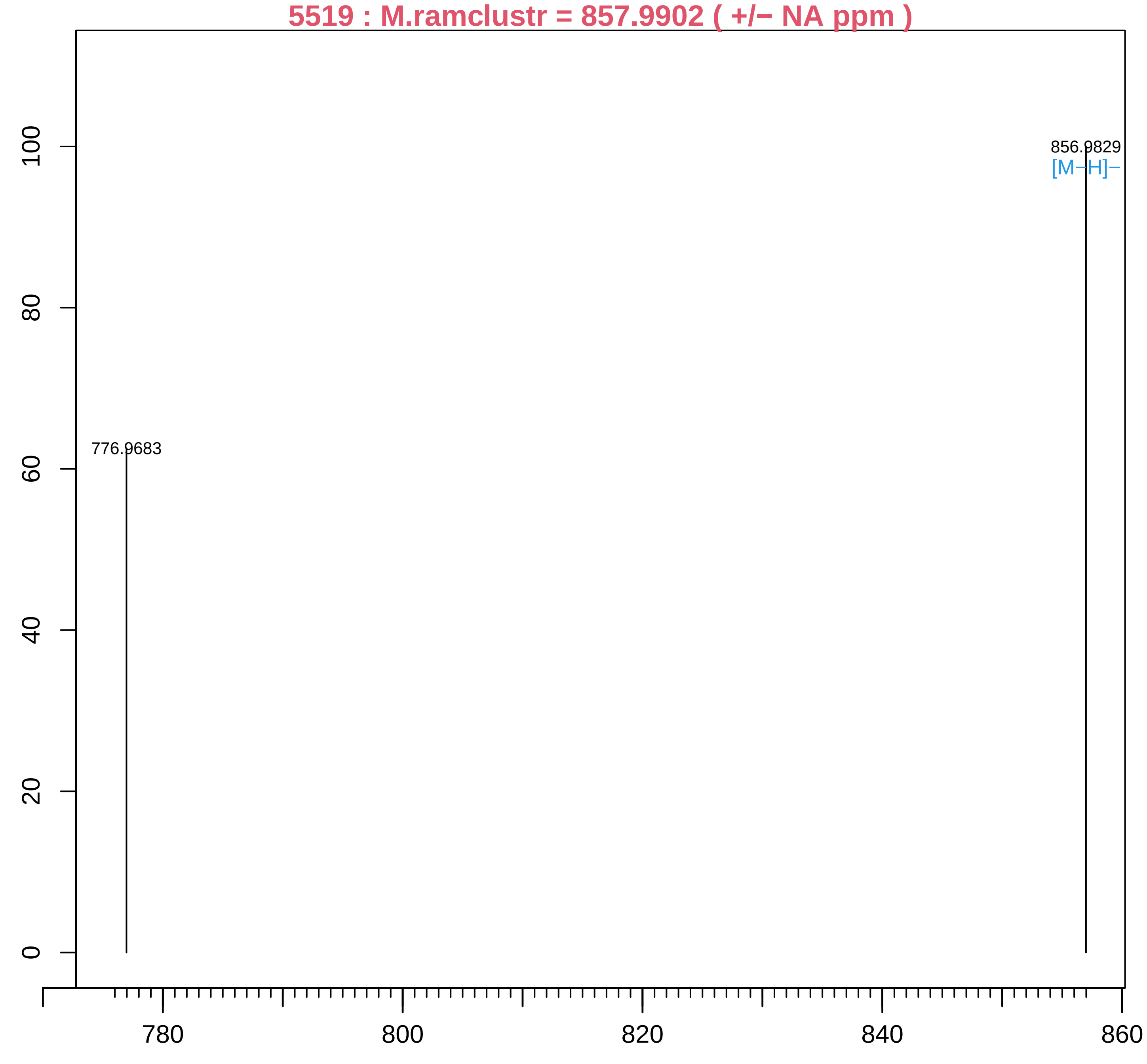
5519 : M.ramclustr = 857.9902 (+/− NA ppm)
776.9683
856.9829
[M−H]−
0
20
40
60
80
100
780
800
820
840
860

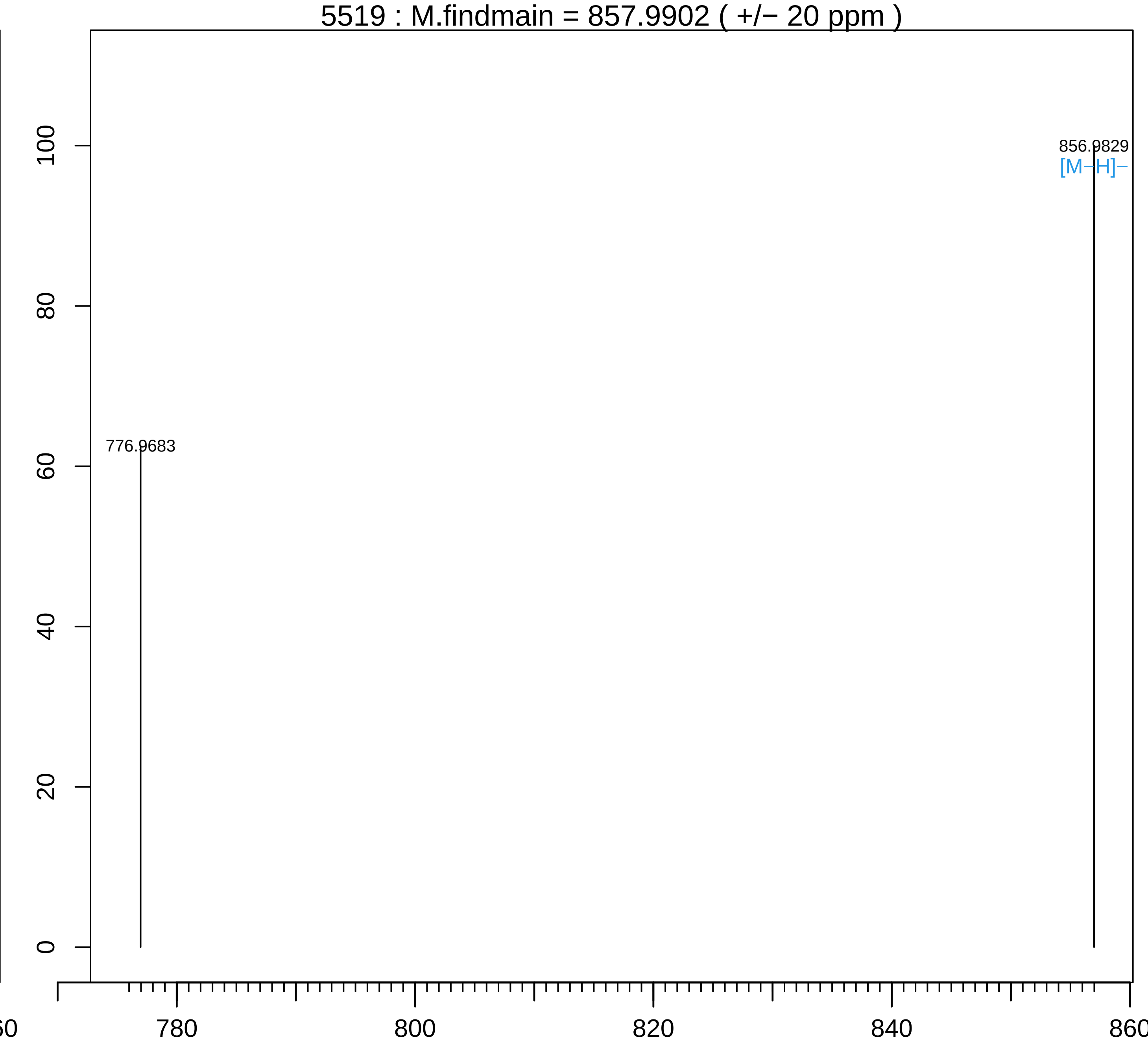
5519 : M.findmain = 857.9902 (+/− 20 ppm)
776.9683
856.9829
[M−H]−
0
20
40
60
80
100
780
800
820
840
860

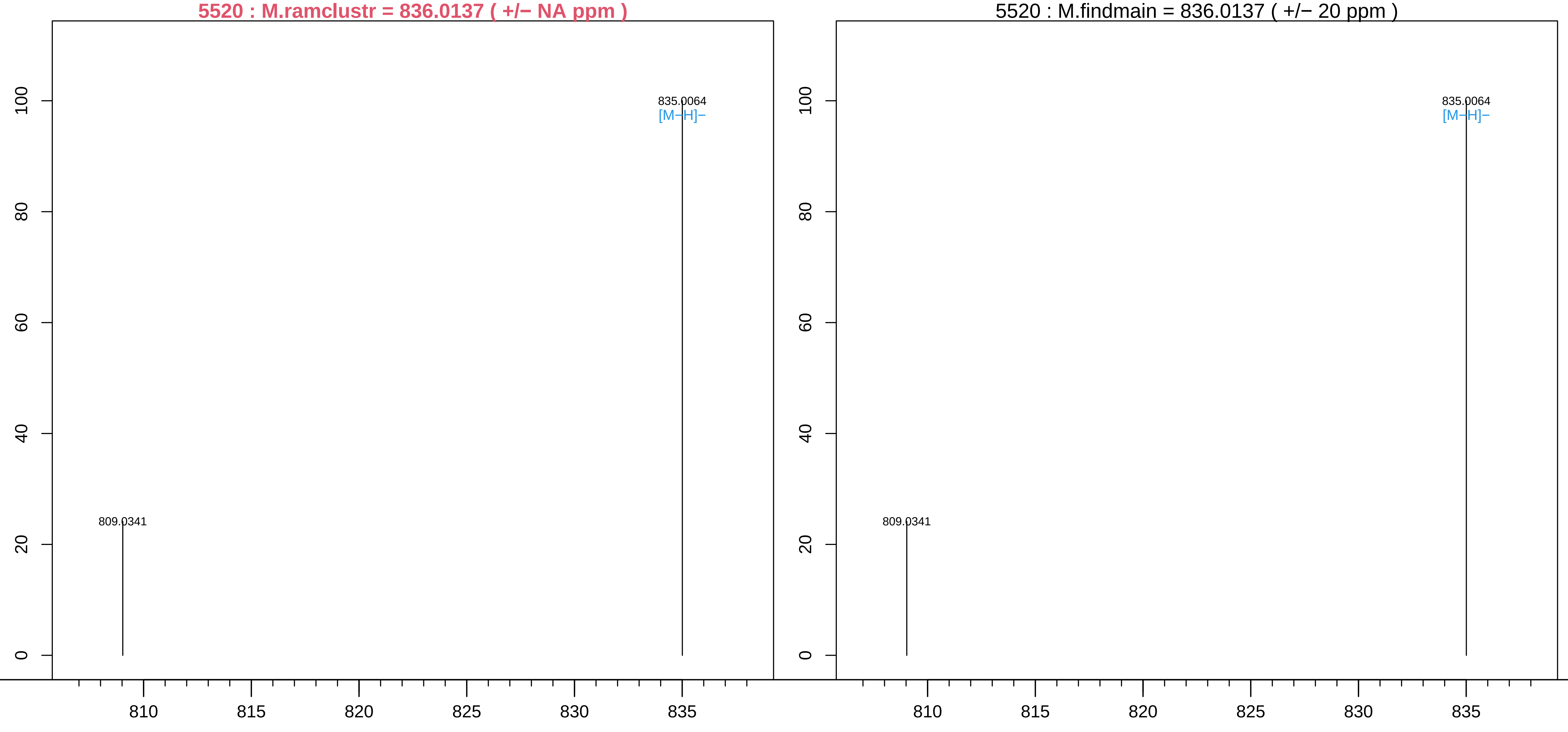

5520 : M.ramclustr = 836.0137 (+/− NA ppm)
5520 : M.findmain = 836.0137 (+/− 20 ppm)
835.0064
[M−H]−
809.0341
835.0064
[M−H]−
809.0341
0
20
40
60
80
100
810
815
820
825
830
835

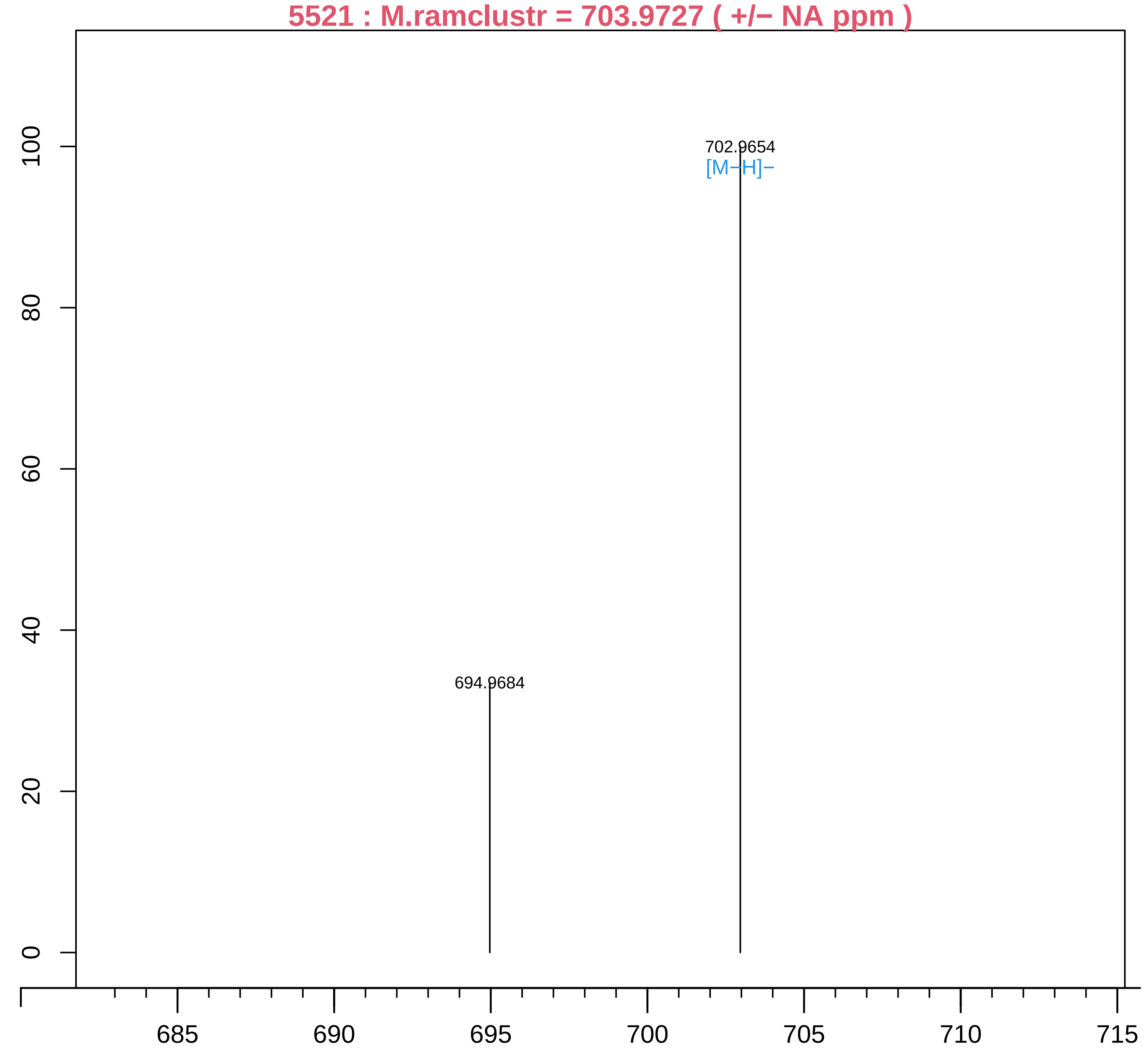
5521 : M.ramclustr = 703.9727 (+/− NA ppm)
702.9654
[M−H]−
694.9684
0
20
40
60
80
100
685
690
695
700
705
710
715

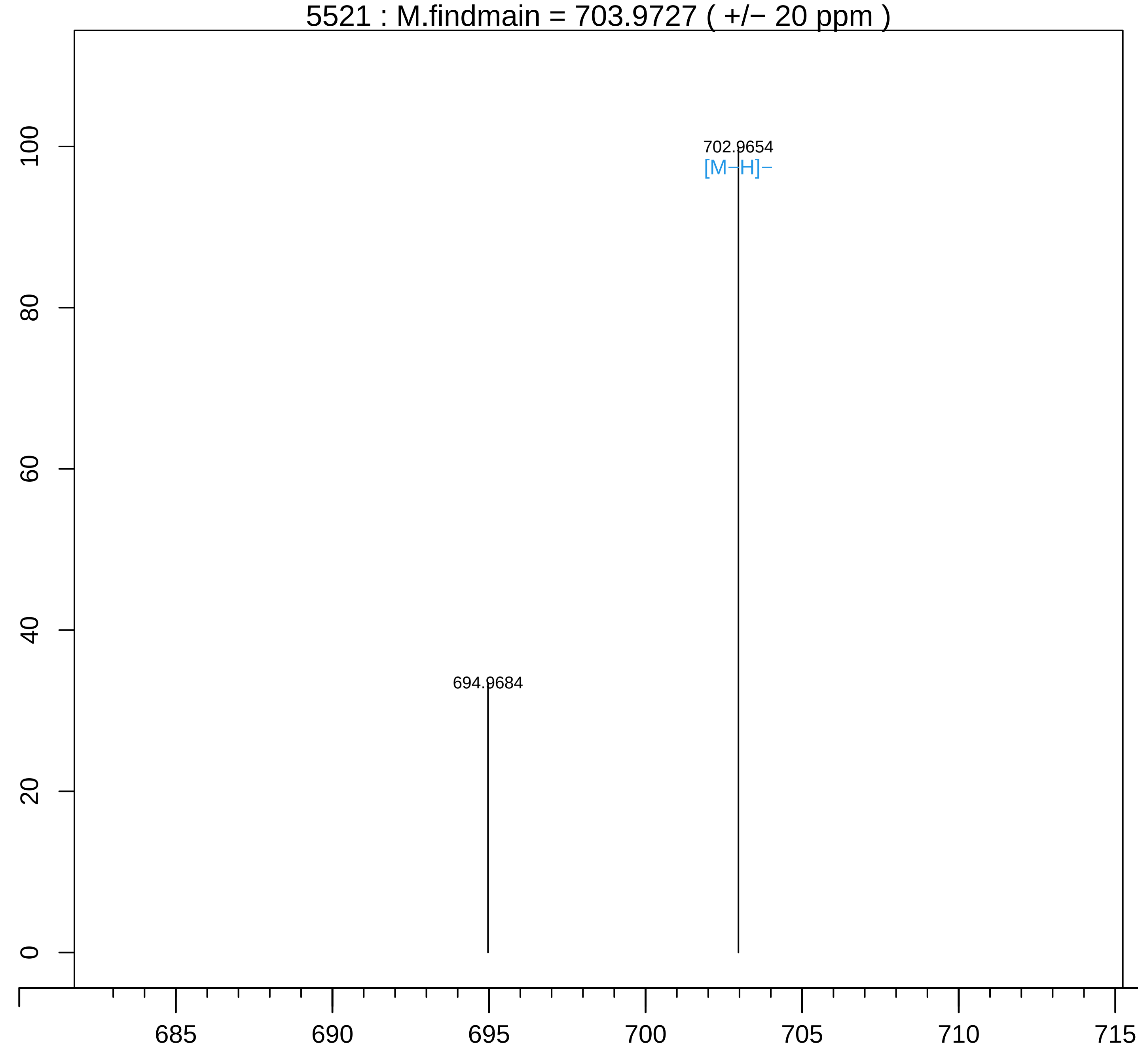
5521 : M.findmain = 703.9727 (+/− 20 ppm)
702.9654
[M−H]−
694.9684
0
20
40
60
80
100
685
690
695
700
705
710
715

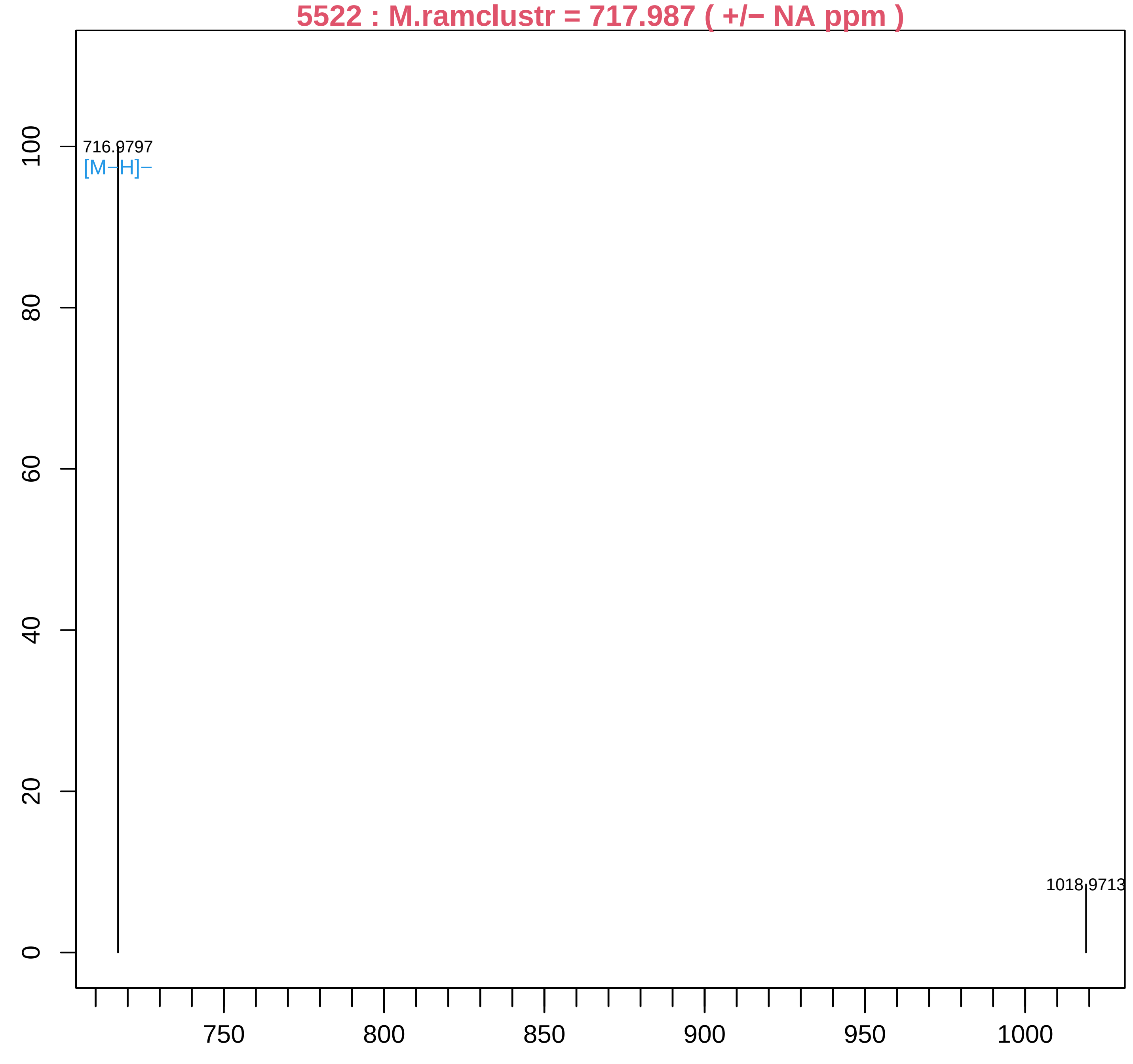

5522 : M.ramclustr = 717.987 (+/− NA ppm)
716.9797
[M−H]−
1018.9713
0
20
40
60
80
100
750
800
850
900
950
1000

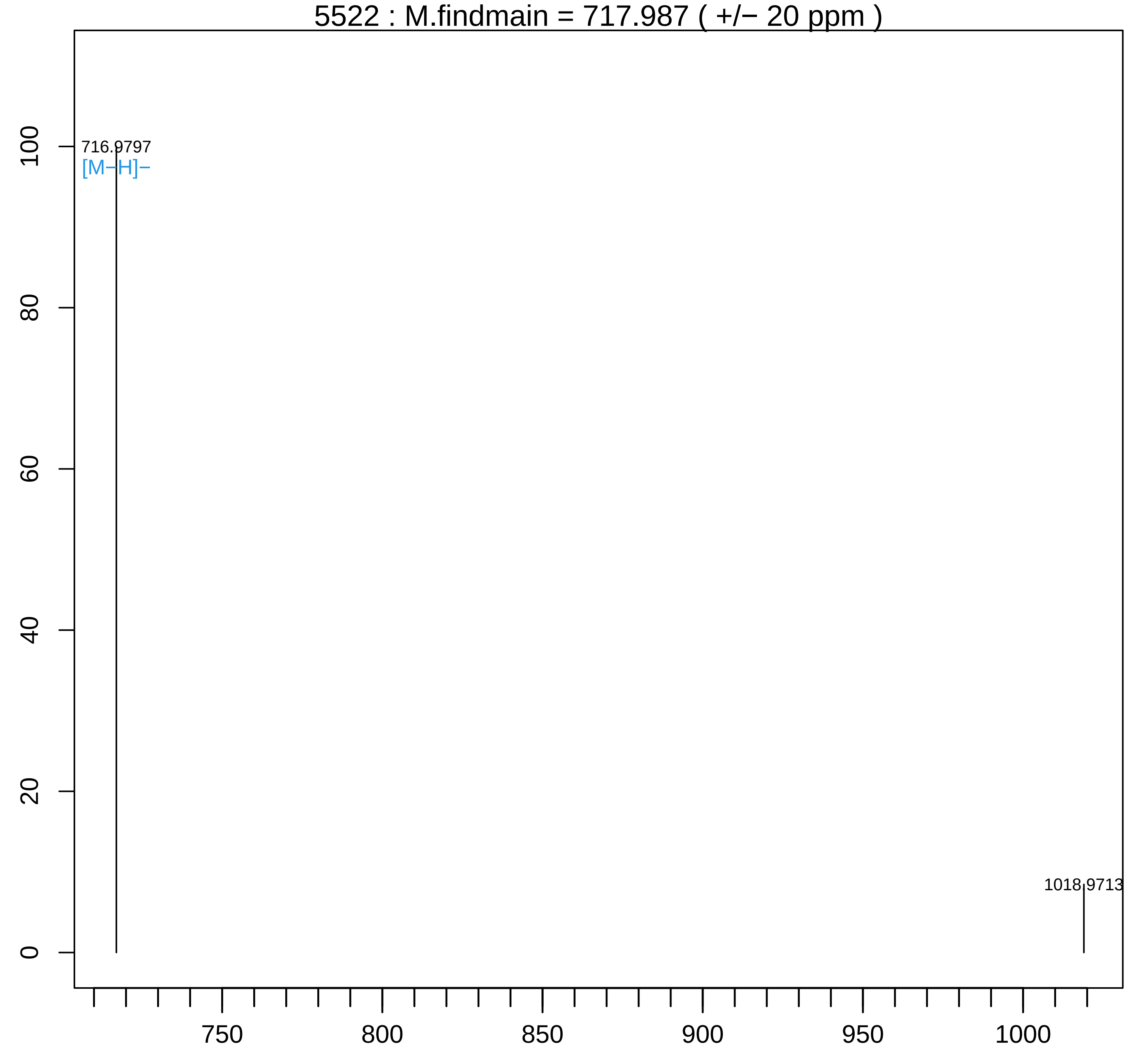

5522 : M.findmain = 717.987 (+/− 20 ppm)
716.9797
[M−H]−
1018.9713
0
20
40
60
80
100
750
800
850
900
950
1000

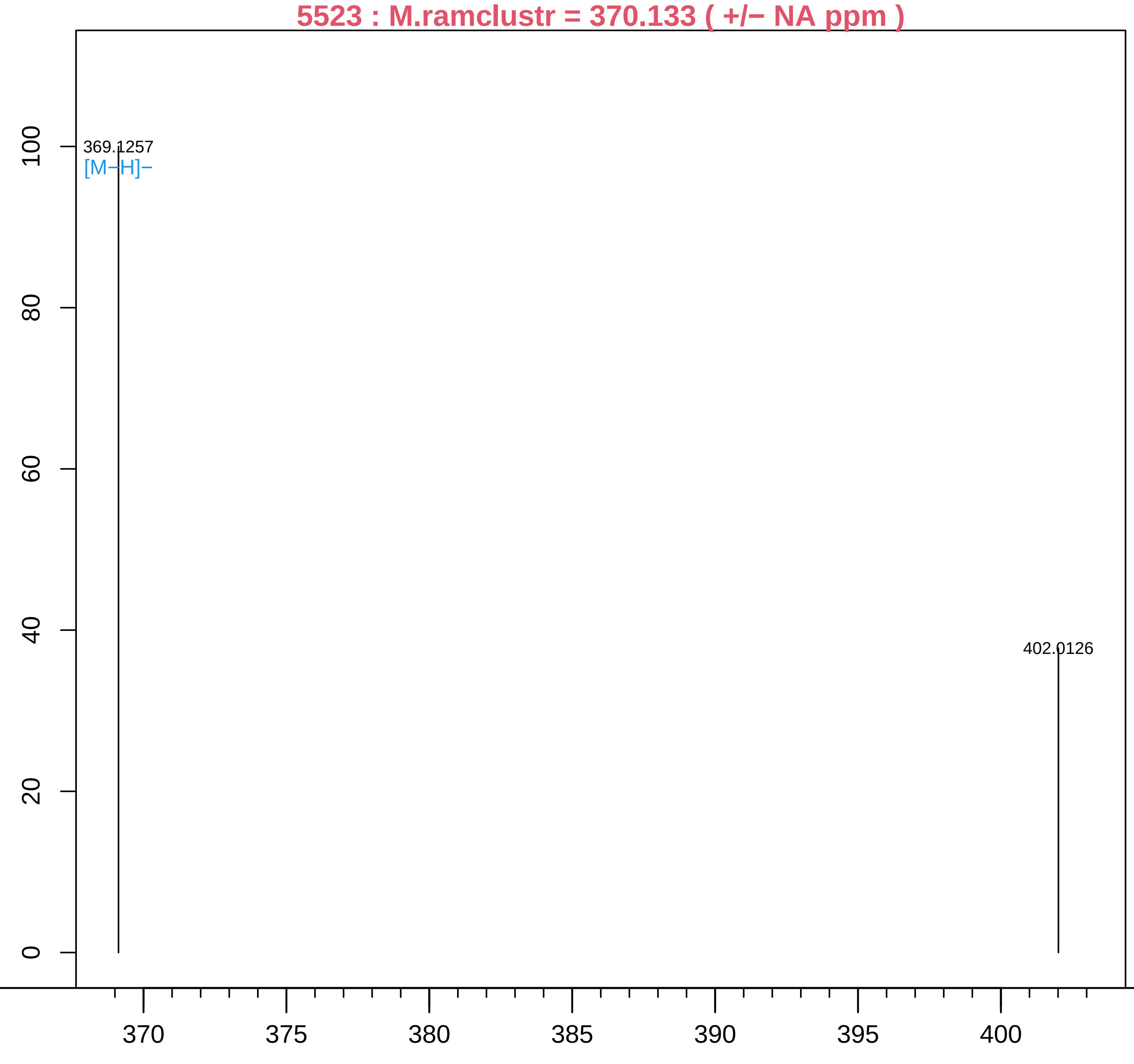
5523 : M.ramclustr = 370.133 (+/− NA ppm)
369.1257
[M−H]−
402.0126
0
20
40
60
80
100
370
375
380
385
390
395
400

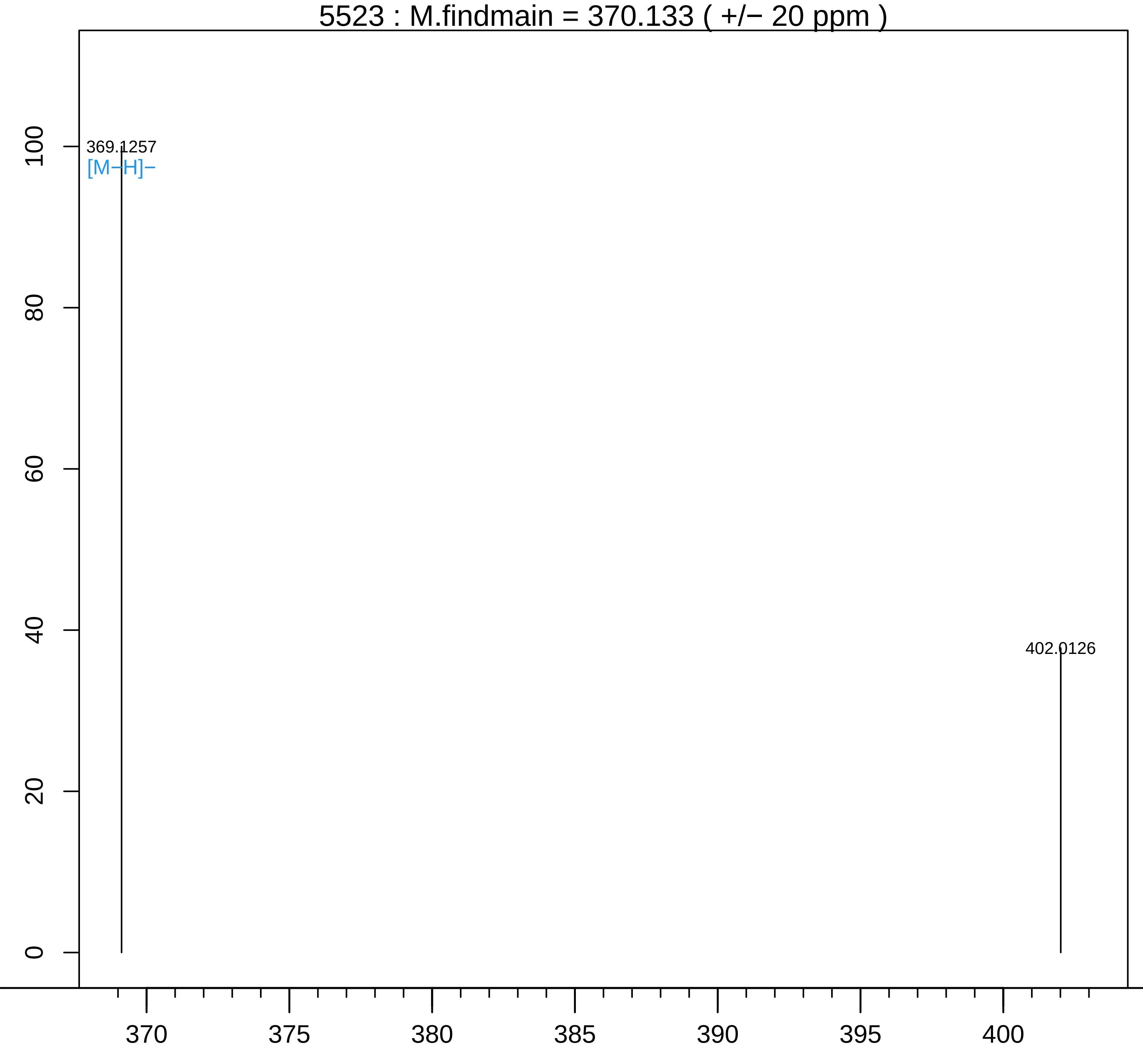
5523 : M.findmain = 370.133 (+/− 20 ppm)
369.1257
[M−H]−
402.0126
0
20
40
60
80
100
370
375
380
385
390
395
400

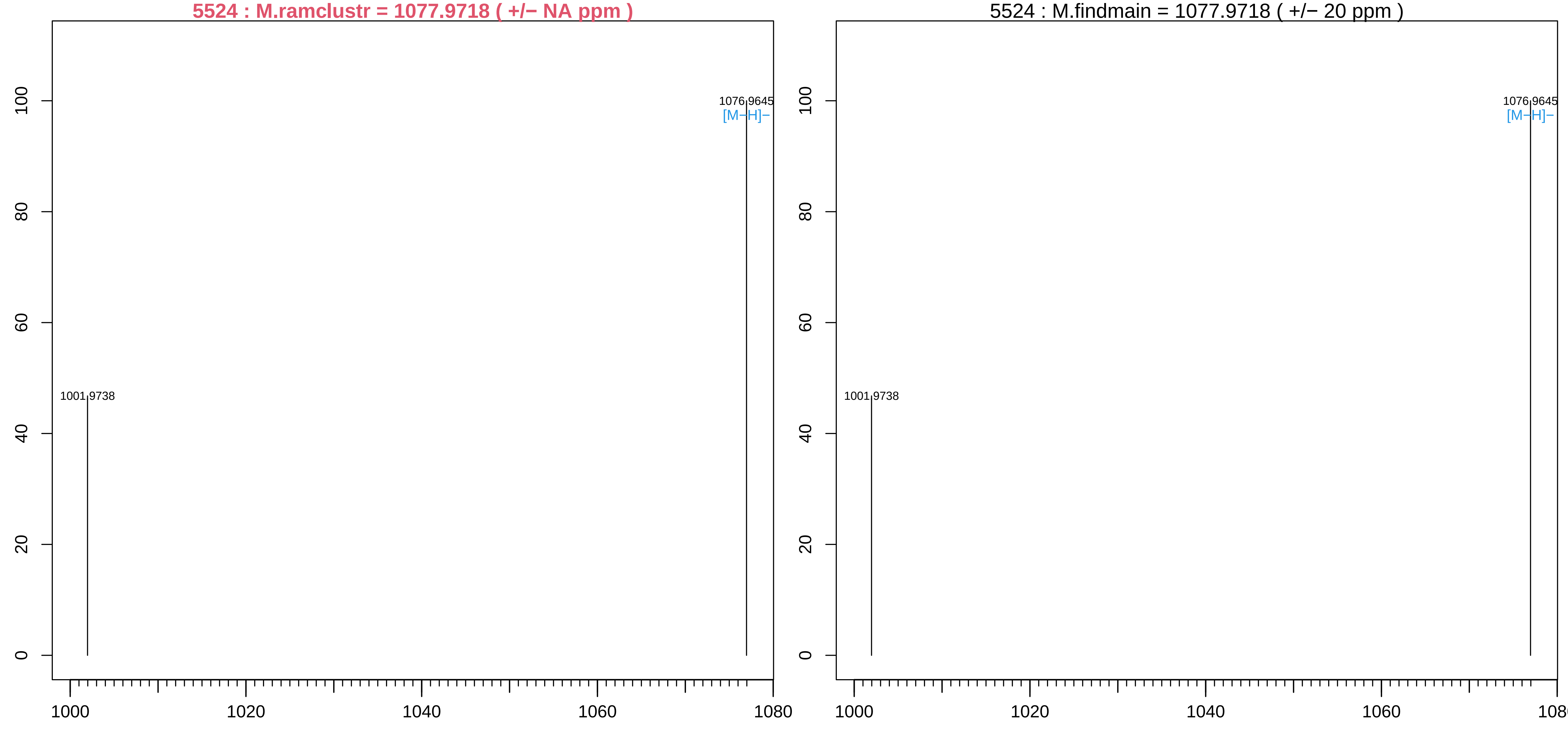
5524 : M.ramclustr = 1077.9718 (+/− NA ppm)
5524 : M.findmain = 1077.9718 (+/− 20 ppm)
1076.9645
[M−H]−
1001.9738
0
20
40
60
80
100
1000
1020
1040
1060
1080

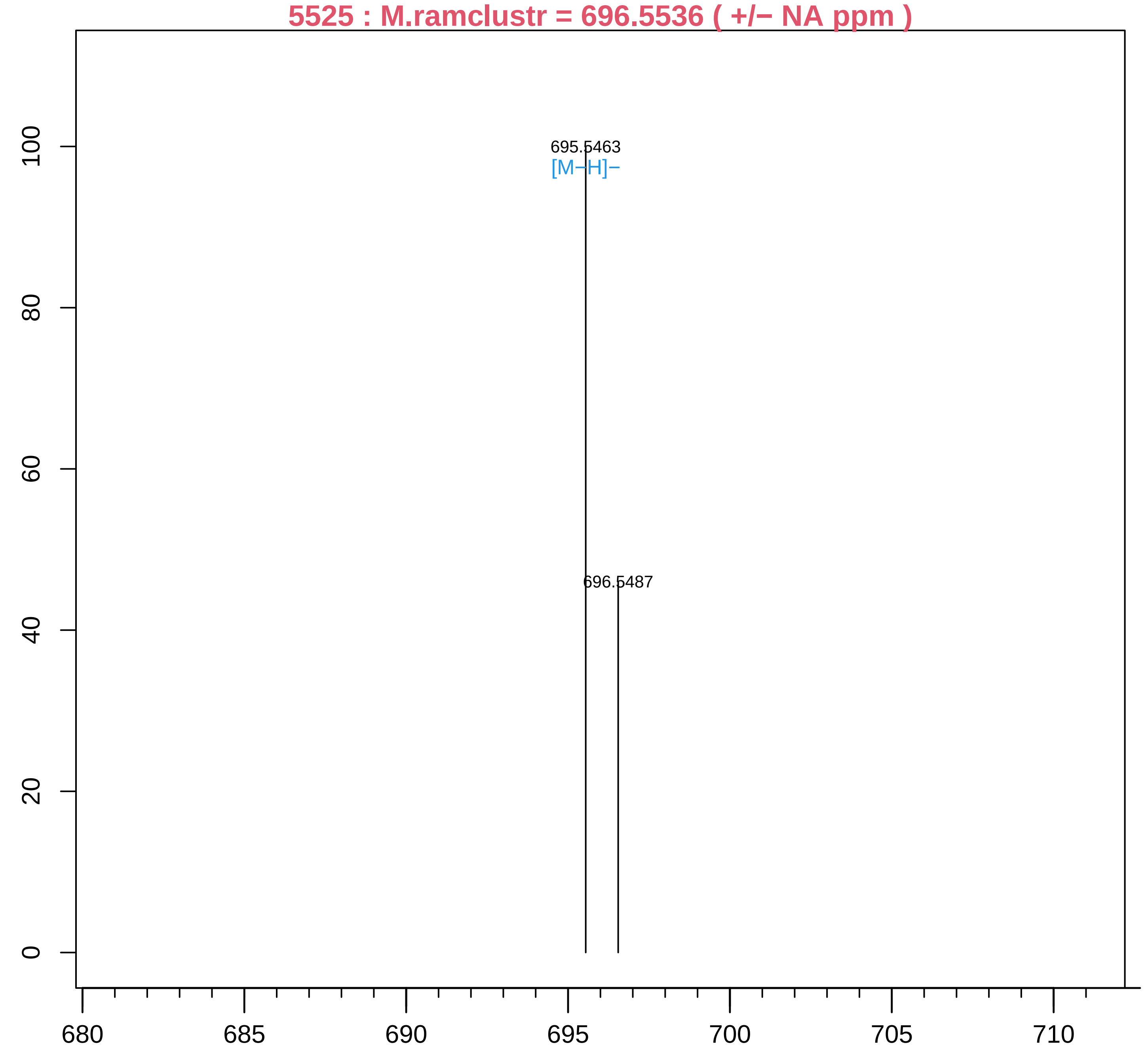
5525 : M.ramclustr = 696.5536 (+/− NA ppm)
695.5463
[M−H]−
696.5487
0
20
40
60
80
100
680
685
690
695
700
705
710

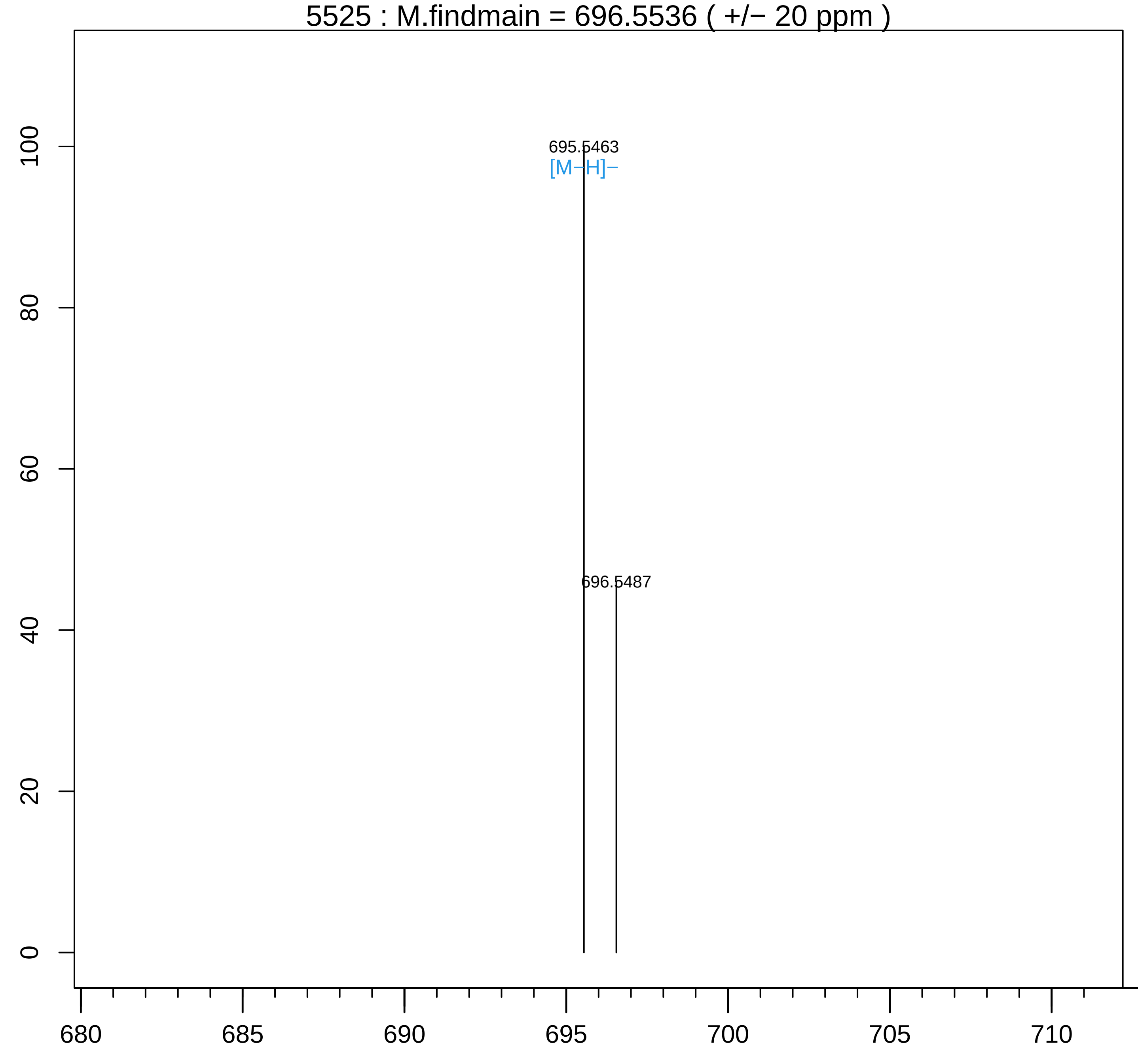
5525 : M.findmain = 696.5536 (+/− 20 ppm)
695.5463
[M−H]−
696.5487
0
20
40
60
80
100
680
685
690
695
700
705
710

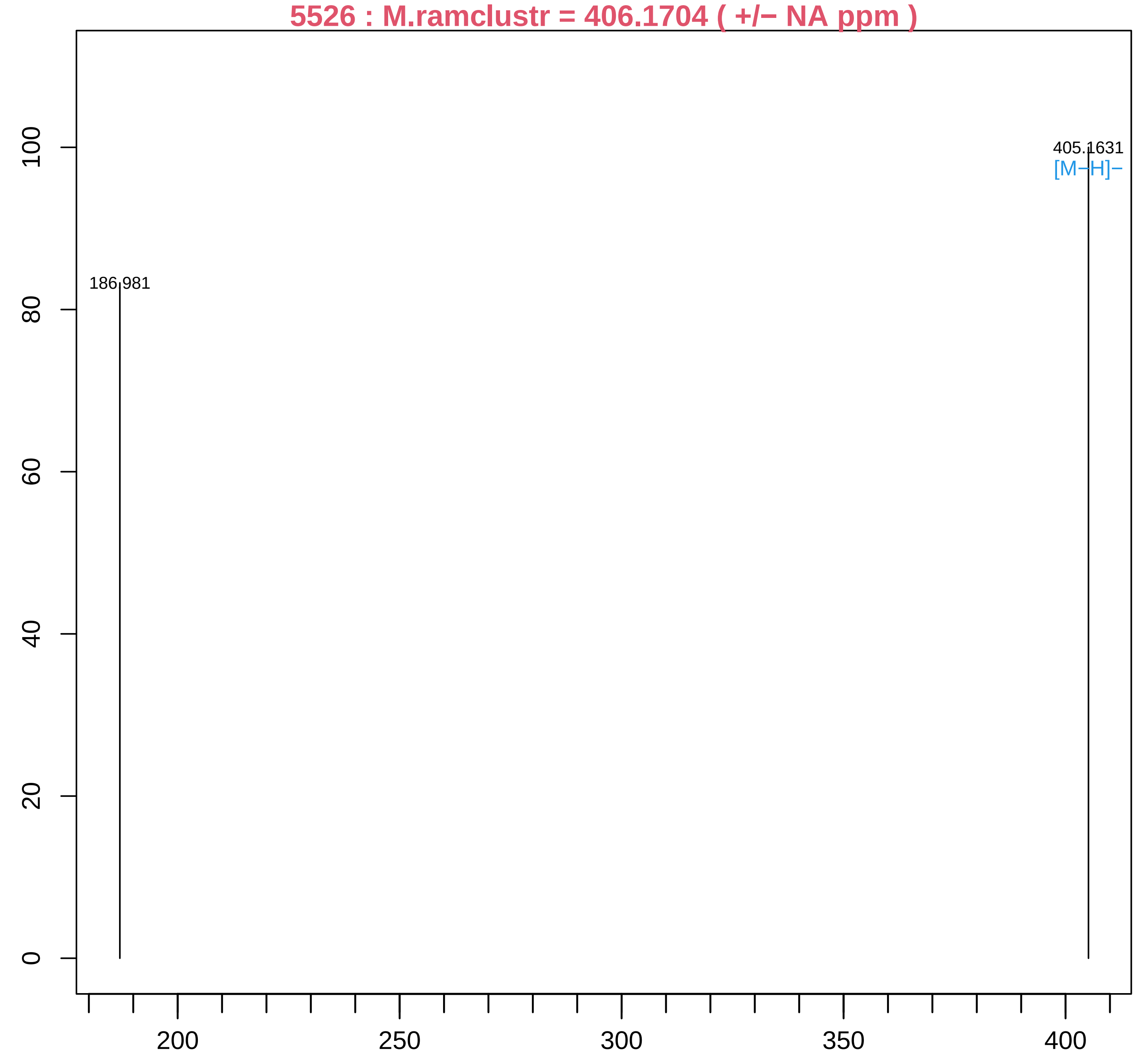
5526 : M.ramclustr = 406.1704 (+/− NA ppm)
405.1631
[M−H]−
186.981
0
20
40
60
80
100
200
250
300
350
400

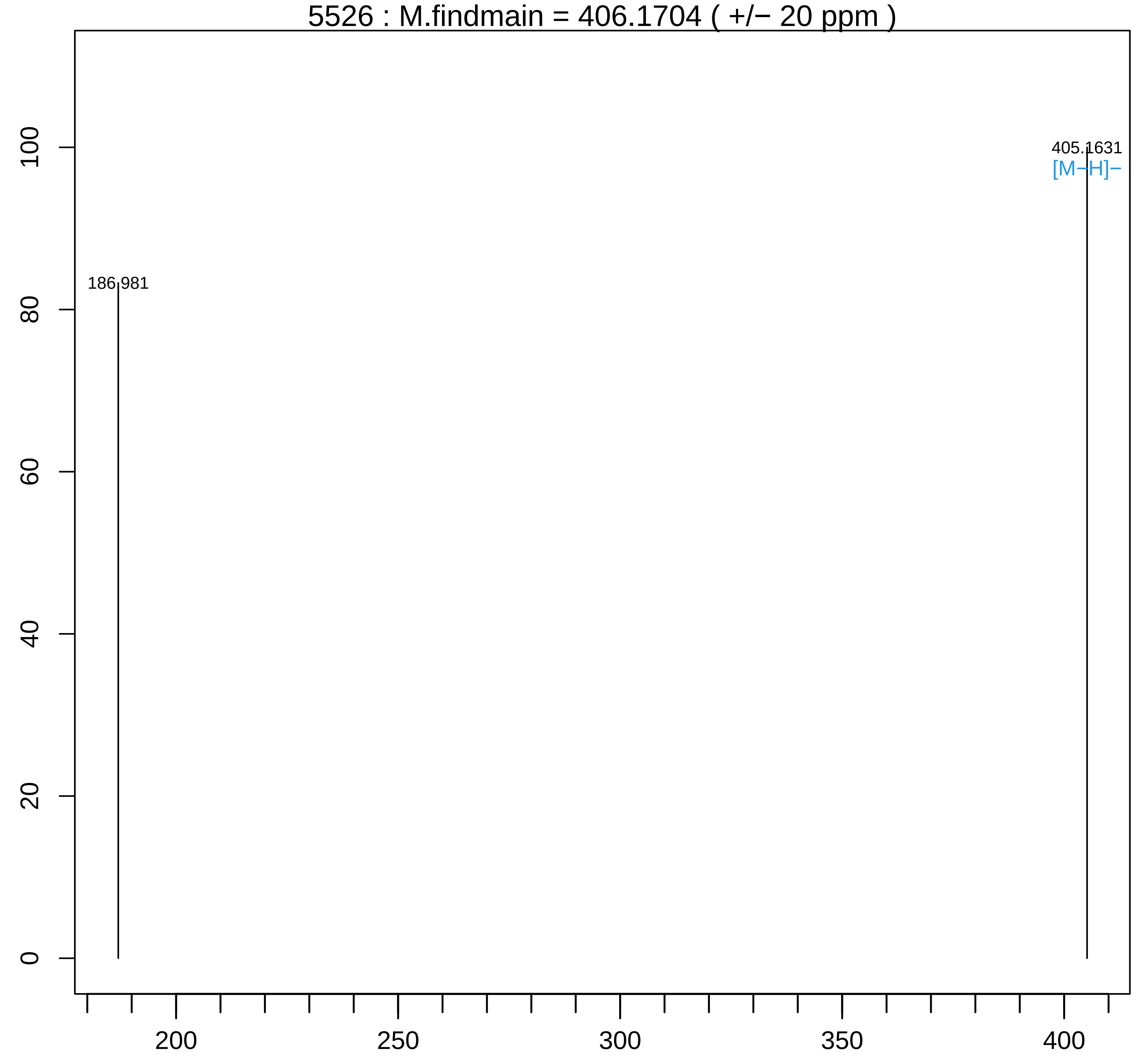
5526 : M.findmain = 406.1704 (+/− 20 ppm)
405.1631
[M−H]−
186.981
0
20
40
60
80
100
200
250
300
350
400

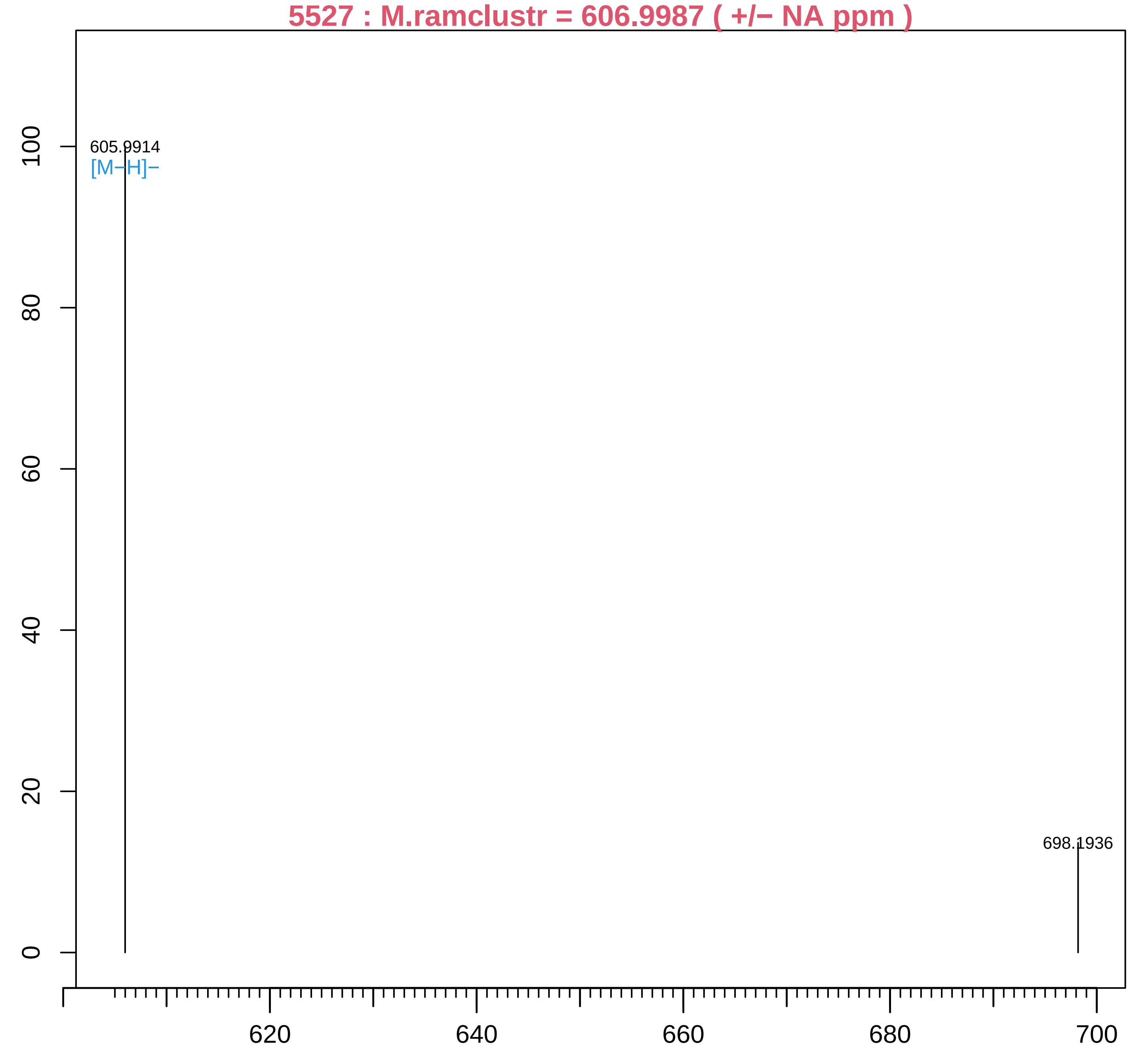

5527 : M.ramclustr = 606.9987 (+/− NA ppm)
605.9914
[M−H]−
698.1936
0
20
40
60
80
100
620
640
660
680
700

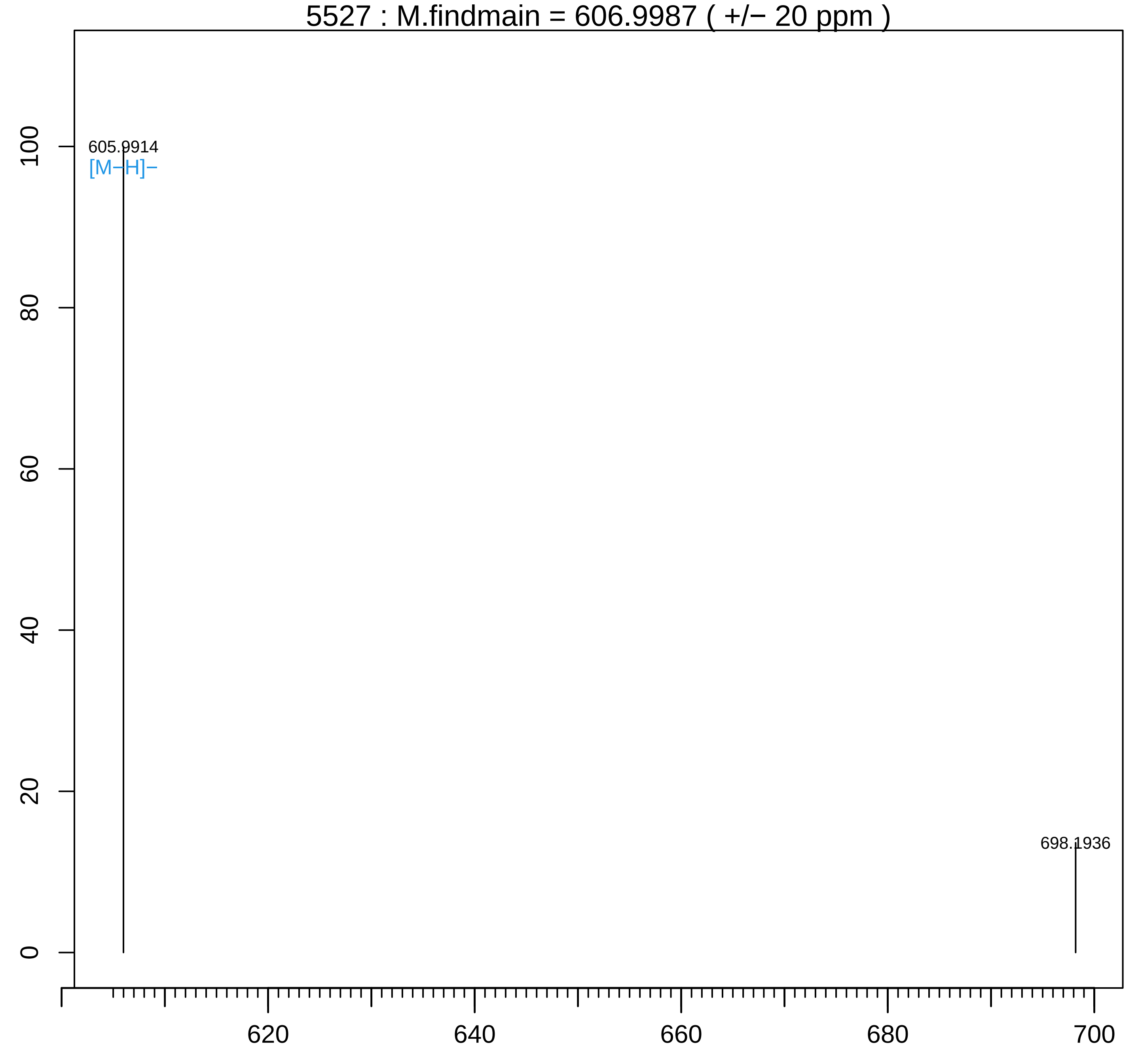

5527 : M.findmain = 606.9987 (+/− 20 ppm)
605.9914
[M−H]−
698.1936
0
20
40
60
80
100
620
640
660
680
700

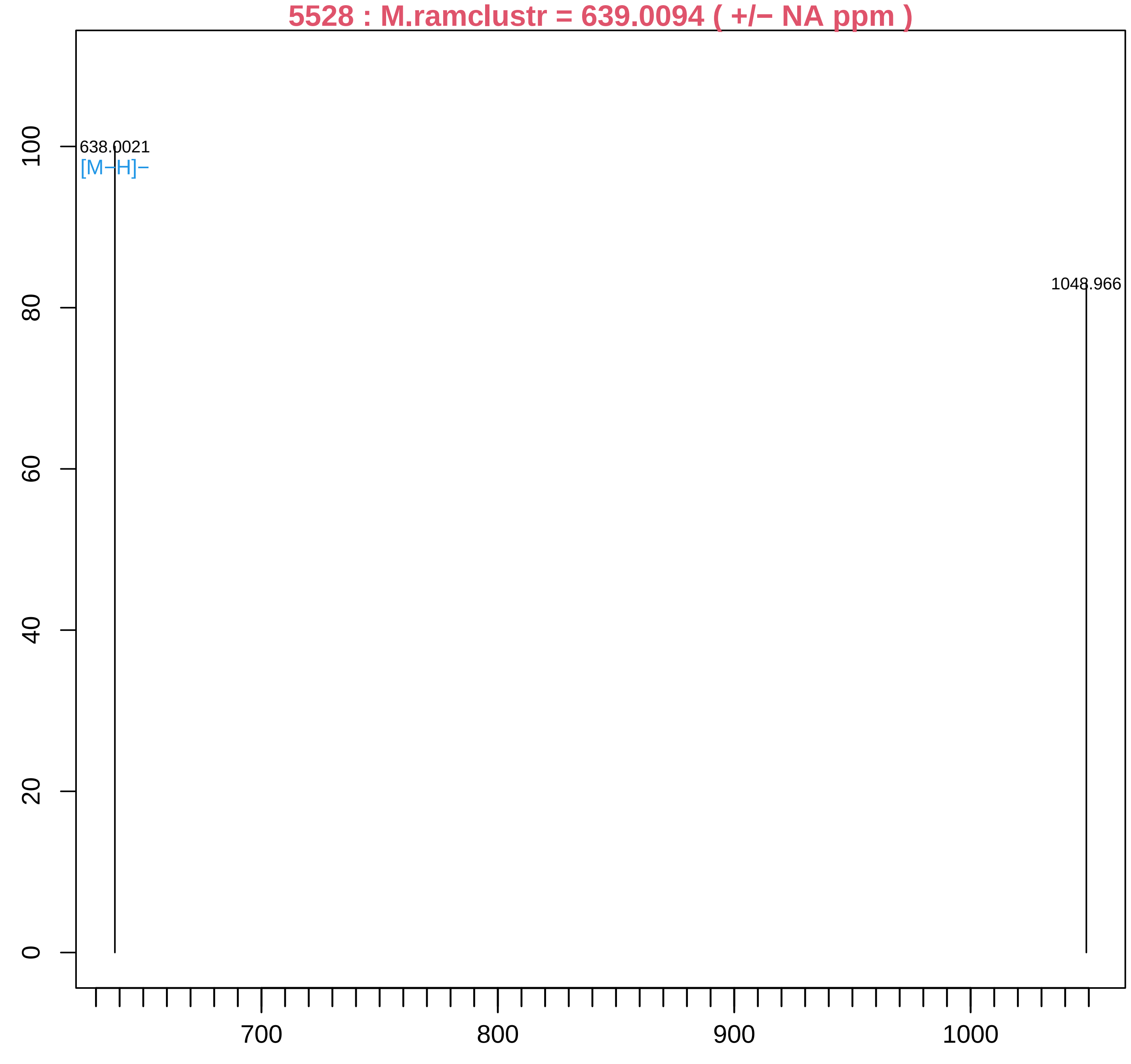
5528 : M.ramclustr = 639.0094 (+/− NA ppm)
638.0021
[M−H]−
1048.966
0
20
40
60
80
100
700
800
900
1000

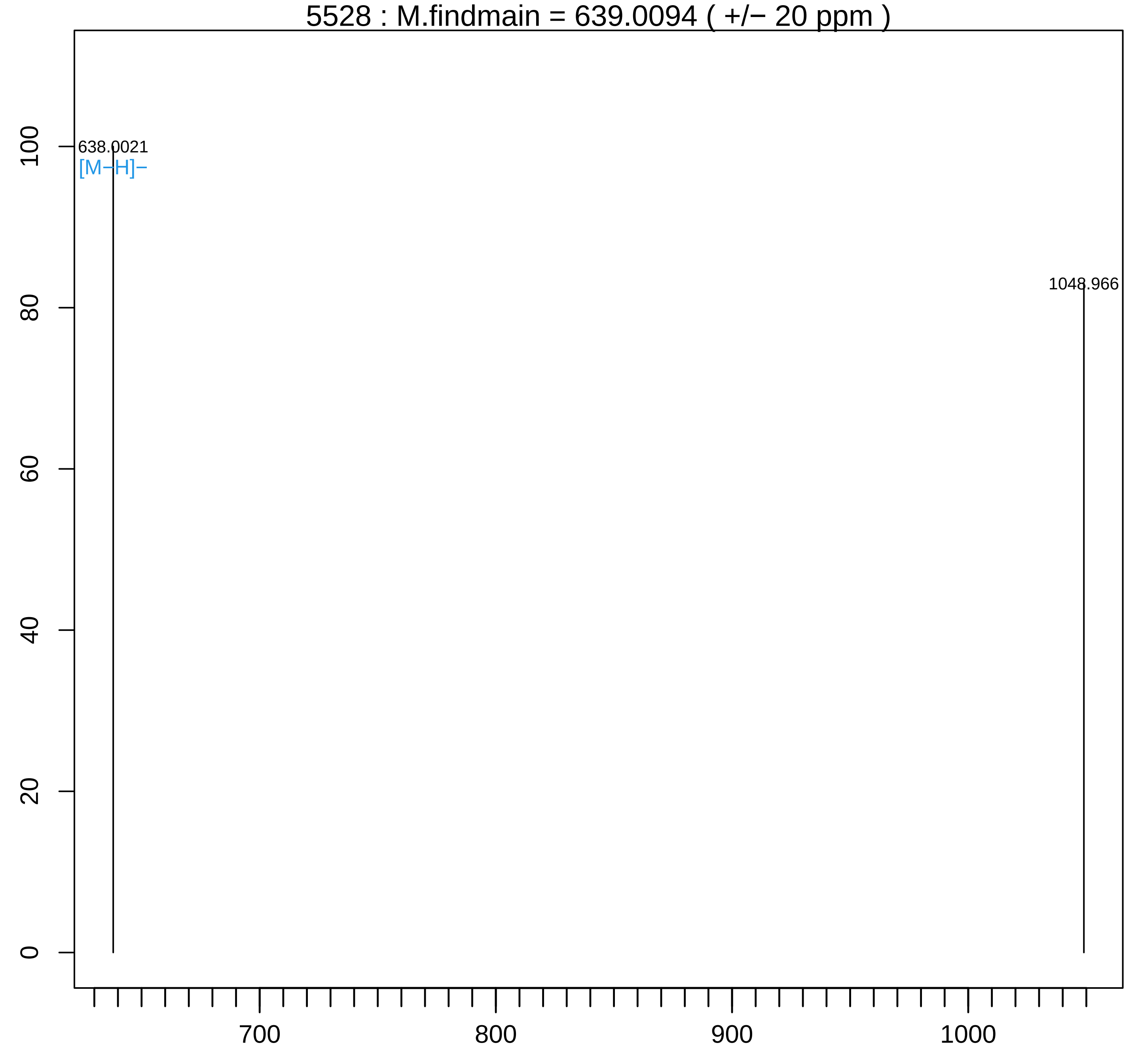
5528 : M.findmain = 639.0094 (+/− 20 ppm)
638.0021
[M−H]−
1048.966
0
20
40
60
80
100
700
800
900
1000

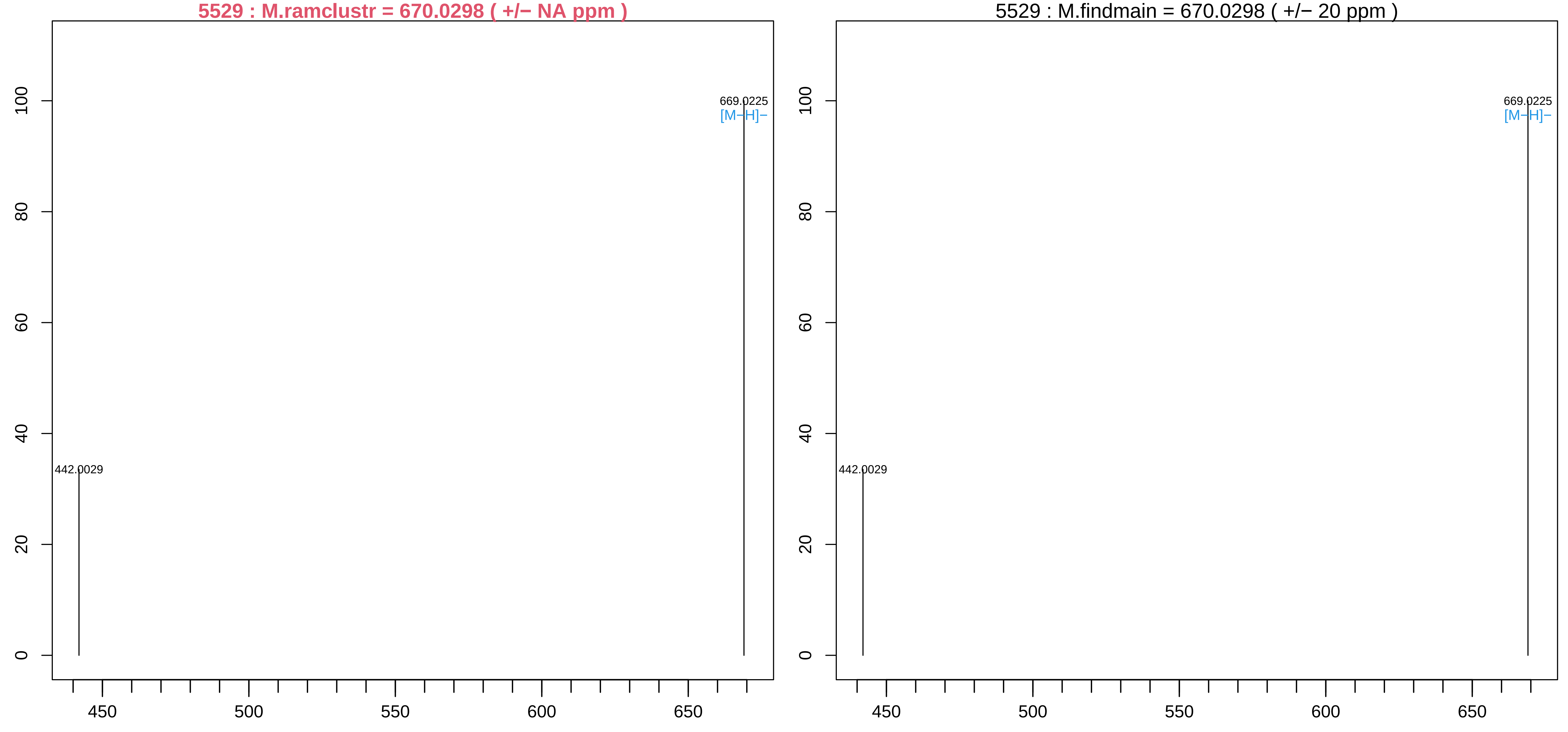
5529 : M.ramclustr = 670.0298 (+/− NA ppm)
669.0225
[M−H]−
442.0029
5529 : M.findmain = 670.0298 (+/− 20 ppm)
669.0225
[M−H]−
442.0029
0
20
40
60
80
100
450
500
550
600
650

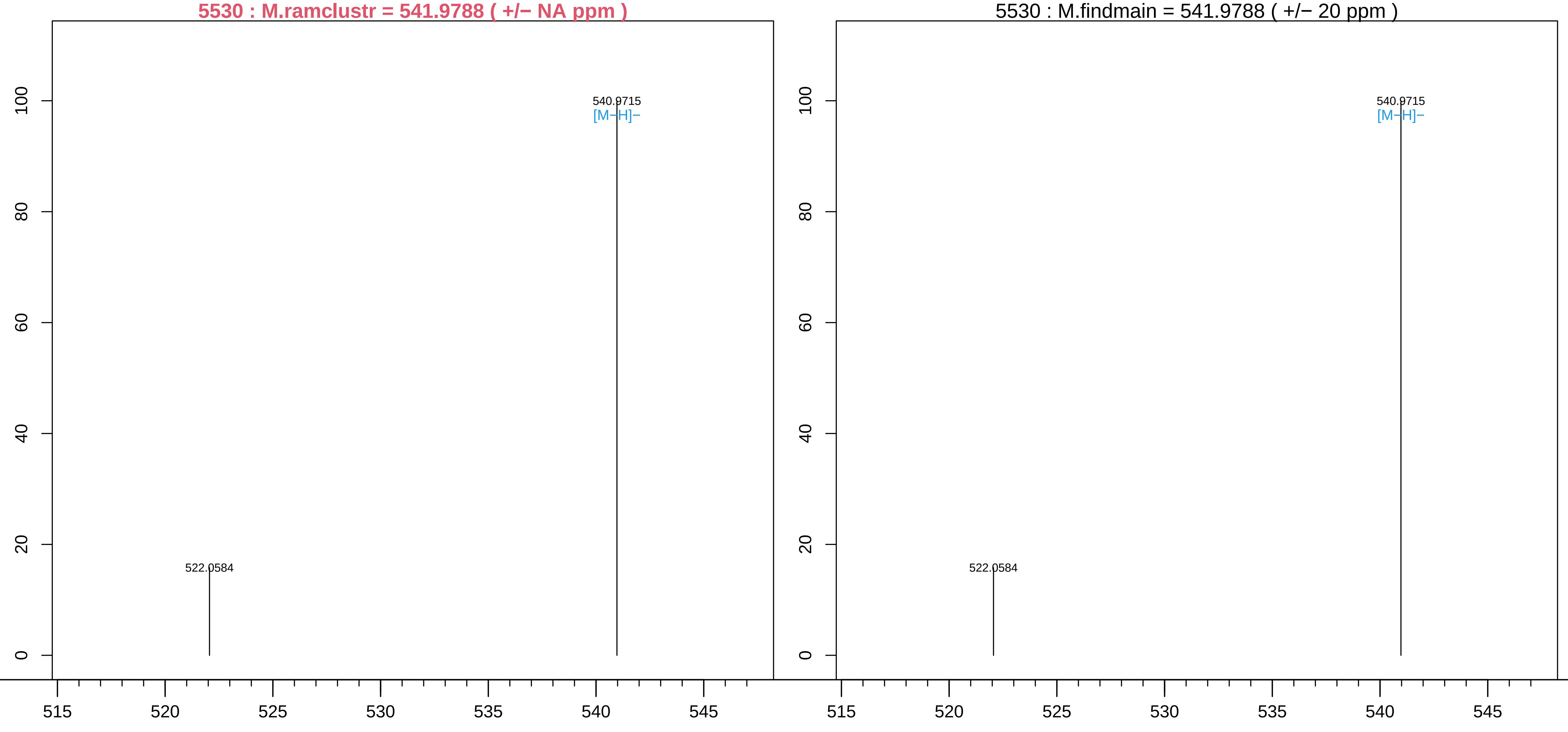
5530 : M.ramclustr = 541.9788 (+/− NA ppm)
540.9715
[M−H]−
522.0584
5530 : M.findmain = 541.9788 (+/− 20 ppm)
540.9715
[M−H]−
522.0584
0
20
40
60
80
100
515
520
525
530
535
540
545

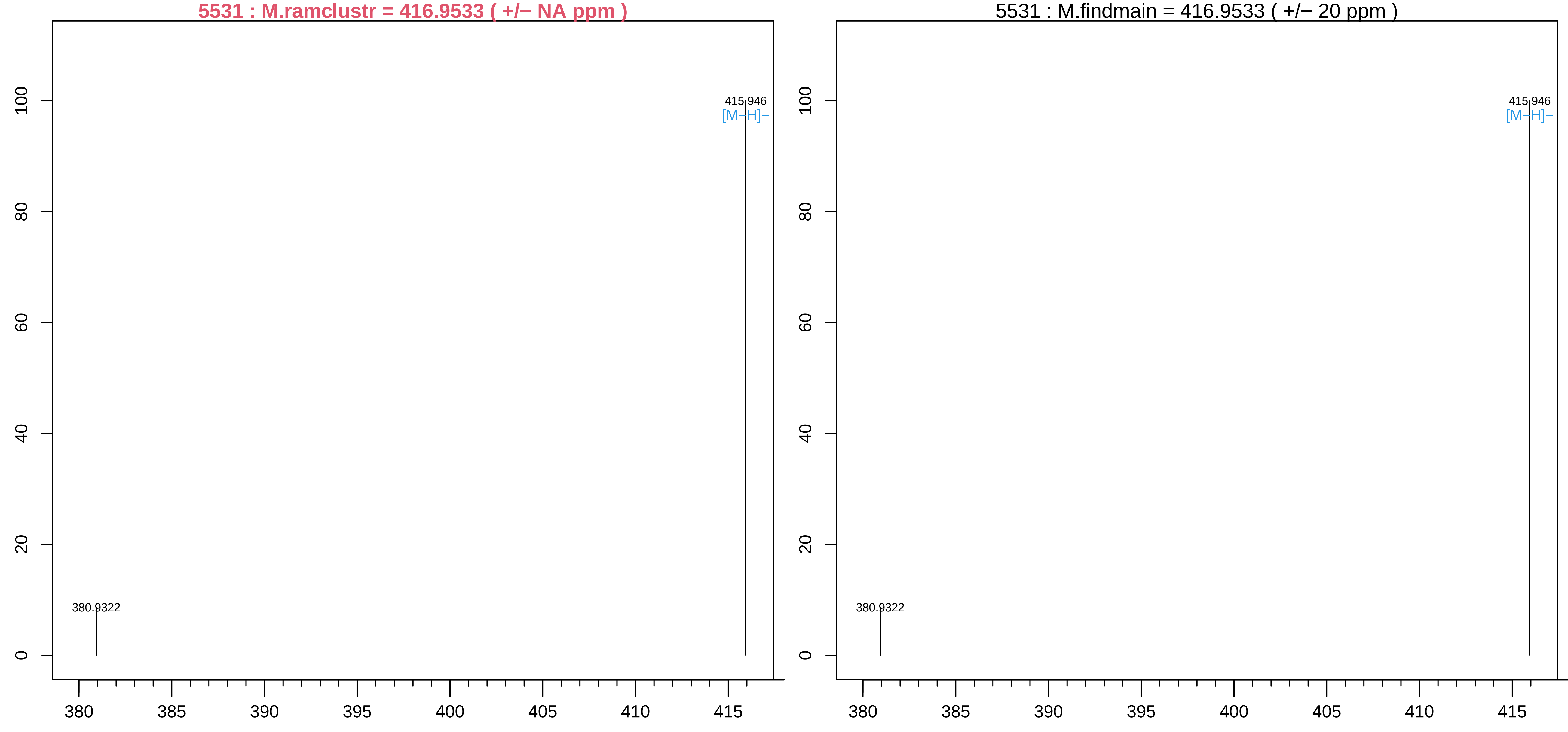

5531 : M.ramclustr = 416.9533 (+/− NA ppm)
5531 : M.findmain = 416.9533 (+/− 20 ppm)
415.946
[M−H]−
380.9322
0
20
40
60
80
100
380
385
390
395
400
405
410
415

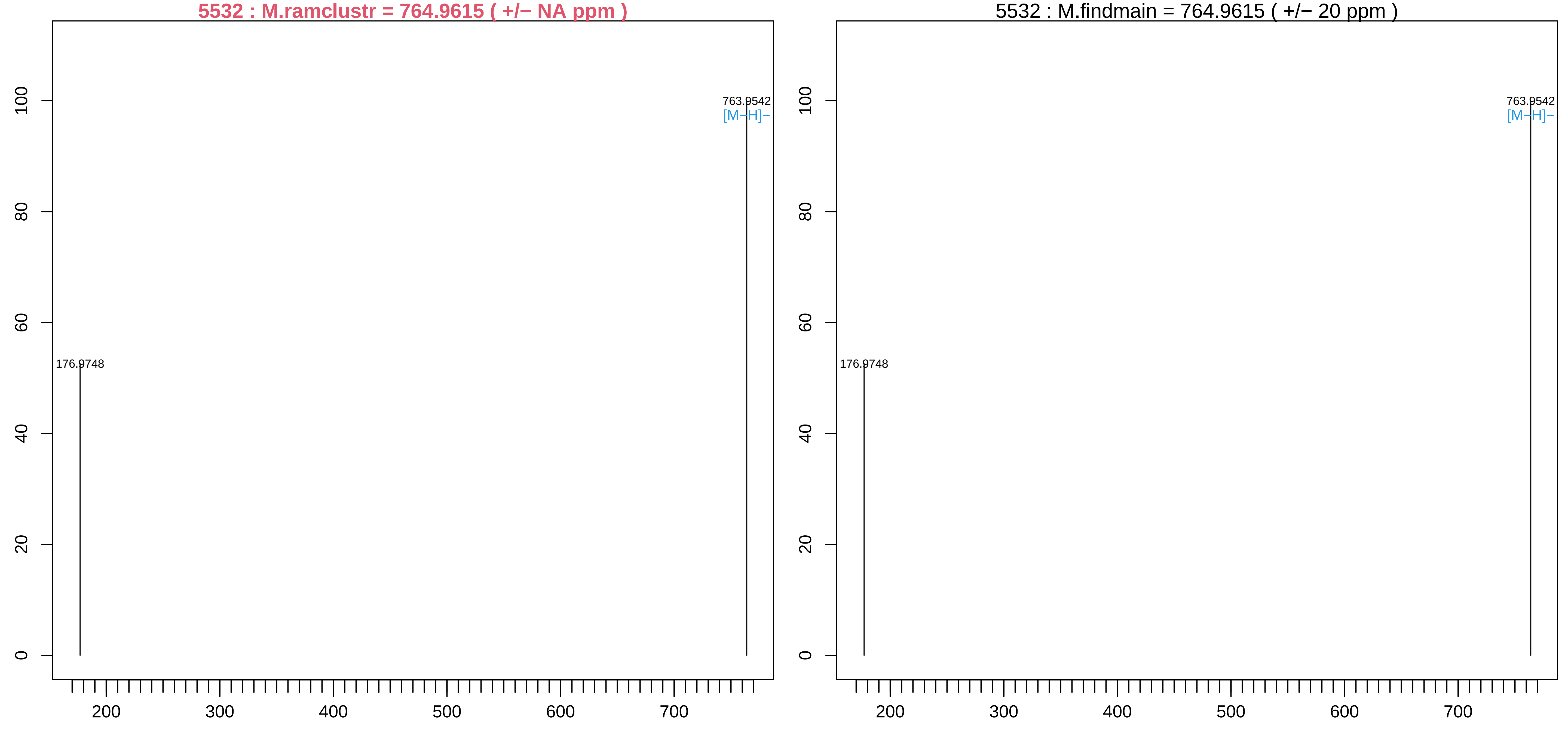
5532 : M.ramclustr = 764.9615 (+/− NA ppm)
763.9542
[M−H]−
176.9748
0
20
40
60
80
100
200
300
400
500
600
700
5532 : M.findmain = 764.9615 (+/− 20 ppm)
763.9542
[M−H]−
176.9748
0
20
40
60
80
100
200
300
400
500
600
700

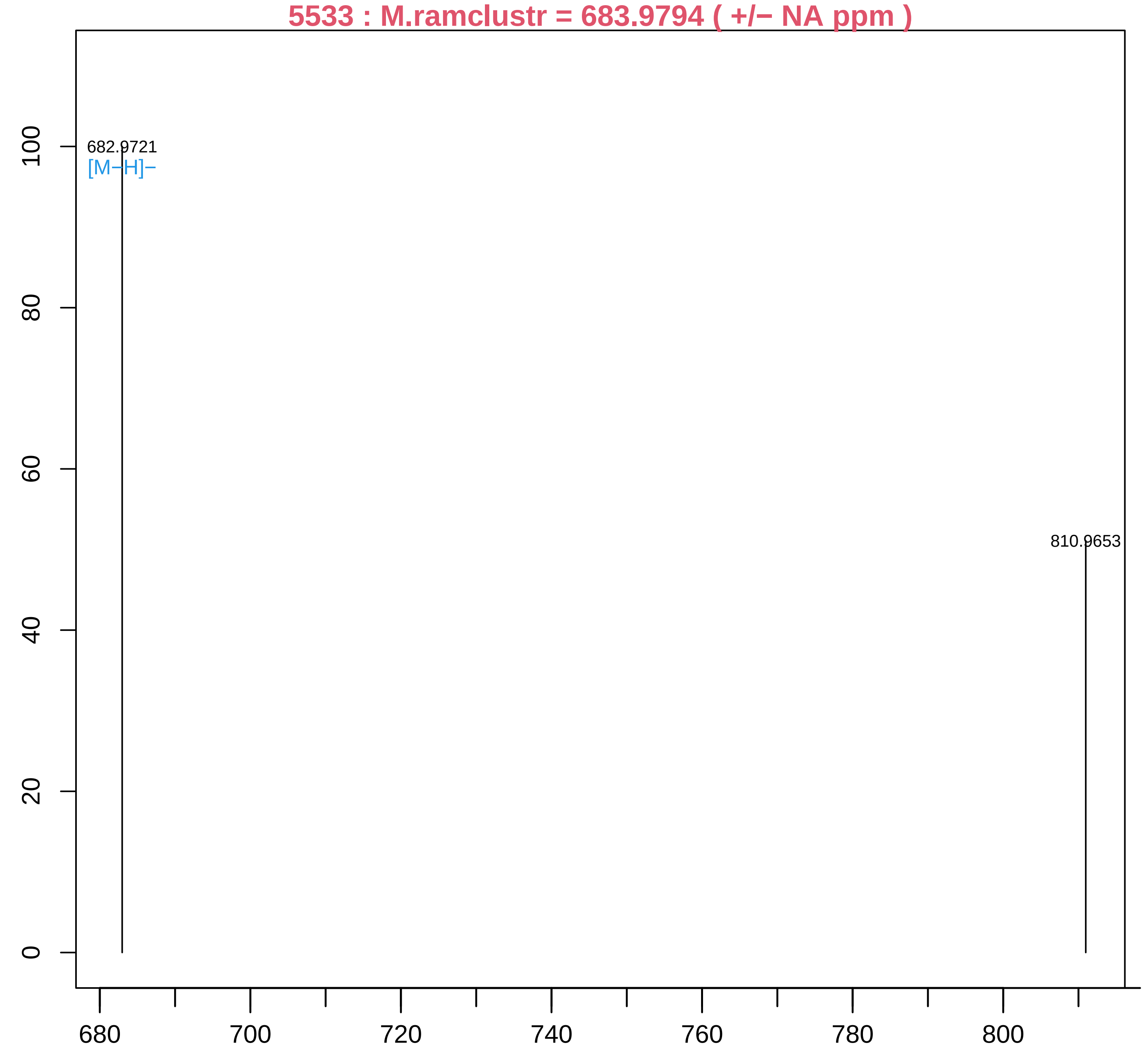

5533 : M.ramclustr = 683.9794 (+/− NA ppm)
682.9721
[M−H]−
810.9653
0
20
40
60
80
100
680
700
720
740
760
780
800

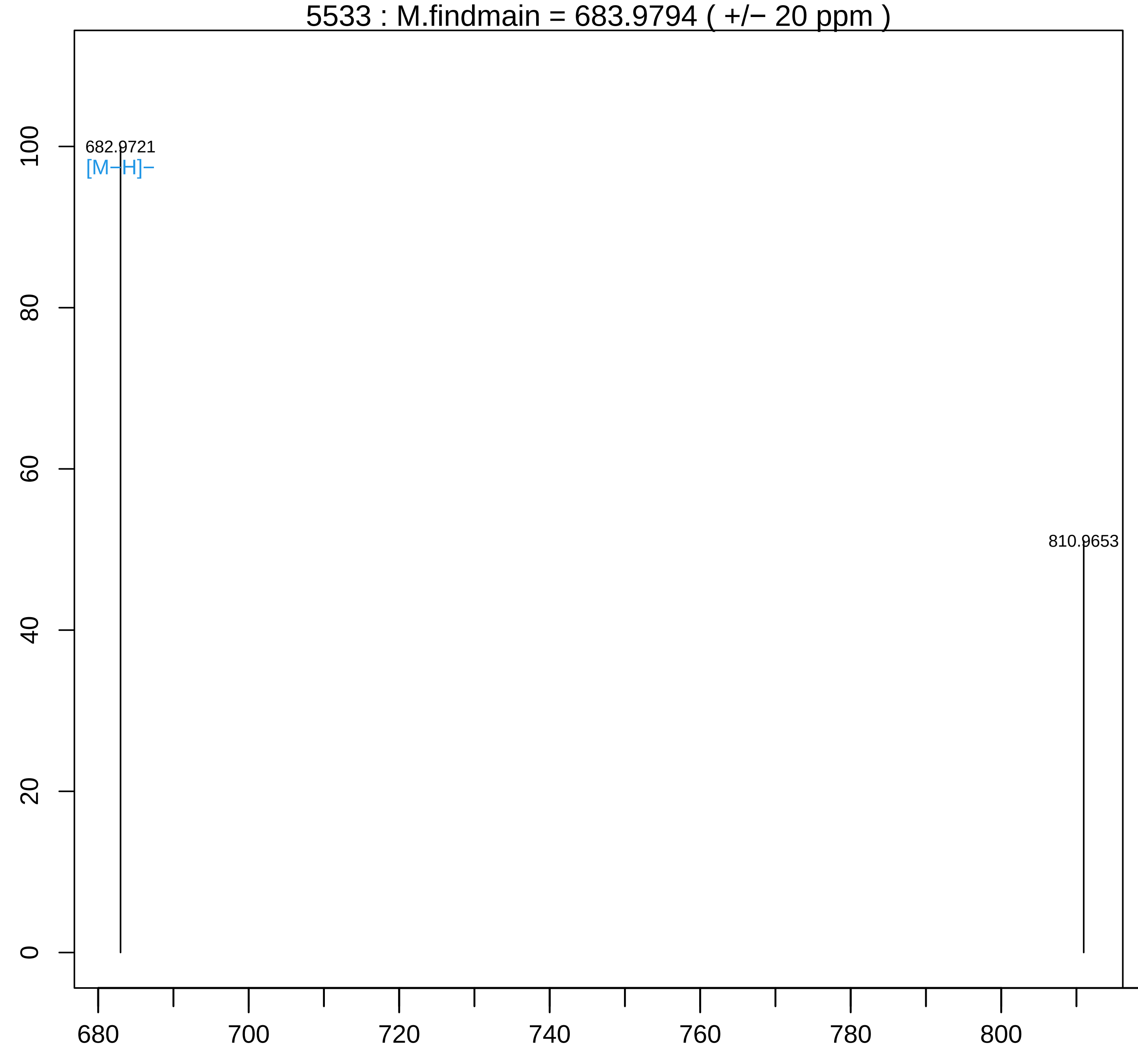

5533 : M.findmain = 683.9794 (+/− 20 ppm)
682.9721
[M−H]−
810.9653
0
20
40
60
80
100
680
700
720
740
760
780
800

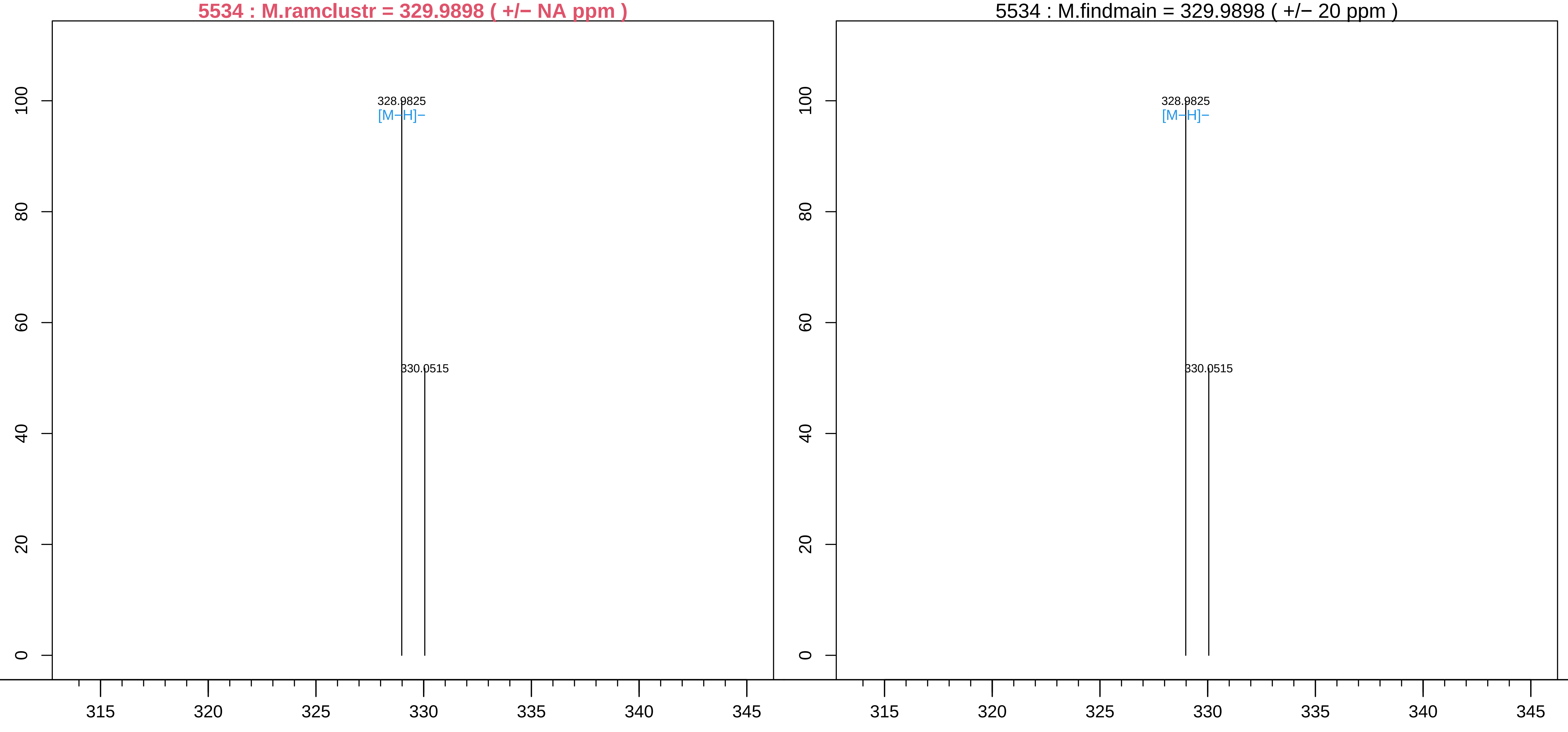
5534 : M.ramclustr = 329.9898 (+/− NA ppm)
328.9825
[M−H]−
330.0515
5534 : M.findmain = 329.9898 (+/− 20 ppm)
328.9825
[M−H]−
330.0515

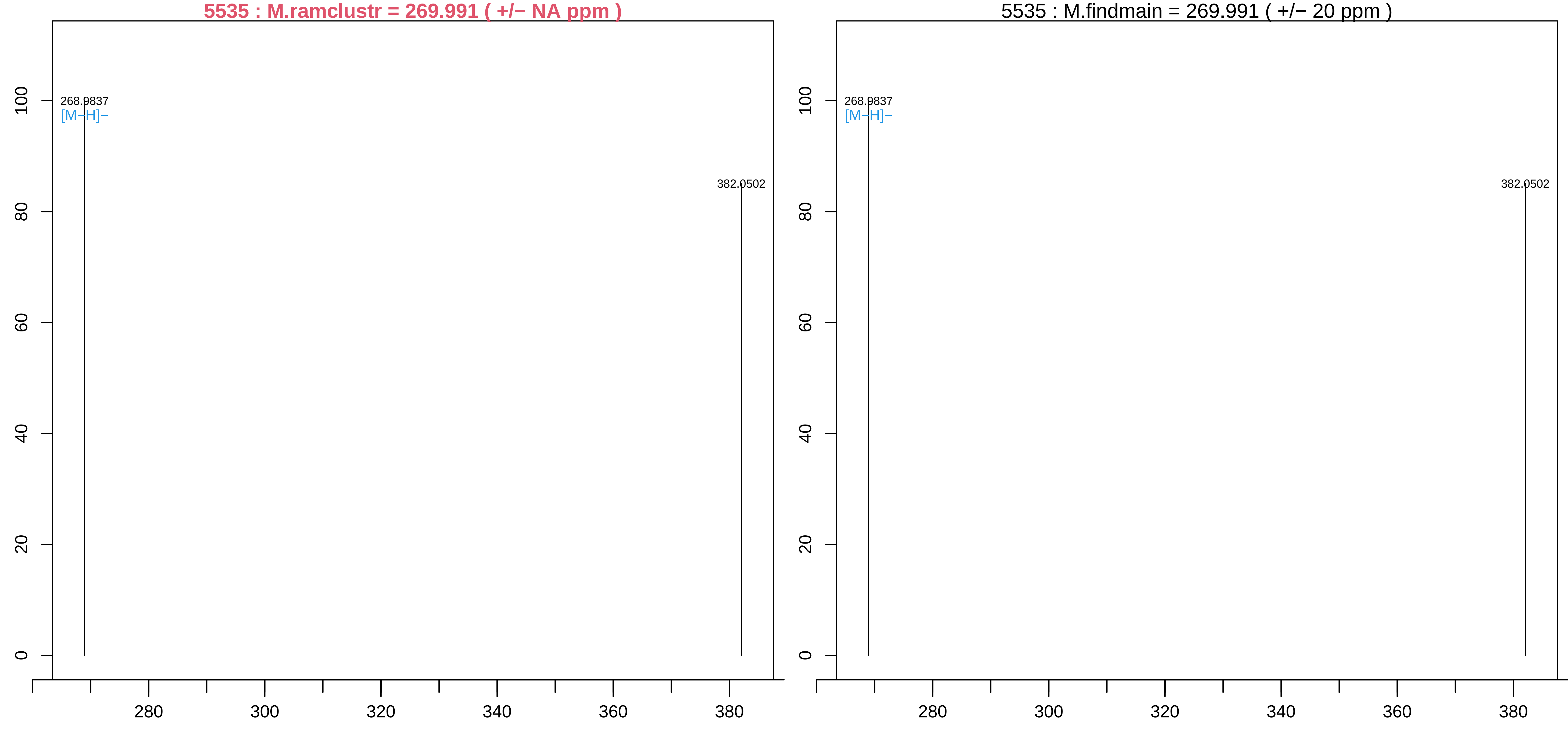
5535 : M.ramclustr = 269.991 (+/− NA ppm)
268.9837
[M−H]−
382.0502
5535 : M.findmain = 269.991 (+/− 20 ppm)
268.9837
[M−H]−
382.0502
0
20
40
60
80
100
280
300
320
340
360
380

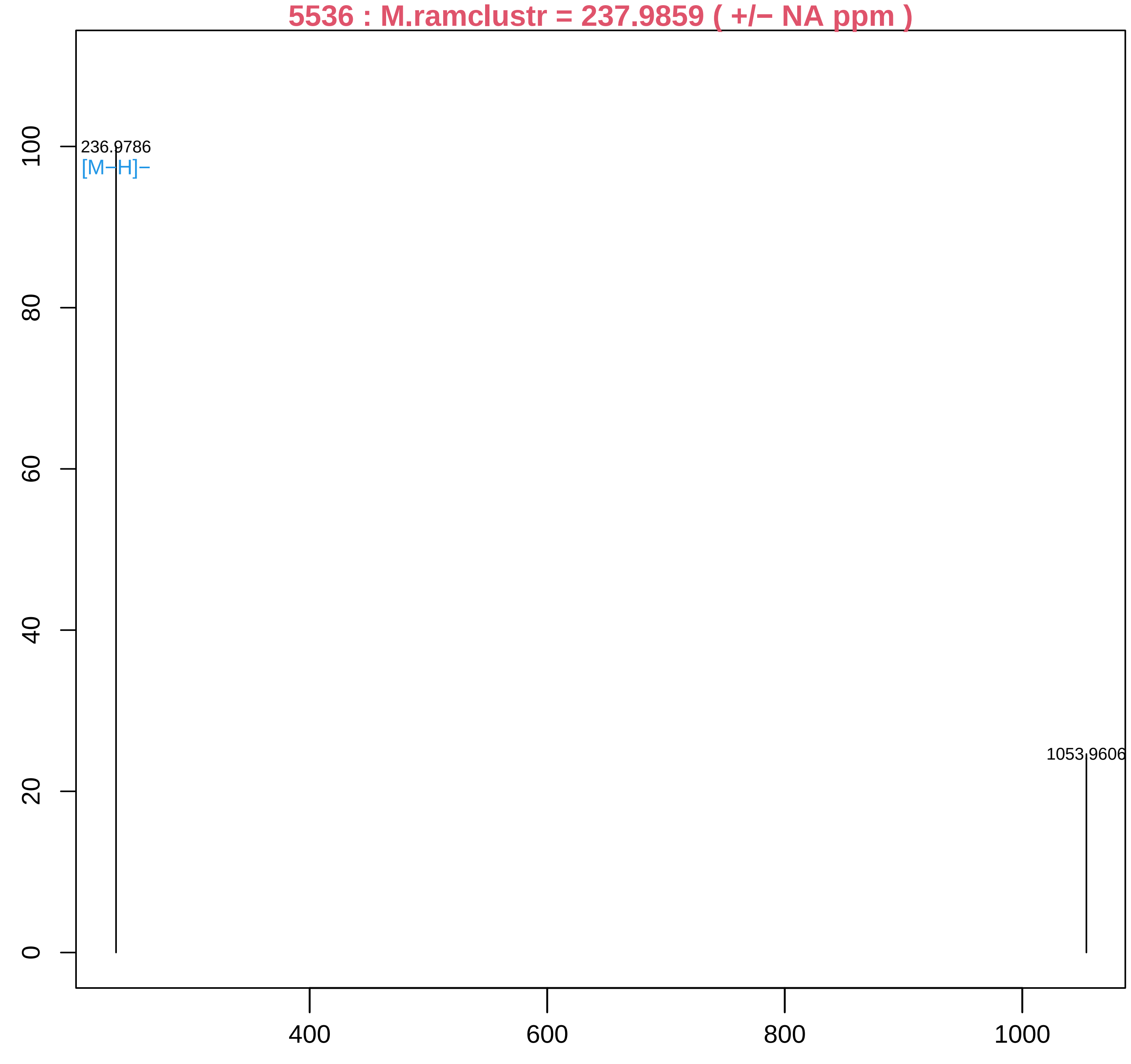
5536 : M.ramclustr = 237.9859 (+/− NA ppm)
236.9786
[M−H]−
1053.9606
0
20
40
60
80
100
400
600
800
1000

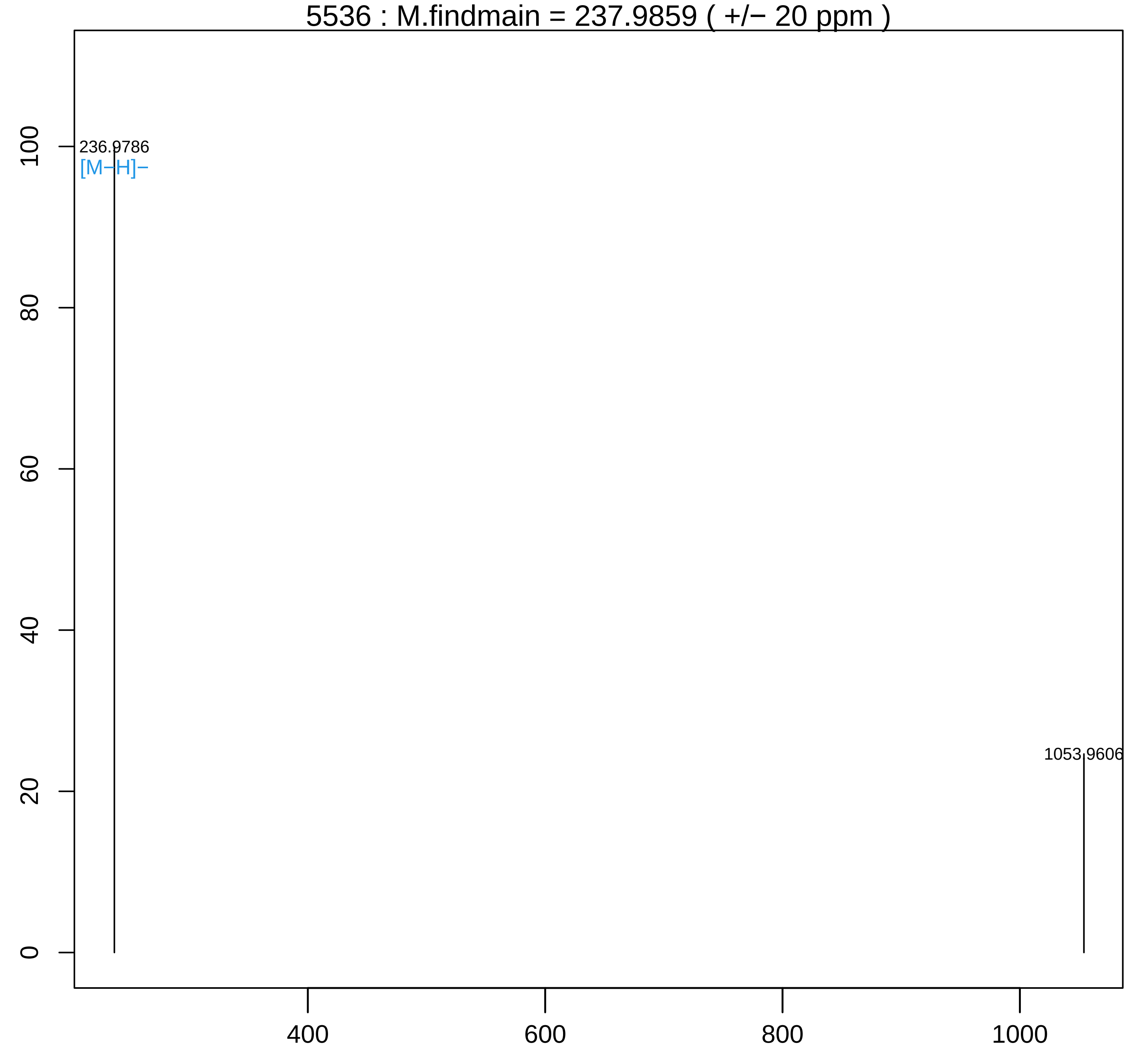
5536 : M.findmain = 237.9859 (+/− 20 ppm)
236.9786
[M−H]−
1053.9606
0
20
40
60
80
100
400
600
800
1000

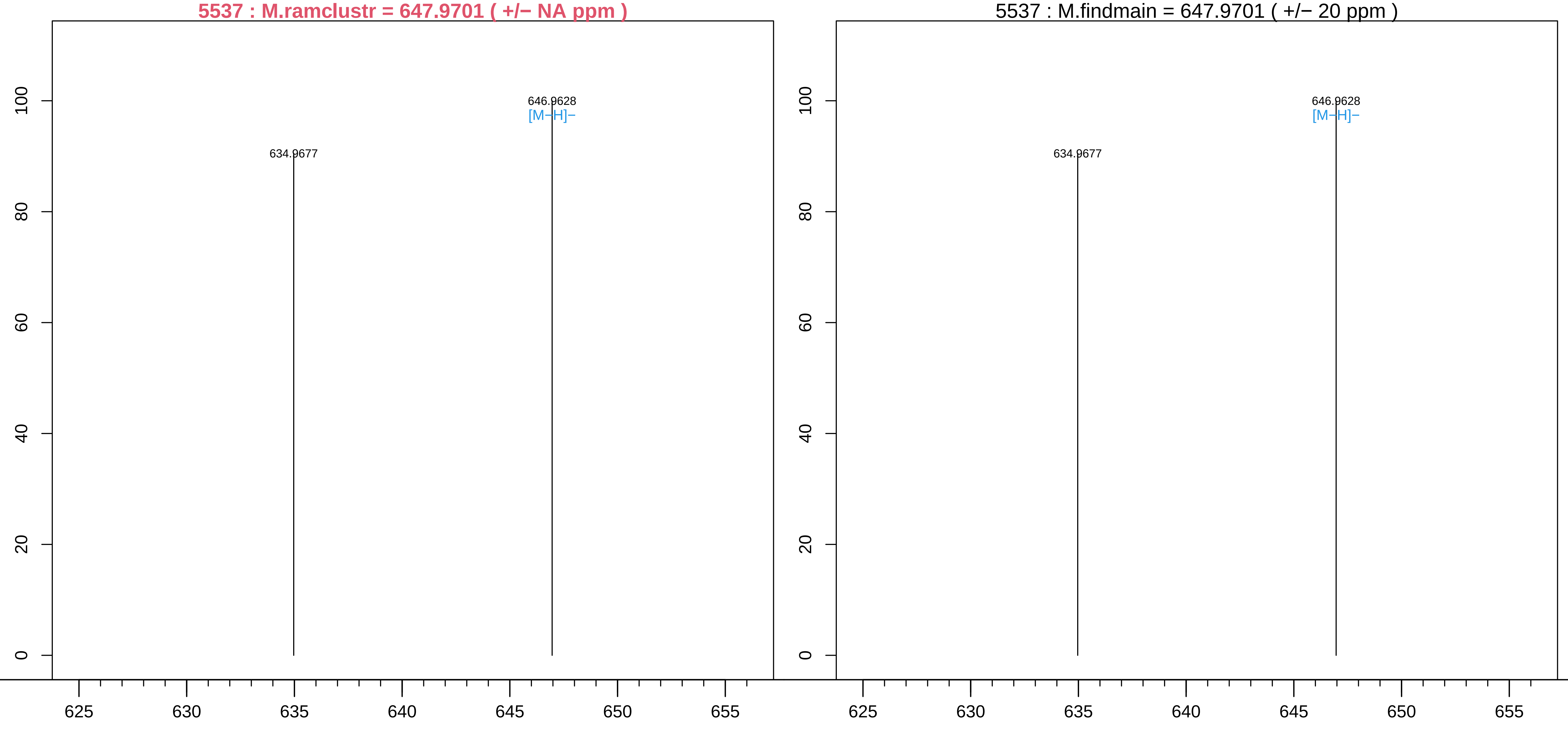

5537 : M.ramclustr = 647.9701 (+/− NA ppm)
5537 : M.findmain = 647.9701 (+/− 20 ppm)
646.9628
[M−H]−
634.9677
0
20
40
60
80
100
625
630
635
640
645
650
655

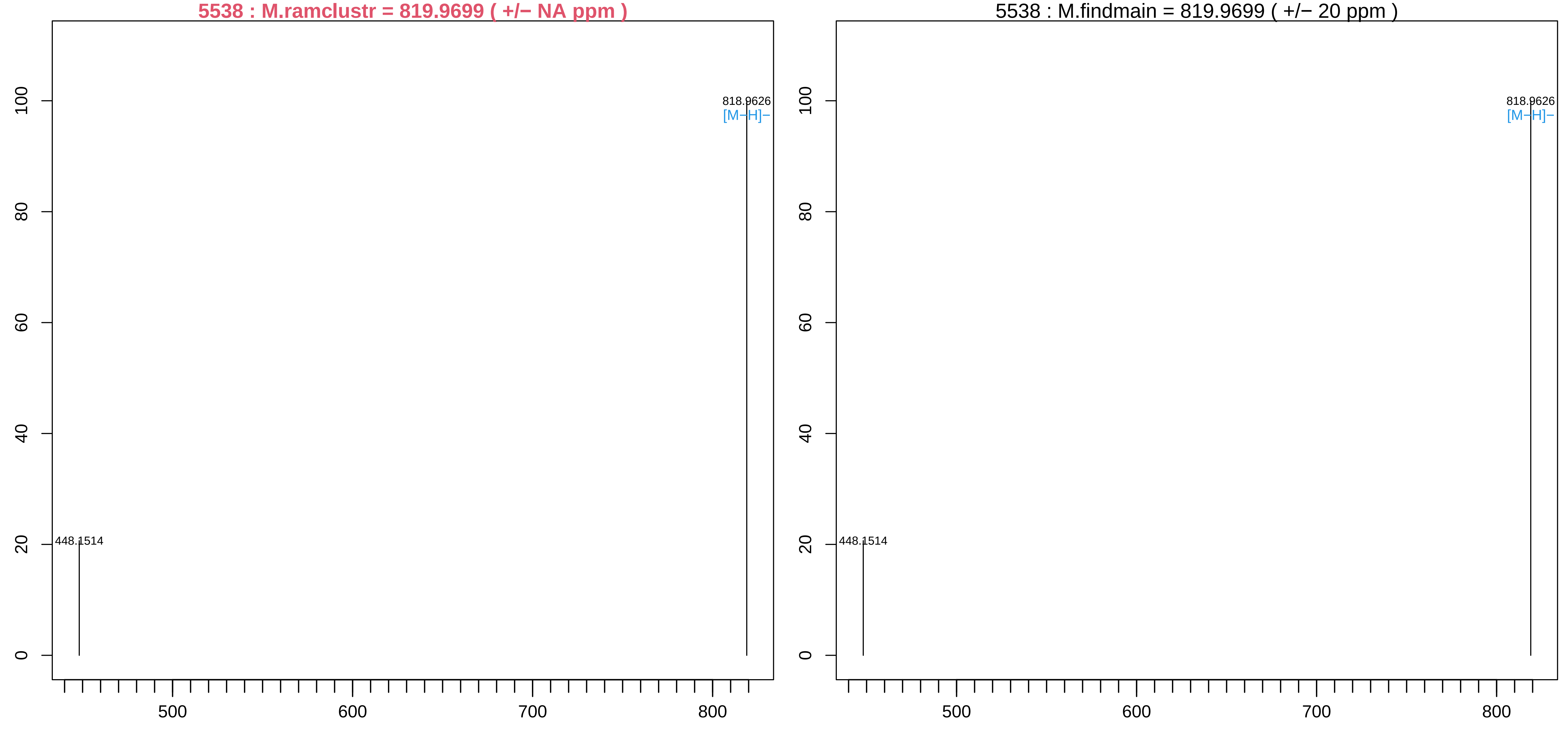
5538 : M.ramclustr = 819.9699 (+/− NA ppm)
818.9626
[M−H]−
448.1514
0
20
40
60
80
100
500
600
700
800
5538 : M.findmain = 819.9699 (+/− 20 ppm)
818.9626
[M−H]−
448.1514
0
20
40
60
80
100
500
600
700
800

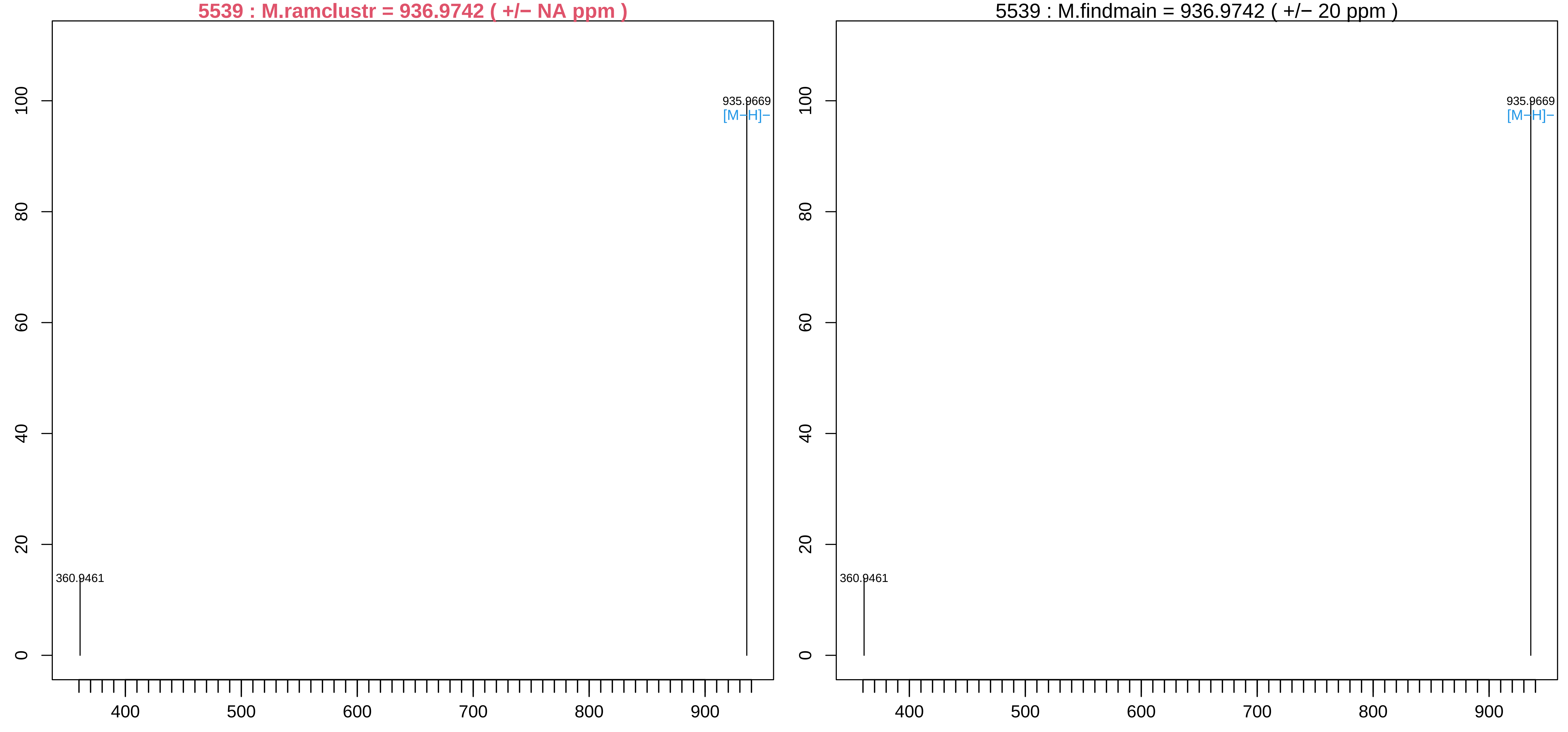

5539 : M.ramclustr = 936.9742 (+/− NA ppm)
935.9669
[M−H]−
360.9461
5539 : M.findmain = 936.9742 (+/− 20 ppm)
935.9669
[M−H]−
360.9461
0
20
40
60
80
100
400
500
600
700
800
900

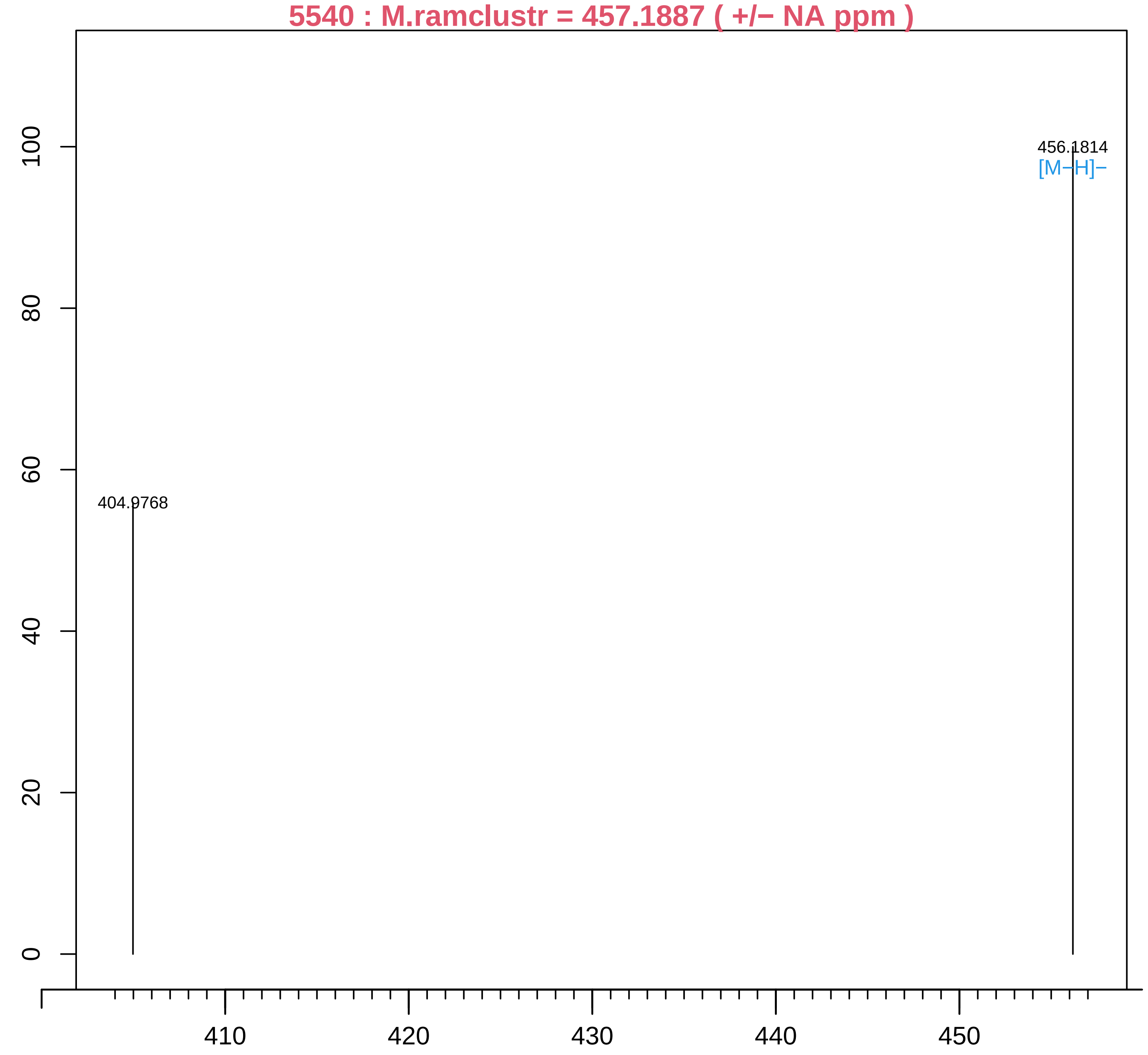

5540 : M.ramclustr = 457.1887 (+/− NA ppm)
456.1814
[M−H]−
404.9768
0
20
40
60
80
100
410
420
430
440
450

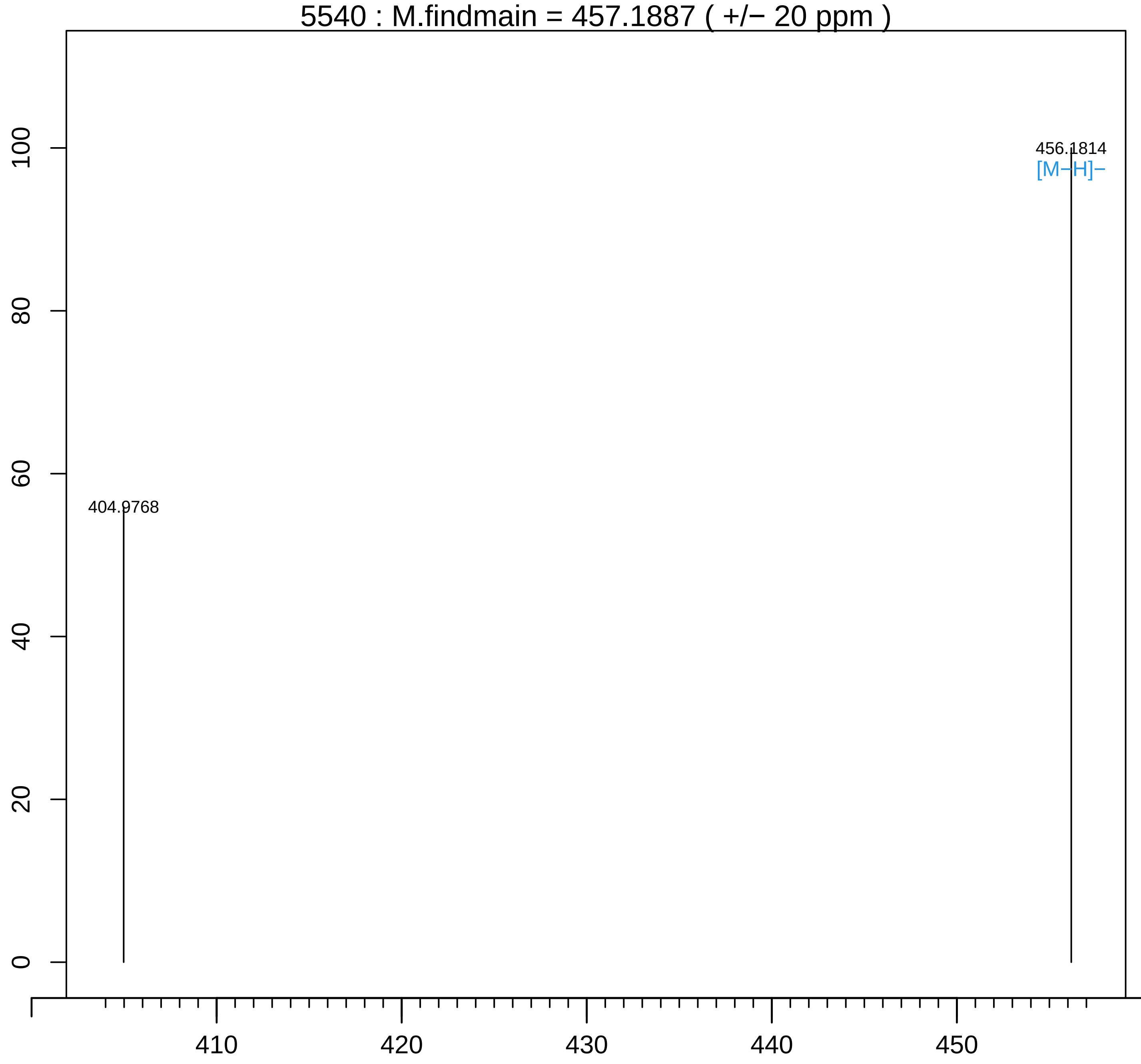

5540 : M.findmain = 457.1887 (+/− 20 ppm)
456.1814
[M−H]−
404.9768
0
20
40
60
80
100
410
420
430
440
450

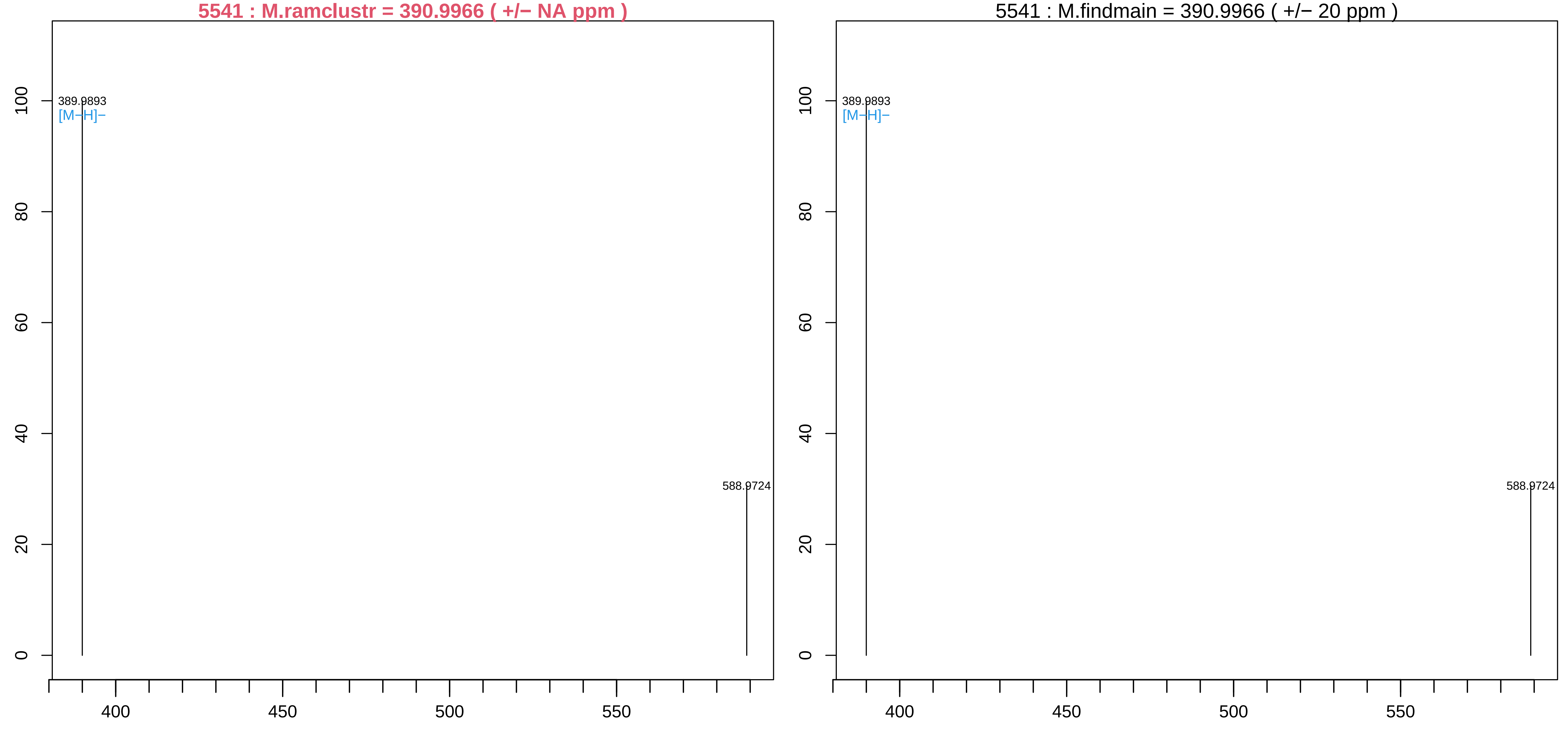

5541 : M.ramclustr = 390.9966 (+/− NA ppm)
389.9893
[M−H]−
588.9724
5541 : M.findmain = 390.9966 (+/− 20 ppm)
389.9893
[M−H]−
588.9724
0
20
40
60
80
100
400
450
500
550

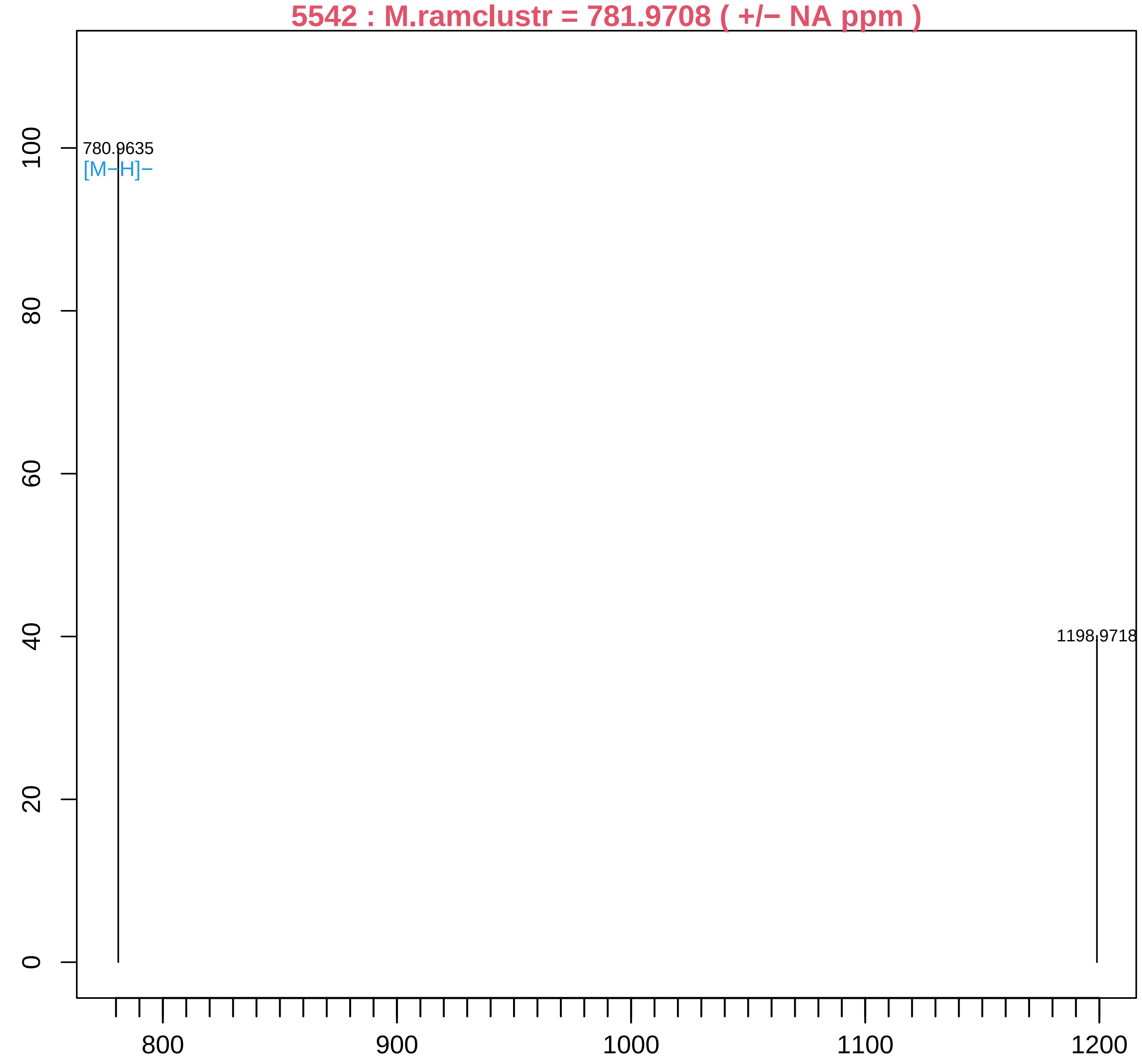

5542 : M.ramclustr = 781.9708 (+/− NA ppm)
780.9635
[M−H]−
1198.9718
0
20
40
60
80
100
800
900
1000
1100
1200

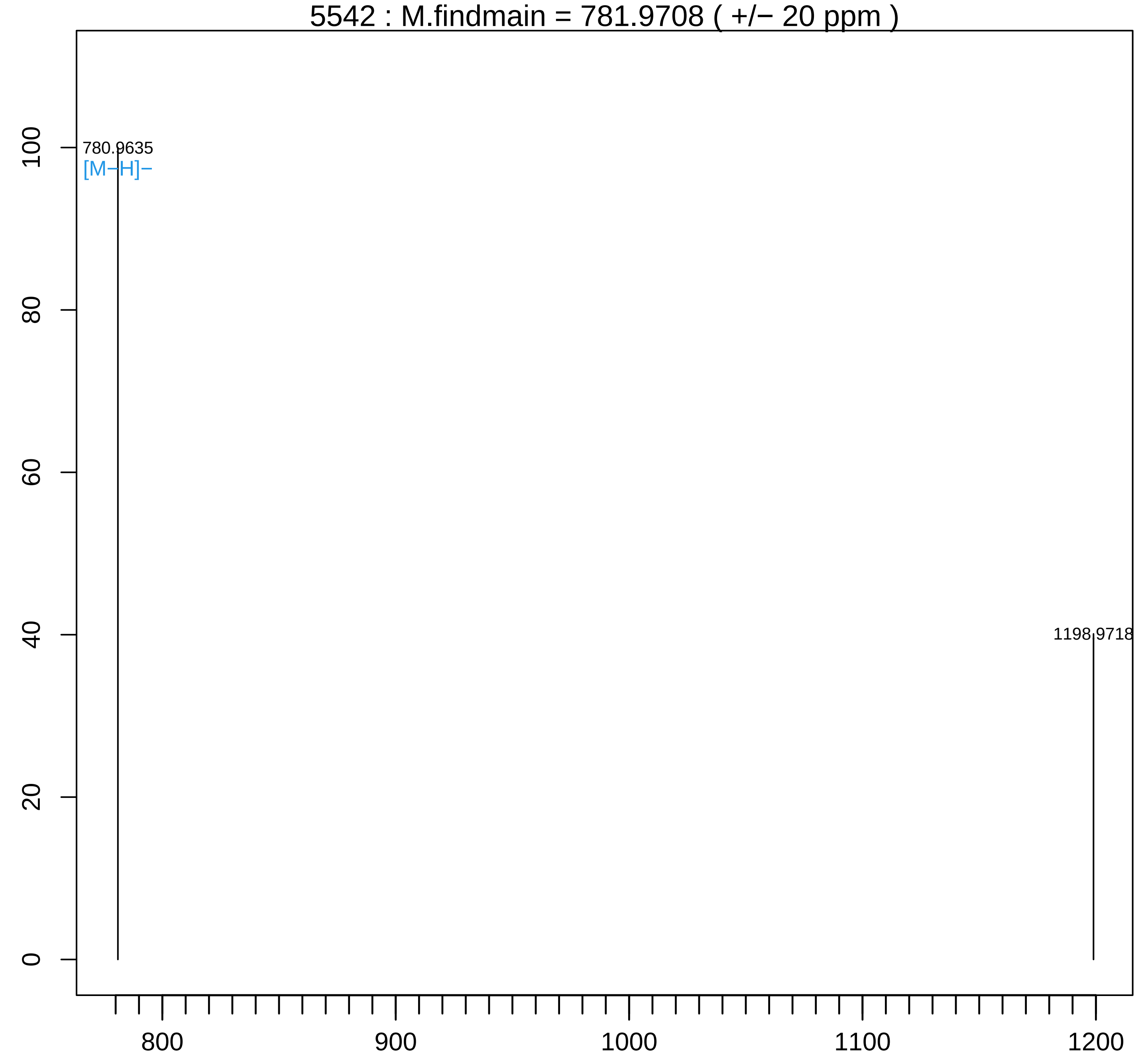

5542 : M.findmain = 781.9708 (+/− 20 ppm)
780.9635
[M−H]−
1198.9718
0
20
40
60
80
100
800
900
1000
1100
1200

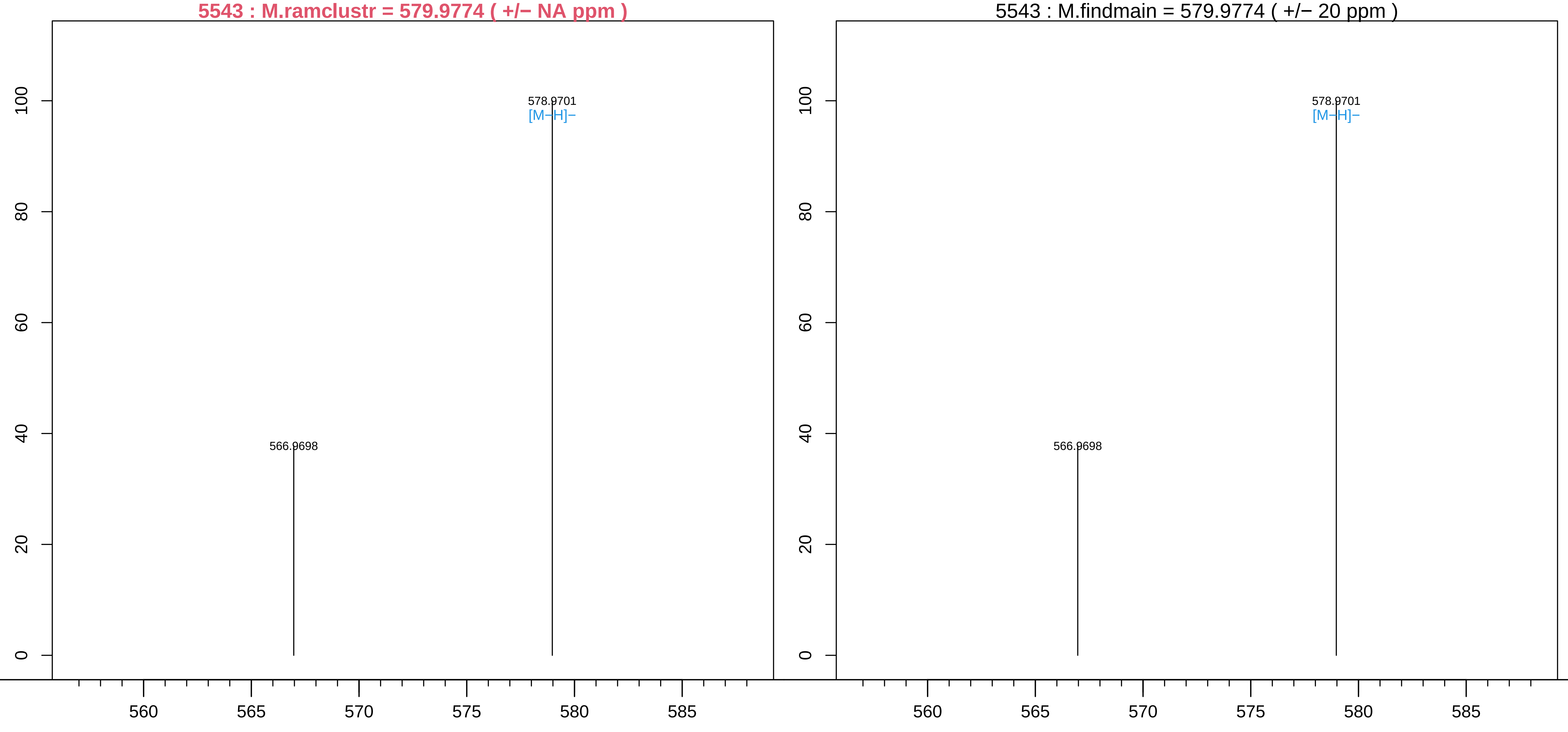

5543 : M.ramclustr = 579.9774 (+/− NA ppm)
5543 : M.findmain = 579.9774 (+/− 20 ppm)
578.9701
[M−H]−
566.9698
0
20
40
60
80
100
560
565
570
575
580
585

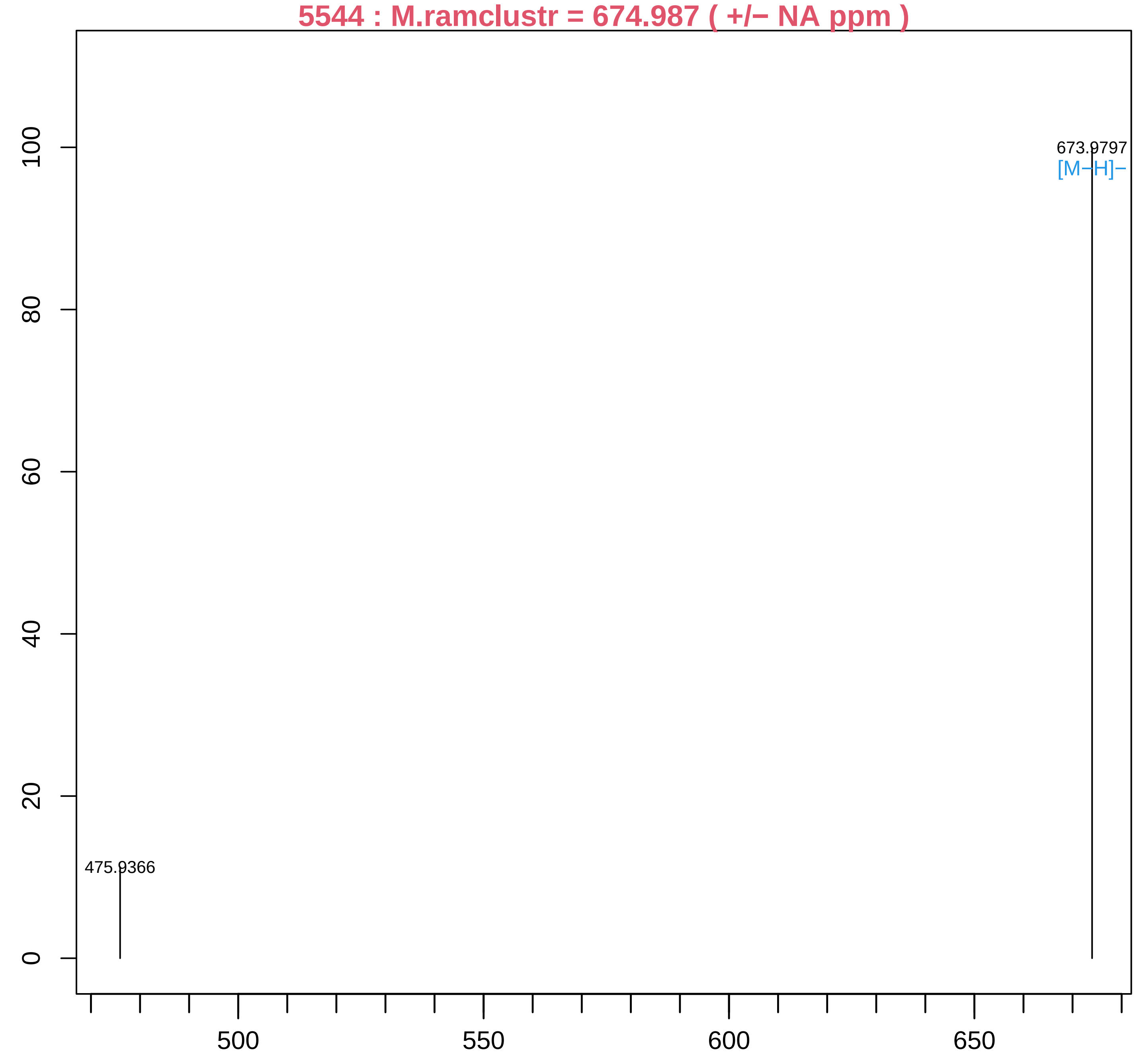
5544 : M.ramclustr = 674.987 (+/− NA ppm)
673.9797
[M−H]−
475.9366
0
20
40
60
80
100
500
550
600
650

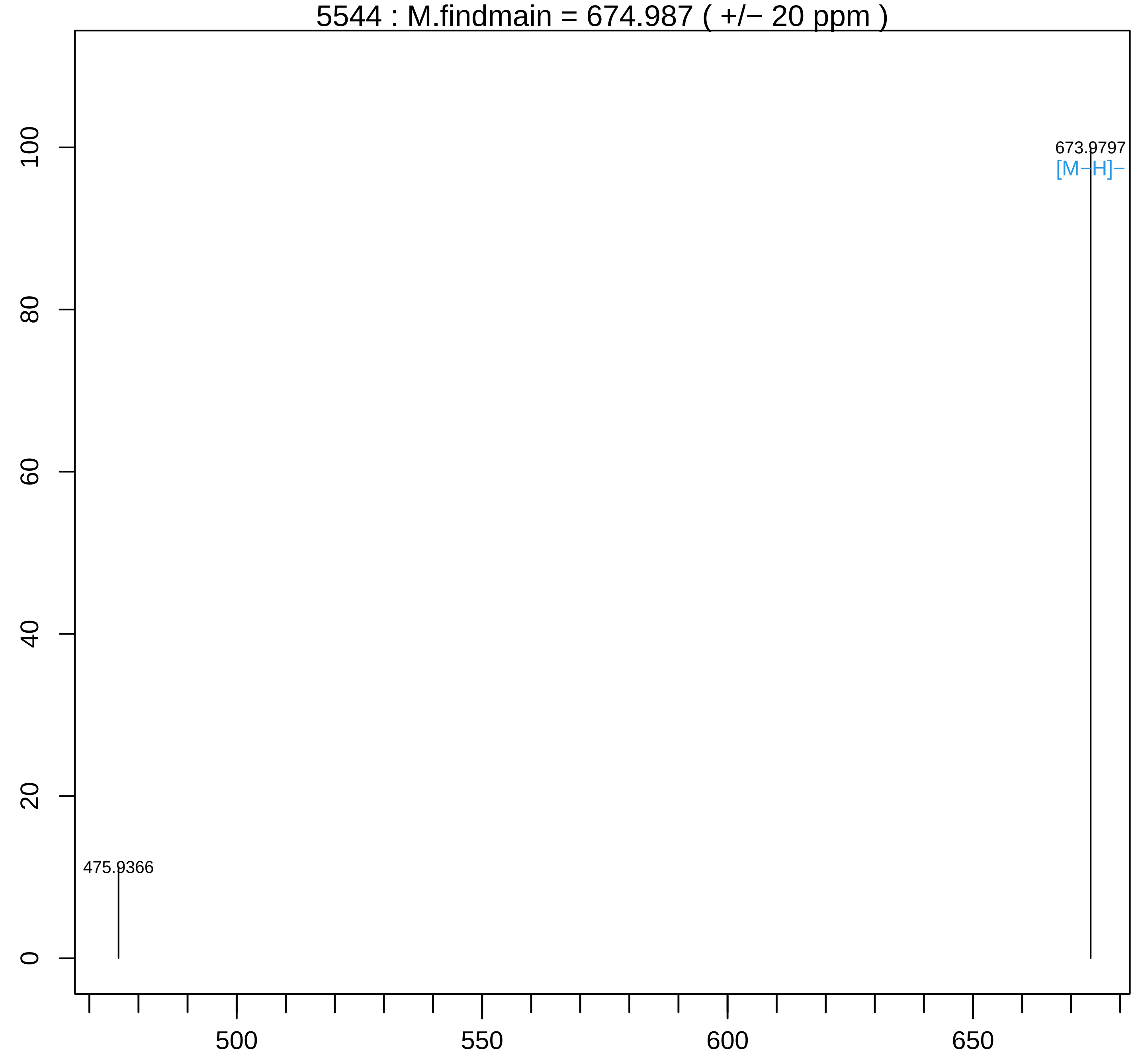
5544 : M.findmain = 674.987 (+/− 20 ppm)
673.9797
[M−H]−
475.9366
0
20
40
60
80
100
500
550
600
650

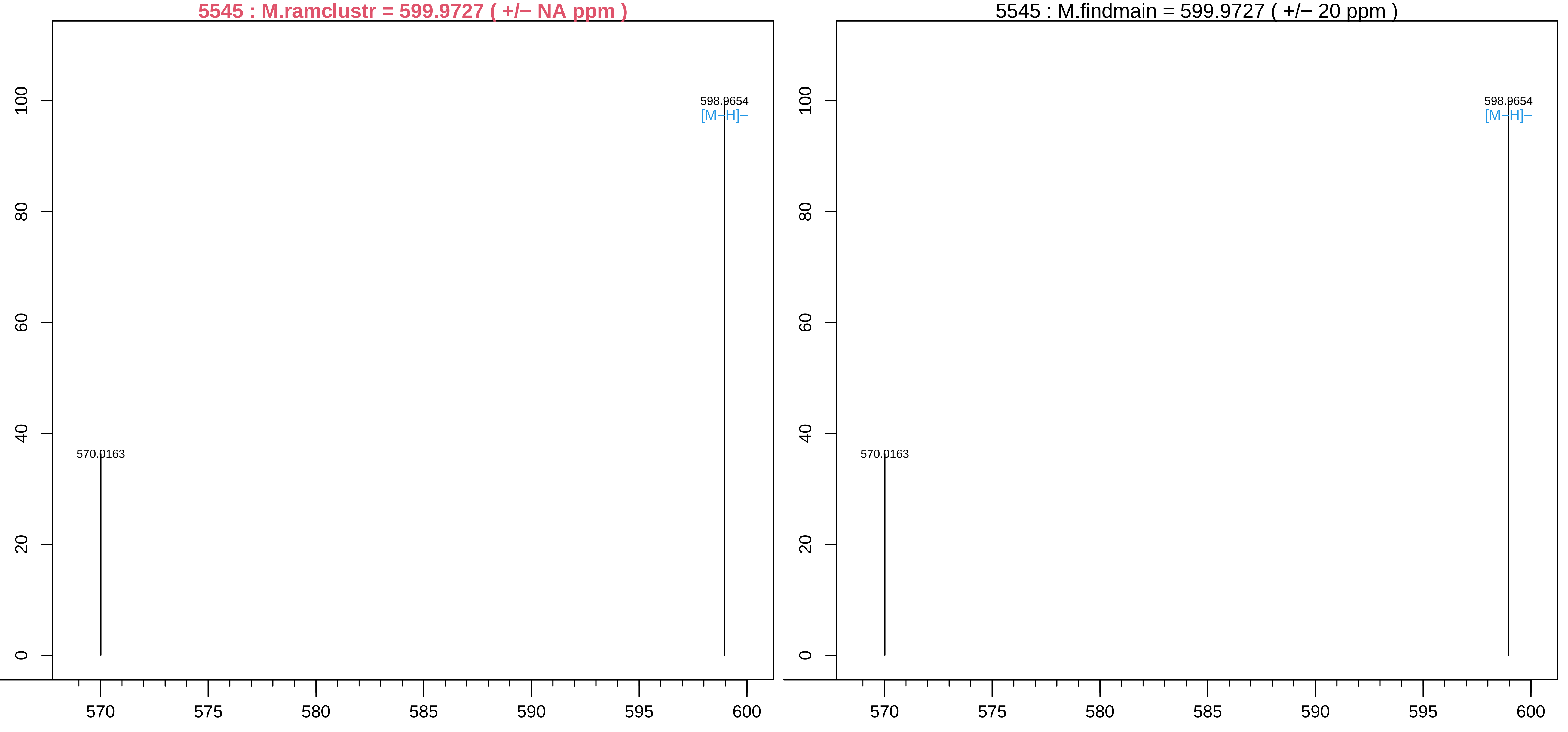

5545 : M.ramclustr = 599.9727 (+/− NA ppm)
598.9654
[M−H]−
570.0163
0
20
40
60
80
100
570
575
580
585
590
595
600
5545 : M.findmain = 599.9727 (+/− 20 ppm)
598.9654
[M−H]−
570.0163
0
20
40
60
80
100
570
575
580
585
590
595
600

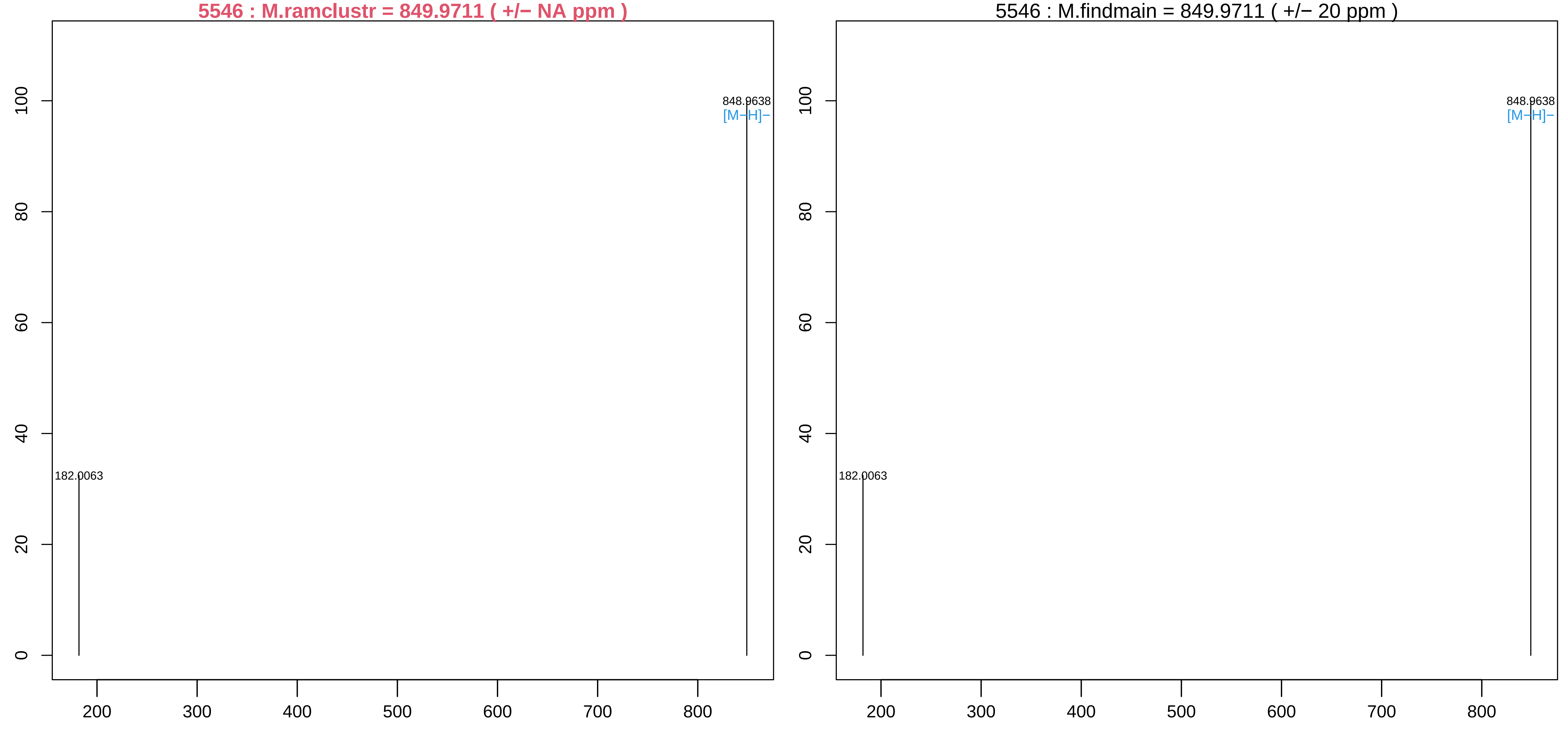

5546 : M.ramclustr = 849.9711 (+/− NA ppm)
848.9638
[M−H]−
182.0063
5546 : M.findmain = 849.9711 (+/− 20 ppm)
848.9638
[M−H]−
182.0063
0
20
40
60
80
100
200
300
400
500
600
700
800

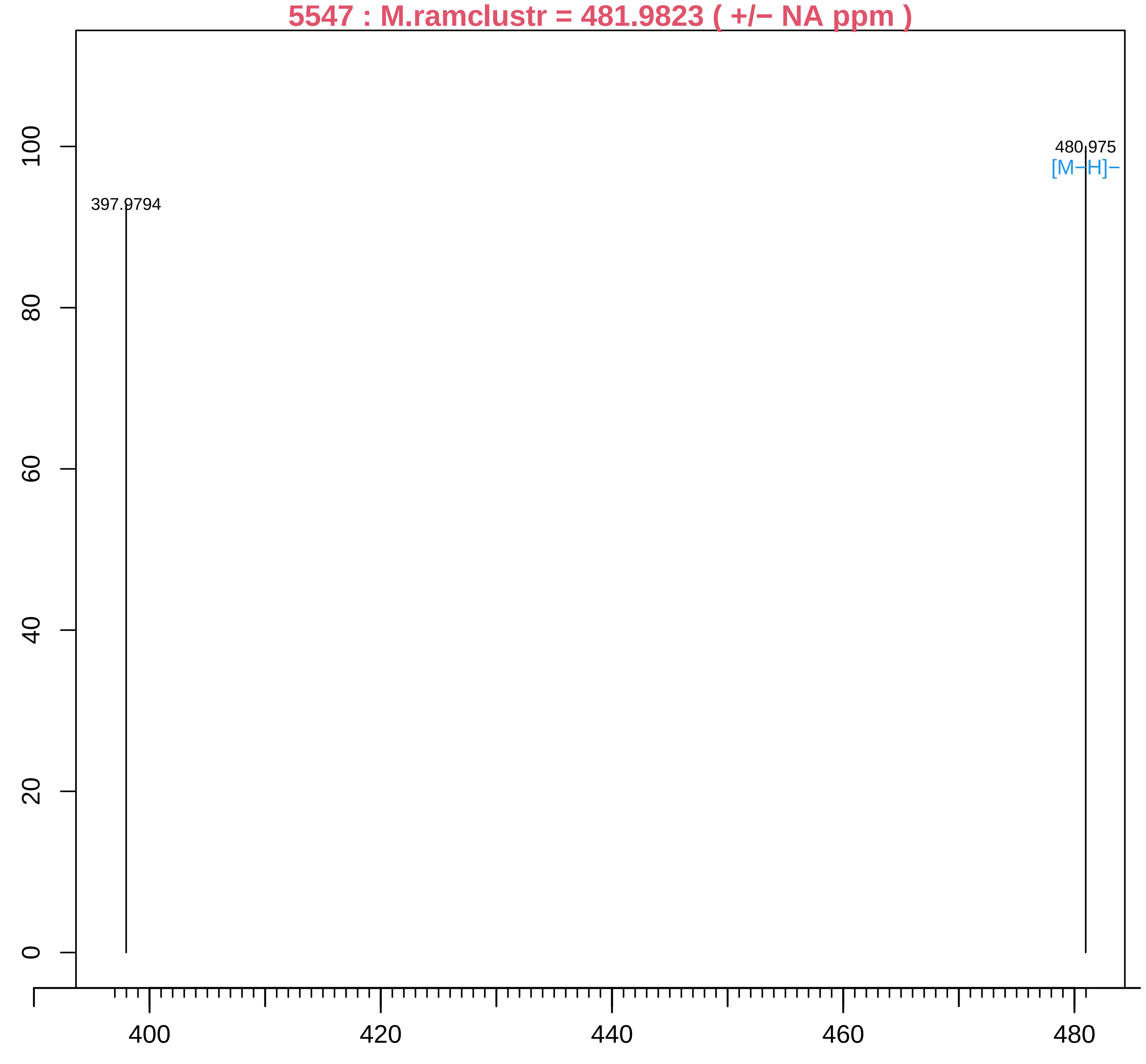
5547 : M.ramclustr = 481.9823 (+/− NA ppm)
480.975
[M−H]−
397.9794
0
20
40
60
80
100
400
420
440
460
480

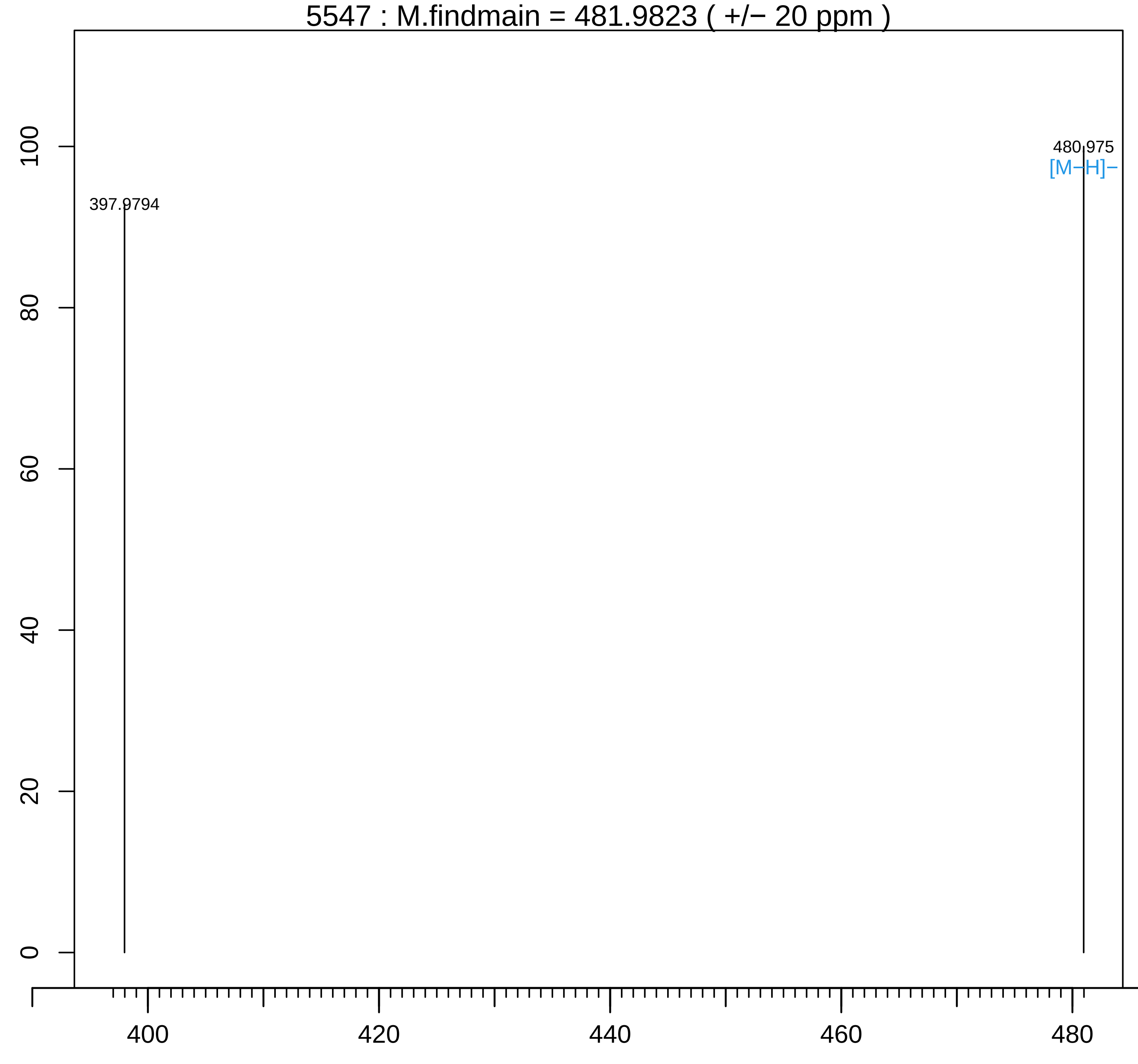
5547 : M.findmain = 481.9823 (+/− 20 ppm)
480.975
[M−H]−
397.9794
0
20
40
60
80
100
400
420
440
460
480

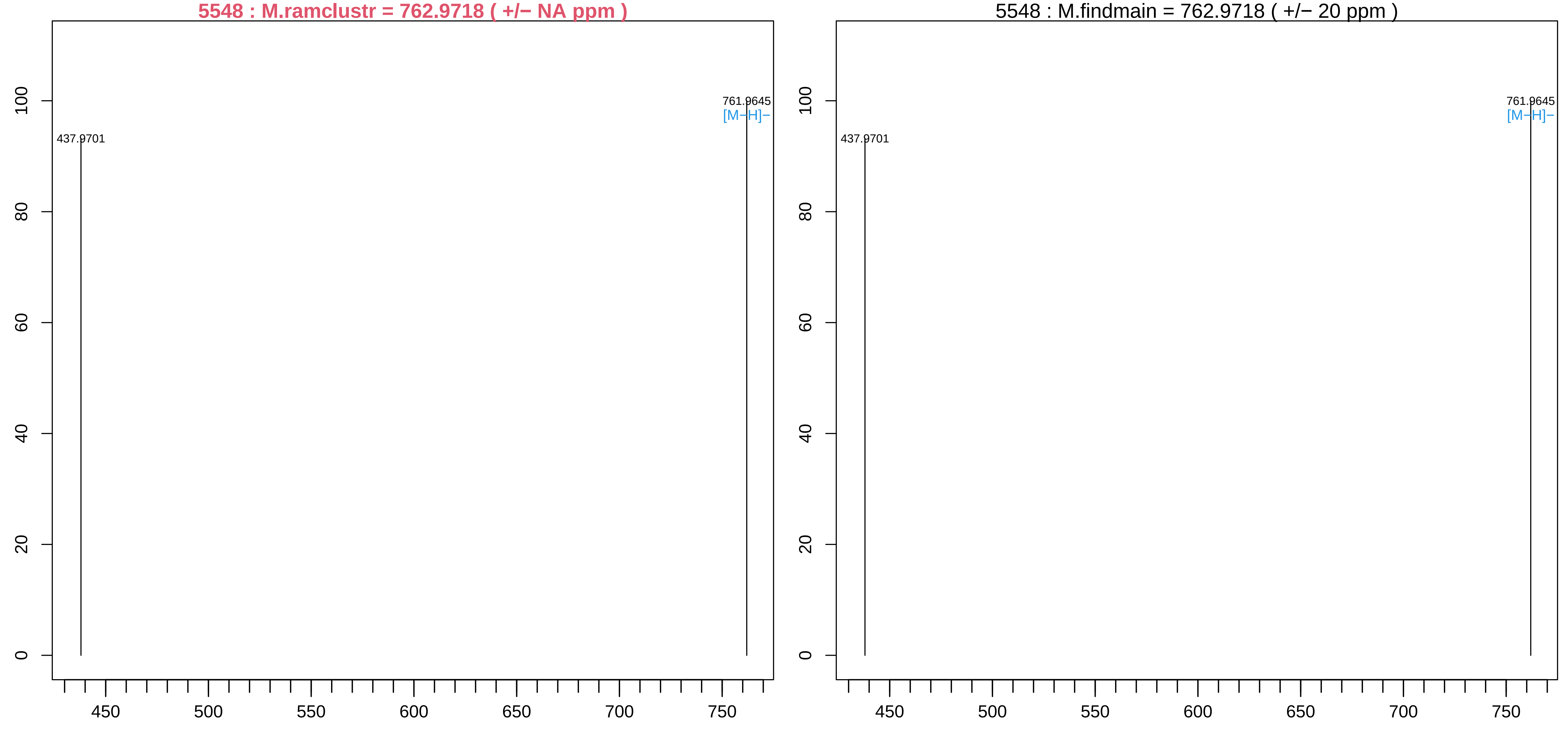
5548 : M.ramclustr = 762.9718 (+/− NA ppm)
5548 : M.findmain = 762.9718 (+/− 20 ppm)
761.9645
[M−H]−
437.9701
761.9645
[M−H]−
437.9701
0
20
40
60
80
100
450
500
550
600
650
700
750

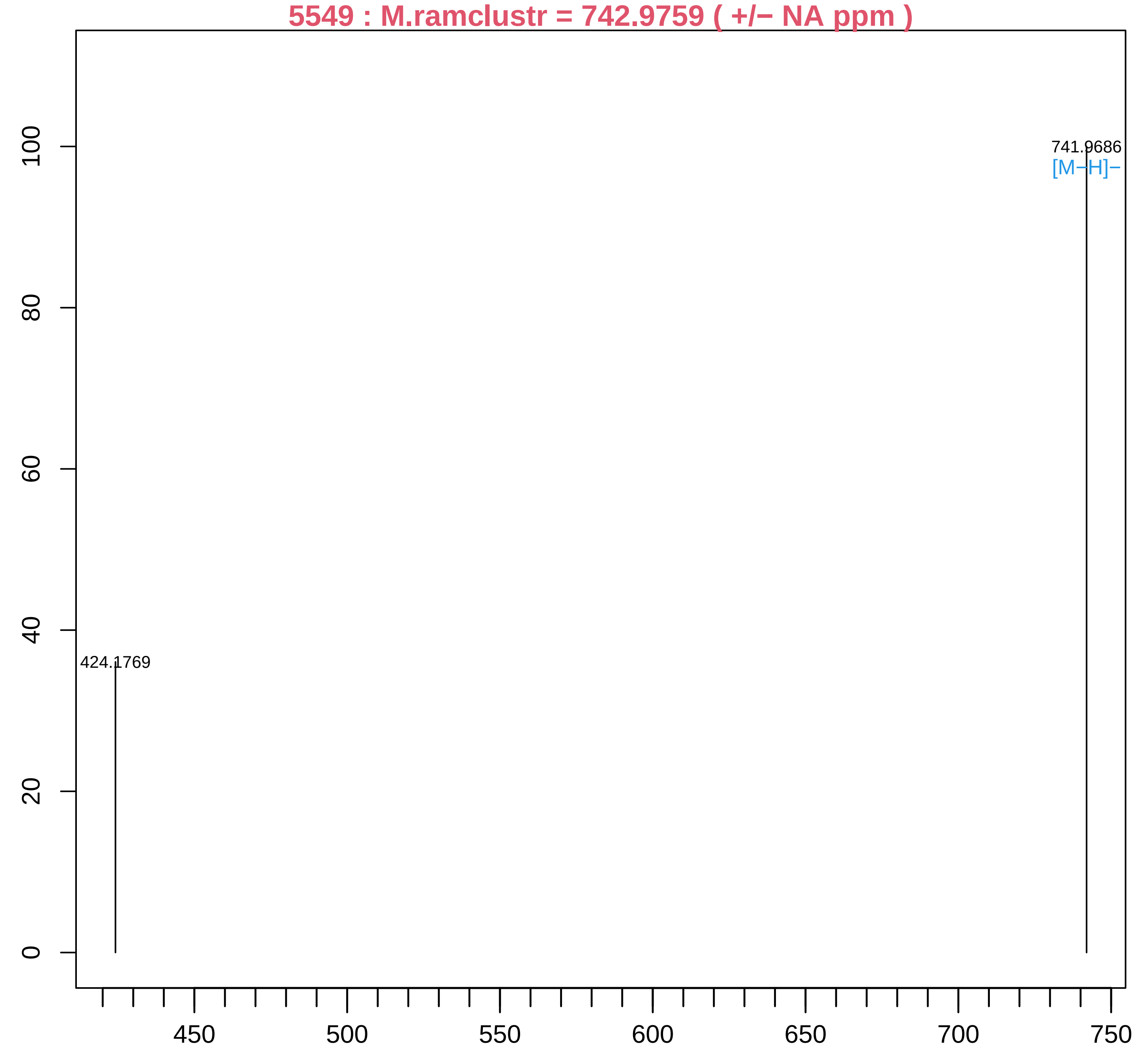
5549 : M.ramclustr = 742.9759 (+/− NA ppm)
741.9686
[M−H]−
424.1769
0
20
40
60
80
100
450
500
550
600
650
700
750

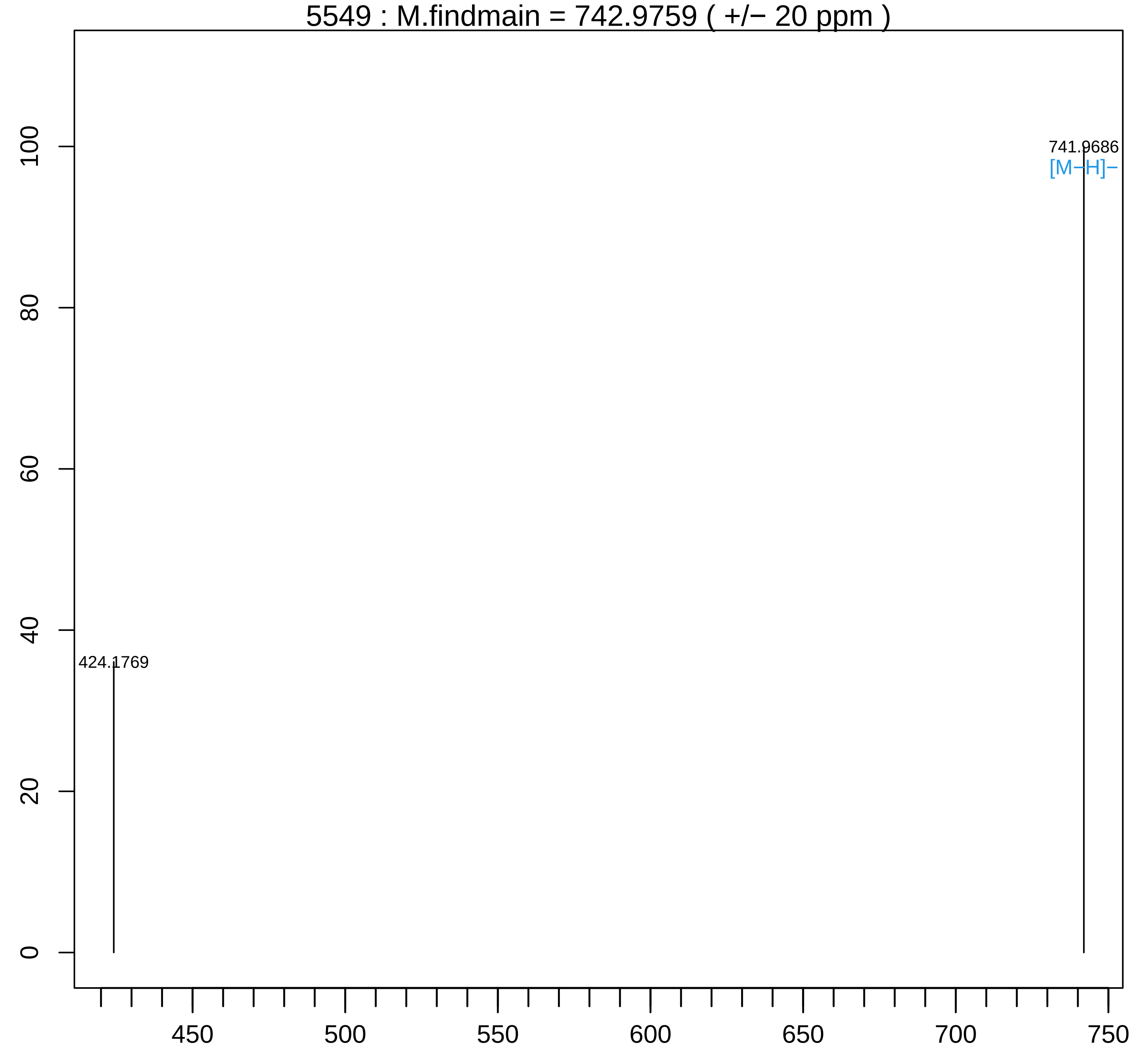
5549 : M.findmain = 742.9759 (+/− 20 ppm)
741.9686
[M−H]−
424.1769
0
20
40
60
80
100
450
500
550
600
650
700
750

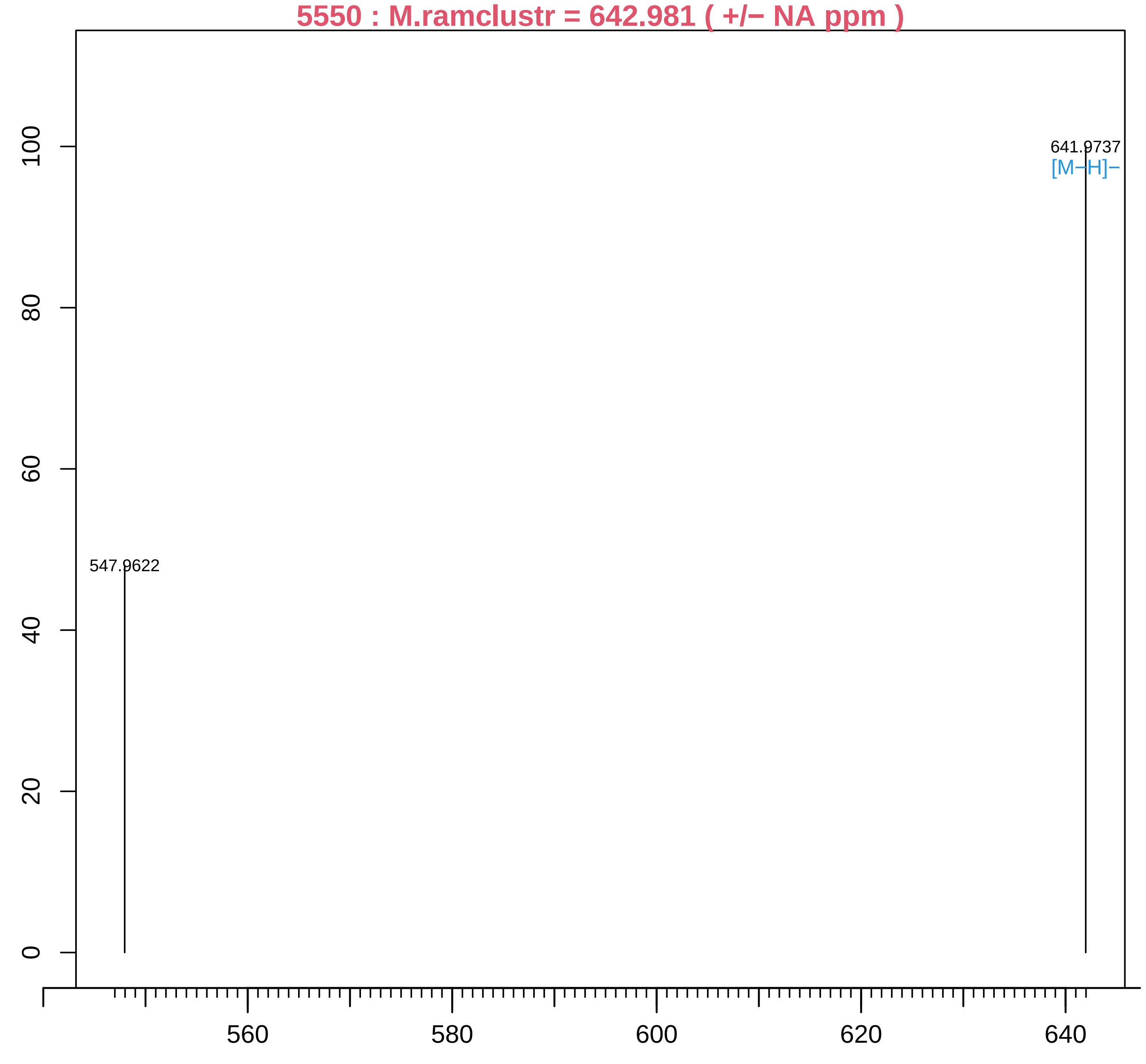
5550 : M.ramclustr = 642.981 (+/− NA ppm)
641.9737
[M−H]−
547.9622
0
20
40
60
80
100
560
580
600
620
640

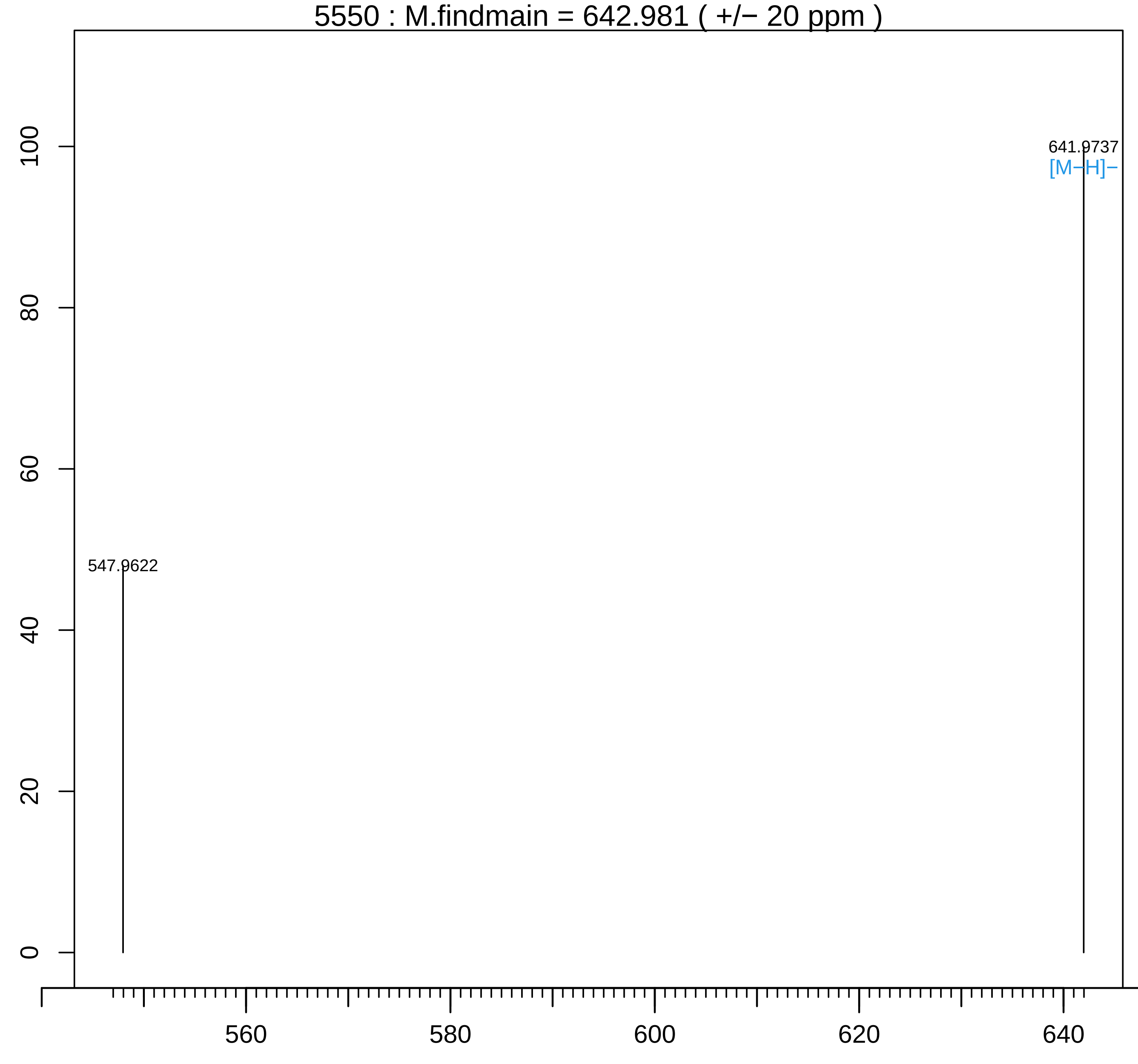
5550 : M.findmain = 642.981 (+/− 20 ppm)
641.9737
[M−H]−
547.9622
0
20
40
60
80
100
560
580
600
620
640

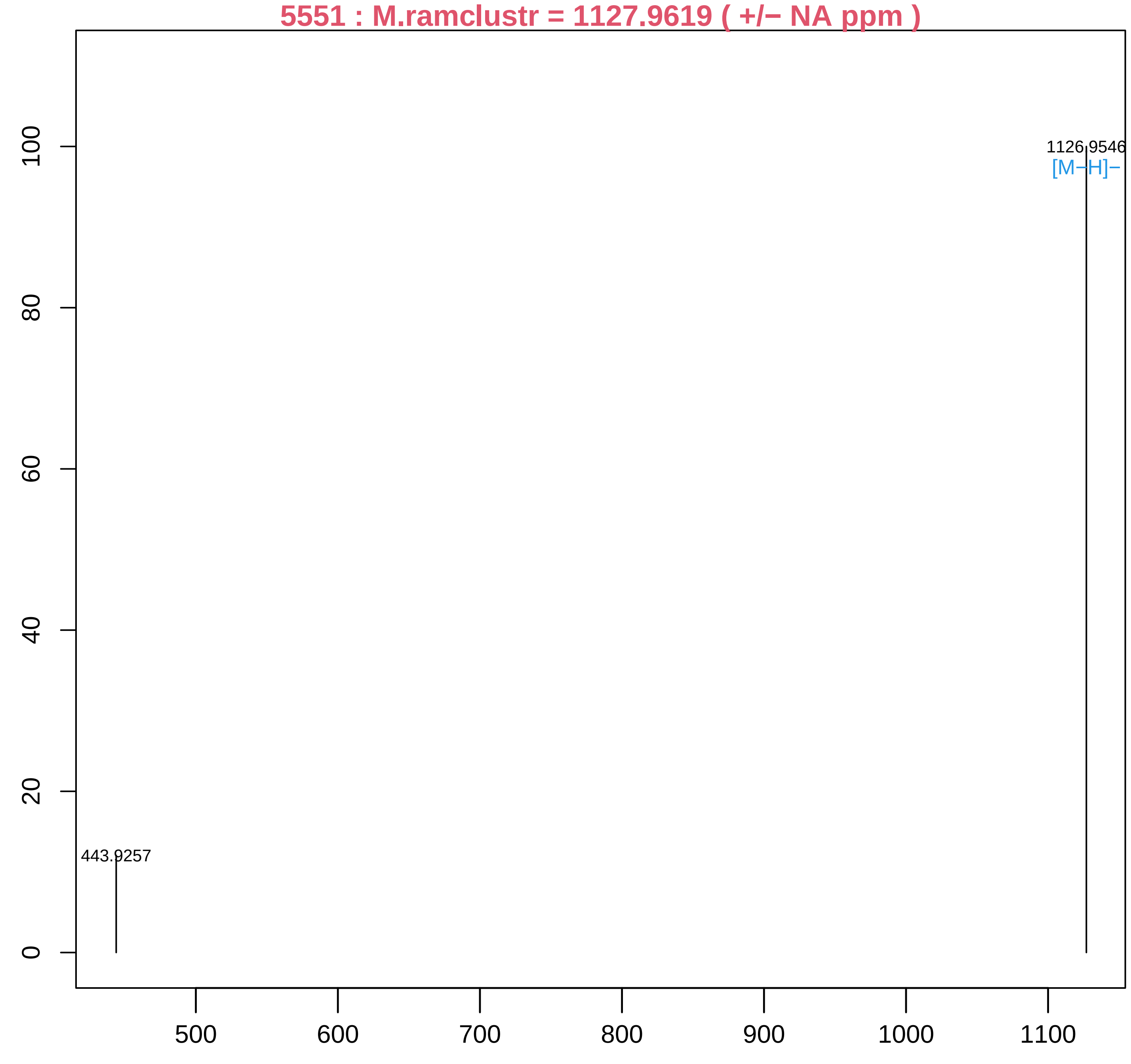
5551 : M.ramclustr = 1127.9619 (+/− NA ppm)
1126.9546
[M−H]−
443.9257
0
20
40
60
80
100
500
600
700
800
900
1000
1100

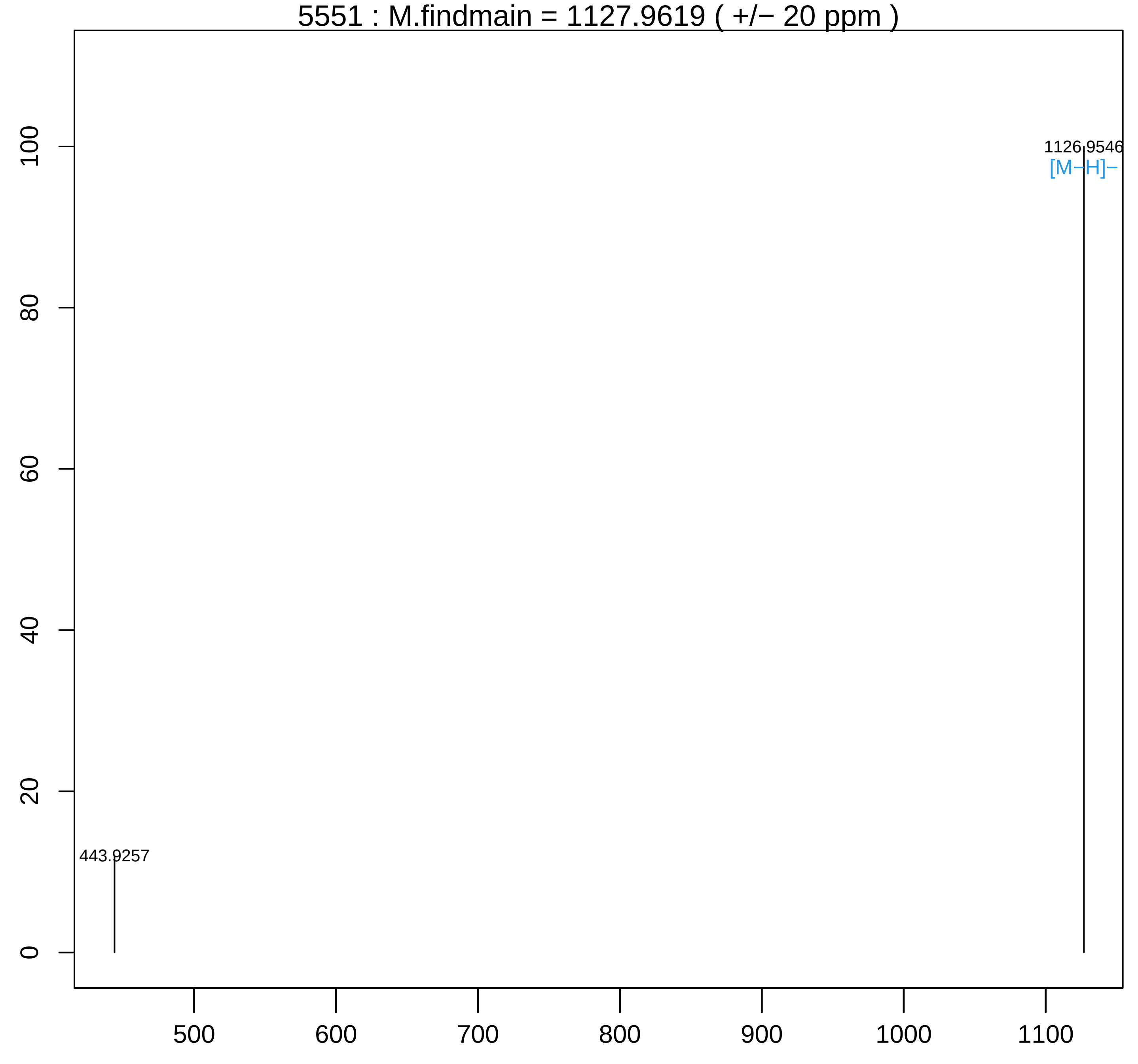
5551 : M.findmain = 1127.9619 (+/− 20 ppm)
1126.9546
[M−H]−
443.9257
0
20
40
60
80
100
500
600
700
800
900
1000
1100

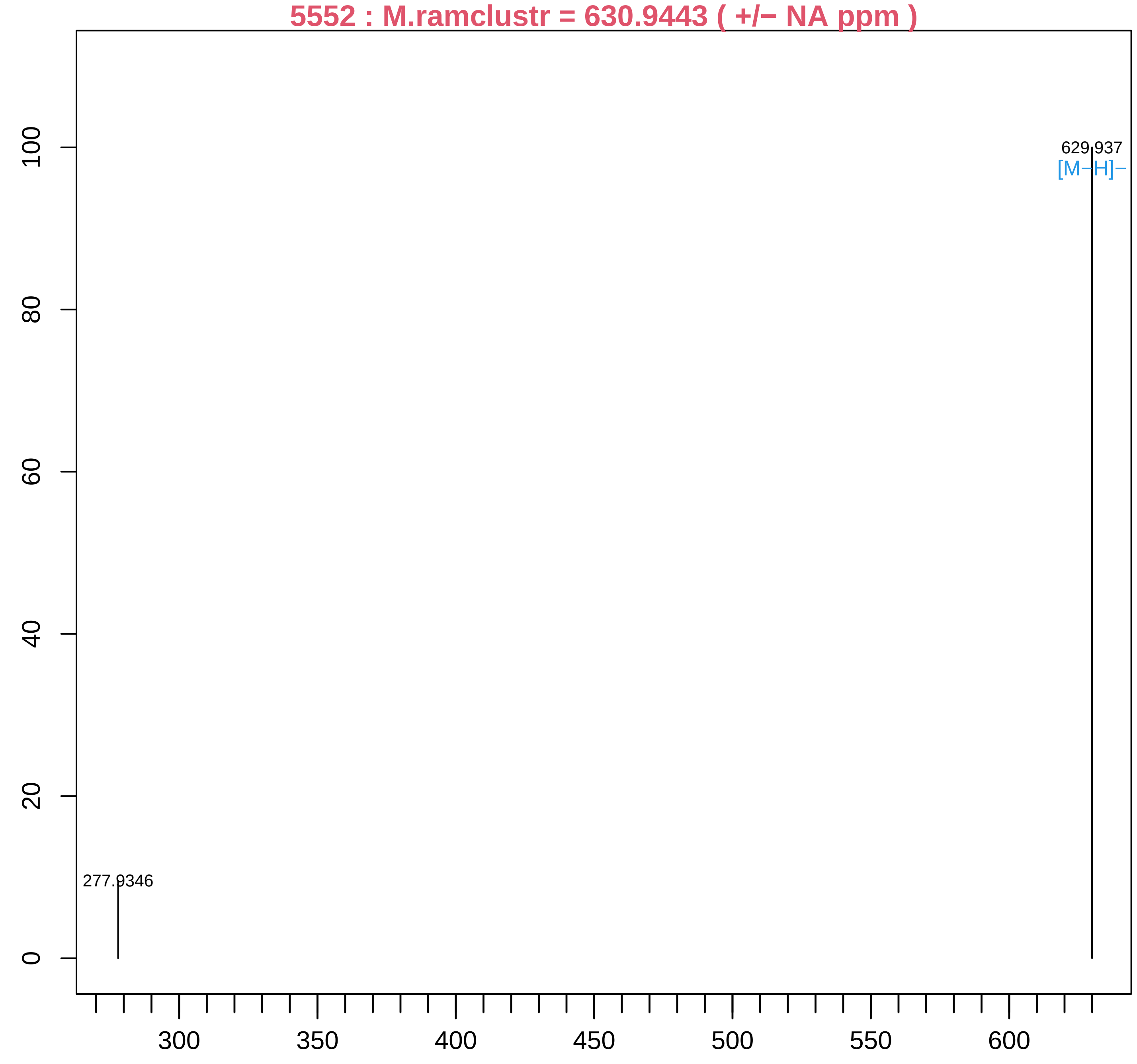

5552 : M.ramclustr = 630.9443 (+/− NA ppm)
629.937
[M−H]−
277.9346
0
20
40
60
80
100
300
350
400
450
500
550
600

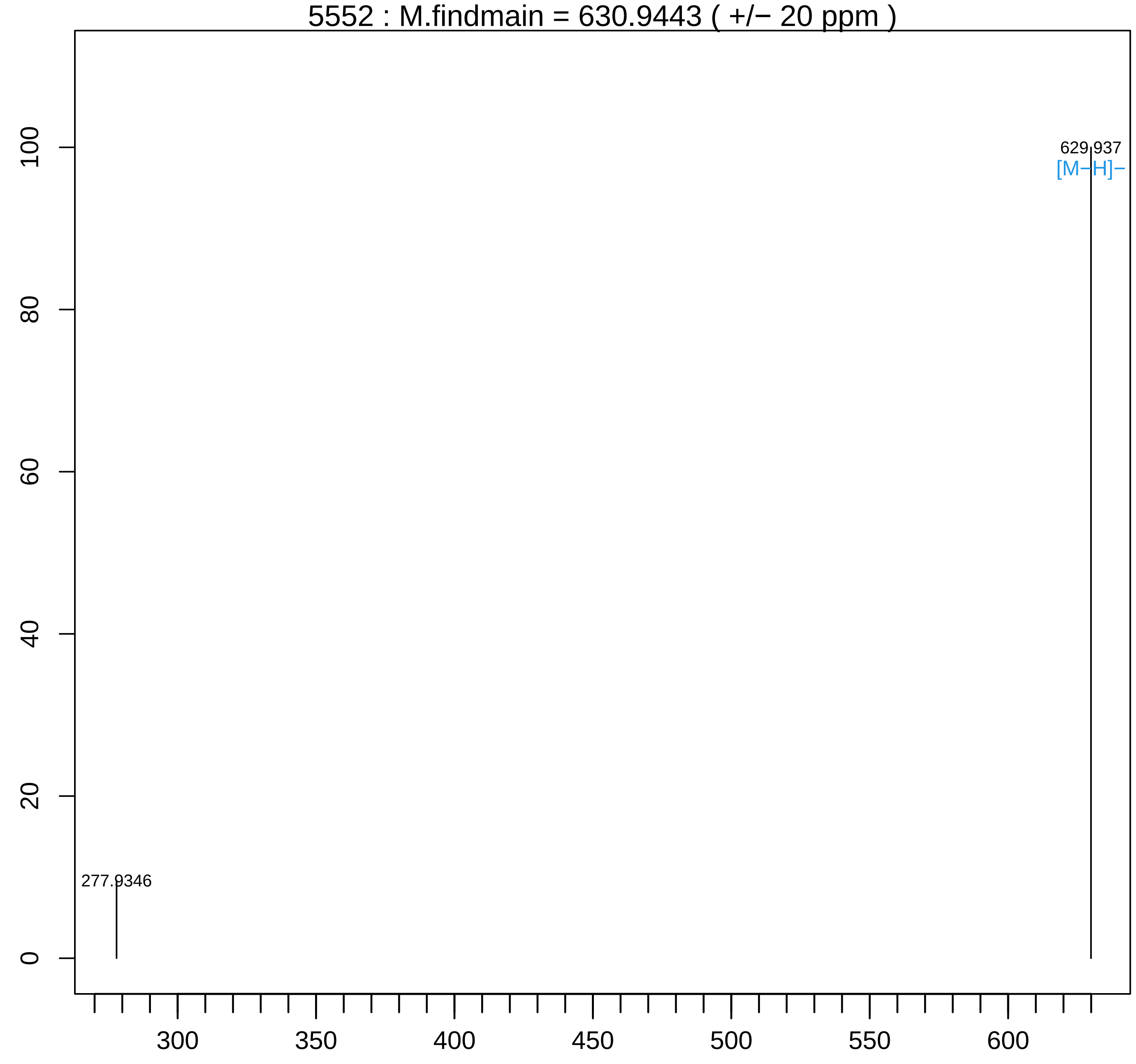

5552 : M.findmain = 630.9443 (+/− 20 ppm)
629.937
[M−H]−
277.9346
0
20
40
60
80
100
300
350
400
450
500
550
600

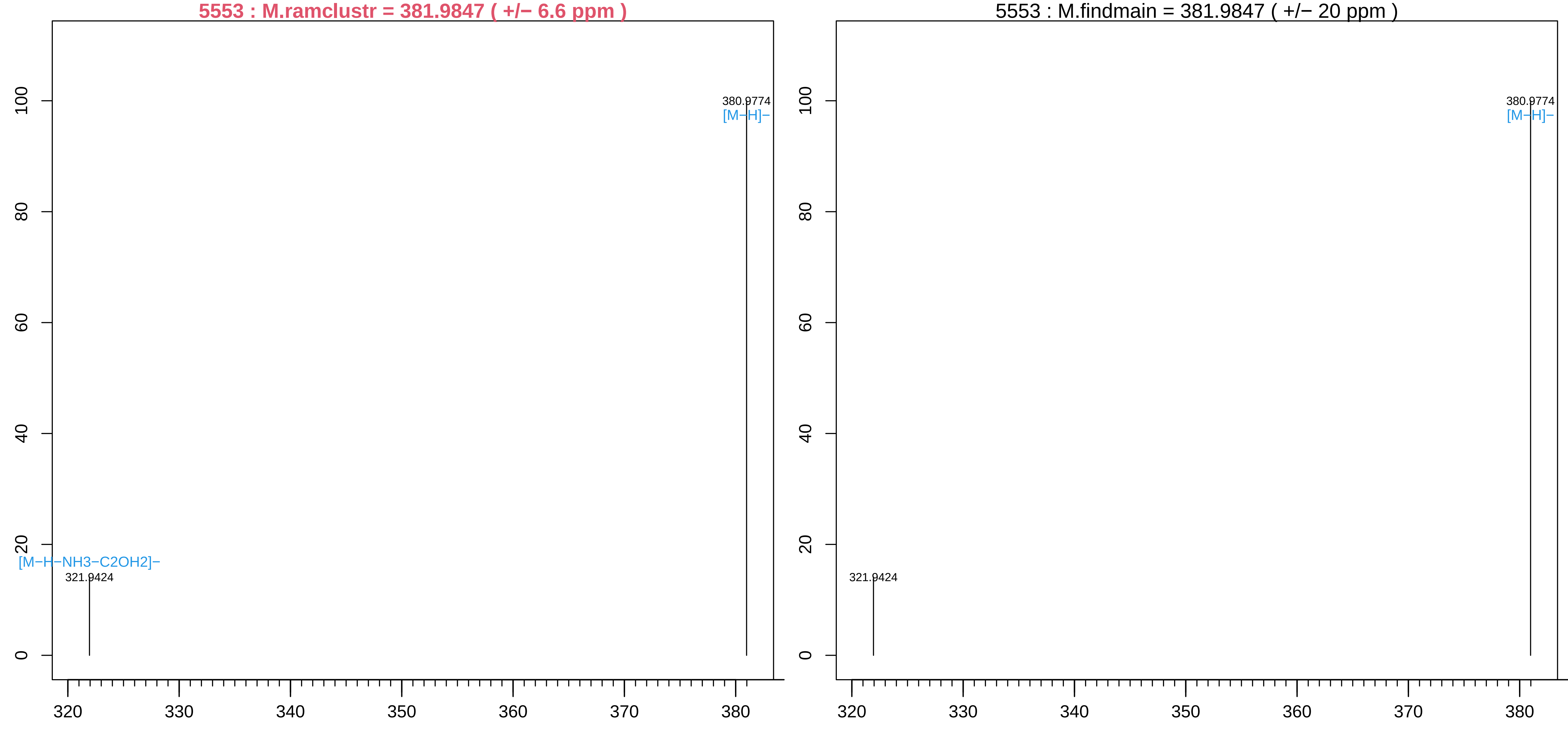
5553 : M.ramclustr = 381.9847 (+/− 6.6 ppm)
5553 : M.findmain = 381.9847 (+/− 20 ppm)
380.9774
[M−H]−
321.9424
[M−H−NH3−C2OH2]−
380.9774
[M−H]−
321.9424
0
20
40
60
80
100
320
330
340
350
360
370
380

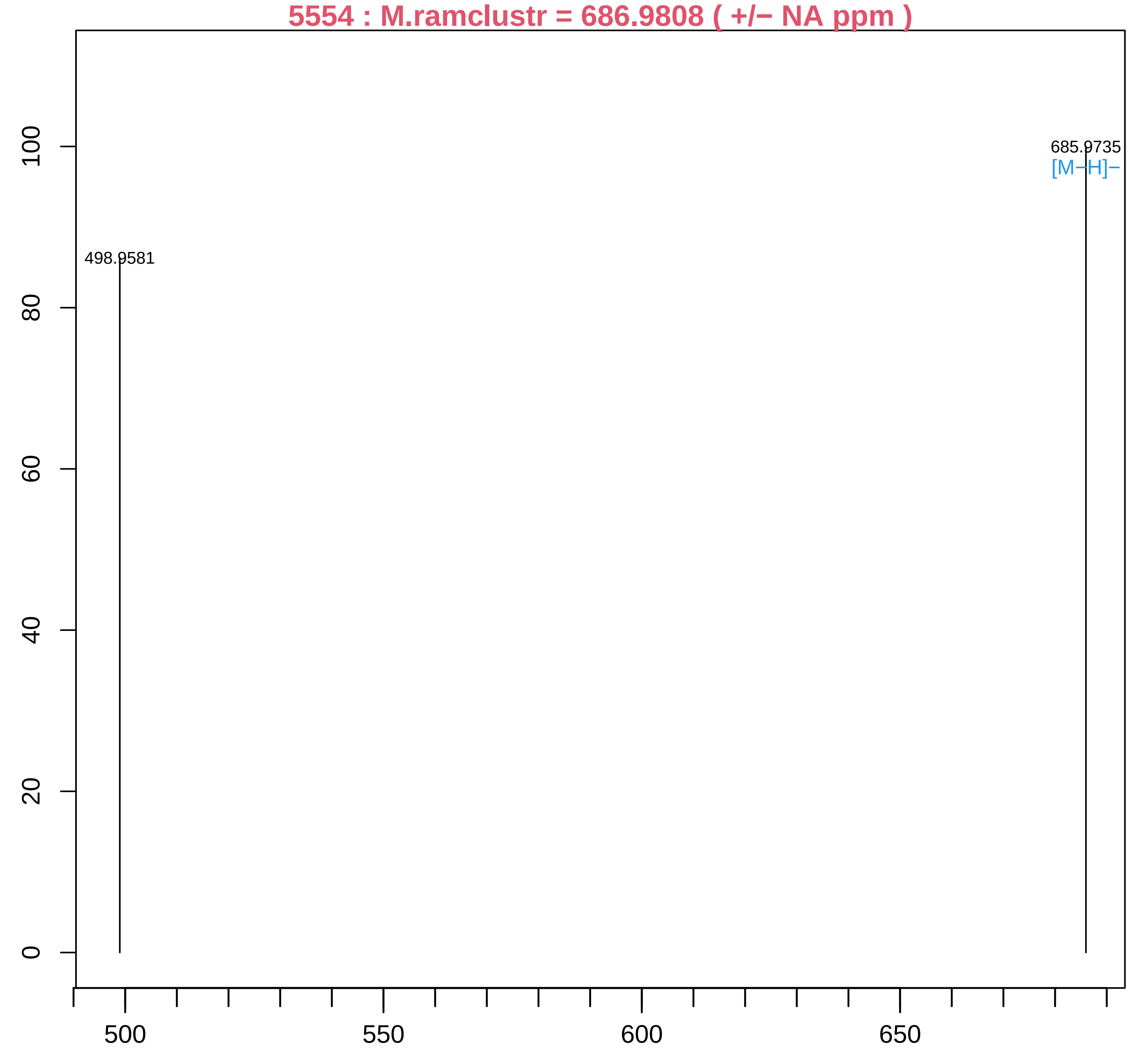
5554 : M.ramclustr = 686.9808 (+/− NA ppm)
685.9735
[M−H]−
498.9581
0
20
40
60
80
100
500
550
600
650

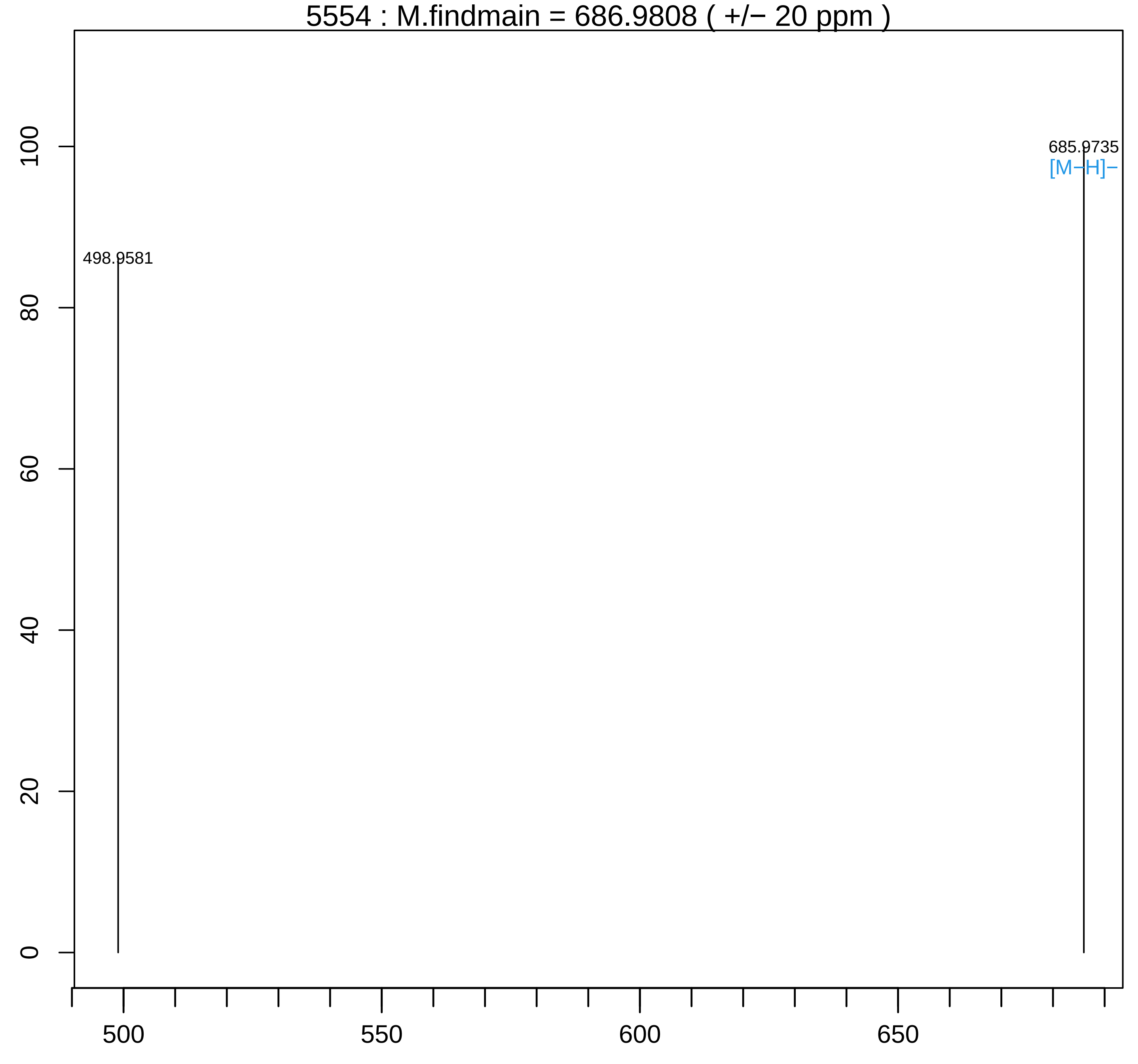
5554 : M.findmain = 686.9808 (+/− 20 ppm)
685.9735
[M−H]−
498.9581
0
20
40
60
80
100
500
550
600
650

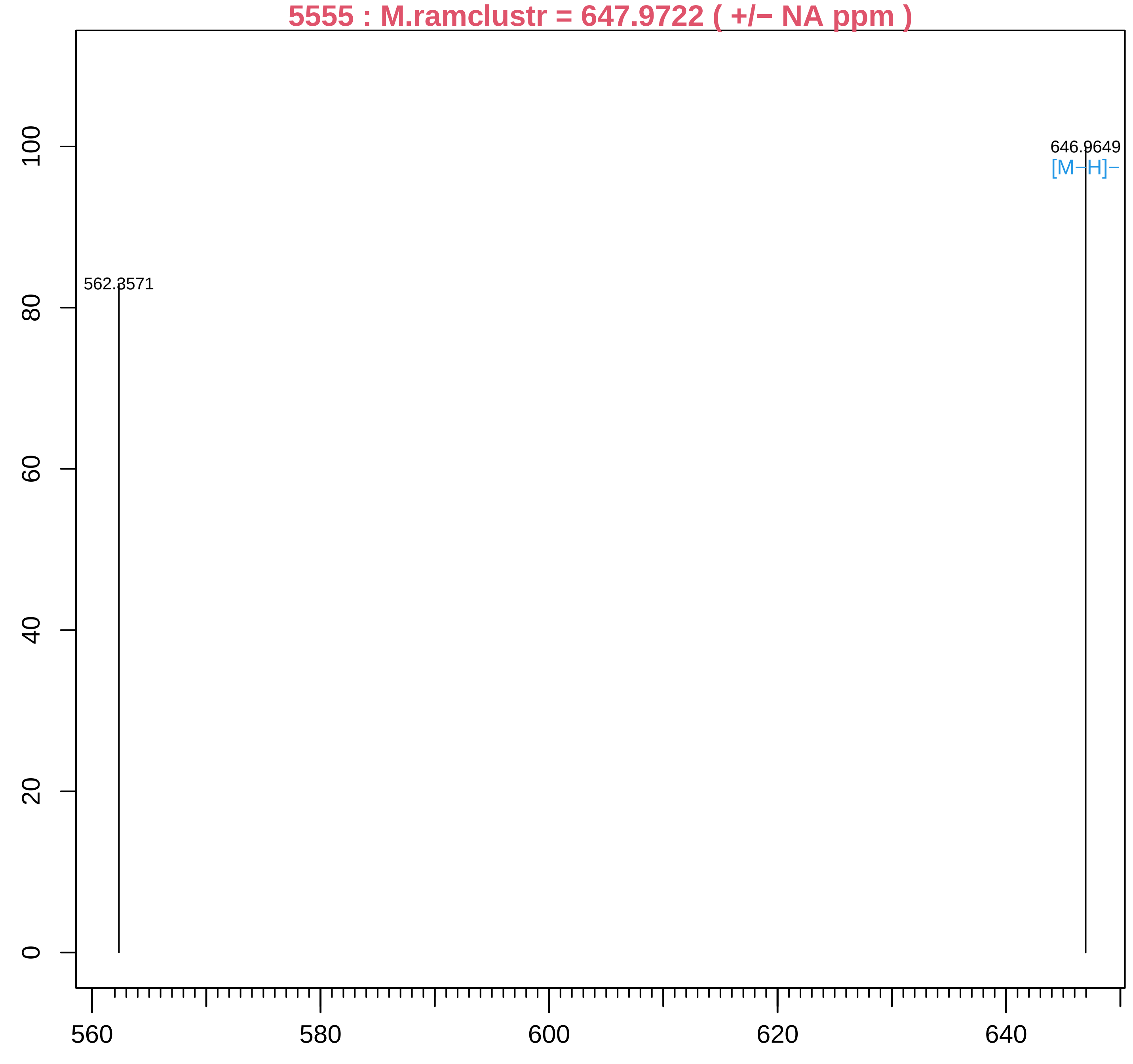
5555 : M.ramclustr = 647.9722 (+/− NA ppm)
646.9649
[M−H]−
562.3571
0
20
40
60
80
100
560
580
600
620
640

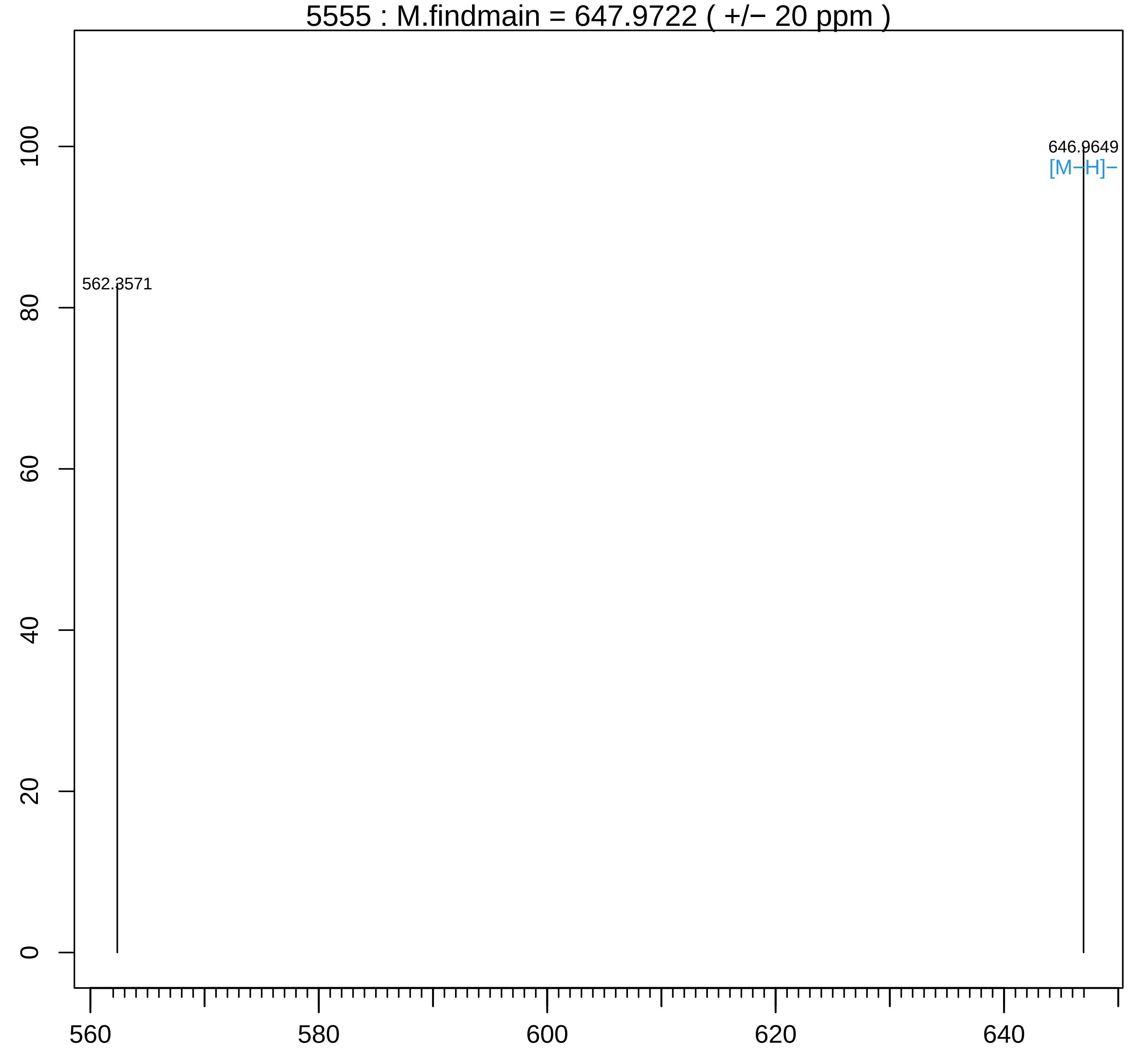
5555 : M.findmain = 647.9722 (+/− 20 ppm)
646.9649
[M−H]−
562.3571
0
20
40
60
80
100
560
580
600
620
640

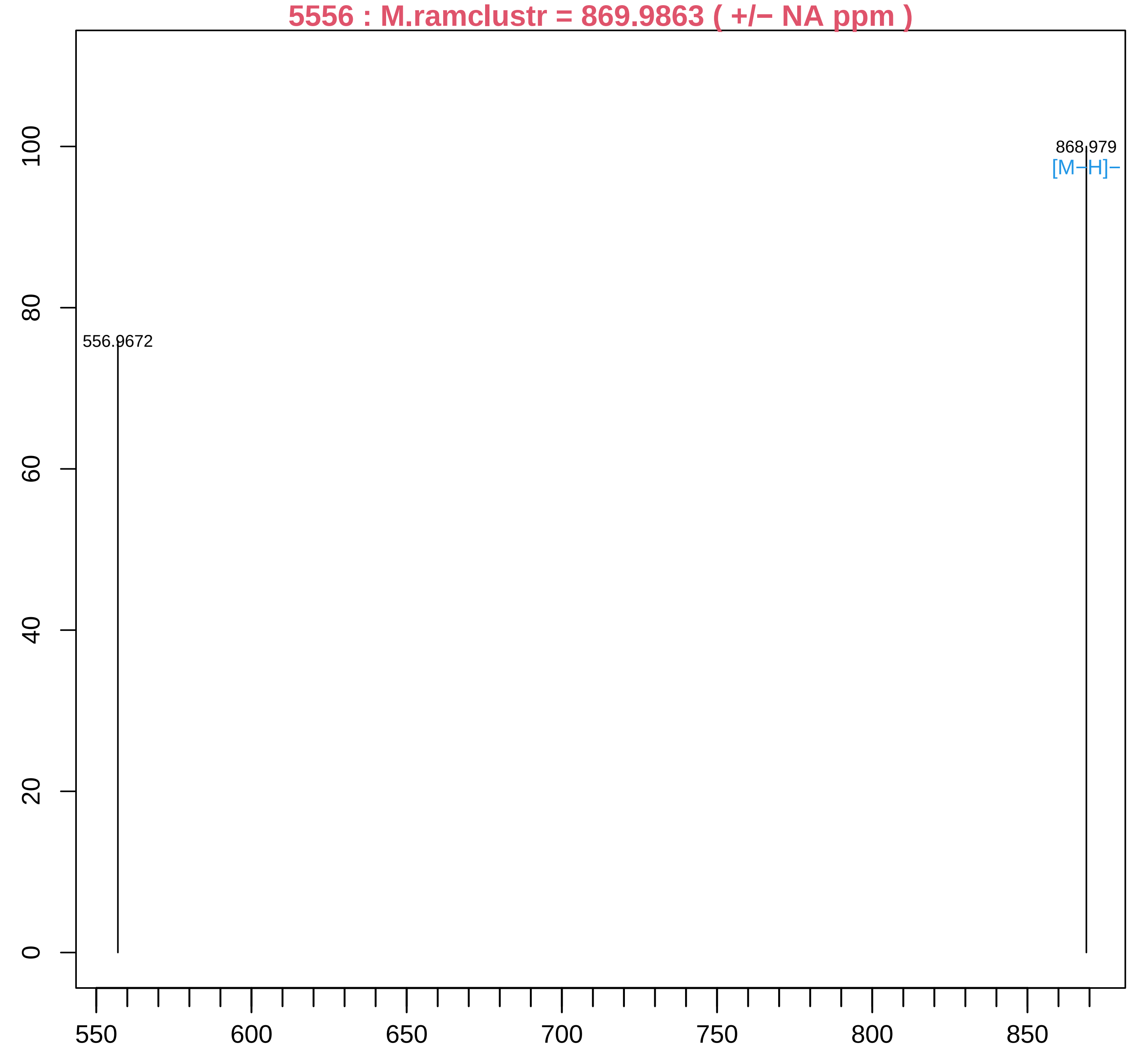
5556 : M.ramclustr = 869.9863 (+/− NA ppm)
868.979
[M−H]−
556.9672
0
20
40
60
80
100
550
600
650
700
750
800
850

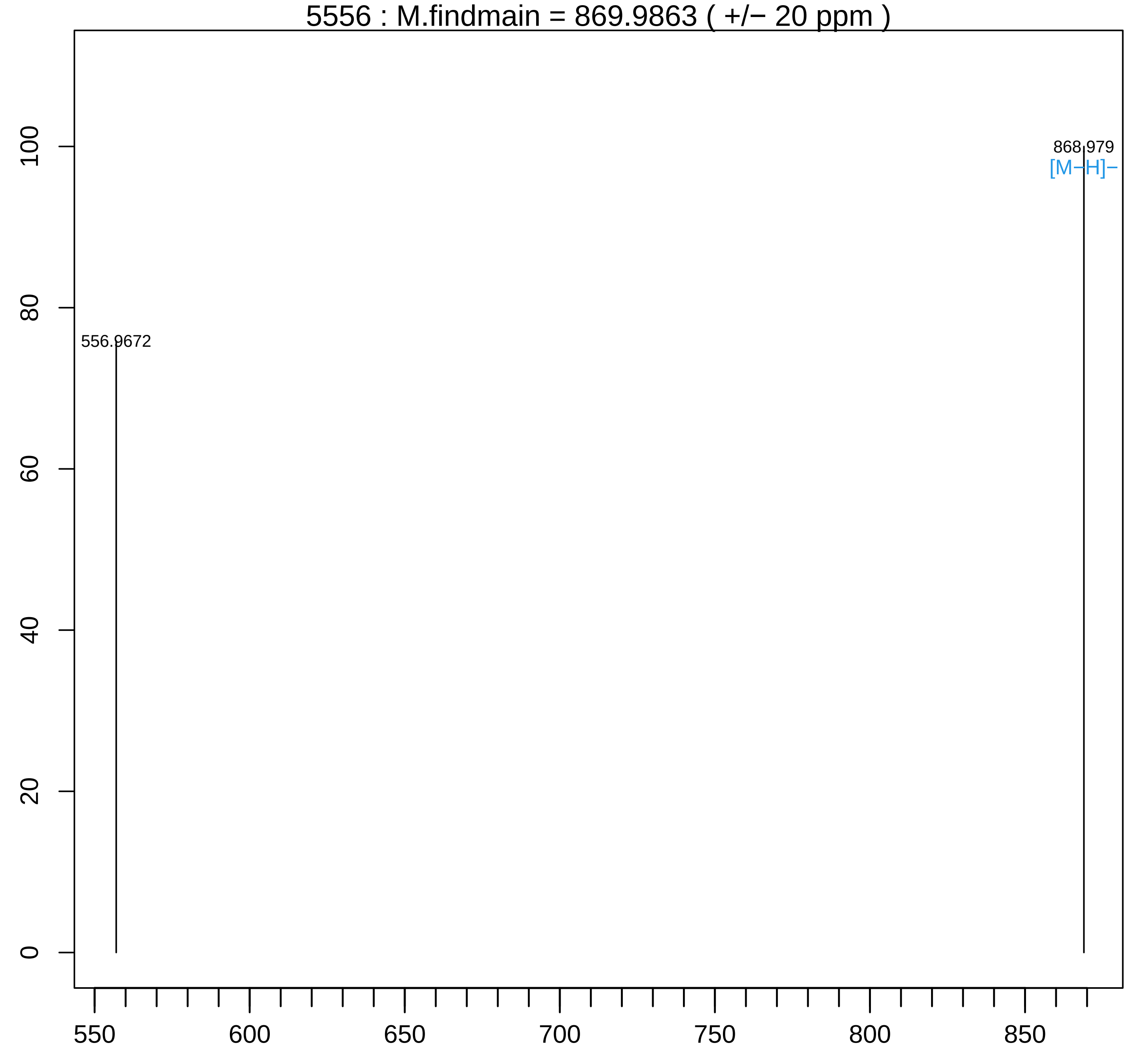
5556 : M.findmain = 869.9863 (+/− 20 ppm)
868.979
[M−H]−
556.9672
0
20
40
60
80
100
550
600
650
700
750
800
850

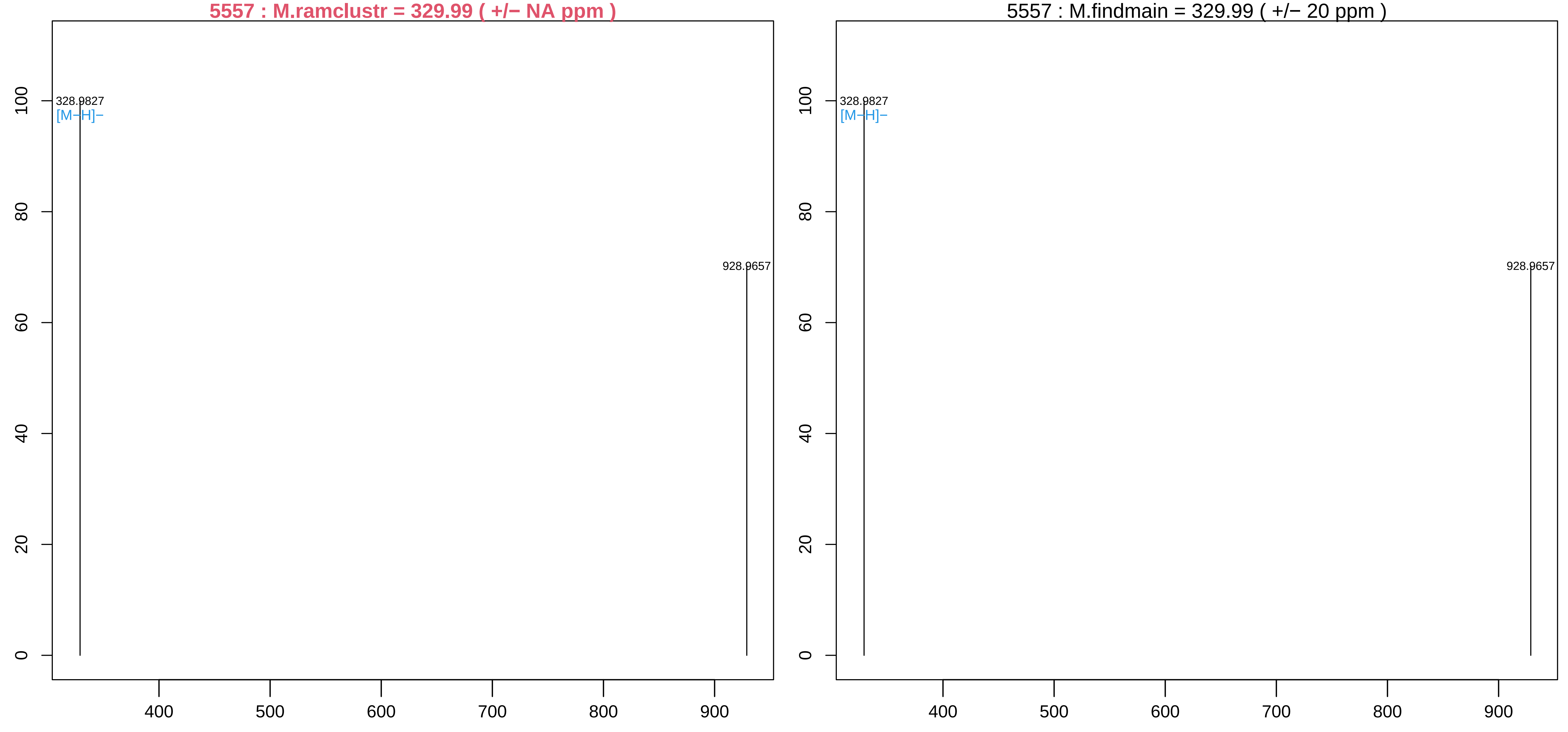
5557 : M.ramclustr = 329.99 (+/− NA ppm)
328.9827
[M−H]−
928.9657
5557 : M.findmain = 329.99 (+/− 20 ppm)
328.9827
[M−H]−
928.9657
0
20
40
60
80
100
400
500
600
700
800
900

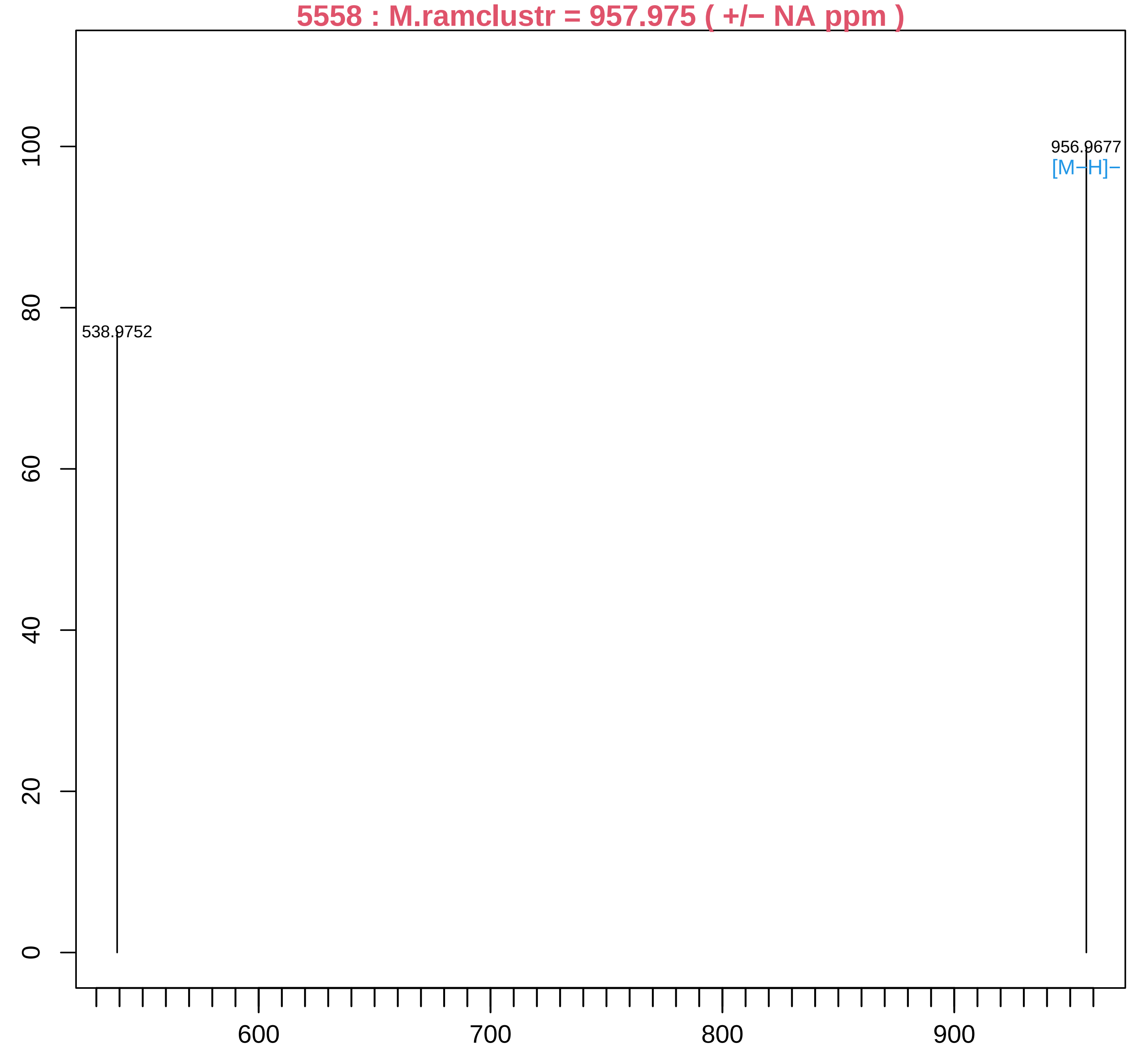

5558 : M.ramclustr = 957.975 (+/− NA ppm)
956.9677
[M−H]−
538.9752
0
20
40
60
80
100
600
700
800
900

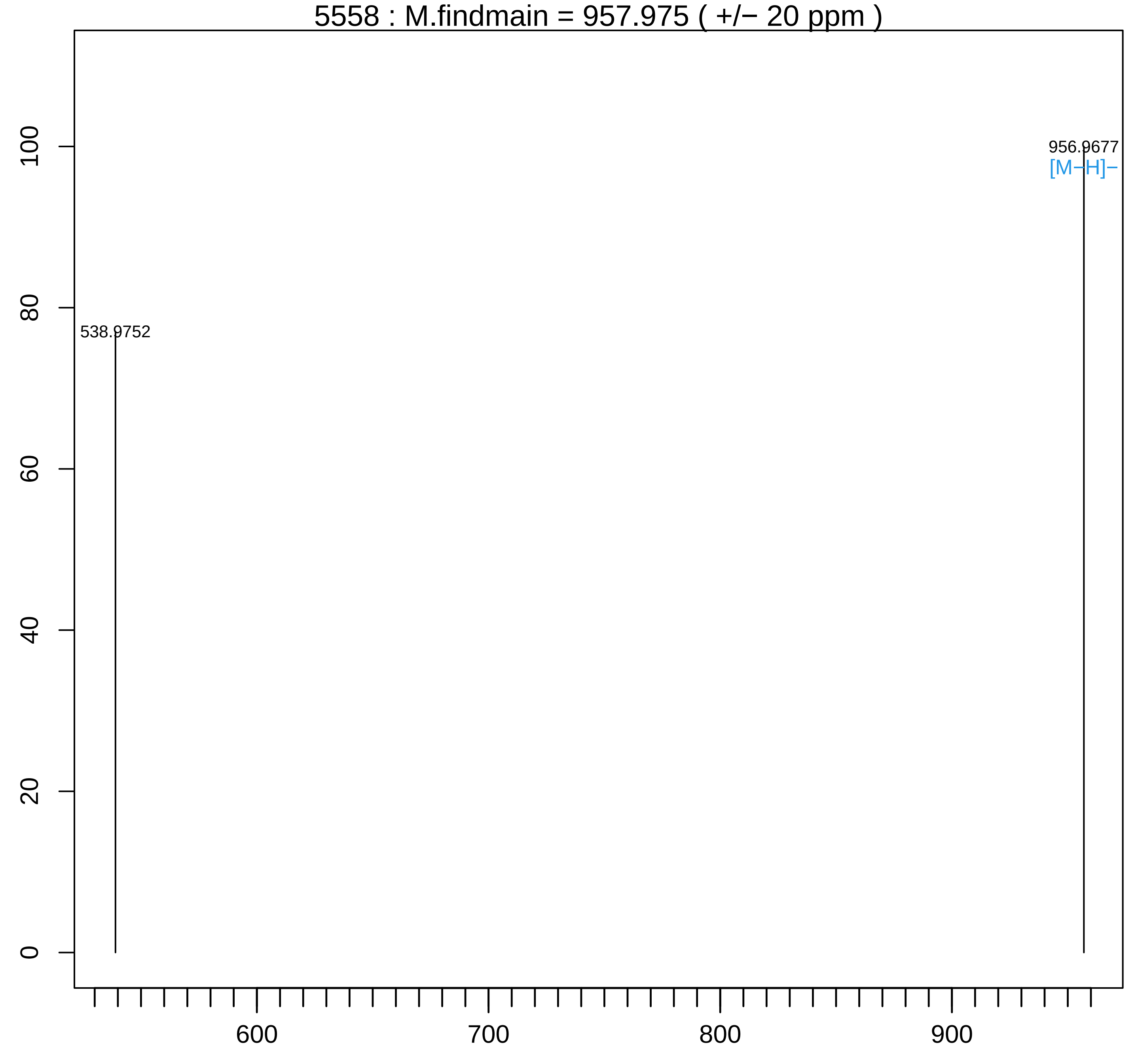

5558 : M.findmain = 957.975 (+/− 20 ppm)
956.9677
[M−H]−
538.9752
0
20
40
60
80
100
600
700
800
900

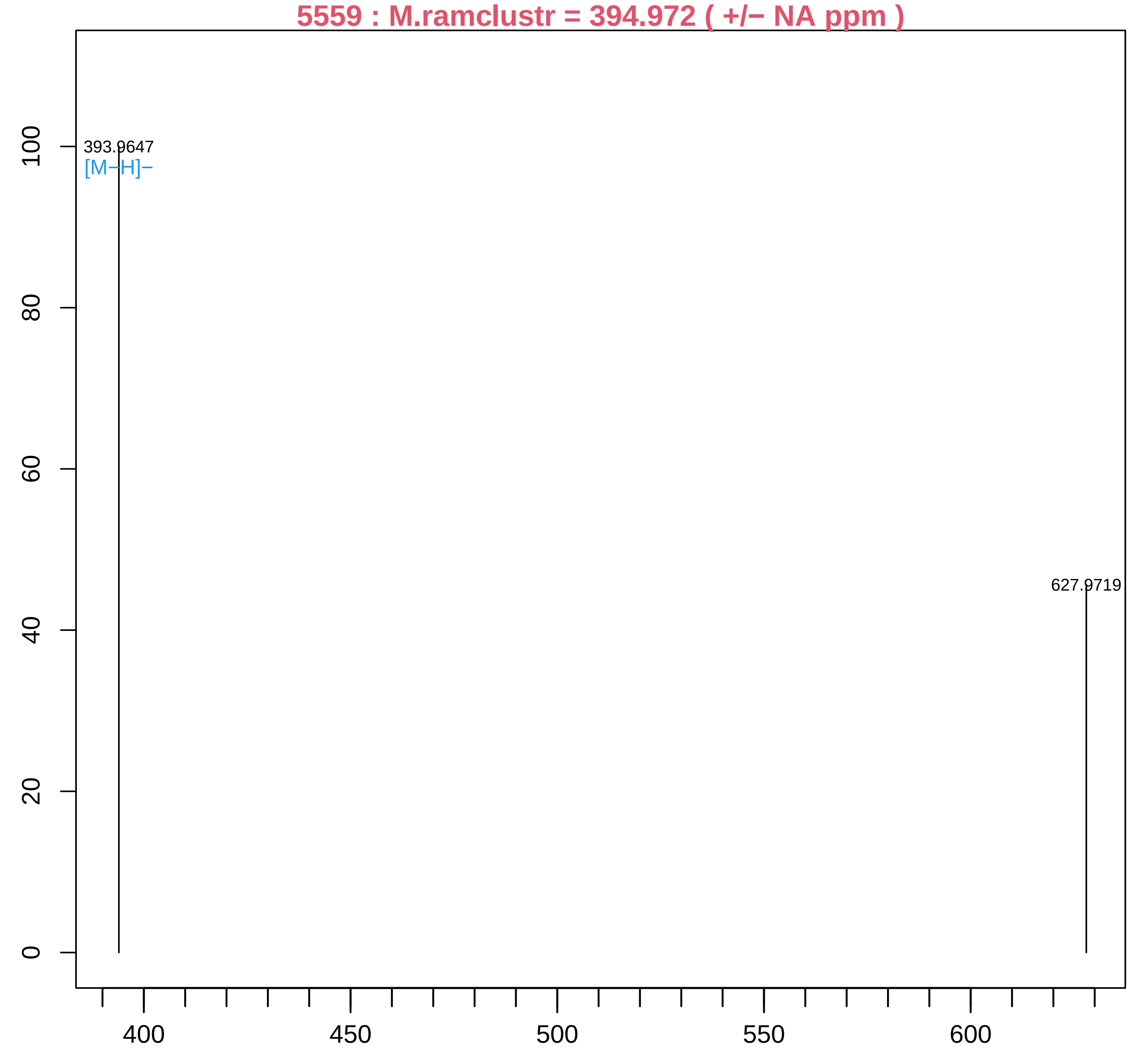
5559 : M.ramclustr = 394.972 (+/− NA ppm)
393.9647
[M−H]−
627.9719
0
20
40
60
80
100
400
450
500
550
600

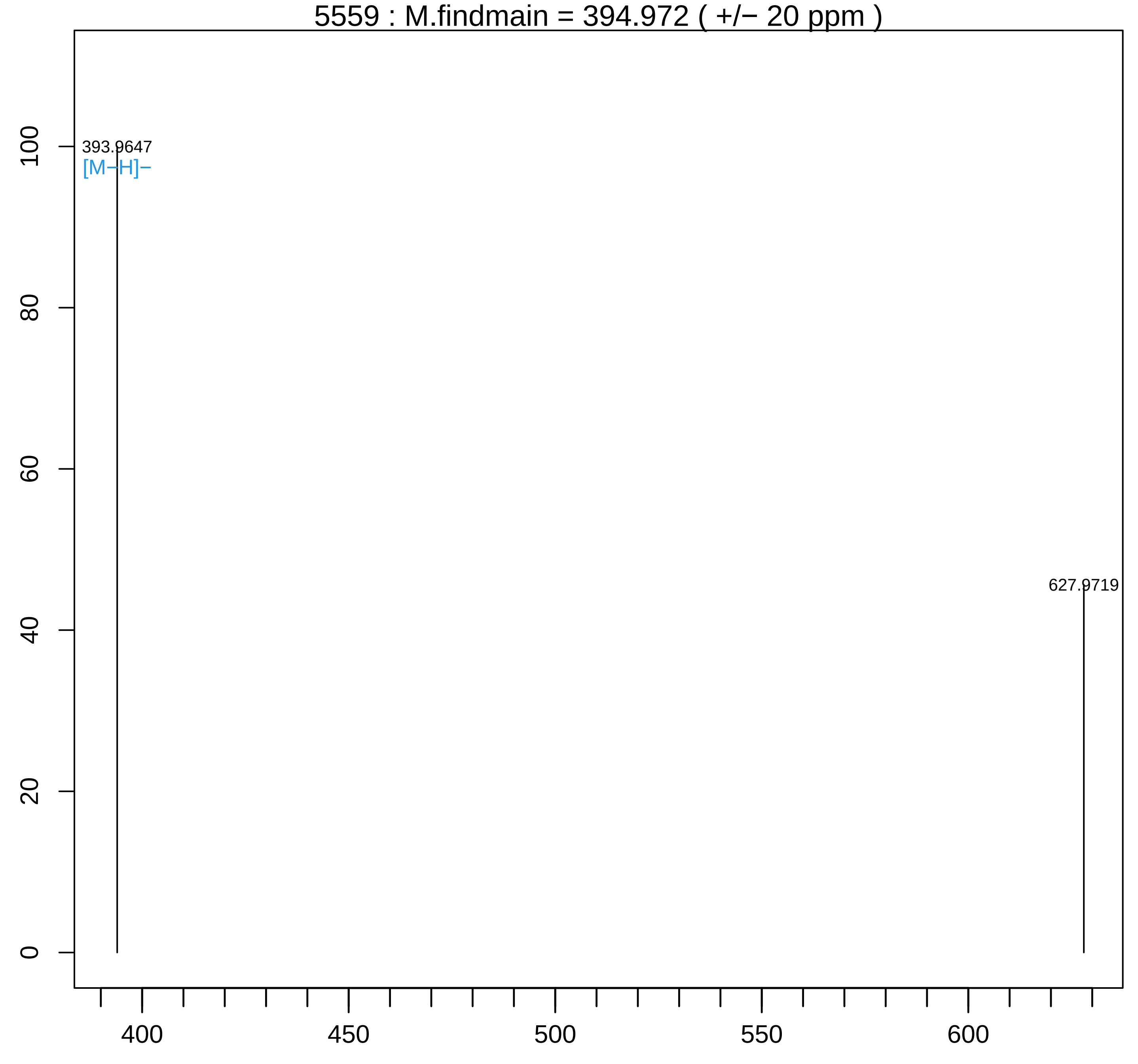
5559 : M.findmain = 394.972 (+/− 20 ppm)
393.9647
[M−H]−
627.9719
0
20
40
60
80
100
400
450
500
550
600

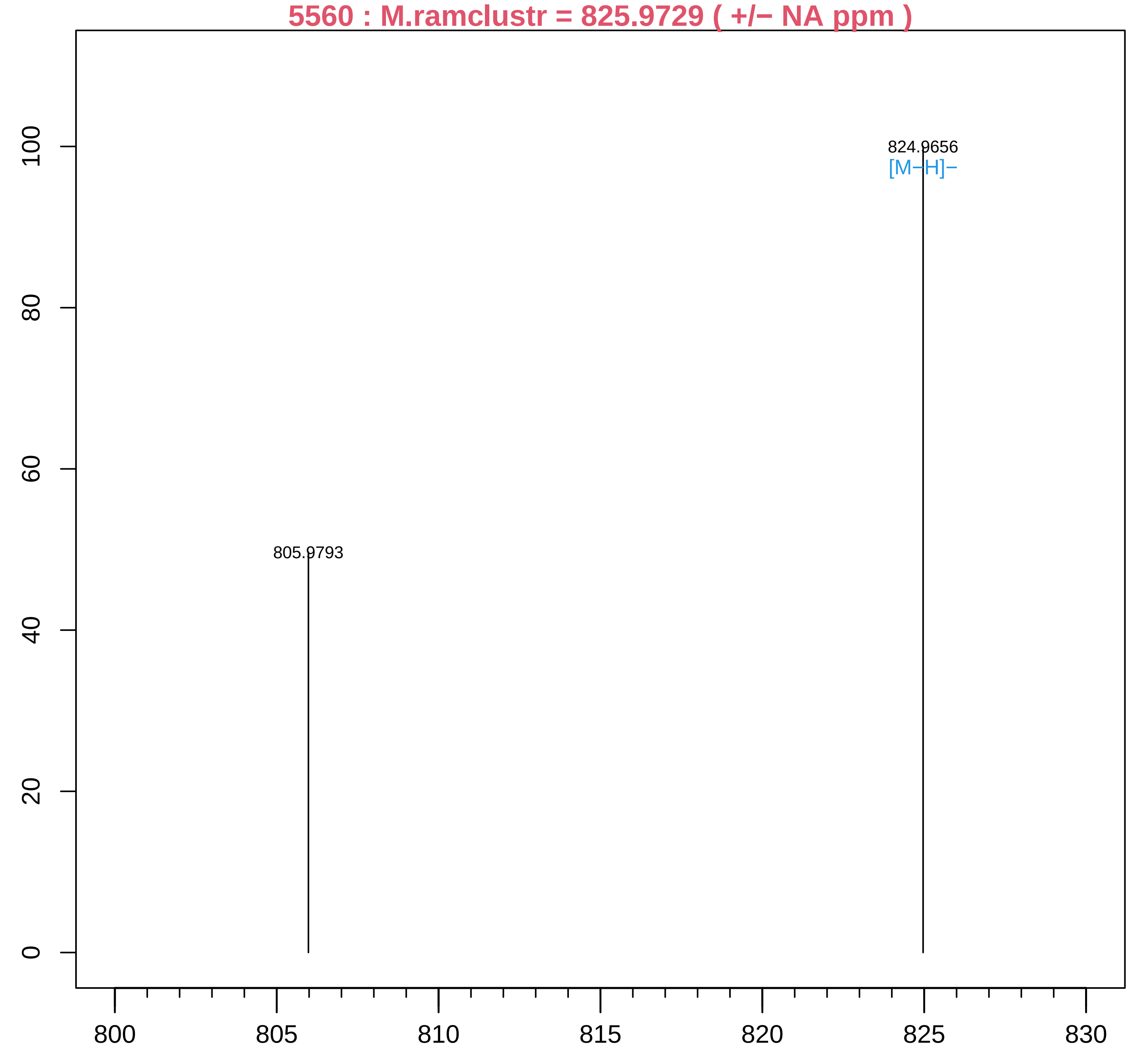

5560 : M.ramclustr = 825.9729 (+/− NA ppm)
824.9656
[M−H]−
805.9793
0
20
40
60
80
100
800
805
810
815
820
825
830

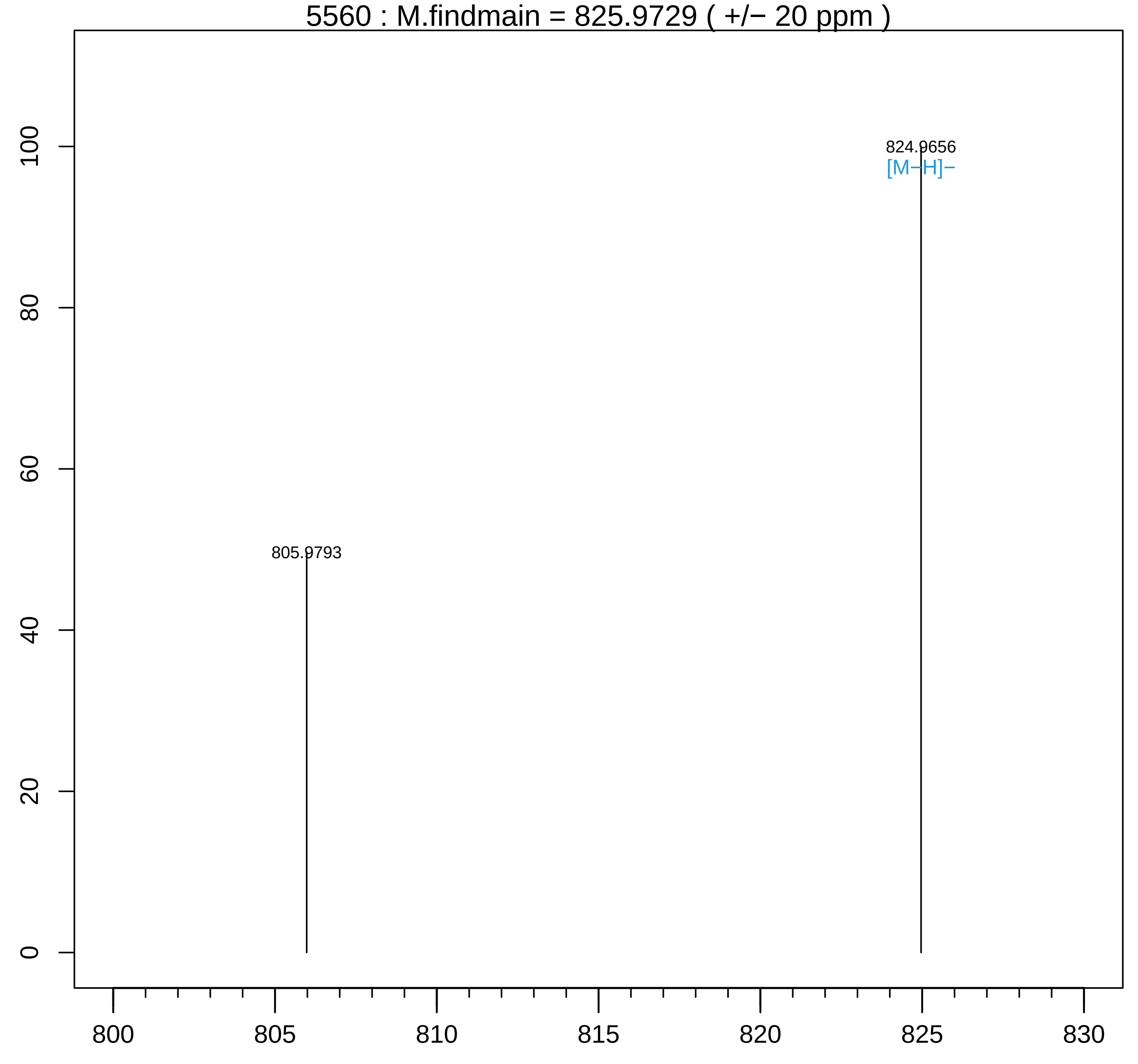

5560 : M.findmain = 825.9729 (+/− 20 ppm)
824.9656
[M−H]−
805.9793
0
20
40
60
80
100
800
805
810
815
820
825
830

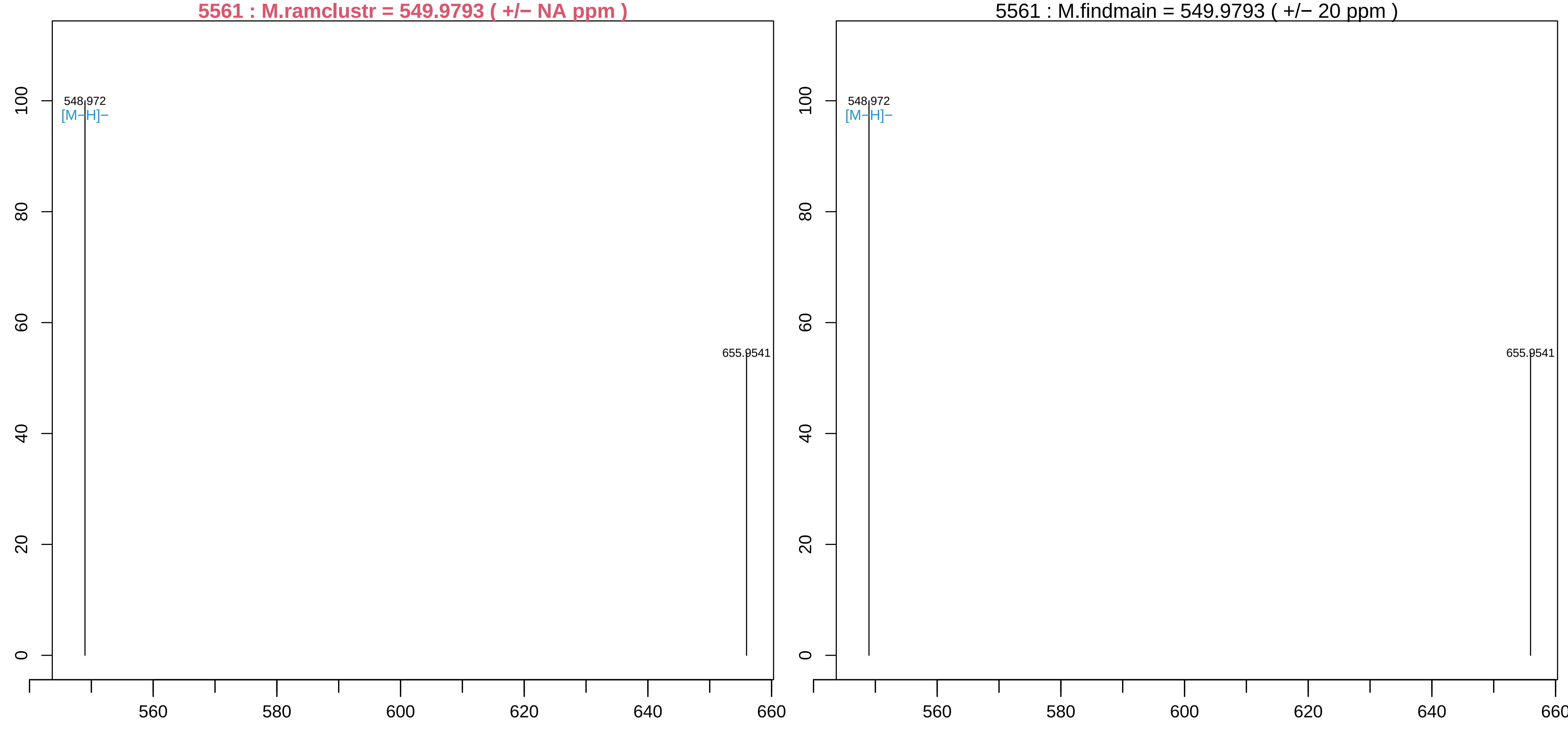

5561 : M.ramclustr = 549.9793 (+/− NA ppm)
548.972
[M−H]−
655.9541
0
20
40
60
80
100
560
580
600
620
640
660
5561 : M.findmain = 549.9793 (+/− 20 ppm)
548.972
[M−H]−
655.9541
0
20
40
60
80
100
560
580
600
620
640
660

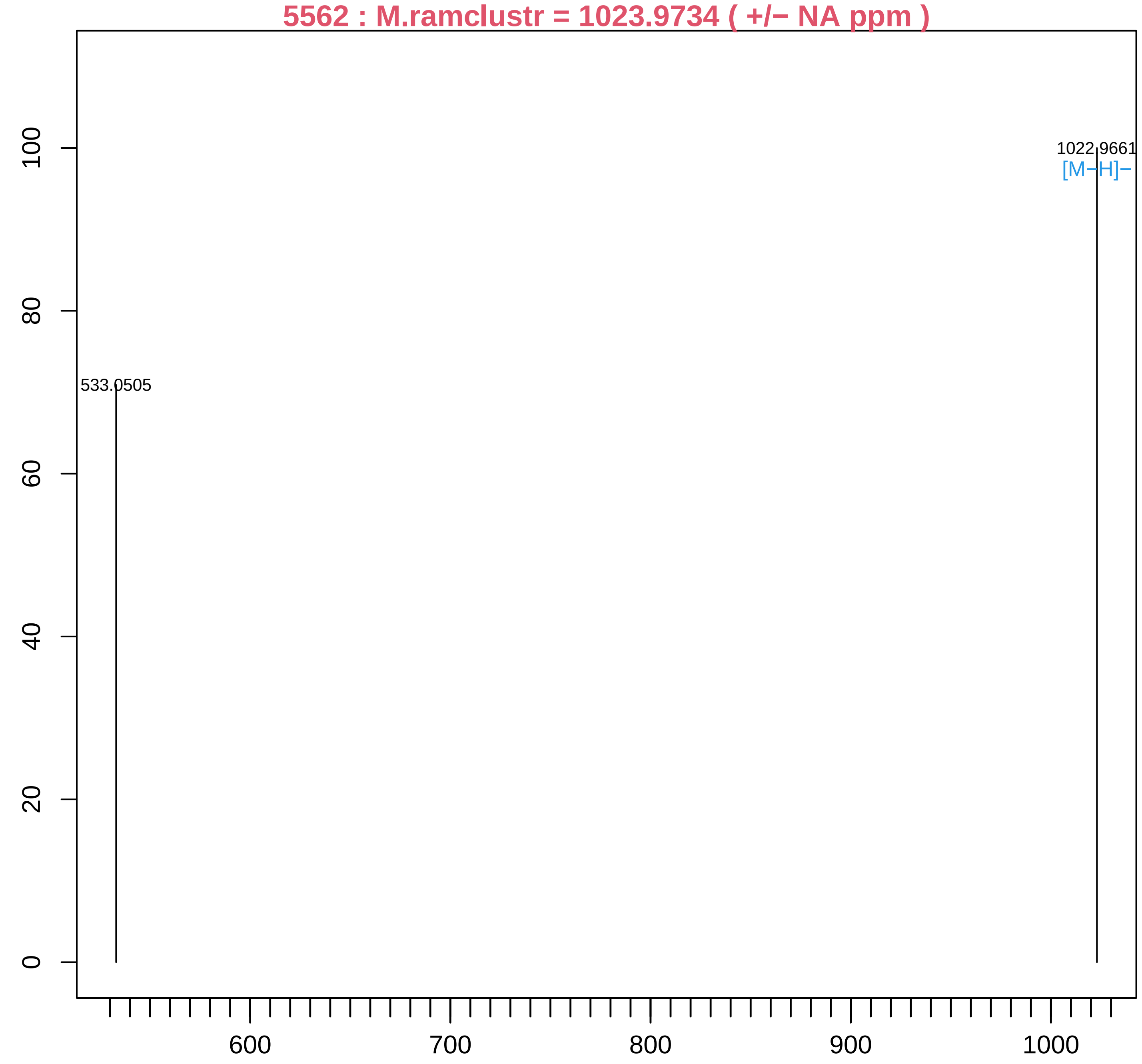
5562 : M.ramclustr = 1023.9734 (+/− NA ppm)
1022.9661
[M−H]−
533.0505
0
20
40
60
80
100
600
700
800
900
1000

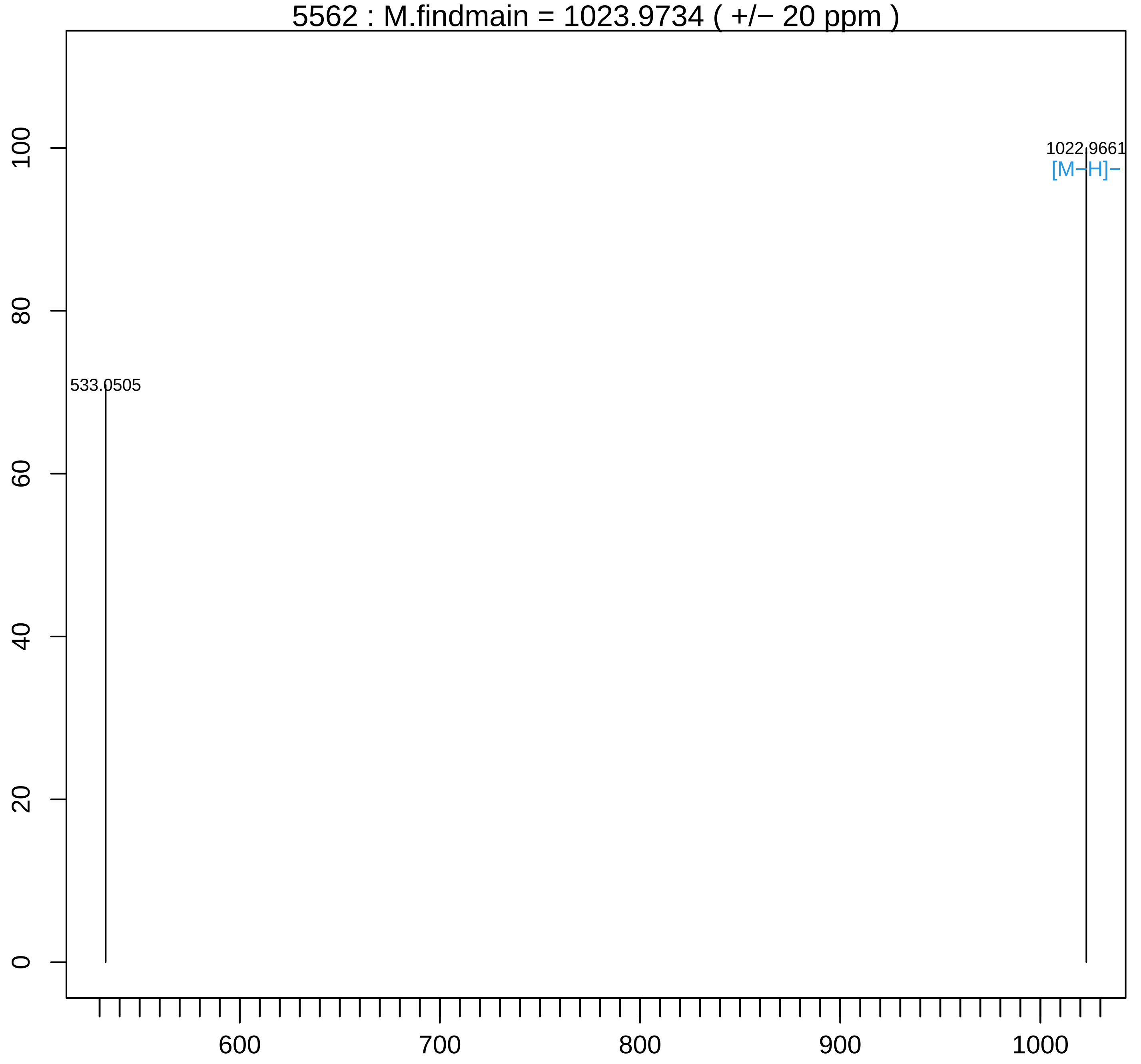
5562 : M.findmain = 1023.9734 (+/− 20 ppm)
1022.9661
[M−H]−
533.0505
0
20
40
60
80
100
600
700
800
900
1000

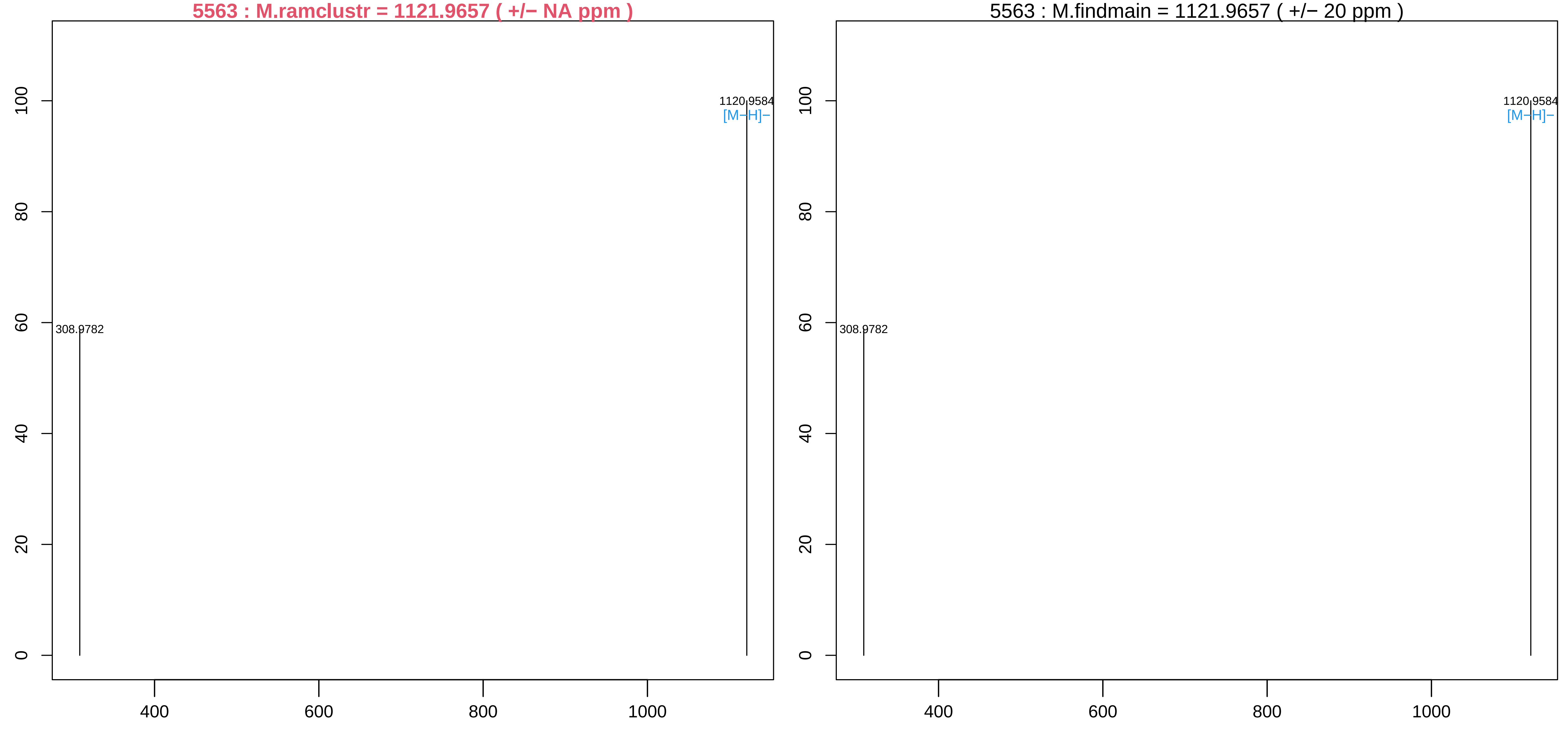
5563 : M.ramclustr = 1121.9657 (+/− NA ppm)
1120.9584
[M−H]−
308.9782
0
20
40
60
80
100
400
600
800
1000
5563 : M.findmain = 1121.9657 (+/− 20 ppm)
1120.9584
[M−H]−
308.9782
0
20
40
60
80
100
400
600
800
1000

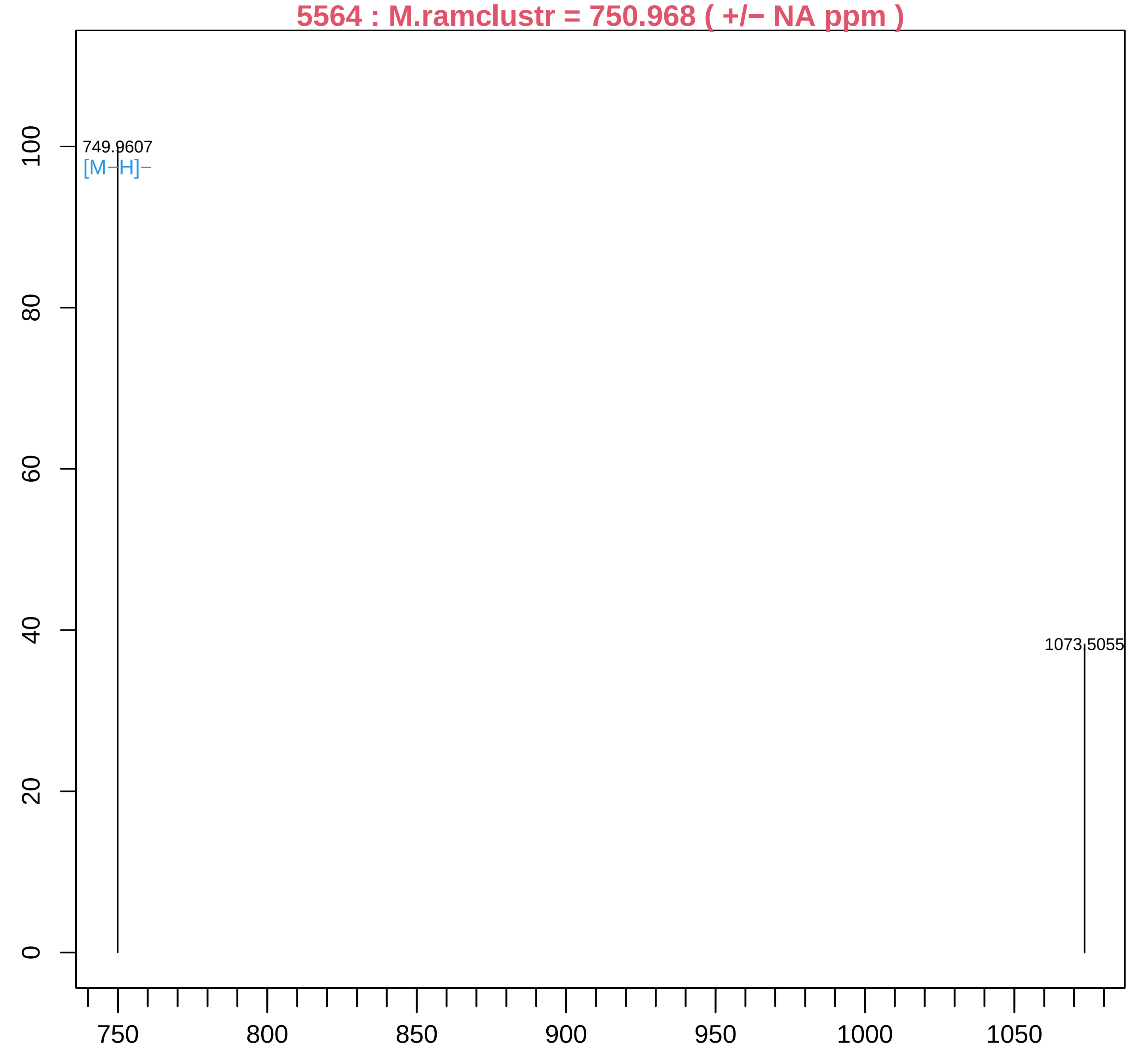
5564 : M.ramclustr = 750.968 (+/− NA ppm)
749.9607
[M−H]−
1073.5055
0
20
40
60
80
100
750
800
850
900
950
1000
1050

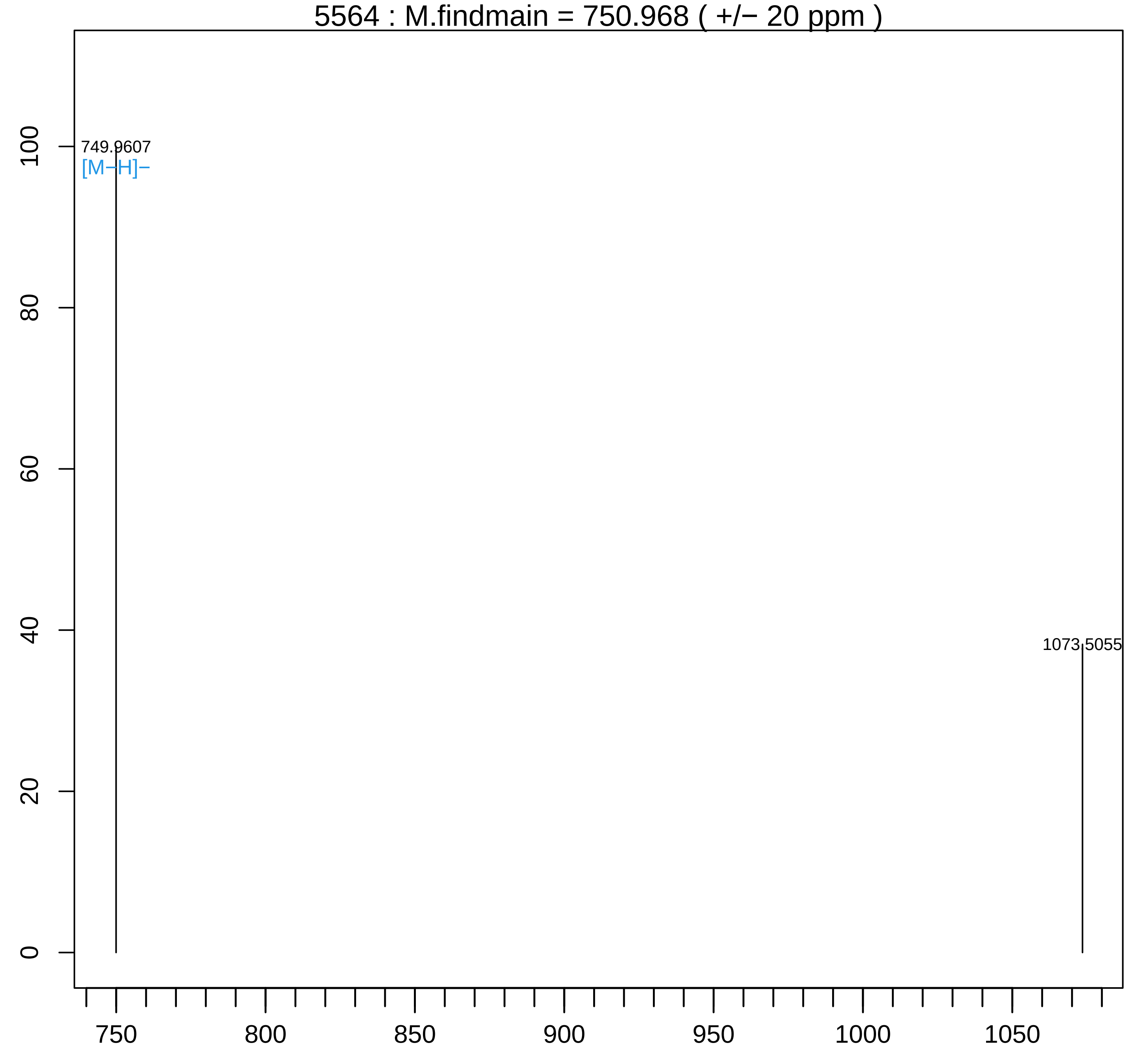
5564 : M.findmain = 750.968 (+/− 20 ppm)
749.9607
[M−H]−
1073.5055
0
20
40
60
80
100
750
800
850
900
950
1000
1050

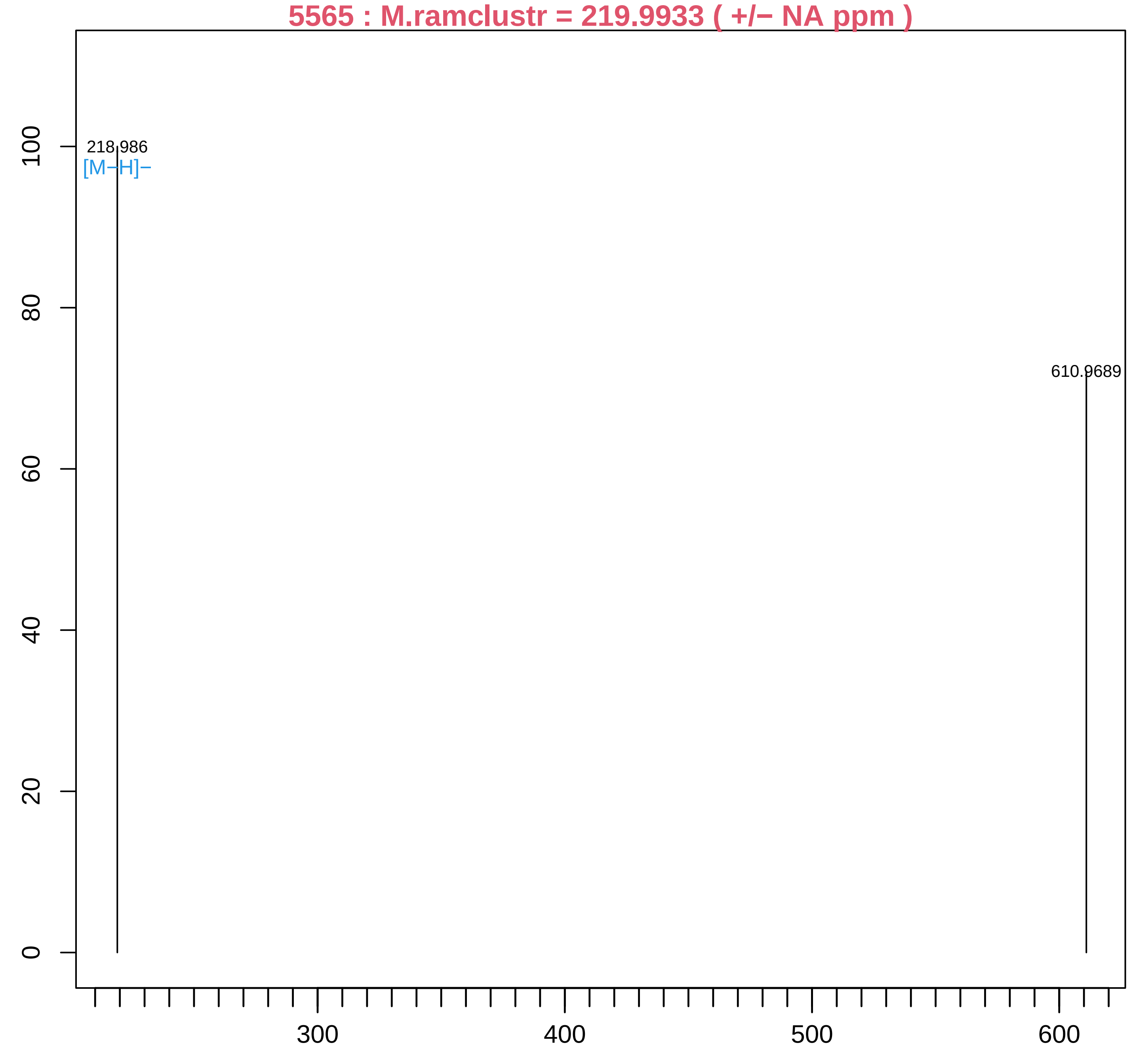

5565 : M.ramclustr = 219.9933 (+/− NA ppm)
218.986
[M−H]−
610.9689
0
20
40
60
80
100
300
400
500
600

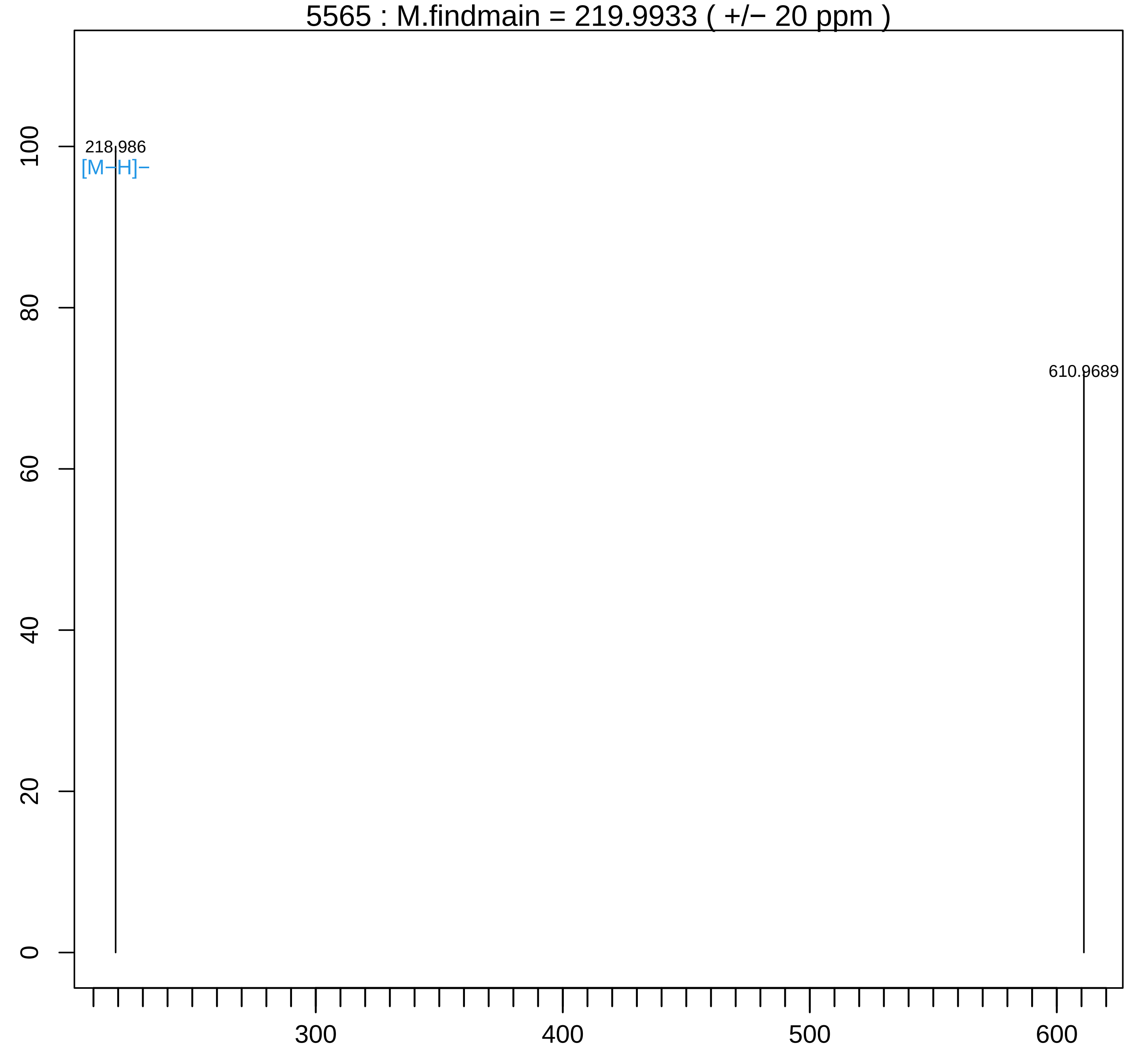

5565 : M.findmain = 219.9933 (+/− 20 ppm)
218.986
[M−H]−
610.9689
0
20
40
60
80
100
300
400
500
600

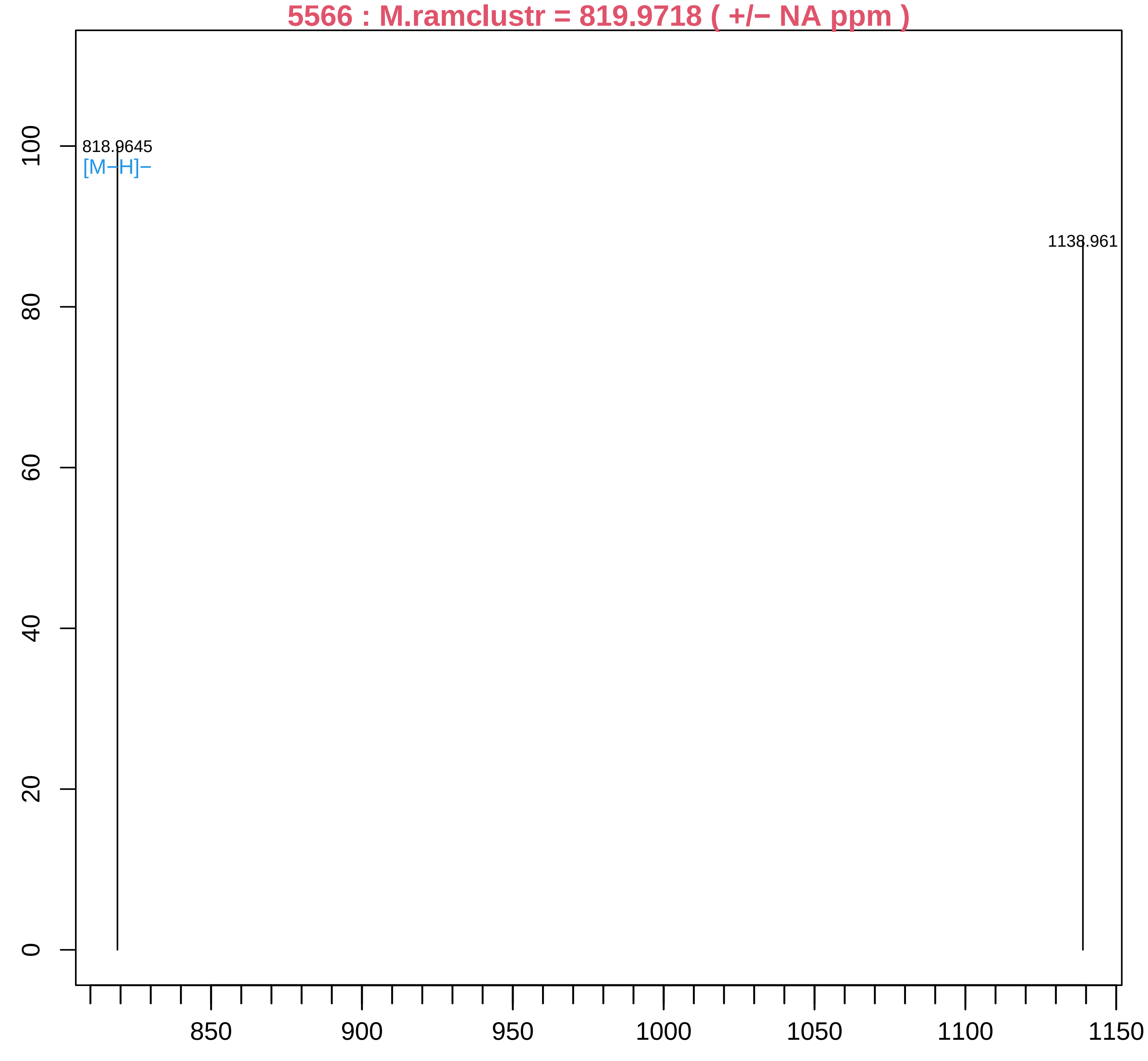
5566 : M.ramclustr = 819.9718 (+/− NA ppm)
818.9645
[M−H]−
1138.961
0
20
40
60
80
100
850
900
950
1000
1050
1100
1150

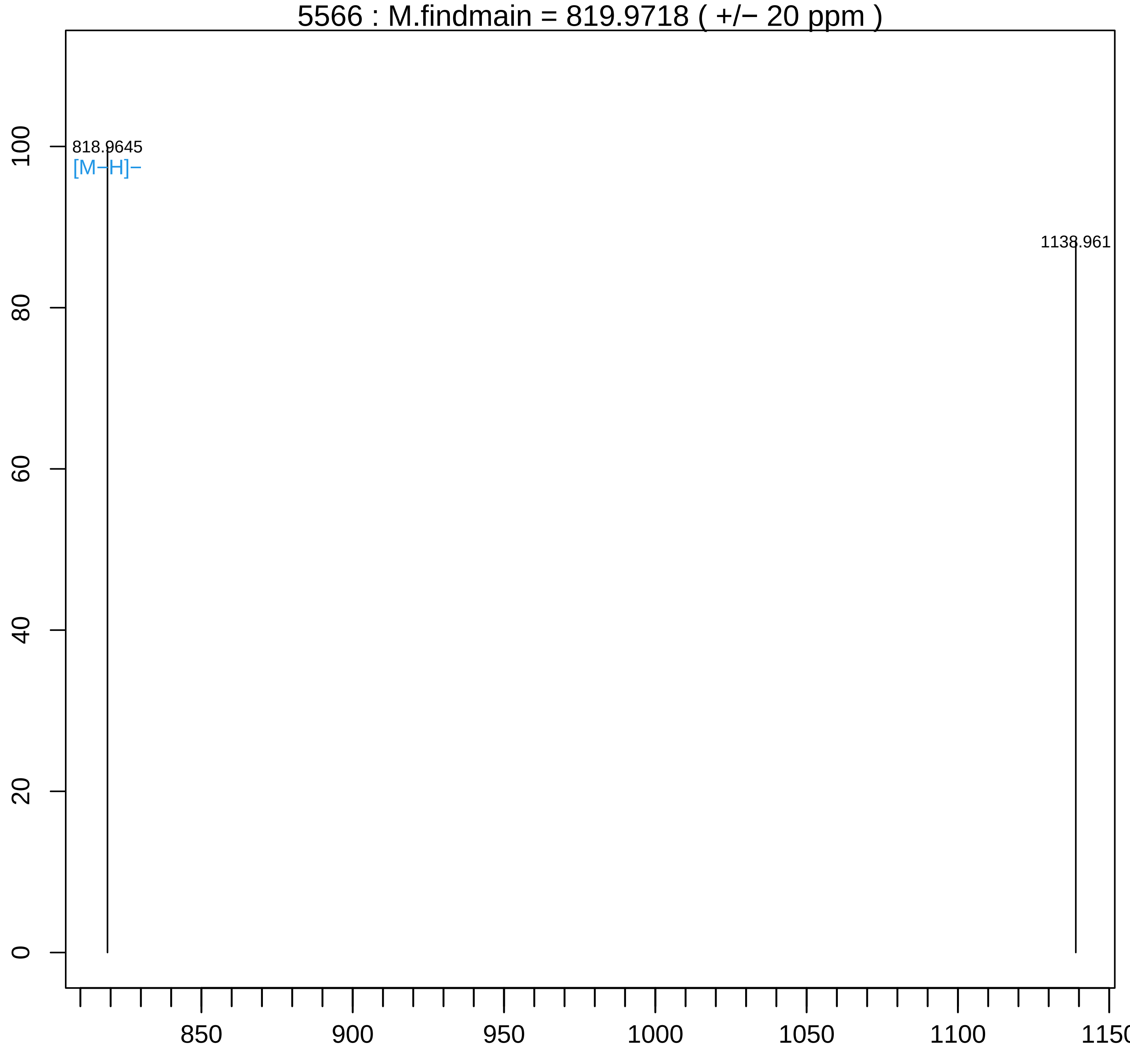
5566 : M.findmain = 819.9718 (+/− 20 ppm)
818.9645
[M−H]−
1138.961
0
20
40
60
80
100
850
900
950
1000
1050
1100
1150

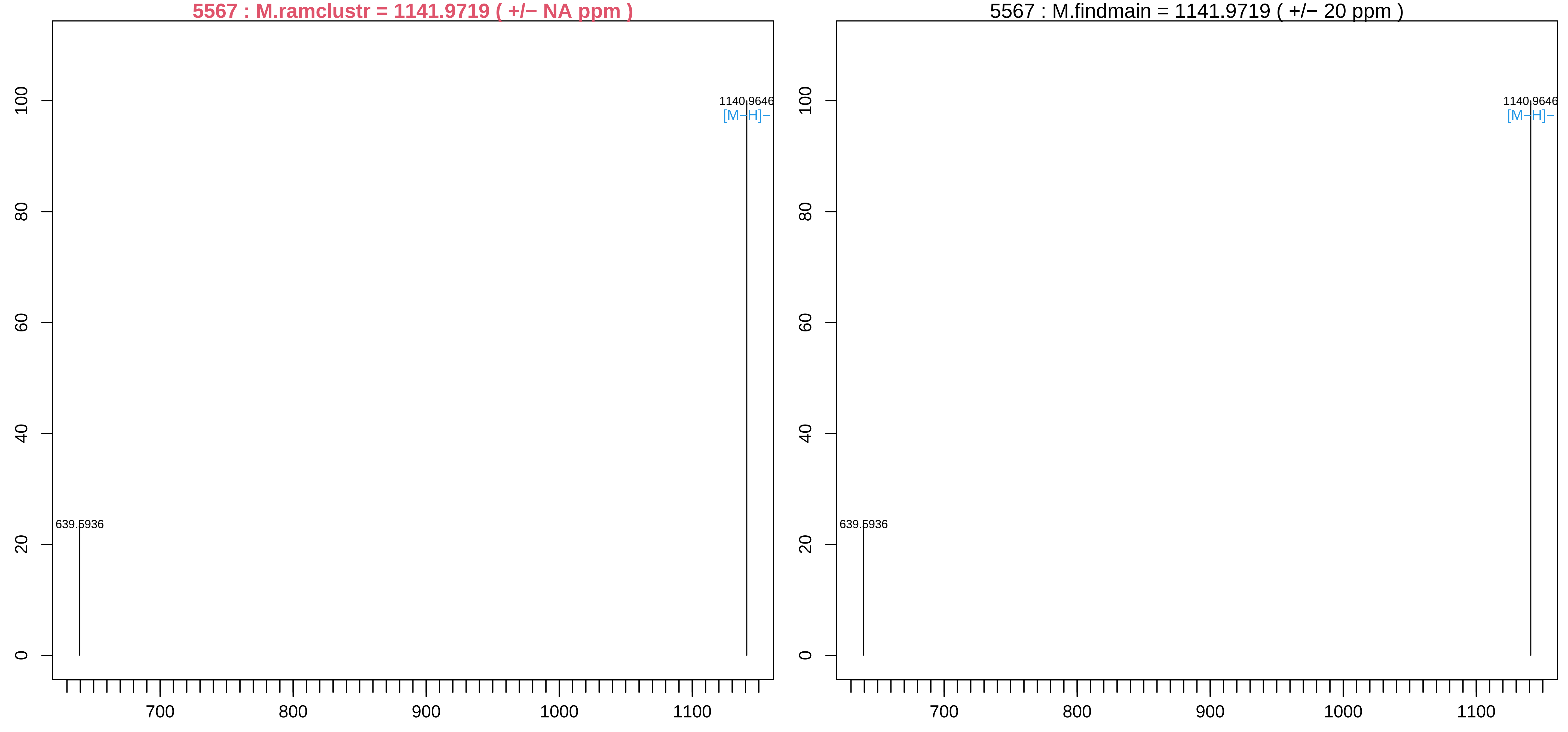
5567 : M.ramclustr = 1141.9719 (+/− NA ppm)
1140.9646
[M−H]−
639.5936
0
20
40
60
80
100
700
800
900
1000
1100
5567 : M.findmain = 1141.9719 (+/− 20 ppm)
1140.9646
[M−H]−
639.5936
0
20
40
60
80
100
700
800
900
1000
1100

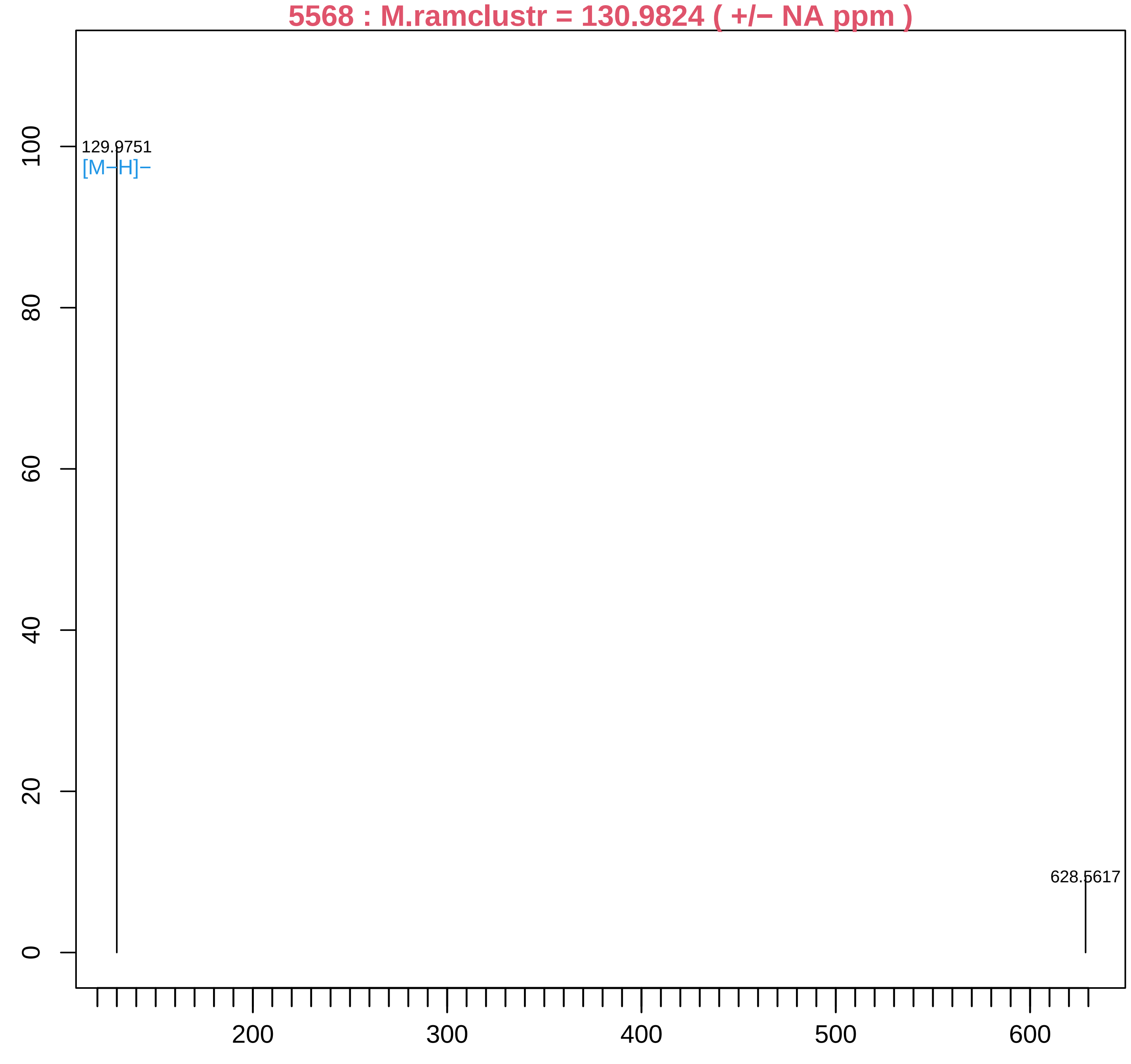
5568 : M.ramclustr = 130.9824 (+/− NA ppm)
129.9751
[M−H]−
628.5617
0
20
40
60
80
100
200
300
400
500
600

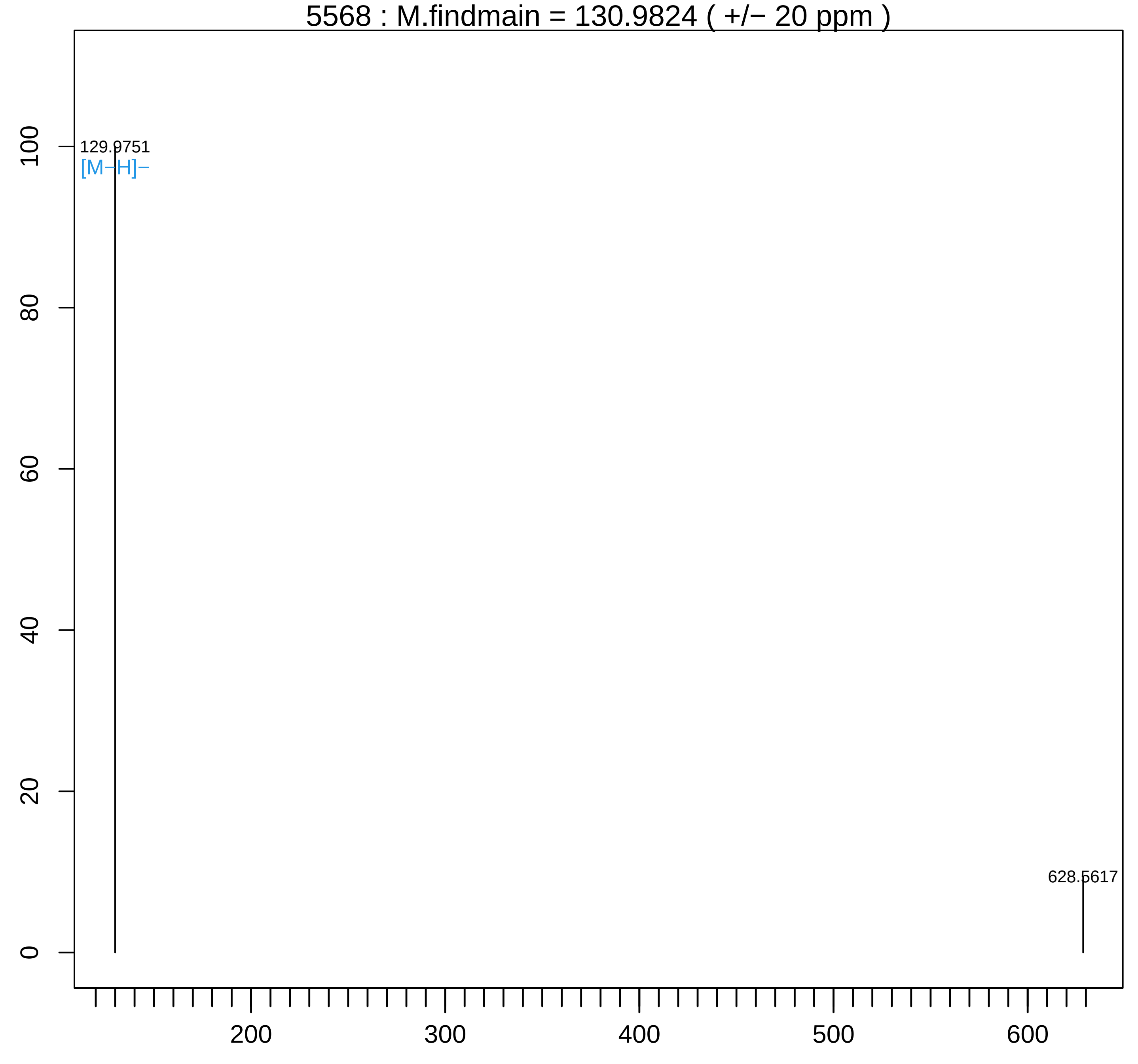
5568 : M.findmain = 130.9824 (+/− 20 ppm)
129.9751
[M−H]−
628.5617
0
20
40
60
80
100
200
300
400
500
600

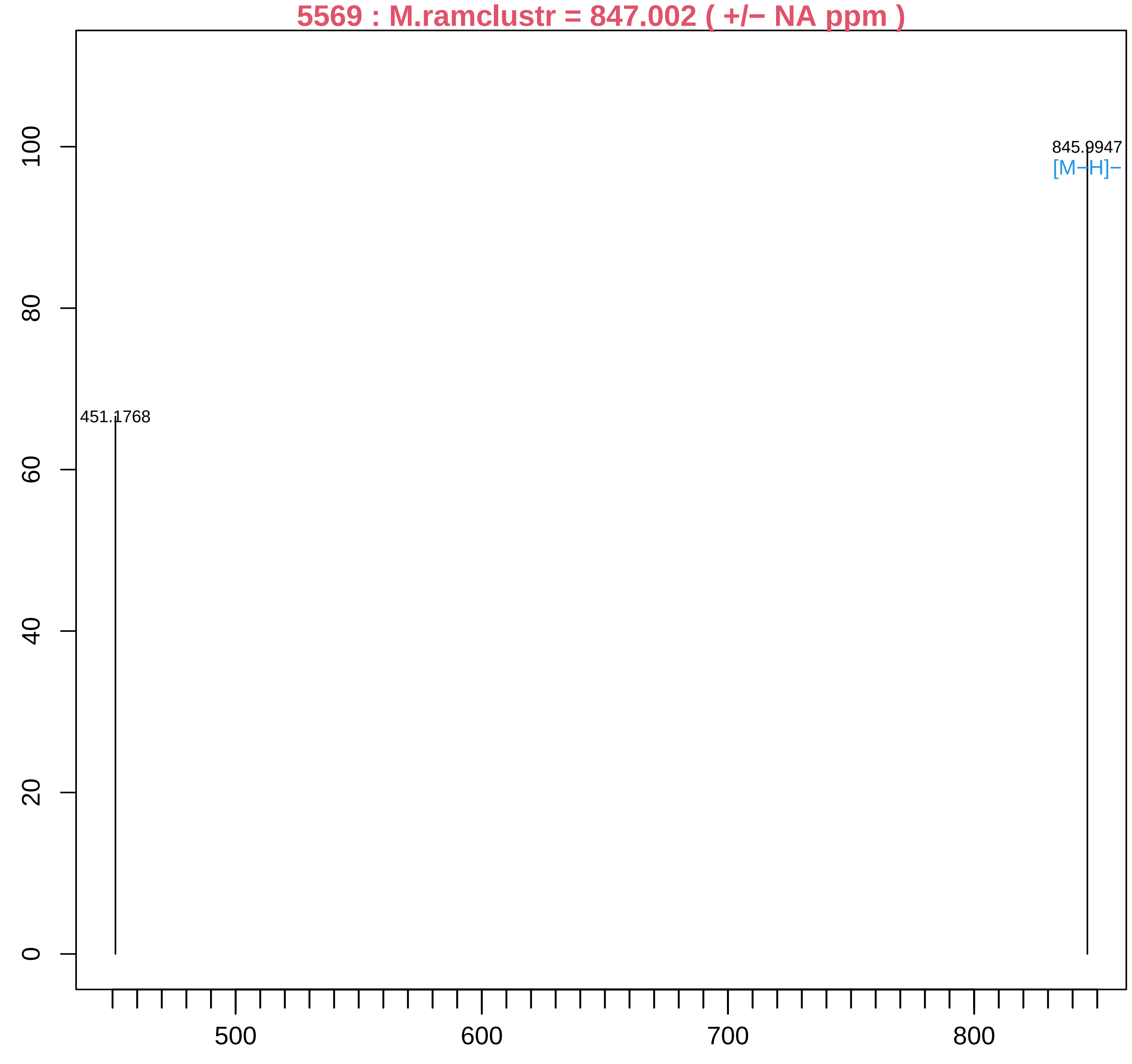
5569 : M.ramclustr = 847.002 (+/− NA ppm)
845.9947
[M−H]−
451.1768
0
20
40
60
80
100
500
600
700
800

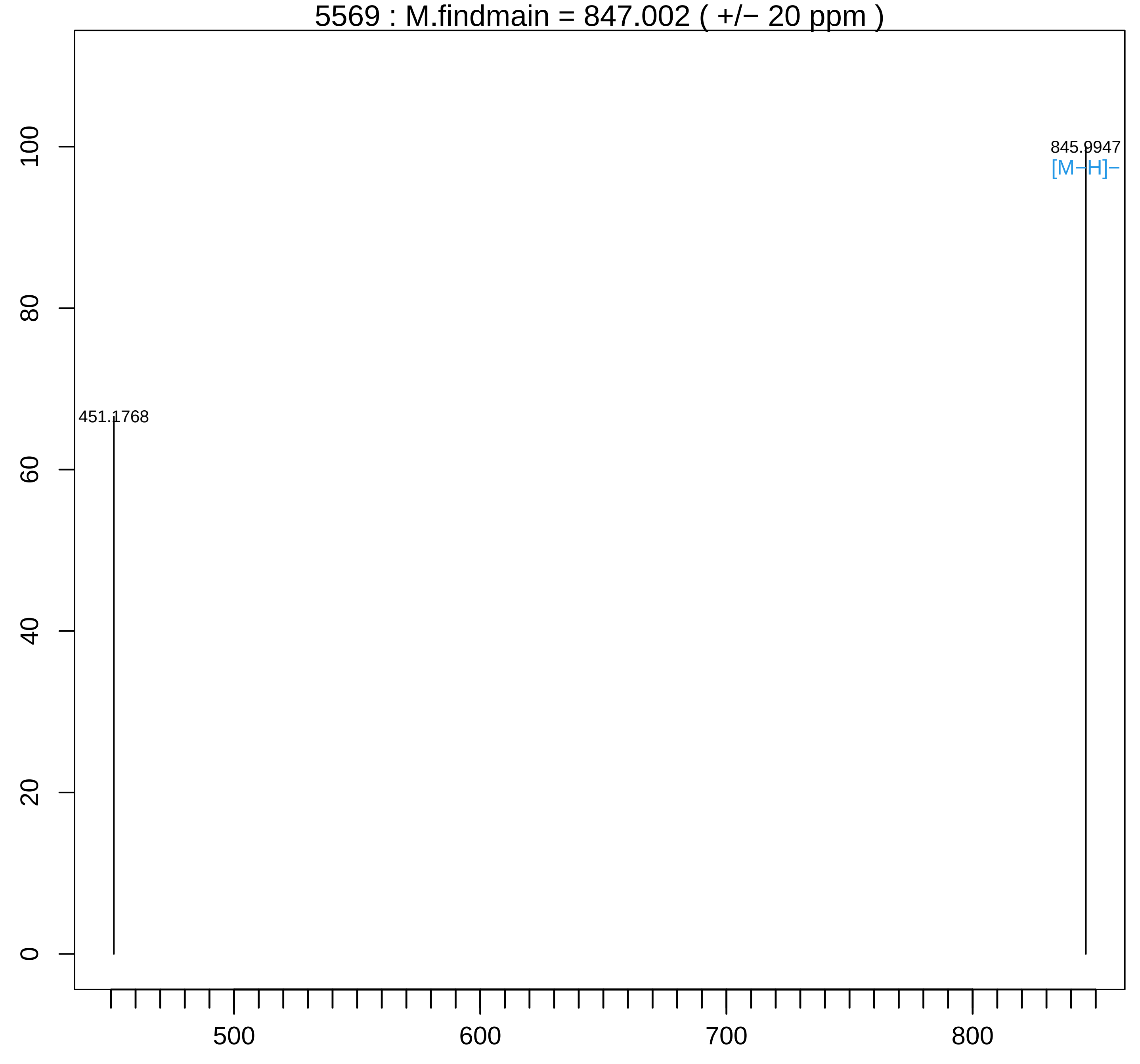
5569 : M.findmain = 847.002 (+/− 20 ppm)
845.9947
[M−H]−
451.1768
0
20
40
60
80
100
500
600
700
800

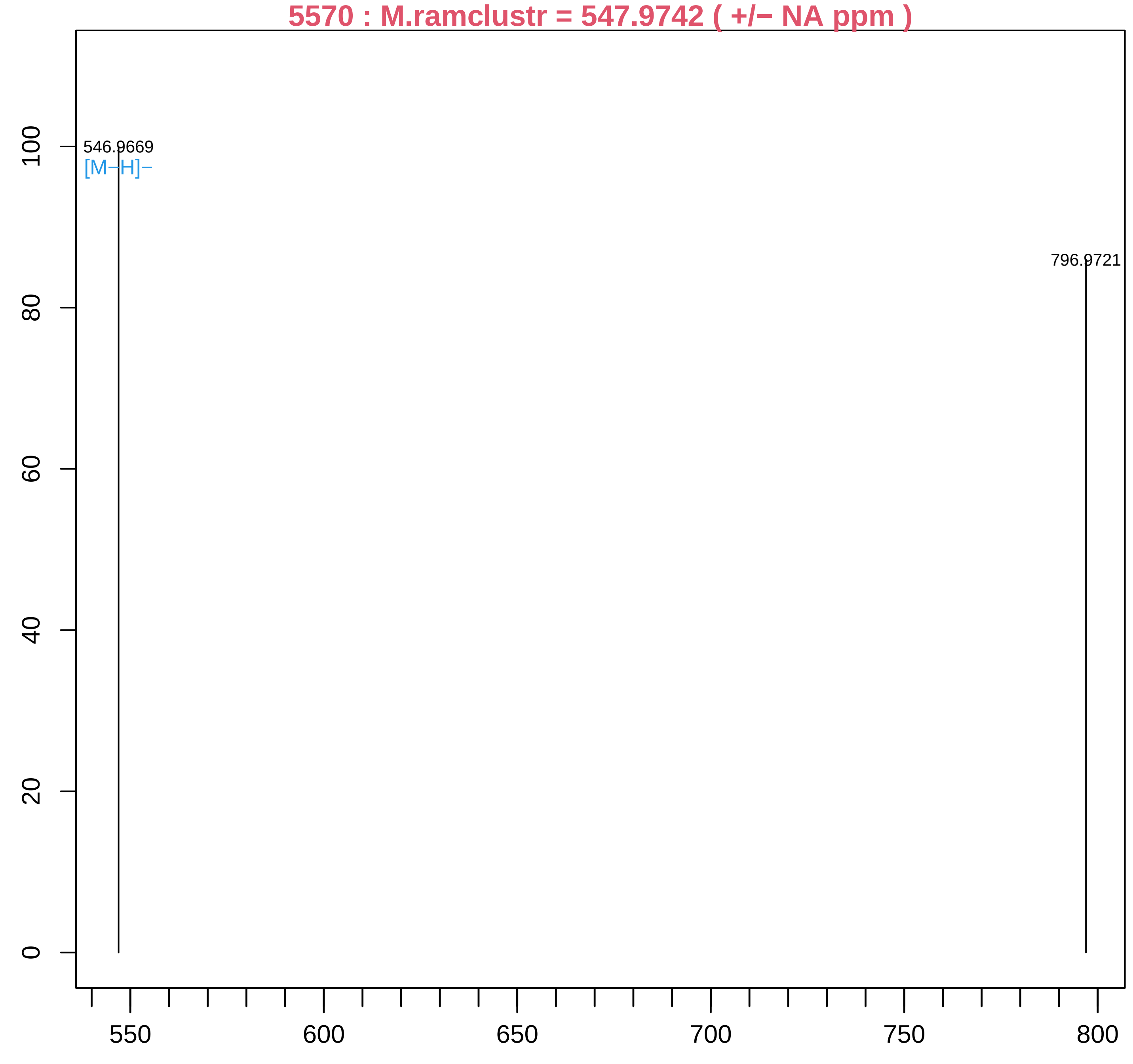
5570 : M.ramclustr = 547.9742 (+/− NA ppm)
546.9669
[M−H]−
796.9721
0
20
40
60
80
100
550
600
650
700
750
800

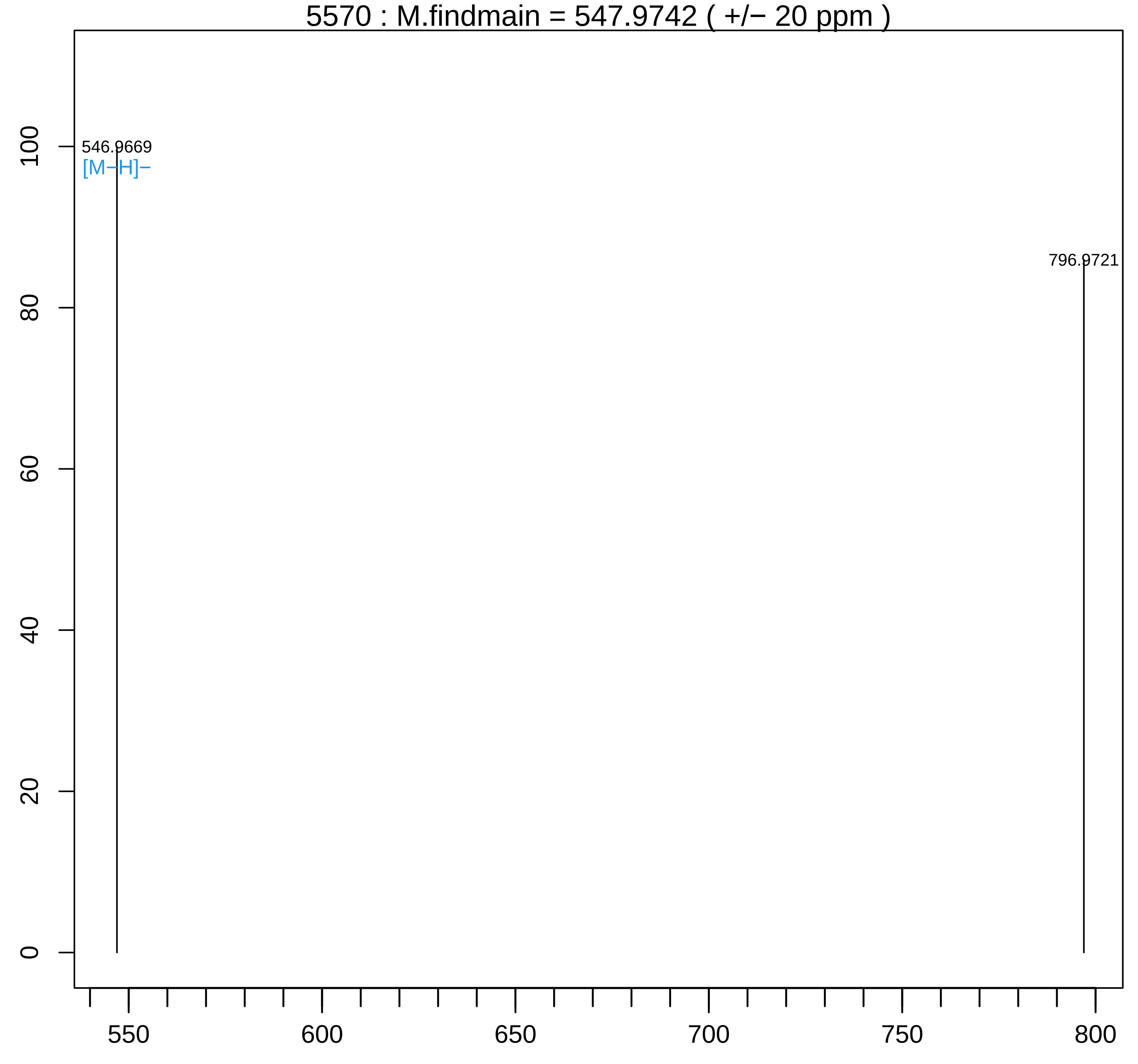
5570 : M.findmain = 547.9742 (+/− 20 ppm)
546.9669
[M−H]−
796.9721
0
20
40
60
80
100
550
600
650
700
750
800

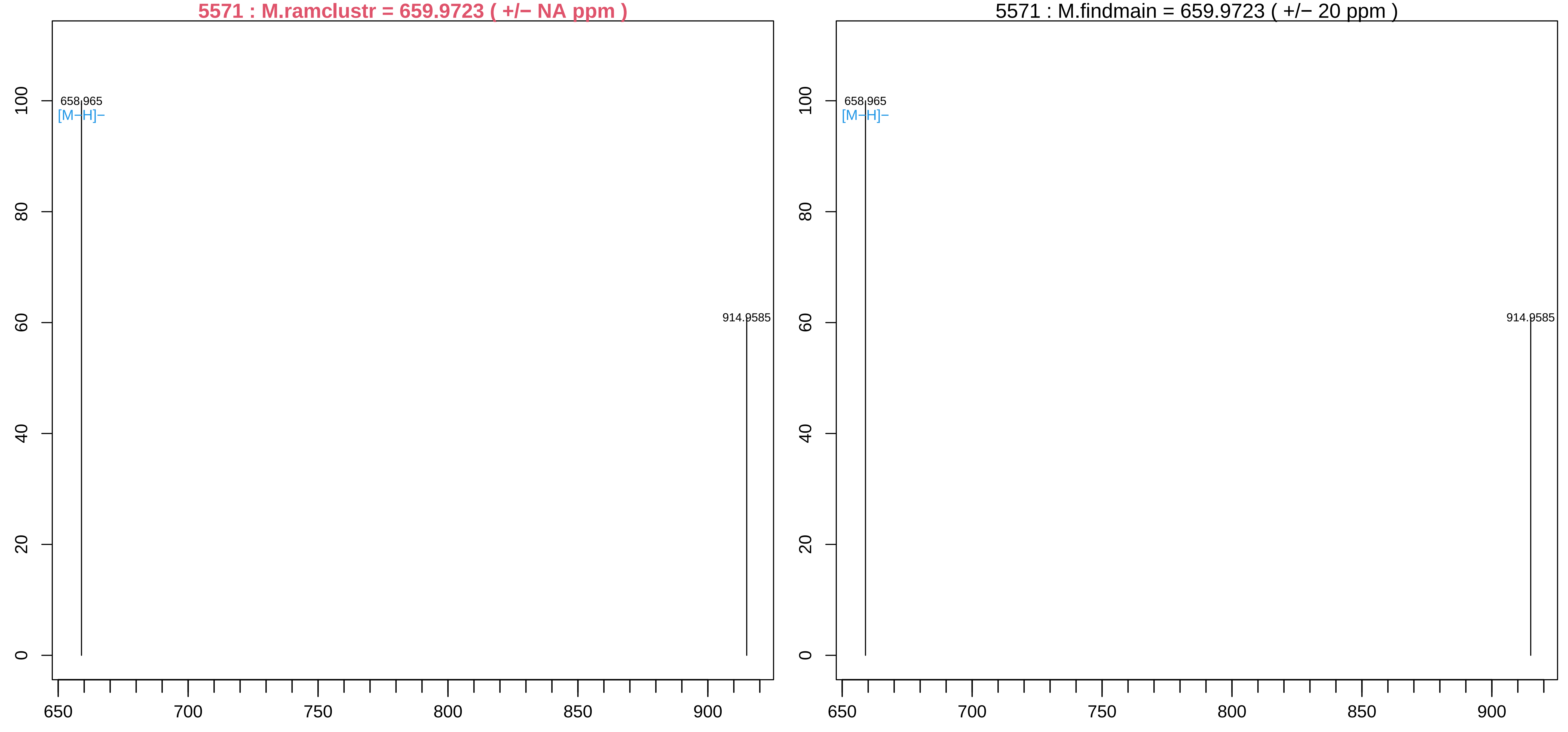
5571 : M.ramclustr = 659.9723 (+/− NA ppm)
658.965
[M−H]−
914.9585
0
20
40
60
80
100
650
700
750
800
850
900
5571 : M.findmain = 659.9723 (+/− 20 ppm)
658.965
[M−H]−
914.9585
0
20
40
60
80
100
650
700
750
800
850
900

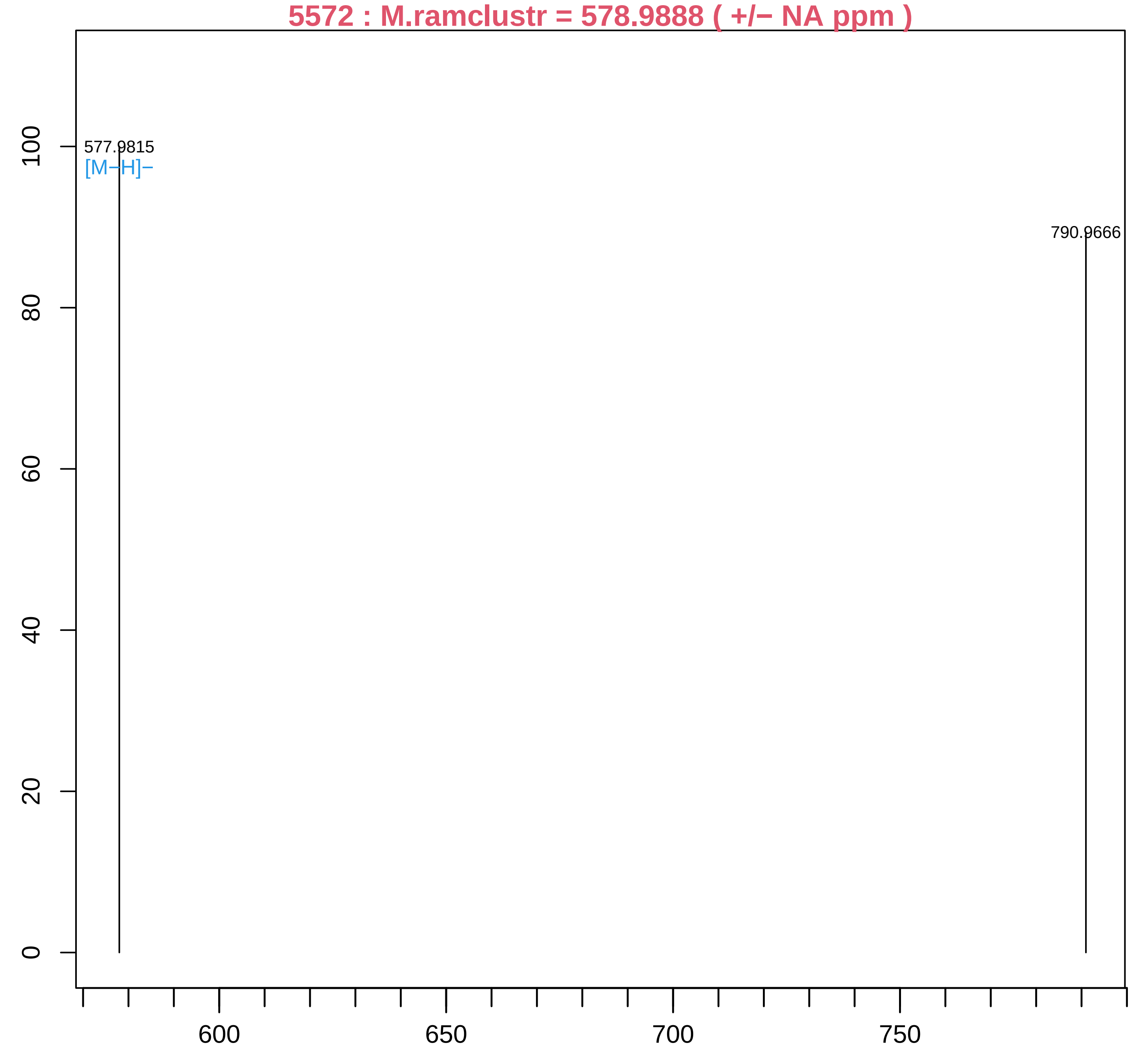
5572 : M.ramclustr = 578.9888 (+/− NA ppm)
577.9815
[M−H]−
790.9666
0
20
40
60
80
100
600
650
700
750

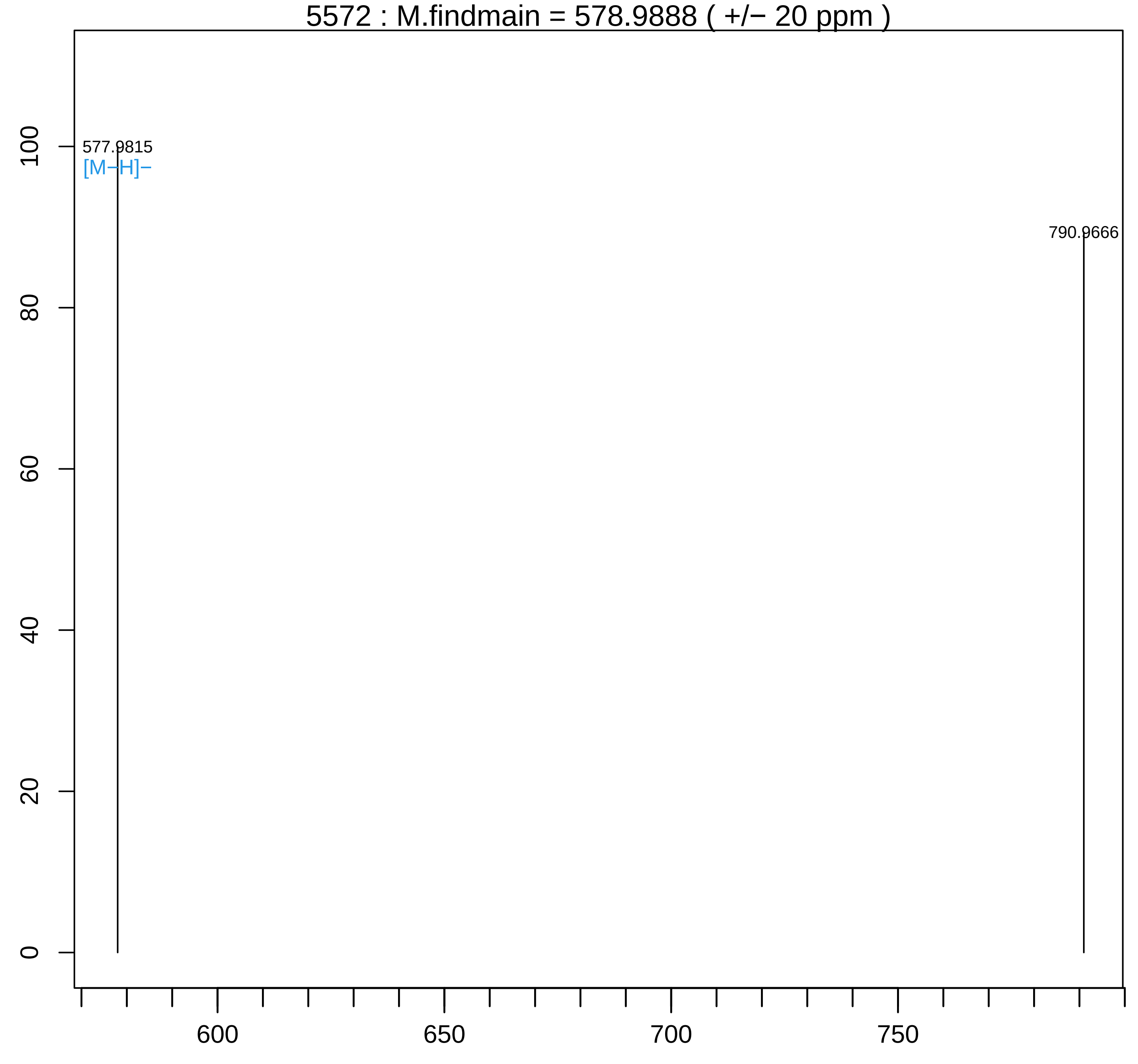
5572 : M.findmain = 578.9888 (+/− 20 ppm)
577.9815
[M−H]−
790.9666
0
20
40
60
80
100
600
650
700
750

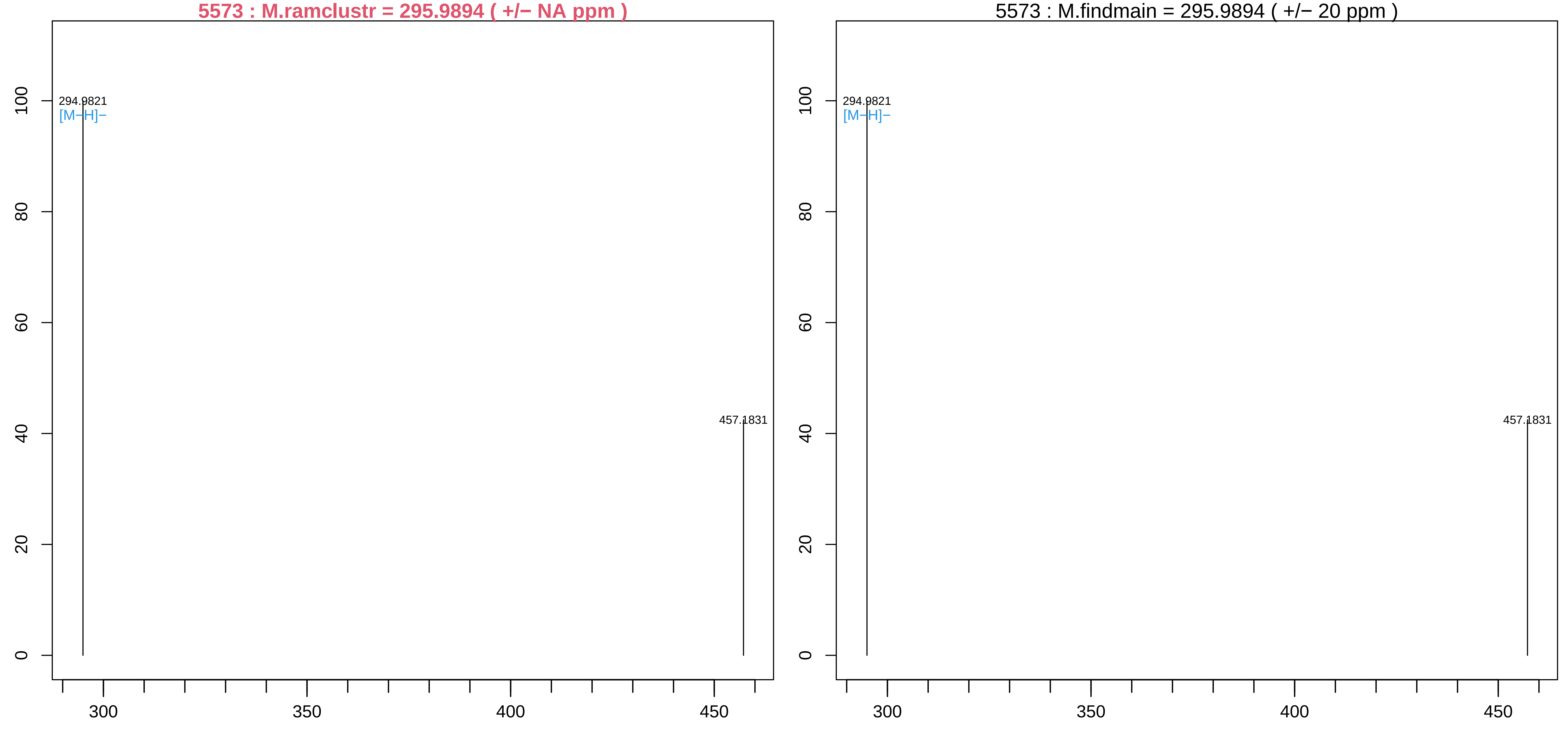

5573 : M.ramclustr = 295.9894 (+/− NA ppm)
294.9821
[M−H]−
457.1831
0
20
40
60
80
100
300
350
400
450
5573 : M.findmain = 295.9894 (+/− 20 ppm)
294.9821
[M−H]−
457.1831
0
20
40
60
80
100
300
350
400
450

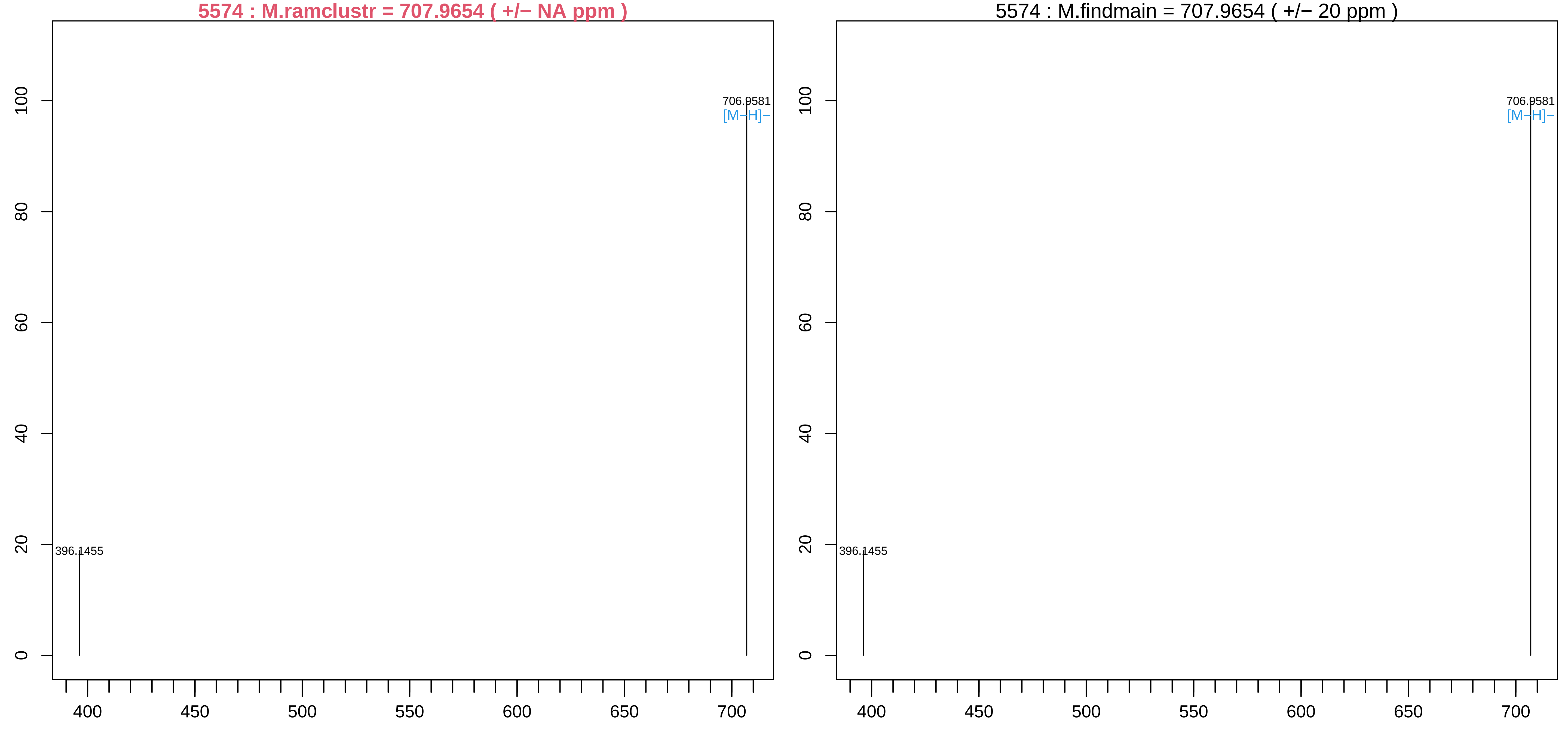
5574 : M.ramclustr = 707.9654 (+/− NA ppm)
706.9581
[M−H]−
396.1455
0
20
40
60
80
100
400
450
500
550
600
650
700
5574 : M.findmain = 707.9654 (+/− 20 ppm)
706.9581
[M−H]−
396.1455
0
20
40
60
80
100
400
450
500
550
600
650
700

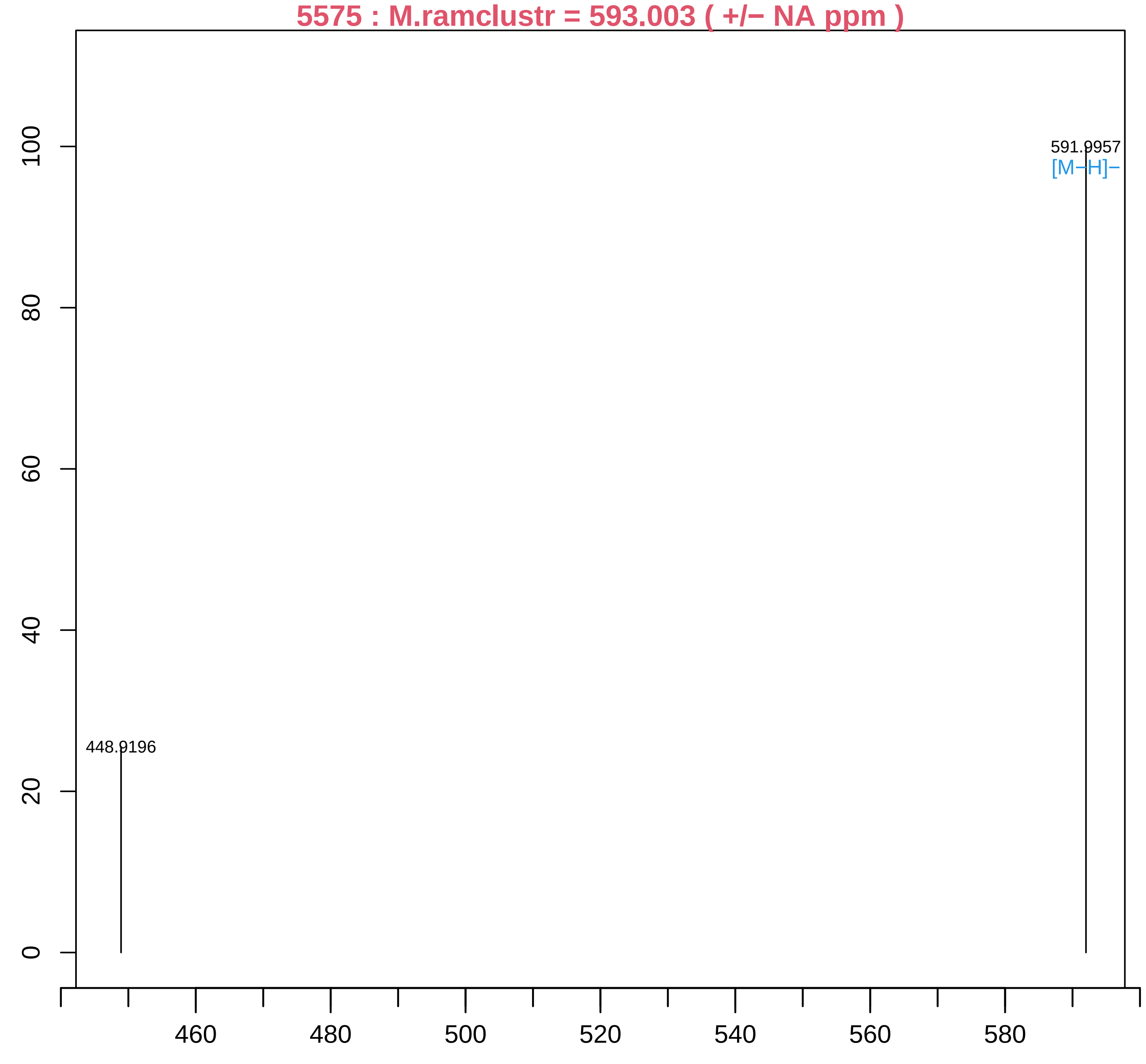
5575 : M.ramclustr = 593.003 (+/− NA ppm)
591.9957
[M−H]−
448.9196
0
20
40
60
80
100
460
480
500
520
540
560
580

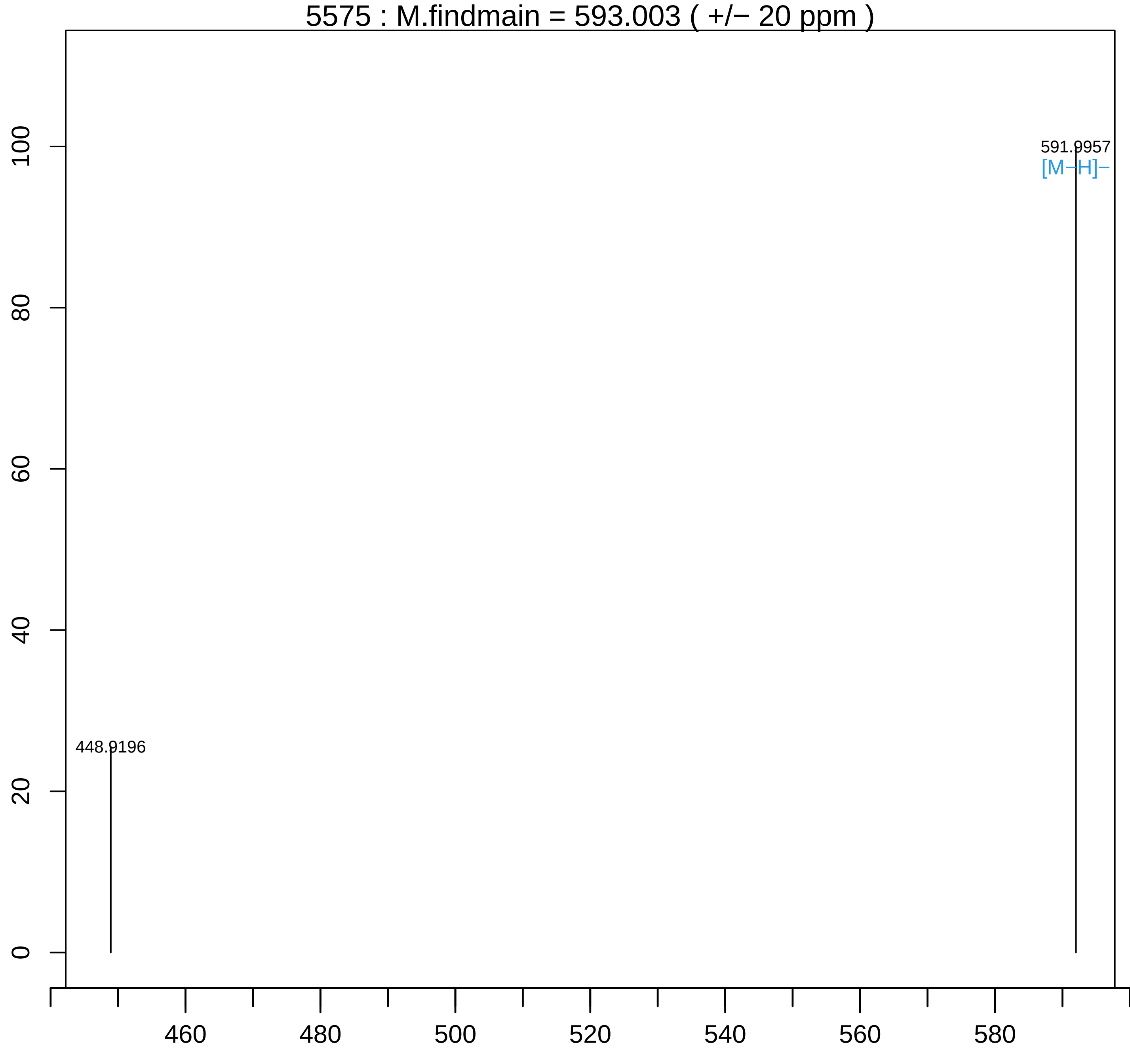
5575 : M.findmain = 593.003 (+/− 20 ppm)
591.9957
[M−H]−
448.9196
0
20
40
60
80
100
460
480
500
520
540
560
580

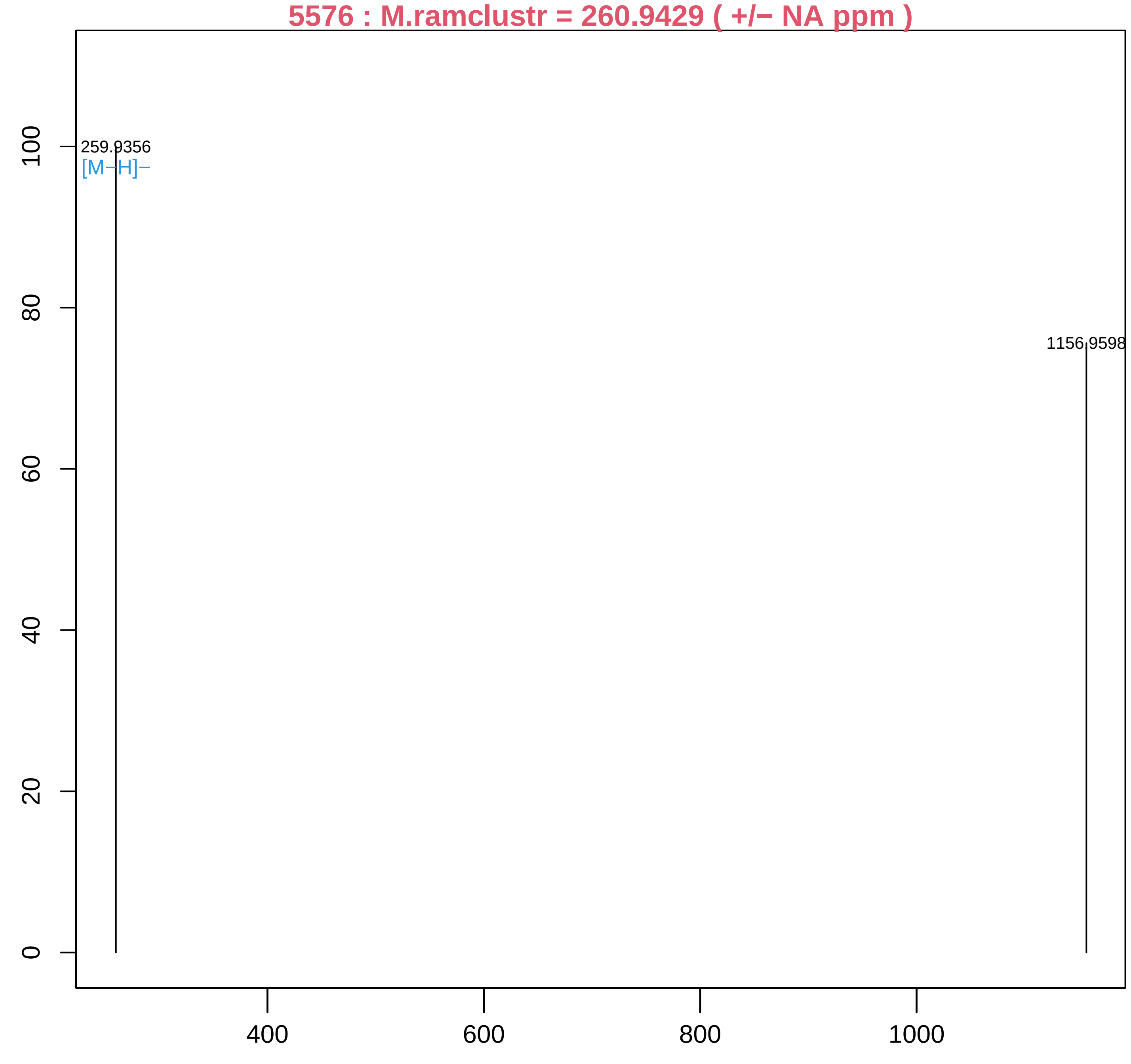
5576 : M.ramclustr = 260.9429 (+/− NA ppm)
259.9356
[M−H]−
1156.9598
0
20
40
60
80
100
400
600
800
1000

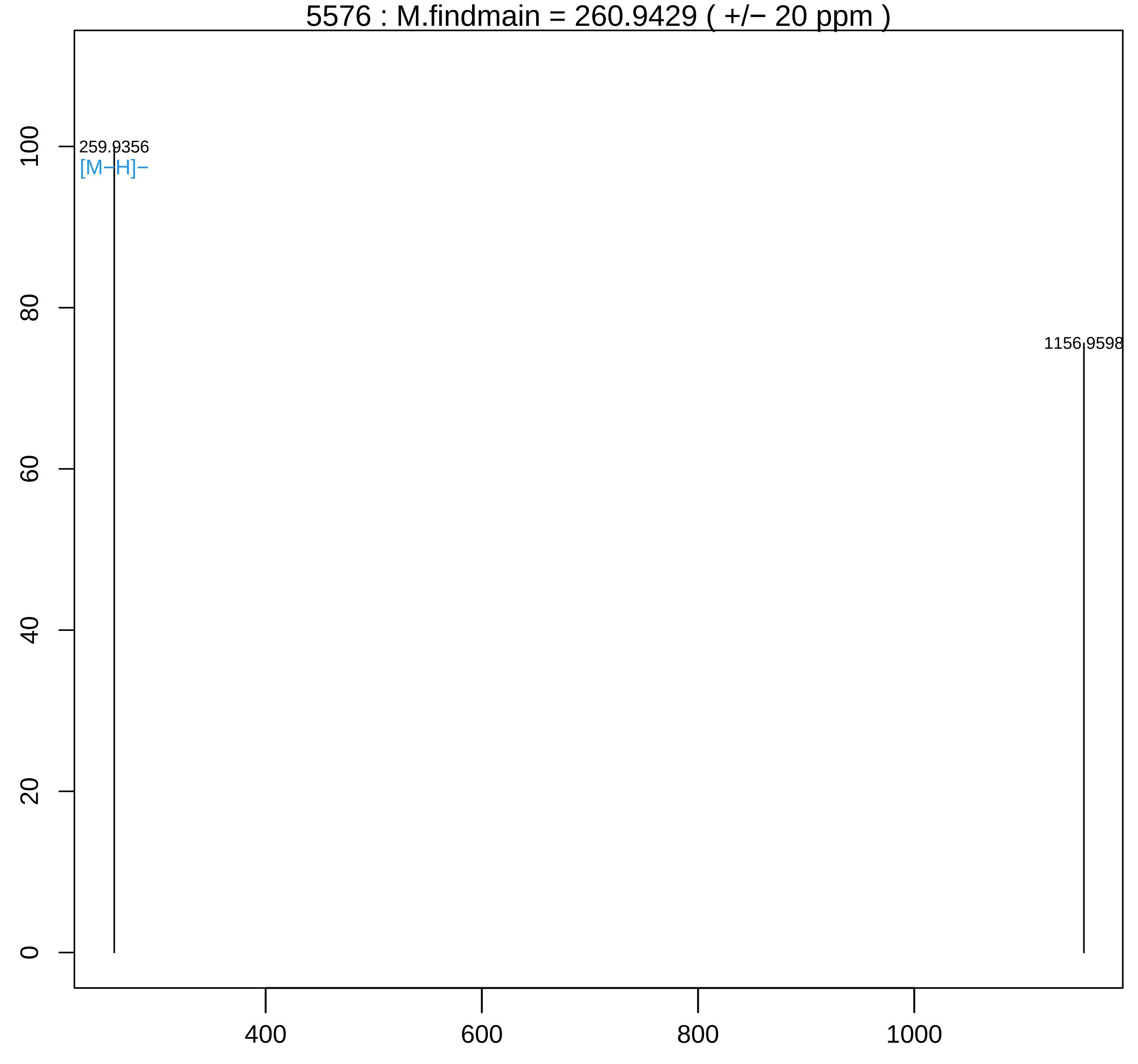
5576 : M.findmain = 260.9429 (+/− 20 ppm)
259.9356
[M−H]−
1156.9598
0
20
40
60
80
100
400
600
800
1000

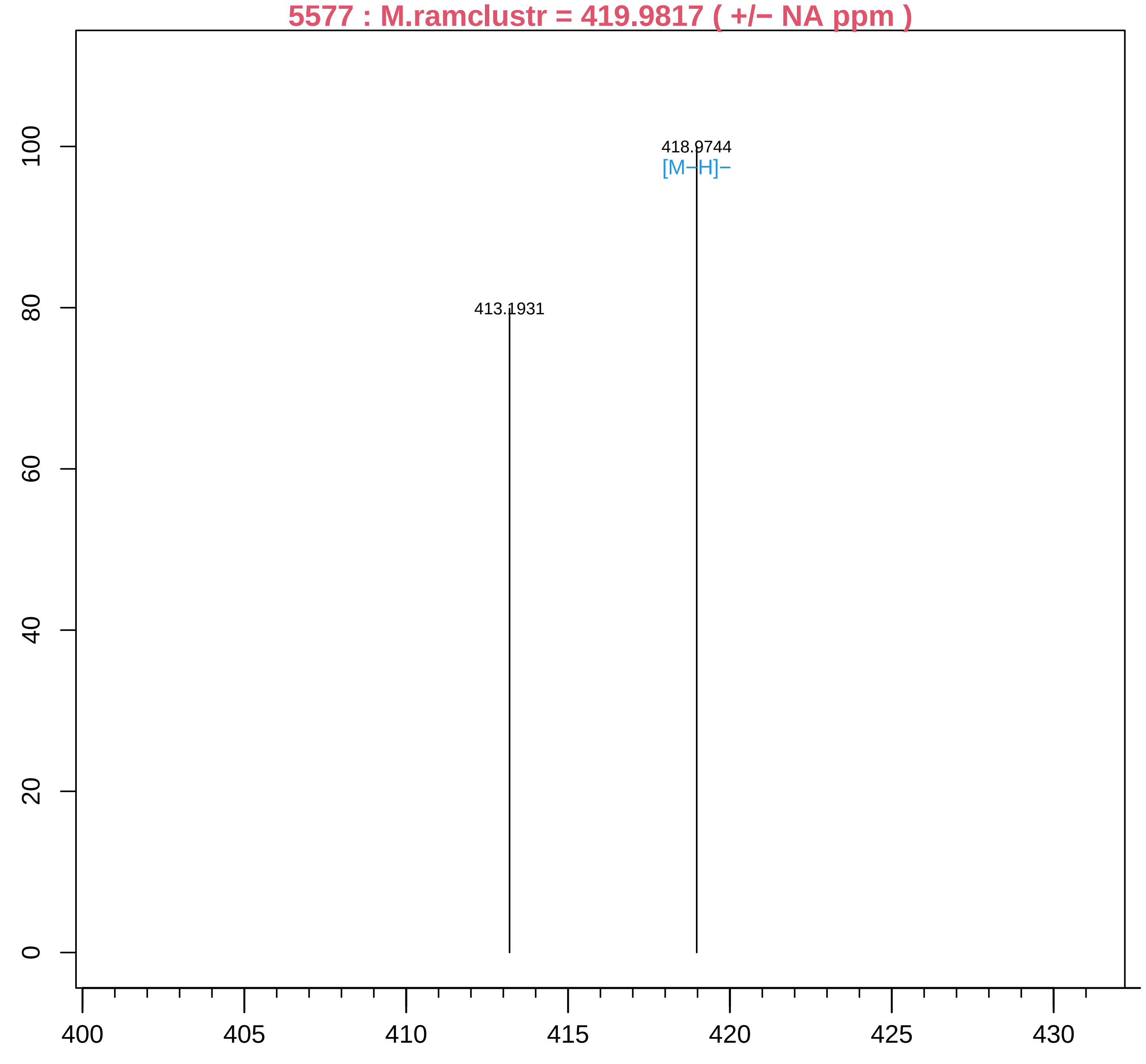
5577 : M.ramclustr = 419.9817 (+/− NA ppm)
418.9744
[M−H]−
413.1931
0
20
40
60
80
100
400
405
410
415
420
425
430

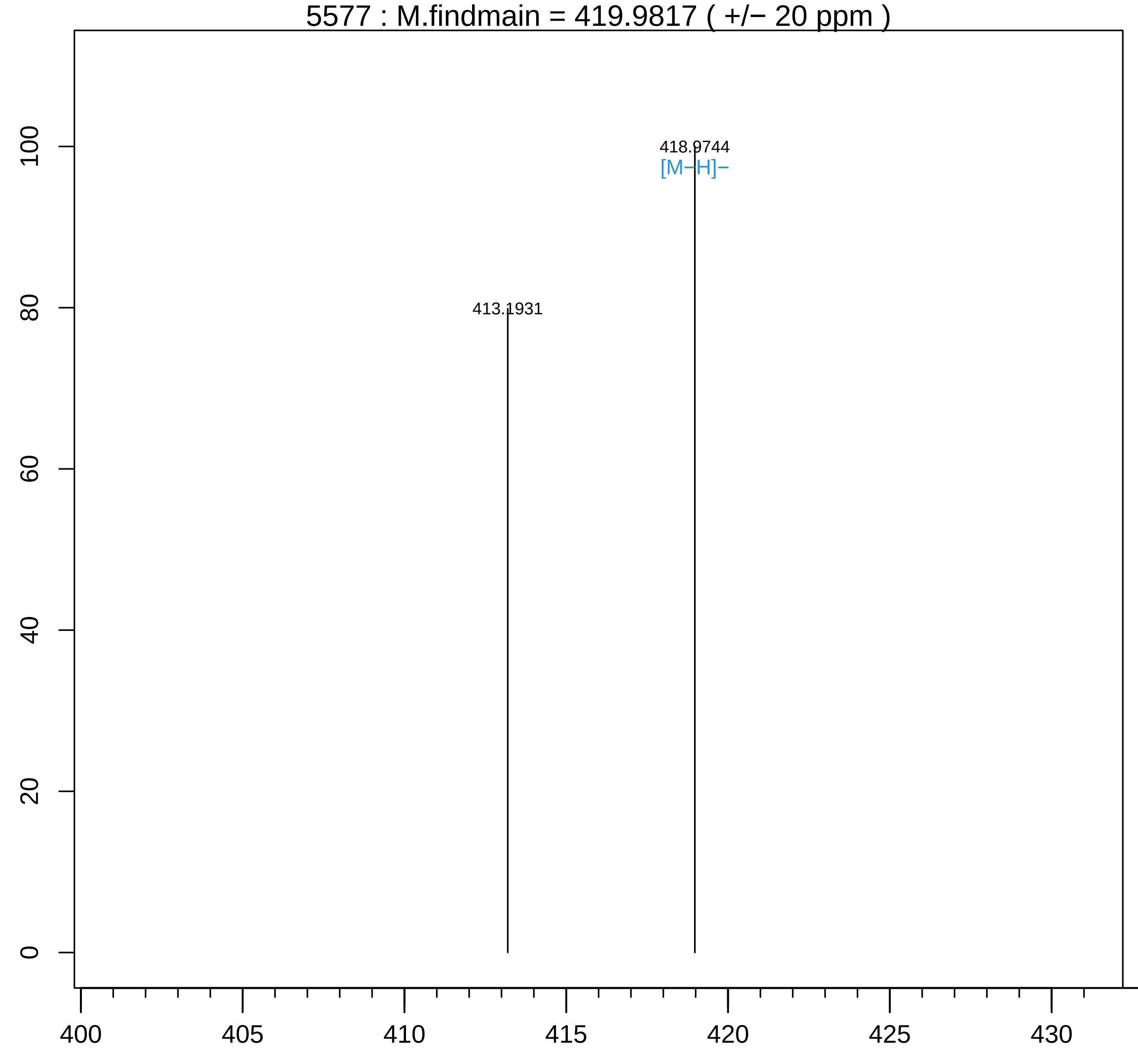
5577 : M.findmain = 419.9817 (+/− 20 ppm)
418.9744
[M−H]−
413.1931
0
20
40
60
80
100
400
405
410
415
420
425
430

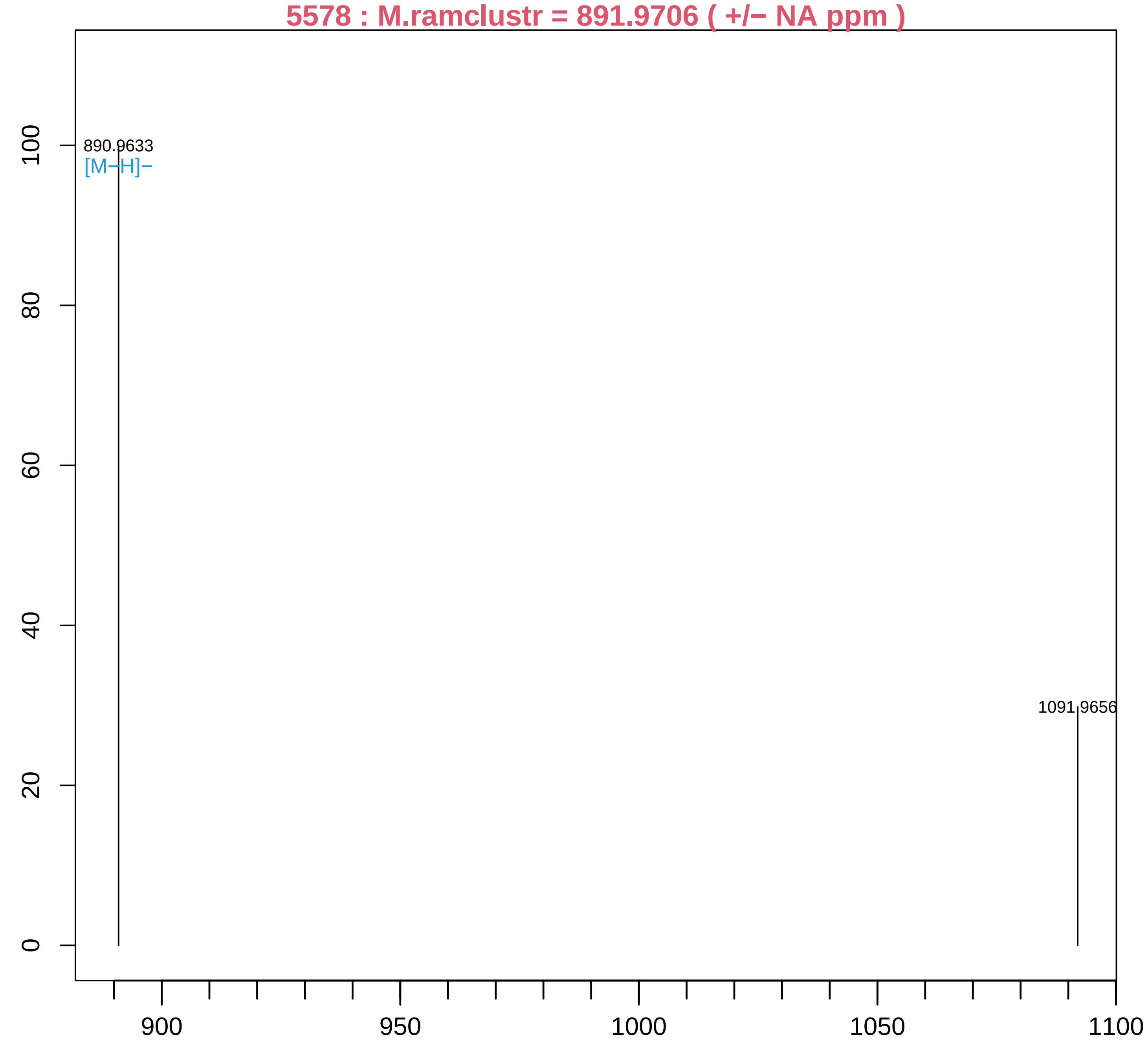

5578 : M.ramclustr = 891.9706 (+/− NA ppm)
890.9633
[M−H]−
1091.9656
0
20
40
60
80
100
900
950
1000
1050
1100

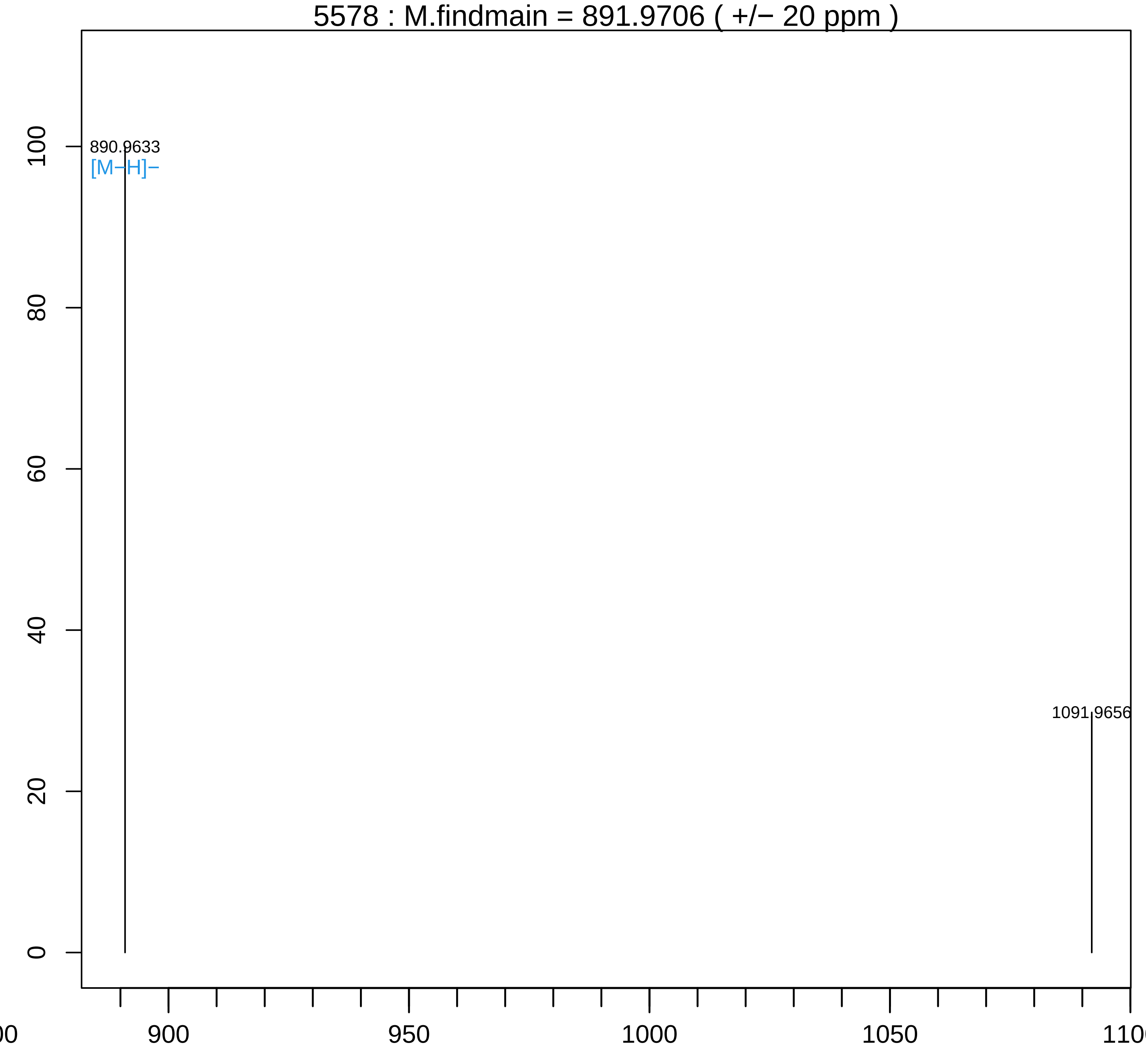

5578 : M.findmain = 891.9706 (+/− 20 ppm)
890.9633
[M−H]−
1091.9656
0
20
40
60
80
100
900
950
1000
1050

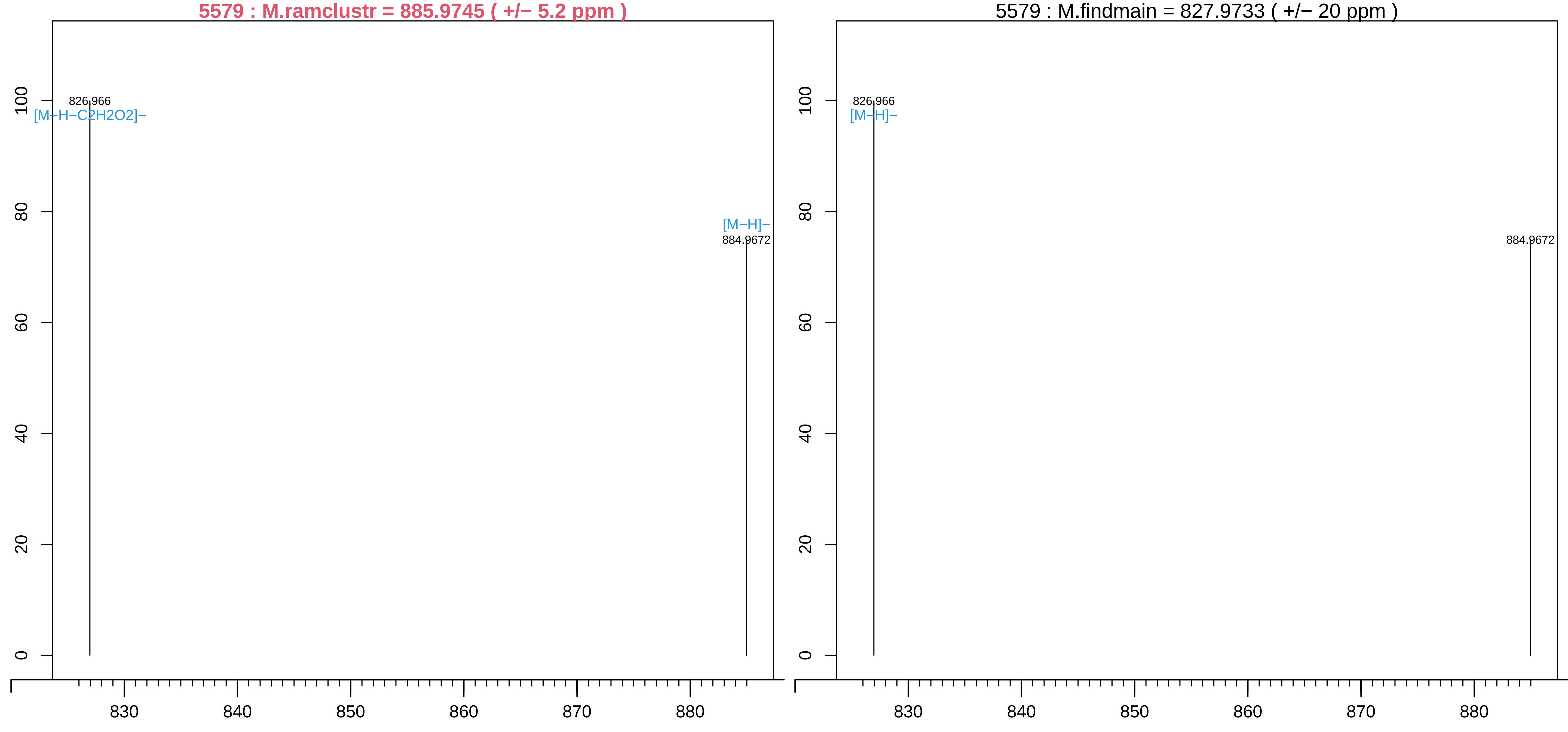
5579 : M.ramclustr = 885.9745 (+/− 5.2 ppm)
826.966
[M−H−C2H2O2]−
[M−H]−
884.9672
5579 : M.findmain = 827.9733 (+/− 20 ppm)
826.966
[M−H]−
884.9672
0
20
40
60
80
100
830
840
850
860
870
880

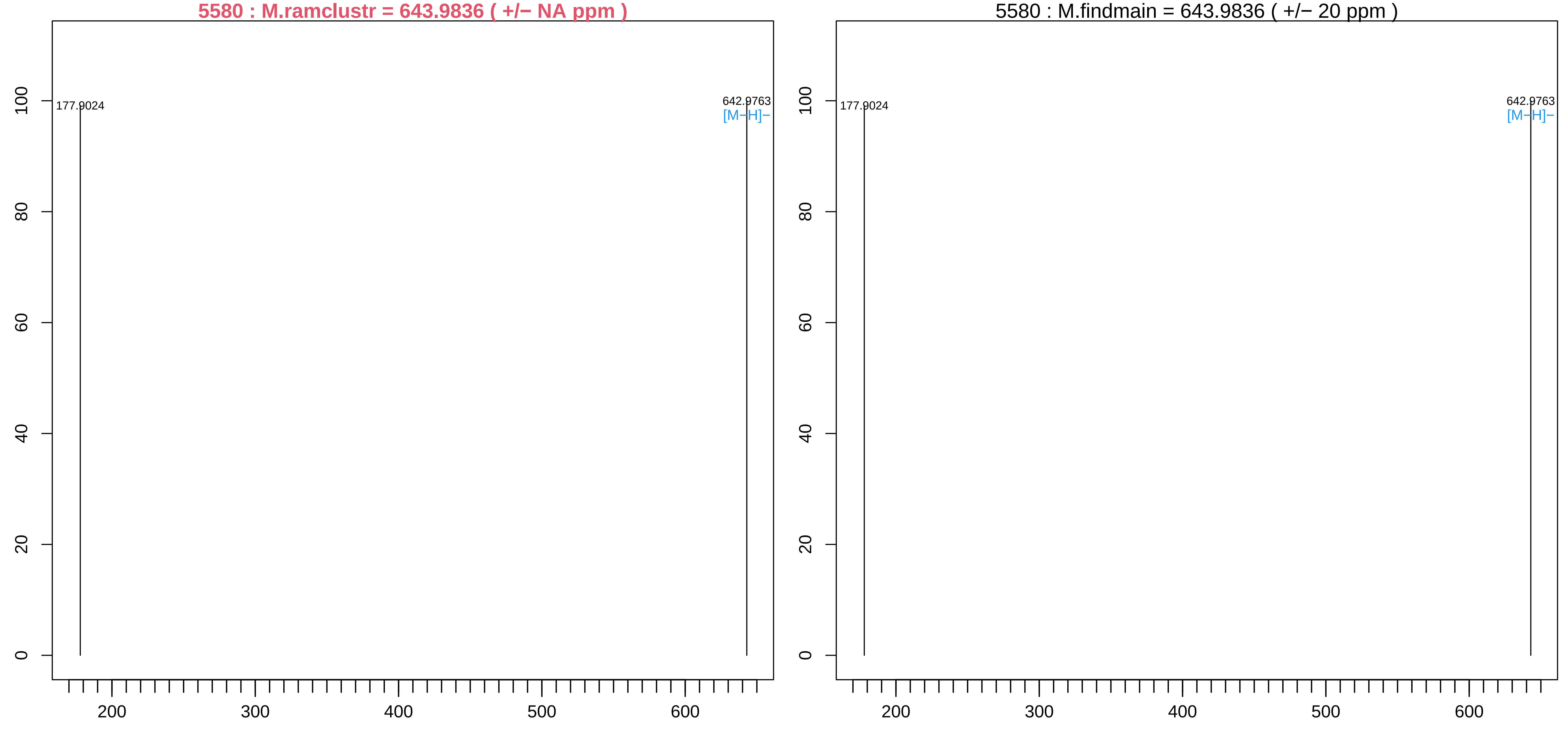
5580 : M.ramclustr = 643.9836 (+/− NA ppm)
177.9024
642.9763
[M−H]−
0
20
40
60
80
100
200
300
400
500
600
5580 : M.findmain = 643.9836 (+/− 20 ppm)
177.9024
642.9763
[M−H]−
0
20
40
60
80
100
200
300
400
500
600

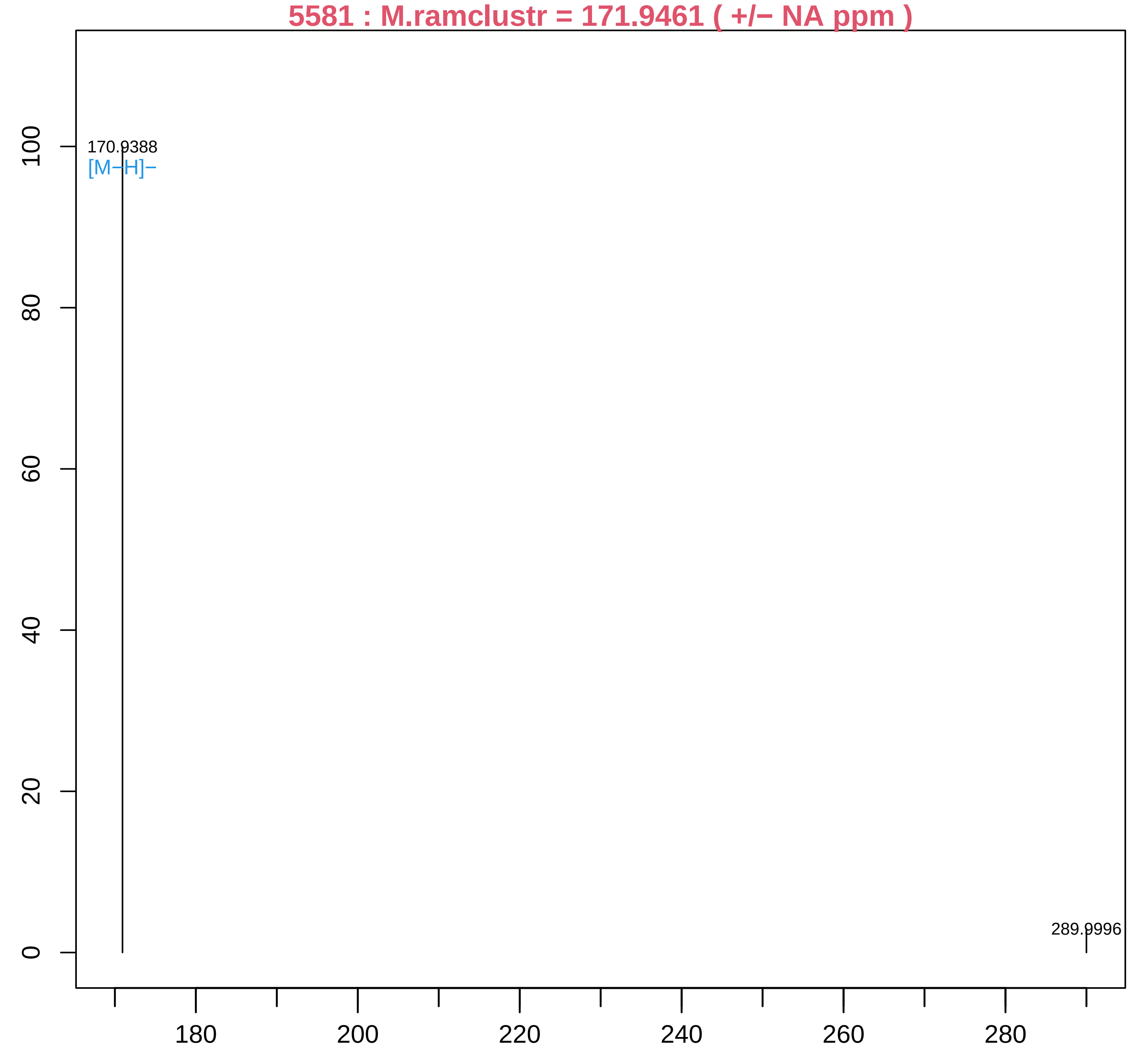

5581 : M.ramclustr = 171.9461 (+/− NA ppm)
170.9388
[M−H]−
289.9996
0
20
40
60
80
100
180
200
220
240
260
280

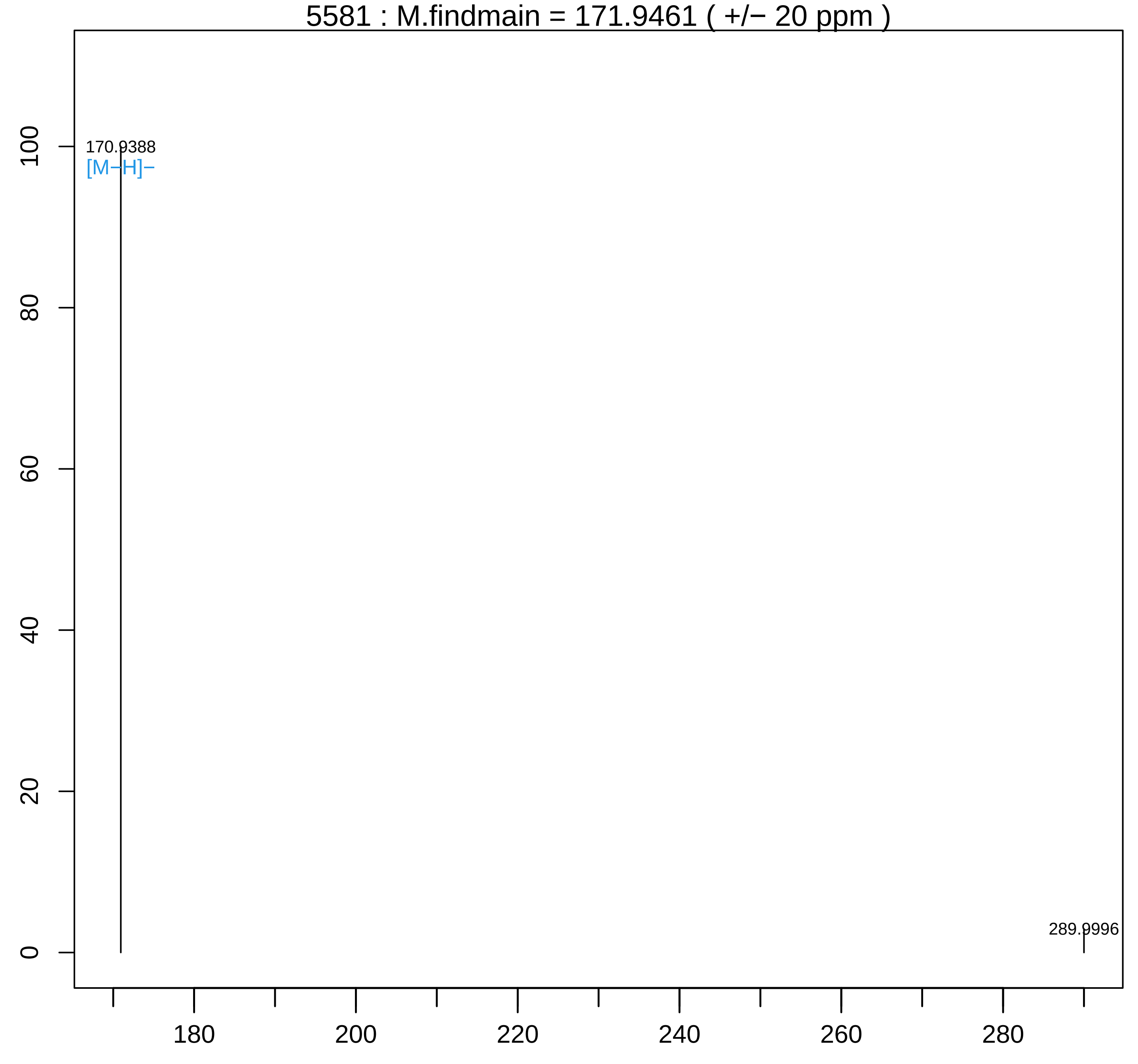

5581 : M.findmain = 171.9461 (+/− 20 ppm)
170.9388
[M−H]−
289.9996
0
20
40
60
80
100
180
200
220
240
260
280

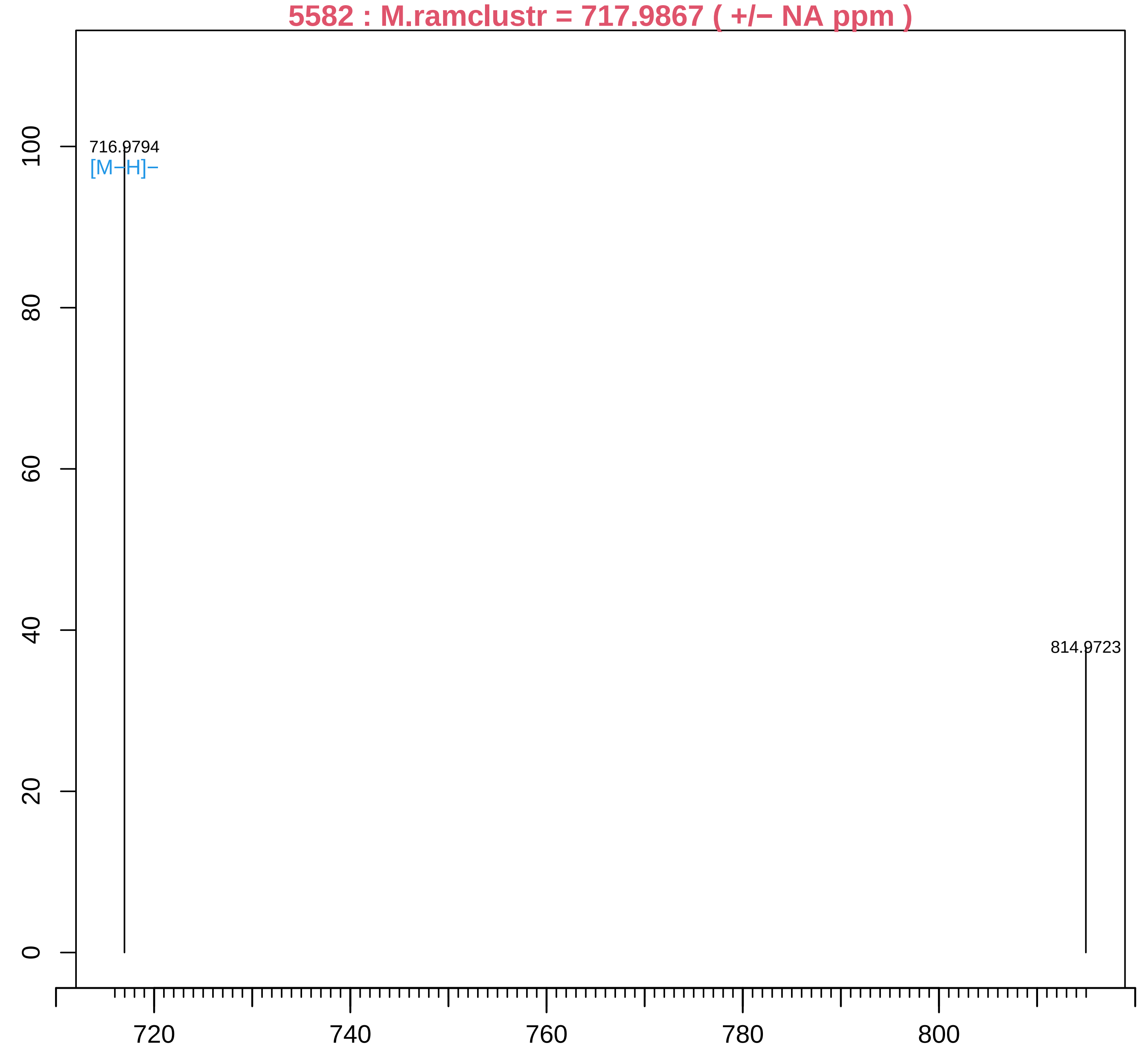

5582 : M.ramclustr = 717.9867 (+/− NA ppm)
716.9794
[M−H]−
814.9723
0
20
40
60
80
100
720
740
760
780
800

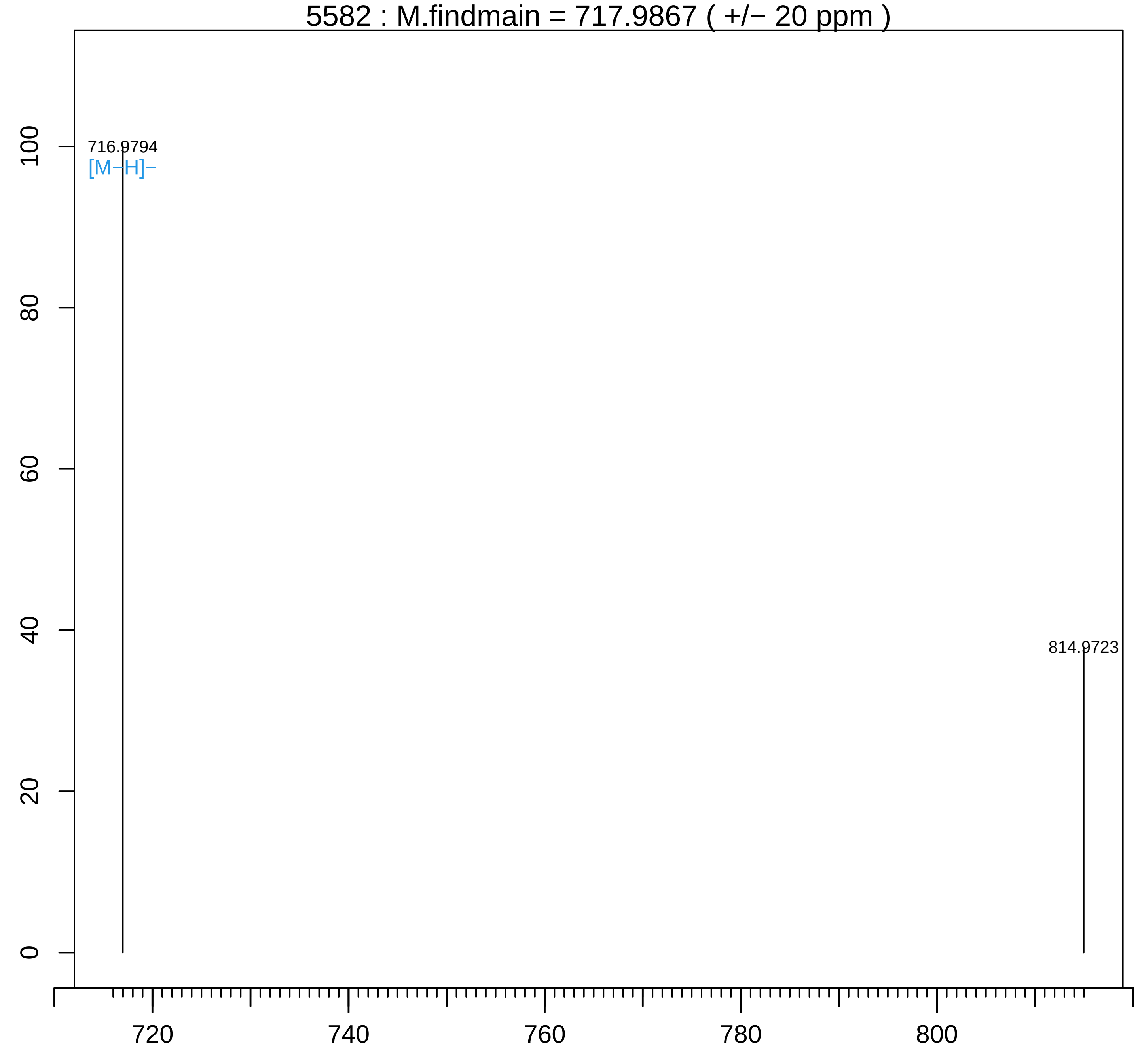

5582 : M.findmain = 717.9867 (+/− 20 ppm)
716.9794
[M−H]−
814.9723
0
20
40
60
80
100
720
740
760
780
800

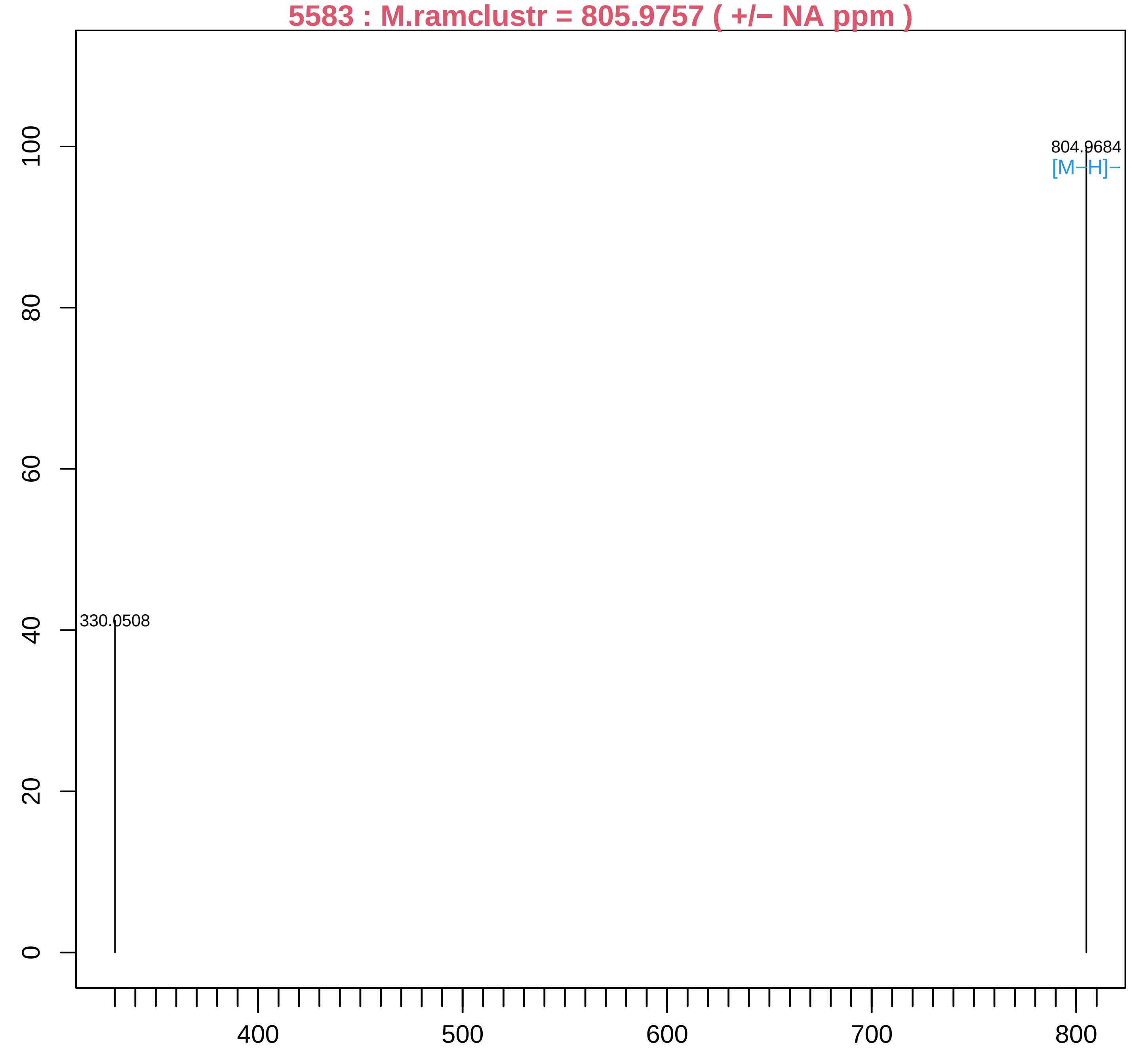
5583 : M.ramclustr = 805.9757 (+/− NA ppm)
804.9684
[M−H]−
330.0508
0
20
40
60
80
100
400
500
600
700
800

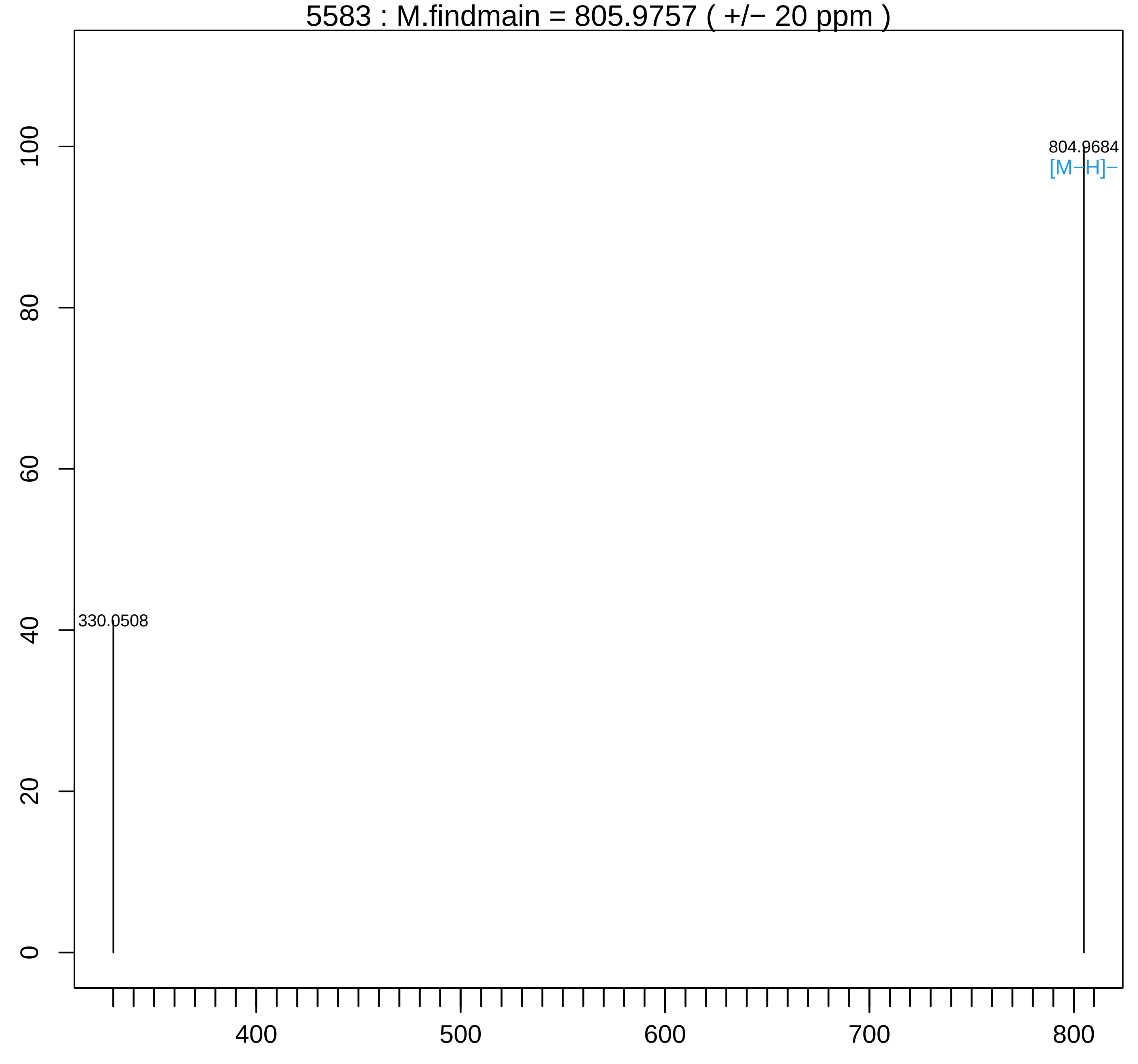
5583 : M.findmain = 805.9757 (+/− 20 ppm)
804.9684
[M−H]−
330.0508
0
20
40
60
80
100
400
500
600
700
800

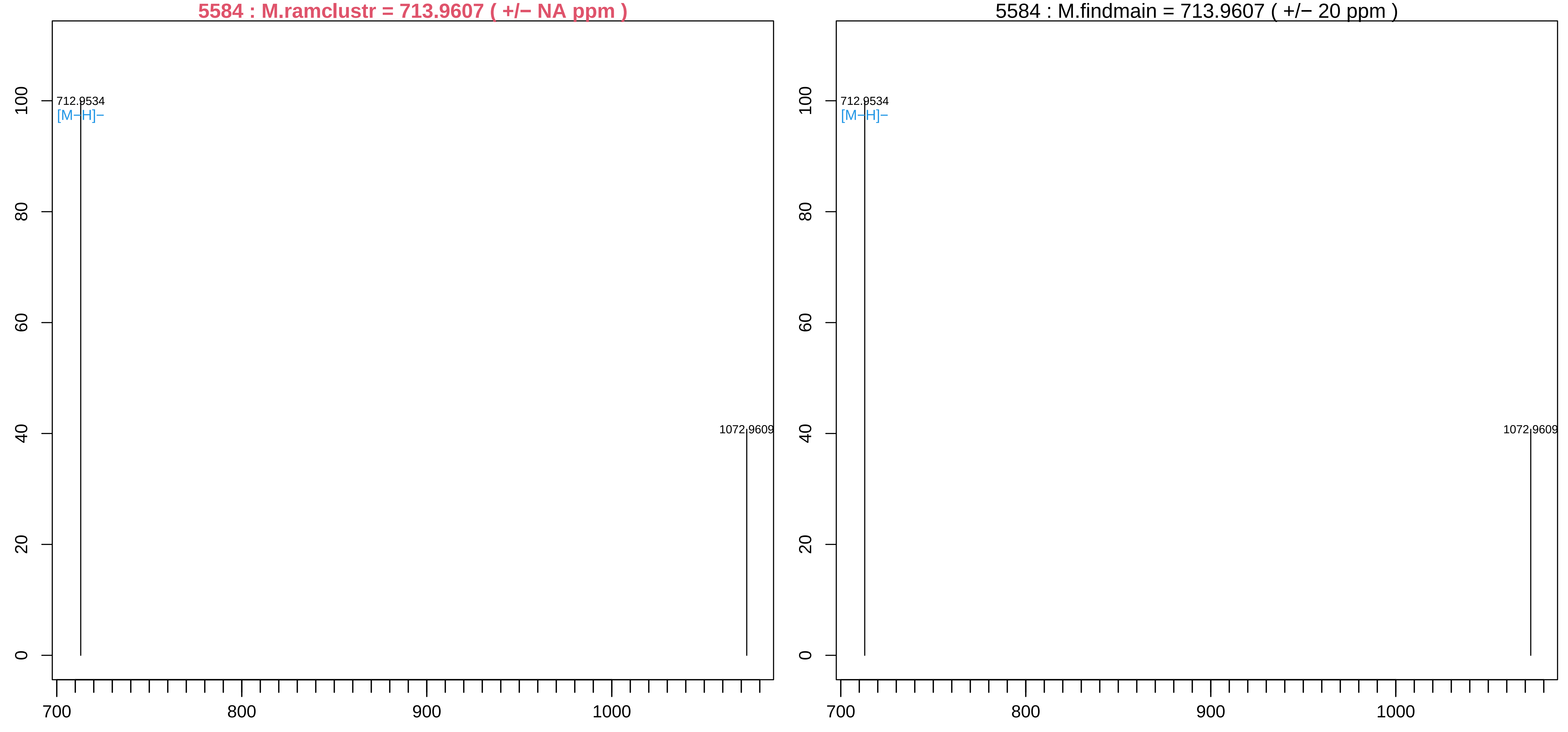

5584 : M.ramclustr = 713.9607 (+/− NA ppm)
712.9534
[M−H]−
1072.9609
5584 : M.findmain = 713.9607 (+/− 20 ppm)
712.9534
[M−H]−
1072.9609
0
20
40
60
80
100
700
800
900
1000

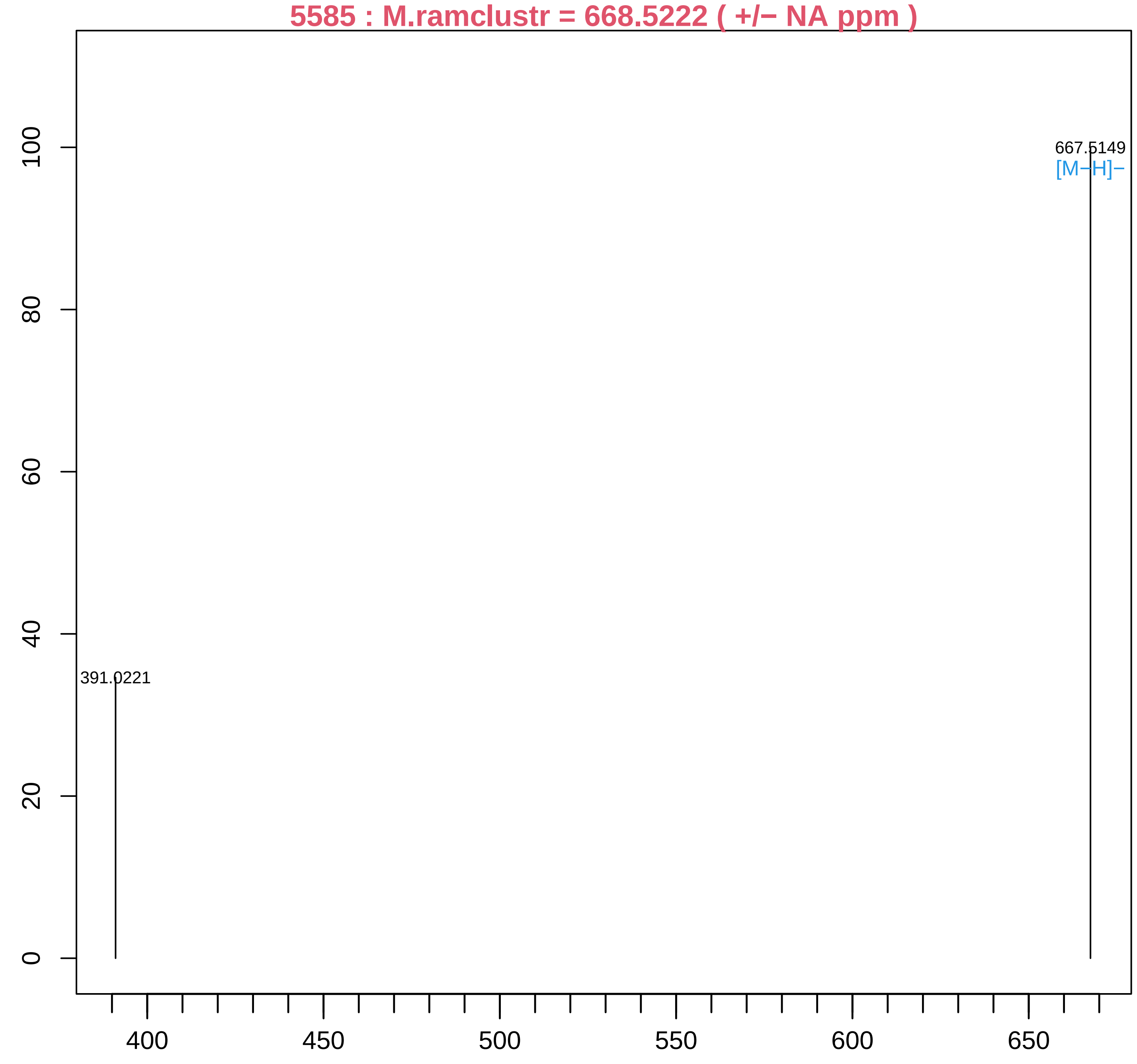
5585 : M.ramclustr = 668.5222 (+/− NA ppm)
667.5149
[M−H]−
391.0221
0
20
40
60
80
100
400
450
500
550
600
650

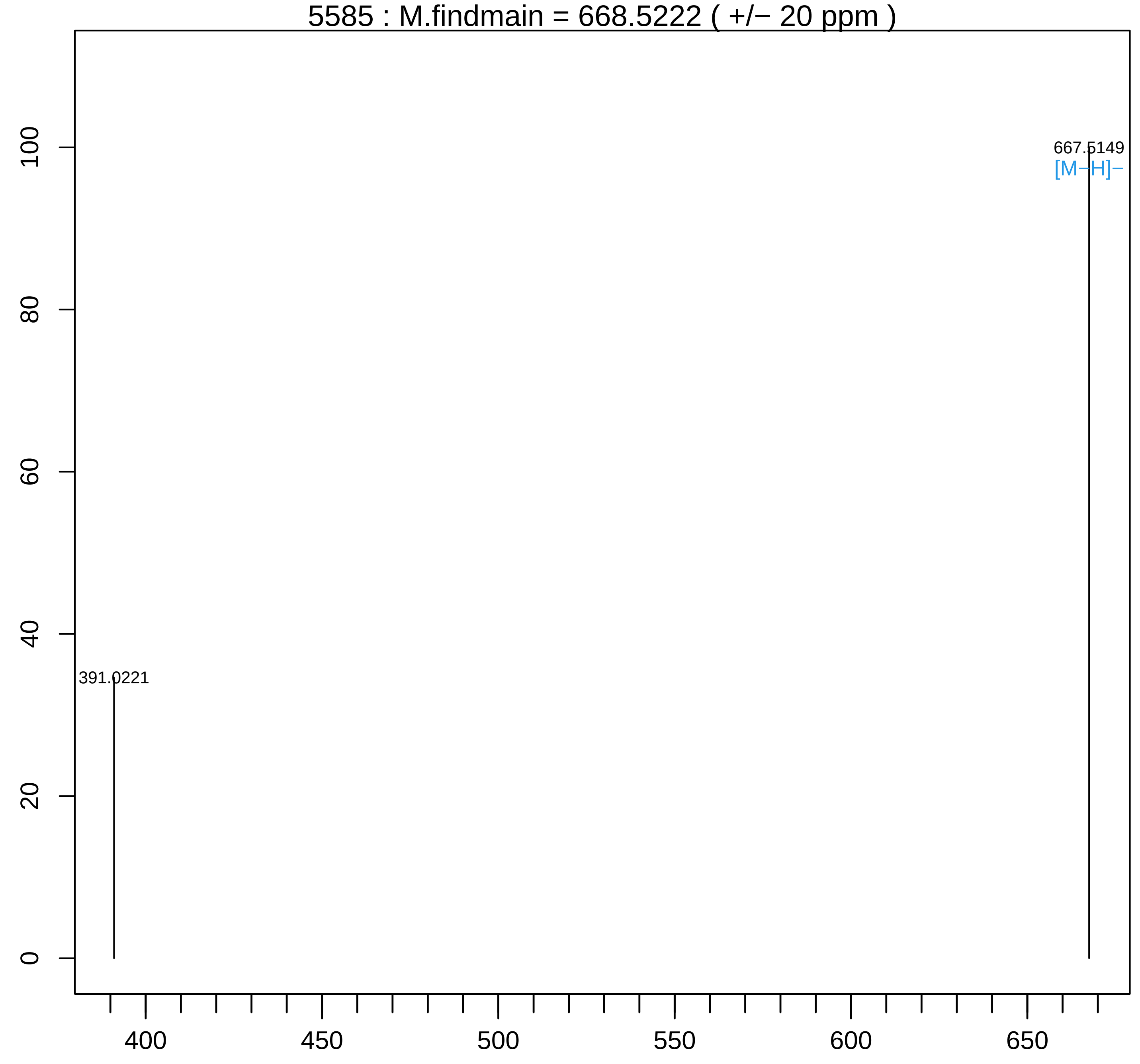
5585 : M.findmain = 668.5222 (+/− 20 ppm)
667.5149
[M−H]−
391.0221
0
20
40
60
80
100
400
450
500
550
600
650

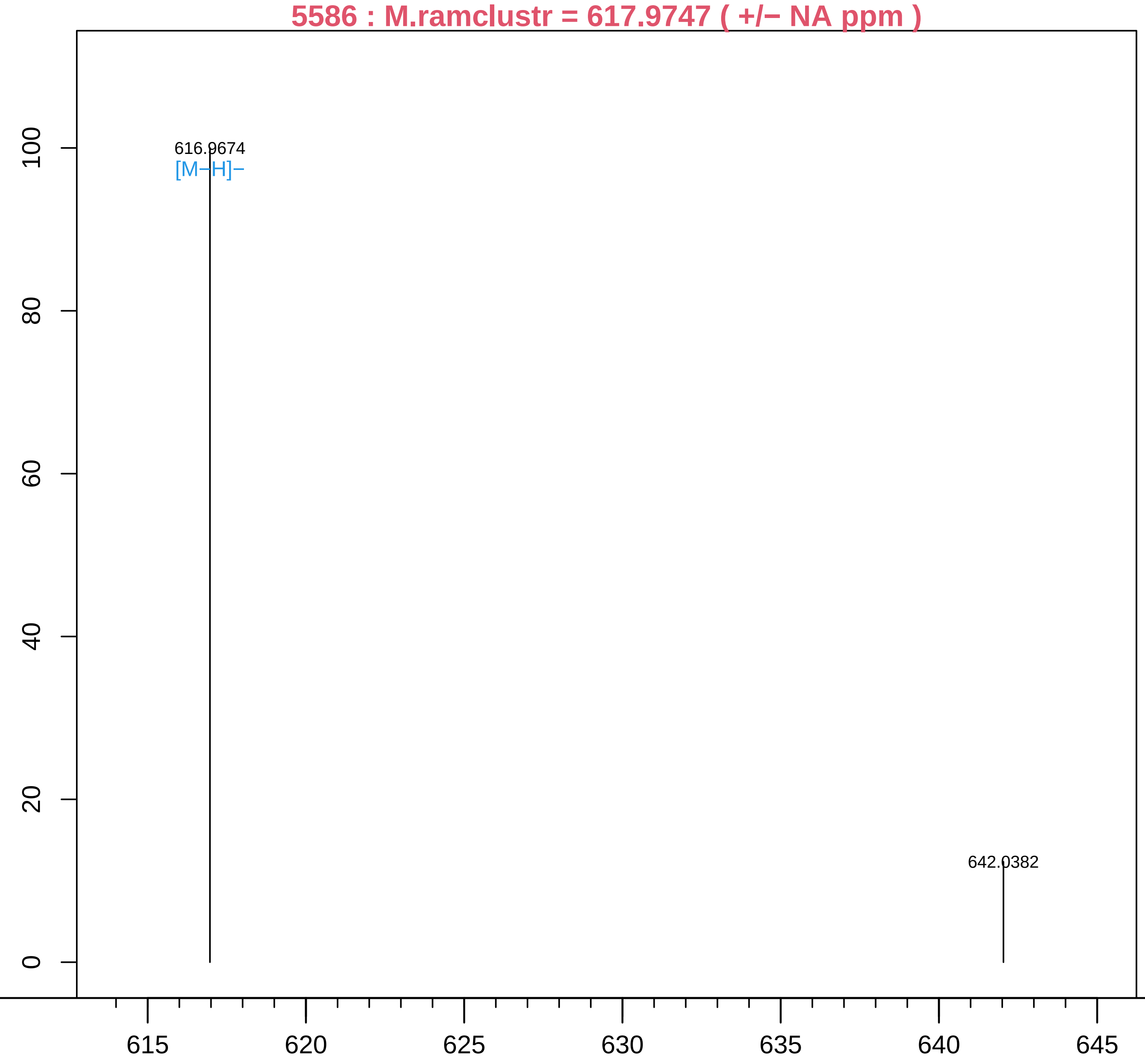
5586 : M.ramclustr = 617.9747 (+/− NA ppm)
616.9674
[M−H]−
642.0382
0
20
40
60
80
100
615
620
625
630
635
640
645
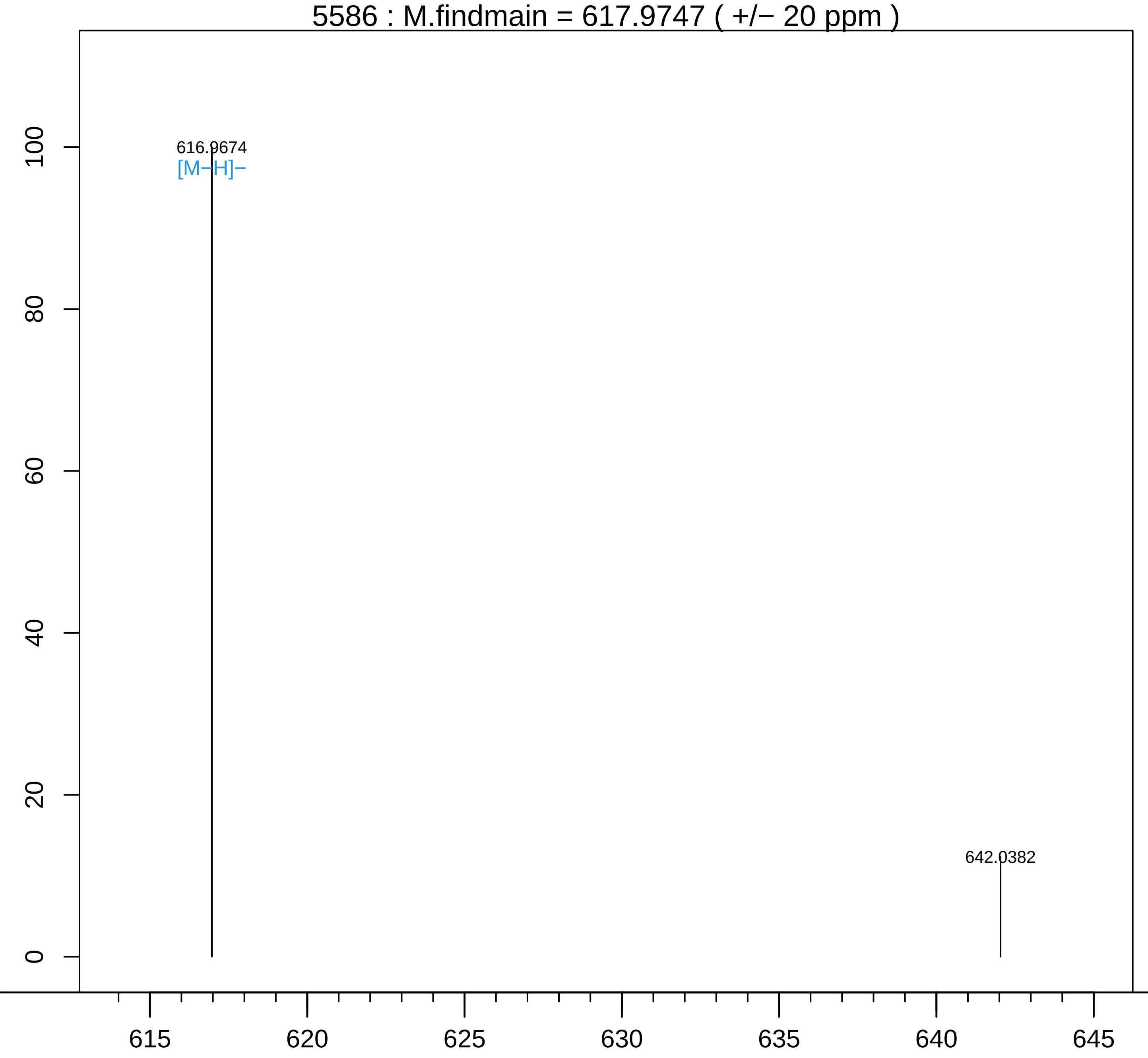
5586 : M.findmain = 617.9747 (+/− 20 ppm)
616.9674
[M−H]−
642.0382
0
20
40
60
80
100
615
620
625
630
635
640
645

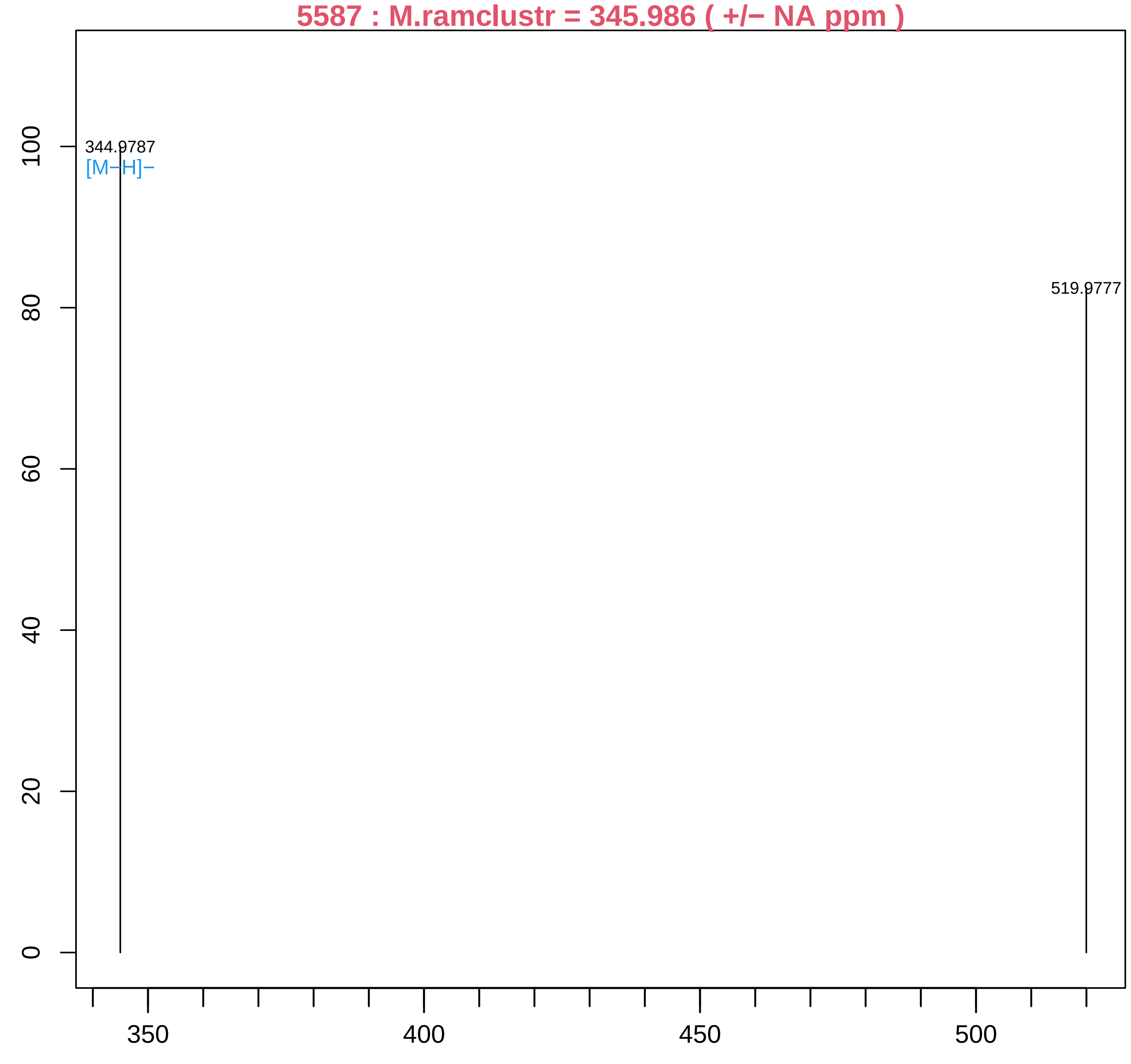
5587 : M.ramclustr = 345.986 (+/− NA ppm)
344.9787
[M−H]−
519.9777
0
20
40
60
80
100
350
400
450
500
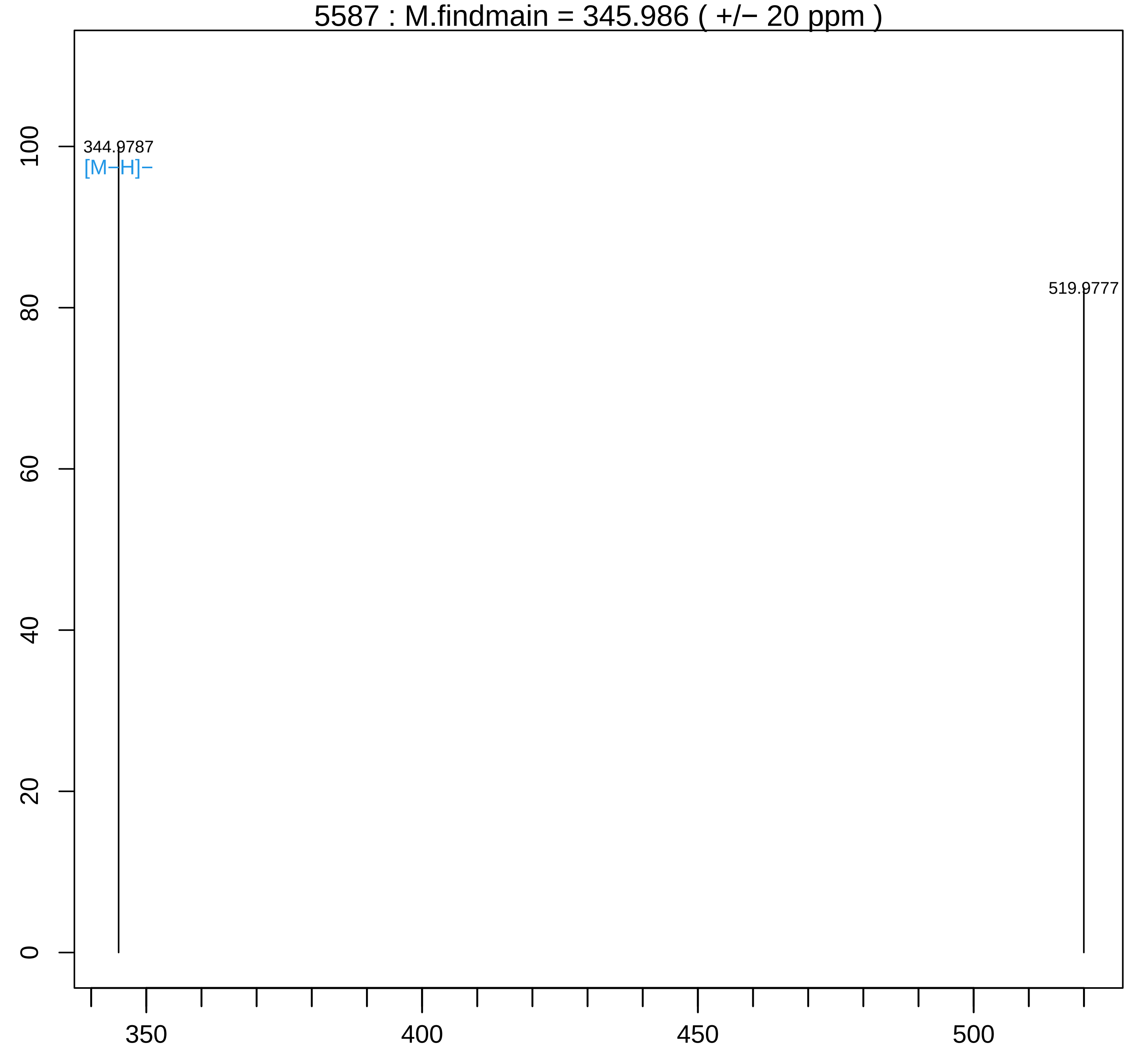
5587 : M.findmain = 345.986 (+/− 20 ppm)
344.9787
[M−H]−
519.9777
0
20
40
60
80
100
350
400
450
500

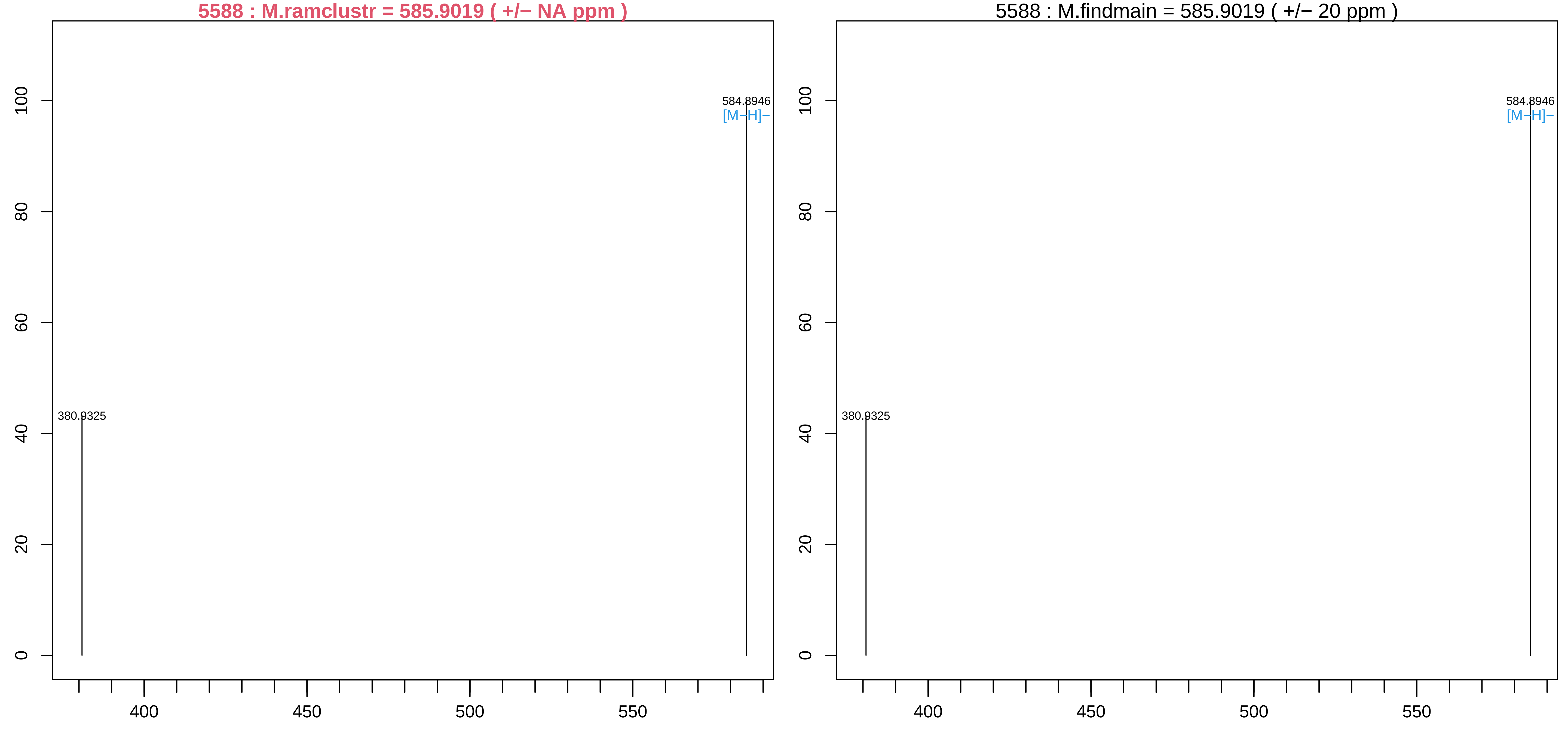

5588 : M.ramclustr = 585.9019 (+/− NA ppm)
584.8946
[M−H]−
380.9325
0
20
40
60
80
100
400
450
500
550
5588 : M.findmain = 585.9019 (+/− 20 ppm)
584.8946
[M−H]−
380.9325
0
20
40
60
80
100
400
450
500
550

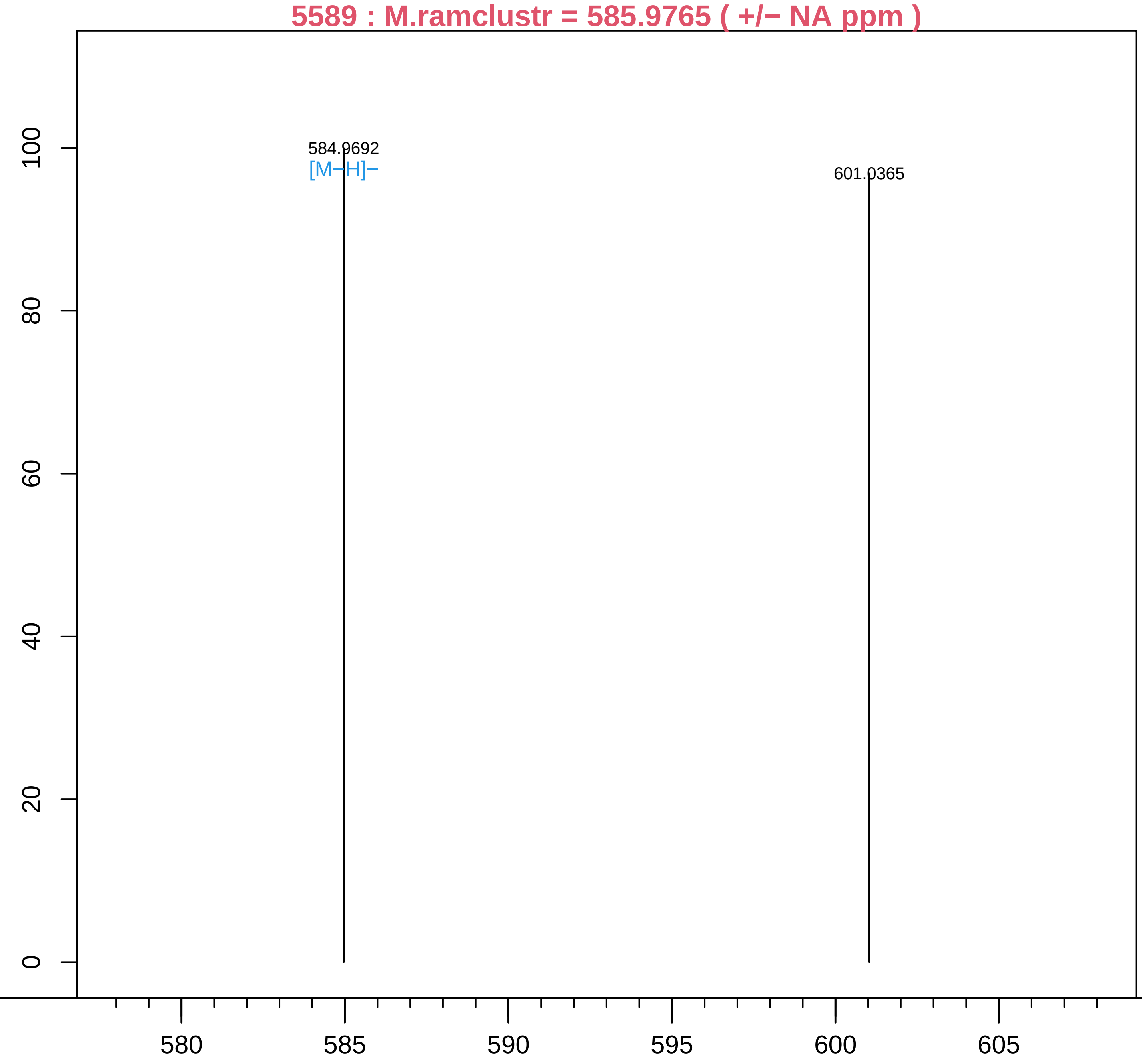
5589 : M.ramclustr = 585.9765 (+/− NA ppm)
584.9692
[M−H]−
601.0365
0
20
40
60
80
100
580
585
590
595
600
605

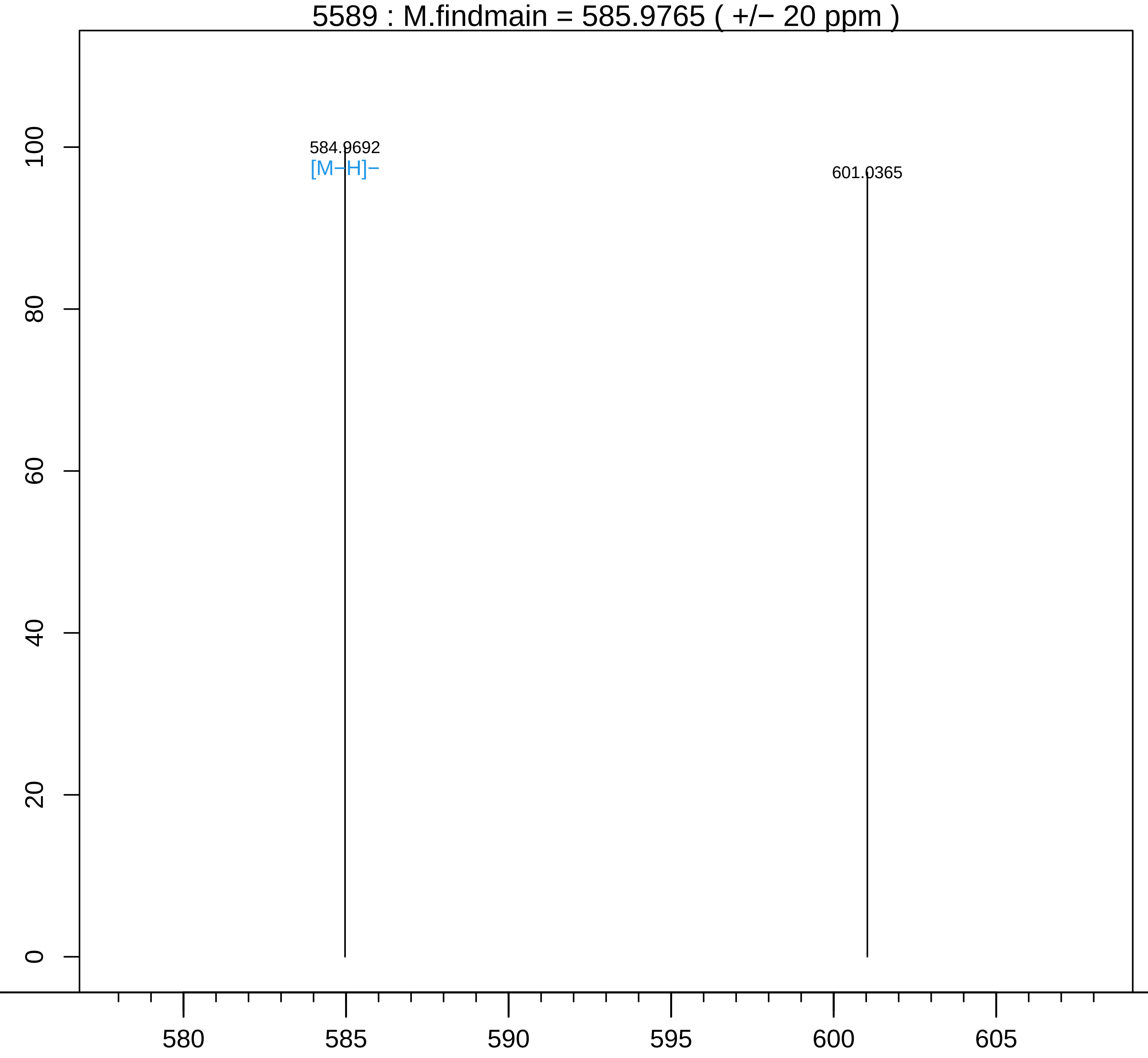
5589 : M.findmain = 585.9765 (+/− 20 ppm)
584.9692
[M−H]−
601.0365
0
20
40
60
80
100
580
585
590
595
600
605

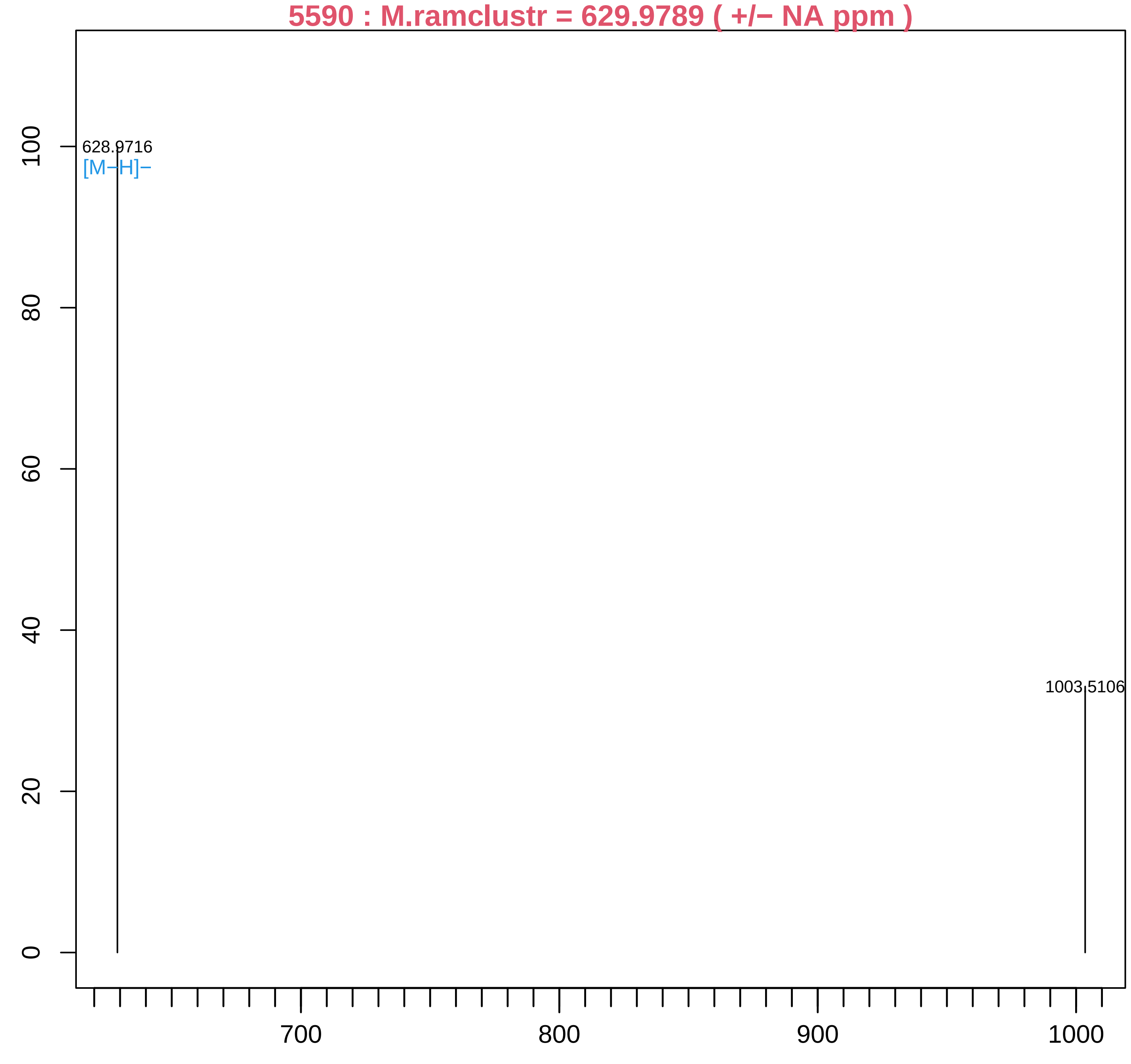
5590 : M.ramclustr = 629.9789 (+/− NA ppm)
628.9716
[M−H]−
1003.5106
0
20
40
60
80
100
700
800
900
1000

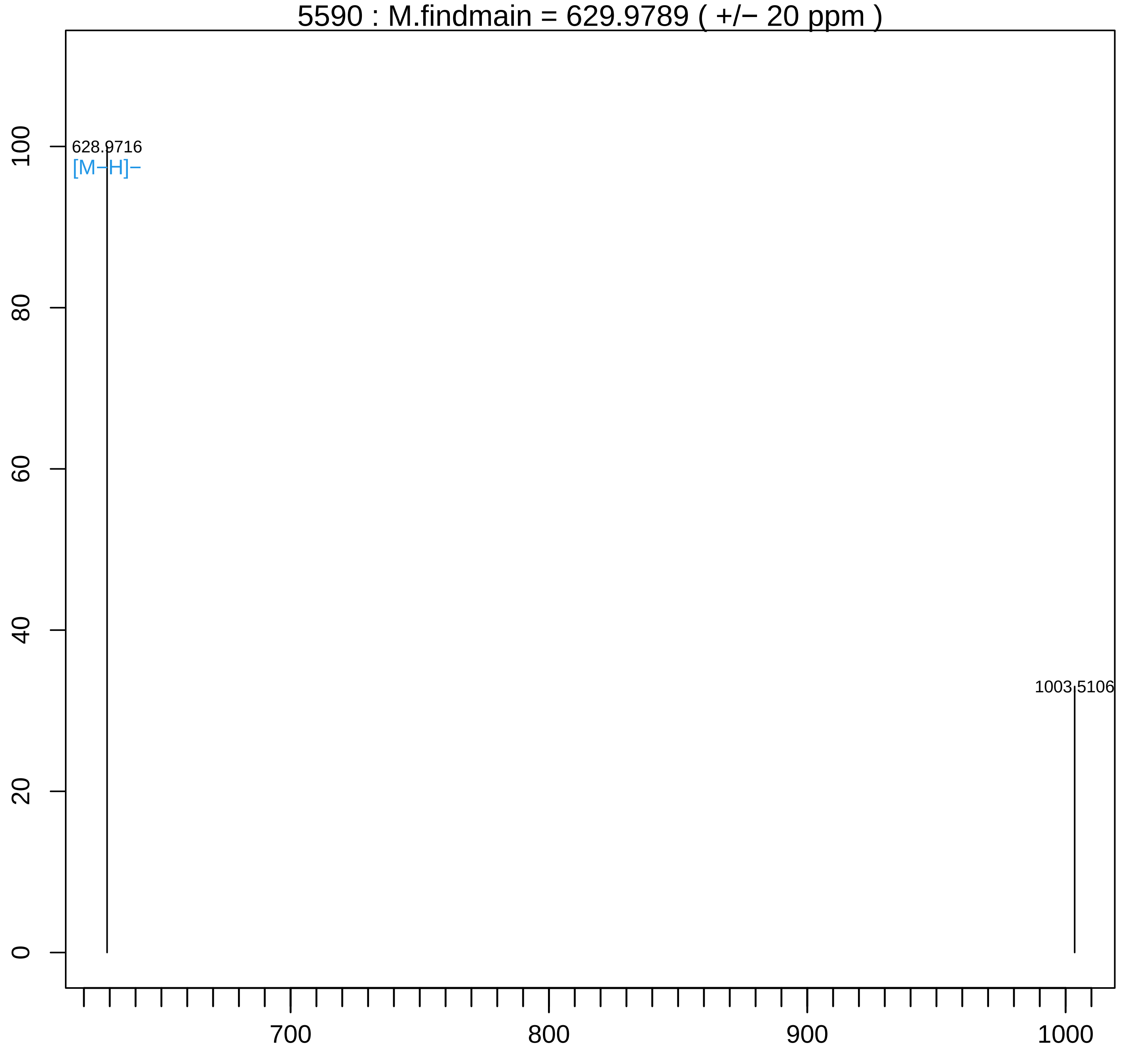
5590 : M.findmain = 629.9789 (+/− 20 ppm)
628.9716
[M−H]−
1003.5106
0
20
40
60
80
100
700
800
900
1000

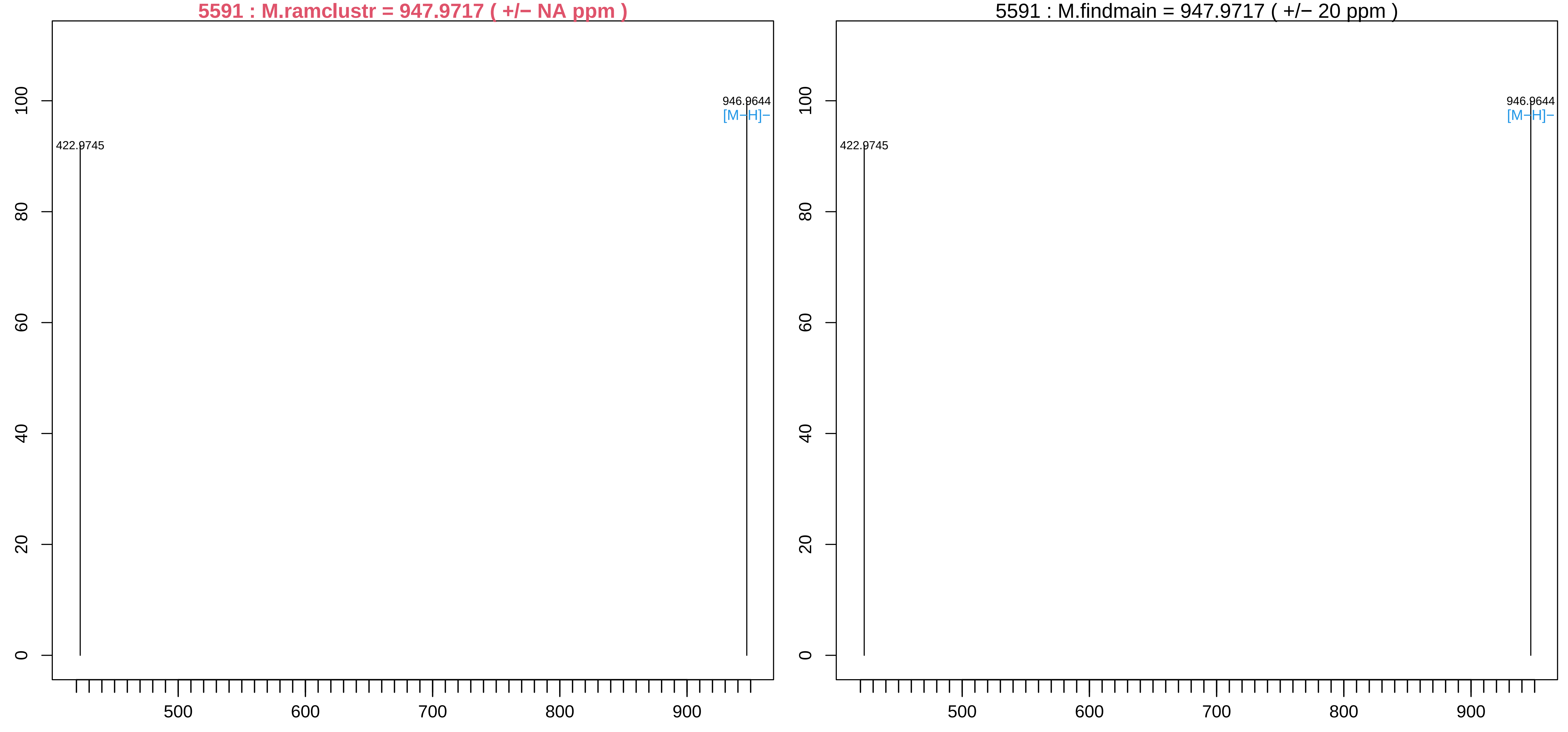
5591 : M.ramclustr = 947.9717 (+/− NA ppm)
422.9745
946.9644
[M−H]−
0
20
40
60
80
100
500
600
700
800
900
5591 : M.findmain = 947.9717 (+/− 20 ppm)
422.9745
946.9644
[M−H]−
0
20
40
60
80
100
500
600
700
800
900

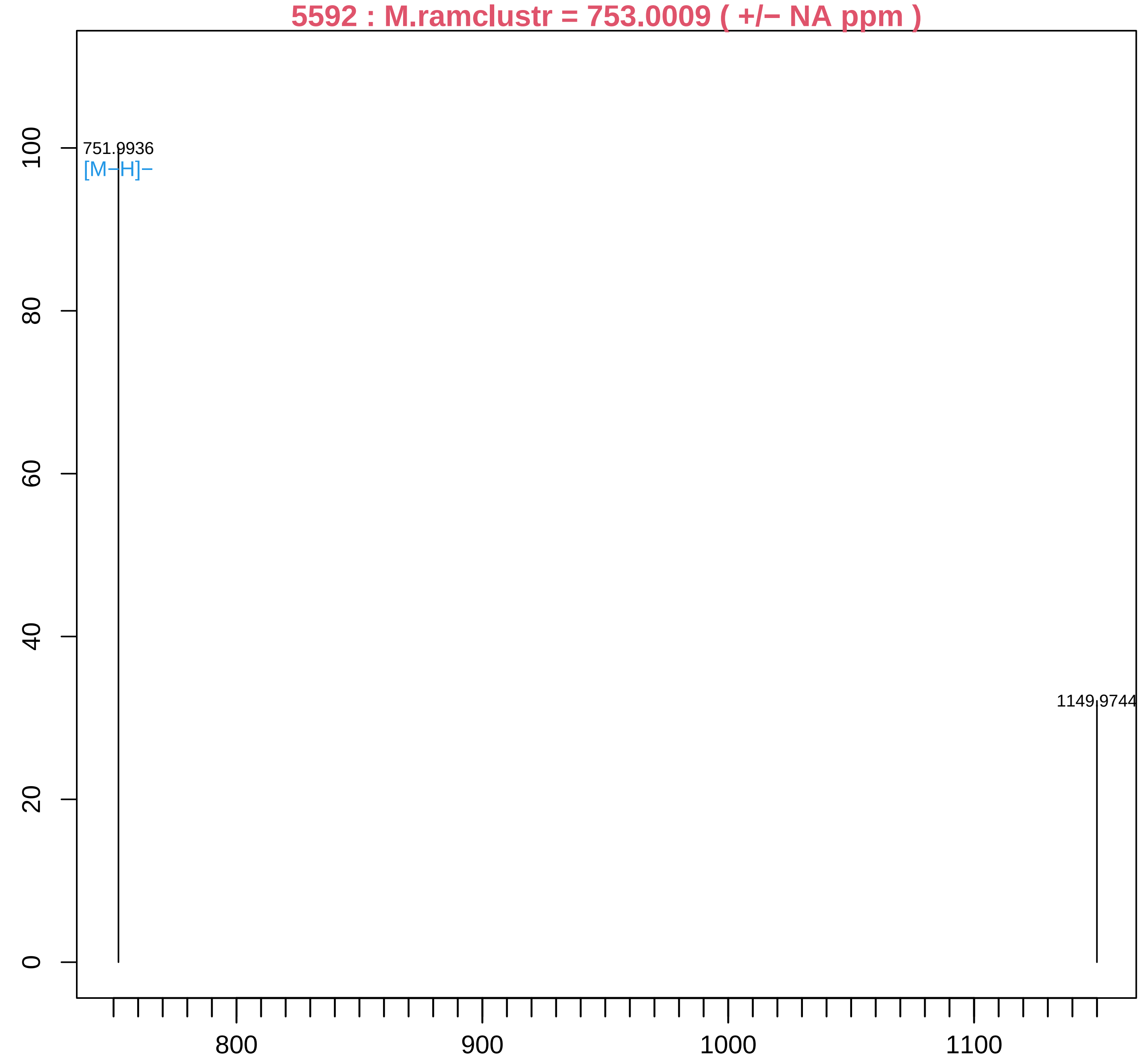

5592 : M.ramclustr = 753.0009 (+/− NA ppm)
751.9936
[M−H]−
1149.9744
0
20
40
60
80
100
800
900
1000
1100

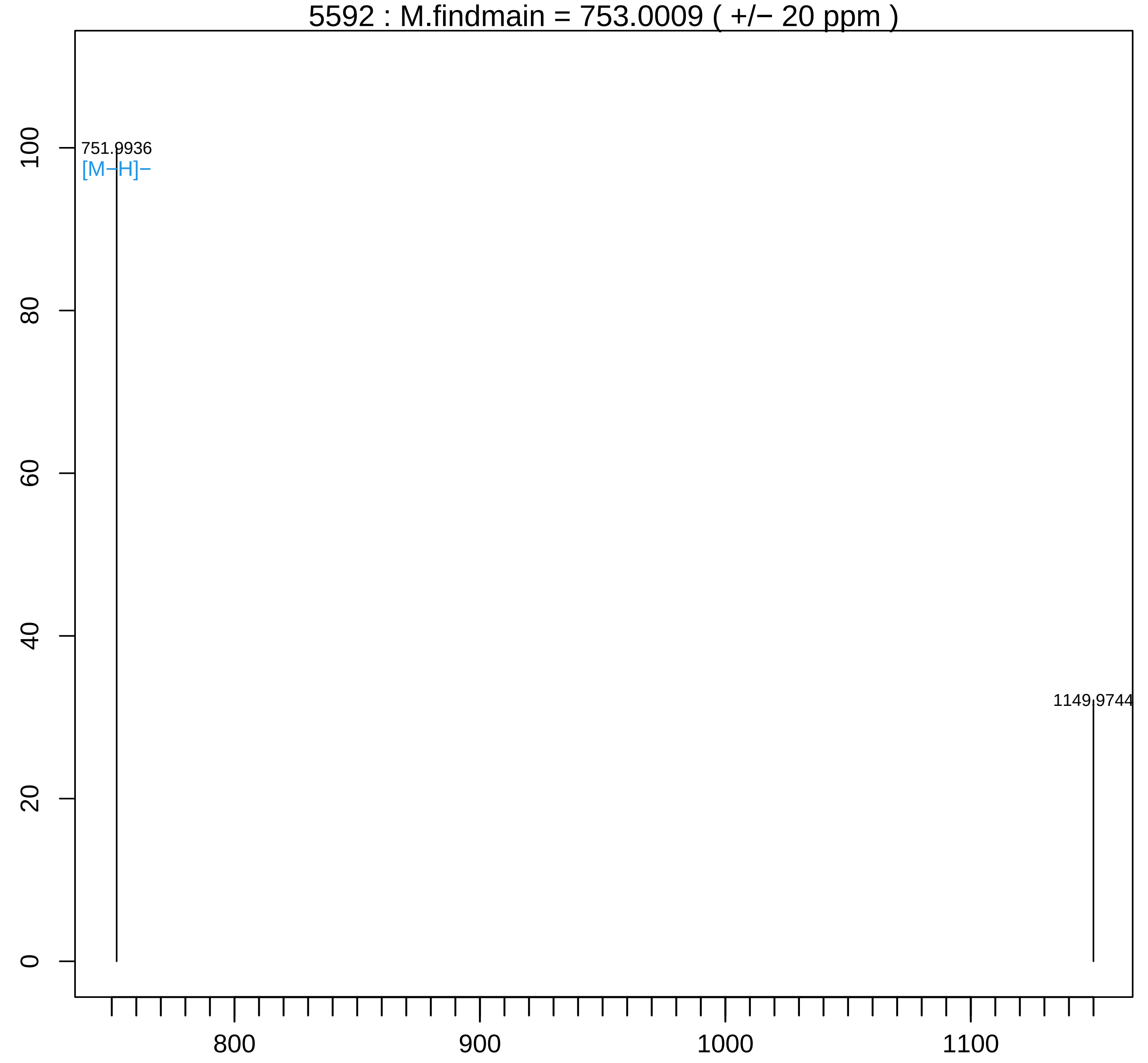

5592 : M.findmain = 753.0009 (+/− 20 ppm)
751.9936
[M−H]−
1149.9744
0
20
40
60
80
100
800
900
1000
1100

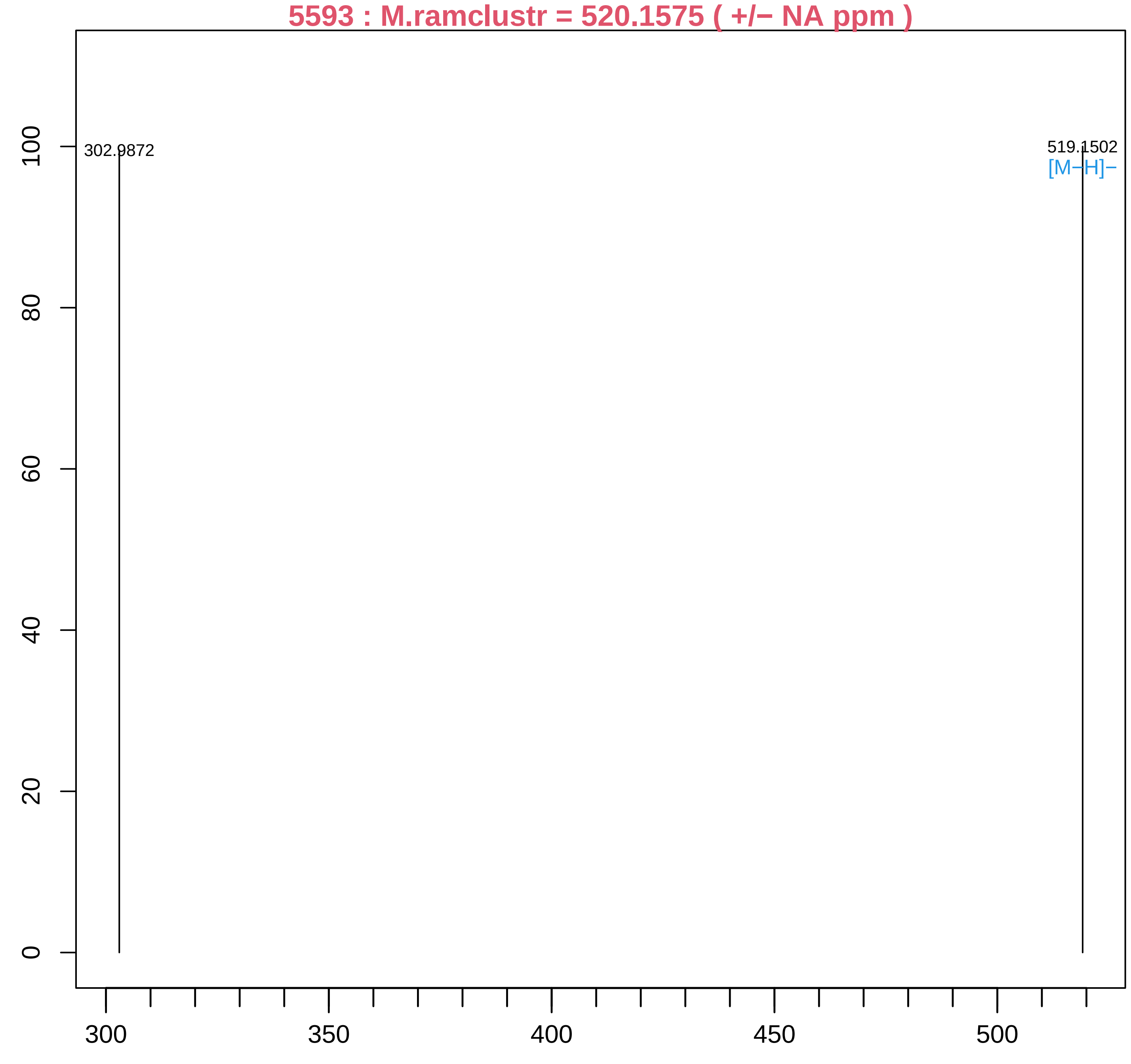
5593 : M.ramclustr = 520.1575 (+/− NA ppm)
302.9872
519.1502
[M−H]−
0
20
40
60
80
100
300
350
400
450
500

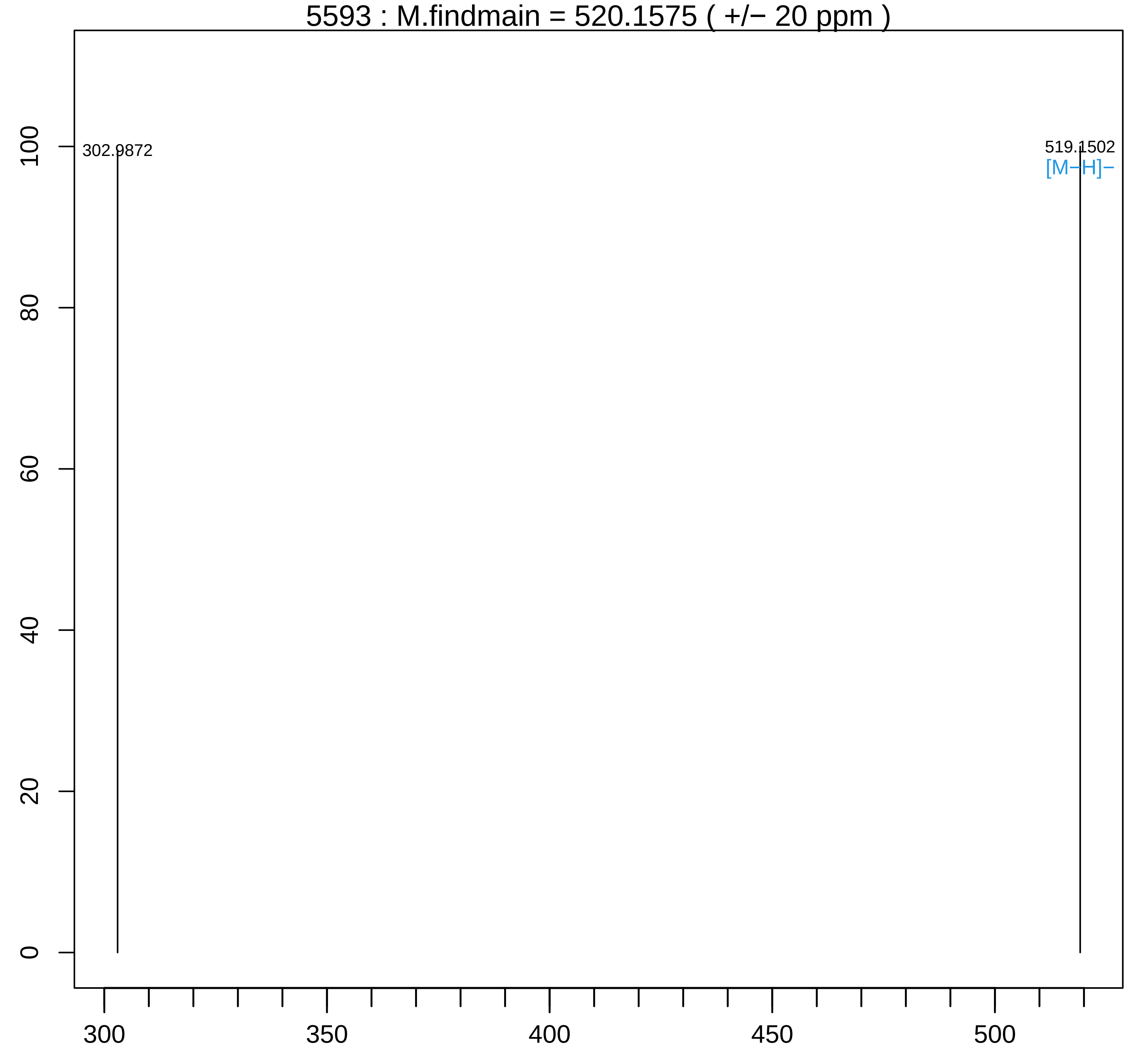
5593 : M.findmain = 520.1575 (+/− 20 ppm)
302.9872
519.1502
[M−H]−
0
20
40
60
80
100
300
350
400
450
500

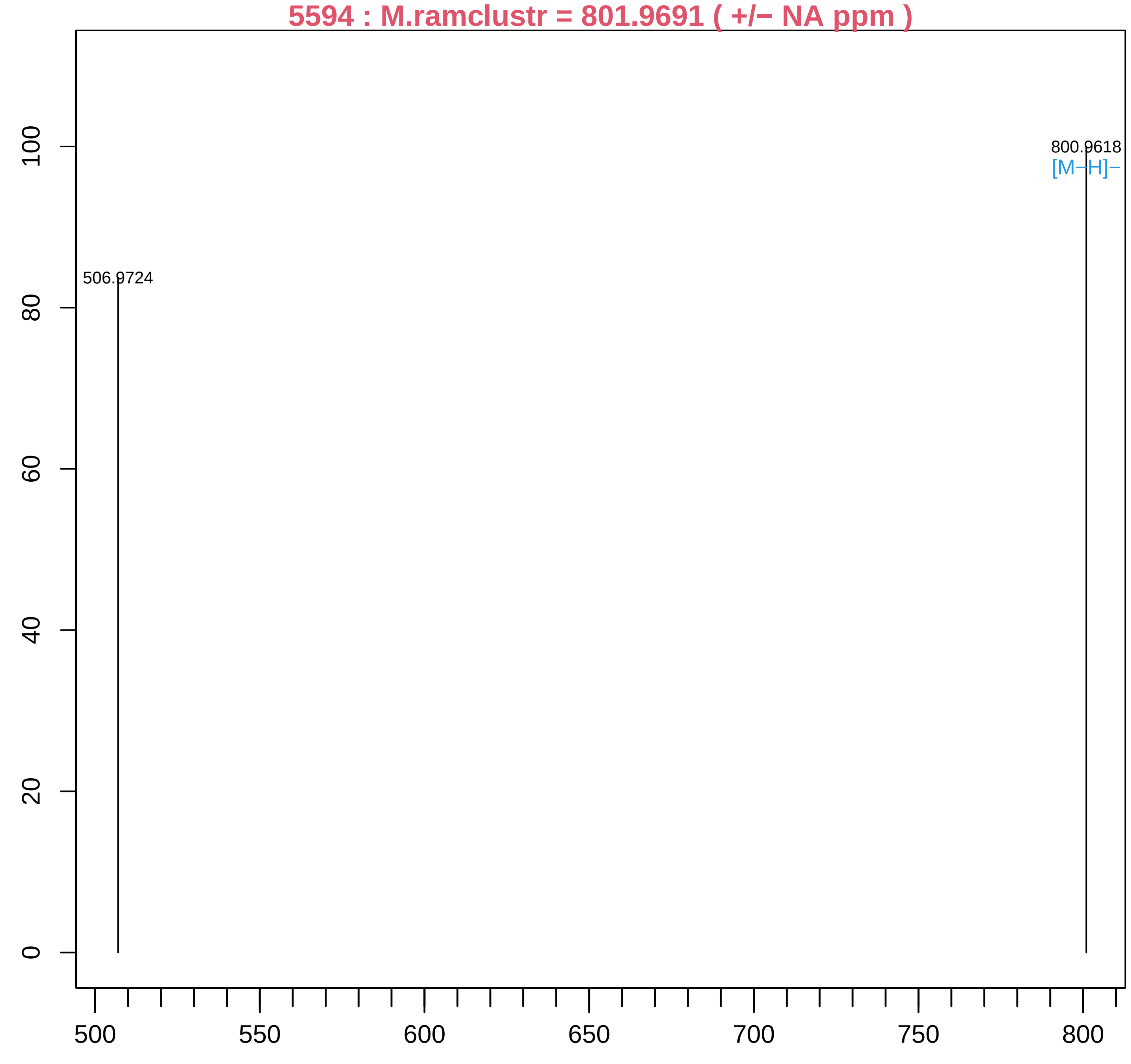
5594 : M.ramclustr = 801.9691 (+/− NA ppm)
800.9618
[M−H]−
506.9724
0
20
40
60
80
100
500
550
600
650
700
750
800

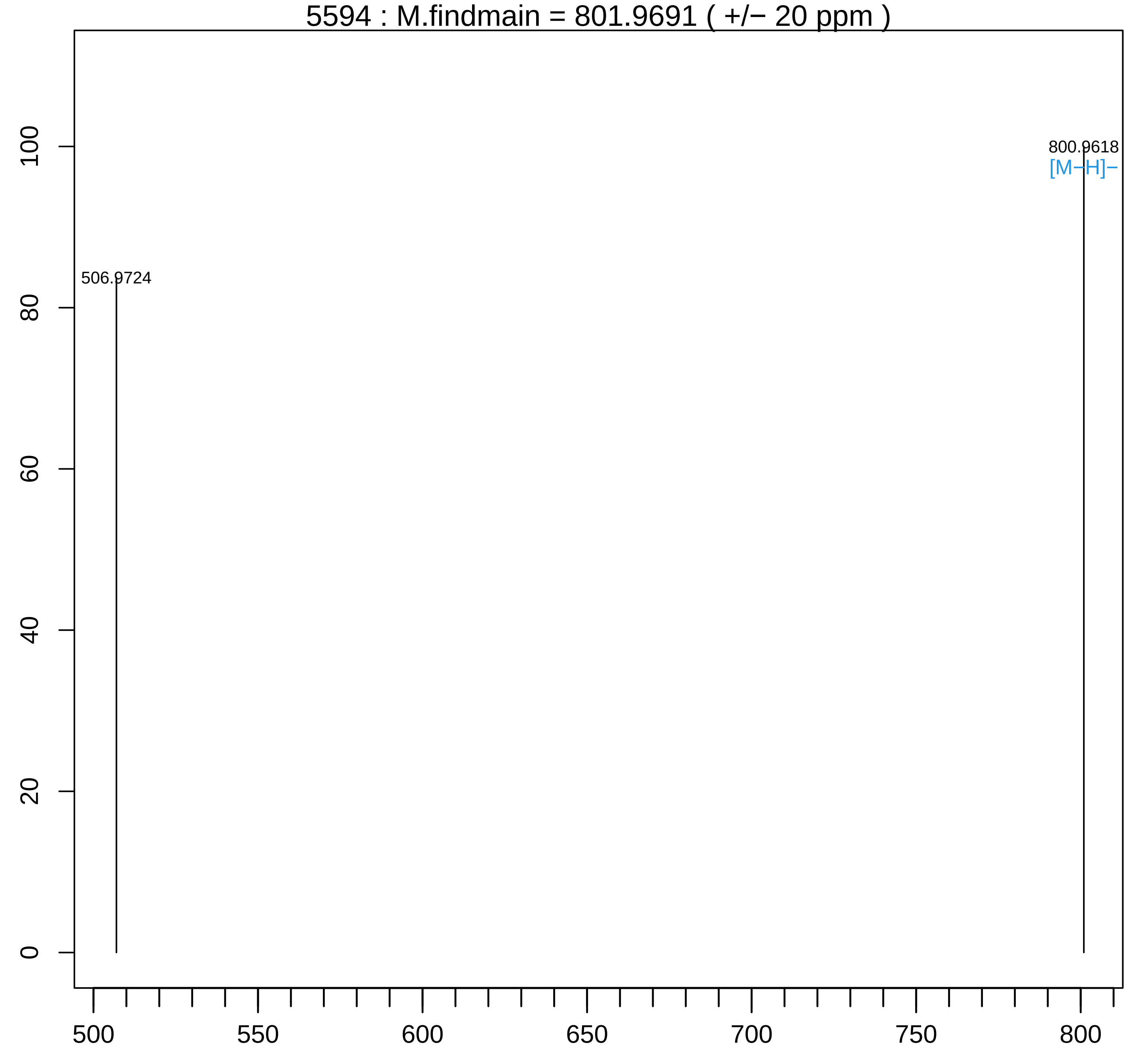
5594 : M.findmain = 801.9691 (+/− 20 ppm)
800.9618
[M−H]−
506.9724
0
20
40
60
80
100
500
550
600
650
700
750
800

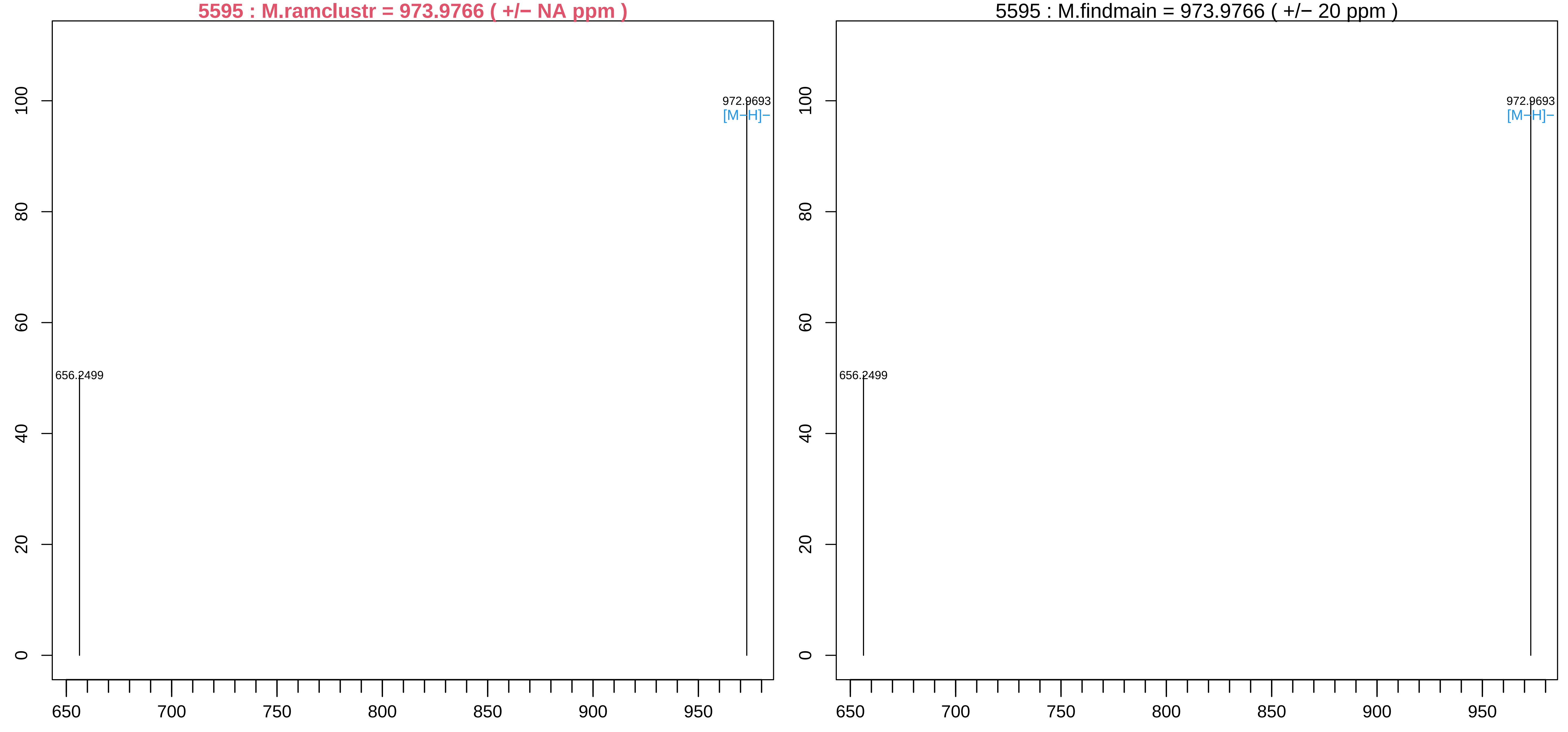

5595 : M.ramclustr = 973.9766 (+/− NA ppm)
5595 : M.findmain = 973.9766 (+/− 20 ppm)
972.9693
[M−H]−
656.2499
0
20
40
60
80
100
650
700
750
800
850
900
950

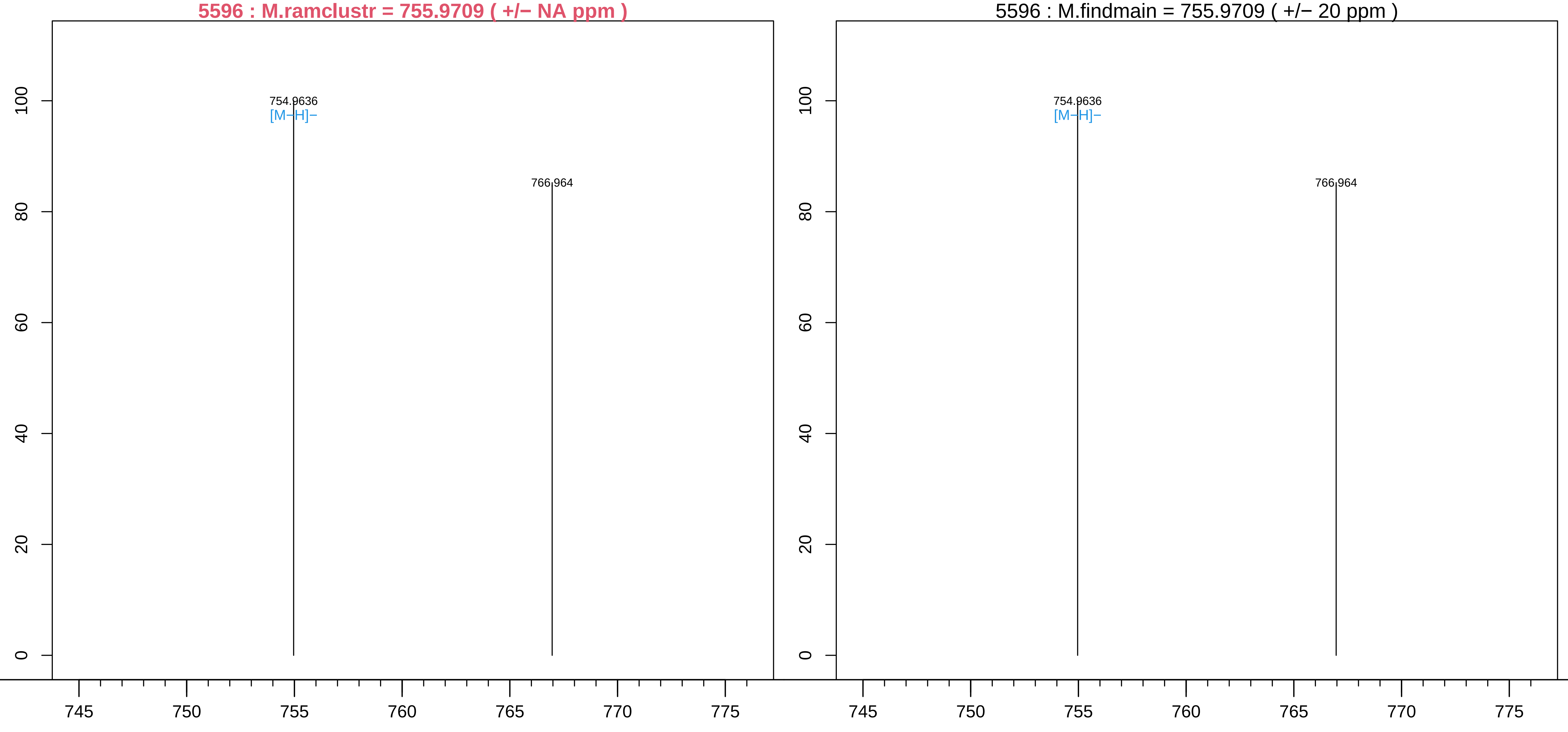
5596 : M.ramclustr = 755.9709 (+/− NA ppm)
754.9636
[M−H]−
766.964
0
20
40
60
80
100
745
750
755
760
765
770
775
5596 : M.findmain = 755.9709 (+/− 20 ppm)
754.9636
[M−H]−
766.964
0
20
40
60
80
100
745
750
755
760
765
770
775

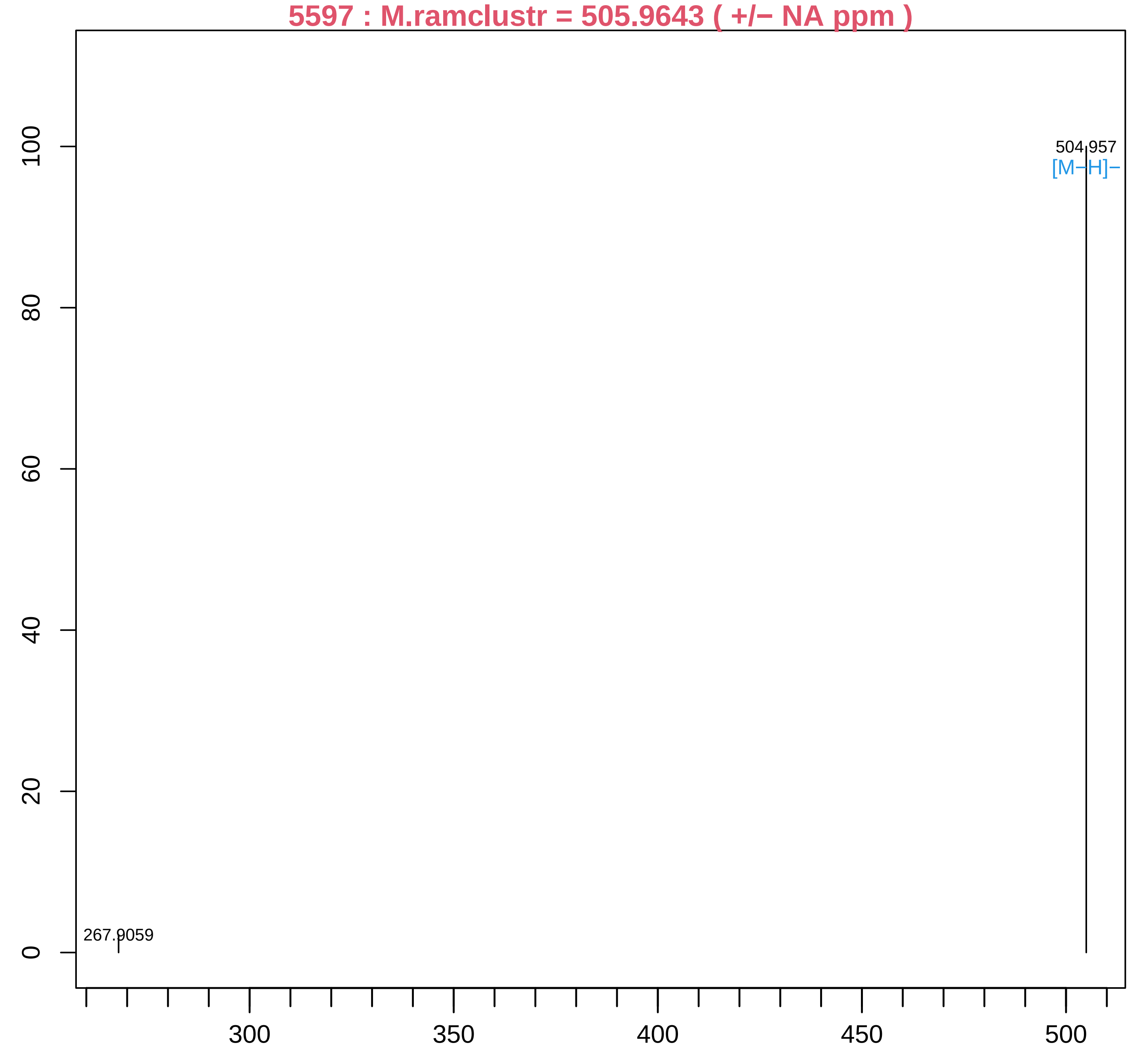
5597 : M.ramclustr = 505.9643 (+/− NA ppm)
504.957
[M−H]−
267.9059
0
20
40
60
80
100
300
350
400
450
500

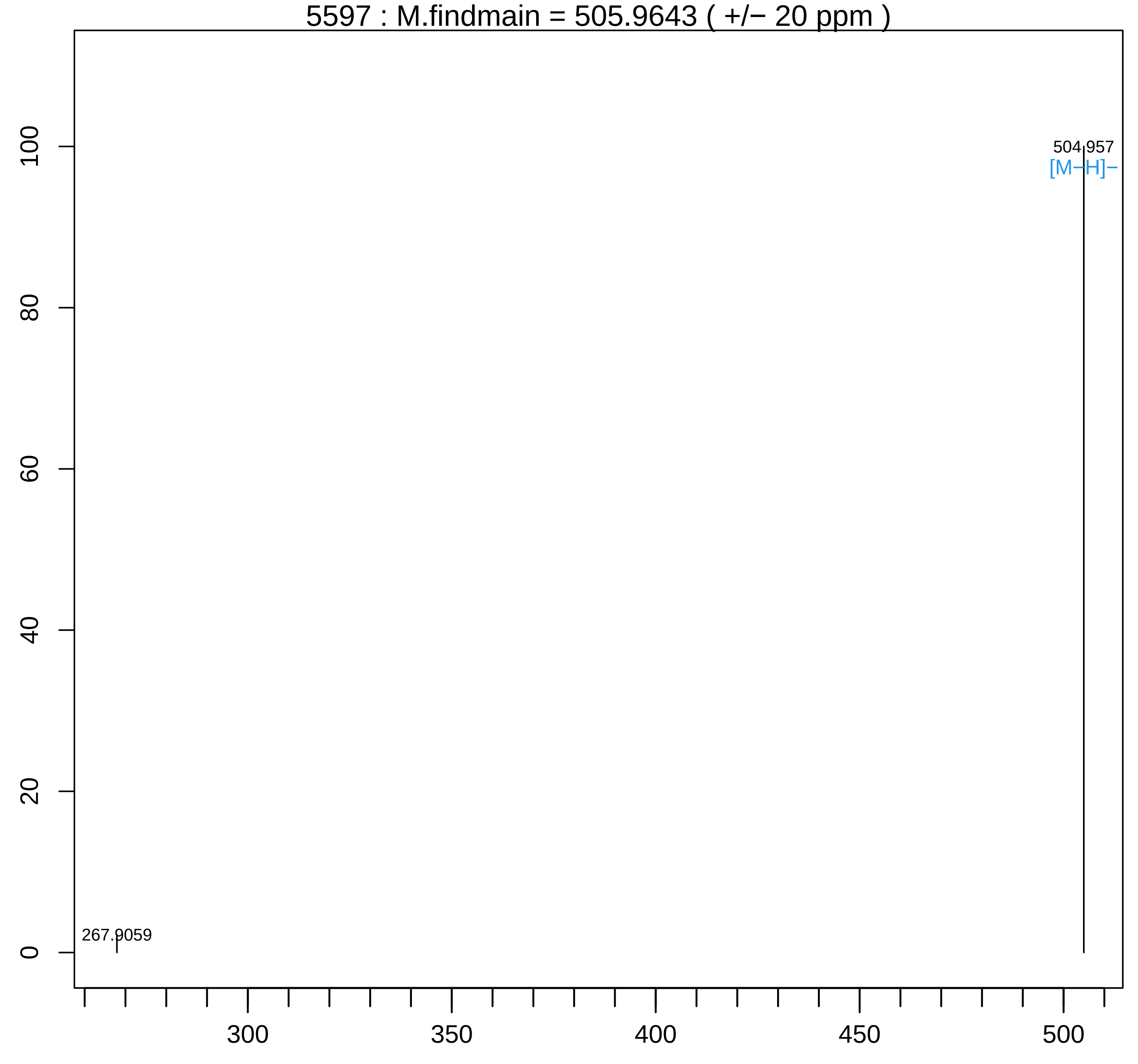
5597 : M.findmain = 505.9643 (+/− 20 ppm)
504.957
[M−H]−
267.9059
0
20
40
60
80
100
300
350
400
450
500

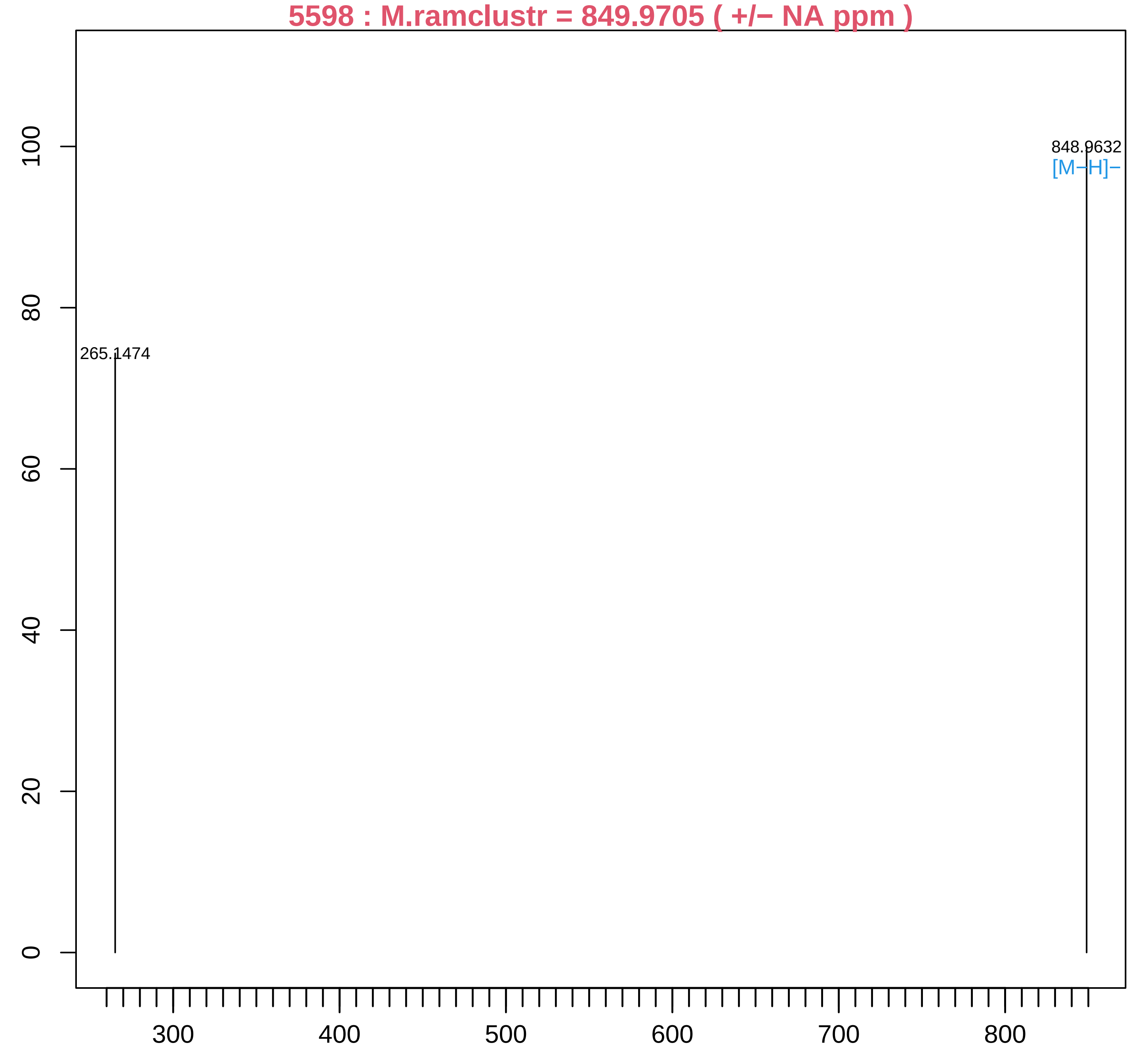
5598 : M.ramclustr = 849.9705 (+/− NA ppm)
848.9632
[M−H]−
265.1474
0
20
40
60
80
100
300
400
500
600
700
800

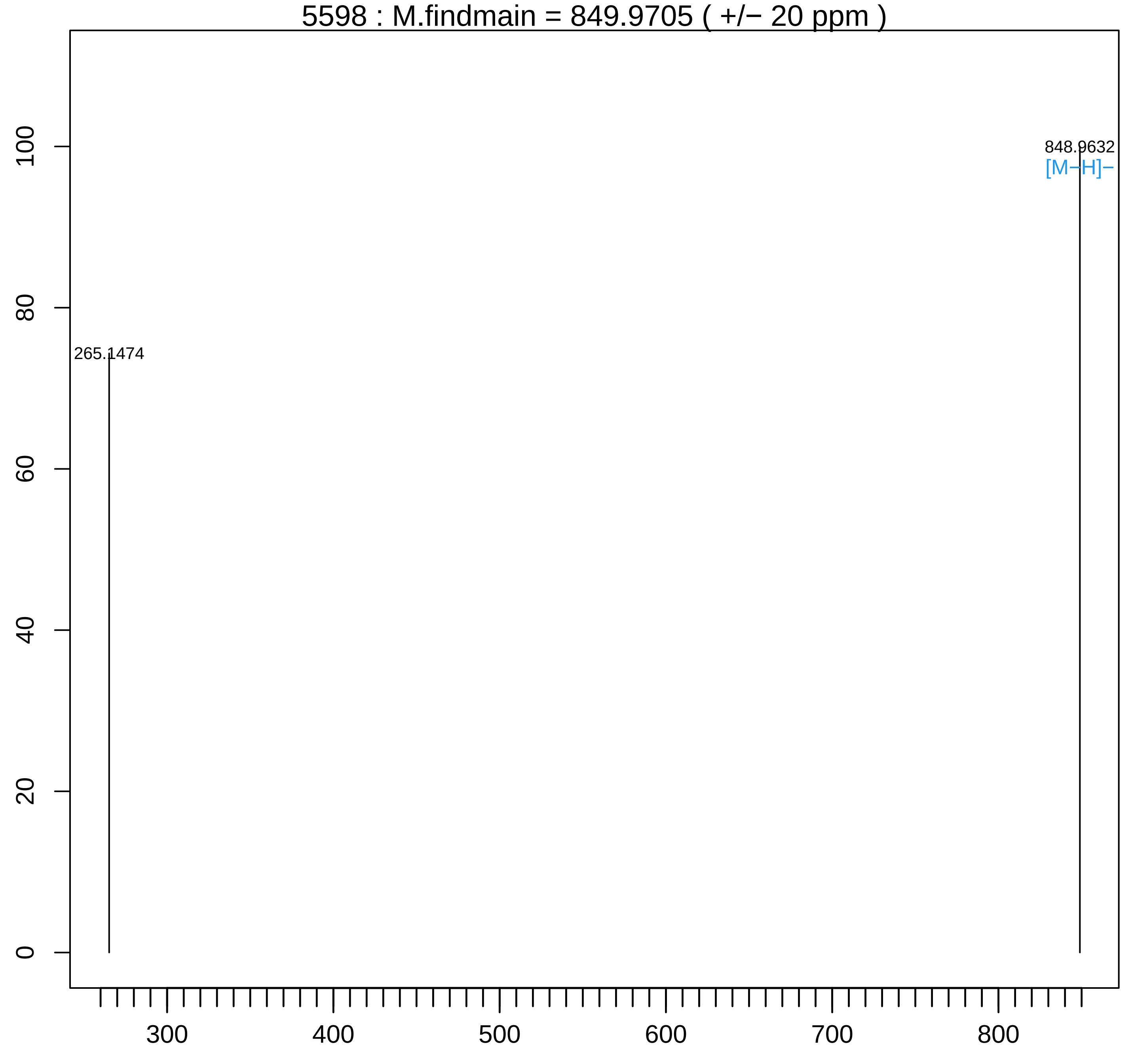
5598 : M.findmain = 849.9705 (+/− 20 ppm)
848.9632
[M−H]−
265.1474
0
20
40
60
80
100
300
400
500
600
700
800

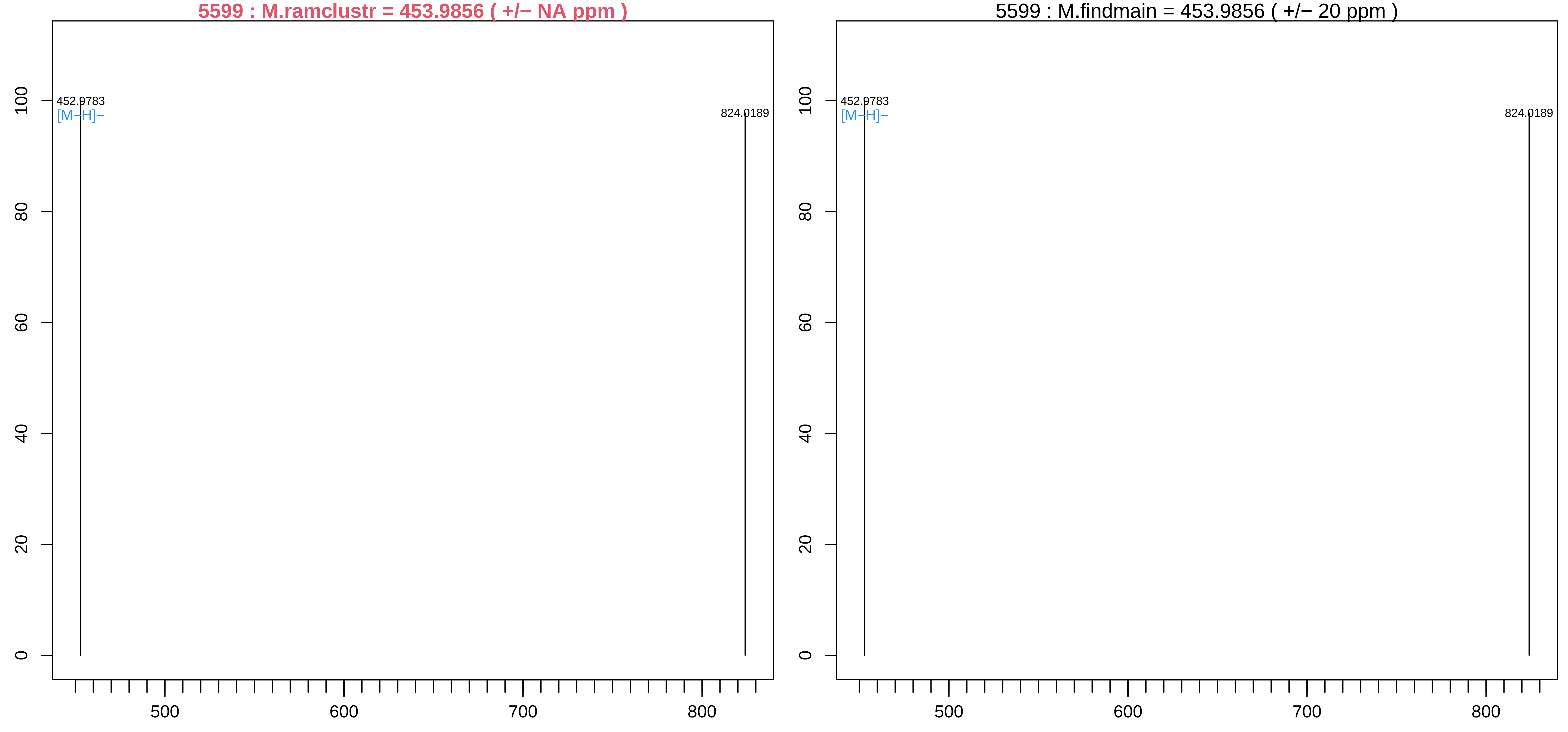

5599 : M.ramclustr = 453.9856 (+/− NA ppm)
452.9783
[M−H]−
824.0189
0
20
40
60
80
100
500
600
700
800
5599 : M.findmain = 453.9856 (+/− 20 ppm)
452.9783
[M−H]−
824.0189
0
20
40
60
80
100
500
600
700
800

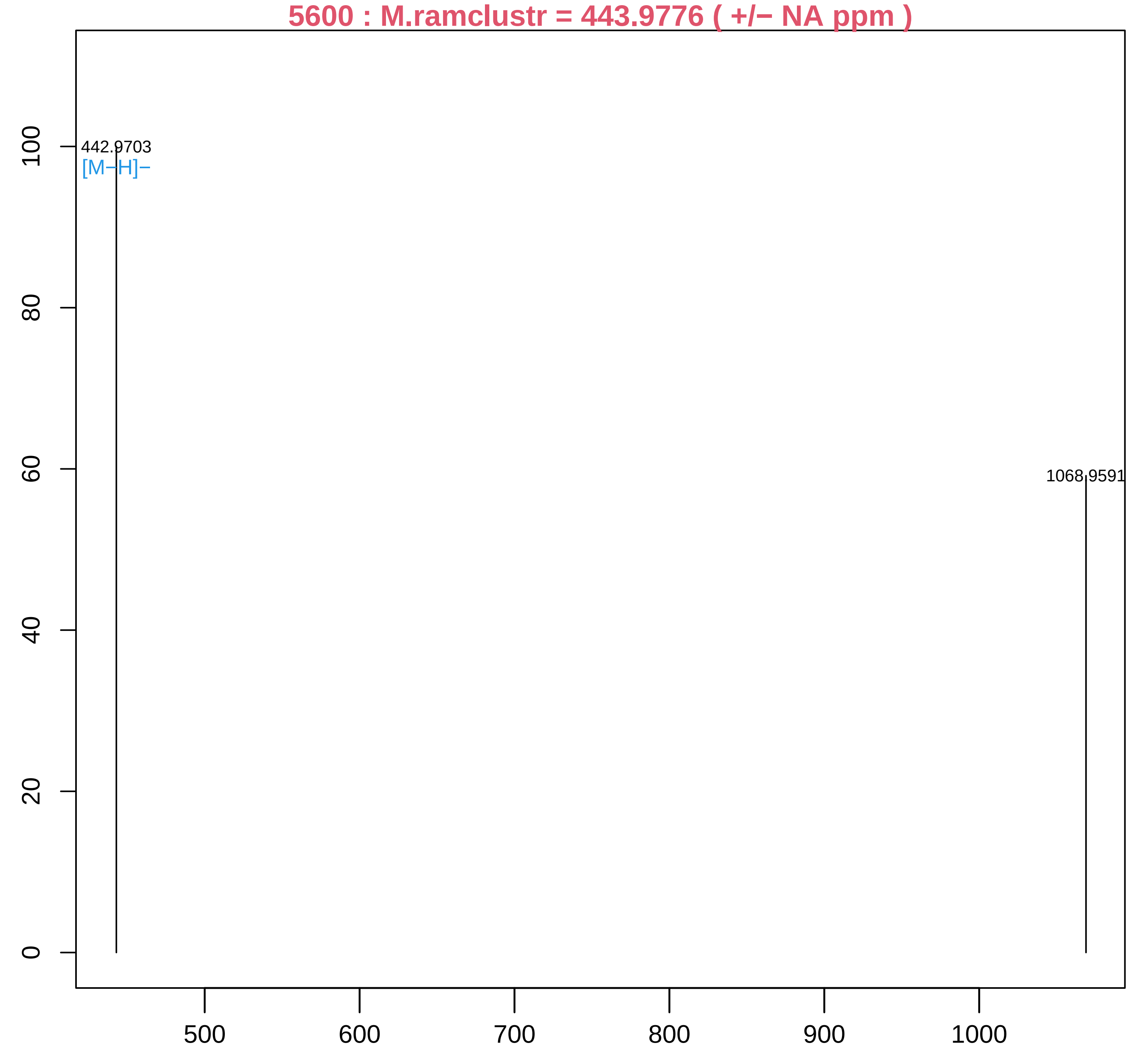
5600 : M.ramclustr = 443.9776 (+/− NA ppm)
442.9703
[M−H]−
1068.9591
0
20
40
60
80
100
500
600
700
800
900
1000

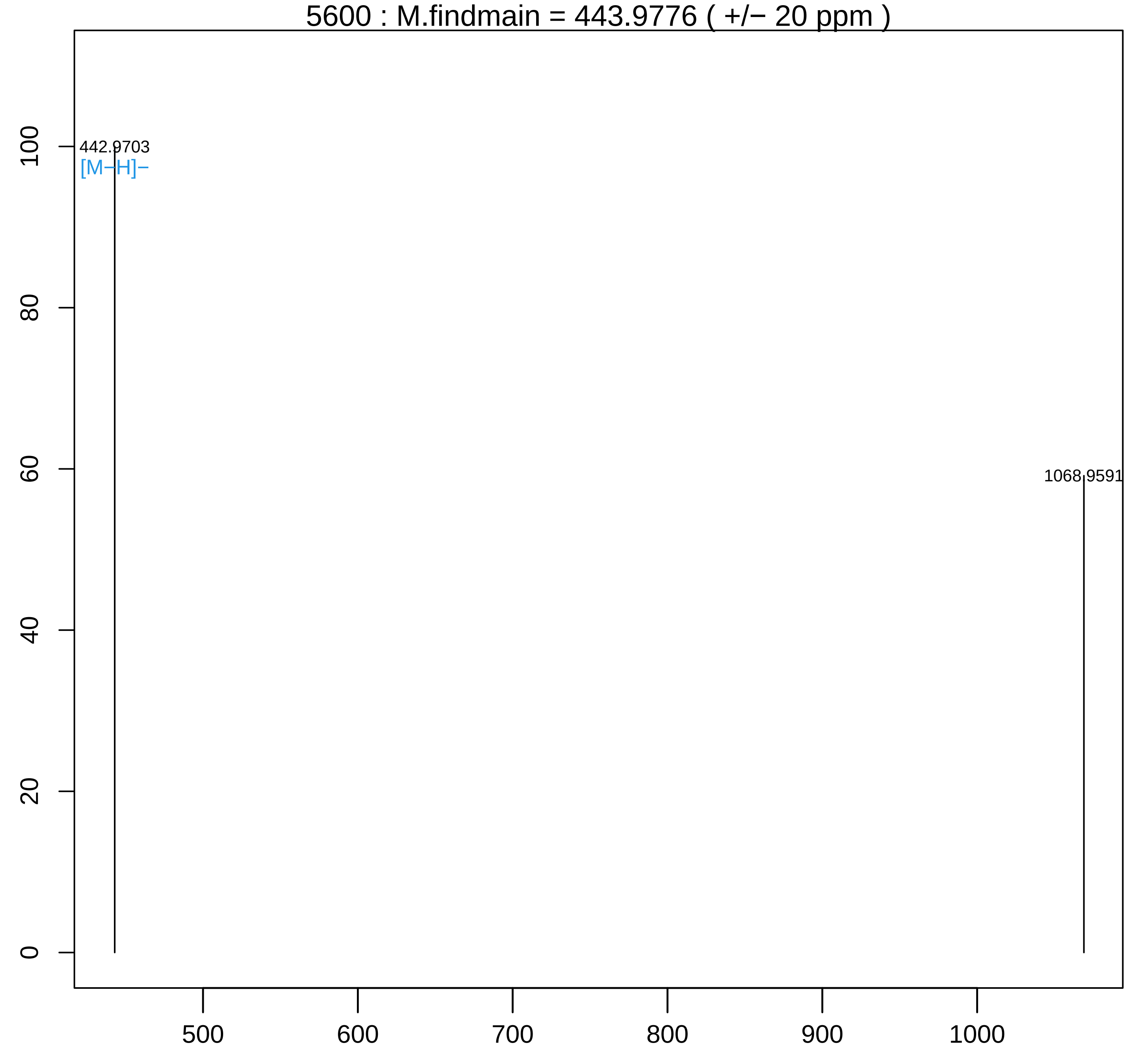
5600 : M.findmain = 443.9776 (+/− 20 ppm)
442.9703
[M−H]−
1068.9591
0
20
40
60
80
100
500
600
700
800
900
1000

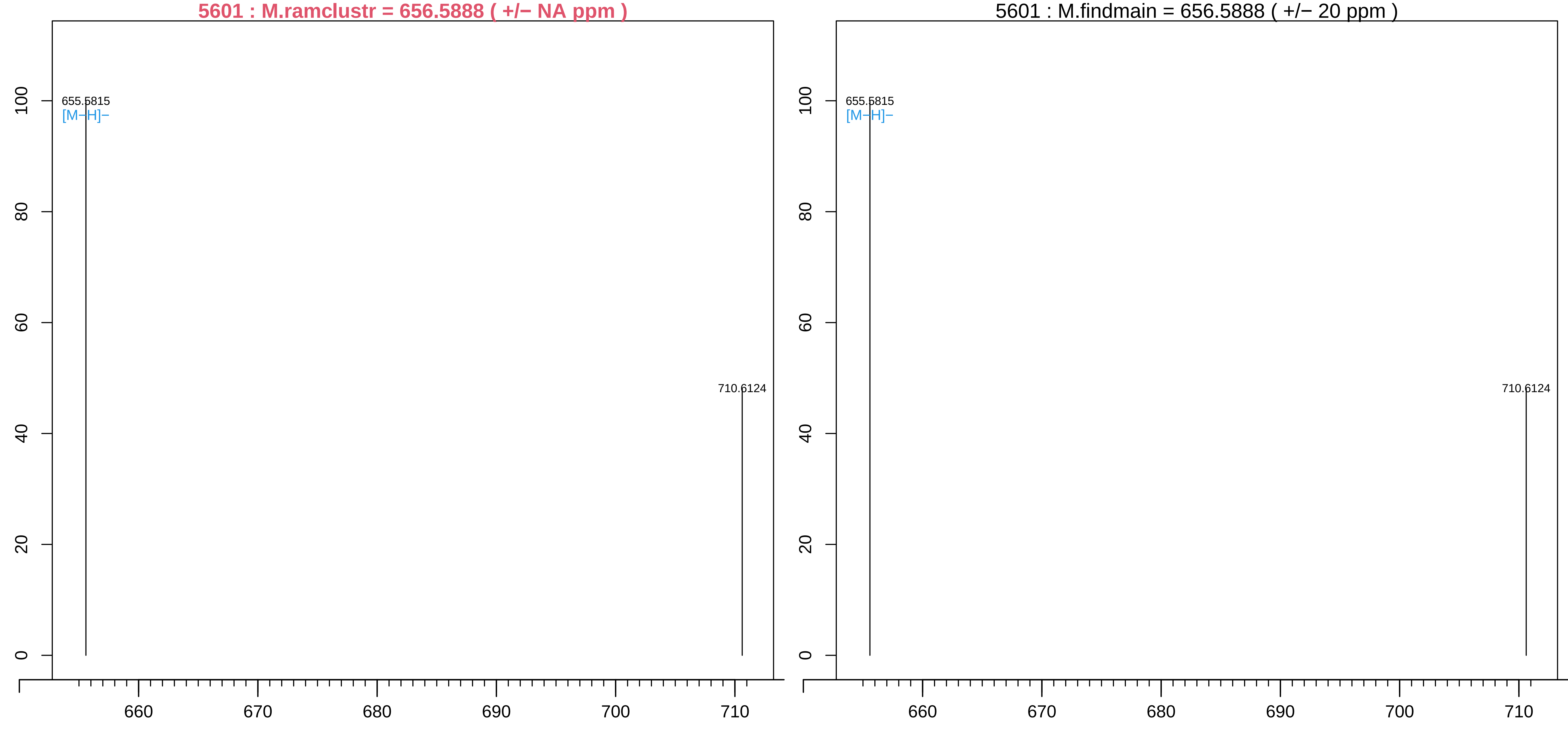
5601 : M.ramclustr = 656.5888 (+/− NA ppm)
655.5815
[M−H]−
710.6124
5601 : M.findmain = 656.5888 (+/− 20 ppm)
655.5815
[M−H]−
710.6124
0
20
40
60
80
100
660
670
680
690
700
710

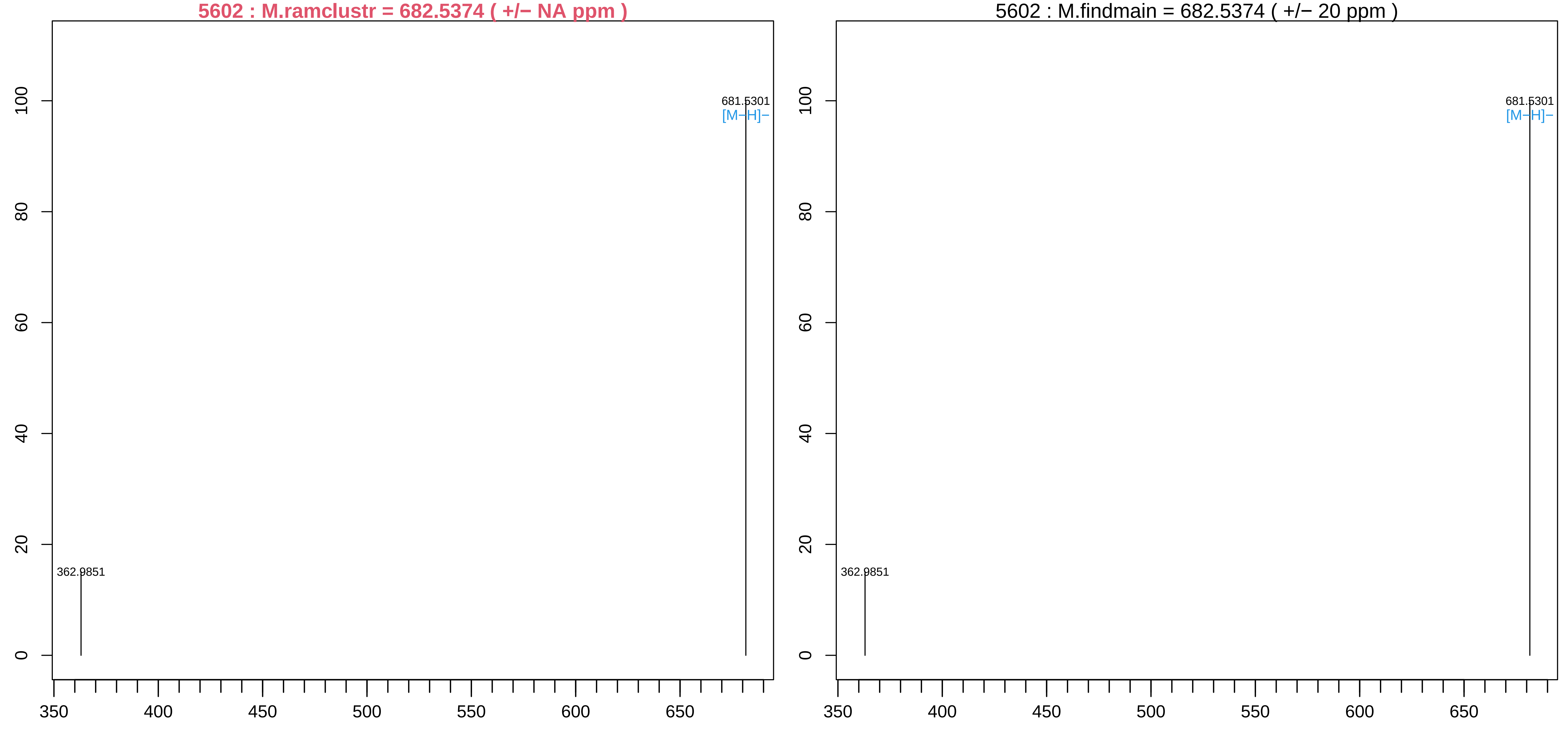
5602 : M.ramclustr = 682.5374 (+/− NA ppm)
681.5301
[M−H]−
362.9851
5602 : M.findmain = 682.5374 (+/− 20 ppm)
681.5301
[M−H]−
362.9851
0
20
40
60
80
100
350
400
450
500
550
600
650

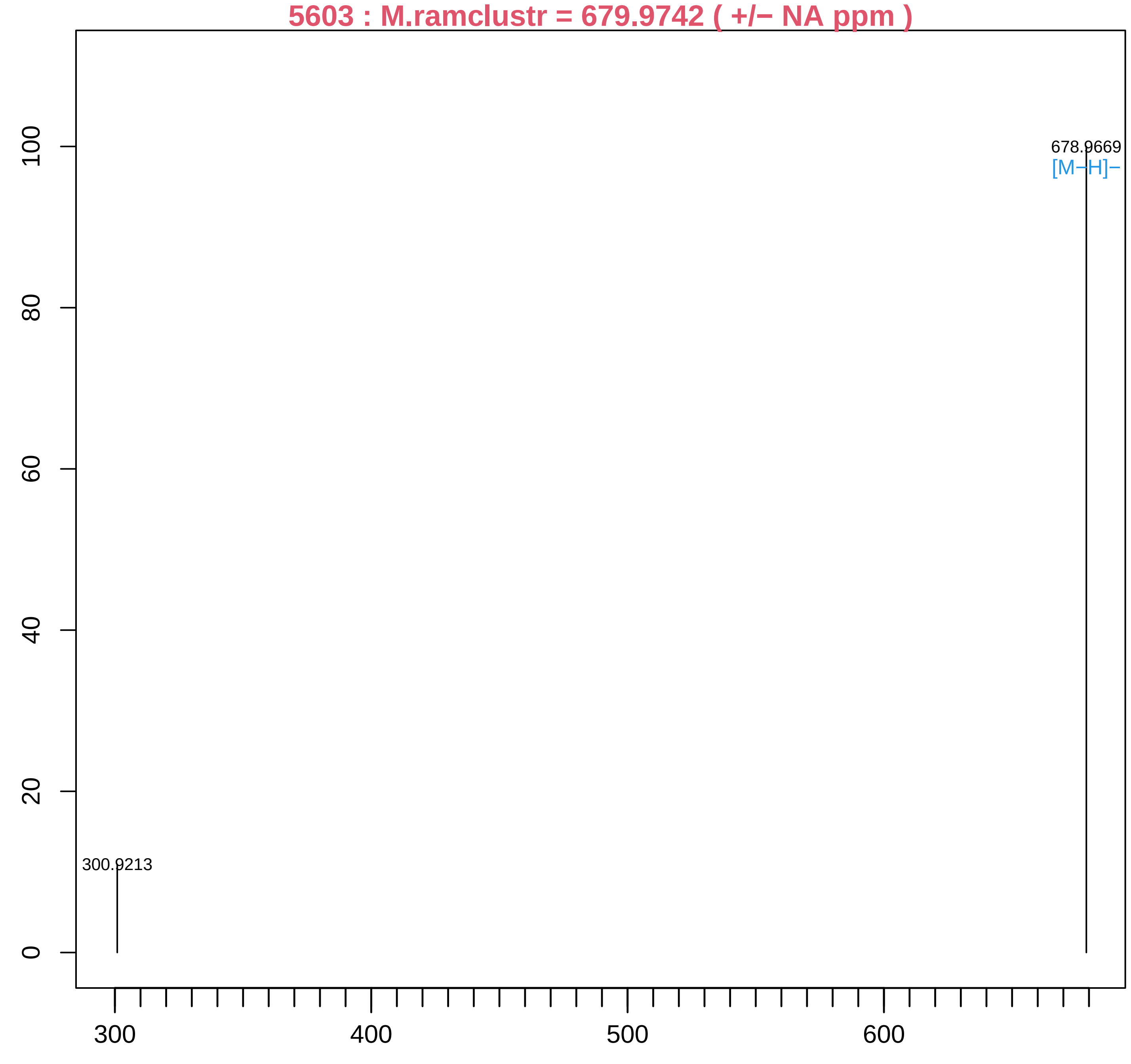

5603 : M.ramclustr = 679.9742 (+/− NA ppm)
678.9669
[M−H]−
300.9213
0
20
40
60
80
100
300
400
500
600

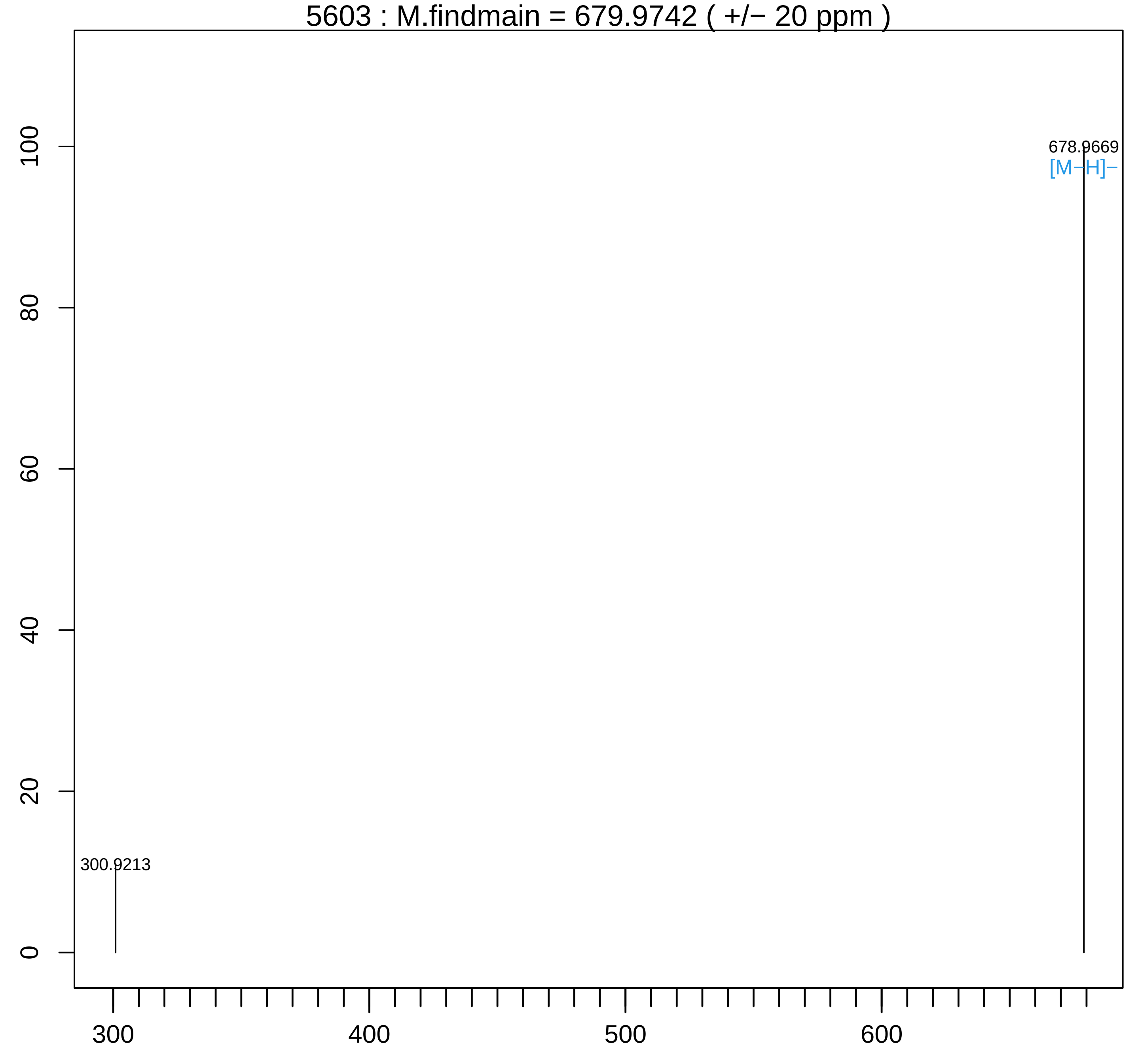

5603 : M.findmain = 679.9742 (+/− 20 ppm)
678.9669
[M−H]−
300.9213
0
20
40
60
80
100
300
400
500
600

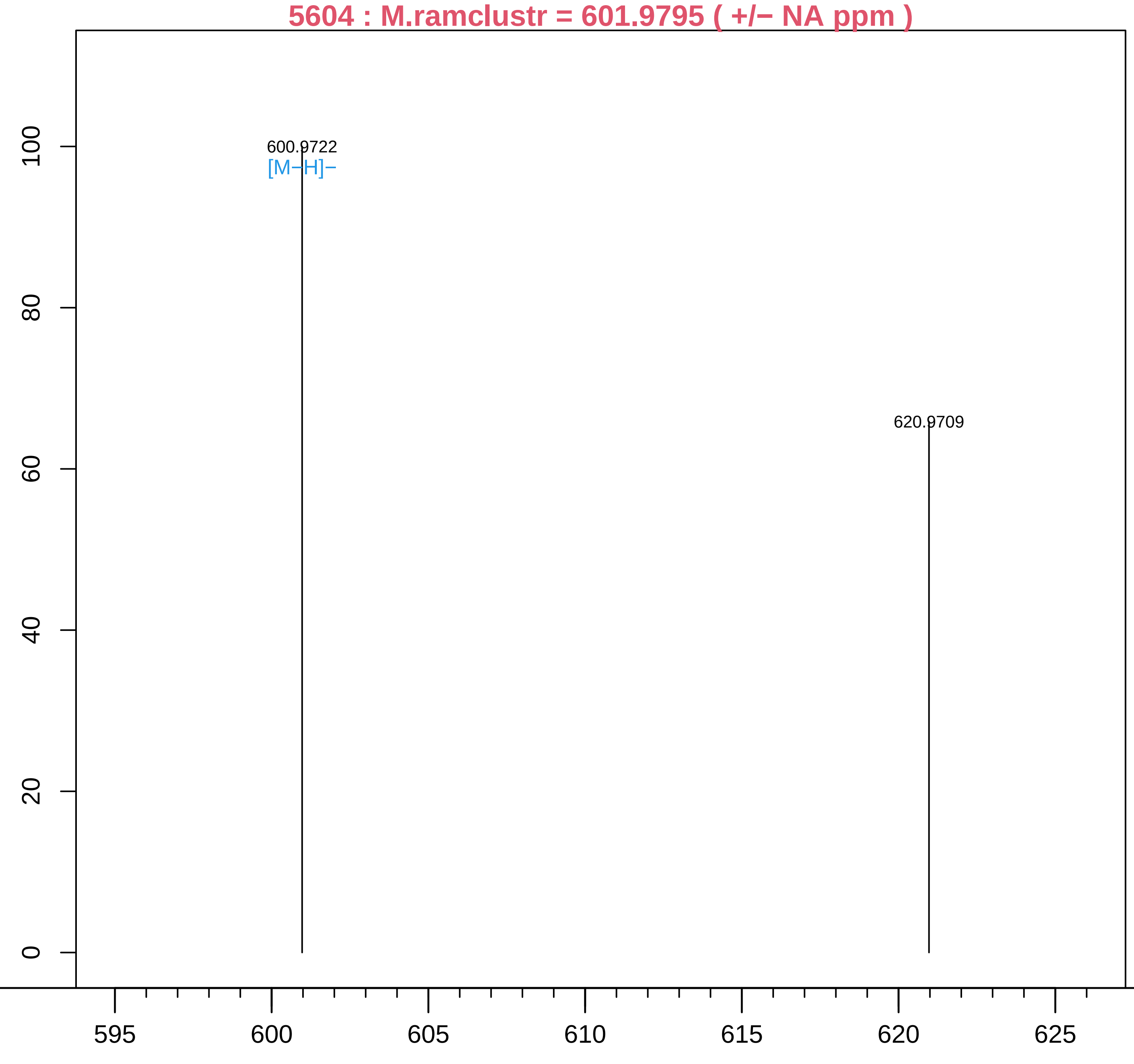
5604 : M.ramclustr = 601.9795 (+/− NA ppm)
600.9722
[M−H]−
620.9709
0
20
40
60
80
100
595
600
605
610
615
620
625

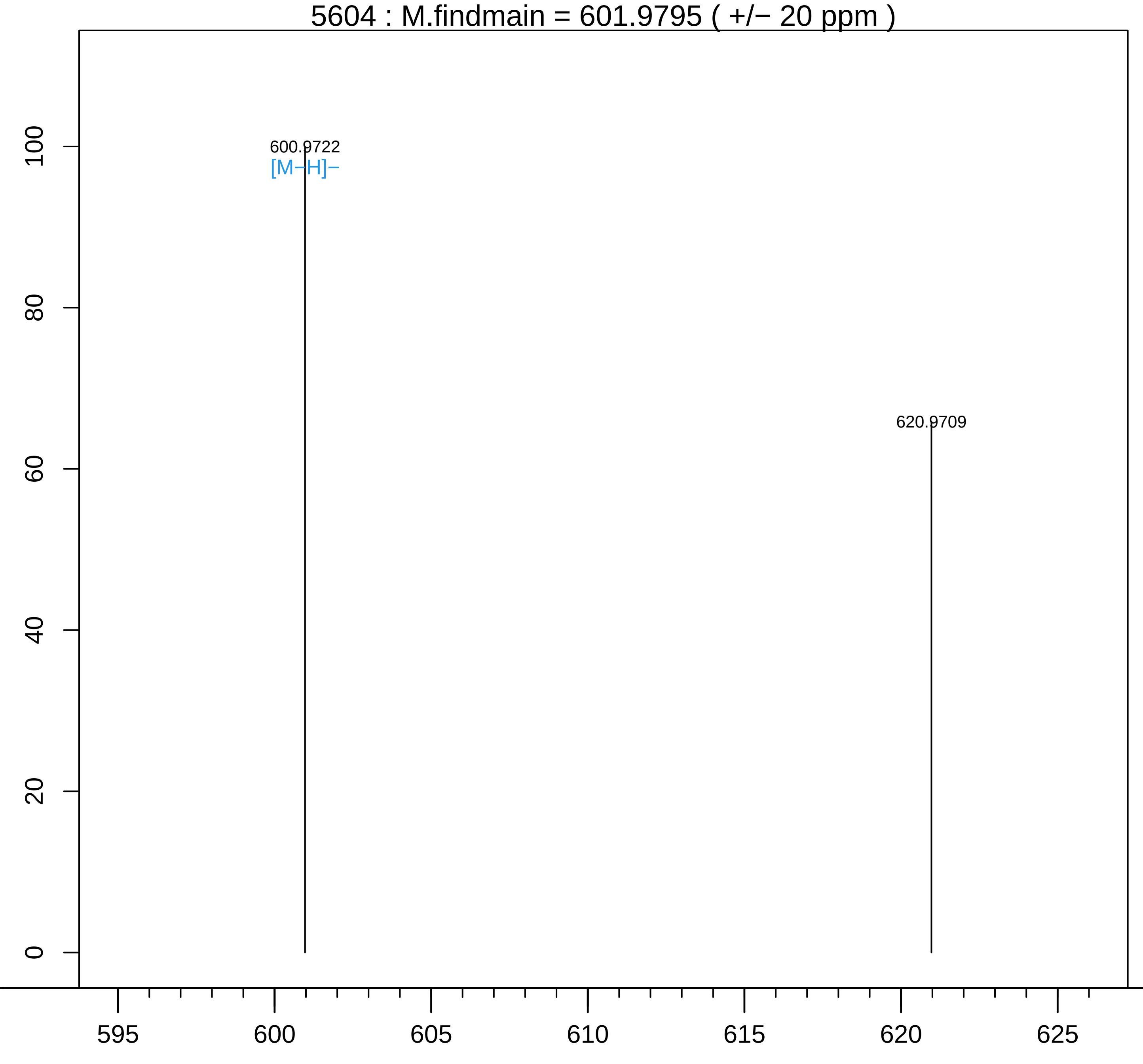
5604 : M.findmain = 601.9795 (+/− 20 ppm)
600.9722
[M−H]−
620.9709
0
20
40
60
80
100
595
600
605
610
615
620
625

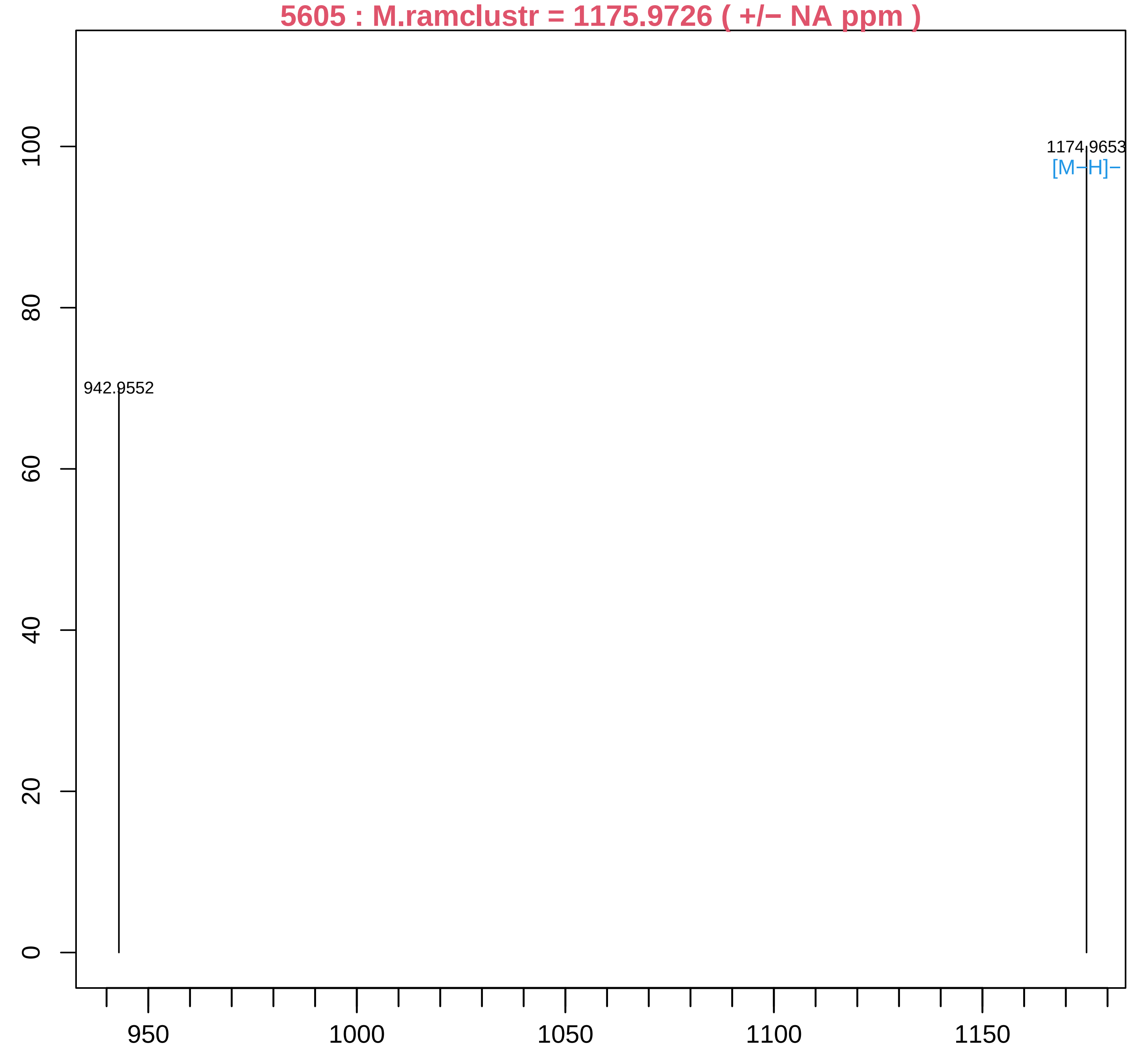
5605 : M.ramclustr = 1175.9726 (+/− NA ppm)
1174.9653
[M−H]−
942.9552
0
20
40
60
80
100
950
1000
1050
1100
1150

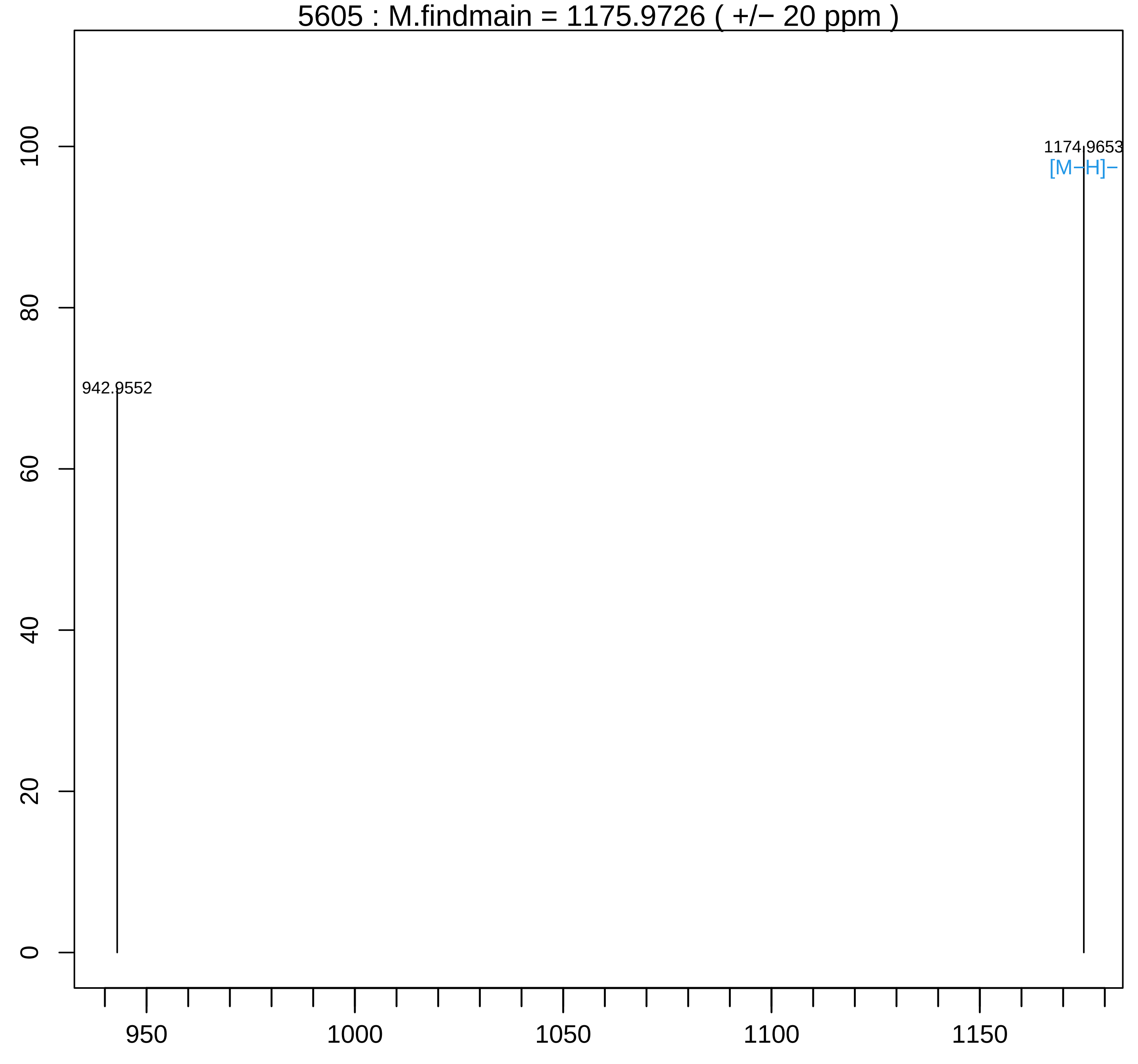
5605 : M.findmain = 1175.9726 (+/− 20 ppm)
1174.9653
[M−H]−
942.9552
0
20
40
60
80
100
950
1000
1050
1100
1150

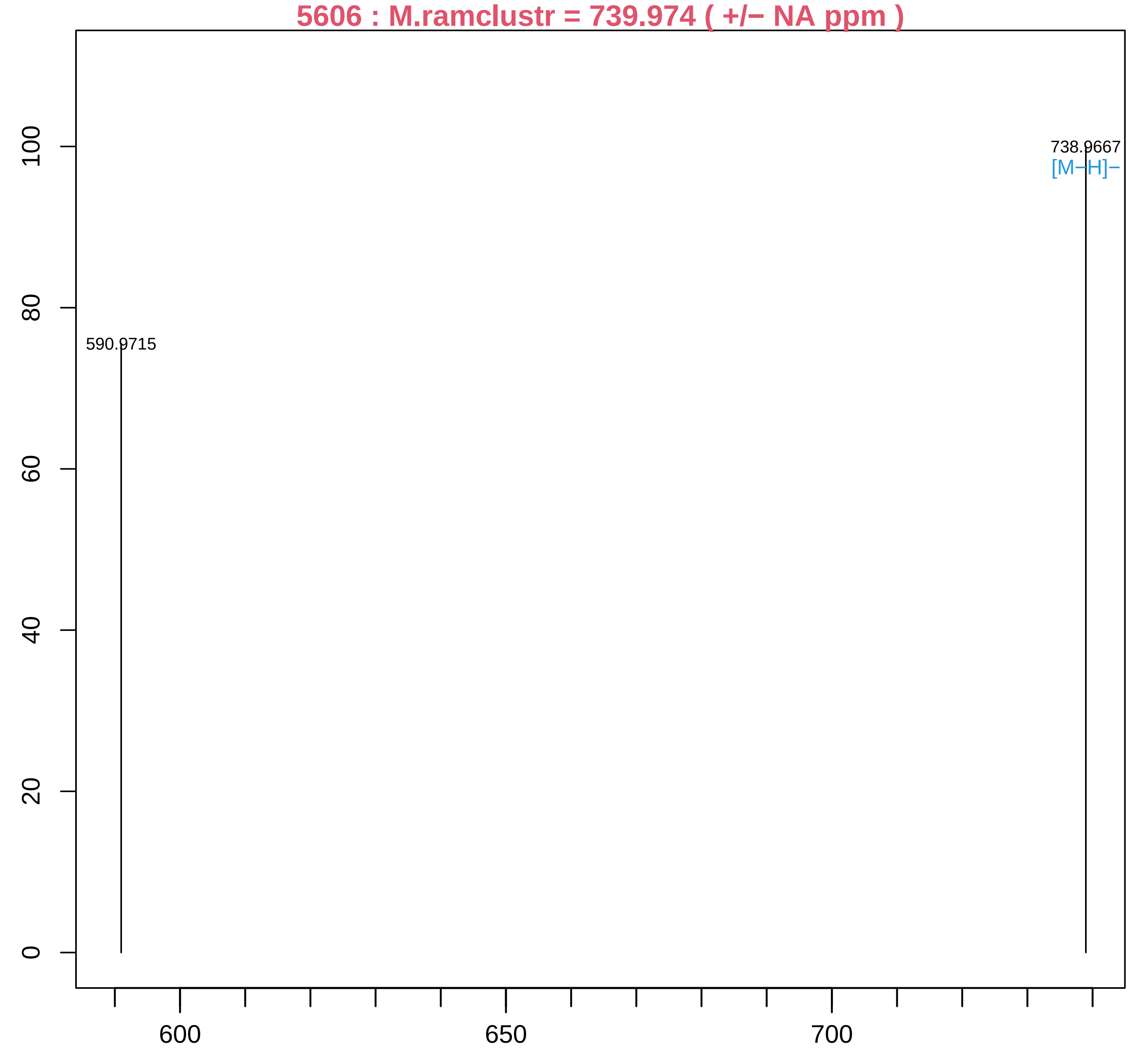
5606 : M.ramclustr = 739.974 (+/− NA ppm)
590.9715
738.9667
[M−H]−
0
20
40
60
80
100
600
650
700

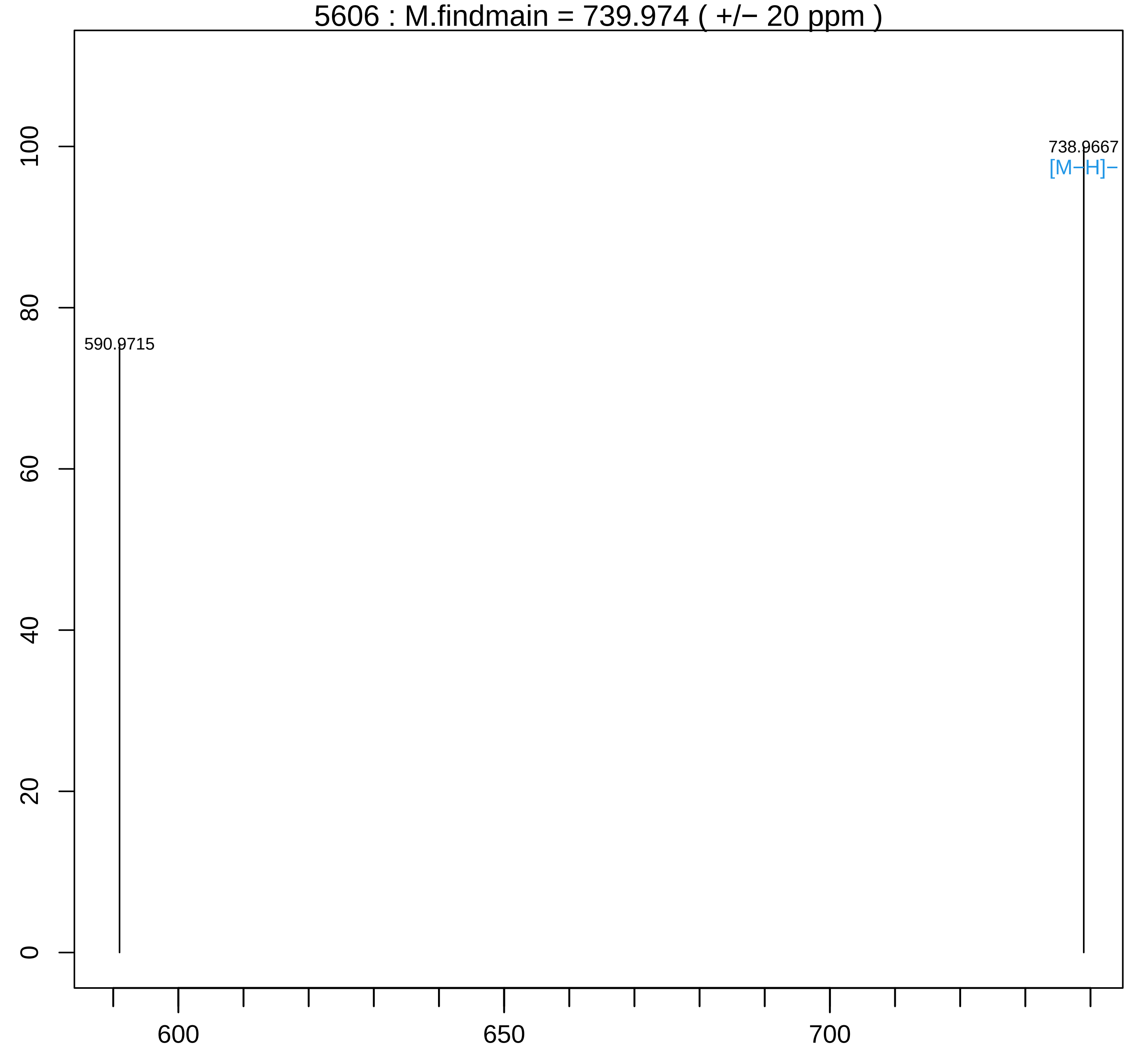
5606 : M.findmain = 739.974 (+/− 20 ppm)
590.9715
738.9667
[M−H]−
0
20
40
60
80
100
600
650
700

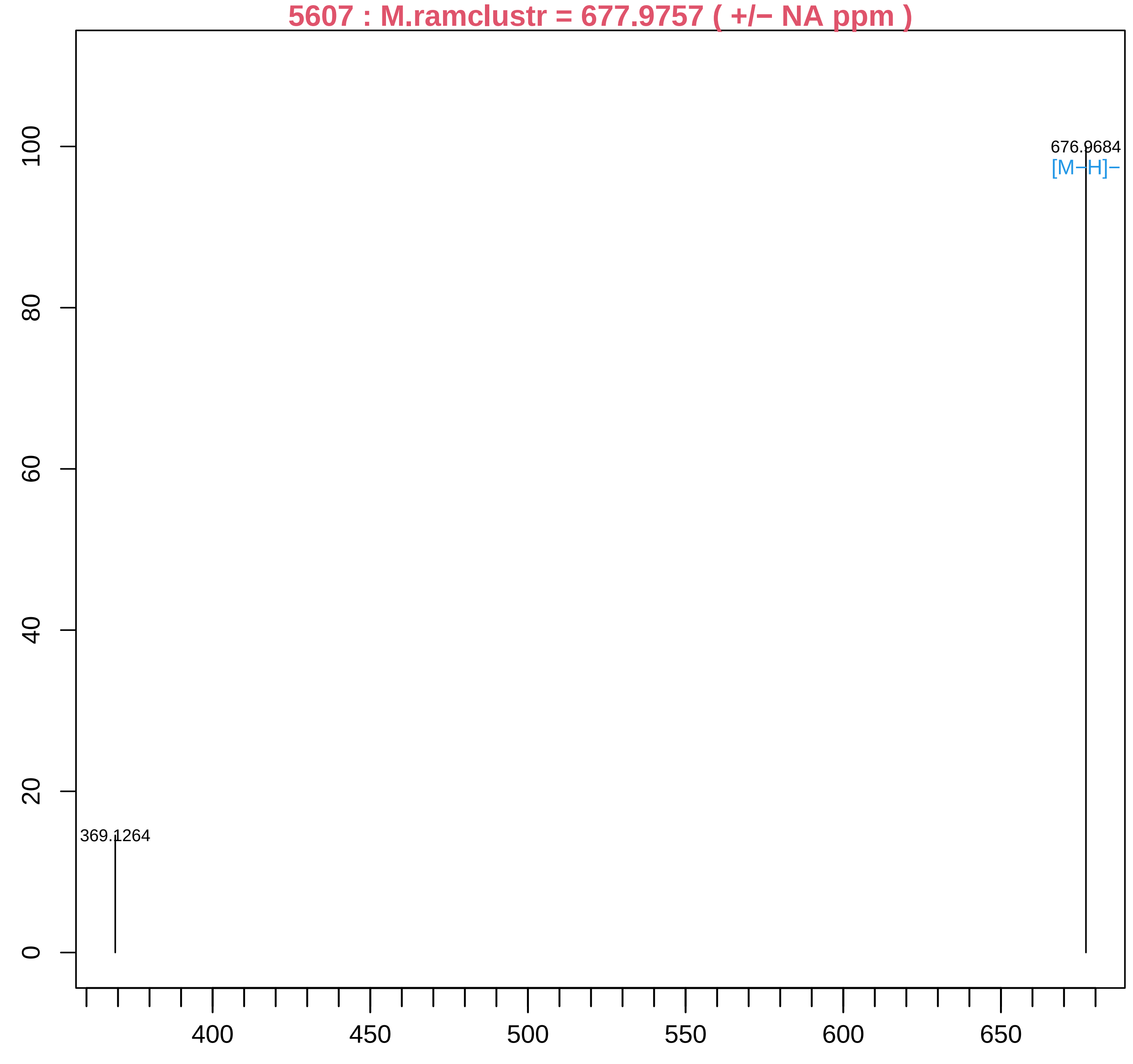
5607 : M.ramclustr = 677.9757 (+/− NA ppm)
676.9684
[M−H]−
369.1264
0
20
40
60
80
100
400
450
500
550
600
650

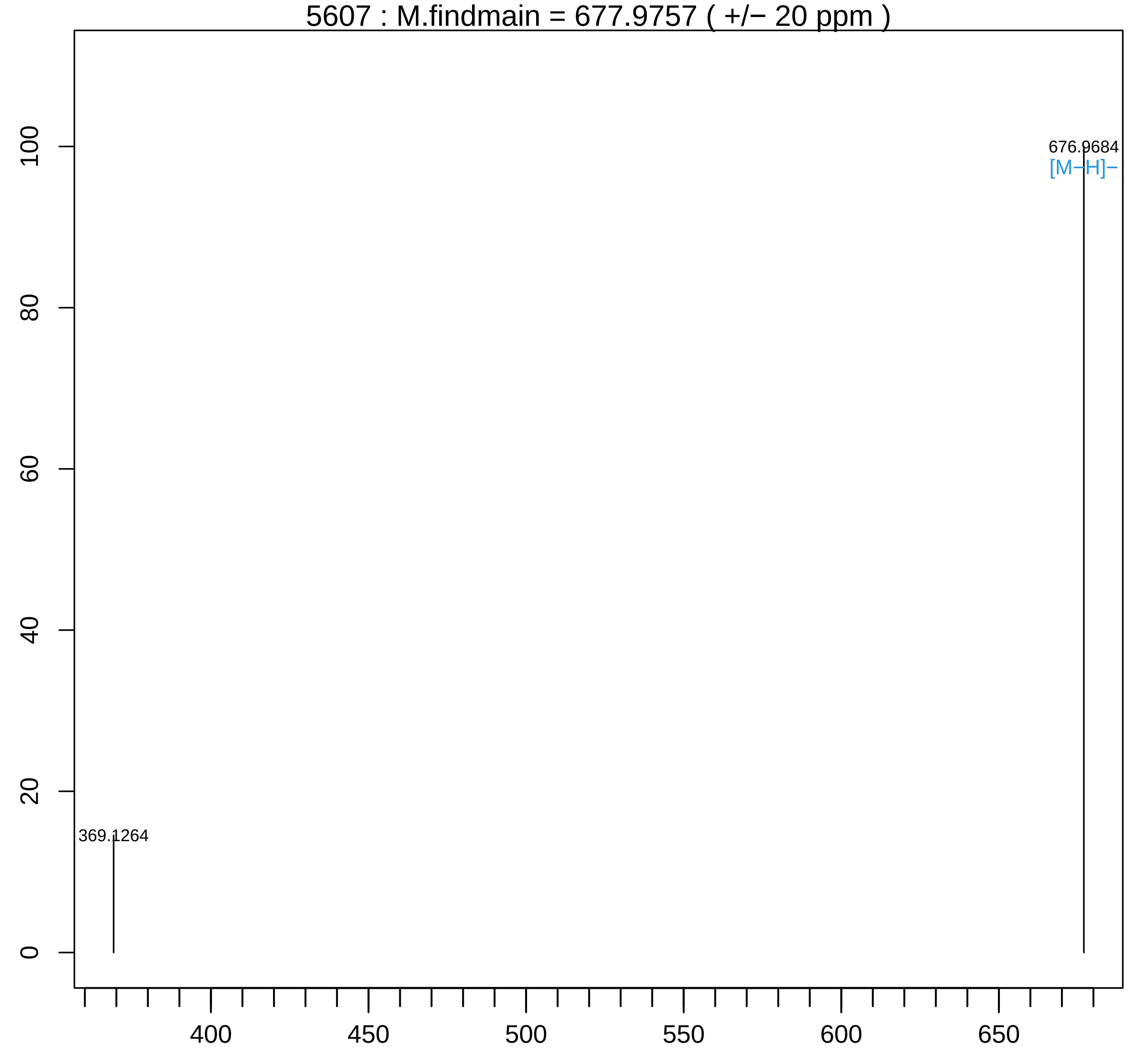
5607 : M.findmain = 677.9757 (+/− 20 ppm)
676.9684
[M−H]−
369.1264
0
20
40
60
80
100
400
450
500
550
600
650

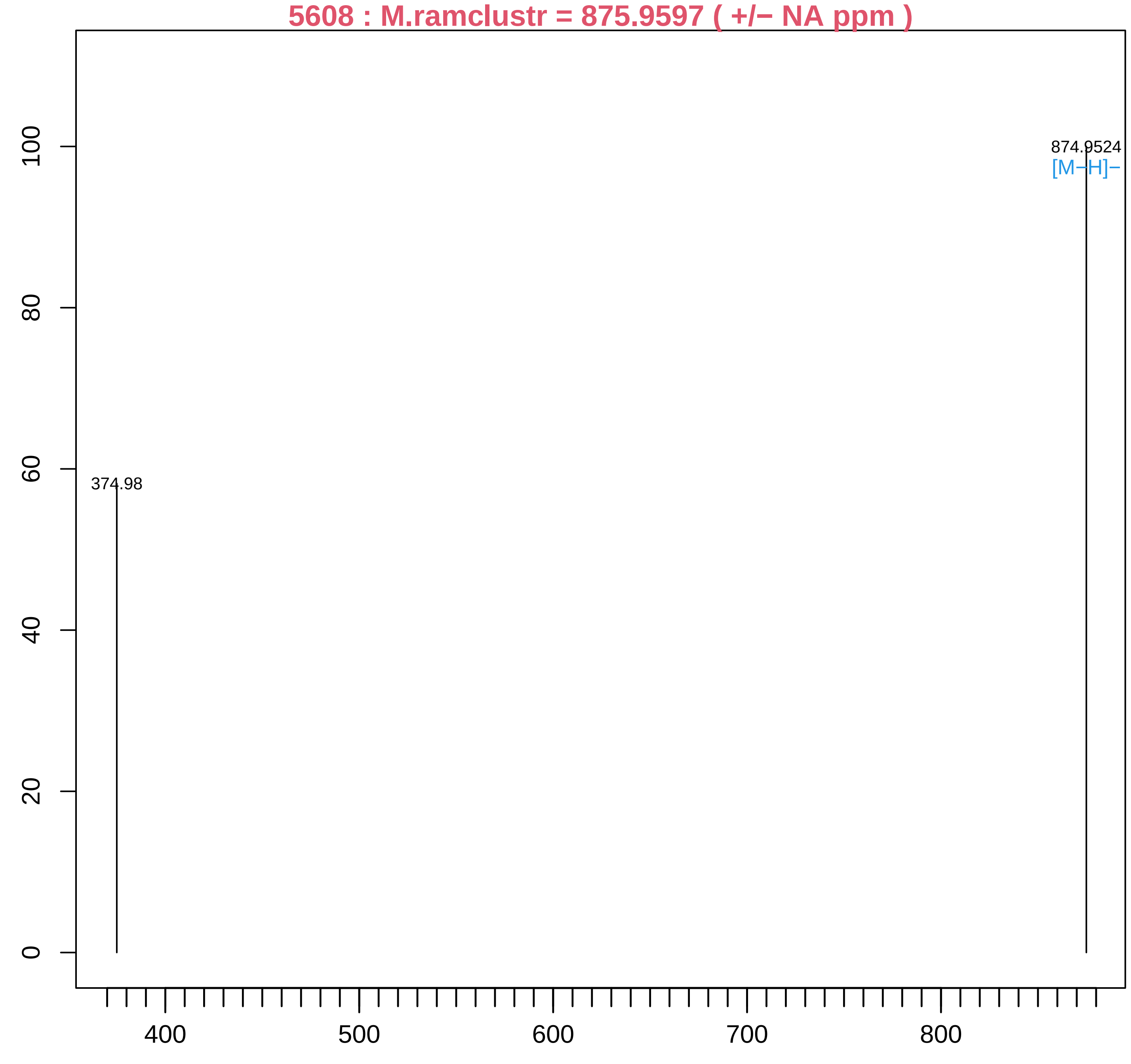
5608 : M.ramclustr = 875.9597 (+/− NA ppm)
874.9524
[M−H]−
374.98
0
20
40
60
80
100
400
500
600
700
800

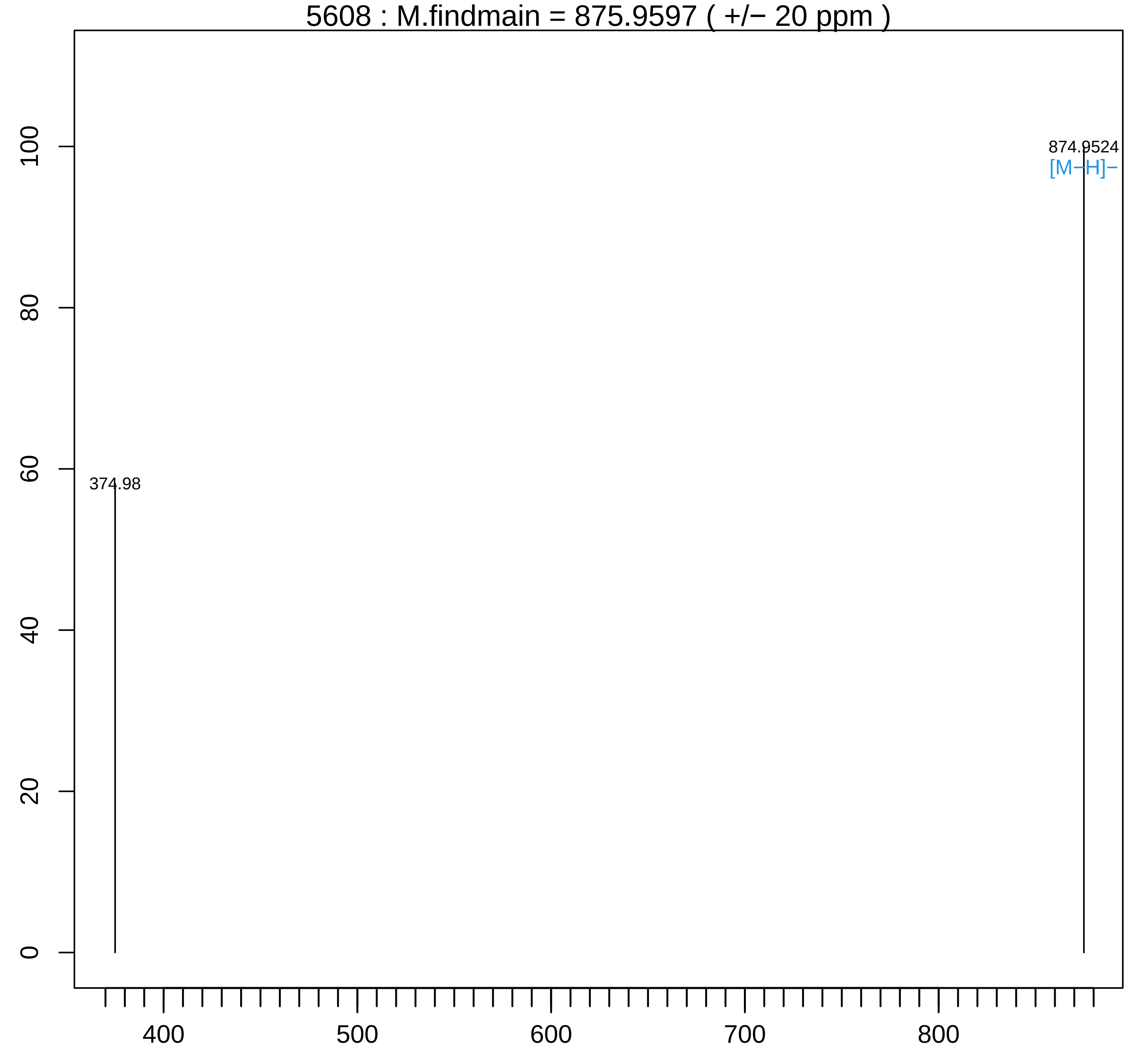
5608 : M.findmain = 875.9597 (+/− 20 ppm)
874.9524
[M−H]−
374.98
0
20
40
60
80
100
400
500
600
700
800

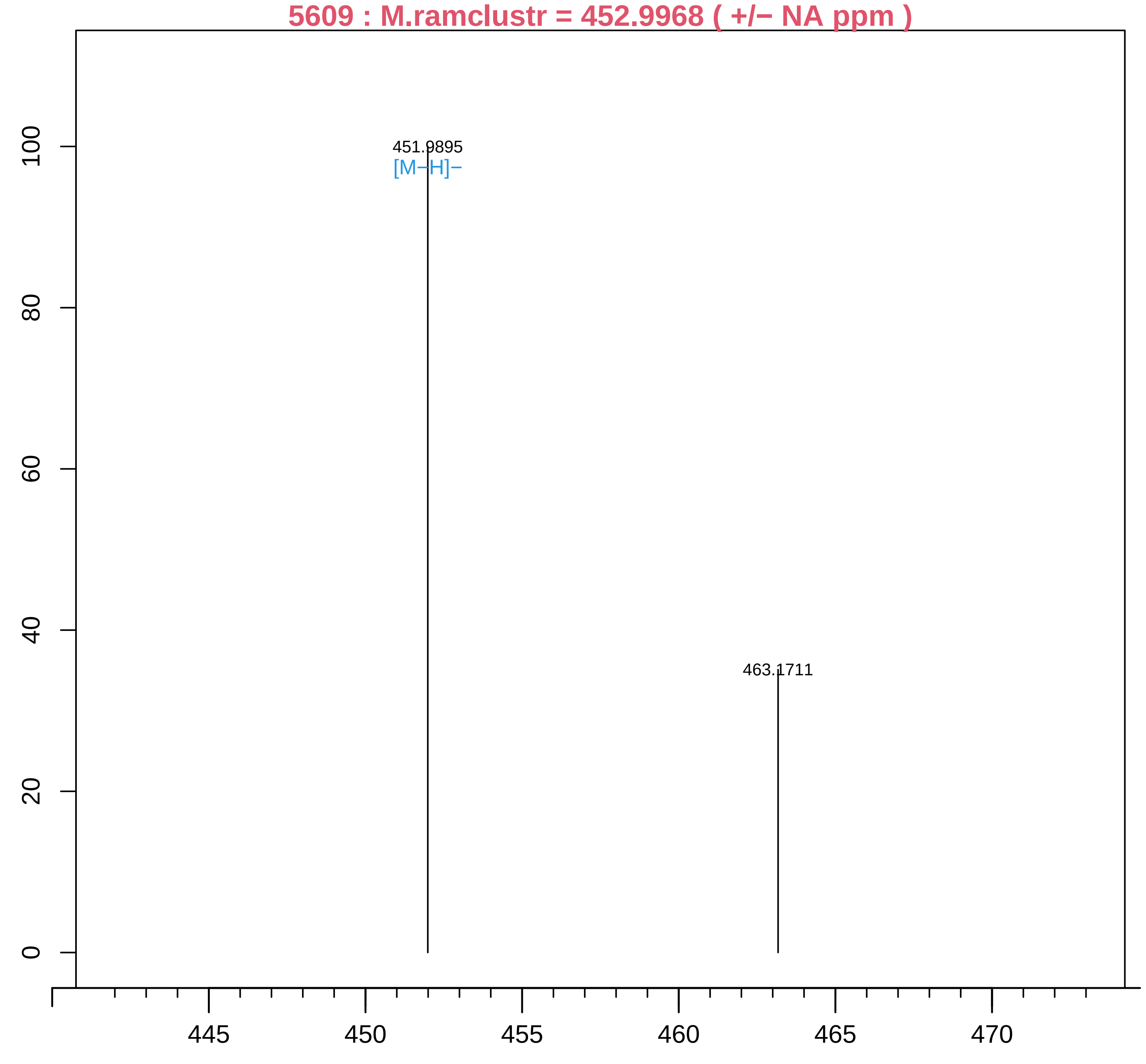
5609 : M.ramclustr = 452.9968 (+/− NA ppm)
451.9895
[M−H]−
463.1711
0
20
40
60
80
100
445
450
455
460
465
470

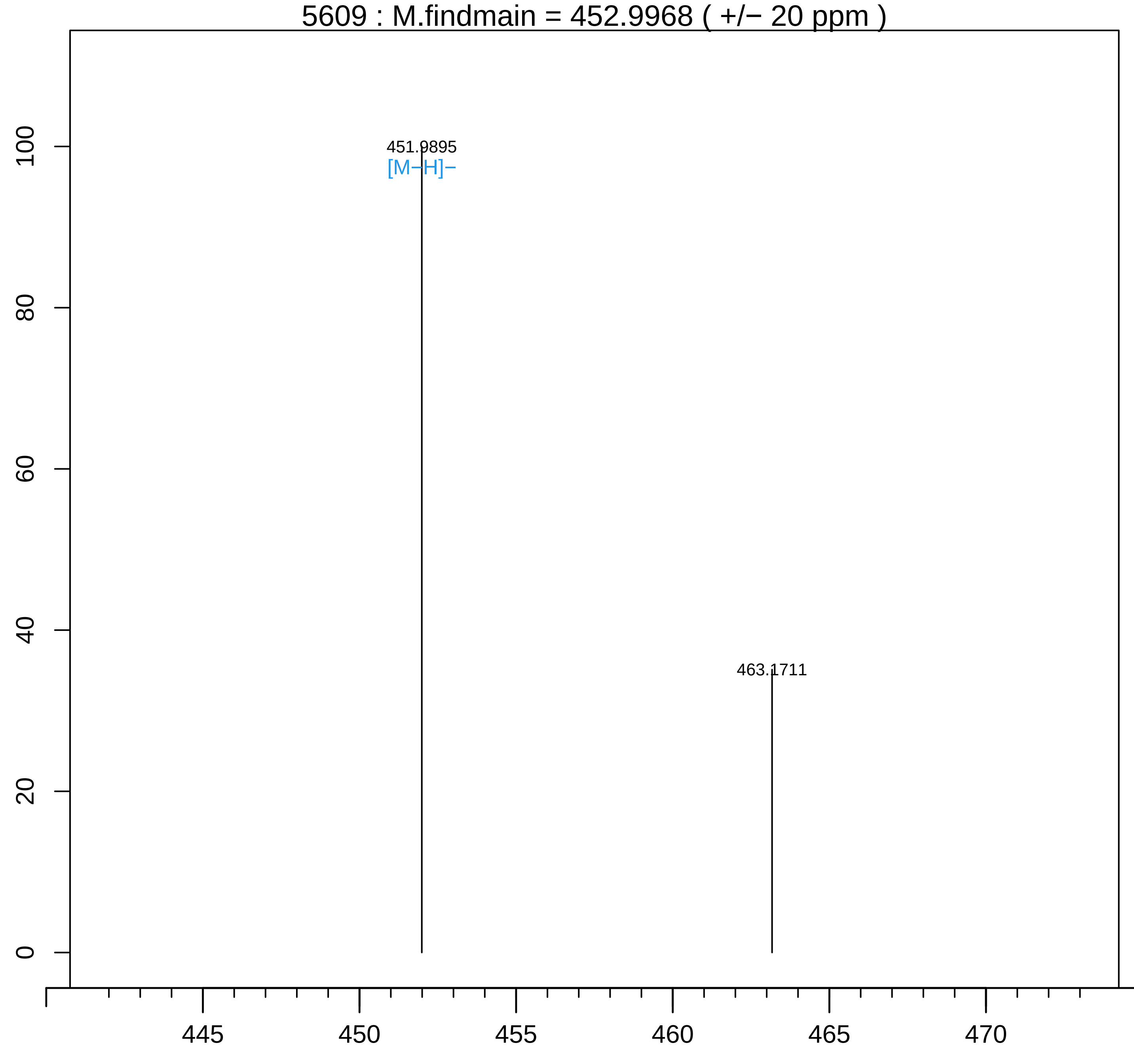
5609 : M.findmain = 452.9968 (+/− 20 ppm)
451.9895
[M−H]−
463.1711
0
20
40
60
80
100
445
450
455
460
465
470

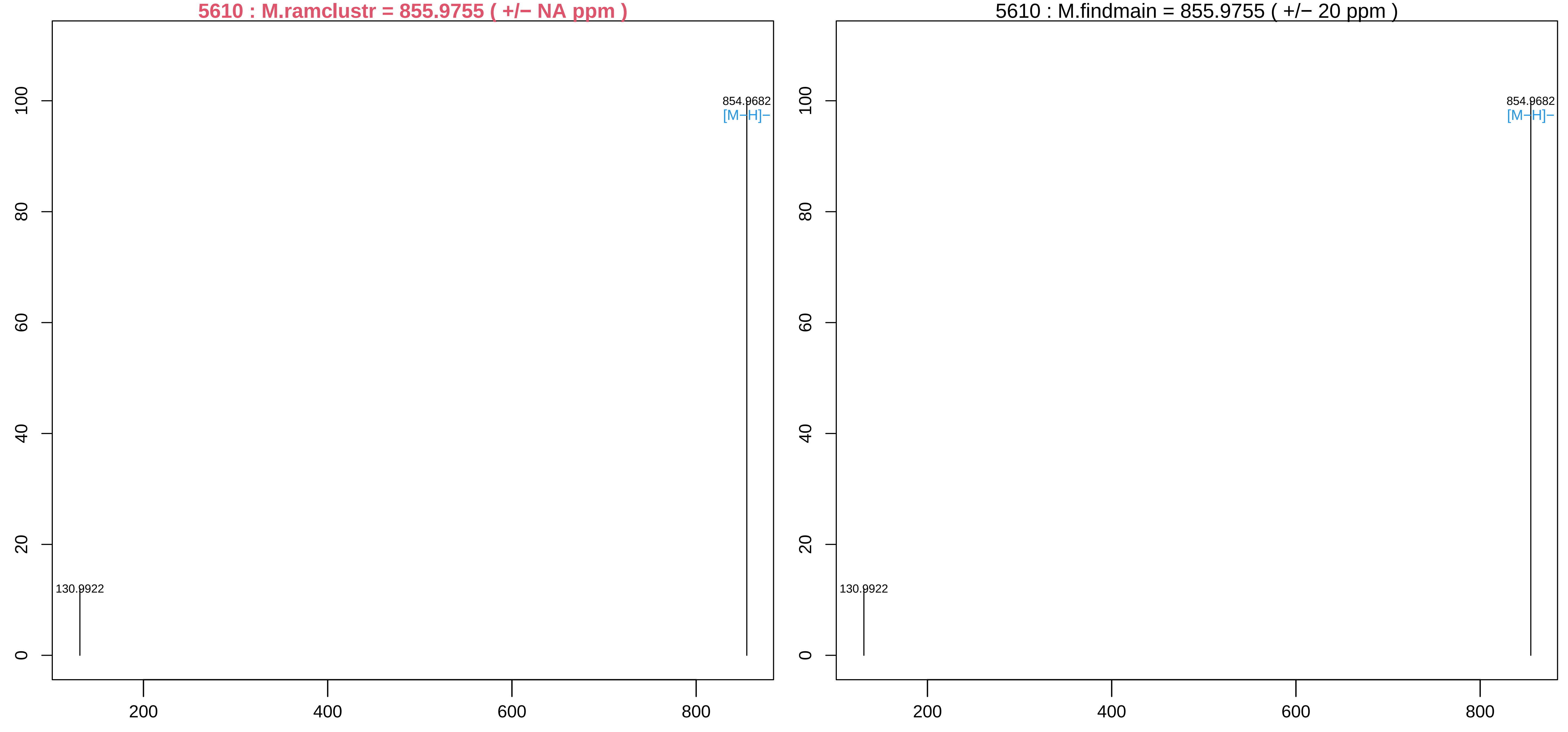
5610 : M.ramclustr = 855.9755 (+/− NA ppm)
854.9682
[M−H]−
130.9922
5610 : M.findmain = 855.9755 (+/− 20 ppm)
854.9682
[M−H]−
130.9922
0
20
40
60
80
100
200
400
600
800

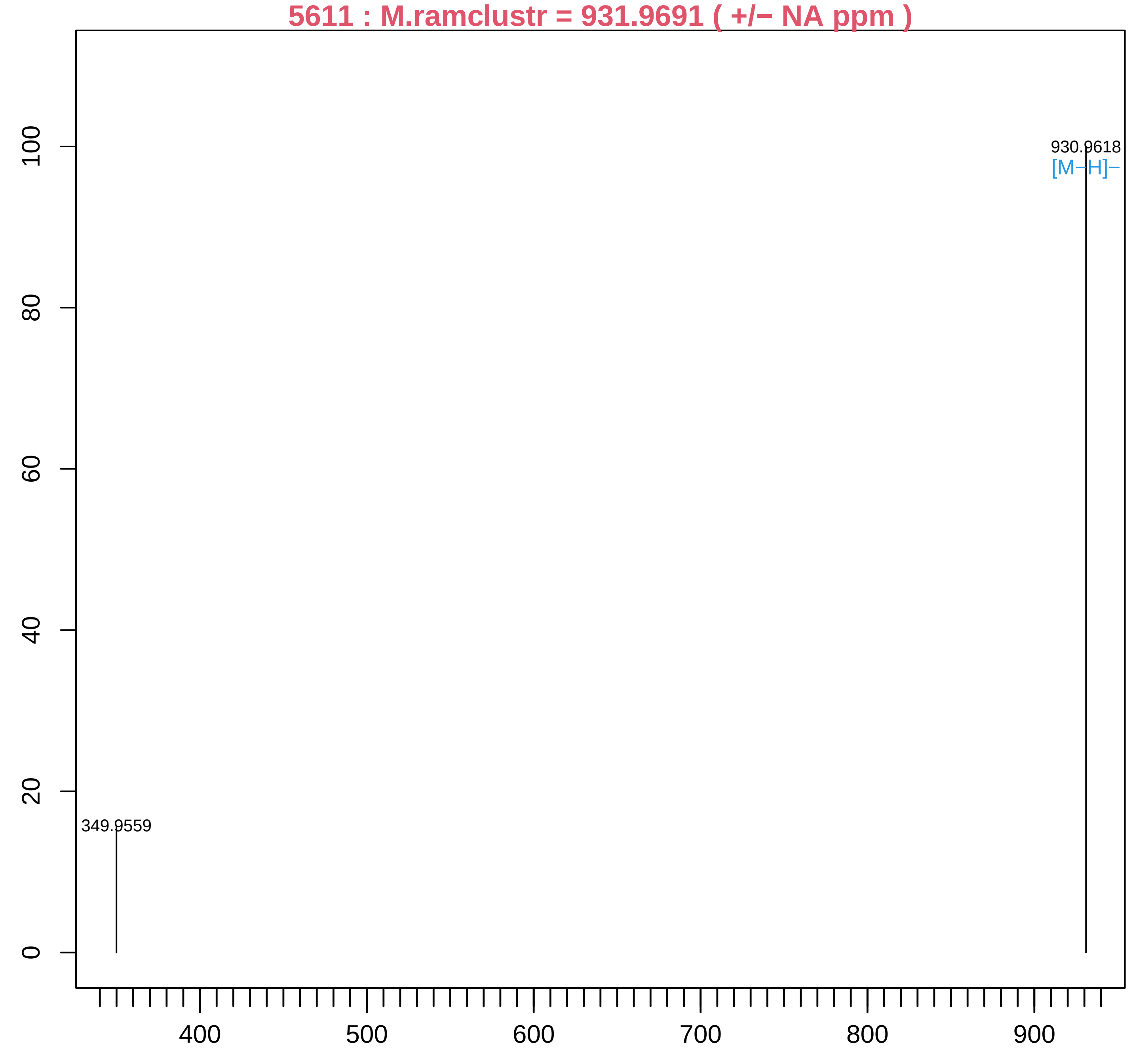
5611 : M.ramclustr = 931.9691 (+/− NA ppm)
930.9618
[M−H]−
349.9559
0
20
40
60
80
100
400
500
600
700
800
900

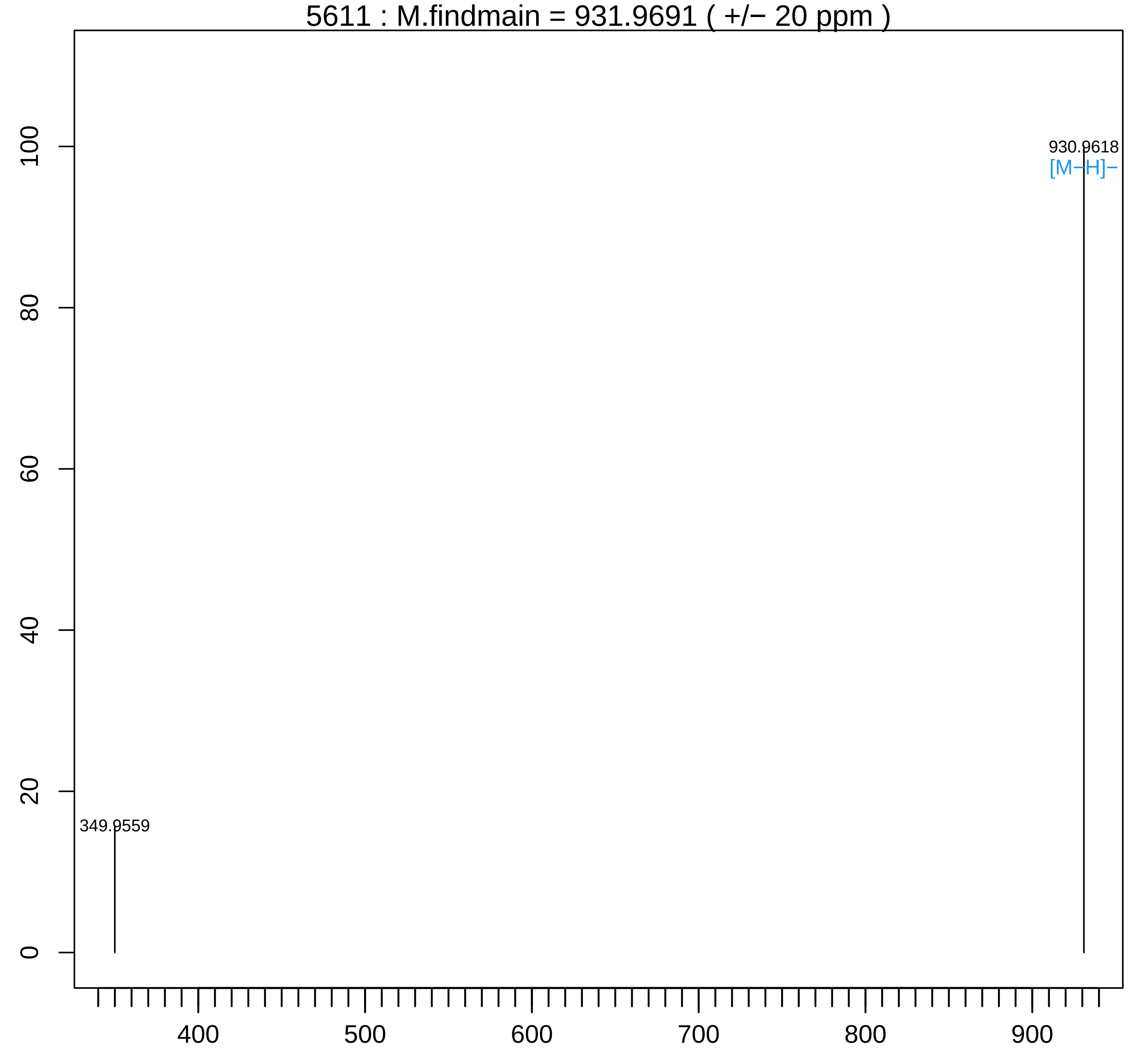
5611 : M.findmain = 931.9691 (+/− 20 ppm)
930.9618
[M−H]−
349.9559
0
20
40
60
80
100
400
500
600
700
800
900

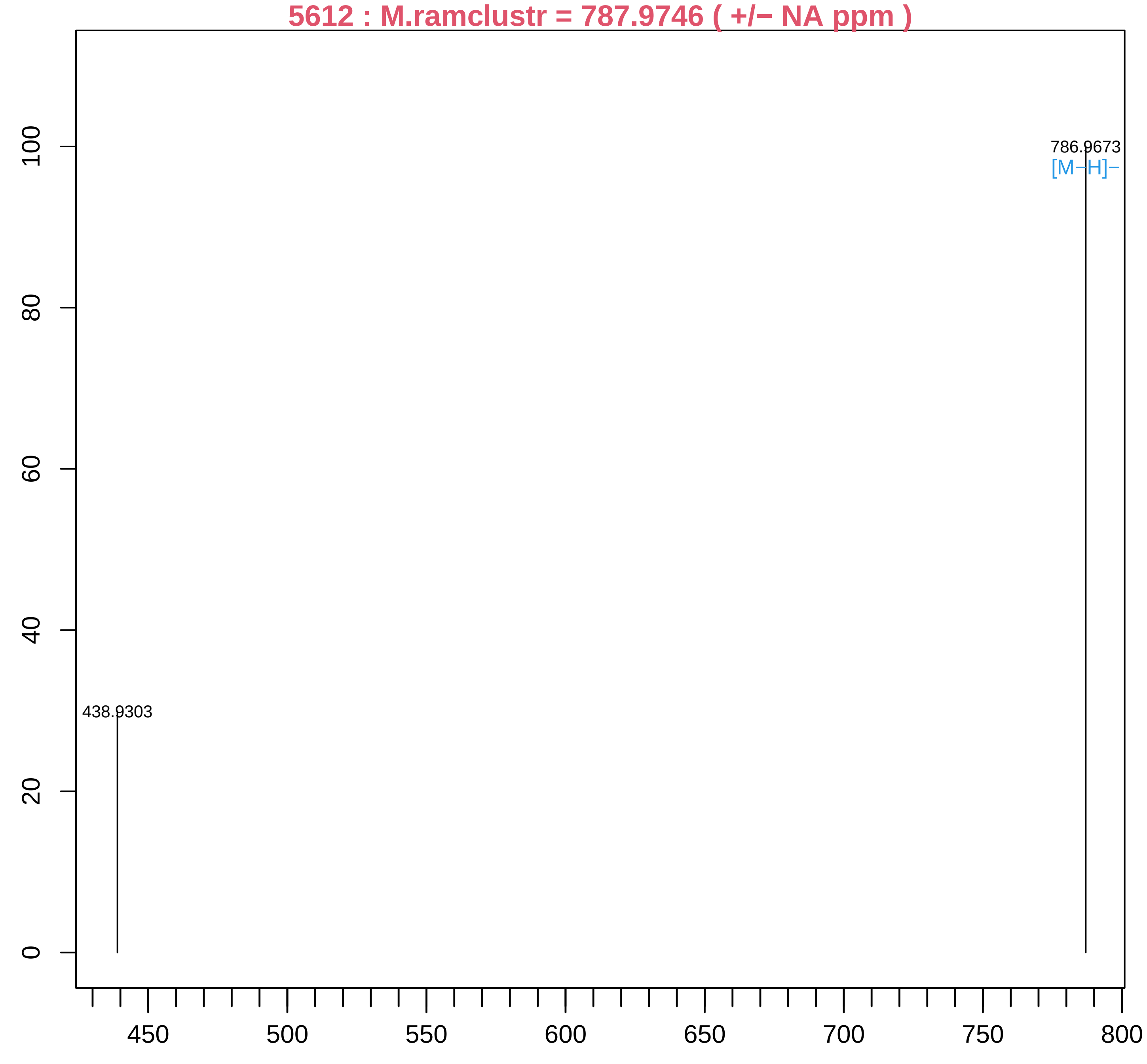
5612 : M.ramclustr = 787.9746 (+/− NA ppm)
786.9673
[M−H]−
438.9303
0
20
40
60
80
100
450
500
550
600
650
700
750
800

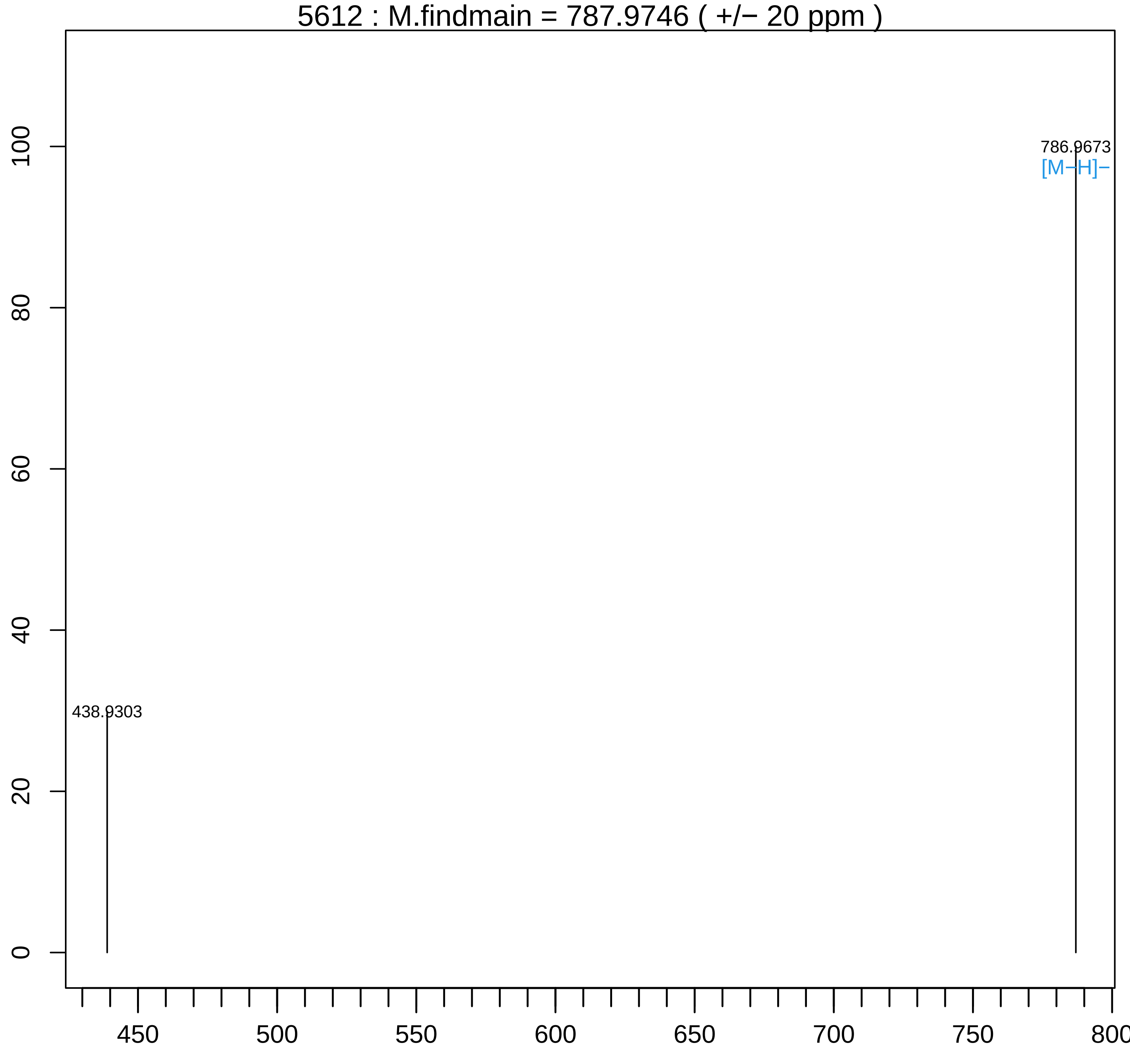
5612 : M.findmain = 787.9746 (+/− 20 ppm)
786.9673
[M−H]−
438.9303
0
20
40
60
80
100
450
500
550
600
650
700
750
800

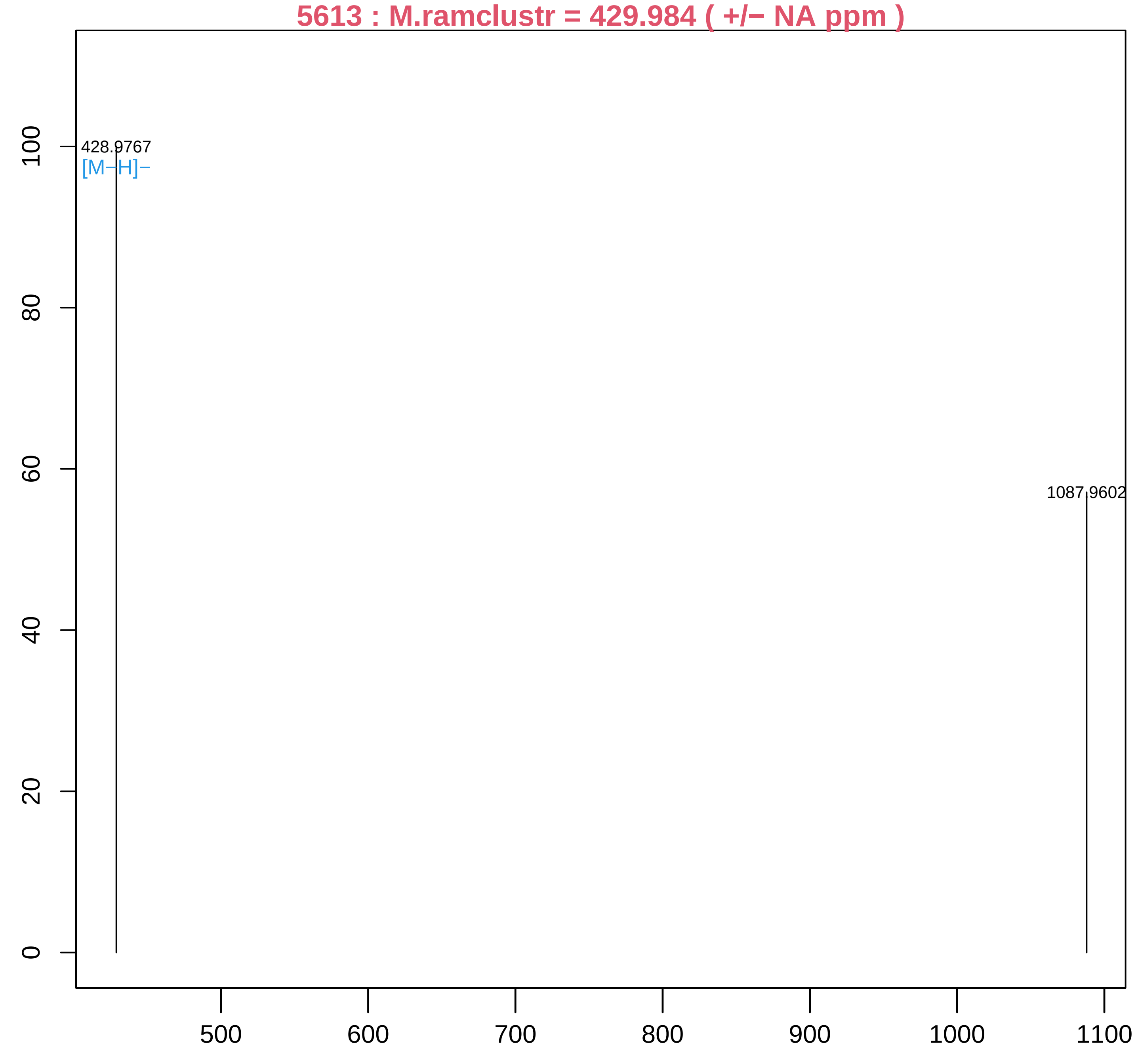
5613 : M.ramclustr = 429.984 (+/− NA ppm)
428.9767
[M−H]−
1087.9602
0
20
40
60
80
100
500
600
700
800
900
1000
1100

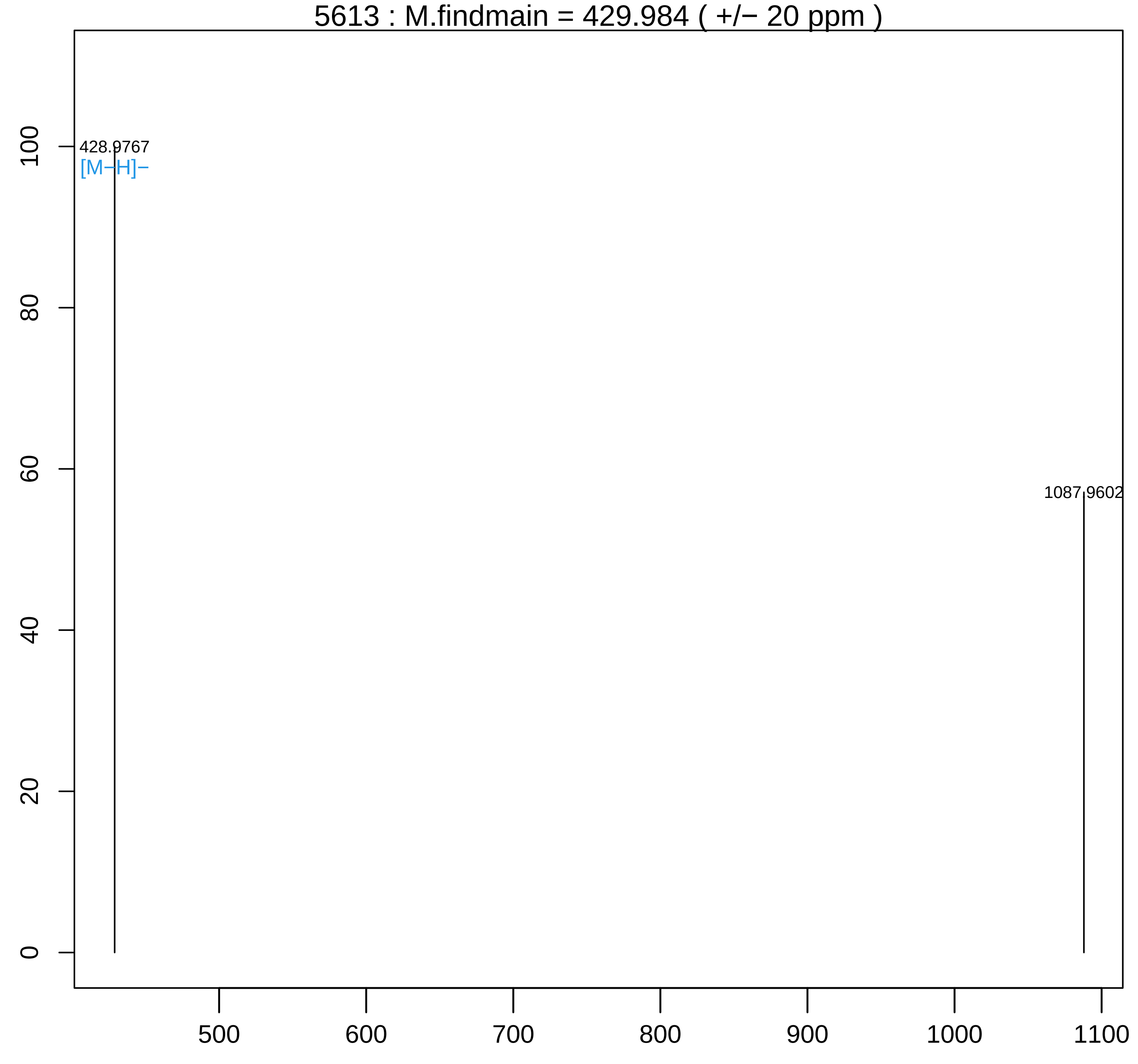
5613 : M.findmain = 429.984 (+/− 20 ppm)
428.9767
[M−H]−
1087.9602
0
20
40
60
80
100
500
600
700
800
900
1000
1100

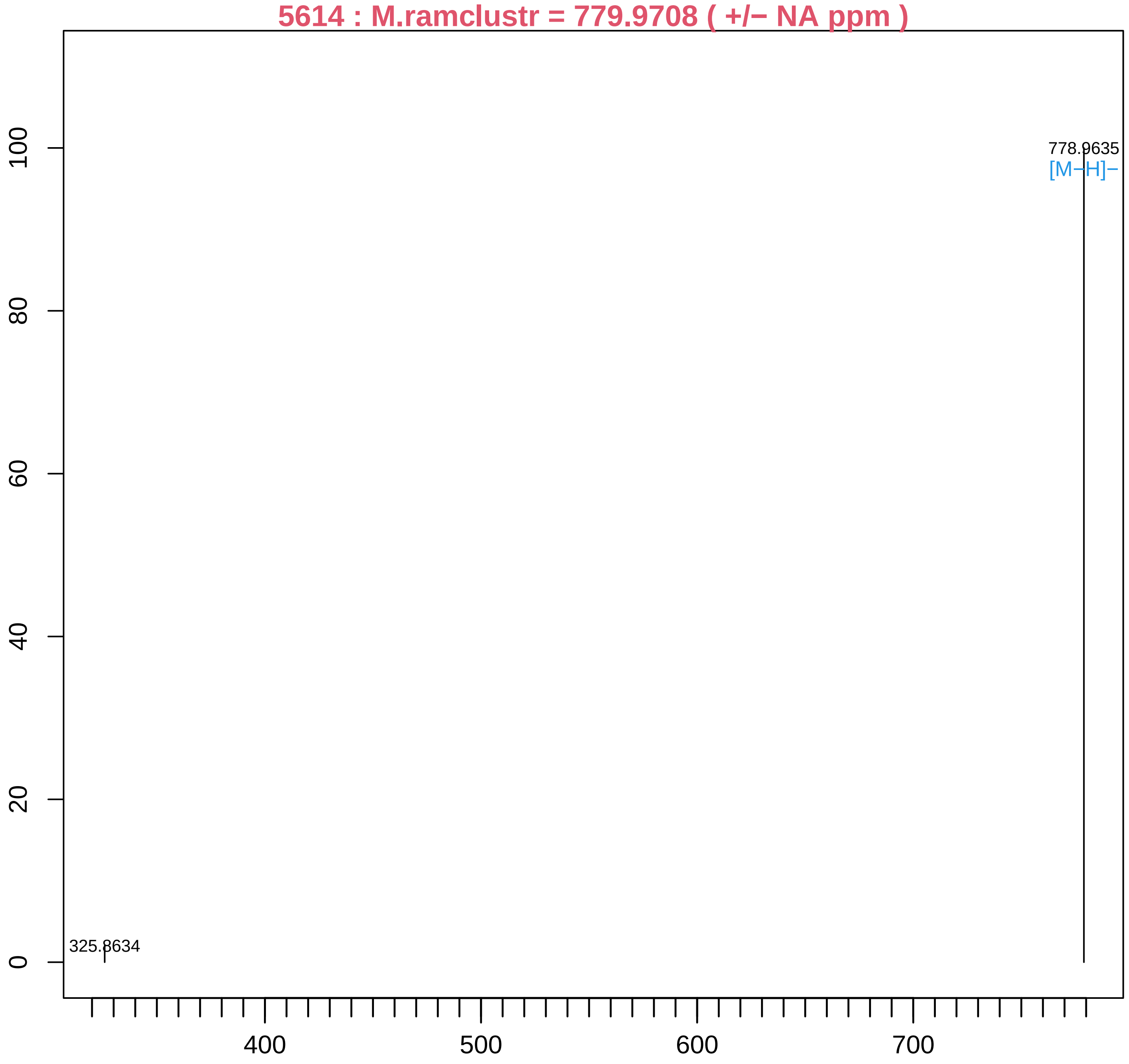
5614 : M.ramclustr = 779.9708 (+/− NA ppm)
778.9635
[M−H]−
325.8634
0
20
40
60
80
100
400
500
600
700

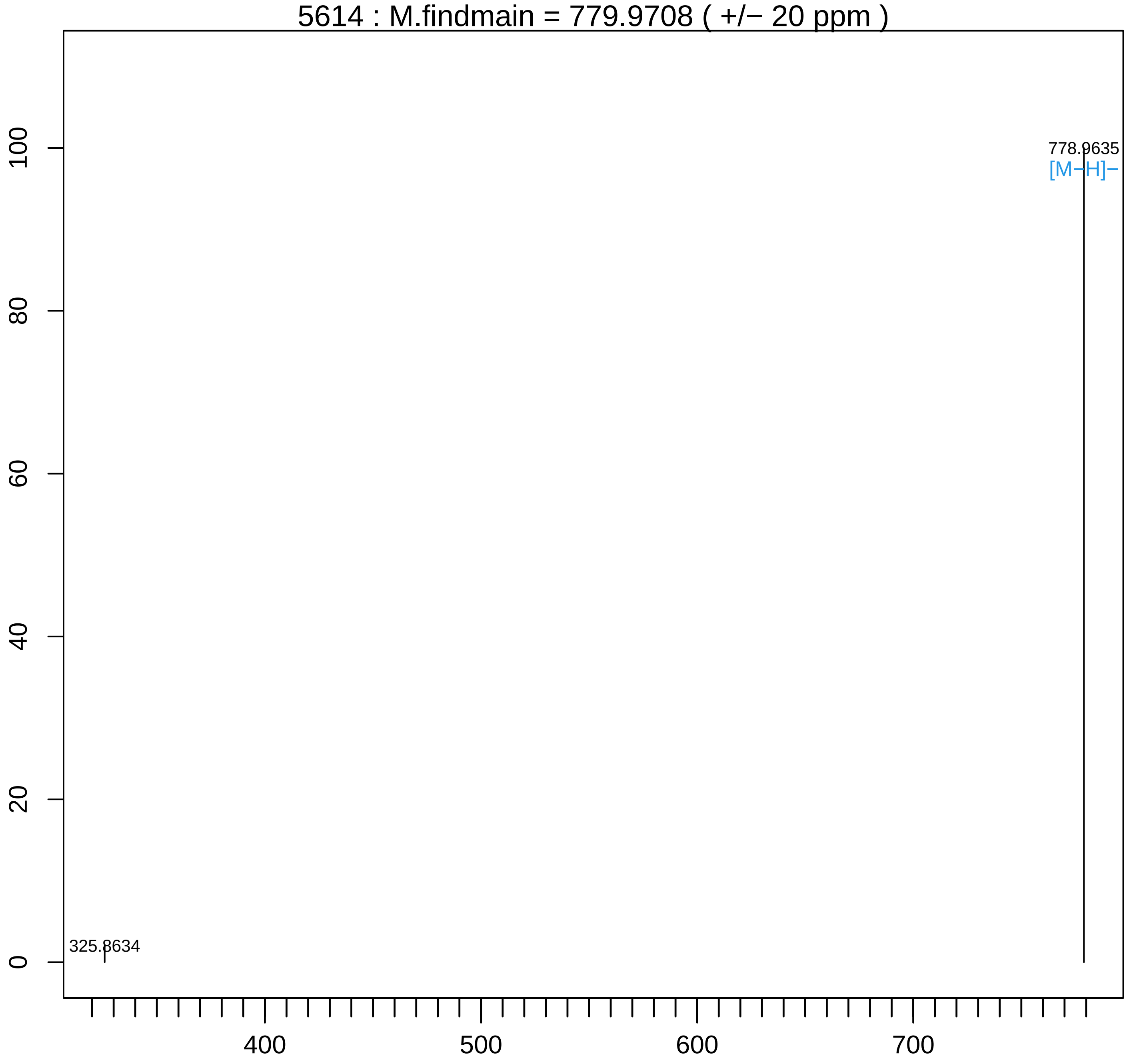
5614 : M.findmain = 779.9708 (+/− 20 ppm)
778.9635
[M−H]−
325.8634
0
20
40
60
80
100
400
500
600
700

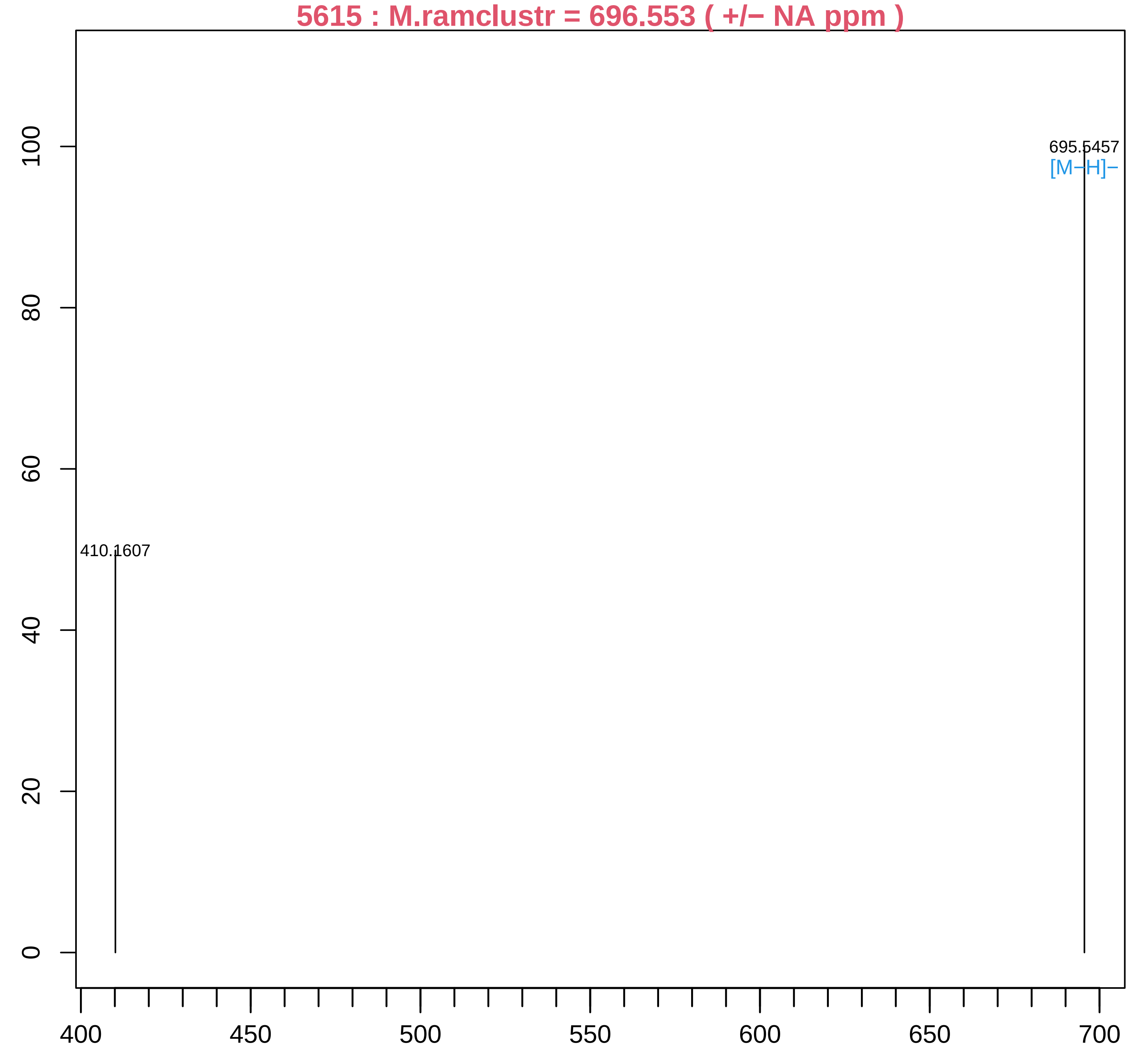
5615 : M.ramclustr = 696.553 (+/− NA ppm)
695.5457
[M−H]−
410.1607
0
20
40
60
80
100
400
450
500
550
600
650
700

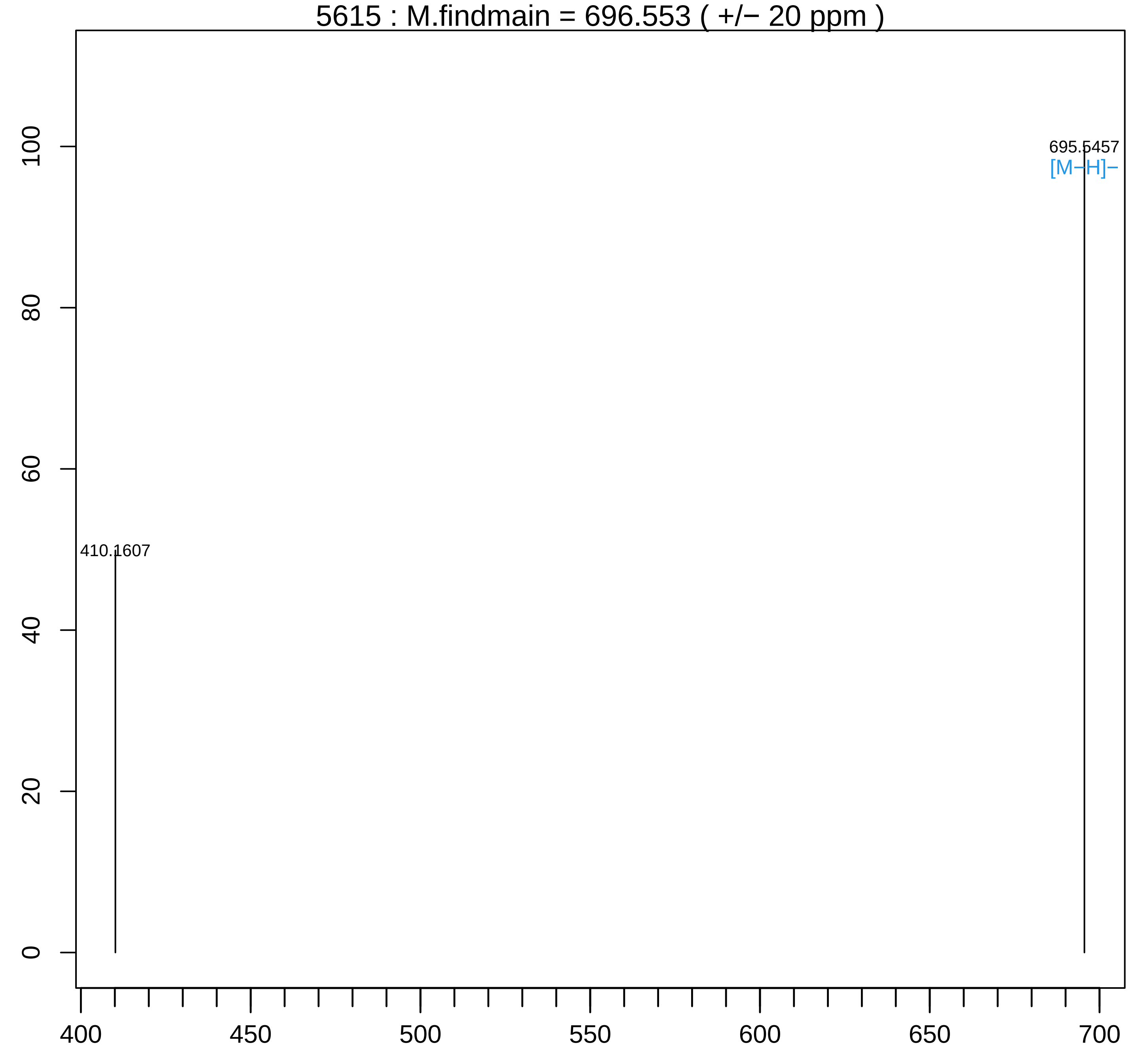
5615 : M.findmain = 696.553 (+/− 20 ppm)
695.5457
[M−H]−
410.1607
0
20
40
60
80
100
400
450
500
550
600
650
700

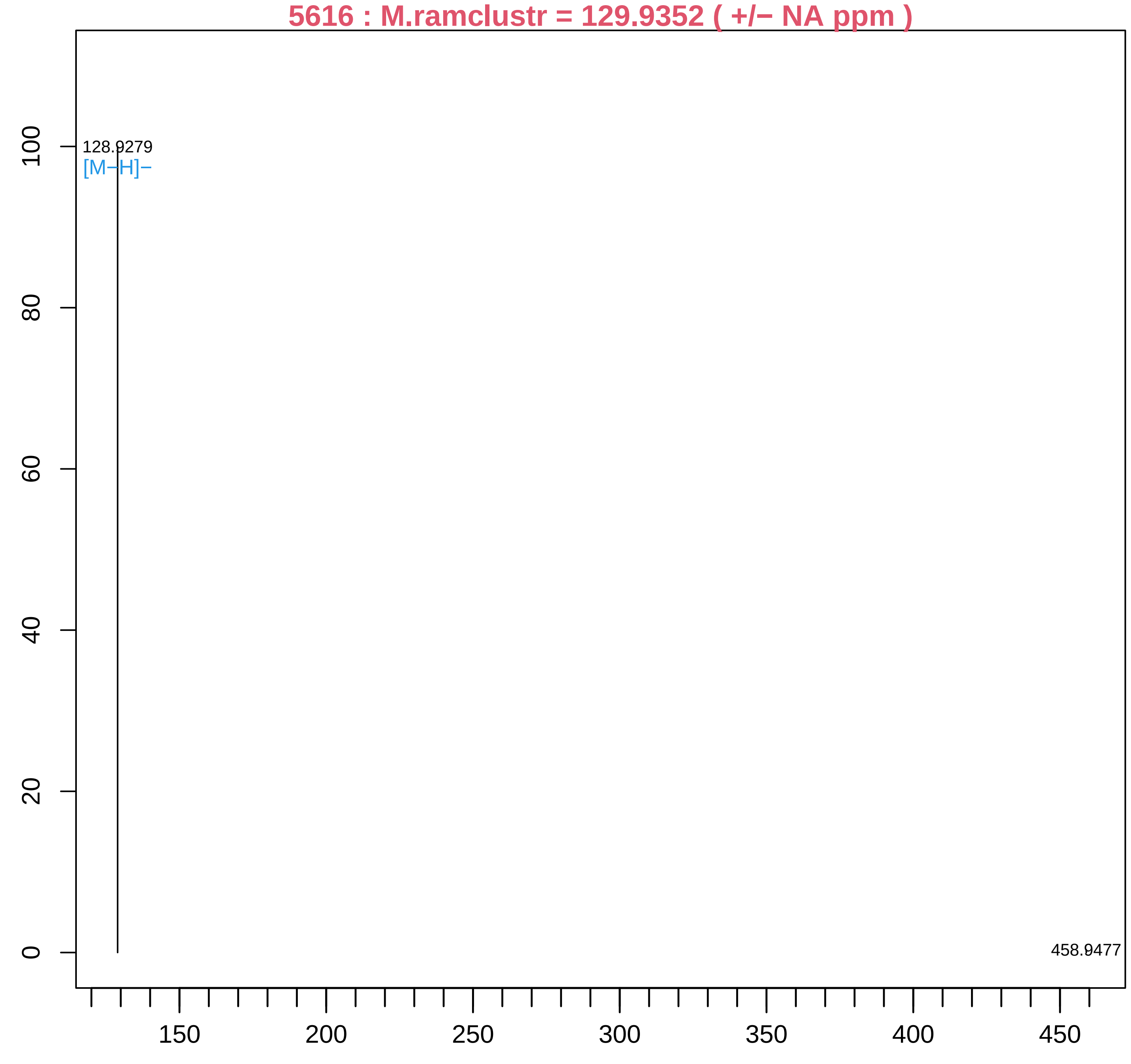
5616 : M.ramclustr = 129.9352 (+/− NA ppm)
128.9279
[M−H]−
458.9477
0
20
40
60
80
100
150
200
250
300
350
400
450

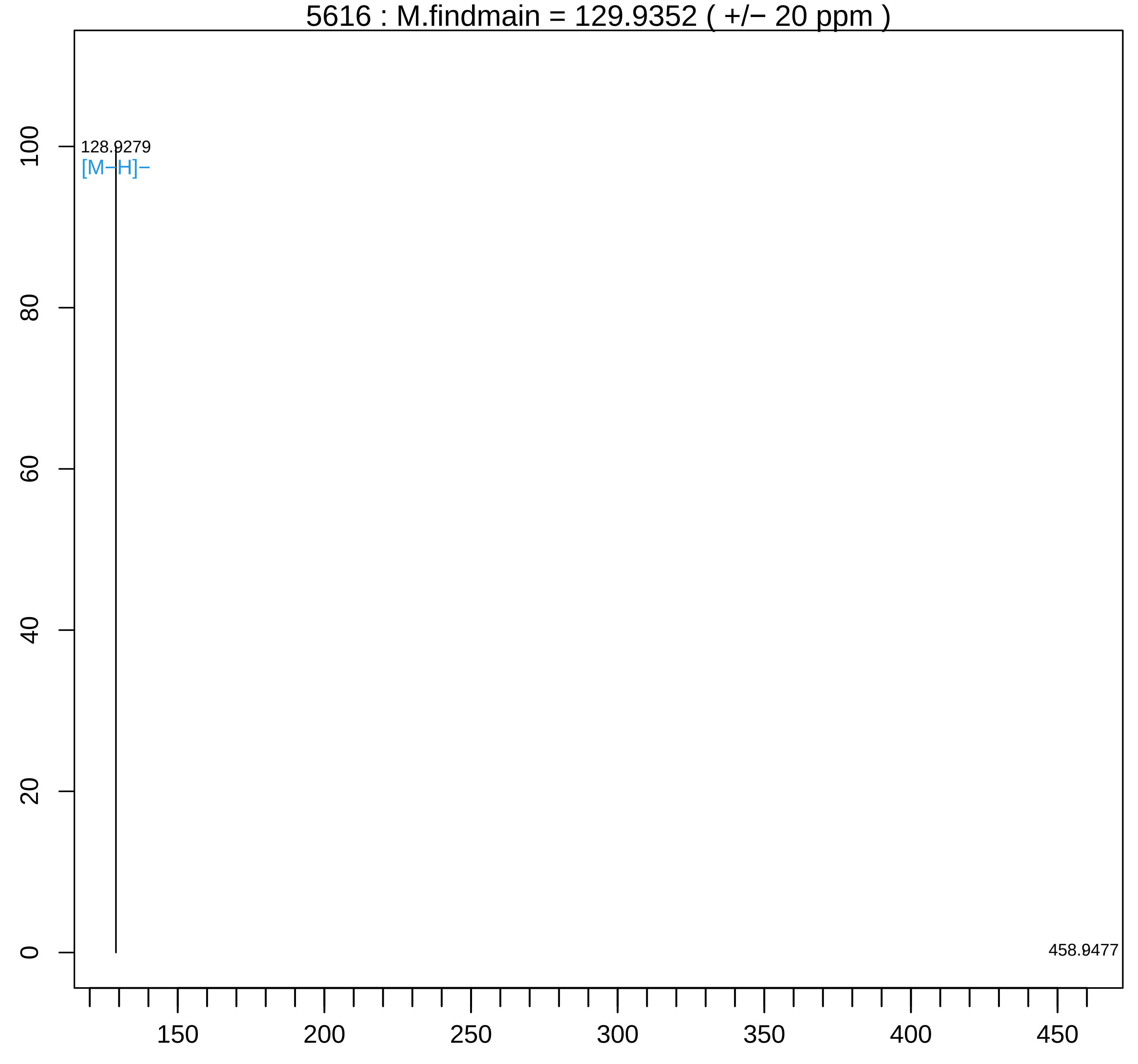
5616 : M.findmain = 129.9352 (+/− 20 ppm)
128.9279
[M−H]−
458.9477
0
20
40
60
80
100
150
200
250
300
350
400
450

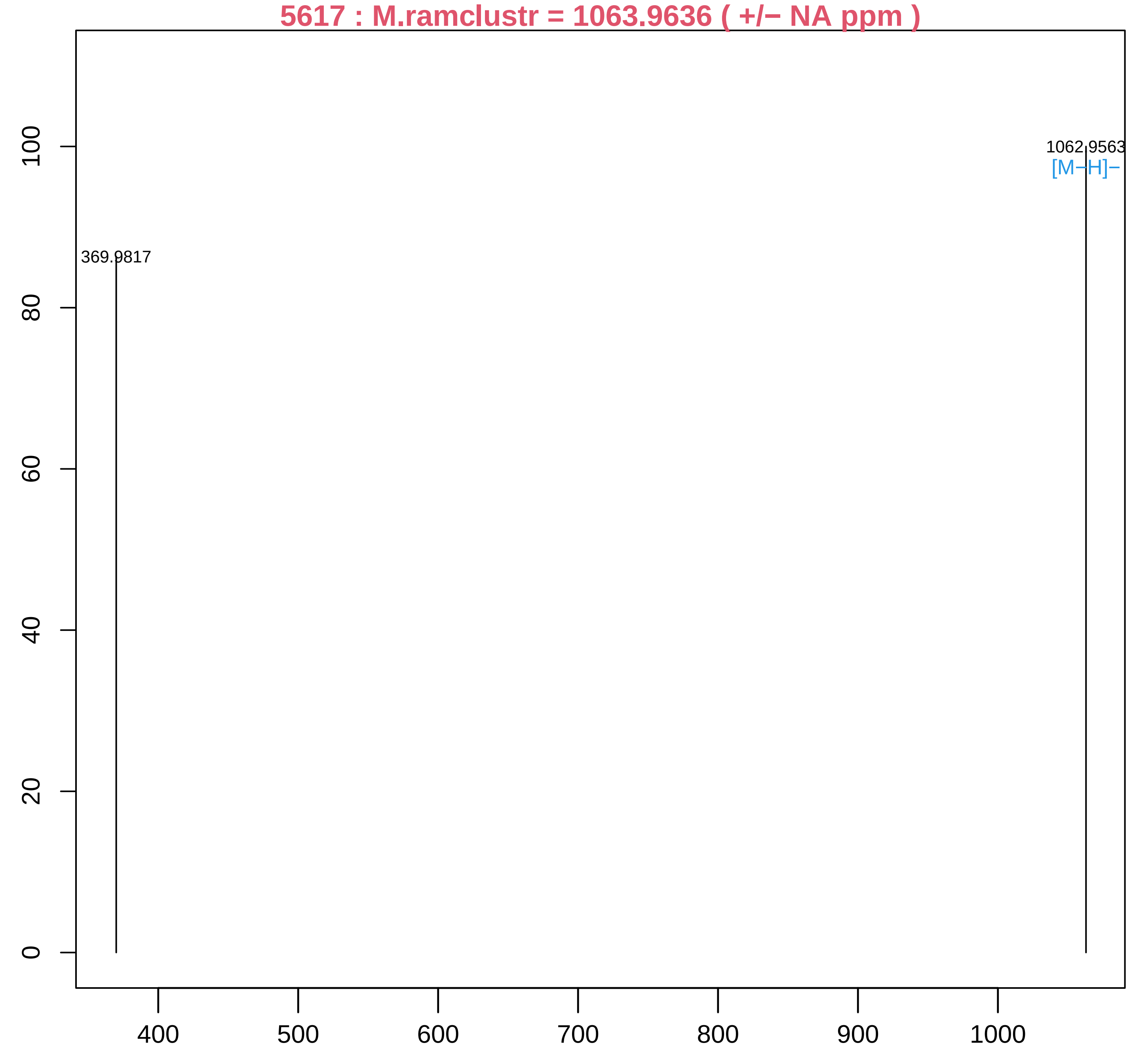
5617 : M.ramclustr = 1063.9636 (+/− NA ppm)
1062.9563
[M−H]−
369.9817
0
20
40
60
80
100
400
500
600
700
800
900
1000

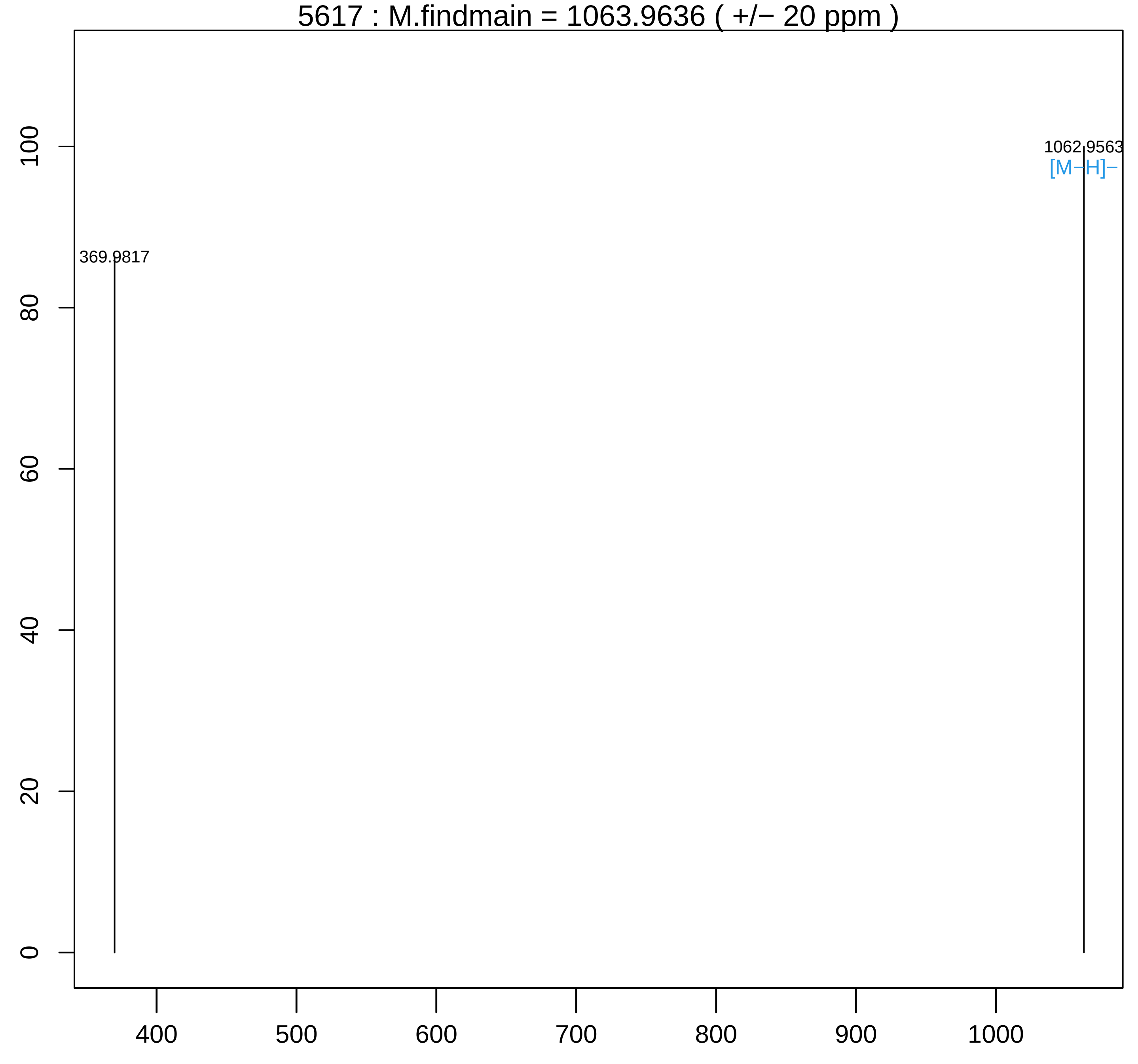
5617 : M.findmain = 1063.9636 (+/− 20 ppm)
1062.9563
[M−H]−
369.9817
0
20
40
60
80
100
400
500
600
700
800
900
1000

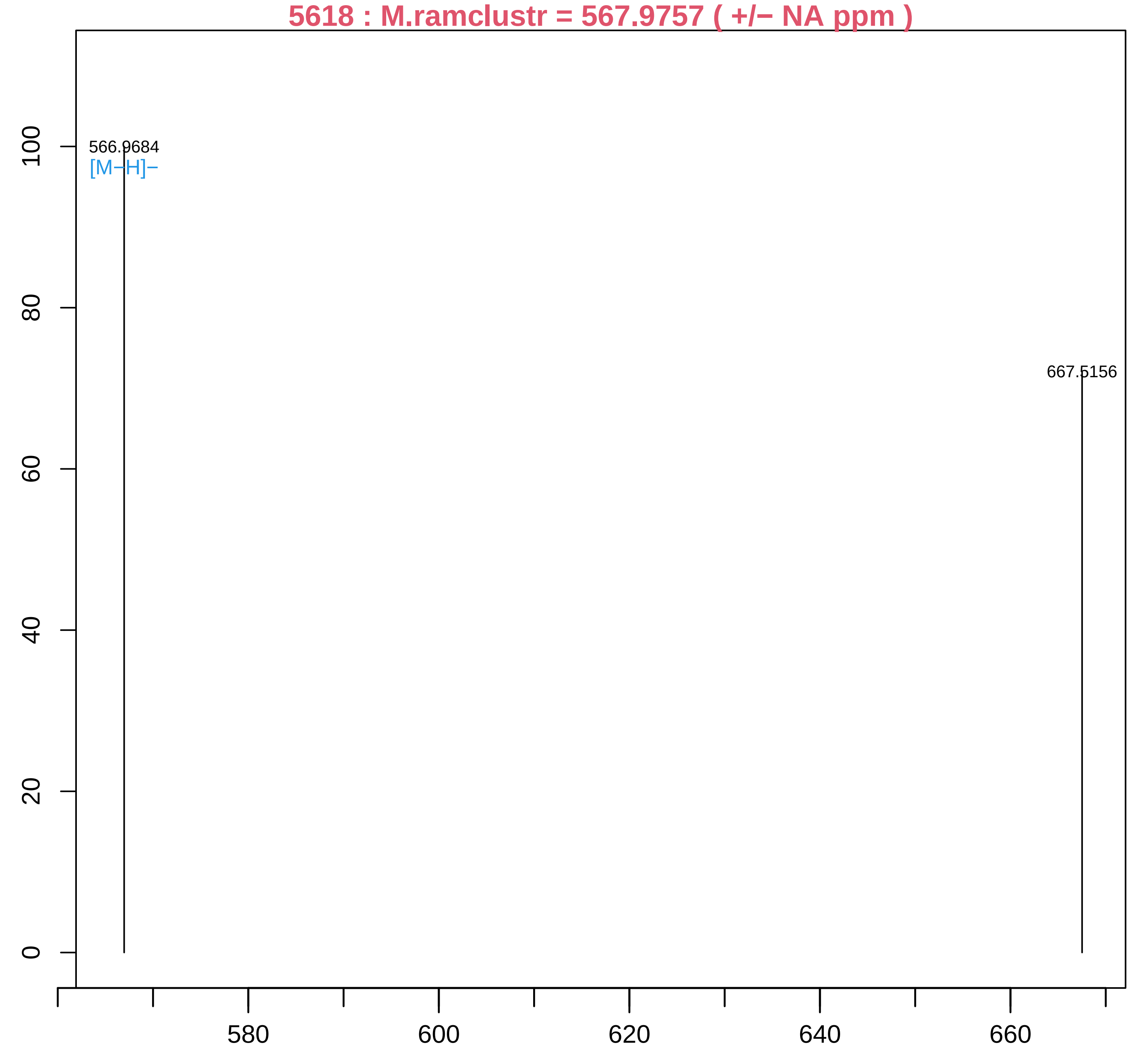
5618 : M.ramclustr = 567.9757 (+/− NA ppm)
566.9684
[M−H]−
667.5156
0
20
40
60
80
100
580
600
620
640
660

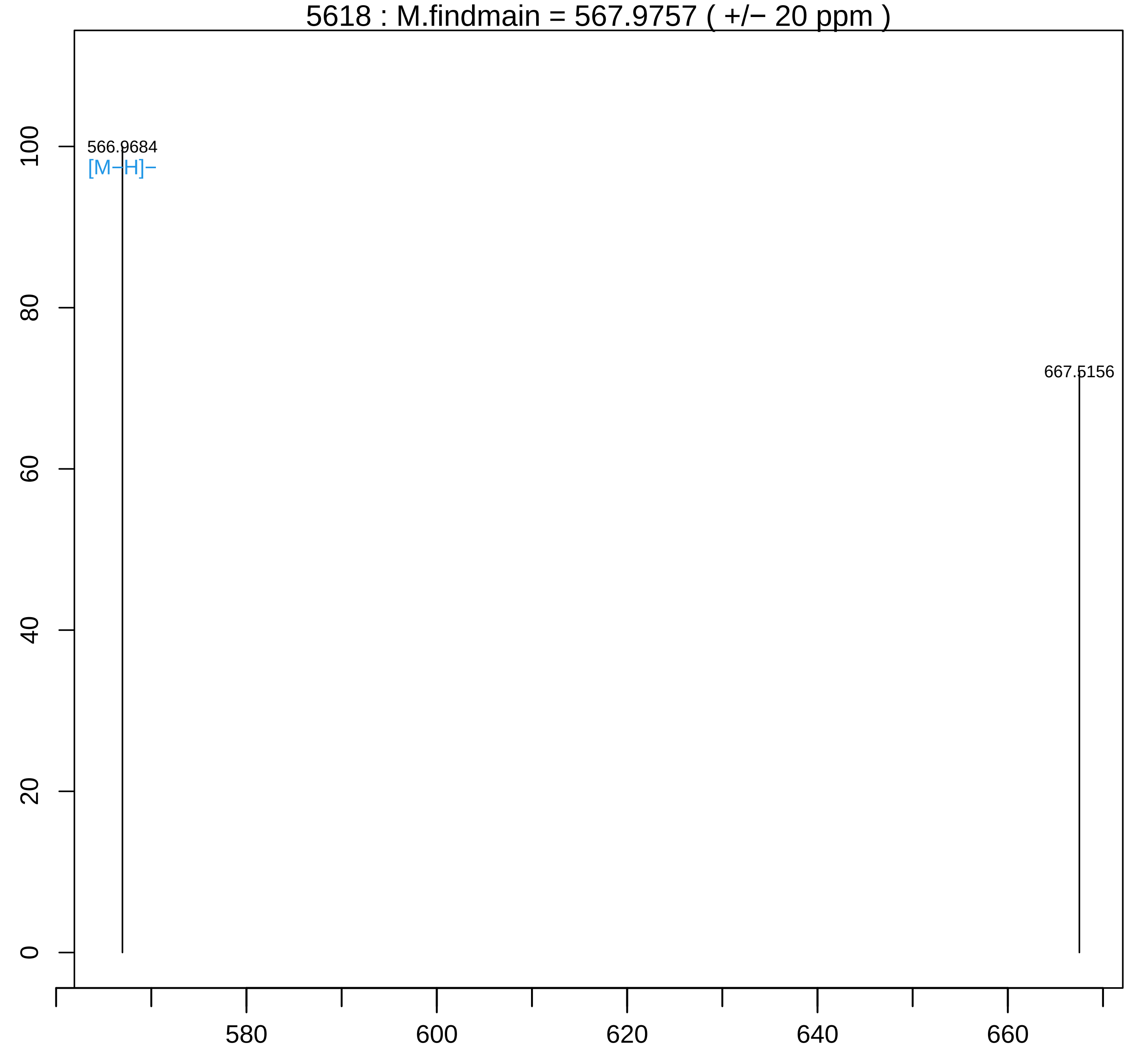
5618 : M.findmain = 567.9757 (+/− 20 ppm)
566.9684
[M−H]−
667.5156
0
20
40
60
80
100
580
600
620
640
660

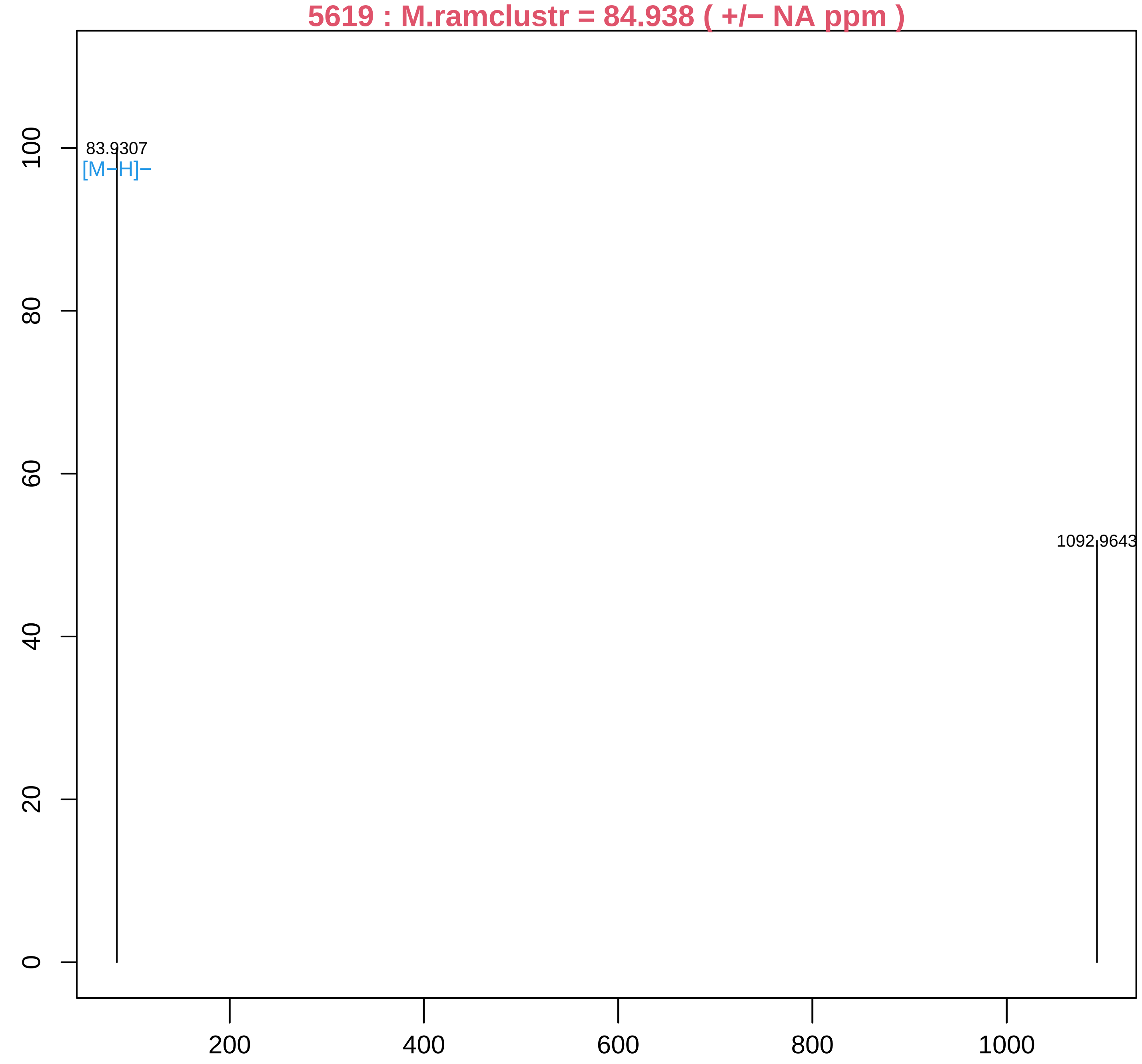
5619 : M.ramclustr = 84.938 (+/− NA ppm)
83.9307
[M−H]−
1092.9643
0
20
40
60
80
100
200
400
600
800
1000

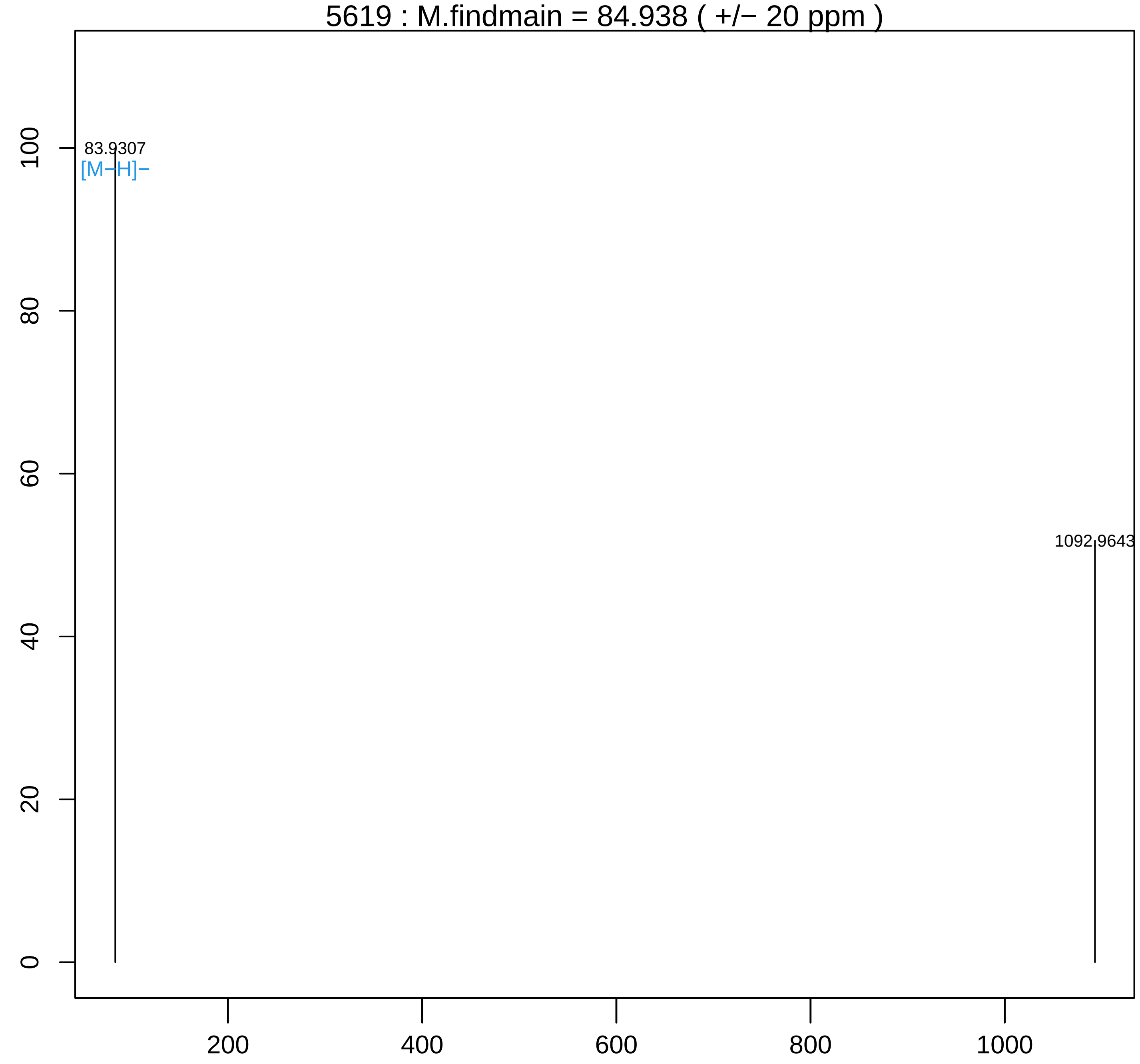
5619 : M.findmain = 84.938 (+/− 20 ppm)
83.9307
[M−H]−
1092.9643
0
20
40
60
80
100
200
400
600
800
1000

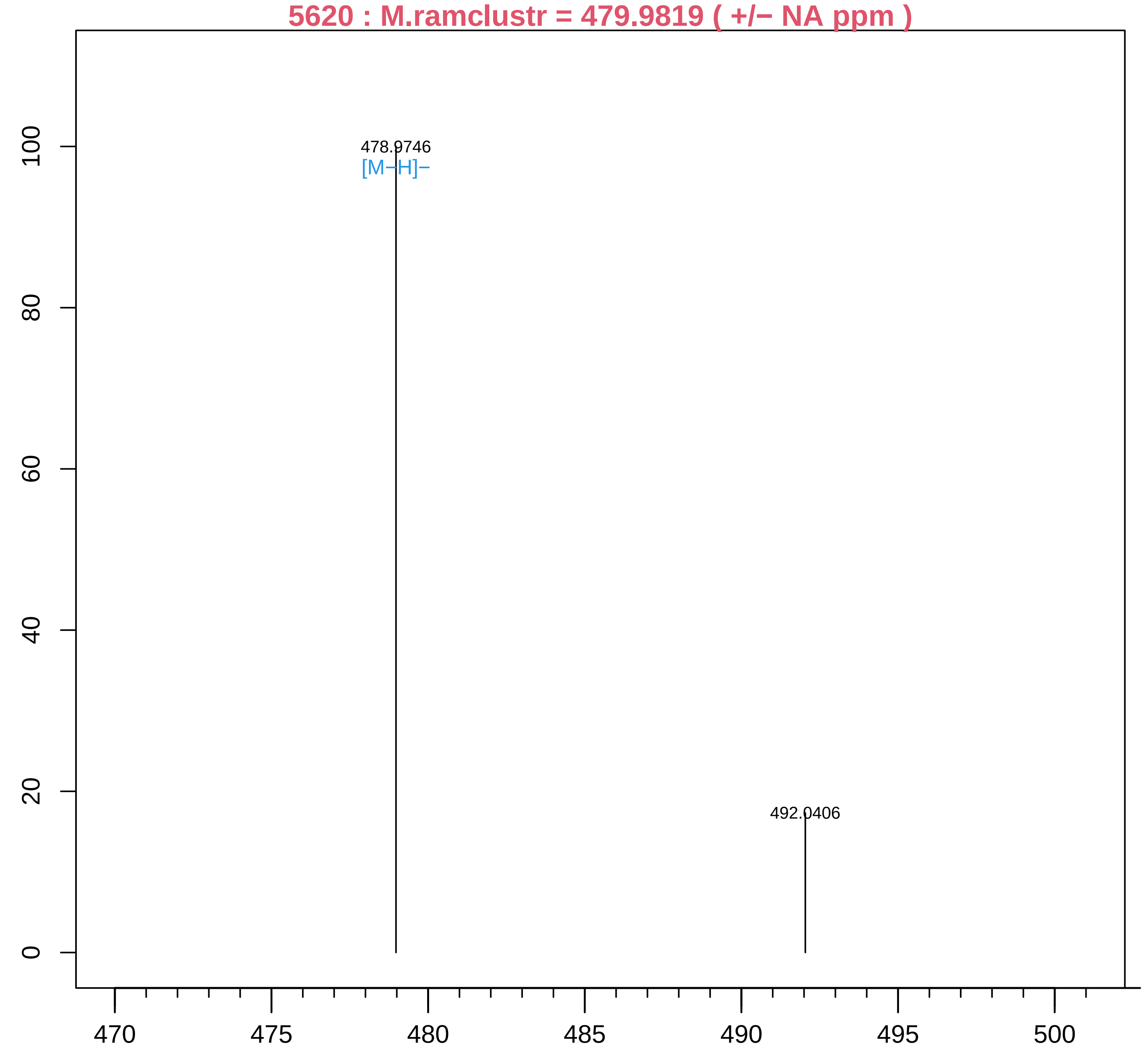
5620 : M.ramclustr = 479.9819 (+/− NA ppm)
478.9746
[M−H]−
492.0406
0
20
40
60
80
100
470
475
480
485
490
495
500

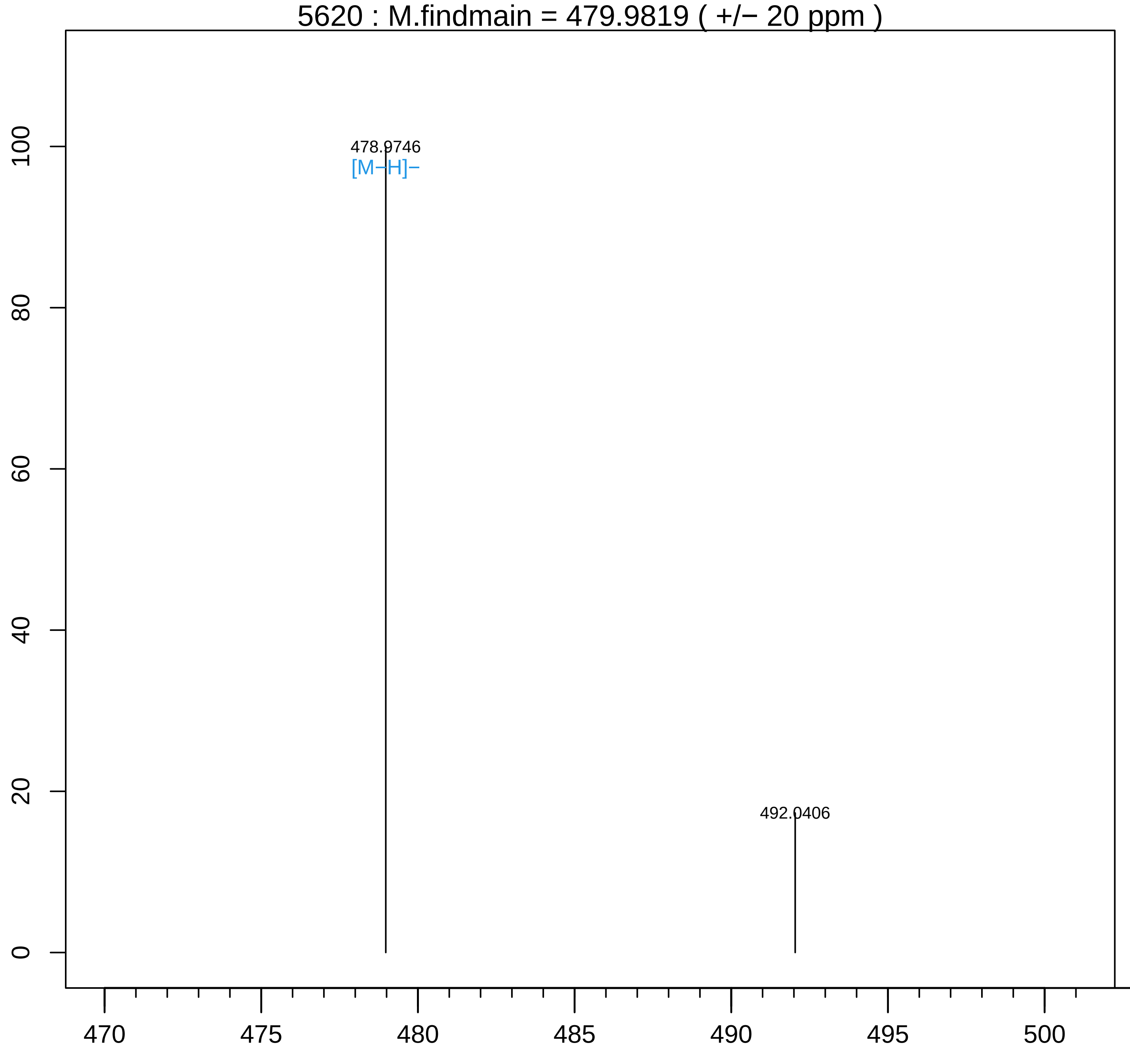
5620 : M.findmain = 479.9819 (+/− 20 ppm)
478.9746
[M−H]−
492.0406
0
20
40
60
80
100
470
475
480
485
490
495
500

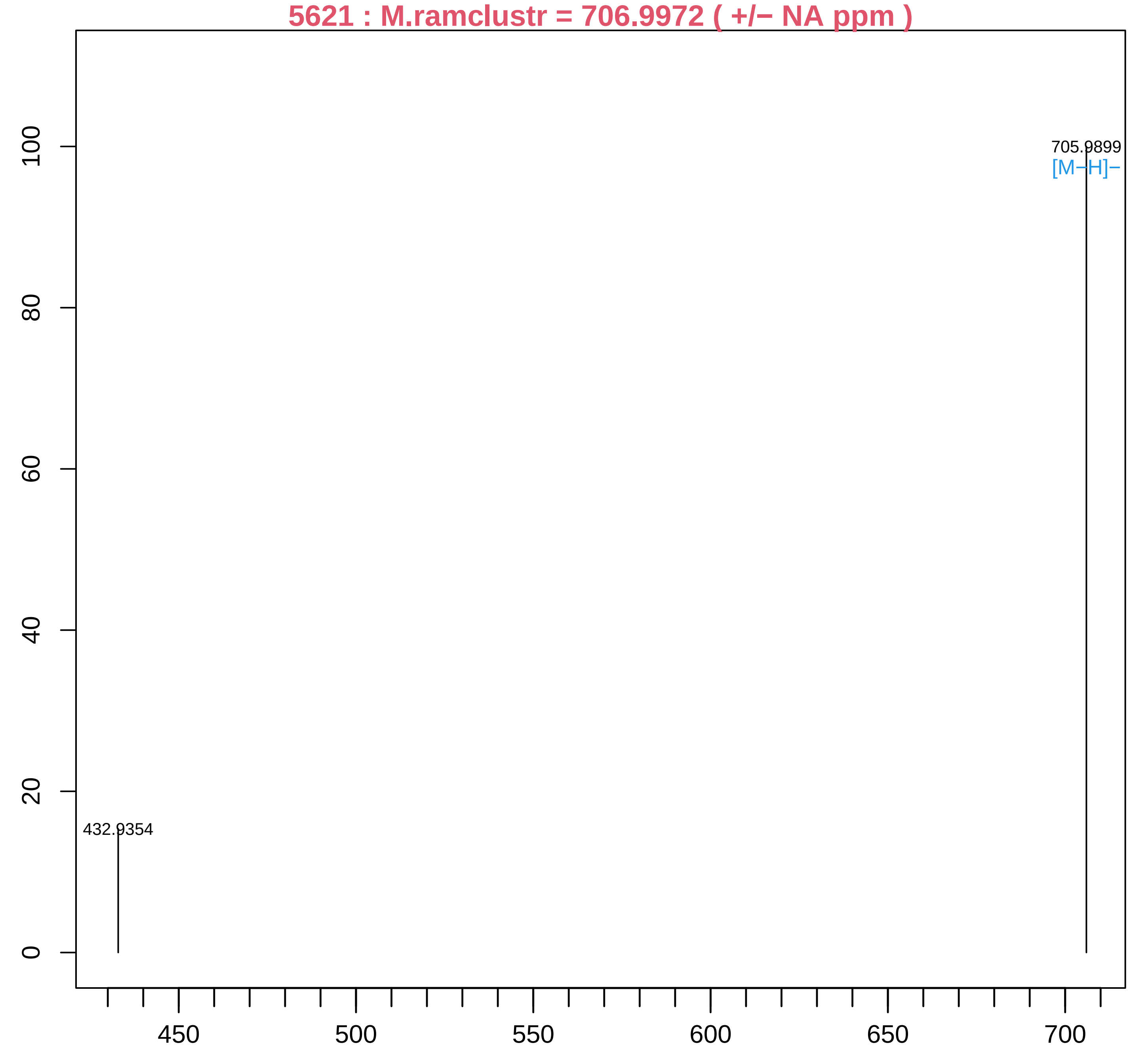
5621 : M.ramclustr = 706.9972 (+/− NA ppm)
705.9899
[M−H]−
432.9354
0
20
40
60
80
100
450
500
550
600
650
700
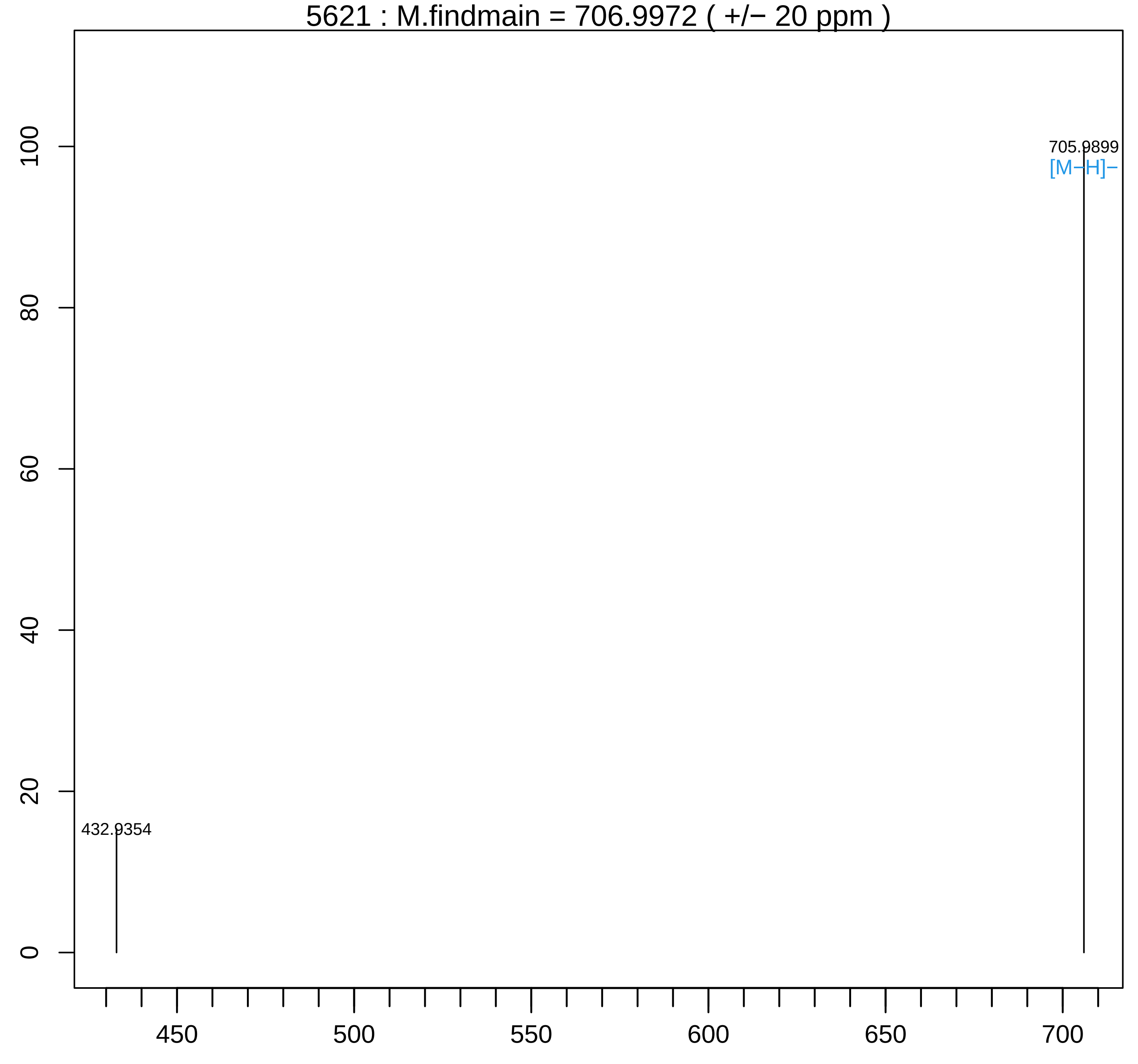
5621 : M.findmain = 706.9972 (+/− 20 ppm)
705.9899
[M−H]−
432.9354
0
20
40
60
80
100
450
500
550
600
650
700

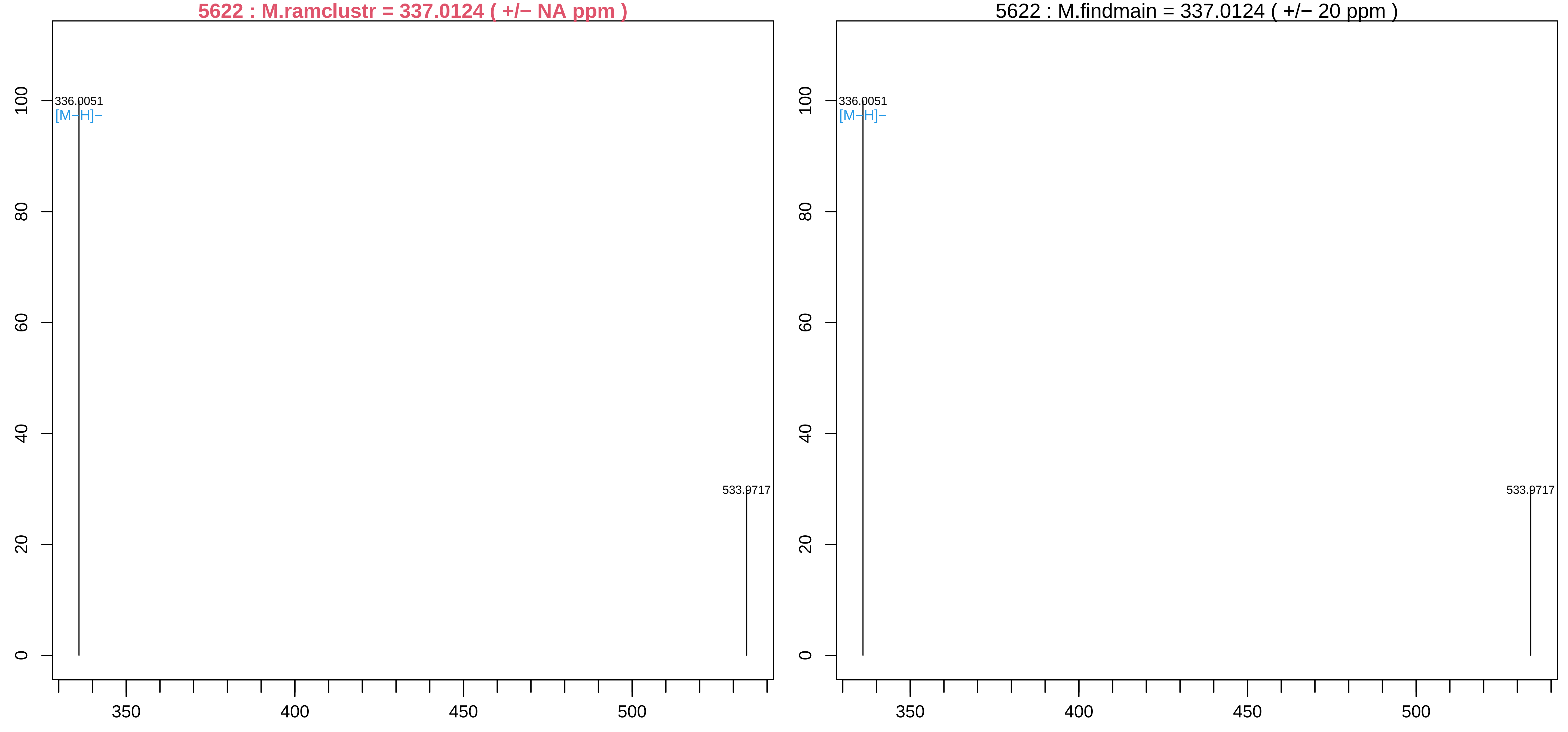
5622 : M.ramclustr = 337.0124 (+/− NA ppm)
336.0051
[M−H]−
533.9717
0
20
40
60
80
100
350
400
450
500
5622 : M.findmain = 337.0124 (+/− 20 ppm)
336.0051
[M−H]−
533.9717
0
20
40
60
80
100
350
400
450
500

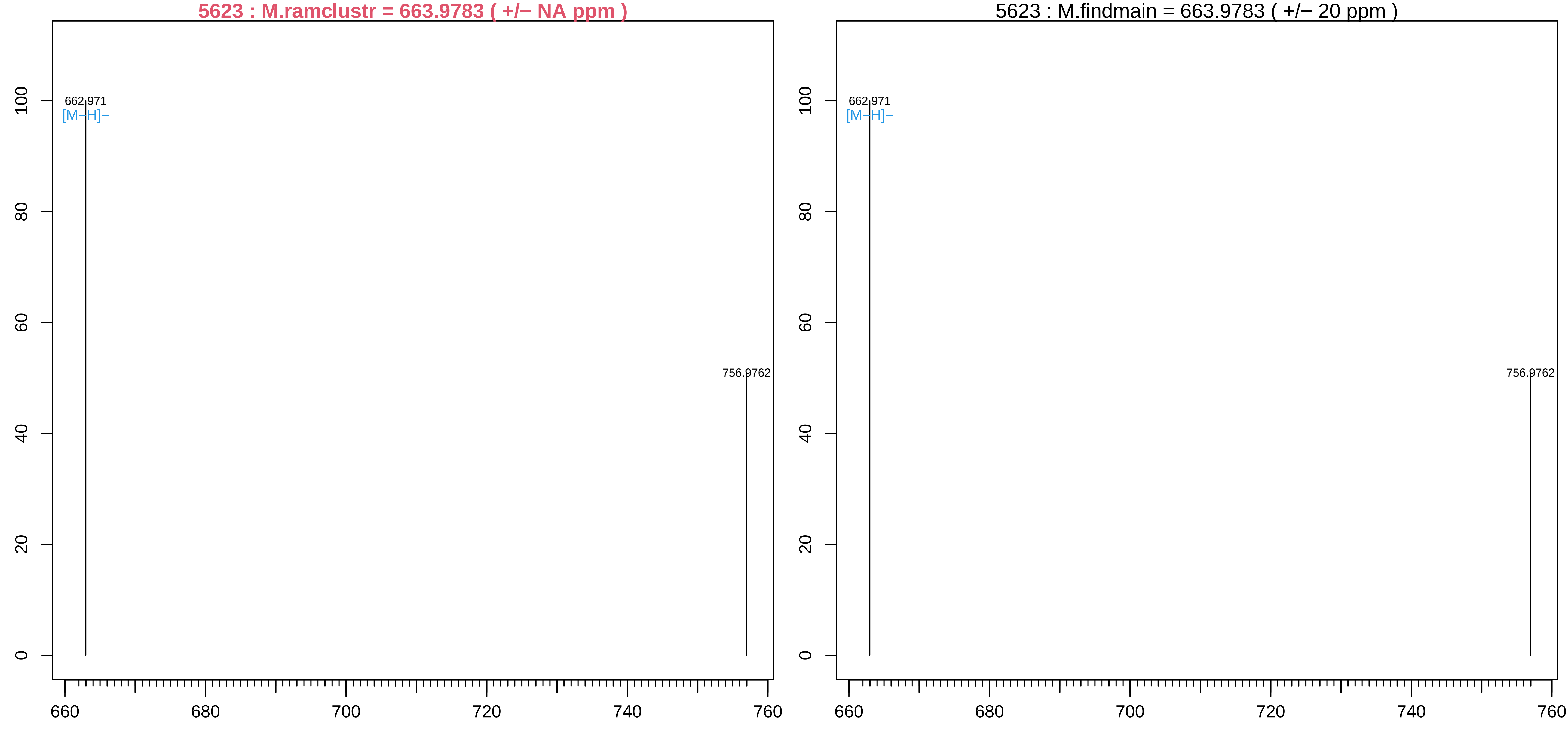
5623 : M.ramclustr = 663.9783 (+/− NA ppm)
662.971
[M−H]−
756.9762
0
20
40
60
80
100
660
680
700
720
740
760
5623 : M.findmain = 663.9783 (+/− 20 ppm)
662.971
[M−H]−
756.9762
0
20
40
60
80
100
660
680
700
720
740
760

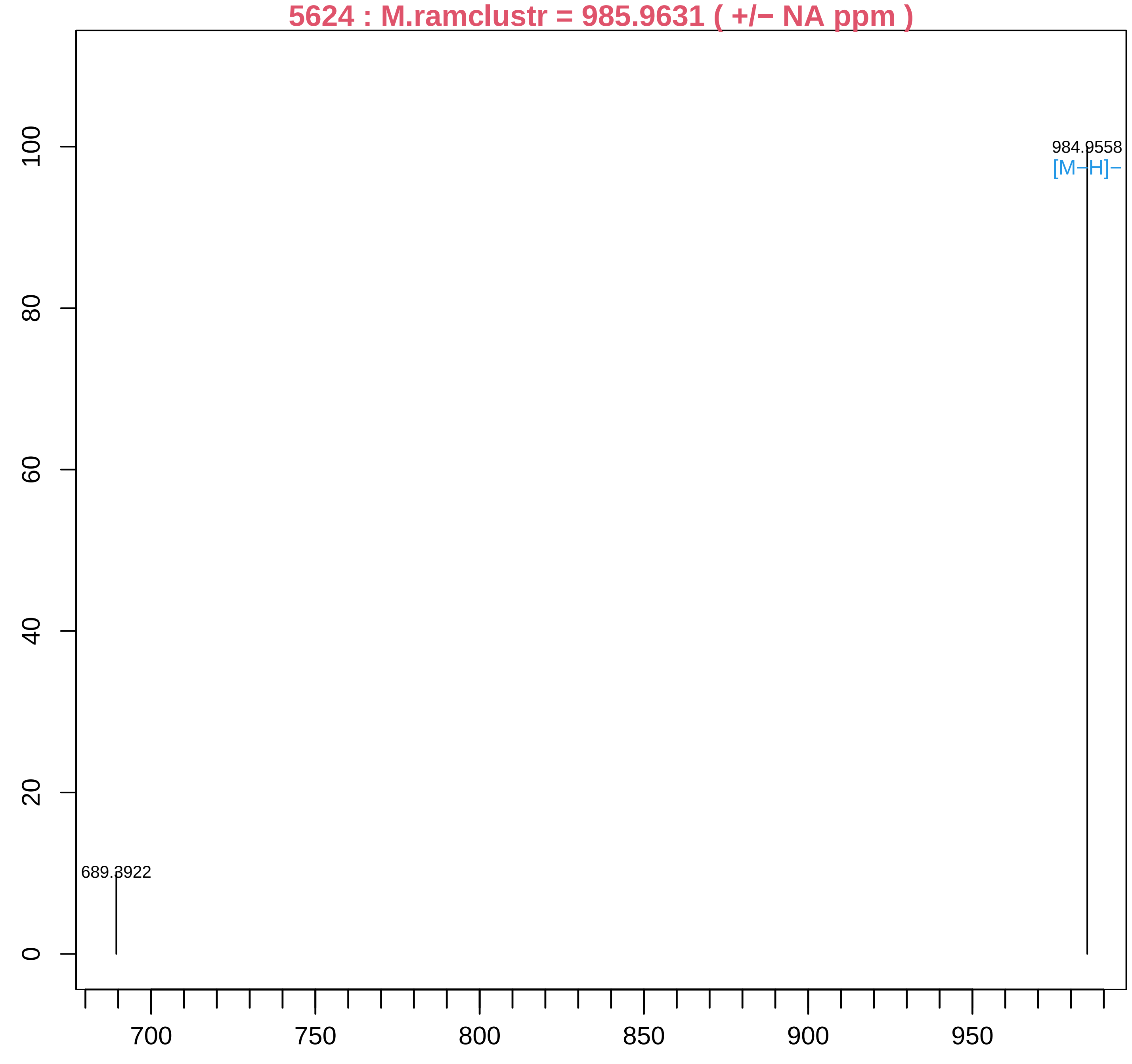
5624 : M.ramclustr = 985.9631 (+/− NA ppm)
984.9558
[M−H]−
689.3922
0
20
40
60
80
100
700
750
800
850
900
950

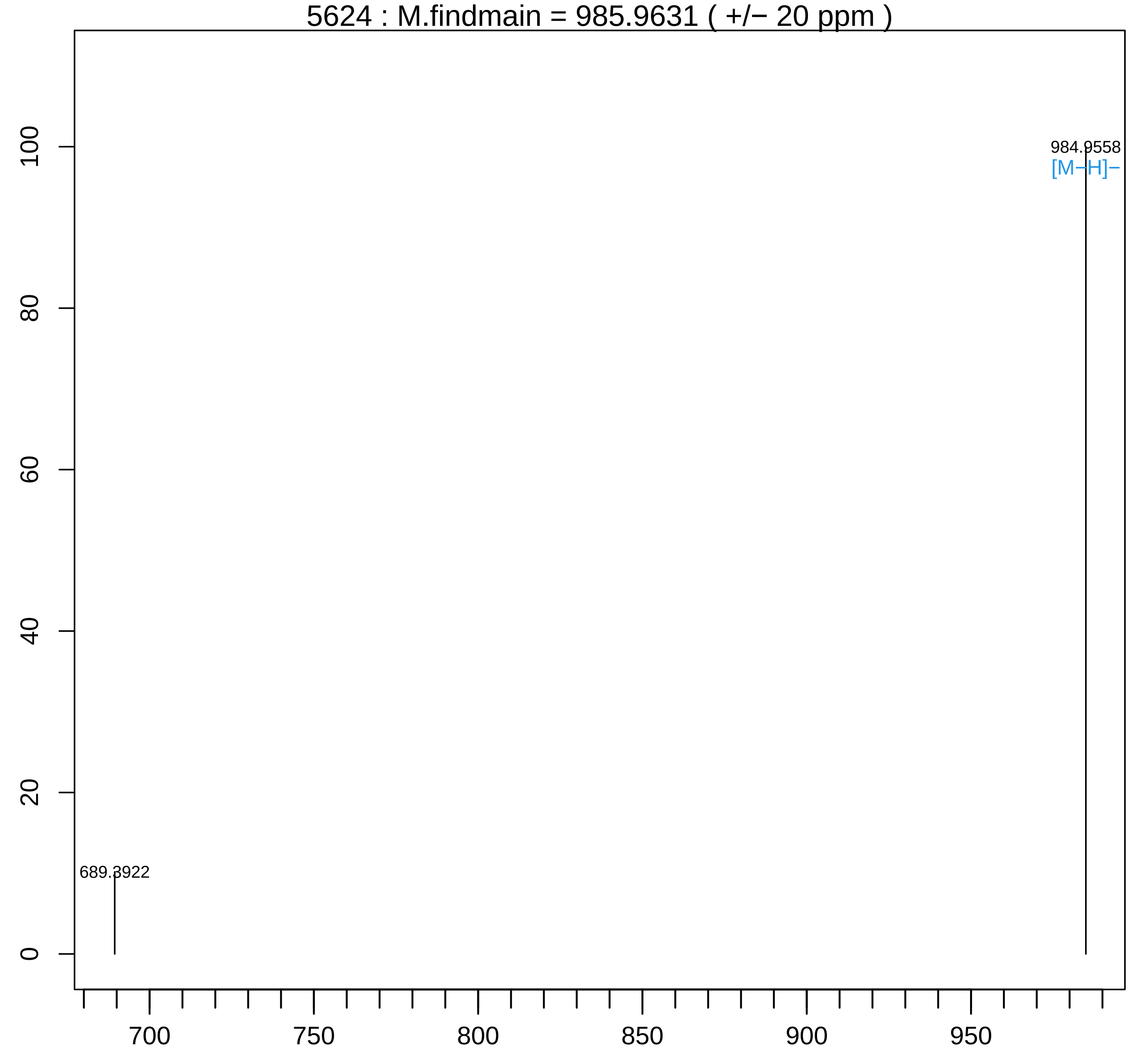
5624 : M.findmain = 985.9631 (+/− 20 ppm)
984.9558
[M−H]−
689.3922
0
20
40
60
80
100
700
750
800
850
900
950

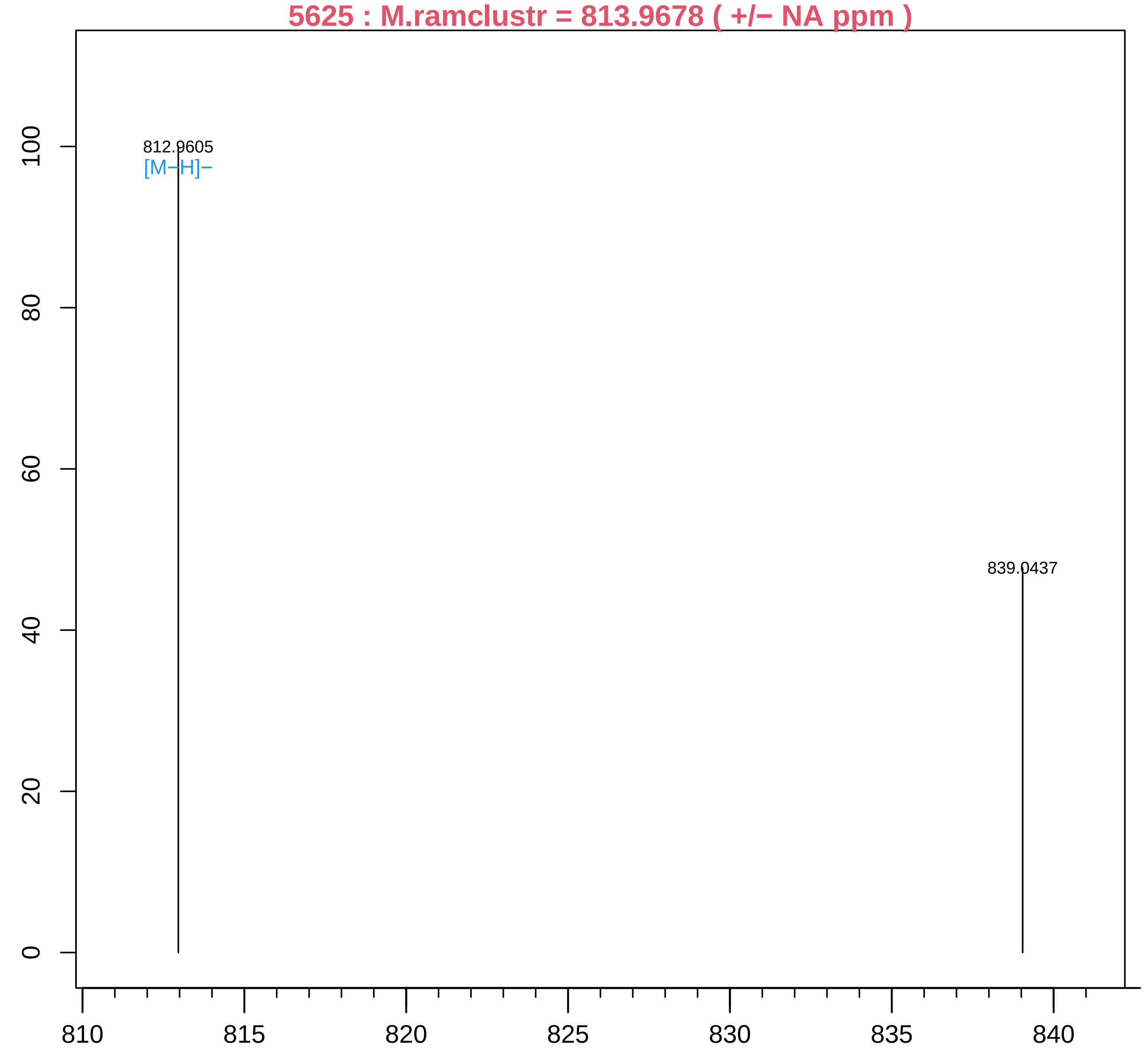

5625 : M.ramclustr = 813.9678 (+/− NA ppm)
812.9605
[M−H]−
839.0437
0
20
40
60
80
100
810
815
820
825
830
835
840

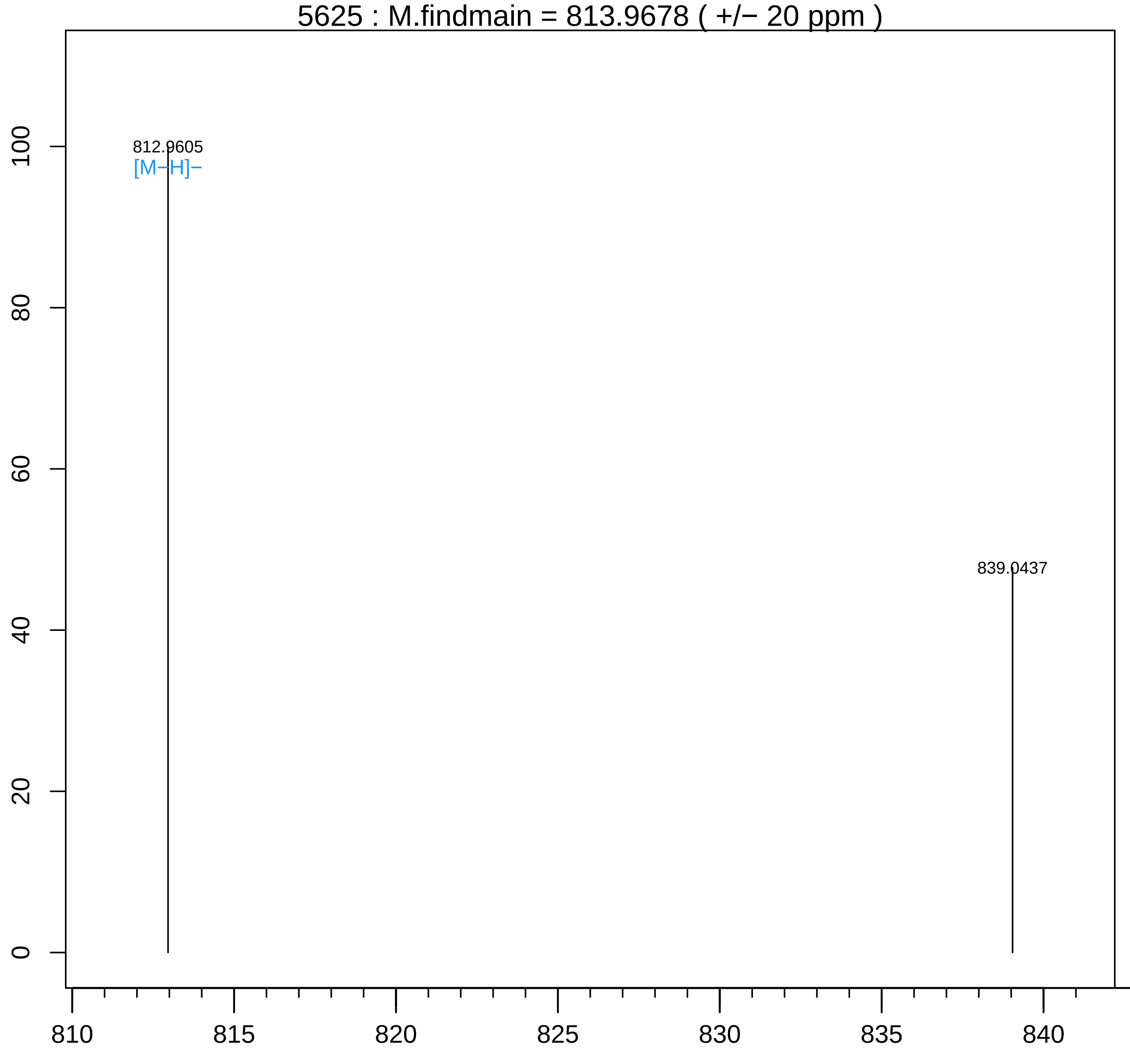

5625 : M.findmain = 813.9678 (+/− 20 ppm)
812.9605
[M−H]−
839.0437
0
20
40
60
80
100
810
815
820
825
830
835
840

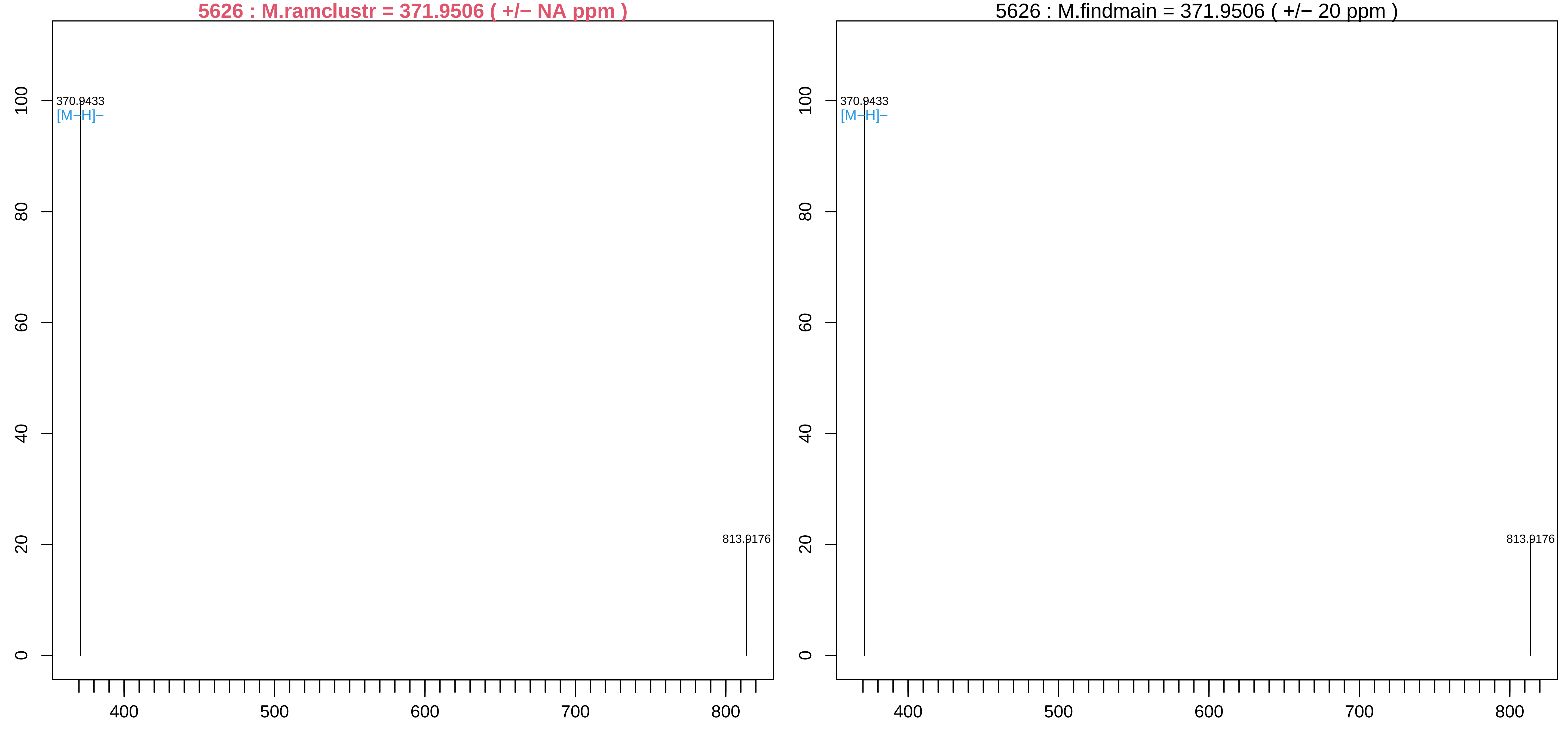
5626 : M.ramclustr = 371.9506 (+/− NA ppm)
370.9433
[M−H]−
813.9176
5626 : M.findmain = 371.9506 (+/− 20 ppm)
370.9433
[M−H]−
813.9176
0
20
40
60
80
100
400
500
600
700
800

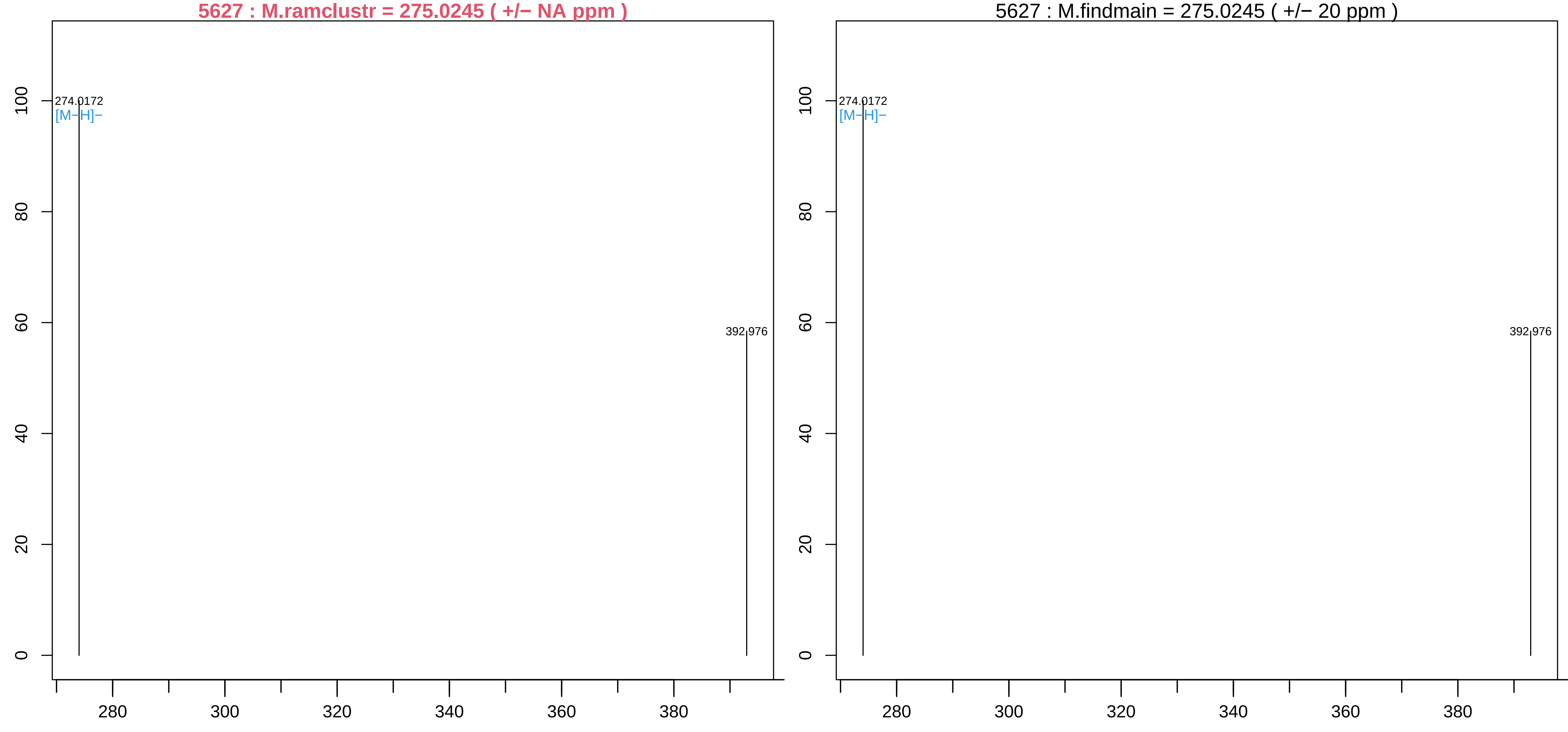

5627 : M.ramclustr = 275.0245 (+/− NA ppm)
274.0172
[M−H]−
392.976
0
20
40
60
80
100
280
300
320
340
360
380
5627 : M.findmain = 275.0245 (+/− 20 ppm)
274.0172
[M−H]−
392.976
0
20
40
60
80
100
280
300
320
340
360
380

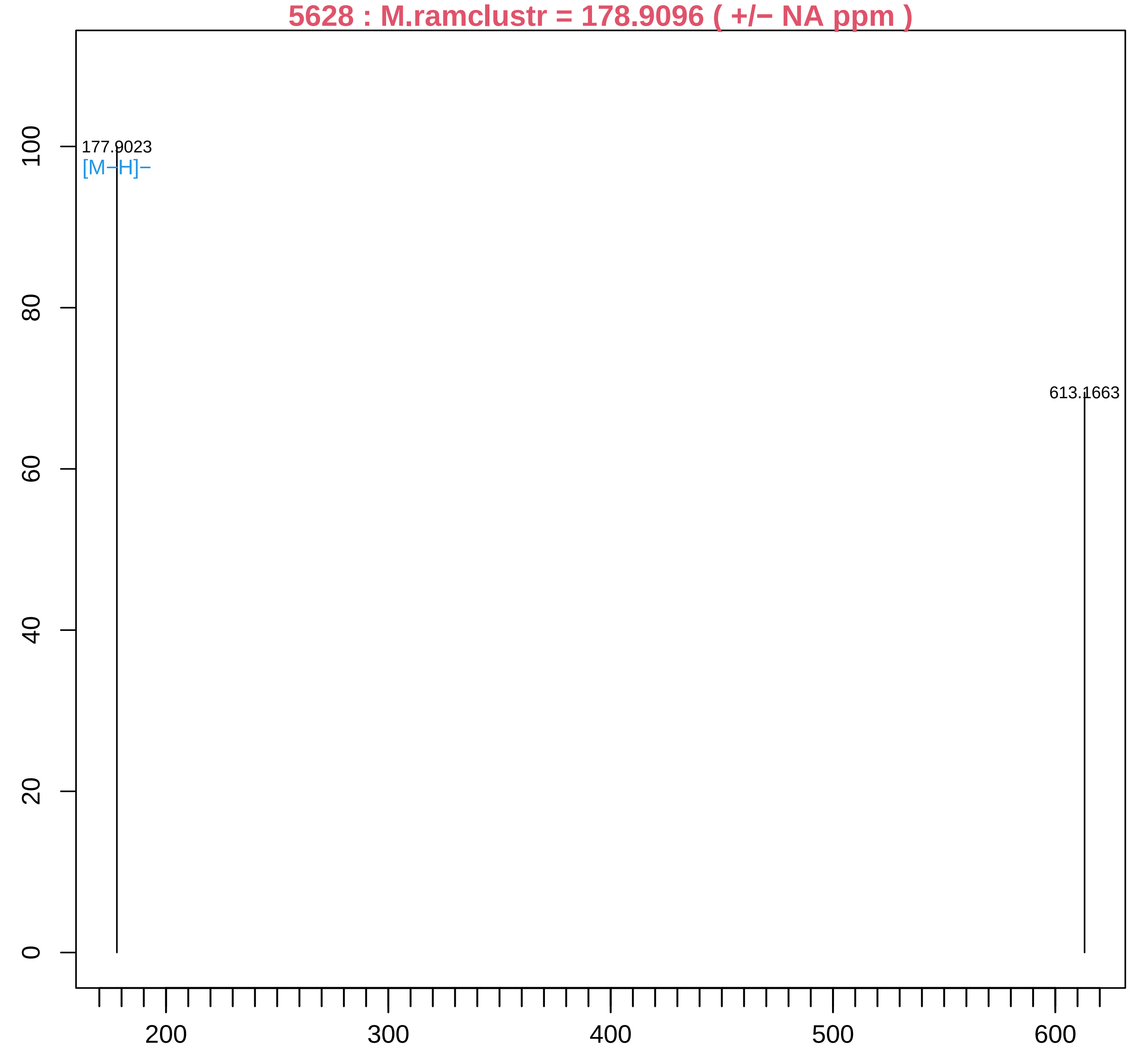
5628 : M.ramclustr = 178.9096 (+/− NA ppm)
177.9023
[M−H]−
613.1663
0
20
40
60
80
100
200
300
400
500
600

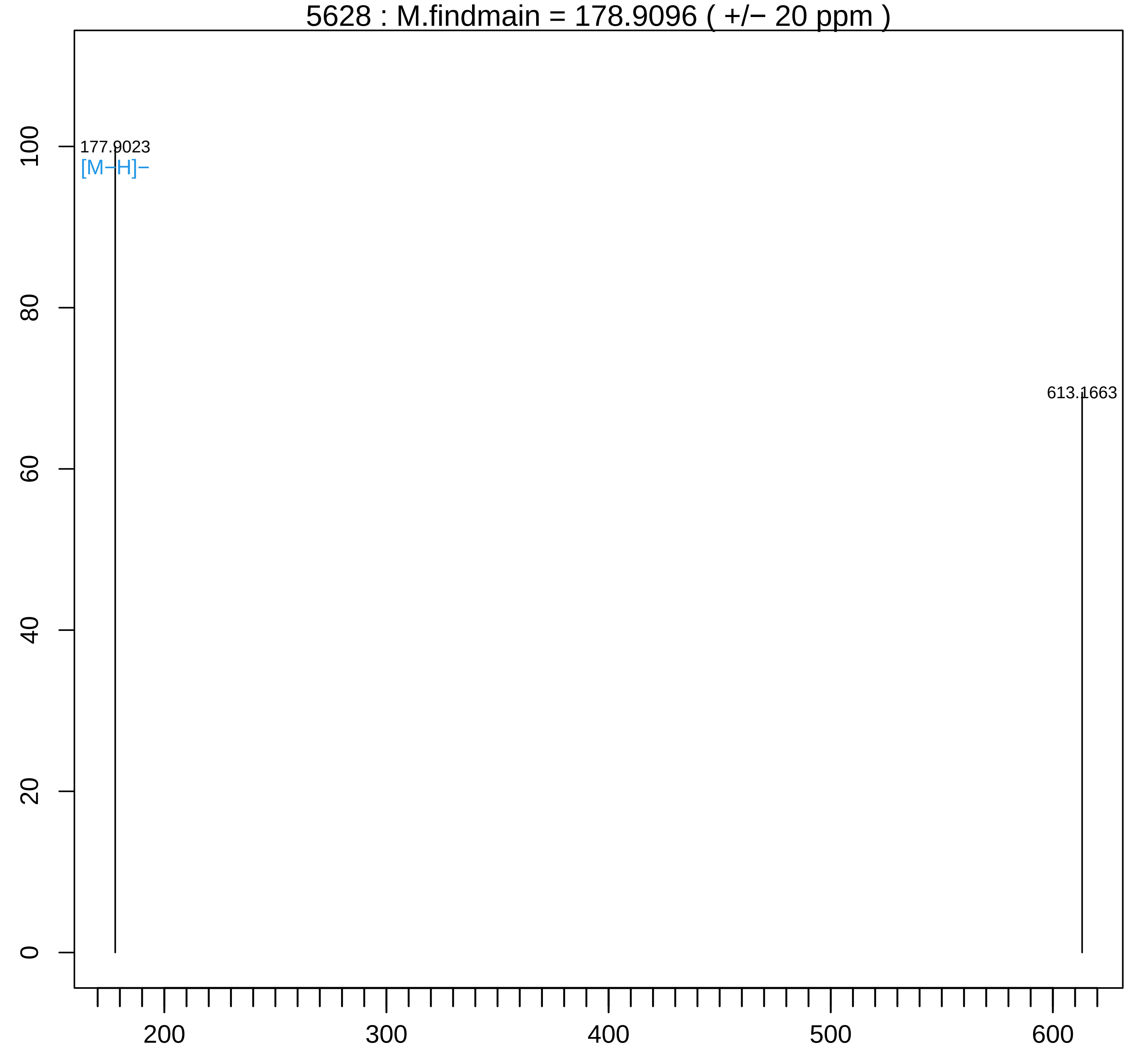
5628 : M.findmain = 178.9096 (+/− 20 ppm)
177.9023
[M−H]−
613.1663
0
20
40
60
80
100
200
300
400
500
600

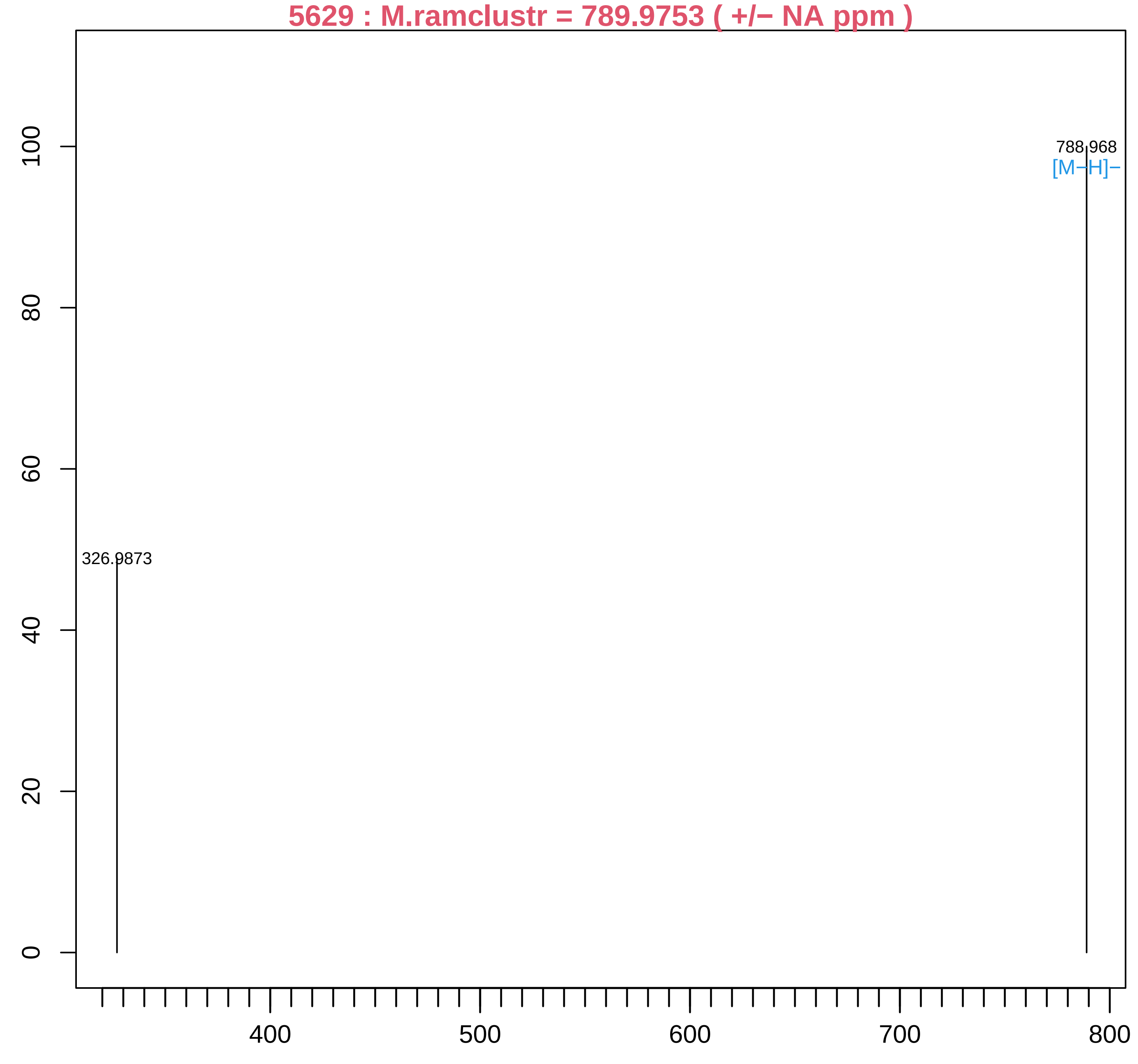
5629 : M.ramclustr = 789.9753 (+/− NA ppm)
788.968
[M−H]−
326.9873
0
20
40
60
80
100
400
500
600
700
800

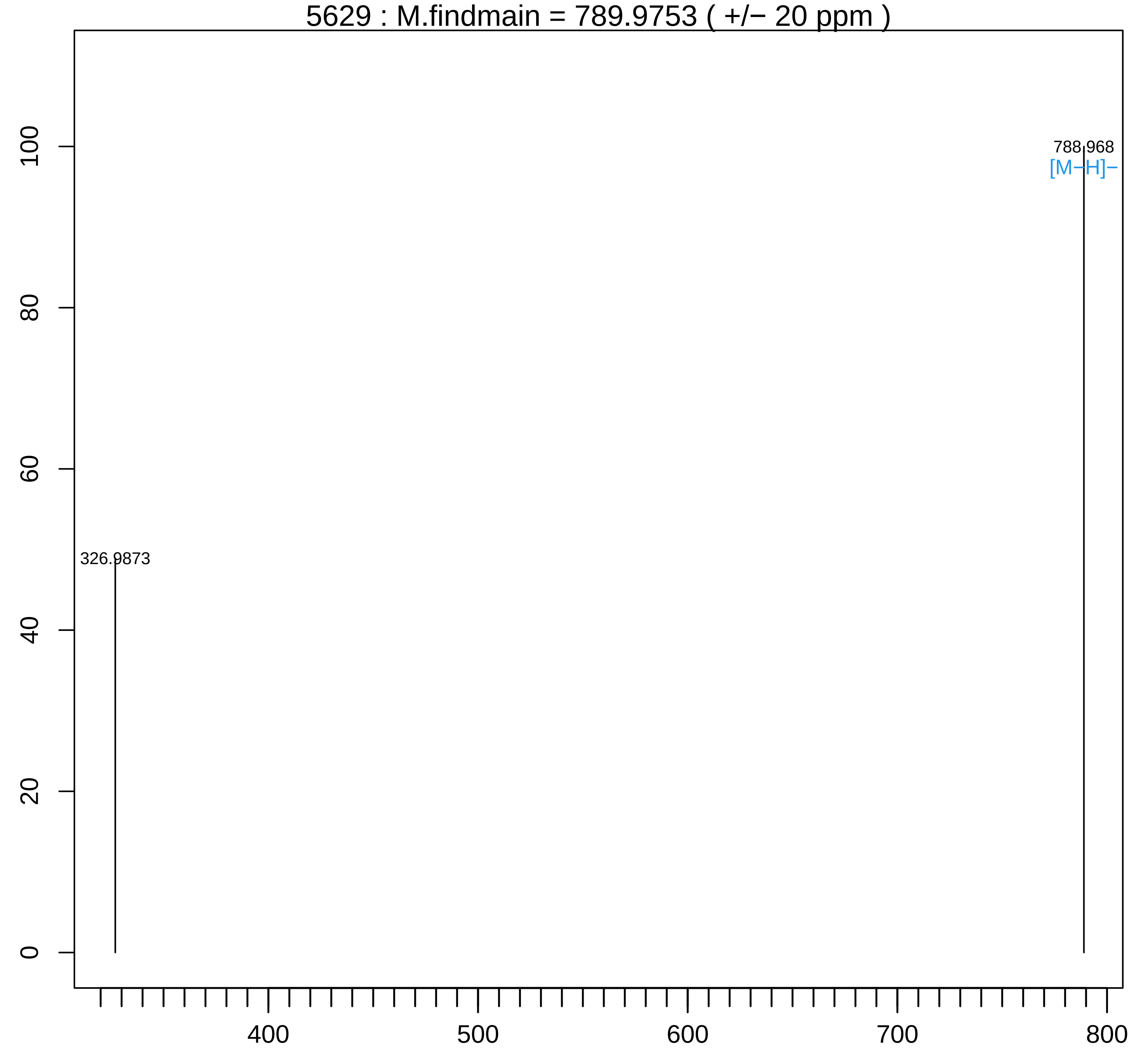
5629 : M.findmain = 789.9753 (+/− 20 ppm)
788.968
[M−H]−
326.9873
0
20
40
60
80
100
400
500
600
700
800

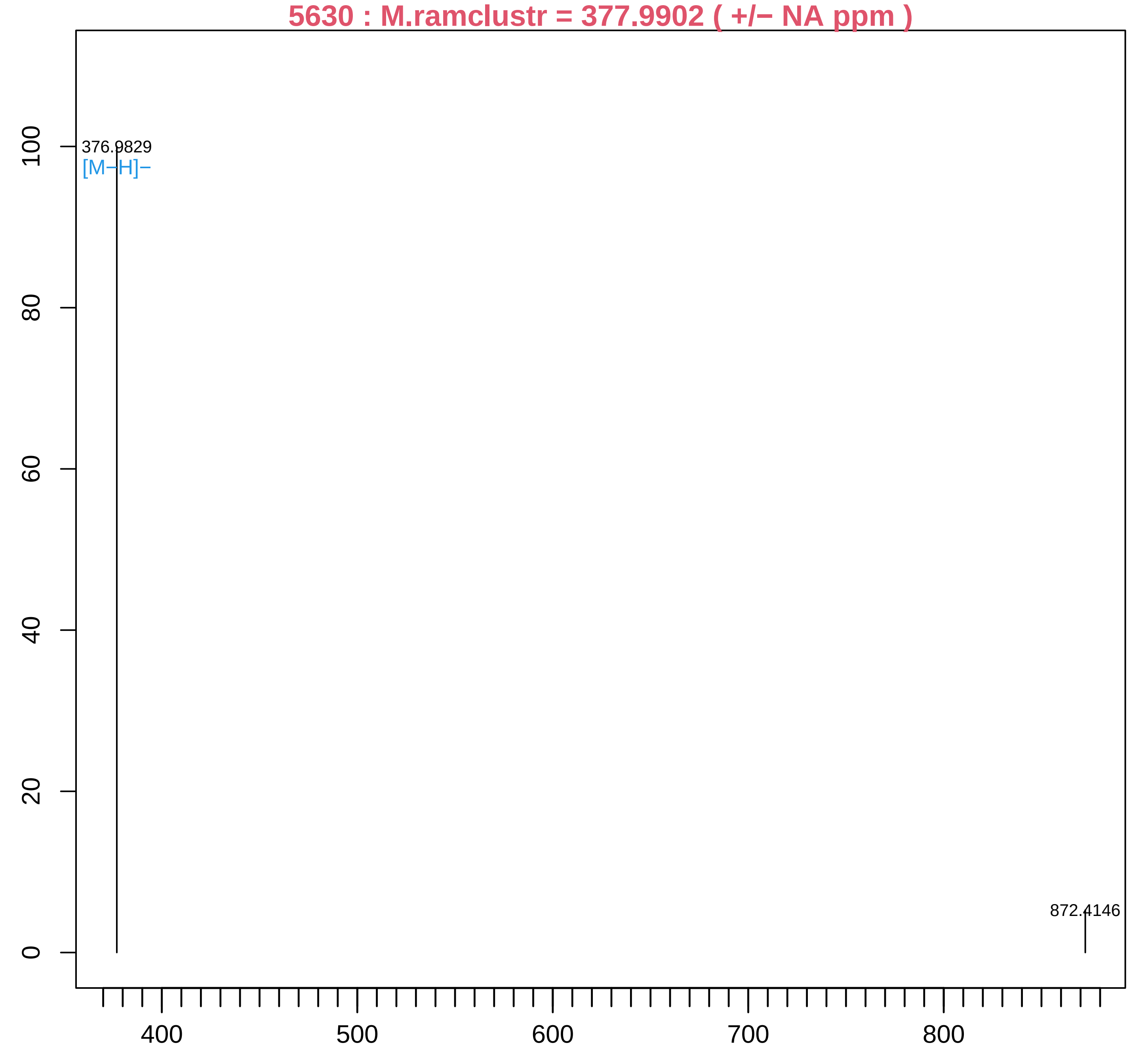
5630 : M.ramclustr = 377.9902 (+/− NA ppm)
376.9829
[M−H]−
872.4146
0
20
40
60
80
100
400
500
600
700
800

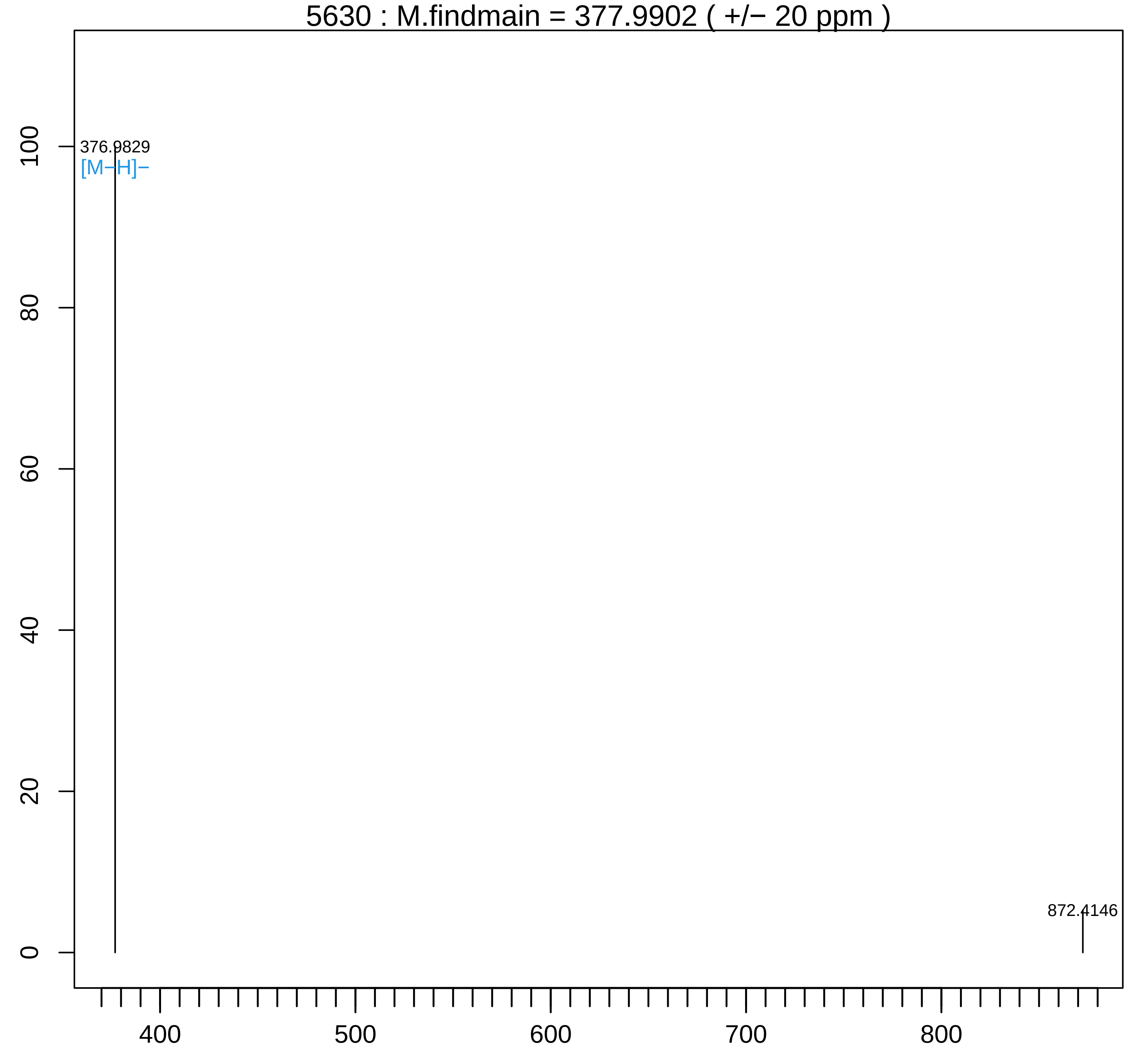
5630 : M.findmain = 377.9902 (+/− 20 ppm)
376.9829
[M−H]−
872.4146
0
20
40
60
80
100
400
500
600
700
800

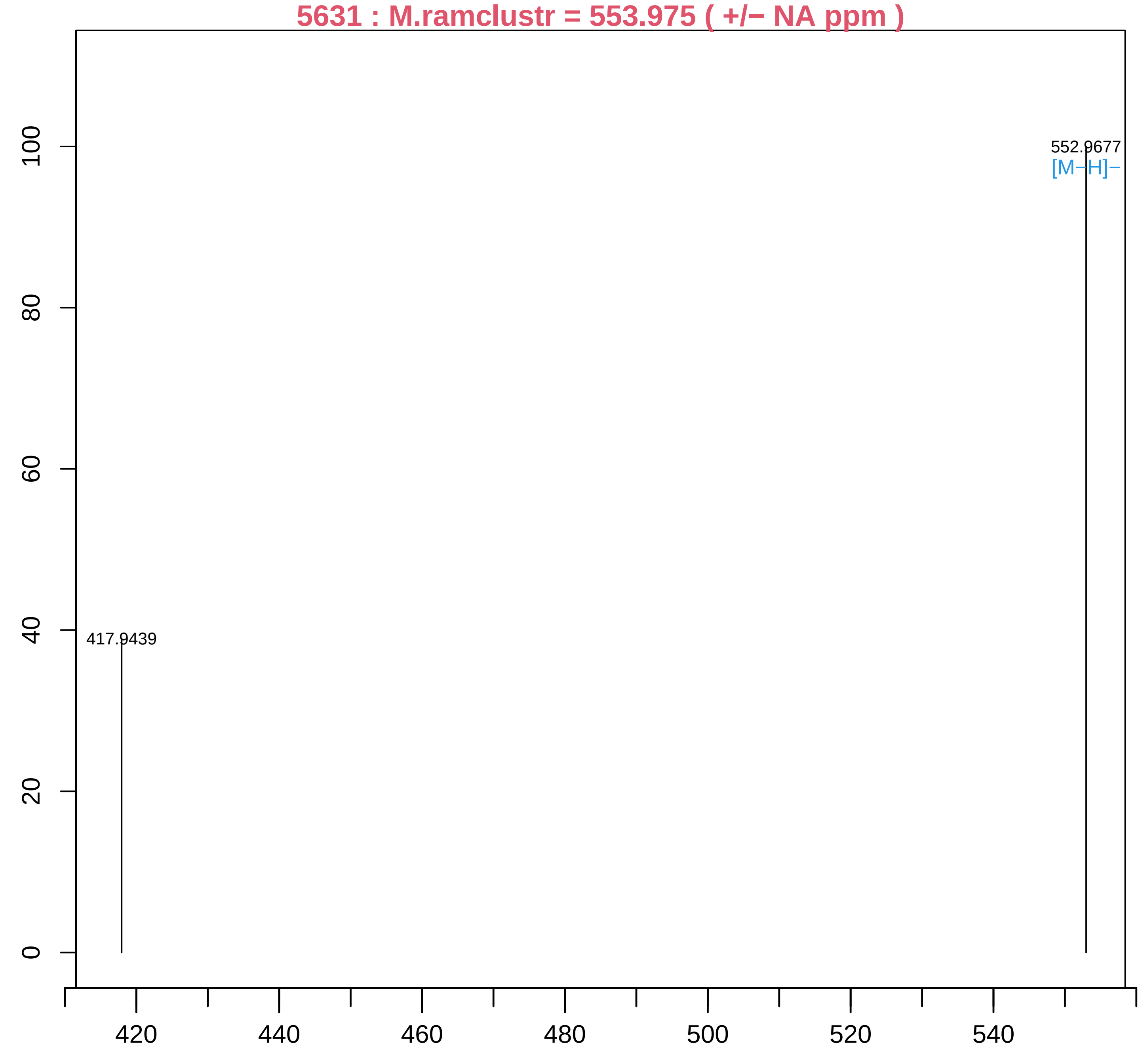
5631 : M.ramclustr = 553.975 (+/− NA ppm)
552.9677
[M−H]−
417.9439
0
20
40
60
80
100
420
440
460
480
500
520
540

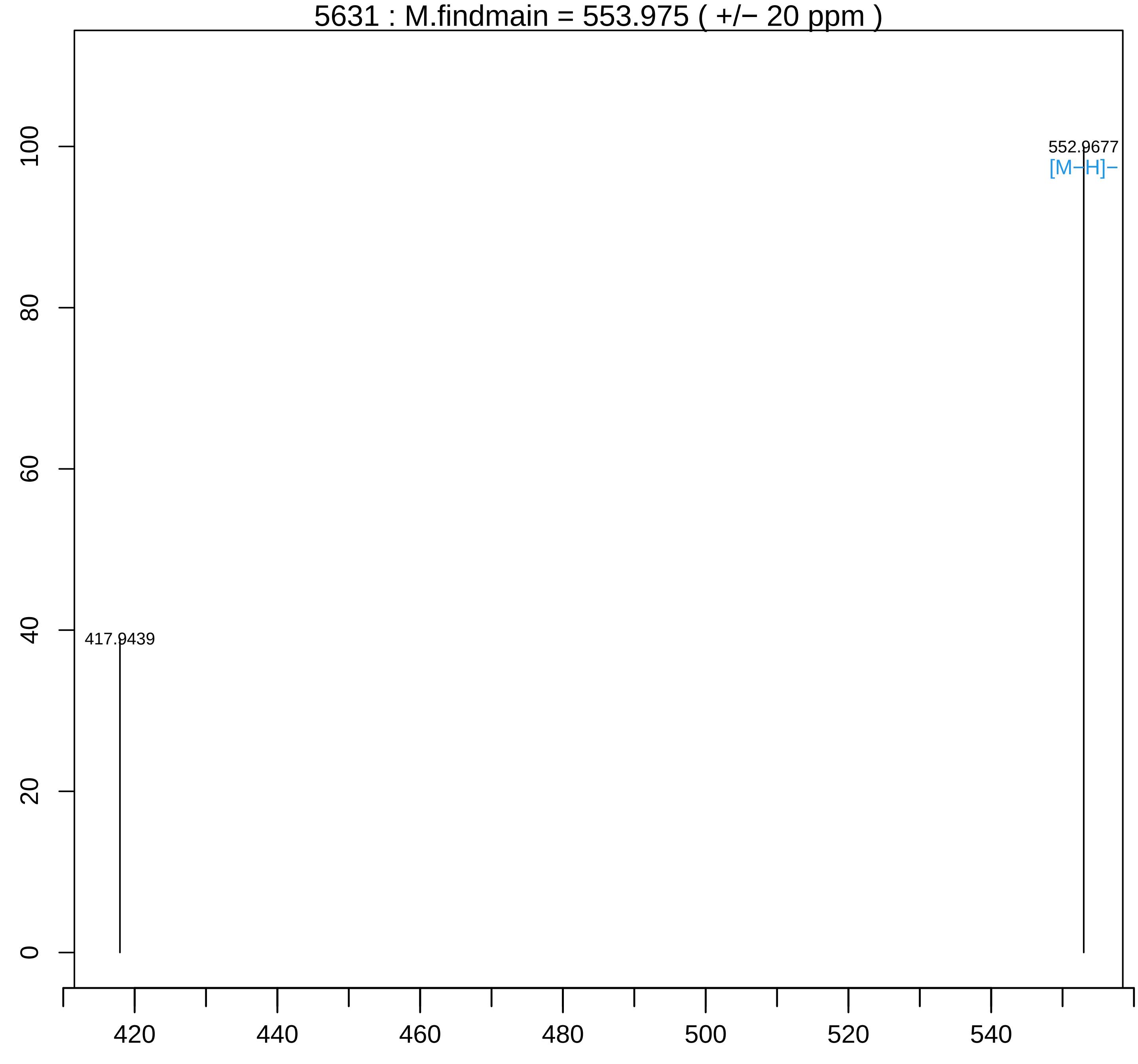
5631 : M.findmain = 553.975 (+/− 20 ppm)
552.9677
[M−H]−
417.9439
0
20
40
60
80
100
420
440
460
480
500
520
540

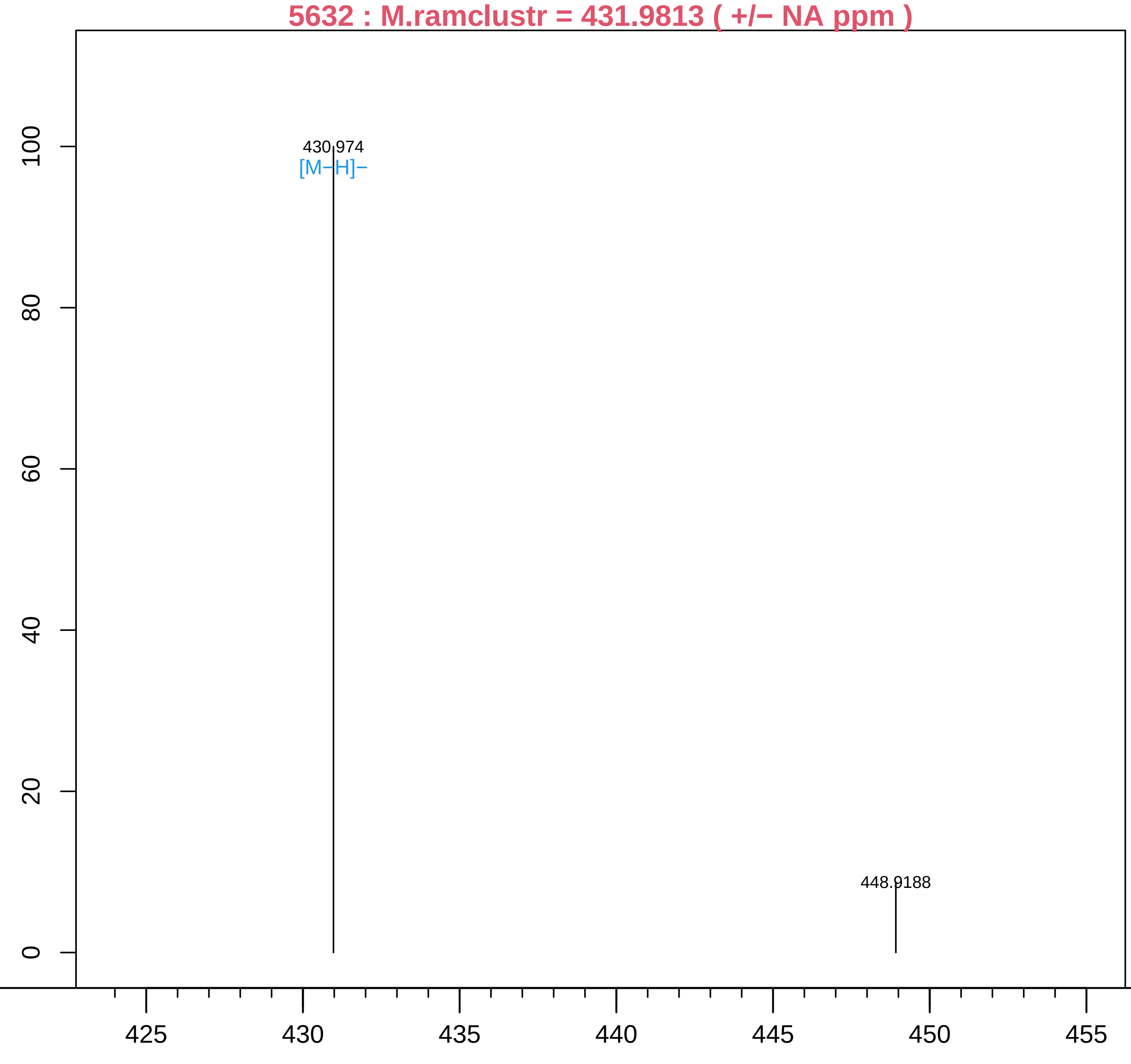
5632 : M.ramclustr = 431.9813 (+/− NA ppm)
430.974
[M−H]−
448.9188
0
20
40
60
80
100
425
430
435
440
445
450
455

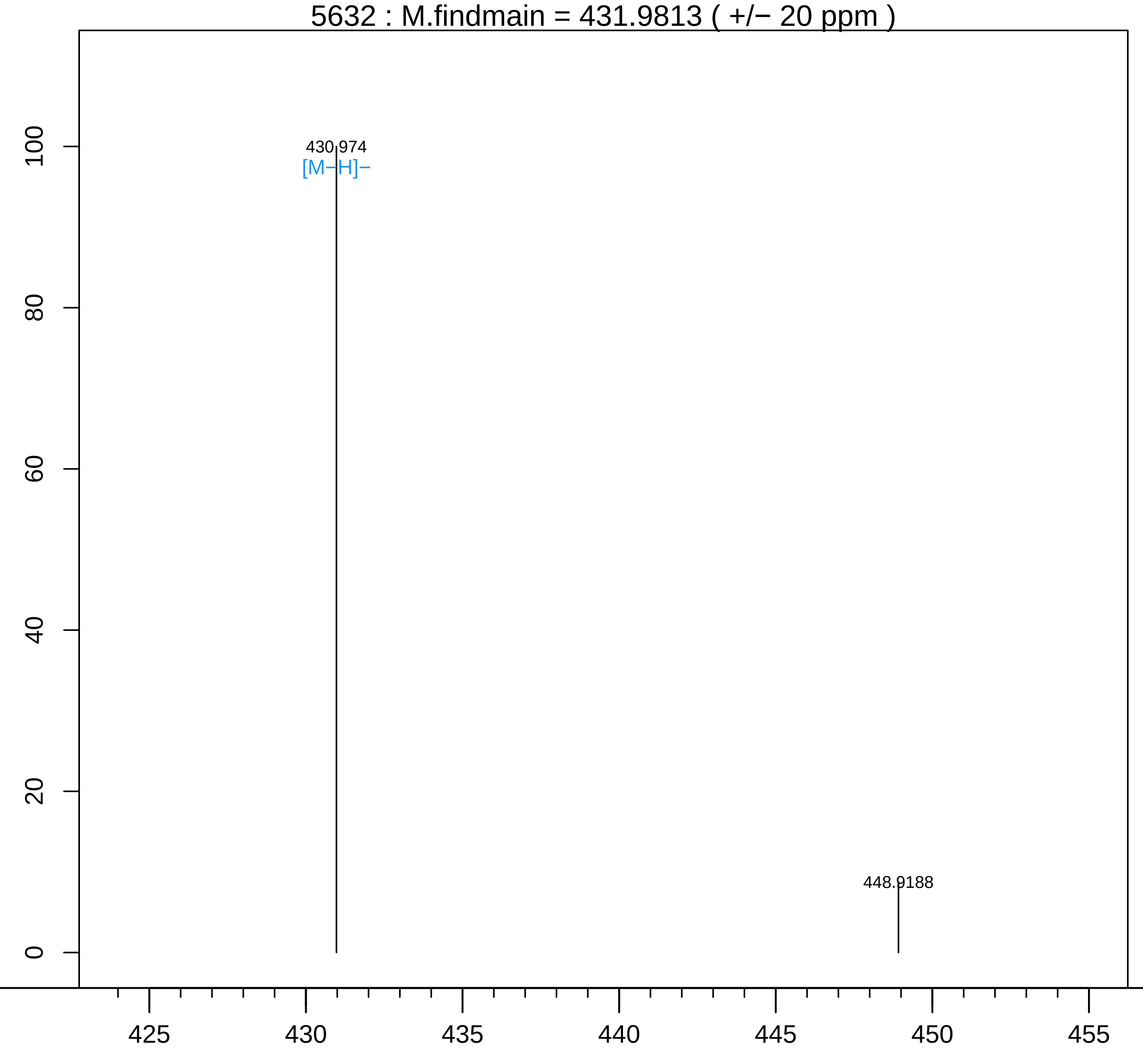
5632 : M.findmain = 431.9813 (+/− 20 ppm)
430.974
[M−H]−
448.9188
0
20
40
60
80
100
425
430
435
440
445
450
455

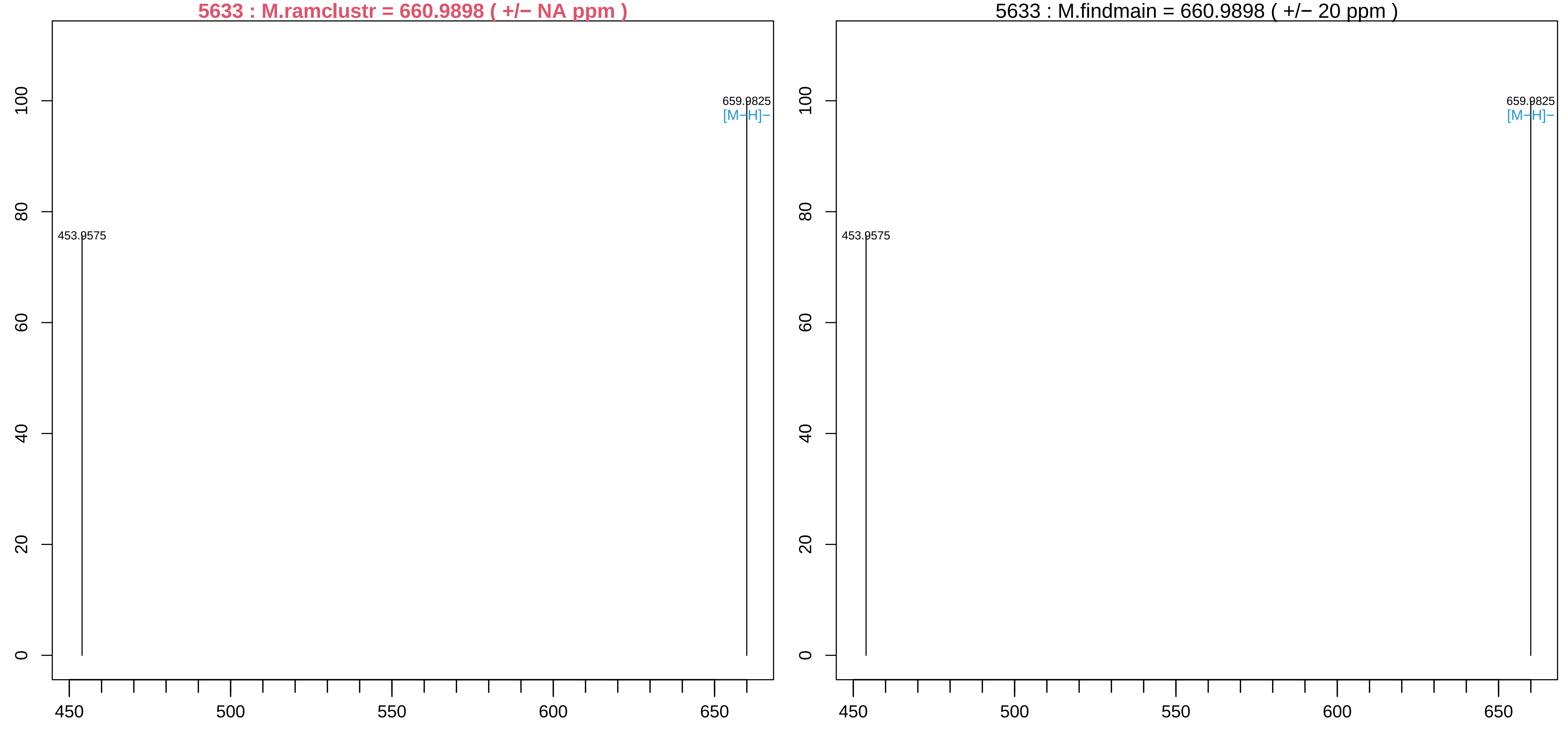
5633 : M.ramclustr = 660.9898 (+/− NA ppm)
659.9825
[M−H]−
453.9575
0
20
40
60
80
100
450
500
550
600
650
5633 : M.findmain = 660.9898 (+/− 20 ppm)
659.9825
[M−H]−
453.9575
0
20
40
60
80
100
450
500
550
600
650

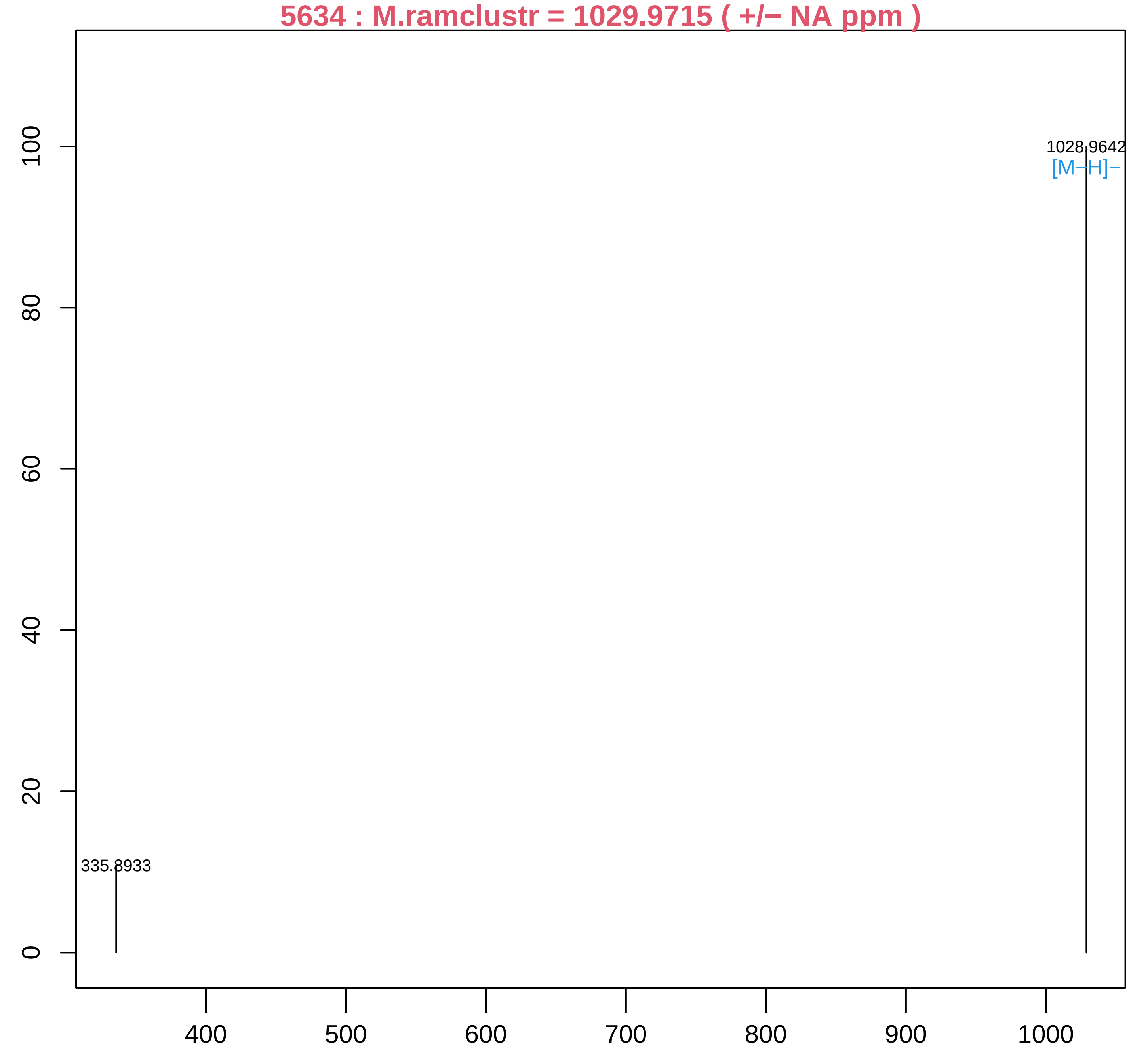

5634 : M.ramclustr = 1029.9715 (+/− NA ppm)
1028.9642
[M−H]−
335.8933
0
20
40
60
80
100
400
500
600
700
800
900
1000

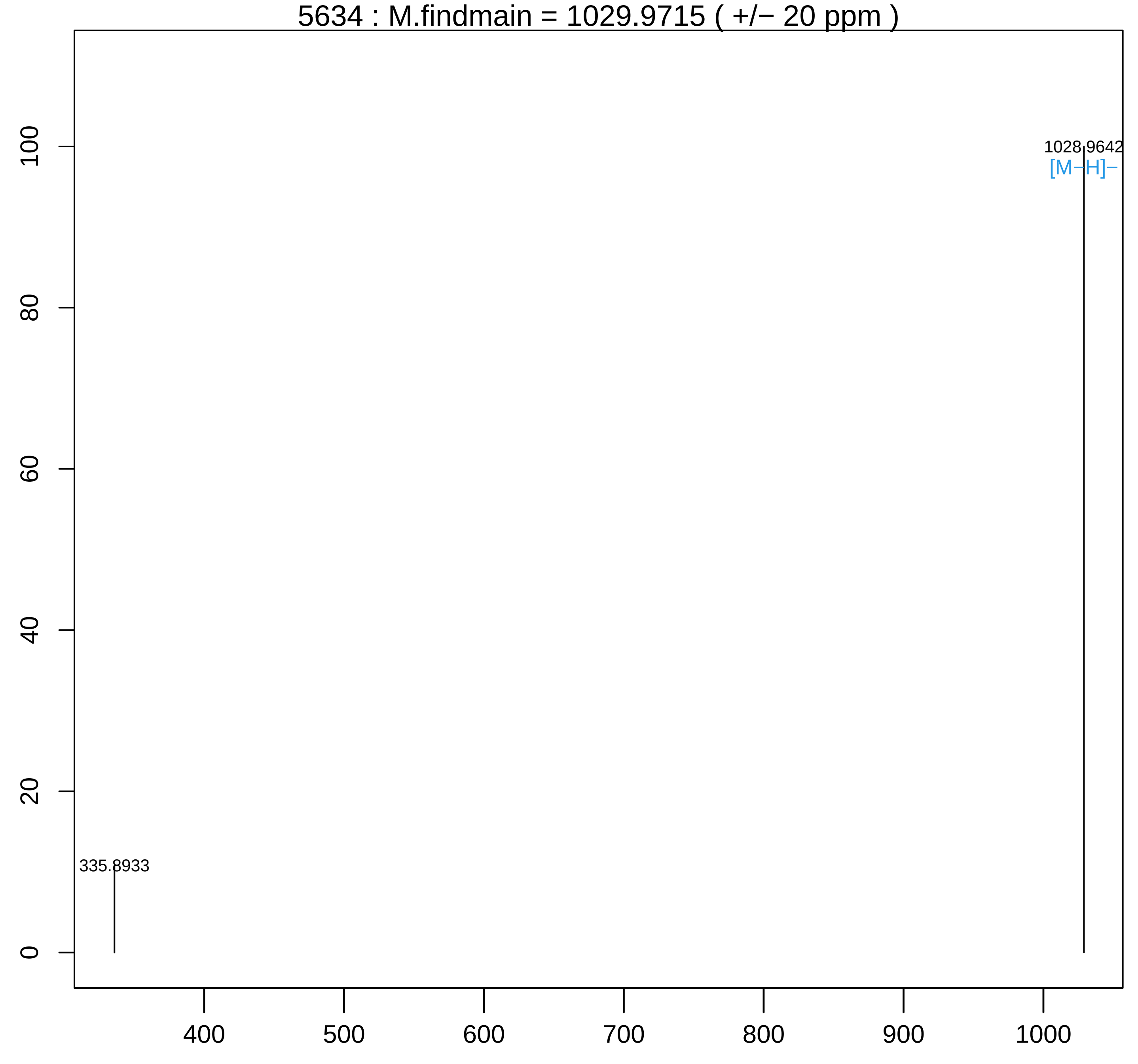

5634 : M.findmain = 1029.9715 (+/− 20 ppm)
1028.9642
[M−H]−
335.8933
0
20
40
60
80
100
400
500
600
700
800
900
1000

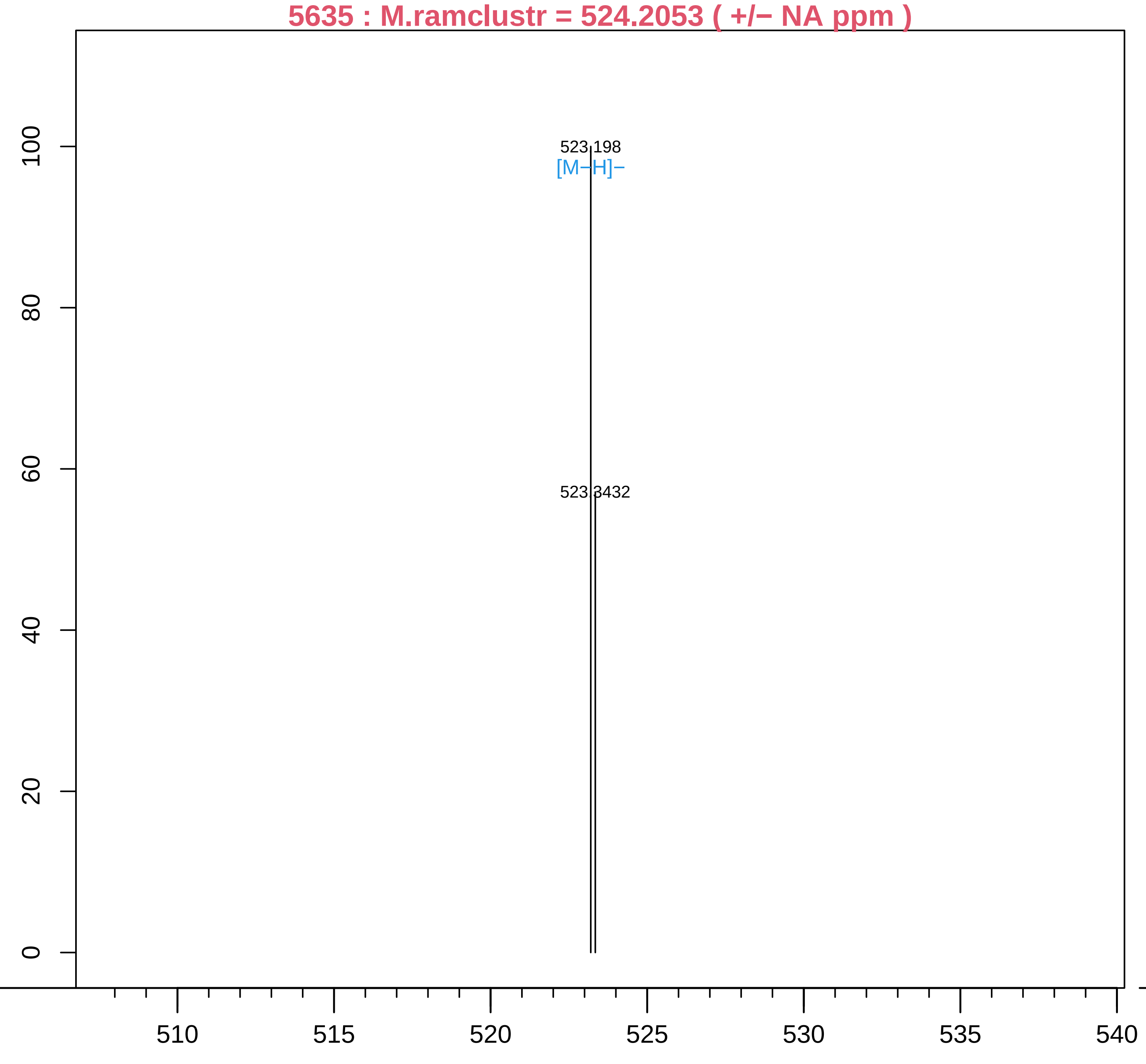
5635 : M.ramclustr = 524.2053 (+/− NA ppm)
523.198
[M−H]−
523.3432
0
20
40
60
80
100
510
515
520
525
530
535
540

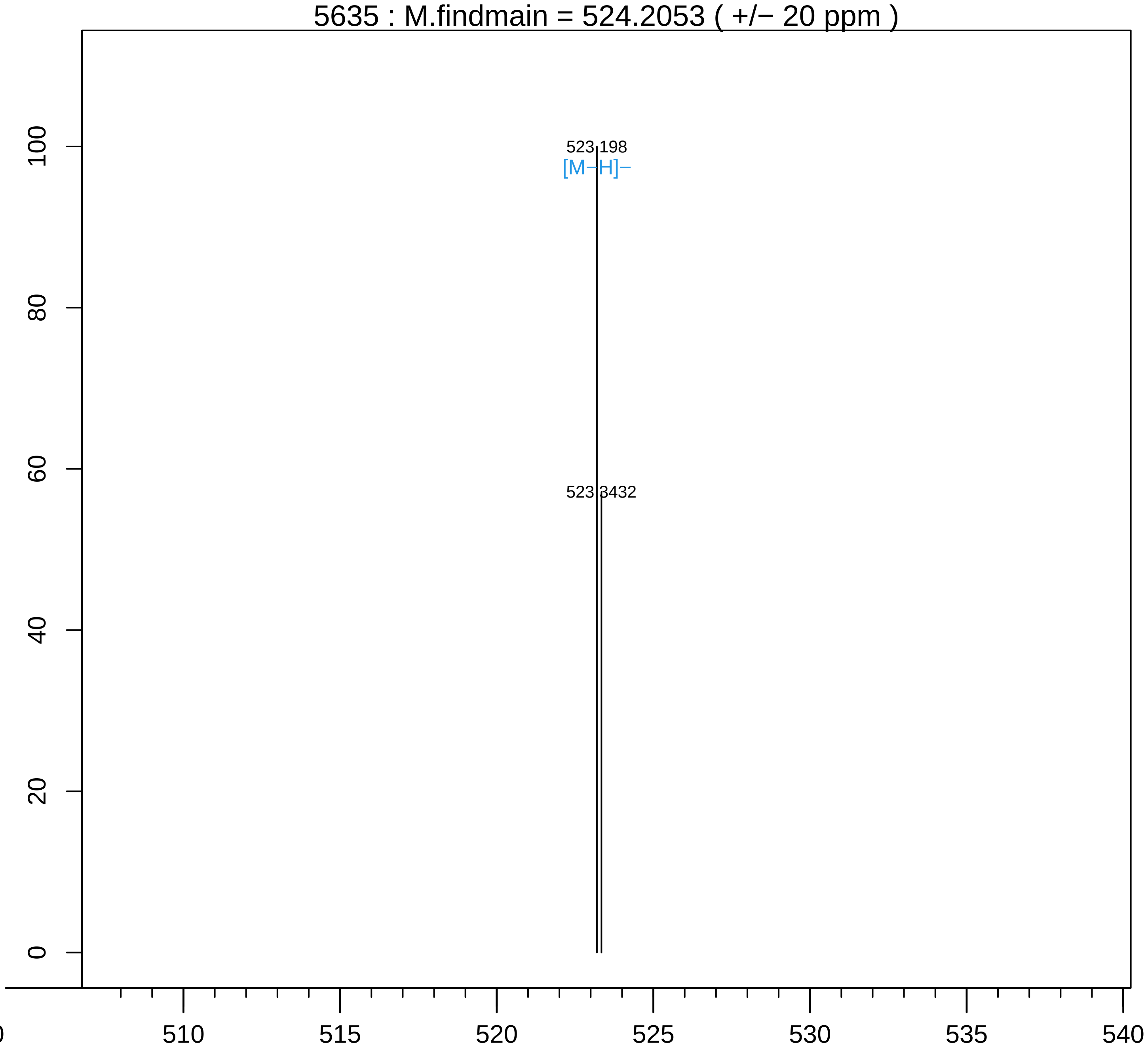
5635 : M.findmain = 524.2053 (+/− 20 ppm)
523.198
[M−H]−
523.3432
0
20
40
60
80
100
510
515
520
525
530
535
540

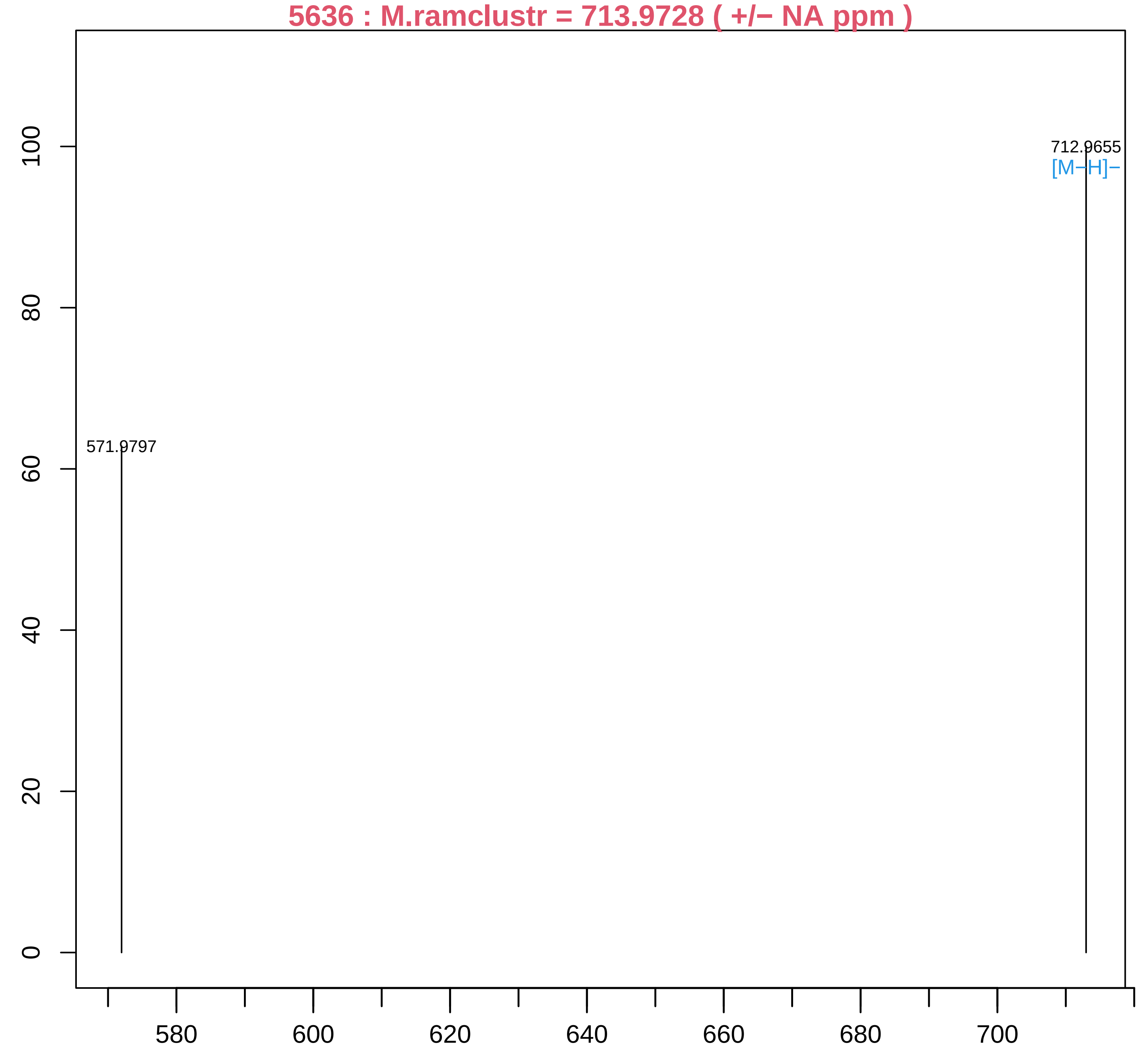
5636 : M.ramclustr = 713.9728 (+/− NA ppm)
712.9655
[M−H]−
571.9797
0
20
40
60
80
100
580
600
620
640
660
680
700

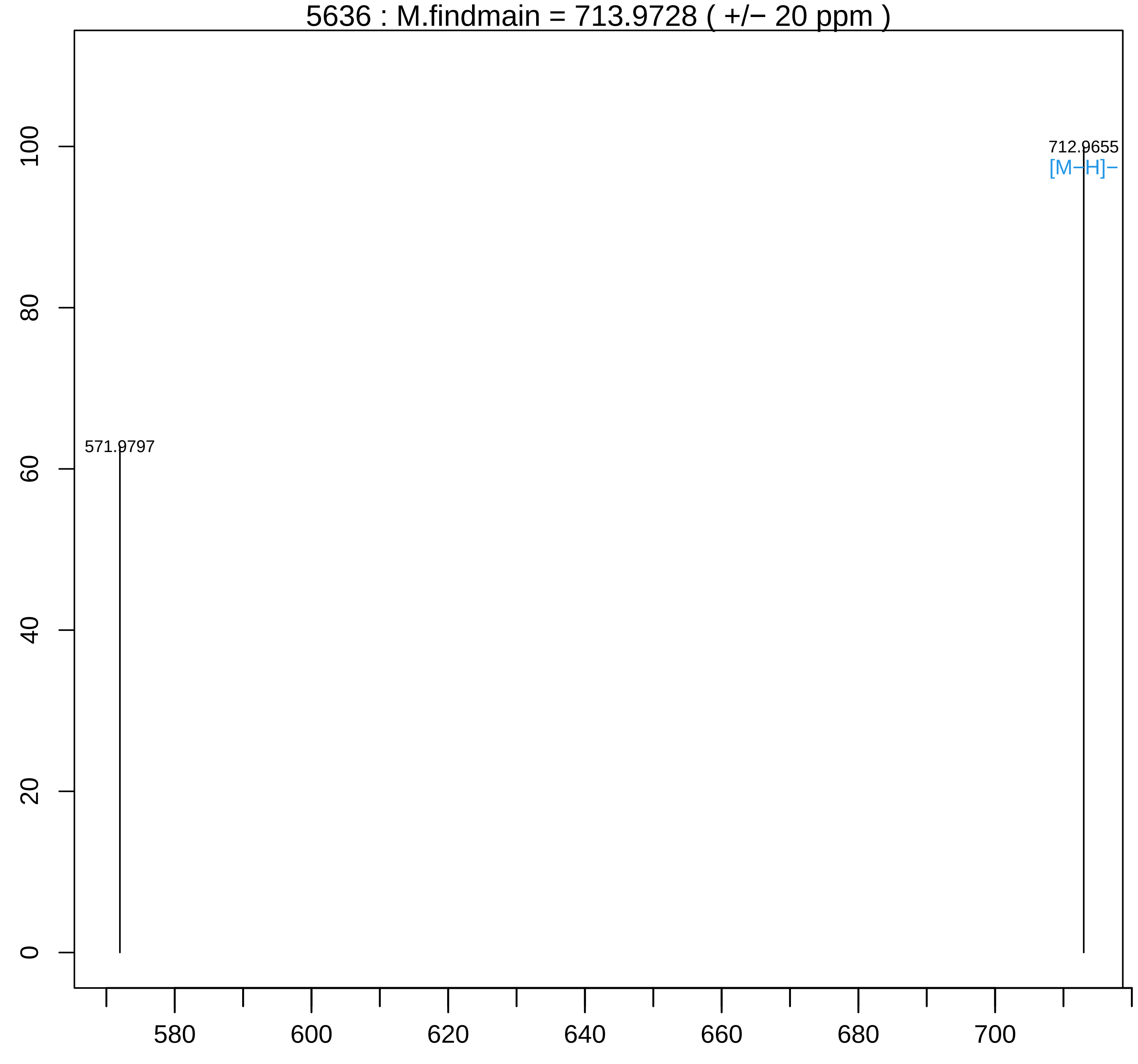
5636 : M.findmain = 713.9728 (+/− 20 ppm)
712.9655
[M−H]−
571.9797
0
20
40
60
80
100
580
600
620
640
660
680
700

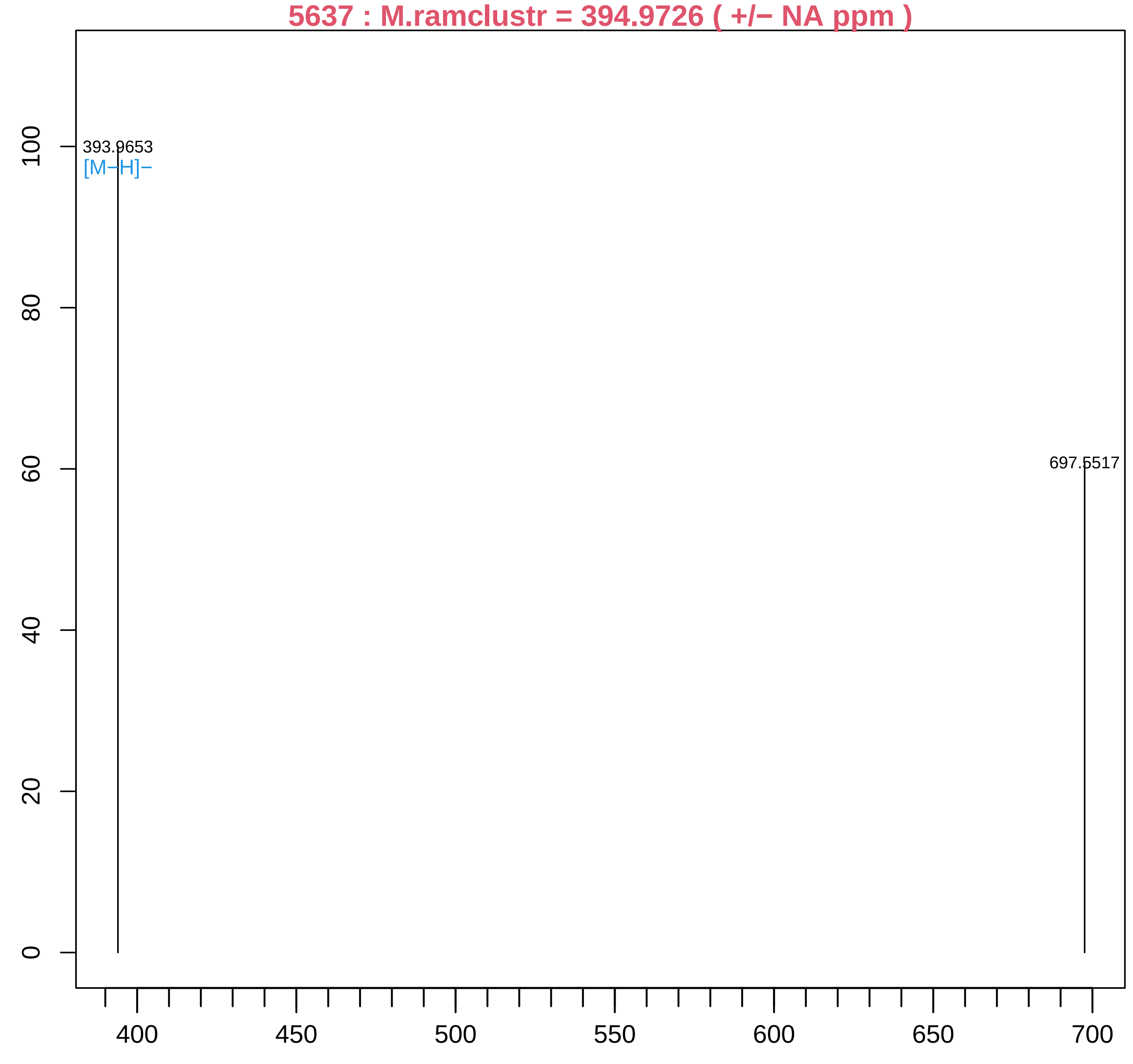
5637 : M.ramclustr = 394.9726 (+/− NA ppm)
393.9653
[M−H]−
697.5517
0
20
40
60
80
100
400
450
500
550
600
650
700

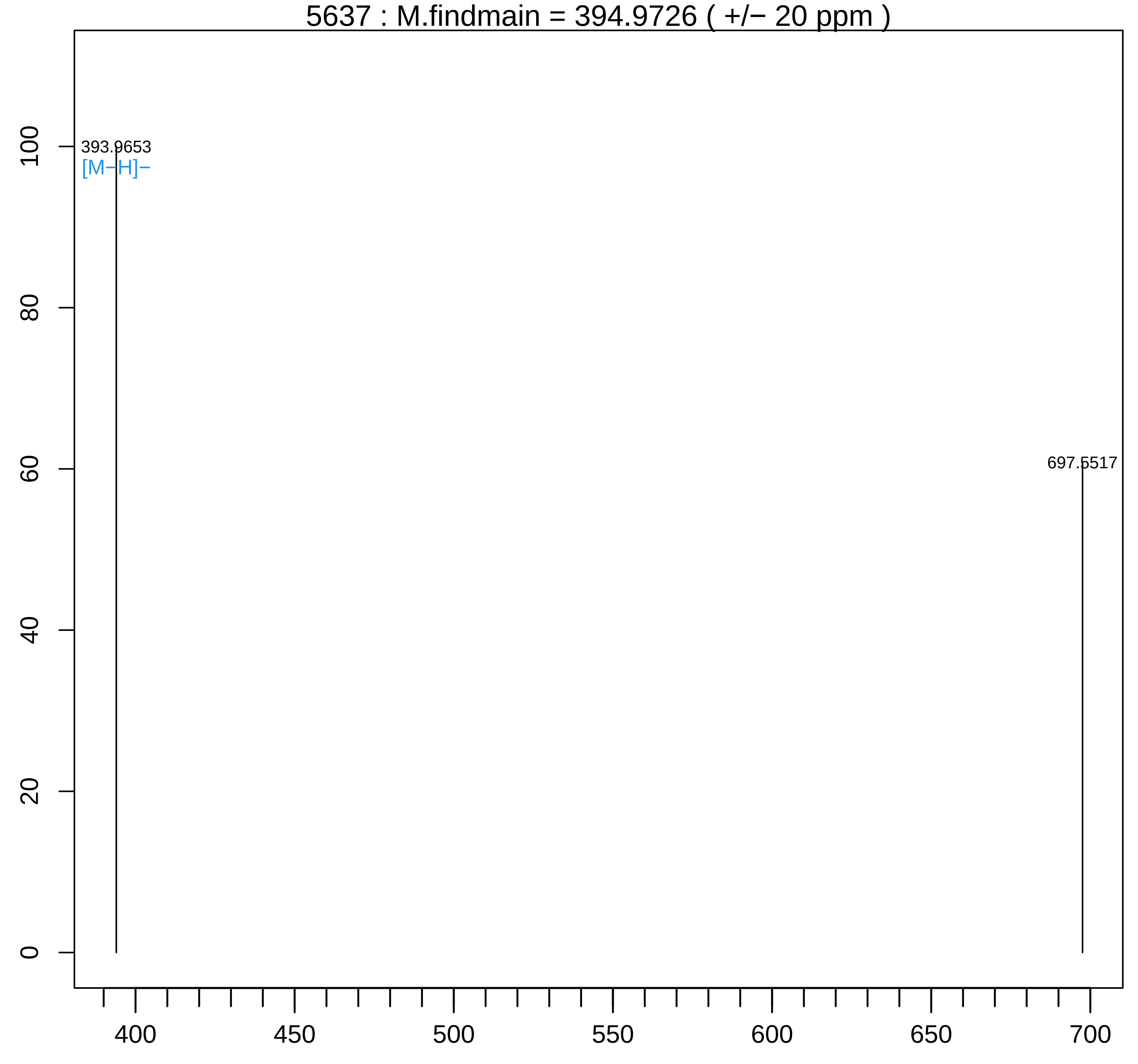
5637 : M.findmain = 394.9726 (+/− 20 ppm)
393.9653
[M−H]−
697.5517
0
20
40
60
80
100
400
450
500
550
600
650
700

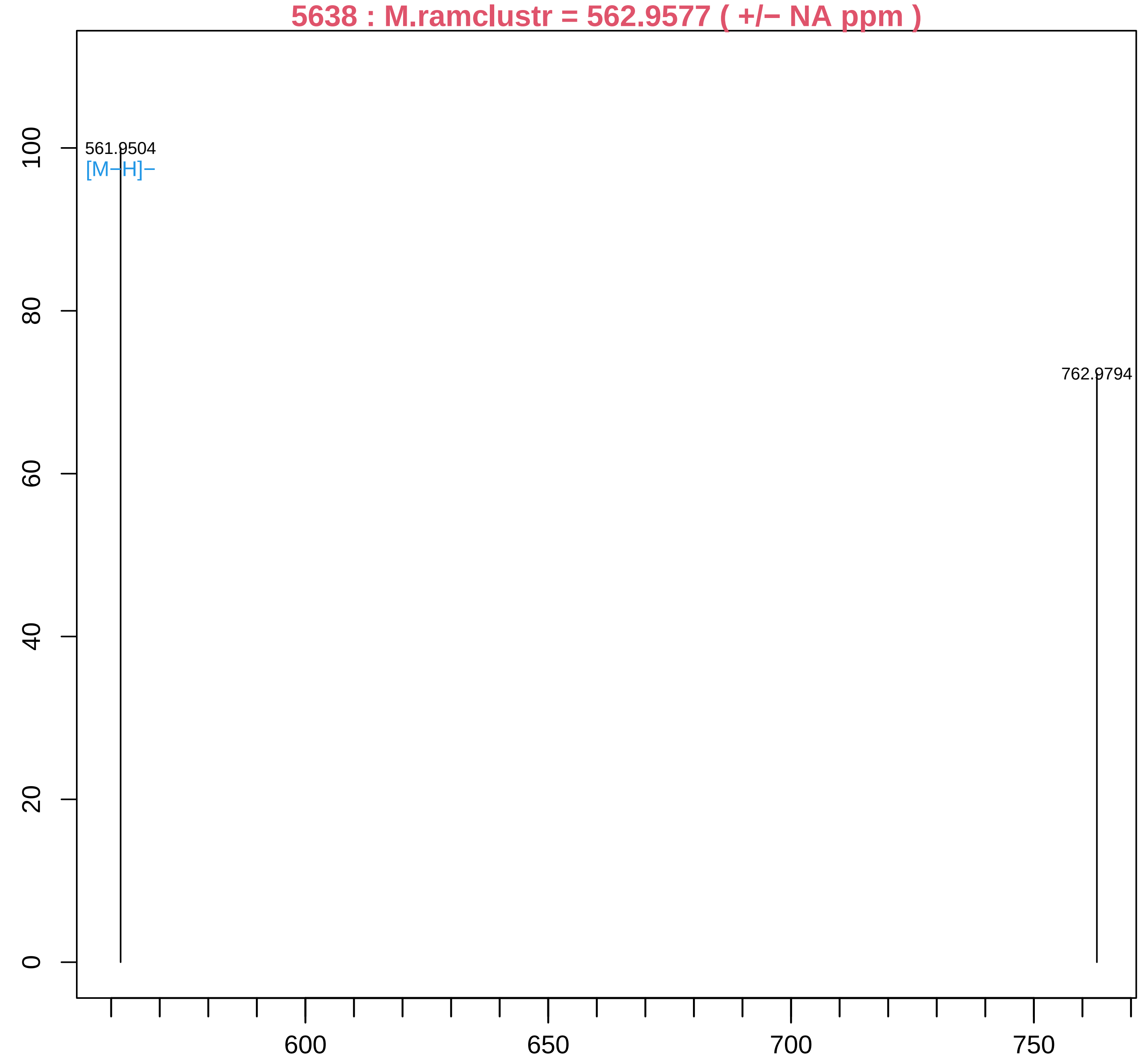

5638 : M.ramclustr = 562.9577 (+/− NA ppm)
561.9504
[M−H]−
762.9794
0
20
40
60
80
100
600
650
700
750

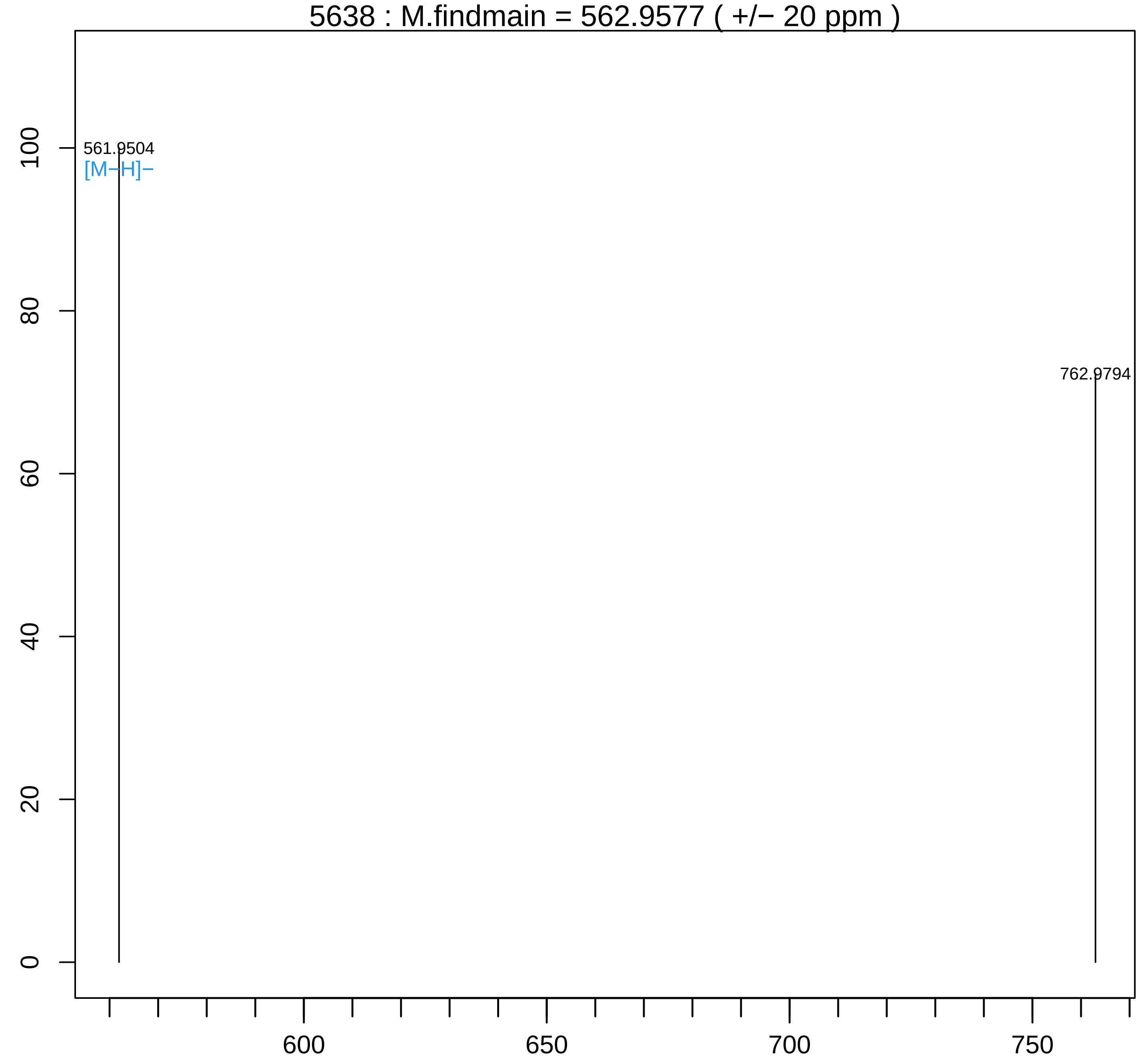

5638 : M.findmain = 562.9577 (+/− 20 ppm)
561.9504
[M−H]−
762.9794
0
20
40
60
80
100
600
650
700
750

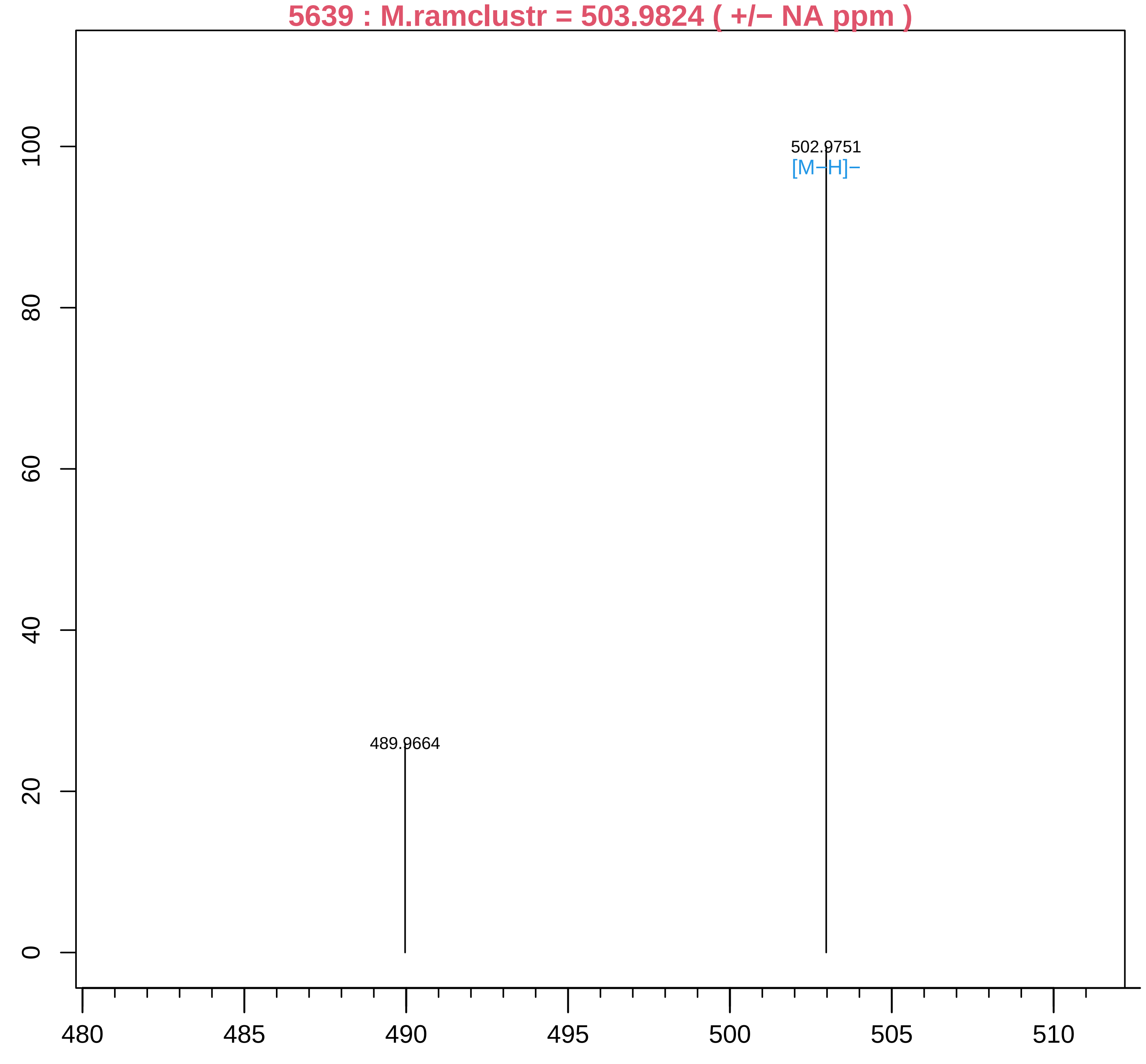
5639 : M.ramclustr = 503.9824 (+/− NA ppm)
502.9751
[M−H]−
489.9664
0
20
40
60
80
100
480
485
490
495
500
505
510

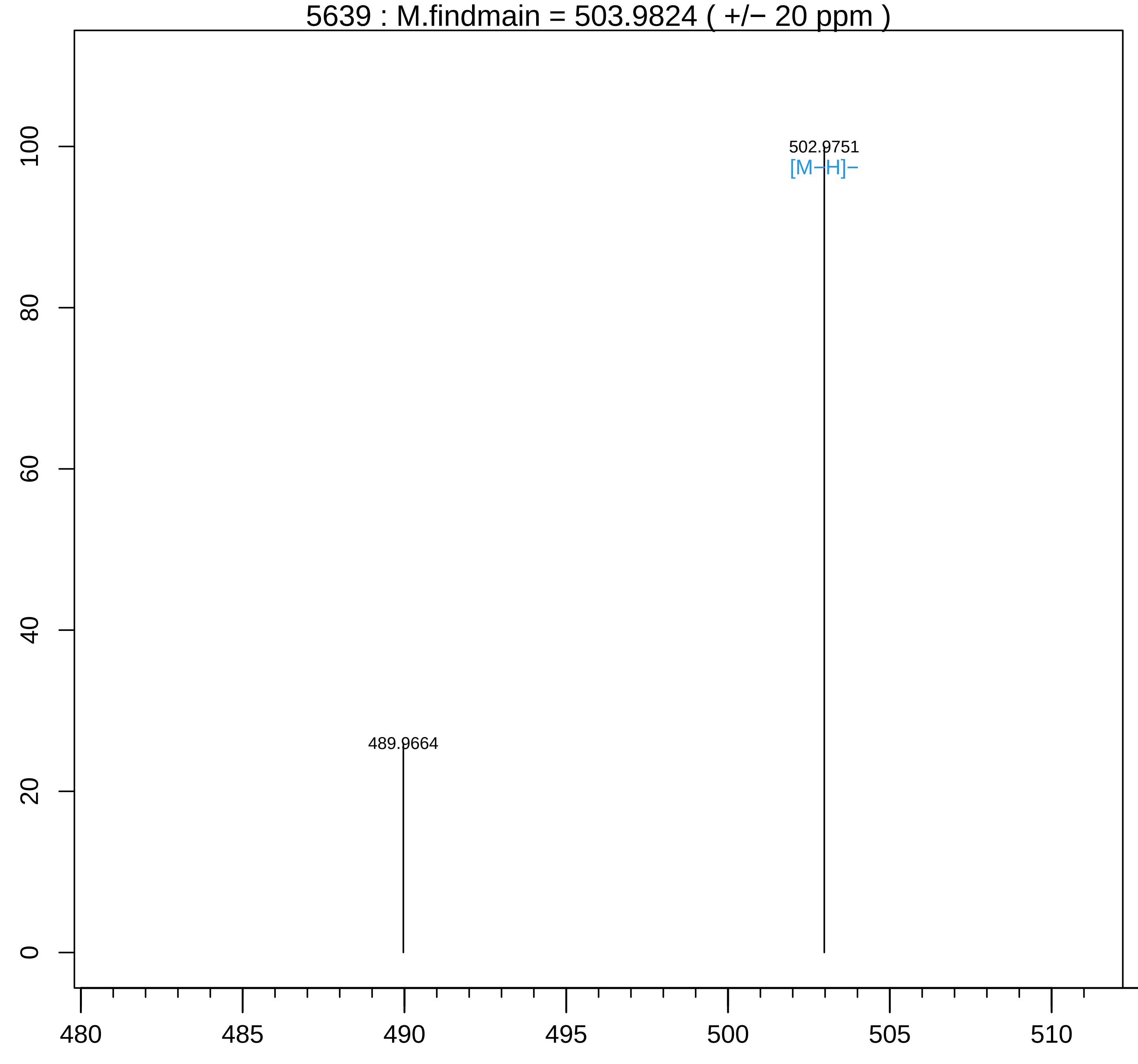
5639 : M.findmain = 503.9824 (+/− 20 ppm)
502.9751
[M−H]−
489.9664
0
20
40
60
80
100
480
485
490
495
500
505
510

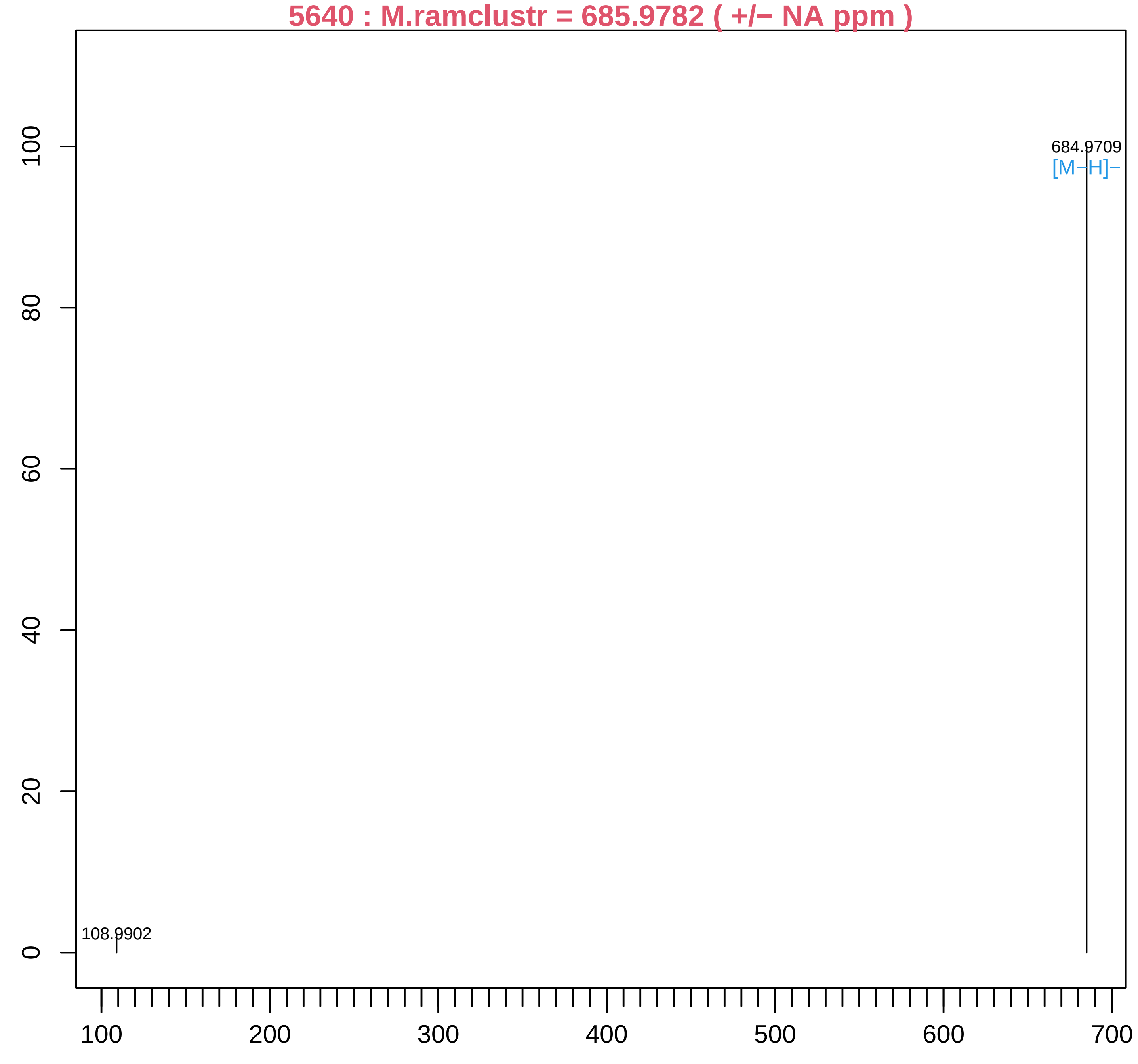

5640 : M.ramclustr = 685.9782 (+/− NA ppm)
684.9709
[M−H]−
108.9902
0
20
40
60
80
100
100
200
300
400
500
600
700

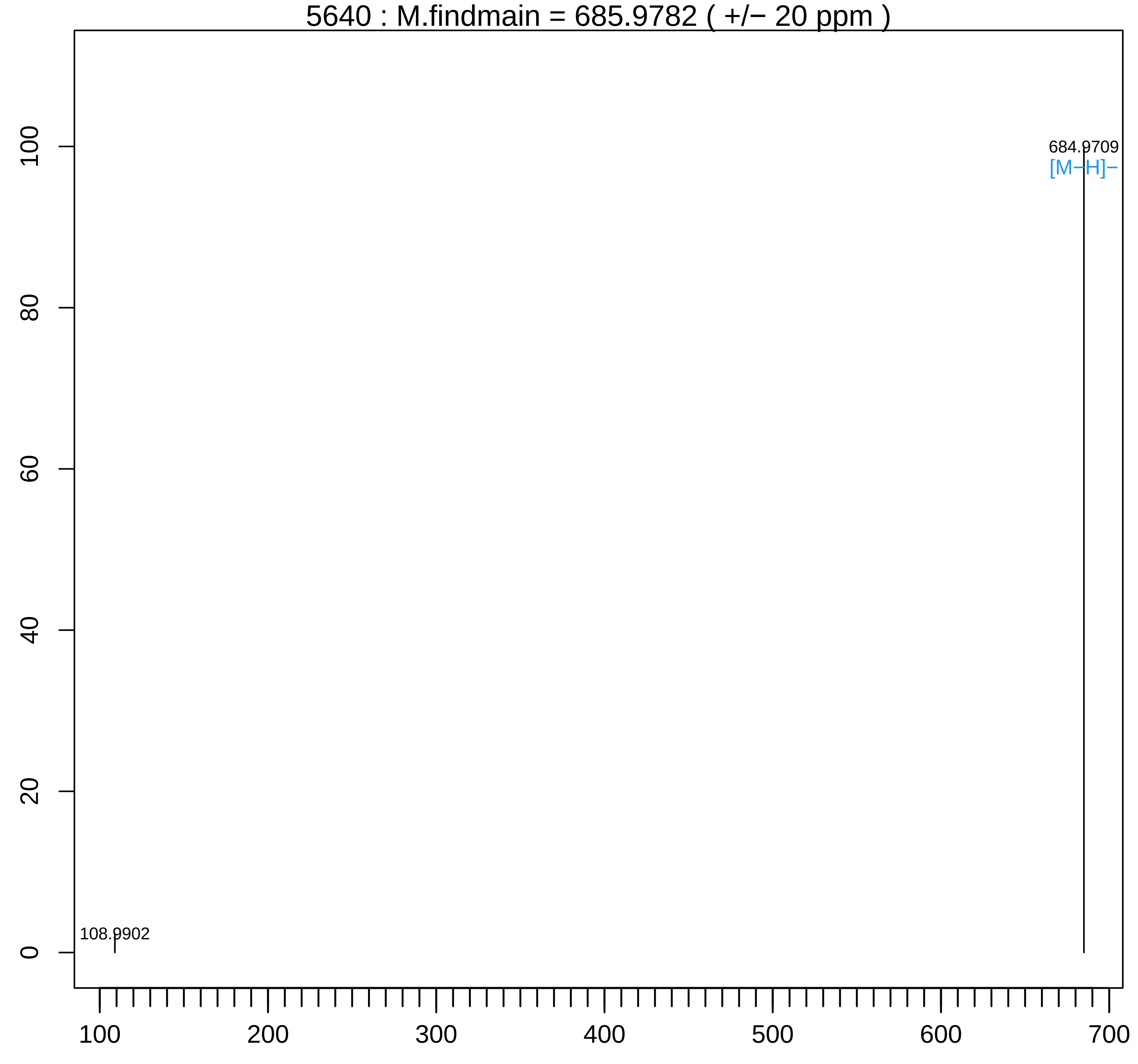

5640 : M.findmain = 685.9782 (+/− 20 ppm)
684.9709
[M−H]−
108.9902
0
20
40
60
80
100
100
200
300
400
500
600
700

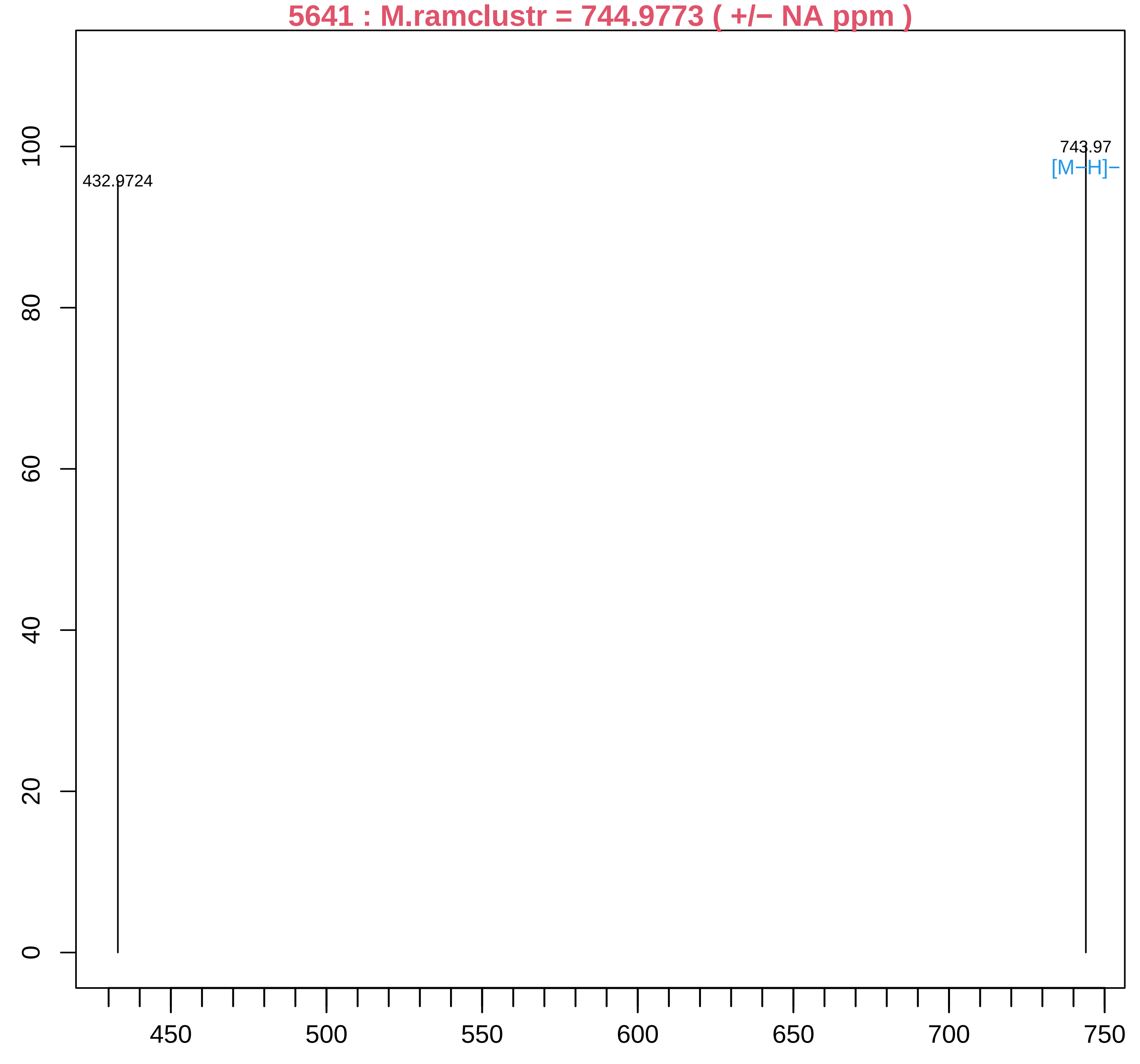
5641 : M.ramclustr = 744.9773 (+/− NA ppm)
432.9724
743.97
[M−H]−
0
20
40
60
80
100
450
500
550
600
650
700
750

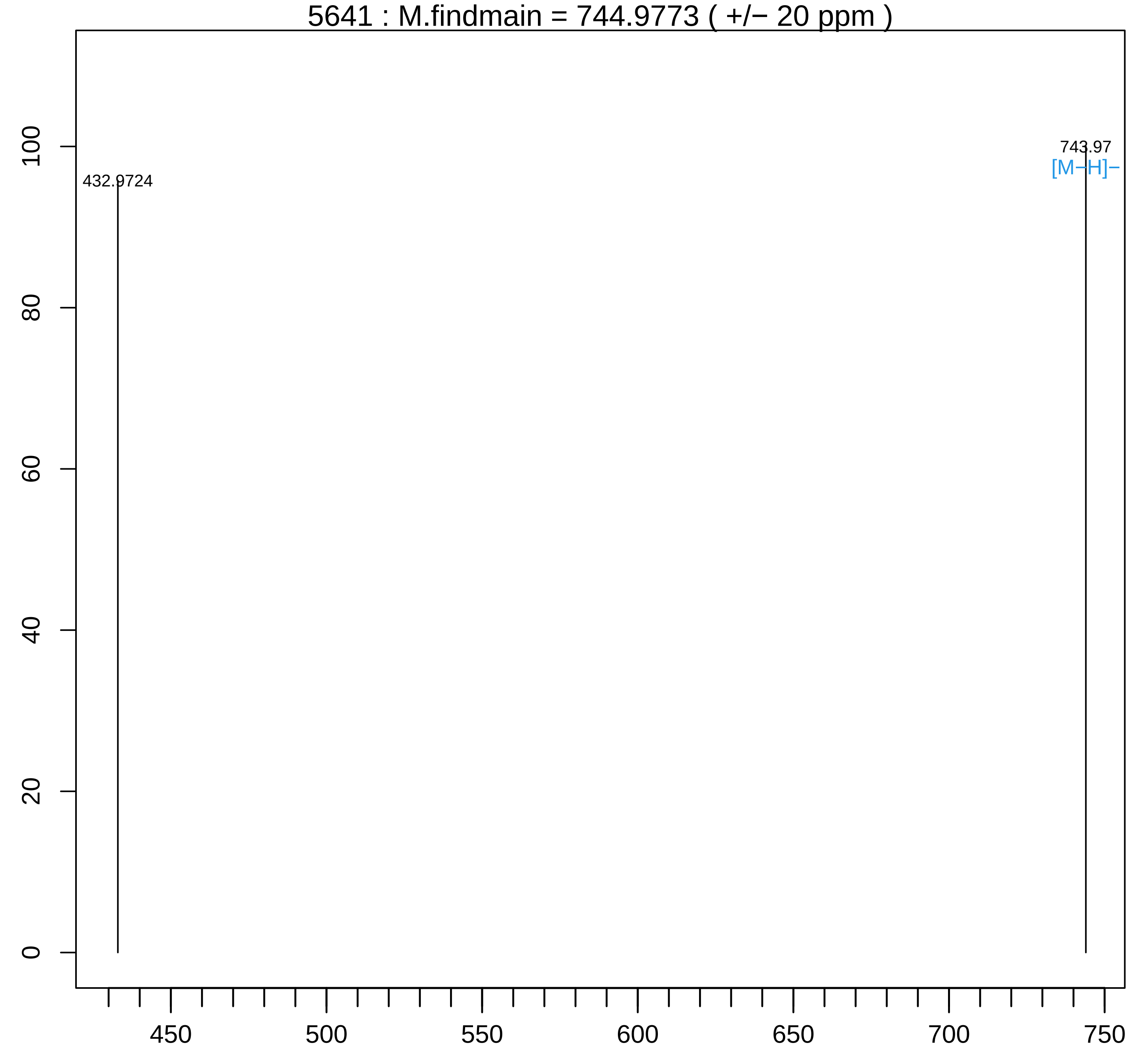
5641 : M.findmain = 744.9773 (+/− 20 ppm)
432.9724
743.97
[M−H]−
0
20
40
60
80
100
450
500
550
600
650
700
750

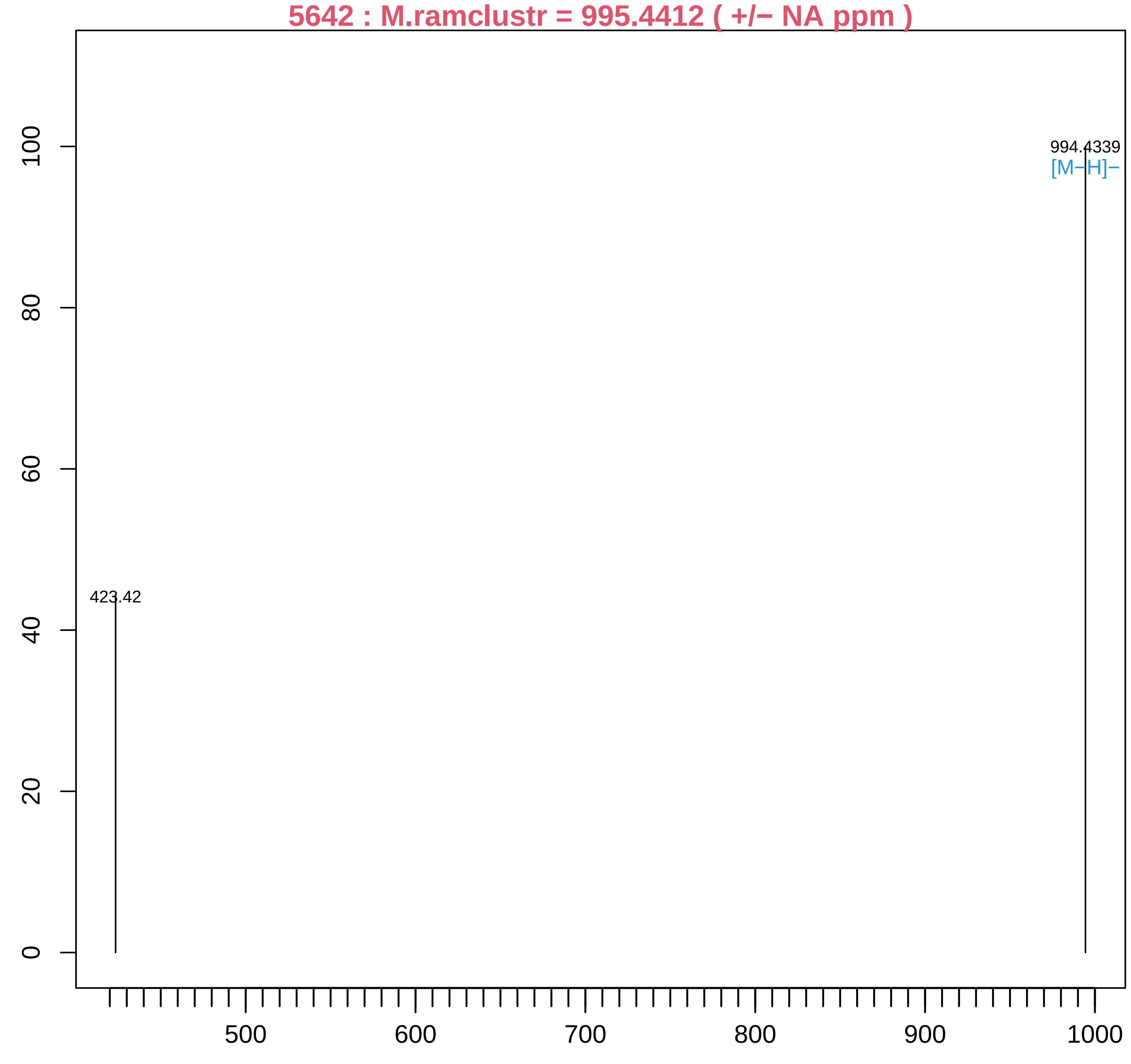
5642 : M.ramclustr = 995.4412 (+/− NA ppm)
994.4339
[M−H]−
423.42
0
20
40
60
80
100
500
600
700
800
900
1000

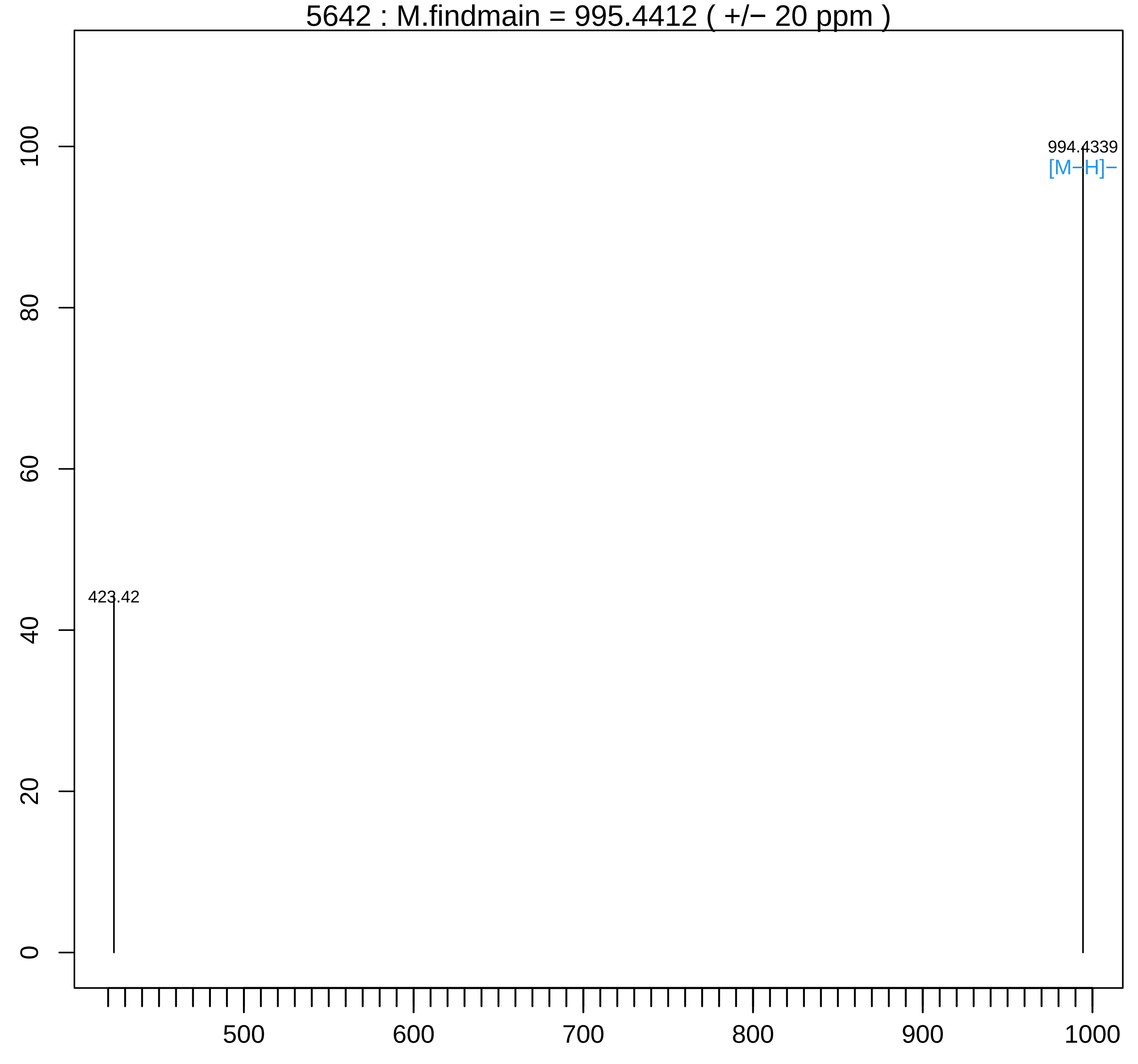
5642 : M.findmain = 995.4412 (+/− 20 ppm)
994.4339
[M−H]−
423.42
0
20
40
60
80
100
500
600
700
800
900
1000

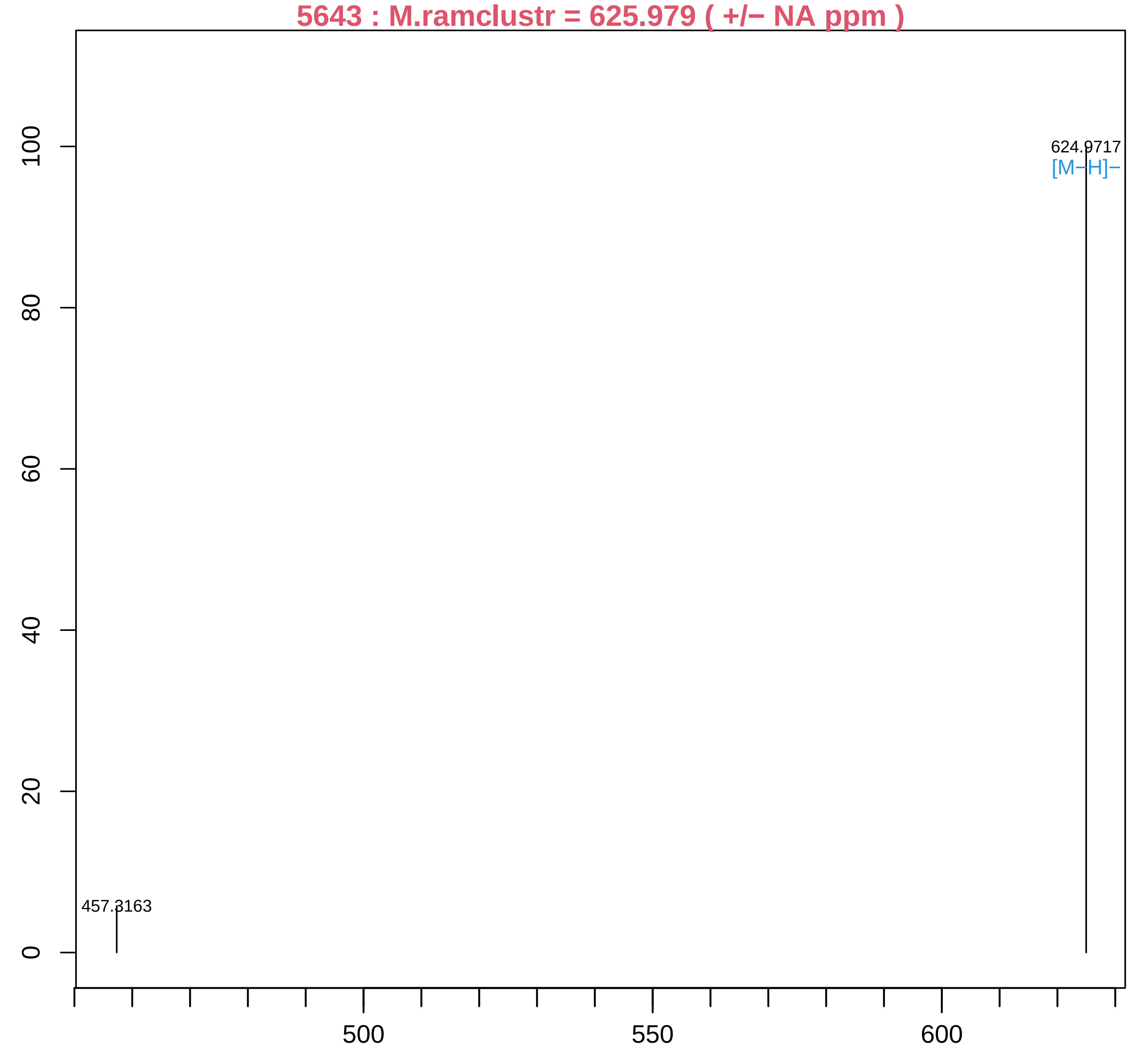
5643 : M.ramclustr = 625.979 (+/− NA ppm)
624.9717
[M−H]−
457.3163
0
20
40
60
80
100
500
550
600

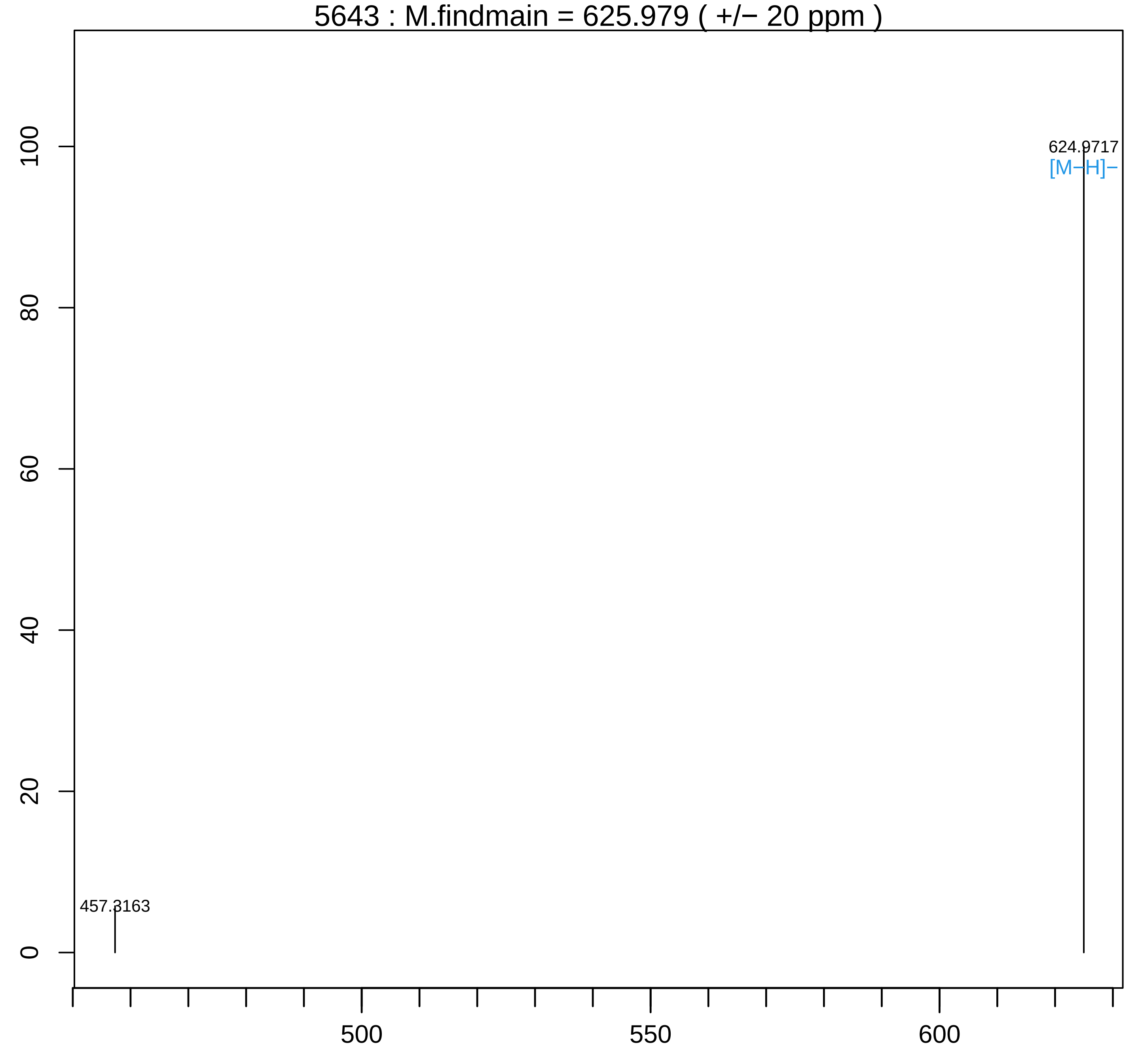
5643 : M.findmain = 625.979 (+/− 20 ppm)
624.9717
[M−H]−
457.3163
0
20
40
60
80
100
500
550
600

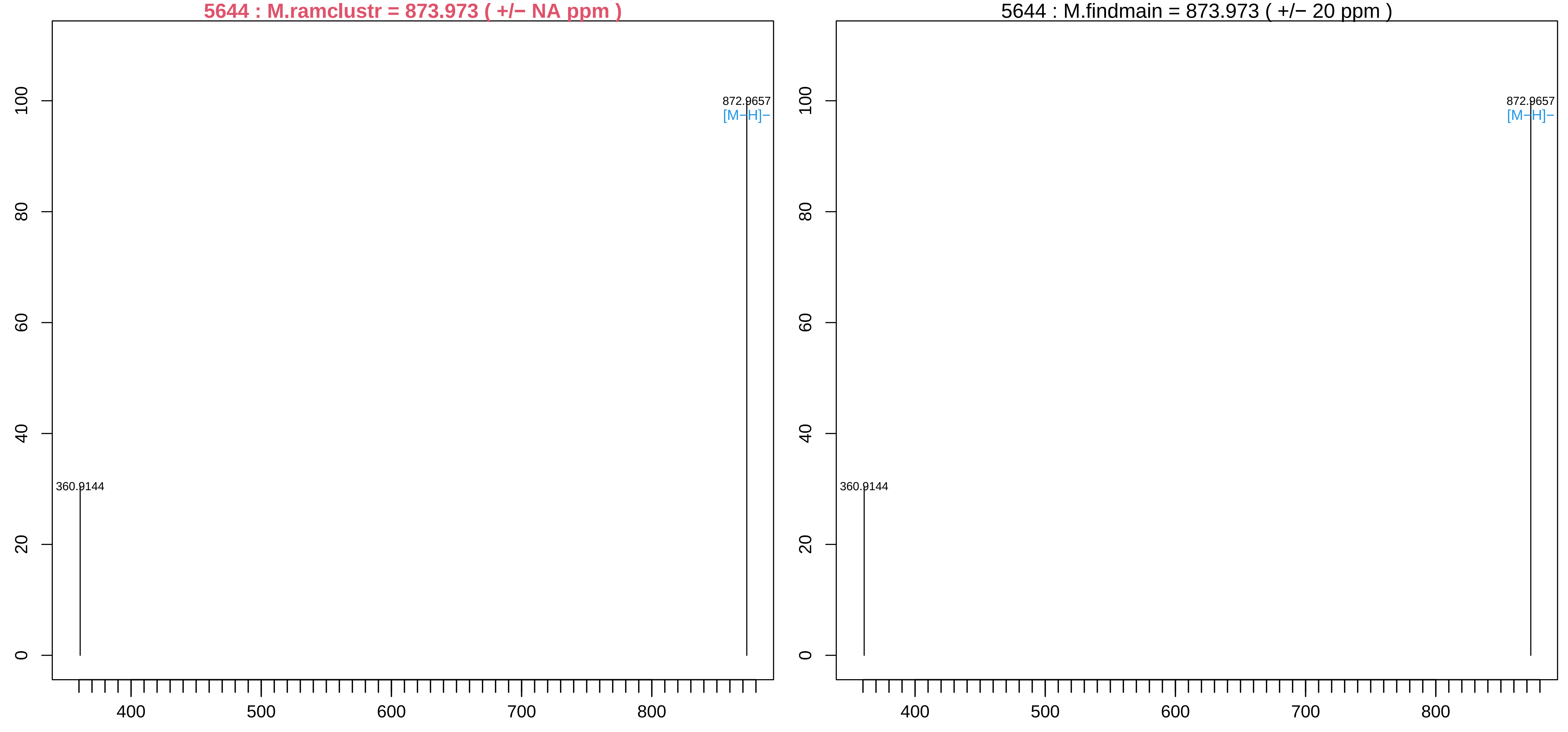

5644 : M.ramclustr = 873.973 (+/− NA ppm)
872.9657
[M−H]−
360.9144
5644 : M.findmain = 873.973 (+/− 20 ppm)
872.9657
[M−H]−
360.9144
0
20
40
60
80
100
400
500
600
700
800

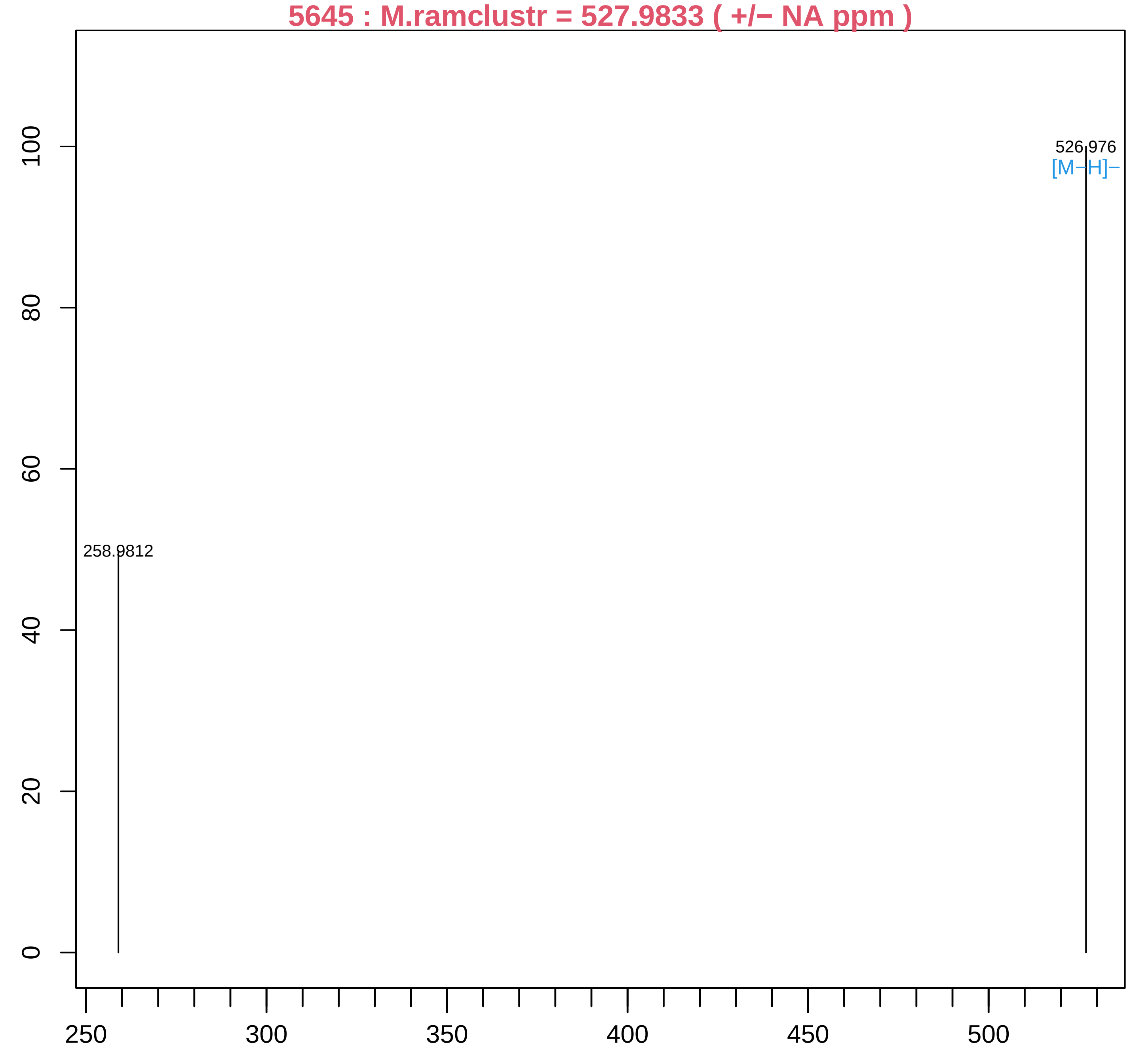

5645 : M.ramclustr = 527.9833 (+/− NA ppm)
526.976
[M−H]−
258.9812
0
20
40
60
80
100
250
300
350
400
450
500

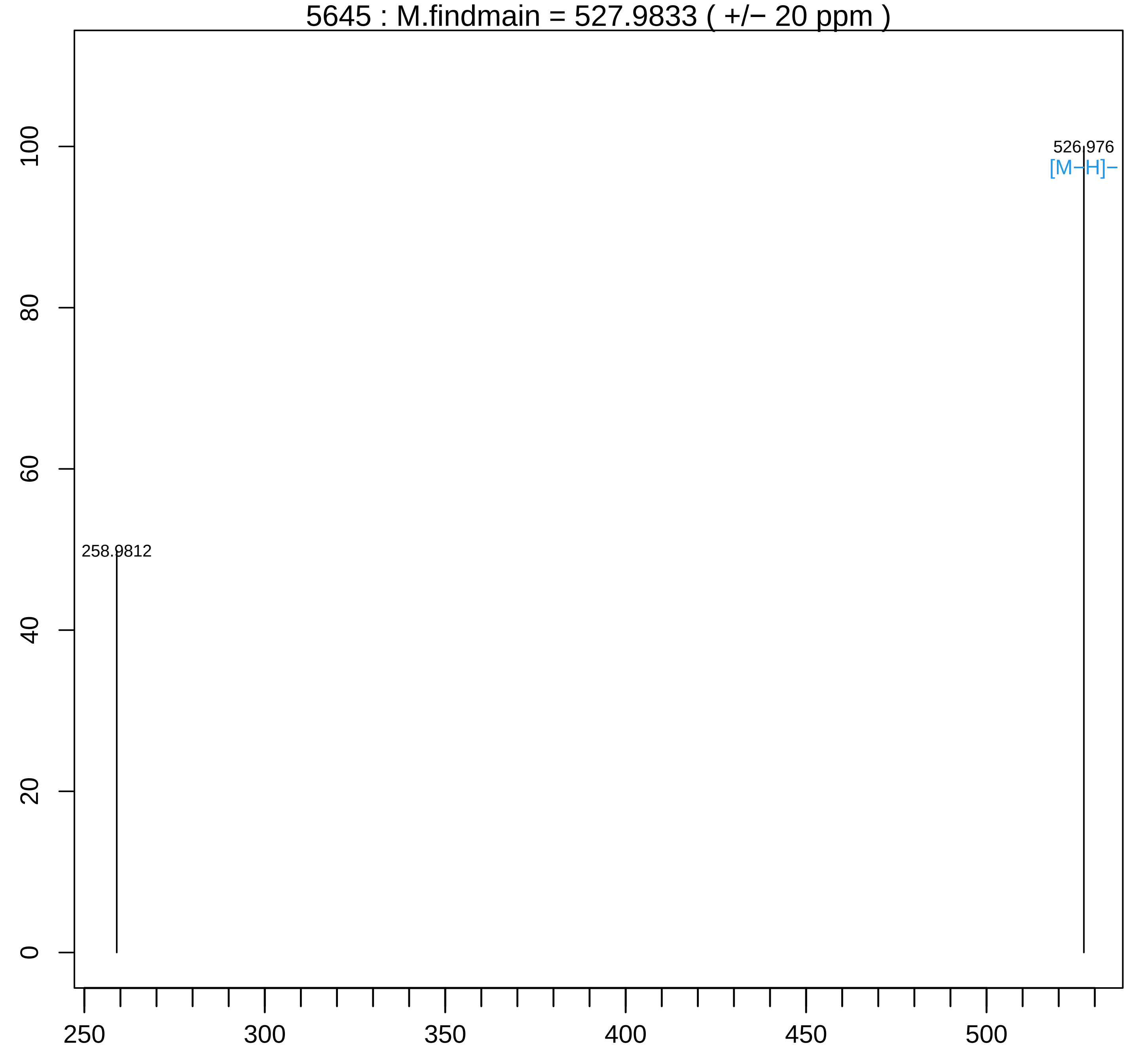

5645 : M.findmain = 527.9833 (+/− 20 ppm)
526.976
[M−H]−
258.9812
0
20
40
60
80
100
250
300
350
400
450
500

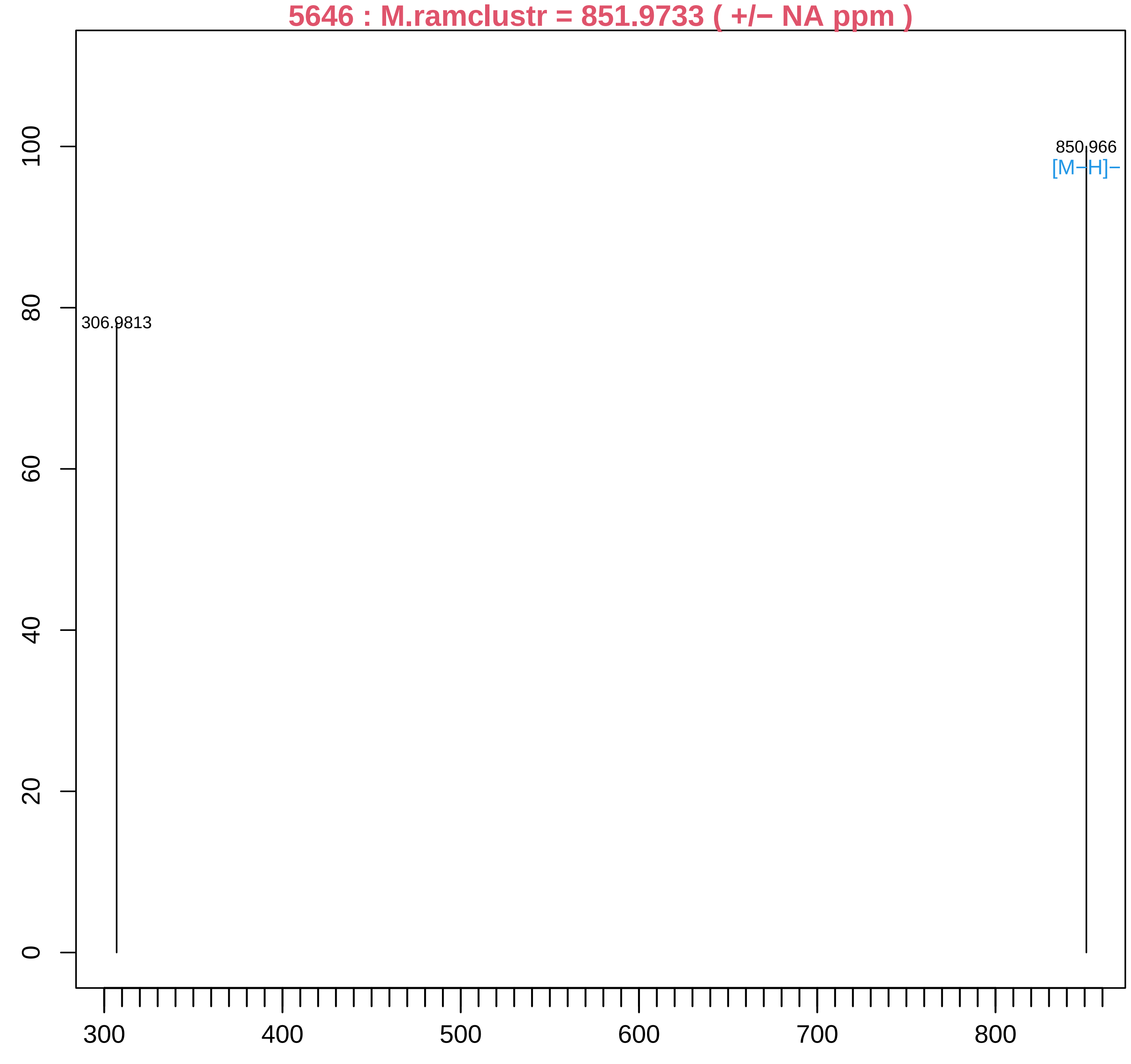

5646 : M.ramclustr = 851.9733 (+/− NA ppm)
306.9813
850.966
[M−H]−
0
20
40
60
80
100
300
400
500
600
700
800

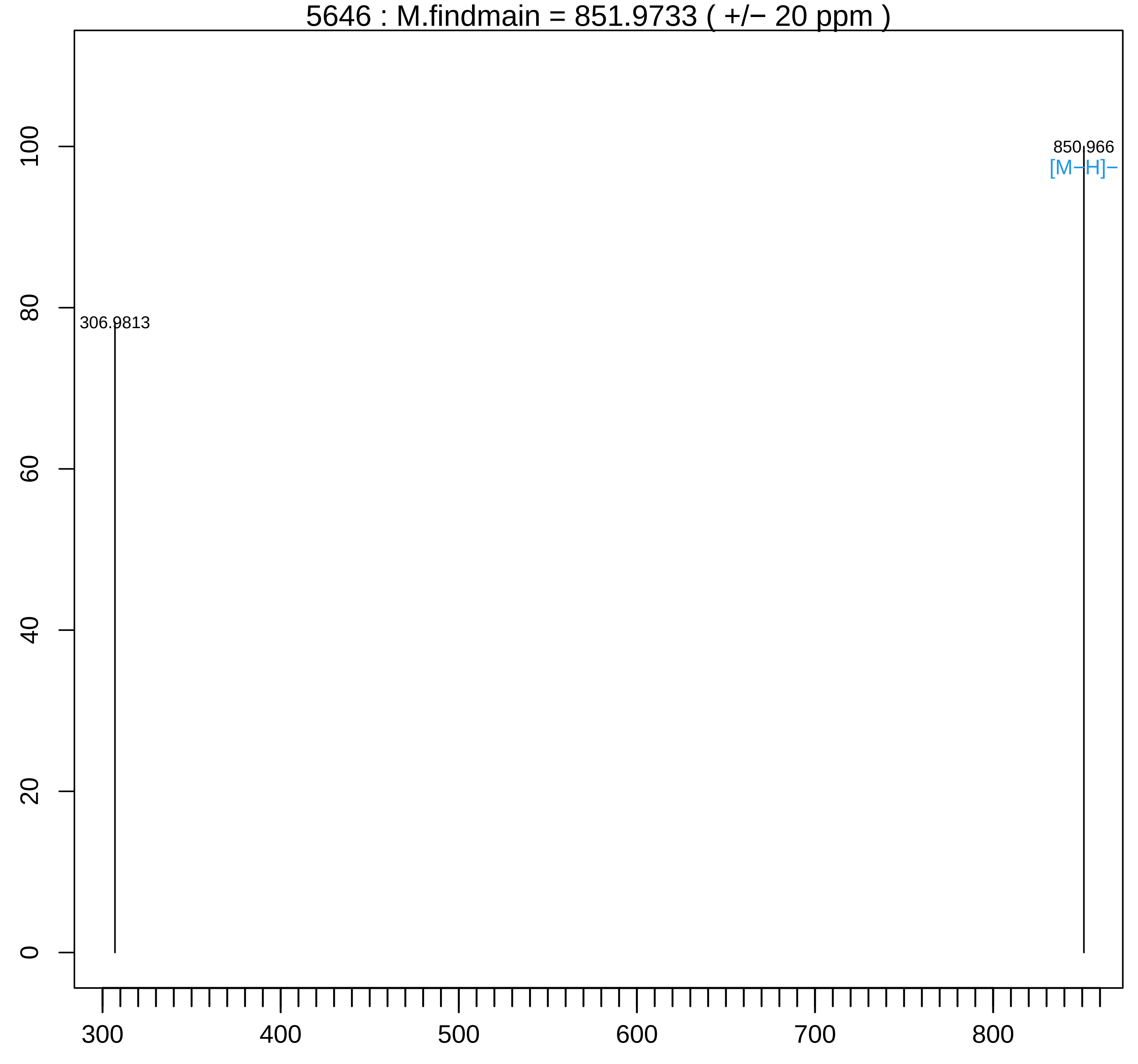

5646 : M.findmain = 851.9733 (+/− 20 ppm)
306.9813
850.966
[M−H]−
0
20
40
60
80
100
300
400
500
600
700
800

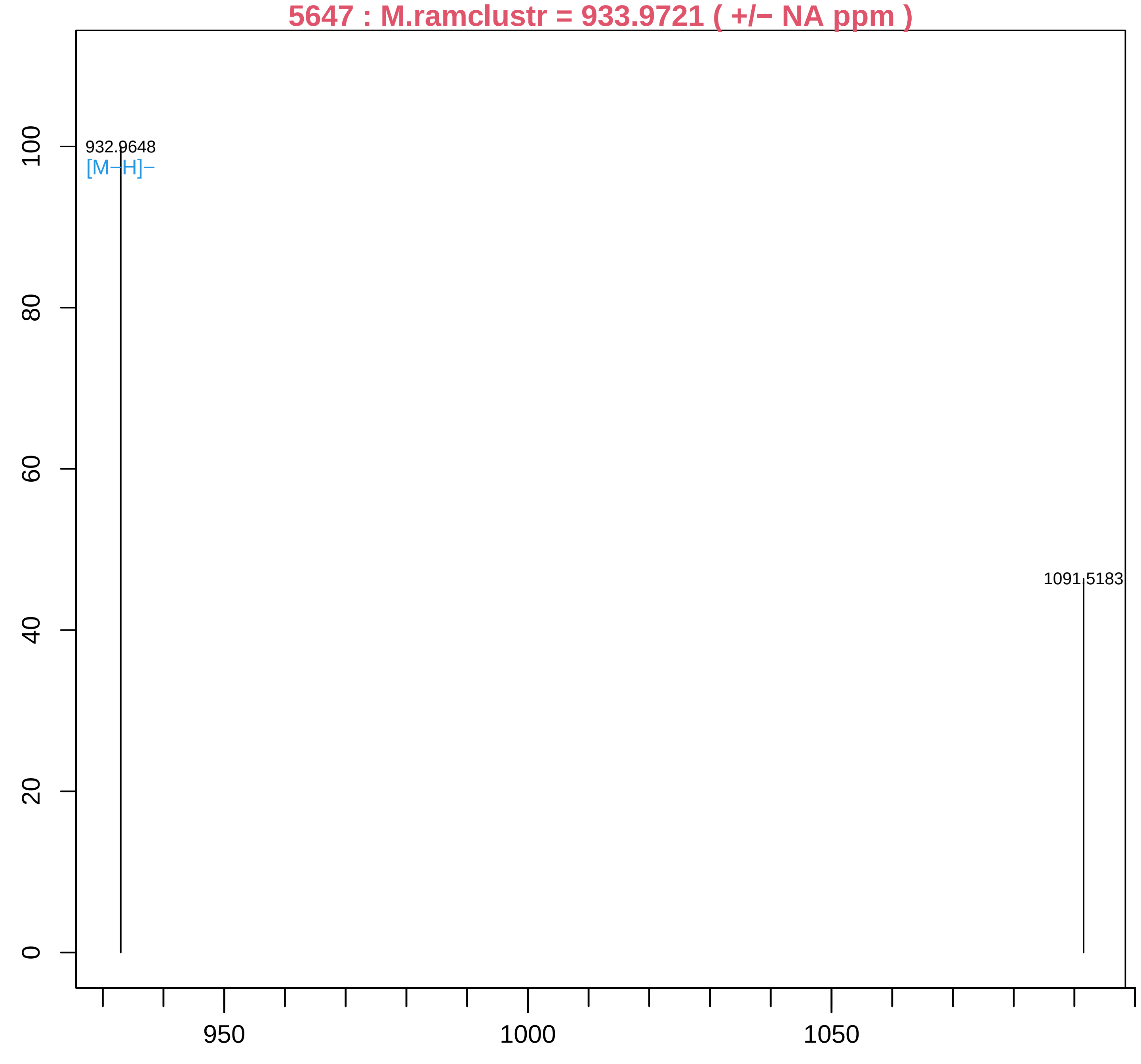
5647 : M.ramclustr = 933.9721 (+/− NA ppm)
932.9648
[M−H]−
1091.5183
0
20
40
60
80
100
950
1000
1050

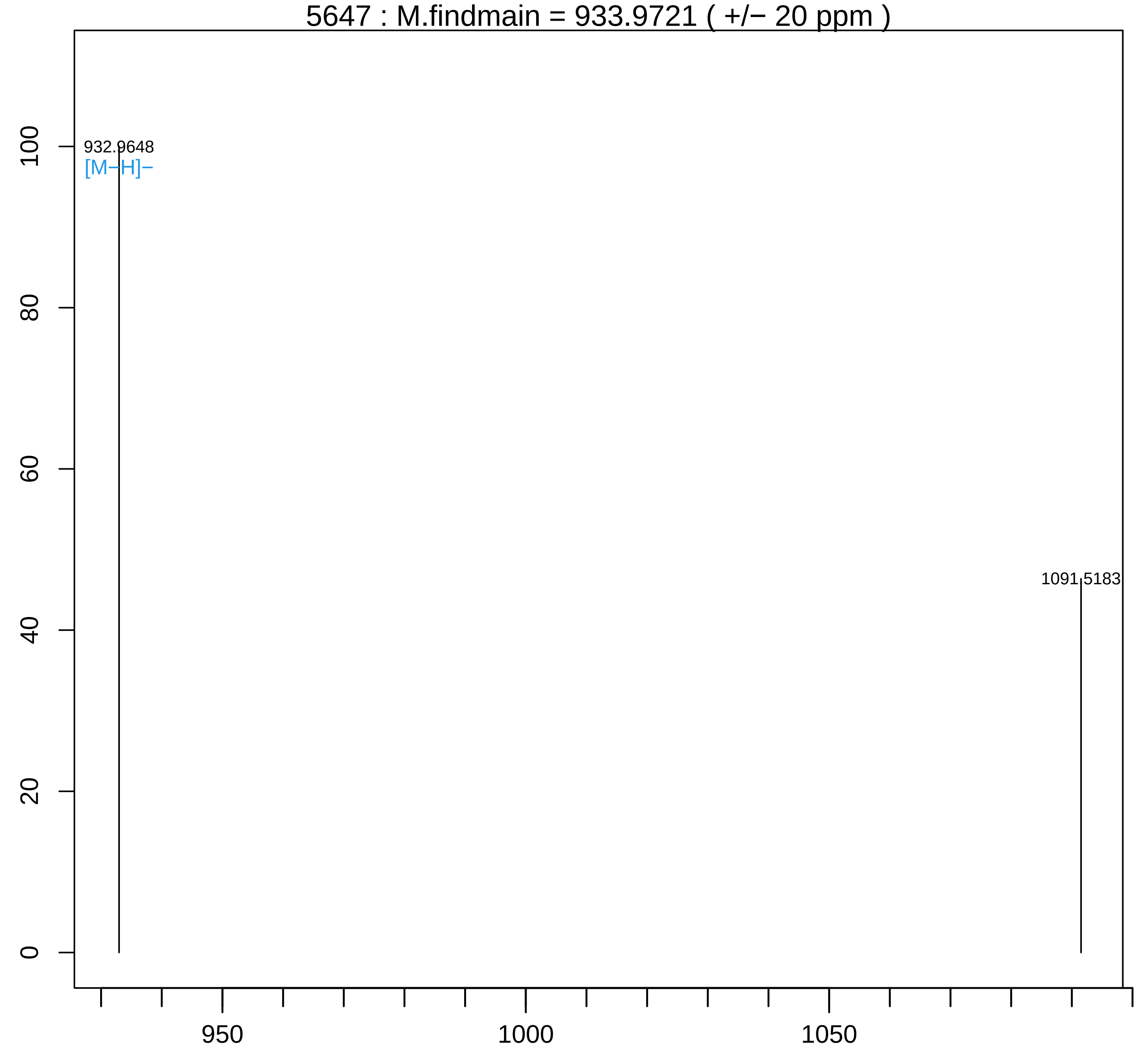
5647 : M.findmain = 933.9721 (+/− 20 ppm)
932.9648
[M−H]−
1091.5183
0
20
40
60
80
100
950
1000
1050

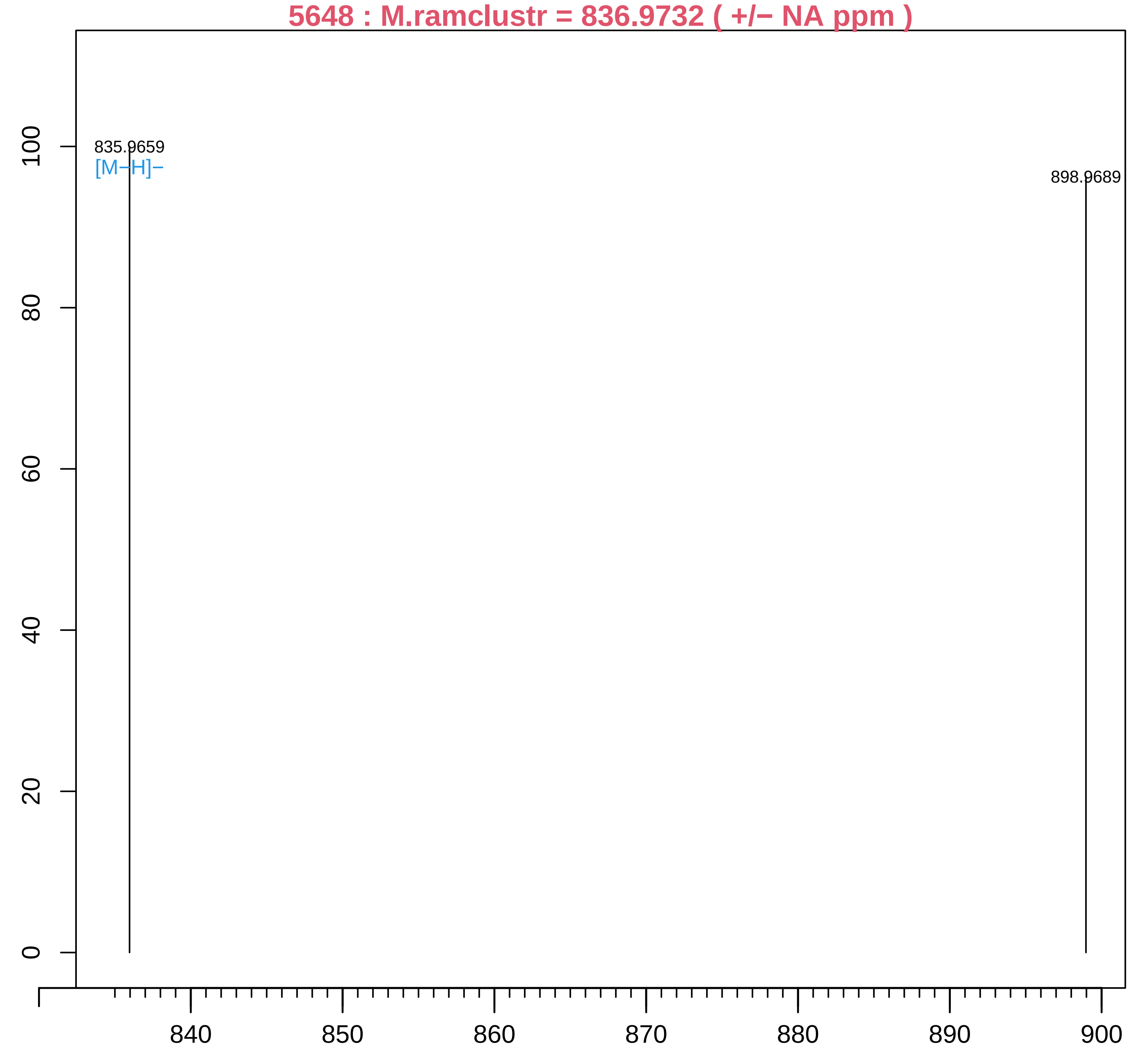
5648 : M.ramclustr = 836.9732 (+/− NA ppm)
835.9659
[M−H]−
898.9689
0
20
40
60
80
100
840
850
860
870
880
890
900
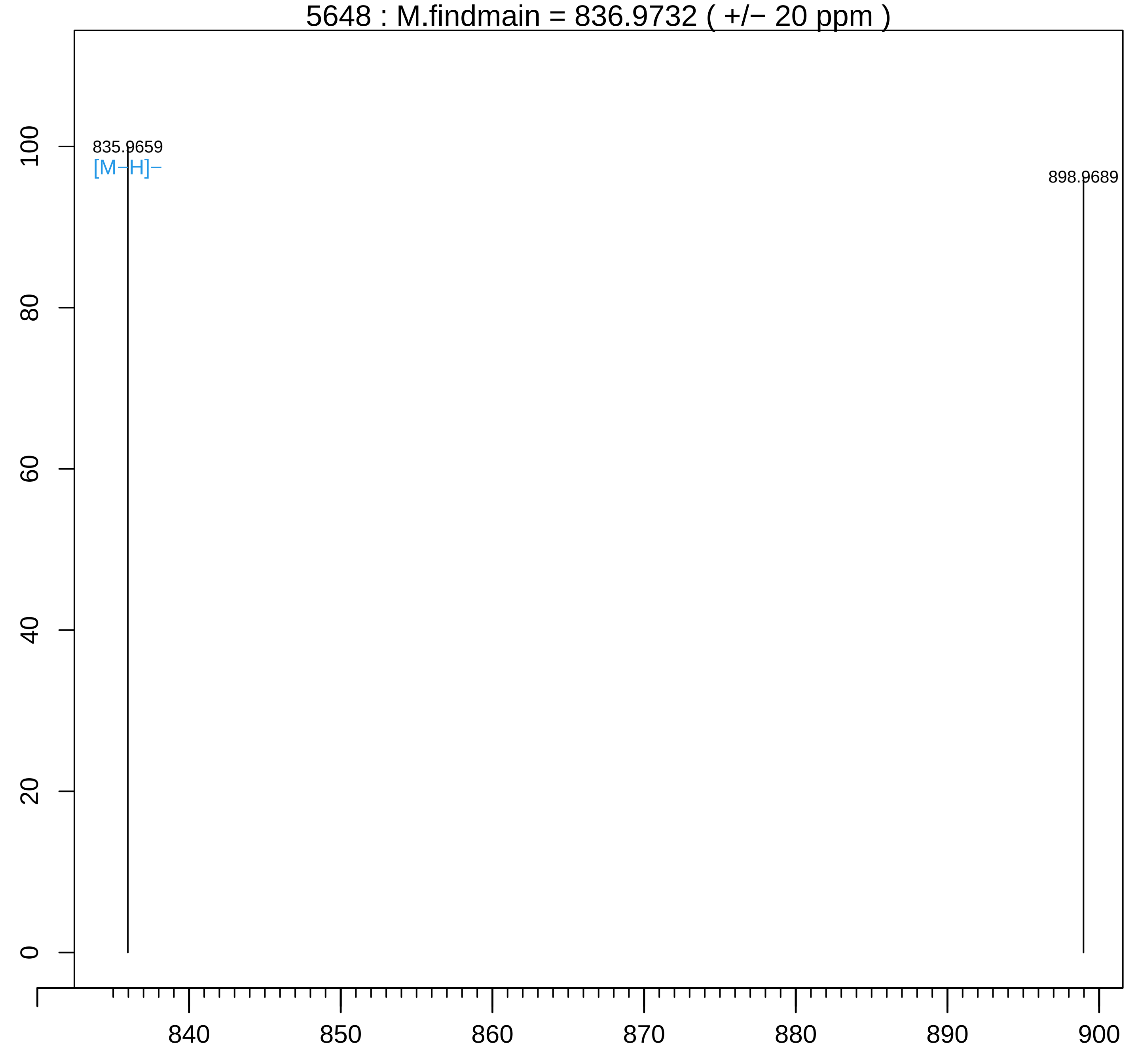
5648 : M.findmain = 836.9732 (+/− 20 ppm)
835.9659
[M−H]−
898.9689
0
20
40
60
80
100
840
850
860
870
880
890
900

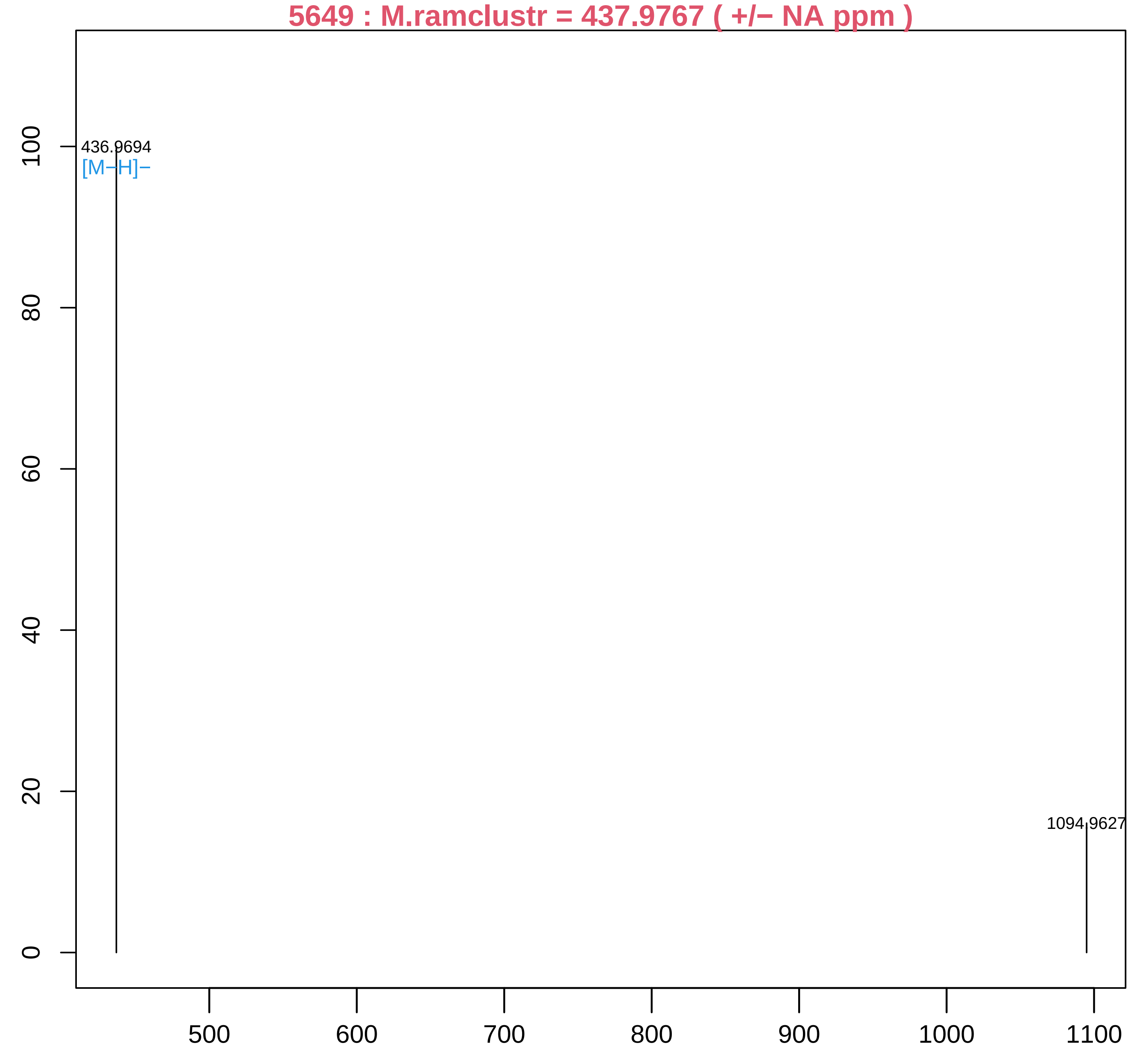
5649 : M.ramclustr = 437.9767 (+/− NA ppm)
436.9694
[M−H]−
1094.9627
0
20
40
60
80
100
500
600
700
800
900
1000
1100

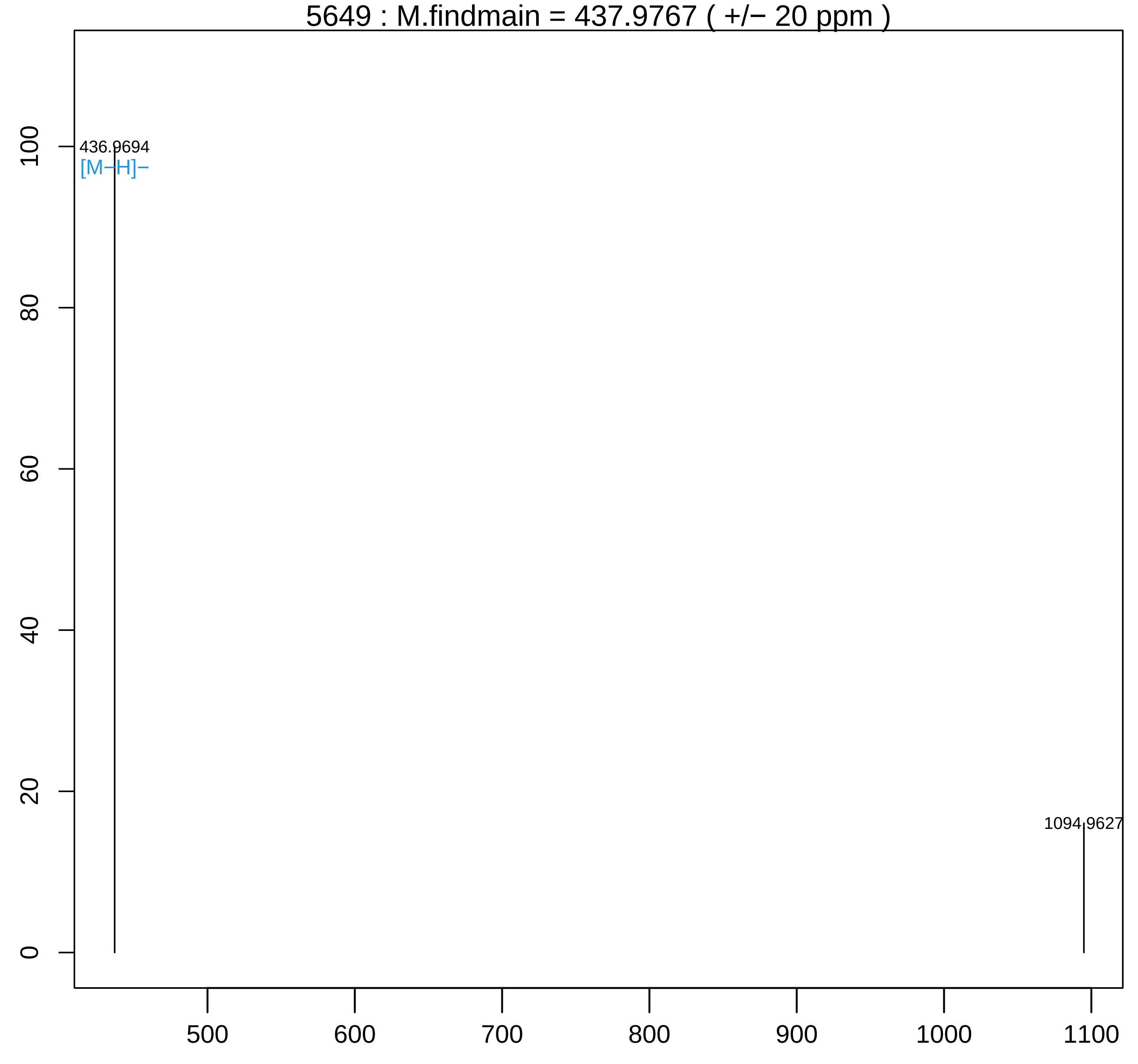
5649 : M.findmain = 437.9767 (+/− 20 ppm)
436.9694
[M−H]−
1094.9627
0
20
40
60
80
100
500
600
700
800
900
1000
1100

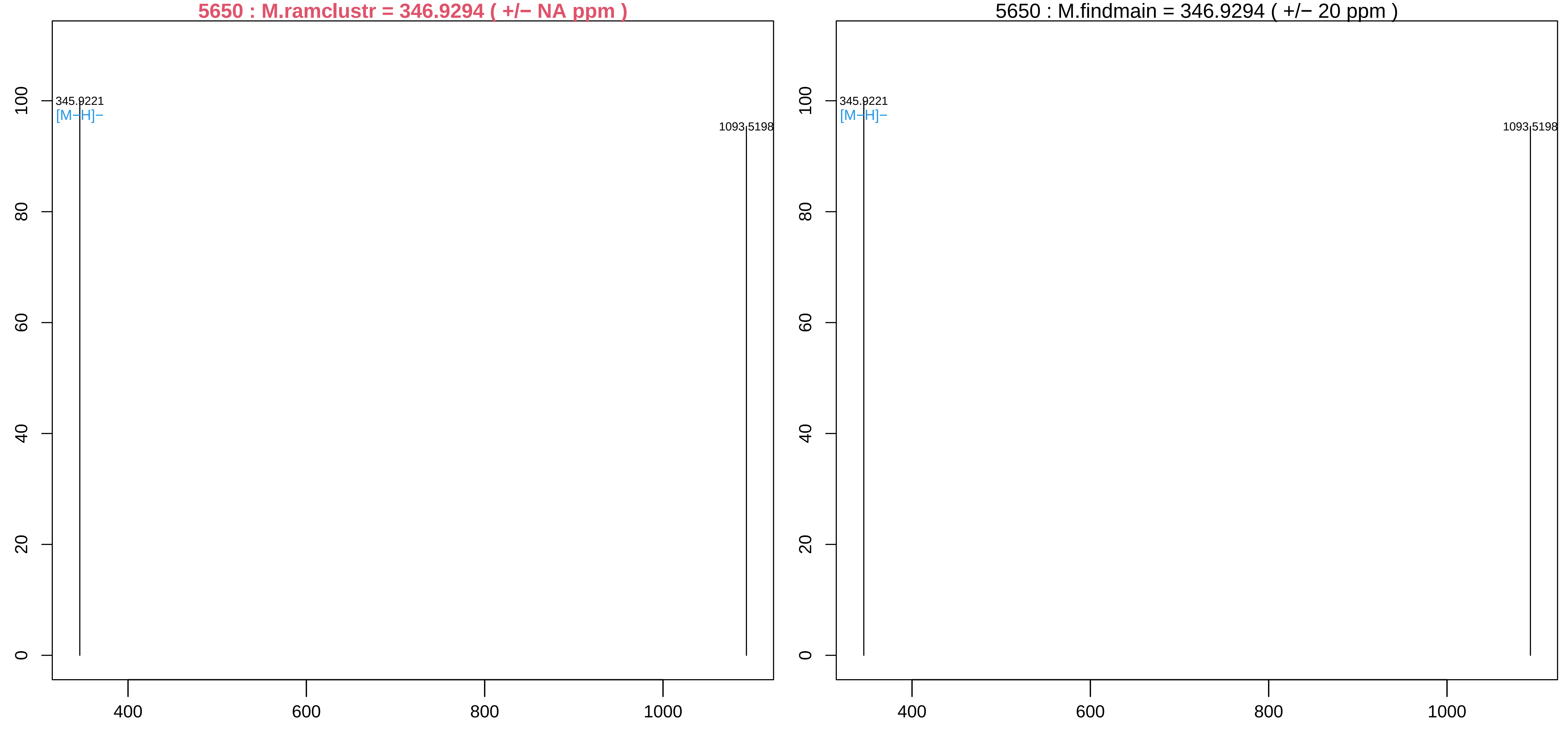
5650 : M.ramclustr = 346.9294 (+/− NA ppm)
5650 : M.findmain = 346.9294 (+/− 20 ppm)
345.9221
[M−H]−
1093.5198
0
20
40
60
80
100
400
600
800
1000

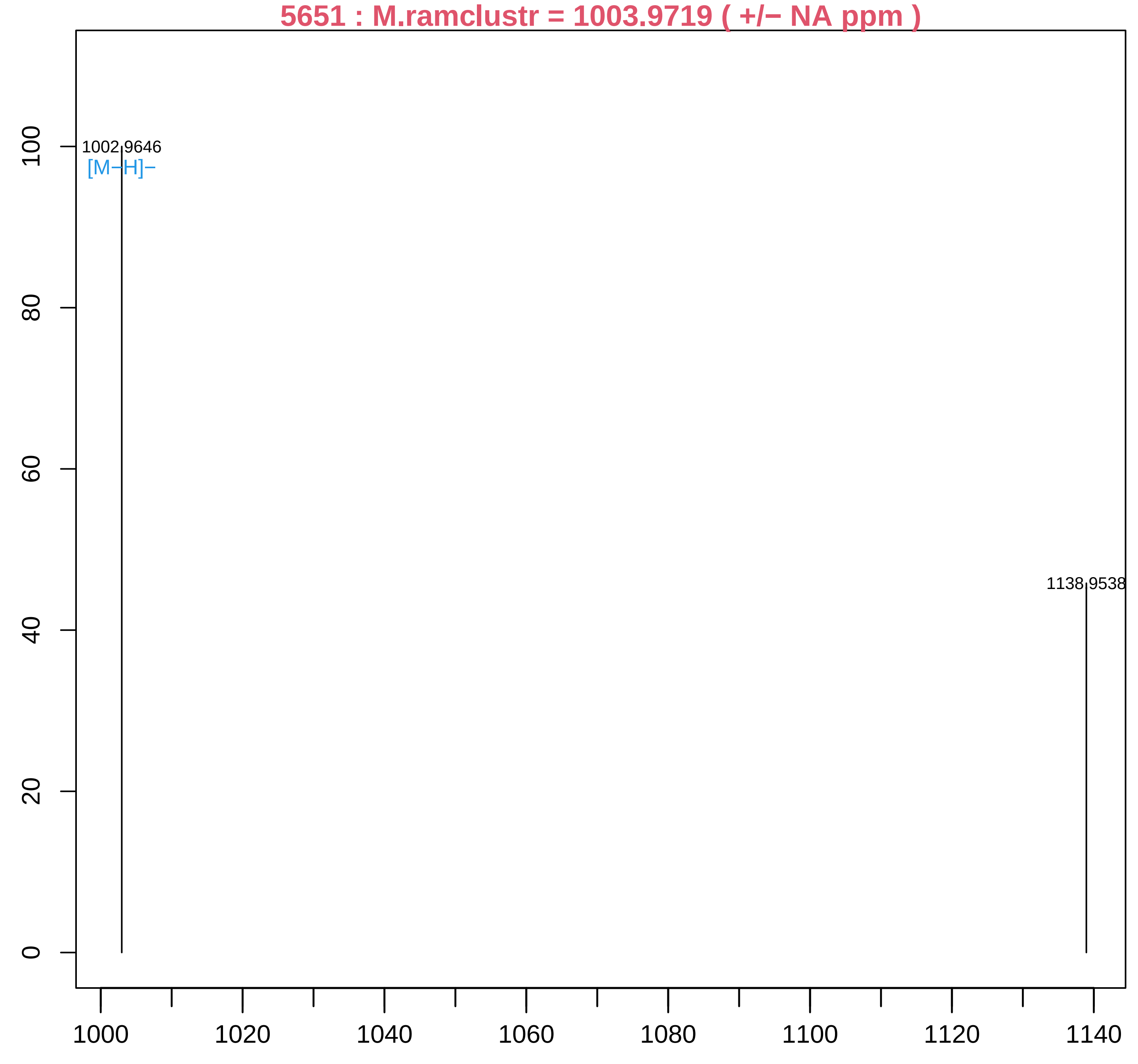
5651 : M.ramclustr = 1003.9719 (+/− NA ppm)
1002.9646
[M−H]−
1138.9538
0
20
40
60
80
100
1000
1020
1040
1060
1080
1100
1120
1140

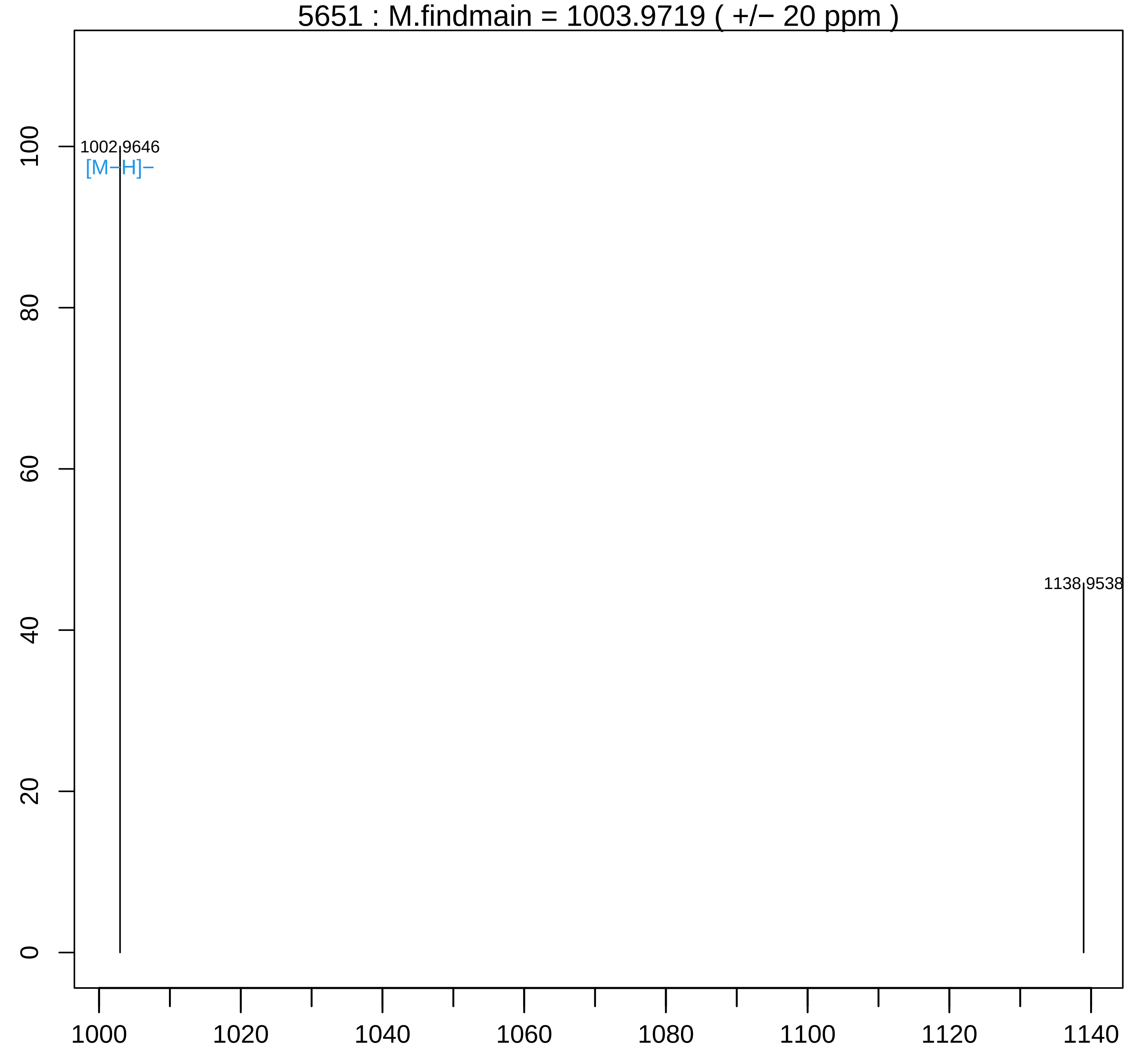
5651 : M.findmain = 1003.9719 (+/− 20 ppm)
1002.9646
[M−H]−
1138.9538
0
20
40
60
80
100
1000
1020
1040
1060
1080
1100
1120
1140

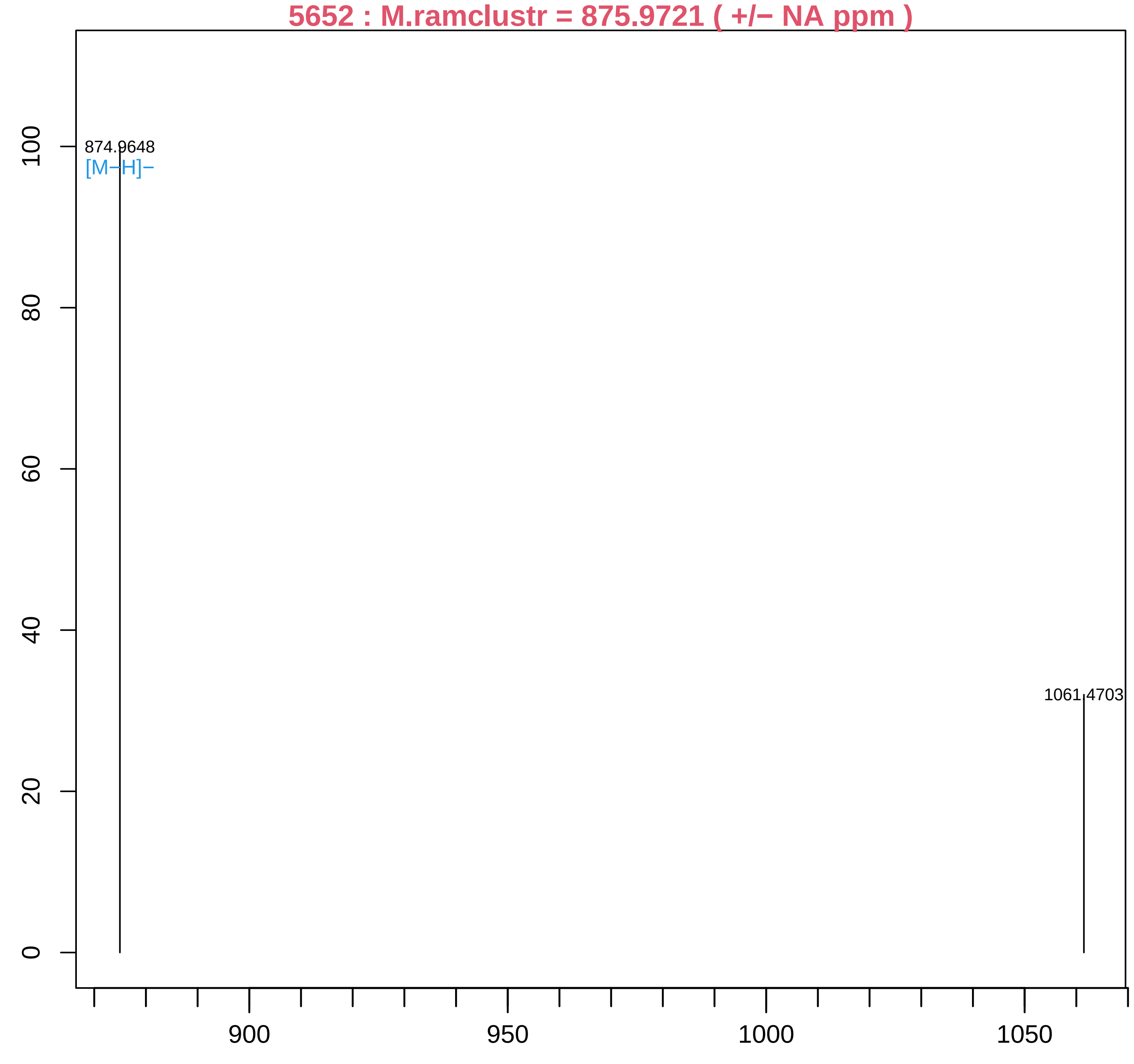
5652 : M.ramclustr = 875.9721 (+/− NA ppm)
874.9648
[M−H]−
1061.4703
0
20
40
60
80
100
900
950
1000
1050

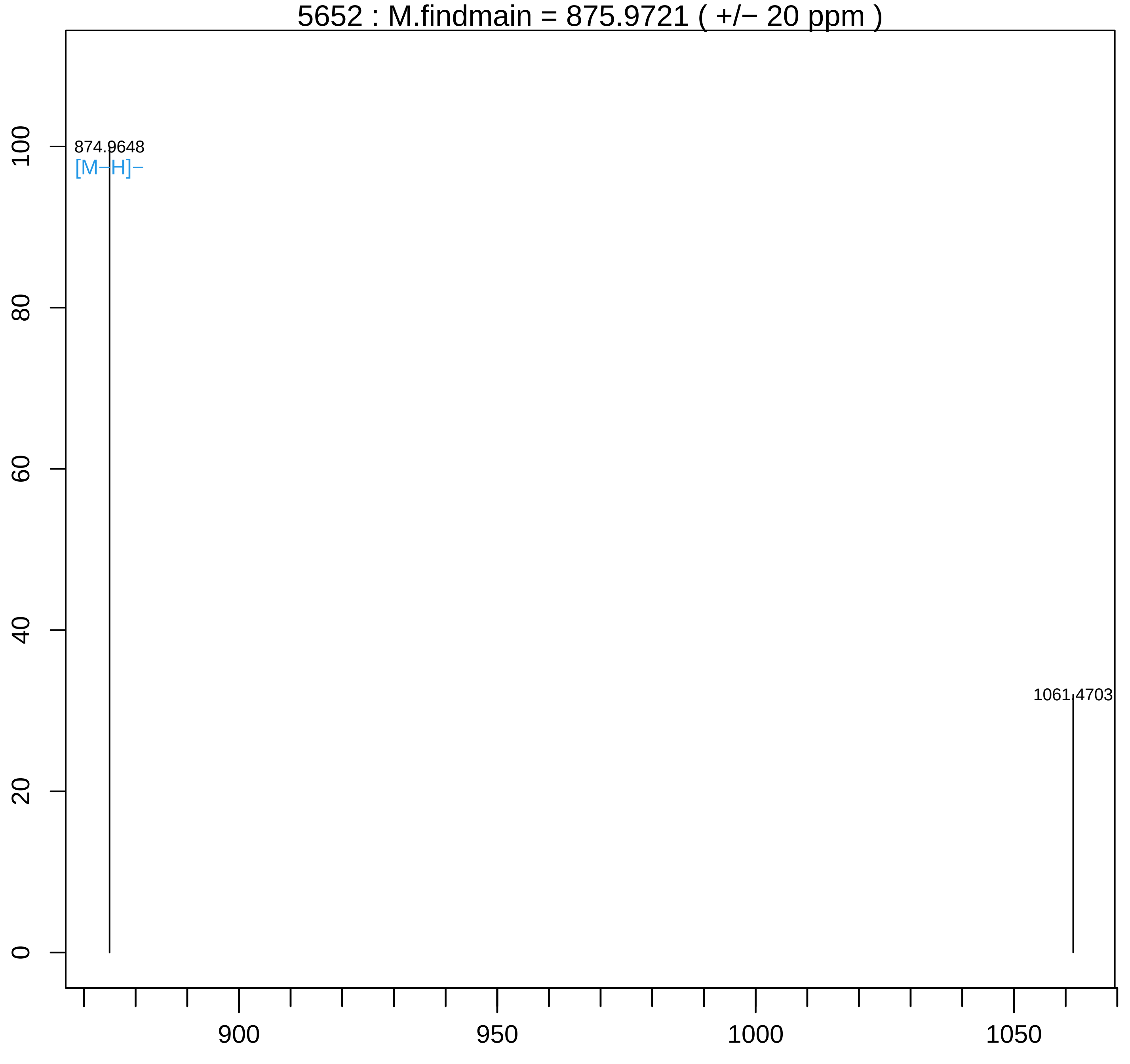
5652 : M.findmain = 875.9721 (+/− 20 ppm)
874.9648
[M−H]−
1061.4703
0
20
40
60
80
100
900
950
1000
1050

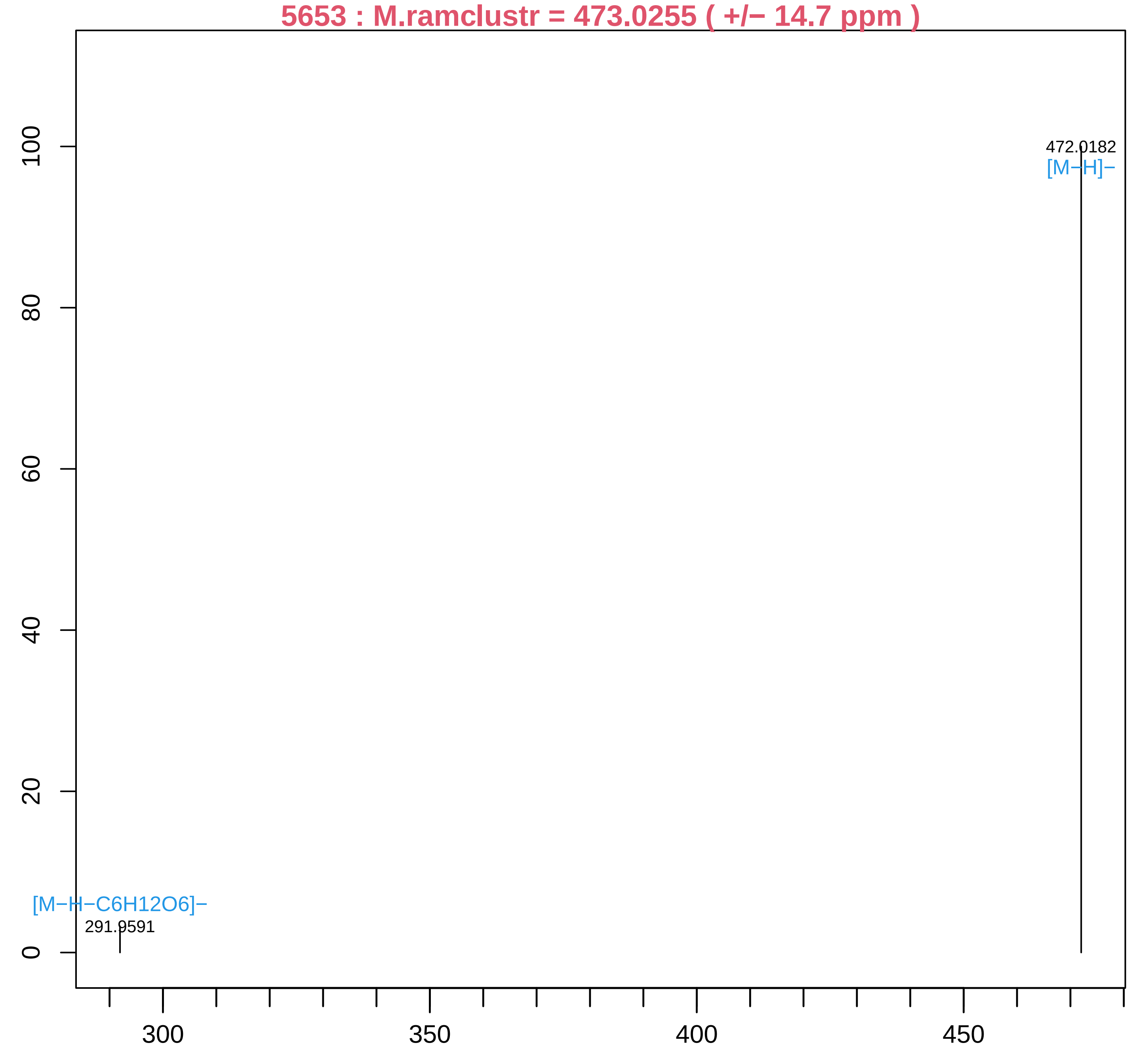
5653 : M.ramclustr = 473.0255 (+/− 14.7 ppm)
472.0182
[M−H]−
[M−H−C6H12O6]−
291.9591
0
20
40
60
80
100
300
350
400
450

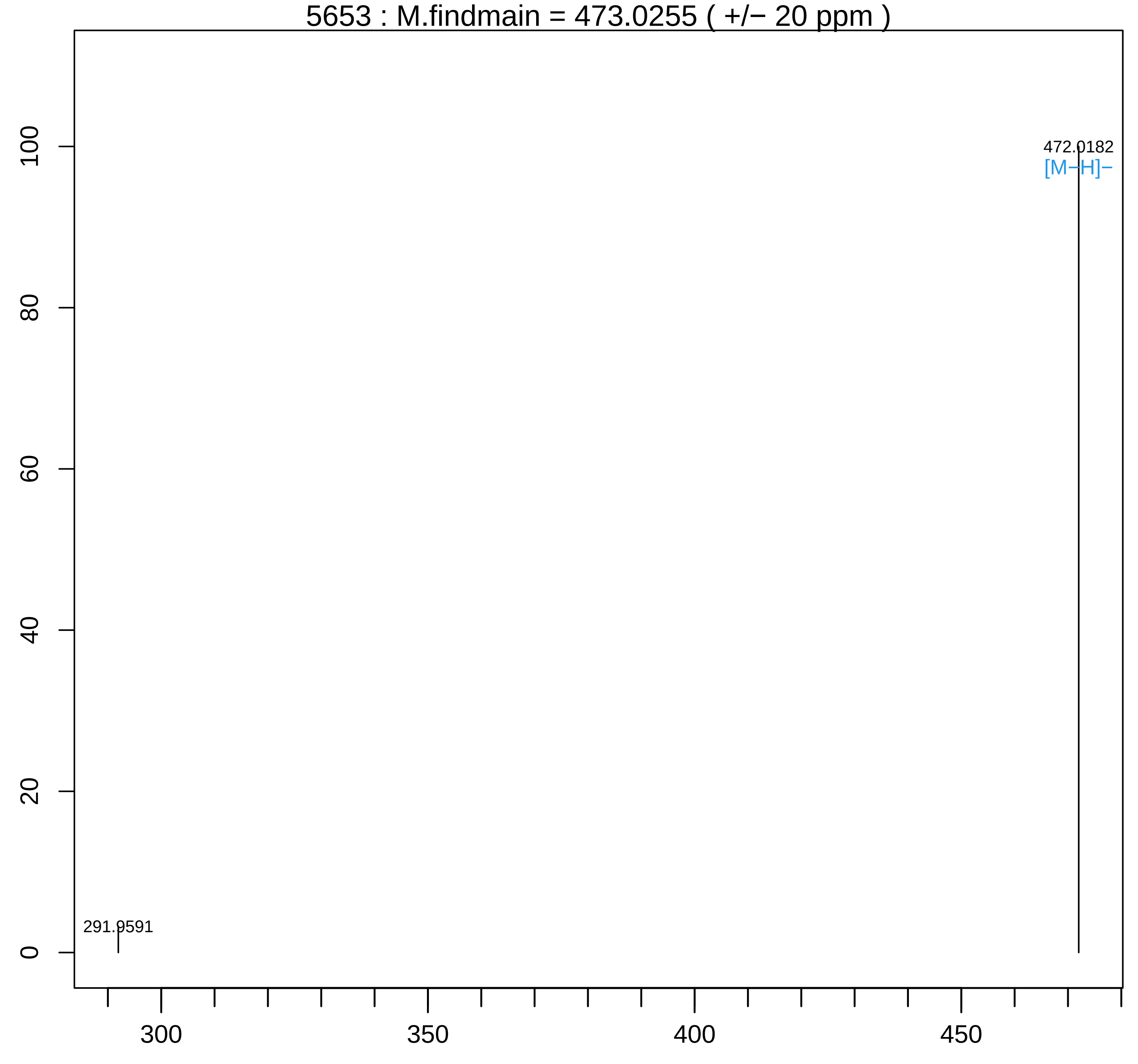
5653 : M.findmain = 473.0255 (+/− 20 ppm)
472.0182
[M−H]−
291.9591
0
20
40
60
80
100
300
350
400
450

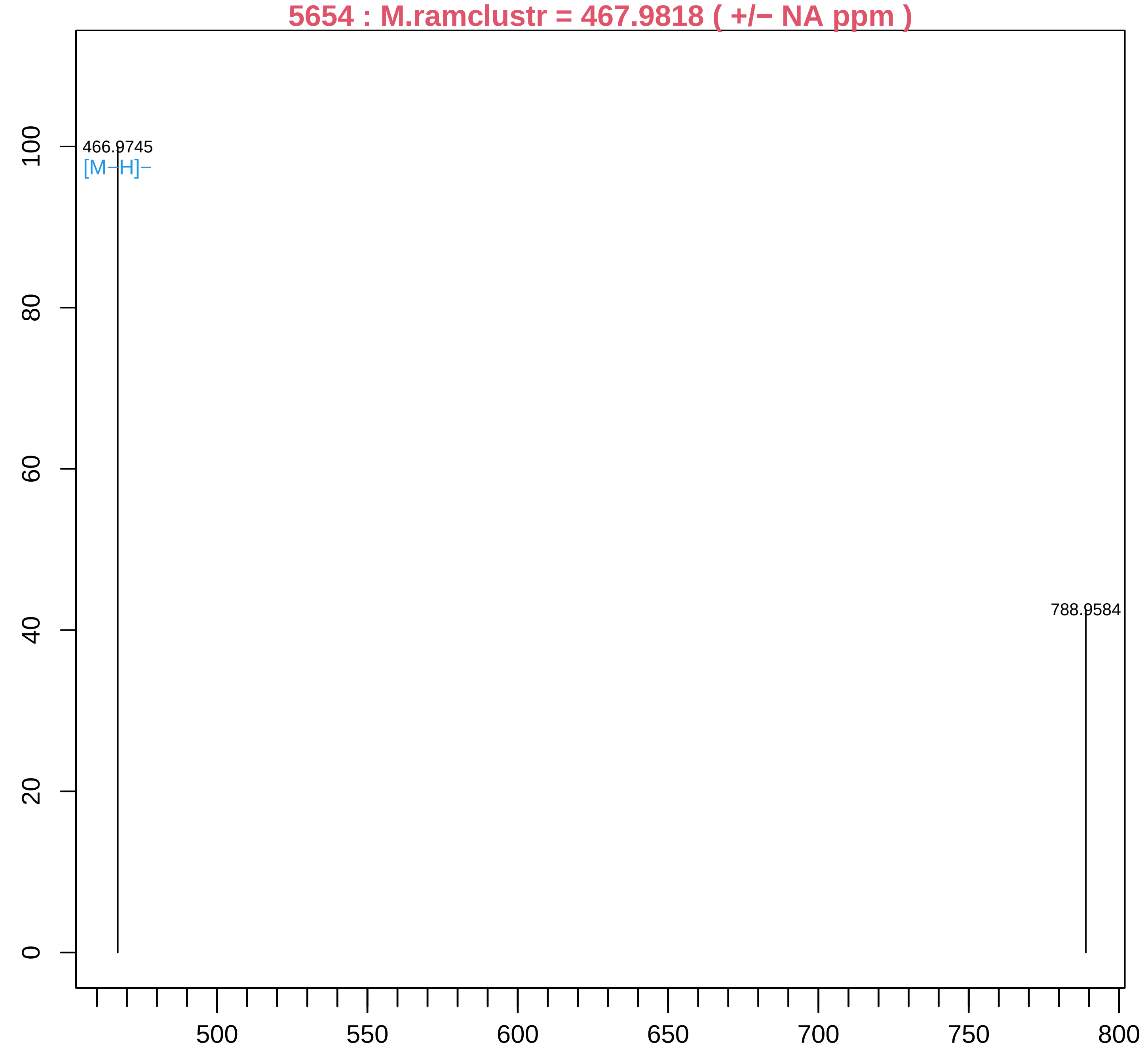
5654 : M.ramclustr = 467.9818 (+/− NA ppm)
466.9745
[M−H]−
788.9584
0
20
40
60
80
100
500
550
600
650
700
750
800

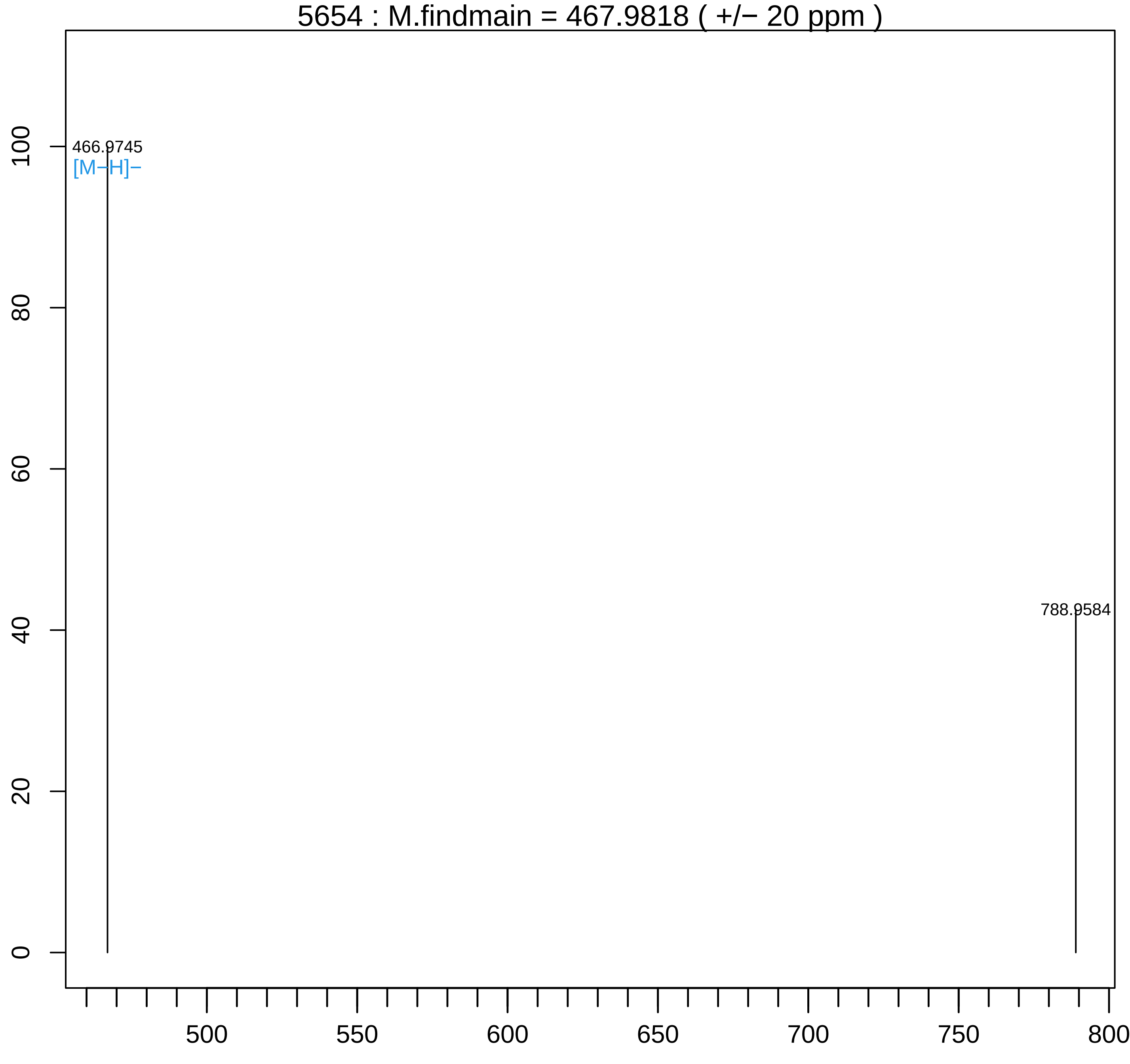
5654 : M.findmain = 467.9818 (+/− 20 ppm)
466.9745
[M−H]−
788.9584
0
20
40
60
80
100
500
550
600
650
700
750
800

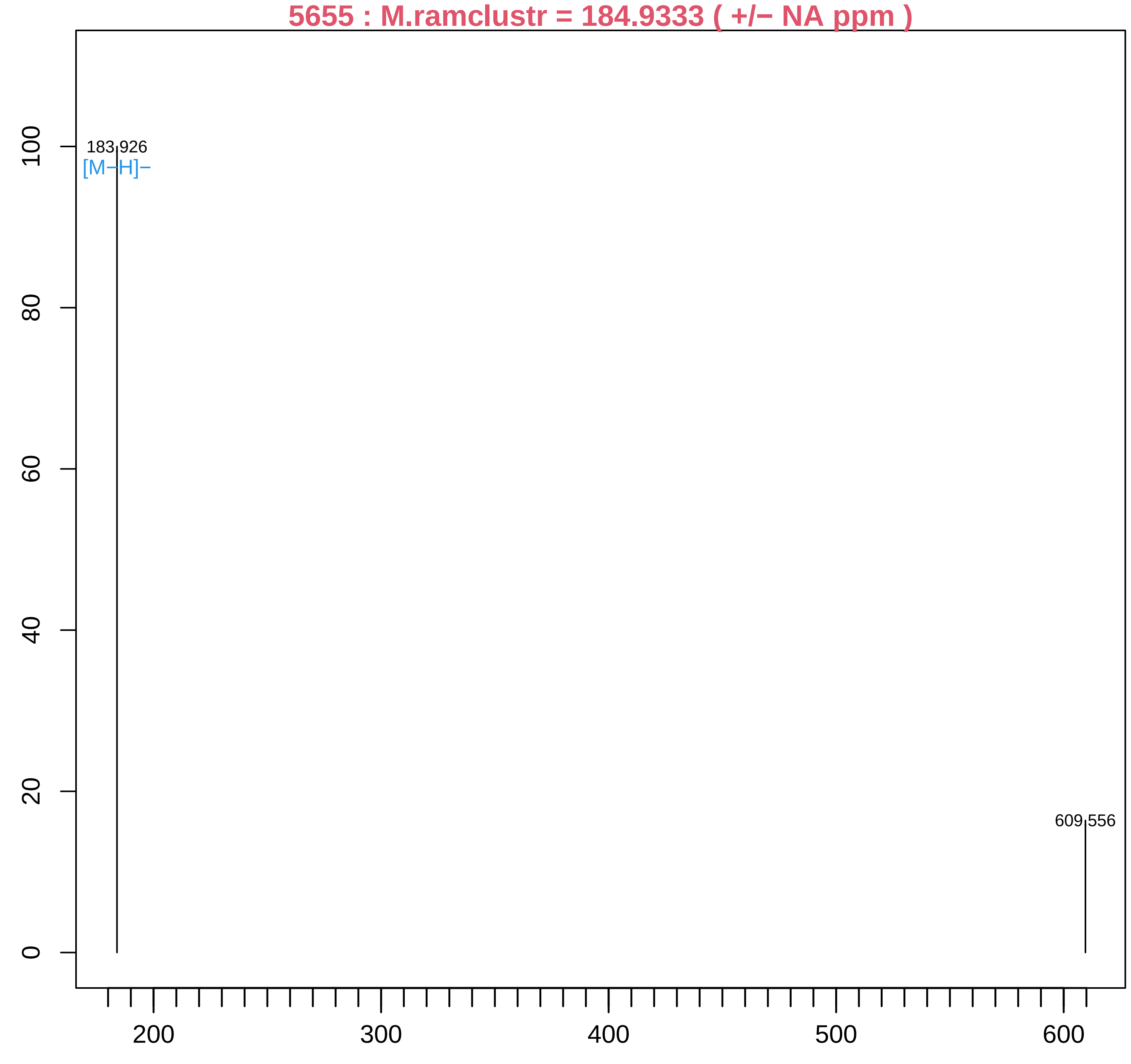

5655 : M.ramclustr = 184.9333 (+/− NA ppm)
183.926
[M−H]−
609.556
0
20
40
60
80
100
200
300
400
500
600

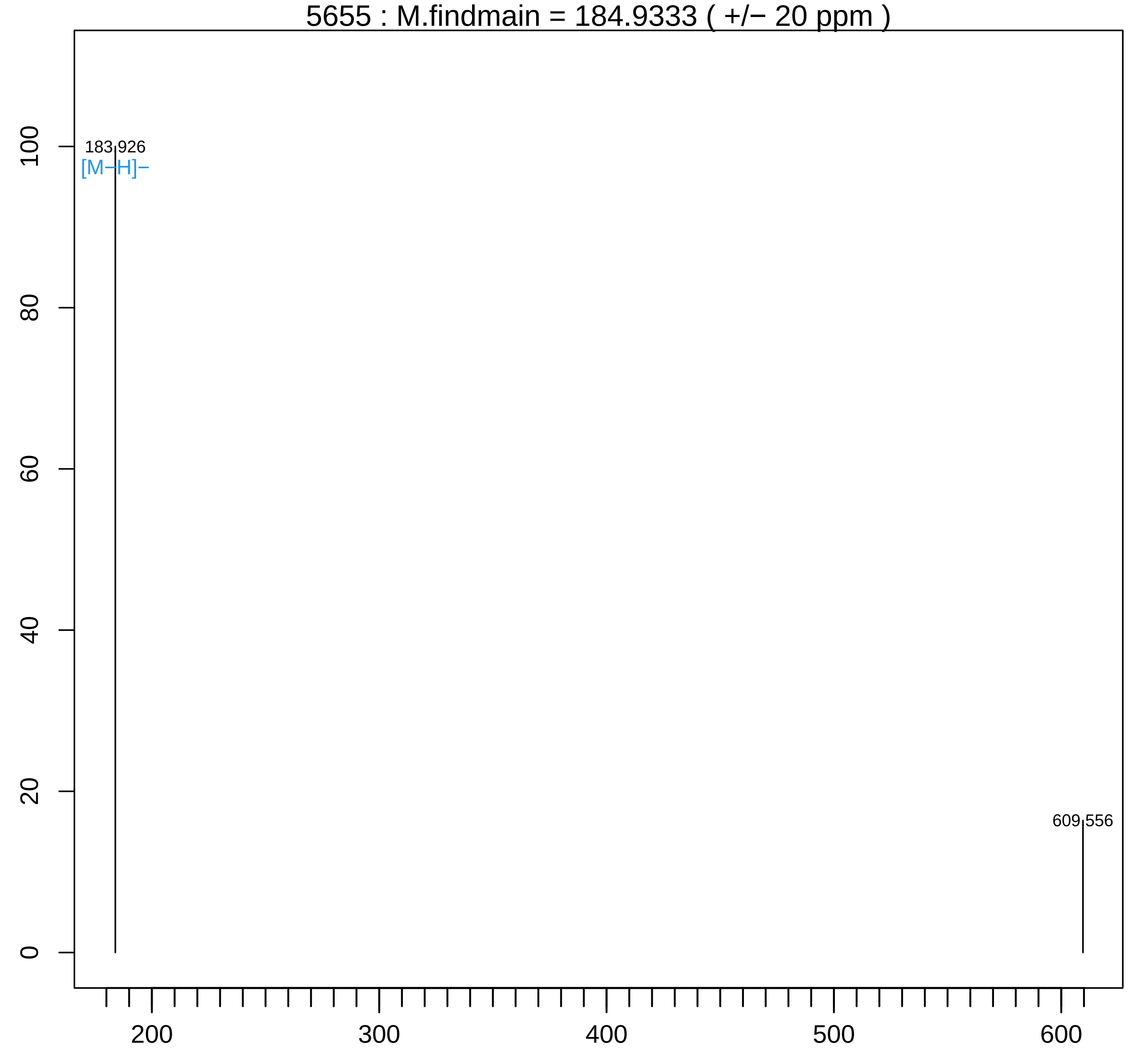

5655 : M.findmain = 184.9333 (+/− 20 ppm)
183.926
[M−H]−
609.556
0
20
40
60
80
100
200
300
400
500
600

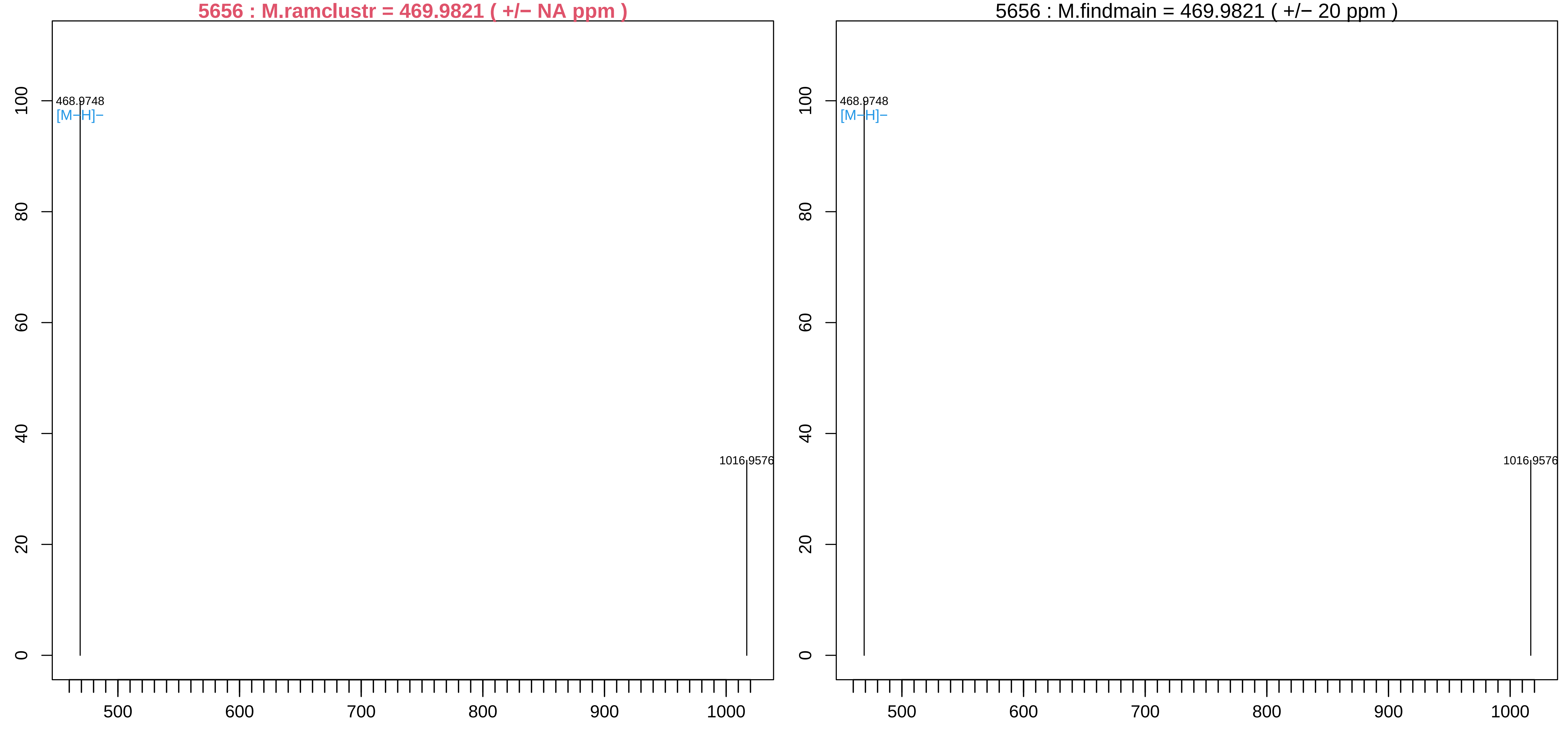
5656 : M.ramclustr = 469.9821 (+/− NA ppm)
468.9748
[M−H]−
1016.9576
0
20
40
60
80
100
500
600
700
800
900
1000
5656 : M.findmain = 469.9821 (+/− 20 ppm)
468.9748
[M−H]−
1016.9576
0
20
40
60
80
100
500
600
700
800
900
1000

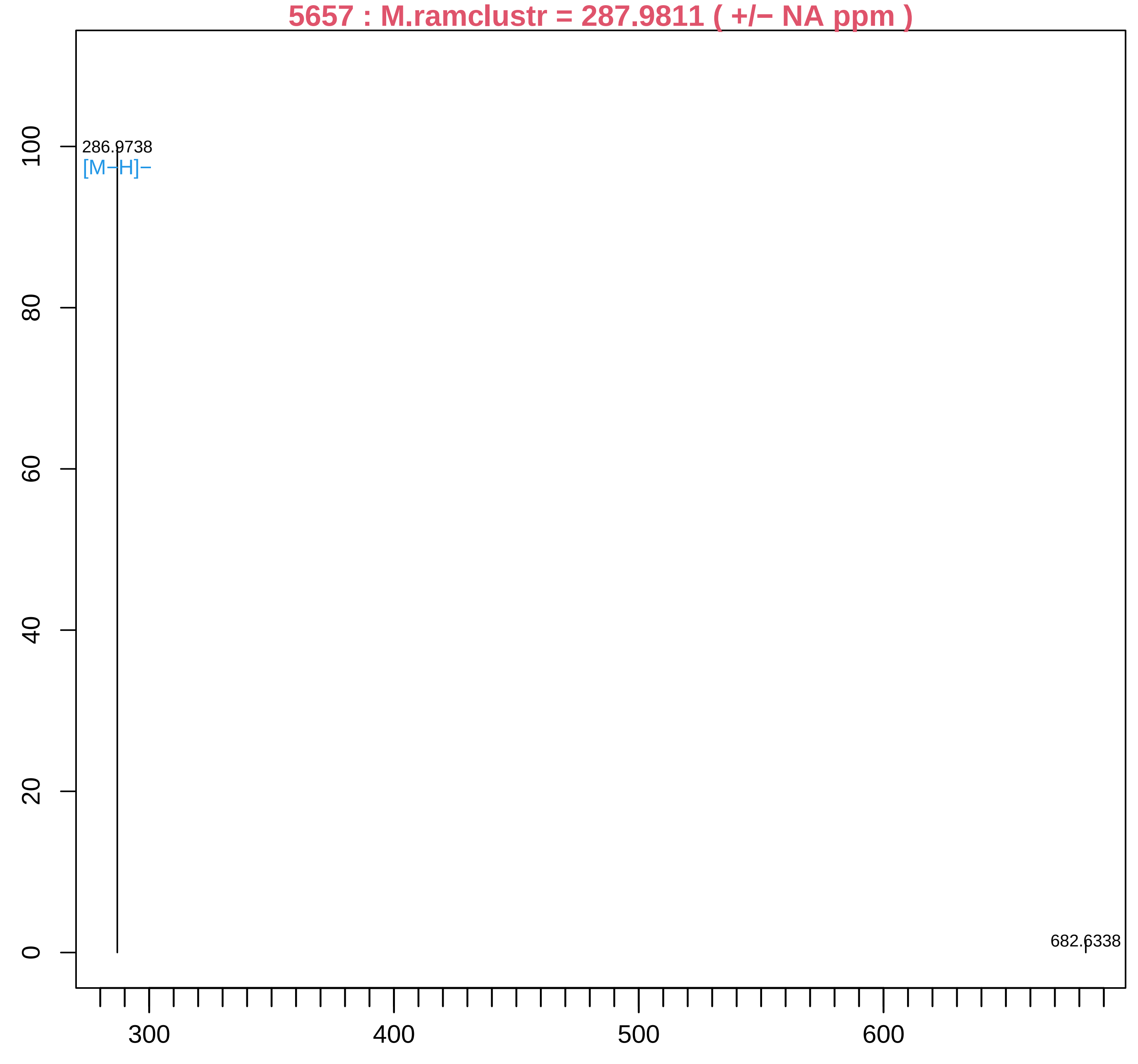
5657 : M.ramclustr = 287.9811 (+/− NA ppm)
286.9738
[M−H]−
682.6338
0
20
40
60
80
100
300
400
500
600

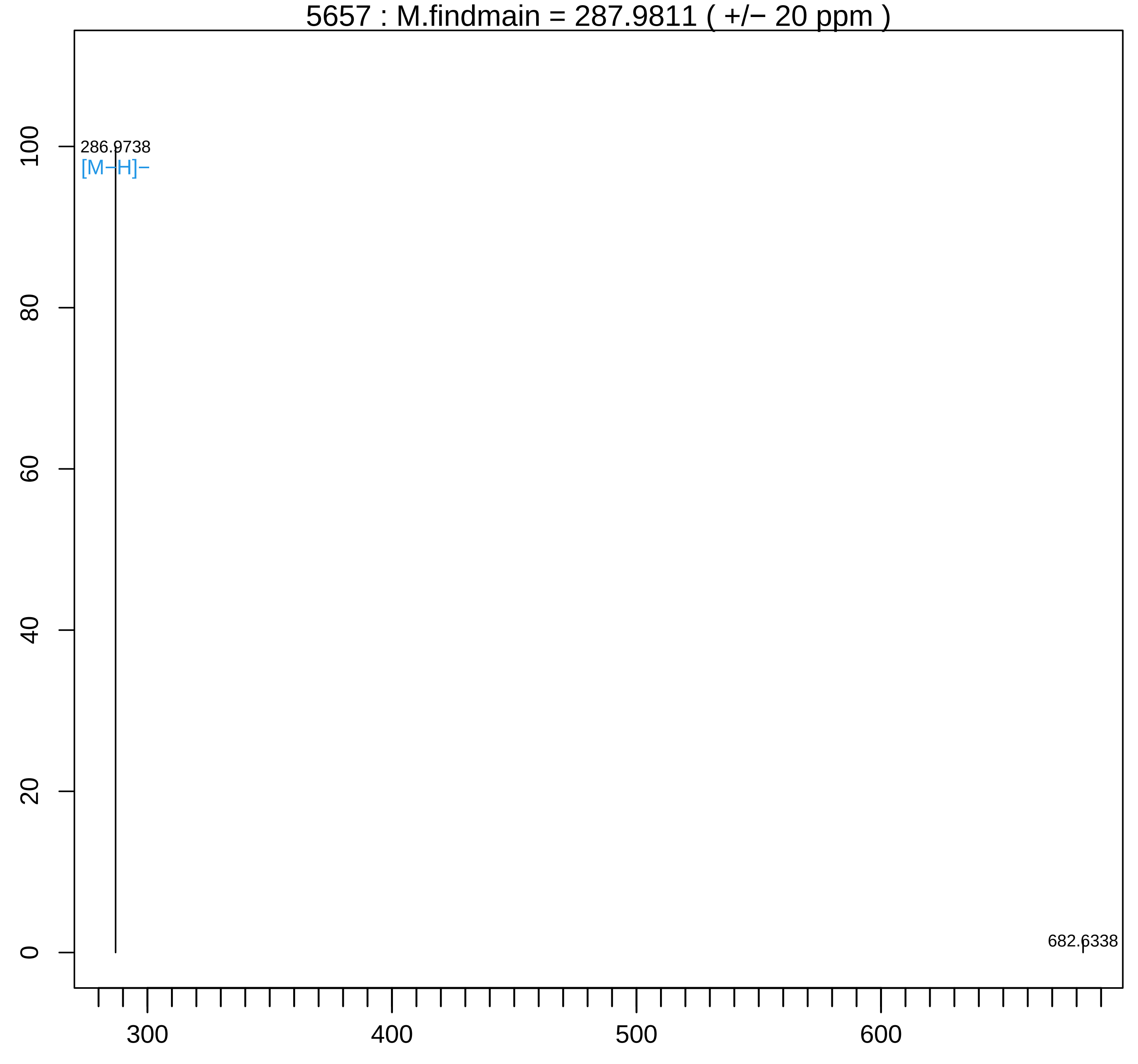
5657 : M.findmain = 287.9811 (+/− 20 ppm)
286.9738
[M−H]−
682.6338
0
20
40
60
80
100
300
400
500
600

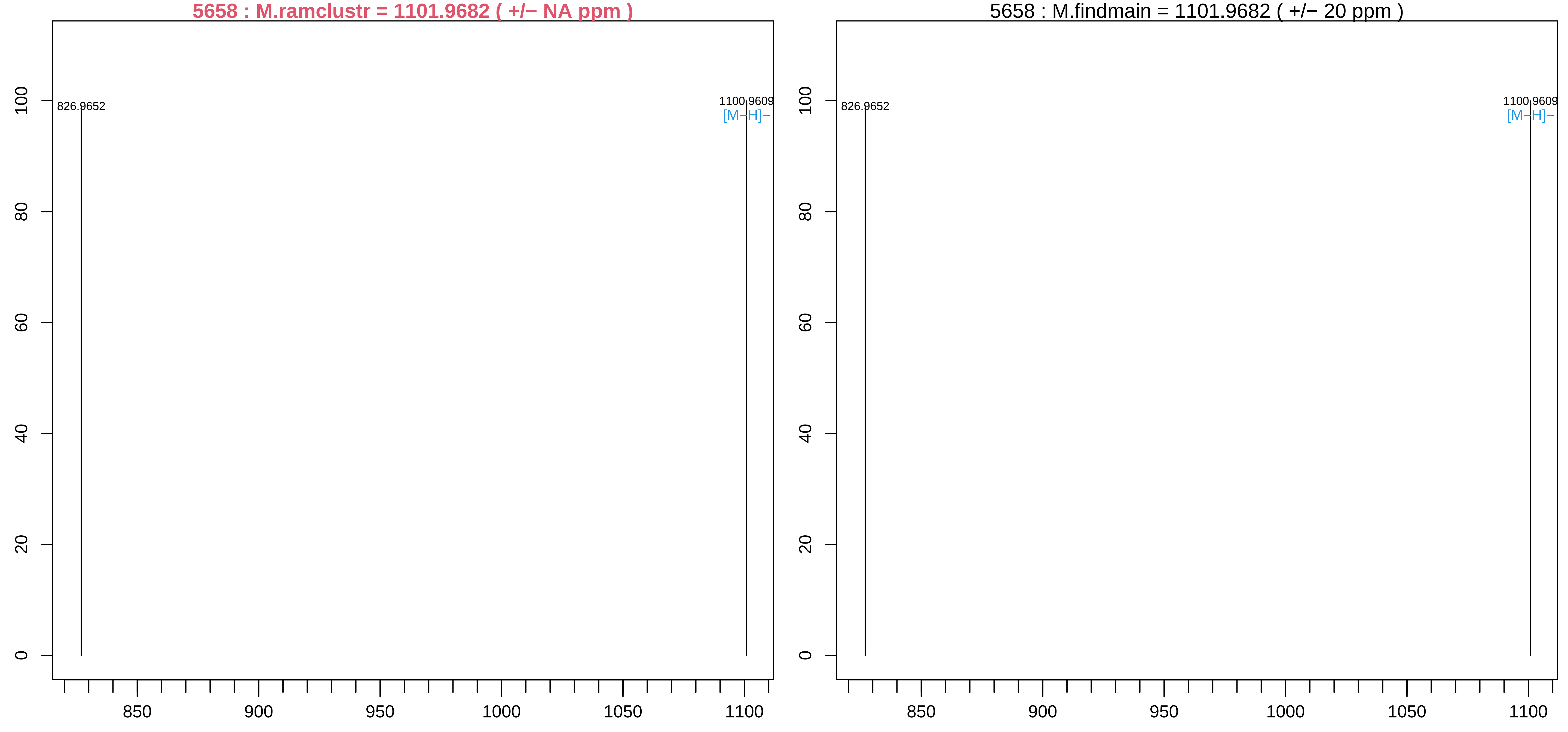

5658 : M.ramclustr = 1101.9682 (+/− NA ppm)
826.9652
1100.9609
[M−H]−
0
20
40
60
80
100
850
900
950
1000
1050
1100
5658 : M.findmain = 1101.9682 (+/− 20 ppm)
826.9652
1100.9609
[M−H]−
0
20
40
60
80
100
850
900
950
1000
1050
1100

5659 : M.ramclustr = 543.9774 (+/− NA ppm)

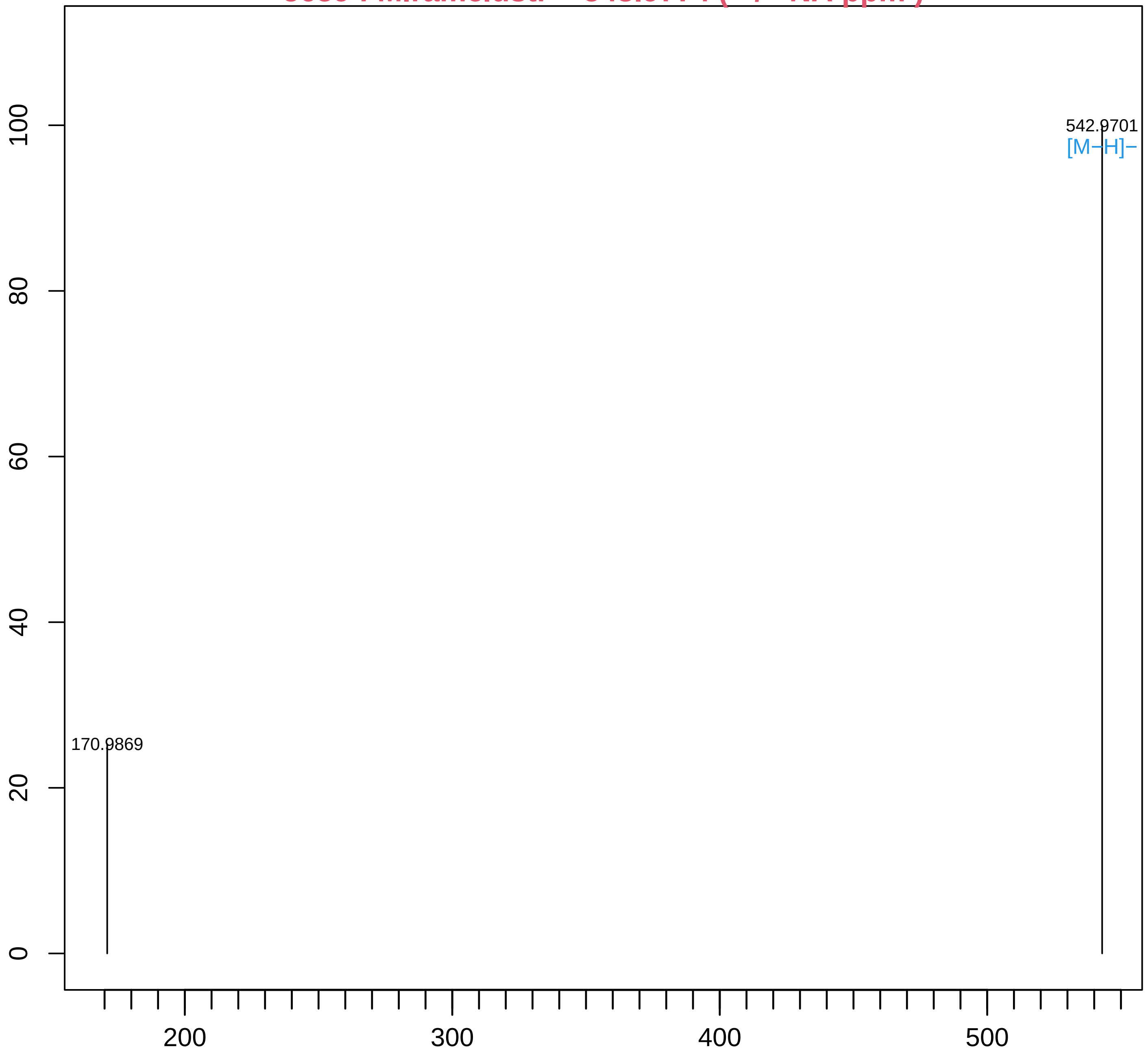

5659 : M.findmain = 543.9774 (+/− 20 ppm)

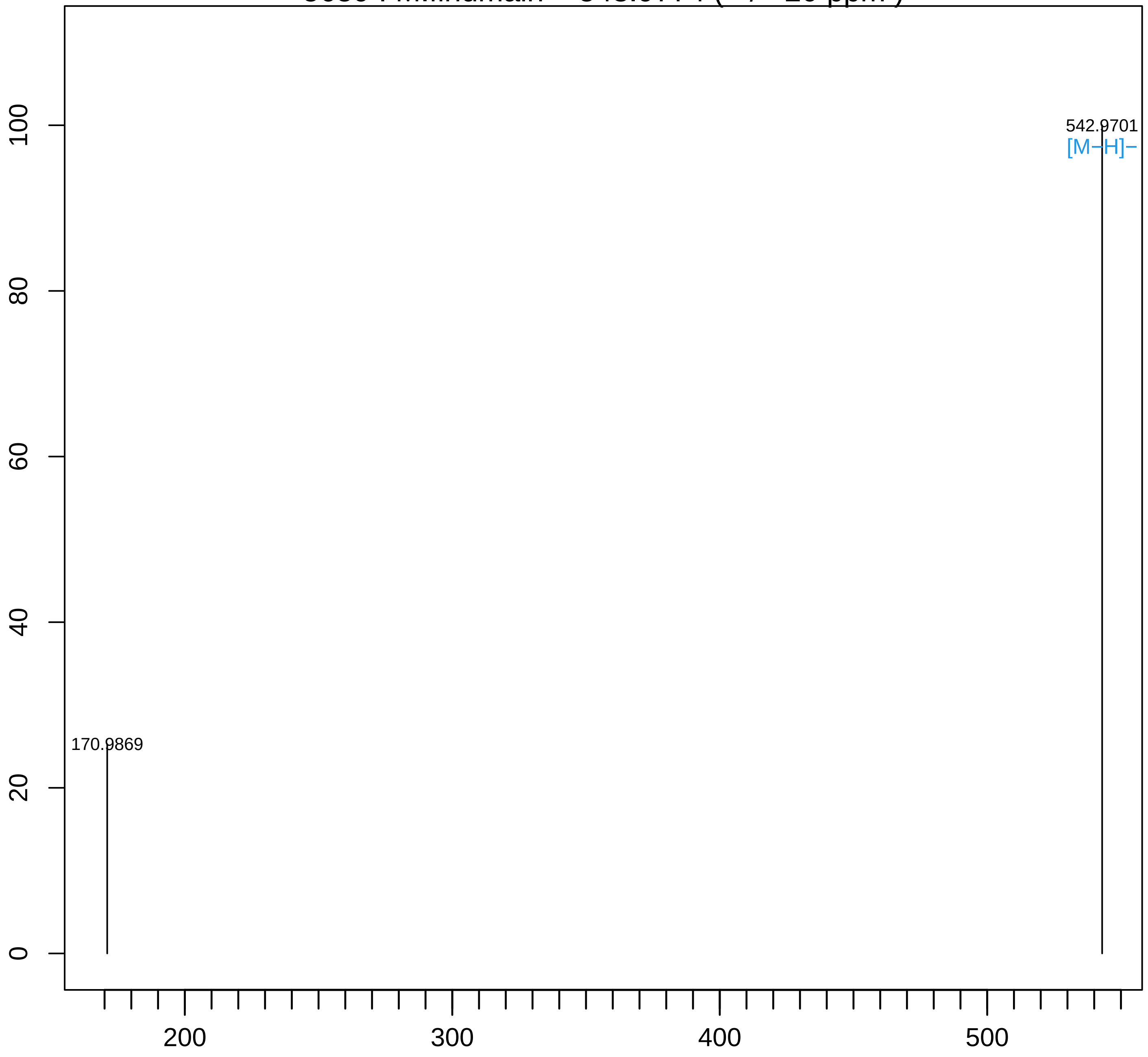

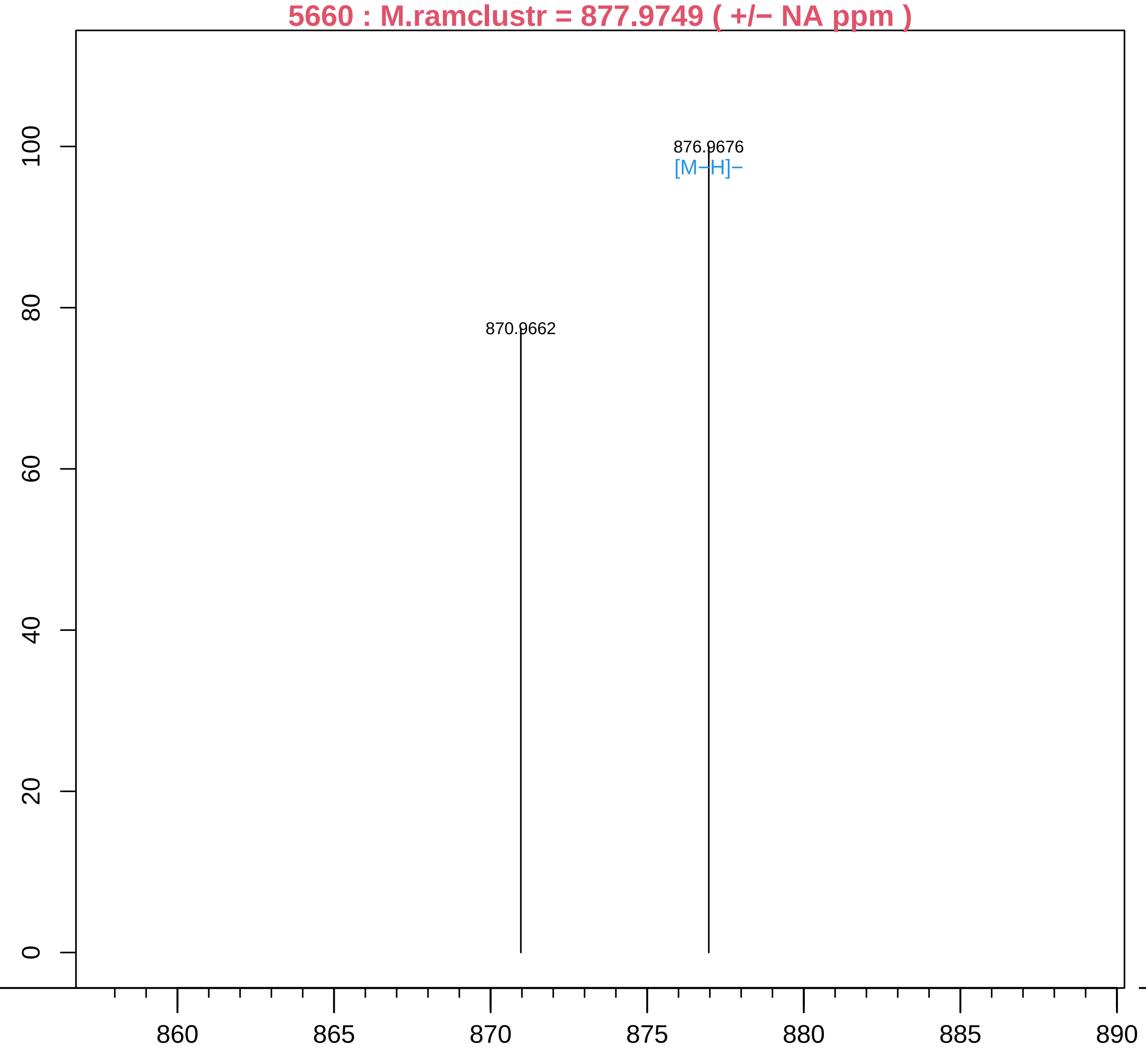
5660 : M.ramclustr = 877.9749 (+/− NA ppm)
876.9676
[M−H]−
870.9662
0
20
40
60
80
100
860
865
870
875
880
885
890

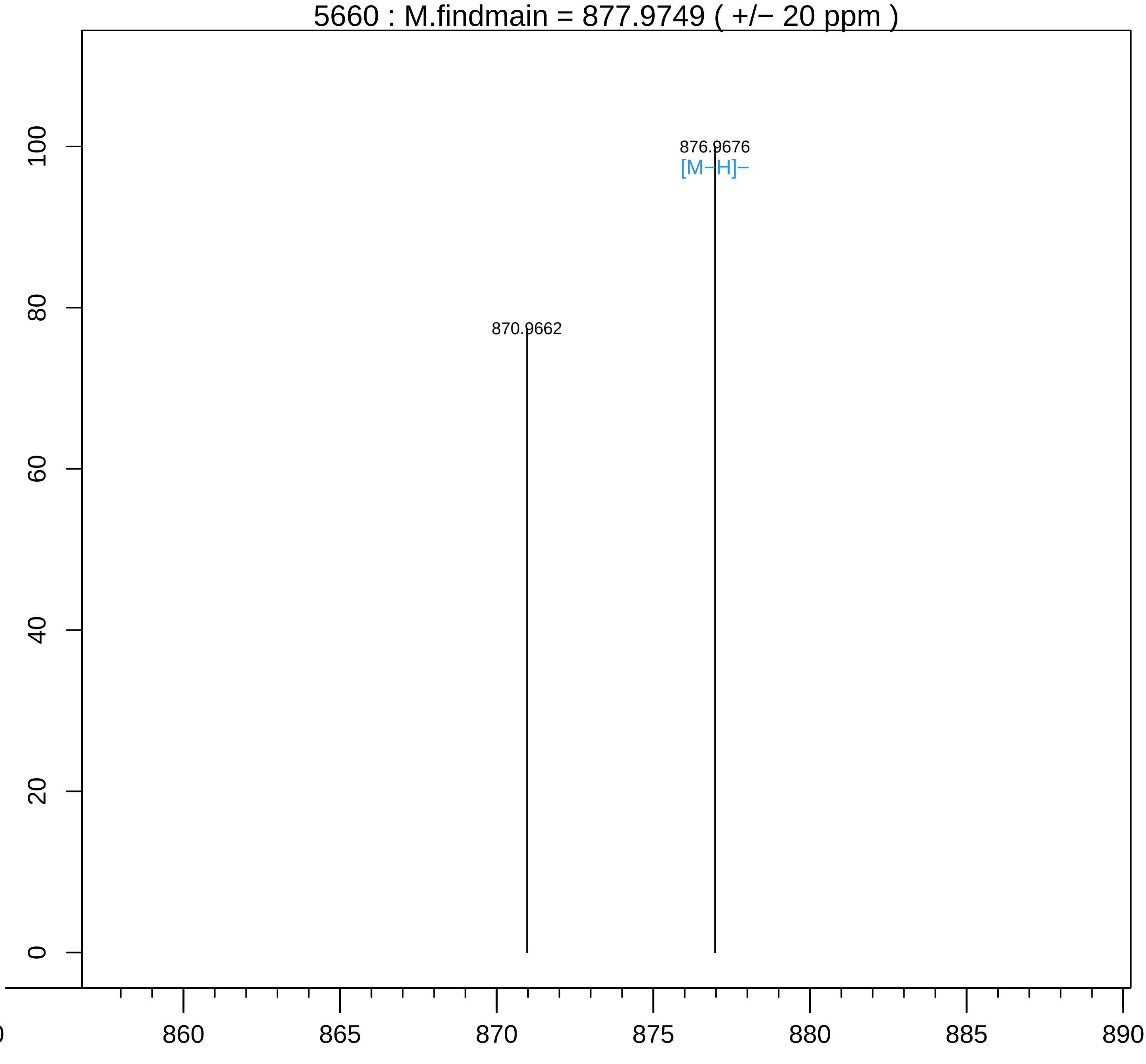
5660 : M.findmain = 877.9749 (+/− 20 ppm)
876.9676
[M−H]−
870.9662
0
20
40
60
80
100
860
865
870
875
880
885
890

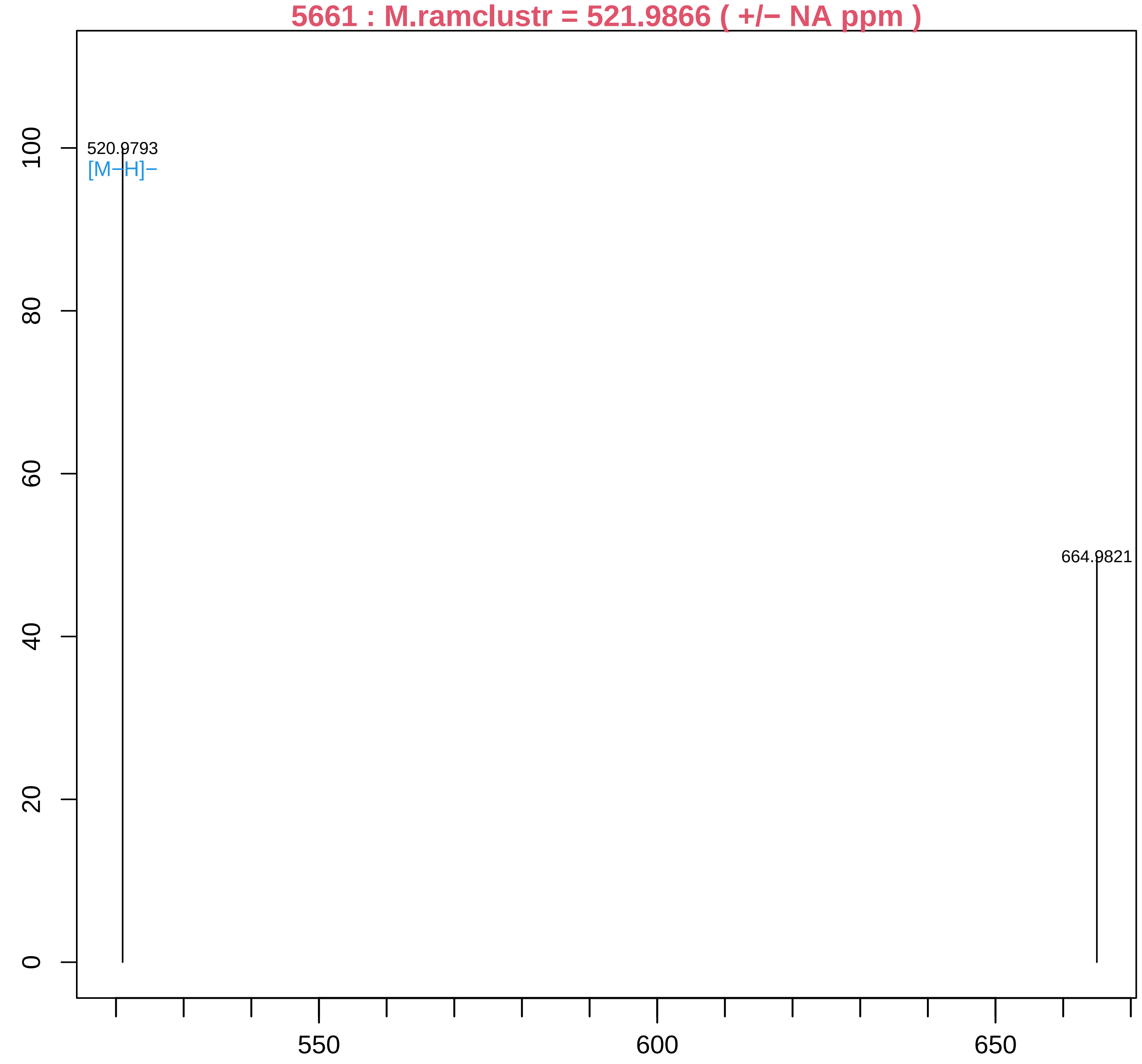

5661 : M.ramclustr = 521.9866 (+/− NA ppm)
520.9793
[M−H]−
664.9821
0
20
40
60
80
100
550
600
650

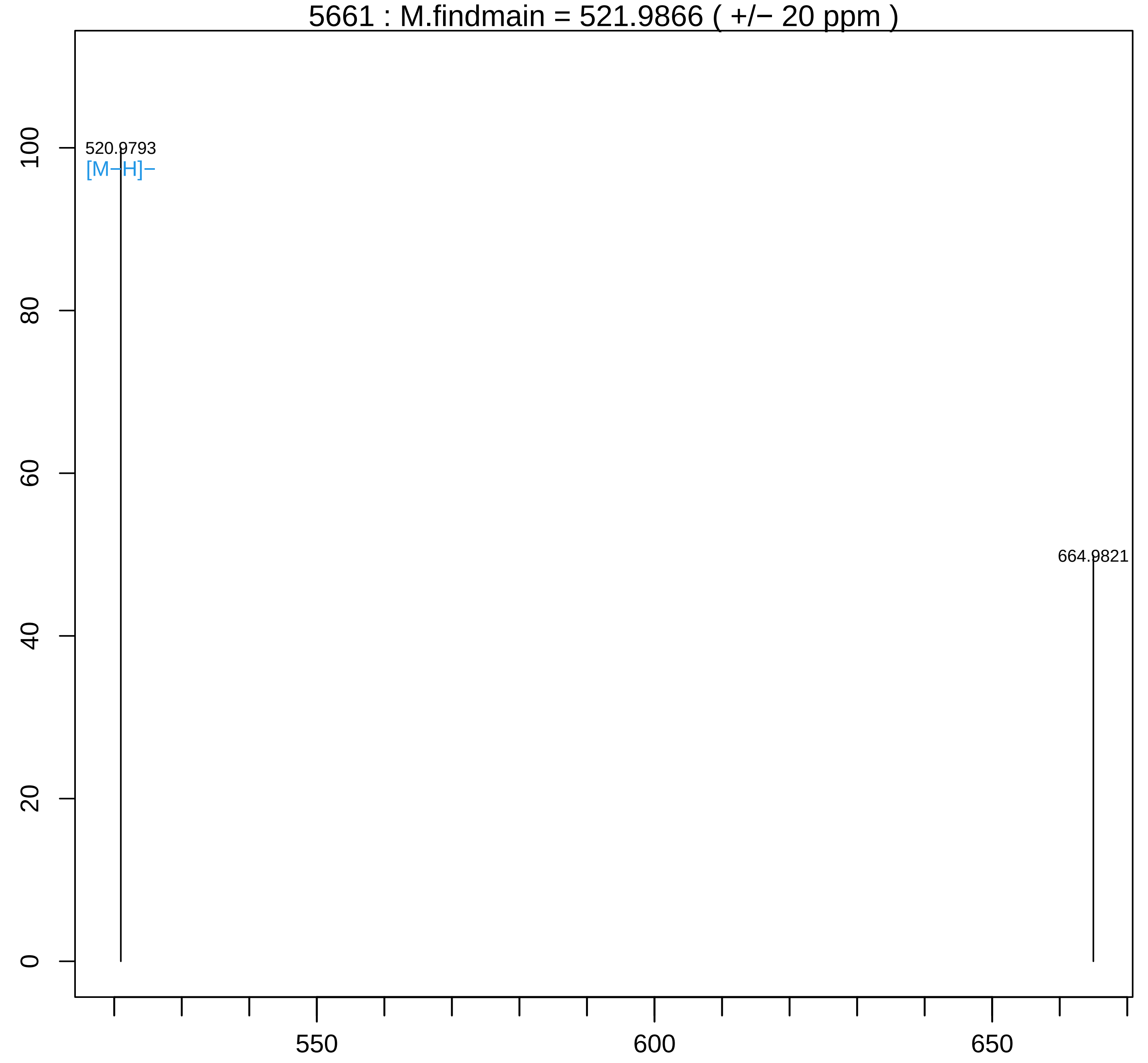

5661 : M.findmain = 521.9866 (+/− 20 ppm)
520.9793
[M−H]−
664.9821
0
20
40
60
80
100
550
600
650

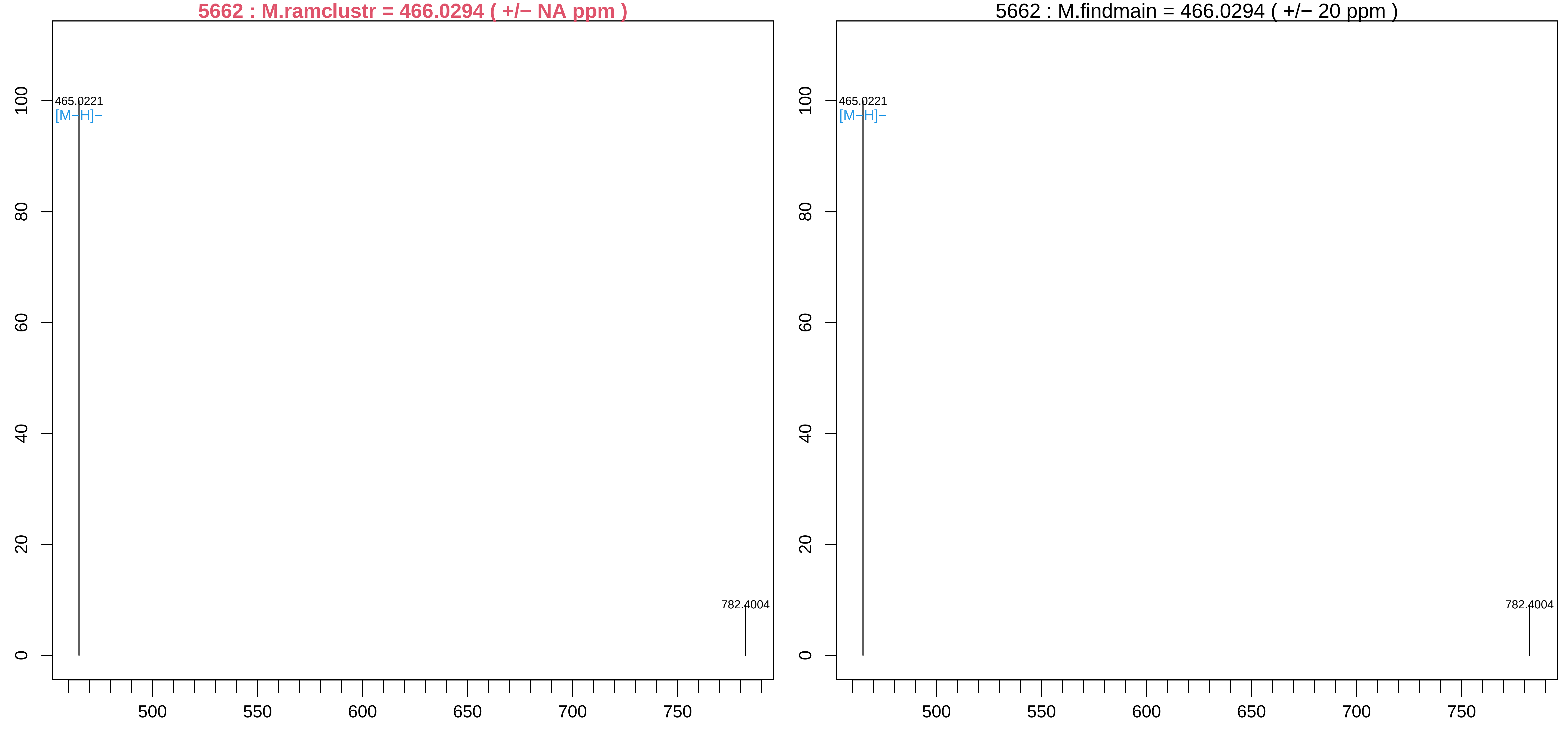
5662 : M.ramclustr = 466.0294 (+/− NA ppm)
465.0221
[M−H]−
782.4004
5662 : M.findmain = 466.0294 (+/− 20 ppm)
465.0221
[M−H]−
782.4004
0
20
40
60
80
100
500
550
600
650
700
750

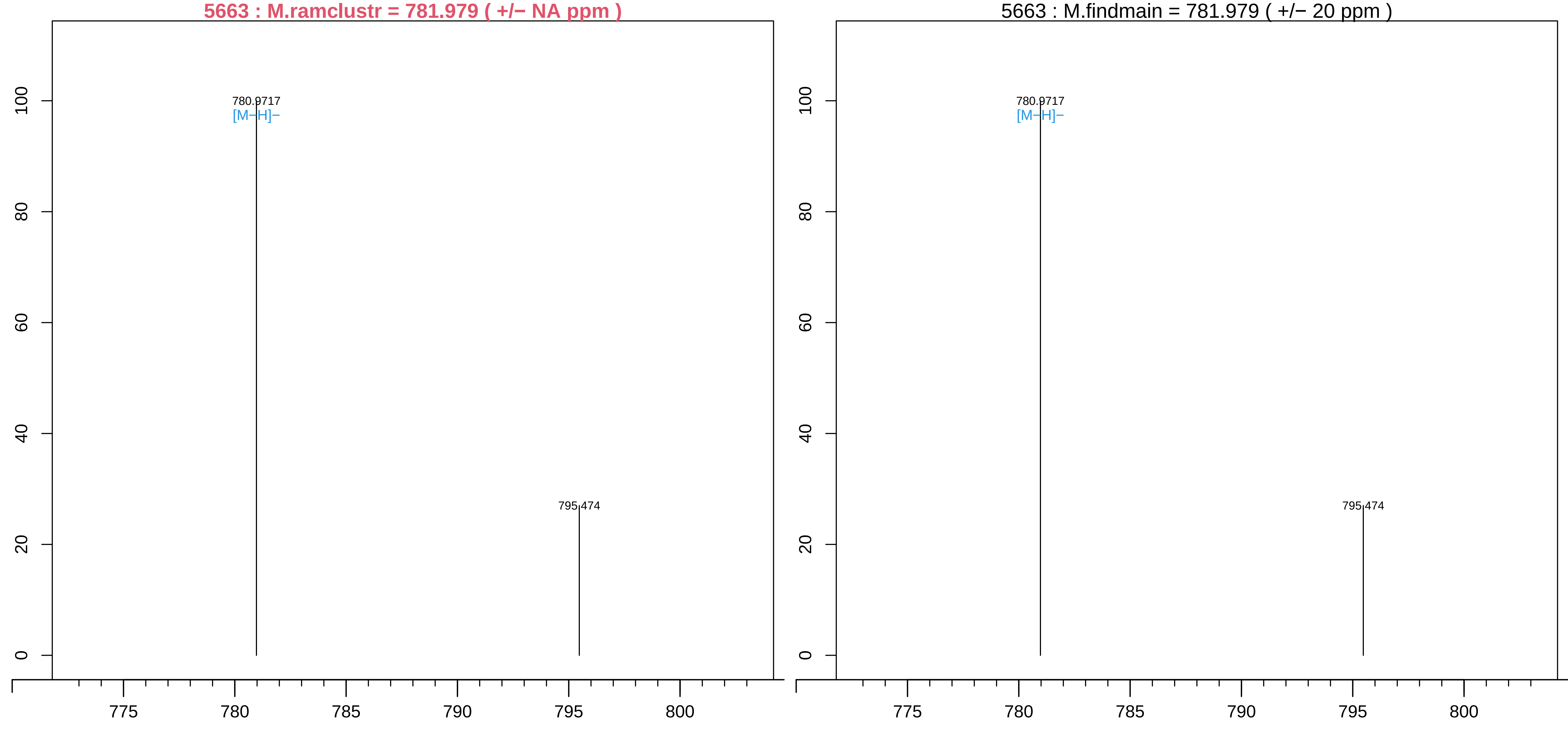
5663 : M.ramclustr = 781.979 (+/− NA ppm)
5663 : M.findmain = 781.979 (+/− 20 ppm)
780.9717
[M−H]−
795.474
0
20
40
60
80
100
775
780
785
790
795
800

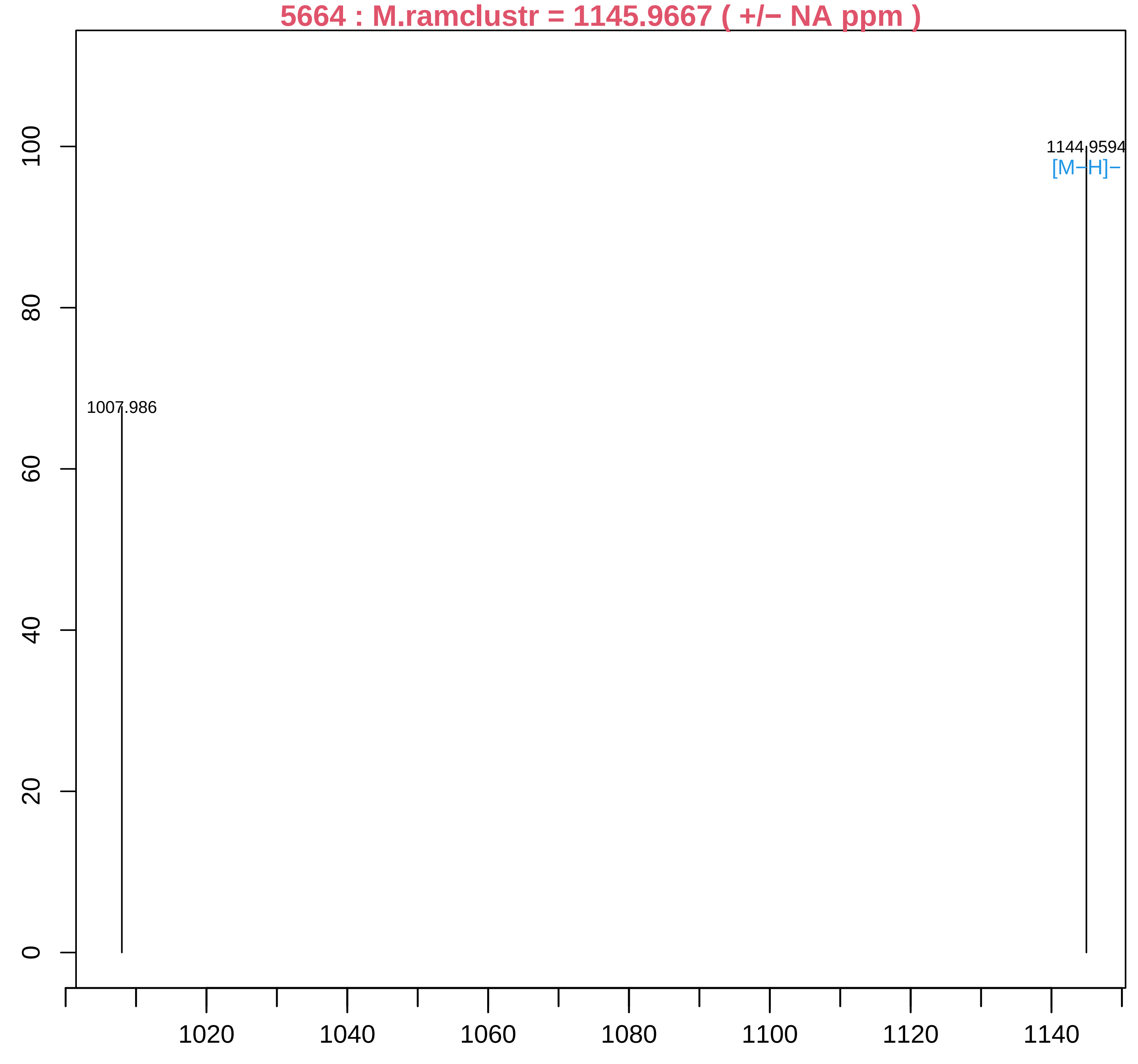
5664 : M.ramclustr = 1145.9667 (+/− NA ppm)
1144.9594
[M−H]−
1007.986
0
20
40
60
80
100
1020
1040
1060
1080
1100
1120
1140

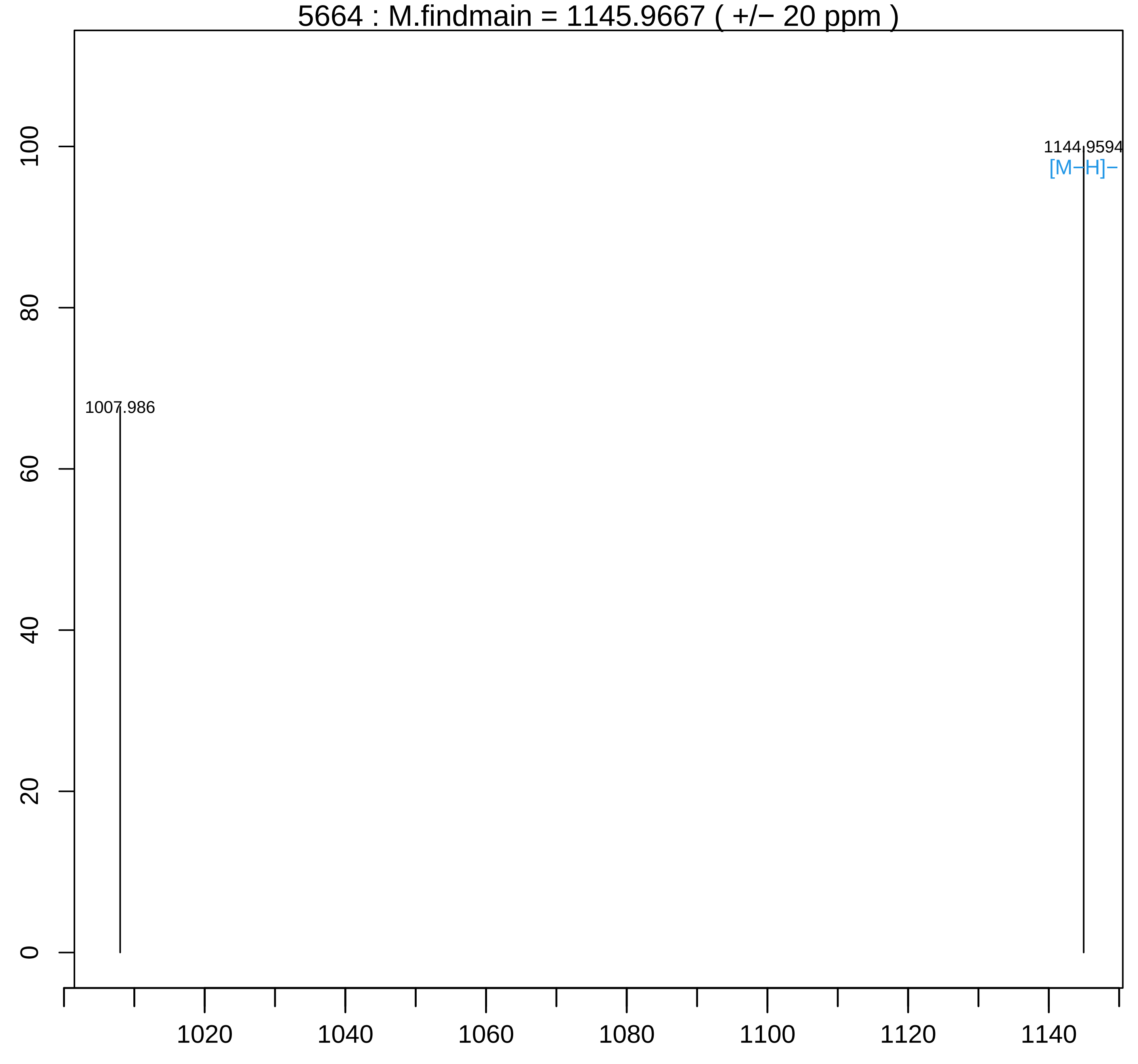
5664 : M.findmain = 1145.9667 (+/− 20 ppm)
1144.9594
[M−H]−
1007.986
0
20
40
60
80
100
1020
1040
1060
1080
1100
1120
1140

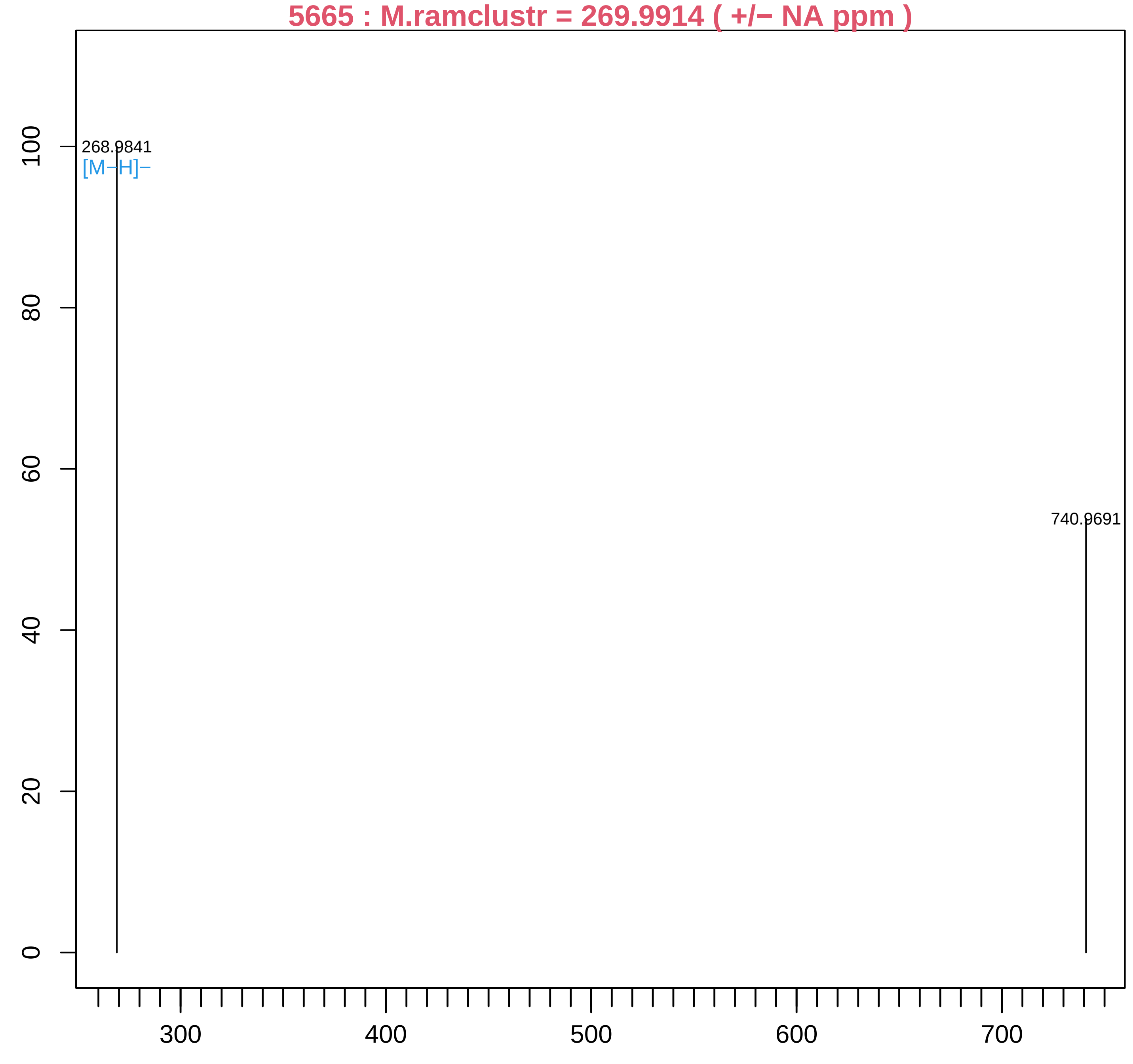

5665 : M.ramclustr = 269.9914 (+/− NA ppm)
268.9841
[M−H]−
740.9691

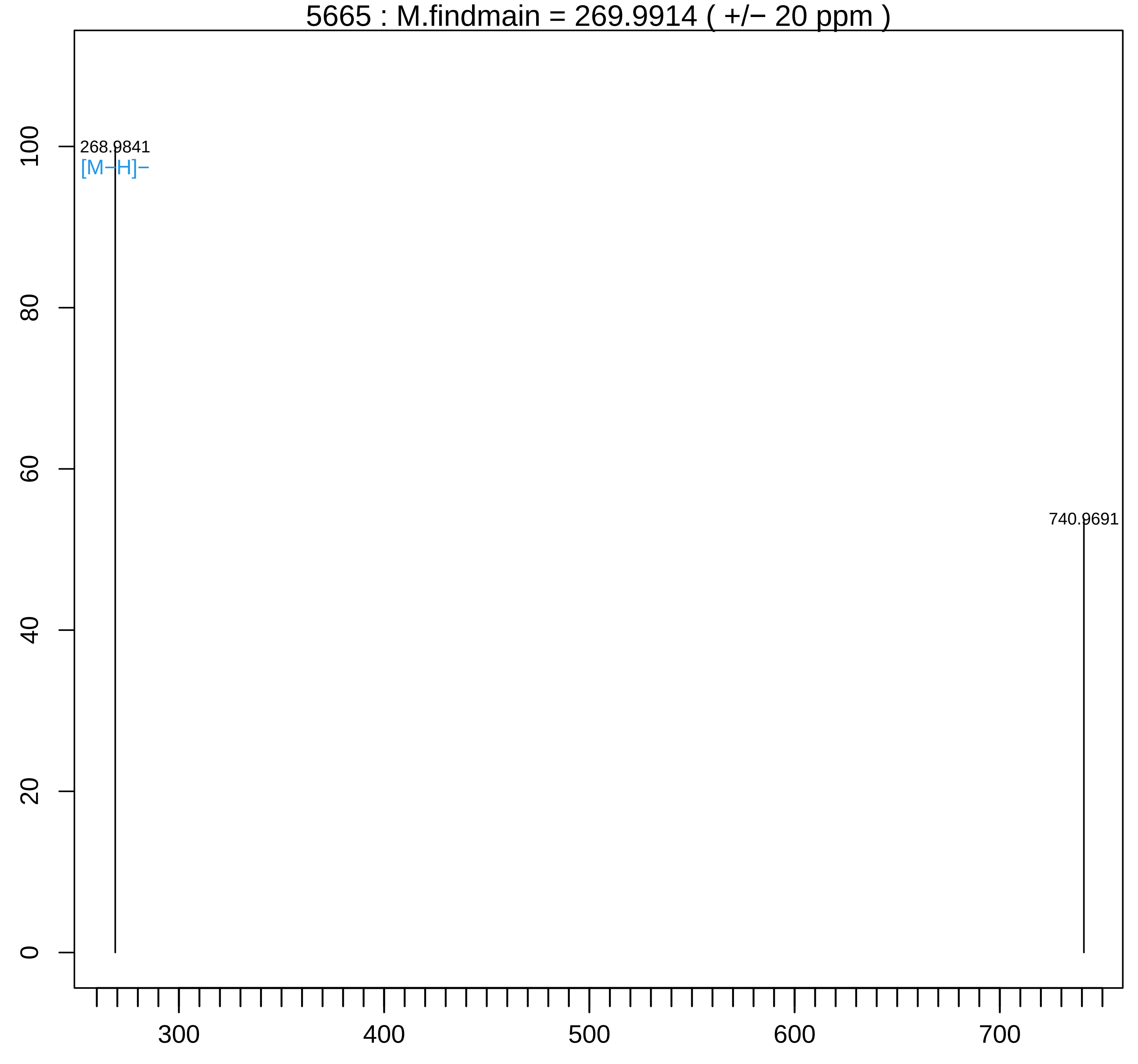

5665 : M.findmain = 269.9914 (+/− 20 ppm)
268.9841
[M−H]−
740.9691

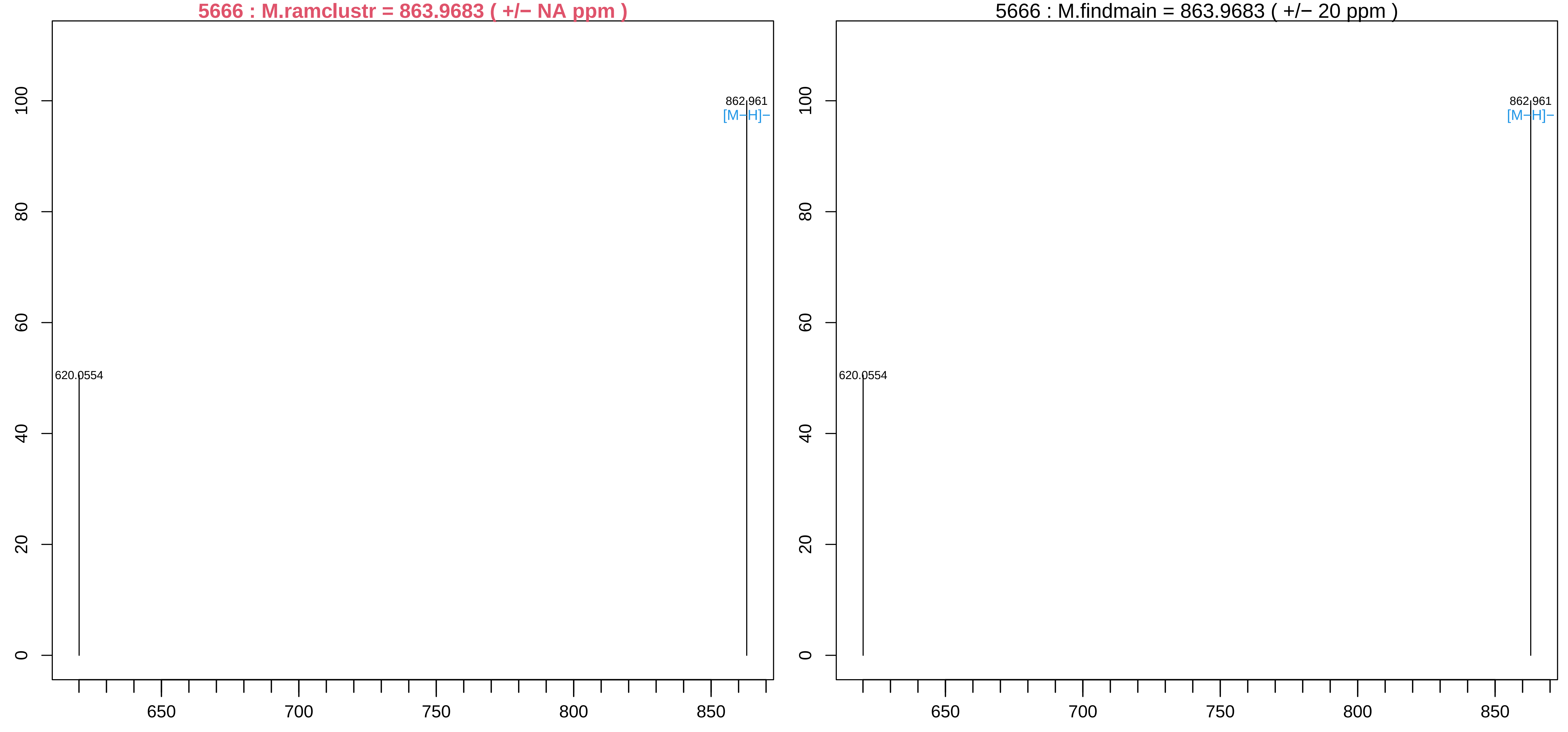
5666 : M.ramclustr = 863.9683 (+/− NA ppm)
862.961
[M−H]−
620.0554
5666 : M.findmain = 863.9683 (+/− 20 ppm)
862.961
[M−H]−
620.0554
0
20
40
60
80
100
650
700
750
800
850

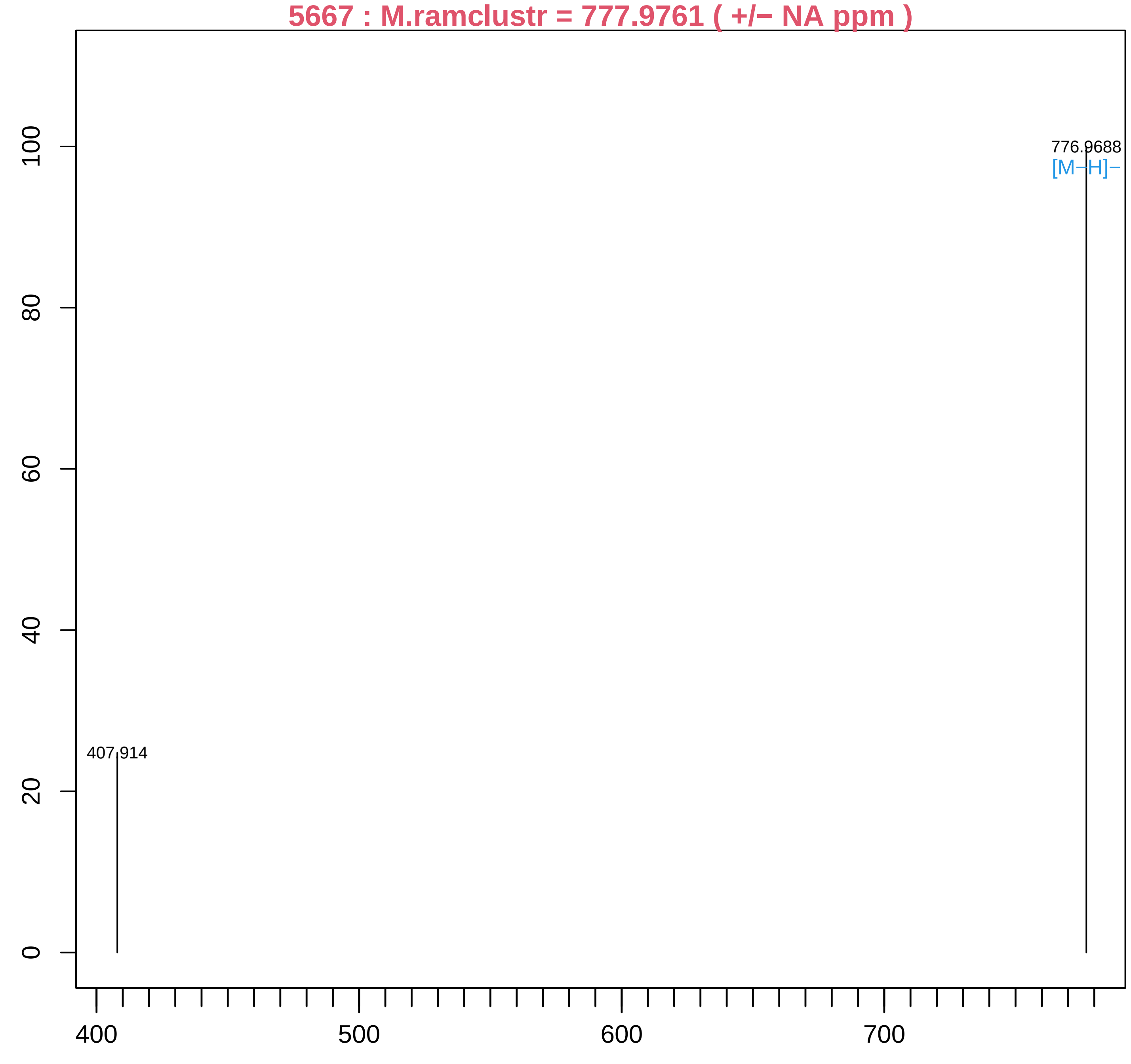

5667 : M.ramclustr = 777.9761 (+/− NA ppm)
776.9688
[M−H]−
407.914
0
20
40
60
80
100
400
500
600
700

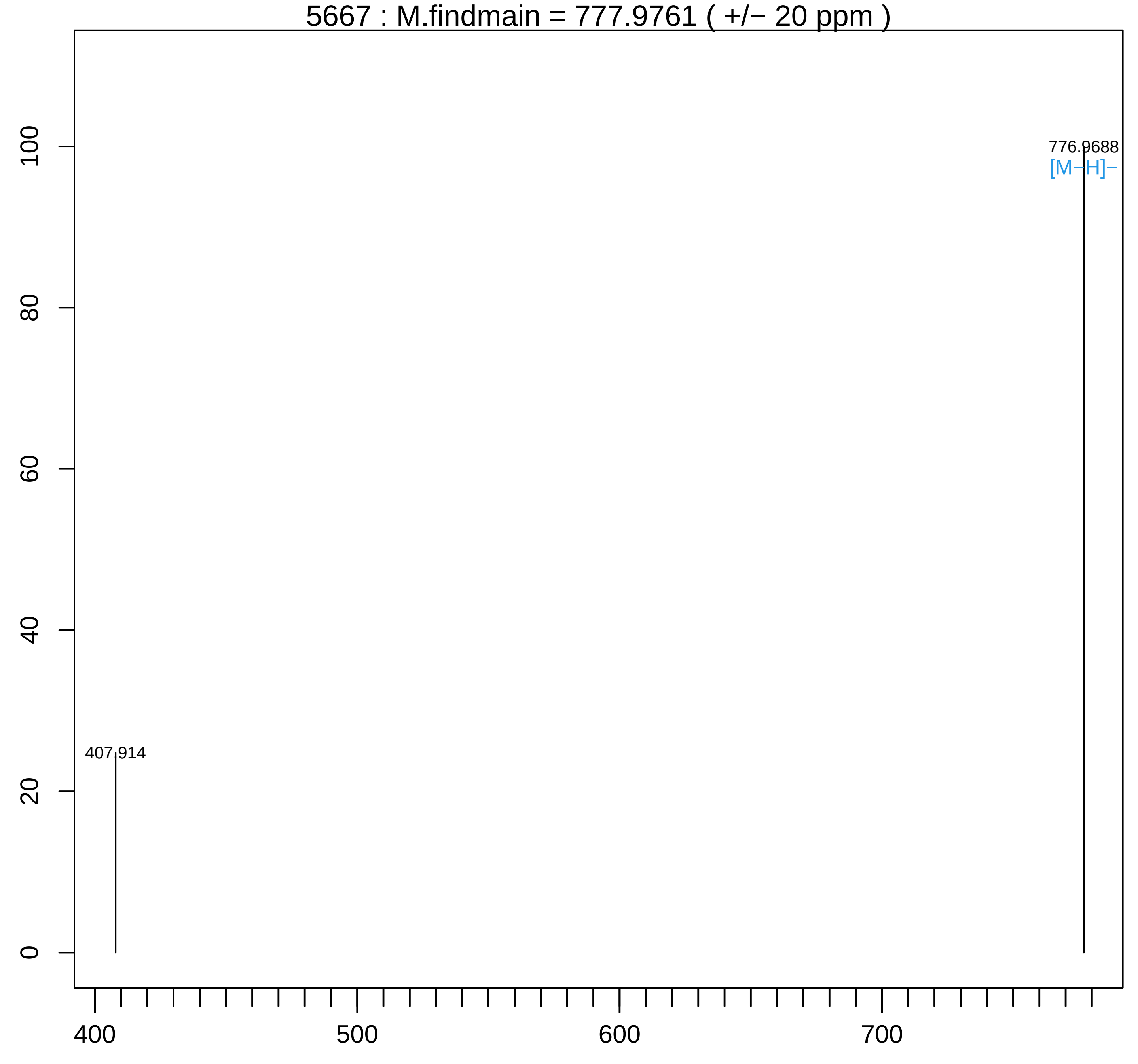

5667 : M.findmain = 777.9761 (+/− 20 ppm)
776.9688
[M−H]−
407.914
0
20
40
60
80
100
400
500
600
700

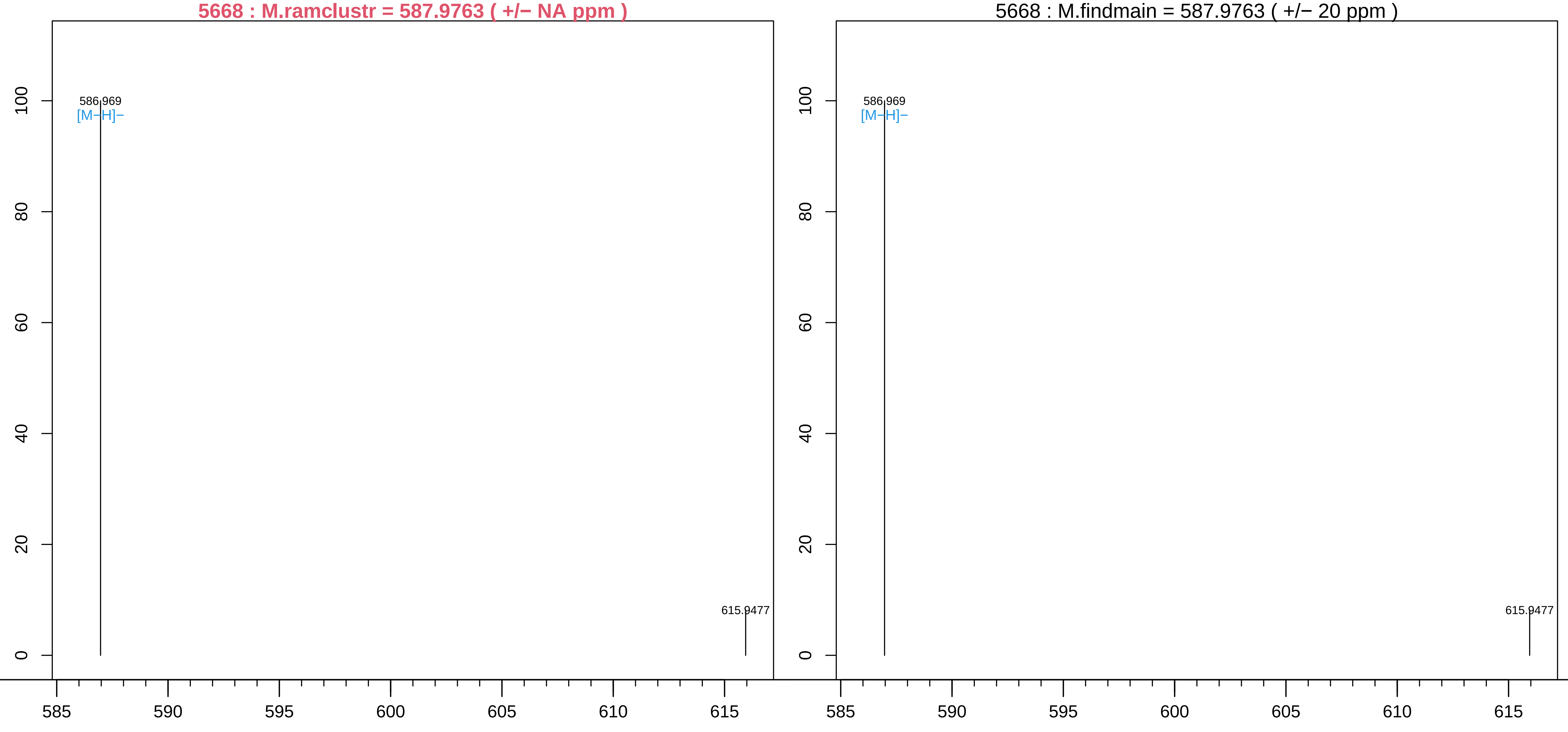

5668 : M.ramclustr = 587.9763 (+/− NA ppm)
5668 : M.findmain = 587.9763 (+/− 20 ppm)
586.969
[M−H]−
615.9477
0
20
40
60
80
100
585
590
595
600
605
610
615

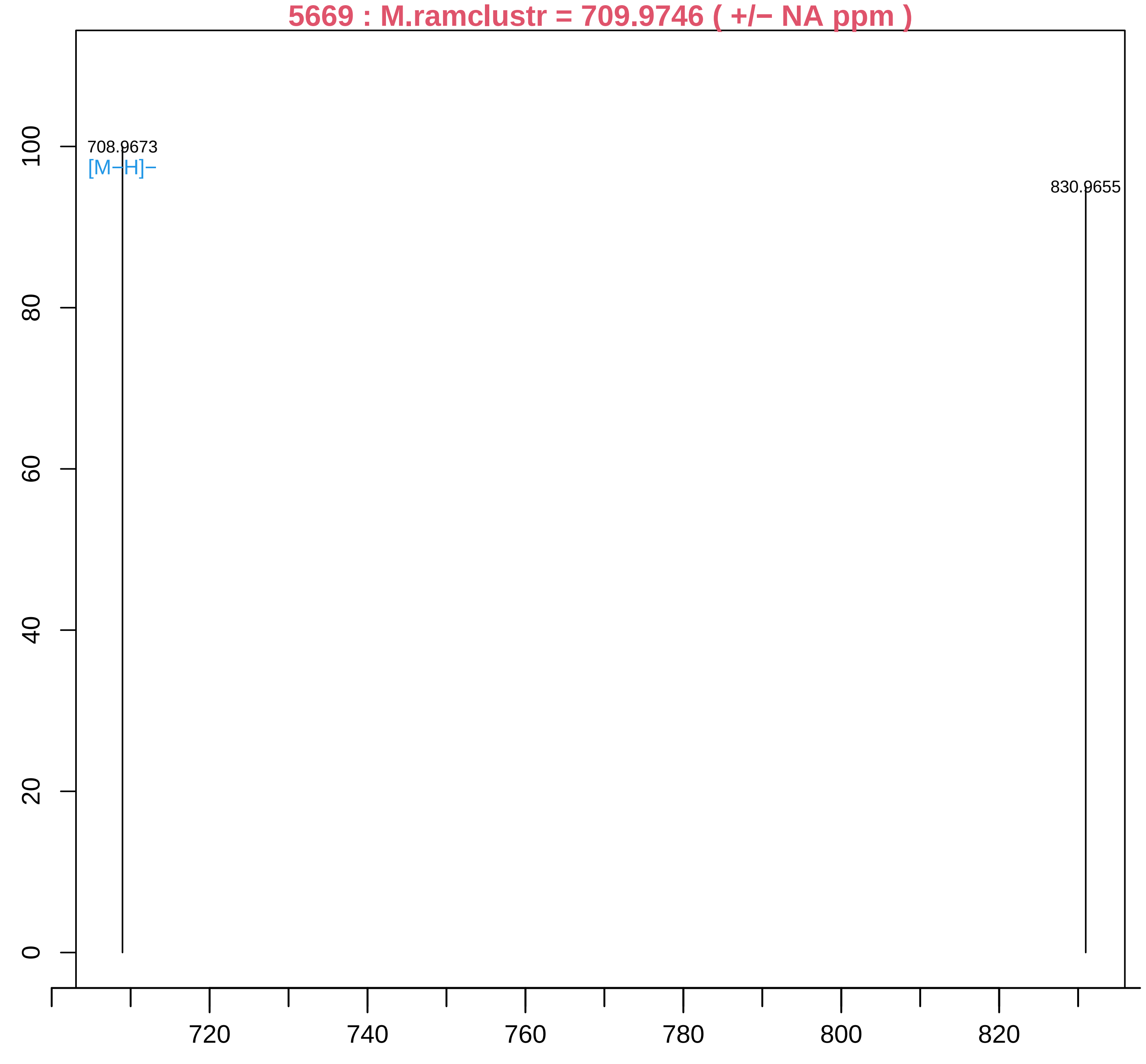
5669 : M.ramclustr = 709.9746 (+/− NA ppm)
708.9673
[M−H]−
830.9655
0
20
40
60
80
100
720
740
760
780
800
820

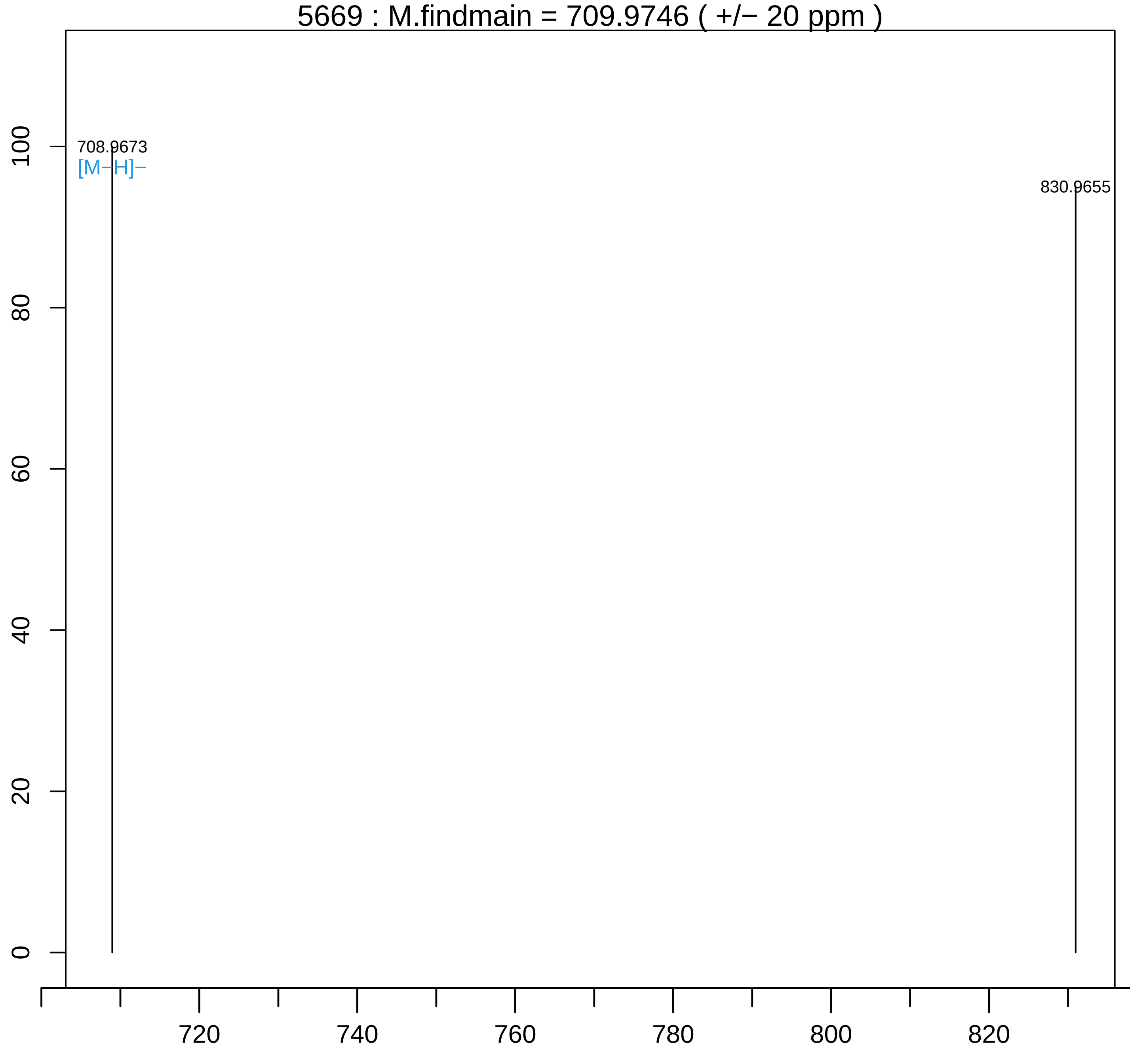
5669 : M.findmain = 709.9746 (+/− 20 ppm)
708.9673
[M−H]−
830.9655
0
20
40
60
80
100
720
740
760
780
800
820

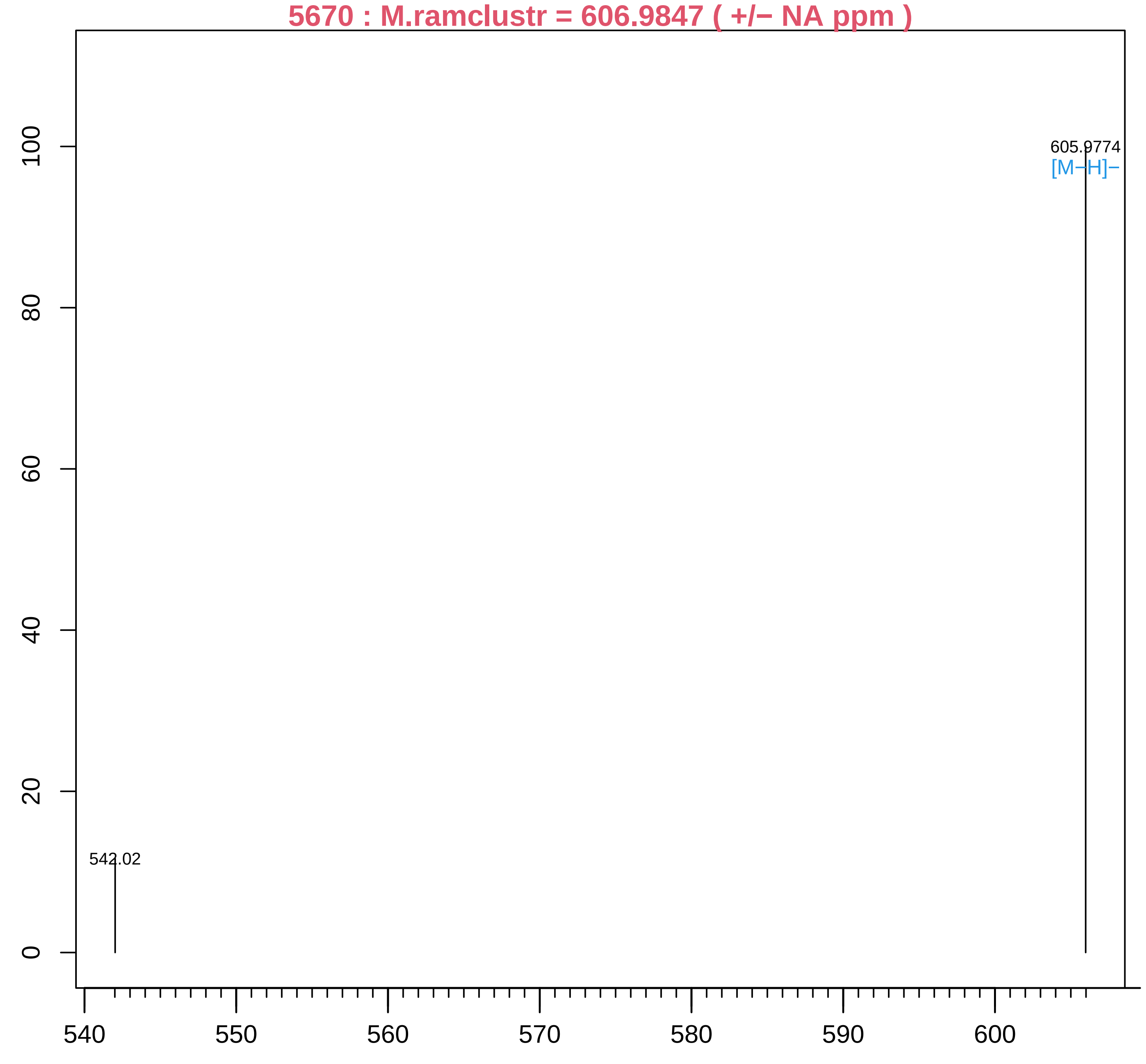

5670 : M.ramclustr = 606.9847 (+/− NA ppm)
605.9774
[M−H]−
542.02
0
20
40
60
80
100
540
550
560
570
580
590
600

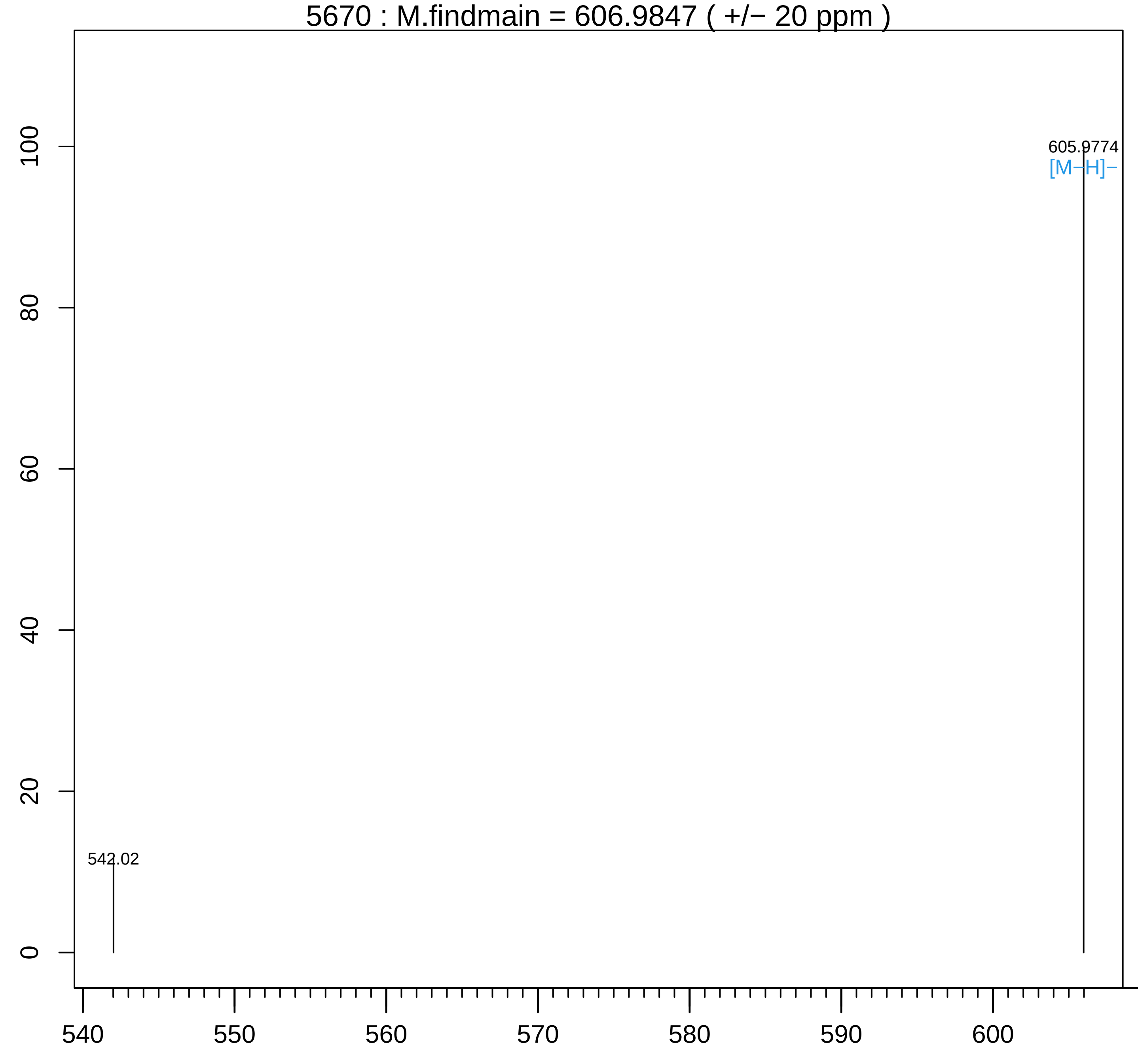

5670 : M.findmain = 606.9847 (+/− 20 ppm)
605.9774
[M−H]−
542.02
0
20
40
60
80
100
540
550
560
570
580
590
600

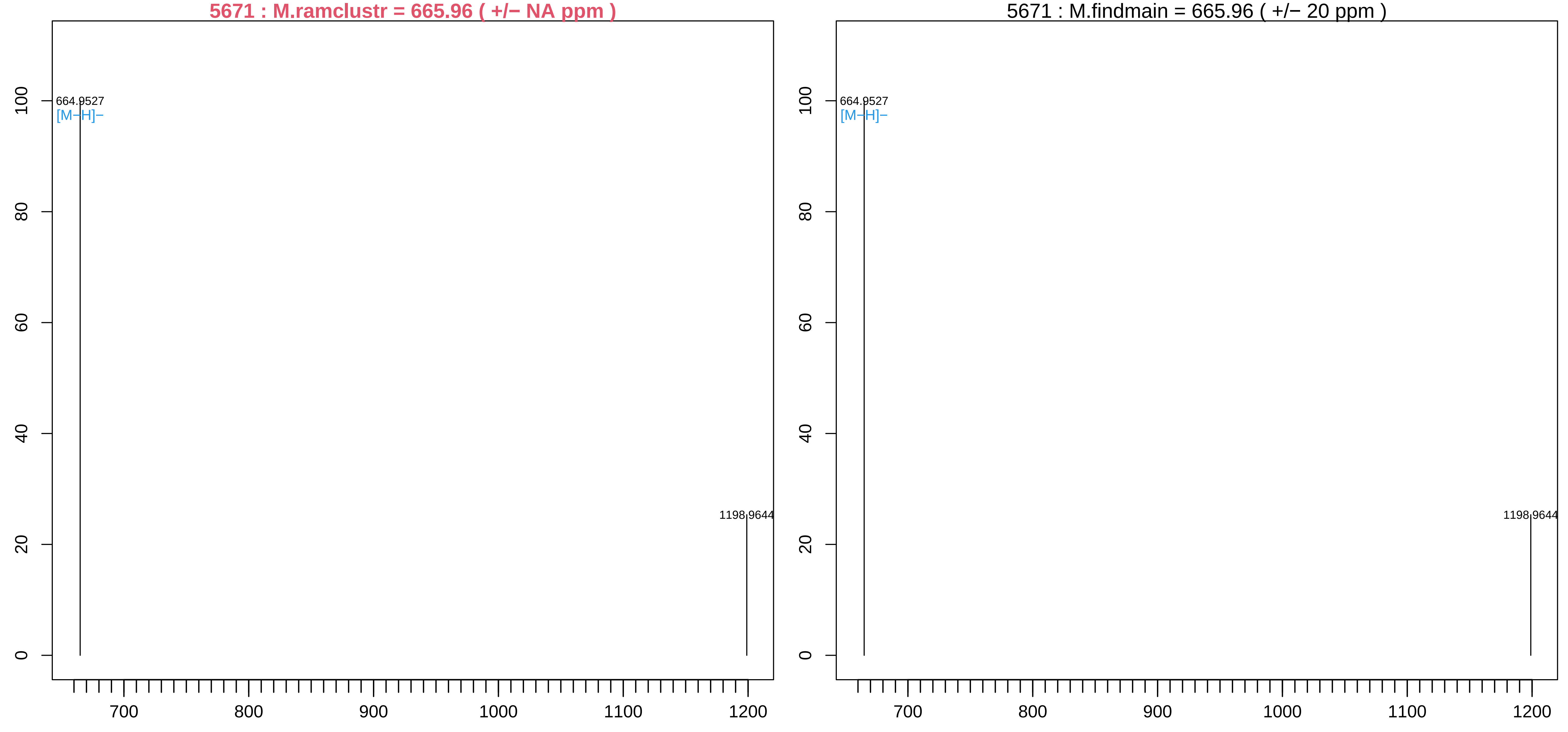

5671 : M.ramclustr = 665.96 (+/− NA ppm)
664.9527
[M−H]−
1198.9644
0
20
40
60
80
100
700
800
900
1000
1100
1200
5671 : M.findmain = 665.96 (+/− 20 ppm)
664.9527
[M−H]−
1198.9644
0
20
40
60
80
100
700
800
900
1000
1100
1200

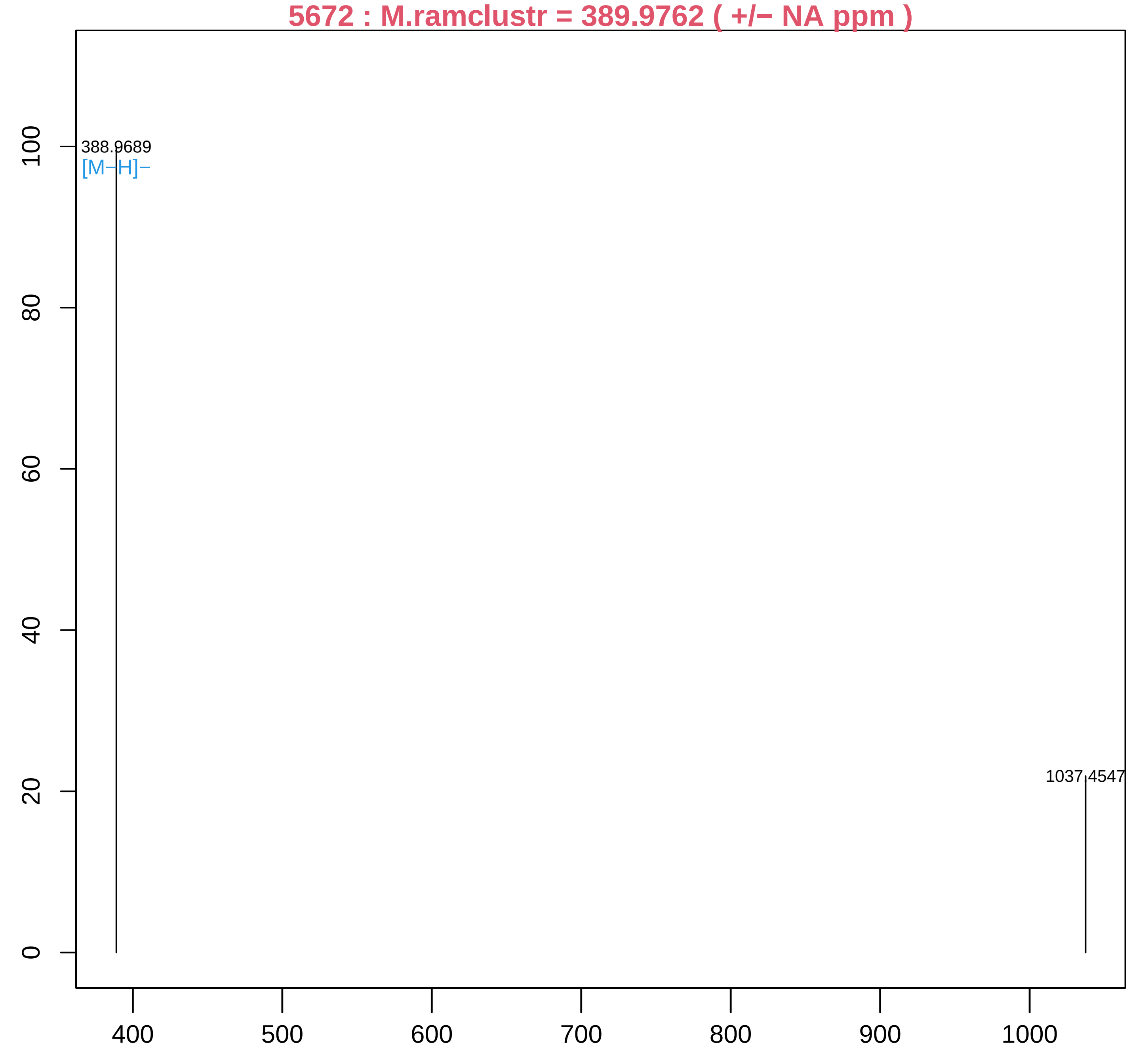
5672 : M.ramclustr = 389.9762 (+/− NA ppm)
388.9689
[M−H]−
1037.4547
0
20
40
60
80
100
400
500
600
700
800
900
1000

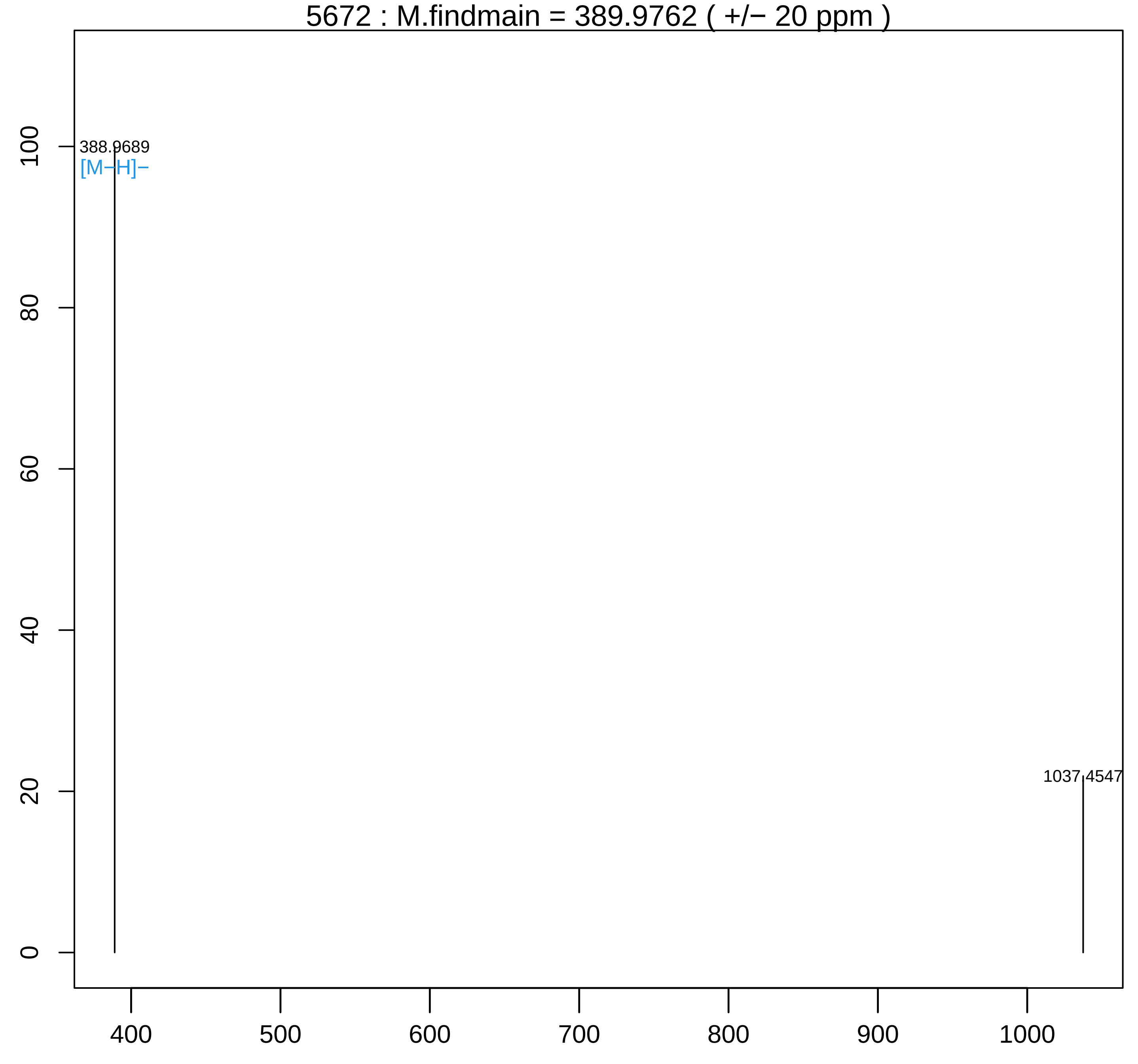
5672 : M.findmain = 389.9762 (+/− 20 ppm)
388.9689
[M−H]−
1037.4547
0
20
40
60
80
100
400
500
600
700
800
900
1000

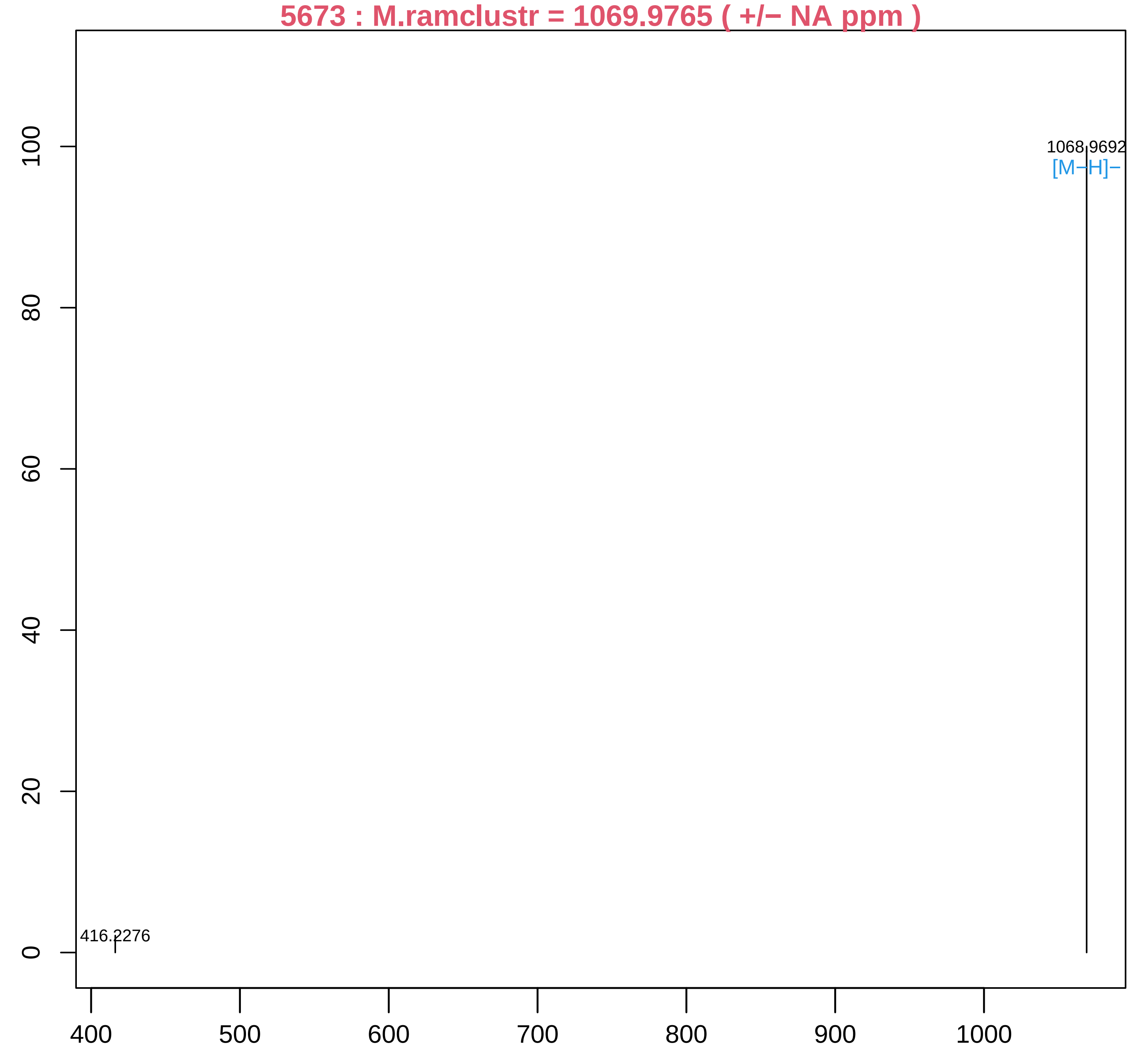
5673 : M.ramclustr = 1069.9765 (+/− NA ppm)
1068.9692
[M−H]−
416.2276
0
20
40
60
80
100
400
500
600
700
800
900
1000

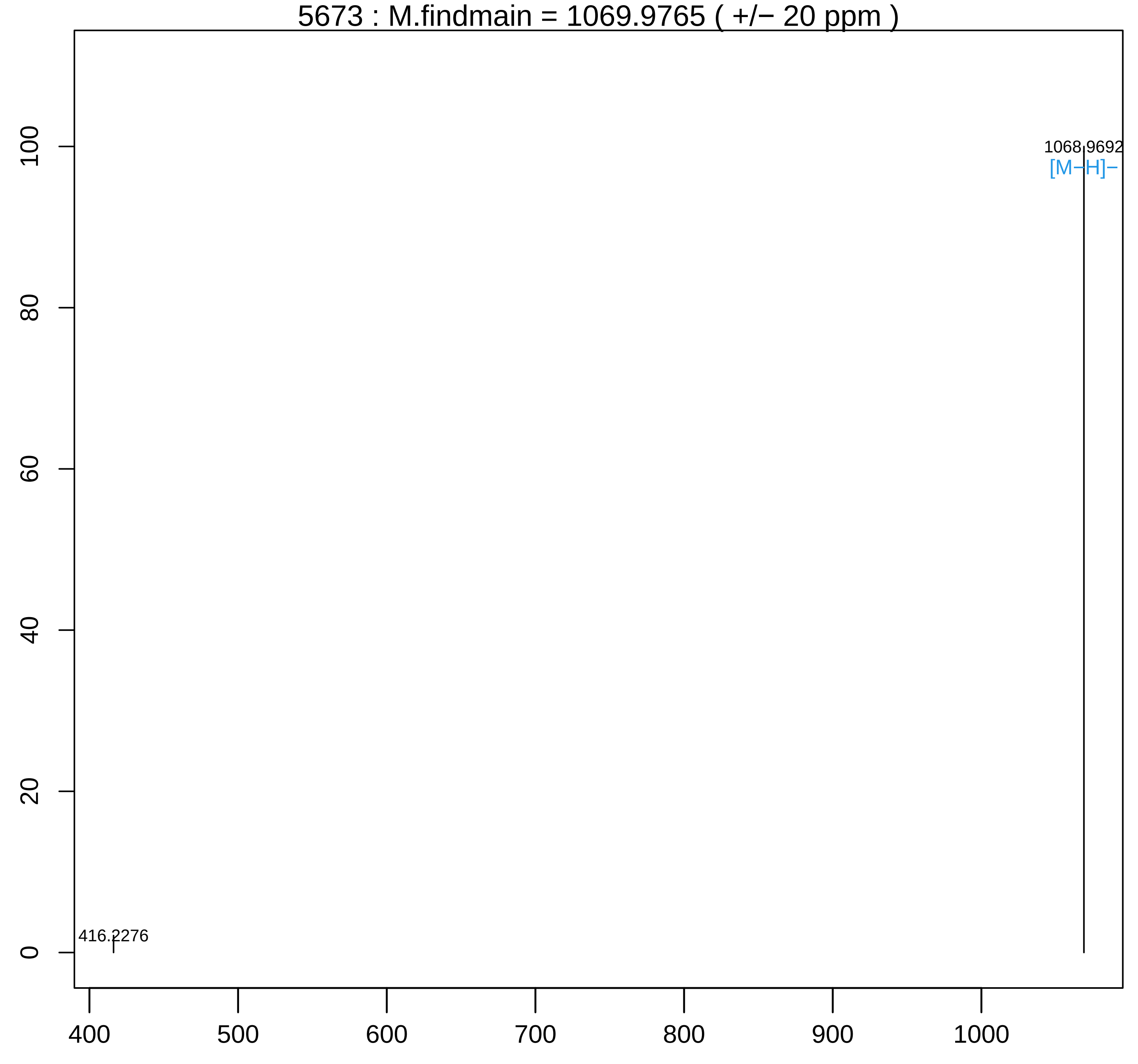
5673 : M.findmain = 1069.9765 (+/− 20 ppm)
1068.9692
[M−H]−
416.2276
0
20
40
60
80
100
400
500
600
700
800
900
1000

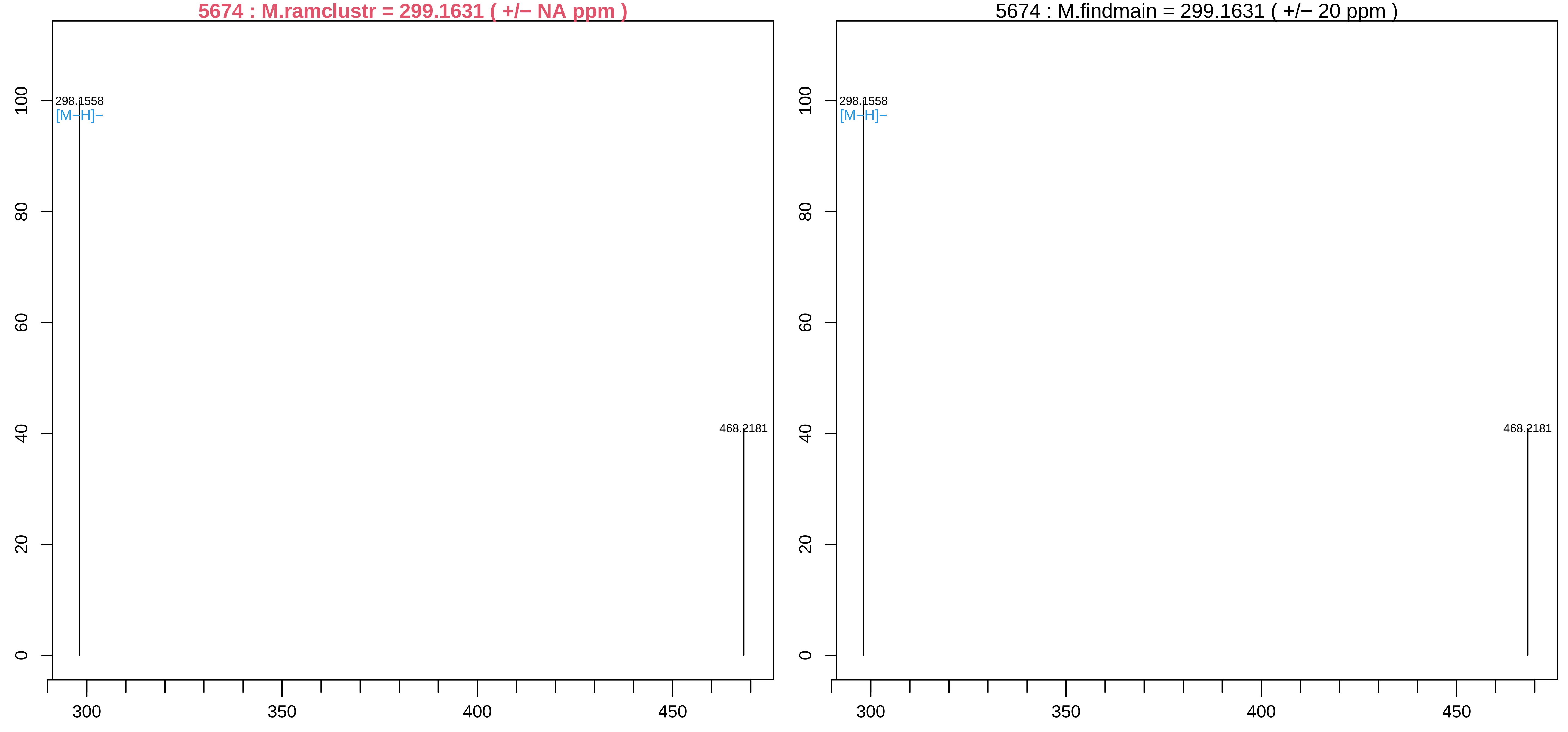

5674 : M.ramclustr = 299.1631 (+/− NA ppm)
298.1558
[M−H]−
468.2181
100
80
60
40
20
0
300
350
400
450
5674 : M.findmain = 299.1631 (+/− 20 ppm)
298.1558
[M−H]−
468.2181
100
80
60
40
20
0
300
350
400
450

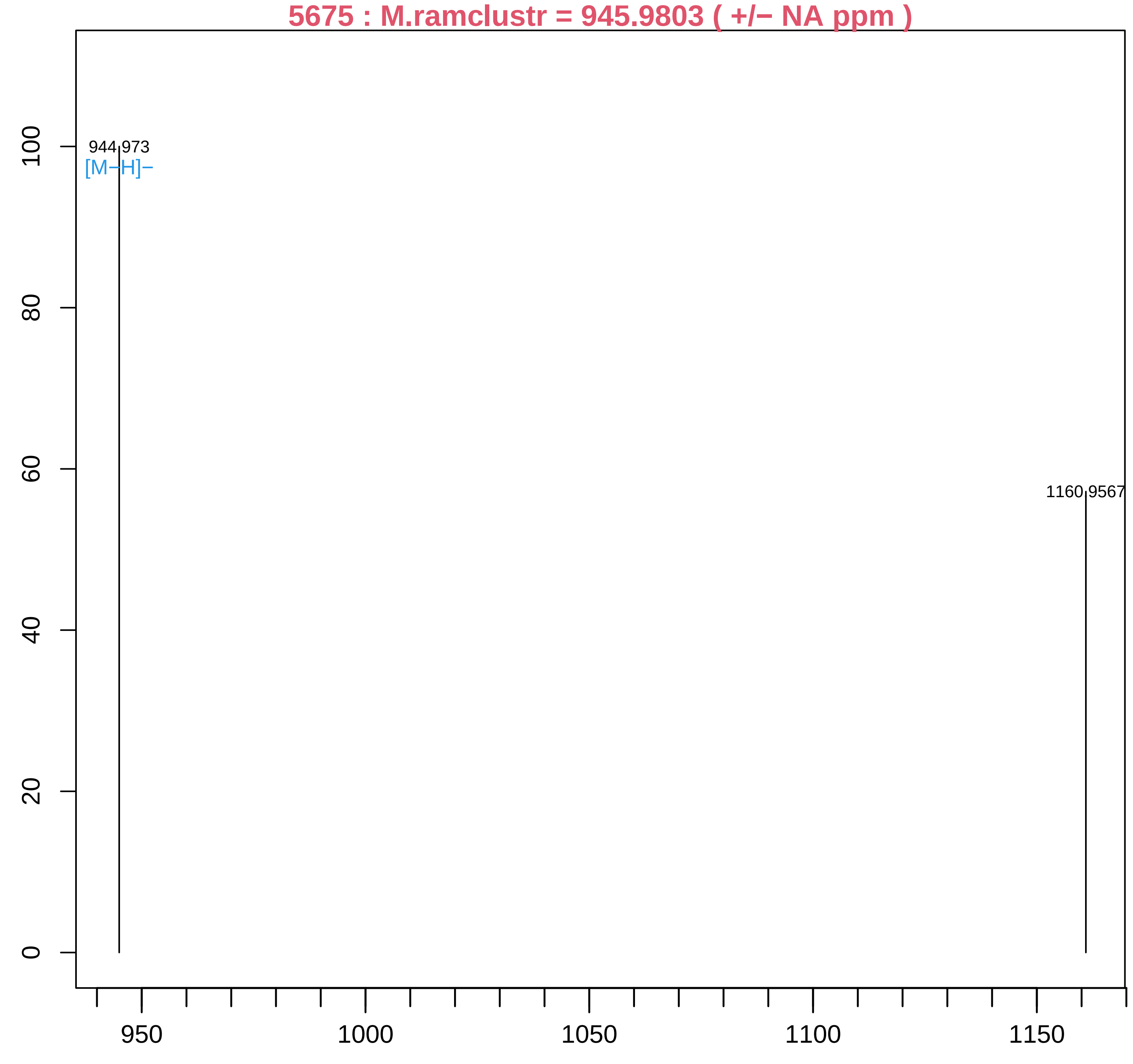

5675 : M.ramclustr = 945.9803 (+/− NA ppm)
944.973
[M−H]−
1160.9567
0
20
40
60
80
100
950
1000
1050
1100
1150

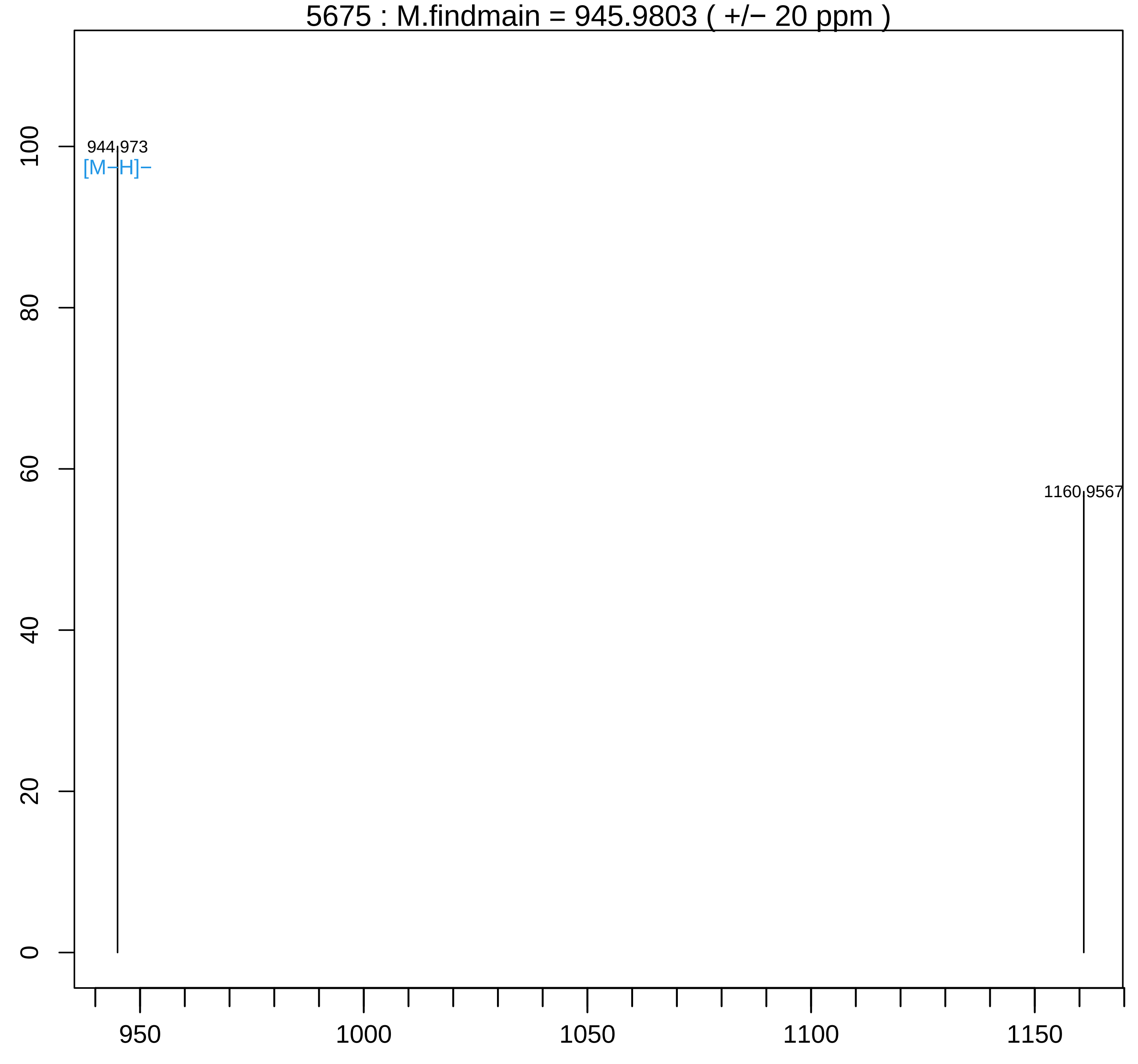

5675 : M.findmain = 945.9803 (+/− 20 ppm)
944.973
[M−H]−
1160.9567
0
20
40
60
80
100
950
1000
1050
1100
1150

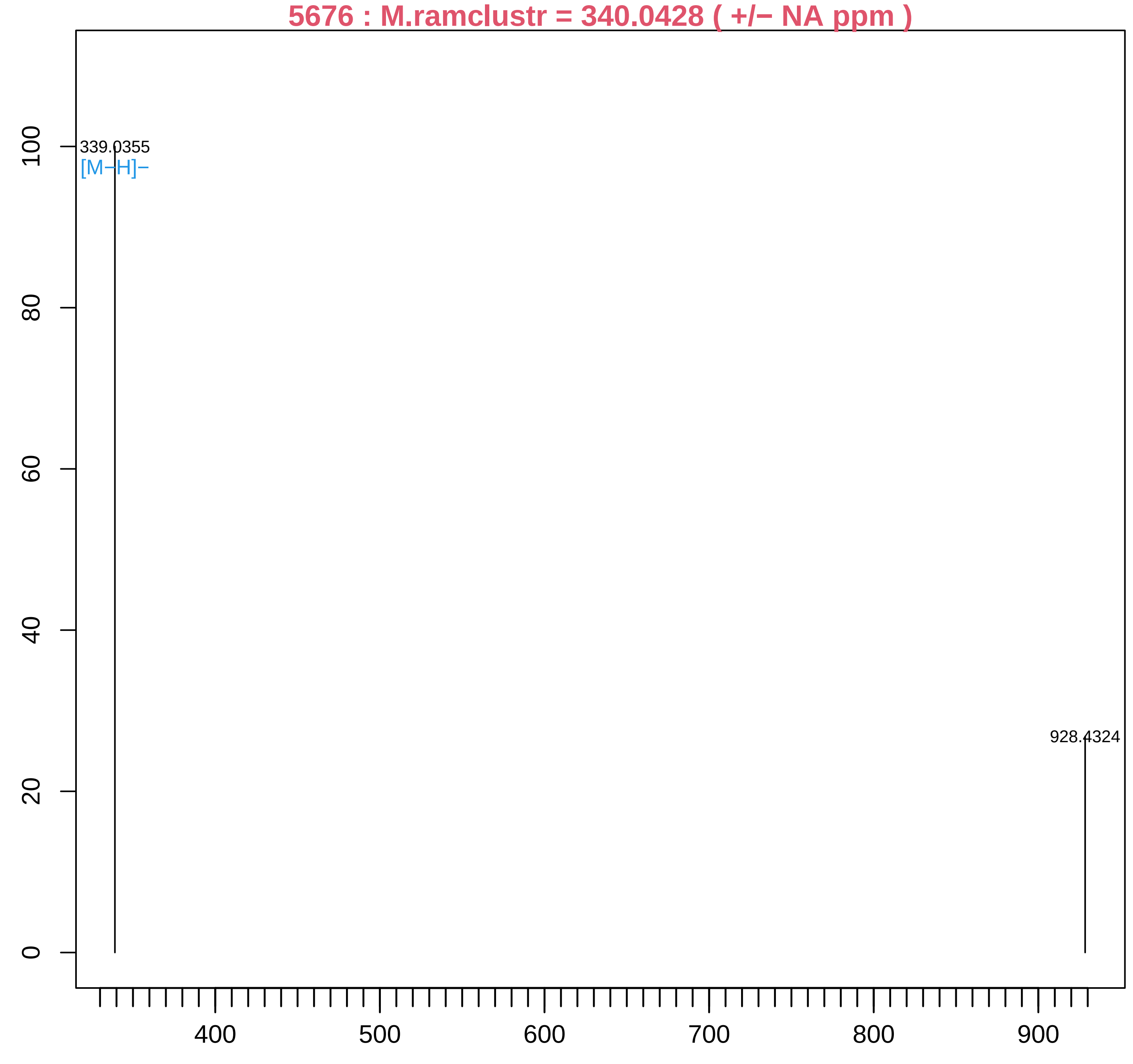
5676 : M.ramclustr = 340.0428 (+/− NA ppm)
339.0355
[M−H]−
928.4324
0
20
40
60
80
100
400
500
600
700
800
900

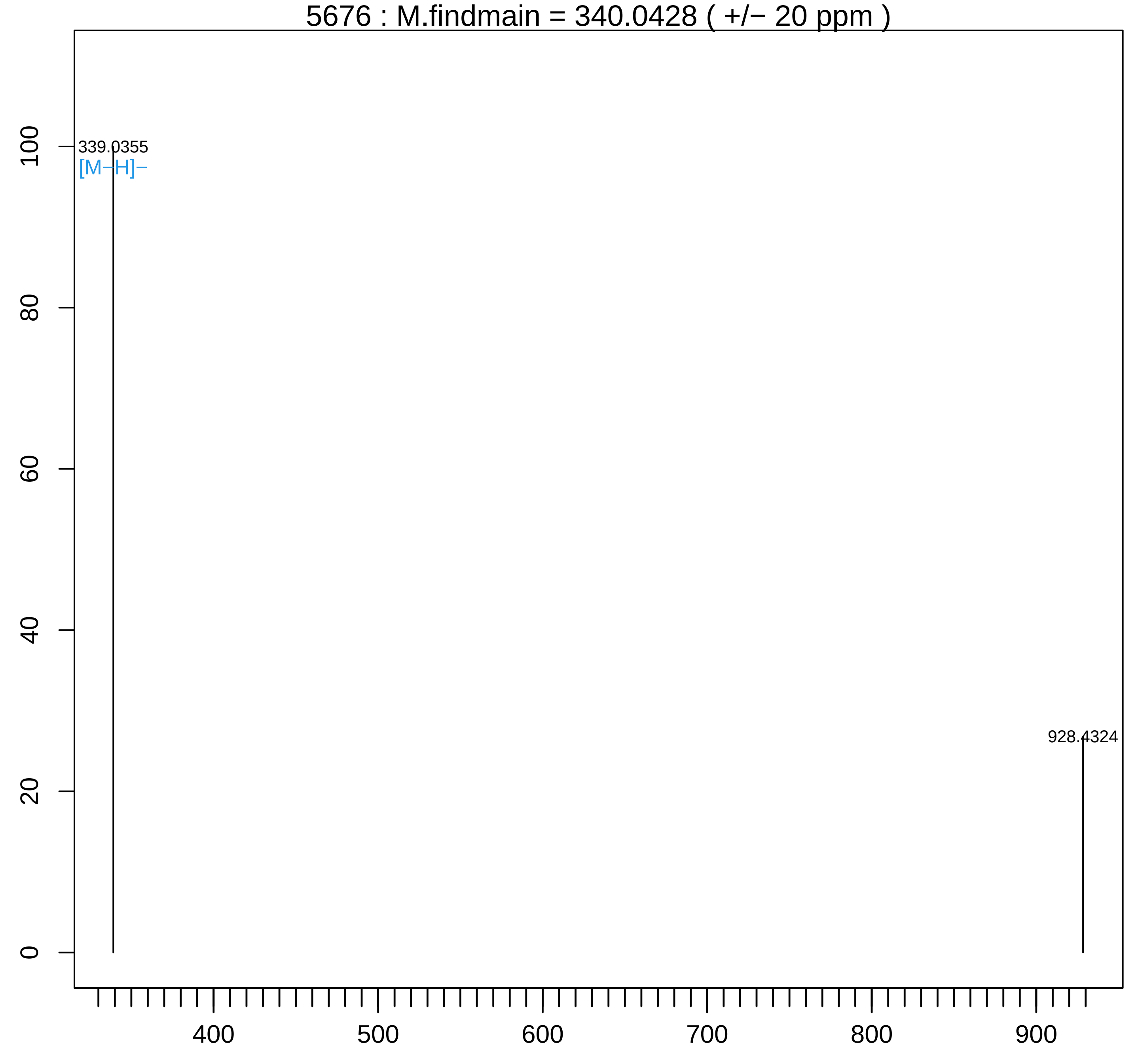
5676 : M.findmain = 340.0428 (+/− 20 ppm)
339.0355
[M−H]−
928.4324
0
20
40
60
80
100
400
500
600
700
800
900

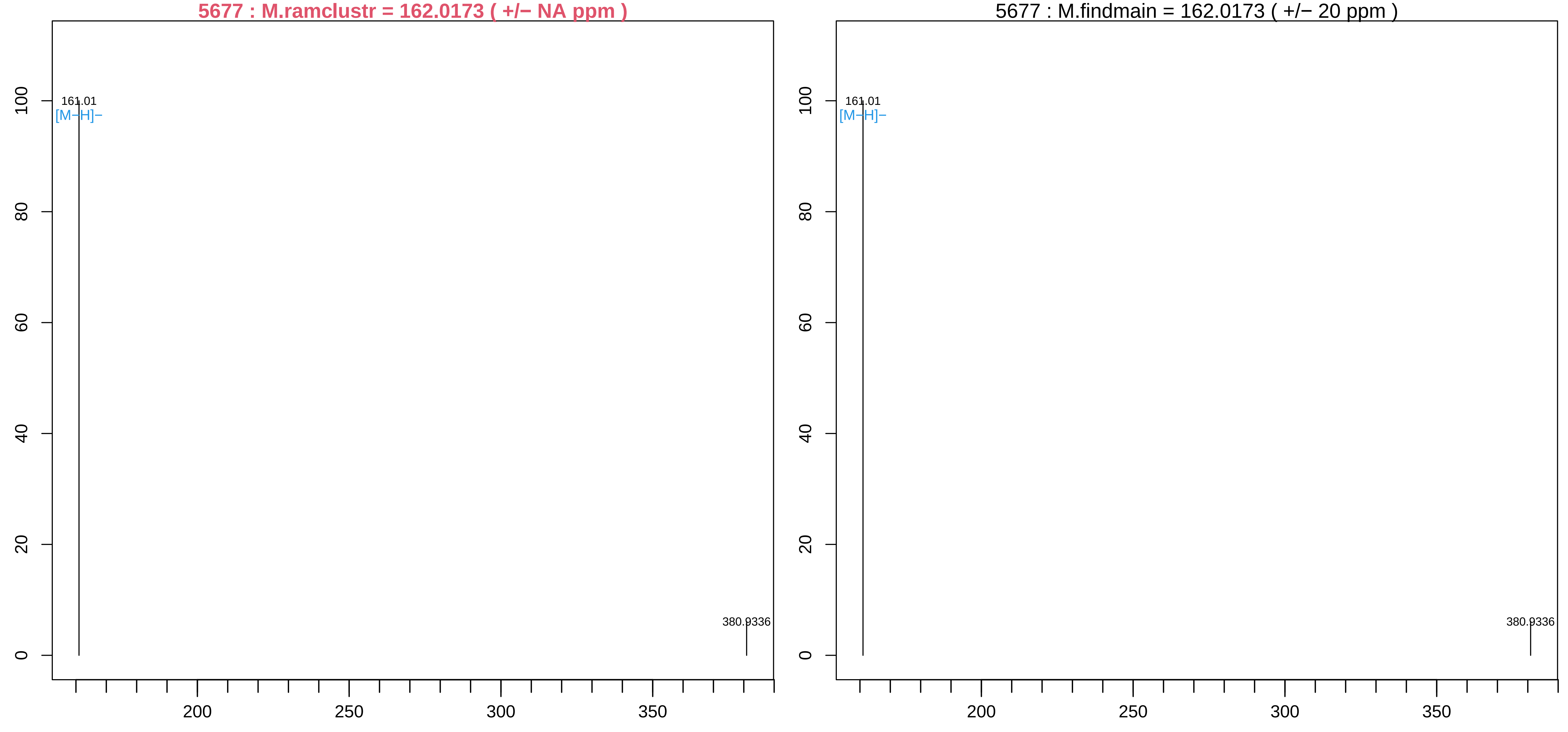
5677 : M.ramclustr = 162.0173 (+/− NA ppm)
161.01
[M−H]−
380.9336
5677 : M.findmain = 162.0173 (+/− 20 ppm)
161.01
[M−H]−
380.9336
0
20
40
60
80
100
200
250
300
350

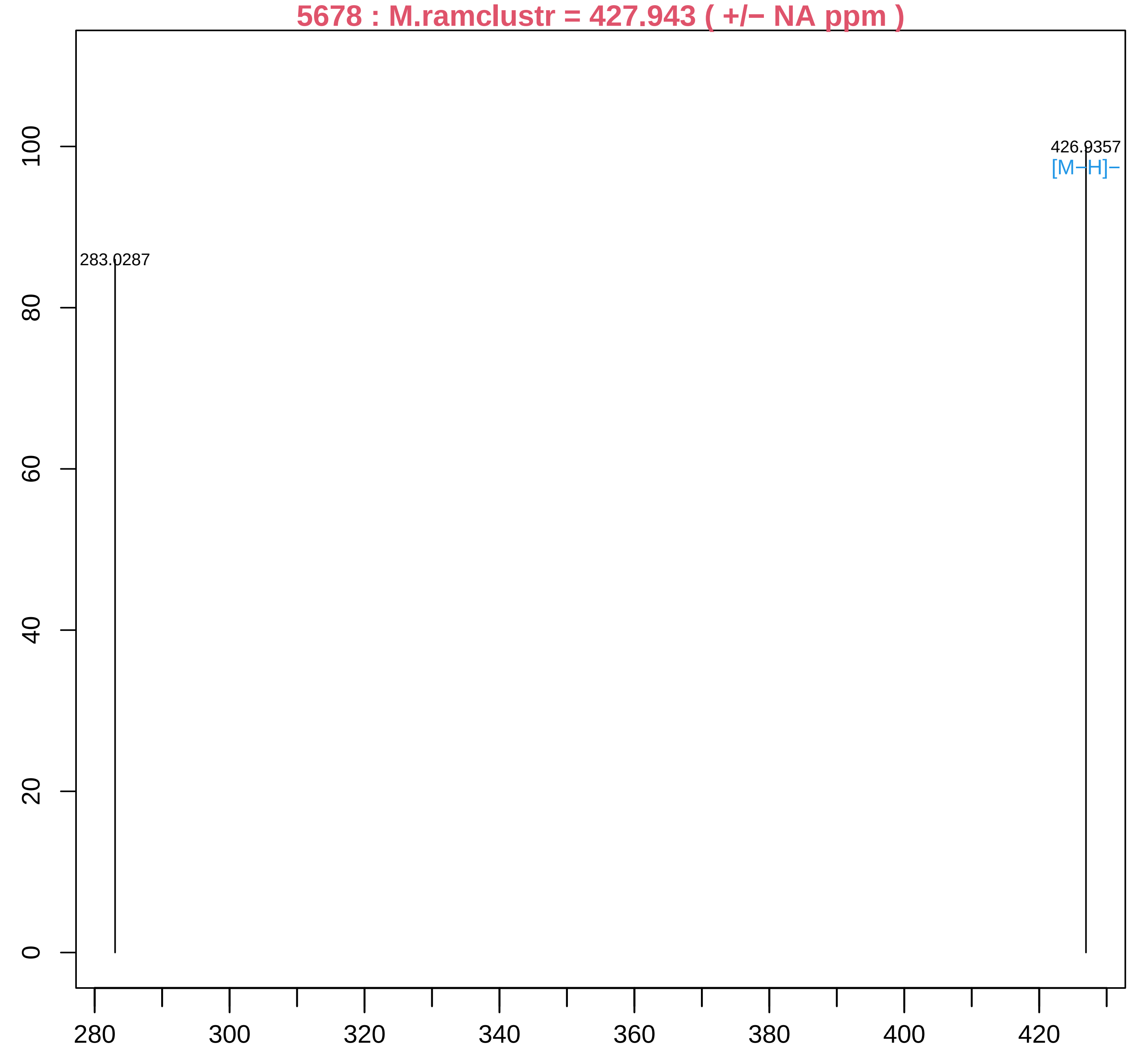
5678 : M.ramclustr = 427.943 (+/− NA ppm)
283.0287
426.9357
[M−H]−
0
20
40
60
80
100
280
300
320
340
360
380
400
420

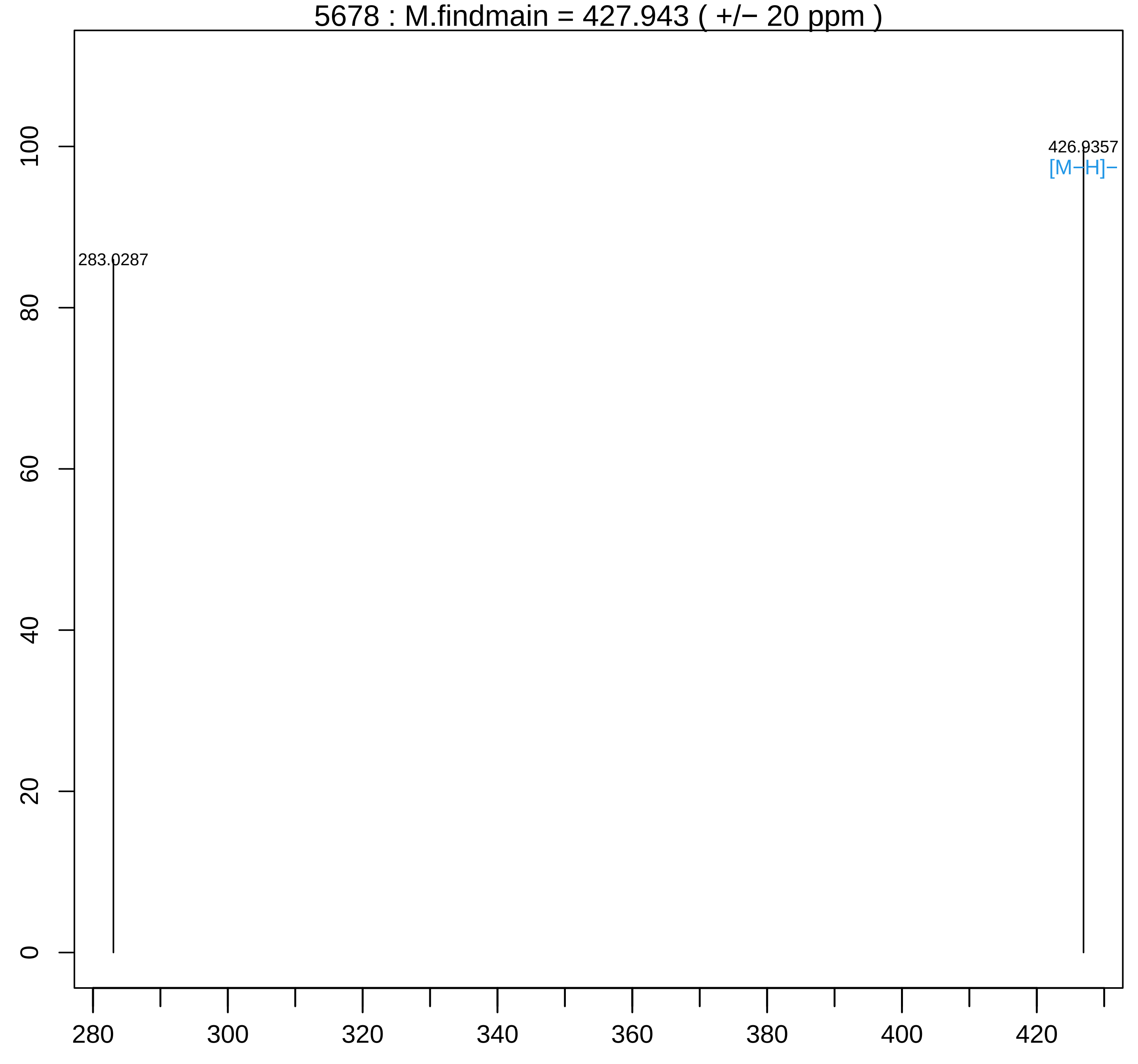
5678 : M.findmain = 427.943 (+/− 20 ppm)
283.0287
426.9357
[M−H]−
0
20
40
60
80
100
280
300
320
340
360
380
400
420

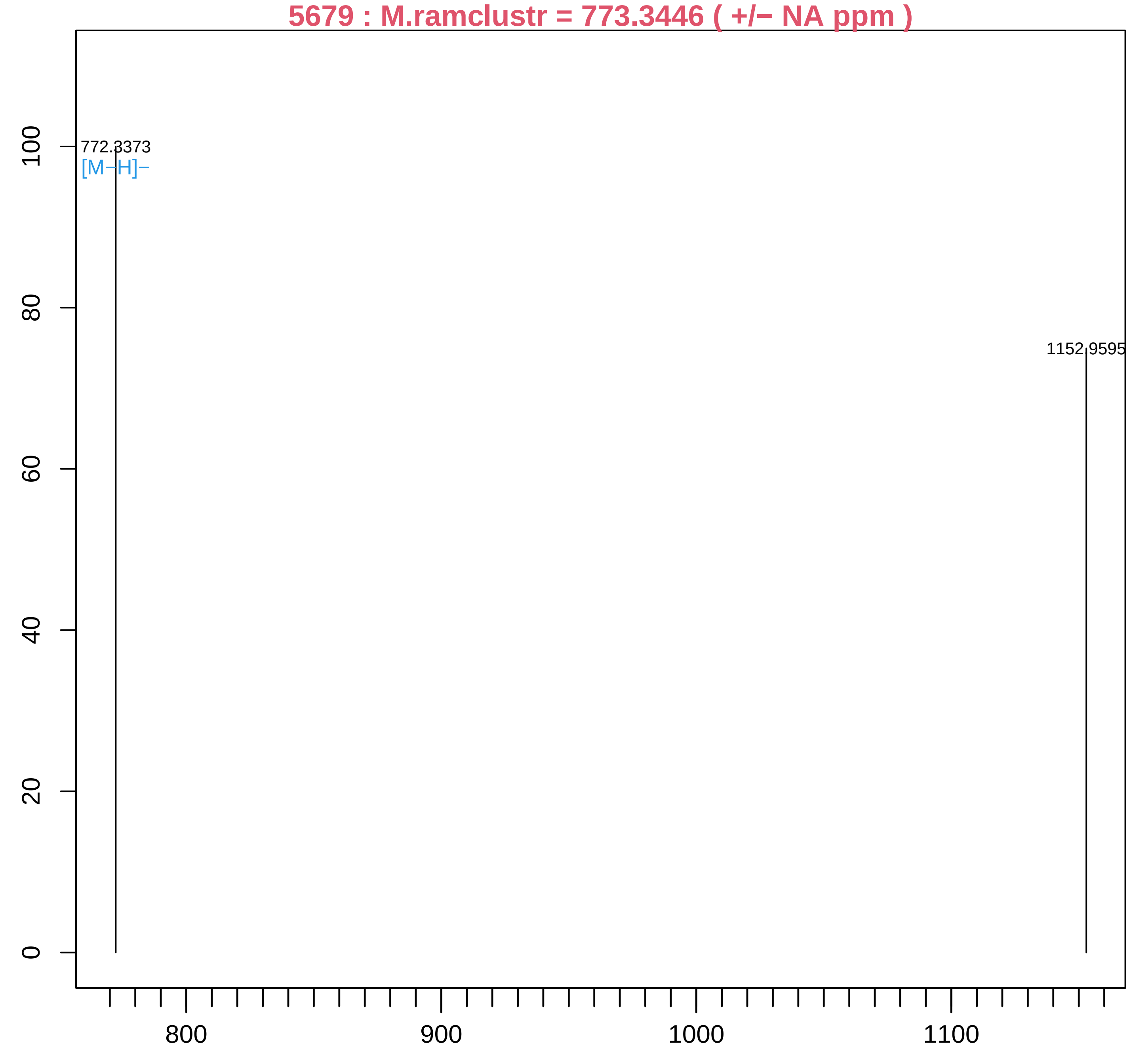
5679 : M.ramclustr = 773.3446 (+/− NA ppm)
772.3373
[M−H]−
1152.9595
0
20
40
60
80
100
800
900
1000
1100

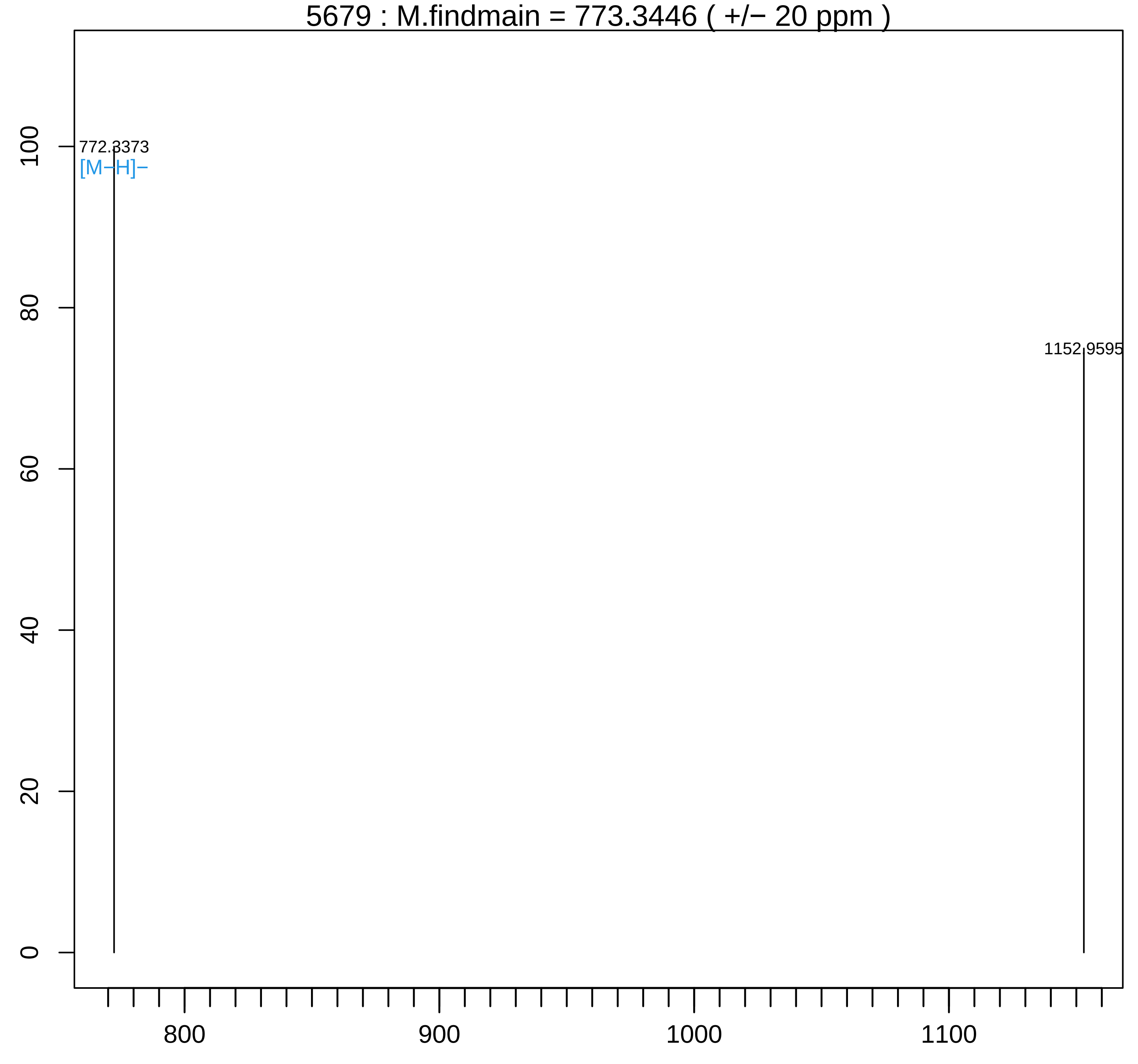
5679 : M.findmain = 773.3446 (+/− 20 ppm)
772.3373
[M−H]−
1152.9595
0
20
40
60
80
100
800
900
1000
1100

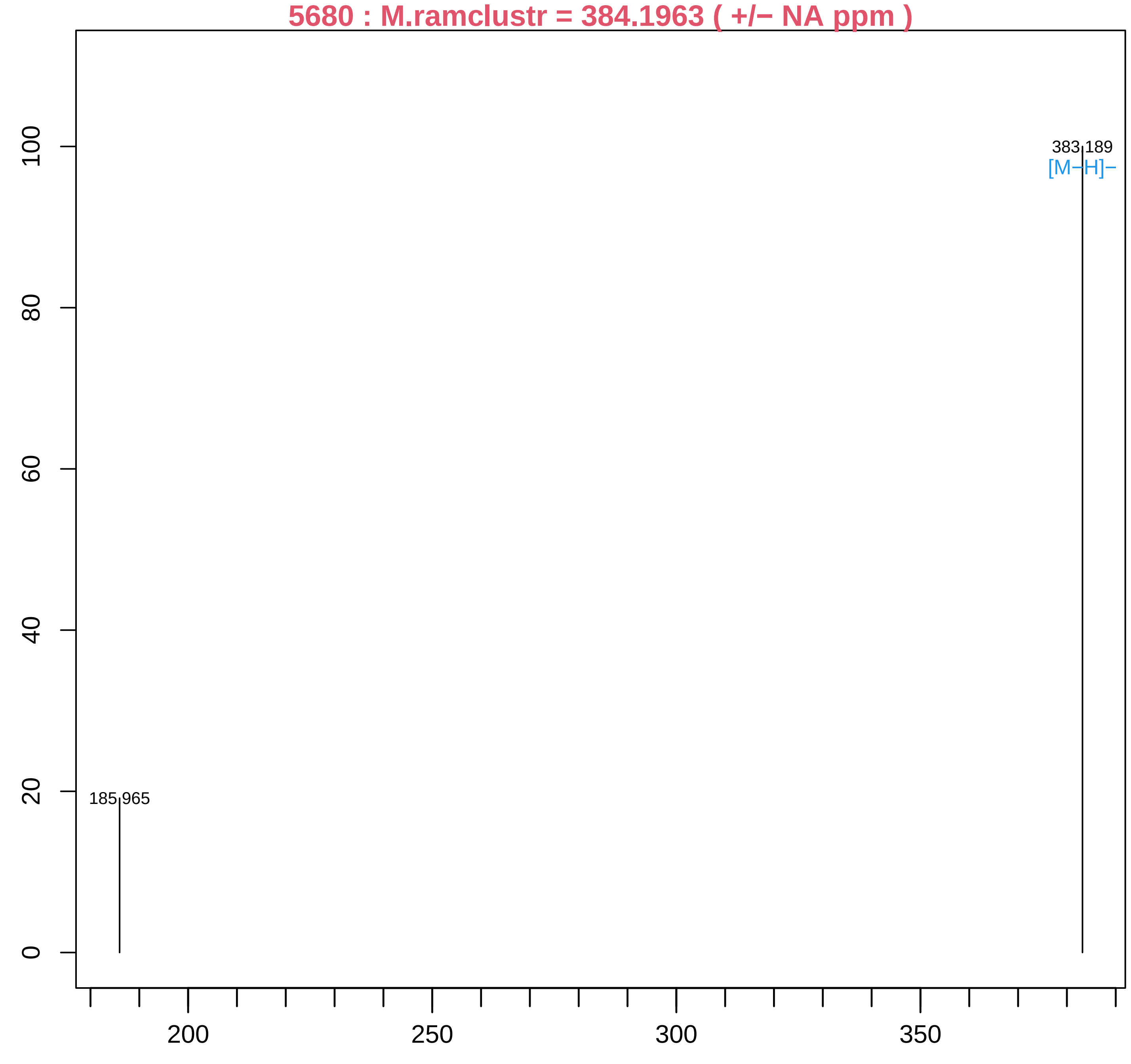
5680 : M.ramclustr = 384.1963 (+/− NA ppm)
0
20
40
60
80
100
200
250
300
350
185.965
383.189
[M−H]−

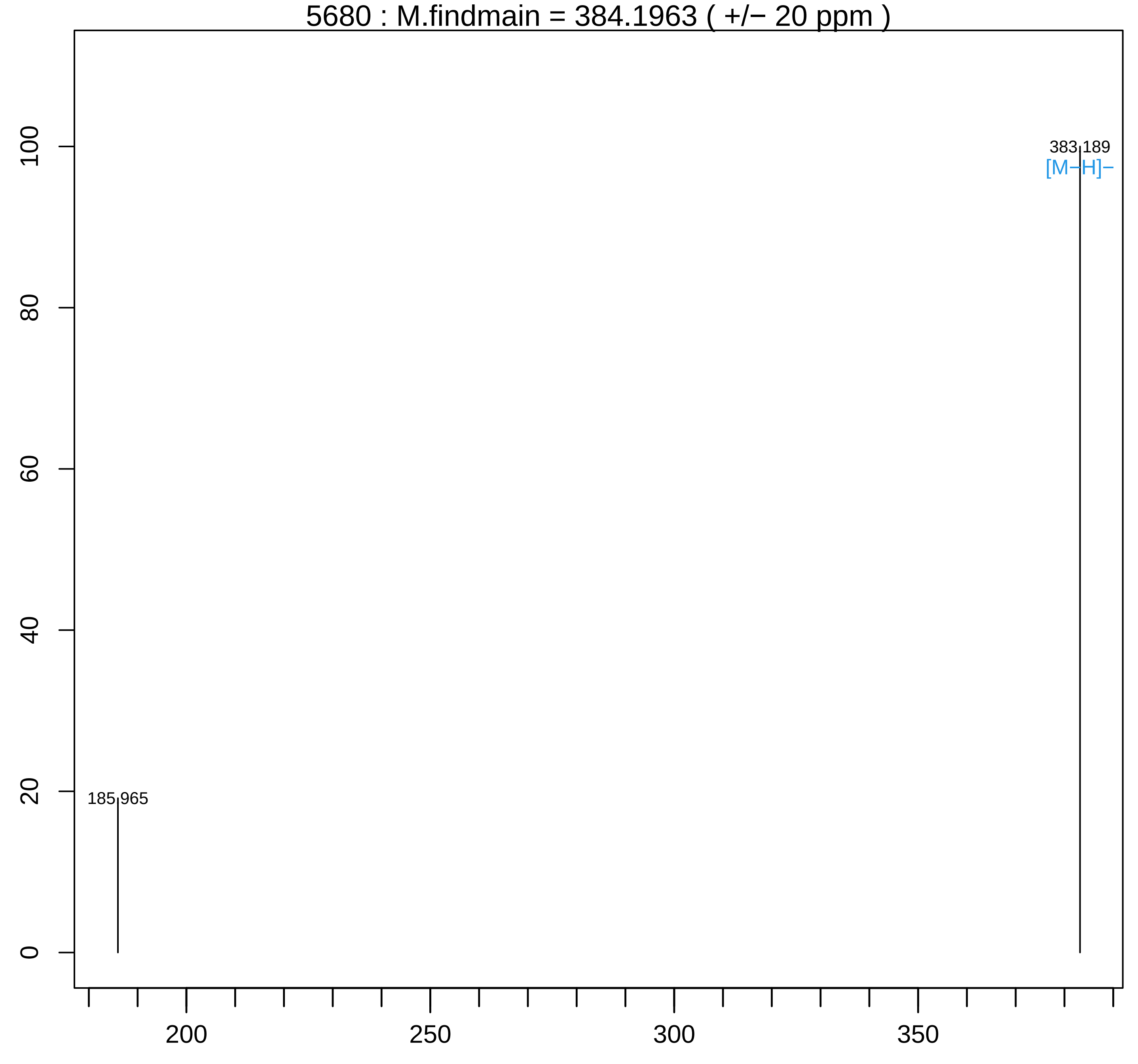
5680 : M.findmain = 384.1963 (+/− 20 ppm)
0
20
40
60
80
100
200
250
300
350
185.965
383.189
[M−H]−

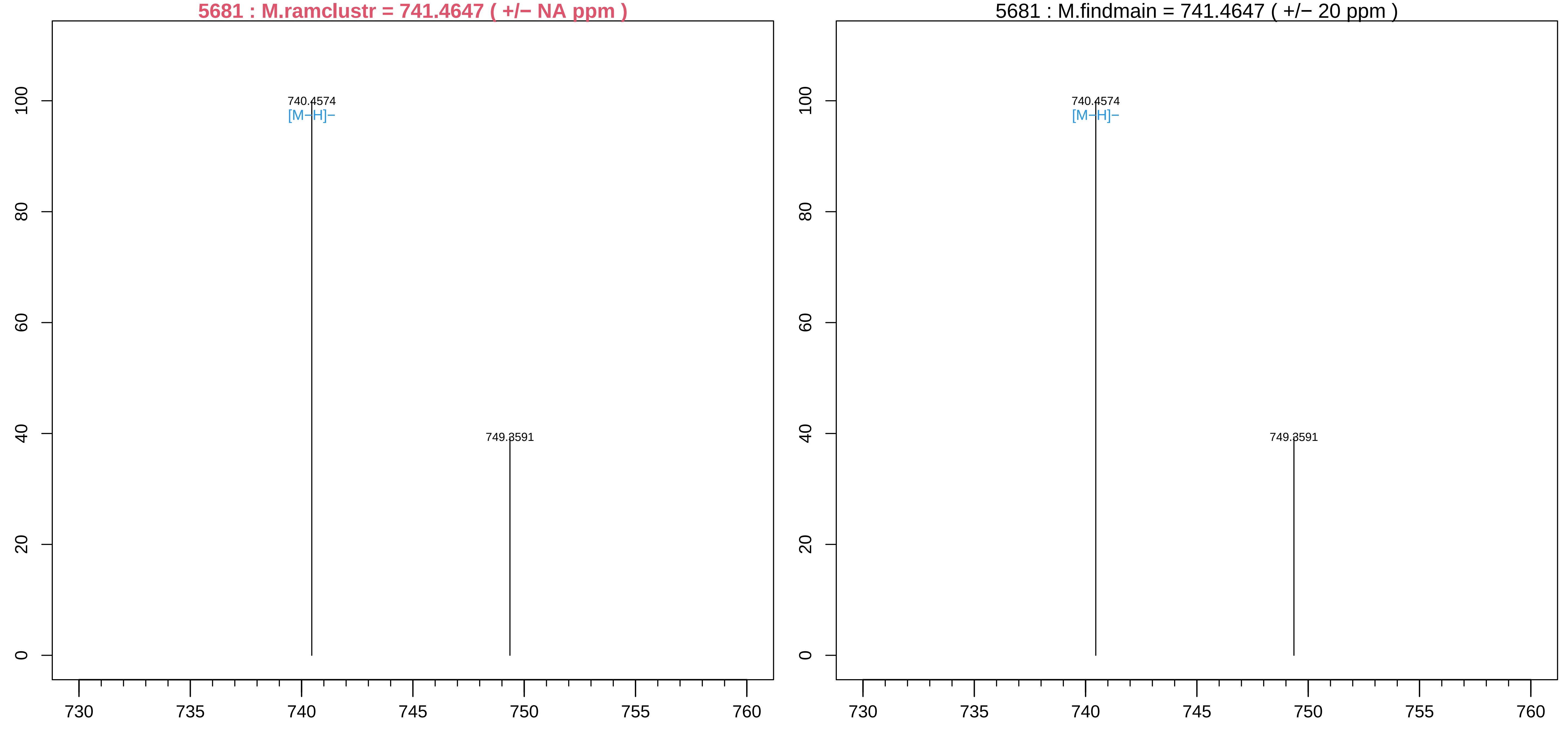
5681 : M.ramclustr = 741.4647 (+/− NA ppm)
740.4574
[M−H]−
749.3591
5681 : M.findmain = 741.4647 (+/− 20 ppm)
740.4574
[M−H]−
749.3591
0
20
40
60
80
100
730
735
740
745
750
755
760

5682 : M.ramclustr = 355.9871 (+/− NA ppm)

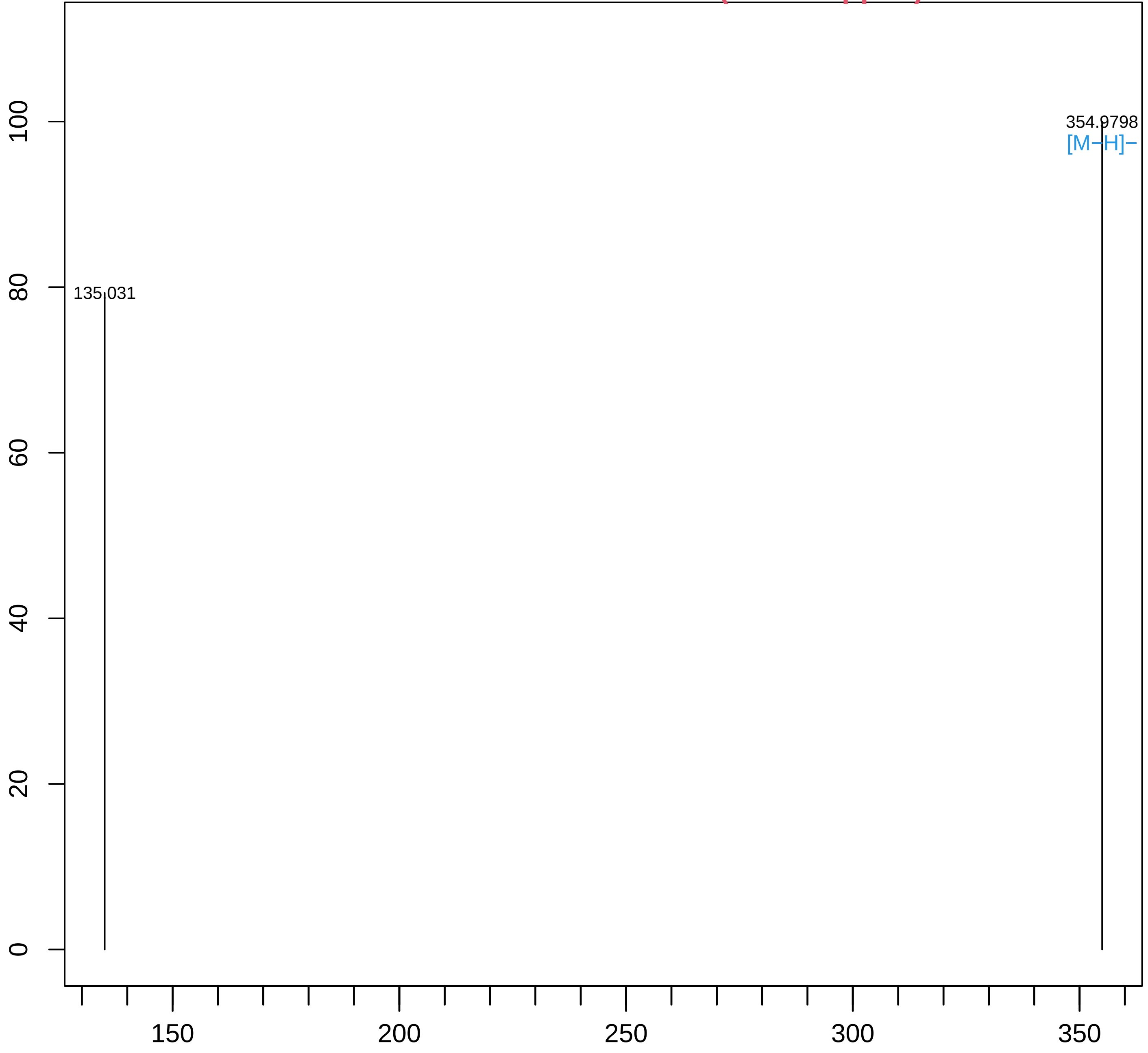

5682 : M.findmain = 355.9871 (+/− 20 ppm)

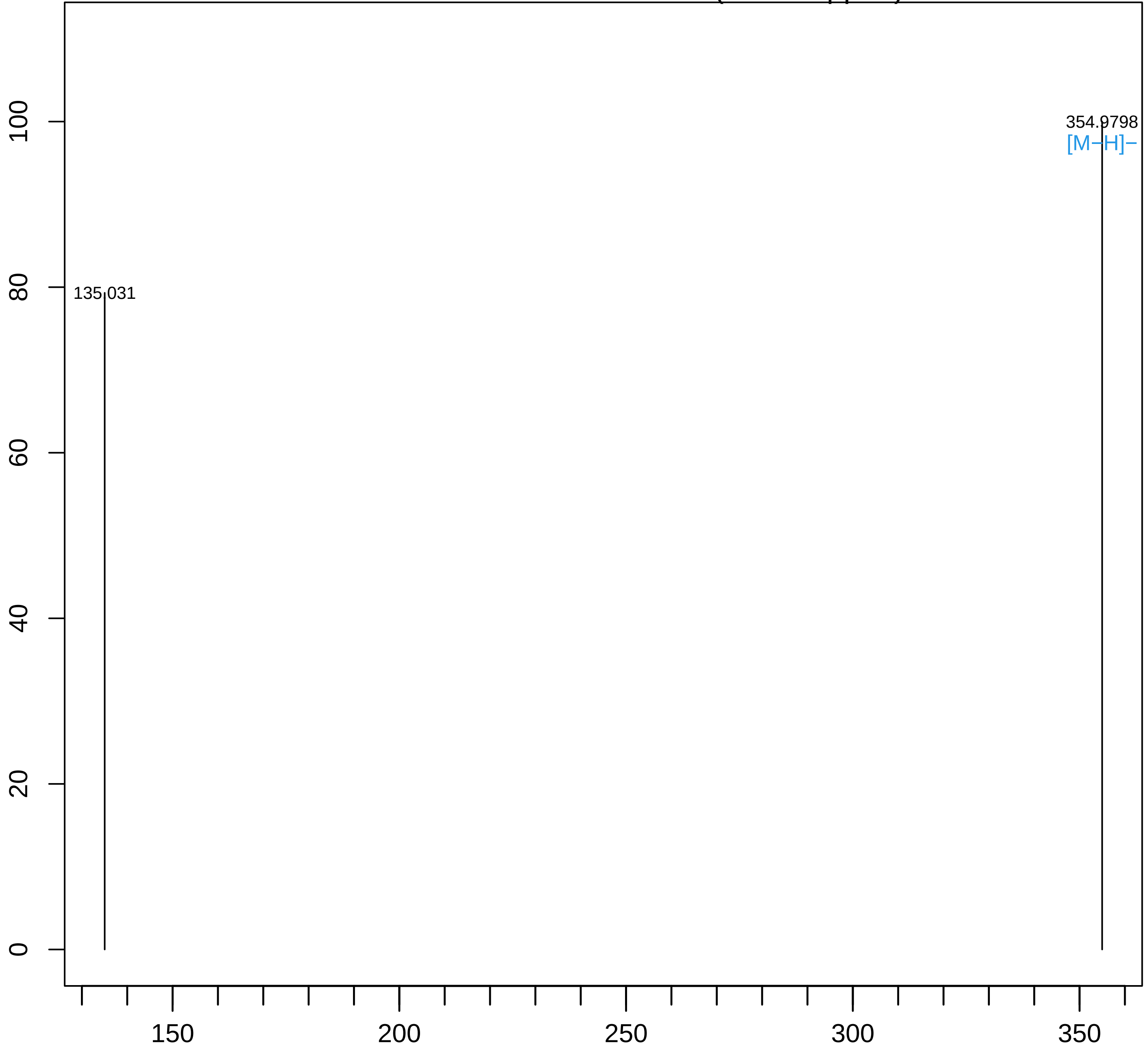

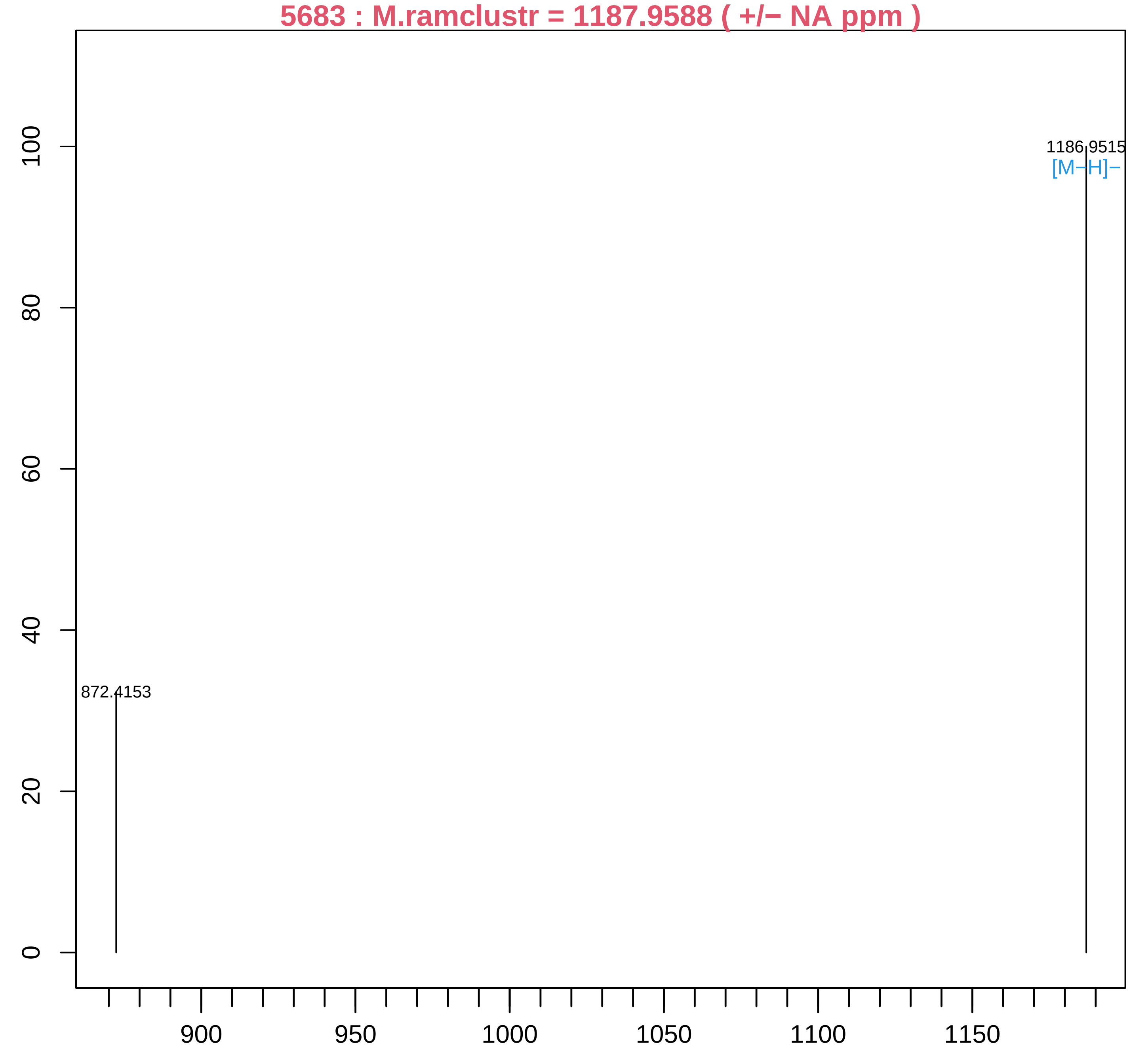

5683 : M.ramclustr = 1187.9588 (+/− NA ppm)
1186.9515
[M−H]−
872.4153
0
20
40
60
80
100
900
950
1000
1050
1100
1150

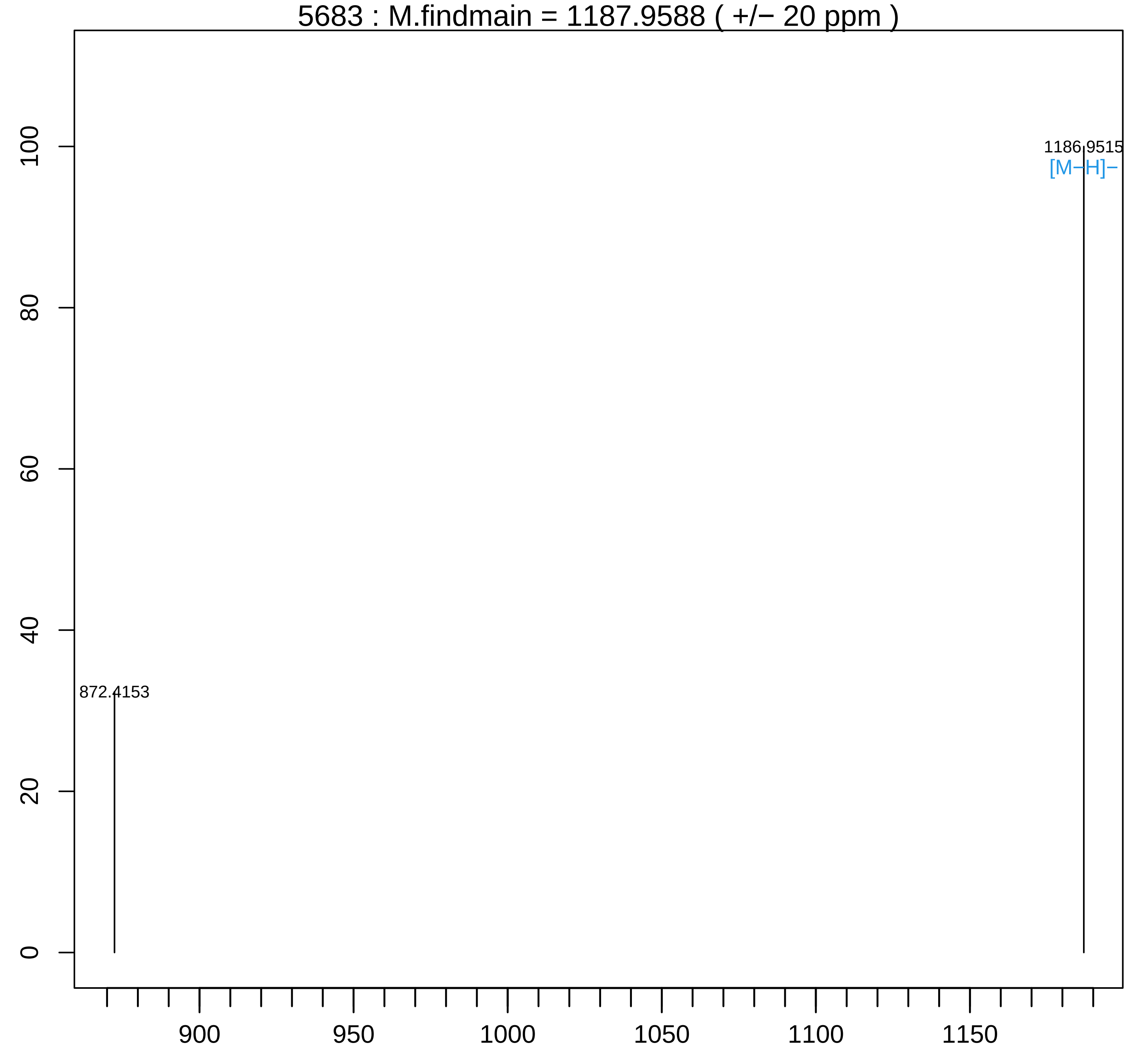

5683 : M.findmain = 1187.9588 (+/− 20 ppm)
1186.9515
[M−H]−
872.4153
0
20
40
60
80
100
900
950
1000
1050
1100
1150

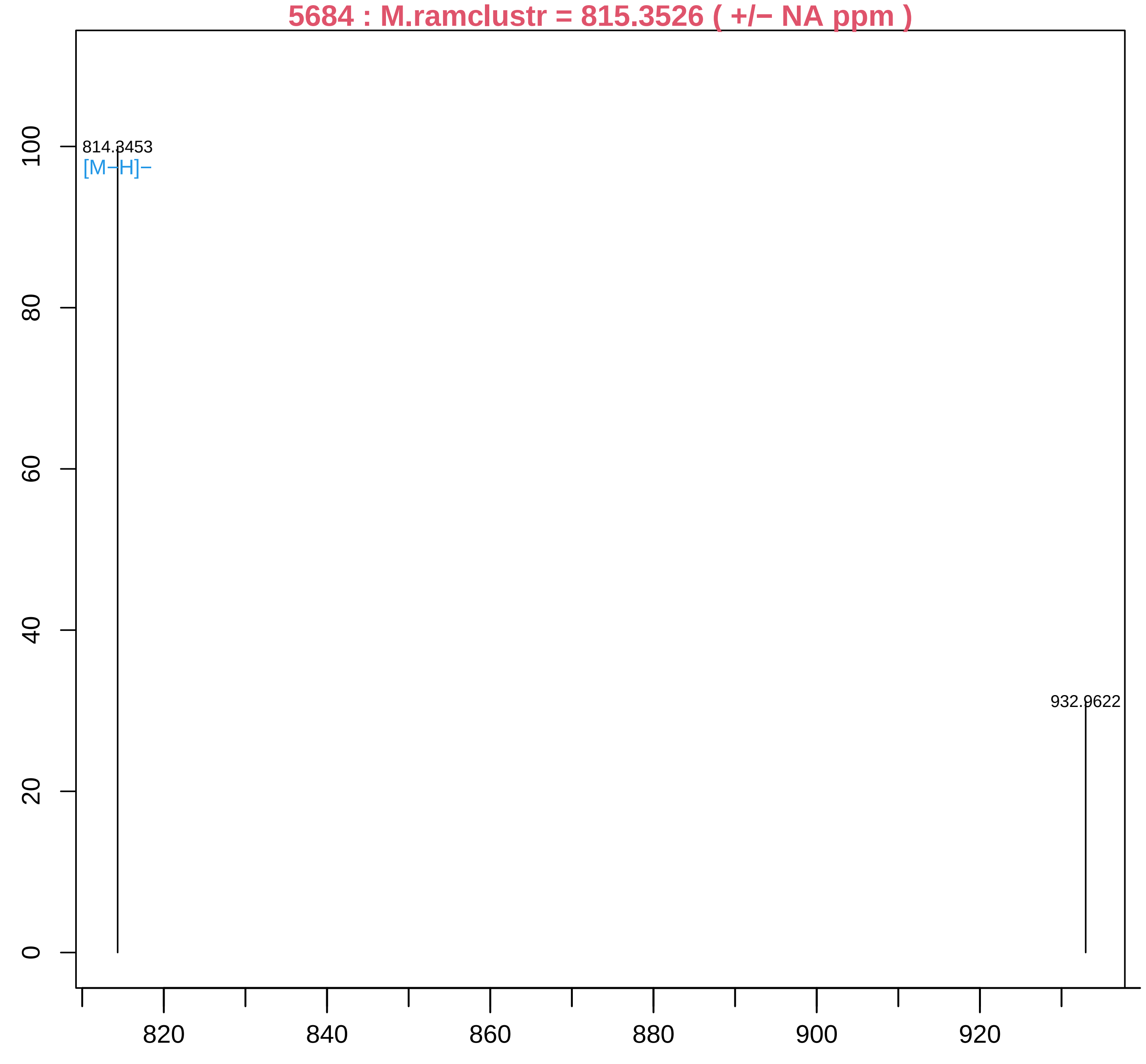

5684 : M.ramclustr = 815.3526 (+/− NA ppm)
814.3453
[M−H]−
932.9622
0
20
40
60
80
100
820
840
860
880
900
920

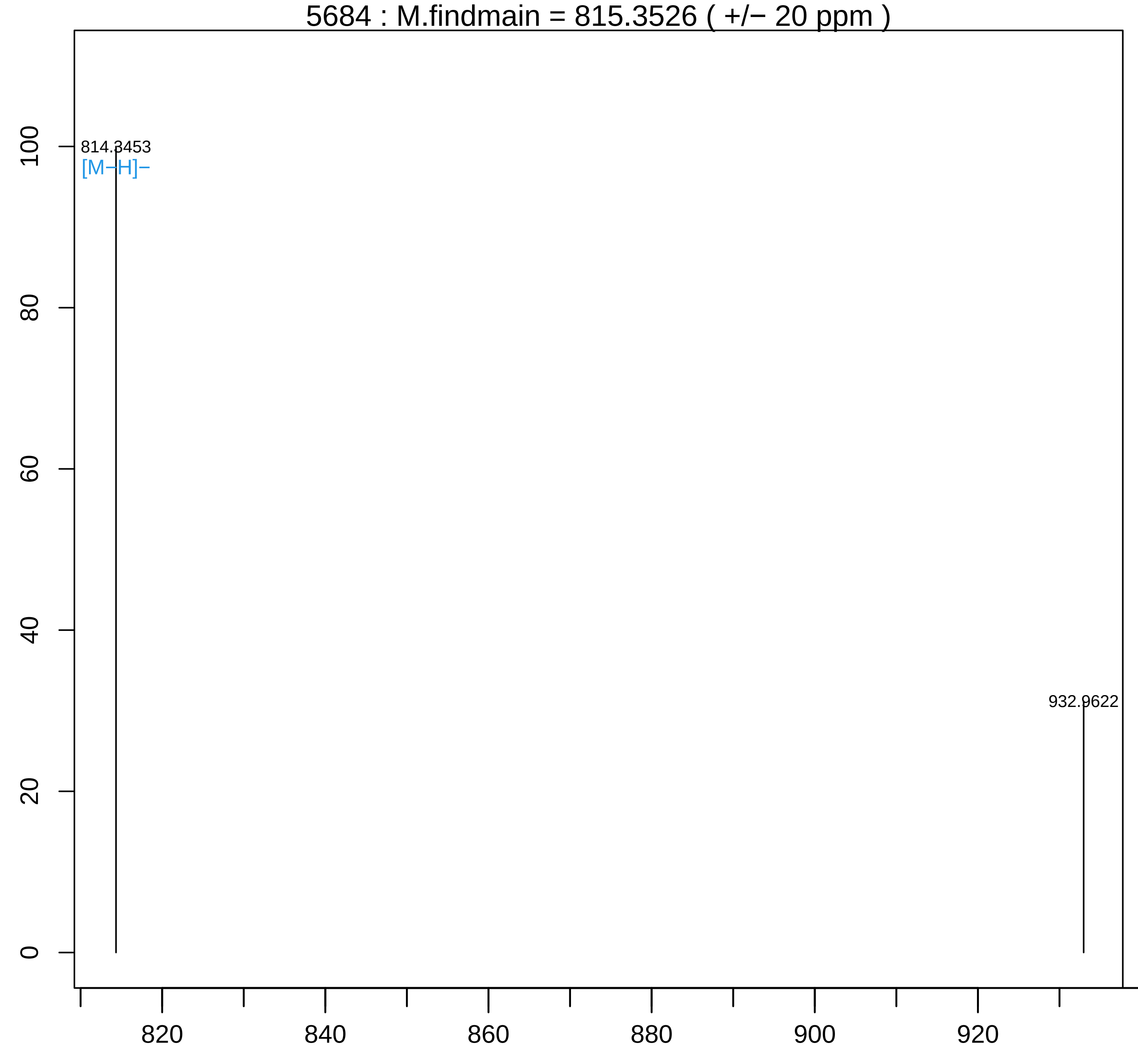

5684 : M.findmain = 815.3526 (+/− 20 ppm)
814.3453
[M−H]−
932.9622
0
20
40
60
80
100
820
840
860
880
900
920

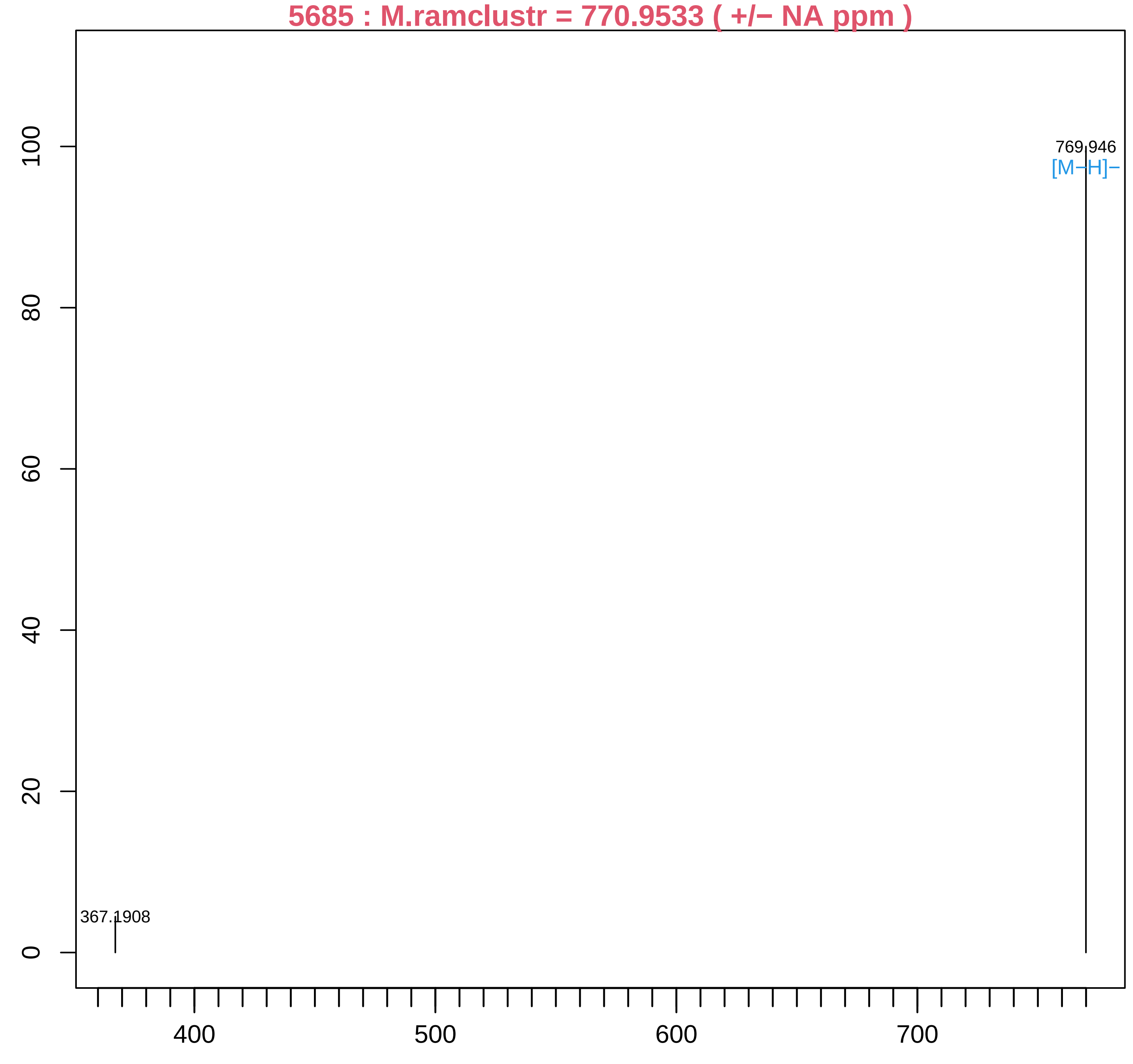
5685 : M.ramclustr = 770.9533 (+/− NA ppm)
769.946
[M−H]−
367.1908
0
20
40
60
80
100
400
500
600
700

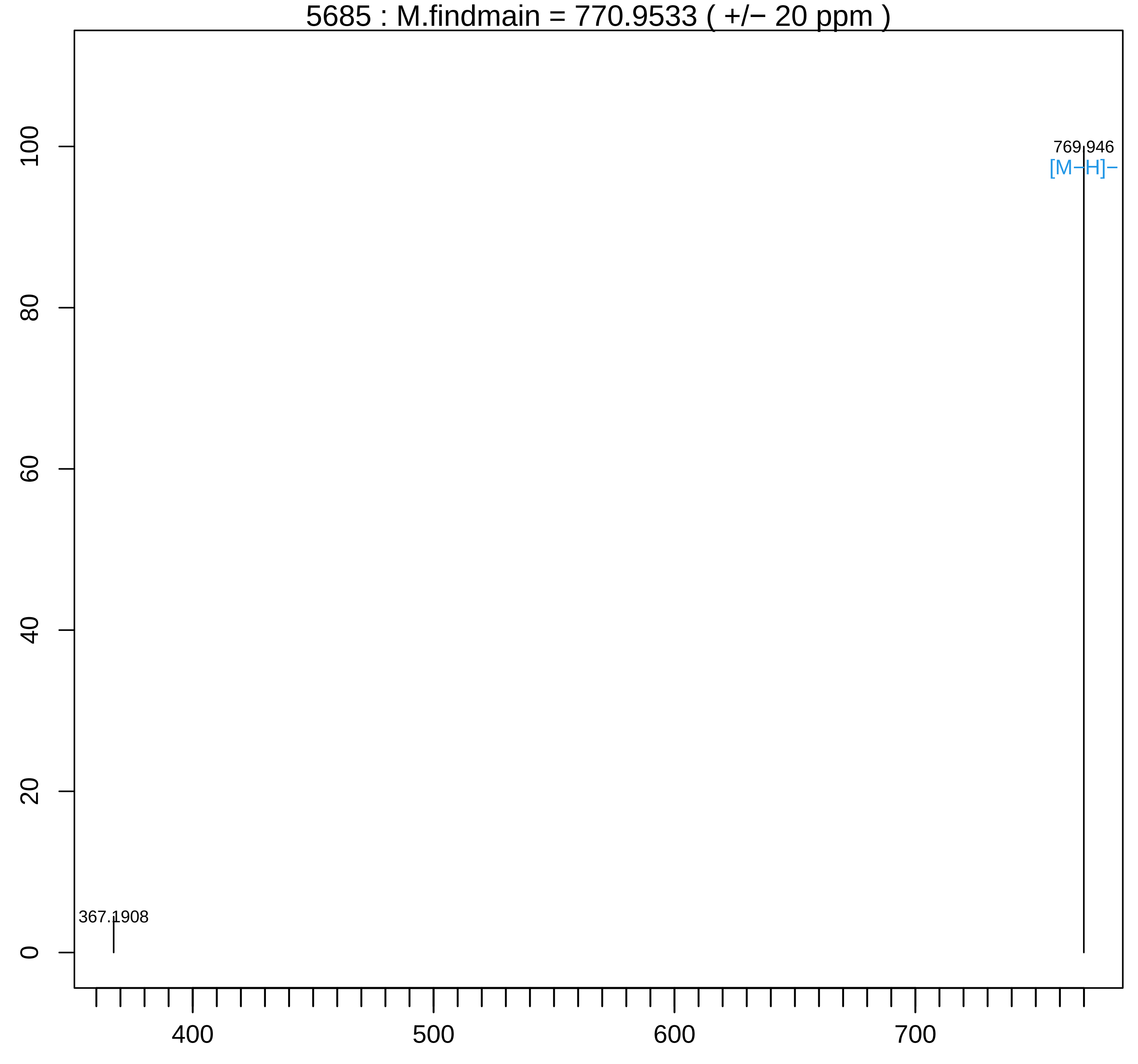
5685 : M.findmain = 770.9533 (+/− 20 ppm)
769.946
[M−H]−
367.1908
0
20
40
60
80
100
400
500
600
700

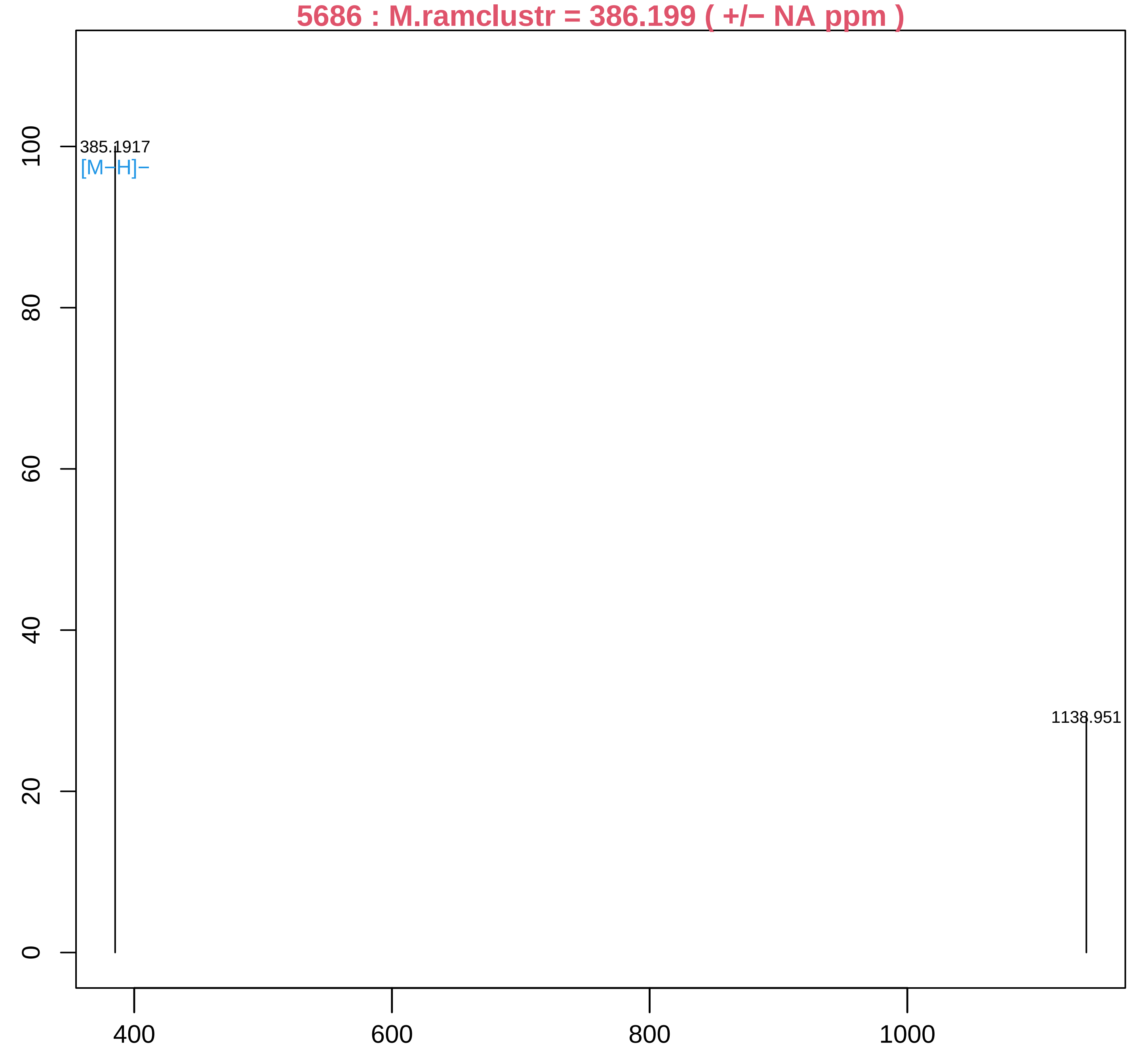
5686 : M.ramclustr = 386.199 (+/− NA ppm)
385.1917
[M−H]−
1138.951
0
20
40
60
80
100
400
600
800
1000

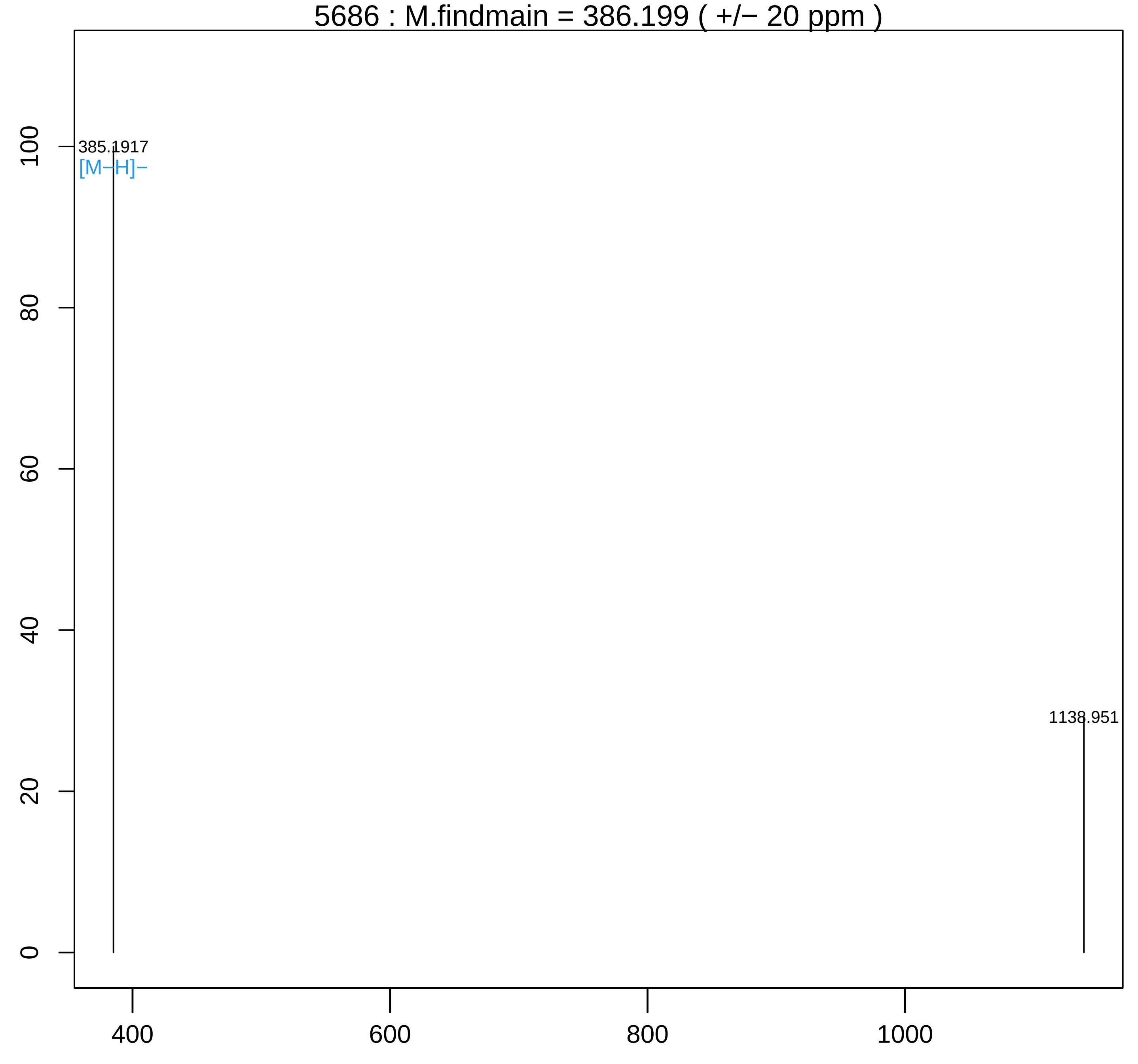
5686 : M.findmain = 386.199 (+/− 20 ppm)
385.1917
[M−H]−
1138.951
0
20
40
60
80
100
400
600
800
1000

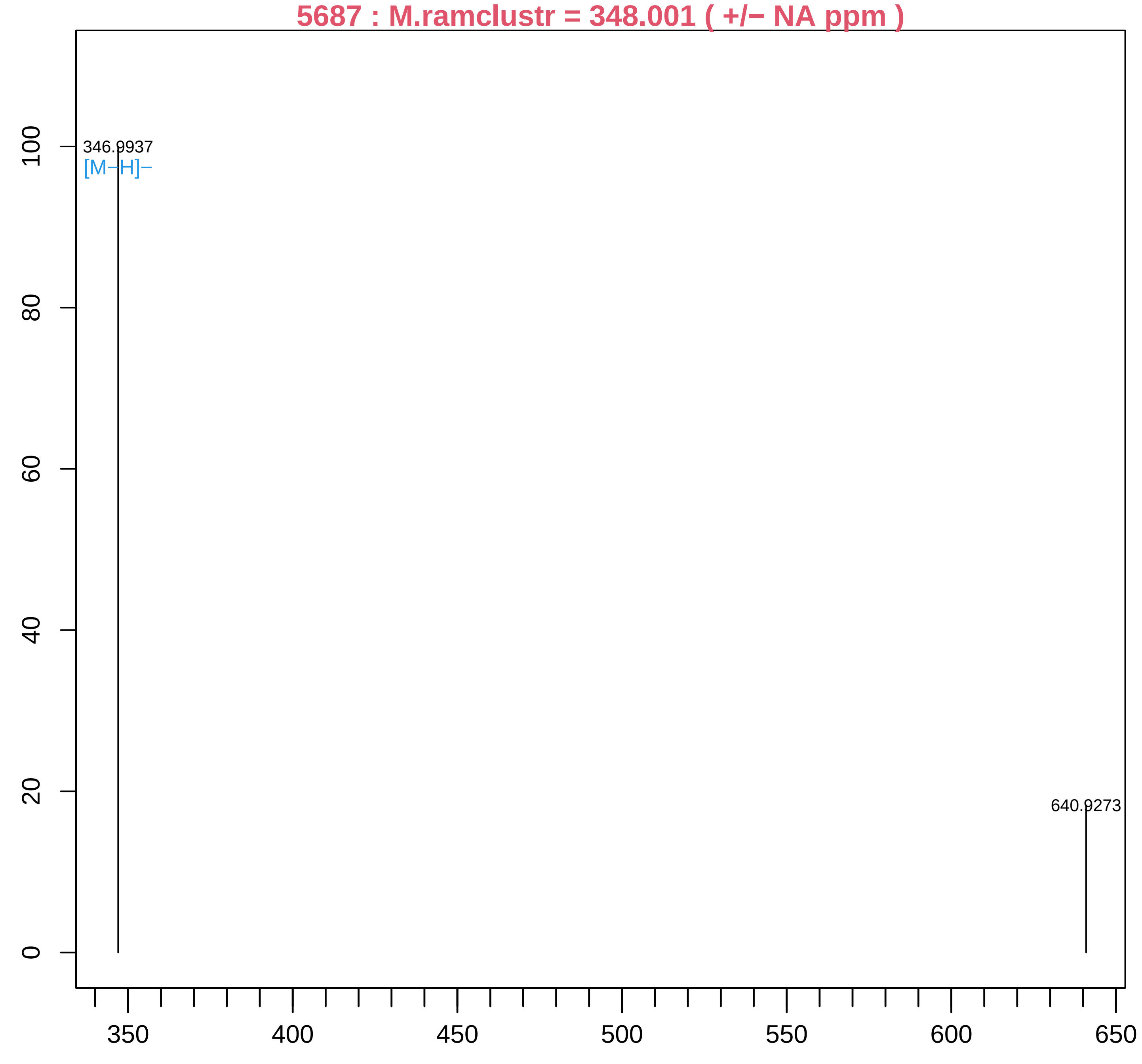
5687 : M.ramclustr = 348.001 (+/− NA ppm)
346.9937
[M−H]−
640.9273
0
20
40
60
80
100
350
400
450
500
550
600
650

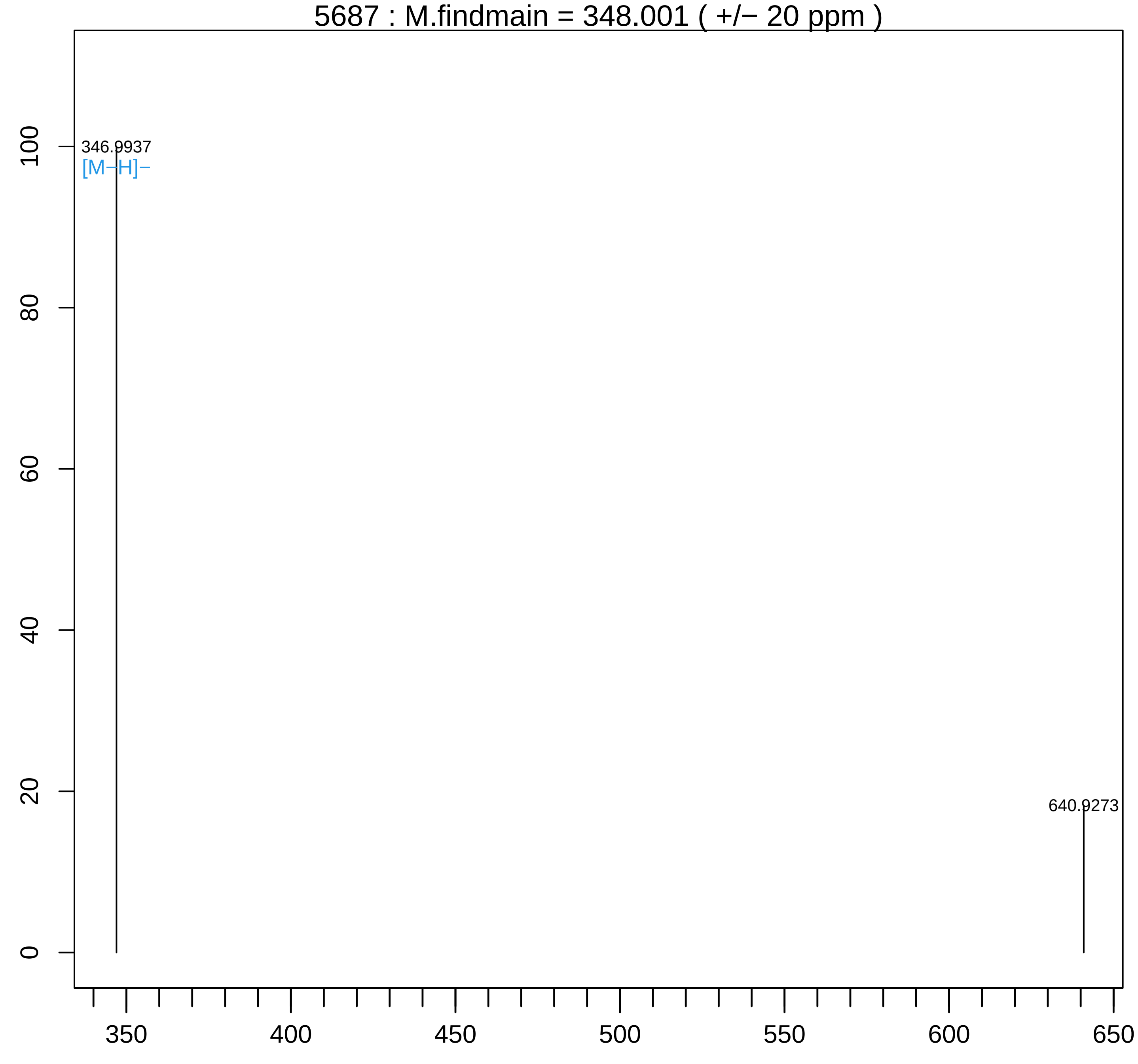
5687 : M.findmain = 348.001 (+/− 20 ppm)
346.9937
[M−H]−
640.9273
0
20
40
60
80
100
350
400
450
500
550
600
650

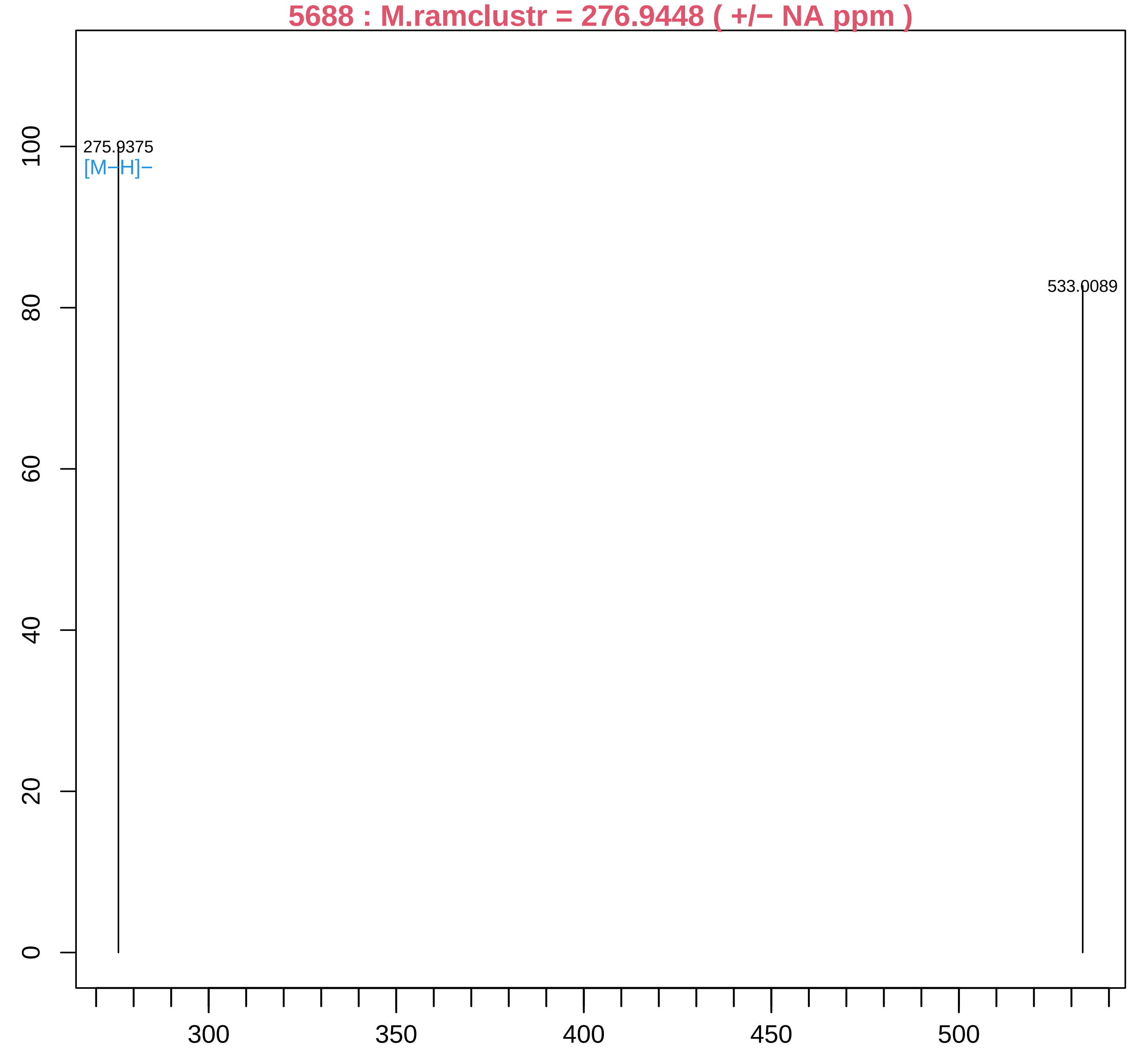

5688 : M.ramclustr = 276.9448 (+/− NA ppm)
275.9375
[M−H]−
533.0089
0
20
40
60
80
100
300
350
400
450
500

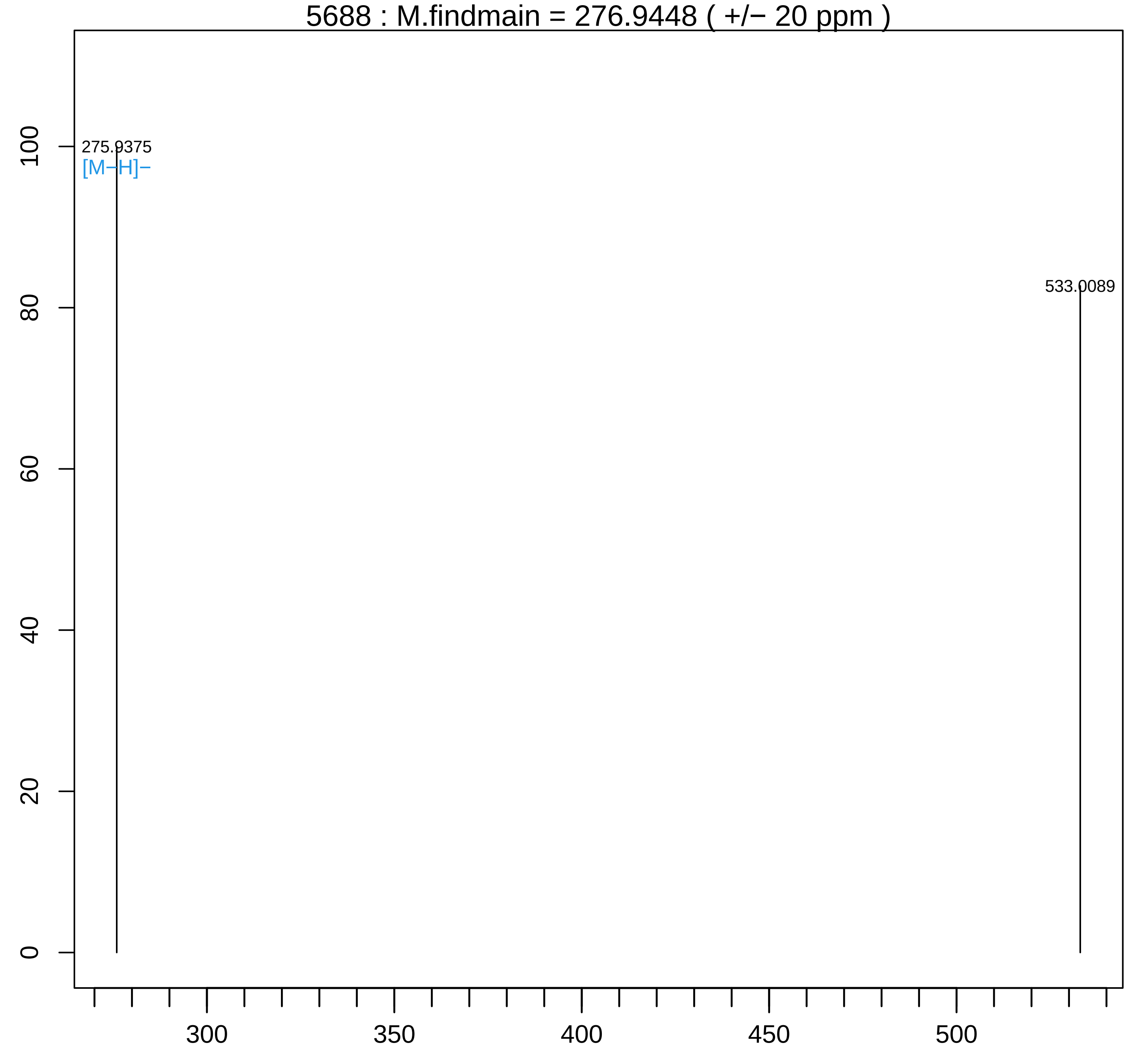

5688 : M.findmain = 276.9448 (+/− 20 ppm)
275.9375
[M−H]−
533.0089
0
20
40
60
80
100
300
350
400
450
500

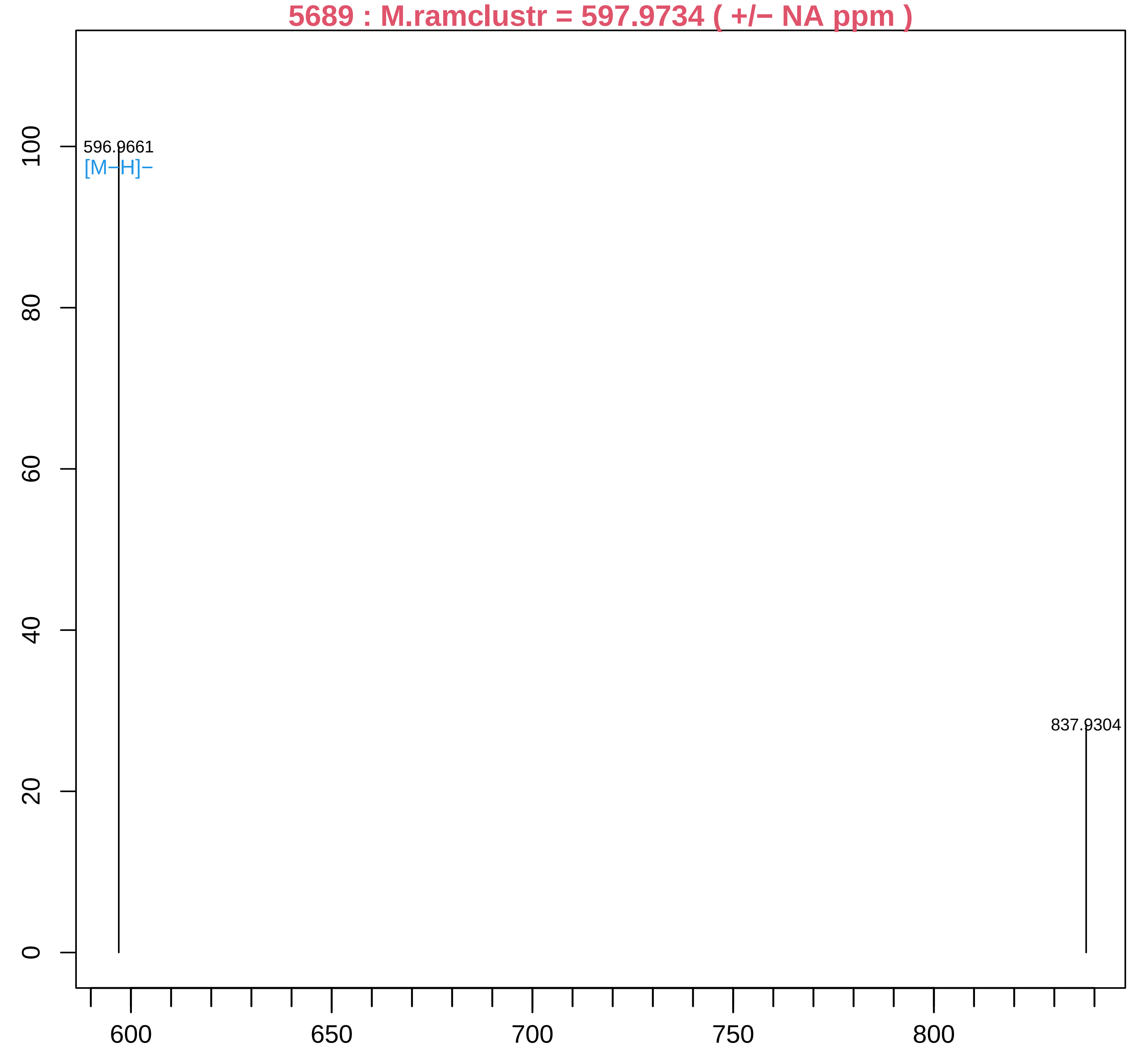
5689 : M.ramclustr = 597.9734 (+/− NA ppm)
596.9661
[M−H]−
837.9304
0
20
40
60
80
100
600
650
700
750
800

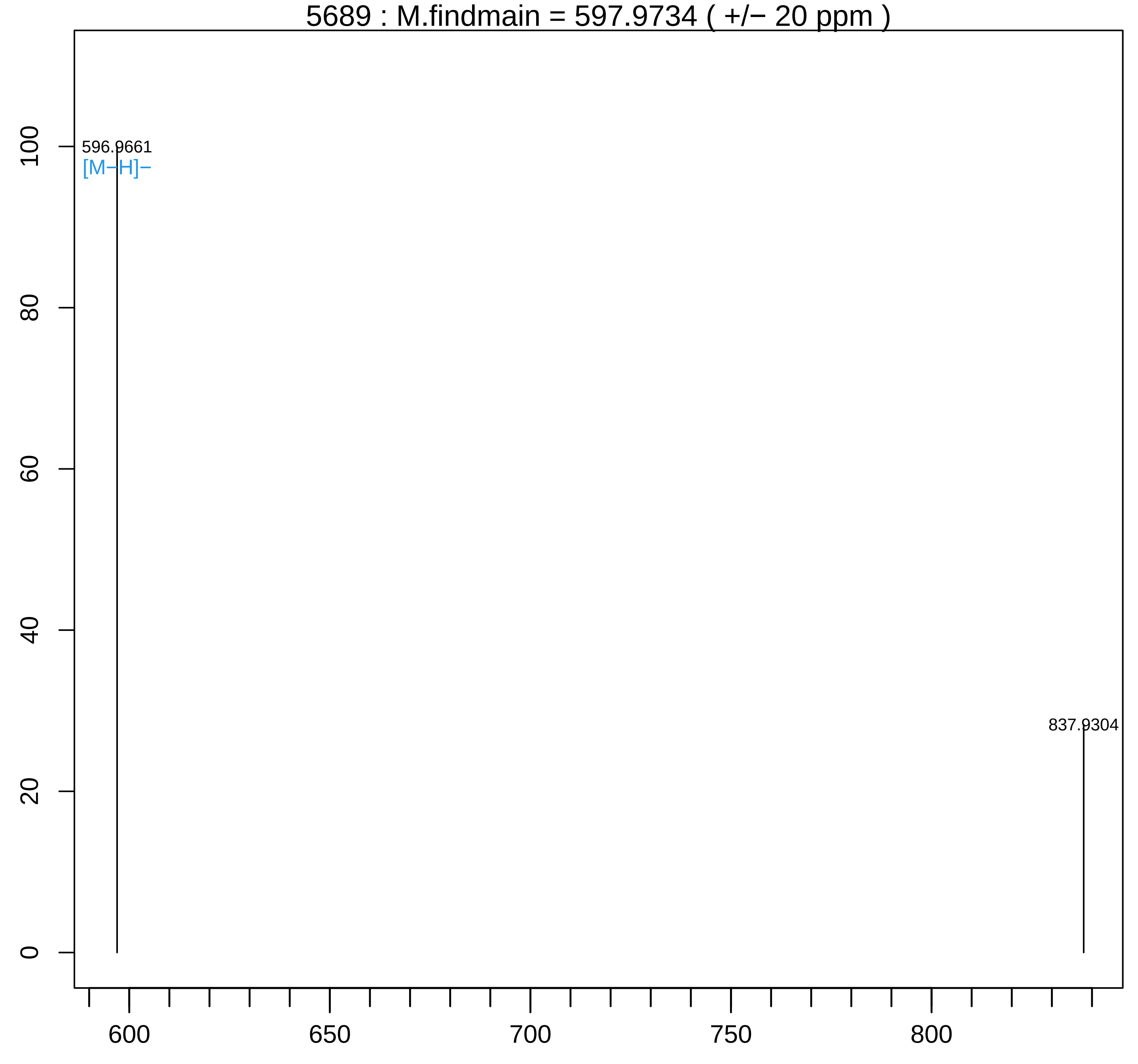
5689 : M.findmain = 597.9734 (+/− 20 ppm)
596.9661
[M−H]−
837.9304
0
20
40
60
80
100
600
650
700
750
800

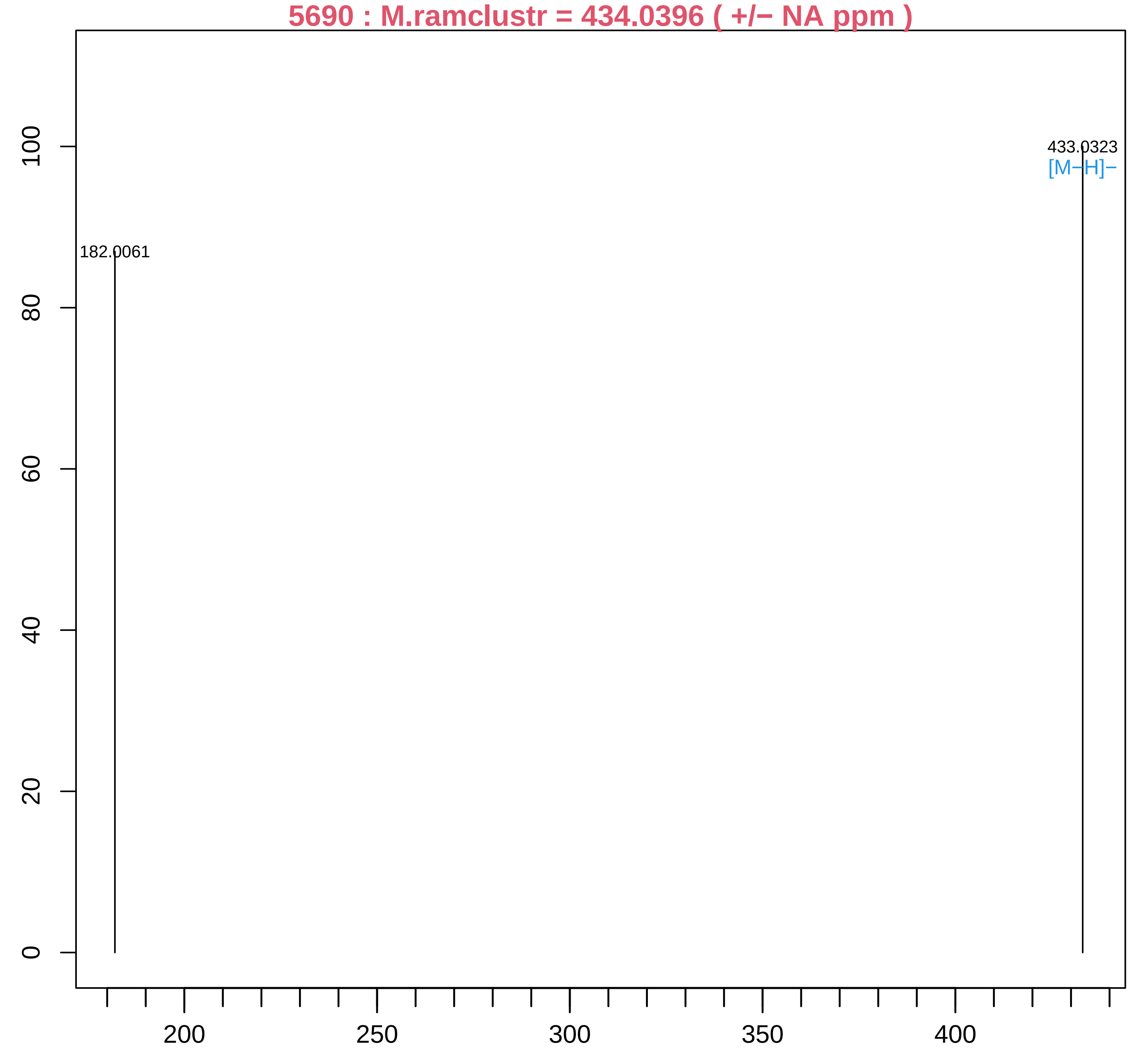
5690 : M.ramclustr = 434.0396 (+/− NA ppm)
182.0061
433.0323
[M−H]−
0
20
40
60
80
100
200
250
300
350
400

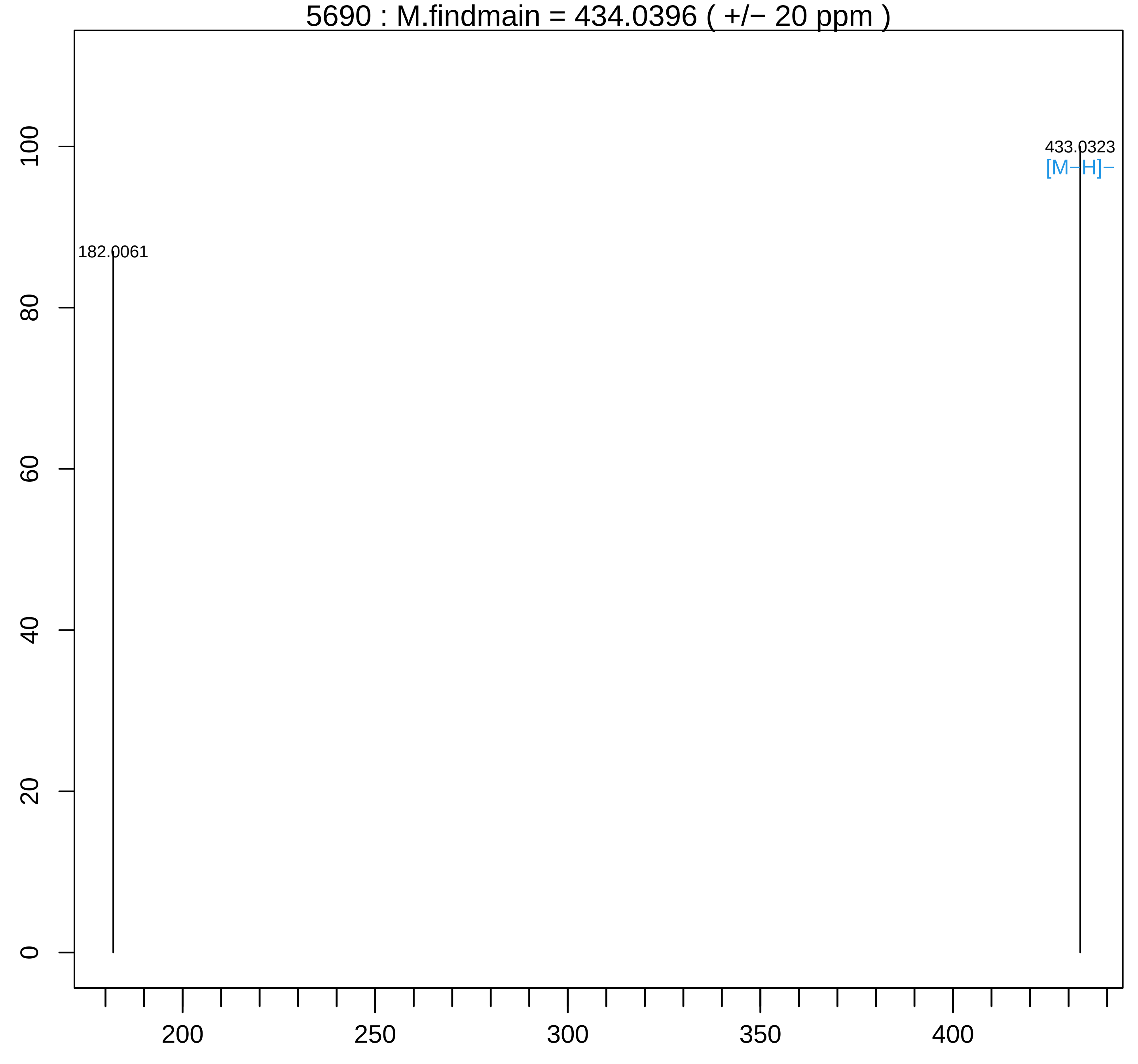
5690 : M.findmain = 434.0396 (+/− 20 ppm)
182.0061
433.0323
[M−H]−
0
20
40
60
80
100
200
250
300
350
400

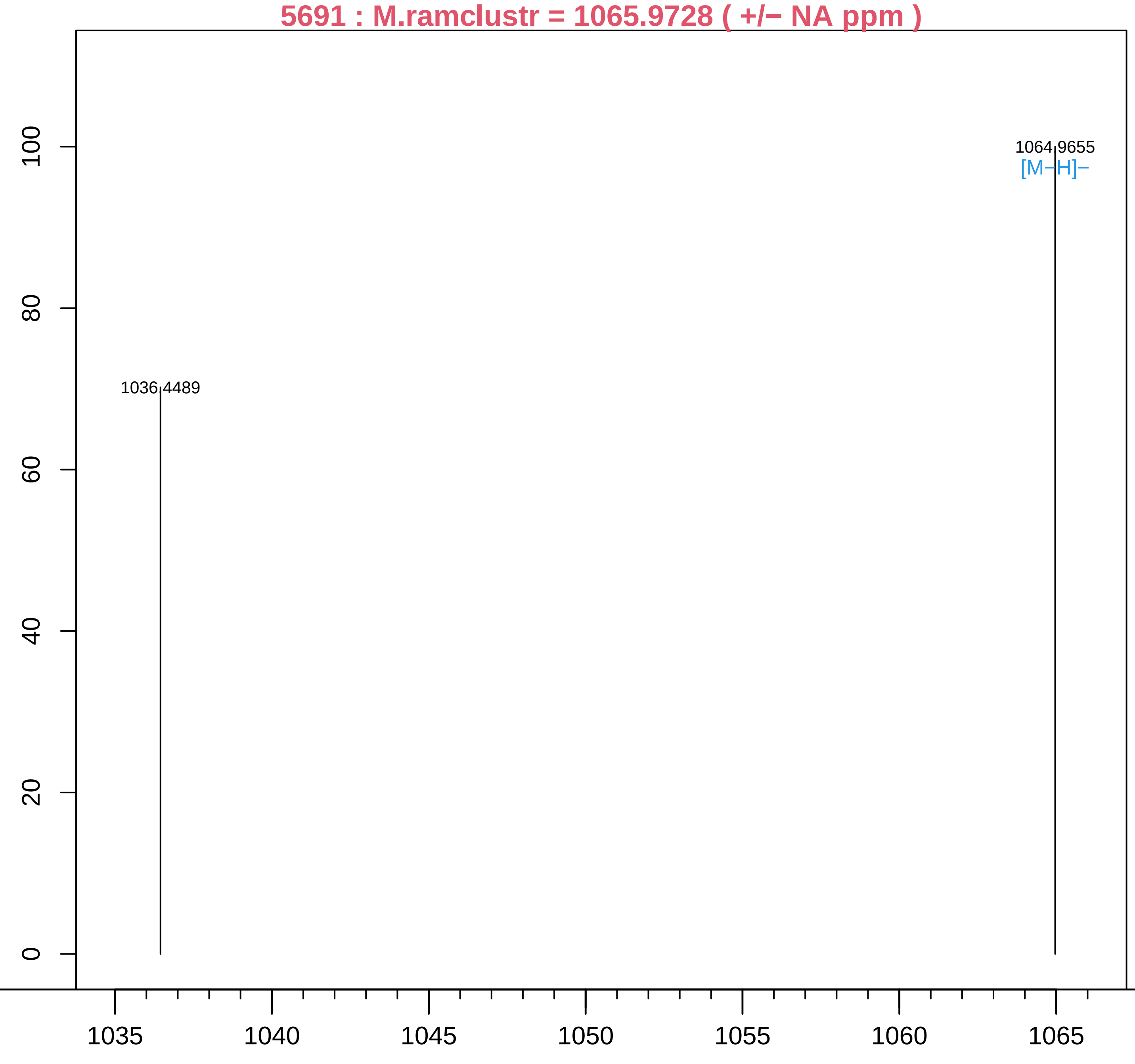

5691 : M.ramclustr = 1065.9728 (+/− NA ppm)
1064.9655
[M−H]−
1036.4489
0
20
40
60
80
100
1035
1040
1045
1050
1055
1060
1065

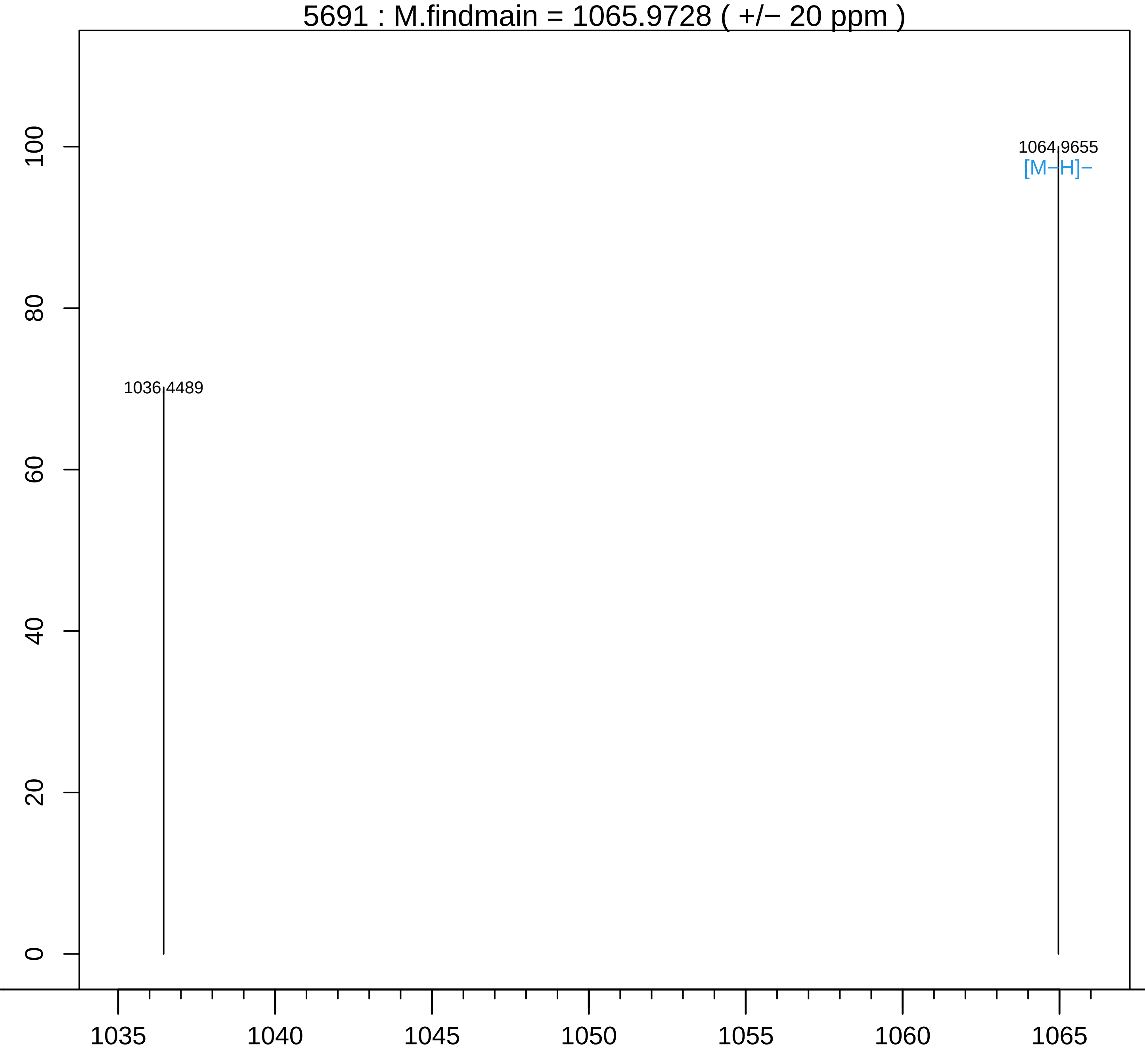

5691 : M.findmain = 1065.9728 (+/− 20 ppm)
1064.9655
[M−H]−
1036.4489
0
20
40
60
80
100
1035
1040
1045
1050
1055
1060
1065

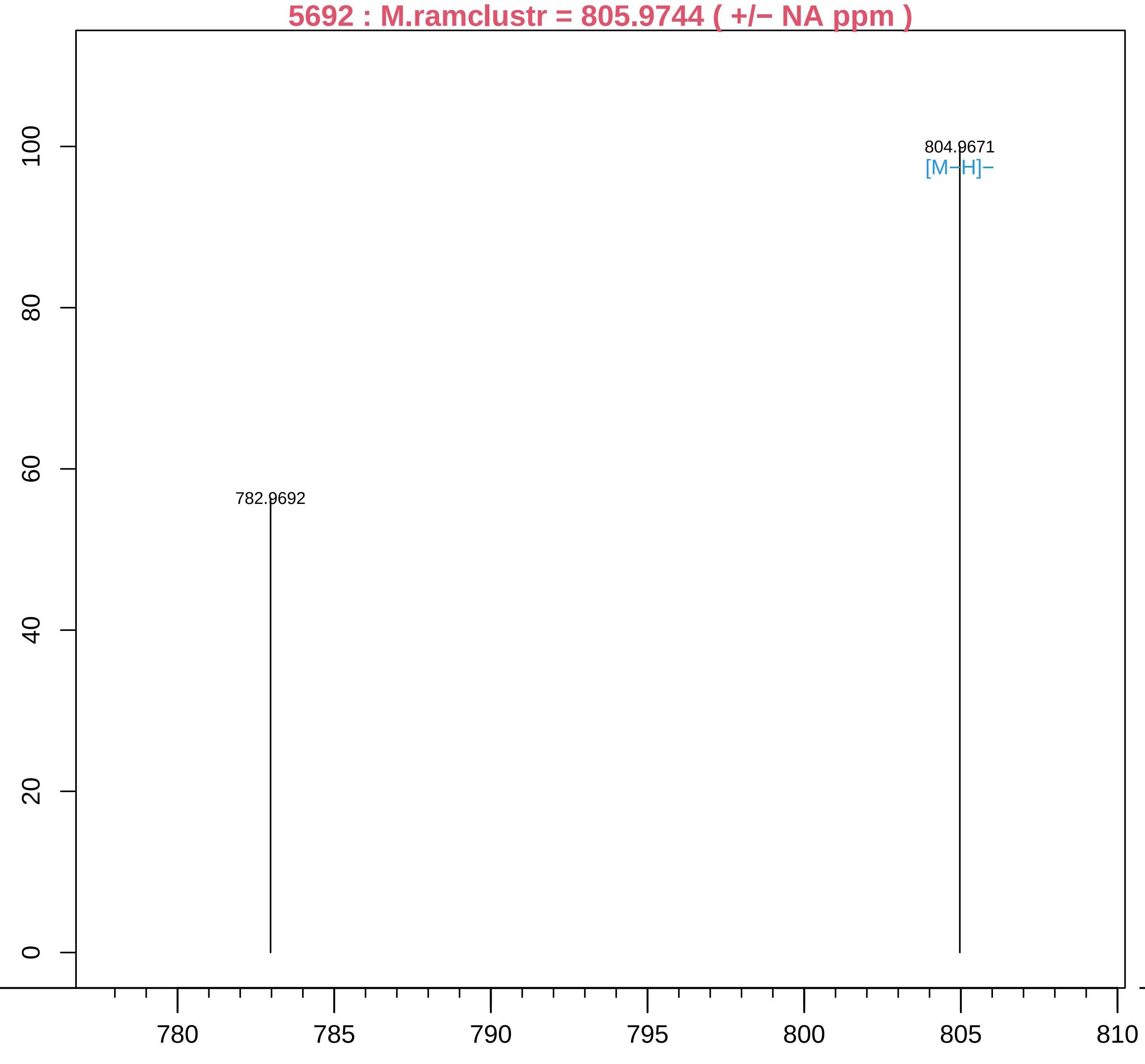
5692 : M.ramclustr = 805.9744 (+/− NA ppm)
804.9671
[M−H]−
782.9692
0
20
40
60
80
100
780
785
790
795
800
805
810

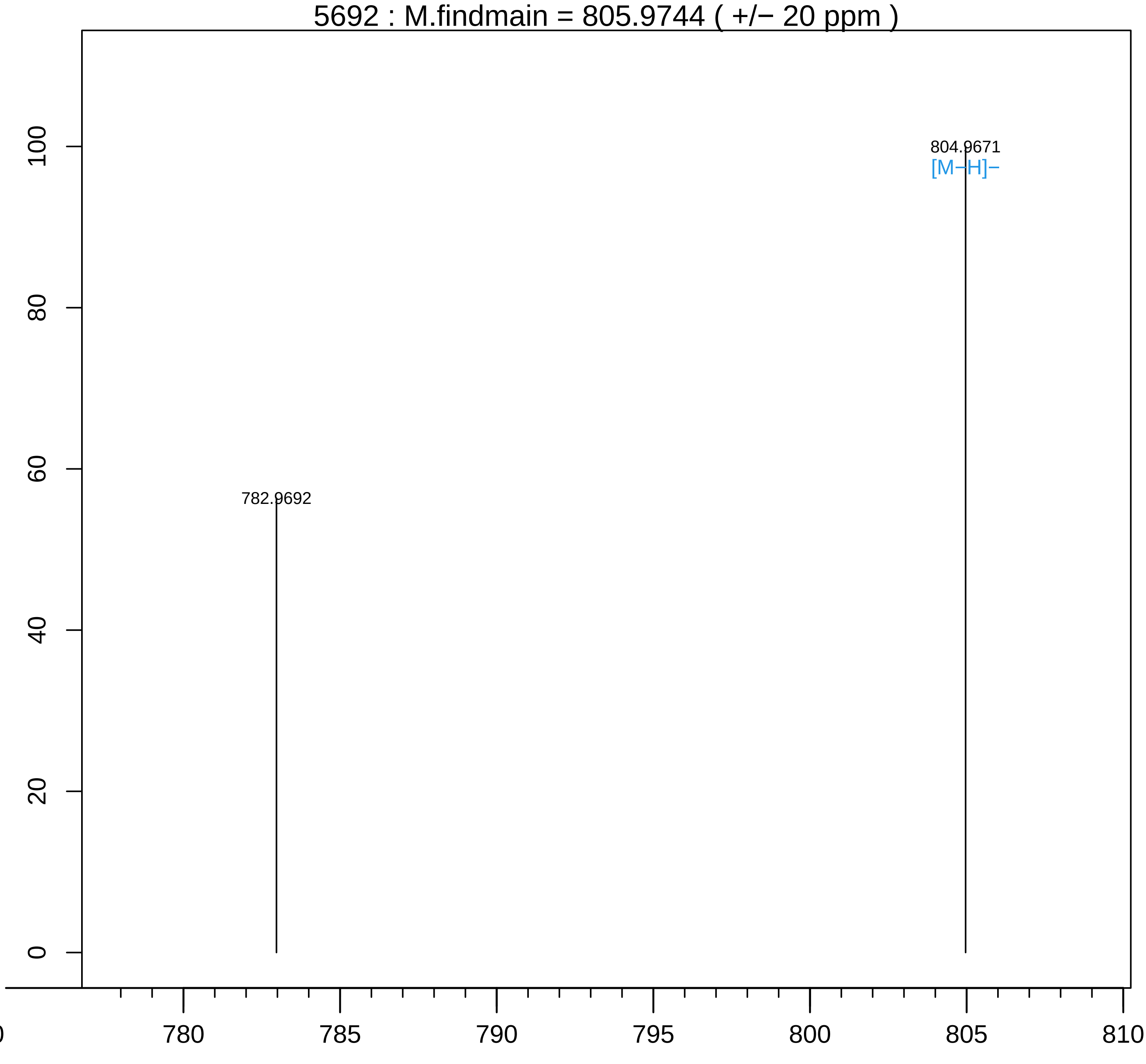
5692 : M.findmain = 805.9744 (+/− 20 ppm)
804.9671
[M−H]−
782.9692
0
20
40
60
80
100
780
785
790
795
800
805
810

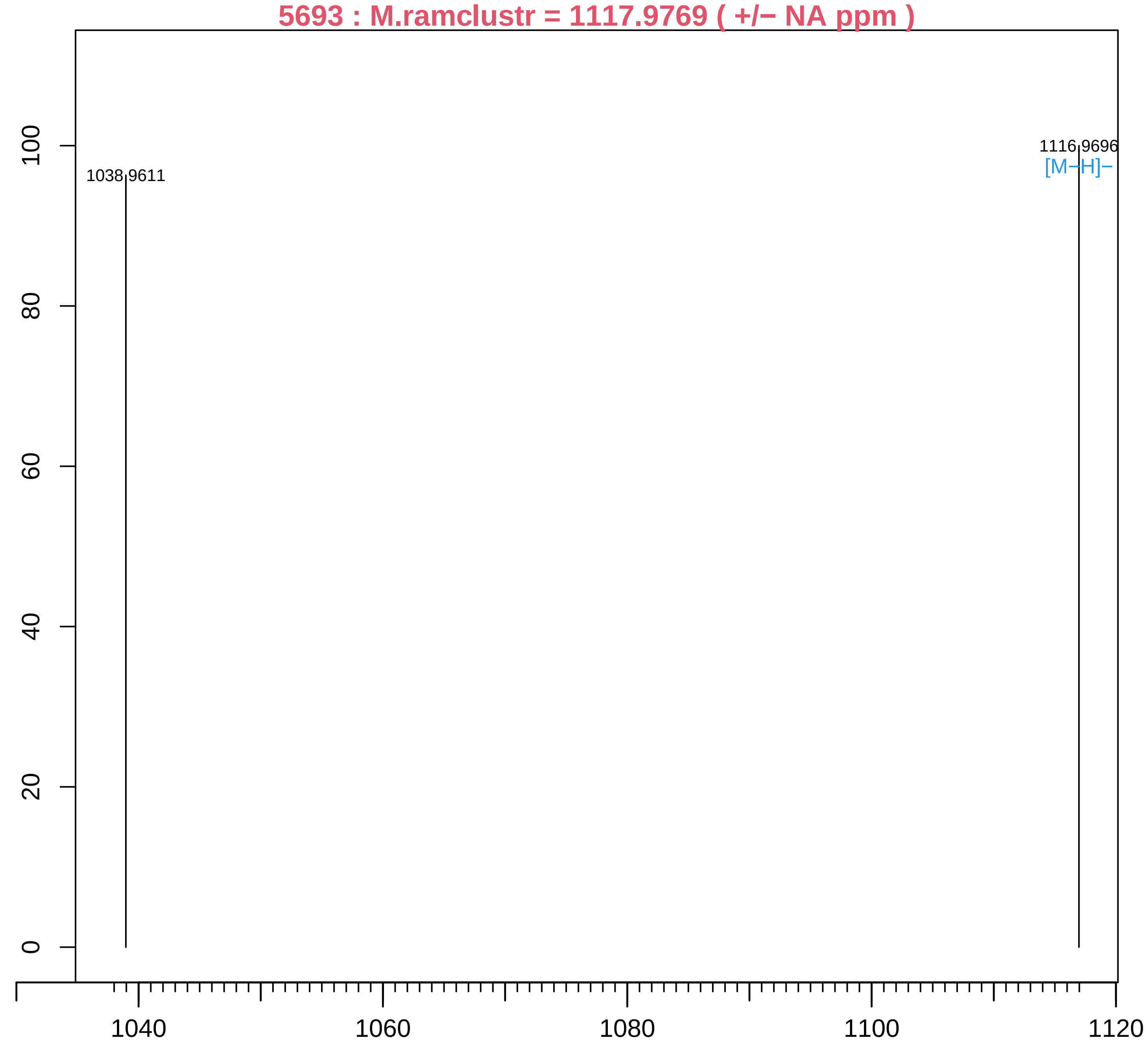
5693 : M.ramclustr = 1117.9769 (+/− NA ppm)
1038.9611
1116.9696
[M−H]−
0
20
40
60
80
100
1040
1060
1080
1100
1120

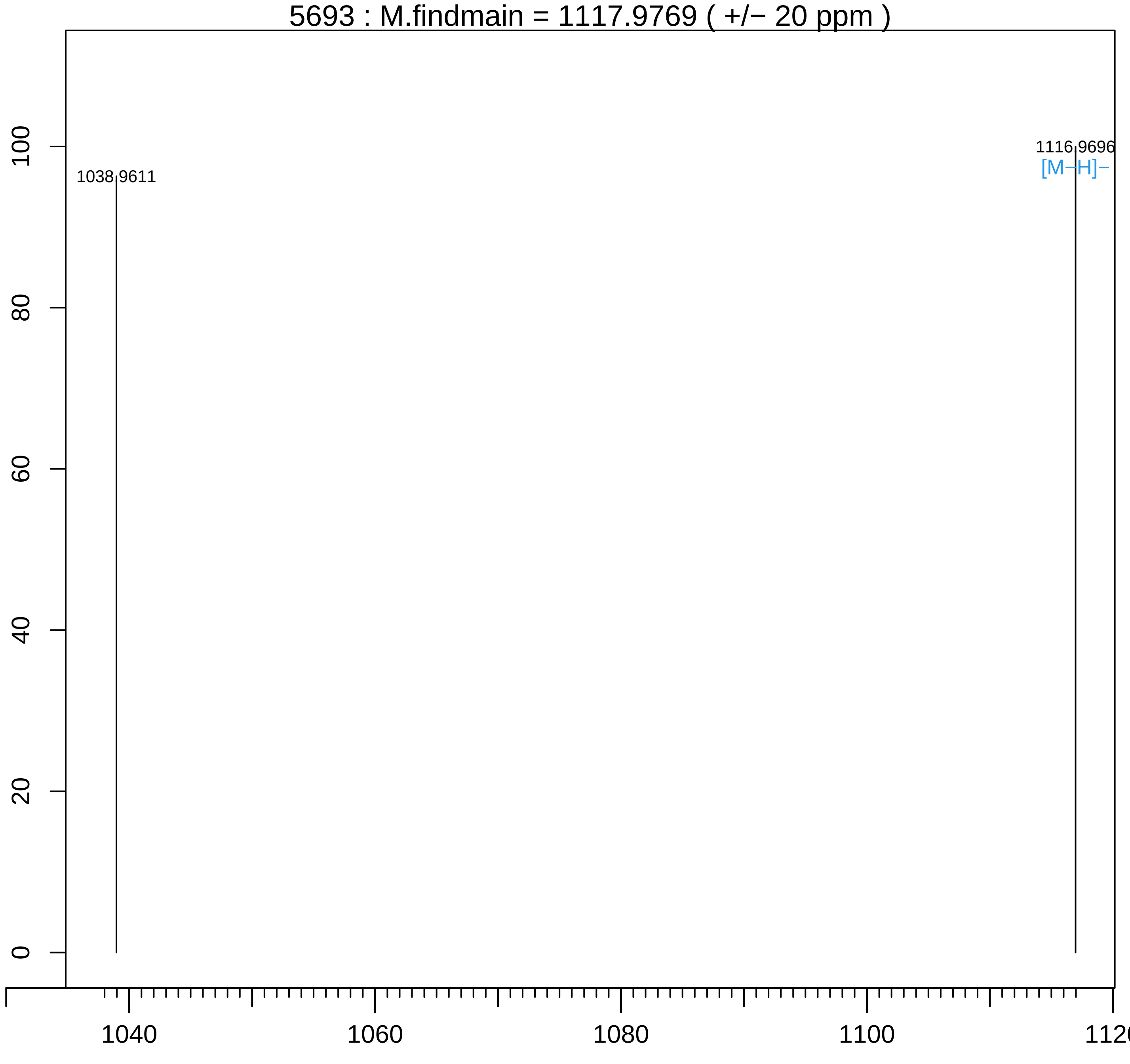
5693 : M.findmain = 1117.9769 (+/− 20 ppm)
1038.9611
1116.9696
[M−H]−
0
20
40
60
80
100
1040
1060
1080
1100

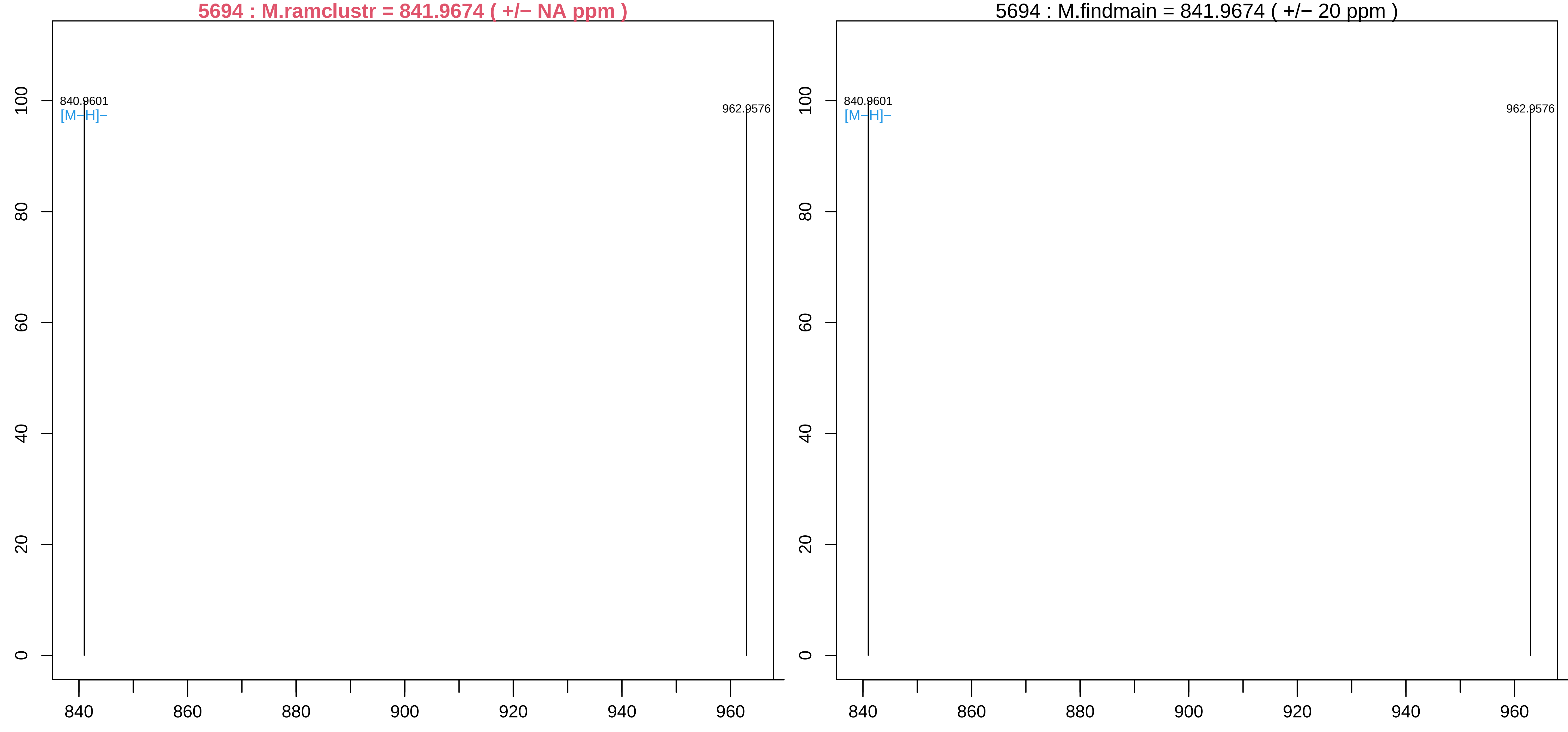
5694 : M.ramclustr = 841.9674 (+/− NA ppm)
840.9601
[M−H]−
962.9576
5694 : M.findmain = 841.9674 (+/− 20 ppm)
840.9601
[M−H]−
962.9576
0
20
40
60
80
100
840
860
880
900
920
940
960

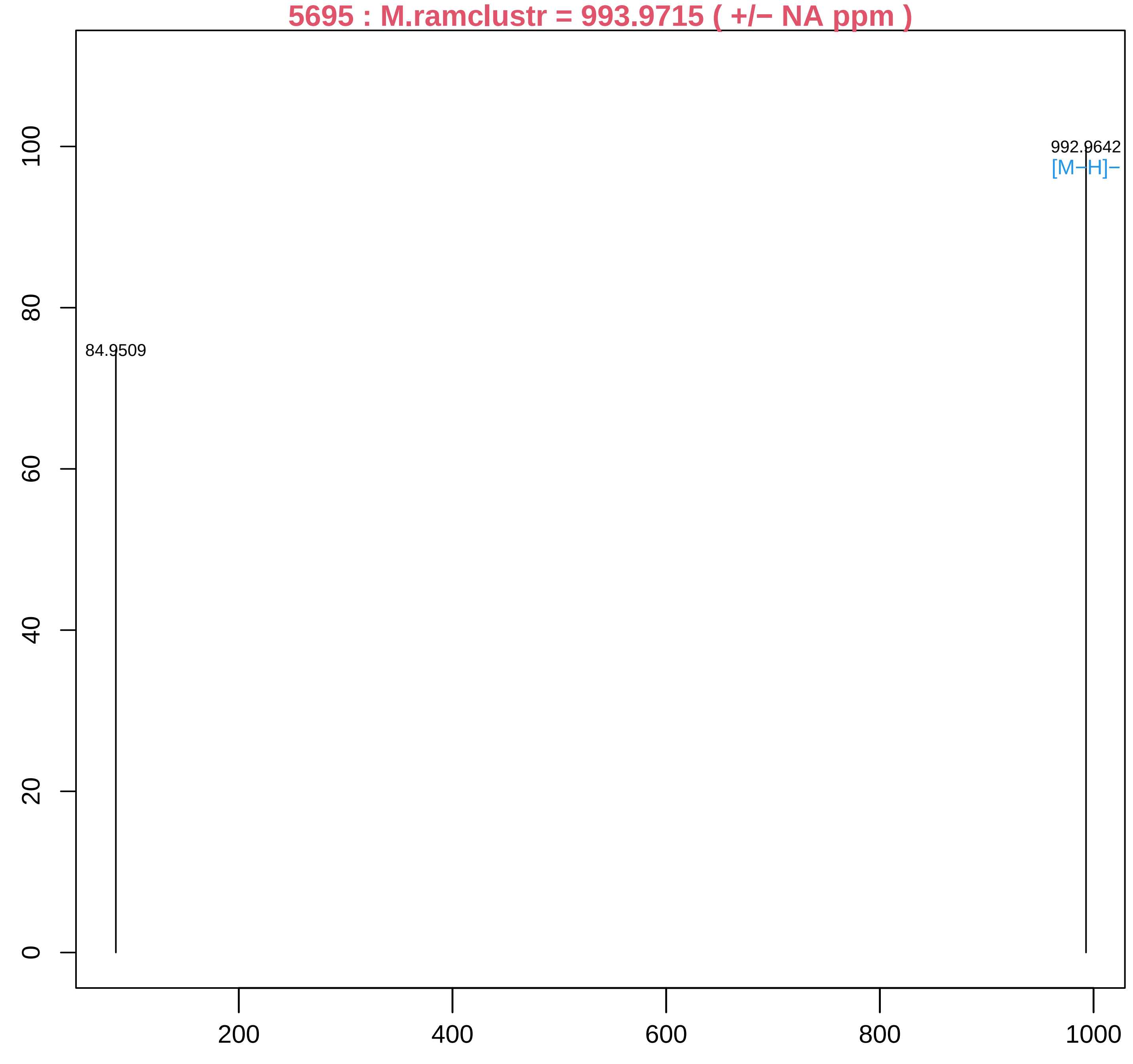
5695 : M.ramclustr = 993.9715 (+/− NA ppm)
992.9642
[M−H]−
84.9509
0
20
40
60
80
100
200
400
600
800
1000

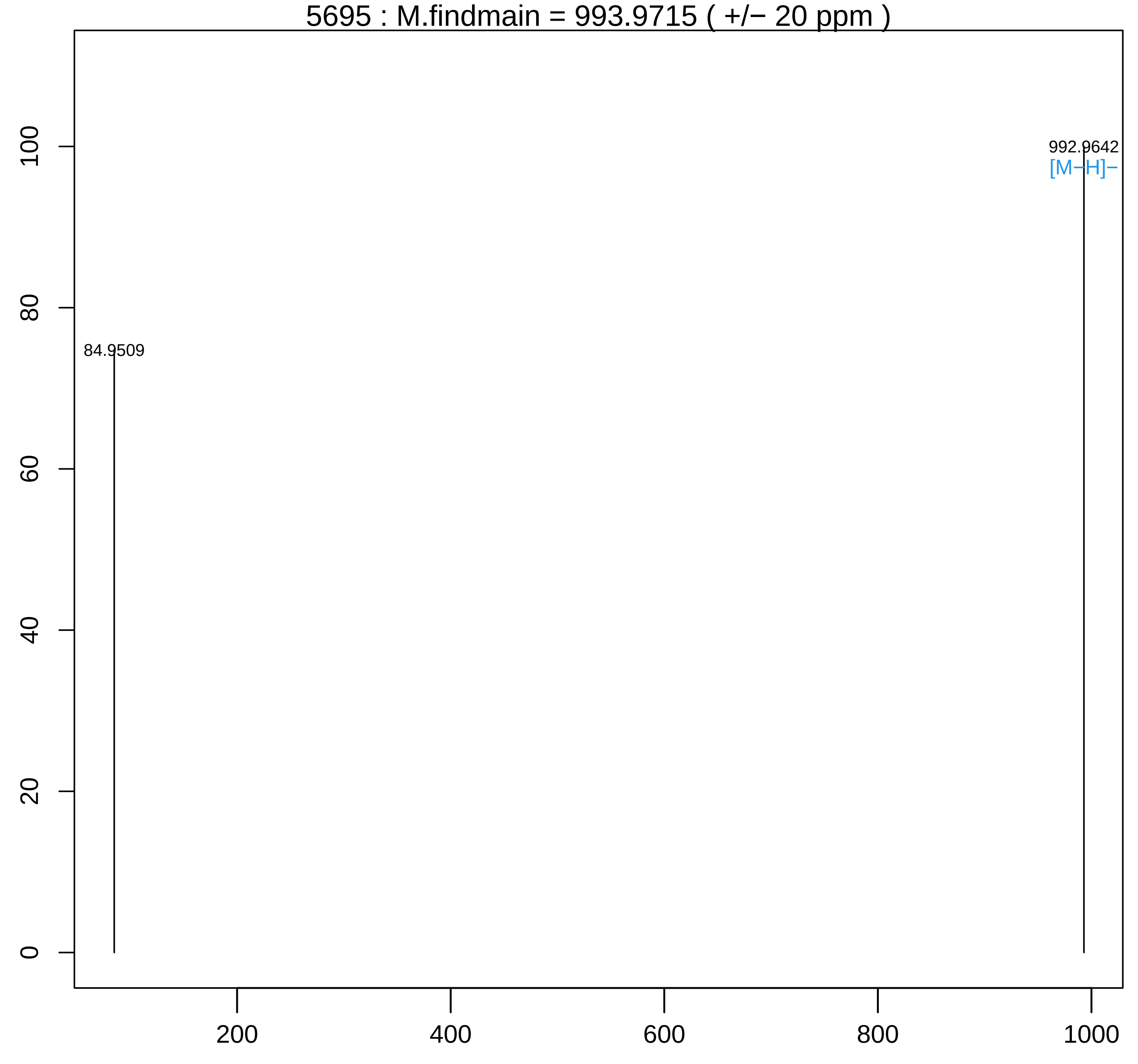
5695 : M.findmain = 993.9715 (+/− 20 ppm)
992.9642
[M−H]−
84.9509
0
20
40
60
80
100
200
400
600
800
1000

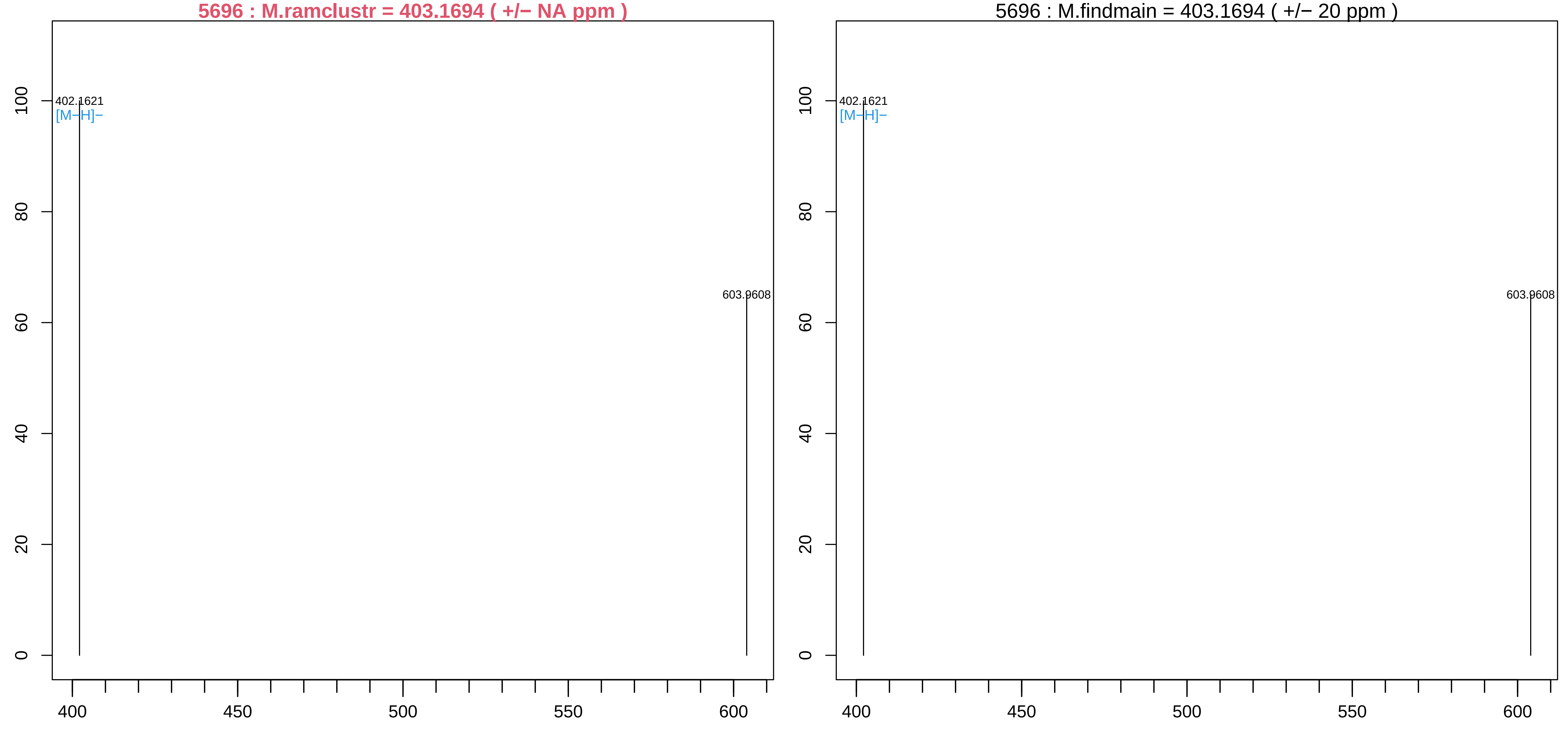

5696 : M.ramclustr = 403.1694 (+/− NA ppm)
402.1621
[M−H]−
603.9608
5696 : M.findmain = 403.1694 (+/− 20 ppm)
402.1621
[M−H]−
603.9608
0
20
40
60
80
100
400
450
500
550
600

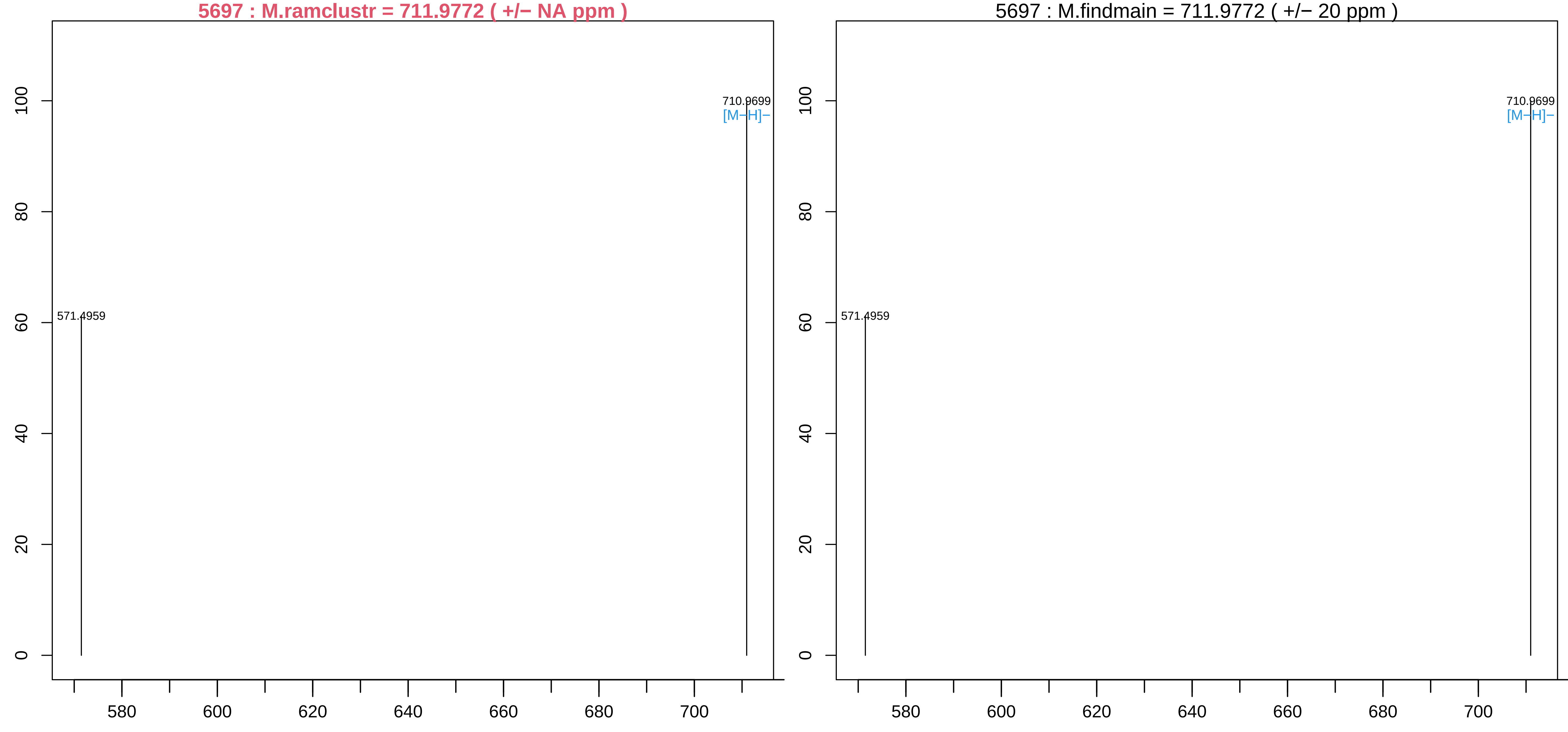
5697 : M.ramclustr = 711.9772 (+/− NA ppm)
5697 : M.findmain = 711.9772 (+/− 20 ppm)
710.9699
[M−H]−
571.4959
0
20
40
60
80
100
580
600
620
640
660
680
700

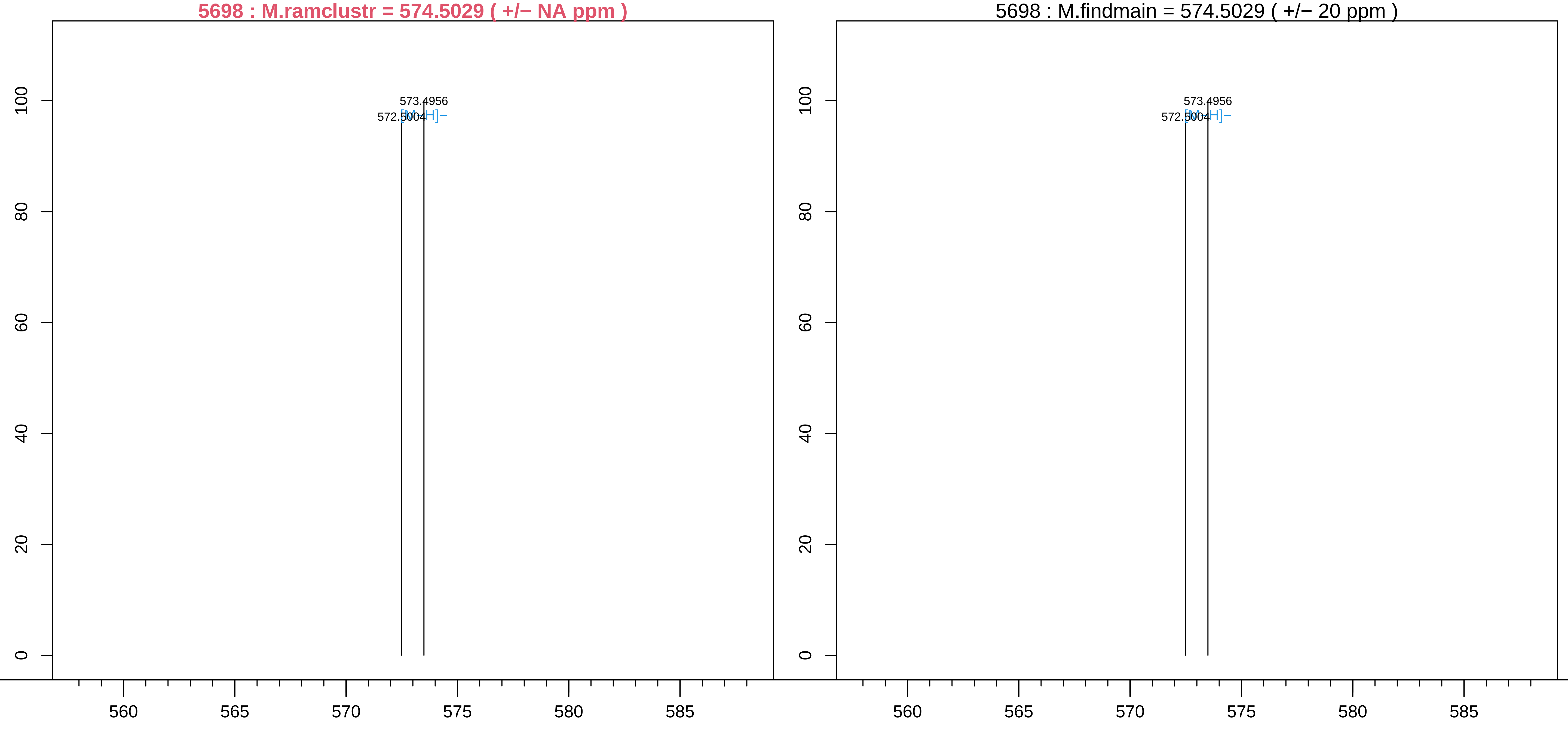

5698 : M.ramclustr = 574.5029 (+/− NA ppm)
573.4956
572.5004
[M−H]−
5698 : M.findmain = 574.5029 (+/− 20 ppm)
573.4956
572.5004
[M−H]−
0
20
40
60
80
100
560
565
570
575
580
585

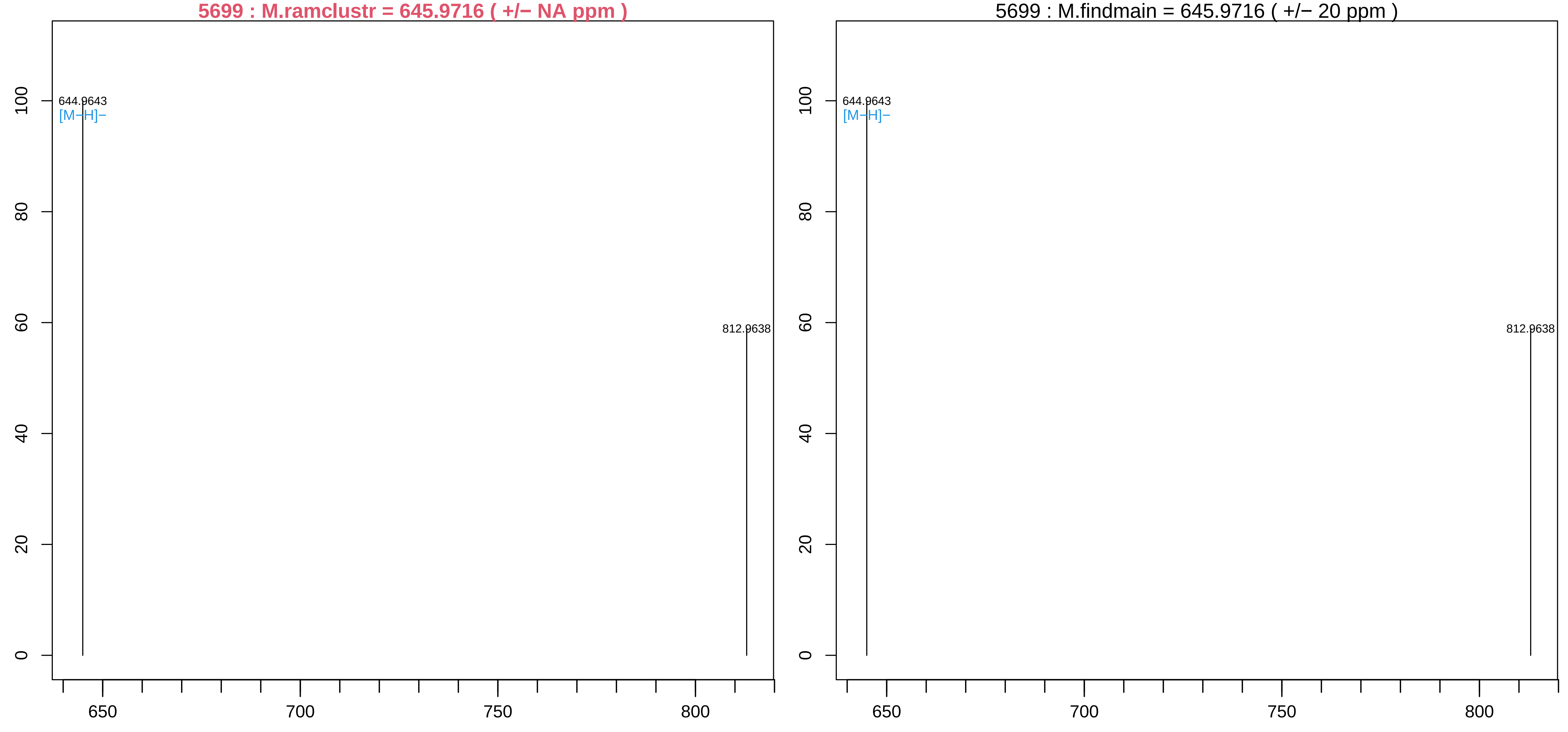
5699 : M.ramclustr = 645.9716 (+/− NA ppm)
644.9643
[M−H]−
812.9638
0
20
40
60
80
100
650
700
750
800
5699 : M.findmain = 645.9716 (+/− 20 ppm)
644.9643
[M−H]−
812.9638
0
20
40
60
80
100
650
700
750
800

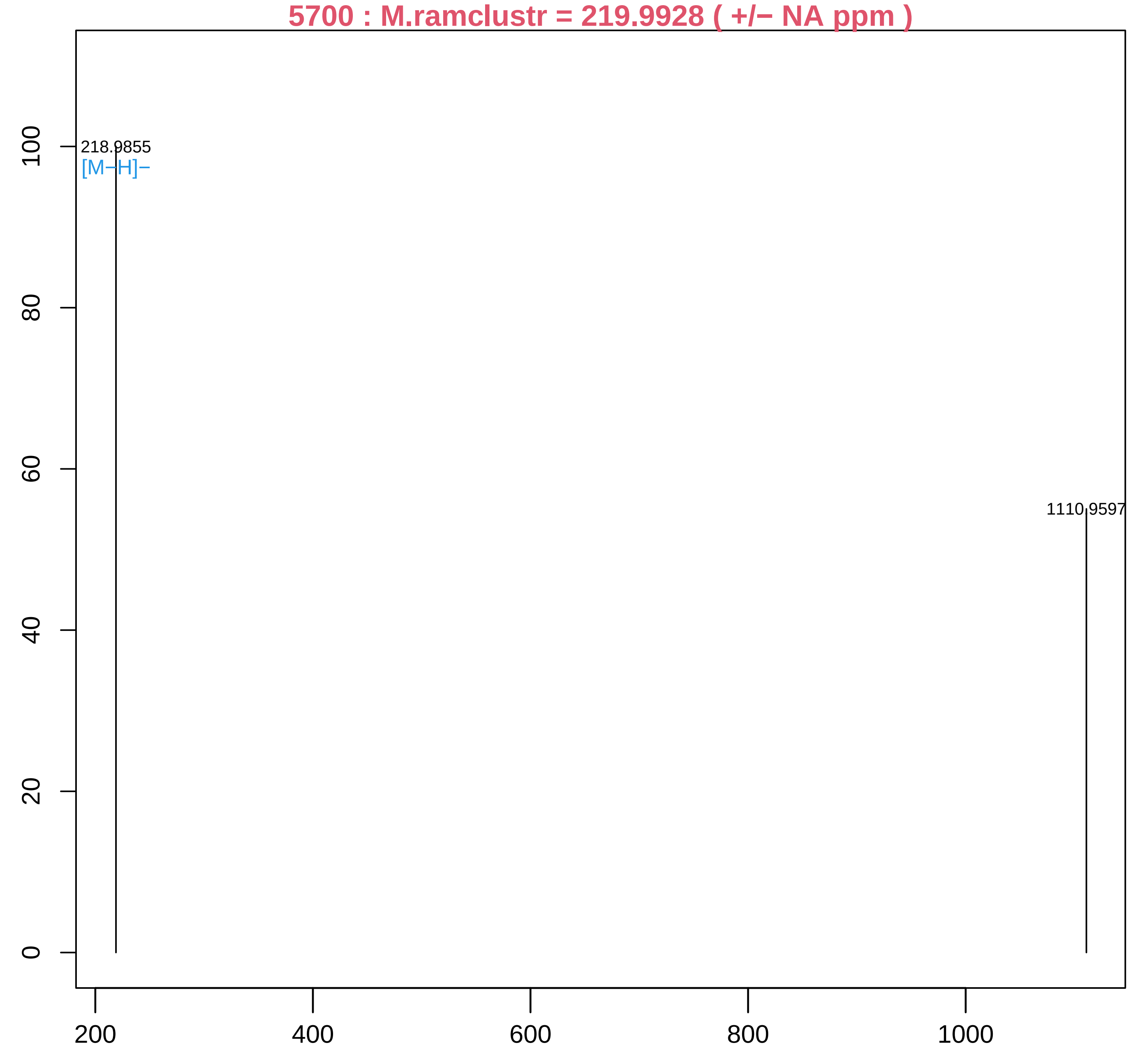
5700 : M.ramclustr = 219.9928 (+/− NA ppm)
218.9855
[M−H]−
1110.9597
0
20
40
60
80
100
200
400
600
800
1000

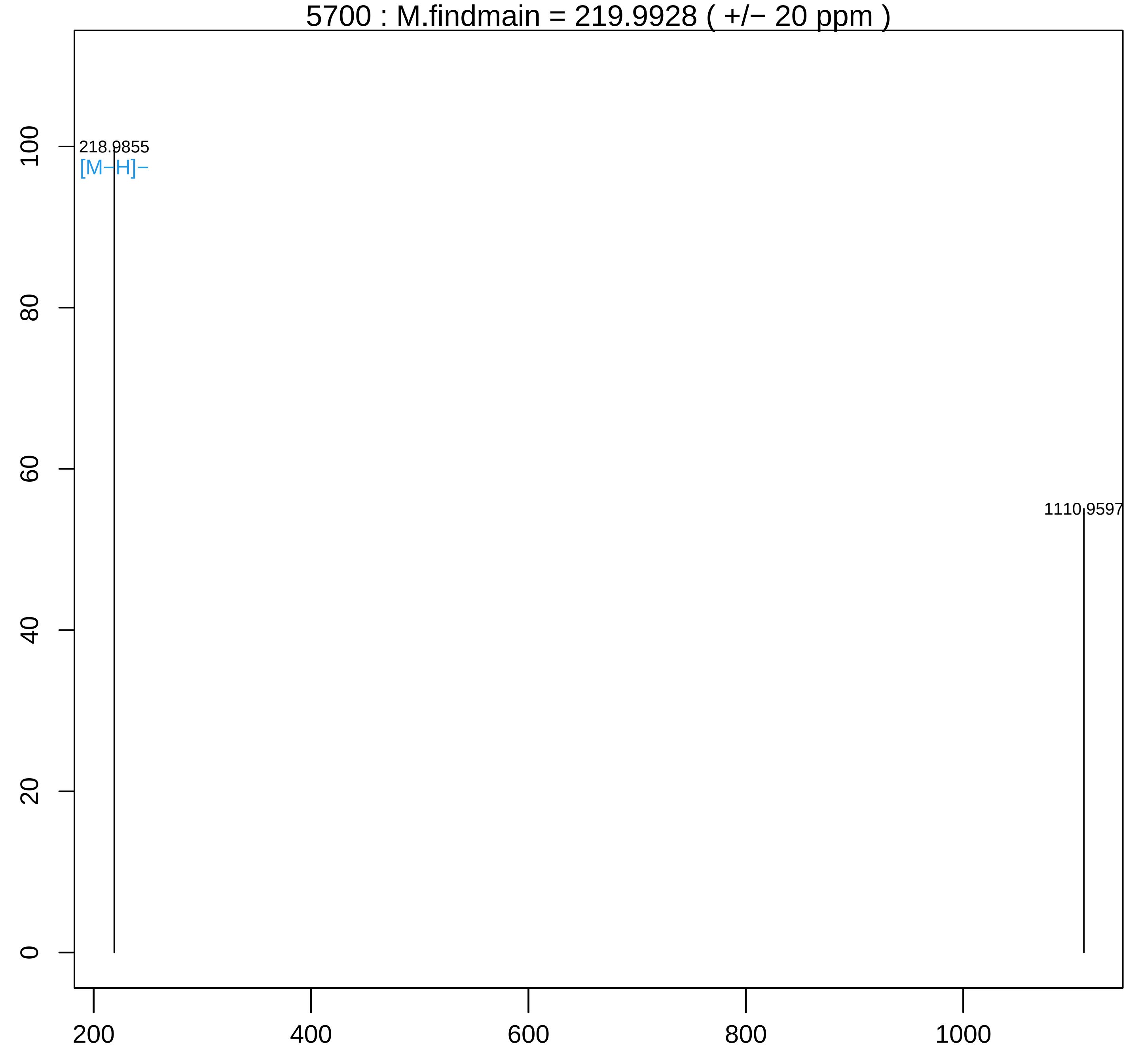
5700 : M.findmain = 219.9928 (+/− 20 ppm)
218.9855
[M−H]−
1110.9597
0
20
40
60
80
100
200
400
600
800
1000

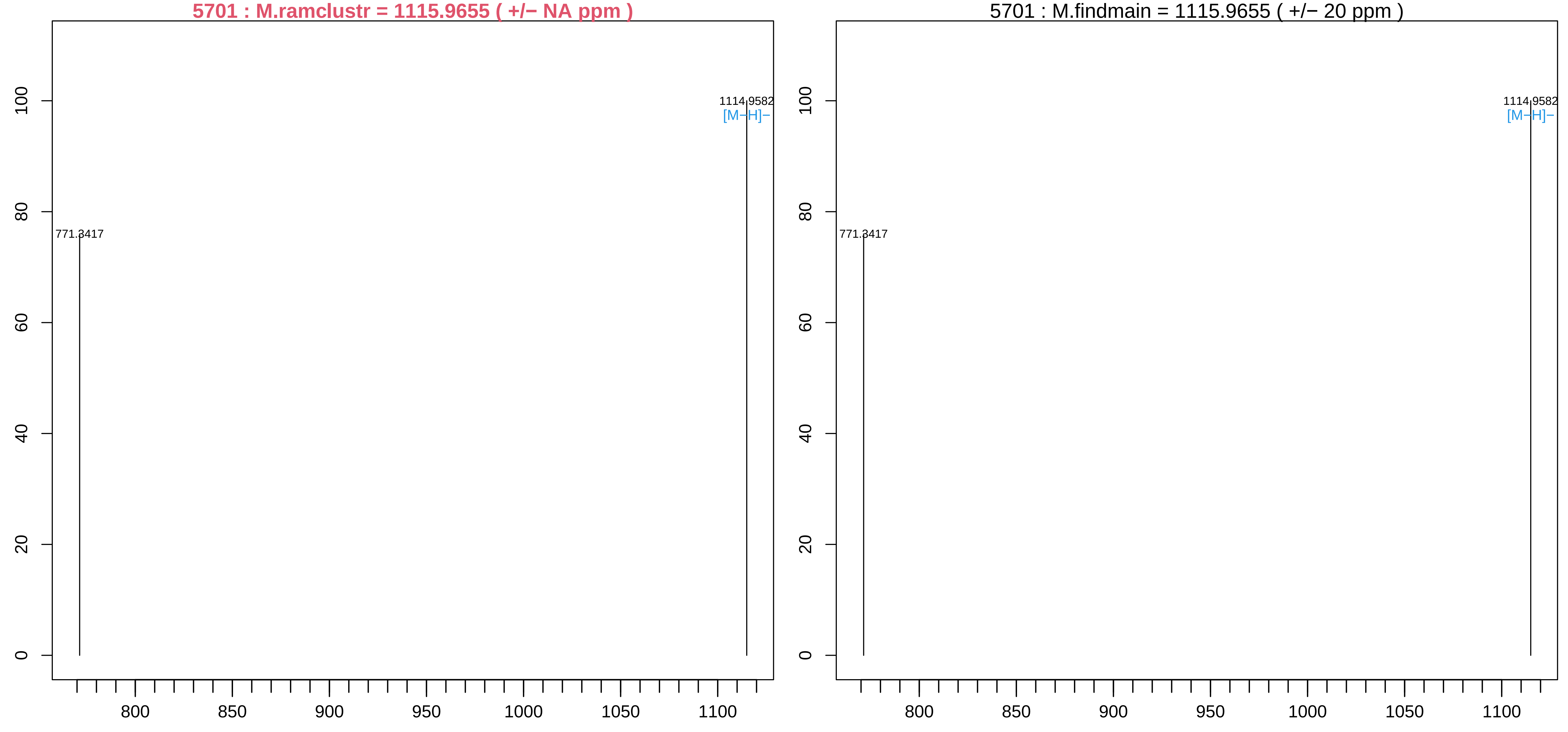
5701 : M.ramclustr = 1115.9655 (+/− NA ppm)
5701 : M.findmain = 1115.9655 (+/− 20 ppm)
1114.9582
[M−H]−
771.3417
0
20
40
60
80
100
800
850
900
950
1000
1050
1100

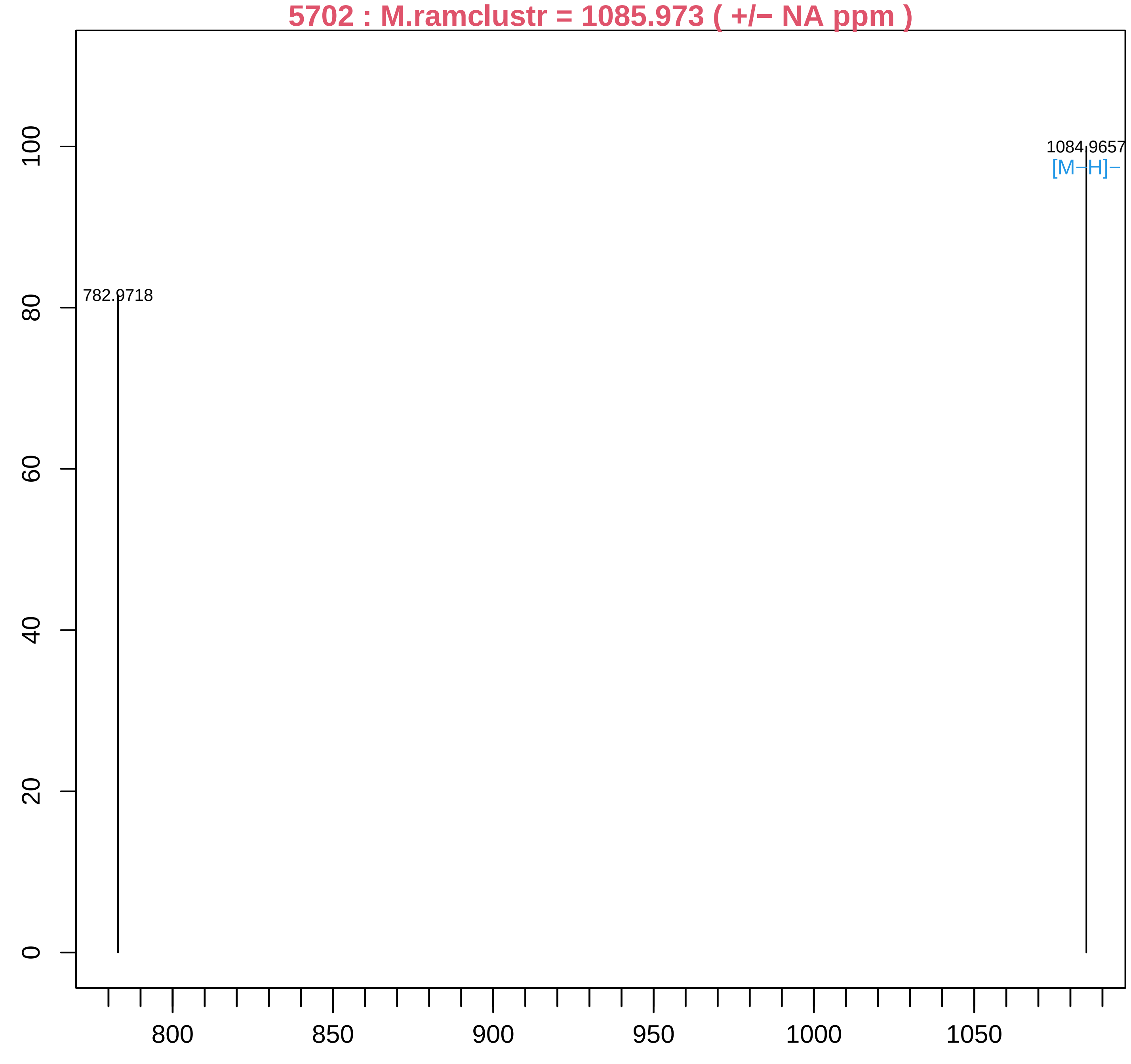
5702 : M.ramclustr = 1085.973 (+/− NA ppm)
1084.9657
[M−H]−
782.9718
0
20
40
60
80
100
800
850
900
950
1000
1050
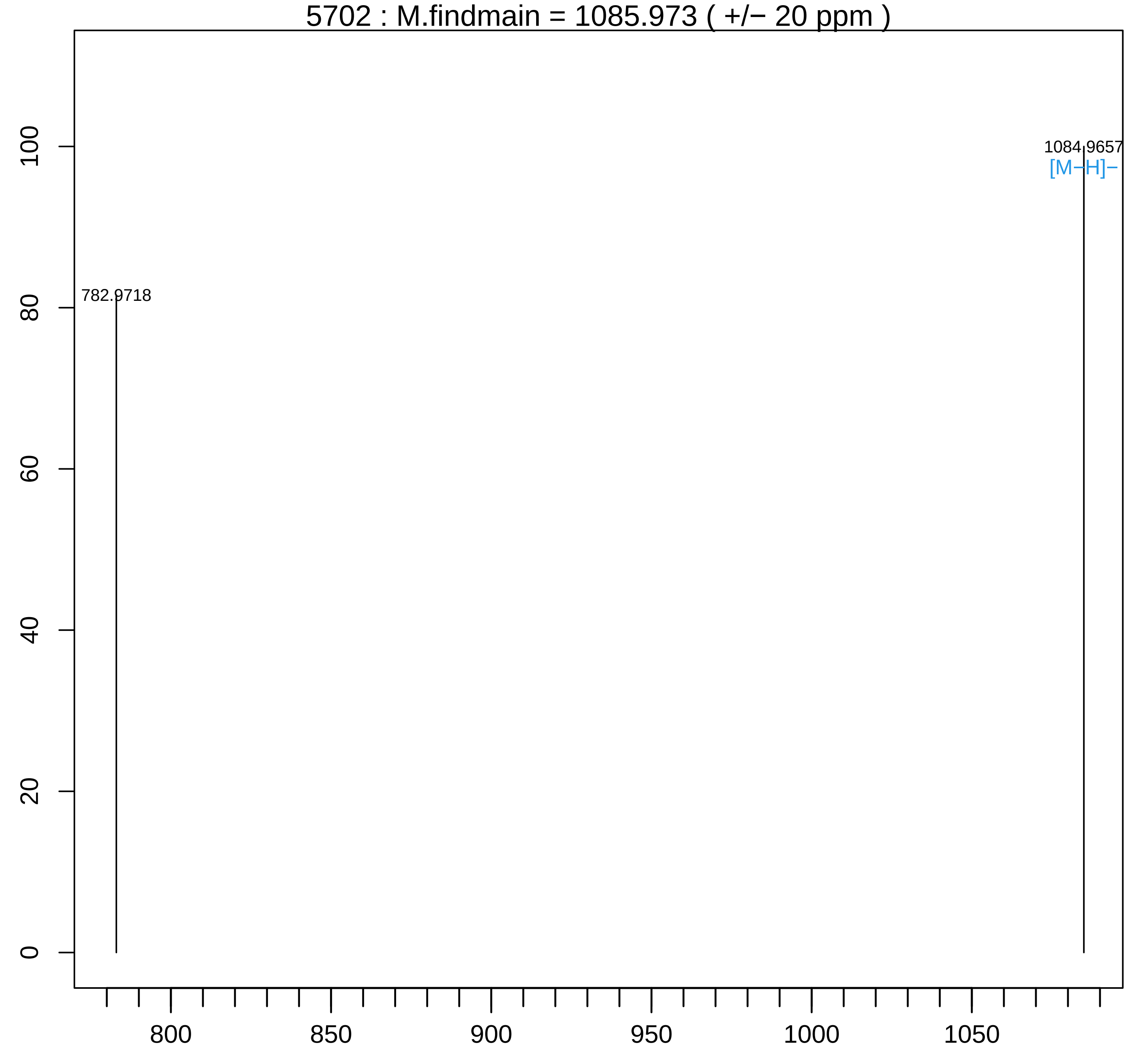
5702 : M.findmain = 1085.973 (+/− 20 ppm)
1084.9657
[M−H]−
782.9718
0
20
40
60
80
100
800
850
900
950
1000
1050

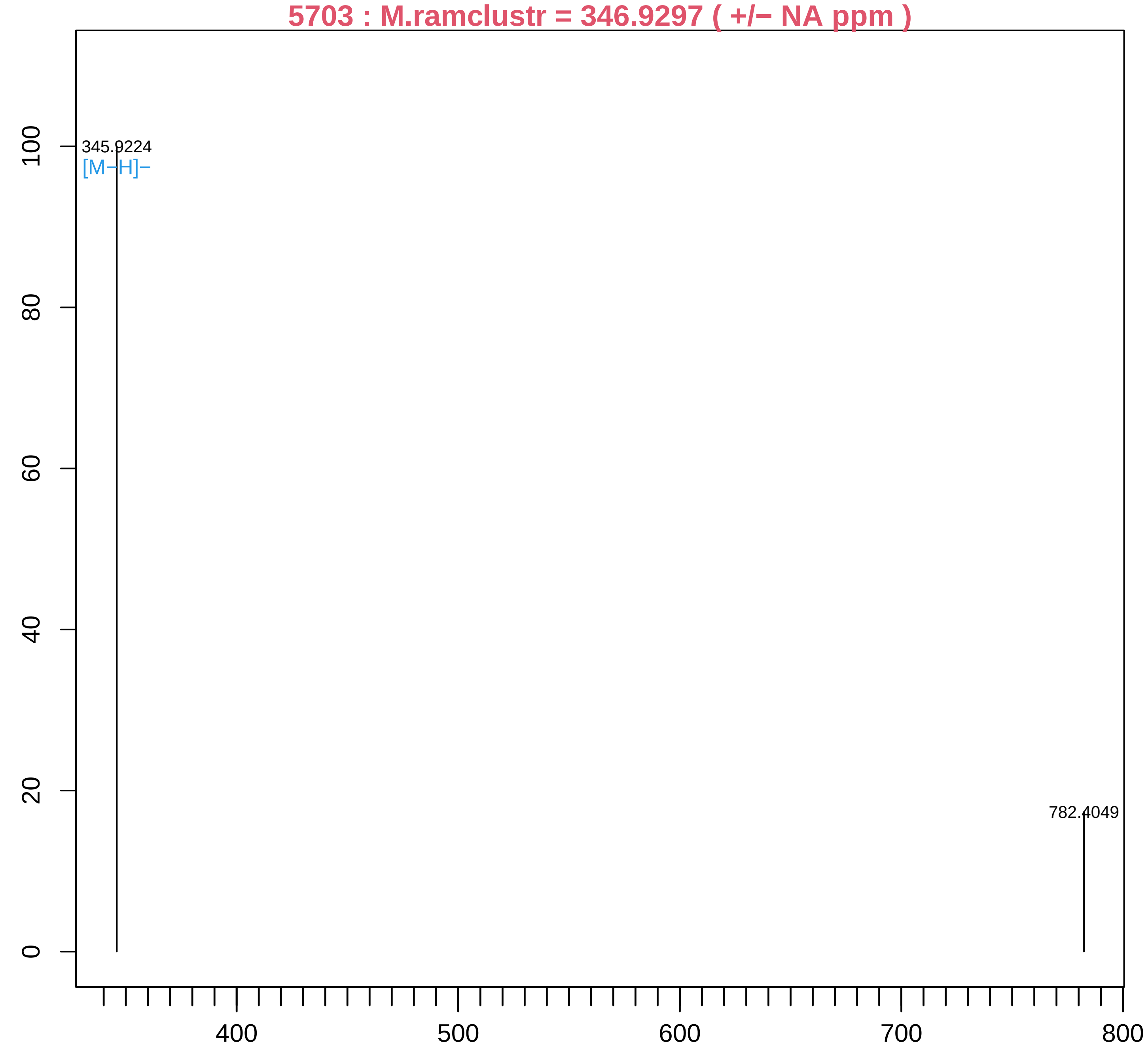

5703 : M.ramclustr = 346.9297 (+/− NA ppm)
345.9224
[M−H]−
782.4049
0
20
40
60
80
100
400
500
600
700
800

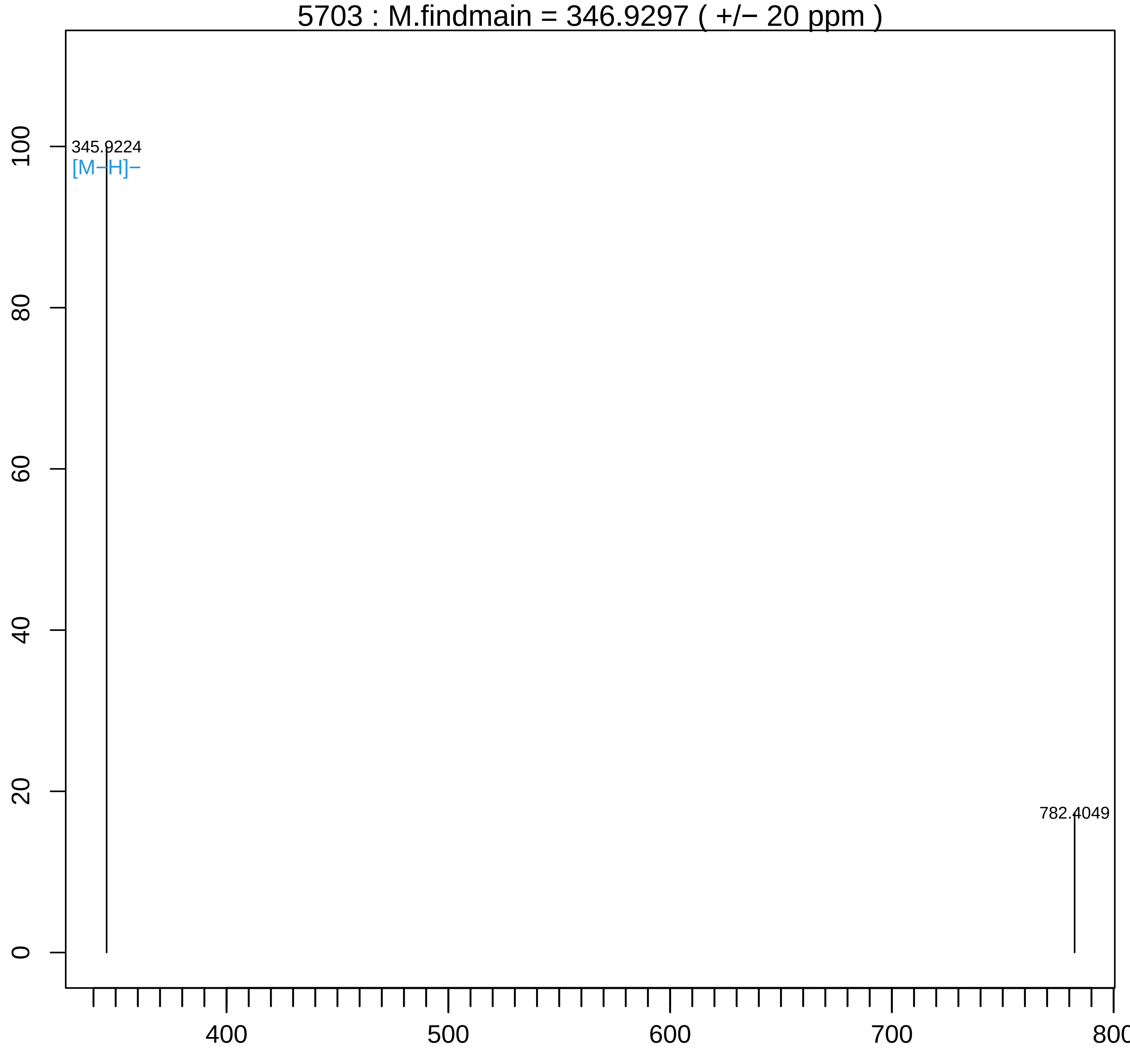

5703 : M.findmain = 346.9297 (+/− 20 ppm)
345.9224
[M−H]−
782.4049
0
20
40
60
80
100
400
500
600
700
800

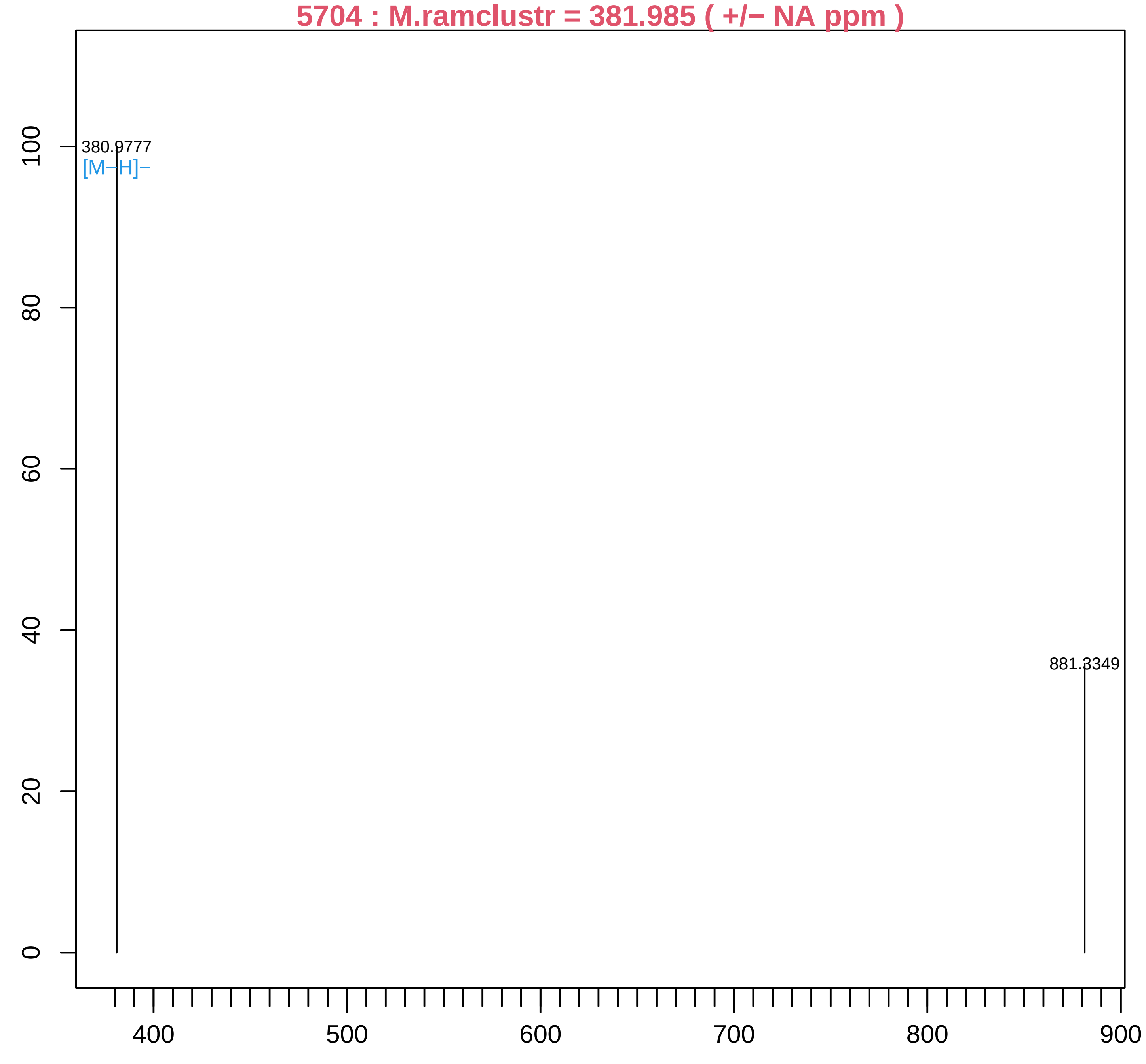

5704 : M.ramclustr = 381.985 (+/− NA ppm)
380.9777
[M−H]−
881.3349
0
20
40
60
80
100
400
500
600
700
800
900

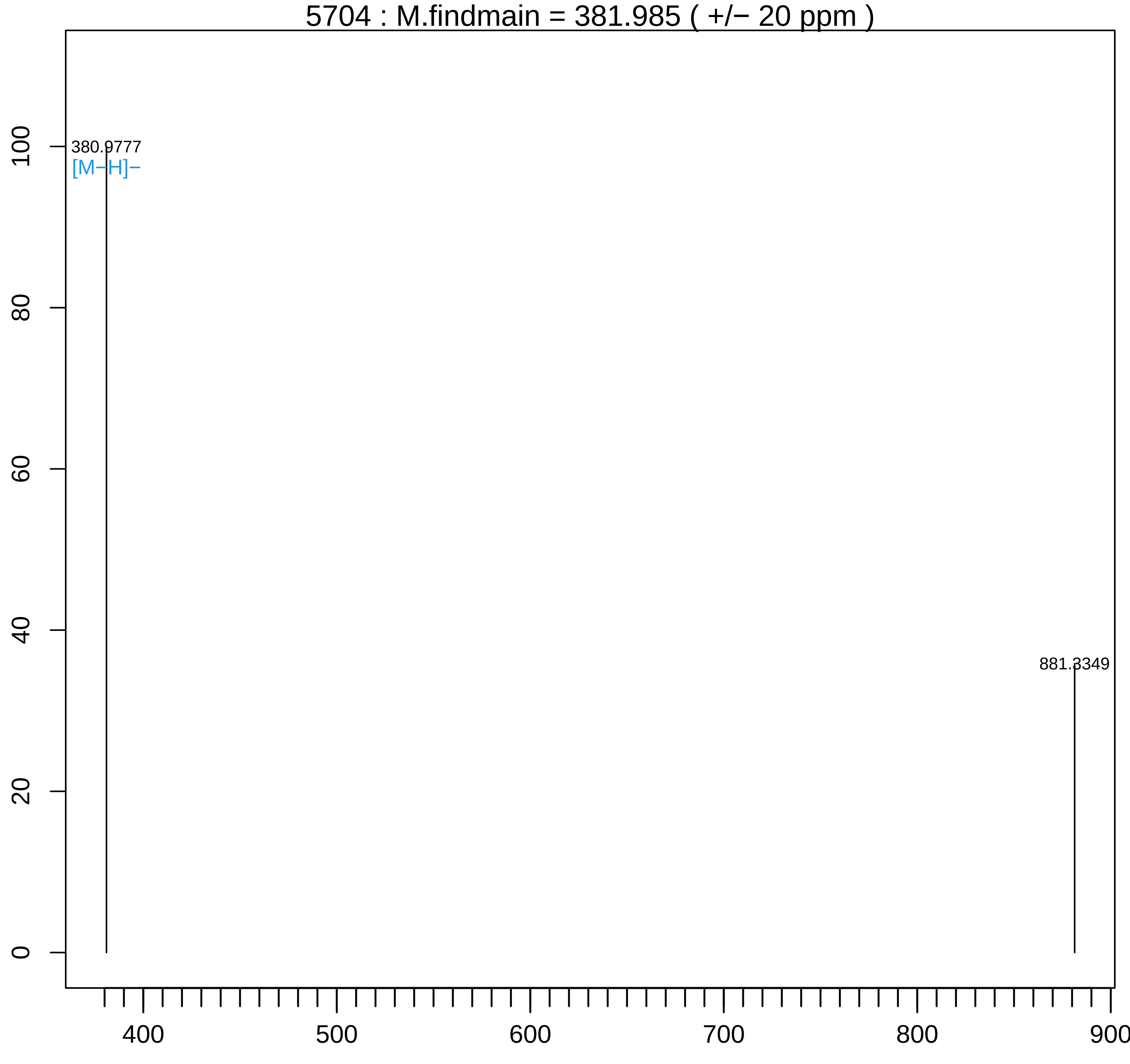

5704 : M.findmain = 381.985 (+/− 20 ppm)
380.9777
[M−H]−
881.3349
0
20
40
60
80
100
400
500
600
700
800
900

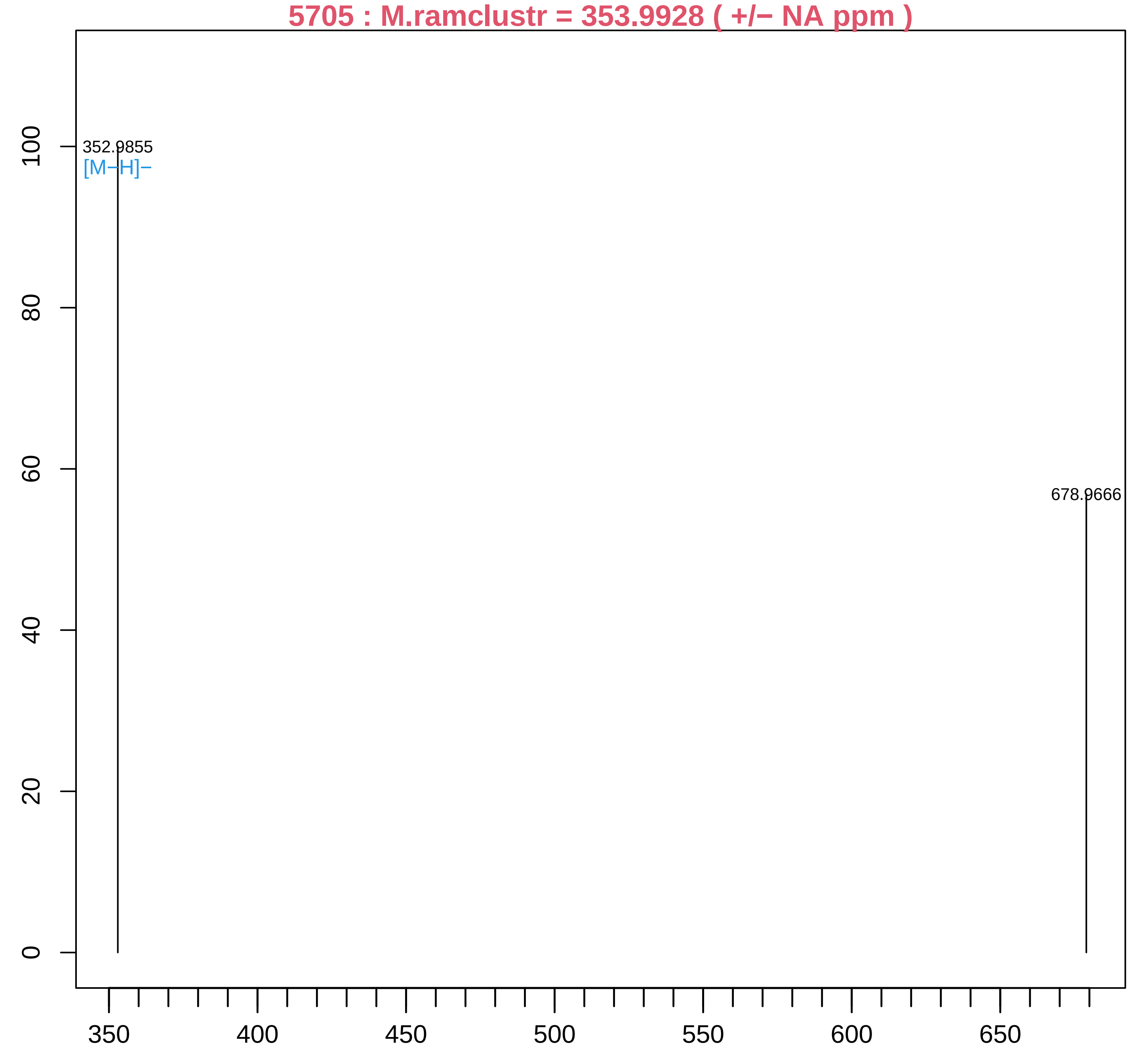

5705 : M.ramclustr = 353.9928 (+/− NA ppm)
352.9855
[M−H]−
678.9666
0
20
40
60
80
100
350
400
450
500
550
600
650

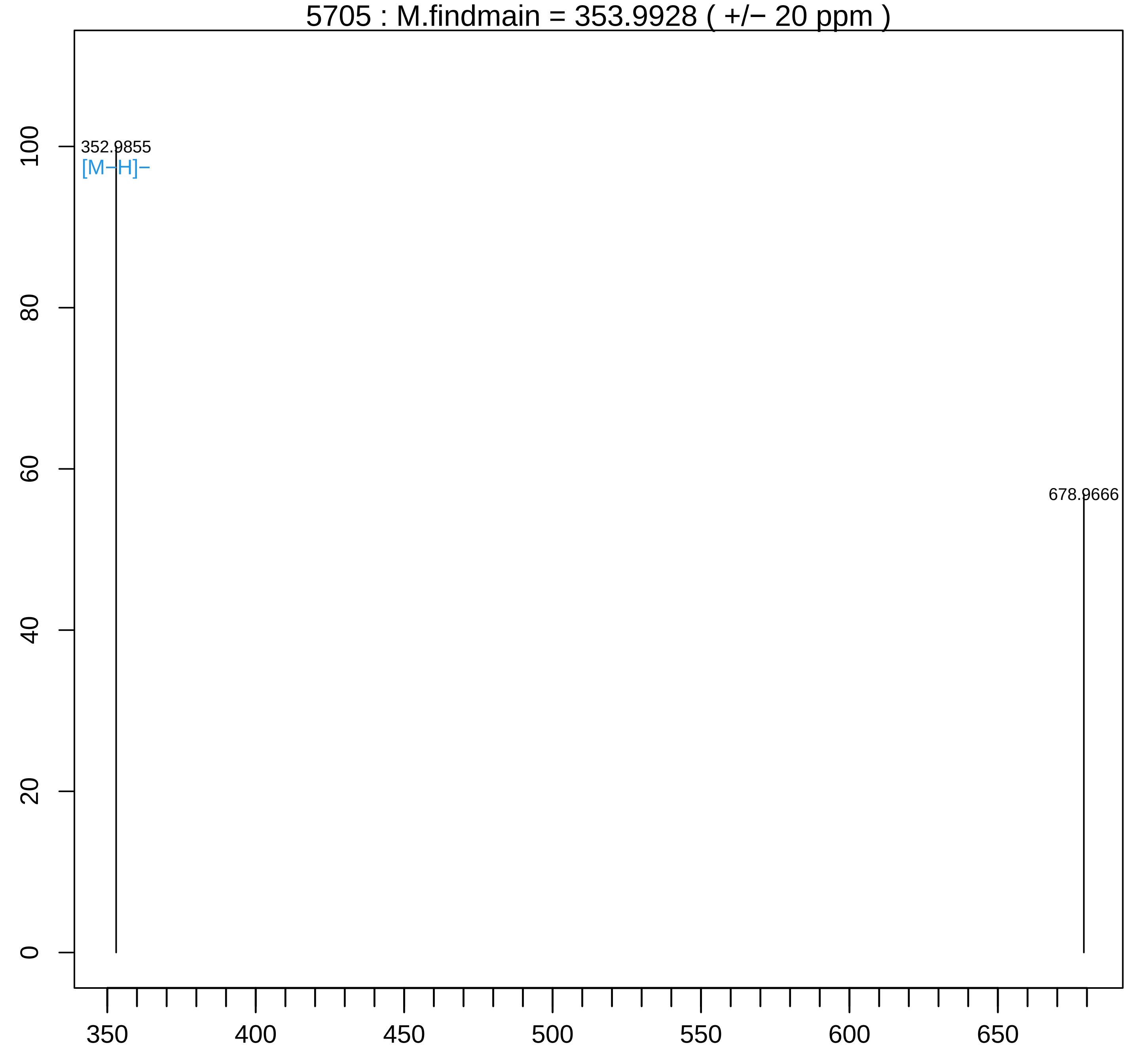

5705 : M.findmain = 353.9928 (+/− 20 ppm)
352.9855
[M−H]−
678.9666
0
20
40
60
80
100
350
400
450
500
550
600
650

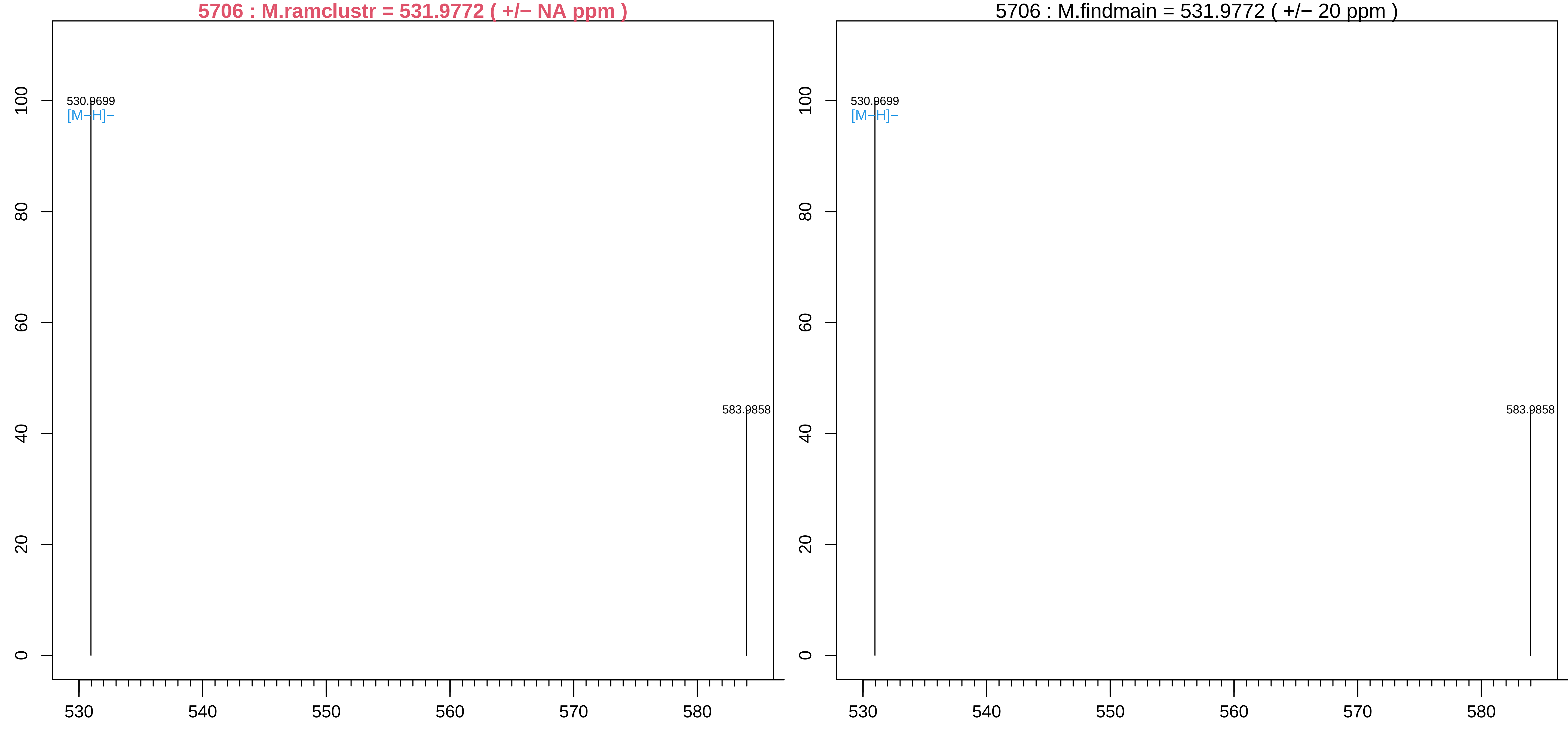
5706 : M.ramclustr = 531.9772 (+/− NA ppm)
530.9699
[M−H]−
583.9858
5706 : M.findmain = 531.9772 (+/− 20 ppm)
530.9699
[M−H]−
583.9858
0
20
40
60
80
100
530
540
550
560
570
580

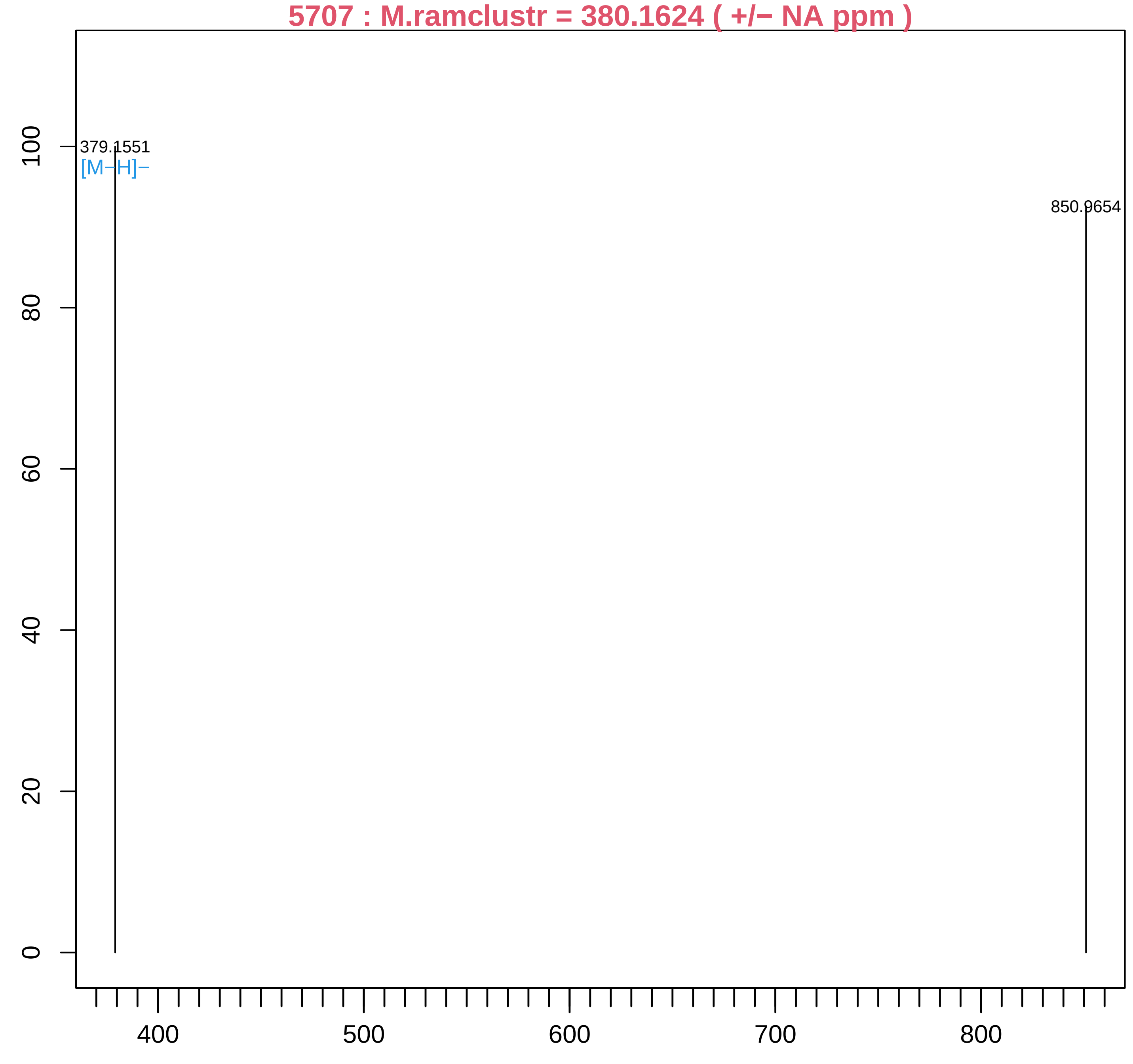
5707 : M.ramclustr = 380.1624 (+/− NA ppm)
379.1551
[M−H]−
850.9654
0
20
40
60
80
100
400
500
600
700
800

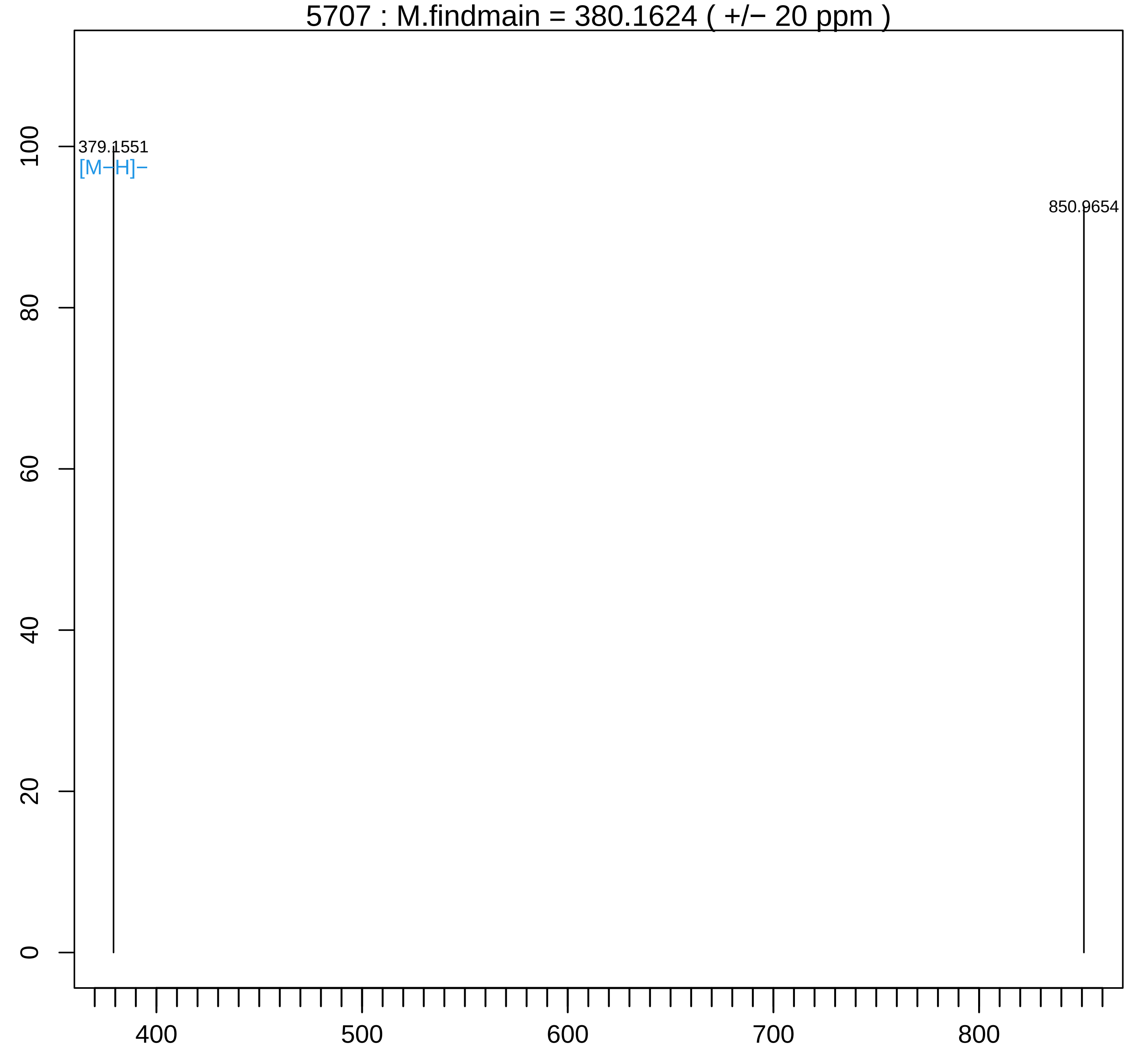
5707 : M.findmain = 380.1624 (+/− 20 ppm)
379.1551
[M−H]−
850.9654
0
20
40
60
80
100
400
500
600
700
800

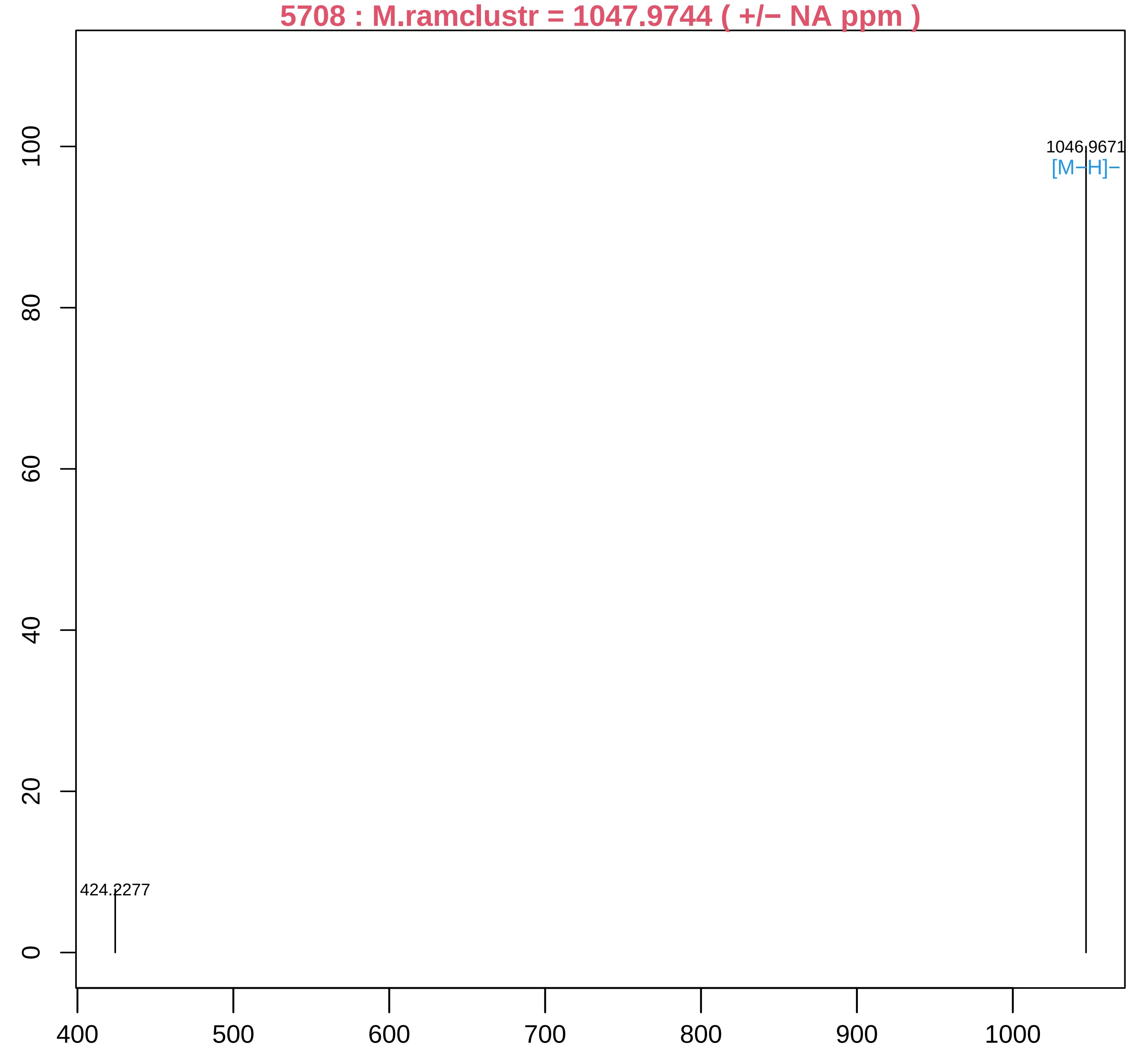
5708 : M.ramclustr = 1047.9744 (+/− NA ppm)
1046.9671
[M−H]−
424.2277
0
20
40
60
80
100
400
500
600
700
800
900
1000

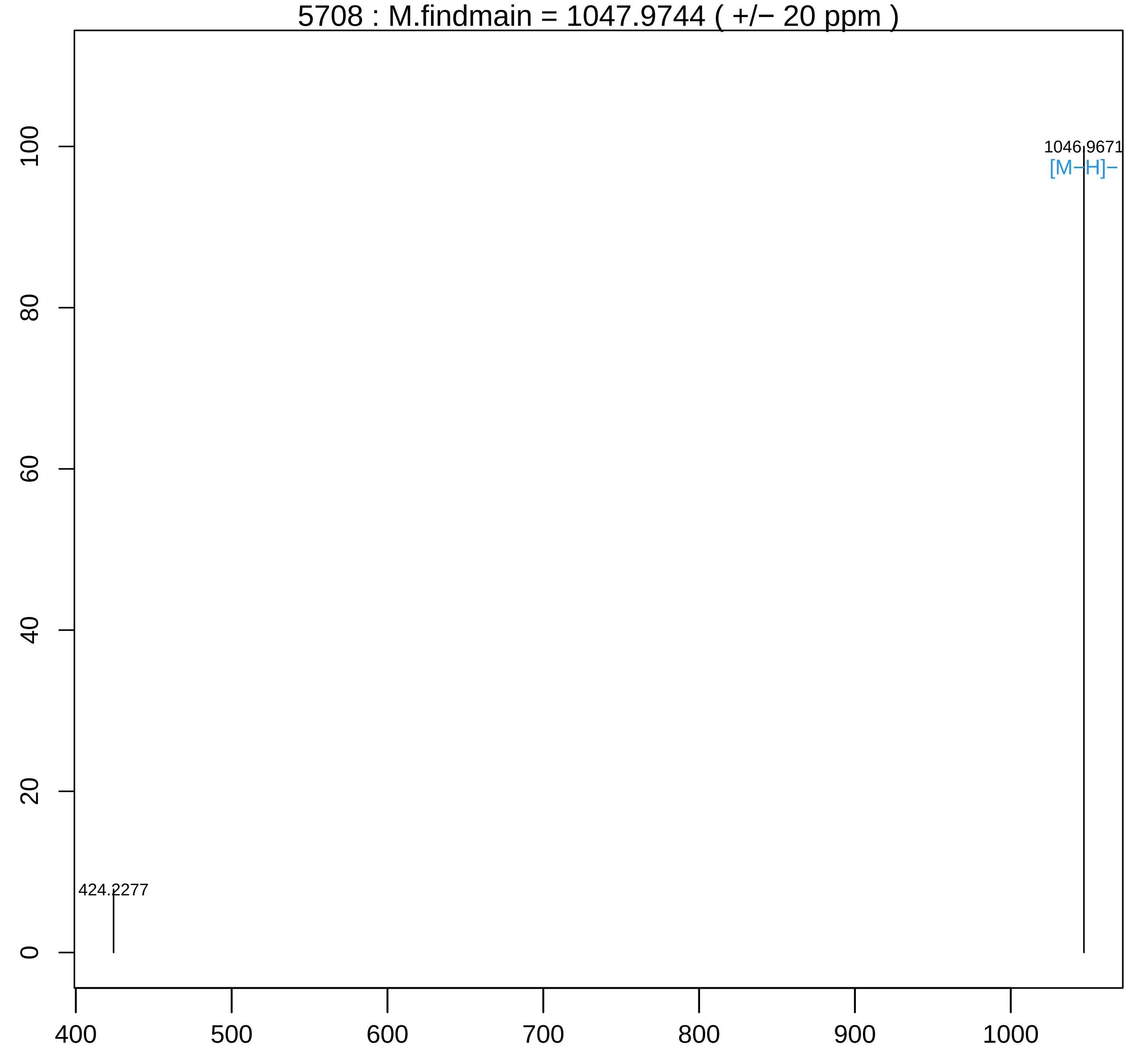
5708 : M.findmain = 1047.9744 (+/− 20 ppm)
1046.9671
[M−H]−
424.2277
0
20
40
60
80
100
400
500
600
700
800
900
1000

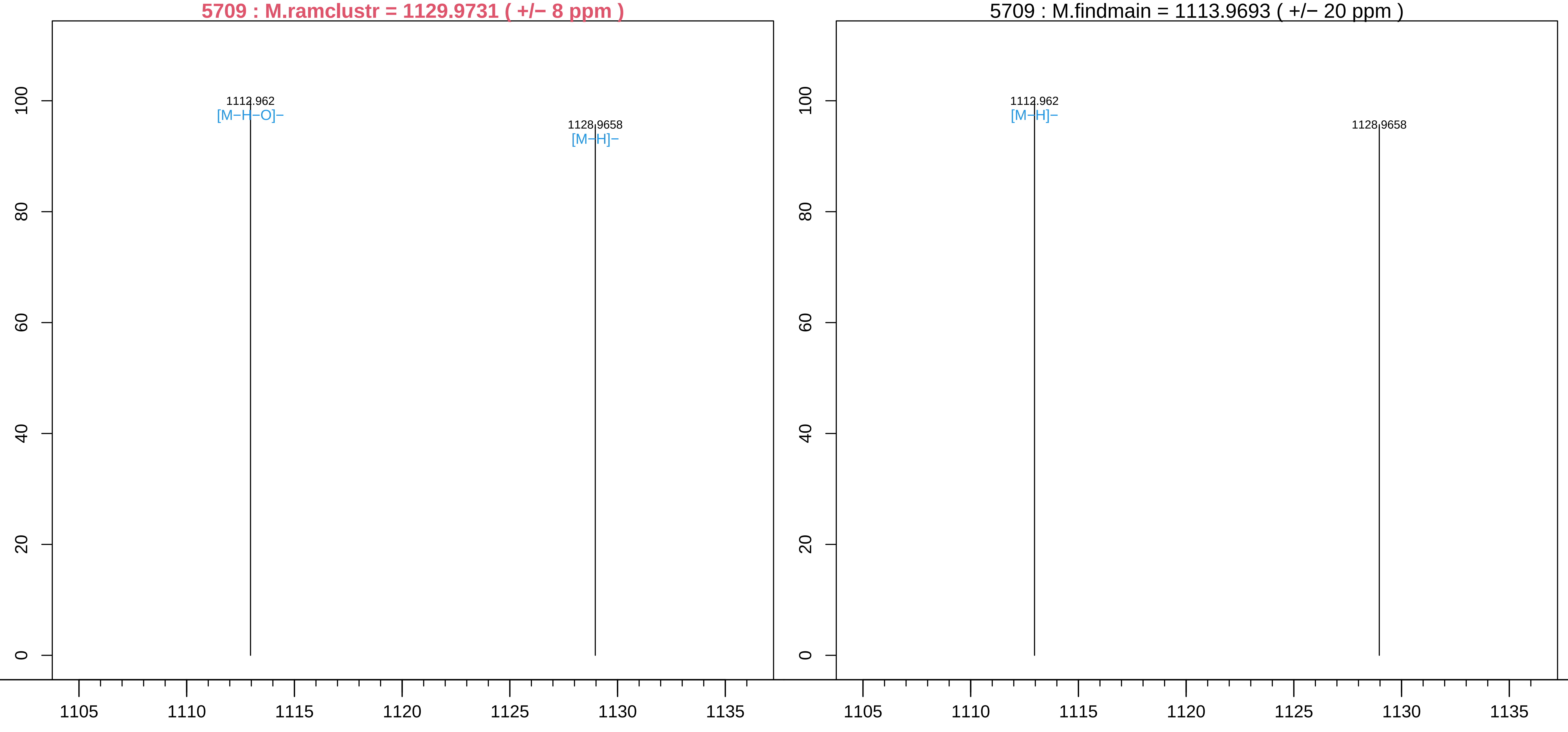
5709 : M.ramclustr = 1129.9731 (+/− 8 ppm)
1112.962
[M−H−O]−
1128.9658
[M−H]−
5709 : M.findmain = 1113.9693 (+/− 20 ppm)
1112.962
[M−H]−
1128.9658
0
20
40
60
80
100
1105
1110
1115
1120
1125
1130
1135

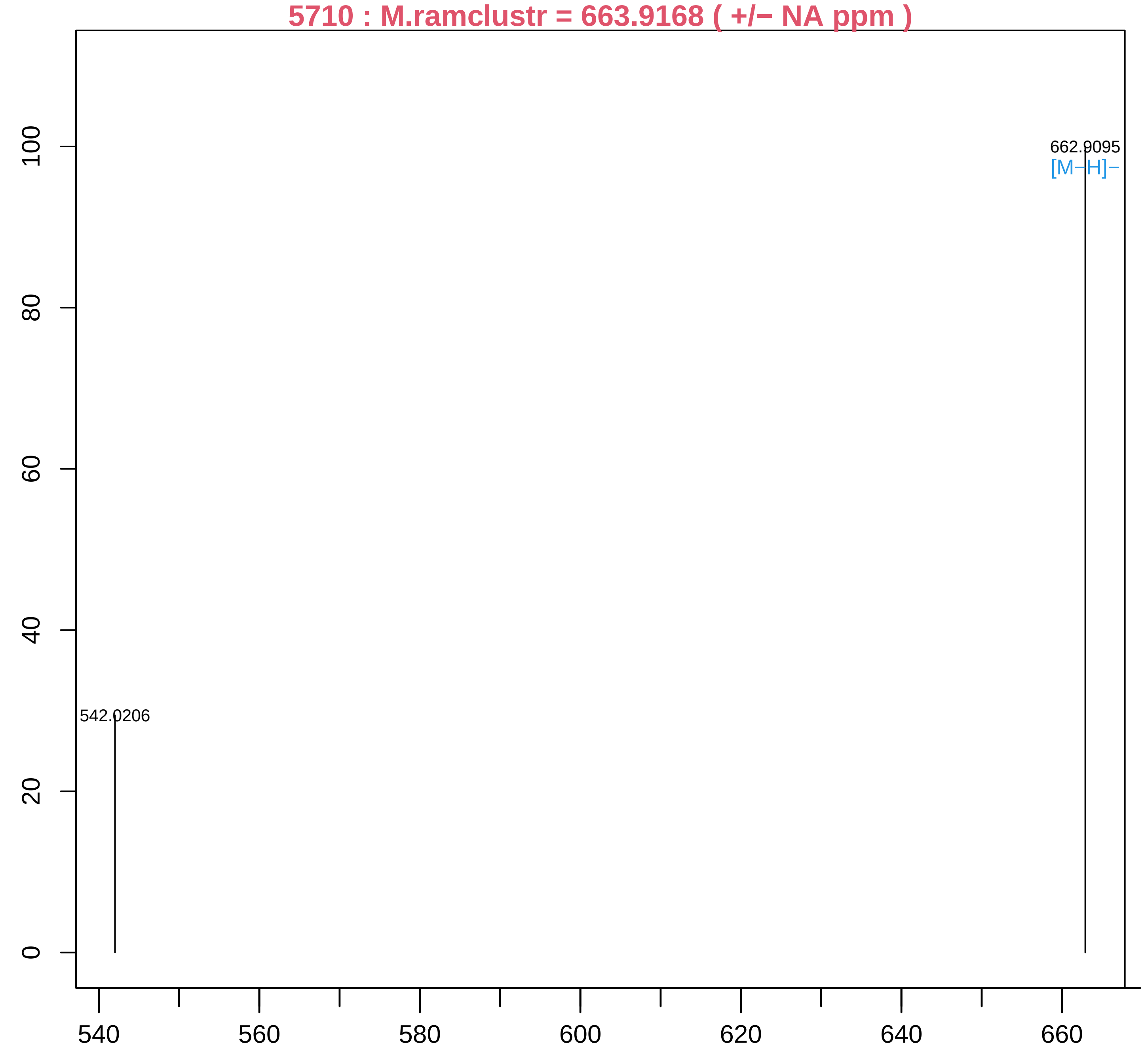
5710 : M.ramclustr = 663.9168 (+/− NA ppm)
662.9095
[M−H]−
542.0206
0
20
40
60
80
100
540
560
580
600
620
640
660

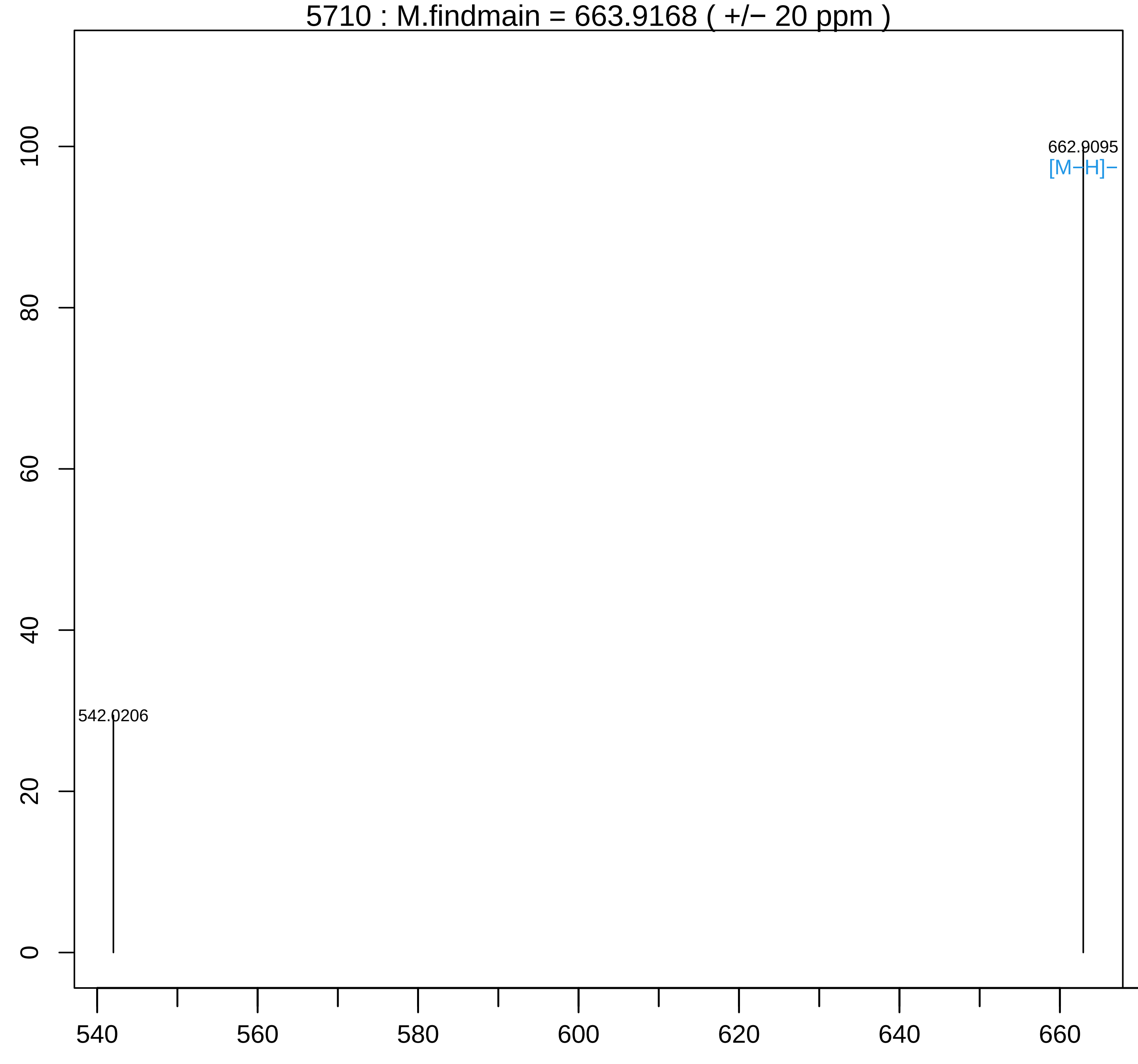
5710 : M.findmain = 663.9168 (+/− 20 ppm)
662.9095
[M−H]−
542.0206
0
20
40
60
80
100
540
560
580
600
620
640
660

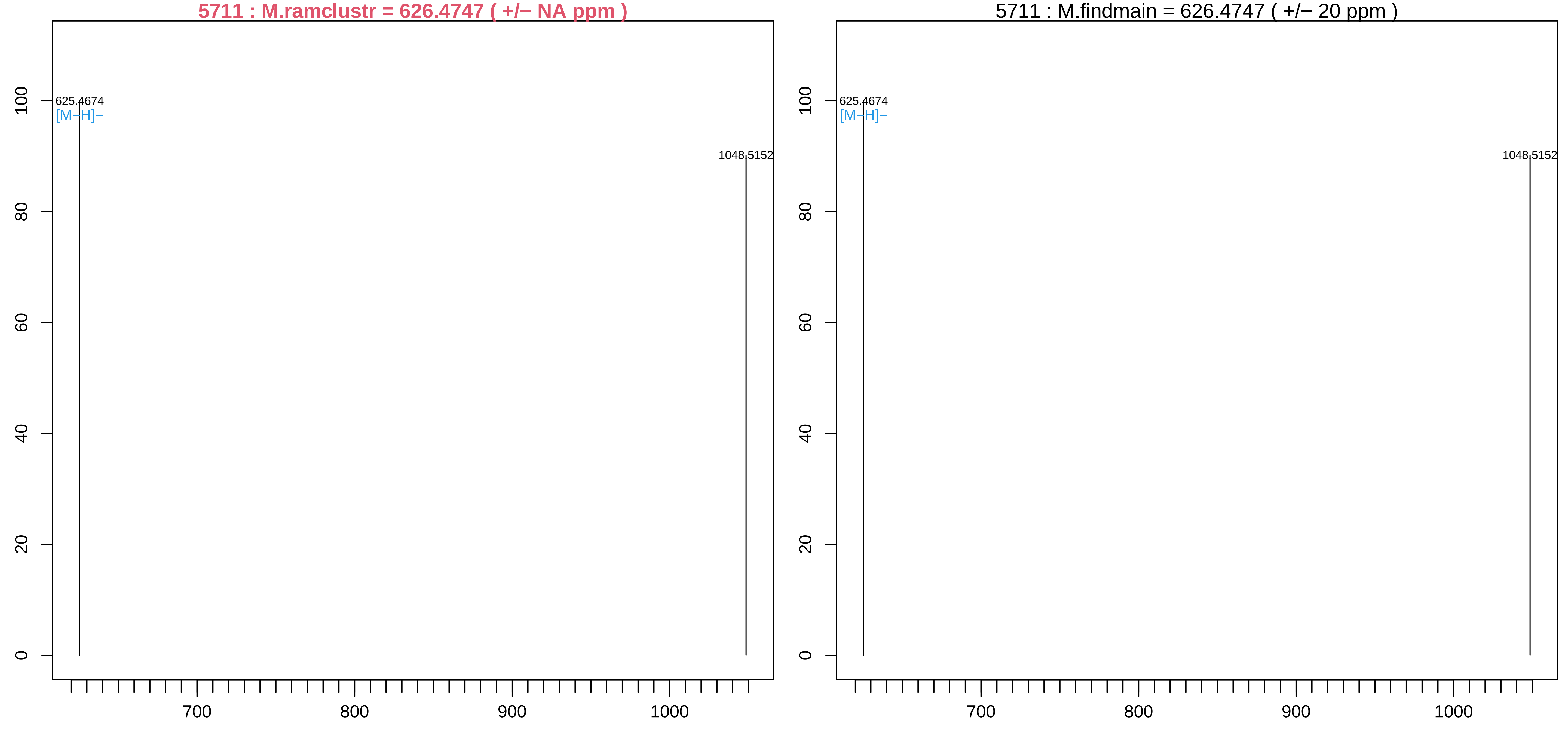

5711 : M.ramclustr = 626.4747 (+/− NA ppm)
625.4674
[M−H]−
1048.5152
5711 : M.findmain = 626.4747 (+/− 20 ppm)
625.4674
[M−H]−
1048.5152
0
20
40
60
80
100
700
800
900
1000

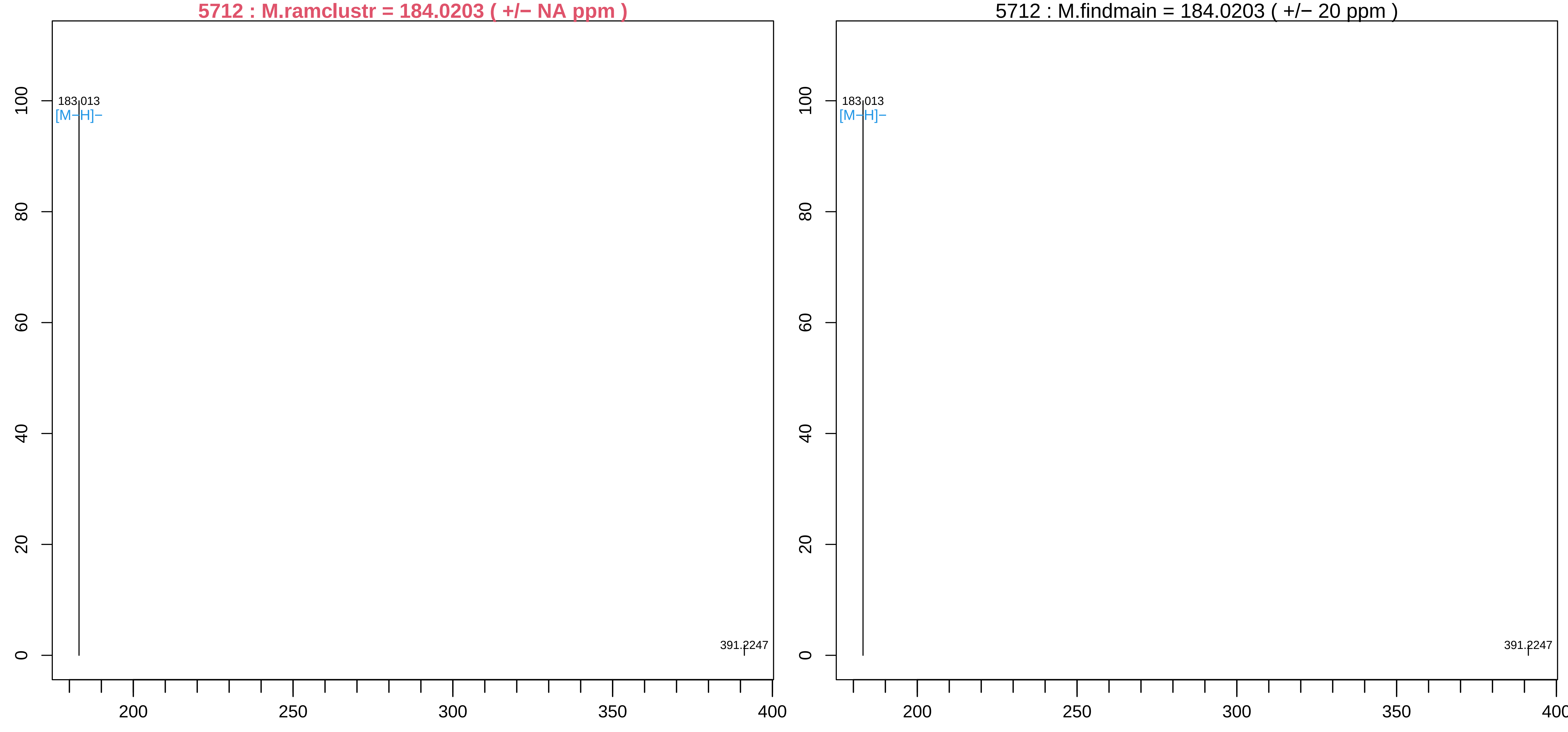
5712 : M.ramclustr = 184.0203 (+/− NA ppm)
183.013
[M−H]−
391.2247
5712 : M.findmain = 184.0203 (+/− 20 ppm)
183.013
[M−H]−
391.2247

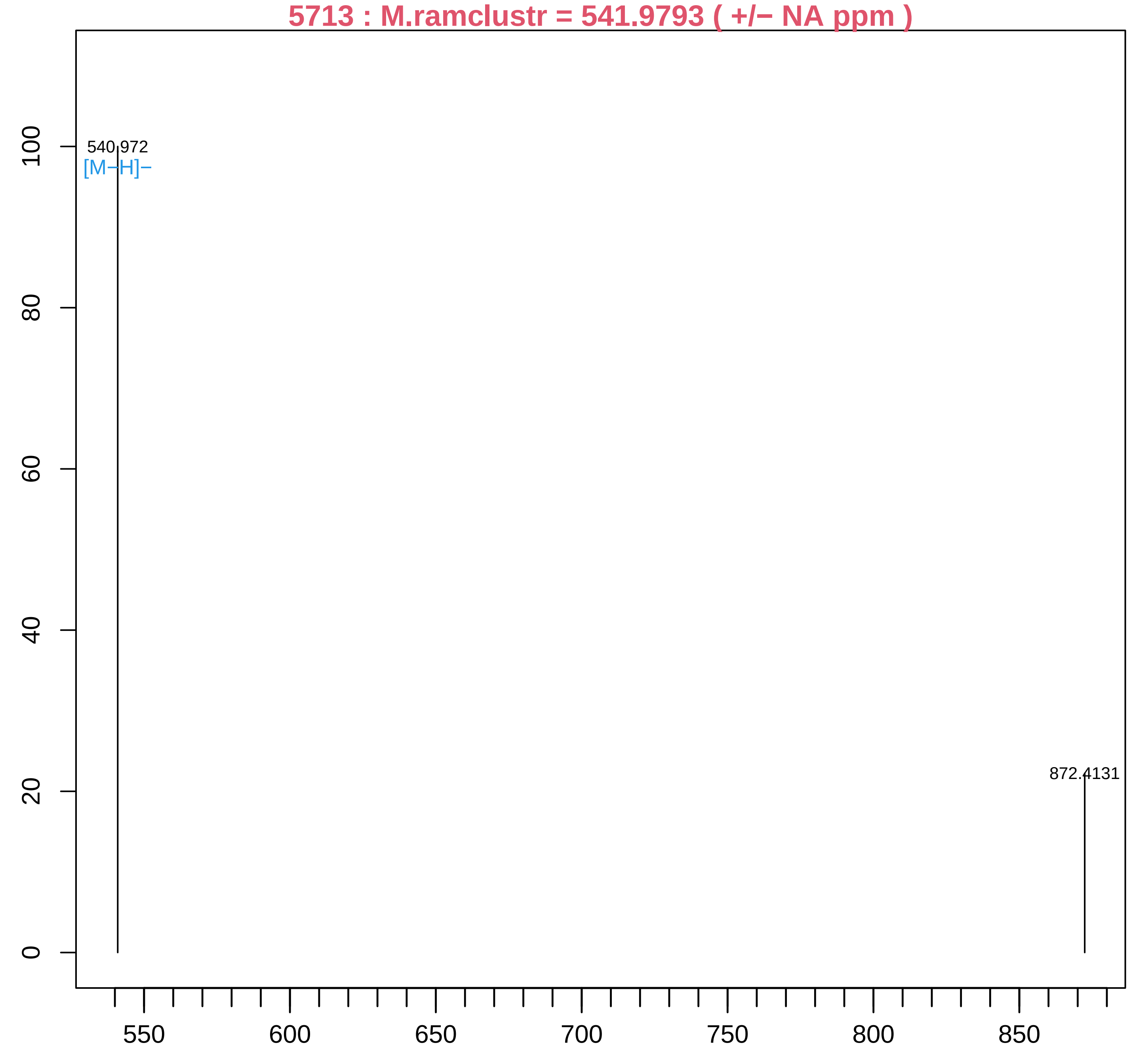
5713 : M.ramclustr = 541.9793 (+/− NA ppm)
540.972
[M−H]−
872.4131
0
20
40
60
80
100
550
600
650
700
750
800
850

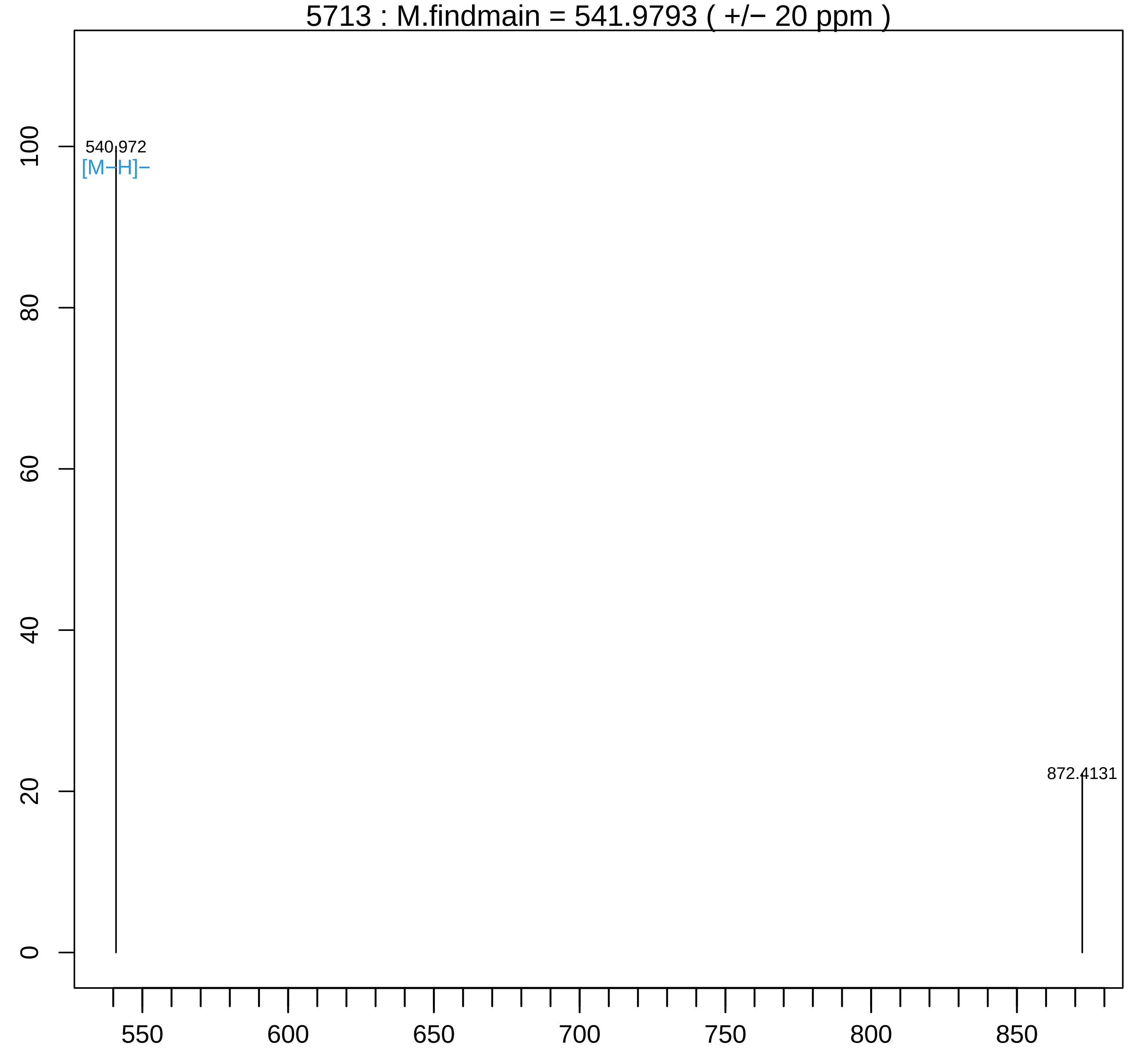
5713 : M.findmain = 541.9793 (+/− 20 ppm)
540.972
[M−H]−
872.4131
0
20
40
60
80
100
550
600
650
700
750
800
850

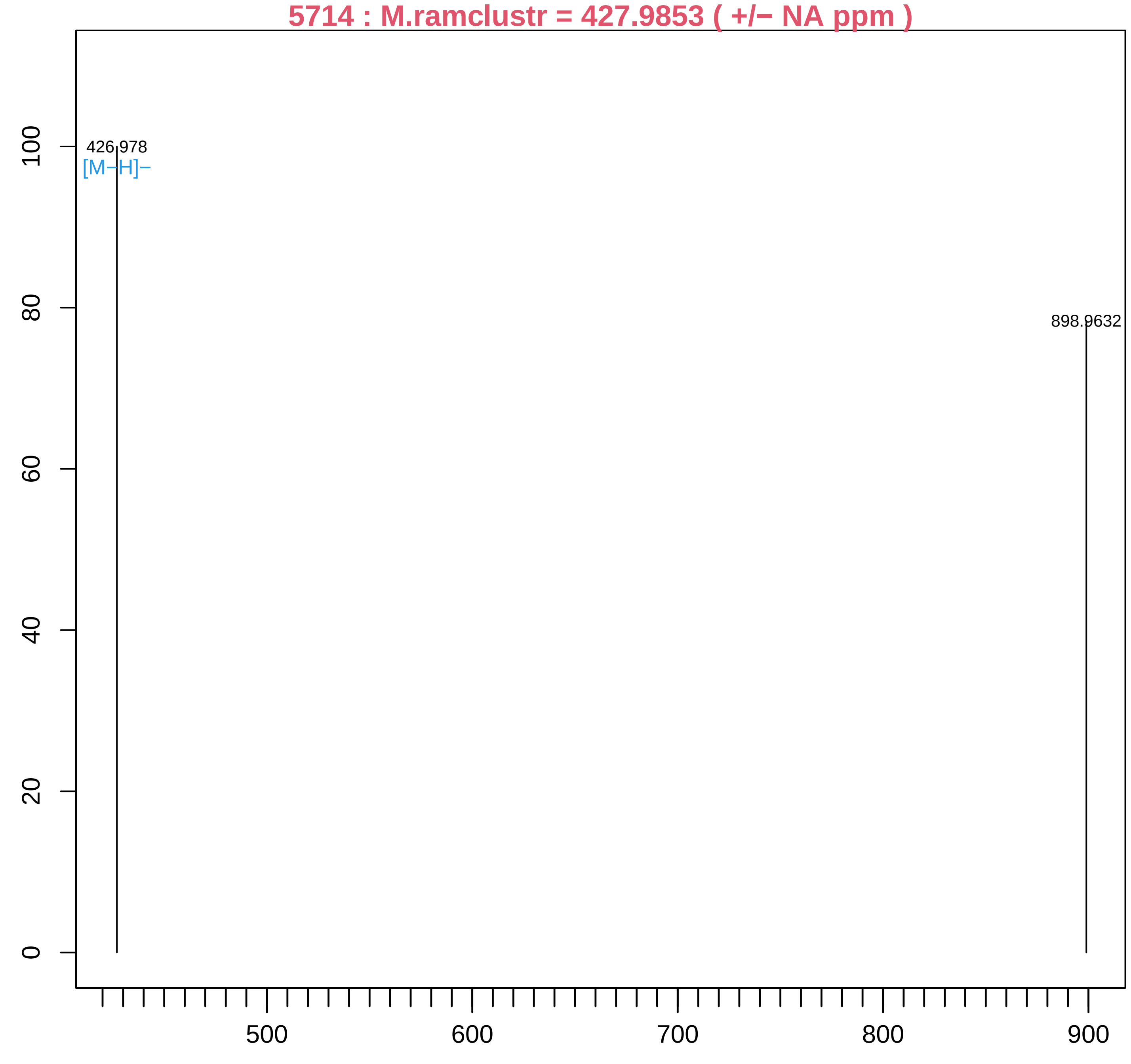
5714 : M.ramclustr = 427.9853 (+/− NA ppm)
426.978
[M−H]−
898.9632
0
20
40
60
80
100
500
600
700
800
900

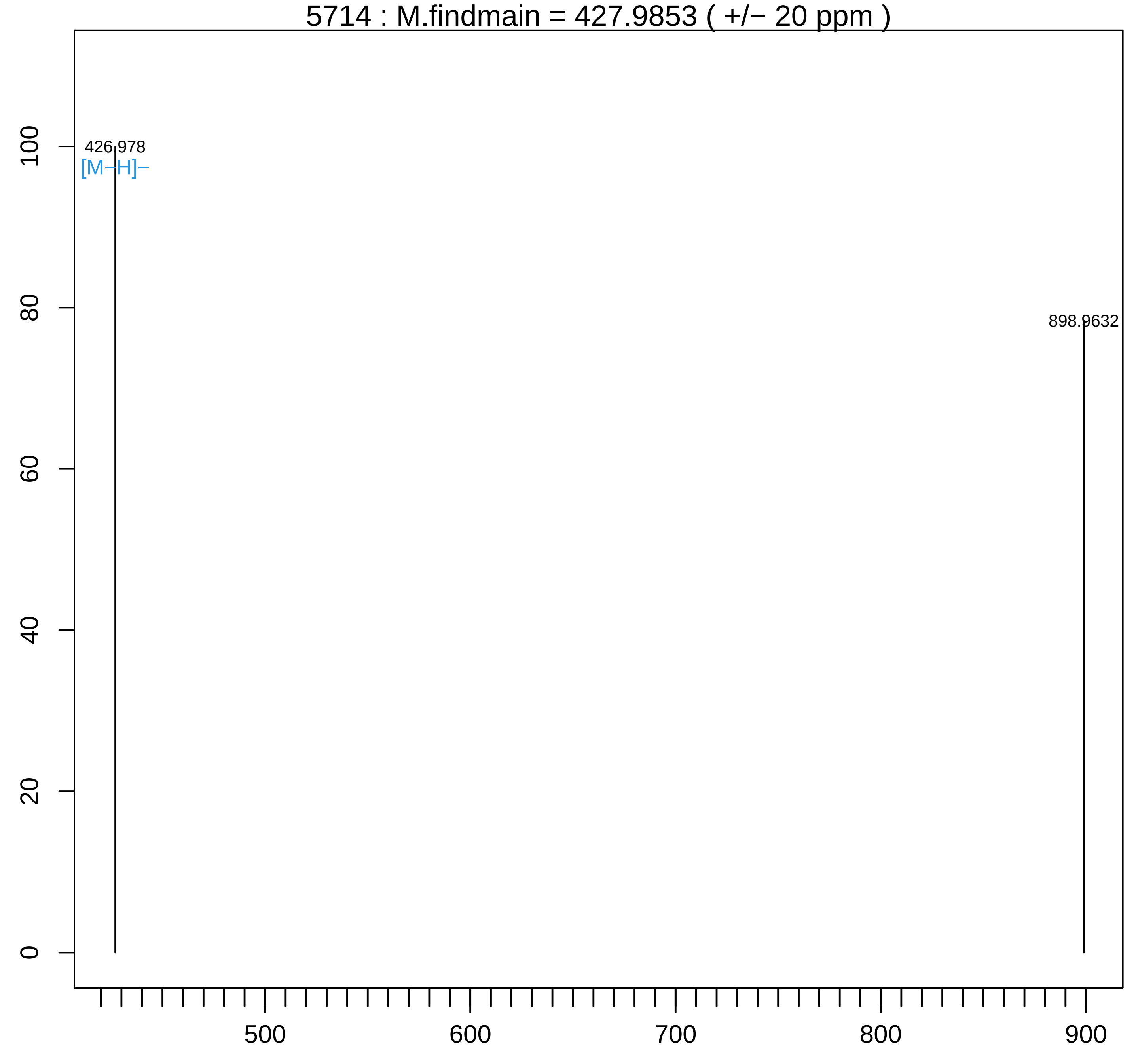
5714 : M.findmain = 427.9853 (+/− 20 ppm)
426.978
[M−H]−
898.9632
0
20
40
60
80
100
500
600
700
800
900

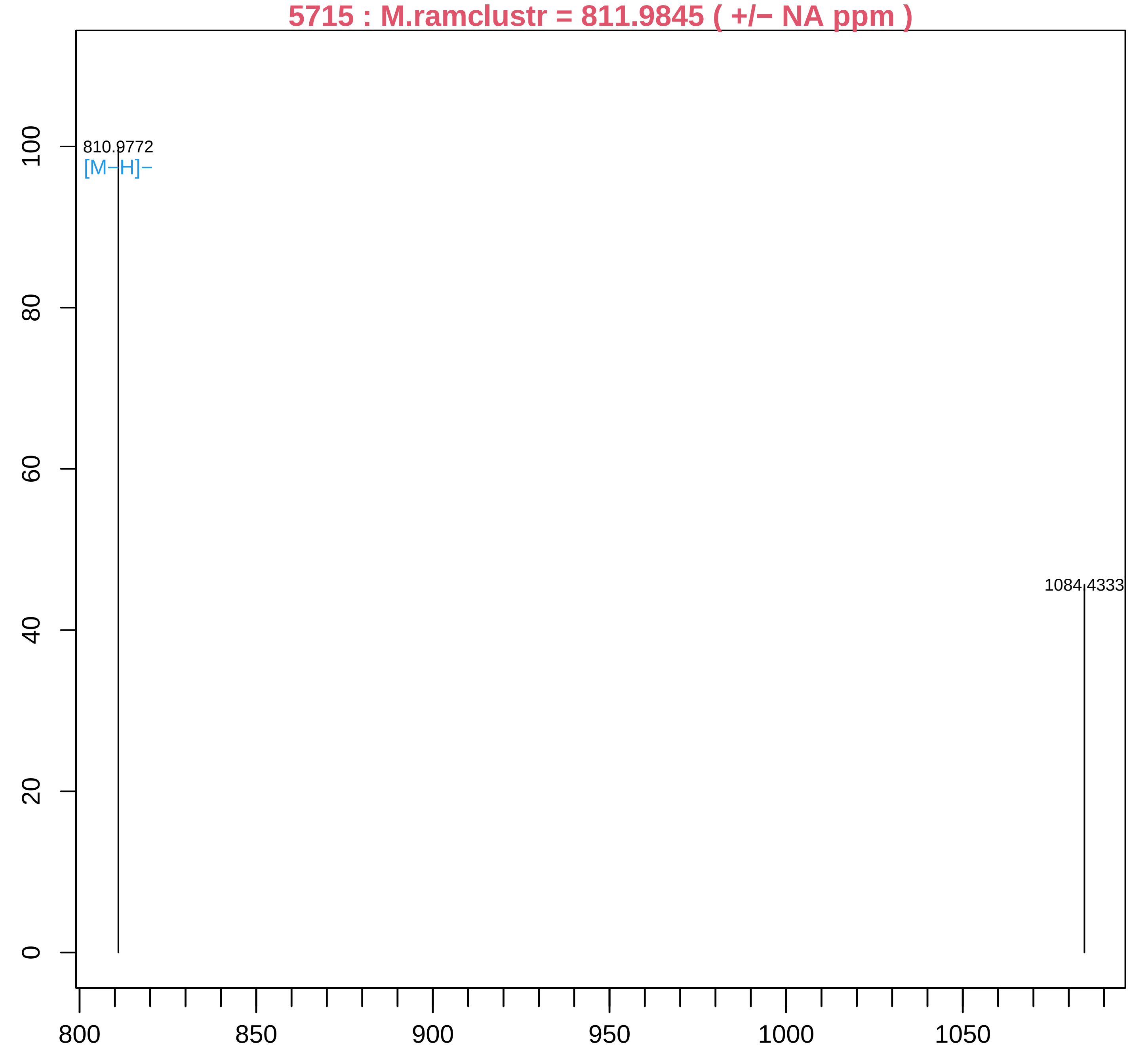
5715 : M.ramclustr = 811.9845 (+/− NA ppm)
810.9772
[M−H]−
1084.4333
0
20
40
60
80
100
800
850
900
950
1000
1050

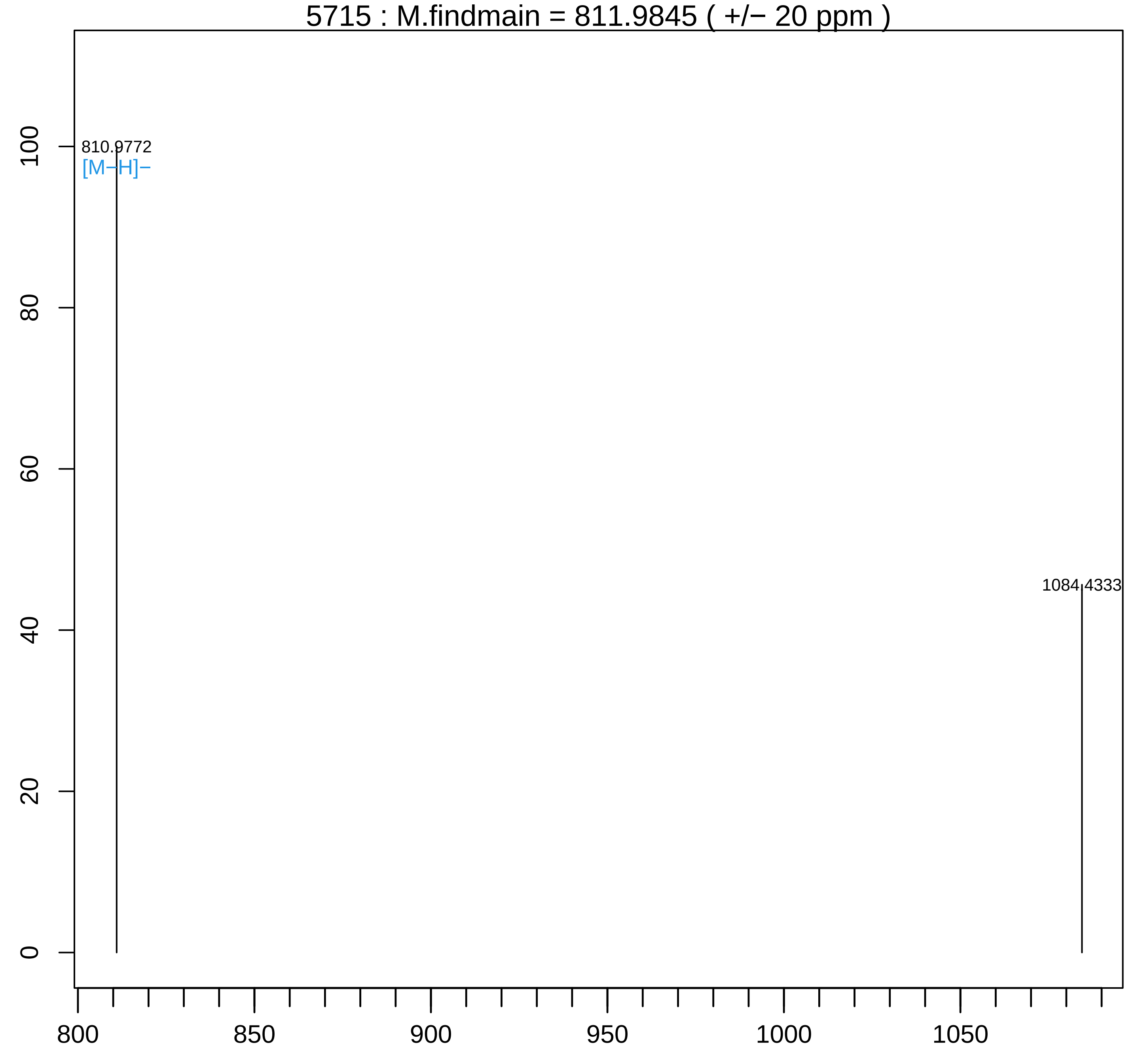
5715 : M.findmain = 811.9845 (+/− 20 ppm)
810.9772
[M−H]−
1084.4333
0
20
40
60
80
100
800
850
900
950
1000
1050

5716 : M.ramclustr = 293.9905 (+/− NA ppm)

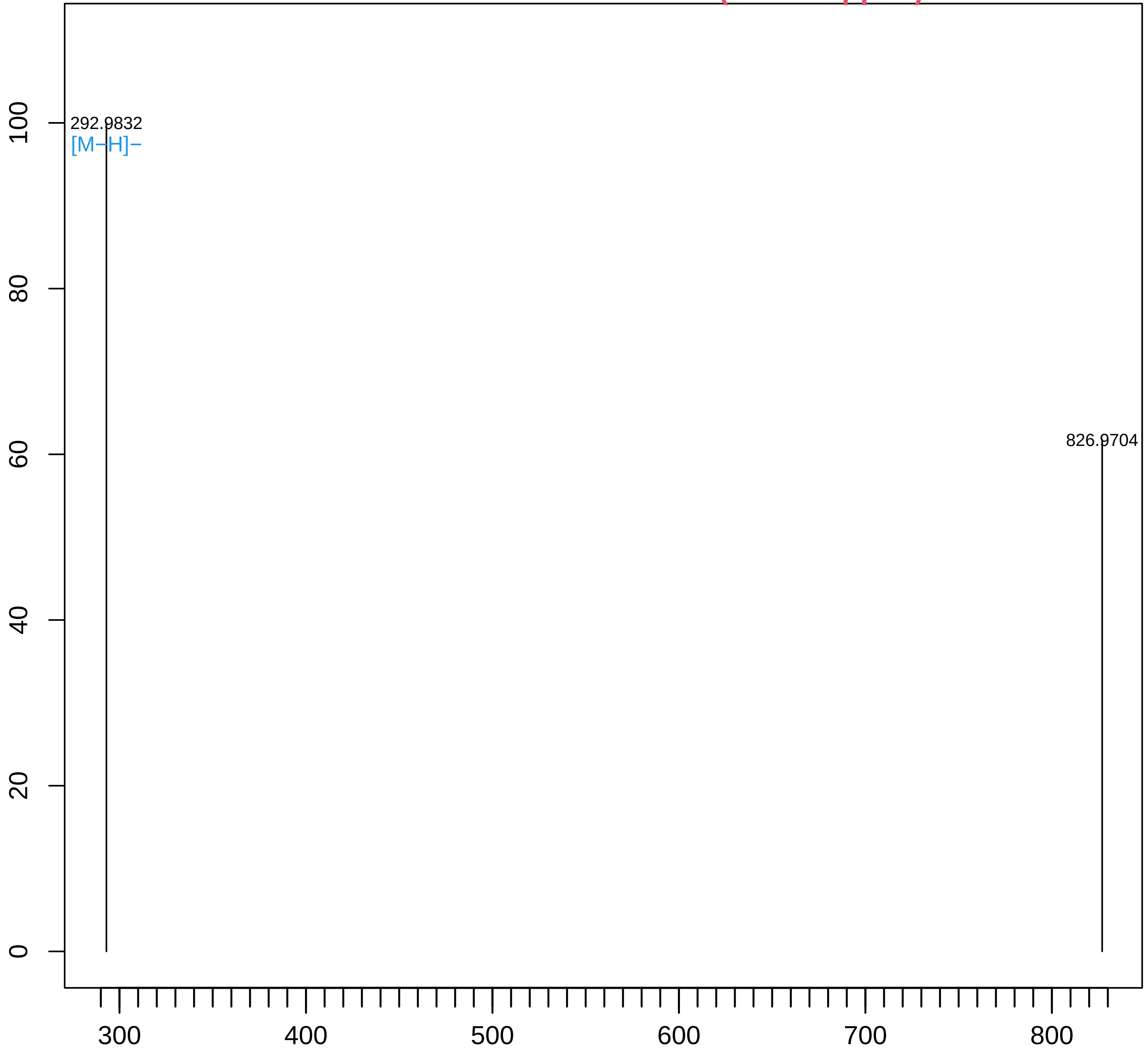

5716 : M.findmain = 293.9905 (+/− 20 ppm)

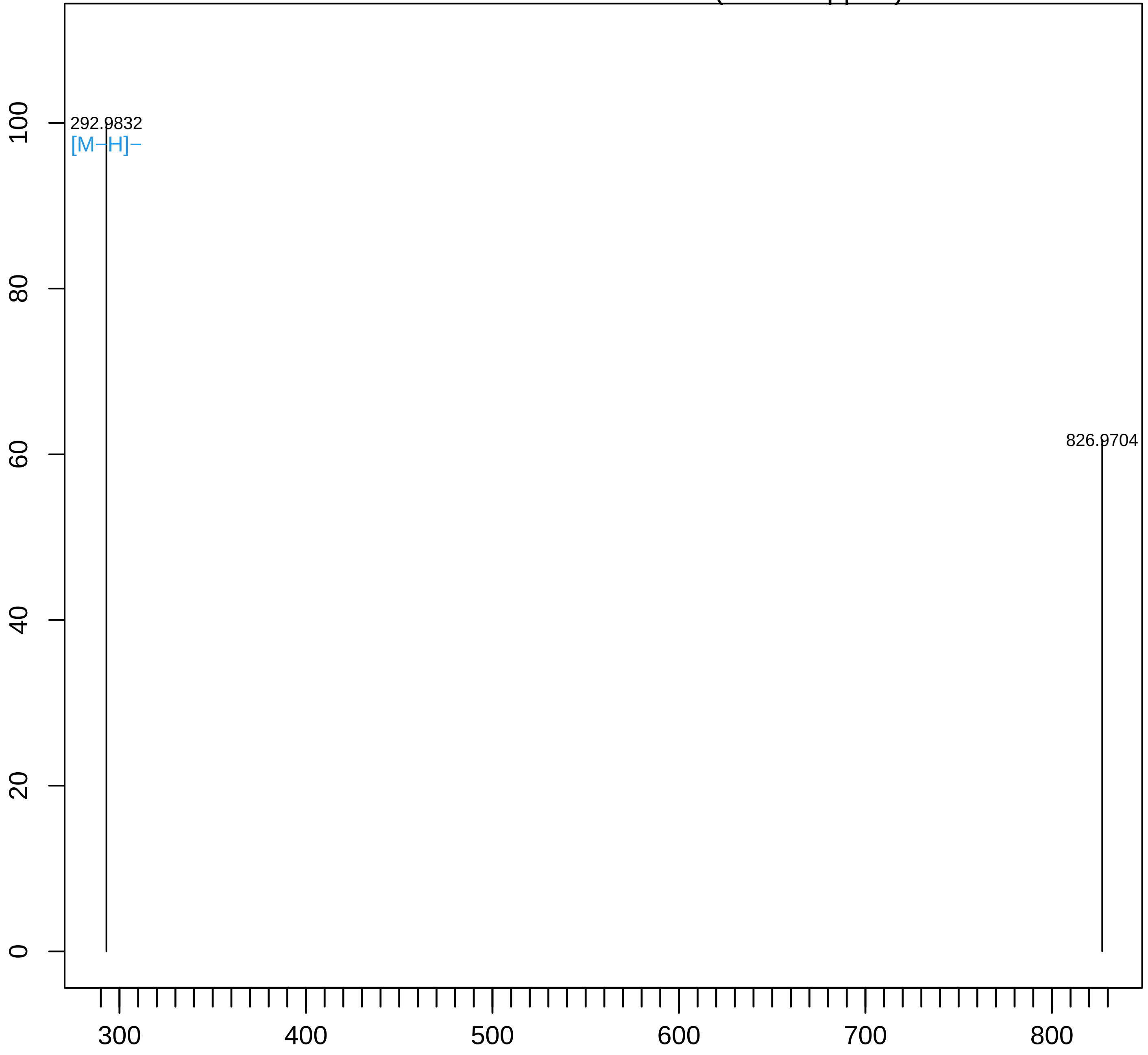

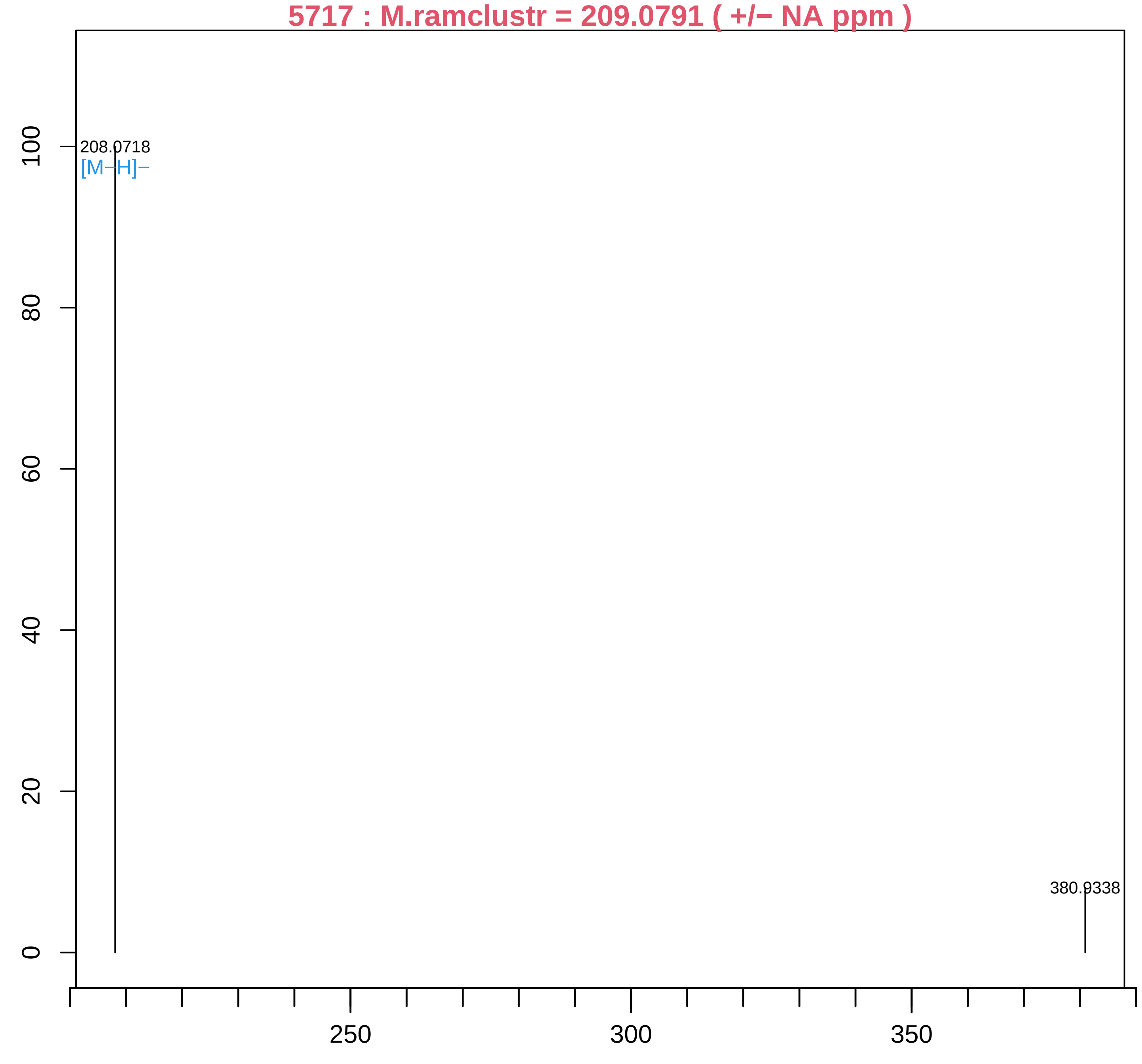
5717 : M.ramclustr = 209.0791 (+/− NA ppm)
208.0718
[M−H]−
380.9338
0
20
40
60
80
100
250
300
350

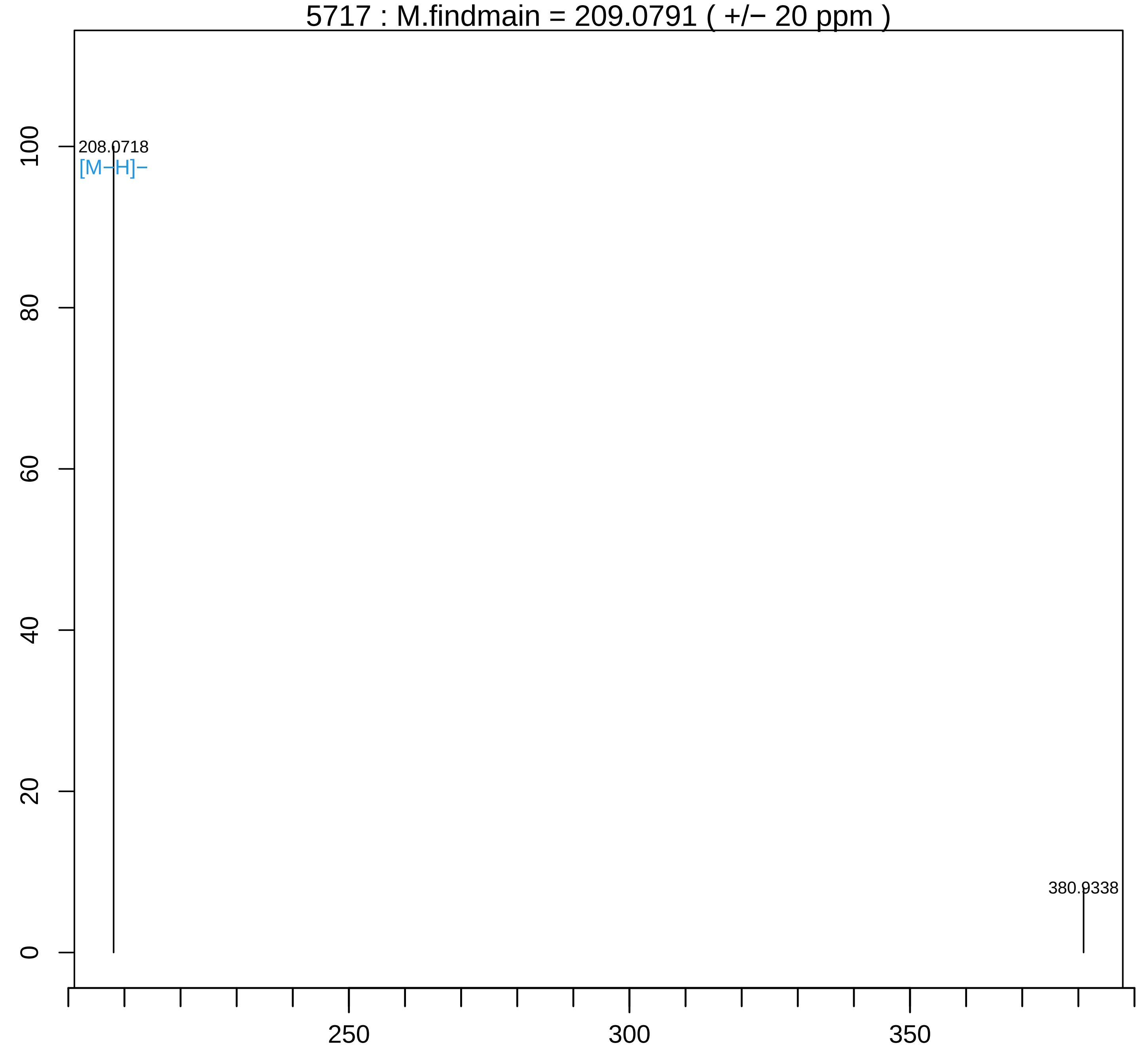
5717 : M.findmain = 209.0791 (+/− 20 ppm)
208.0718
[M−H]−
380.9338
0
20
40
60
80
100
250
300
350

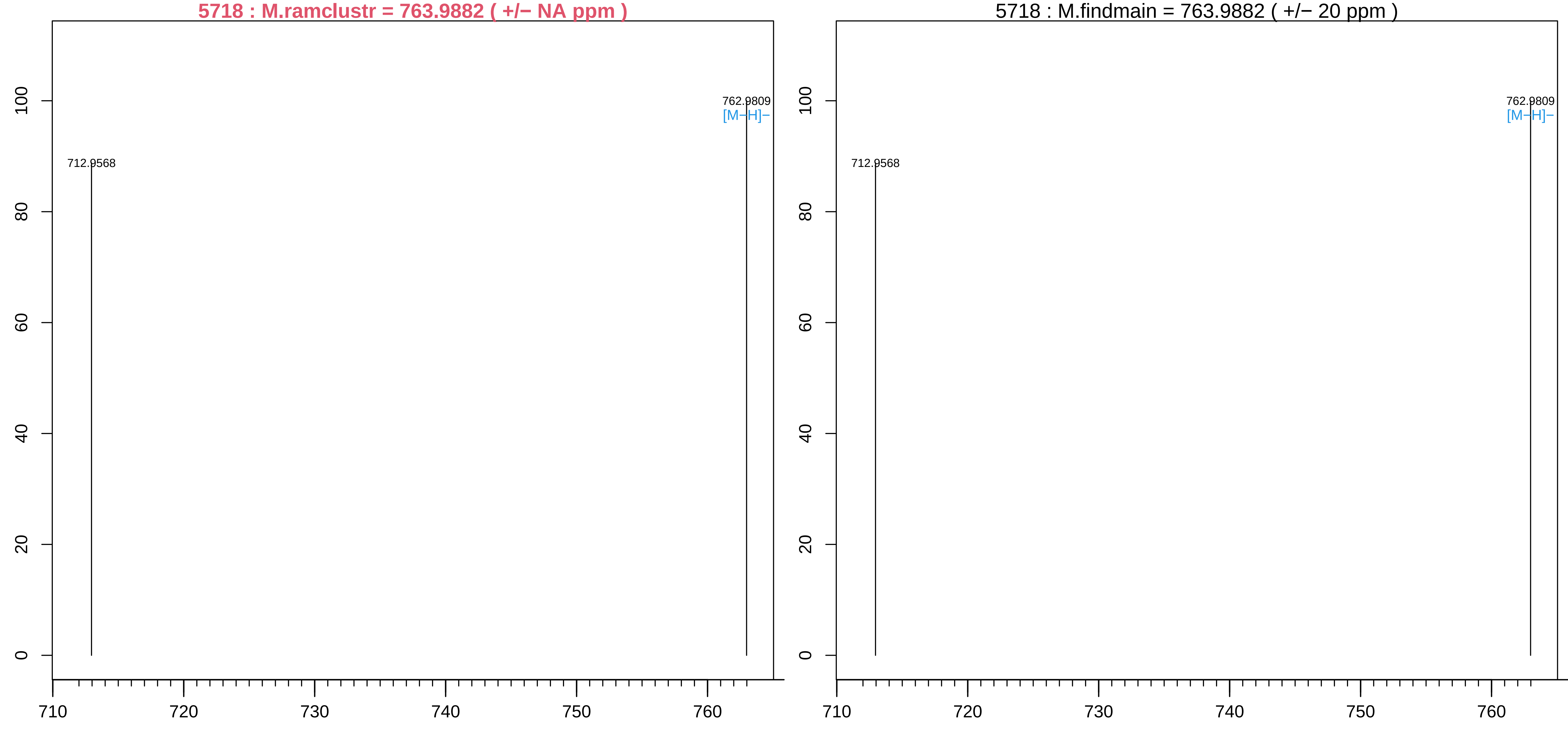

5718 : M.ramclustr = 763.9882 (+/− NA ppm)
5718 : M.findmain = 763.9882 (+/− 20 ppm)
762.9809
[M−H]−
712.9568
0
20
40
60
80
100
710
720
730
740
750
760

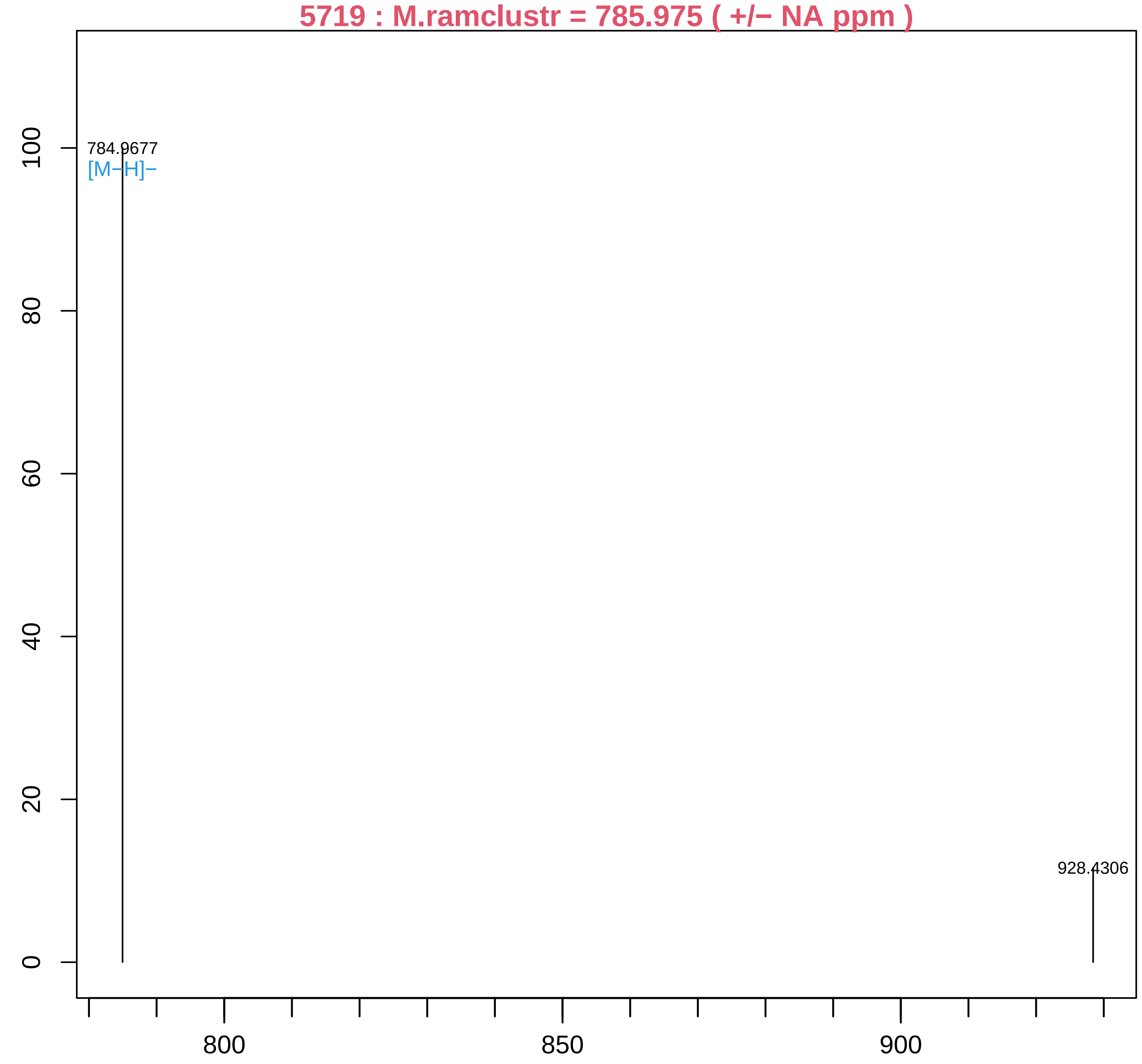
5719 : M.ramclustr = 785.975 (+/− NA ppm)
784.9677
[M−H]−
928.4306
0
20
40
60
80
100
800
850
900

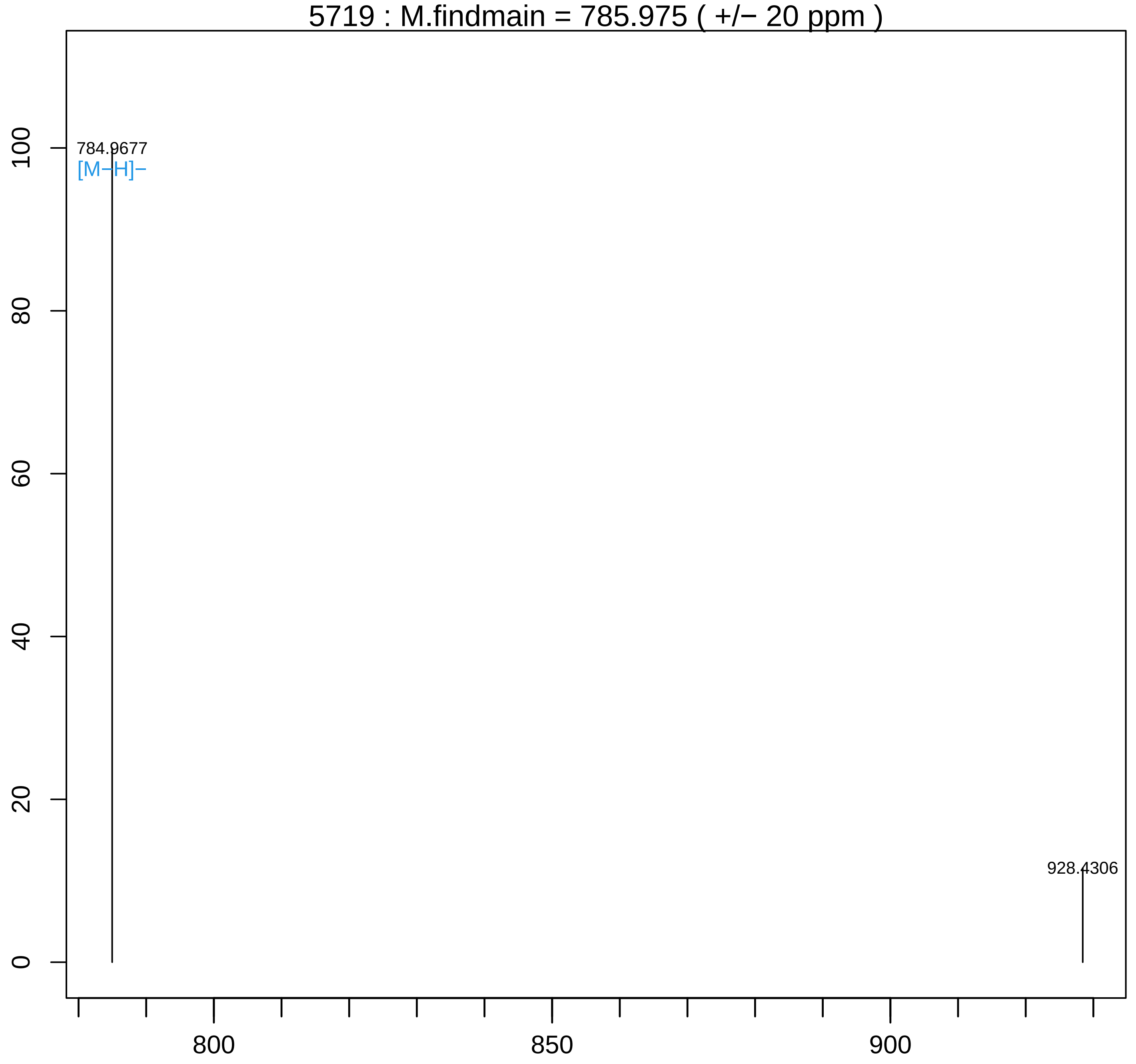
5719 : M.findmain = 785.975 (+/− 20 ppm)
784.9677
[M−H]−
928.4306
0
20
40
60
80
100
800
850
900

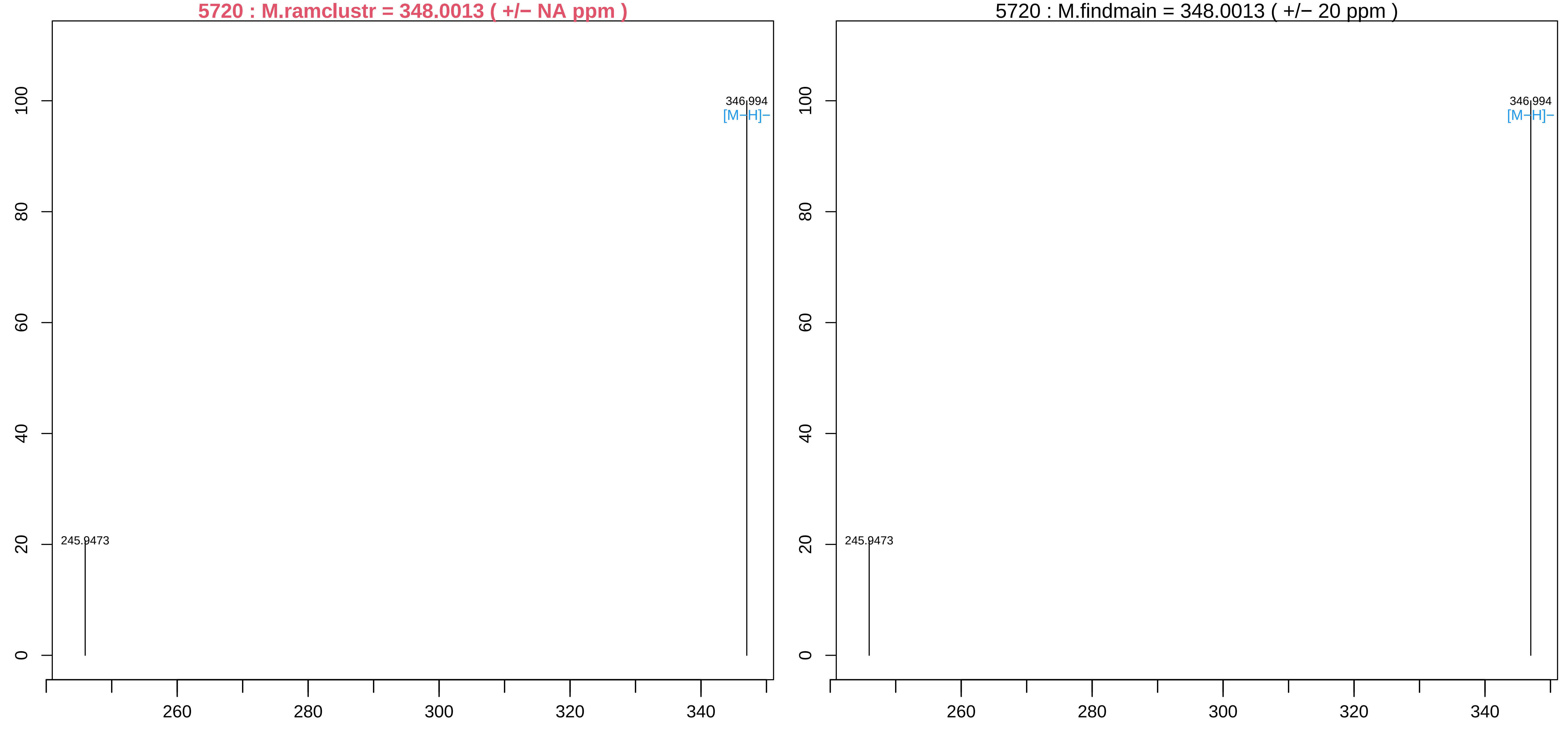
5720 : M.ramclustr = 348.0013 (+/− NA ppm)
5720 : M.findmain = 348.0013 (+/− 20 ppm)
346.994
[M−H]−
245.9473
0
20
40
60
80
100
260
280
300
320
340

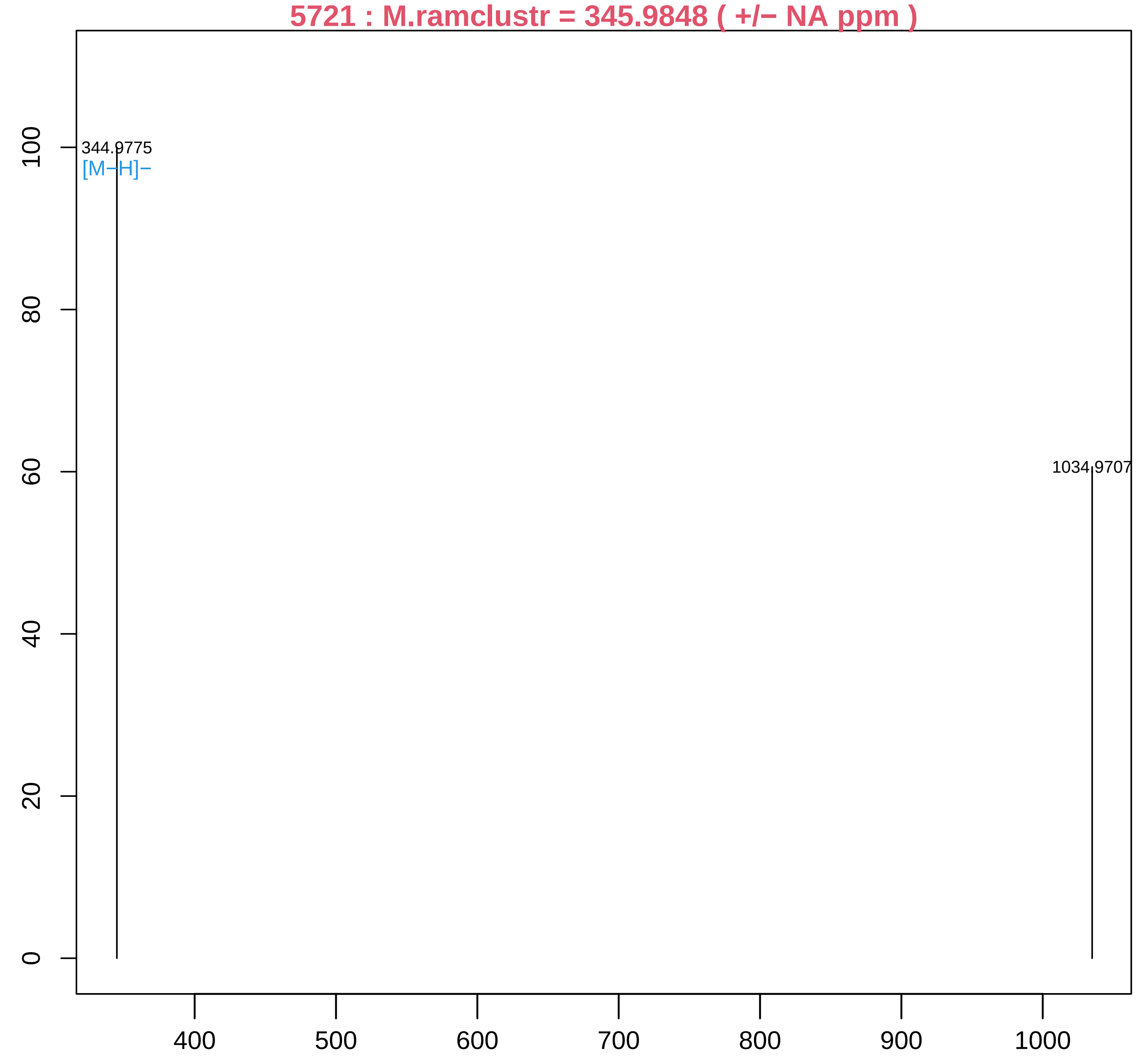
5721 : M.ramclustr = 345.9848 (+/− NA ppm)
344.9775
[M−H]−
1034.9707
0
20
40
60
80
100
400
500
600
700
800
900
1000

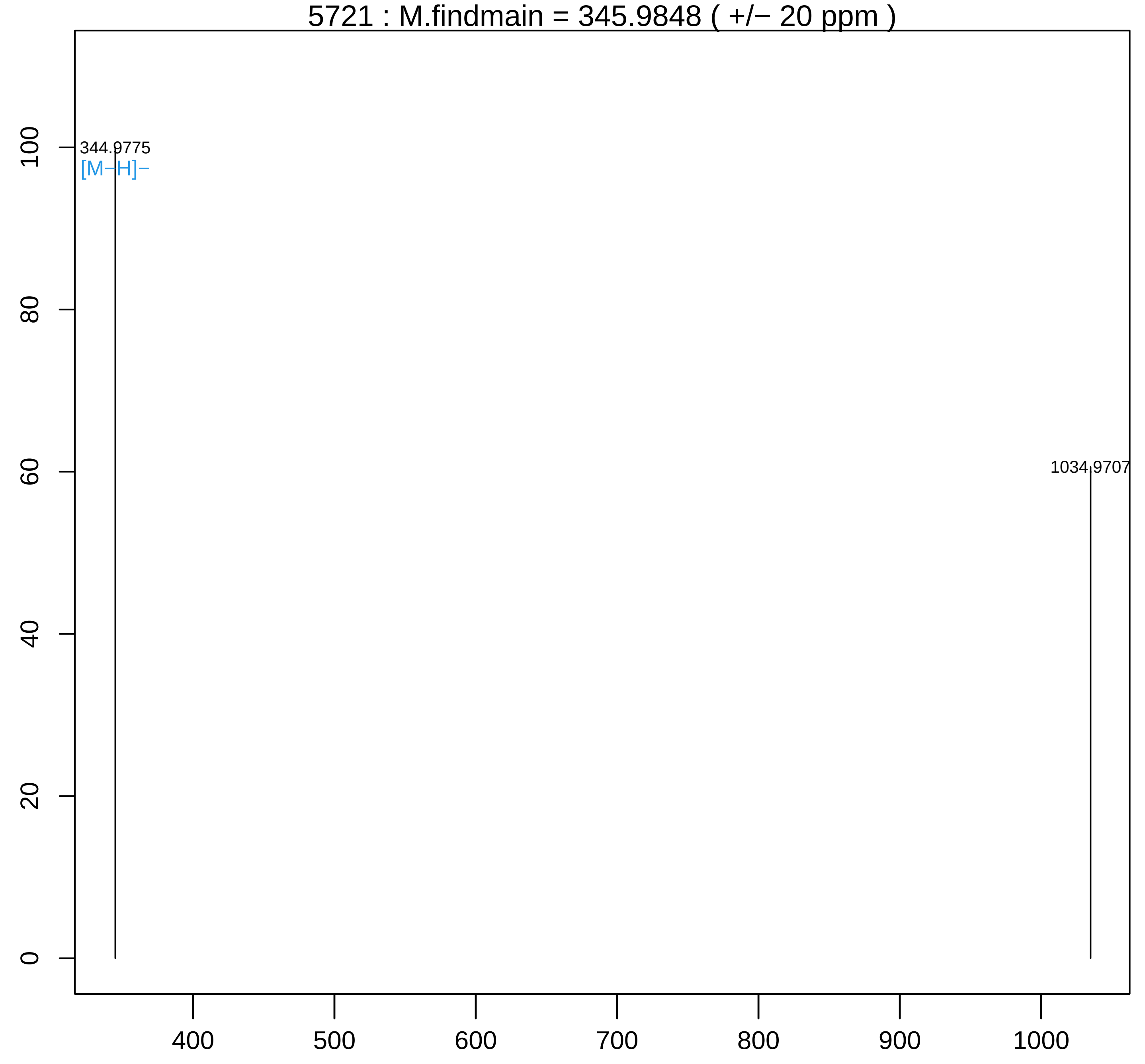
5721 : M.findmain = 345.9848 (+/− 20 ppm)
344.9775
[M−H]−
1034.9707
0
20
40
60
80
100
400
500
600
700
800
900
1000

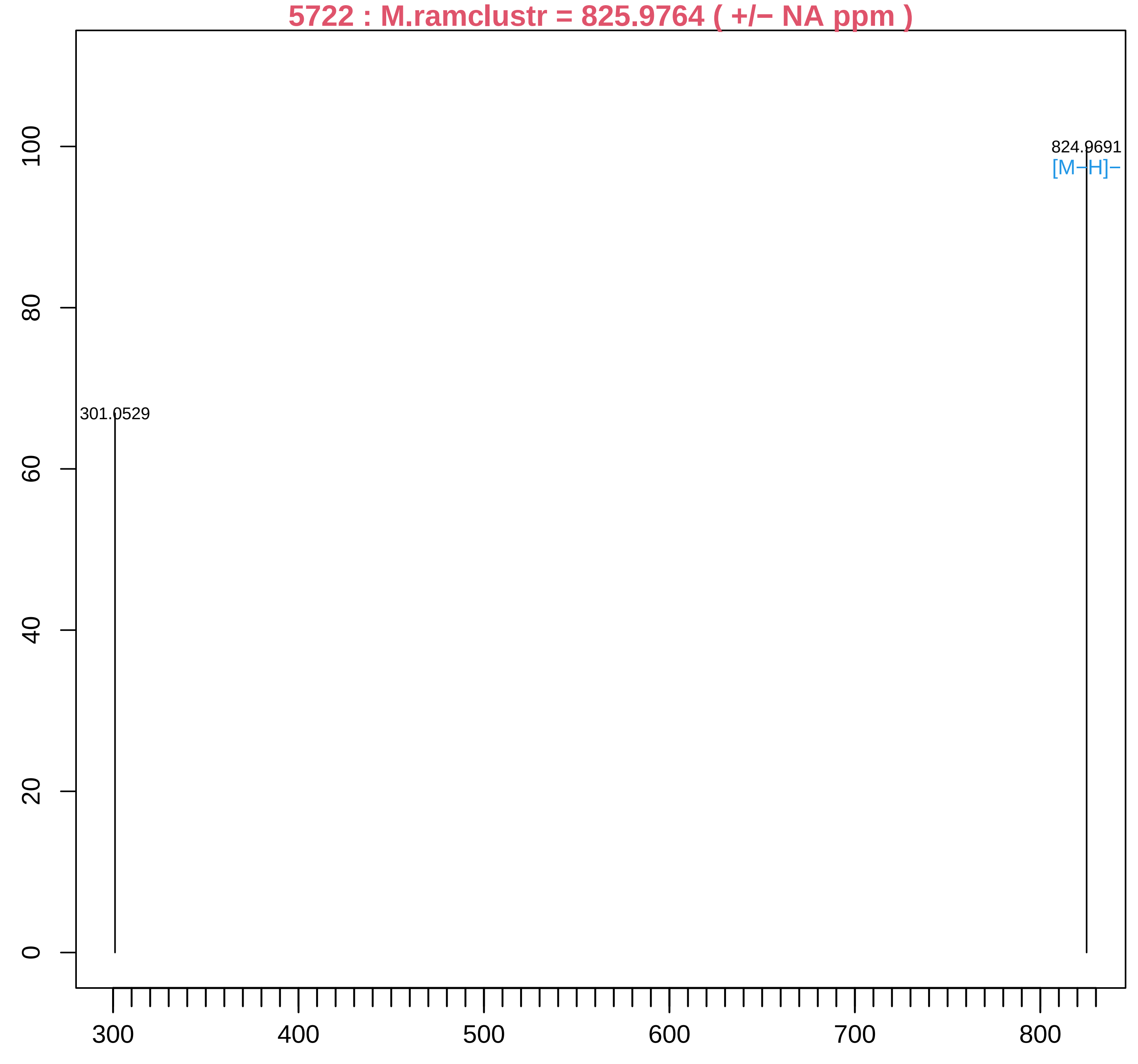
5722 : M.ramclustr = 825.9764 (+/− NA ppm)
824.9691
[M−H]−
301.0529
0
20
40
60
80
100
300
400
500
600
700
800

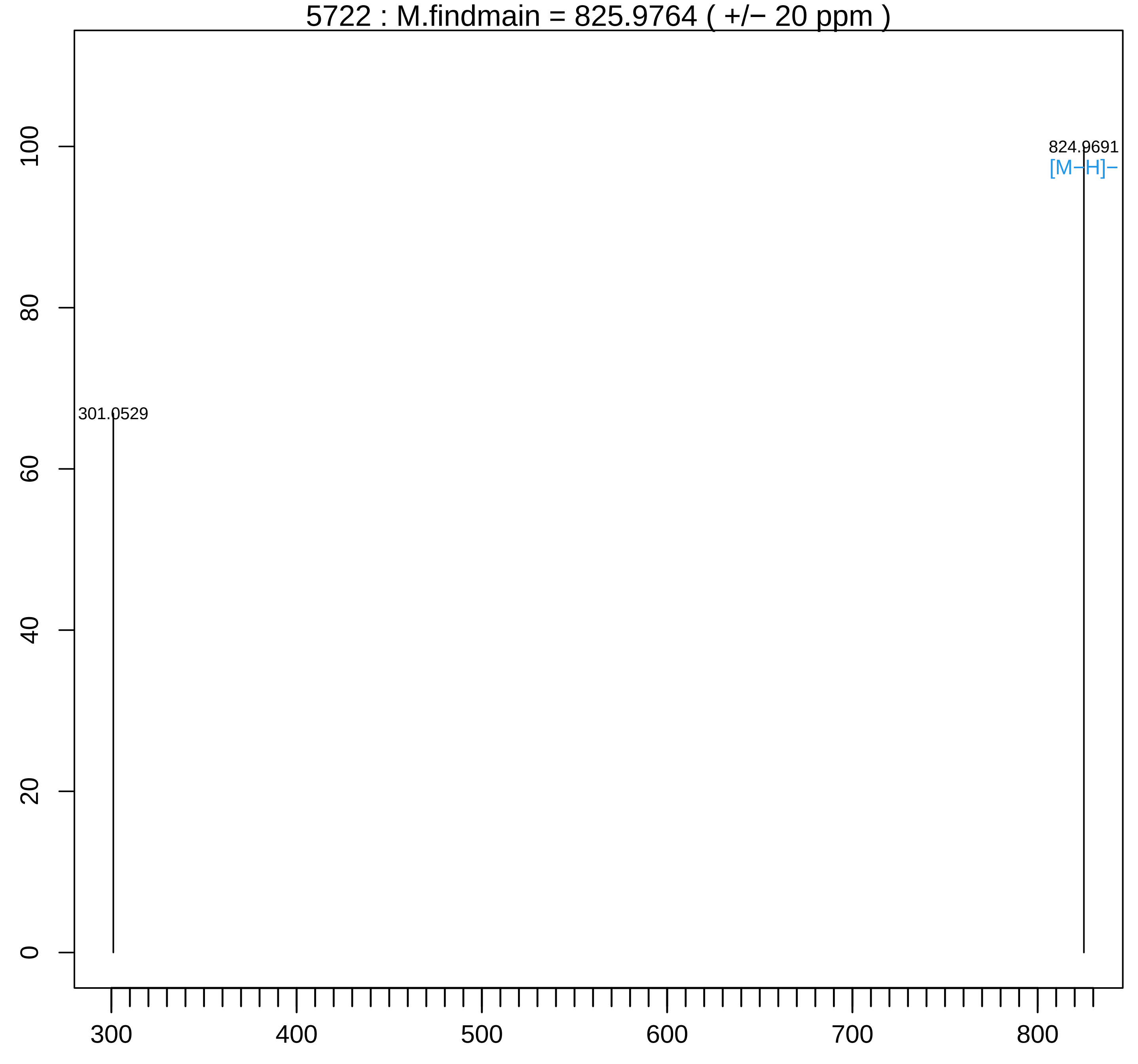
5722 : M.findmain = 825.9764 (+/− 20 ppm)
824.9691
[M−H]−
301.0529
0
20
40
60
80
100
300
400
500
600
700
800

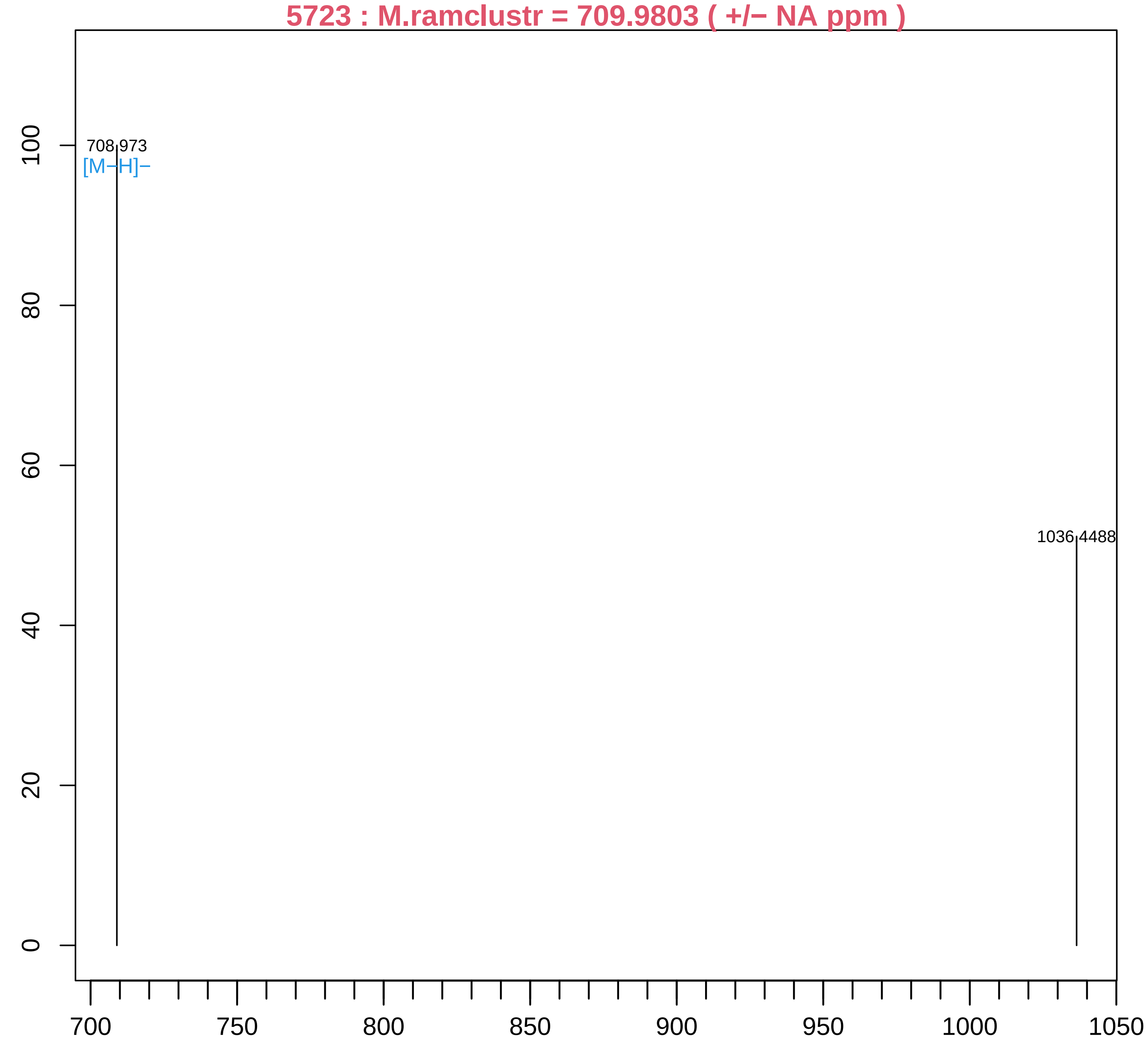
5723 : M.ramclustr = 709.9803 (+/− NA ppm)
708.973
[M−H]−
1036.4488
0
20
40
60
80
100
700
750
800
850
900
950
1000
1050

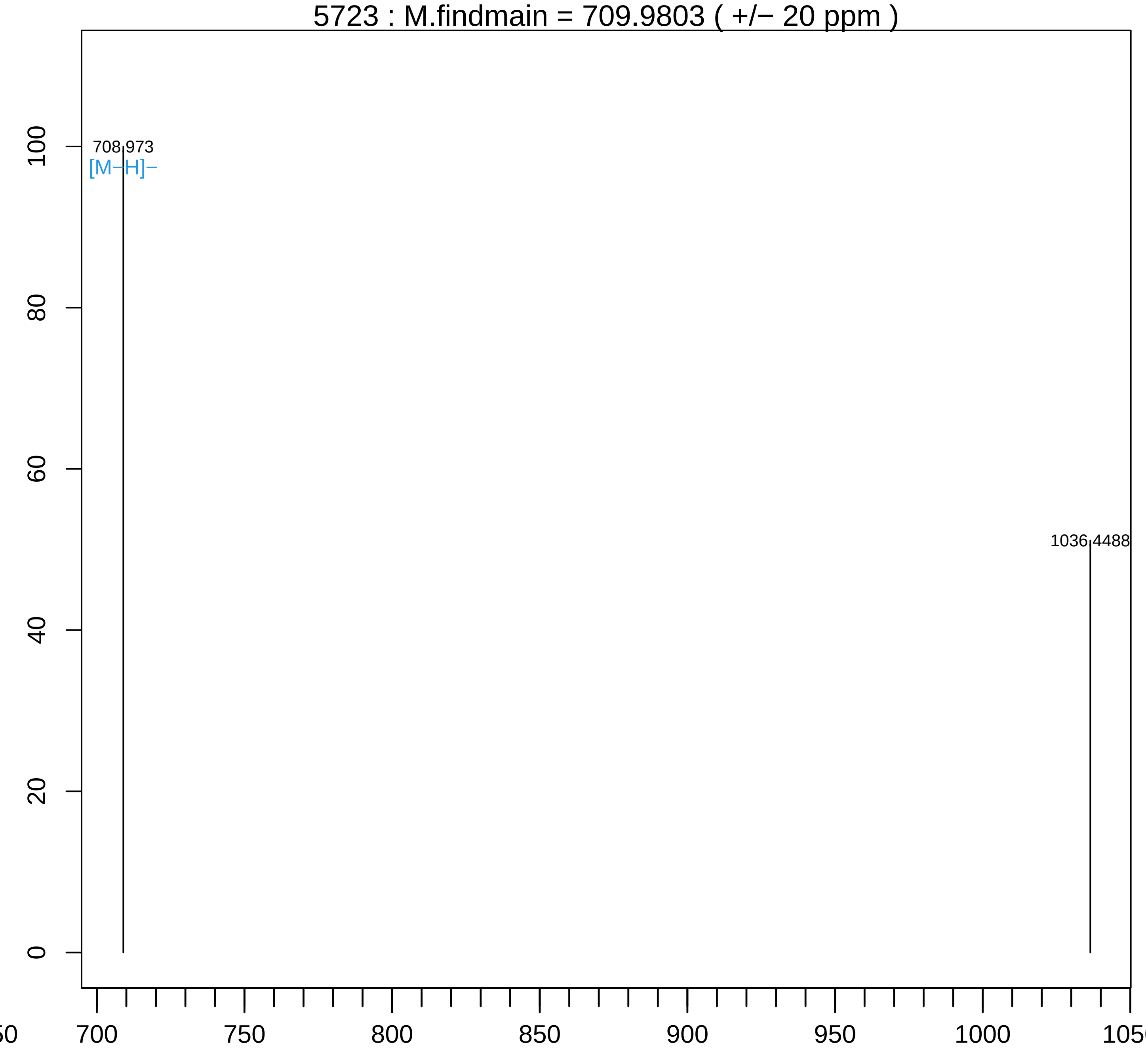
5723 : M.findmain = 709.9803 (+/− 20 ppm)
708.973
[M−H]−
1036.4488
0
20
40
60
80
100
700
750
800
850
900
950
1000

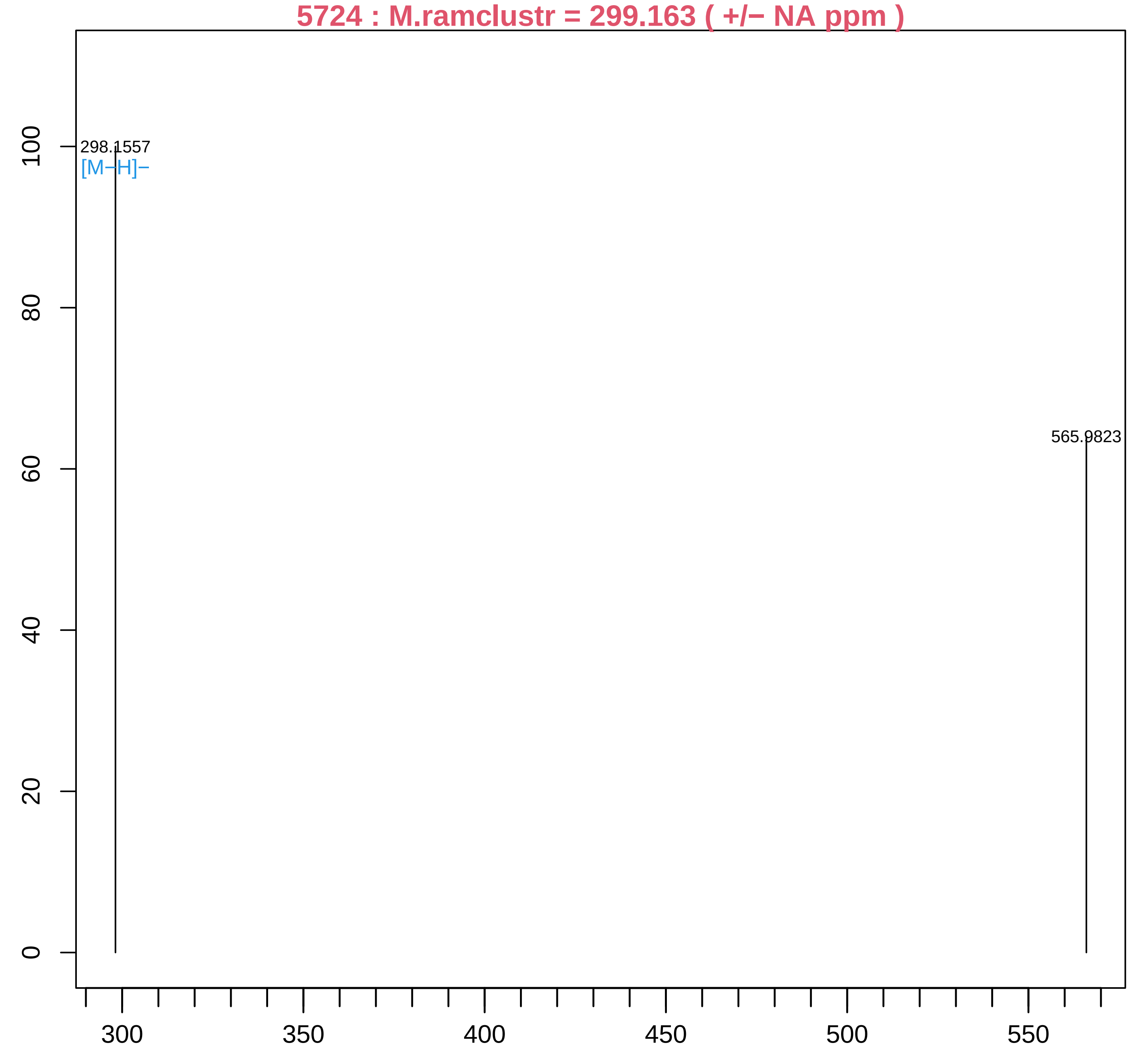
5724 : M.ramclustr = 299.163 (+/− NA ppm)
298.1557
[M−H]−
565.9823
0
20
40
60
80
100
300
350
400
450
500
550

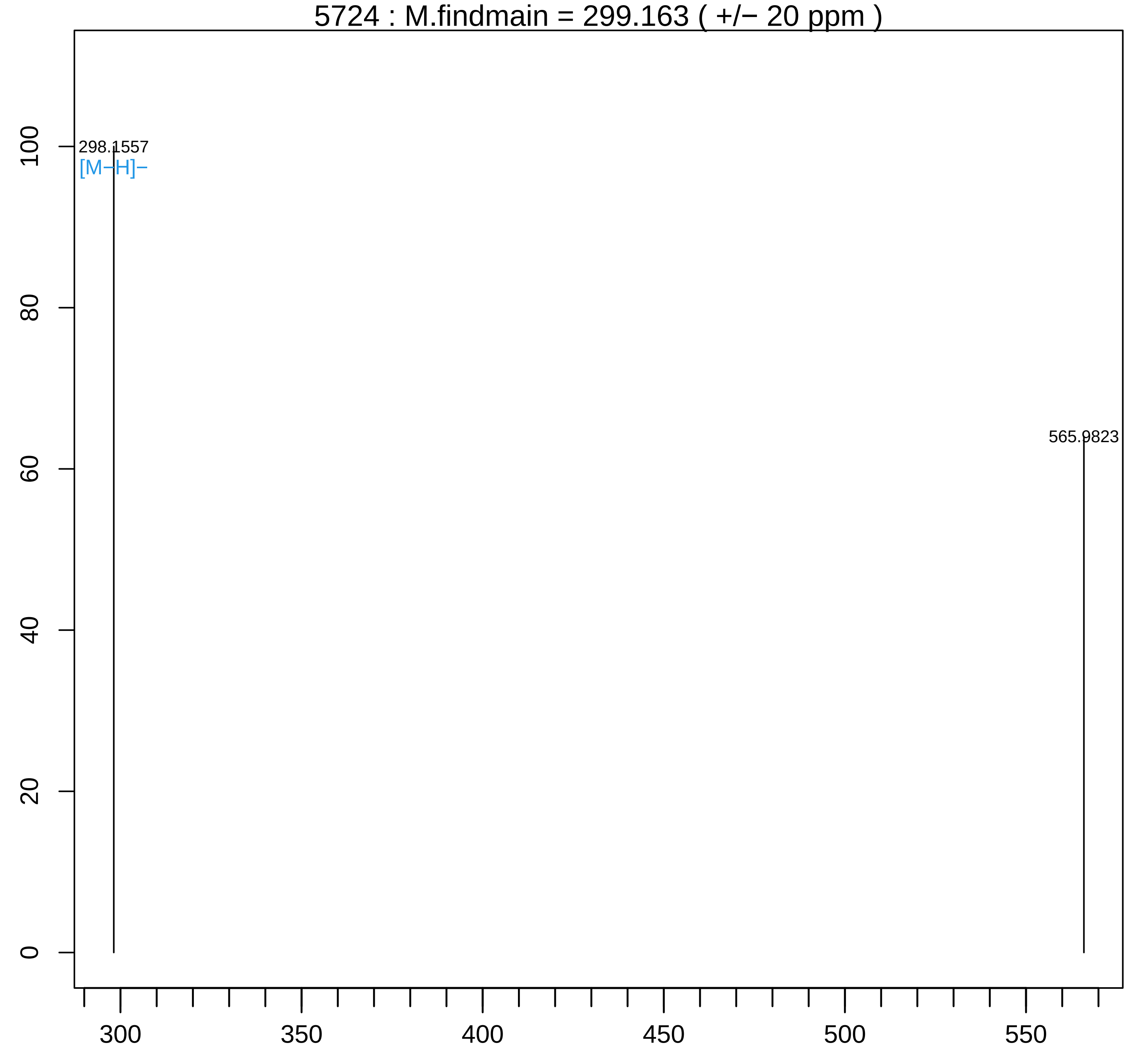
5724 : M.findmain = 299.163 (+/− 20 ppm)
298.1557
[M−H]−
565.9823
0
20
40
60
80
100
300
350
400
450
500
550

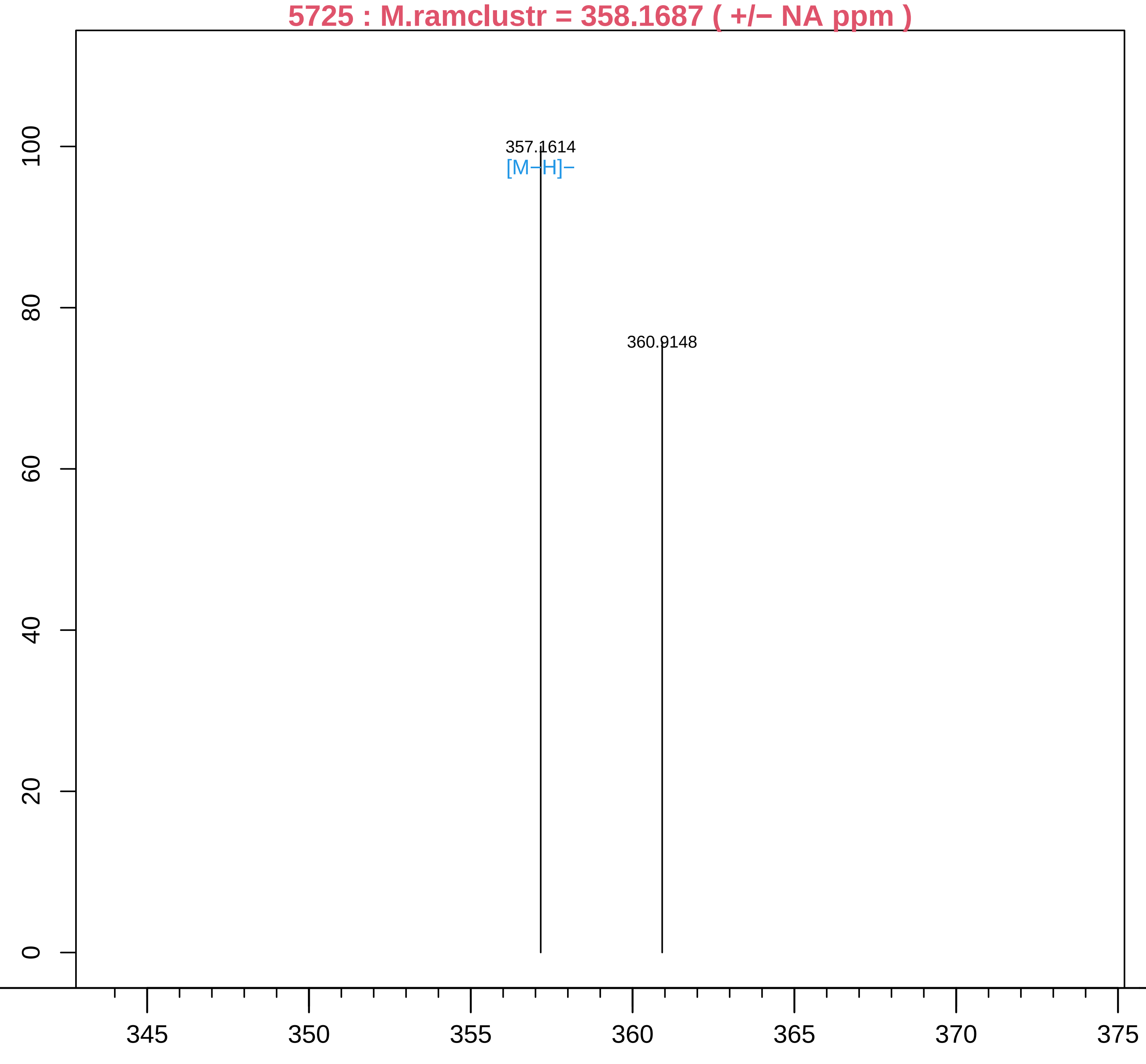

5725 : M.ramclustr = 358.1687 (+/− NA ppm)
357.1614
[M−H]−
360.9148
0
20
40
60
80
100
345
350
355
360
365
370
375

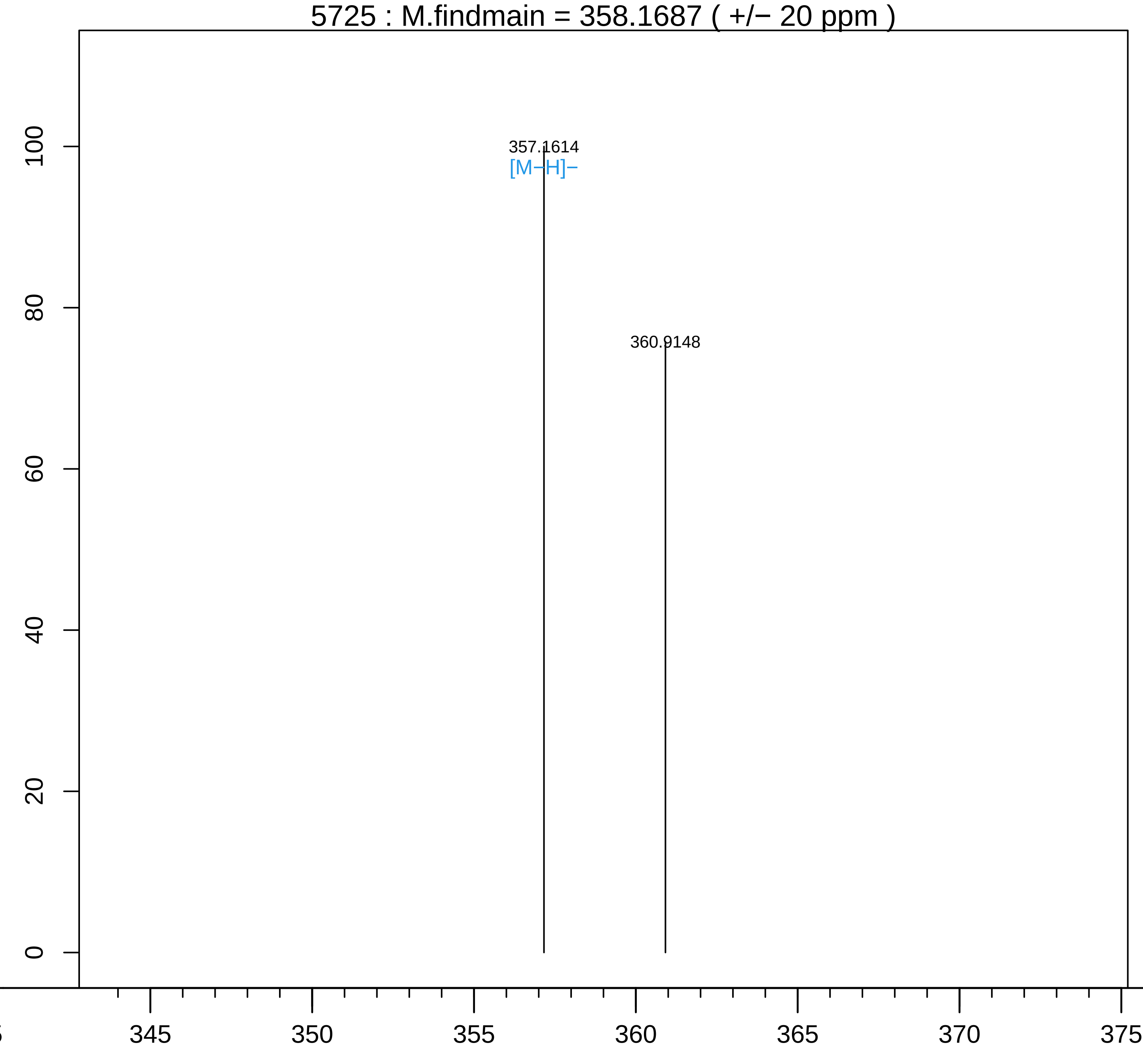

5725 : M.findmain = 358.1687 (+/− 20 ppm)
357.1614
[M−H]−
360.9148
0
20
40
60
80
100
345
350
355
360
365
370
375

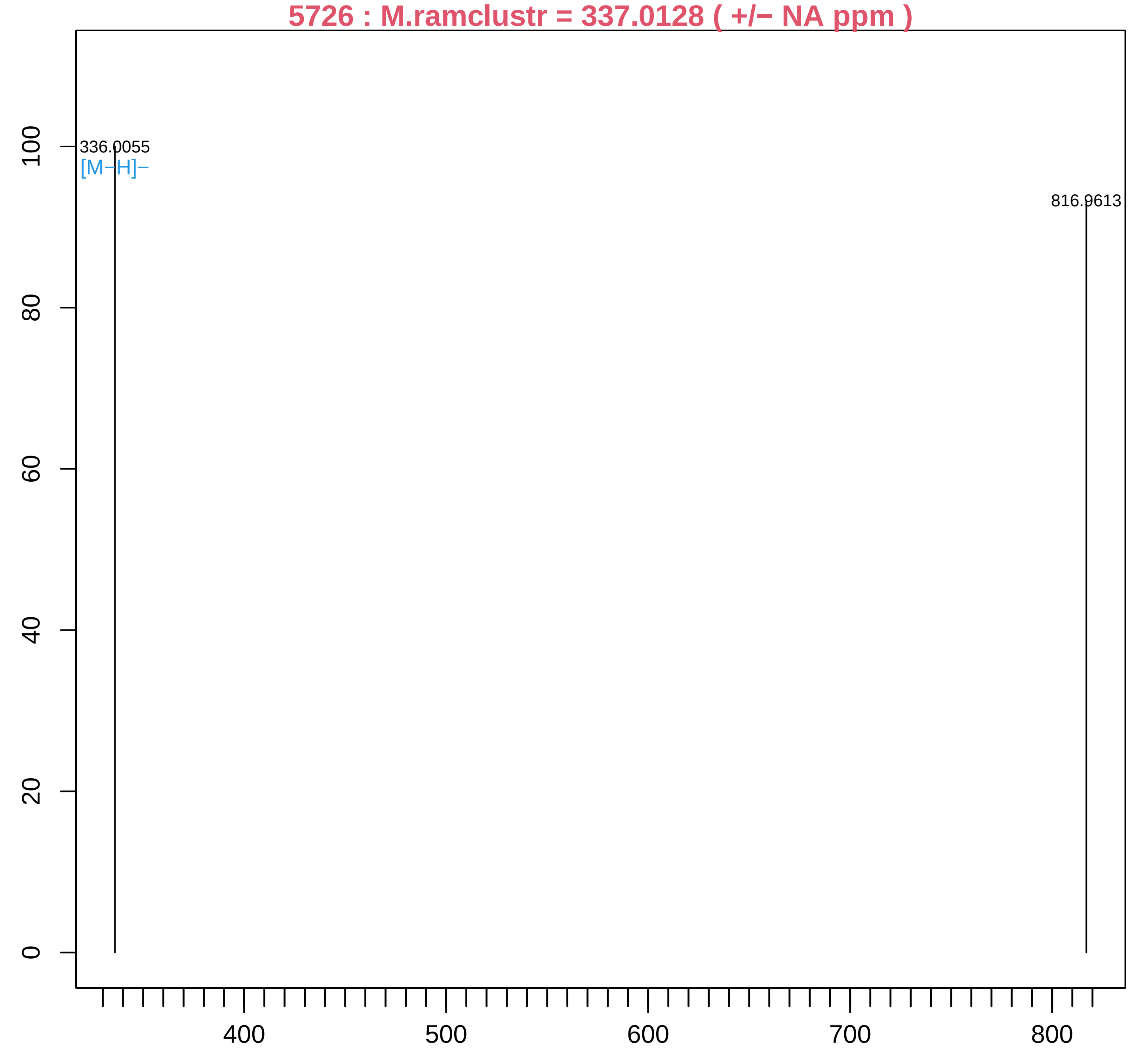
5726 : M.ramclustr = 337.0128 (+/− NA ppm)
336.0055
[M−H]−
816.9613
0
20
40
60
80
100
400
500
600
700
800
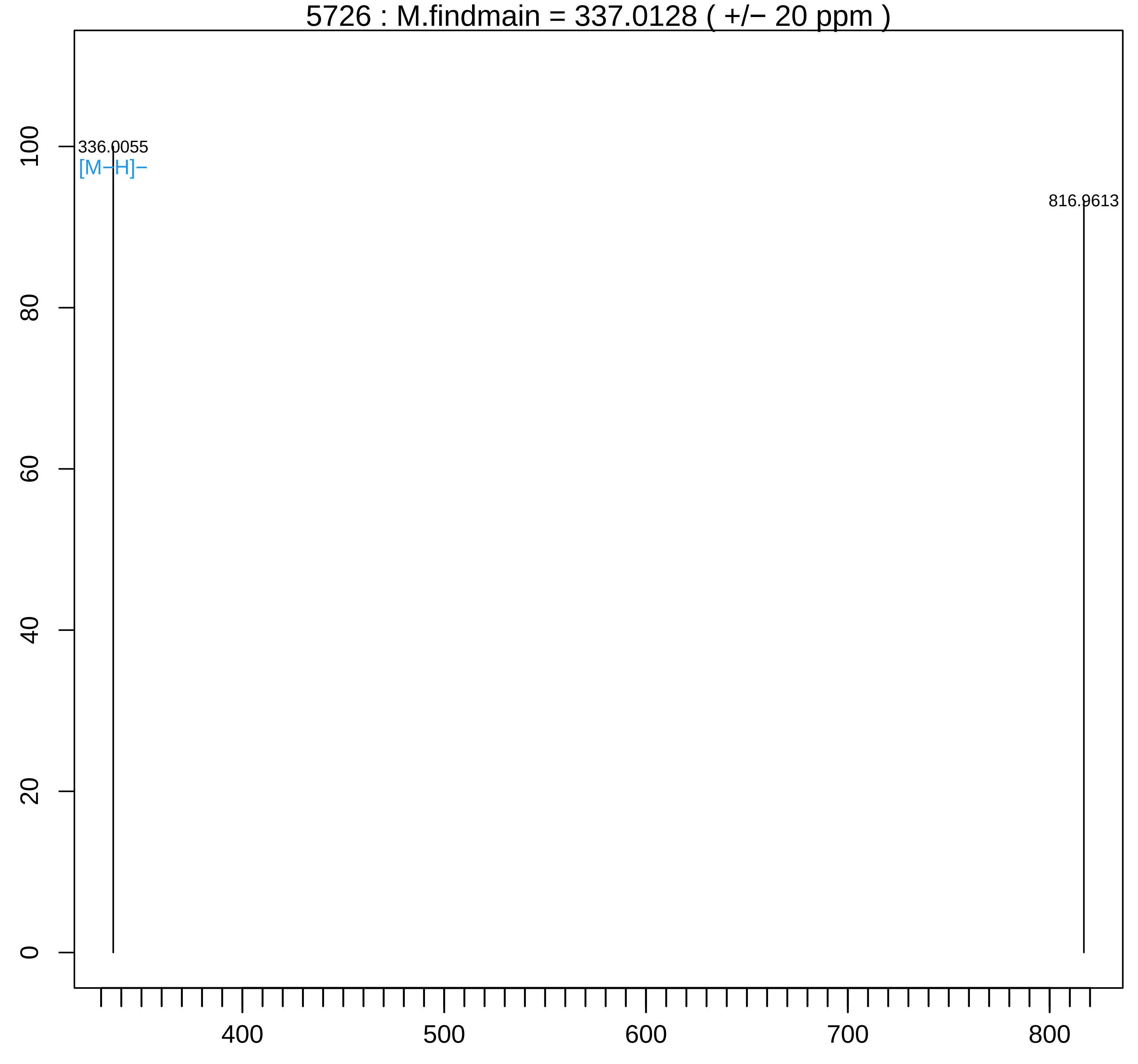
5726 : M.findmain = 337.0128 (+/− 20 ppm)
336.0055
[M−H]−
816.9613
0
20
40
60
80
100
400
500
600
700
800

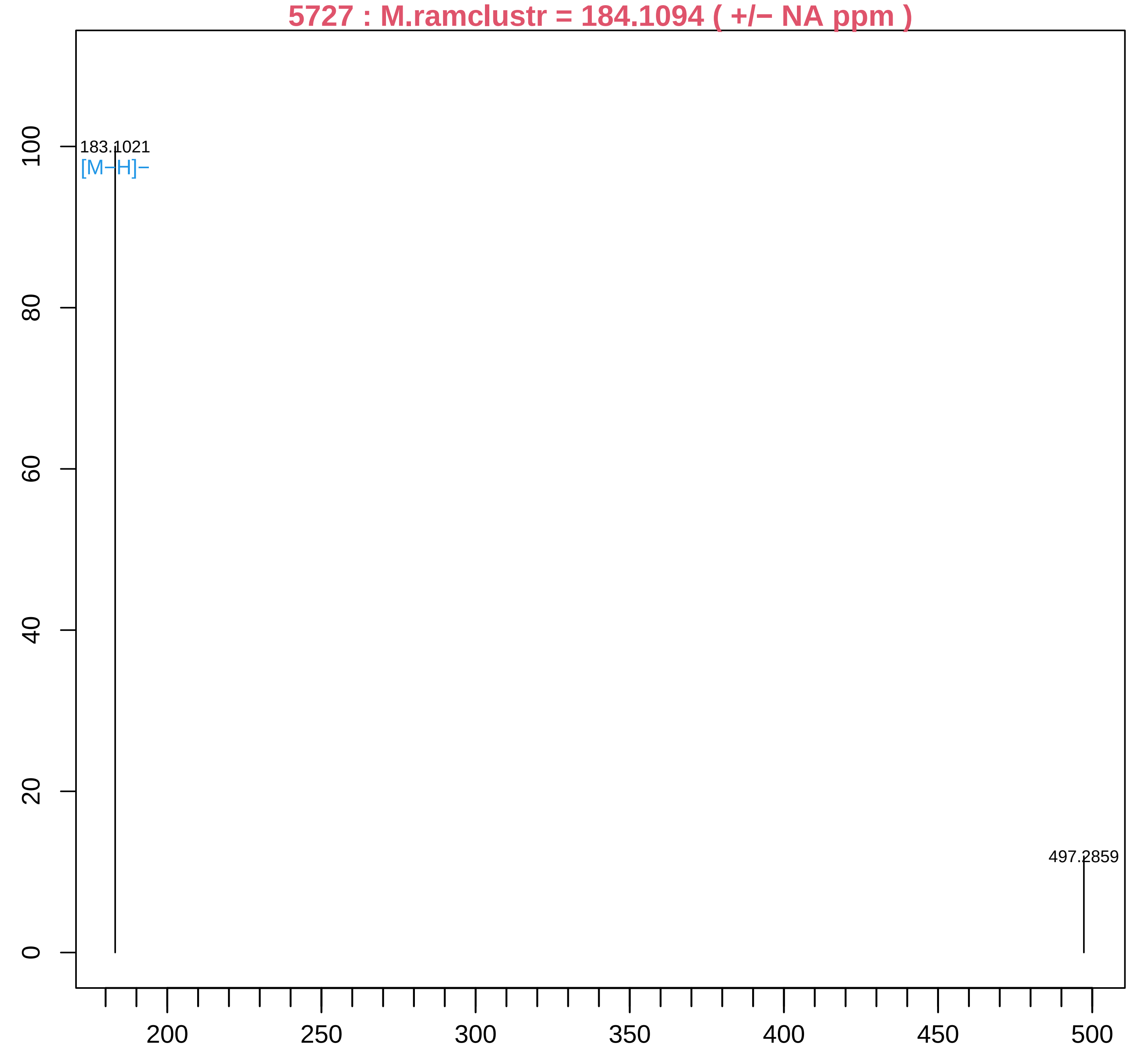
5727 : M.ramclustr = 184.1094 (+/− NA ppm)
183.1021
[M−H]−
497.2859
0
20
40
60
80
100
200
250
300
350
400
450
500

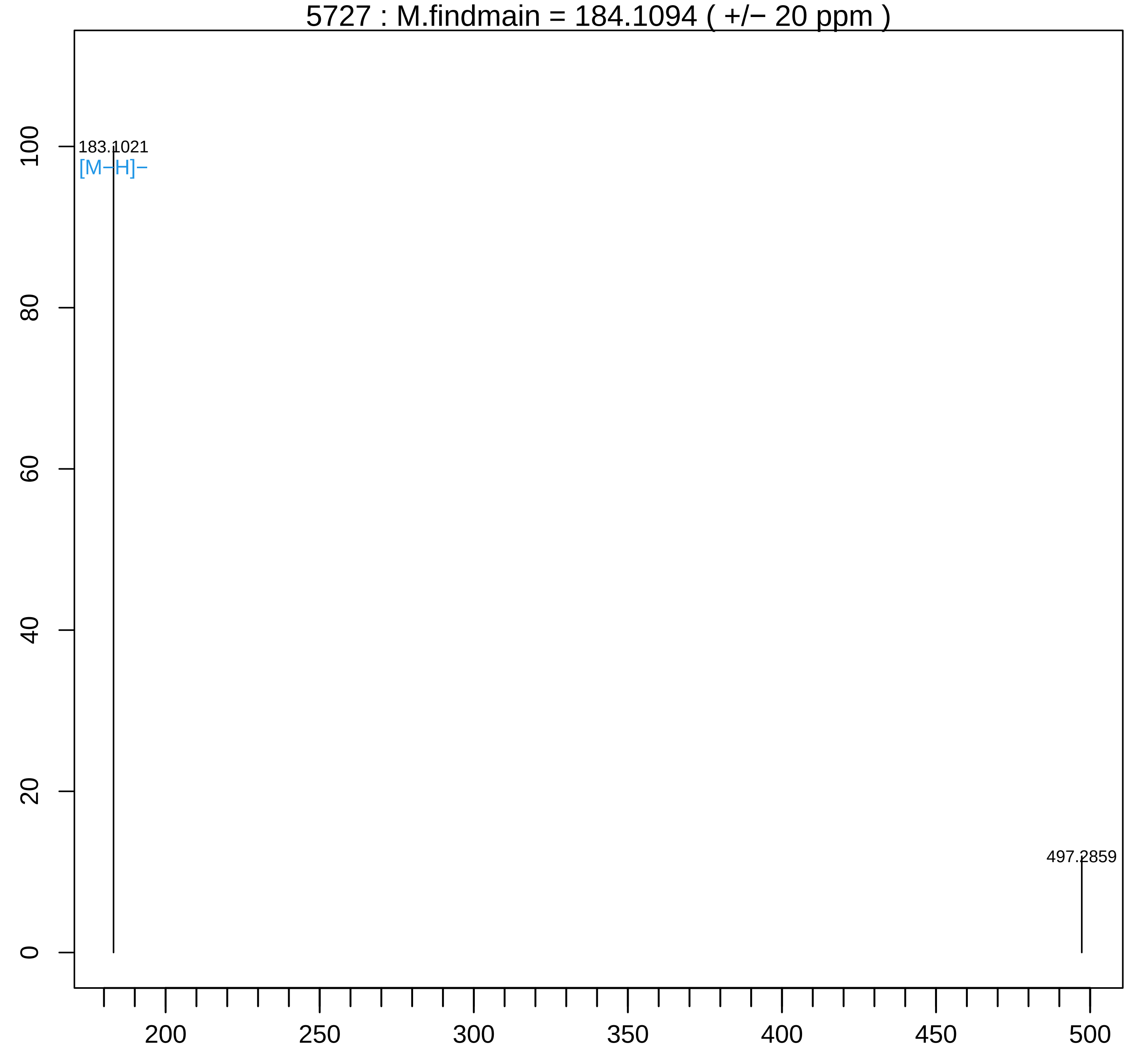
5727 : M.findmain = 184.1094 (+/− 20 ppm)
183.1021
[M−H]−
497.2859
0
20
40
60
80
100
200
250
300
350
400
450
500

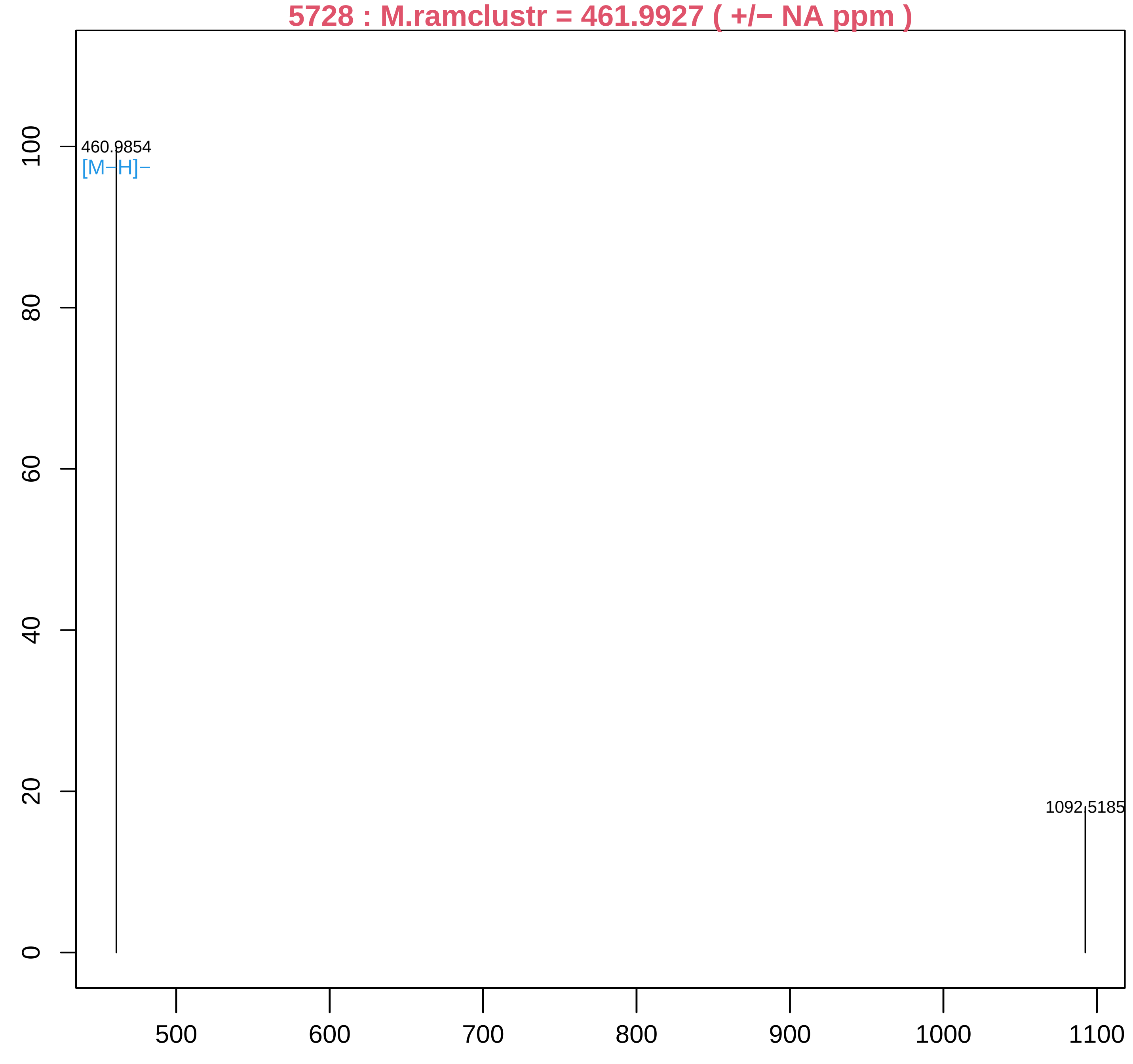

5728 : M.ramclustr = 461.9927 (+/− NA ppm)
460.9854
[M−H]−
1092.5185
0
20
40
60
80
100
500
600
700
800
900
1000
1100

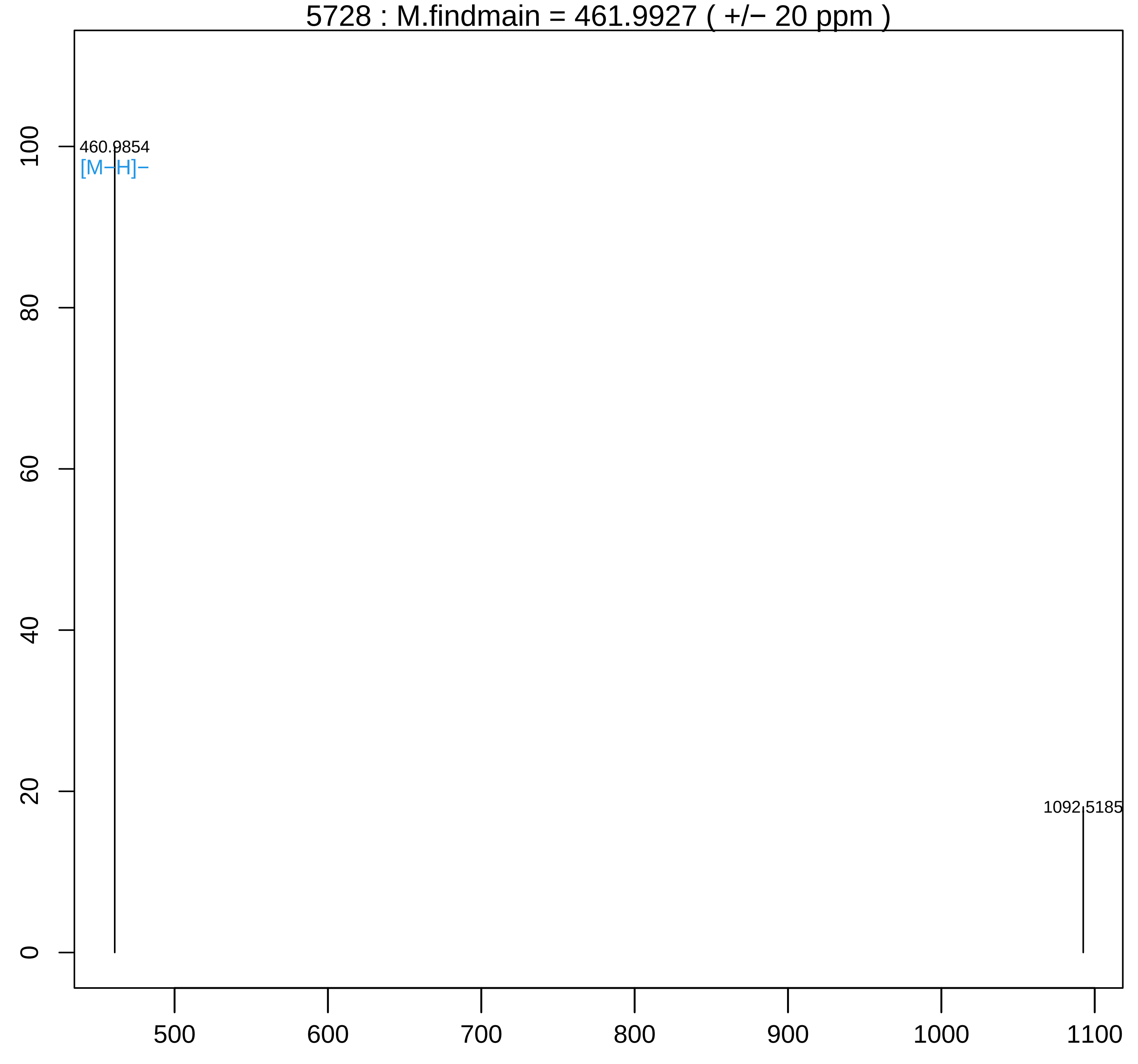

5728 : M.findmain = 461.9927 (+/− 20 ppm)
460.9854
[M−H]−
1092.5185
0
20
40
60
80
100
500
600
700
800
900
1000
1100

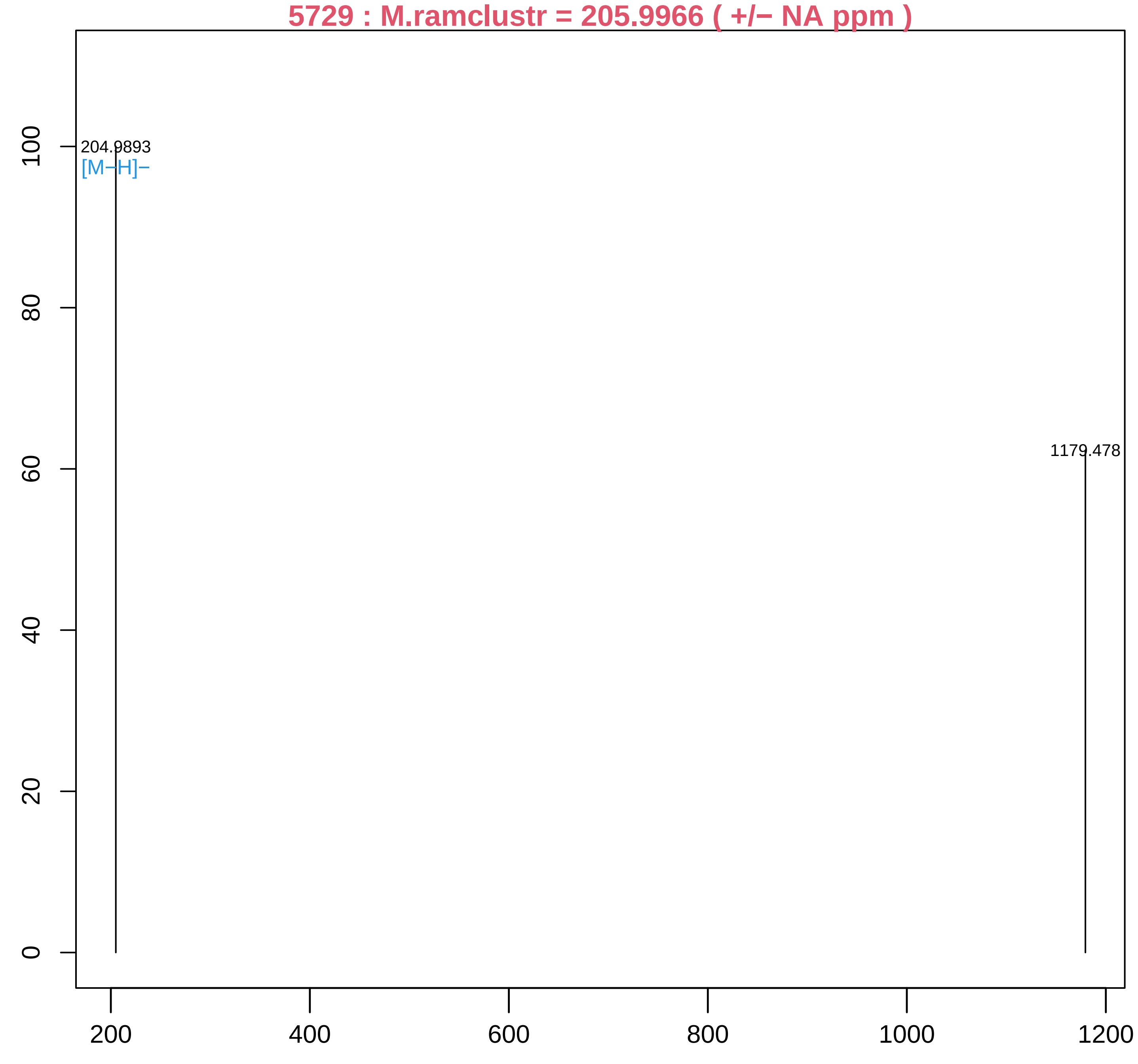
5729 : M.ramclustr = 205.9966 (+/− NA ppm)
204.9893
[M−H]−
1179.478
0
20
40
60
80
100
200
400
600
800
1000
1200

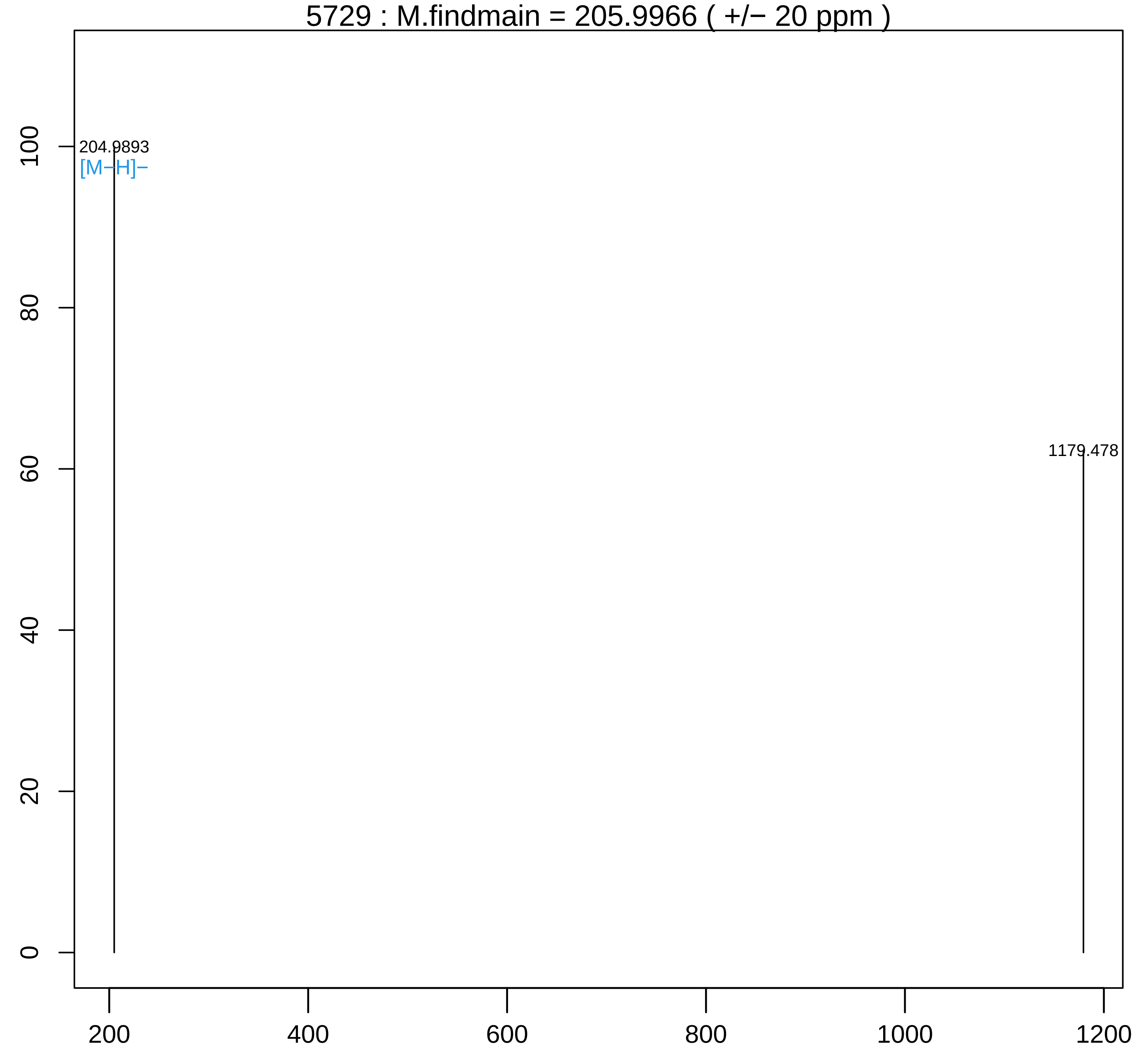
5729 : M.findmain = 205.9966 (+/− 20 ppm)
204.9893
[M−H]−
1179.478
0
20
40
60
80
100
200
400
600
800
1000
1200

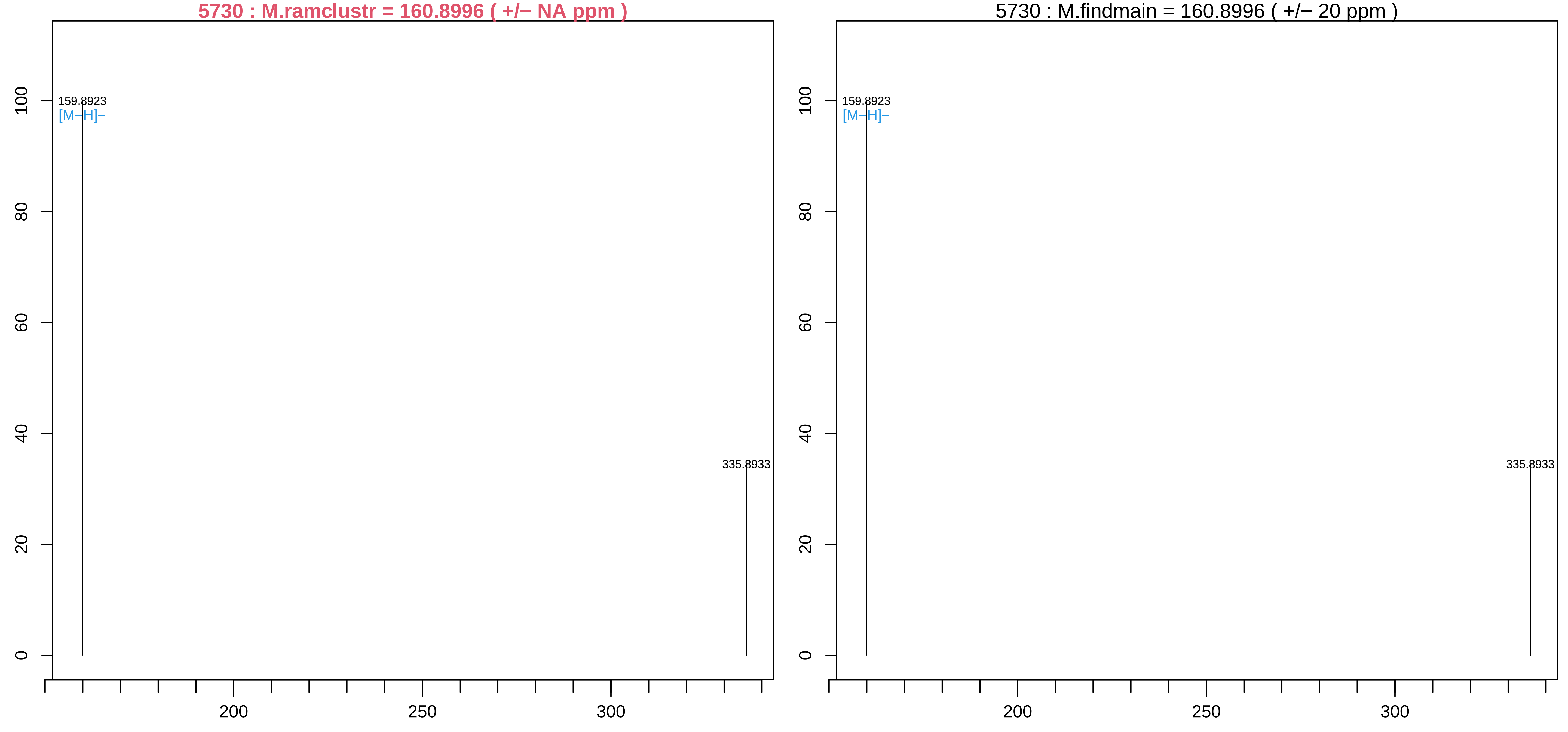
5730 : M.ramclustr = 160.8996 (+/− NA ppm)
159.8923
[M−H]−
335.8933
0
20
40
60
80
100
200
250
300
5730 : M.findmain = 160.8996 (+/− 20 ppm)
159.8923
[M−H]−
335.8933
0
20
40
60
80
100
200
250
300

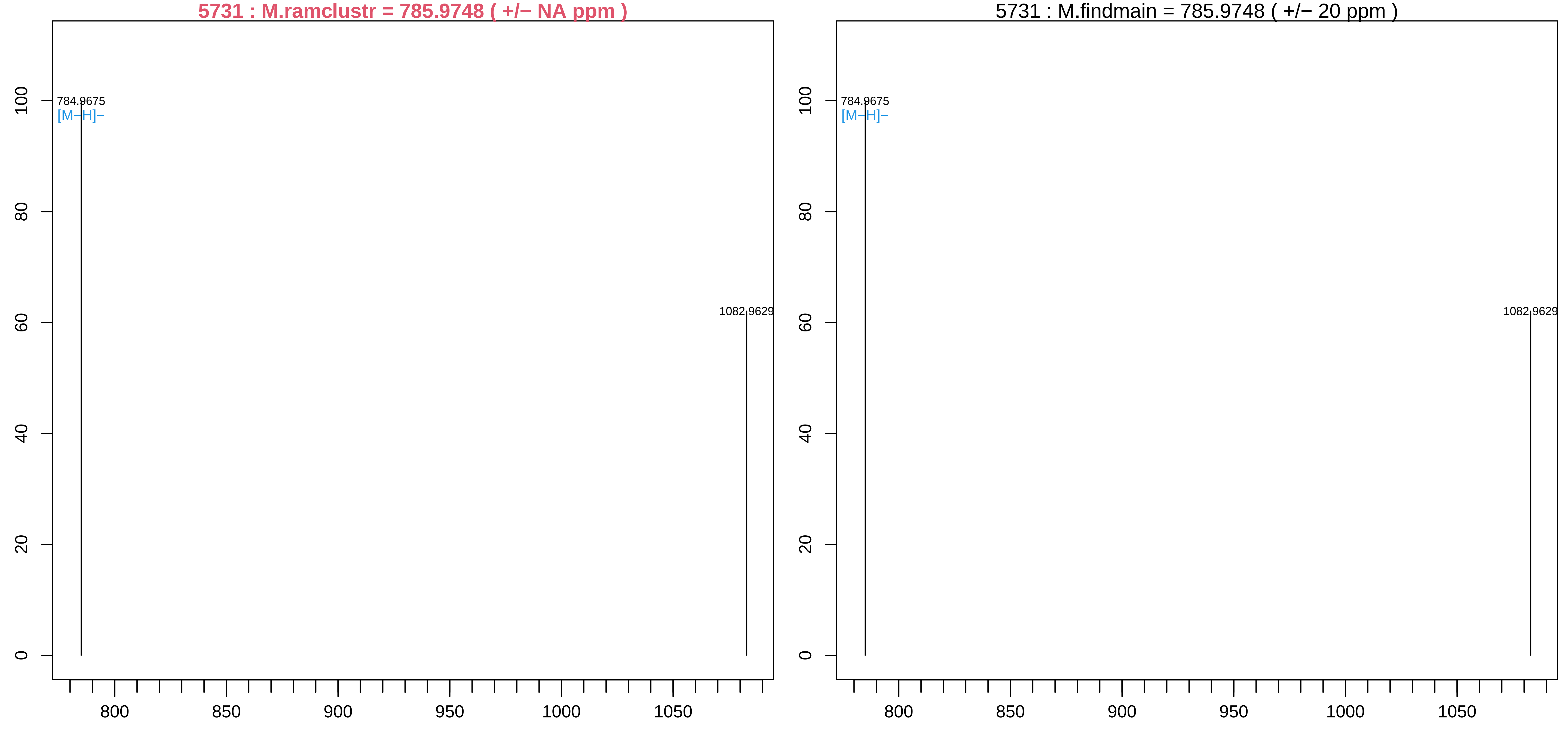
5731 : M.ramclustr = 785.9748 (+/− NA ppm)
784.9675
[M−H]−
1082.9629
0
20
40
60
80
100
800
850
900
950
1000
1050
5731 : M.findmain = 785.9748 (+/− 20 ppm)
784.9675
[M−H]−
1082.9629
0
20
40
60
80
100
800
850
900
950
1000
1050

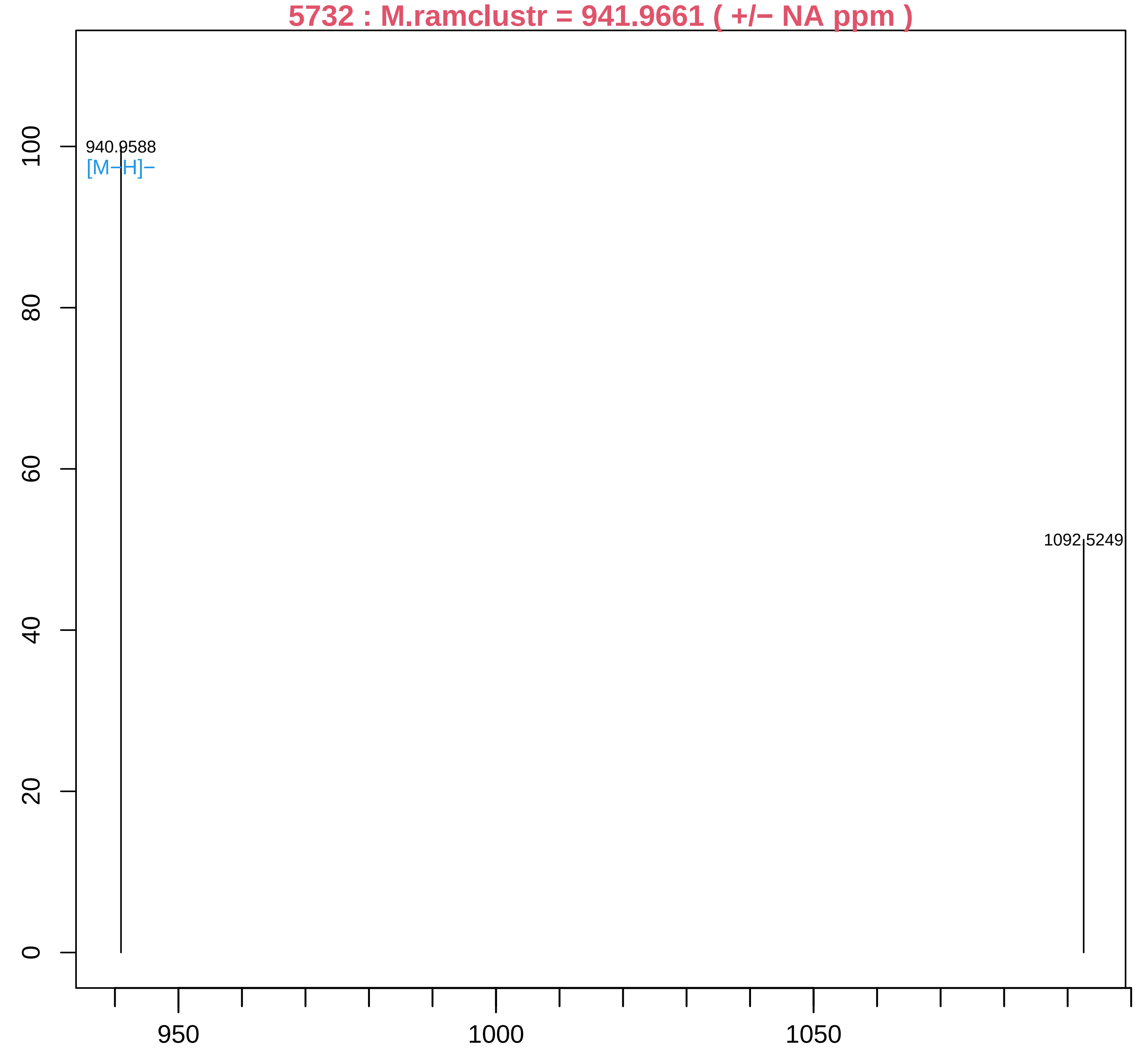

5732 : M.ramclustr = 941.9661 (+/− NA ppm)
940.9588
[M−H]−
1092.5249
0
20
40
60
80
100
950
1000
1050

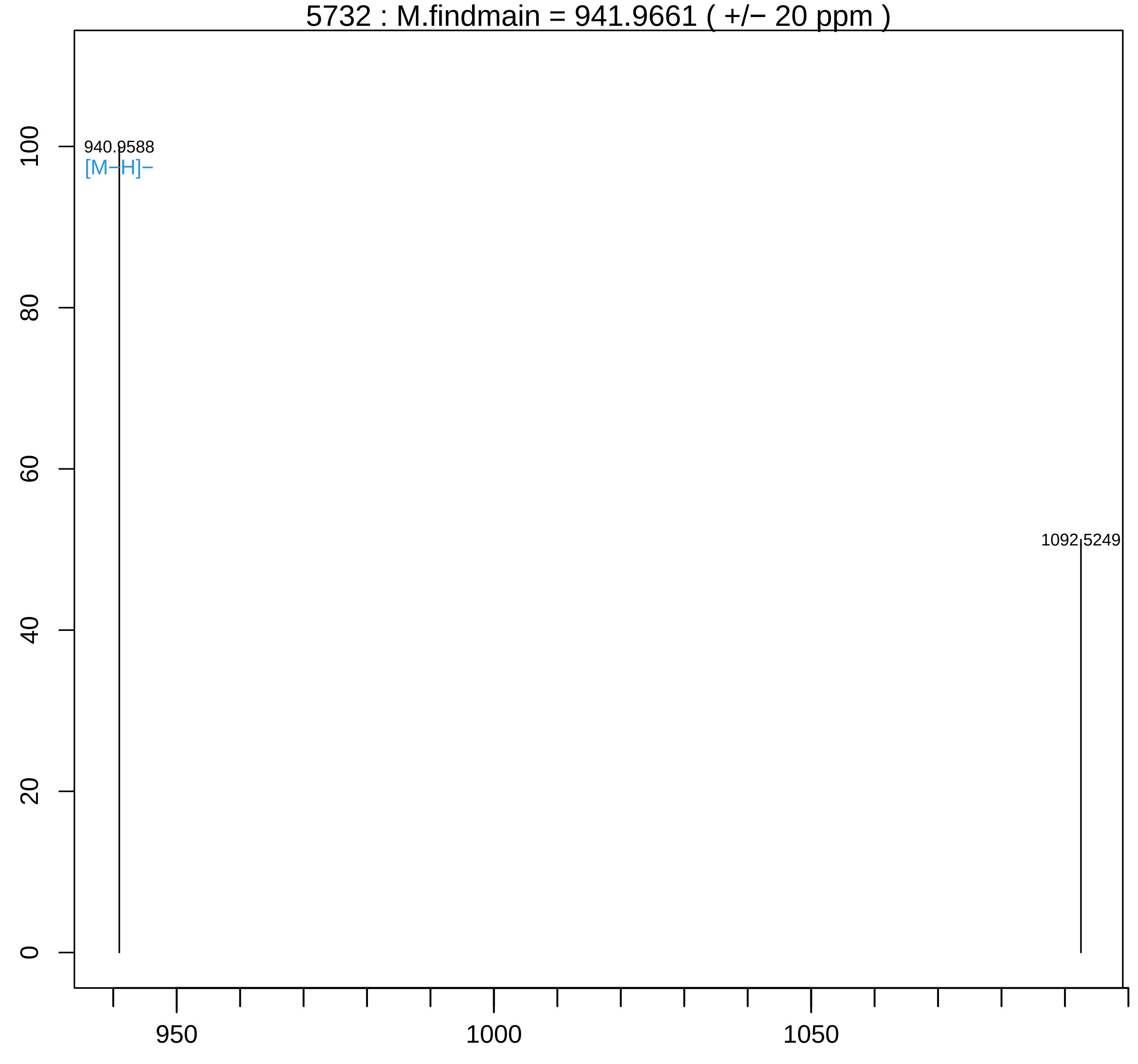

5732 : M.findmain = 941.9661 (+/− 20 ppm)
940.9588
[M−H]−
1092.5249
0
20
40
60
80
100
950
1000
1050

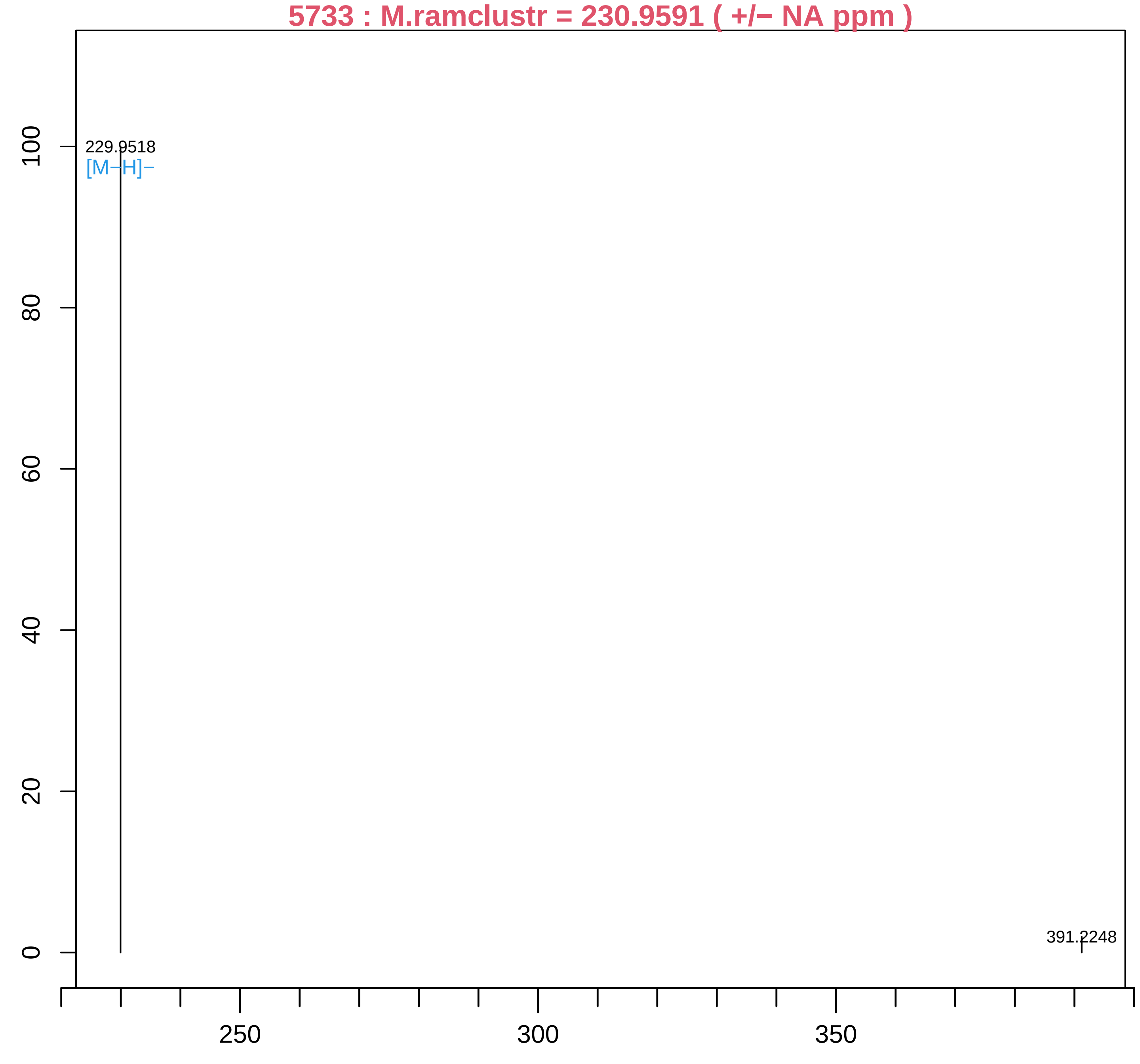
5733 : M.ramclustr = 230.9591 (+/− NA ppm)
229.9518
[M−H]−
391.2248
0
20
40
60
80
100
250
300
350

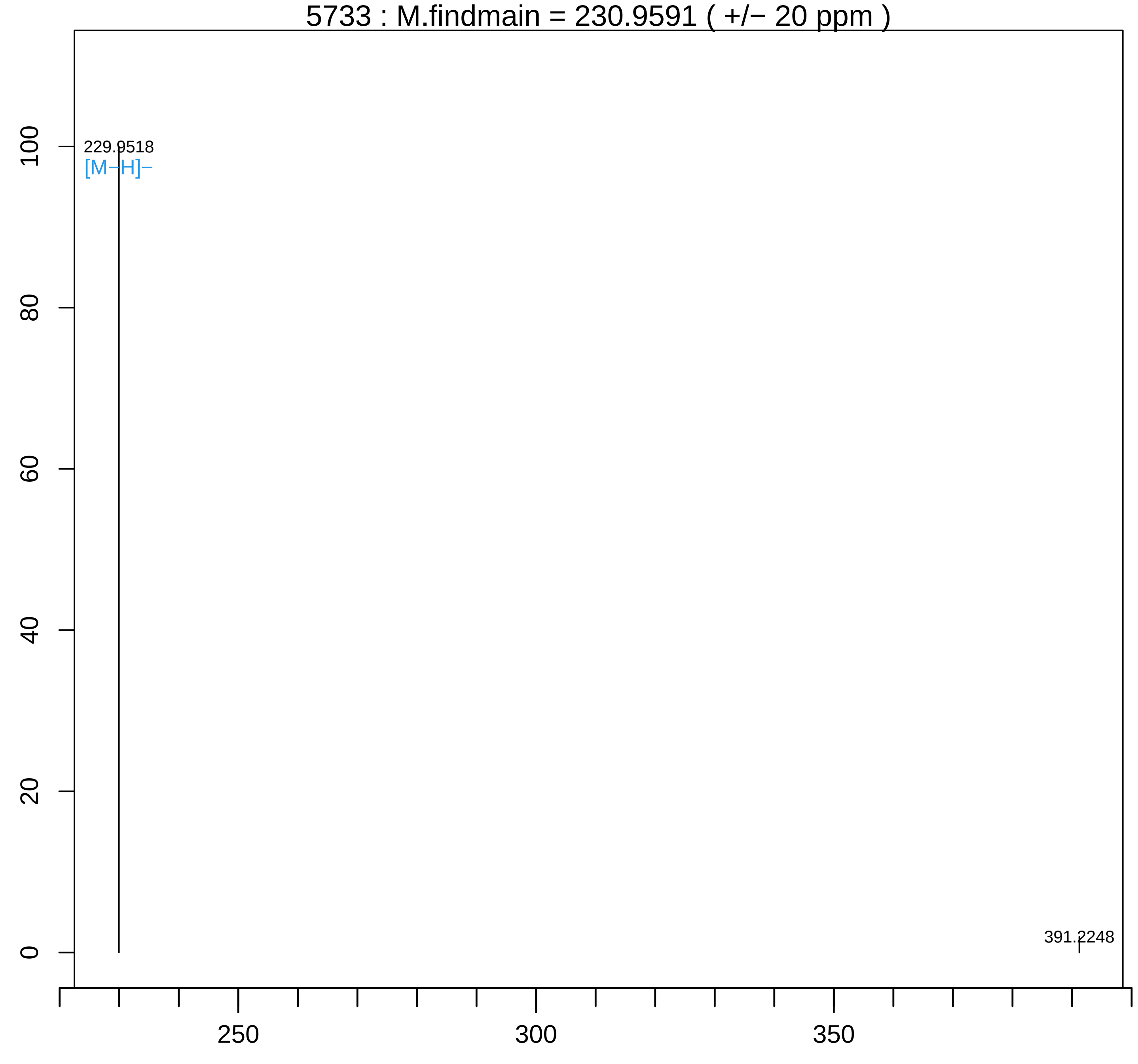
5733 : M.findmain = 230.9591 (+/− 20 ppm)
229.9518
[M−H]−
391.2248
0
20
40
60
80
100
250
300
350

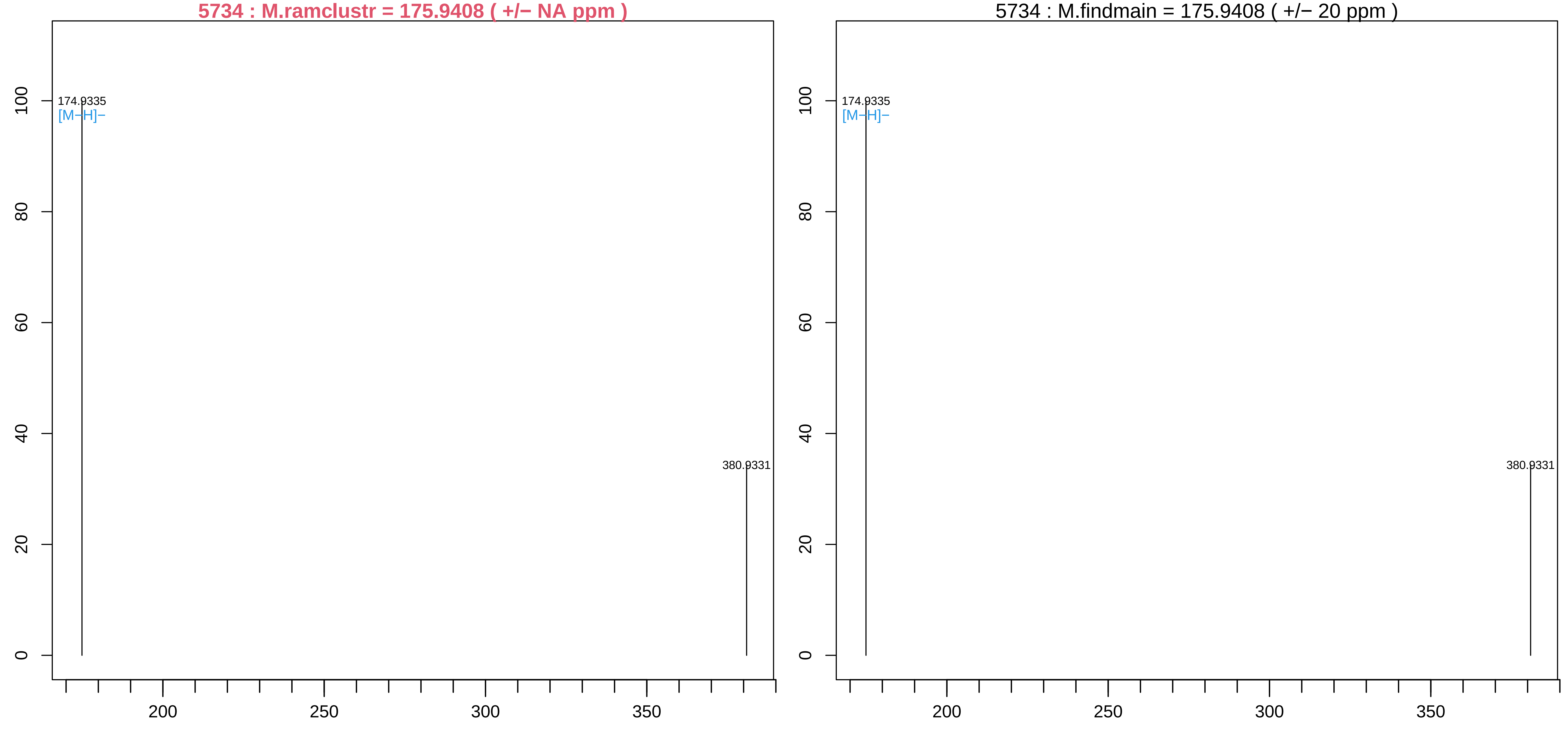

5734 : M.ramclustr = 175.9408 (+/− NA ppm)
174.9335
[M−H]−
380.9331
0
20
40
60
80
100
200
250
300
350
5734 : M.findmain = 175.9408 (+/− 20 ppm)
174.9335
[M−H]−
380.9331
0
20
40
60
80
100
200
250
300
350

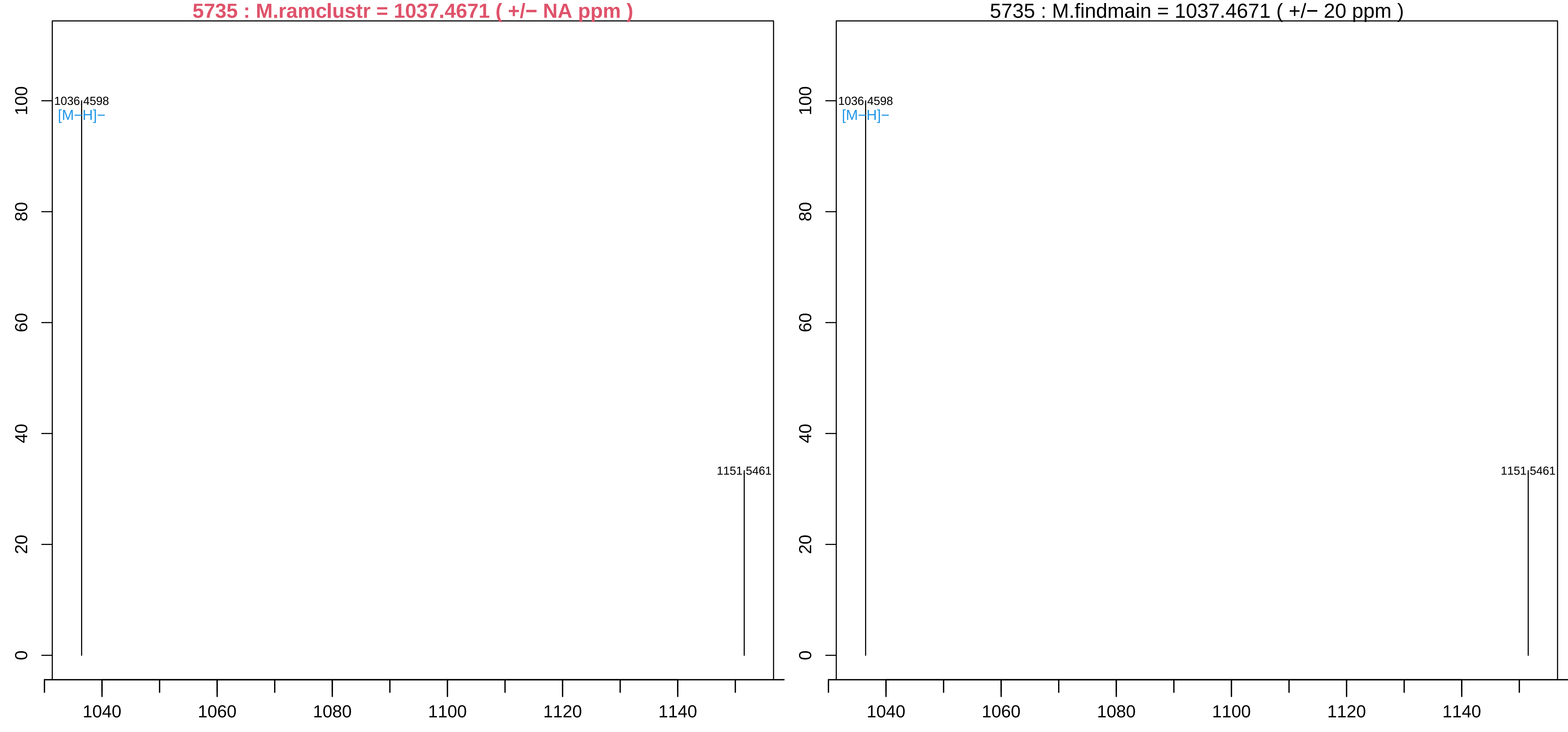
5735 : M.ramclustr = 1037.4671 (+/− NA ppm)
1036.4598
[M−H]−
1151.5461
5735 : M.findmain = 1037.4671 (+/− 20 ppm)
1036.4598
[M−H]−
1151.5461
0
20
40
60
80
100
1040
1060
1080
1100
1120
1140

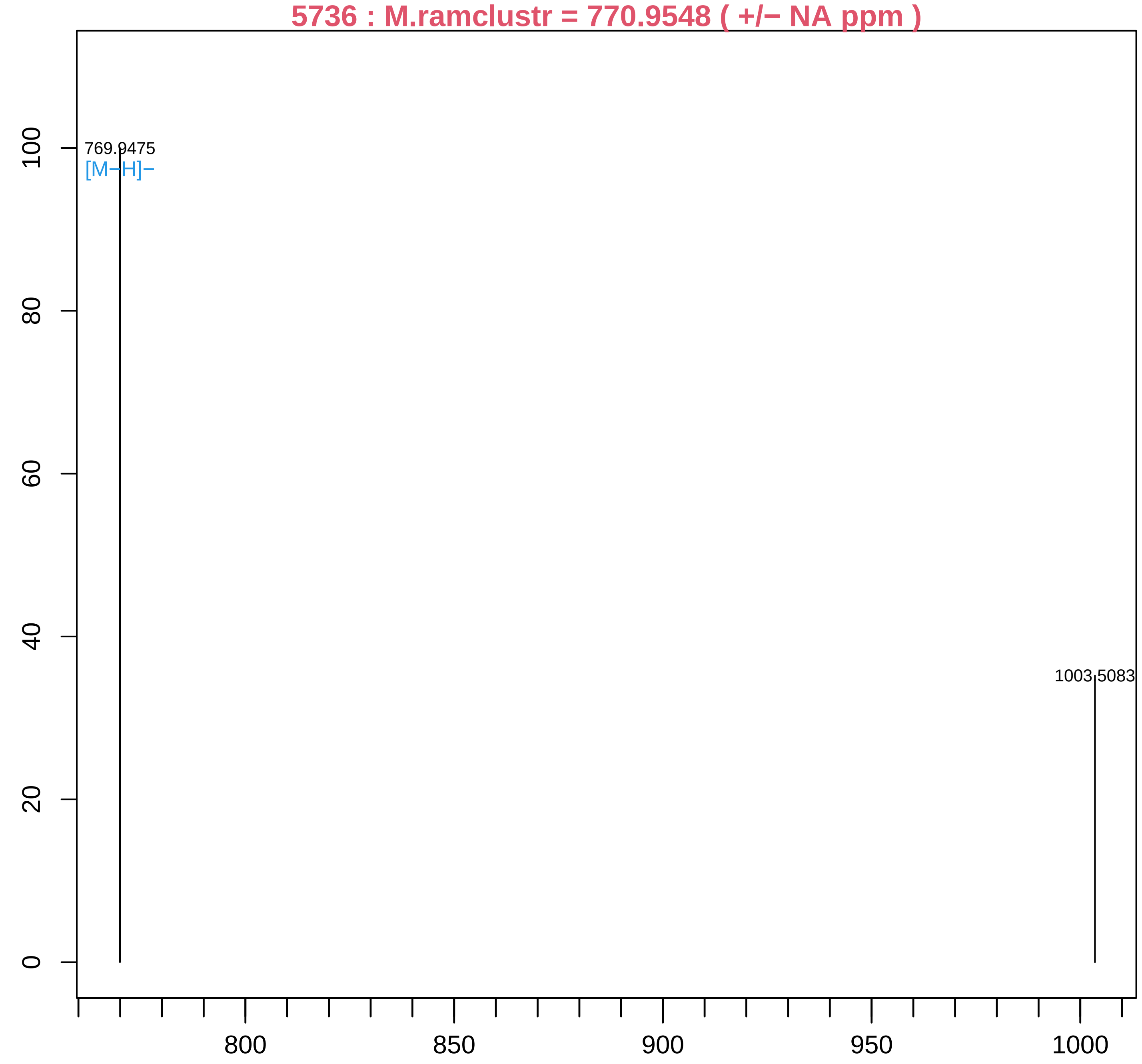
5736 : M.ramclustr = 770.9548 (+/− NA ppm)
769.9475
[M−H]−
1003.5083
0
20
40
60
80
100
800
850
900
950
1000

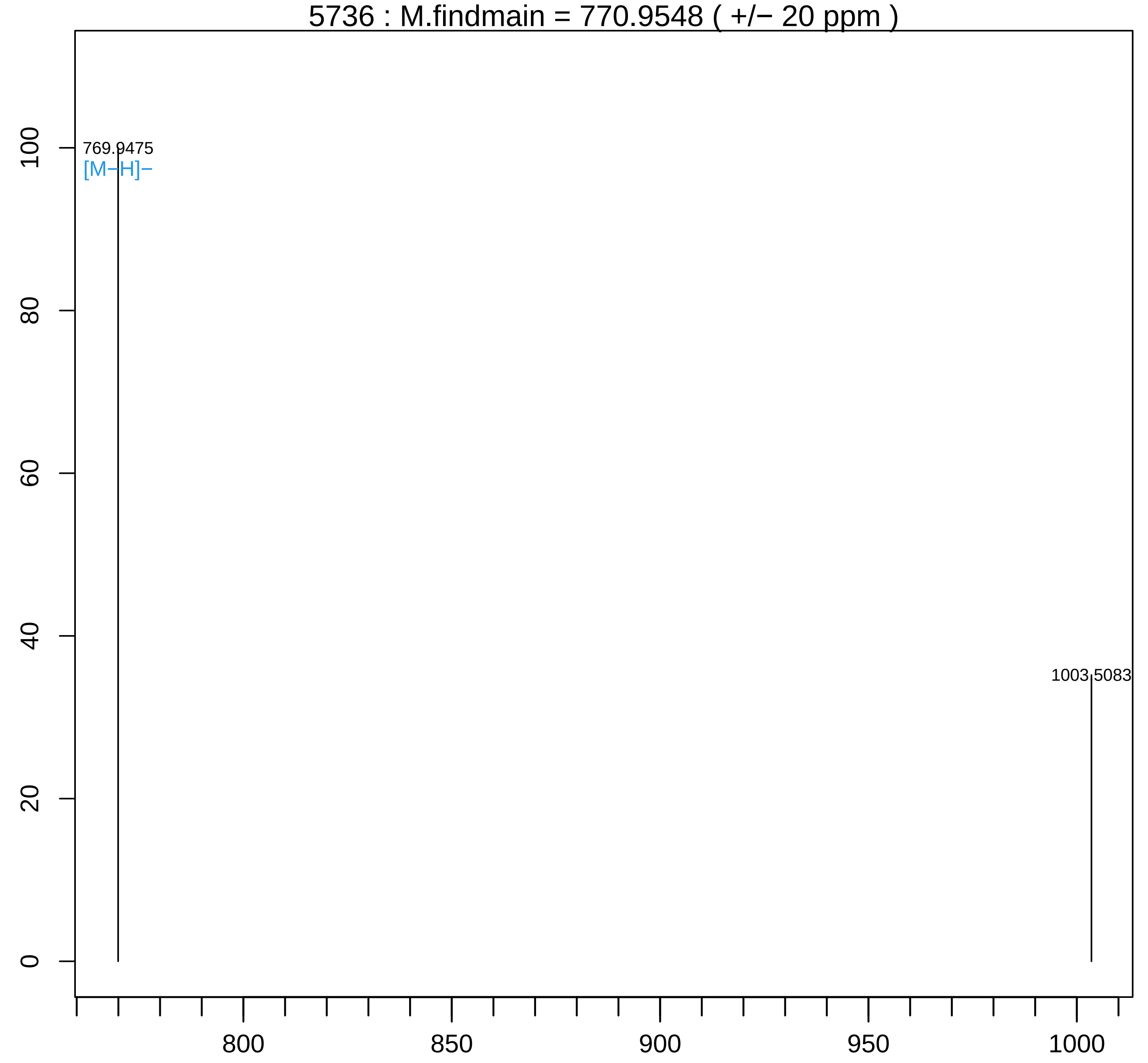
5736 : M.findmain = 770.9548 (+/− 20 ppm)
769.9475
[M−H]−
1003.5083
0
20
40
60
80
100
800
850
900
950
1000

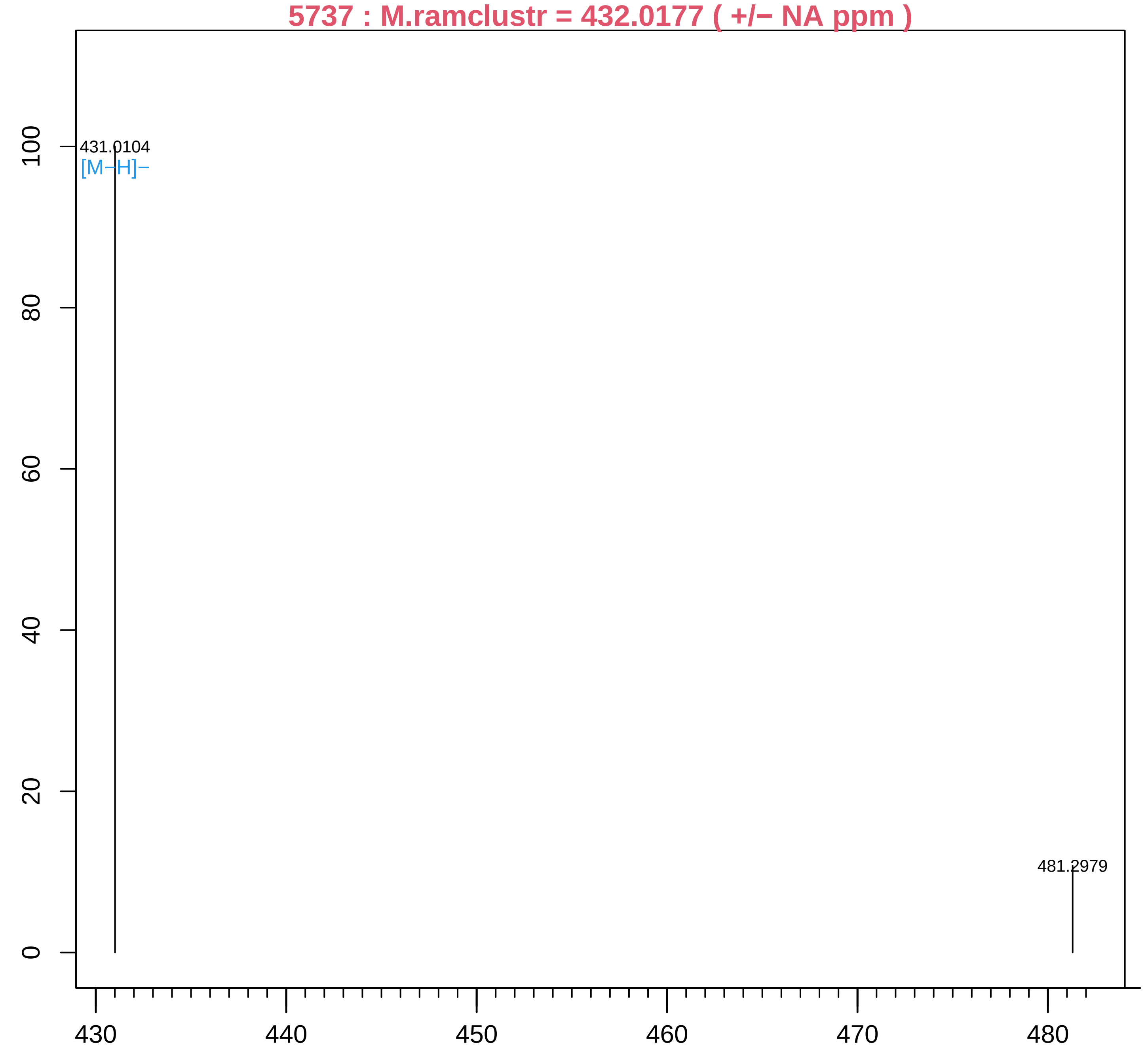
5737 : M.ramclustr = 432.0177 (+/− NA ppm)
431.0104
[M−H]−
481.2979
0
20
40
60
80
100
430
440
450
460
470
480

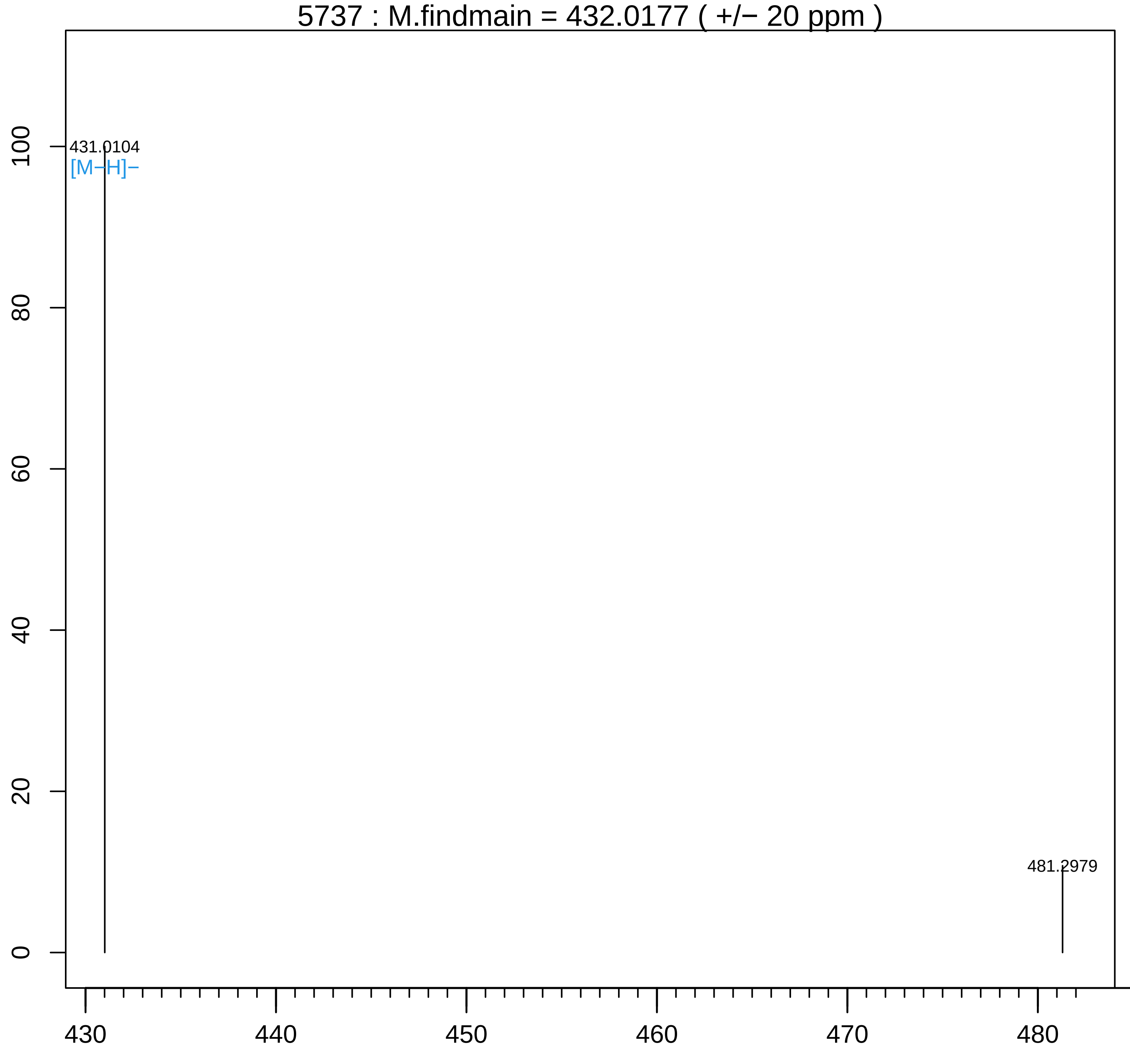
5737 : M.findmain = 432.0177 (+/− 20 ppm)
431.0104
[M−H]−
481.2979
0
20
40
60
80
100
430
440
450
460
470
480

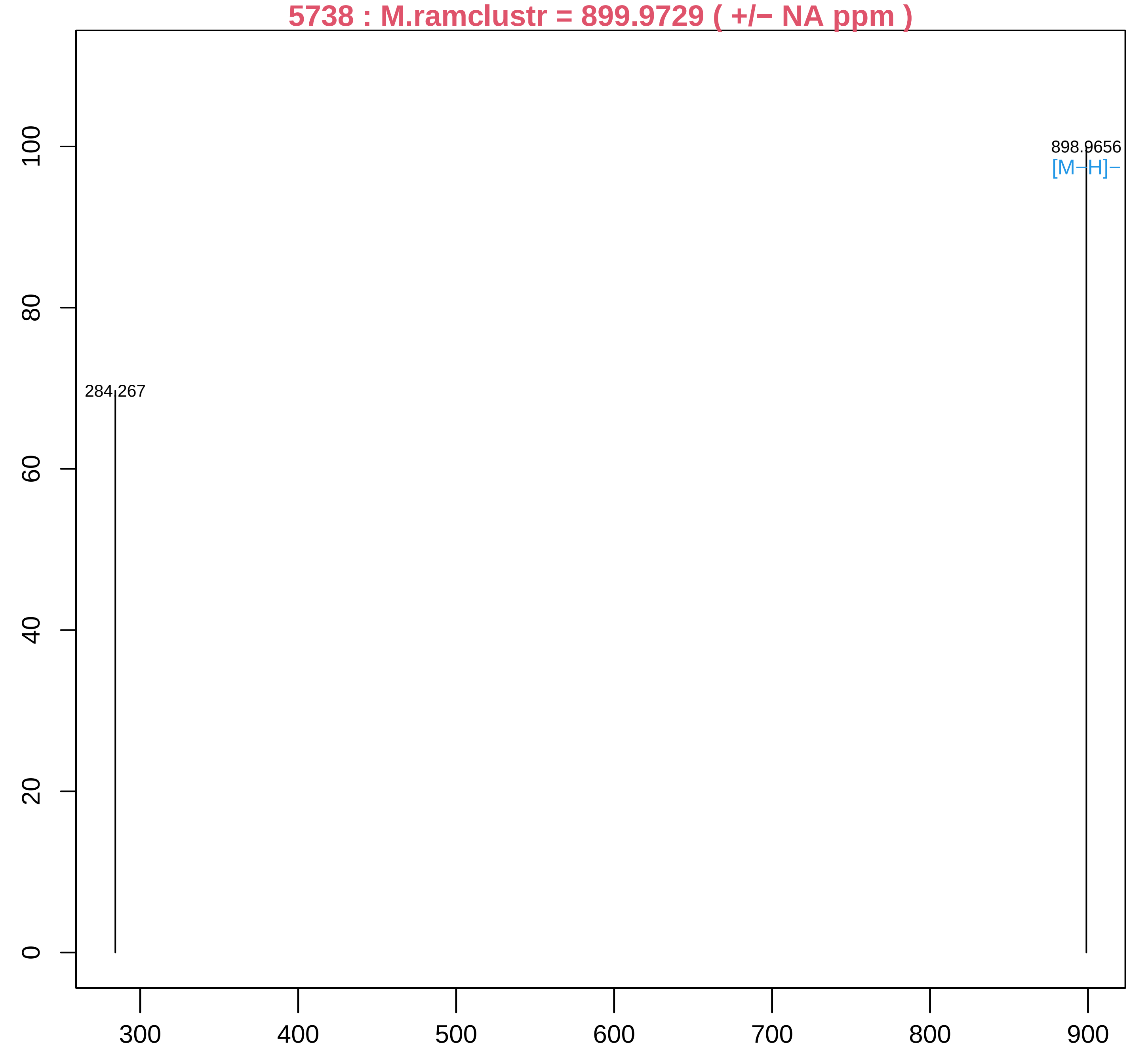
5738 : M.ramclustr = 899.9729 (+/− NA ppm)
898.9656
[M−H]−
284.267
0
20
40
60
80
100
300
400
500
600
700
800
900

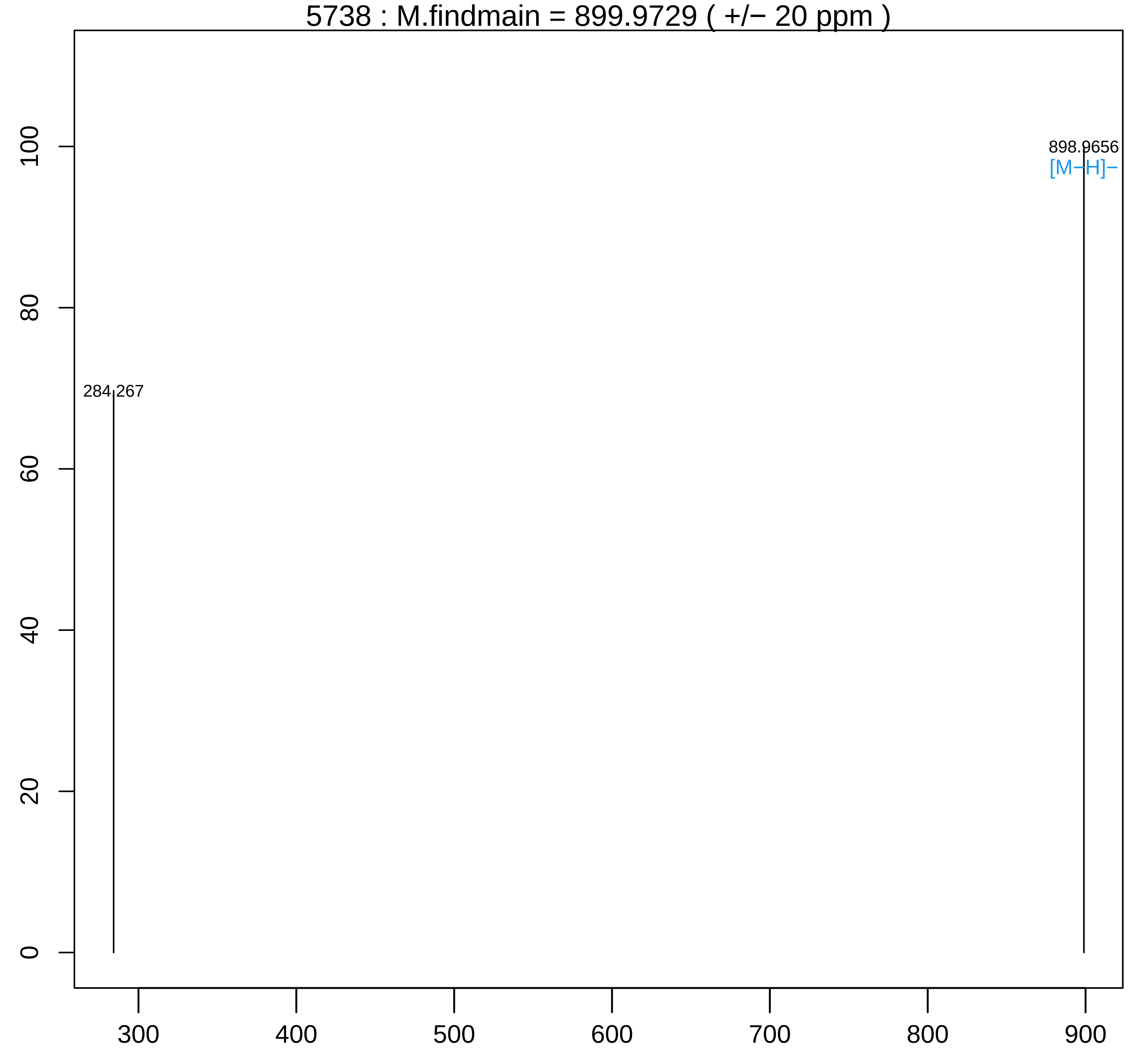
5738 : M.findmain = 899.9729 (+/− 20 ppm)
898.9656
[M−H]−
284.267
0
20
40
60
80
100
300
400
500
600
700
800
900

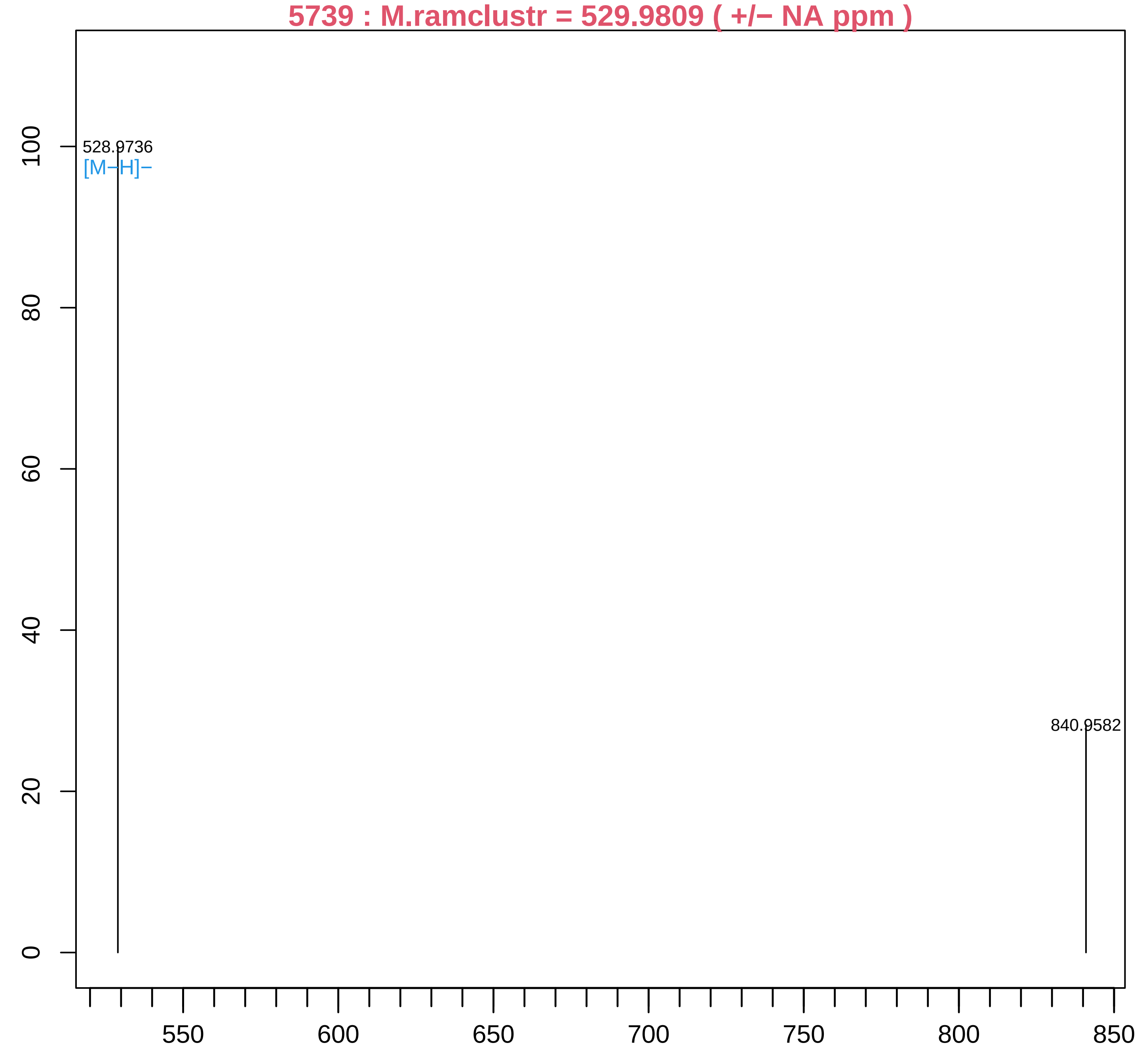
5739 : M.ramclustr = 529.9809 (+/− NA ppm)
528.9736
[M−H]−
840.9582
0
20
40
60
80
100
550
600
650
700
750
800
850

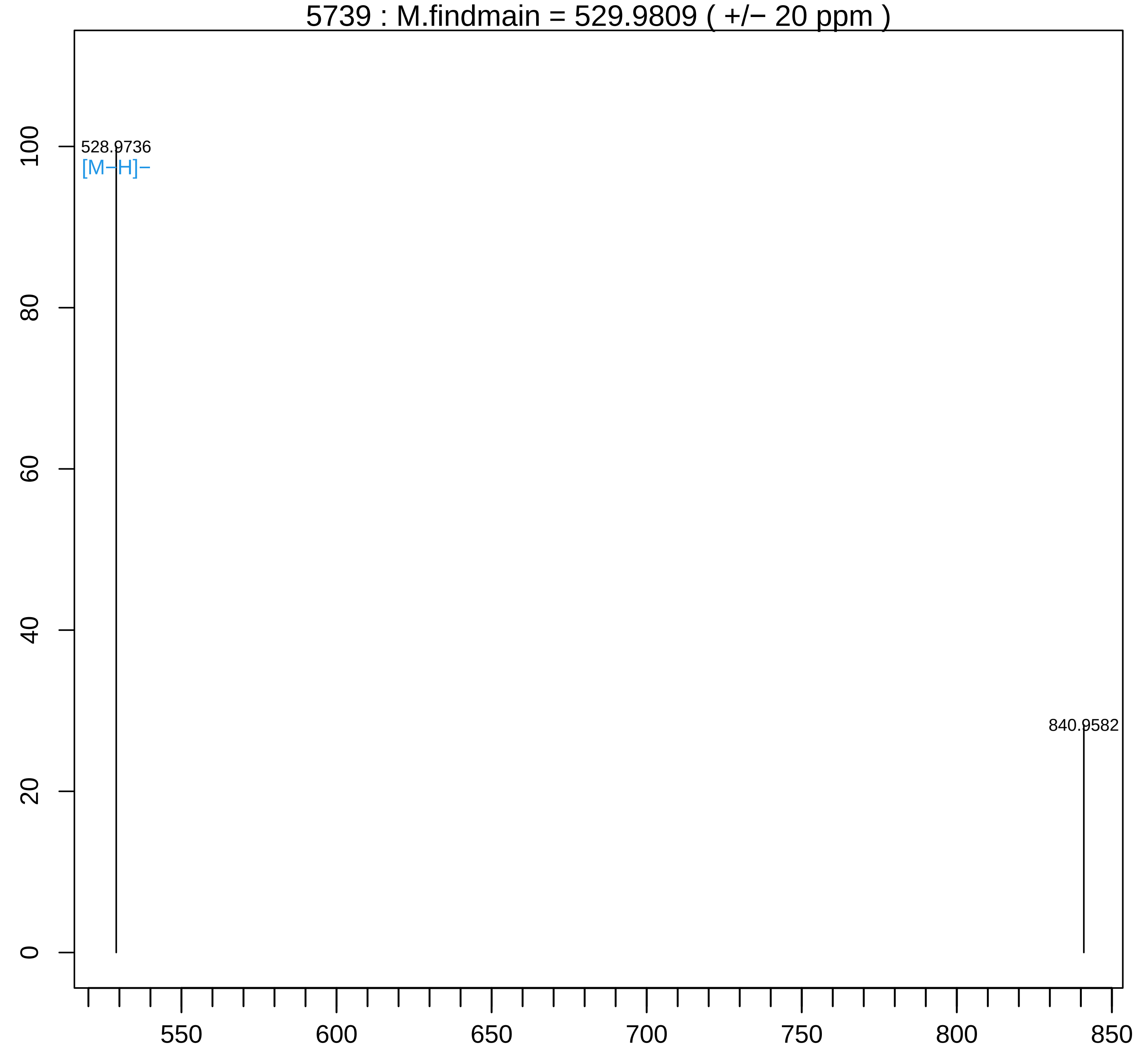
5739 : M.findmain = 529.9809 (+/− 20 ppm)
528.9736
[M−H]−
840.9582
0
20
40
60
80
100
550
600
650
700
750
800
850

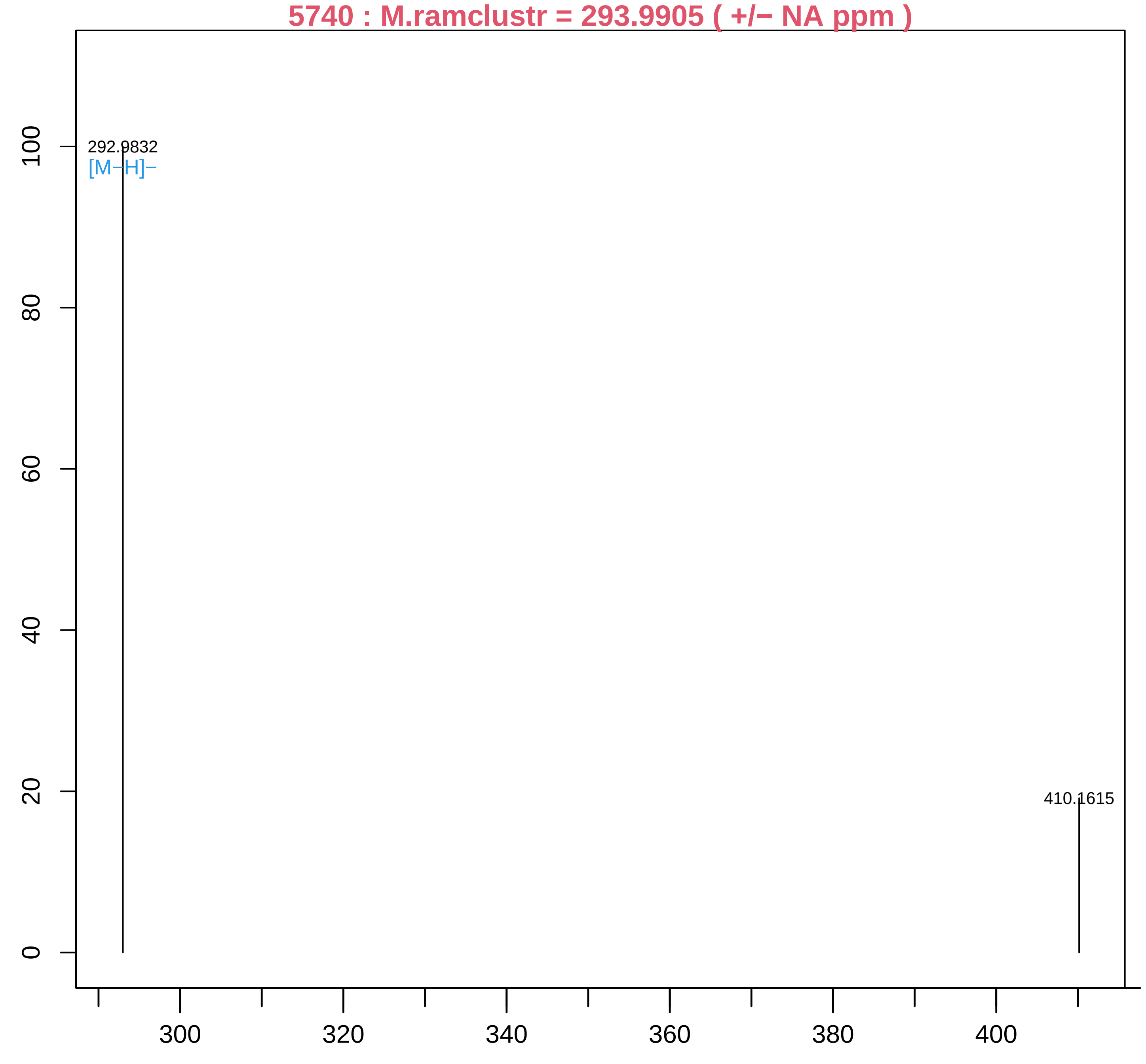
5740 : M.ramclustr = 293.9905 (+/− NA ppm)
292.9832
[M−H]−
410.1615
0
20
40
60
80
100
300
320
340
360
380
400

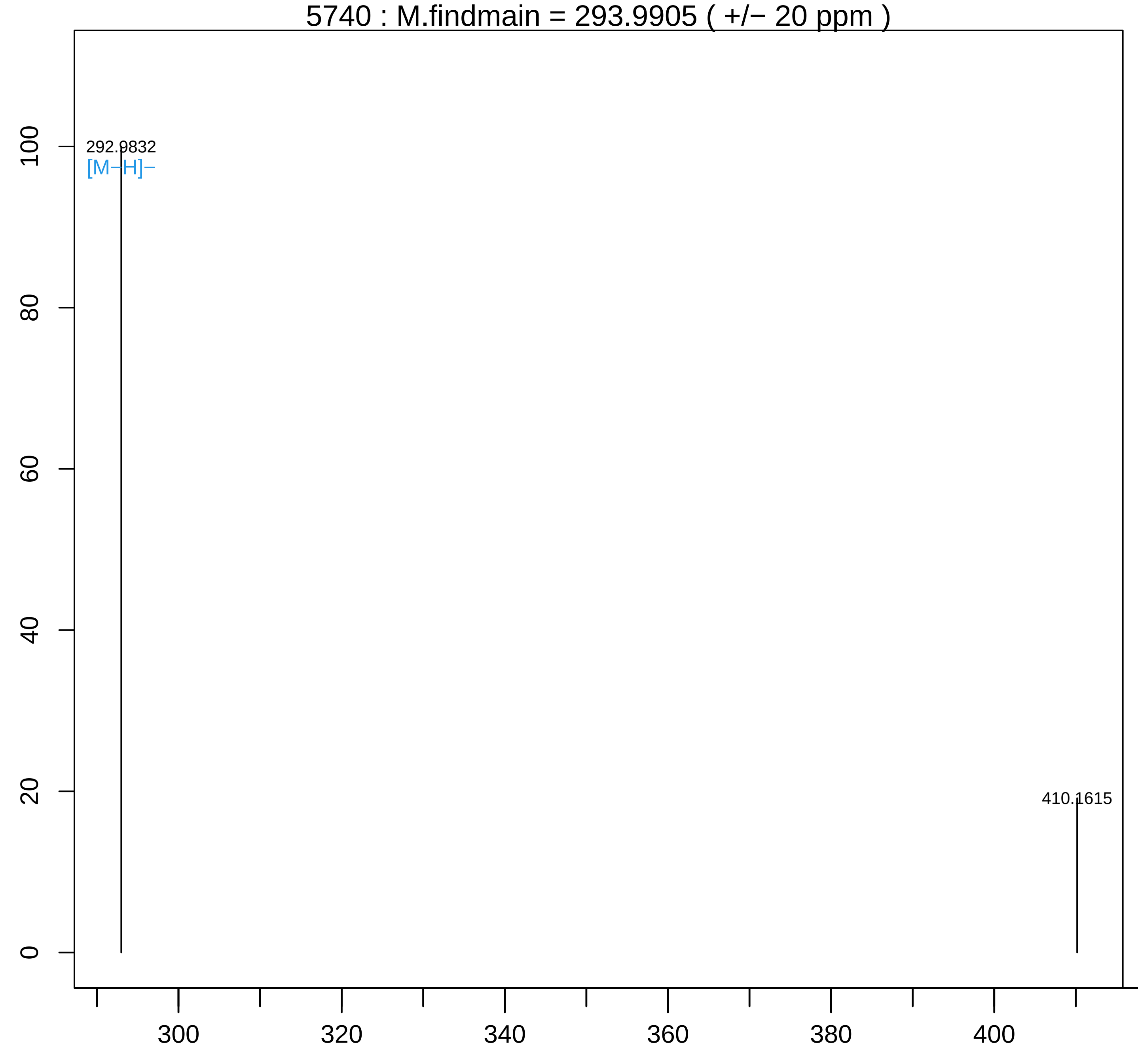
5740 : M.findmain = 293.9905 (+/− 20 ppm)
292.9832
[M−H]−
410.1615
0
20
40
60
80
100
300
320
340
360
380
400

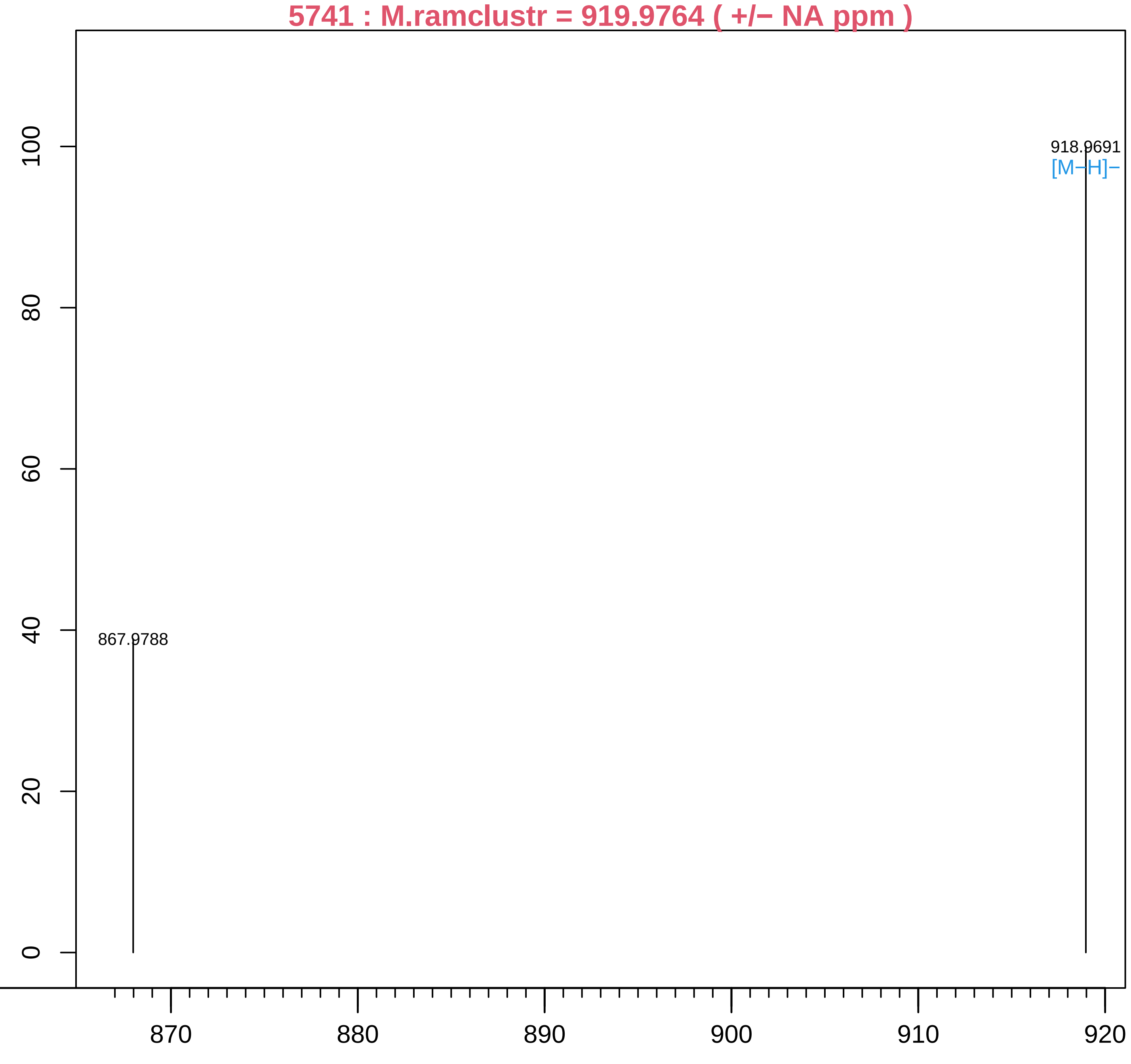
5741 : M.ramclustr = 919.9764 (+/− NA ppm)
918.9691
[M−H]−
867.9788
0
20
40
60
80
100
870
880
890
900
910
920

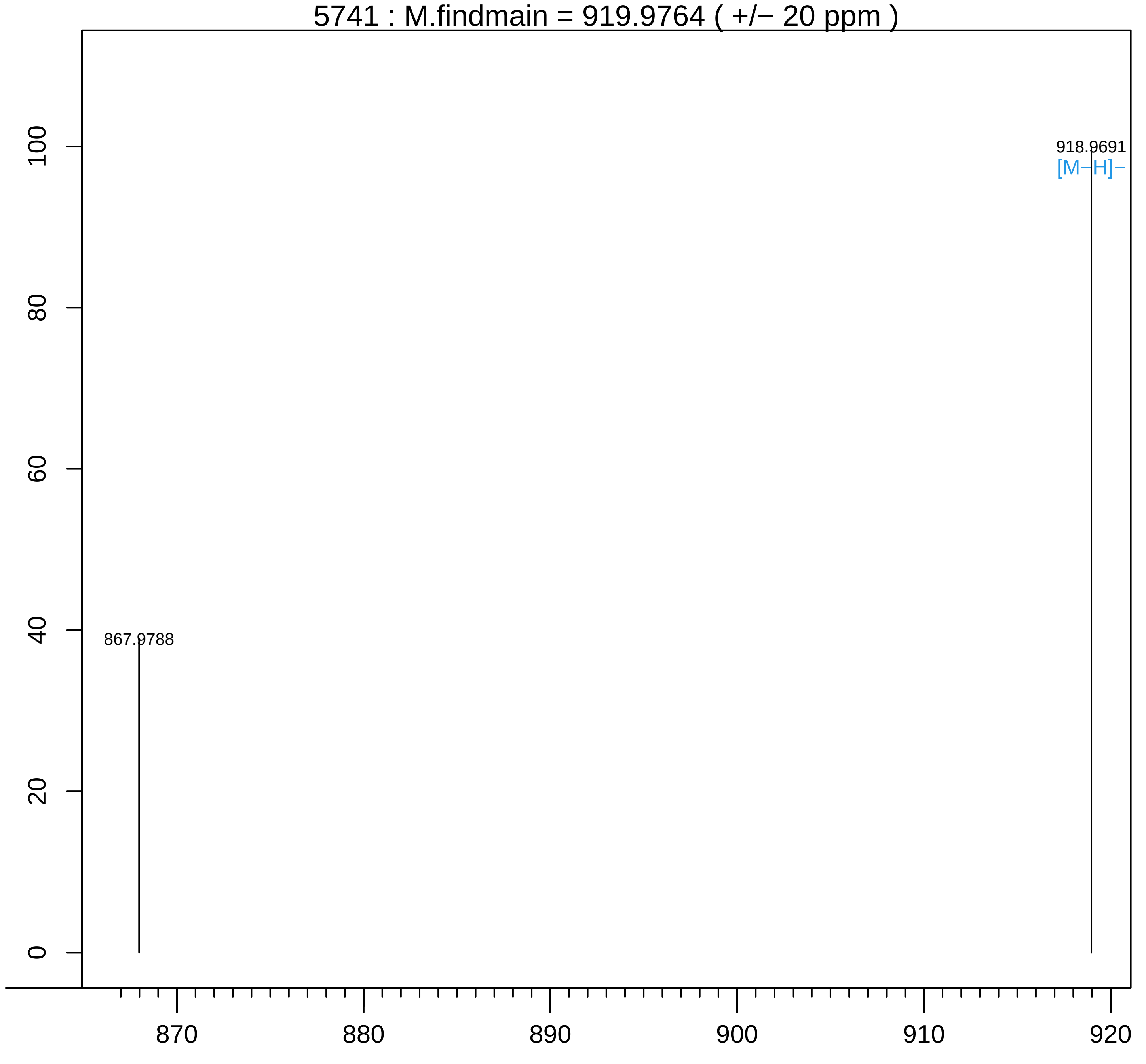
5741 : M.findmain = 919.9764 (+/− 20 ppm)
918.9691
[M−H]−
867.9788
0
20
40
60
80
100
870
880
890
900
910
920

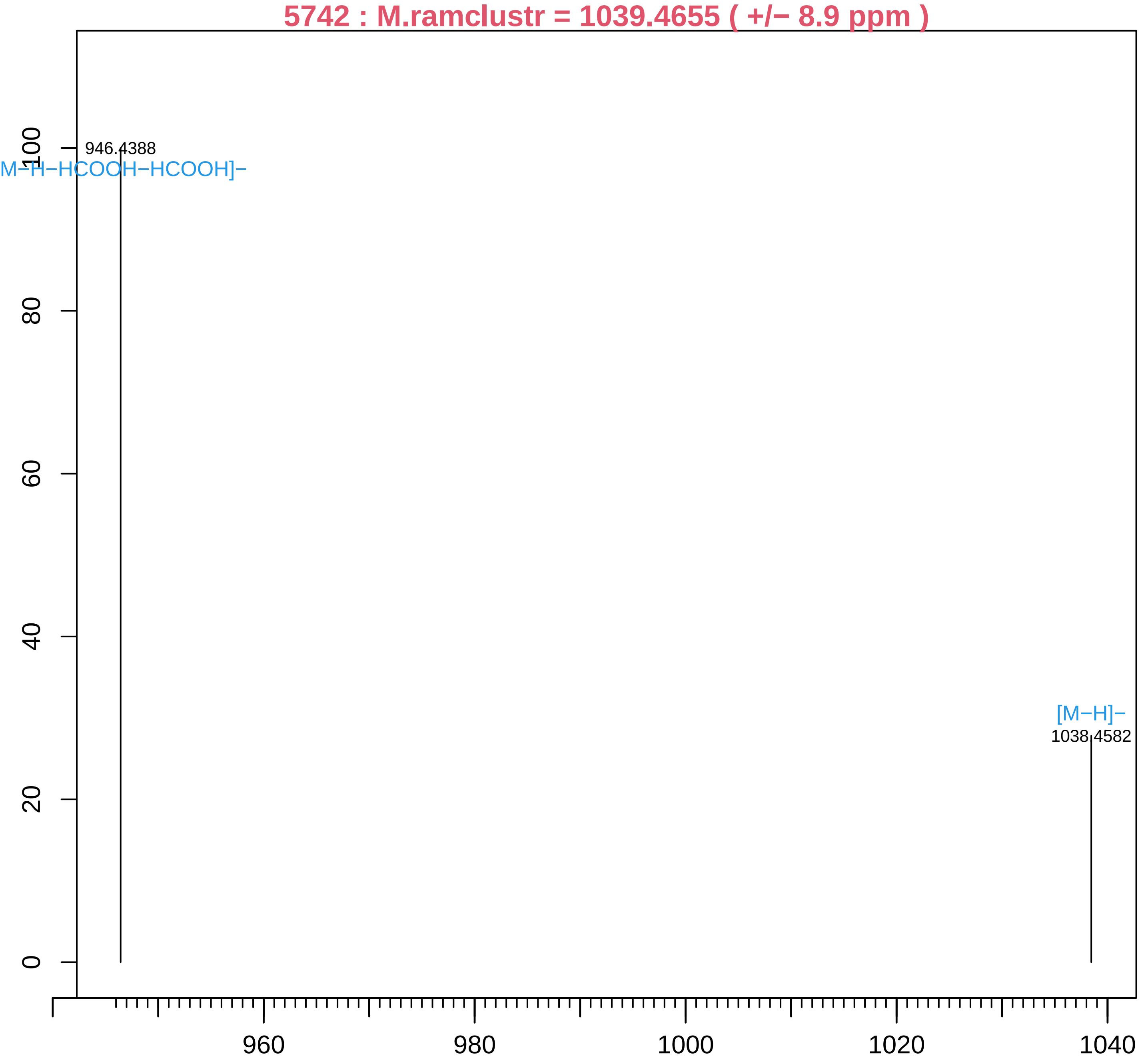

5742 : M.ramclustr = 1039.4655 (+/− 8.9 ppm)
946.4388
[M−H−HCOOH−HCOOH]−
[M−H]−
1038.4582
0
20
40
60
80
100
960
980
1000
1020
1040

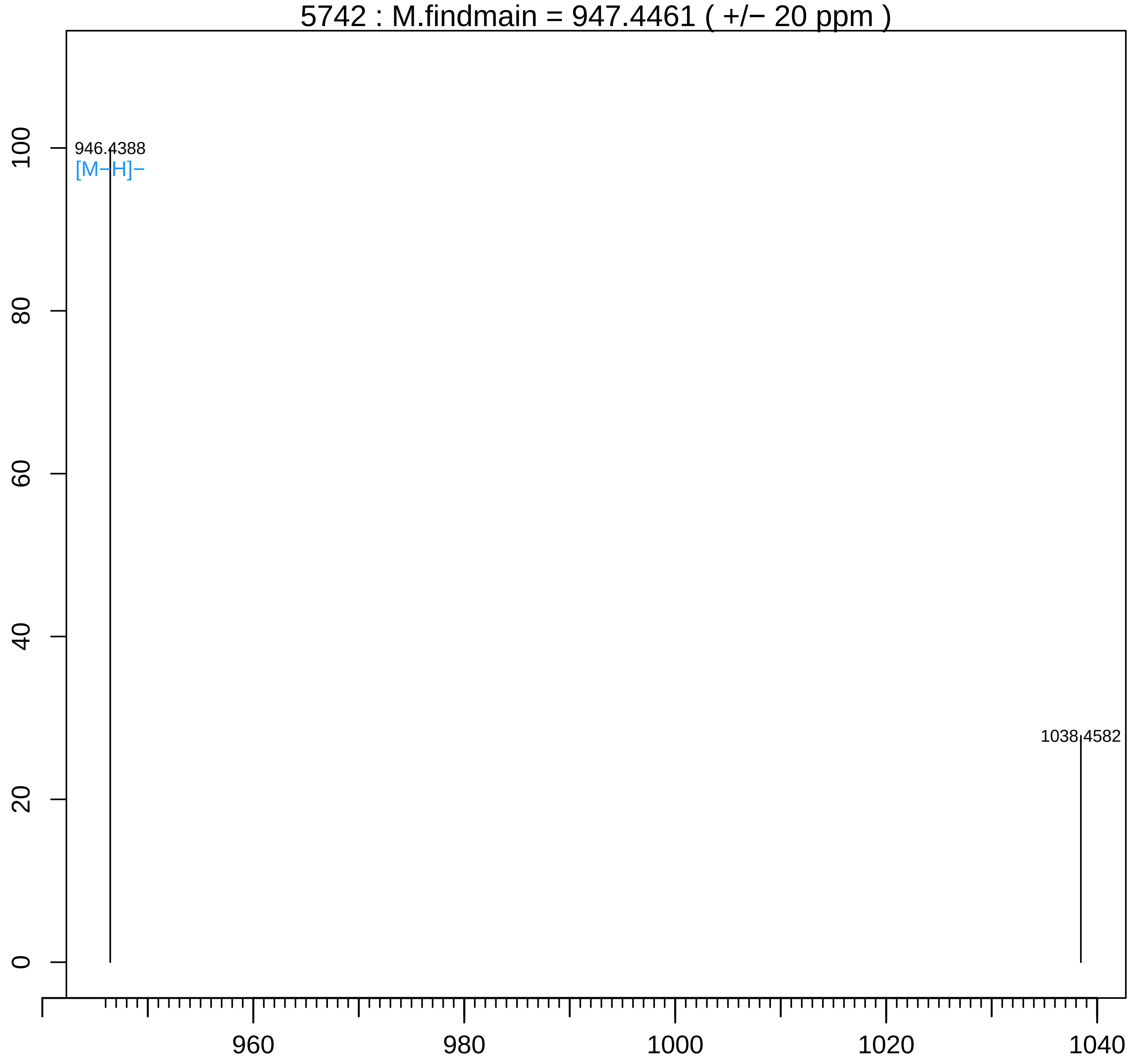

5742 : M.findmain = 947.4461 (+/− 20 ppm)
946.4388
[M−H]−
1038.4582
0
20
40
60
80
100
960
980
1000
1020
1040

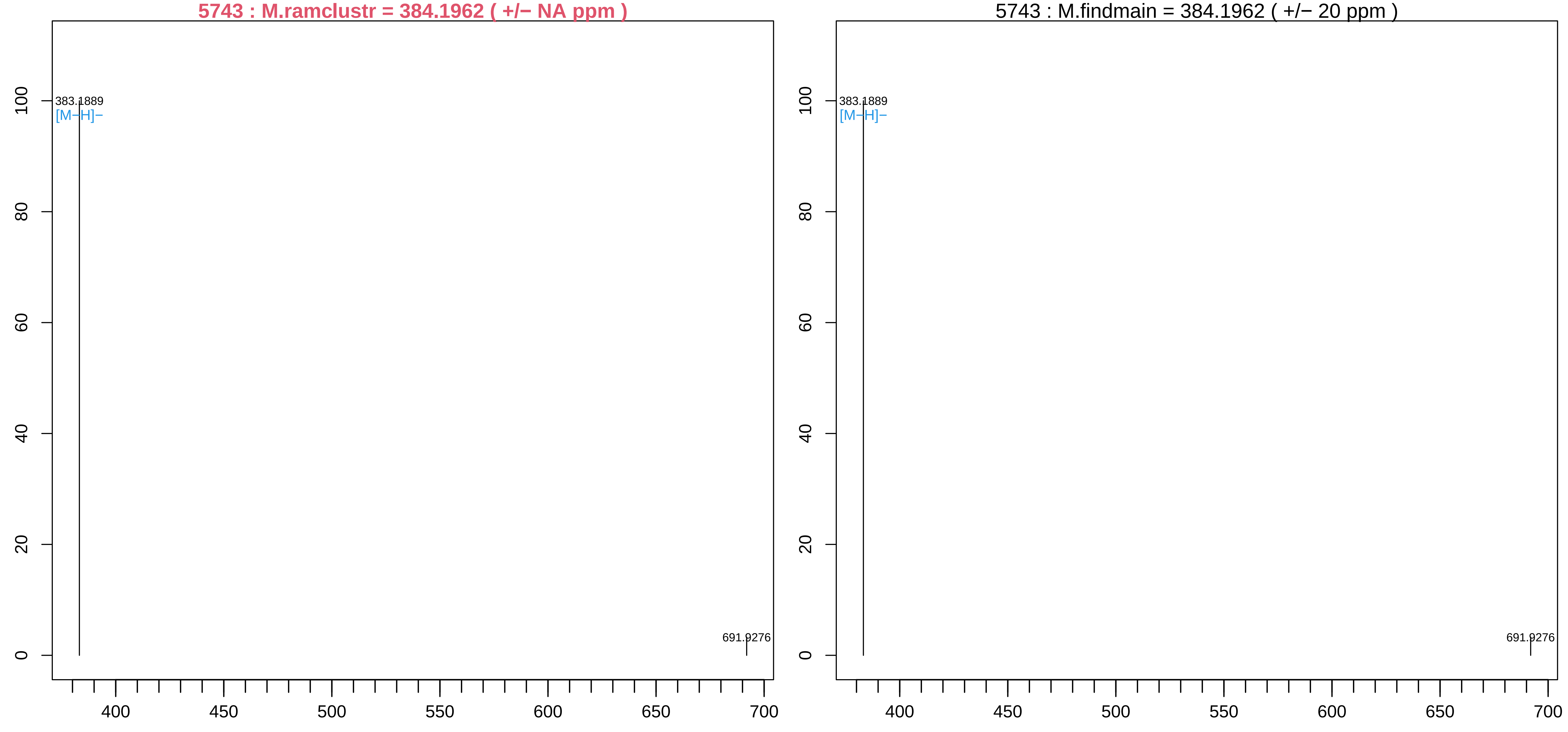
5743 : M.ramclustr = 384.1962 (+/− NA ppm)
383.1889
[M−H]−
691.9276
0
20
40
60
80
100
400
450
500
550
600
650
700
5743 : M.findmain = 384.1962 (+/− 20 ppm)
383.1889
[M−H]−
691.9276
0
20
40
60
80
100
400
450
500
550
600
650
700

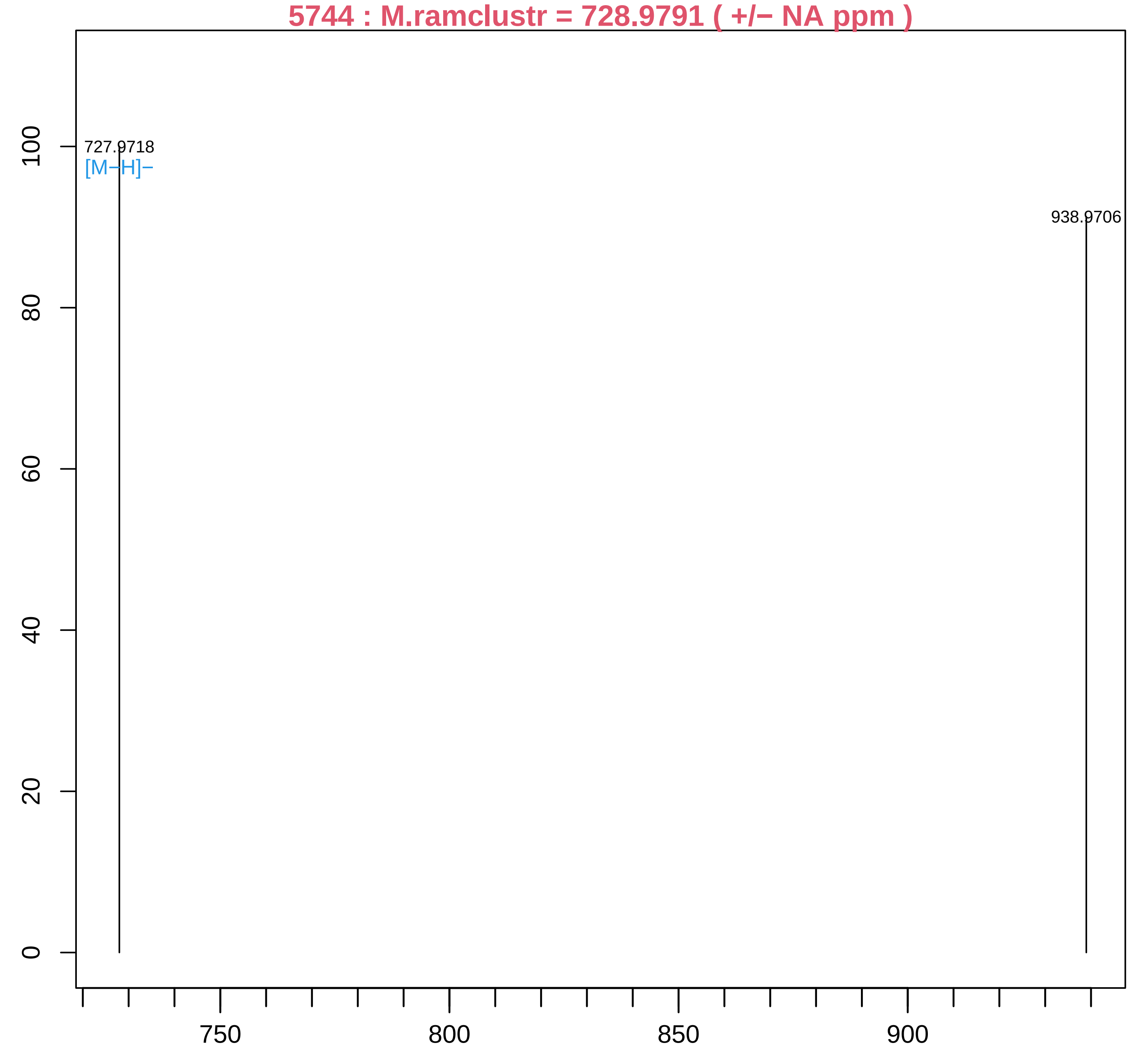
5744 : M.ramclustr = 728.9791 (+/− NA ppm)
727.9718
[M−H]−
938.9706
0
20
40
60
80
100
750
800
850
900

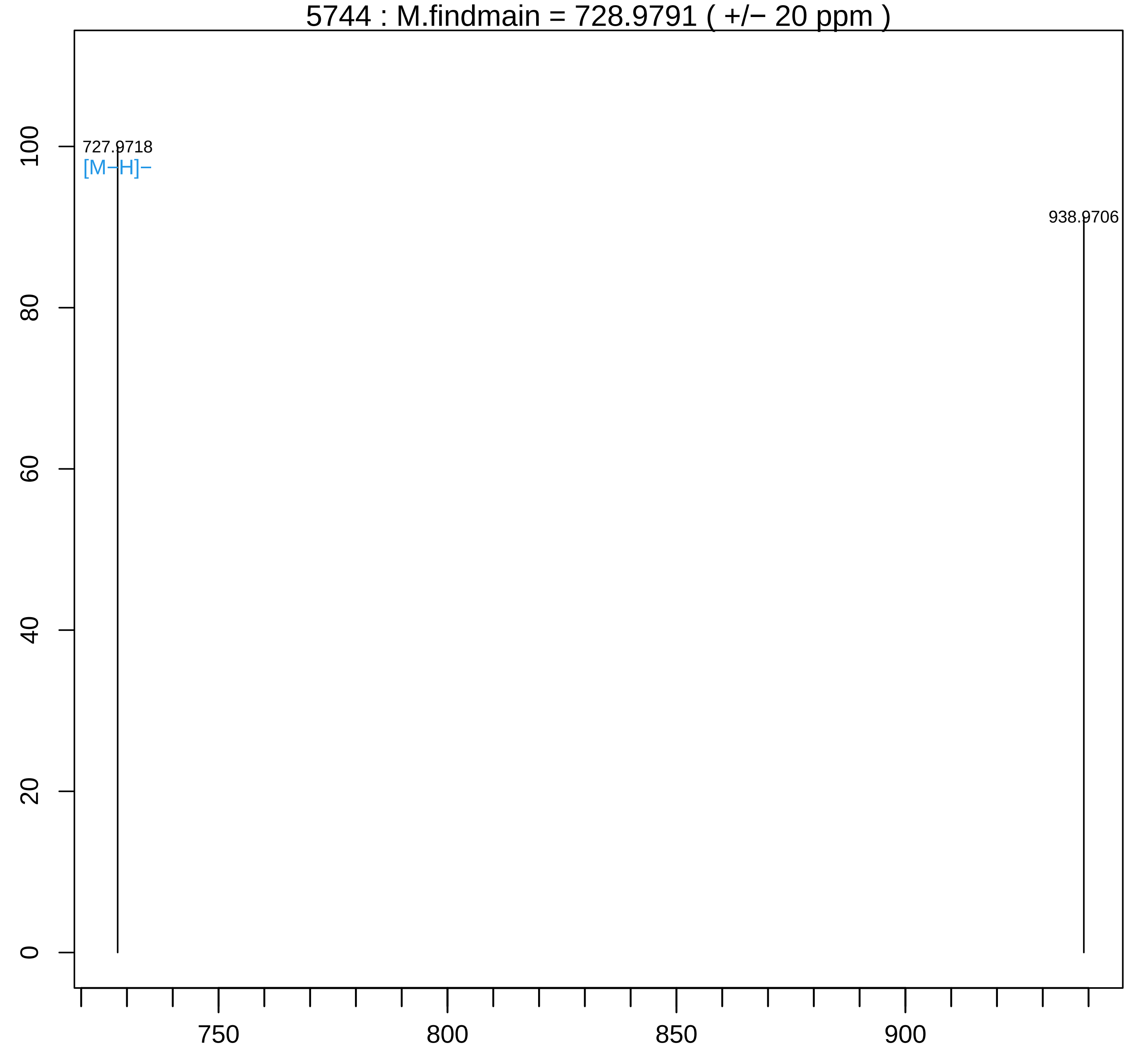
5744 : M.findmain = 728.9791 (+/− 20 ppm)
727.9718
[M−H]−
938.9706
0
20
40
60
80
100
750
800
850
900

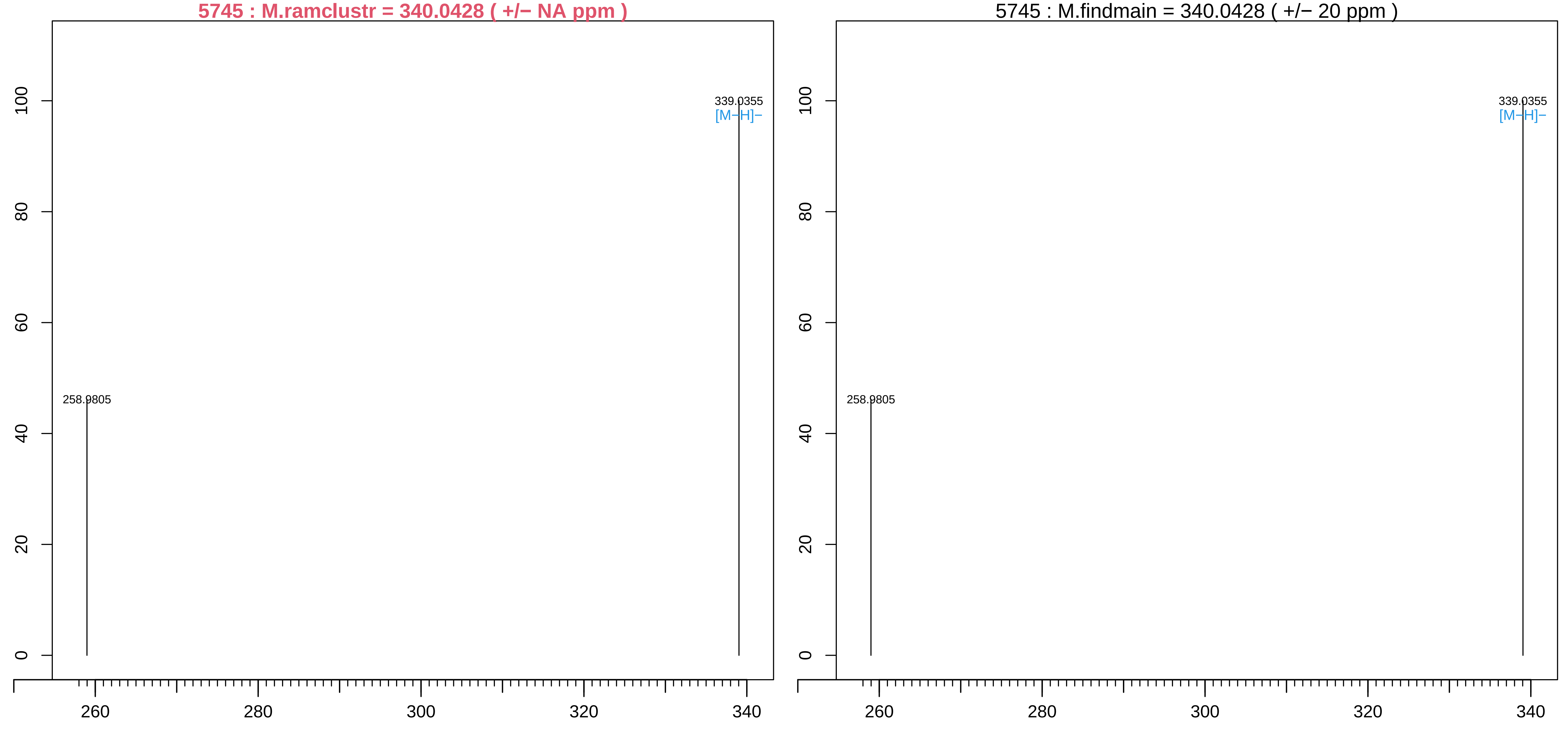

5745 : M.ramclustr = 340.0428 (+/− NA ppm)
258.9805
339.0355
[M−H]−
5745 : M.findmain = 340.0428 (+/− 20 ppm)
258.9805
339.0355
[M−H]−

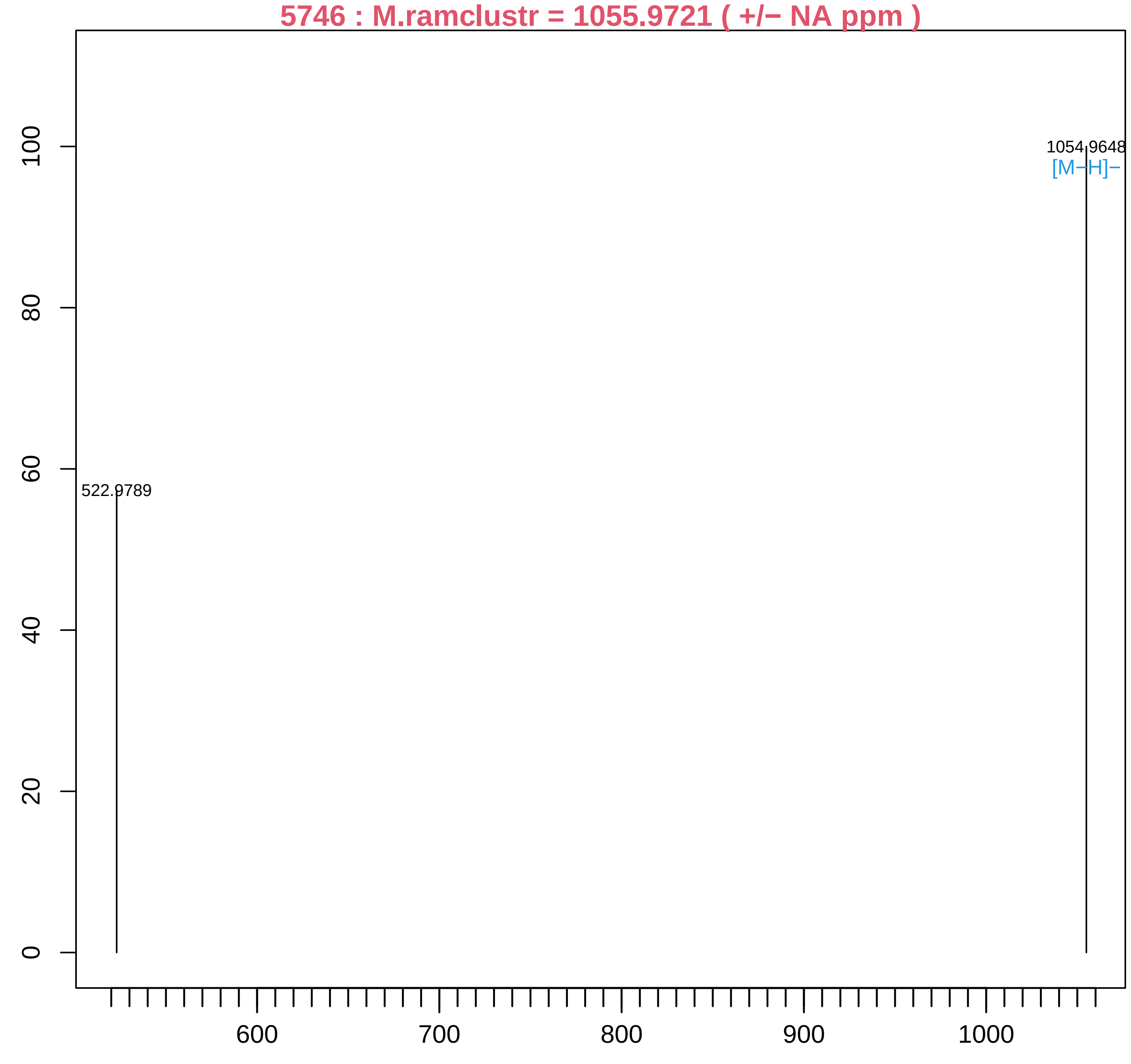
5746 : M.ramclustr = 1055.9721 (+/− NA ppm)
1054.9648
[M−H]−
522.9789
0
20
40
60
80
100
600
700
800
900
1000

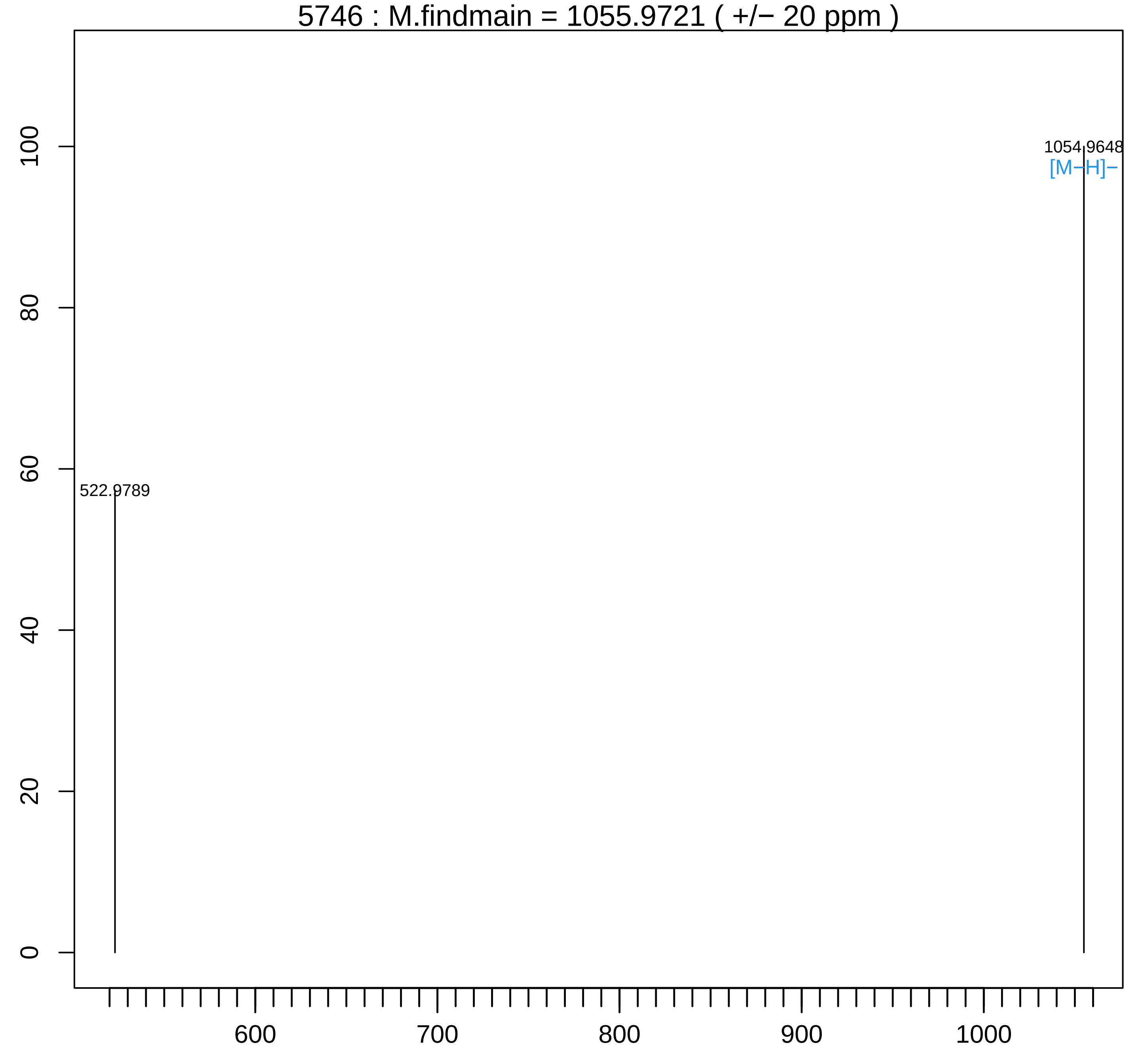
5746 : M.findmain = 1055.9721 (+/− 20 ppm)
1054.9648
[M−H]−
522.9789
0
20
40
60
80
100
600
700
800
900
1000

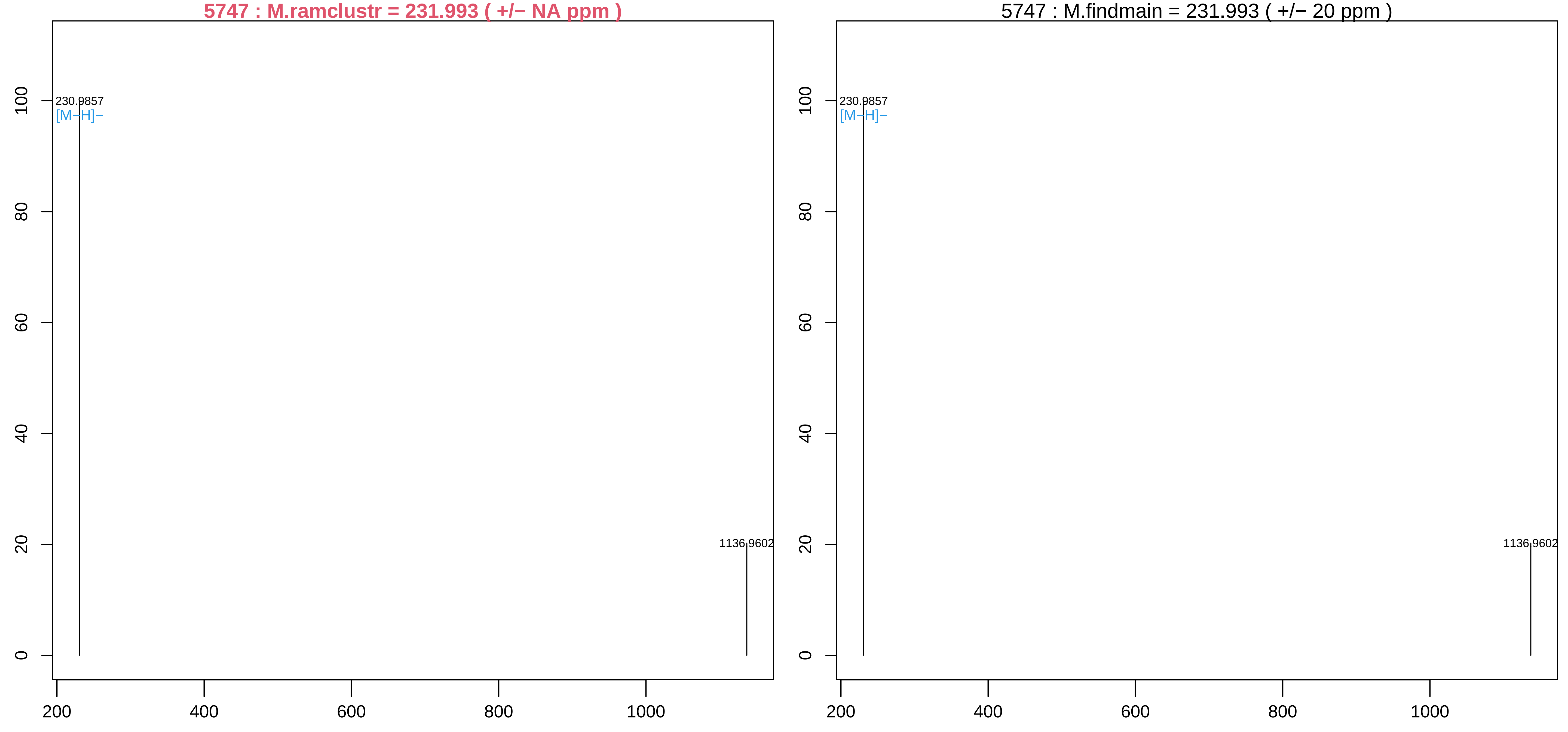
5747 : M.ramclustr = 231.993 (+/− NA ppm)
230.9857
[M−H]−
1136.9602
5747 : M.findmain = 231.993 (+/− 20 ppm)
230.9857
[M−H]−
1136.9602
0
20
40
60
80
100
200
400
600
800
1000

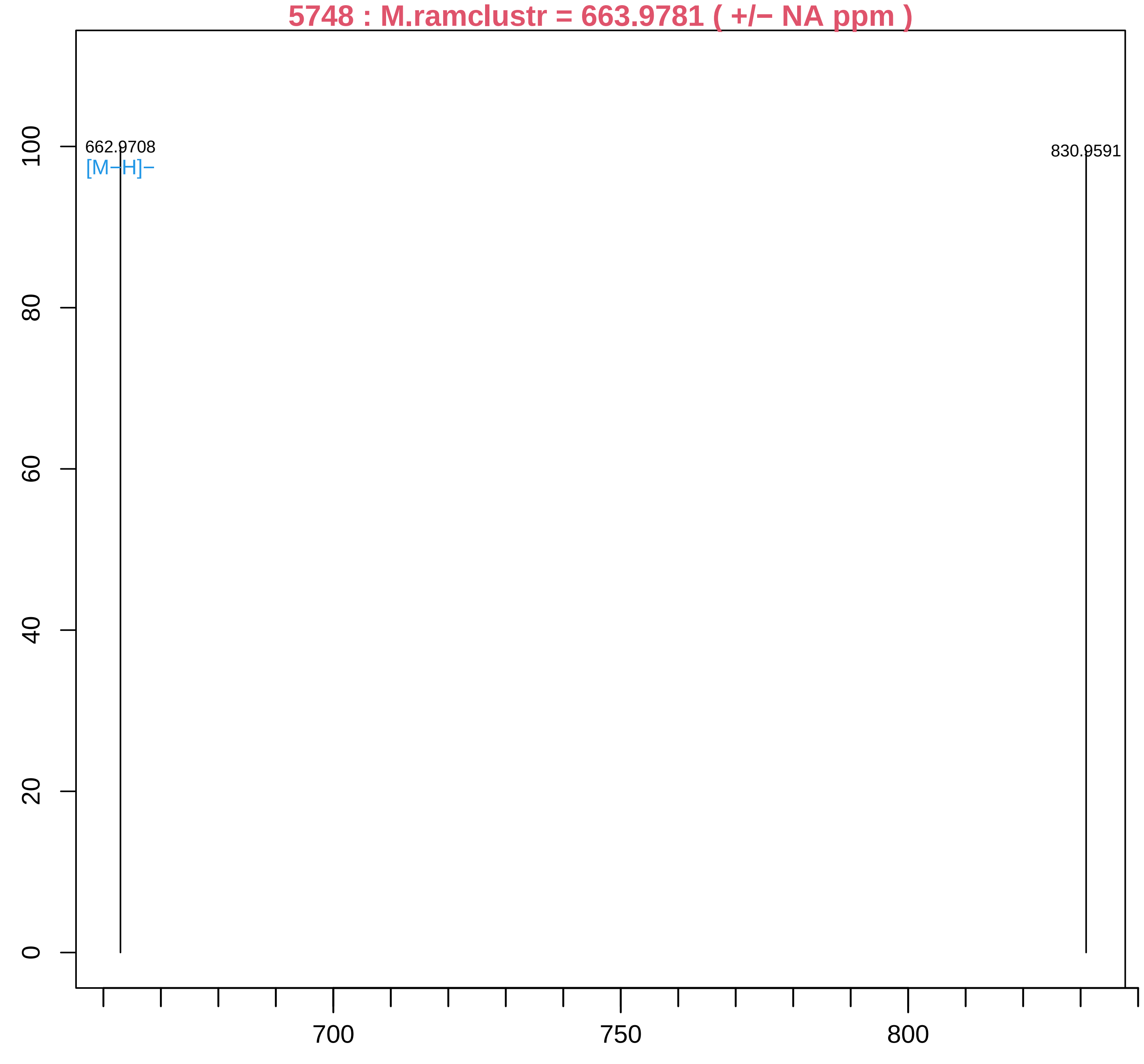

5748 : M.ramclustr = 663.9781 (+/− NA ppm)
662.9708
[M−H]−
830.9591
0
20
40
60
80
100
700
750
800

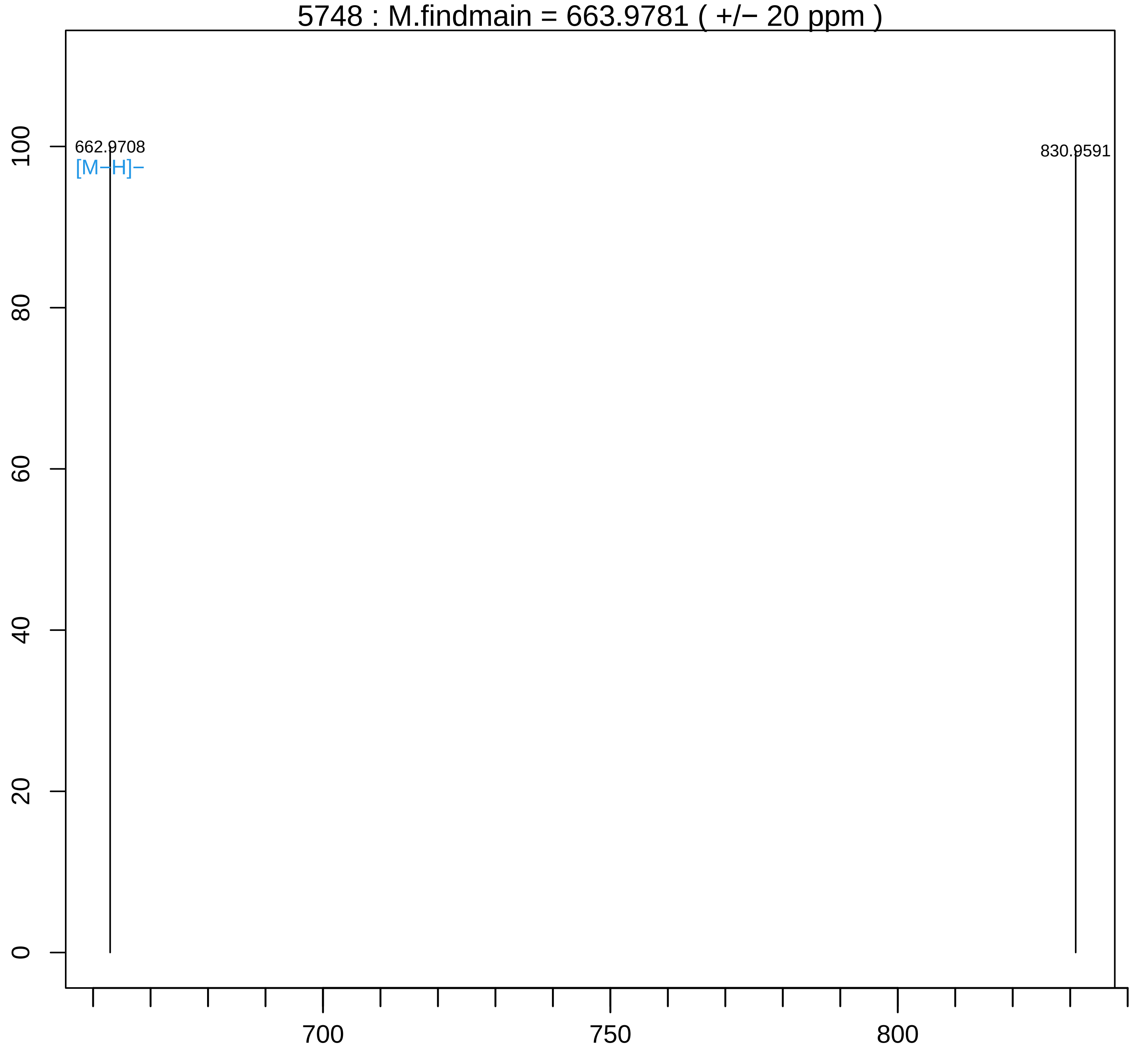

5748 : M.findmain = 663.9781 (+/− 20 ppm)
662.9708
[M−H]−
830.9591
0
20
40
60
80
100
700
750
800

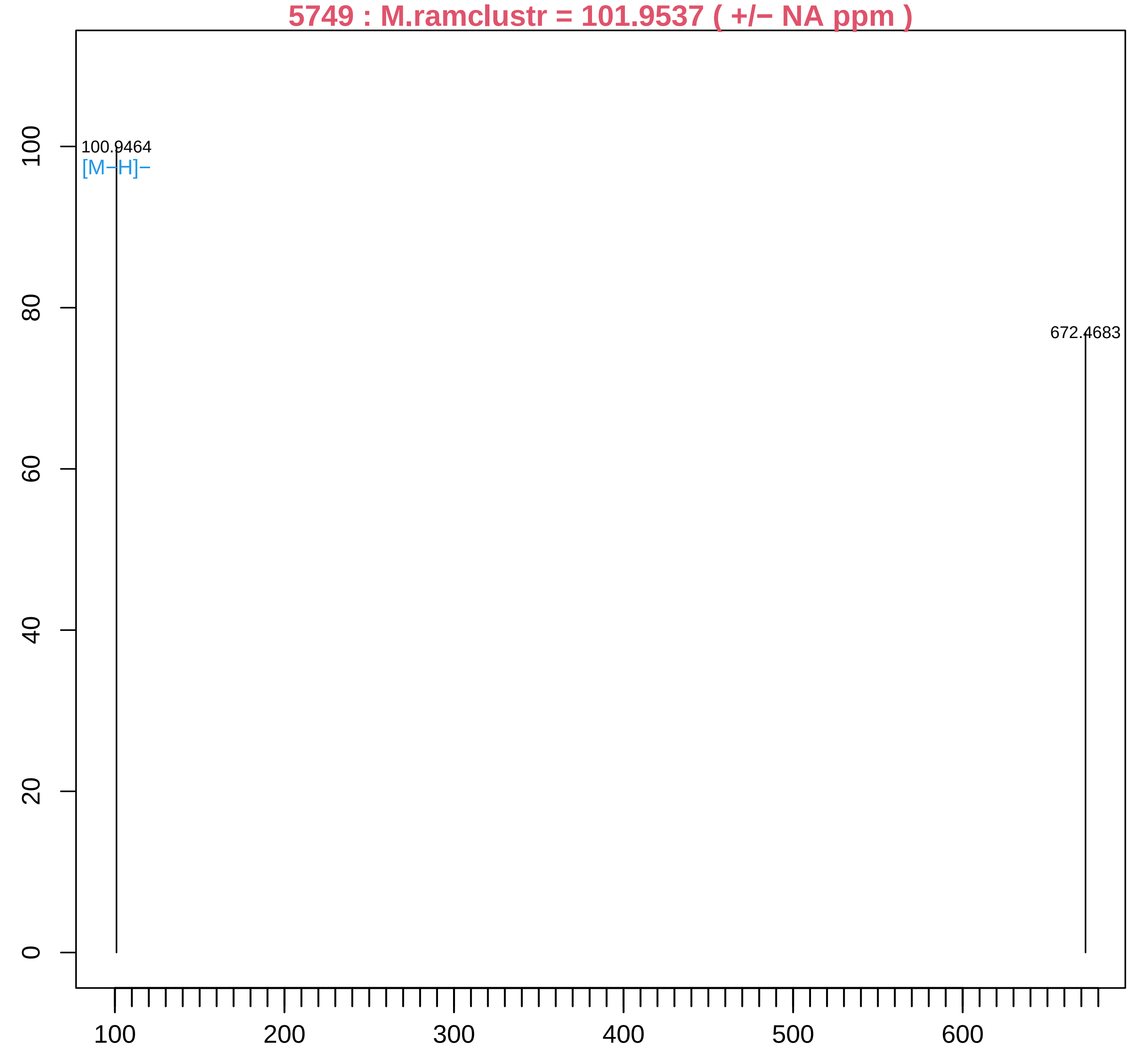
5749 : M.ramclustr = 101.9537 (+/− NA ppm)
100.9464
[M−H]−
672.4683
0
20
40
60
80
100
100
200
300
400
500
600

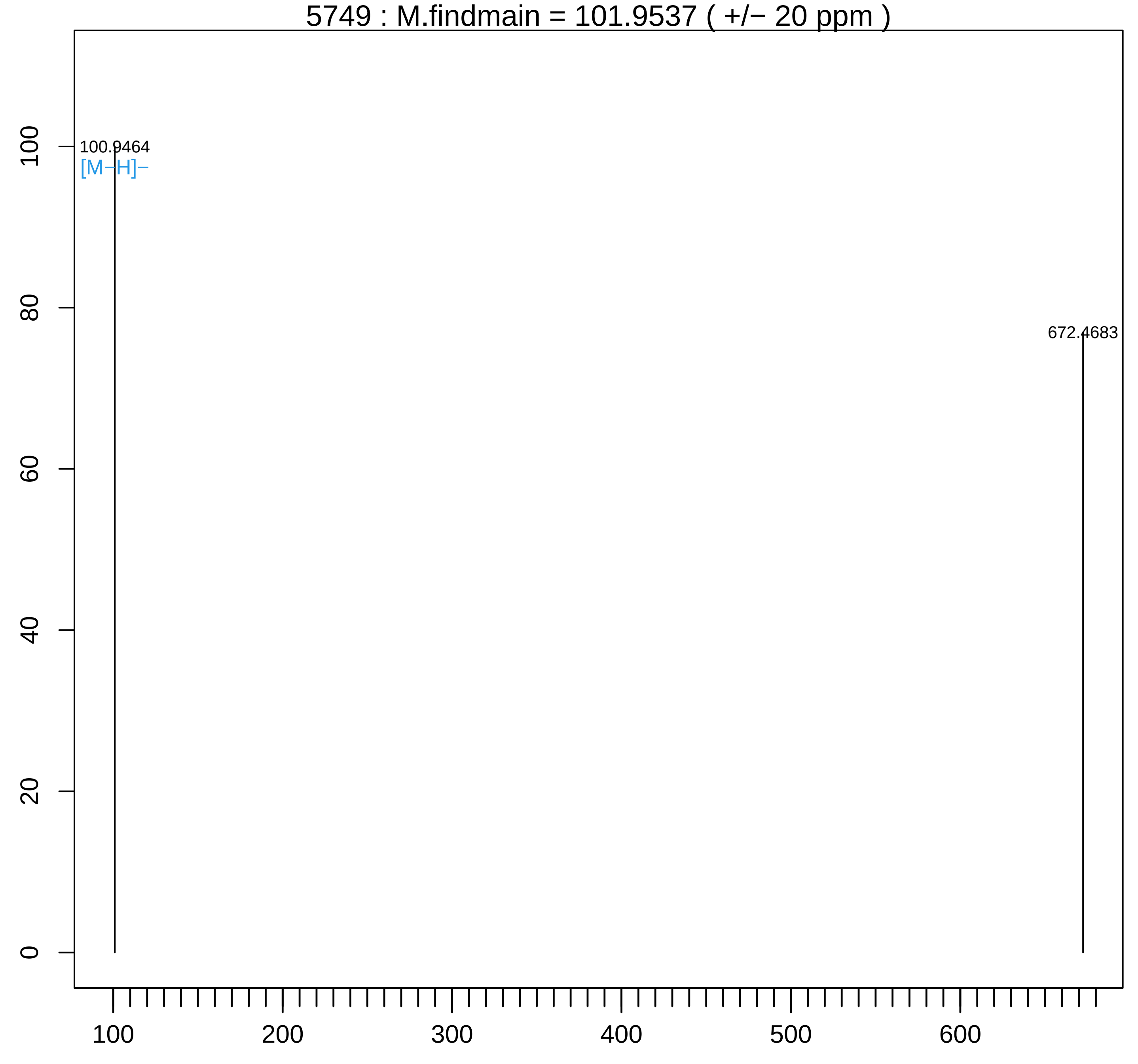
5749 : M.findmain = 101.9537 (+/− 20 ppm)
100.9464
[M−H]−
672.4683
0
20
40
60
80
100
100
200
300
400
500
600

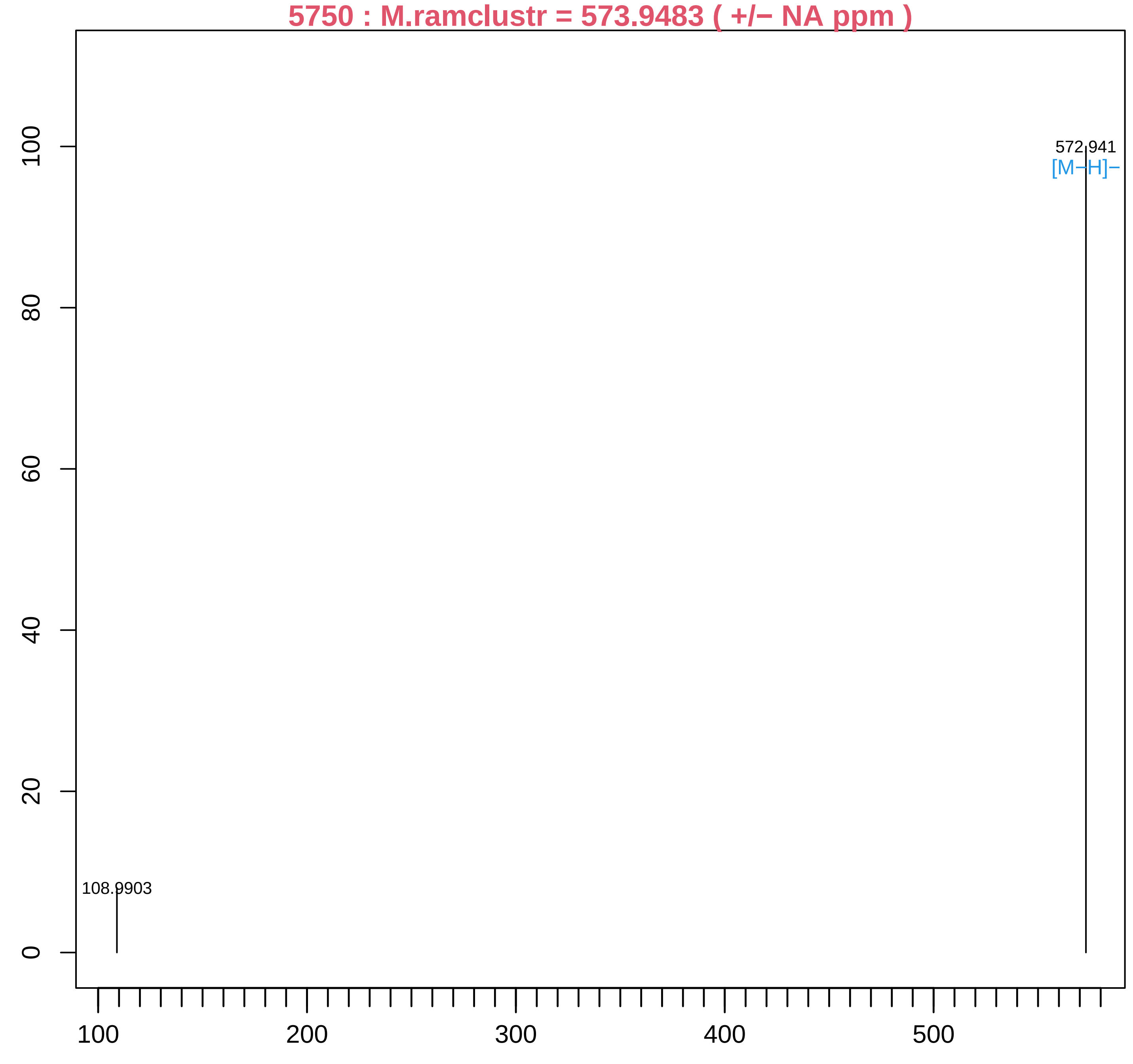

5750 : M.ramclustr = 573.9483 (+/− NA ppm)
572.941
[M−H]−
108.9903

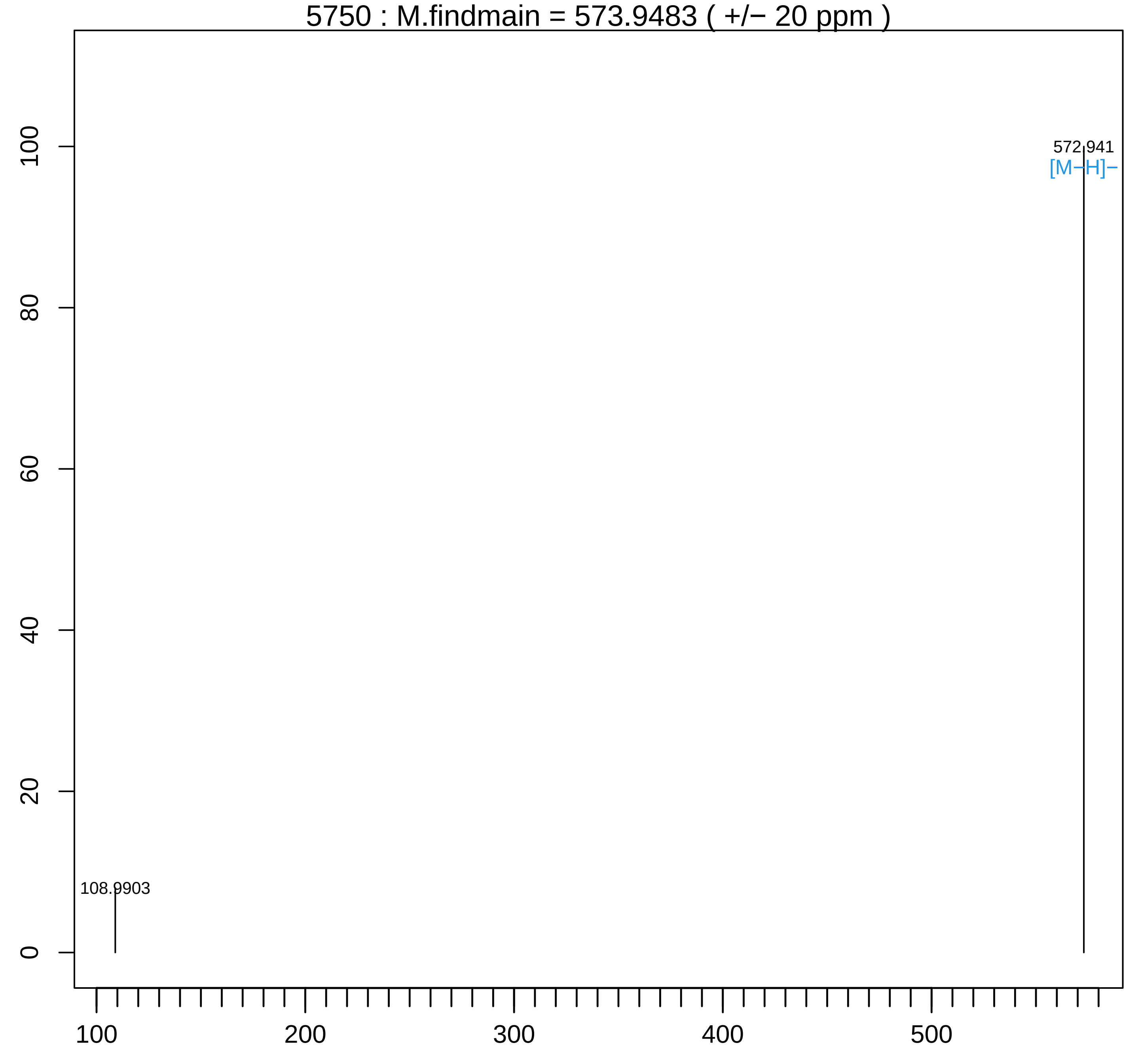

5750 : M.findmain = 573.9483 (+/− 20 ppm)
572.941
[M−H]−
108.9903

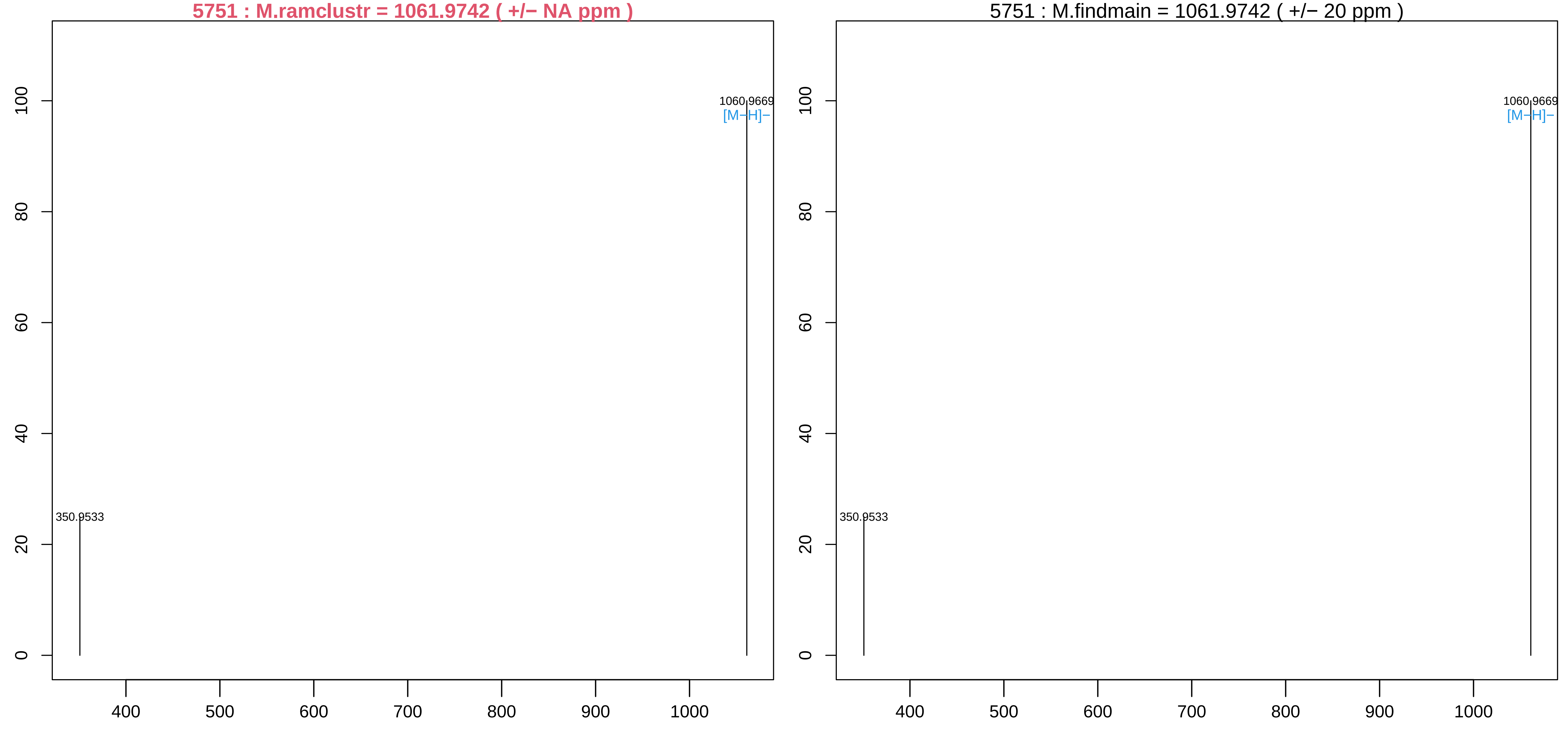
5751 : M.ramclustr = 1061.9742 (+/− NA ppm)
1060.9669
[M−H]−
350.9533
0
20
40
60
80
100
400
500
600
700
800
900
1000
5751 : M.findmain = 1061.9742 (+/− 20 ppm)
1060.9669
[M−H]−
350.9533
0
20
40
60
80
100
400
500
600
700
800
900
1000

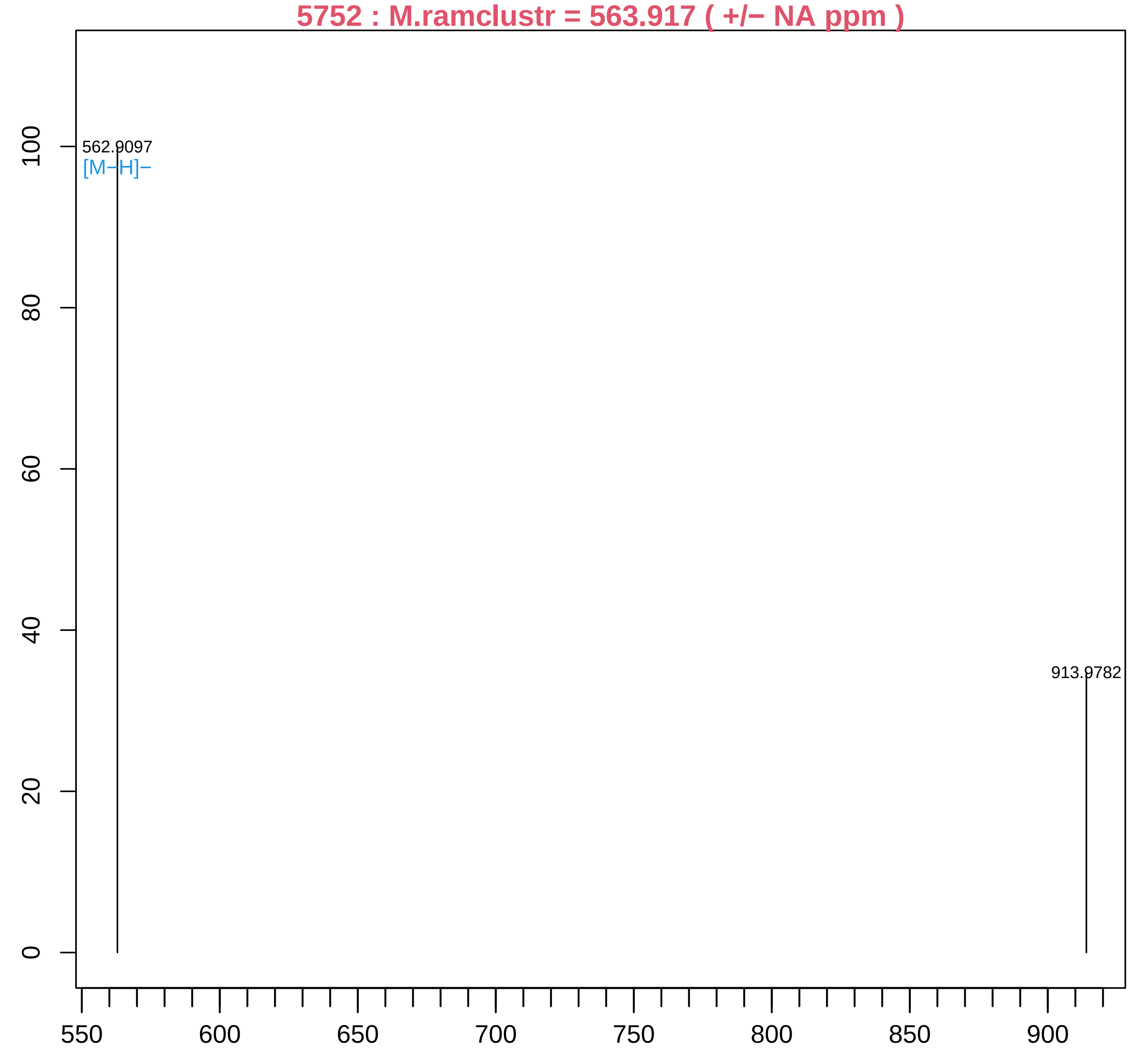
5752 : M.ramclustr = 563.917 (+/− NA ppm)
562.9097
[M−H]−
913.9782
0
20
40
60
80
100
550
600
650
700
750
800
850
900

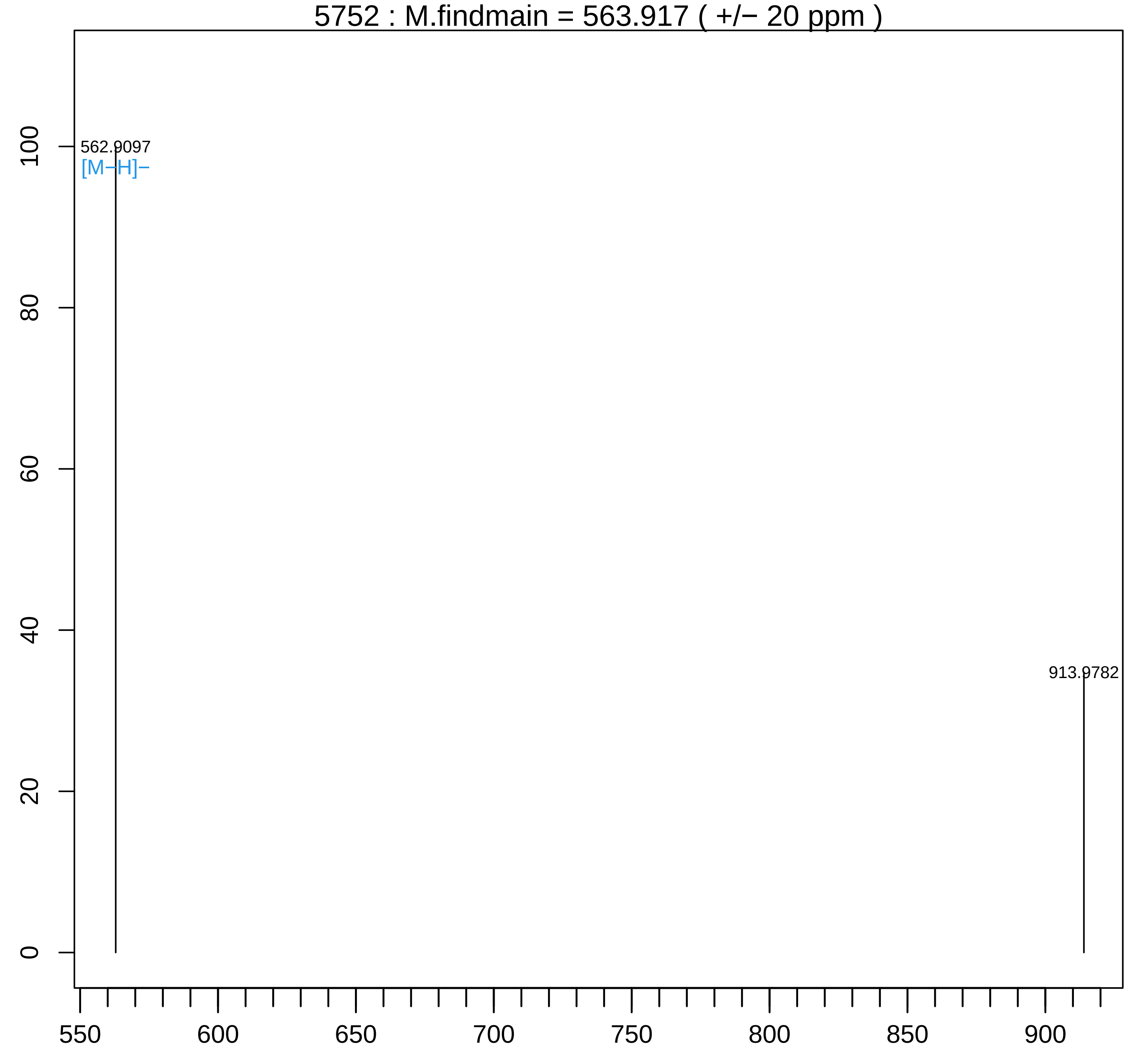
5752 : M.findmain = 563.917 (+/− 20 ppm)
562.9097
[M−H]−
913.9782
0
20
40
60
80
100
550
600
650
700
750
800
850
900

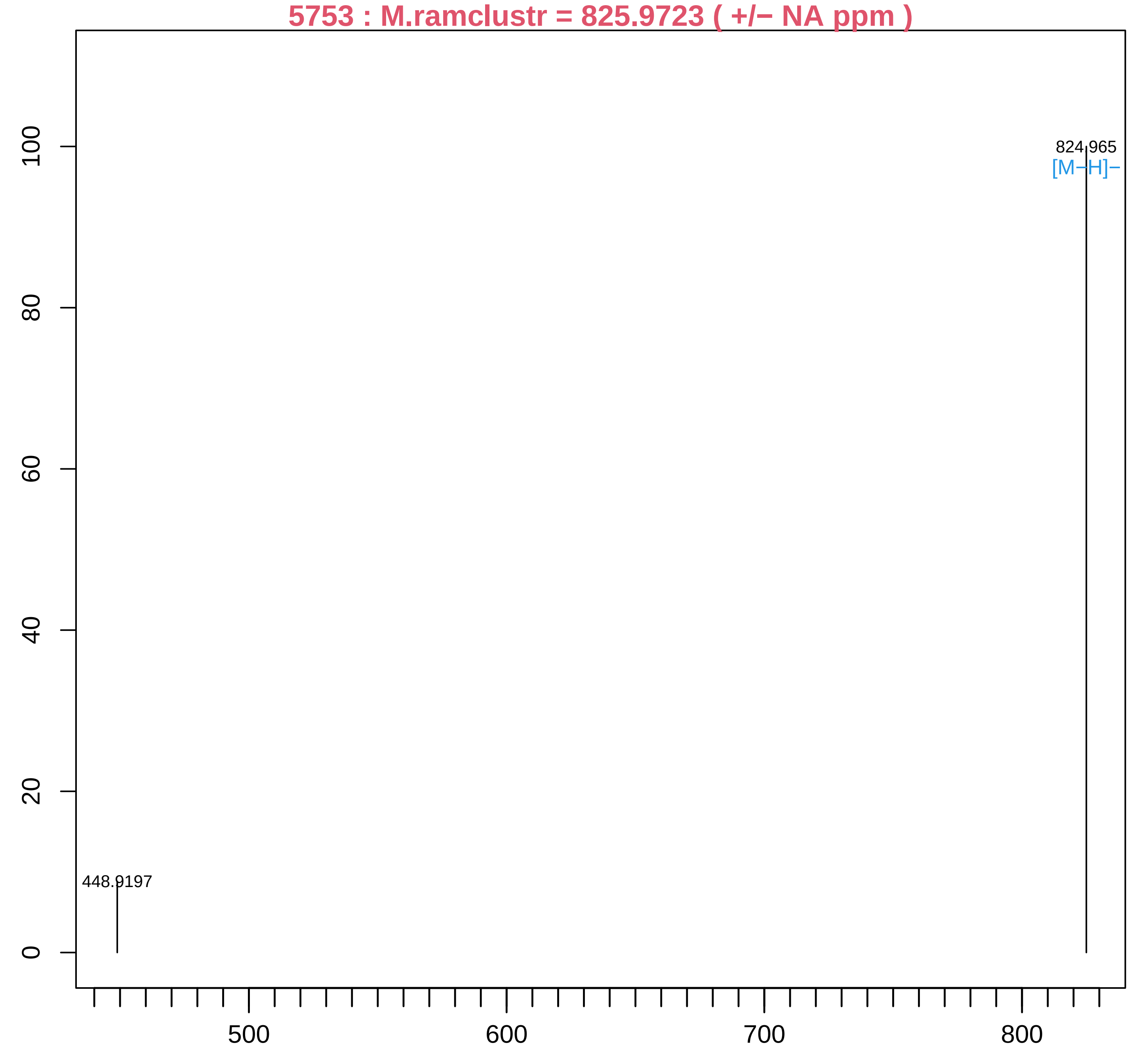
5753 : M.ramclustr = 825.9723 (+/− NA ppm)
824.965
[M−H]−
448.9197
0
20
40
60
80
100
500
600
700
800

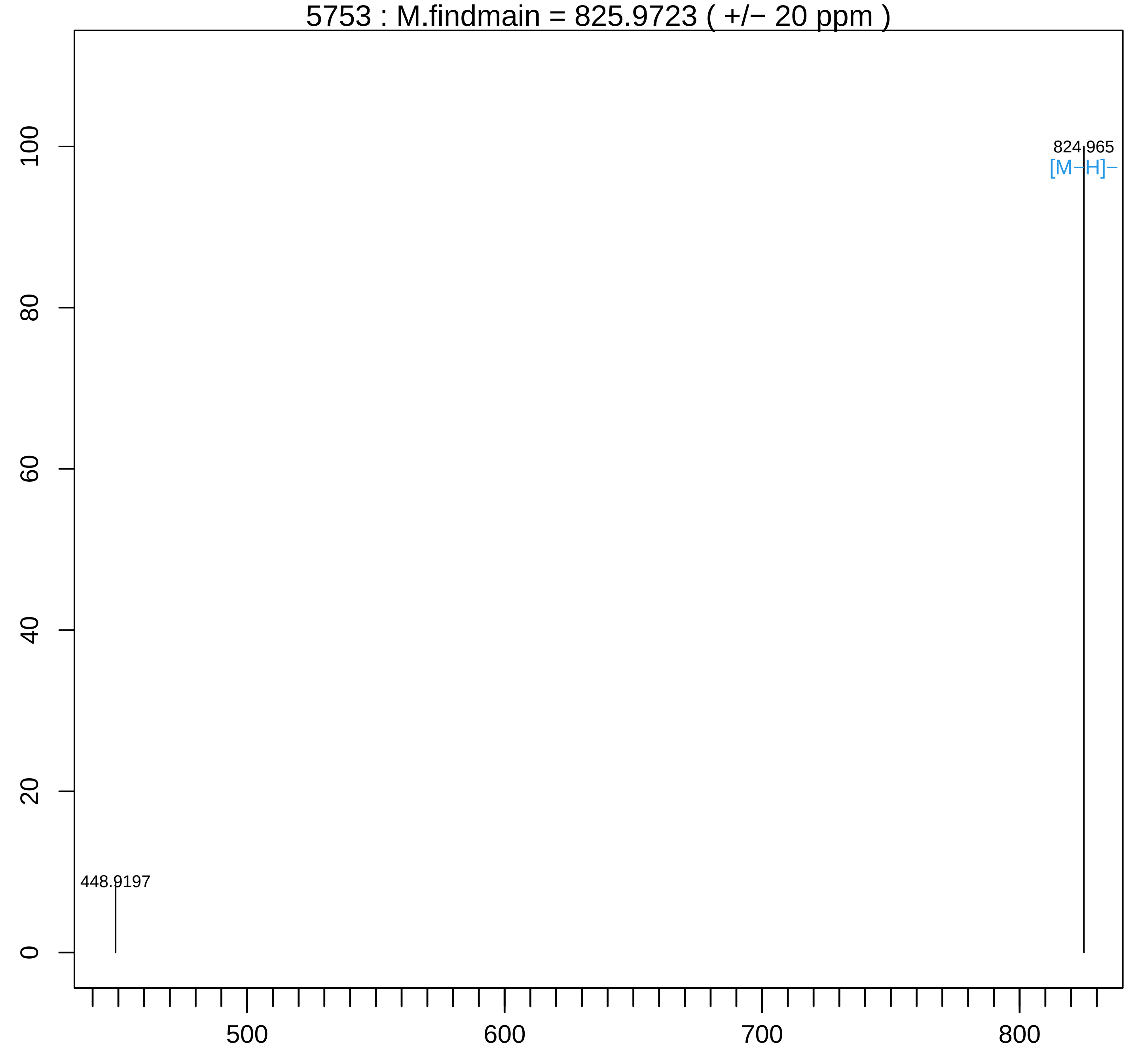
5753 : M.findmain = 825.9723 (+/− 20 ppm)
824.965
[M−H]−
448.9197
0
20
40
60
80
100
500
600
700
800

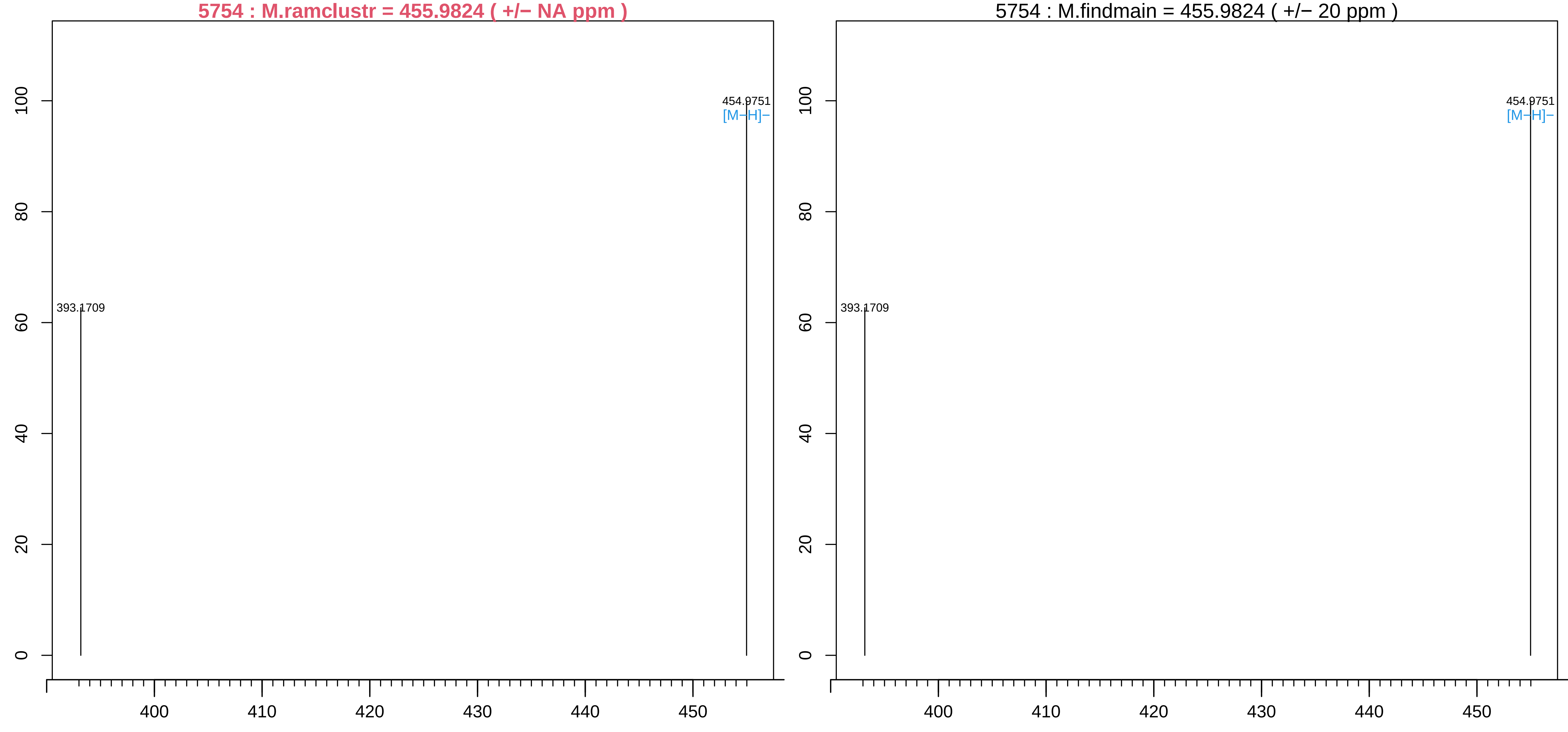
5754 : M.ramclustr = 455.9824 (+/− NA ppm)
5754 : M.findmain = 455.9824 (+/− 20 ppm)
454.9751
[M−H]−
393.1709
0
20
40
60
80
100
400
410
420
430
440
450

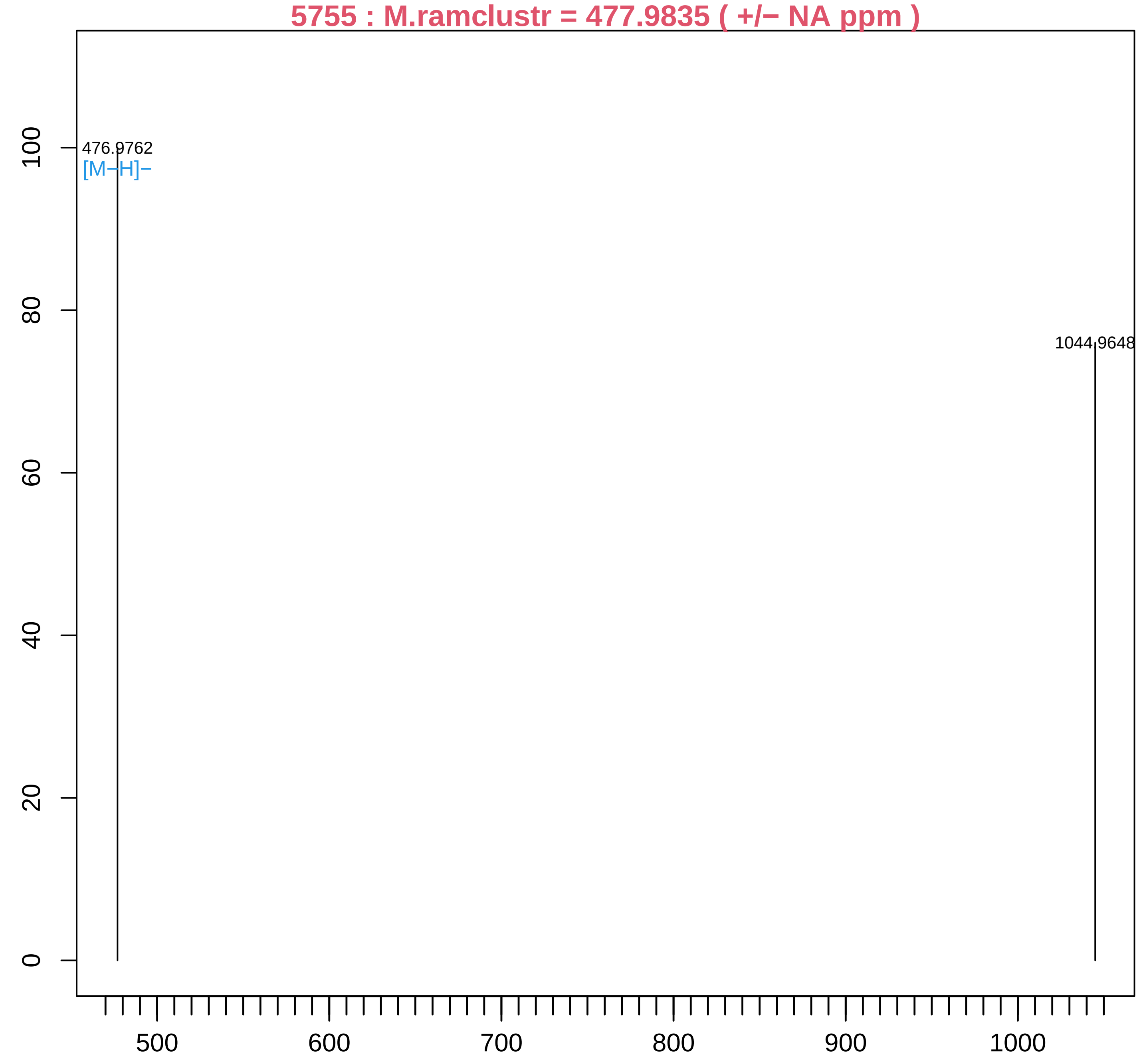
5755 : M.ramclustr = 477.9835 (+/− NA ppm)
476.9762
[M−H]−
1044.9648
0
20
40
60
80
100
500
600
700
800
900
1000

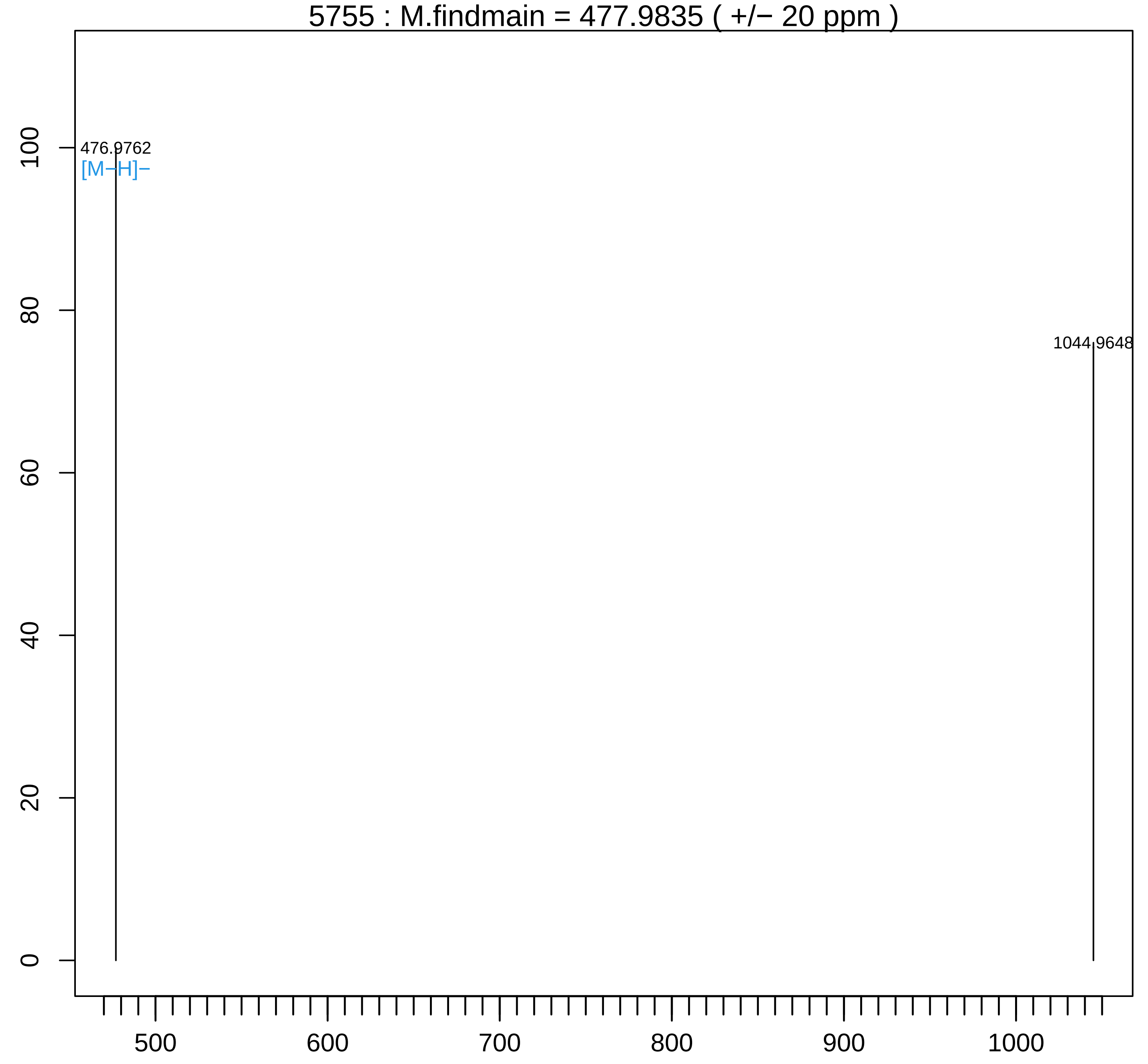
5755 : M.findmain = 477.9835 (+/− 20 ppm)
476.9762
[M−H]−
1044.9648
0
20
40
60
80
100
500
600
700
800
900
1000

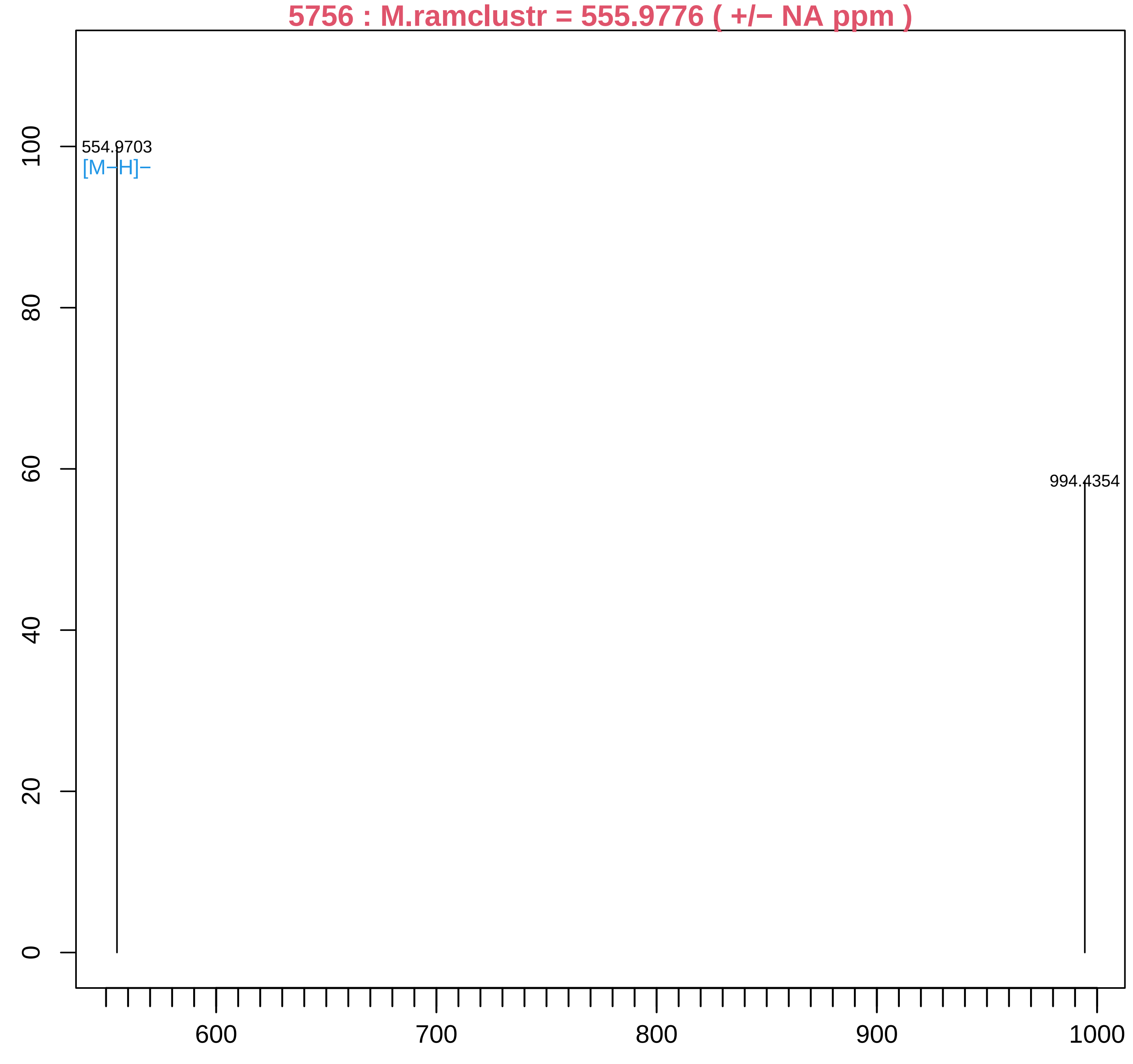
5756 : M.ramclustr = 555.9776 (+/− NA ppm)
554.9703
[M−H]−
994.4354
0
20
40
60
80
100
600
700
800
900
1000

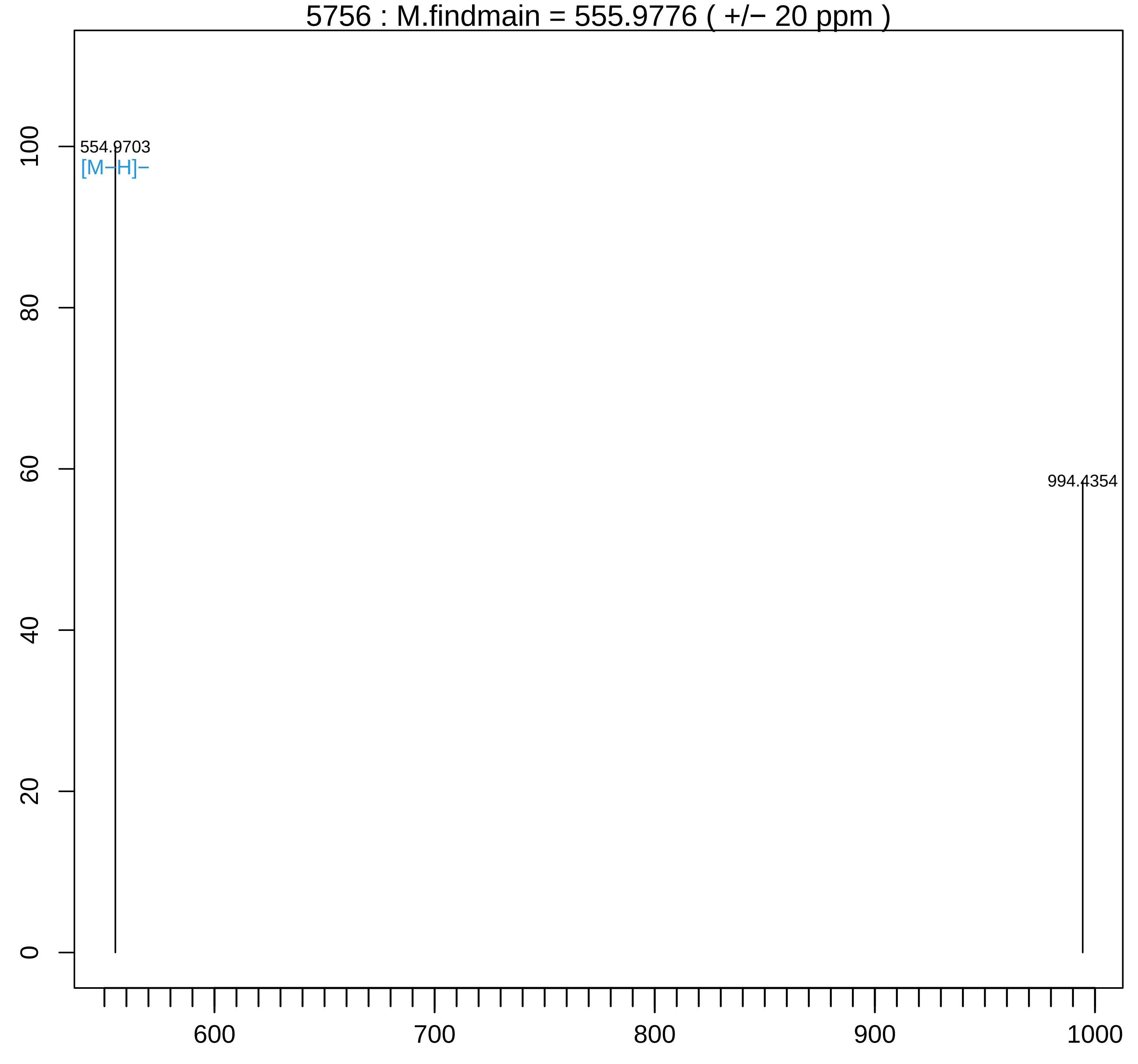
5756 : M.findmain = 555.9776 (+/− 20 ppm)
554.9703
[M−H]−
994.4354
0
20
40
60
80
100
600
700
800
900
1000

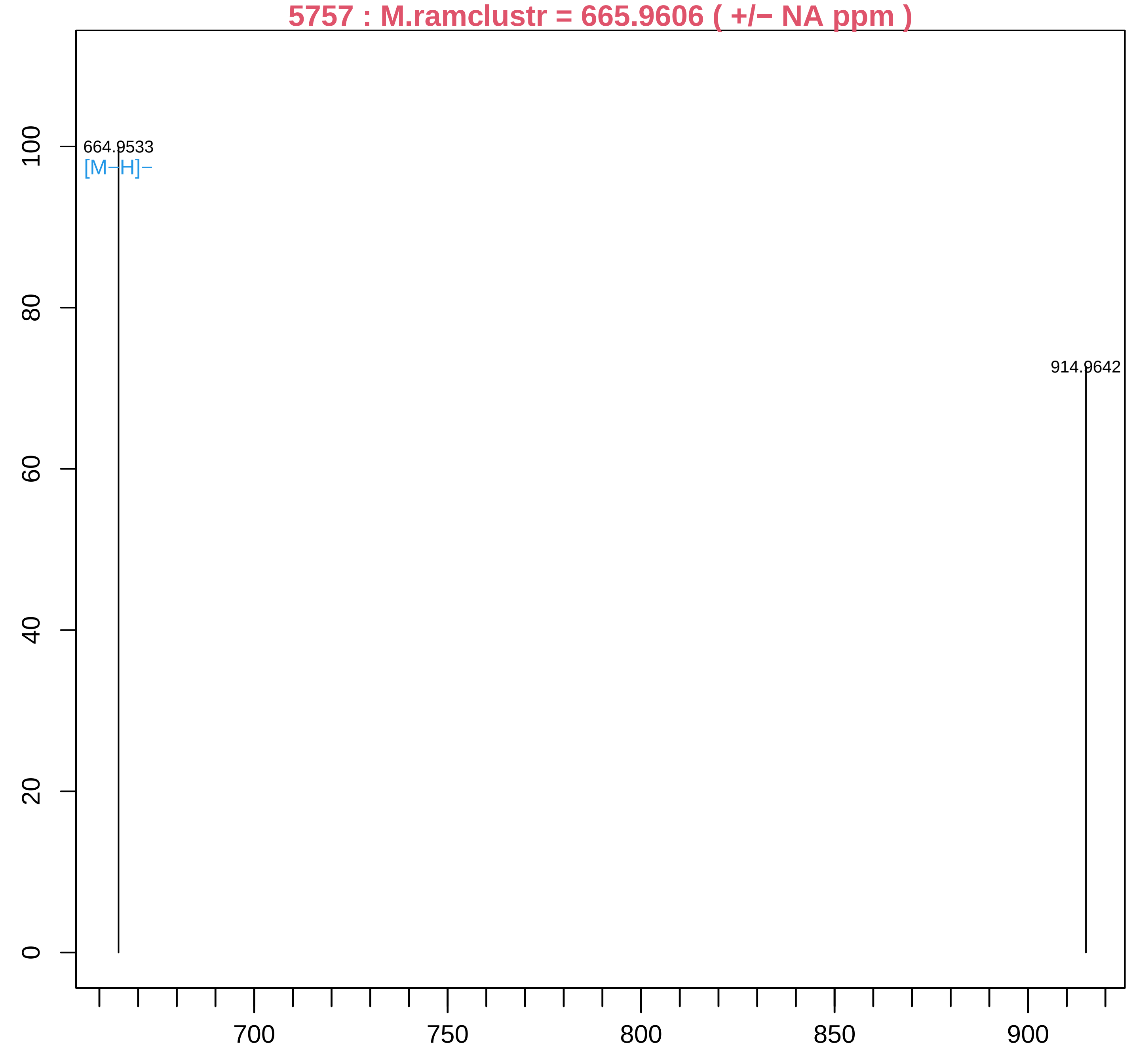
5757 : M.ramclustr = 665.9606 (+/− NA ppm)
664.9533
[M−H]−
914.9642
0
20
40
60
80
100
700
750
800
850
900

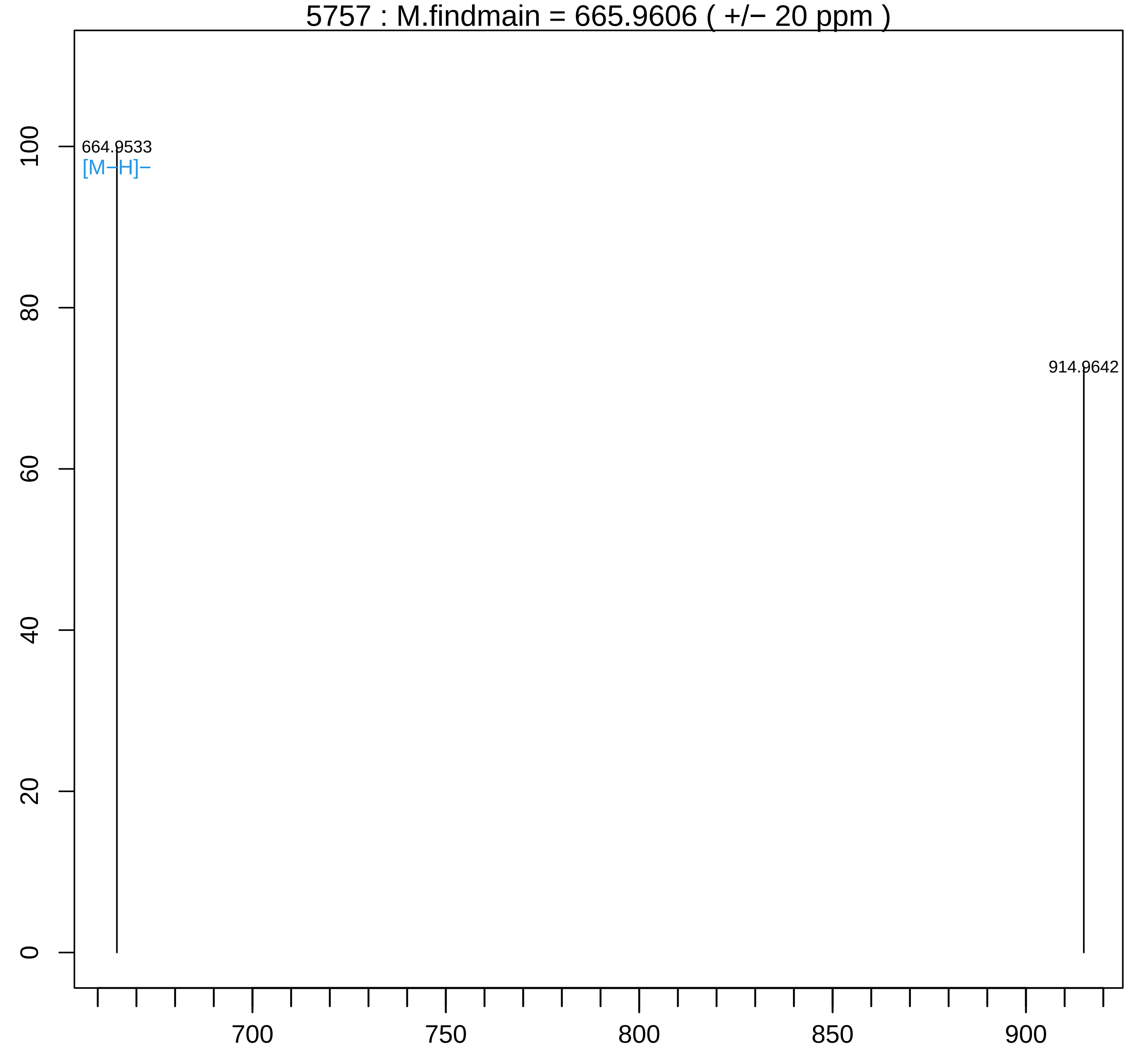
5757 : M.findmain = 665.9606 (+/− 20 ppm)
664.9533
[M−H]−
914.9642
0
20
40
60
80
100
700
750
800
850
900

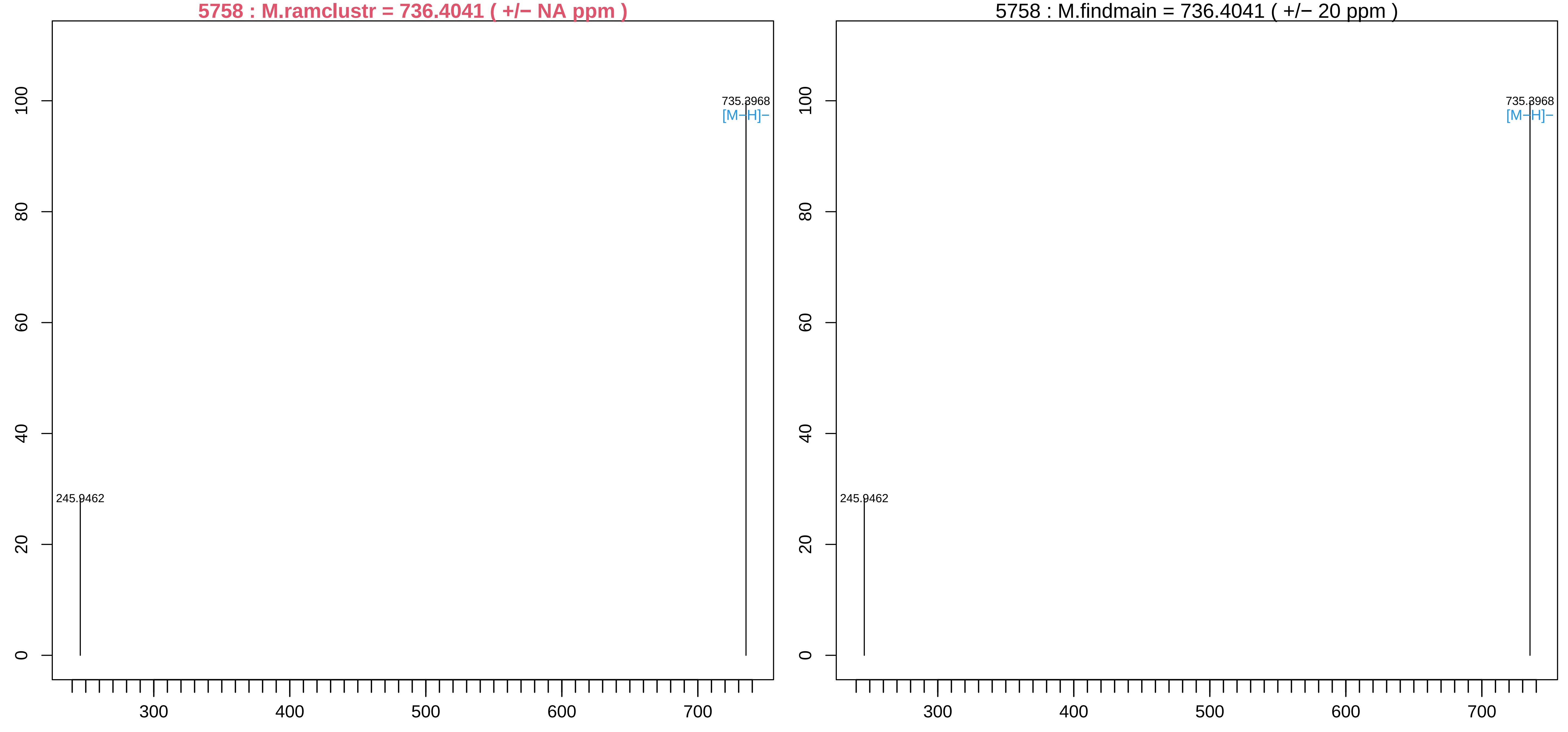
5758 : M.ramclustr = 736.4041 (+/− NA ppm)
5758 : M.findmain = 736.4041 (+/− 20 ppm)
735.3968
[M−H]−
245.9462
0
20
40
60
80
100
300
400
500
600
700

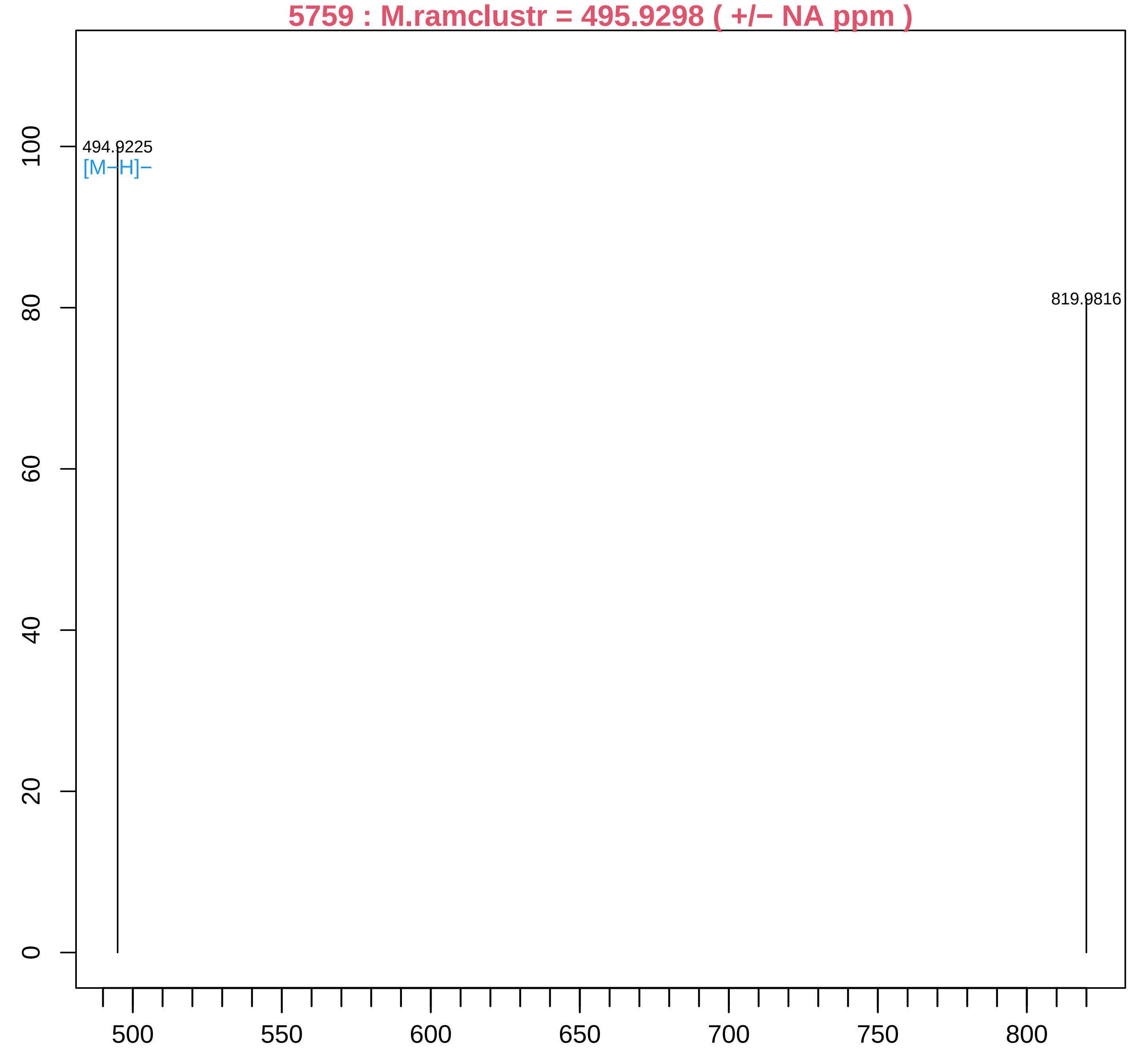

5759 : M.ramclustr = 495.9298 (+/− NA ppm)
494.9225
[M−H]−
819.9816
0
20
40
60
80
100
500
550
600
650
700
750
800

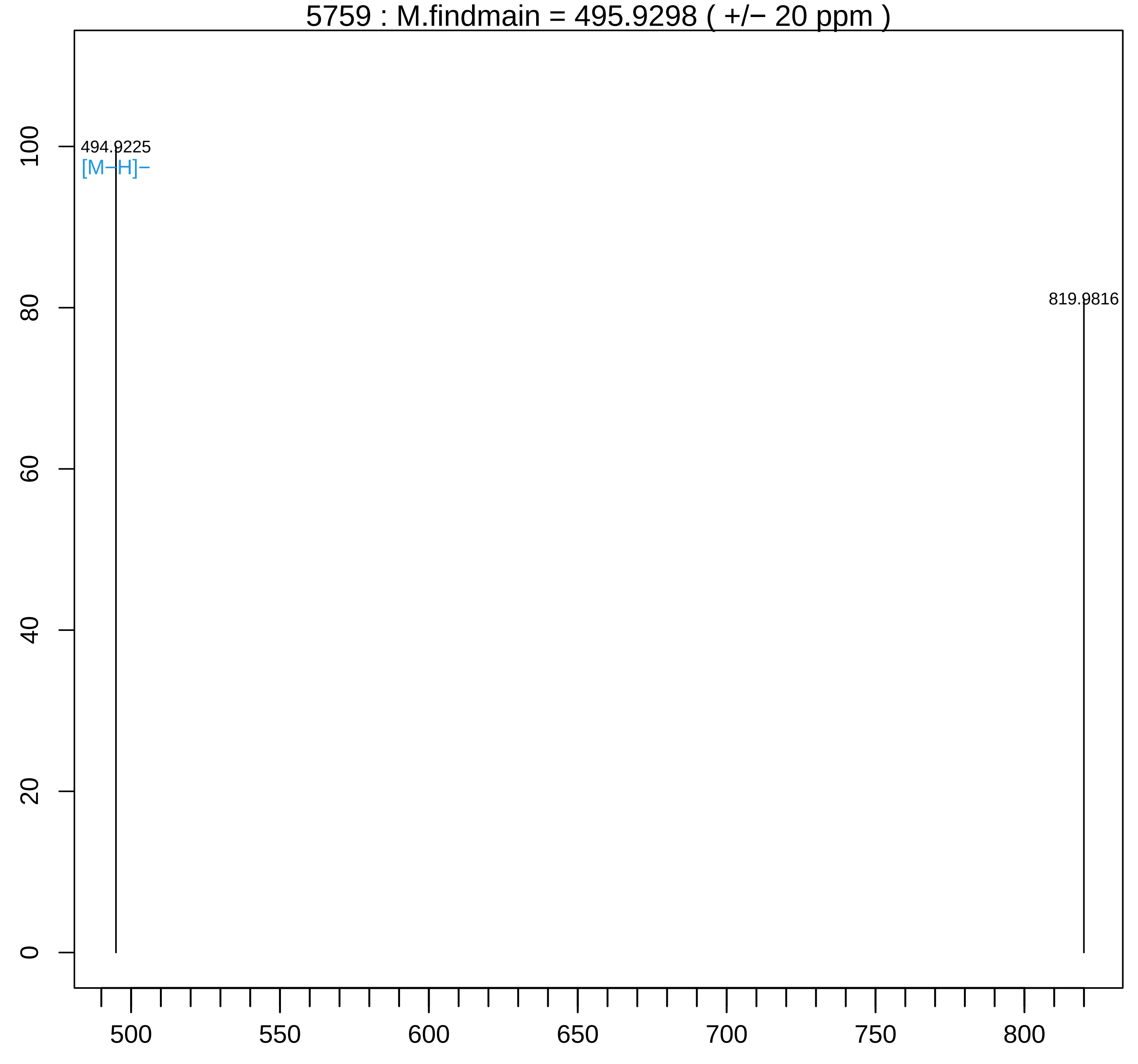

5759 : M.findmain = 495.9298 (+/− 20 ppm)
494.9225
[M−H]−
819.9816
0
20
40
60
80
100
500
550
600
650
700
750
800

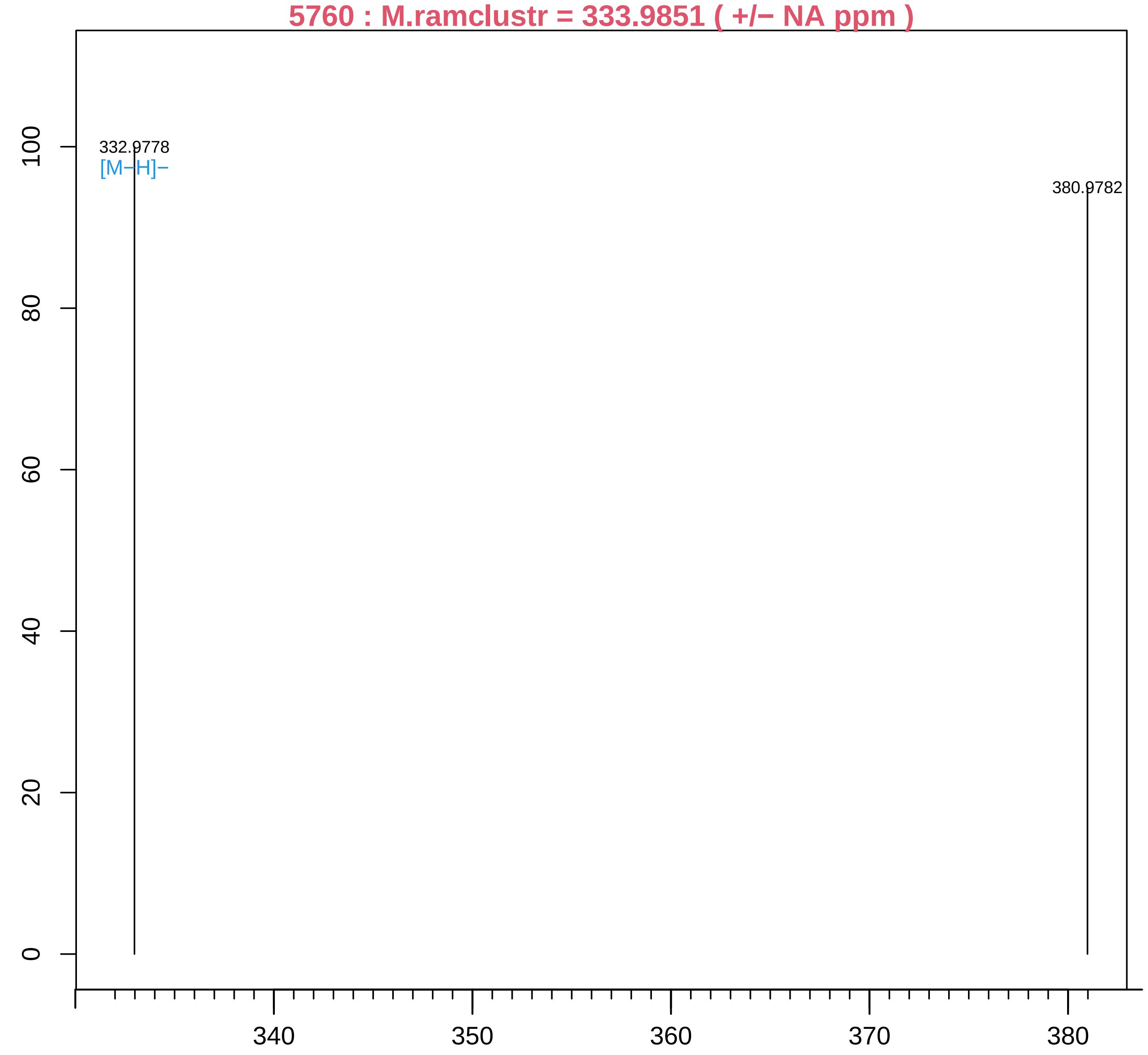
5760 : M.ramclustr = 333.9851 (+/− NA ppm)
332.9778
[M−H]−
380.9782
0
20
40
60
80
100
340
350
360
370
380

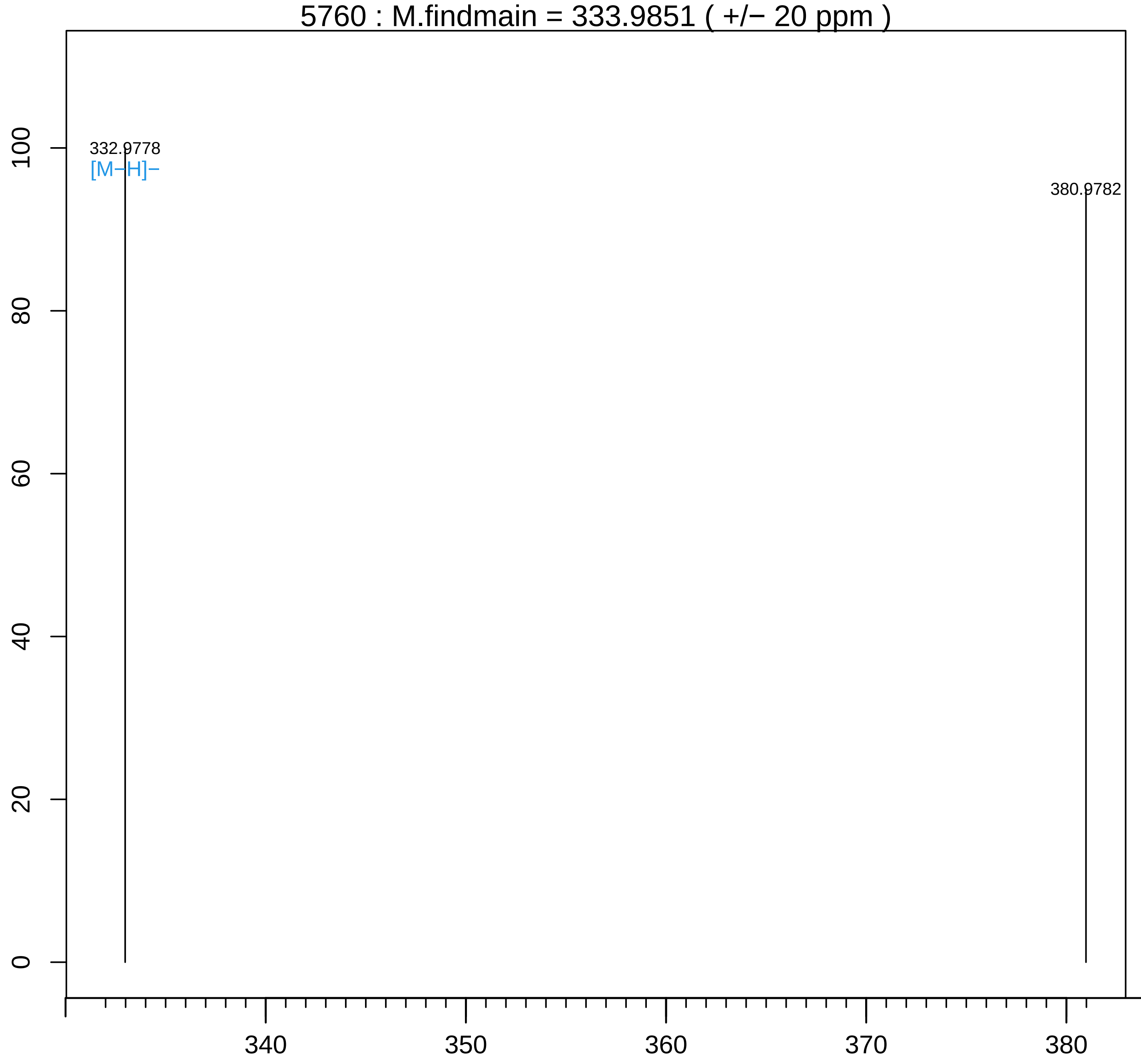
5760 : M.findmain = 333.9851 (+/− 20 ppm)
332.9778
[M−H]−
380.9782
0
20
40
60
80
100
340
350
360
370
380

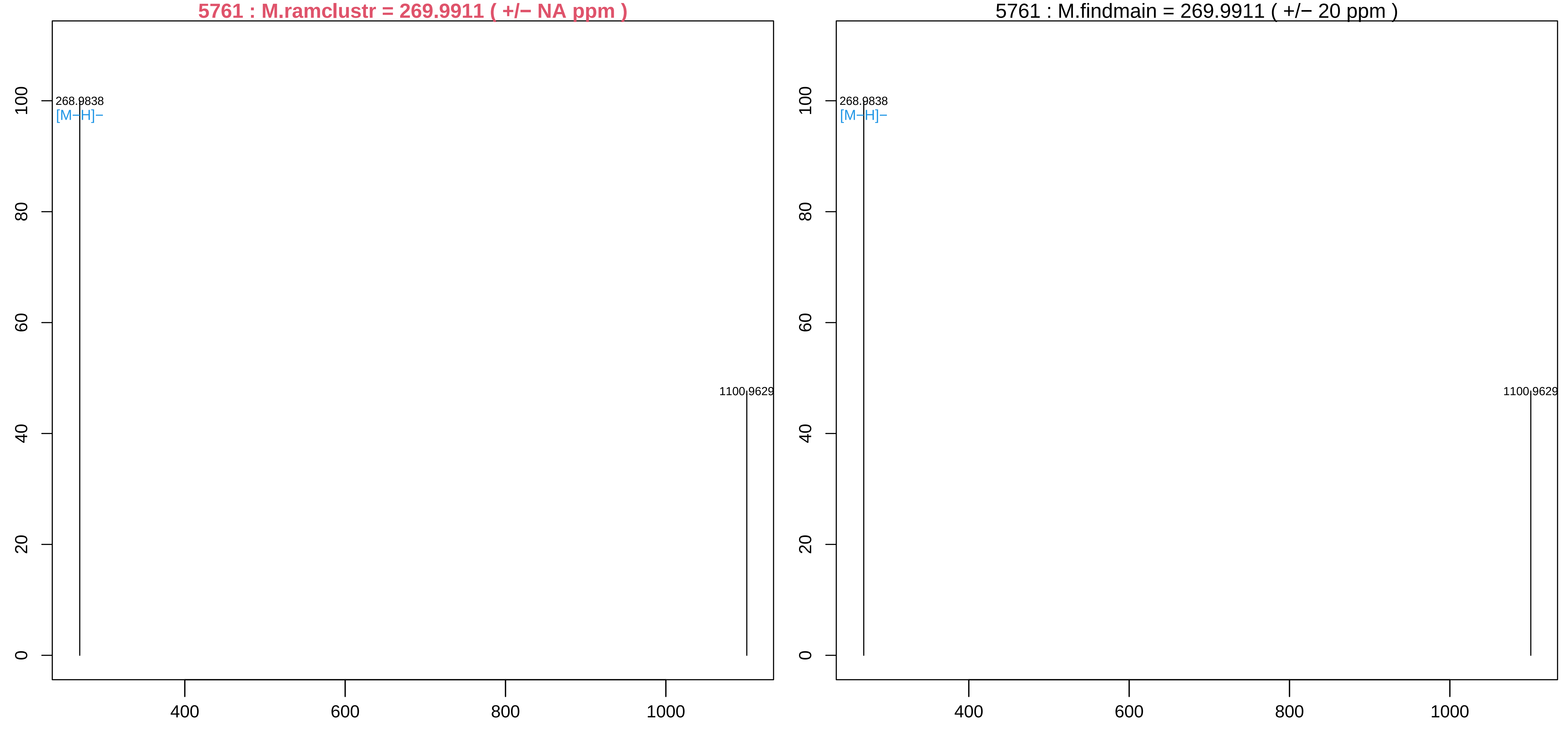
5761 : M.ramclustr = 269.9911 (+/− NA ppm)
268.9838
[M−H]−
1100.9629
0
20
40
60
80
100
400
600
800
1000
5761 : M.findmain = 269.9911 (+/− 20 ppm)
268.9838
[M−H]−
1100.9629
0
20
40
60
80
100
400
600
800
1000

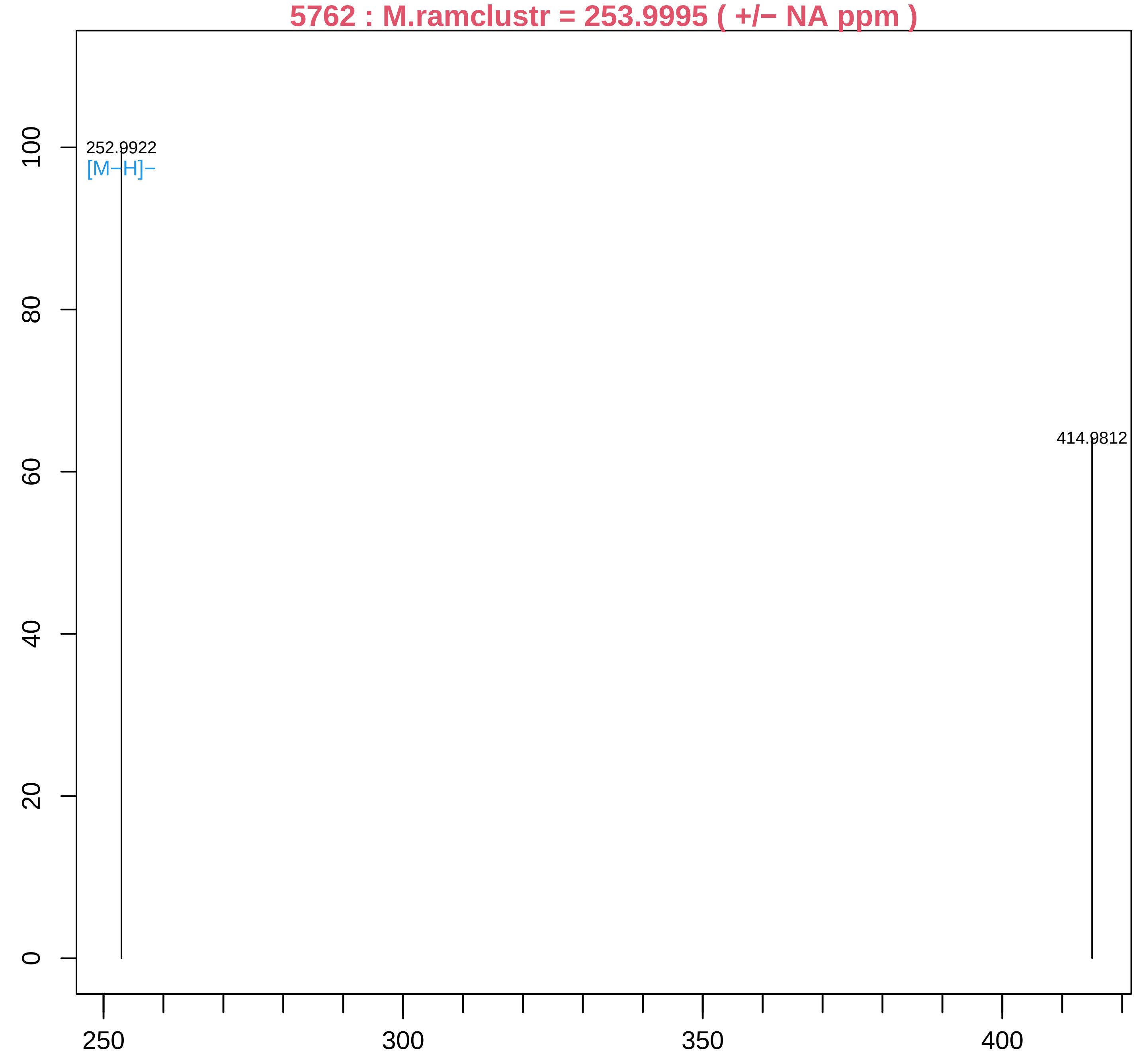
5762 : M.ramclustr = 253.9995 (+/− NA ppm)
252.9922
[M−H]−
414.9812
0
20
40
60
80
100
250
300
350
400

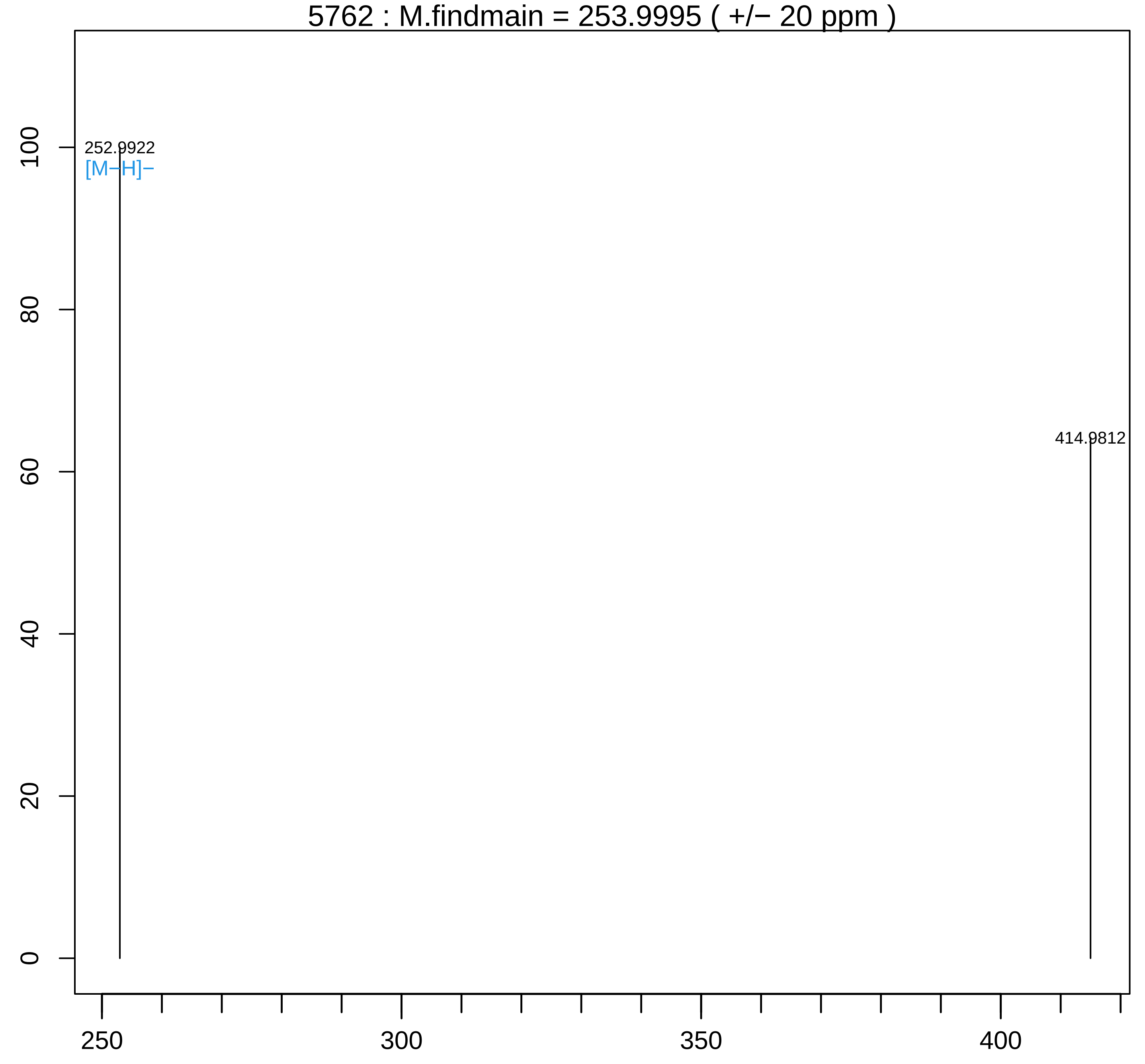
5762 : M.findmain = 253.9995 (+/− 20 ppm)
252.9922
[M−H]−
414.9812
0
20
40
60
80
100
250
300
350
400

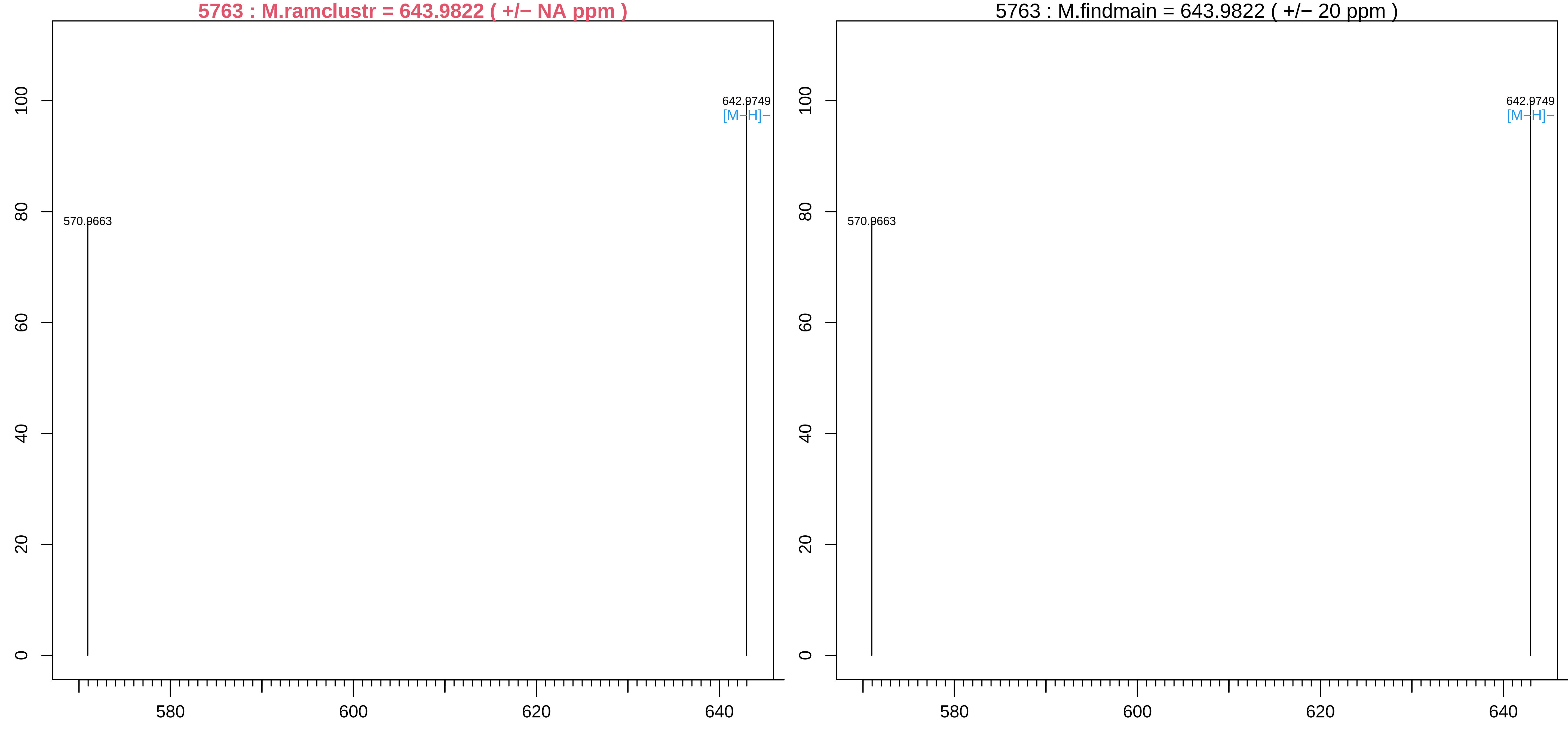
5763 : M.ramclustr = 643.9822 (+/− NA ppm)
642.9749
[M−H]−
570.9663
5763 : M.findmain = 643.9822 (+/− 20 ppm)
642.9749
[M−H]−
570.9663
0
20
40
60
80
100
580
600
620
640

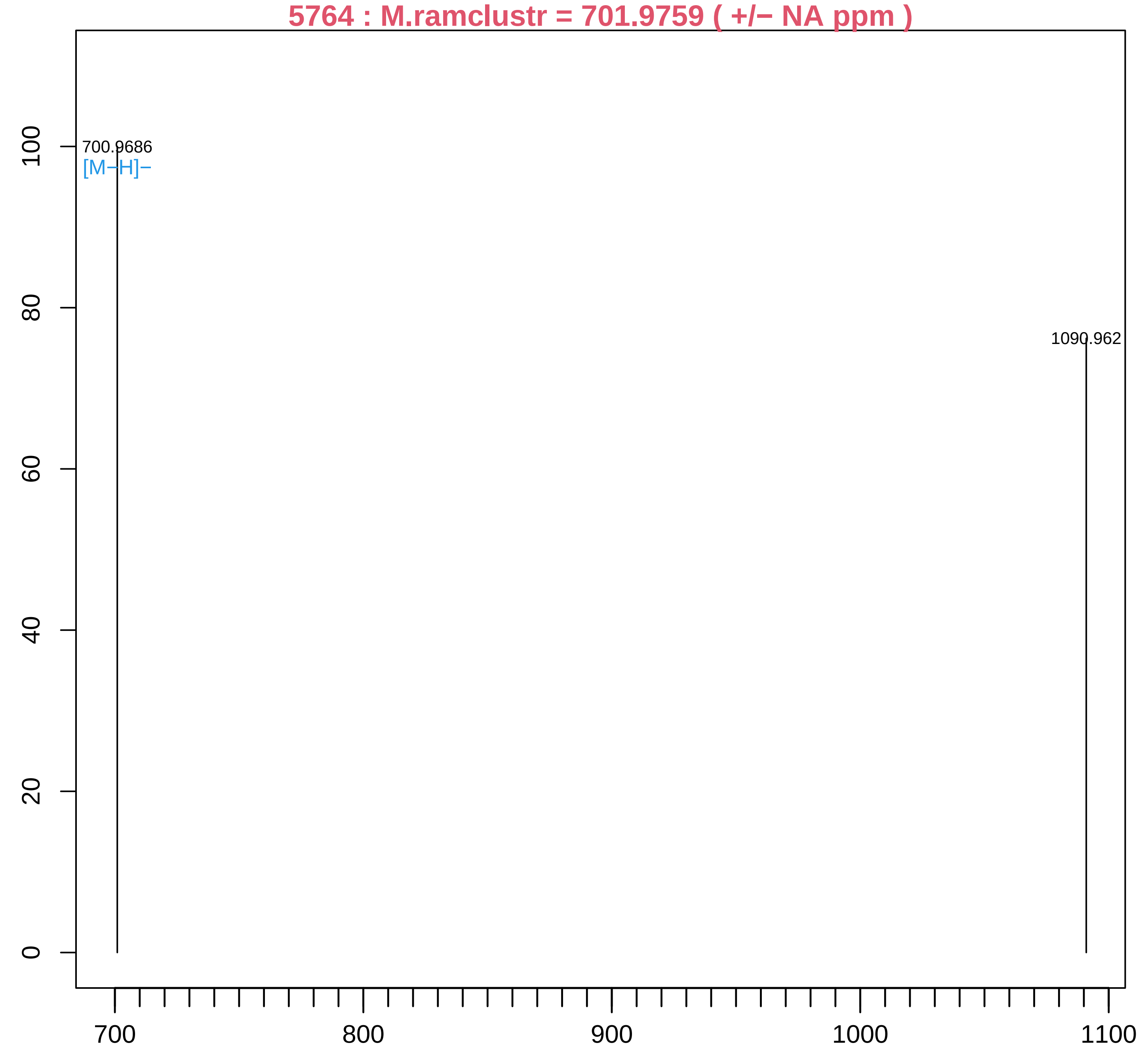
5764 : M.ramclustr = 701.9759 (+/− NA ppm)
700.9686
[M−H]−
1090.962
0
20
40
60
80
100
700
800
900
1000
1100

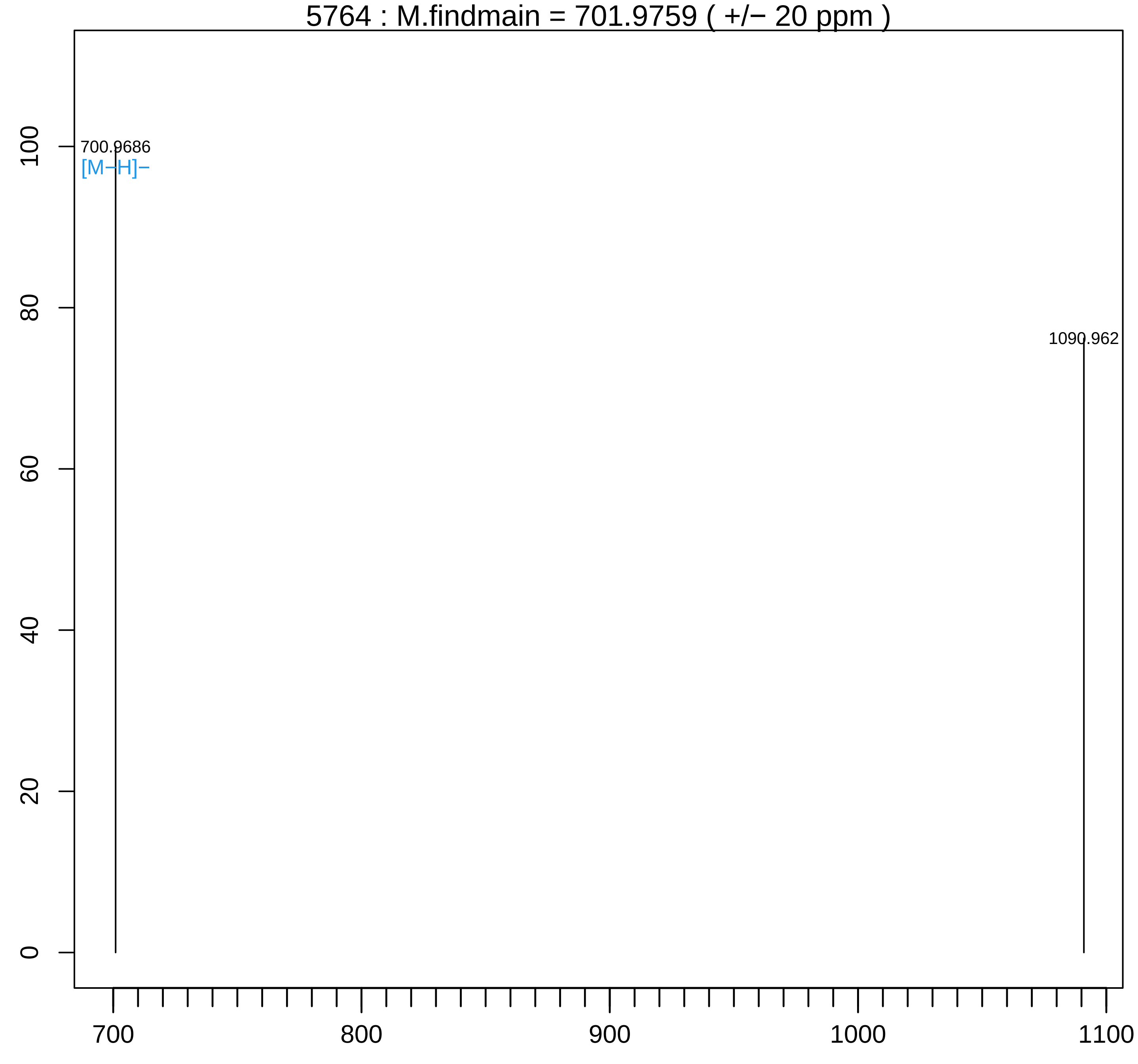
5764 : M.findmain = 701.9759 (+/− 20 ppm)
700.9686
[M−H]−
1090.962
0
20
40
60
80
100
700
800
900
1000
1100

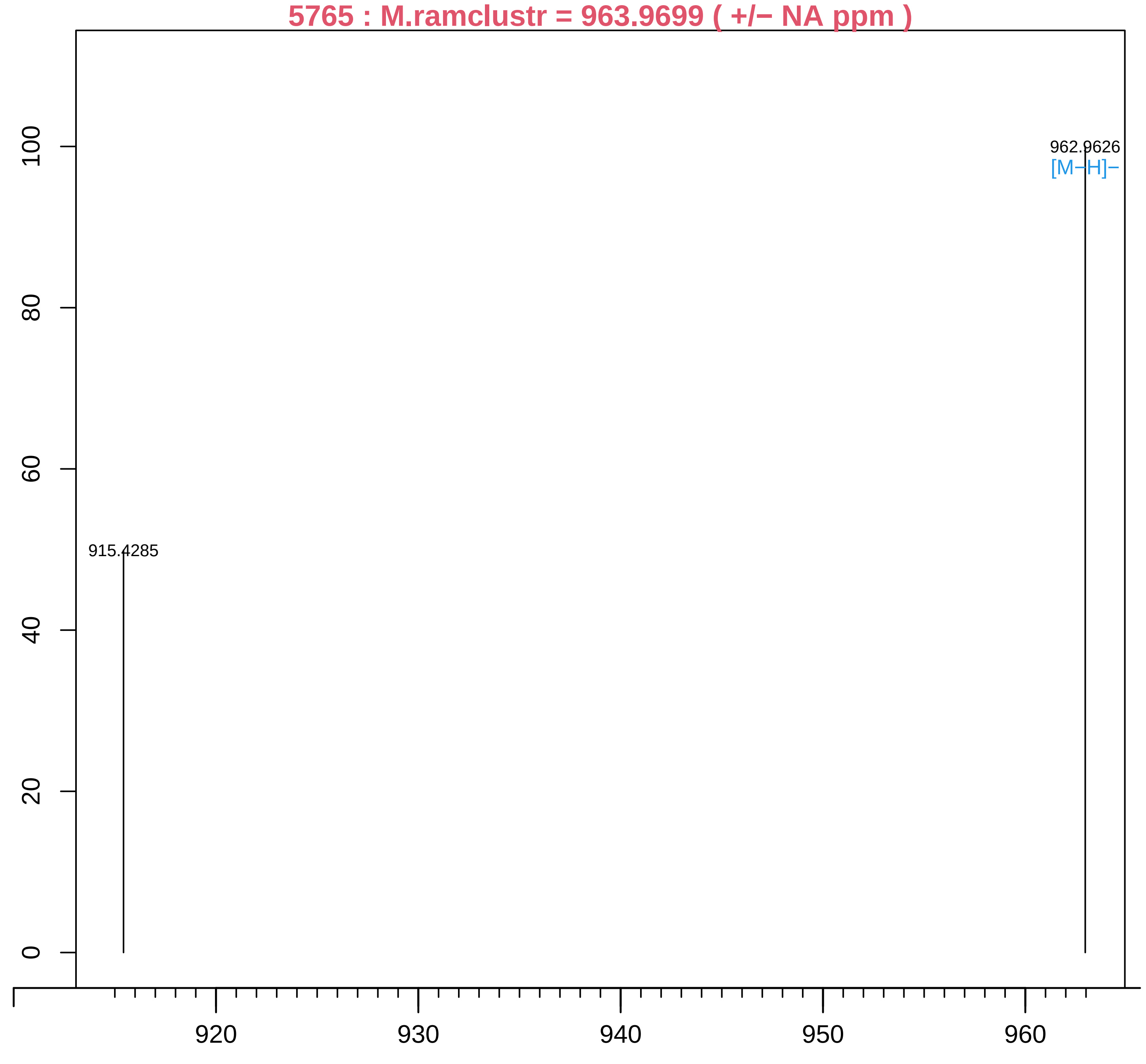

5765 : M.ramclustr = 963.9699 (+/− NA ppm)
915.4285
962.9626
[M−H]−
0
20
40
60
80
100
920
930
940
950
960

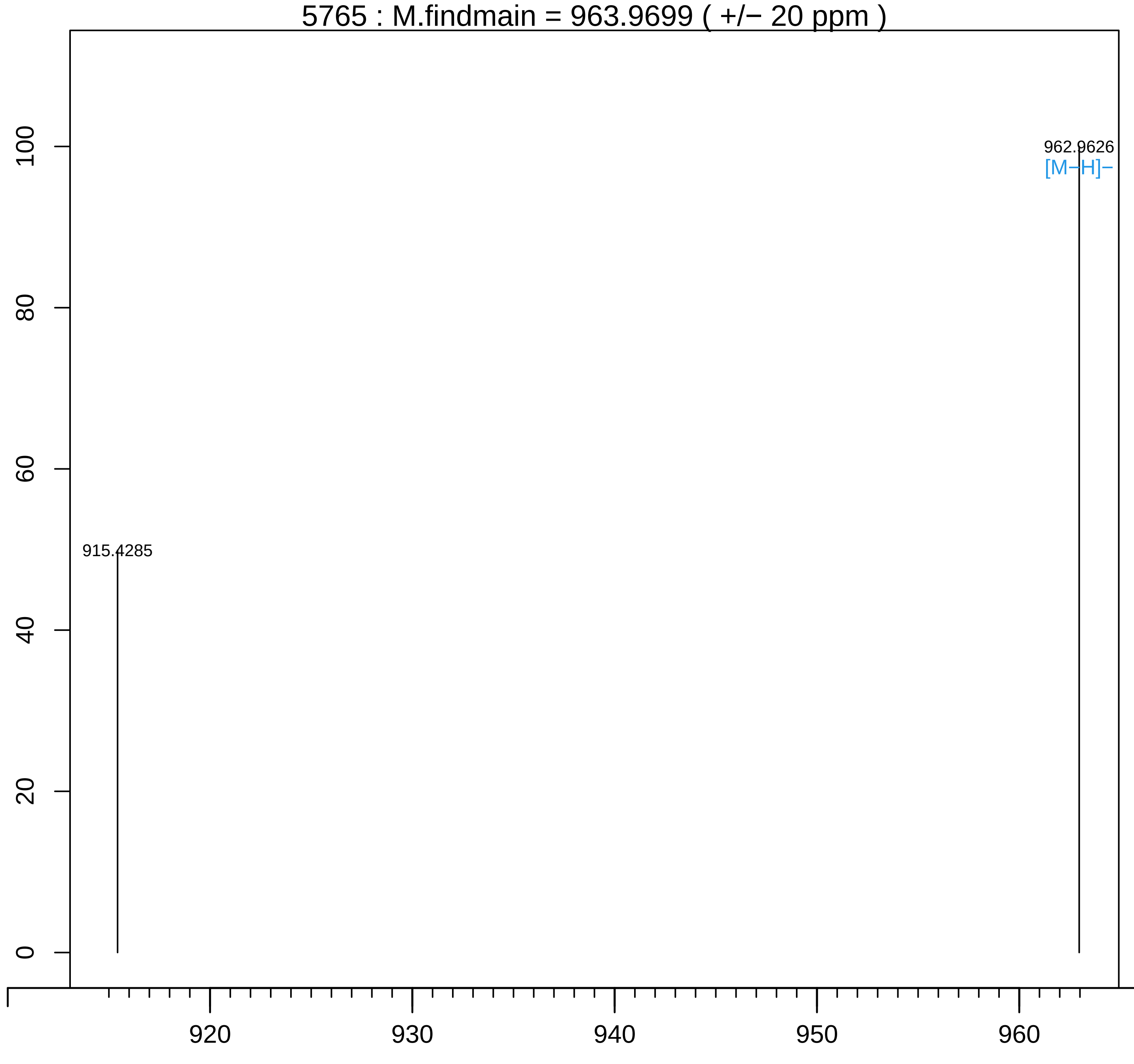

5765 : M.findmain = 963.9699 (+/− 20 ppm)
915.4285
962.9626
[M−H]−
0
20
40
60
80
100
920
930
940
950
960

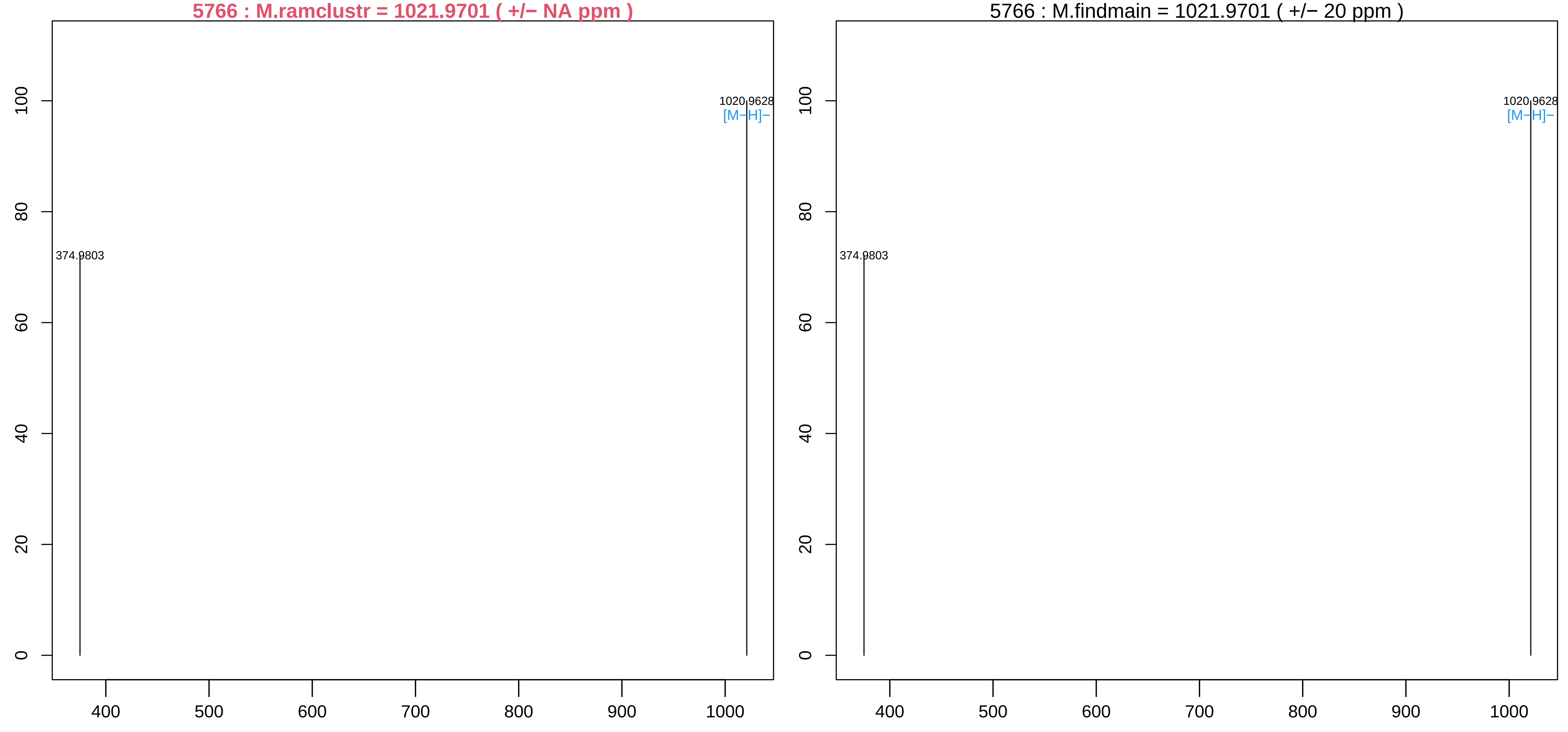

5766 : M.ramclustr = 1021.9701 (+/− NA ppm)
5766 : M.findmain = 1021.9701 (+/− 20 ppm)
1020.9628
[M−H]−
374.9803
1020.9628
[M−H]−
374.9803

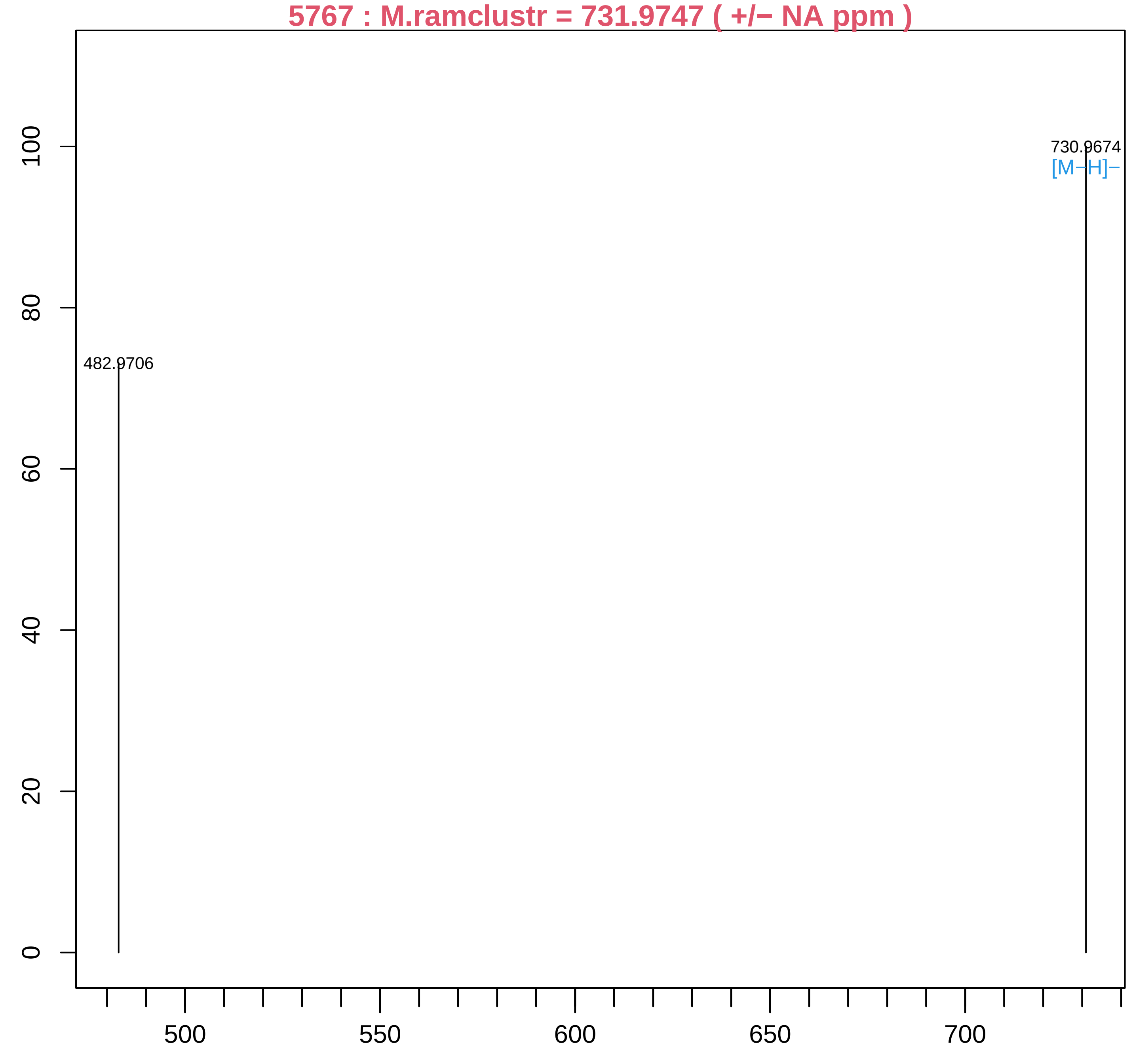
5767 : M.ramclustr = 731.9747 (+/− NA ppm)
482.9706
730.9674
[M−H]−
0
20
40
60
80
100
500
550
600
650
700

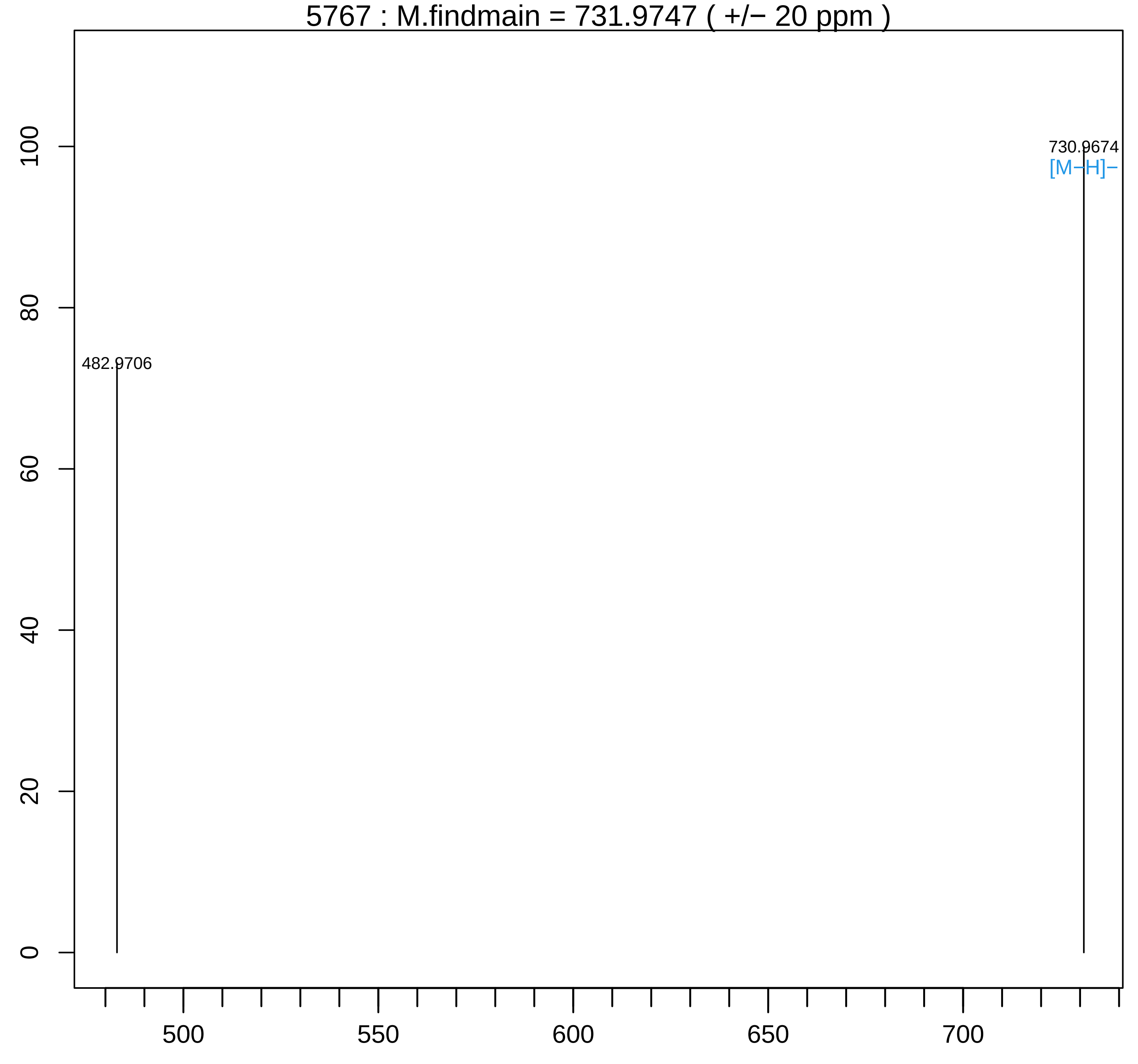
5767 : M.findmain = 731.9747 (+/− 20 ppm)
482.9706
730.9674
[M−H]−
0
20
40
60
80
100
500
550
600
650
700

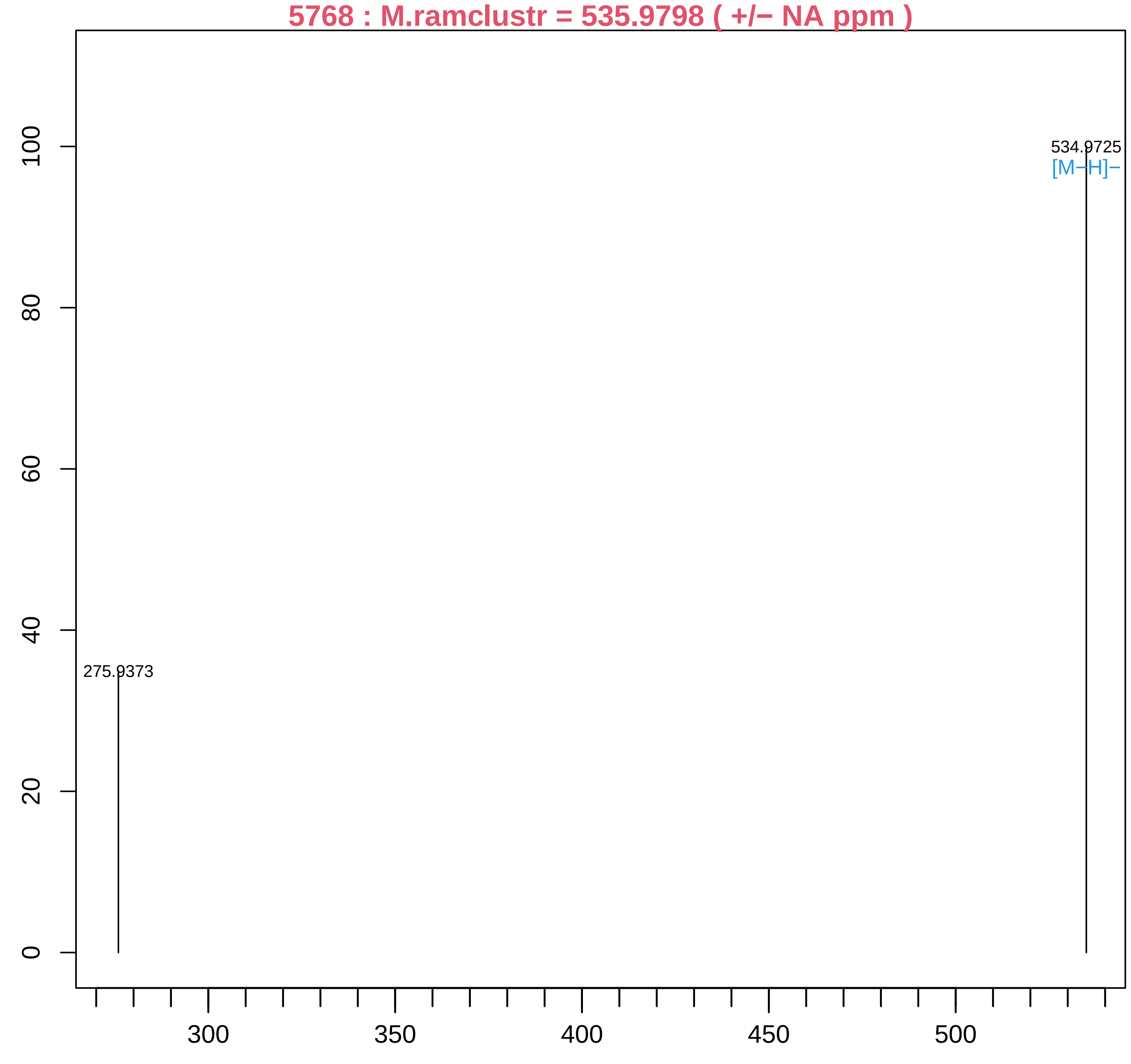
5768 : M.ramclustr = 535.9798 (+/− NA ppm)
534.9725
[M−H]−
275.9373
0
20
40
60
80
100
300
350
400
450
500

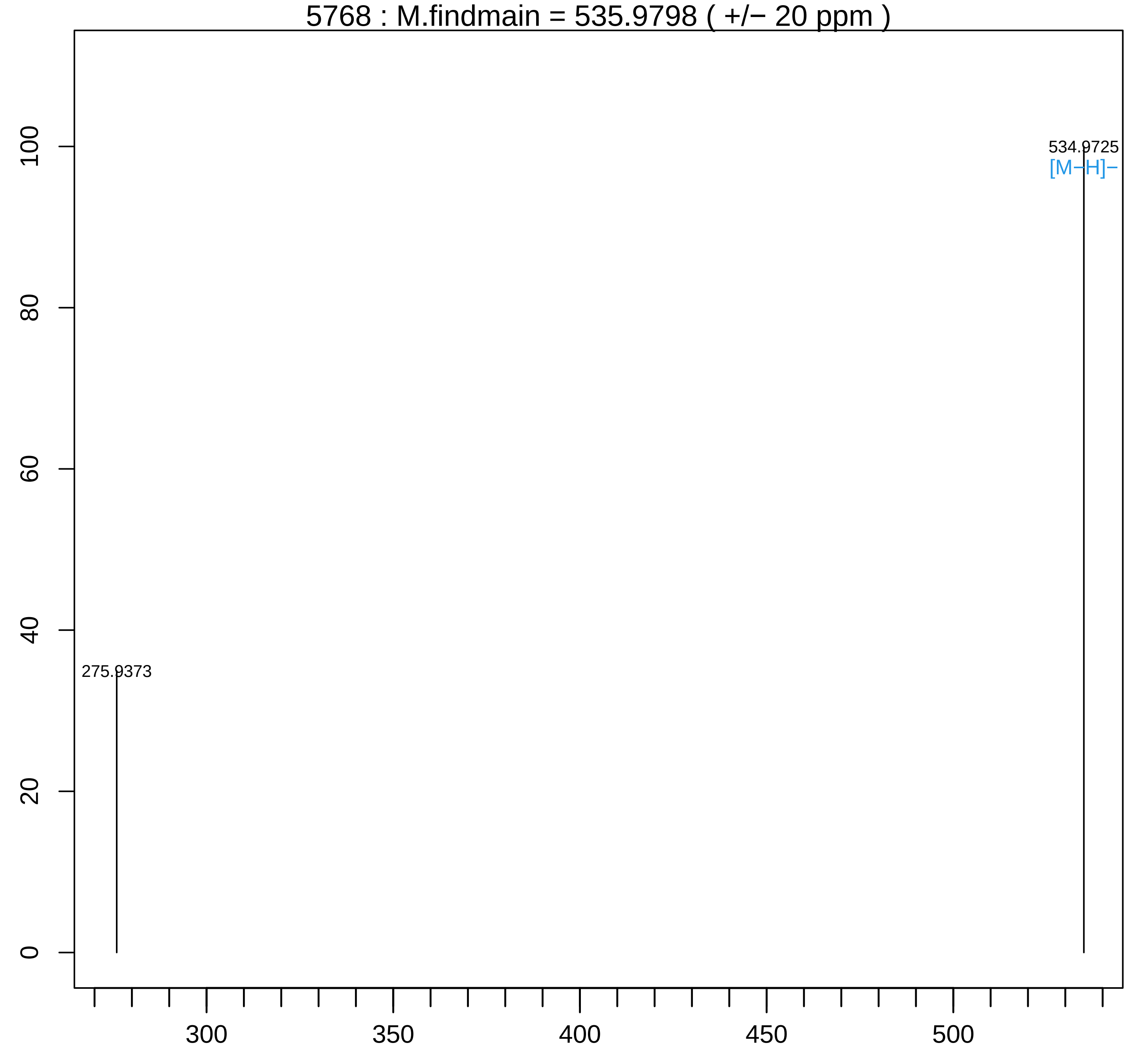
5768 : M.findmain = 535.9798 (+/− 20 ppm)
534.9725
[M−H]−
275.9373
0
20
40
60
80
100
300
350
400
450
500

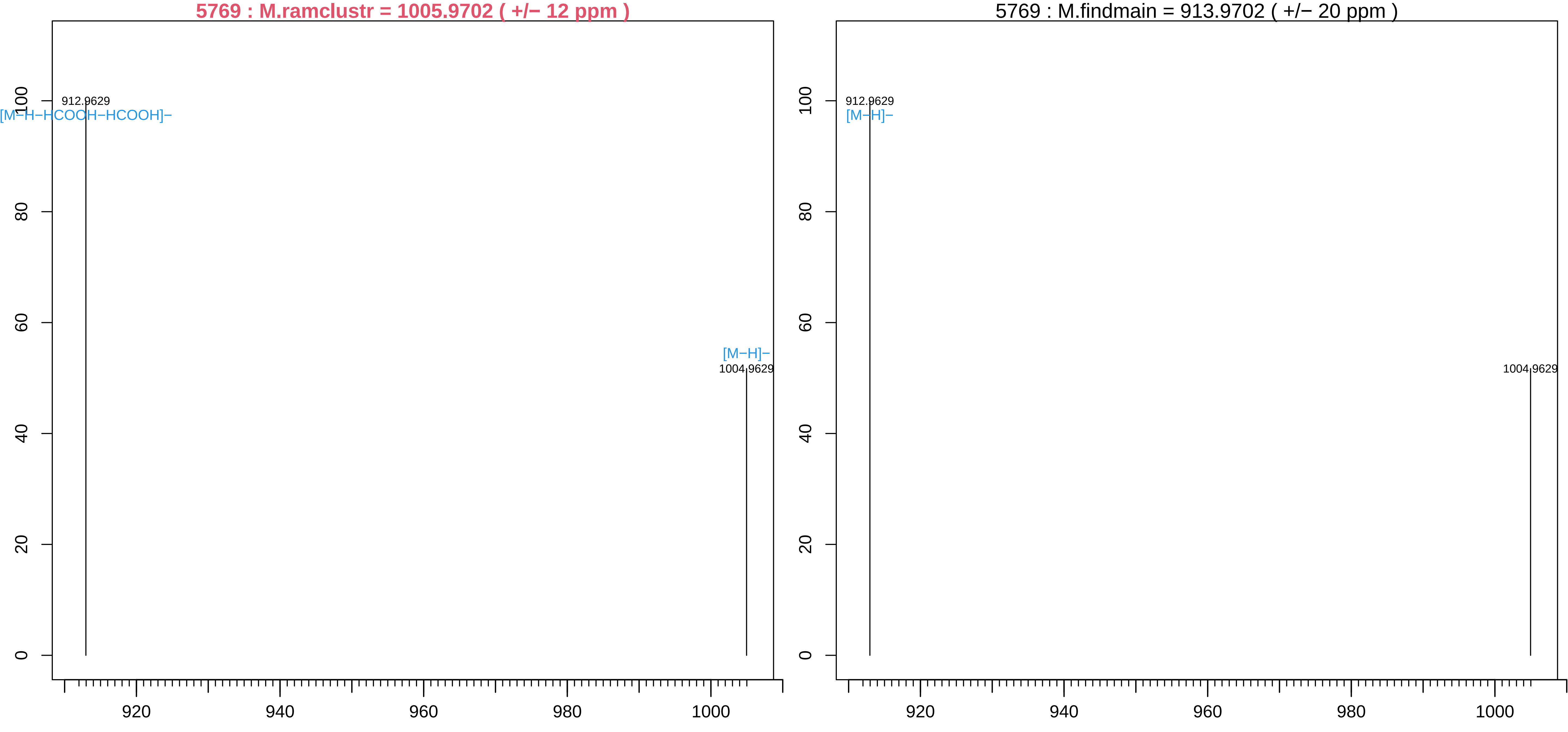
5769 : M.ramclustr = 1005.9702 (+/− 12 ppm)
912.9629
[M−H−HCOOH−HCOOH]−
[M−H]−
1004.9629
5769 : M.findmain = 913.9702 (+/− 20 ppm)
912.9629
[M−H]−
1004.9629
0
20
40
60
80
100
920
940
960
980
1000

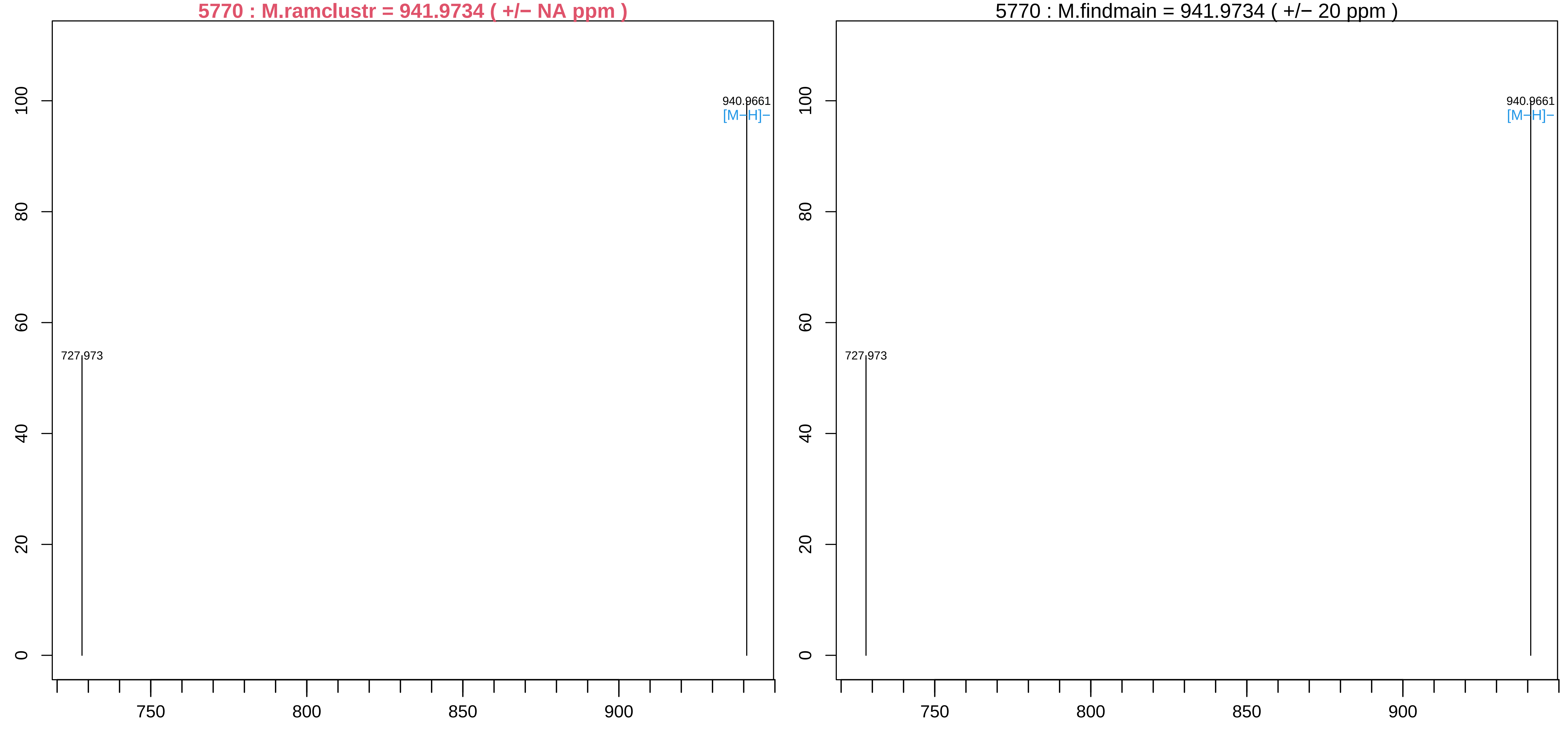
5770 : M.ramclustr = 941.9734 (+/− NA ppm)
5770 : M.findmain = 941.9734 (+/− 20 ppm)
940.9661
[M−H]−
727.973
0
20
40
60
80
100
750
800
850
900

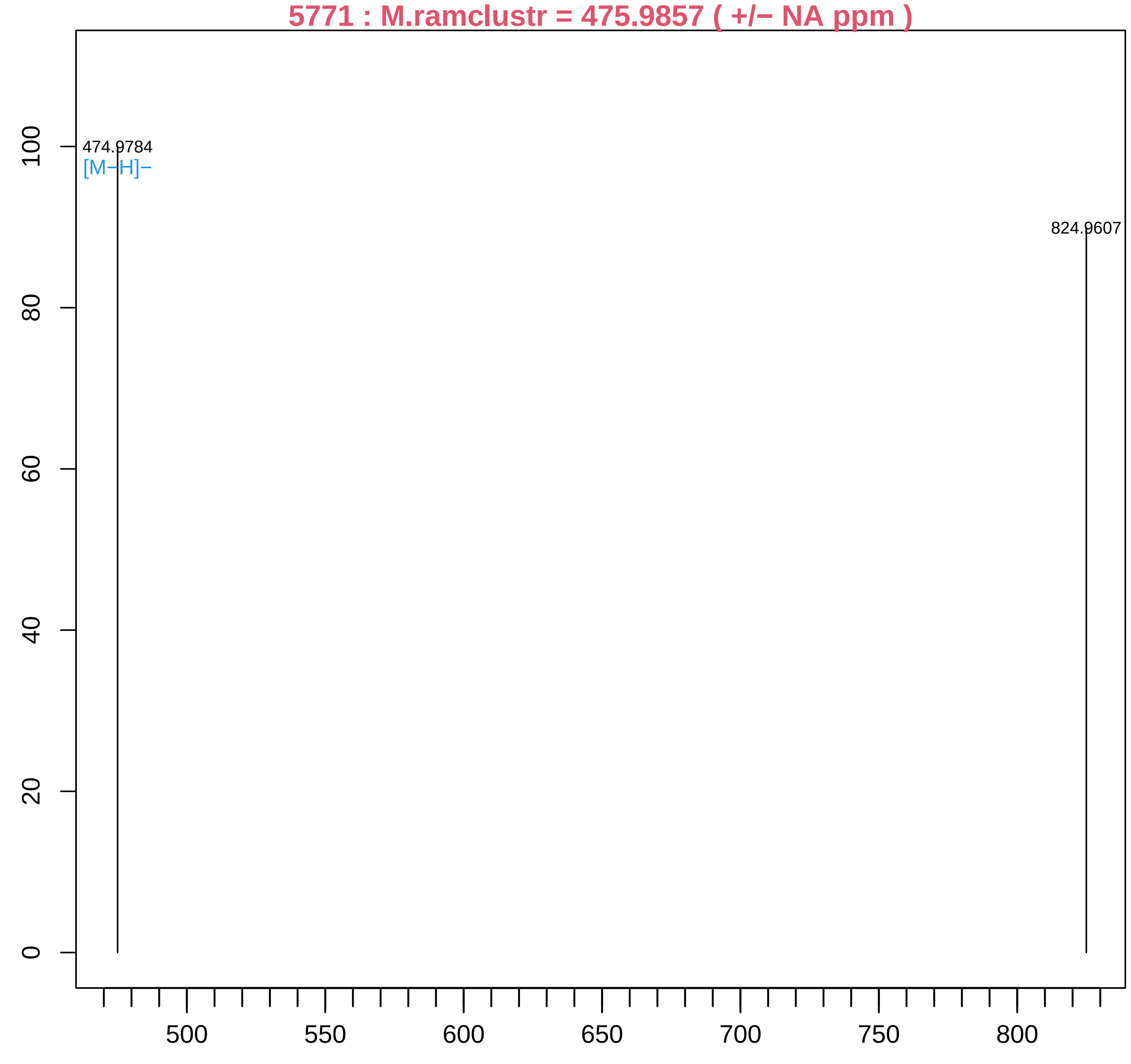
5771 : M.ramclustr = 475.9857 (+/− NA ppm)
474.9784
[M−H]−
824.9607
0
20
40
60
80
100
500
550
600
650
700
750
800
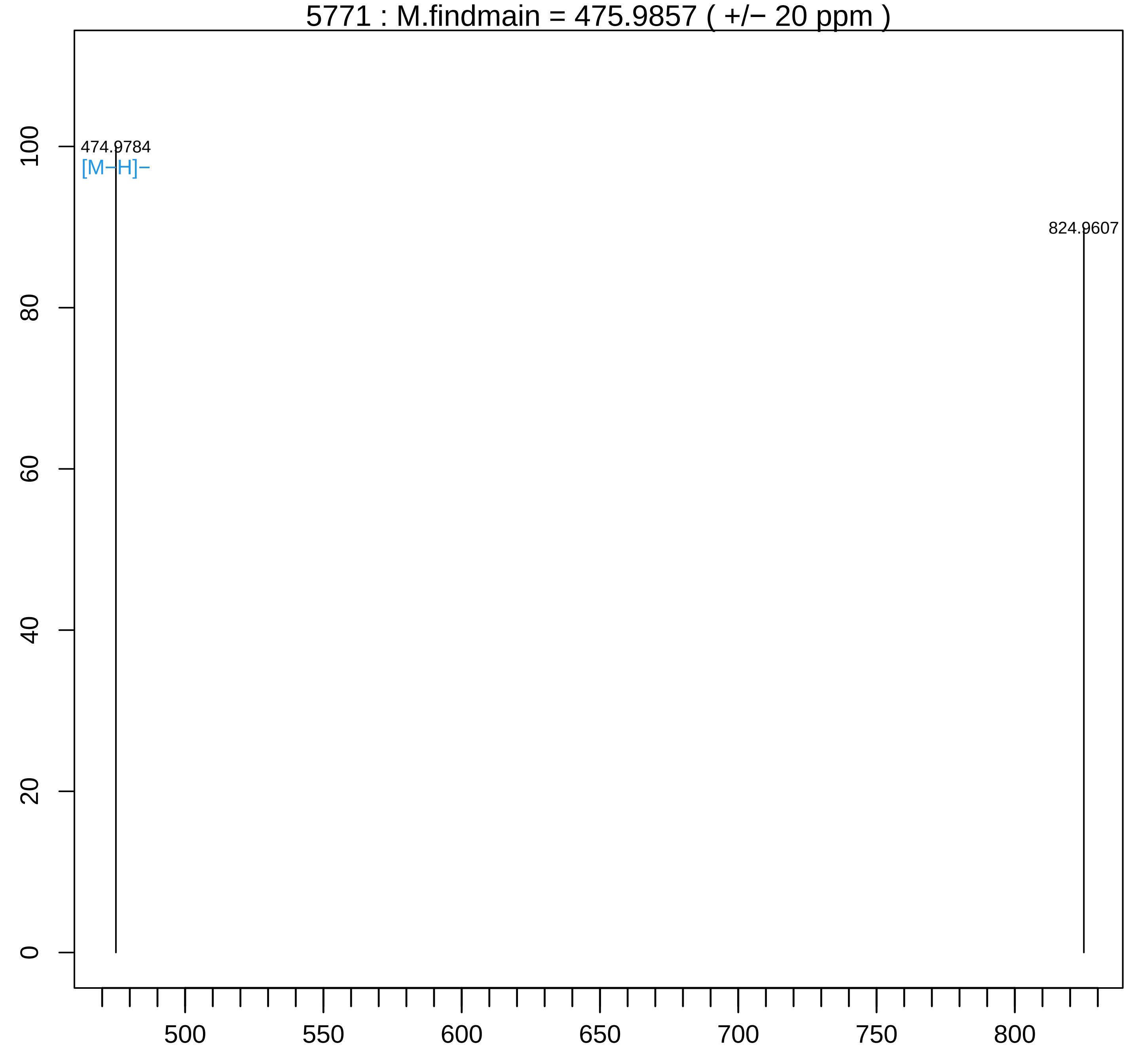
5771 : M.findmain = 475.9857 (+/− 20 ppm)
474.9784
[M−H]−
824.9607
0
20
40
60
80
100
500
550
600
650
700
750
800

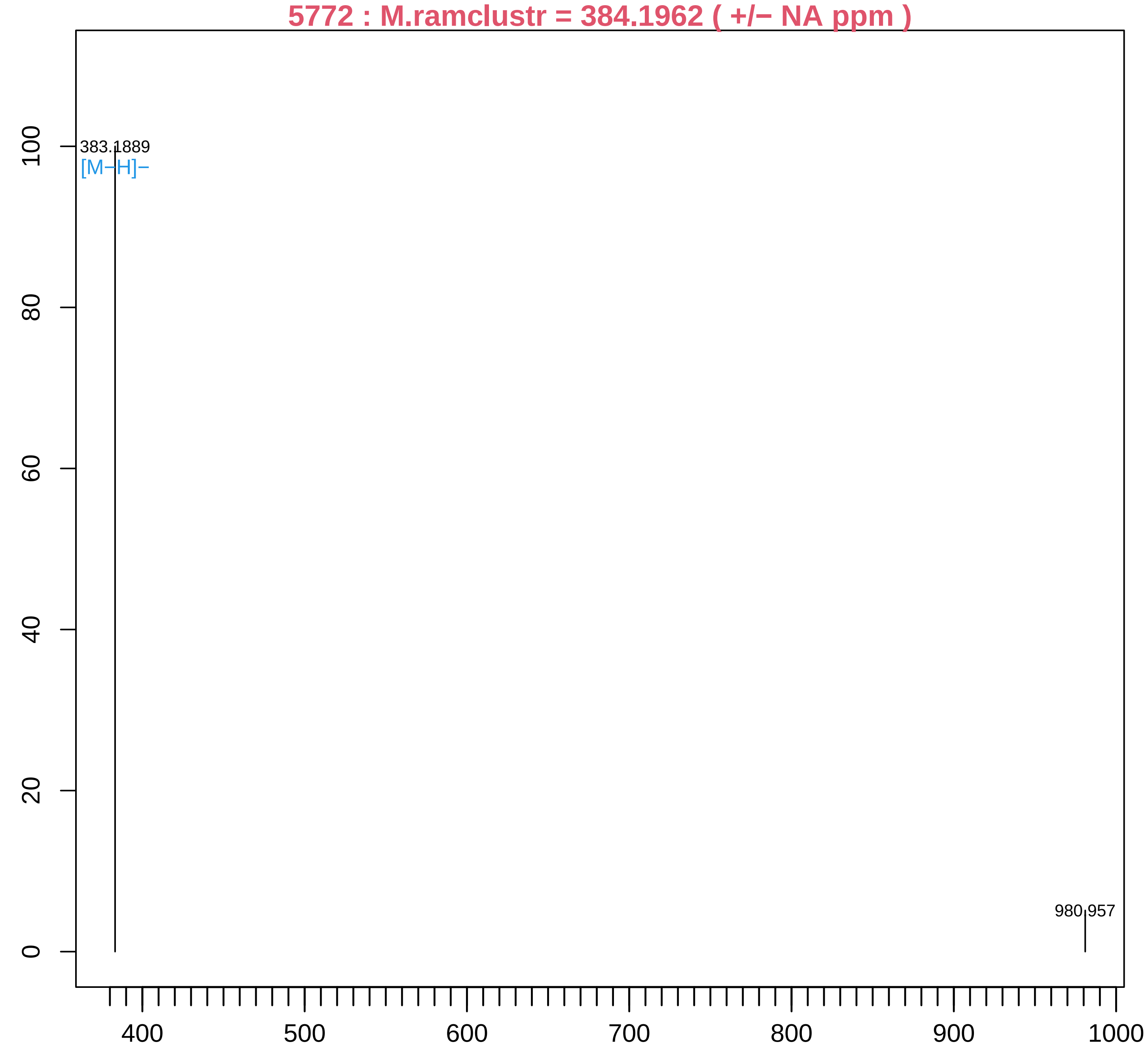
5772 : M.ramclustr = 384.1962 (+/− NA ppm)
383.1889
[M−H]−
980.957
0
20
40
60
80
100
400
500
600
700
800
900
1000

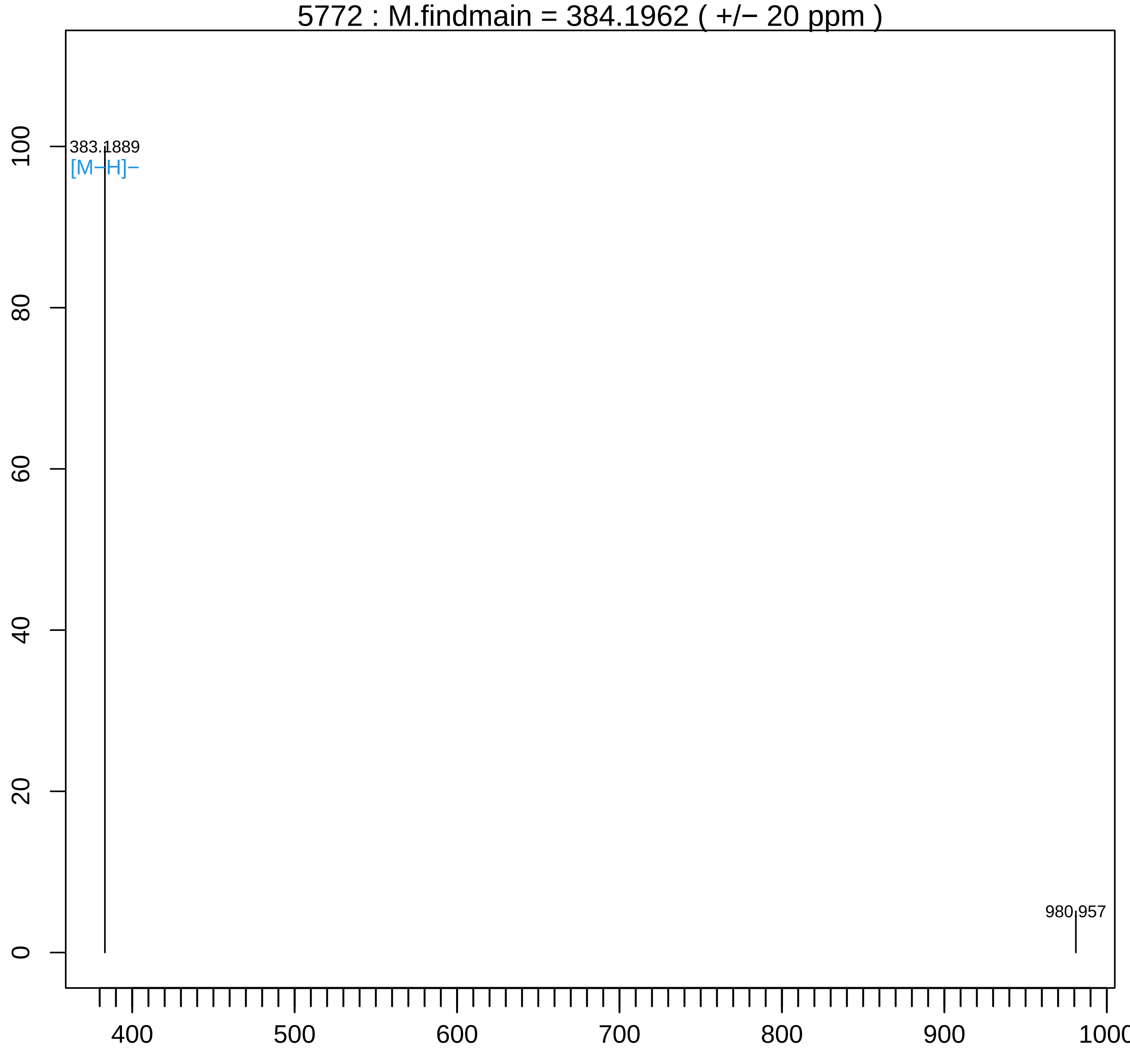
5772 : M.findmain = 384.1962 (+/− 20 ppm)
383.1889
[M−H]−
980.957
0
20
40
60
80
100
400
500
600
700
800
900
1000

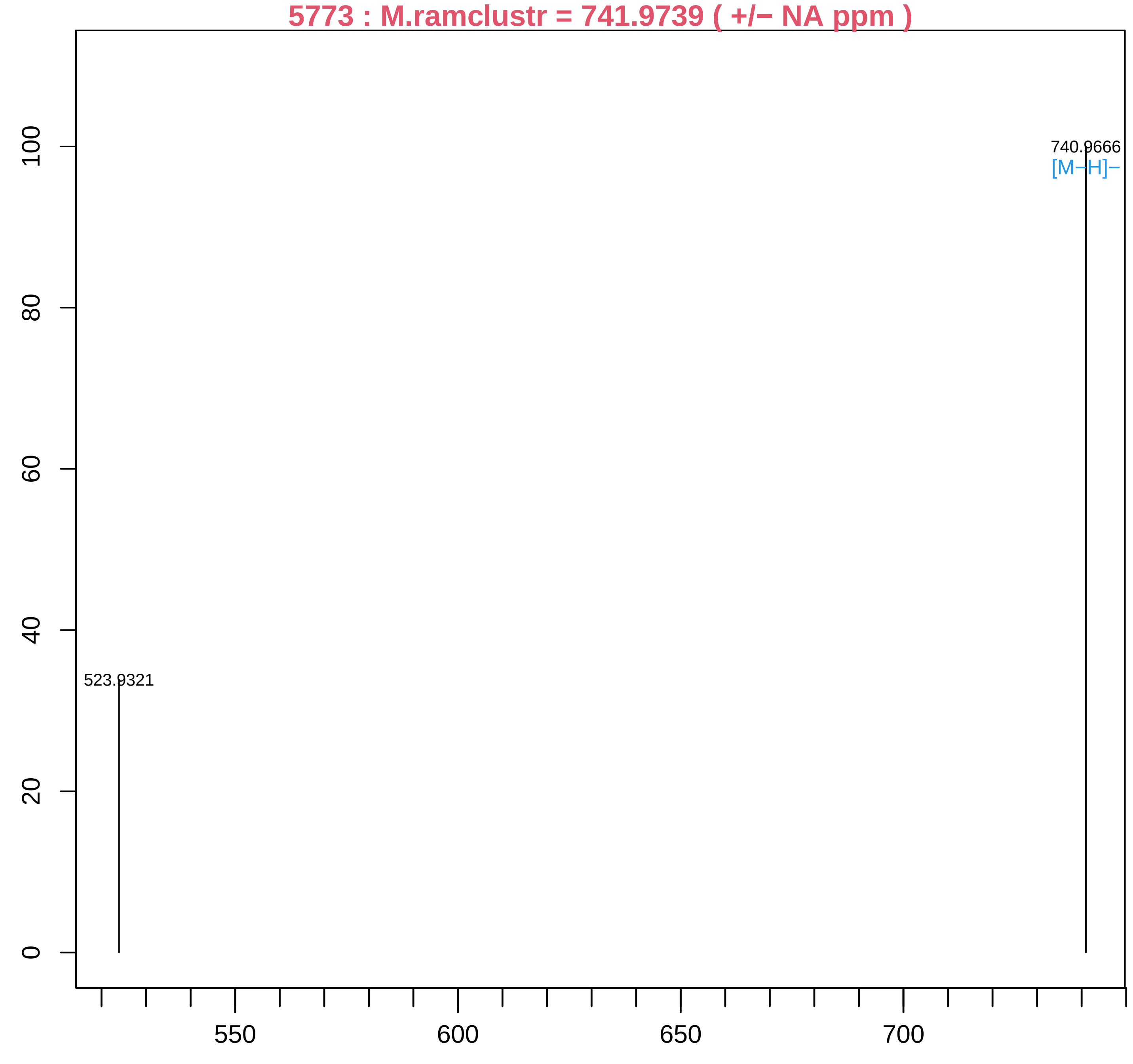
5773 : M.ramclustr = 741.9739 (+/− NA ppm)
740.9666
[M−H]−
523.9321
0
20
40
60
80
100
550
600
650
700

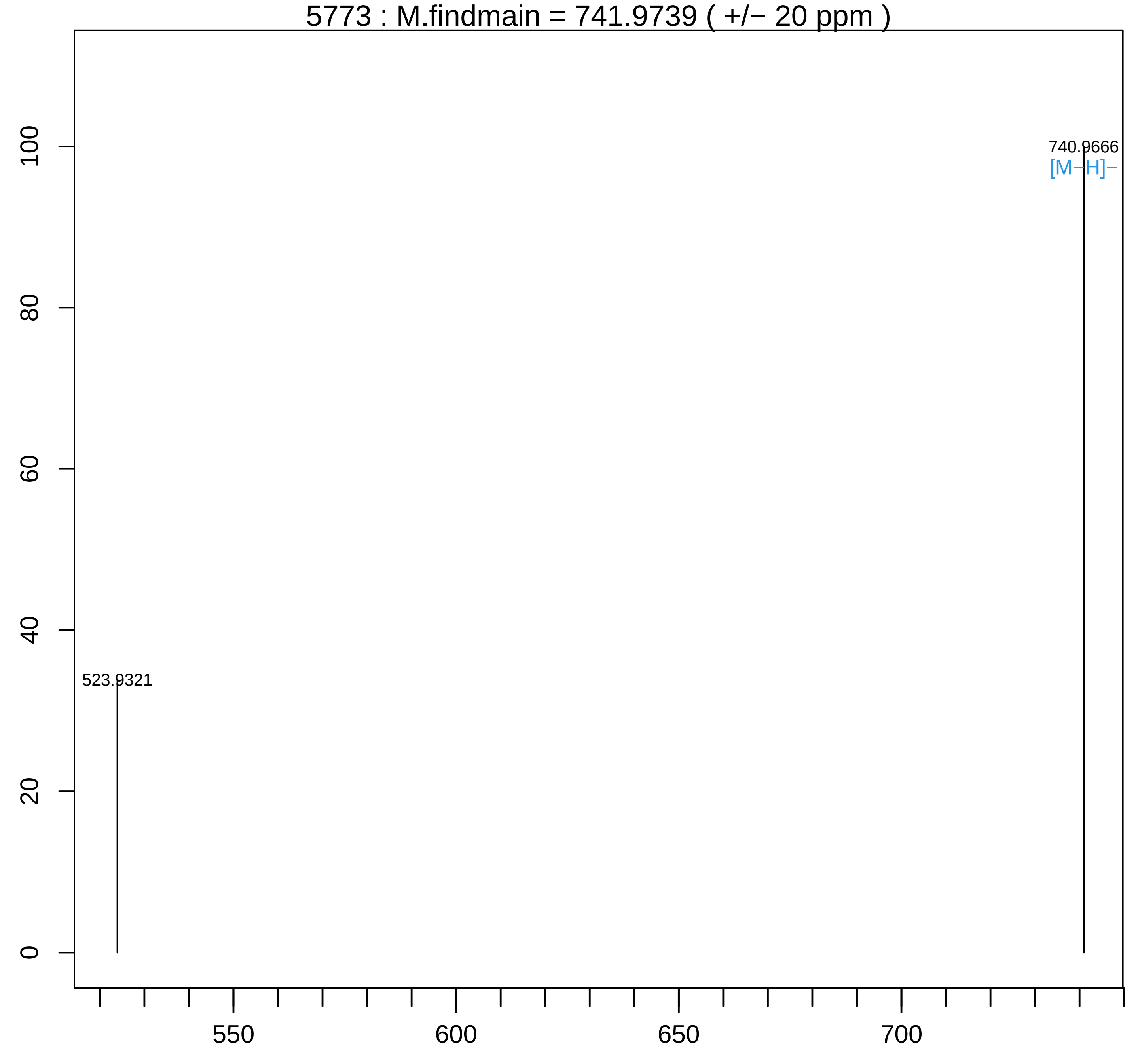
5773 : M.findmain = 741.9739 (+/− 20 ppm)
740.9666
[M−H]−
523.9321
0
20
40
60
80
100
550
600
650
700

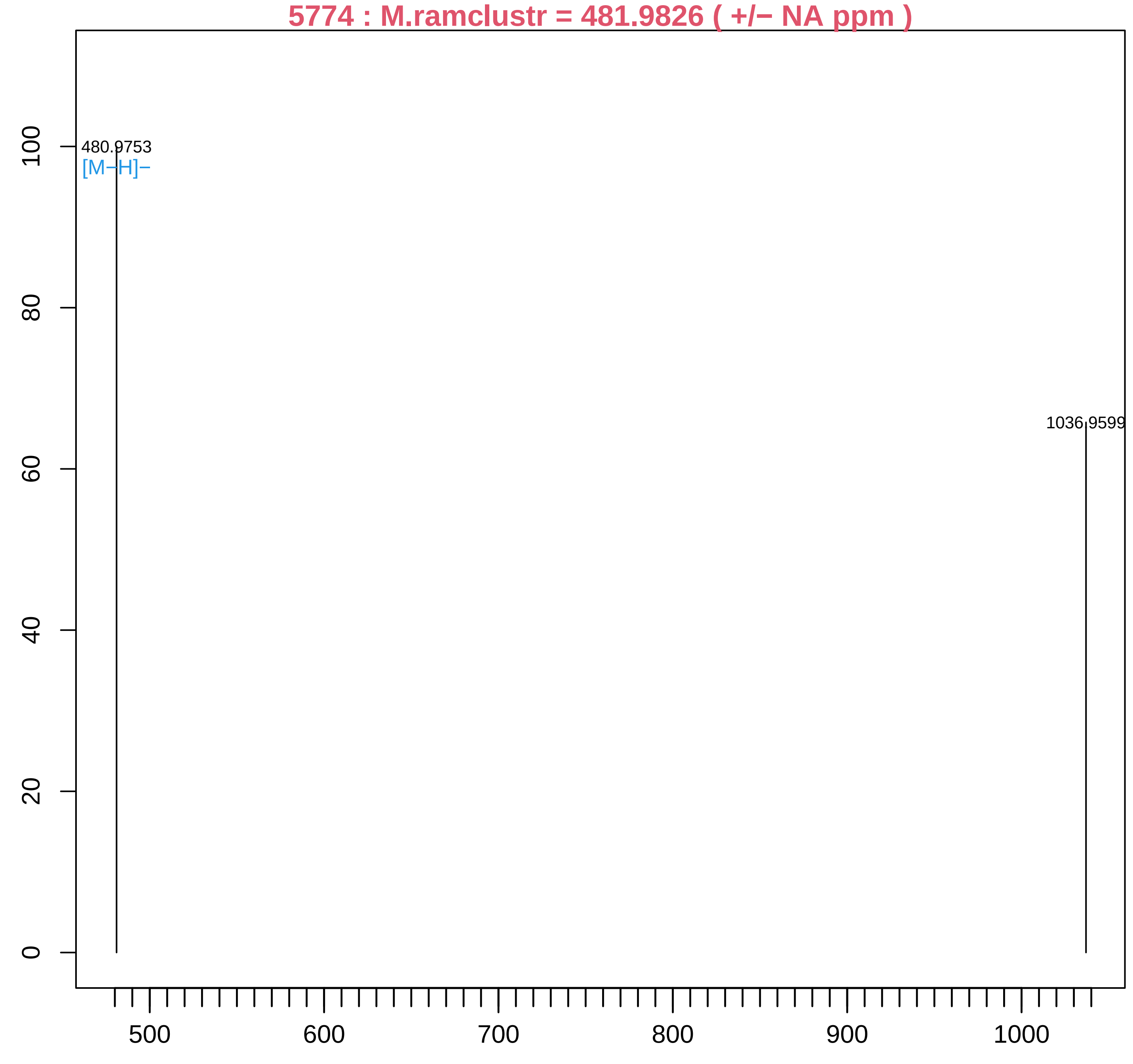
5774 : M.ramclustr = 481.9826 (+/− NA ppm)
480.9753
[M−H]−
1036.9599
0
20
40
60
80
100
500
600
700
800
900
1000
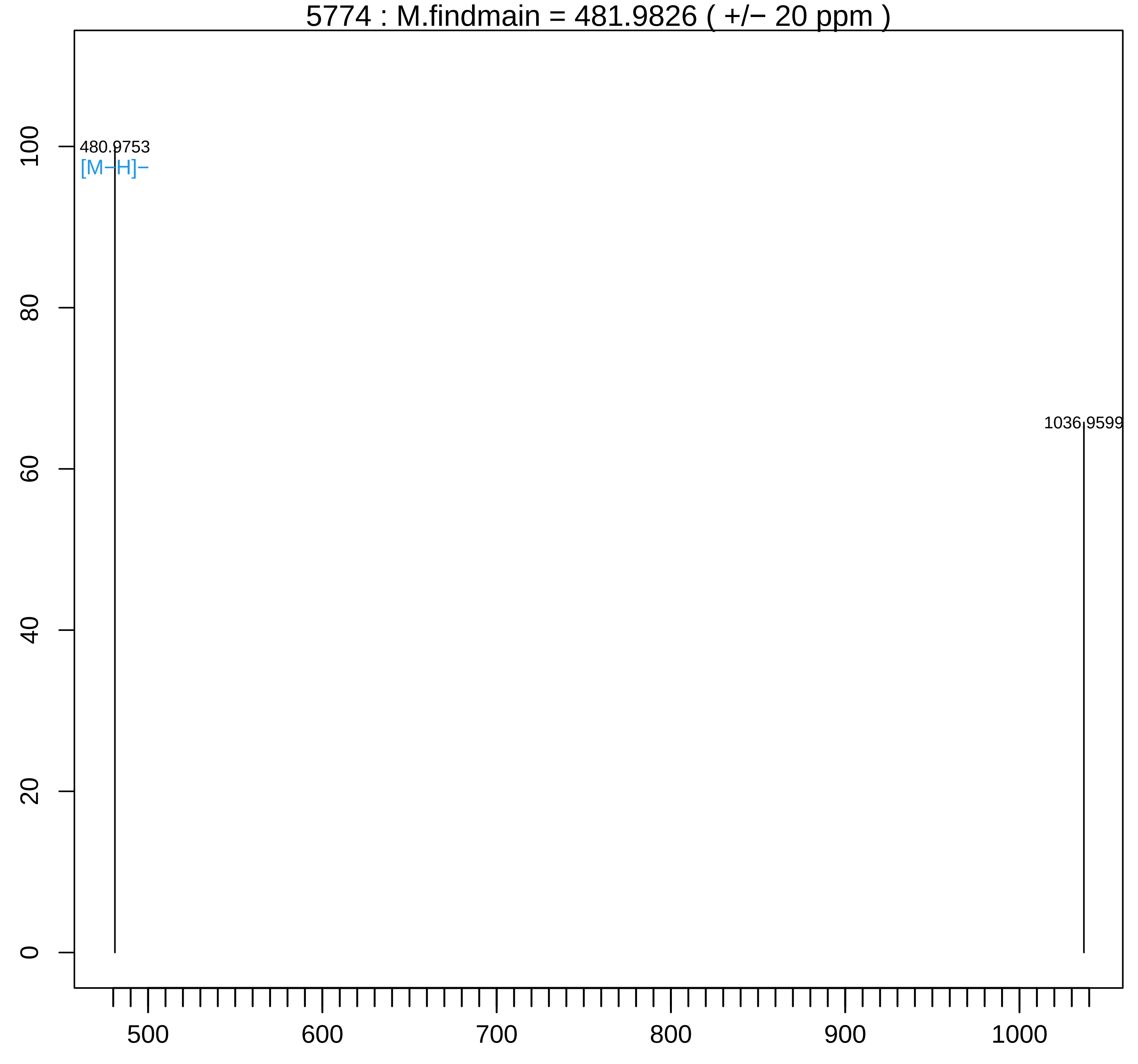
5774 : M.findmain = 481.9826 (+/− 20 ppm)
480.9753
[M−H]−
1036.9599
0
20
40
60
80
100
500
600
700
800
900
1000

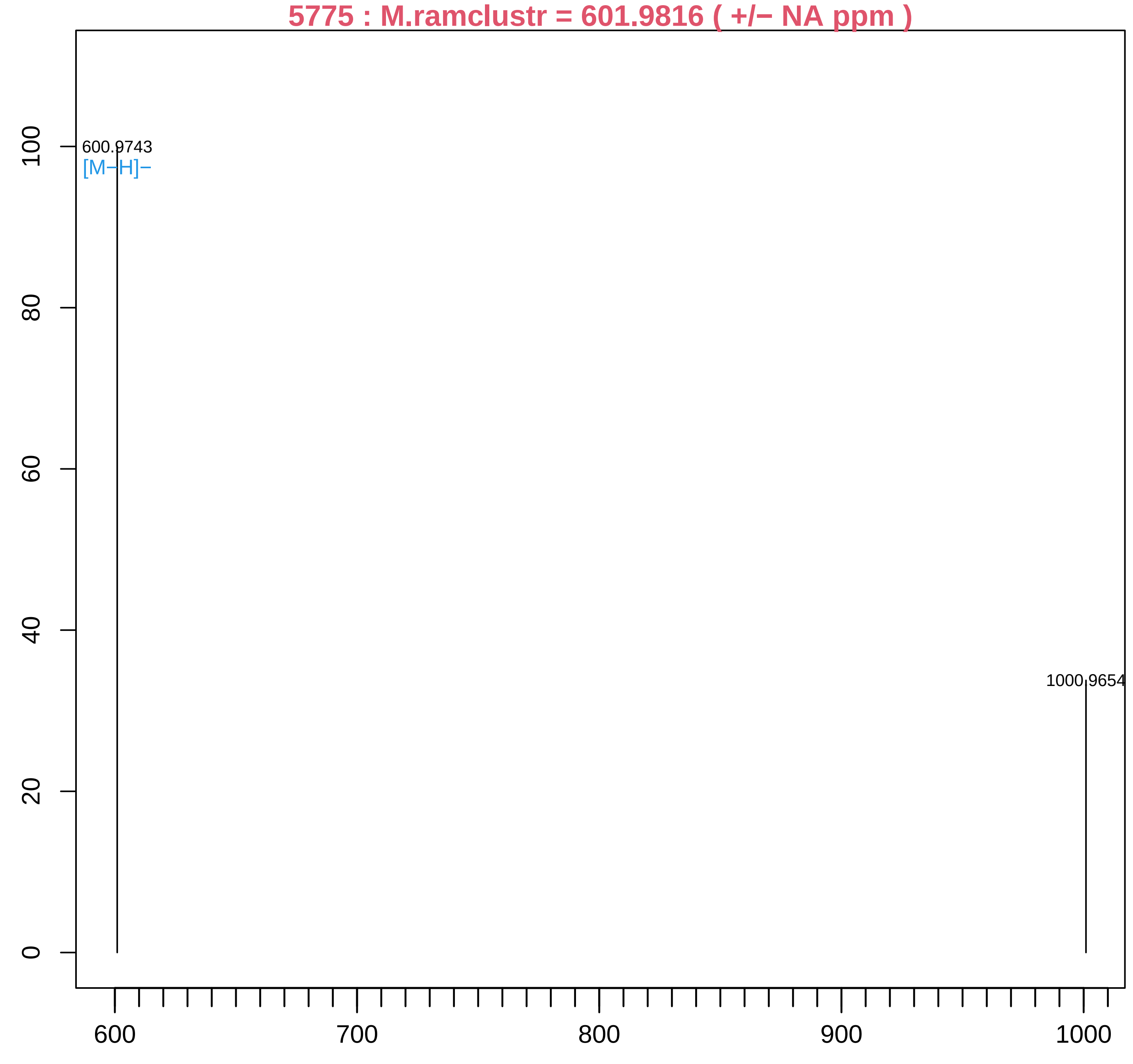
5775 : M.ramclustr = 601.9816 (+/− NA ppm)
600.9743
[M−H]−
1000.9654
0
20
40
60
80
100
600
700
800
900
1000

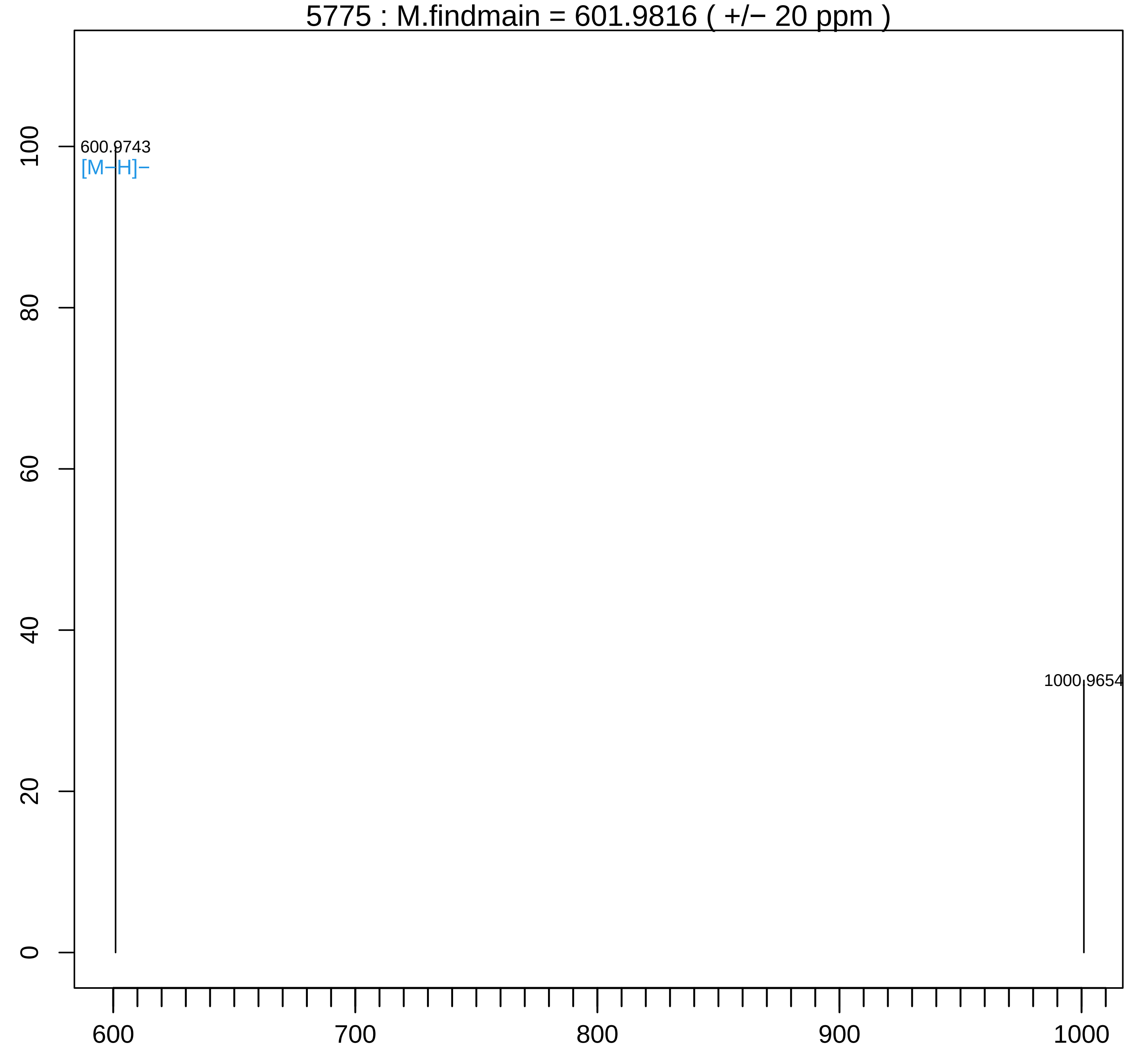
5775 : M.findmain = 601.9816 (+/− 20 ppm)
600.9743
[M−H]−
1000.9654
0
20
40
60
80
100
600
700
800
900
1000

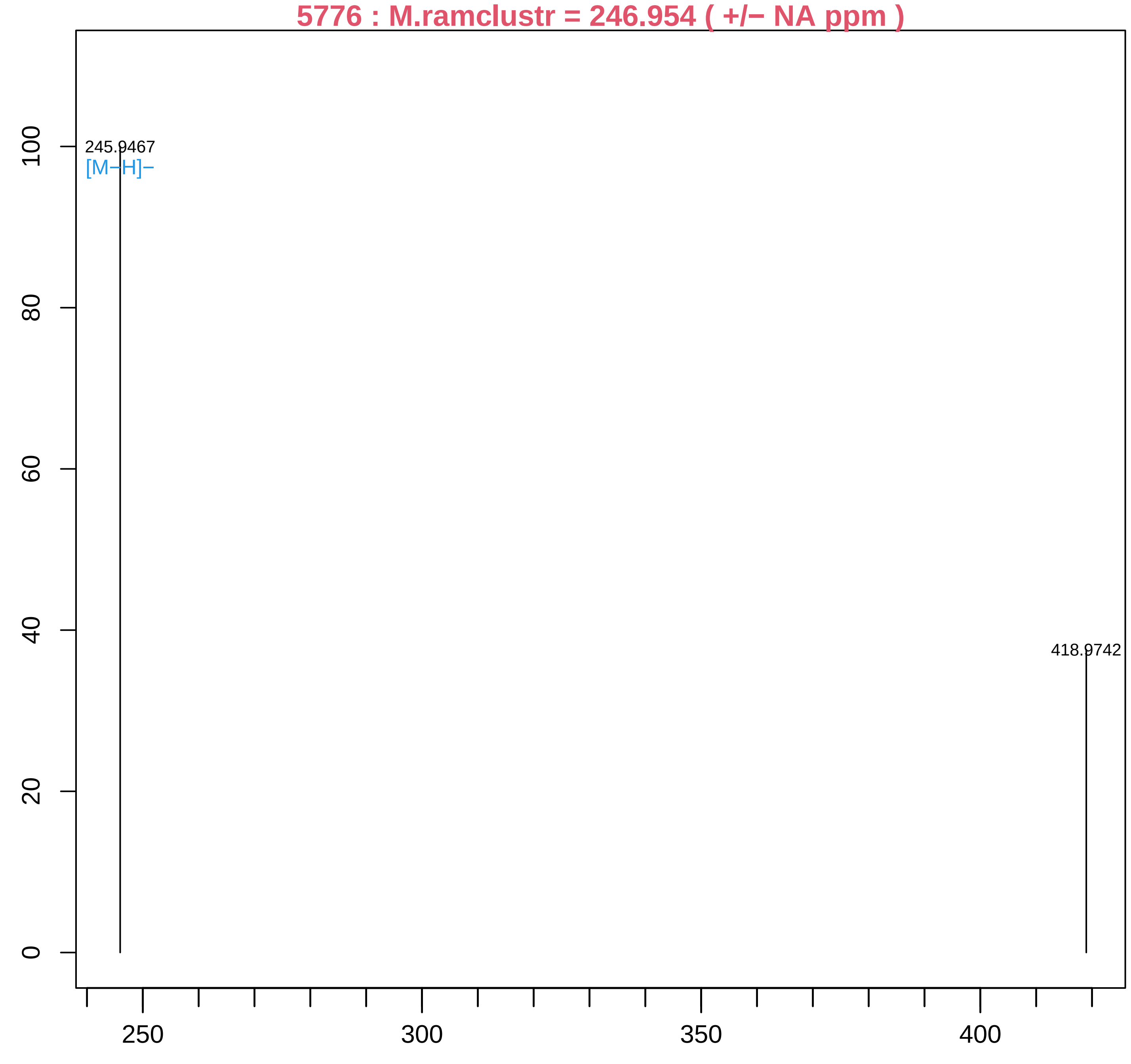

5776 : M.ramclustr = 246.954 (+/− NA ppm)
245.9467
[M−H]−
418.9742
0
20
40
60
80
100
250
300
350
400

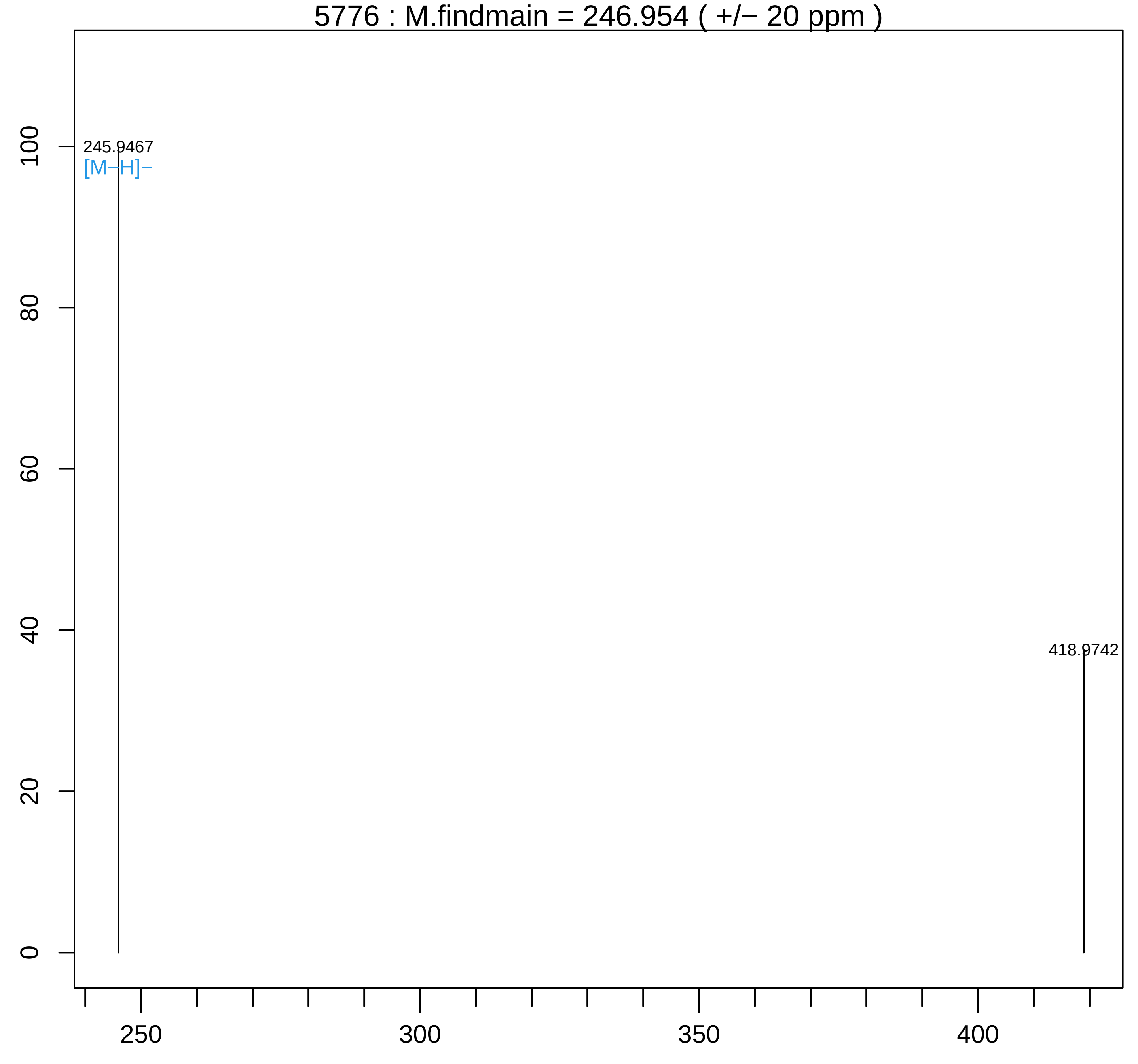

5776 : M.findmain = 246.954 (+/− 20 ppm)
245.9467
[M−H]−
418.9742
0
20
40
60
80
100
250
300
350
400

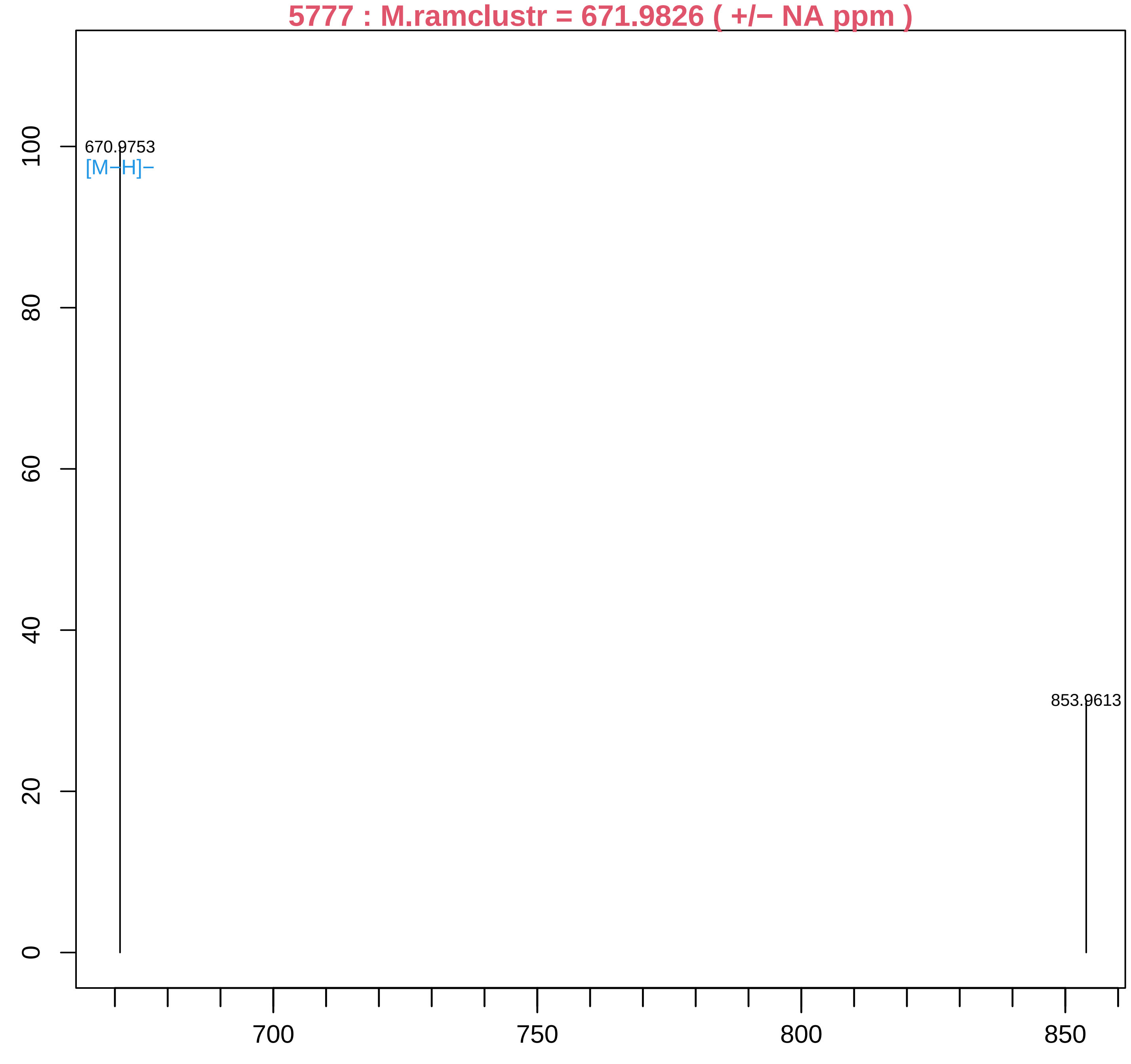
5777 : M.ramclustr = 671.9826 (+/− NA ppm)
670.9753
[M−H]−
853.9613
0
20
40
60
80
100
700
750
800
850

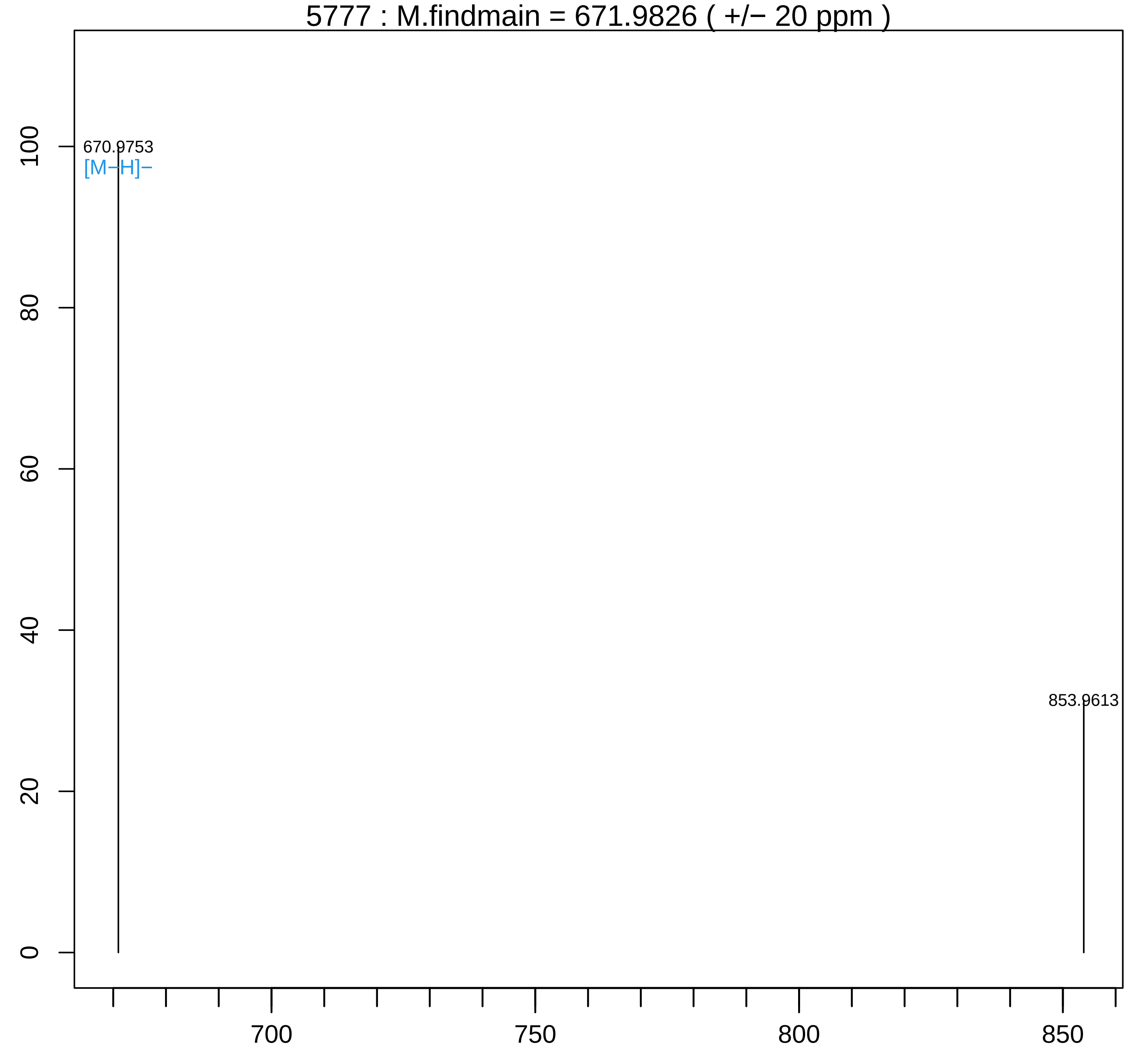
5777 : M.findmain = 671.9826 (+/− 20 ppm)
670.9753
[M−H]−
853.9613
0
20
40
60
80
100
700
750
800
850

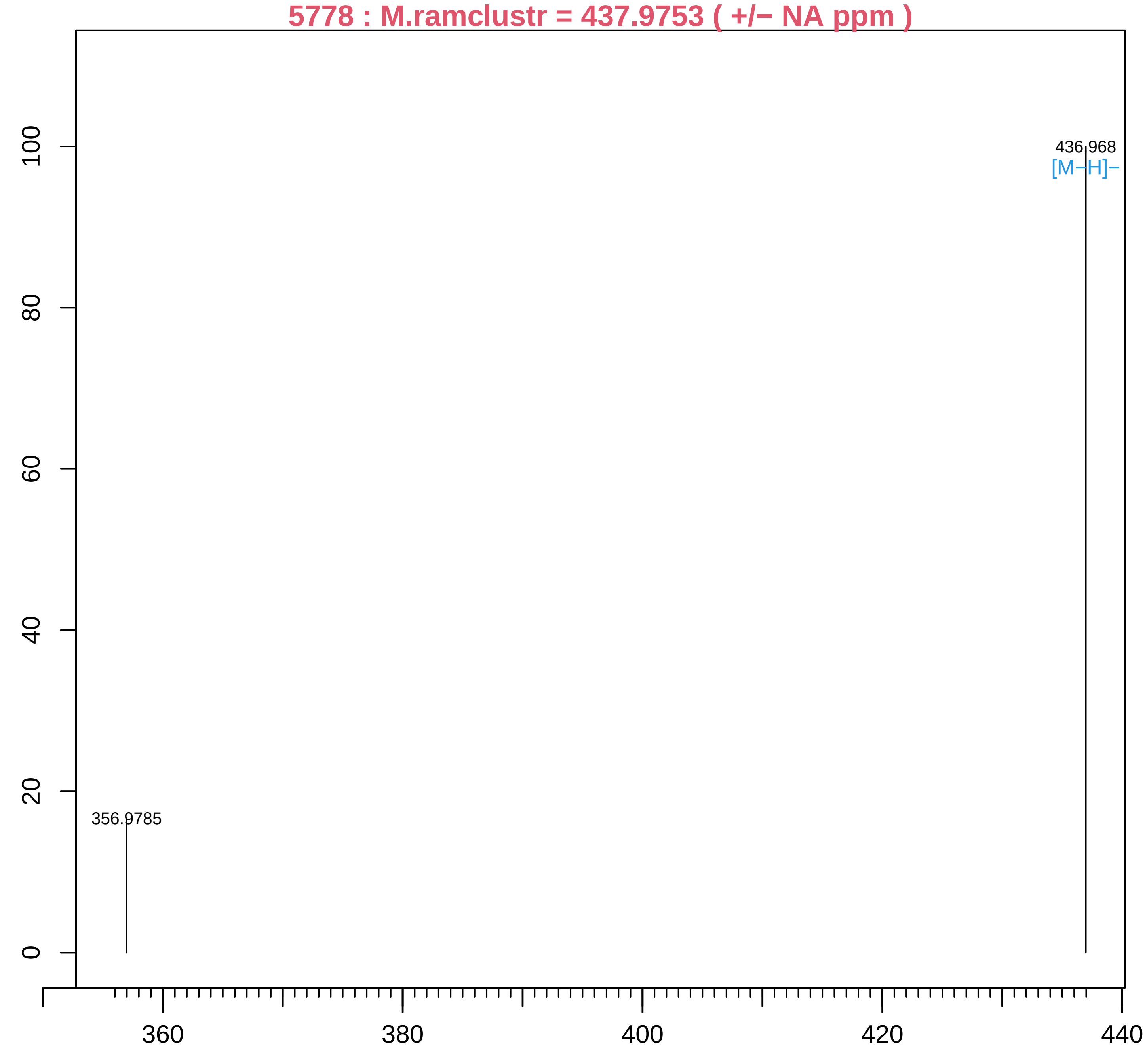
5778 : M.ramclustr = 437.9753 (+/− NA ppm)
436.968
[M−H]−
356.9785
0
20
40
60
80
100
360
380
400
420
440

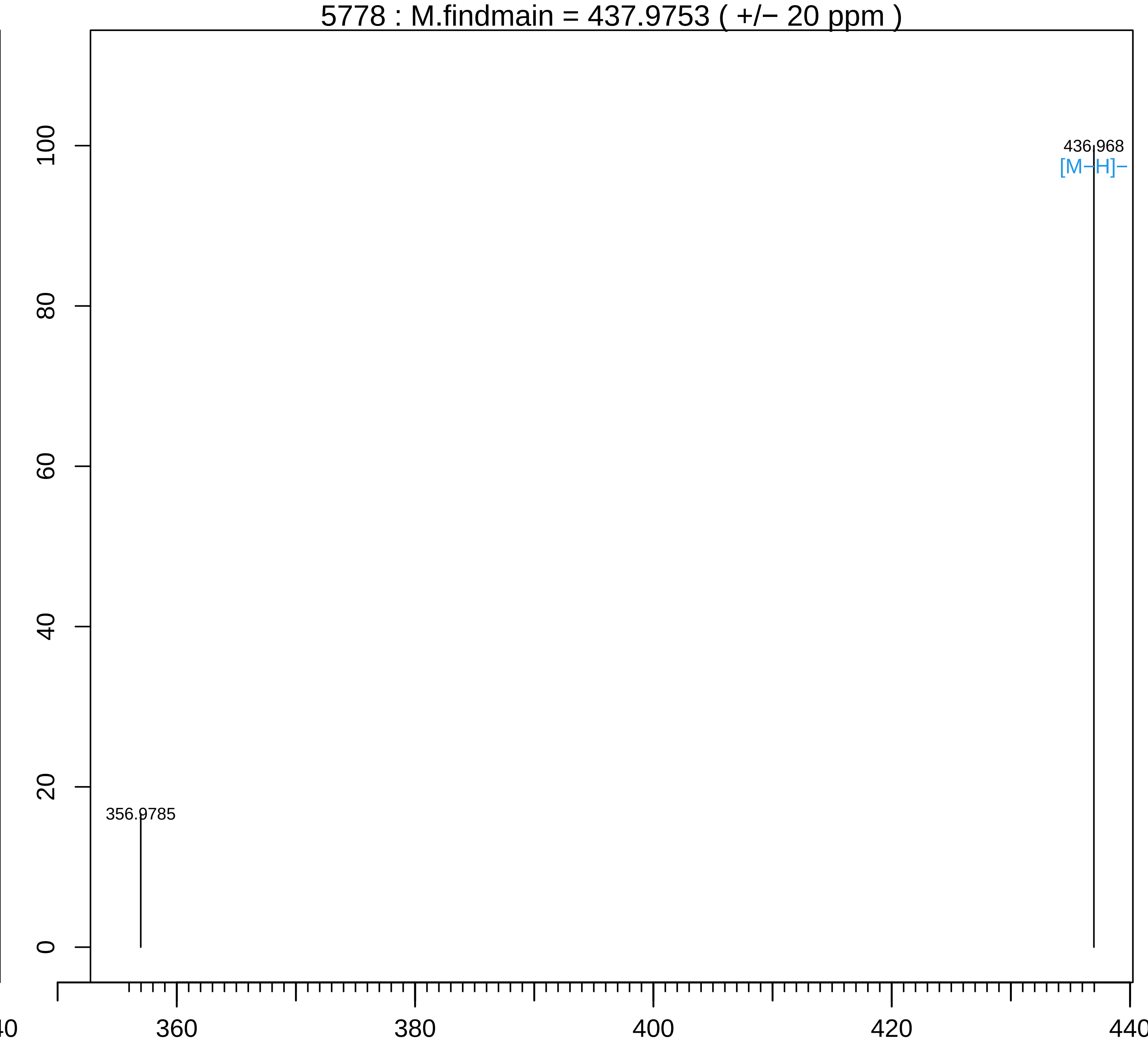
5778 : M.findmain = 437.9753 (+/− 20 ppm)
436.968
[M−H]−
356.9785
0
20
40
60
80
100
360
380
400
420
440

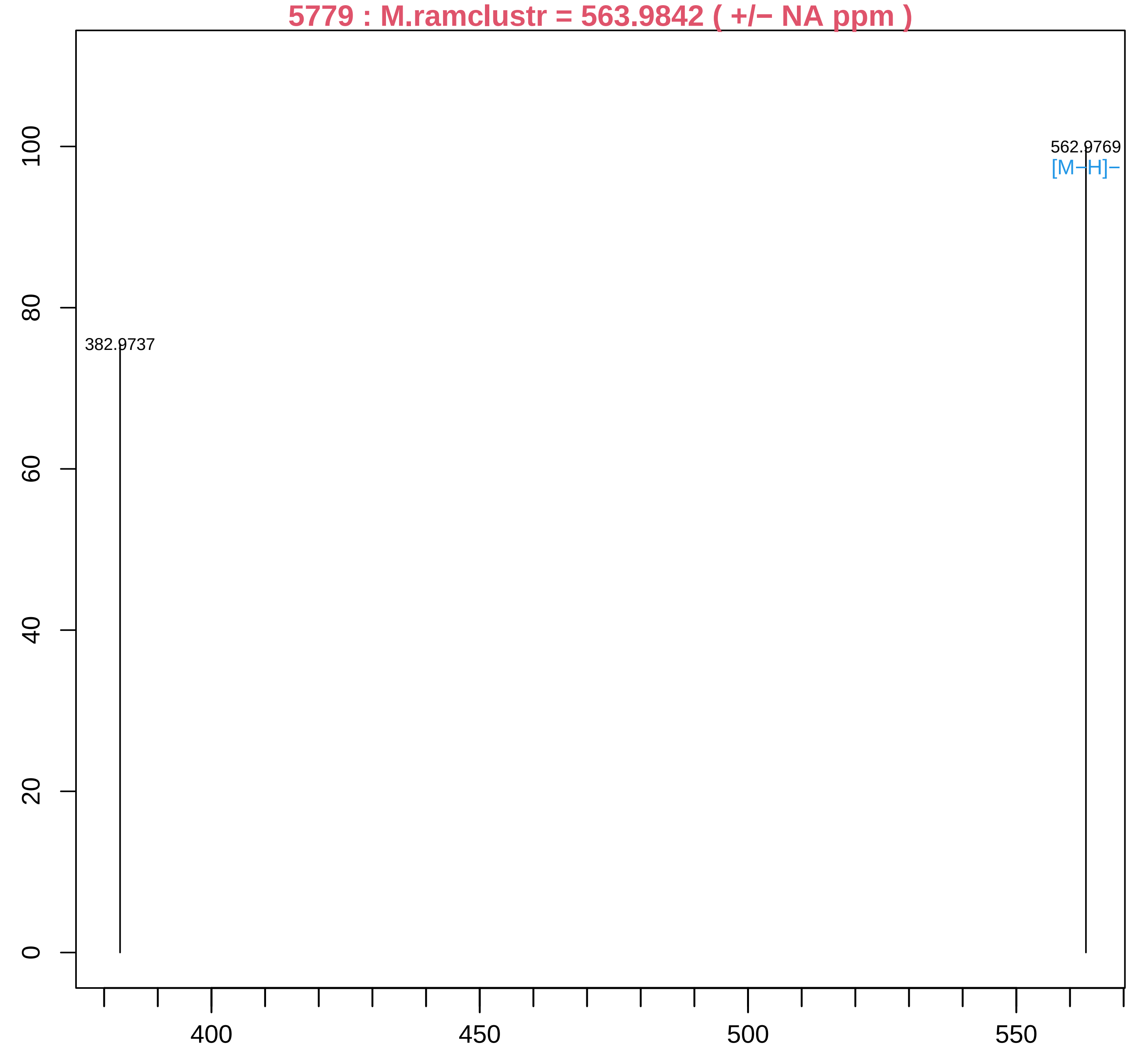

5779 : M.ramclustr = 563.9842 (+/− NA ppm)
562.9769
[M−H]−
382.9737
0
20
40
60
80
100
400
450
500
550

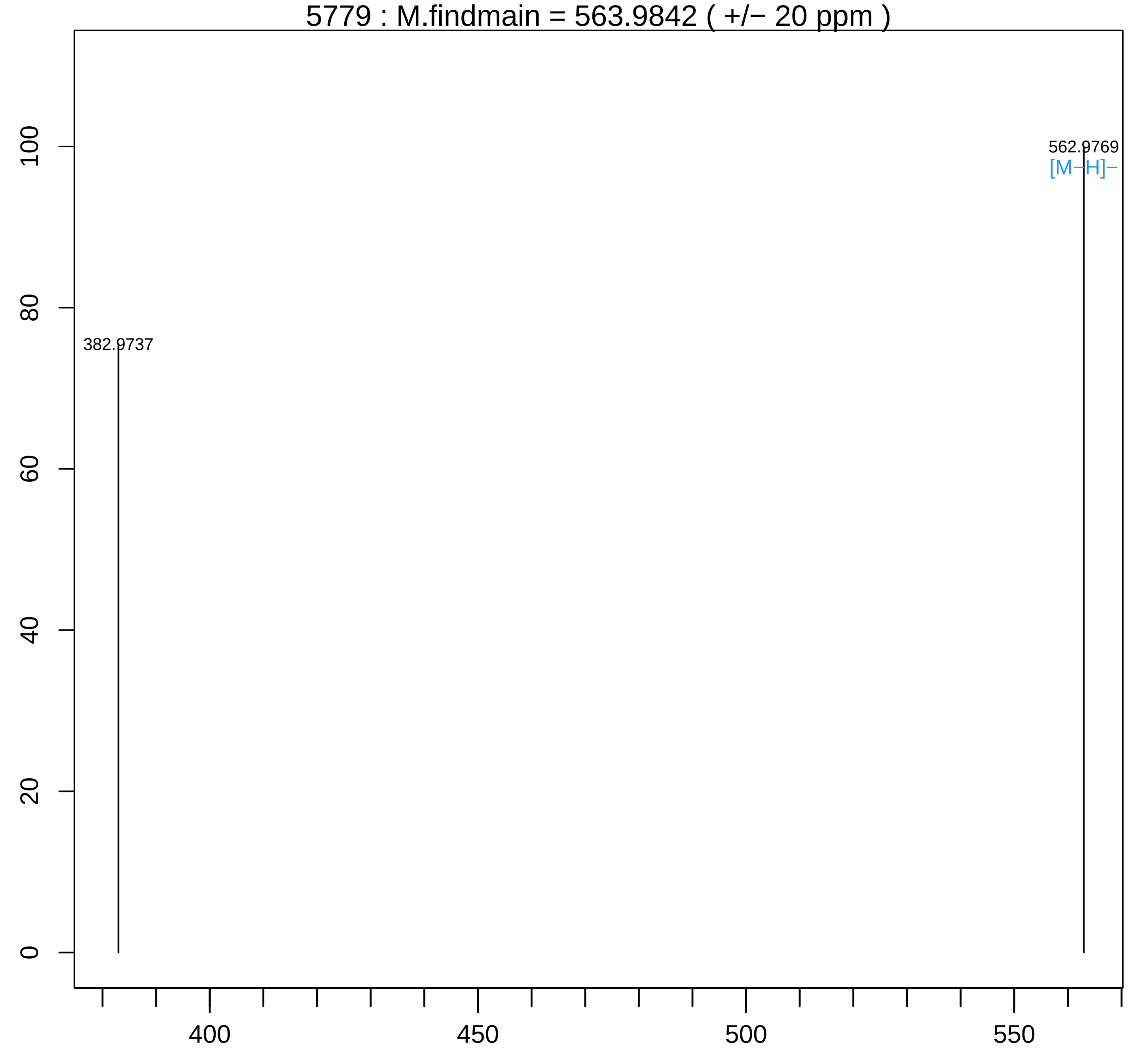

5779 : M.findmain = 563.9842 (+/− 20 ppm)
562.9769
[M−H]−
382.9737
0
20
40
60
80
100
400
450
500
550

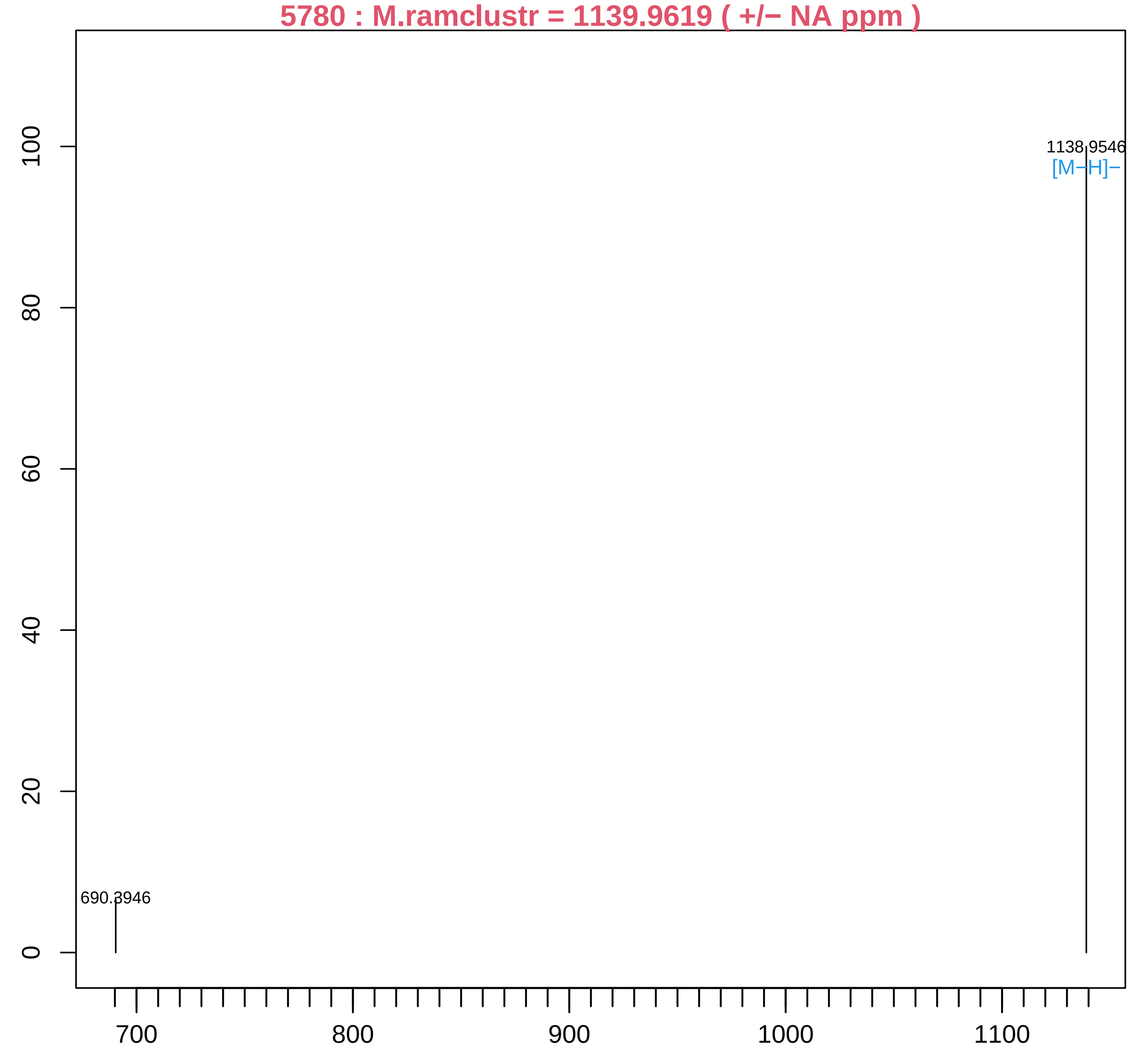
5780 : M.ramclustr = 1139.9619 (+/− NA ppm)
1138.9546
[M−H]−
690.3946
0
20
40
60
80
100
700
800
900
1000
1100

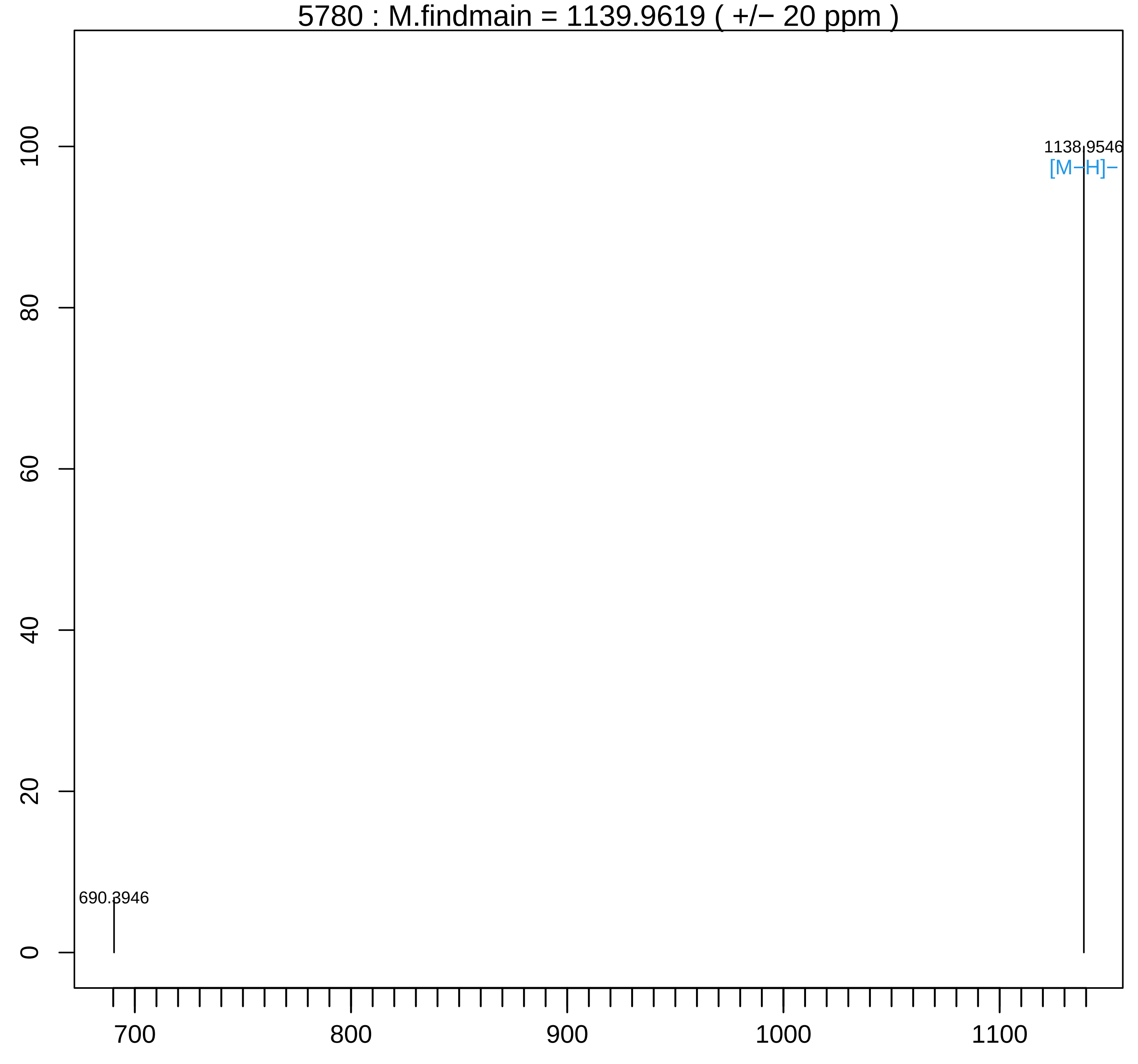
5780 : M.findmain = 1139.9619 (+/− 20 ppm)
1138.9546
[M−H]−
690.3946
0
20
40
60
80
100
700
800
900
1000
1100

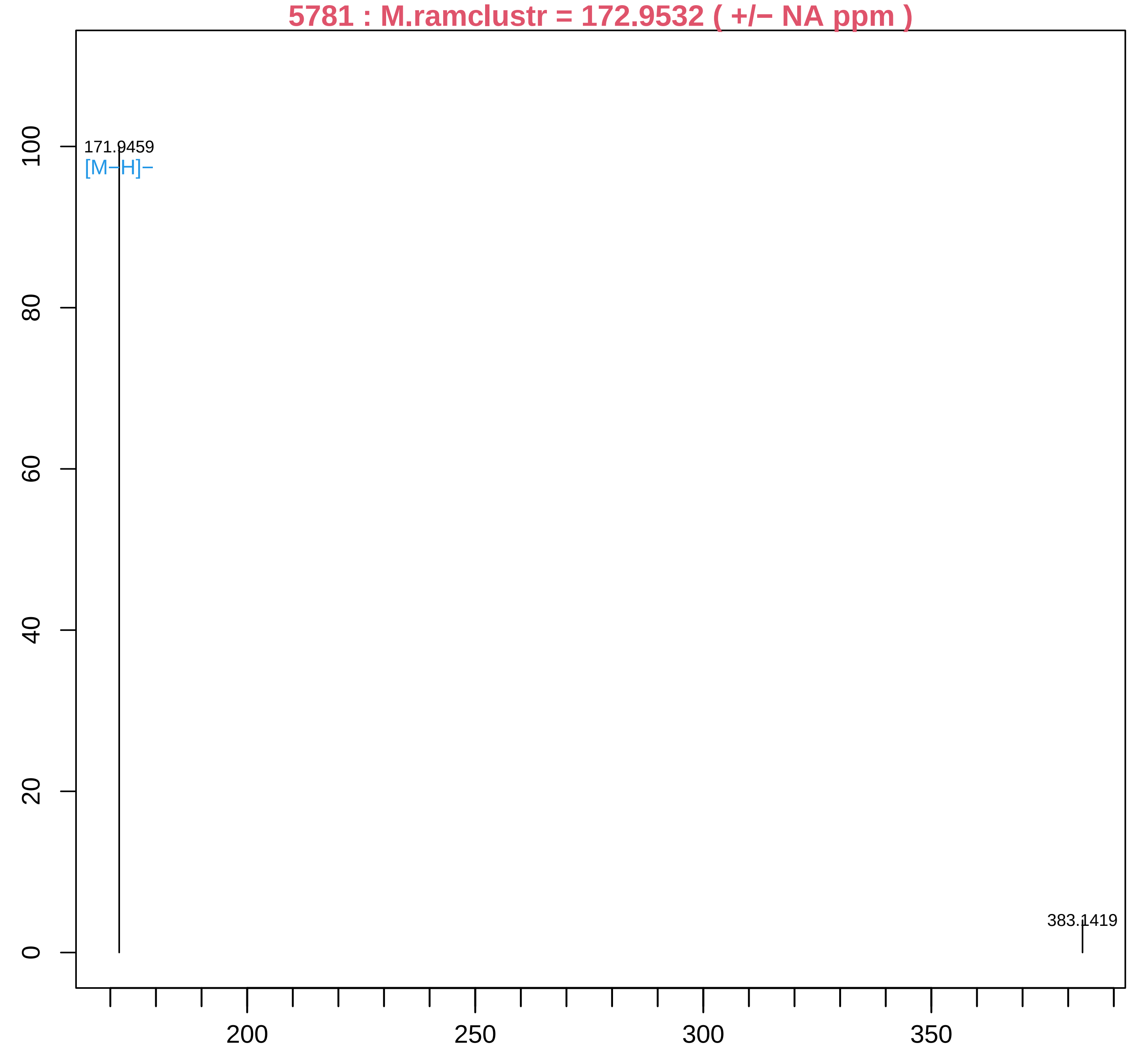
5781 : M.ramclustr = 172.9532 (+/− NA ppm)
171.9459
[M−H]−
383.1419
0
20
40
60
80
100
200
250
300
350

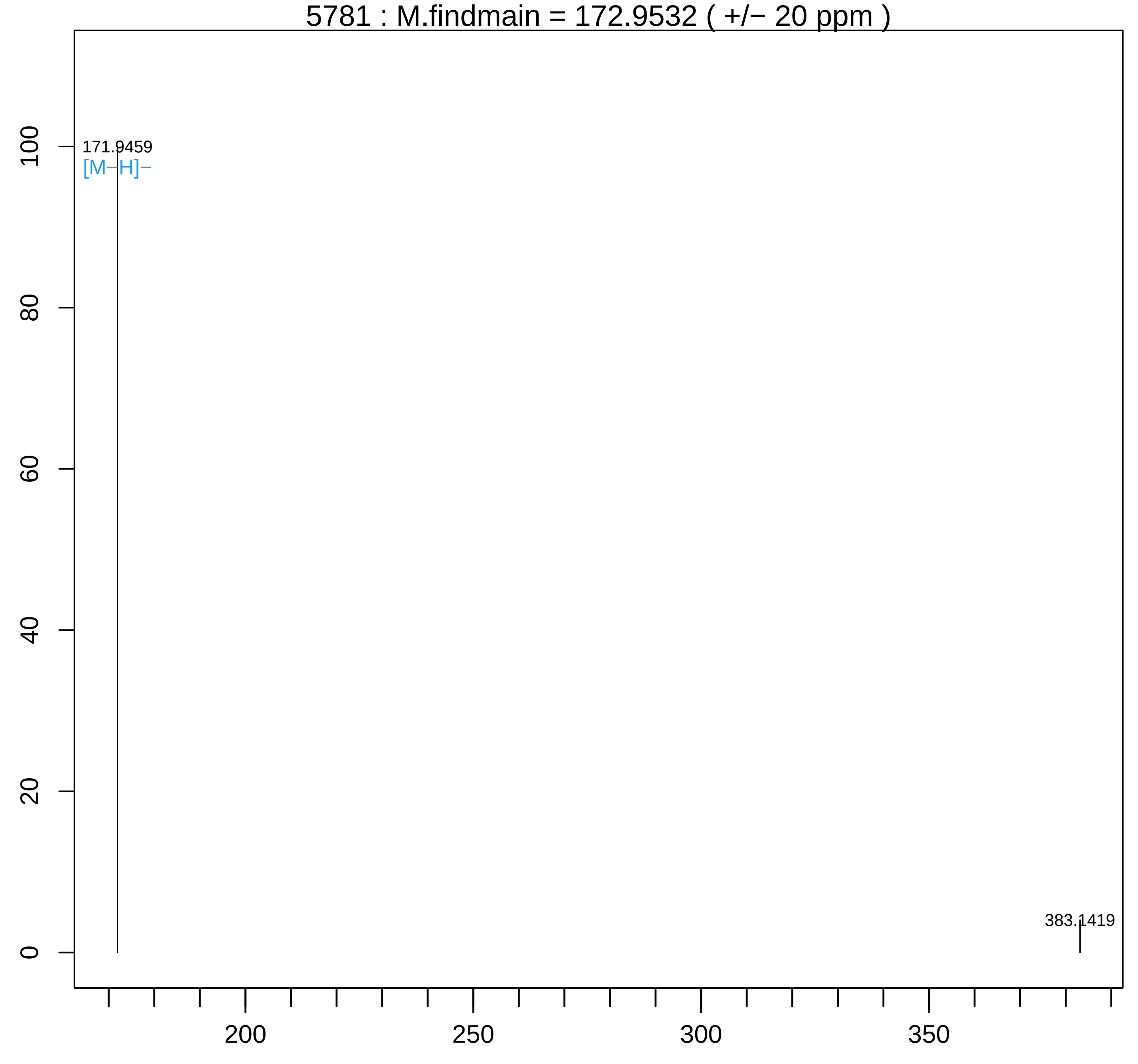
5781 : M.findmain = 172.9532 (+/− 20 ppm)
171.9459
[M−H]−
383.1419
0
20
40
60
80
100
200
250
300
350

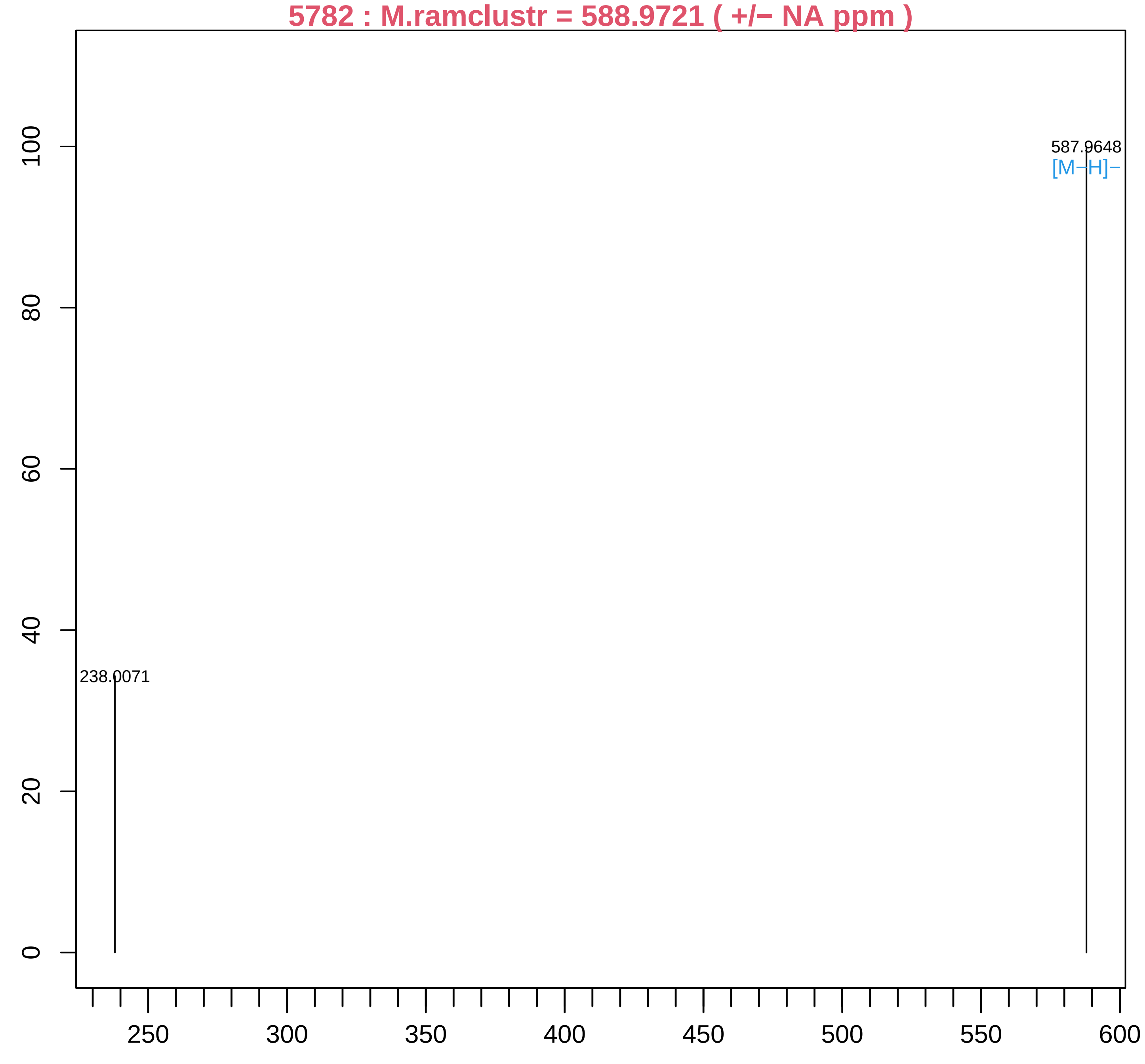
5782 : M.ramclustr = 588.9721 (+/− NA ppm)
587.9648
[M−H]−
238.0071
0
20
40
60
80
100
250
300
350
400
450
500
550
600

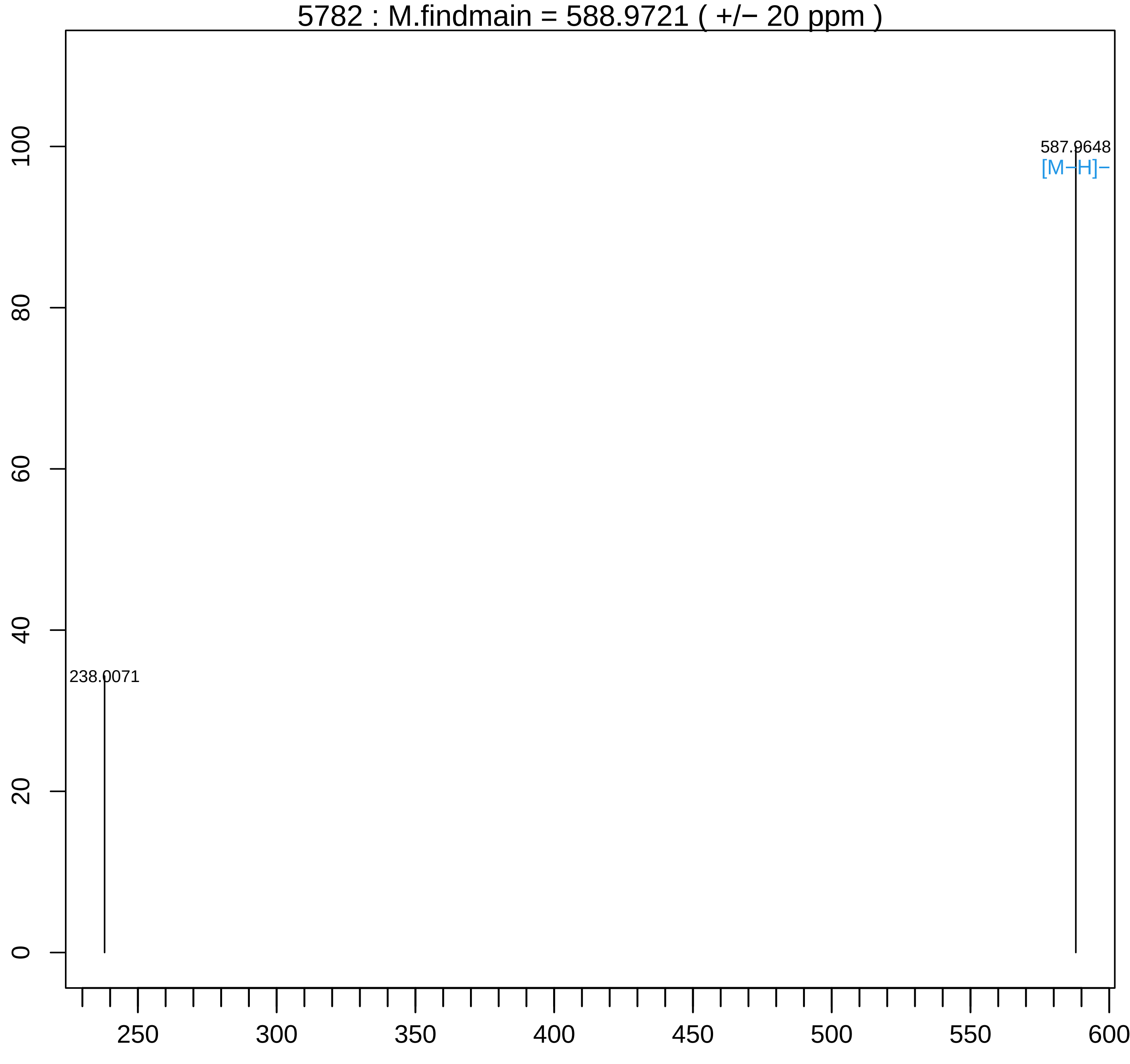
5782 : M.findmain = 588.9721 (+/− 20 ppm)
587.9648
[M−H]−
238.0071
0
20
40
60
80
100
250
300
350
400
450
500
550
600

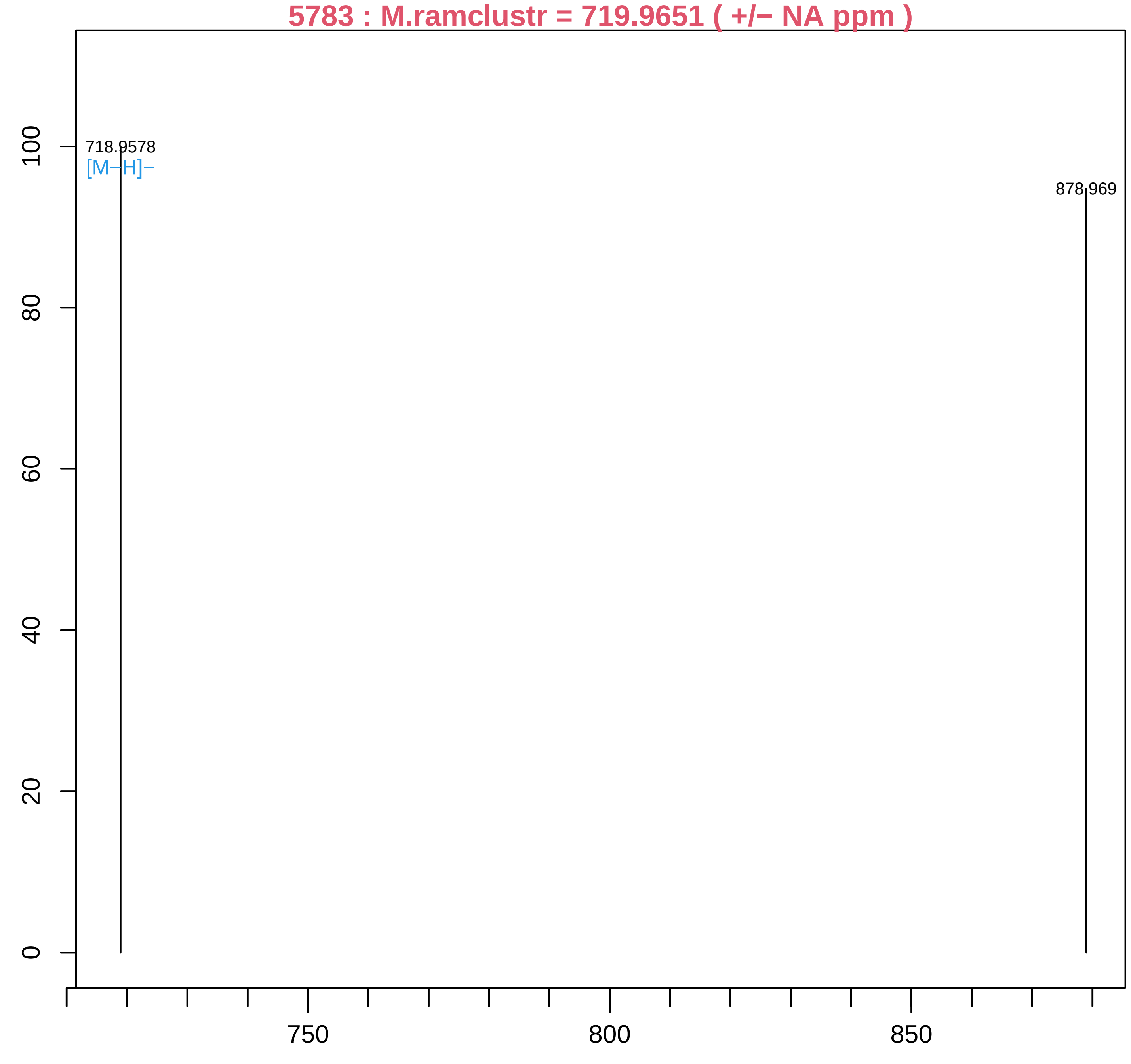
5783 : M.ramclustr = 719.9651 (+/− NA ppm)
718.9578
[M−H]−
878.969
0
20
40
60
80
100
750
800
850

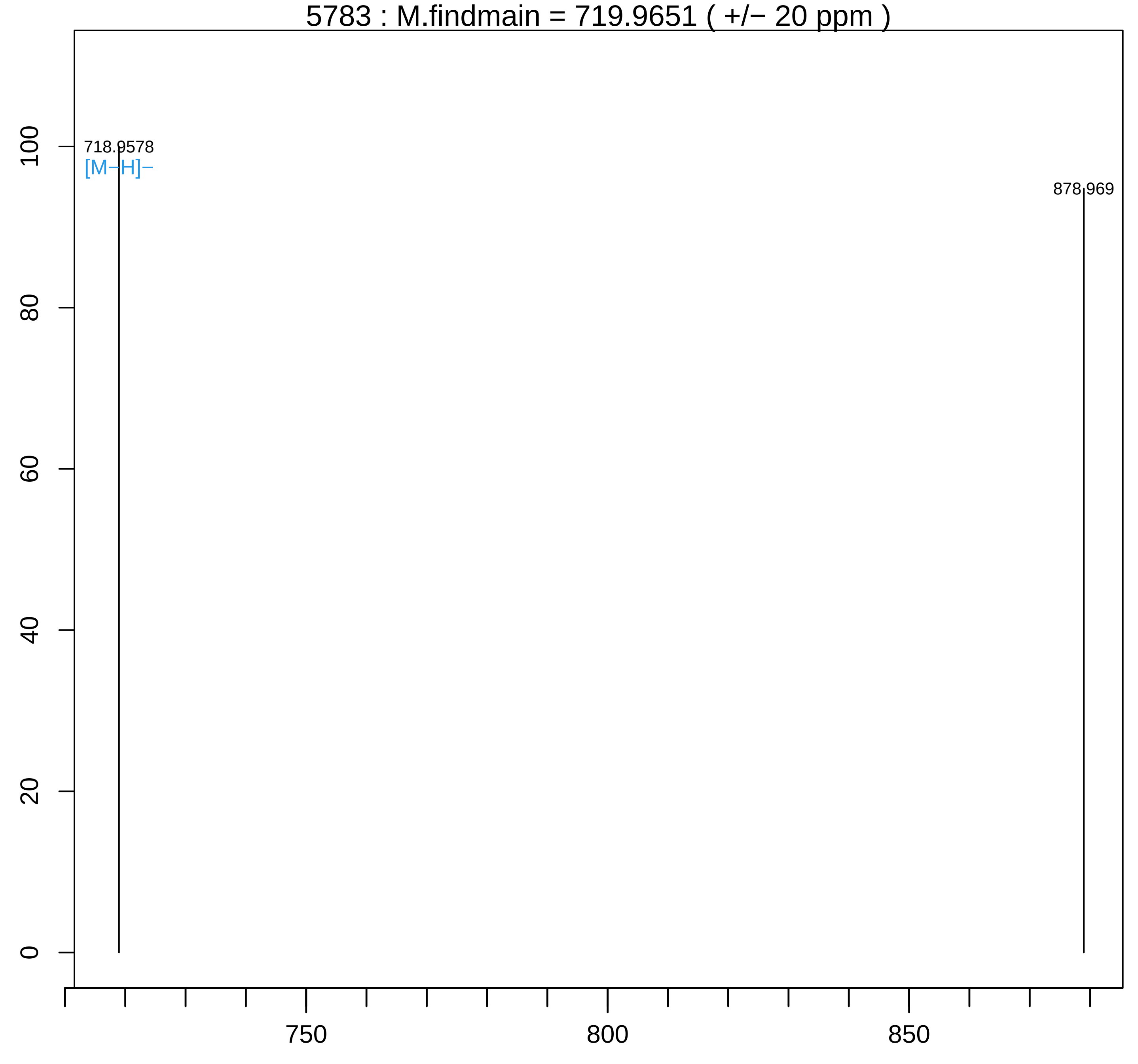
5783 : M.findmain = 719.9651 (+/− 20 ppm)
718.9578
[M−H]−
878.969
0
20
40
60
80
100
750
800
850

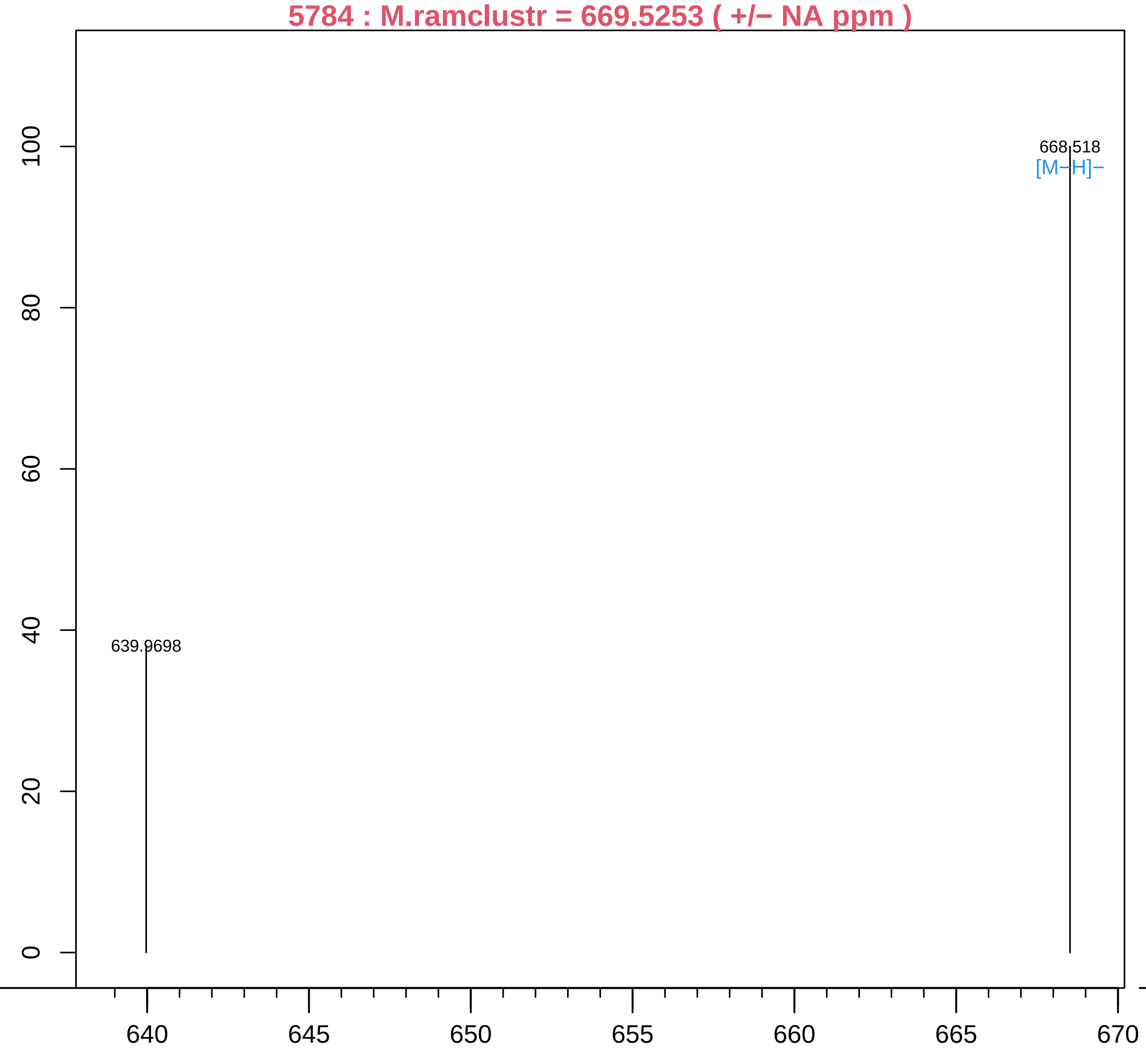
5784 : M.ramclustr = 669.5253 (+/− NA ppm)
668.518
[M−H]−
639.9698
0
20
40
60
80
100
640
645
650
655
660
665
670

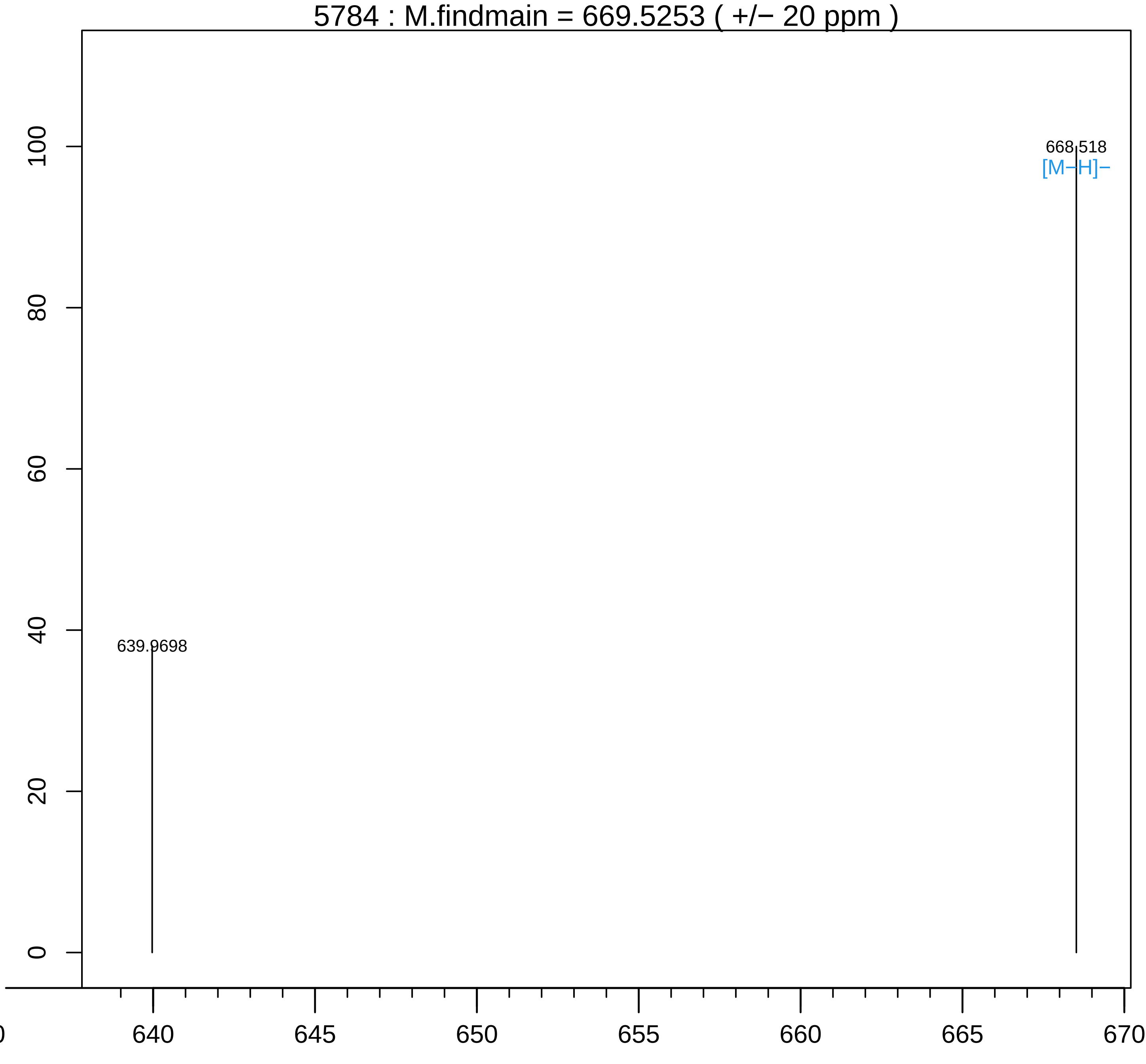
5784 : M.findmain = 669.5253 (+/− 20 ppm)
668.518
[M−H]−
639.9698
0
20
40
60
80
100
640
645
650
655
660
665
670

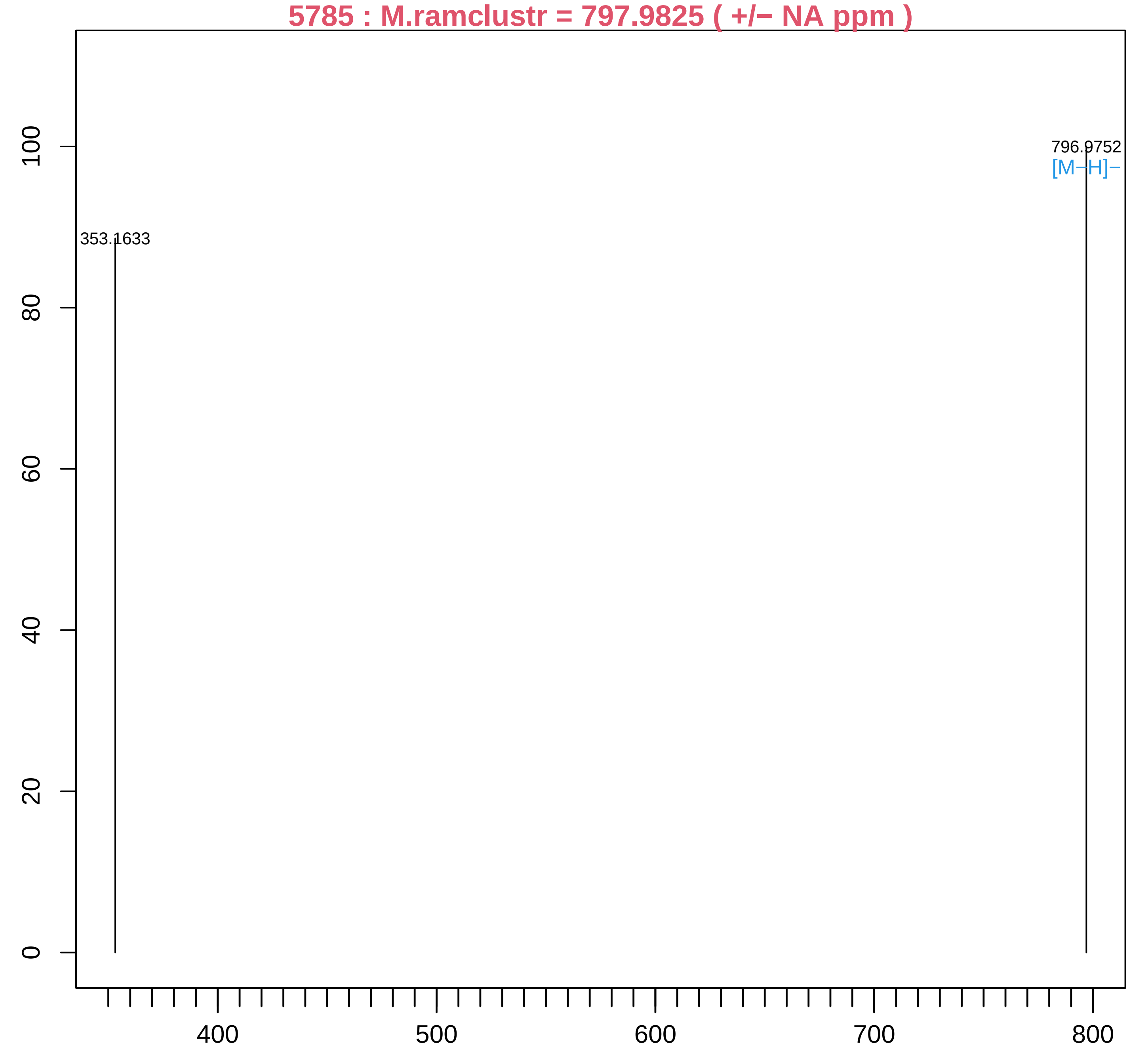

5785 : M.ramclustr = 797.9825 (+/− NA ppm)
796.9752
[M−H]−
353.1633
0
20
40
60
80
100
400
500
600
700
800

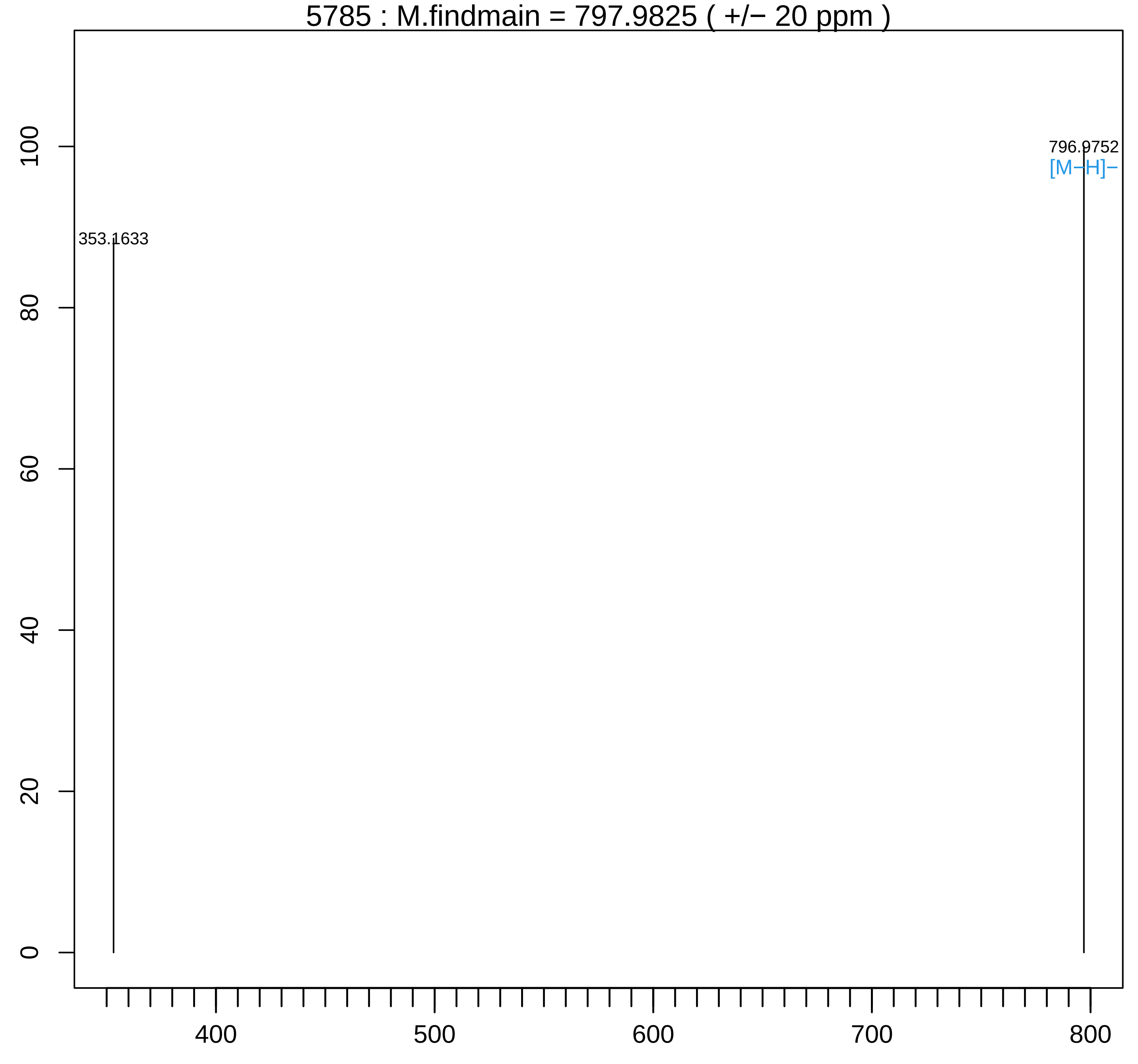

5785 : M.findmain = 797.9825 (+/− 20 ppm)
796.9752
[M−H]−
353.1633
0
20
40
60
80
100
400
500
600
700
800

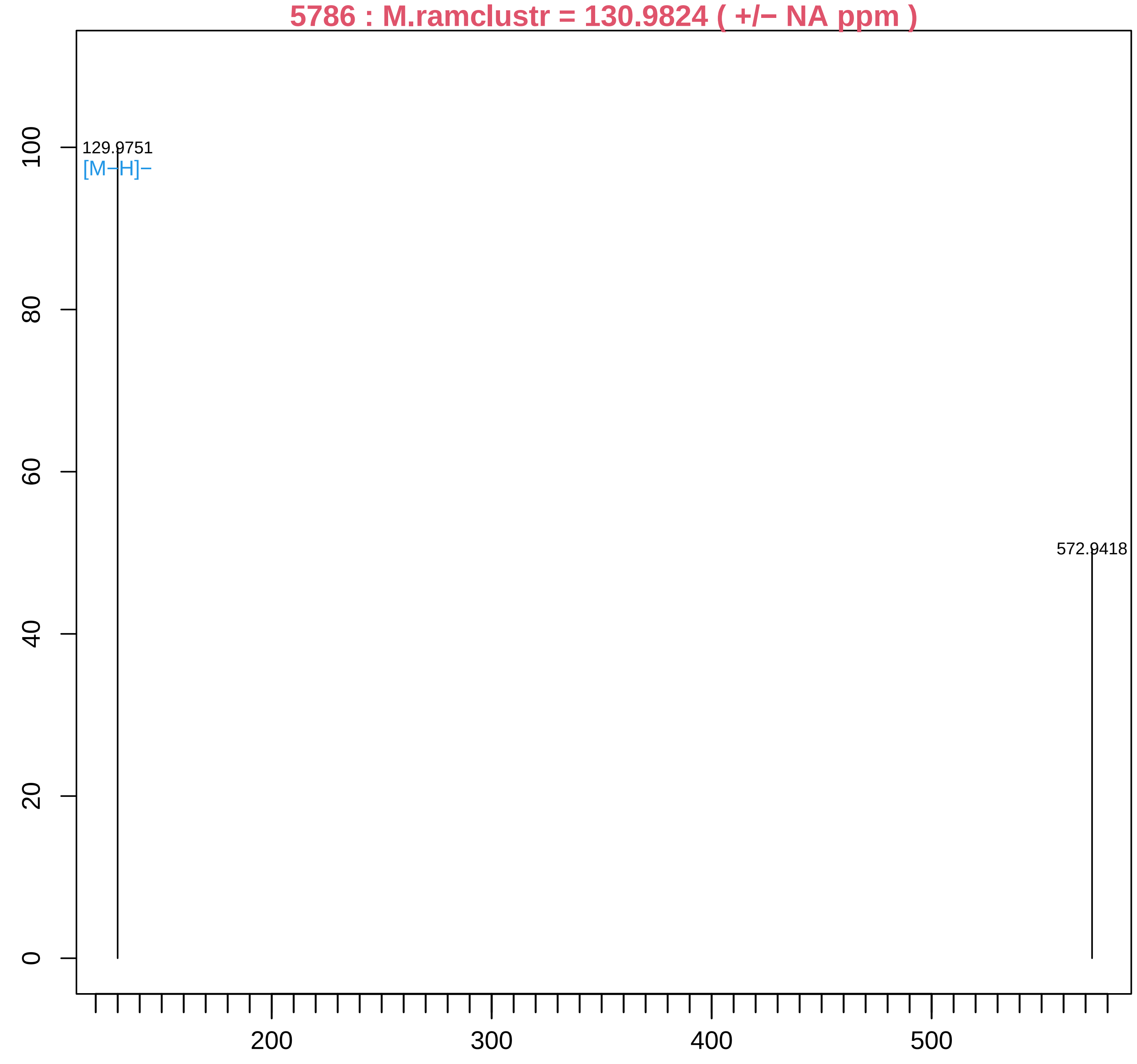
5786 : M.ramclustr = 130.9824 (+/− NA ppm)
129.9751
[M−H]−
572.9418
0
20
40
60
80
100
200
300
400
500

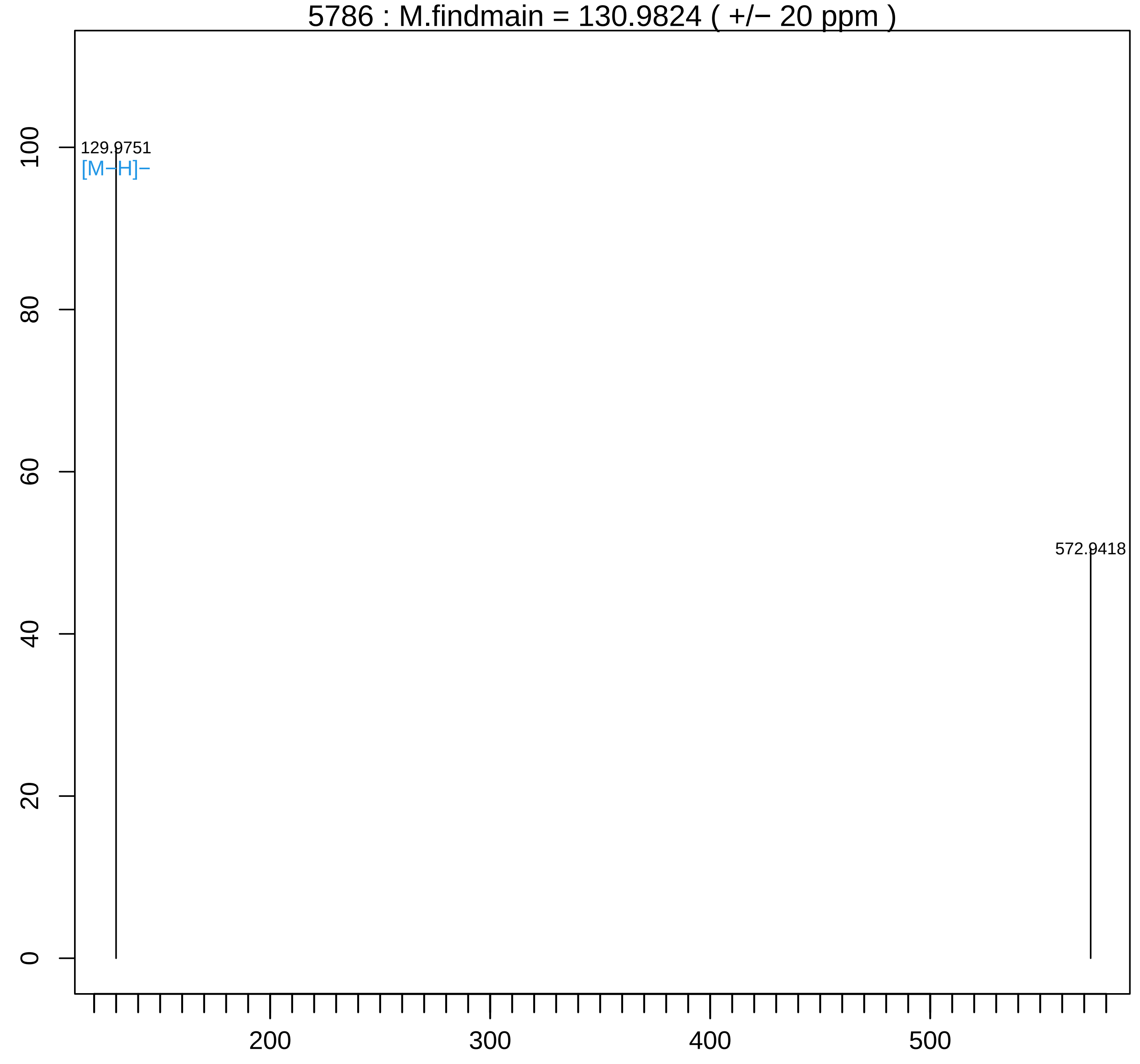
5786 : M.findmain = 130.9824 (+/− 20 ppm)
129.9751
[M−H]−
572.9418
0
20
40
60
80
100
200
300
400
500

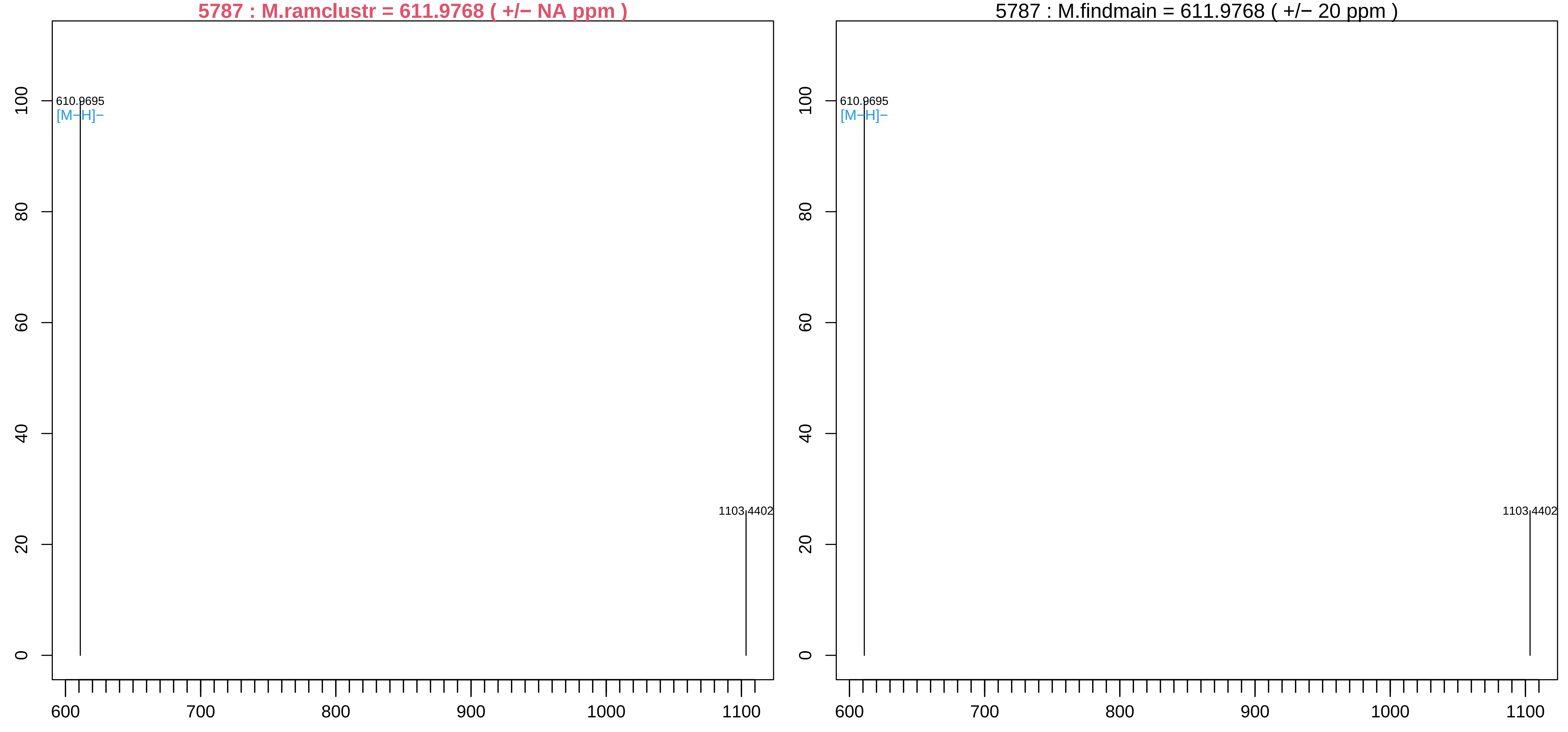
5787 : M.ramclustr = 611.9768 (+/− NA ppm)
610.9695
[M−H]−
1103.4402
5787 : M.findmain = 611.9768 (+/− 20 ppm)
610.9695
[M−H]−
1103.4402
0
20
40
60
80
100
600
700
800
900
1000
1100

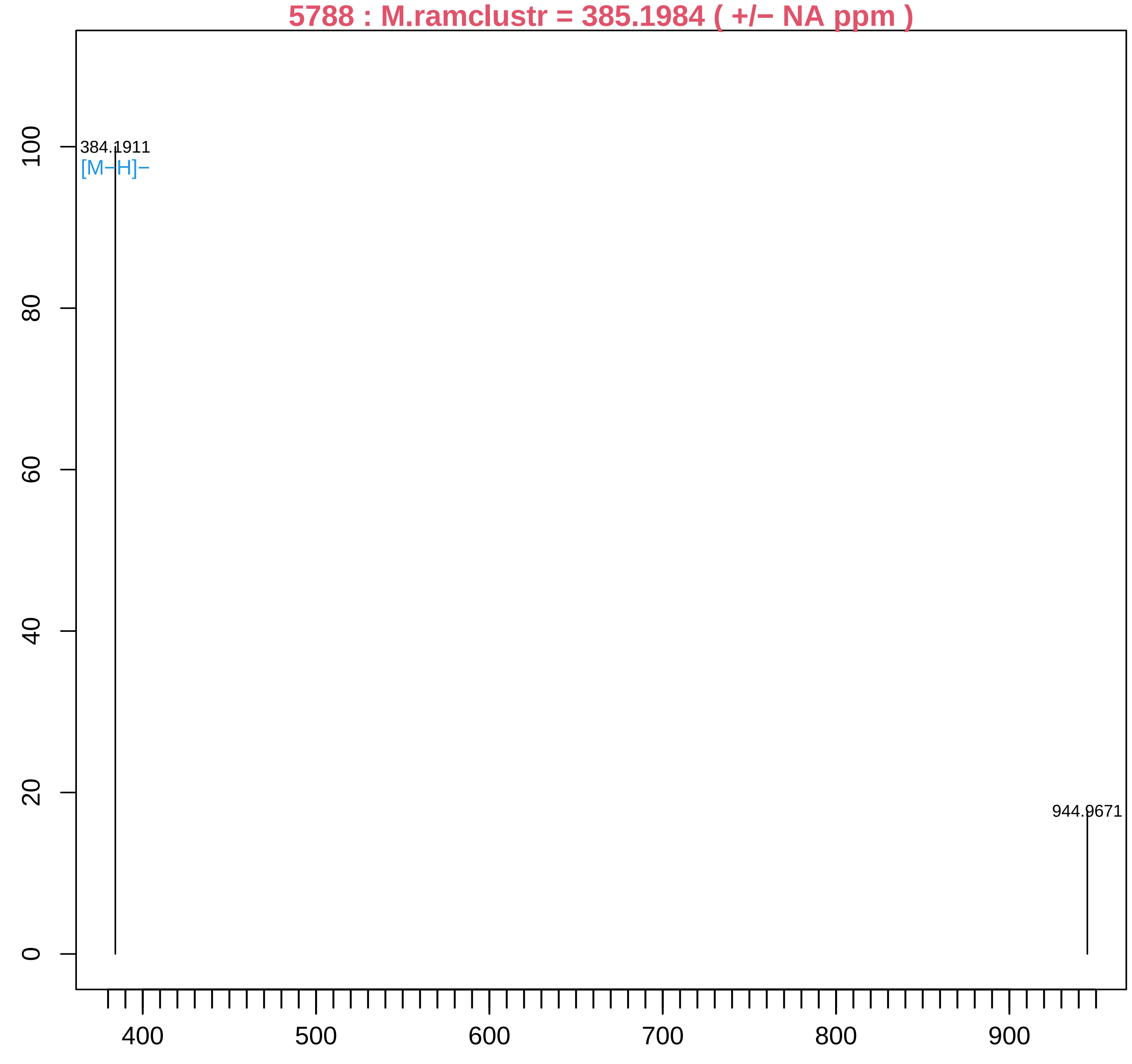

5788 : M.ramclustr = 385.1984 (+/− NA ppm)
384.1911
[M−H]−
944.9671
0
20
40
60
80
100
400
500
600
700
800
900

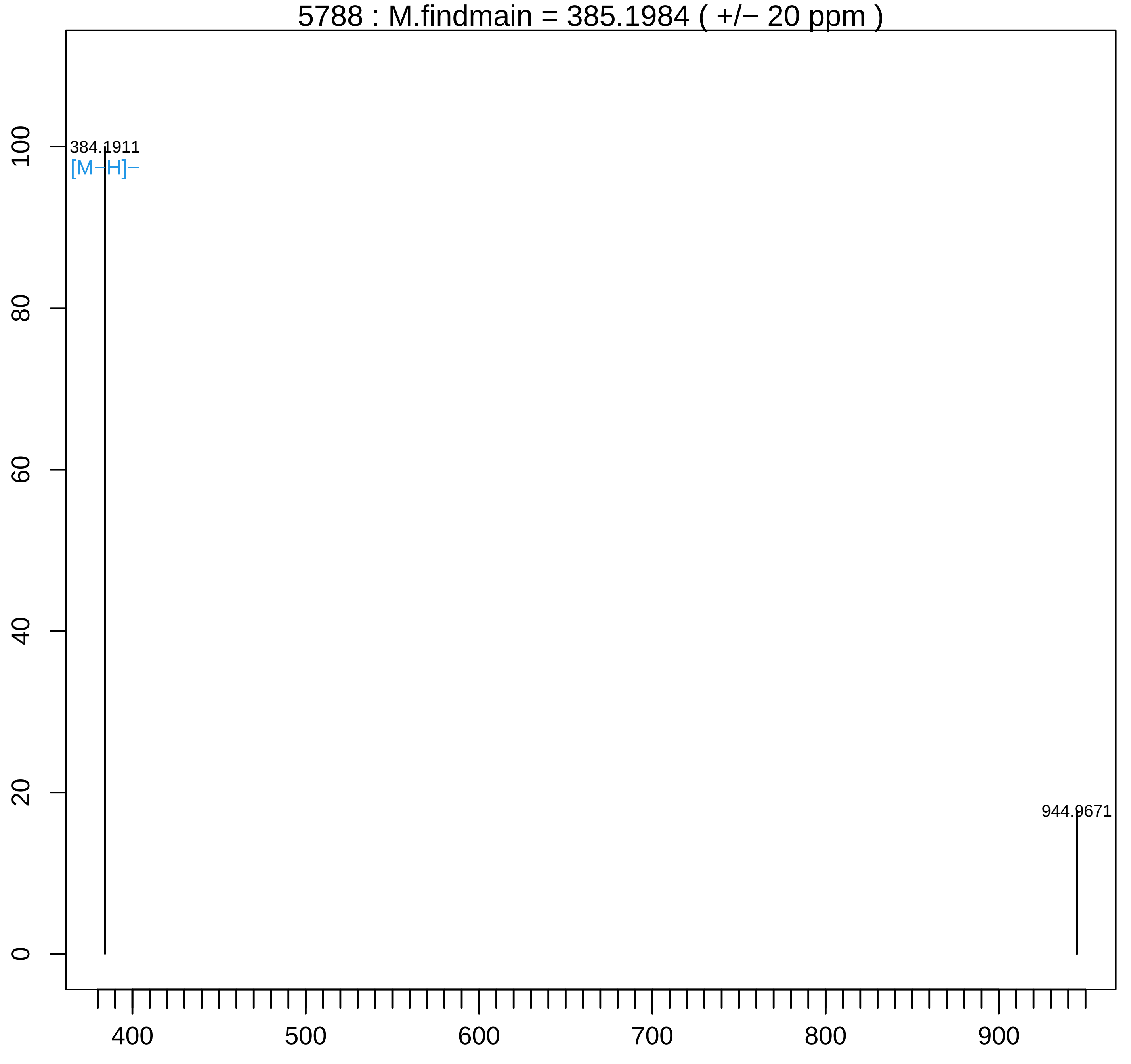

5788 : M.findmain = 385.1984 (+/− 20 ppm)
384.1911
[M−H]−
944.9671
0
20
40
60
80
100
400
500
600
700
800
900

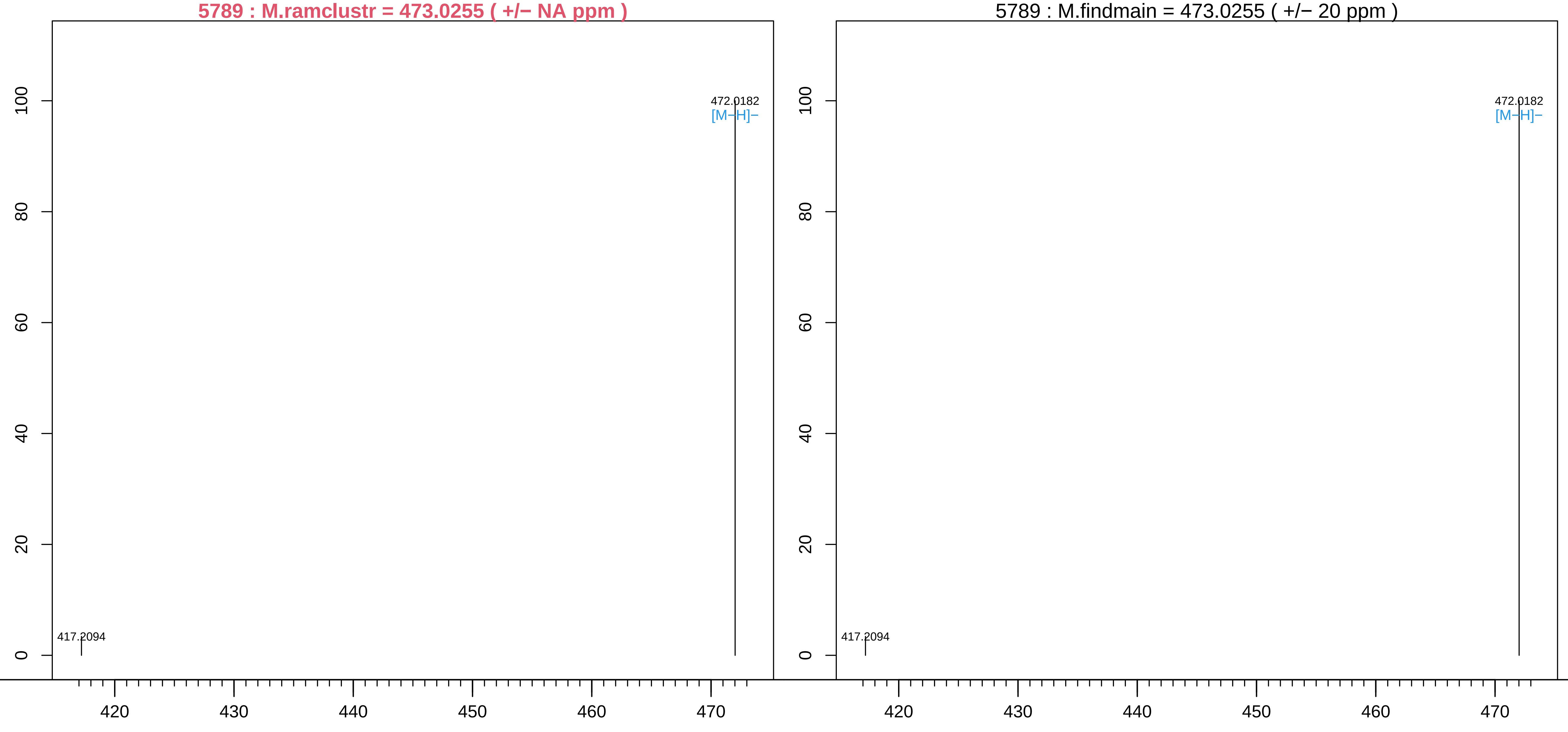
5789 : M.ramclustr = 473.0255 (+/− NA ppm)
472.0182
[M−H]−
417.2094
0
20
40
60
80
100
420
430
440
450
460
470
5789 : M.findmain = 473.0255 (+/− 20 ppm)
472.0182
[M−H]−
417.2094
0
20
40
60
80
100
420
430
440
450
460
470

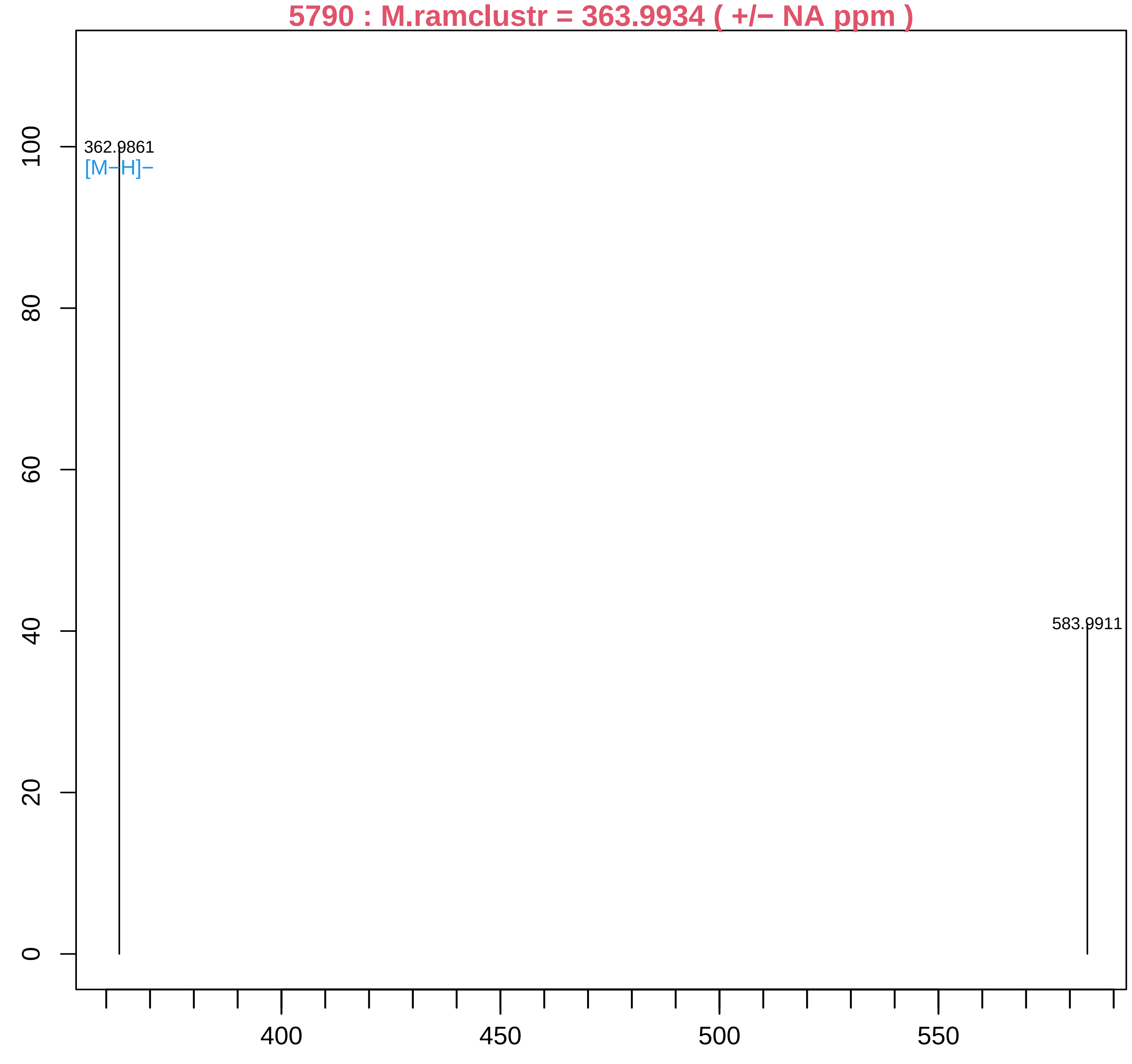
5790 : M.ramclustr = 363.9934 (+/− NA ppm)
362.9861
[M−H]−
583.9911
0
20
40
60
80
100
400
450
500
550

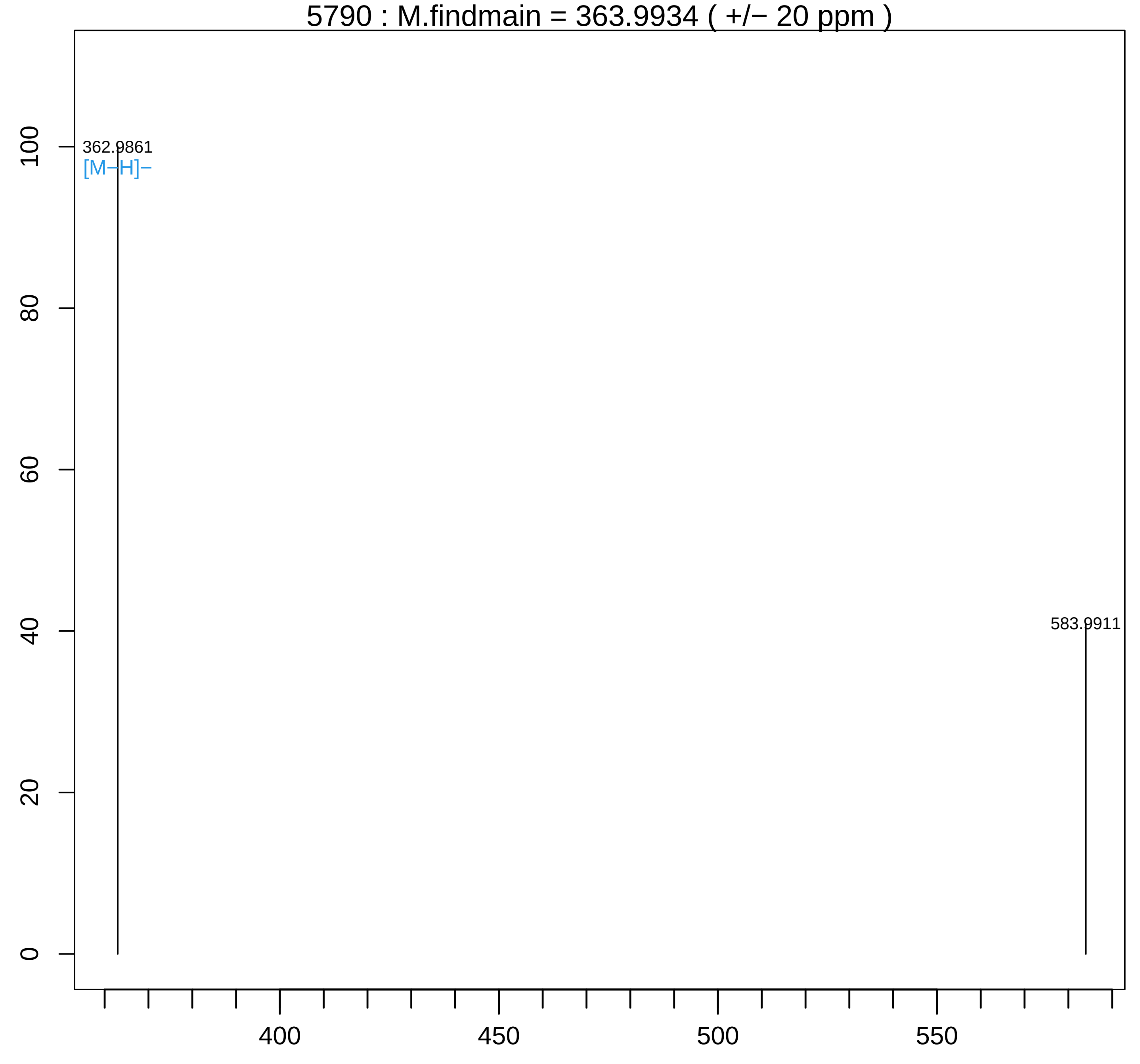
5790 : M.findmain = 363.9934 (+/− 20 ppm)
362.9861
[M−H]−
583.9911
0
20
40
60
80
100
400
450
500
550

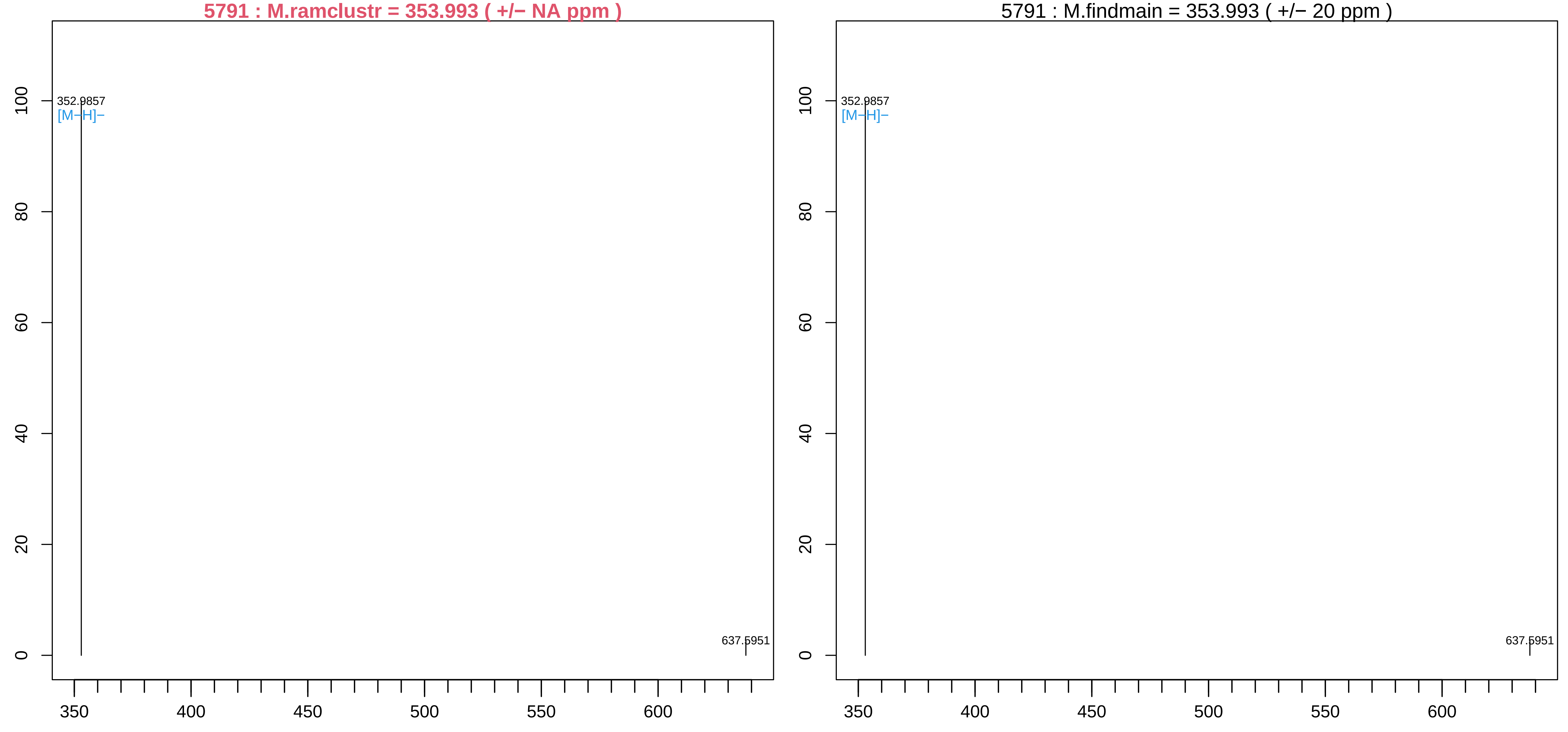
5791 : M.ramclustr = 353.993 (+/− NA ppm)
5791 : M.findmain = 353.993 (+/− 20 ppm)
352.9857
[M−H]−
637.5951
0
20
40
60
80
100
350
400
450
500
550
600

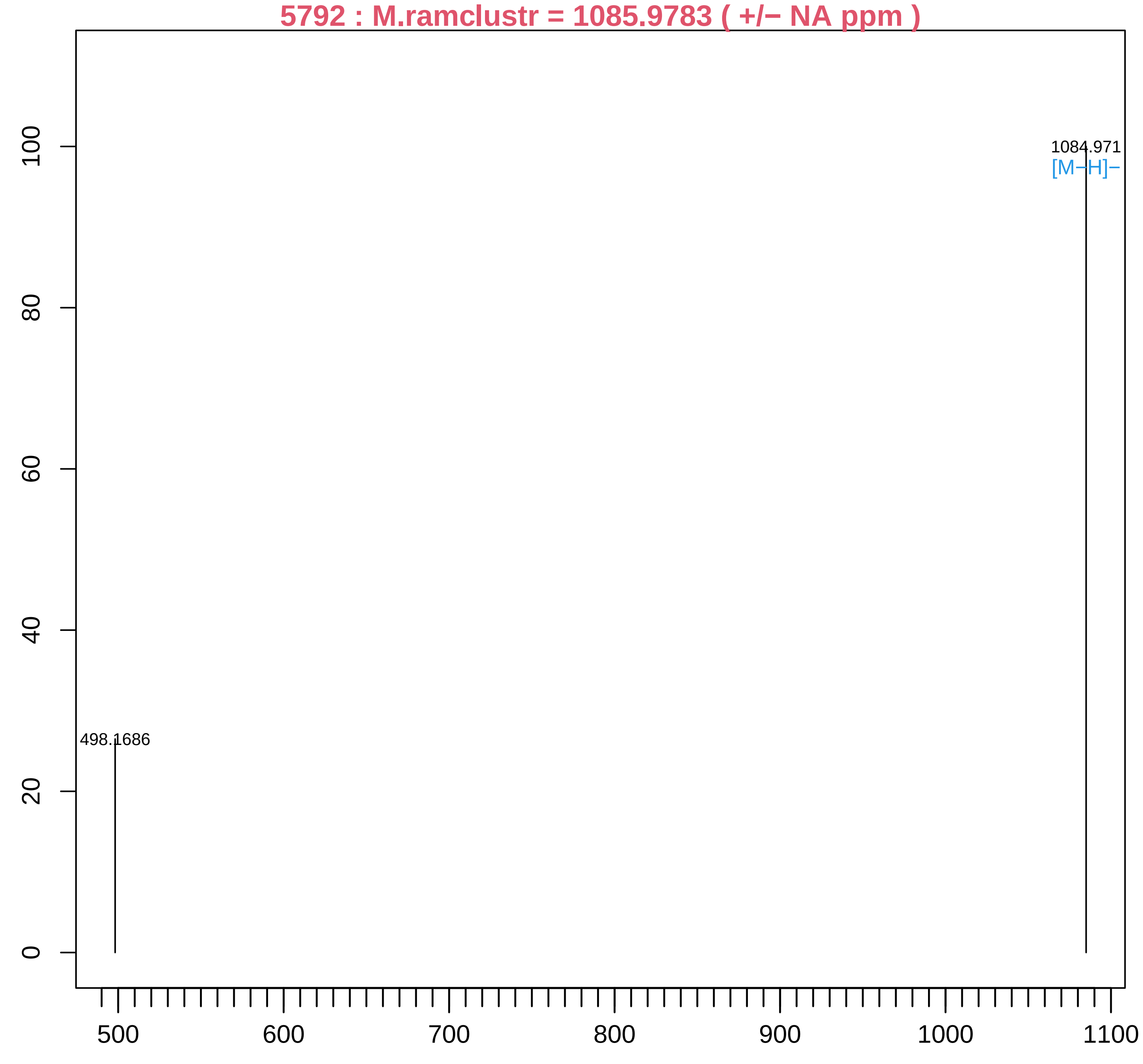
5792 : M.ramclustr = 1085.9783 (+/− NA ppm)
1084.971
[M−H]−
498.1686
0
20
40
60
80
100
500
600
700
800
900
1000
1100

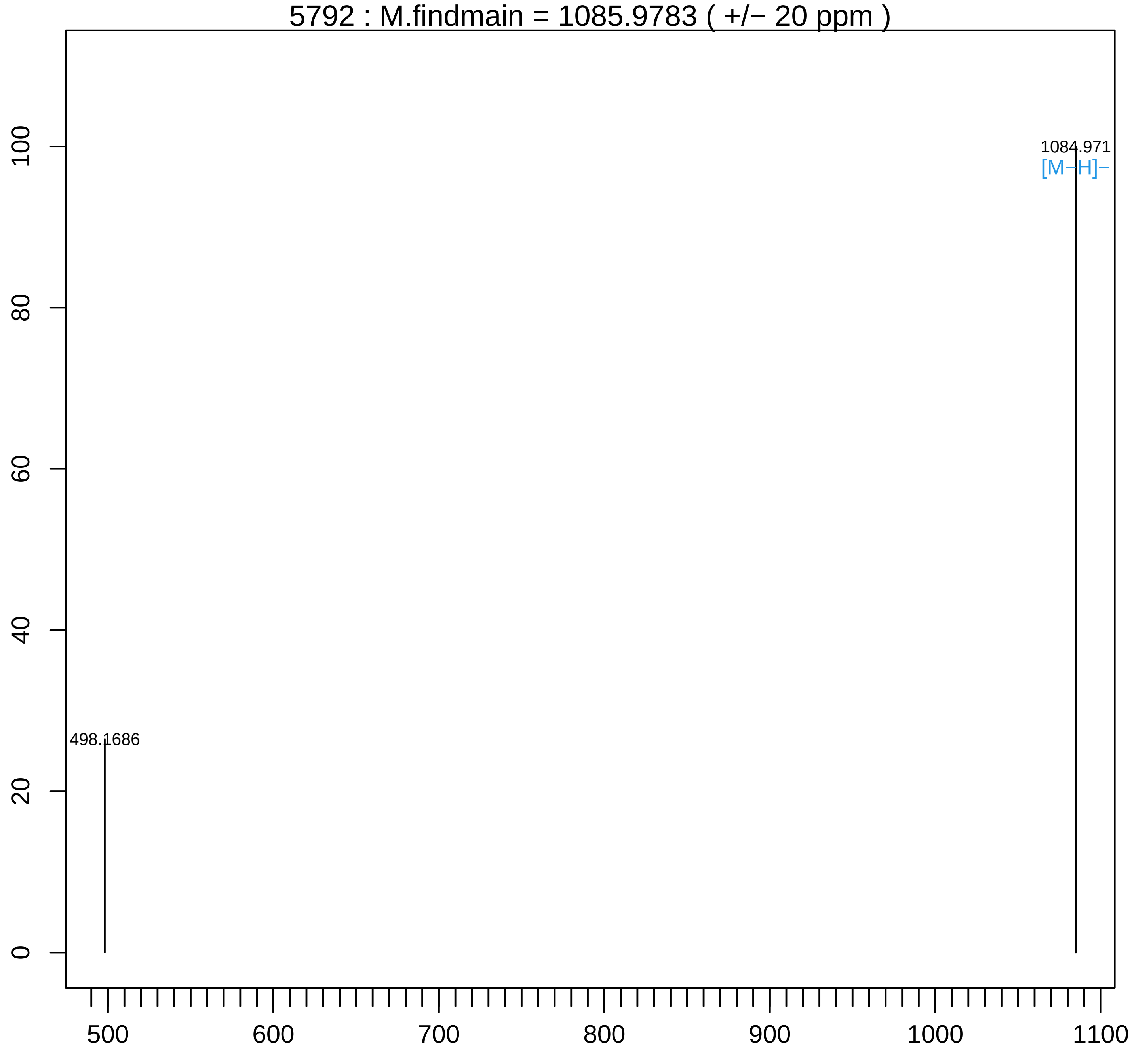
5792 : M.findmain = 1085.9783 (+/− 20 ppm)
1084.971
[M−H]−
498.1686
0
20
40
60
80
100
500
600
700
800
900
1000
1100

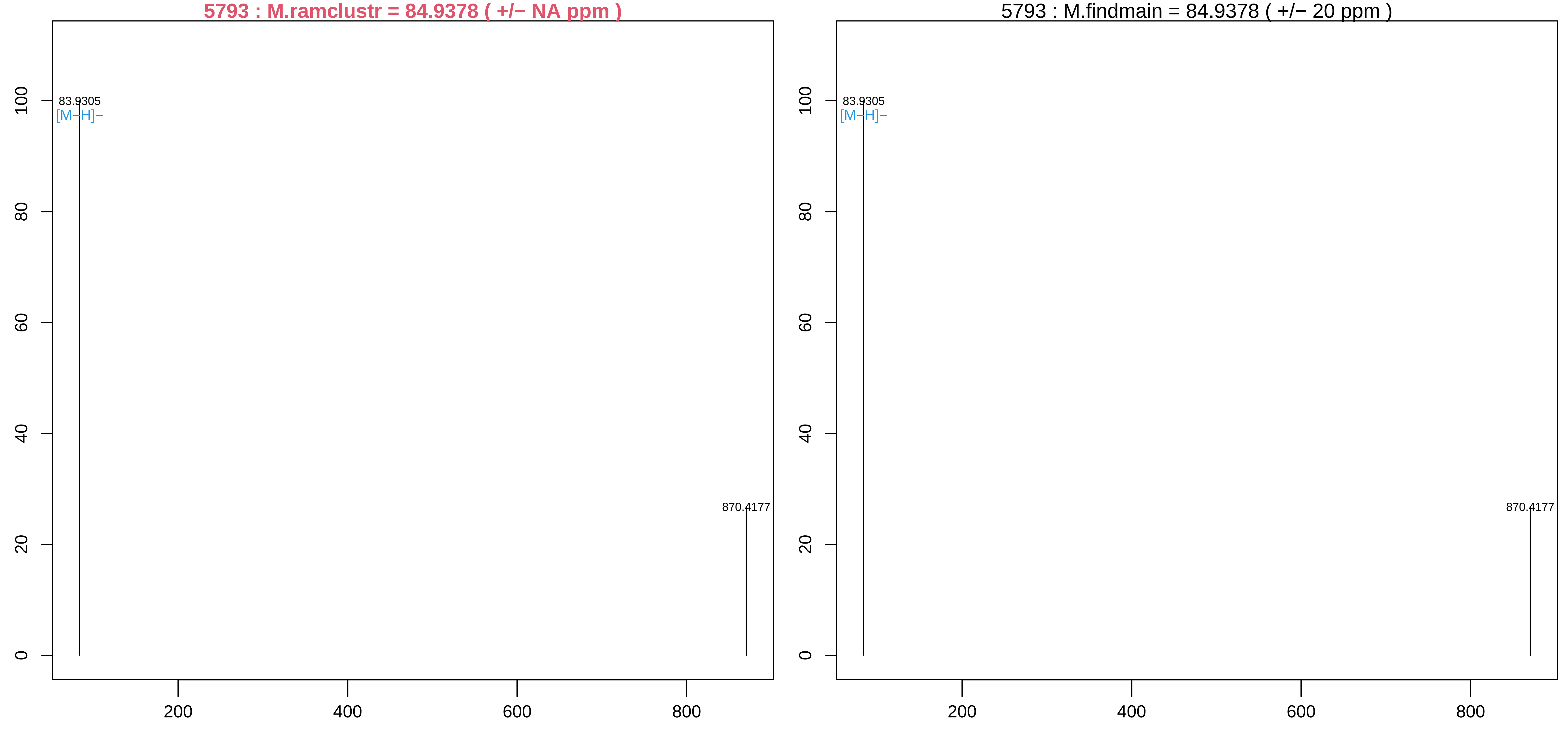
5793 : M.ramclustr = 84.9378 (+/− NA ppm)
83.9305
[M−H]−
870.4177
0
20
40
60
80
100
200
400
600
800
5793 : M.findmain = 84.9378 (+/− 20 ppm)
83.9305
[M−H]−
870.4177
0
20
40
60
80
100
200
400
600
800

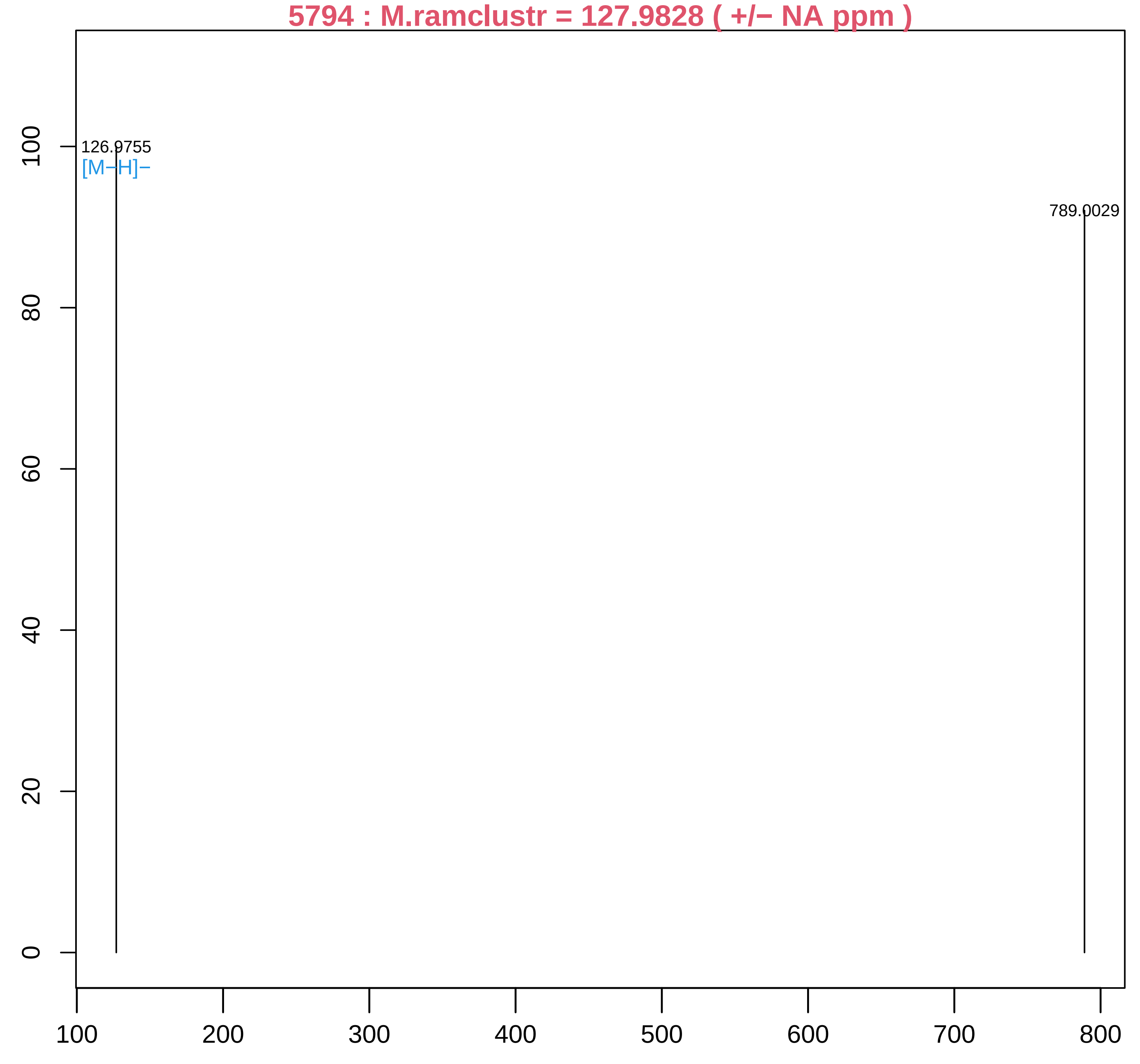
5794 : M.ramclustr = 127.9828 (+/− NA ppm)
126.9755
[M−H]−
789.0029
0
20
40
60
80
100
100
200
300
400
500
600
700
800
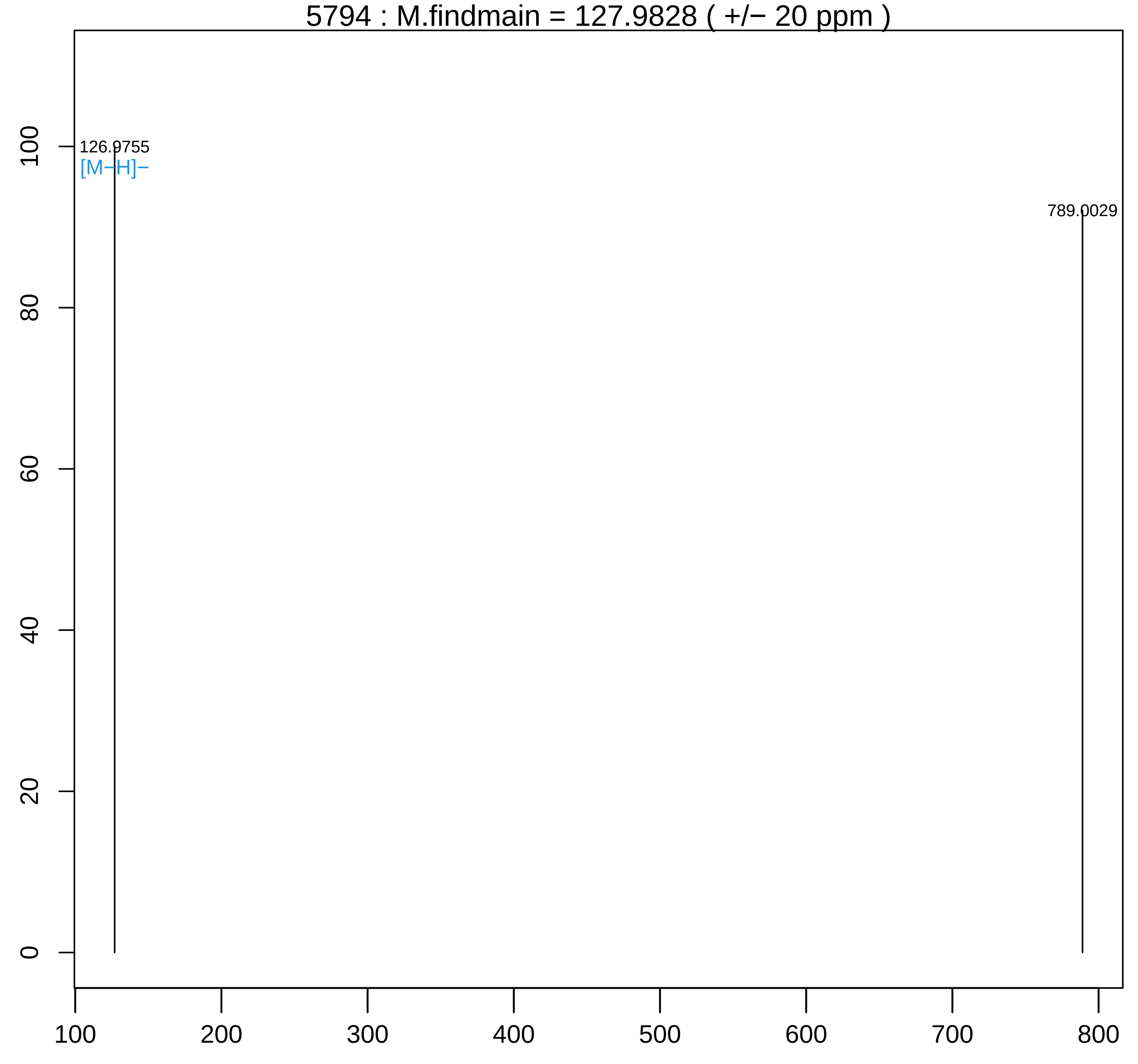
5794 : M.findmain = 127.9828 (+/− 20 ppm)
126.9755
[M−H]−
789.0029
0
20
40
60
80
100
100
200
300
400
500
600
700
800

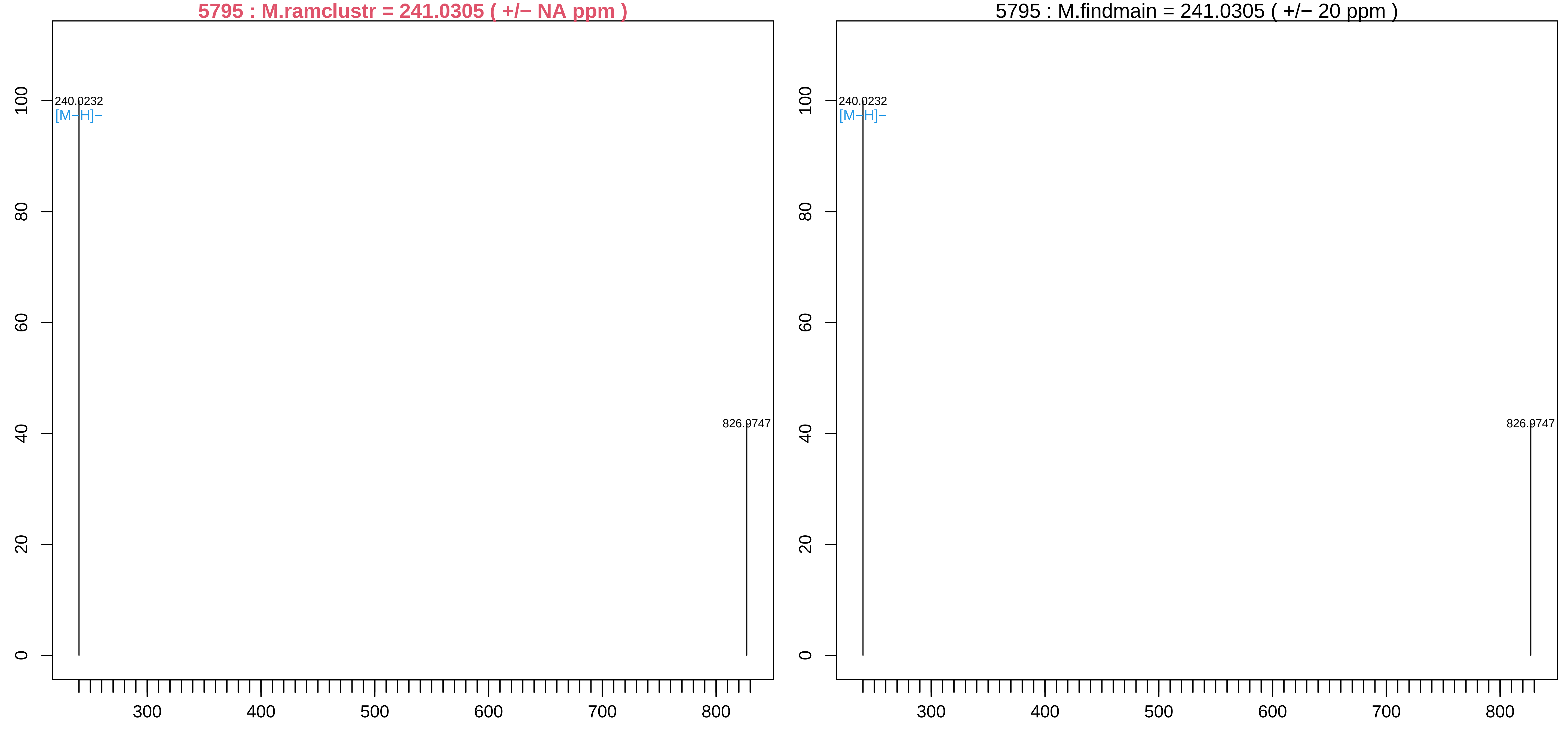
5795 : M.ramclustr = 241.0305 (+/− NA ppm)
240.0232
[M−H]−
826.9747
0
20
40
60
80
100
300
400
500
600
700
800
5795 : M.findmain = 241.0305 (+/− 20 ppm)
240.0232
[M−H]−
826.9747
0
20
40
60
80
100
300
400
500
600
700
800

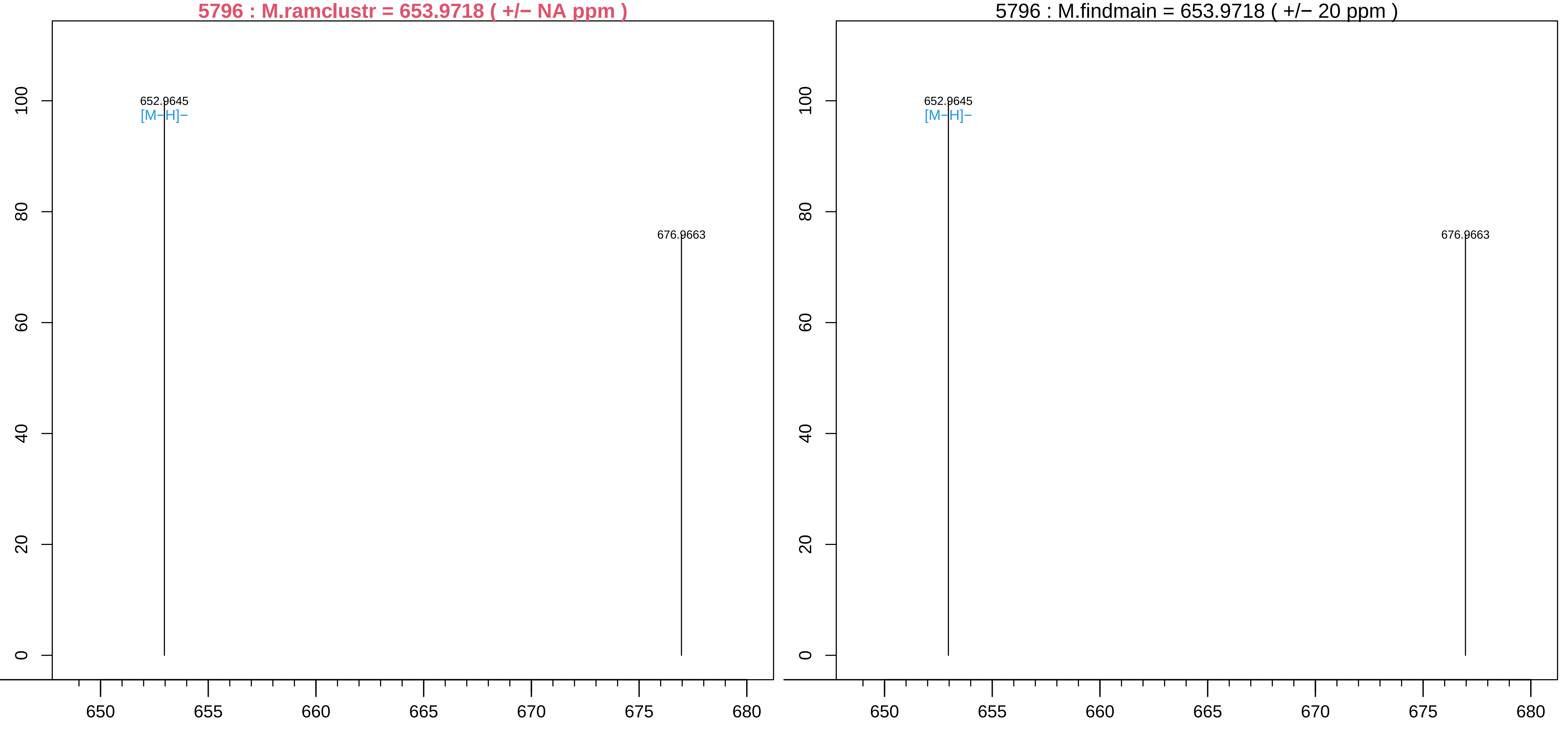
5796 : M.ramclustr = 653.9718 (+/− NA ppm)
652.9645
[M−H]−
676.9663
5796 : M.findmain = 653.9718 (+/− 20 ppm)
652.9645
[M−H]−
676.9663
0
20
40
60
80
100
650
655
660
665
670
675
680

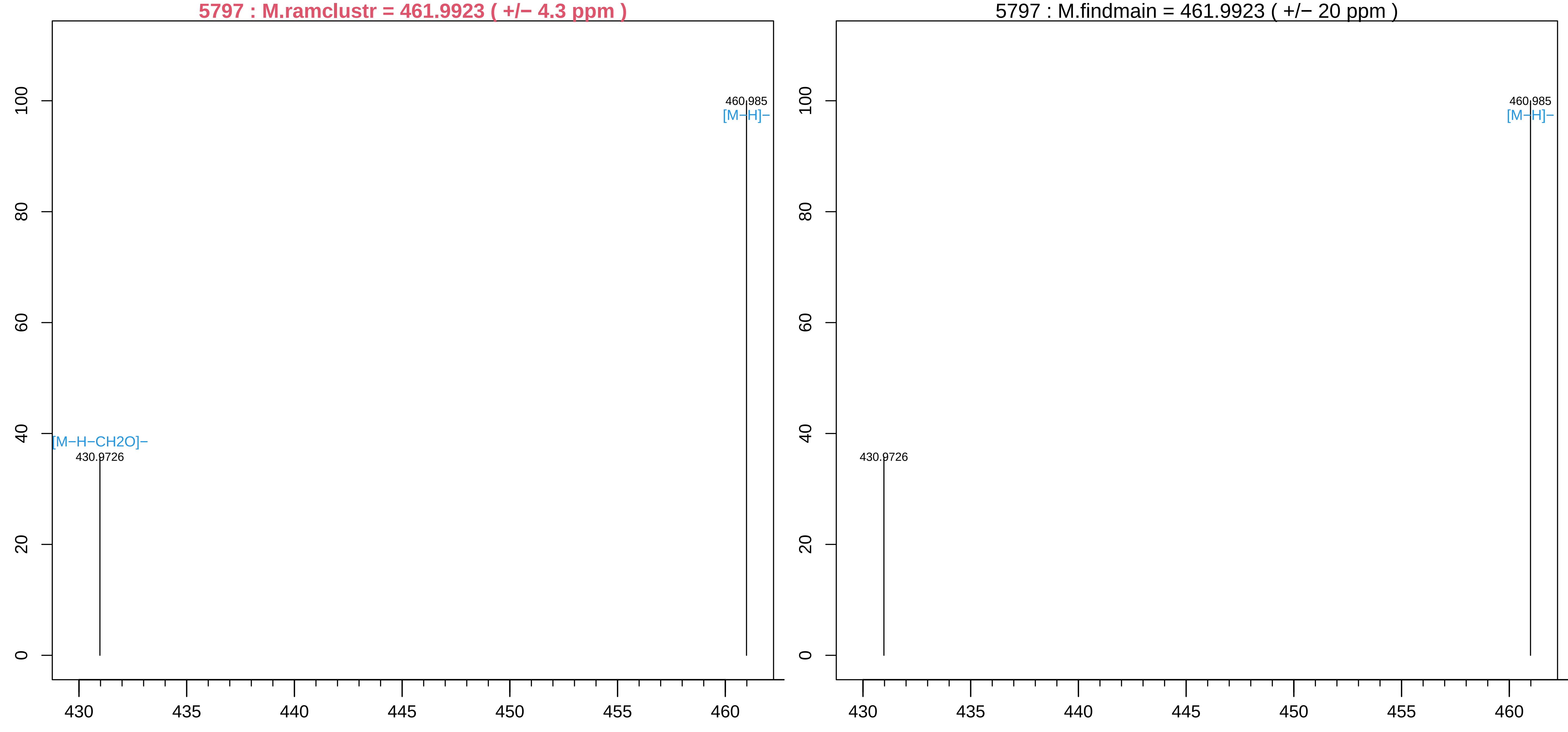
5797 : M.ramclustr = 461.9923 (+/− 4.3 ppm)
460.985
[M−H]−
[M−H−CH2O]−
430.9726
5797 : M.findmain = 461.9923 (+/− 20 ppm)
460.985
[M−H]−
430.9726
0
20
40
60
80
100
430
435
440
445
450
455
460

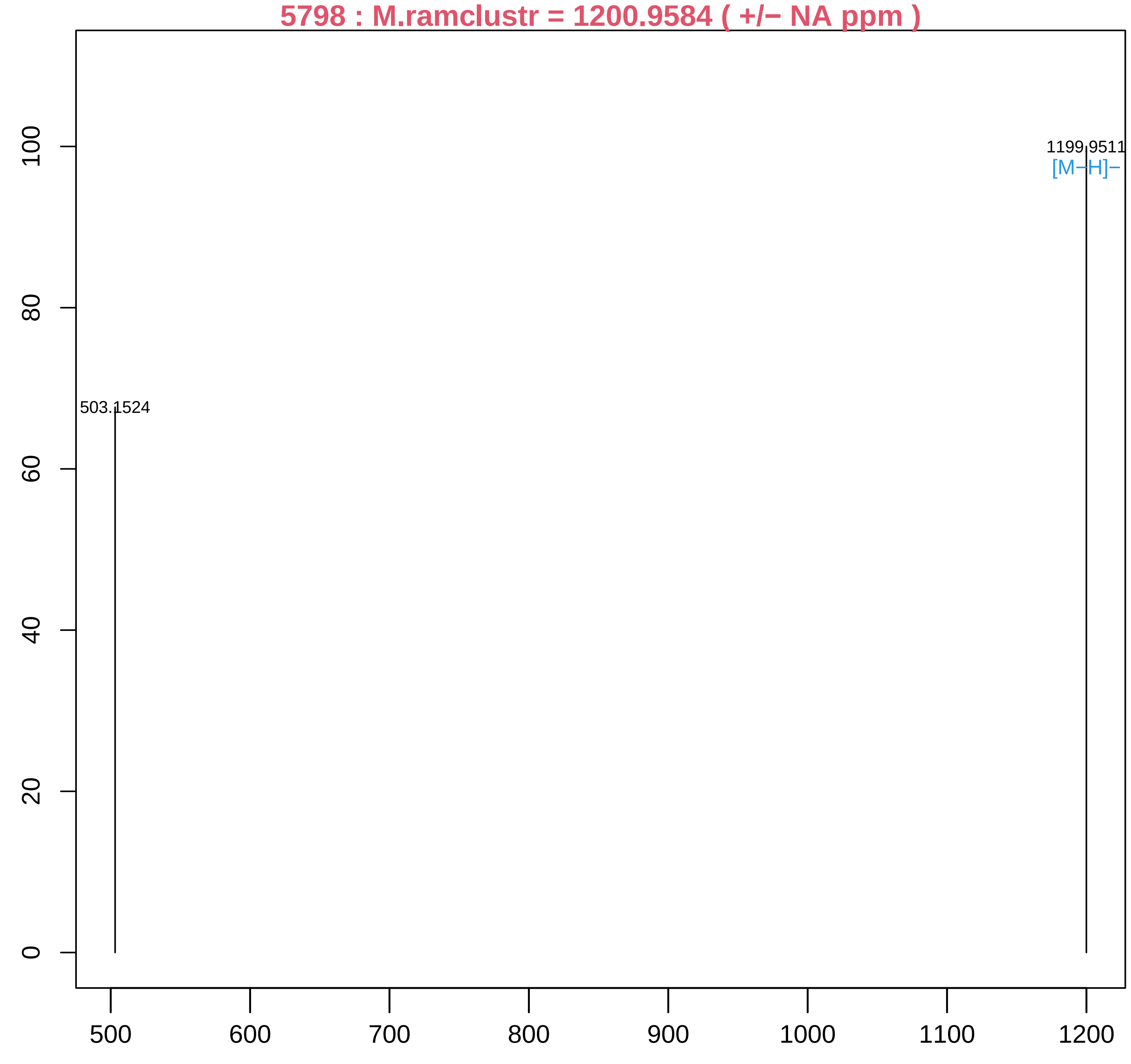

5798 : M.ramclustr = 1200.9584 (+/− NA ppm)
1199.9511
[M−H]−
503.1524
0
20
40
60
80
100
500
600
700
800
900
1000
1100
1200

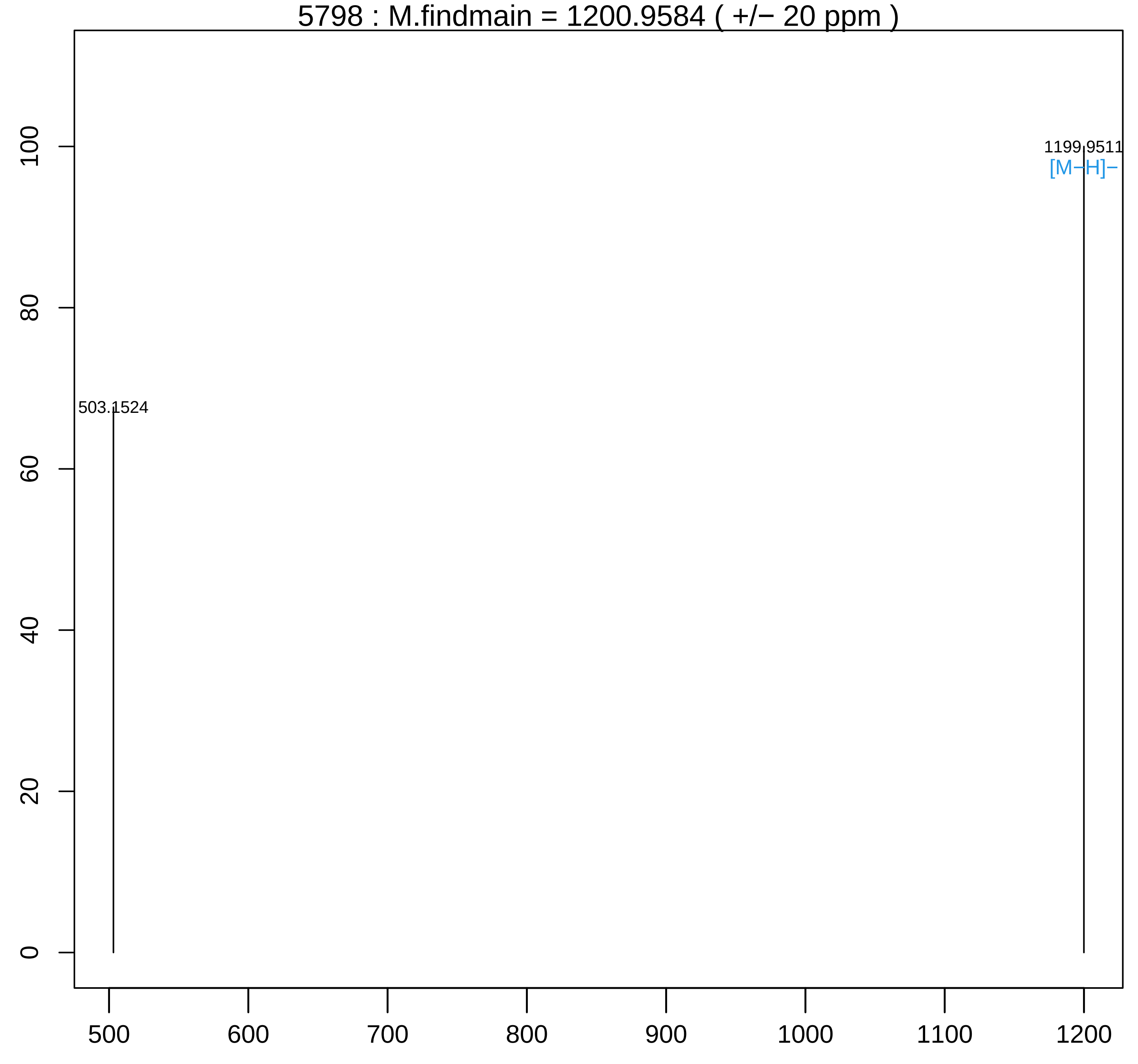

5798 : M.findmain = 1200.9584 (+/− 20 ppm)
1199.9511
[M−H]−
503.1524
0
20
40
60
80
100
500
600
700
800
900
1000
1100
1200

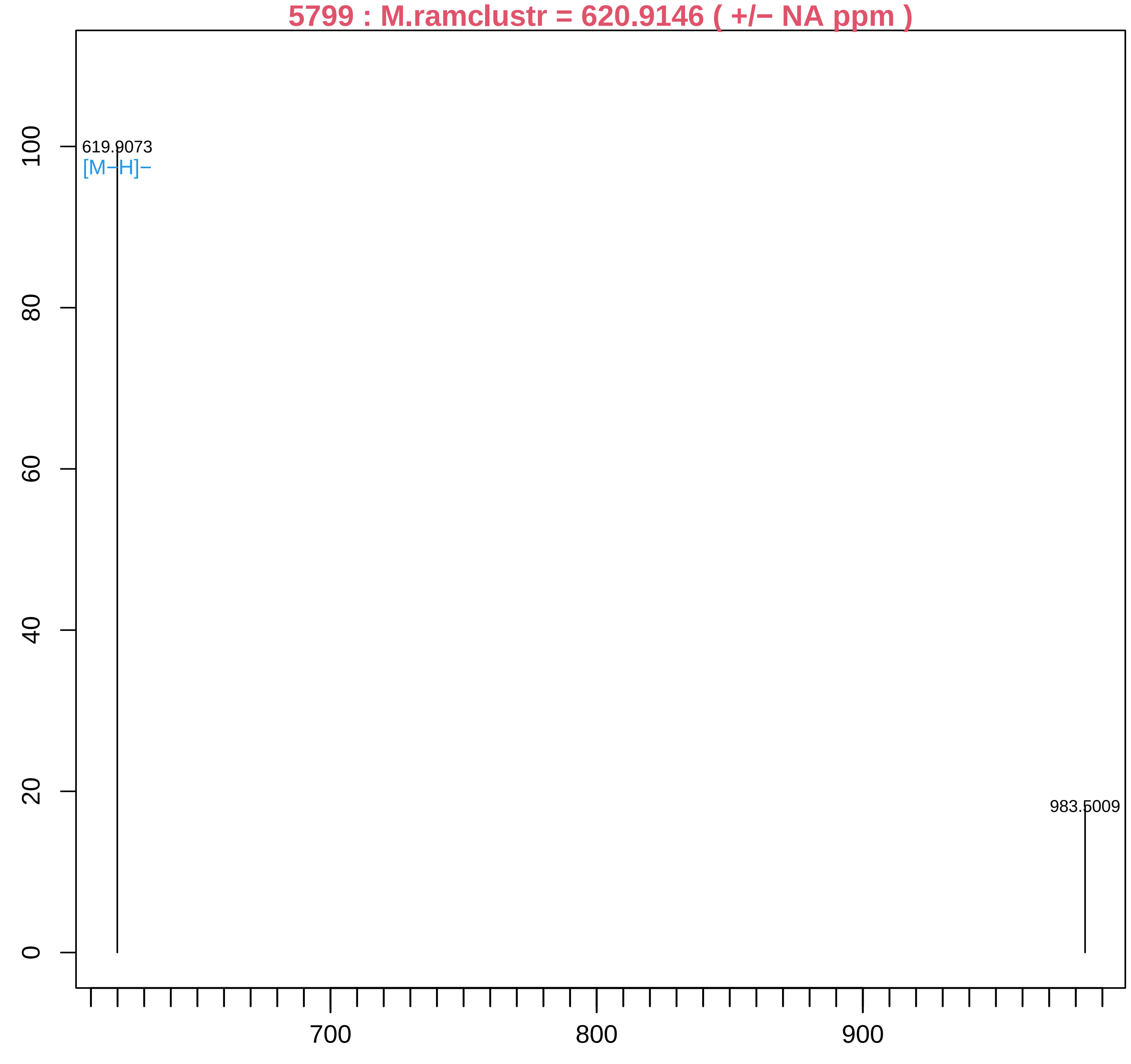

5799 : M.ramclustr = 620.9146 (+/− NA ppm)
619.9073
[M−H]−
983.5009
0
20
40
60
80
100
700
800
900

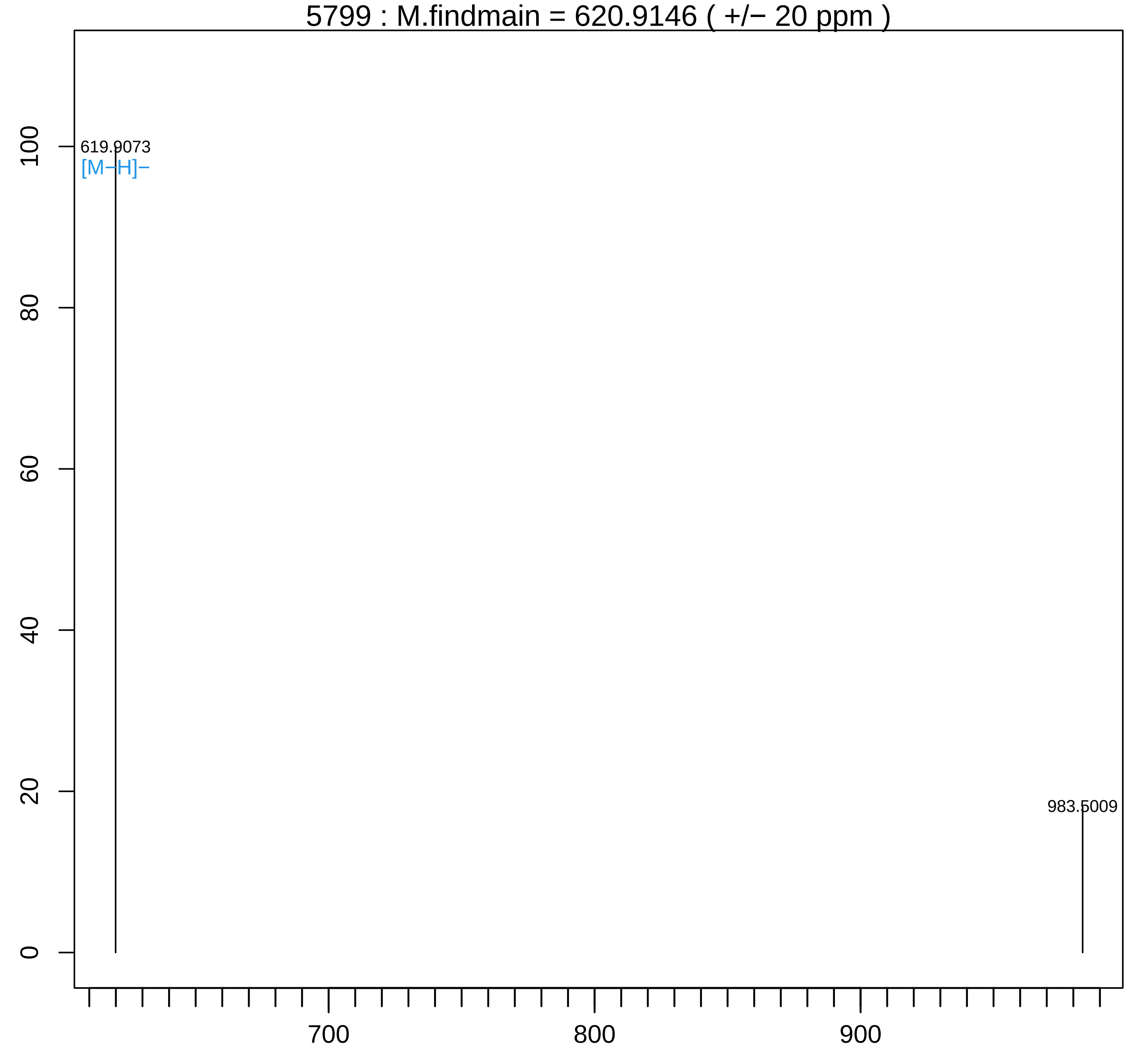

5799 : M.findmain = 620.9146 (+/− 20 ppm)
619.9073
[M−H]−
983.5009
0
20
40
60
80
100
700
800
900

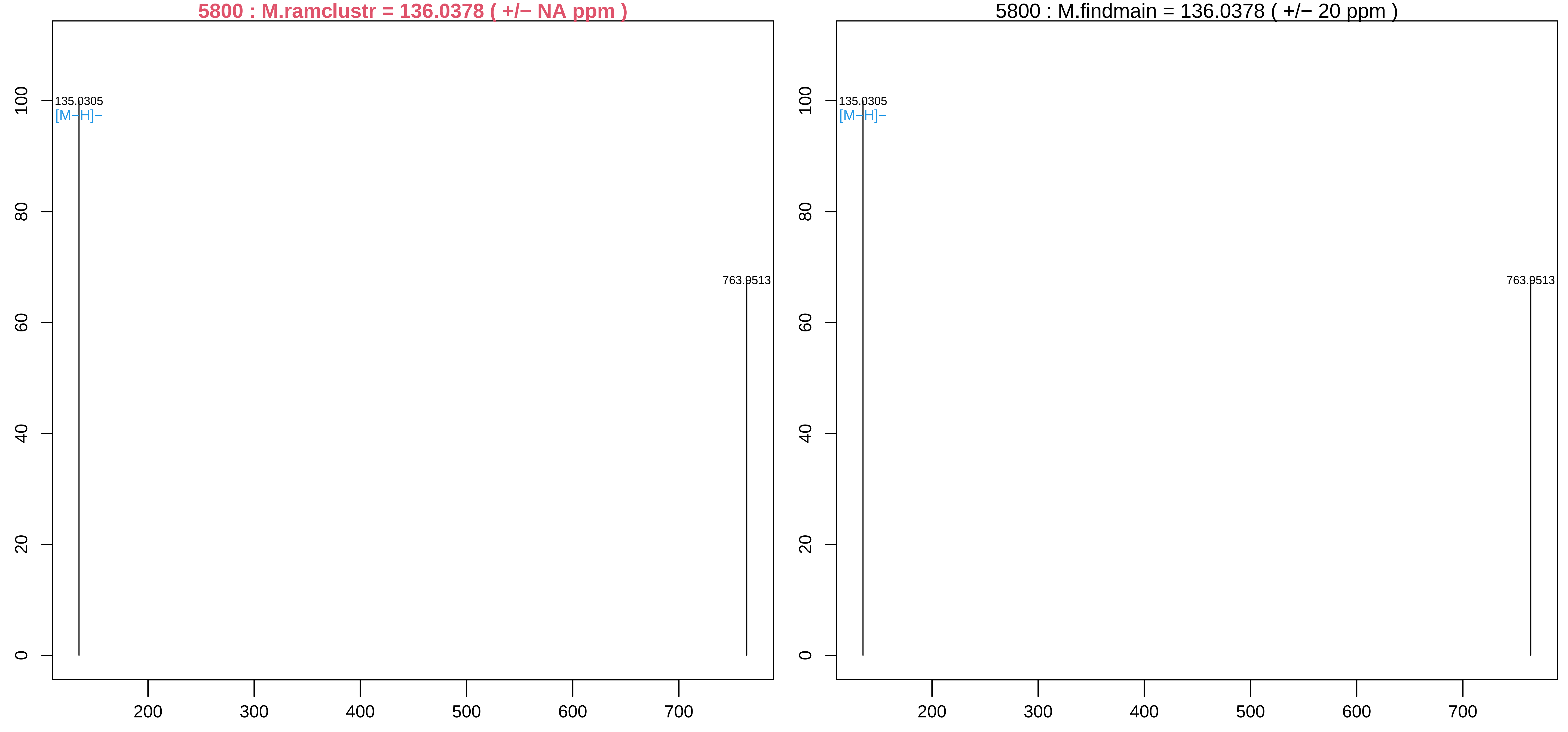

5800 : M.ramclustr = 136.0378 (+/− NA ppm)
135.0305
[M−H]−
763.9513
0
20
40
60
80
100
200
300
400
500
600
700
5800 : M.findmain = 136.0378 (+/− 20 ppm)
135.0305
[M−H]−
763.9513
0
20
40
60
80
100
200
300
400
500
600
700

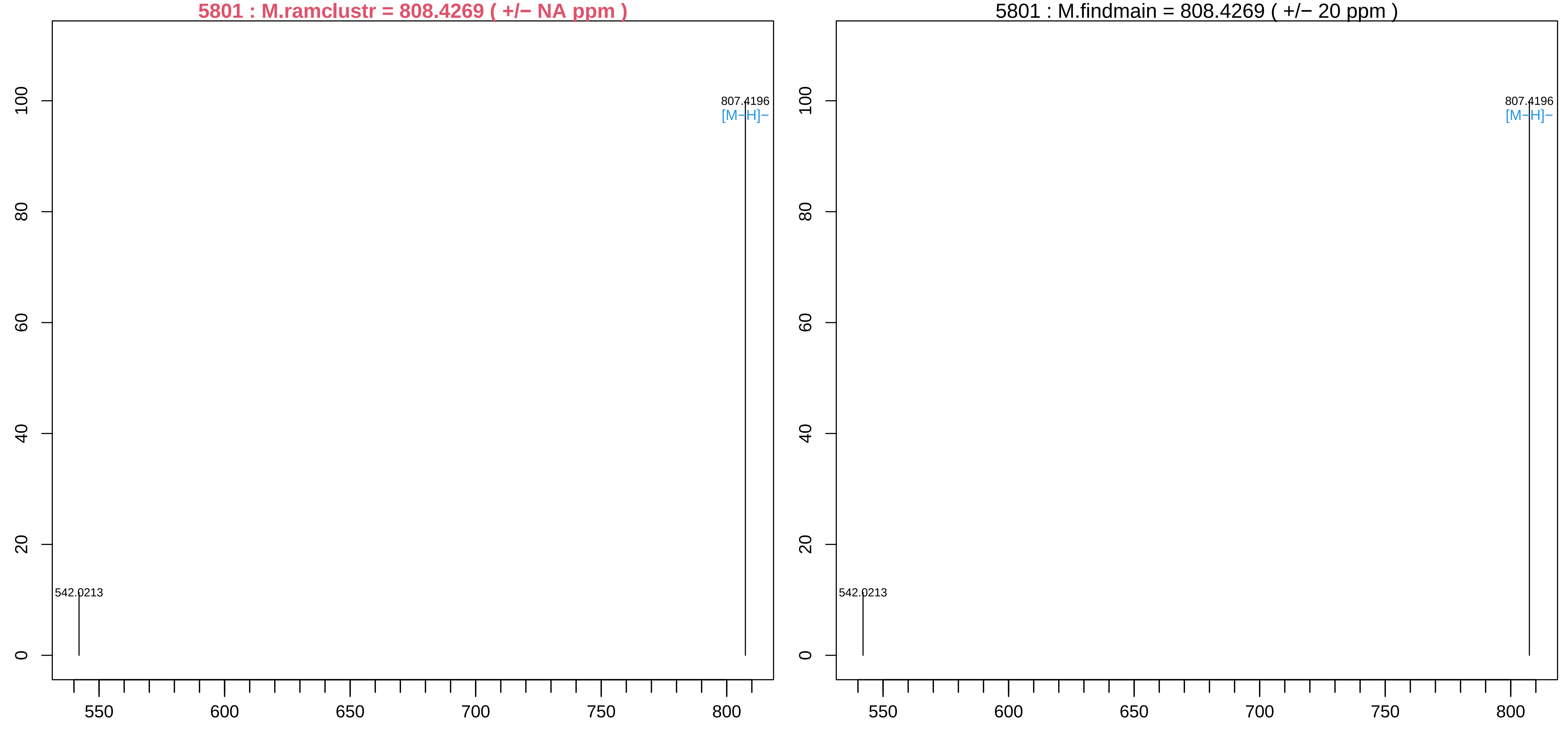
5801 : M.ramclustr = 808.4269 (+/− NA ppm)
807.4196
[M−H]−
542.0213
5801 : M.findmain = 808.4269 (+/− 20 ppm)
807.4196
[M−H]−
542.0213

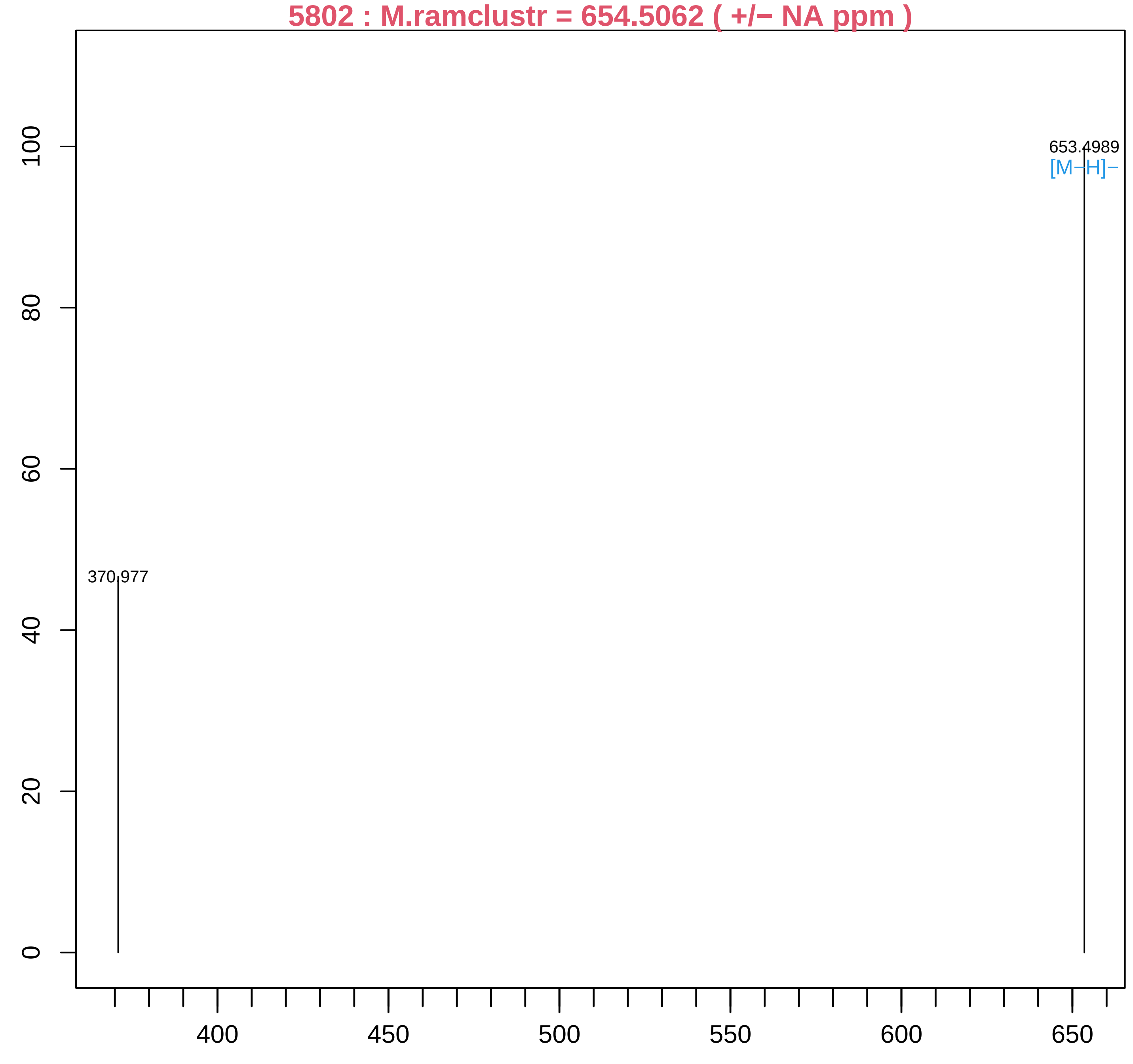
5802 : M.ramclustr = 654.5062 (+/− NA ppm)
653.4989
[M−H]−
370.977

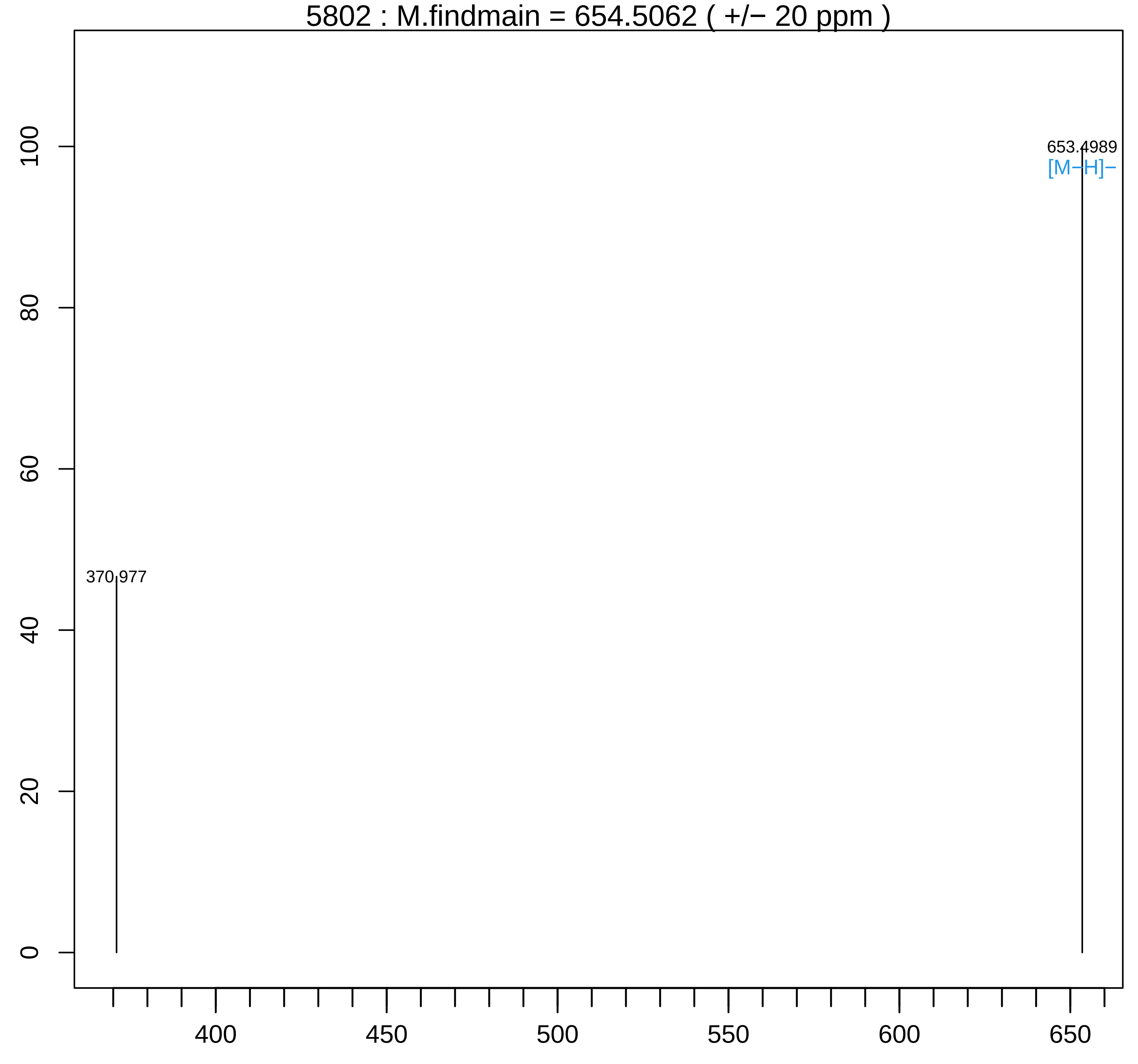
5802 : M.findmain = 654.5062 (+/− 20 ppm)
653.4989
[M−H]−
370.977

5803 : M.ramclustr = 612.5707 (+/− NA ppm)

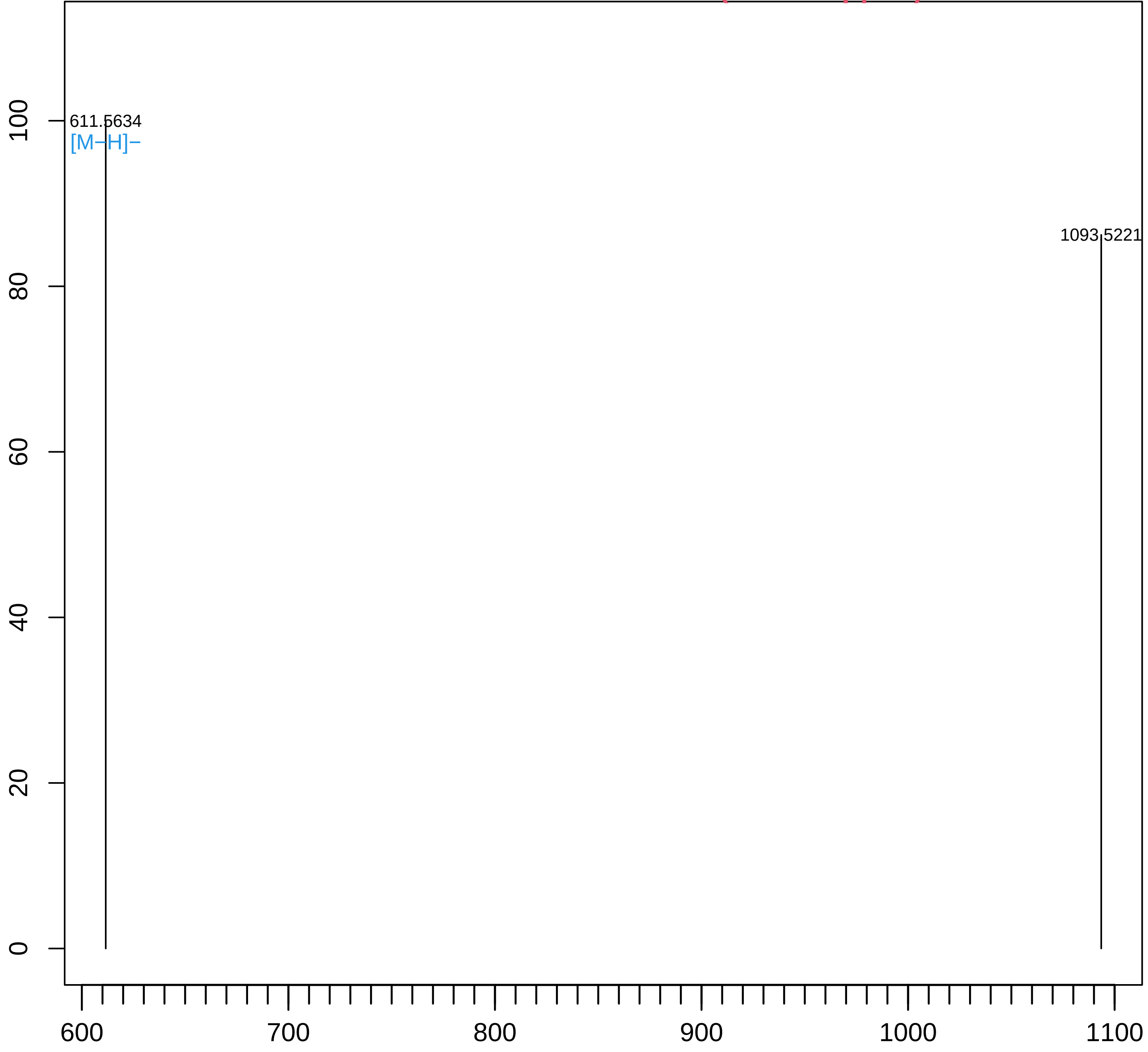

5803 : M.findmain = 612.5707 (+/− 20 ppm)

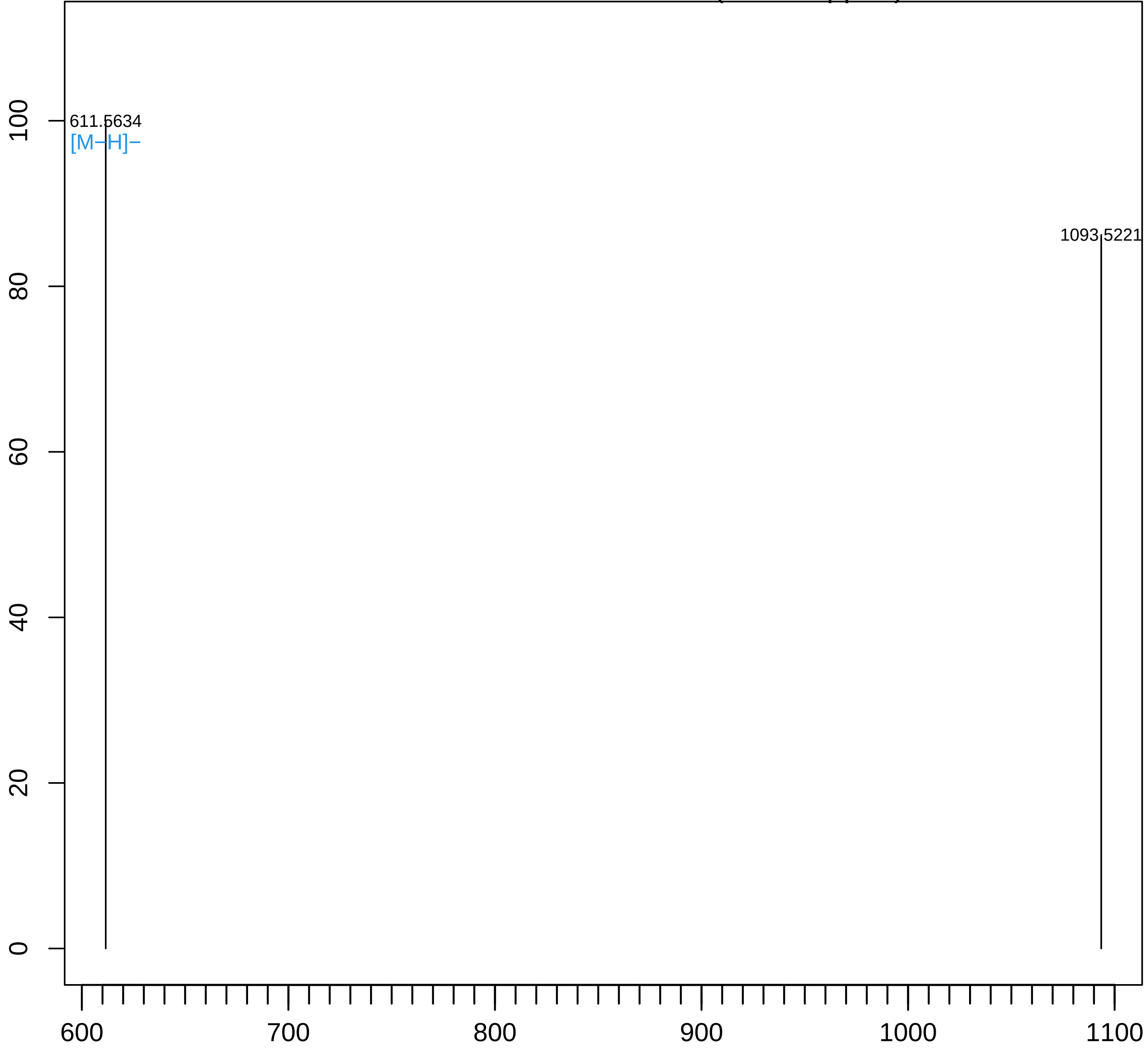

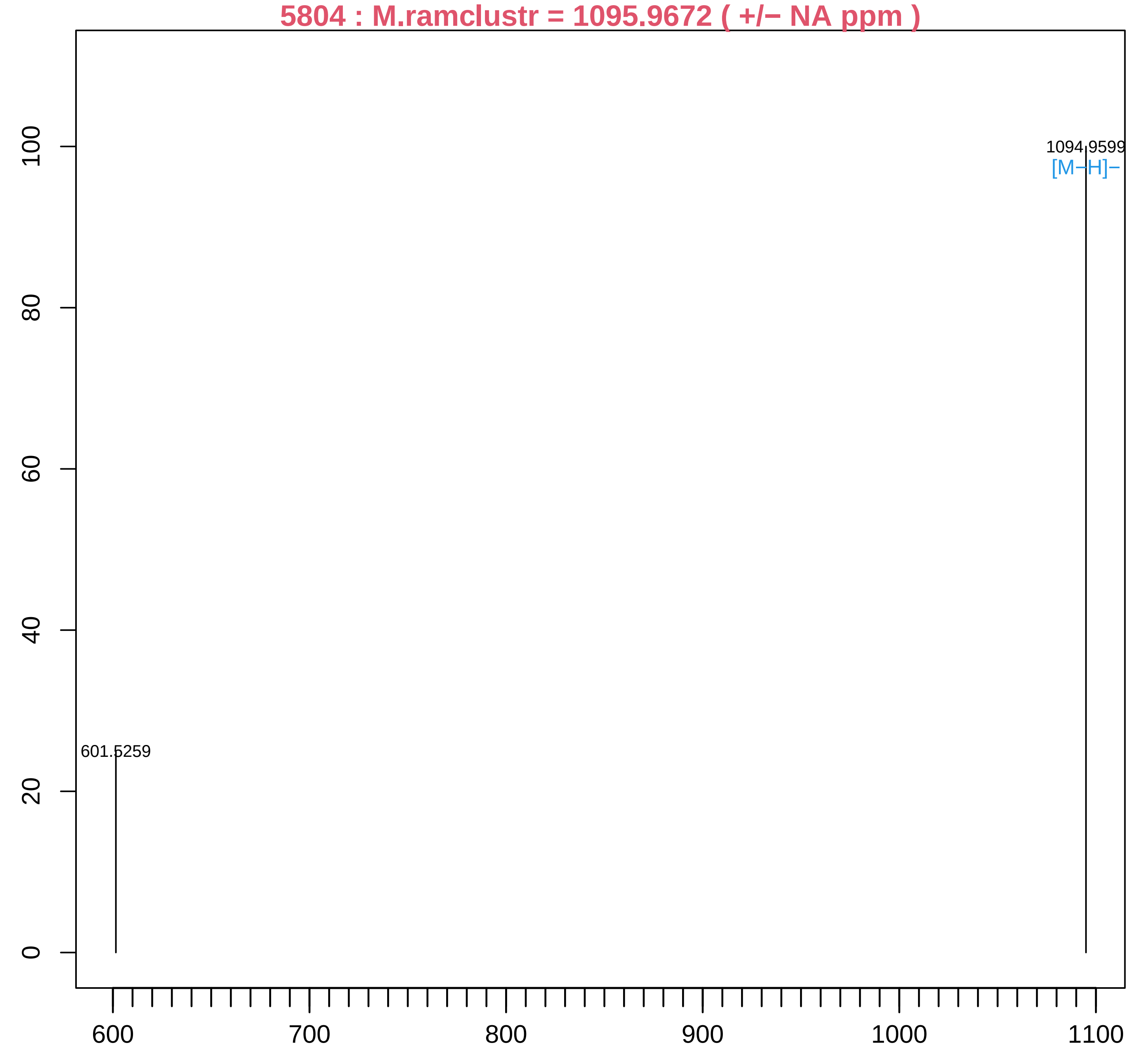
5804 : M.ramclustr = 1095.9672 (+/− NA ppm)
1094.9599
[M−H]−
601.5259
0
20
40
60
80
100
600
700
800
900
1000
1100

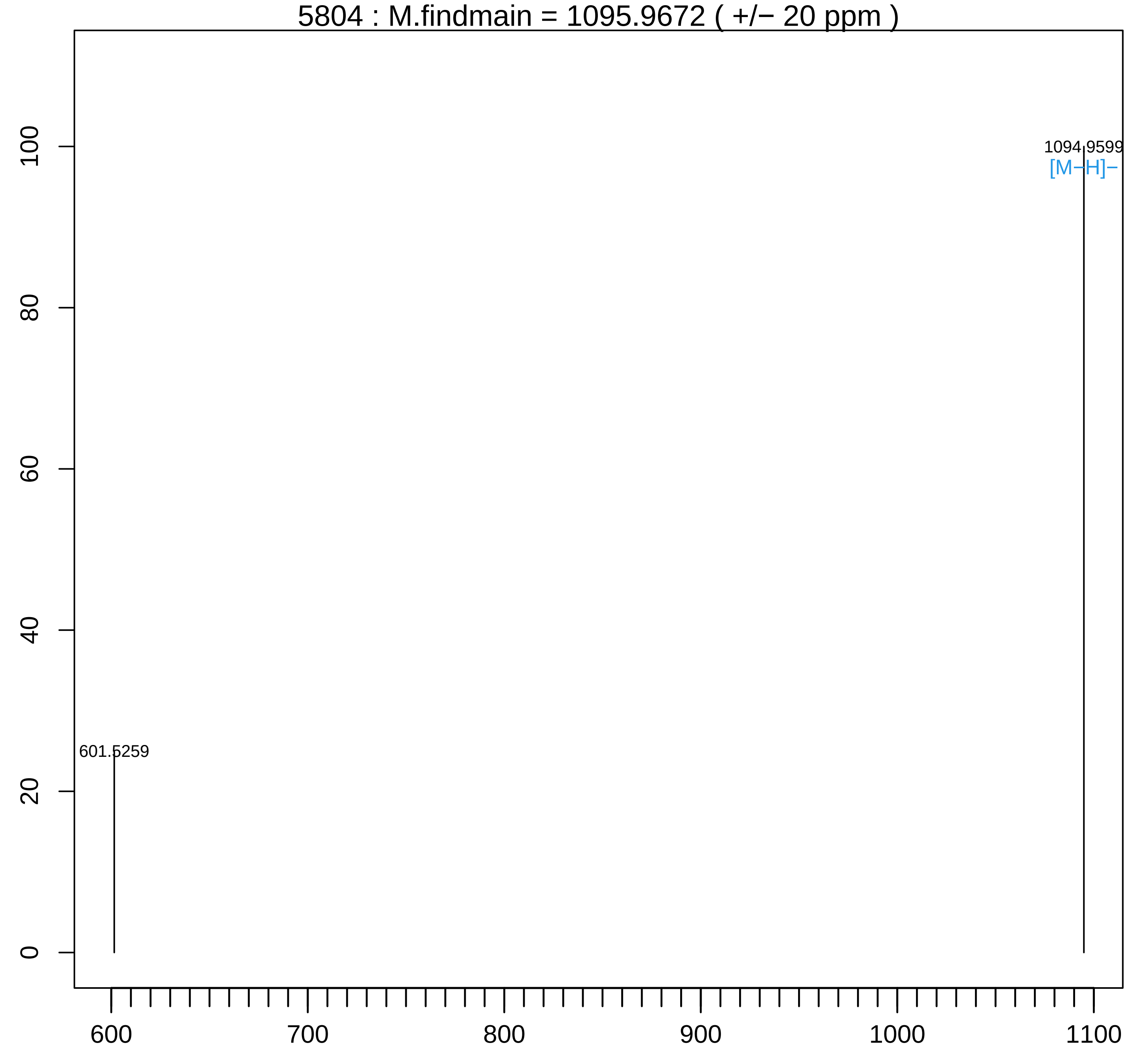
5804 : M.findmain = 1095.9672 (+/− 20 ppm)
1094.9599
[M−H]−
601.5259
0
20
40
60
80
100
600
700
800
900
1000
1100

5805 : M.ramclustr = 859.9681 (+/− NA ppm)

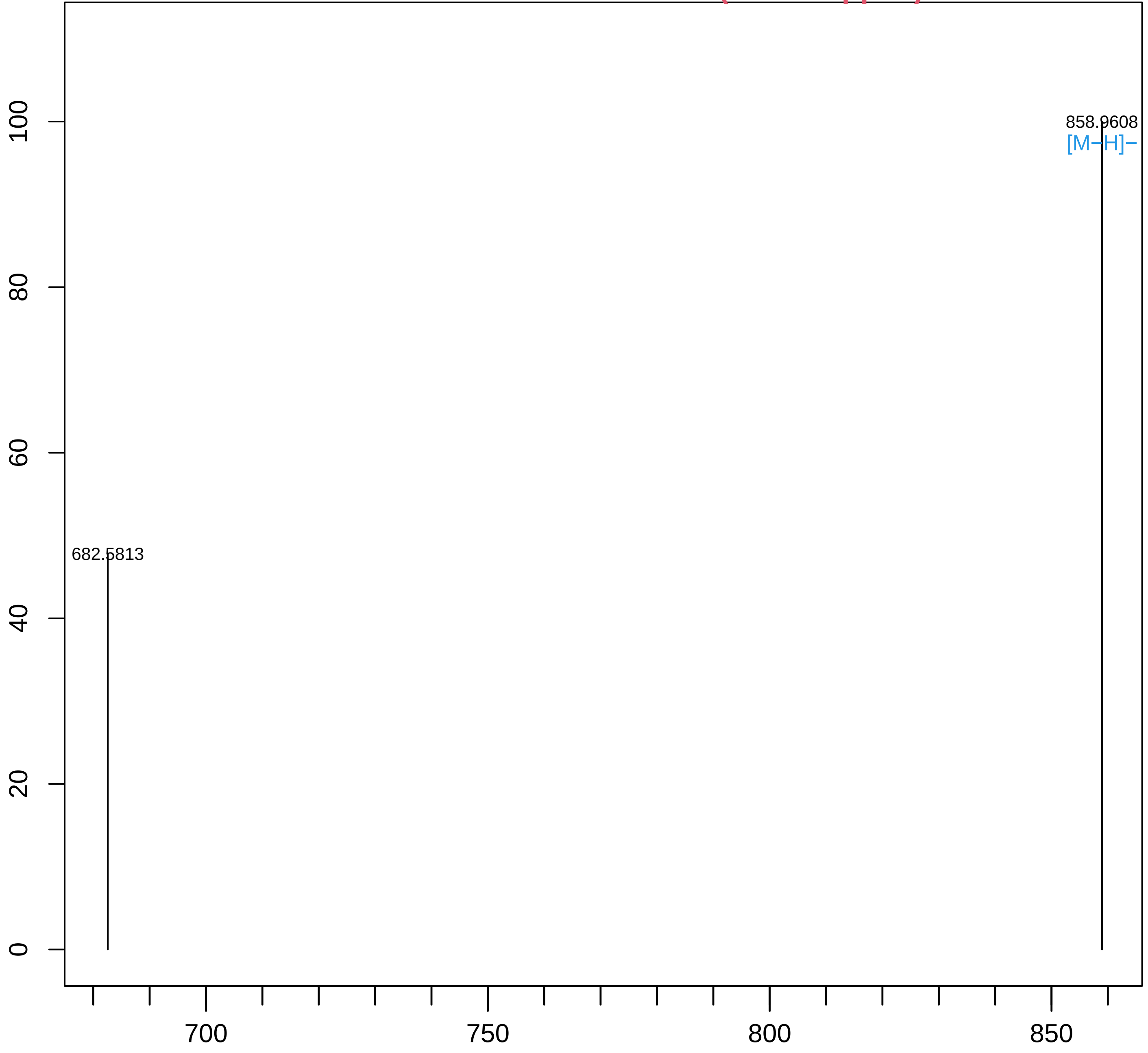

5805 : M.findmain = 859.9681 (+/− 20 ppm)

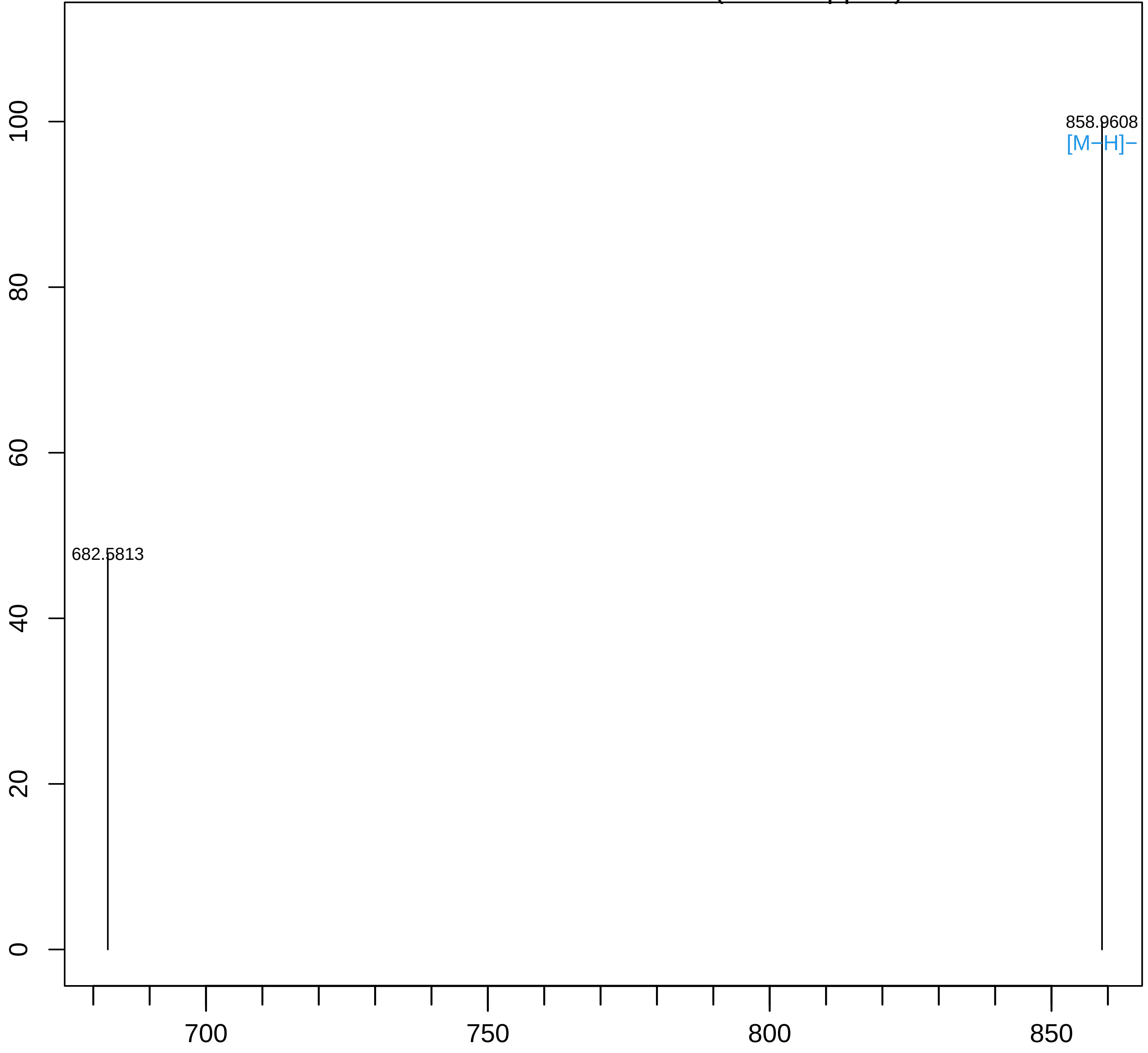

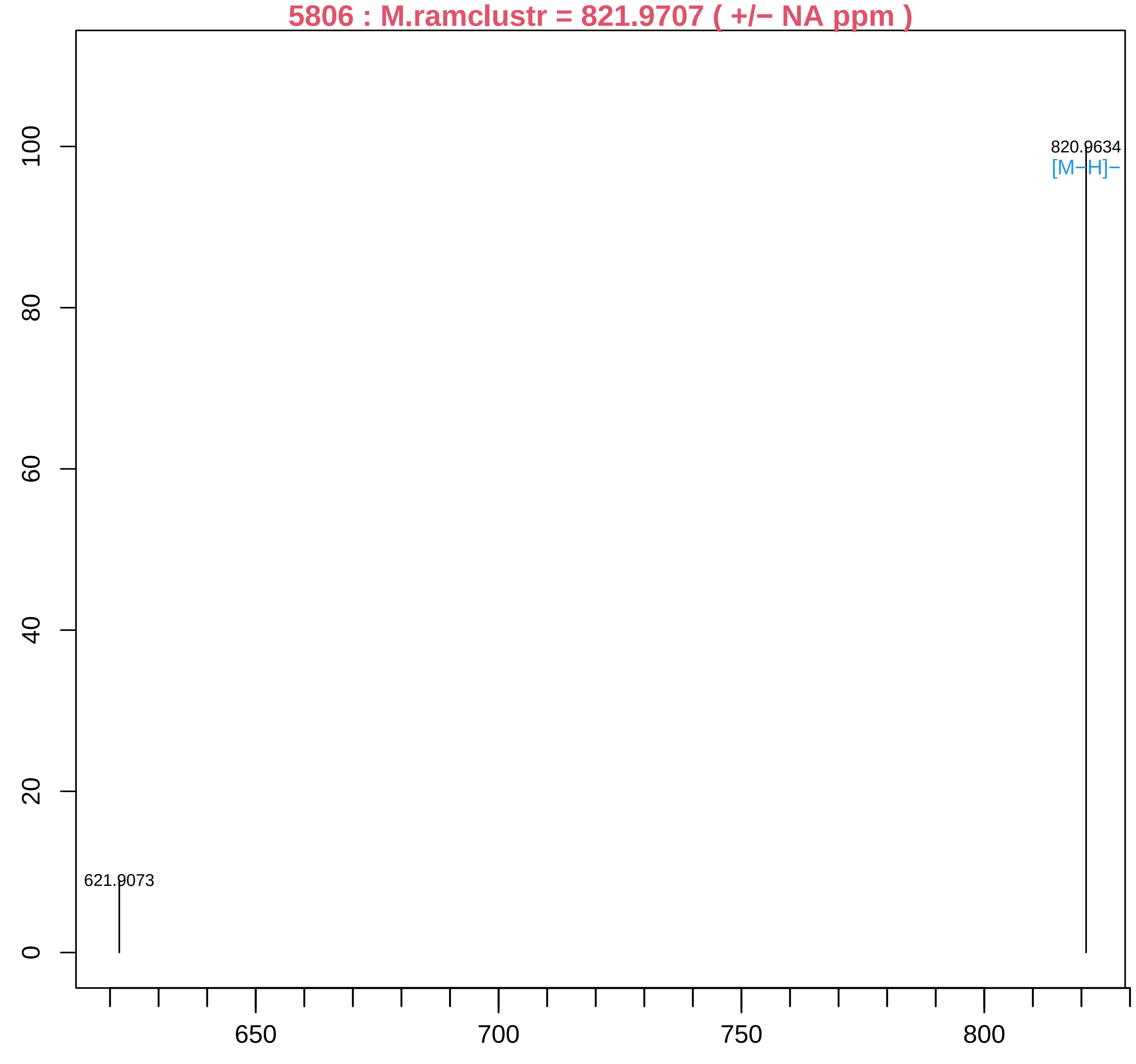
5806 : M.ramclustr = 821.9707 (+/− NA ppm)
820.9634
[M−H]−
621.9073
0
20
40
60
80
100
650
700
750
800

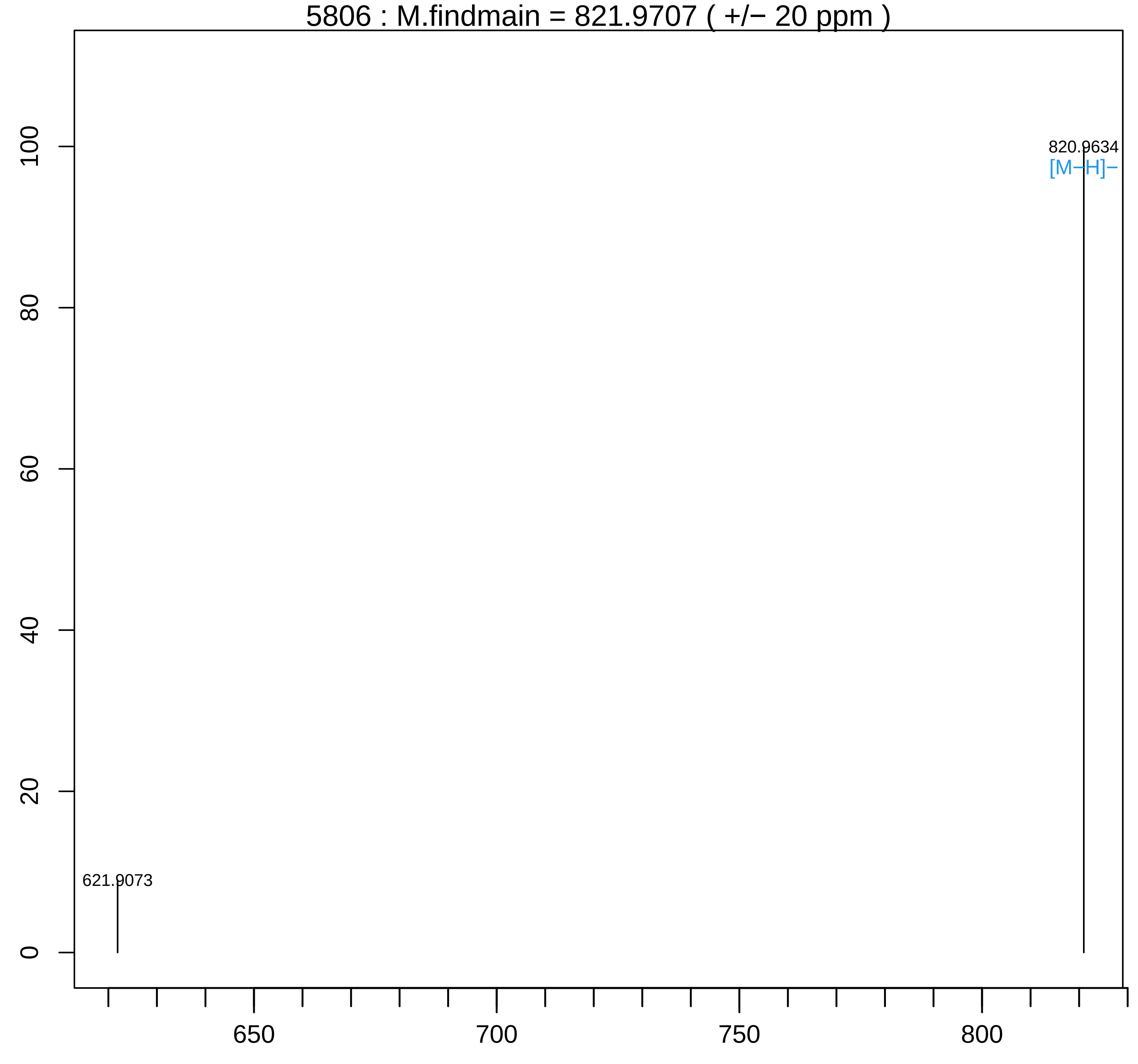
5806 : M.findmain = 821.9707 (+/− 20 ppm)
820.9634
[M−H]−
621.9073
0
20
40
60
80
100
650
700
750
800

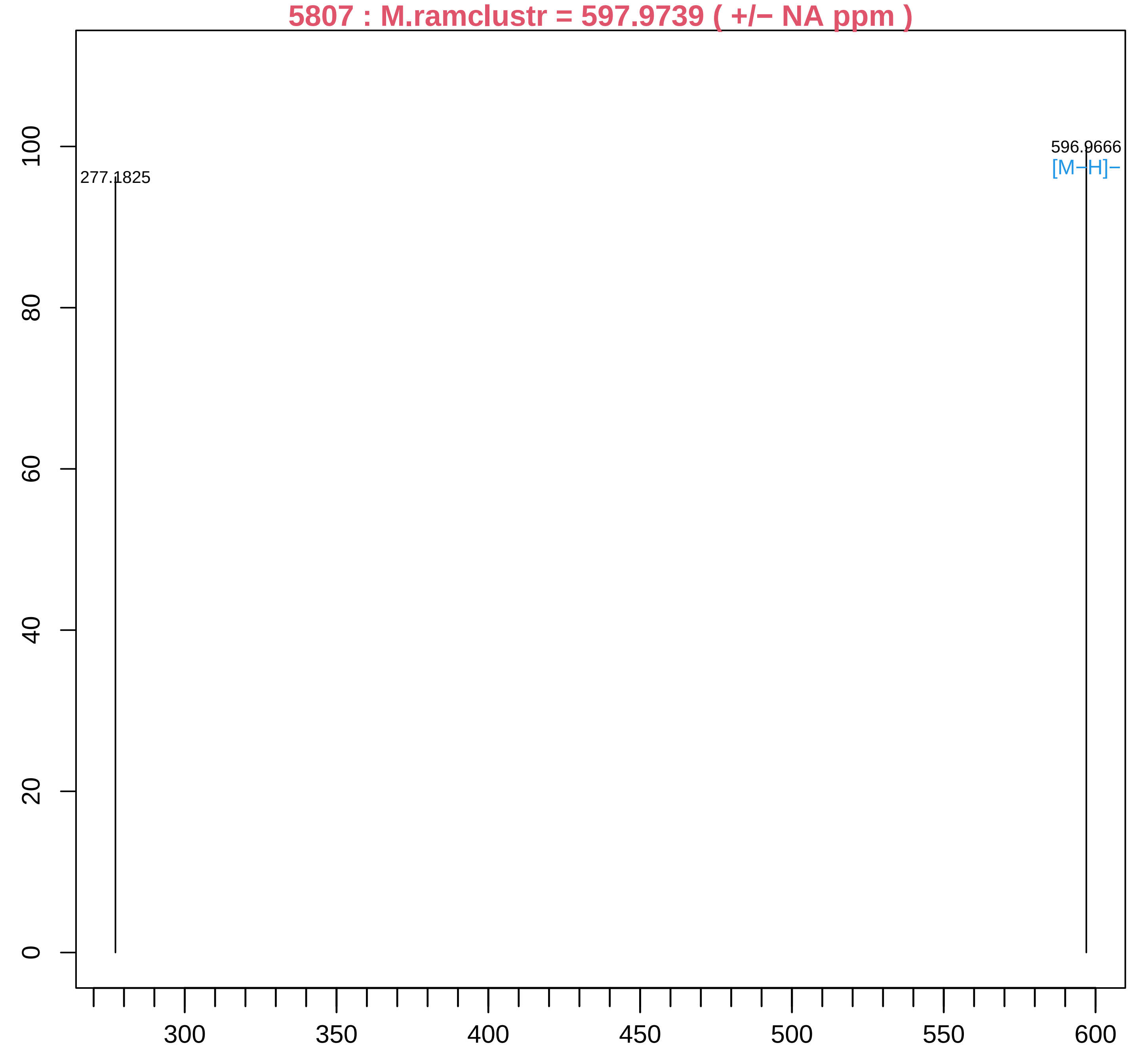
5807 : M.ramclustr = 597.9739 (+/− NA ppm)
277.1825
596.9666
[M−H]−
0
20
40
60
80
100
300
350
400
450
500
550
600

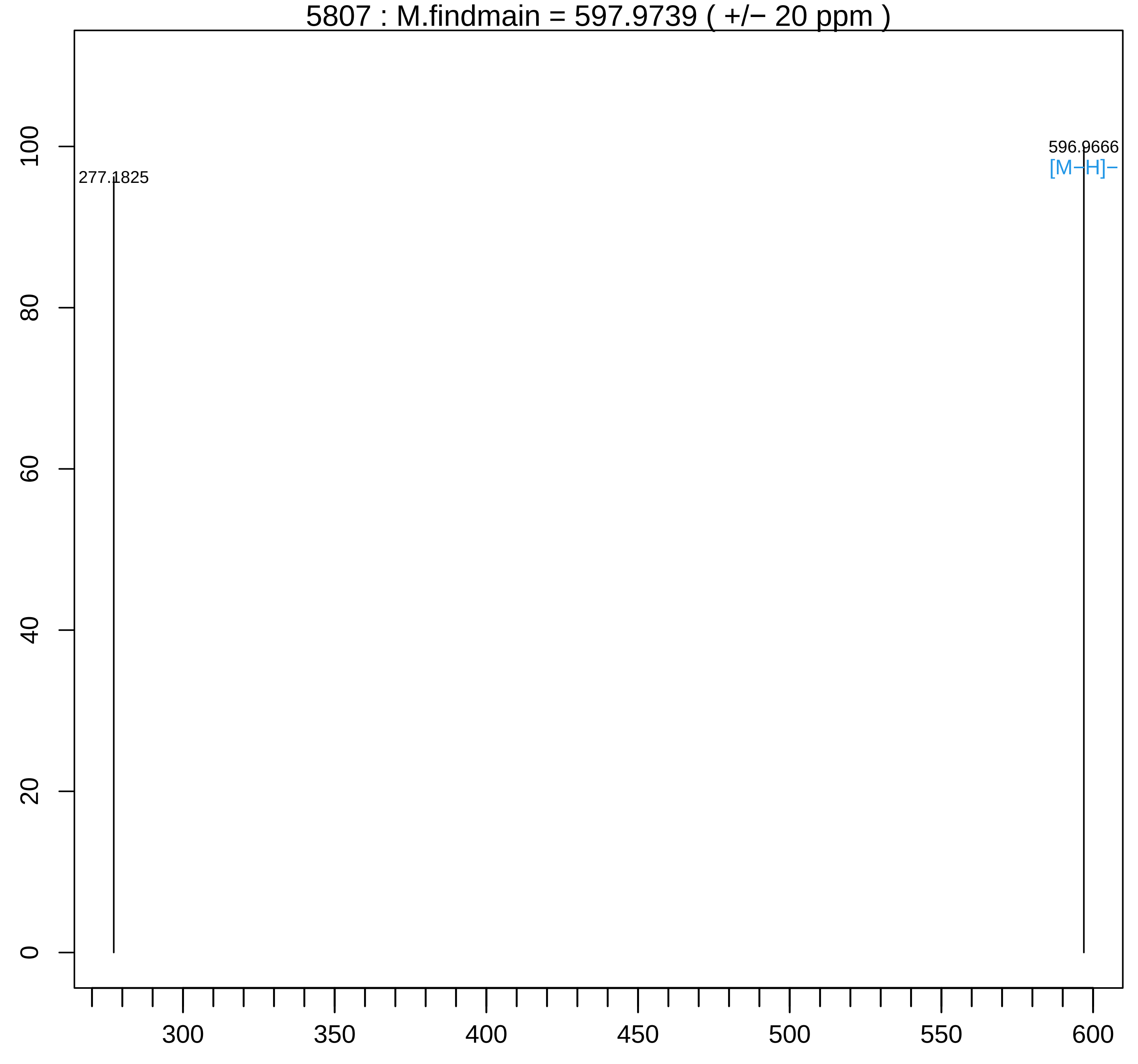
5807 : M.findmain = 597.9739 (+/− 20 ppm)
277.1825
596.9666
[M−H]−
0
20
40
60
80
100
300
350
400
450
500
550
600

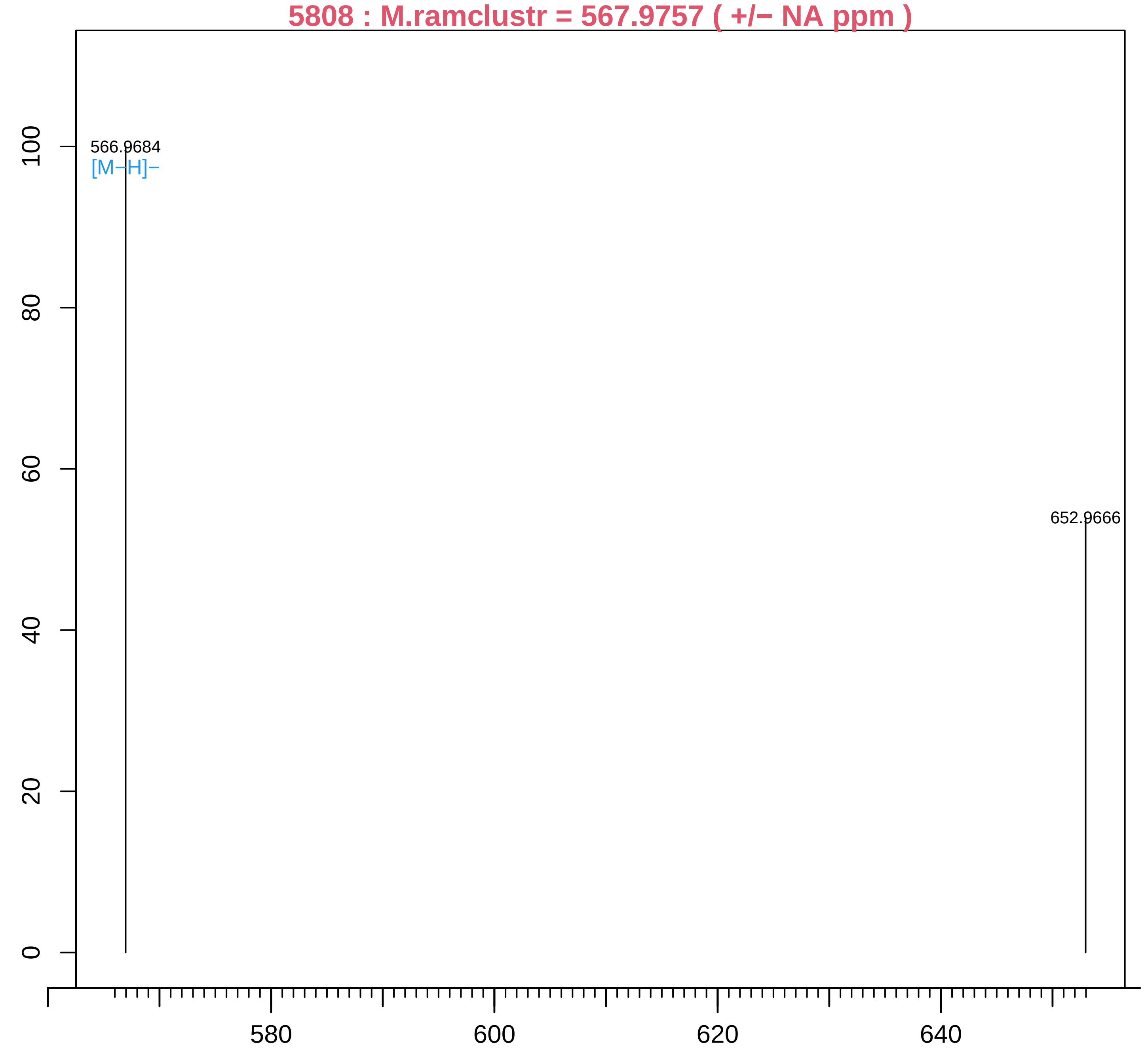
5808 : M.ramclustr = 567.9757 (+/− NA ppm)
566.9684
[M−H]−
652.9666
0
20
40
60
80
100
580
600
620
640

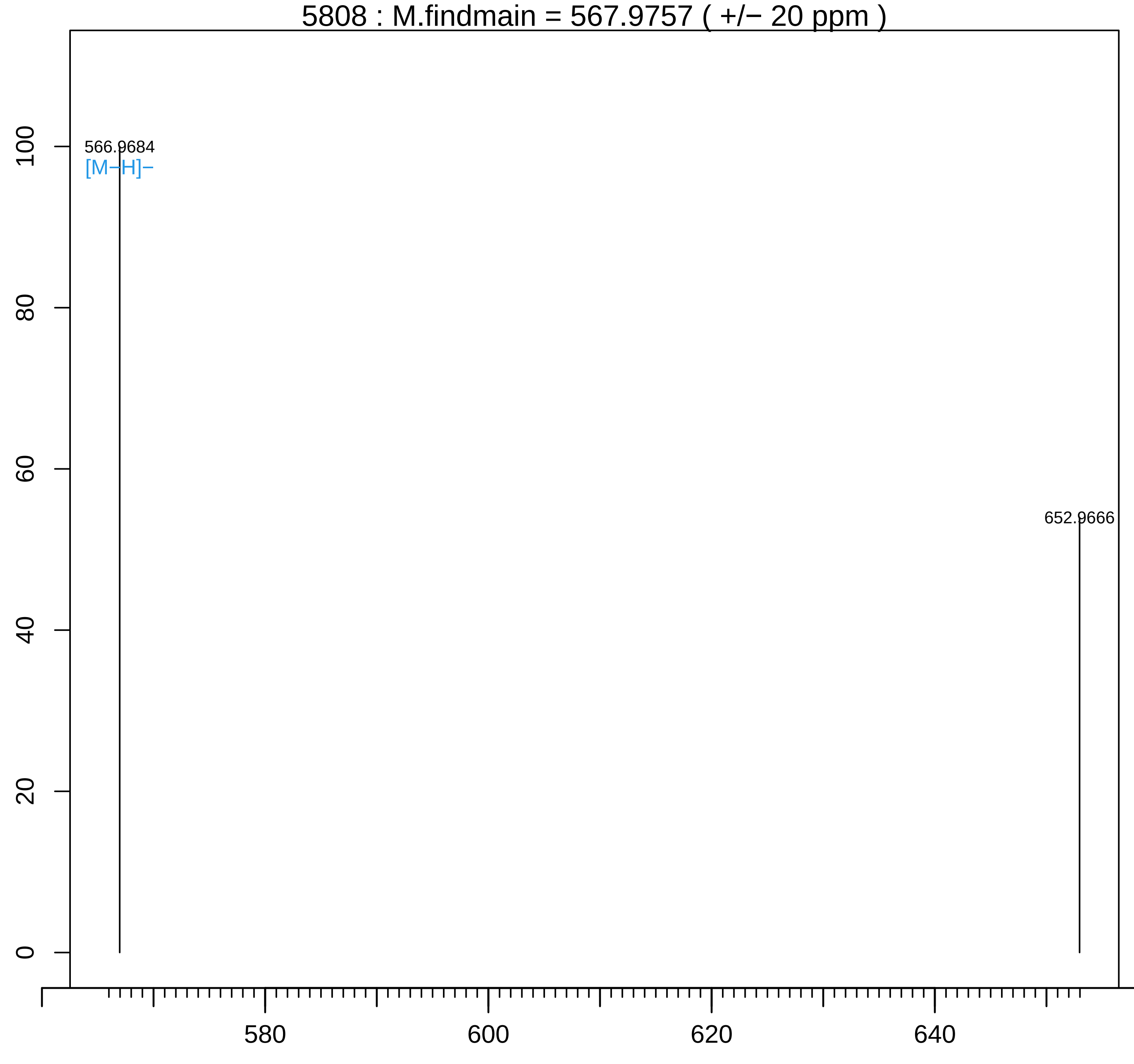
5808 : M.findmain = 567.9757 (+/− 20 ppm)
566.9684
[M−H]−
652.9666
0
20
40
60
80
100
580
600
620
640

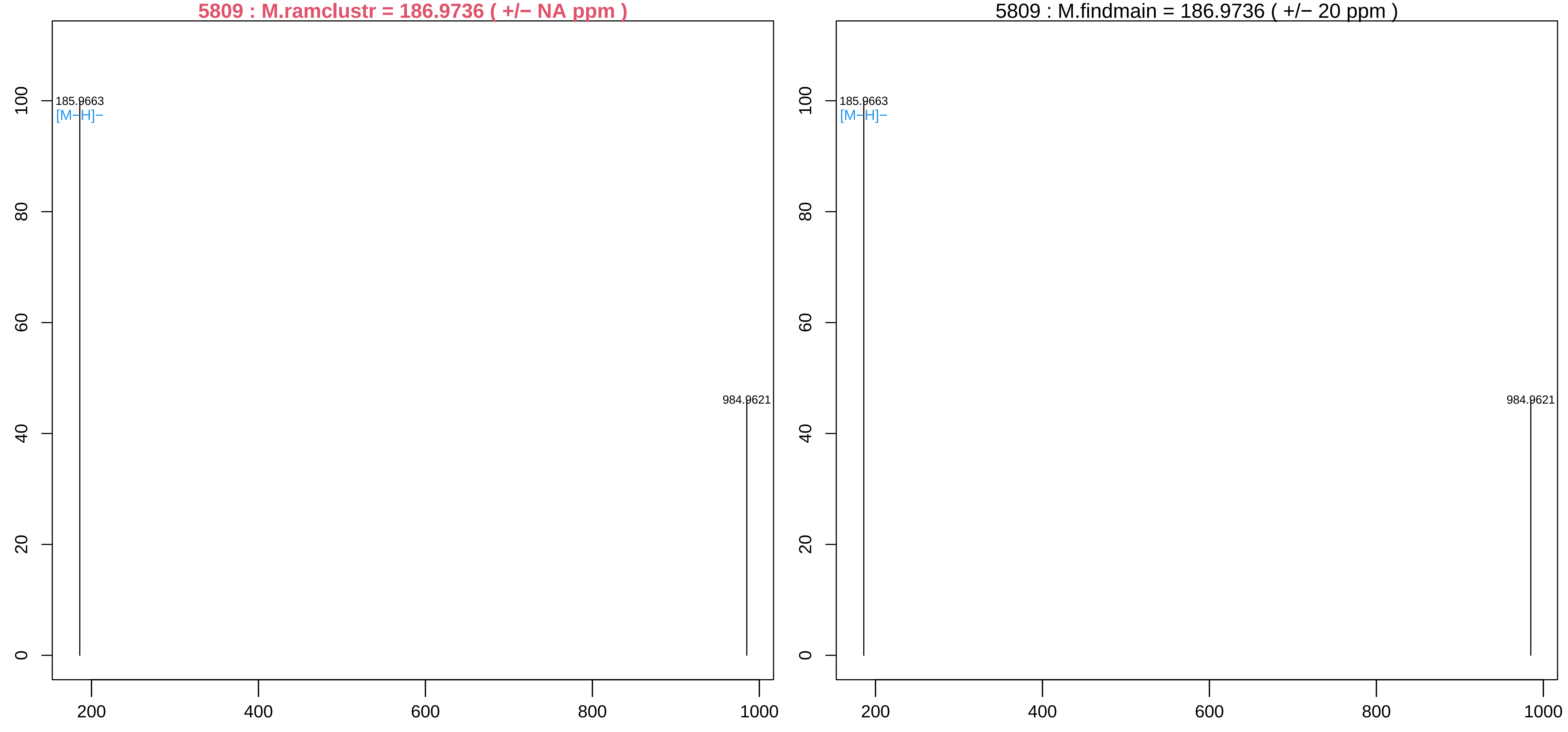
5809 : M.ramclustr = 186.9736 (+/− NA ppm)
5809 : M.findmain = 186.9736 (+/− 20 ppm)
185.9663
[M−H]−
984.9621
185.9663
[M−H]−
984.9621
0
20
40
60
80
100
200
400
600
800
1000

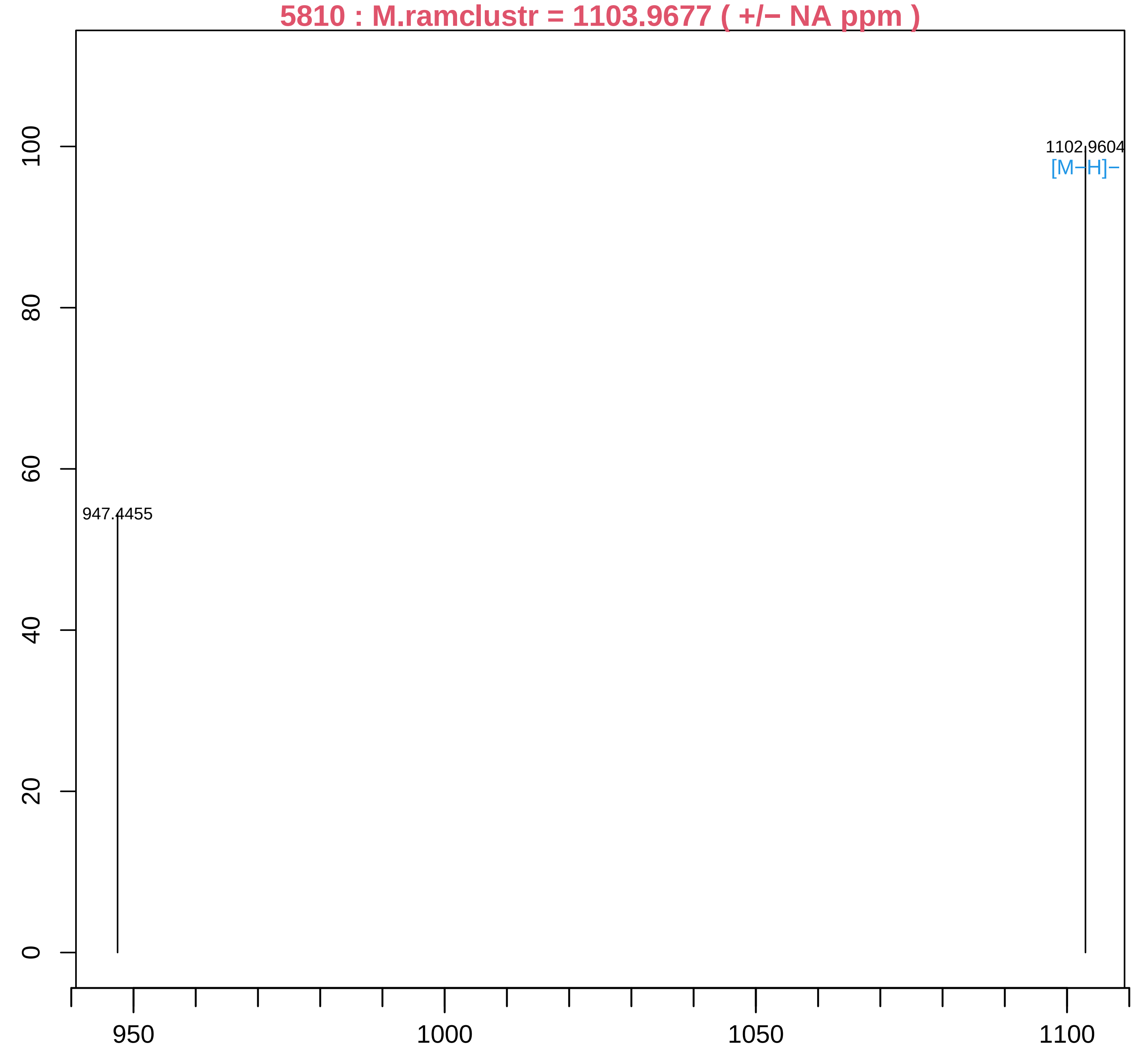
5810 : M.ramclustr = 1103.9677 (+/− NA ppm)
1102.9604
[M−H]−
947.4455
0
20
40
60
80
100
950
1000
1050
1100

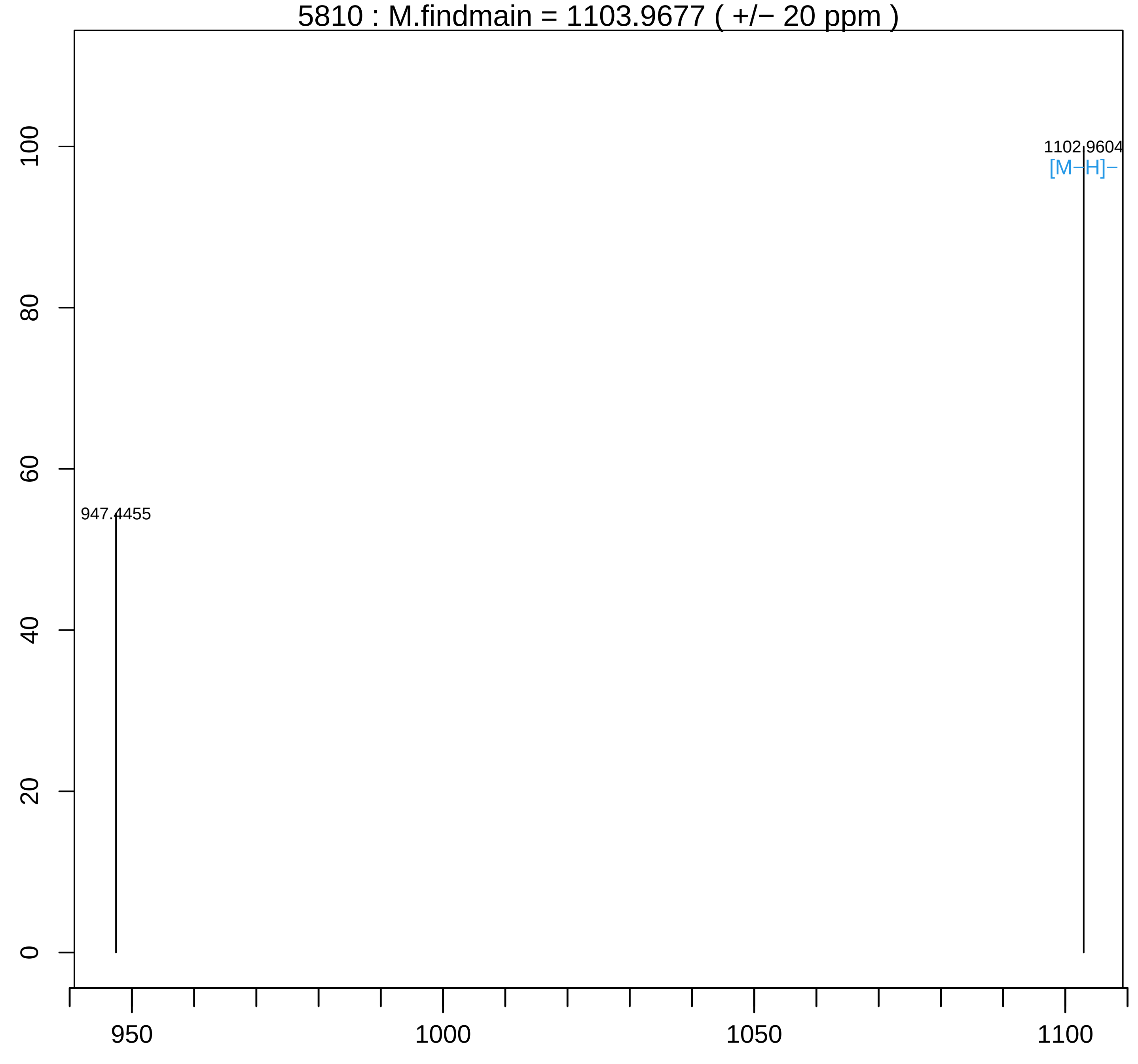
5810 : M.findmain = 1103.9677 (+/− 20 ppm)
1102.9604
[M−H]−
947.4455
0
20
40
60
80
100
950
1000
1050
1100

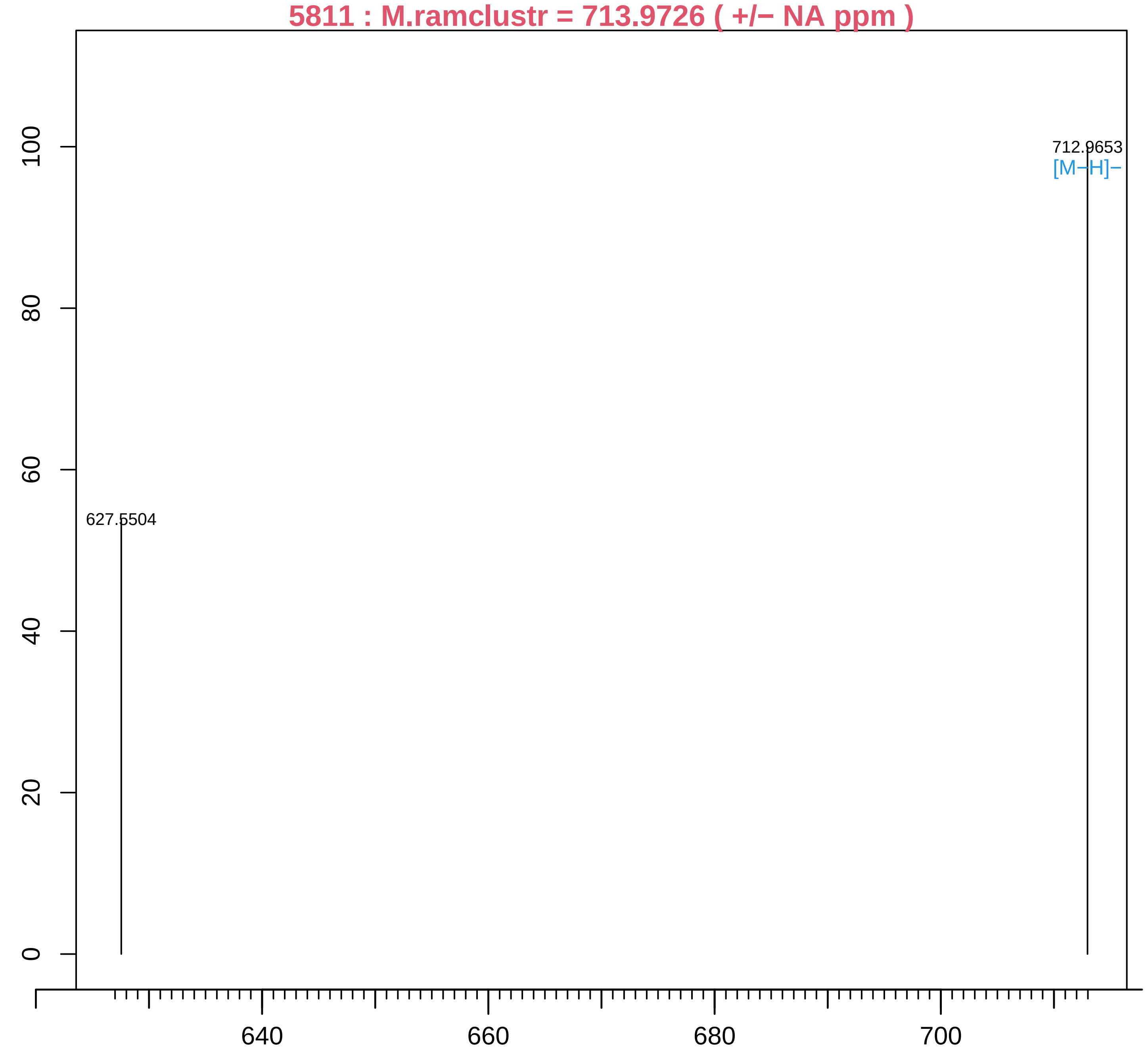
5811 : M.ramclustr = 713.9726 (+/− NA ppm)
712.9653
[M−H]−
627.5504
0
20
40
60
80
100
640
660
680
700

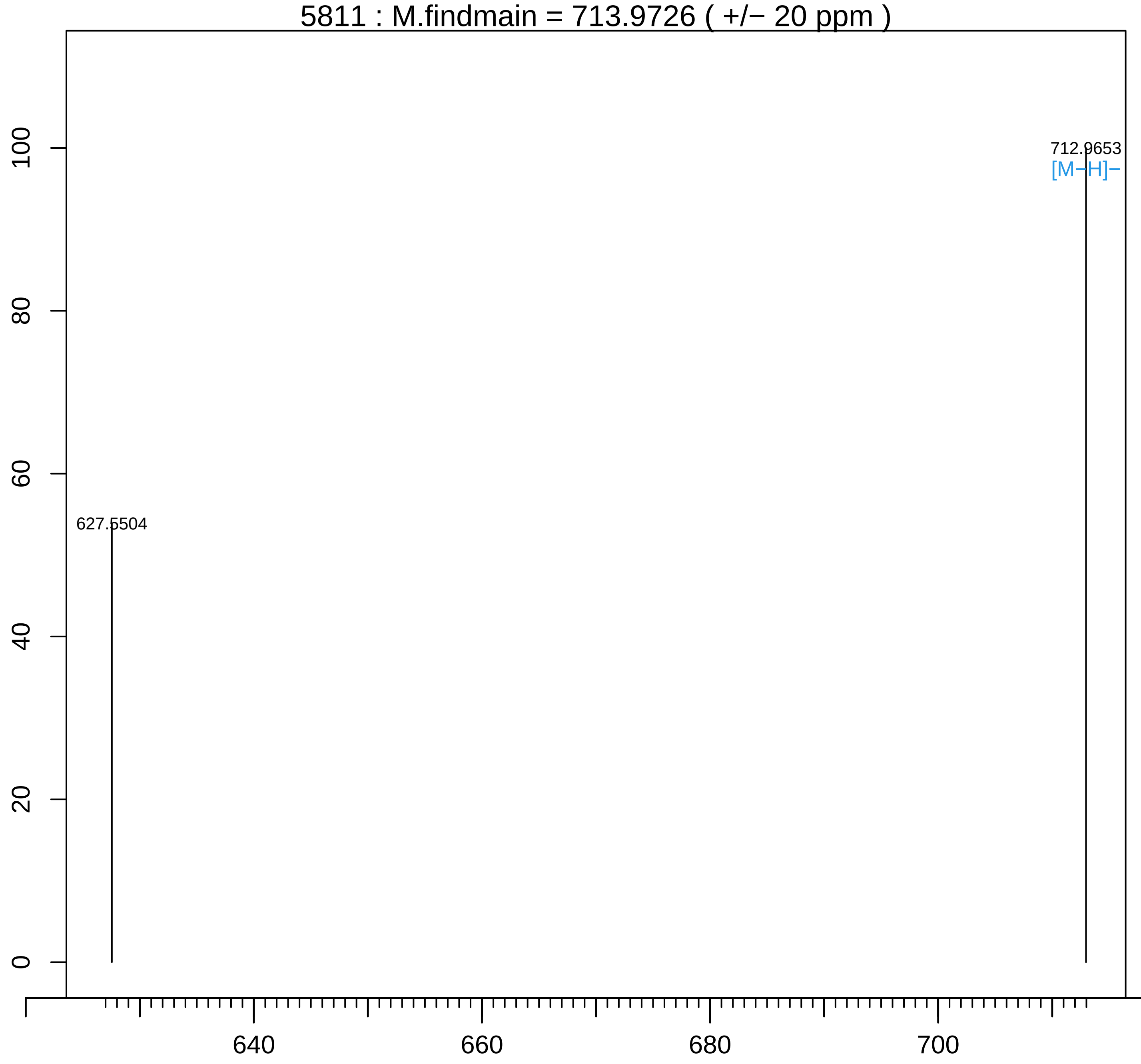
5811 : M.findmain = 713.9726 (+/− 20 ppm)
712.9653
[M−H]−
627.5504
0
20
40
60
80
100
640
660
680
700

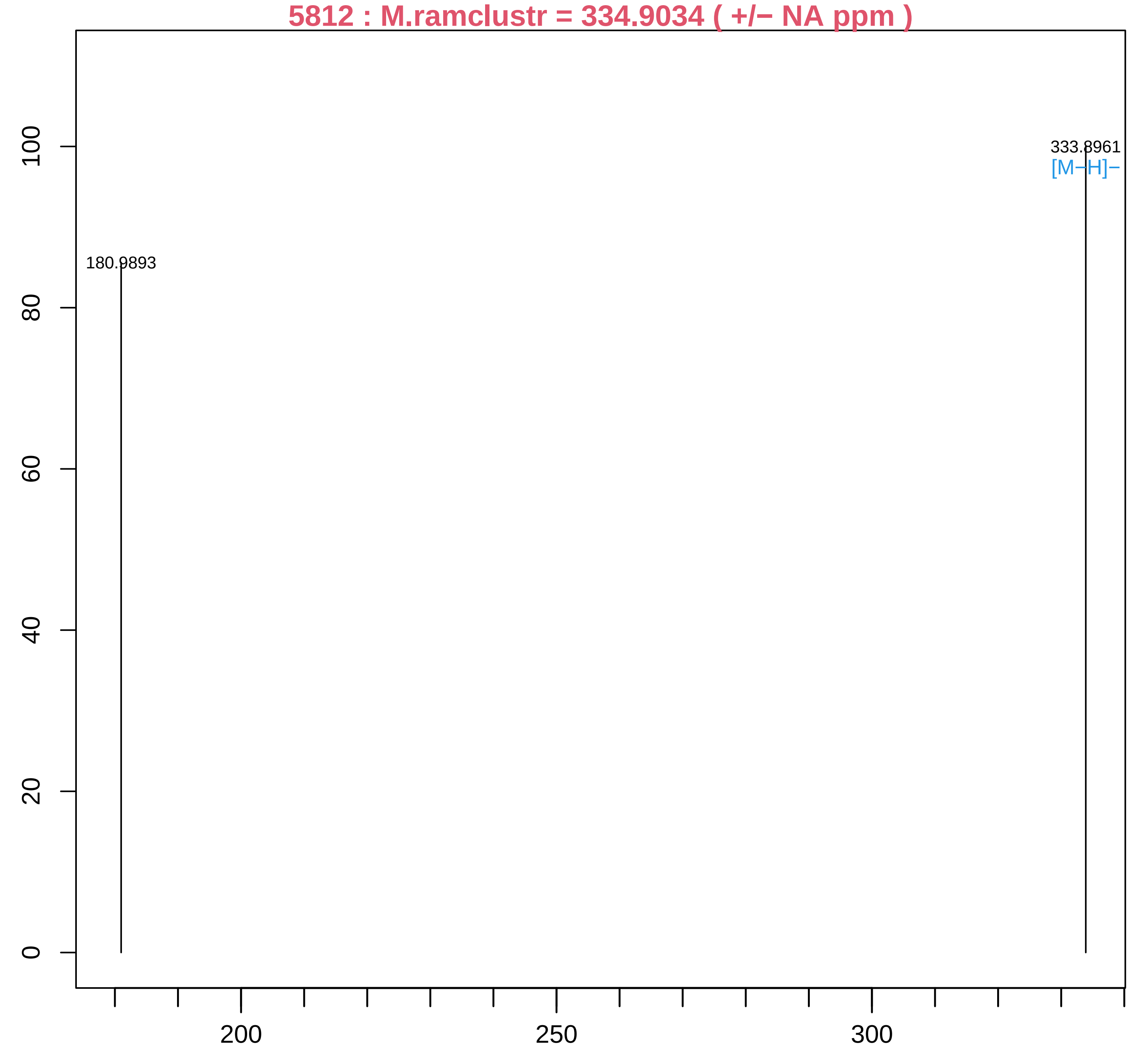
5812 : M.ramclustr = 334.9034 (+/− NA ppm)
333.8961
[M−H]−
180.9893
0
20
40
60
80
100
200
250
300

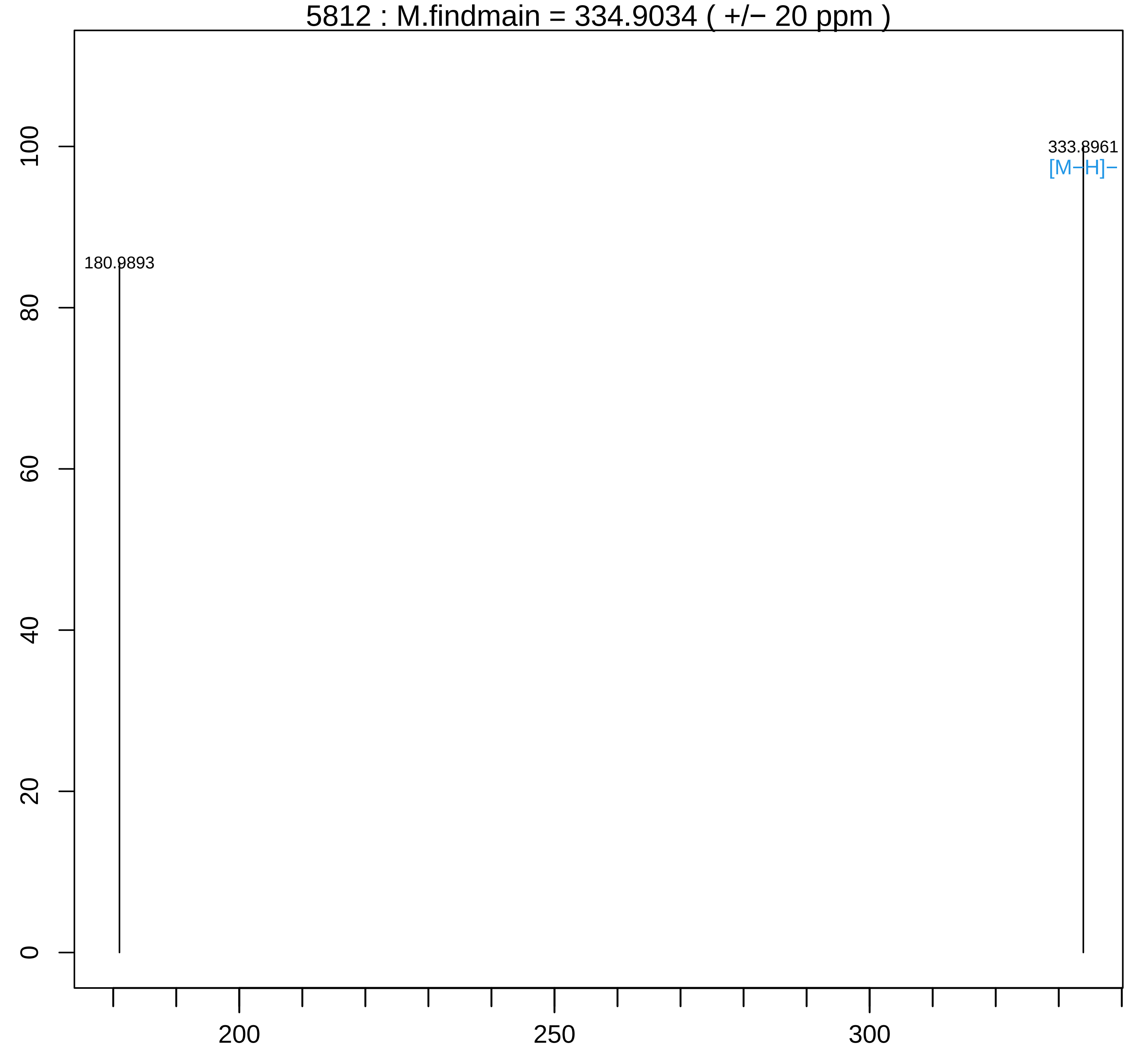
5812 : M.findmain = 334.9034 (+/− 20 ppm)
333.8961
[M−H]−
180.9893
0
20
40
60
80
100
200
250
300

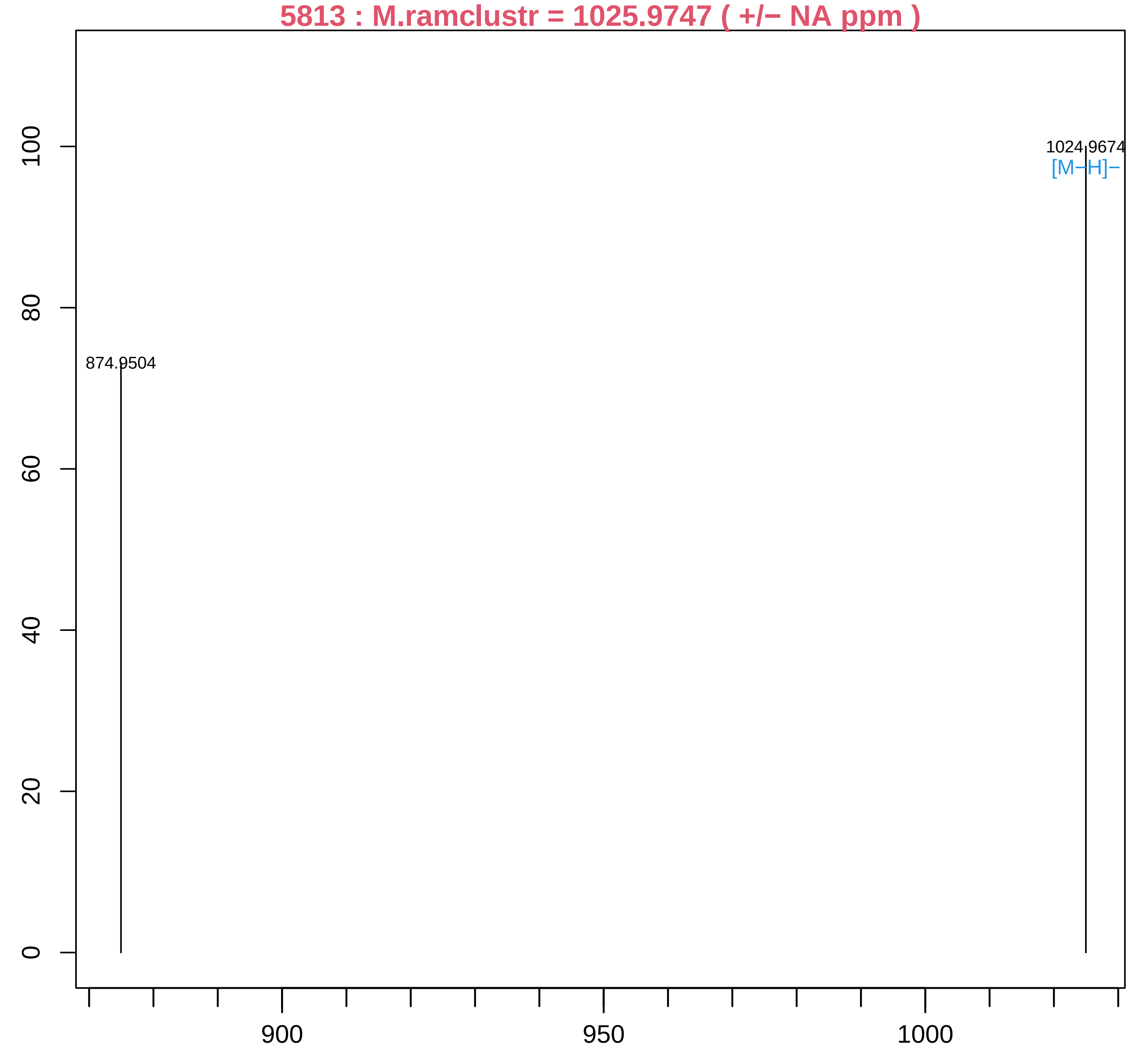
5813 : M.ramclustr = 1025.9747 (+/− NA ppm)
100
80
60
40
20
0
900
950
1000
874.9504
1024.9674
[M−H]−

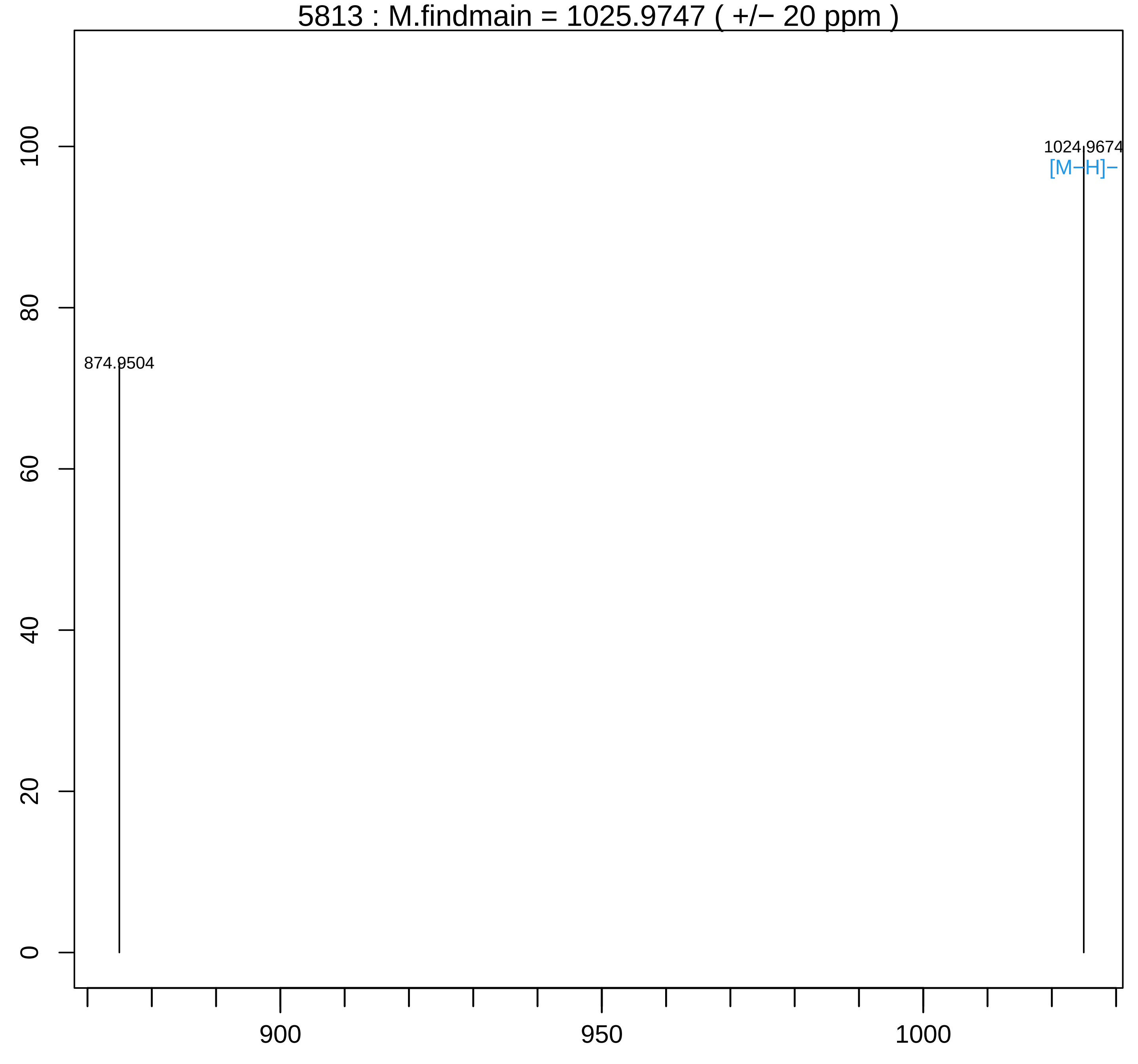
5813 : M.findmain = 1025.9747 (+/− 20 ppm)
100
80
60
40
20
0
900
950
1000
874.9504
1024.9674
[M−H]−

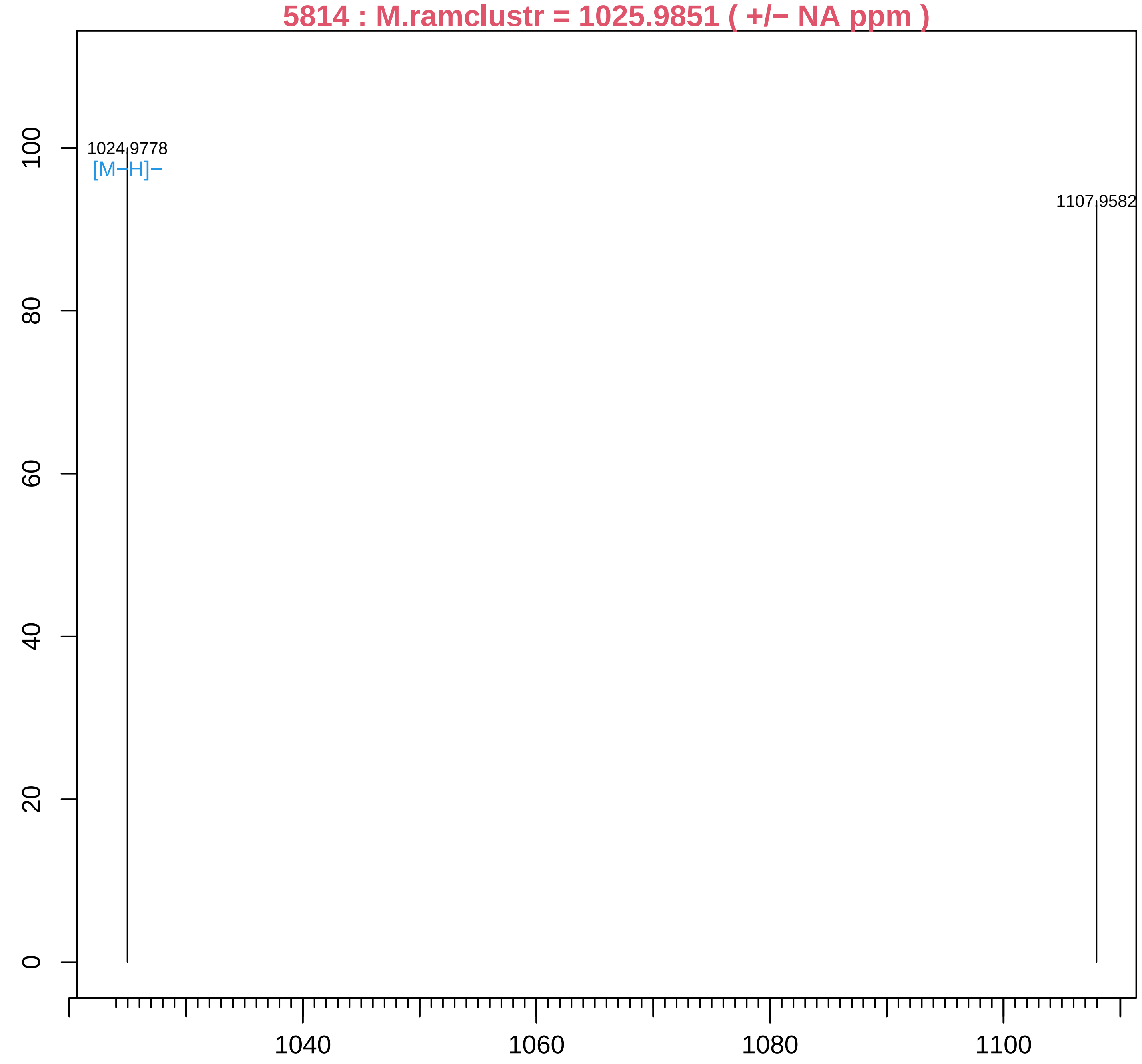
5814 : M.ramclustr = 1025.9851 (+/− NA ppm)
1024.9778
[M−H]−
1107.9582
0
20
40
60
80
100
1040
1060
1080
1100

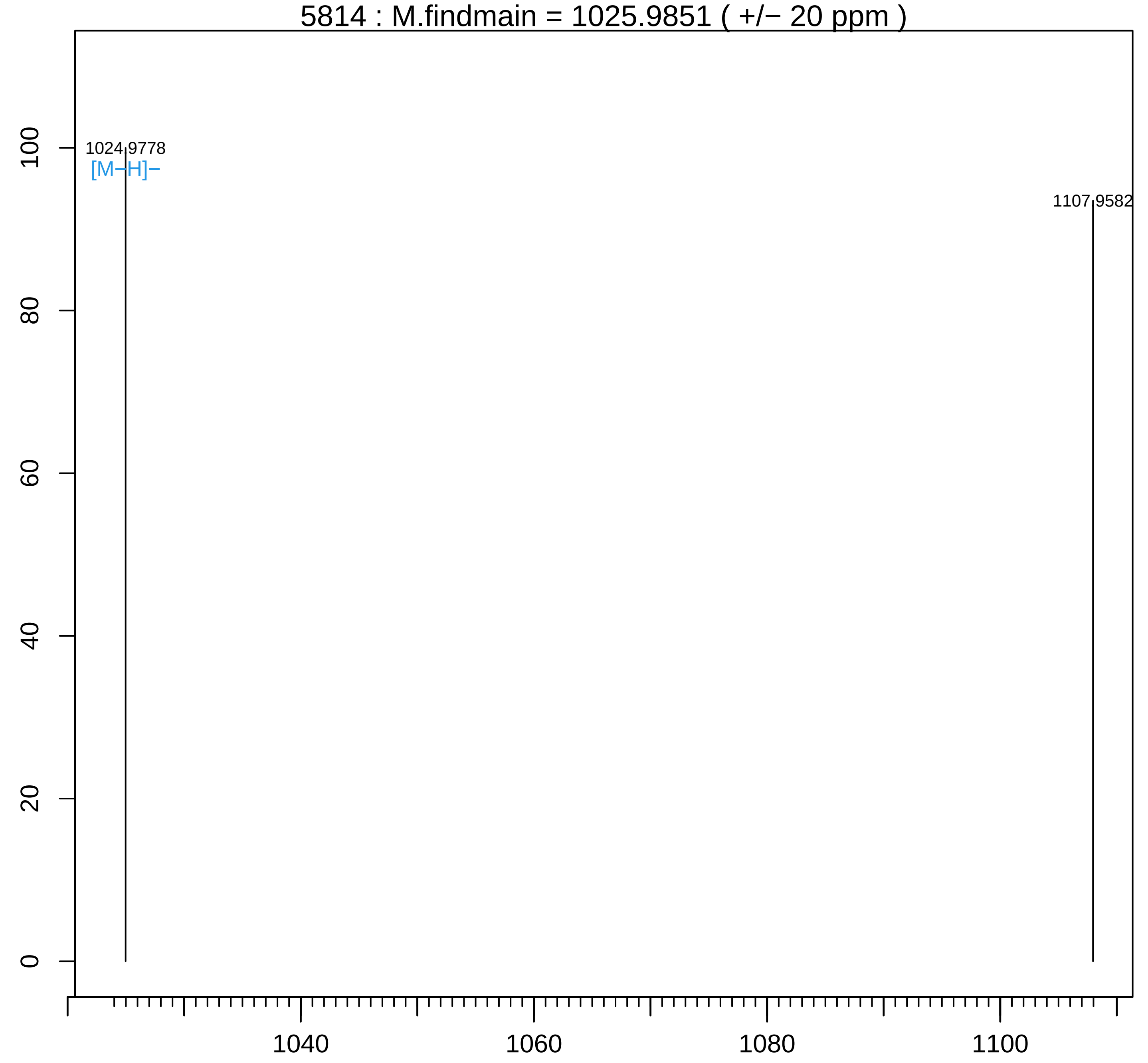
5814 : M.findmain = 1025.9851 (+/− 20 ppm)
1024.9778
[M−H]−
1107.9582
0
20
40
60
80
100
1040
1060
1080
1100

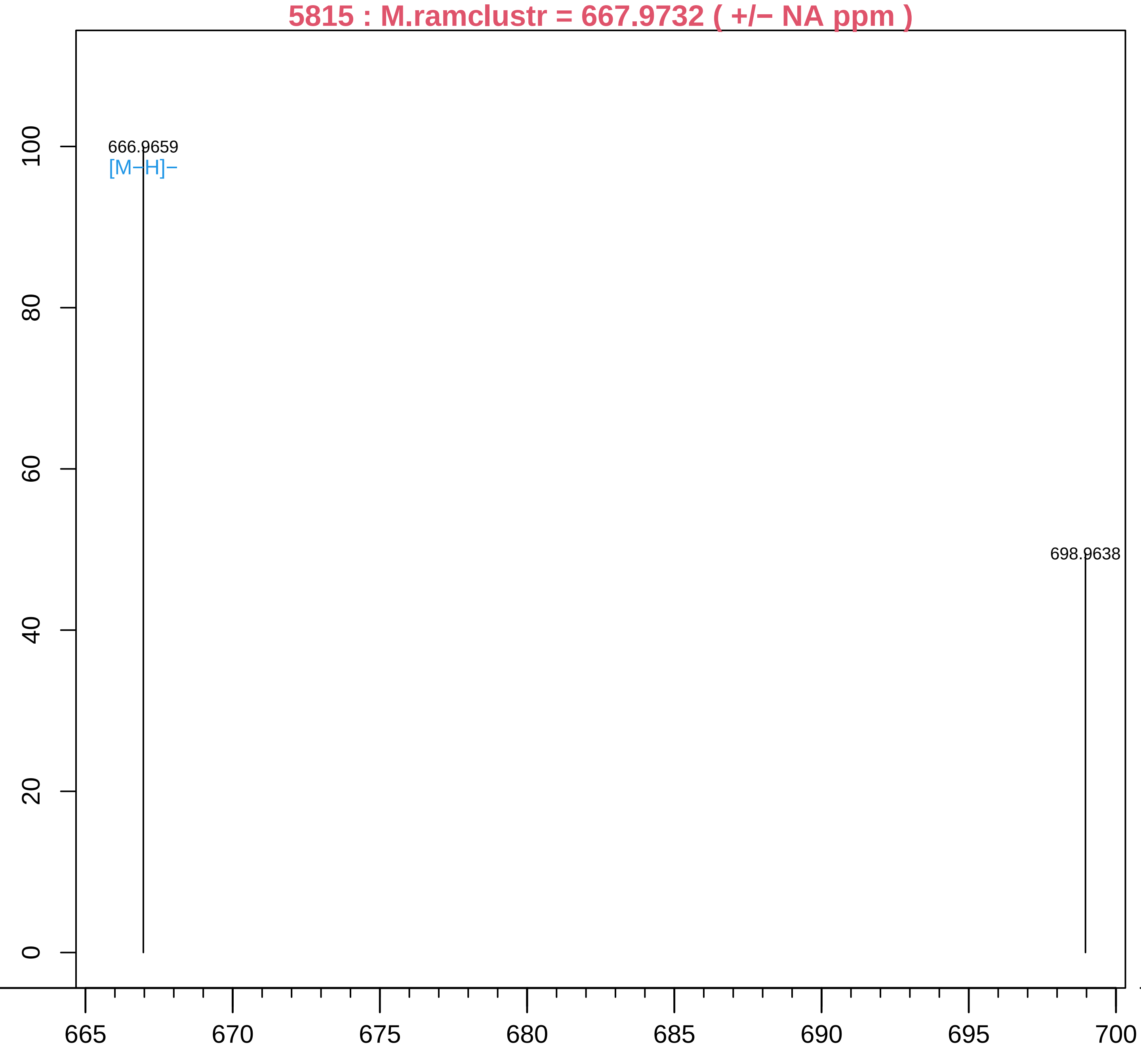
5815 : M.ramclustr = 667.9732 (+/− NA ppm)
666.9659
[M−H]−
698.9638
0
20
40
60
80
100
665
670
675
680
685
690
695
700

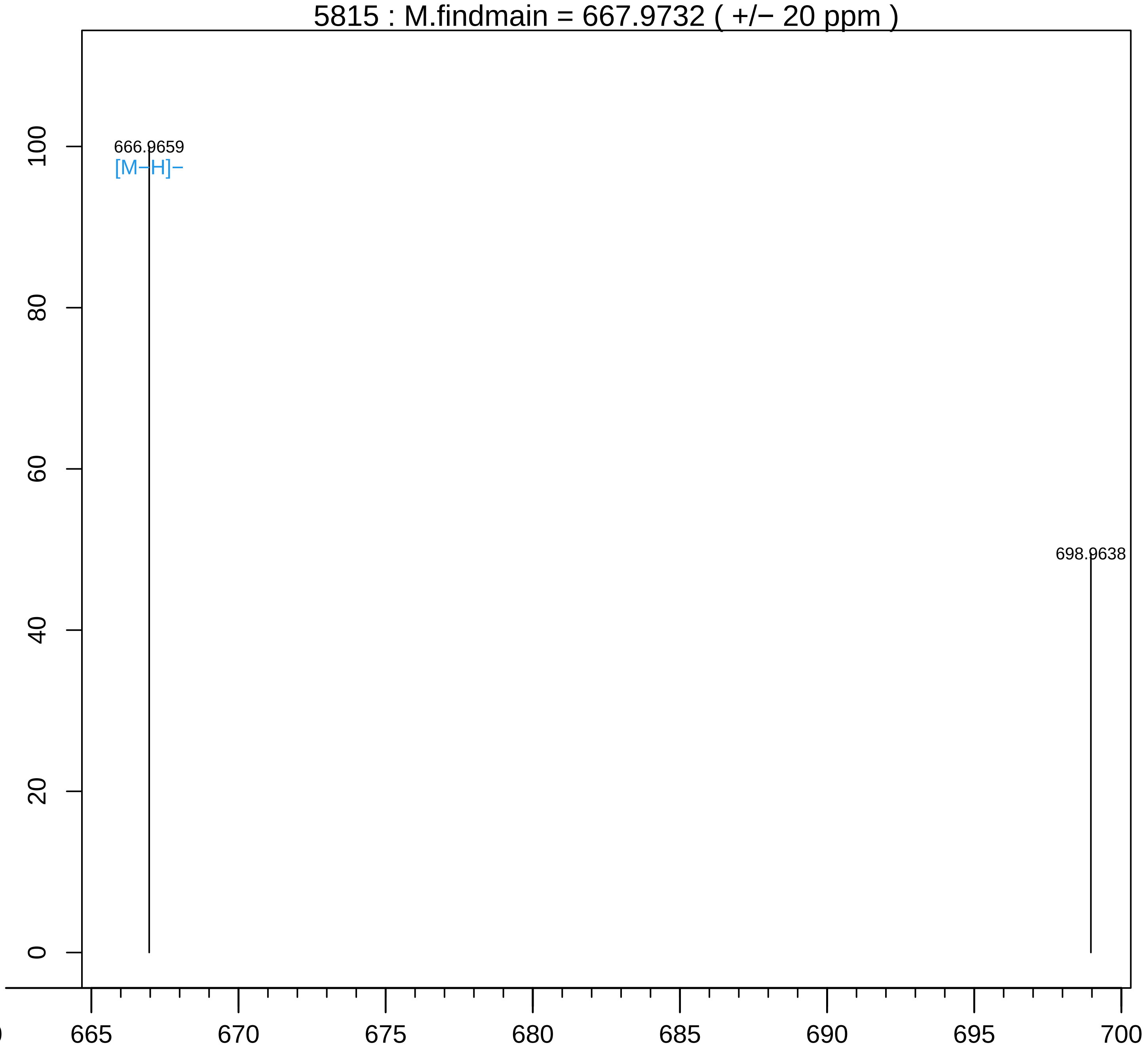
5815 : M.findmain = 667.9732 (+/− 20 ppm)
666.9659
[M−H]−
698.9638
0
20
40
60
80
100
665
670
675
680
685
690
695
700

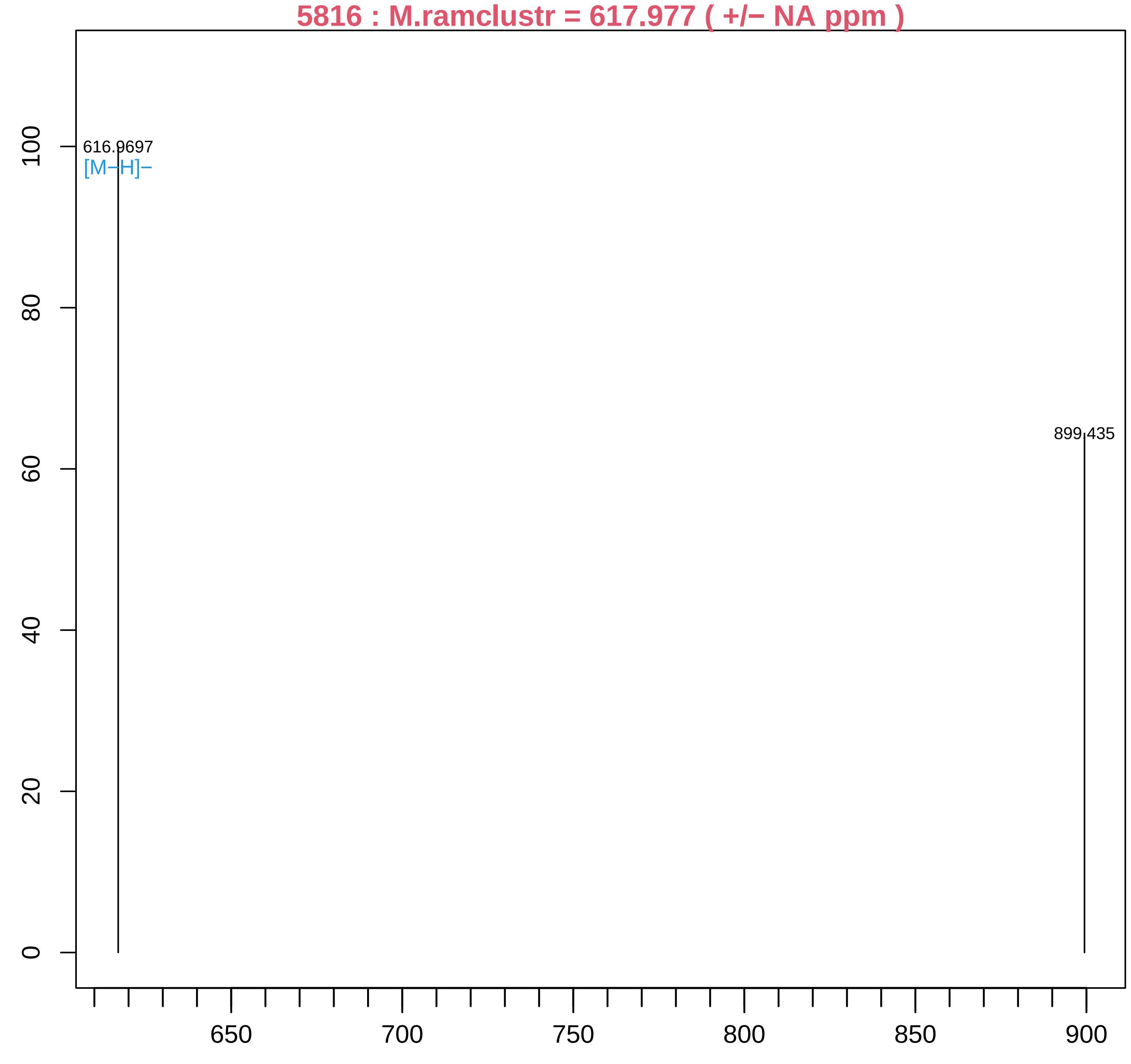
5816 : M.ramclustr = 617.977 (+/− NA ppm)
616.9697
[M−H]−
899.435
0
20
40
60
80
100
650
700
750
800
850
900

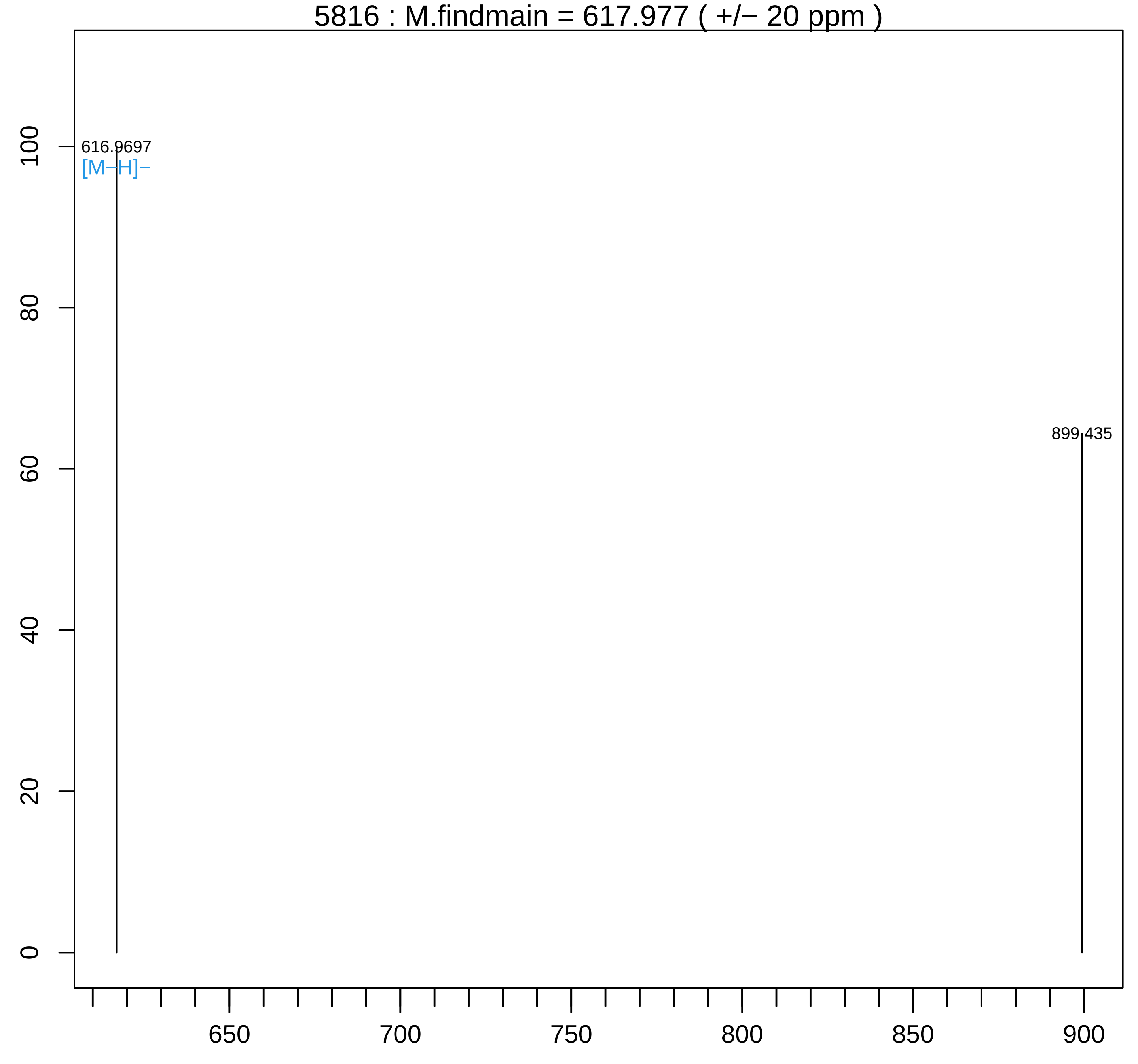
5816 : M.findmain = 617.977 (+/− 20 ppm)
616.9697
[M−H]−
899.435
0
20
40
60
80
100
650
700
750
800
850
900

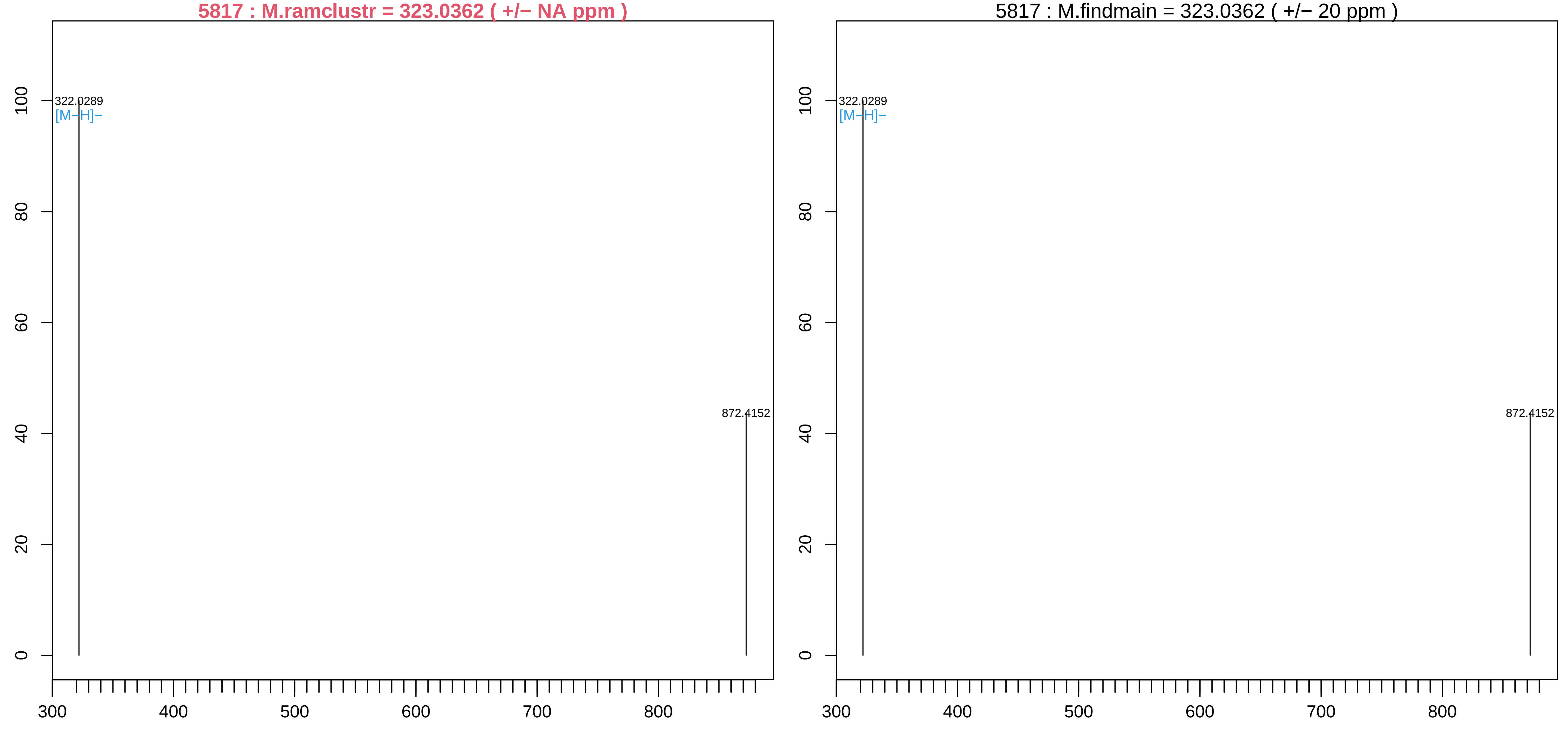

5817 : M.ramclustr = 323.0362 (+/− NA ppm)
322.0289
[M−H]−
872.4152
0
20
40
60
80
100
300
400
500
600
700
800
5817 : M.findmain = 323.0362 (+/− 20 ppm)
322.0289
[M−H]−
872.4152
0
20
40
60
80
100
300
400
500
600
700
800

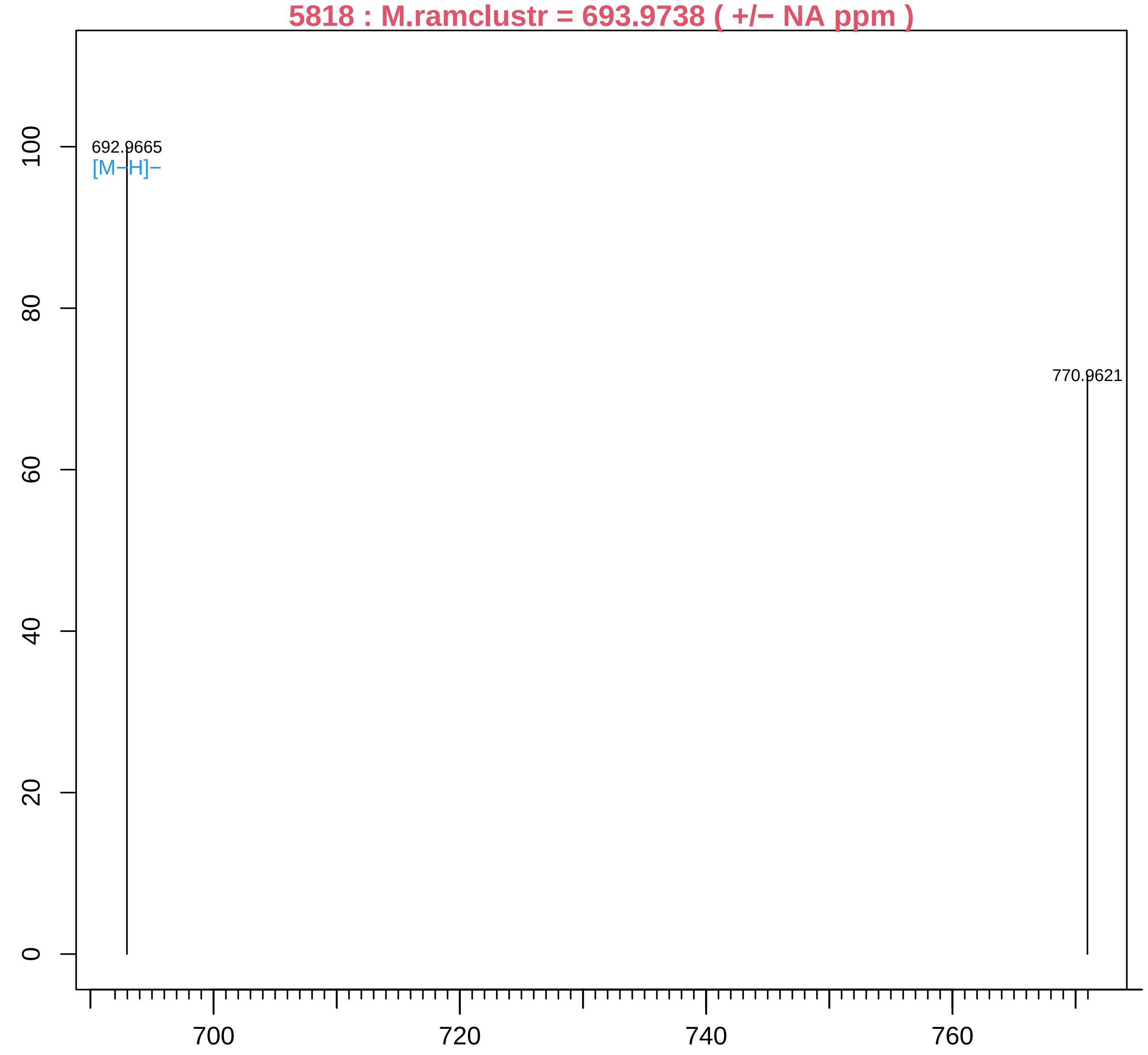
5818 : M.ramclustr = 693.9738 (+/− NA ppm)
692.9665
[M−H]−
770.9621
0
20
40
60
80
100
700
720
740
760

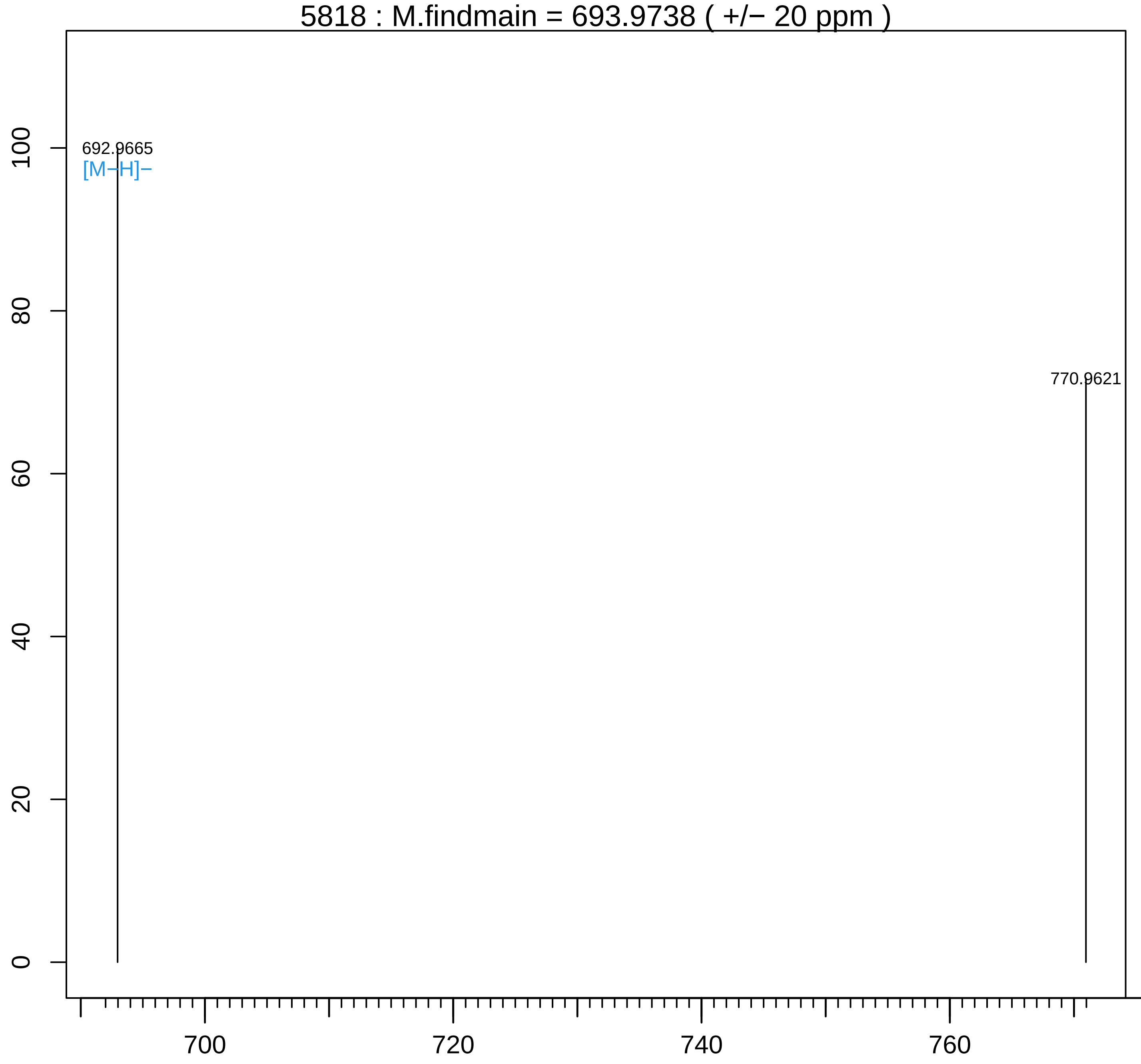
5818 : M.findmain = 693.9738 (+/− 20 ppm)
692.9665
[M−H]−
770.9621
0
20
40
60
80
100
700
720
740
760

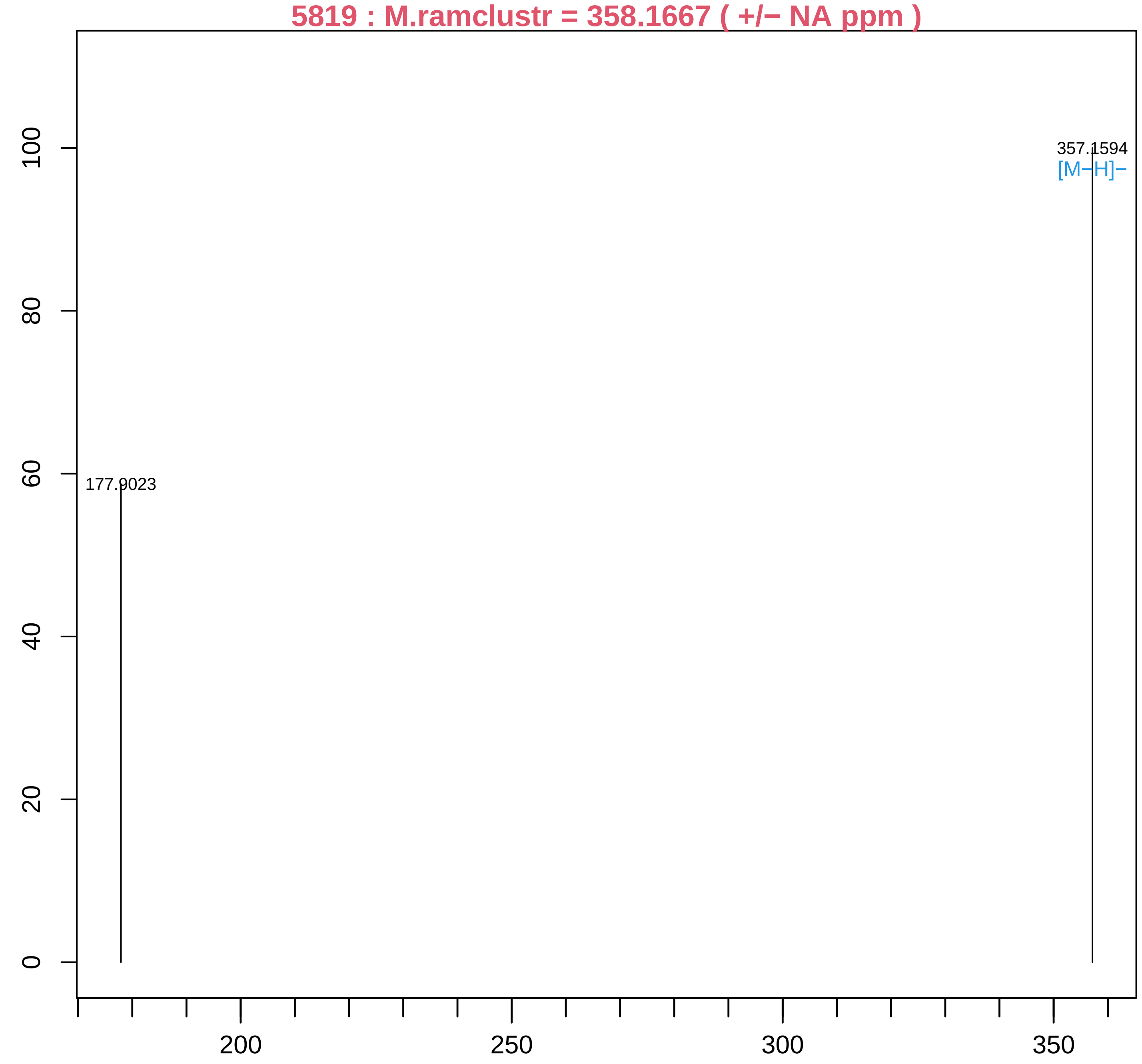

5819 : M.ramclustr = 358.1667 (+/− NA ppm)
357.1594
[M−H]−
177.9023
0
20
40
60
80
100
200
250
300
350

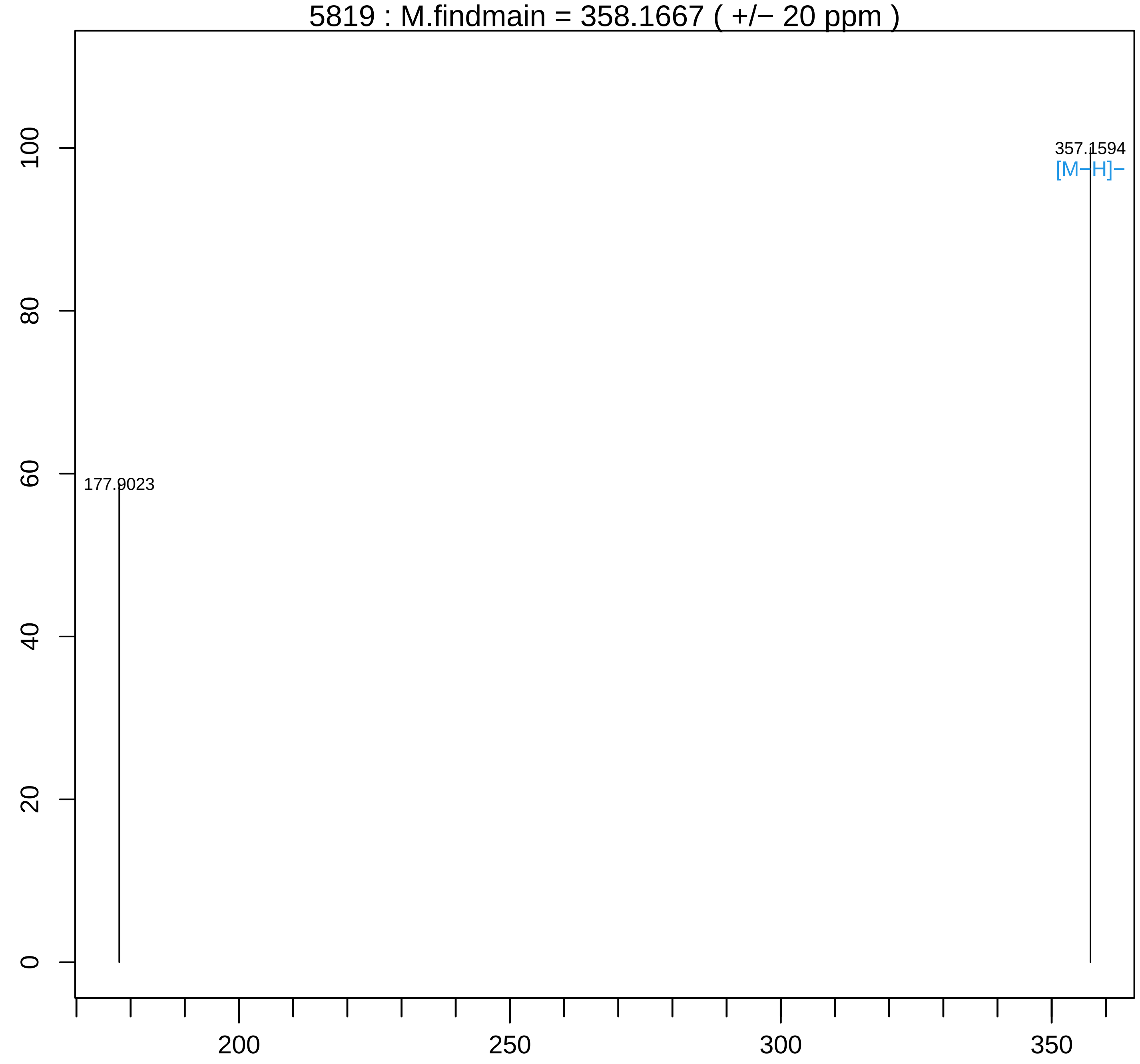

5819 : M.findmain = 358.1667 (+/− 20 ppm)
357.1594
[M−H]−
177.9023
0
20
40
60
80
100
200
250
300
350

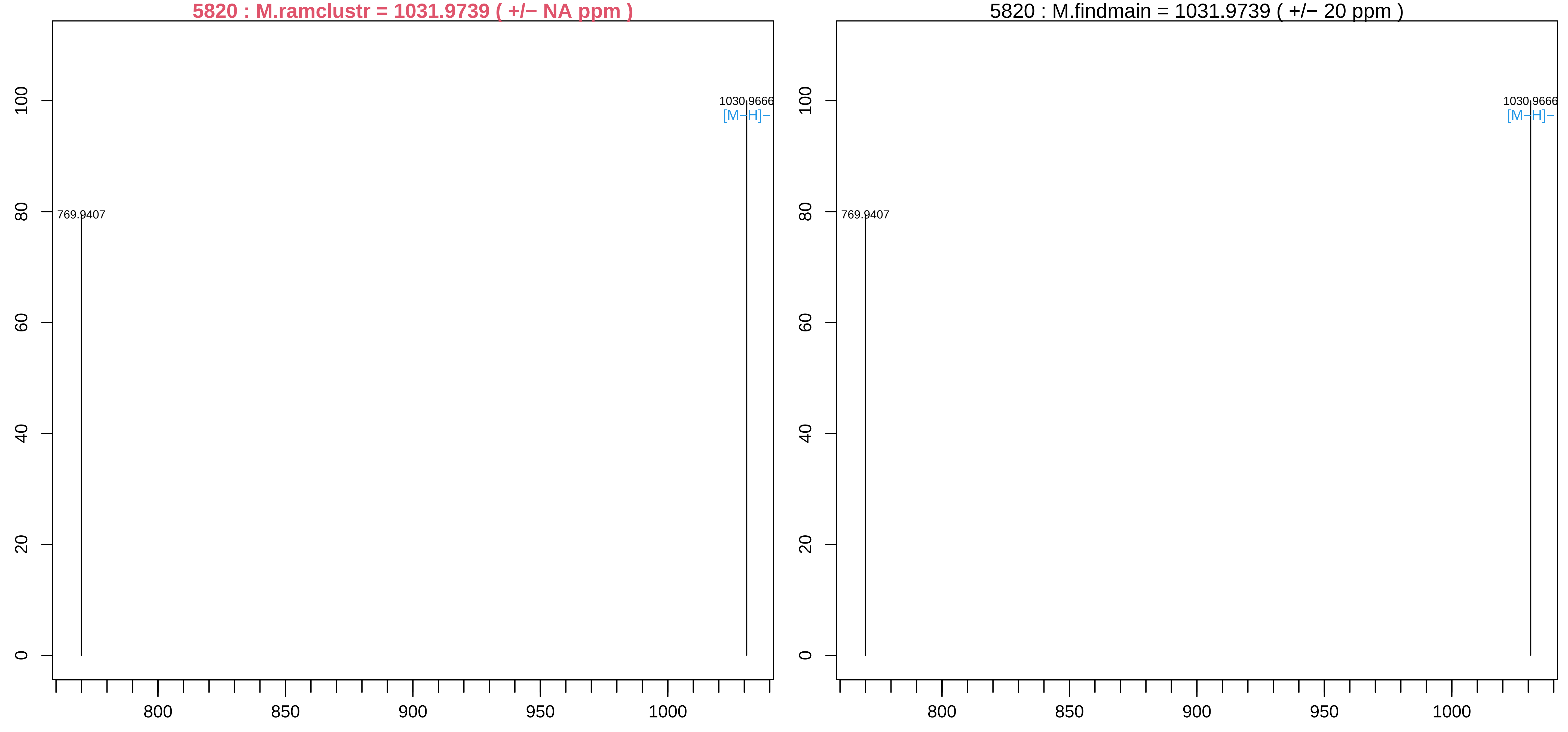
5820 : M.ramclustr = 1031.9739 (+/− NA ppm)
1030.9666
[M−H]−
769.9407
0
20
40
60
80
100
800
850
900
950
1000
5820 : M.findmain = 1031.9739 (+/− 20 ppm)
1030.9666
[M−H]−
769.9407
0
20
40
60
80
100
800
850
900
950
1000

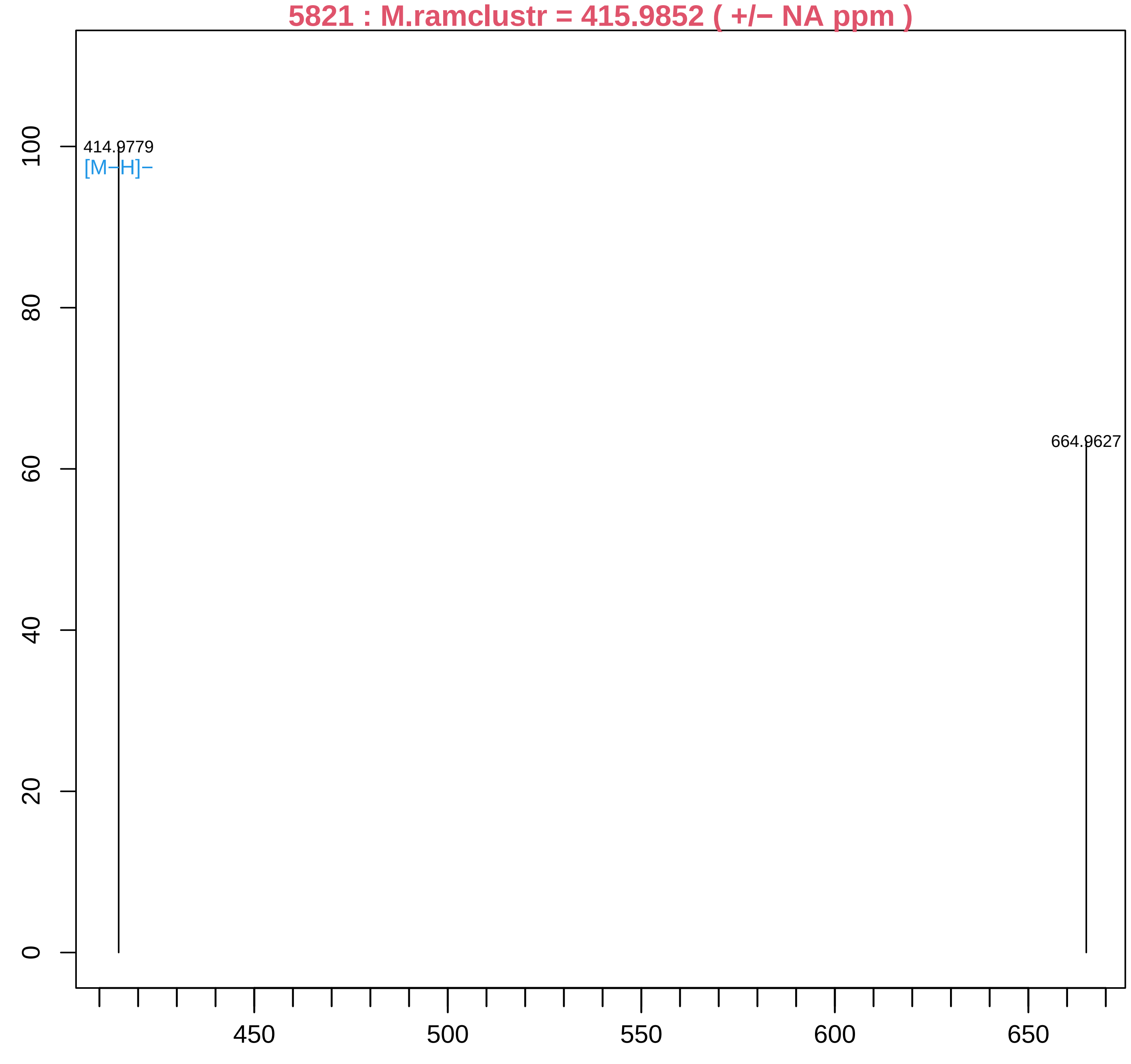
5821 : M.ramclustr = 415.9852 (+/− NA ppm)
414.9779
[M−H]−
664.9627
0
20
40
60
80
100
450
500
550
600
650

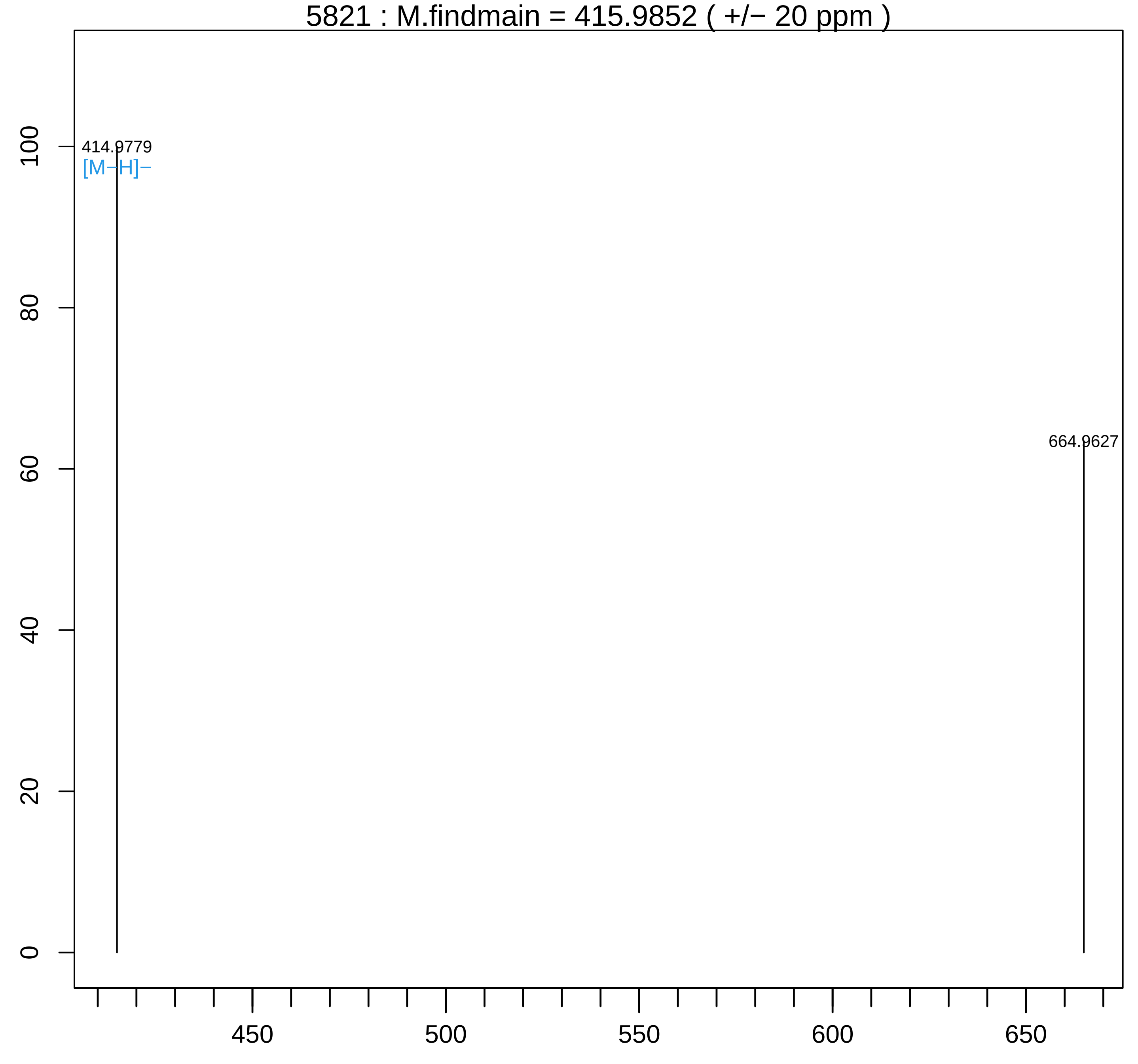
5821 : M.findmain = 415.9852 (+/− 20 ppm)
414.9779
[M−H]−
664.9627
0
20
40
60
80
100
450
500
550
600
650

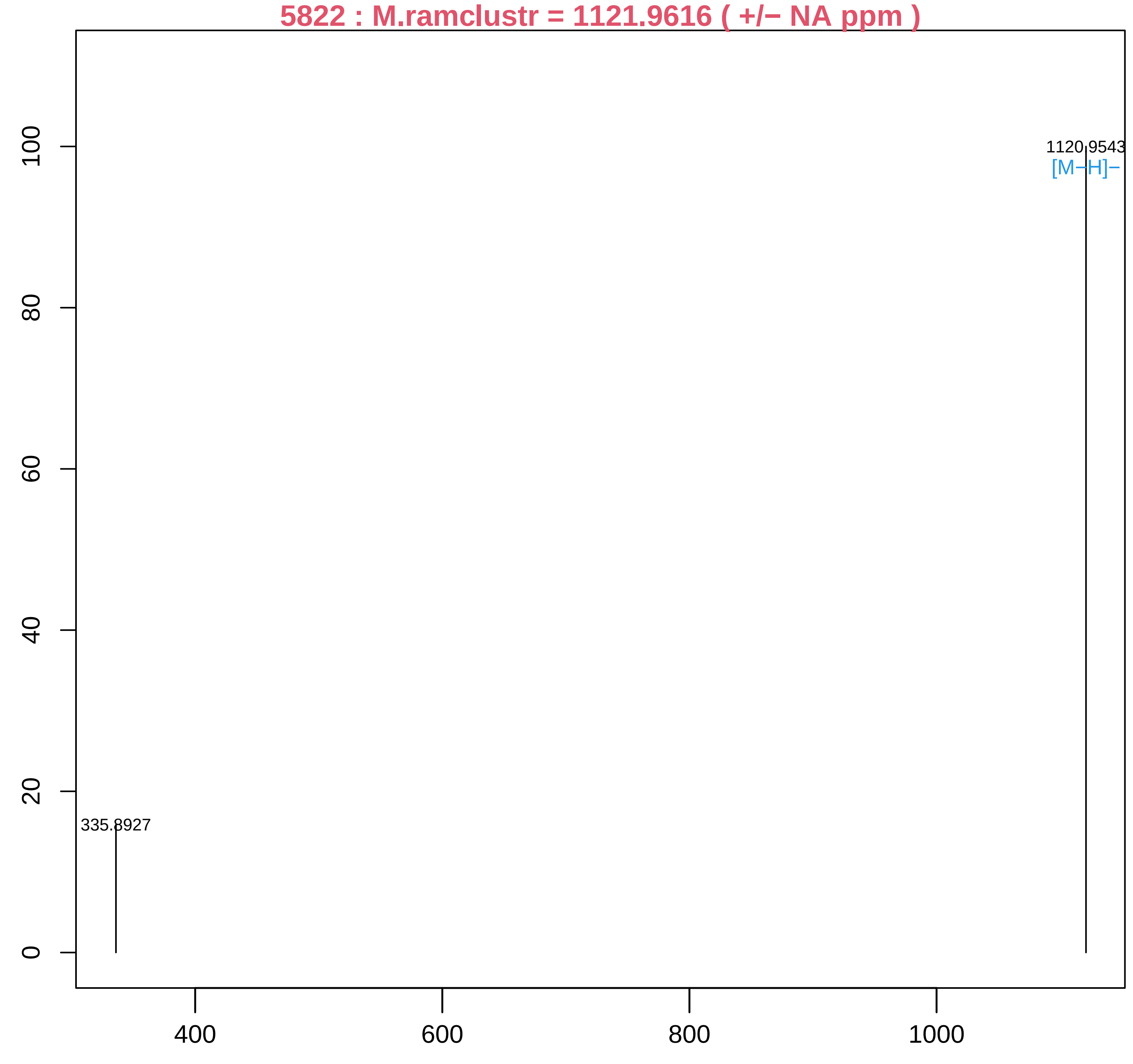
5822 : M.ramclustr = 1121.9616 (+/− NA ppm)
1120.9543
[M−H]−
335.8927
0
20
40
60
80
100
400
600
800
1000

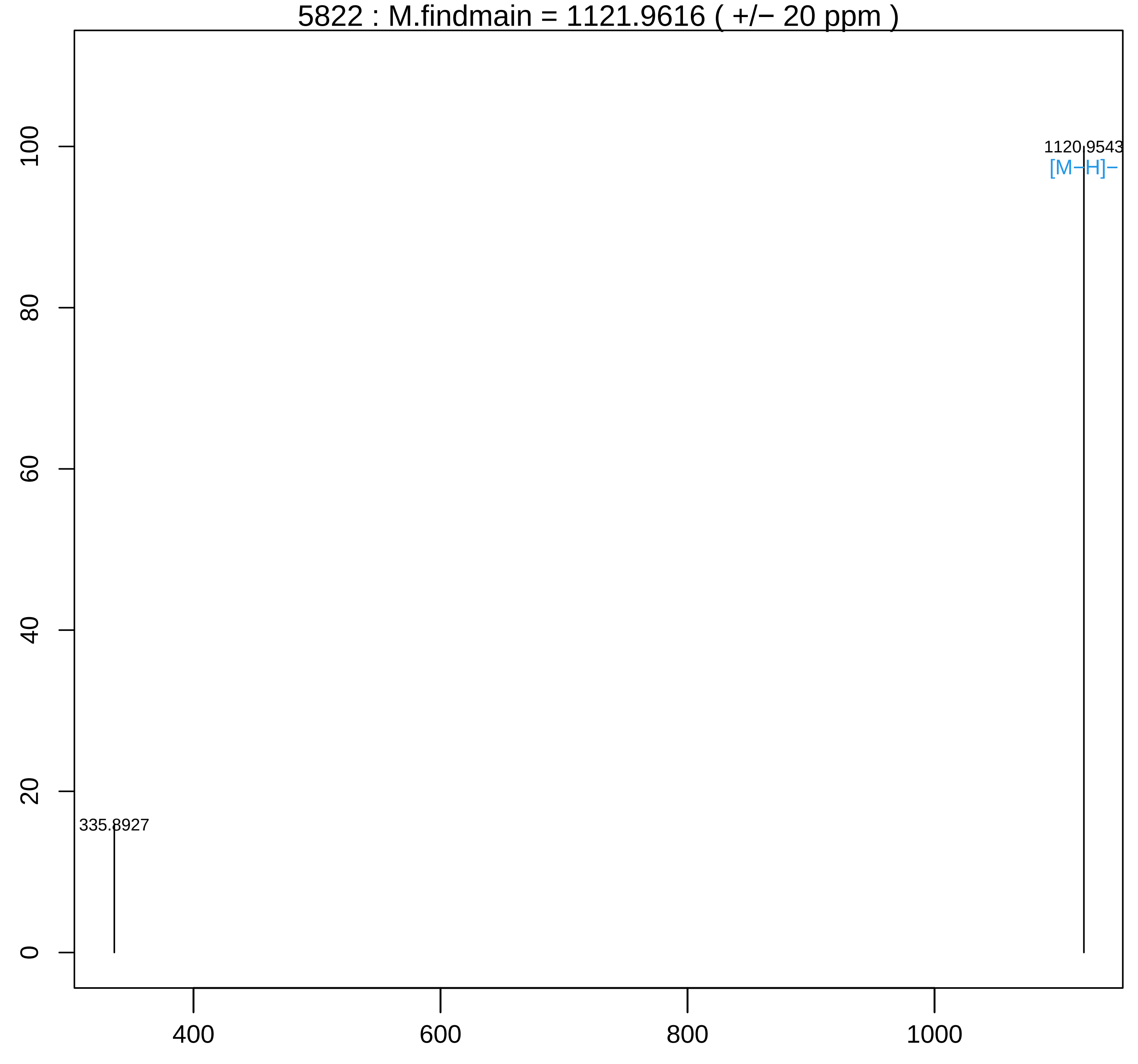
5822 : M.findmain = 1121.9616 (+/− 20 ppm)
1120.9543
[M−H]−
335.8927
0
20
40
60
80
100
400
600
800
1000

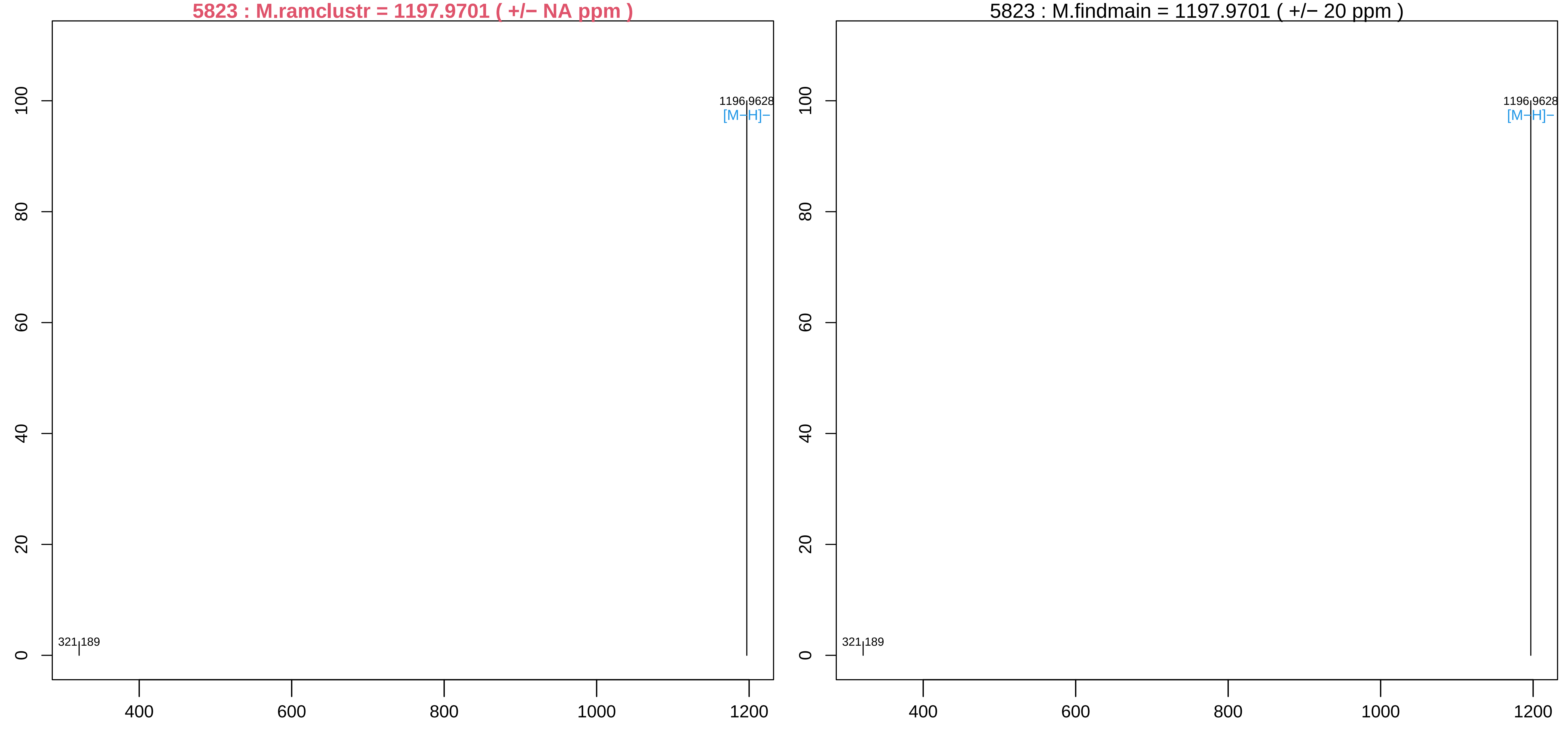

5823 : M.ramclustr = 1197.9701 (+/− NA ppm)
1196.9628
[M−H]−
321.189
5823 : M.findmain = 1197.9701 (+/− 20 ppm)
1196.9628
[M−H]−
321.189
0
20
40
60
80
100
400
600
800
1000
1200

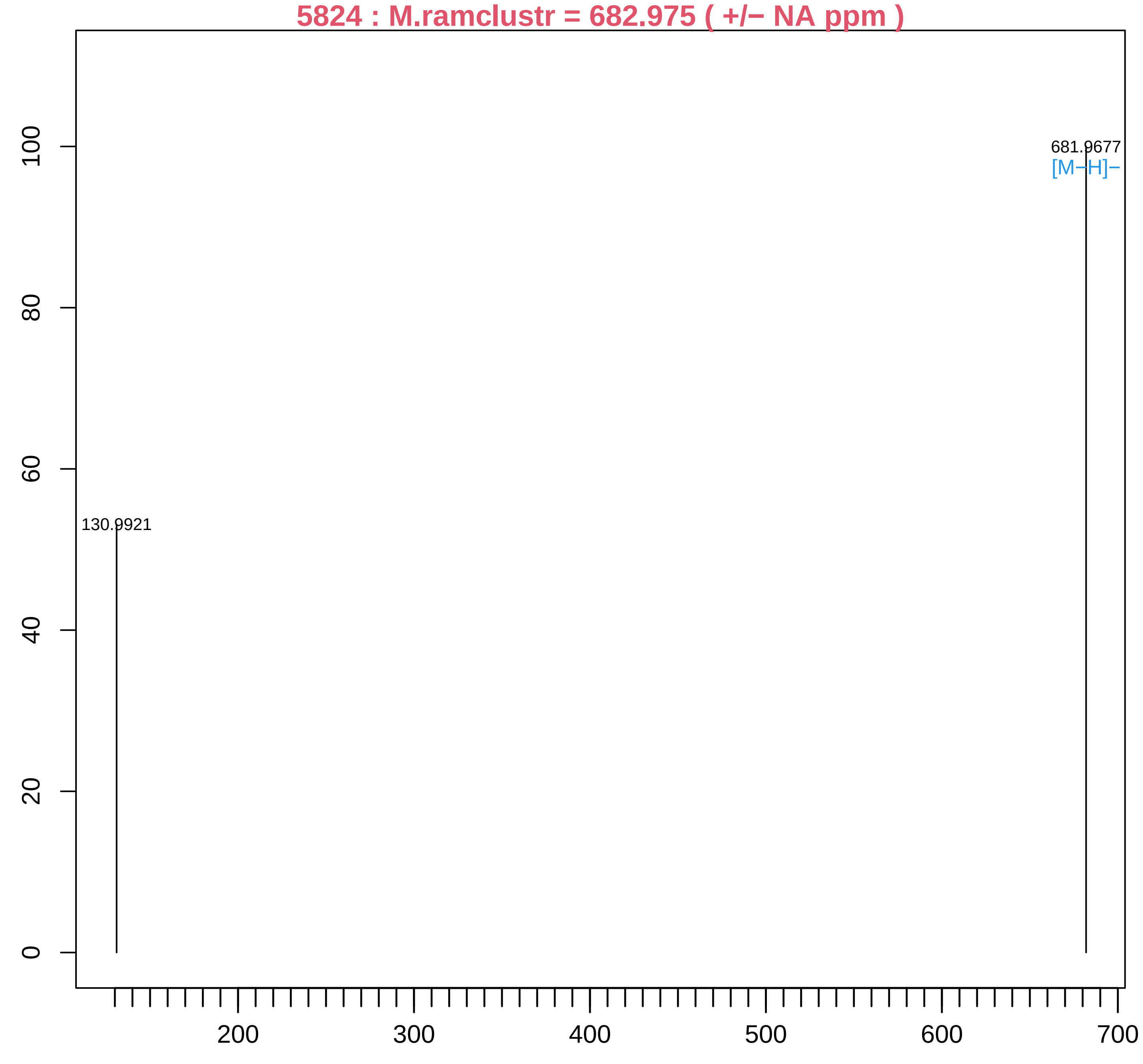
5824 : M.ramclustr = 682.975 (+/− NA ppm)
681.9677
[M−H]−
130.9921
0
20
40
60
80
100
200
300
400
500
600
700

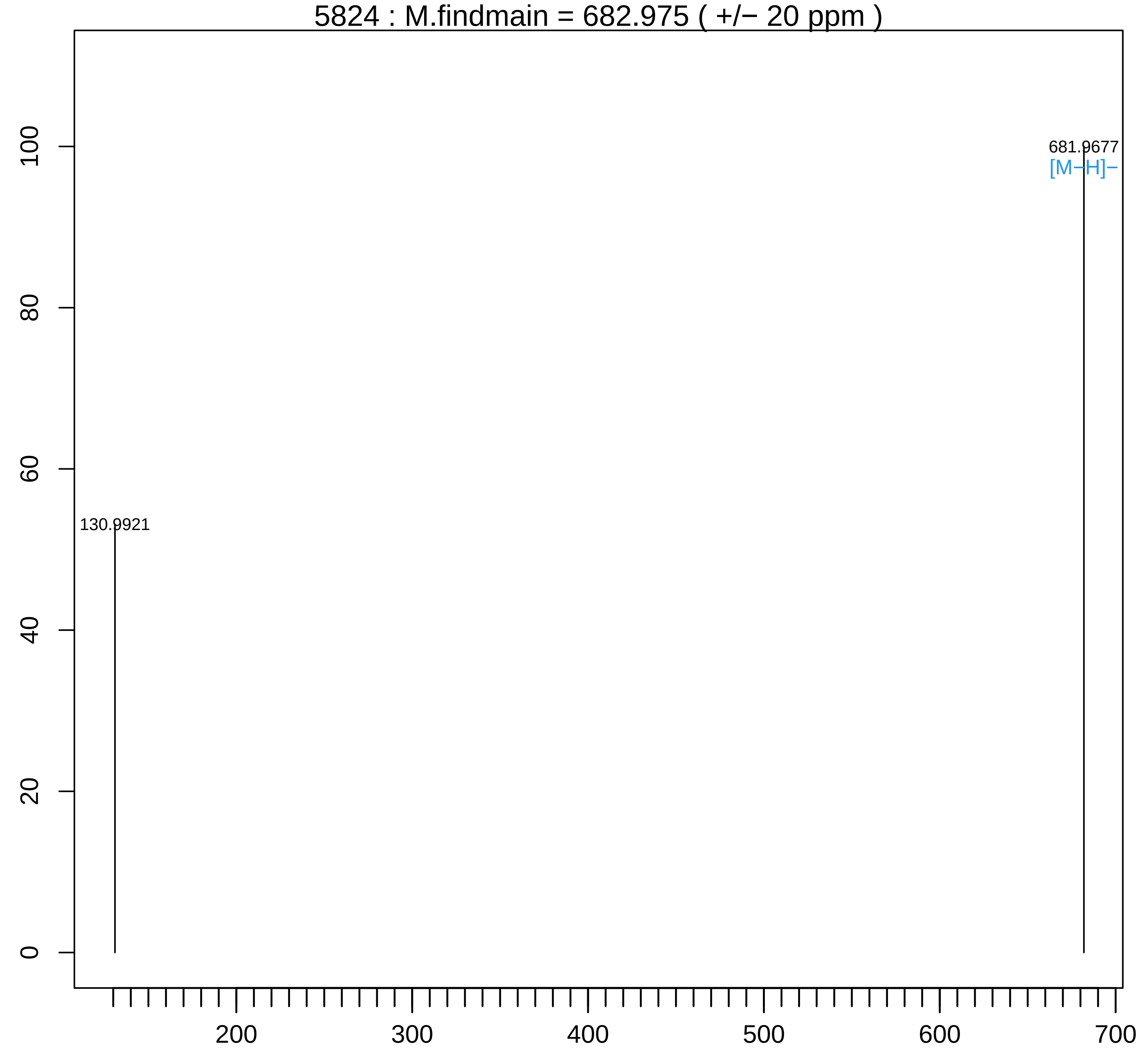
5824 : M.findmain = 682.975 (+/− 20 ppm)
681.9677
[M−H]−
130.9921
0
20
40
60
80
100
200
300
400
500
600
700

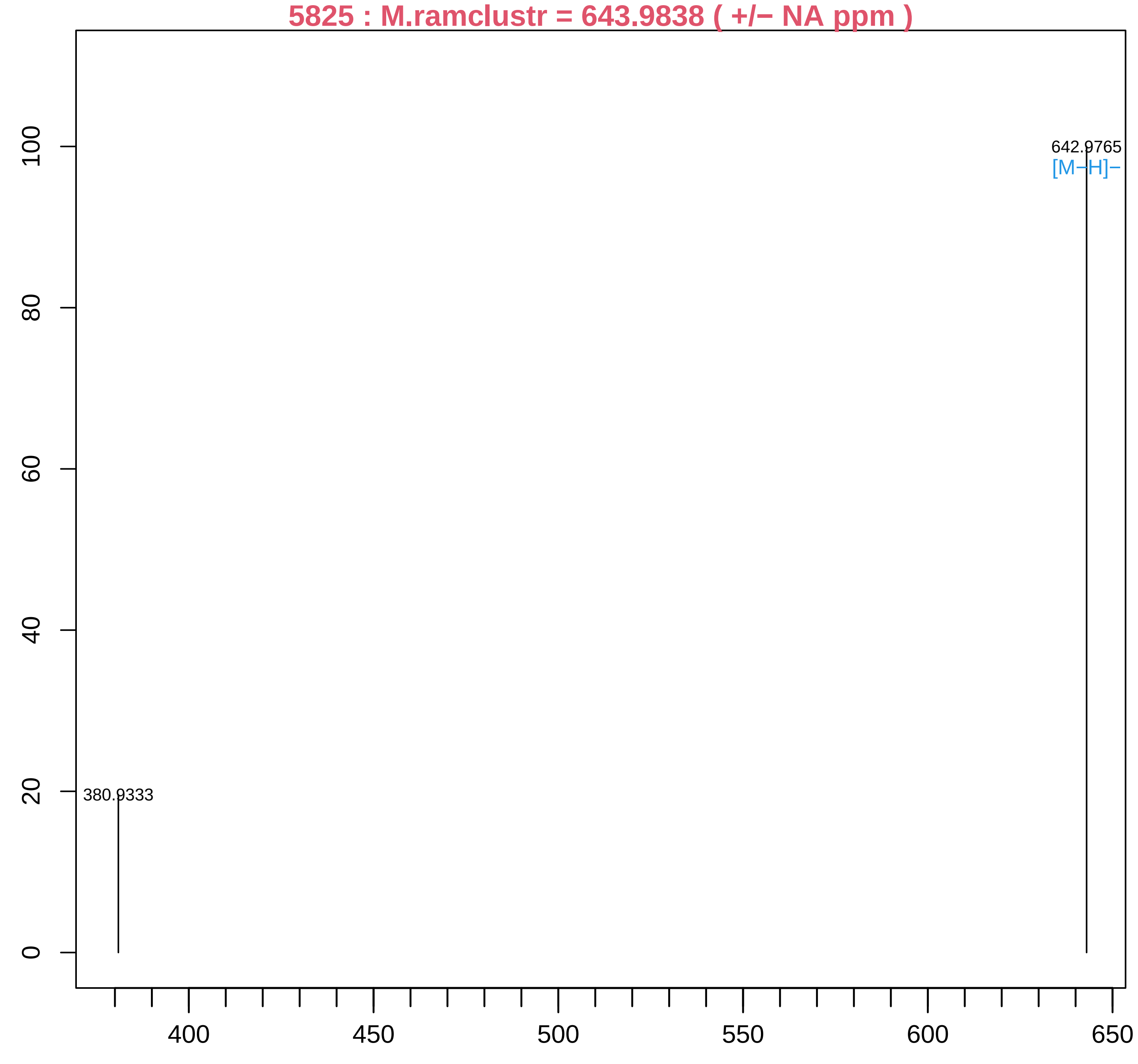

5825 : M.ramclustr = 643.9838 (+/− NA ppm)
642.9765
[M−H]−
380.9333
0
20
40
60
80
100
400
450
500
550
600
650

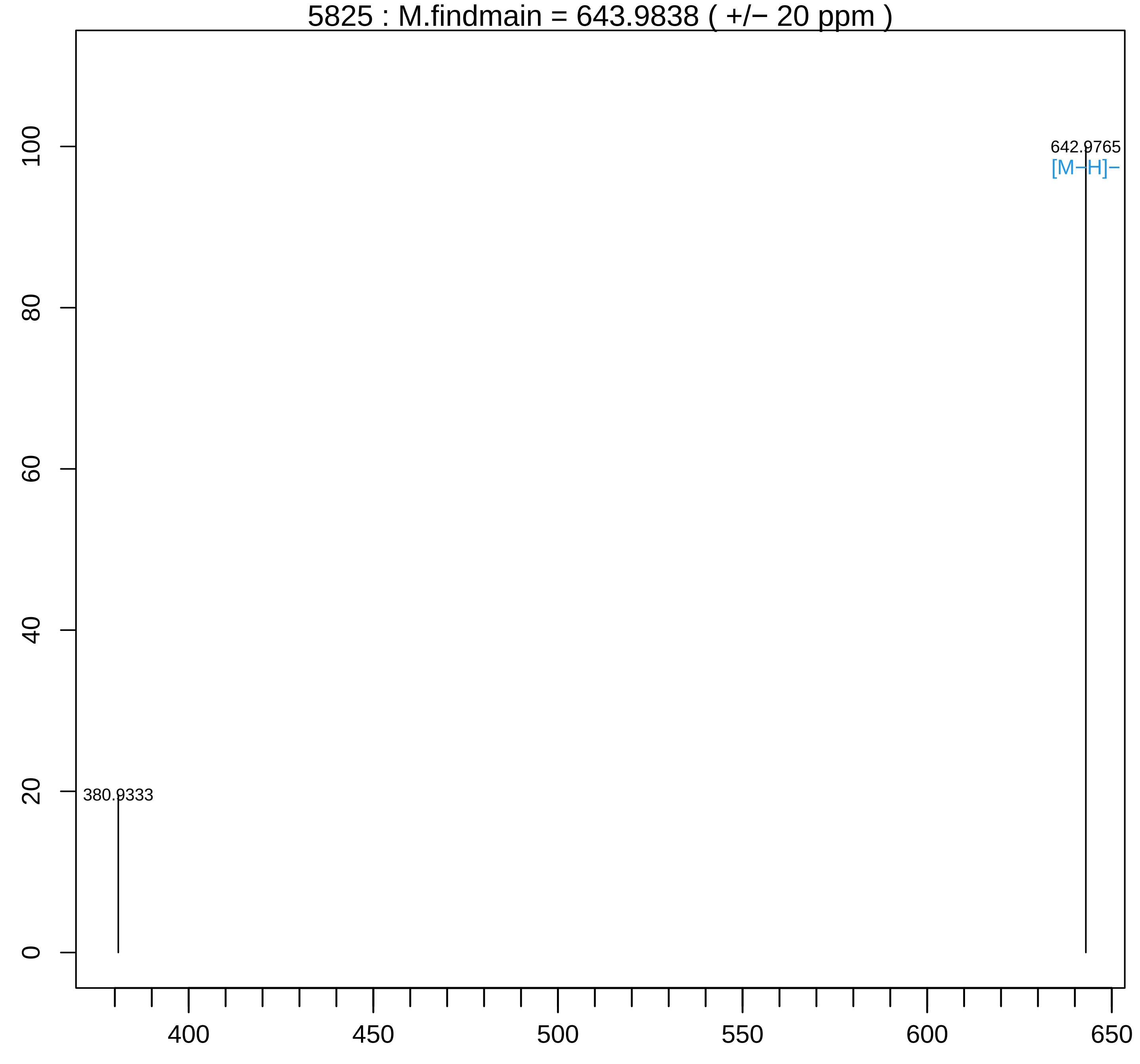

5825 : M.findmain = 643.9838 (+/− 20 ppm)
642.9765
[M−H]−
380.9333
0
20
40
60
80
100
400
450
500
550
600
650

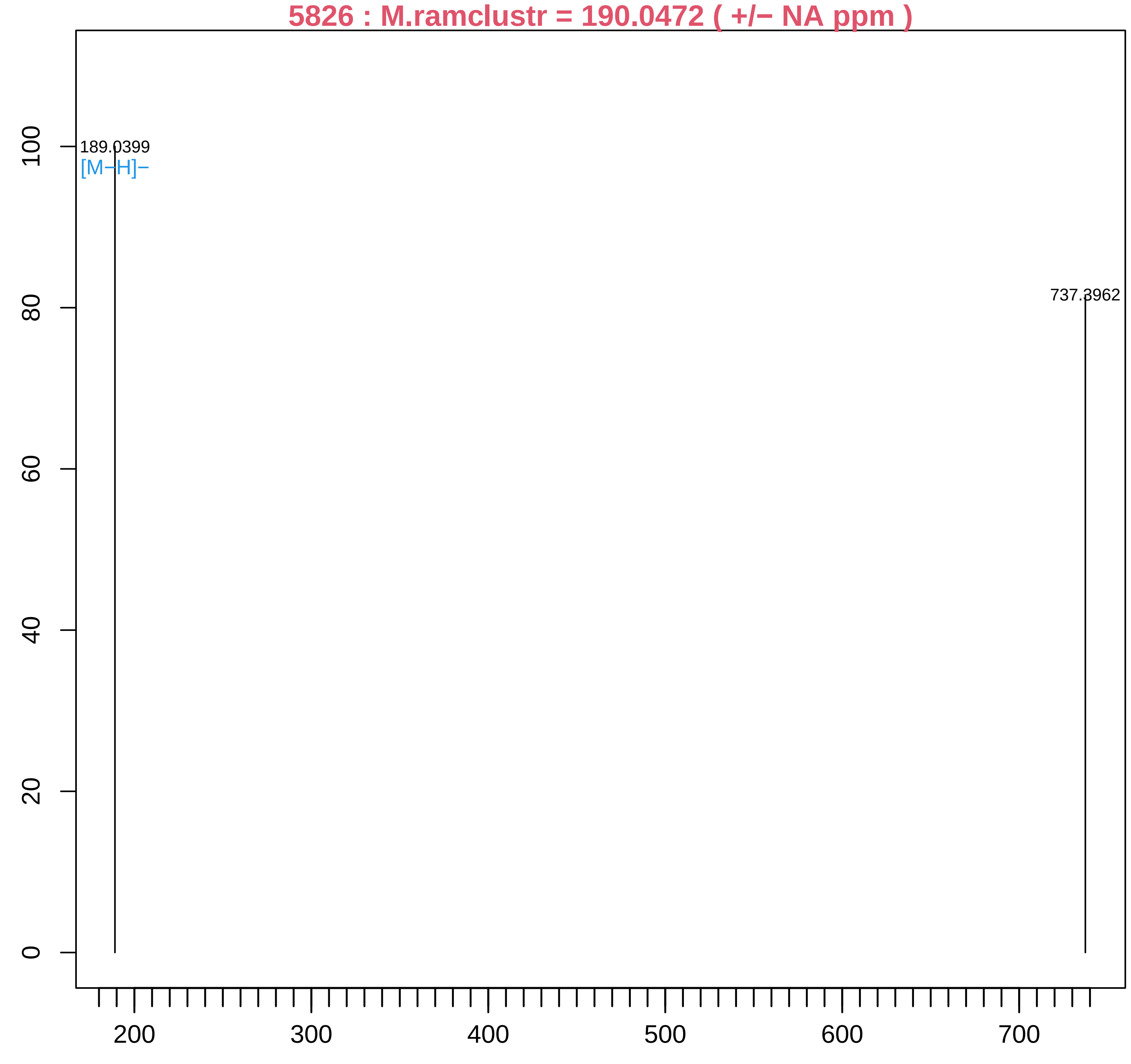
5826 : M.ramclustr = 190.0472 (+/− NA ppm)
189.0399
[M−H]−
737.3962
0
20
40
60
80
100
200
300
400
500
600
700

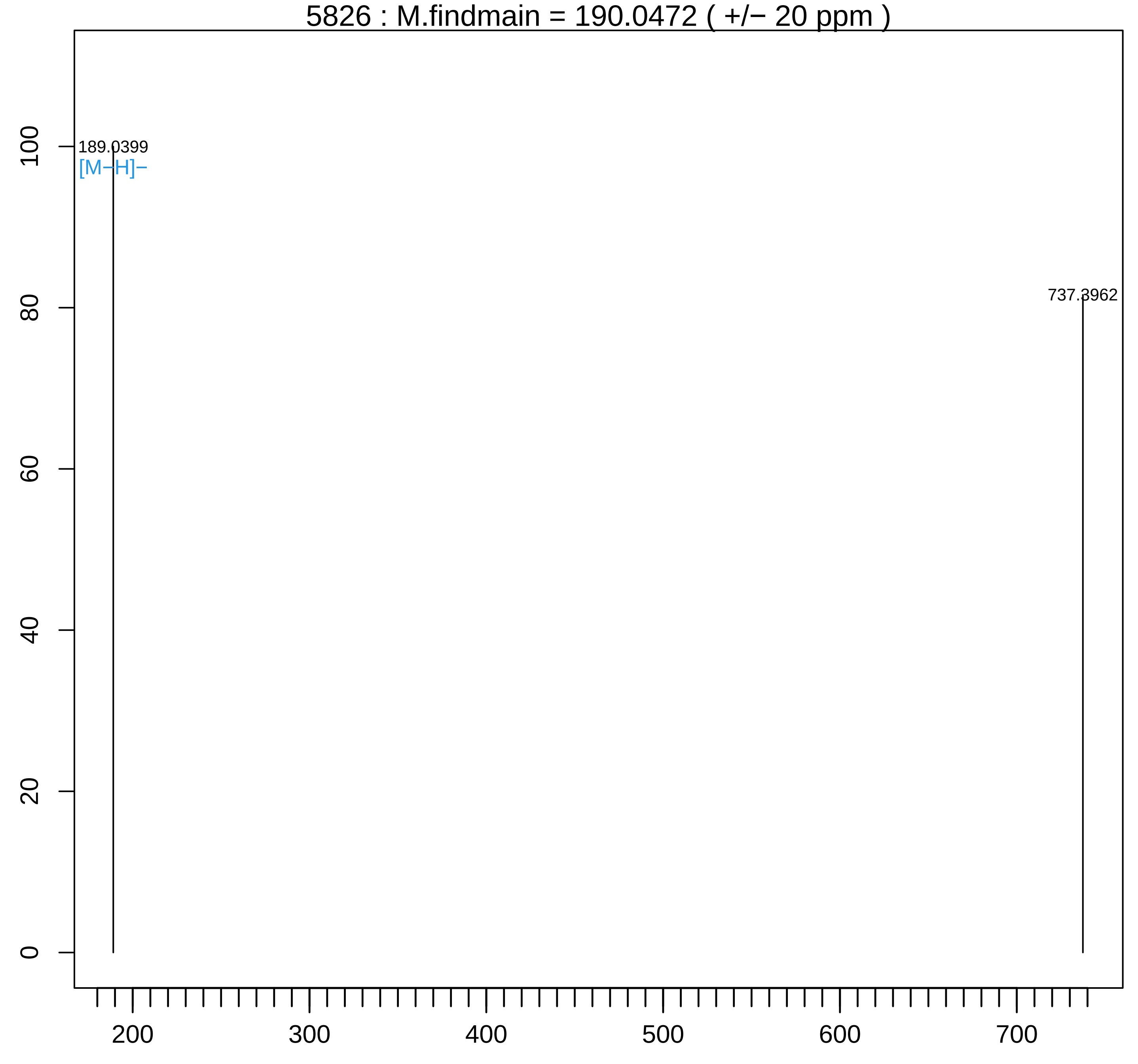
5826 : M.findmain = 190.0472 (+/− 20 ppm)
189.0399
[M−H]−
737.3962
0
20
40
60
80
100
200
300
400
500
600
700

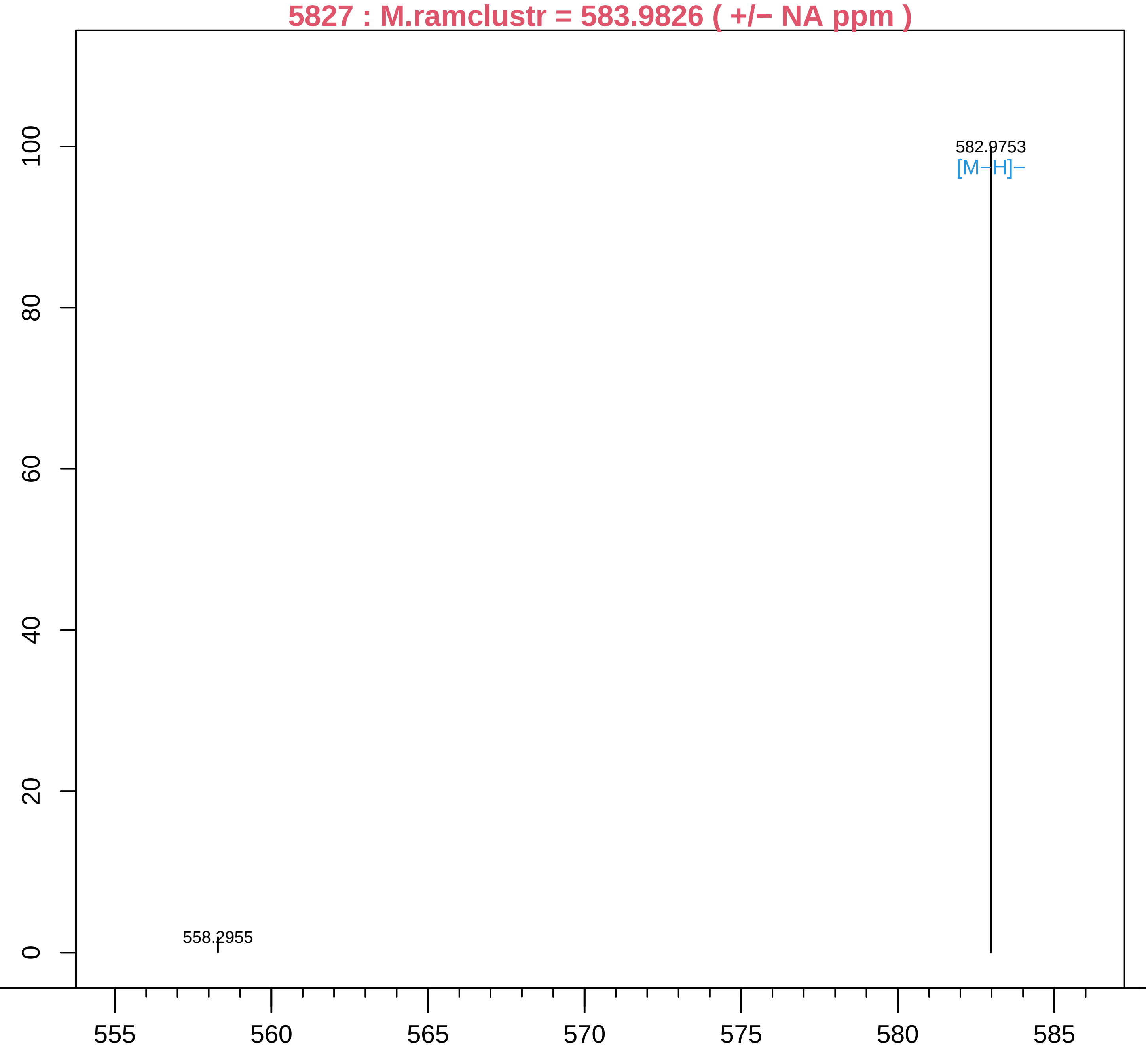
5827 : M.ramclustr = 583.9826 (+/− NA ppm)
582.9753
[M−H]−
558.2955
0
20
40
60
80
100
555
560
565
570
575
580
585

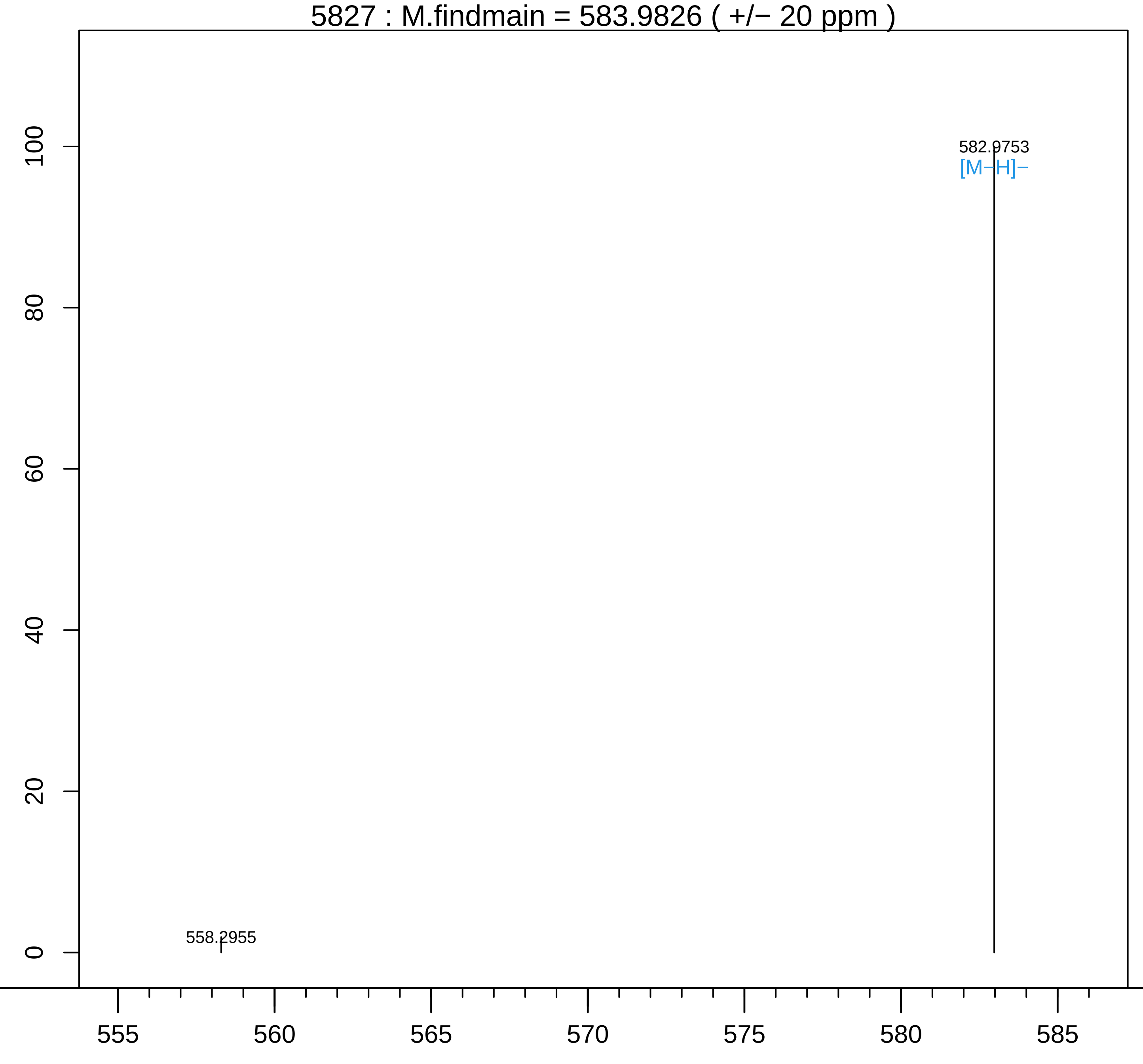
5827 : M.findmain = 583.9826 (+/− 20 ppm)
582.9753
[M−H]−
558.2955
0
20
40
60
80
100
555
560
565
570
575
580
585

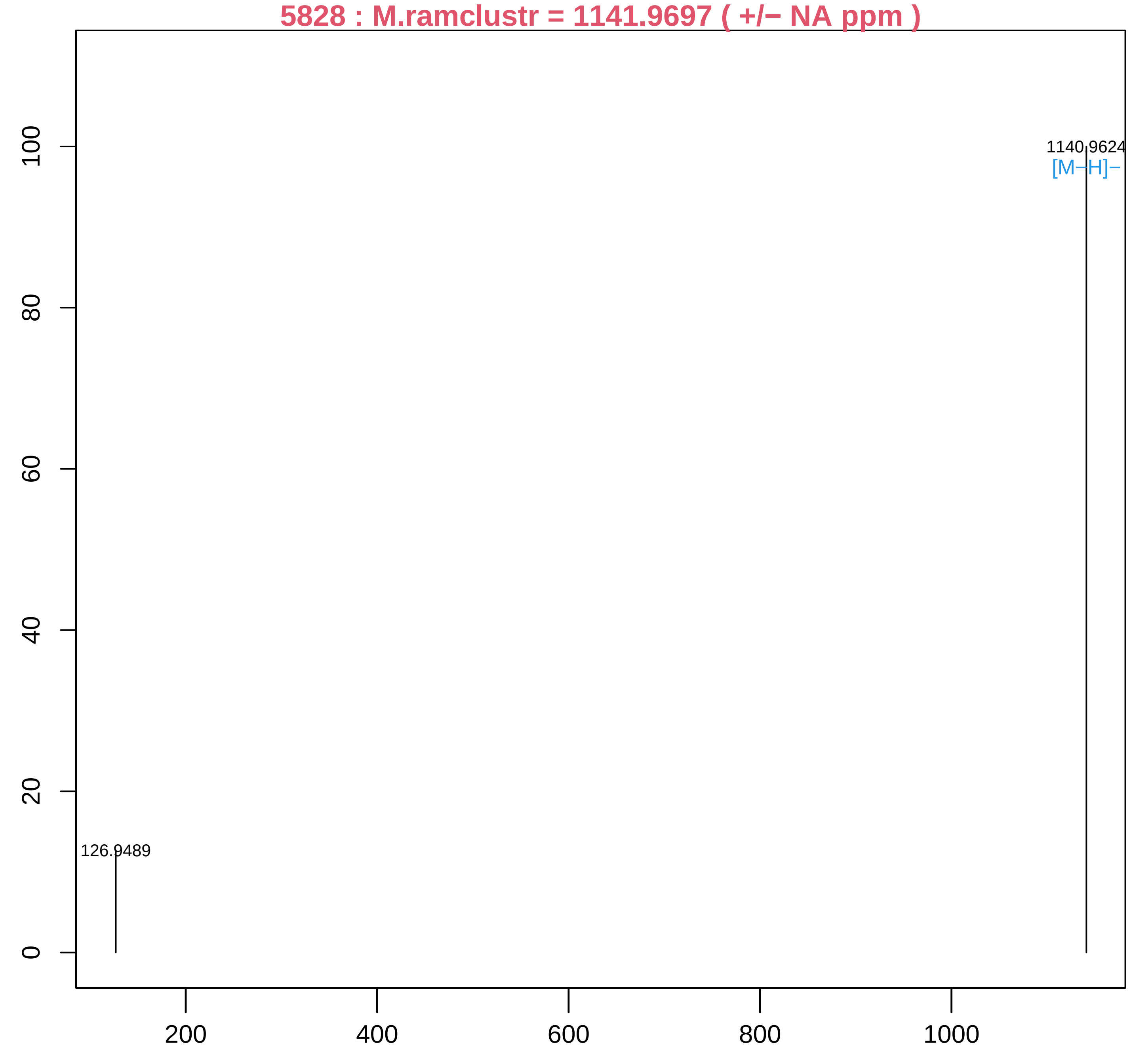
5828 : M.ramclustr = 1141.9697 (+/− NA ppm)
1140.9624
[M−H]−
126.9489
0
20
40
60
80
100
200
400
600
800
1000

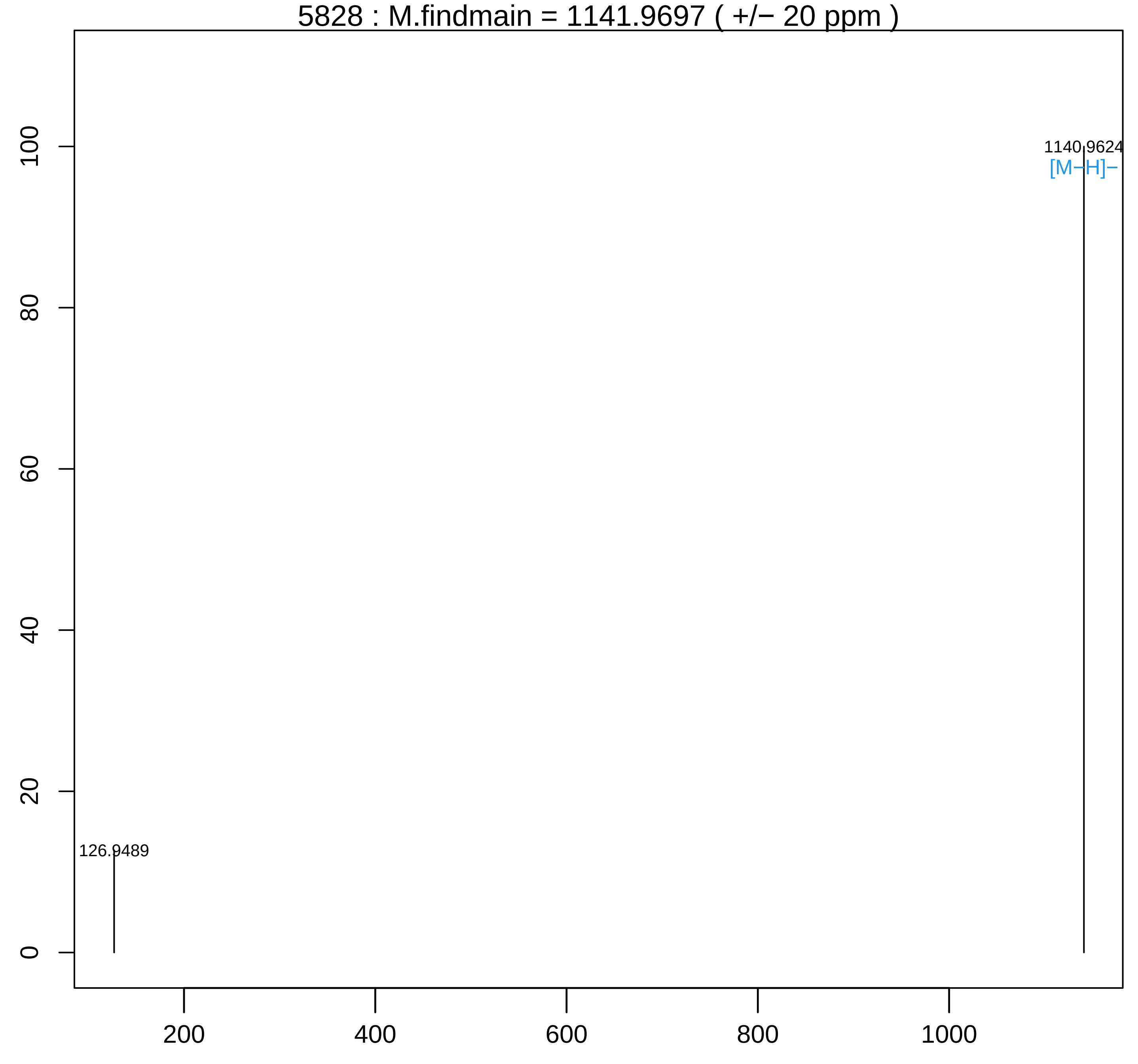
5828 : M.findmain = 1141.9697 (+/− 20 ppm)
1140.9624
[M−H]−
126.9489
0
20
40
60
80
100
200
400
600
800
1000

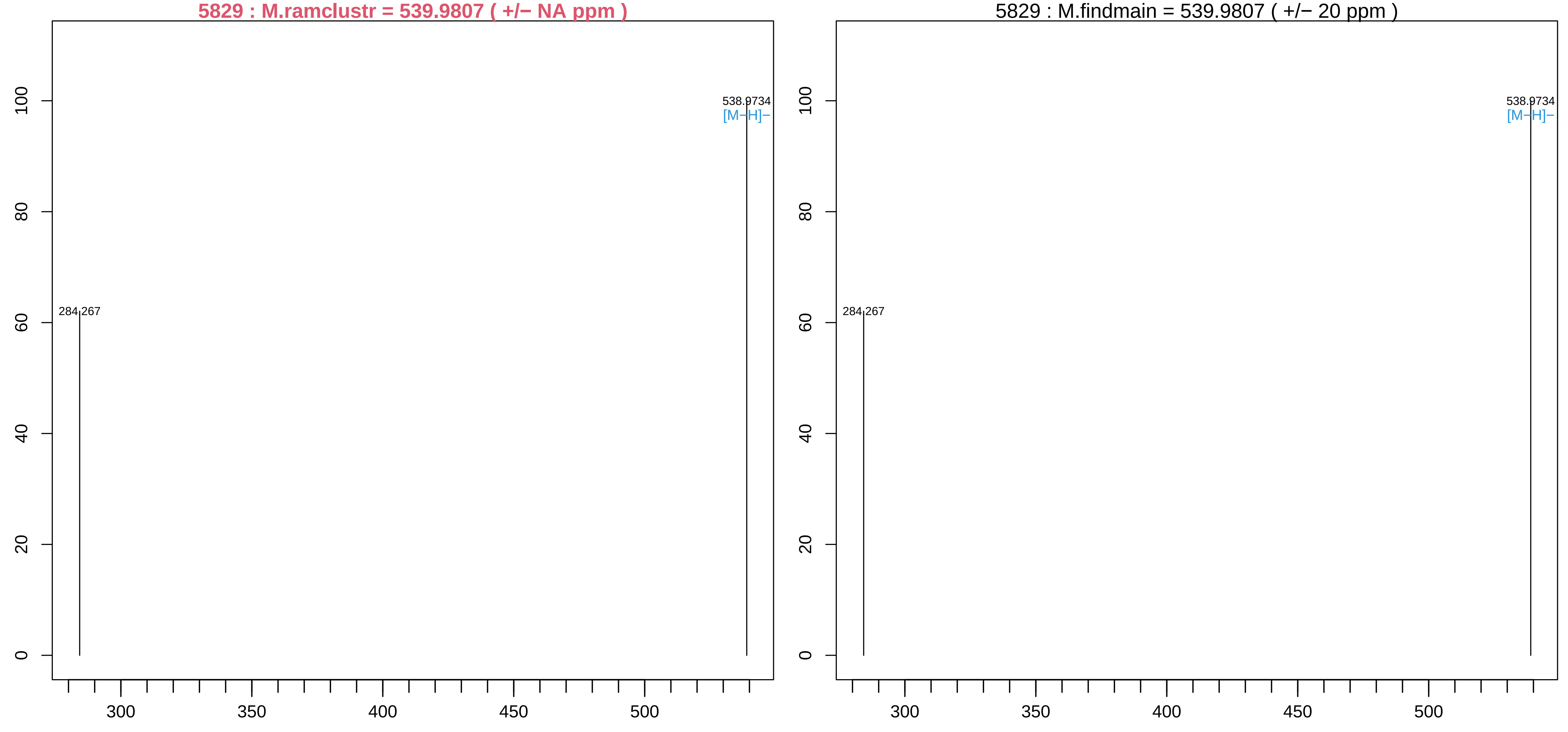
5829 : M.ramclustr = 539.9807 (+/− NA ppm)
538.9734
[M−H]−
284.267
5829 : M.findmain = 539.9807 (+/− 20 ppm)
538.9734
[M−H]−
284.267
0
20
40
60
80
100
300
350
400
450
500

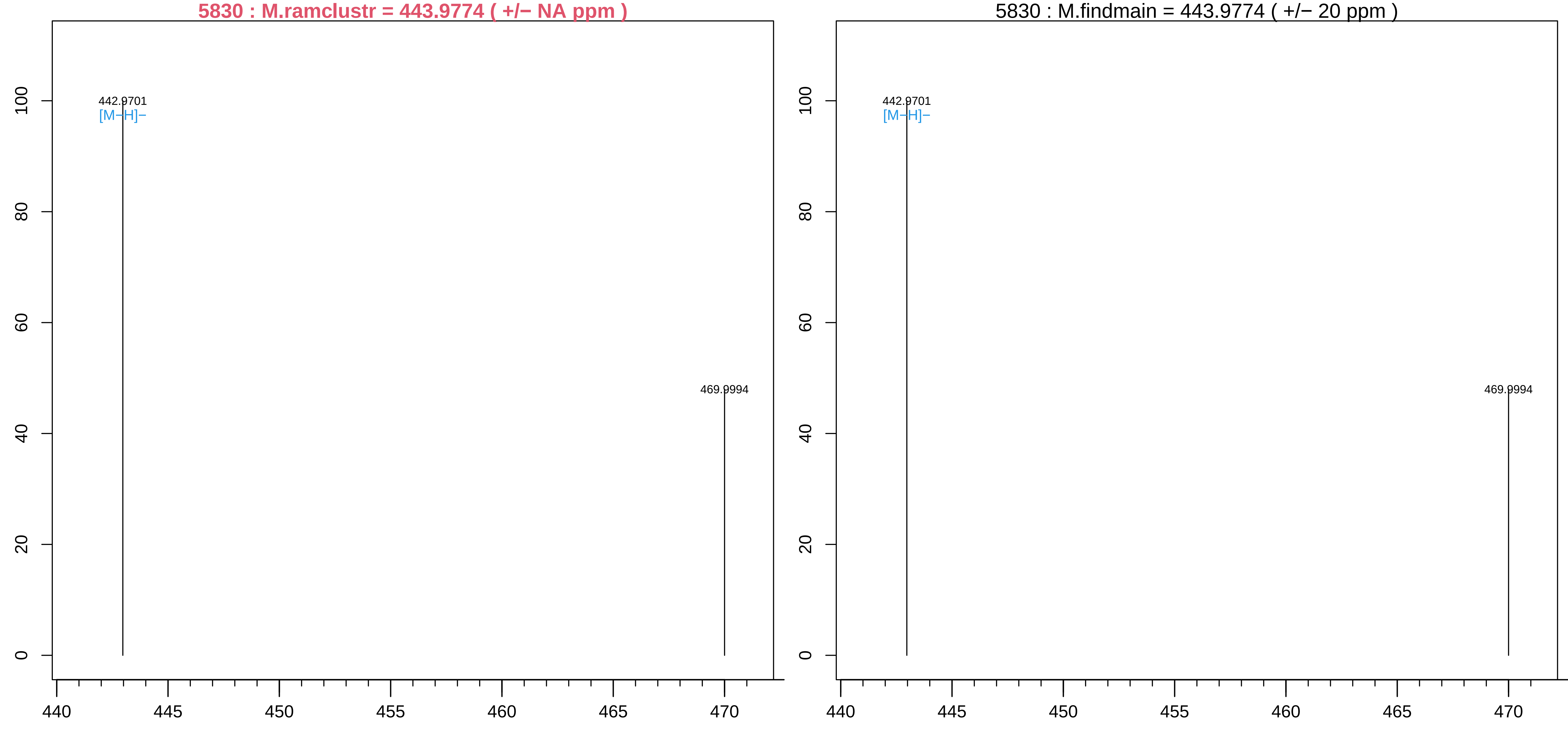
5830 : M.ramclustr = 443.9774 (+/− NA ppm)
442.9701
[M−H]−
469.9994
5830 : M.findmain = 443.9774 (+/− 20 ppm)
442.9701
[M−H]−
469.9994
0
20
40
60
80
100
440
445
450
455
460
465
470

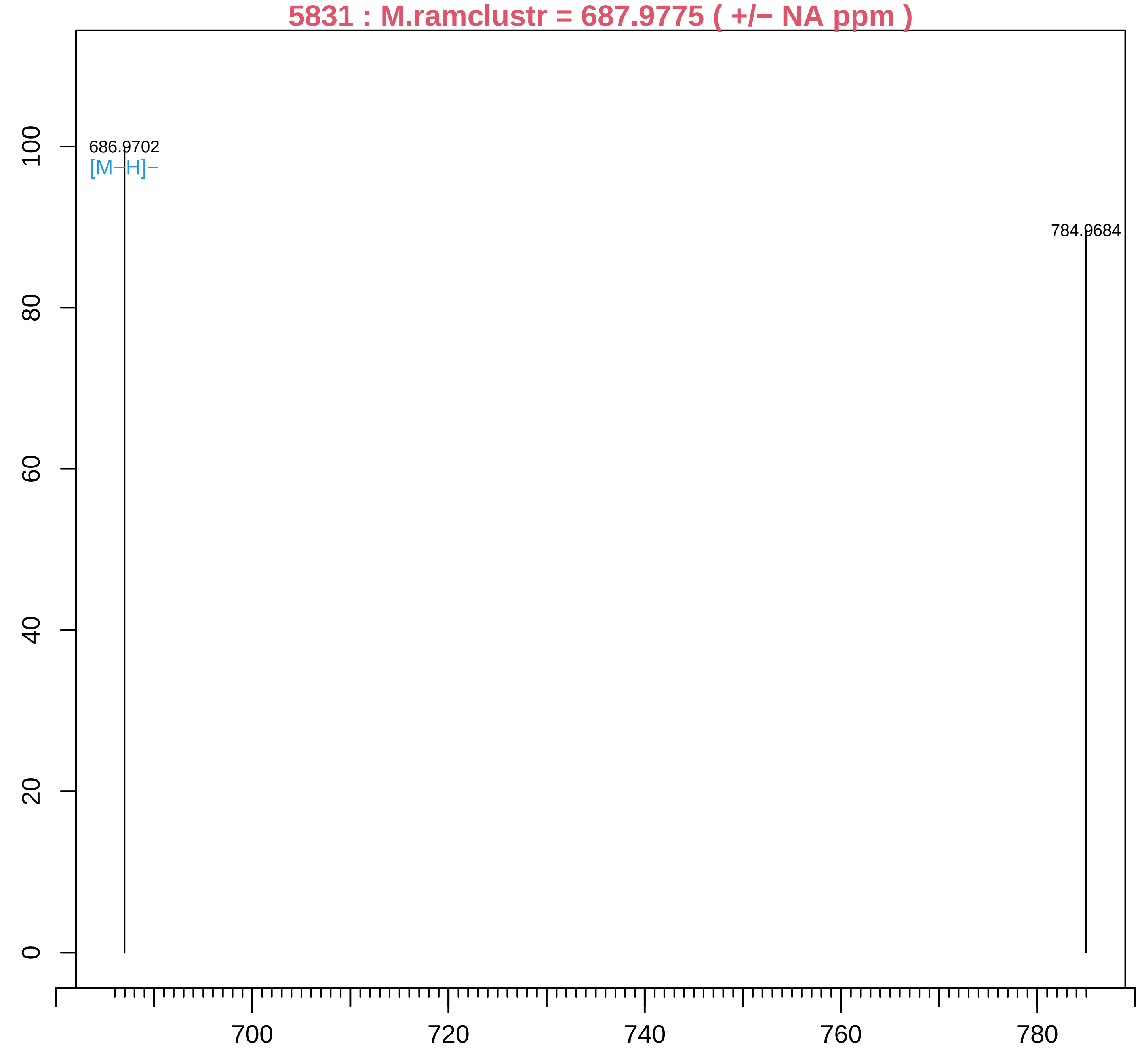
5831 : M.ramclustr = 687.9775 (+/− NA ppm)
686.9702
[M−H]−
784.9684
0
20
40
60
80
100
700
720
740
760
780

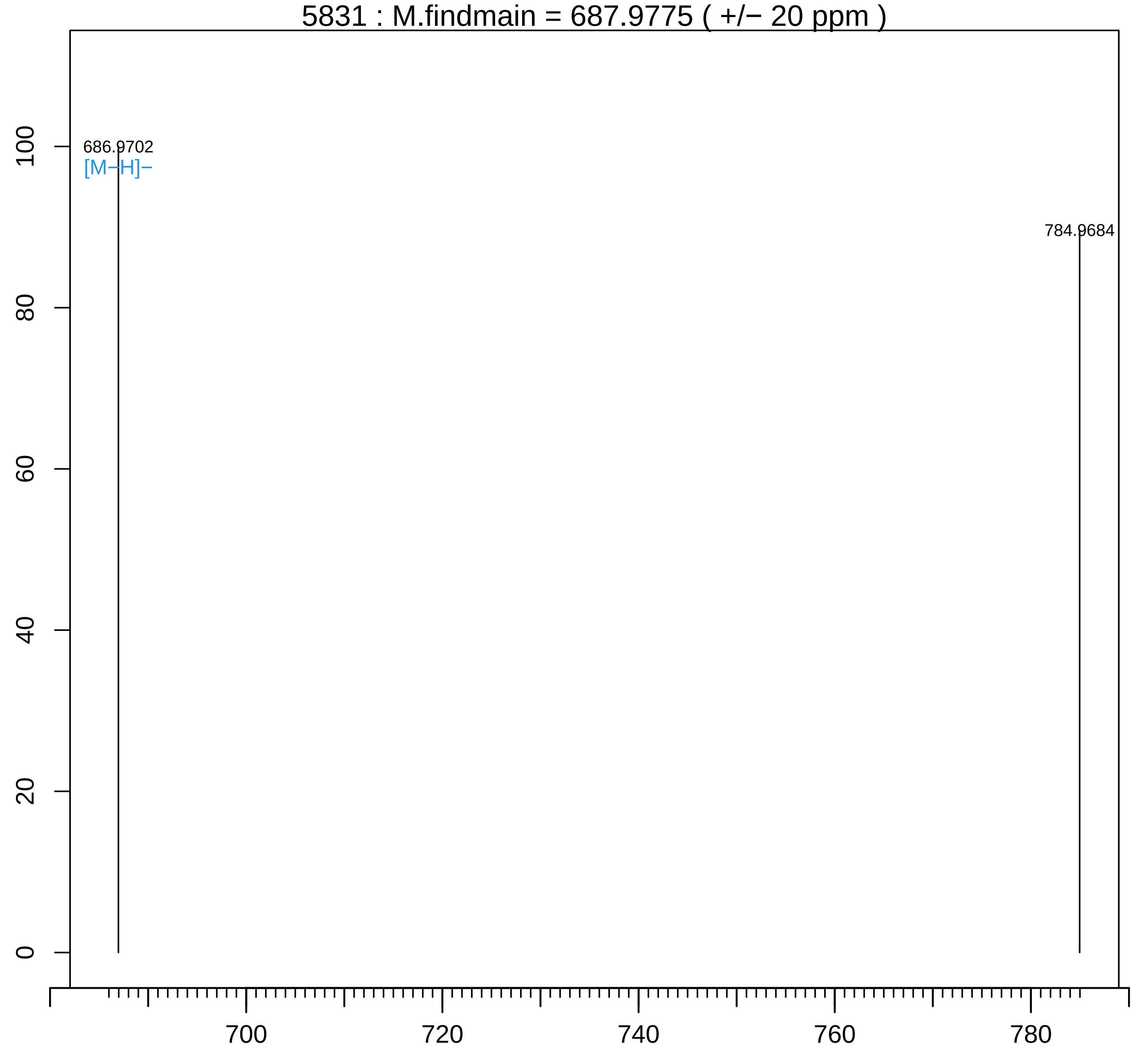
5831 : M.findmain = 687.9775 (+/− 20 ppm)
686.9702
[M−H]−
784.9684
0
20
40
60
80
100
700
720
740
760
780

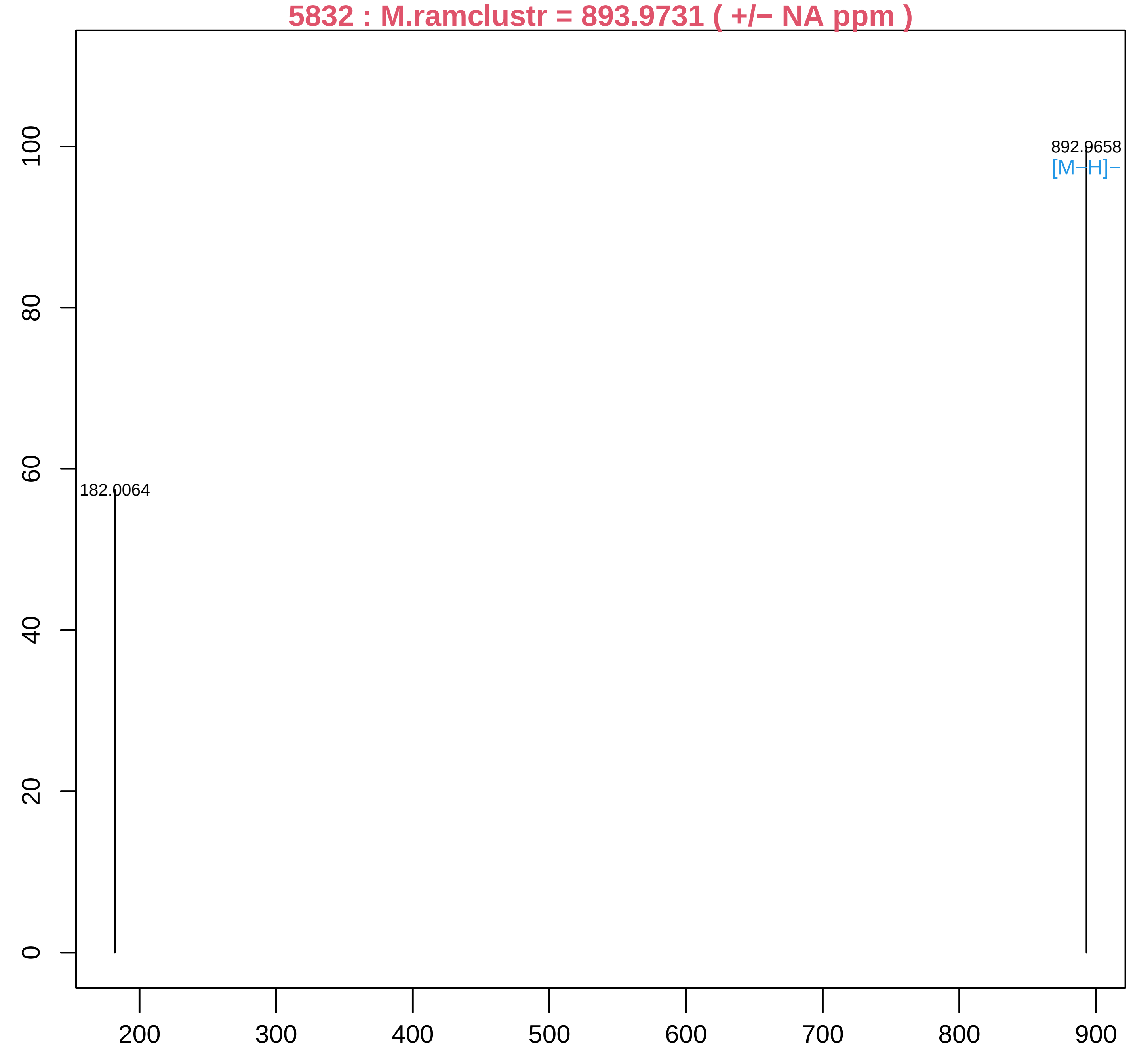

5832 : M.ramclustr = 893.9731 (+/− NA ppm)
892.9658
[M−H]−
182.0064
0
20
40
60
80
100
200
300
400
500
600
700
800
900

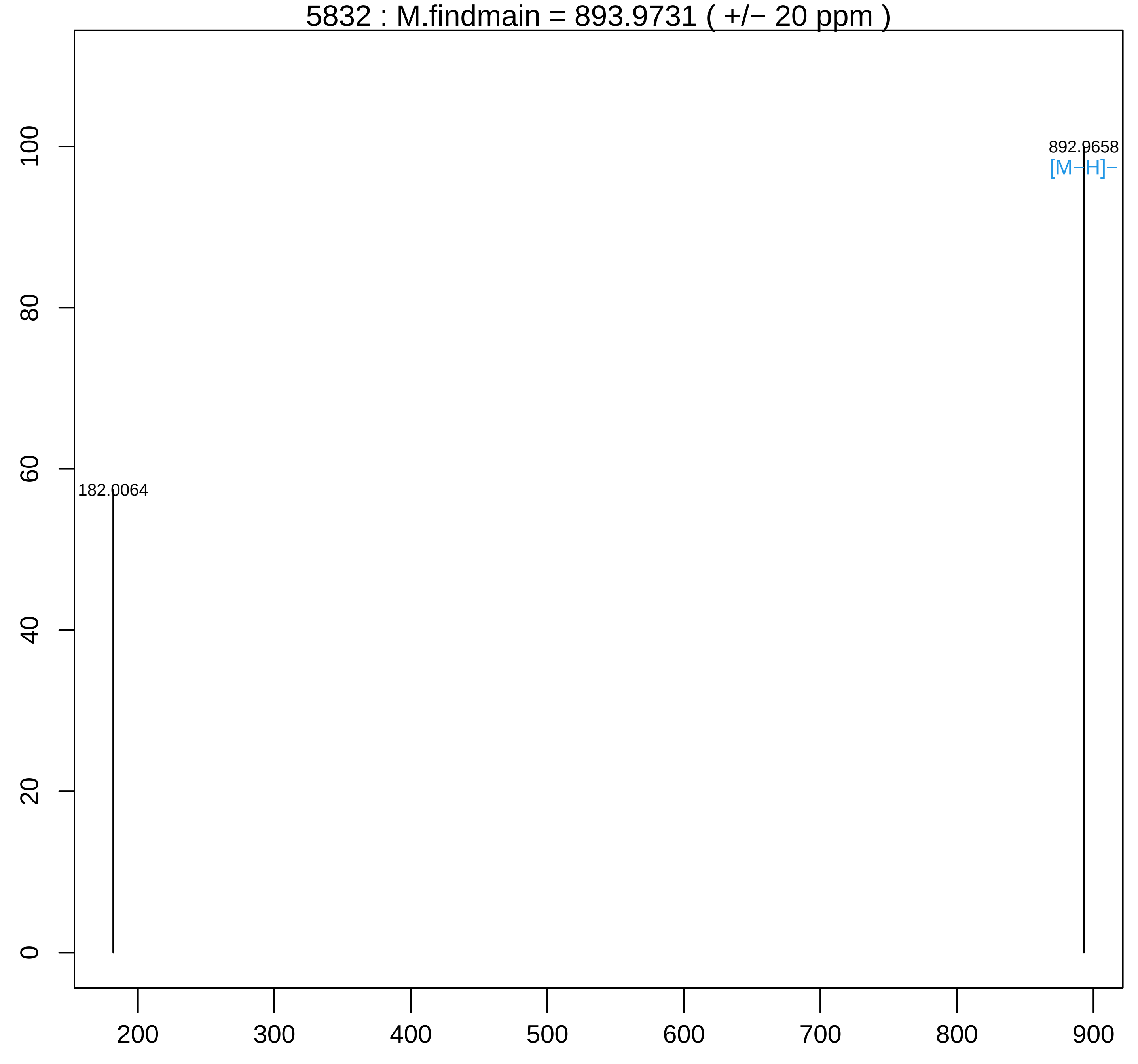

5832 : M.findmain = 893.9731 (+/− 20 ppm)
892.9658
[M−H]−
182.0064
0
20
40
60
80
100
200
300
400
500
600
700
800
900

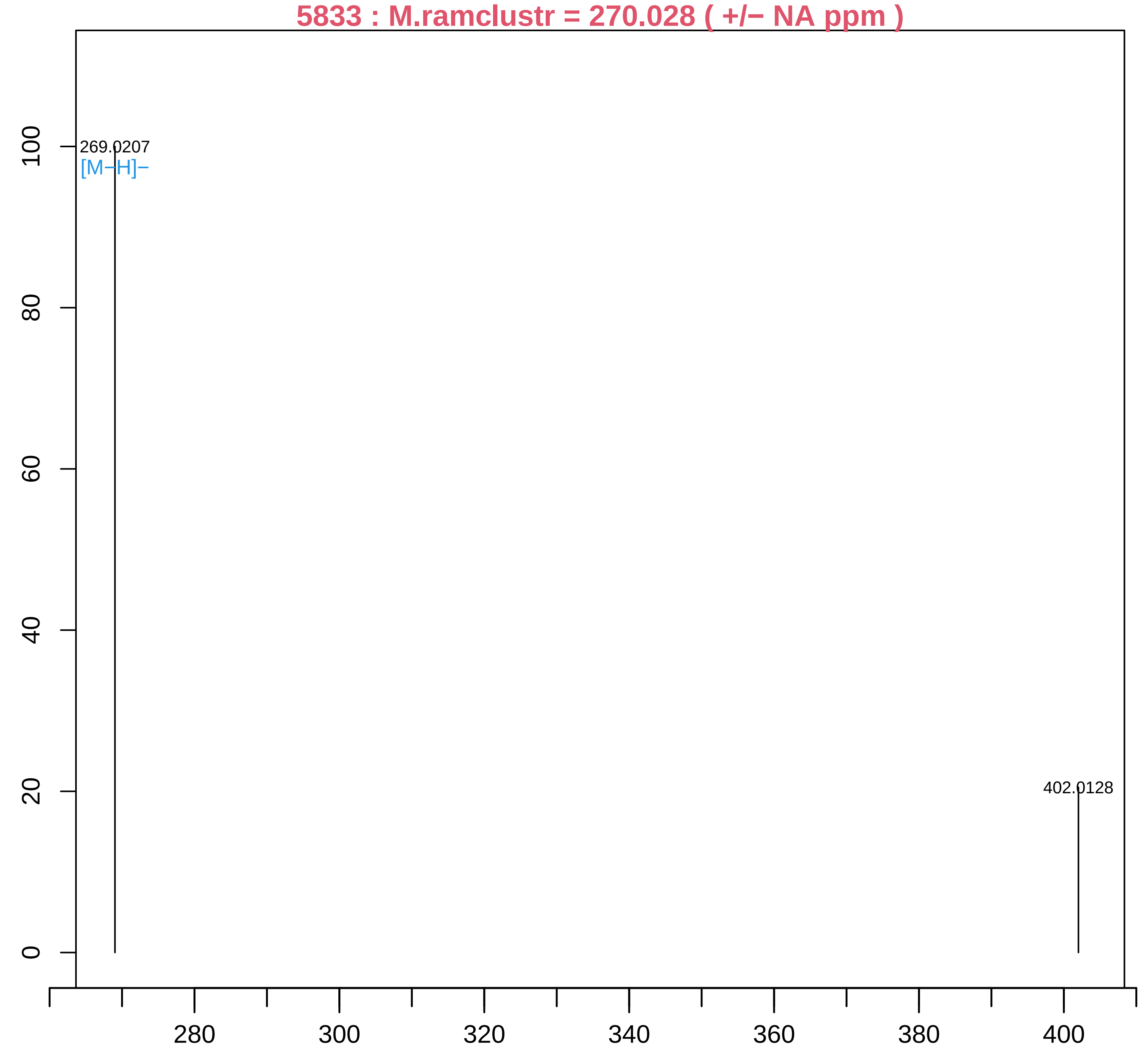
5833 : M.ramclustr = 270.028 (+/− NA ppm)
269.0207
[M−H]−
402.0128
0
20
40
60
80
100
280
300
320
340
360
380
400

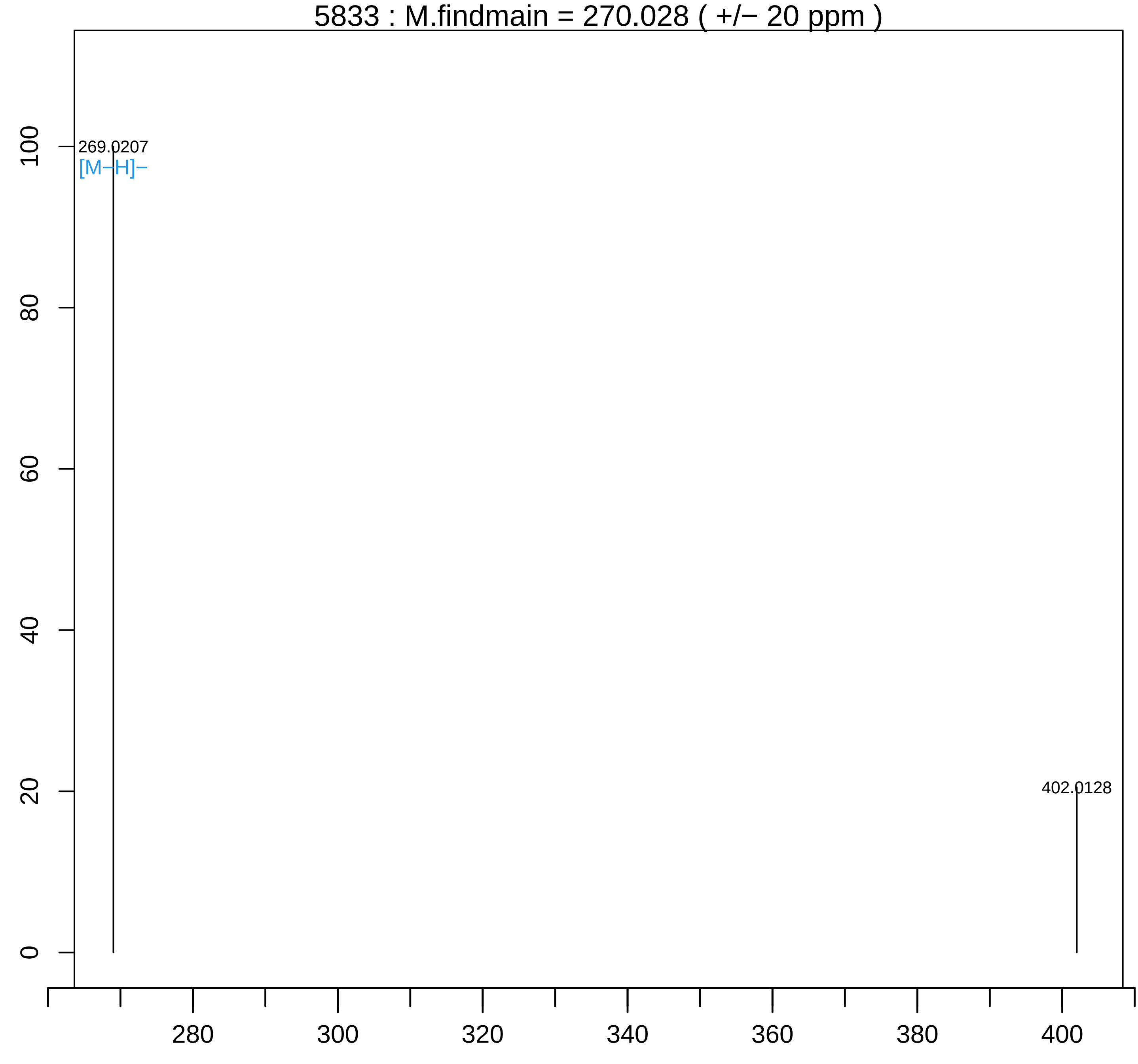
5833 : M.findmain = 270.028 (+/− 20 ppm)
269.0207
[M−H]−
402.0128
0
20
40
60
80
100
280
300
320
340
360
380
400

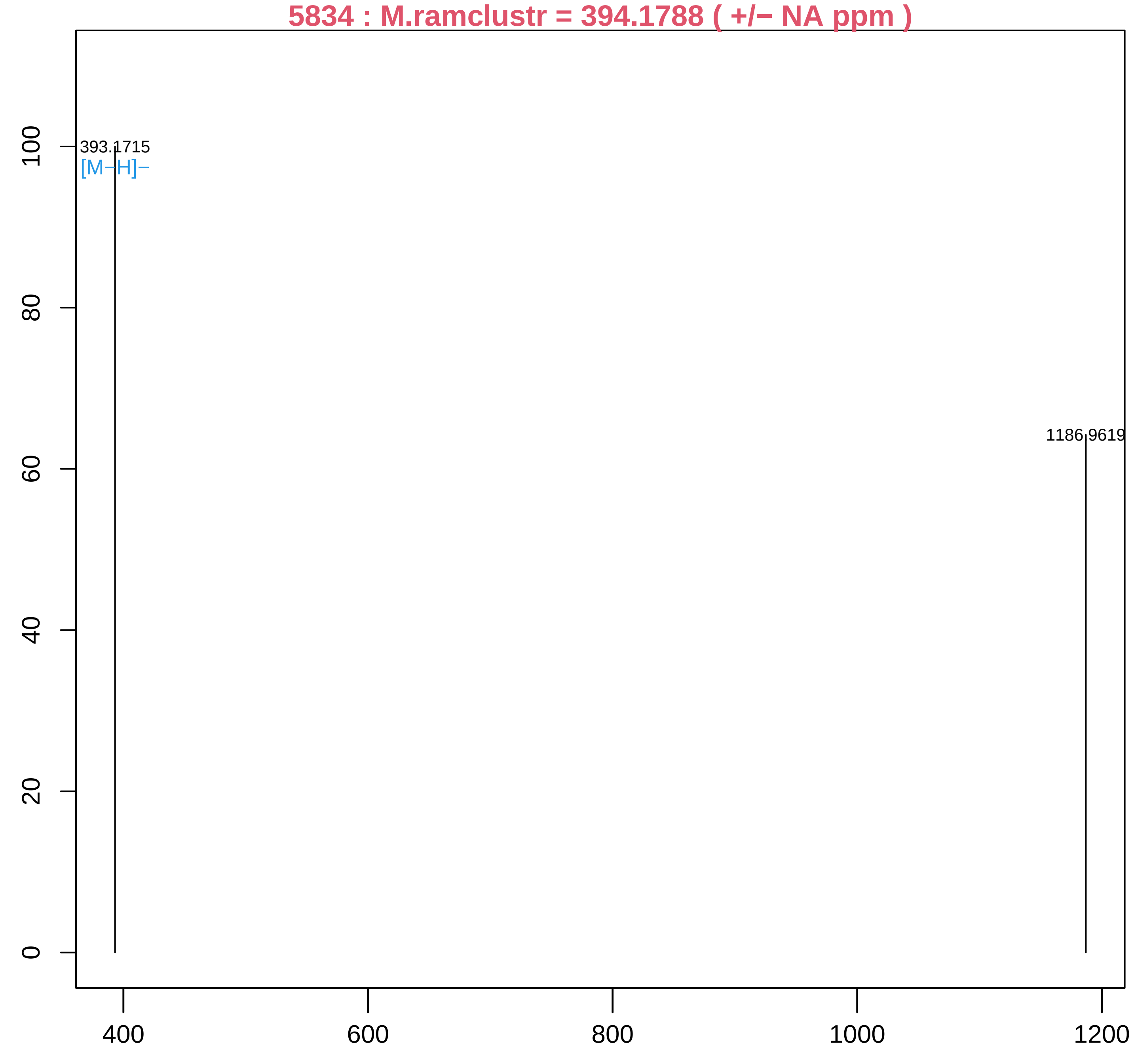

5834 : M.ramclustr = 394.1788 (+/− NA ppm)
393.1715
[M−H]−
1186.9619
0
20
40
60
80
100
400
600
800
1000
1200

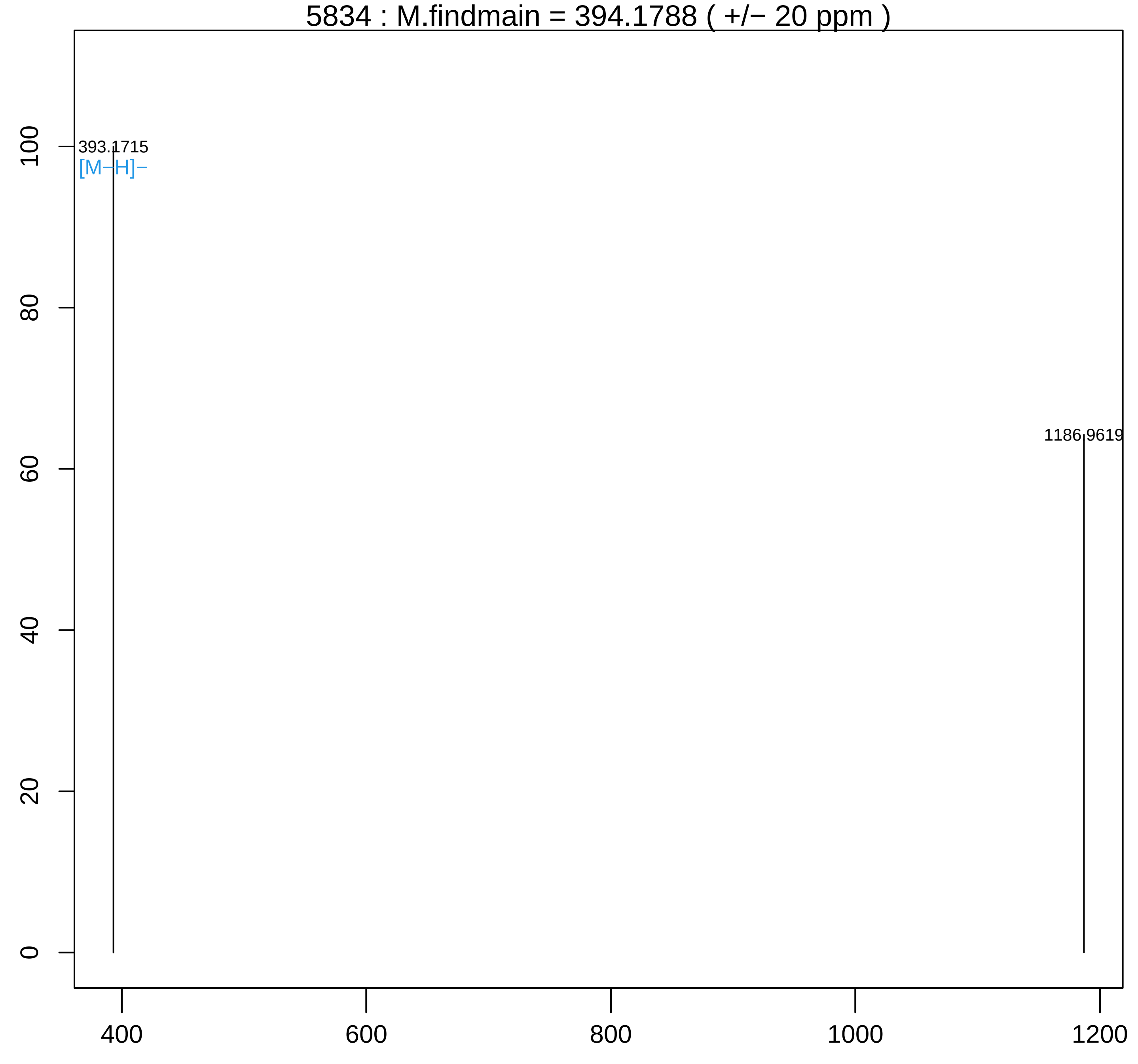

5834 : M.findmain = 394.1788 (+/− 20 ppm)
393.1715
[M−H]−
1186.9619
0
20
40
60
80
100
400
600
800
1000
1200

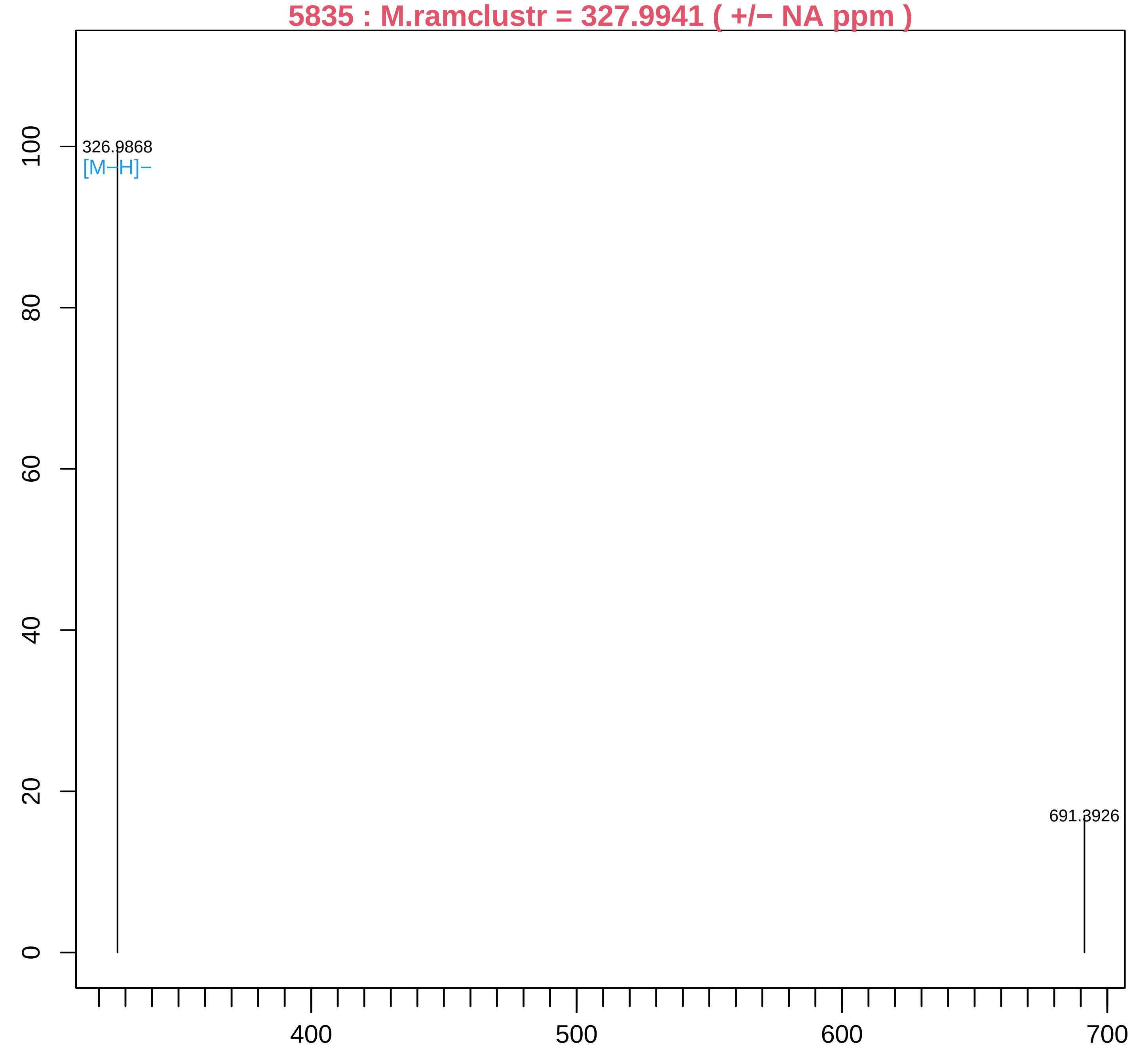
5835 : M.ramclustr = 327.9941 (+/− NA ppm)
326.9868
[M−H]−
691.3926
0
20
40
60
80
100
400
500
600
700

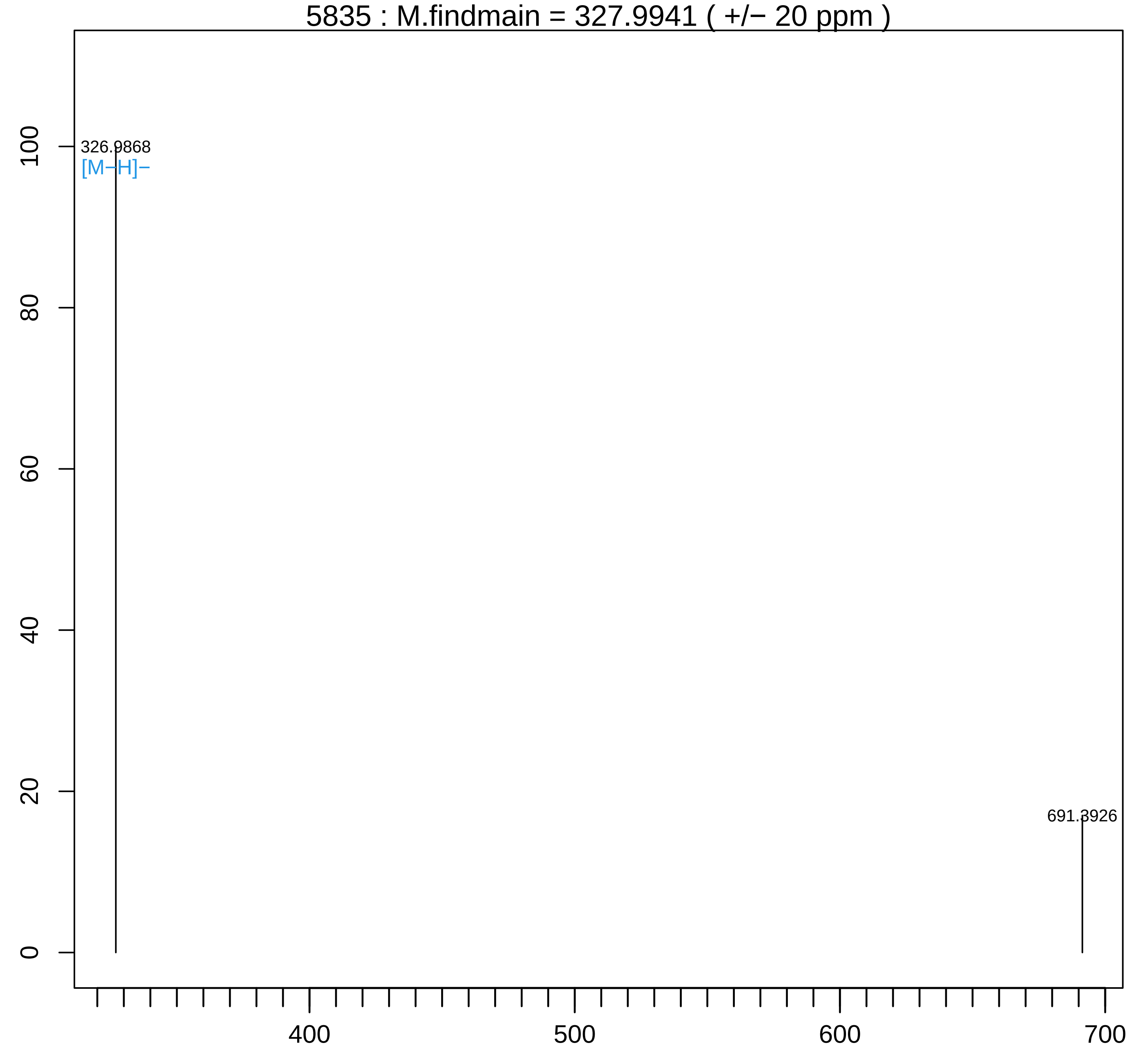
5835 : M.findmain = 327.9941 (+/− 20 ppm)
326.9868
[M−H]−
691.3926
0
20
40
60
80
100
400
500
600
700

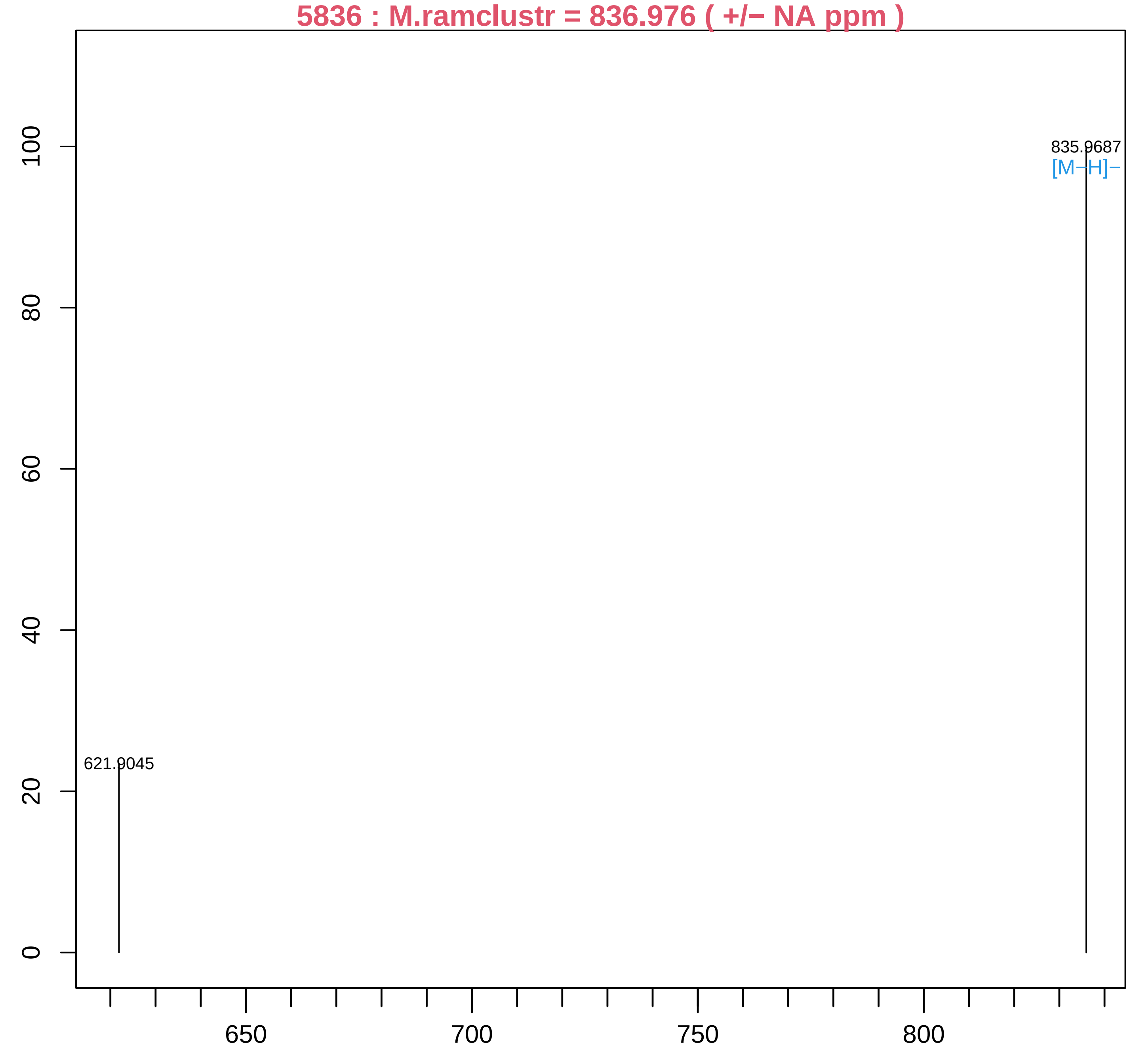
5836 : M.ramclustr = 836.976 (+/− NA ppm)
835.9687
[M−H]−
621.9045
0
20
40
60
80
100
650
700
750
800

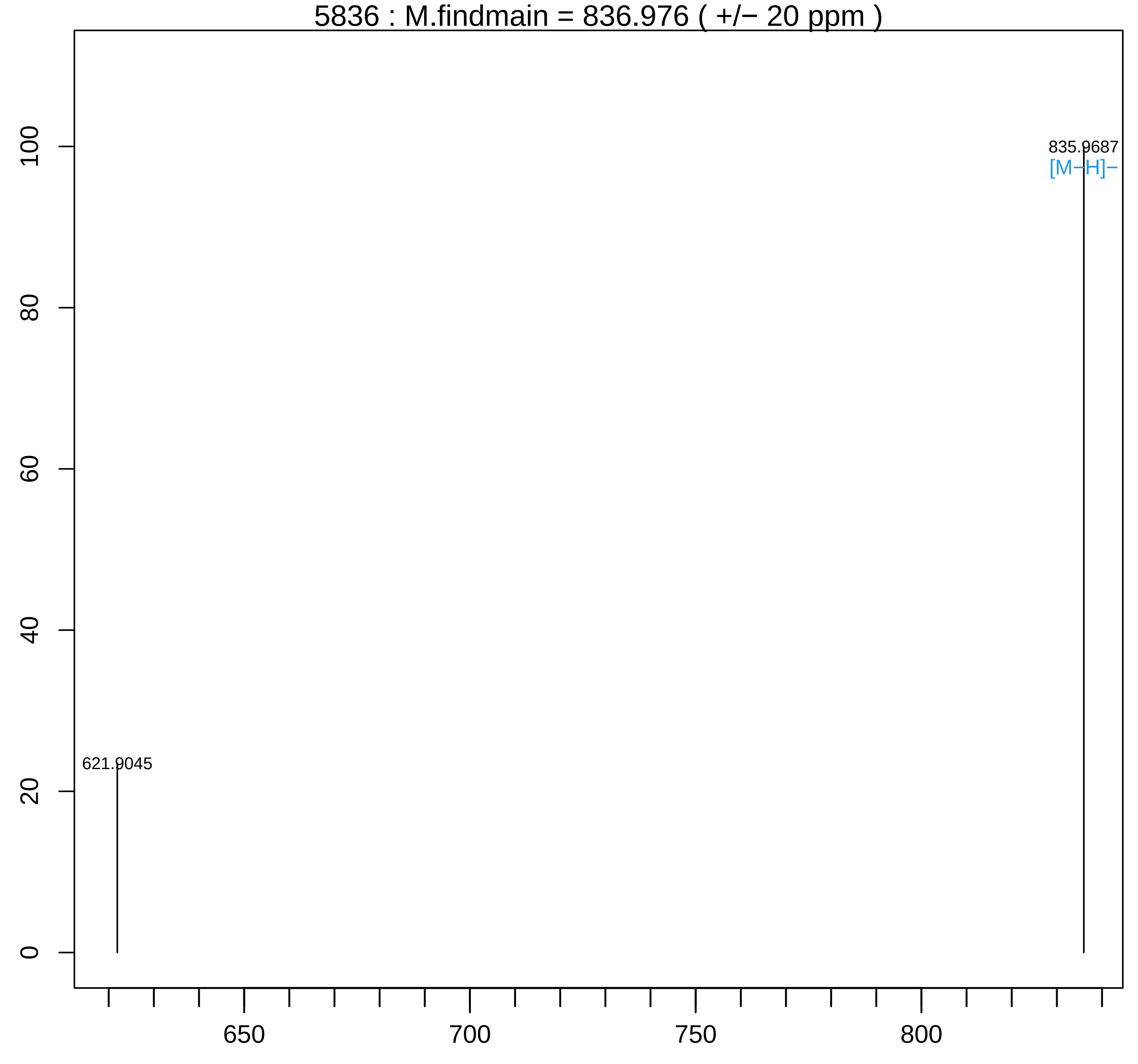
5836 : M.findmain = 836.976 (+/− 20 ppm)
835.9687
[M−H]−
621.9045
0
20
40
60
80
100
650
700
750
800

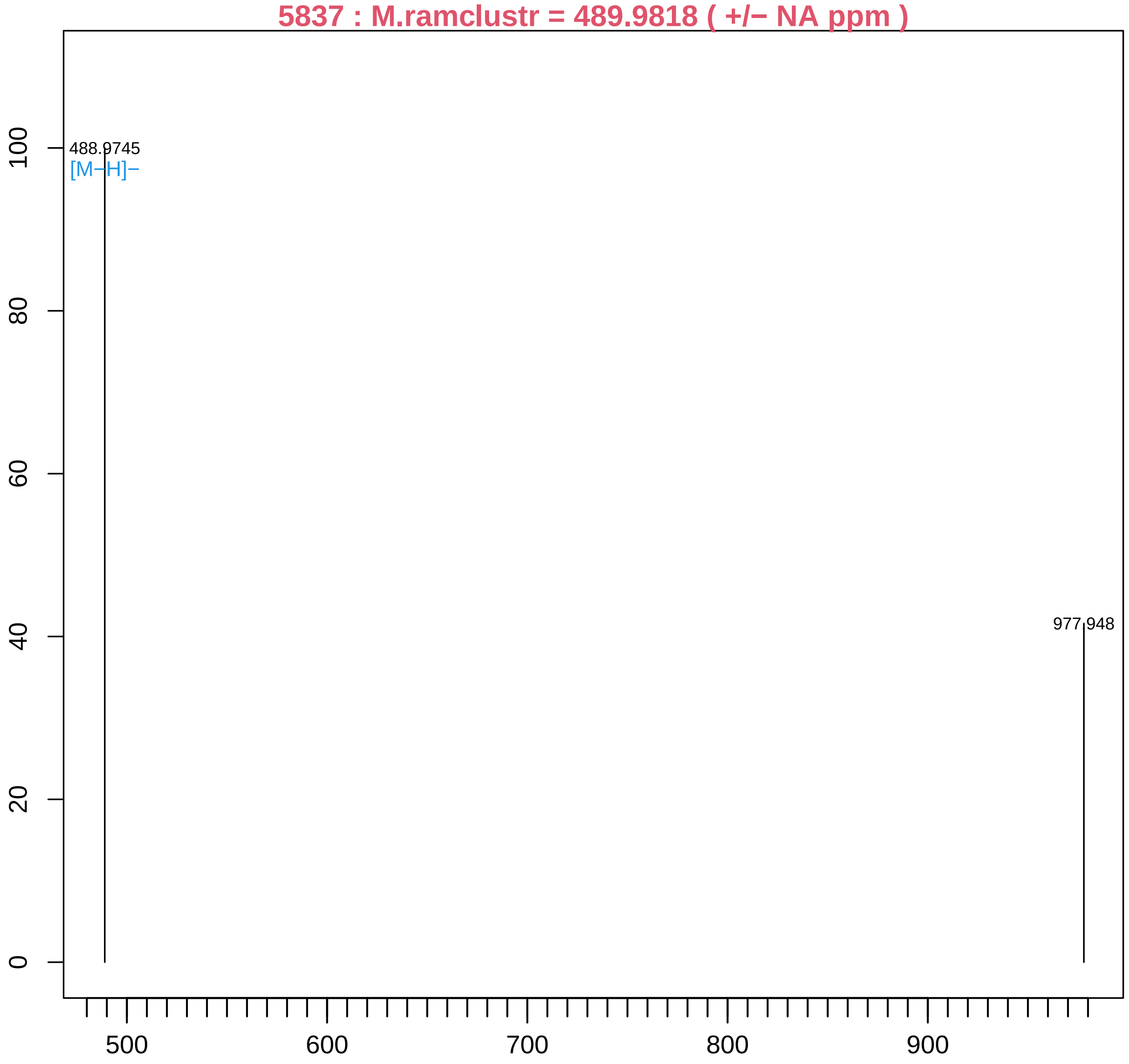
5837 : M.ramclustr = 489.9818 (+/− NA ppm)
488.9745
[M−H]−
977.948
0
20
40
60
80
100
500
600
700
800
900

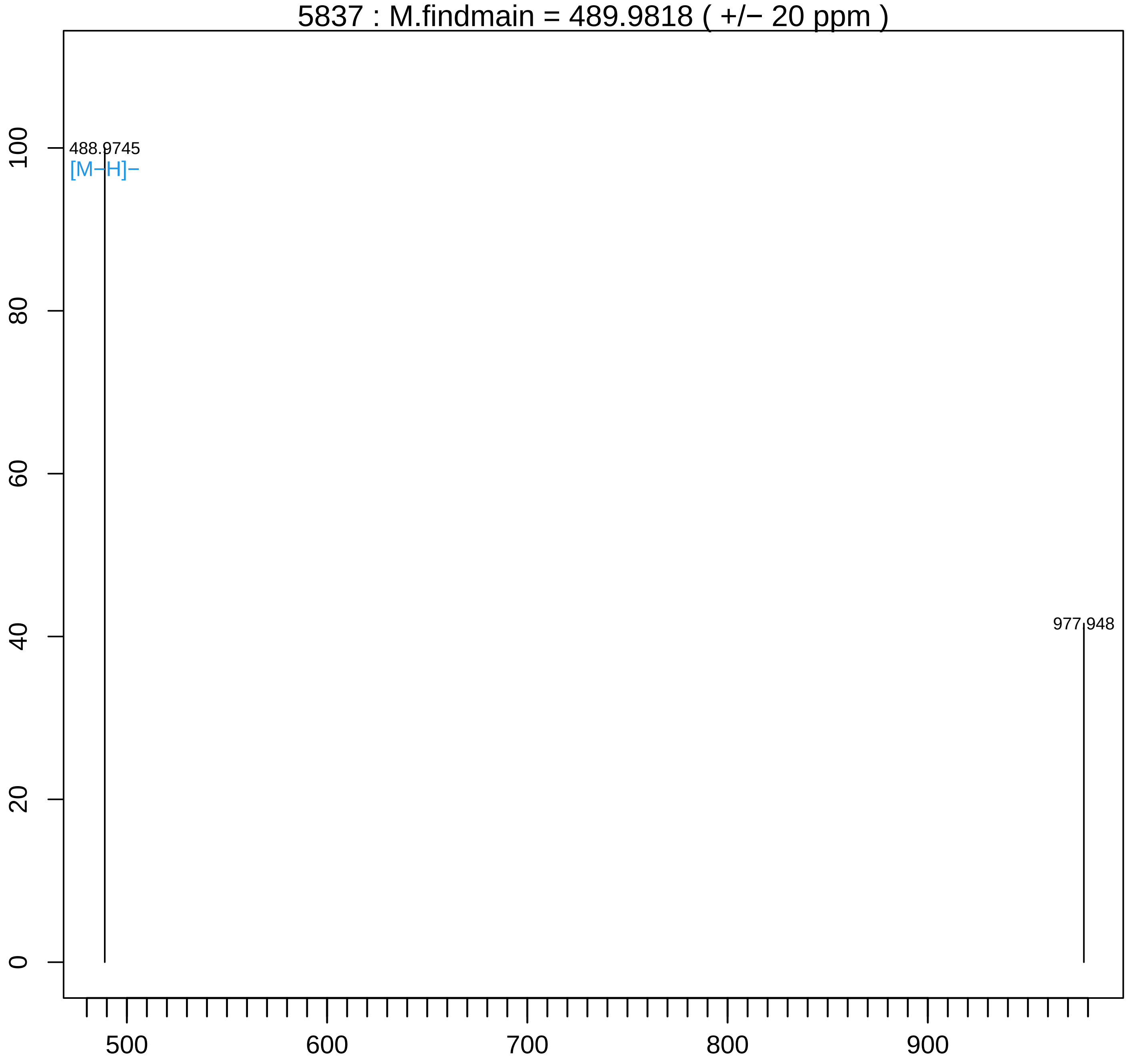
5837 : M.findmain = 489.9818 (+/− 20 ppm)
488.9745
[M−H]−
977.948
0
20
40
60
80
100
500
600
700
800
900

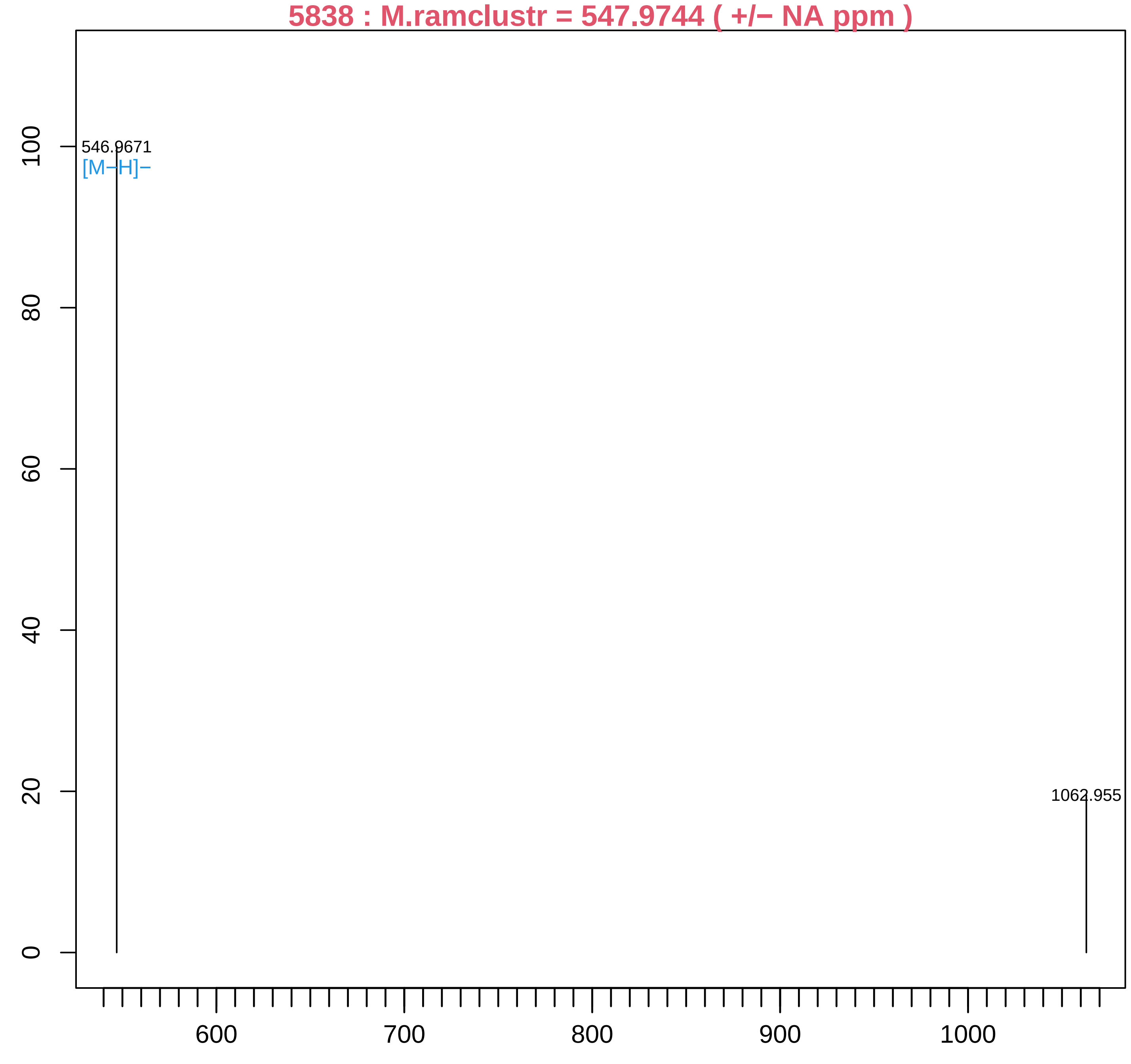
5838 : M.ramclustr = 547.9744 (+/− NA ppm)
546.9671
[M−H]−
1062.955
0
20
40
60
80
100
600
700
800
900
1000

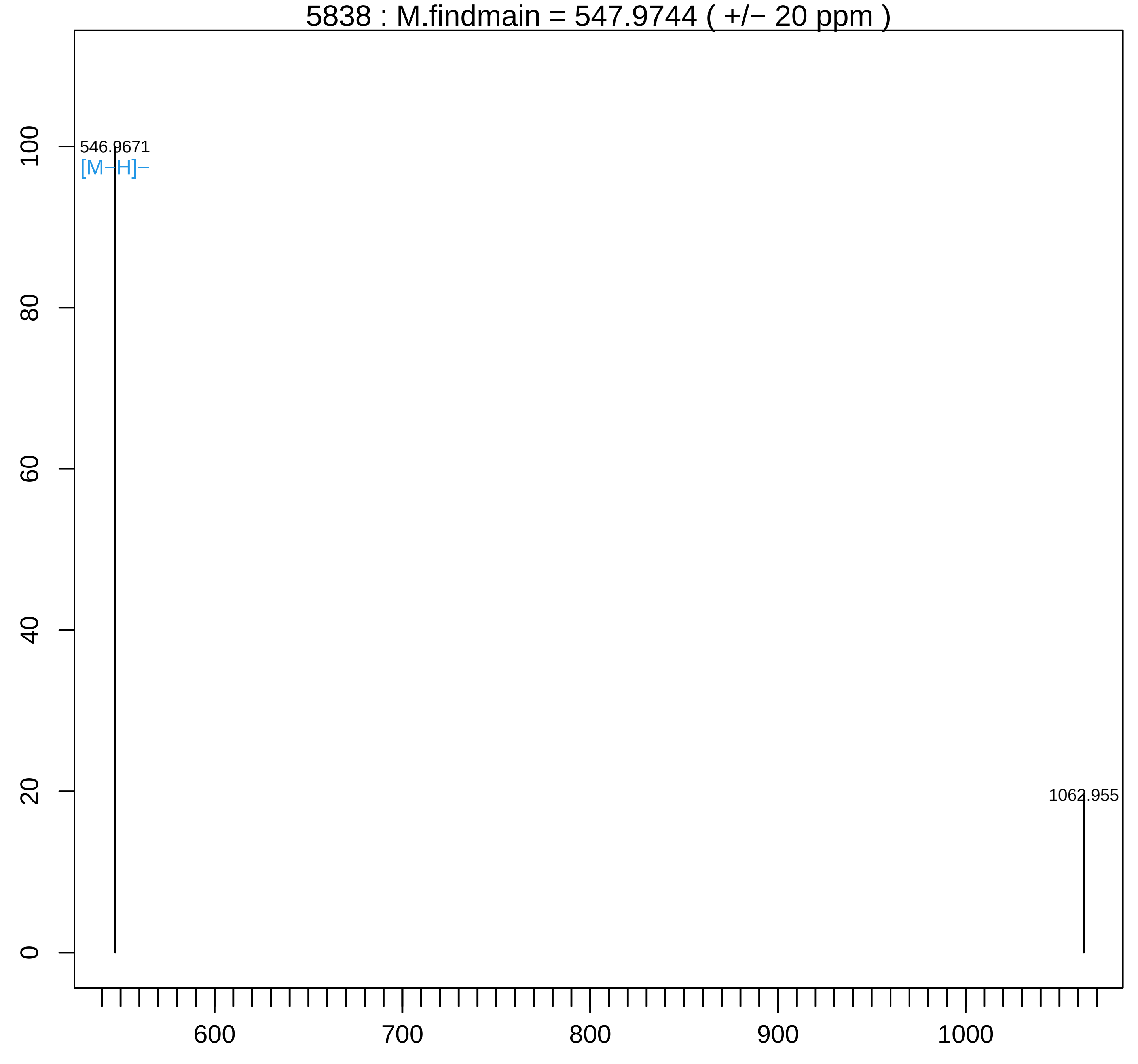
5838 : M.findmain = 547.9744 (+/− 20 ppm)
546.9671
[M−H]−
1062.955
0
20
40
60
80
100
600
700
800
900
1000

5839 : M.ramclustr = 834.9853 (+/− NA ppm)

5839 : M.findmain = 834.9853 (+/− 20 ppm)

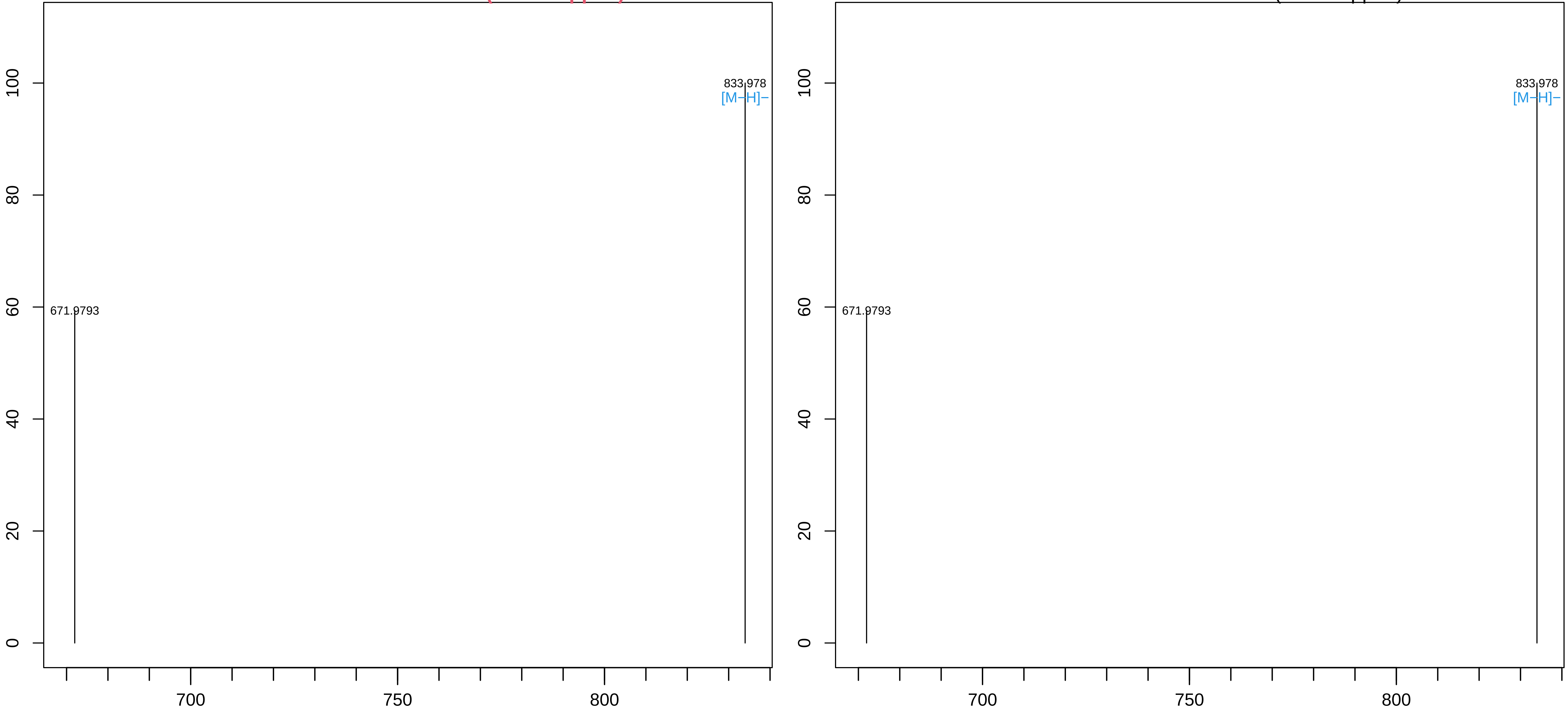

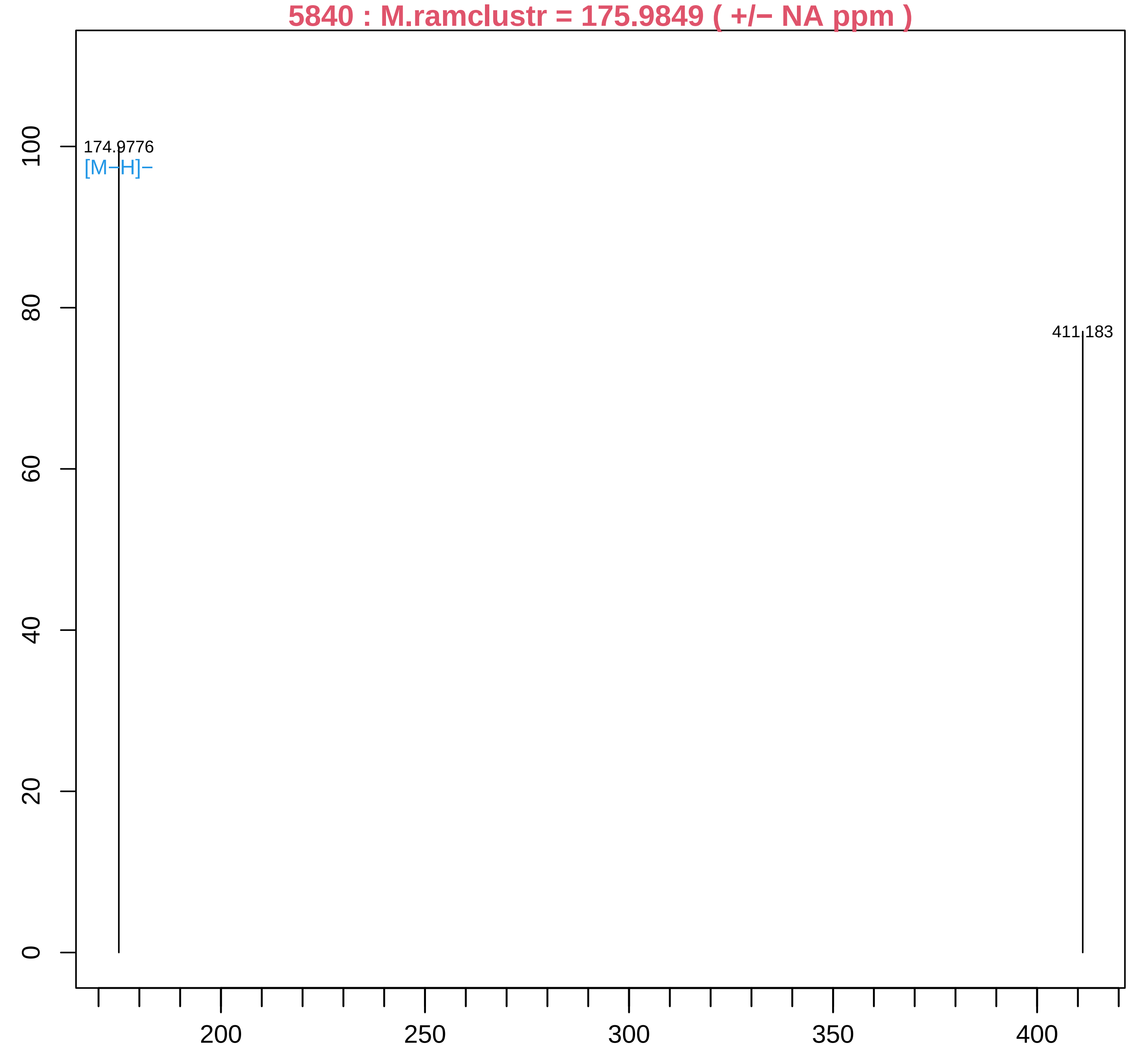
5840 : M.ramclustr = 175.9849 (+/− NA ppm)
174.9776
[M−H]−
411.183
0
20
40
60
80
100
200
250
300
350
400

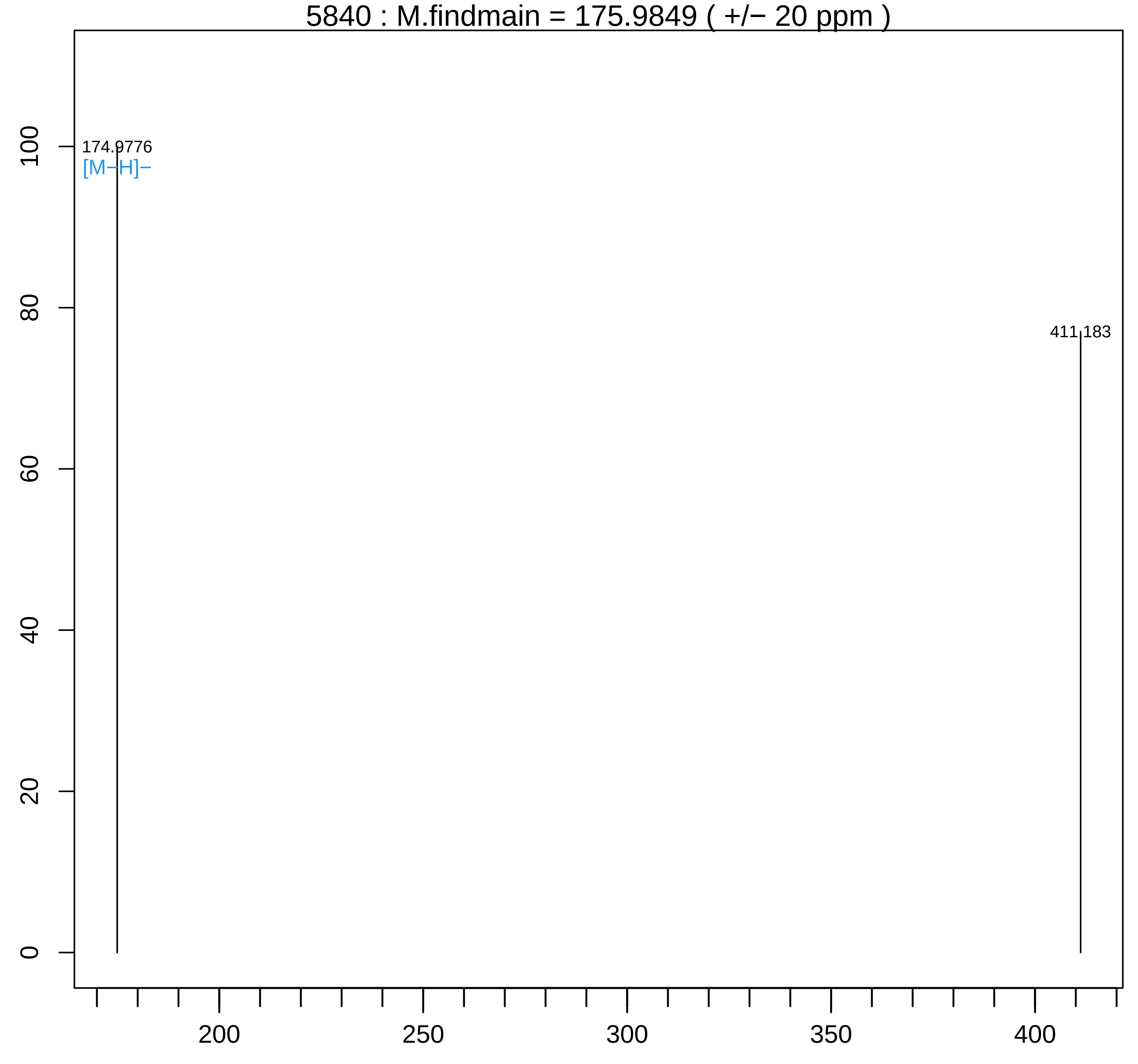
5840 : M.findmain = 175.9849 (+/− 20 ppm)
174.9776
[M−H]−
411.183
0
20
40
60
80
100
200
250
300
350
400

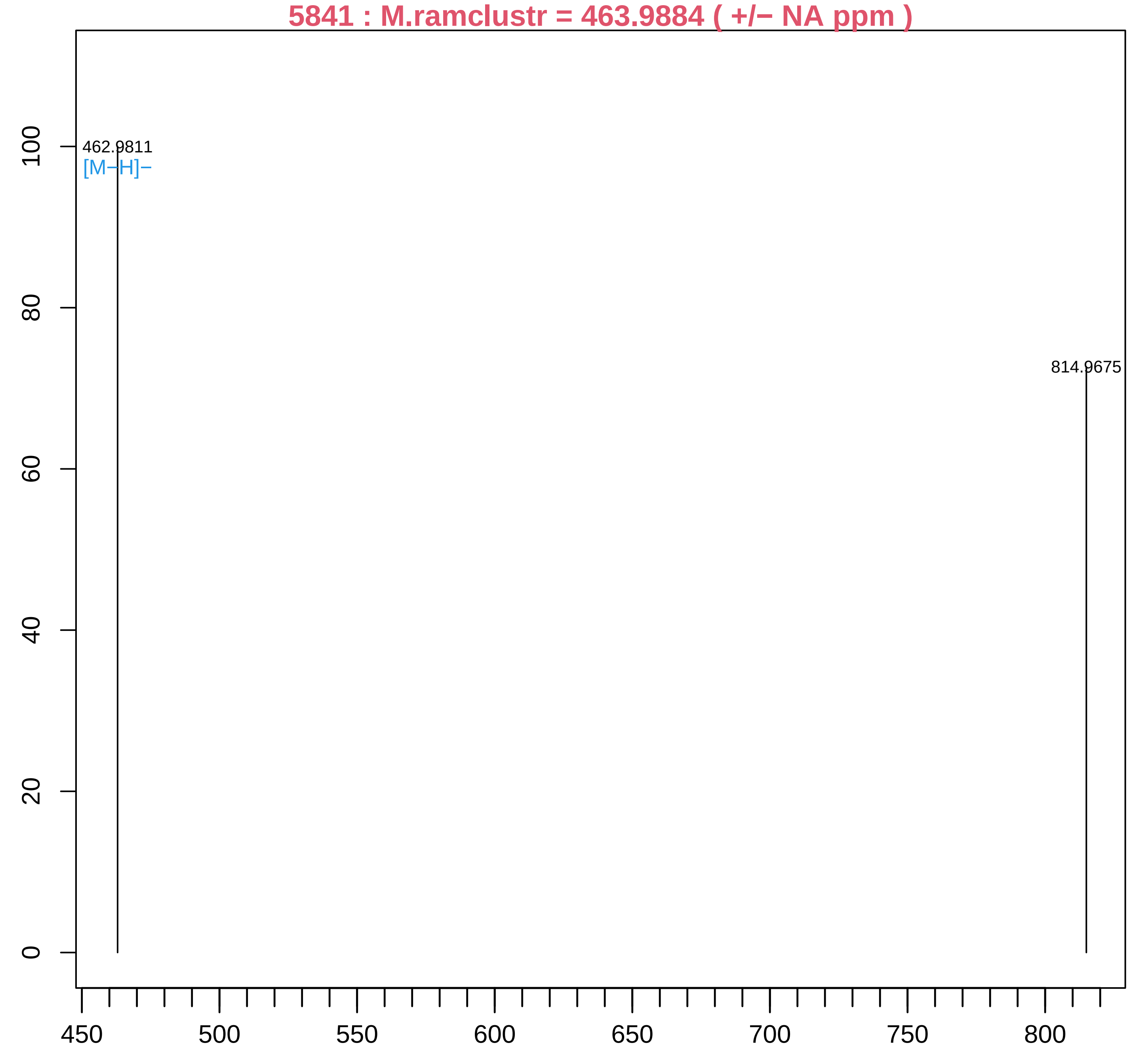
5841 : M.ramclustr = 463.9884 (+/− NA ppm)
462.9811
[M−H]−
814.9675
0
20
40
60
80
100
450
500
550
600
650
700
750
800
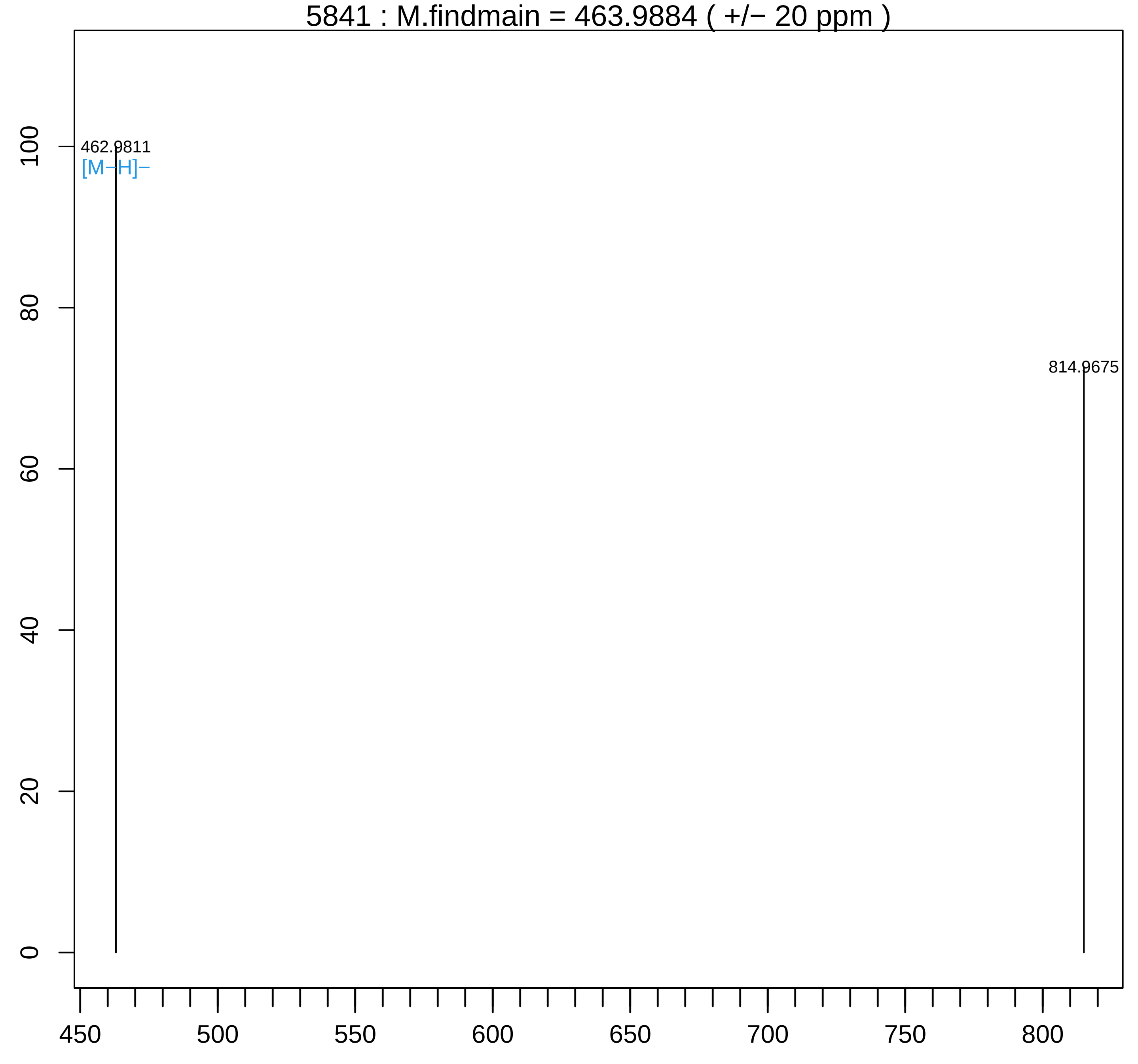
5841 : M.findmain = 463.9884 (+/− 20 ppm)
462.9811
[M−H]−
814.9675
0
20
40
60
80
100
450
500
550
600
650
700
750
800

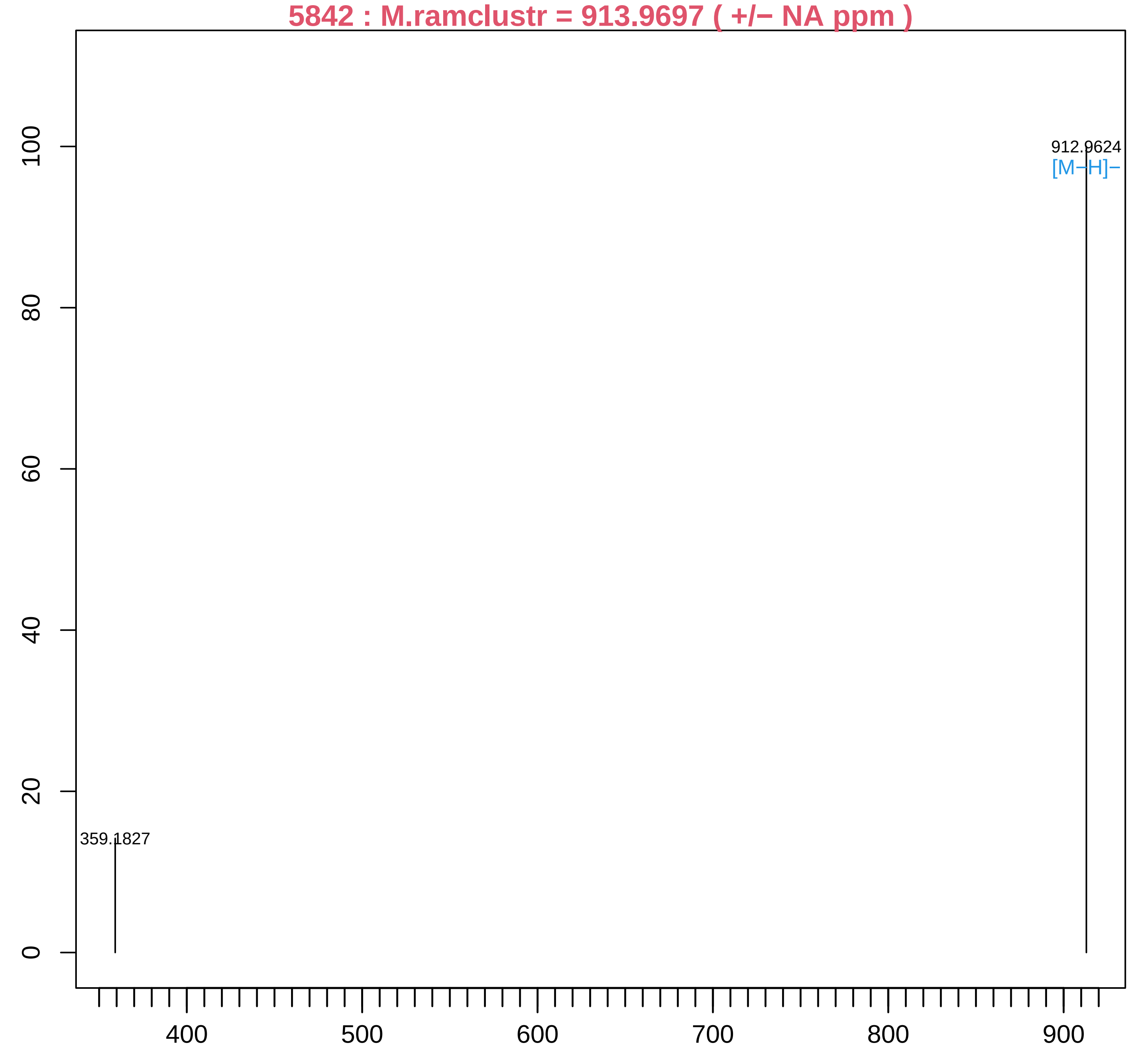

5842 : M.ramclustr = 913.9697 (+/− NA ppm)
912.9624
[M−H]−
359.1827
0
20
40
60
80
100
400
500
600
700
800
900

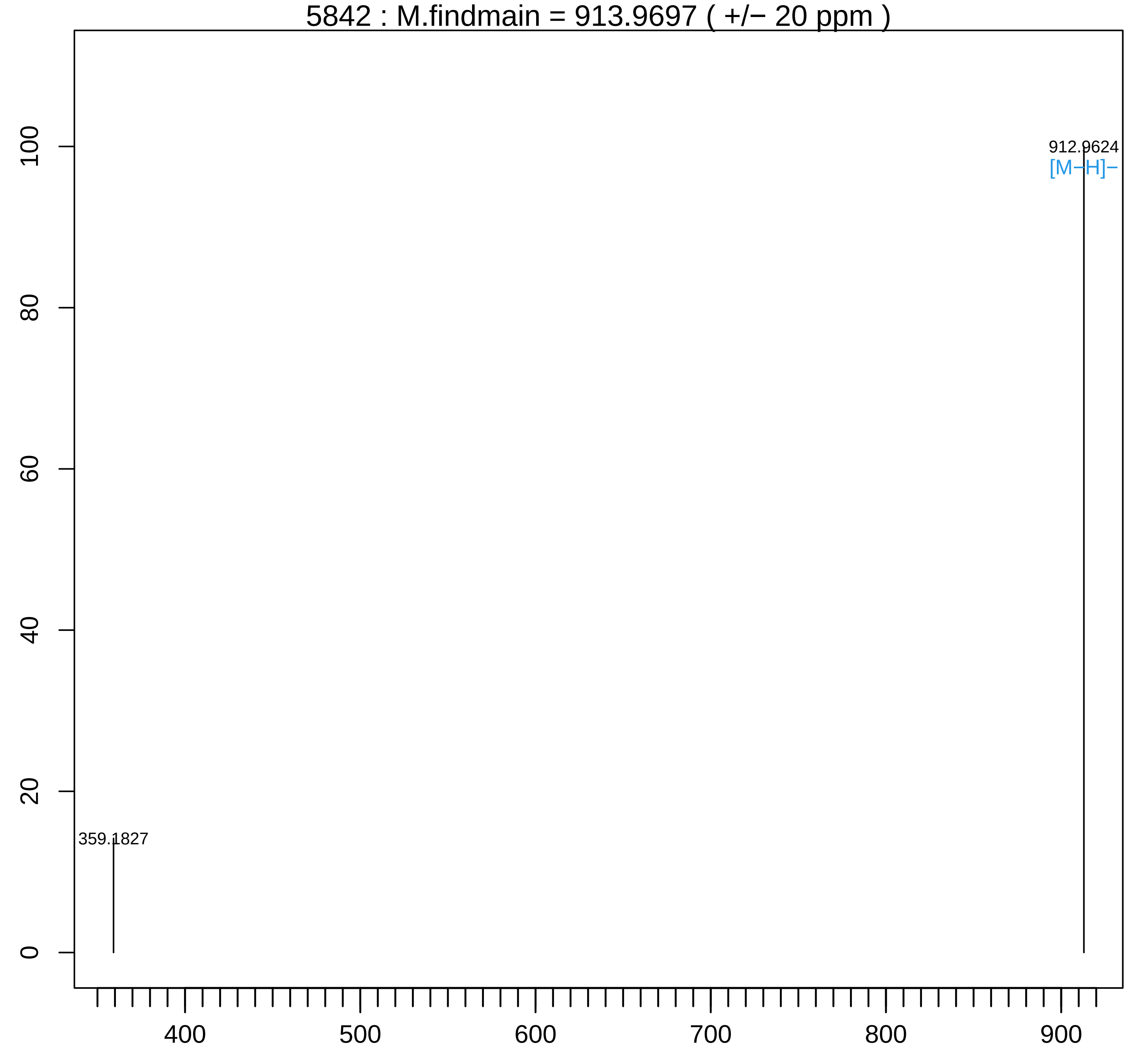

5842 : M.findmain = 913.9697 (+/− 20 ppm)
912.9624
[M−H]−
359.1827
0
20
40
60
80
100
400
500
600
700
800
900

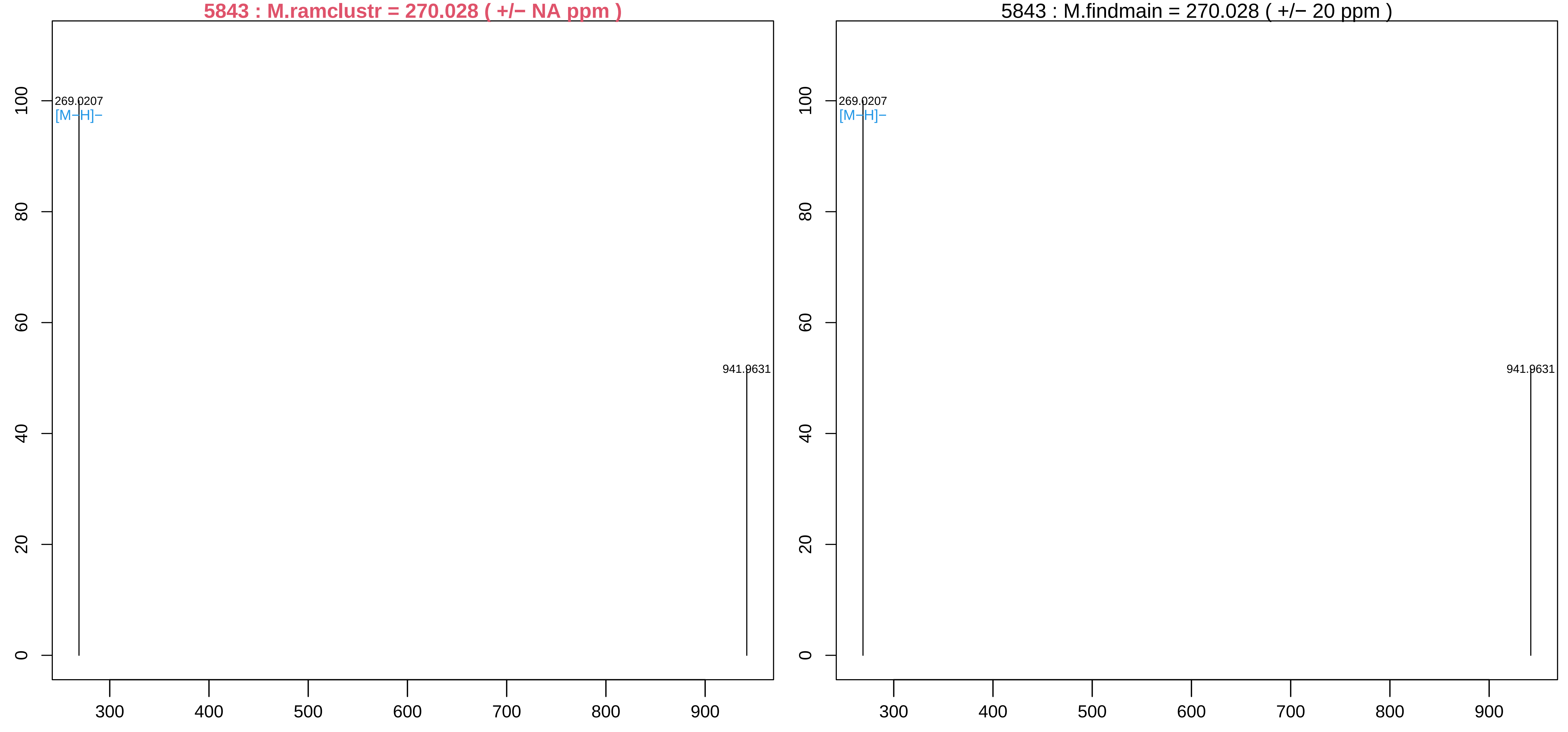
5843 : M.ramclustr = 270.028 (+/− NA ppm)
269.0207
[M−H]−
941.9631
0
20
40
60
80
100
300
400
500
600
700
800
900
5843 : M.findmain = 270.028 (+/− 20 ppm)
269.0207
[M−H]−
941.9631

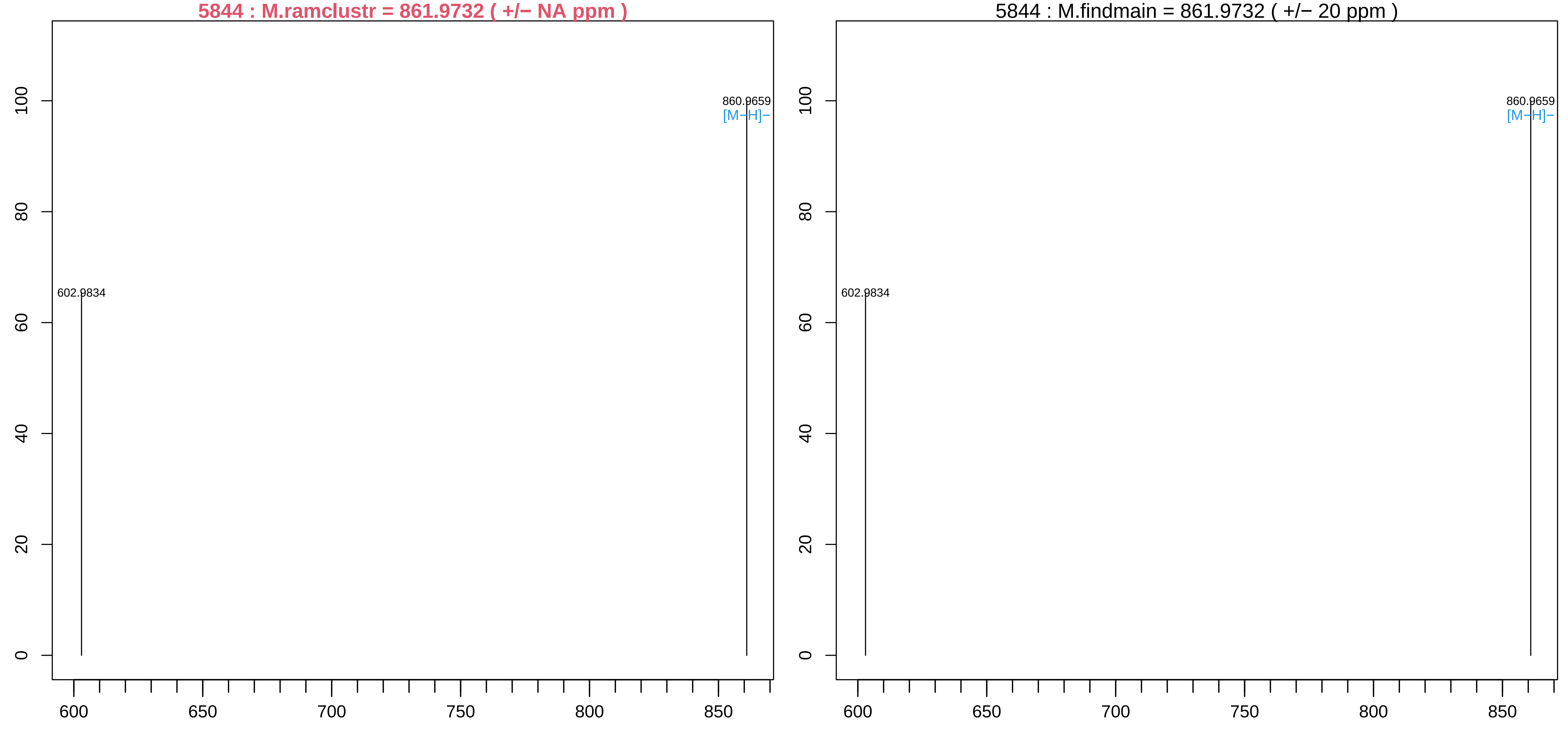
5844 : M.ramclustr = 861.9732 (+/− NA ppm)
602.9834
860.9659
[M−H]−
5844 : M.findmain = 861.9732 (+/− 20 ppm)
602.9834
860.9659
[M−H]−
0
20
40
60
80
100
600
650
700
750
800
850

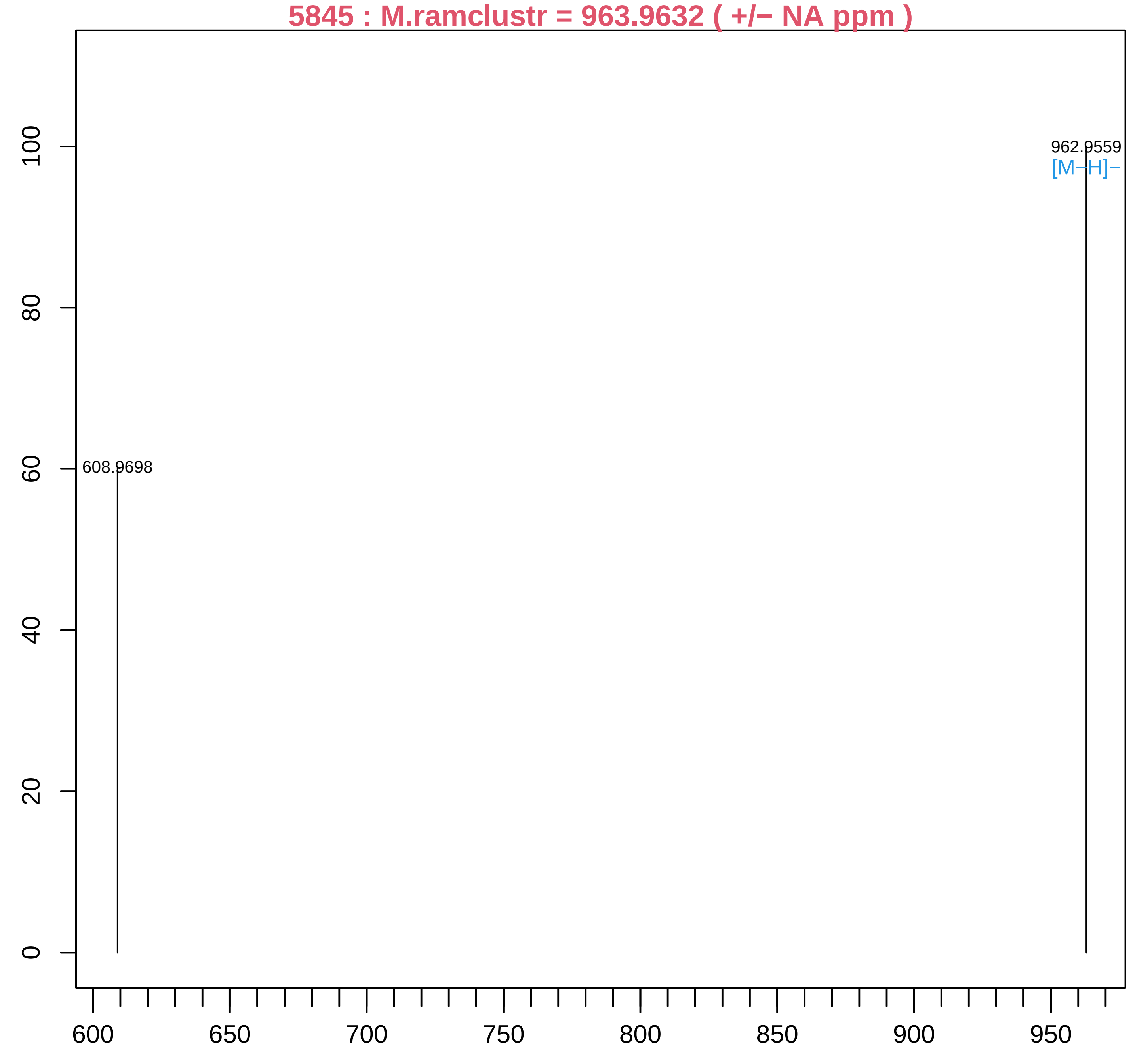
5845 : M.ramclustr = 963.9632 (+/− NA ppm)
962.9559
[M−H]−
608.9698
0
20
40
60
80
100
600
650
700
750
800
850
900
950

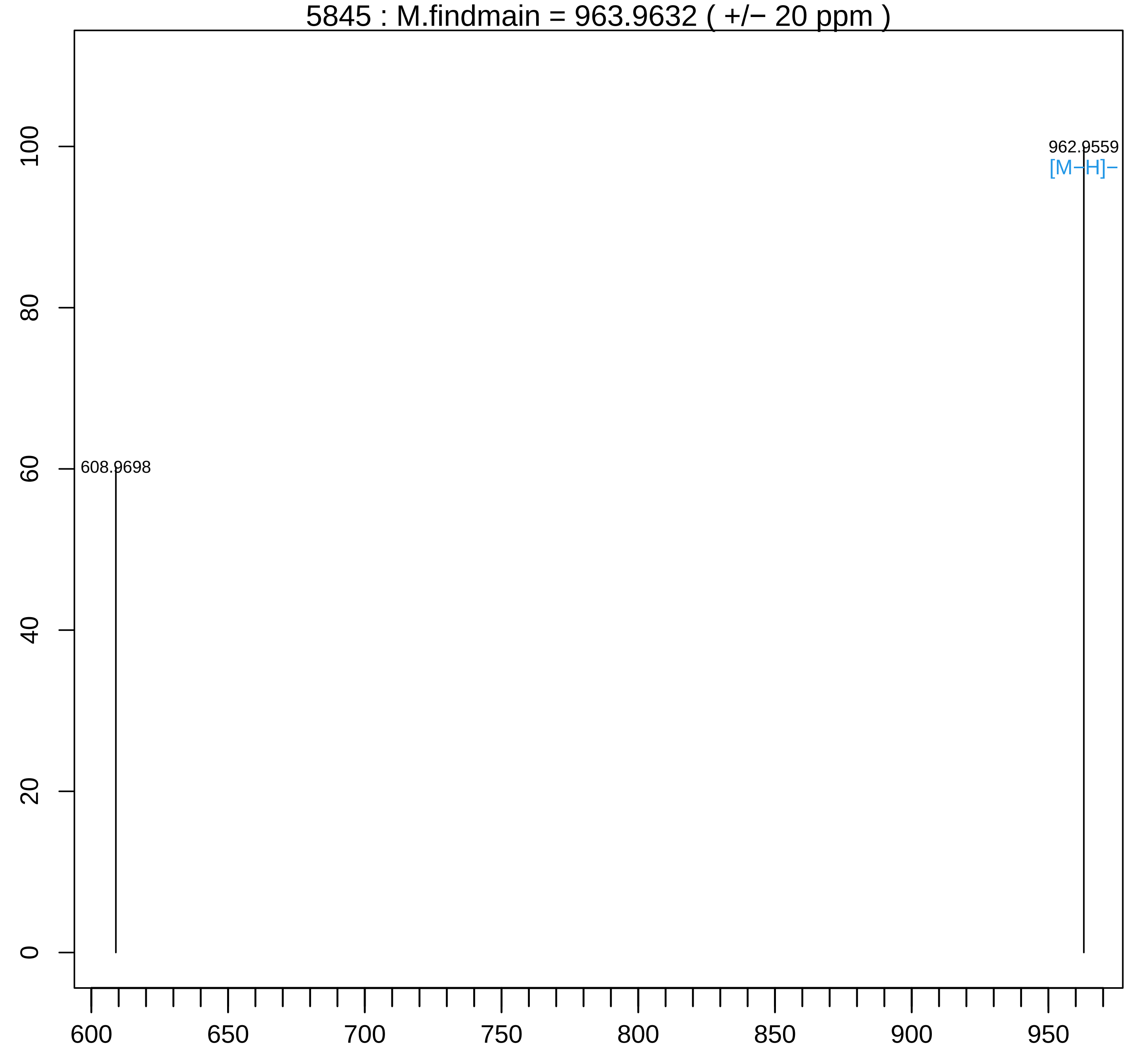
5845 : M.findmain = 963.9632 (+/− 20 ppm)
962.9559
[M−H]−
608.9698
0
20
40
60
80
100
600
650
700
750
800
850
900
950

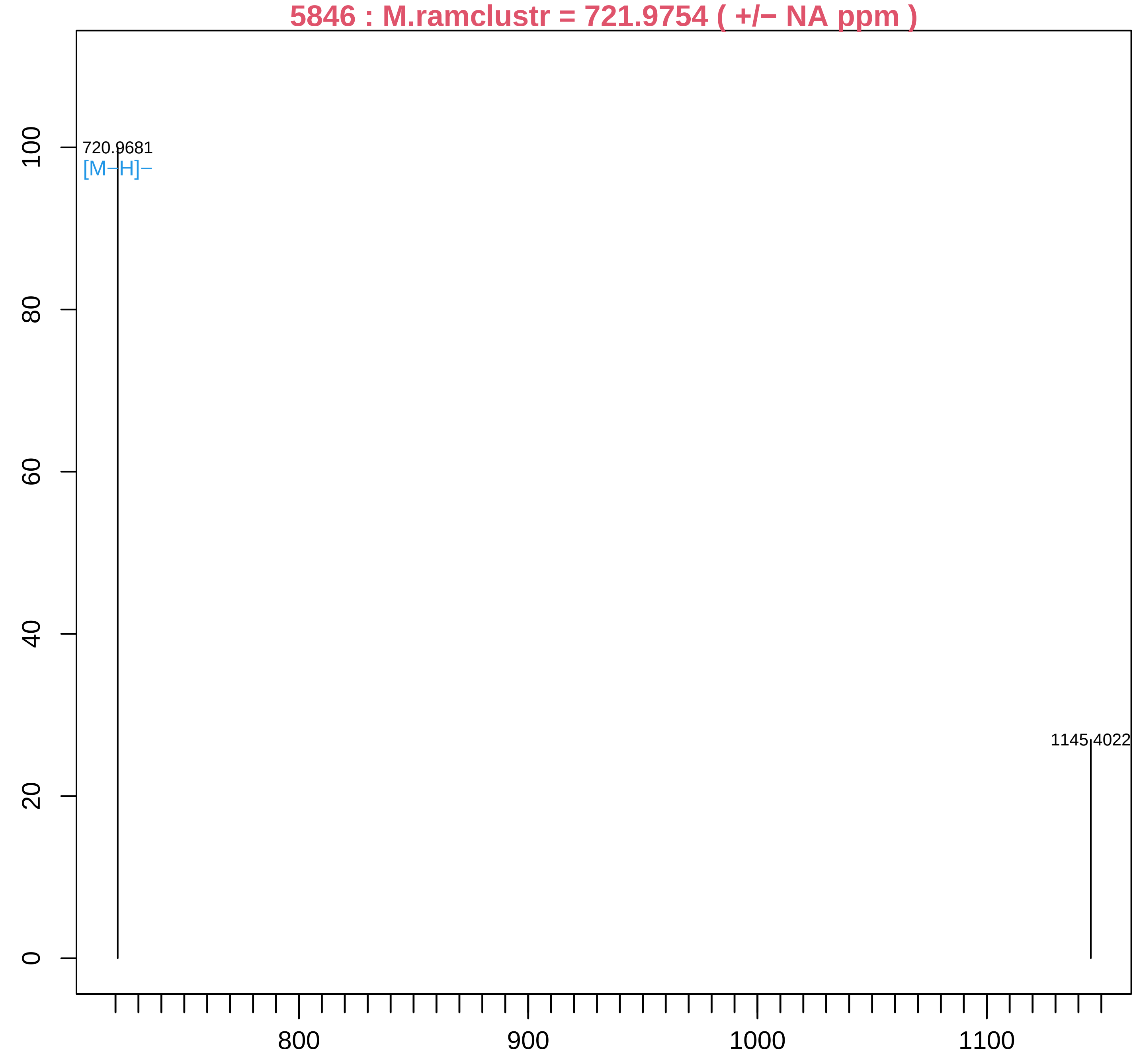
5846 : M.ramclustr = 721.9754 (+/− NA ppm)
720.9681
[M−H]−
1145.4022
0
20
40
60
80
100
800
900
1000
1100

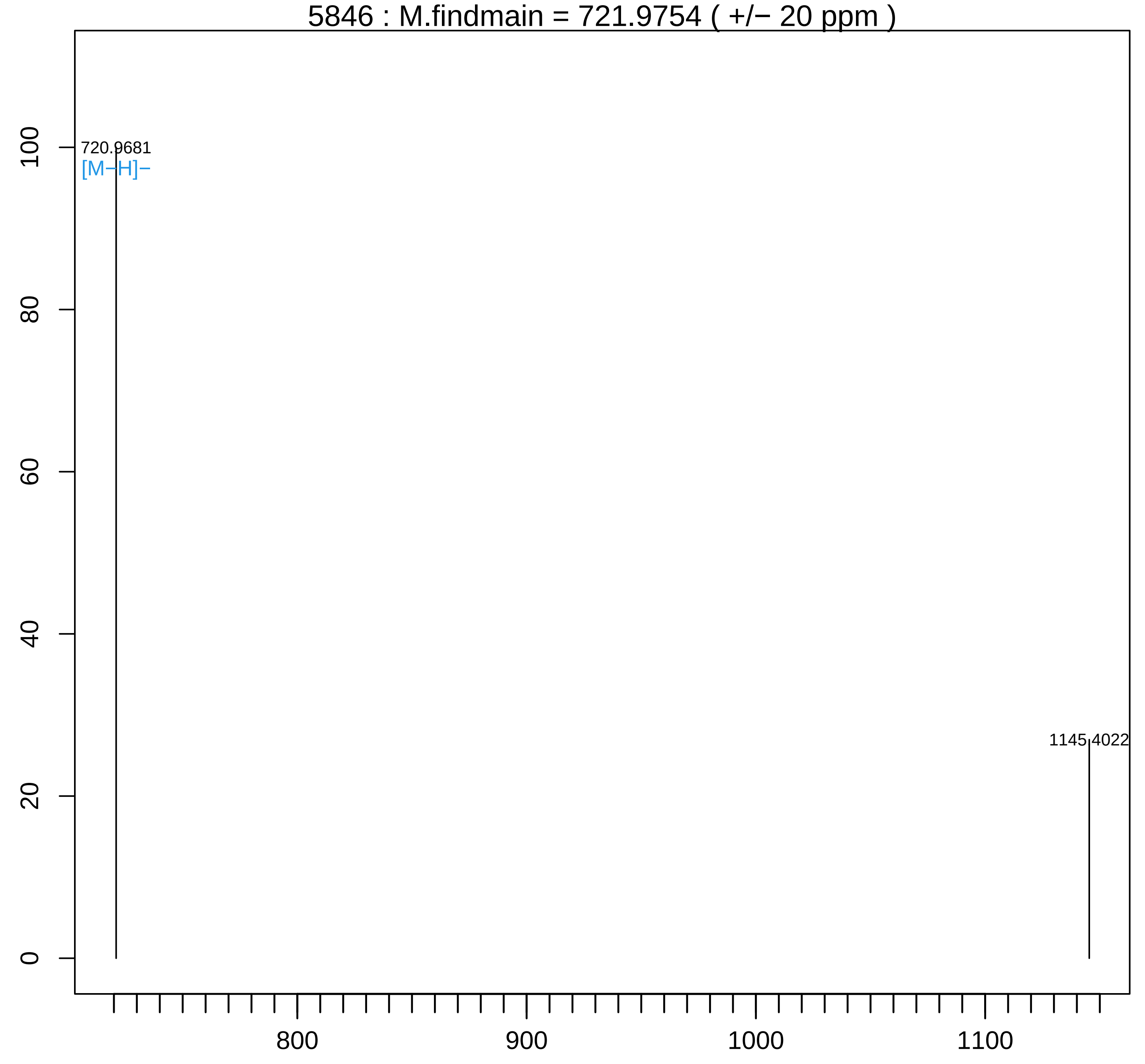
5846 : M.findmain = 721.9754 (+/− 20 ppm)
720.9681
[M−H]−
1145.4022
0
20
40
60
80
100
800
900
1000
1100

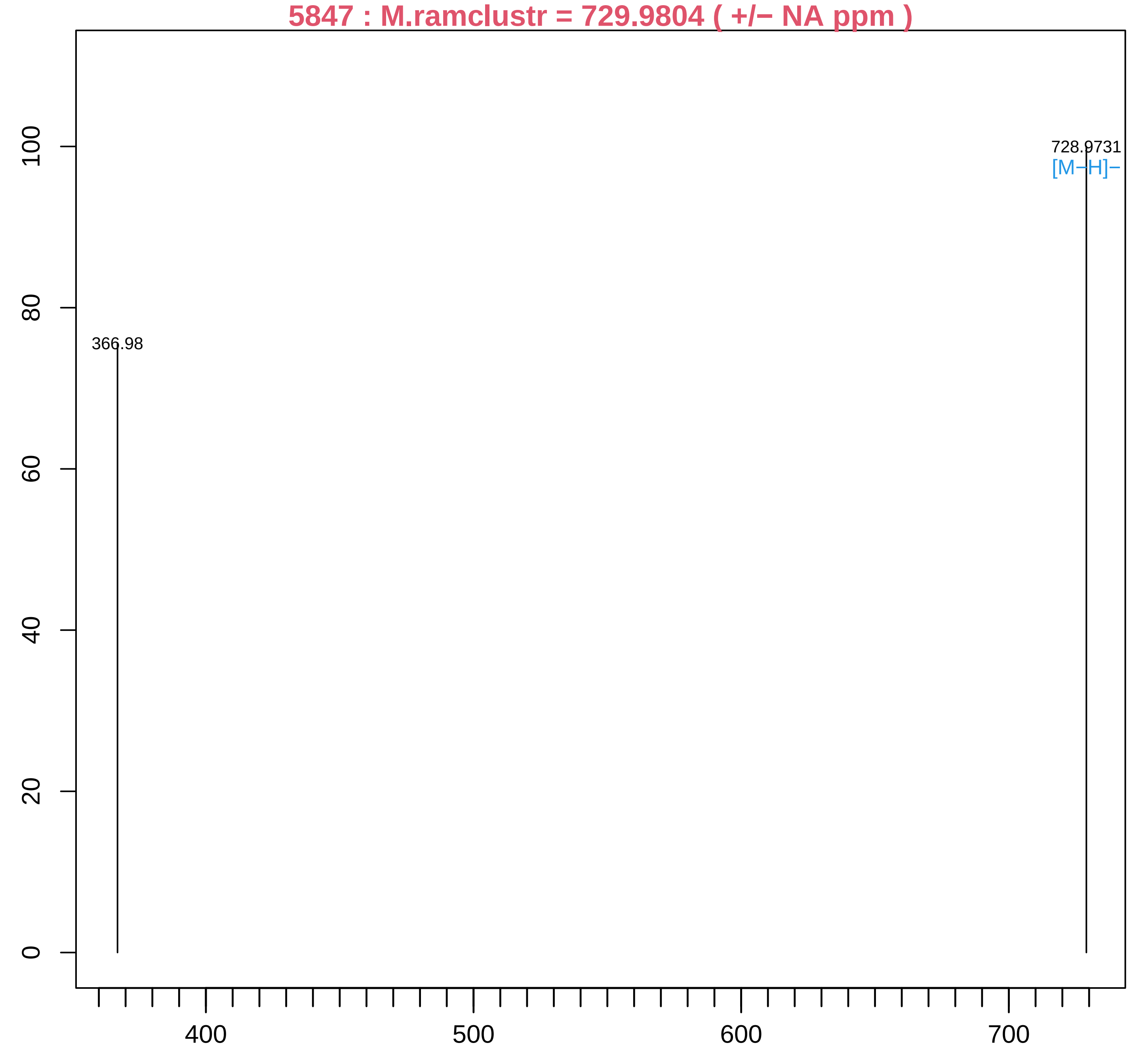
5847 : M.ramclustr = 729.9804 (+/− NA ppm)
728.9731
[M−H]−
366.98
0
20
40
60
80
100
400
500
600
700

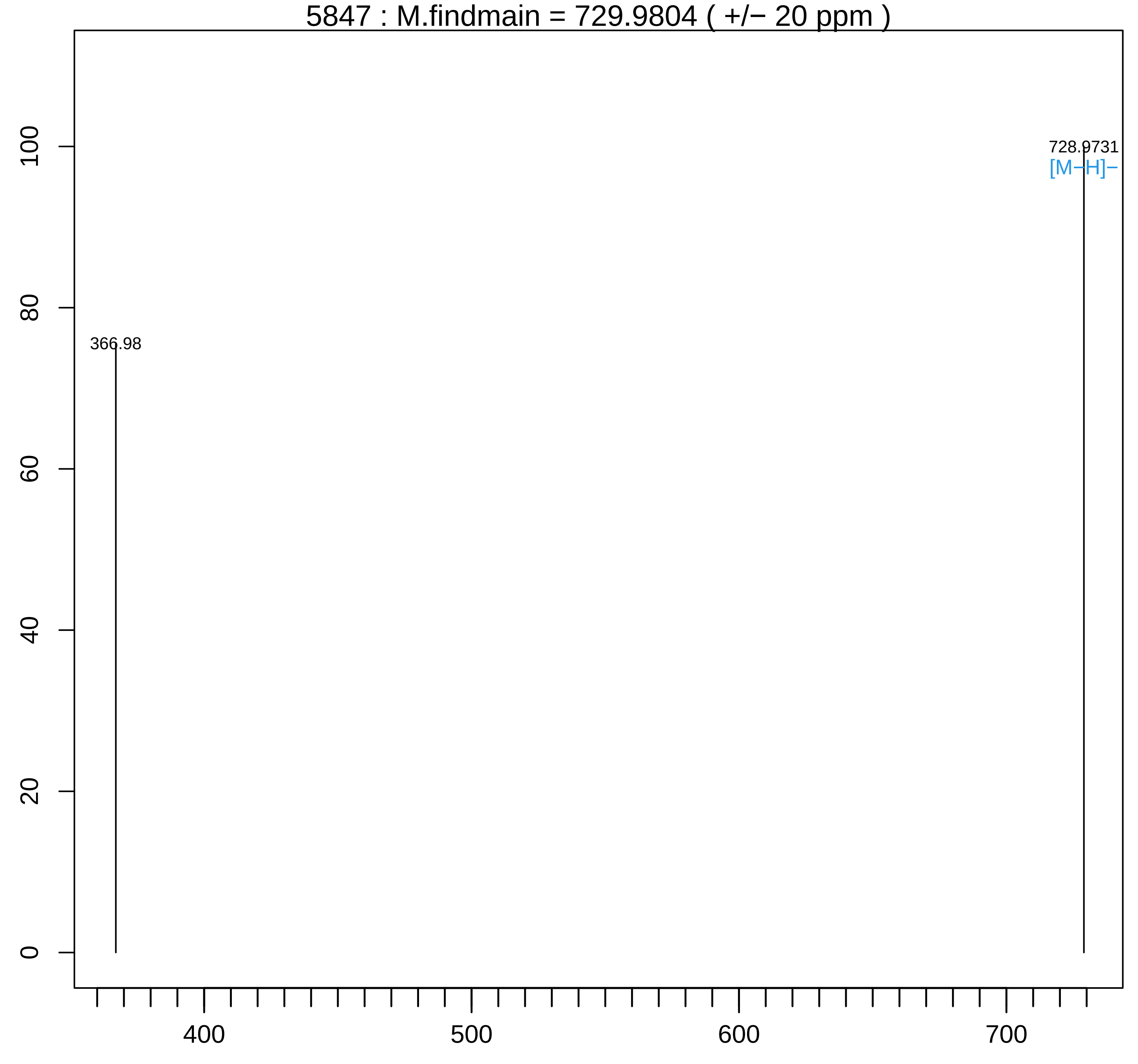
5847 : M.findmain = 729.9804 (+/− 20 ppm)
728.9731
[M−H]−
366.98
0
20
40
60
80
100
400
500
600
700

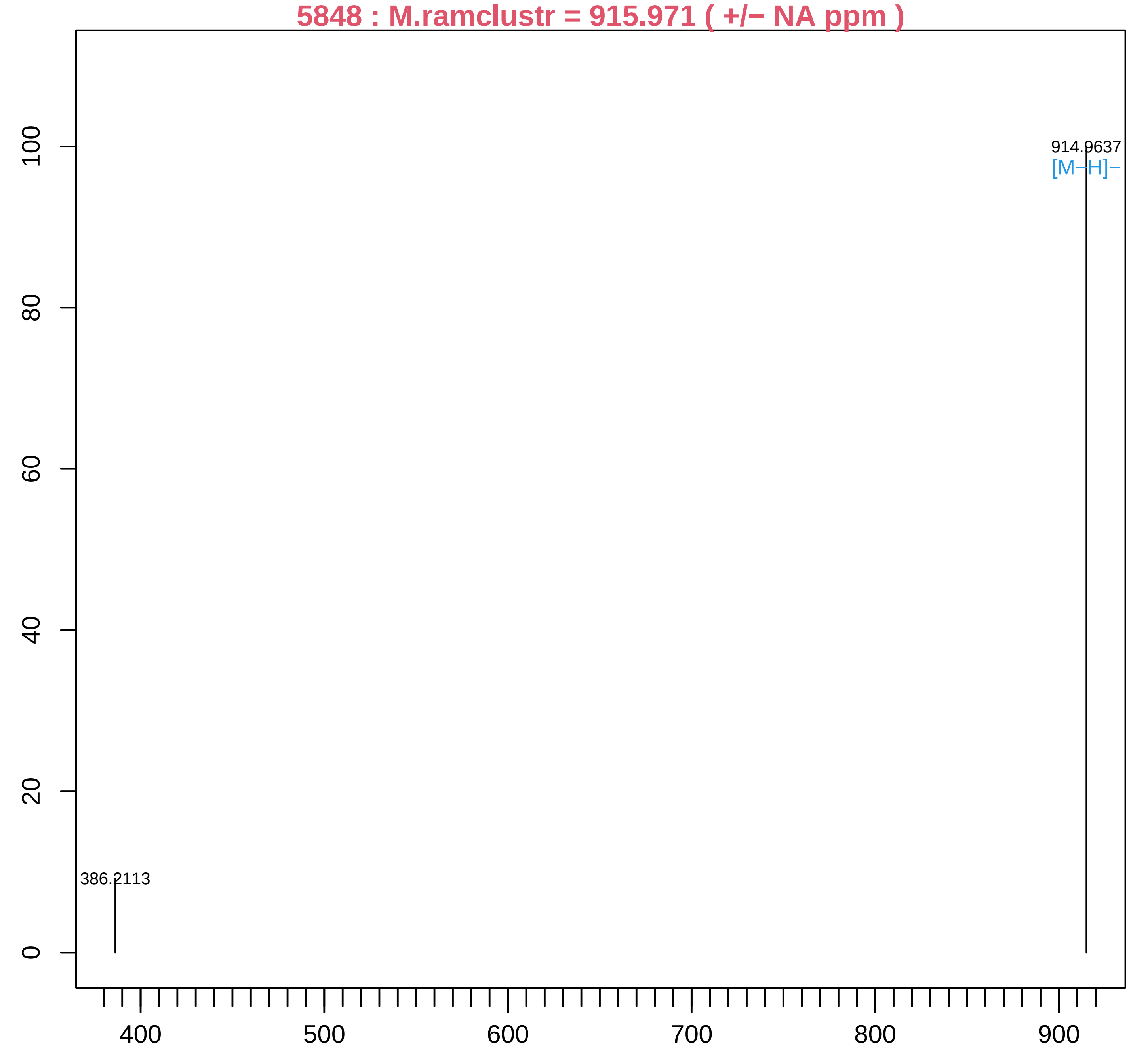
5848 : M.ramclustr = 915.971 (+/− NA ppm)
914.9637
[M−H]−
386.2113
0
20
40
60
80
100
400
500
600
700
800
900
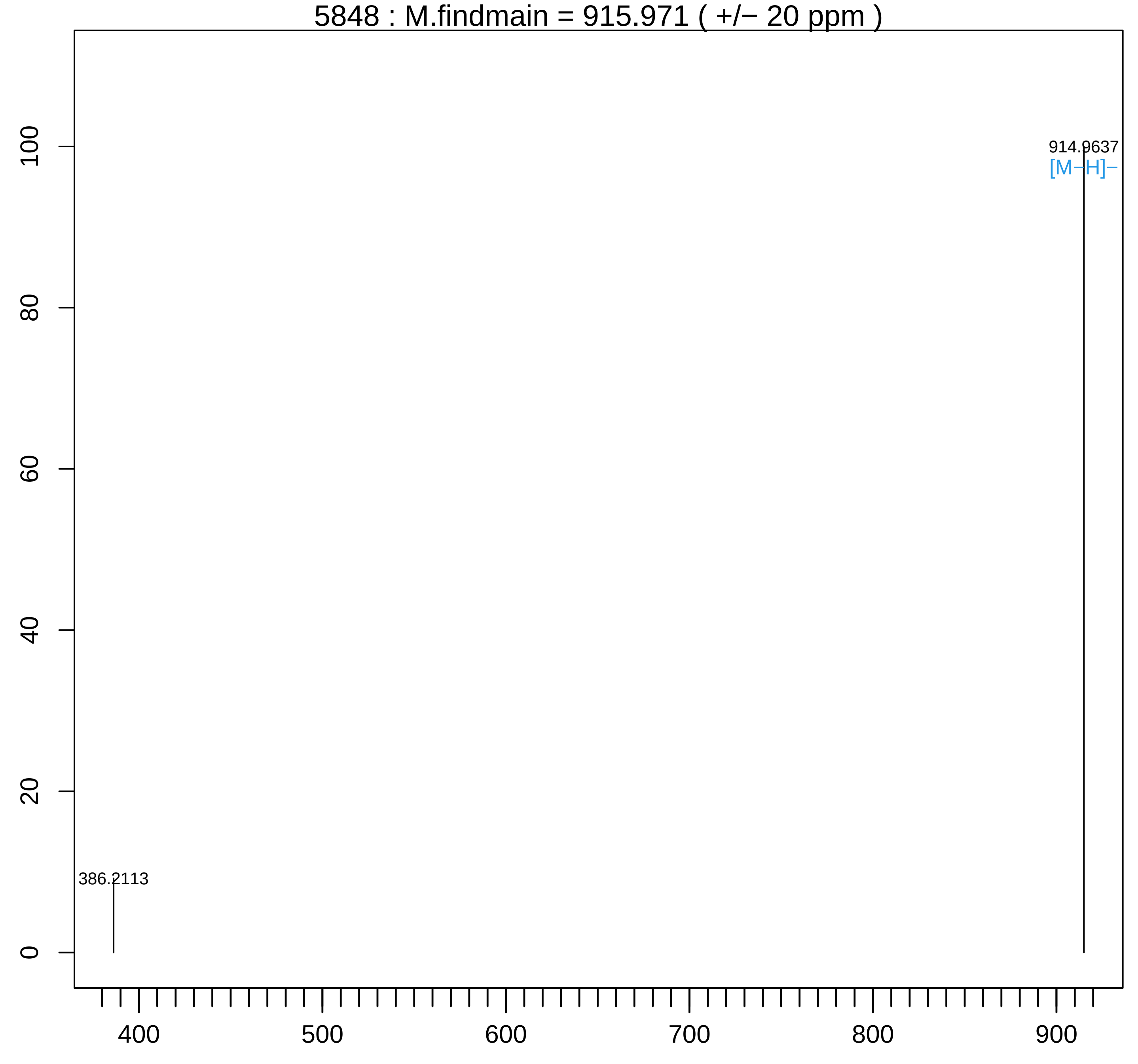
5848 : M.findmain = 915.971 (+/− 20 ppm)
914.9637
[M−H]−
386.2113
0
20
40
60
80
100
400
500
600
700
800
900

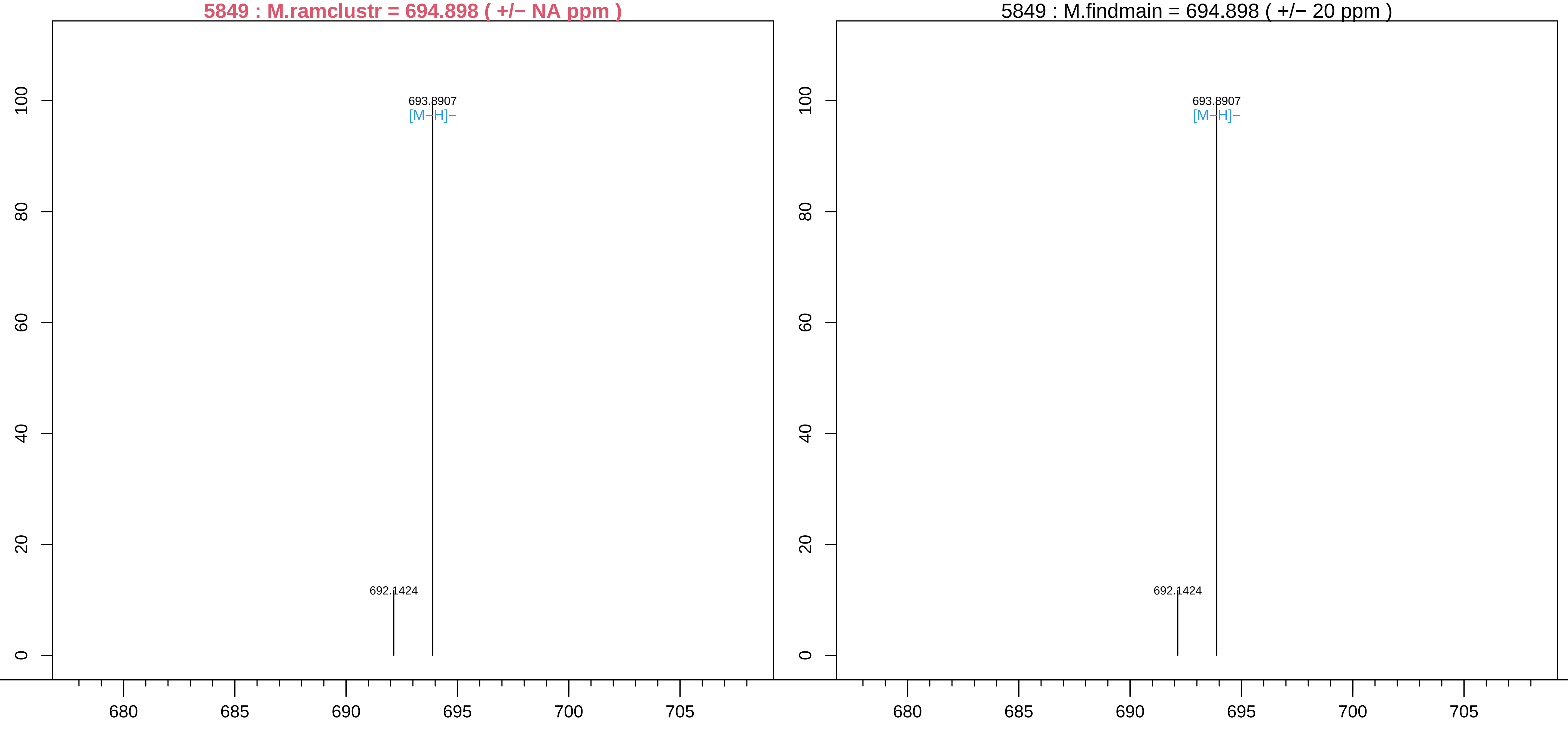
5849 : M.ramclustr = 694.898 (+/− NA ppm)
693.8907
[M−H]−
692.1424
5849 : M.findmain = 694.898 (+/− 20 ppm)
693.8907
[M−H]−
692.1424
0
20
40
60
80
100
680
685
690
695
700
705

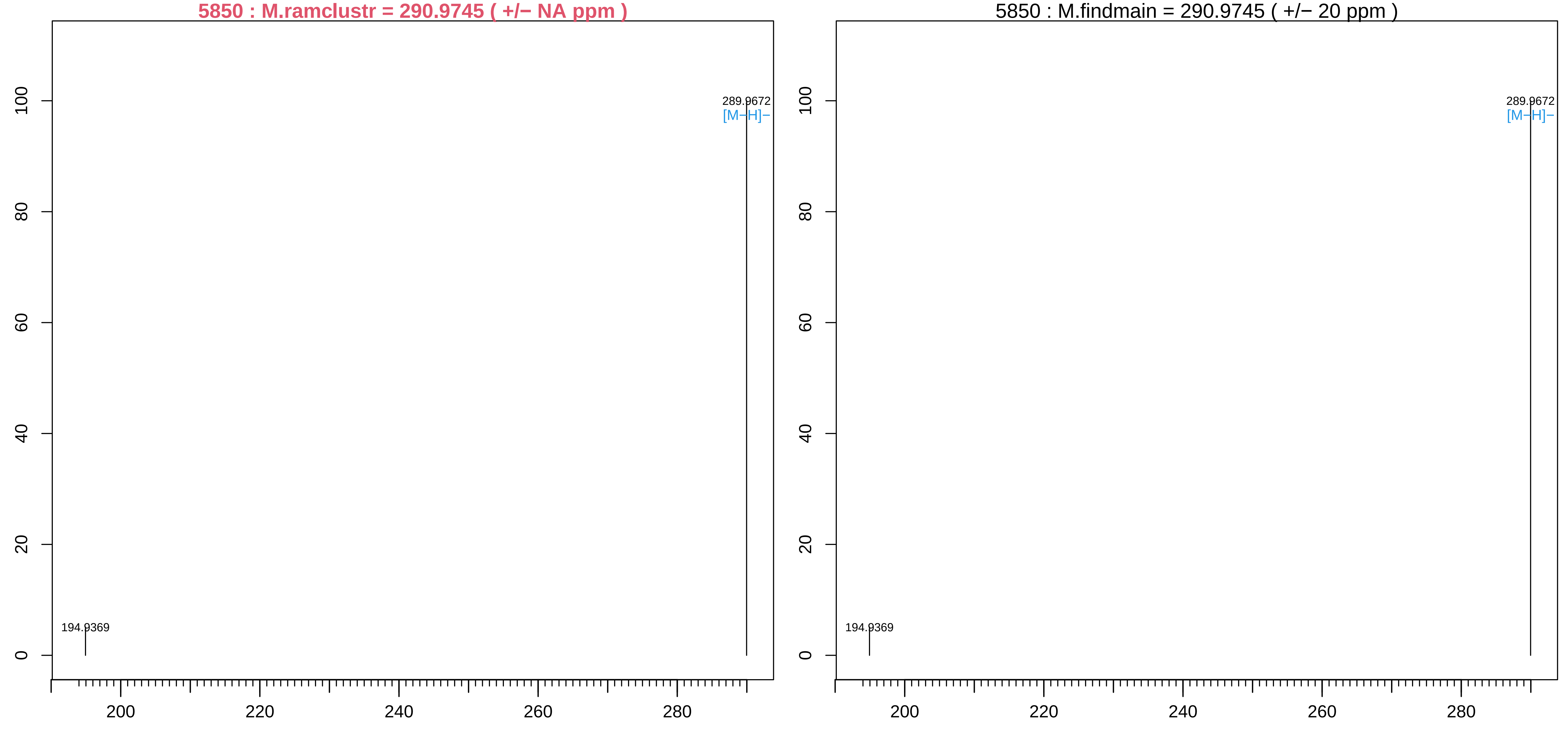

5850 : M.ramclustr = 290.9745 (+/− NA ppm)
289.9672
[M−H]−
194.9369
0
20
40
60
80
100
200
220
240
260
280
5850 : M.findmain = 290.9745 (+/− 20 ppm)
289.9672
[M−H]−
194.9369

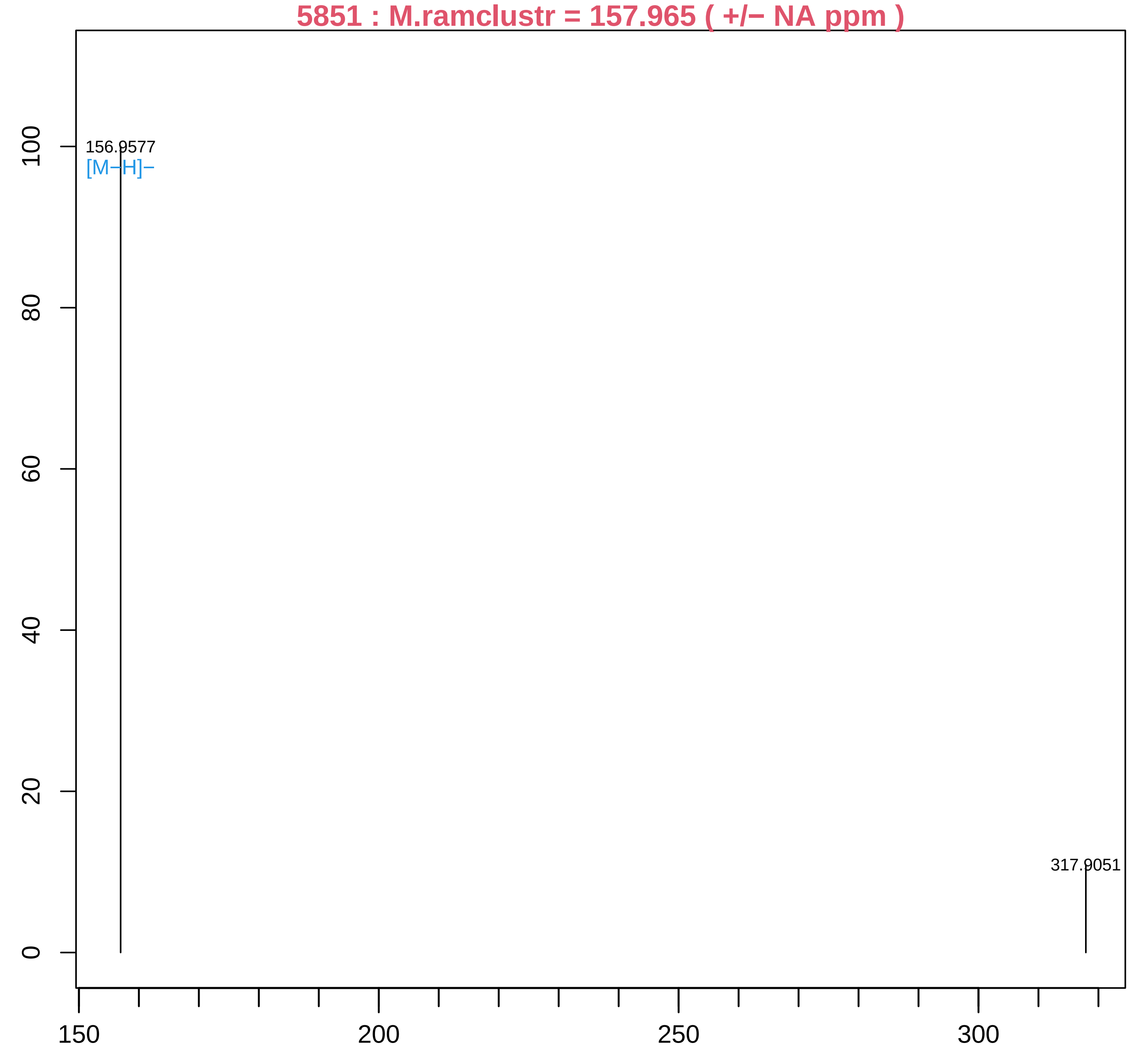

5851 : M.ramclustr = 157.965 (+/− NA ppm)
156.9577
[M−H]−
317.9051
0
20
40
60
80
100
150
200
250
300

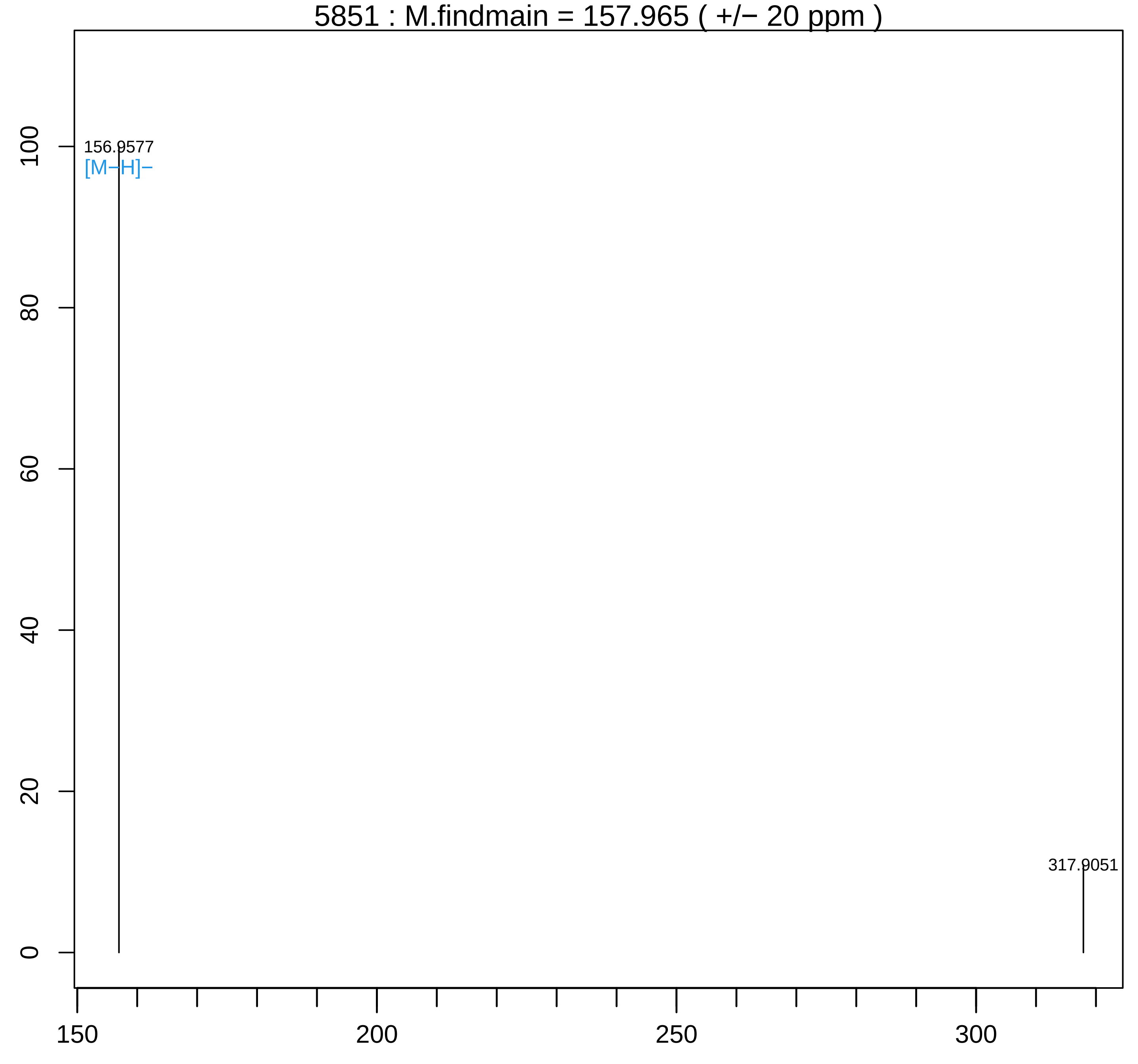

5851 : M.findmain = 157.965 (+/− 20 ppm)
156.9577
[M−H]−
317.9051
0
20
40
60
80
100
150
200
250
300

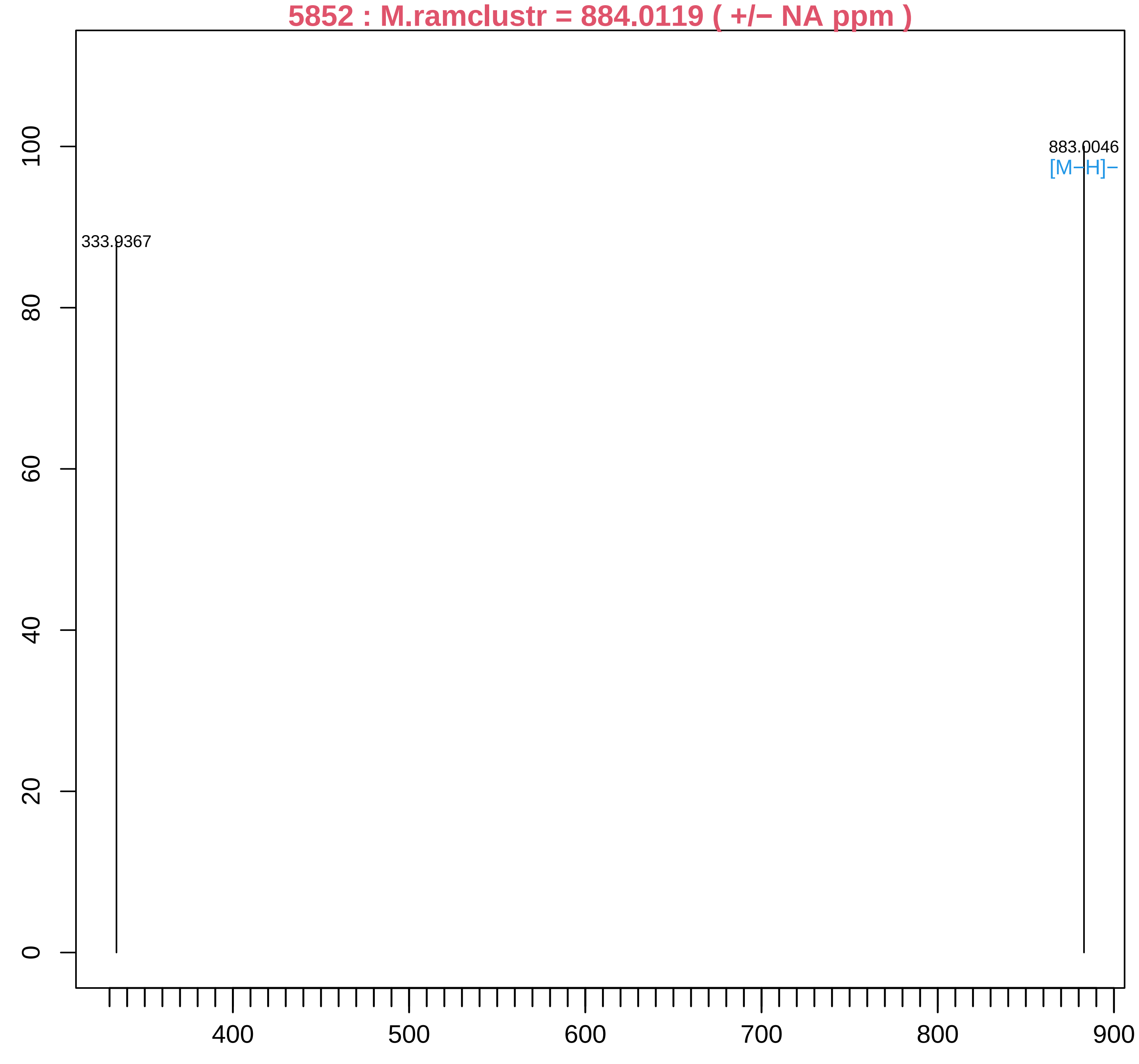
5852 : M.ramclustr = 884.0119 (+/− NA ppm)
883.0046
[M−H]−
333.9367
0
20
40
60
80
100
400
500
600
700
800
900

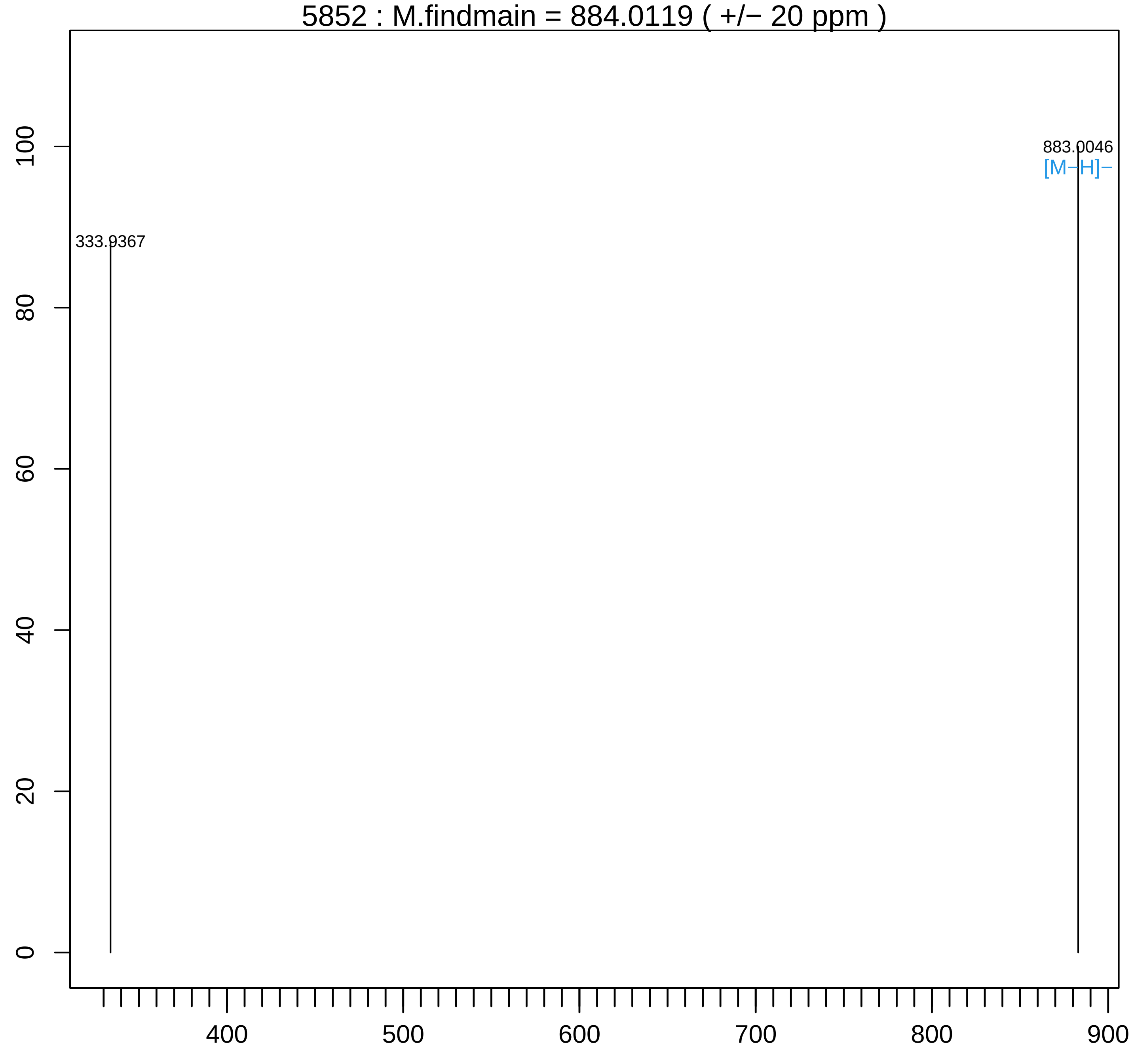
5852 : M.findmain = 884.0119 (+/− 20 ppm)
883.0046
[M−H]−
333.9367
0
20
40
60
80
100
400
500
600
700
800
900

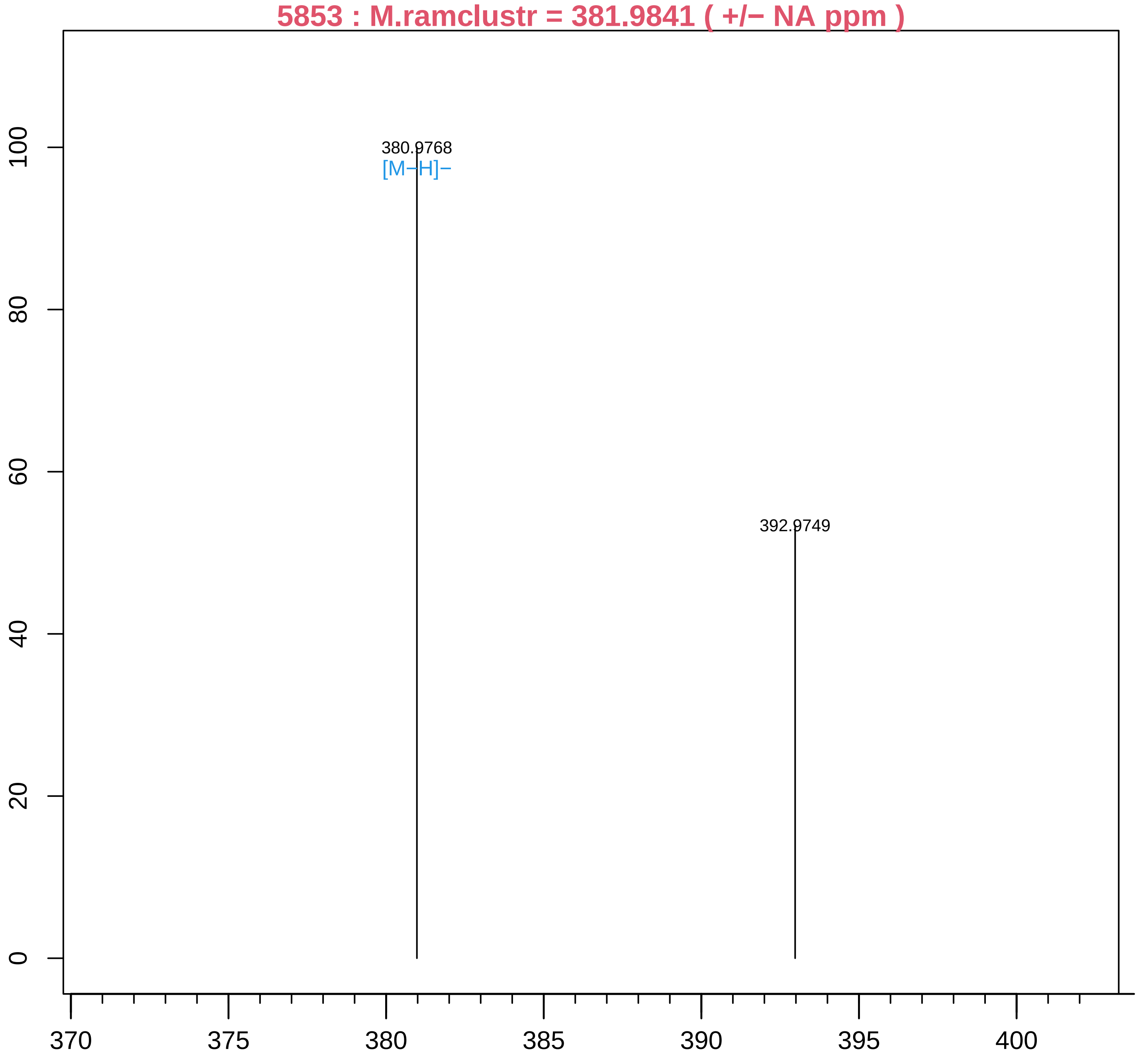
5853 : M.ramclustr = 381.9841 (+/− NA ppm)
380.9768
[M−H]−
392.9749
0
20
40
60
80
100
370
375
380
385
390
395
400

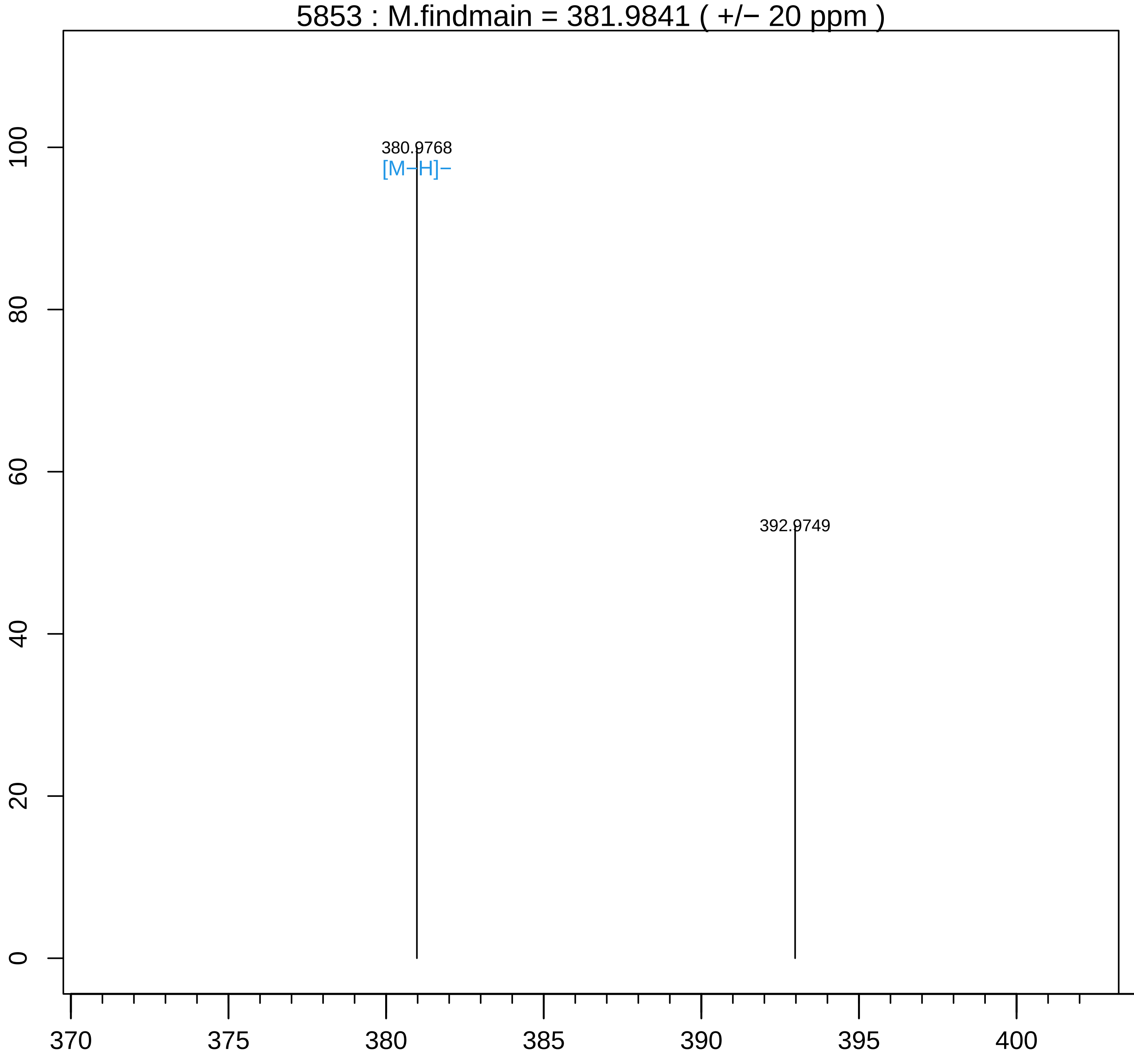
5853 : M.findmain = 381.9841 (+/− 20 ppm)
380.9768
[M−H]−
392.9749
0
20
40
60
80
100
370
375
380
385
390
395
400

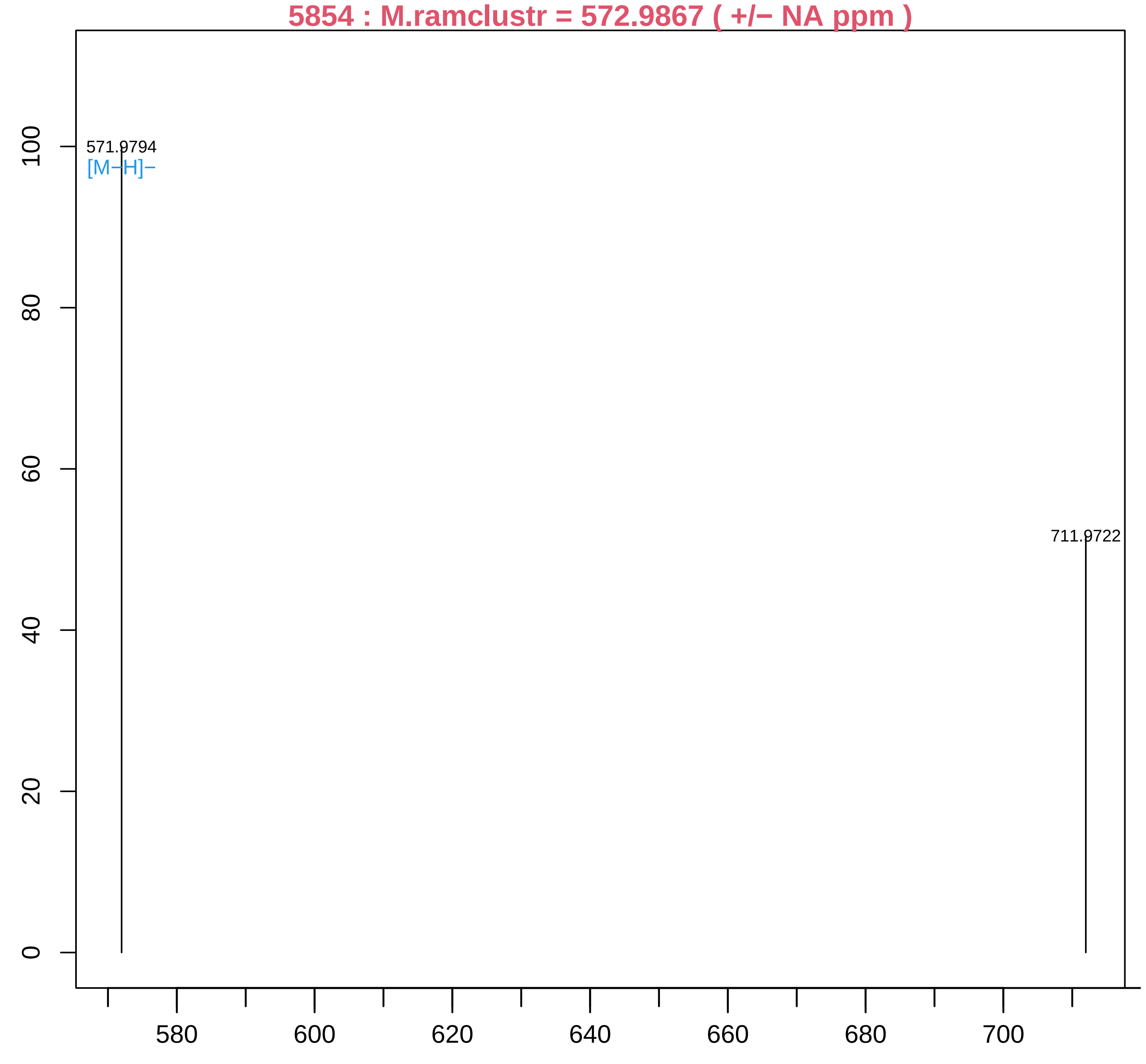

5854 : M.ramclustr = 572.9867 (+/− NA ppm)
571.9794
[M−H]−
711.9722
0
20
40
60
80
100
580
600
620
640
660
680
700

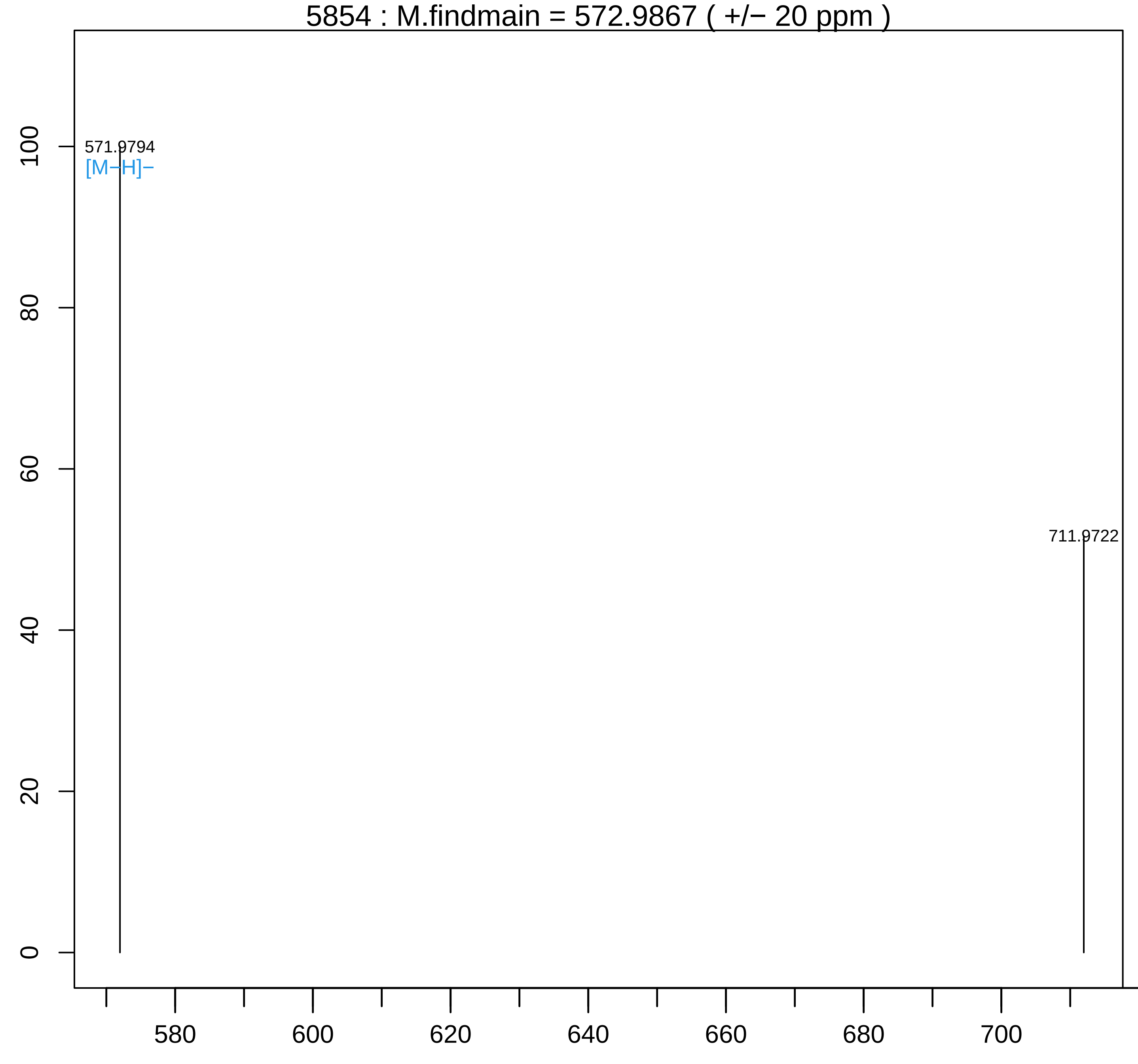

5854 : M.findmain = 572.9867 (+/− 20 ppm)
571.9794
[M−H]−
711.9722
0
20
40
60
80
100
580
600
620
640
660
680
700

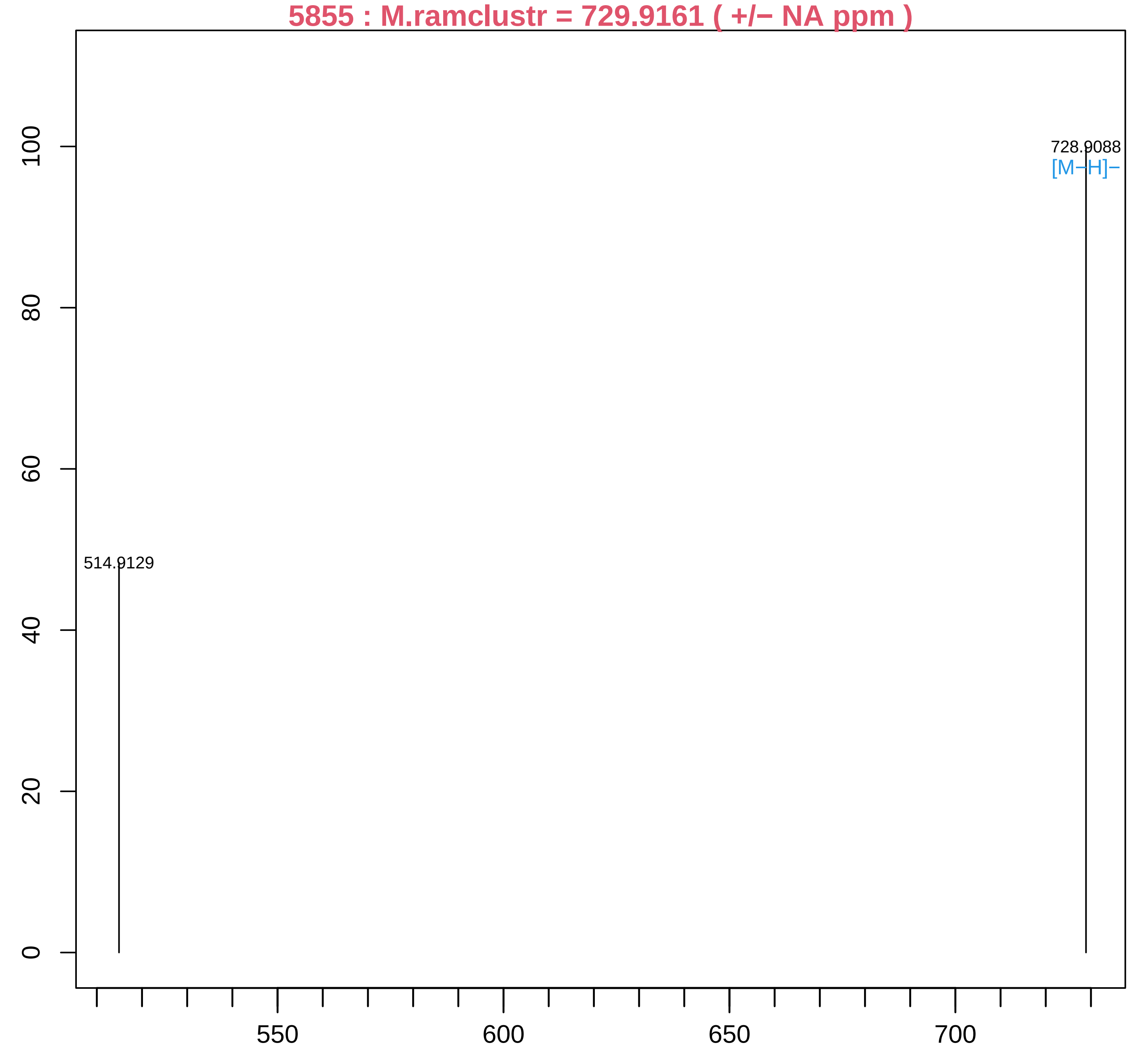
5855 : M.ramclustr = 729.9161 (+/− NA ppm)
728.9088
[M−H]−
514.9129
0
20
40
60
80
100
550
600
650
700

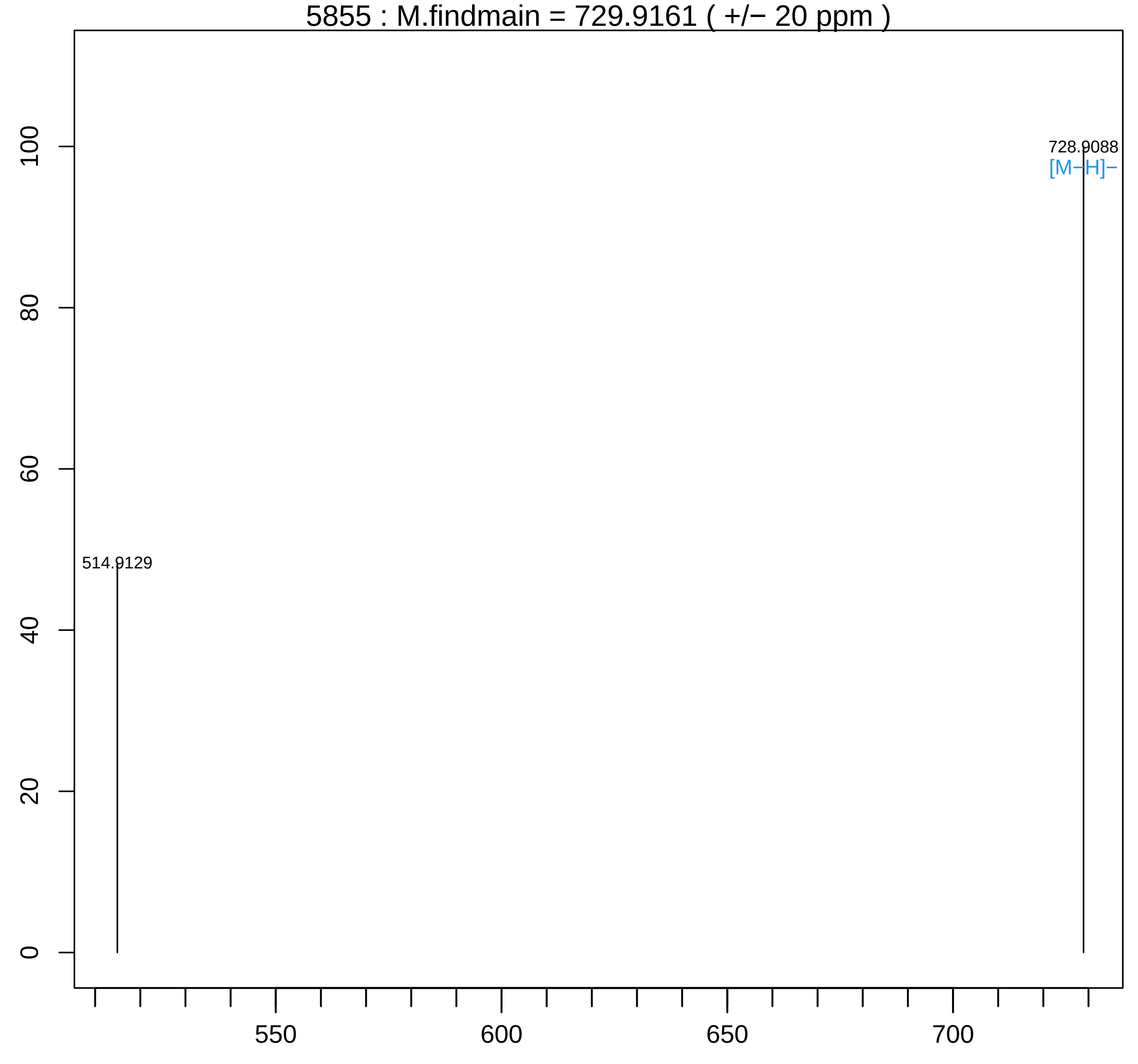
5855 : M.findmain = 729.9161 (+/− 20 ppm)
728.9088
[M−H]−
514.9129
0
20
40
60
80
100
550
600
650
700

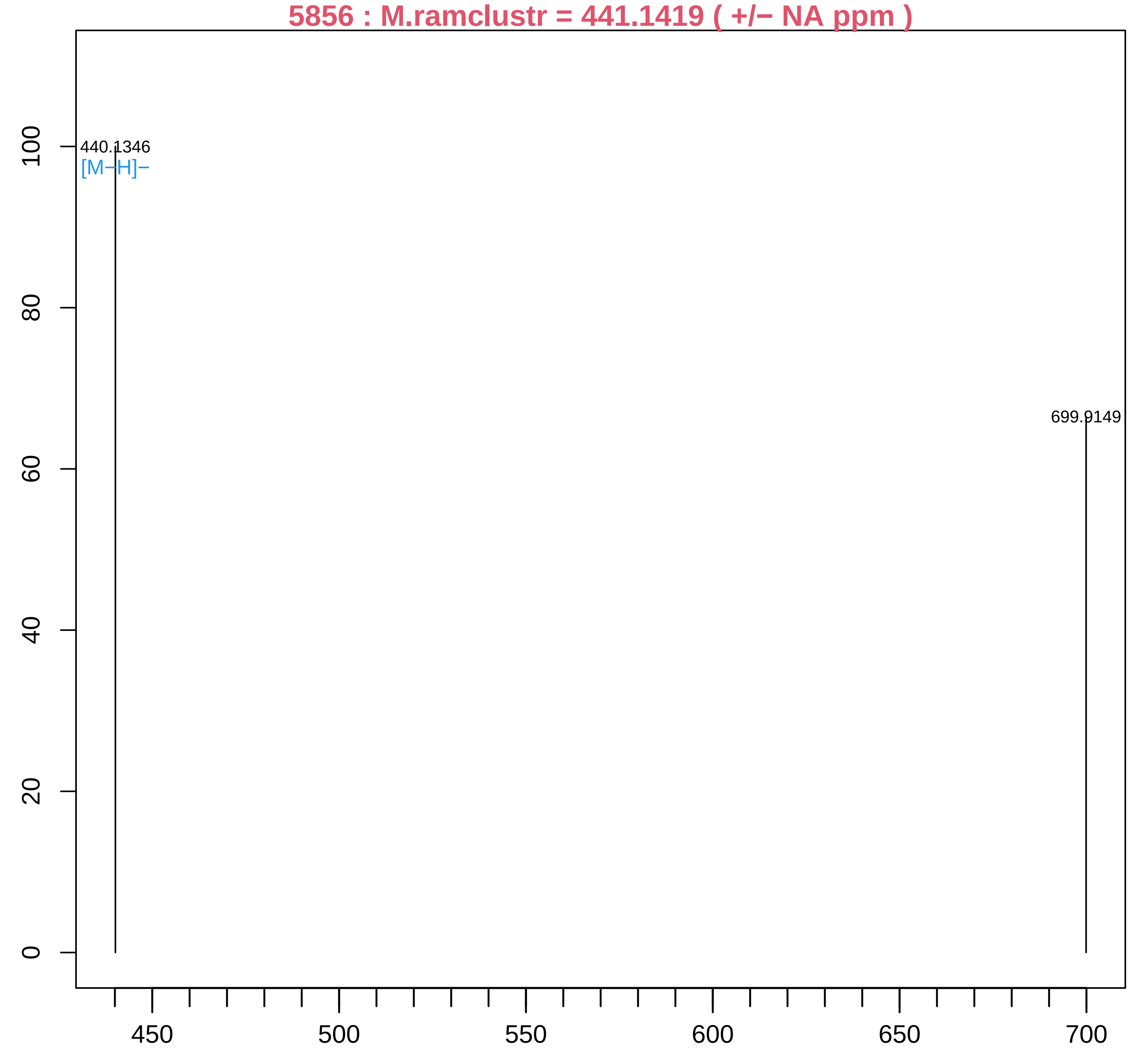
5856 : M.ramclustr = 441.1419 (+/− NA ppm)
440.1346
[M−H]−
699.9149
0
20
40
60
80
100
450
500
550
600
650
700

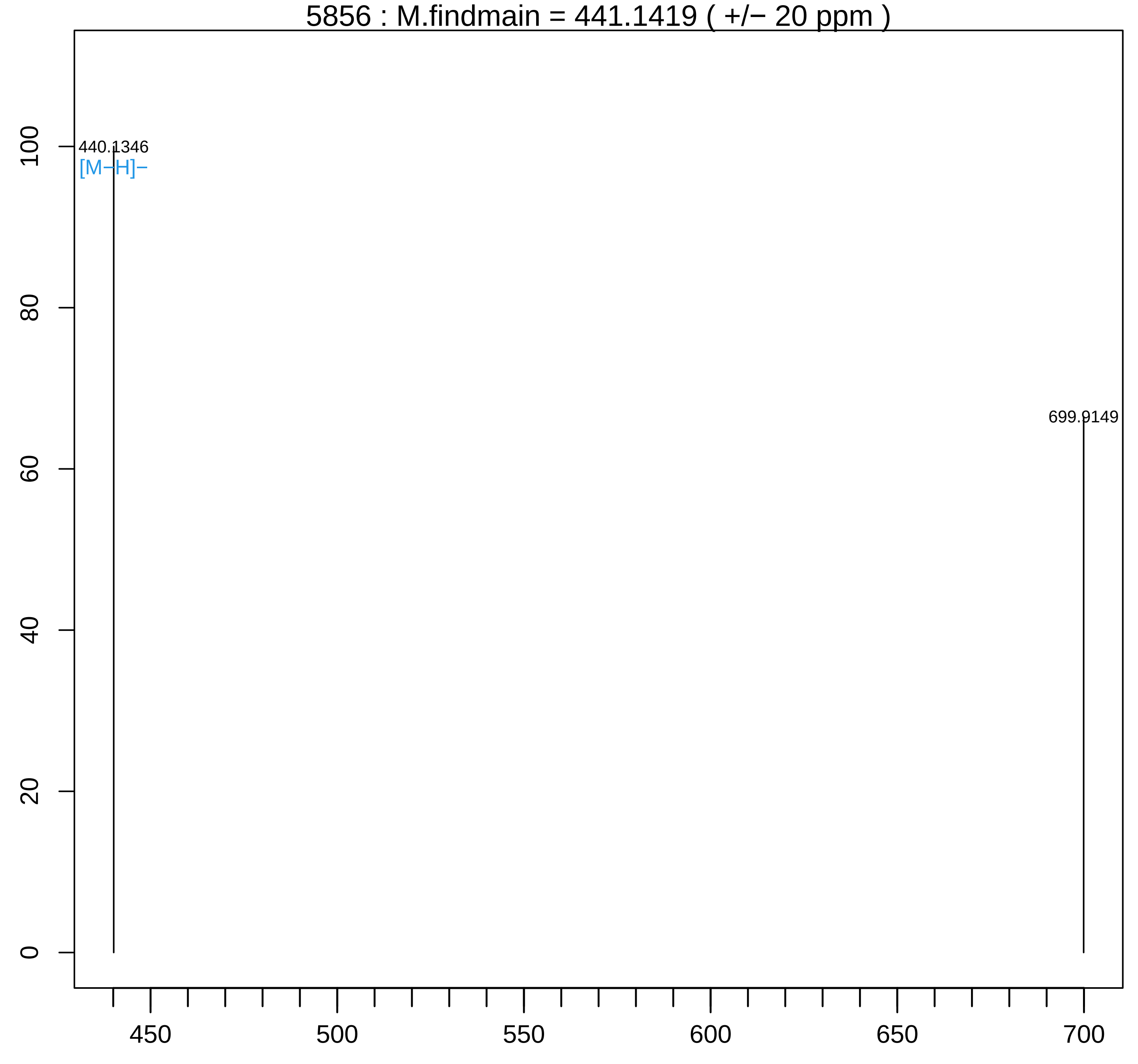
5856 : M.findmain = 441.1419 (+/− 20 ppm)
440.1346
[M−H]−
699.9149
0
20
40
60
80
100
450
500
550
600
650
700

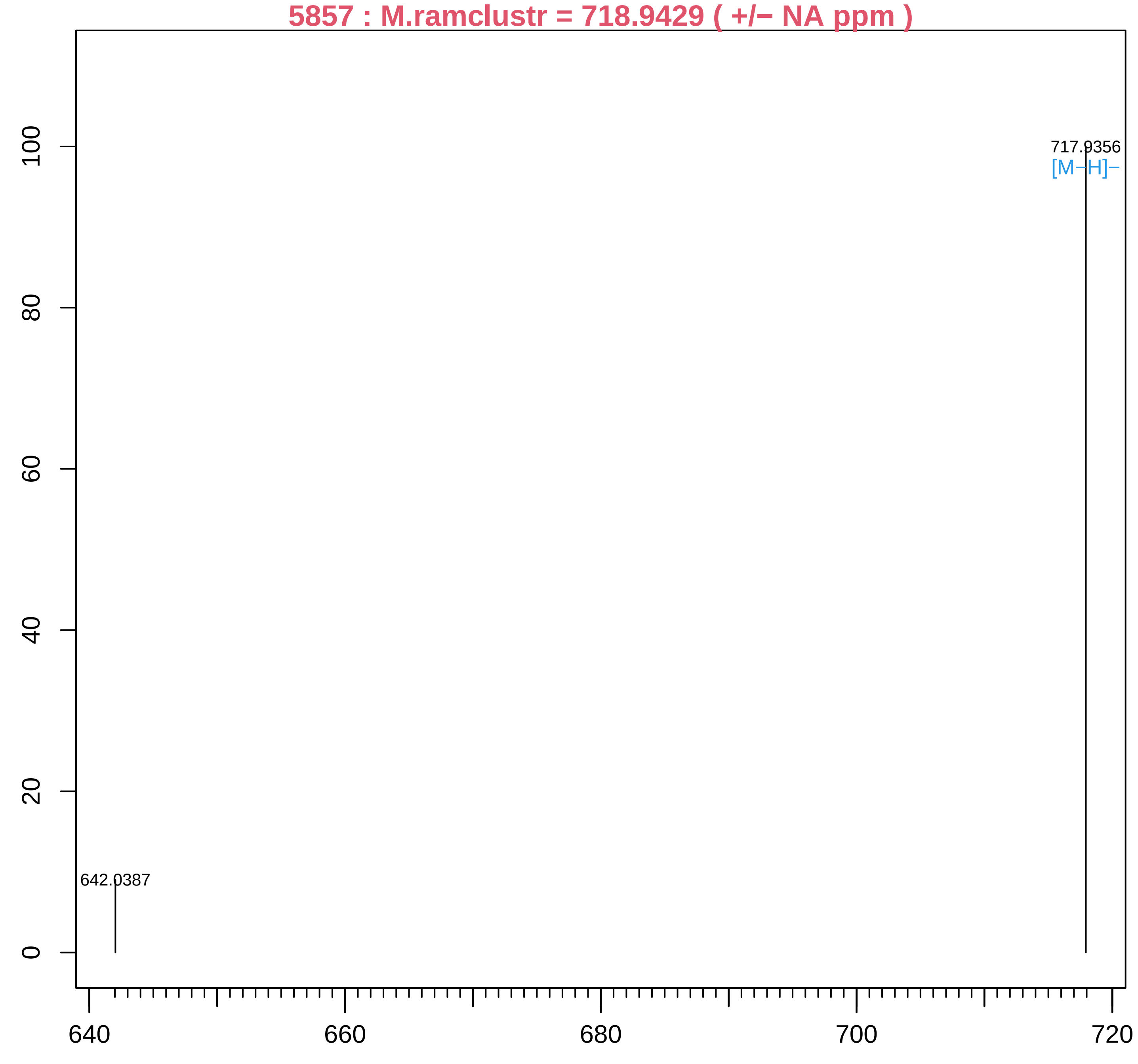

5857 : M.ramclustr = 718.9429 (+/− NA ppm)
717.9356
[M−H]−
642.0387
0
20
40
60
80
100
640
660
680
700
720

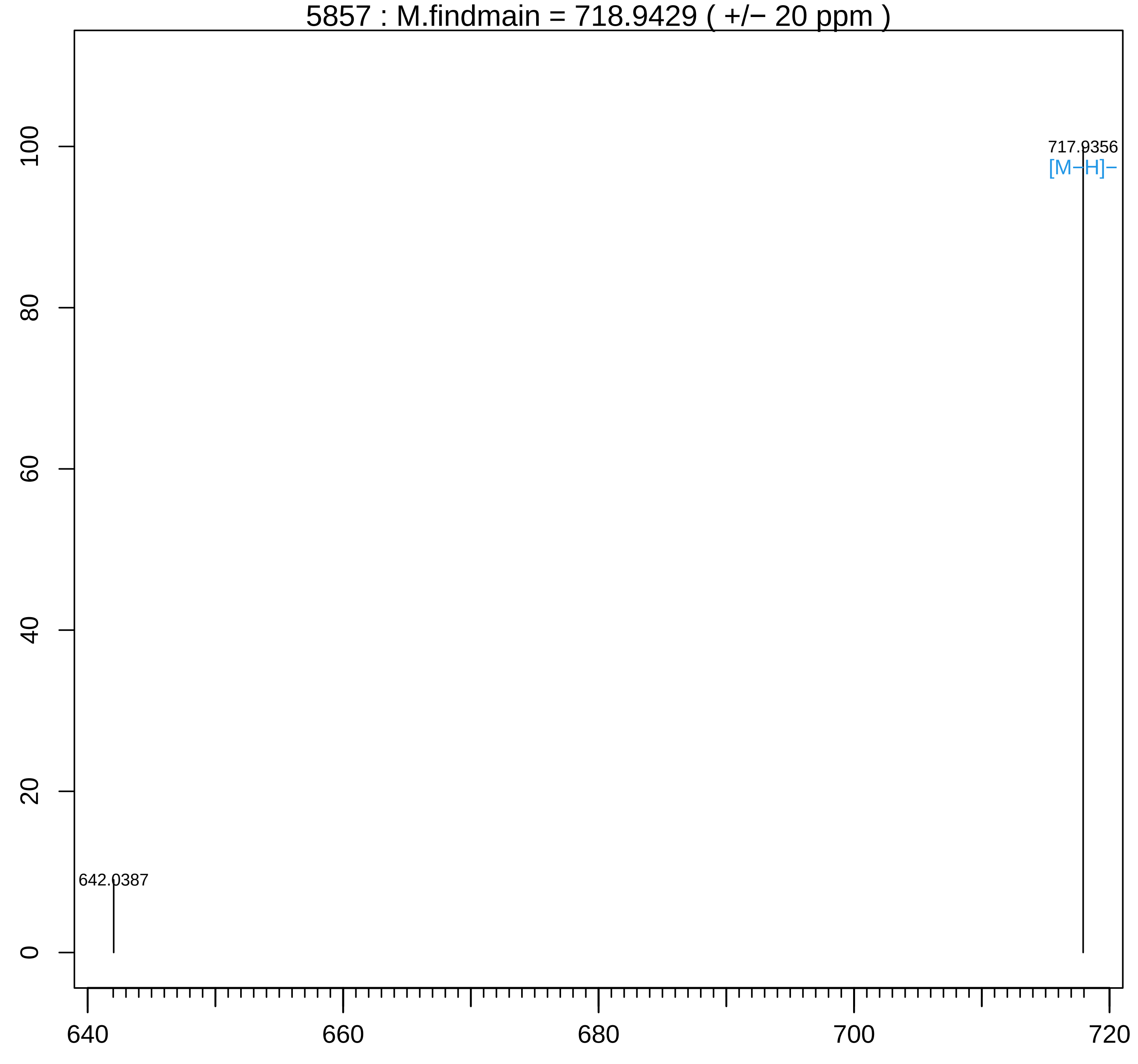

5857 : M.findmain = 718.9429 (+/− 20 ppm)
717.9356
[M−H]−
642.0387
0
20
40
60
80
100
640
660
680
700
720

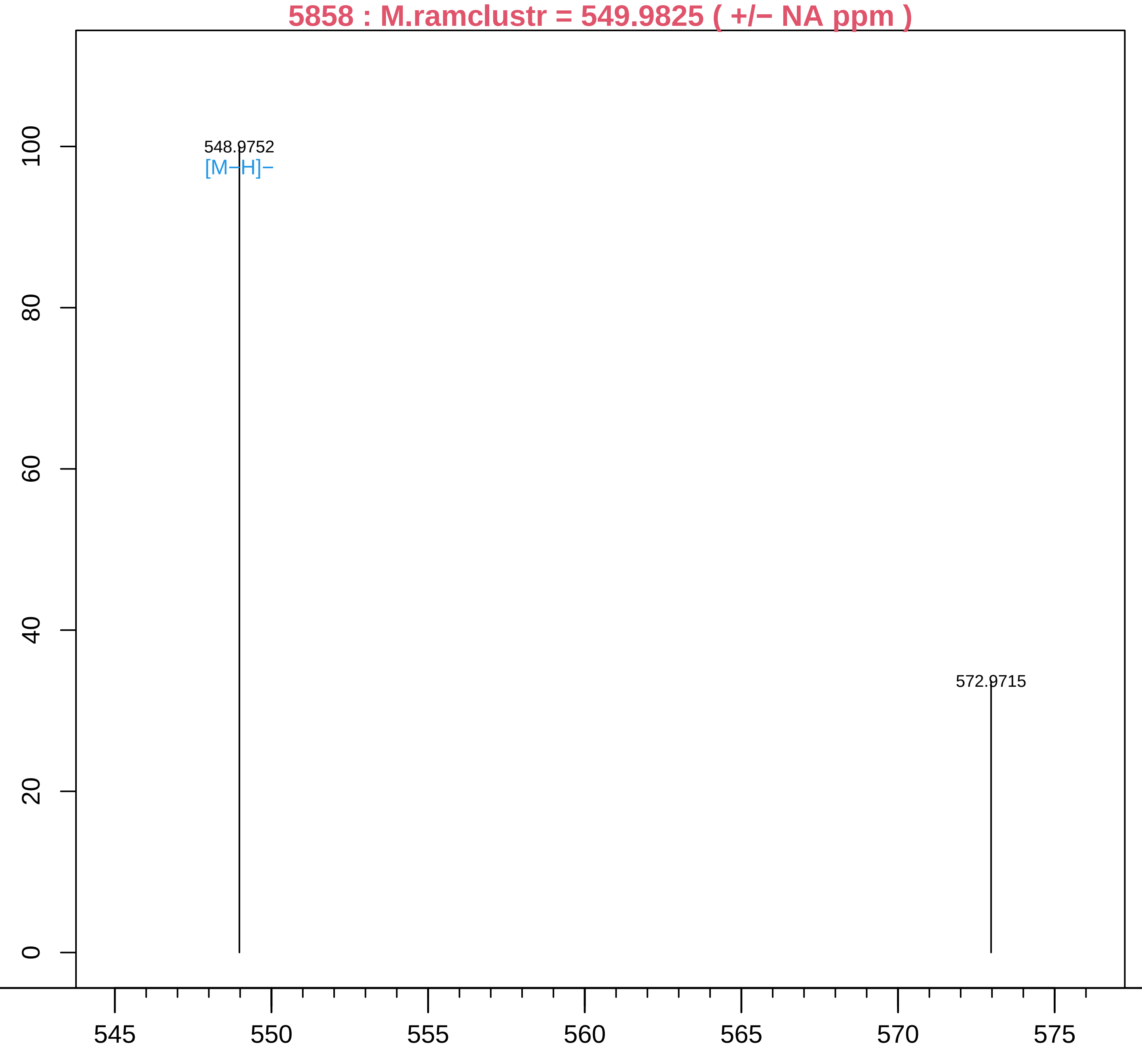
5858 : M.ramclustr = 549.9825 (+/− NA ppm)
548.9752
[M−H]−
572.9715
0
20
40
60
80
100
545
550
555
560
565
570
575
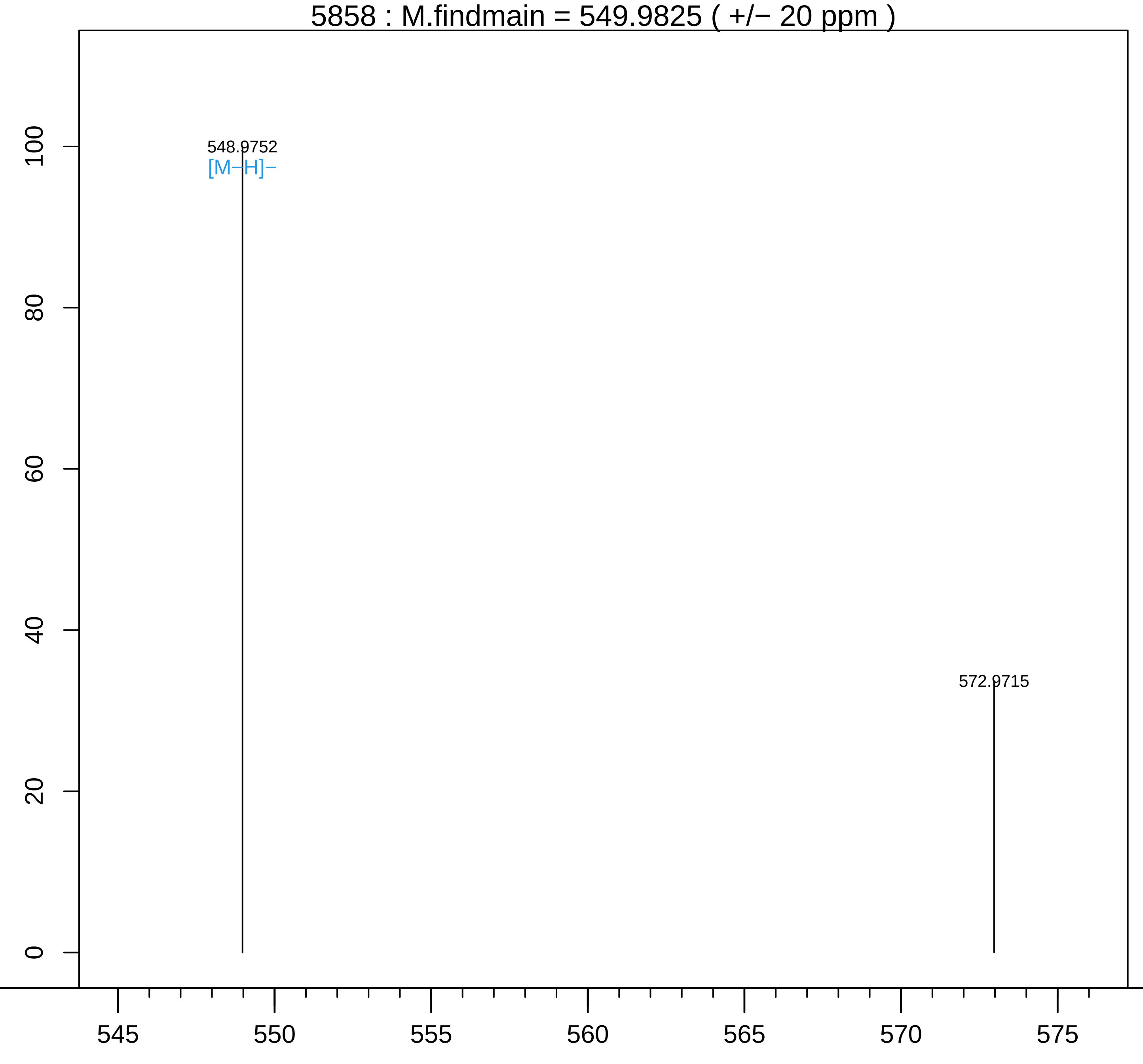
5858 : M.findmain = 549.9825 (+/− 20 ppm)
548.9752
[M−H]−
572.9715
0
20
40
60
80
100
545
550
555
560
565
570
575

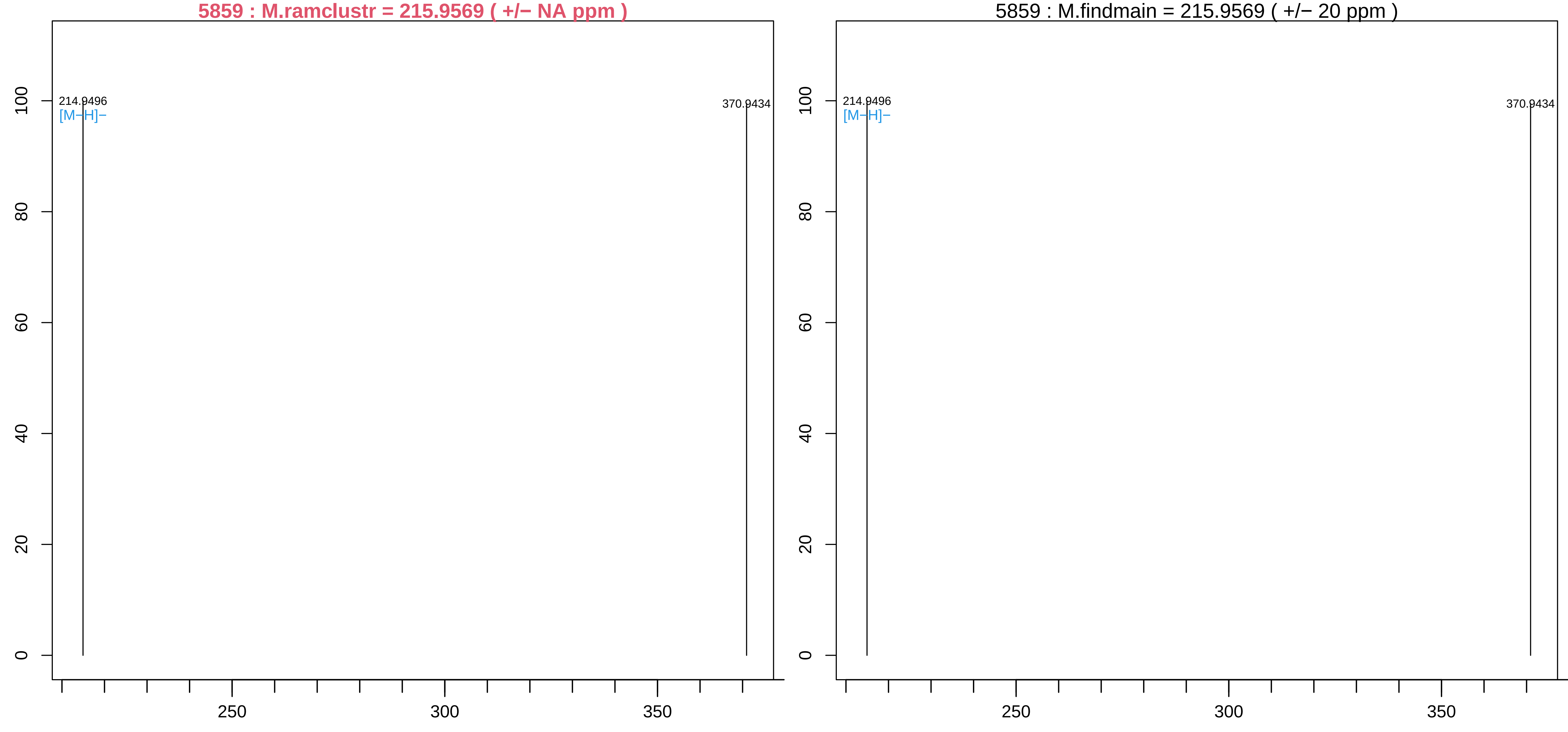
5859 : M.ramclustr = 215.9569 (+/− NA ppm)
214.9496
[M−H]−
370.9434
5859 : M.findmain = 215.9569 (+/− 20 ppm)
214.9496
[M−H]−
370.9434
0
20
40
60
80
100
250
300
350

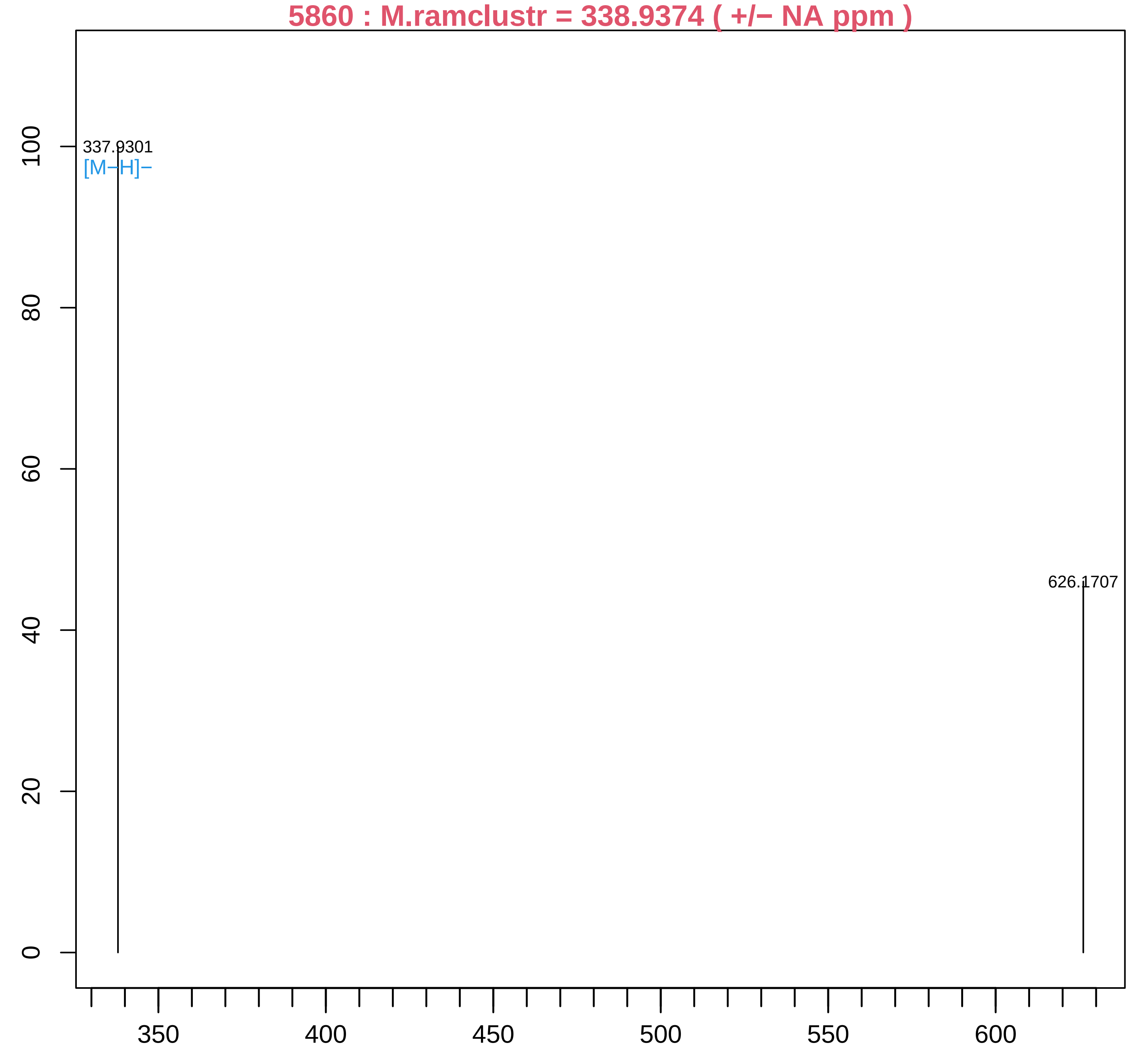
5860 : M.ramclustr = 338.9374 (+/− NA ppm)
337.9301
[M−H]−
626.1707
0
20
40
60
80
100
350
400
450
500
550
600

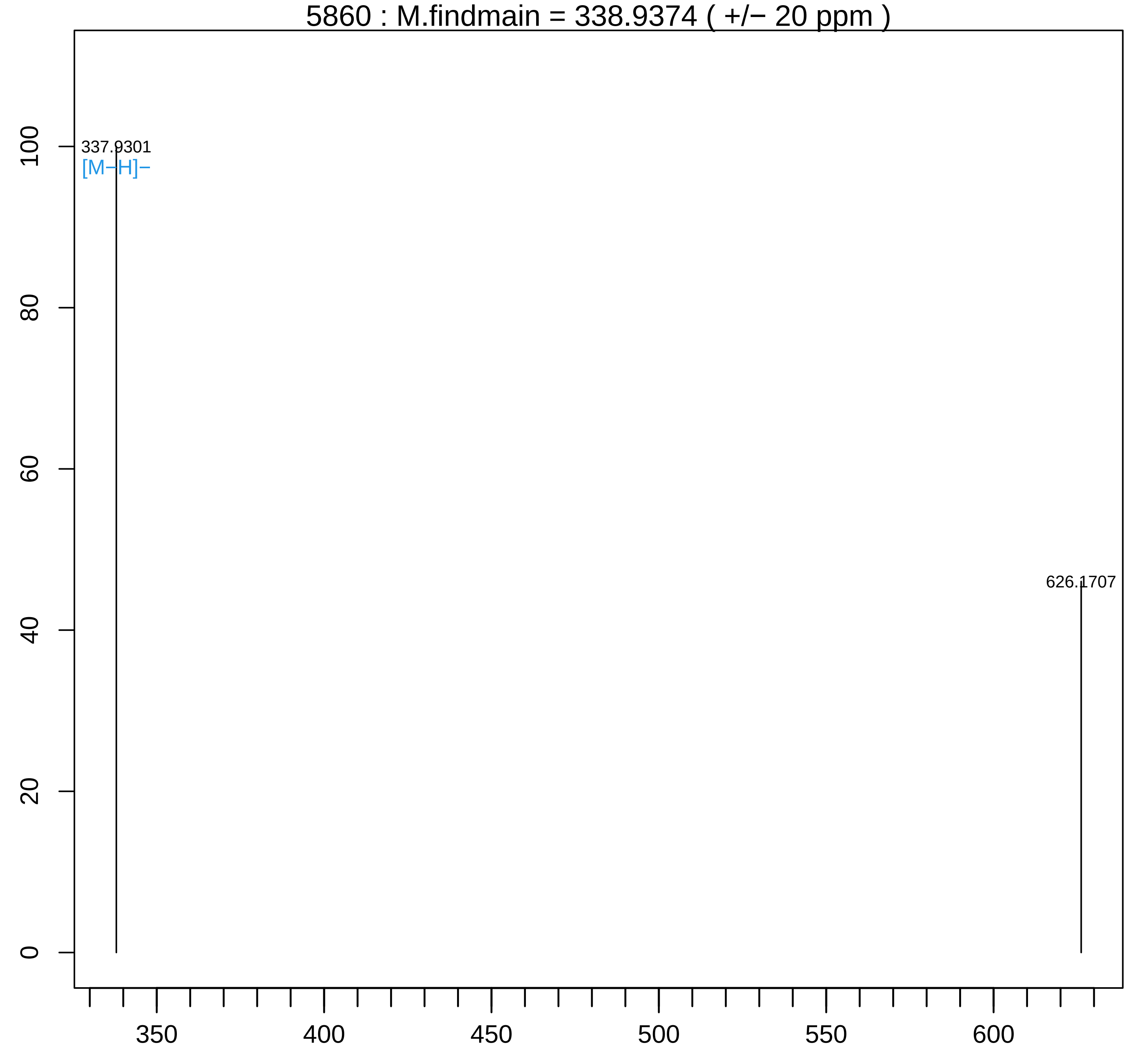
5860 : M.findmain = 338.9374 (+/− 20 ppm)
337.9301
[M−H]−
626.1707
0
20
40
60
80
100
350
400
450
500
550
600

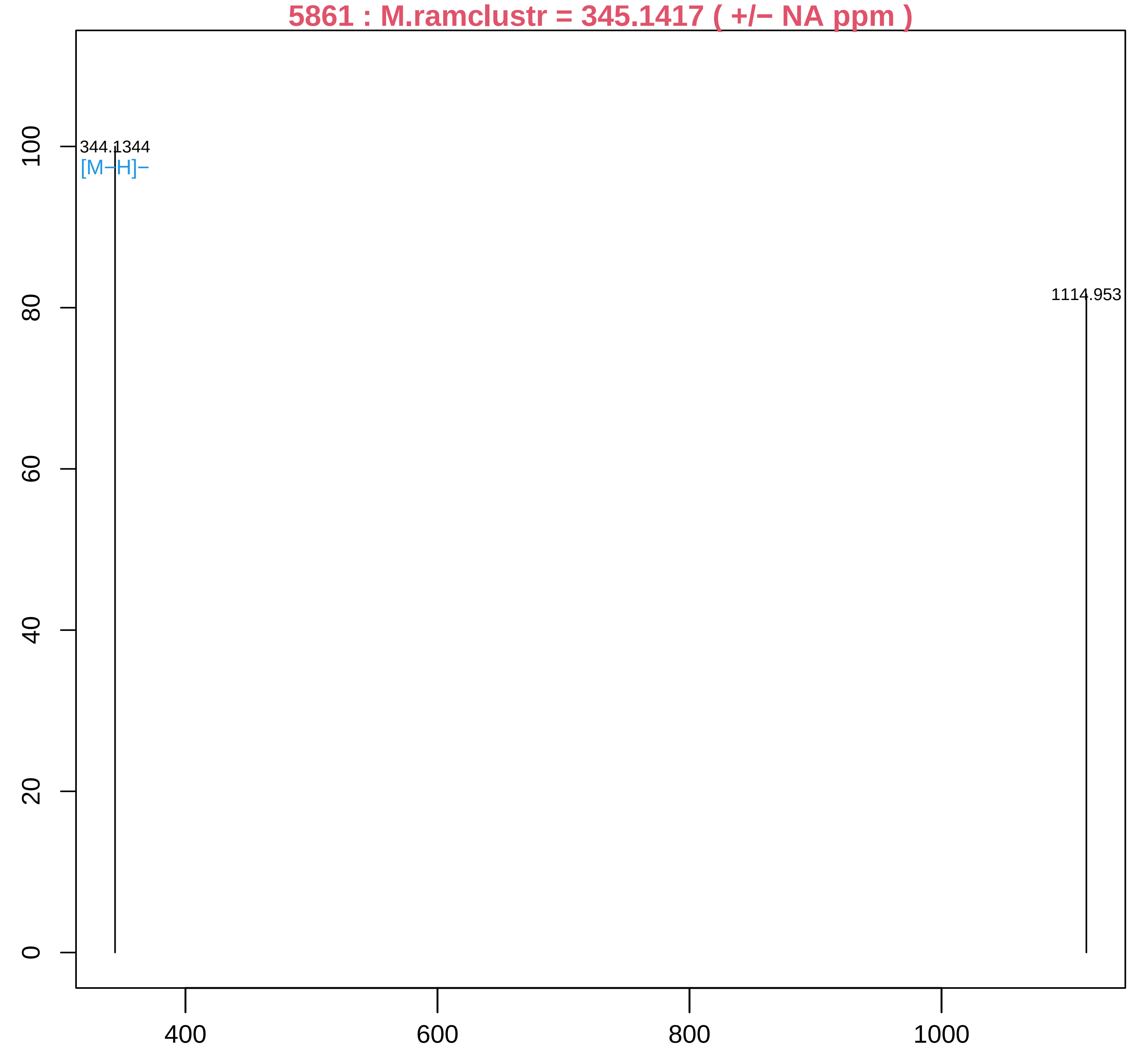
5861 : M.ramclustr = 345.1417 (+/− NA ppm)
344.1344
[M−H]−
1114.953
0
20
40
60
80
100
400
600
800
1000

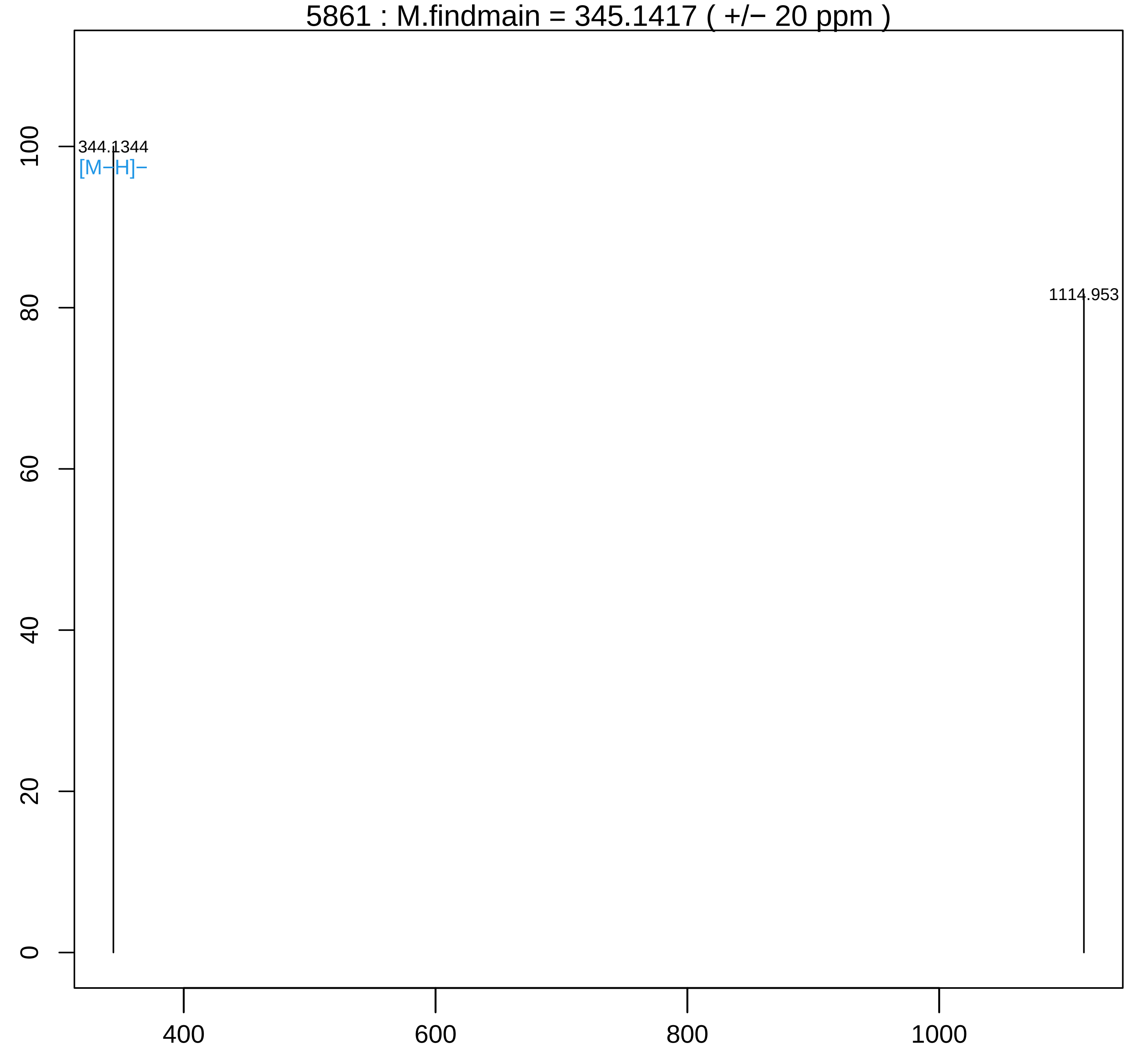
5861 : M.findmain = 345.1417 (+/− 20 ppm)
344.1344
[M−H]−
1114.953
0
20
40
60
80
100
400
600
800
1000

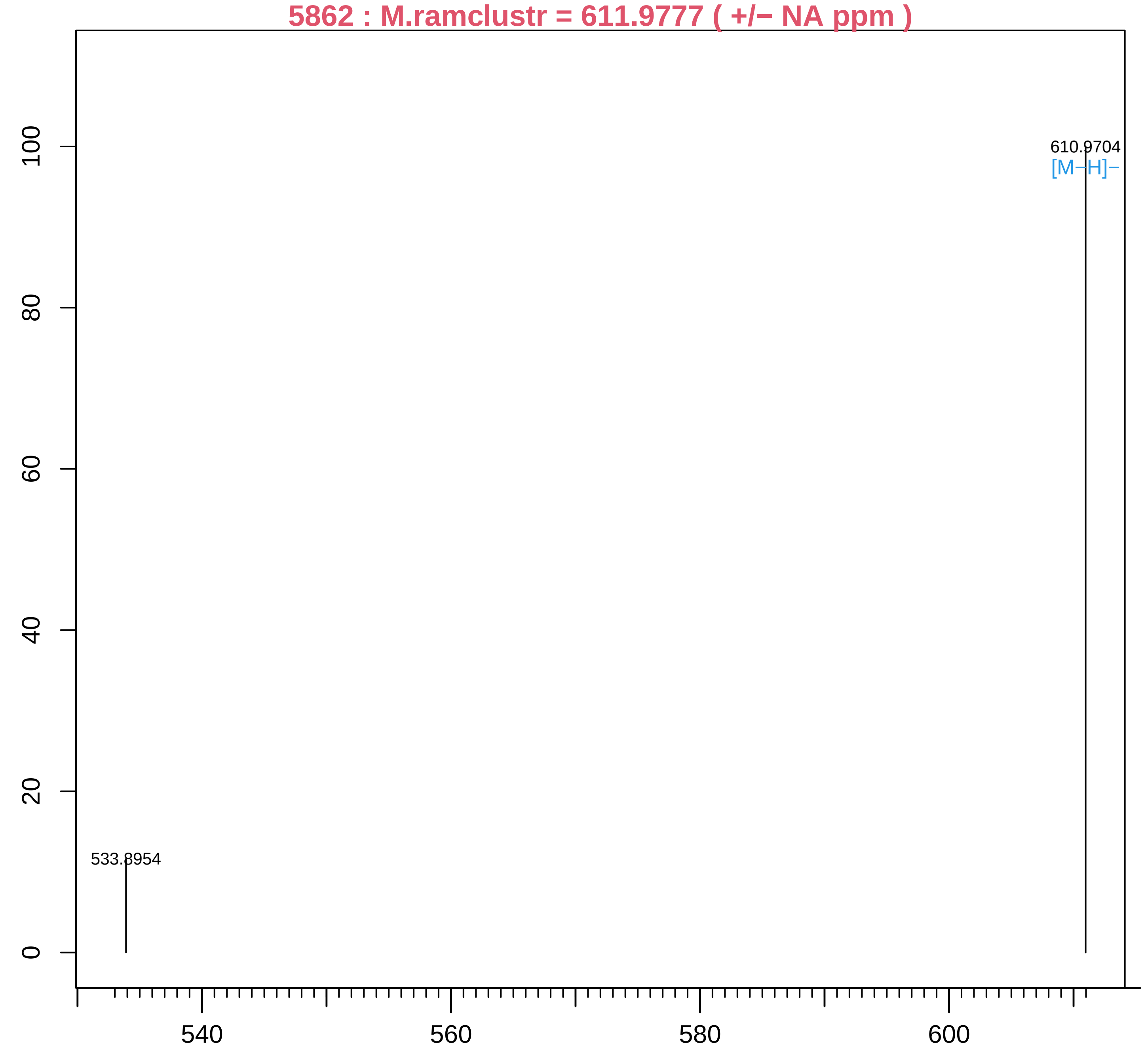
5862 : M.ramclustr = 611.9777 (+/− NA ppm)
610.9704
[M−H]−
533.8954
0
20
40
60
80
100
540
560
580
600

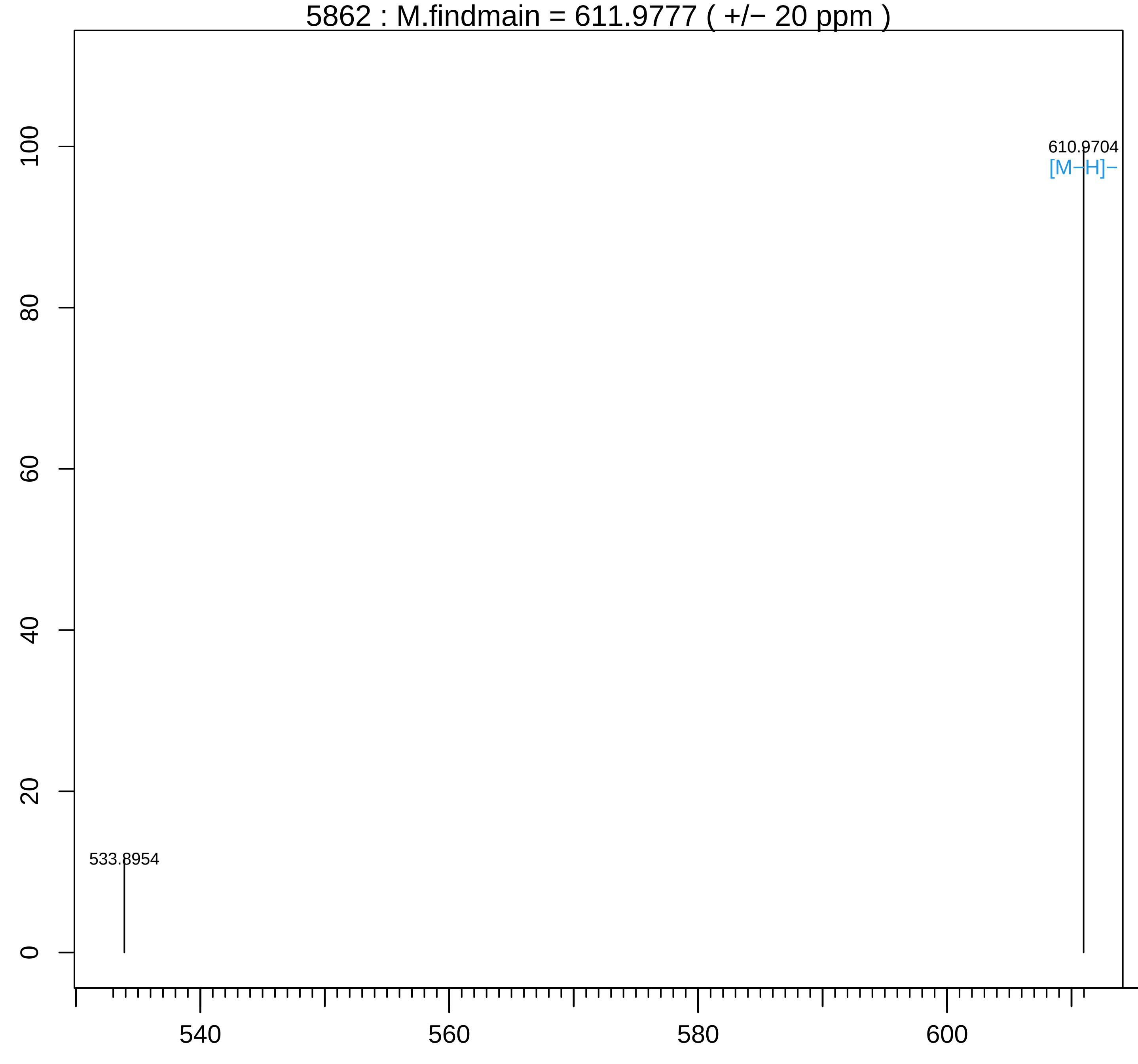
5862 : M.findmain = 611.9777 (+/− 20 ppm)
610.9704
[M−H]−
533.8954
0
20
40
60
80
100
540
560
580
600

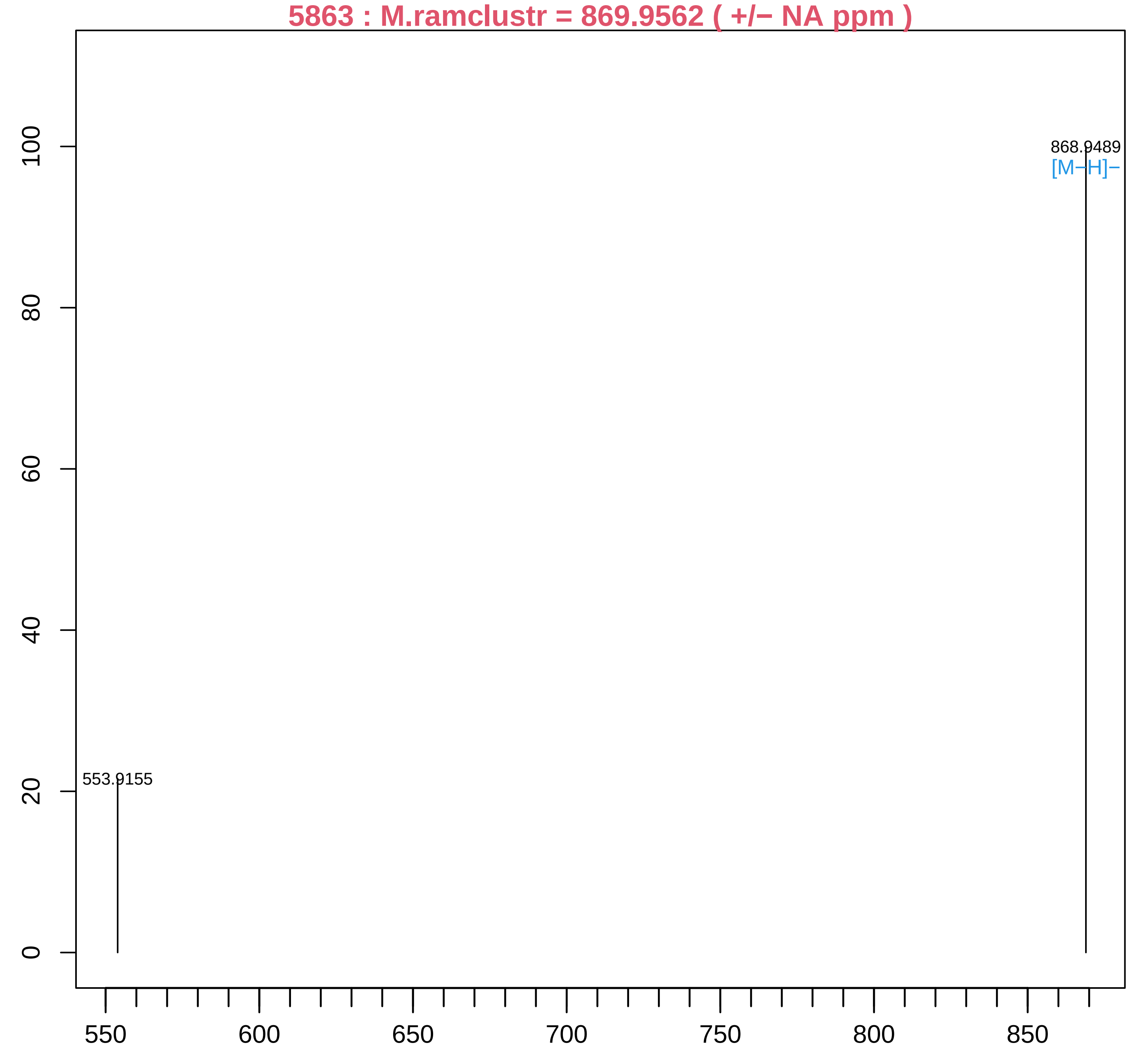
5863 : M.ramclustr = 869.9562 (+/− NA ppm)
868.9489
[M−H]−
553.9155
0
20
40
60
80
100
550
600
650
700
750
800
850

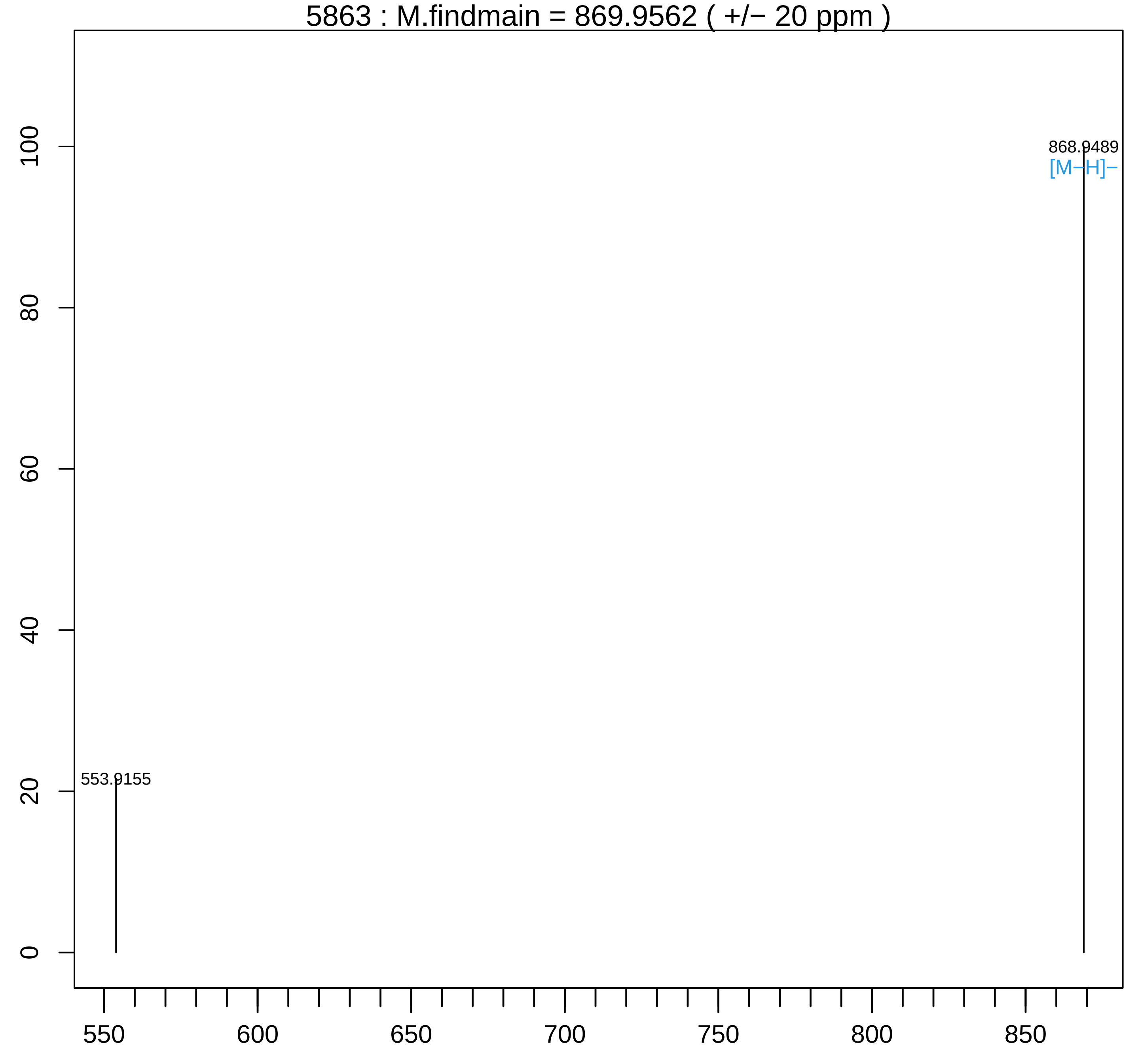
5863 : M.findmain = 869.9562 (+/− 20 ppm)
868.9489
[M−H]−
553.9155
0
20
40
60
80
100
550
600
650
700
750
800
850

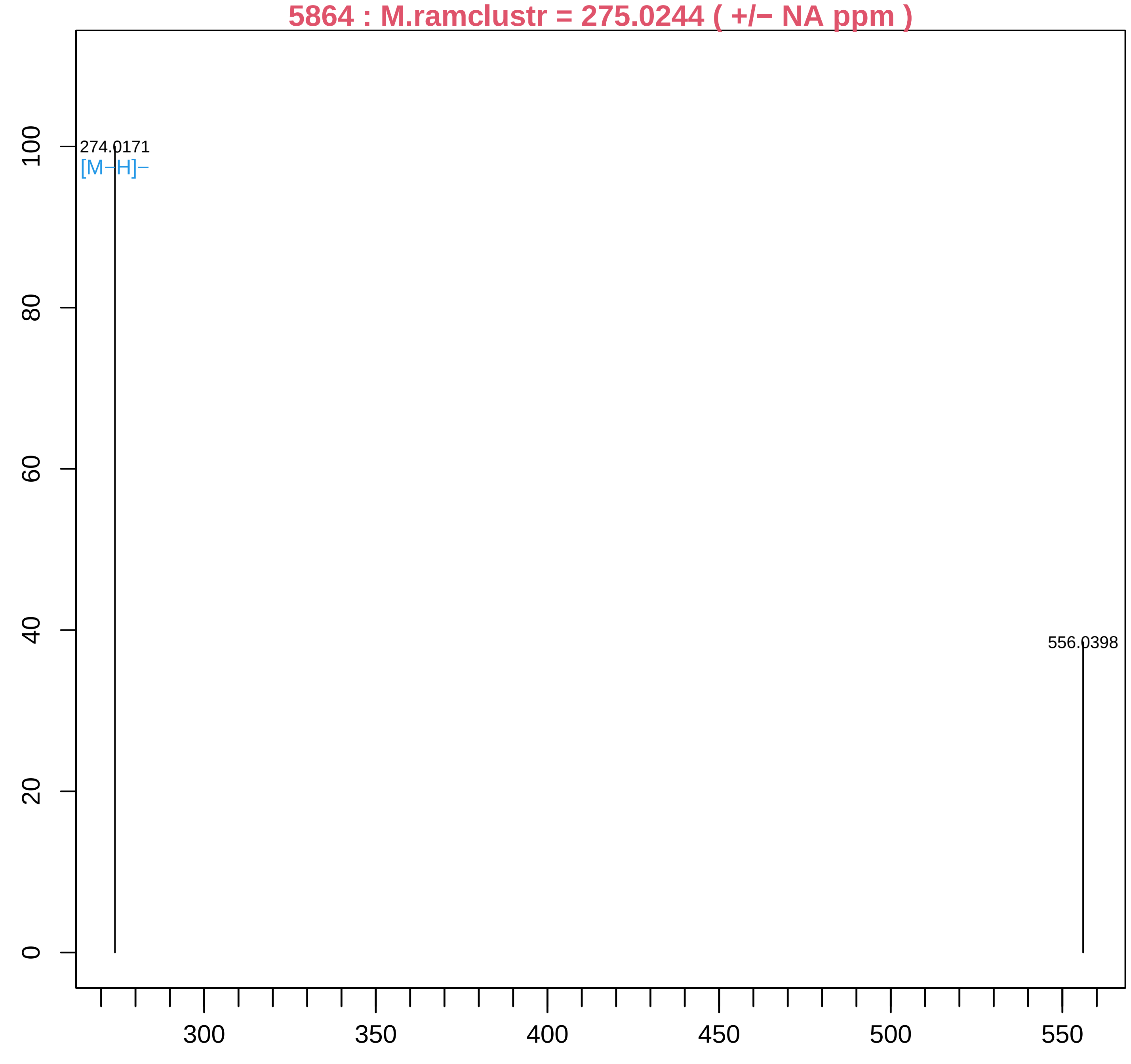
5864 : M.ramclustr = 275.0244 (+/− NA ppm)
274.0171
[M−H]−
556.0398
0
20
40
60
80
100
300
350
400
450
500
550

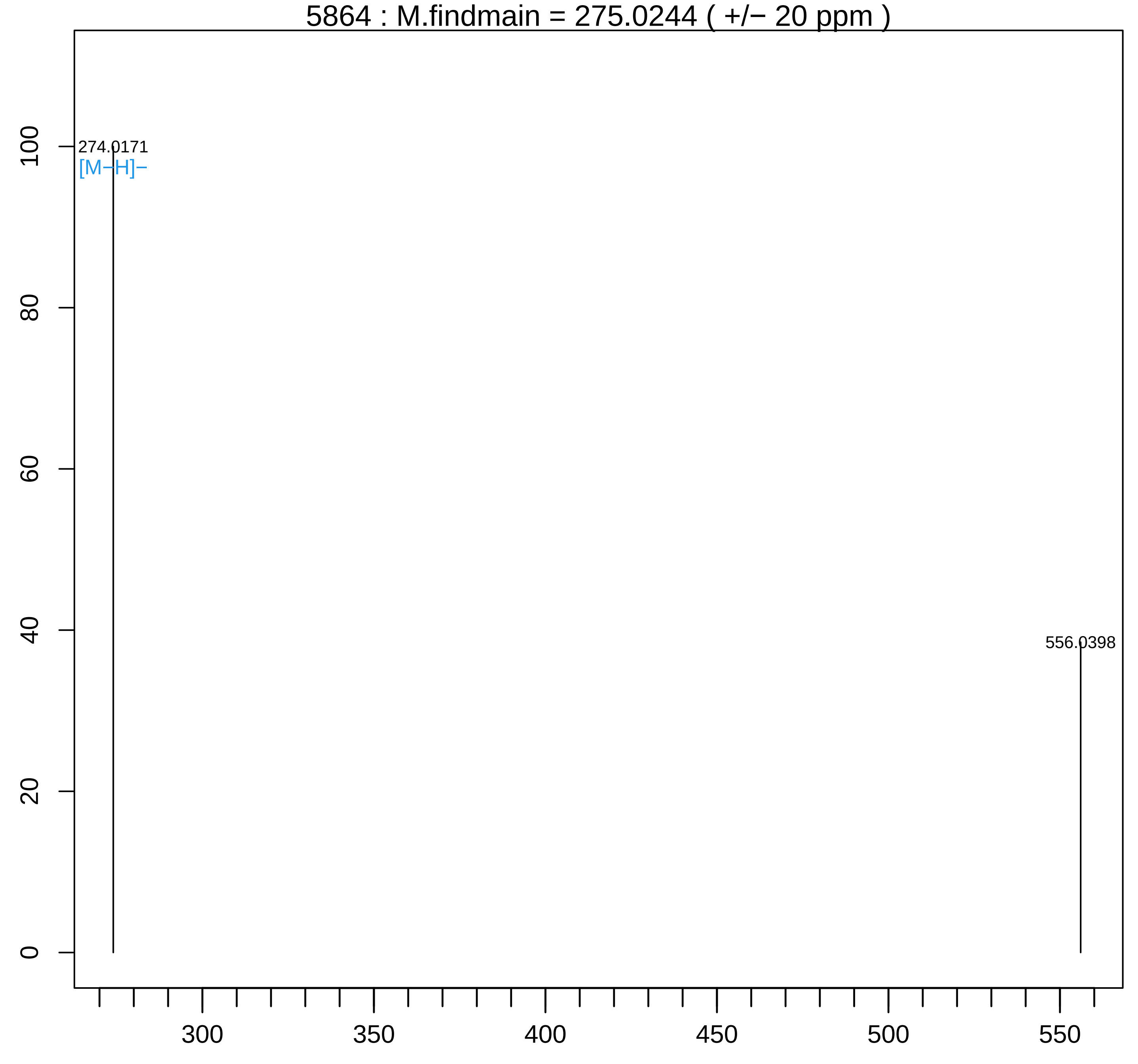
5864 : M.findmain = 275.0244 (+/− 20 ppm)
274.0171
[M−H]−
556.0398
0
20
40
60
80
100
300
350
400
450
500
550

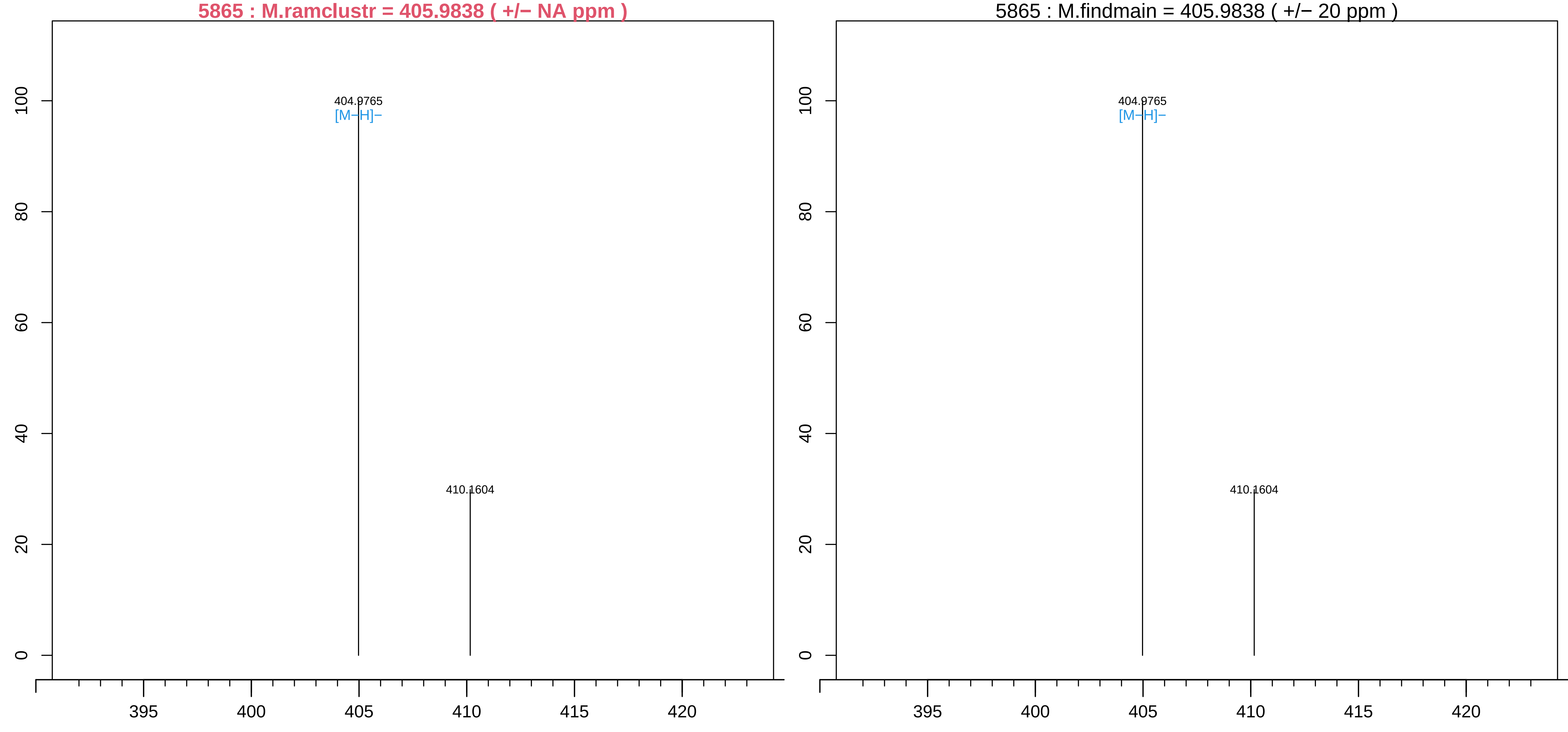
5865 : M.ramclustr = 405.9838 (+/− NA ppm)
404.9765
[M−H]−
410.1604
5865 : M.findmain = 405.9838 (+/− 20 ppm)
404.9765
[M−H]−
410.1604
0
20
40
60
80
100
395
400
405
410
415
420

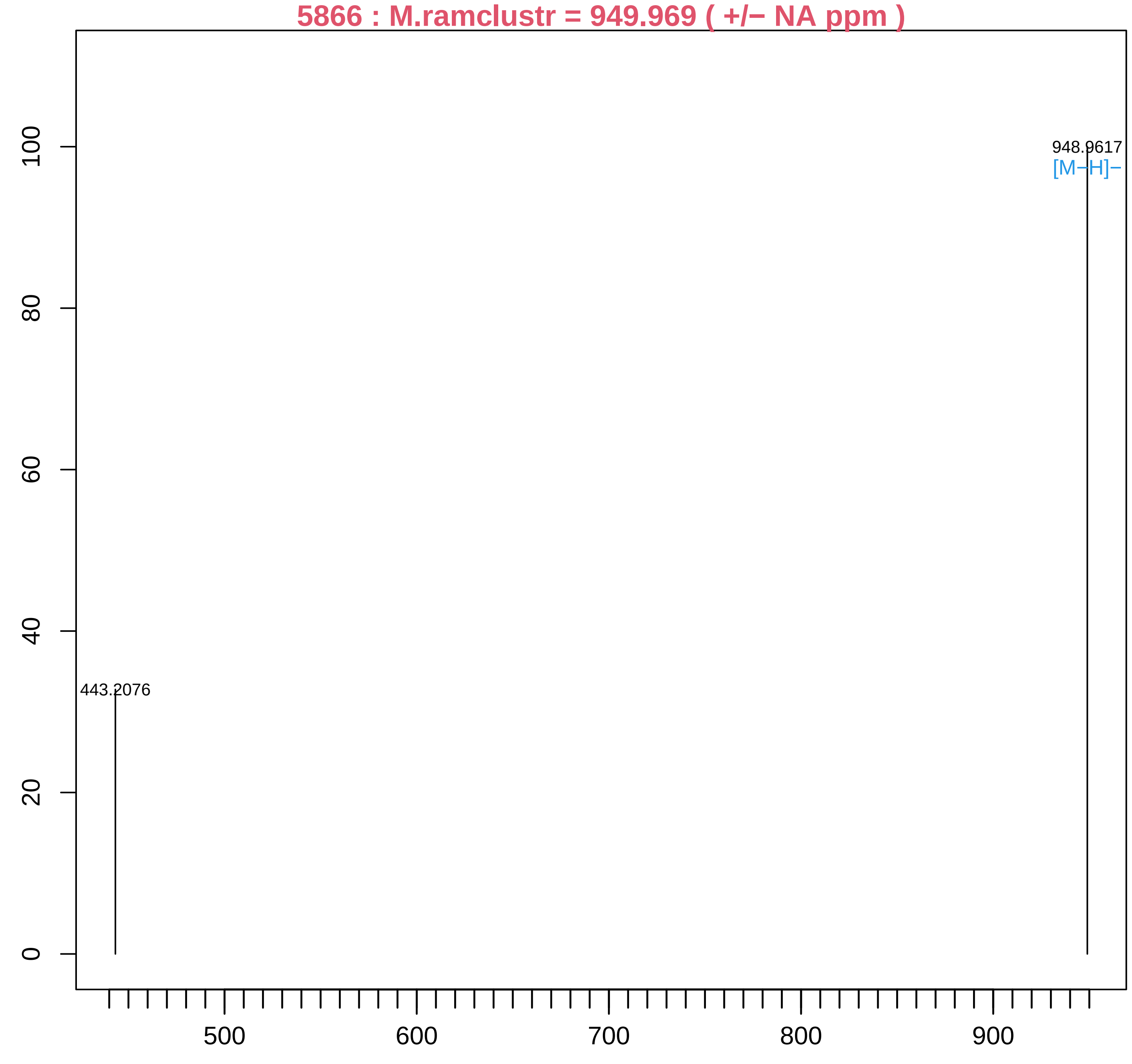
5866 : M.ramclustr = 949.969 (+/− NA ppm)
948.9617
[M−H]−
443.2076
0
20
40
60
80
100
500
600
700
800
900

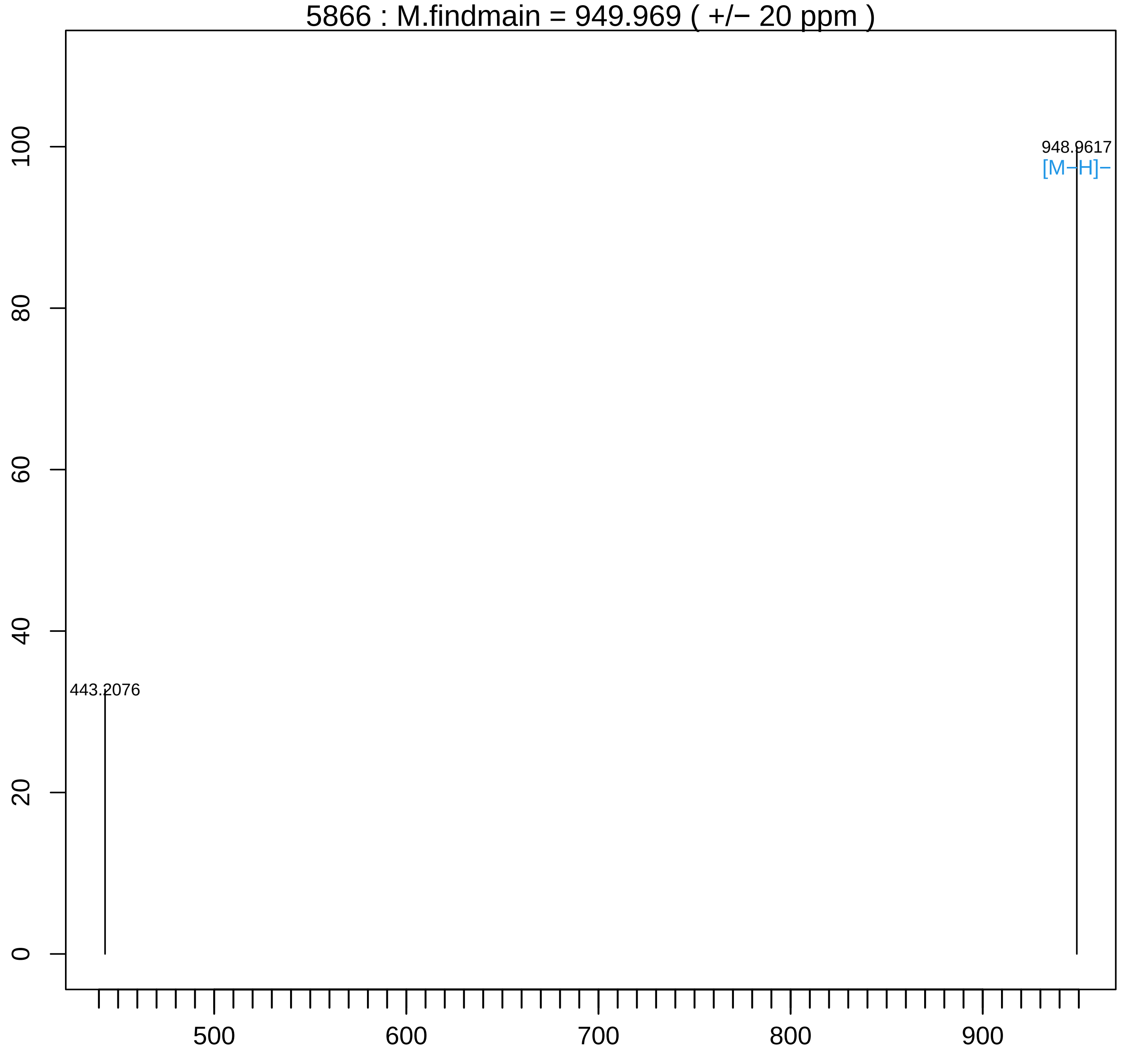
5866 : M.findmain = 949.969 (+/− 20 ppm)
948.9617
[M−H]−
443.2076
0
20
40
60
80
100
500
600
700
800
900

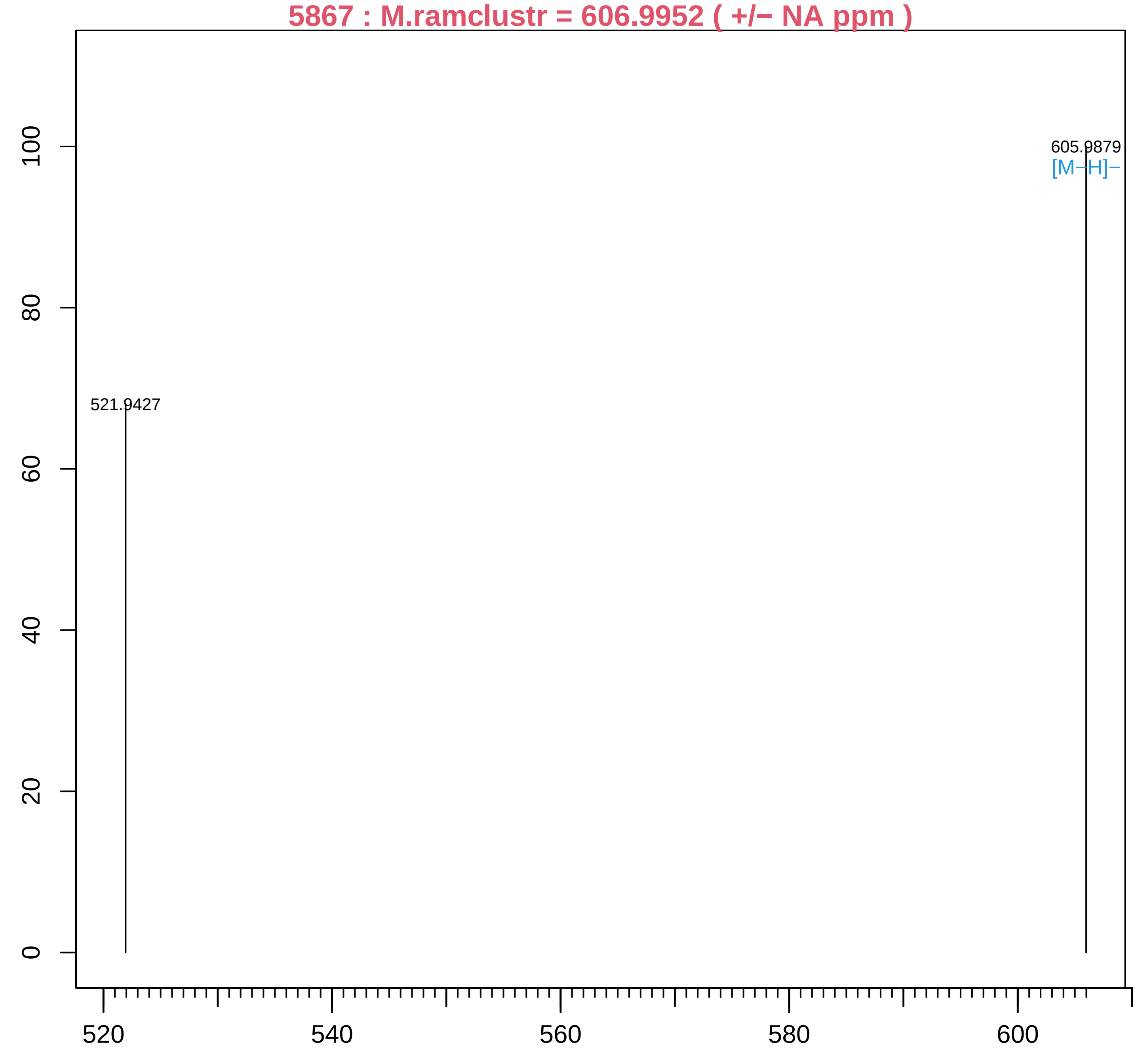
5867 : M.ramclustr = 606.9952 (+/− NA ppm)
605.9879
[M−H]−
521.9427
0
20
40
60
80
100
520
540
560
580
600

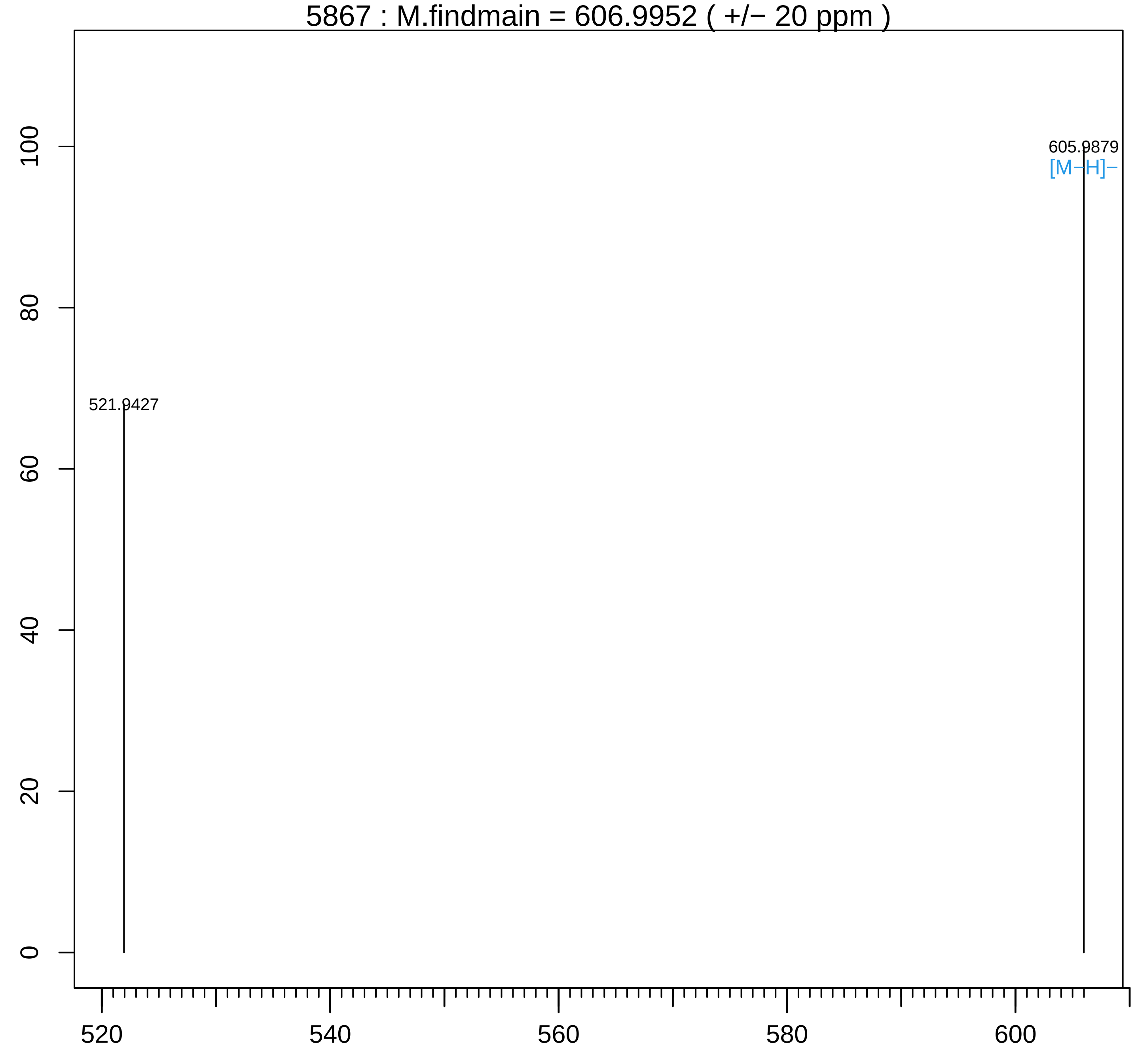
5867 : M.findmain = 606.9952 (+/− 20 ppm)
605.9879
[M−H]−
521.9427
0
20
40
60
80
100
520
540
560
580
600

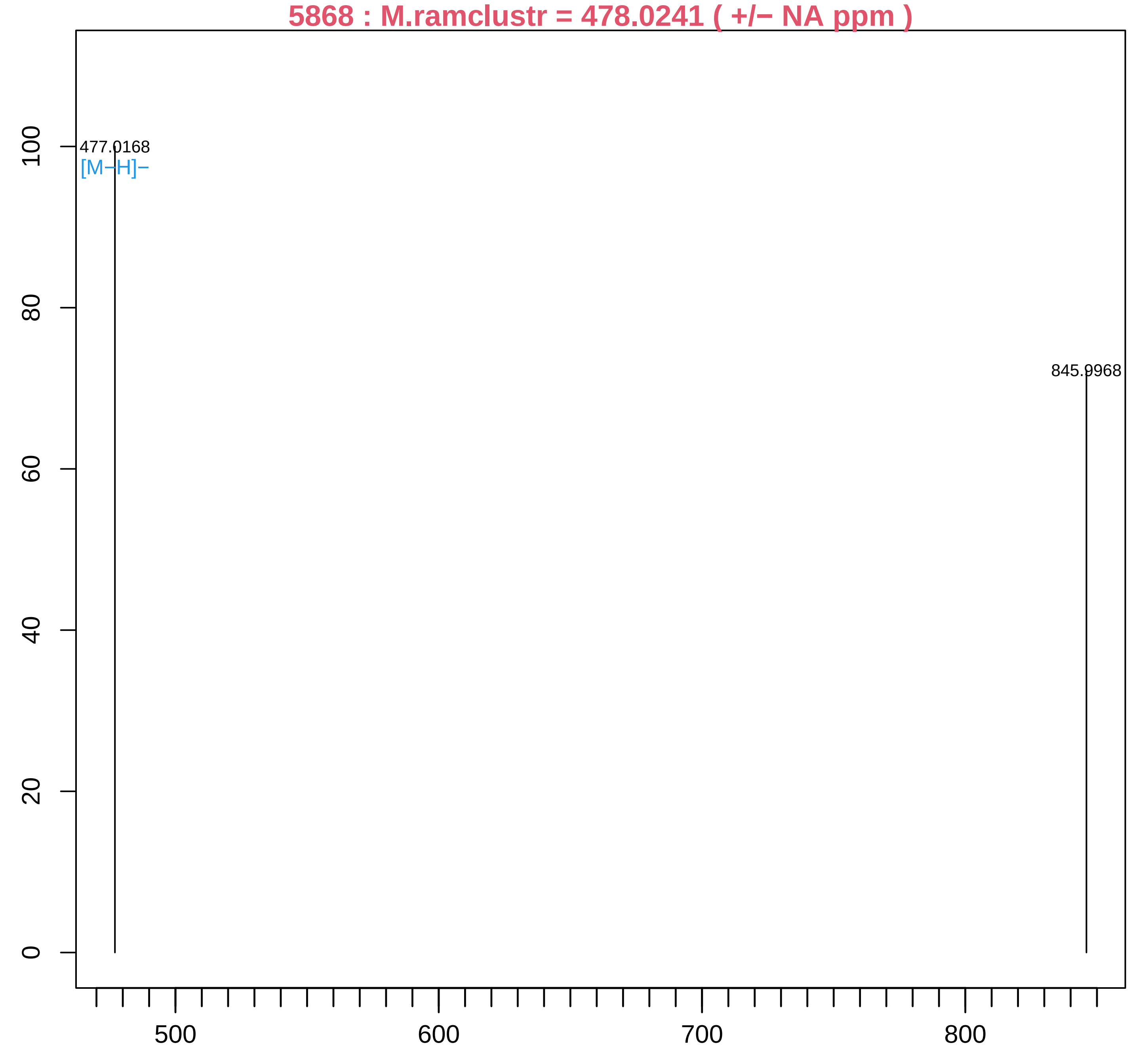
5868 : M.ramclustr = 478.0241 (+/− NA ppm)
477.0168
[M−H]−
845.9968
0
20
40
60
80
100
500
600
700
800

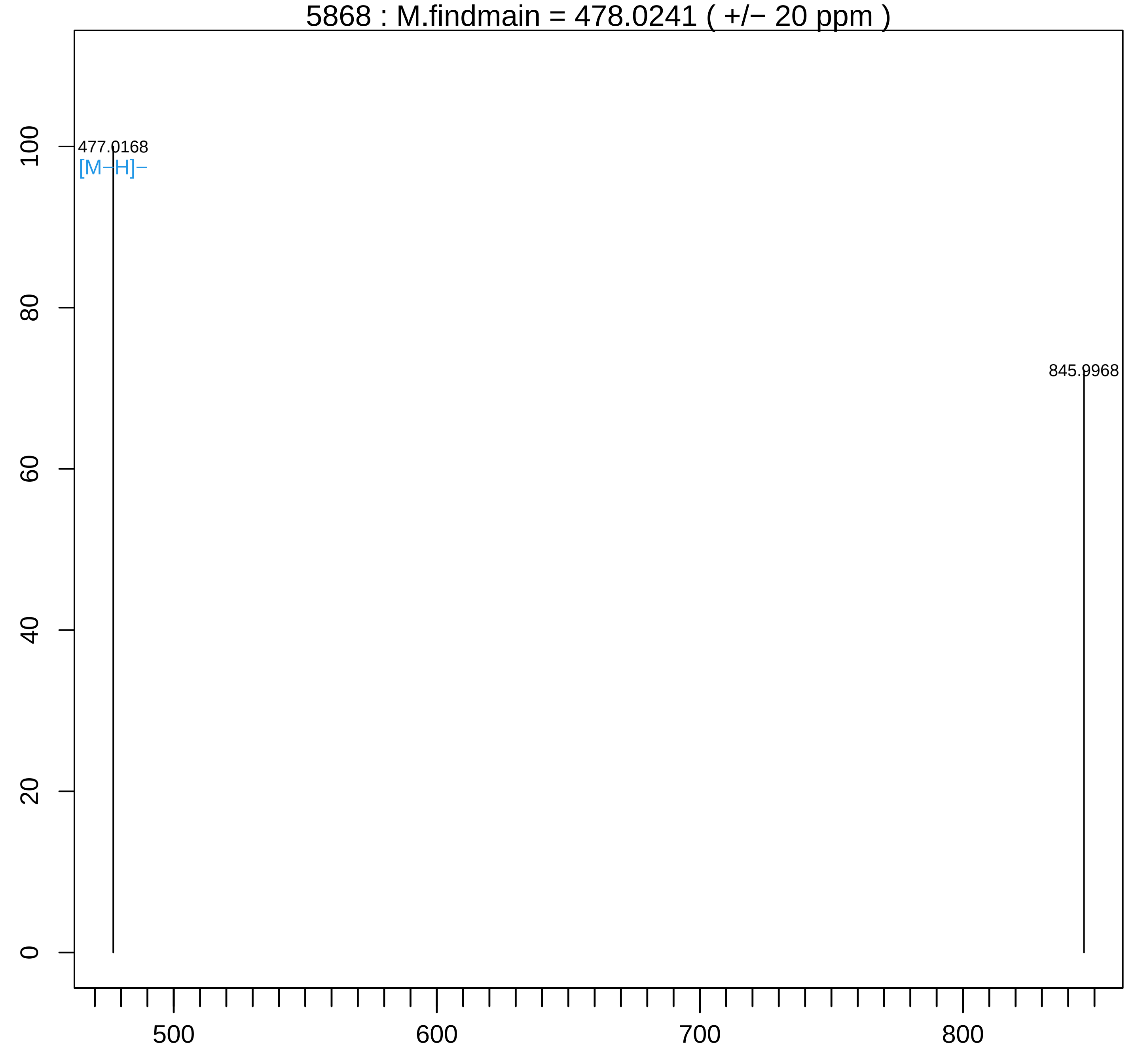
5868 : M.findmain = 478.0241 (+/− 20 ppm)
477.0168
[M−H]−
845.9968
0
20
40
60
80
100
500
600
700
800

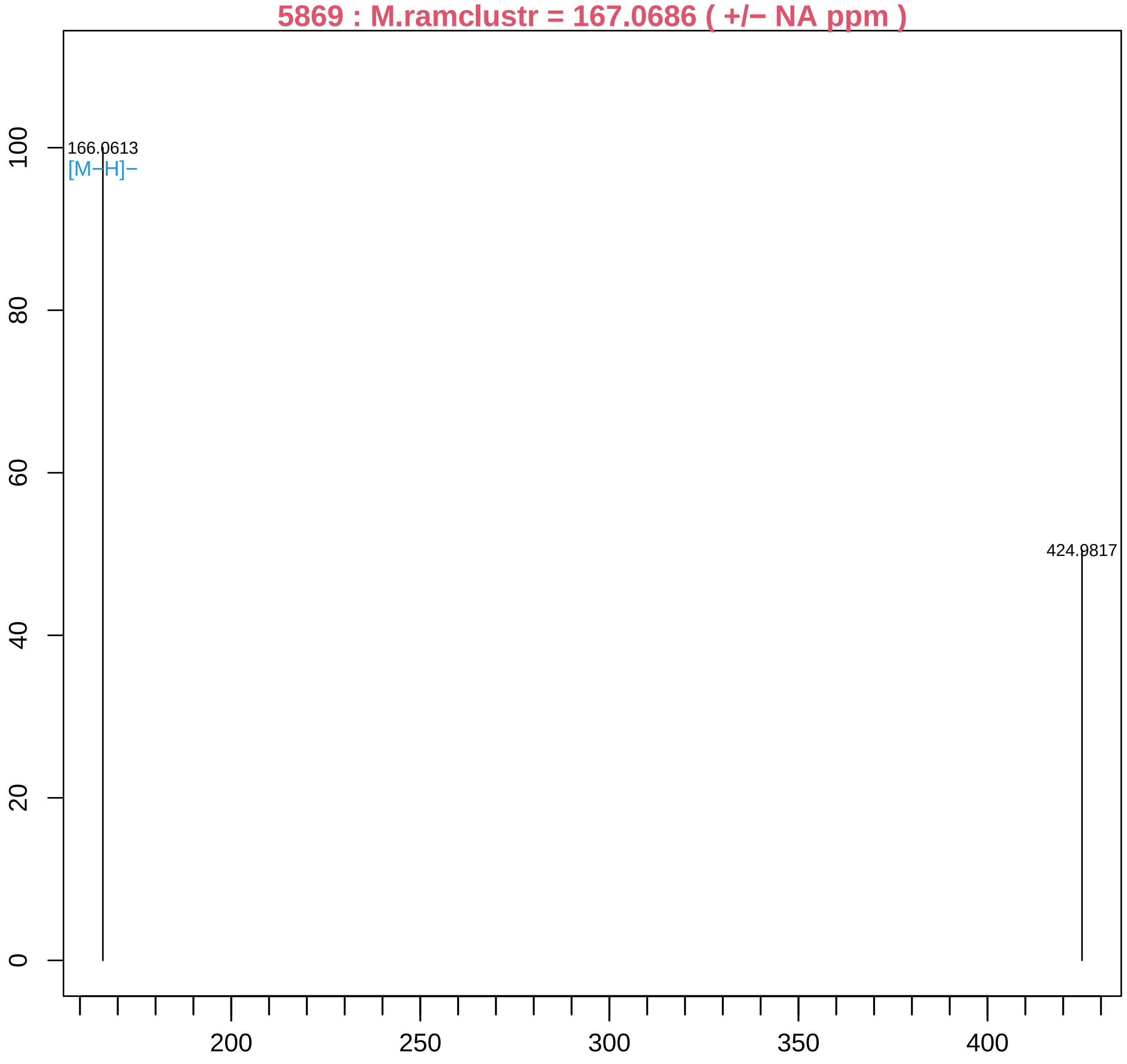
5869 : M.ramclustr = 167.0686 (+/− NA ppm)
166.0613
[M−H]−
424.9817
0
20
40
60
80
100
200
250
300
350
400

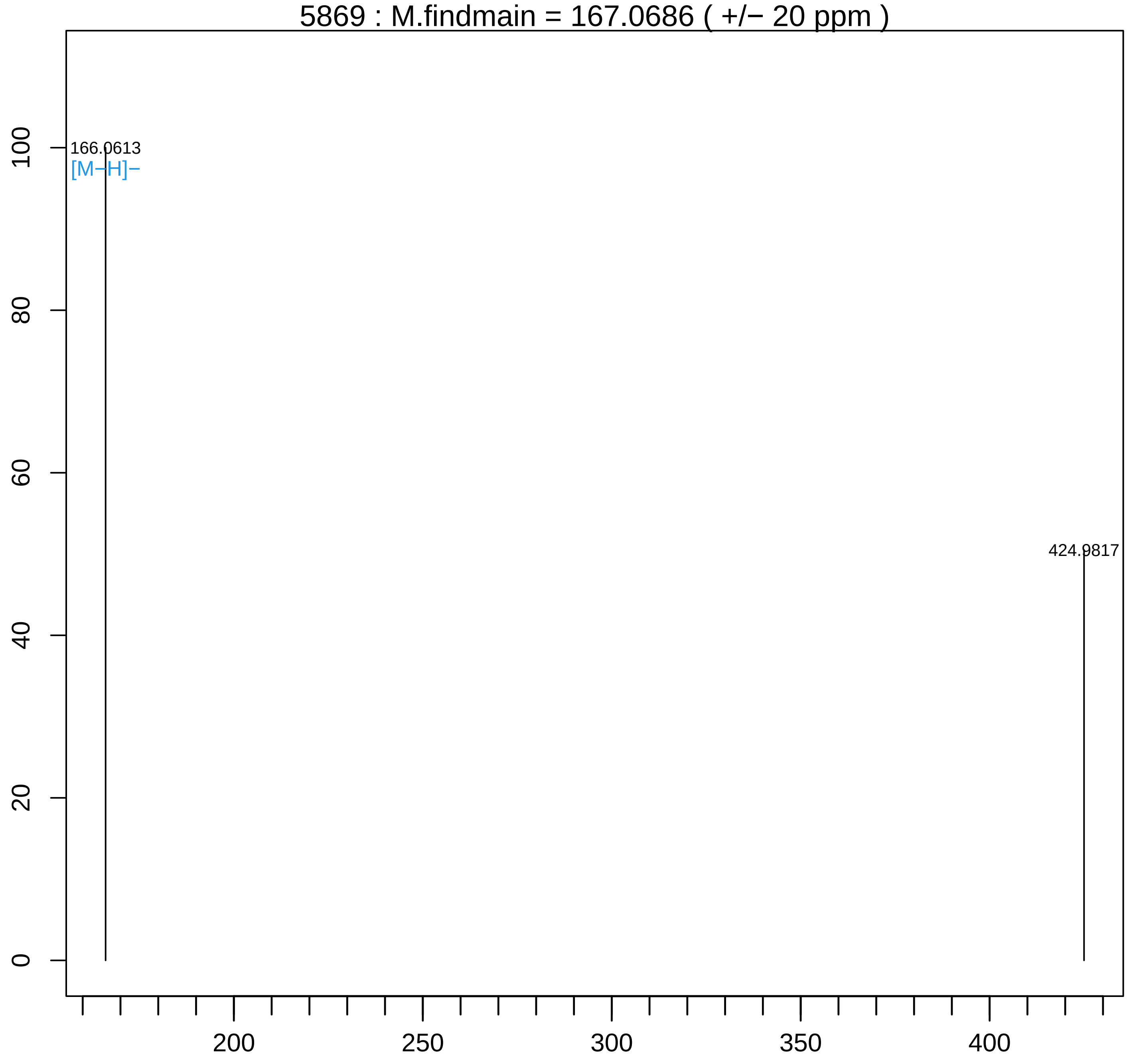
5869 : M.findmain = 167.0686 (+/− 20 ppm)
166.0613
[M−H]−
424.9817
0
20
40
60
80
100
200
250
300
350
400

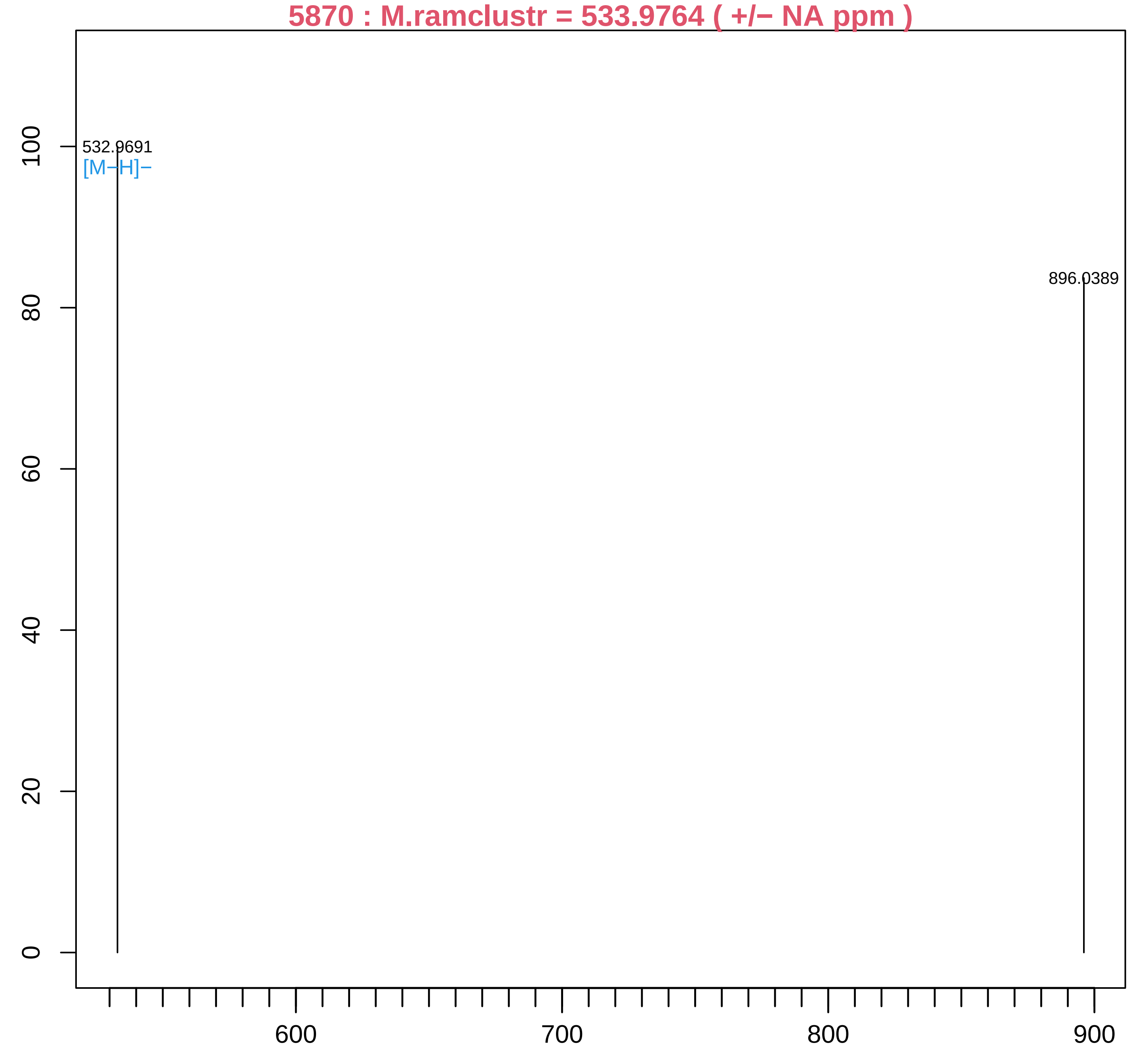
5870 : M.ramclustr = 533.9764 (+/− NA ppm)
532.9691
[M−H]−
896.0389
0
20
40
60
80
100
600
700
800
900

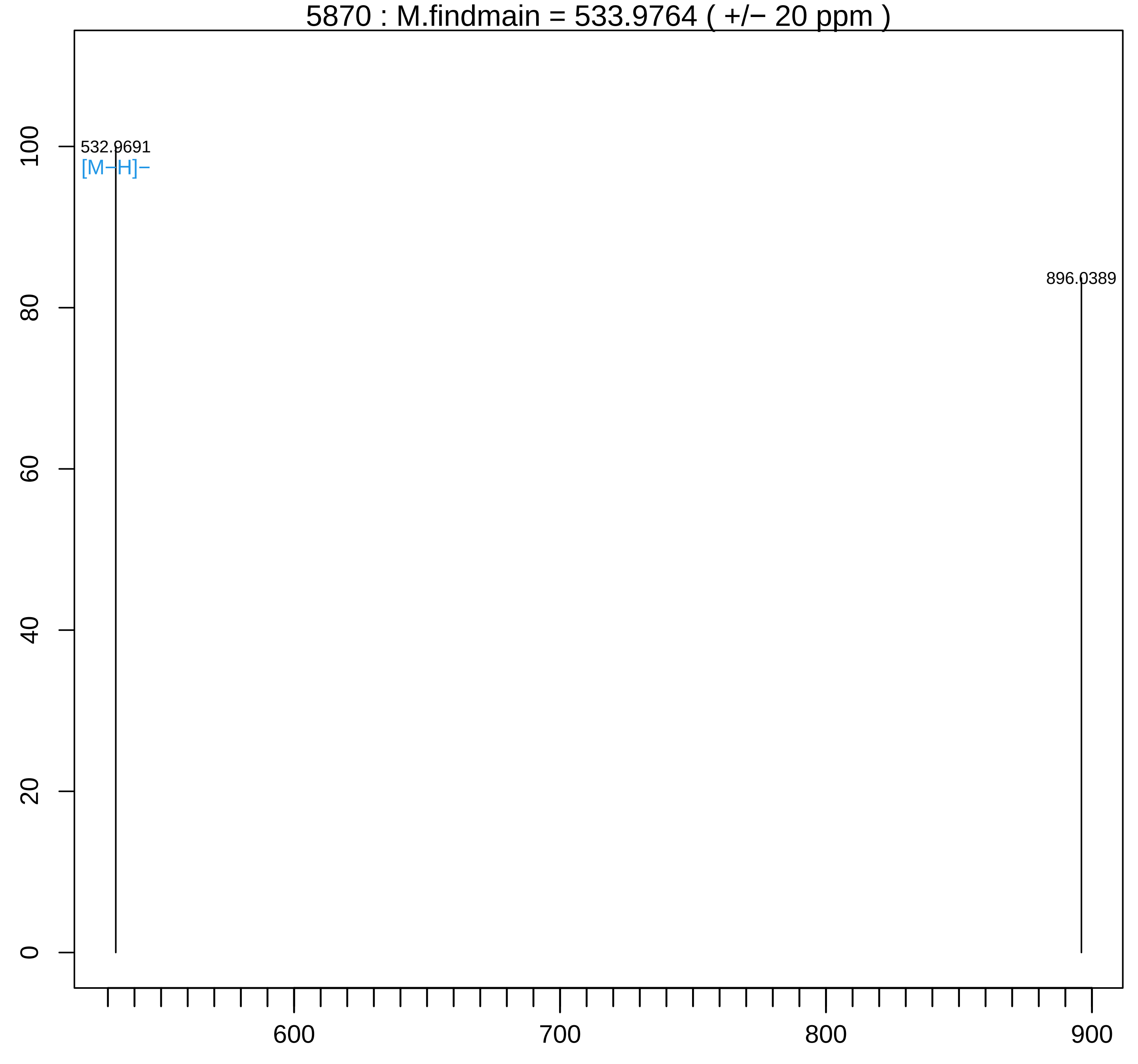
5870 : M.findmain = 533.9764 (+/− 20 ppm)
532.9691
[M−H]−
896.0389
0
20
40
60
80
100
600
700
800
900

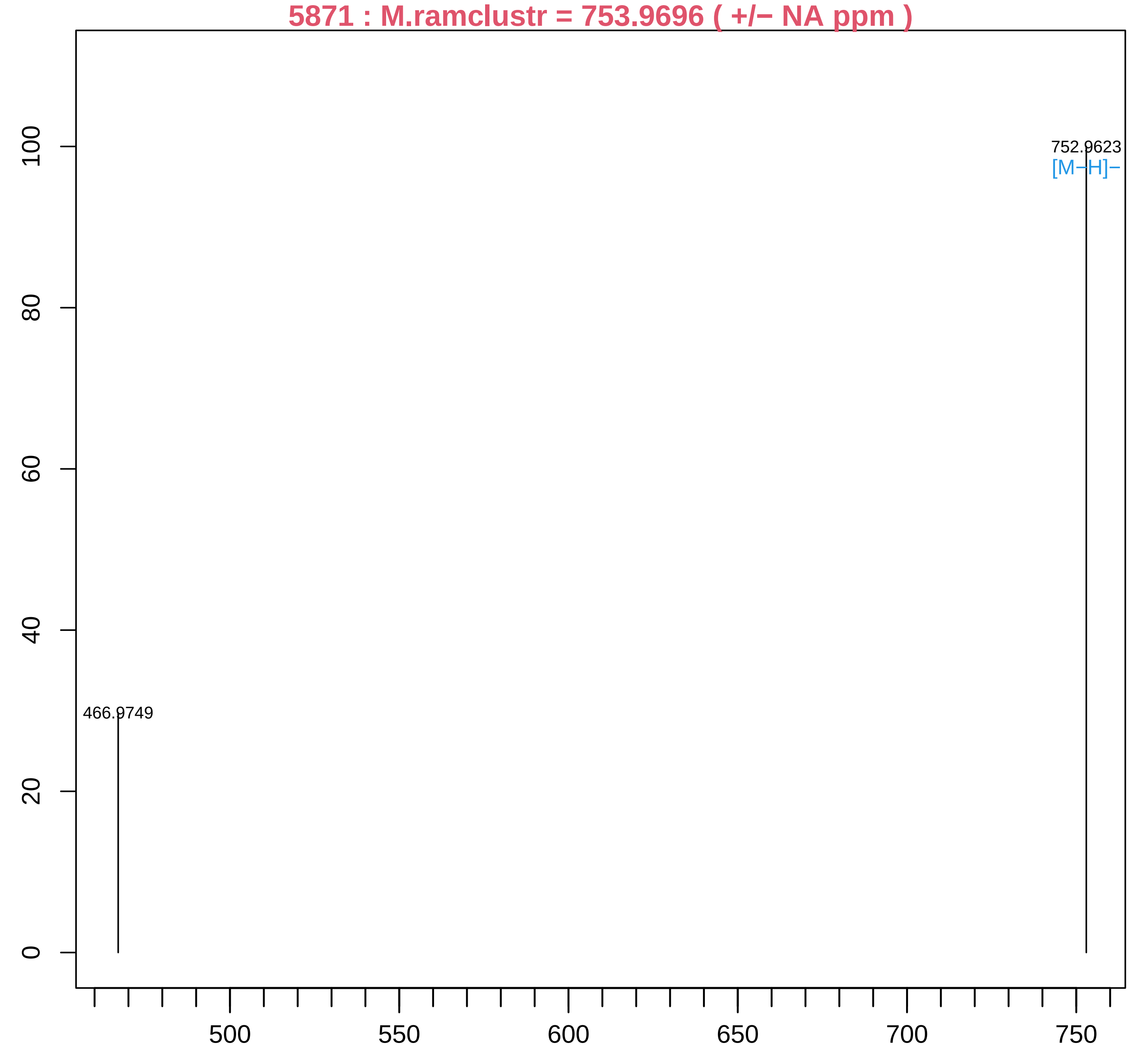
5871 : M.ramclustr = 753.9696 (+/− NA ppm)
752.9623
[M−H]−
466.9749
0
20
40
60
80
100
500
550
600
650
700
750

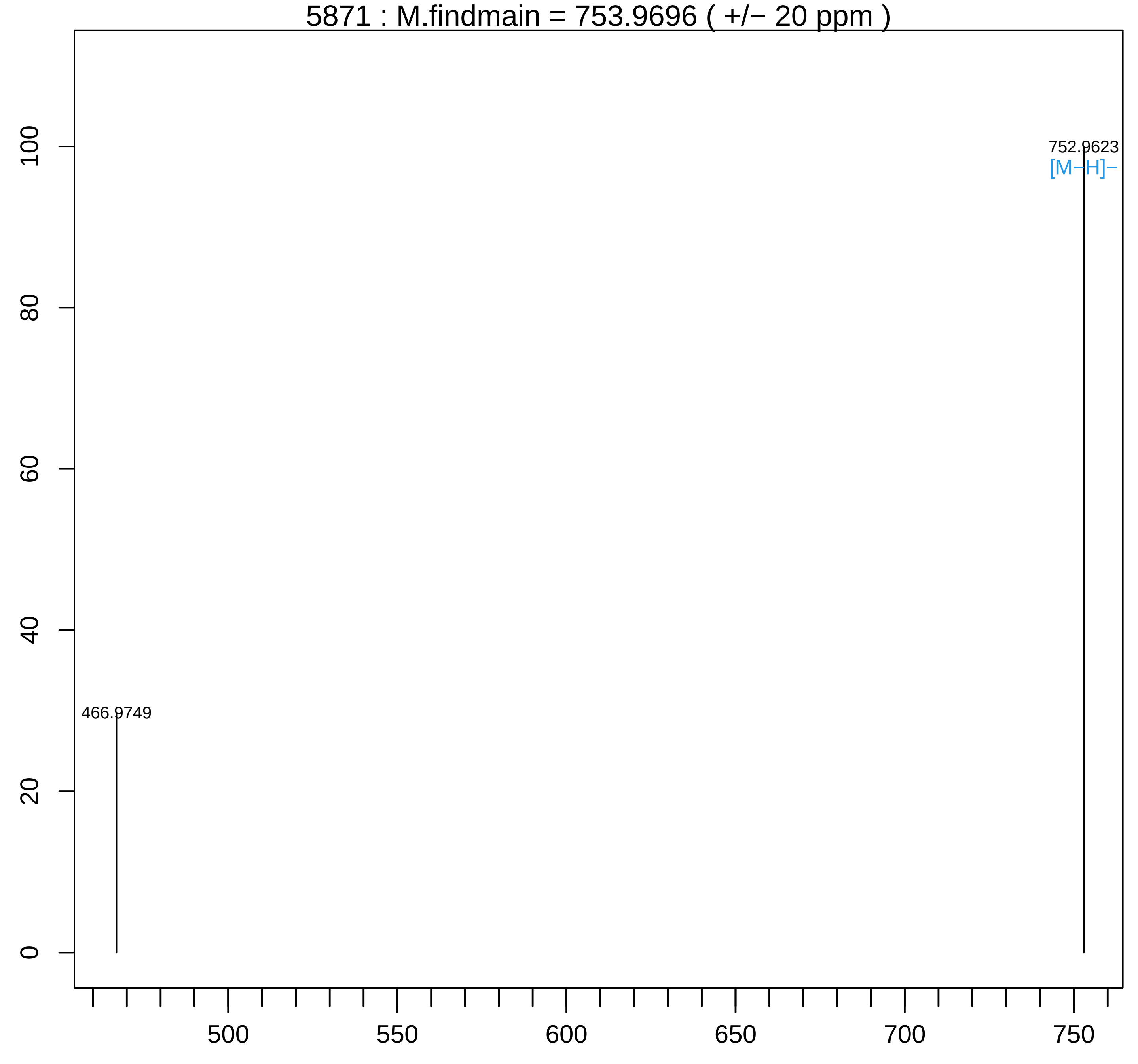
5871 : M.findmain = 753.9696 (+/− 20 ppm)
752.9623
[M−H]−
466.9749
0
20
40
60
80
100
500
550
600
650
700
750

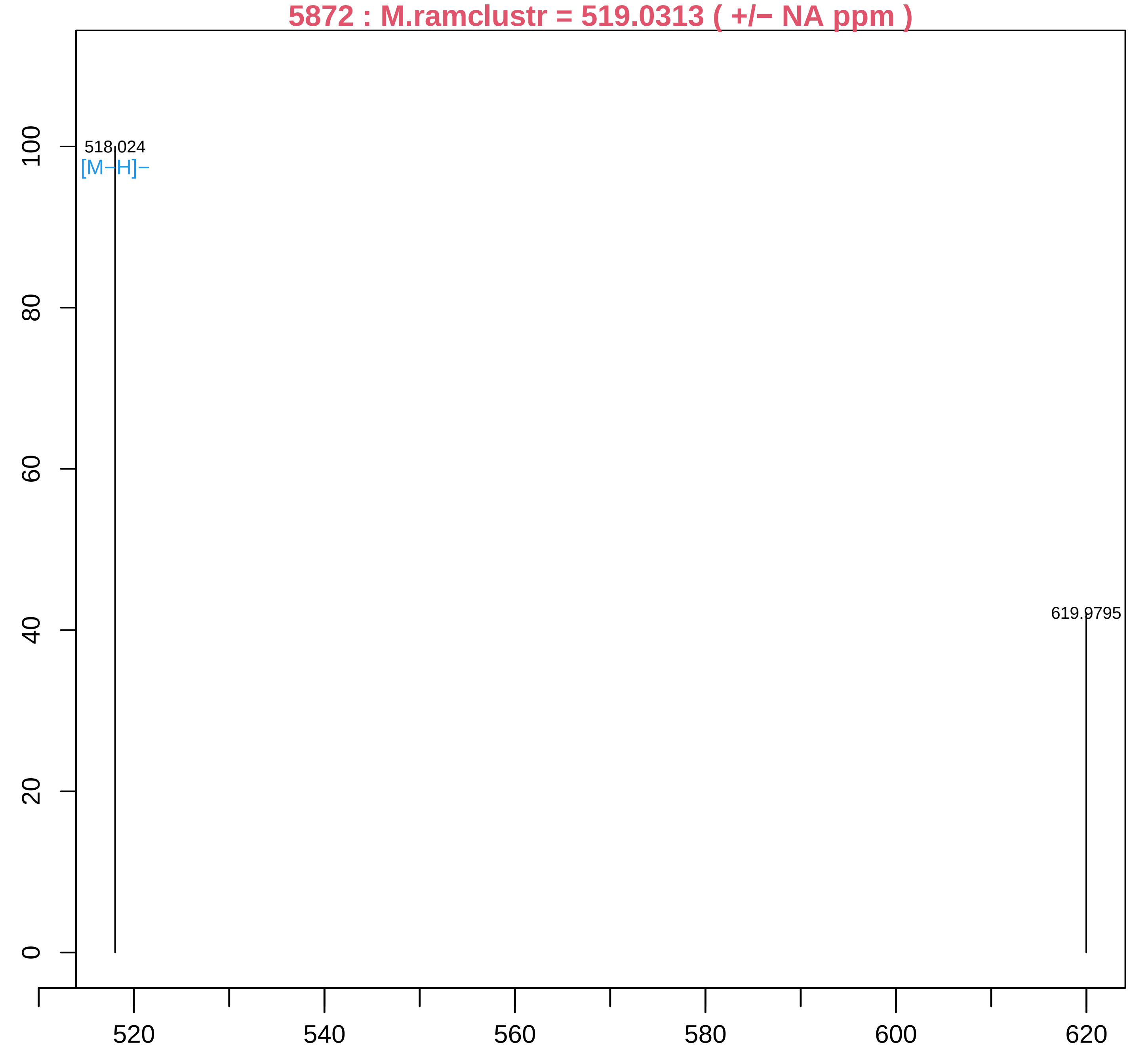
5872 : M.ramclustr = 519.0313 (+/− NA ppm)
518.024
[M−H]−
619.9795
0
20
40
60
80
100
520
540
560
580
600
620

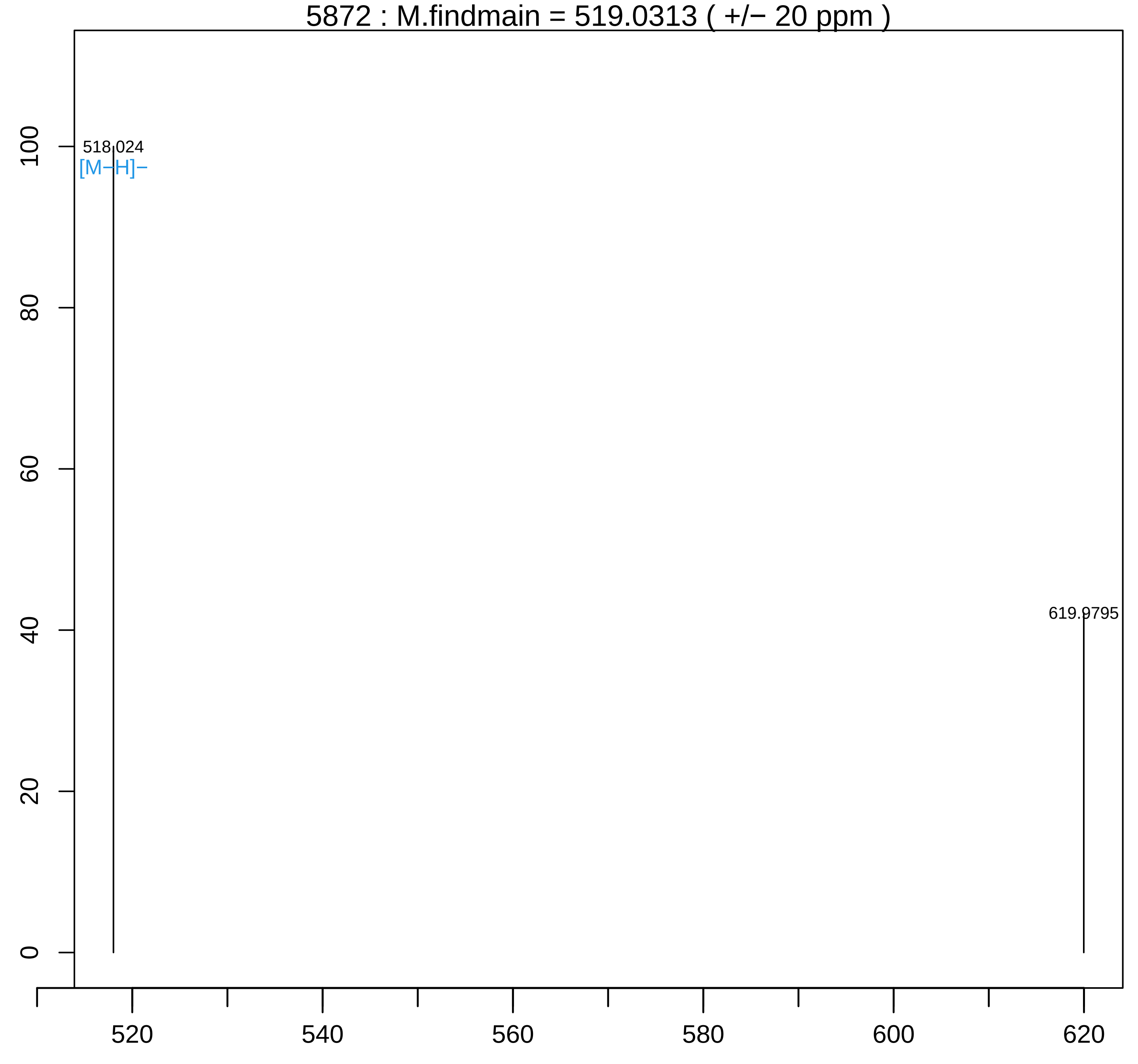
5872 : M.findmain = 519.0313 (+/− 20 ppm)
518.024
[M−H]−
619.9795
0
20
40
60
80
100
520
540
560
580
600
620

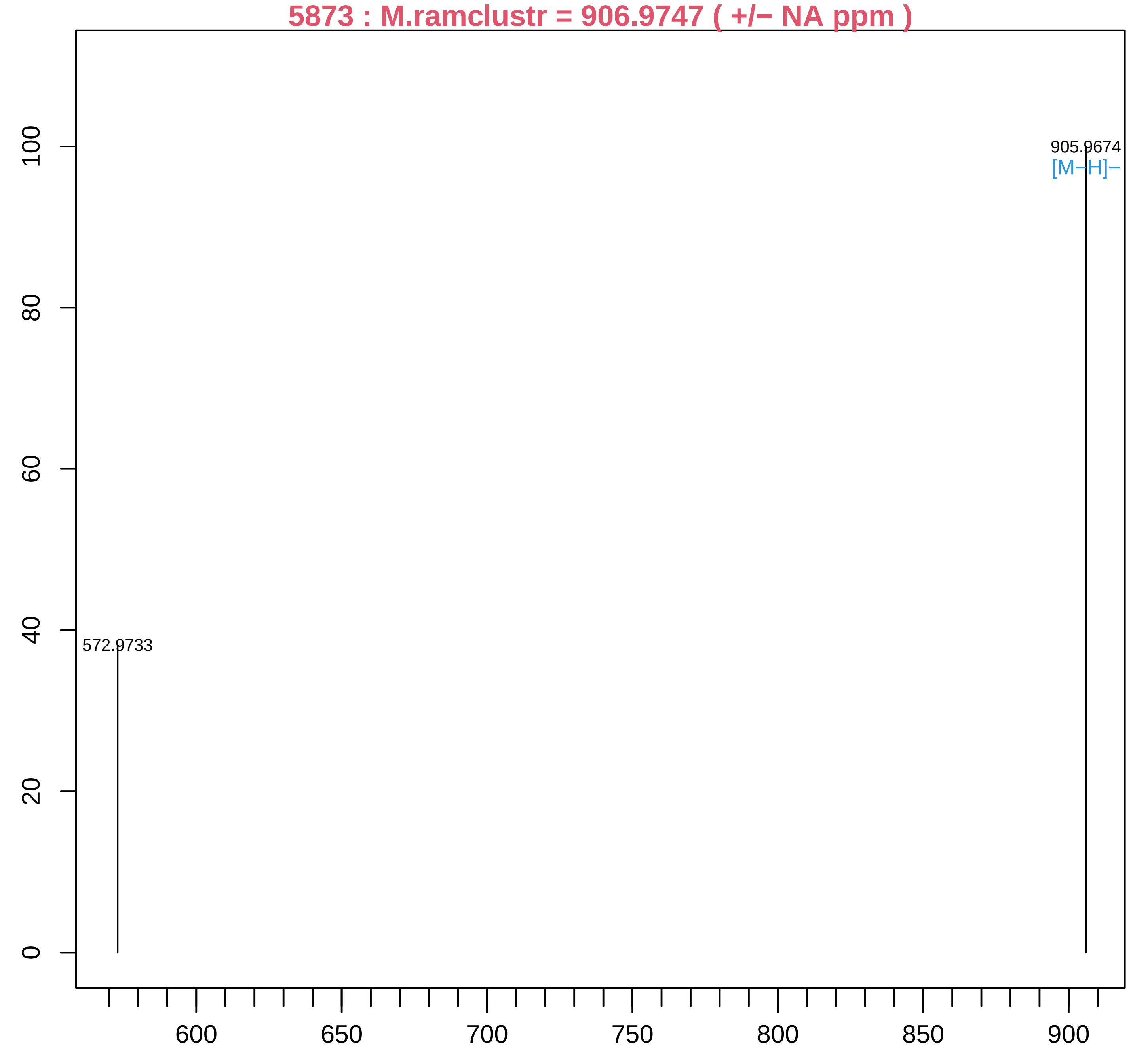
5873 : M.ramclustr = 906.9747 (+/− NA ppm)
905.9674
[M−H]−
572.9733
0
20
40
60
80
100
600
650
700
750
800
850
900

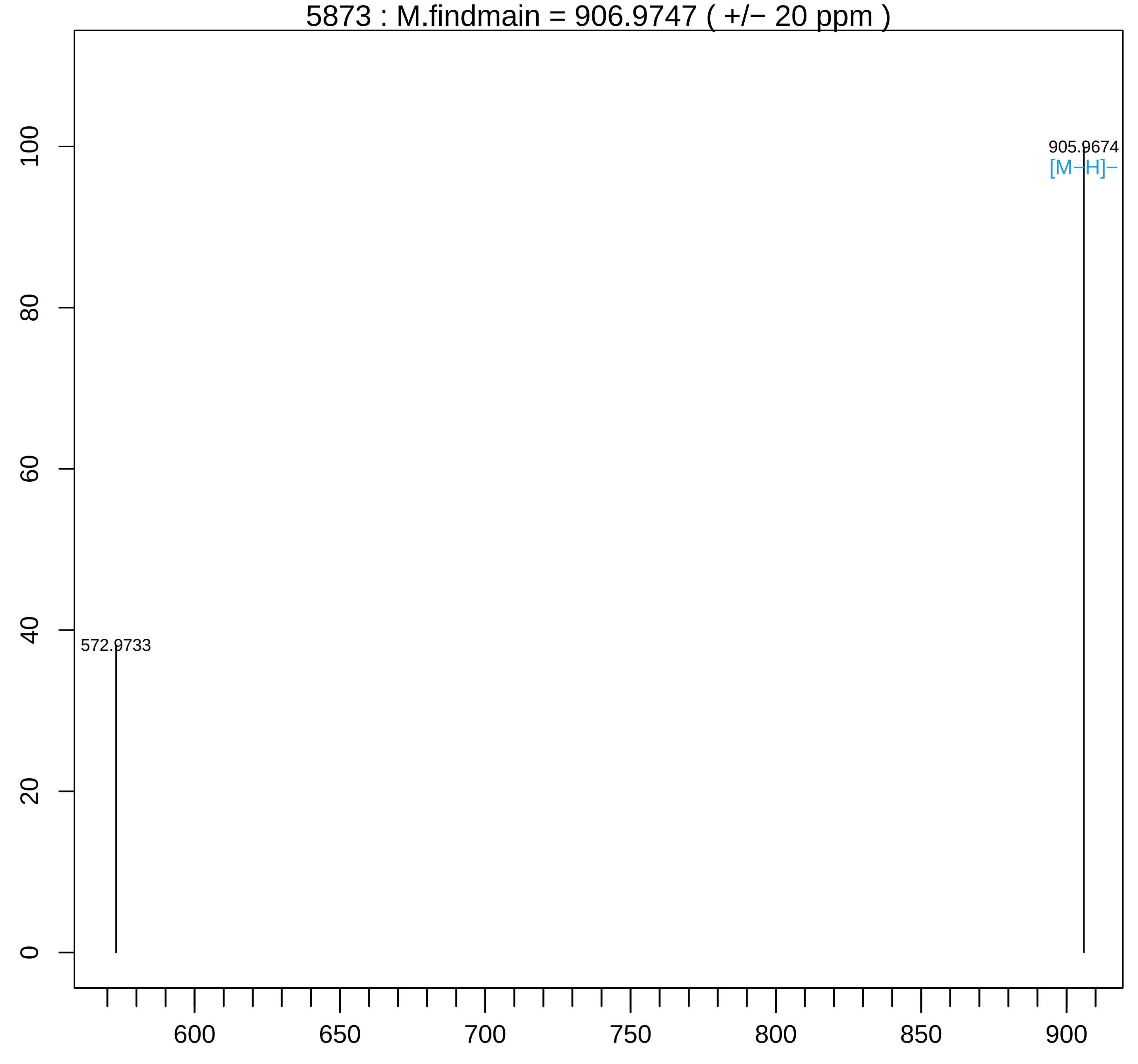
5873 : M.findmain = 906.9747 (+/− 20 ppm)
905.9674
[M−H]−
572.9733
0
20
40
60
80
100
600
650
700
750
800
850
900

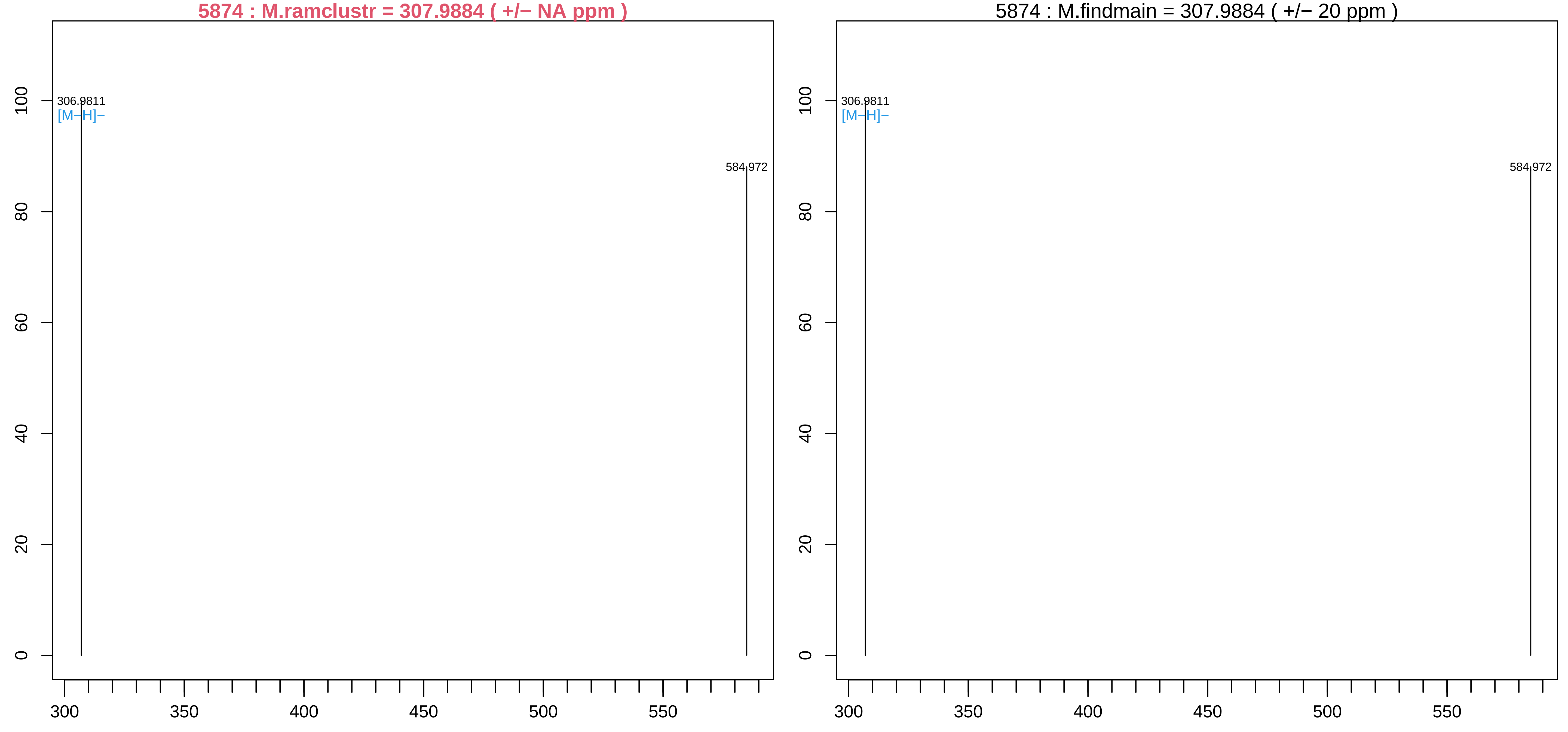

5874 : M.ramclustr = 307.9884 (+/− NA ppm)
306.9811
[M−H]−
584.972
5874 : M.findmain = 307.9884 (+/− 20 ppm)
306.9811
[M−H]−
584.972

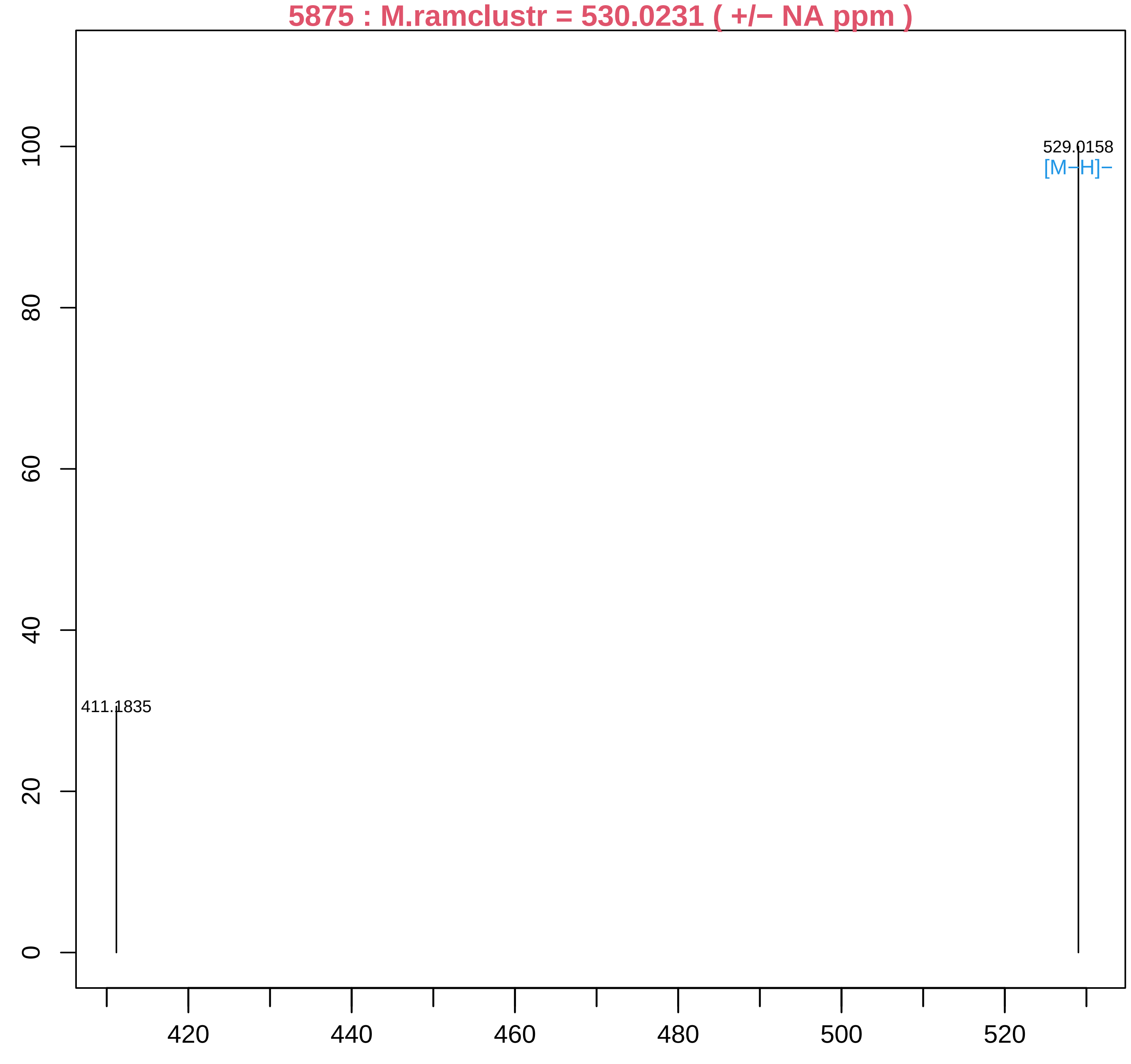

5875 : M.ramclustr = 530.0231 (+/− NA ppm)
529.0158
[M−H]−
411.1835
0
20
40
60
80
100
420
440
460
480
500
520

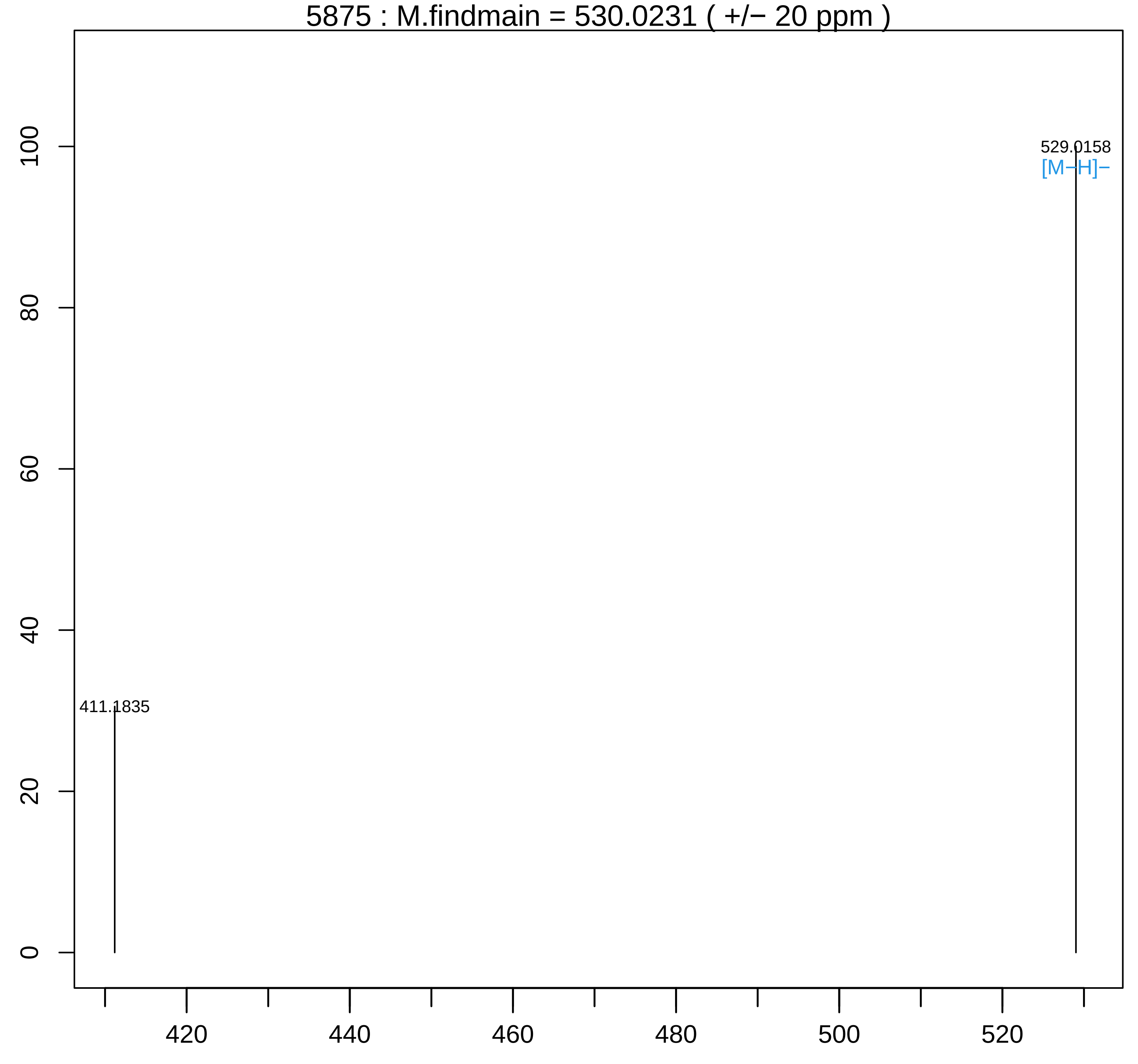

5875 : M.findmain = 530.0231 (+/− 20 ppm)
529.0158
[M−H]−
411.1835
0
20
40
60
80
100
420
440
460
480
500
520

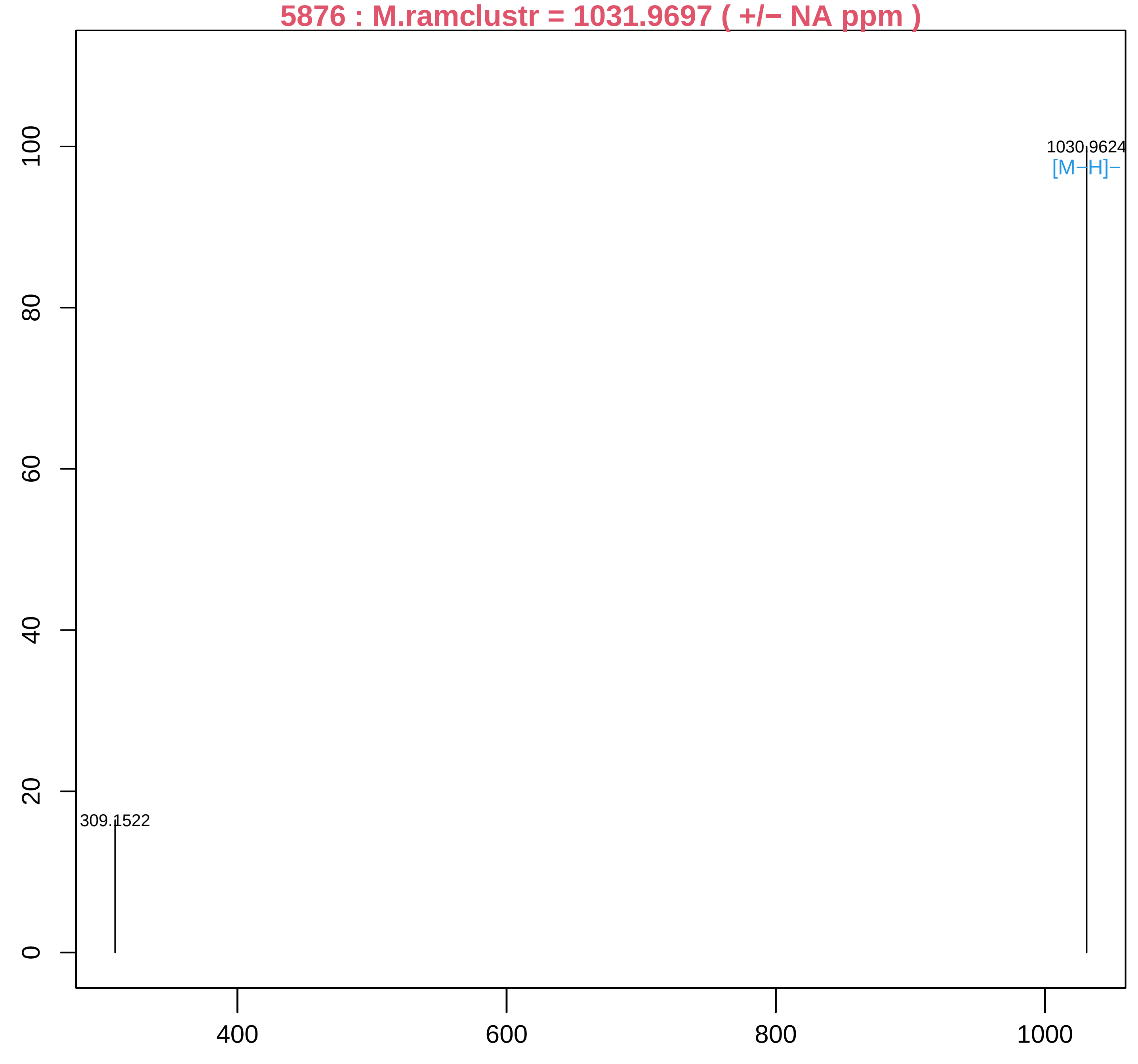
5876 : M.ramclustr = 1031.9697 (+/− NA ppm)
1030.9624
[M−H]−
309.1522
0
20
40
60
80
100
400
600
800
1000
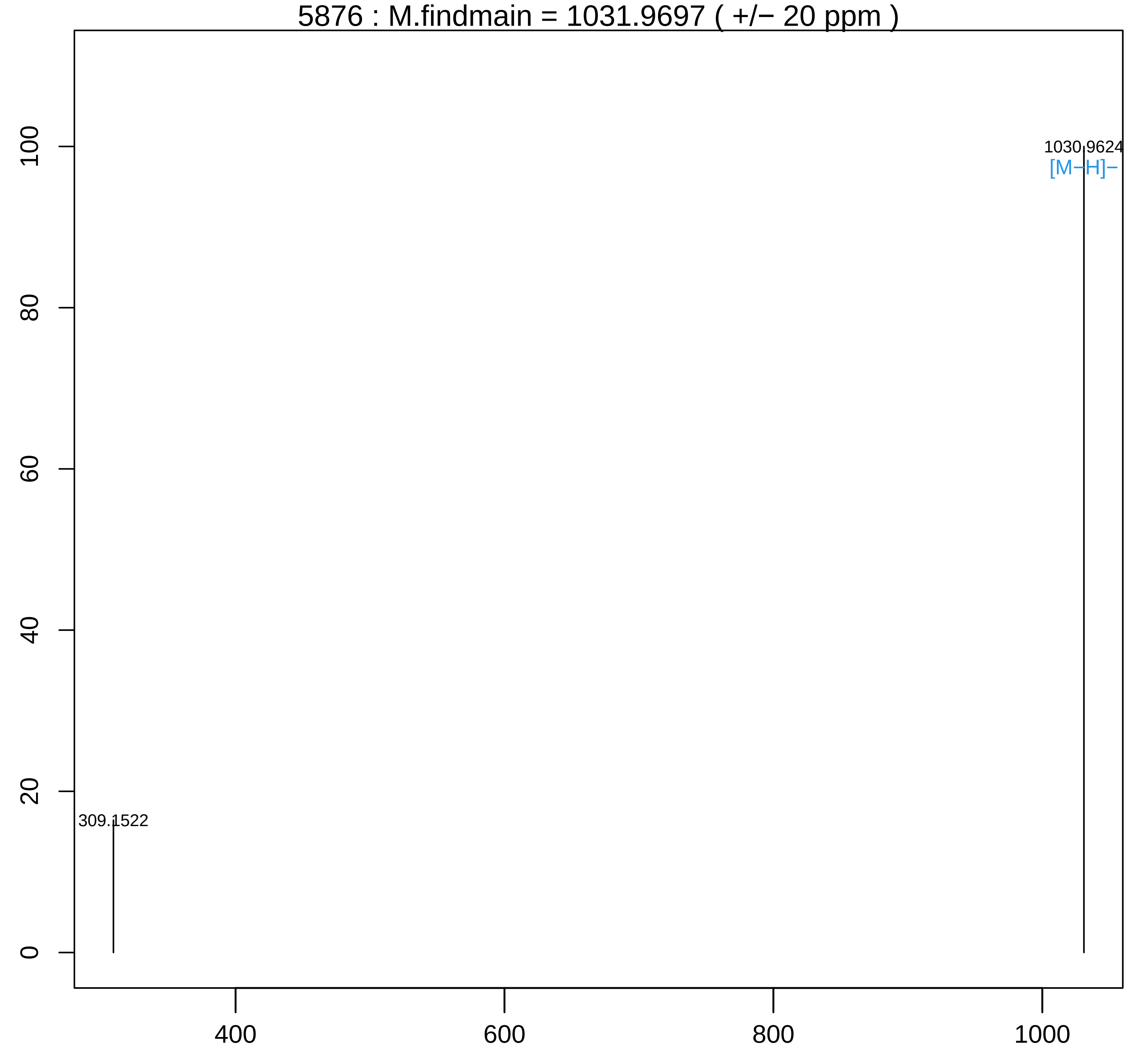
5876 : M.findmain = 1031.9697 (+/− 20 ppm)
1030.9624
[M−H]−
309.1522
0
20
40
60
80
100
400
600
800
1000

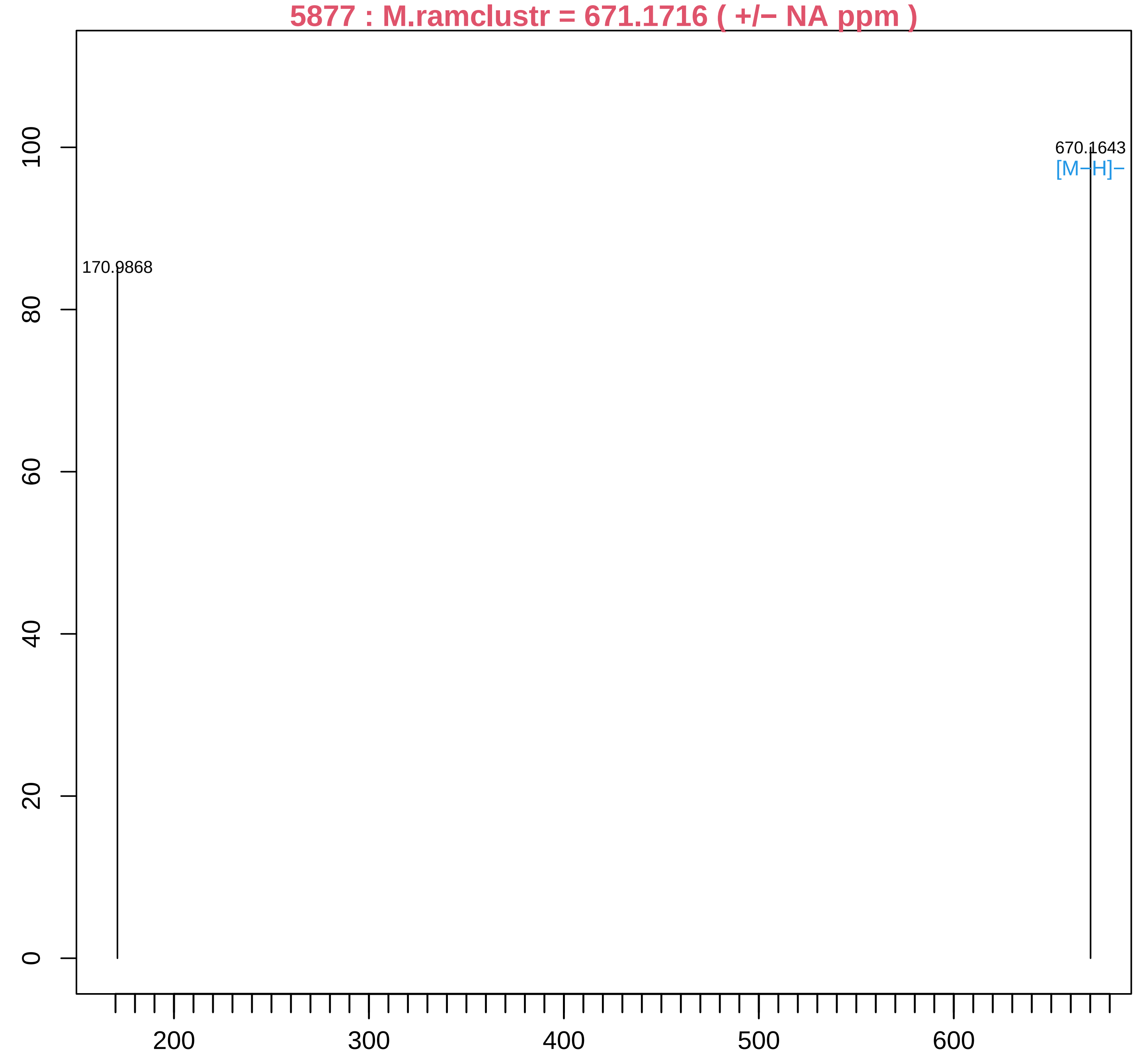
5877 : M.ramclustr = 671.1716 (+/− NA ppm)
170.9868
670.1643
[M−H]−
0
20
40
60
80
100
200
300
400
500
600

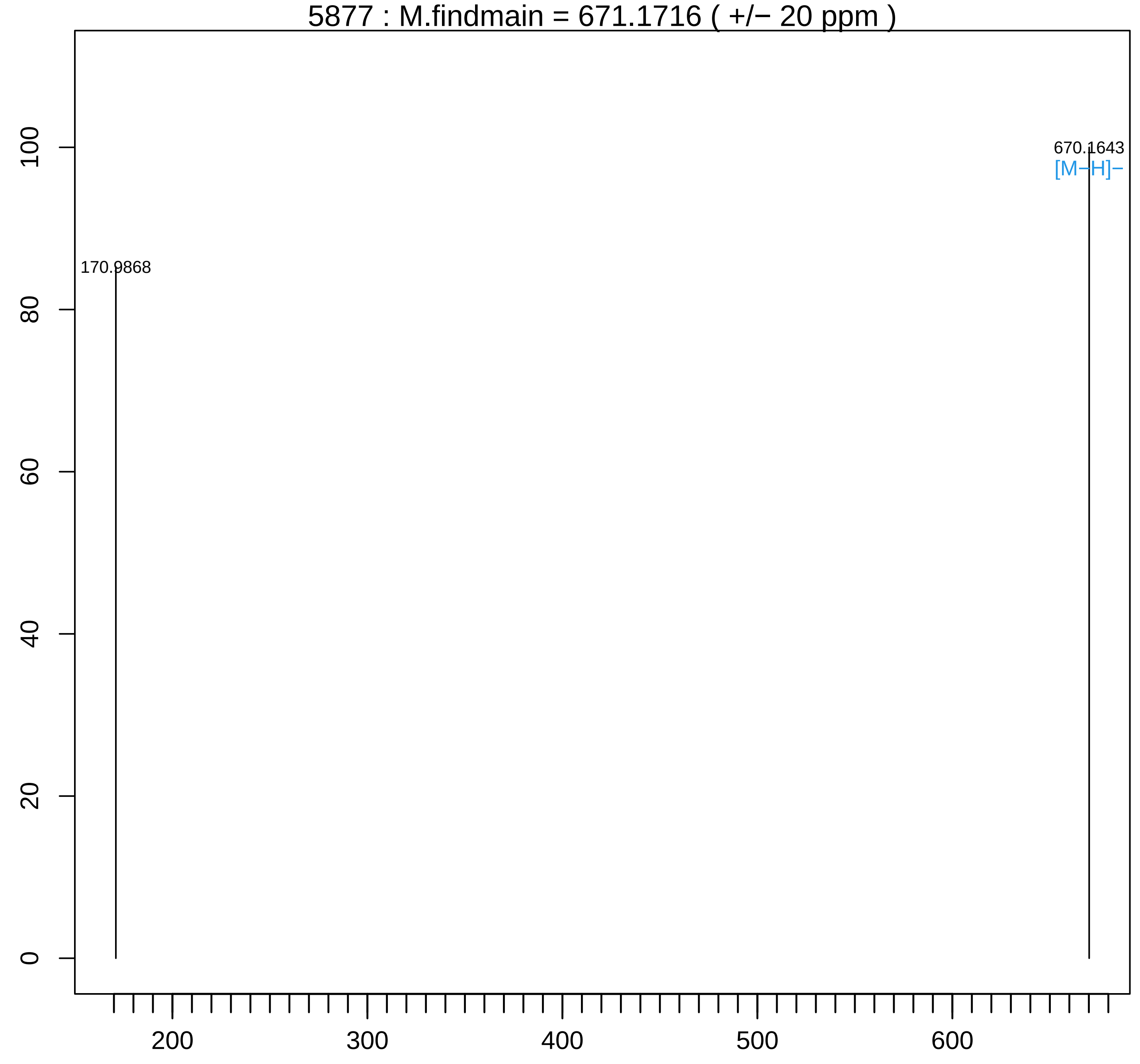
5877 : M.findmain = 671.1716 (+/− 20 ppm)
170.9868
670.1643
[M−H]−
0
20
40
60
80
100
200
300
400
500
600

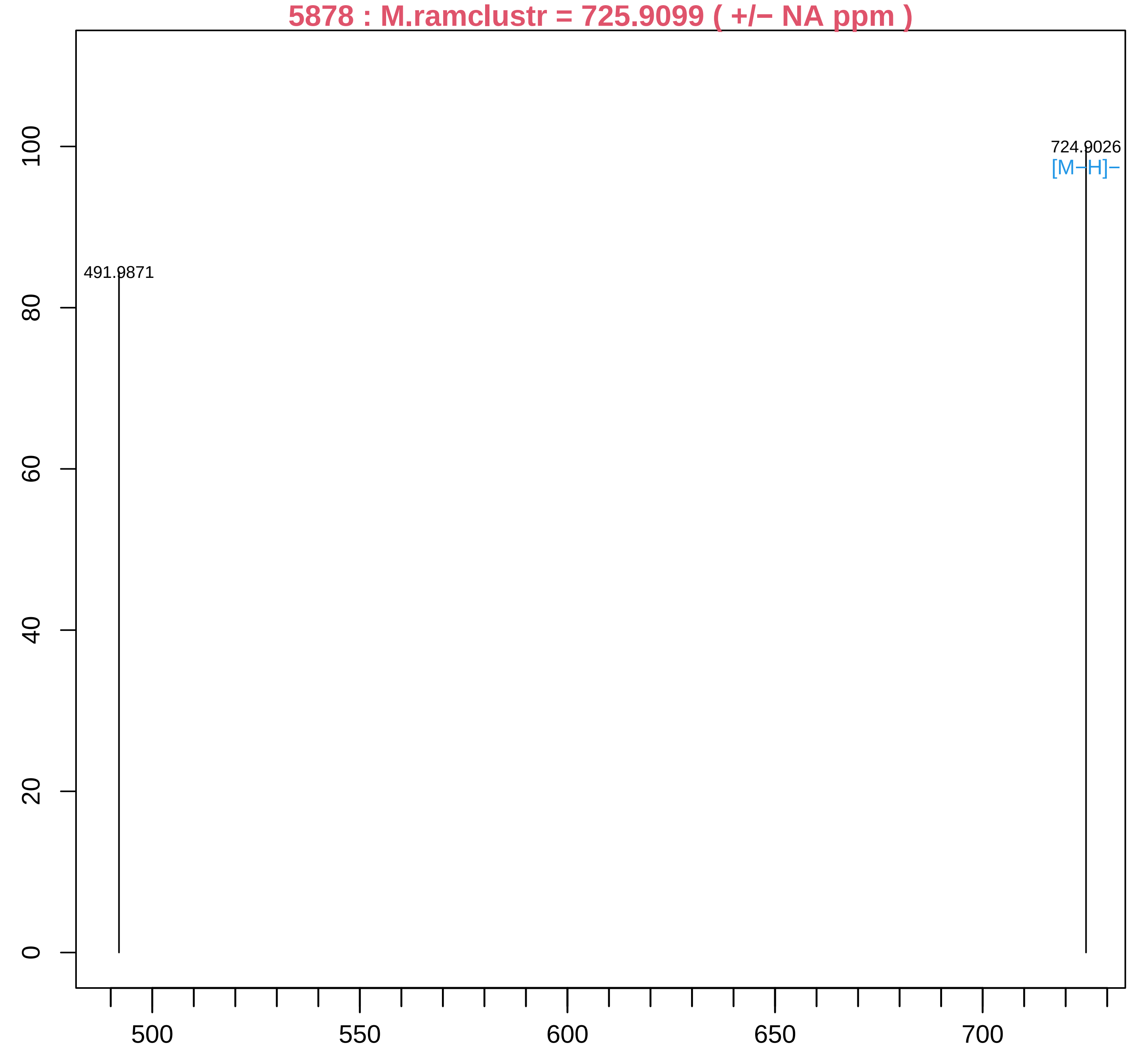
5878 : M.ramclustr = 725.9099 (+/− NA ppm)
724.9026
[M−H]−
491.9871
0
20
40
60
80
100
500
550
600
650
700

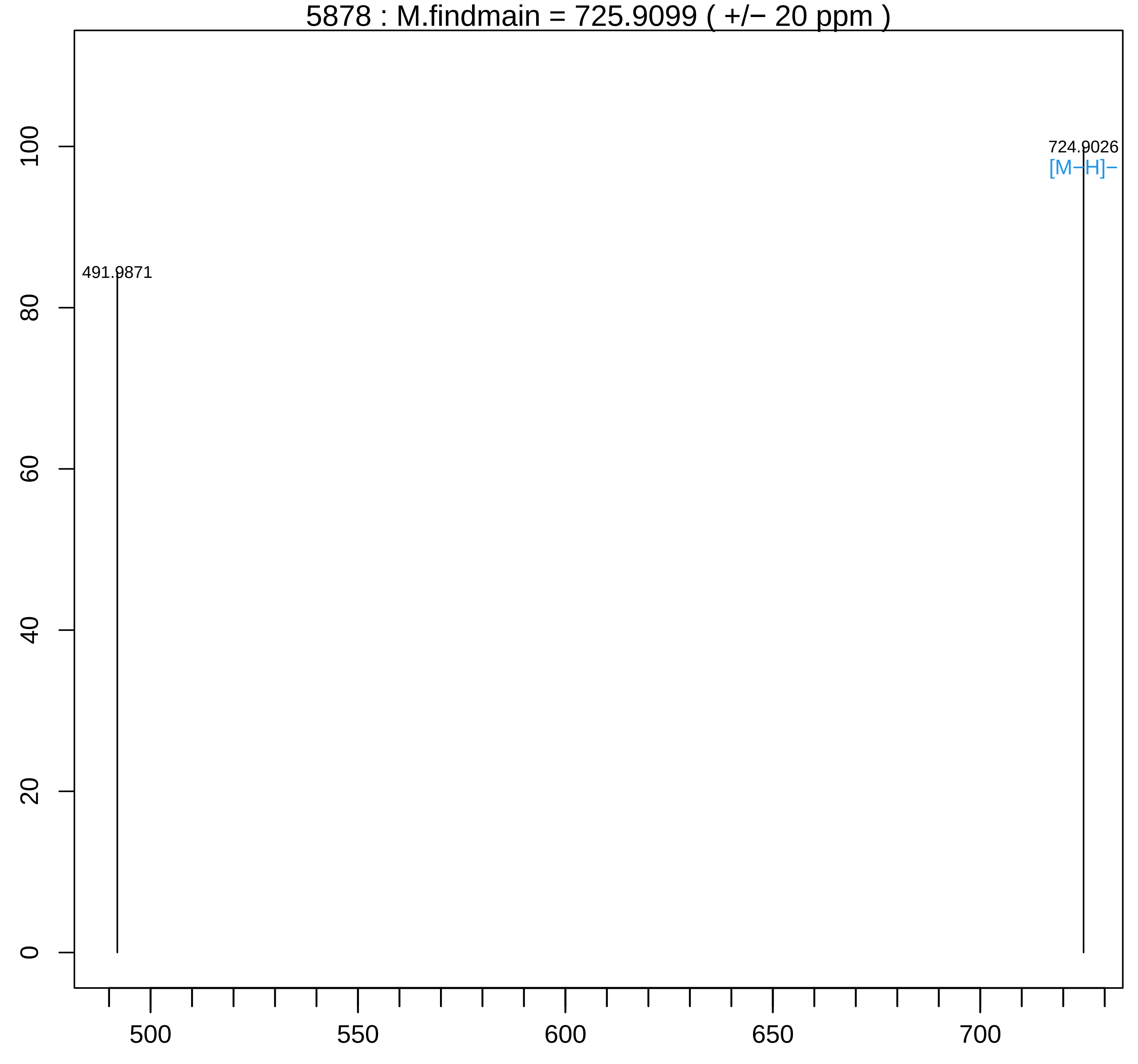
5878 : M.findmain = 725.9099 (+/− 20 ppm)
724.9026
[M−H]−
491.9871
0
20
40
60
80
100
500
550
600
650
700

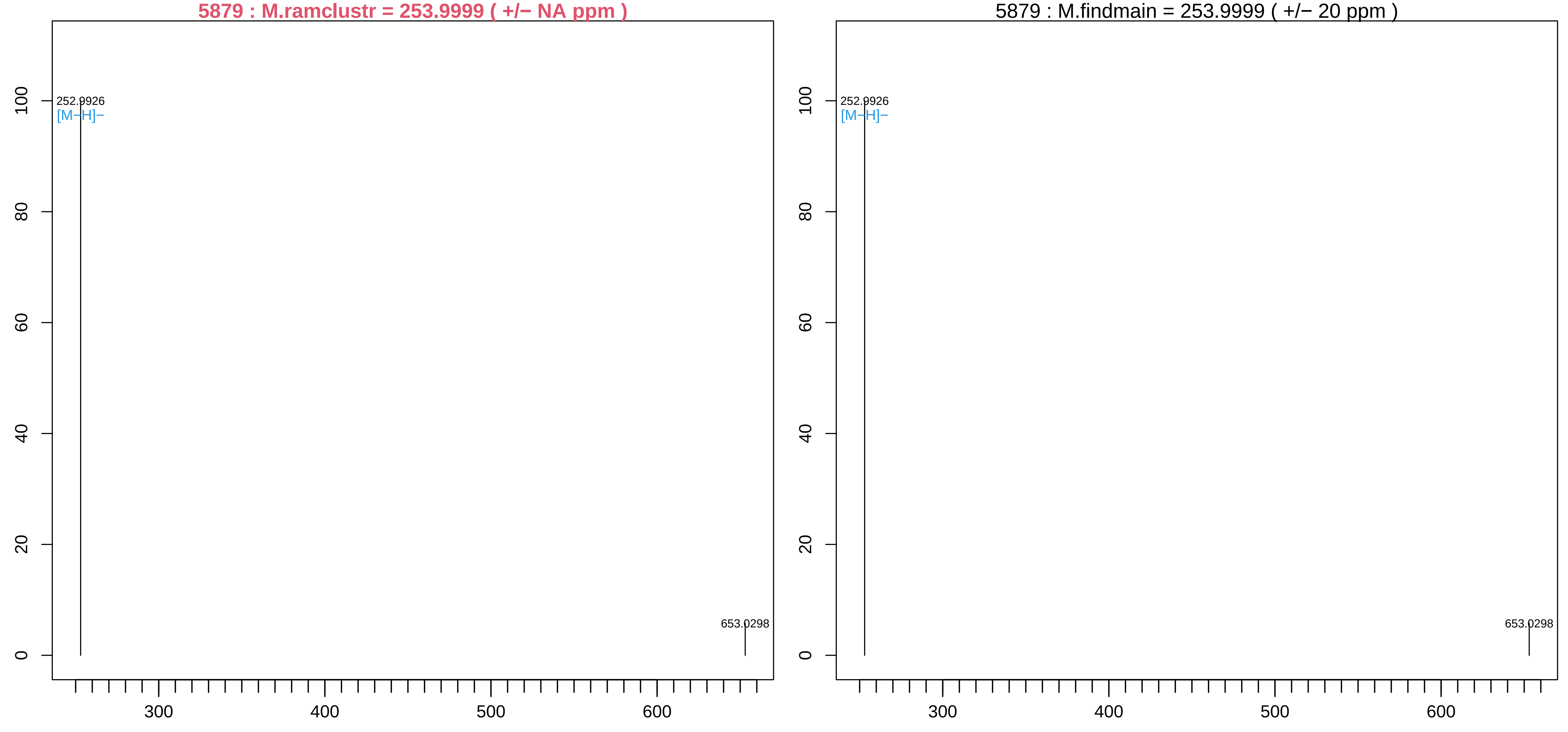

5879 : M.ramclustr = 253.9999 (+/− NA ppm)
252.9926
[M−H]−
653.0298
5879 : M.findmain = 253.9999 (+/− 20 ppm)
252.9926
[M−H]−
653.0298
0
20
40
60
80
100
300
400
500
600

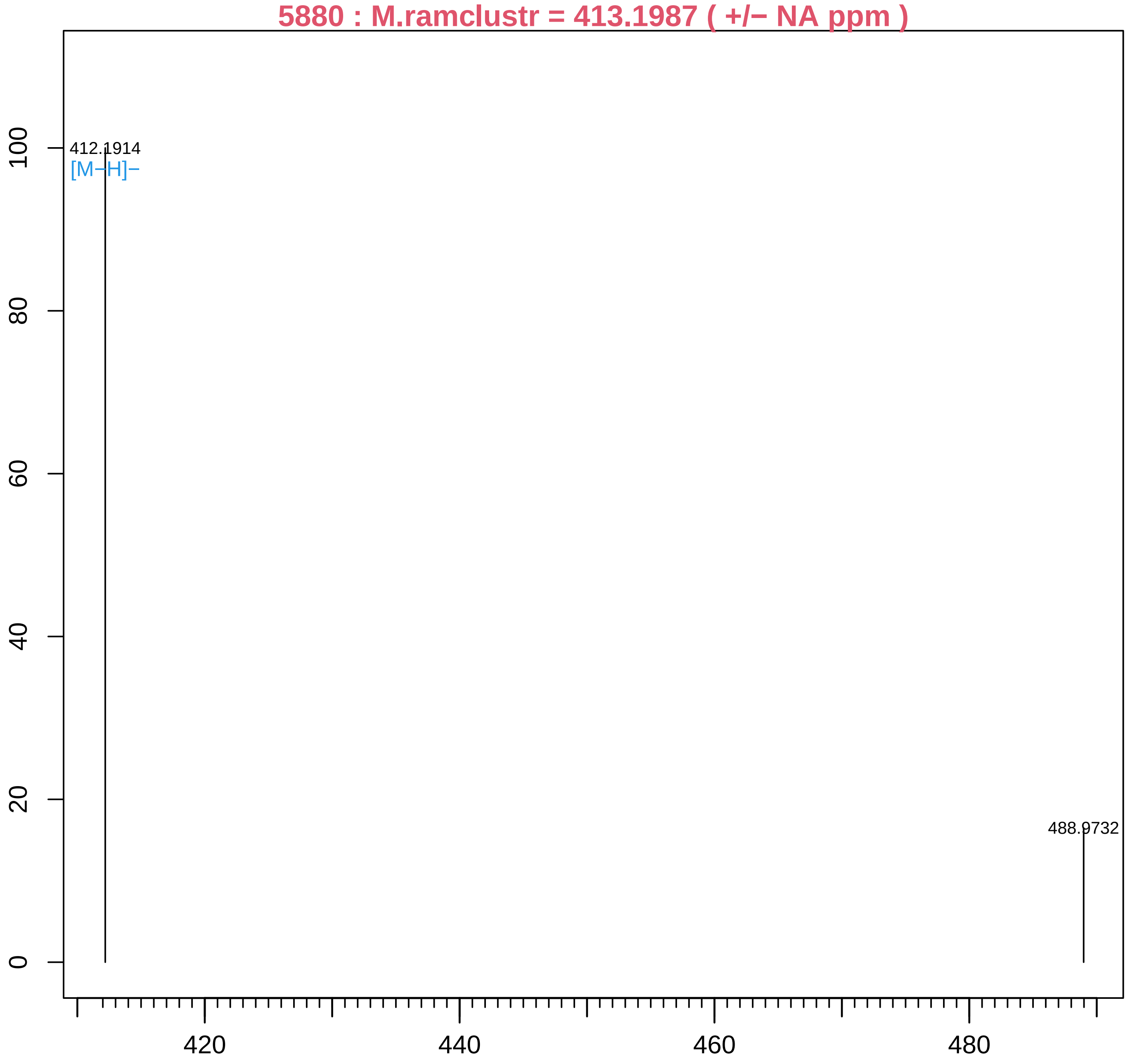
5880 : M.ramclustr = 413.1987 (+/− NA ppm)
412.1914
[M−H]−
488.9732
0
20
40
60
80
100
420
440
460
480

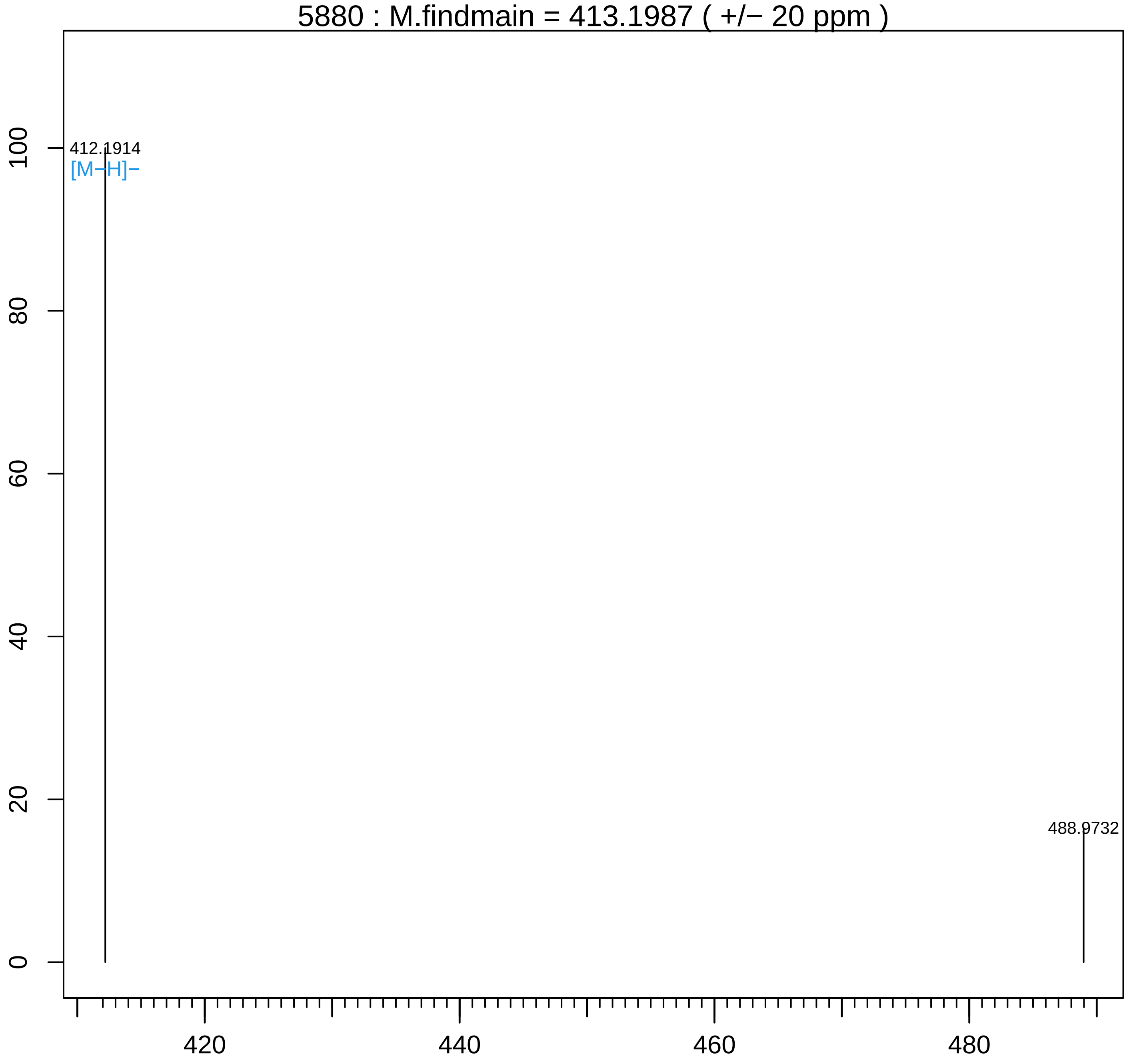
5880 : M.findmain = 413.1987 (+/− 20 ppm)
412.1914
[M−H]−
488.9732
0
20
40
60
80
100
420
440
460
480

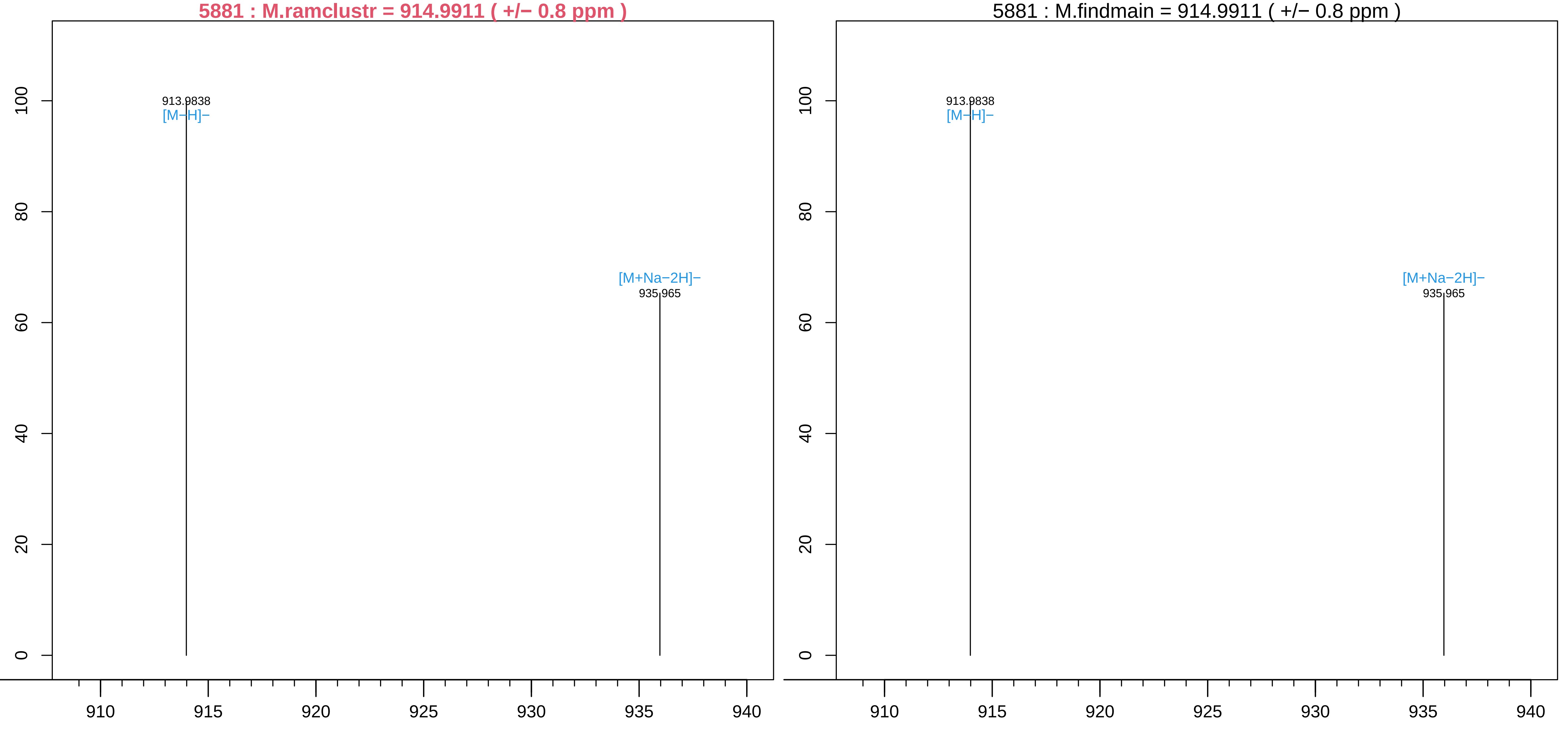

5881 : M.ramclustr = 914.9911 (+/− 0.8 ppm)
913.9838
[M−H]−
[M+Na−2H]−
935.965
5881 : M.findmain = 914.9911 (+/− 0.8 ppm)
913.9838
[M−H]−
[M+Na−2H]−
935.965
0
20
40
60
80
100
910
915
920
925
930
935
940

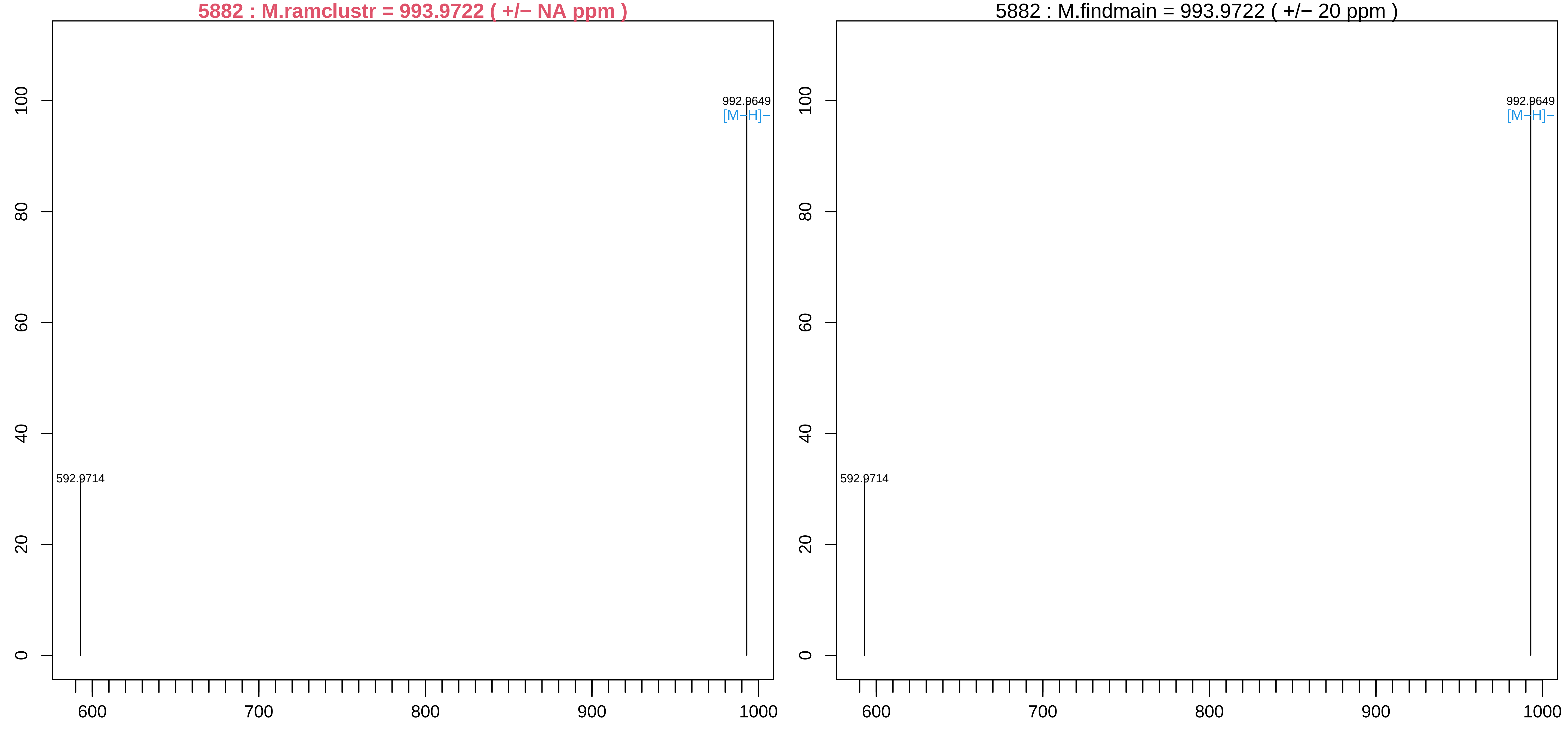

5882 : M.ramclustr = 993.9722 (+/− NA ppm)
992.9649
[M−H]−
592.9714
0
20
40
60
80
100
600
700
800
900
1000
5882 : M.findmain = 993.9722 (+/− 20 ppm)
992.9649
[M−H]−
592.9714
0
20
40
60
80
100
600
700
800
900
1000

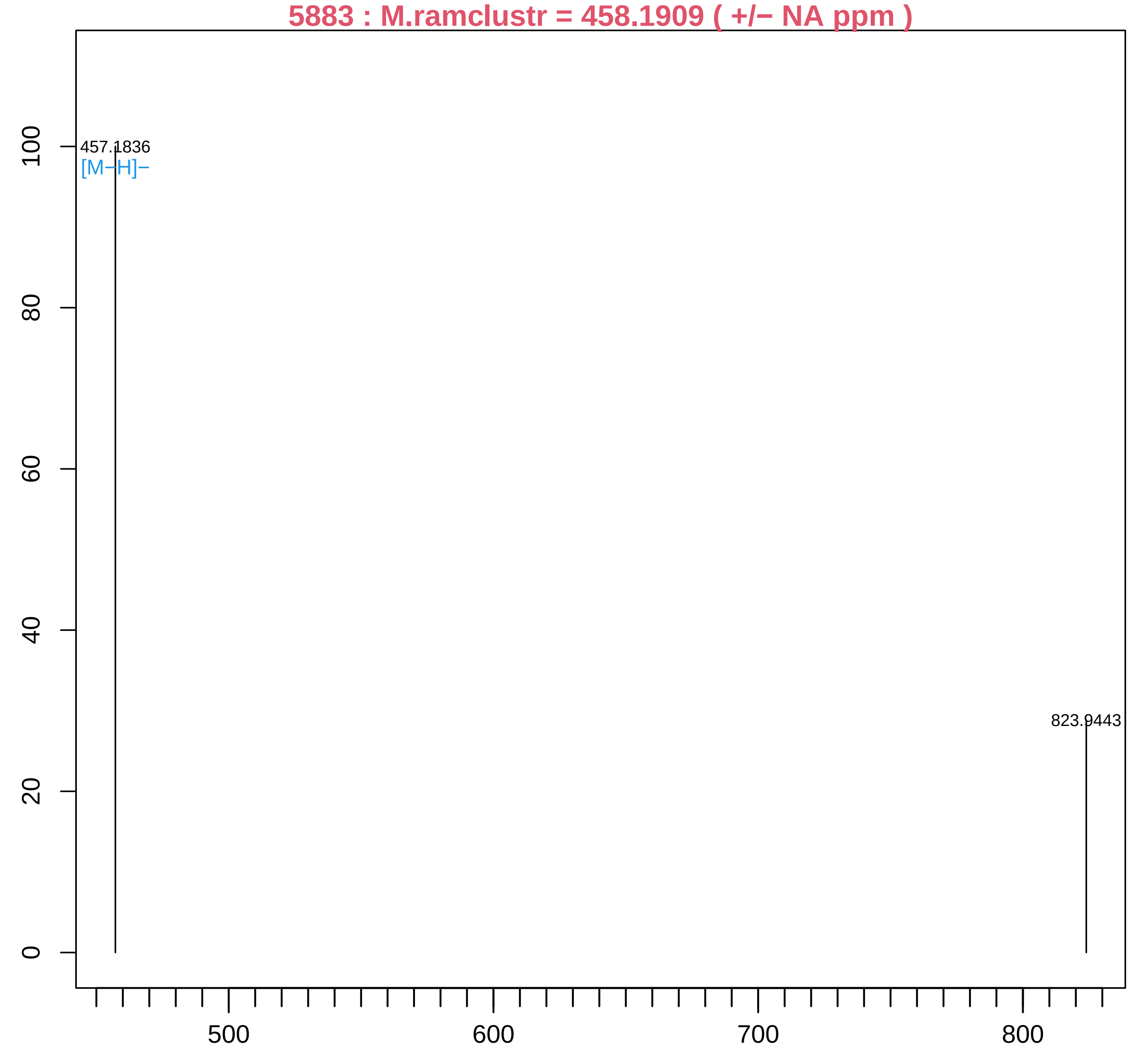
5883 : M.ramclustr = 458.1909 (+/− NA ppm)
457.1836
[M−H]−
823.9443
0
20
40
60
80
100
500
600
700
800

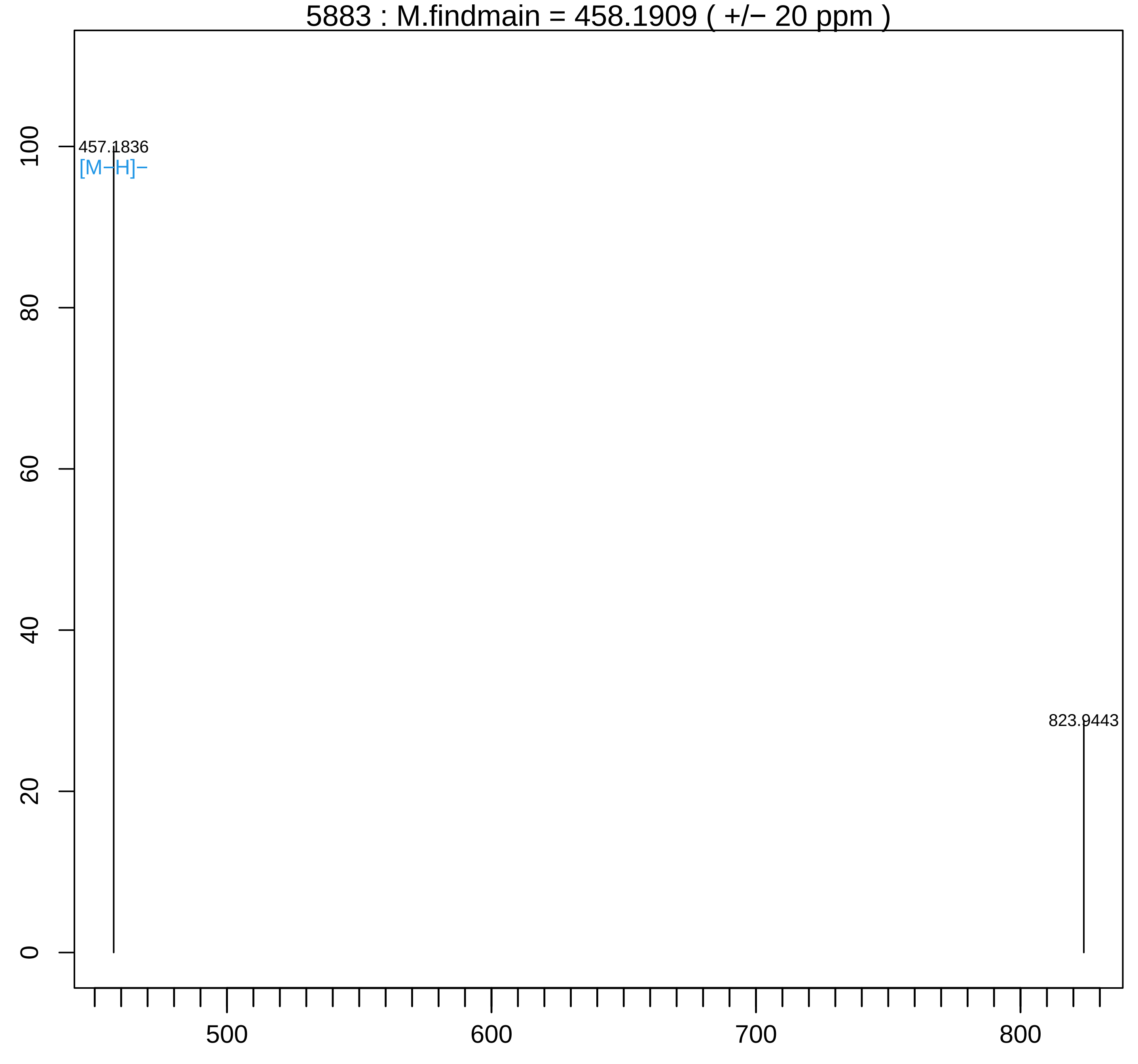
5883 : M.findmain = 458.1909 (+/− 20 ppm)
457.1836
[M−H]−
823.9443
0
20
40
60
80
100
500
600
700
800

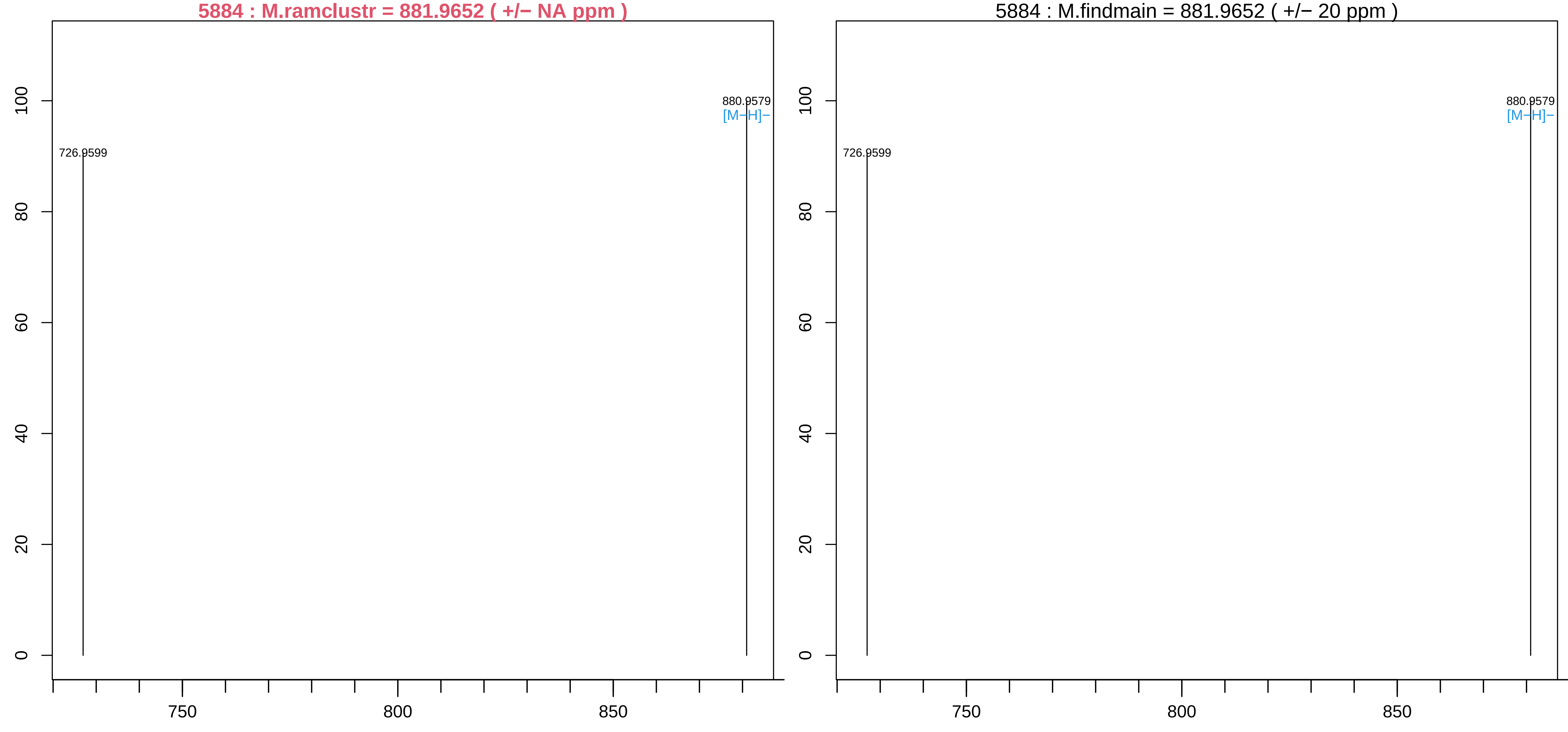
5884 : M.ramclustr = 881.9652 (+/− NA ppm)
5884 : M.findmain = 881.9652 (+/− 20 ppm)
880.9579
[M−H]−
726.9599
0
20
40
60
80
100
750
800
850

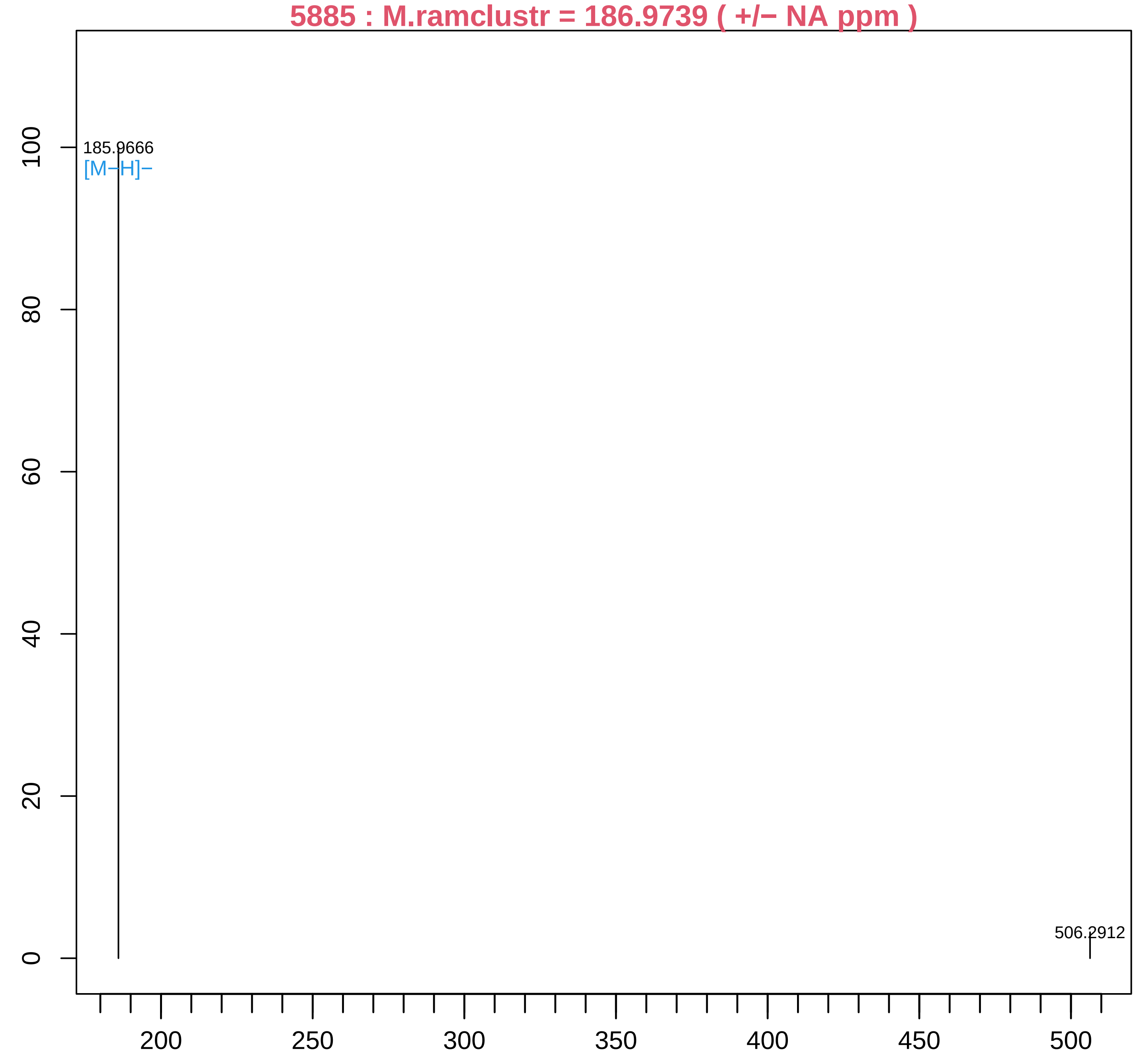
5885 : M.ramclustr = 186.9739 (+/− NA ppm)
185.9666
[M−H]−
506.2912
0
20
40
60
80
100
200
250
300
350
400
450
500
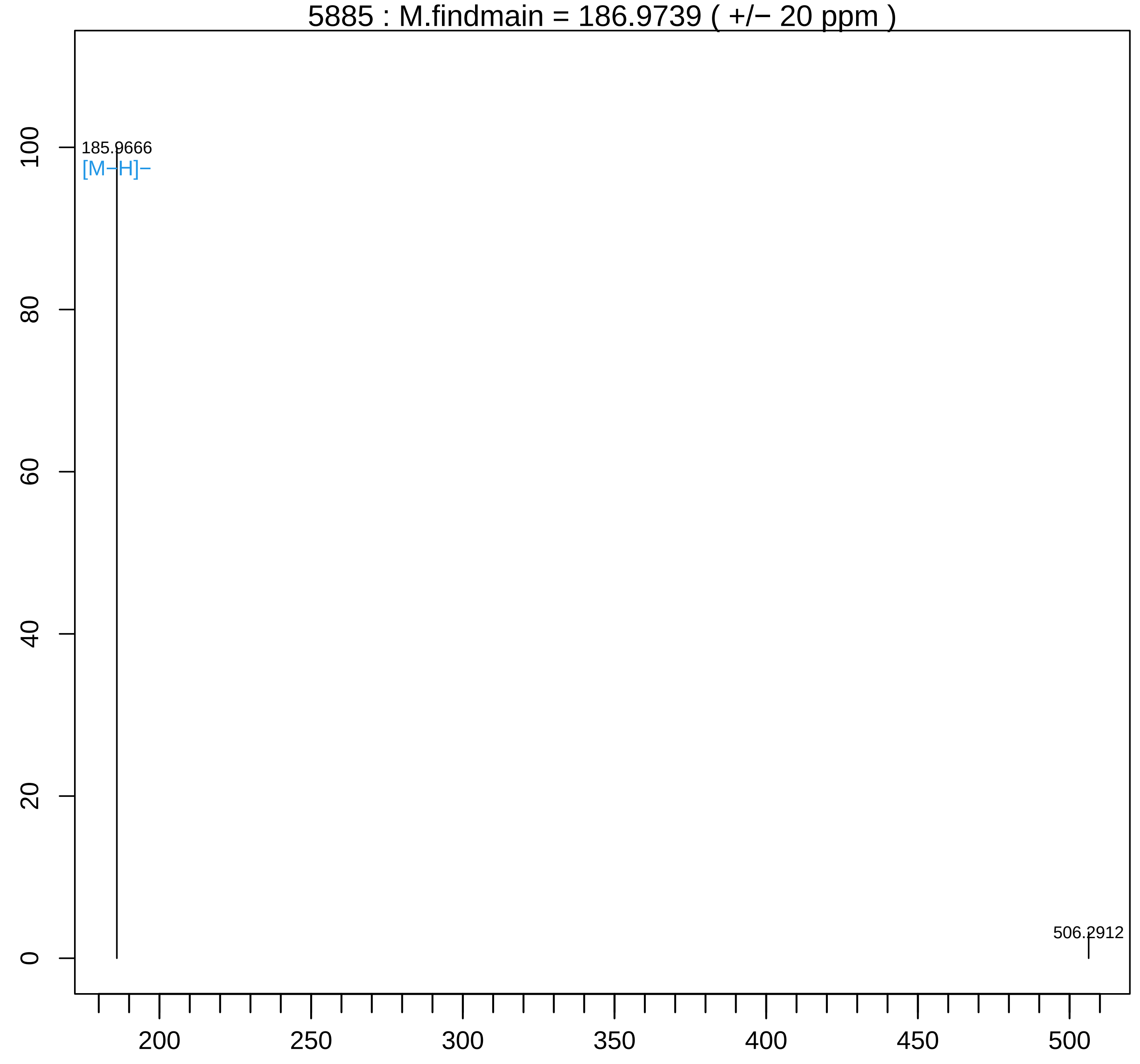
5885 : M.findmain = 186.9739 (+/− 20 ppm)
185.9666
[M−H]−
506.2912
0
20
40
60
80
100
200
250
300
350
400
450
500

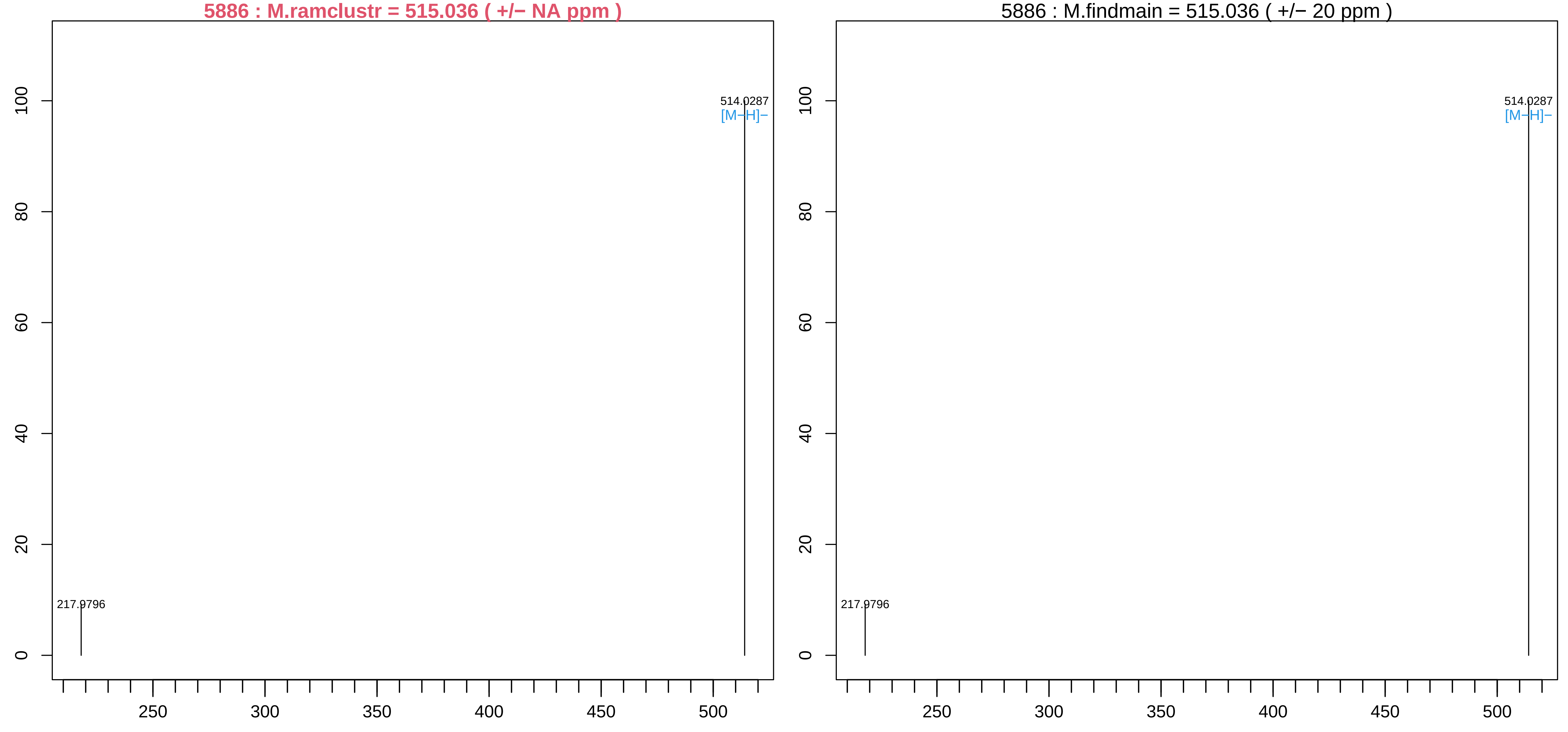

5886 : M.ramclustr = 515.036 (+/− NA ppm)
514.0287
[M−H]−
217.9796
5886 : M.findmain = 515.036 (+/− 20 ppm)
514.0287
[M−H]−
217.9796
0
20
40
60
80
100
250
300
350
400
450
500

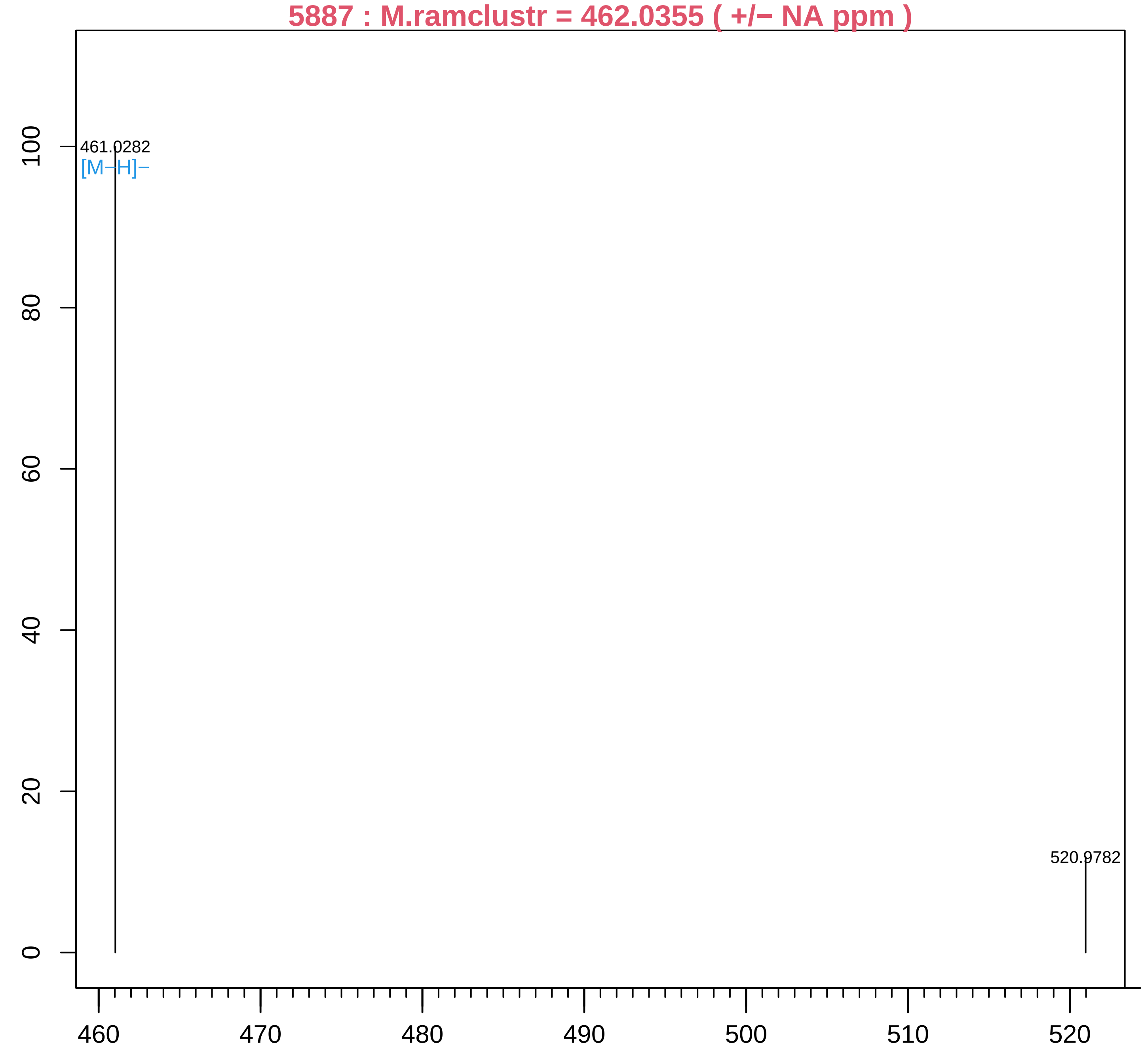
5887 : M.ramclustr = 462.0355 (+/− NA ppm)
461.0282
[M−H]−
520.9782
0
20
40
60
80
100
460
470
480
490
500
510
520

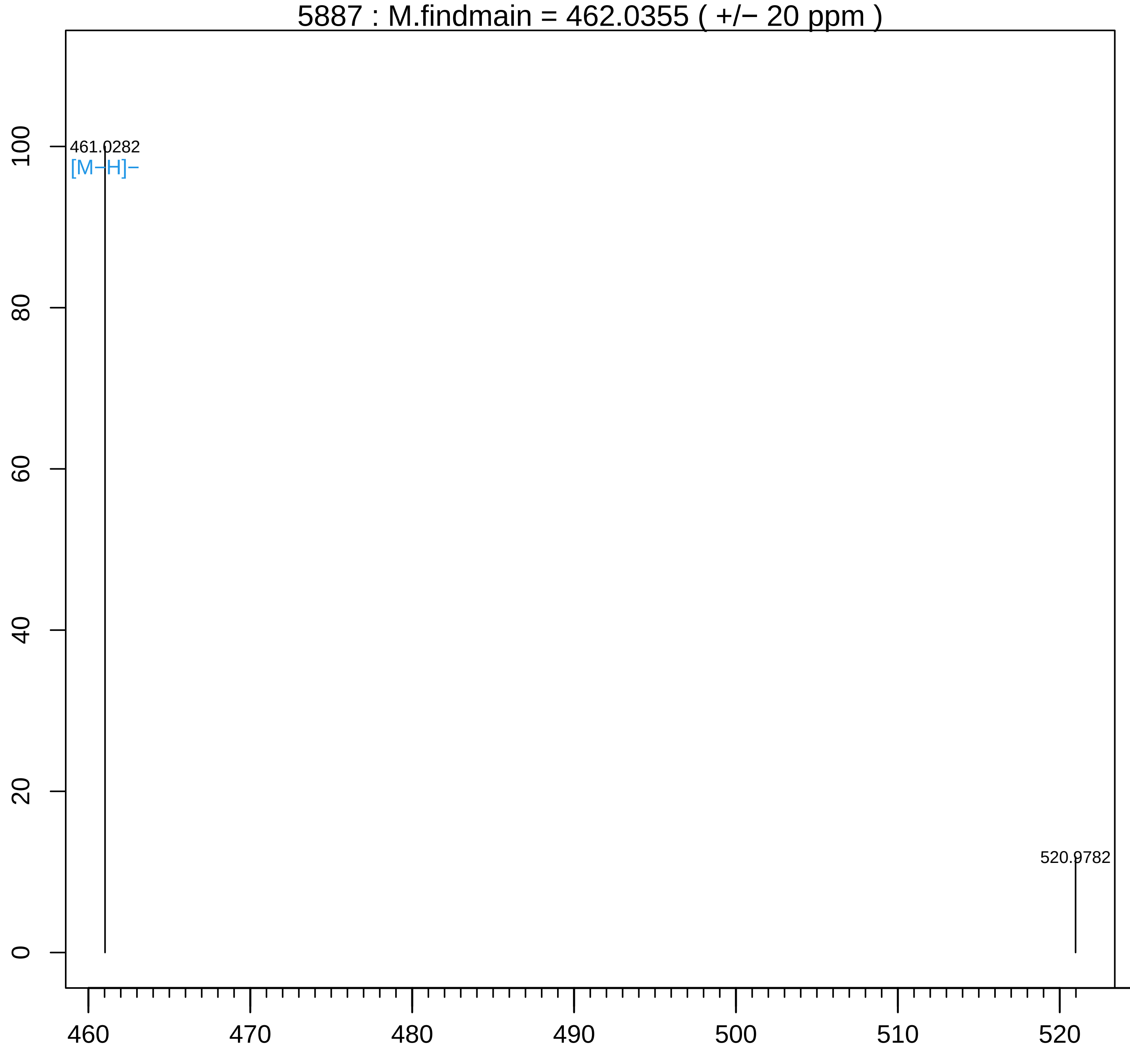
5887 : M.findmain = 462.0355 (+/− 20 ppm)
461.0282
[M−H]−
520.9782
0
20
40
60
80
100
460
470
480
490
500
510
520

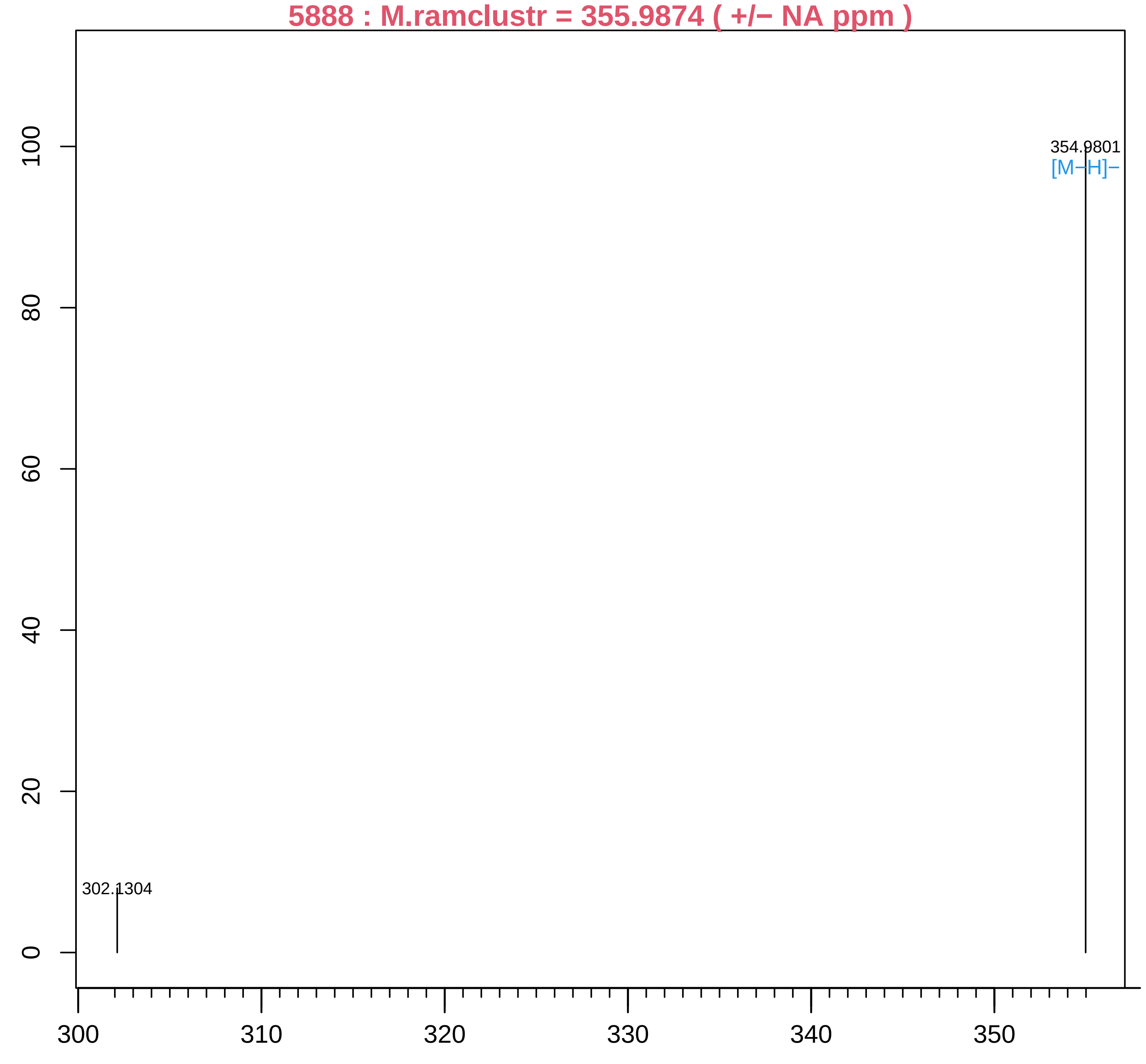

5888 : M.ramclustr = 355.9874 (+/− NA ppm)
354.9801
[M−H]−
302.1304
0
20
40
60
80
100
300
310
320
330
340
350

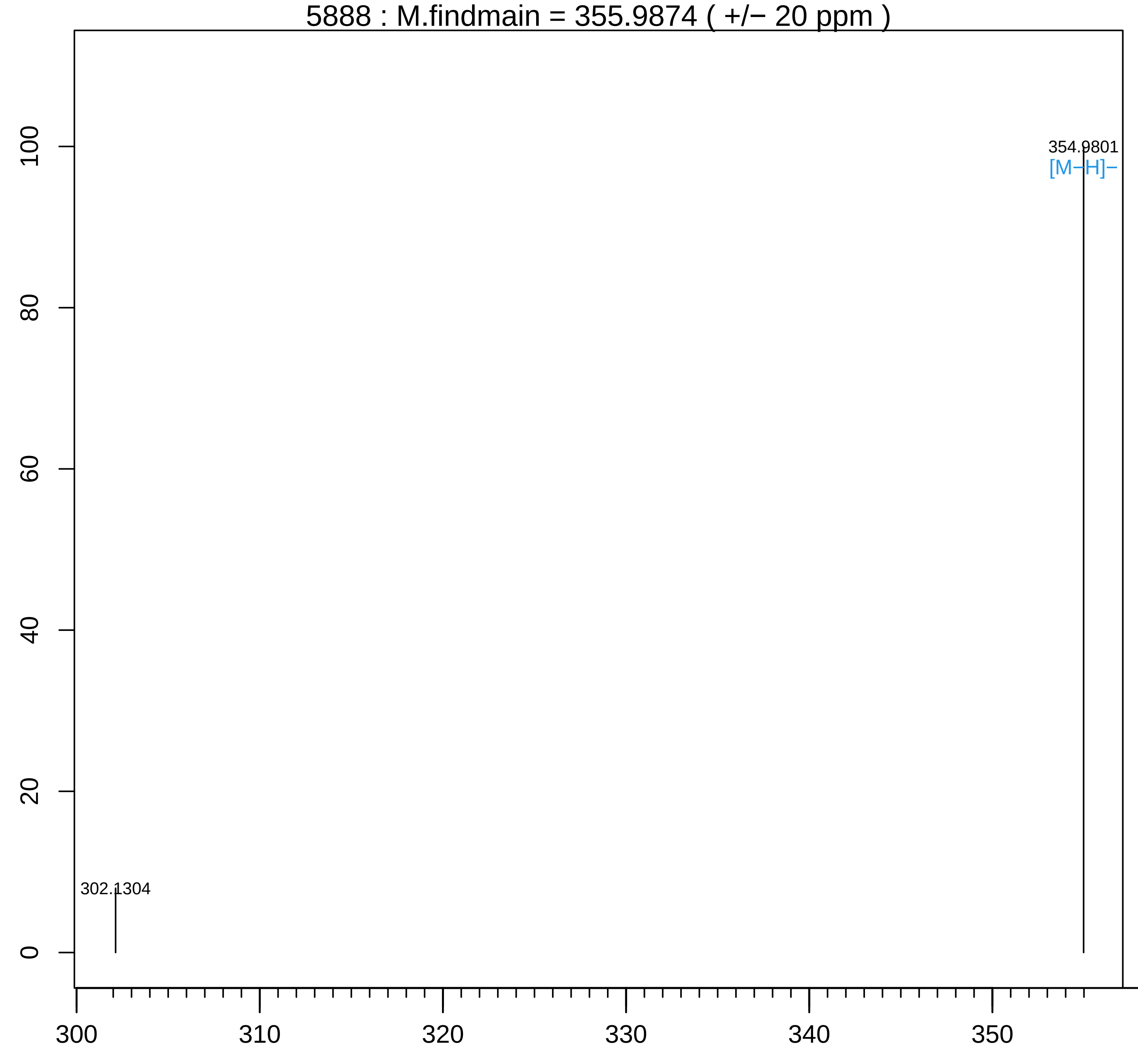

5888 : M.findmain = 355.9874 (+/− 20 ppm)
354.9801
[M−H]−
302.1304
0
20
40
60
80
100
300
310
320
330
340
350

5889 : M.ramclustr = 535.9821 (+/− NA ppm)

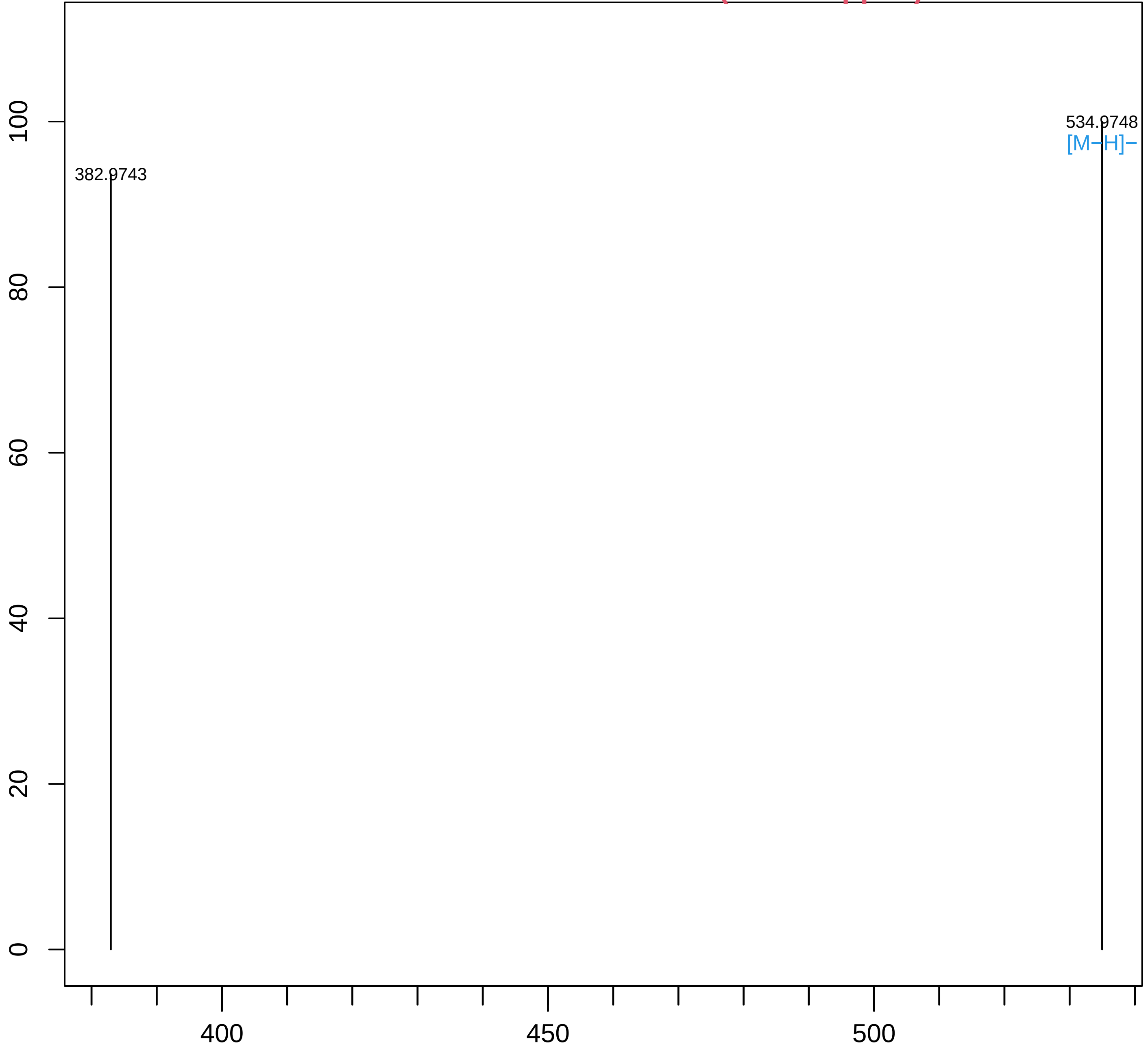

5889 : M.findmain = 535.9821 (+/− 20 ppm)

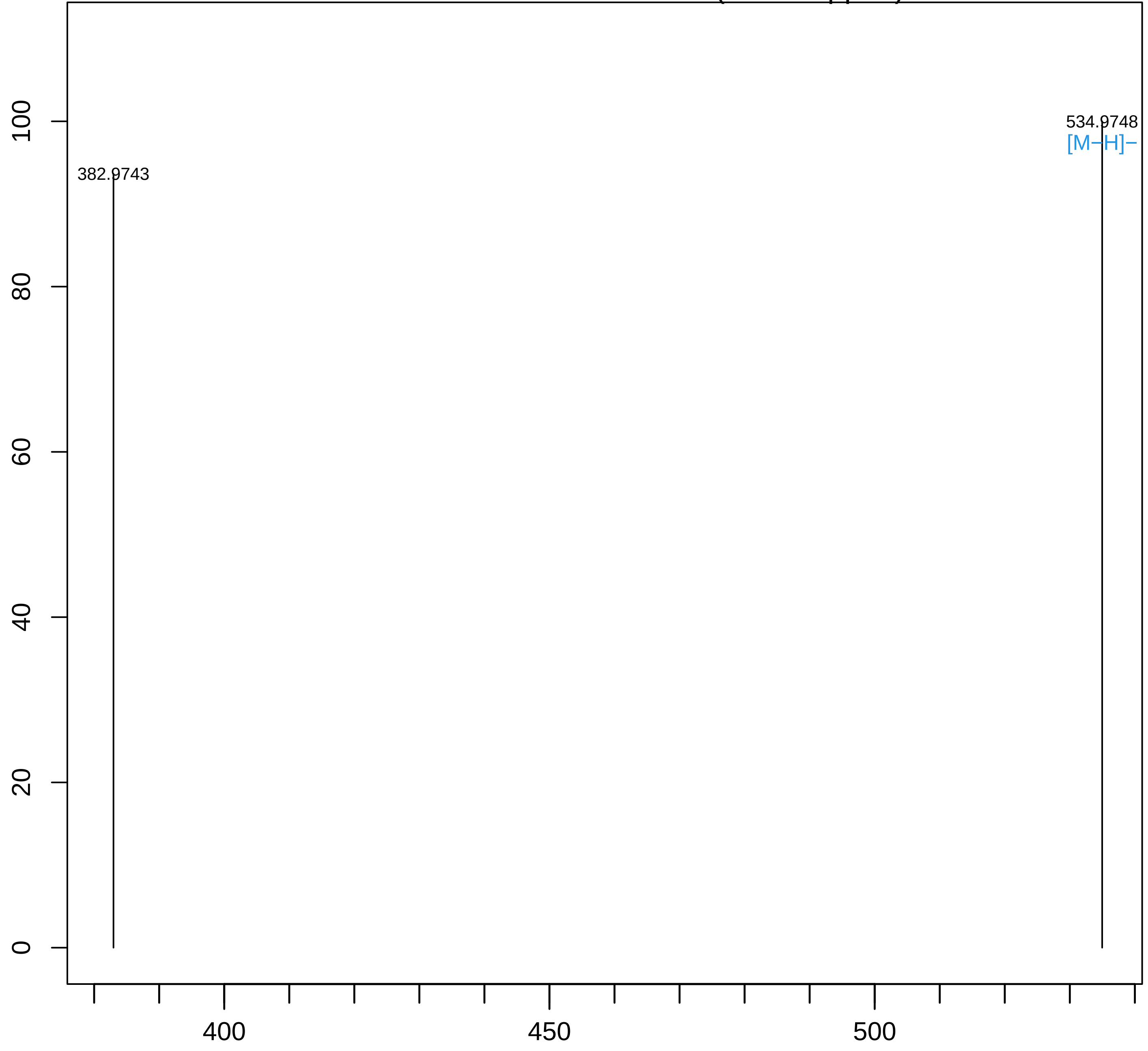

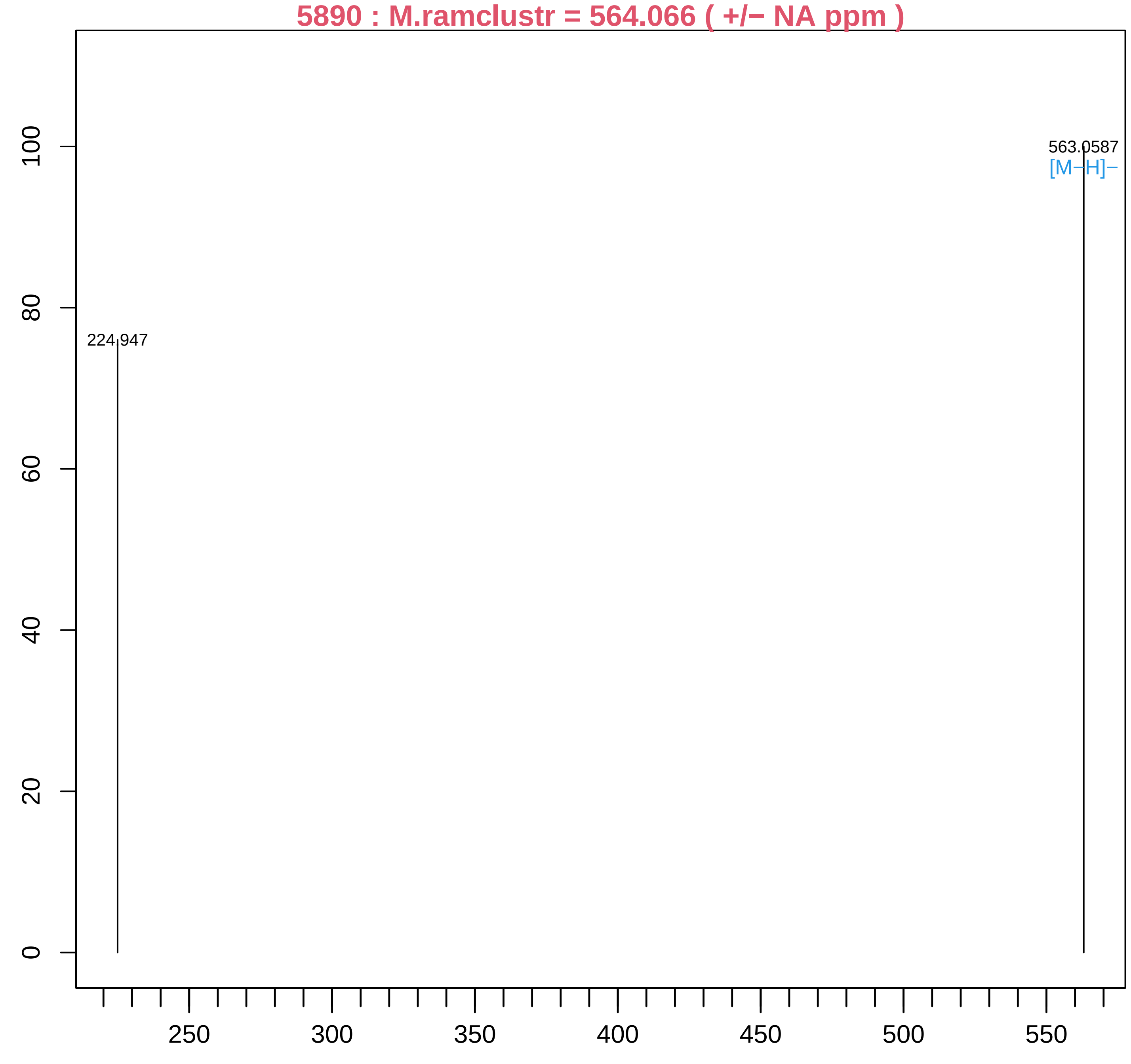

5890 : M.ramclustr = 564.066 (+/− NA ppm)
224.947
563.0587
[M−H]−
0
20
40
60
80
100
250
300
350
400
450
500
550

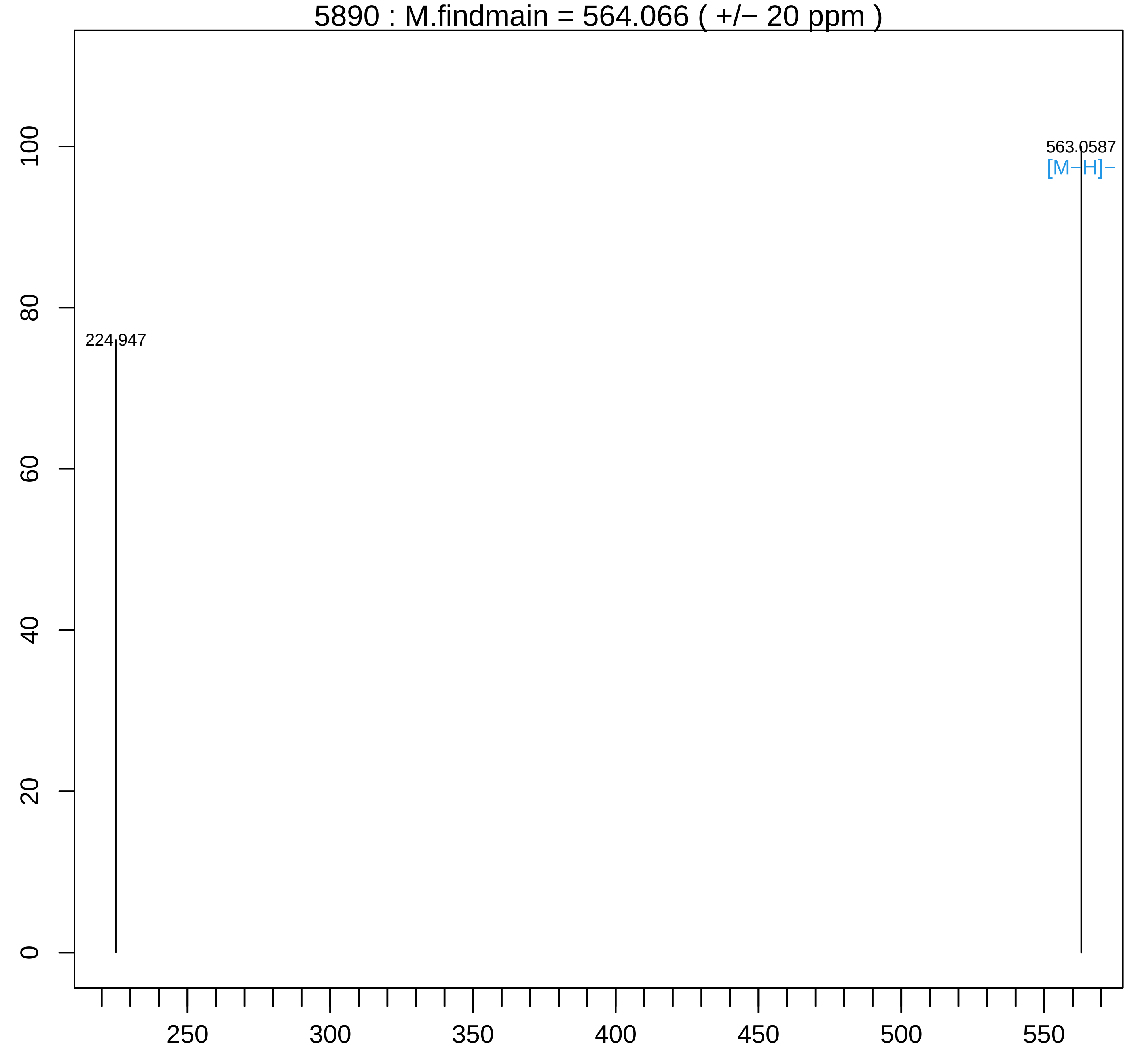

5890 : M.findmain = 564.066 (+/− 20 ppm)
224.947
563.0587
[M−H]−
0
20
40
60
80
100
250
300
350
400
450
500
550

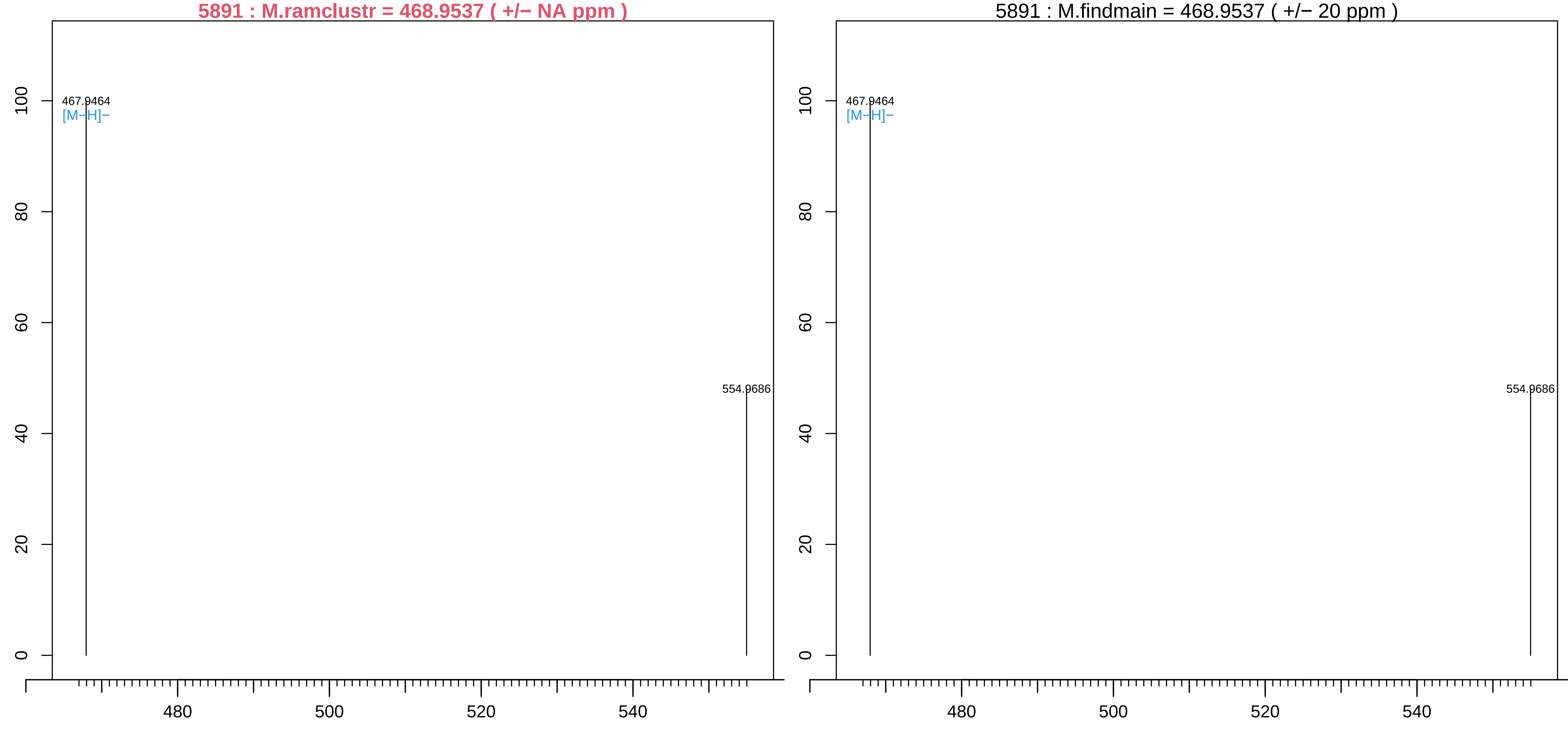
5891 : M.ramclustr = 468.9537 (+/− NA ppm)
467.9464
[M−H]−
554.9686
0
20
40
60
80
100
480
500
520
540
5891 : M.findmain = 468.9537 (+/− 20 ppm)
467.9464
[M−H]−
554.9686
0
20
40
60
80
100
480
500
520
540

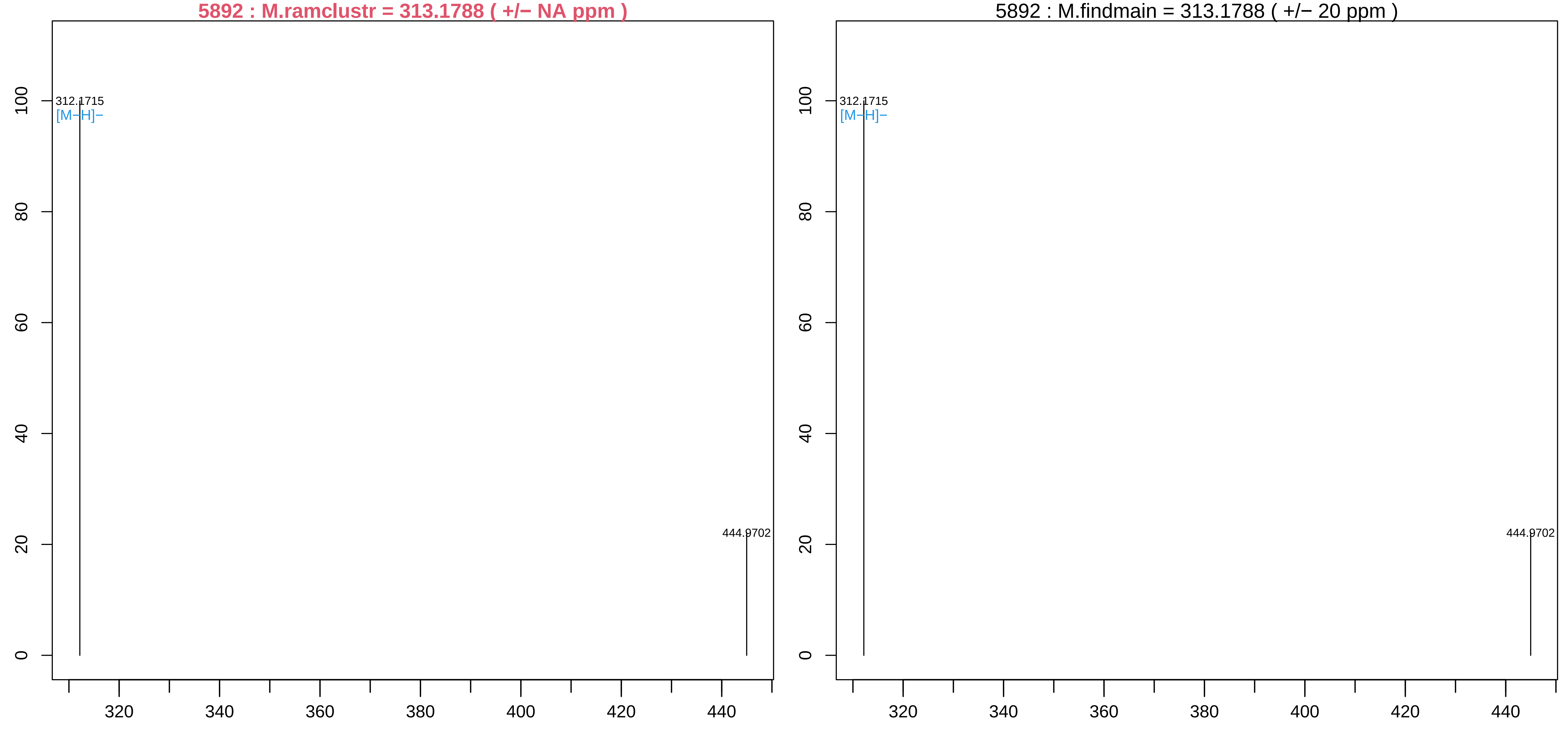
5892 : M.ramclustr = 313.1788 (+/− NA ppm)
5892 : M.findmain = 313.1788 (+/− 20 ppm)
312.1715
[M−H]−
444.9702
0
20
40
60
80
100
320
340
360
380
400
420
440

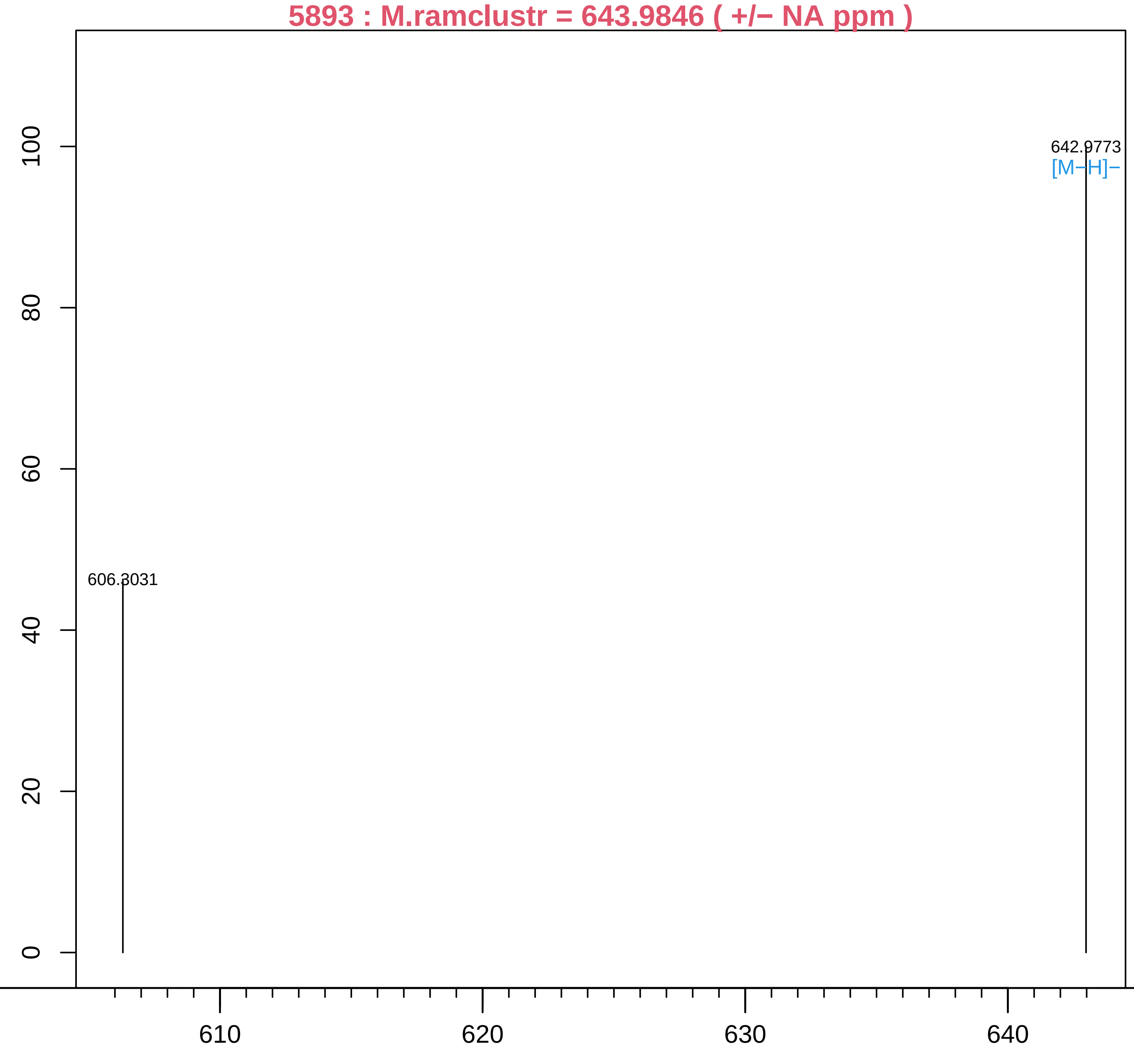
5893 : M.ramclustr = 643.9846 (+/− NA ppm)
642.9773
[M−H]−
606.3031
0
20
40
60
80
100
610
620
630
640

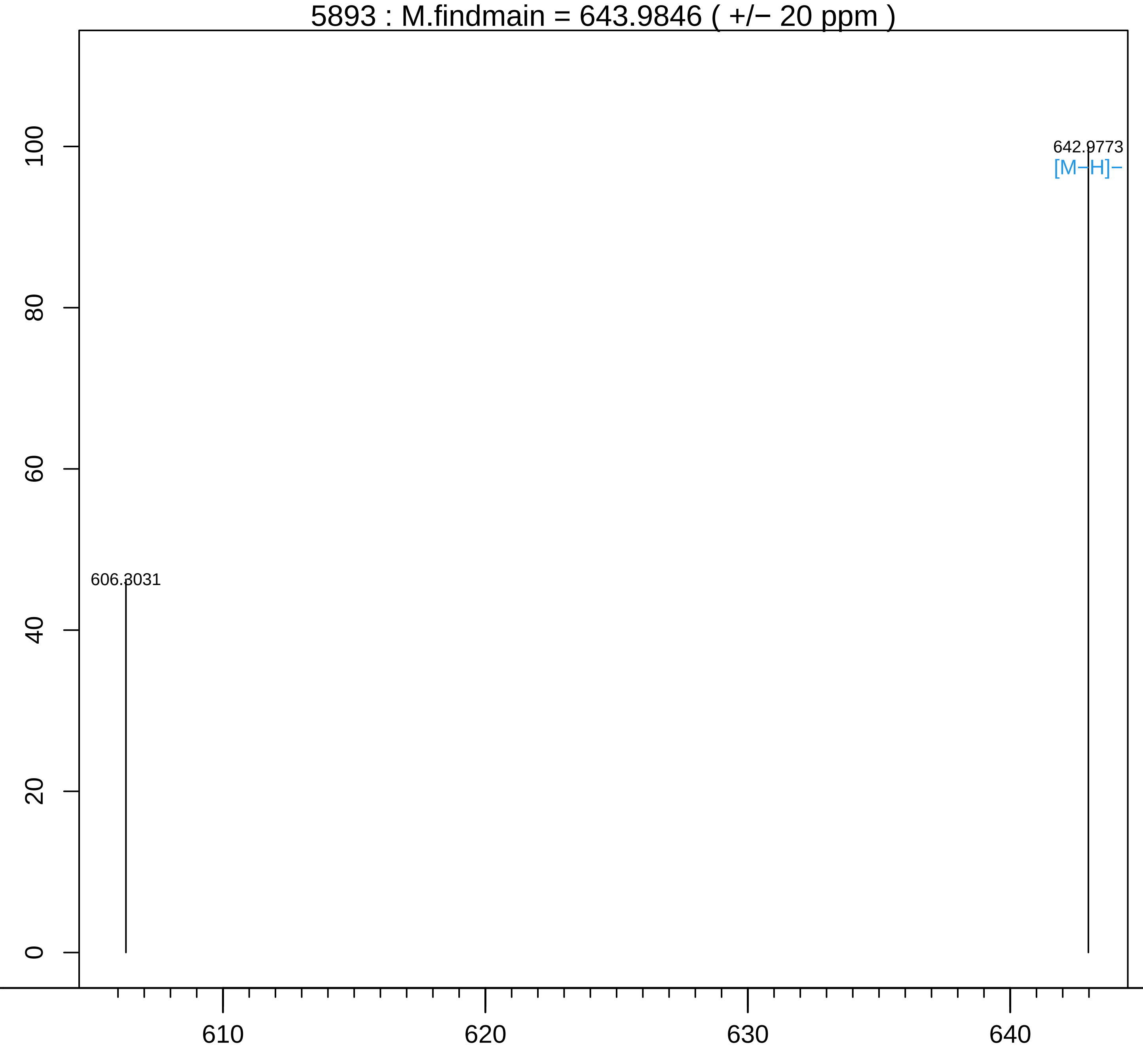
5893 : M.findmain = 643.9846 (+/− 20 ppm)
642.9773
[M−H]−
606.3031
0
20
40
60
80
100
610
620
630
640

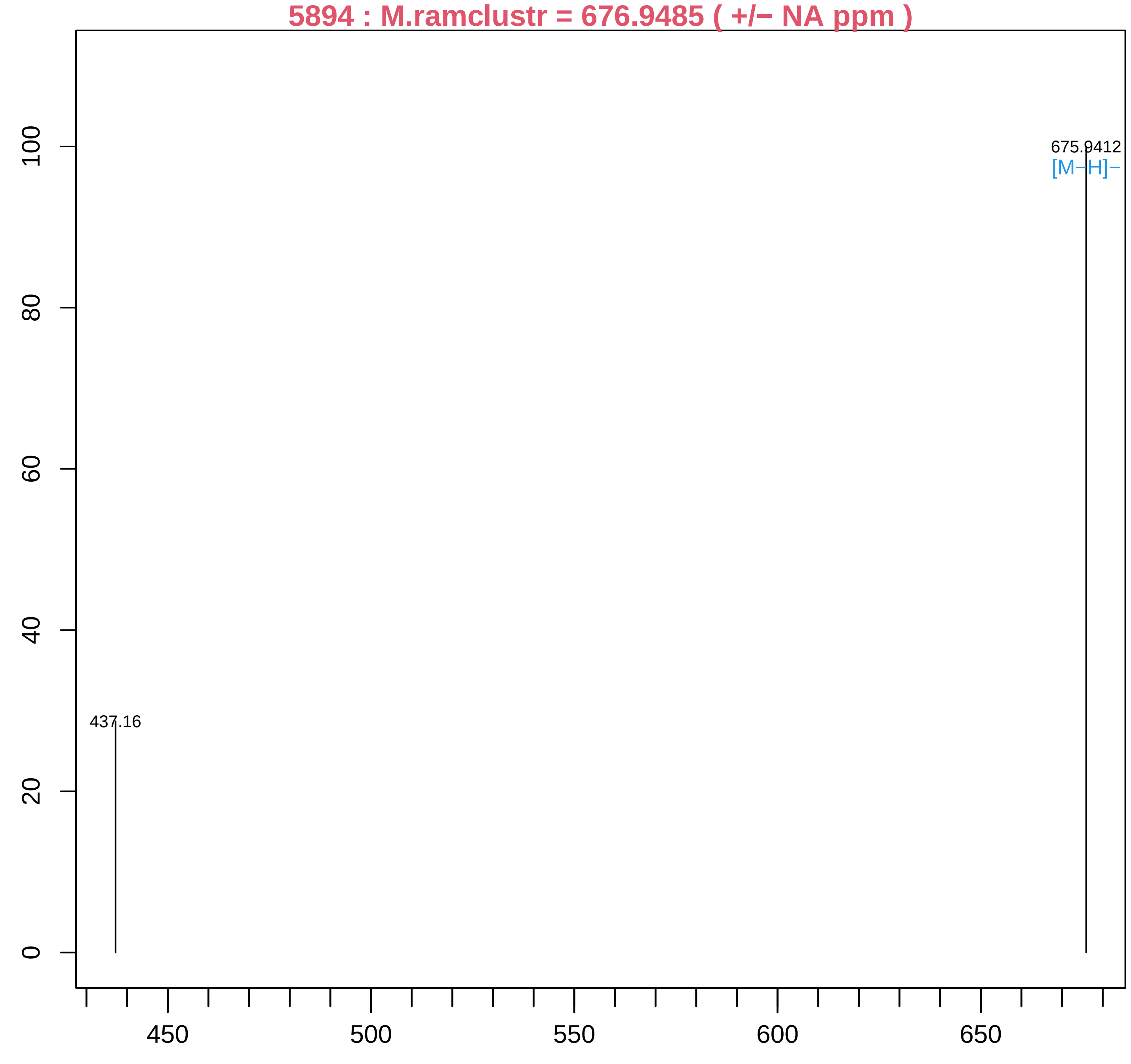
5894 : M.ramclustr = 676.9485 (+/− NA ppm)
675.9412
[M−H]−
437.16
0
20
40
60
80
100
450
500
550
600
650

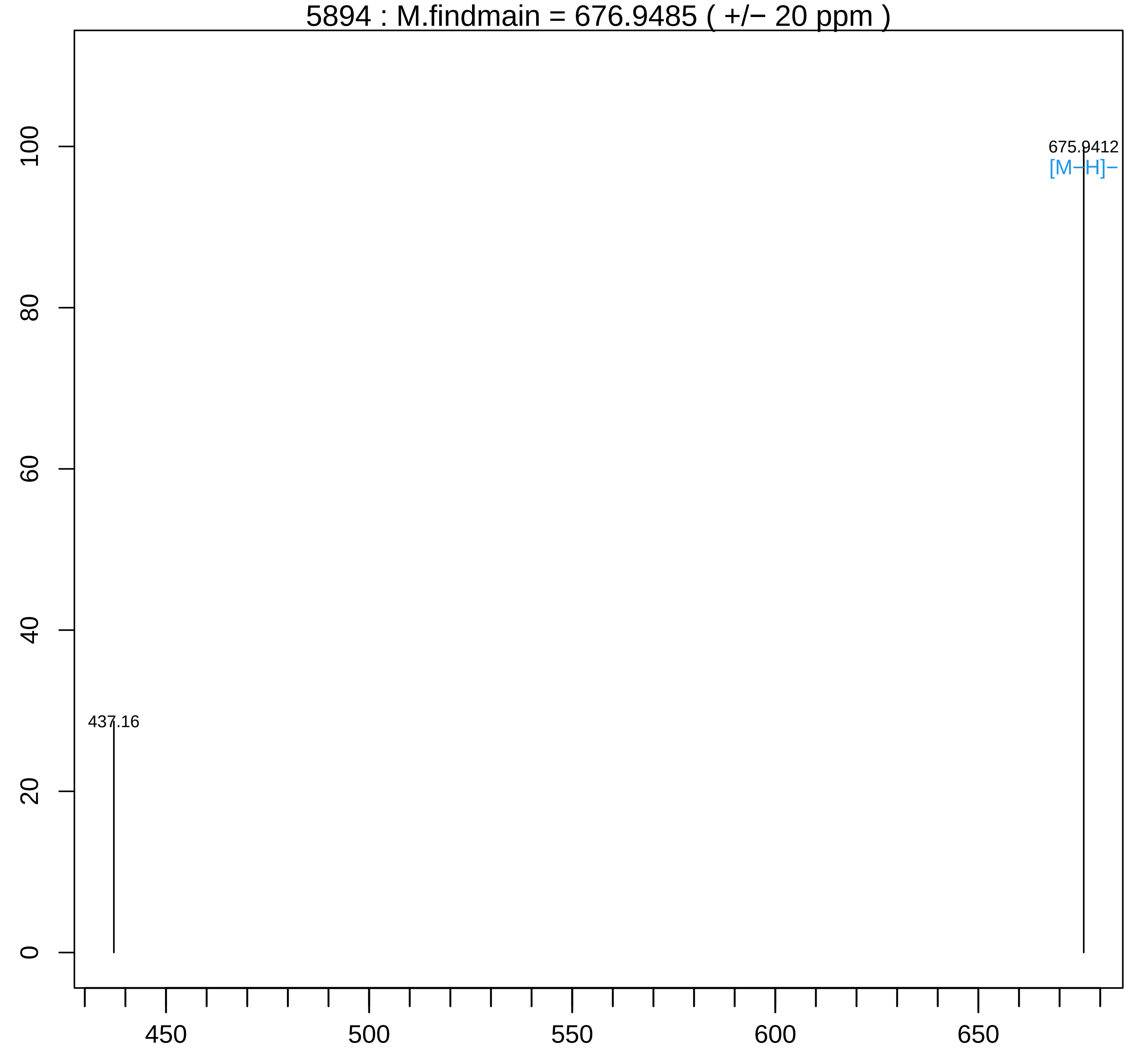
5894 : M.findmain = 676.9485 (+/− 20 ppm)
675.9412
[M−H]−
437.16
0
20
40
60
80
100
450
500
550
600
650

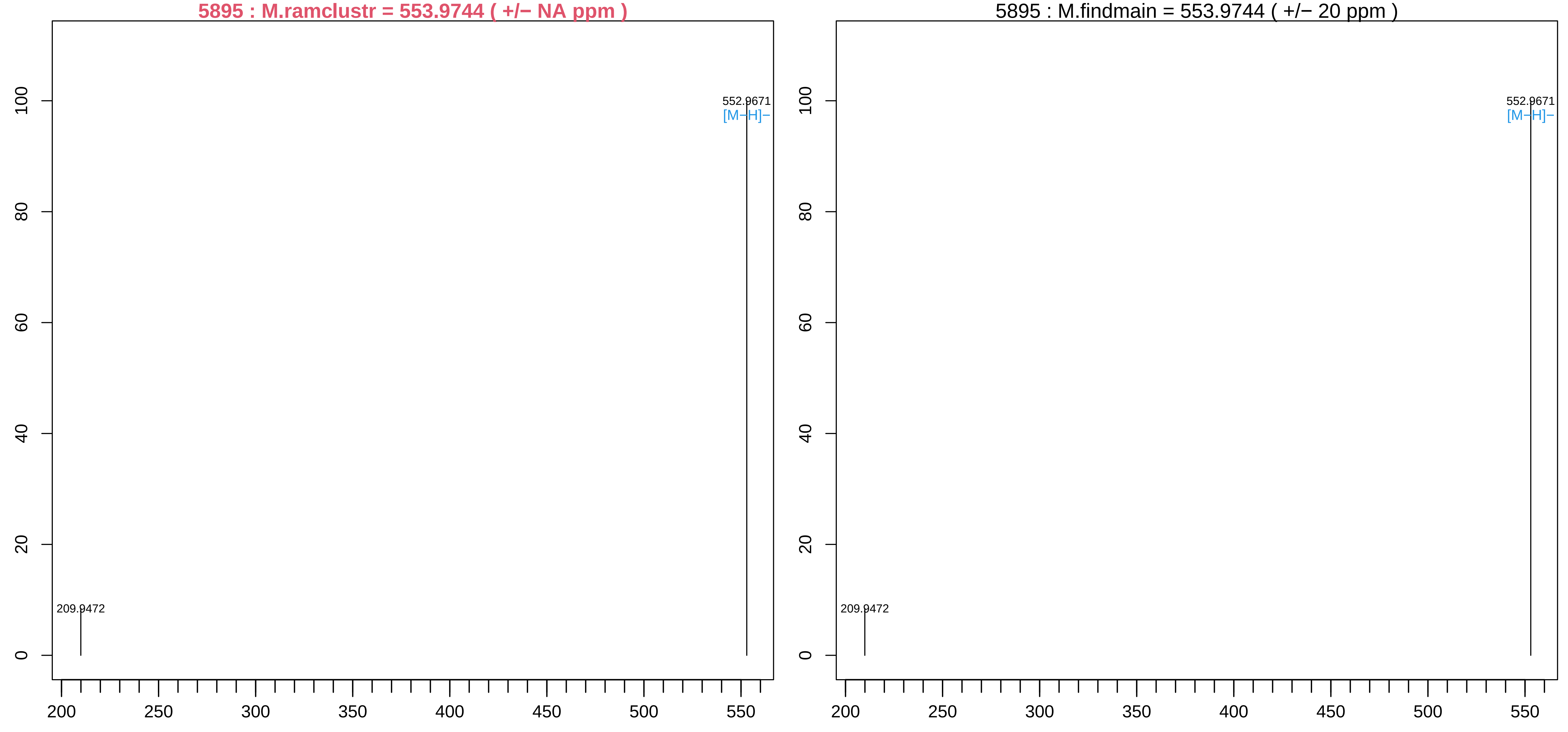

5895 : M.ramclustr = 553.9744 (+/− NA ppm)
552.9671
[M−H]−
209.9472
0
20
40
60
80
100
200
250
300
350
400
450
500
550
5895 : M.findmain = 553.9744 (+/− 20 ppm)
552.9671
[M−H]−
209.9472
0
20
40
60
80
100
200
250
300
350
400
450
500
550

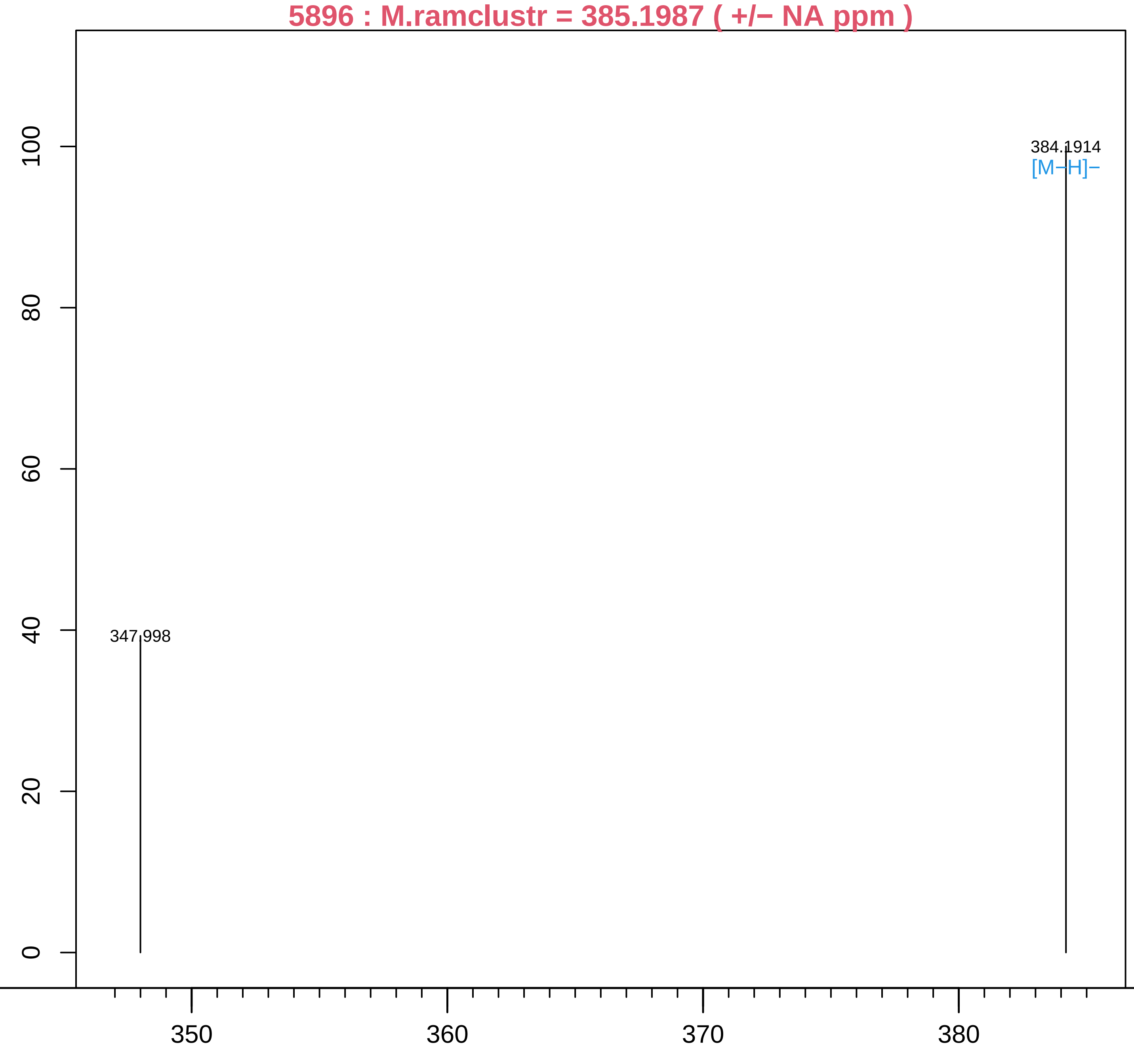
5896 : M.ramclustr = 385.1987 (+/− NA ppm)
100
80
60
40
20
0
350
360
370
380
384.1914
[M−H]−
347.998

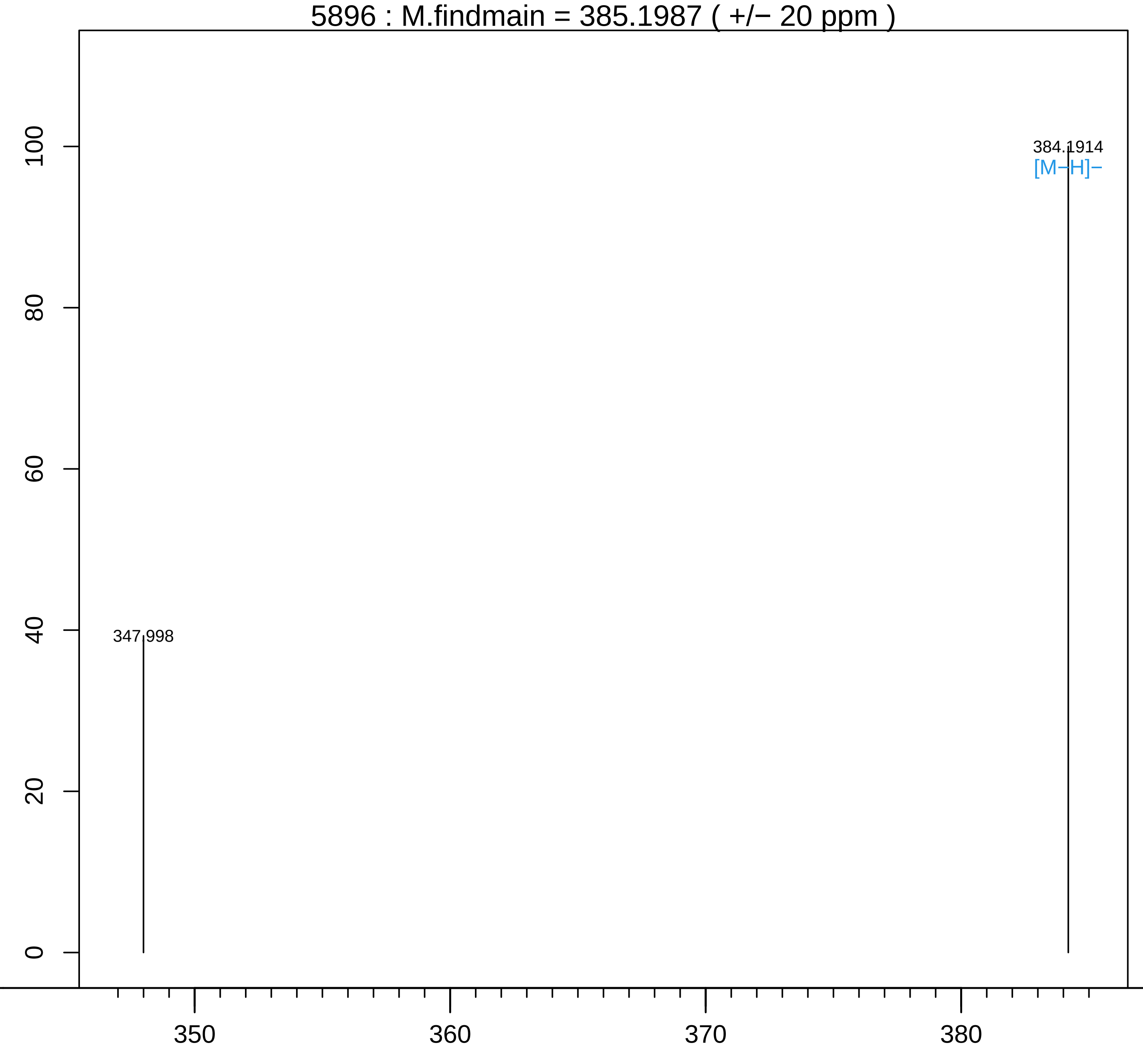
5896 : M.findmain = 385.1987 (+/− 20 ppm)
100
80
60
40
20
0
350
360
370
380
384.1914
[M−H]−
347.998

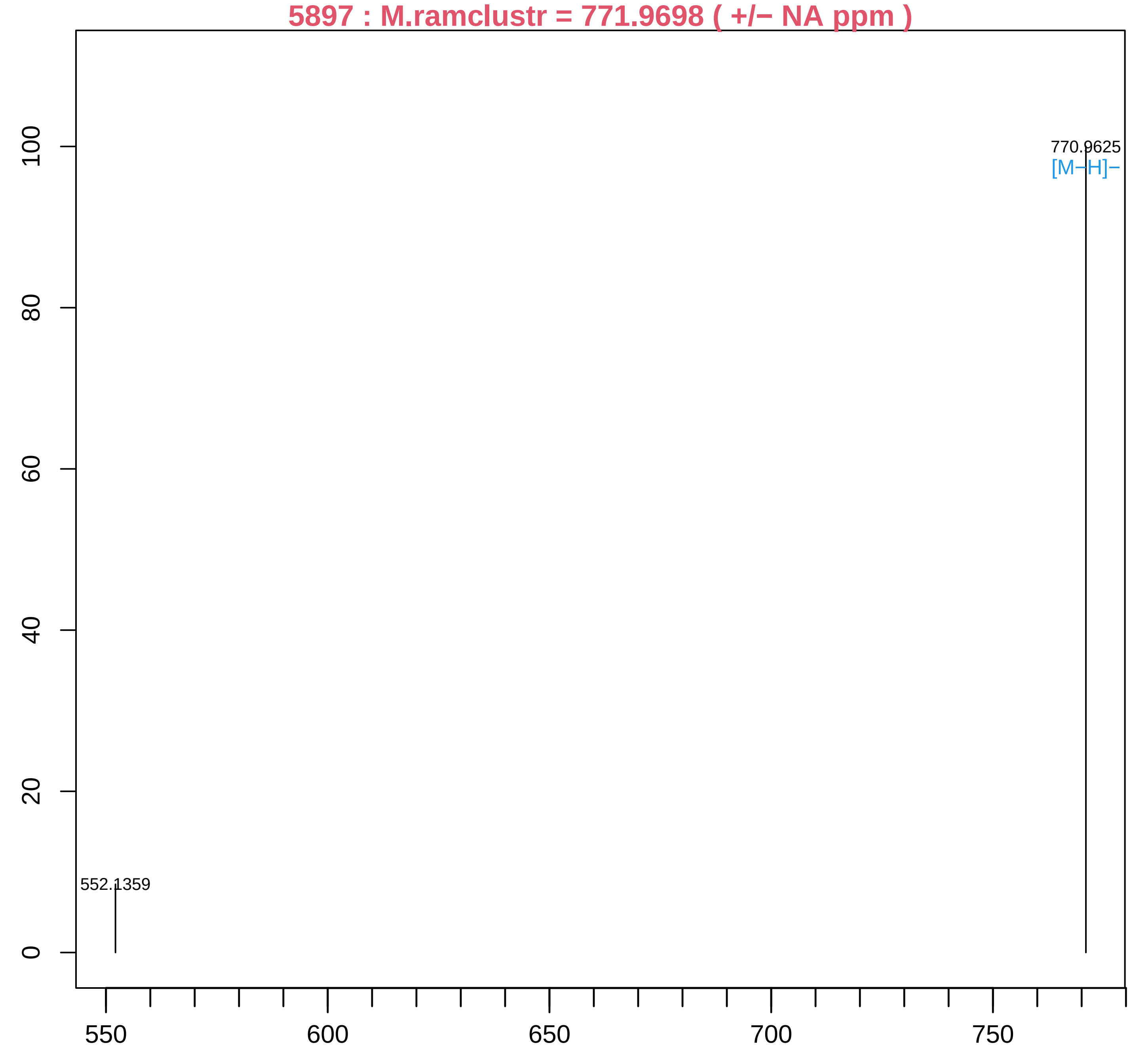
5897 : M.ramclustr = 771.9698 (+/− NA ppm)
770.9625
[M−H]−
552.1359
0
20
40
60
80
100
550
600
650
700
750

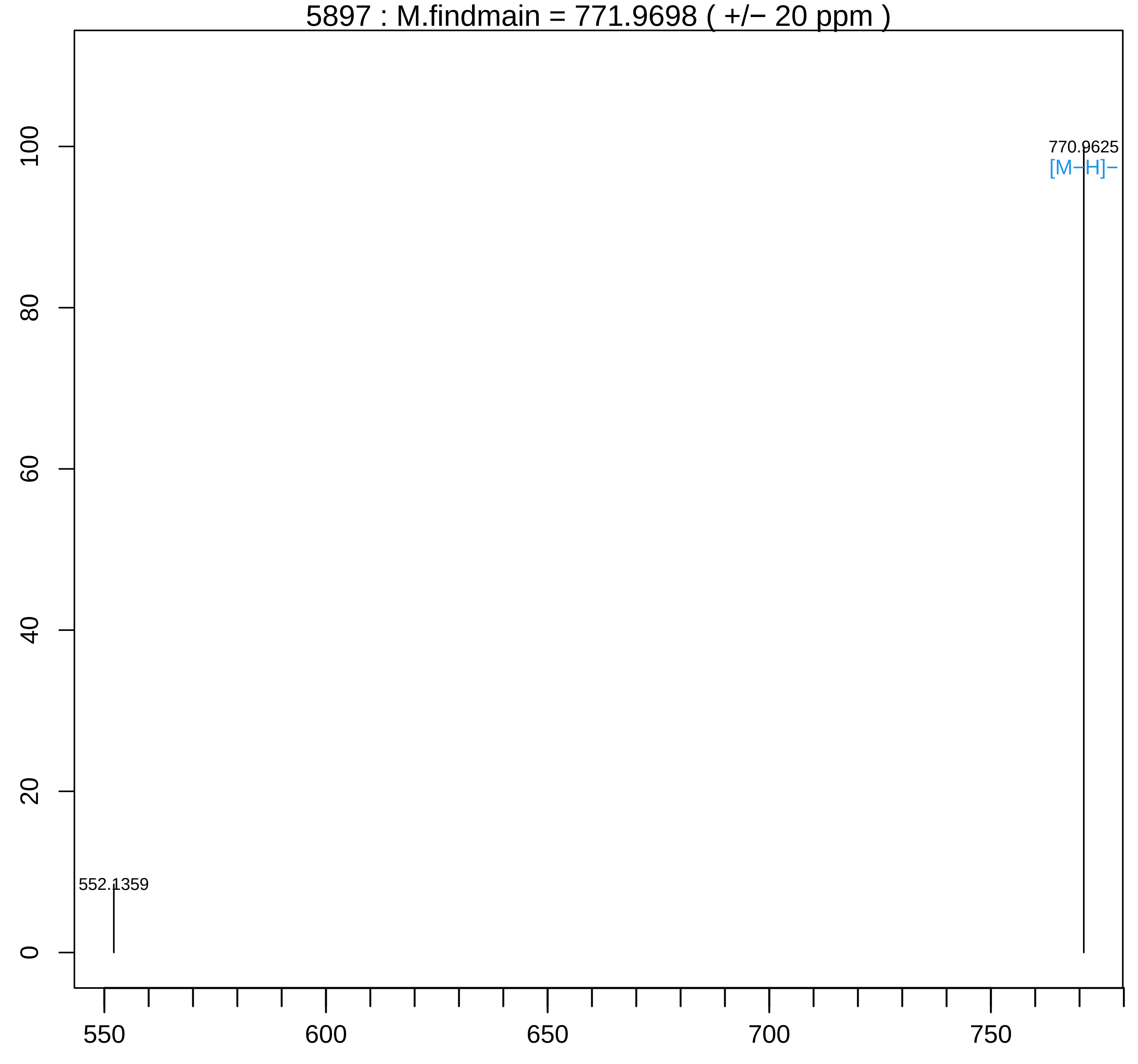
5897 : M.findmain = 771.9698 (+/− 20 ppm)
770.9625
[M−H]−
552.1359
0
20
40
60
80
100
550
600
650
700
750

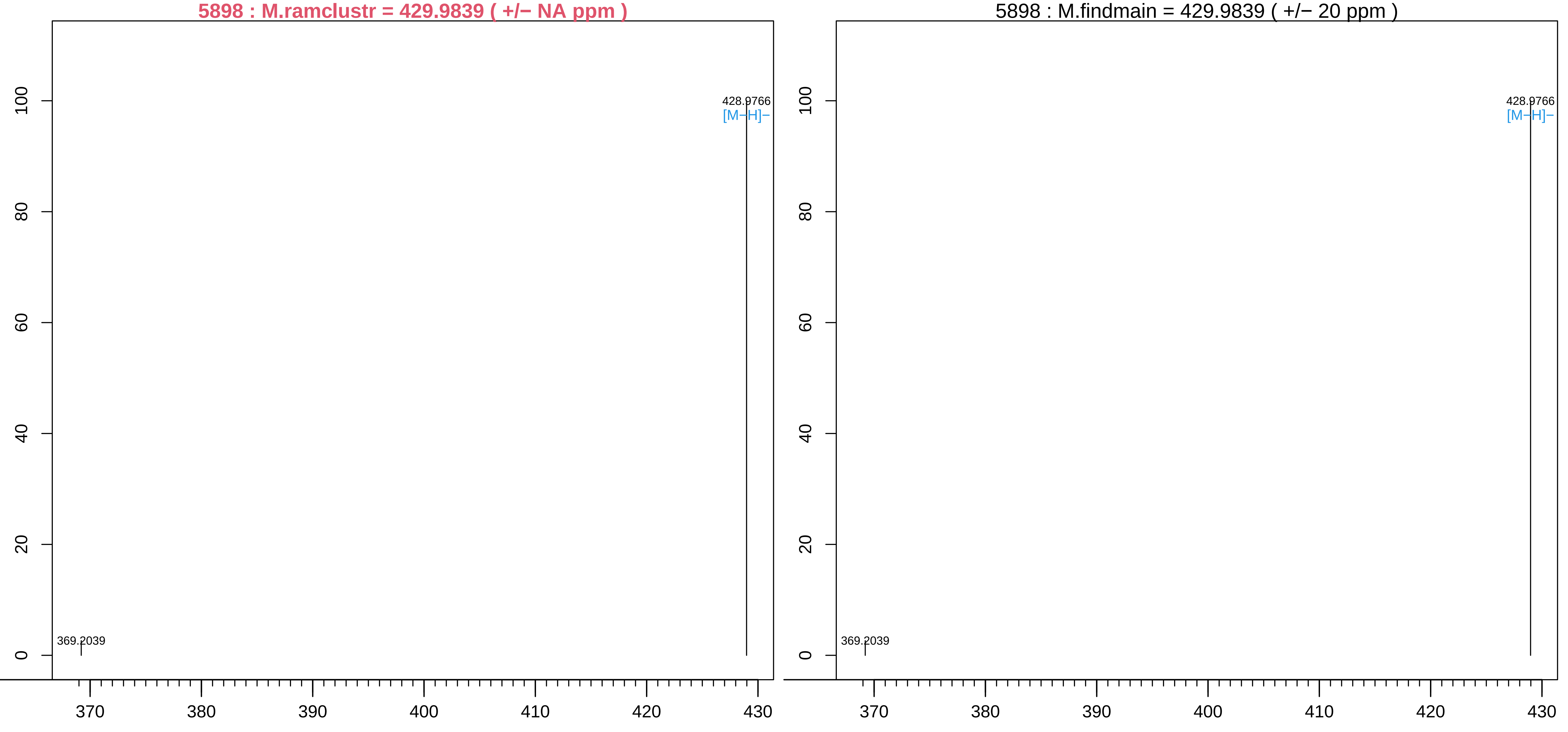
5898 : M.ramclustr = 429.9839 (+/− NA ppm)
428.9766
[M−H]−
369.2039
5898 : M.findmain = 429.9839 (+/− 20 ppm)
428.9766
[M−H]−
369.2039
0
20
40
60
80
100
370
380
390
400
410
420
430

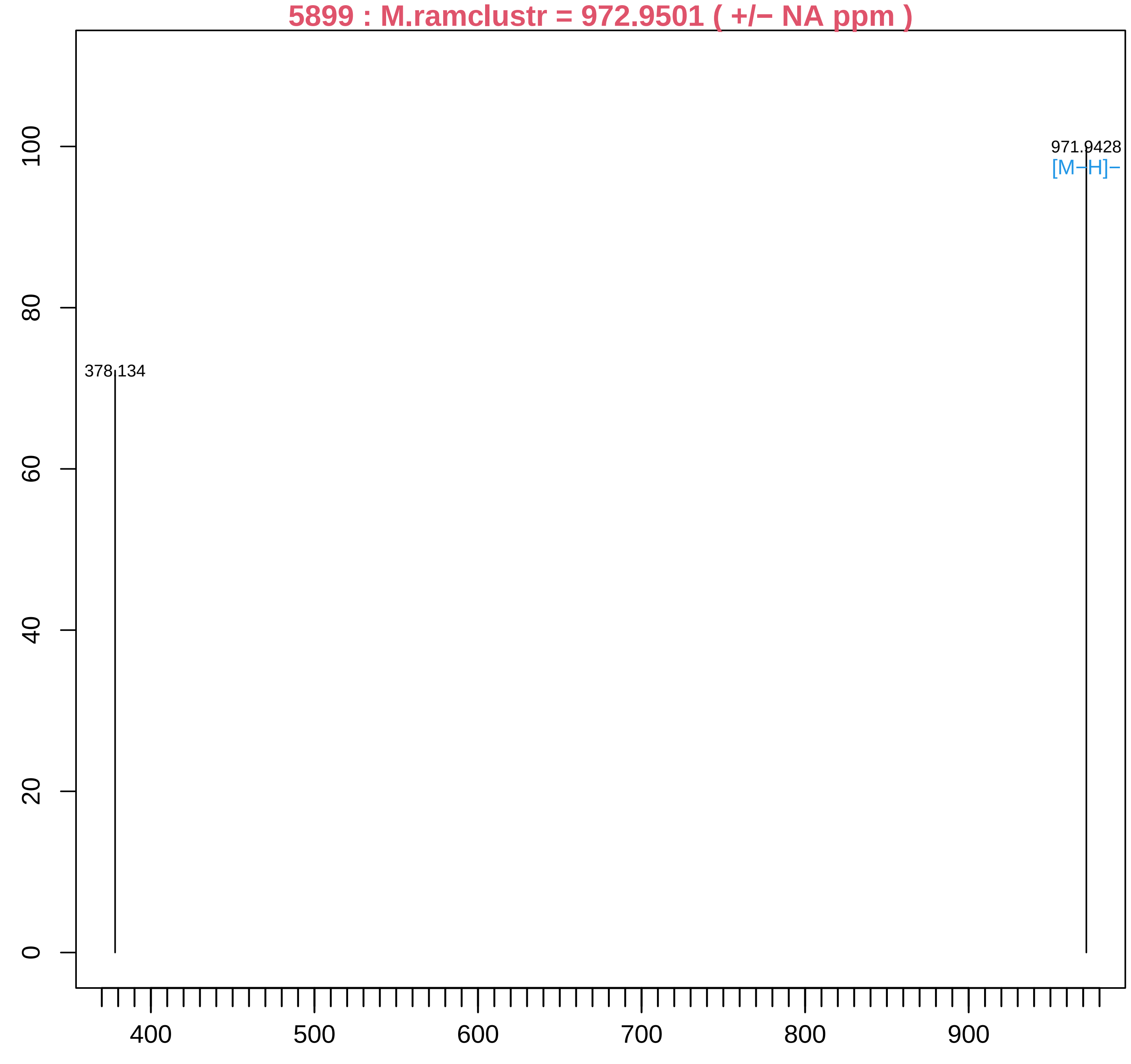
5899 : M.ramclustr = 972.9501 (+/− NA ppm)
971.9428
[M−H]−
378.134
0
20
40
60
80
100
400
500
600
700
800
900

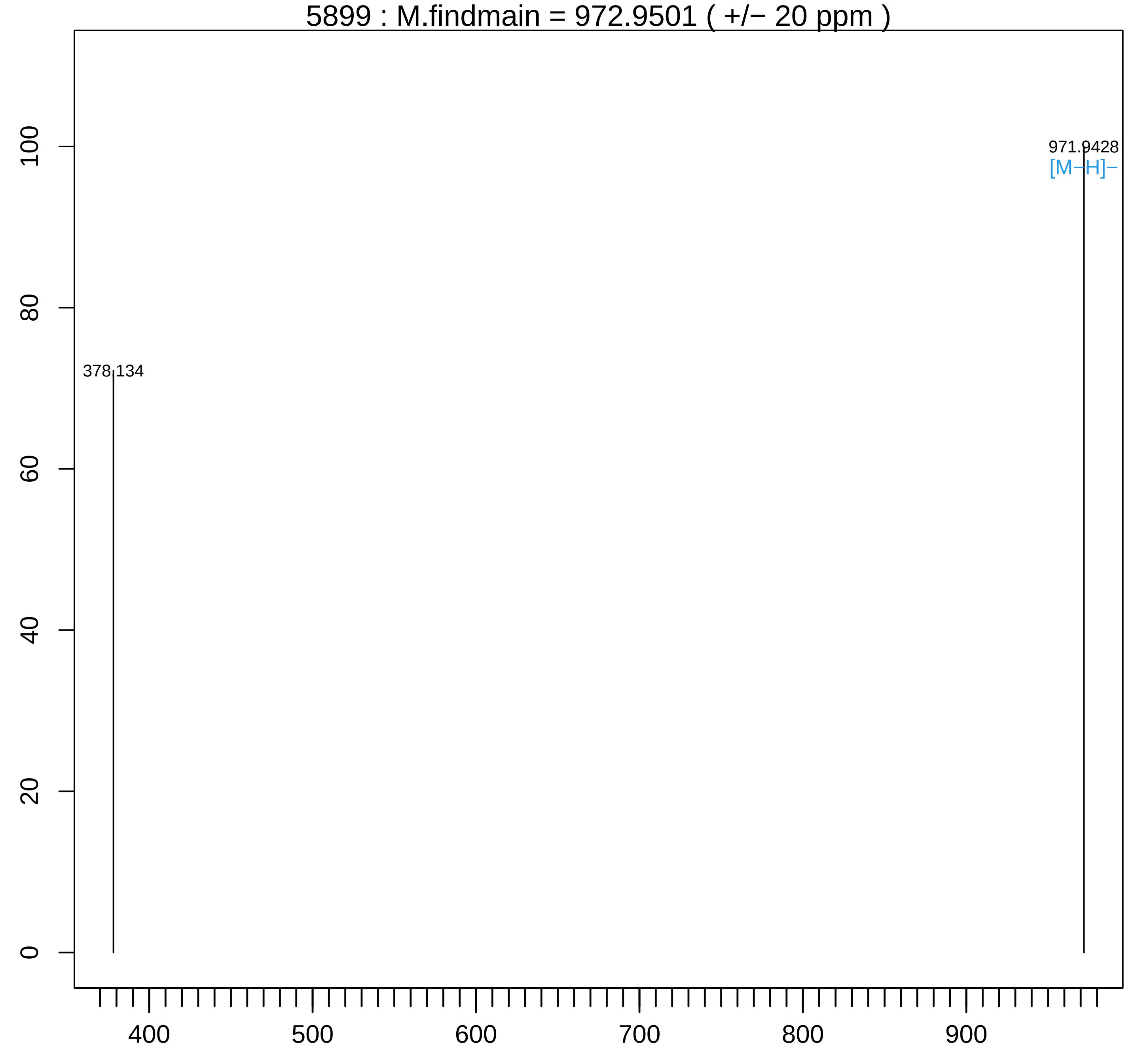
5899 : M.findmain = 972.9501 (+/− 20 ppm)
971.9428
[M−H]−
378.134
0
20
40
60
80
100
400
500
600
700
800
900

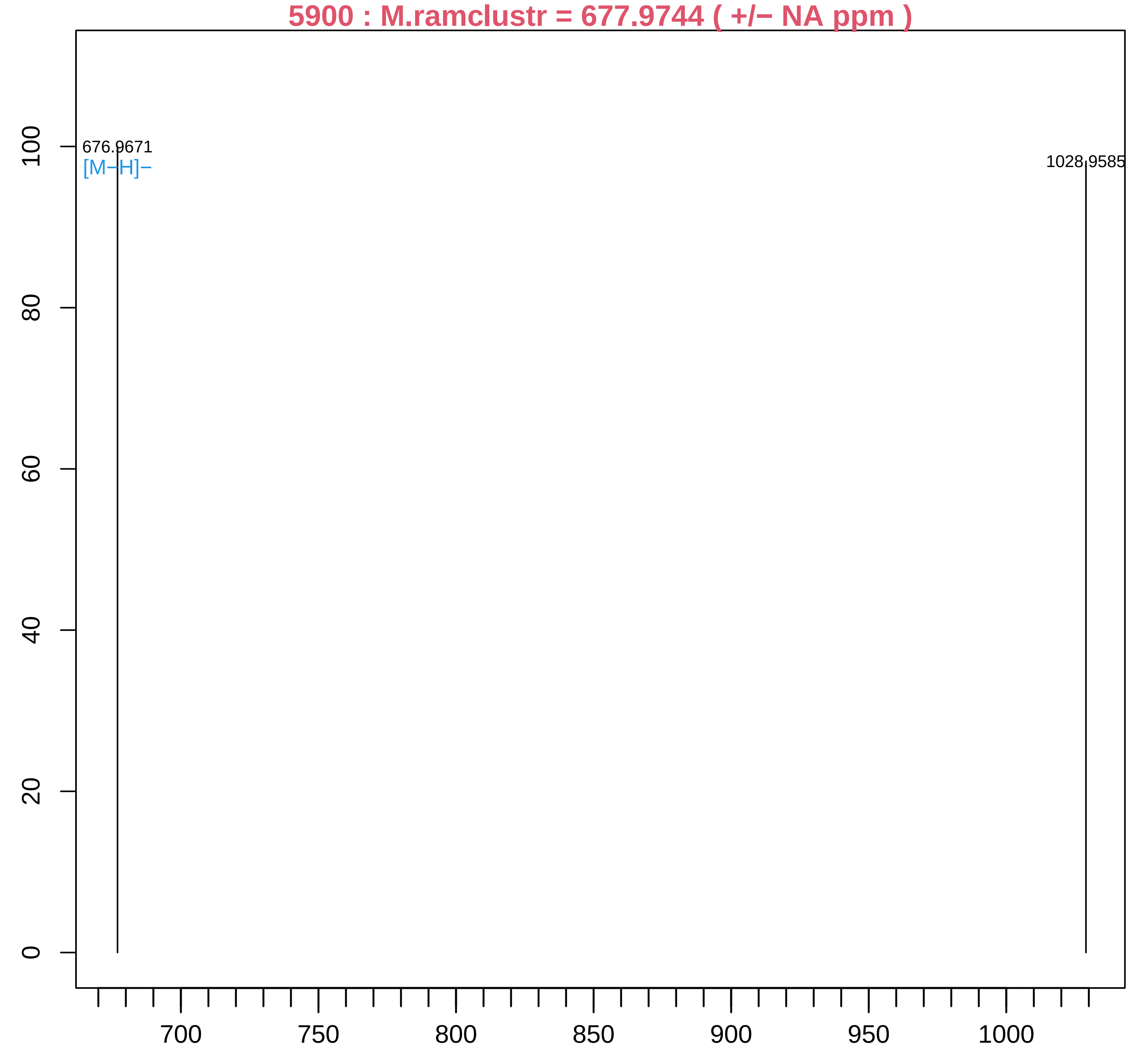
5900 : M.ramclustr = 677.9744 (+/− NA ppm)
676.9671
[M−H]−
1028.9585
0
20
40
60
80
100
700
750
800
850
900
950
1000

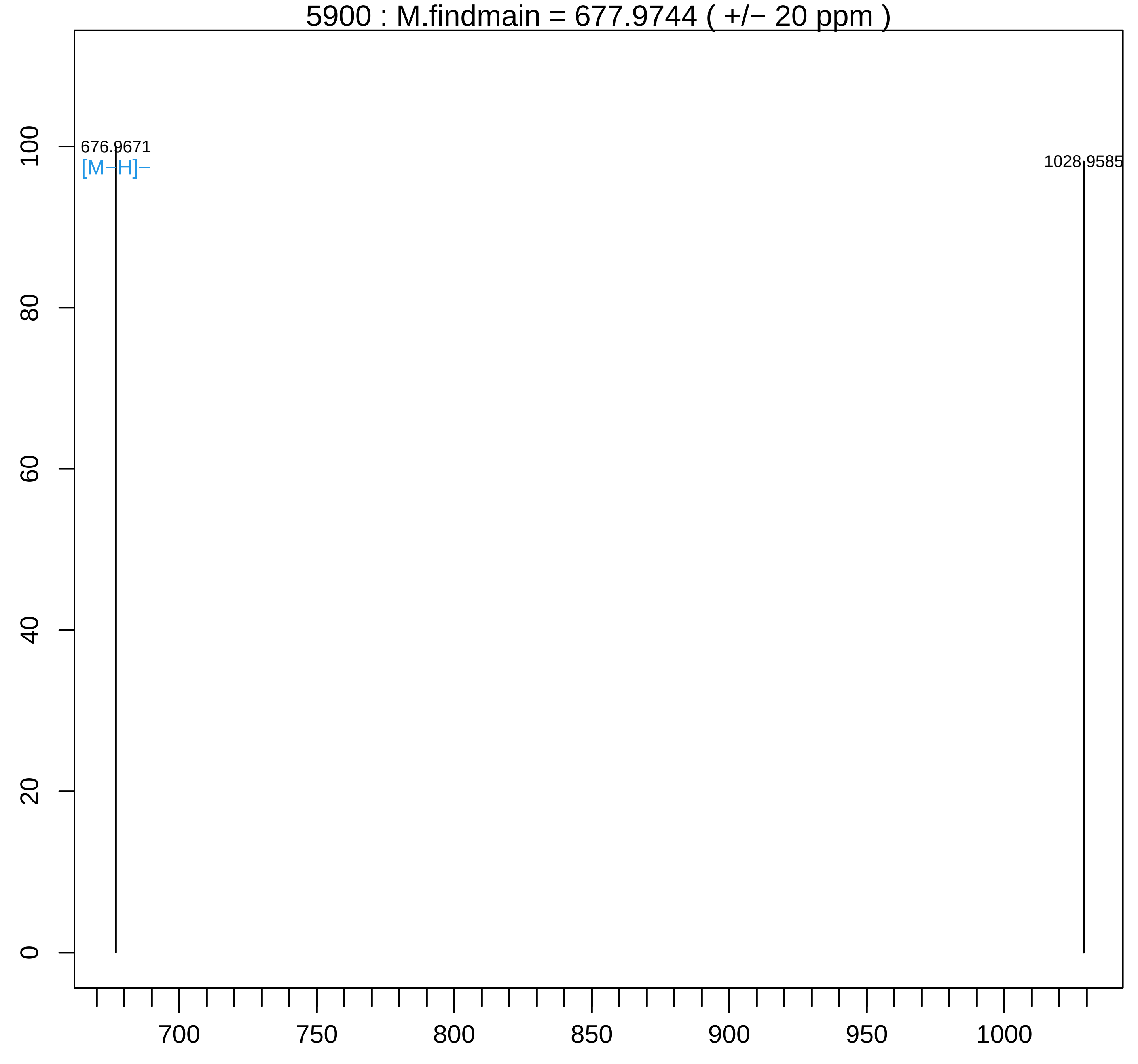
5900 : M.findmain = 677.9744 (+/− 20 ppm)
676.9671
[M−H]−
1028.9585
0
20
40
60
80
100
700
750
800
850
900
950
1000

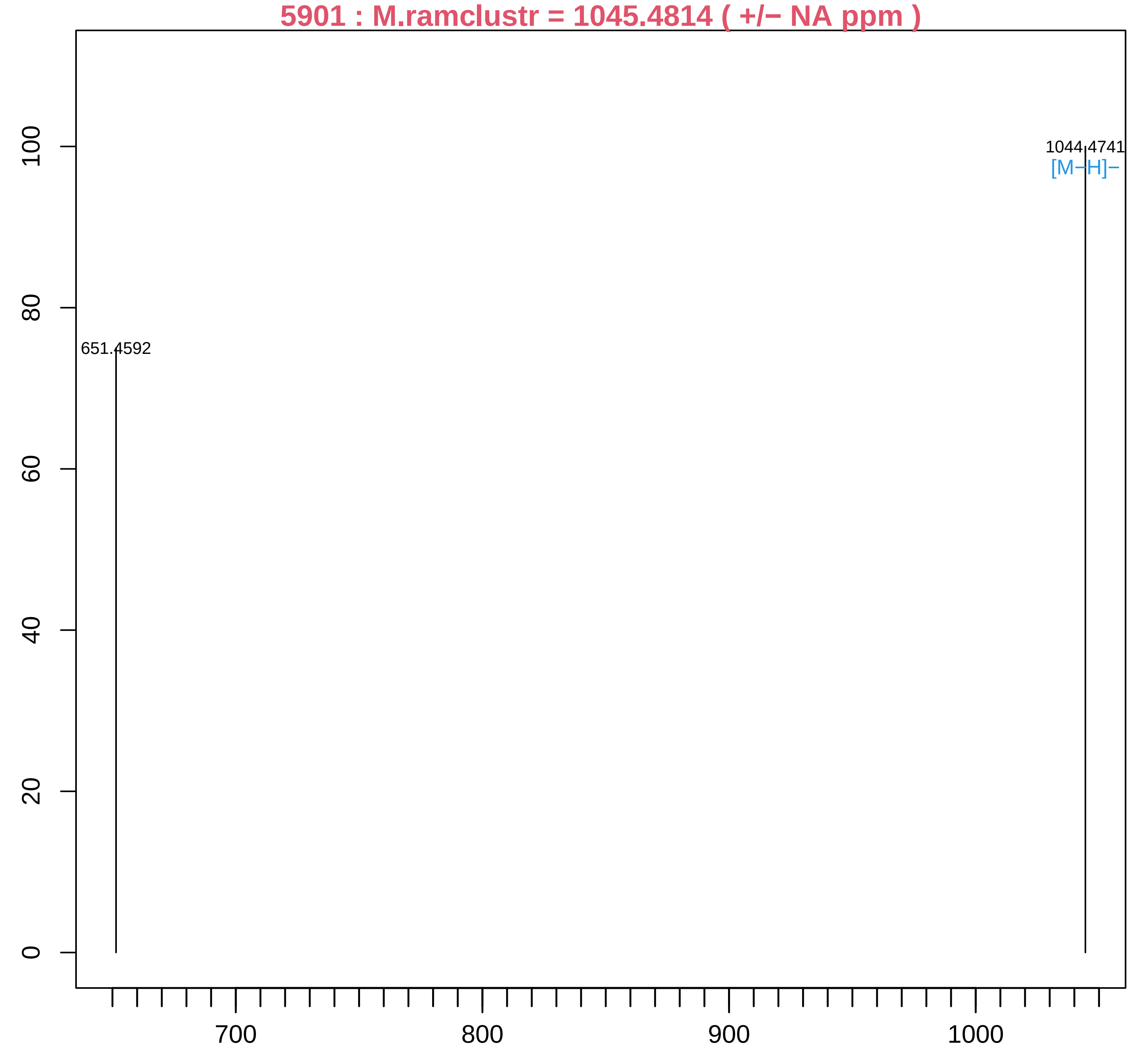
5901 : M.ramclustr = 1045.4814 (+/− NA ppm)
651.4592
1044.4741
[M−H]−
0
20
40
60
80
100
700
800
900
1000

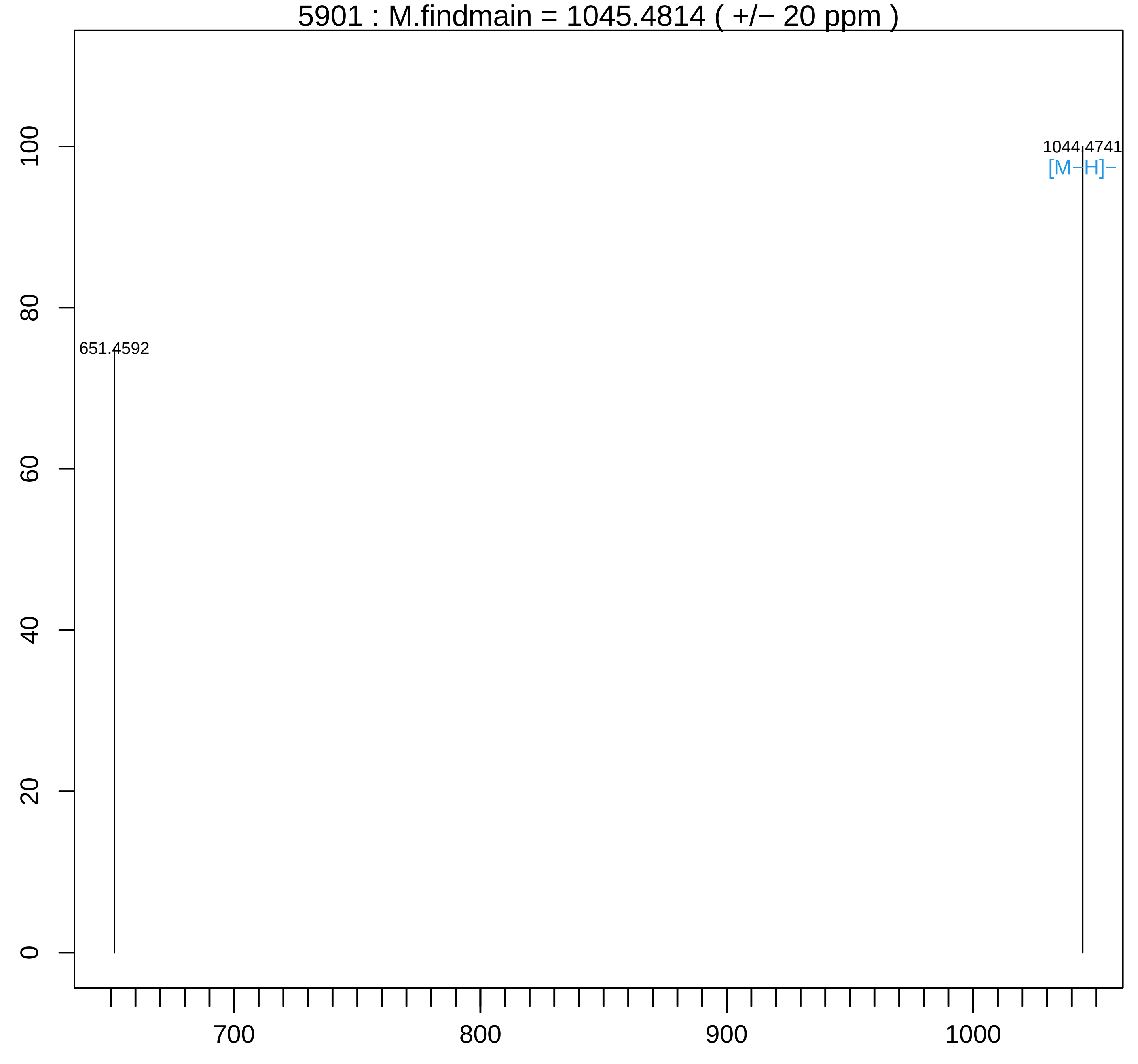
5901 : M.findmain = 1045.4814 (+/− 20 ppm)
651.4592
1044.4741
[M−H]−
0
20
40
60
80
100
700
800
900
1000

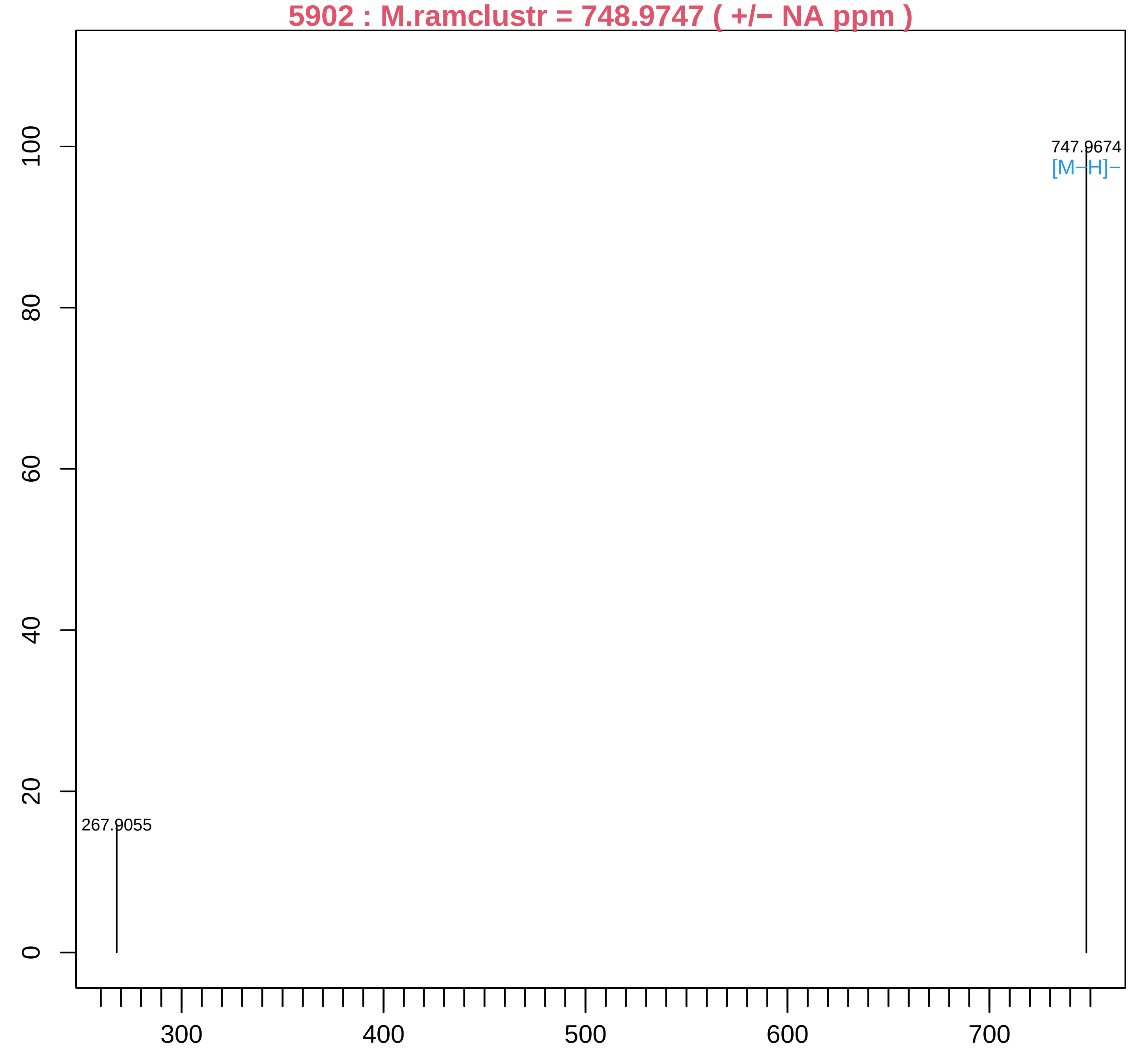

5902 : M.ramclustr = 748.9747 (+/− NA ppm)
747.9674
[M−H]−
267.9055
0
20
40
60
80
100
300
400
500
600
700

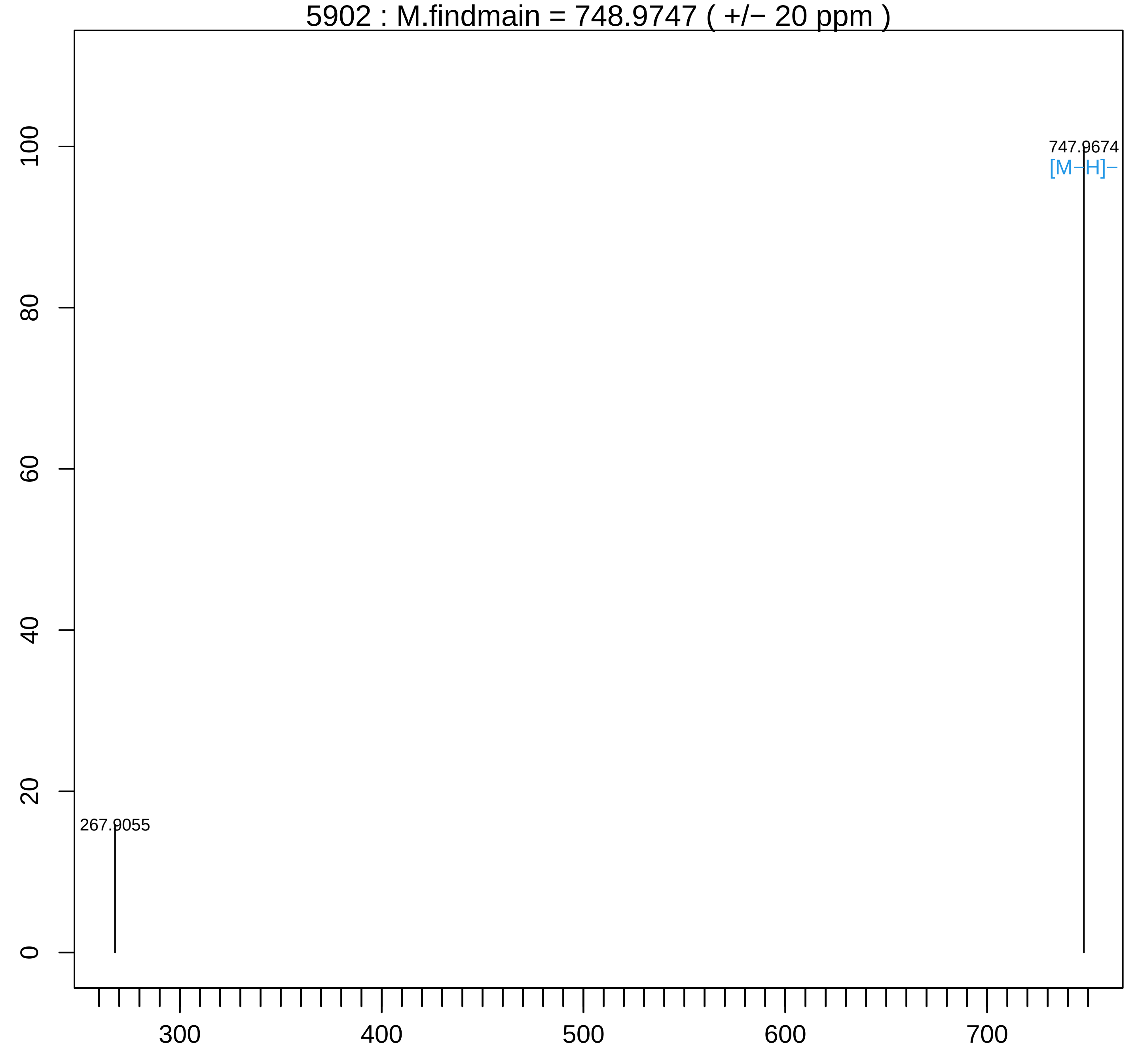

5902 : M.findmain = 748.9747 (+/− 20 ppm)
747.9674
[M−H]−
267.9055
0
20
40
60
80
100
300
400
500
600
700

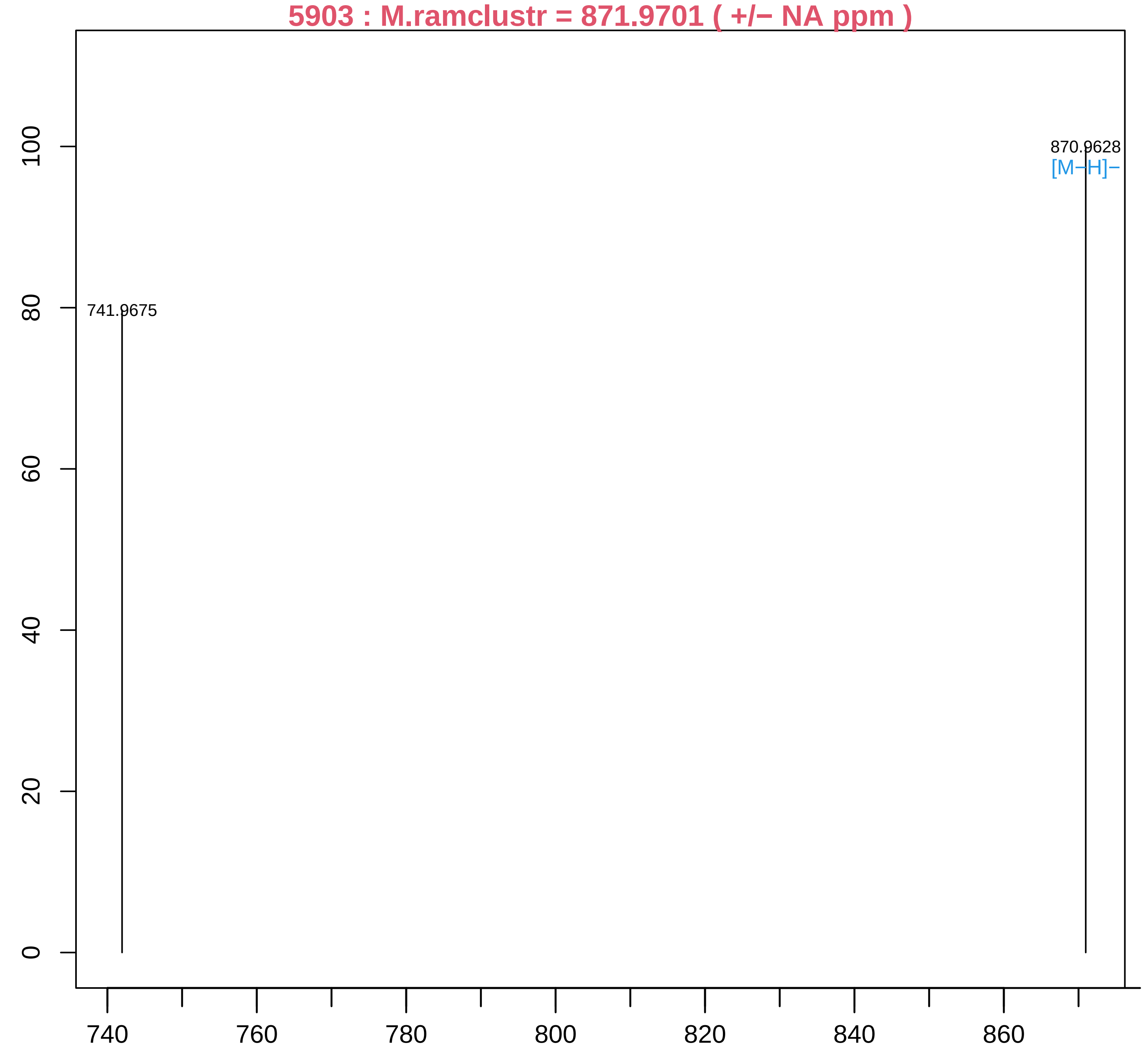

5903 : M.ramclustr = 871.9701 (+/− NA ppm)
870.9628
[M−H]−
741.9675
0
20
40
60
80
100
740
760
780
800
820
840
860

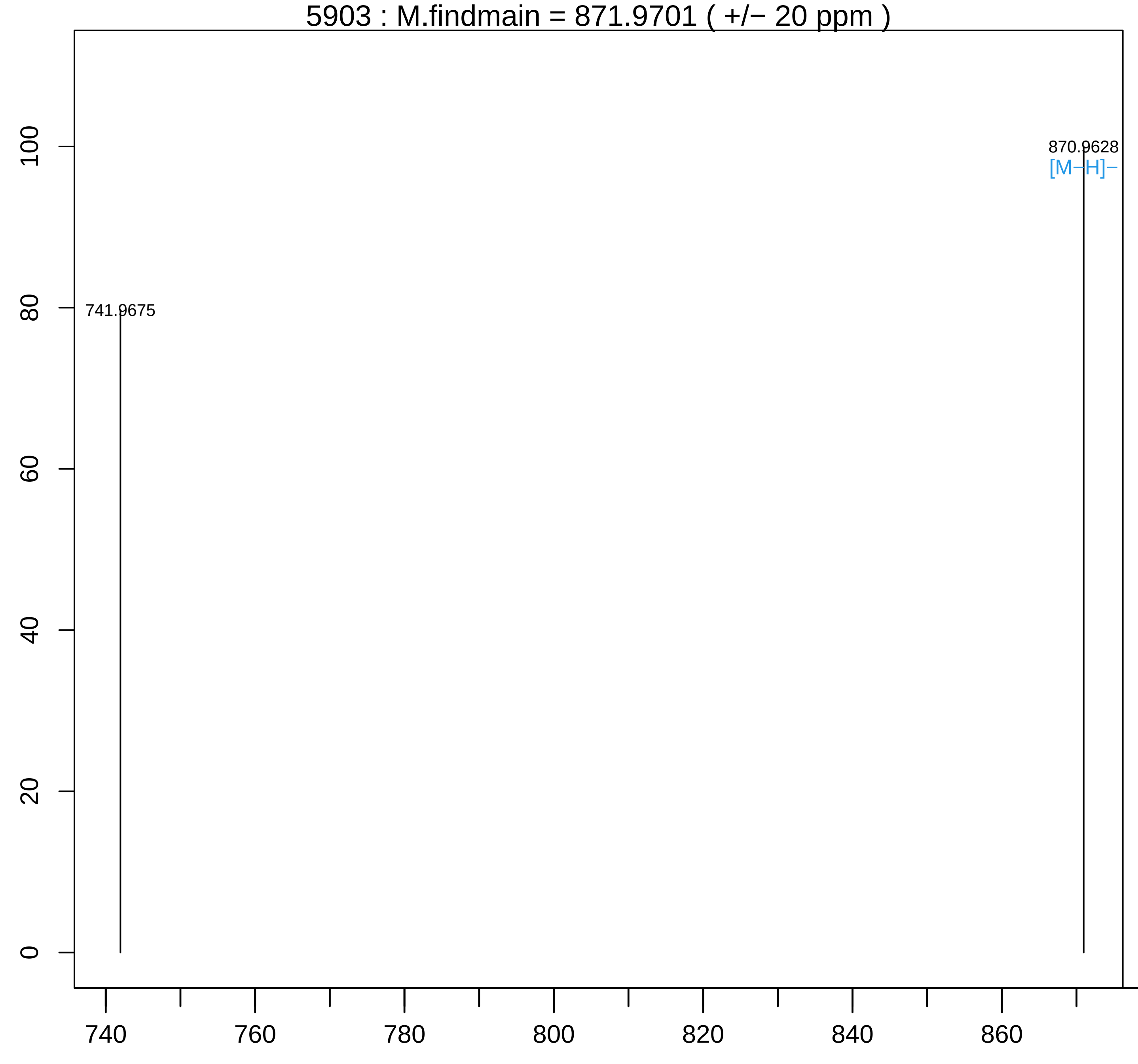

5903 : M.findmain = 871.9701 (+/− 20 ppm)
870.9628
[M−H]−
741.9675
0
20
40
60
80
100
740
760
780
800
820
840
860

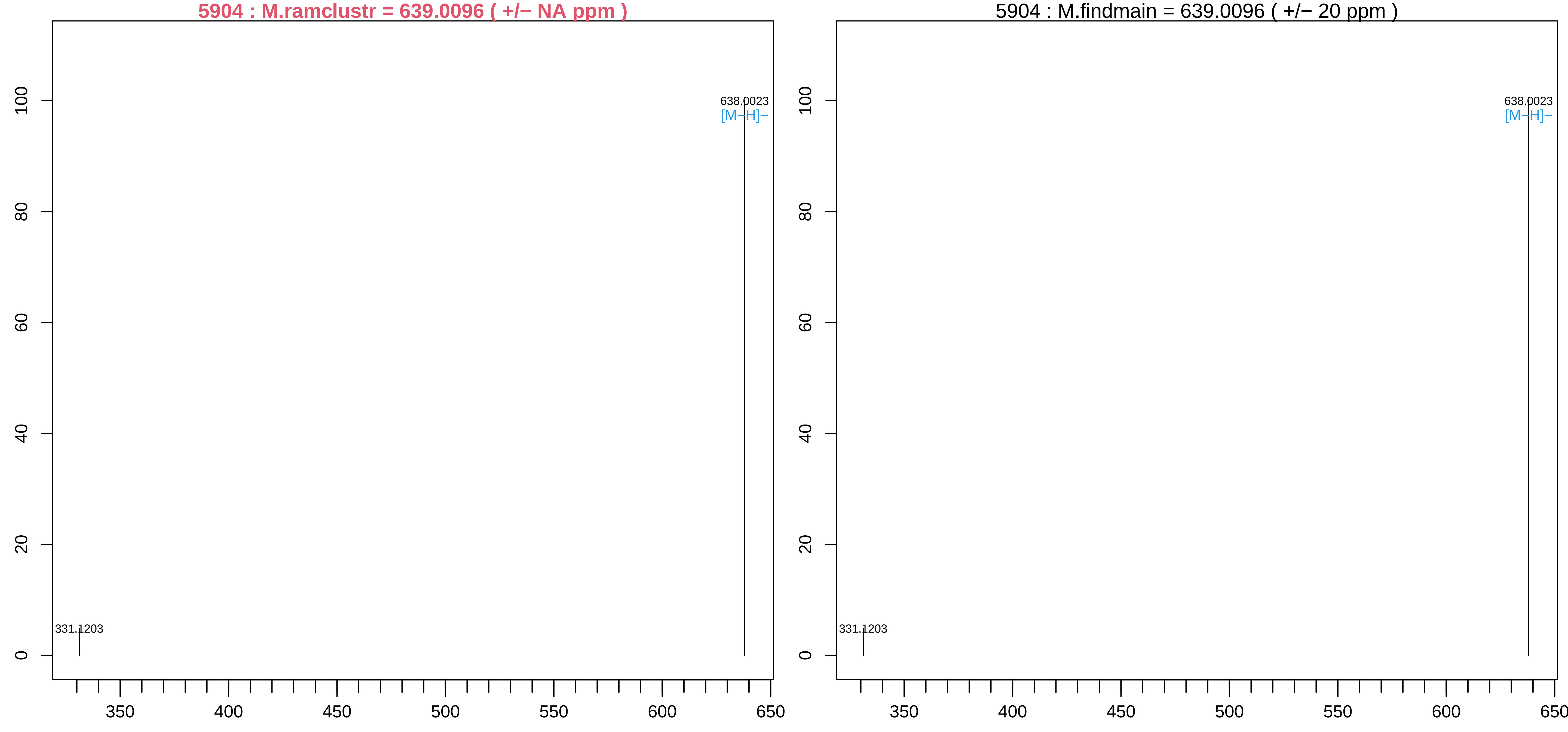
5904 : M.ramclustr = 639.0096 (+/− NA ppm)
638.0023
[M−H]−
331.1203
5904 : M.findmain = 639.0096 (+/− 20 ppm)
638.0023
[M−H]−
331.1203

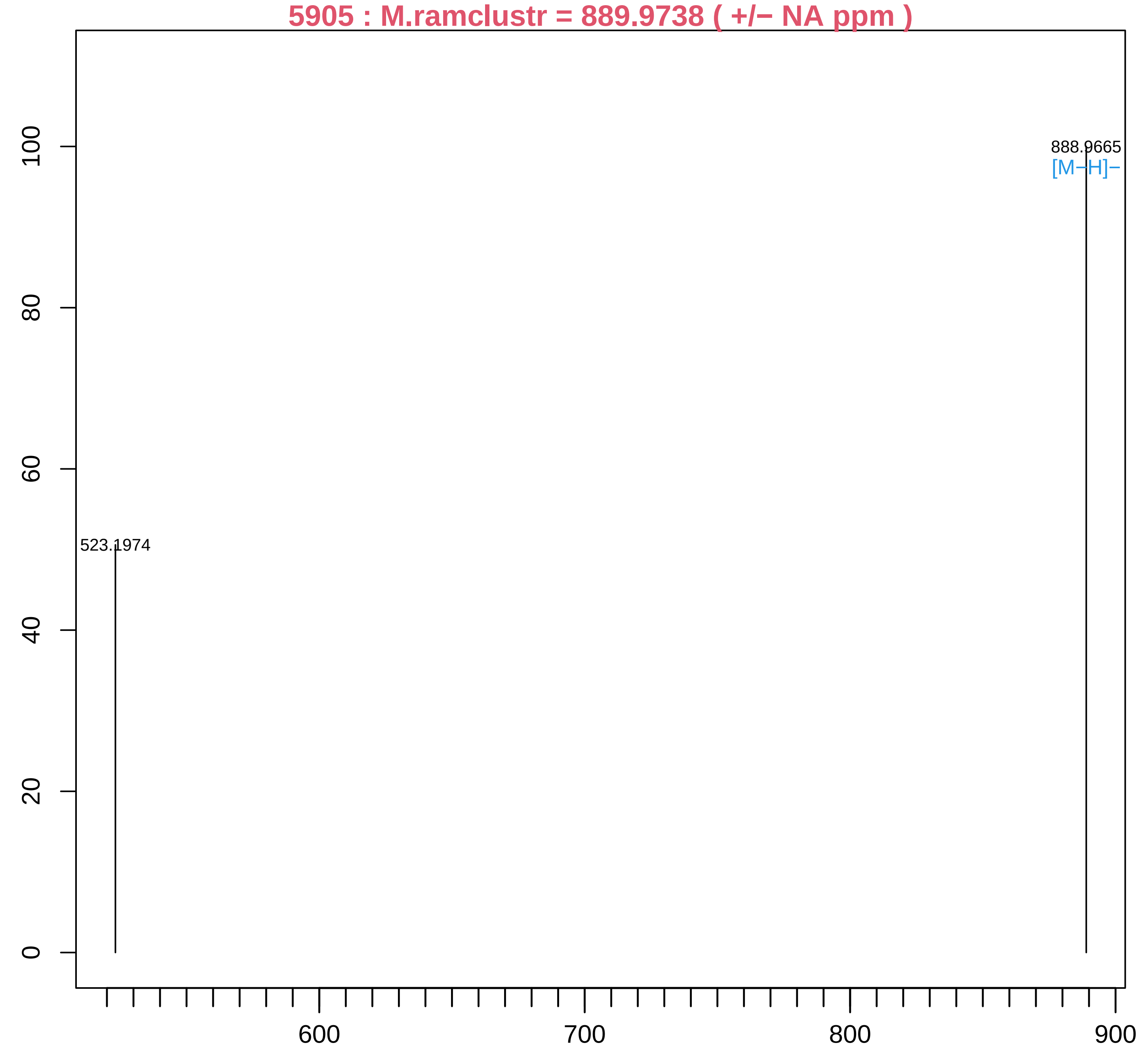
5905 : M.ramclustr = 889.9738 (+/− NA ppm)
888.9665
[M−H]−
523.1974
0
20
40
60
80
100
600
700
800
900

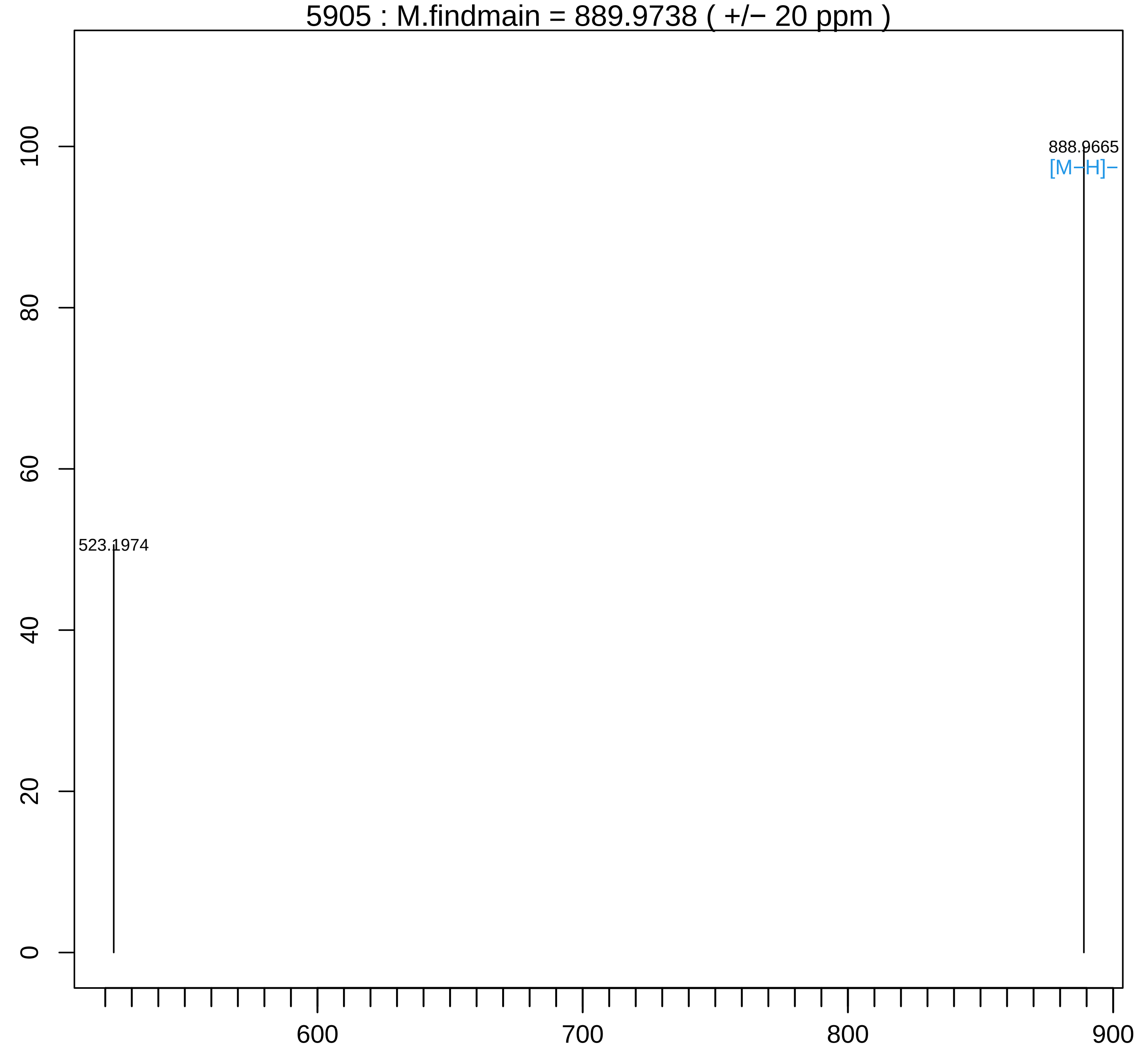
5905 : M.findmain = 889.9738 (+/− 20 ppm)
888.9665
[M−H]−
523.1974
0
20
40
60
80
100
600
700
800
900

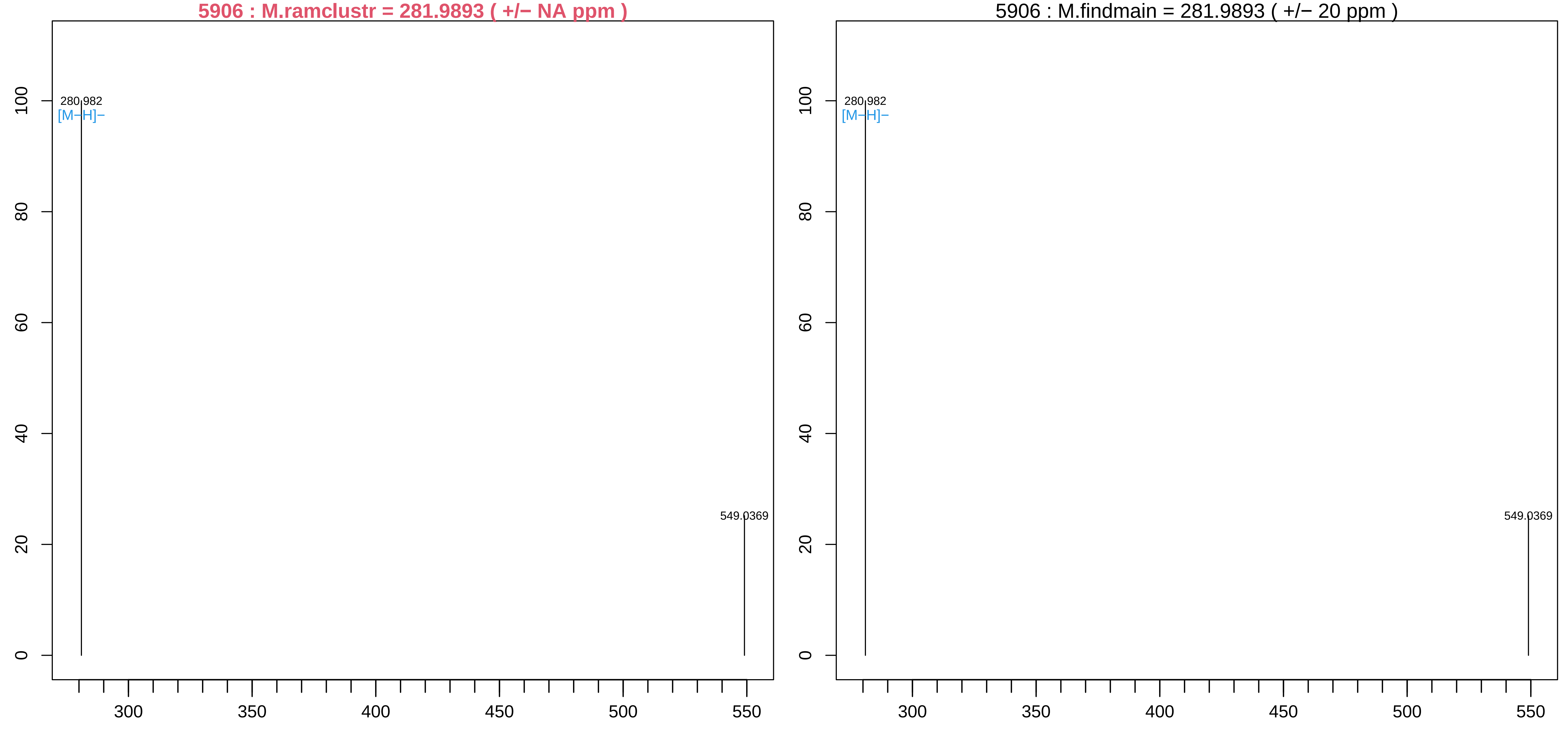
5906 : M.ramclustr = 281.9893 (+/− NA ppm)
280.982
[M−H]−
549.0369
5906 : M.findmain = 281.9893 (+/− 20 ppm)
280.982
[M−H]−
549.0369

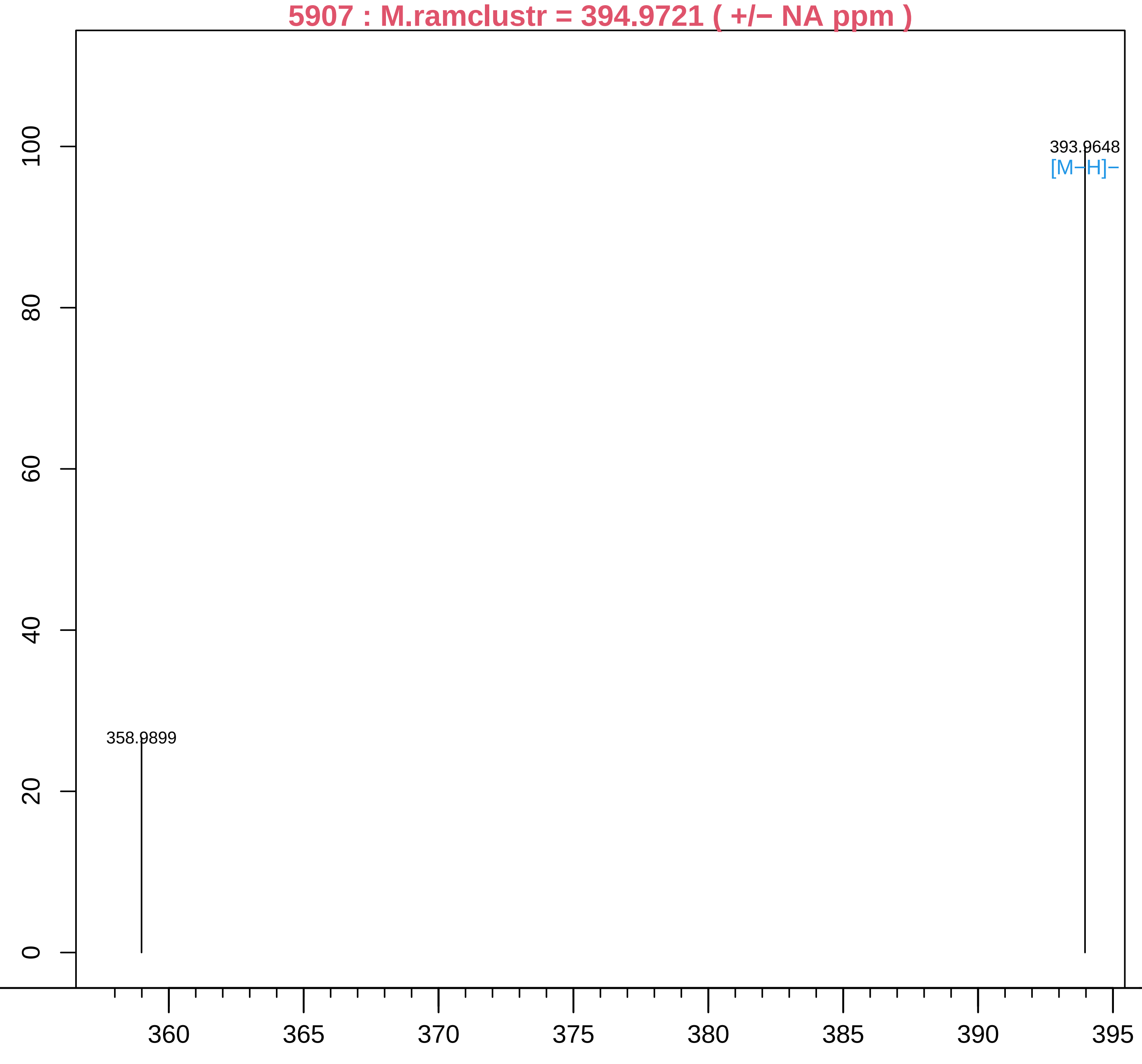
5907 : M.ramclustr = 394.9721 (+/− NA ppm)
393.9648
[M−H]−
358.9899
0
20
40
60
80
100
360
365
370
375
380
385
390
395

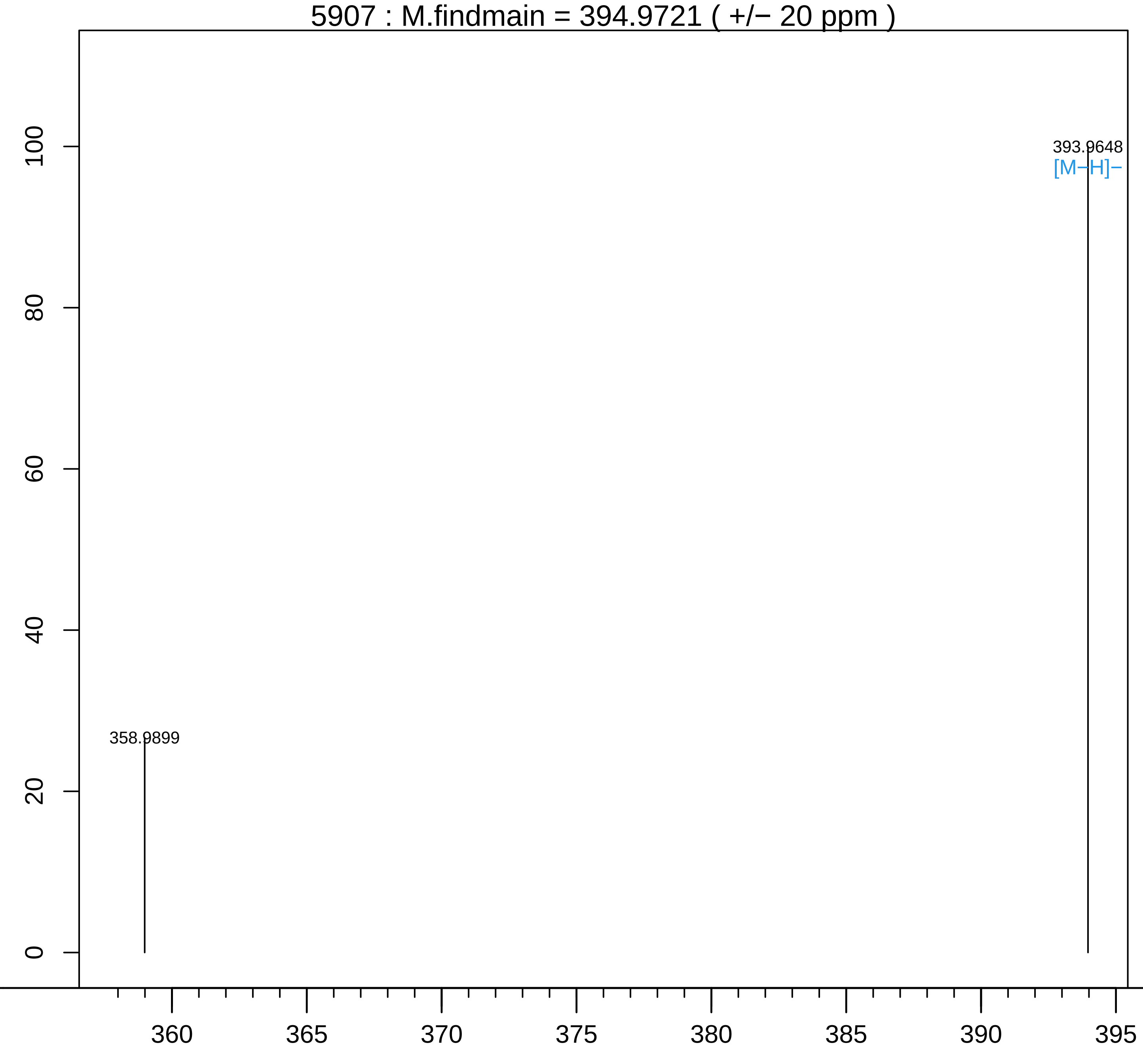
5907 : M.findmain = 394.9721 (+/− 20 ppm)
393.9648
[M−H]−
358.9899
0
20
40
60
80
100
360
365
370
375
380
385
390
395

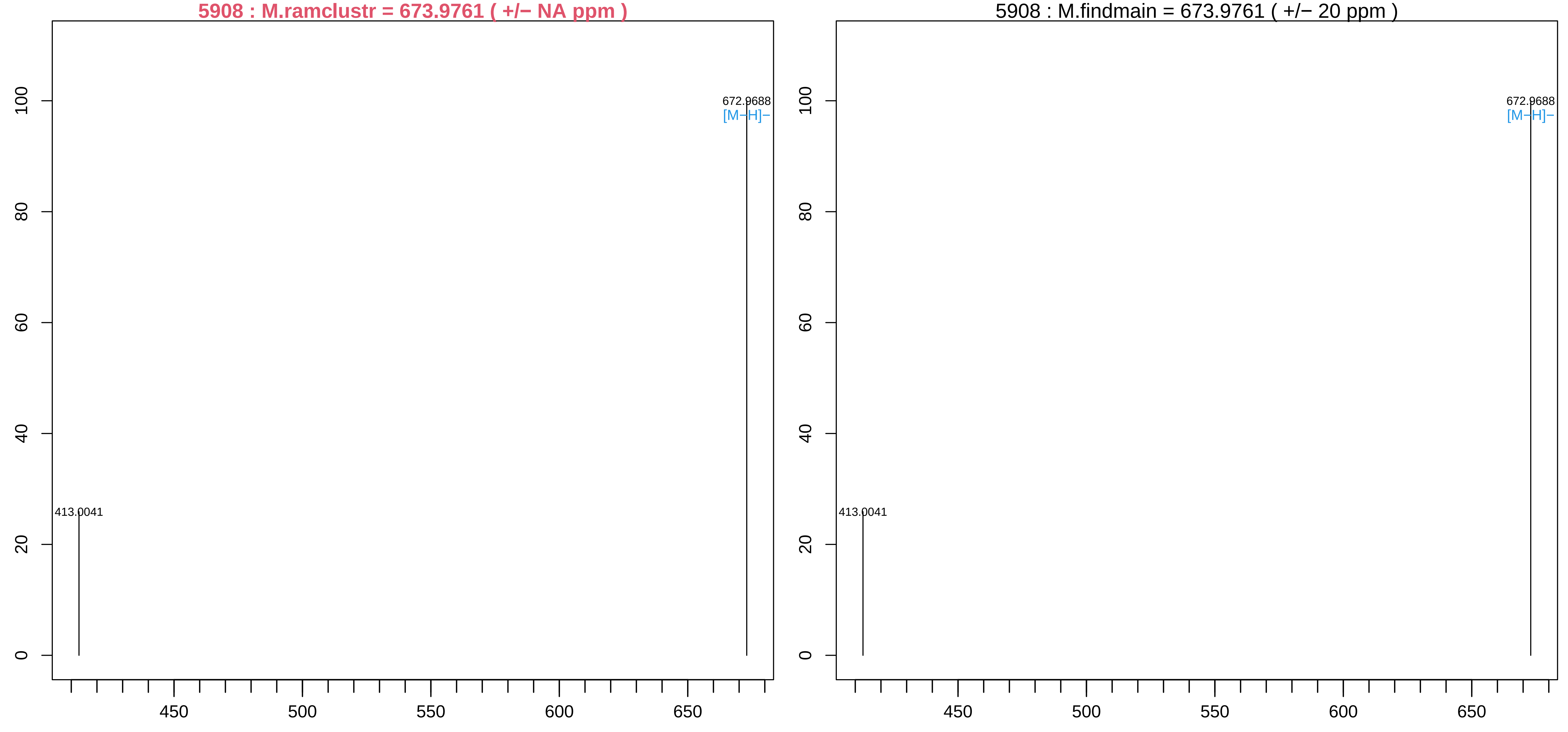
5908 : M.ramclustr = 673.9761 (+/− NA ppm)
672.9688
[M−H]−
413.0041
0
20
40
60
80
100
450
500
550
600
650
5908 : M.findmain = 673.9761 (+/− 20 ppm)
672.9688
[M−H]−
413.0041
0
20
40
60
80
100
450
500
550
600
650

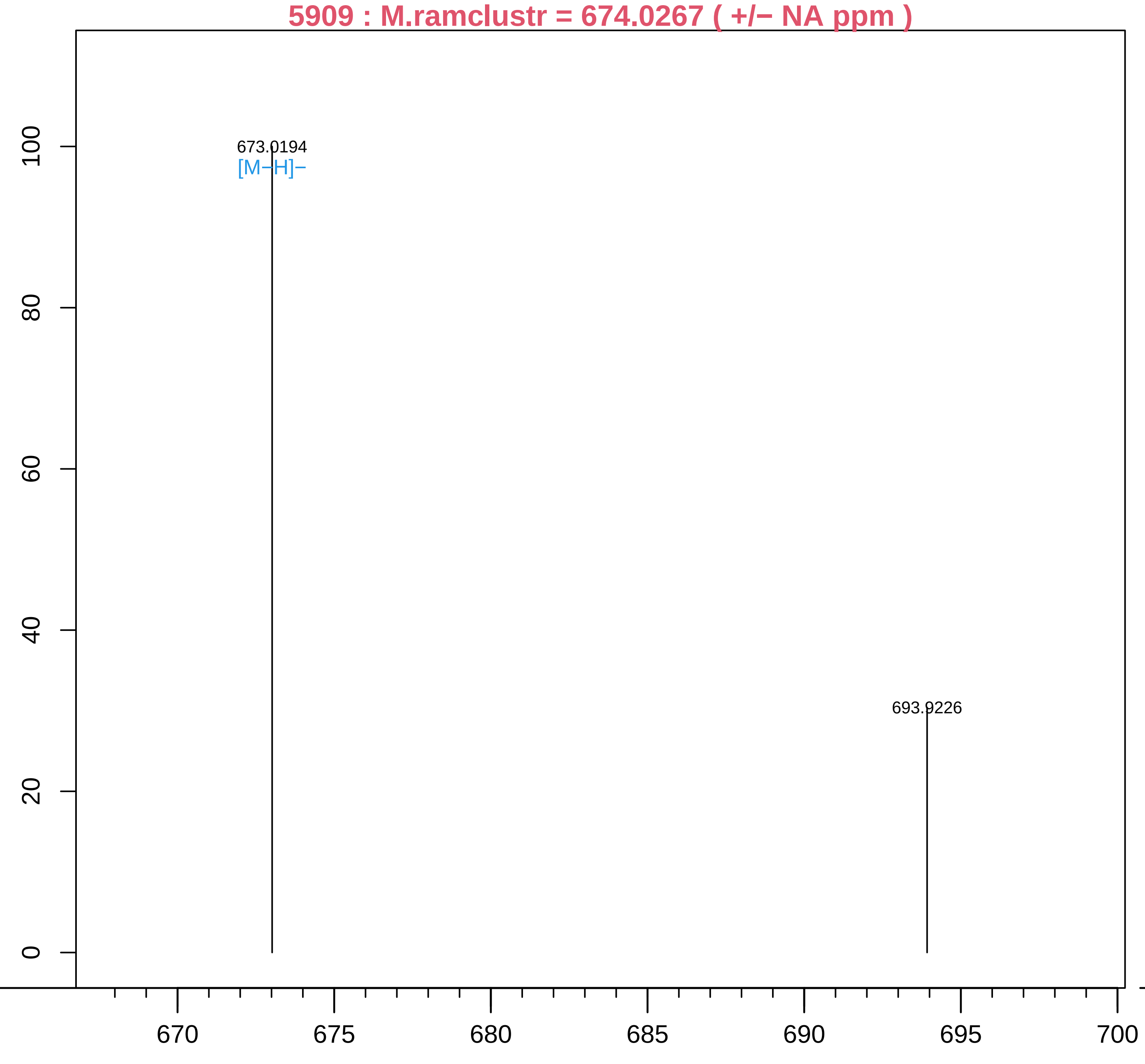
5909 : M.ramclustr = 674.0267 (+/− NA ppm)
673.0194
[M−H]−
693.9226
0
20
40
60
80
100
670
675
680
685
690
695
700

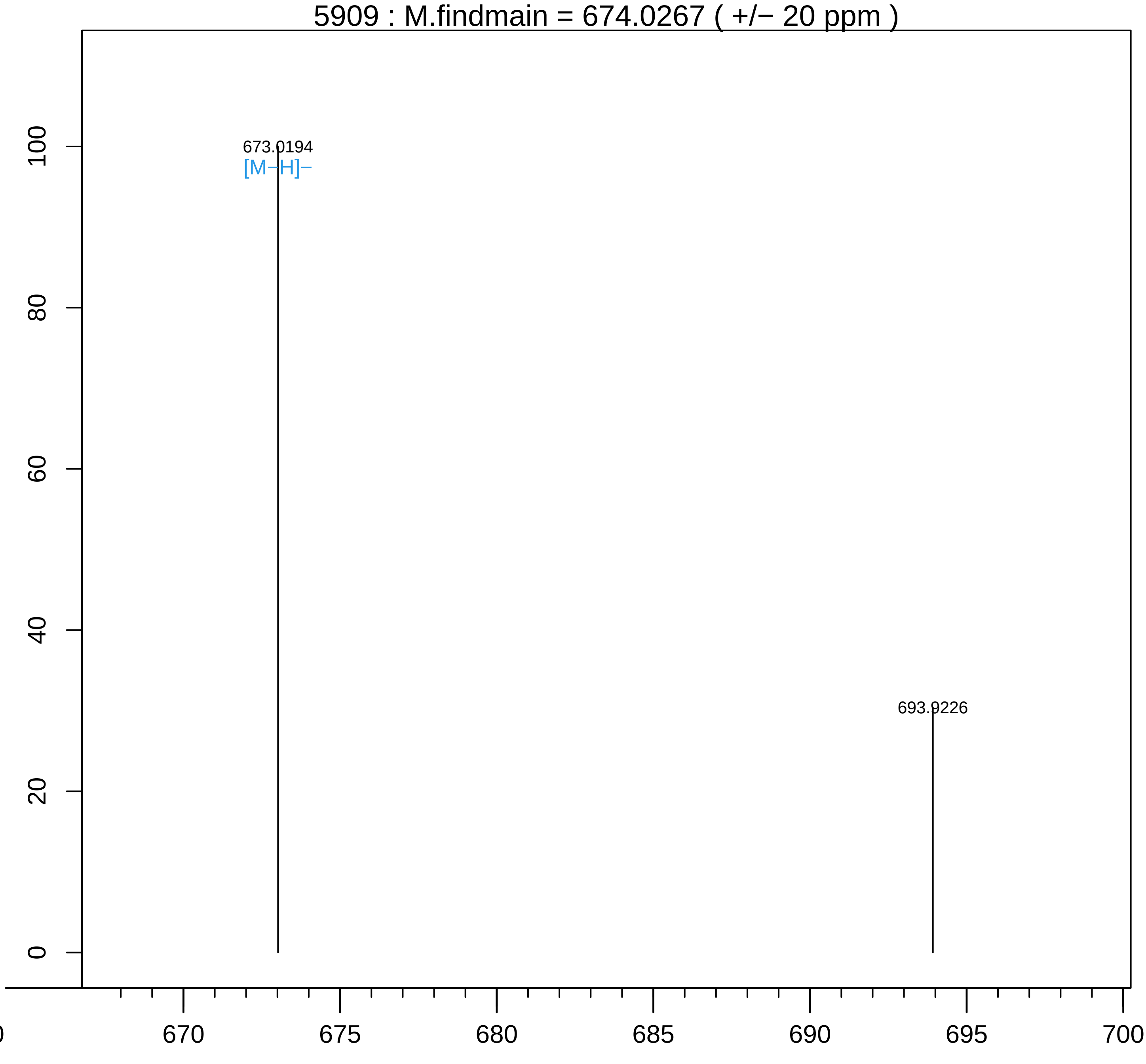
5909 : M.findmain = 674.0267 (+/− 20 ppm)
673.0194
[M−H]−
693.9226
0
20
40
60
80
100
670
675
680
685
690
695
700

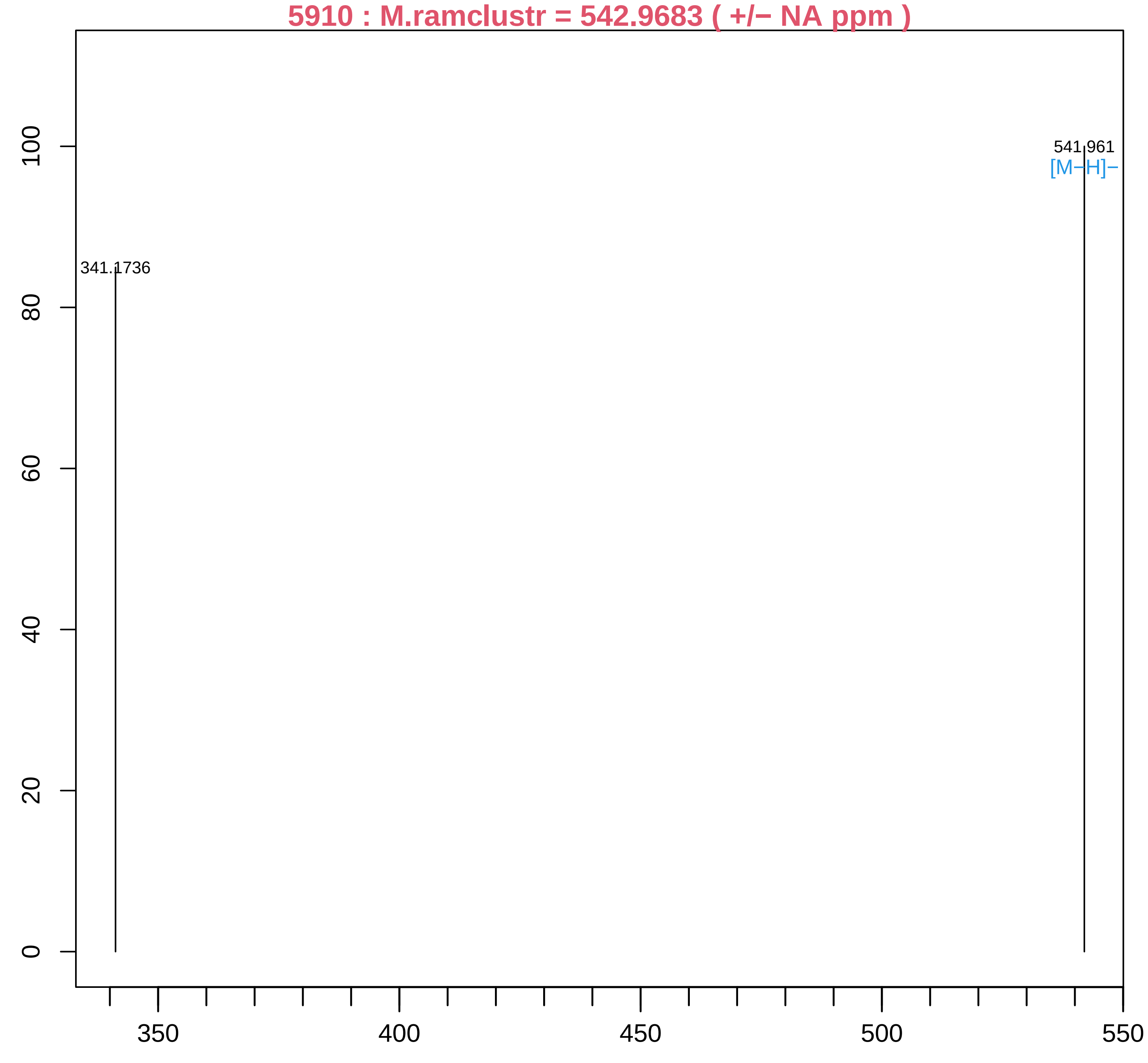
5910 : M.ramclustr = 542.9683 (+/− NA ppm)
541.961
[M−H]−
341.1736
0
20
40
60
80
100
350
400
450
500
550

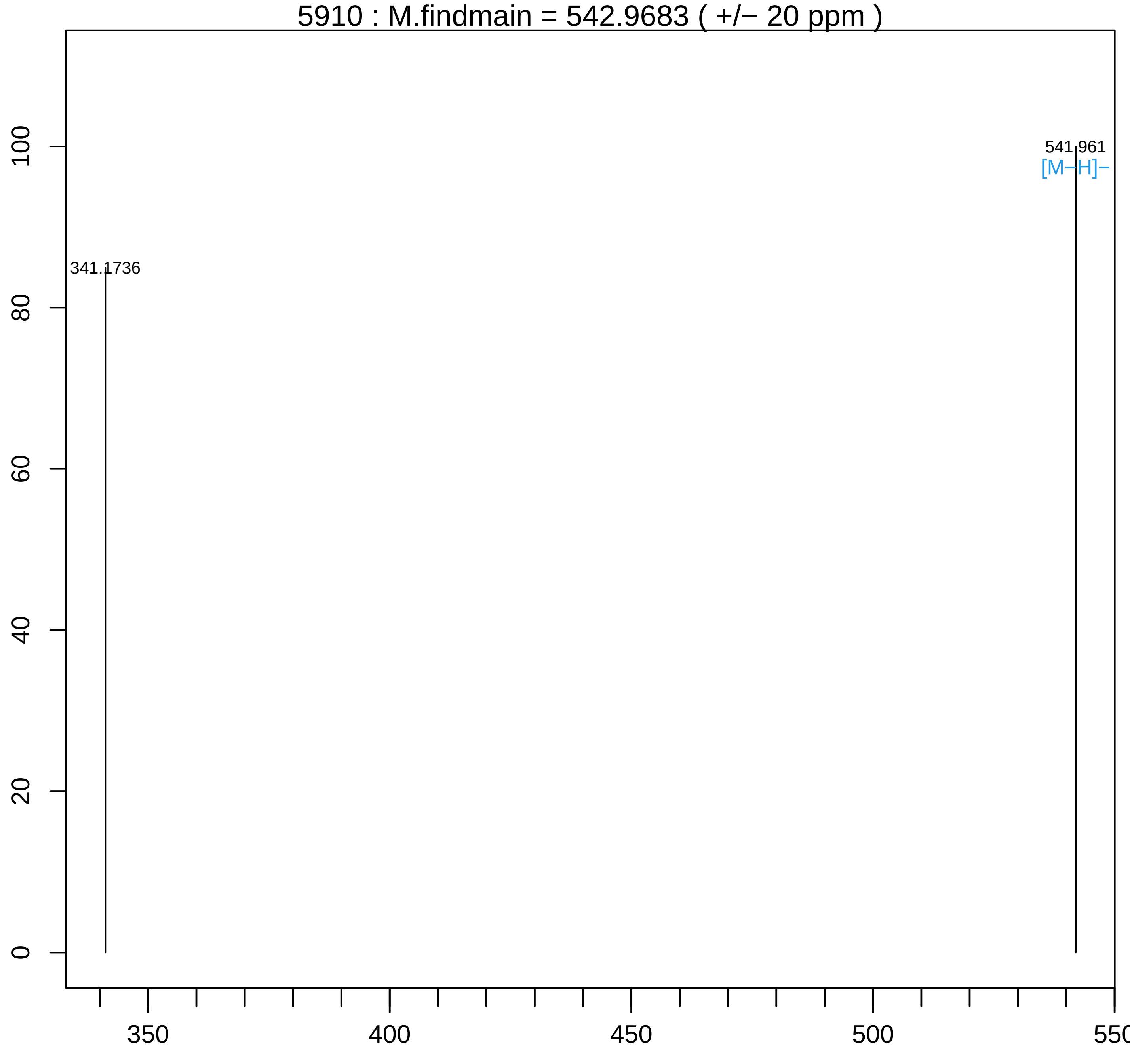
5910 : M.findmain = 542.9683 (+/− 20 ppm)
541.961
[M−H]−
341.1736
0
20
40
60
80
100
350
400
450
500
550

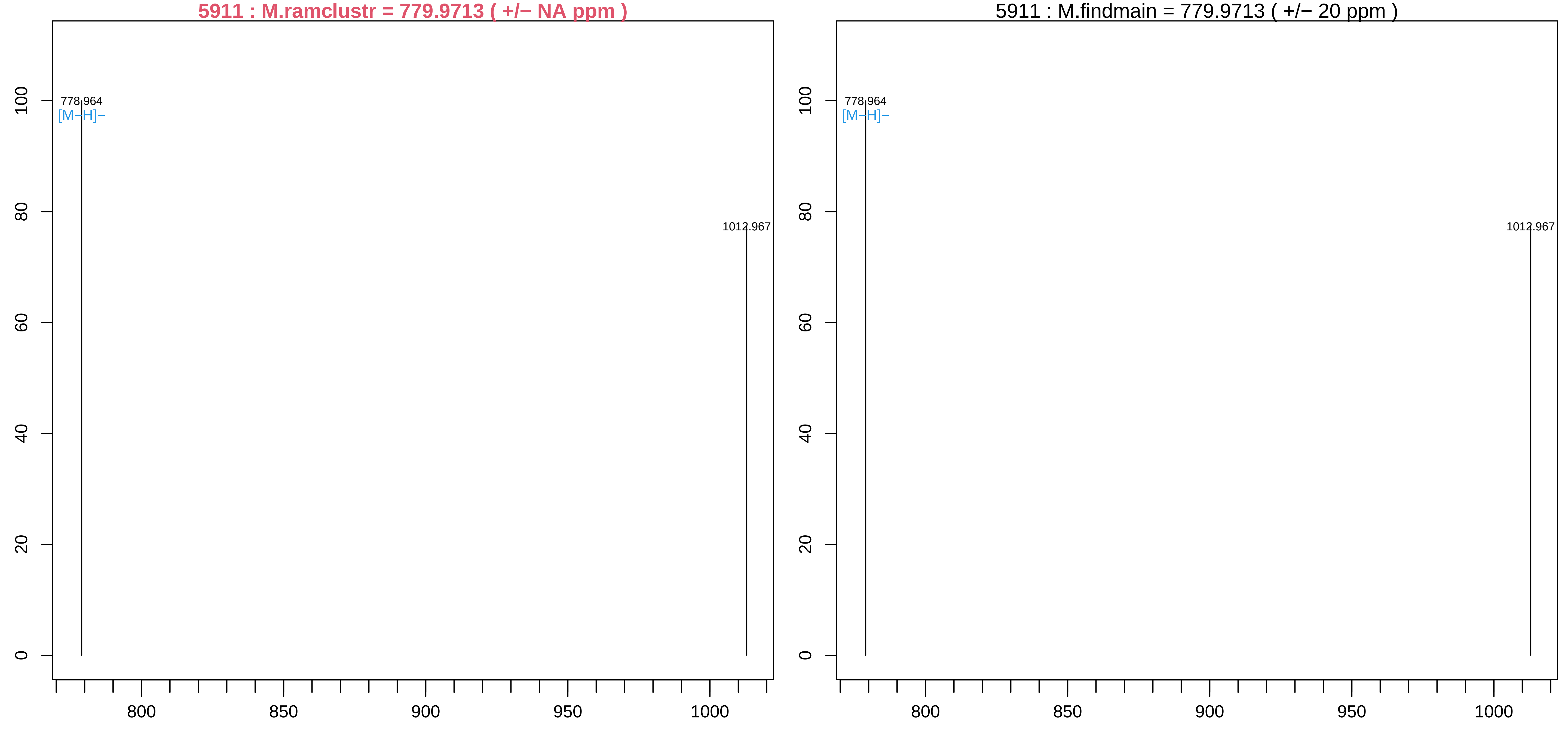

5911 : M.ramclustr = 779.9713 (+/− NA ppm)
778.964
[M−H]−
1012.967
0
20
40
60
80
100
800
850
900
950
1000
5911 : M.findmain = 779.9713 (+/− 20 ppm)
778.964
[M−H]−
1012.967
0
20
40
60
80
100
800
850
900
950
1000

5912 : M.ramclustr = 905.9743 (+/− NA ppm)

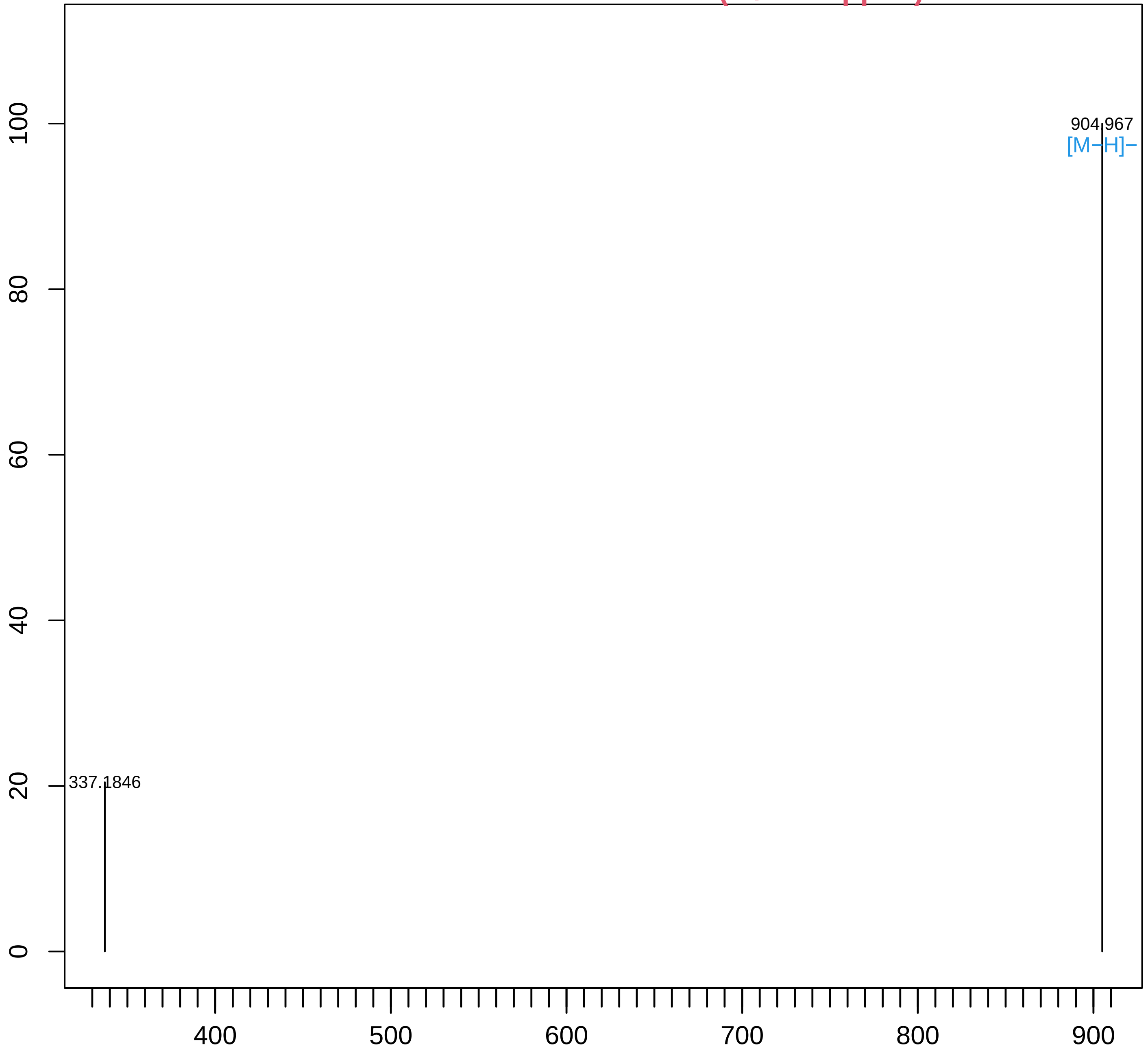

5912 : M.findmain = 905.9743 (+/− 20 ppm)

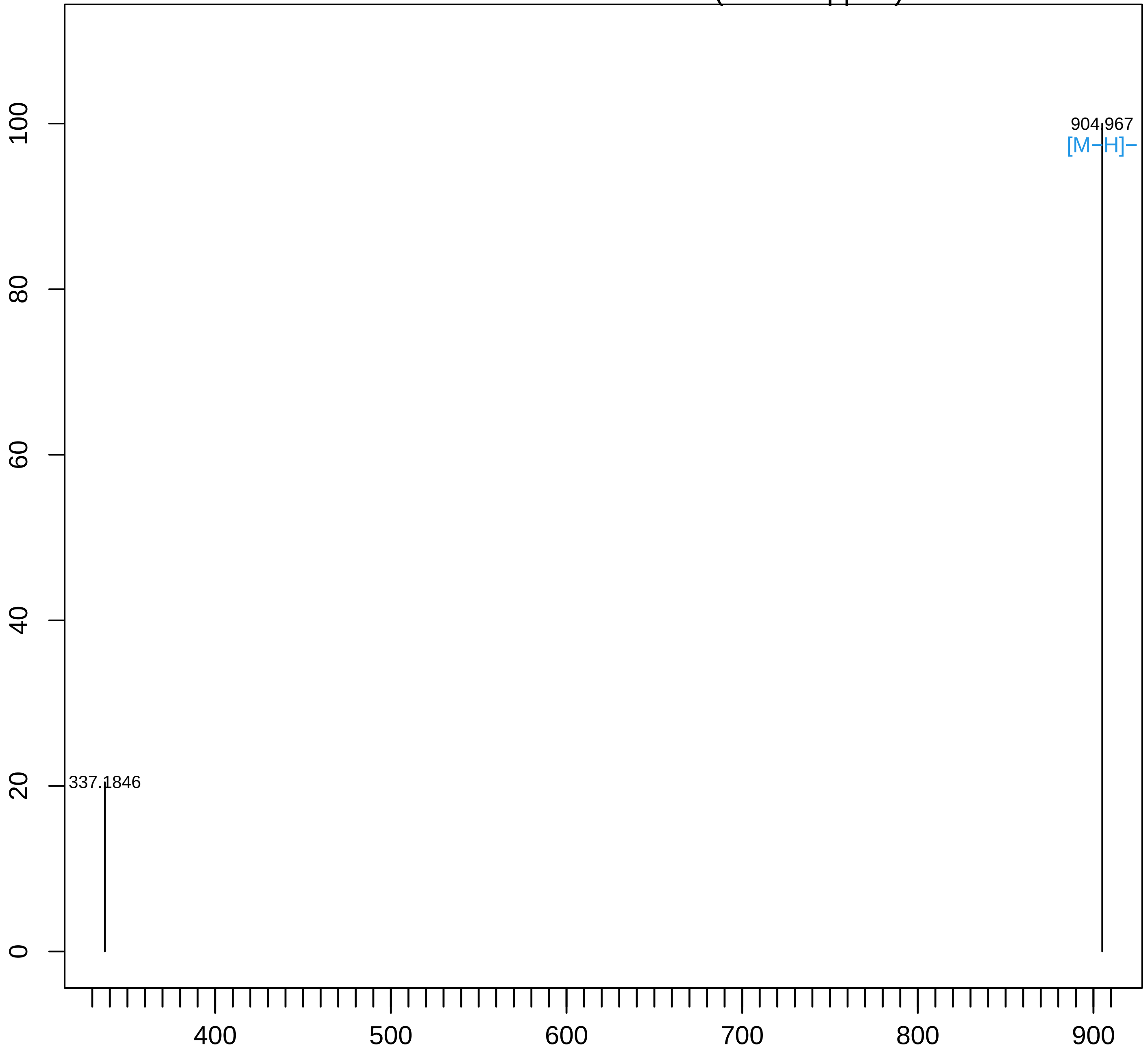

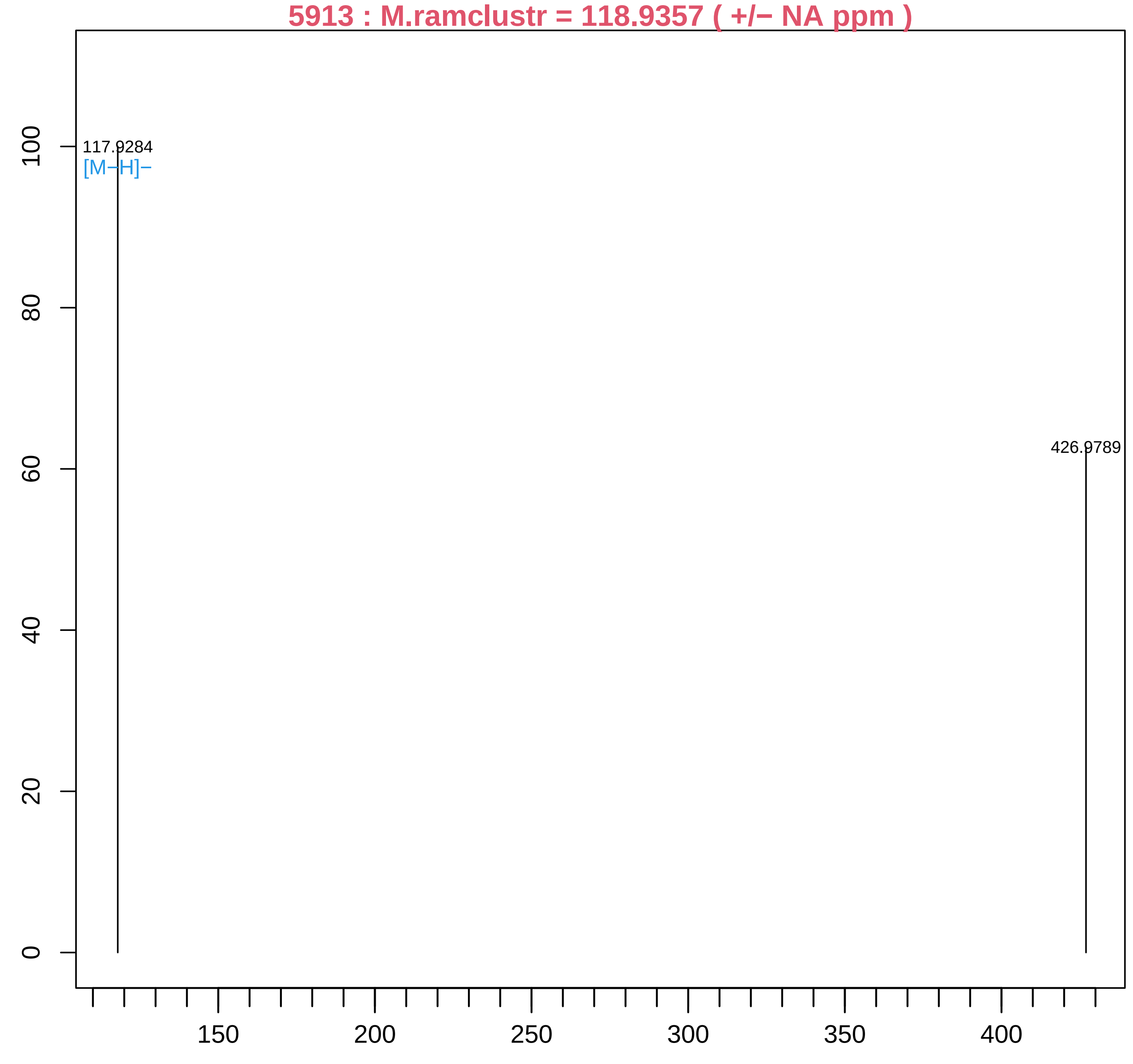
5913 : M.ramclustr = 118.9357 (+/− NA ppm)
117.9284
[M−H]−
426.9789
0
20
40
60
80
100
150
200
250
300
350
400

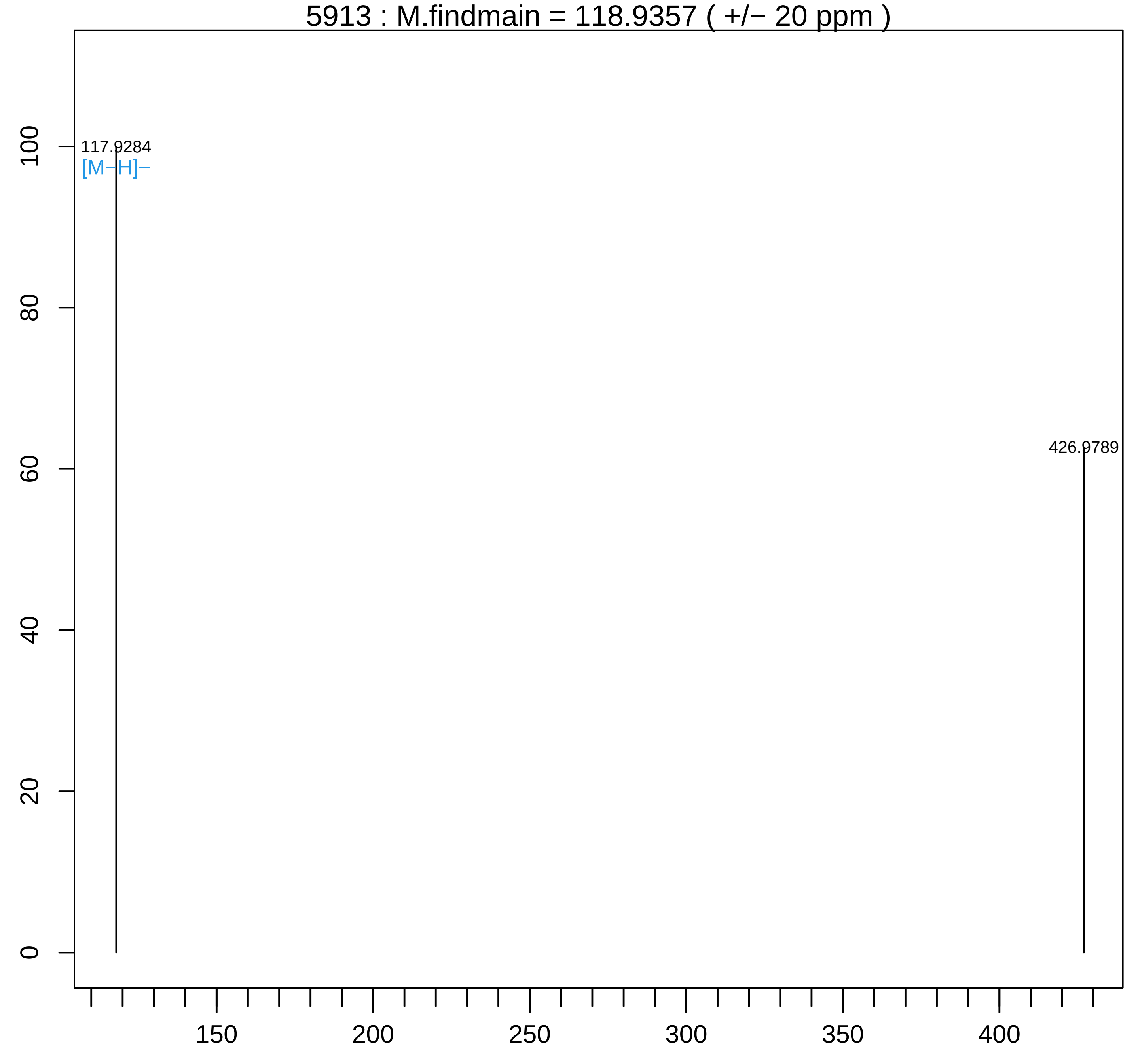
5913 : M.findmain = 118.9357 (+/− 20 ppm)
117.9284
[M−H]−
426.9789
0
20
40
60
80
100
150
200
250
300
350
400

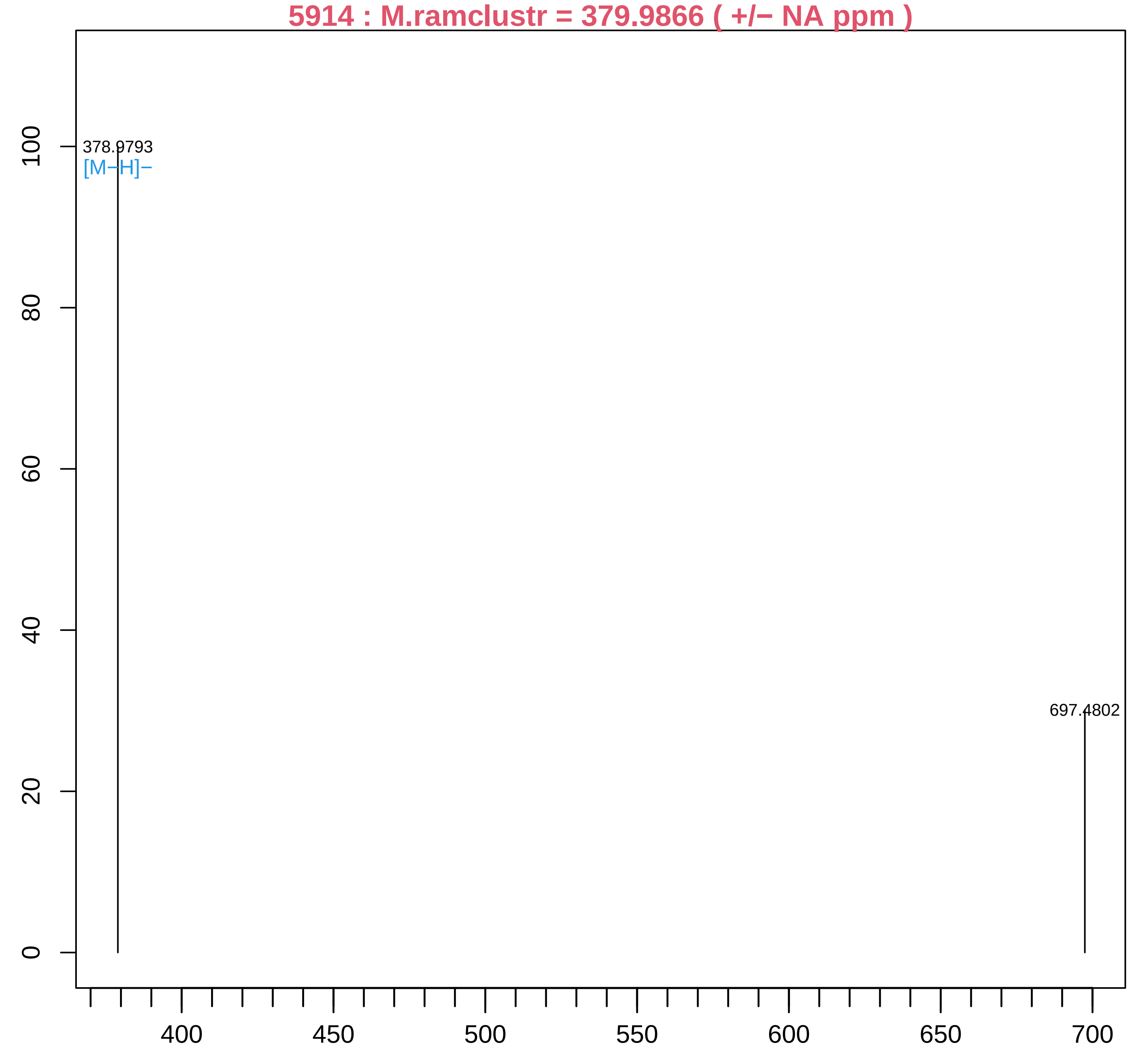
5914 : M.ramclustr = 379.9866 (+/− NA ppm)
378.9793
[M−H]−
697.4802
0
20
40
60
80
100
400
450
500
550
600
650
700

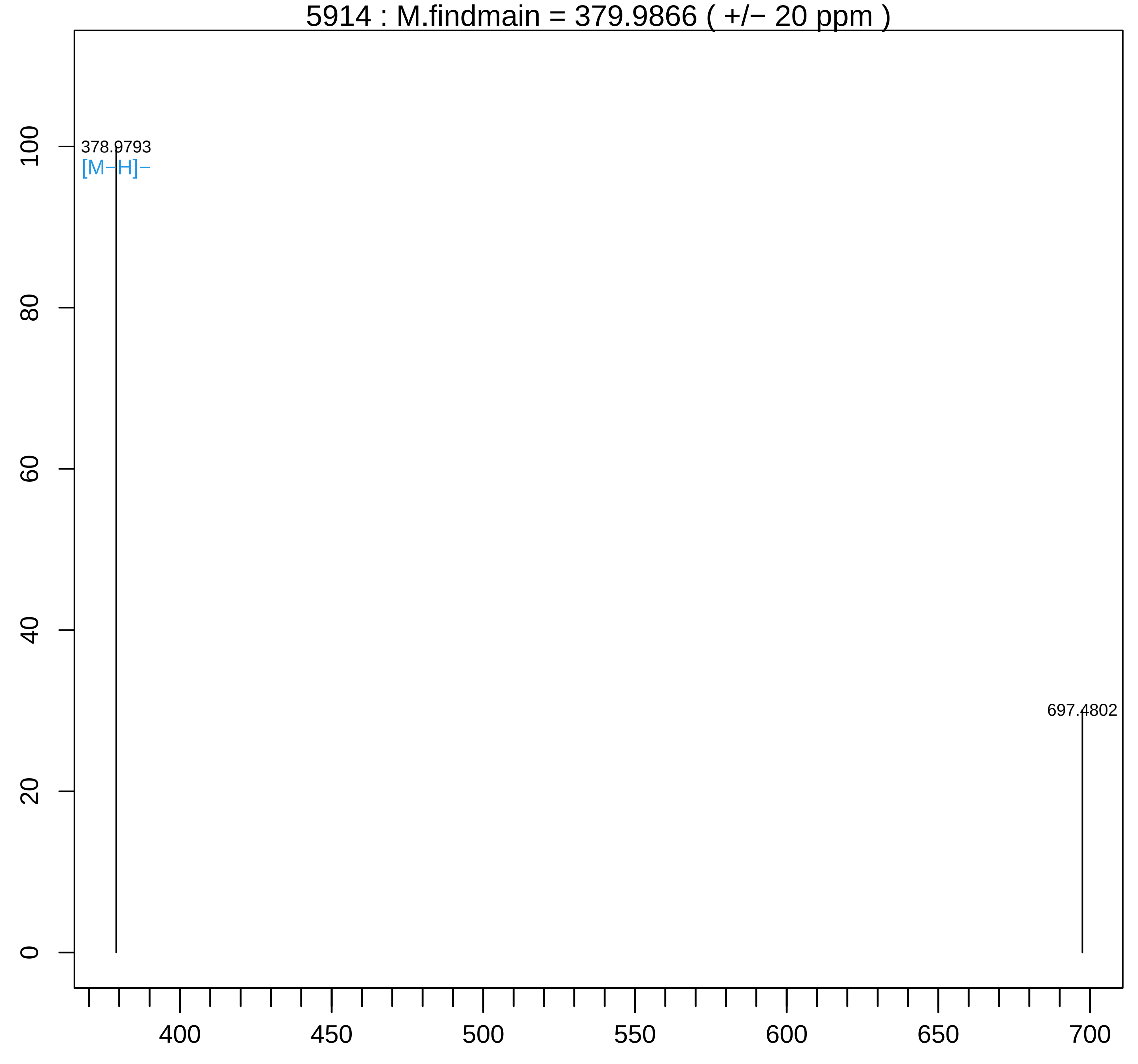
5914 : M.findmain = 379.9866 (+/− 20 ppm)
378.9793
[M−H]−
697.4802
0
20
40
60
80
100
400
450
500
550
600
650
700

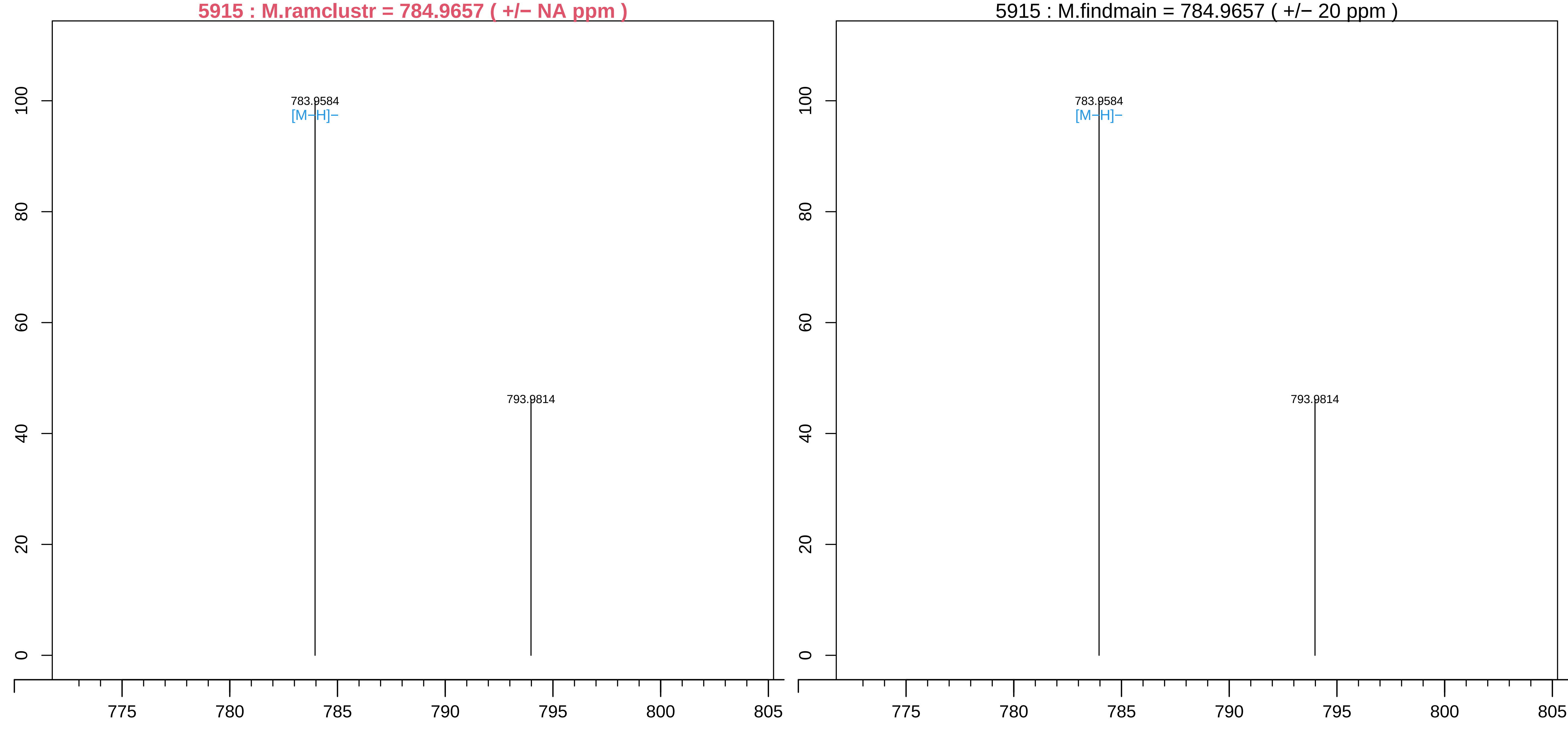
5915 : M.ramclustr = 784.9657 (+/− NA ppm)
783.9584
[M−H]−
793.9814
0
20
40
60
80
100
775
780
785
790
795
800
805
5915 : M.findmain = 784.9657 (+/− 20 ppm)
783.9584
[M−H]−
793.9814
0
20
40
60
80
100
775
780
785
790
795
800
805

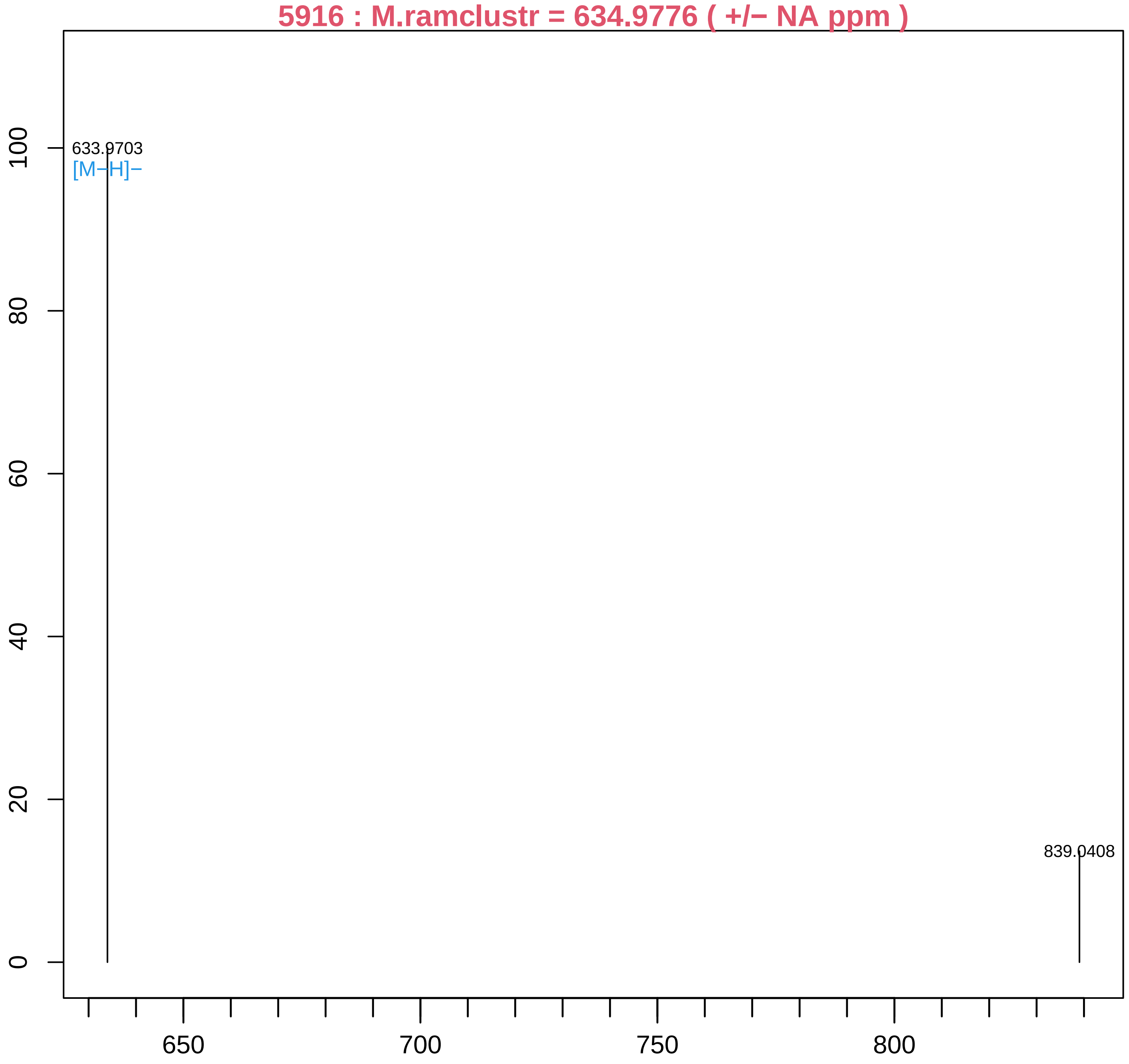

5916 : M.ramclustr = 634.9776 (+/− NA ppm)
633.9703
[M−H]−
839.0408
0
20
40
60
80
100
650
700
750
800

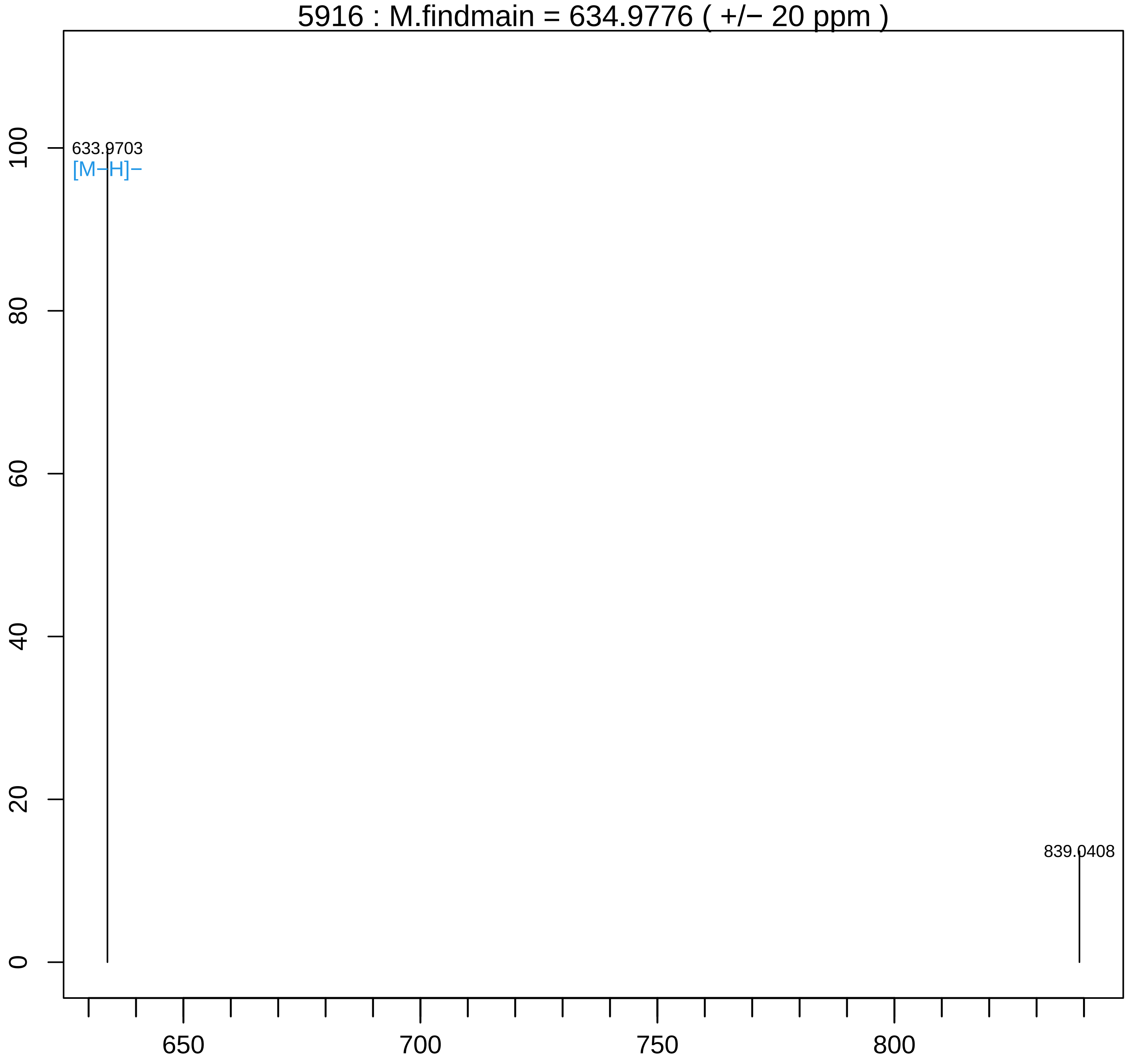

5916 : M.findmain = 634.9776 (+/− 20 ppm)
633.9703
[M−H]−
839.0408
0
20
40
60
80
100
650
700
750
800

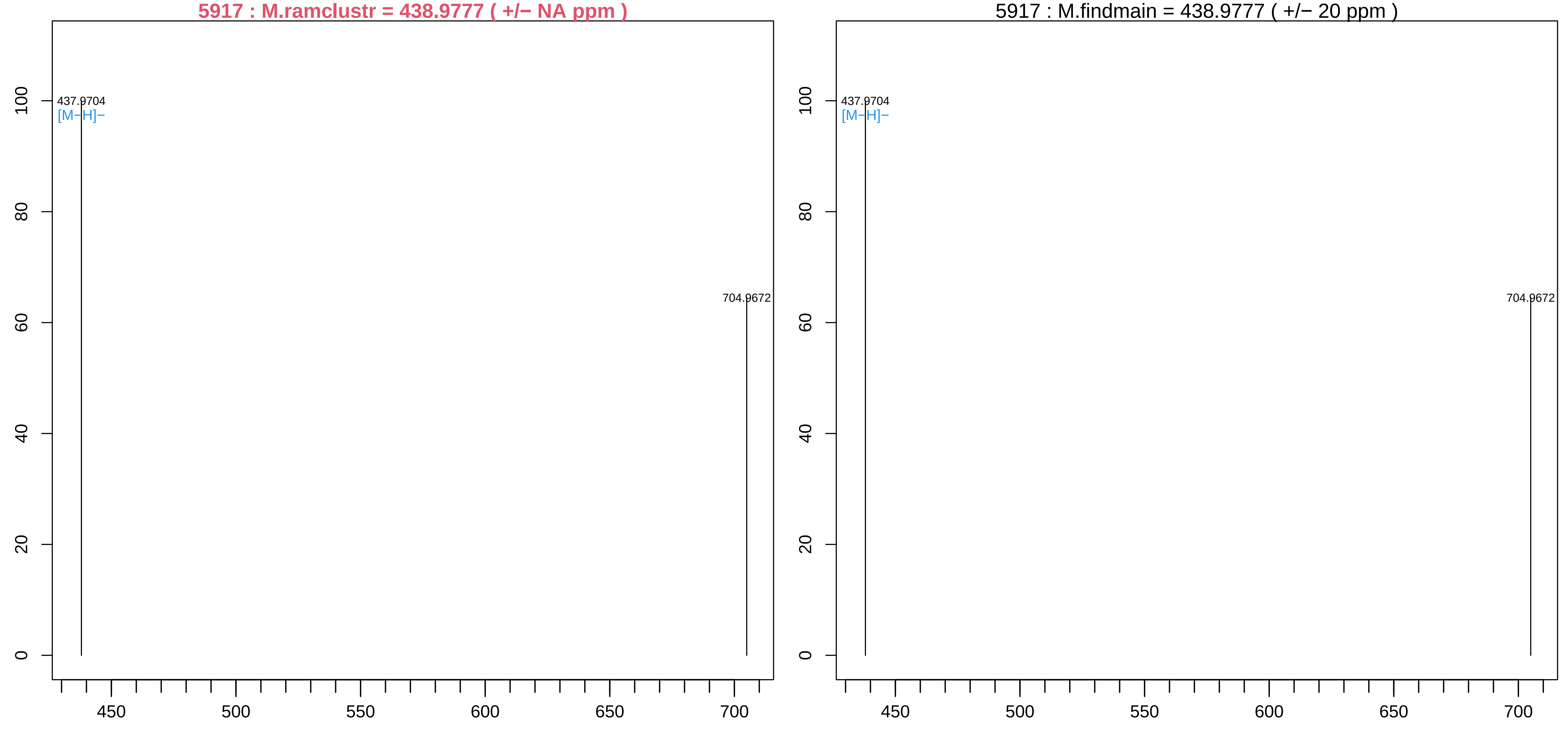
5917 : M.ramclustr = 438.9777 (+/− NA ppm)
437.9704
[M−H]−
704.9672
5917 : M.findmain = 438.9777 (+/− 20 ppm)
437.9704
[M−H]−
704.9672
0
20
40
60
80
100
450
500
550
600
650
700

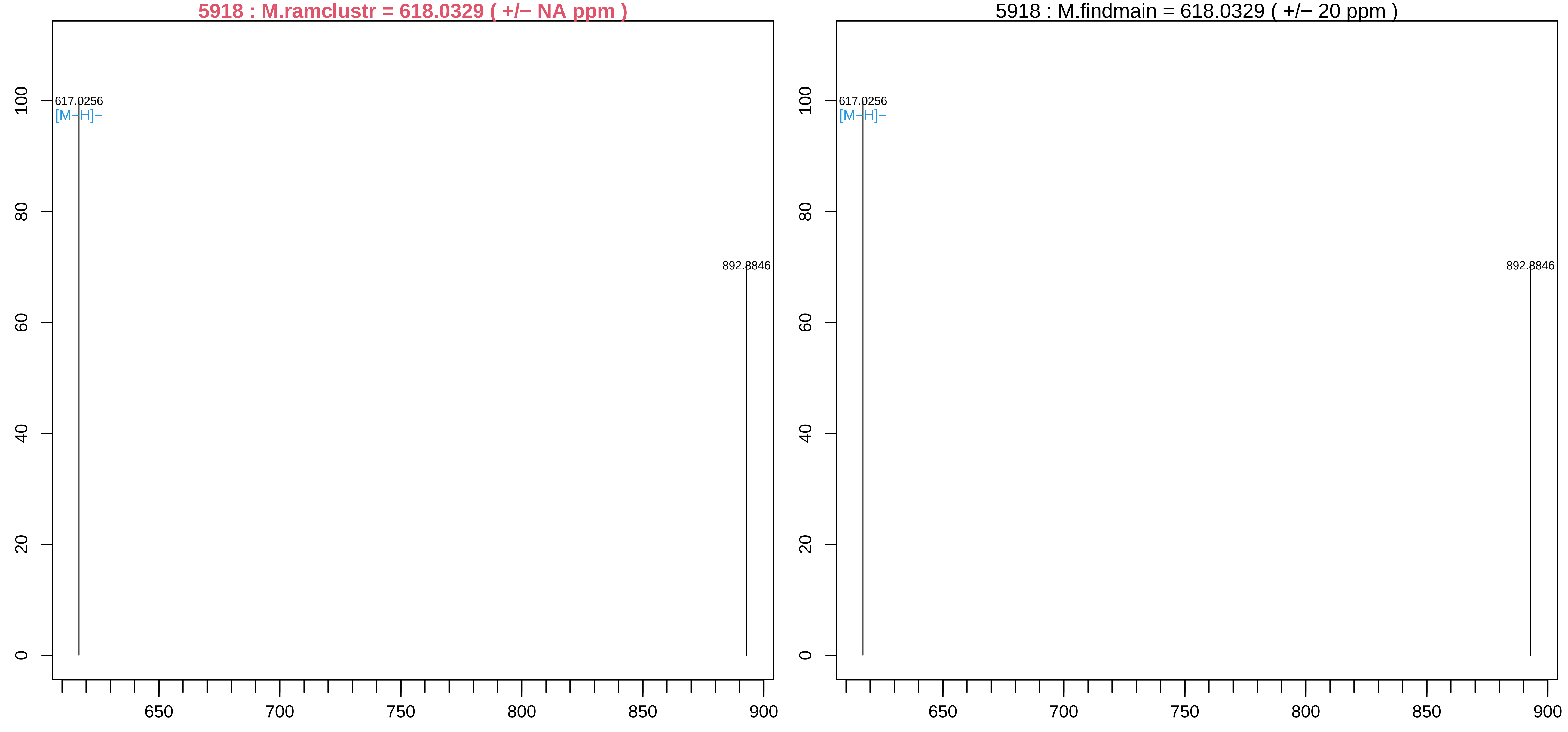
5918 : M.ramclustr = 618.0329 (+/− NA ppm)
617.0256
[M−H]−
892.8846
0
20
40
60
80
100
650
700
750
800
850
900
5918 : M.findmain = 618.0329 (+/− 20 ppm)
617.0256
[M−H]−
892.8846
0
20
40
60
80
100
650
700
750
800
850
900

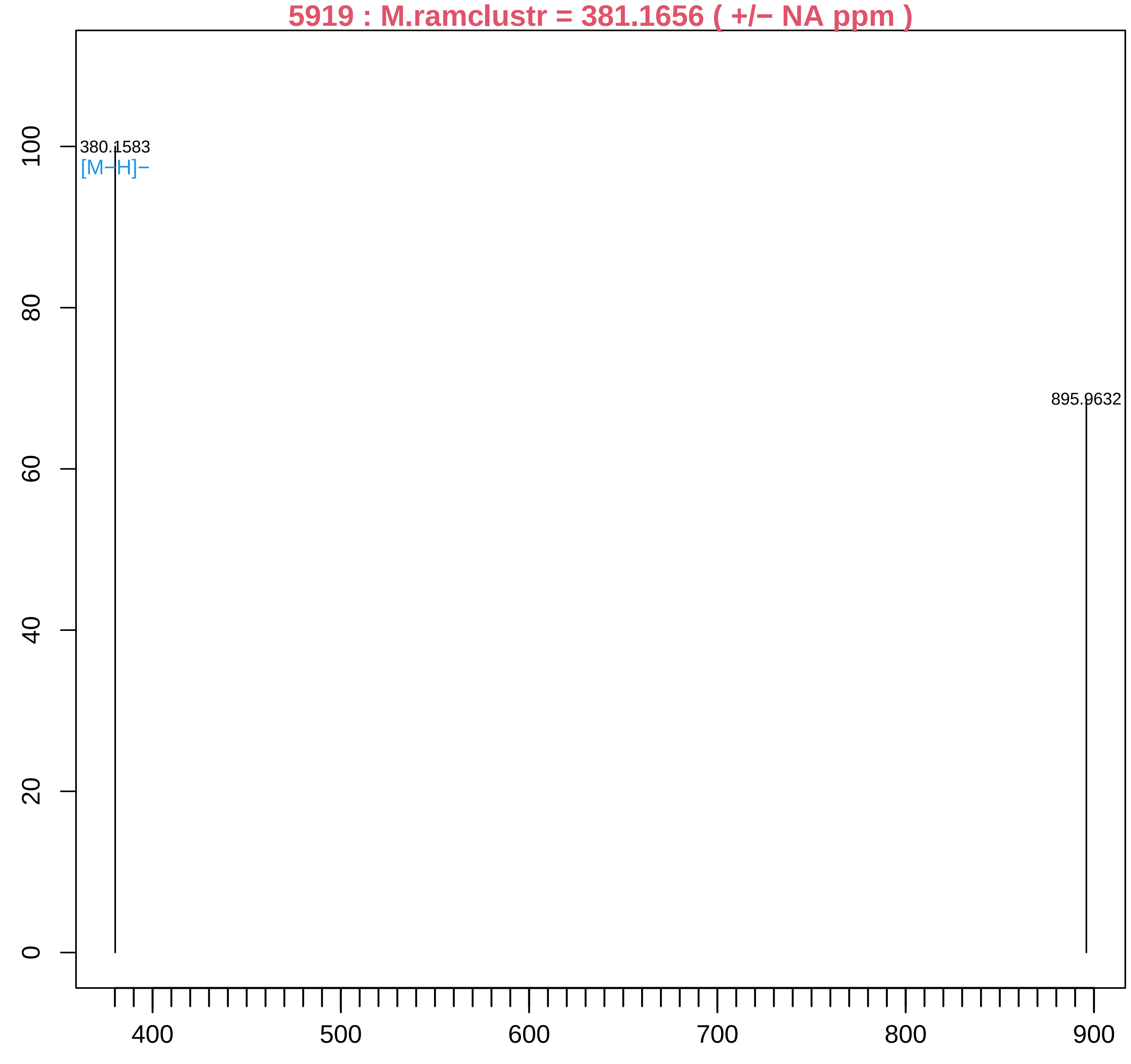
5919 : M.ramclustr = 381.1656 (+/− NA ppm)
380.1583
[M−H]−
895.9632
0
20
40
60
80
100
400
500
600
700
800
900

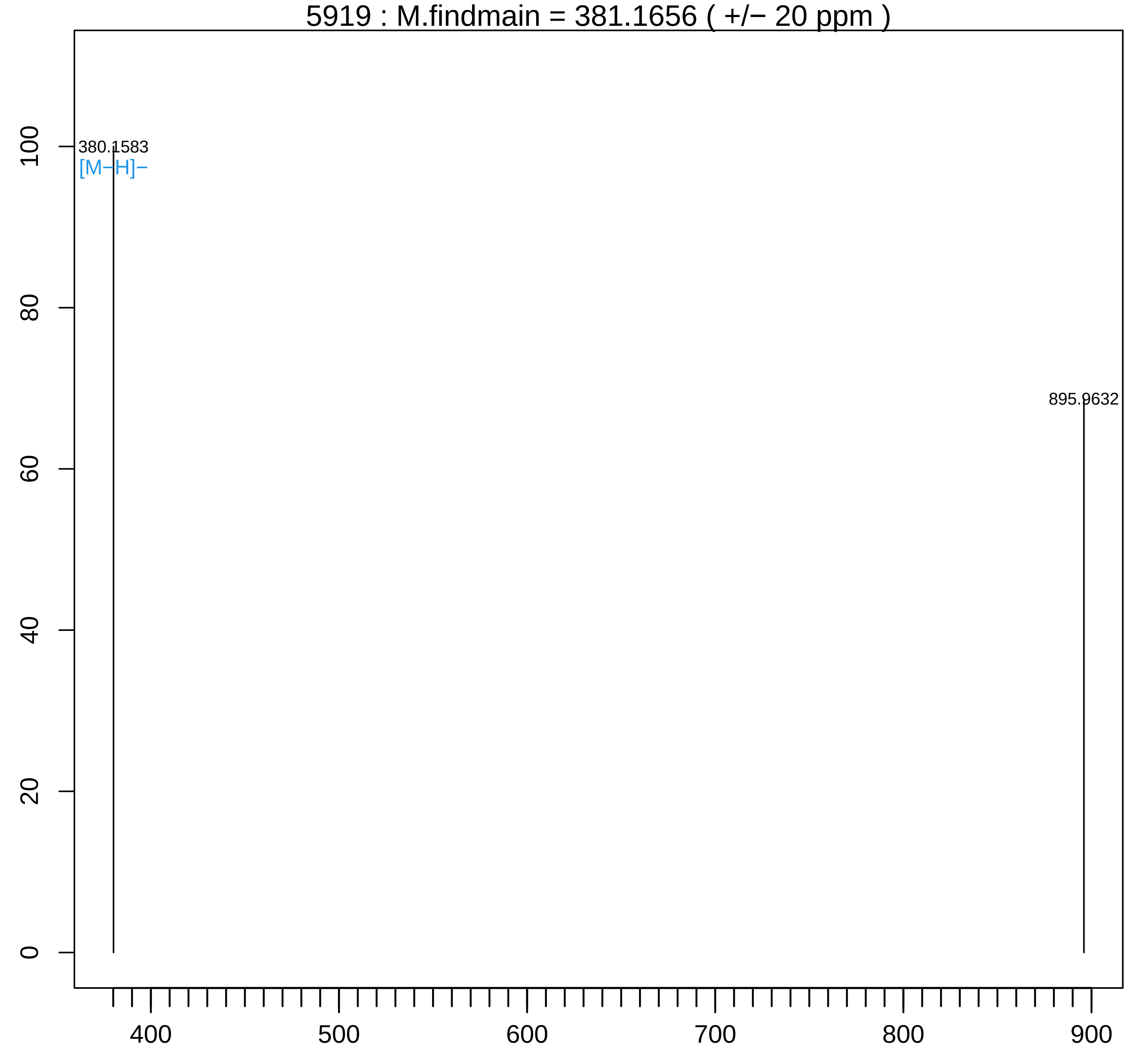
5919 : M.findmain = 381.1656 (+/− 20 ppm)
380.1583
[M−H]−
895.9632
0
20
40
60
80
100
400
500
600
700
800
900

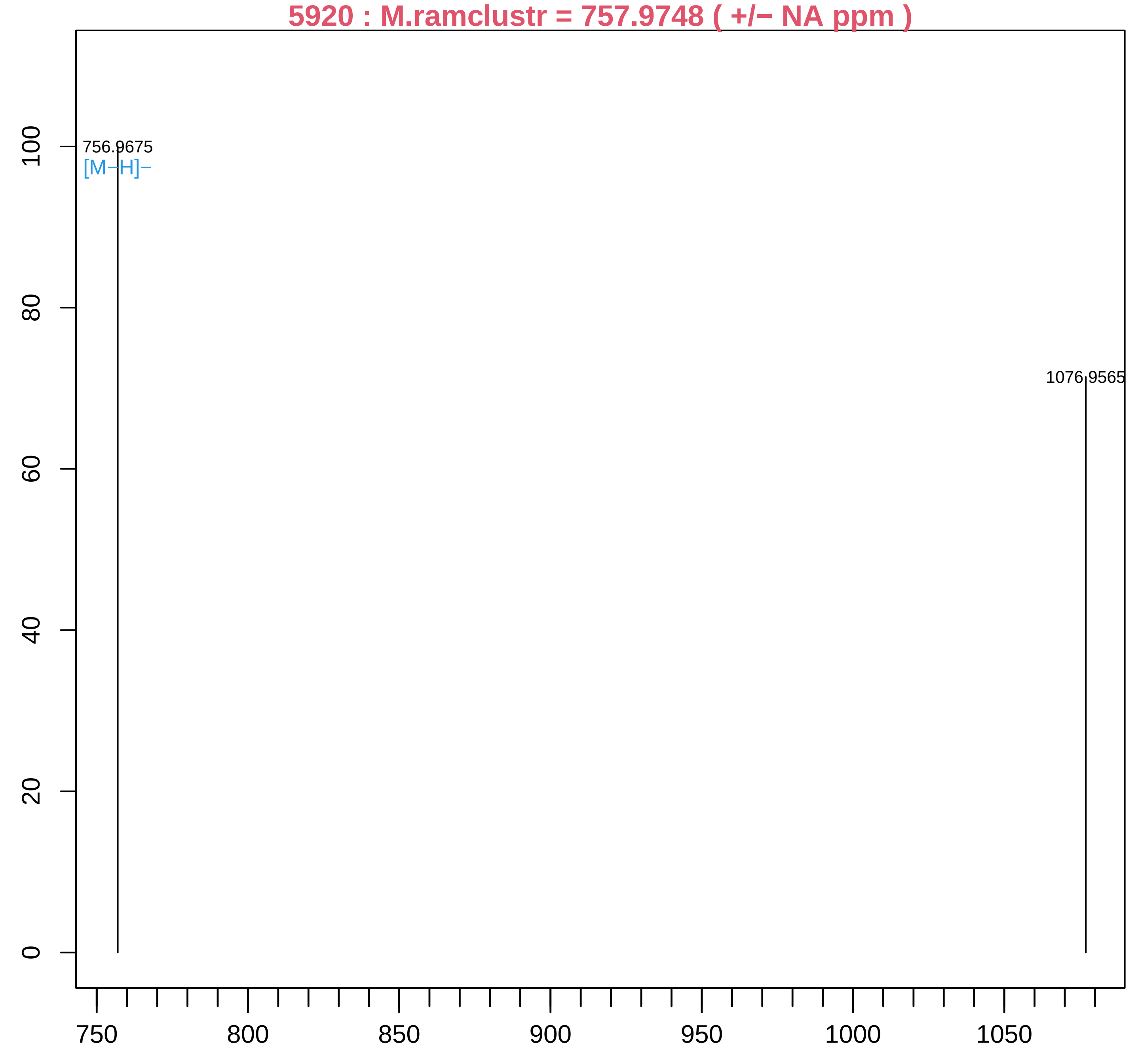
5920 : M.ramclustr = 757.9748 (+/− NA ppm)
756.9675
[M−H]−
1076.9565
0
20
40
60
80
100
750
800
850
900
950
1000
1050

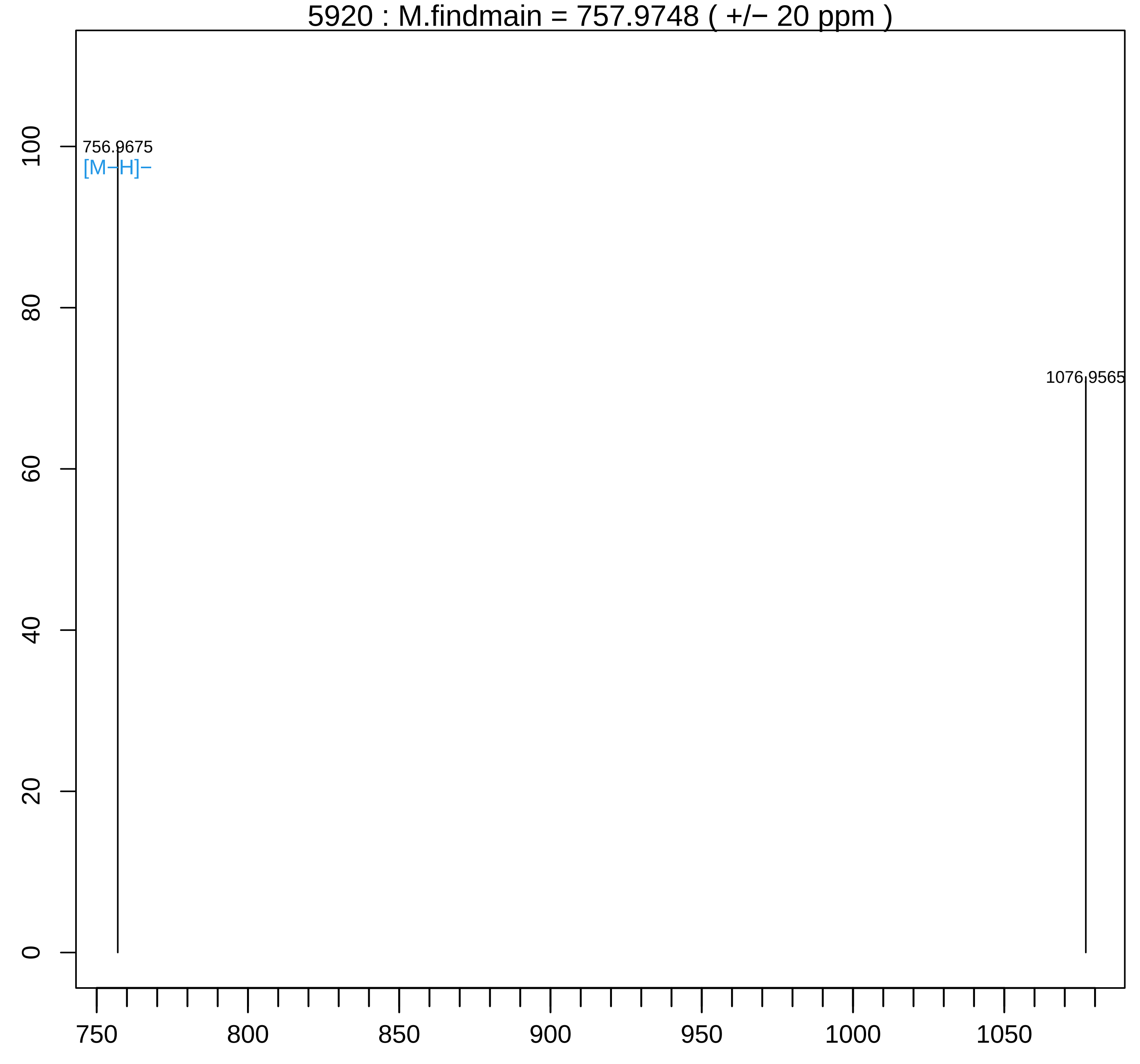
5920 : M.findmain = 757.9748 (+/− 20 ppm)
756.9675
[M−H]−
1076.9565
0
20
40
60
80
100
750
800
850
900
950
1000
1050

5921 : M.ramclustr = 617.9757 (+/− NA ppm)

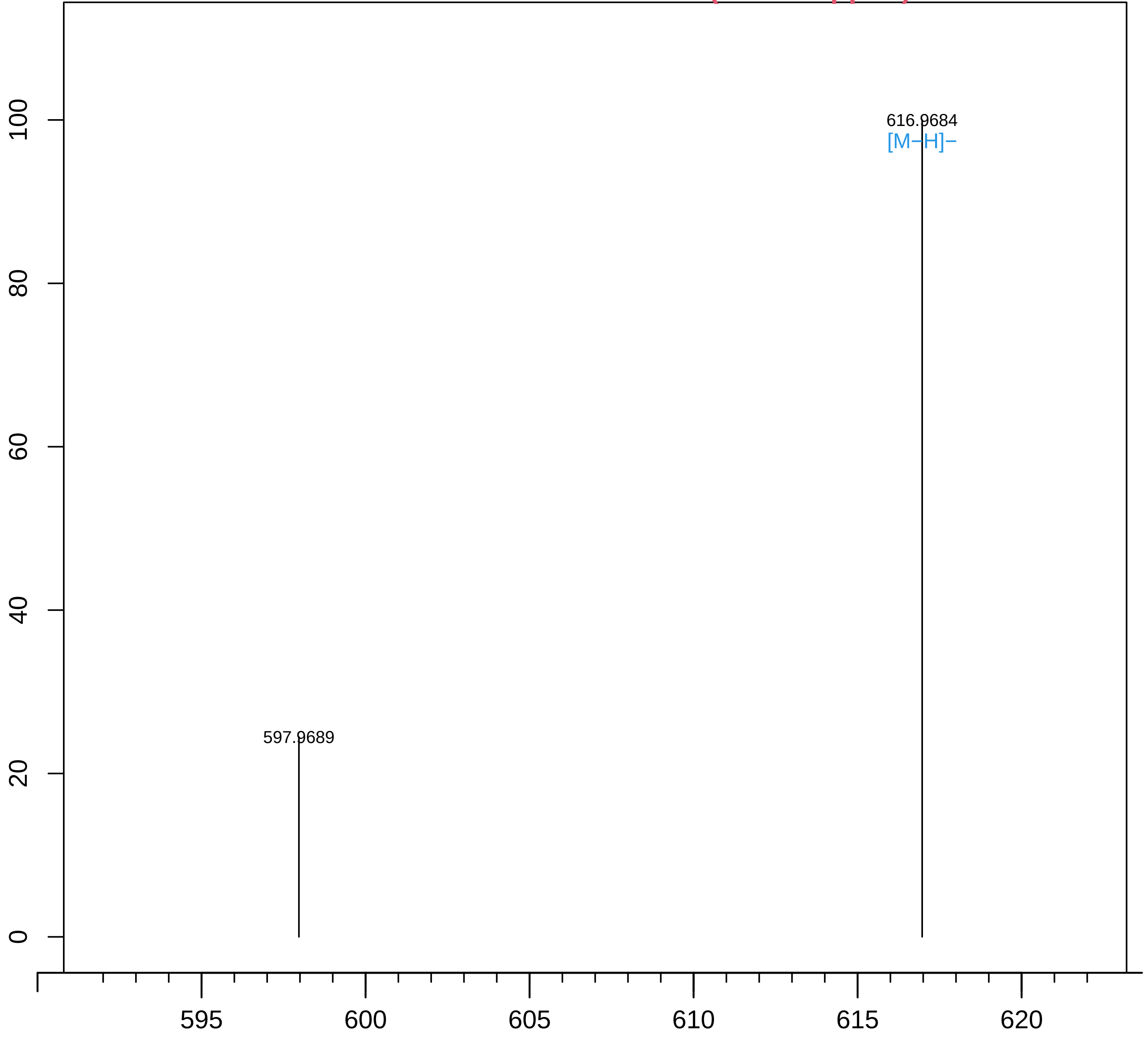

5921 : M.findmain = 617.9757 (+/− 20 ppm)

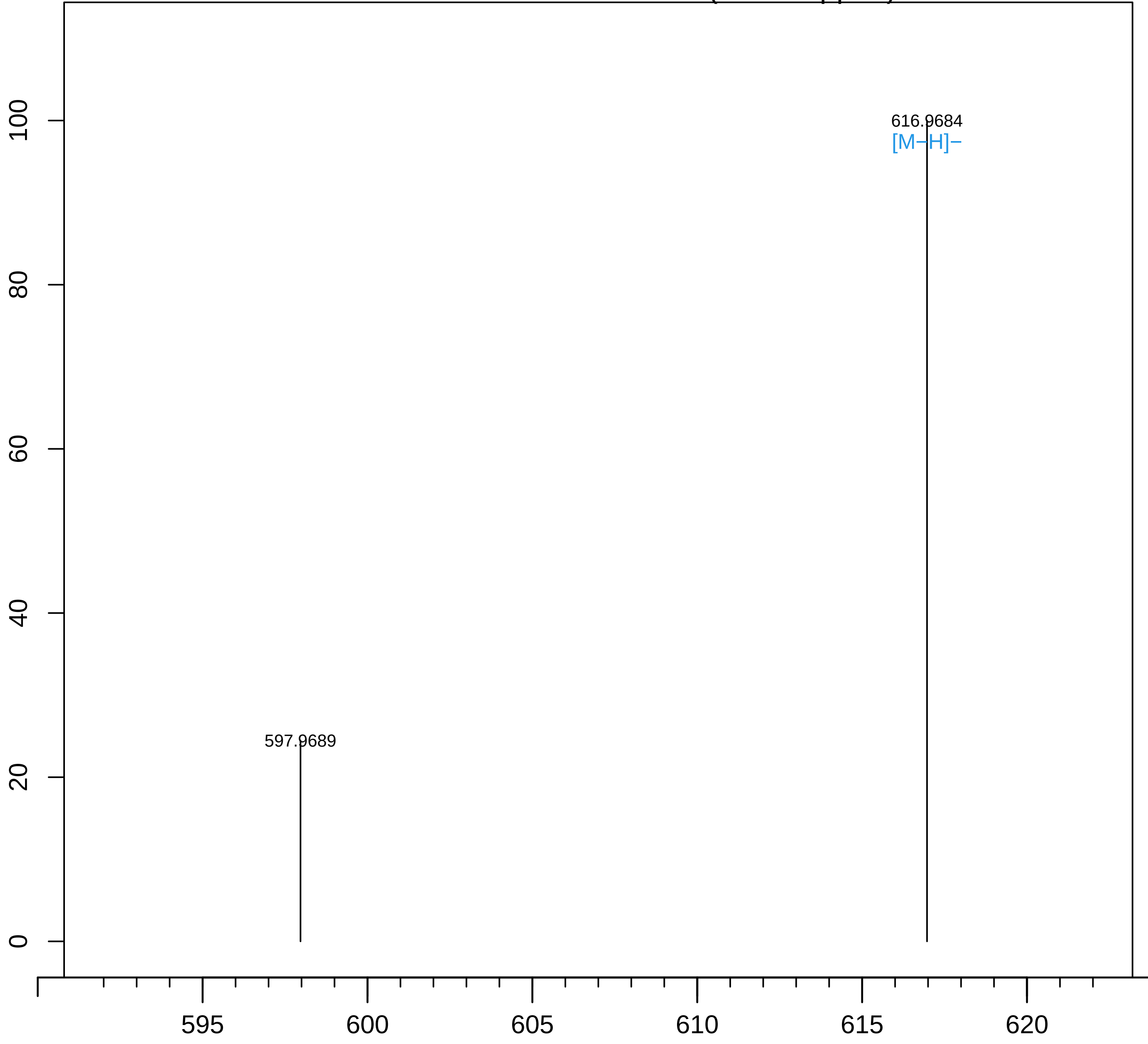

5922 : M.ramclustr = 845.9645 (+/− NA ppm)

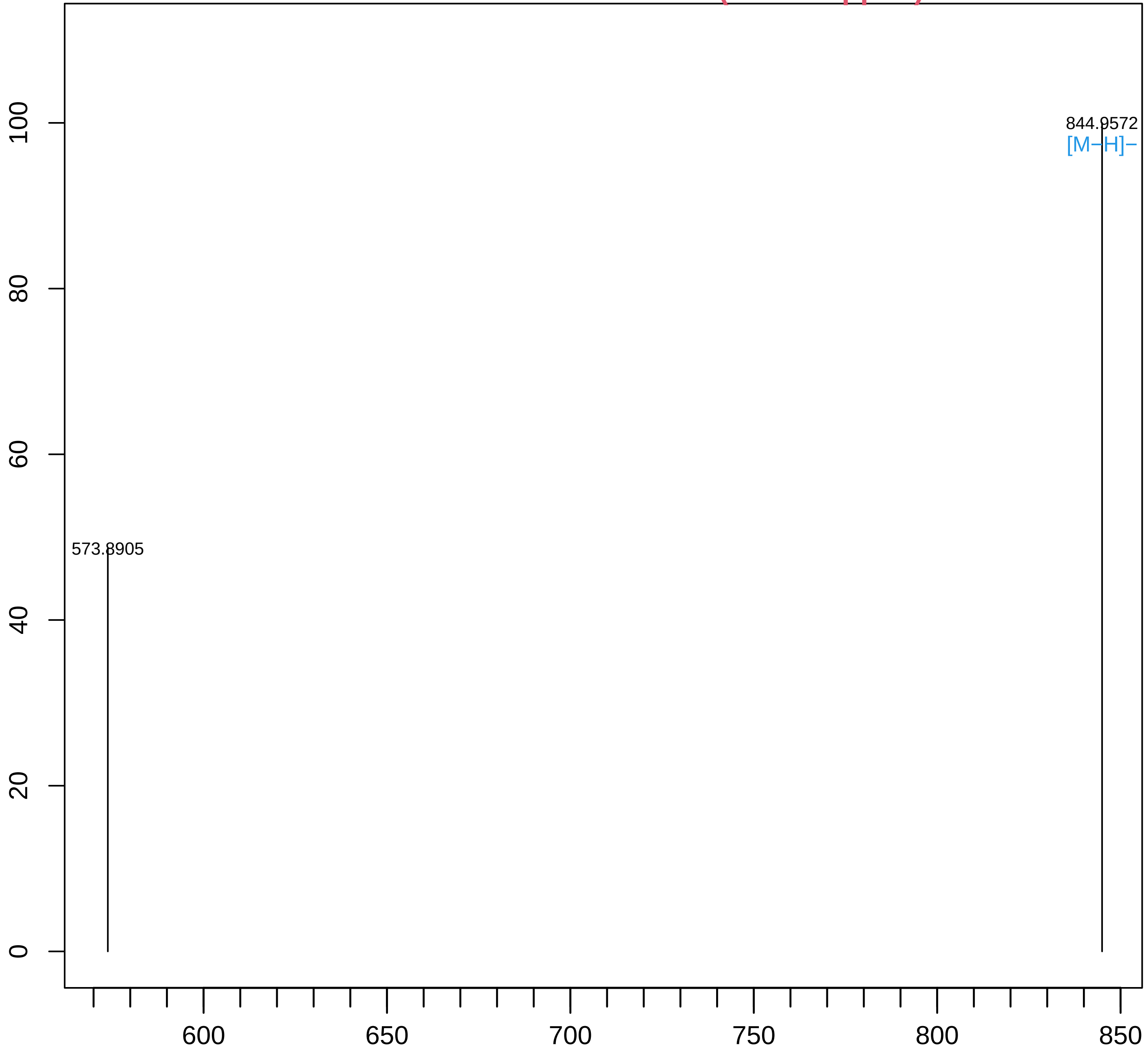

5922 : M.findmain = 845.9645 (+/− 20 ppm)

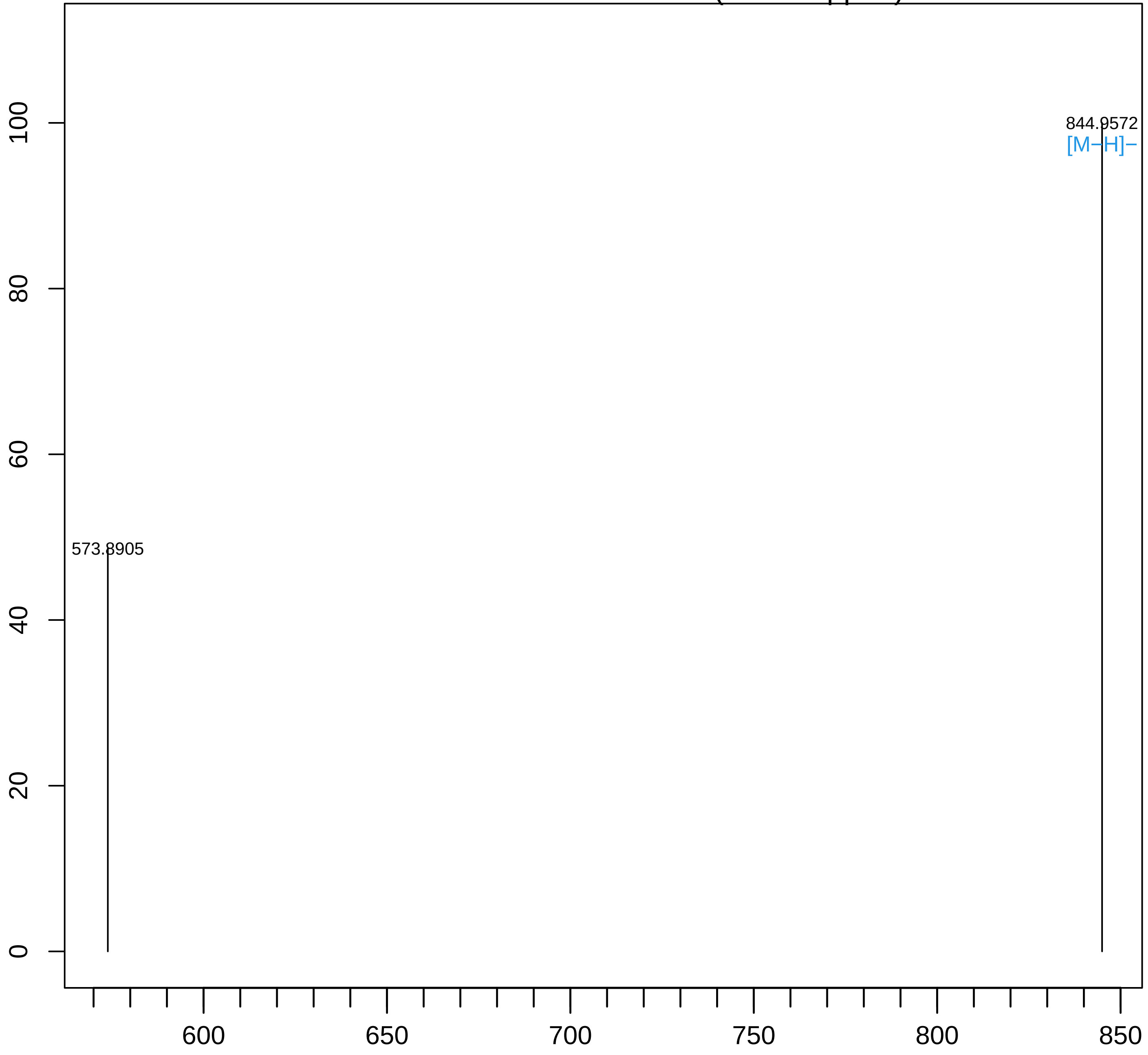

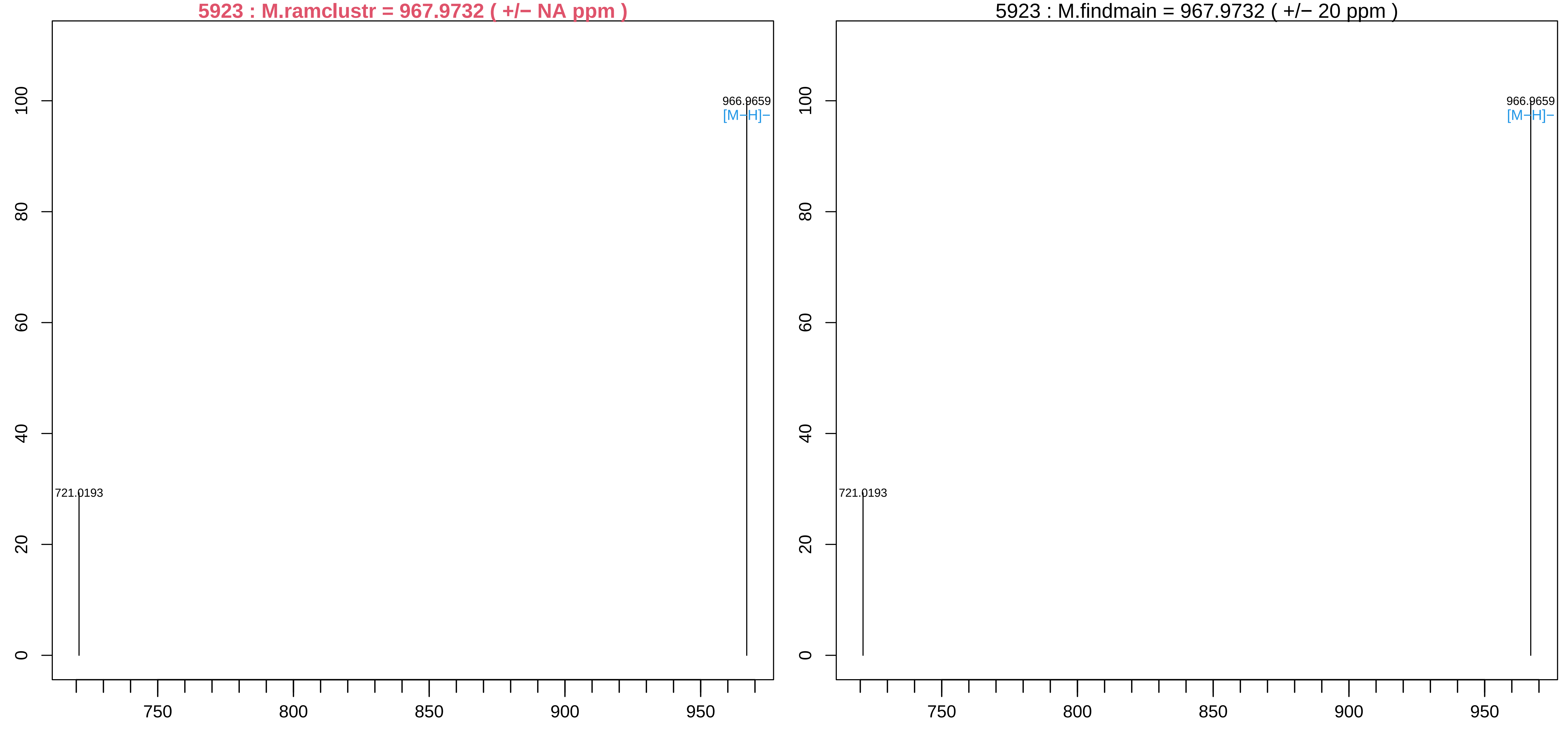

5923 : M.ramclustr = 967.9732 (+/− NA ppm)
966.9659
[M−H]−
721.0193
5923 : M.findmain = 967.9732 (+/− 20 ppm)
966.9659
[M−H]−
721.0193
0
20
40
60
80
100
750
800
850
900
950

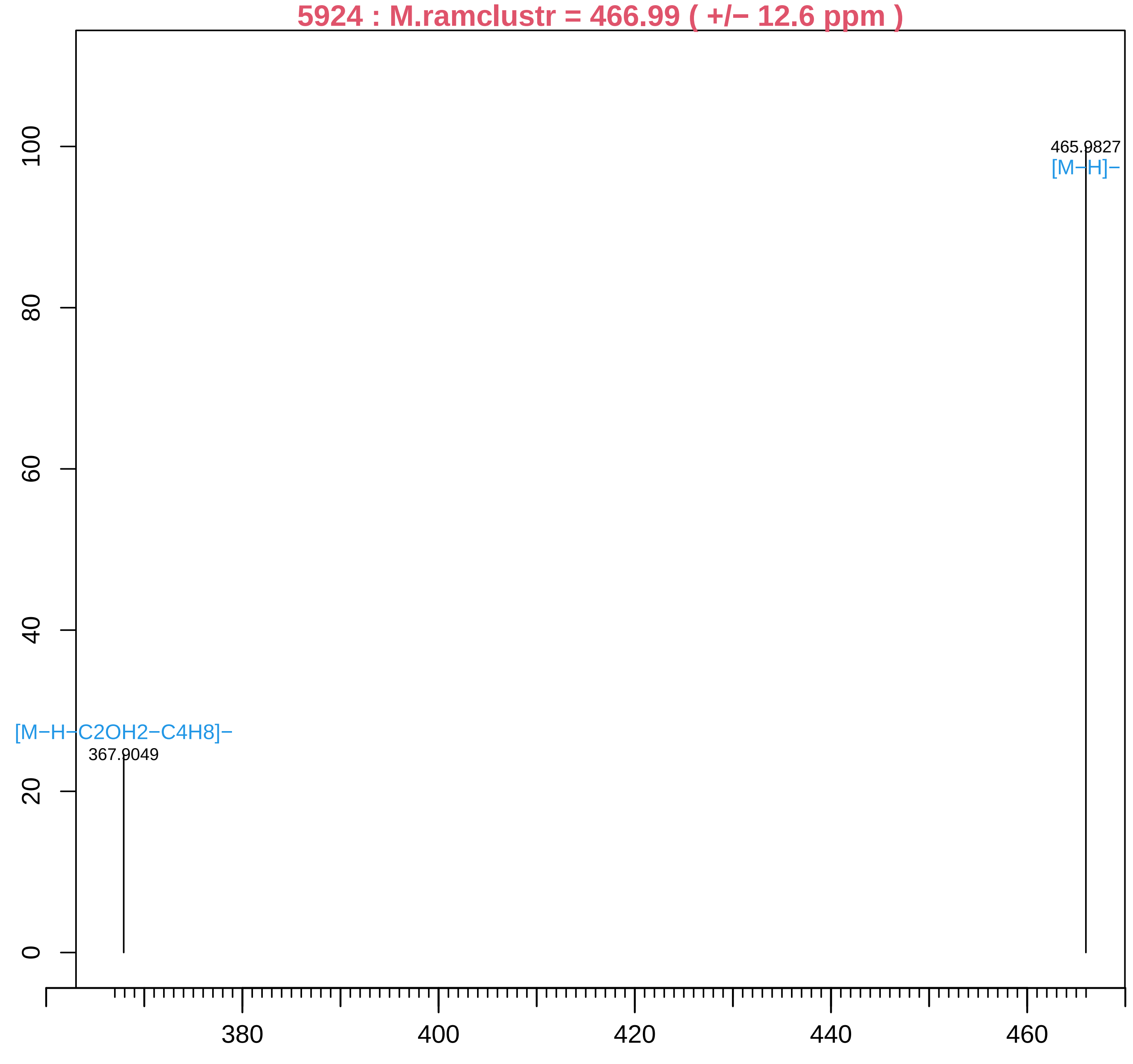
5924 : M.ramclustr = 466.99 (+/− 12.6 ppm)
465.9827
[M−H]−
[M−H−C2OH2−C4H8]−
367.9049
0
20
40
60
80
100
380
400
420
440
460

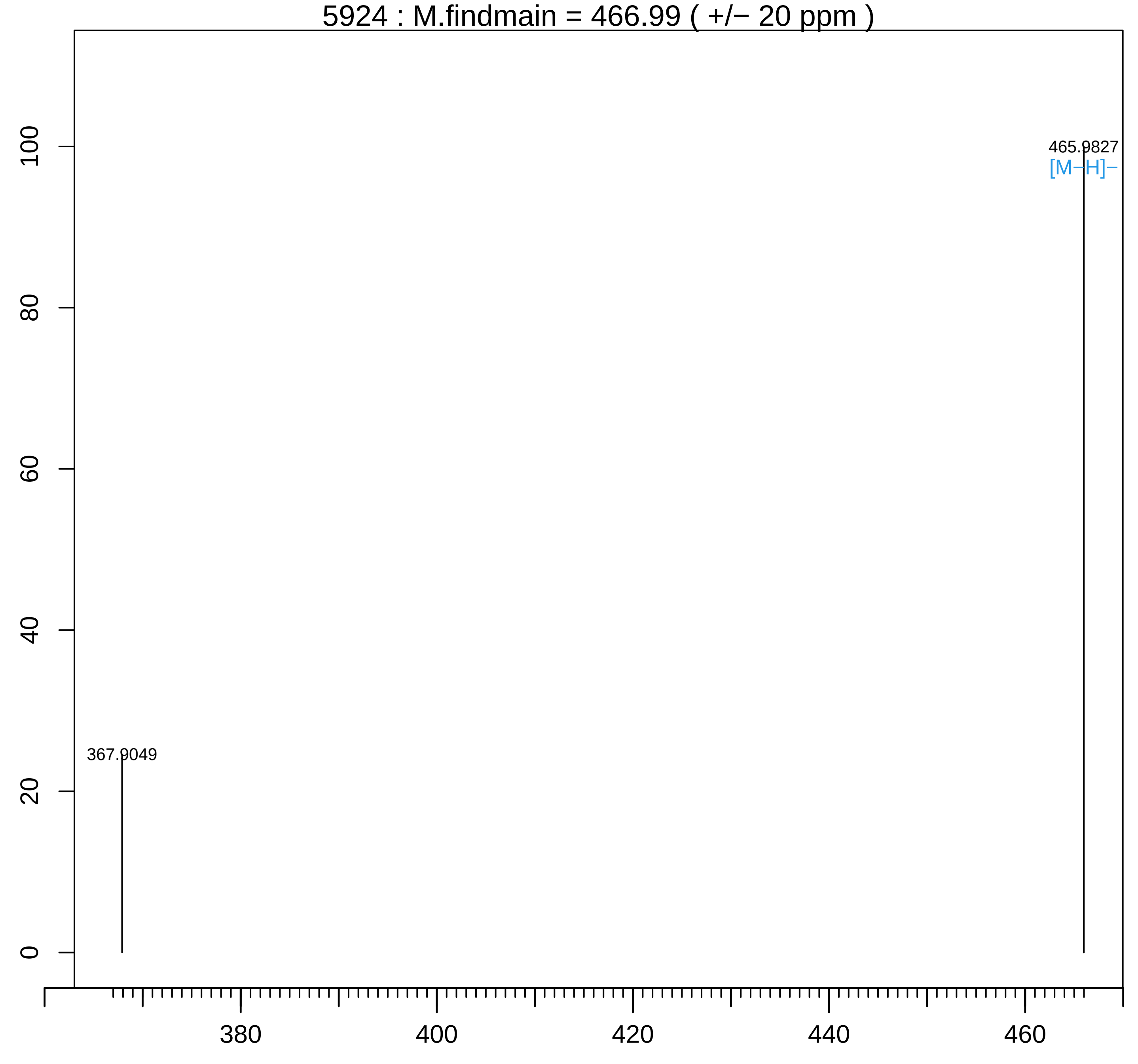
5924 : M.findmain = 466.99 (+/− 20 ppm)
465.9827
[M−H]−
367.9049
0
20
40
60
80
100
380
400
420
440
460

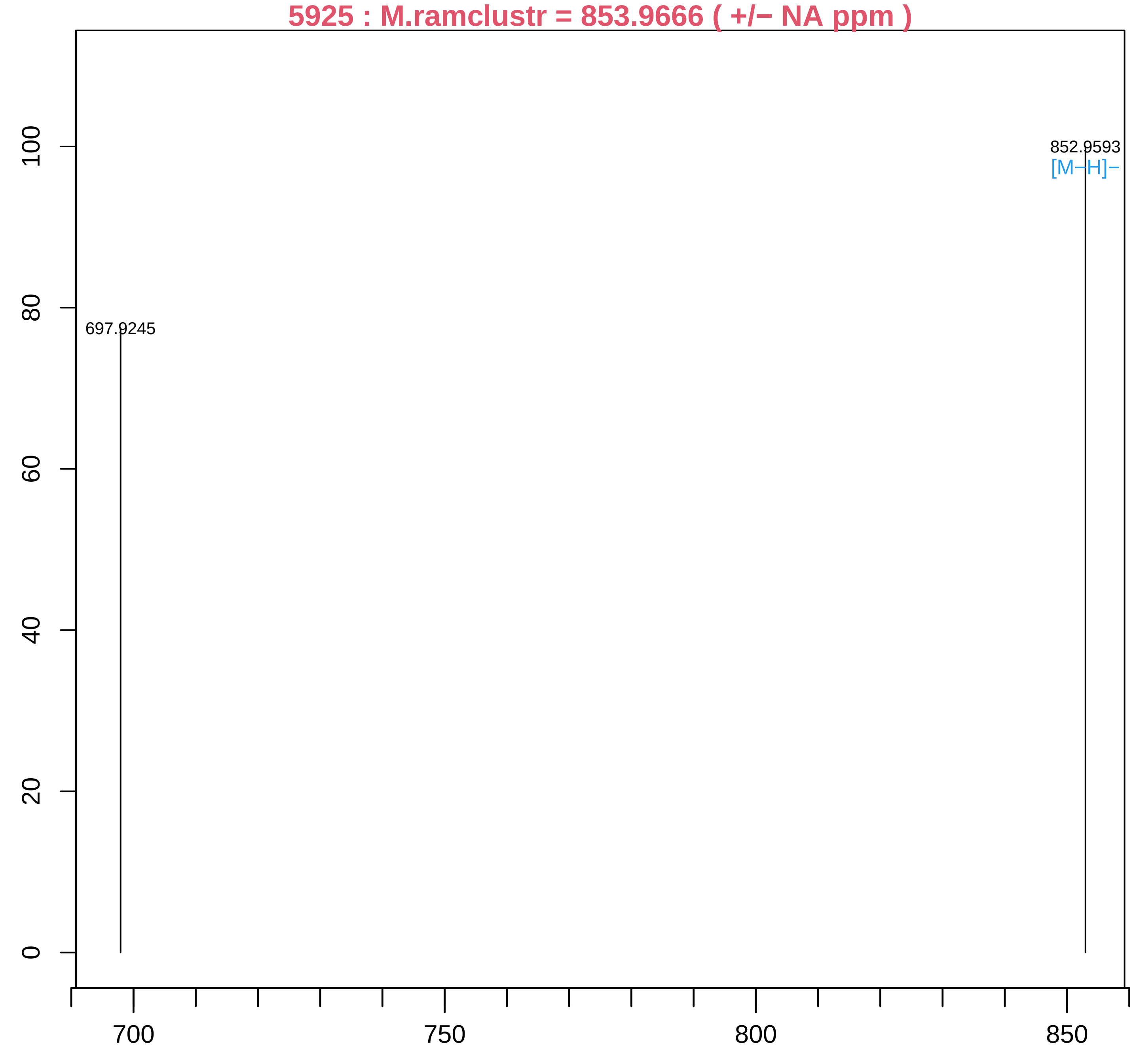
5925 : M.ramclustr = 853.9666 (+/− NA ppm)
852.9593
[M−H]−
697.9245
0
20
40
60
80
100
700
750
800
850
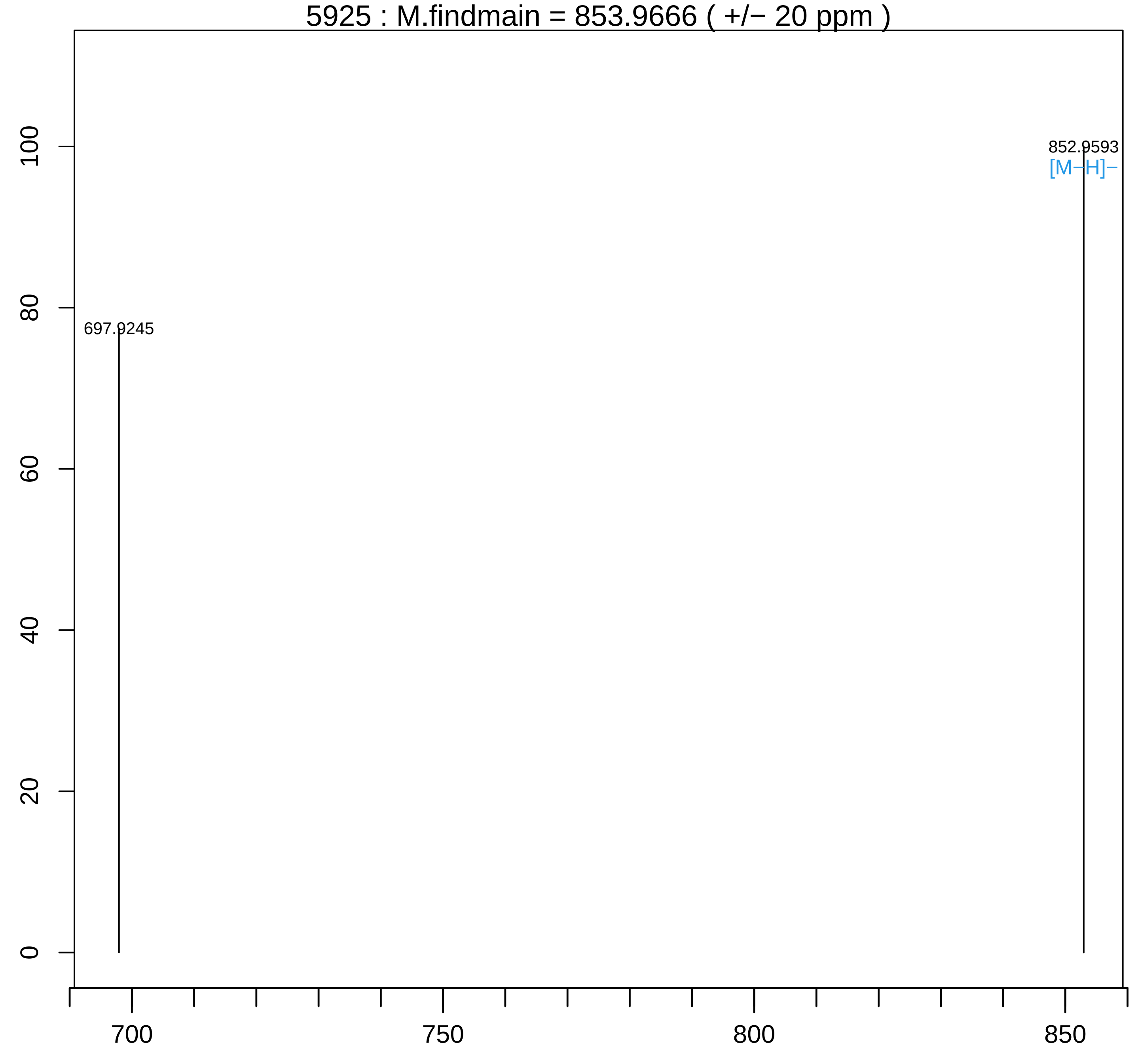
5925 : M.findmain = 853.9666 (+/− 20 ppm)
852.9593
[M−H]−
697.9245
0
20
40
60
80
100
700
750
800
850

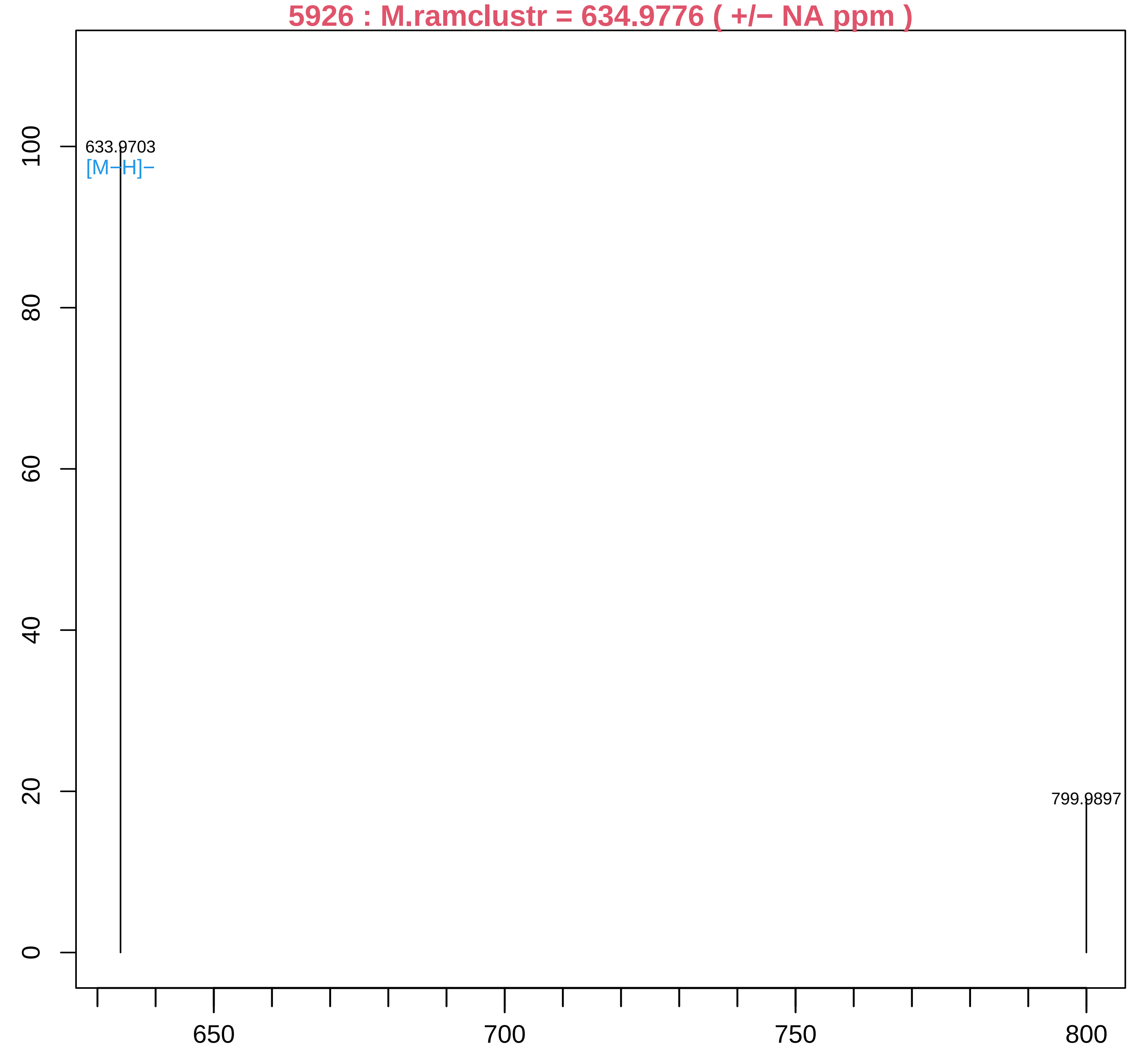
5926 : M.ramclustr = 634.9776 (+/− NA ppm)
633.9703
[M−H]−
799.9897
0
20
40
60
80
100
650
700
750
800

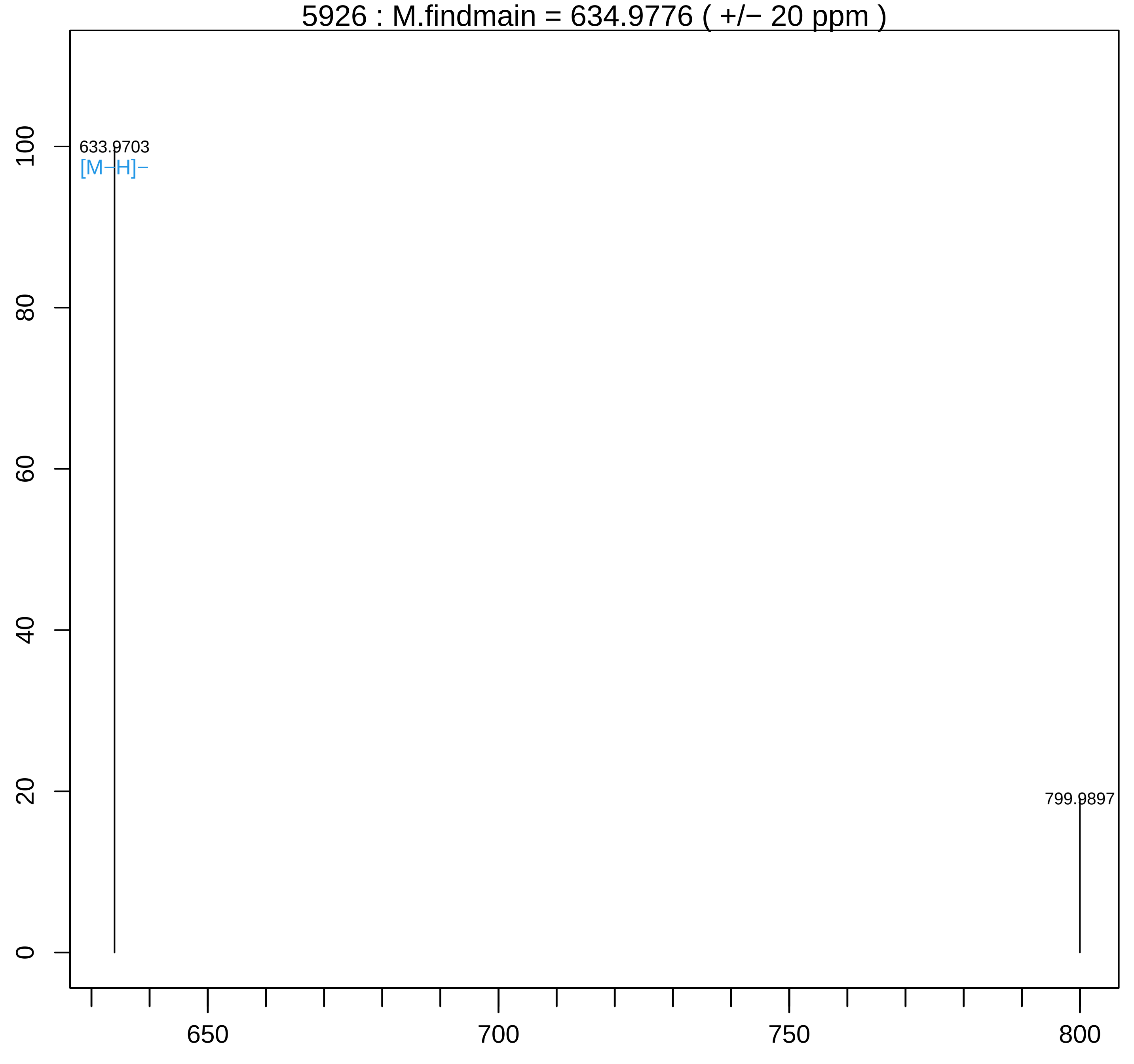
5926 : M.findmain = 634.9776 (+/− 20 ppm)
633.9703
[M−H]−
799.9897
0
20
40
60
80
100
650
700
750
800

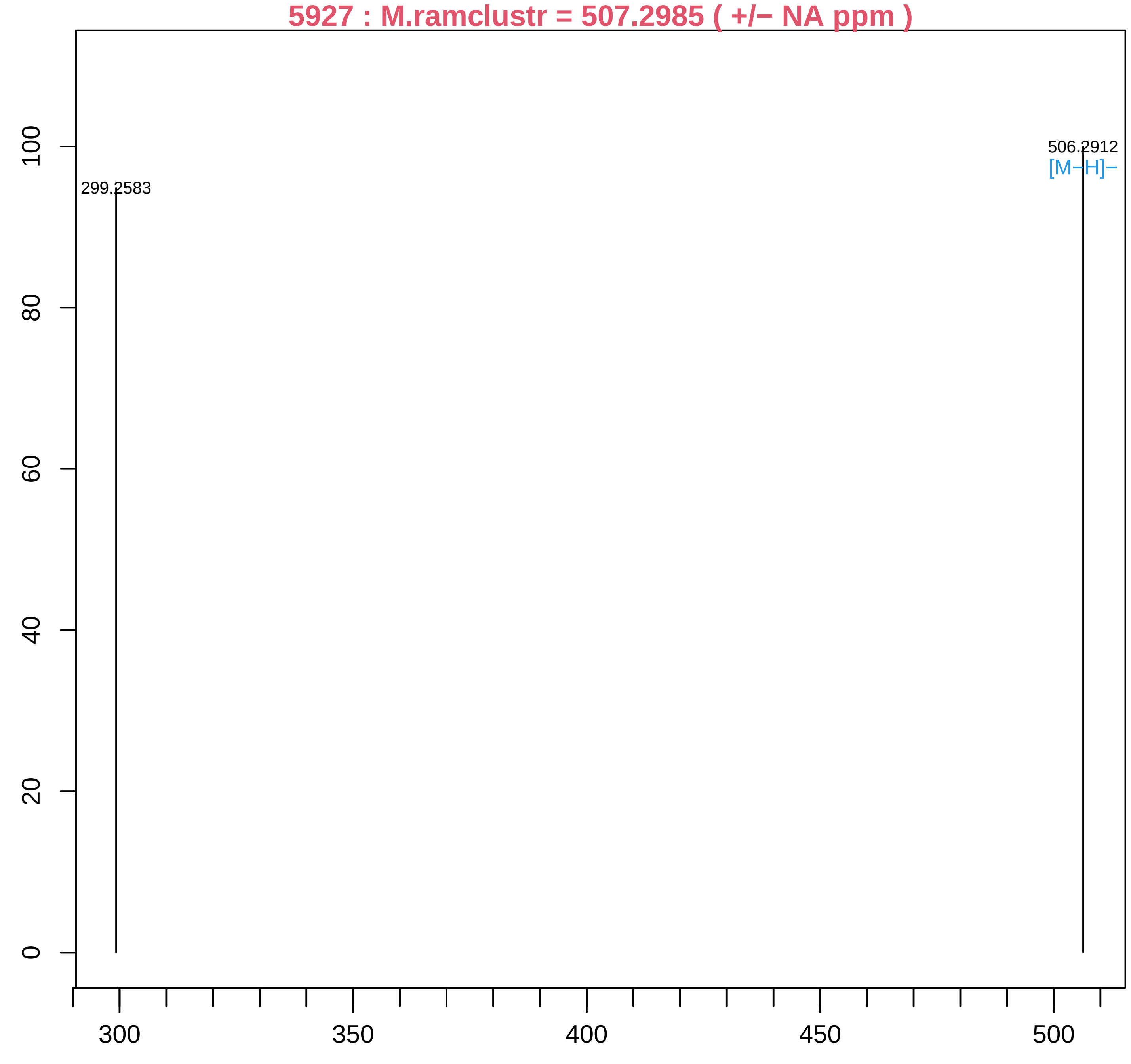
5927 : M.ramclustr = 507.2985 (+/− NA ppm)
299.2583
506.2912
[M−H]−
0
20
40
60
80
100
300
350
400
450
500

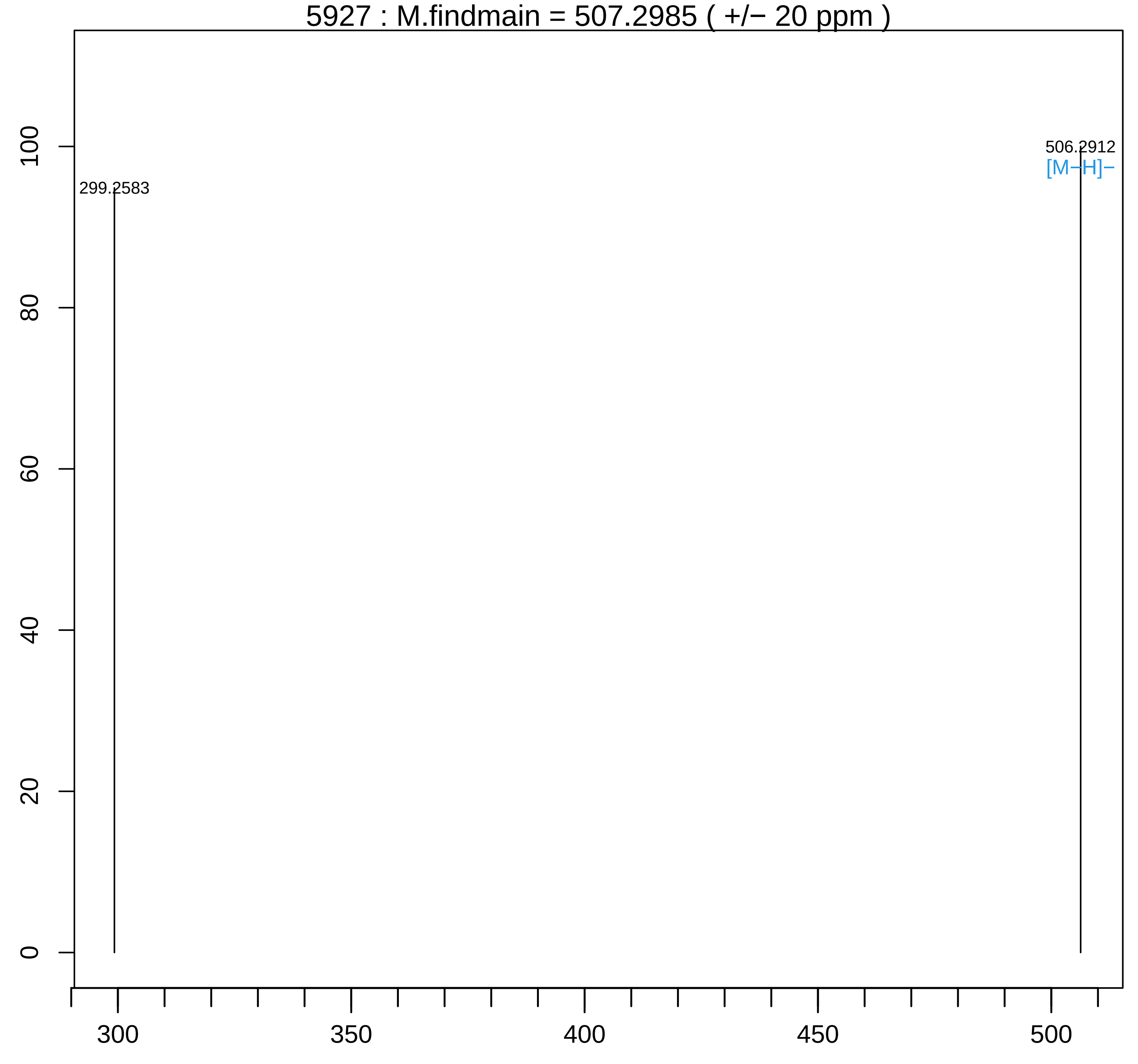
5927 : M.findmain = 507.2985 (+/− 20 ppm)
299.2583
506.2912
[M−H]−
0
20
40
60
80
100
300
350
400
450
500

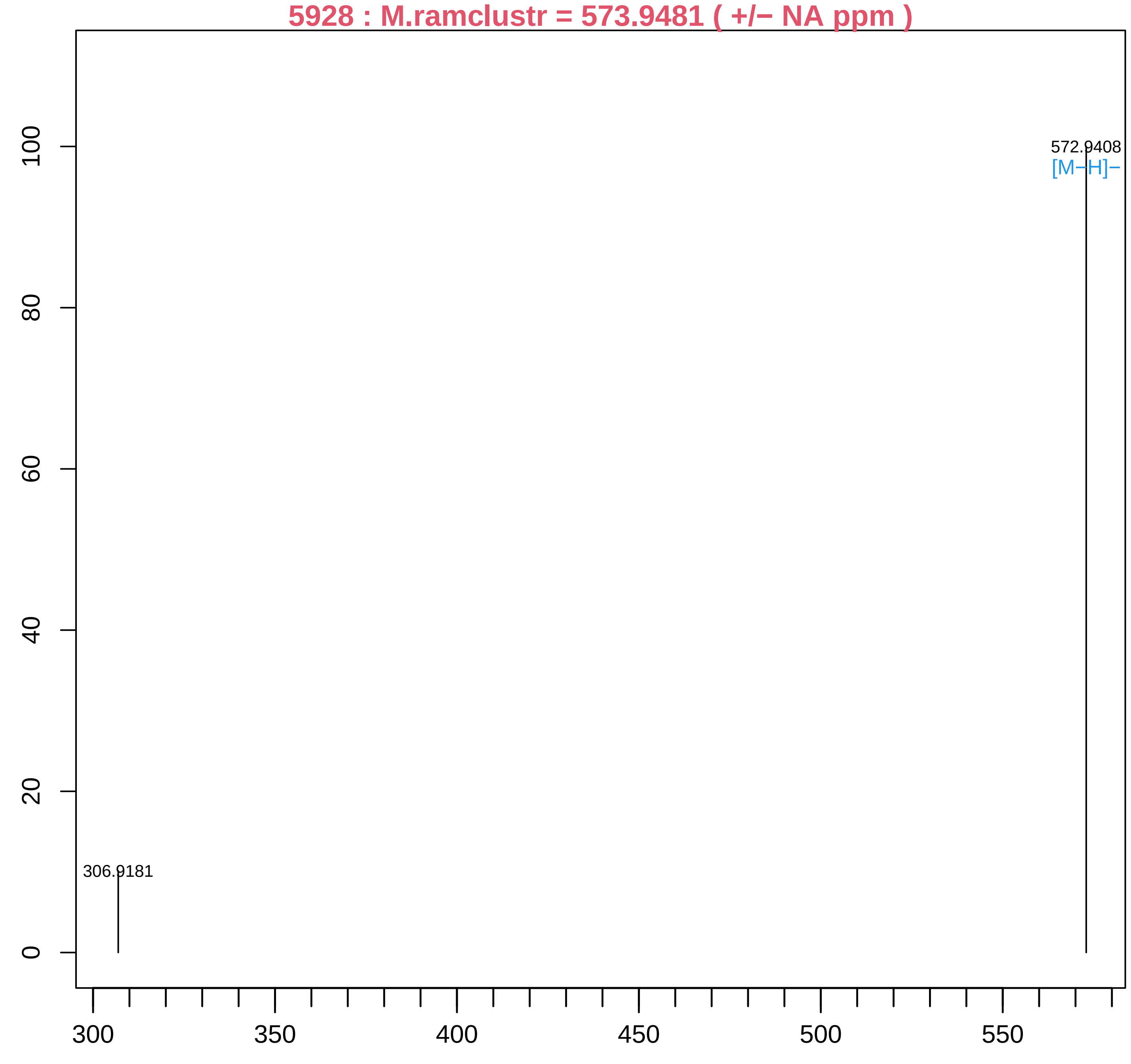
5928 : M.ramclustr = 573.9481 (+/− NA ppm)
572.9408
[M−H]−
306.9181
0
20
40
60
80
100
300
350
400
450
500
550

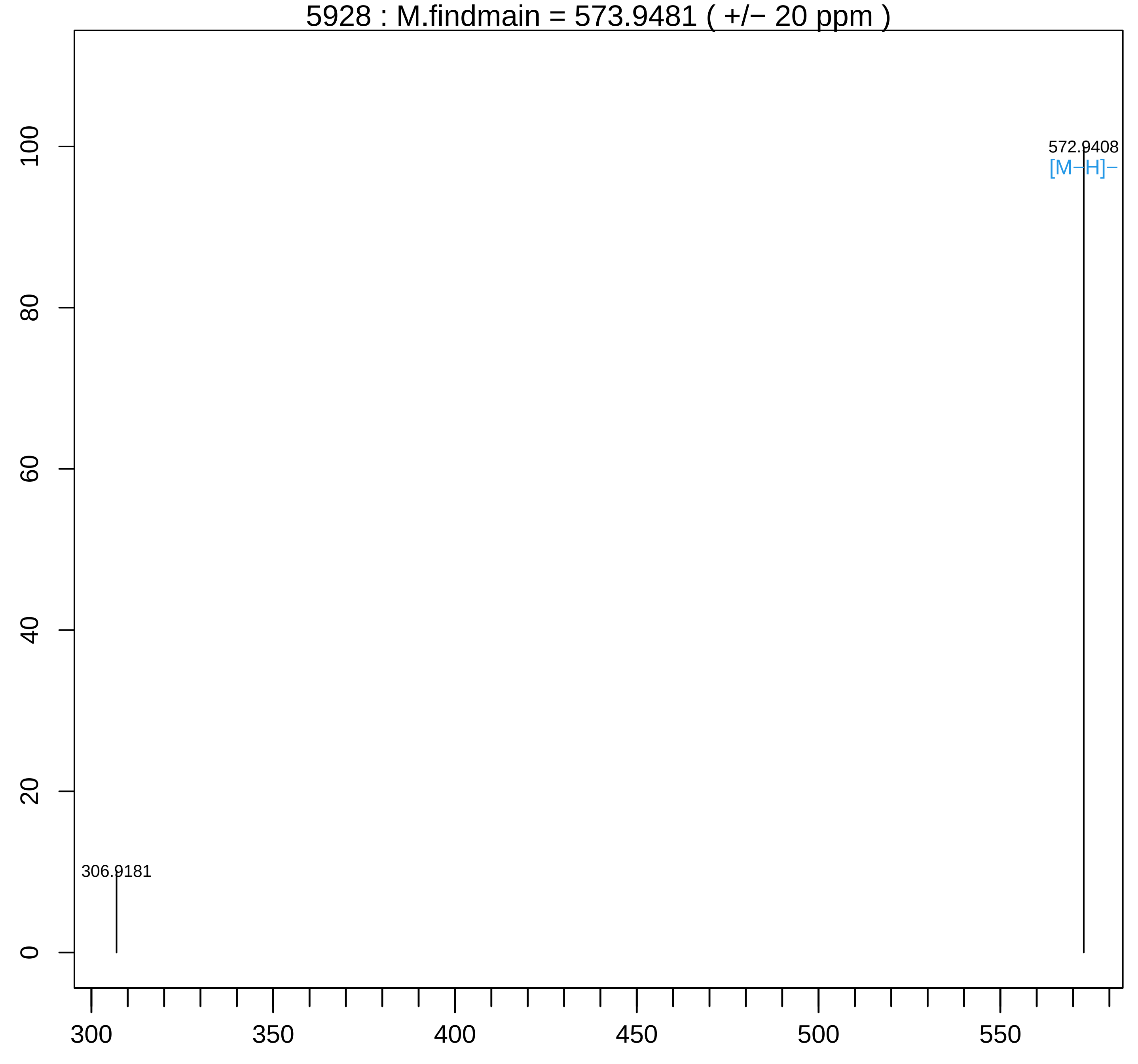
5928 : M.findmain = 573.9481 (+/− 20 ppm)
572.9408
[M−H]−
306.9181
0
20
40
60
80
100
300
350
400
450
500
550

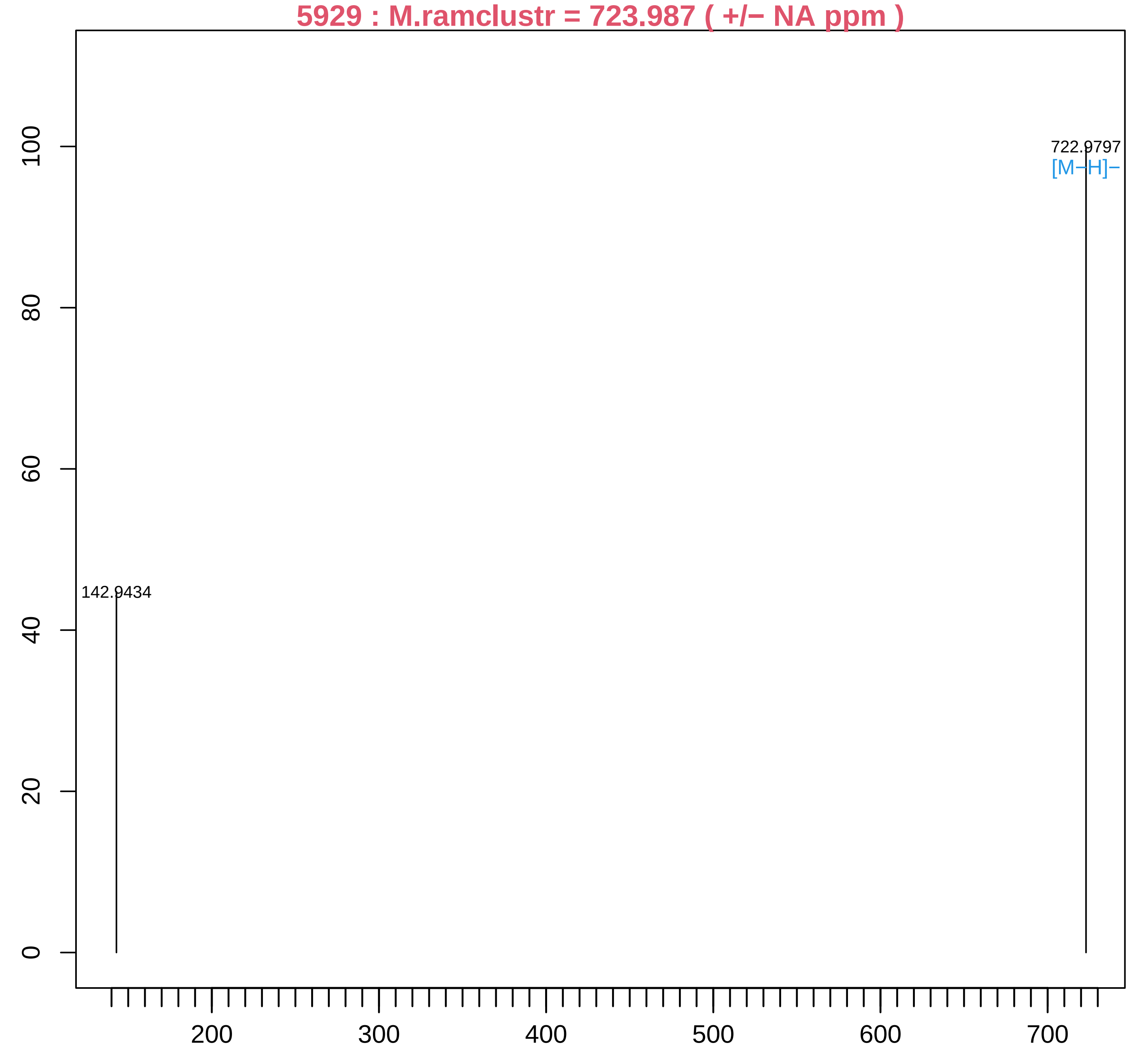

5929 : M.ramclustr = 723.987 (+/− NA ppm)
142.9434
722.9797
[M−H]−

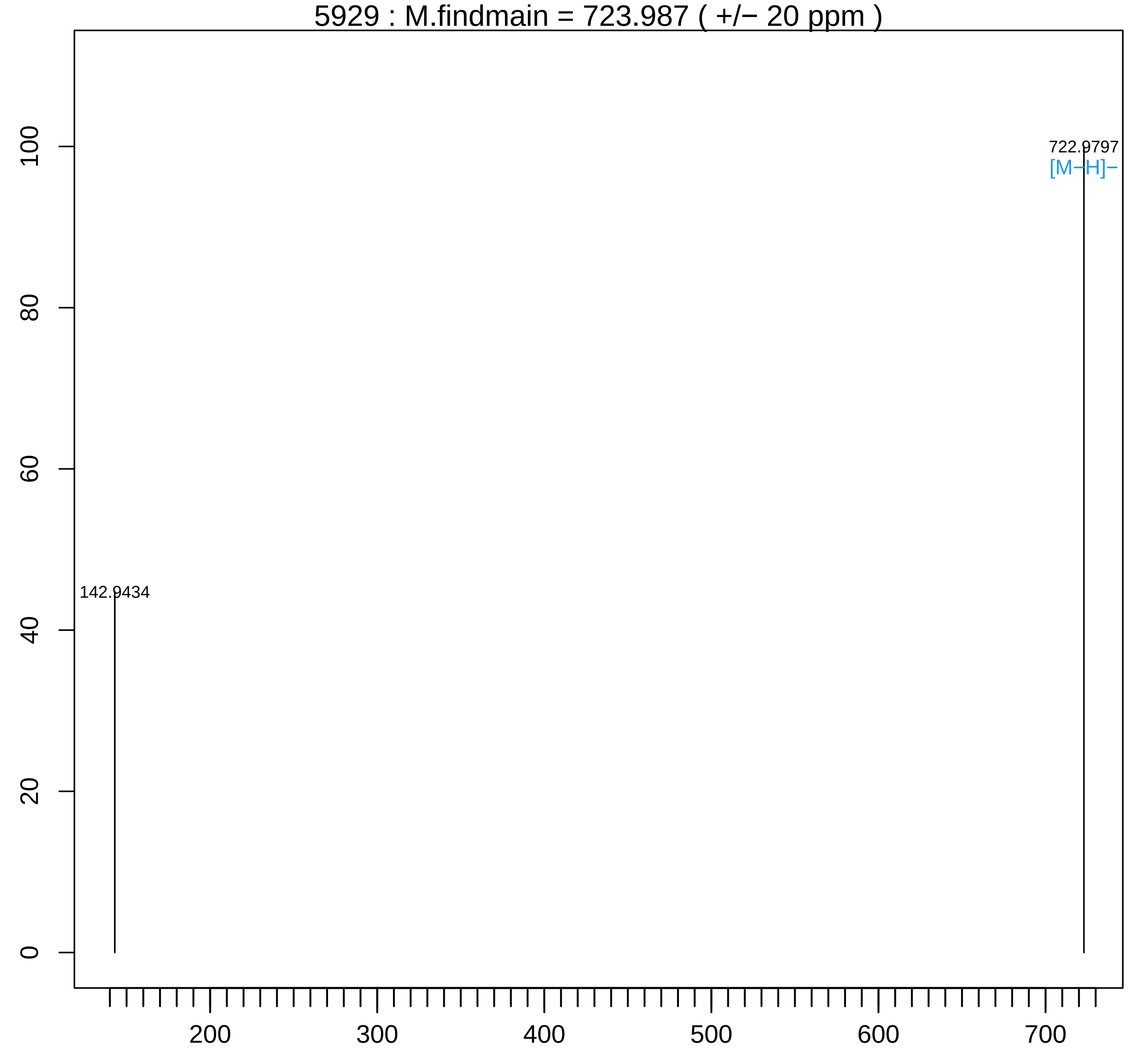

5929 : M.findmain = 723.987 (+/− 20 ppm)
142.9434
722.9797
[M−H]−

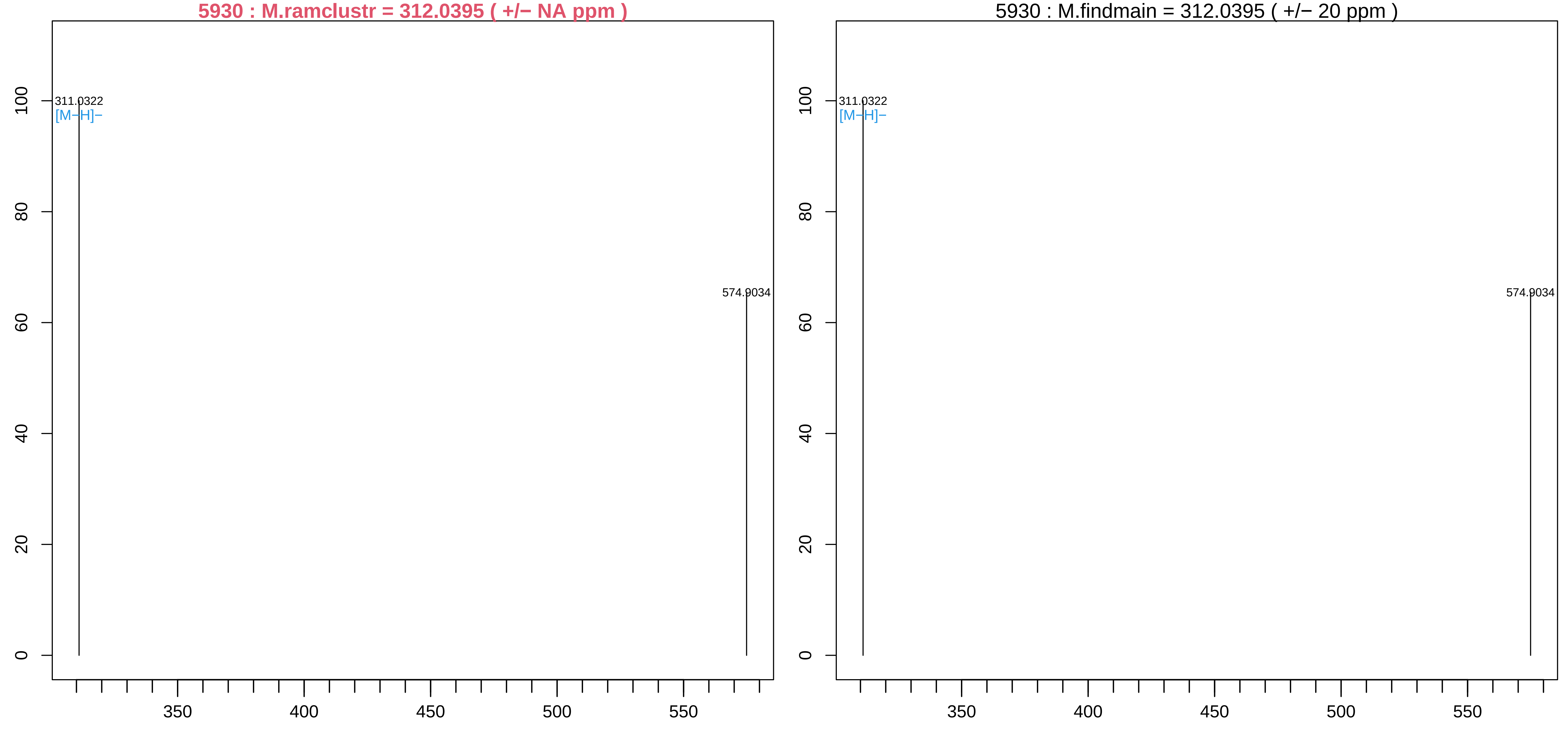

5930 : M.ramclustr = 312.0395 (+/− NA ppm)
311.0322
[M−H]−
574.9034
5930 : M.findmain = 312.0395 (+/− 20 ppm)
311.0322
[M−H]−
574.9034
0
20
40
60
80
100
350
400
450
500
550

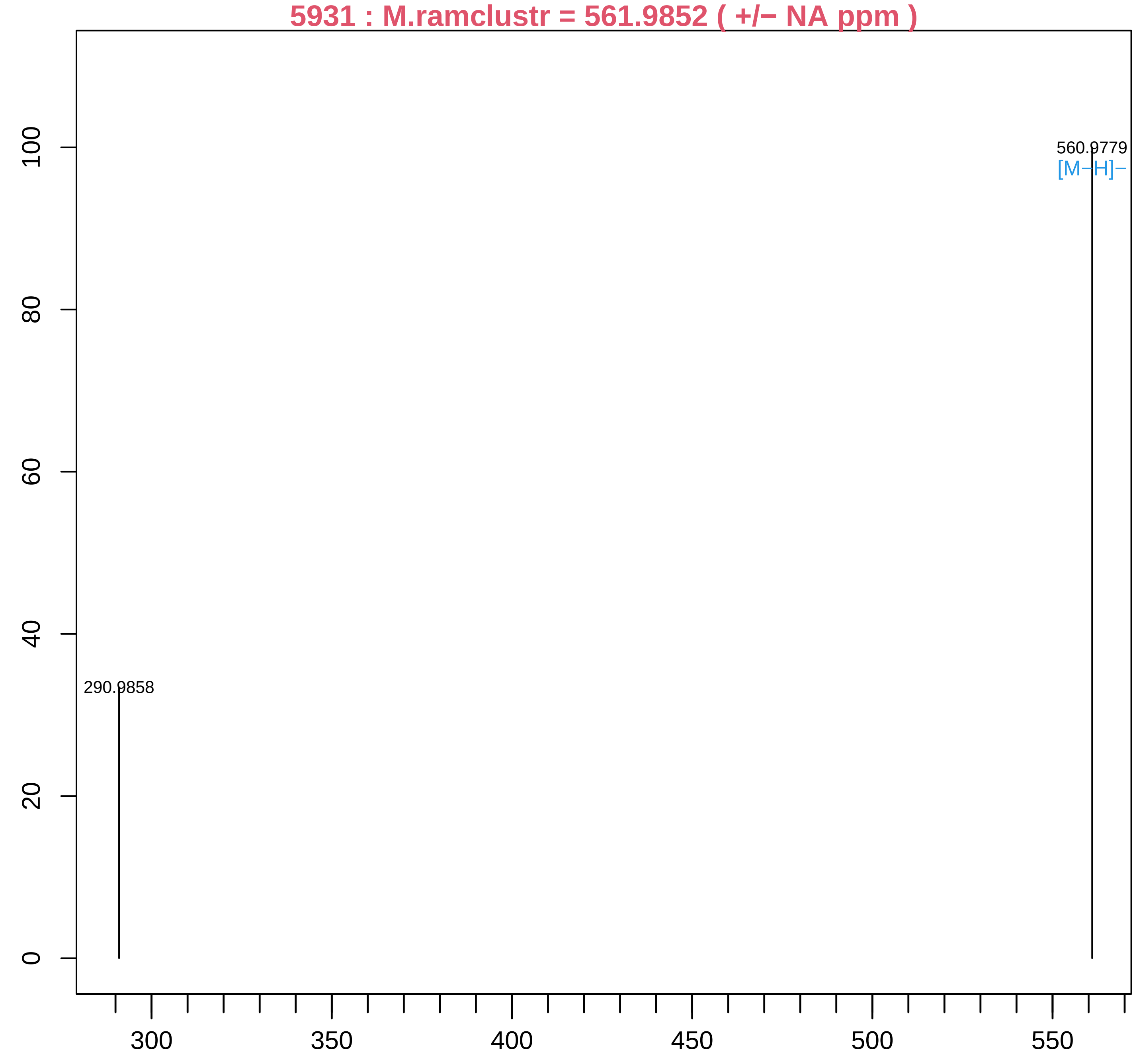

5931 : M.ramclustr = 561.9852 (+/− NA ppm)
560.9779
[M−H]−
290.9858
0
20
40
60
80
100
300
350
400
450
500
550

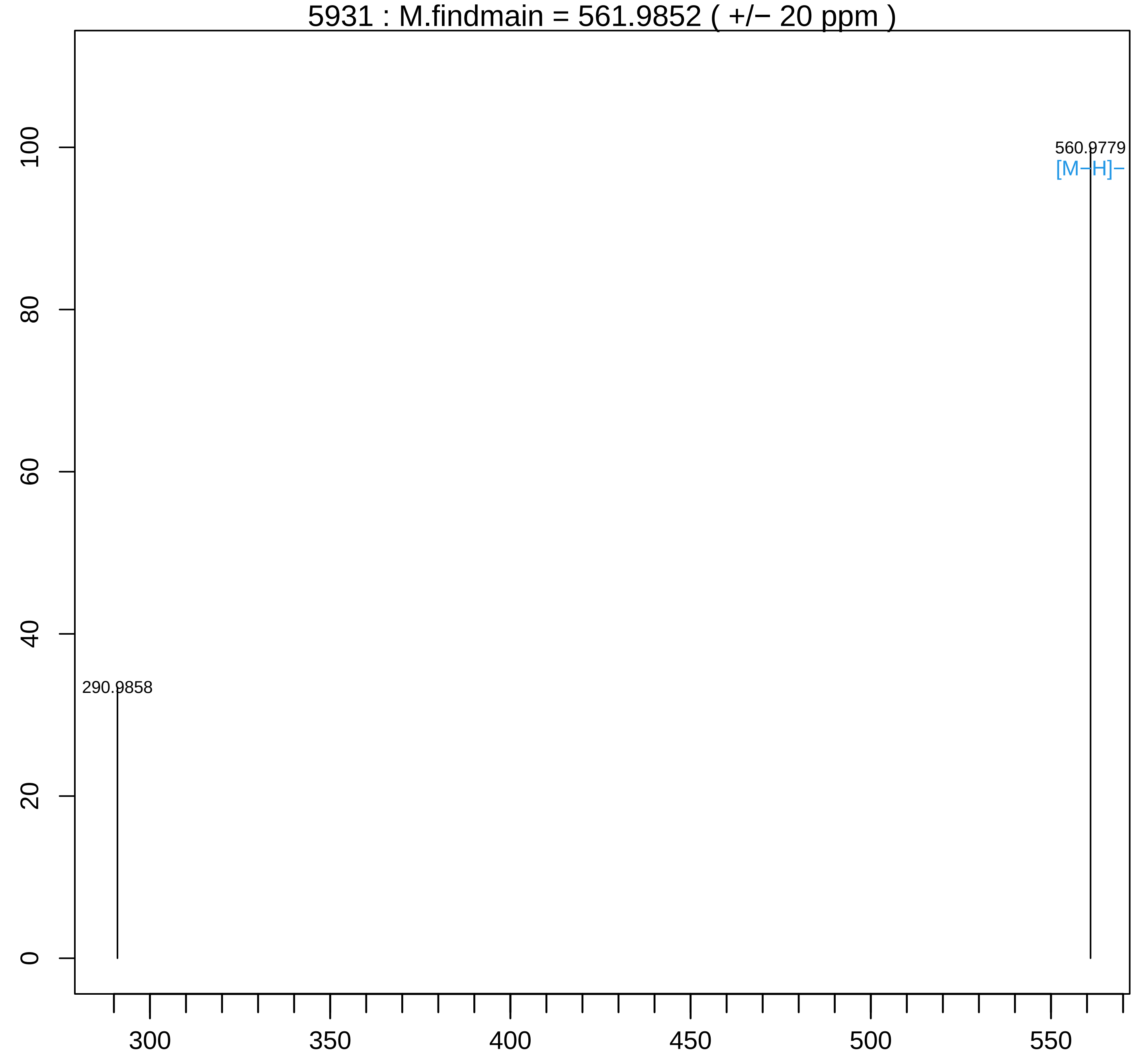

5931 : M.findmain = 561.9852 (+/− 20 ppm)
560.9779
[M−H]−
290.9858
0
20
40
60
80
100
300
350
400
450
500
550

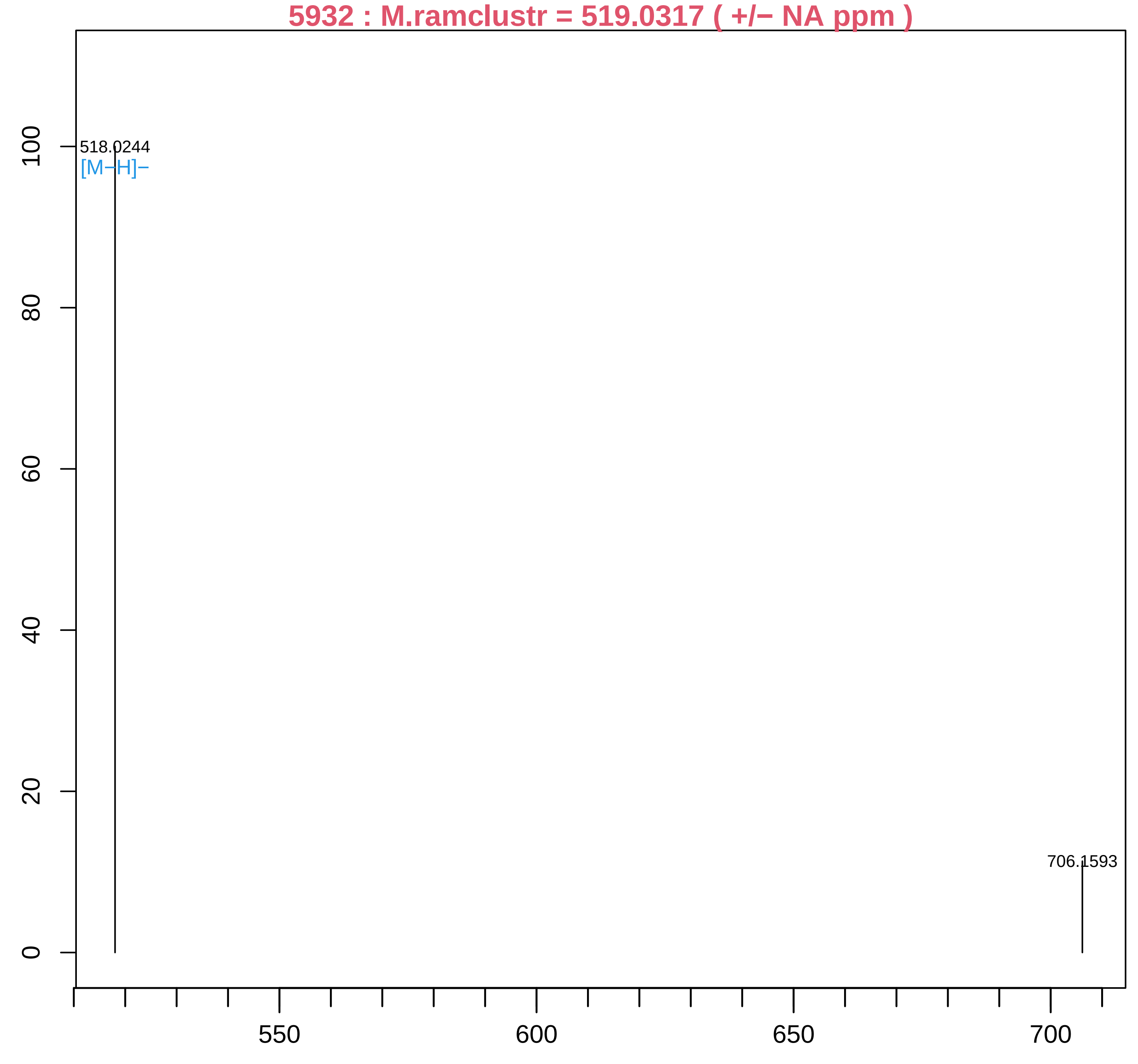
5932 : M.ramclustr = 519.0317 (+/− NA ppm)
518.0244
[M−H]−
706.1593
0
20
40
60
80
100
550
600
650
700

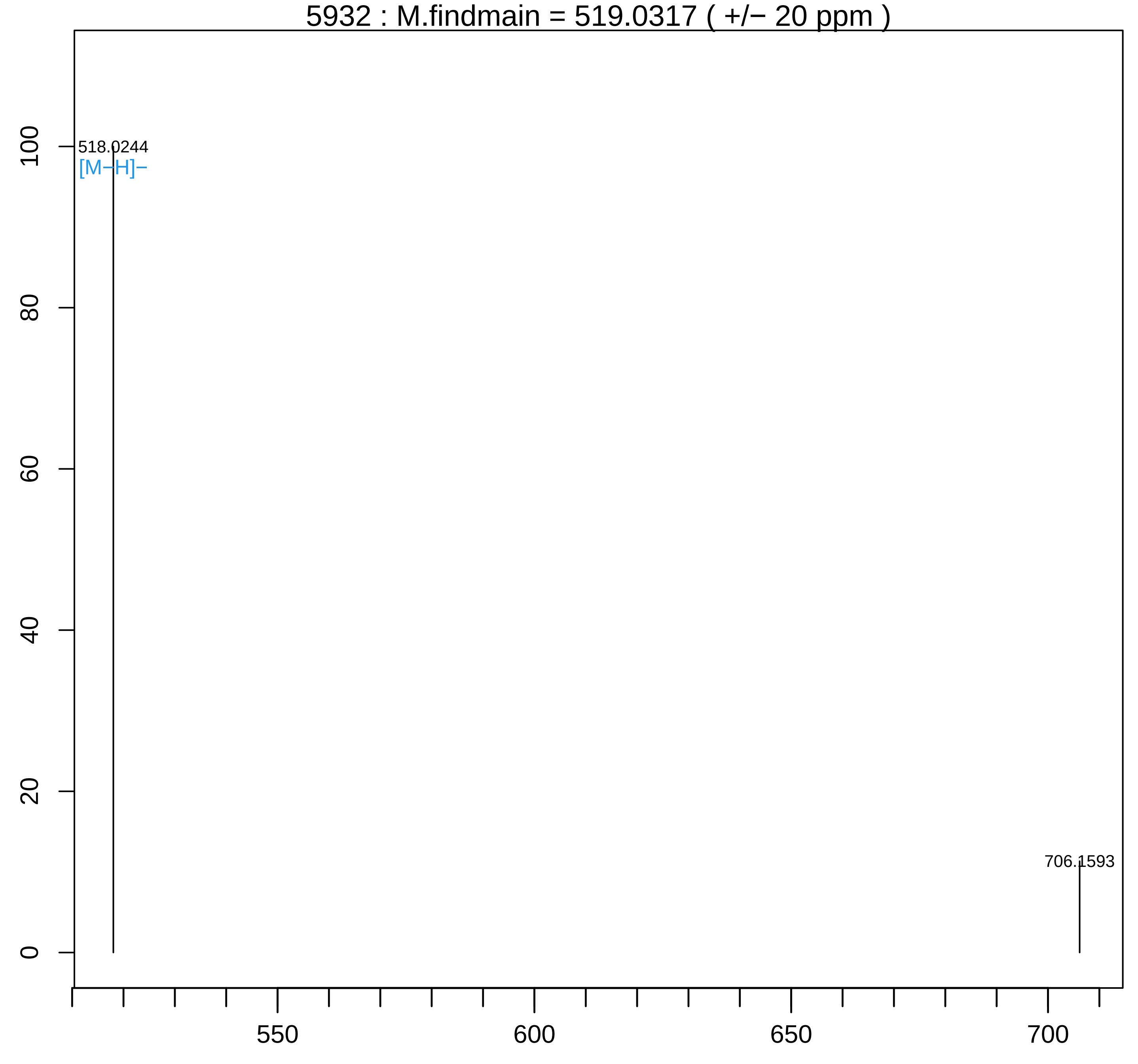
5932 : M.findmain = 519.0317 (+/− 20 ppm)
518.0244
[M−H]−
706.1593
0
20
40
60
80
100
550
600
650
700

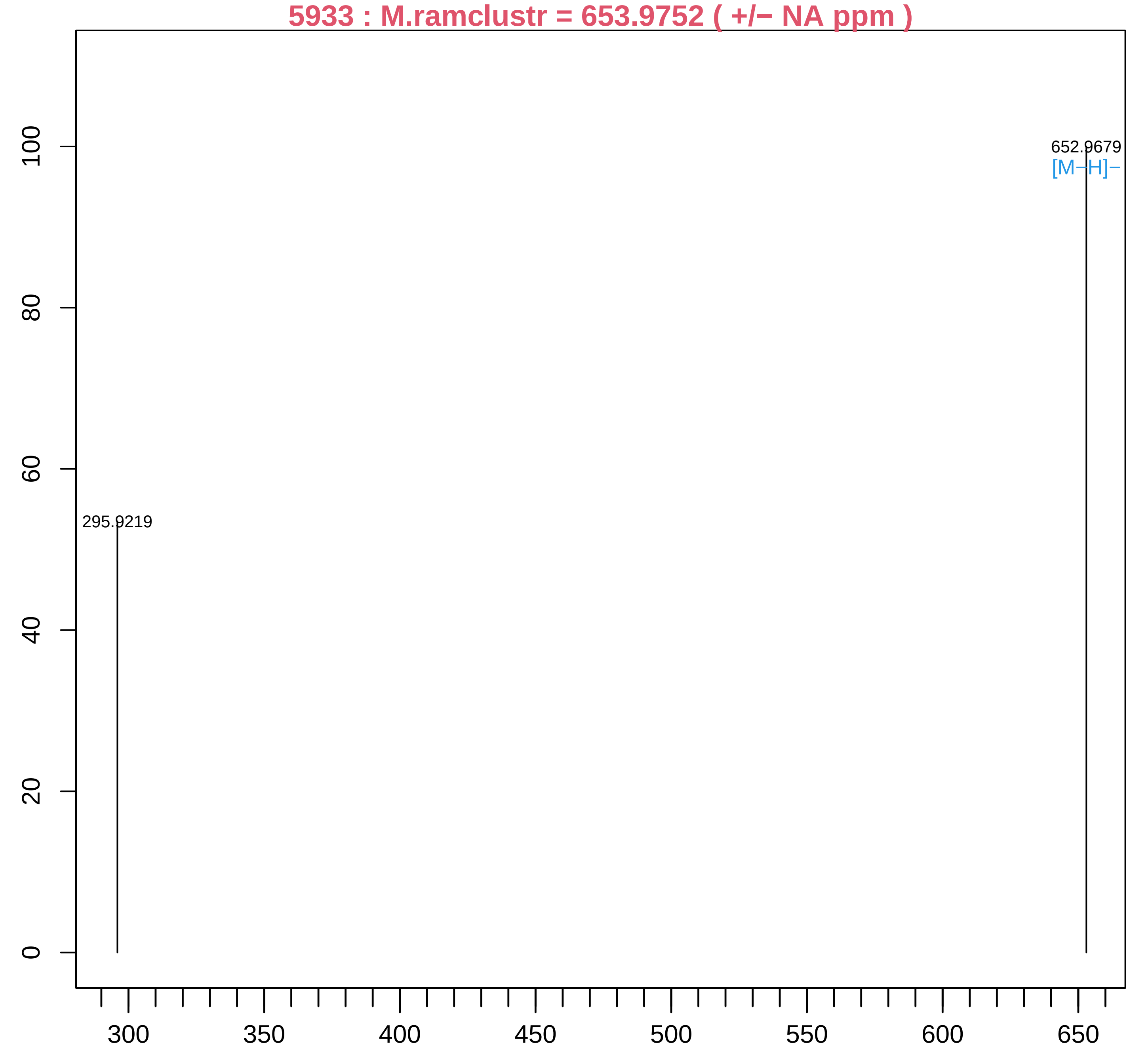
5933 : M.ramclustr = 653.9752 (+/− NA ppm)
652.9679
[M−H]−
295.9219
0
20
40
60
80
100
300
350
400
450
500
550
600
650

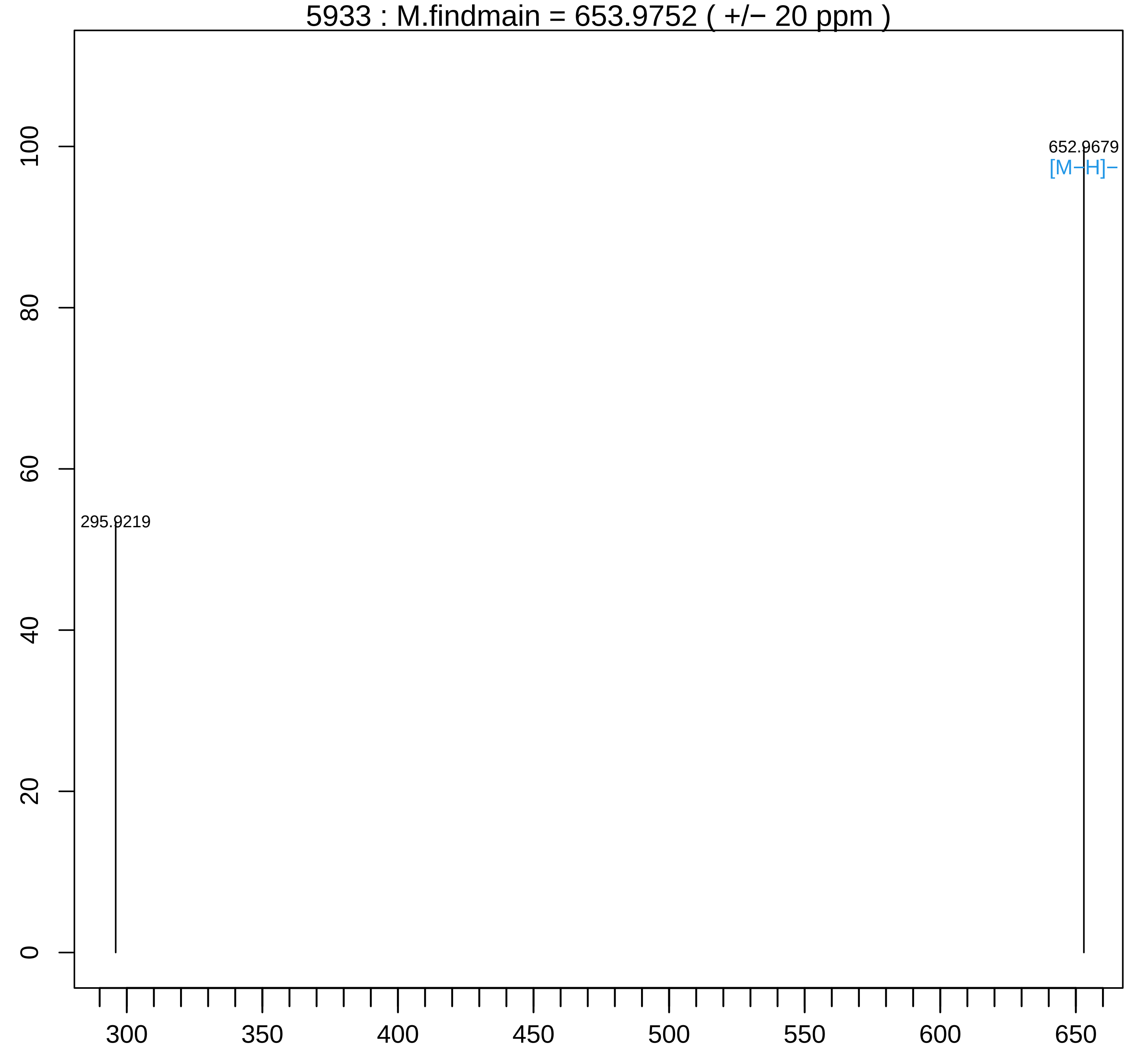
5933 : M.findmain = 653.9752 (+/− 20 ppm)
652.9679
[M−H]−
295.9219
0
20
40
60
80
100
300
350
400
450
500
550
600
650

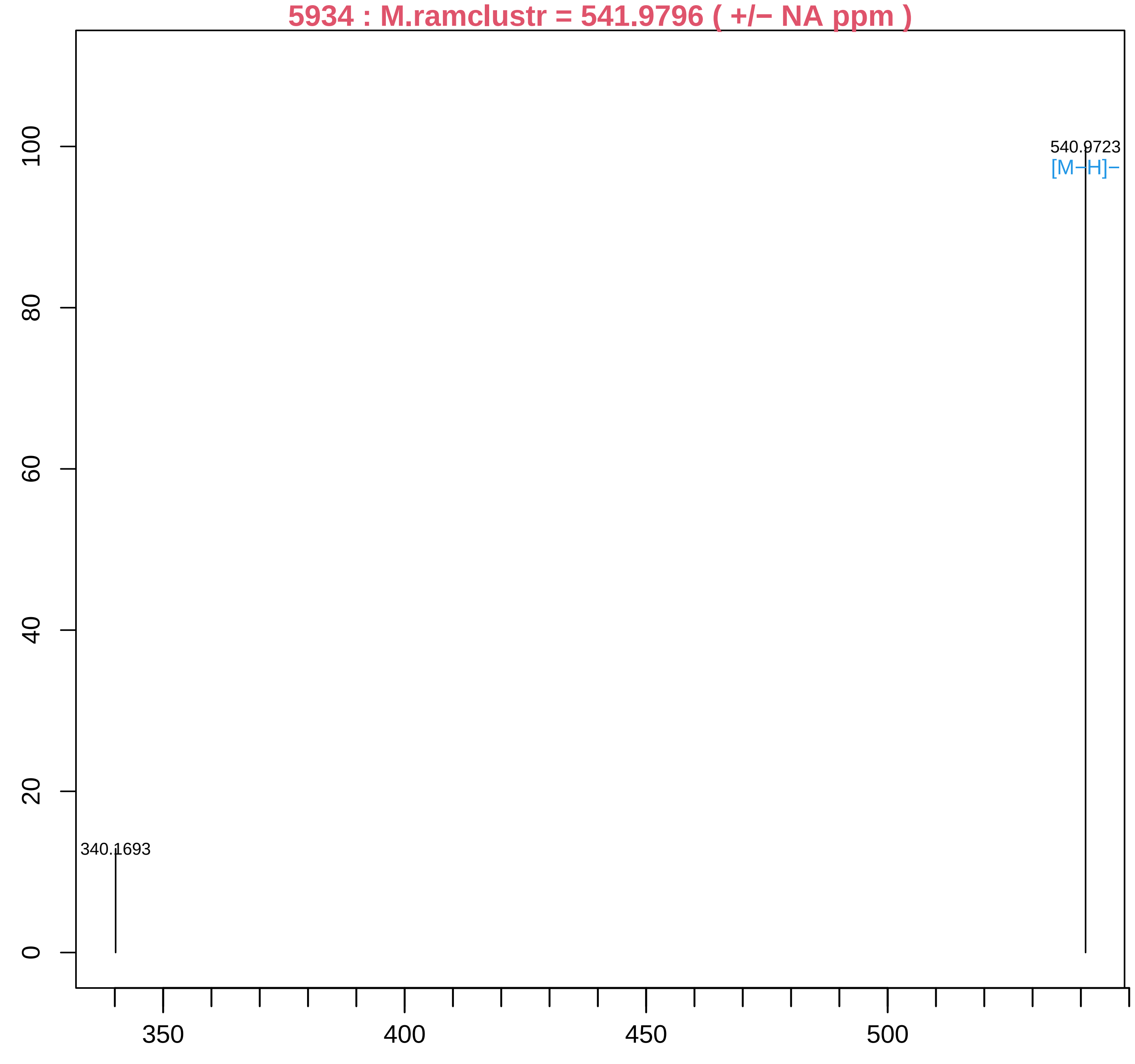
5934 : M.ramclustr = 541.9796 (+/− NA ppm)
540.9723
[M−H]−
340.1693
0
20
40
60
80
100
350
400
450
500

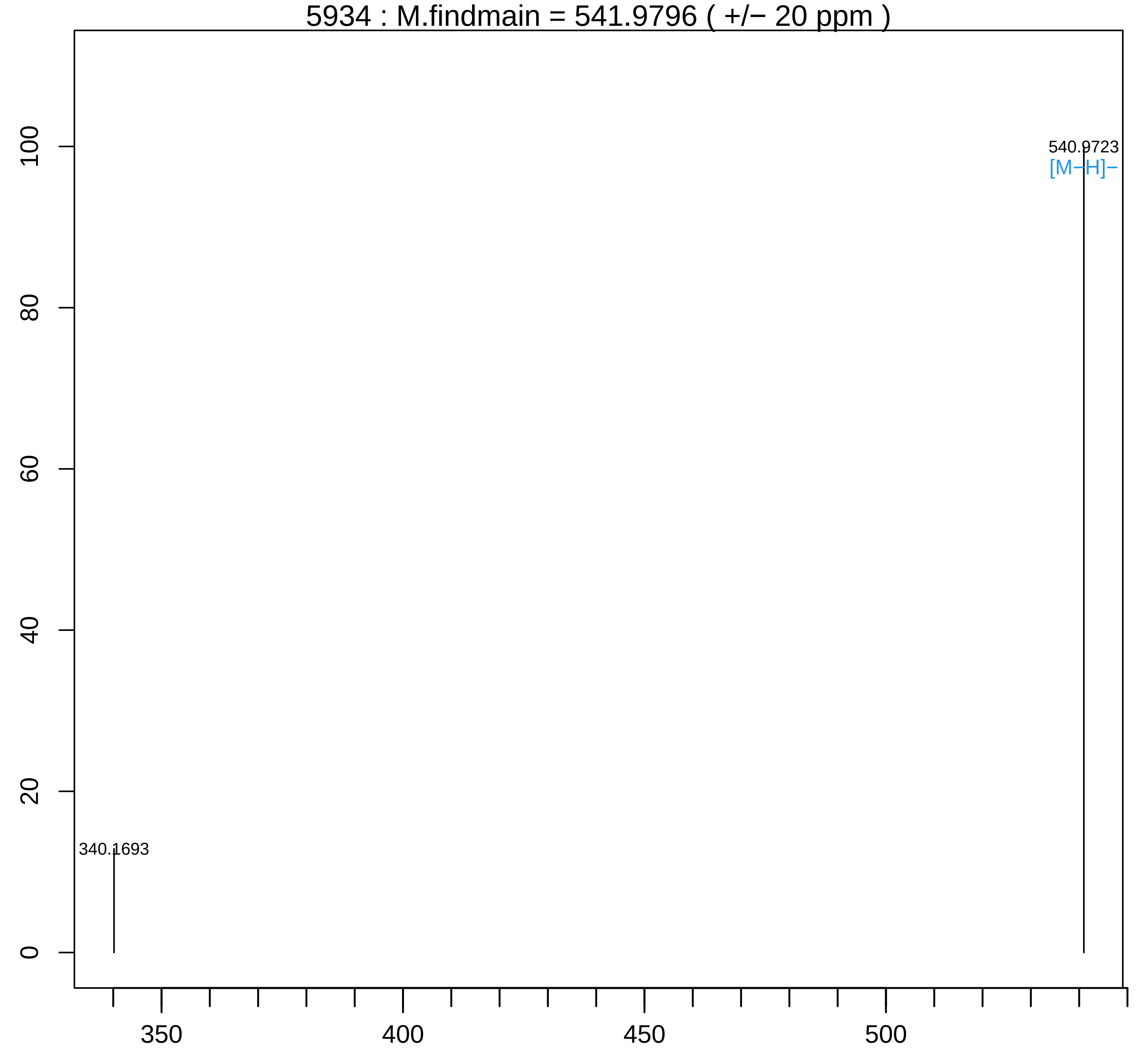
5934 : M.findmain = 541.9796 (+/− 20 ppm)
540.9723
[M−H]−
340.1693
0
20
40
60
80
100
350
400
450
500

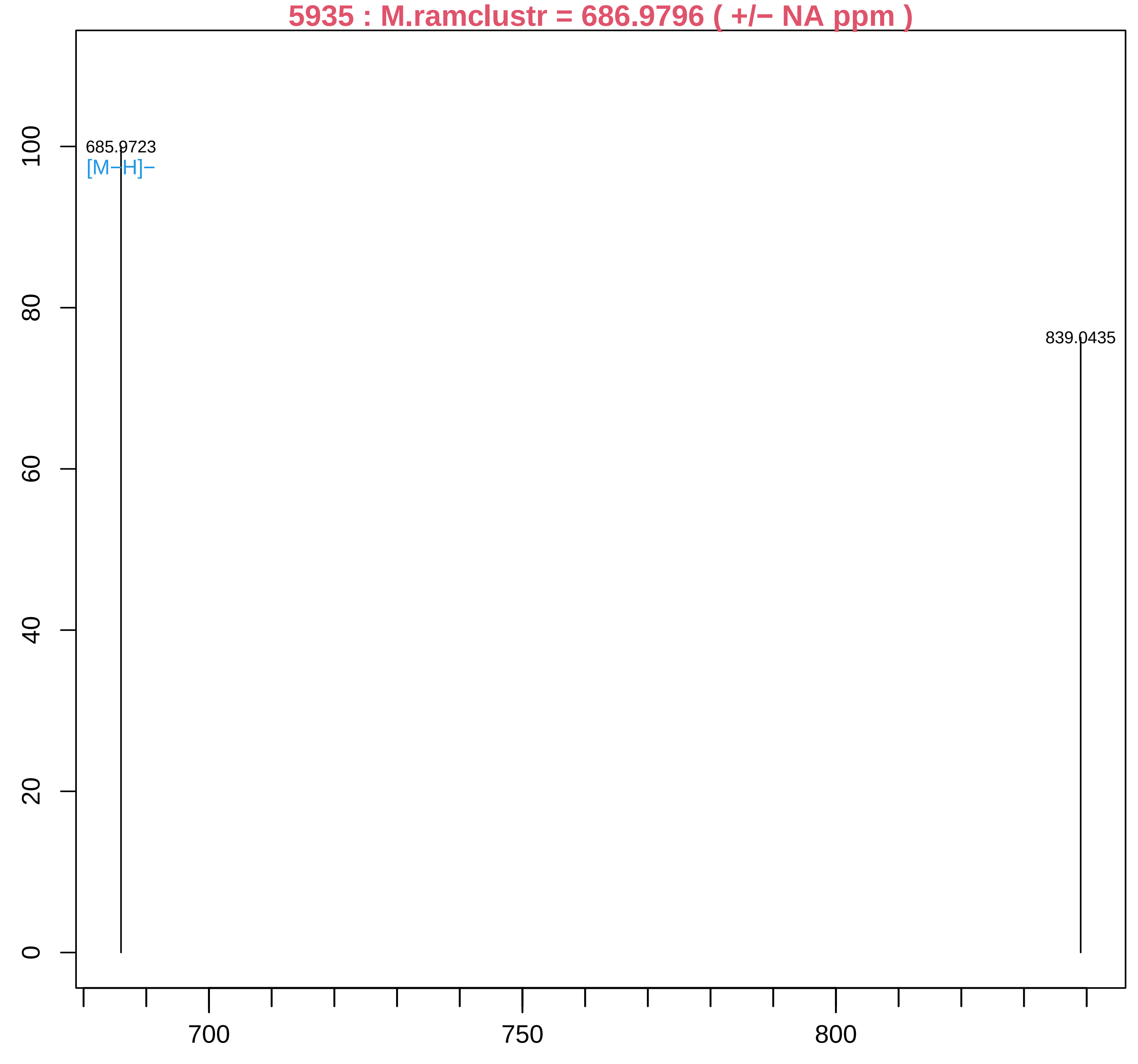
5935 : M.ramclustr = 686.9796 (+/− NA ppm)
685.9723
[M−H]−
839.0435
0
20
40
60
80
100
700
750
800

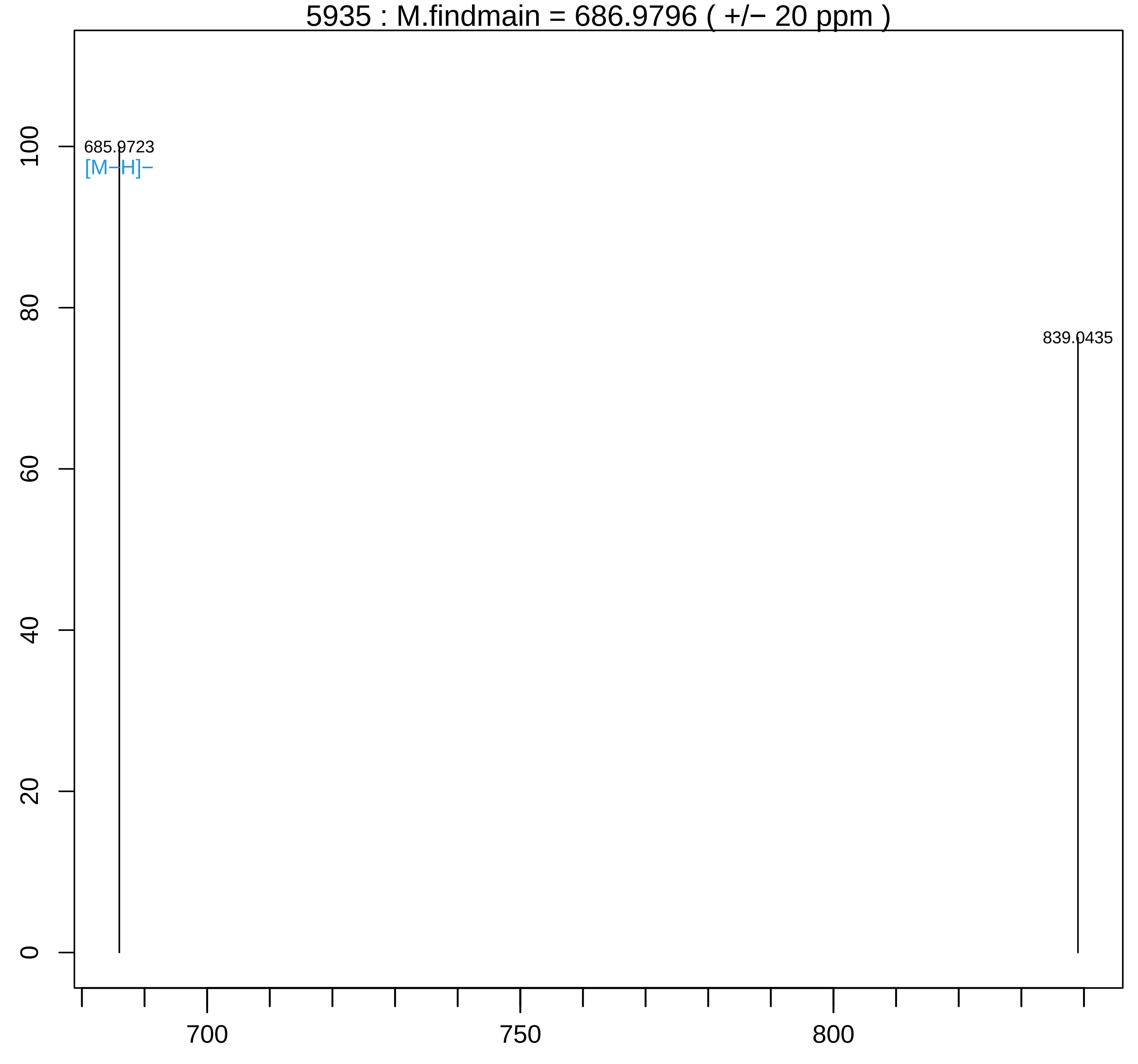
5935 : M.findmain = 686.9796 (+/− 20 ppm)
685.9723
[M−H]−
839.0435
0
20
40
60
80
100
700
750
800

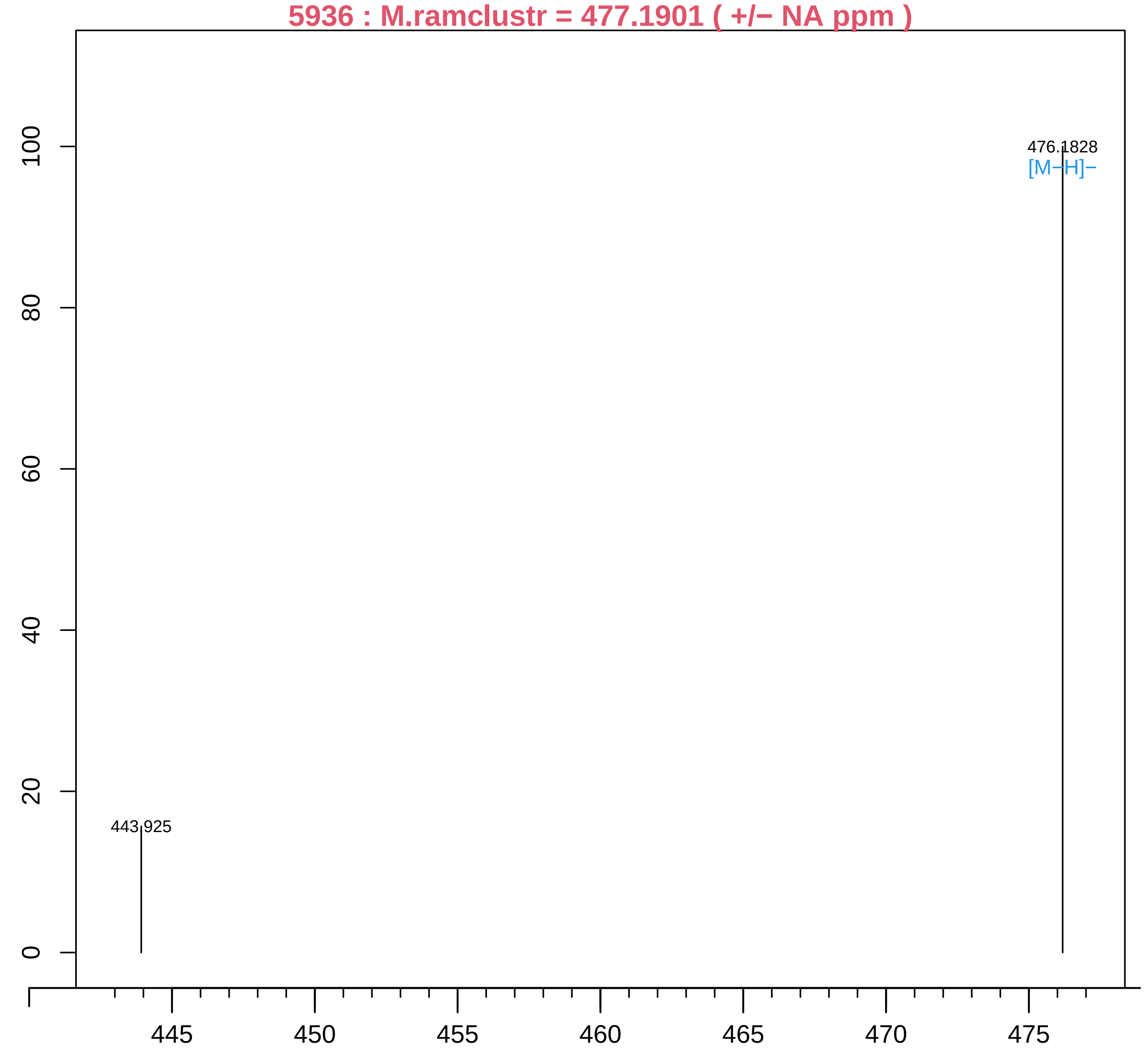
5936 : M.ramclustr = 477.1901 (+/− NA ppm)
476.1828
[M−H]−
443.925
0
20
40
60
80
100
445
450
455
460
465
470
475

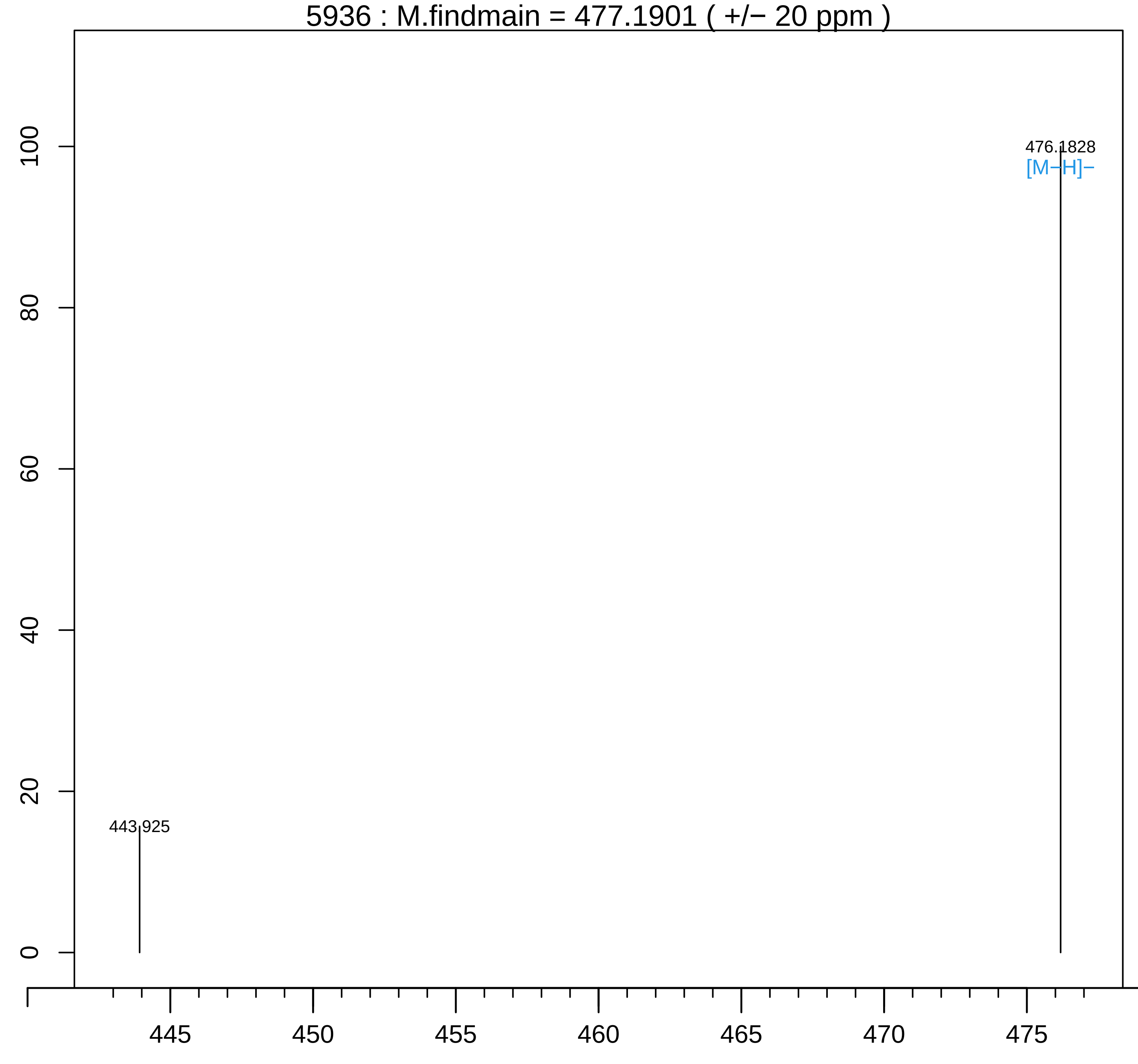
5936 : M.findmain = 477.1901 (+/− 20 ppm)
476.1828
[M−H]−
443.925
0
20
40
60
80
100
445
450
455
460
465
470
475

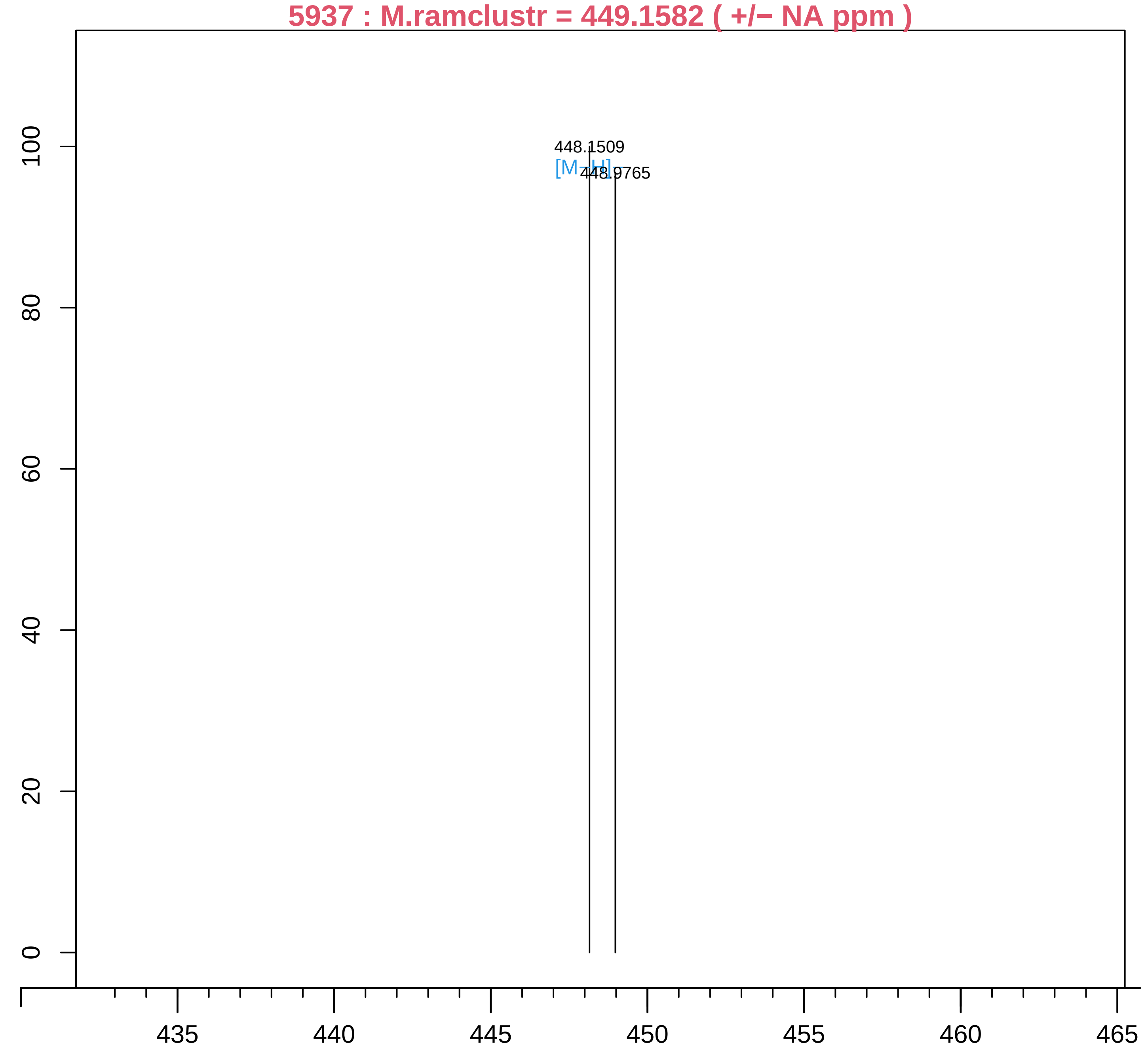
5937 : M.ramclustr = 449.1582 (+/− NA ppm)
448.1509
[M−H]−
448.9765
0
20
40
60
80
100
435
440
445
450
455
460
465

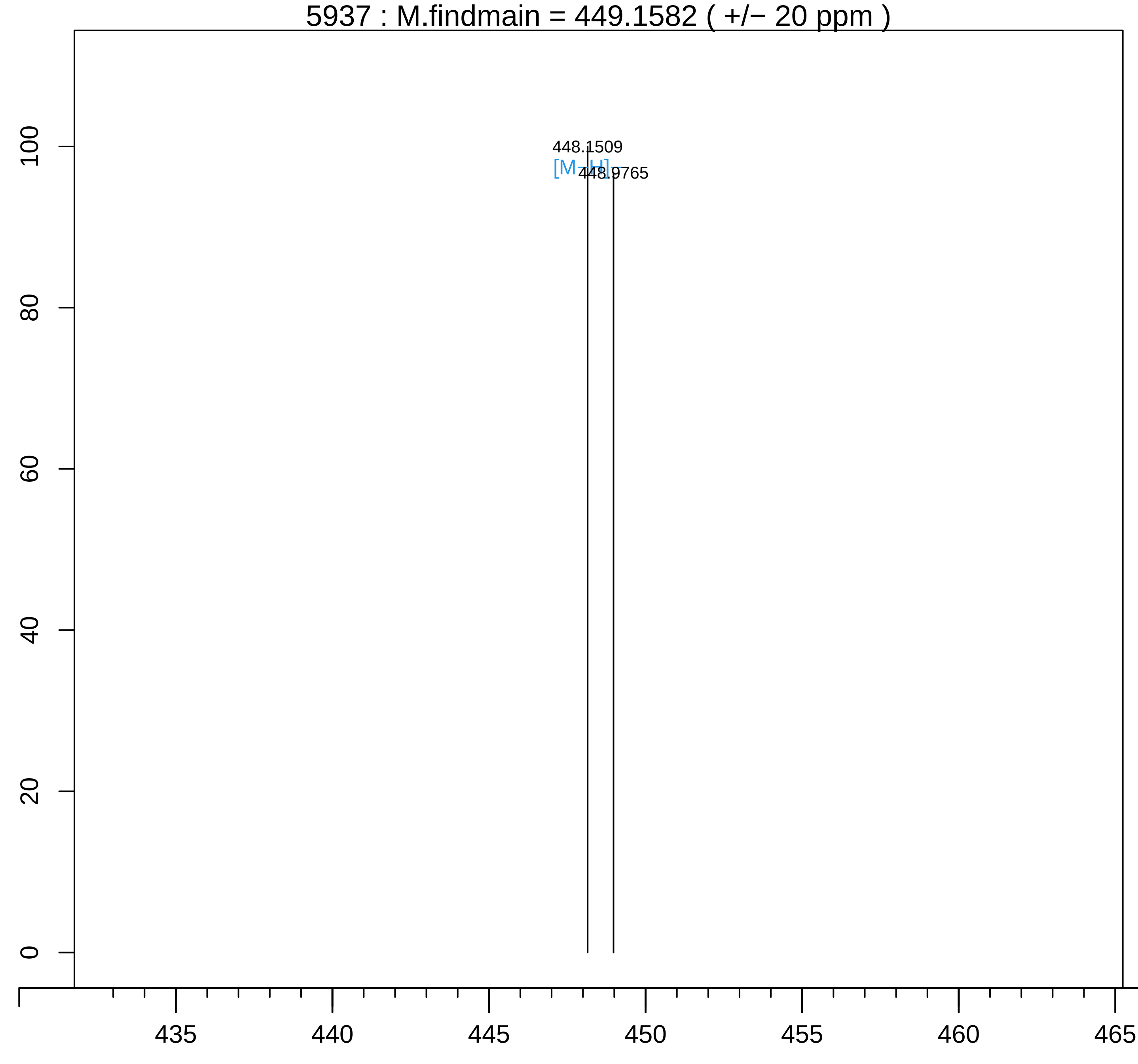
5937 : M.findmain = 449.1582 (+/− 20 ppm)
448.1509
[M−H]−
448.9765
0
20
40
60
80
100
435
440
445
450
455
460
465

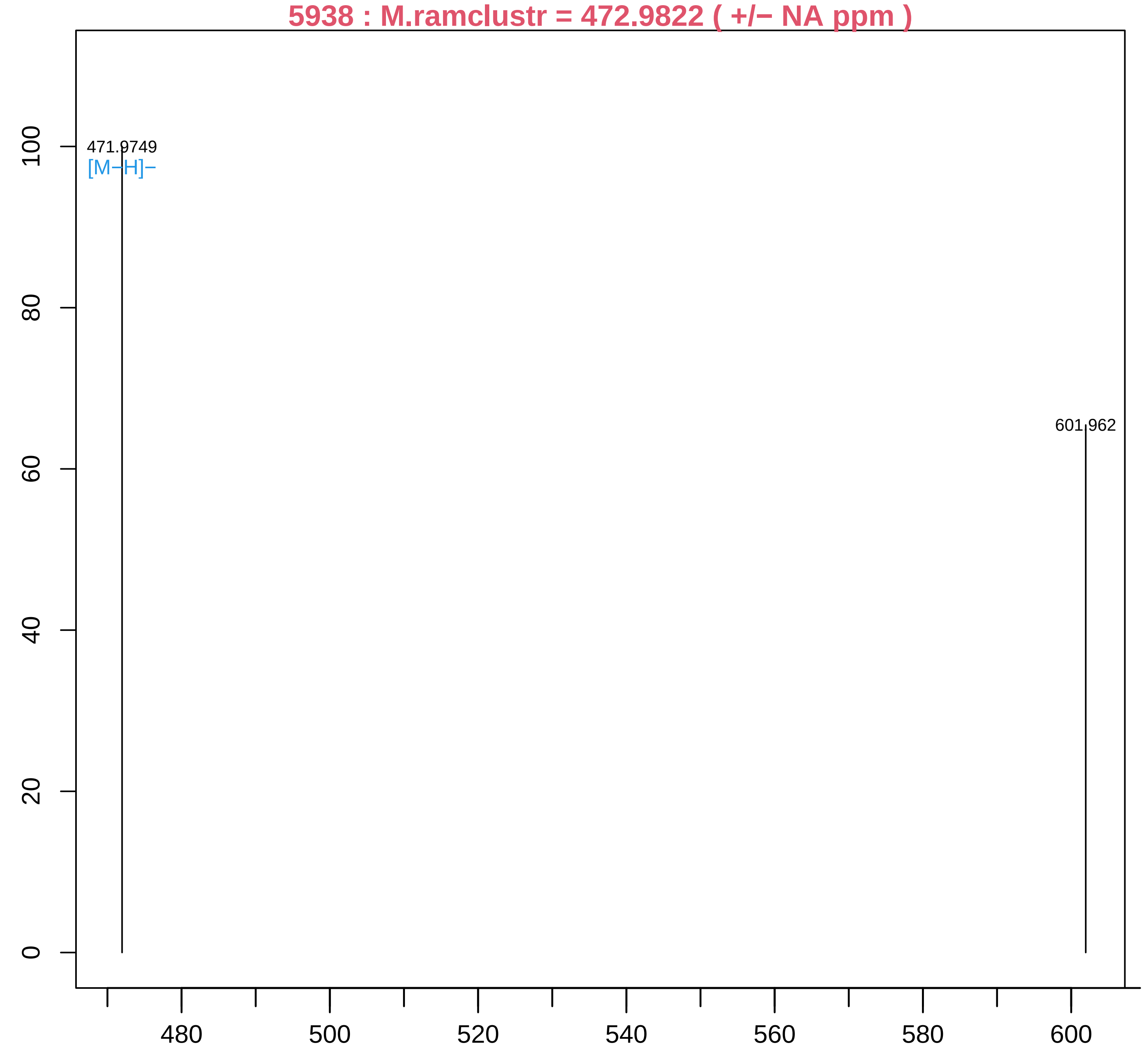
5938 : M.ramclustr = 472.9822 (+/− NA ppm)
471.9749
[M−H]−
601.962
0
20
40
60
80
100
480
500
520
540
560
580
600

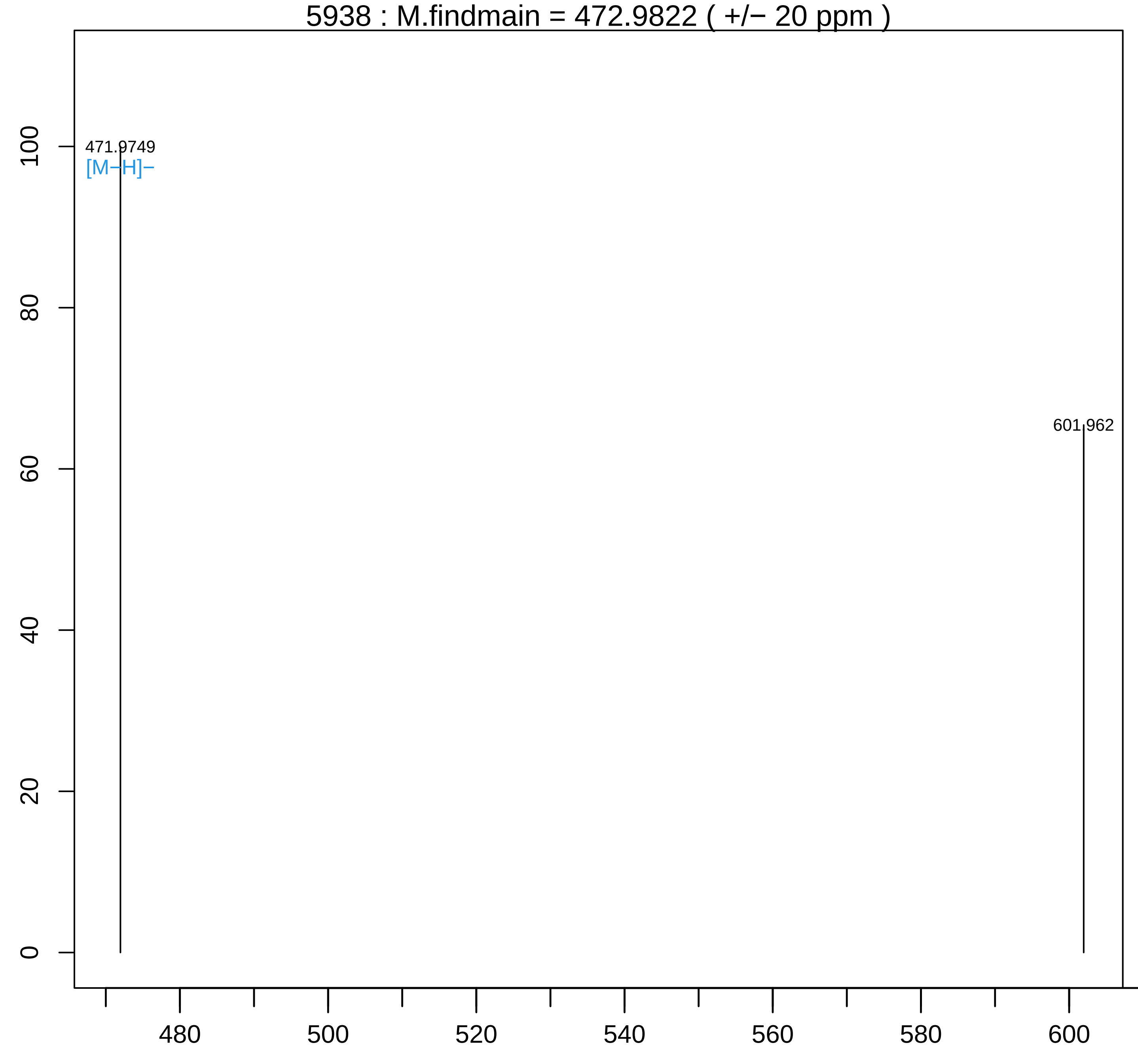
5938 : M.findmain = 472.9822 (+/− 20 ppm)
471.9749
[M−H]−
601.962
0
20
40
60
80
100
480
500
520
540
560
580
600

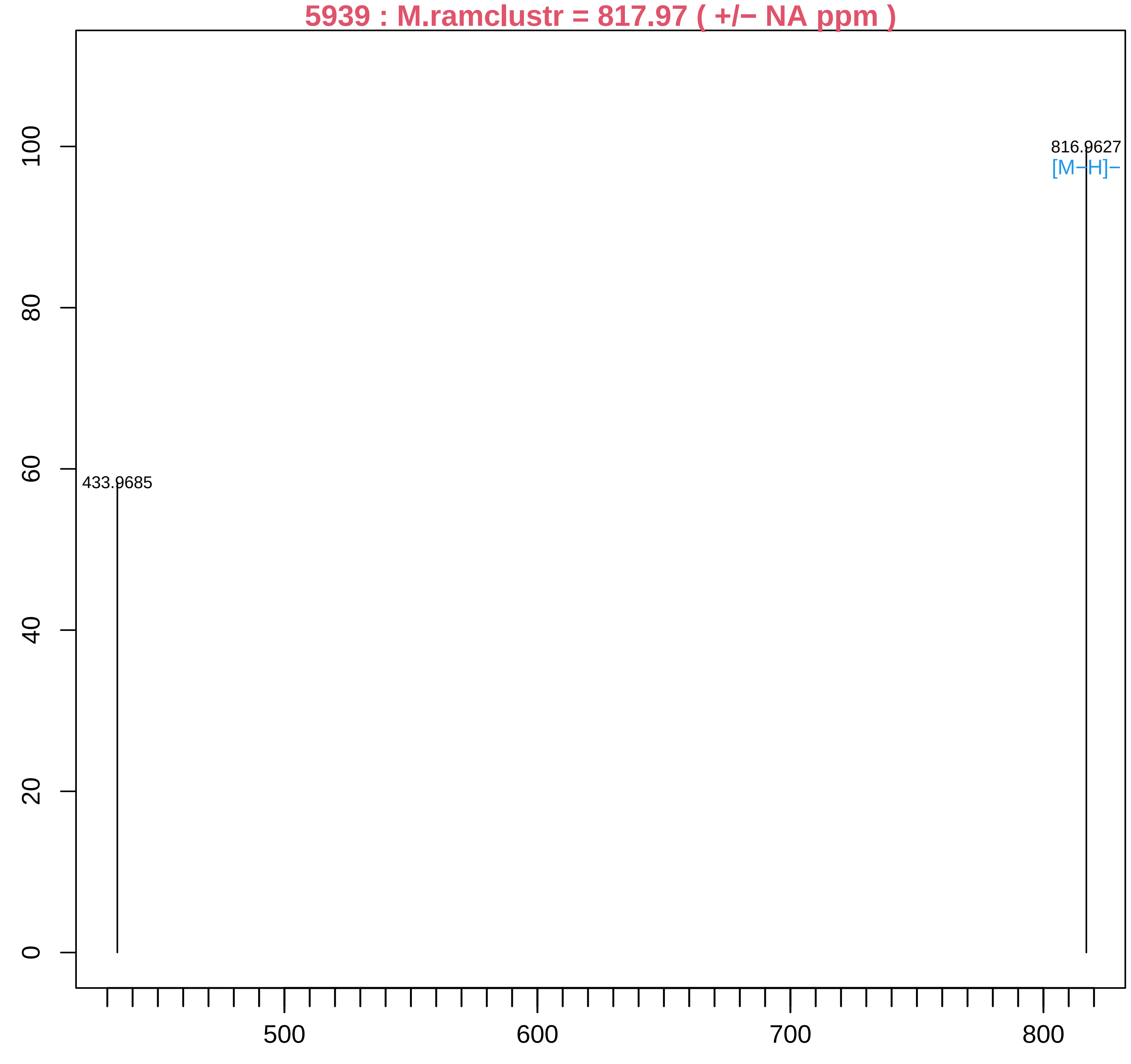
5939 : M.ramclustr = 817.97 (+/− NA ppm)
816.9627
[M−H]−
433.9685
0
20
40
60
80
100
500
600
700
800
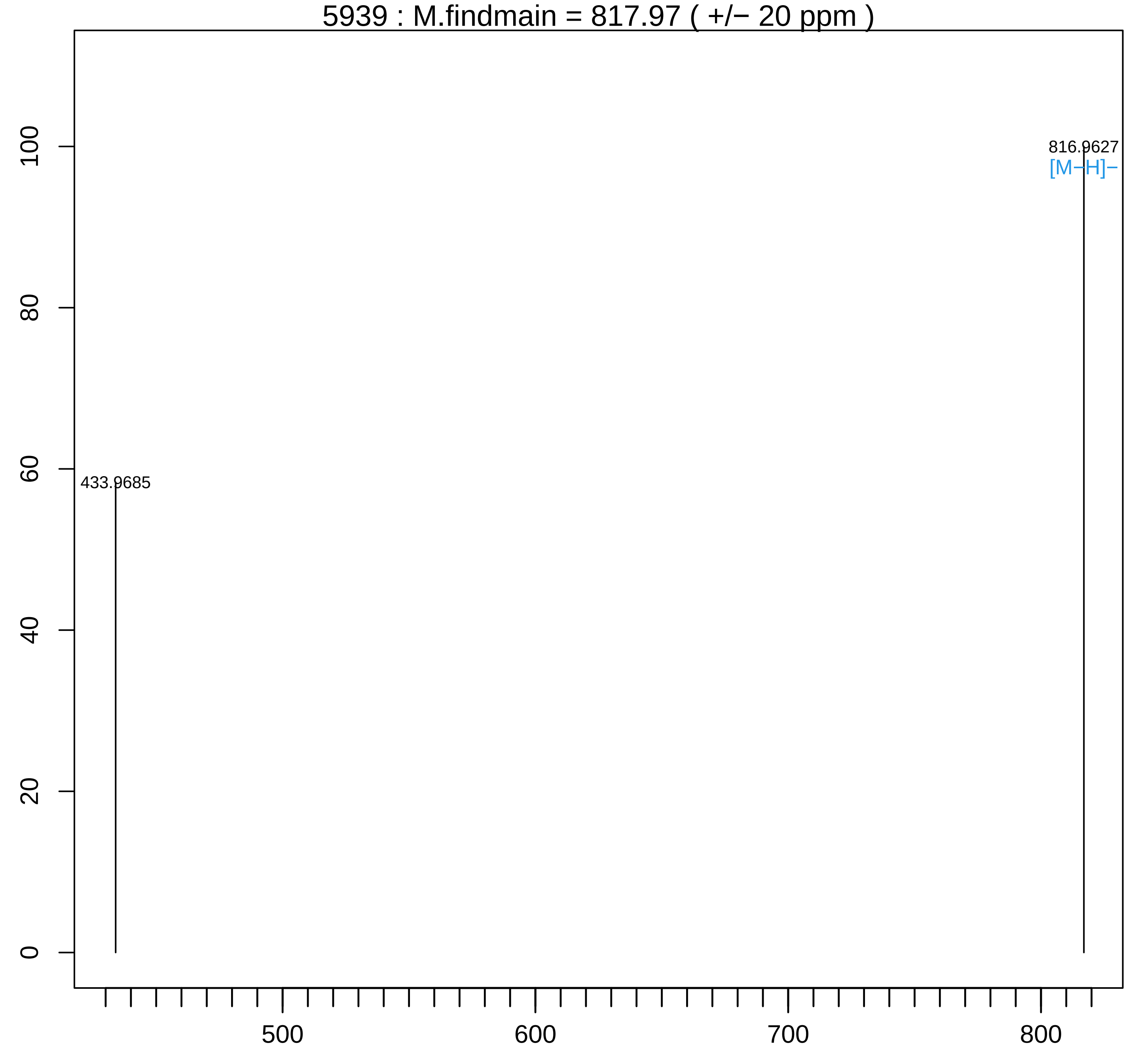
5939 : M.findmain = 817.97 (+/− 20 ppm)
816.9627
[M−H]−
433.9685
0
20
40
60
80
100
500
600
700
800

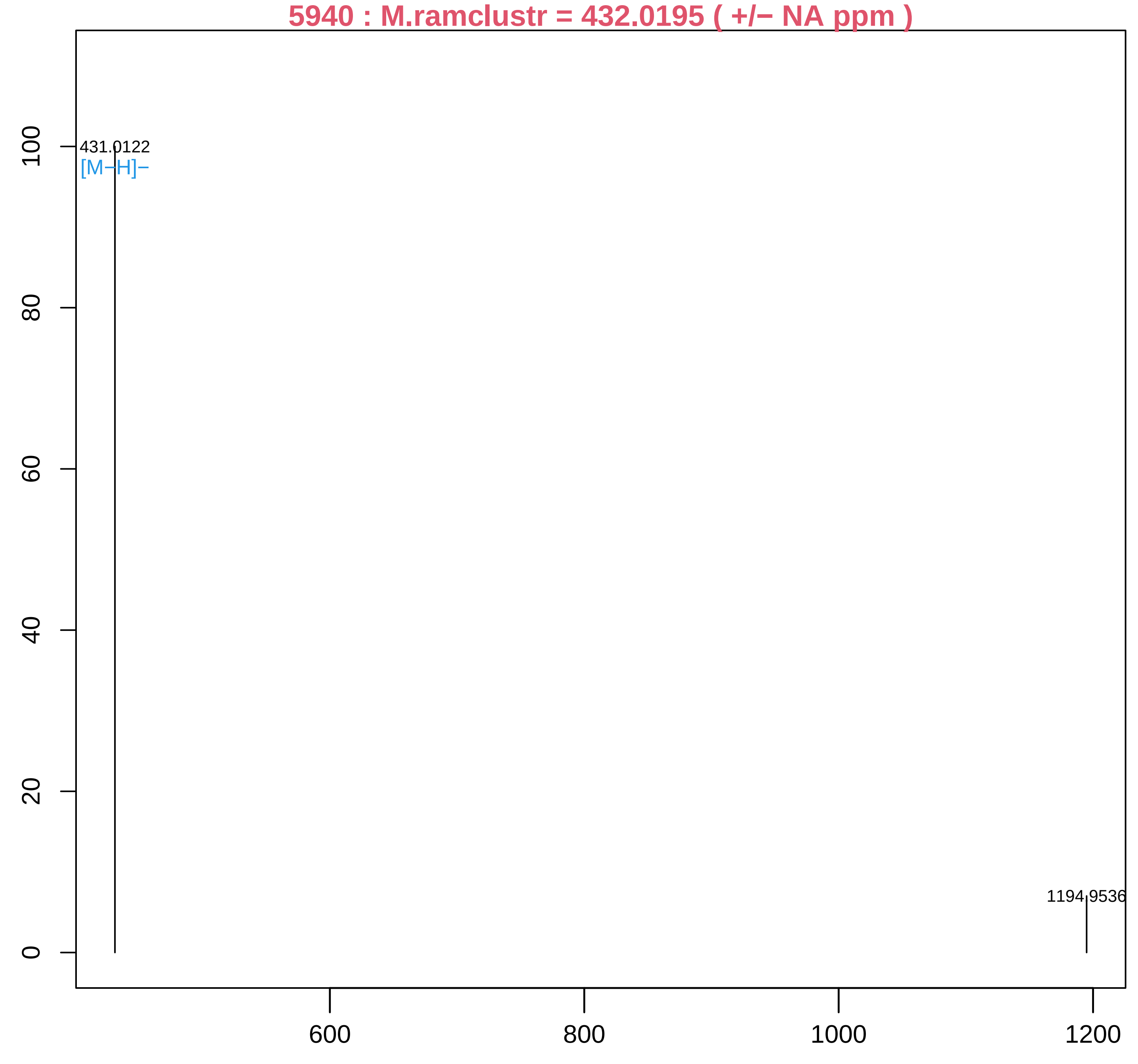
5940 : M.ramclustr = 432.0195 (+/− NA ppm)
431.0122
[M−H]−
1194.9536
0
20
40
60
80
100
600
800
1000
1200

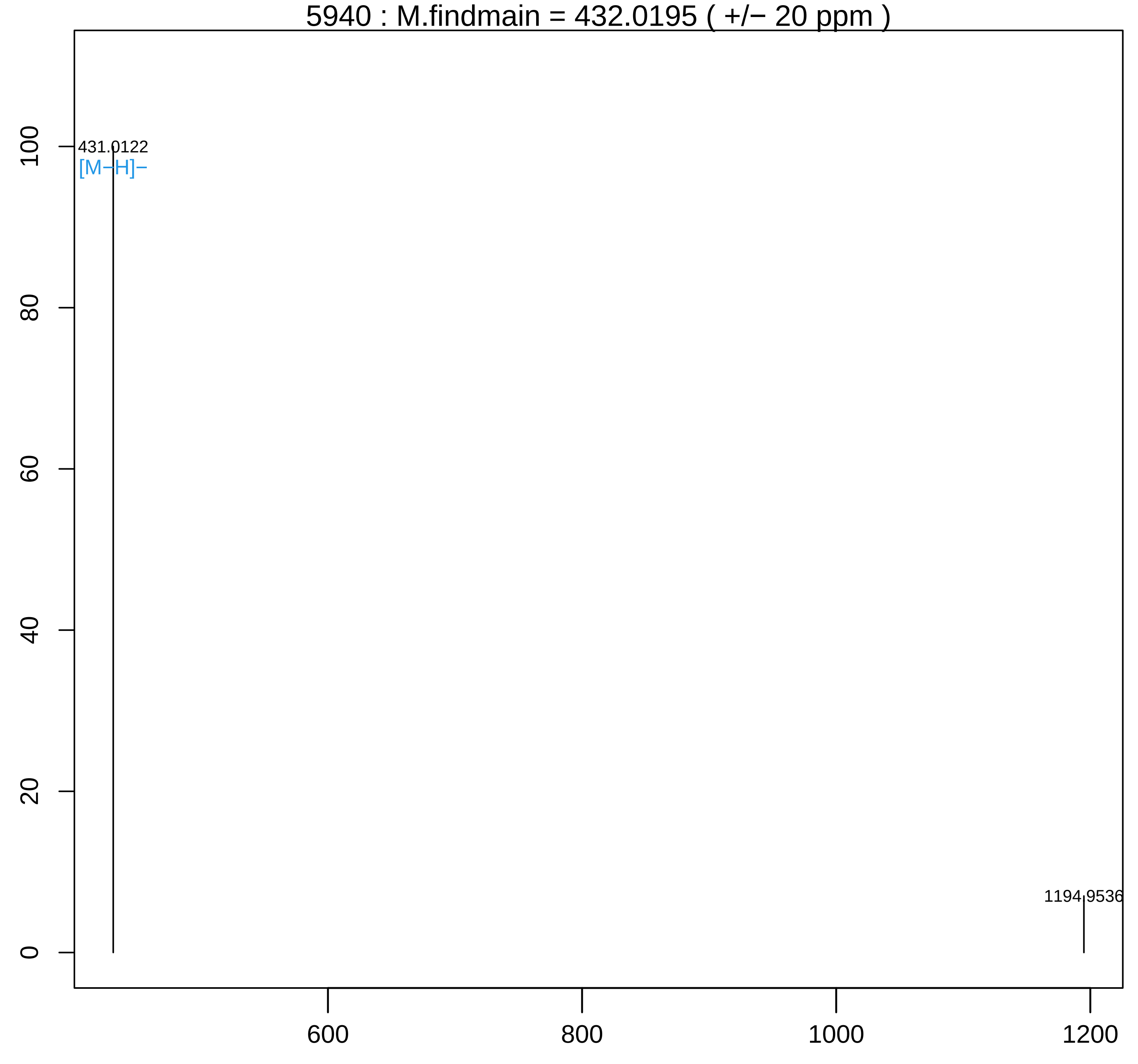
5940 : M.findmain = 432.0195 (+/− 20 ppm)
431.0122
[M−H]−
1194.9536
0
20
40
60
80
100
600
800
1000
1200

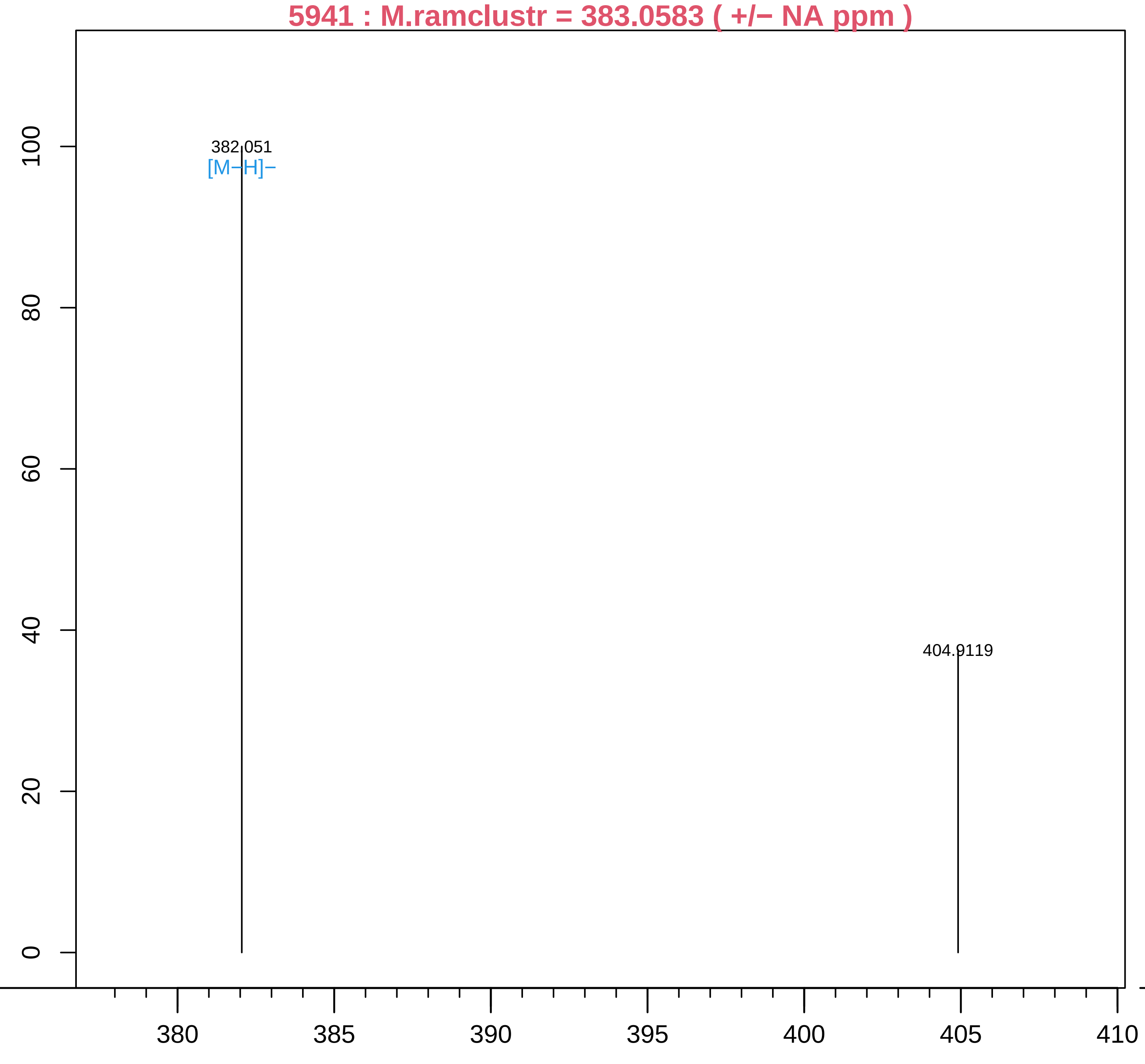
5941 : M.ramclustr = 383.0583 (+/− NA ppm)
382.051
[M−H]−
404.9119

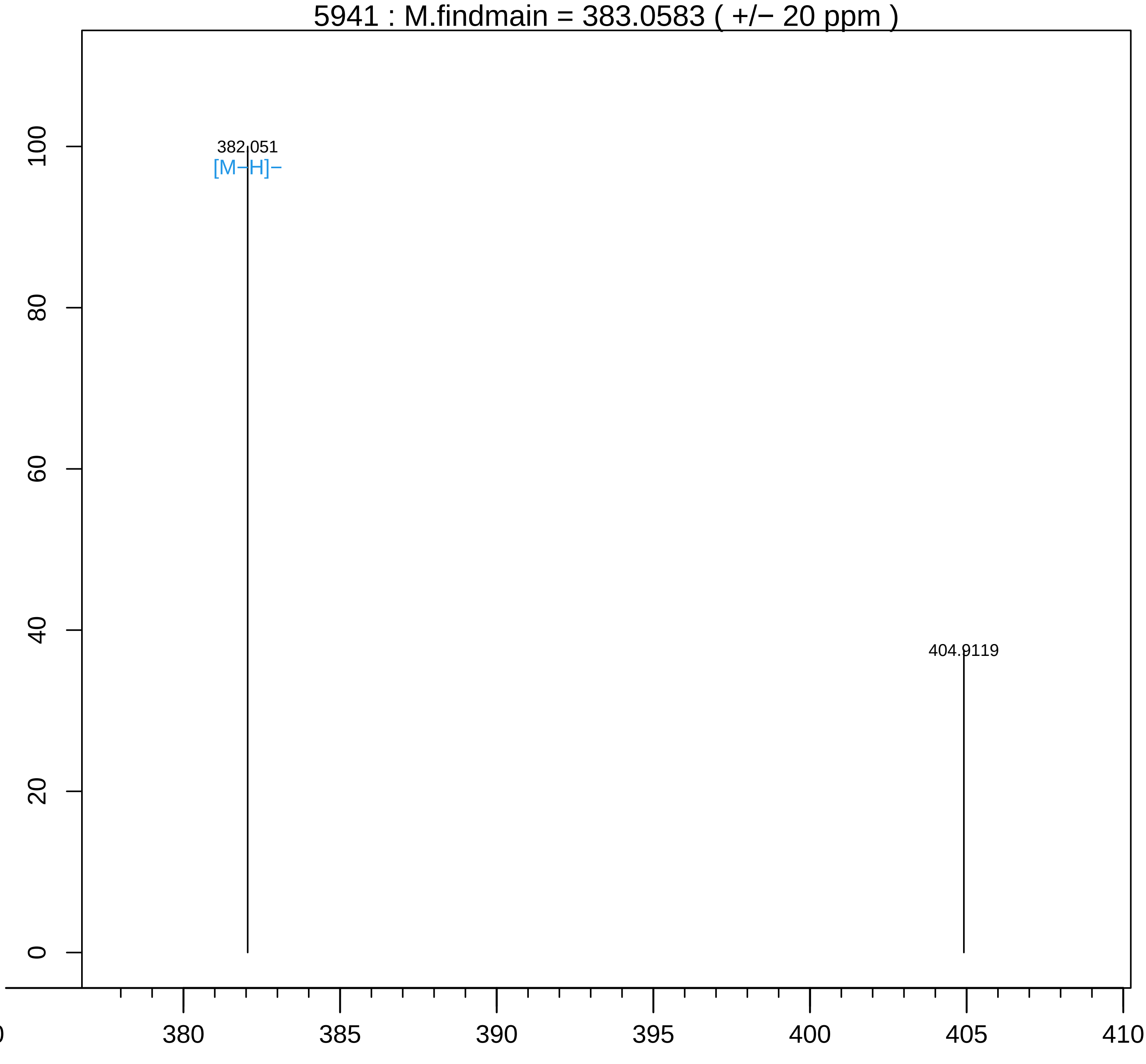
5941 : M.findmain = 383.0583 (+/− 20 ppm)
382.051
[M−H]−
404.9119

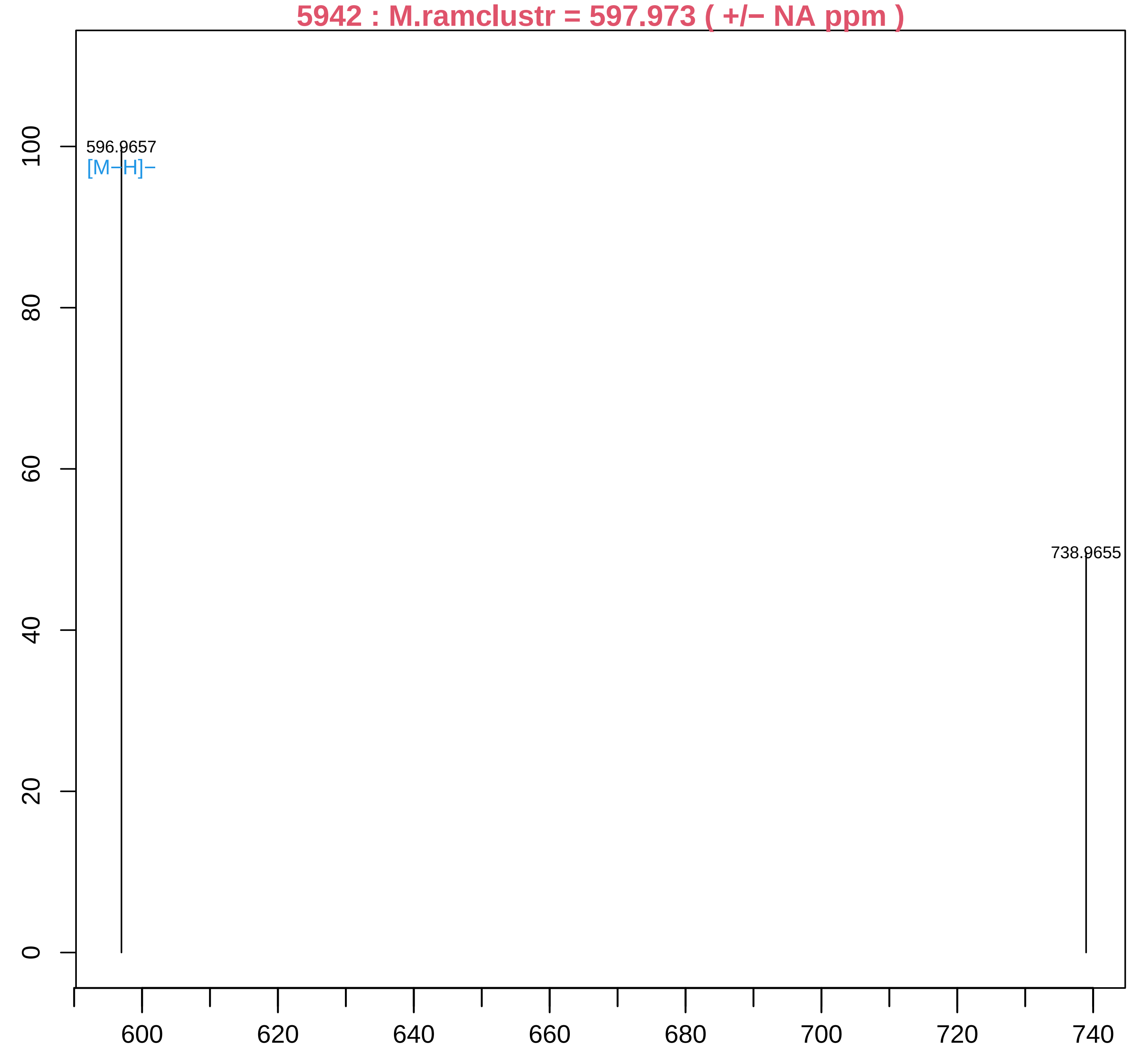
5942 : M.ramclustr = 597.973 (+/− NA ppm)
596.9657
[M−H]−
738.9655
0
20
40
60
80
100
600
620
640
660
680
700
720
740

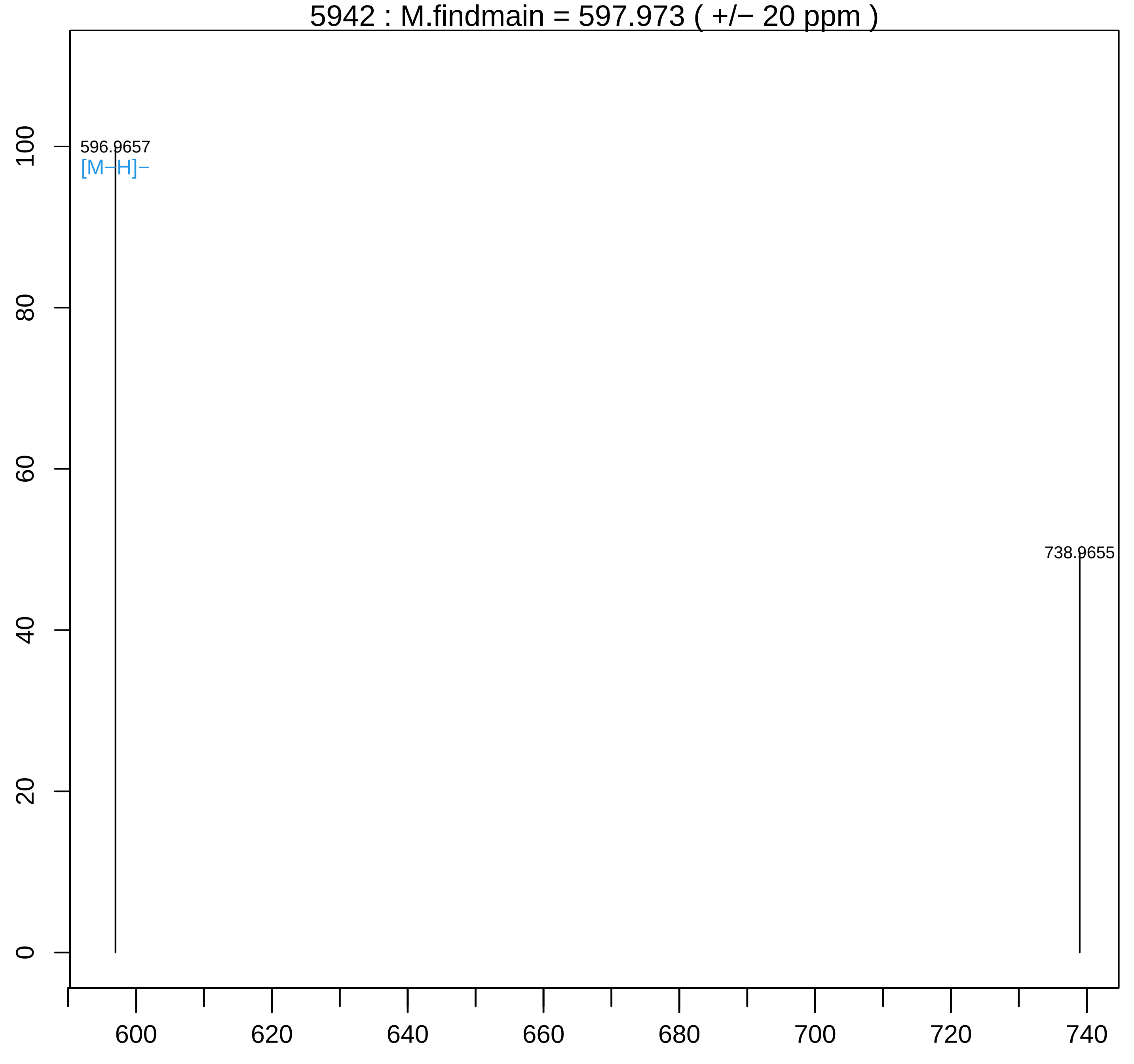
5942 : M.findmain = 597.973 (+/− 20 ppm)
596.9657
[M−H]−
738.9655
0
20
40
60
80
100
600
620
640
660
680
700
720
740

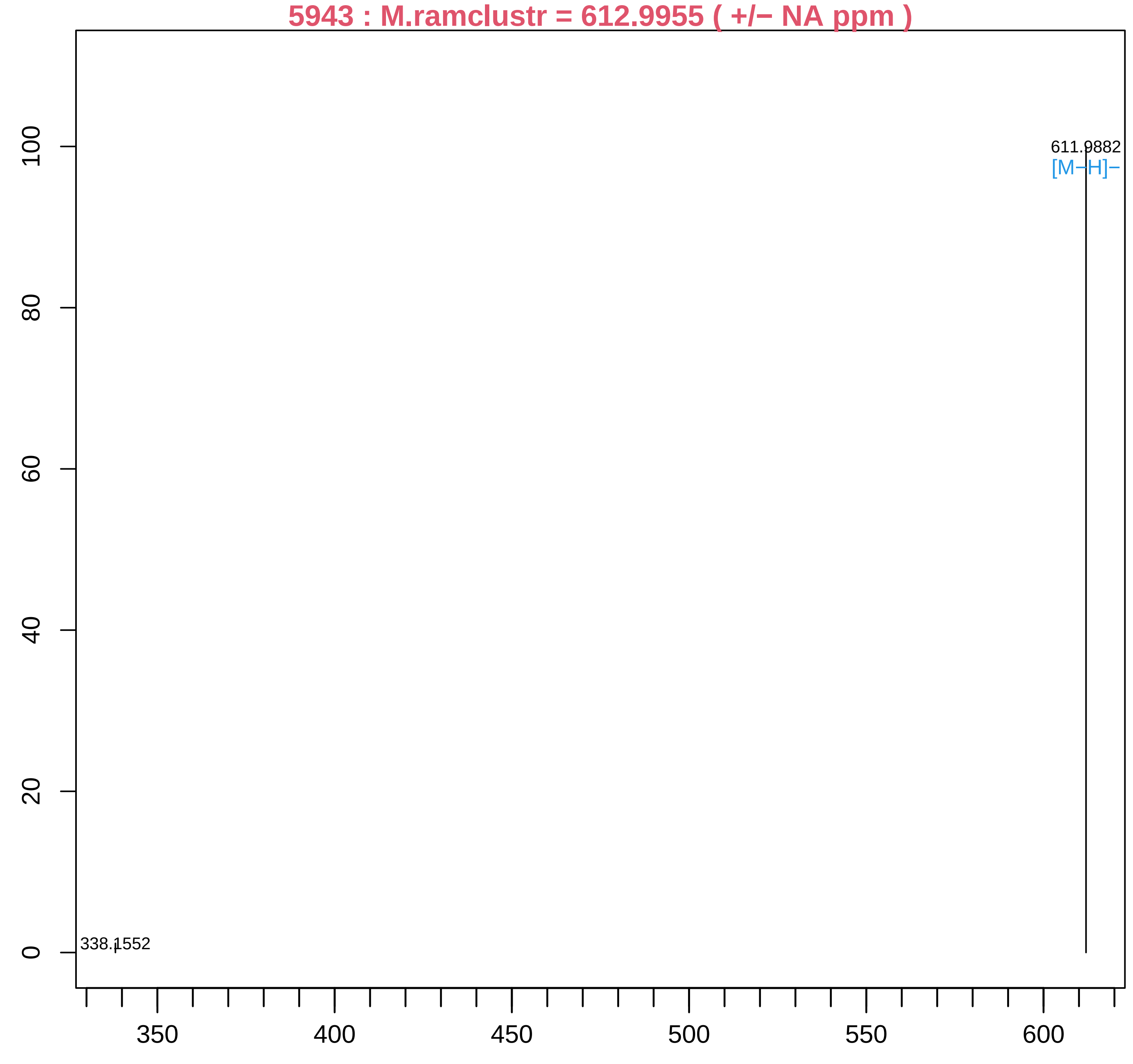
5943 : M.ramclustr = 612.9955 (+/− NA ppm)
611.9882
[M−H]−
338.1552
0
20
40
60
80
100
350
400
450
500
550
600

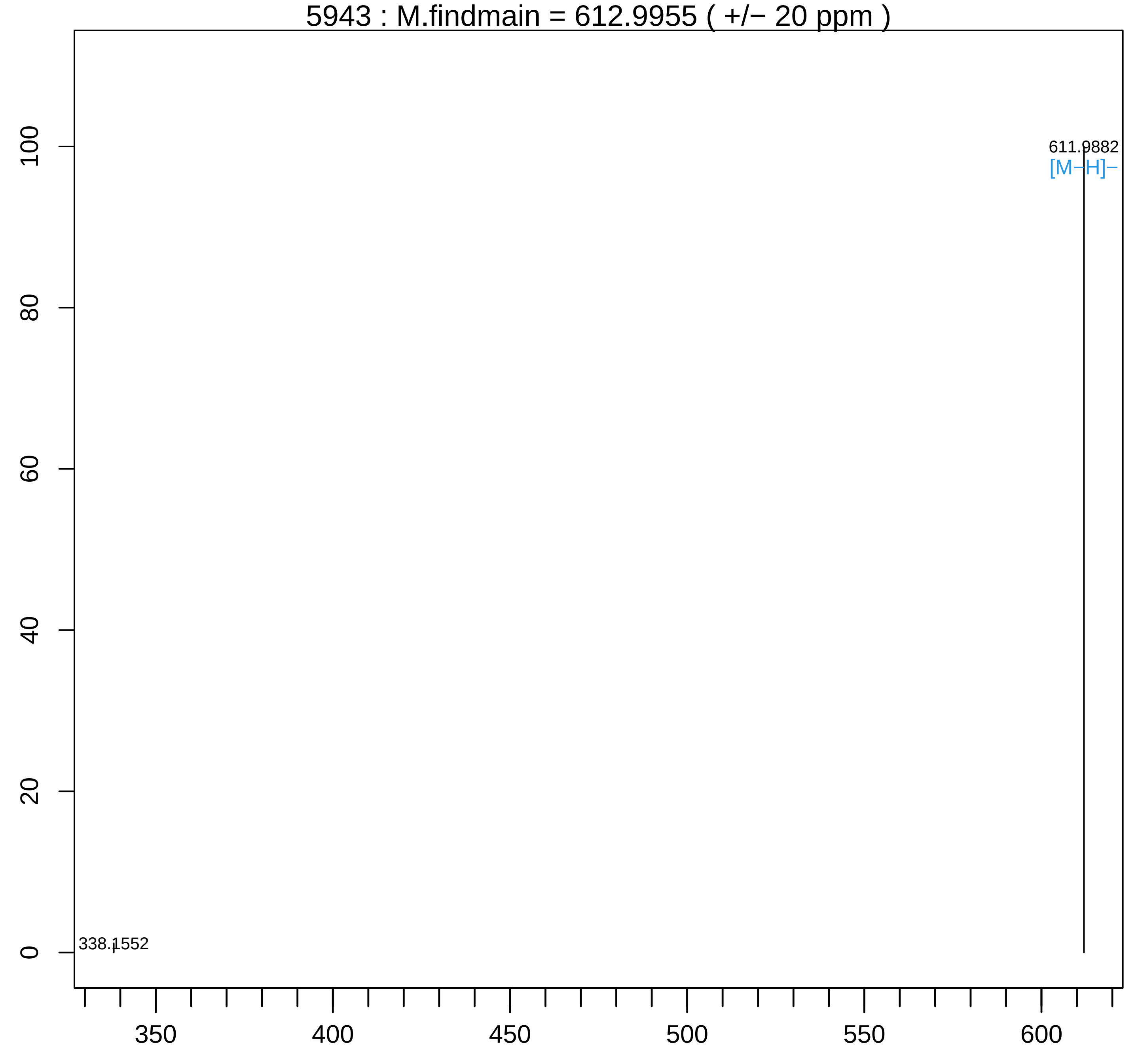
5943 : M.findmain = 612.9955 (+/− 20 ppm)
611.9882
[M−H]−
338.1552
0
20
40
60
80
100
350
400
450
500
550
600

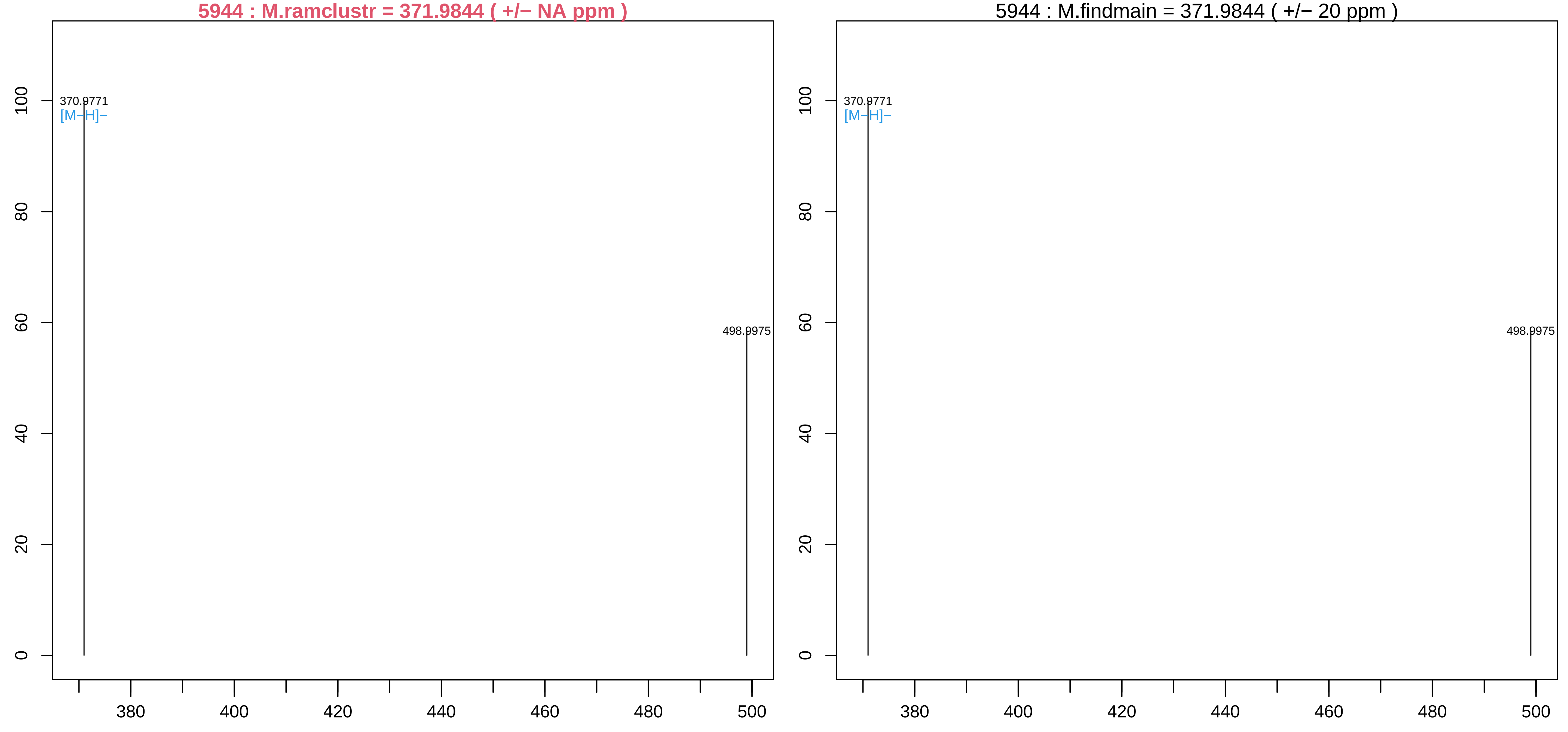
5944 : M.ramclustr = 371.9844 (+/− NA ppm)
370.9771
[M−H]−
498.9975
5944 : M.findmain = 371.9844 (+/− 20 ppm)
370.9771
[M−H]−
498.9975
0
20
40
60
80
100
380
400
420
440
460
480
500

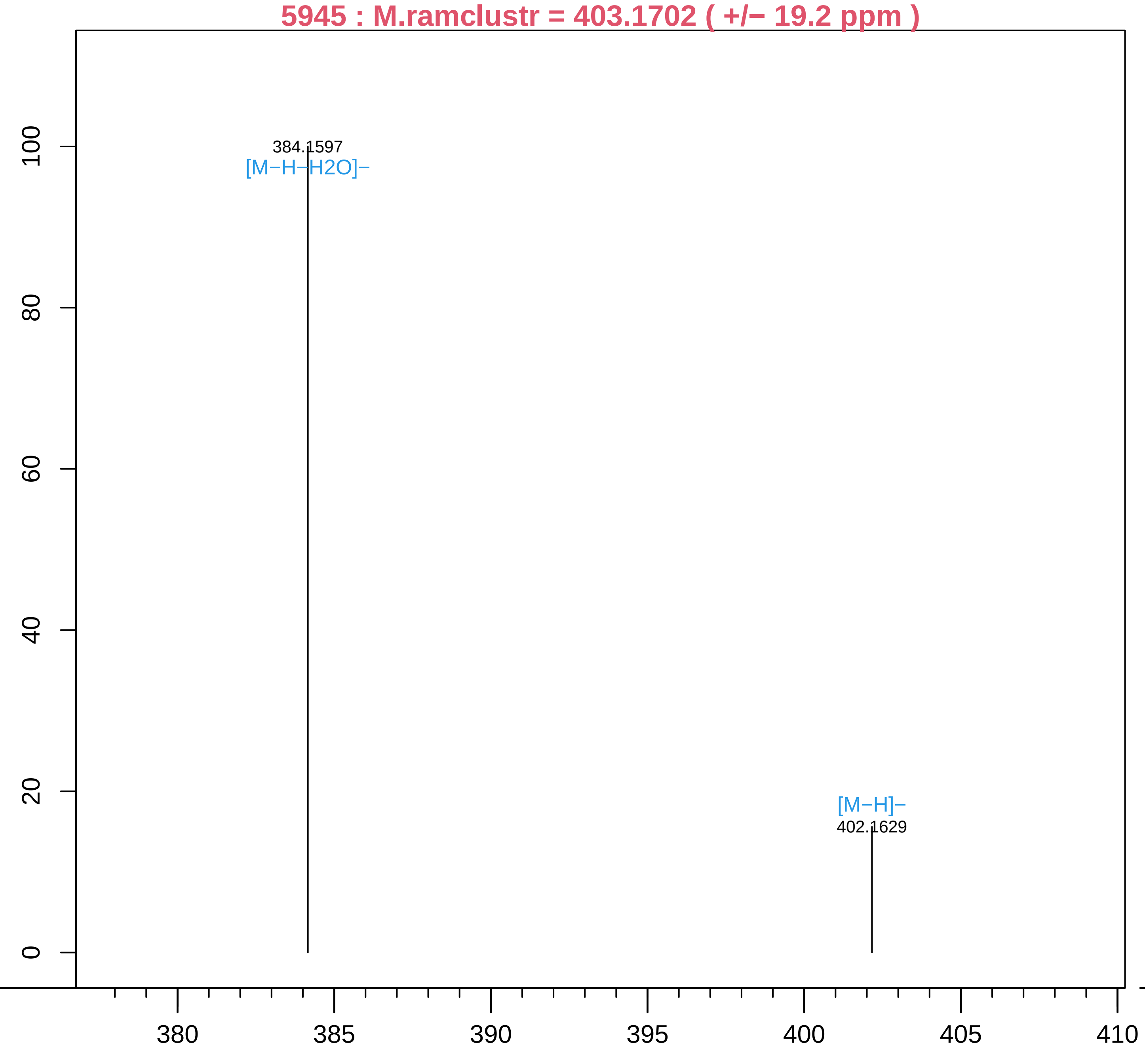
5945 : M.ramclustr = 403.1702 (+/− 19.2 ppm)
384.1597
[M−H−H2O]−
[M−H]−
402.1629
0
20
40
60
80
100
380
385
390
395
400
405
410

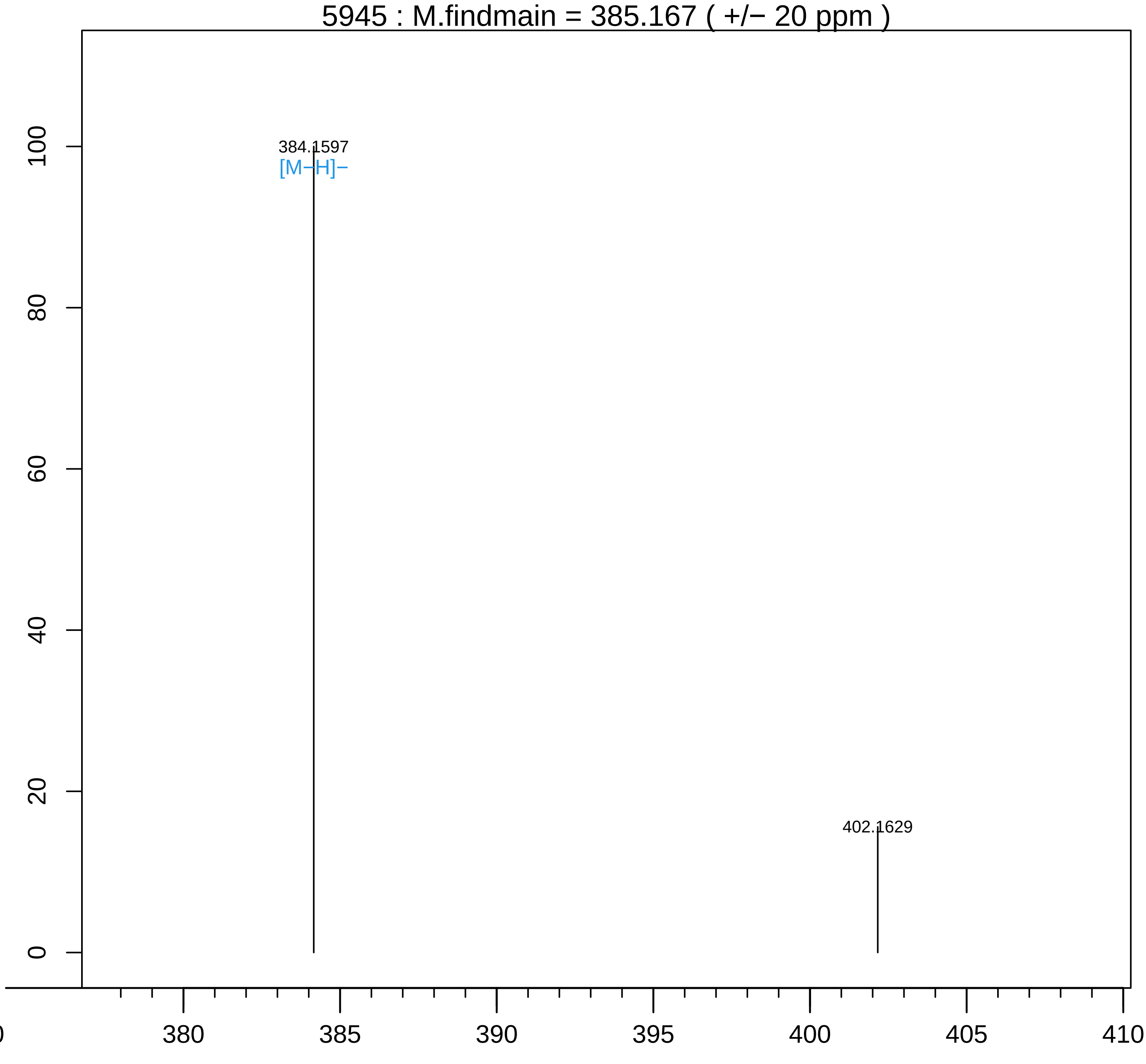
5945 : M.findmain = 385.167 (+/− 20 ppm)
384.1597
[M−H]−
402.1629
0
20
40
60
80
100
380
385
390
395
400
405
410

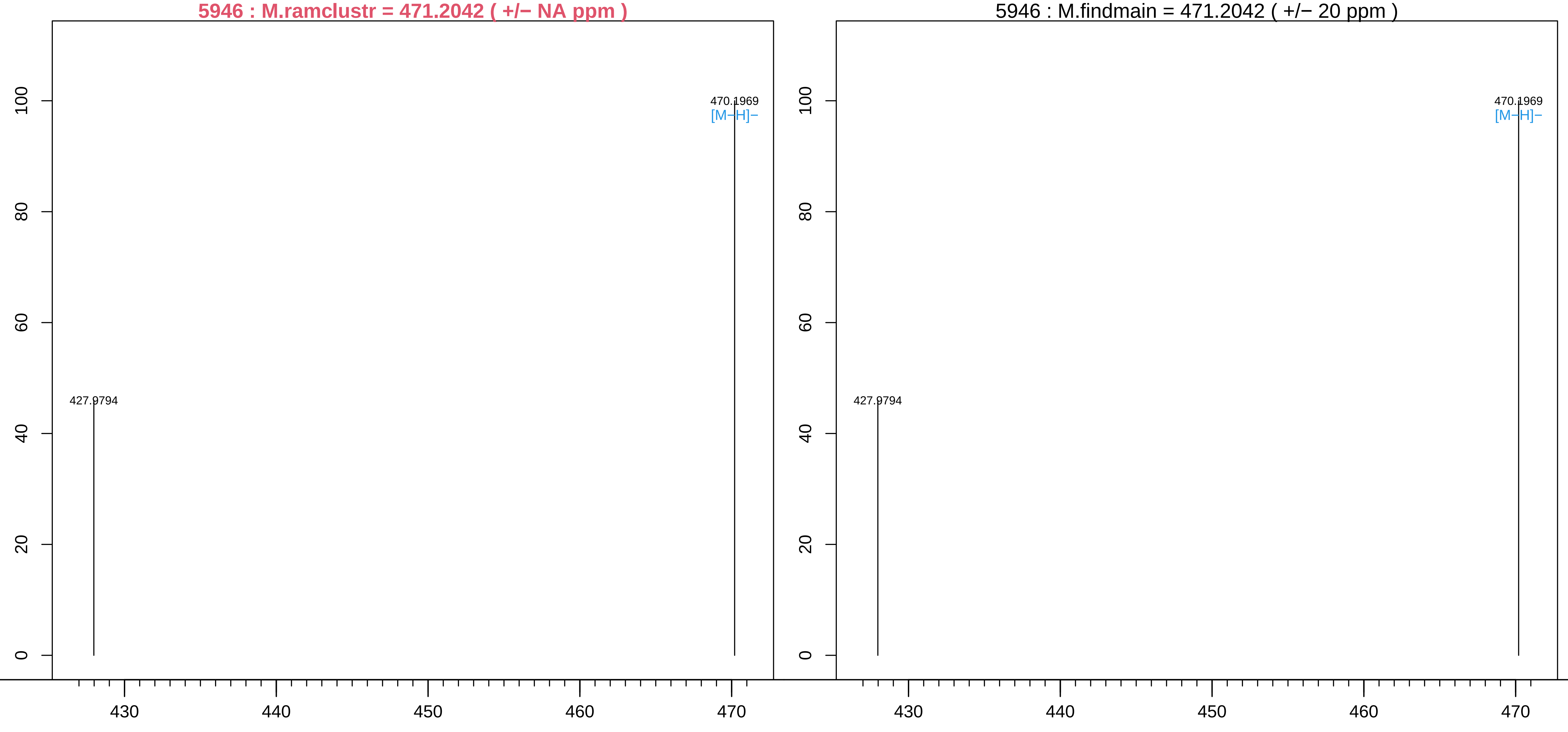
5946 : M.ramclustr = 471.2042 (+/− NA ppm)
5946 : M.findmain = 471.2042 (+/− 20 ppm)
470.1969
[M−H]−
427.9794
470.1969
[M−H]−
427.9794
0
20
40
60
80
100
430
440
450
460
470

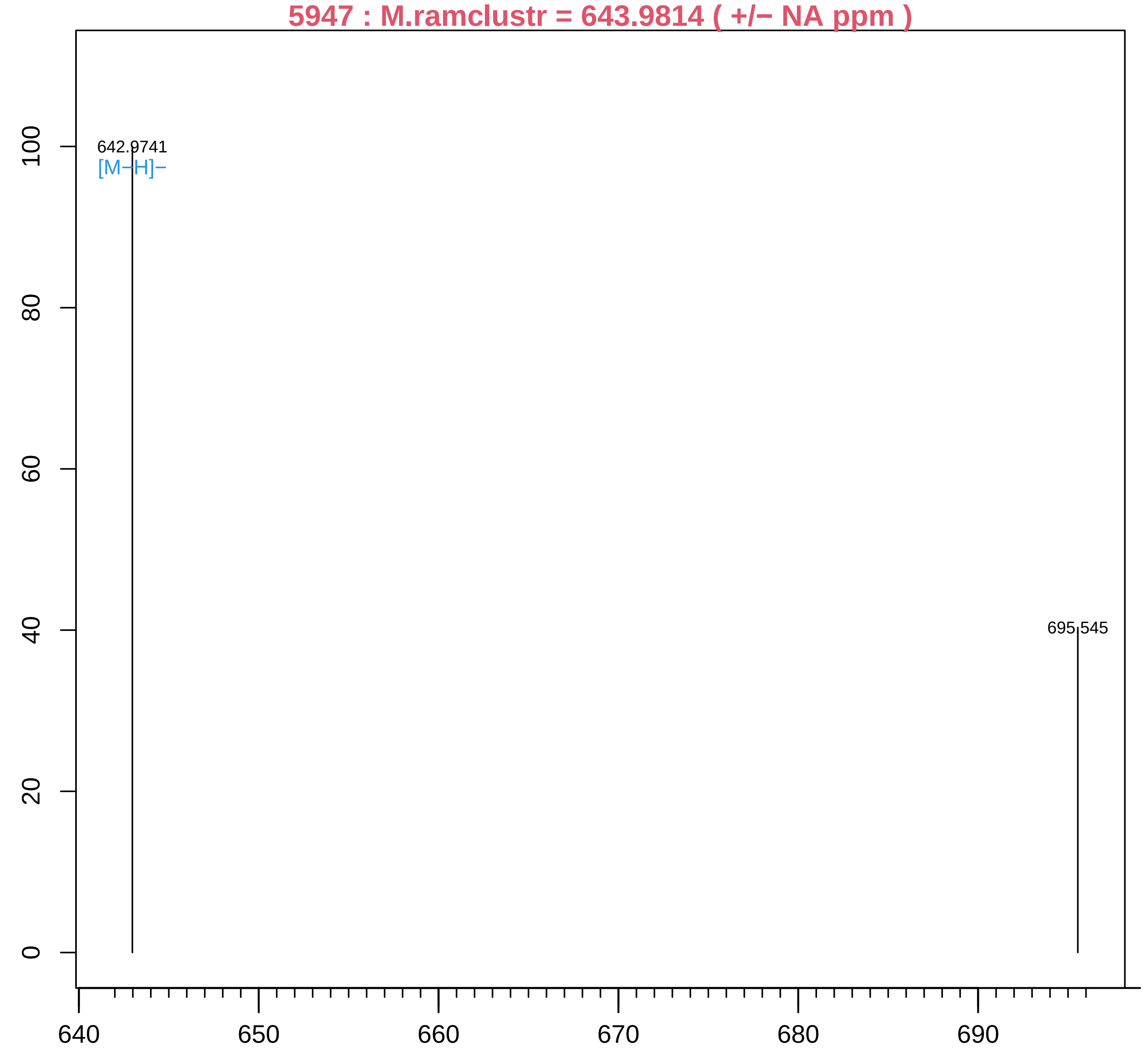
5947 : M.ramclustr = 643.9814 (+/− NA ppm)
642.9741
[M−H]−
695.545
0
20
40
60
80
100
640
650
660
670
680
690

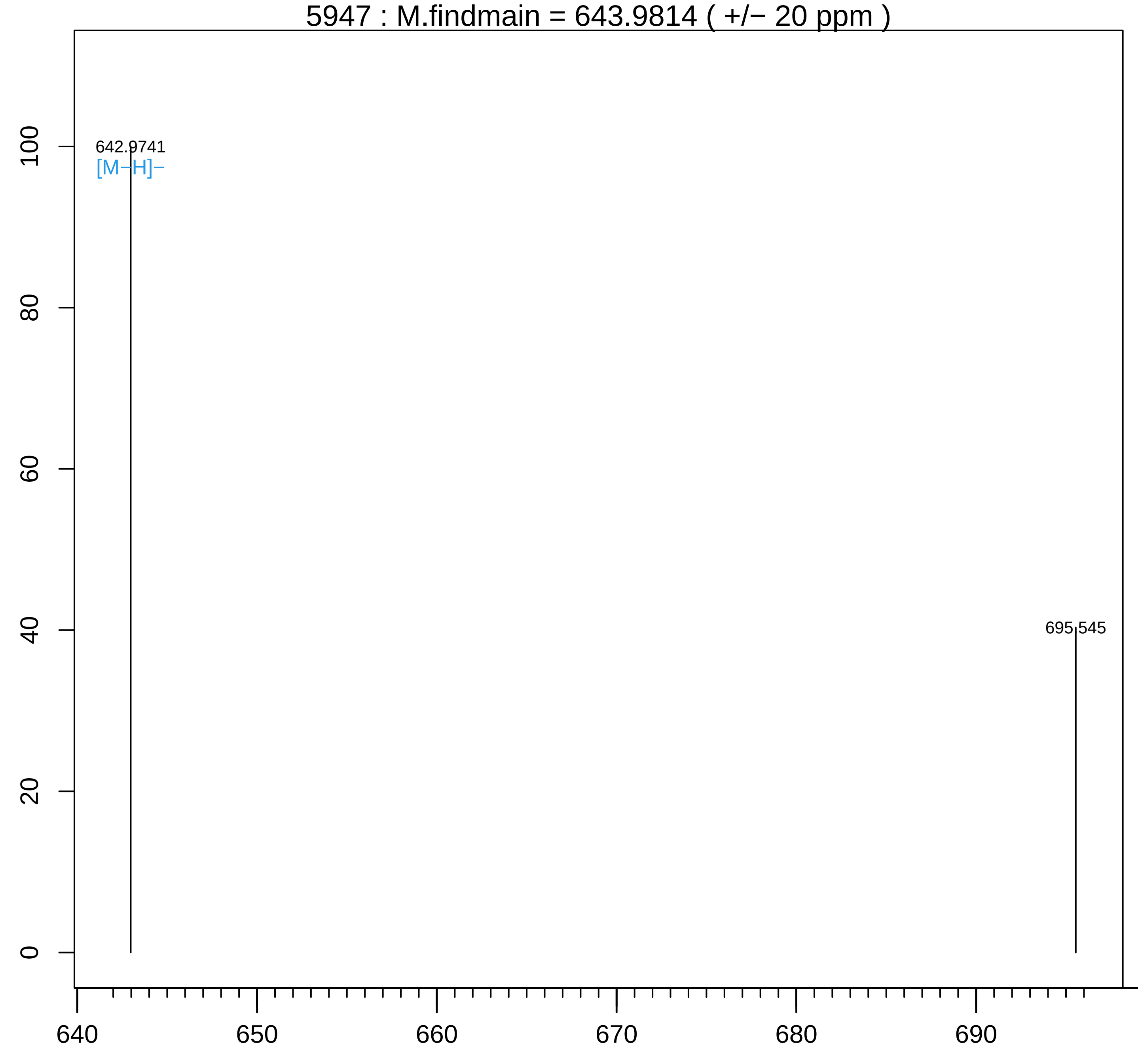
5947 : M.findmain = 643.9814 (+/− 20 ppm)
642.9741
[M−H]−
695.545
0
20
40
60
80
100
640
650
660
670
680
690

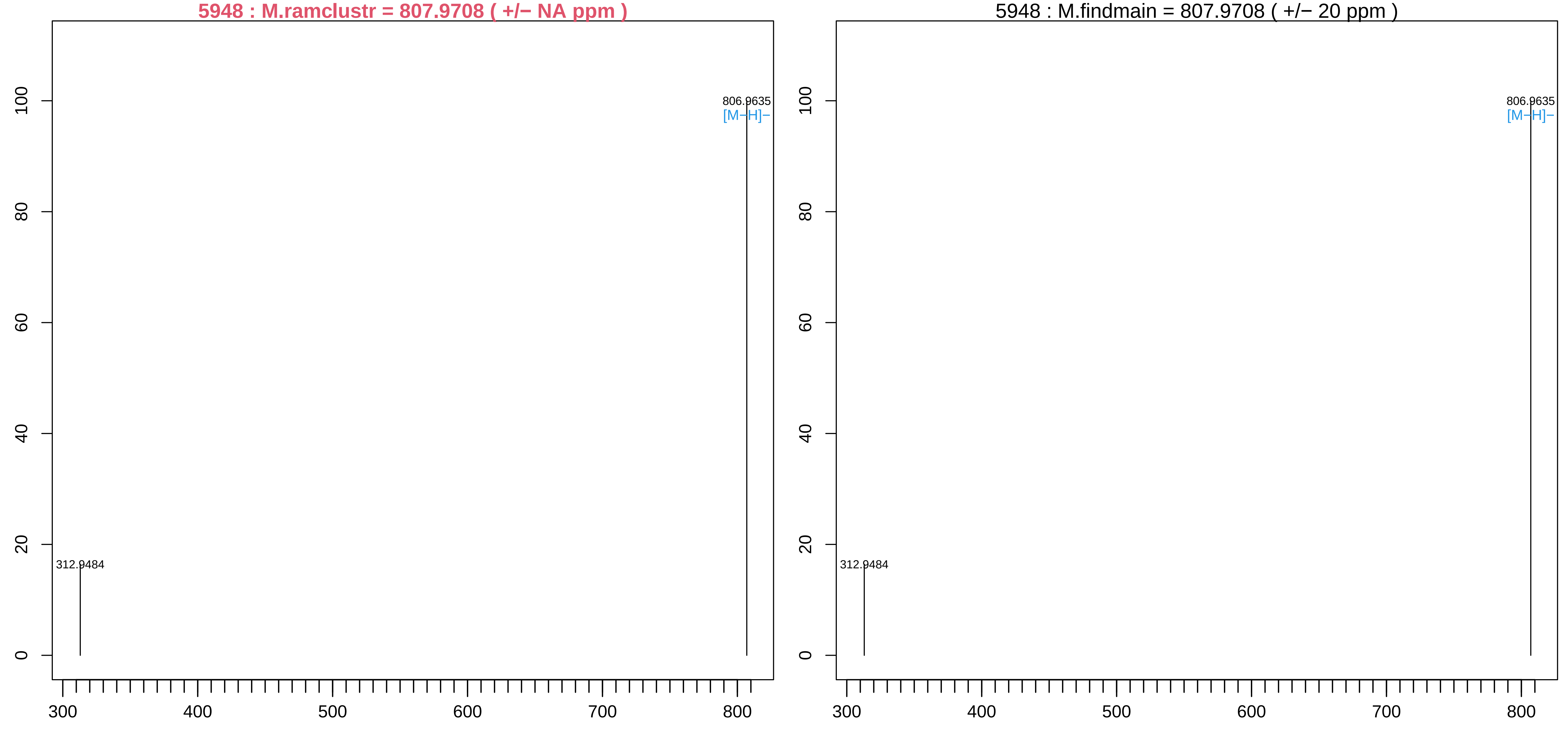
5948 : M.ramclustr = 807.9708 (+/− NA ppm)
5948 : M.findmain = 807.9708 (+/− 20 ppm)
806.9635
[M−H]−
312.9484
806.9635
[M−H]−
312.9484
0
20
40
60
80
100
300
400
500
600
700
800

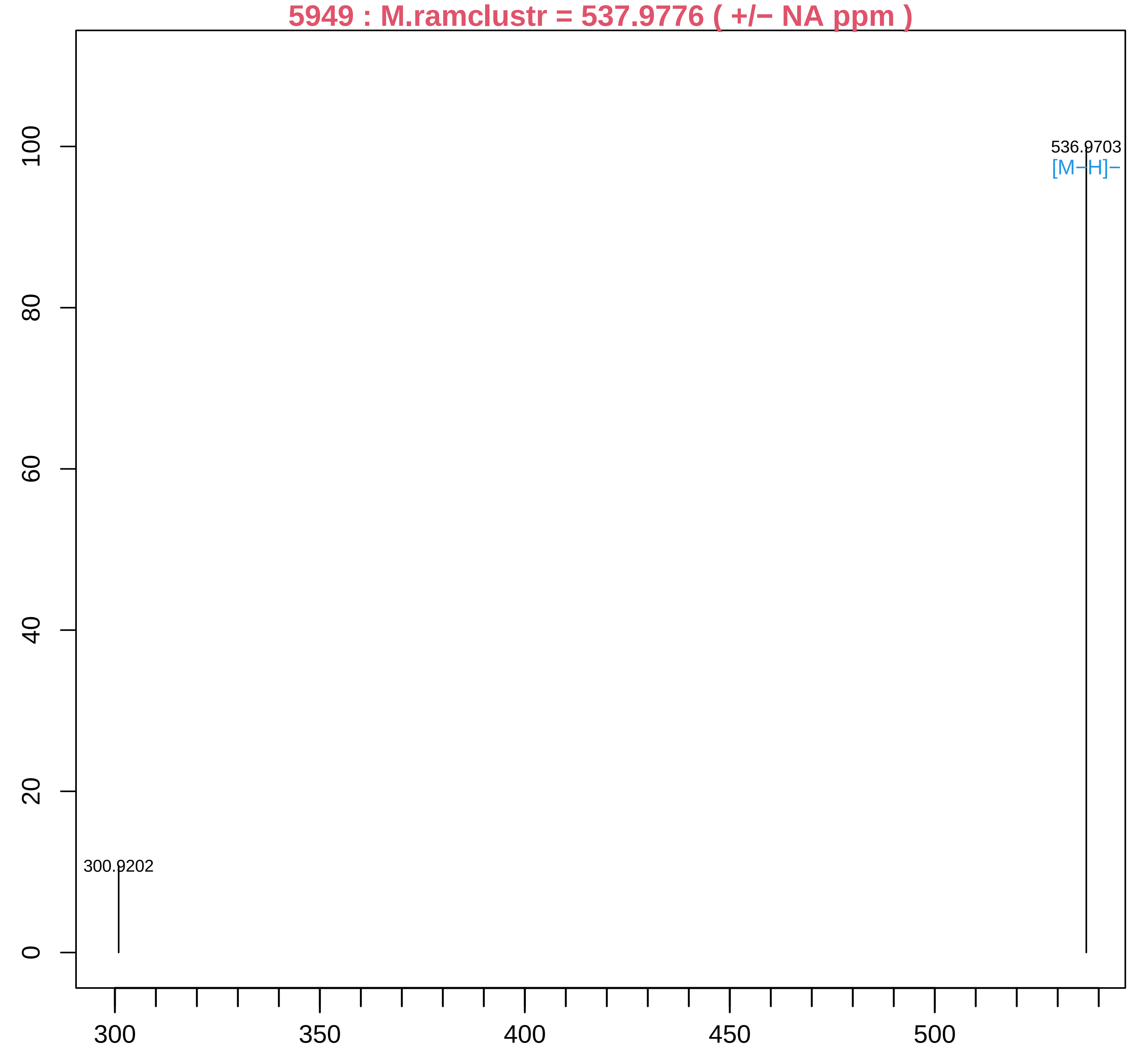
5949 : M.ramclustr = 537.9776 (+/− NA ppm)
536.9703
[M−H]−
300.9202
0
20
40
60
80
100
300
350
400
450
500

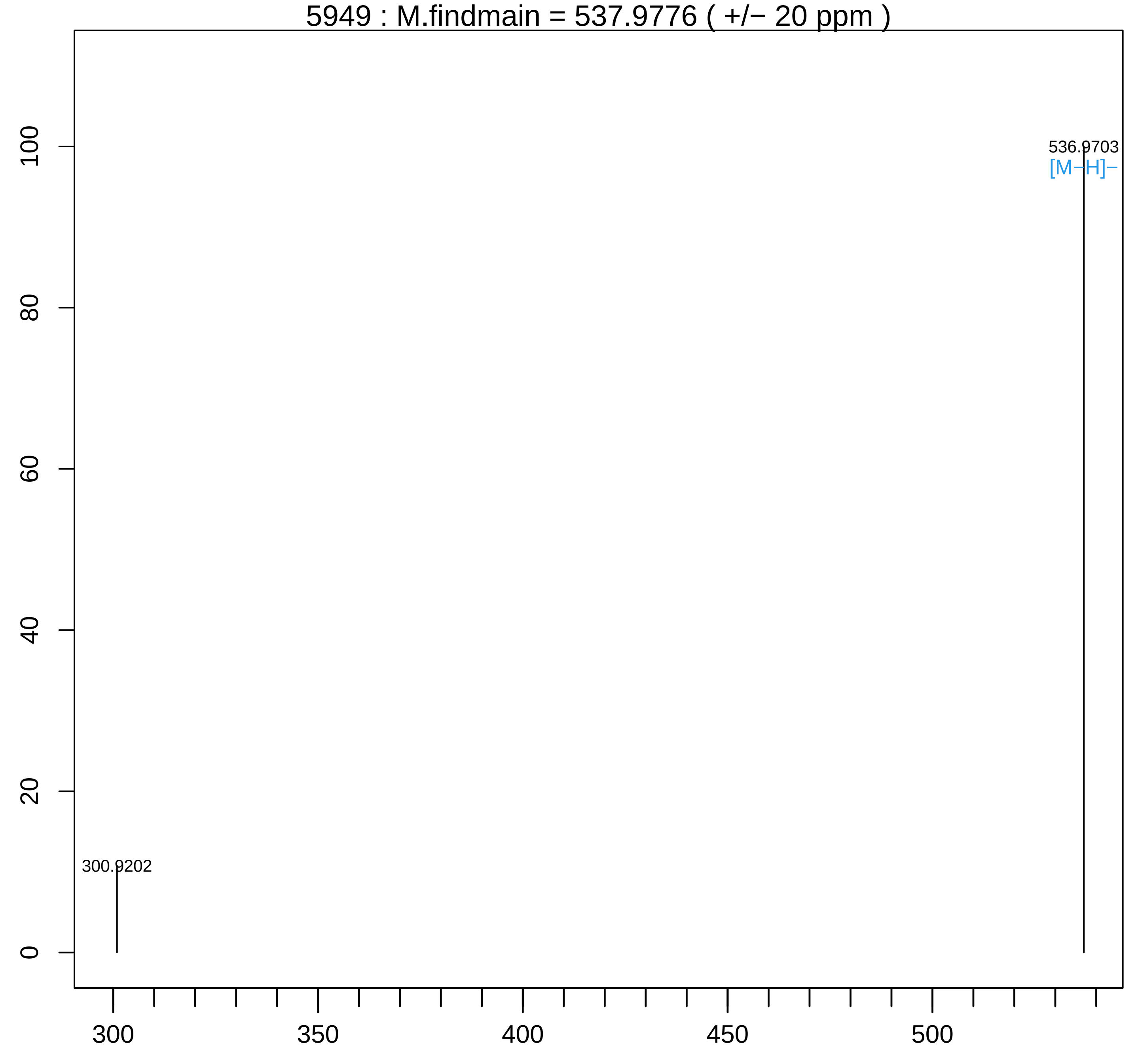
5949 : M.findmain = 537.9776 (+/− 20 ppm)
536.9703
[M−H]−
300.9202
0
20
40
60
80
100
300
350
400
450
500

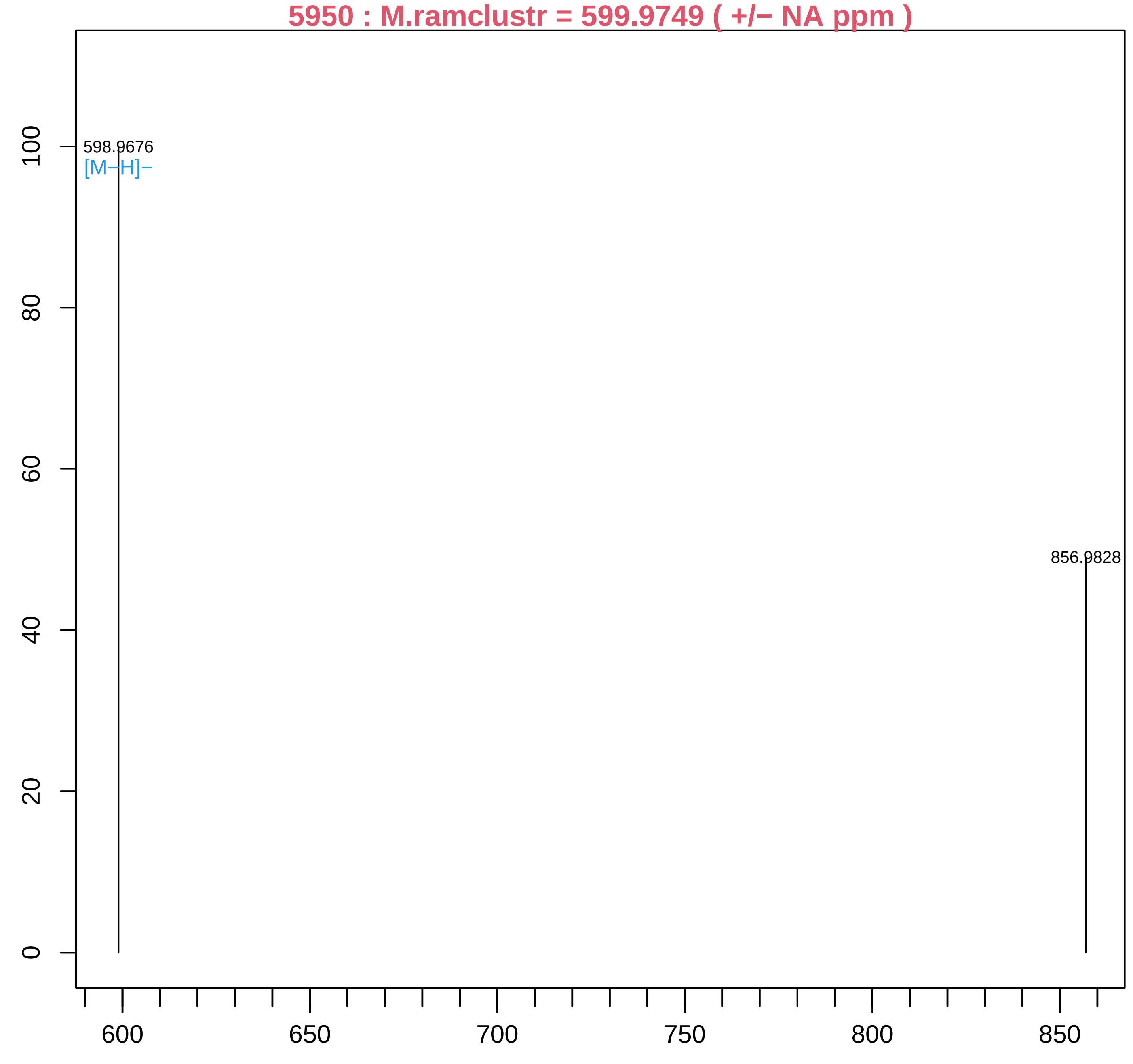
5950 : M.ramclustr = 599.9749 (+/− NA ppm)
598.9676
[M−H]−
856.9828
0
20
40
60
80
100
600
650
700
750
800
850

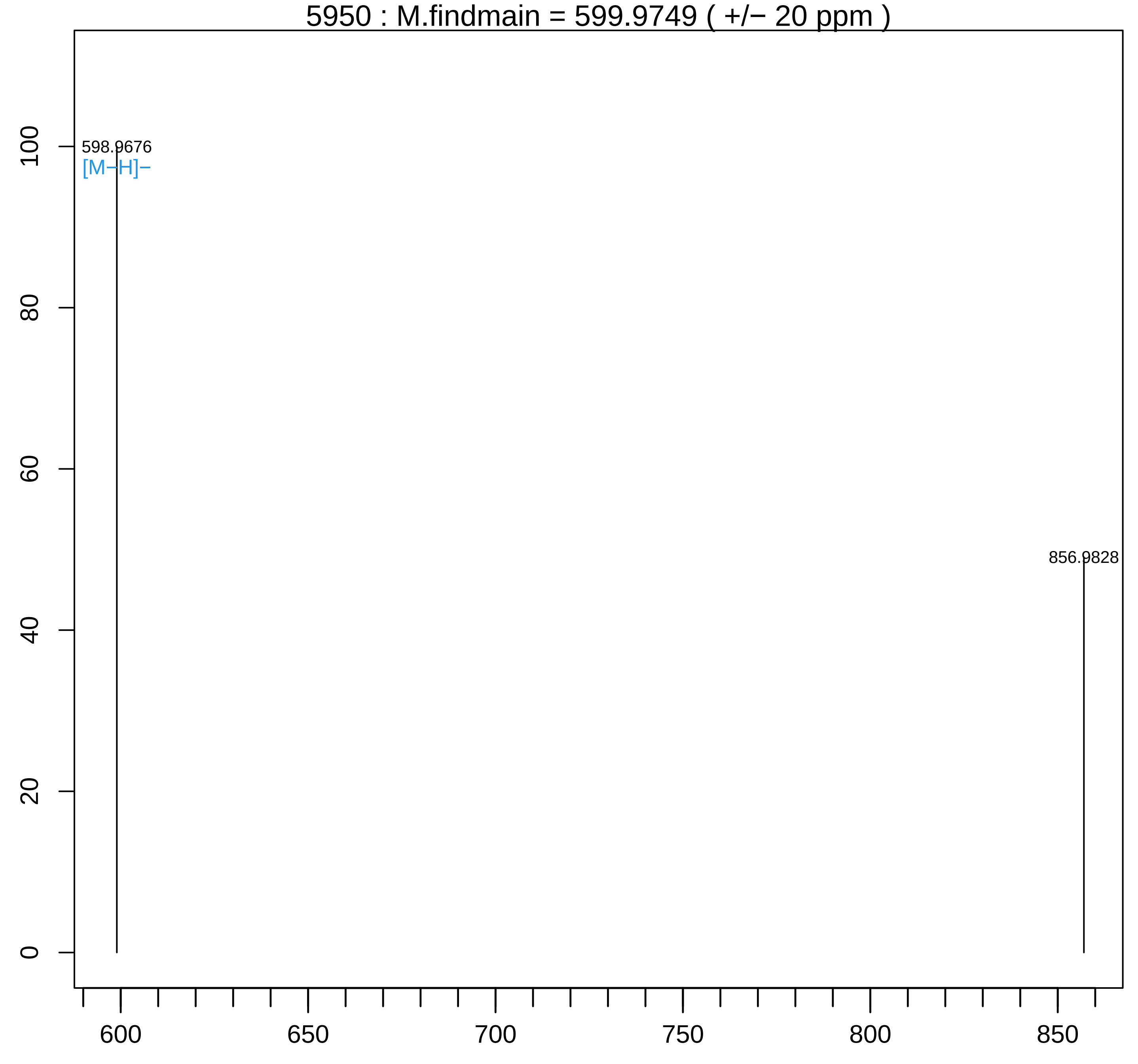
5950 : M.findmain = 599.9749 (+/− 20 ppm)
598.9676
[M−H]−
856.9828
0
20
40
60
80
100
600
650
700
750
800
850

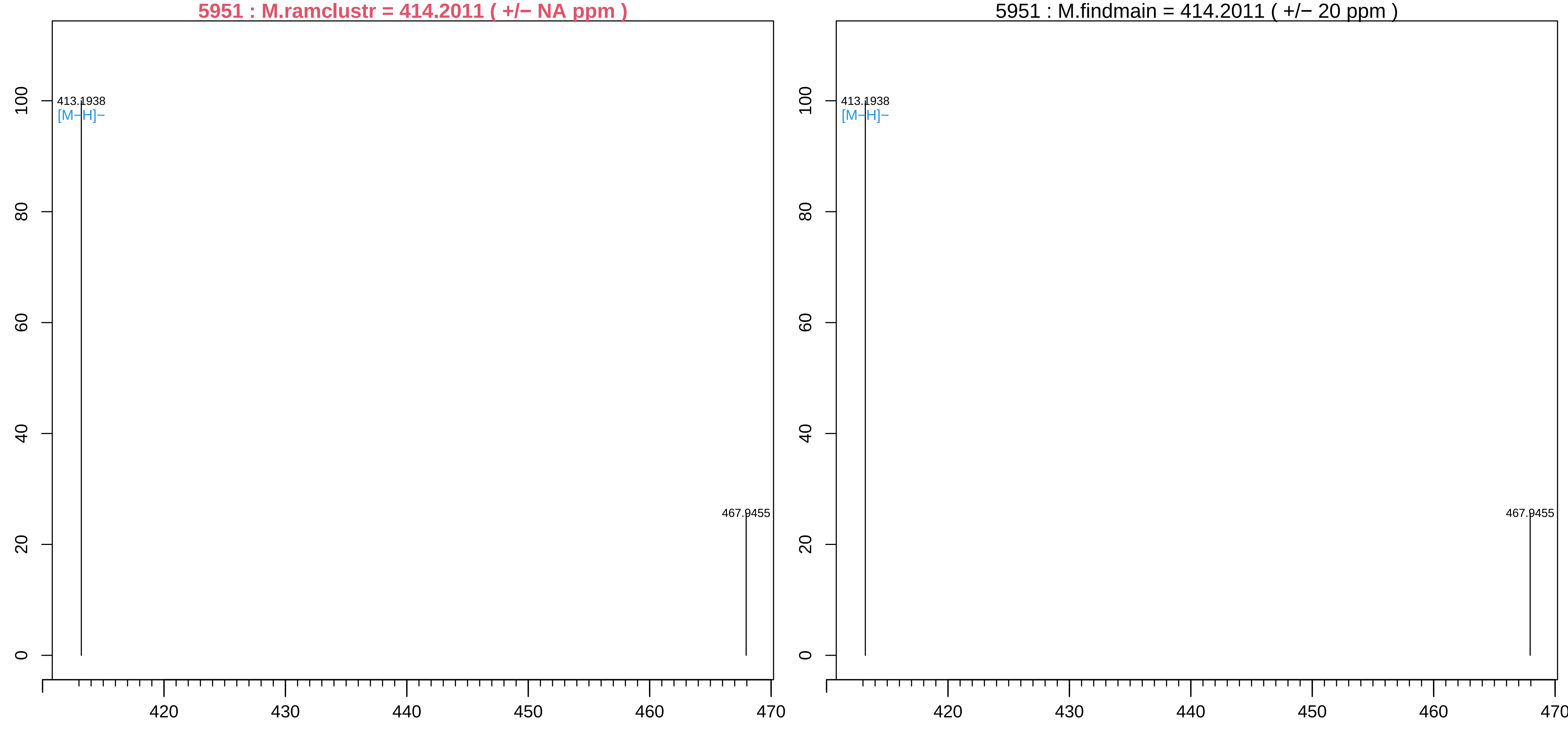
5951 : M.ramclustr = 414.2011 (+/− NA ppm)
413.1938
[M−H]−
467.9455
5951 : M.findmain = 414.2011 (+/− 20 ppm)
413.1938
[M−H]−
467.9455
0
20
40
60
80
100
420
430
440
450
460
470

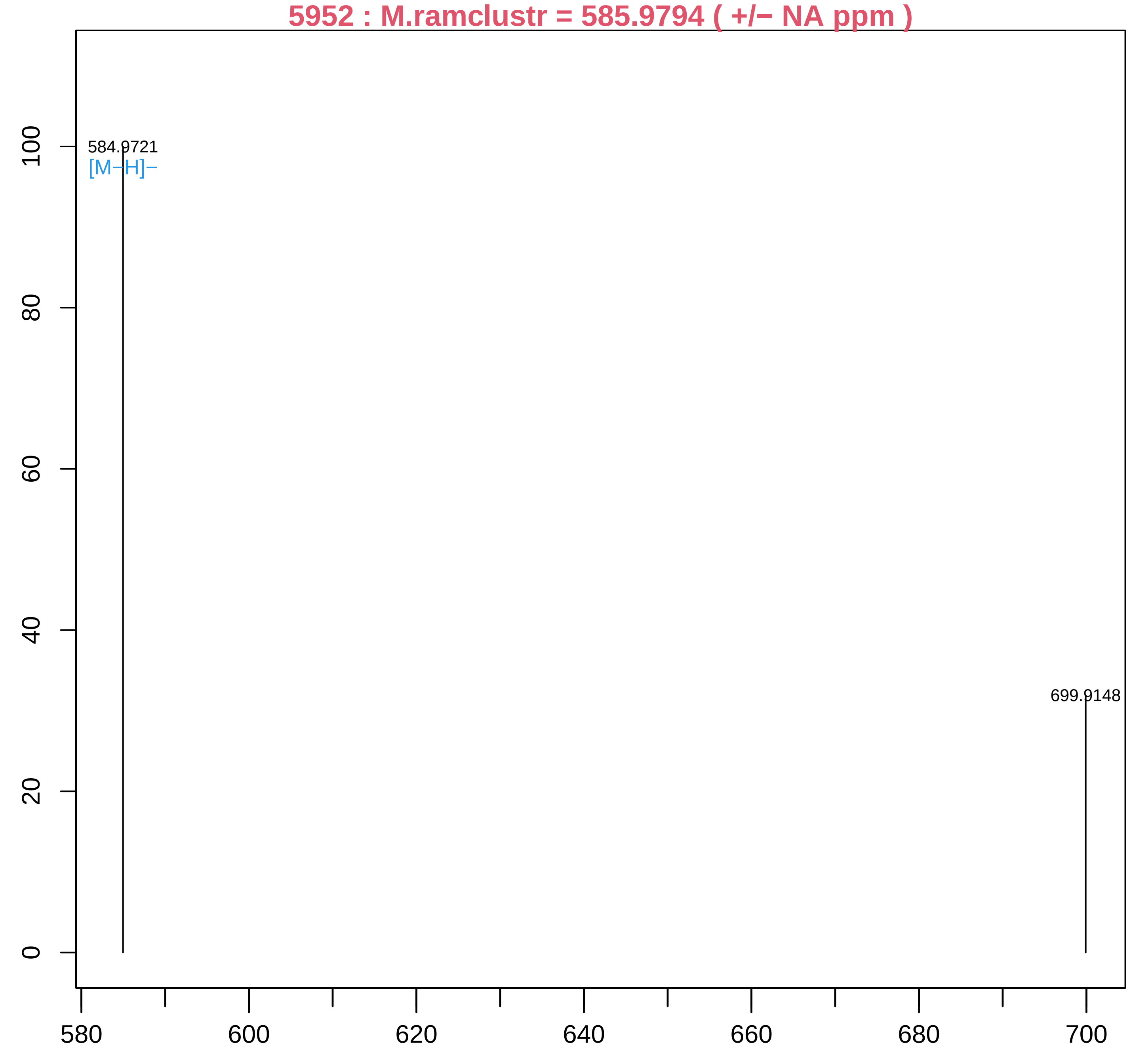

5952 : M.ramclustr = 585.9794 (+/− NA ppm)
584.9721
[M−H]−
699.9148
0
20
40
60
80
100
580
600
620
640
660
680
700

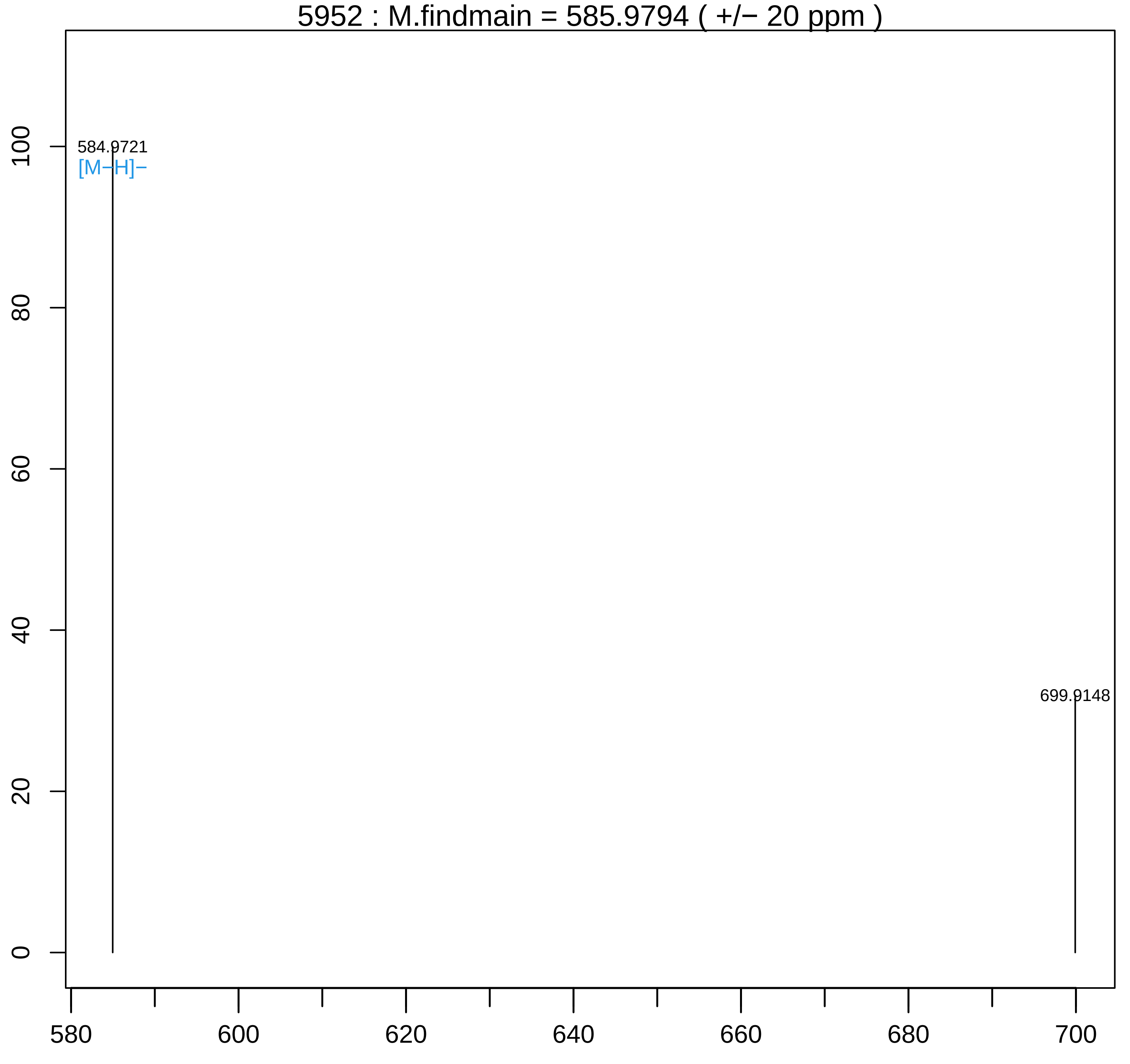

5952 : M.findmain = 585.9794 (+/− 20 ppm)
584.9721
[M−H]−
699.9148
0
20
40
60
80
100
580
600
620
640
660
680
700

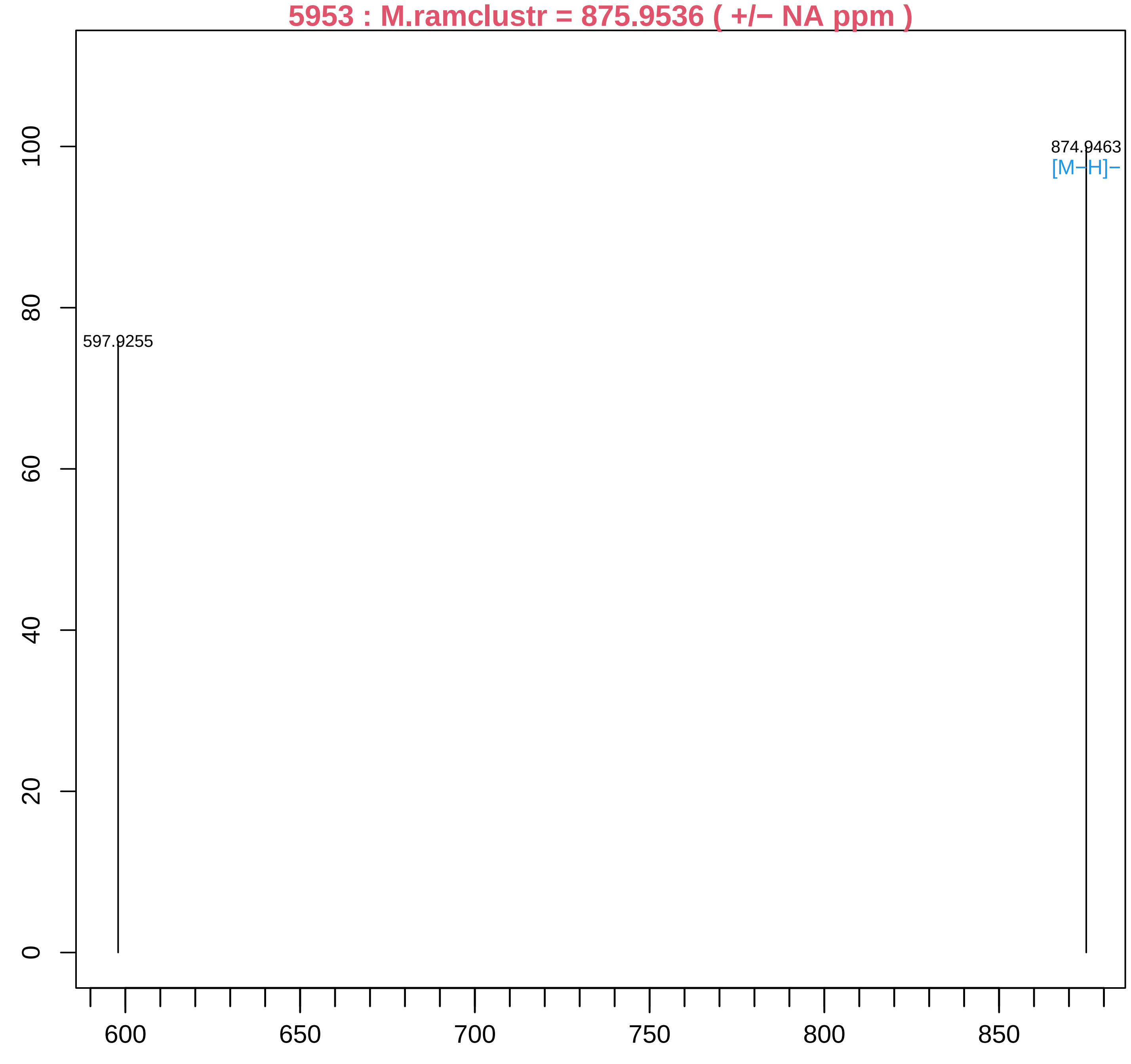
5953 : M.ramclustr = 875.9536 (+/− NA ppm)
597.9255
874.9463
[M−H]−
0
20
40
60
80
100
600
650
700
750
800
850

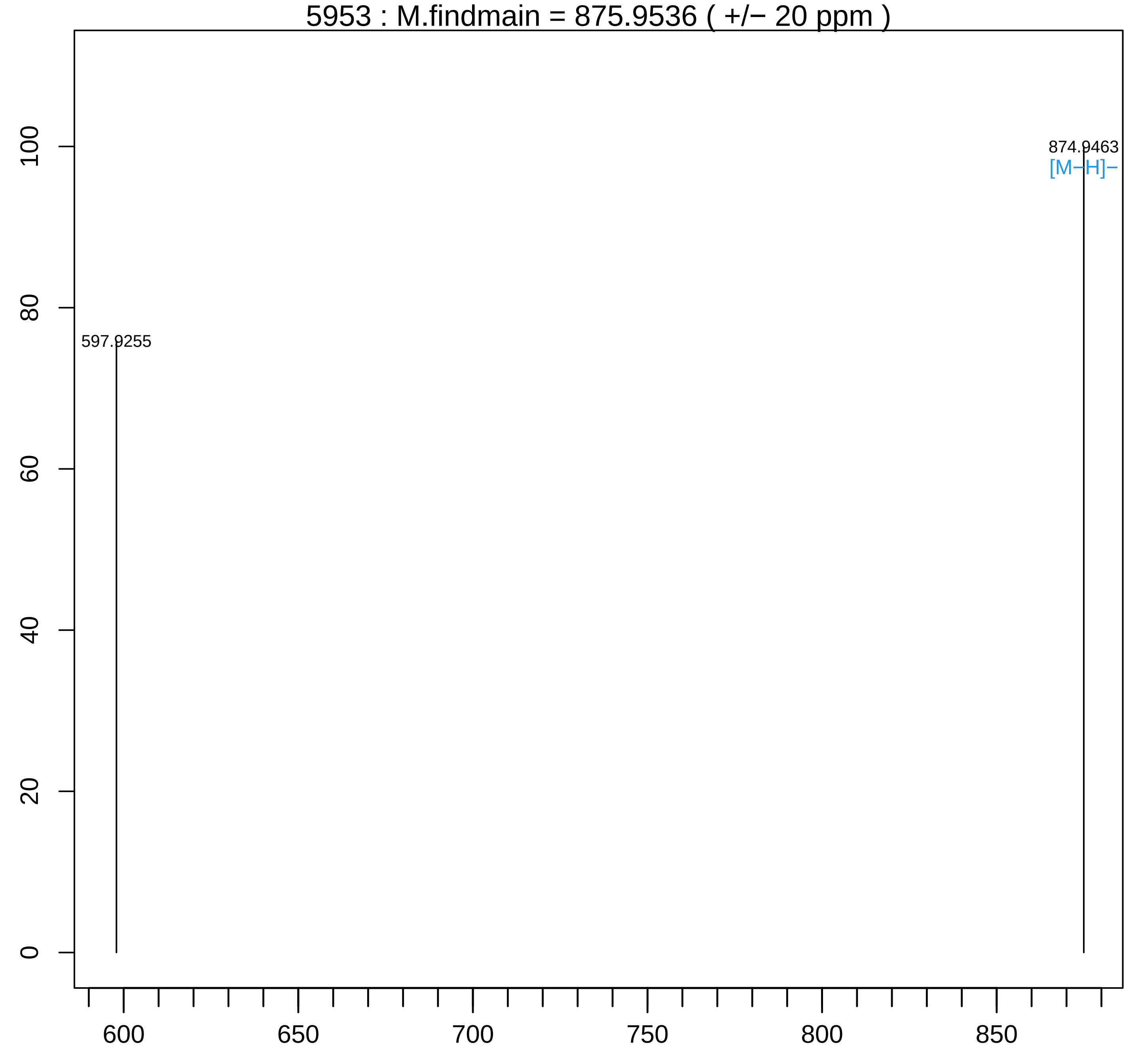
5953 : M.findmain = 875.9536 (+/− 20 ppm)
597.9255
874.9463
[M−H]−
0
20
40
60
80
100
600
650
700
750
800
850

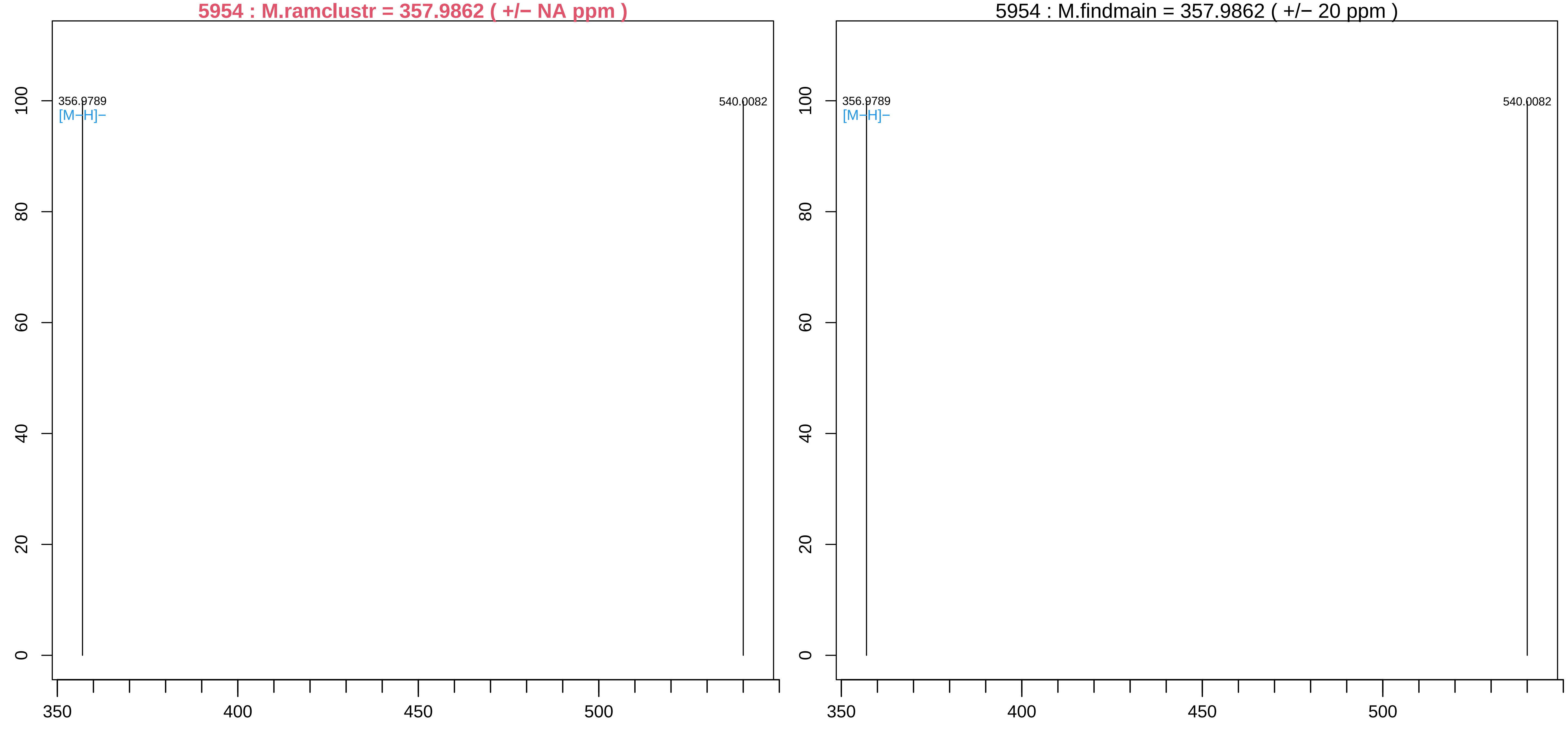
5954 : M.ramclustr = 357.9862 (+/− NA ppm)
356.9789
[M−H]−
540.0082
0
20
40
60
80
100
350
400
450
500
5954 : M.findmain = 357.9862 (+/− 20 ppm)
356.9789
[M−H]−
540.0082
0
20
40
60
80
100
350
400
450
500

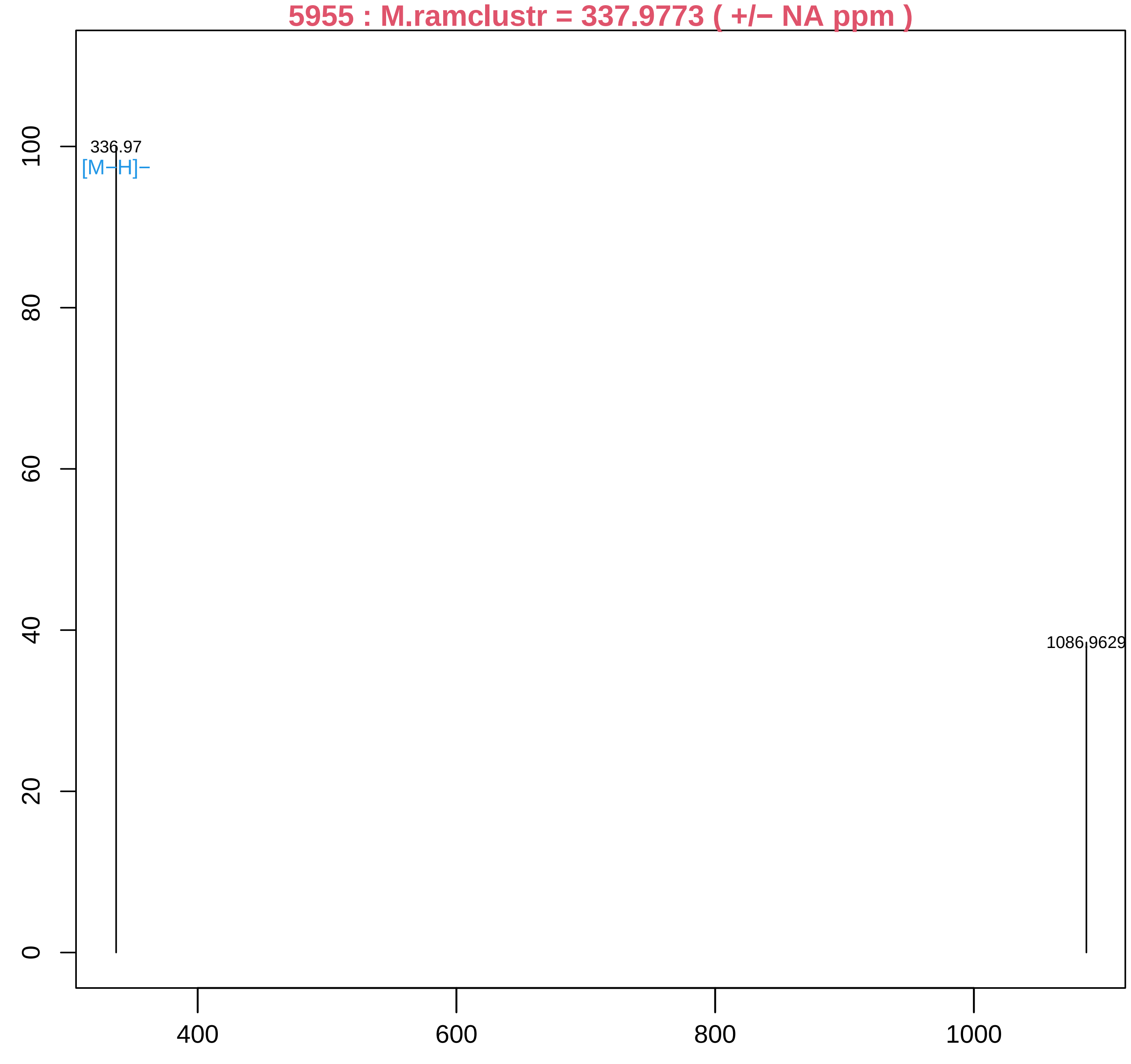

5955 : M.ramclustr = 337.9773 (+/− NA ppm)
336.97
[M−H]−
1086.9629
0
20
40
60
80
100
400
600
800
1000

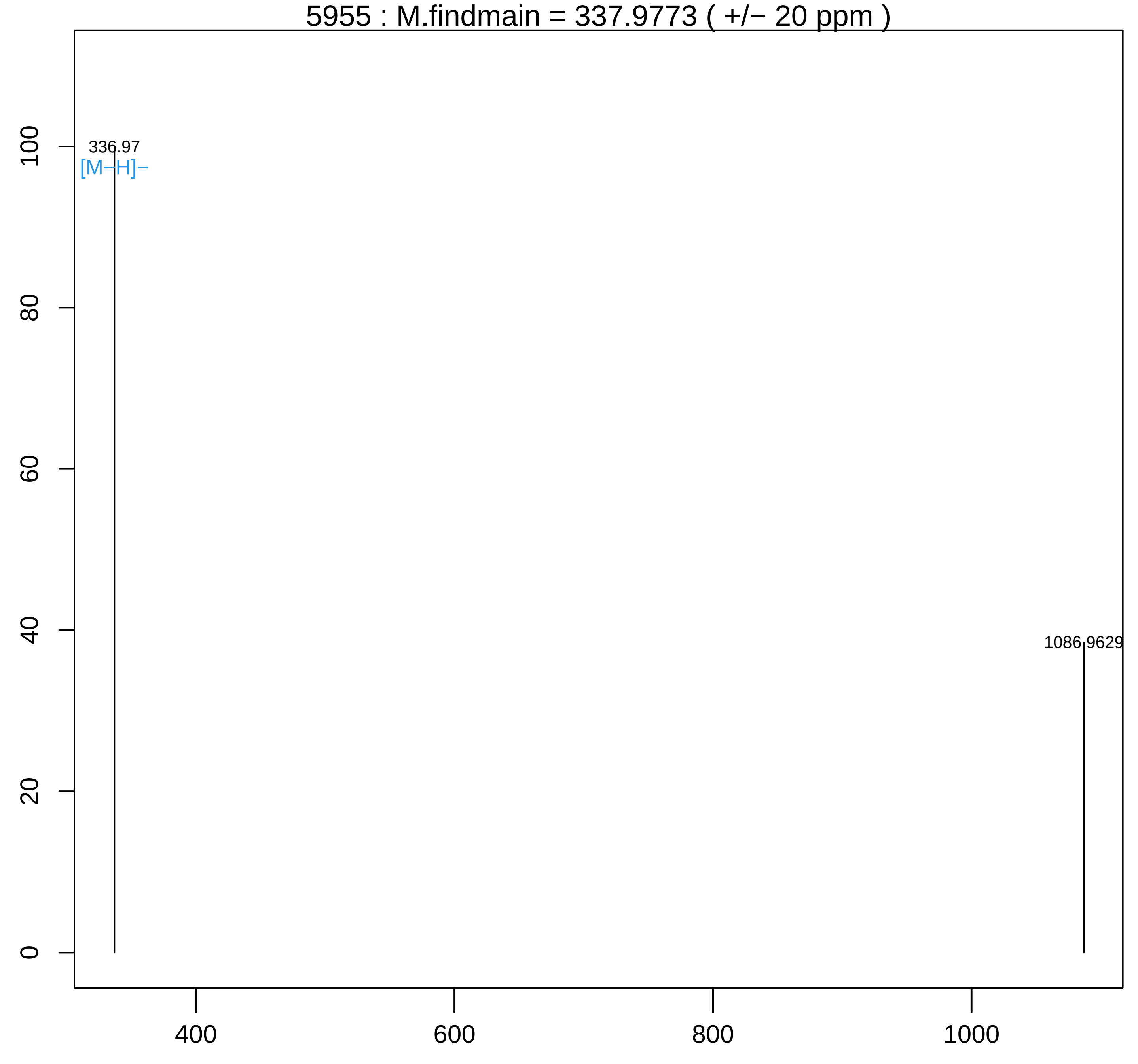

5955 : M.findmain = 337.9773 (+/− 20 ppm)
336.97
[M−H]−
1086.9629
0
20
40
60
80
100
400
600
800
1000

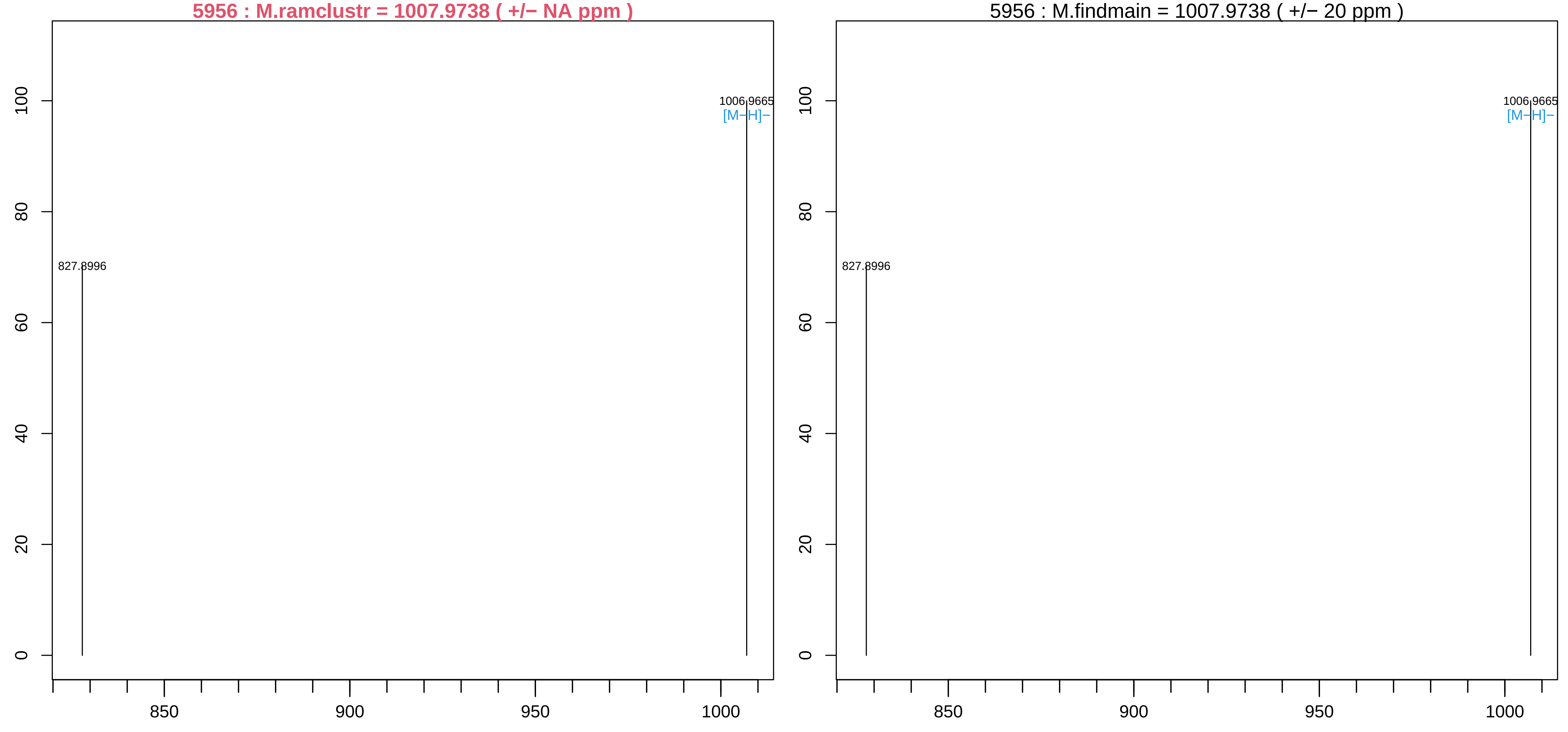
5956 : M.ramclustr = 1007.9738 (+/− NA ppm)
5956 : M.findmain = 1007.9738 (+/− 20 ppm)
1006.9665
[M−H]−
827.8996
0
20
40
60
80
100
850
900
950
1000

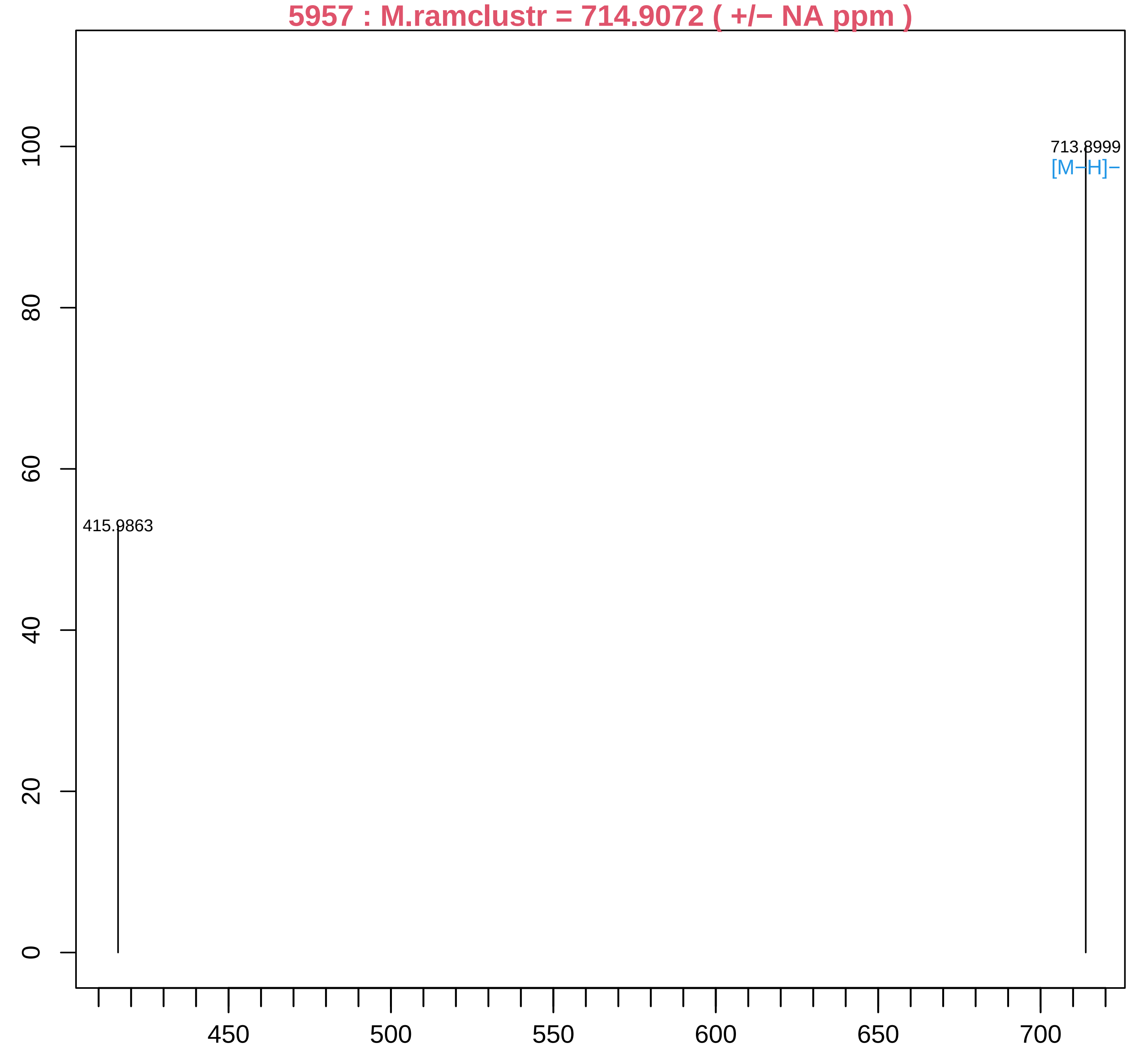
5957 : M.ramclustr = 714.9072 (+/− NA ppm)
713.8999
[M−H]−
415.9863
0
20
40
60
80
100
450
500
550
600
650
700

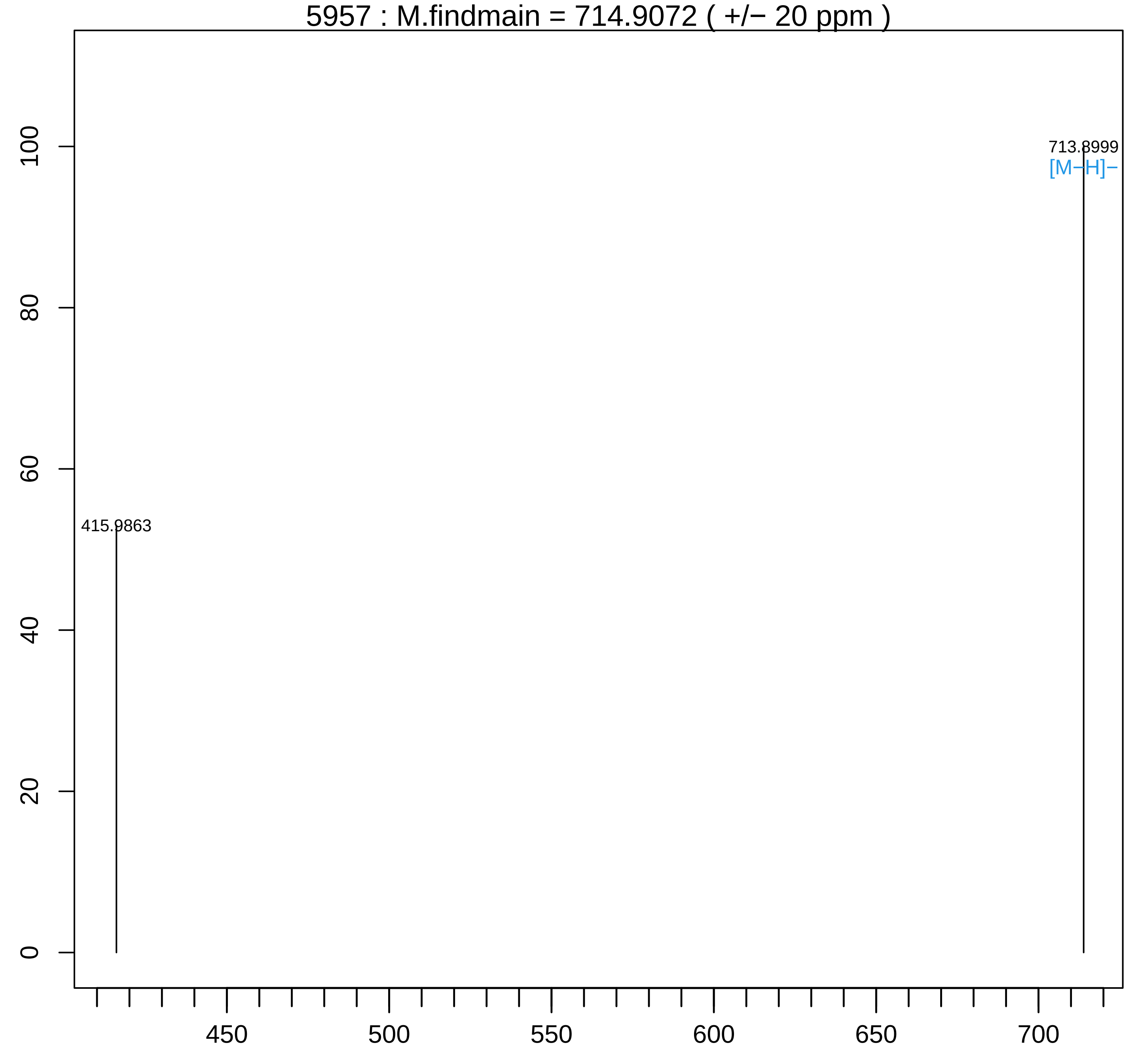
5957 : M.findmain = 714.9072 (+/− 20 ppm)
713.8999
[M−H]−
415.9863
0
20
40
60
80
100
450
500
550
600
650
700

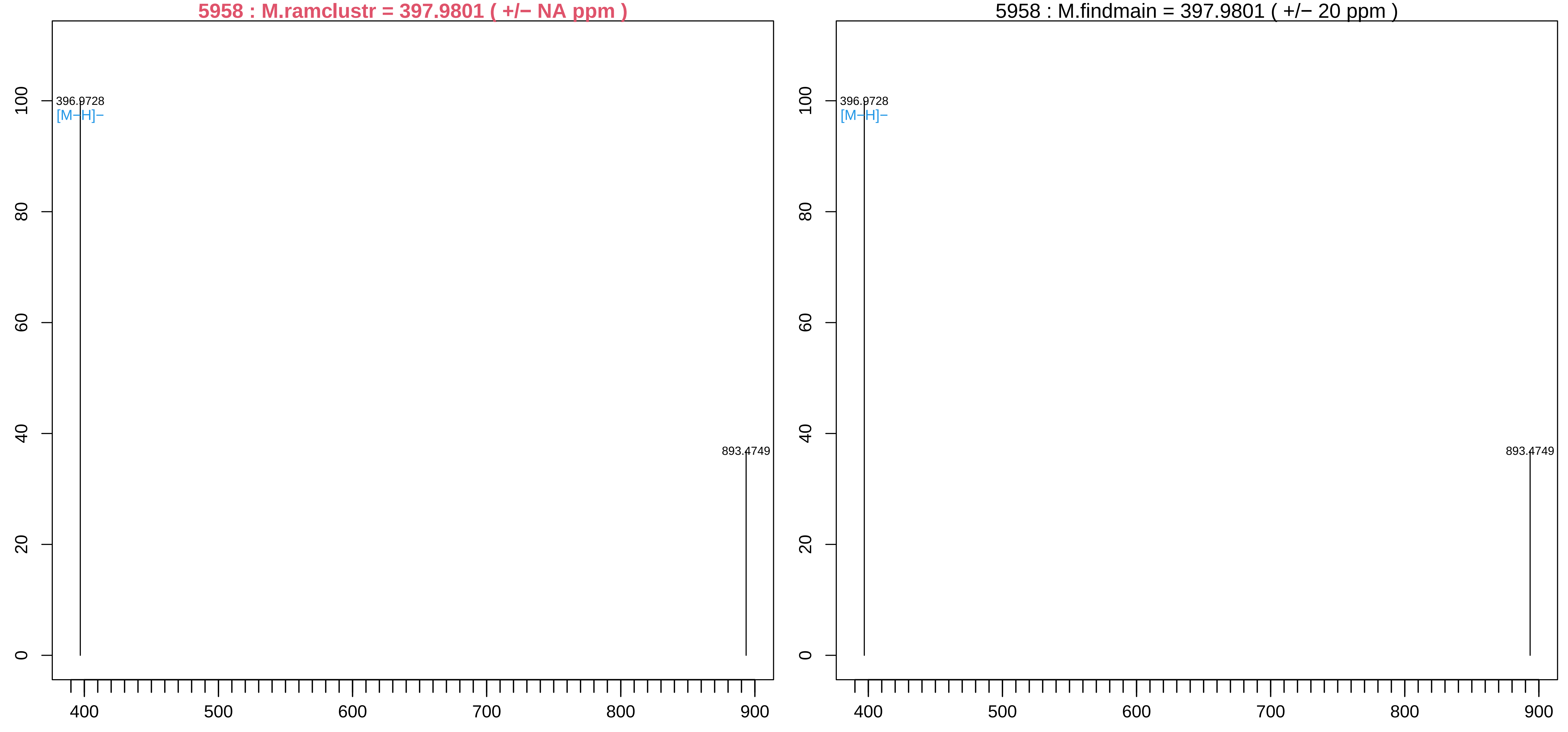
5958 : M.ramclustr = 397.9801 (+/− NA ppm)
396.9728
[M−H]−
893.4749
5958 : M.findmain = 397.9801 (+/− 20 ppm)
396.9728
[M−H]−
893.4749

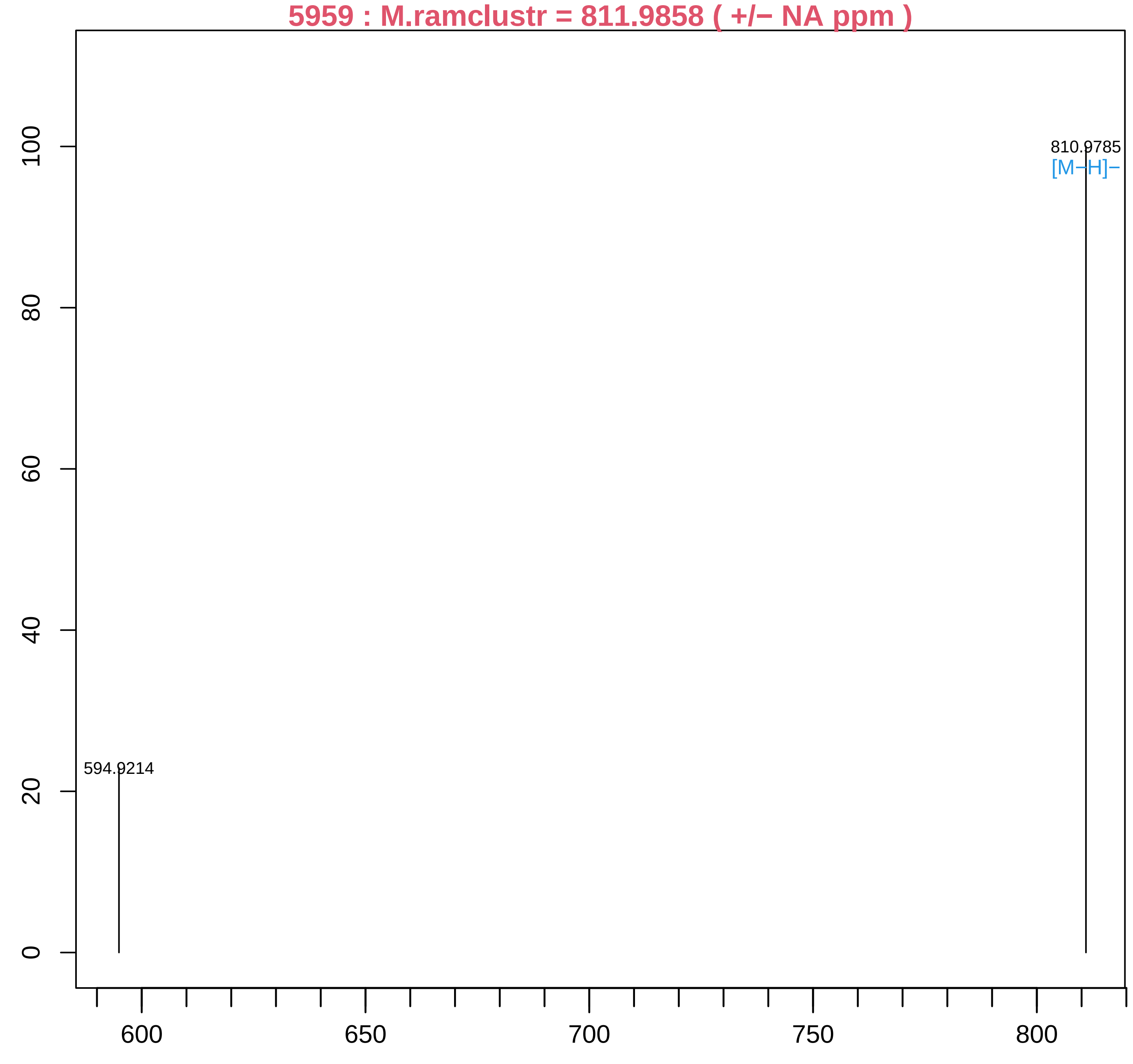
5959 : M.ramclustr = 811.9858 (+/− NA ppm)
810.9785
[M−H]−
594.9214
0
20
40
60
80
100
600
650
700
750
800

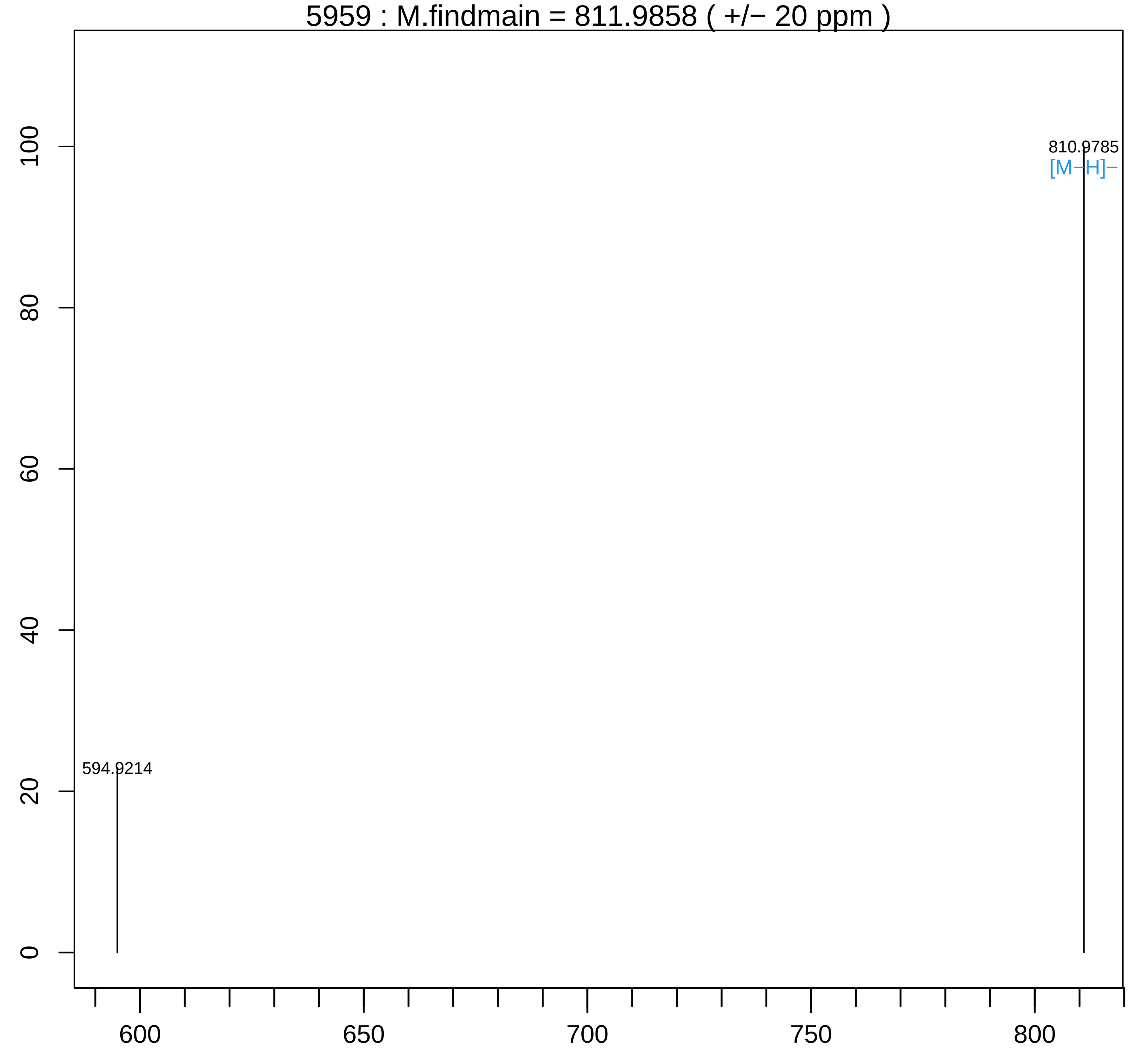
5959 : M.findmain = 811.9858 (+/− 20 ppm)
810.9785
[M−H]−
594.9214
0
20
40
60
80
100
600
650
700
750
800

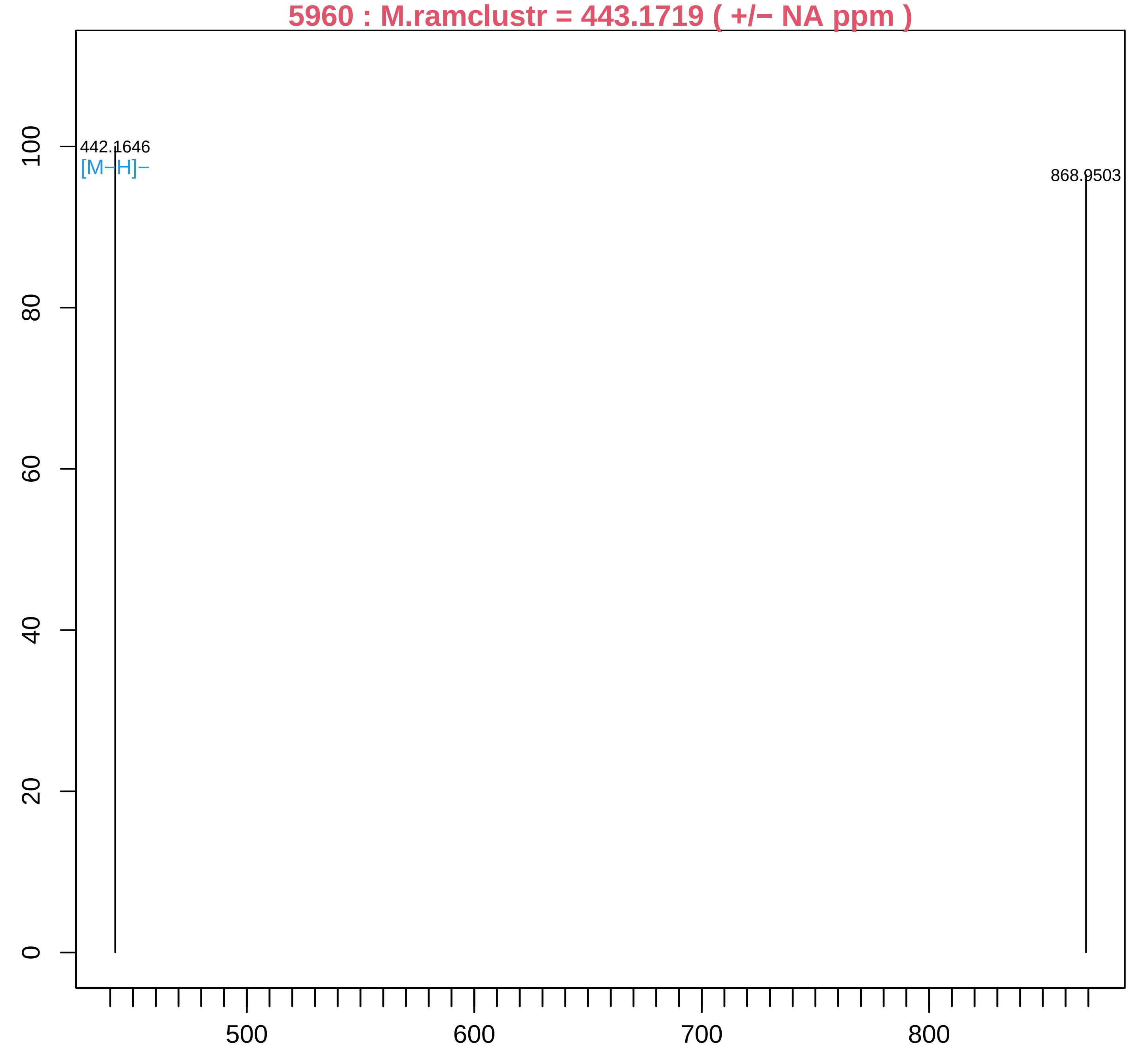
5960 : M.ramclustr = 443.1719 (+/− NA ppm)
442.1646
[M−H]−
868.9503
0
20
40
60
80
100
500
600
700
800

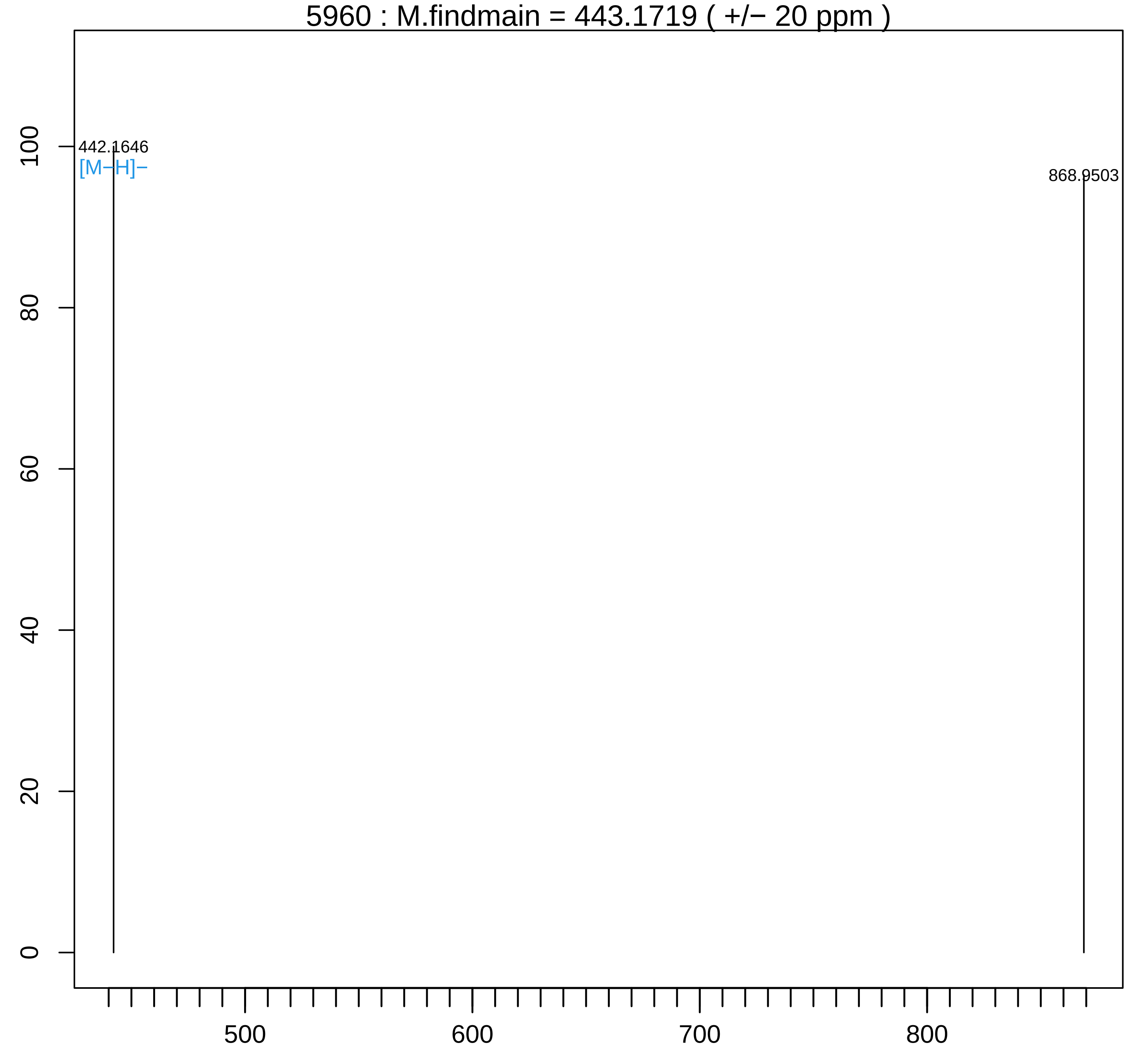
5960 : M.findmain = 443.1719 (+/− 20 ppm)
442.1646
[M−H]−
868.9503
0
20
40
60
80
100
500
600
700
800

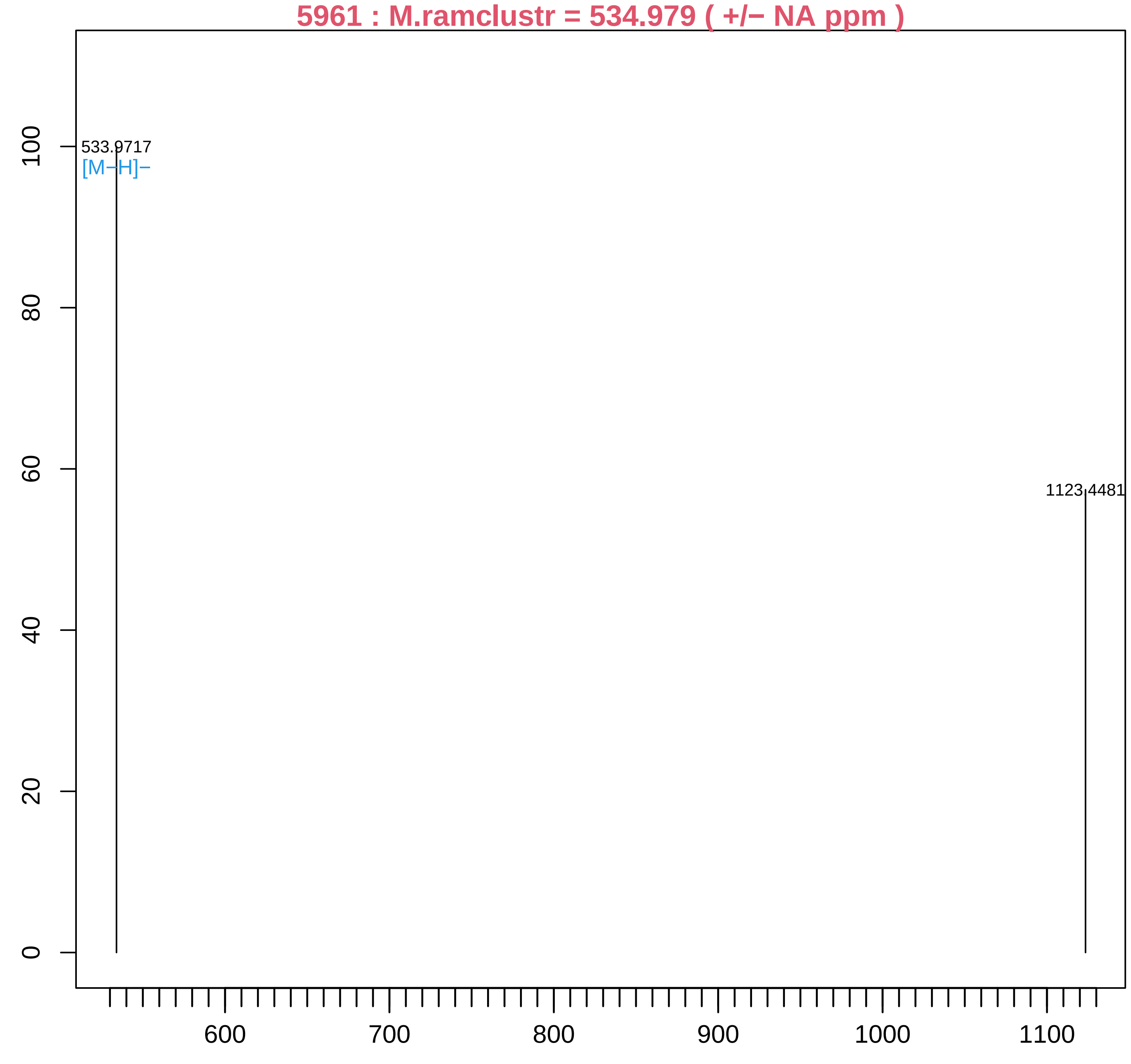
5961 : M.ramclustr = 534.979 (+/− NA ppm)
533.9717
[M−H]−
1123.4481
0
20
40
60
80
100
600
700
800
900
1000
1100

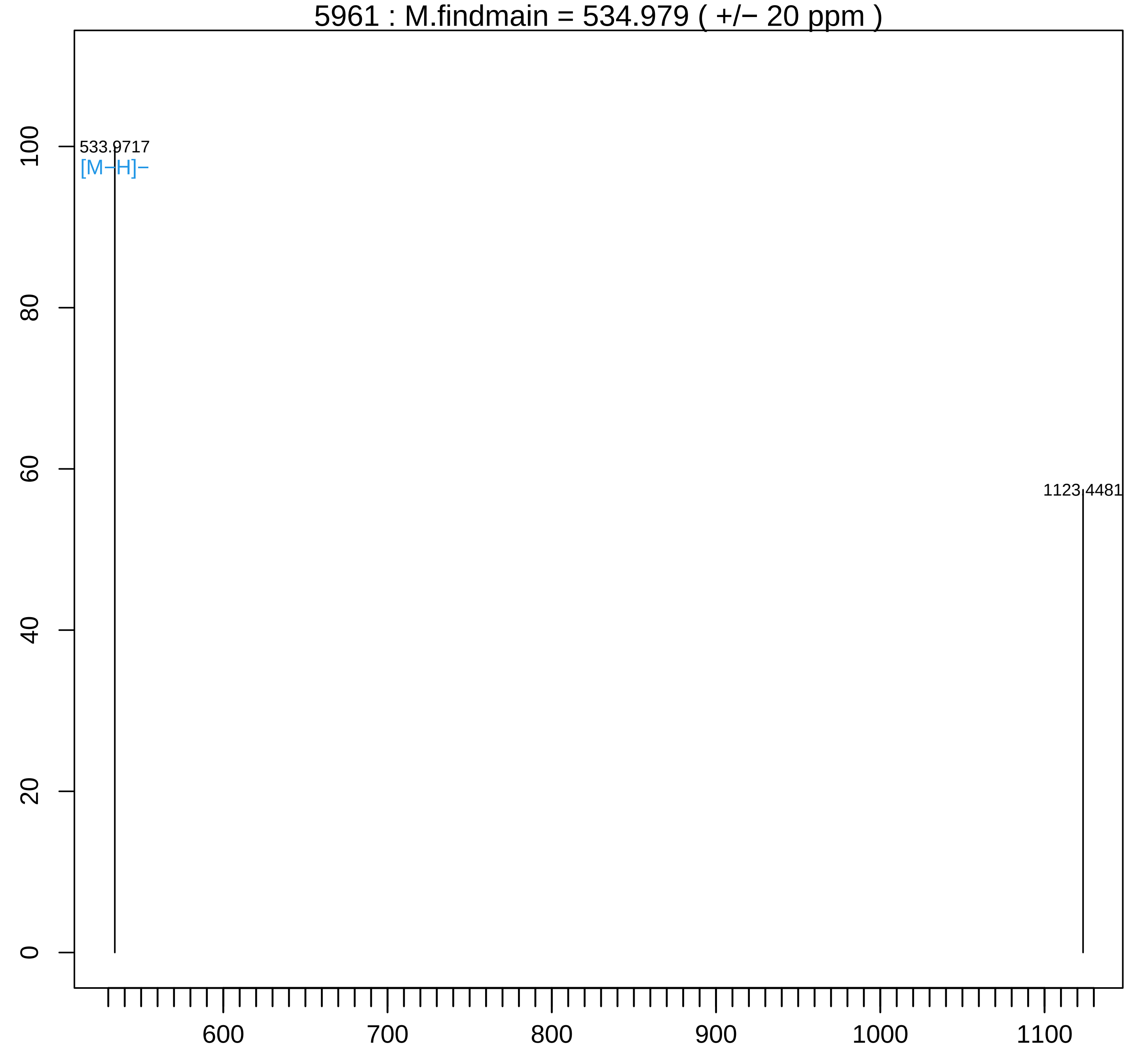
5961 : M.findmain = 534.979 (+/− 20 ppm)
533.9717
[M−H]−
1123.4481
0
20
40
60
80
100
600
700
800
900
1000
1100

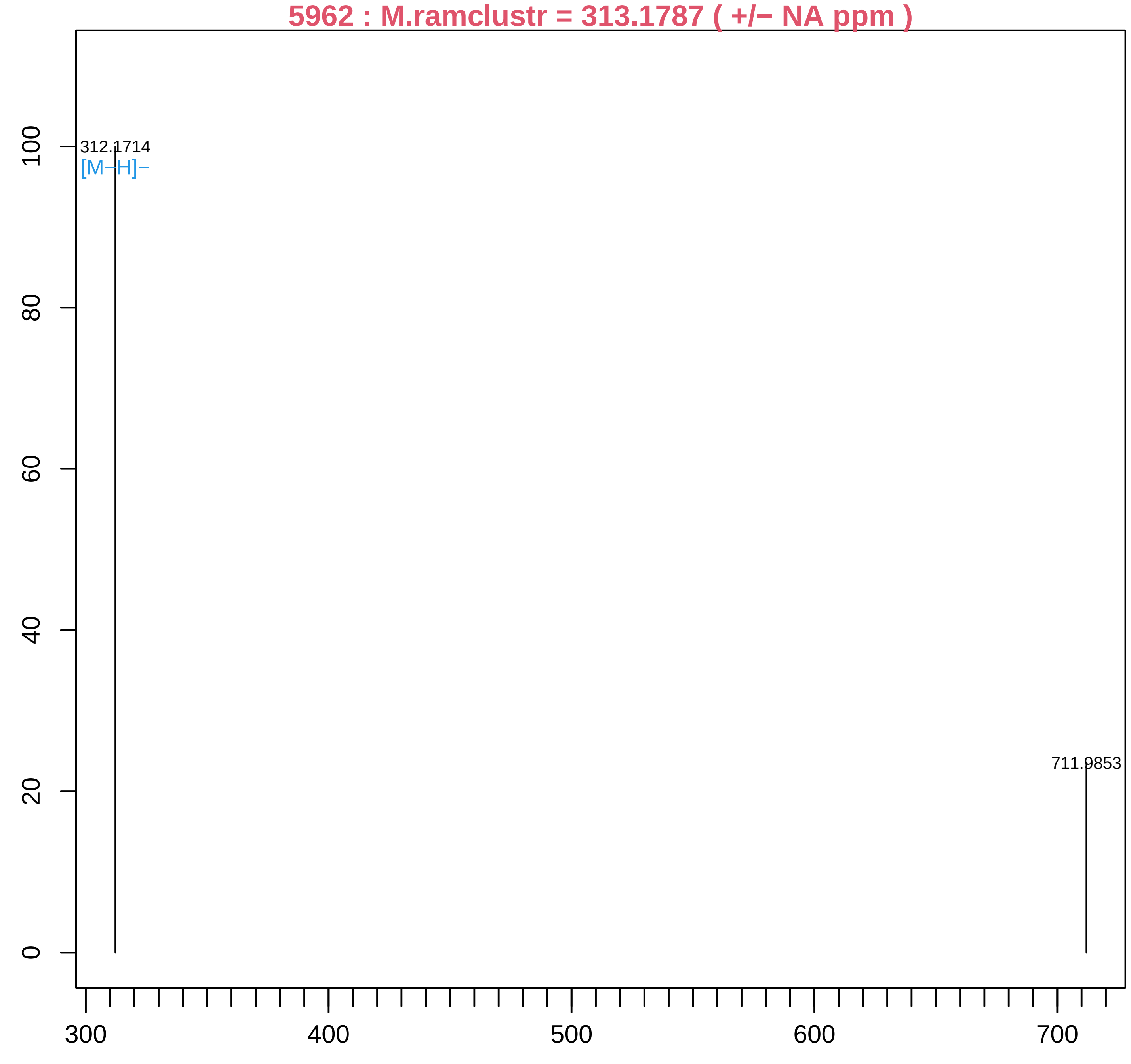
5962 : M.ramclustr = 313.1787 (+/− NA ppm)
312.1714
[M−H]−
711.9853
0
20
40
60
80
100
300
400
500
600
700

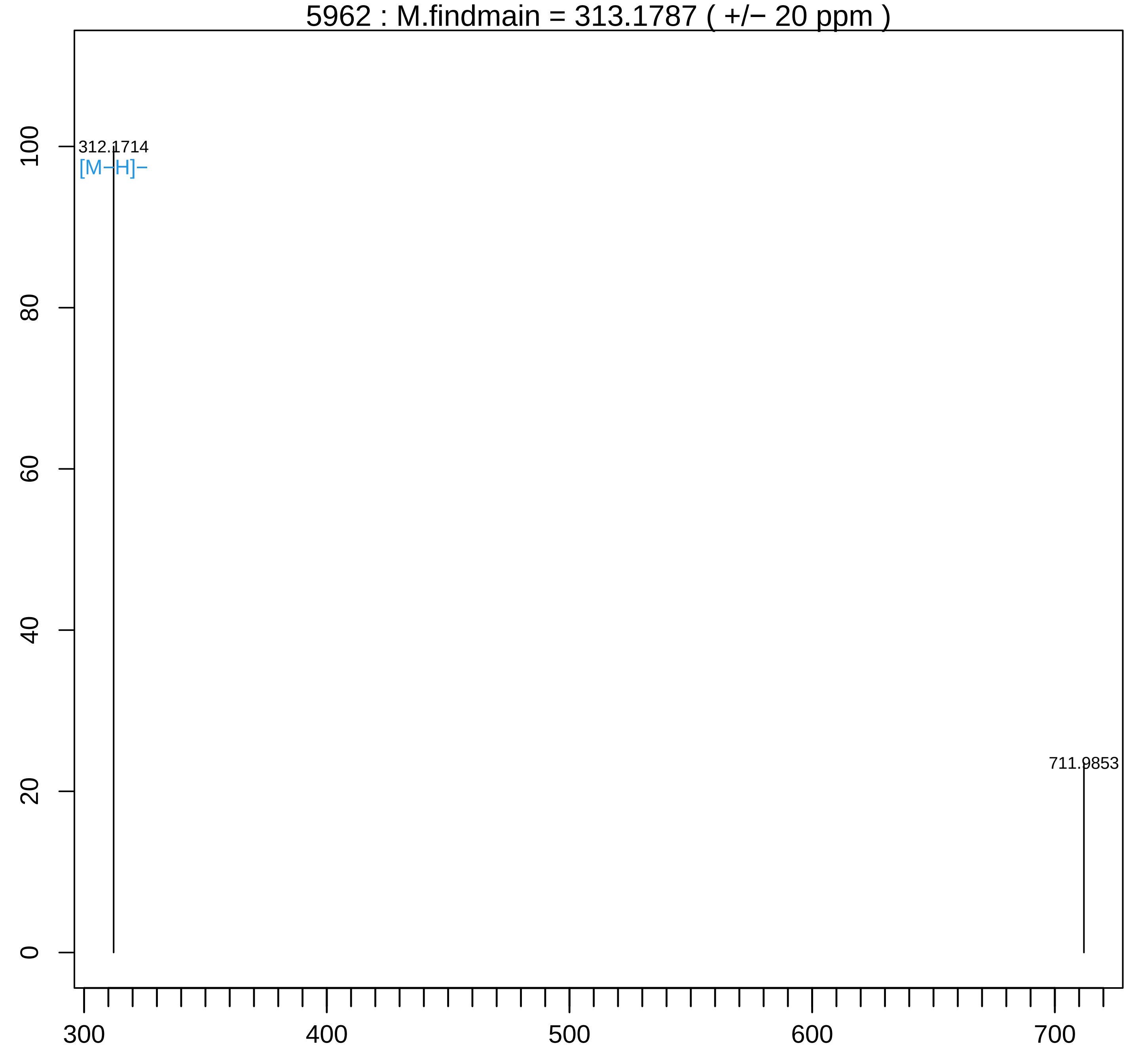
5962 : M.findmain = 313.1787 (+/− 20 ppm)
312.1714
[M−H]−
711.9853
0
20
40
60
80
100
300
400
500
600
700

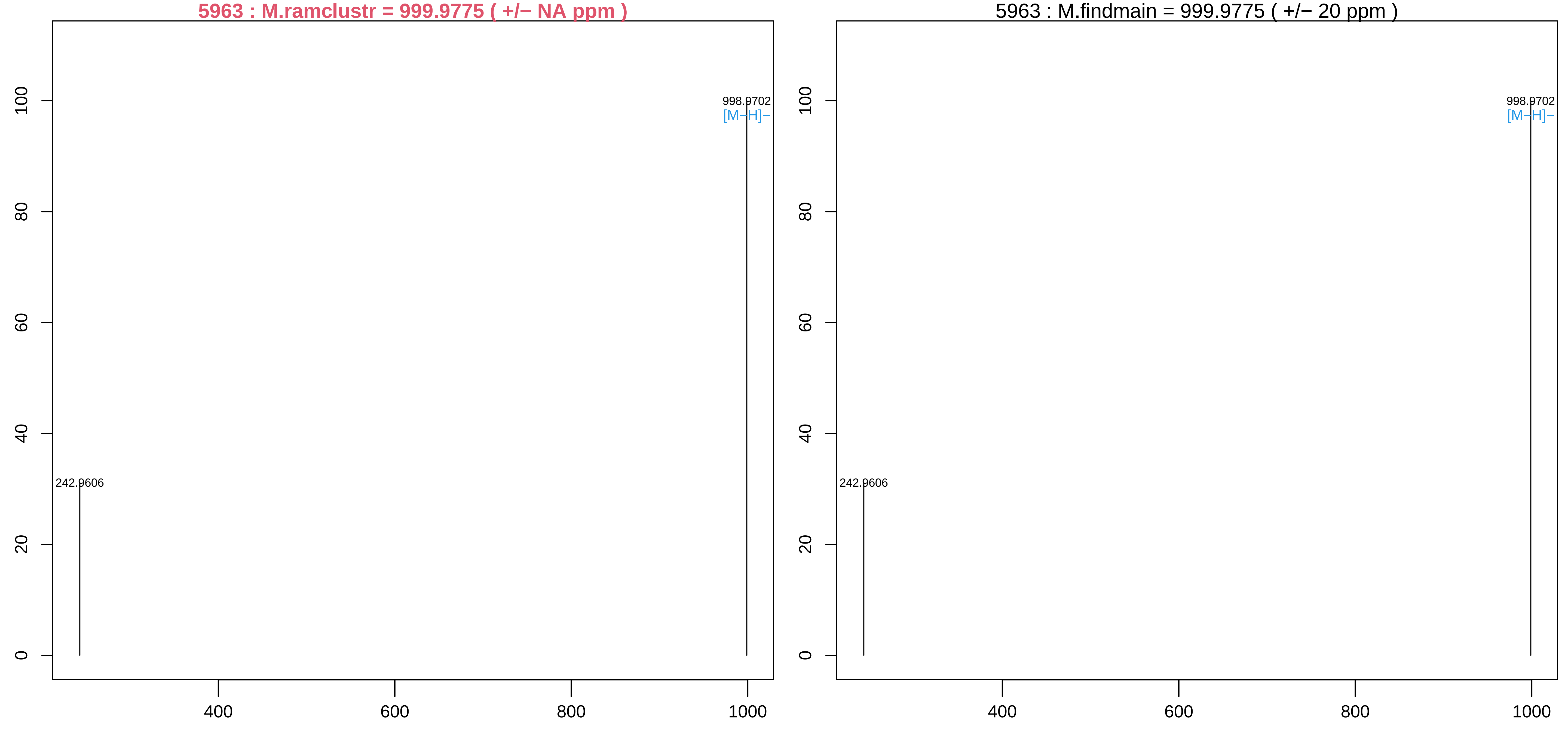
5963 : M.ramclustr = 999.9775 (+/− NA ppm)
998.9702
[M−H]−
242.9606
0
20
40
60
80
100
400
600
800
1000
5963 : M.findmain = 999.9775 (+/− 20 ppm)
998.9702
[M−H]−
242.9606
0
20
40
60
80
100
400
600
800
1000

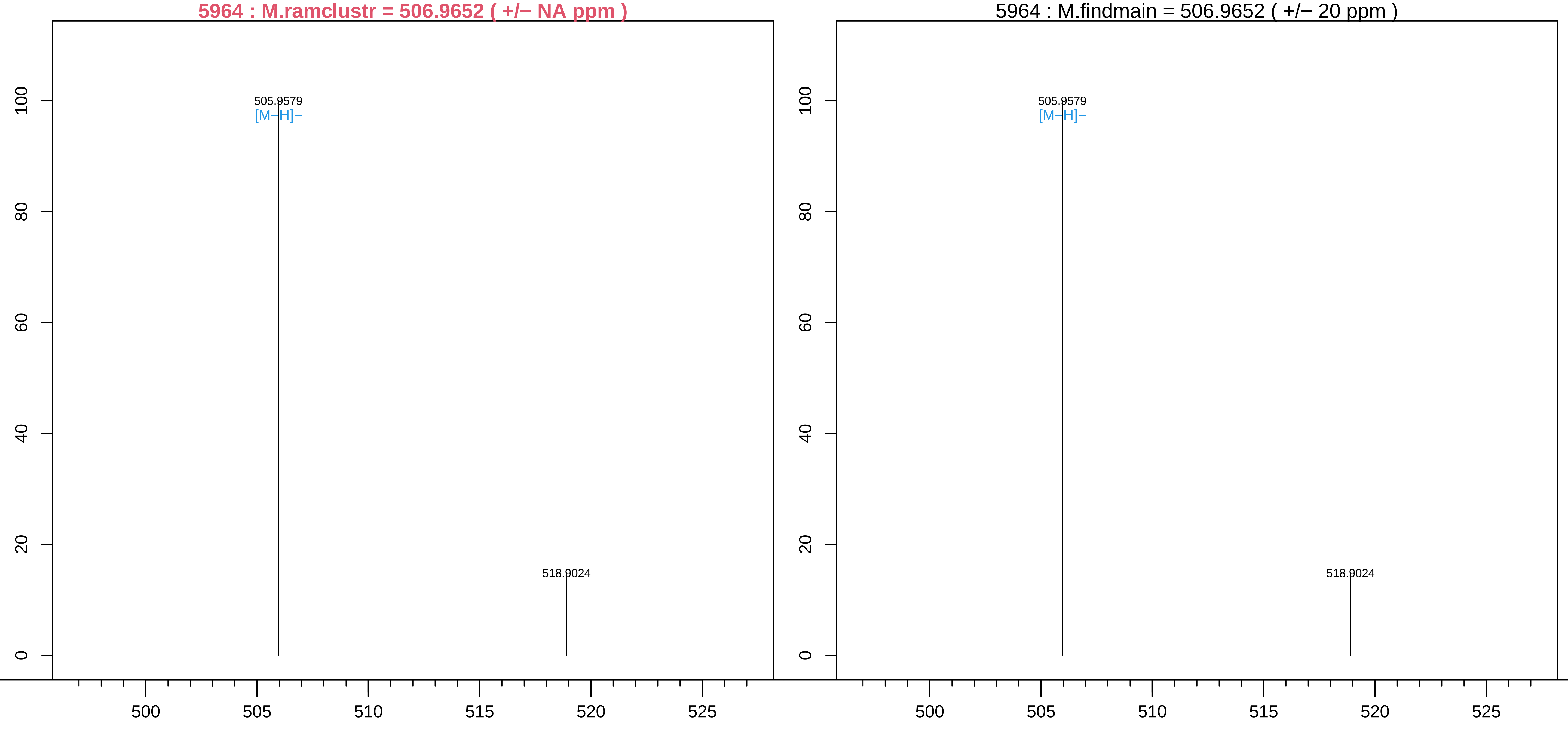
5964 : M.ramclustr = 506.9652 (+/− NA ppm)
5964 : M.findmain = 506.9652 (+/− 20 ppm)
505.9579
[M−H]−
518.9024
0
20
40
60
80
100
500
505
510
515
520
525

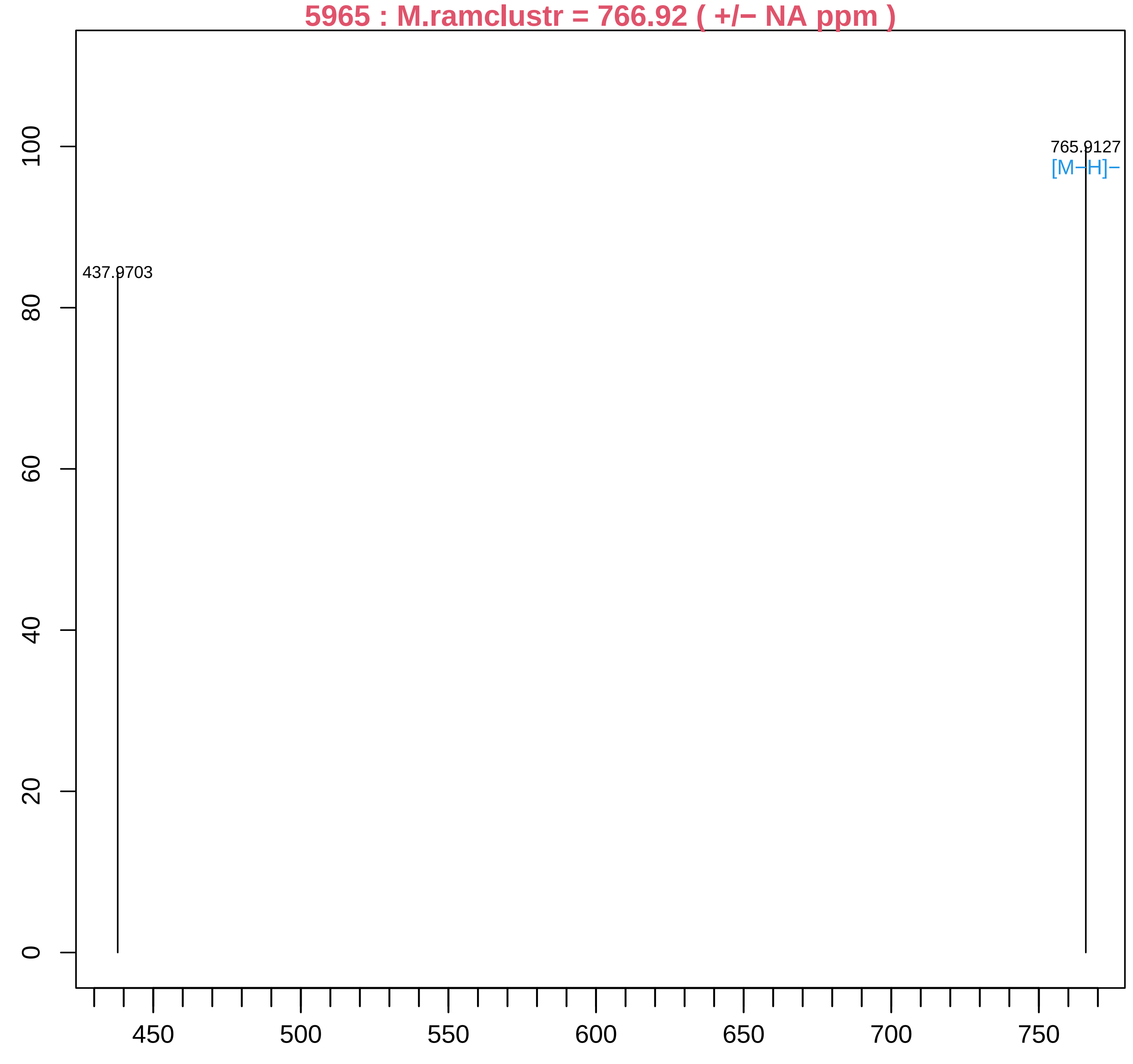
5965 : M.ramclustr = 766.92 (+/− NA ppm)
765.9127
[M−H]−
437.9703
0
20
40
60
80
100
450
500
550
600
650
700
750

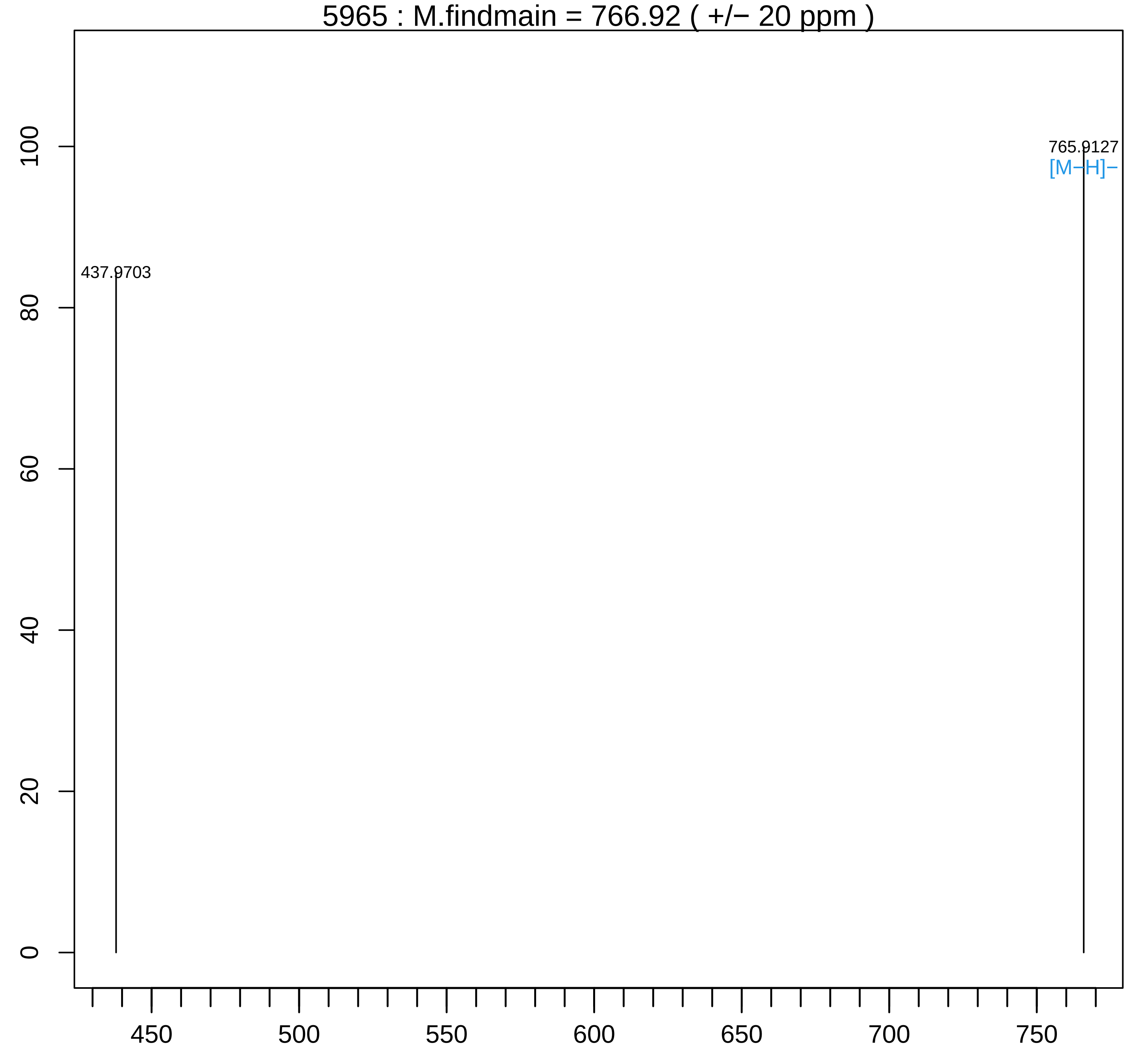
5965 : M.findmain = 766.92 (+/− 20 ppm)
765.9127
[M−H]−
437.9703
0
20
40
60
80
100
450
500
550
600
650
700
750

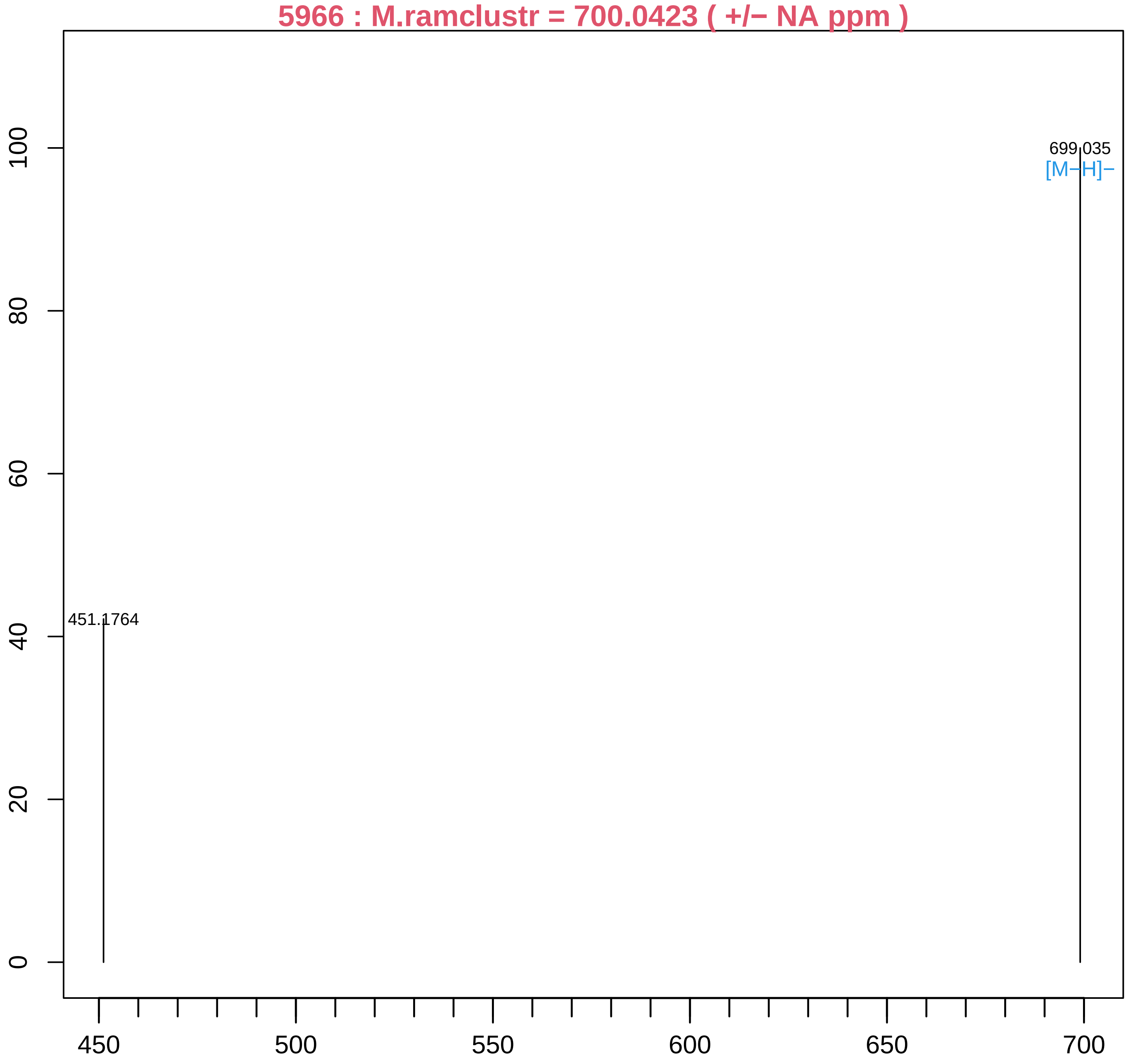
5966 : M.ramclustr = 700.0423 (+/− NA ppm)
699.035
[M−H]−
451.1764
0
20
40
60
80
100
450
500
550
600
650
700

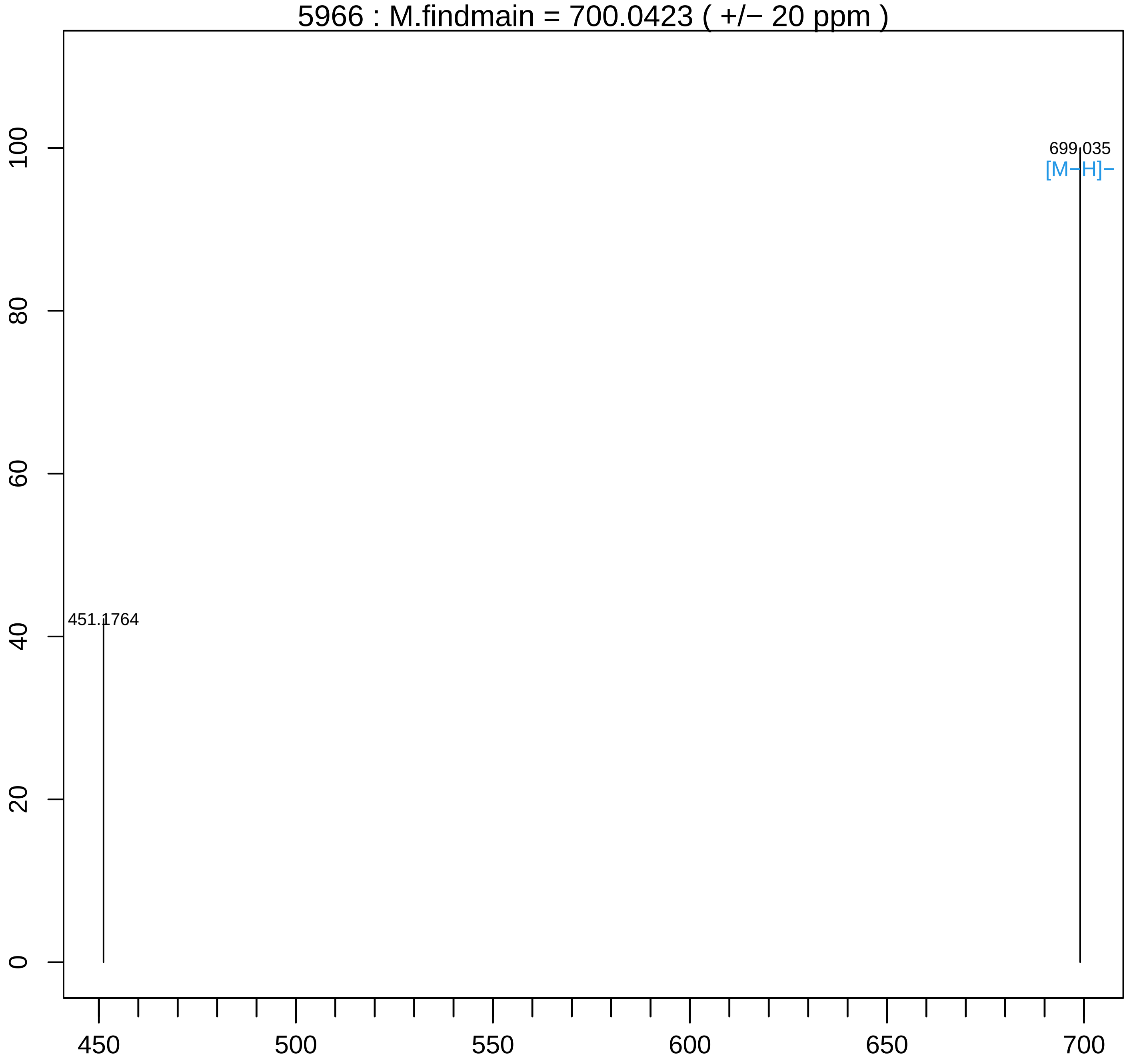
5966 : M.findmain = 700.0423 (+/− 20 ppm)
699.035
[M−H]−
451.1764
0
20
40
60
80
100
450
500
550
600
650
700

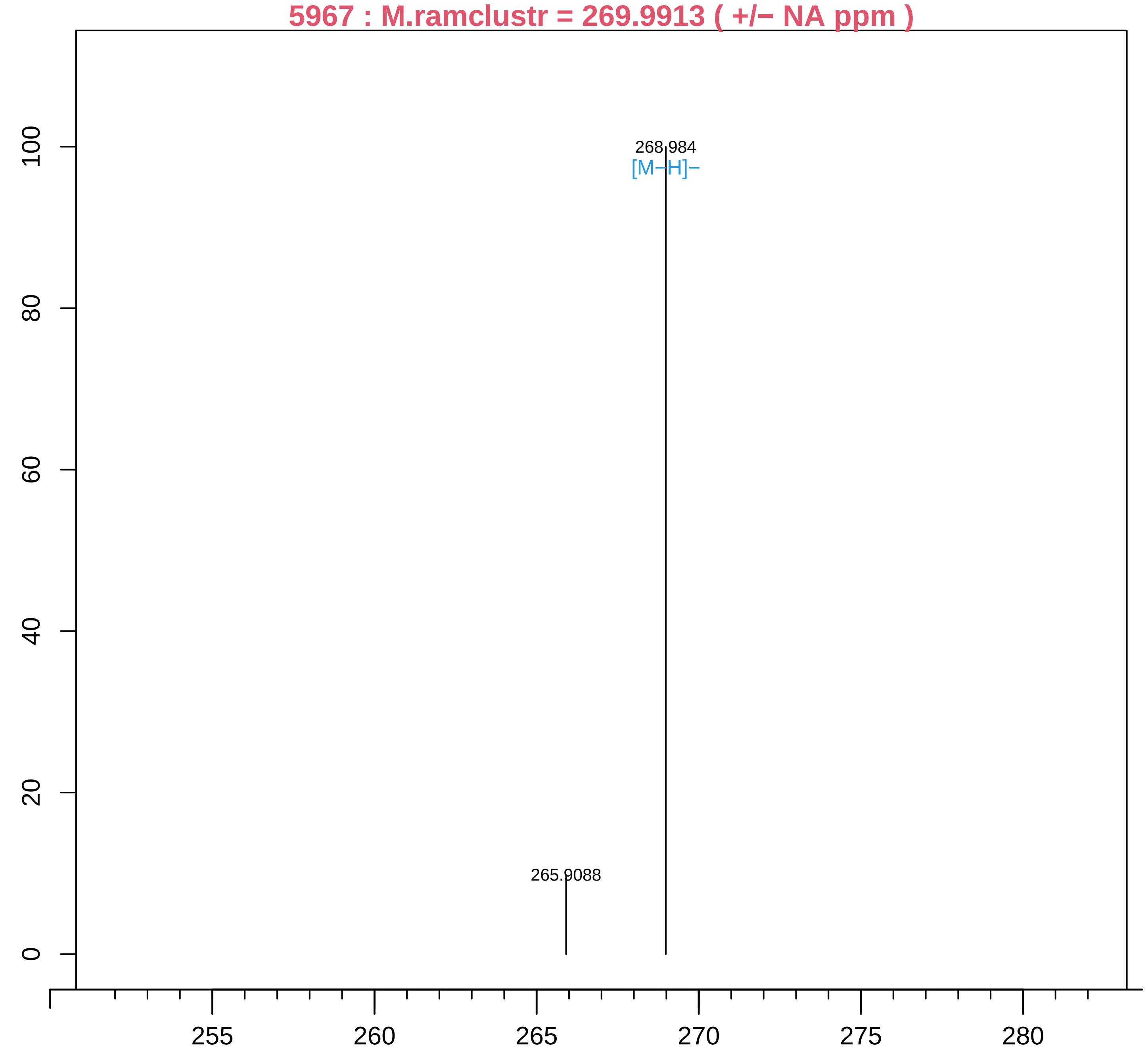
5967 : M.ramclustr = 269.9913 (+/− NA ppm)
268.984
[M−H]−
265.9088
0
20
40
60
80
100
255
260
265
270
275
280

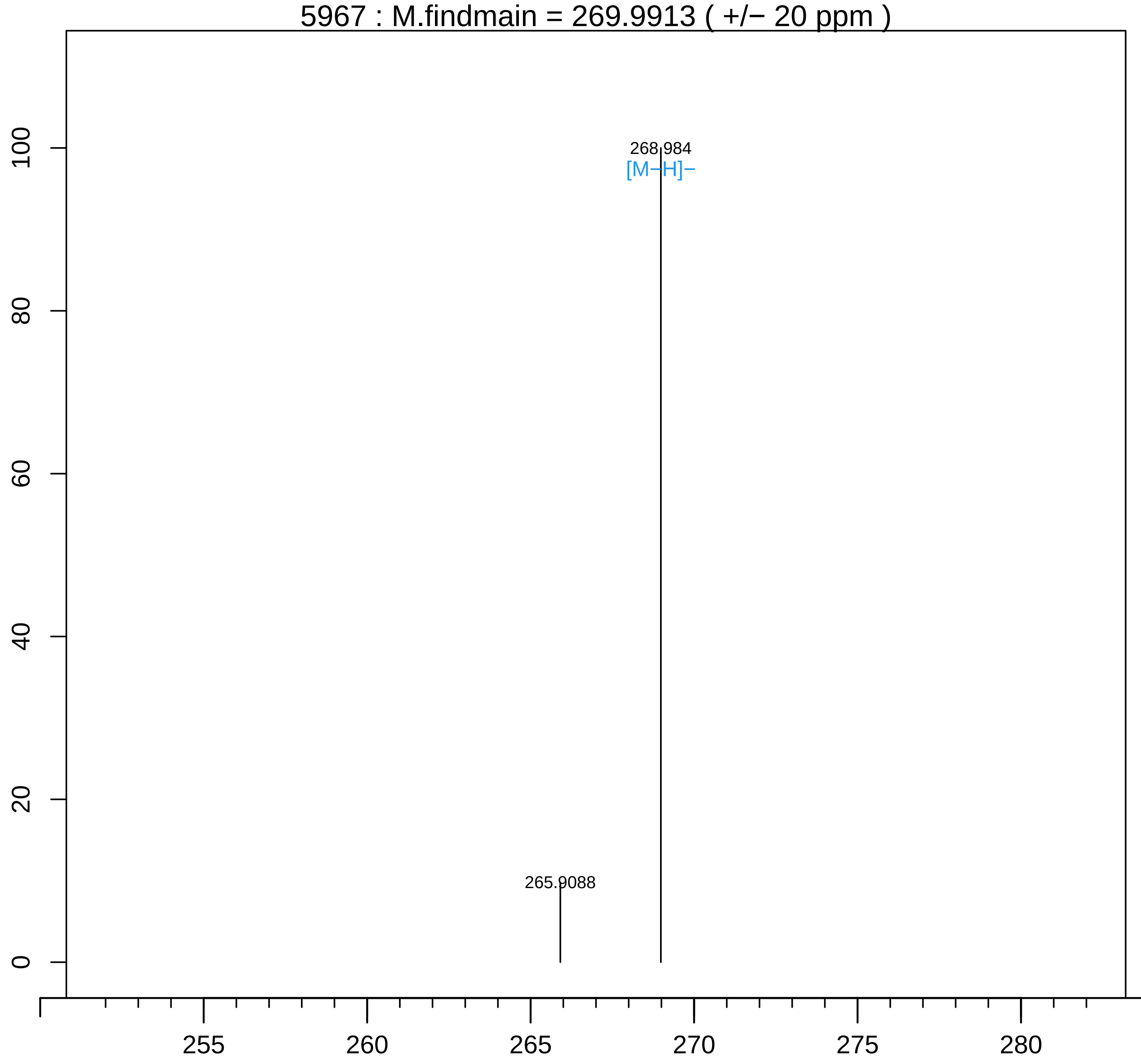
5967 : M.findmain = 269.9913 (+/− 20 ppm)
268.984
[M−H]−
265.9088
0
20
40
60
80
100
255
260
265
270
275
280

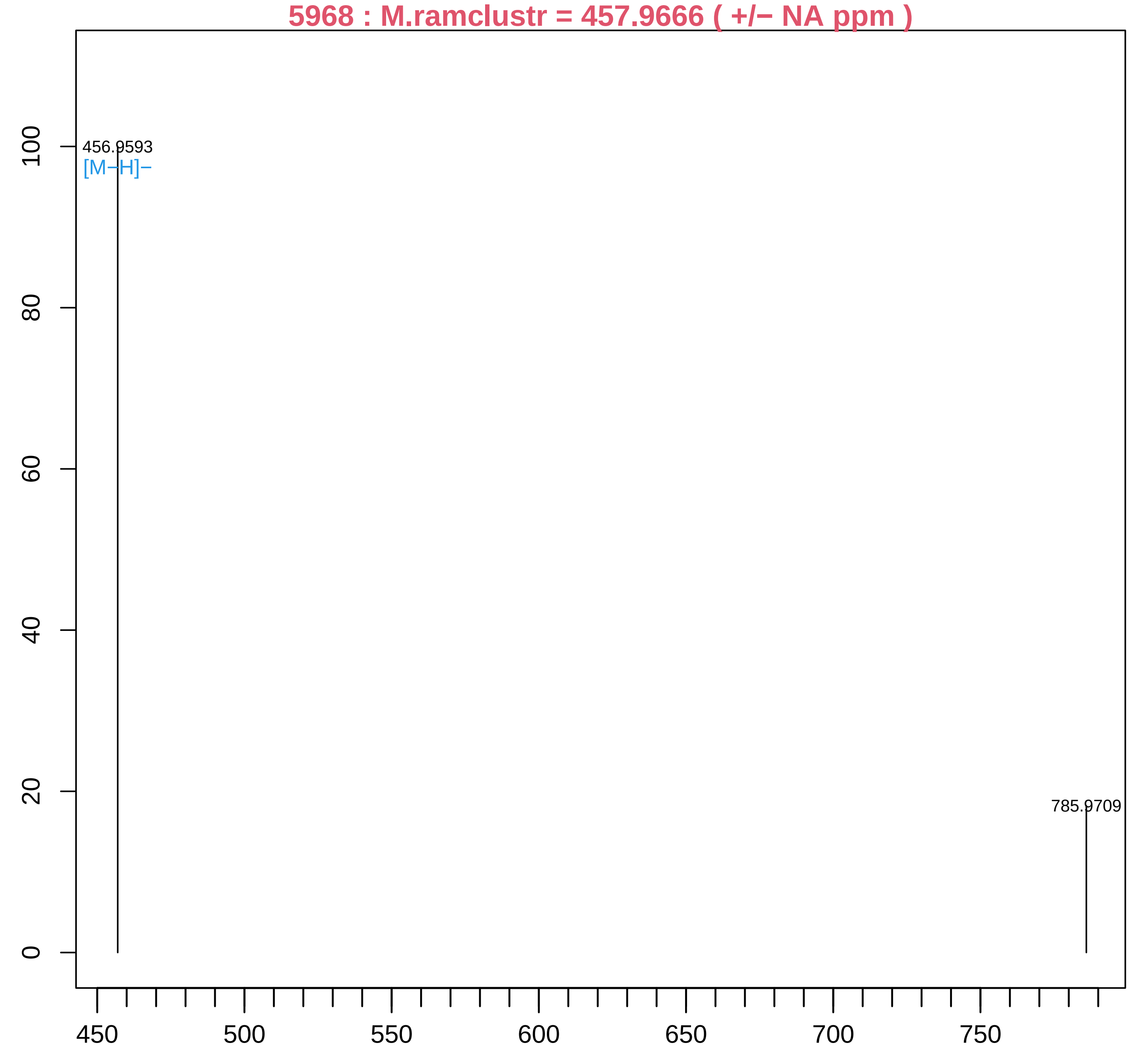
5968 : M.ramclustr = 457.9666 (+/− NA ppm)
456.9593
[M−H]−
785.9709
0
20
40
60
80
100
450
500
550
600
650
700
750

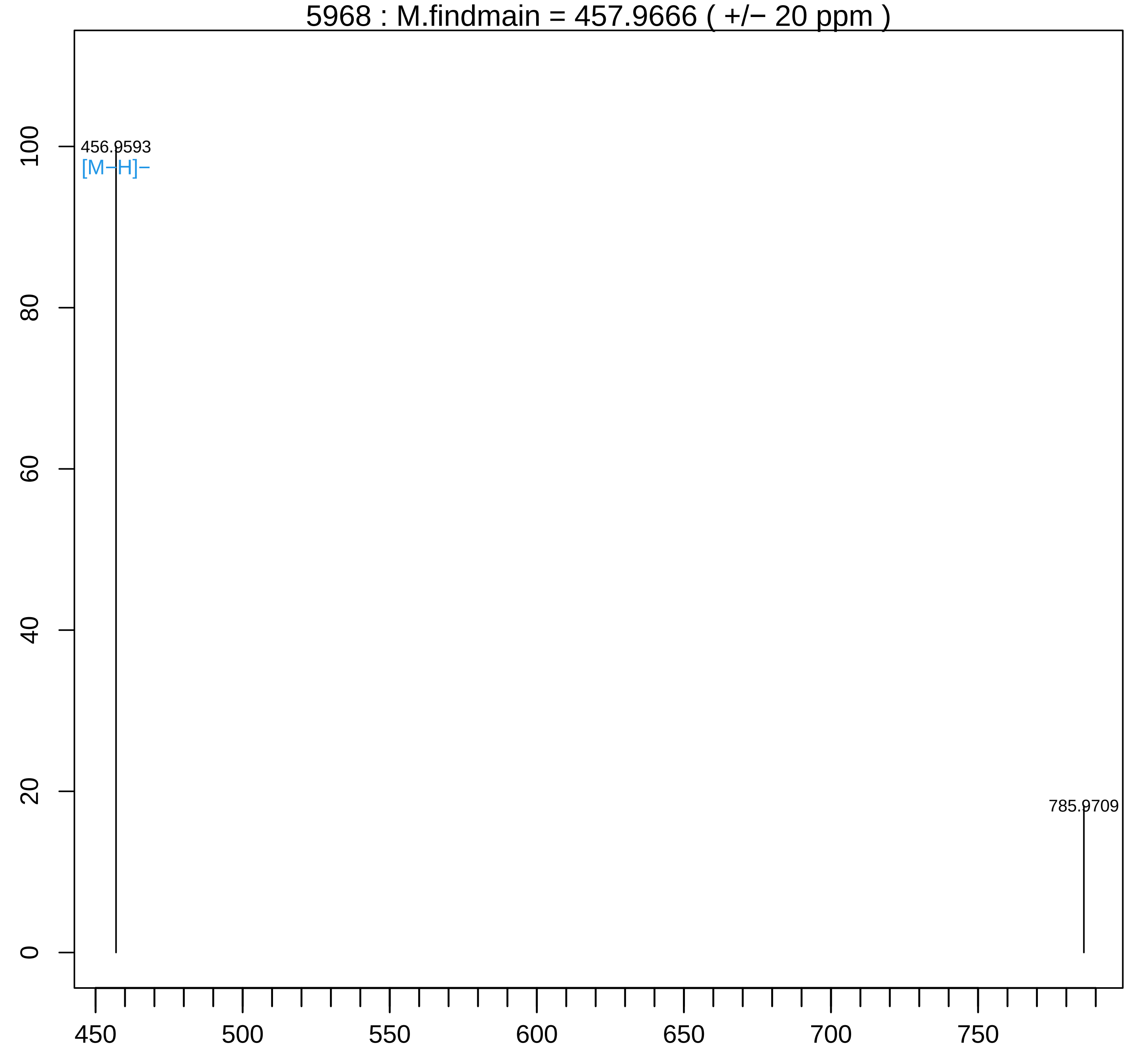
5968 : M.findmain = 457.9666 (+/− 20 ppm)
456.9593
[M−H]−
785.9709
0
20
40
60
80
100
450
500
550
600
650
700
750

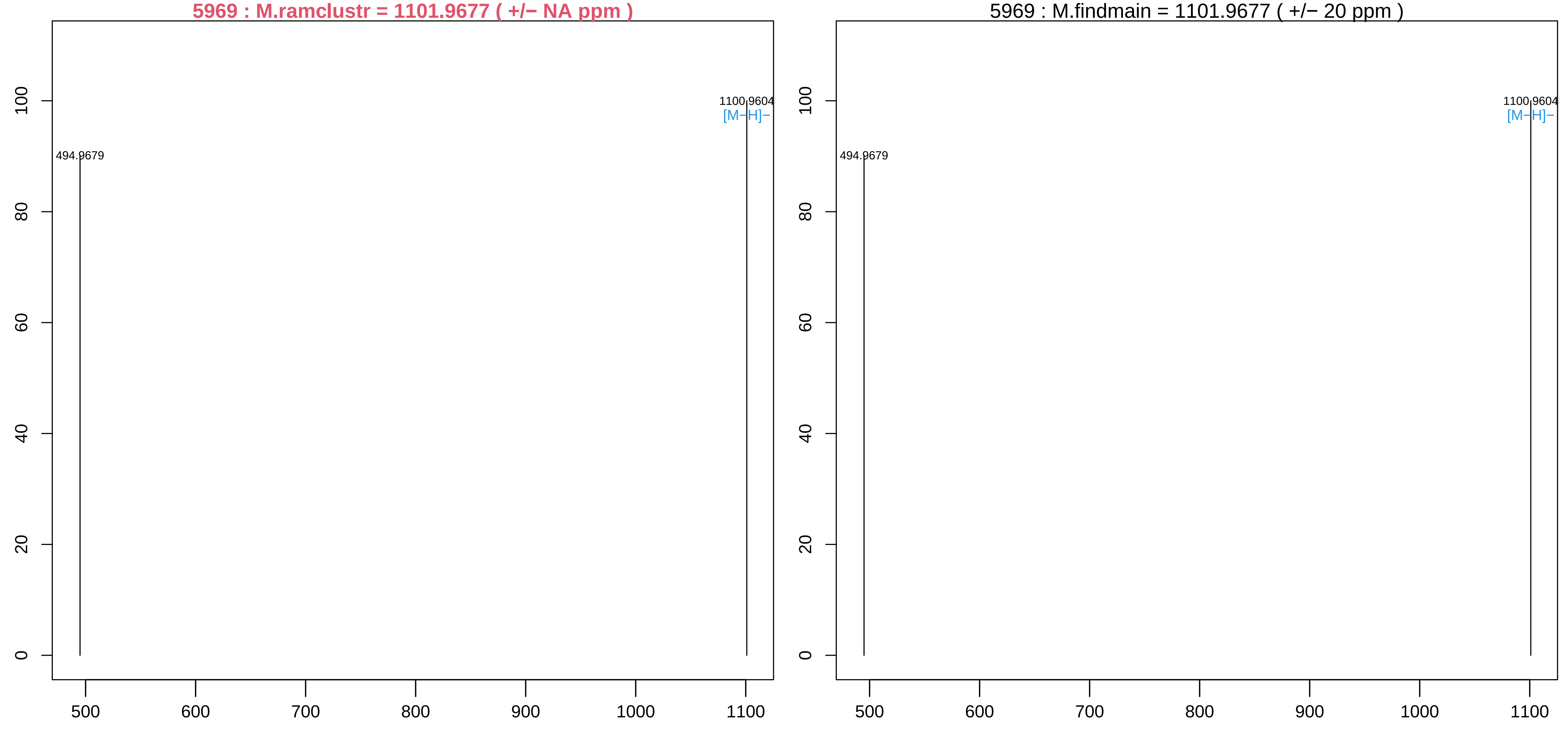

5969 : M.ramclustr = 1101.9677 (+/− NA ppm)
5969 : M.findmain = 1101.9677 (+/− 20 ppm)
494.9679
1100.9604
[M−H]−
494.9679
1100.9604
[M−H]−
0
20
40
60
80
100
500
600
700
800
900
1000
1100

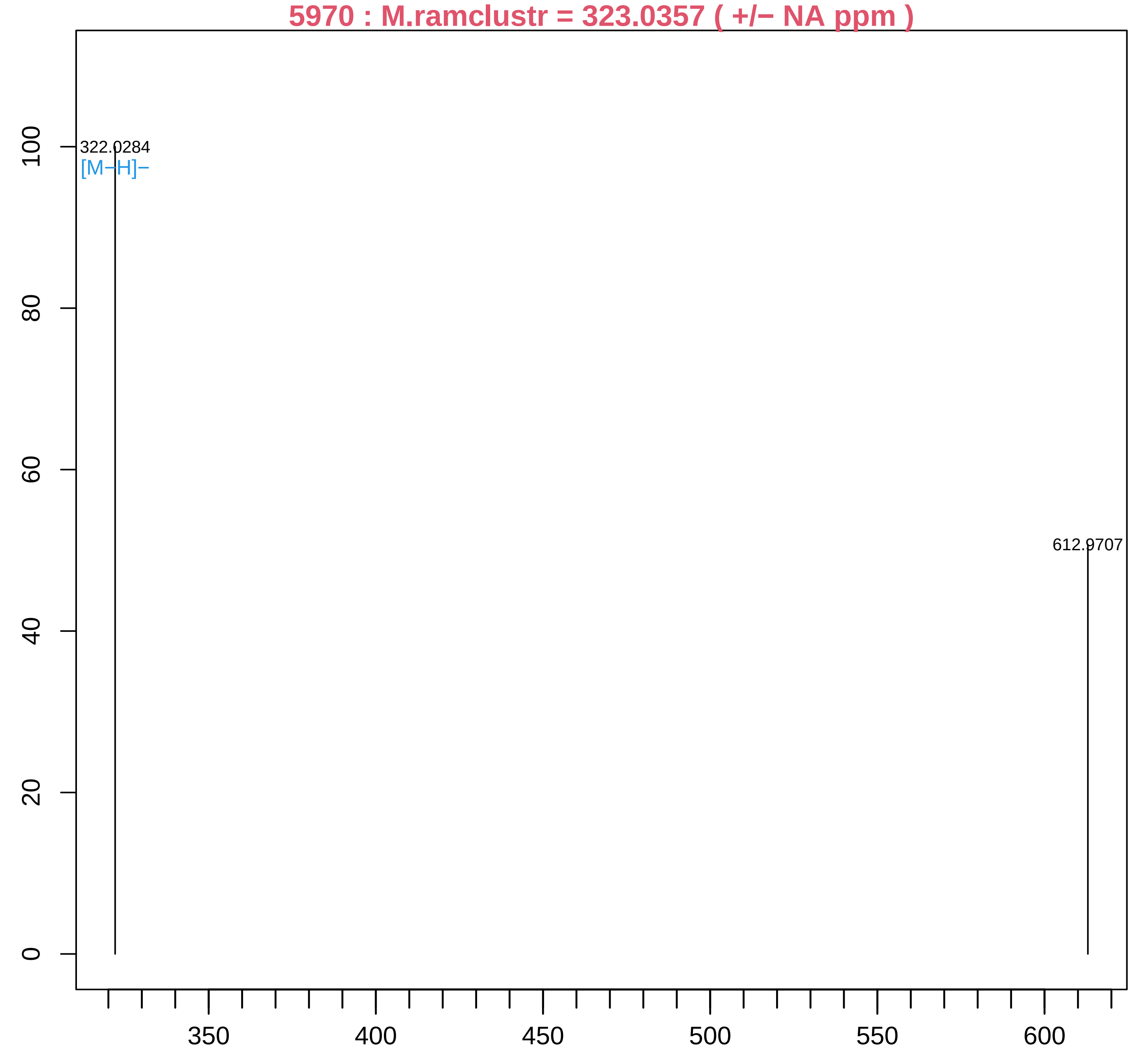

5970 : M.ramclustr = 323.0357 (+/− NA ppm)
322.0284
[M−H]−
612.9707
0
20
40
60
80
100
350
400
450
500
550
600

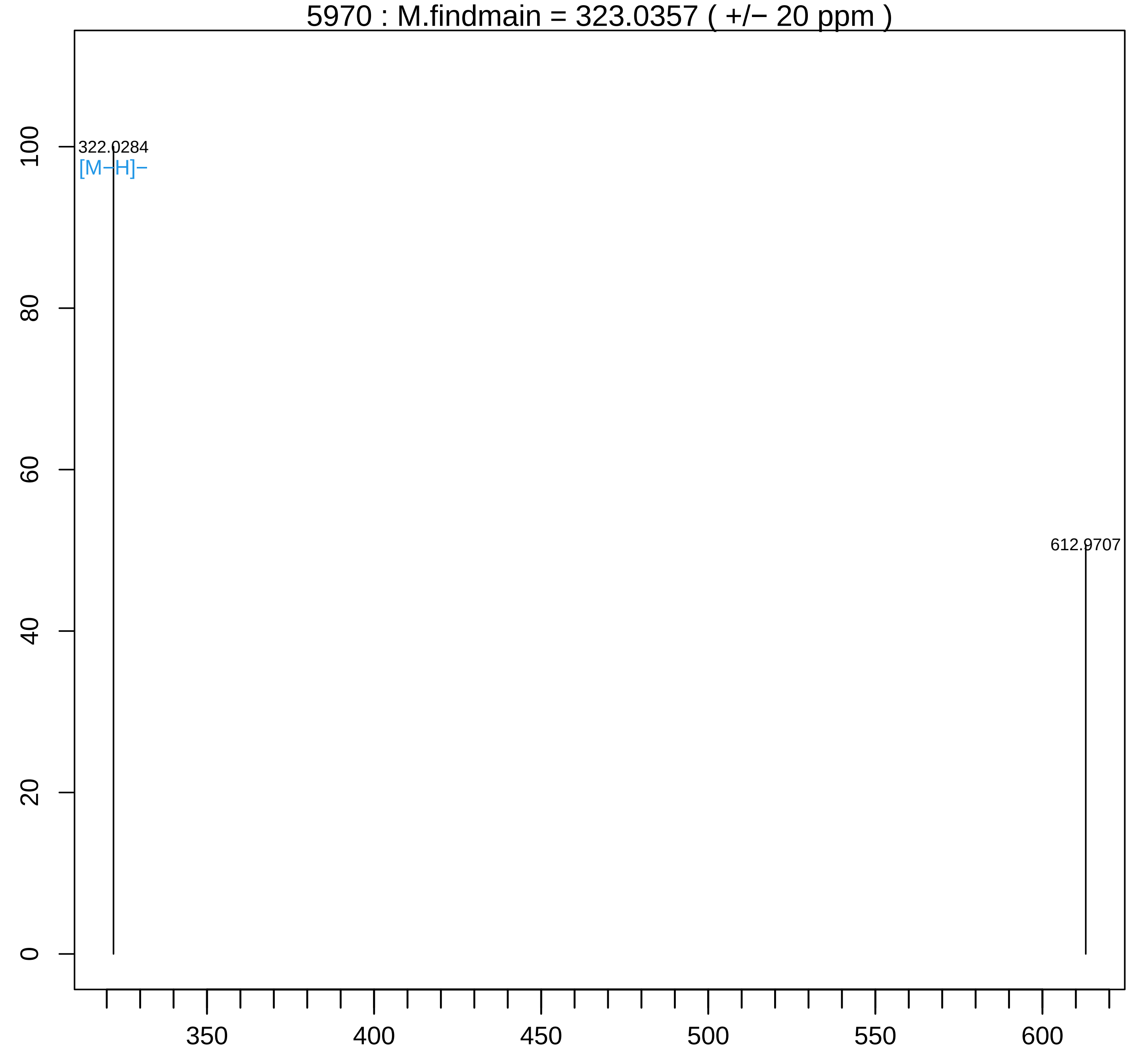

5970 : M.findmain = 323.0357 (+/− 20 ppm)
322.0284
[M−H]−
612.9707
0
20
40
60
80
100
350
400
450
500
550
600

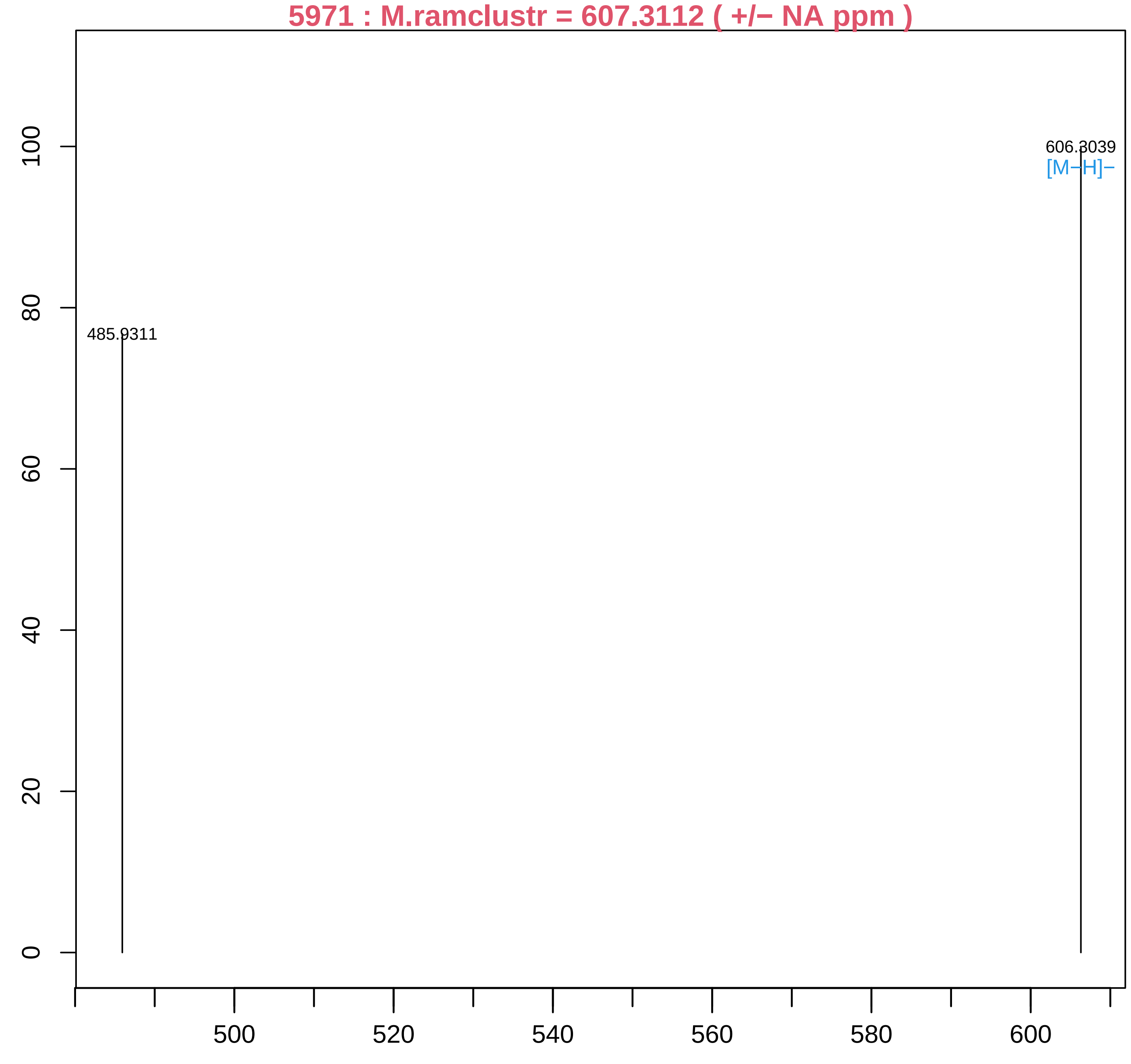
5971 : M.ramclustr = 607.3112 (+/− NA ppm)
606.3039
[M−H]−
485.9311
0
20
40
60
80
100
500
520
540
560
580
600

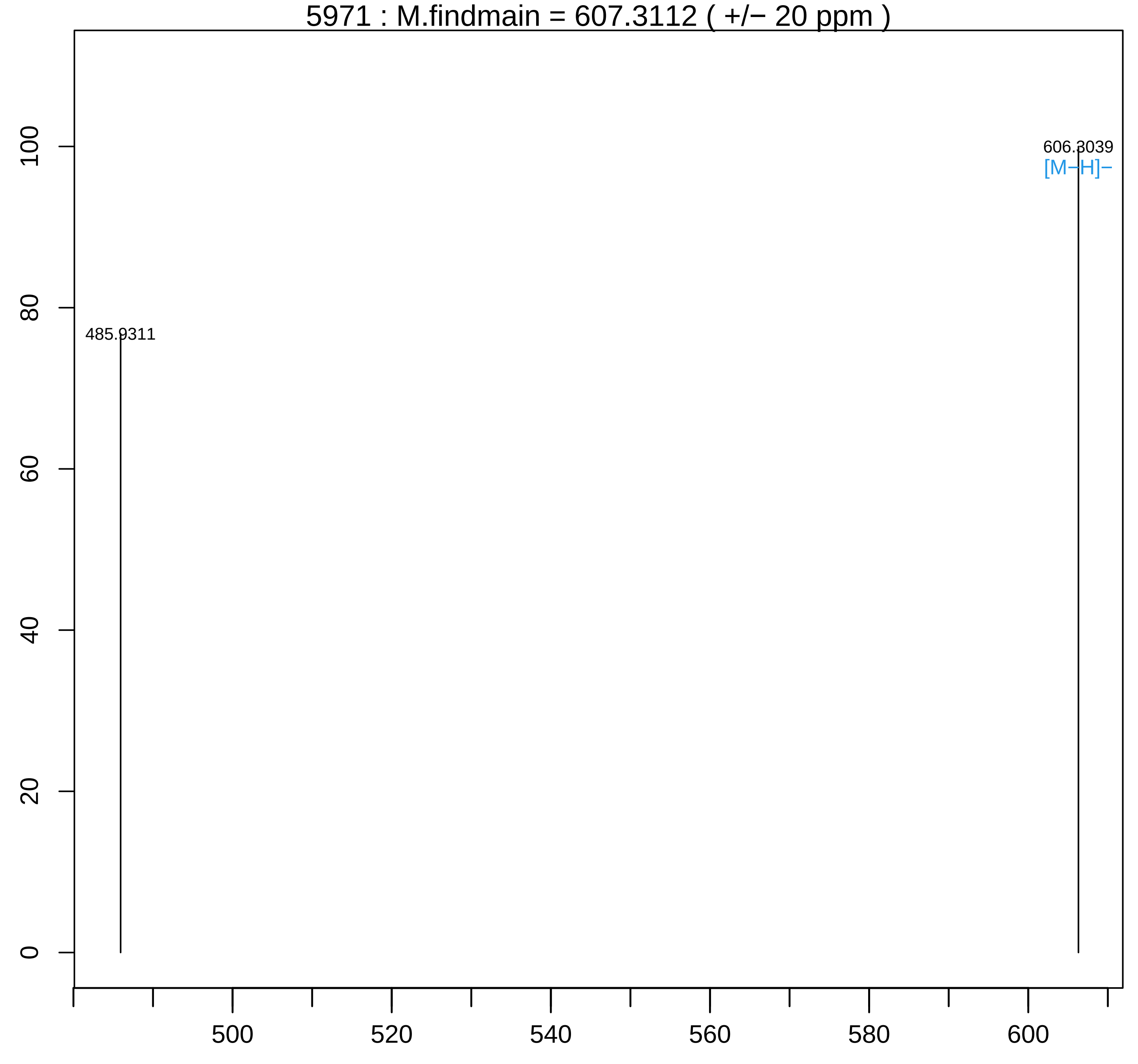
5971 : M.findmain = 607.3112 (+/− 20 ppm)
606.3039
[M−H]−
485.9311
0
20
40
60
80
100
500
520
540
560
580
600

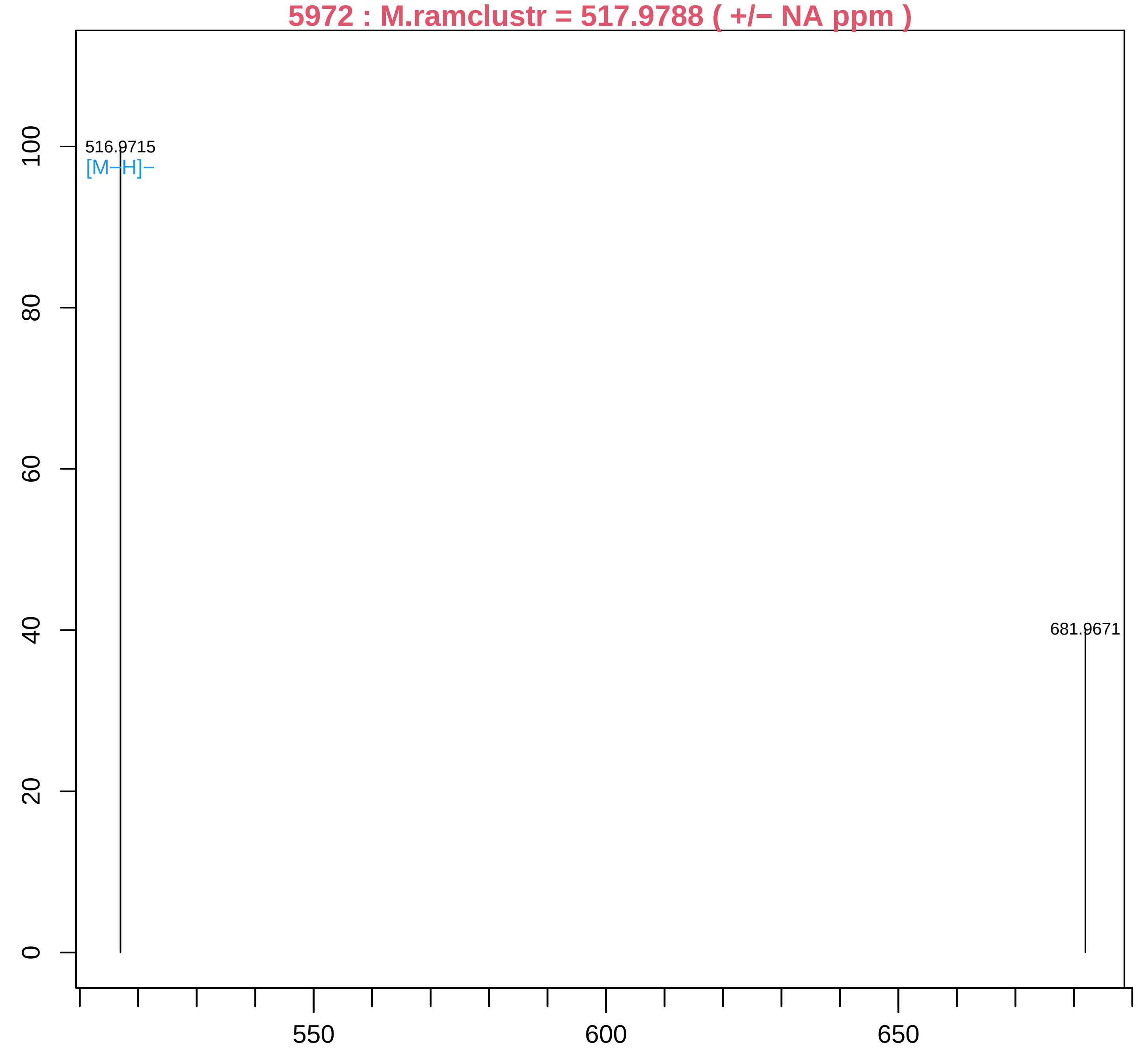
5972 : M.ramclustr = 517.9788 (+/− NA ppm)
516.9715
[M−H]−
681.9671
0
20
40
60
80
100
550
600
650

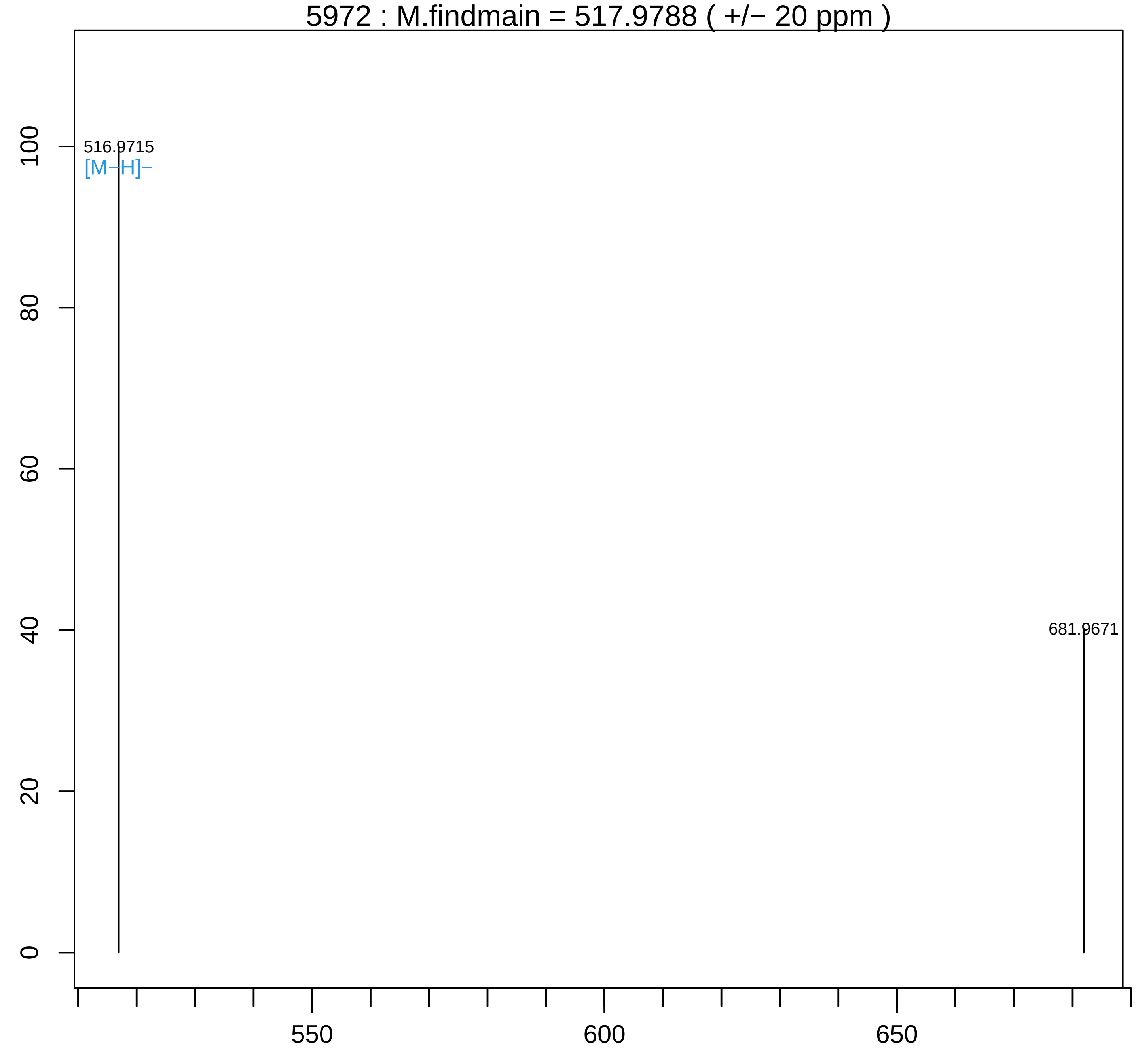
5972 : M.findmain = 517.9788 (+/− 20 ppm)
516.9715
[M−H]−
681.9671
0
20
40
60
80
100
550
600
650

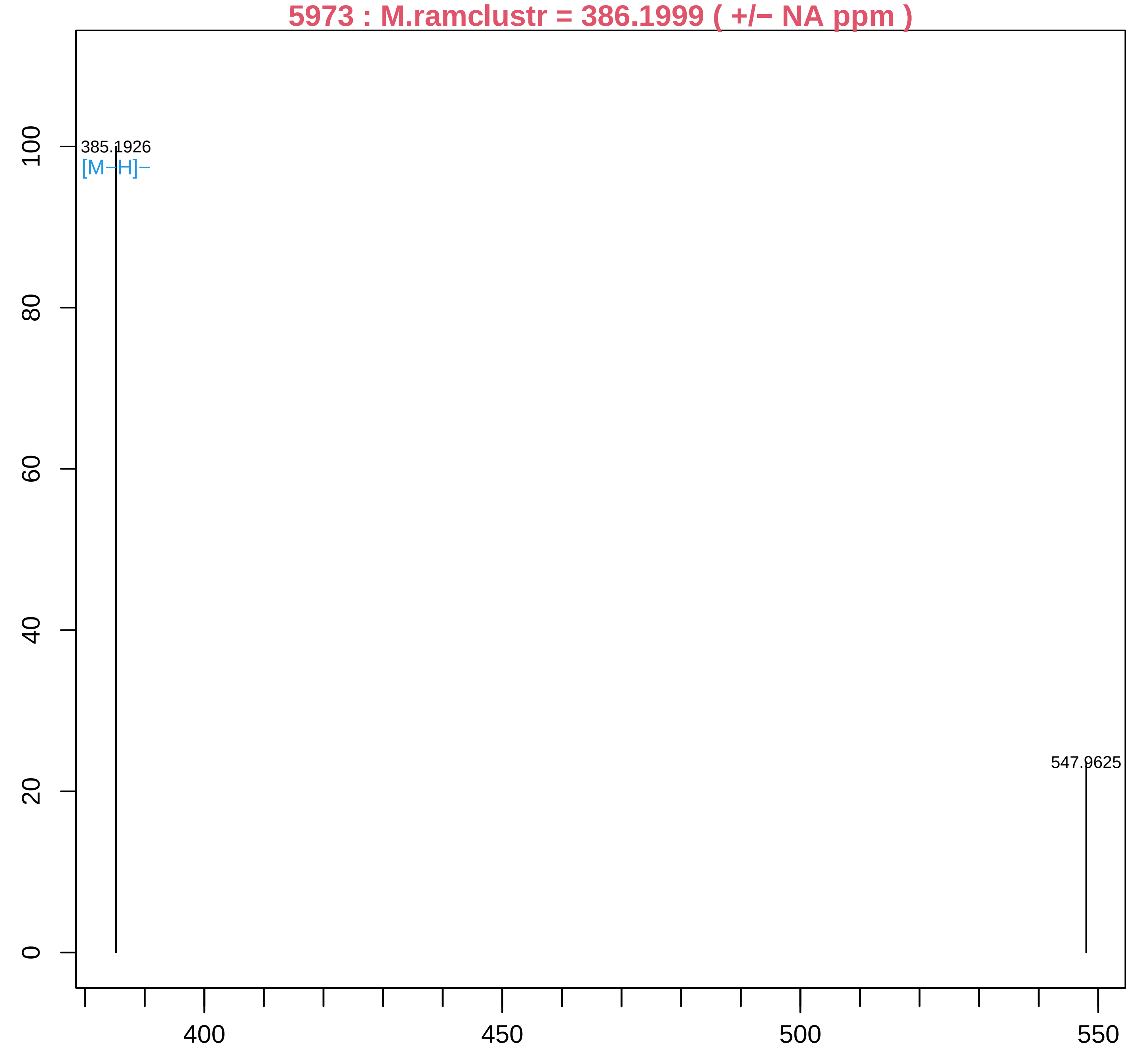
5973 : M.ramclustr = 386.1999 (+/− NA ppm)
385.1926
[M−H]−
547.9625
0
20
40
60
80
100
400
450
500
550

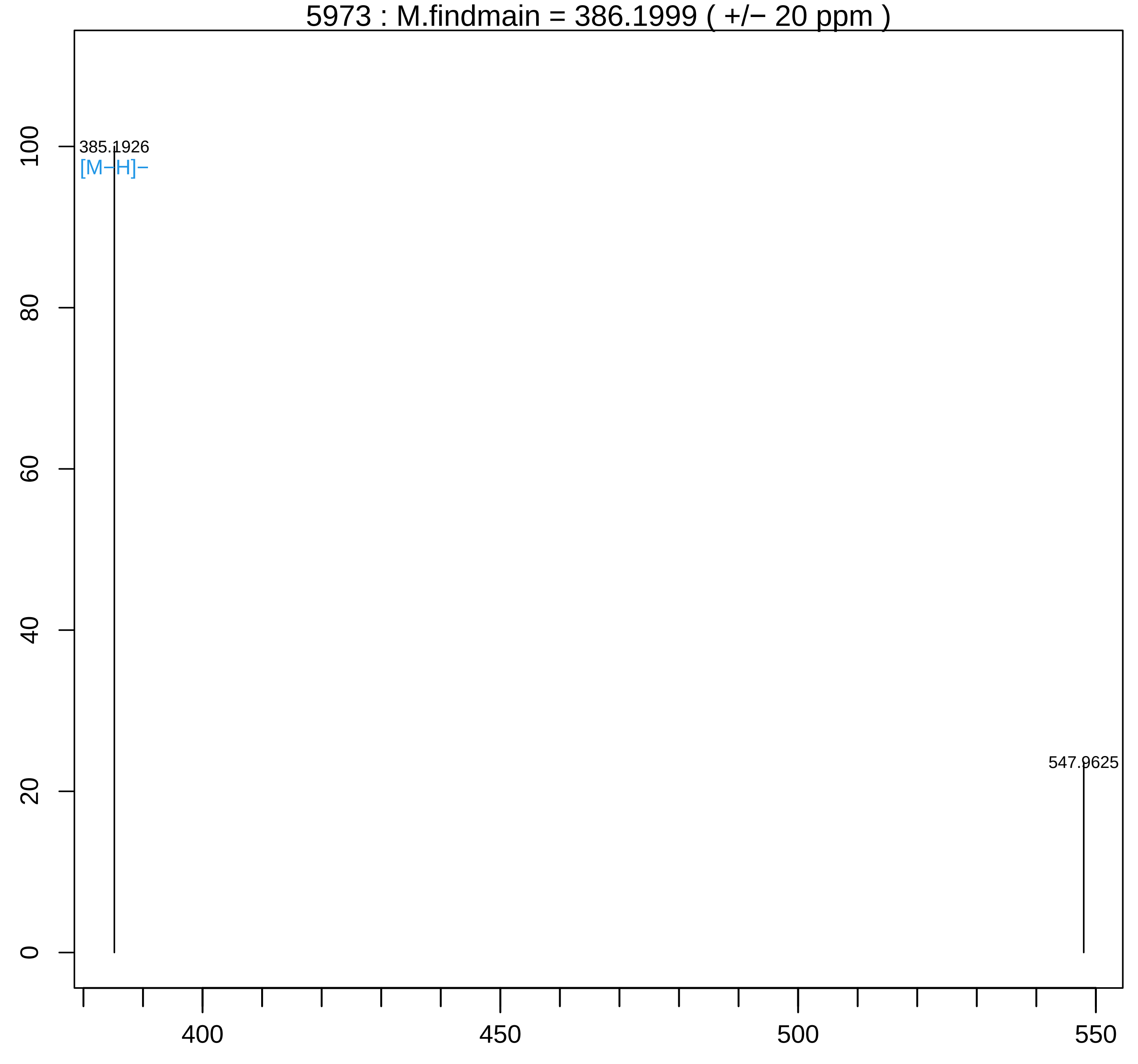
5973 : M.findmain = 386.1999 (+/− 20 ppm)
385.1926
[M−H]−
547.9625
0
20
40
60
80
100
400
450
500
550

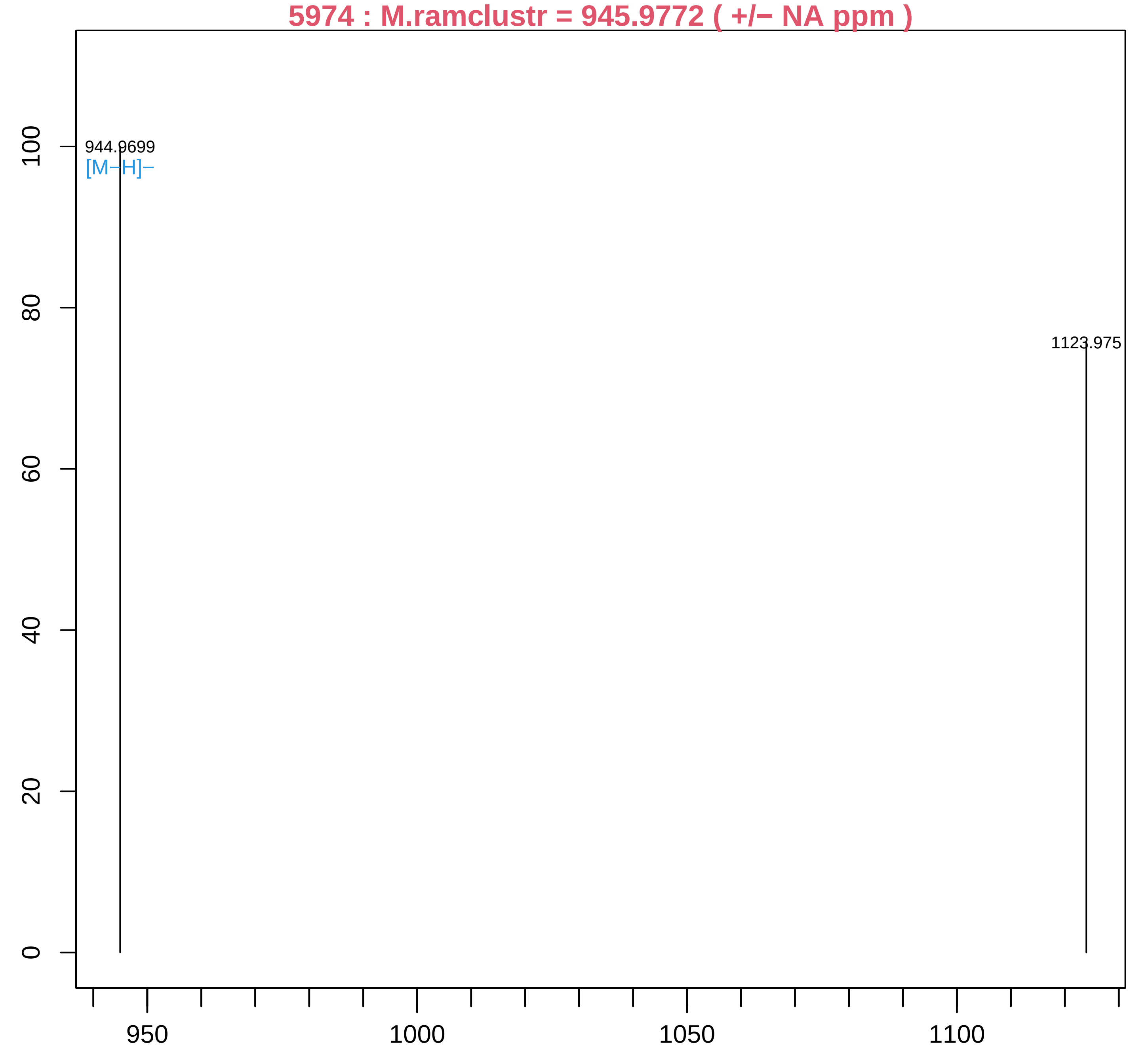

5974 : M.ramclustr = 945.9772 (+/− NA ppm)
944.9699
[M−H]−
1123.975
0
20
40
60
80
100
950
1000
1050
1100

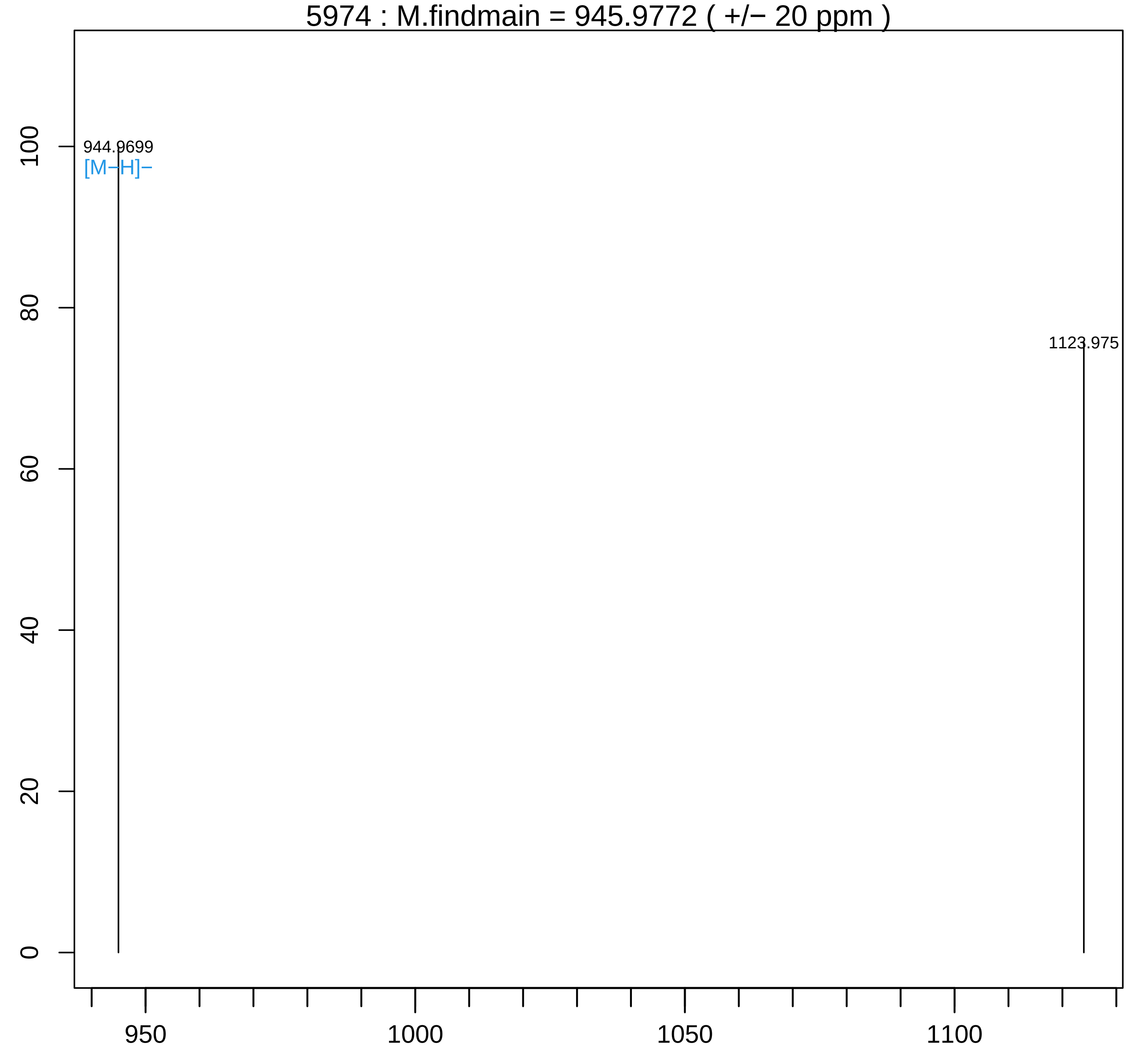

5974 : M.findmain = 945.9772 (+/− 20 ppm)
944.9699
[M−H]−
1123.975
0
20
40
60
80
100
950
1000
1050
1100

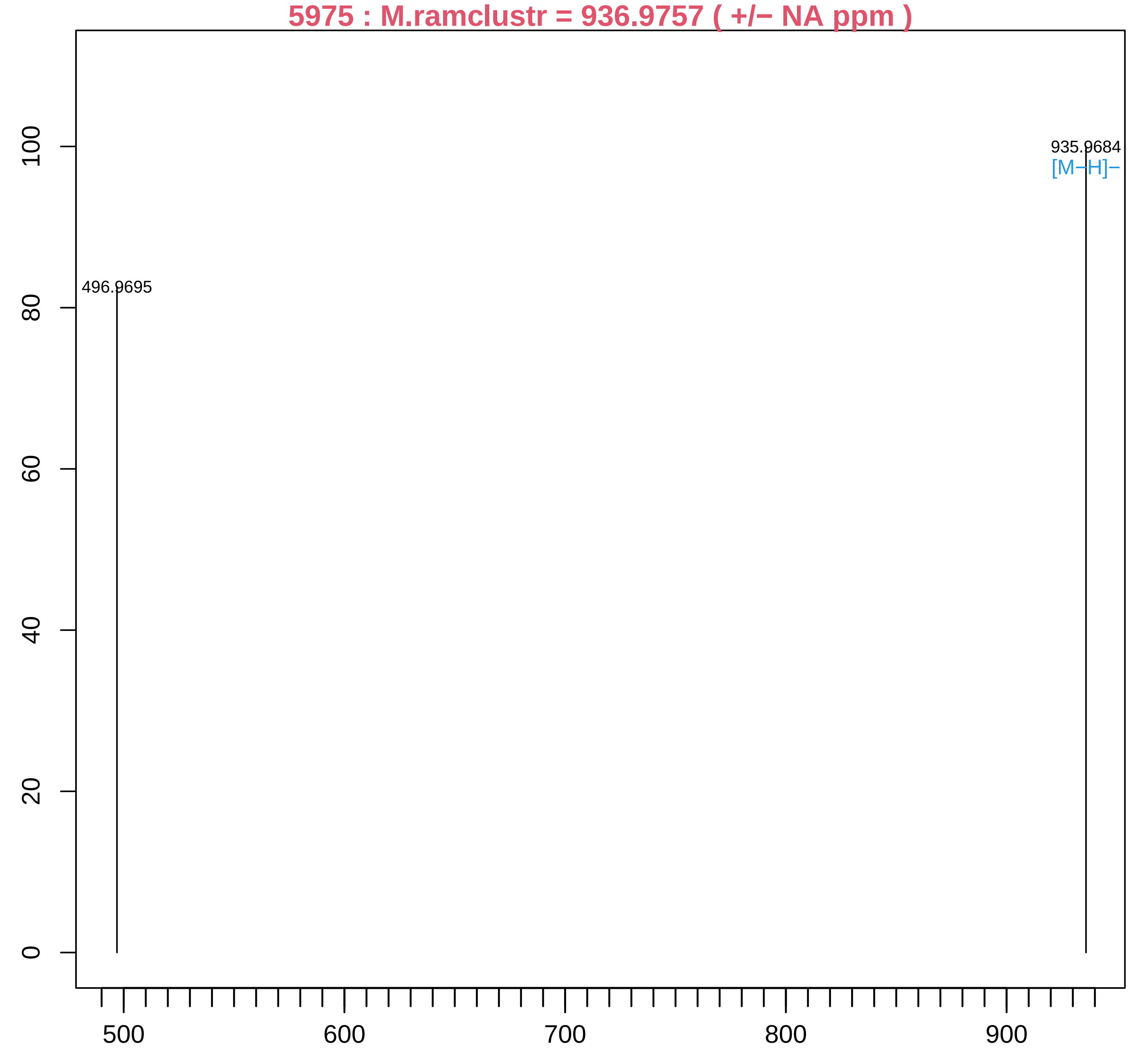

5975 : M.ramclustr = 936.9757 (+/− NA ppm)
496.9695
935.9684
[M−H]−
0
20
40
60
80
100
500
600
700
800
900

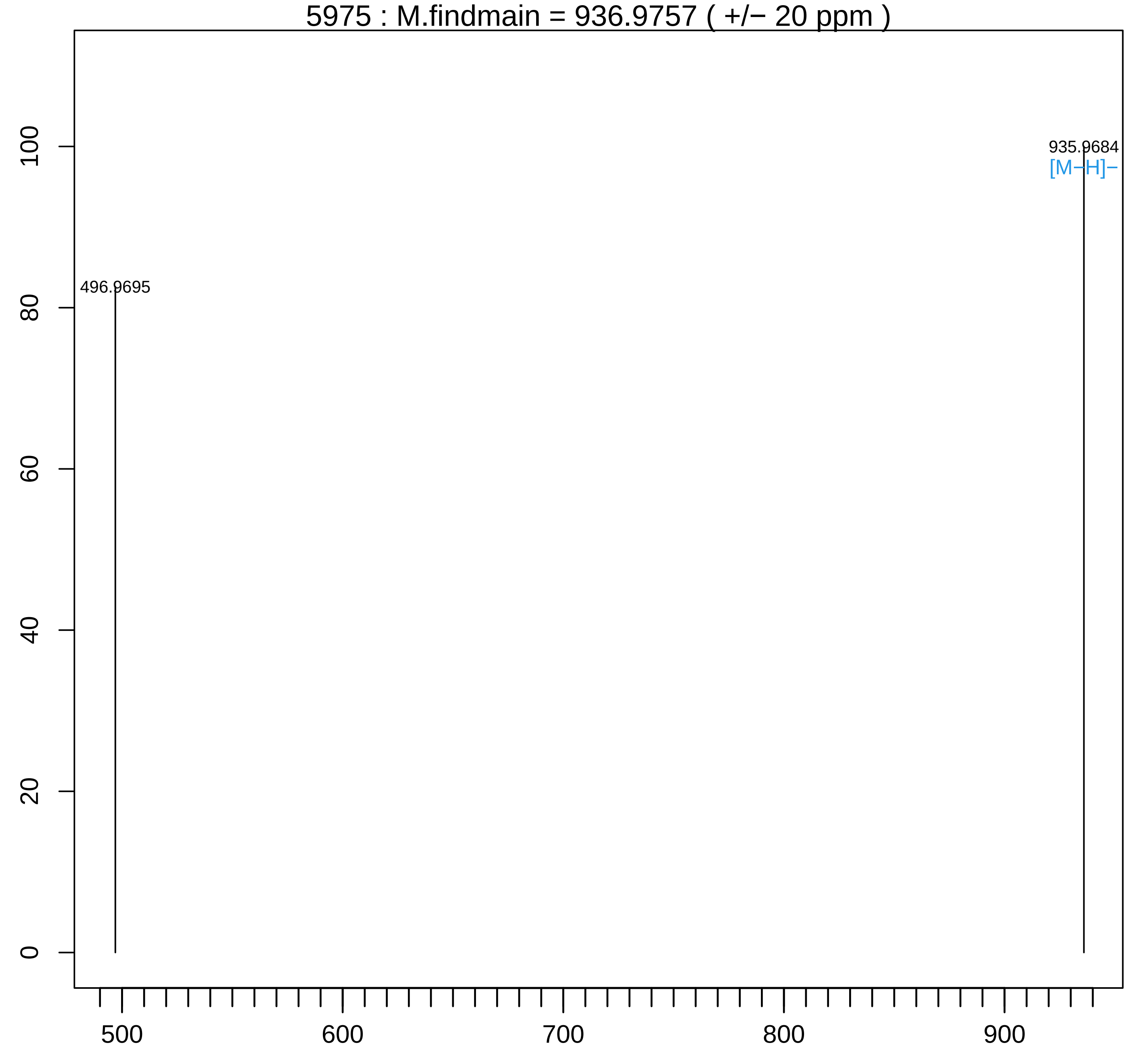

5975 : M.findmain = 936.9757 (+/− 20 ppm)
496.9695
935.9684
[M−H]−
0
20
40
60
80
100
500
600
700
800
900

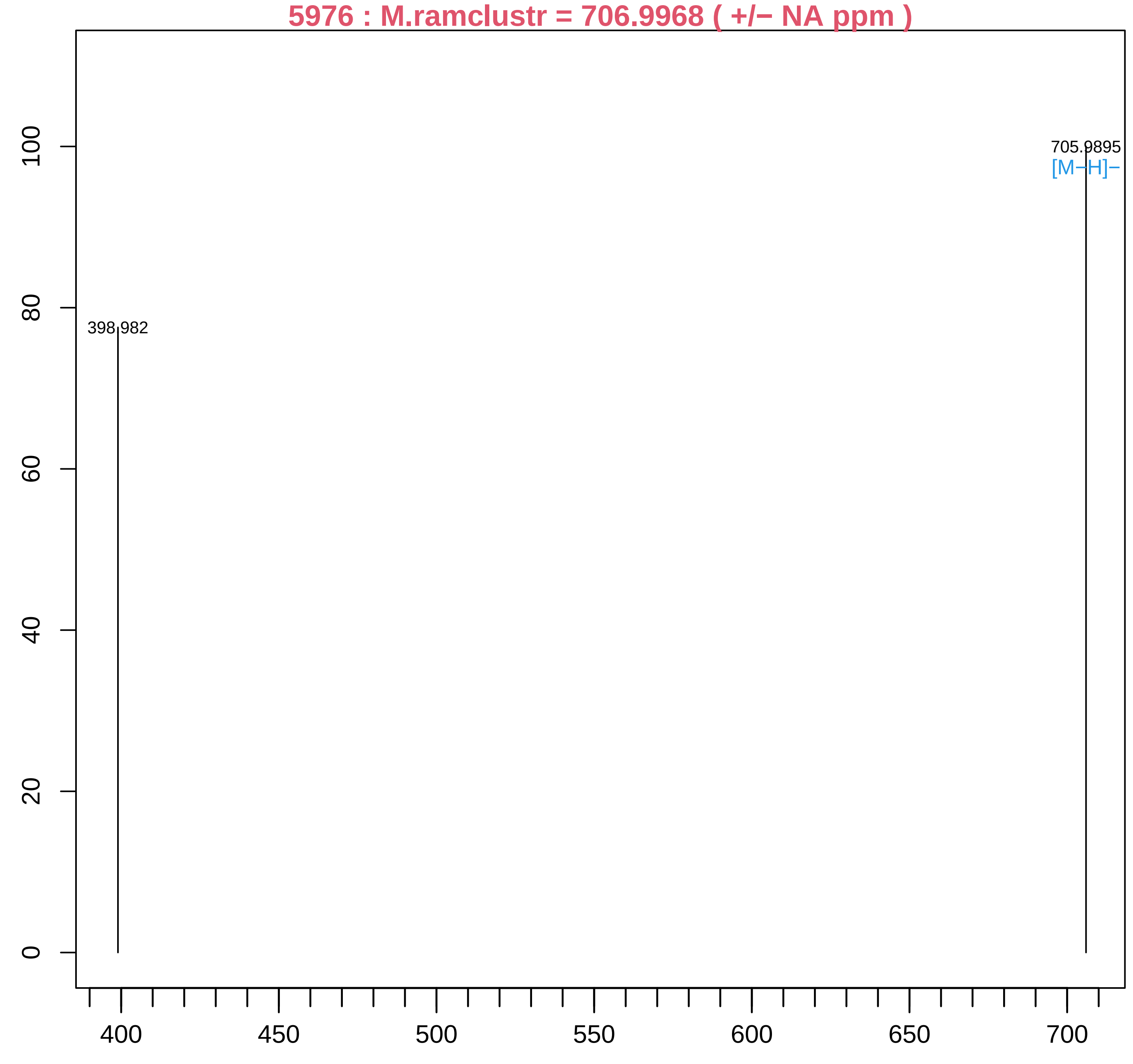
5976 : M.ramclustr = 706.9968 (+/− NA ppm)
705.9895
[M−H]−
398.982
0
20
40
60
80
100
400
450
500
550
600
650
700

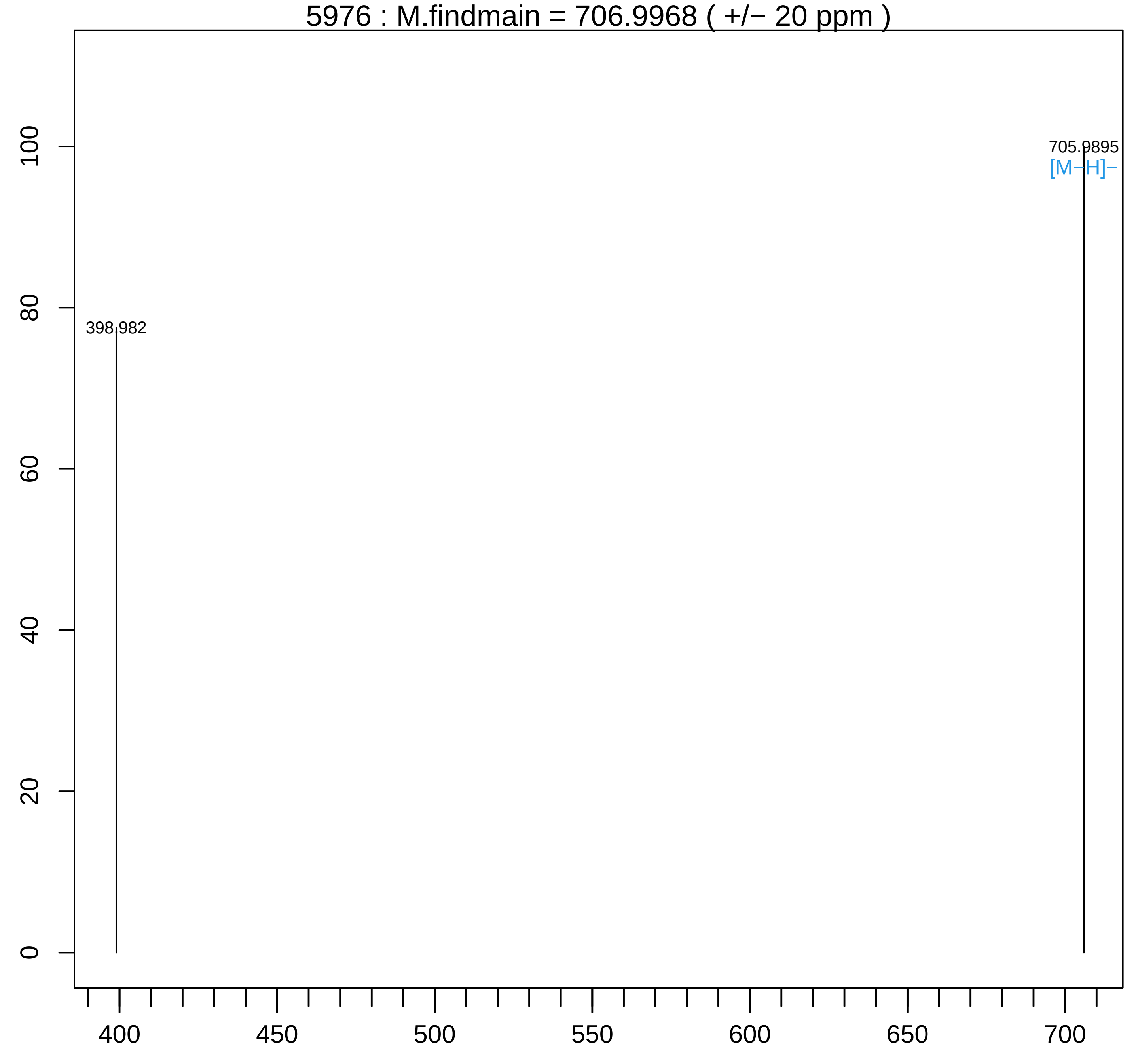
5976 : M.findmain = 706.9968 (+/− 20 ppm)
705.9895
[M−H]−
398.982
0
20
40
60
80
100
400
450
500
550
600
650
700

5977 : M.ramclustr = 889.974 (+/− NA ppm)

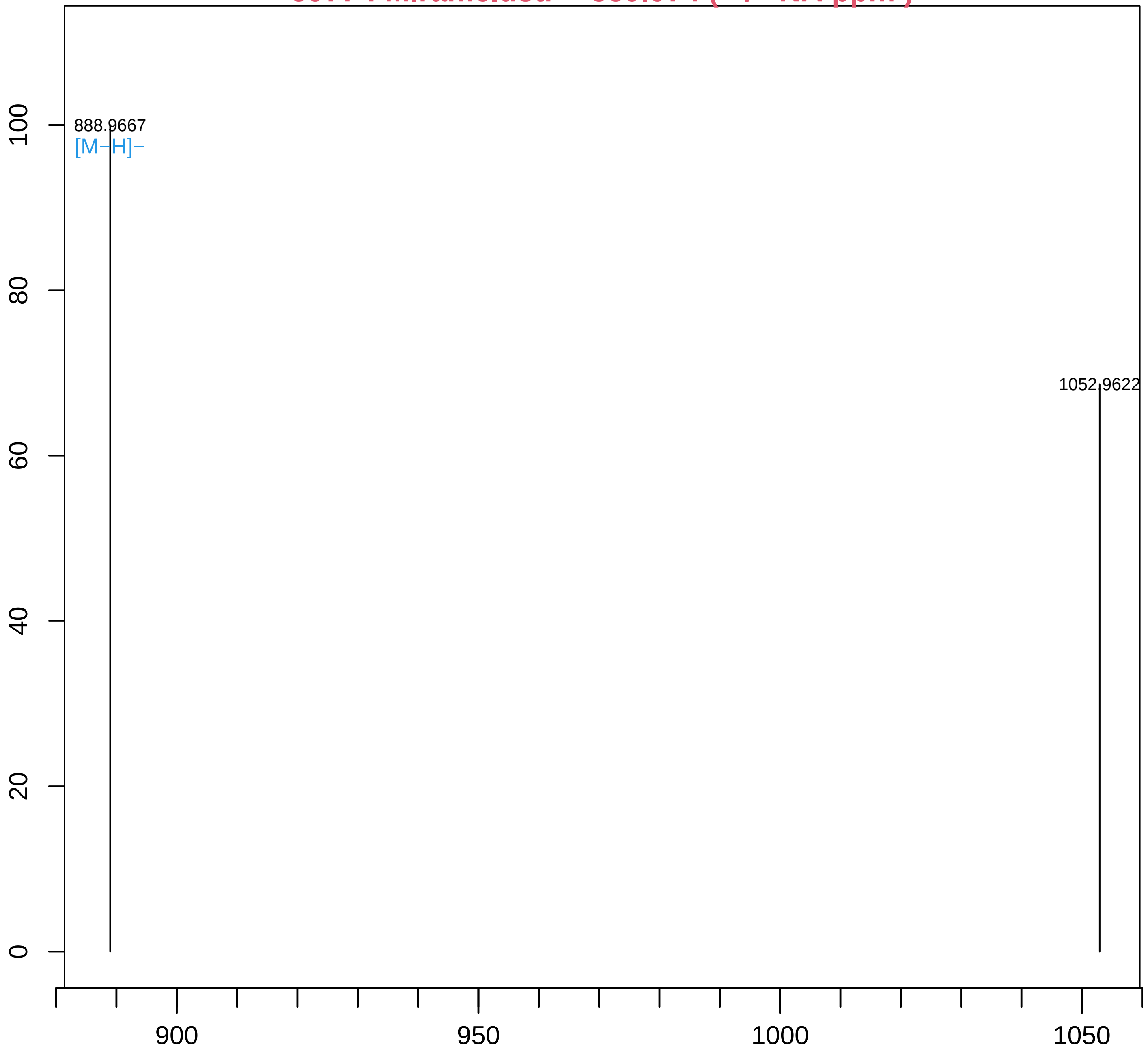

5977 : M.findmain = 889.974 (+/− 20 ppm)

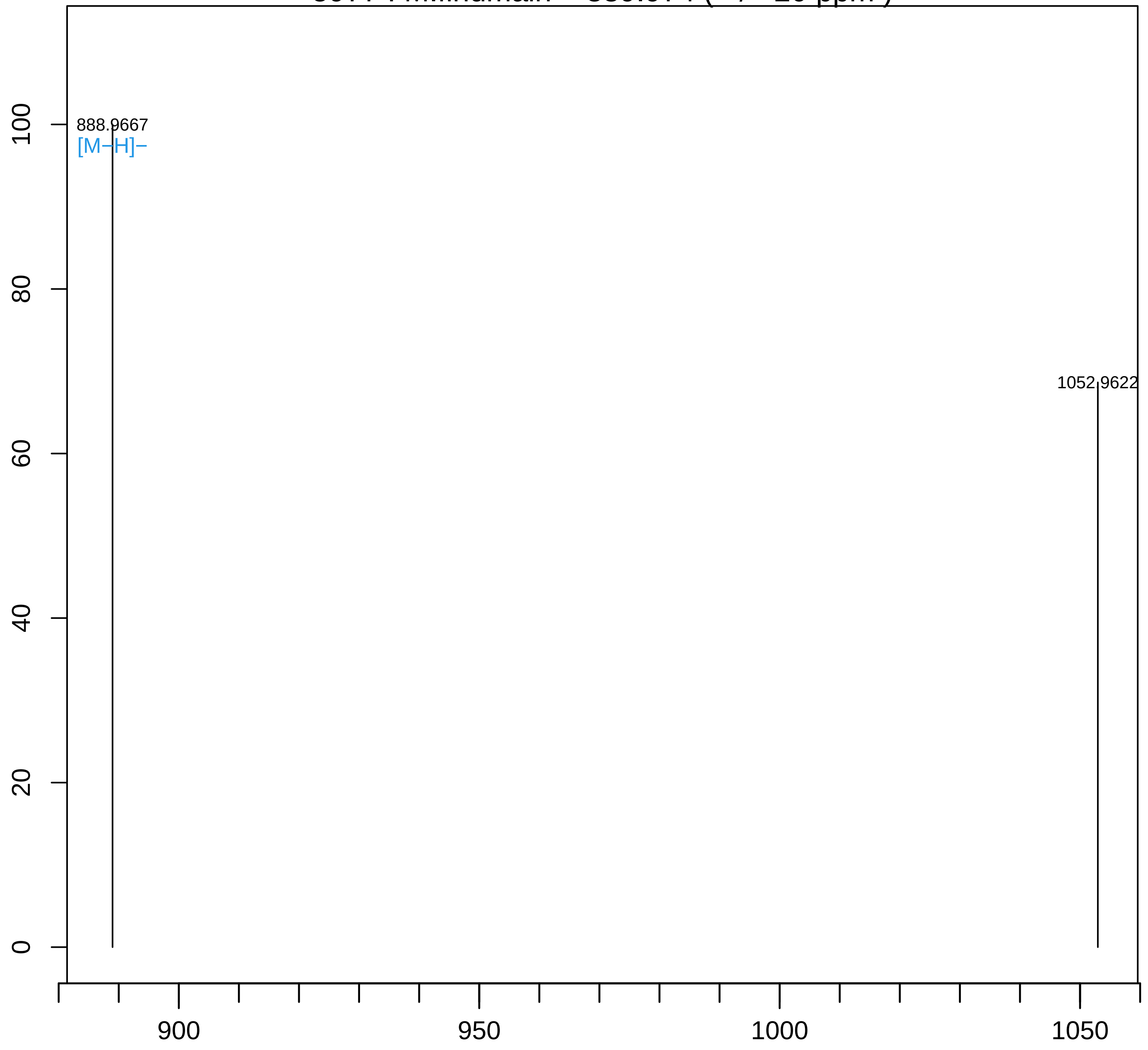

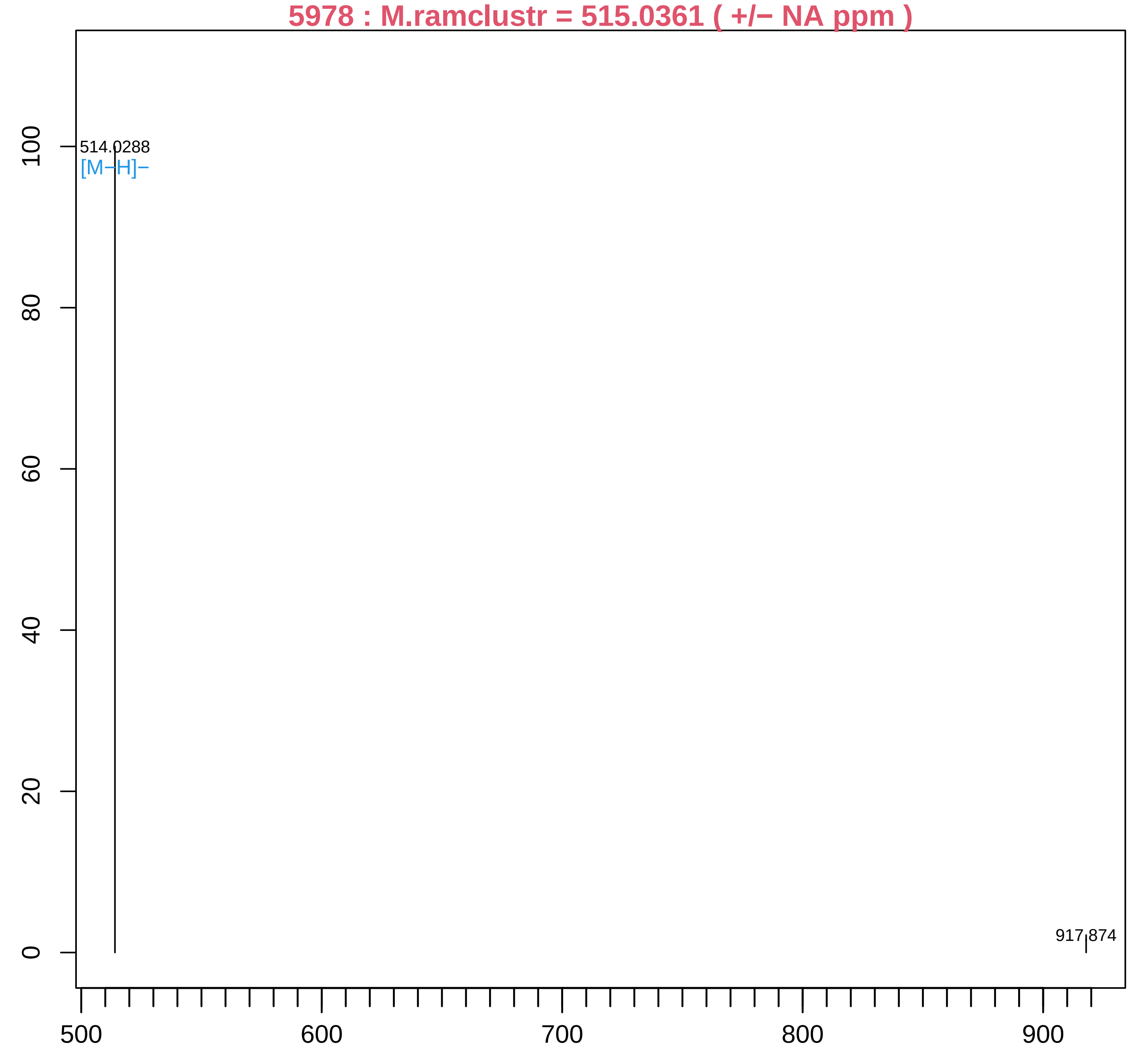
5978 : M.ramclustr = 515.0361 (+/− NA ppm)
514.0288
[M−H]−
917.874
0
20
40
60
80
100
500
600
700
800
900

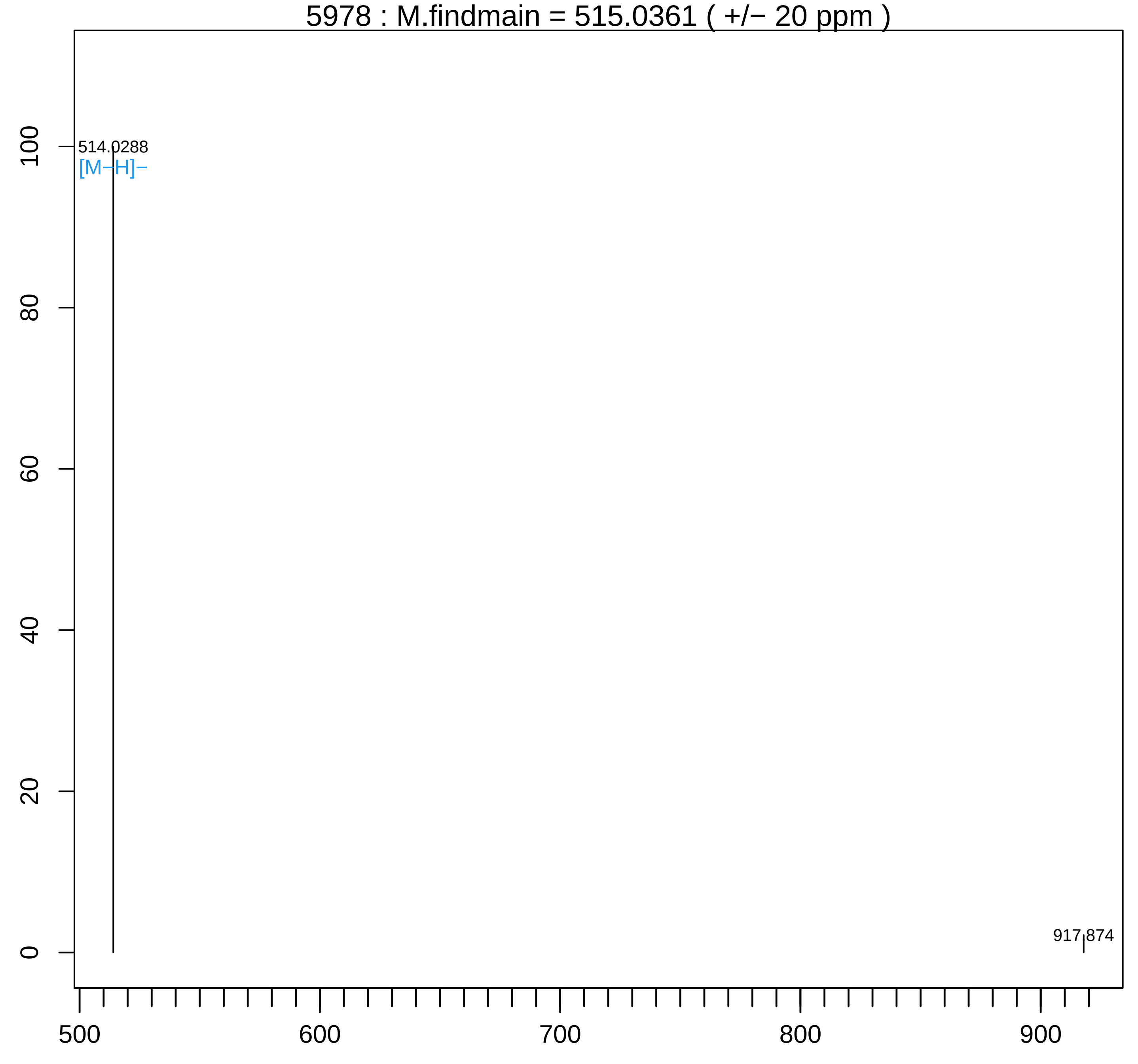
5978 : M.findmain = 515.0361 (+/− 20 ppm)
514.0288
[M−H]−
917.874
0
20
40
60
80
100
500
600
700
800
900

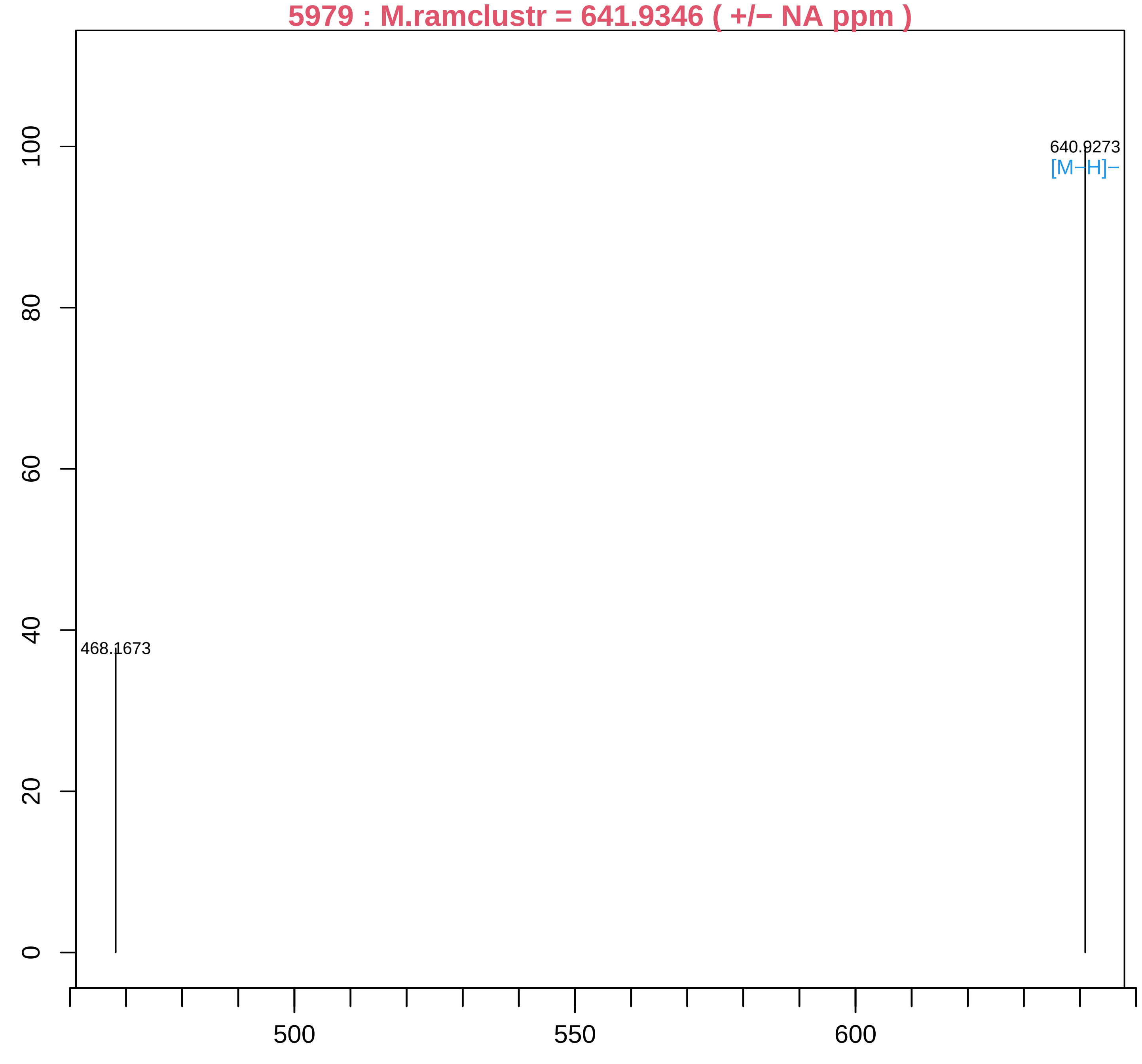
5979 : M.ramclustr = 641.9346 (+/− NA ppm)
640.9273
[M−H]−
468.1673
0
20
40
60
80
100
500
550
600

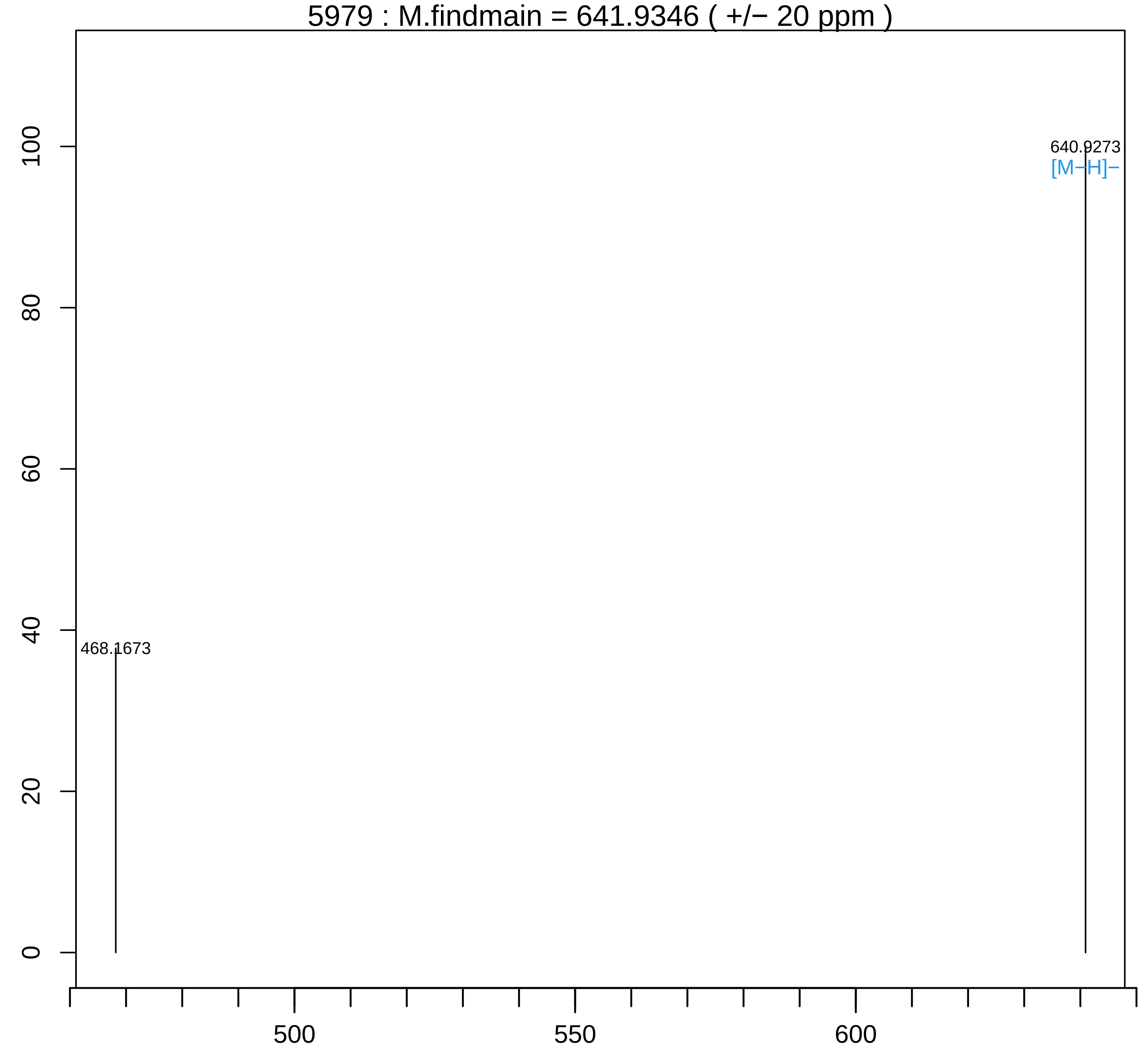
5979 : M.findmain = 641.9346 (+/− 20 ppm)
640.9273
[M−H]−
468.1673
0
20
40
60
80
100
500
550
600

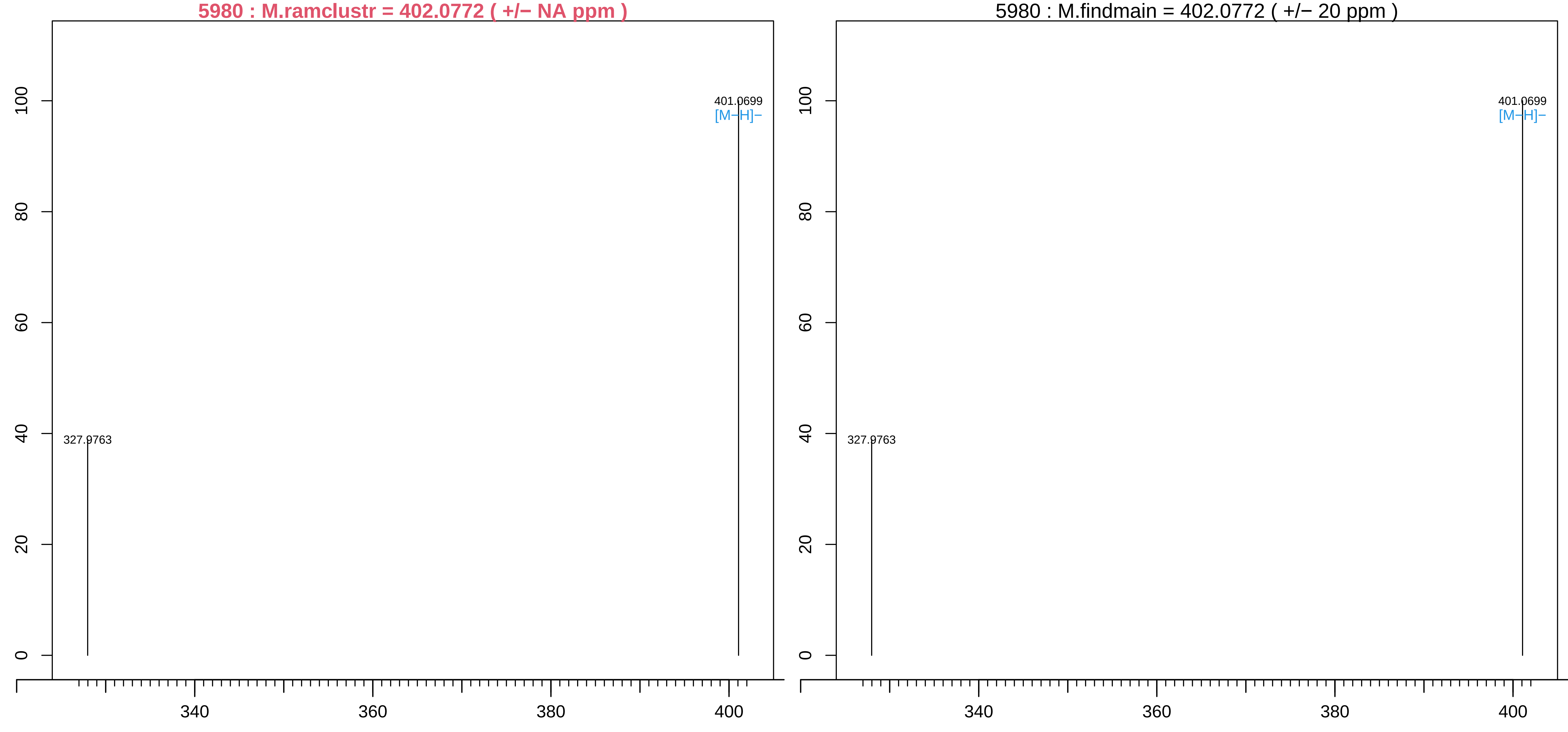
5980 : M.ramclustr = 402.0772 (+/− NA ppm)
401.0699
[M−H]−
327.9763
0
20
40
60
80
100
340
360
380
400
5980 : M.findmain = 402.0772 (+/− 20 ppm)
401.0699
[M−H]−
327.9763
0
20
40
60
80
100
340
360
380
400

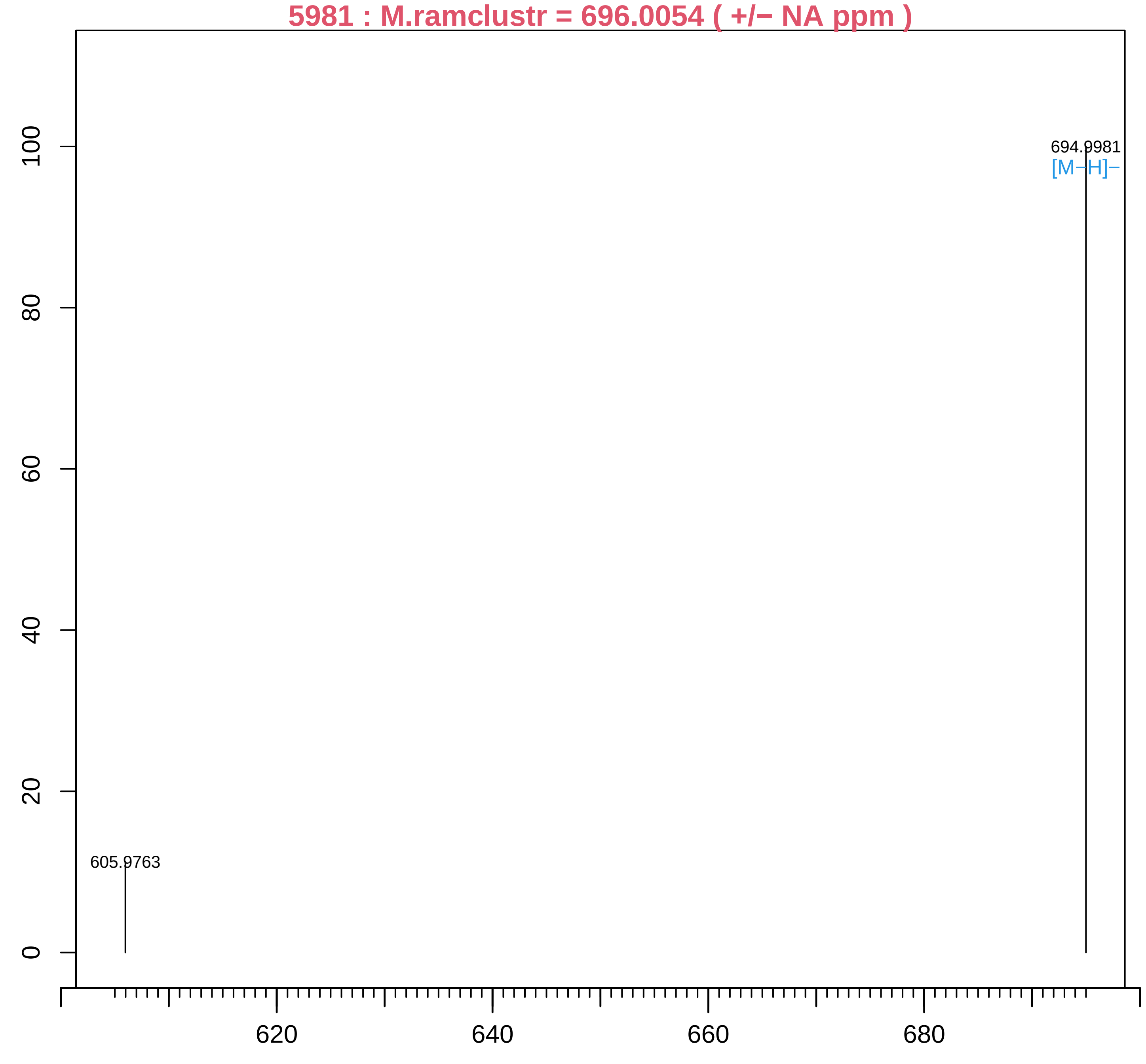
5981 : M.ramclustr = 696.0054 (+/− NA ppm)
694.9981
[M−H]−
605.9763
0
20
40
60
80
100
620
640
660
680

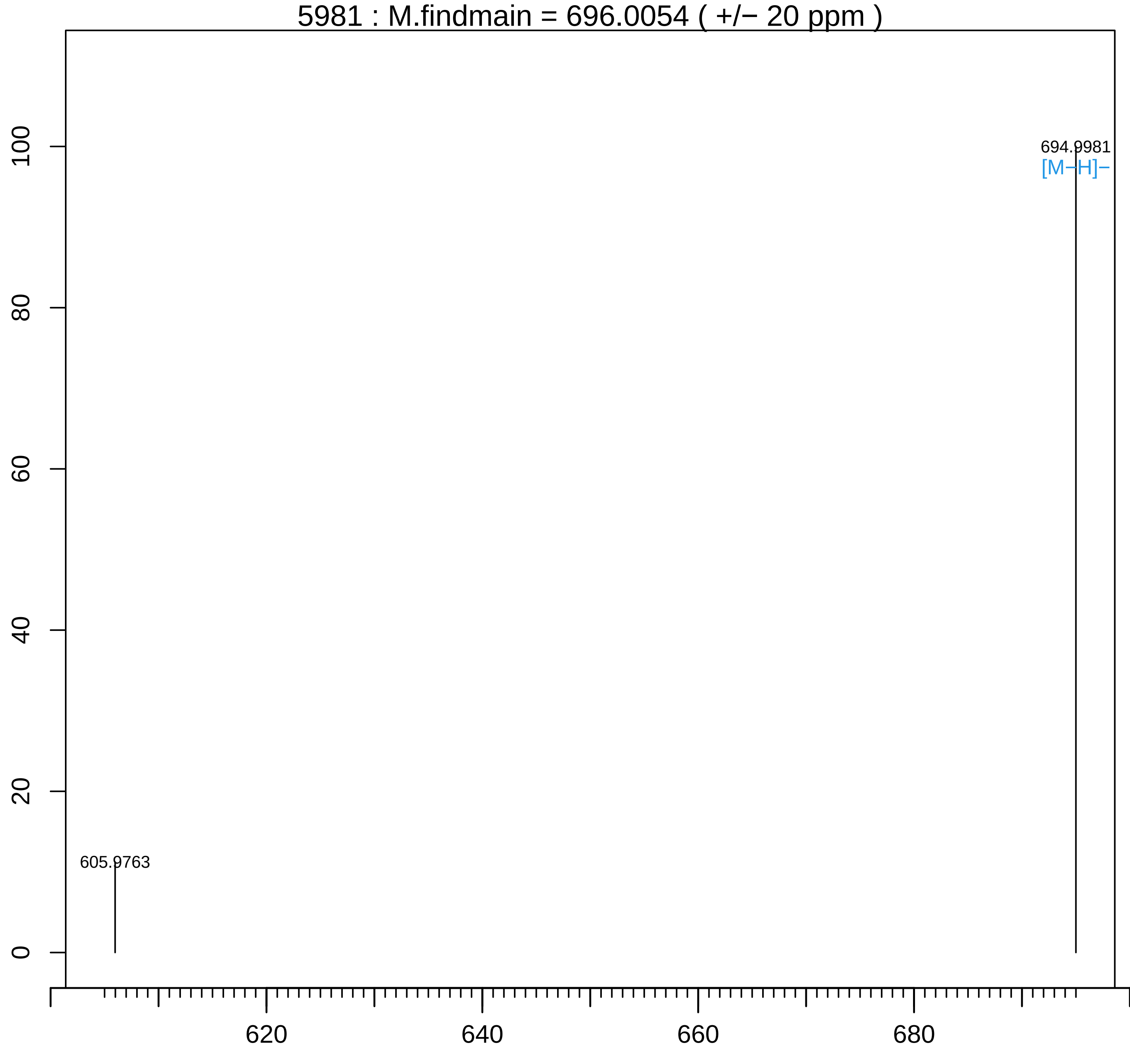
5981 : M.findmain = 696.0054 (+/− 20 ppm)
694.9981
[M−H]−
605.9763
0
20
40
60
80
100
620
640
660
680

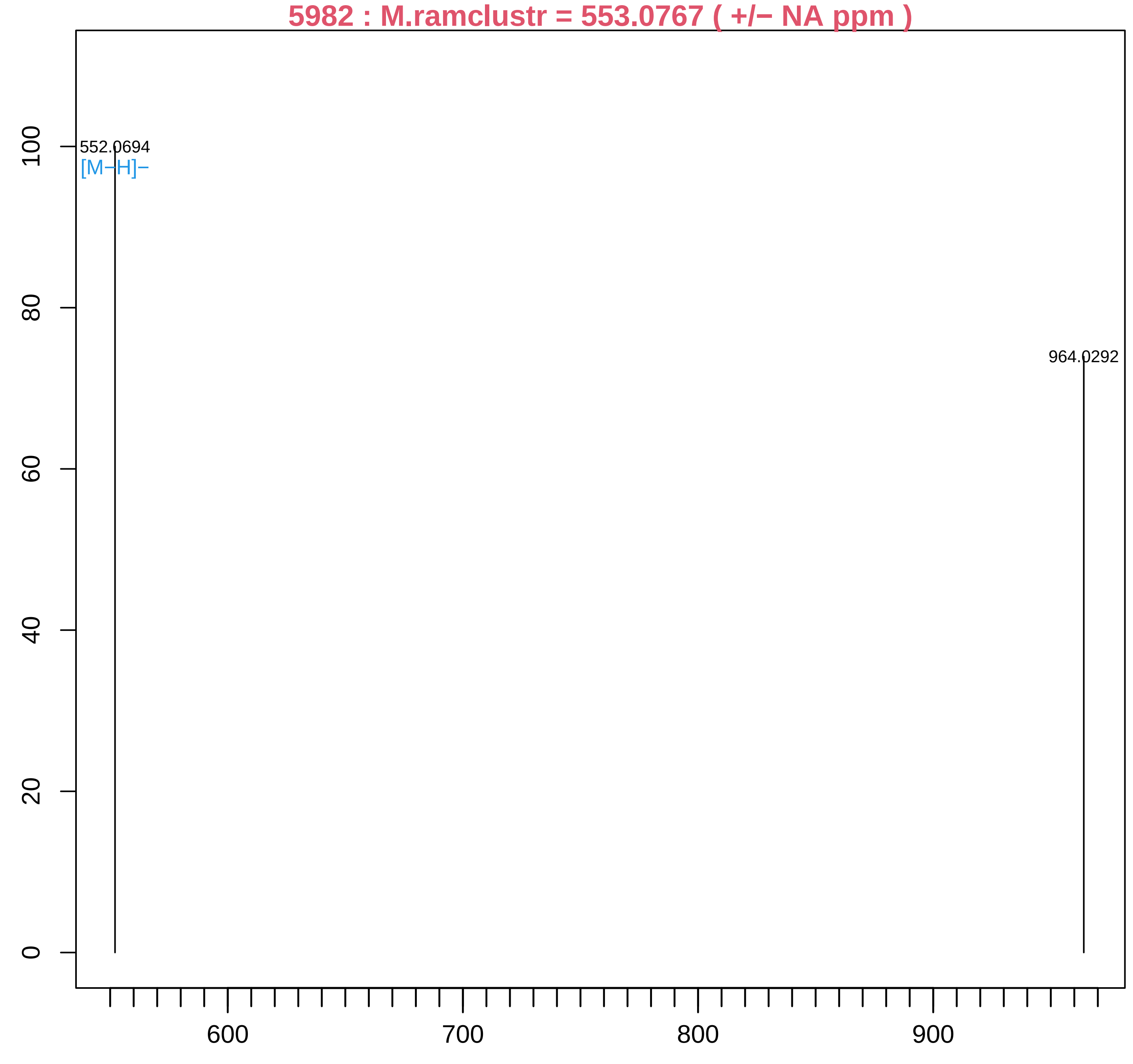

5982 : M.ramclustr = 553.0767 (+/− NA ppm)
552.0694
[M−H]−
964.0292
0
20
40
60
80
100
600
700
800
900

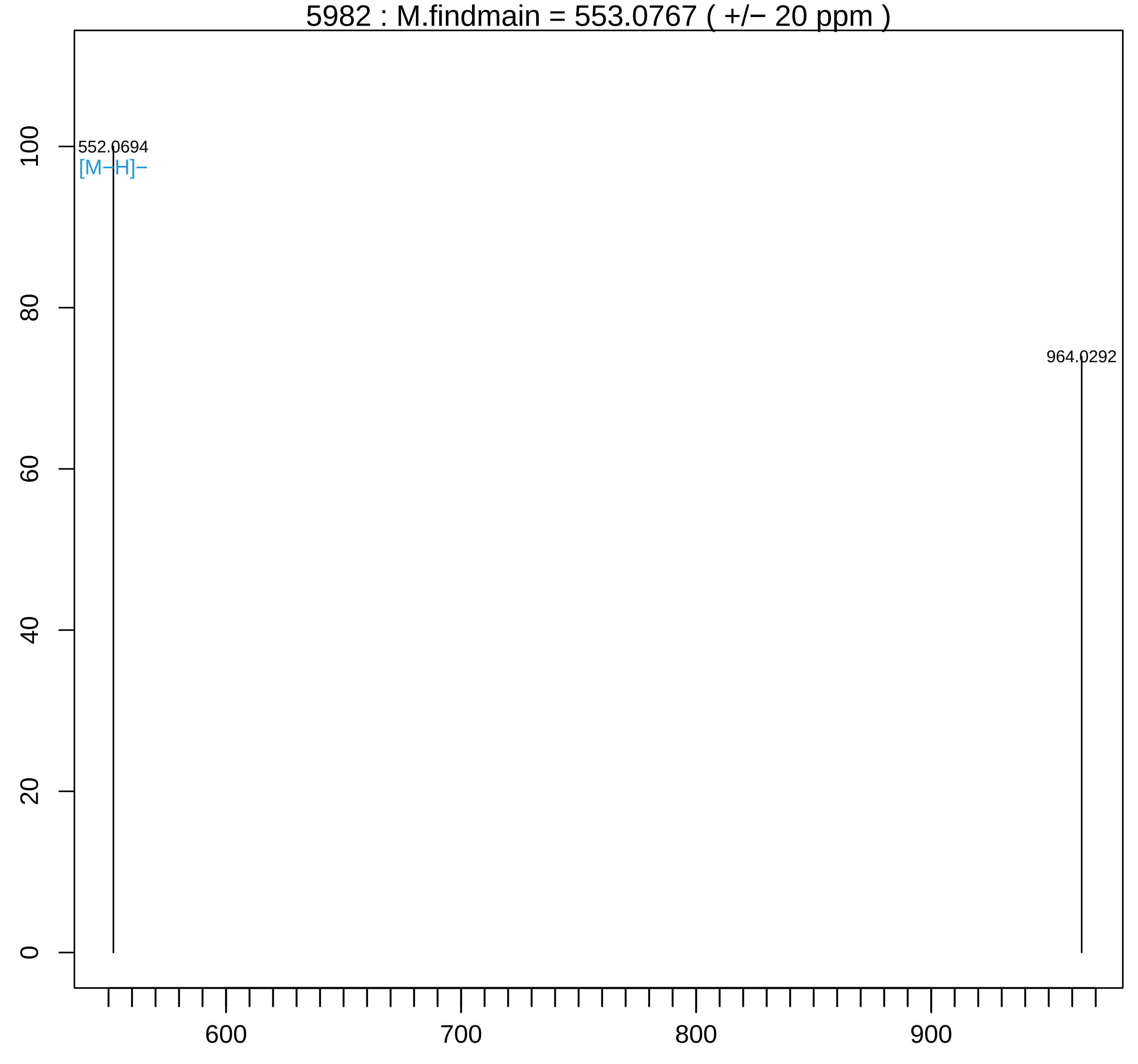

5982 : M.findmain = 553.0767 (+/− 20 ppm)
552.0694
[M−H]−
964.0292
0
20
40
60
80
100
600
700
800
900

5983 : M.ramclustr = 783.9765 (+/− NA ppm)

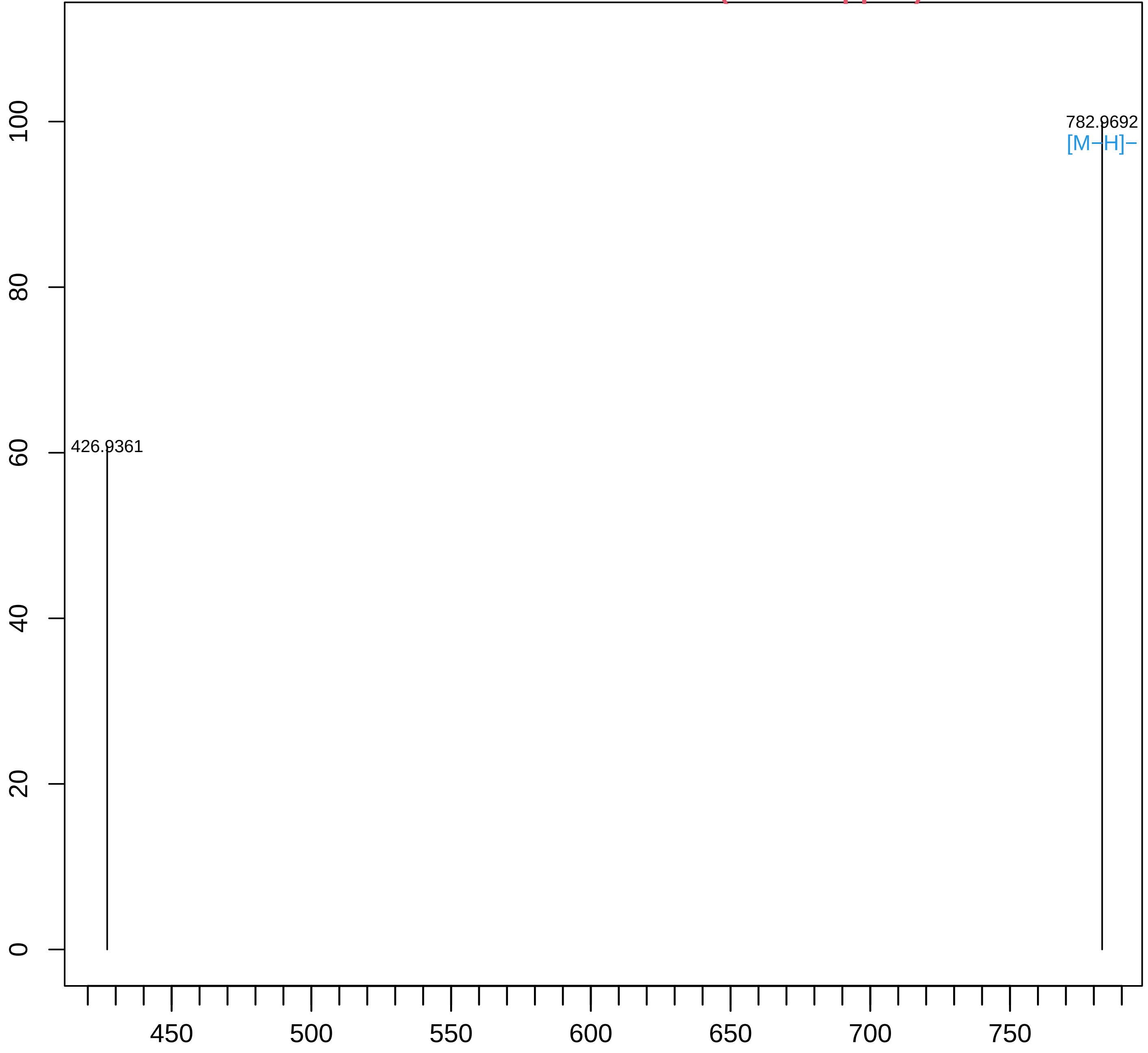

5983 : M.findmain = 783.9765 (+/− 20 ppm)

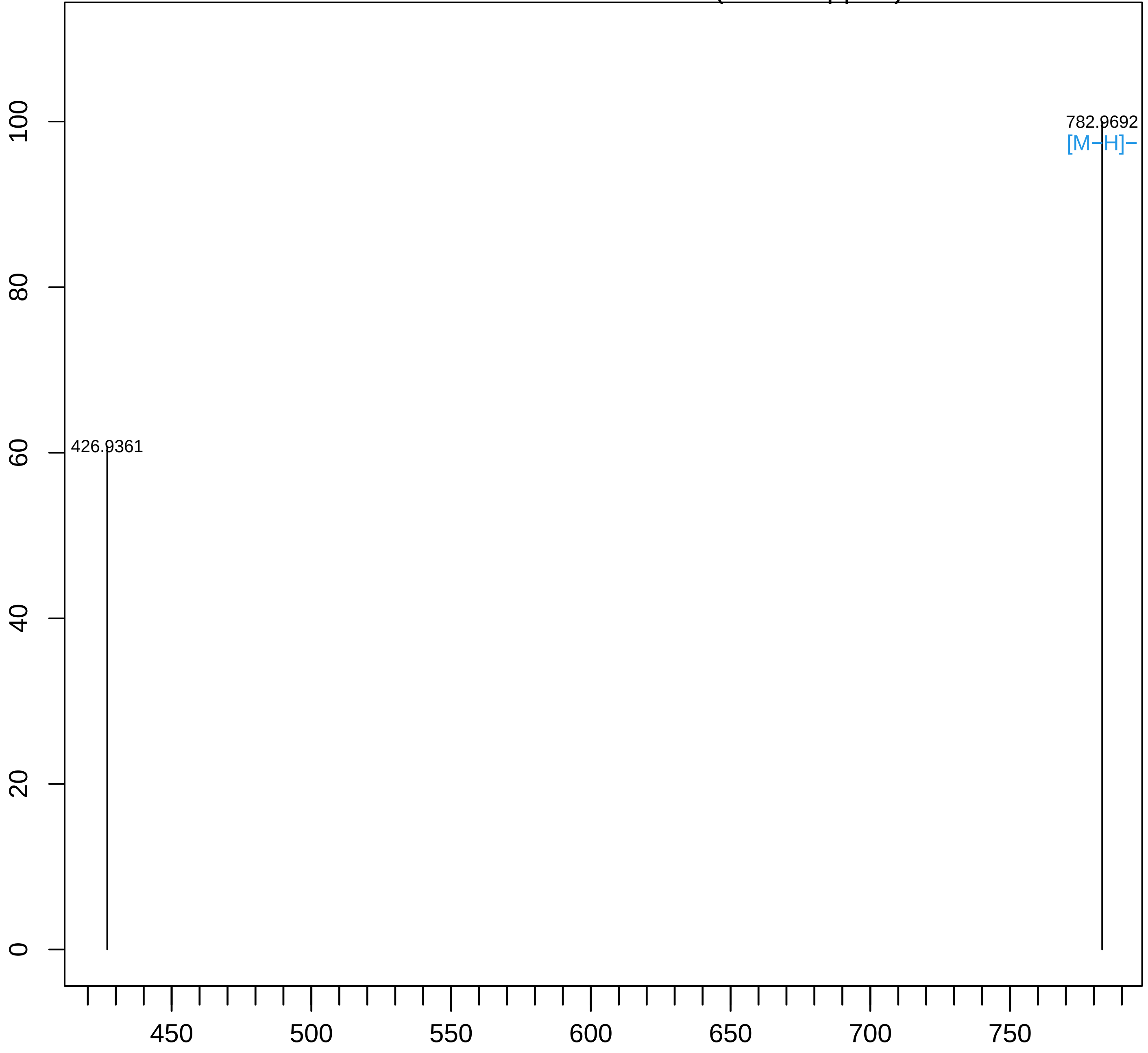

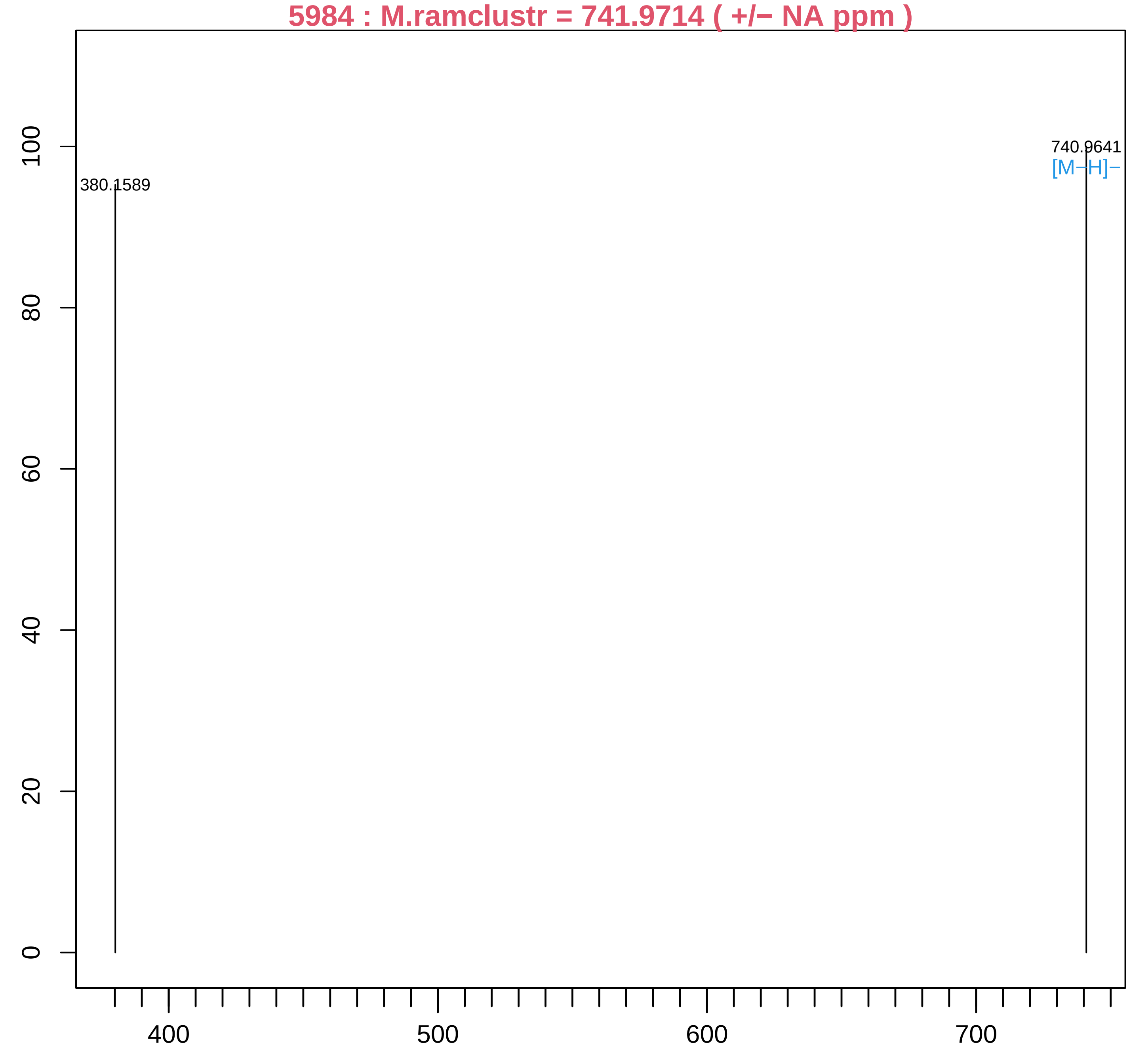

5984 : M.ramclustr = 741.9714 (+/− NA ppm)
740.9641
[M−H]−
380.1589
0
20
40
60
80
100
400
500
600
700

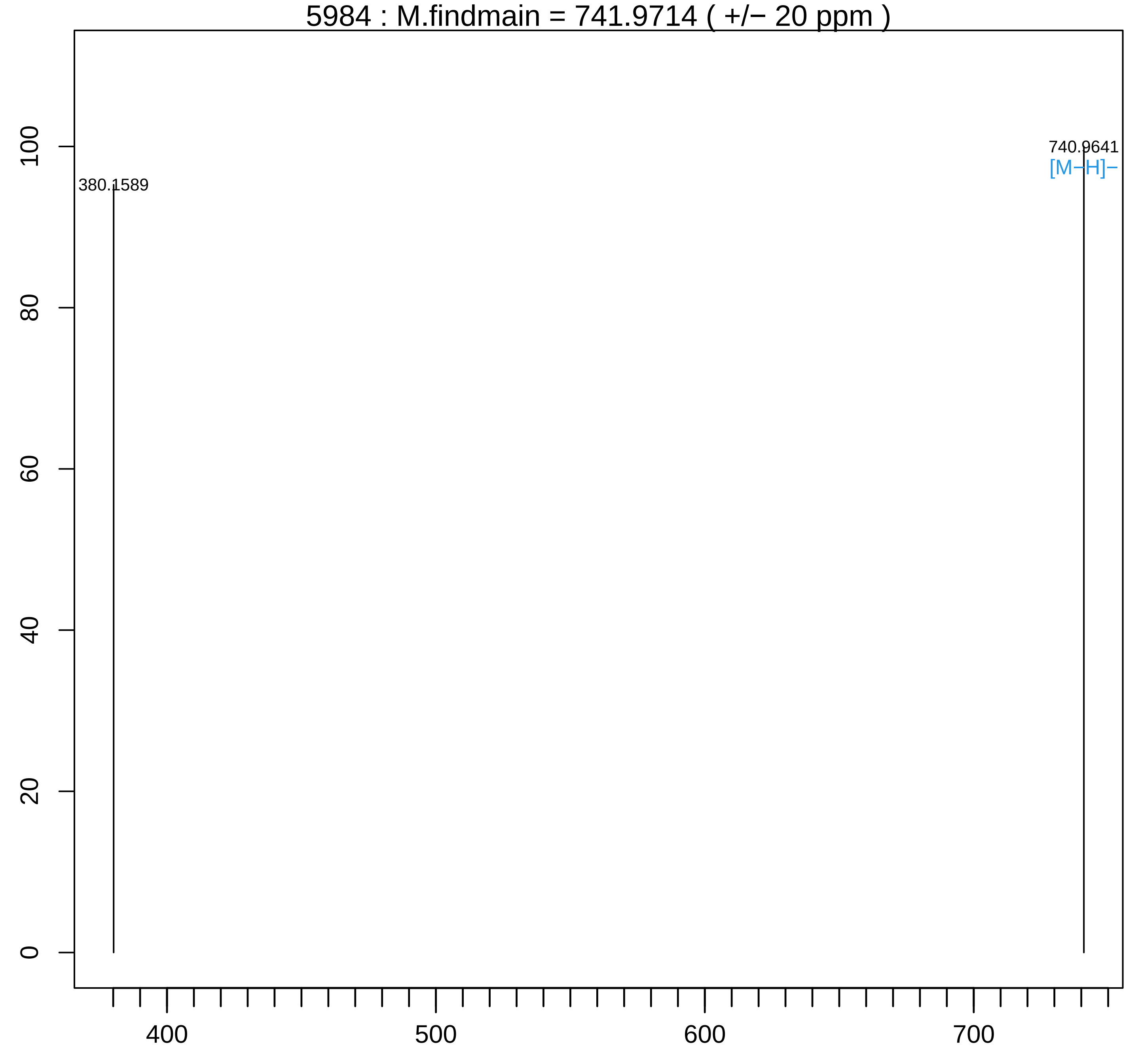

5984 : M.findmain = 741.9714 (+/− 20 ppm)
740.9641
[M−H]−
380.1589
0
20
40
60
80
100
400
500
600
700

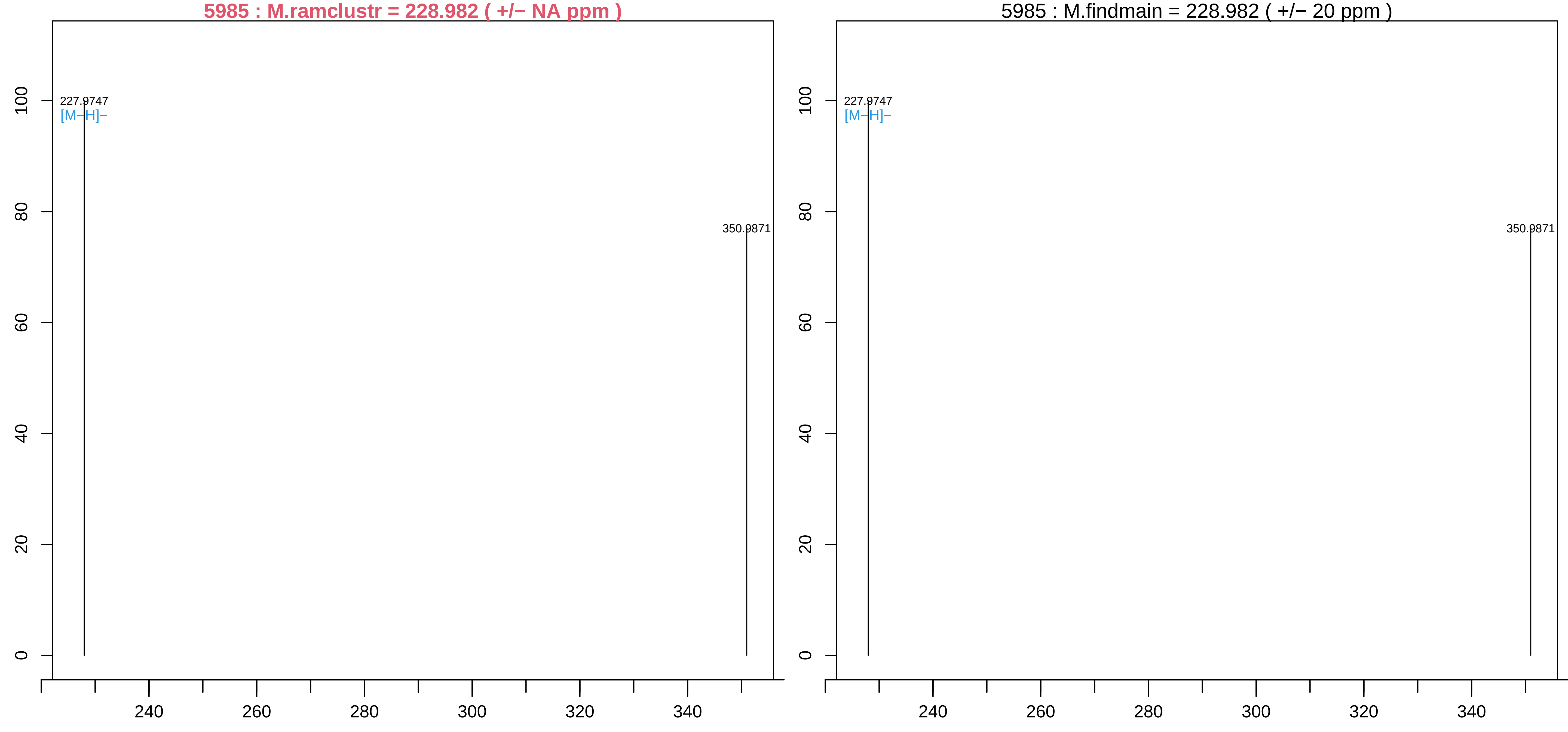
5985 : M.ramclustr = 228.982 (+/− NA ppm)
227.9747
[M−H]−
350.9871
0
20
40
60
80
100
240
260
280
300
320
340
5985 : M.findmain = 228.982 (+/− 20 ppm)
227.9747
[M−H]−
350.9871
0
20
40
60
80
100
240
260
280
300
320
340

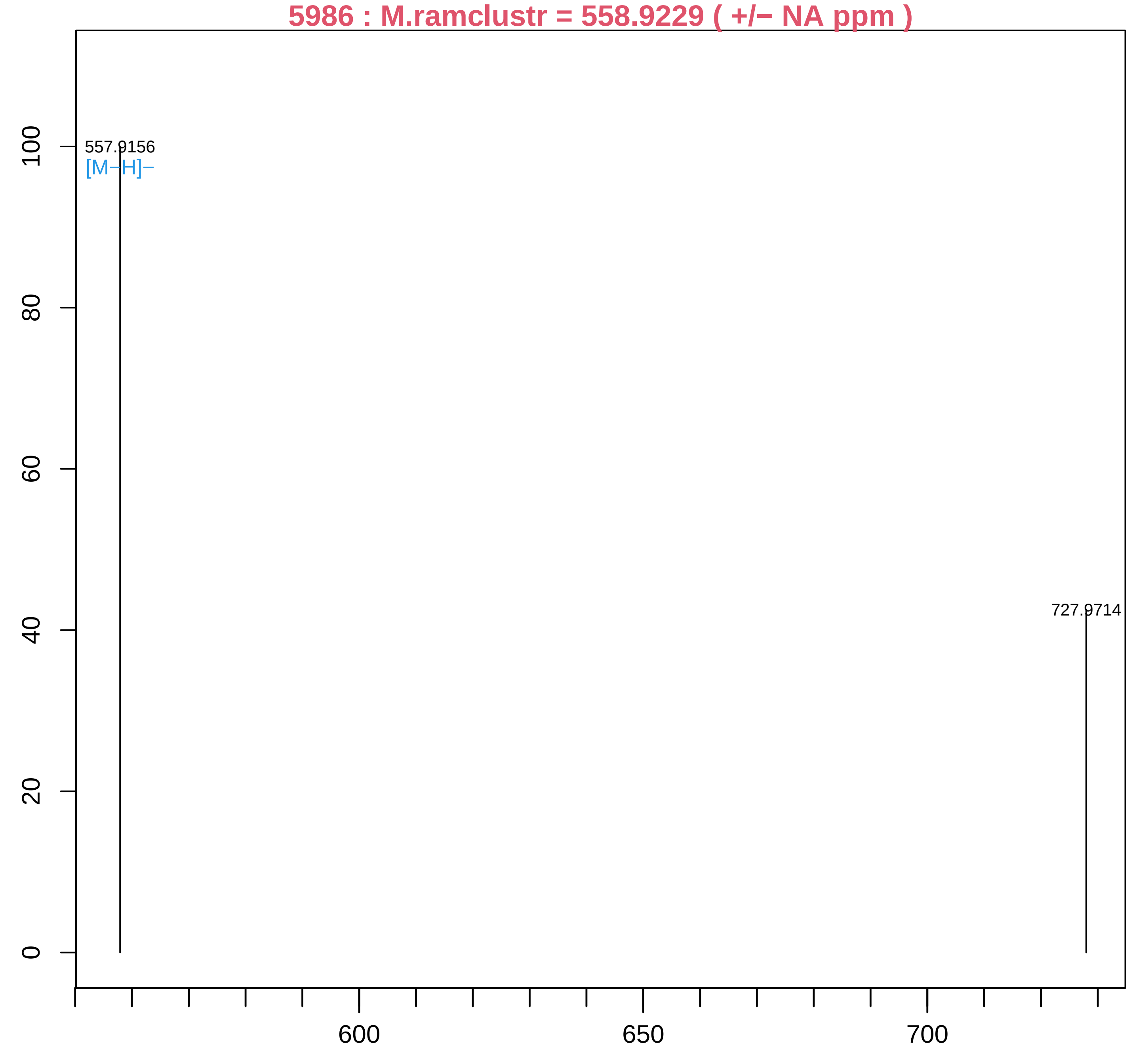

5986 : M.ramclustr = 558.9229 (+/− NA ppm)
557.9156
[M−H]−
727.9714
0
20
40
60
80
100
600
650
700

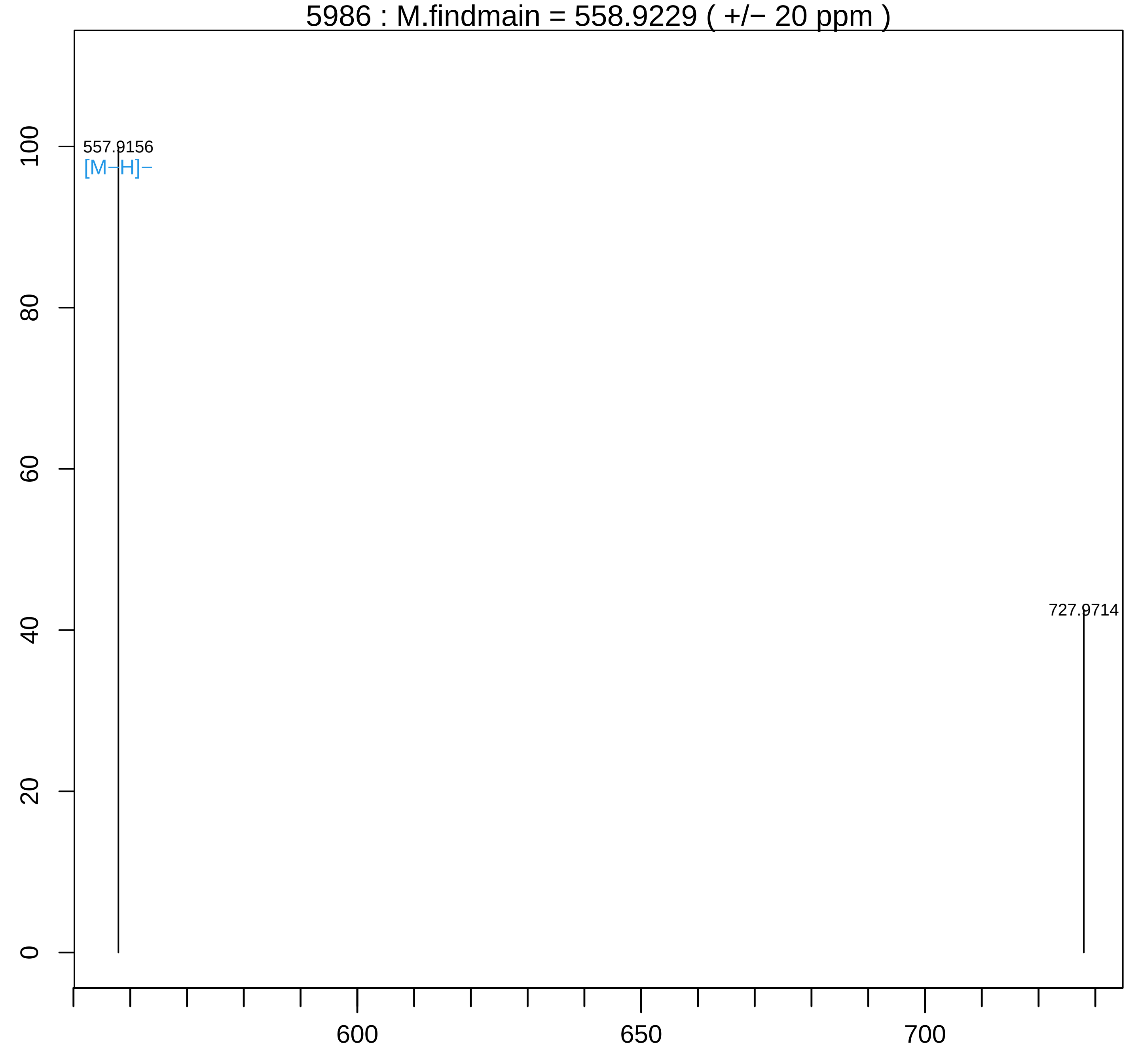

5986 : M.findmain = 558.9229 (+/− 20 ppm)
557.9156
[M−H]−
727.9714
0
20
40
60
80
100
600
650
700

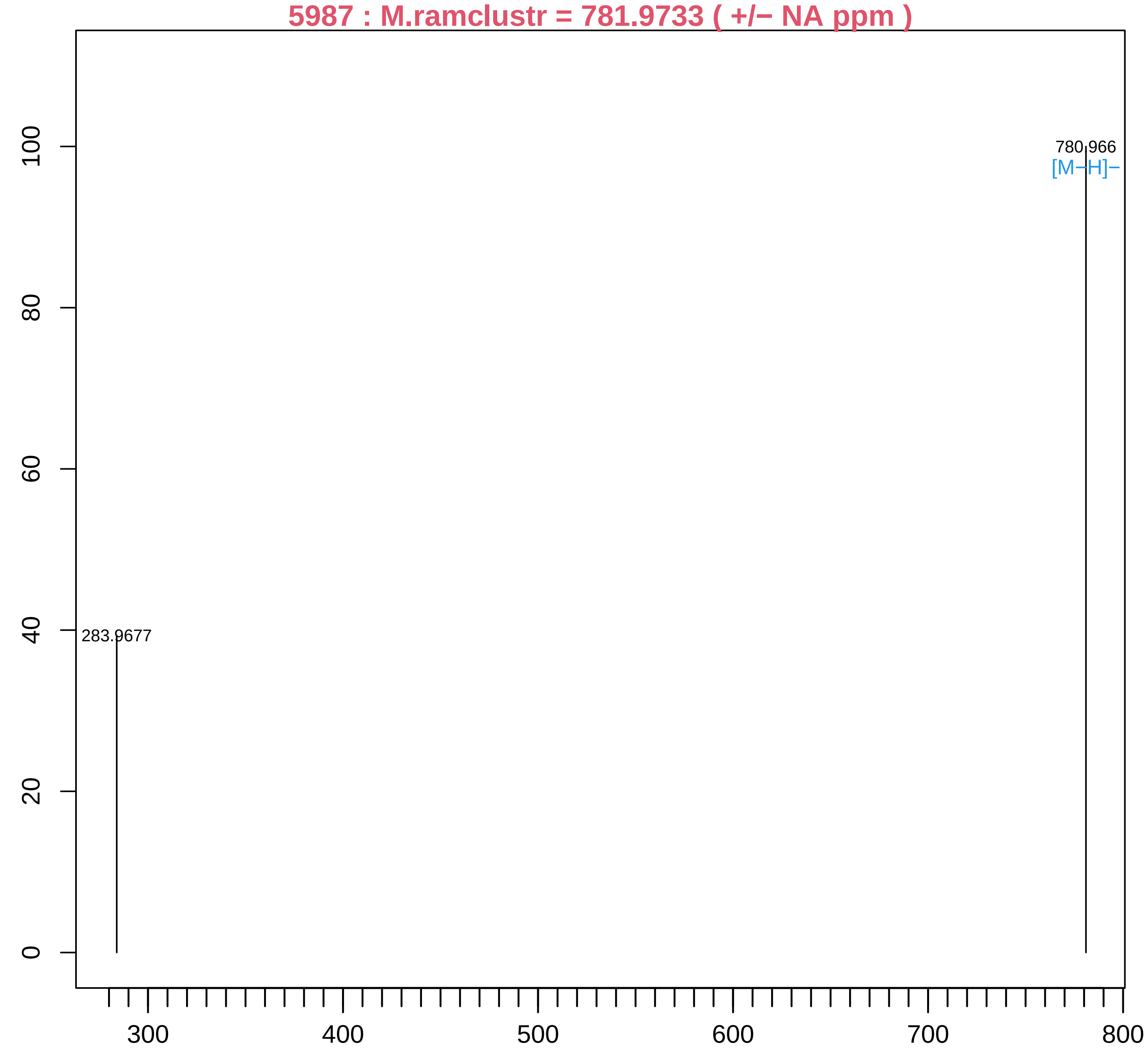

5987 : M.ramclustr = 781.9733 (+/− NA ppm)
780.966
[M−H]−
283.9677
0
20
40
60
80
100
300
400
500
600
700
800

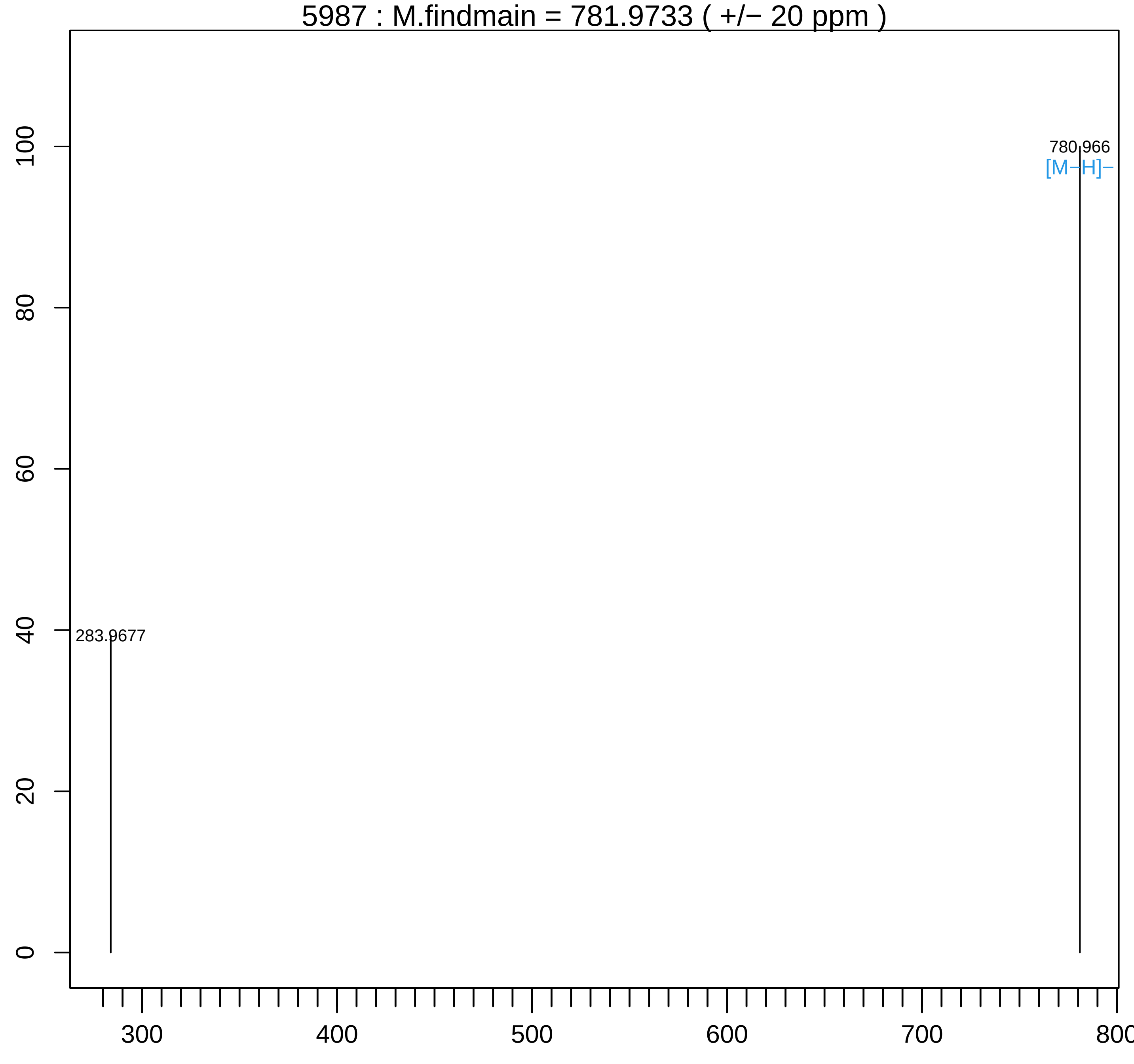

5987 : M.findmain = 781.9733 (+/− 20 ppm)
780.966
[M−H]−
283.9677
0
20
40
60
80
100
300
400
500
600
700
800

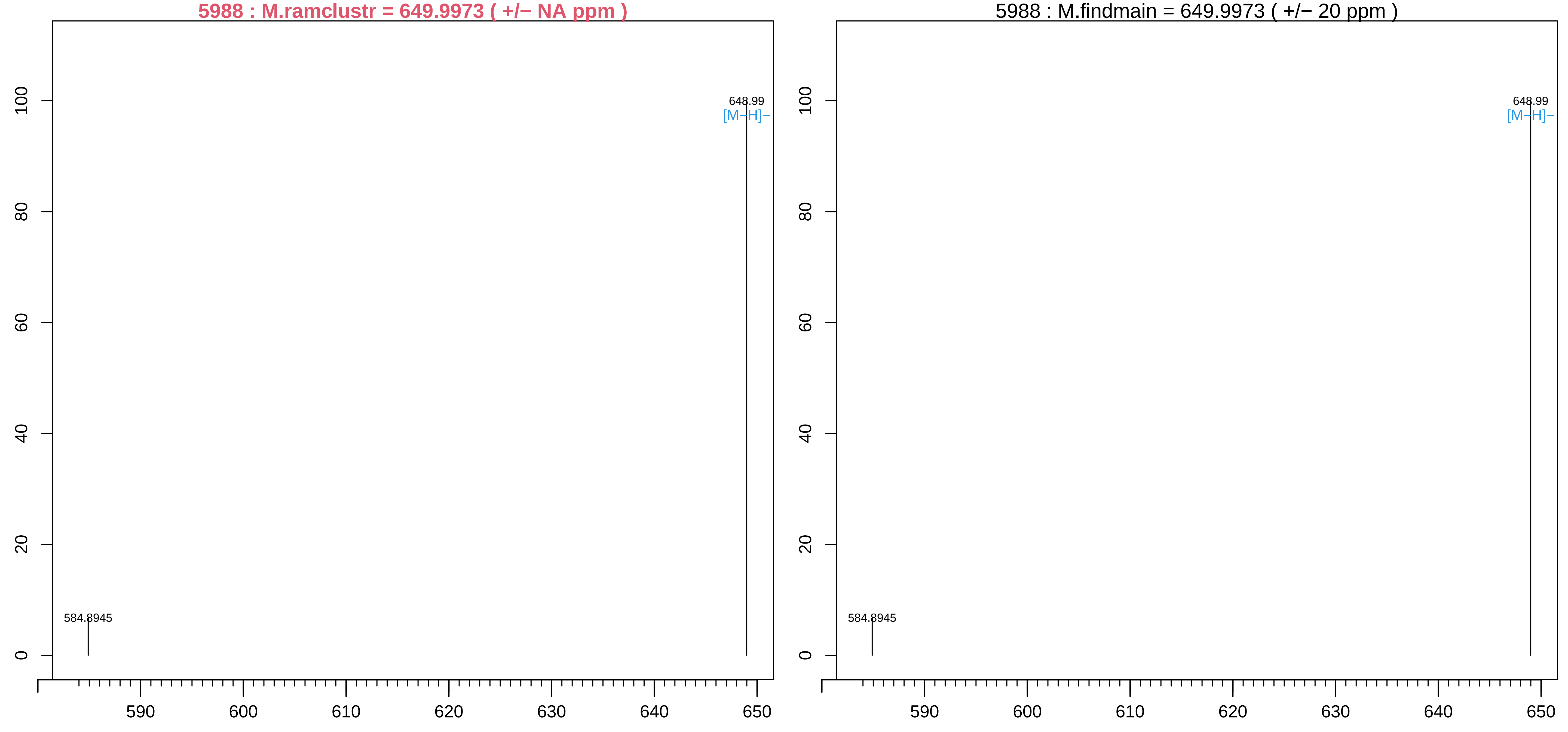
5988 : M.ramclustr = 649.9973 (+/− NA ppm)
5988 : M.findmain = 649.9973 (+/− 20 ppm)
648.99
[M−H]−
584.8945
0
20
40
60
80
100
590
600
610
620
630
640
650

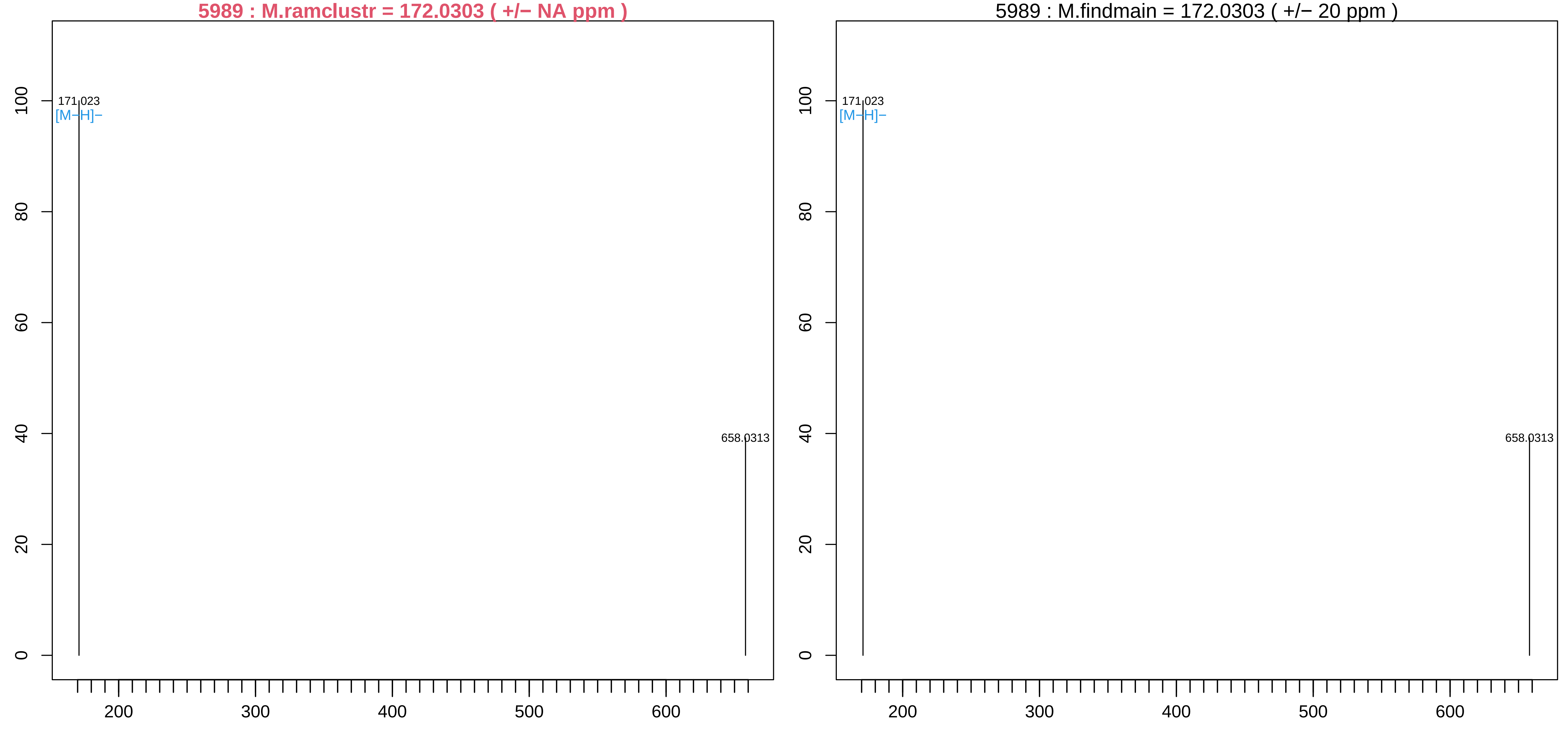
5989 : M.ramclustr = 172.0303 (+/− NA ppm)
171.023
[M−H]−
658.0313
0
20
40
60
80
100
200
300
400
500
600
5989 : M.findmain = 172.0303 (+/− 20 ppm)
171.023
[M−H]−
658.0313
0
20
40
60
80
100
200
300
400
500
600

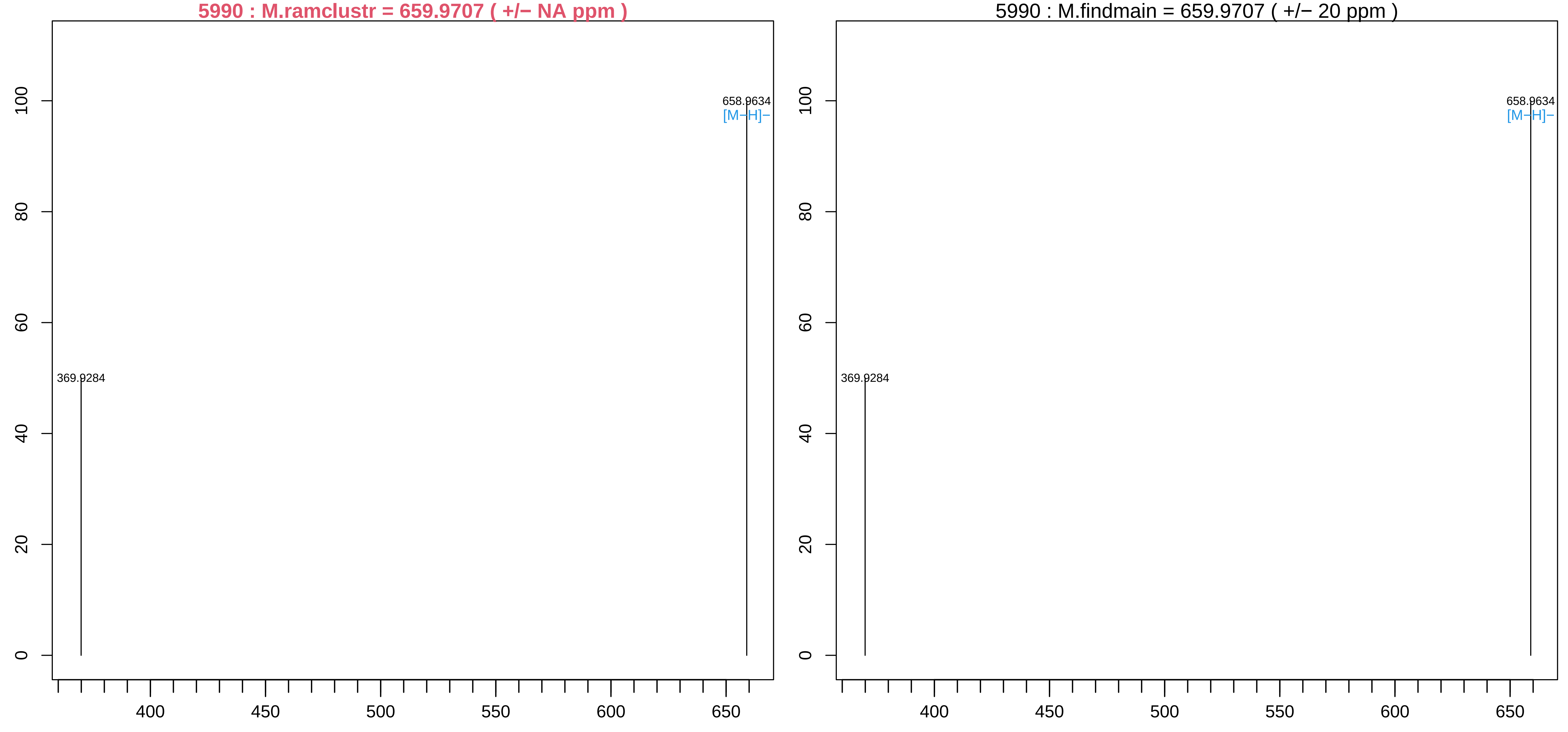
5990 : M.ramclustr = 659.9707 (+/− NA ppm)
658.9634
[M−H]−
369.9284
5990 : M.findmain = 659.9707 (+/− 20 ppm)
658.9634
[M−H]−
369.9284
0
20
40
60
80
100
400
450
500
550
600
650

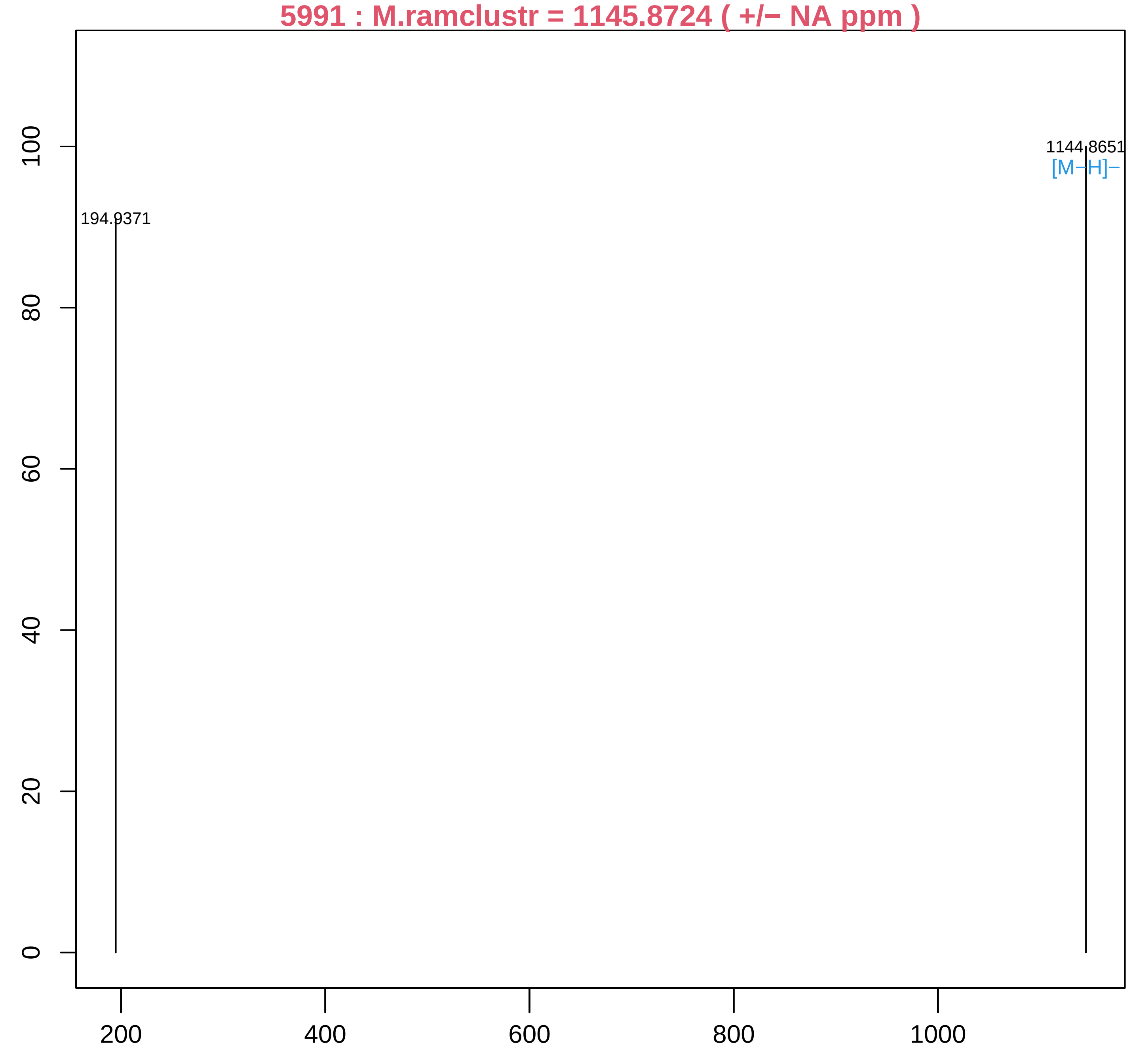
5991 : M.ramclustr = 1145.8724 (+/− NA ppm)
194.9371
1144.8651
[M−H]−
0
20
40
60
80
100
200
400
600
800
1000

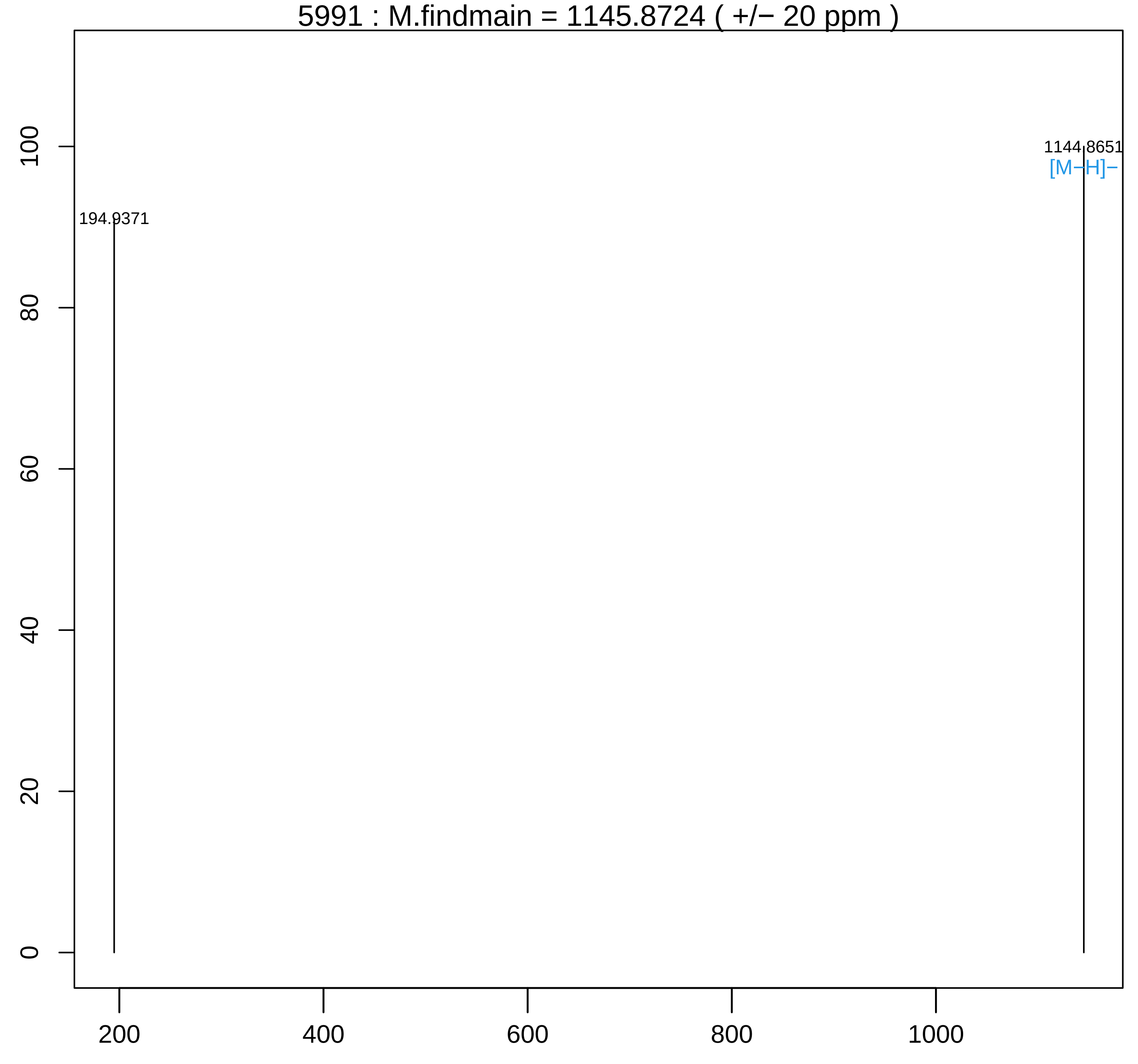
5991 : M.findmain = 1145.8724 (+/− 20 ppm)
194.9371
1144.8651
[M−H]−
0
20
40
60
80
100
200
400
600
800
1000

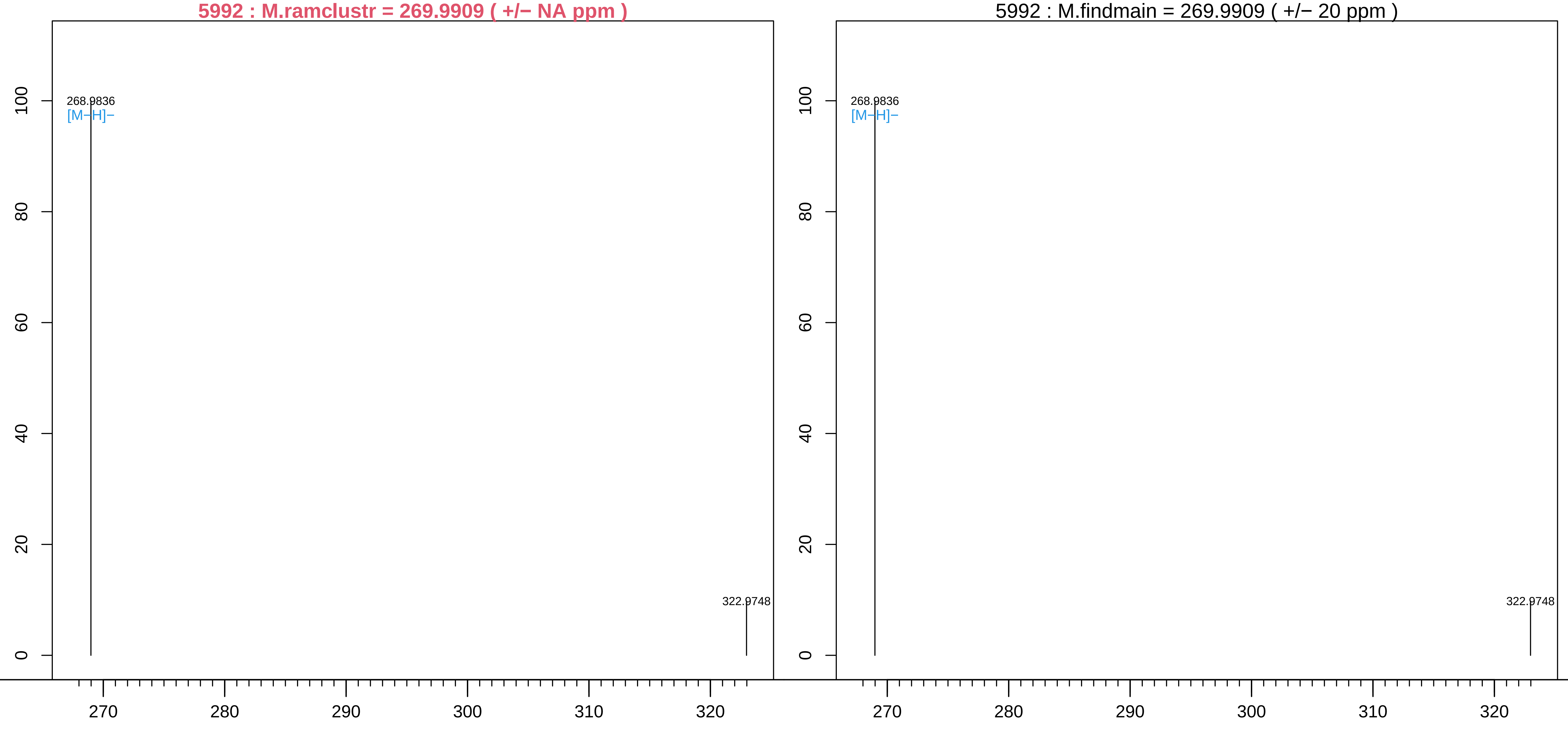
5992 : M.ramclustr = 269.9909 (+/− NA ppm)
268.9836
[M−H]−
322.9748
5992 : M.findmain = 269.9909 (+/− 20 ppm)
268.9836
[M−H]−
322.9748

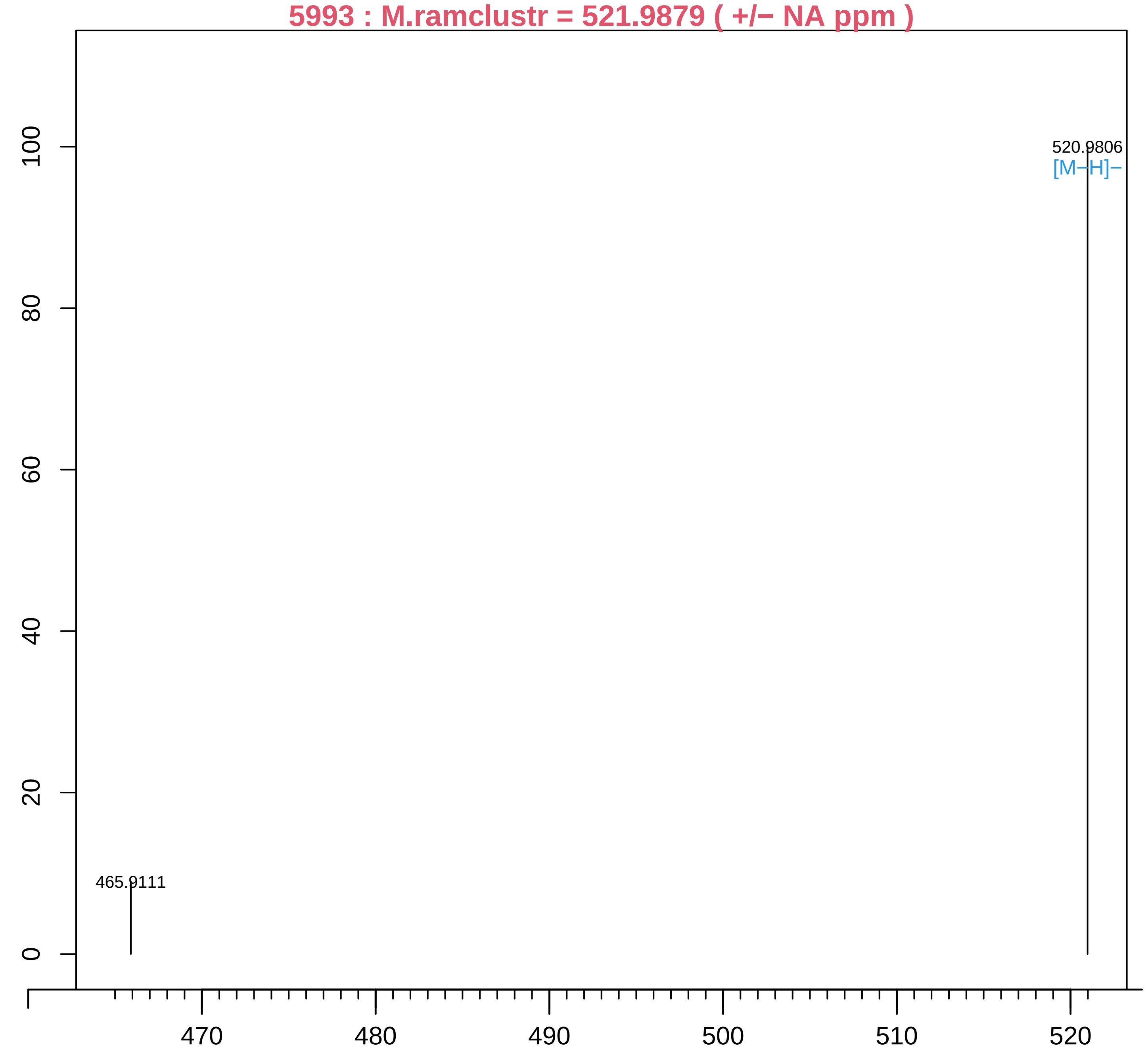
5993 : M.ramclustr = 521.9879 (+/− NA ppm)
520.9806
[M−H]−
465.9111
0
20
40
60
80
100
470
480
490
500
510
520

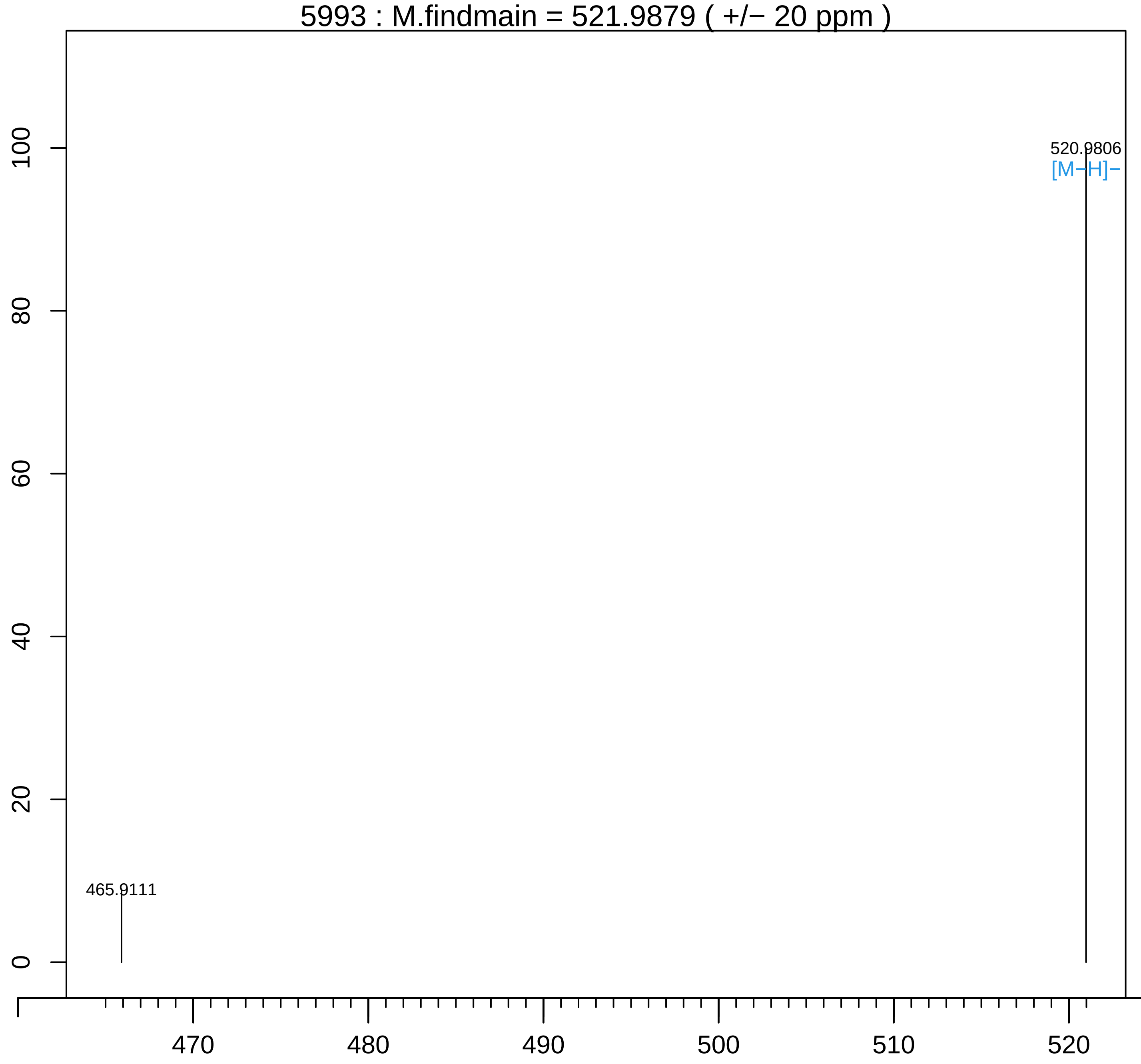
5993 : M.findmain = 521.9879 (+/− 20 ppm)
520.9806
[M−H]−
465.9111
0
20
40
60
80
100
470
480
490
500
510
520

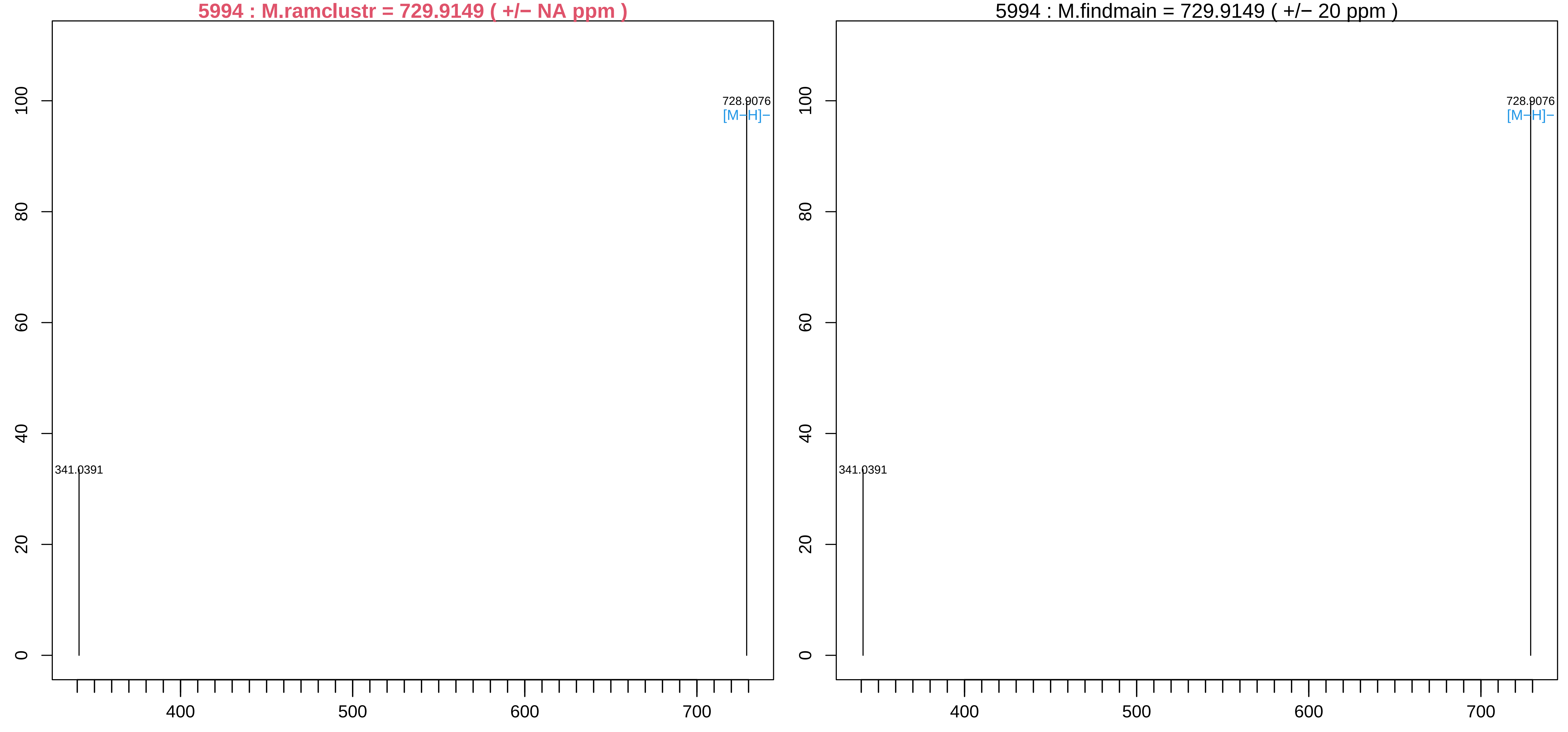
5994 : M.ramclustr = 729.9149 (+/− NA ppm)
728.9076
[M−H]−
341.0391
5994 : M.findmain = 729.9149 (+/− 20 ppm)
728.9076
[M−H]−
341.0391
0
20
40
60
80
100
400
500
600
700

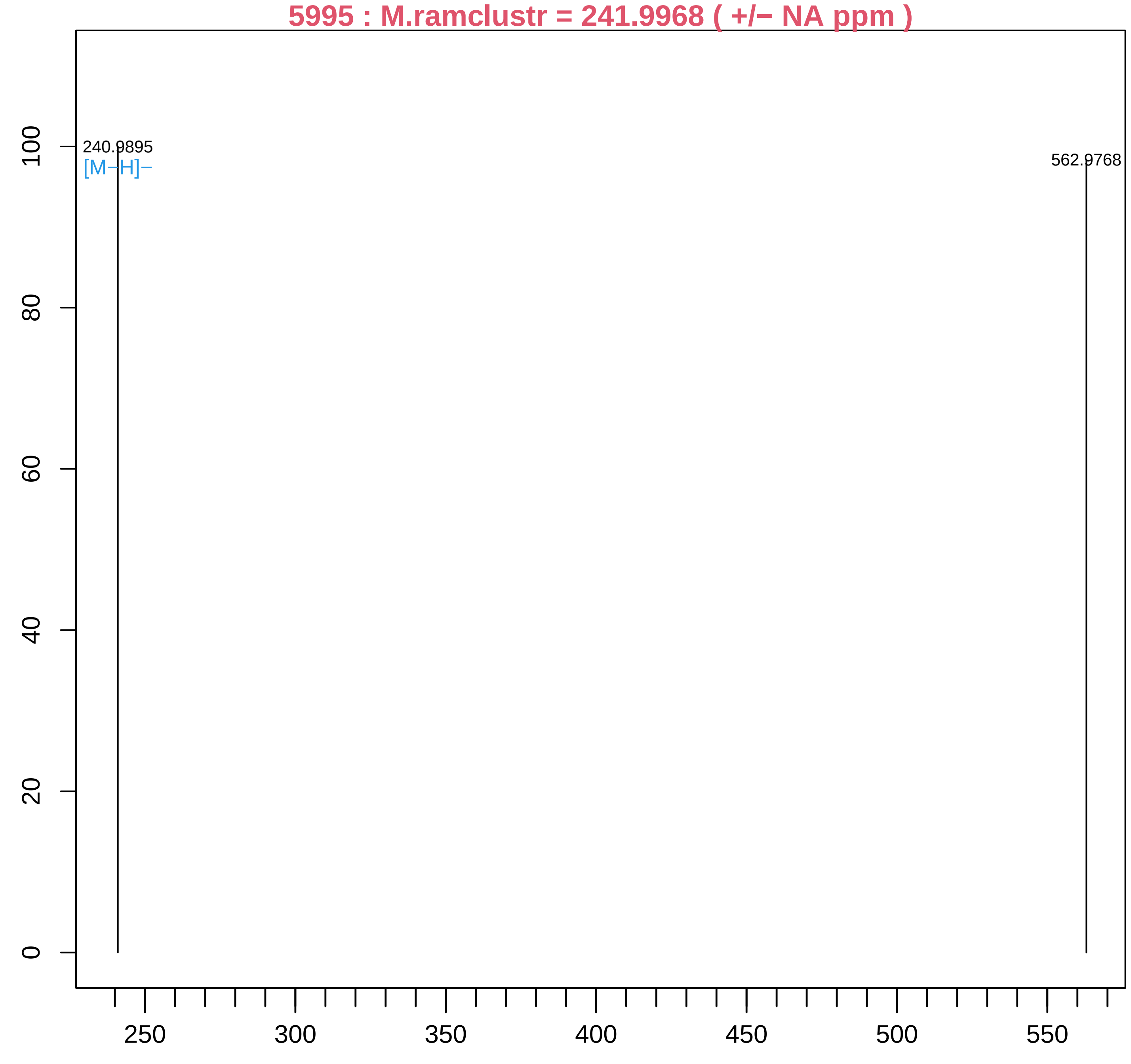
5995 : M.ramclustr = 241.9968 (+/− NA ppm)
240.9895
[M−H]−
562.9768
0
20
40
60
80
100
250
300
350
400
450
500
550

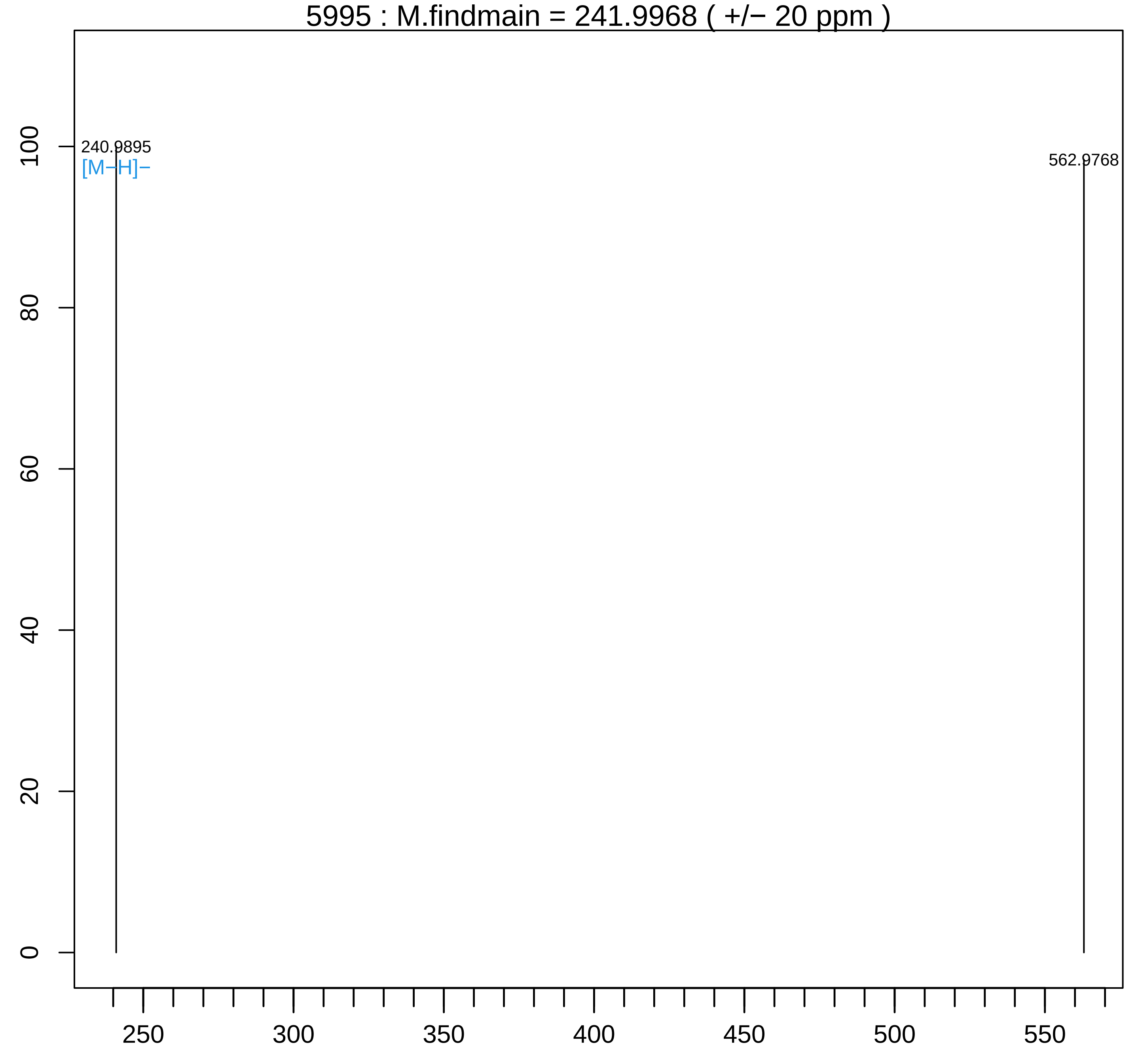
5995 : M.findmain = 241.9968 (+/− 20 ppm)
240.9895
[M−H]−
562.9768
0
20
40
60
80
100
250
300
350
400
450
500
550

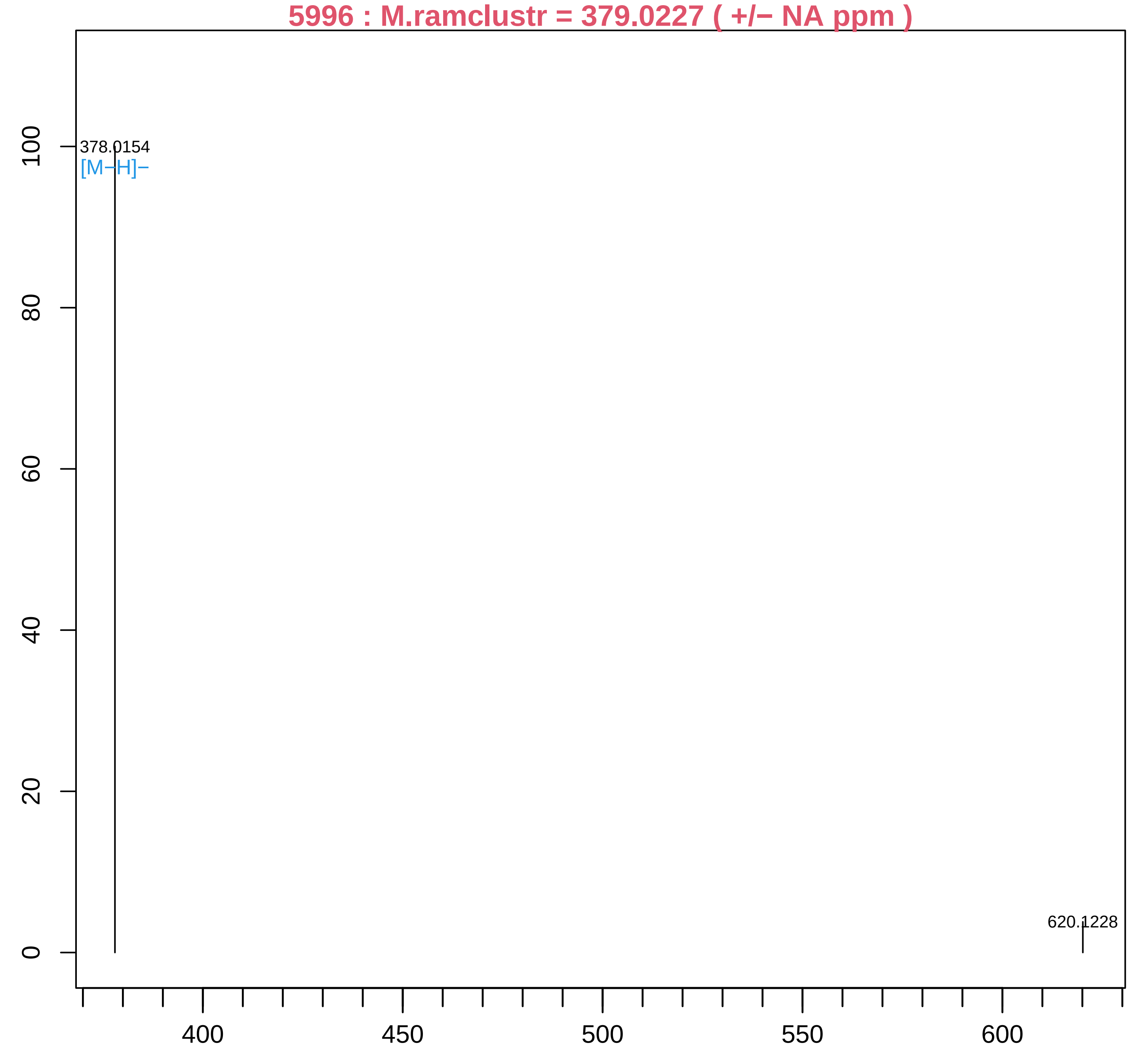

5996 : M.ramclustr = 379.0227 (+/− NA ppm)
378.0154
[M−H]−
620.1228
0
20
40
60
80
100
400
450
500
550
600

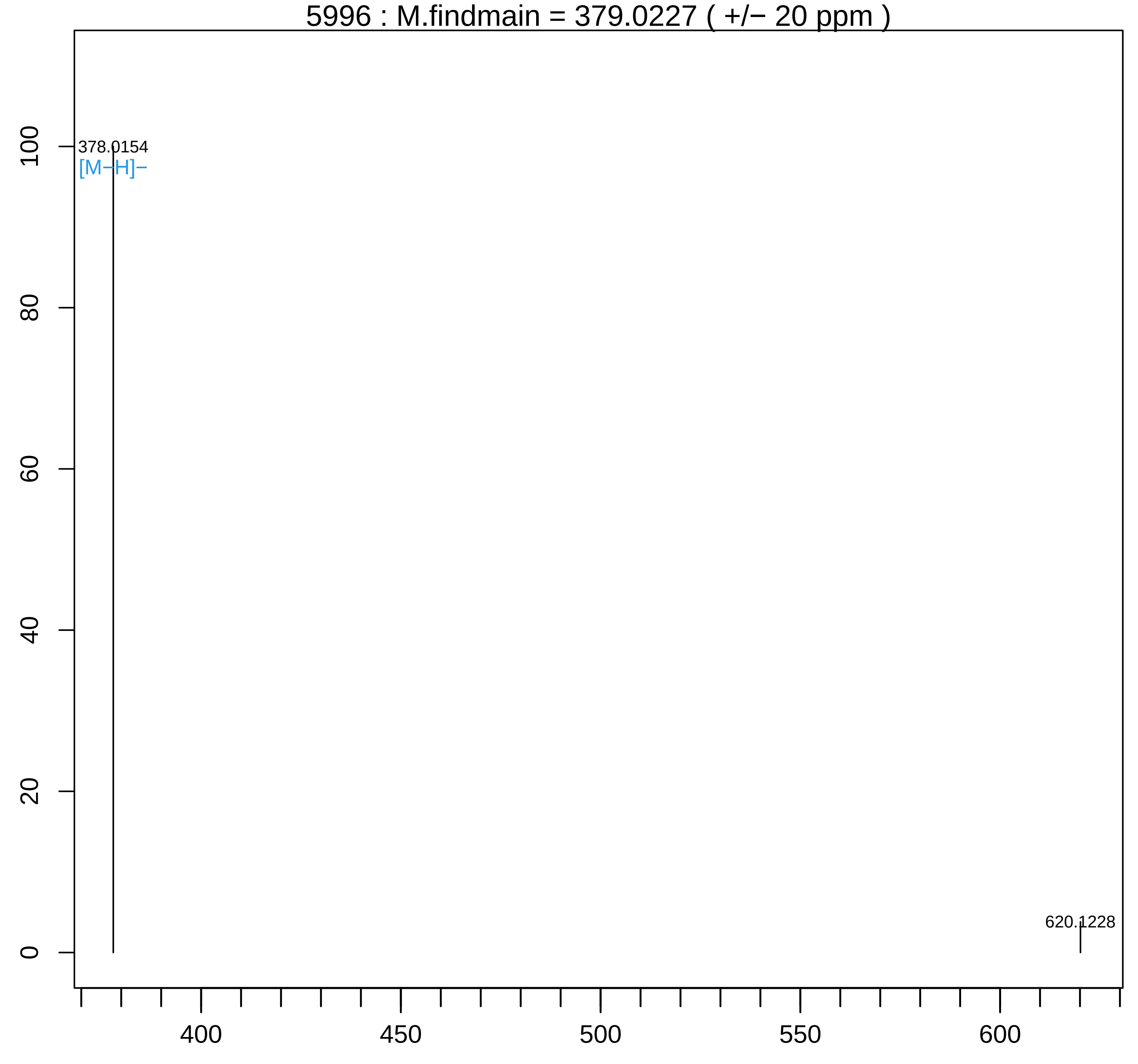

5996 : M.findmain = 379.0227 (+/− 20 ppm)
378.0154
[M−H]−
620.1228
0
20
40
60
80
100
400
450
500
550
600

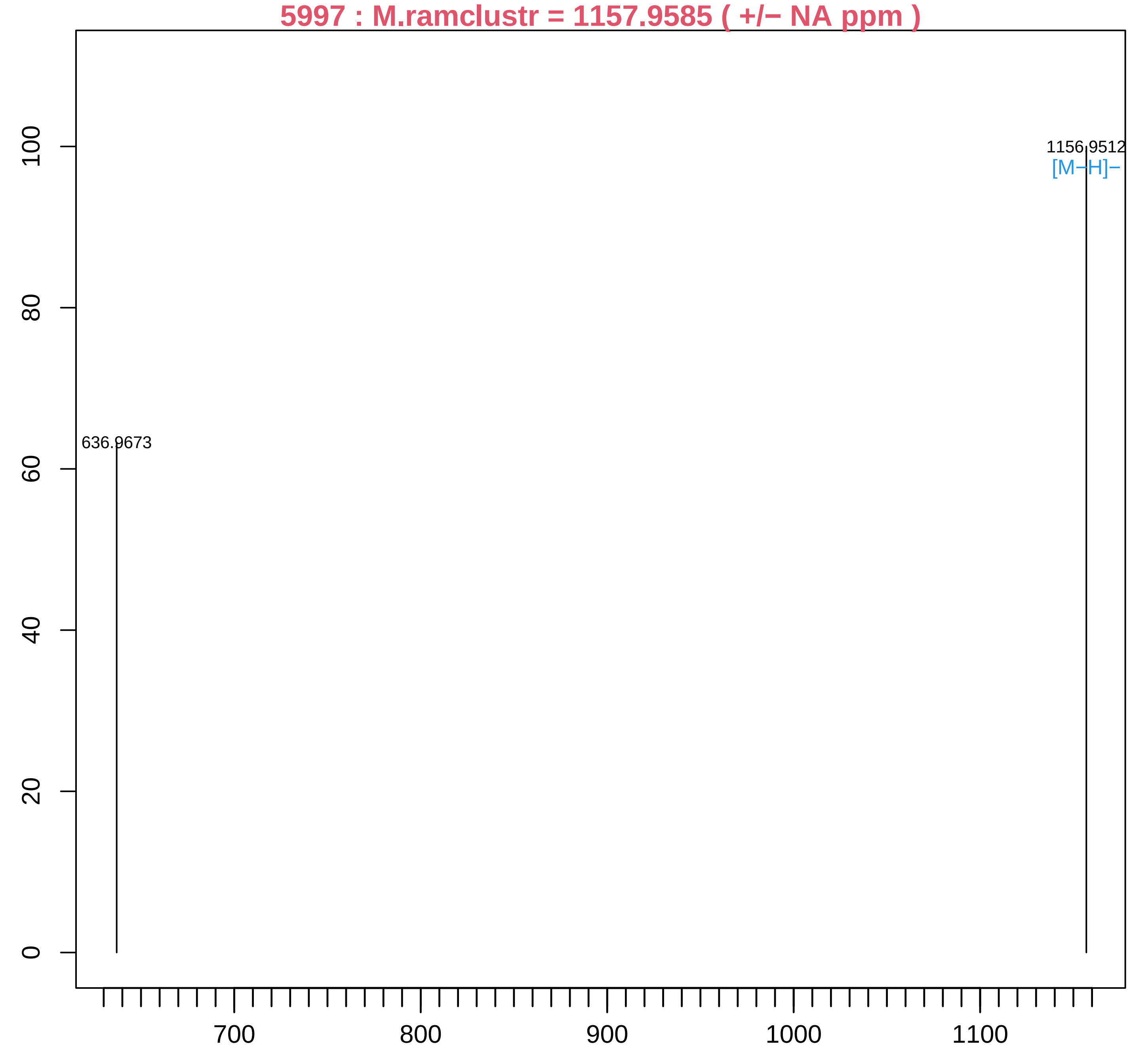
5997 : M.ramclustr = 1157.9585 (+/− NA ppm)
1156.9512
[M−H]−
636.9673
0
20
40
60
80
100
700
800
900
1000
1100

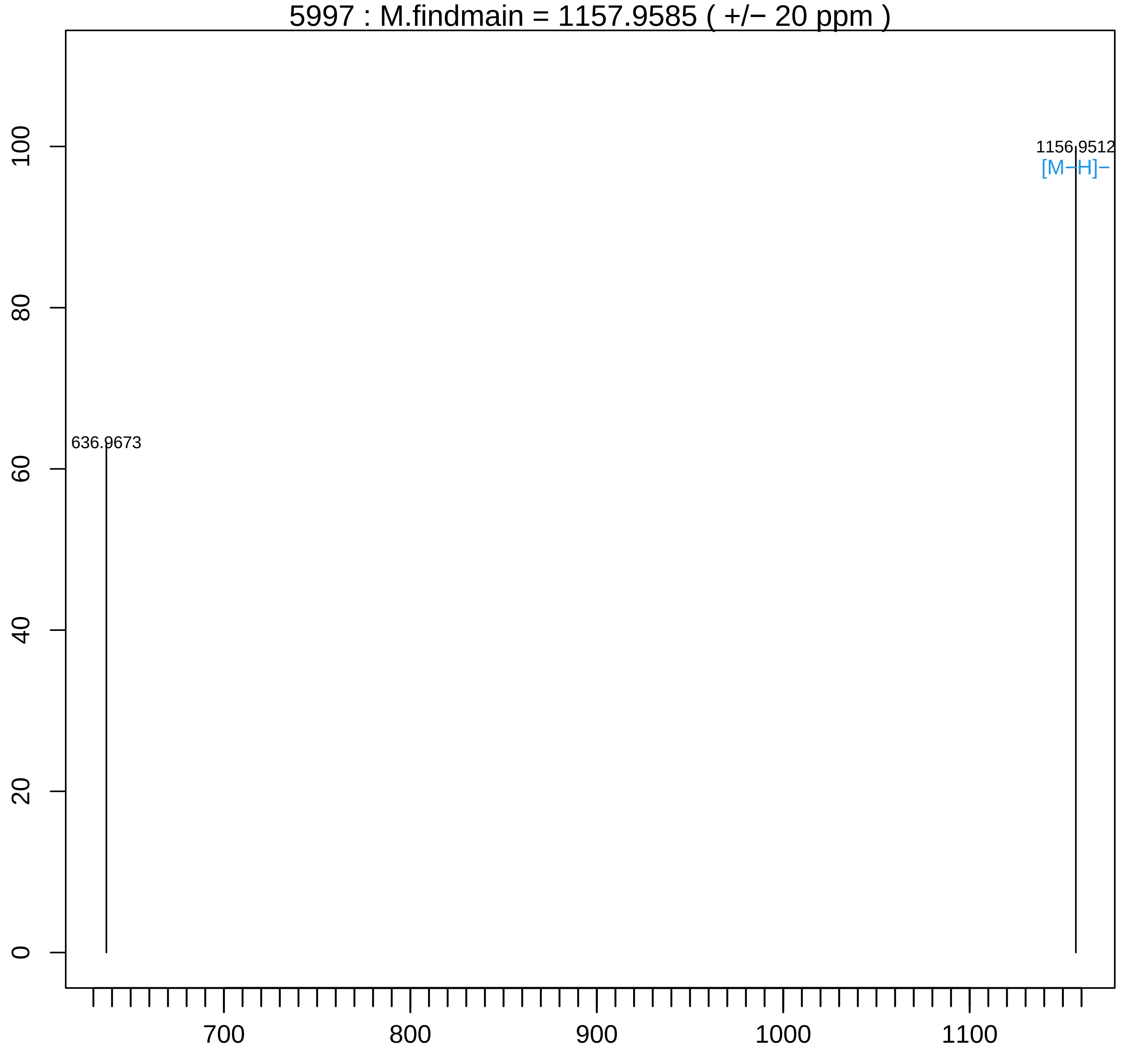
5997 : M.findmain = 1157.9585 (+/− 20 ppm)
1156.9512
[M−H]−
636.9673
0
20
40
60
80
100
700
800
900
1000
1100

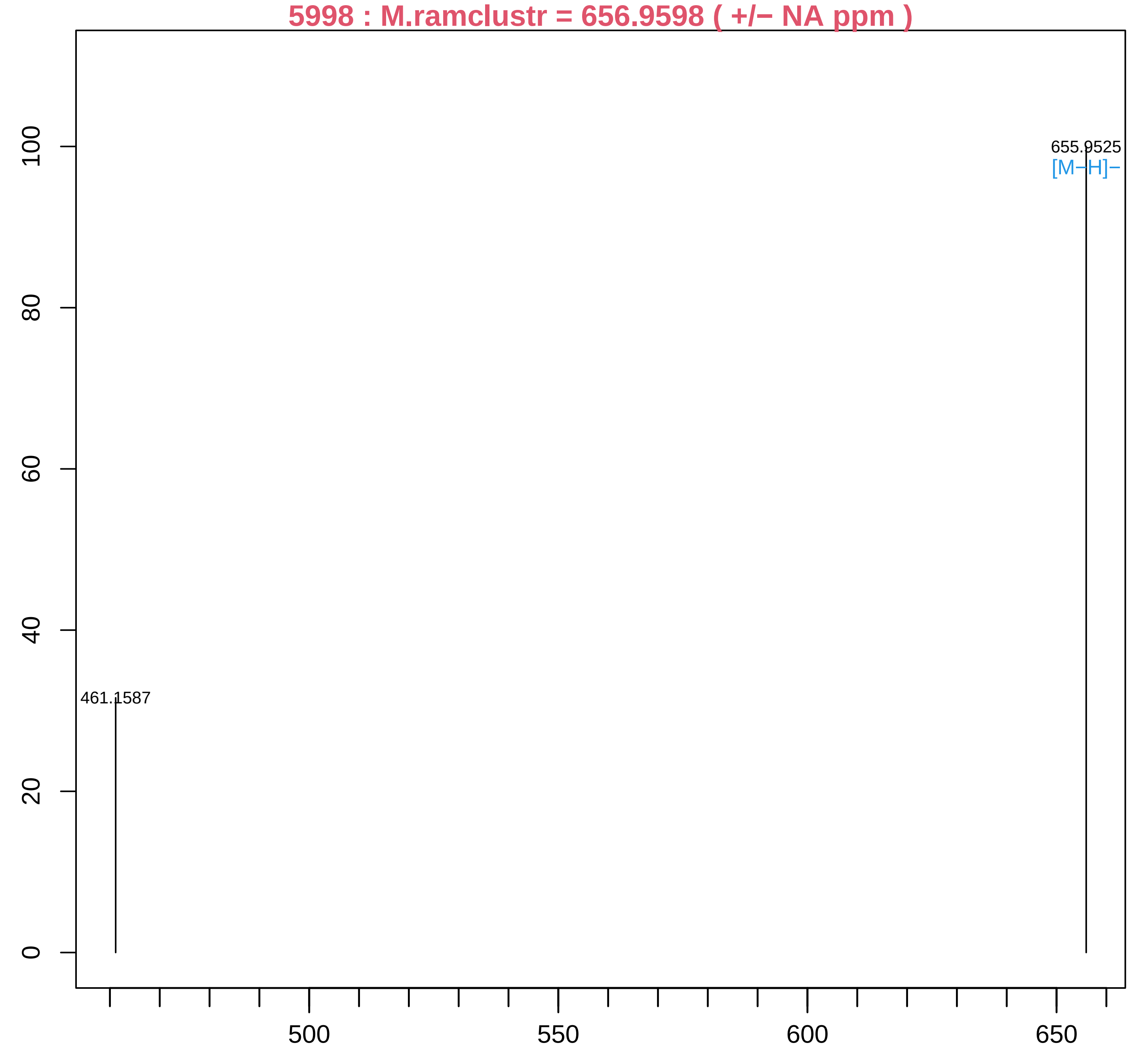
5998 : M.ramclustr = 656.9598 (+/− NA ppm)
655.9525
[M−H]−
461.1587
0
20
40
60
80
100
500
550
600
650

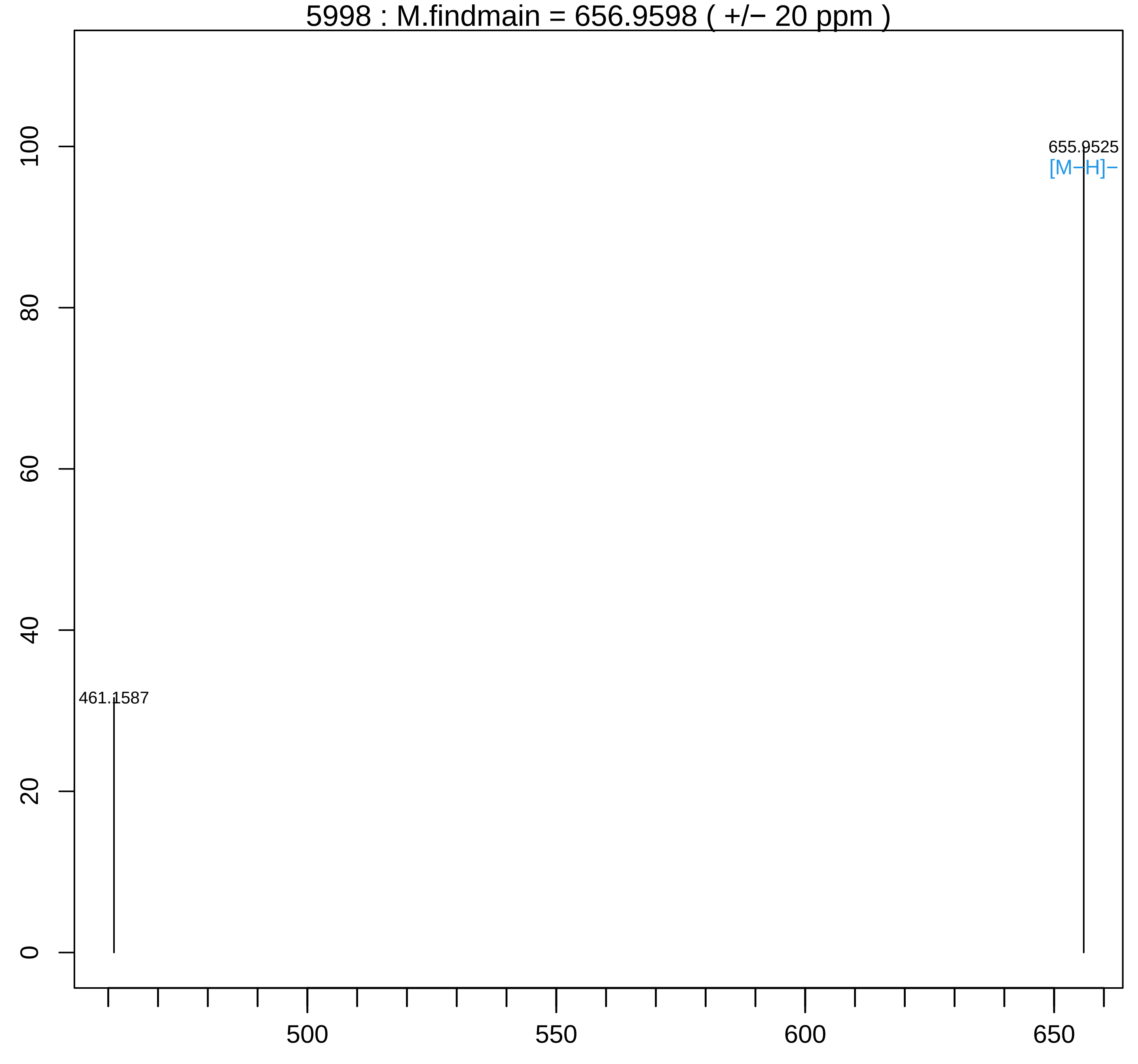
5998 : M.findmain = 656.9598 (+/− 20 ppm)
655.9525
[M−H]−
461.1587
0
20
40
60
80
100
500
550
600
650

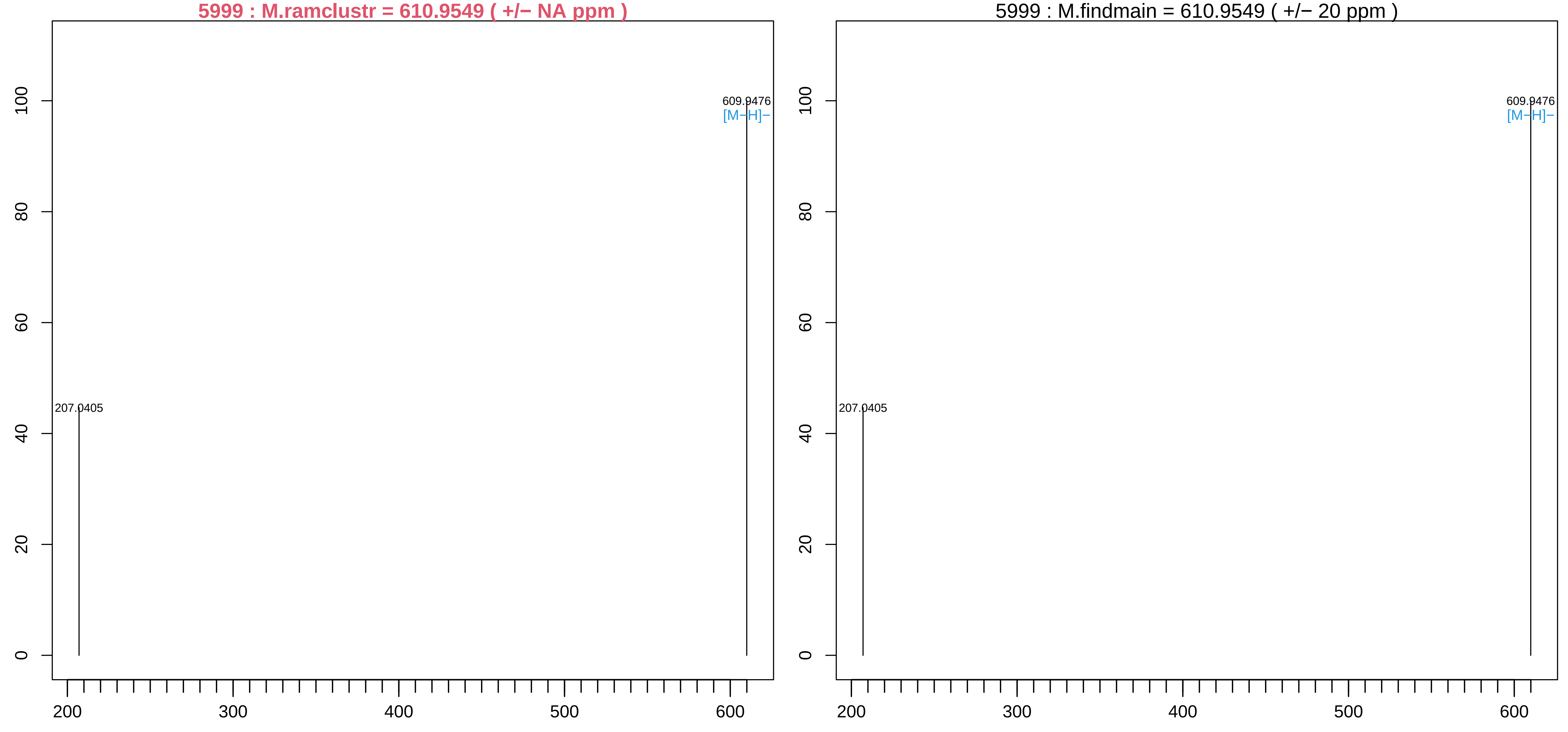
5999 : M.ramclustr = 610.9549 (+/− NA ppm)
609.9476
[M−H]−
207.0405
5999 : M.findmain = 610.9549 (+/− 20 ppm)
609.9476
[M−H]−
207.0405
0
20
40
60
80
100
200
300
400
500
600

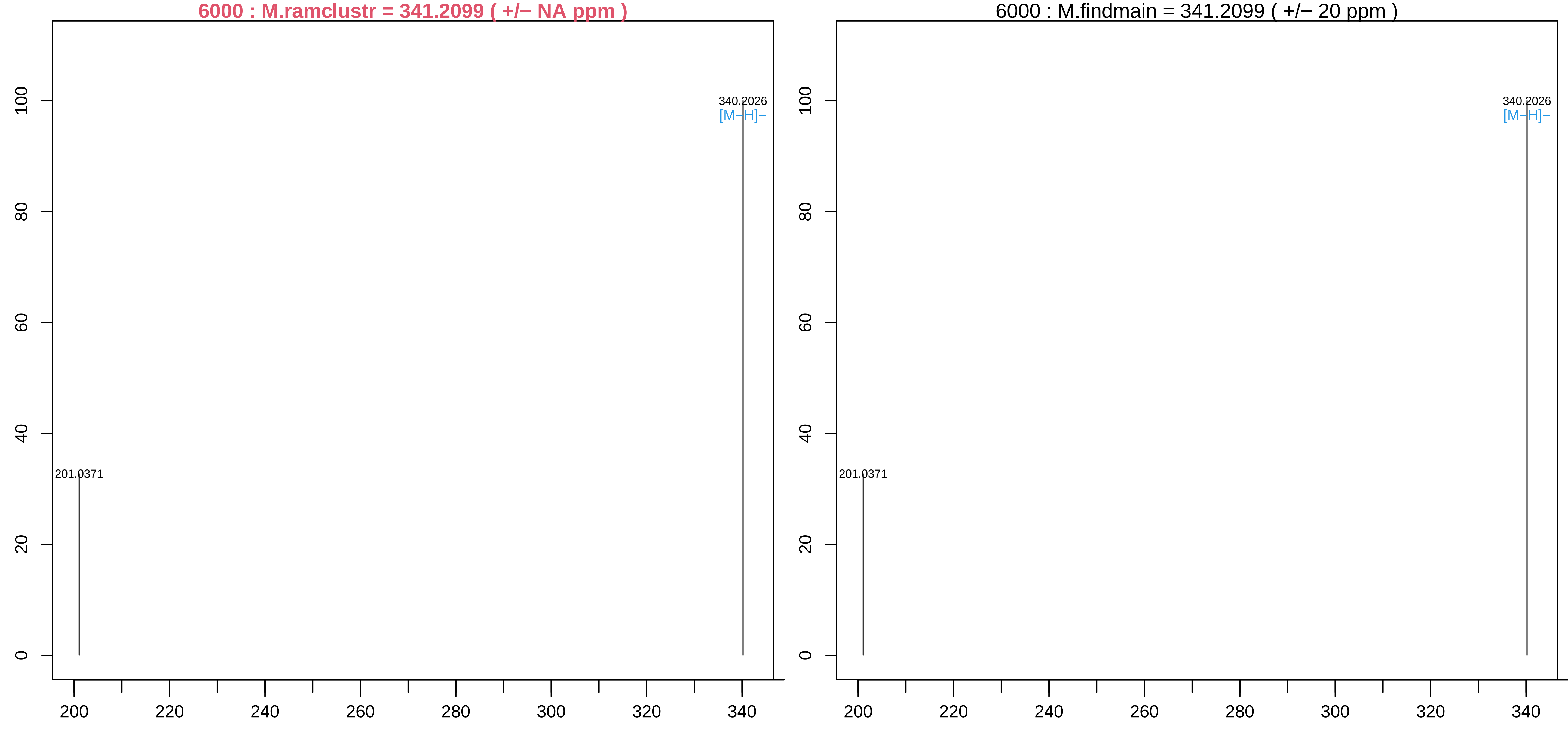
6000 : M.ramclustr = 341.2099 (+/− NA ppm)
340.2026
[M−H]−
201.0371
6000 : M.findmain = 341.2099 (+/− 20 ppm)
340.2026
[M−H]−
201.0371
0
20
40
60
80
100
200
220
240
260
280
300
320
340

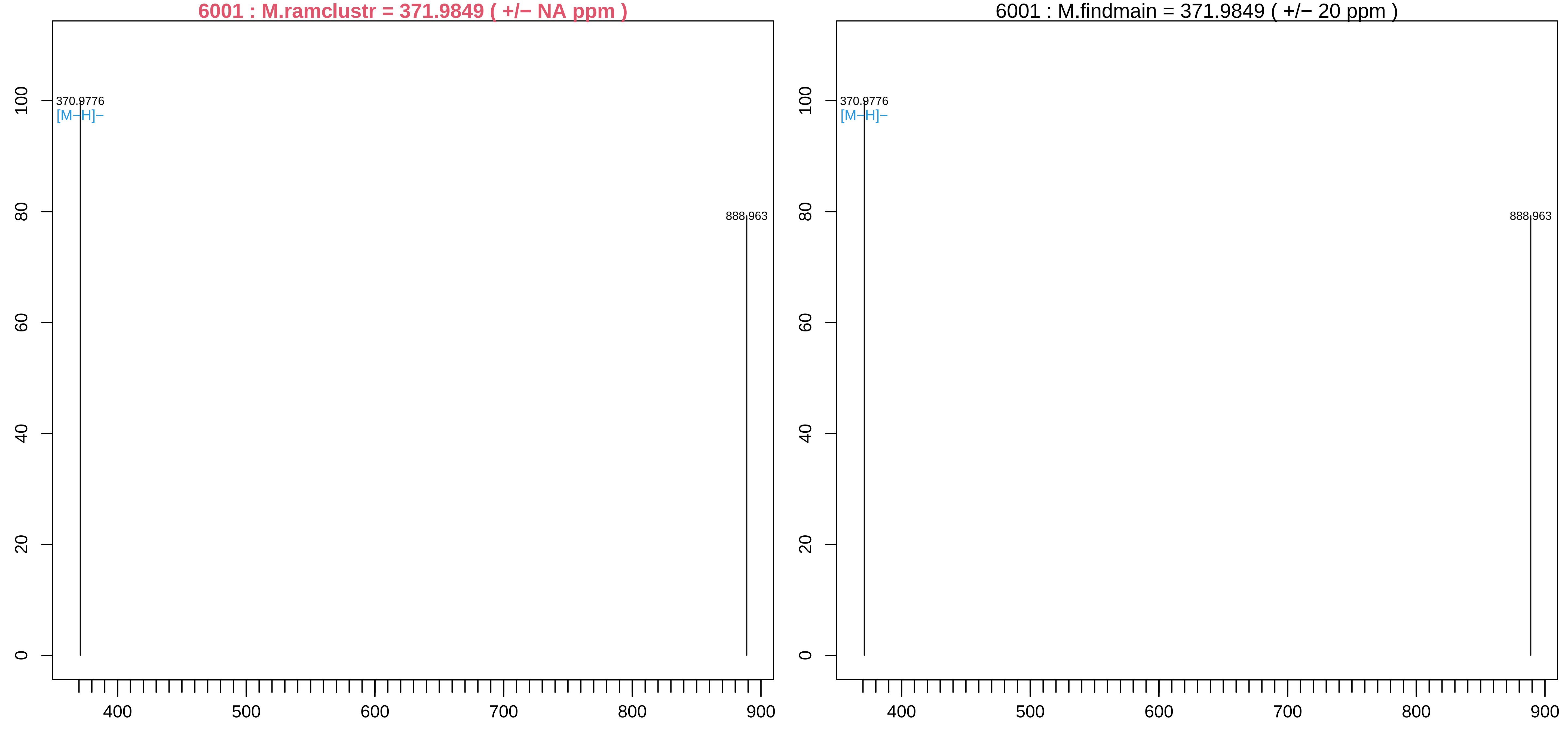
6001 : M.ramclustr = 371.9849 (+/− NA ppm)
370.9776
[M−H]−
888.963
6001 : M.findmain = 371.9849 (+/− 20 ppm)
370.9776
[M−H]−
888.963
0
20
40
60
80
100
400
500
600
700
800
900

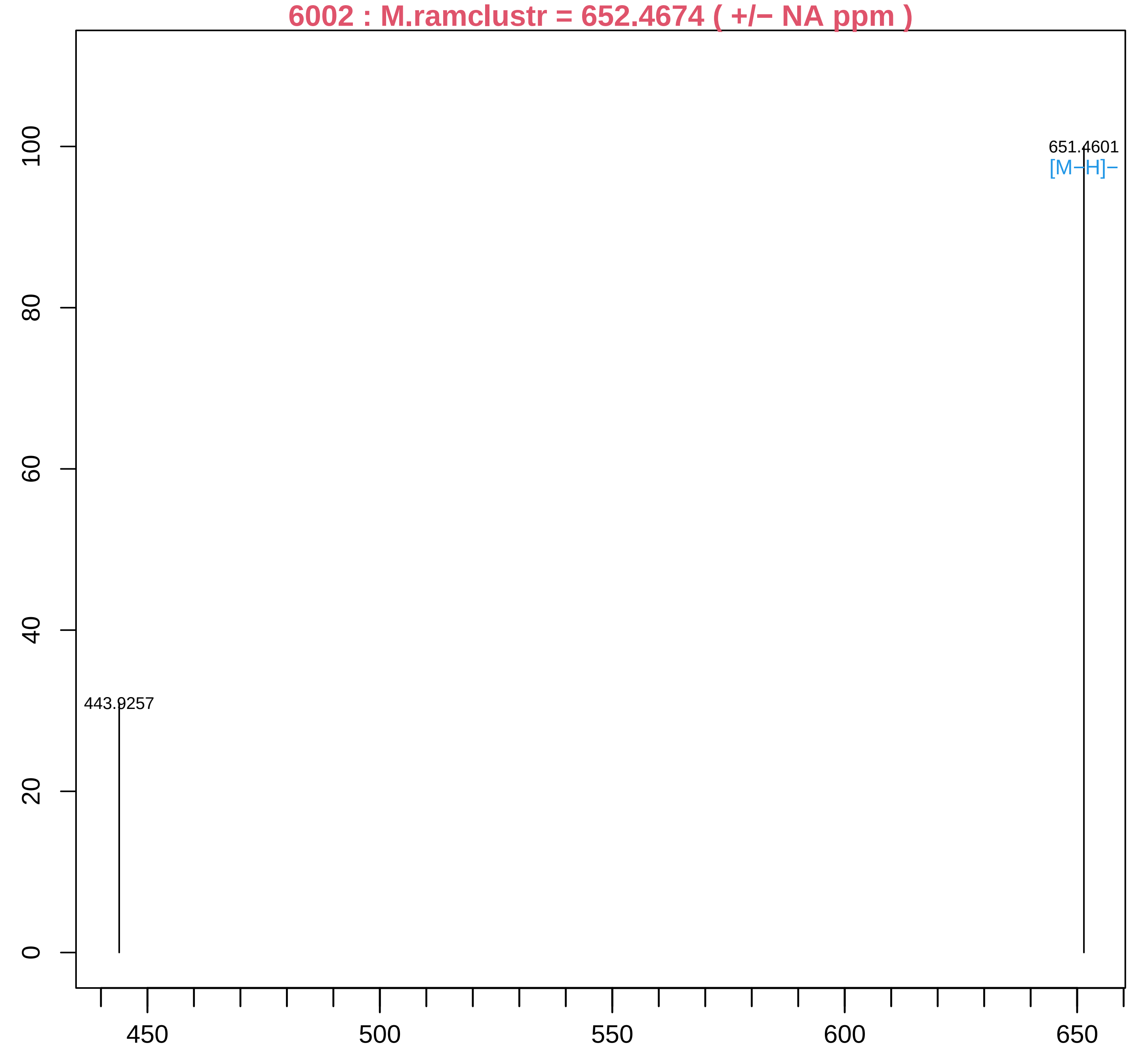

6002 : M.ramclustr = 652.4674 (+/− NA ppm)
651.4601
[M−H]−
443.9257
0
20
40
60
80
100
450
500
550
600
650

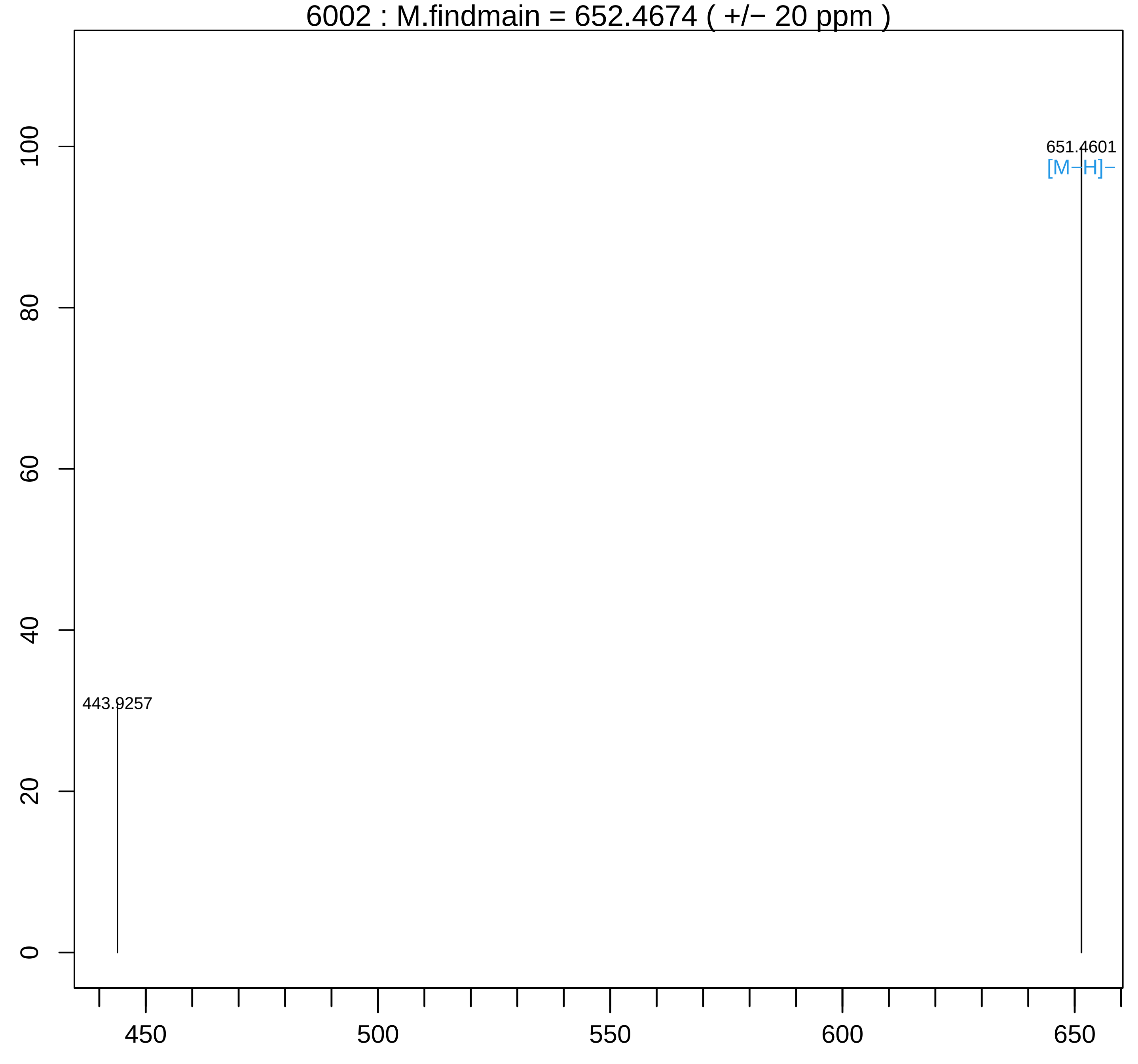

6002 : M.findmain = 652.4674 (+/− 20 ppm)
651.4601
[M−H]−
443.9257
0
20
40
60
80
100
450
500
550
600
650

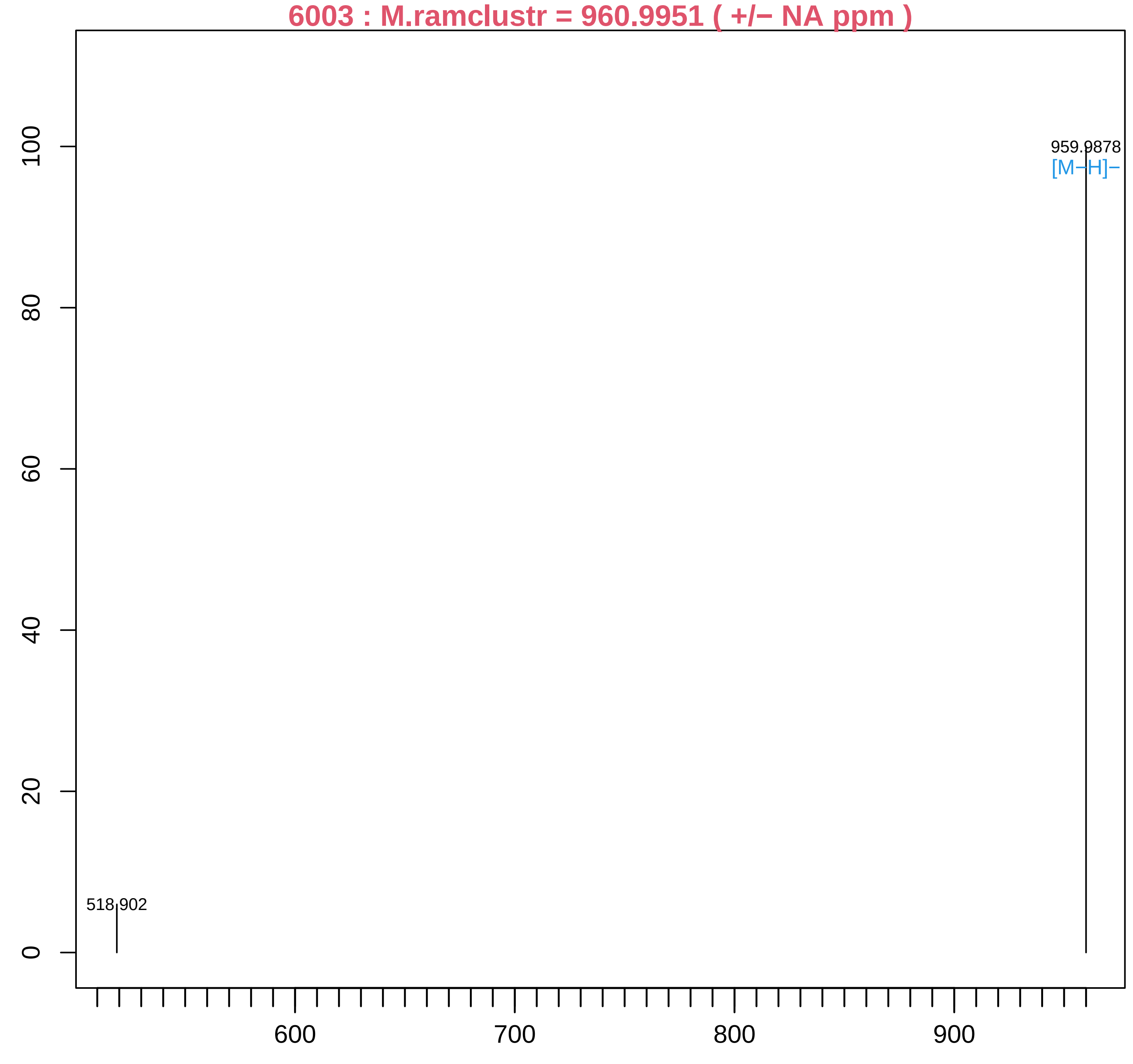

6003 : M.ramclustr = 960.9951 (+/− NA ppm)
959.9878
[M−H]−
518.902

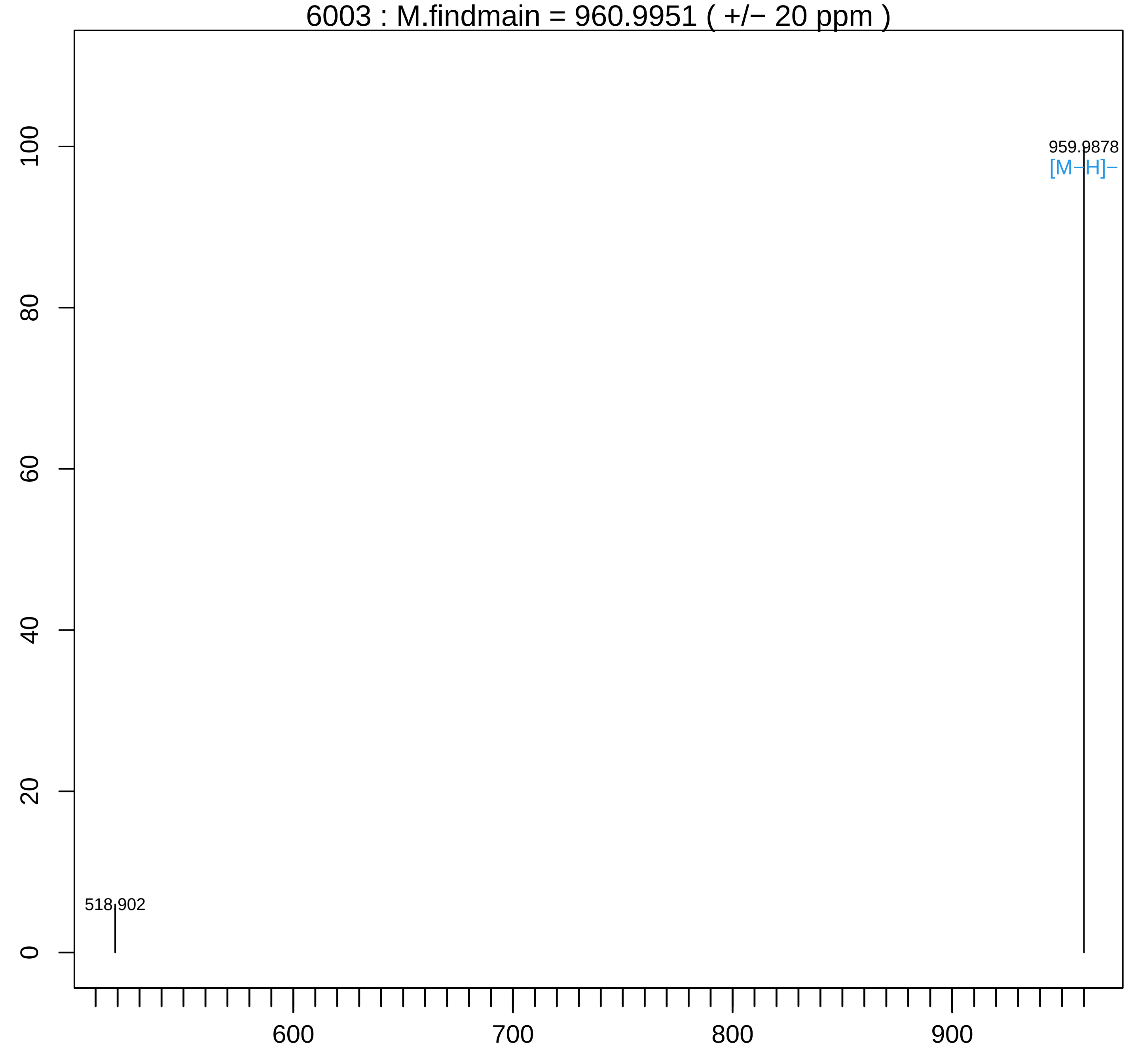

6003 : M.findmain = 960.9951 (+/− 20 ppm)
959.9878
[M−H]−
518.902

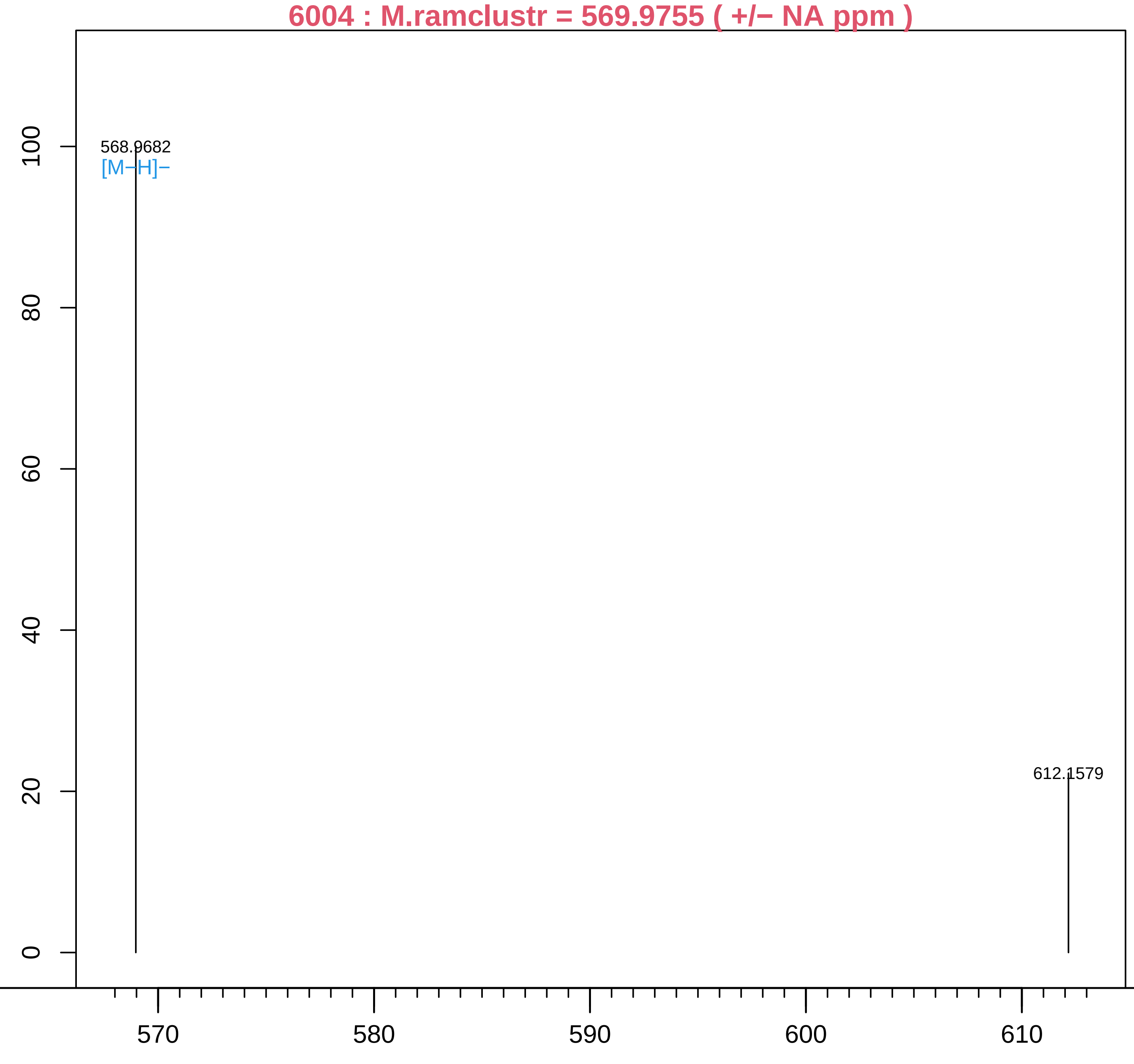
6004 : M.ramclustr = 569.9755 (+/− NA ppm)
568.9682
[M−H]−
612.1579
0
20
40
60
80
100
570
580
590
600
610
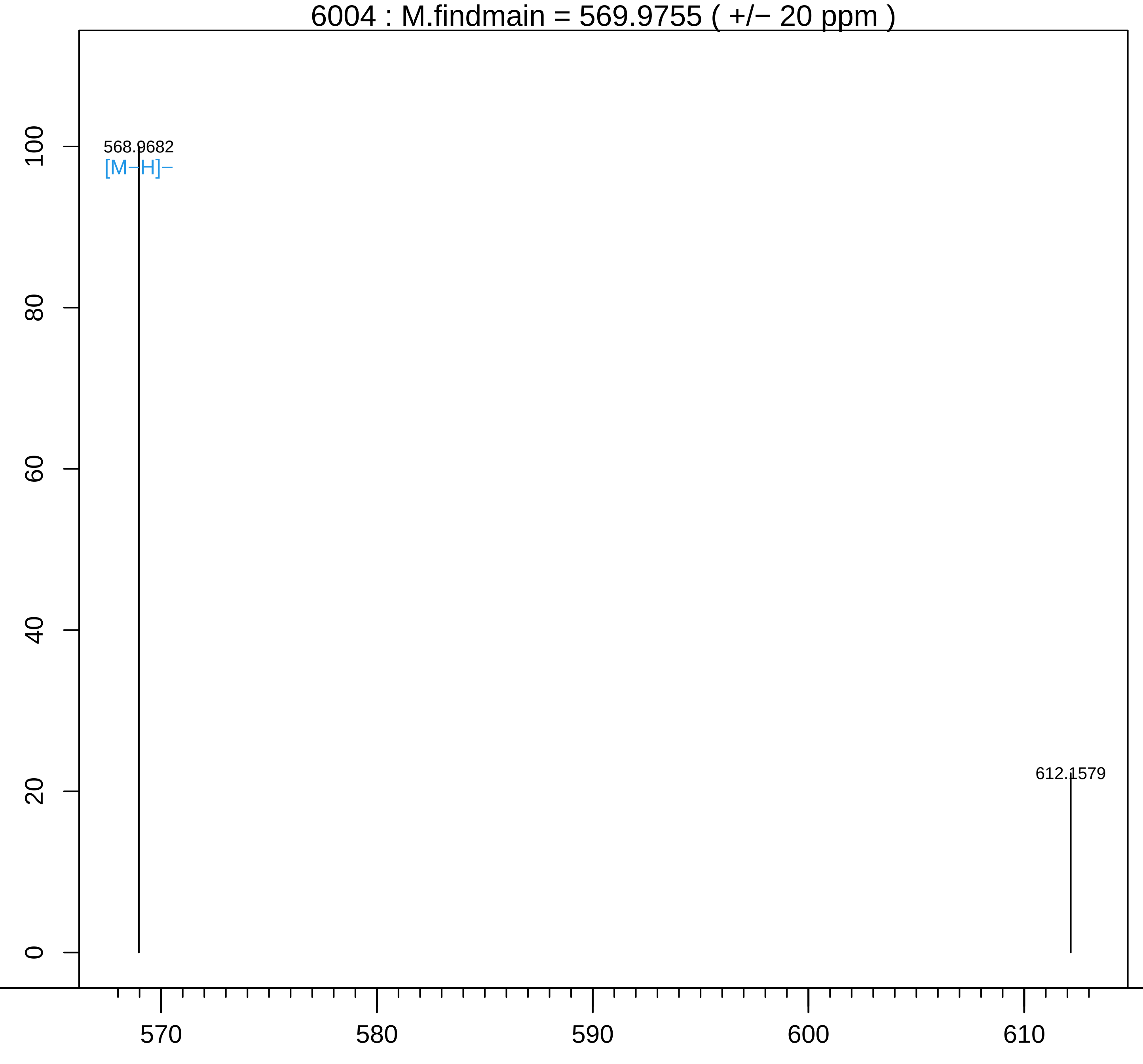
6004 : M.findmain = 569.9755 (+/− 20 ppm)
568.9682
[M−H]−
612.1579
0
20
40
60
80
100
570
580
590
600
610

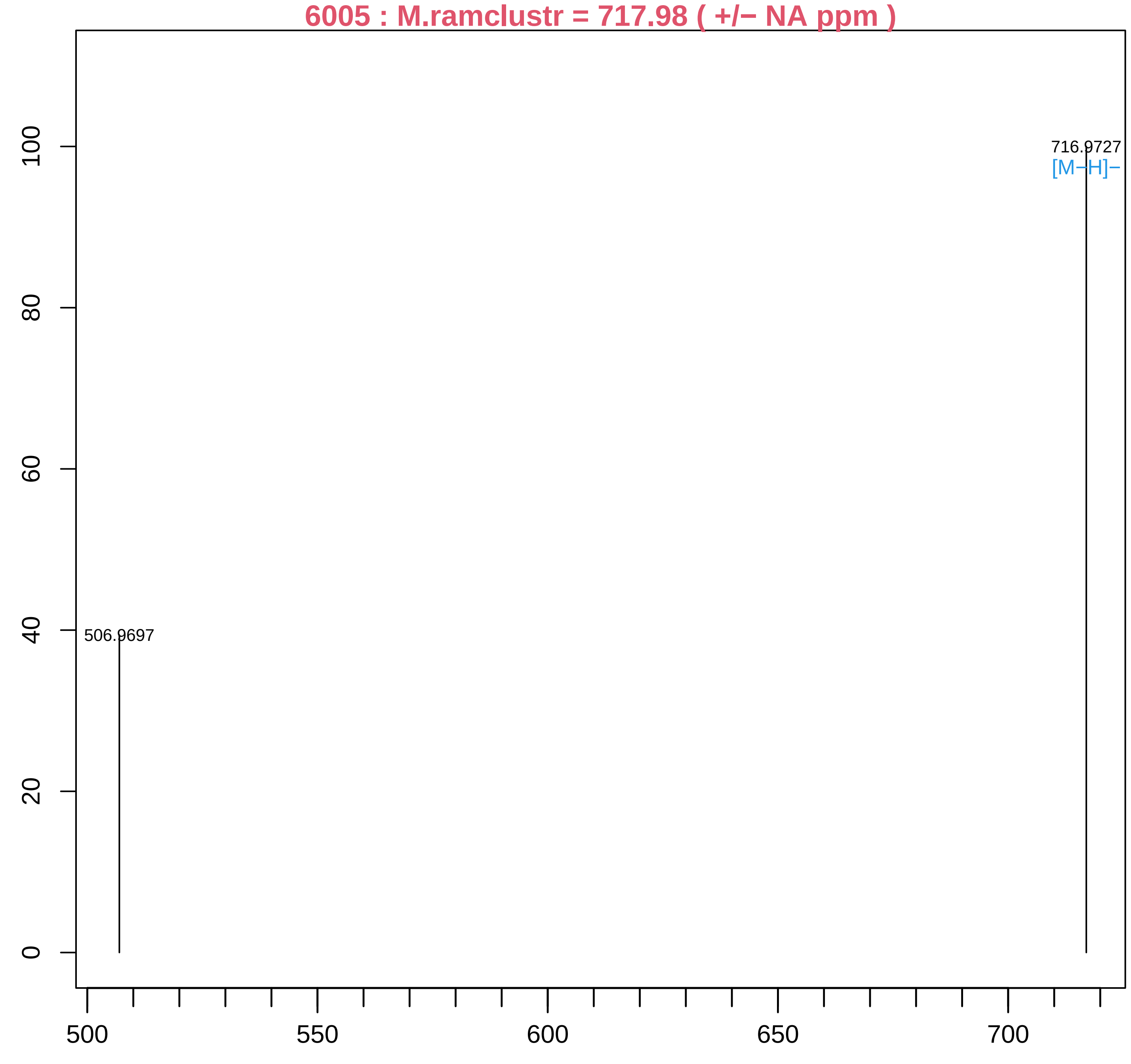
6005 : M.ramclustr = 717.98 (+/− NA ppm)
716.9727
[M−H]−
506.9697
0
20
40
60
80
100
500
550
600
650
700

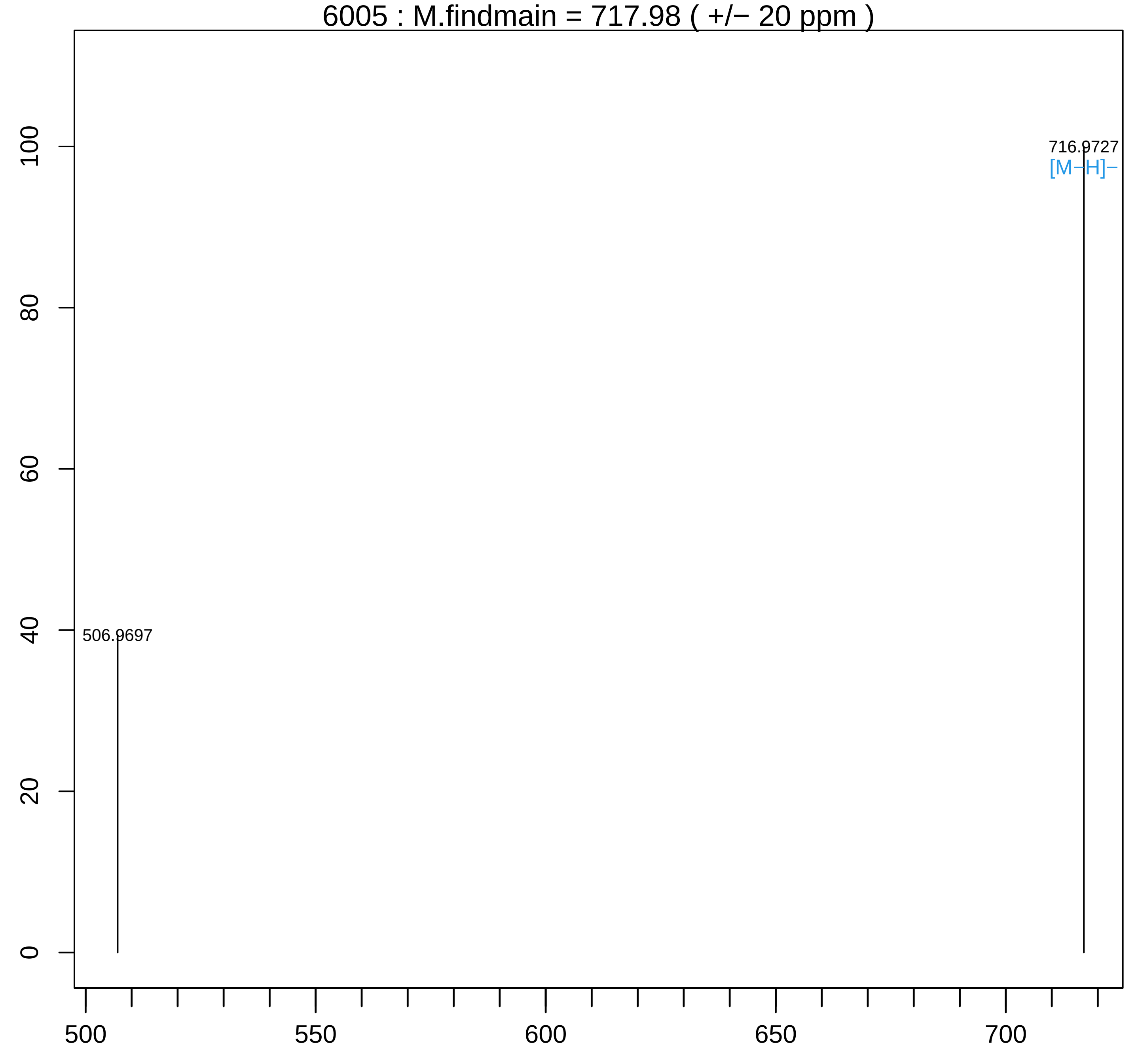
6005 : M.findmain = 717.98 (+/− 20 ppm)
716.9727
[M−H]−
506.9697
0
20
40
60
80
100
500
550
600
650
700

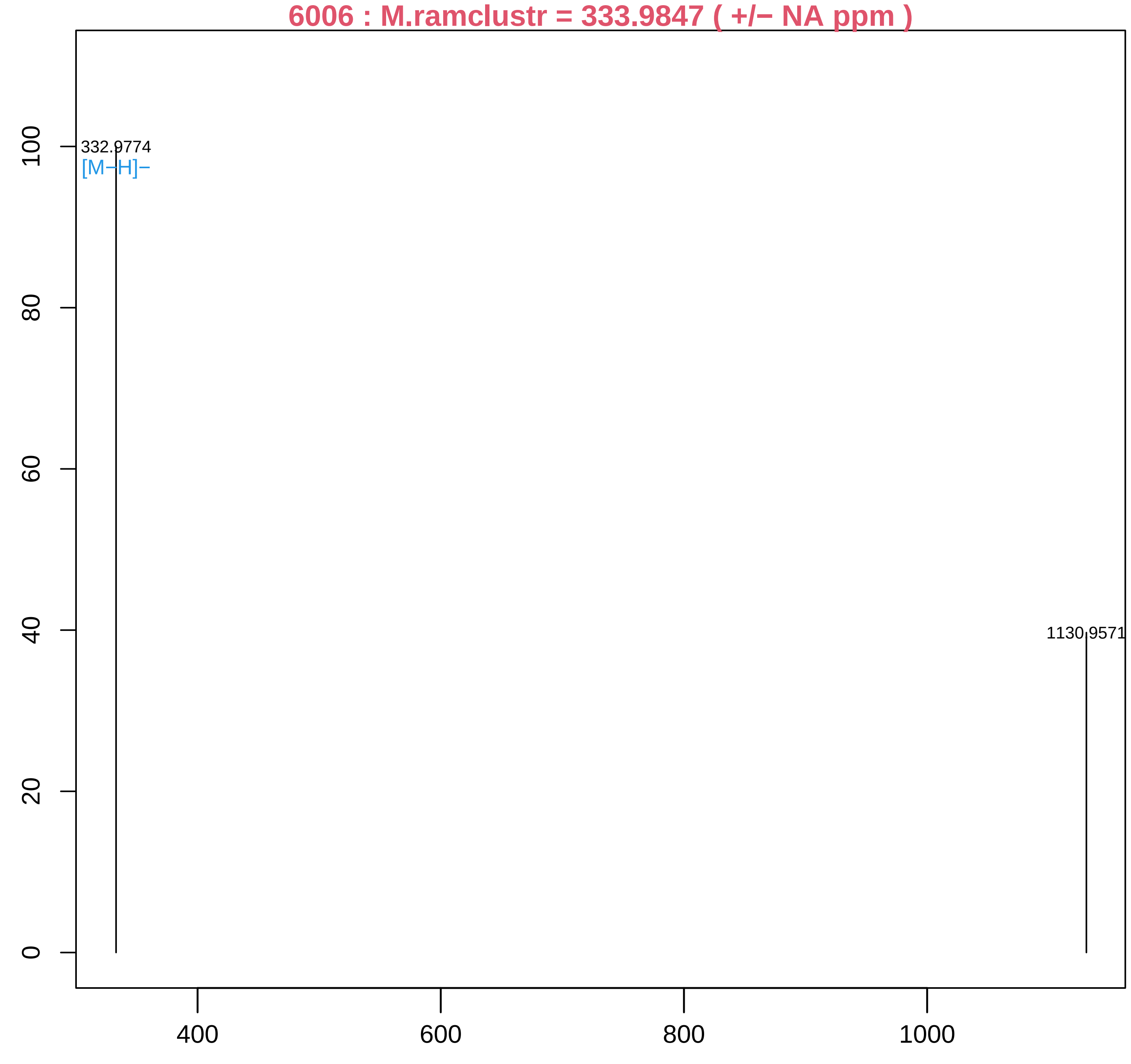
6006 : M.ramclustr = 333.9847 (+/− NA ppm)
332.9774
[M−H]−
1130.9571
0
20
40
60
80
100
400
600
800
1000

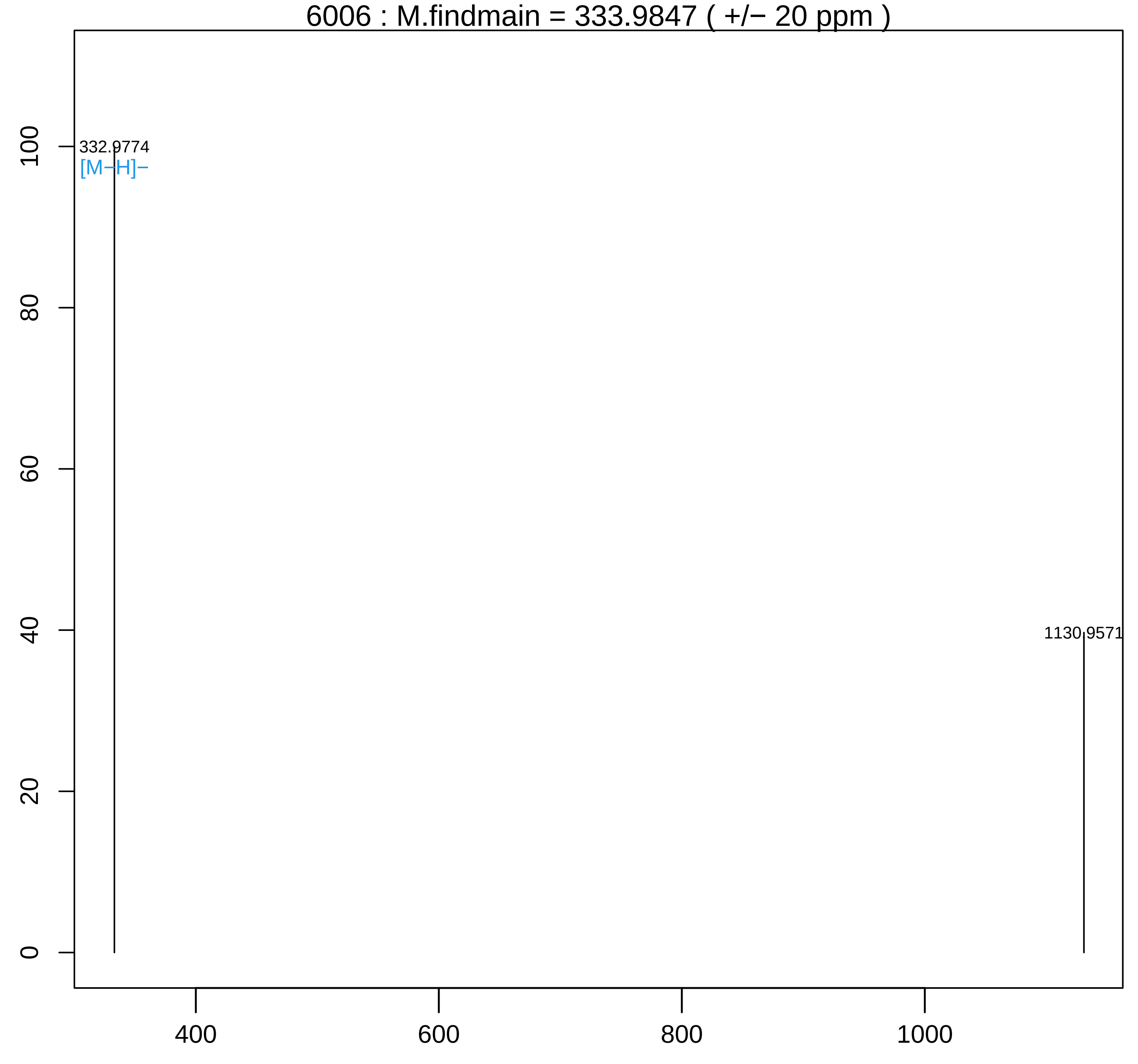
6006 : M.findmain = 333.9847 (+/− 20 ppm)
332.9774
[M−H]−
1130.9571
0
20
40
60
80
100
400
600
800
1000

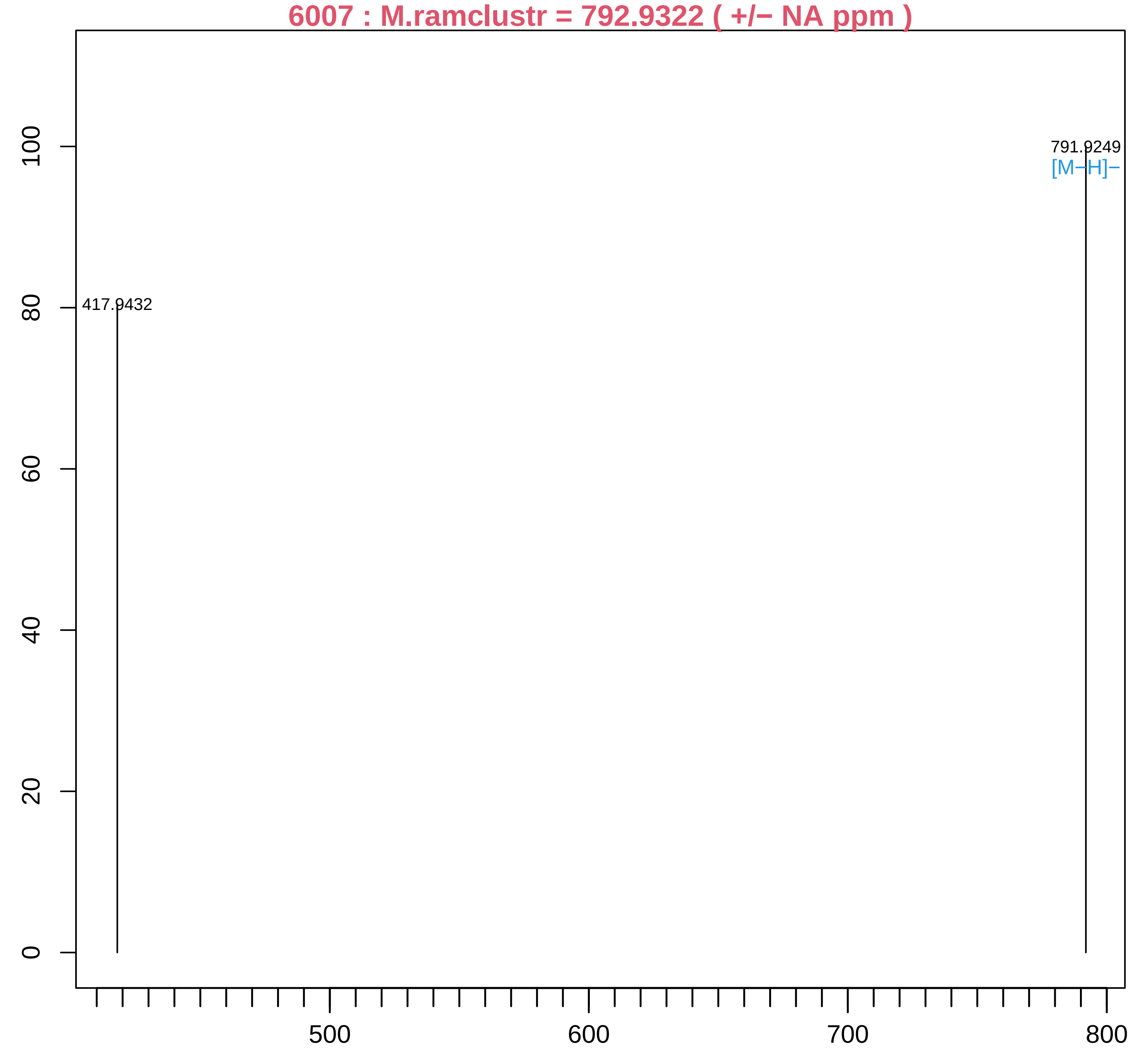
6007 : M.ramclustr = 792.9322 (+/− NA ppm)
791.9249
[M−H]−
417.9432
0
20
40
60
80
100
500
600
700
800

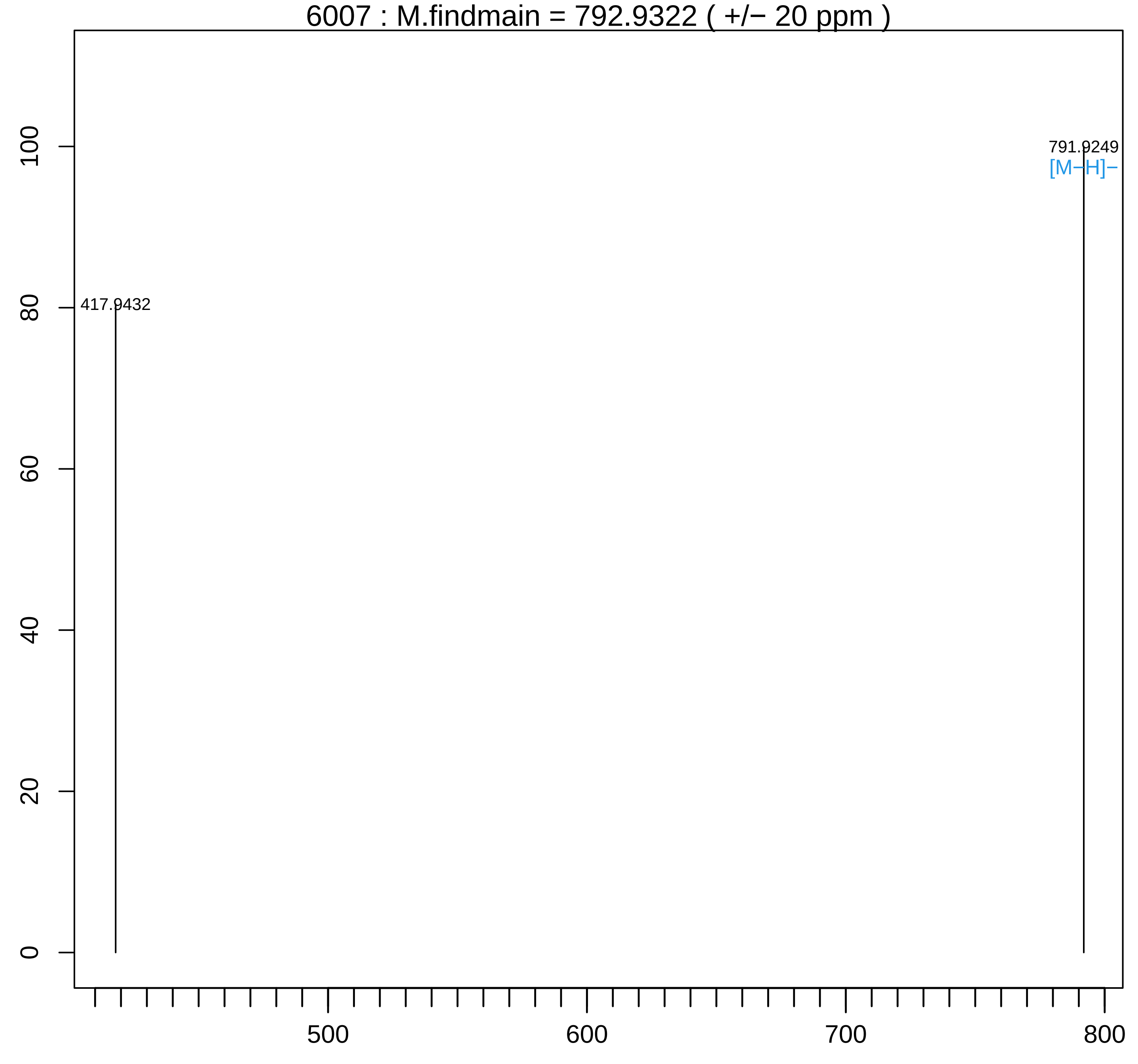
6007 : M.findmain = 792.9322 (+/− 20 ppm)
791.9249
[M−H]−
417.9432
0
20
40
60
80
100
500
600
700
800

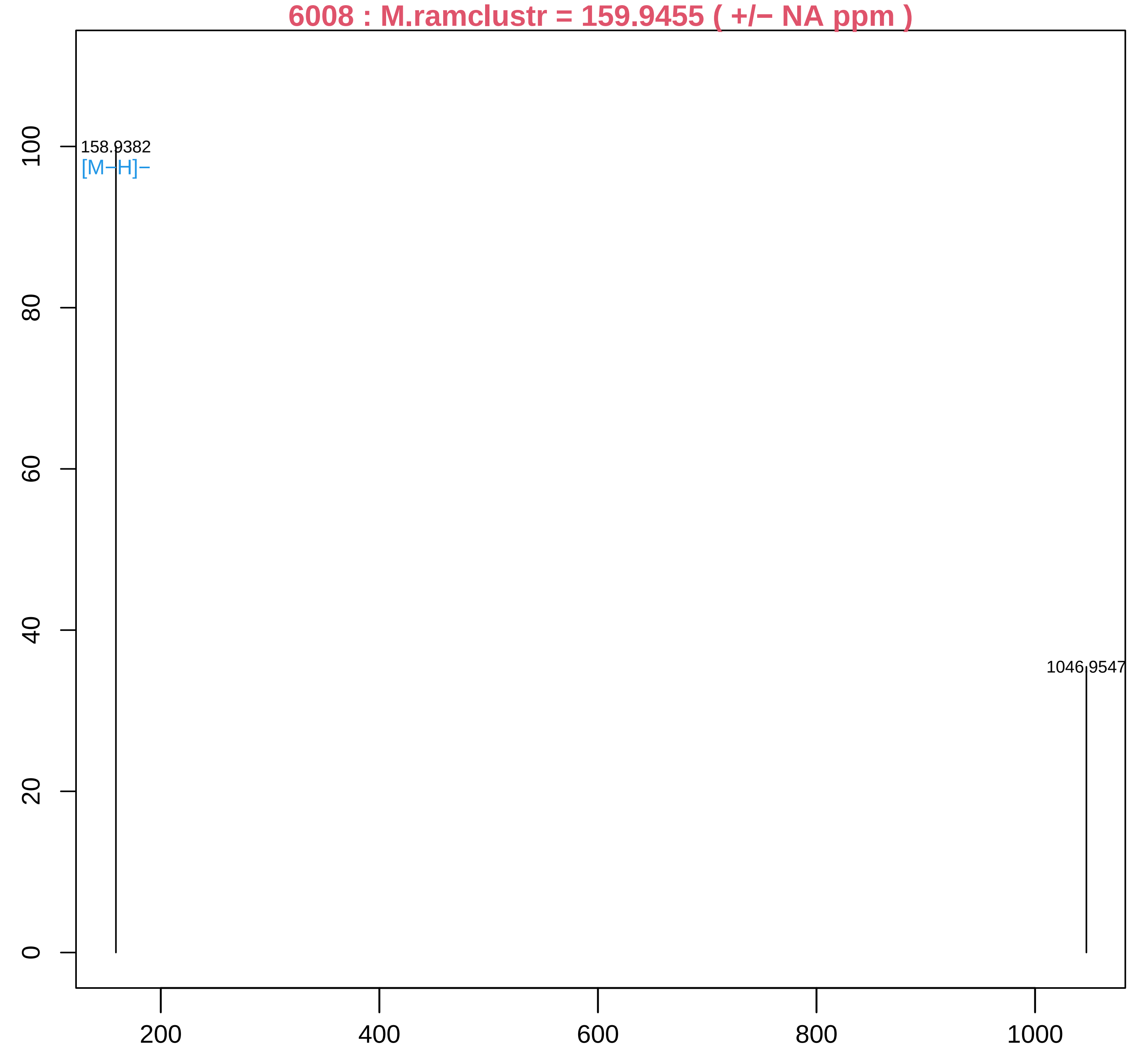
6008 : M.ramclustr = 159.9455 (+/− NA ppm)
158.9382
[M−H]−
1046.9547
0
20
40
60
80
100
200
400
600
800
1000

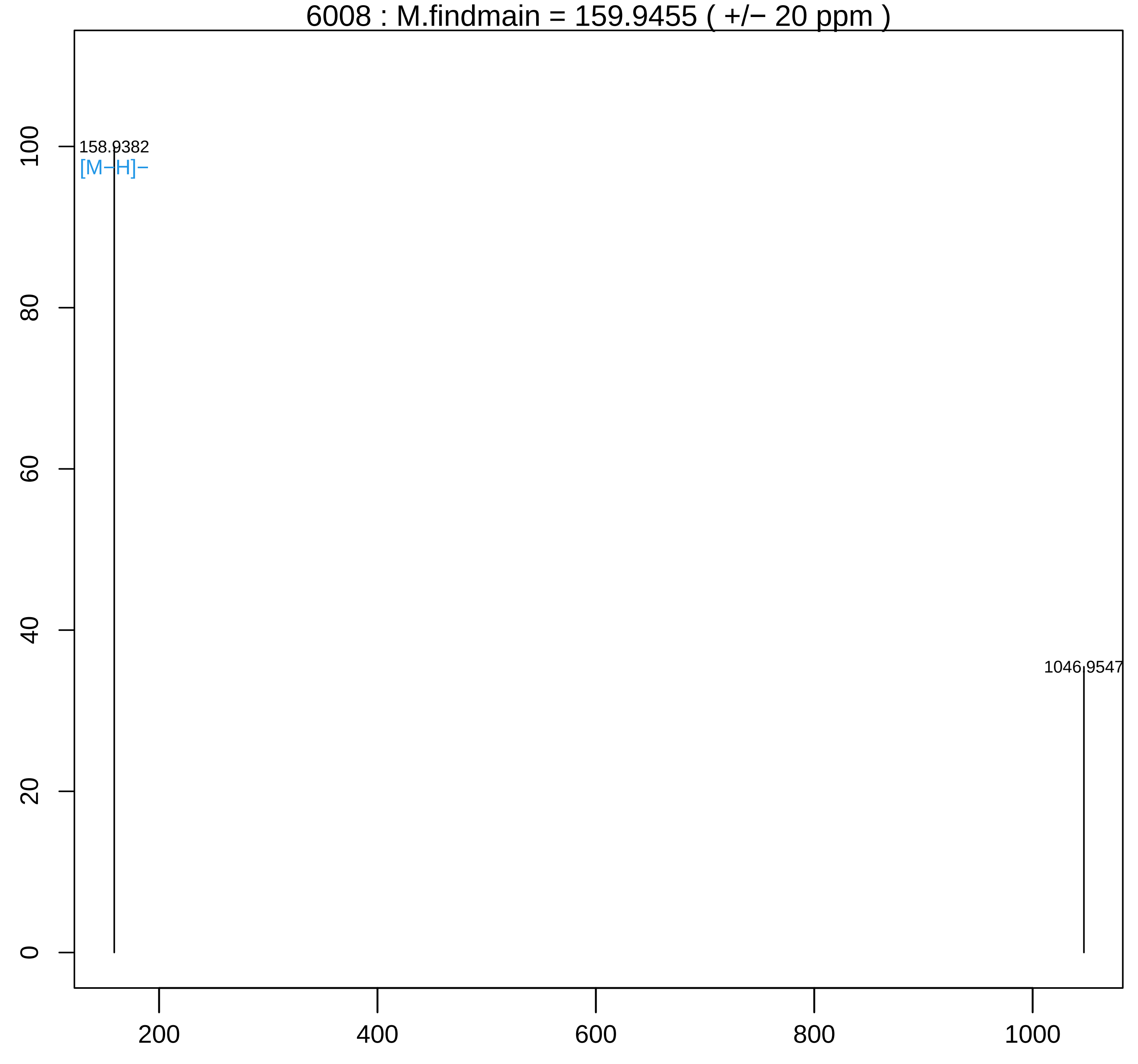
6008 : M.findmain = 159.9455 (+/− 20 ppm)
158.9382
[M−H]−
1046.9547
0
20
40
60
80
100
200
400
600
800
1000

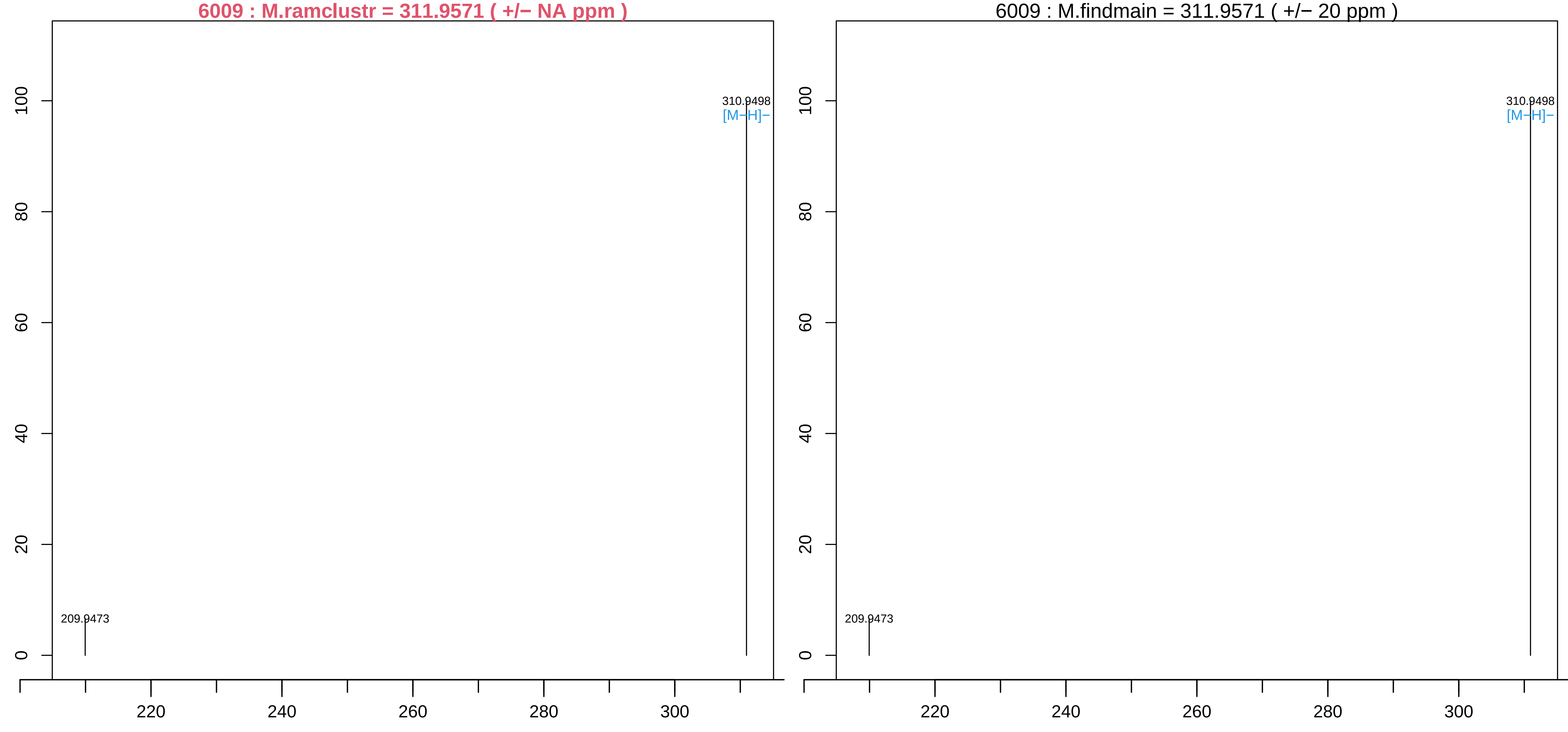

6009 : M.ramclustr = 311.9571 (+/− NA ppm)
310.9498
[M−H]−
209.9473
6009 : M.findmain = 311.9571 (+/− 20 ppm)
310.9498
[M−H]−
209.9473

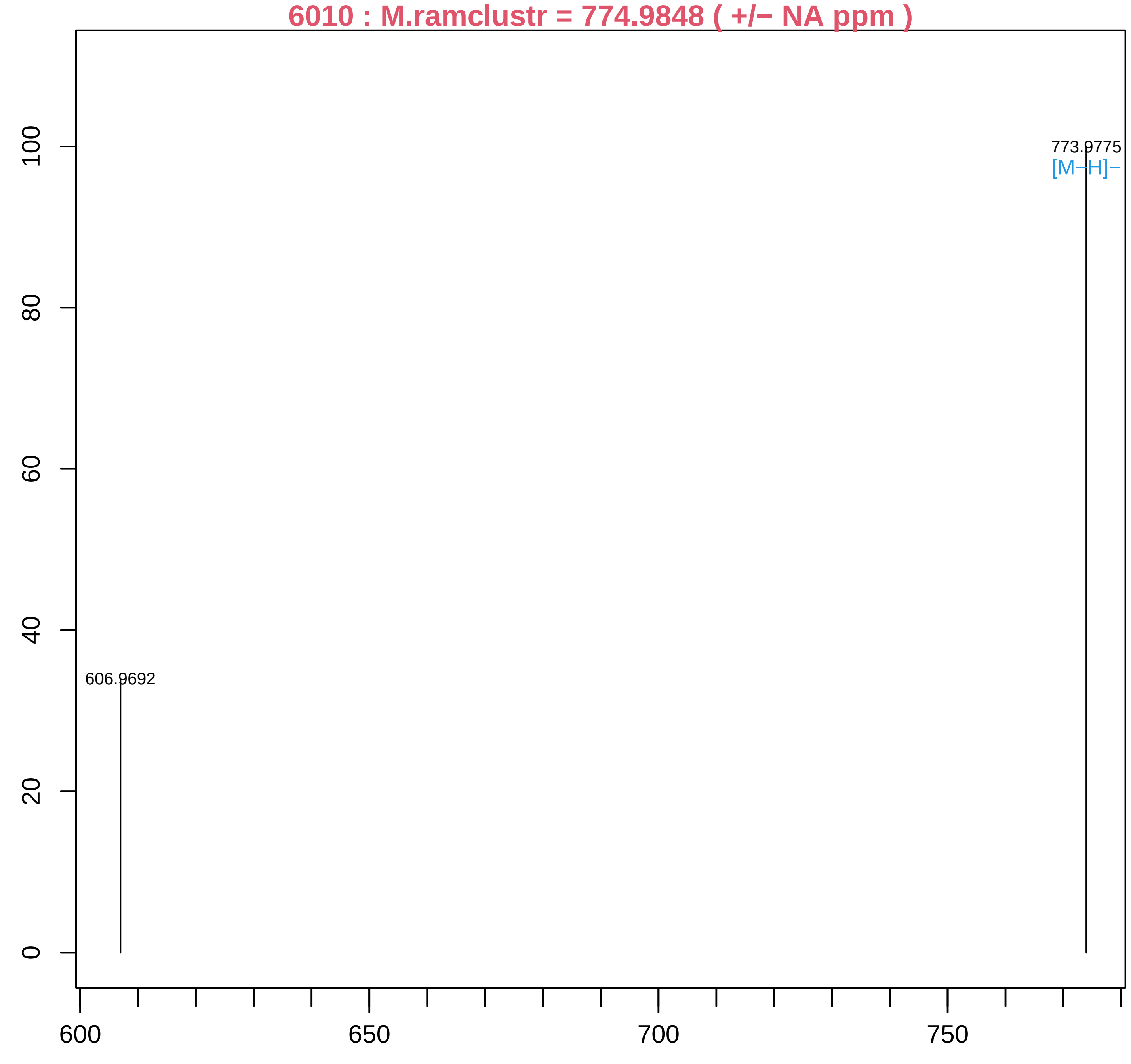

6010 : M.ramclustr = 774.9848 (+/− NA ppm)
773.9775
[M−H]−
606.9692
0
20
40
60
80
100
600
650
700
750

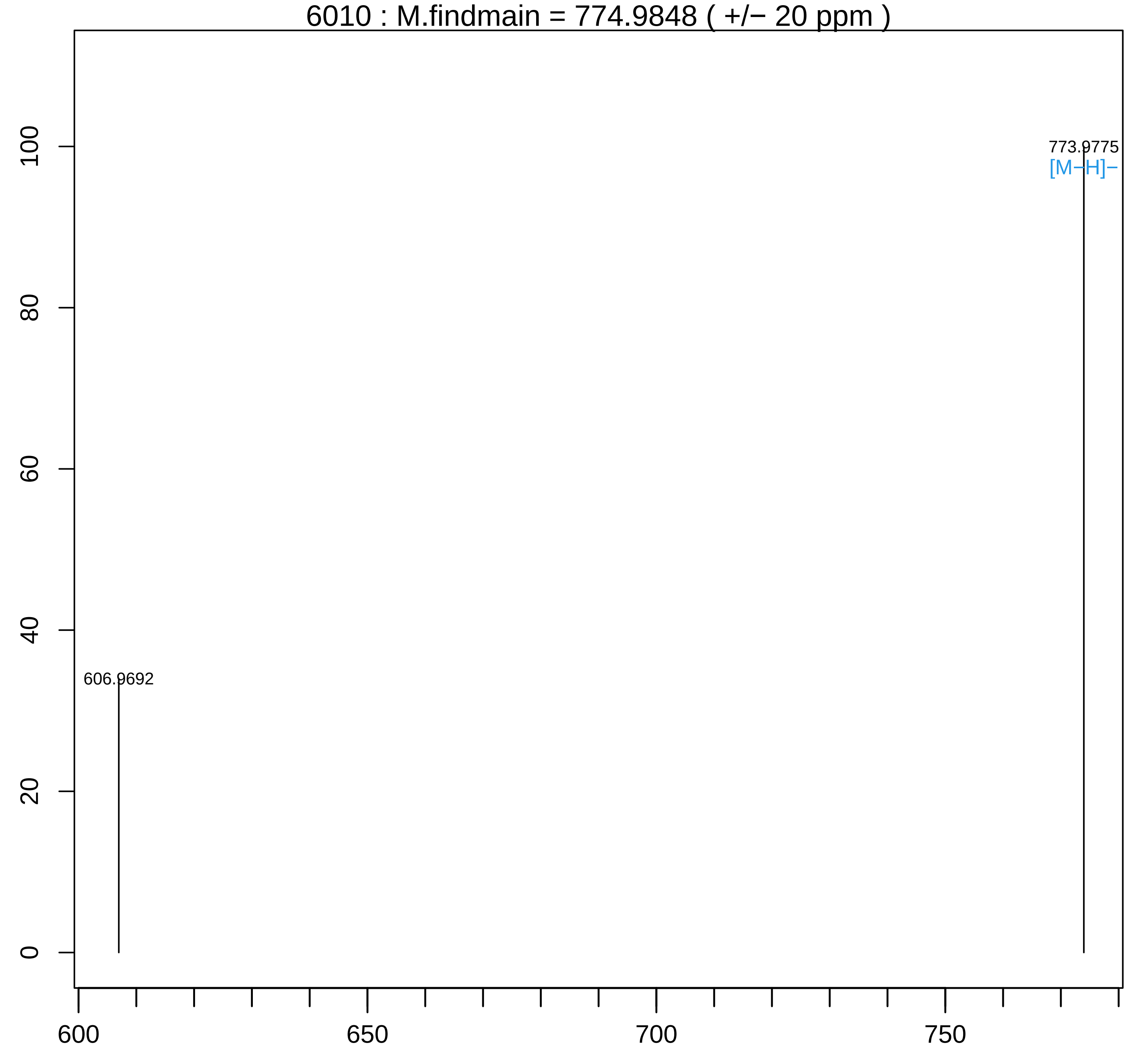

6010 : M.findmain = 774.9848 (+/− 20 ppm)
773.9775
[M−H]−
606.9692
0
20
40
60
80
100
600
650
700
750

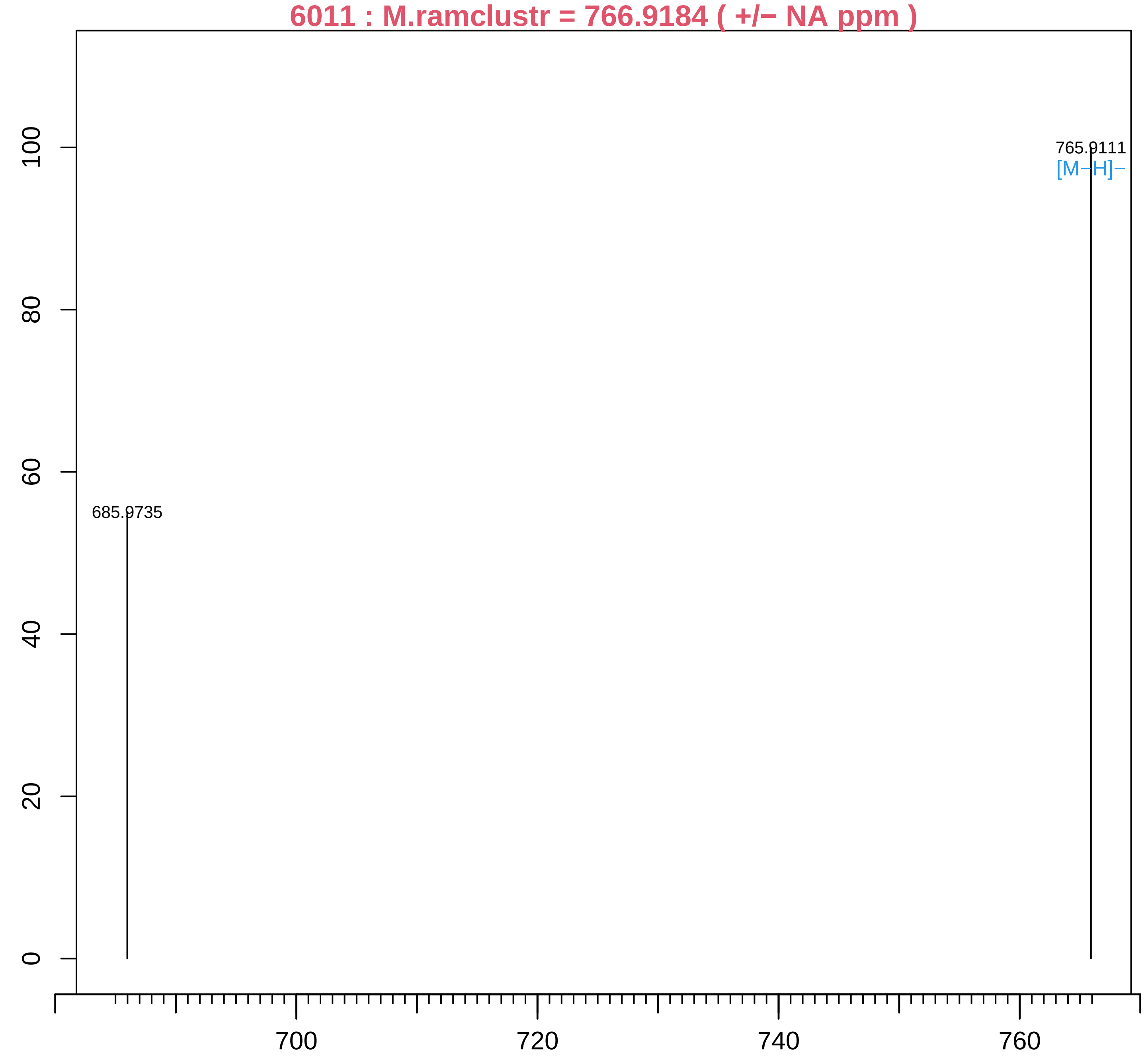
6011 : M.ramclustr = 766.9184 (+/− NA ppm)
765.9111
[M−H]−
685.9735
0
20
40
60
80
100
700
720
740
760

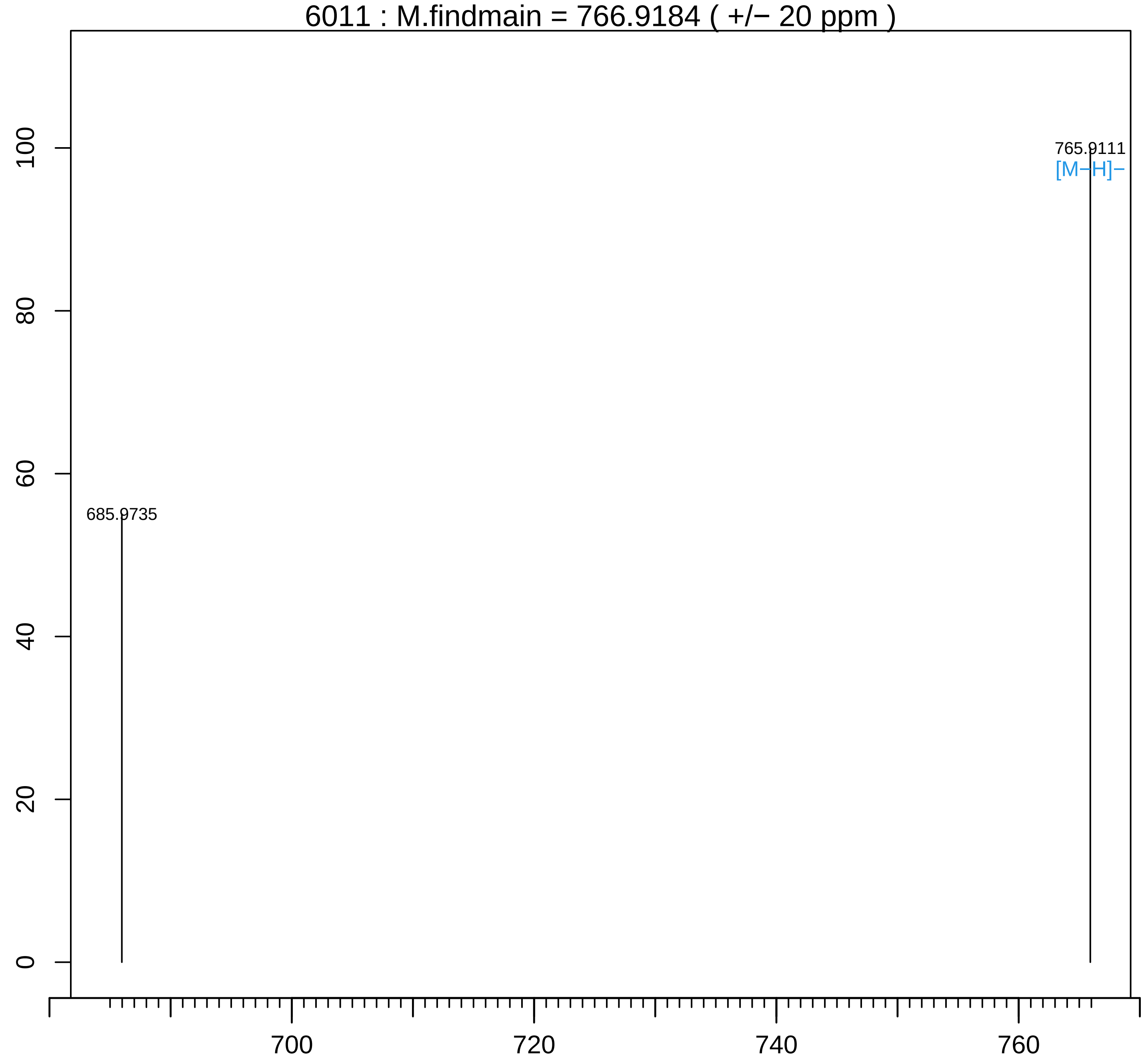
6011 : M.findmain = 766.9184 (+/− 20 ppm)
765.9111
[M−H]−
685.9735
0
20
40
60
80
100
700
720
740
760

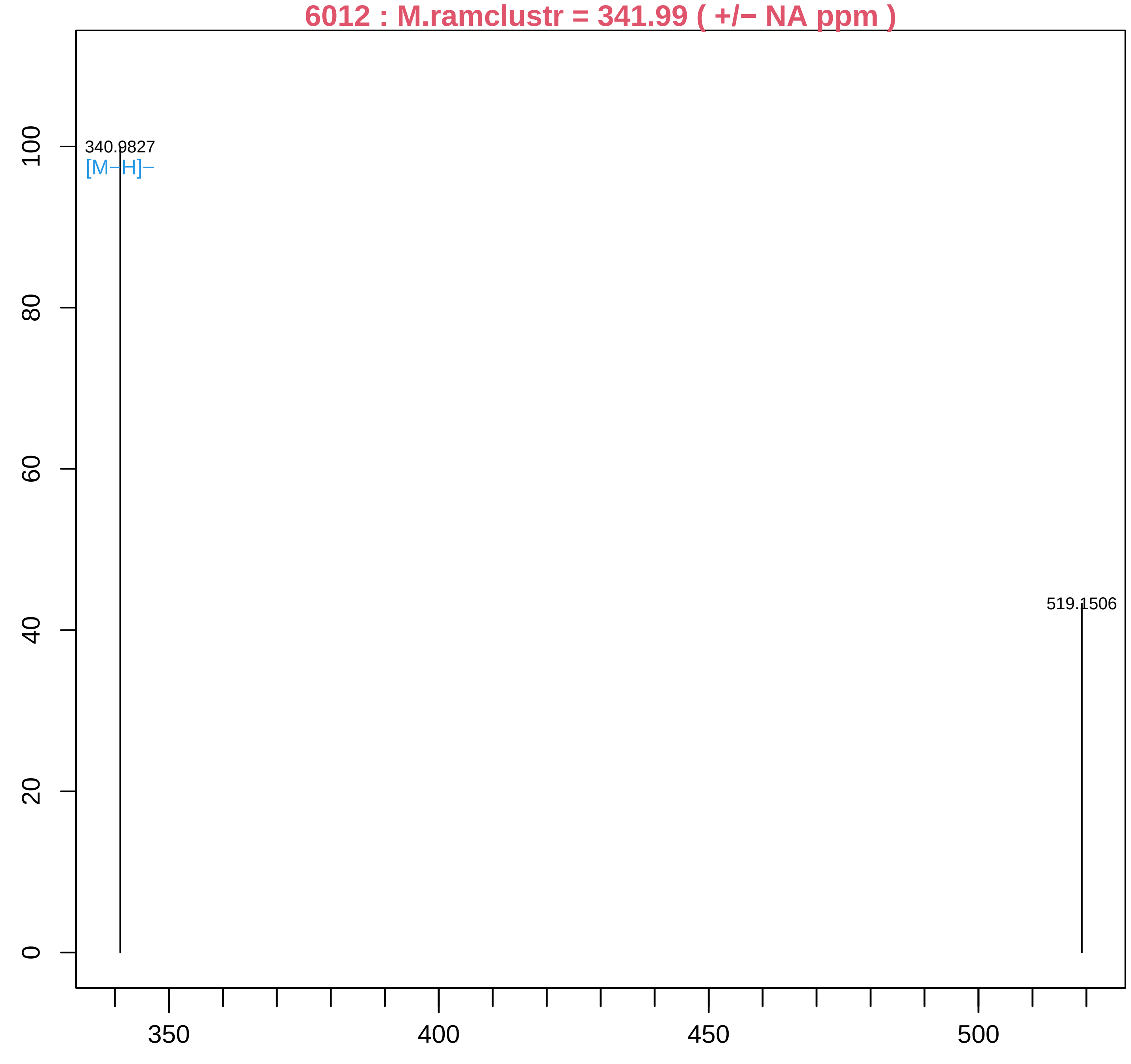
6012 : M.ramclustr = 341.99 (+/− NA ppm)
340.9827
[M−H]−
519.1506
0
20
40
60
80
100
350
400
450
500

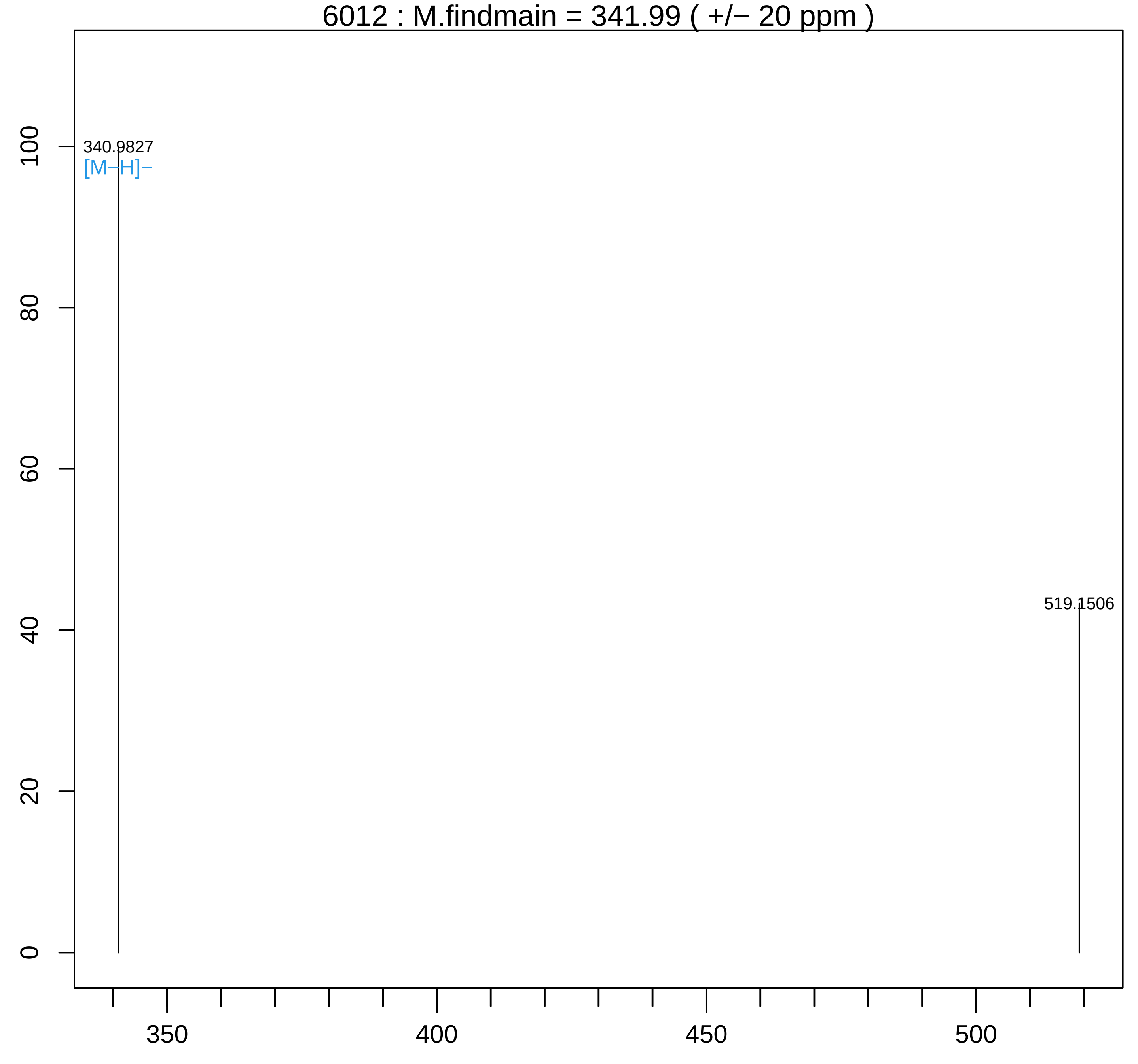
6012 : M.findmain = 341.99 (+/− 20 ppm)
340.9827
[M−H]−
519.1506
0
20
40
60
80
100
350
400
450
500

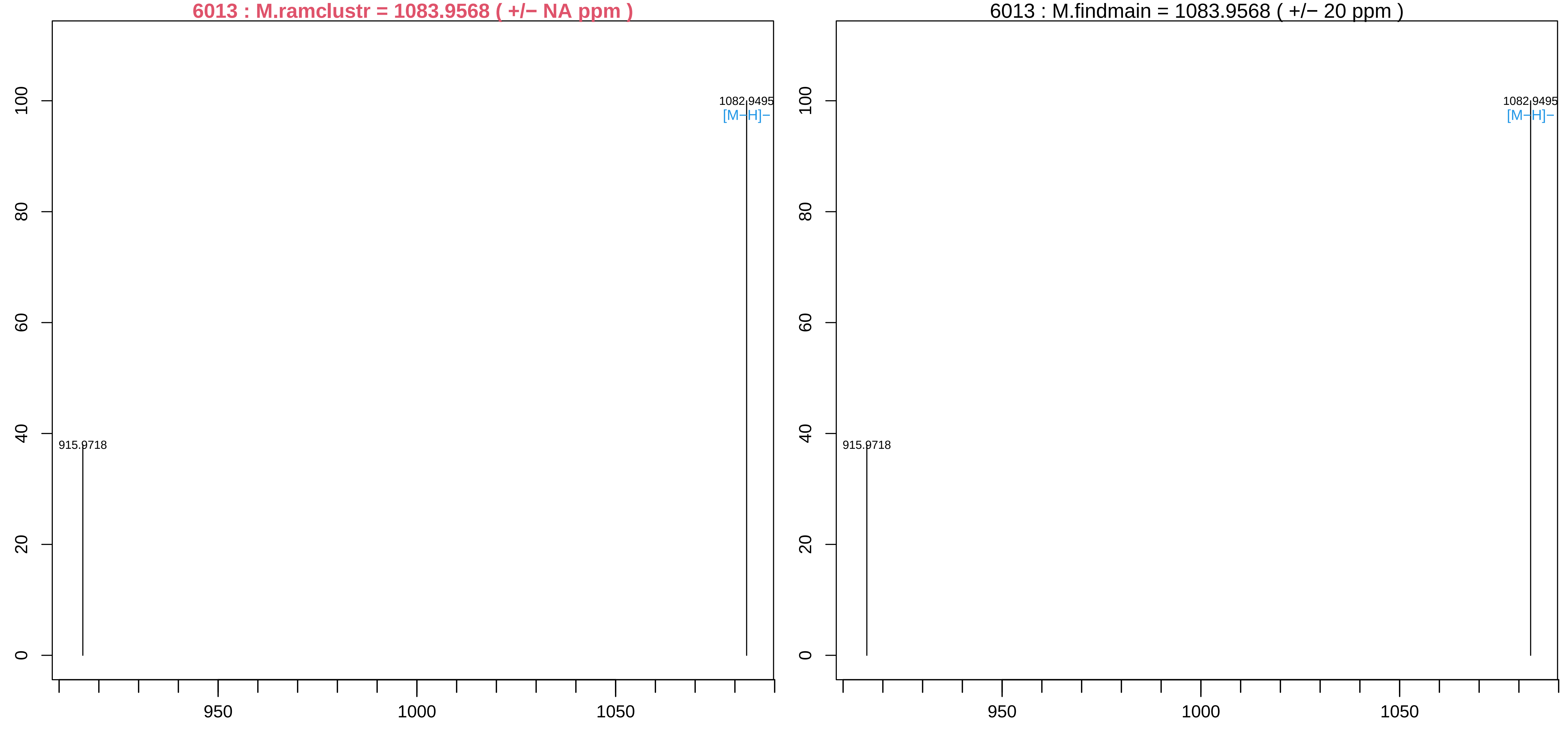

6013 : M.ramclustr = 1083.9568 (+/− NA ppm)
6013 : M.findmain = 1083.9568 (+/− 20 ppm)
1082.9495
[M−H]−
915.9718
0
20
40
60
80
100
950
1000
1050

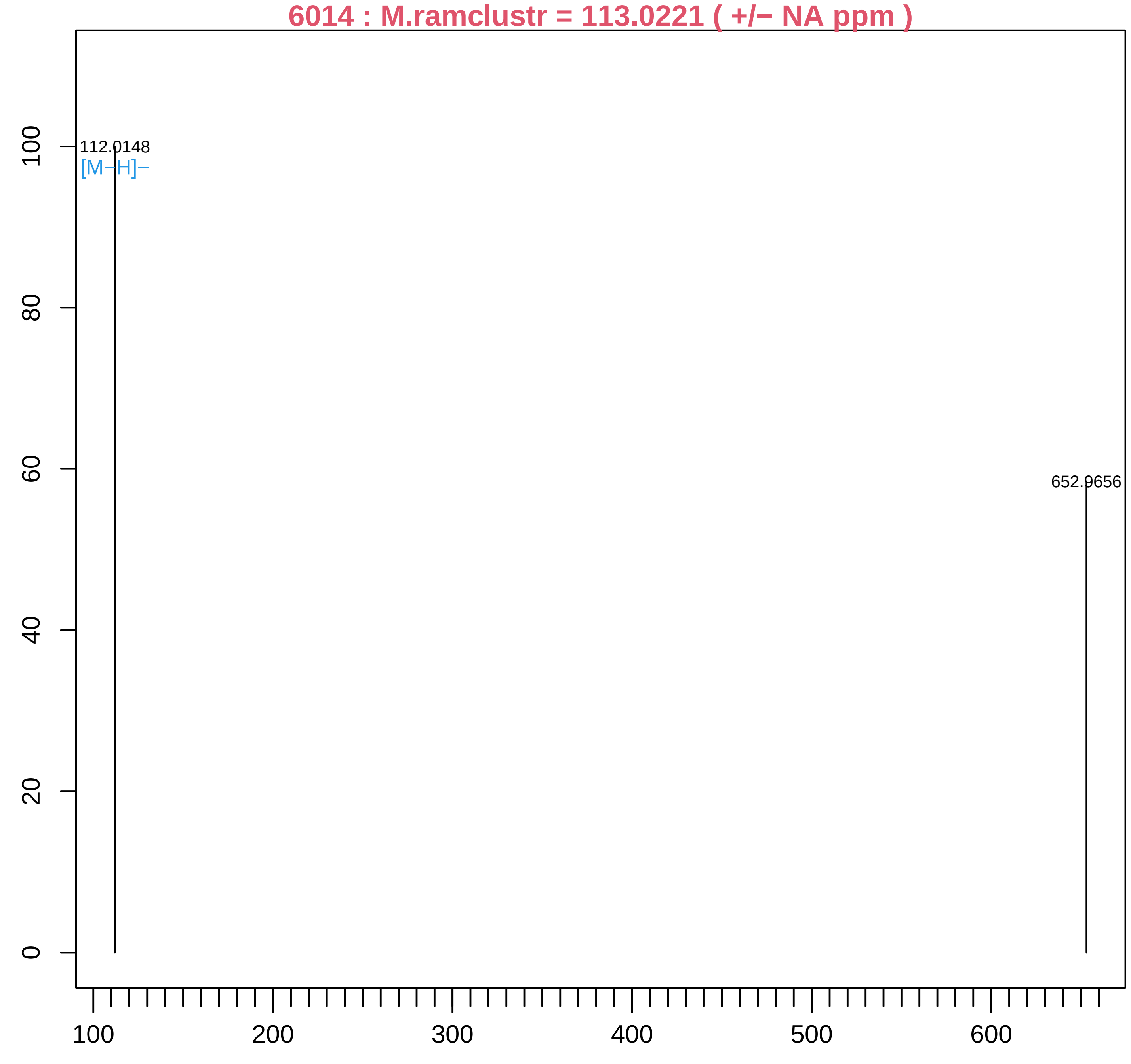
6014 : M.ramclustr = 113.0221 (+/− NA ppm)
112.0148
[M−H]−
652.9656
0
20
40
60
80
100
100
200
300
400
500
600

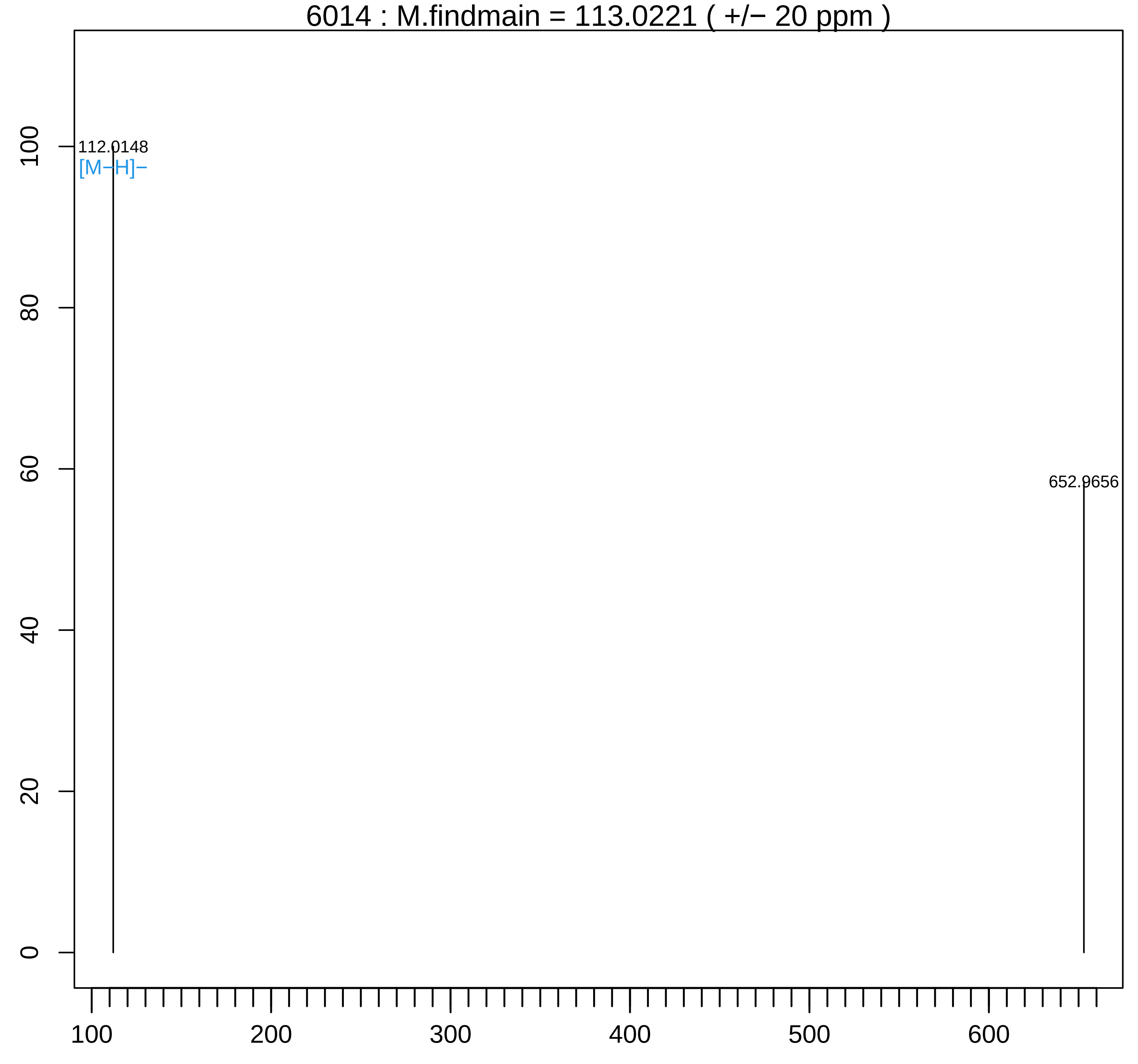
6014 : M.findmain = 113.0221 (+/− 20 ppm)
112.0148
[M−H]−
652.9656
0
20
40
60
80
100
100
200
300
400
500
600

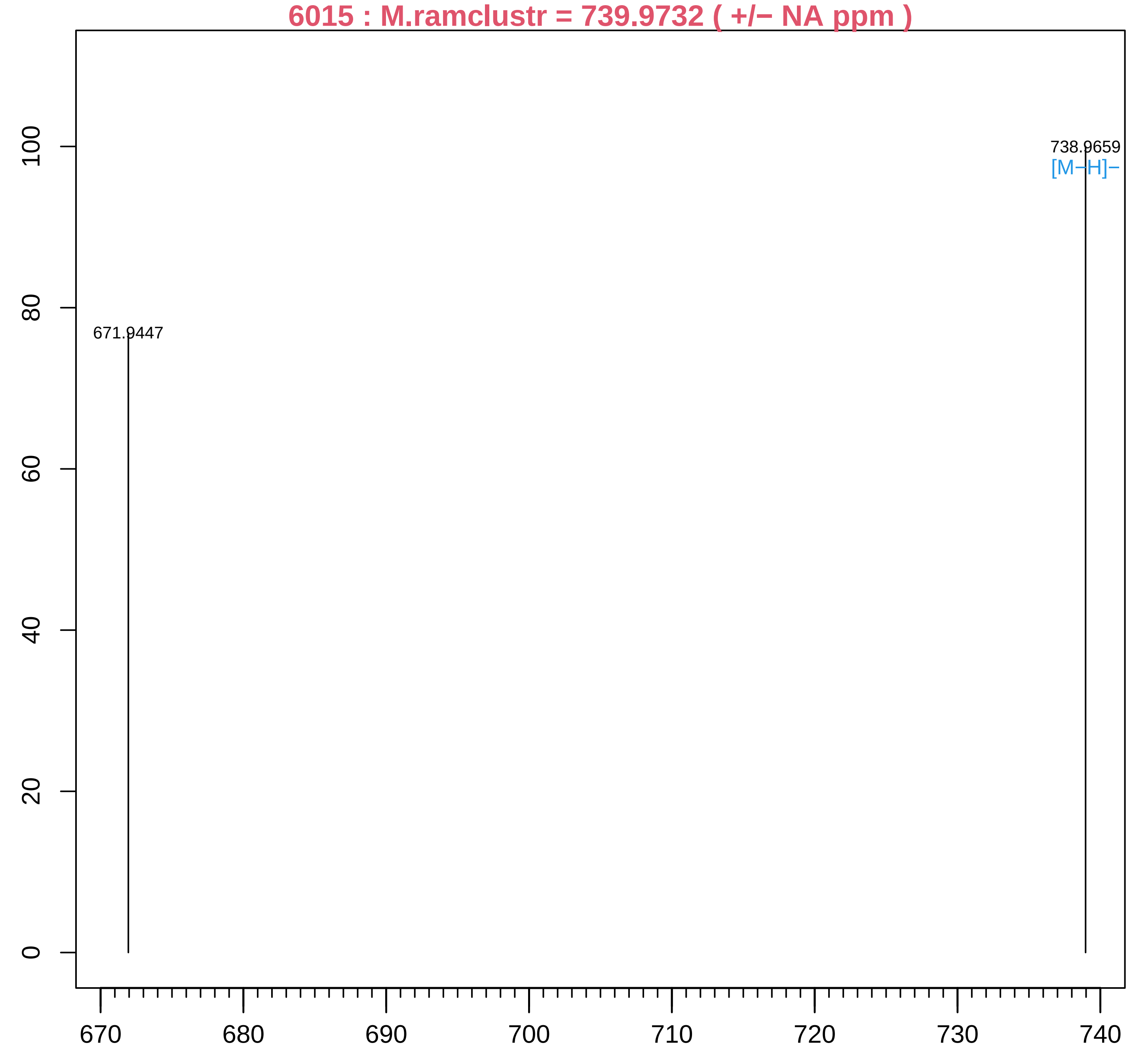
6015 : M.ramclustr = 739.9732 (+/− NA ppm)
738.9659
[M−H]−
671.9447
0
20
40
60
80
100
670
680
690
700
710
720
730
740

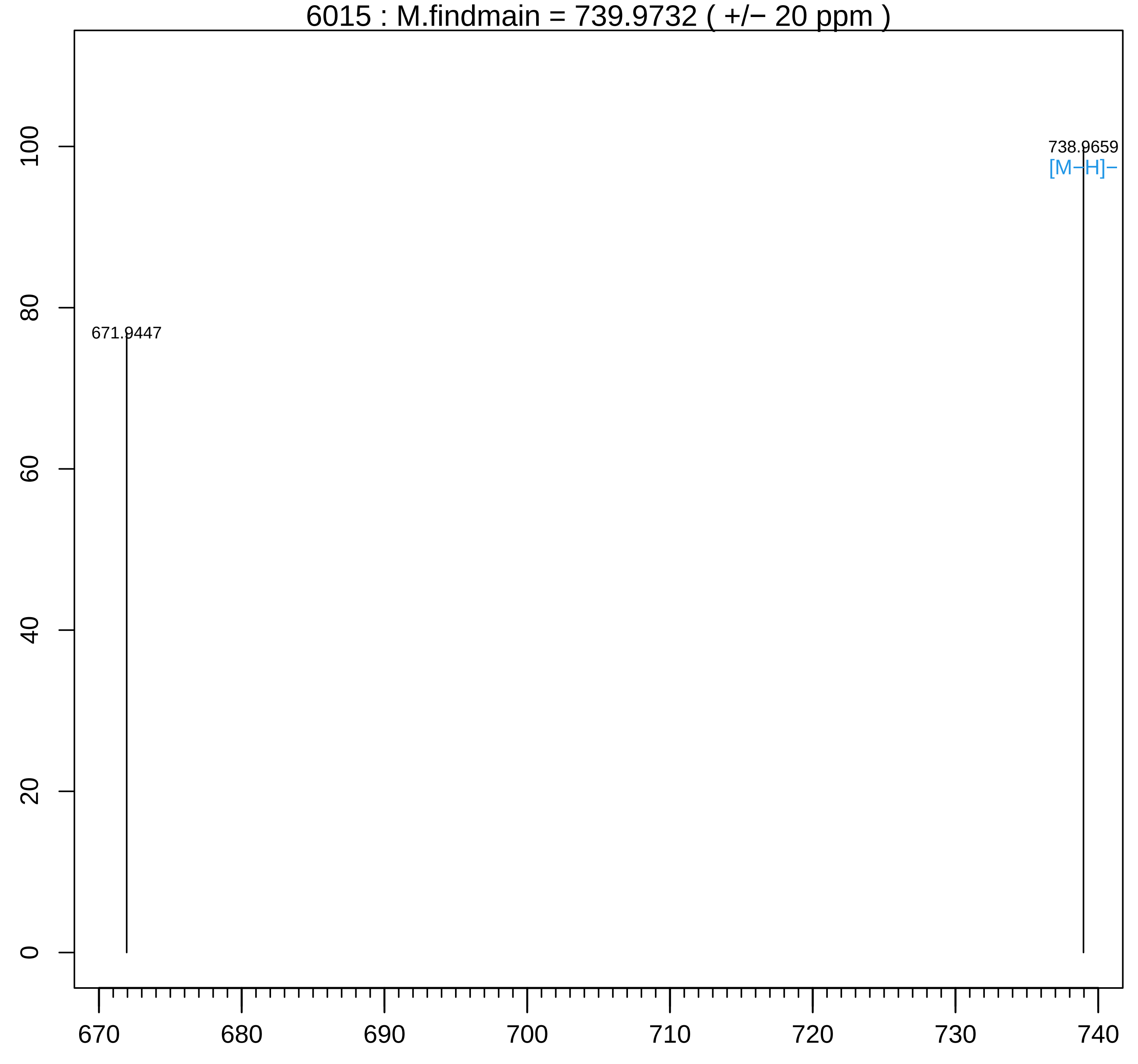
6015 : M.findmain = 739.9732 (+/− 20 ppm)
738.9659
[M−H]−
671.9447
0
20
40
60
80
100
670
680
690
700
710
720
730
740

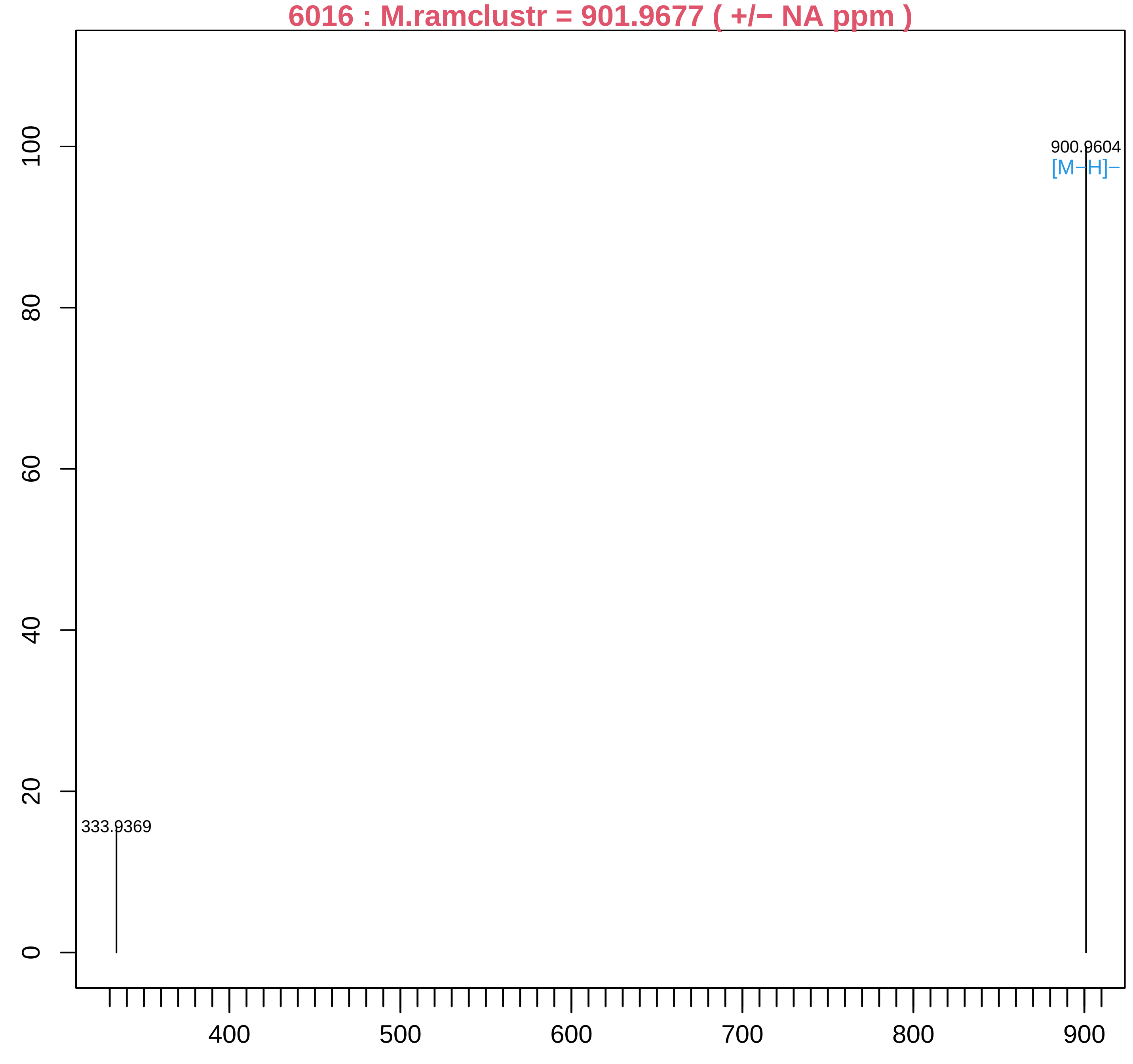

6016 : M.ramclustr = 901.9677 (+/− NA ppm)
900.9604
[M−H]−
333.9369
0
20
40
60
80
100
400
500
600
700
800
900

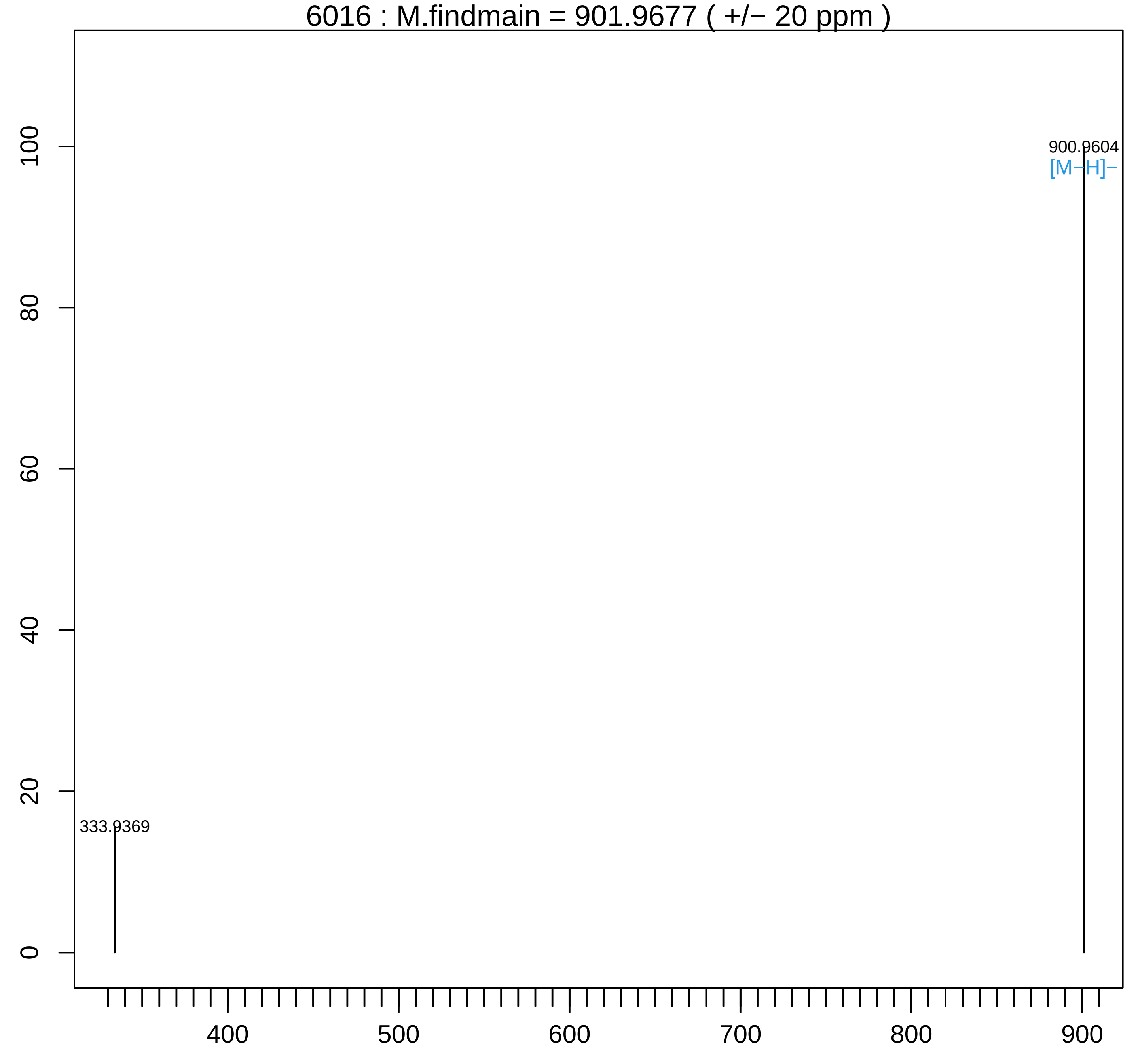

6016 : M.findmain = 901.9677 (+/− 20 ppm)
900.9604
[M−H]−
333.9369
0
20
40
60
80
100
400
500
600
700
800
900

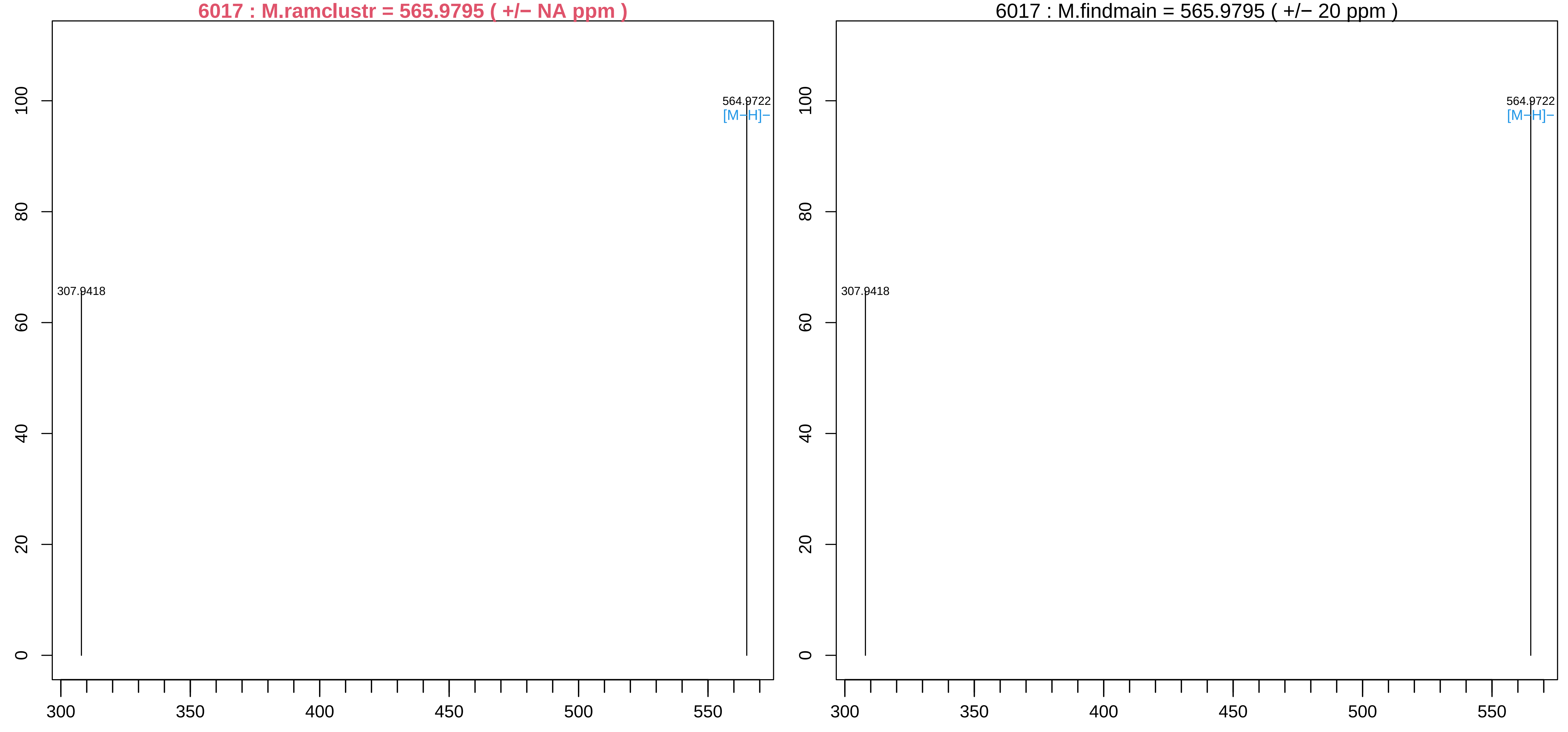
6017 : M.ramclustr = 565.9795 (+/− NA ppm)
6017 : M.findmain = 565.9795 (+/− 20 ppm)
564.9722
[M−H]−
307.9418
0
20
40
60
80
100
300
350
400
450
500
550

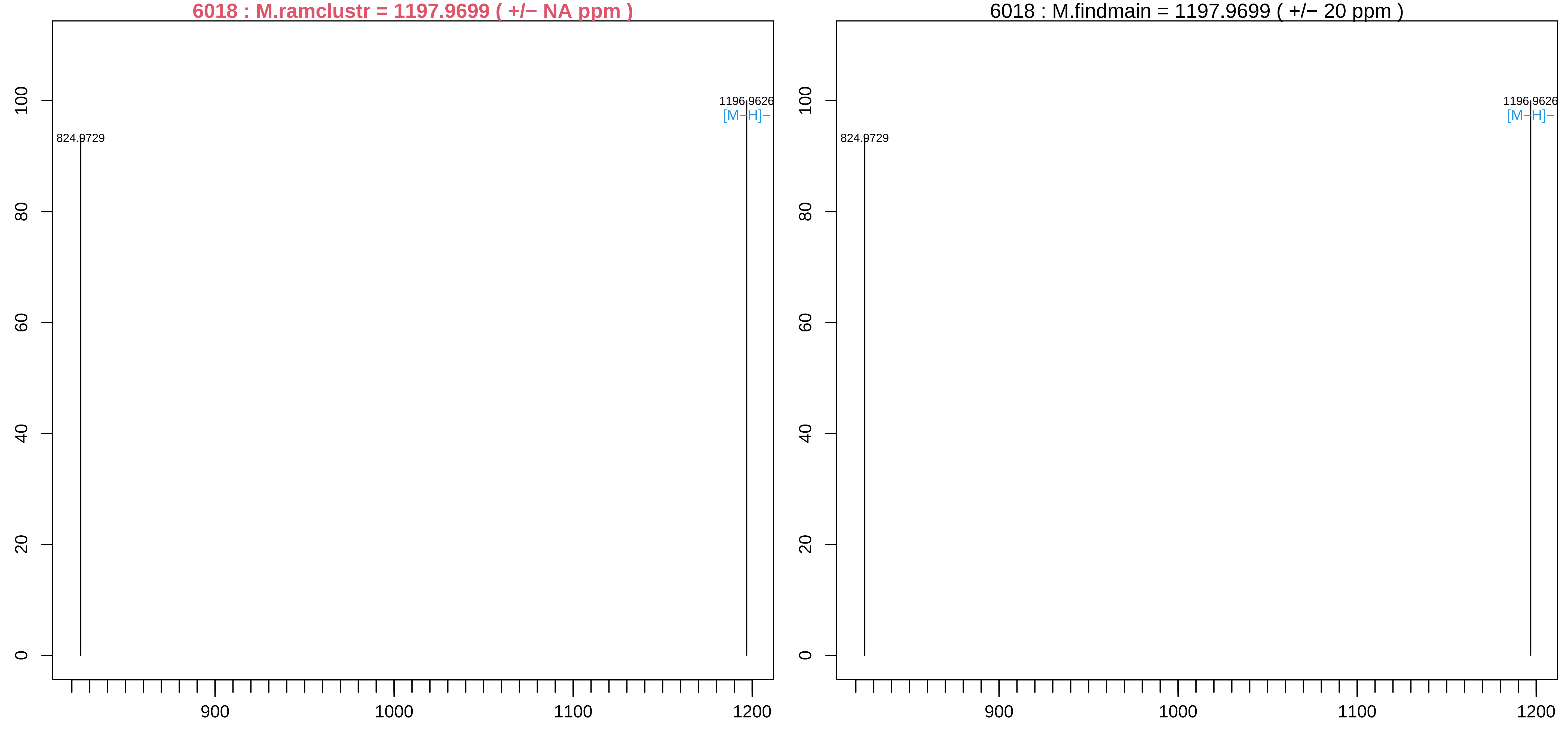
6018 : M.ramclustr = 1197.9699 (+/− NA ppm)
6018 : M.findmain = 1197.9699 (+/− 20 ppm)
1196.9626
[M−H]−
824.9729
0
20
40
60
80
100
900
1000
1100
1200

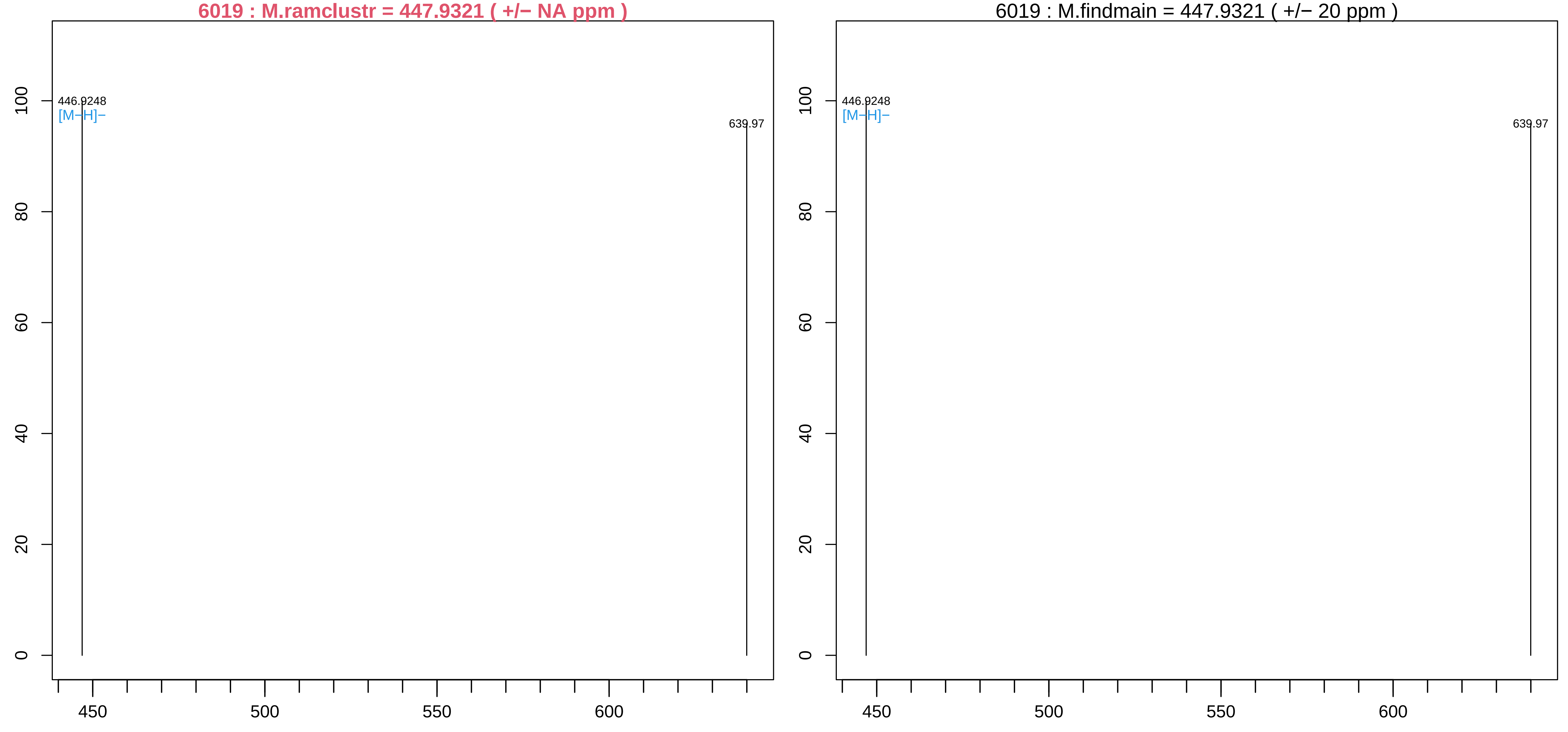

6019 : M.ramclustr = 447.9321 (+/− NA ppm)
446.9248
[M−H]−
639.97
6019 : M.findmain = 447.9321 (+/− 20 ppm)
446.9248
[M−H]−
639.97
0
20
40
60
80
100
450
500
550
600

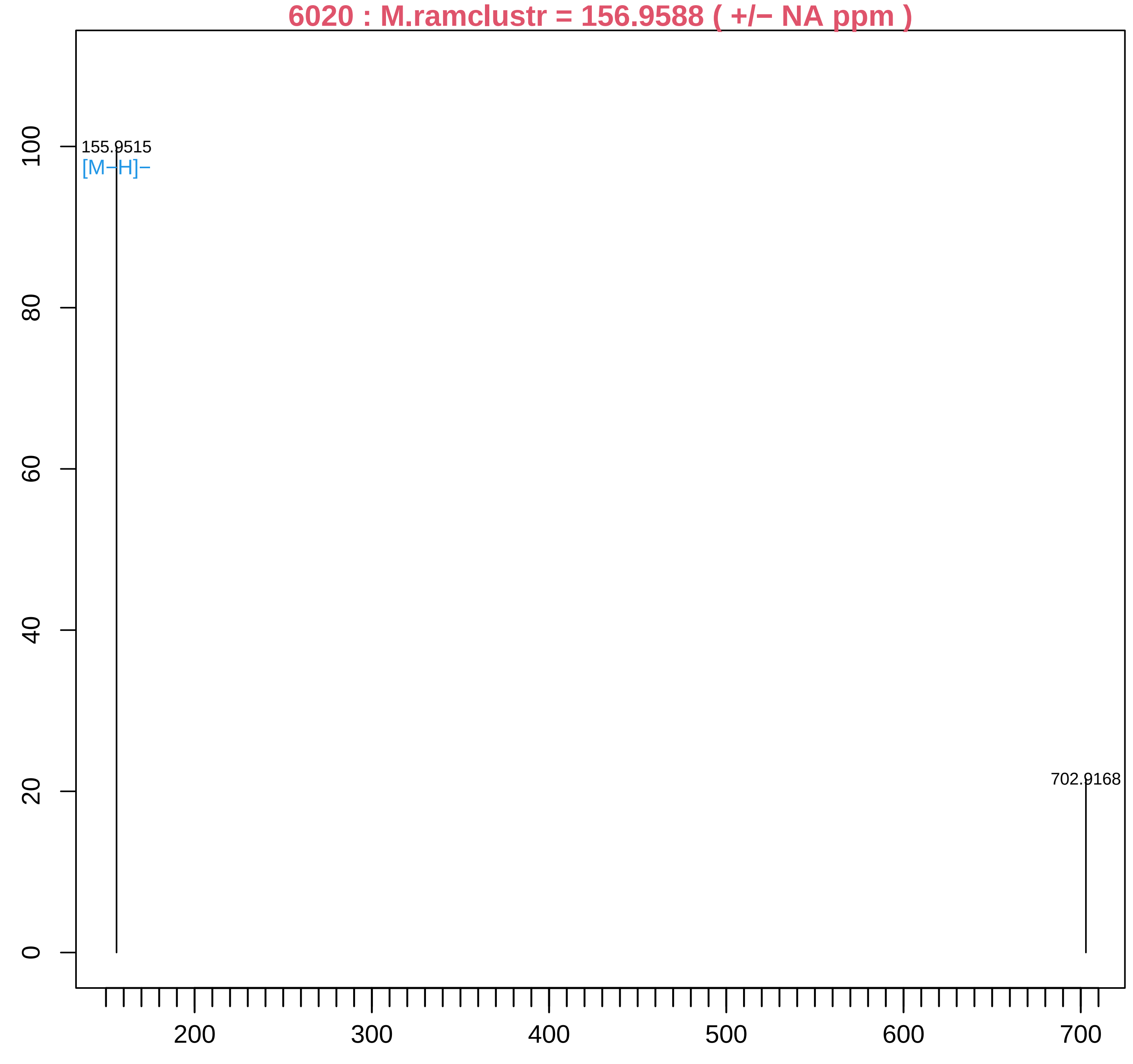
6020 : M.ramclustr = 156.9588 (+/− NA ppm)
155.9515
[M−H]−
702.9168
0
20
40
60
80
100
200
300
400
500
600
700

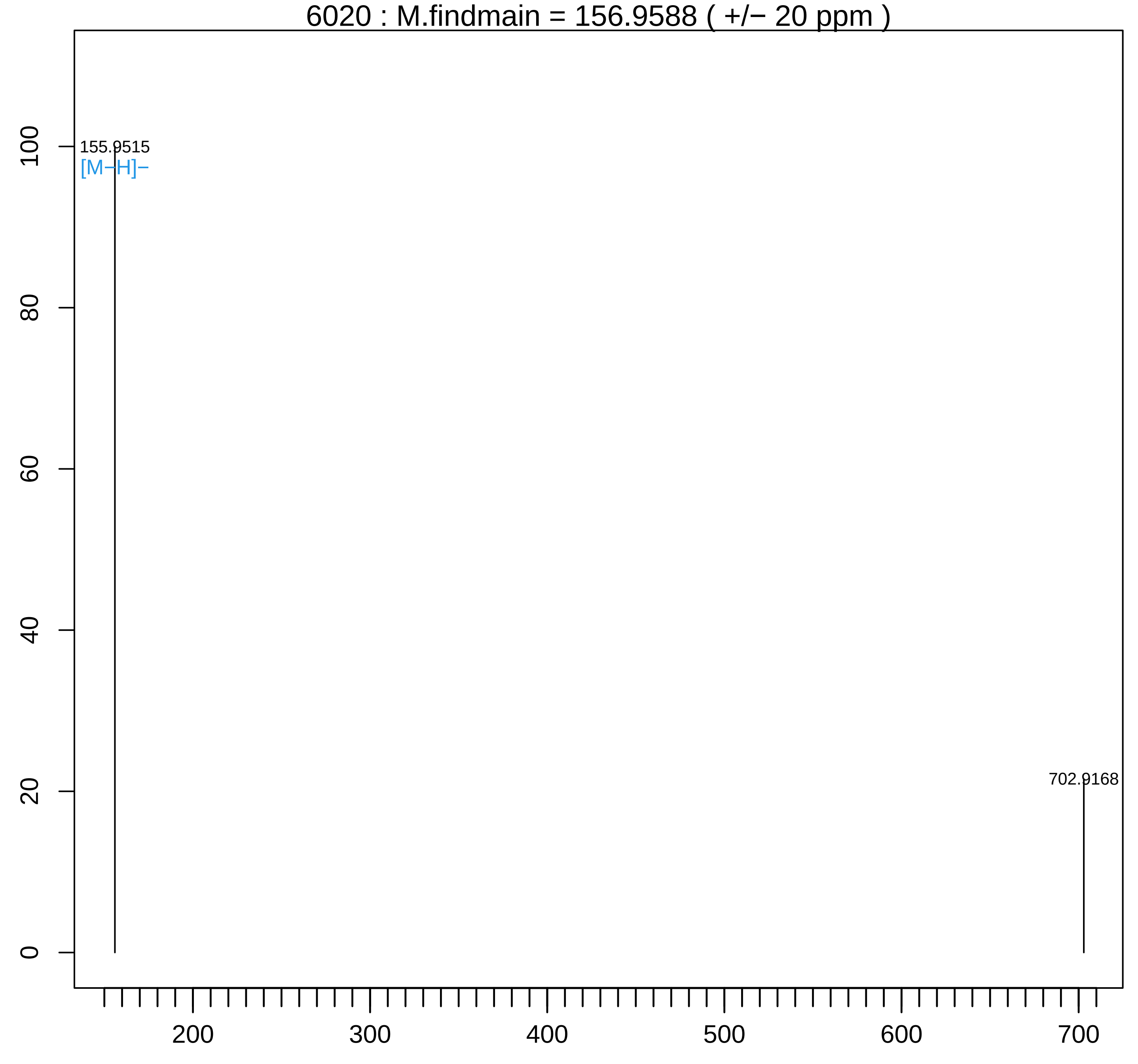
6020 : M.findmain = 156.9588 (+/− 20 ppm)
155.9515
[M−H]−
702.9168
0
20
40
60
80
100
200
300
400
500
600
700

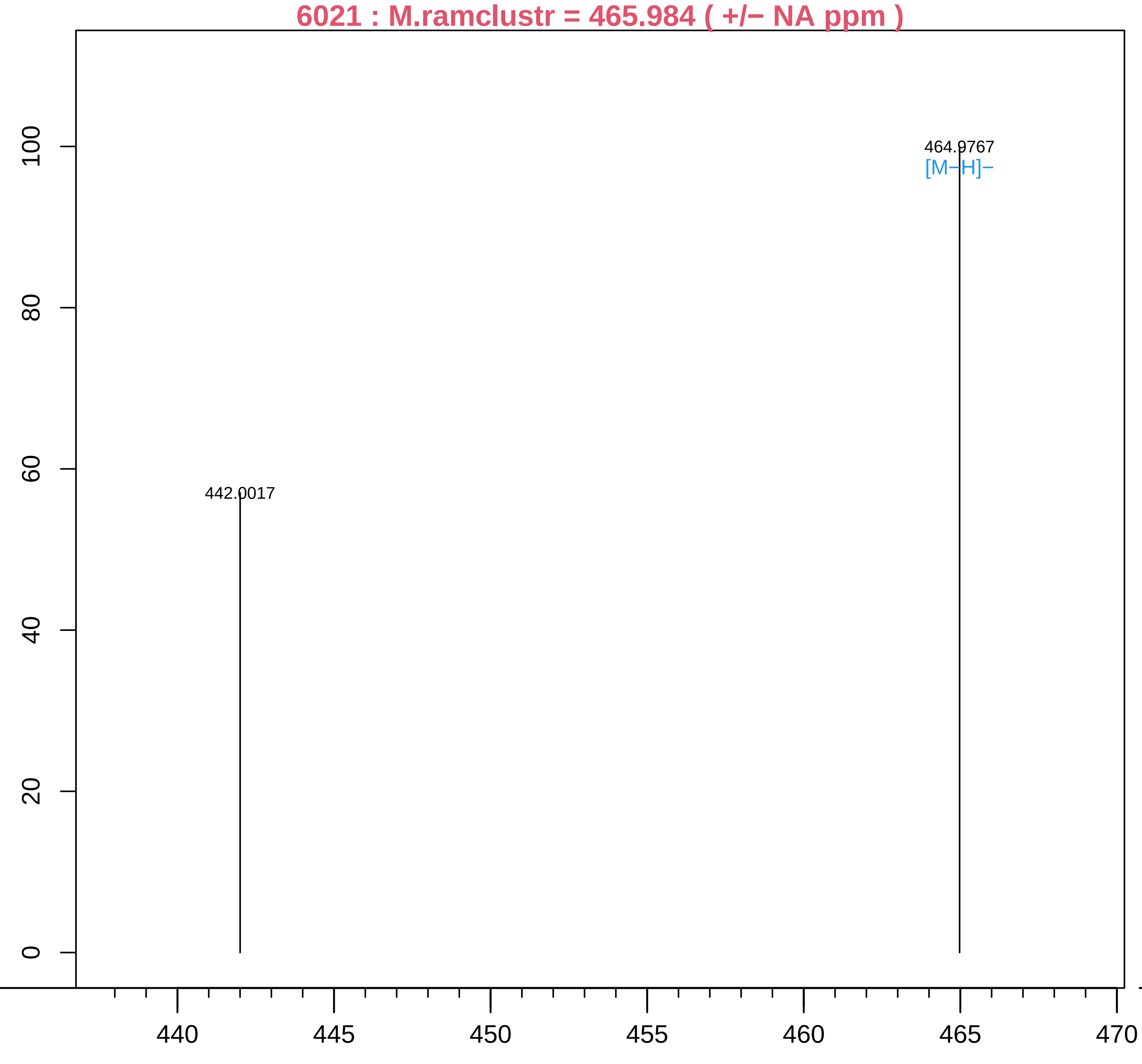
6021 : M.ramclustr = 465.984 (+/− NA ppm)
464.9767
[M−H]−
442.0017
0
20
40
60
80
100
440
445
450
455
460
465
470

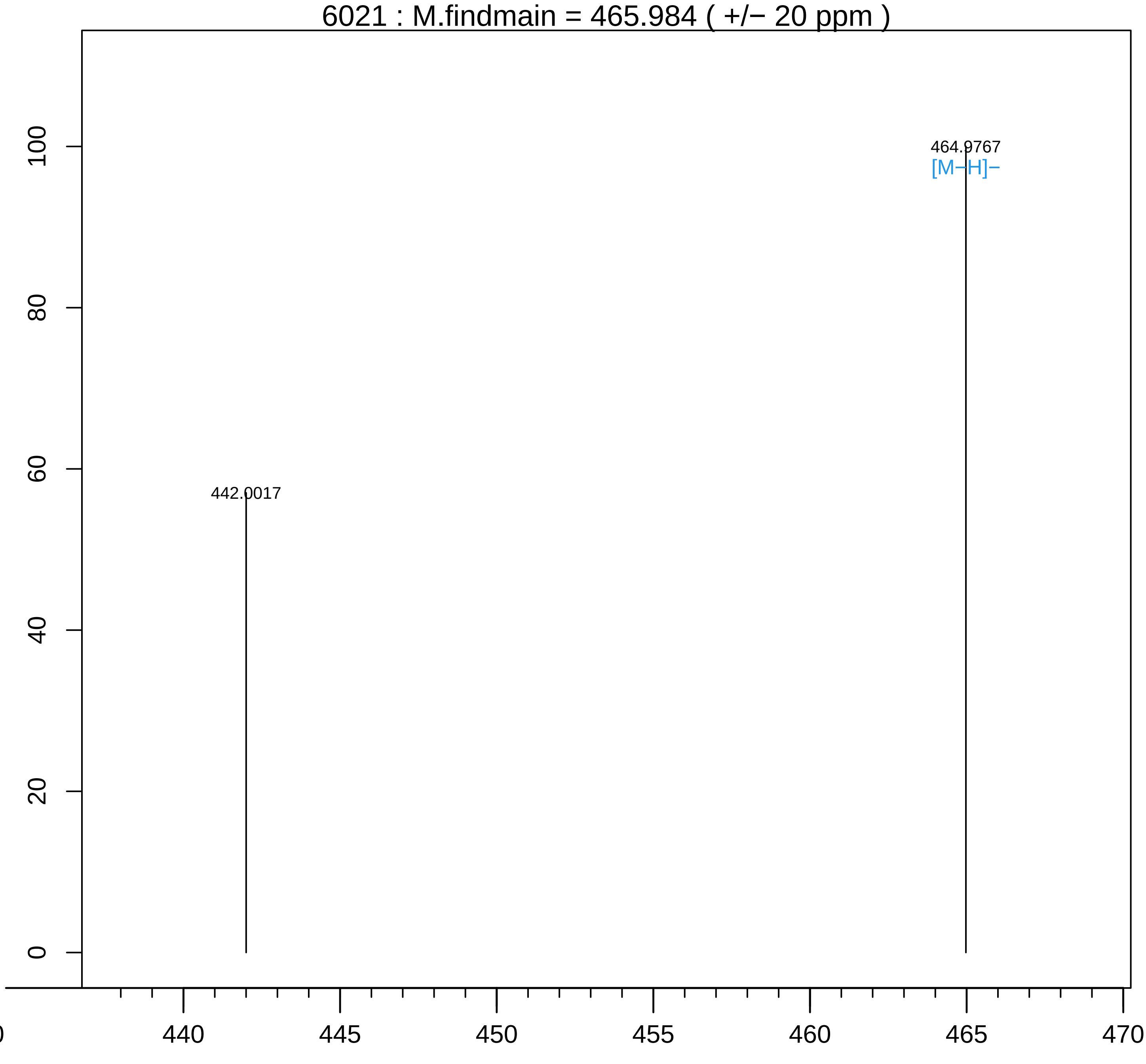
6021 : M.findmain = 465.984 (+/− 20 ppm)
464.9767
[M−H]−
442.0017
0
20
40
60
80
100
440
445
450
455
460
465
470

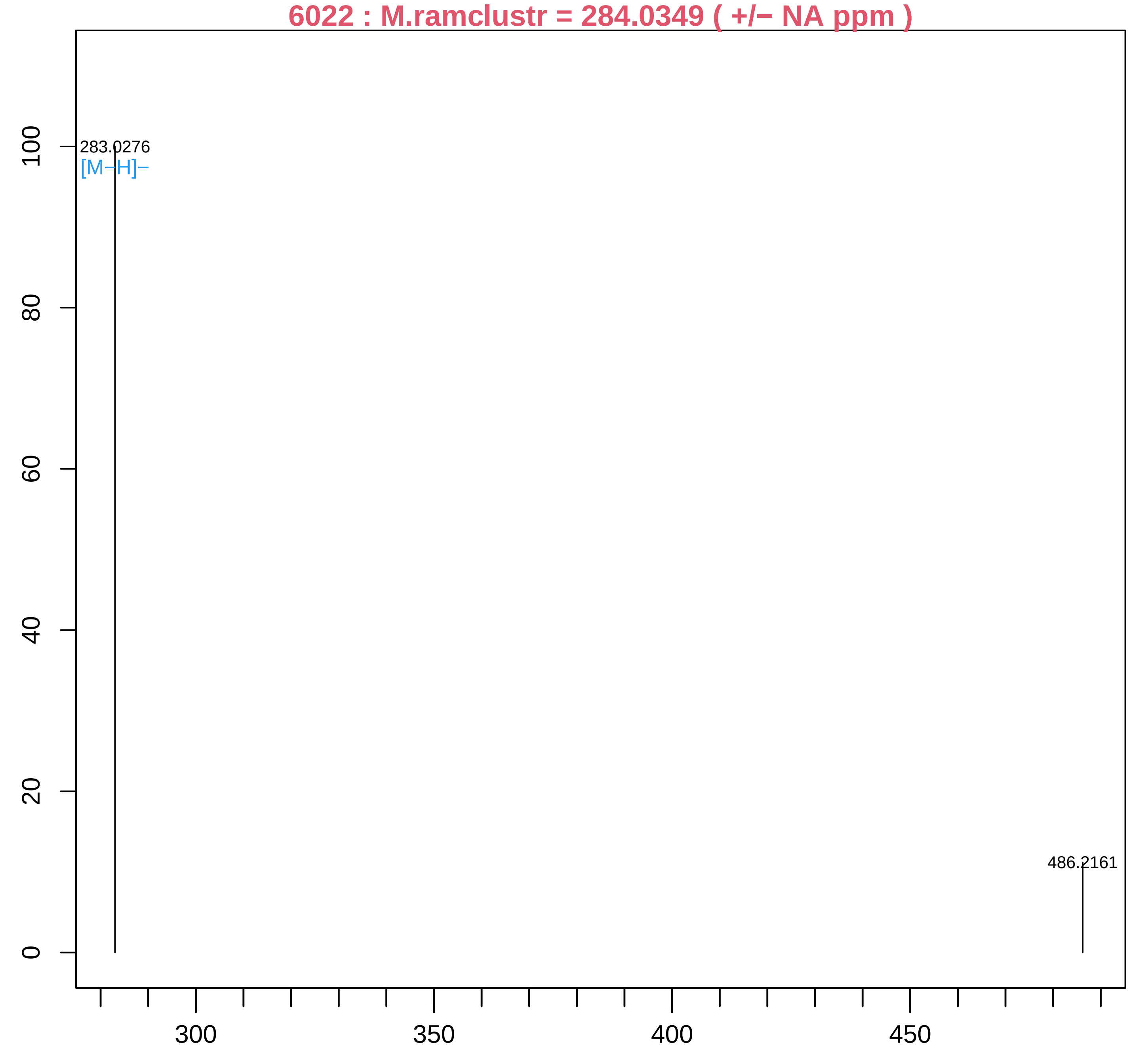
6022 : M.ramclustr = 284.0349 (+/− NA ppm)
283.0276
[M−H]−
486.2161
0
20
40
60
80
100
300
350
400
450

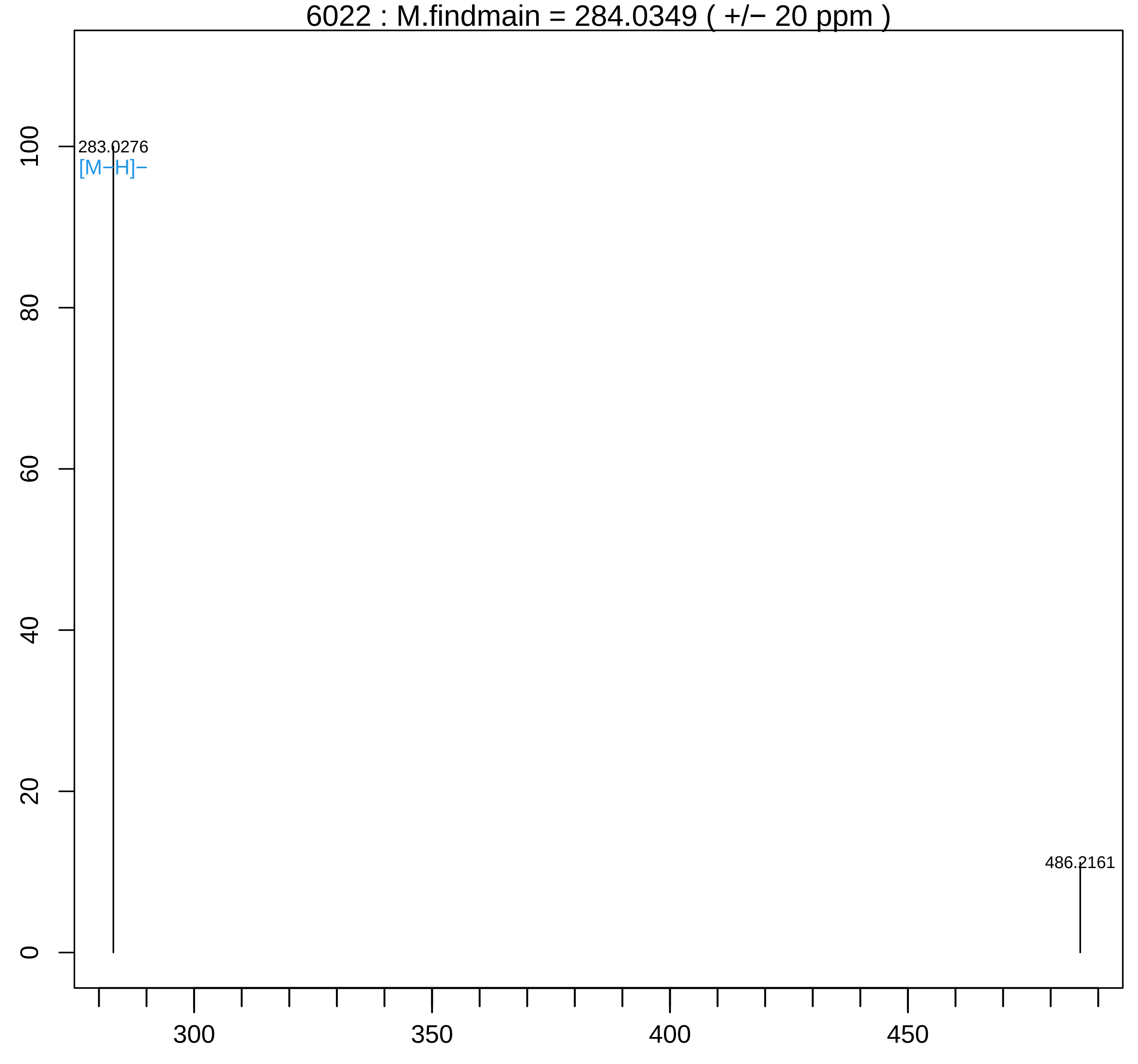
6022 : M.findmain = 284.0349 (+/− 20 ppm)
283.0276
[M−H]−
486.2161
0
20
40
60
80
100
300
350
400
450

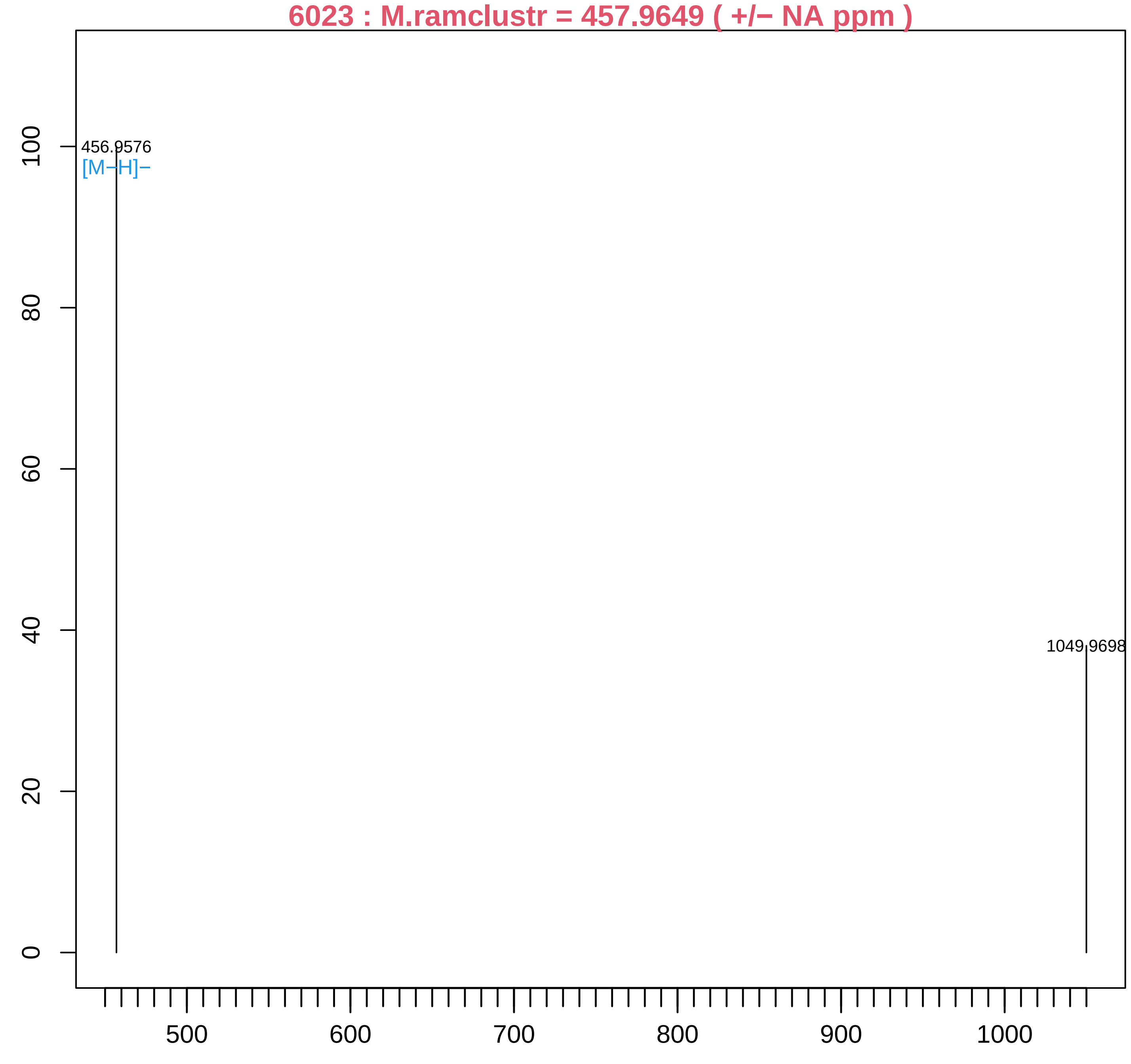
6023 : M.ramclustr = 457.9649 (+/− NA ppm)
456.9576
[M−H]−
1049.9698
0
20
40
60
80
100
500
600
700
800
900
1000

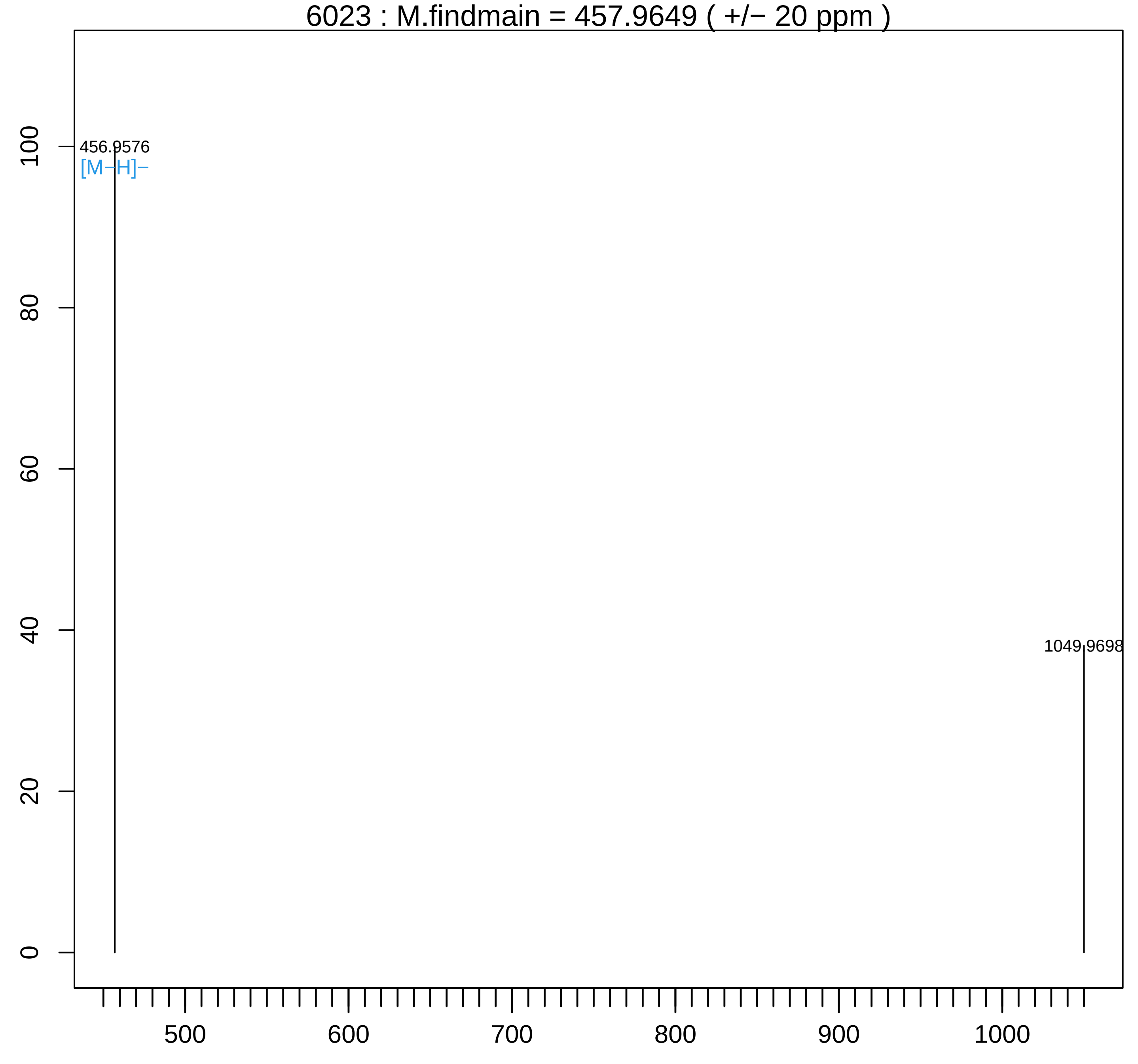
6023 : M.findmain = 457.9649 (+/− 20 ppm)
456.9576
[M−H]−
1049.9698
0
20
40
60
80
100
500
600
700
800
900
1000

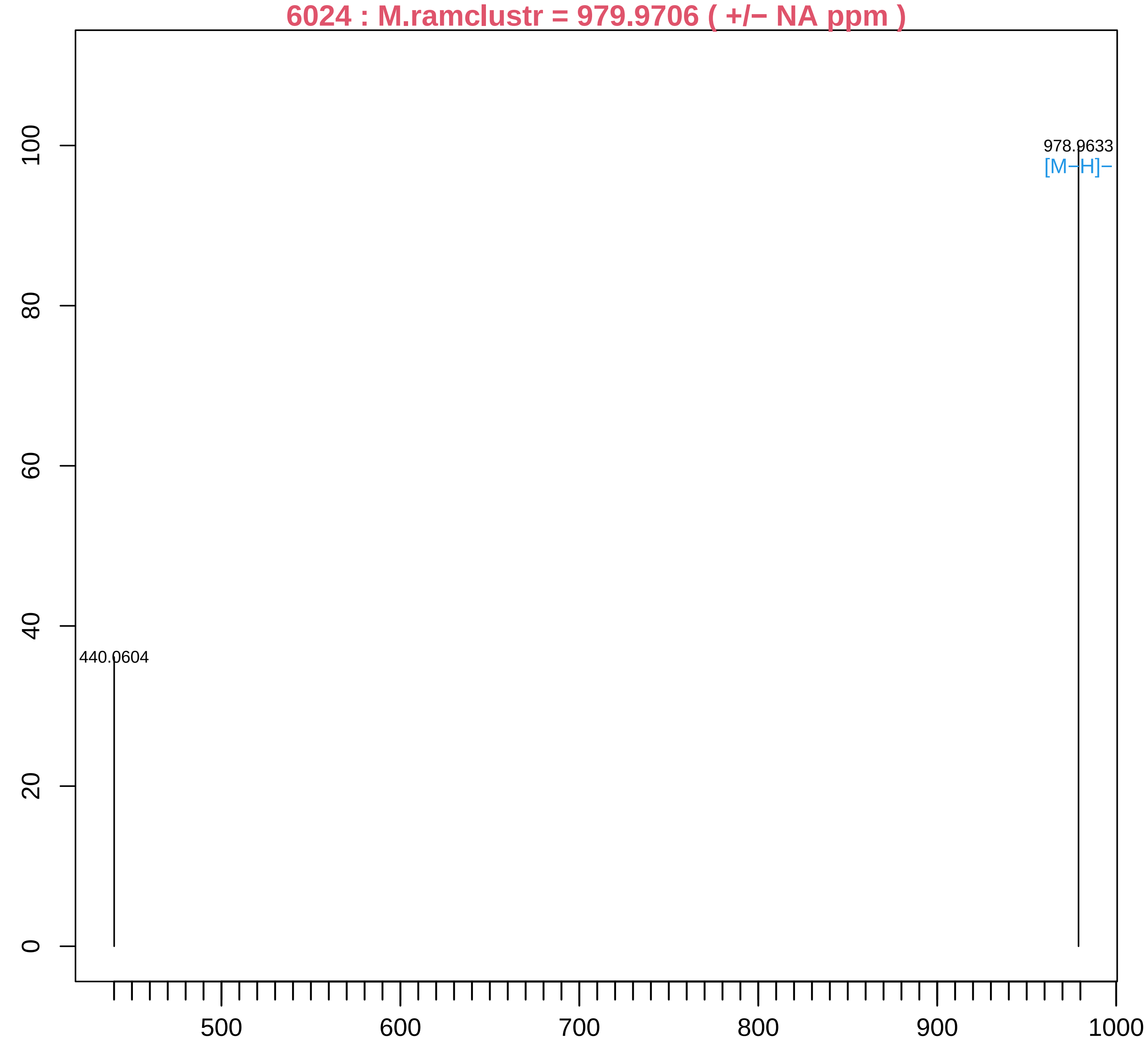
6024 : M.ramclustr = 979.9706 (+/− NA ppm)
978.9633
[M−H]−
440.0604
0
20
40
60
80
100
500
600
700
800
900
1000

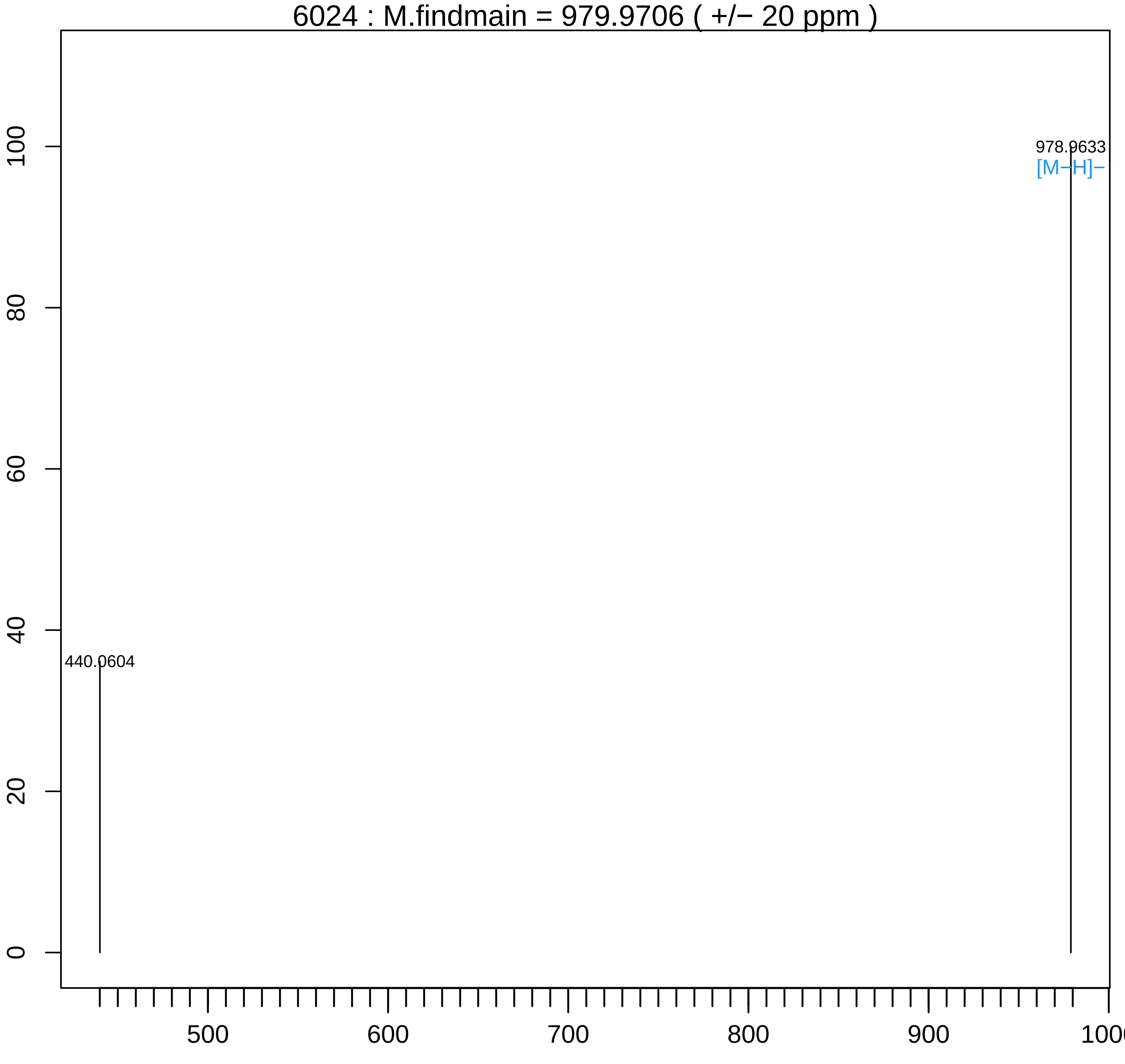
6024 : M.findmain = 979.9706 (+/− 20 ppm)
978.9633
[M−H]−
440.0604
0
20
40
60
80
100
500
600
700
800
900
1000

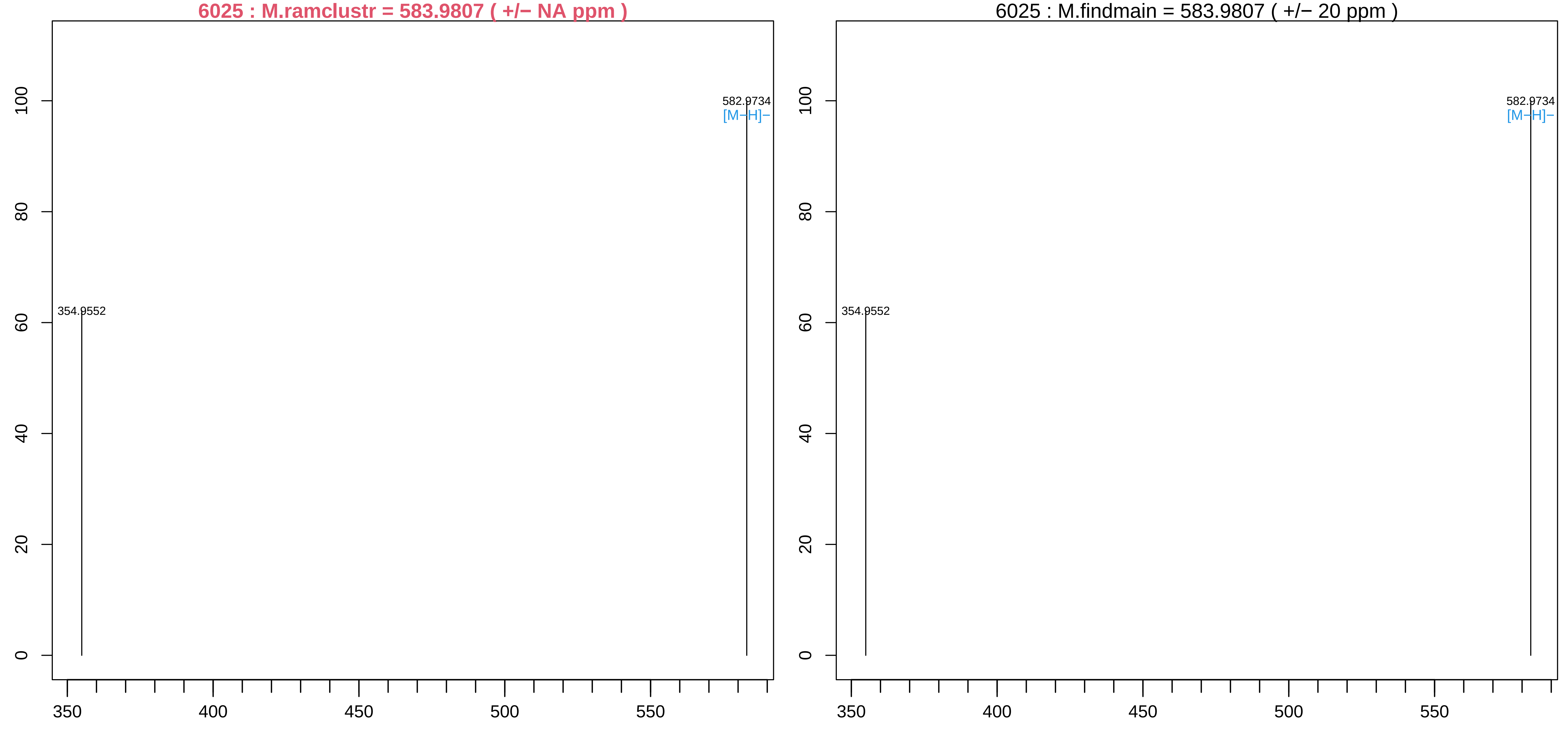
6025 : M.ramclustr = 583.9807 (+/− NA ppm)
6025 : M.findmain = 583.9807 (+/− 20 ppm)
582.9734
[M−H]−
354.9552
0
20
40
60
80
100
350
400
450
500
550

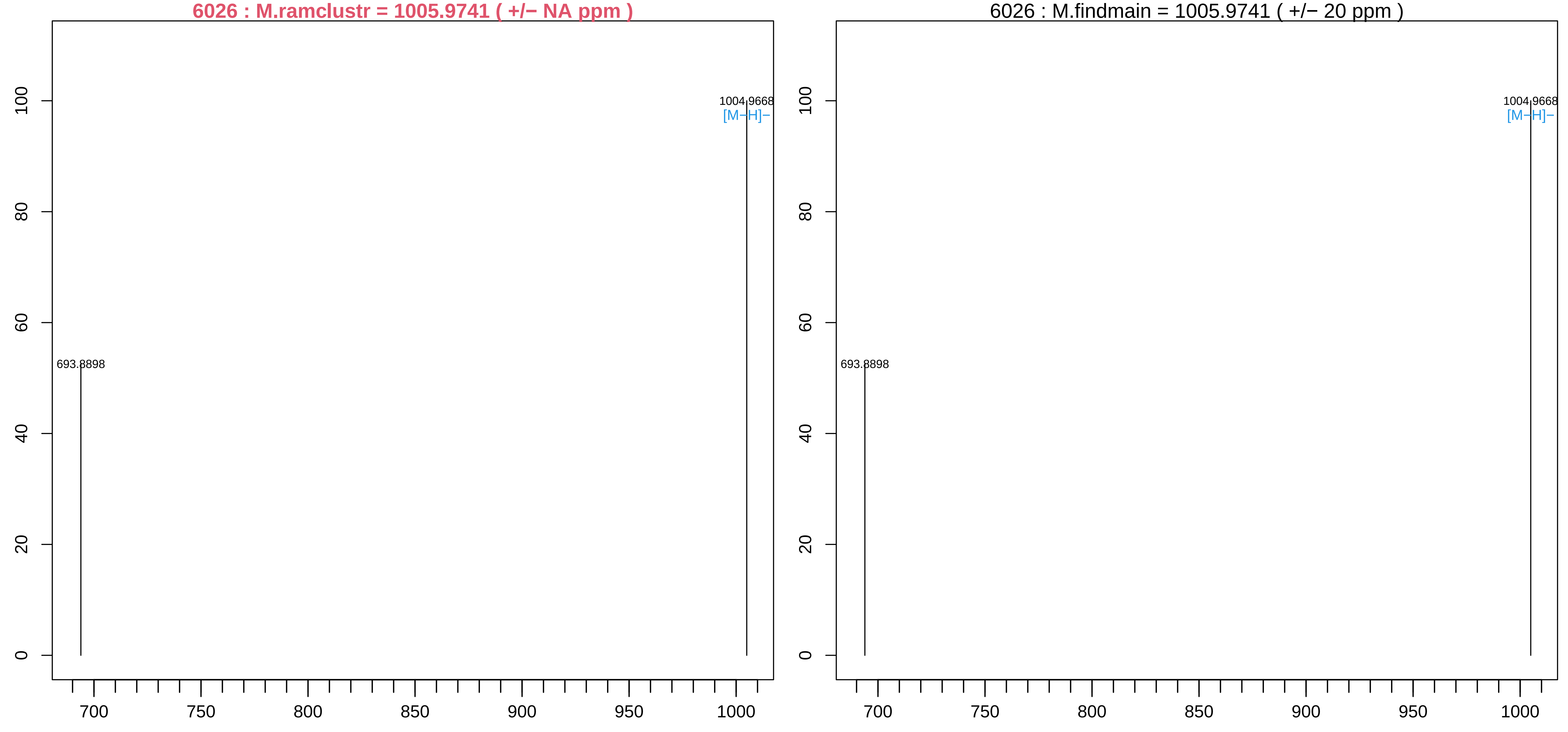
6026 : M.ramclustr = 1005.9741 (+/− NA ppm)
6026 : M.findmain = 1005.9741 (+/− 20 ppm)
1004.9668
[M−H]−
693.8898
1004.9668
[M−H]−
693.8898
0
20
40
60
80
100
700
750
800
850
900
950
1000

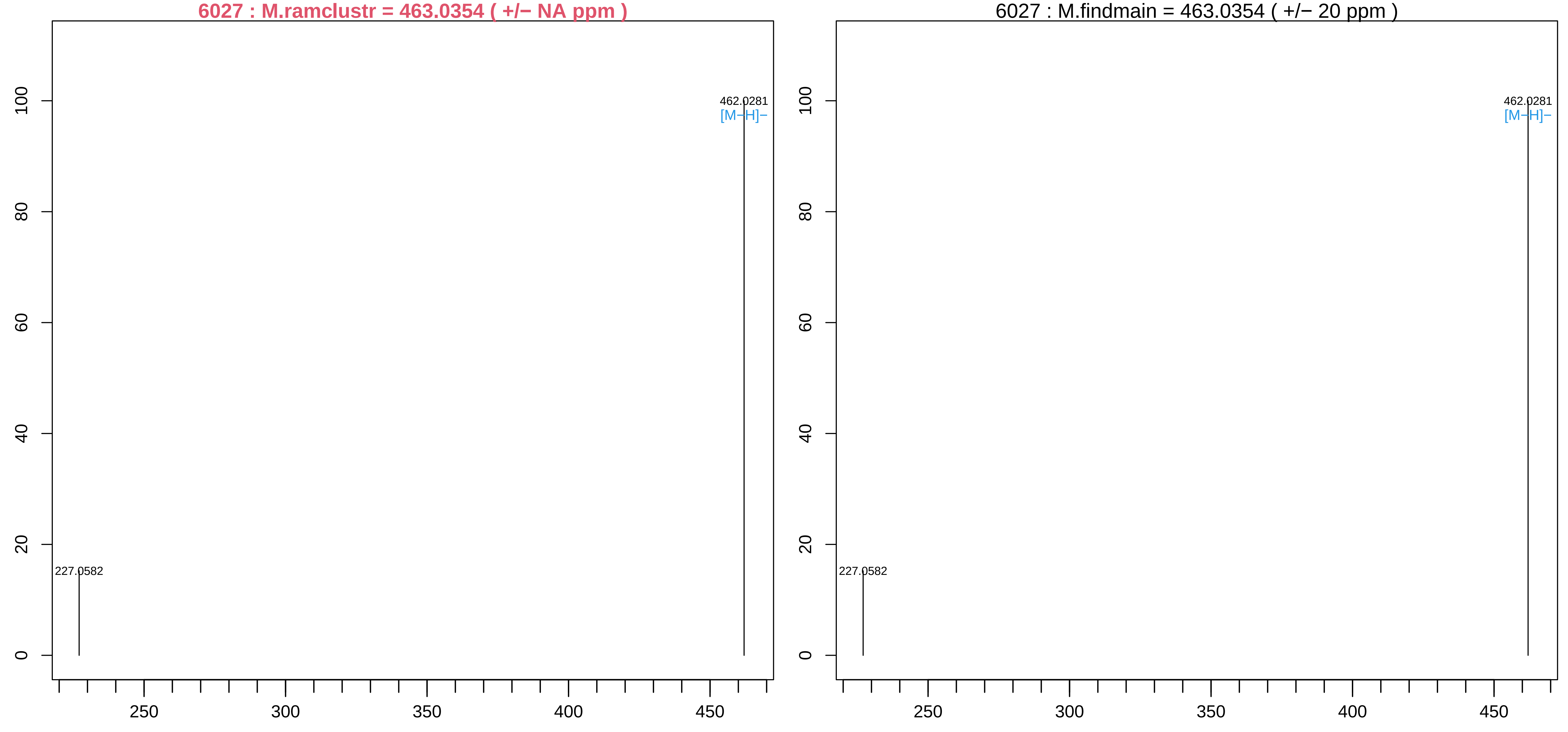

6027 : M.ramclustr = 463.0354 (+/− NA ppm)
462.0281
[M−H]−
227.0582
0
20
40
60
80
100
250
300
350
400
450
6027 : M.findmain = 463.0354 (+/− 20 ppm)
462.0281
[M−H]−
227.0582
0
20
40
60
80
100
250
300
350
400
450

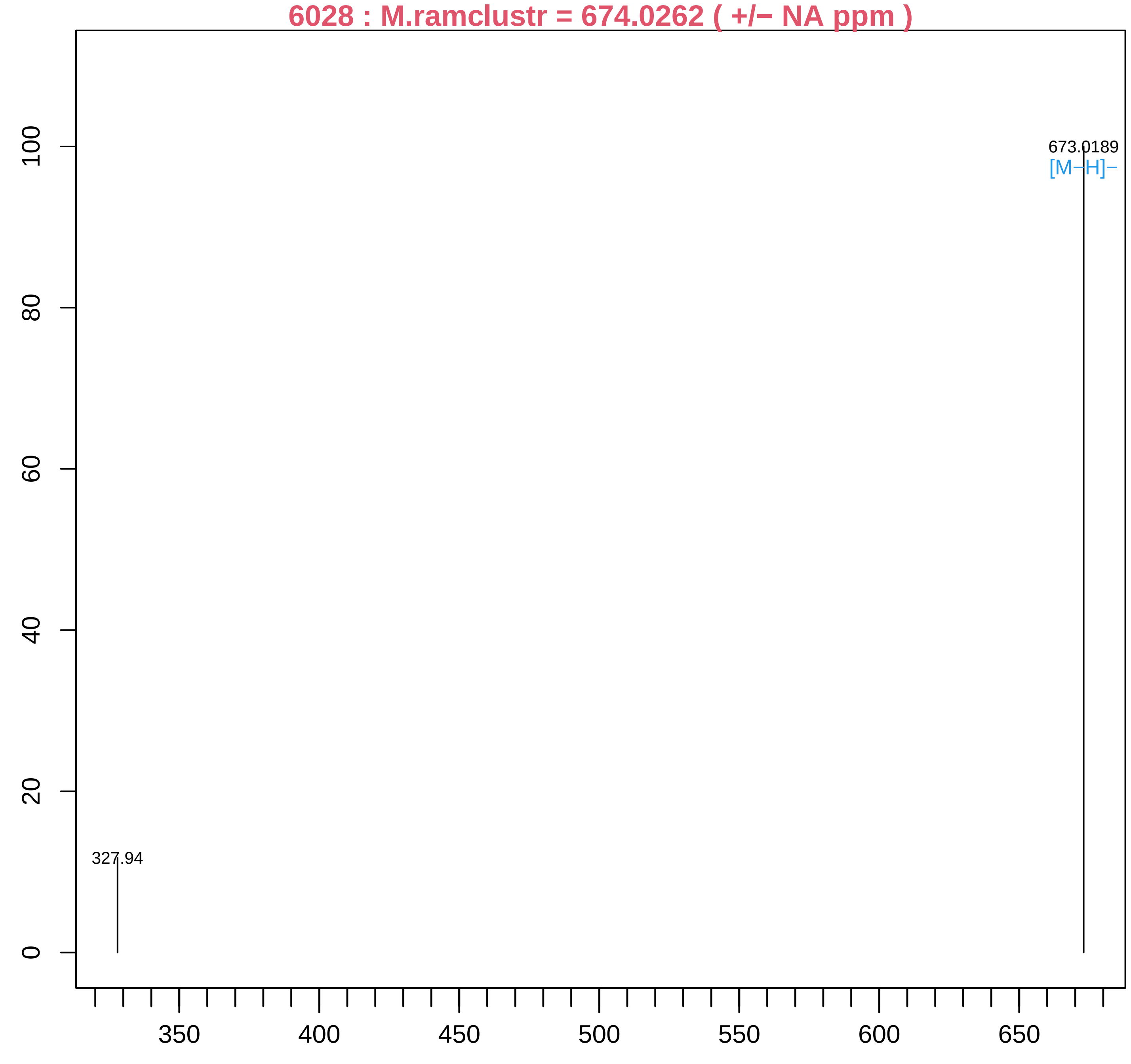
6028 : M.ramclustr = 674.0262 (+/− NA ppm)
673.0189
[M−H]−
327.94
0
20
40
60
80
100
350
400
450
500
550
600
650

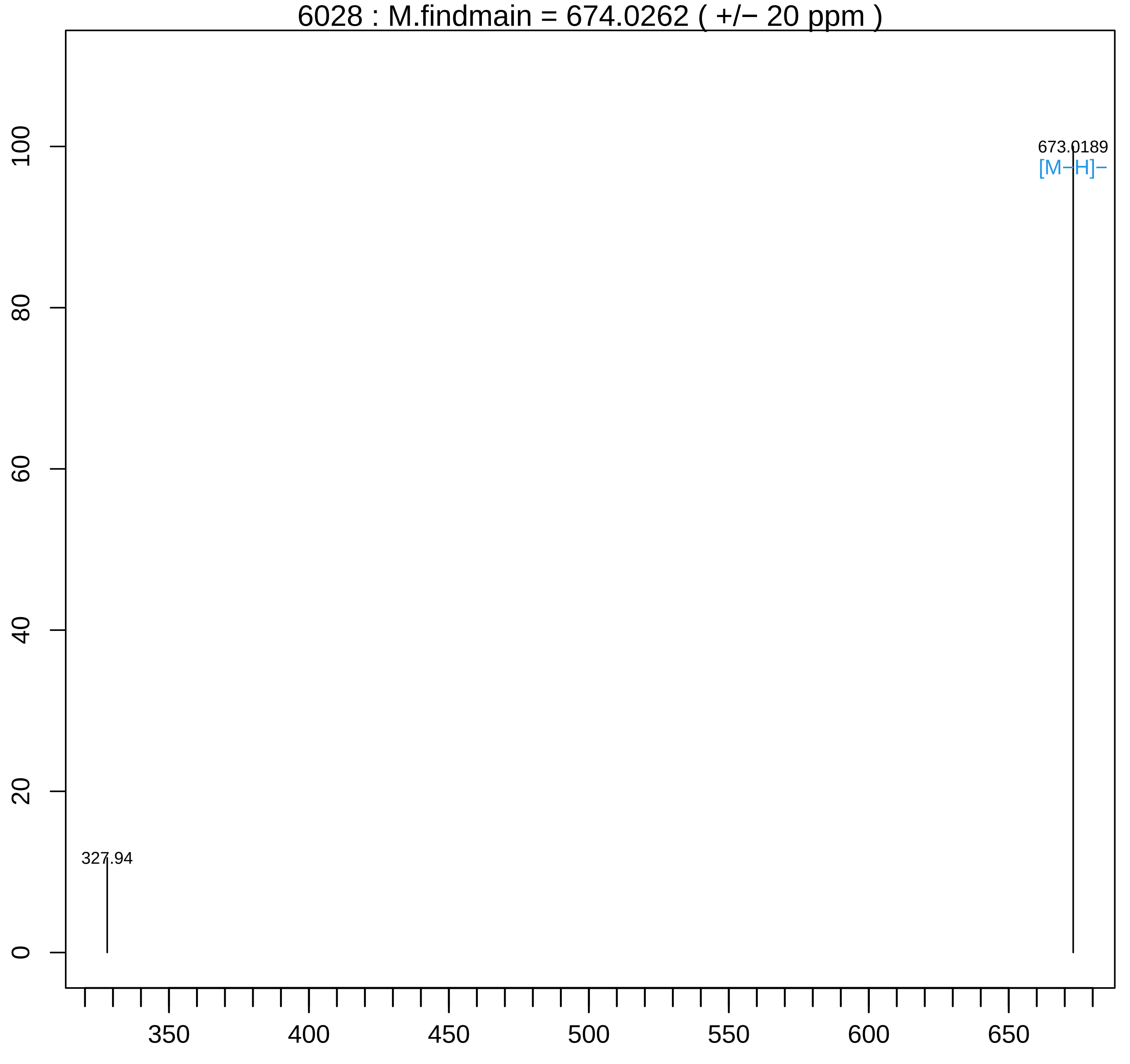
6028 : M.findmain = 674.0262 (+/− 20 ppm)
673.0189
[M−H]−
327.94
0
20
40
60
80
100
350
400
450
500
550
600
650

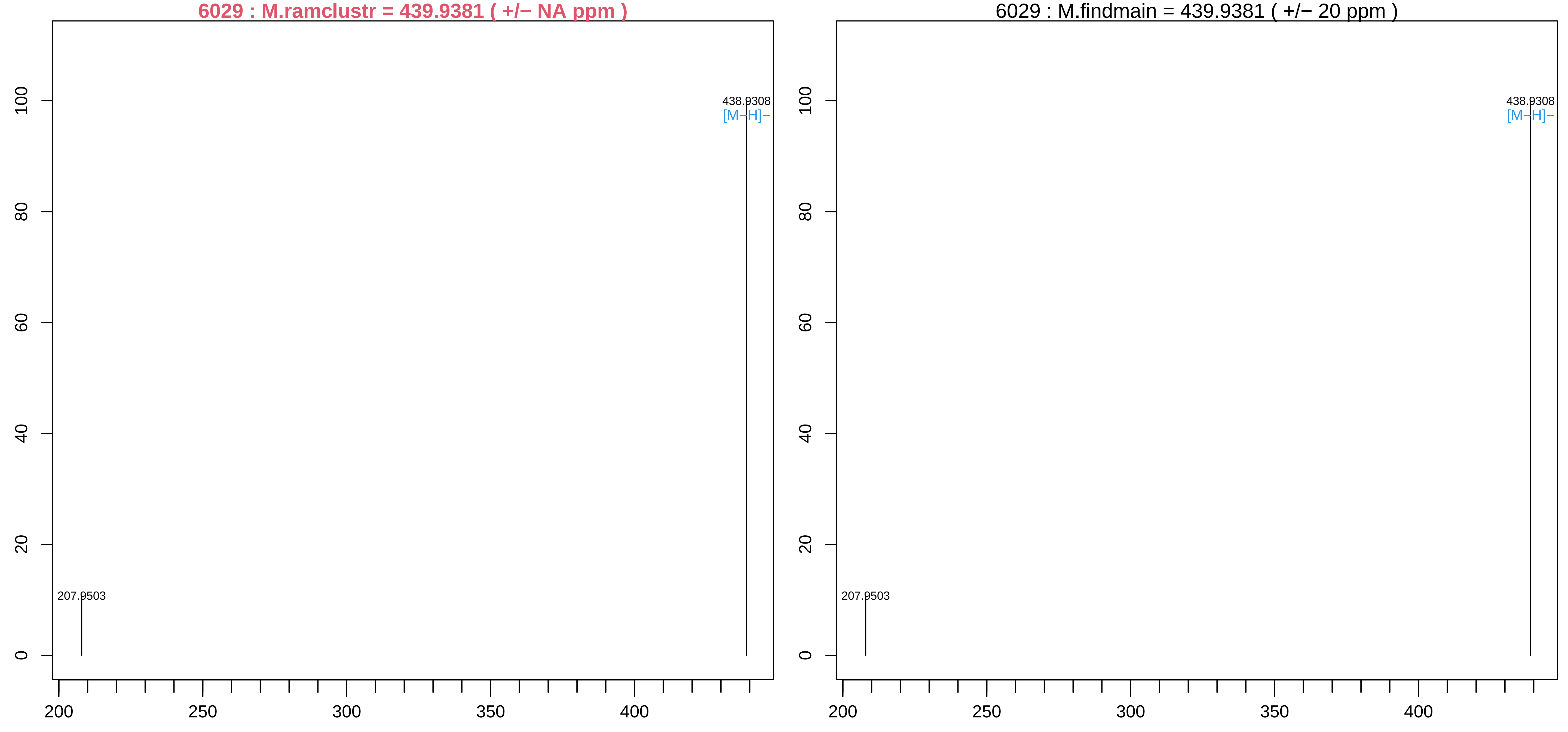

6029 : M.ramclustr = 439.9381 (+/− NA ppm)
438.9308
[M−H]−
207.9503
6029 : M.findmain = 439.9381 (+/− 20 ppm)
438.9308
[M−H]−
207.9503
0
20
40
60
80
100
200
250
300
350
400

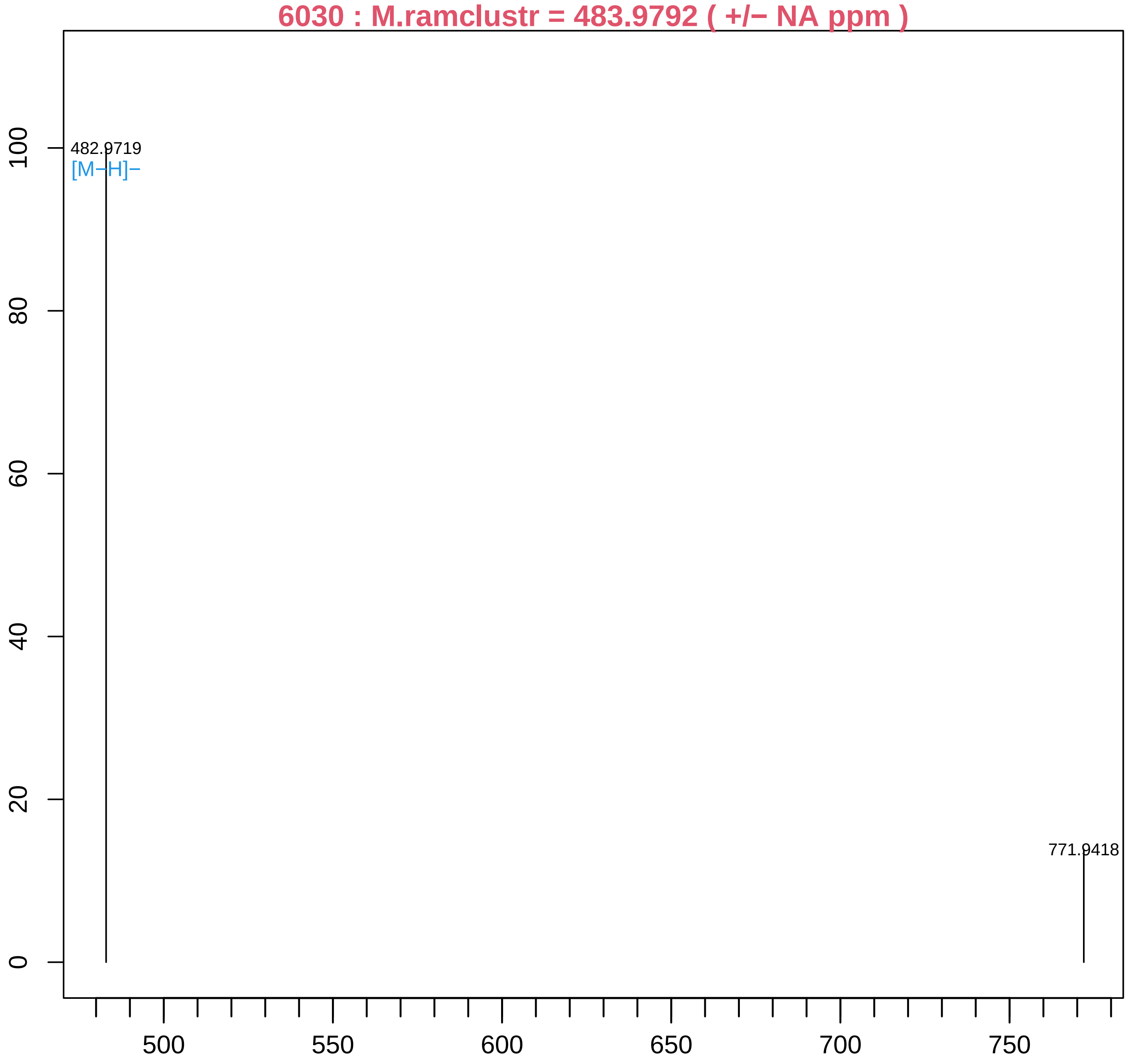
6030 : M.ramclustr = 483.9792 (+/− NA ppm)
482.9719
[M−H]−
771.9418
0
20
40
60
80
100
500
550
600
650
700
750

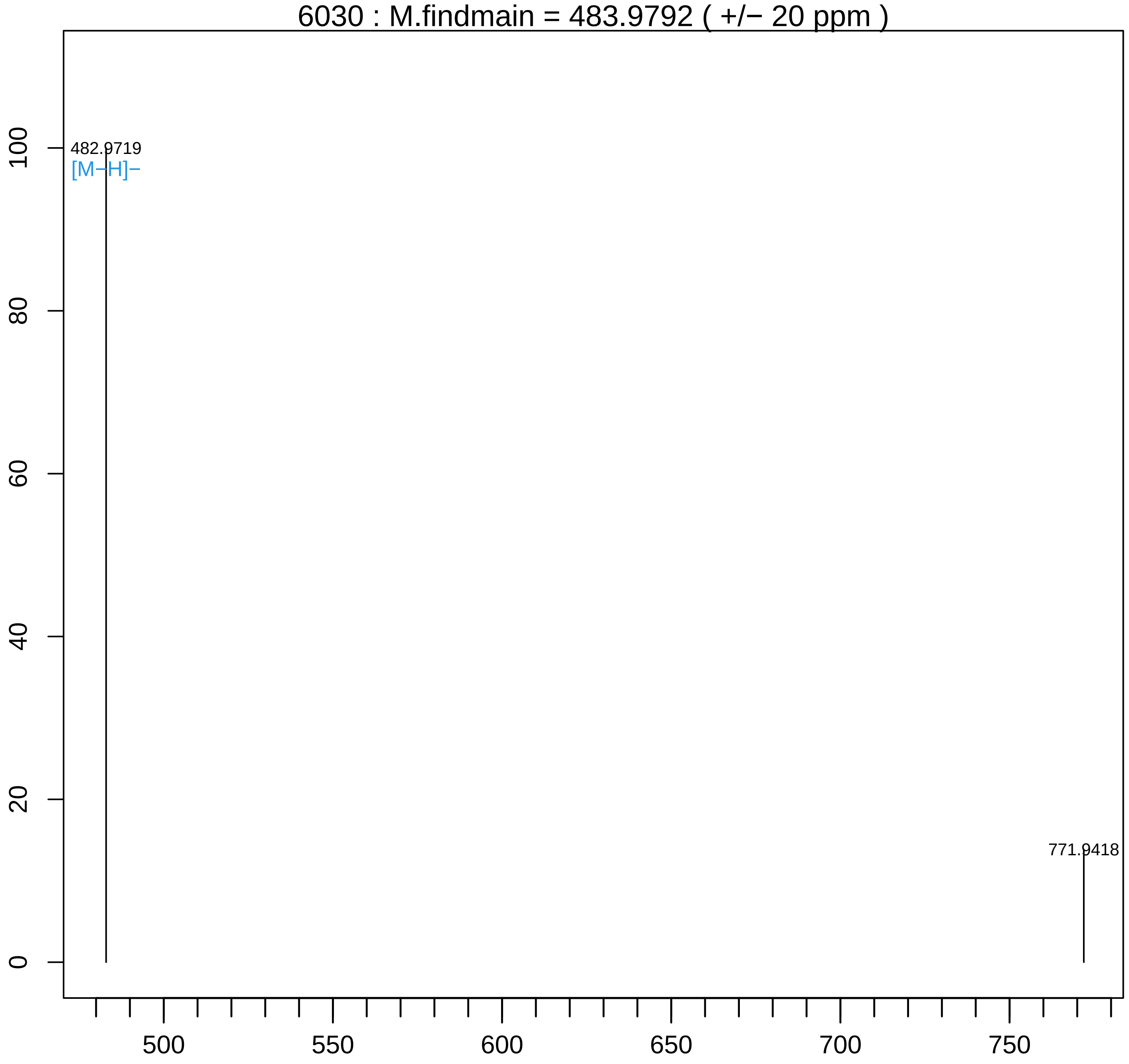
6030 : M.findmain = 483.9792 (+/− 20 ppm)
482.9719
[M−H]−
771.9418
0
20
40
60
80
100
500
550
600
650
700
750

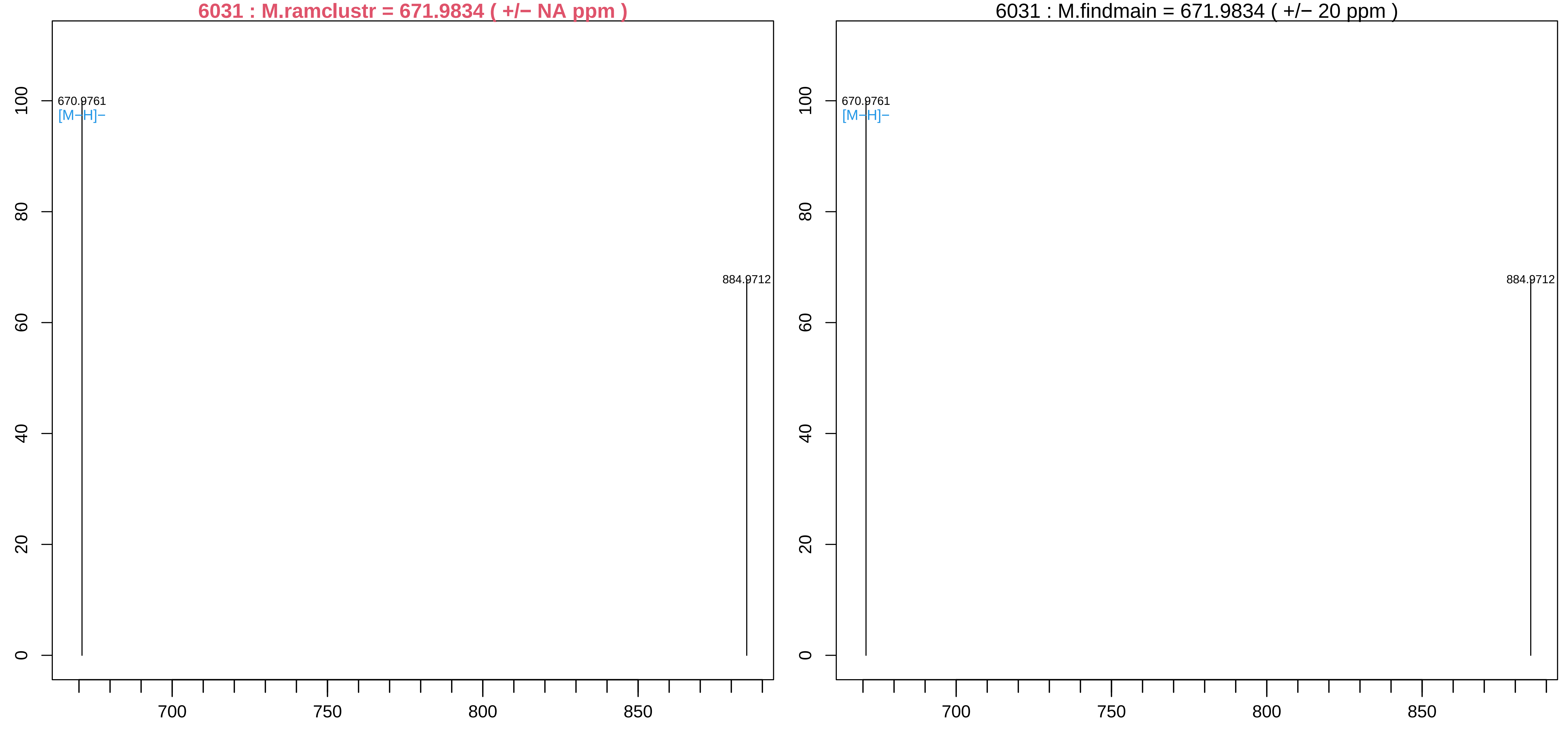
6031 : M.ramclustr = 671.9834 (+/− NA ppm)
670.9761
[M−H]−
884.9712
0
20
40
60
80
100
700
750
800
850
6031 : M.findmain = 671.9834 (+/− 20 ppm)
670.9761
[M−H]−
884.9712
0
20
40
60
80
100
700
750
800
850

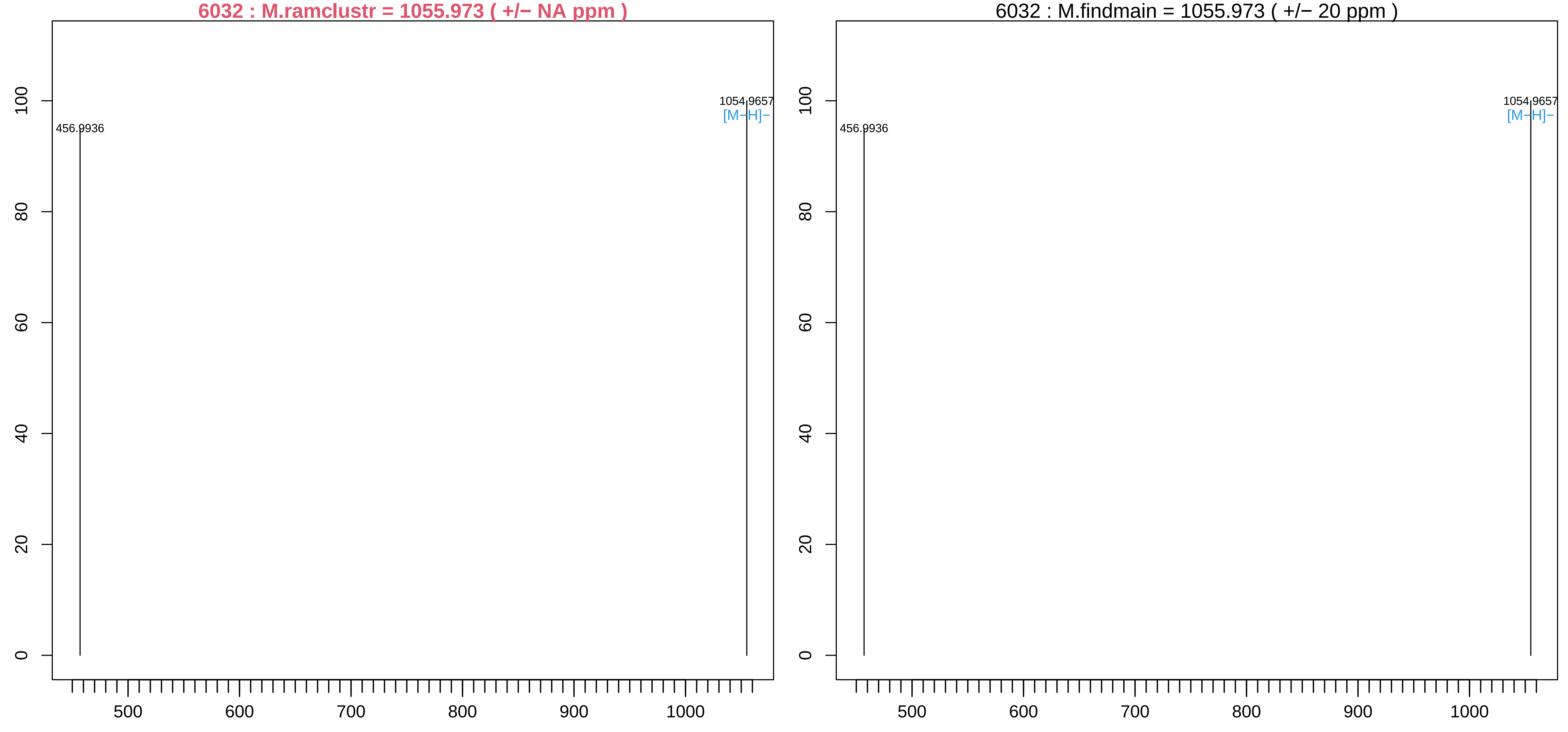

6032 : M.ramclustr = 1055.973 (+/− NA ppm)
456.9936
1054.9657
[M−H]−
6032 : M.findmain = 1055.973 (+/− 20 ppm)
456.9936
1054.9657
[M−H]−
0
20
40
60
80
100
500
600
700
800
900
1000

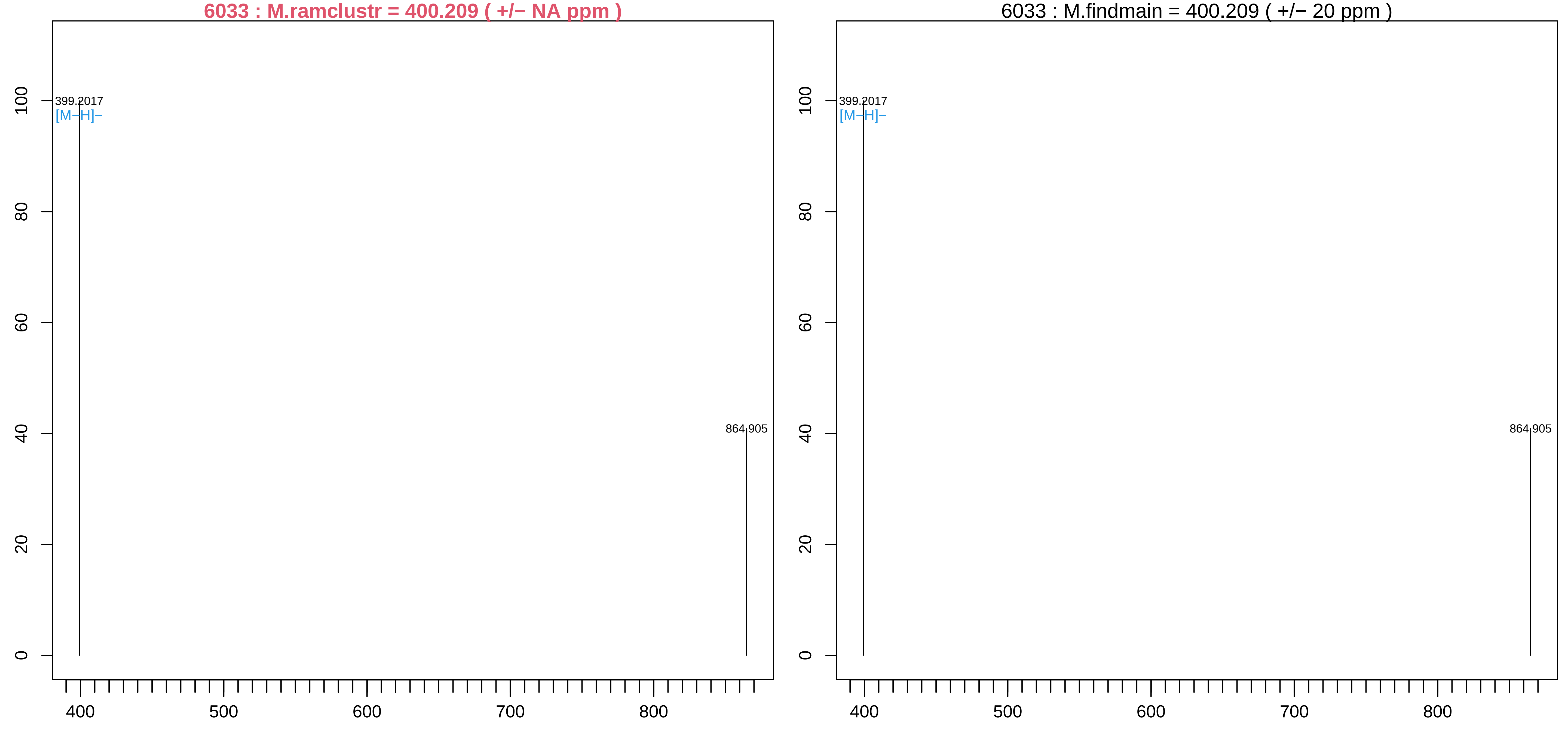

6033 : M.ramclustr = 400.209 (+/− NA ppm)
399.2017
[M−H]−
864.905
0
20
40
60
80
100
400
500
600
700
800
6033 : M.findmain = 400.209 (+/− 20 ppm)
399.2017
[M−H]−
864.905
0
20
40
60
80
100
400
500
600
700
800

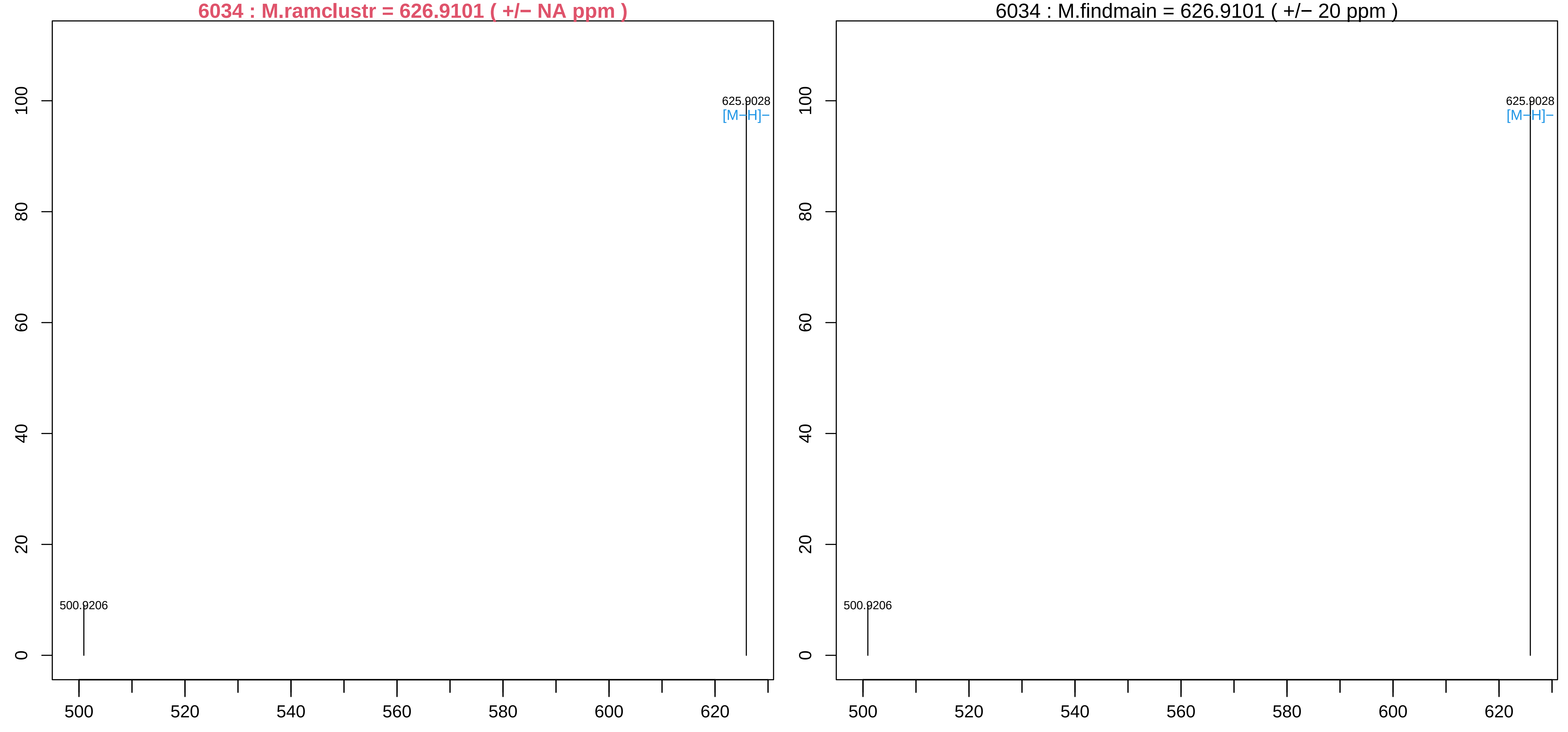
6034 : M.ramclustr = 626.9101 (+/− NA ppm)
6034 : M.findmain = 626.9101 (+/− 20 ppm)
625.9028
[M−H]−
500.9206
0
20
40
60
80
100
500
520
540
560
580
600
620

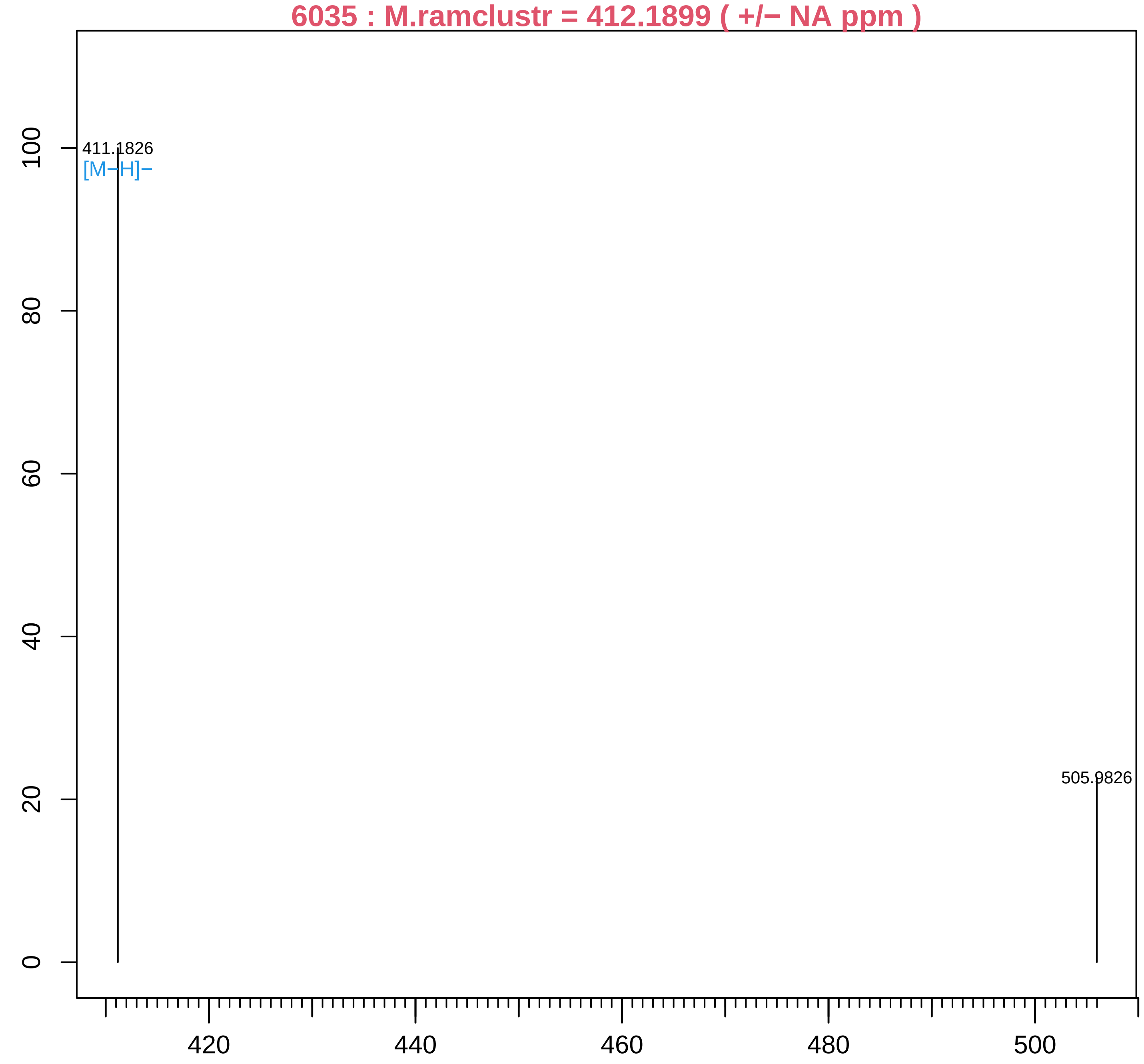

6035 : M.ramclustr = 412.1899 (+/− NA ppm)
411.1826
[M−H]−
505.9826
0
20
40
60
80
100
420
440
460
480
500

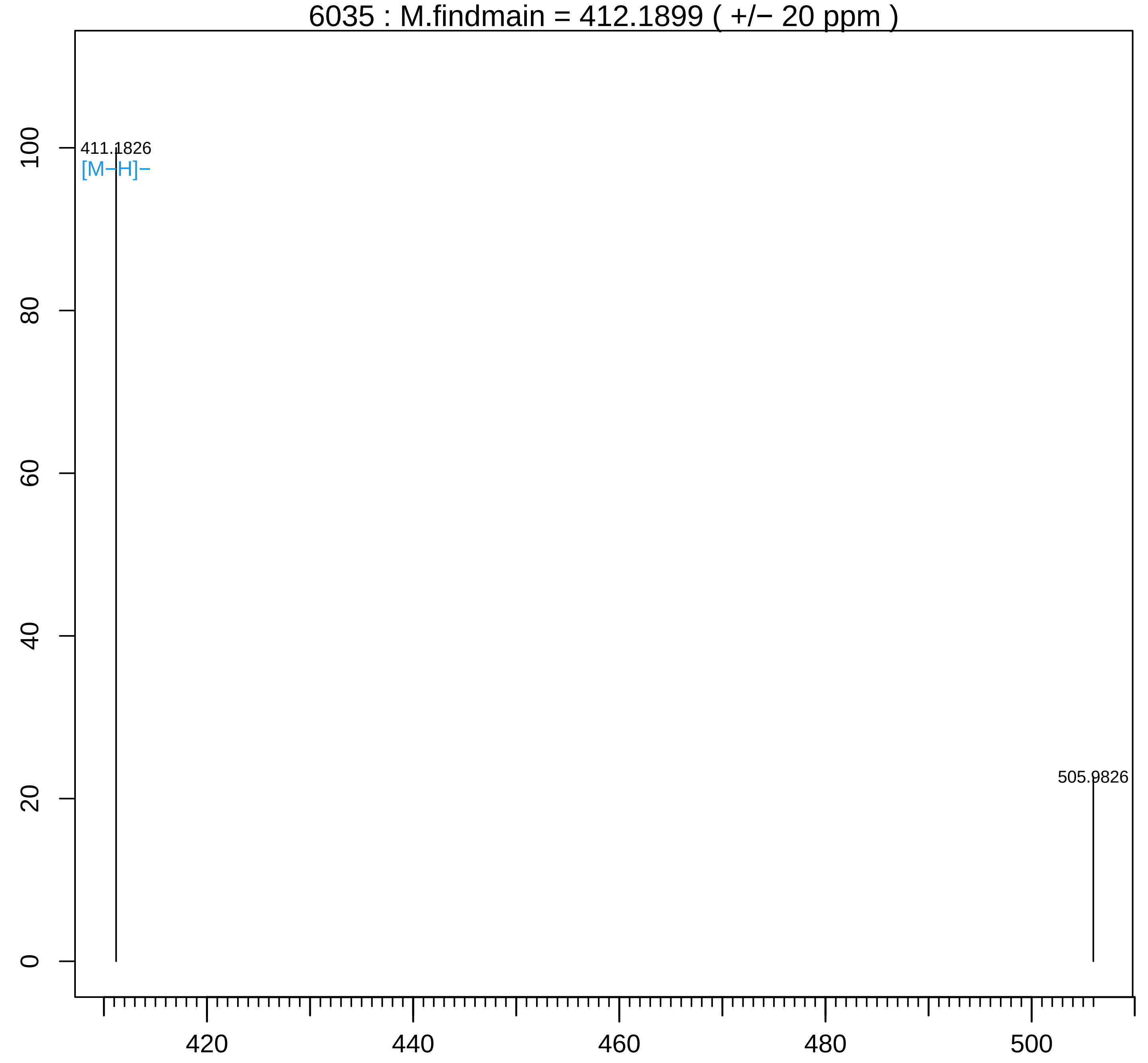

6035 : M.findmain = 412.1899 (+/− 20 ppm)
411.1826
[M−H]−
505.9826
0
20
40
60
80
100
420
440
460
480
500

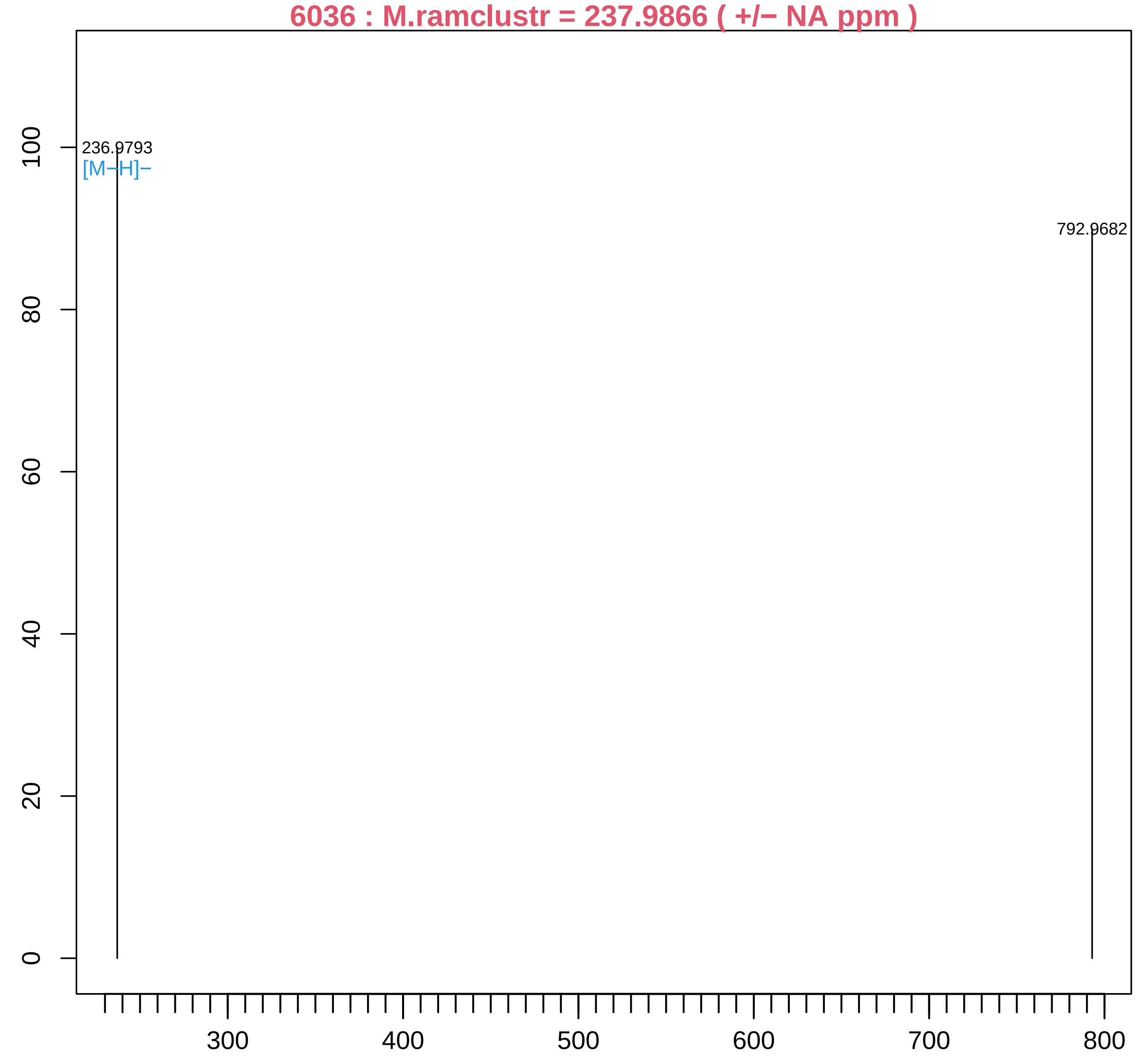
6036 : M.ramclustr = 237.9866 (+/− NA ppm)
236.9793
[M−H]−
792.9682
0
20
40
60
80
100
300
400
500
600
700
800

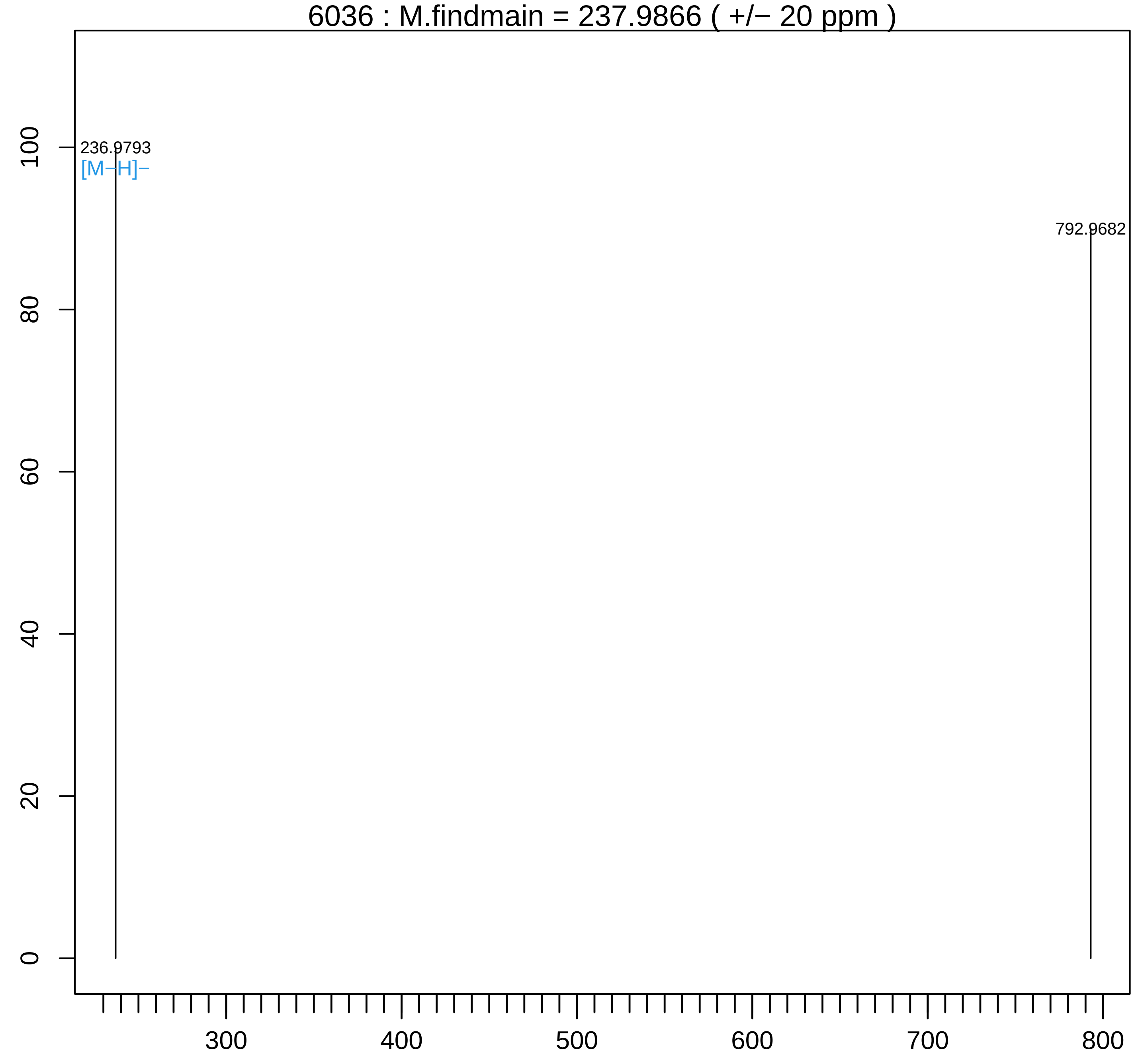
6036 : M.findmain = 237.9866 (+/− 20 ppm)
236.9793
[M−H]−
792.9682
0
20
40
60
80
100
300
400
500
600
700
800

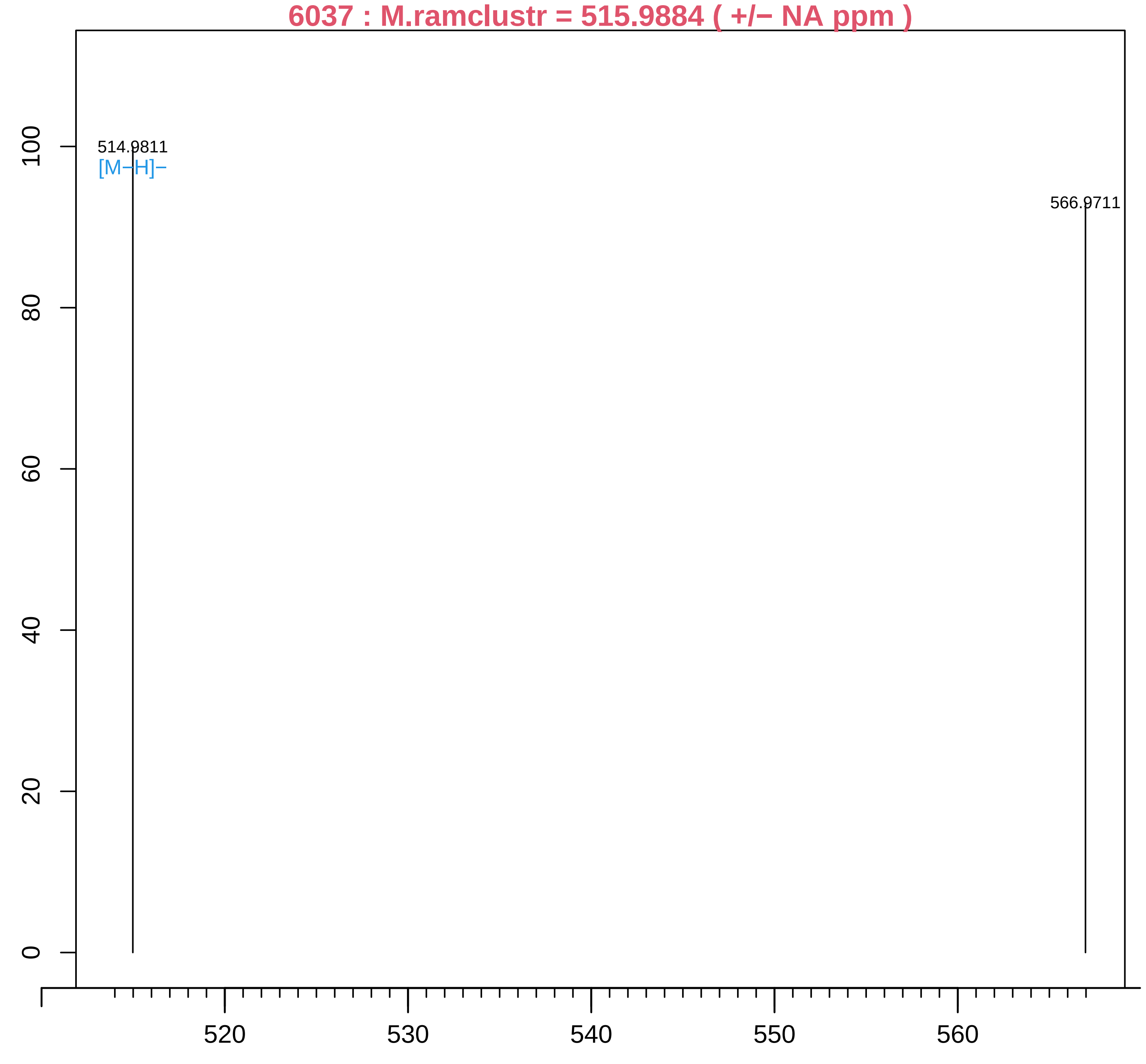
6037 : M.ramclustr = 515.9884 (+/− NA ppm)
514.9811
[M−H]−
566.9711
0
20
40
60
80
100
520
530
540
550
560

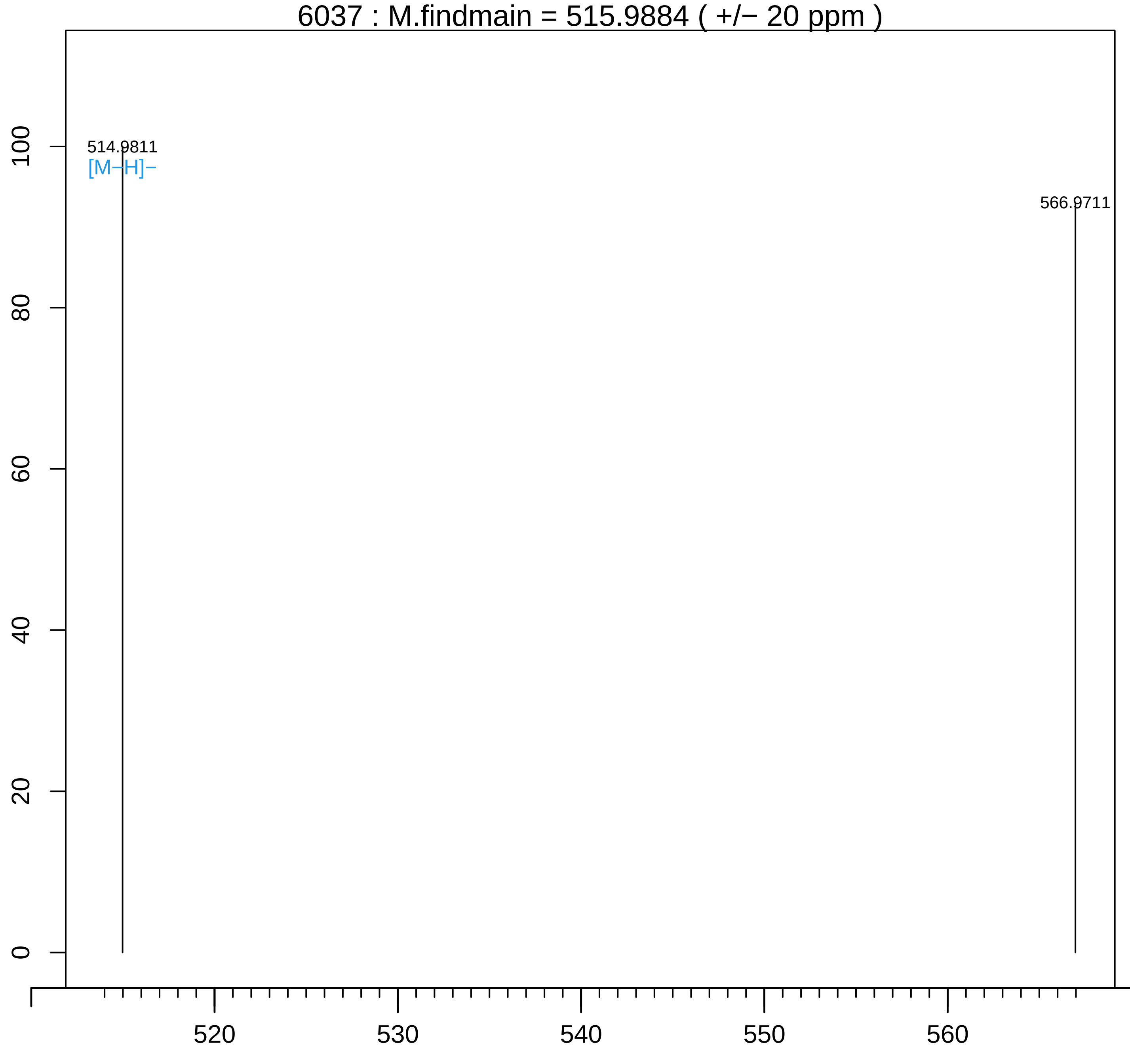
6037 : M.findmain = 515.9884 (+/− 20 ppm)
514.9811
[M−H]−
566.9711
0
20
40
60
80
100
520
530
540
550
560

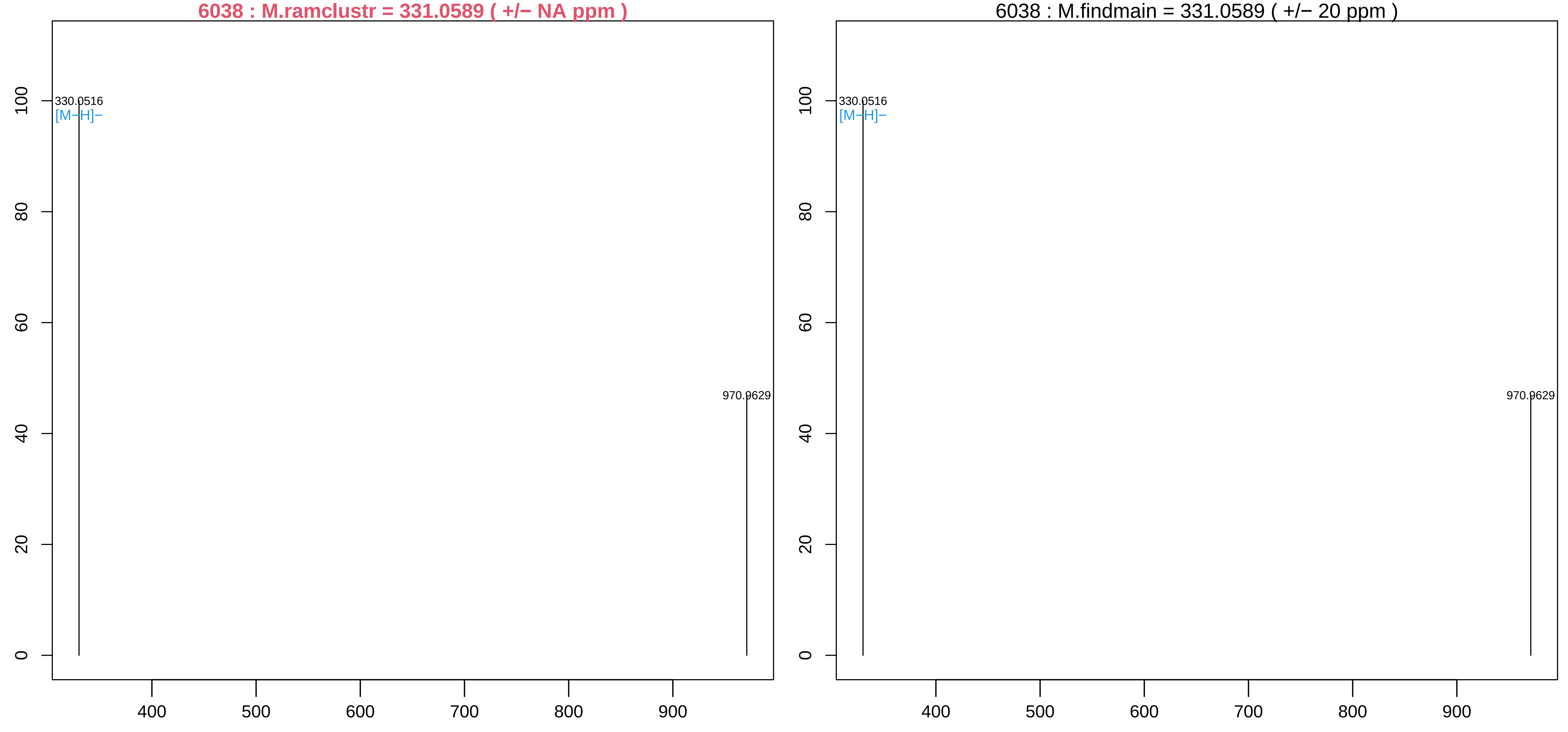
6038 : M.ramclustr = 331.0589 (+/− NA ppm)
330.0516
[M−H]−
970.9629
6038 : M.findmain = 331.0589 (+/− 20 ppm)
330.0516
[M−H]−
970.9629
0
20
40
60
80
100
400
500
600
700
800
900

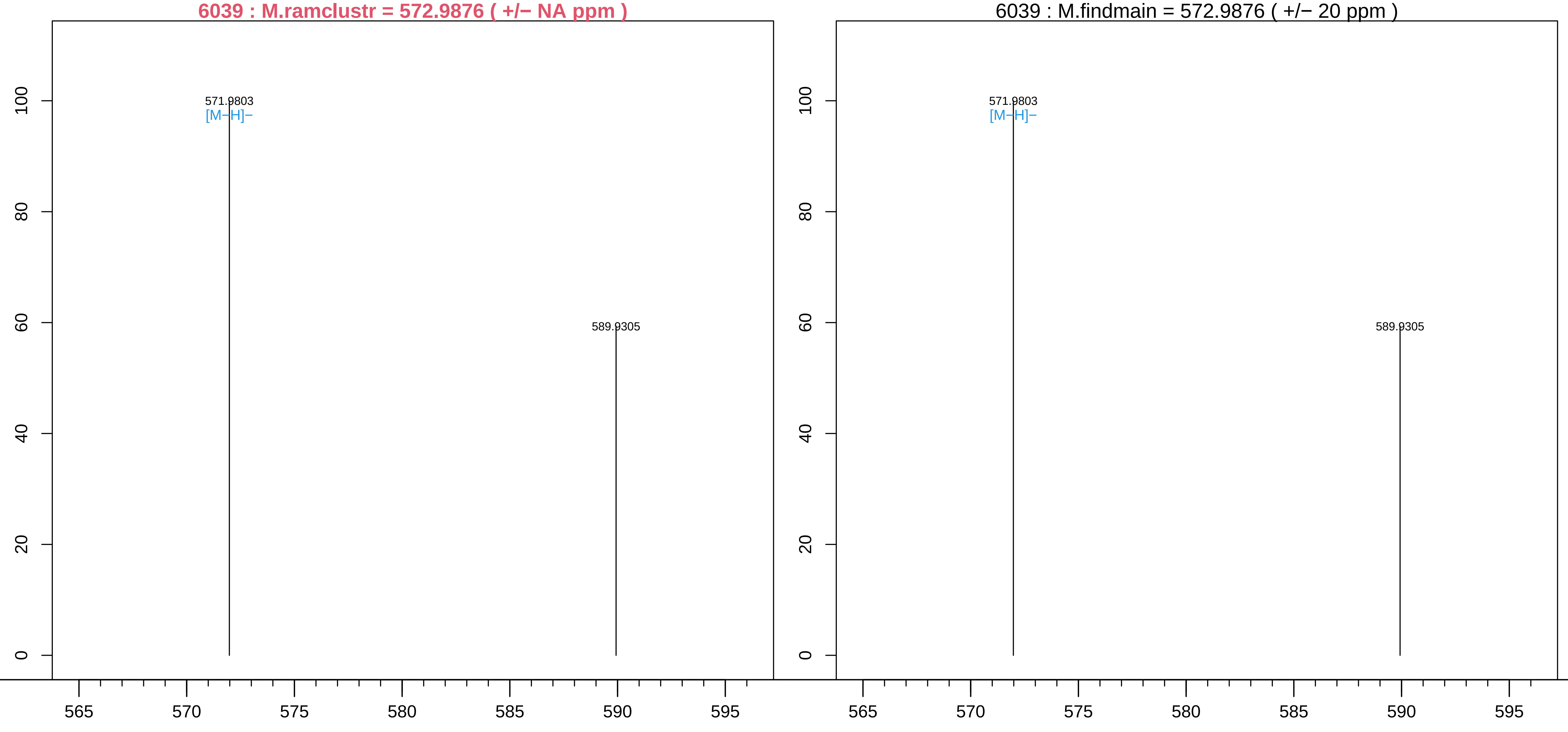
6039 : M.ramclustr = 572.9876 (+/− NA ppm)
571.9803
[M−H]−
589.9305
6039 : M.findmain = 572.9876 (+/− 20 ppm)
571.9803
[M−H]−
589.9305
0
20
40
60
80
100
565
570
575
580
585
590
595

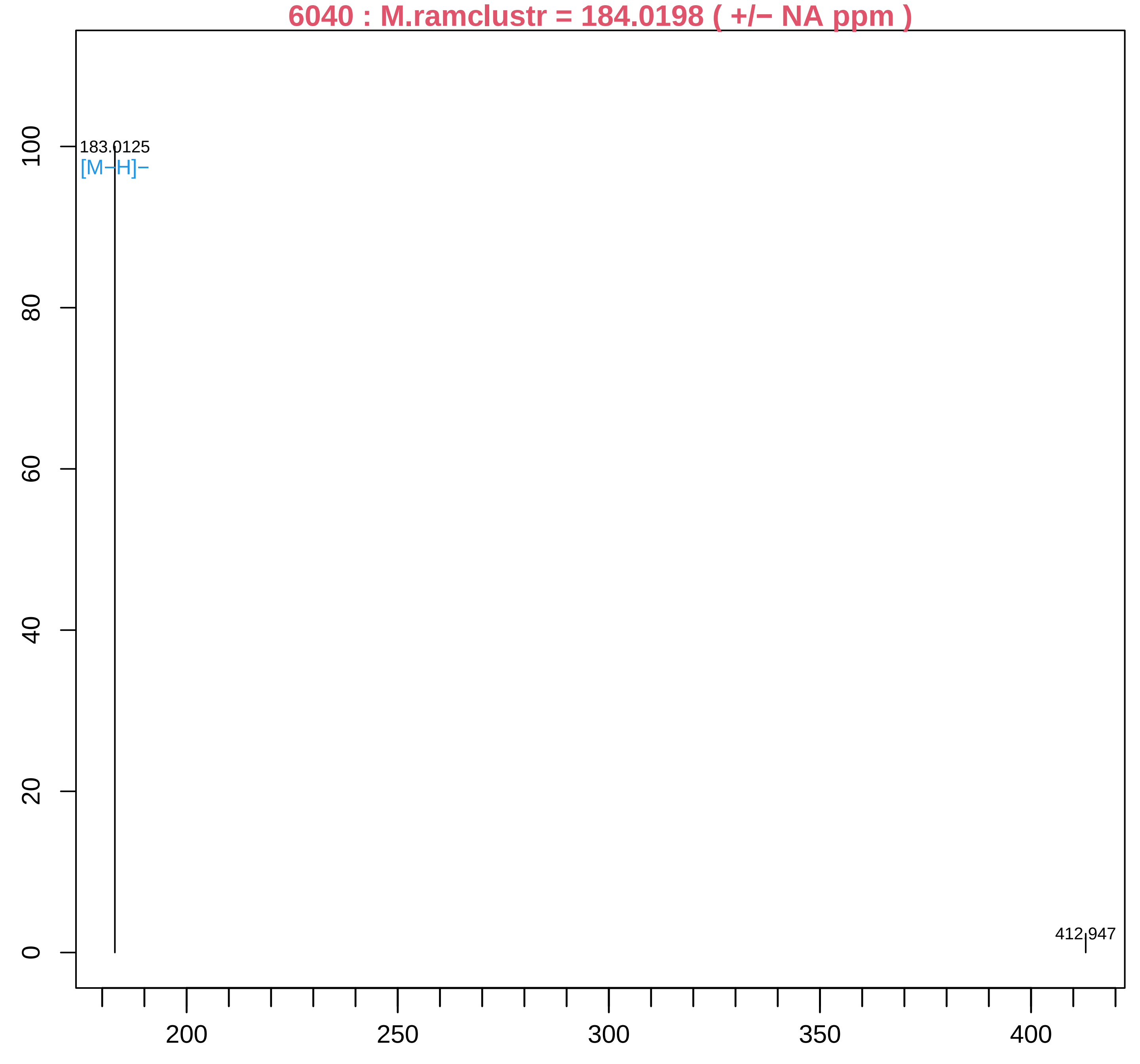
6040 : M.ramclustr = 184.0198 (+/− NA ppm)
183.0125
[M−H]−
412.947
0
20
40
60
80
100
200
250
300
350
400

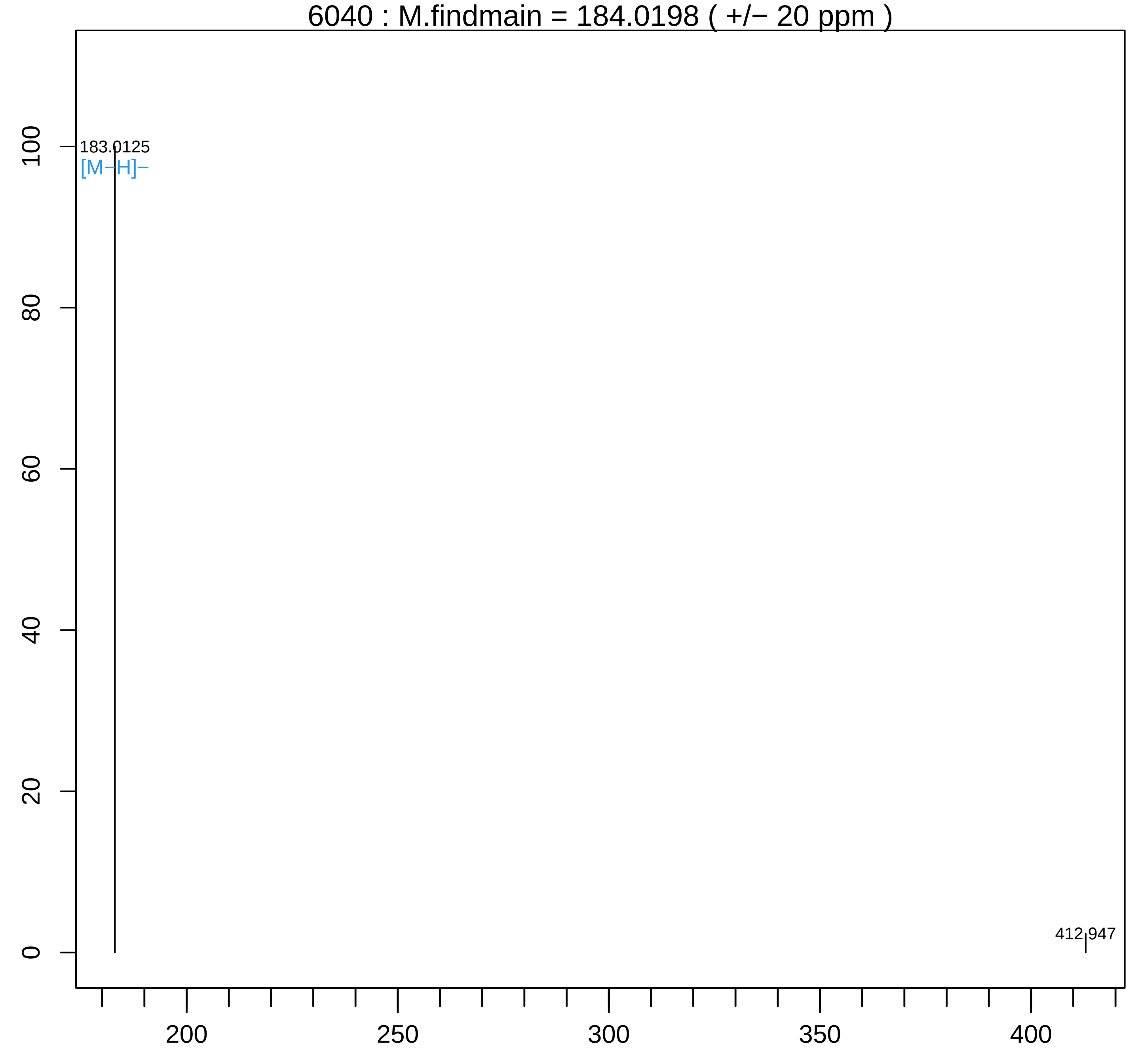
6040 : M.findmain = 184.0198 (+/− 20 ppm)
183.0125
[M−H]−
412.947
0
20
40
60
80
100
200
250
300
350
400

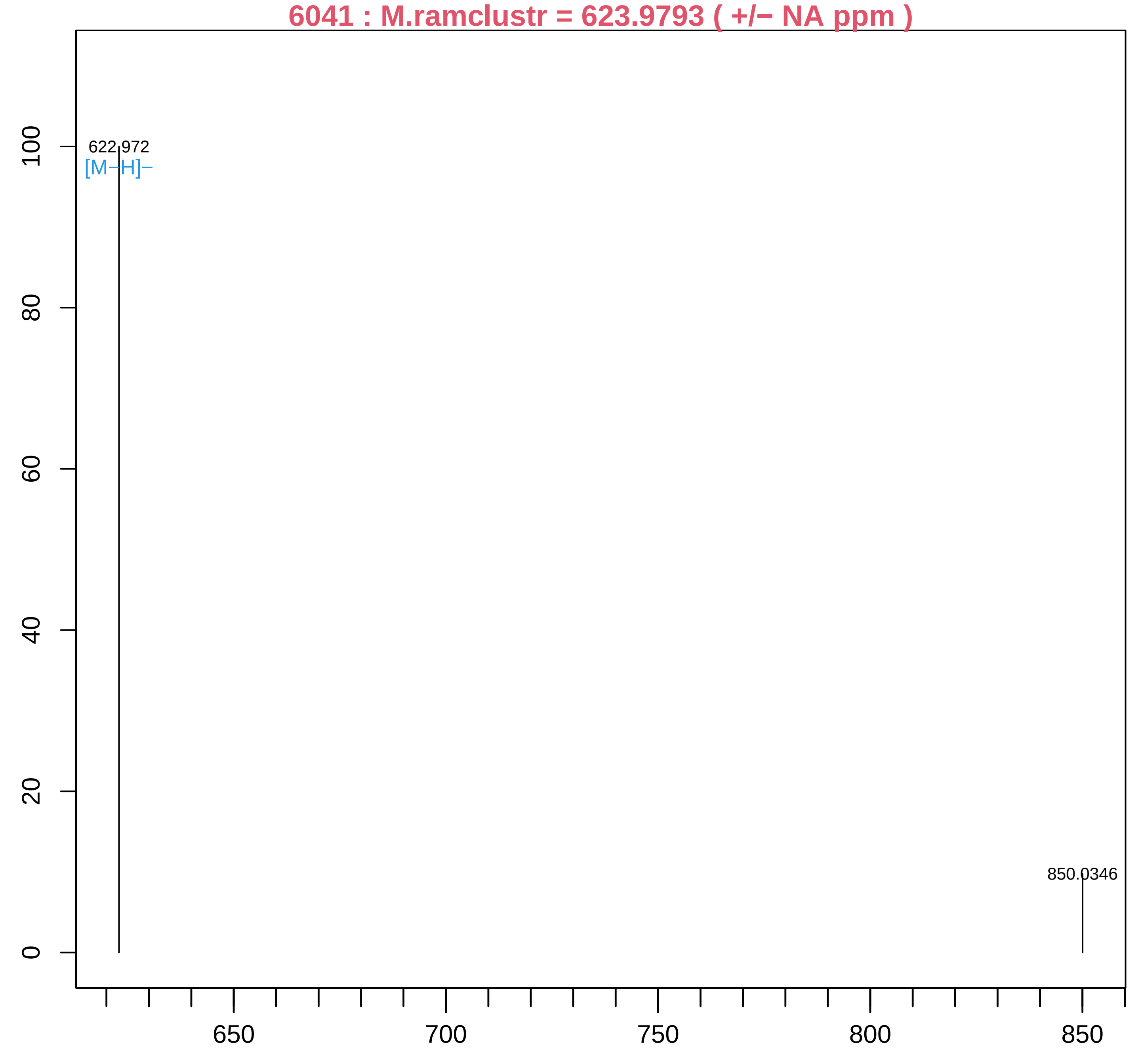
6041 : M.ramclustr = 623.9793 (+/− NA ppm)
622.972
[M−H]−
850.0346
0
20
40
60
80
100
650
700
750
800
850

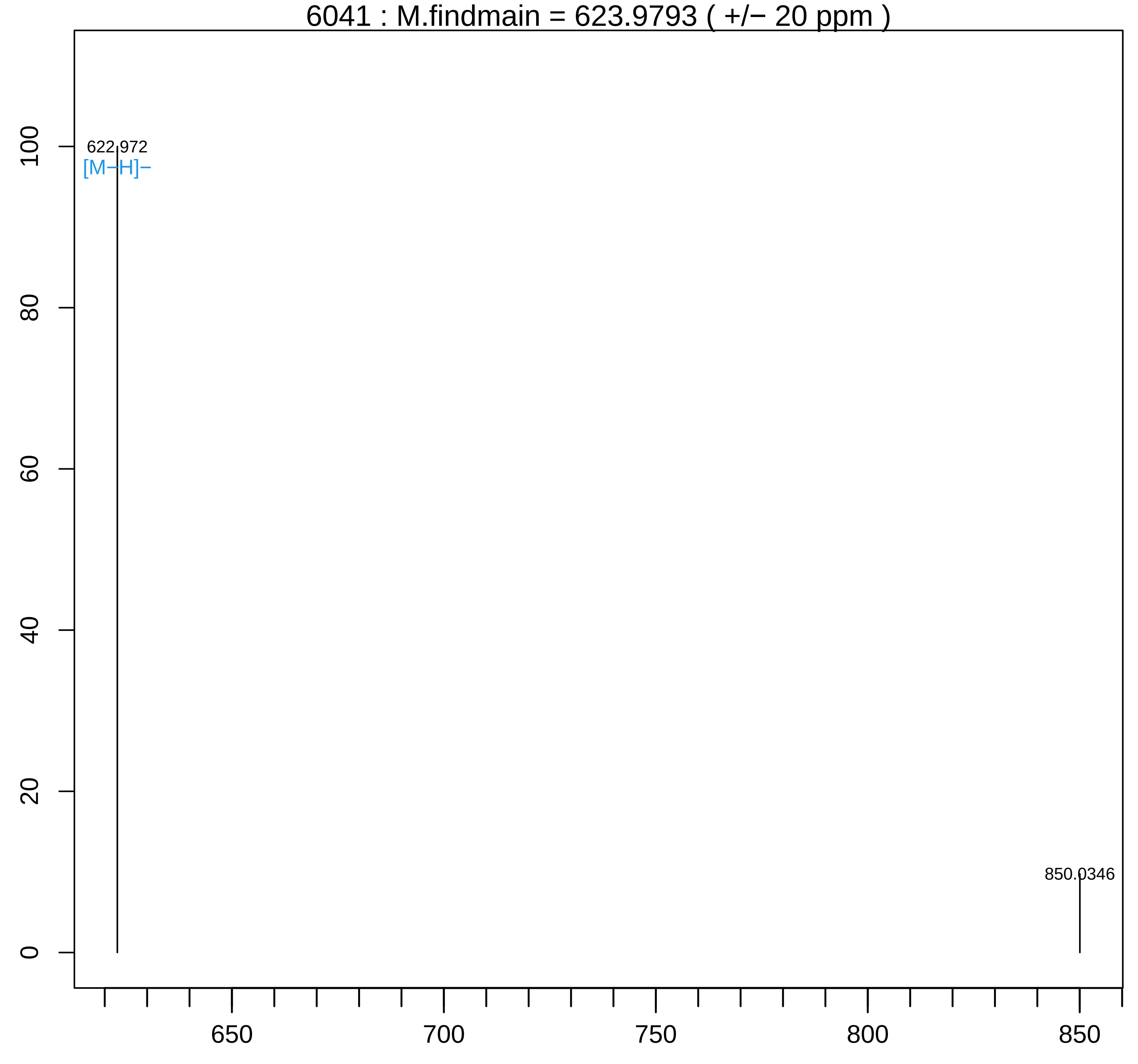
6041 : M.findmain = 623.9793 (+/− 20 ppm)
622.972
[M−H]−
850.0346
0
20
40
60
80
100
650
700
750
800
850

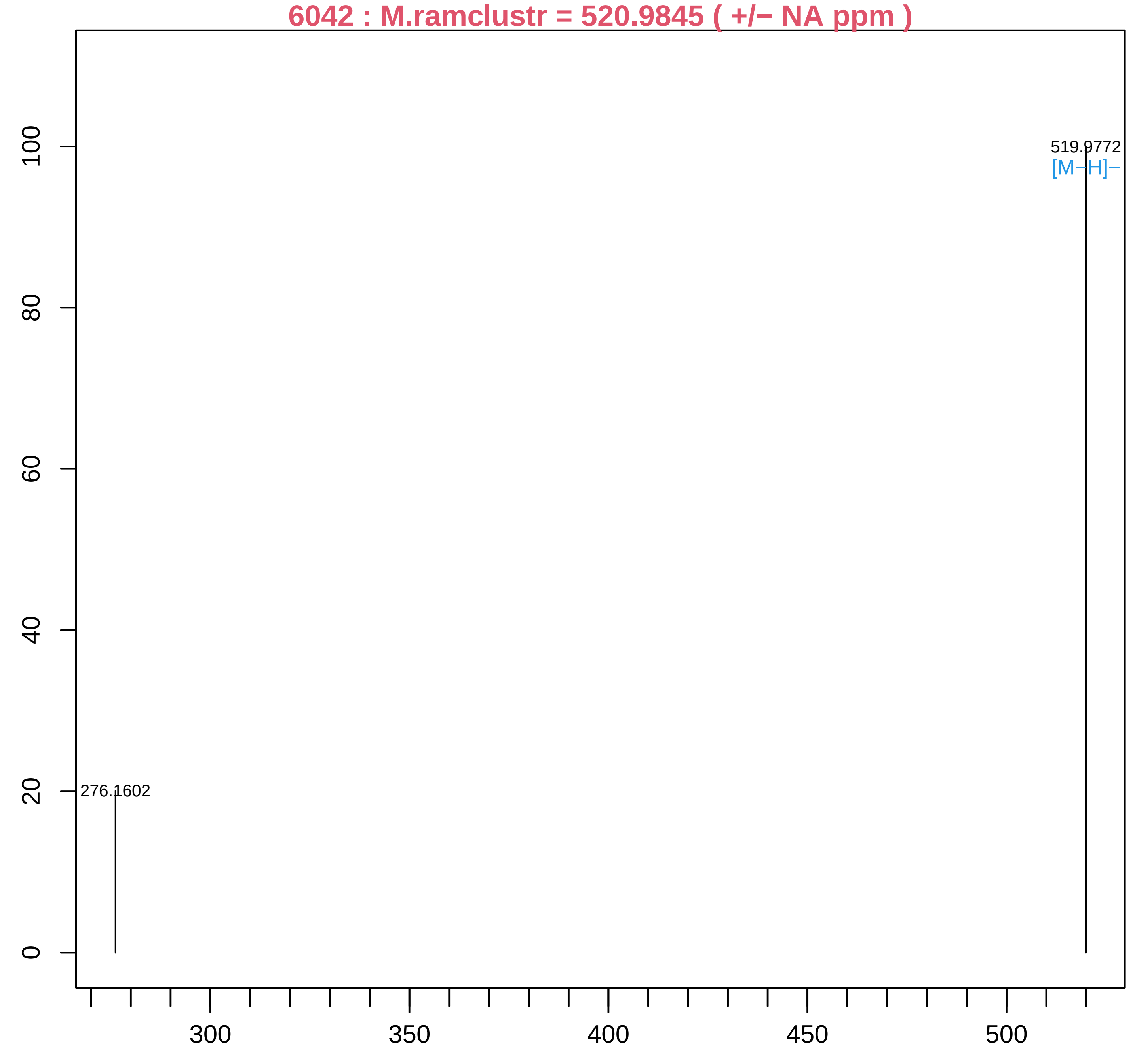

6042 : M.ramclustr = 520.9845 (+/− NA ppm)
519.9772
[M−H]−
276.1602
0
20
40
60
80
100
300
350
400
450
500

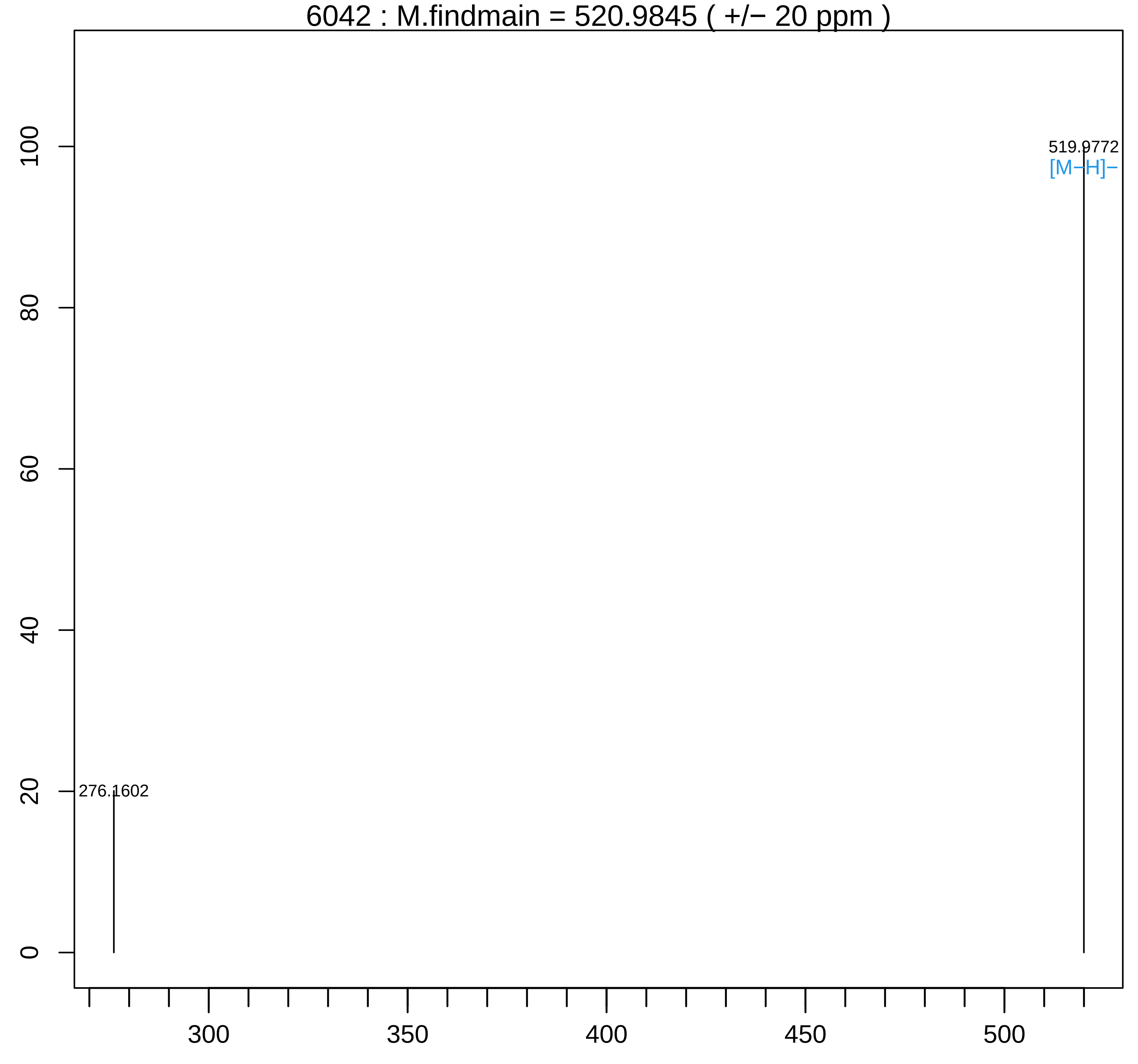

6042 : M.findmain = 520.9845 (+/− 20 ppm)
519.9772
[M−H]−
276.1602
0
20
40
60
80
100
300
350
400
450
500

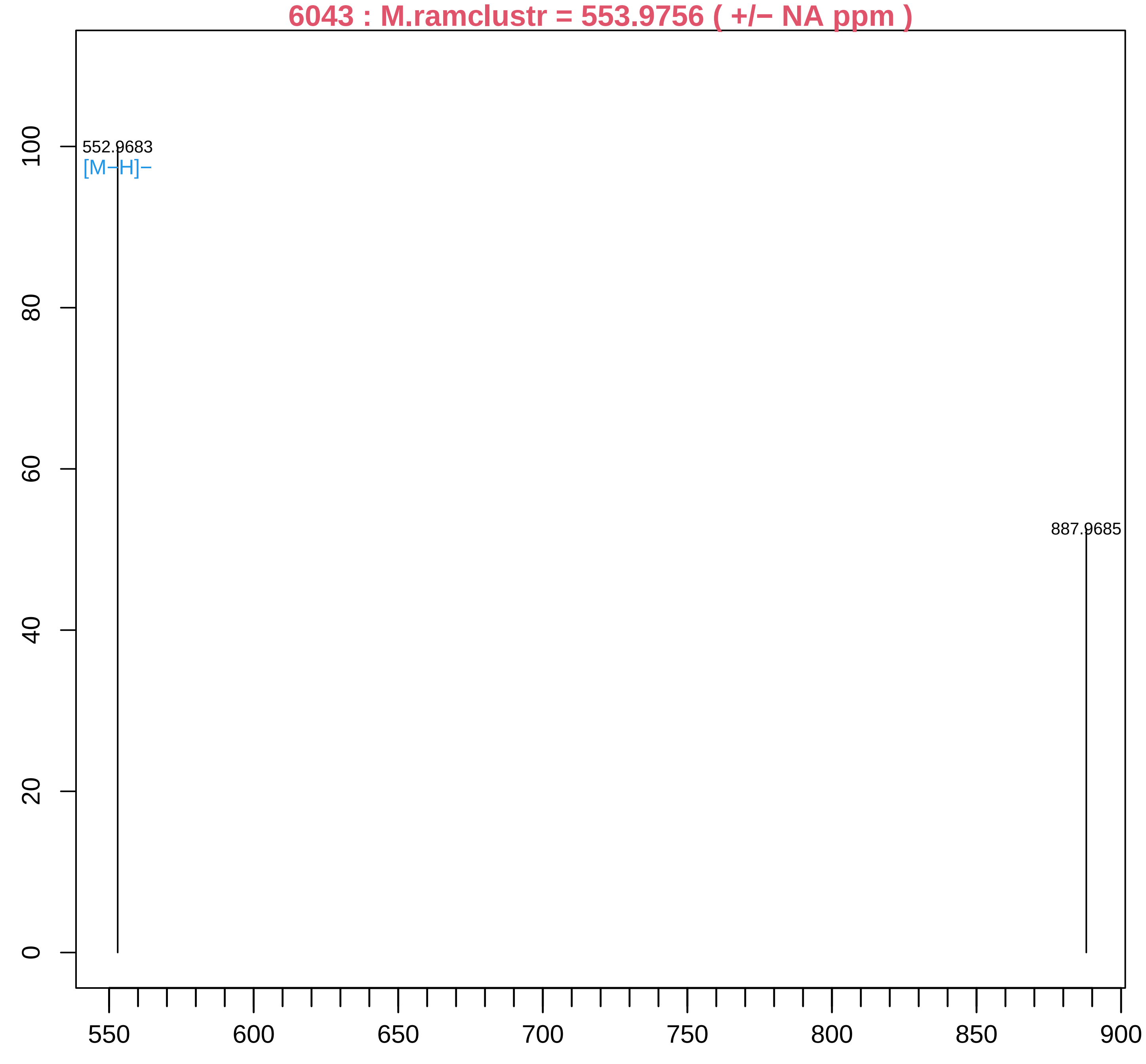
6043 : M.ramclustr = 553.9756 (+/− NA ppm)
552.9683
[M−H]−
887.9685
0
20
40
60
80
100
550
600
650
700
750
800
850
900

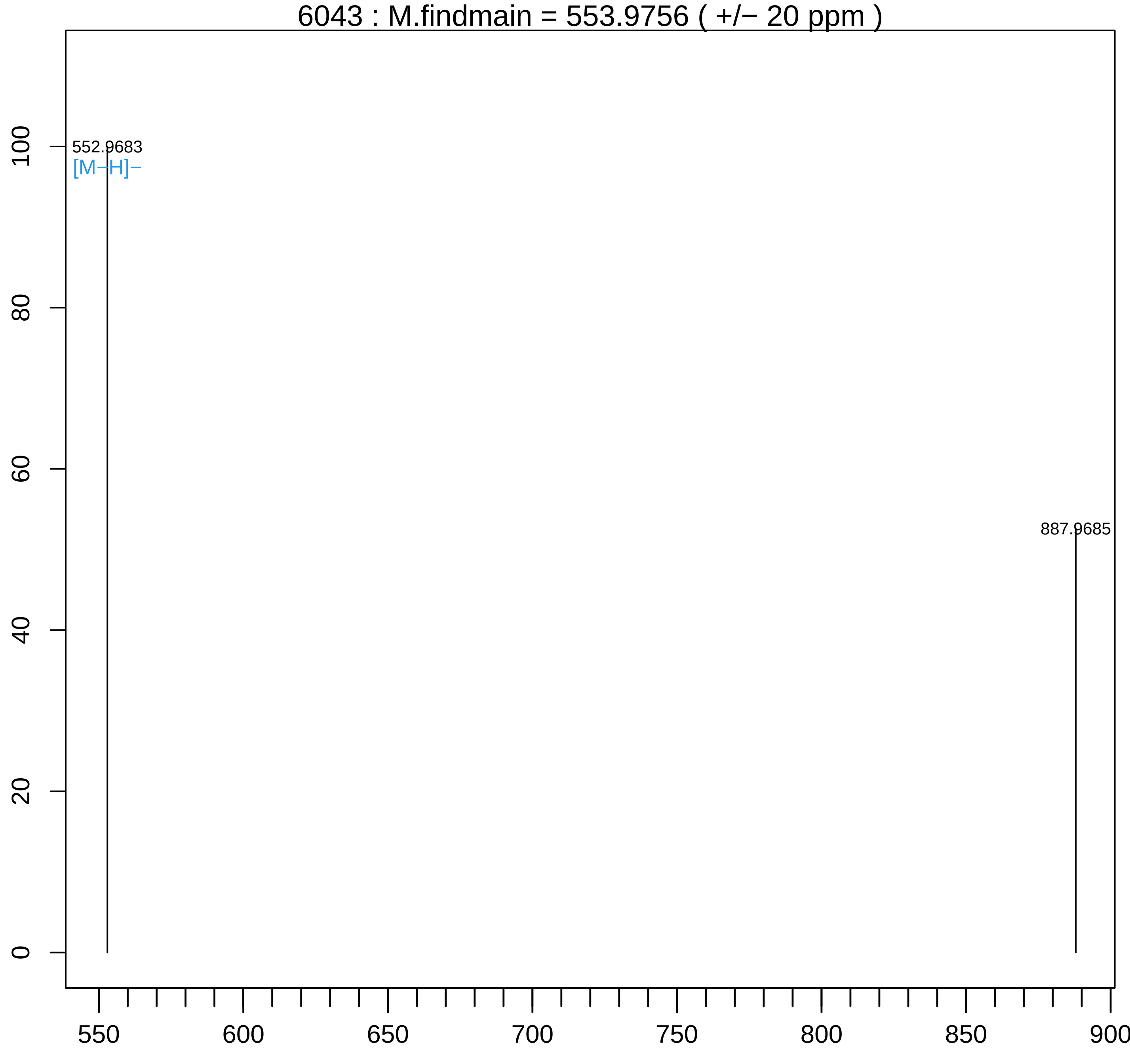
6043 : M.findmain = 553.9756 (+/− 20 ppm)
552.9683
[M−H]−
887.9685
0
20
40
60
80
100
550
600
650
700
750
800
850
900

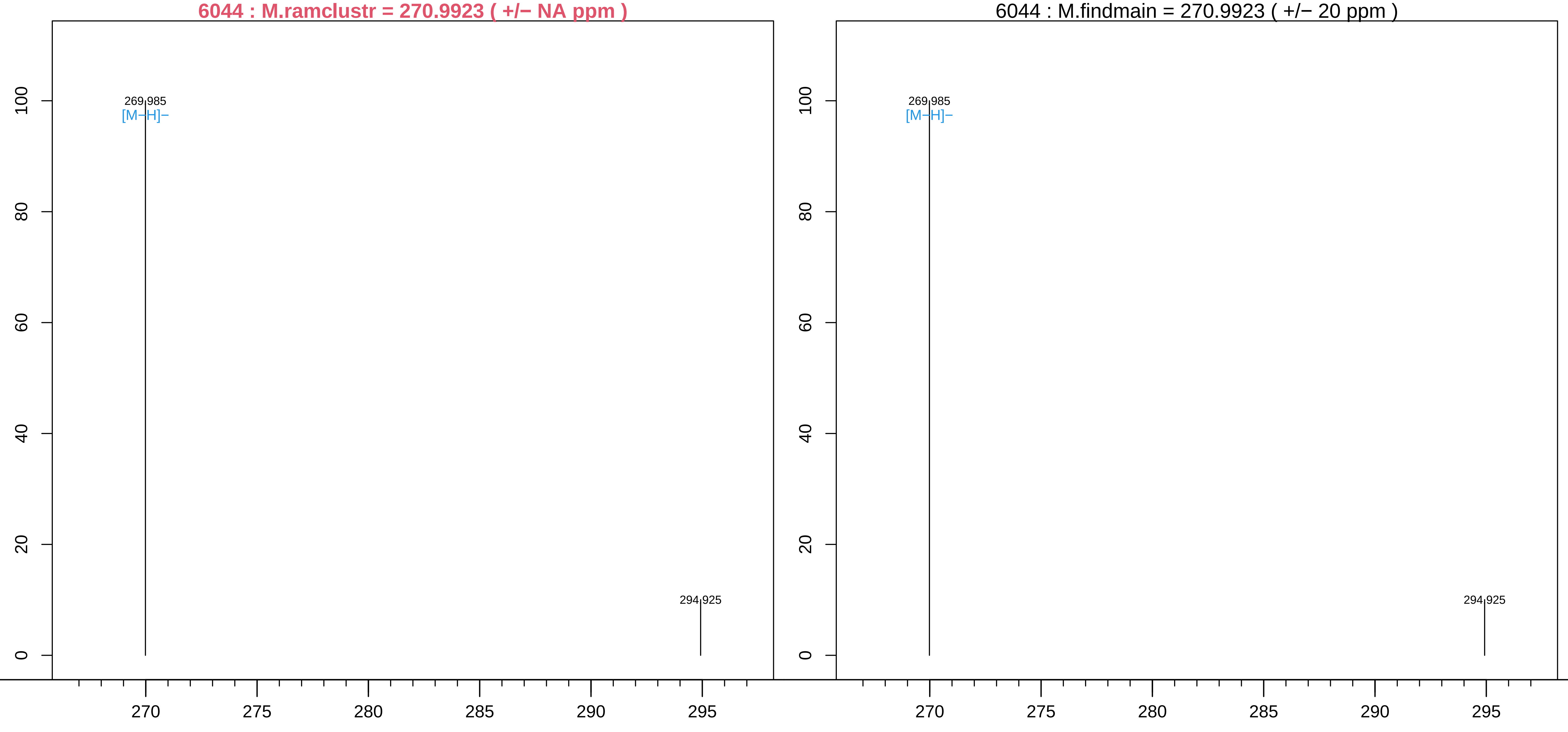
6044 : M.ramclustr = 270.9923 (+/− NA ppm)
6044 : M.findmain = 270.9923 (+/− 20 ppm)
269.985
[M−H]−
294.925
0
20
40
60
80
100
270
275
280
285
290
295

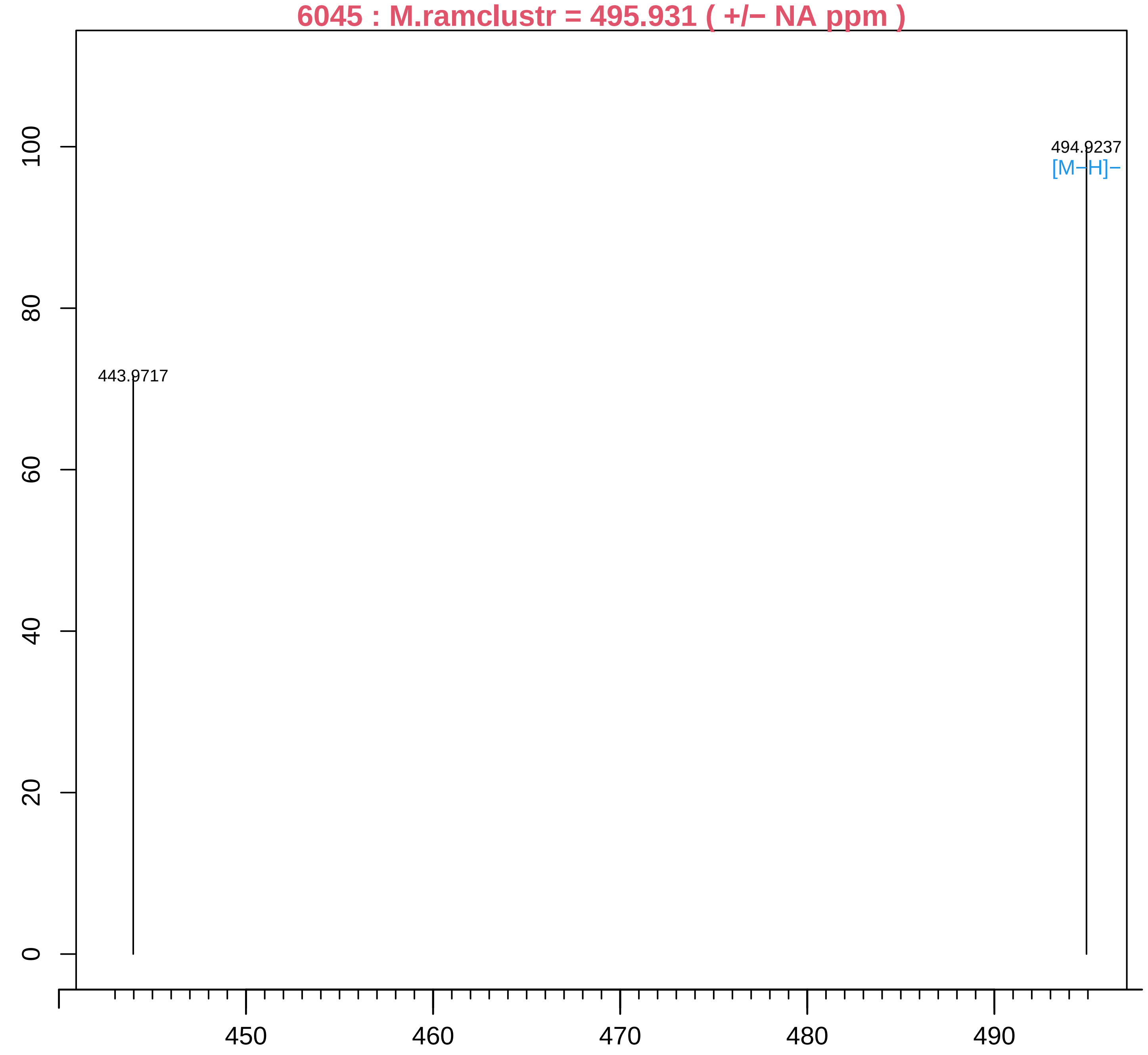

6045 : M.ramclustr = 495.931 (+/− NA ppm)
494.9237
[M−H]−
443.9717
0
20
40
60
80
100
450
460
470
480
490

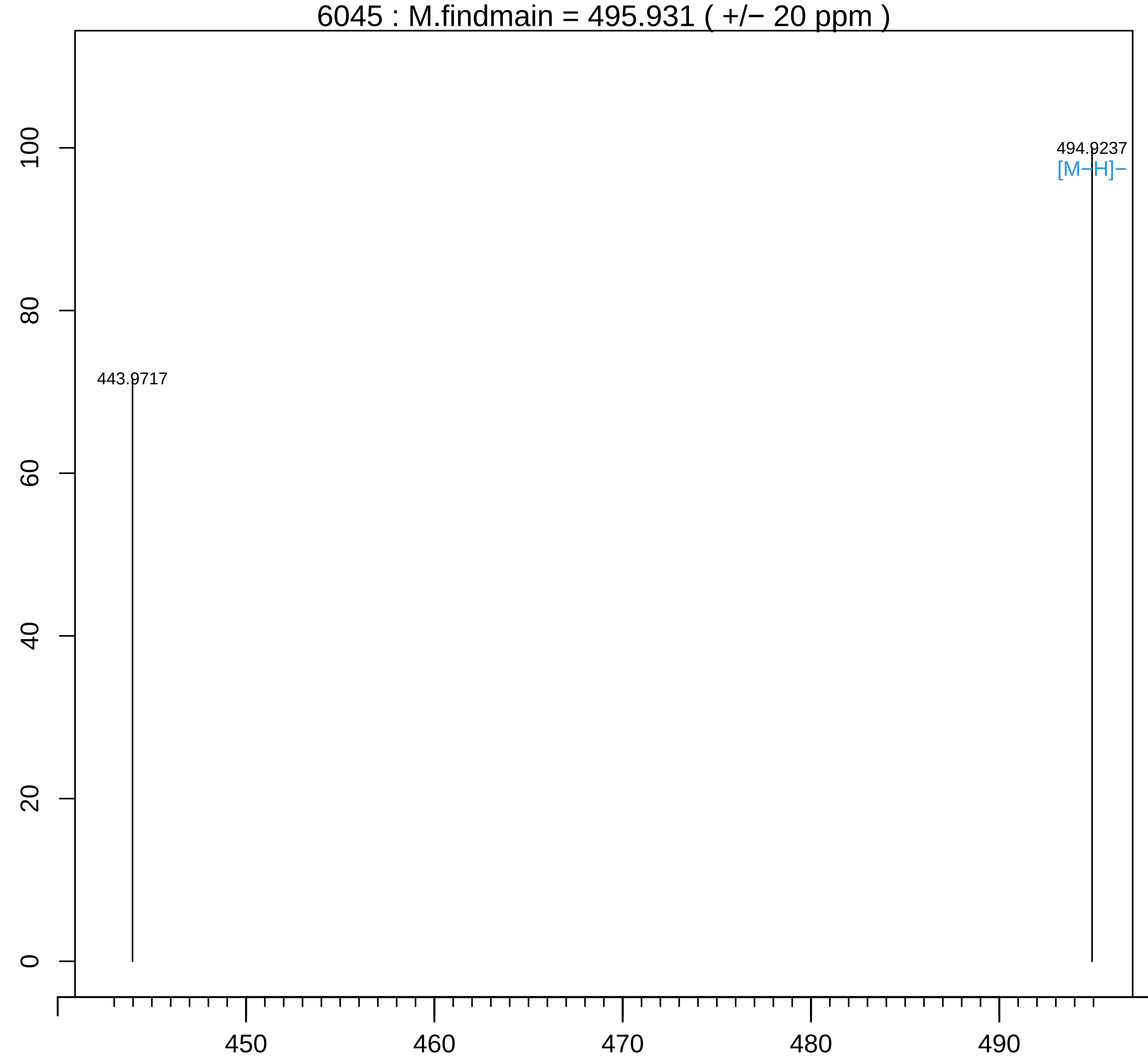

6045 : M.findmain = 495.931 (+/− 20 ppm)
494.9237
[M−H]−
443.9717
0
20
40
60
80
100
450
460
470
480
490

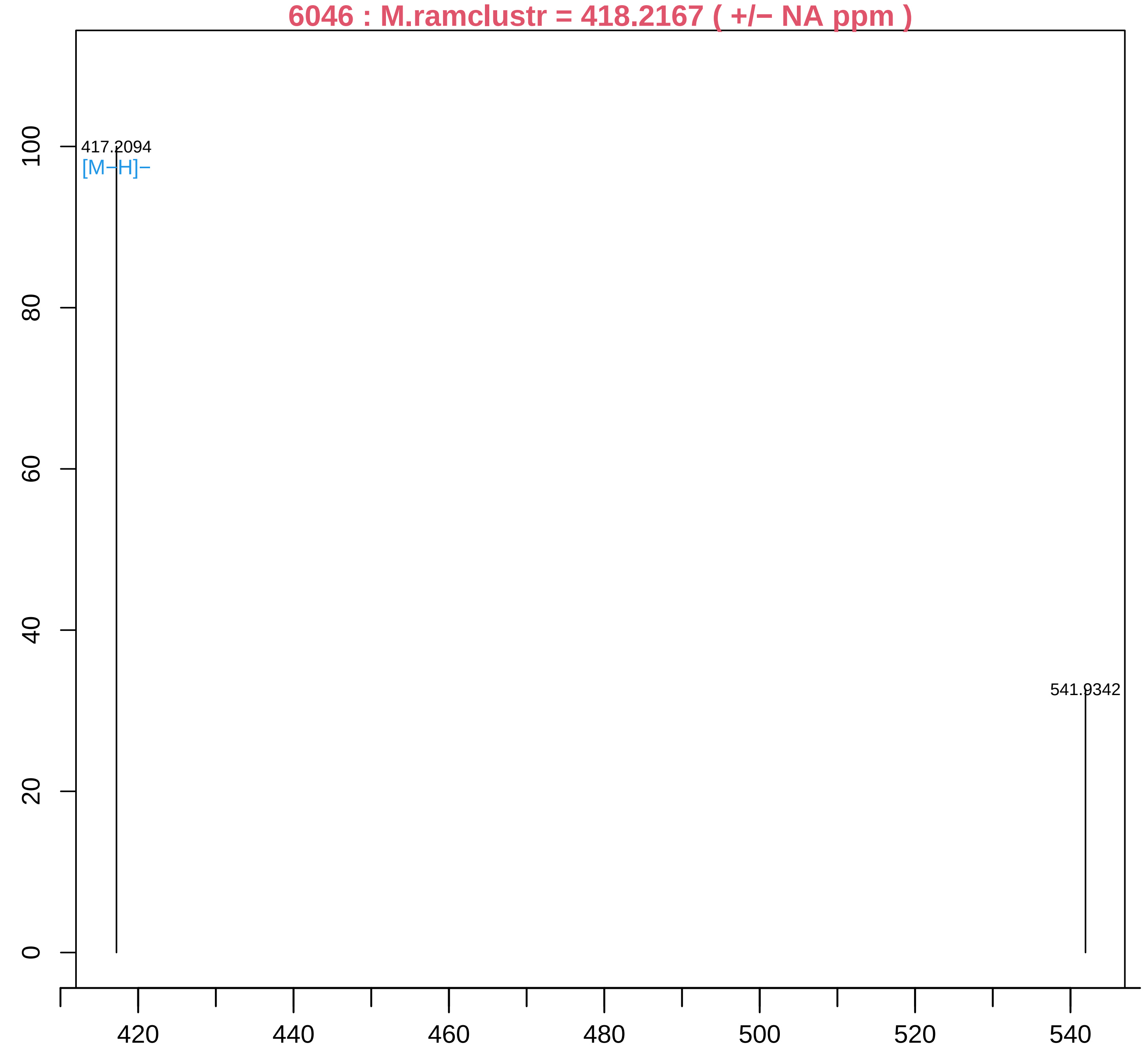
6046 : M.ramclustr = 418.2167 (+/− NA ppm)
417.2094
[M−H]−
541.9342
0
20
40
60
80
100
420
440
460
480
500
520
540

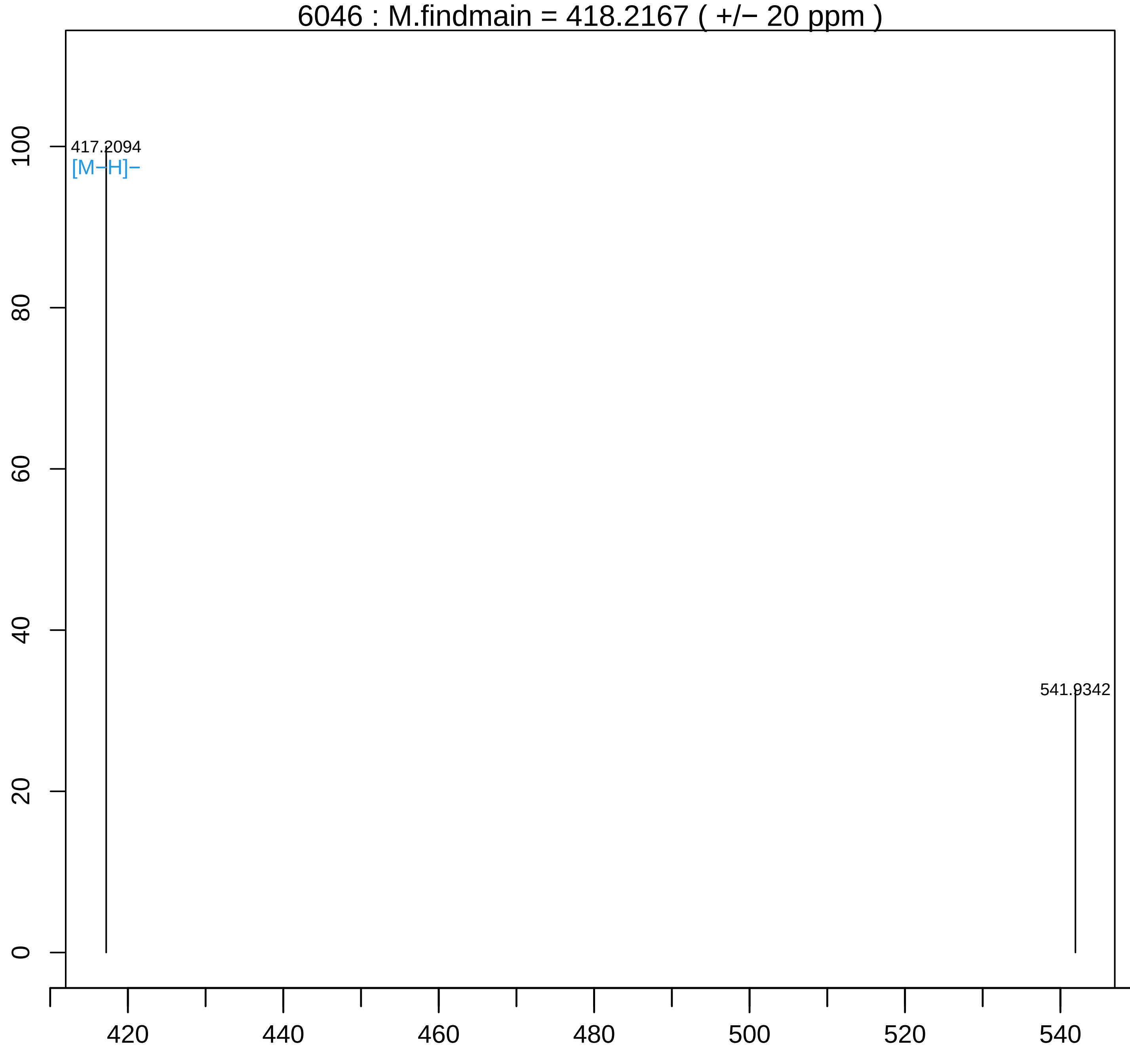
6046 : M.findmain = 418.2167 (+/− 20 ppm)
417.2094
[M−H]−
541.9342
0
20
40
60
80
100
420
440
460
480
500
520
540

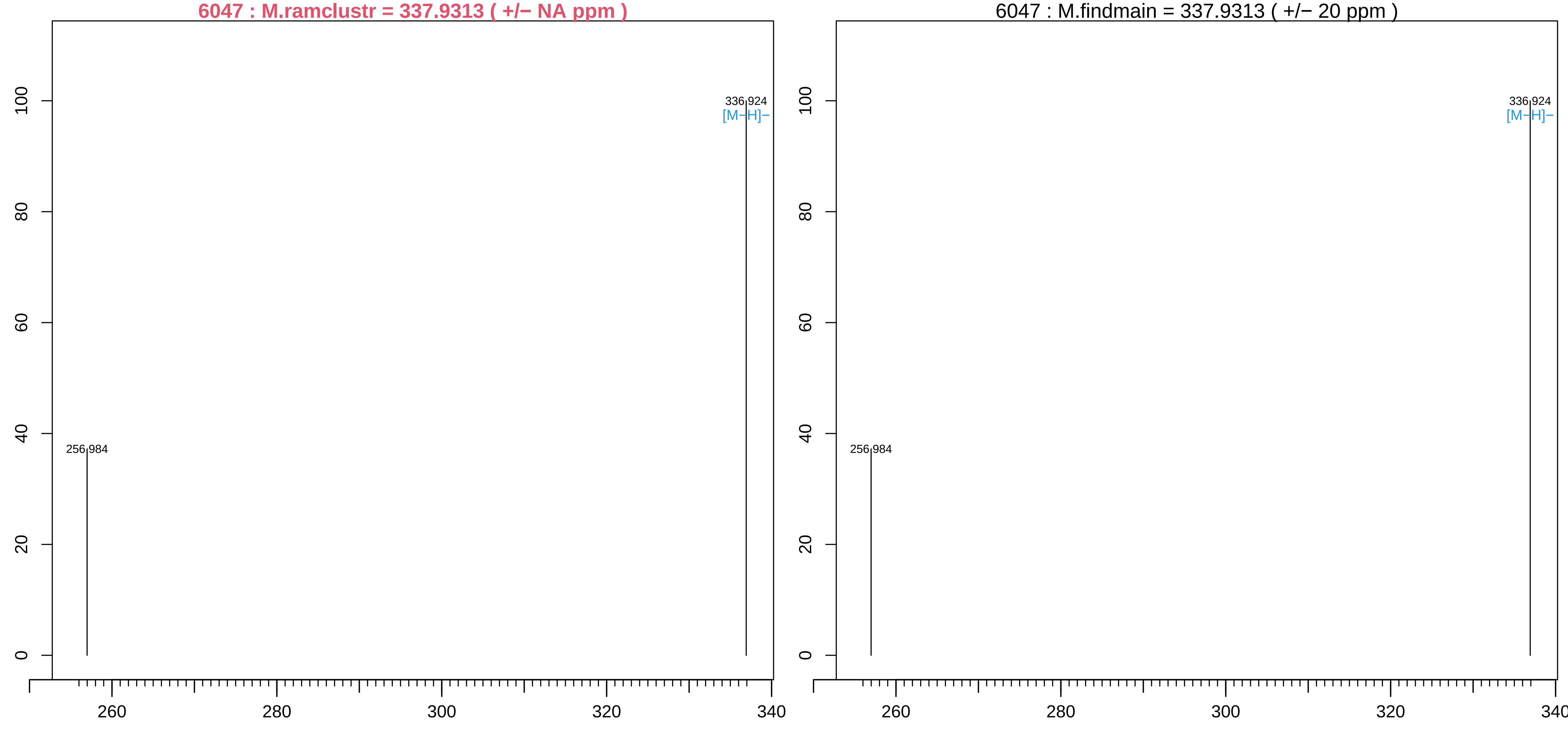
6047 : M.ramclustr = 337.9313 (+/− NA ppm)
6047 : M.findmain = 337.9313 (+/− 20 ppm)
336.924
[M−H]−
256.984
0
20
40
60
80
100
260
280
300
320
340

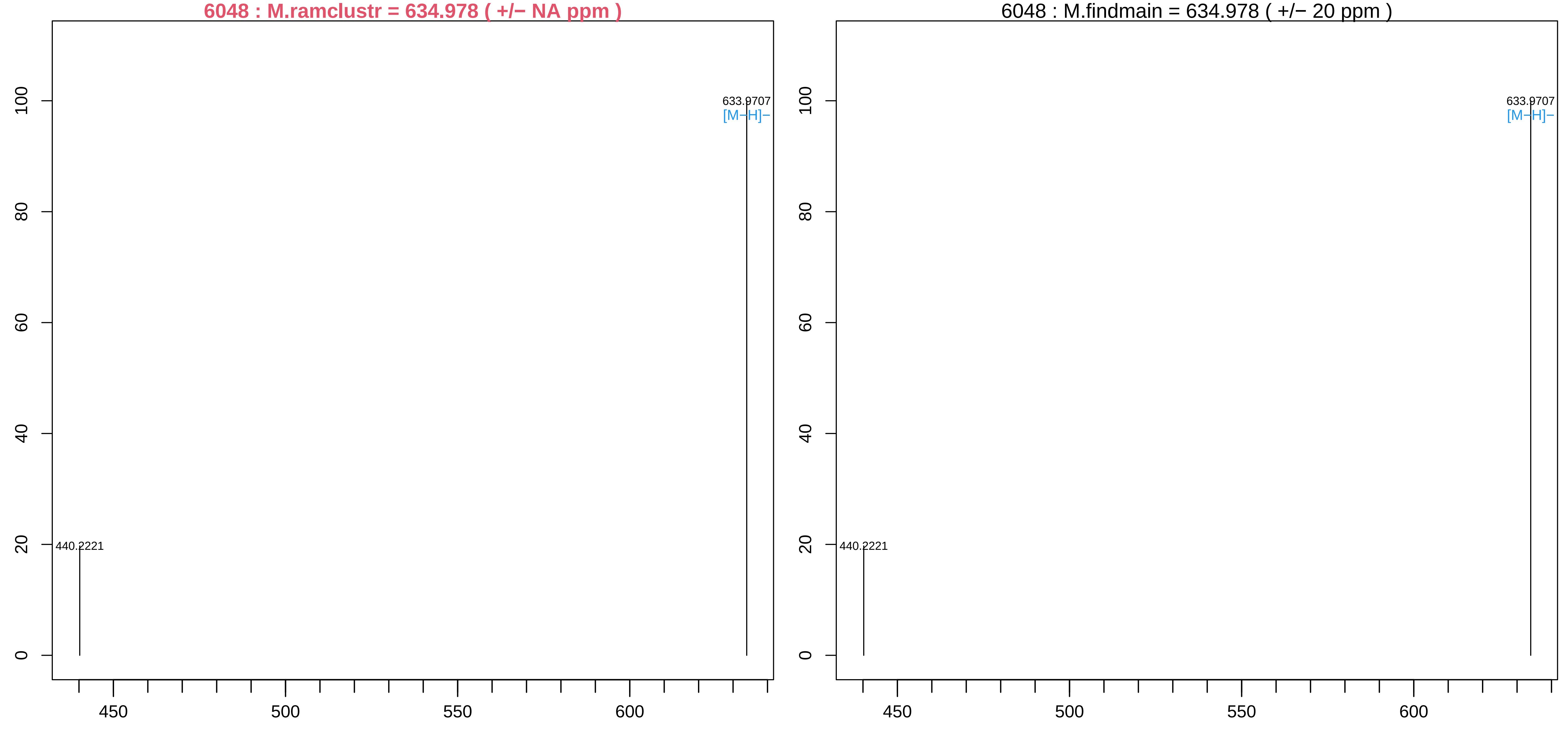
6048 : M.ramclustr = 634.978 (+/− NA ppm)
633.9707
[M−H]−
440.2221
6048 : M.findmain = 634.978 (+/− 20 ppm)
633.9707
[M−H]−
440.2221
0
20
40
60
80
100
450
500
550
600

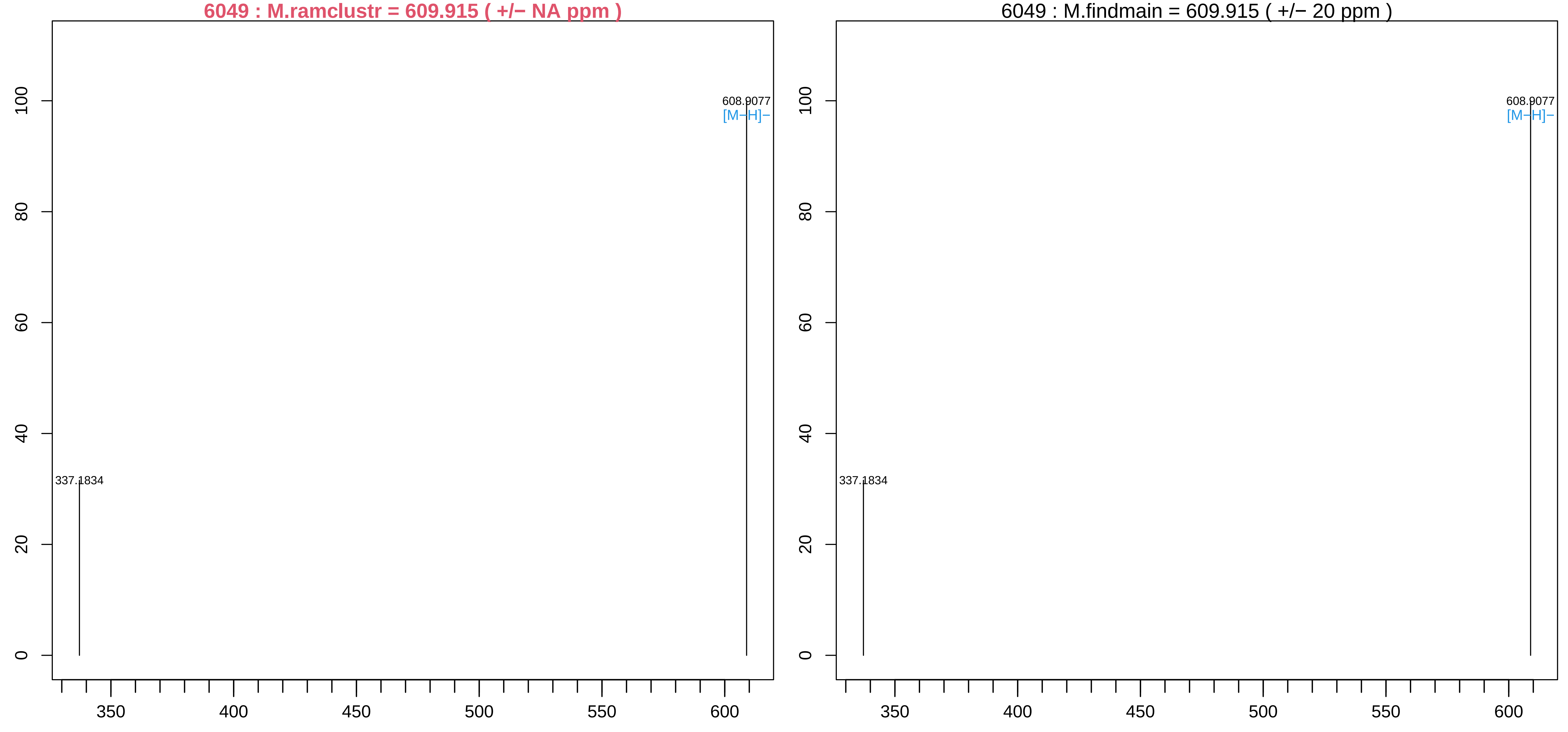
6049 : M.ramclustr = 609.915 (+/− NA ppm)
608.9077
[M−H]−
337.1834
6049 : M.findmain = 609.915 (+/− 20 ppm)
608.9077
[M−H]−
337.1834

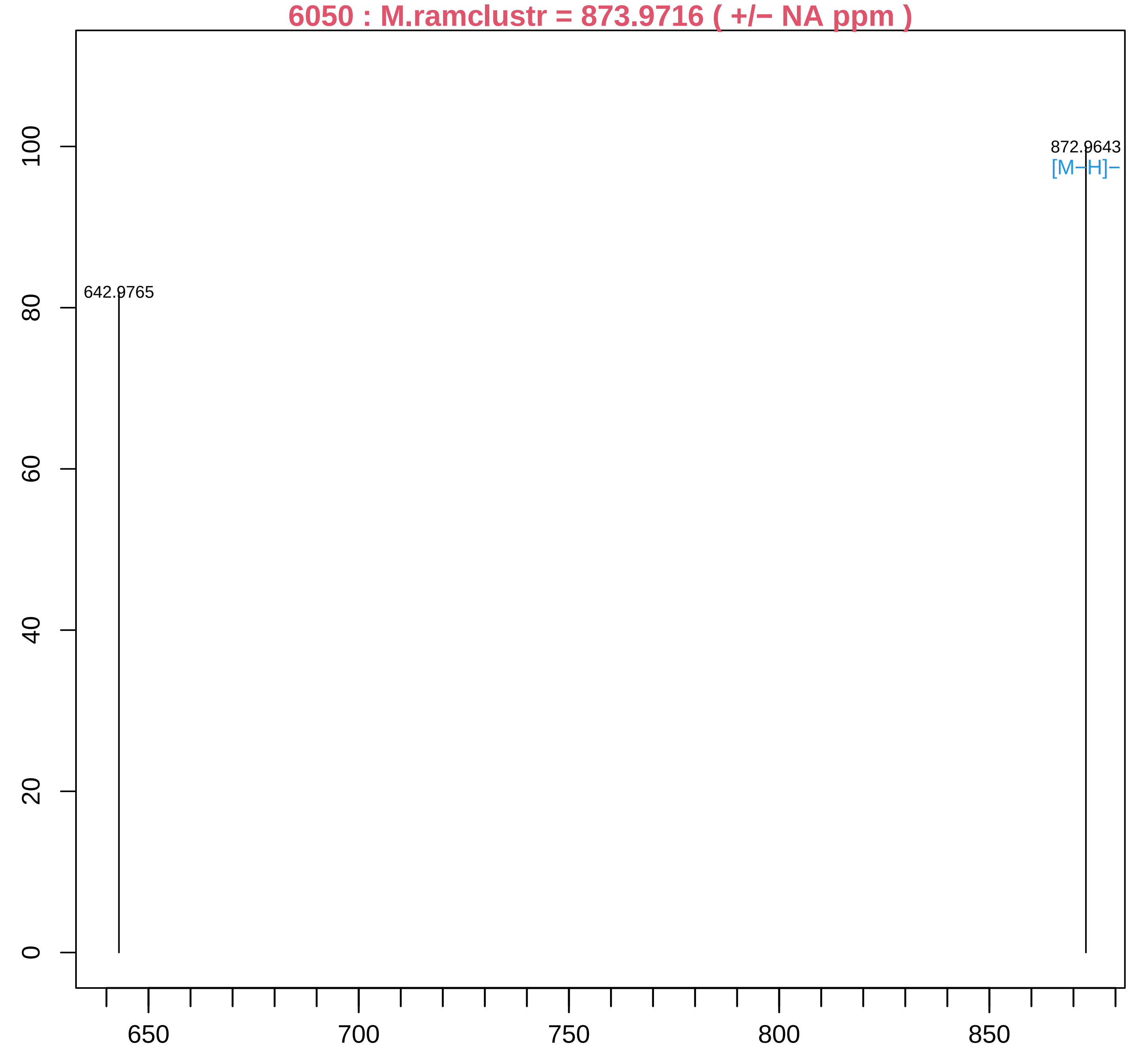

6050 : M.ramclustr = 873.9716 (+/− NA ppm)
872.9643
[M−H]−
642.9765
0
20
40
60
80
100
650
700
750
800
850

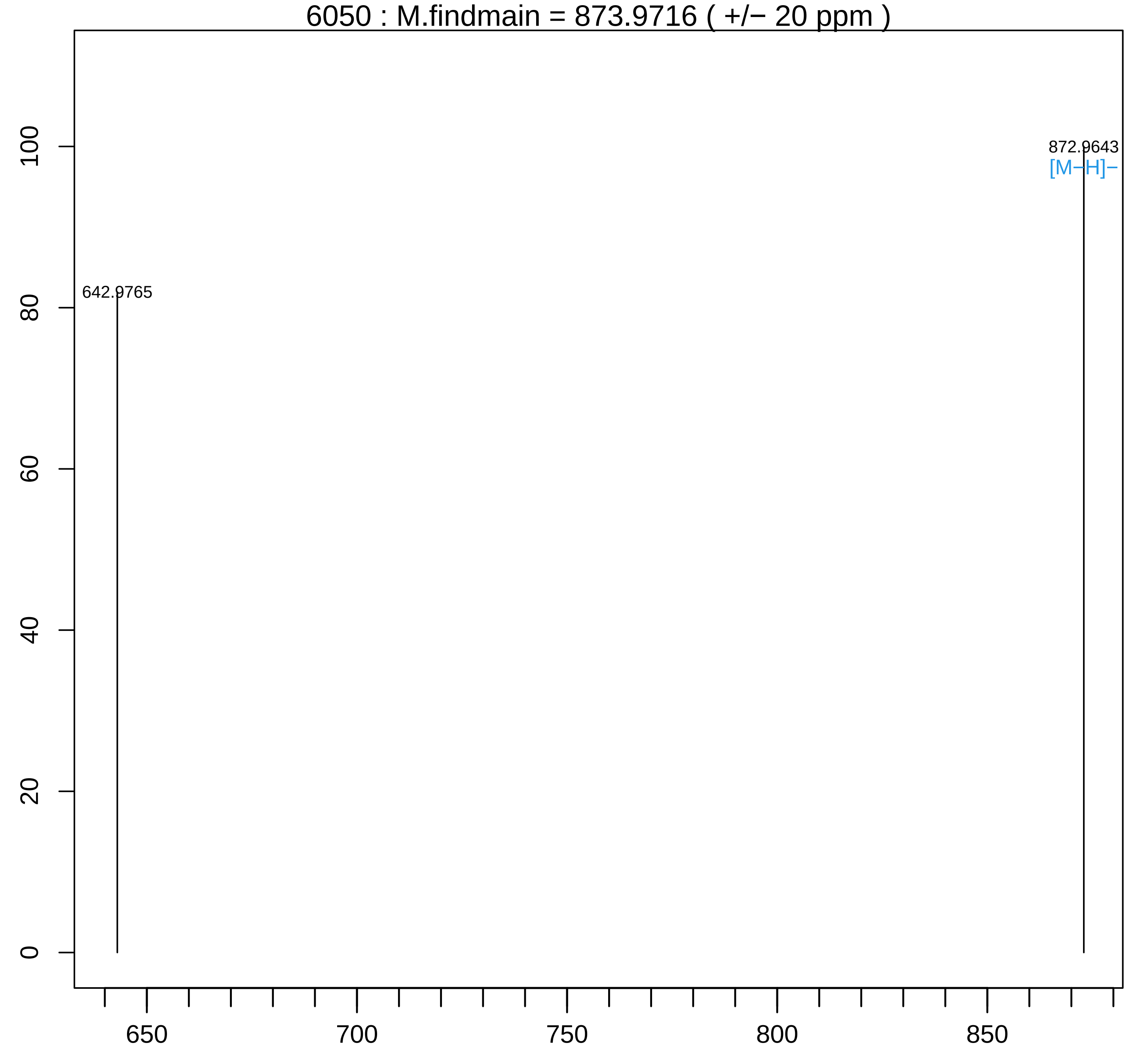

6050 : M.findmain = 873.9716 (+/− 20 ppm)
872.9643
[M−H]−
642.9765
0
20
40
60
80
100
650
700
750
800
850

6051 : M.ramclustr = 836.9755 (+/− NA ppm)

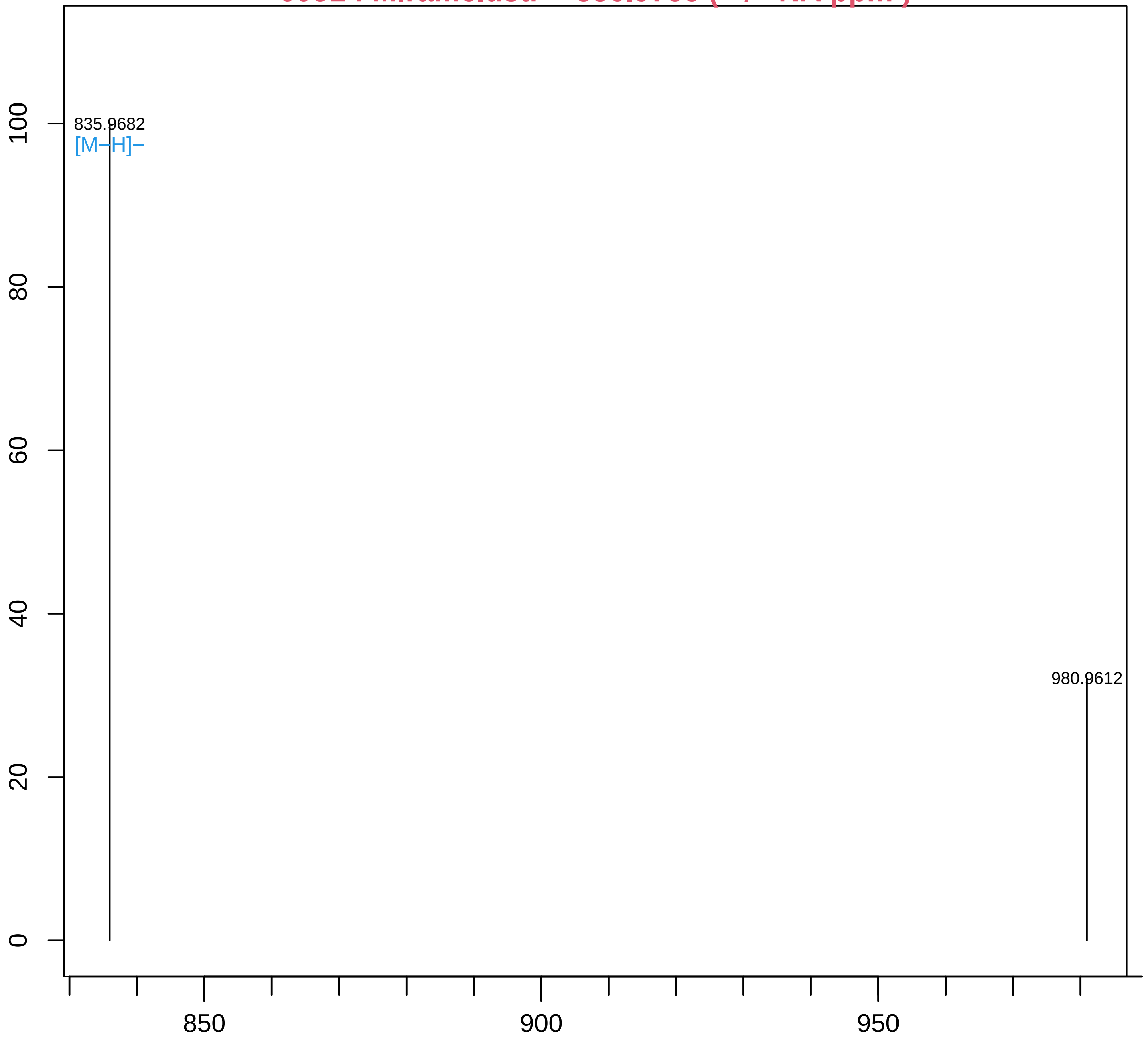

6051 : M.findmain = 836.9755 (+/− 20 ppm)

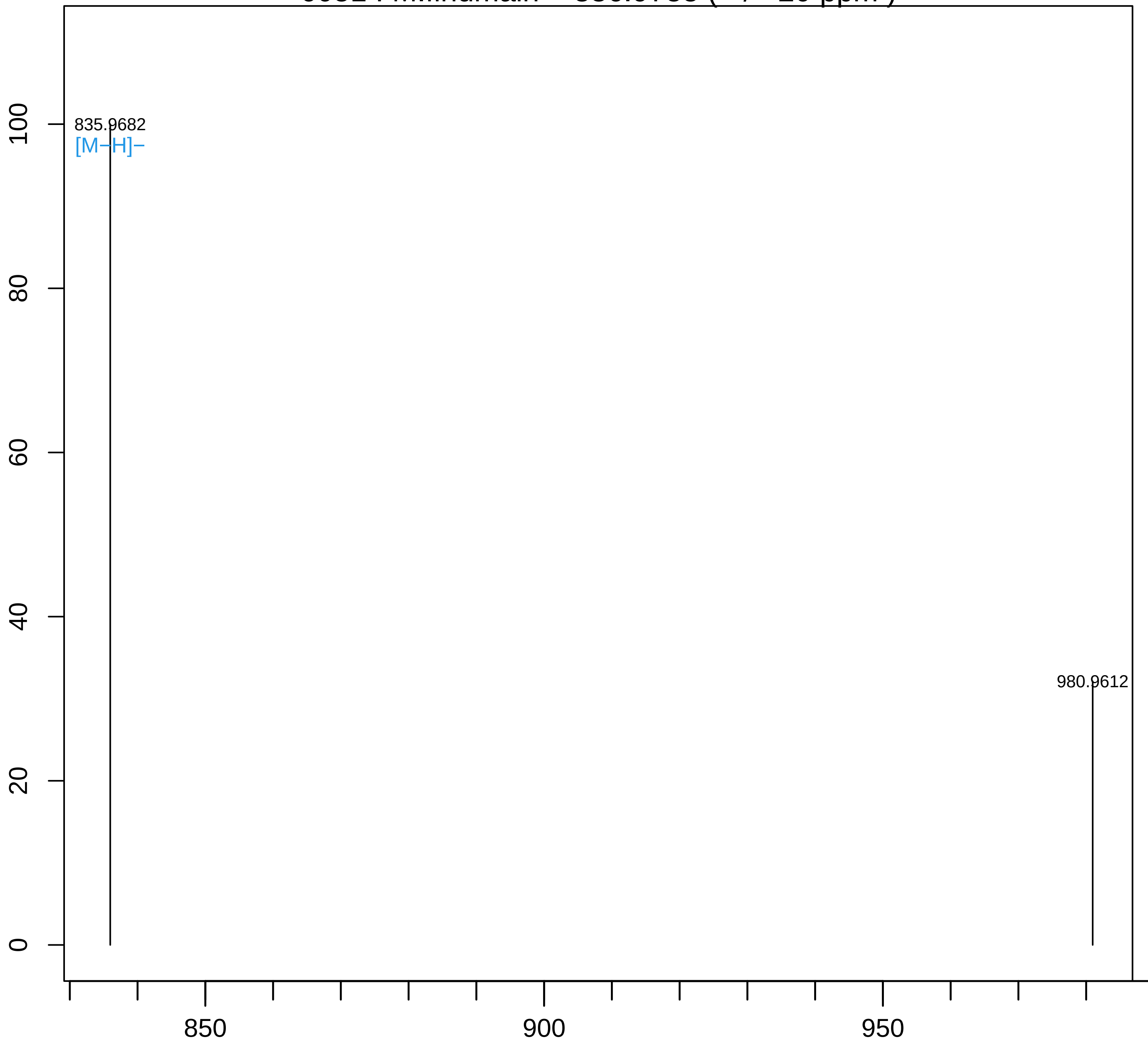

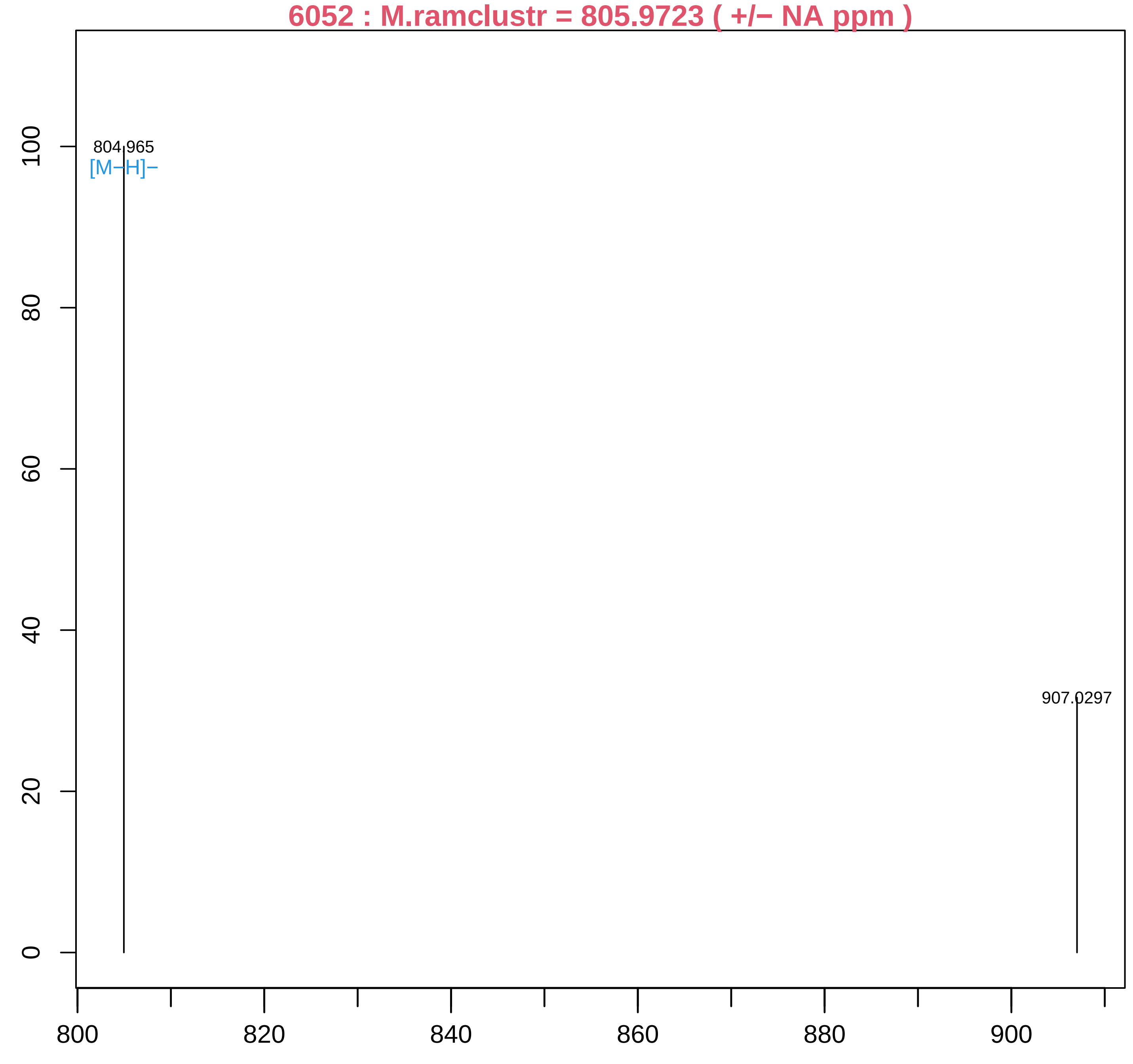
6052 : M.ramclustr = 805.9723 (+/− NA ppm)
804.965
[M−H]−
907.0297
0
20
40
60
80
100
800
820
840
860
880
900

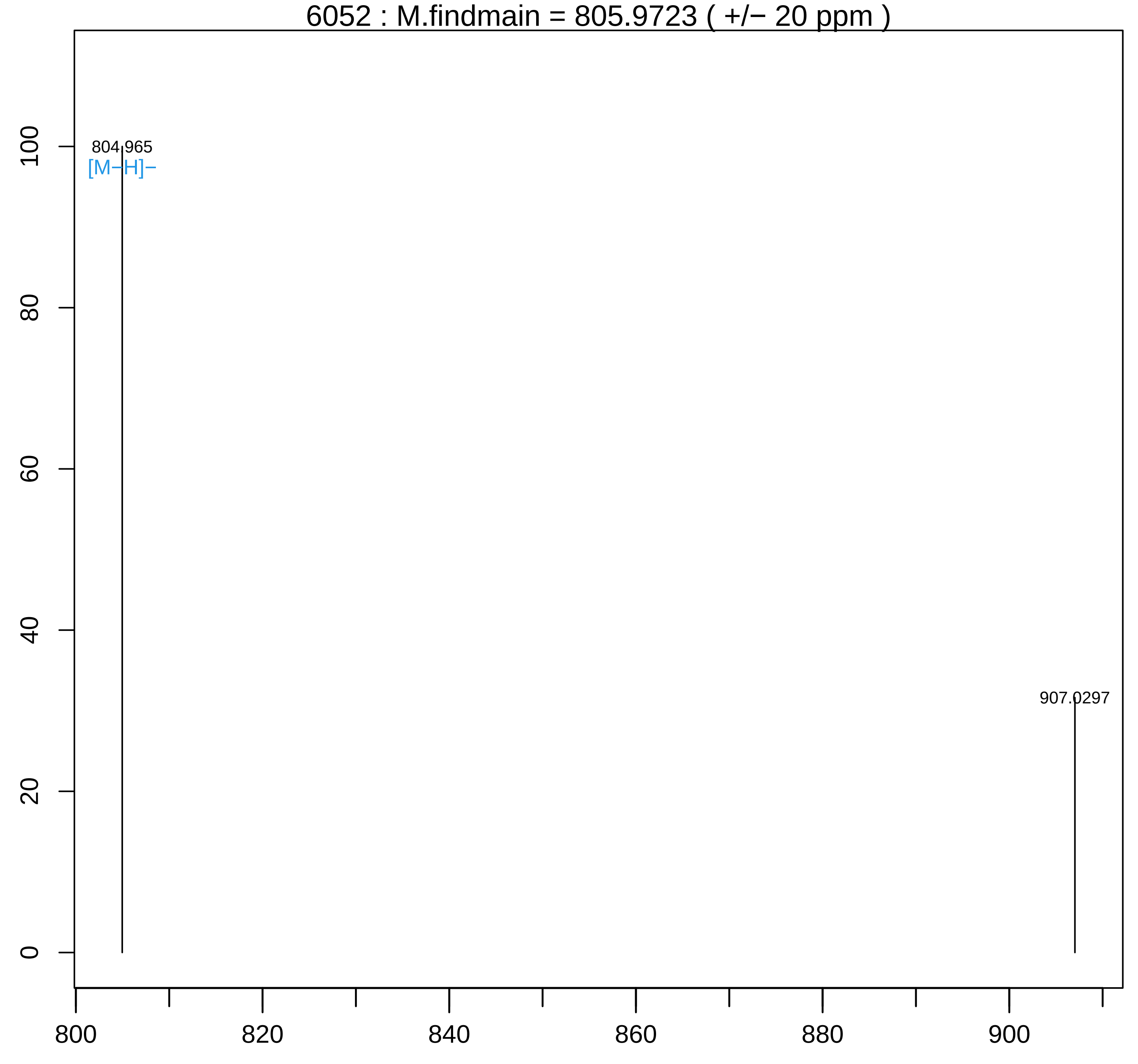
6052 : M.findmain = 805.9723 (+/− 20 ppm)
804.965
[M−H]−
907.0297
0
20
40
60
80
100
800
820
840
860
880
900

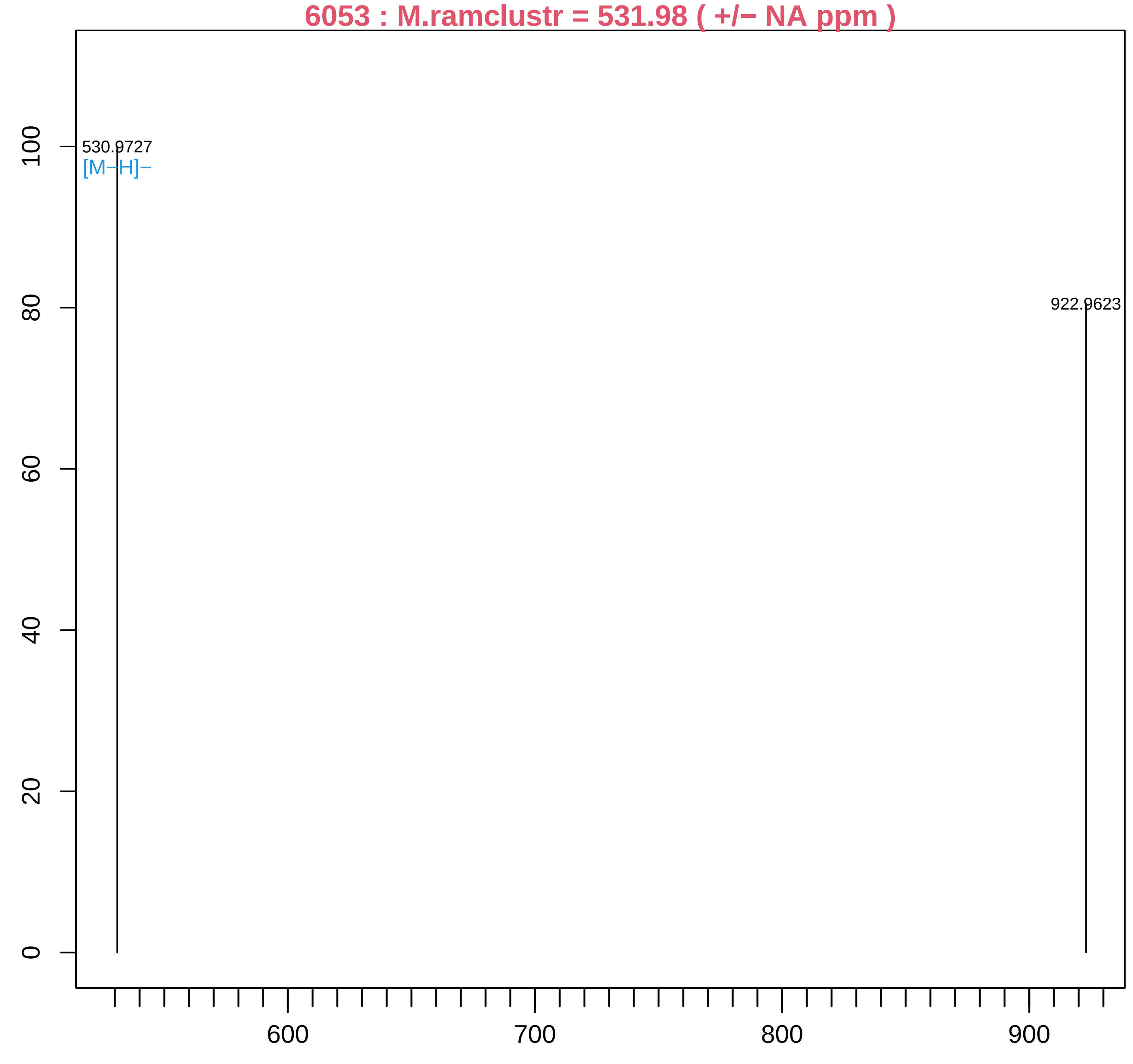

6053 : M.ramclustr = 531.98 (+/− NA ppm)
530.9727
[M−H]−
922.9623
0
20
40
60
80
100
600
700
800
900

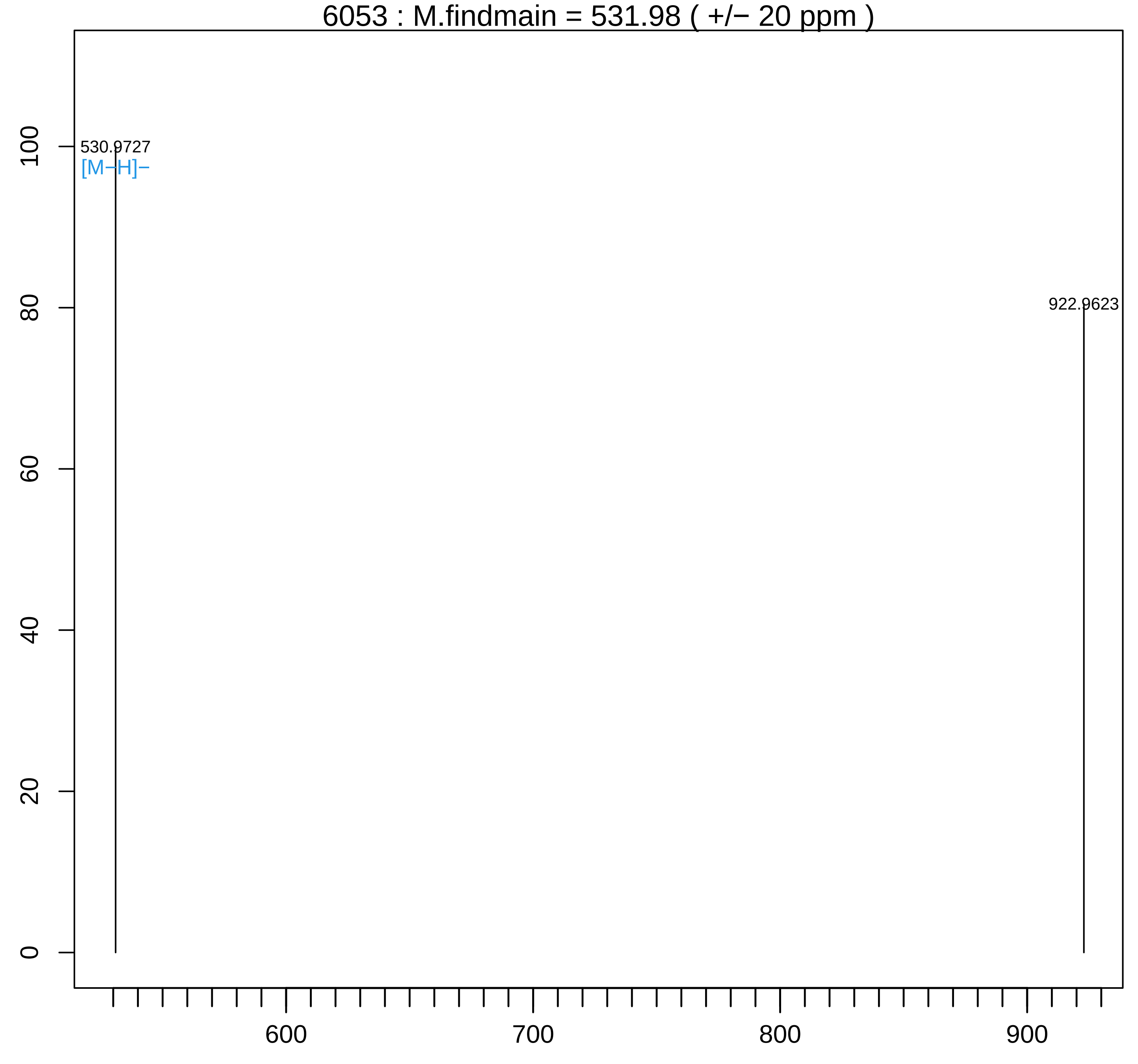

6053 : M.findmain = 531.98 (+/− 20 ppm)
530.9727
[M−H]−
922.9623
0
20
40
60
80
100
600
700
800
900

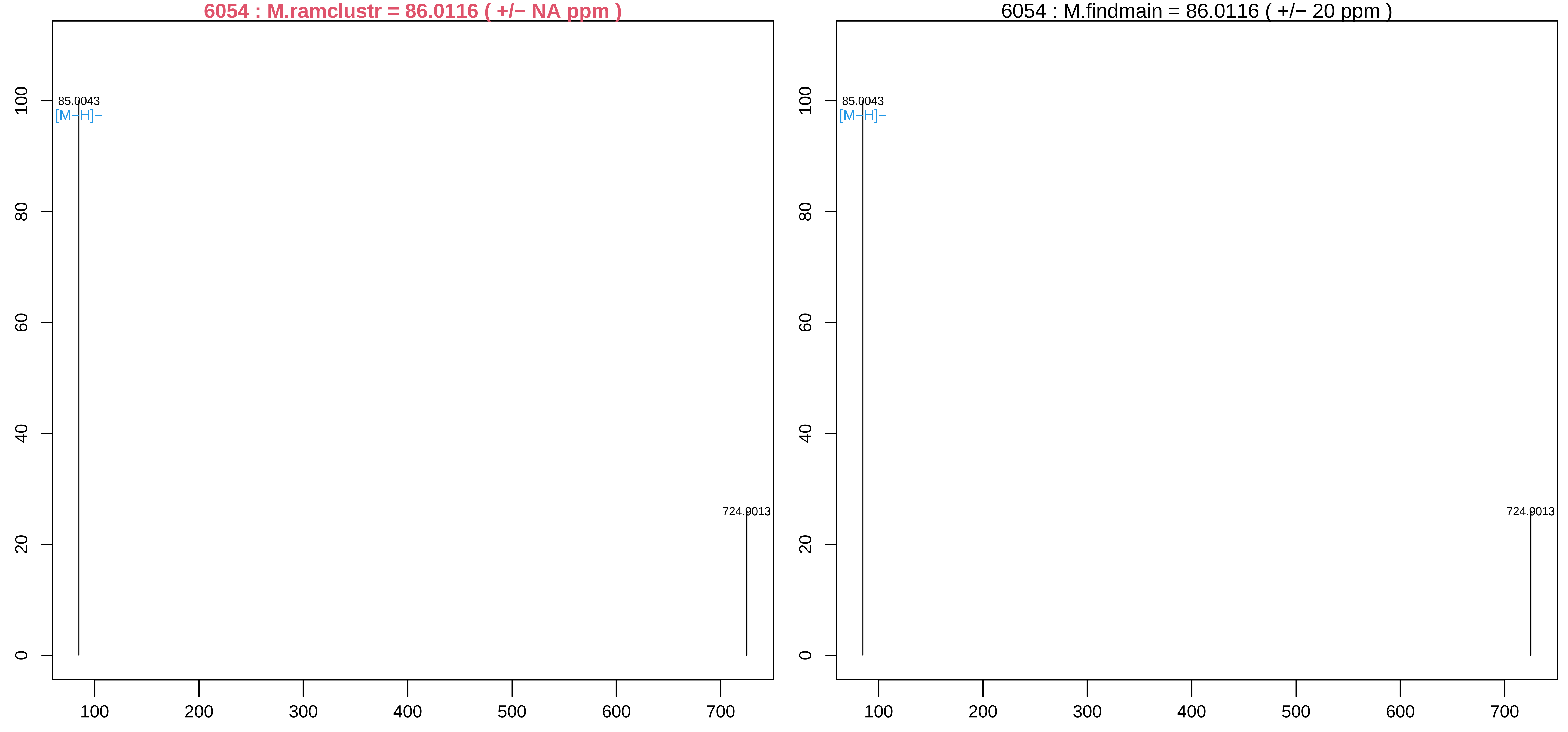

6054 : M.ramclustr = 86.0116 (+/− NA ppm)
85.0043
[M−H]−
724.9013
0
20
40
60
80
100
100
200
300
400
500
600
700
6054 : M.findmain = 86.0116 (+/− 20 ppm)
85.0043
[M−H]−
724.9013
0
20
40
60
80
100
100
200
300
400
500
600
700

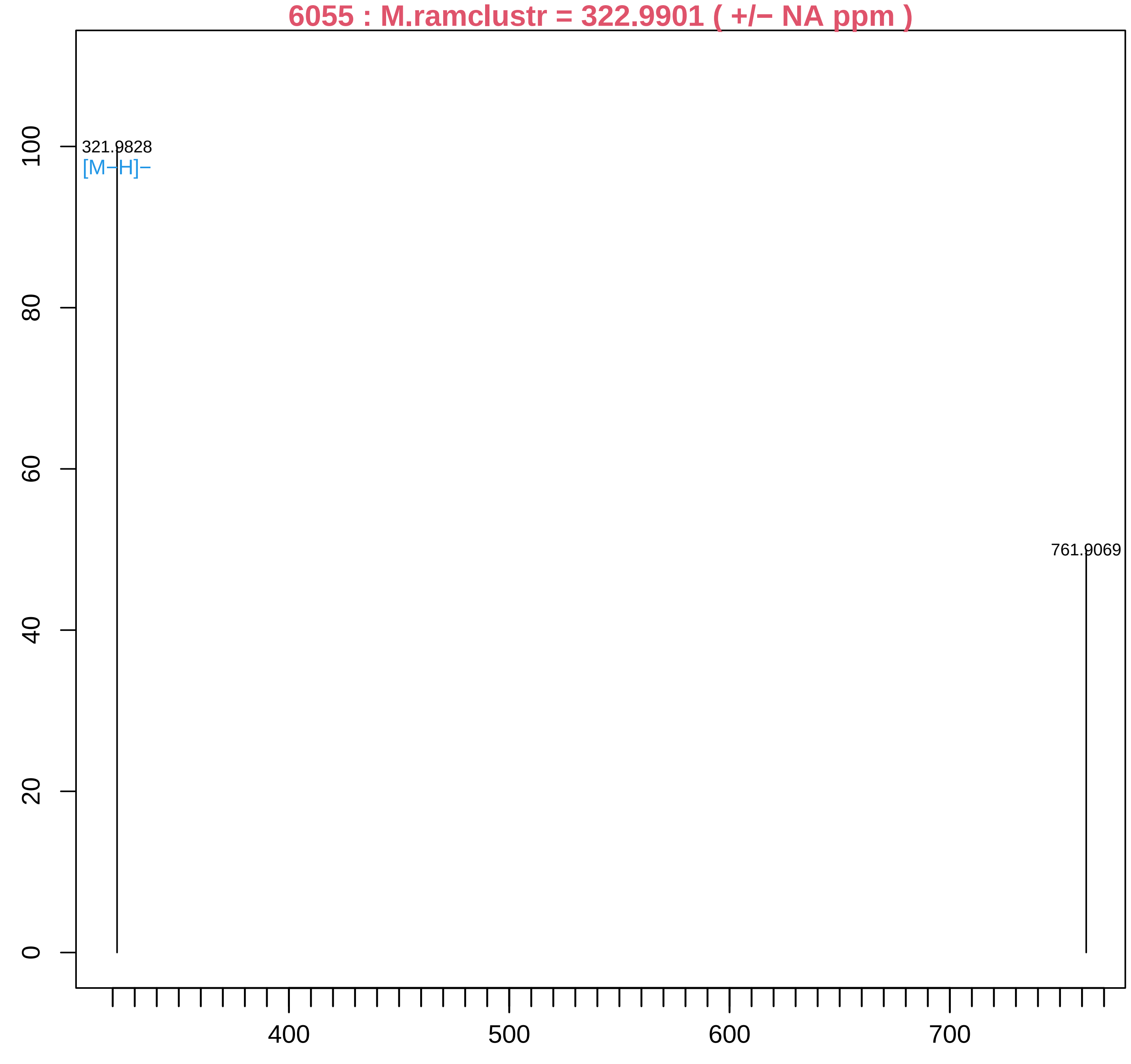
6055 : M.ramclustr = 322.9901 (+/− NA ppm)
321.9828
[M−H]−
761.9069
0
20
40
60
80
100
400
500
600
700

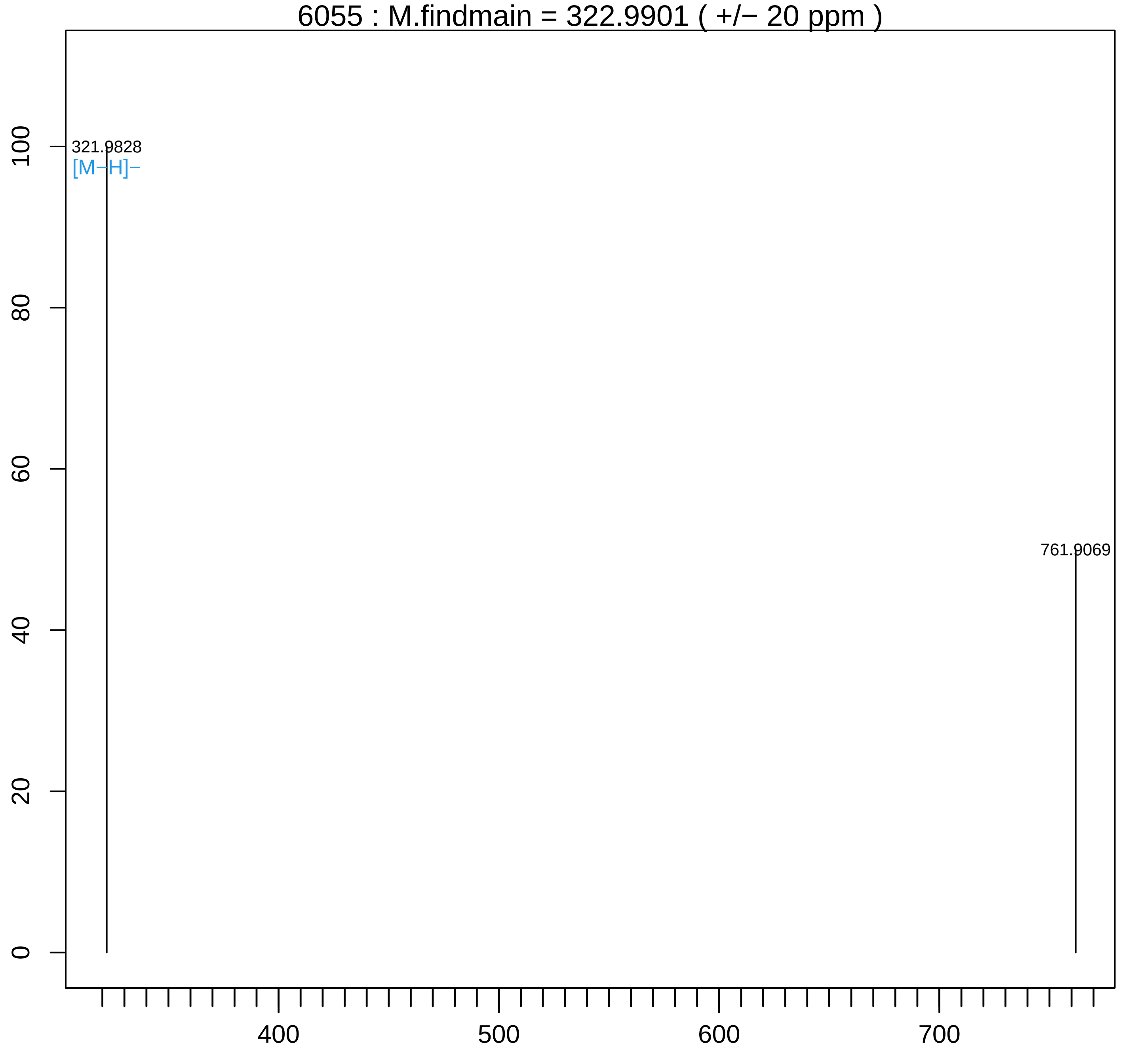
6055 : M.findmain = 322.9901 (+/− 20 ppm)
321.9828
[M−H]−
761.9069
0
20
40
60
80
100
400
500
600
700

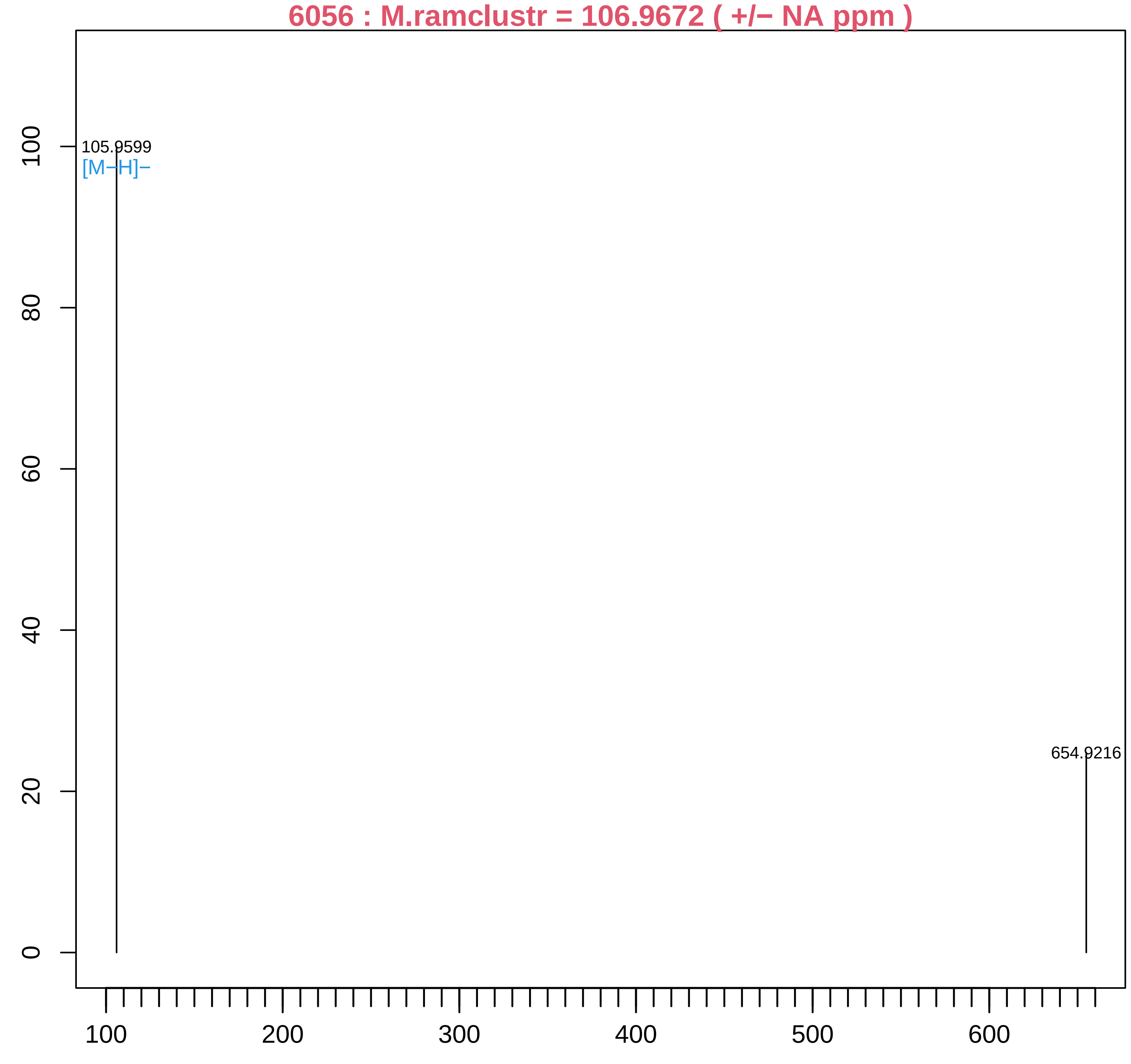
6056 : M.ramclustr = 106.9672 (+/− NA ppm)
105.9599
[M−H]−
654.9216
0
20
40
60
80
100
100
200
300
400
500
600

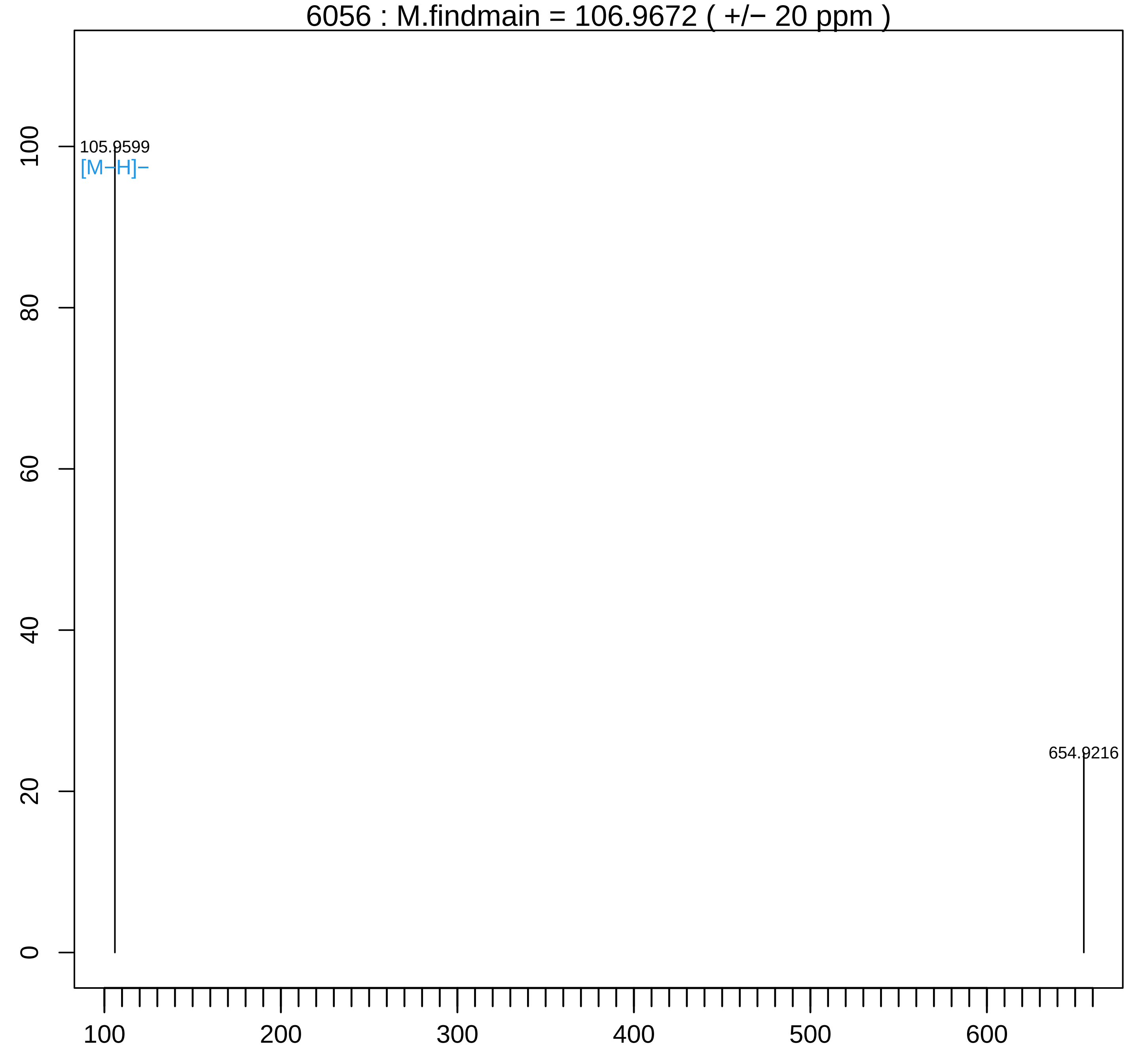
6056 : M.findmain = 106.9672 (+/− 20 ppm)
105.9599
[M−H]−
654.9216
0
20
40
60
80
100
100
200
300
400
500
600

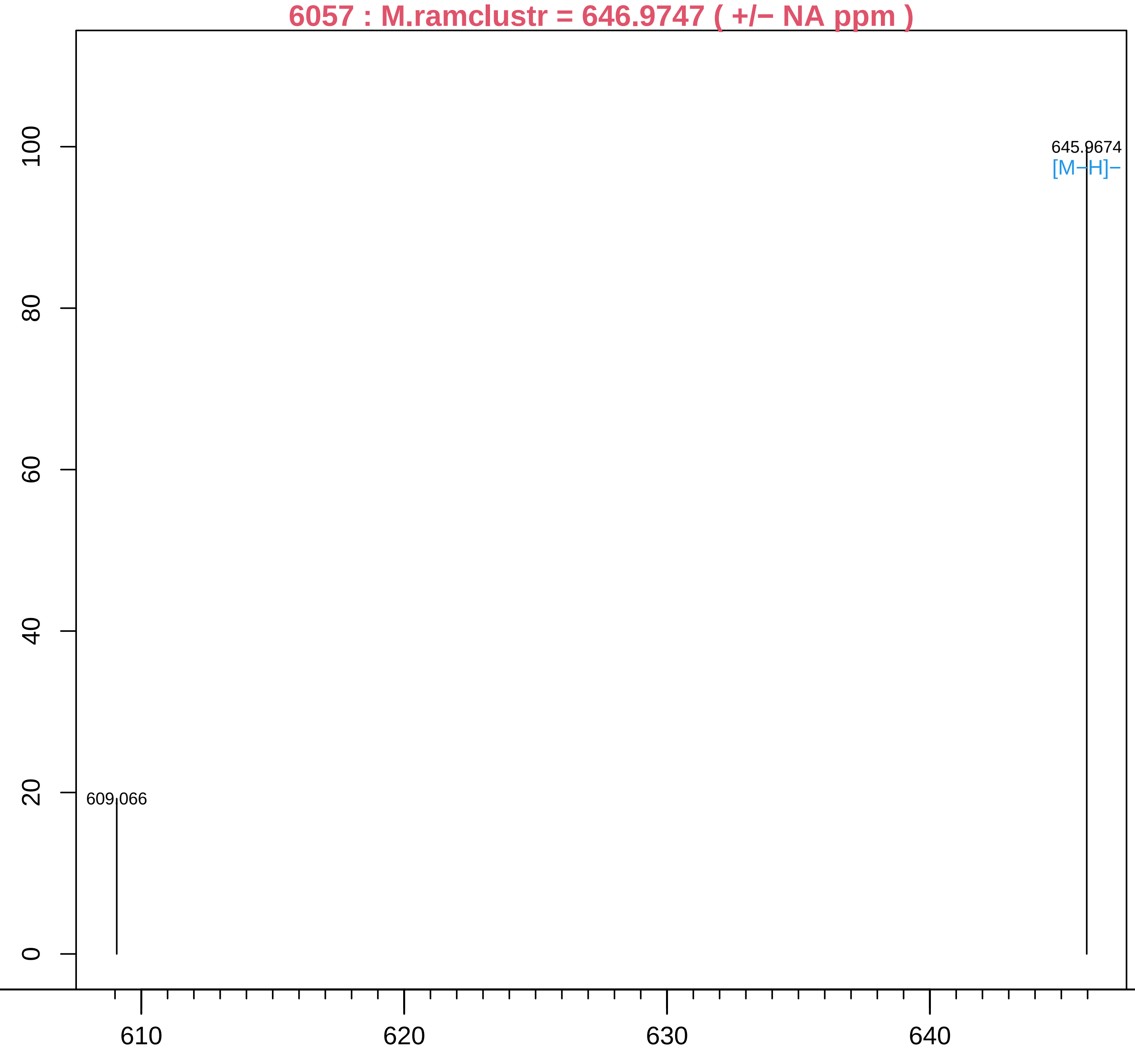
6057 : M.ramclustr = 646.9747 (+/− NA ppm)
645.9674
[M−H]−
609.066
0
20
40
60
80
100
610
620
630
640

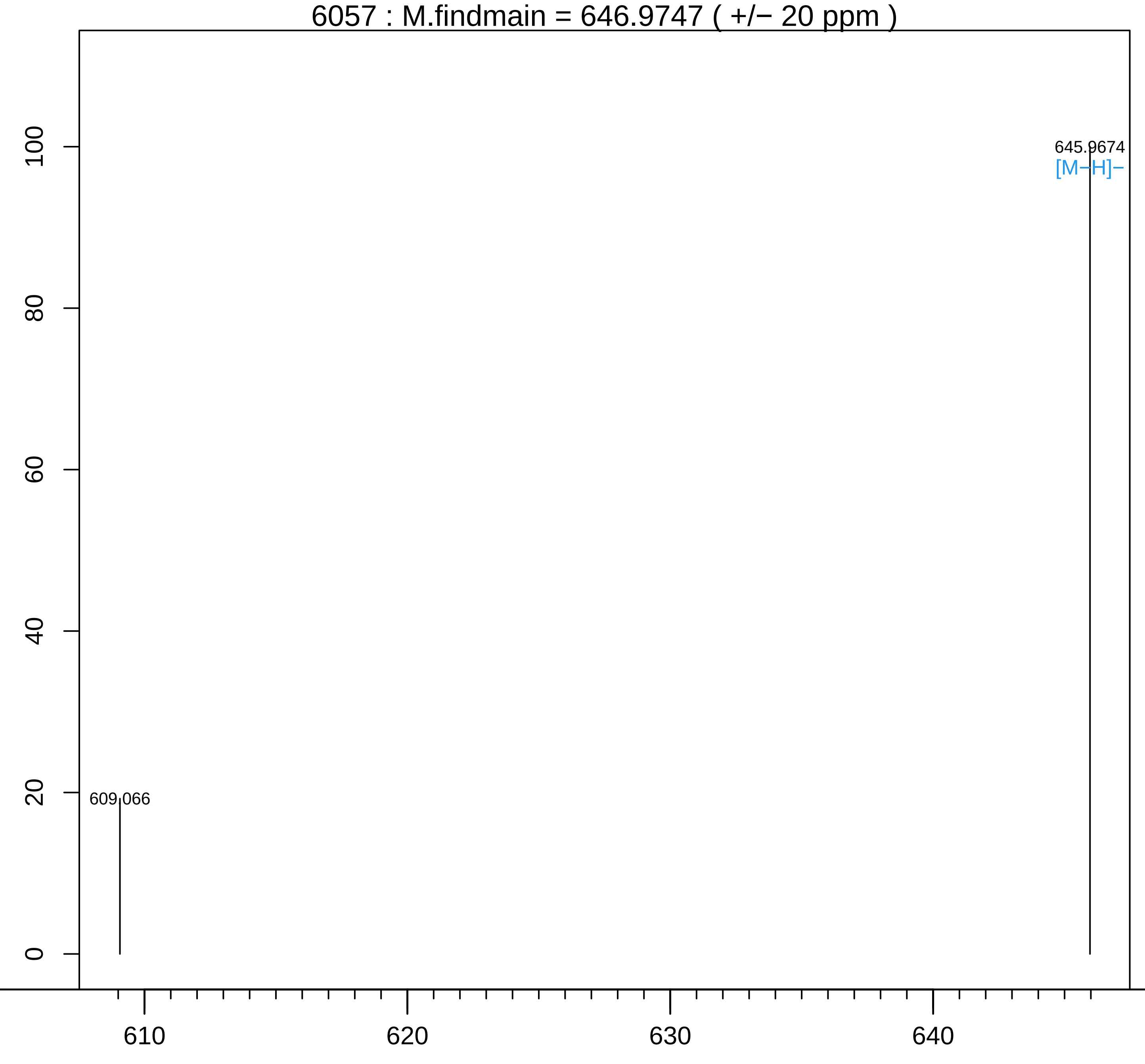
6057 : M.findmain = 646.9747 (+/− 20 ppm)
645.9674
[M−H]−
609.066
0
20
40
60
80
100
610
620
630
640

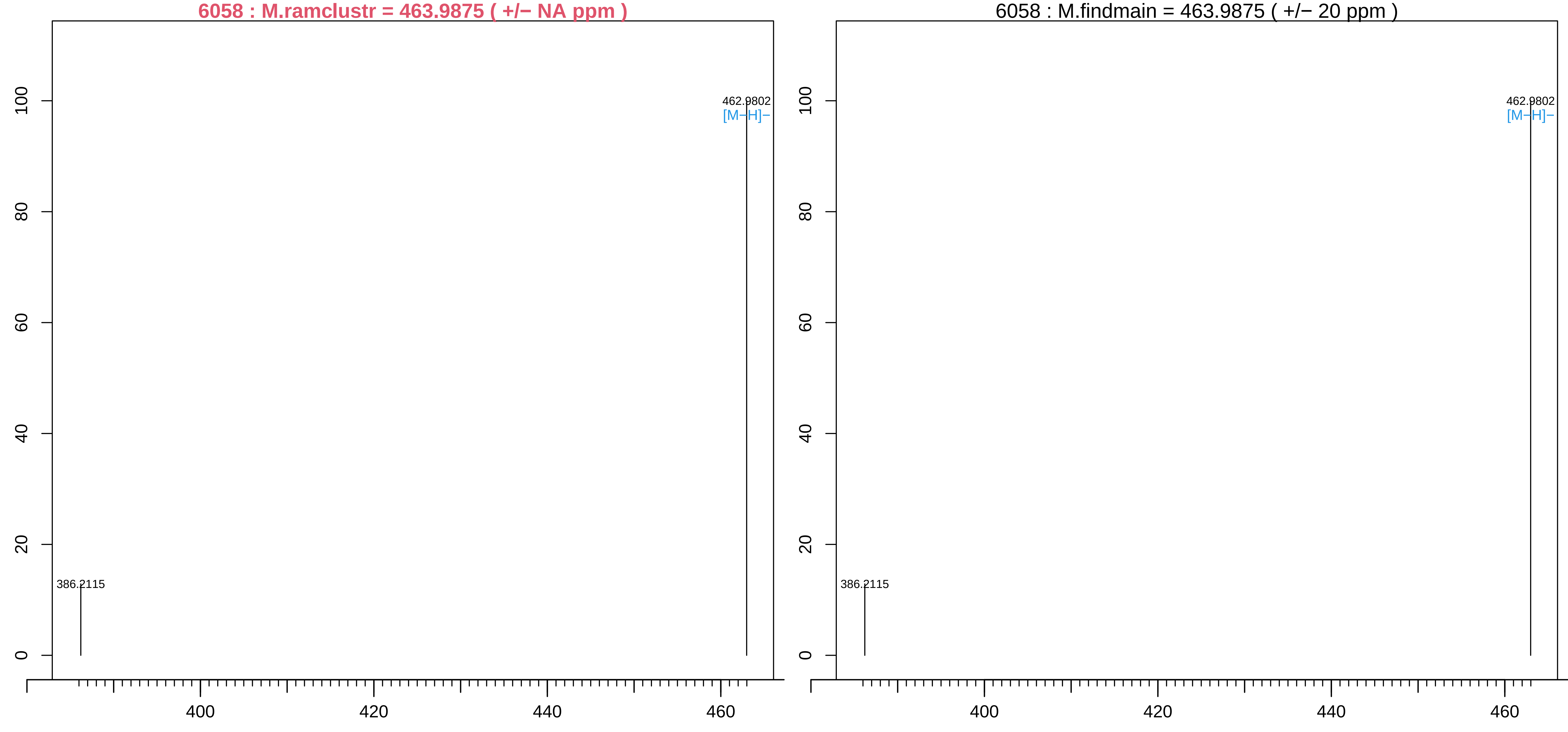

6058 : M.ramclustr = 463.9875 (+/− NA ppm)
462.9802
[M−H]−
386.2115
0
20
40
60
80
100
400
420
440
460
6058 : M.findmain = 463.9875 (+/− 20 ppm)
462.9802
[M−H]−
386.2115
0
20
40
60
80
100
400
420
440
460

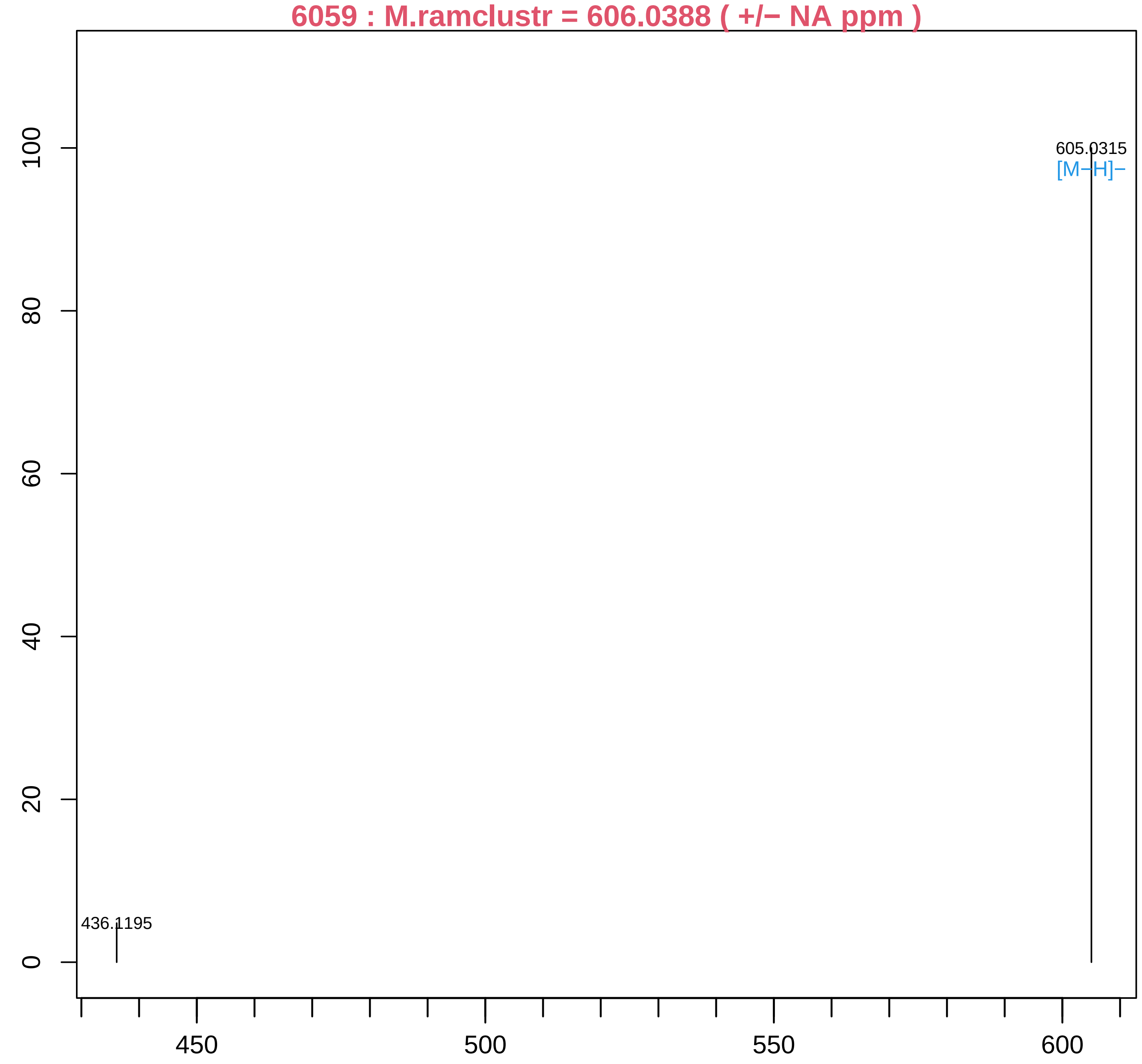
6059 : M.ramclustr = 606.0388 (+/− NA ppm)
605.0315
[M−H]−
436.1195
0
20
40
60
80
100
450
500
550
600

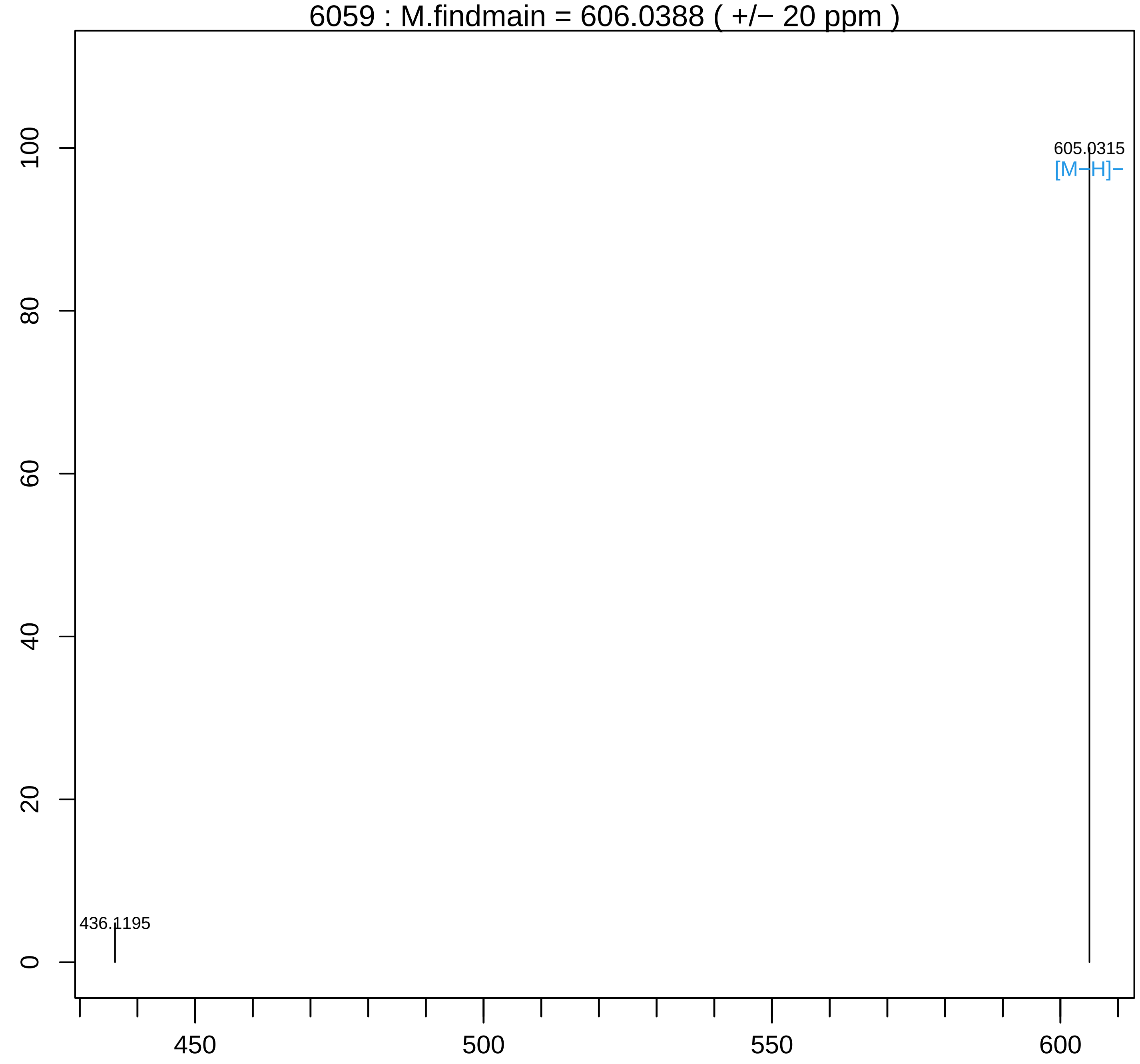
6059 : M.findmain = 606.0388 (+/− 20 ppm)
605.0315
[M−H]−
436.1195
0
20
40
60
80
100
450
500
550
600

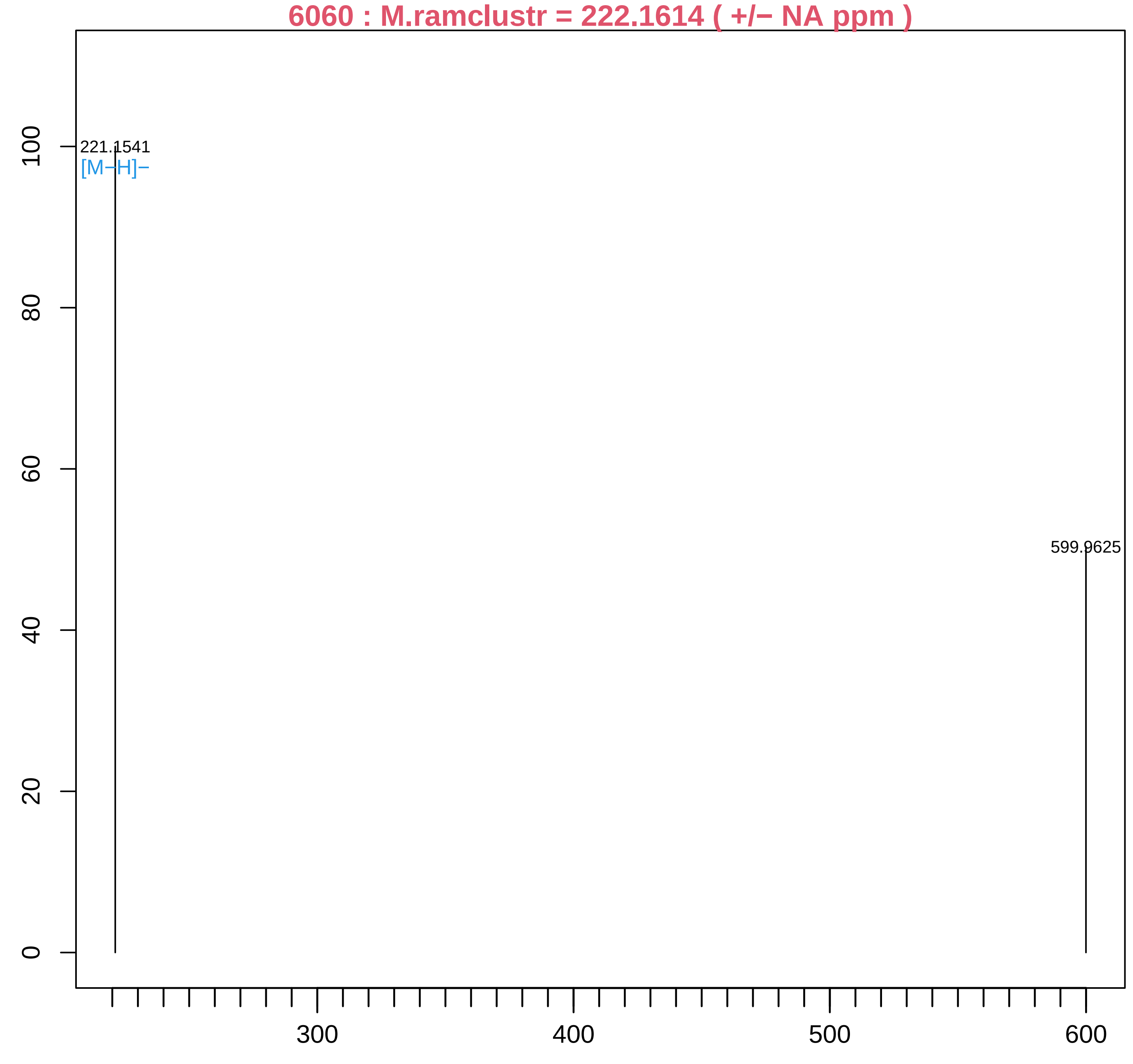
6060 : M.ramclustr = 222.1614 (+/− NA ppm)
221.1541
[M−H]−
599.9625
0
20
40
60
80
100
300
400
500
600

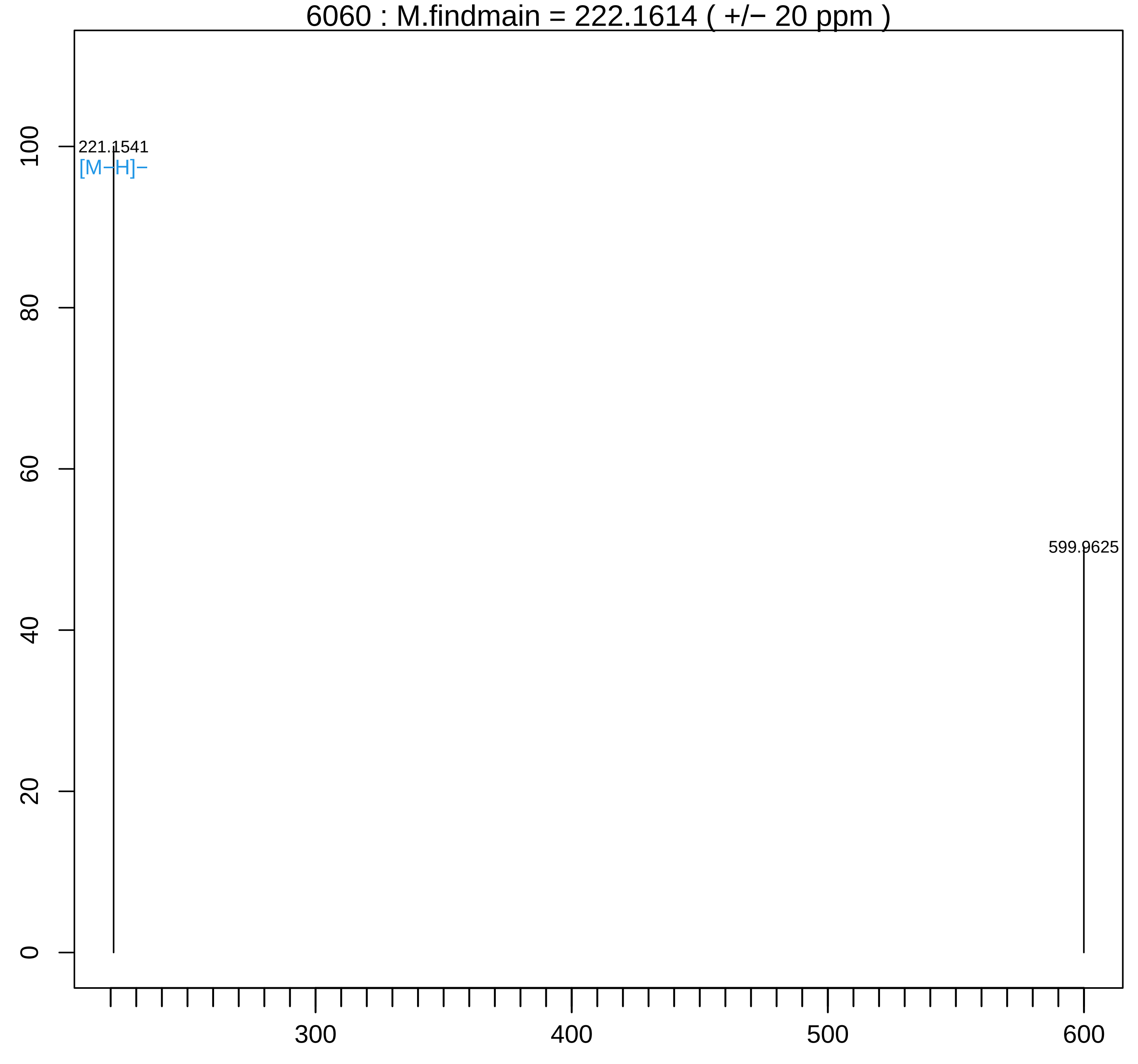
6060 : M.findmain = 222.1614 (+/− 20 ppm)
221.1541
[M−H]−
599.9625
0
20
40
60
80
100
300
400
500
600

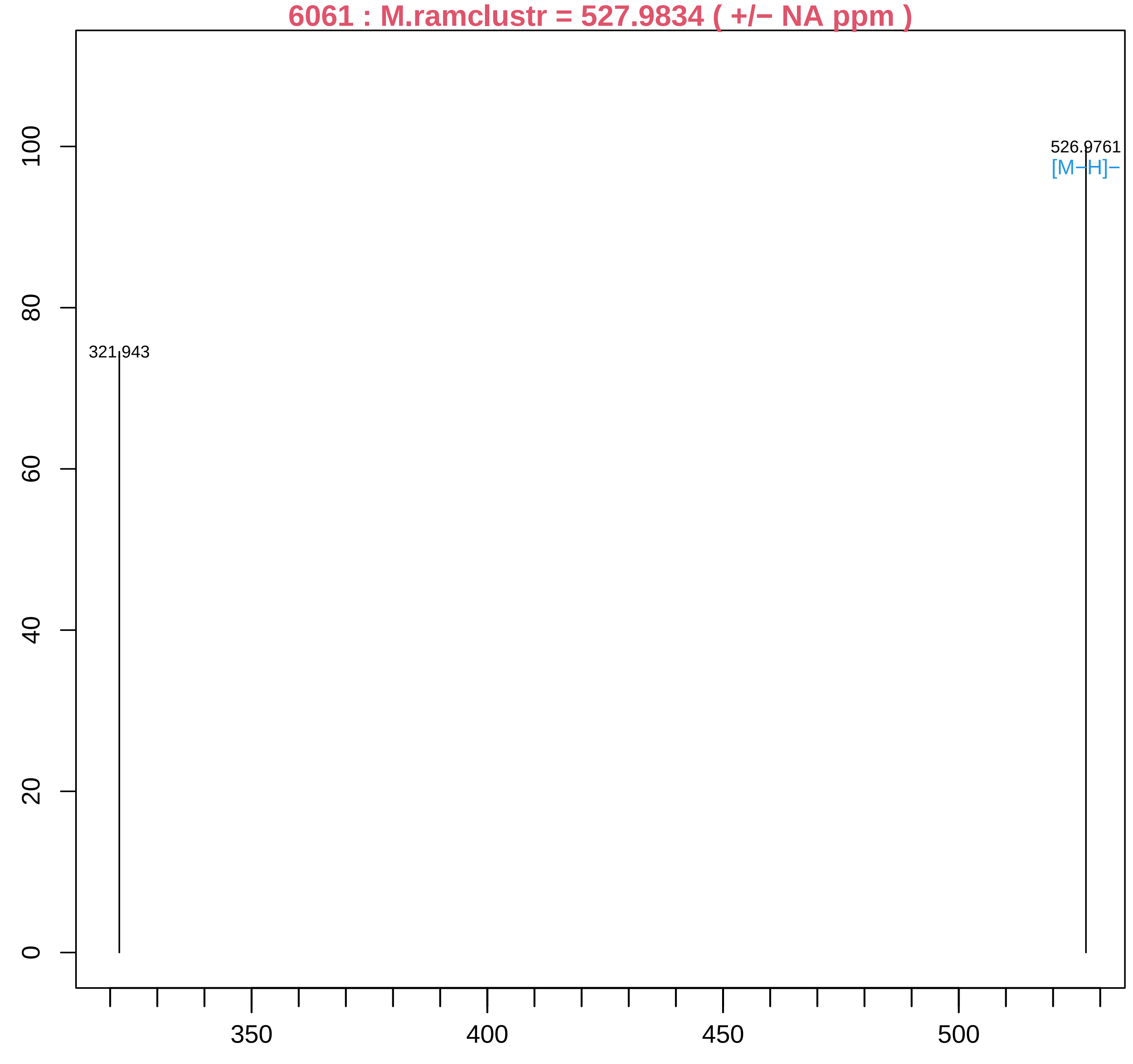
6061 : M.ramclustr = 527.9834 (+/− NA ppm)
526.9761
[M−H]−
321.943
0
20
40
60
80
100
350
400
450
500

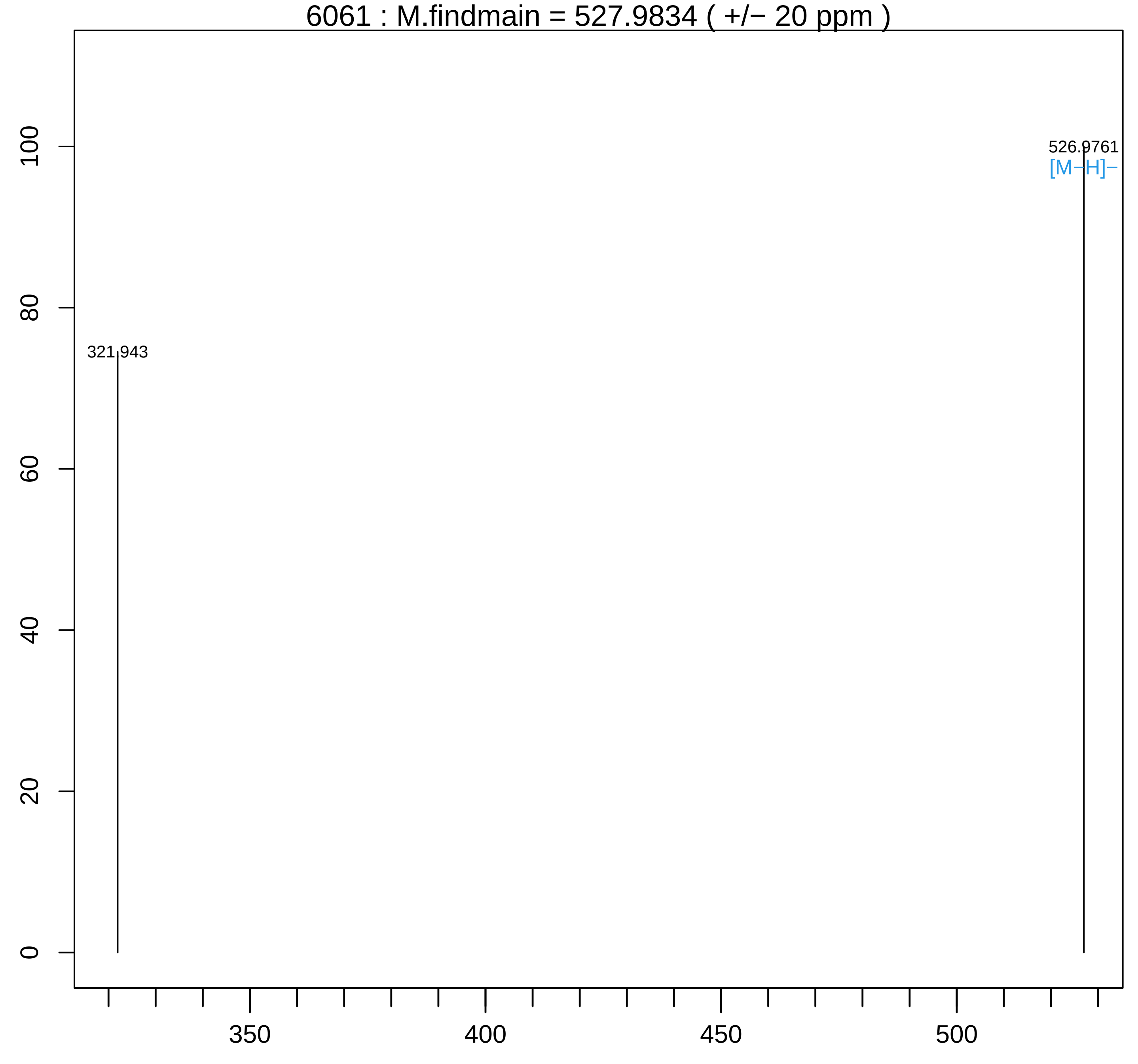
6061 : M.findmain = 527.9834 (+/− 20 ppm)
526.9761
[M−H]−
321.943
0
20
40
60
80
100
350
400
450
500

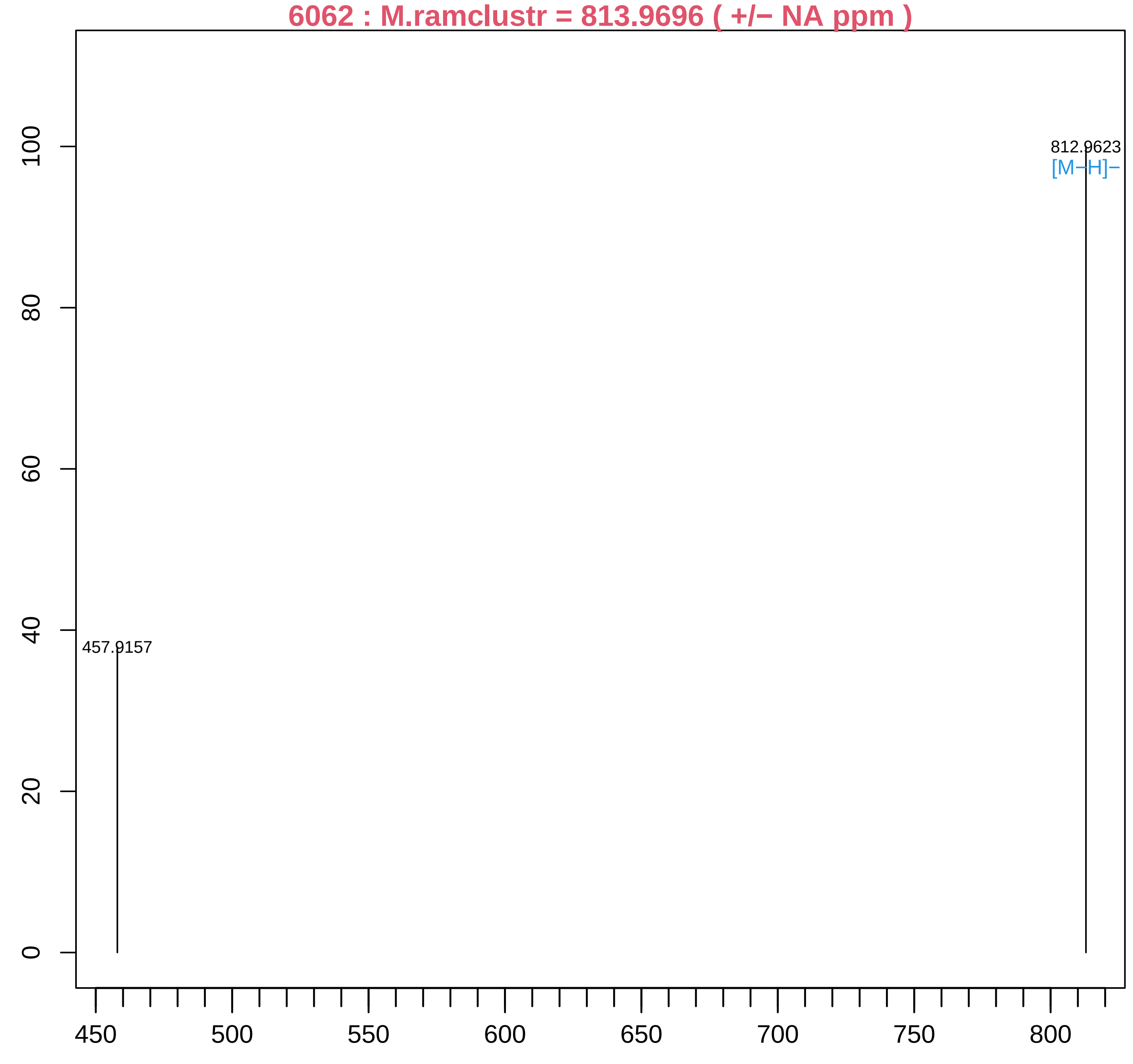

6062 : M.ramclustr = 813.9696 (+/− NA ppm)
812.9623
[M−H]−
457.9157
0
20
40
60
80
100
450
500
550
600
650
700
750
800

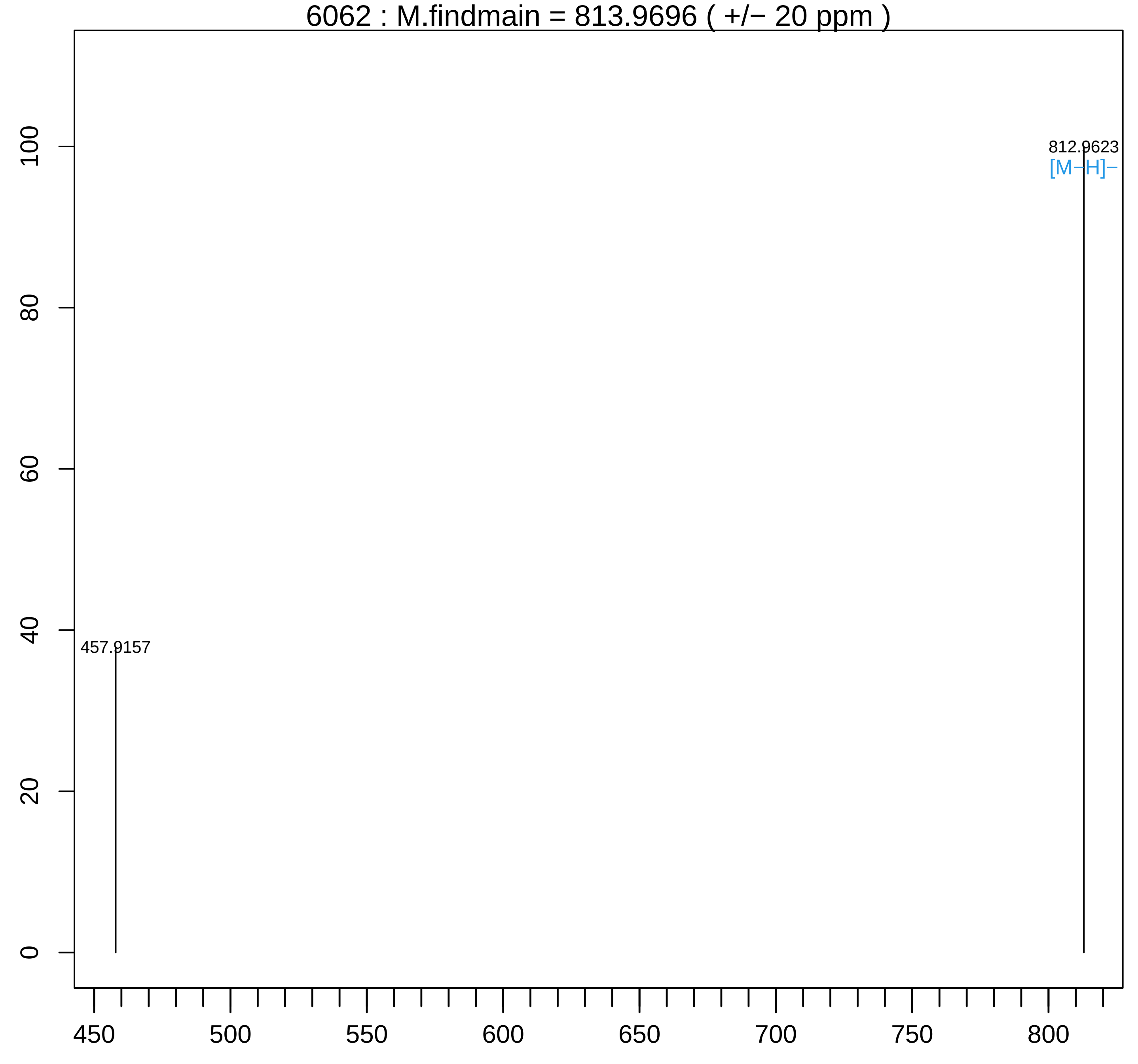

6062 : M.findmain = 813.9696 (+/− 20 ppm)
812.9623
[M−H]−
457.9157
0
20
40
60
80
100
450
500
550
600
650
700
750
800

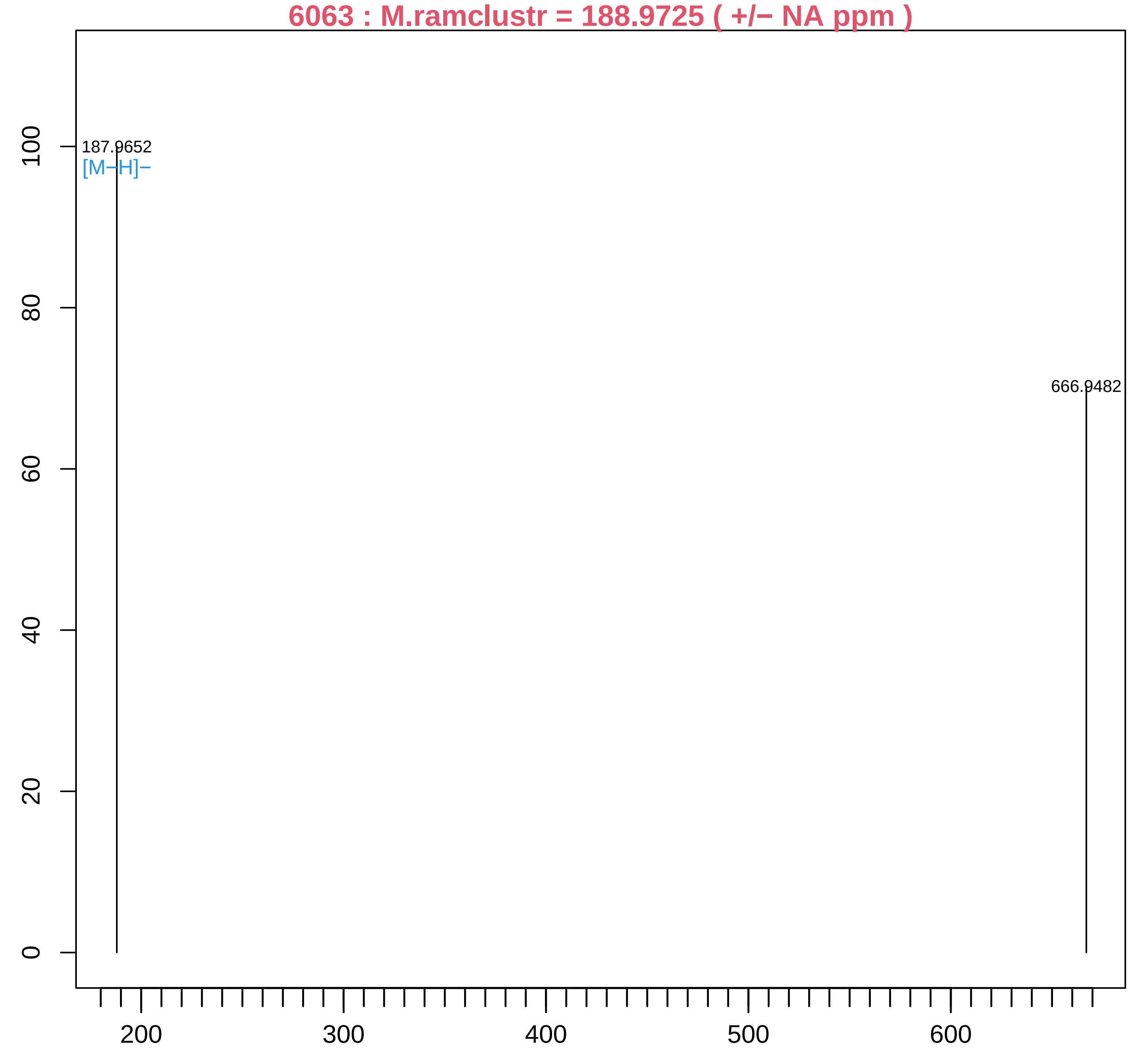
6063 : M.ramclustr = 188.9725 (+/− NA ppm)
187.9652
[M−H]−
666.9482
0
20
40
60
80
100
200
300
400
500
600

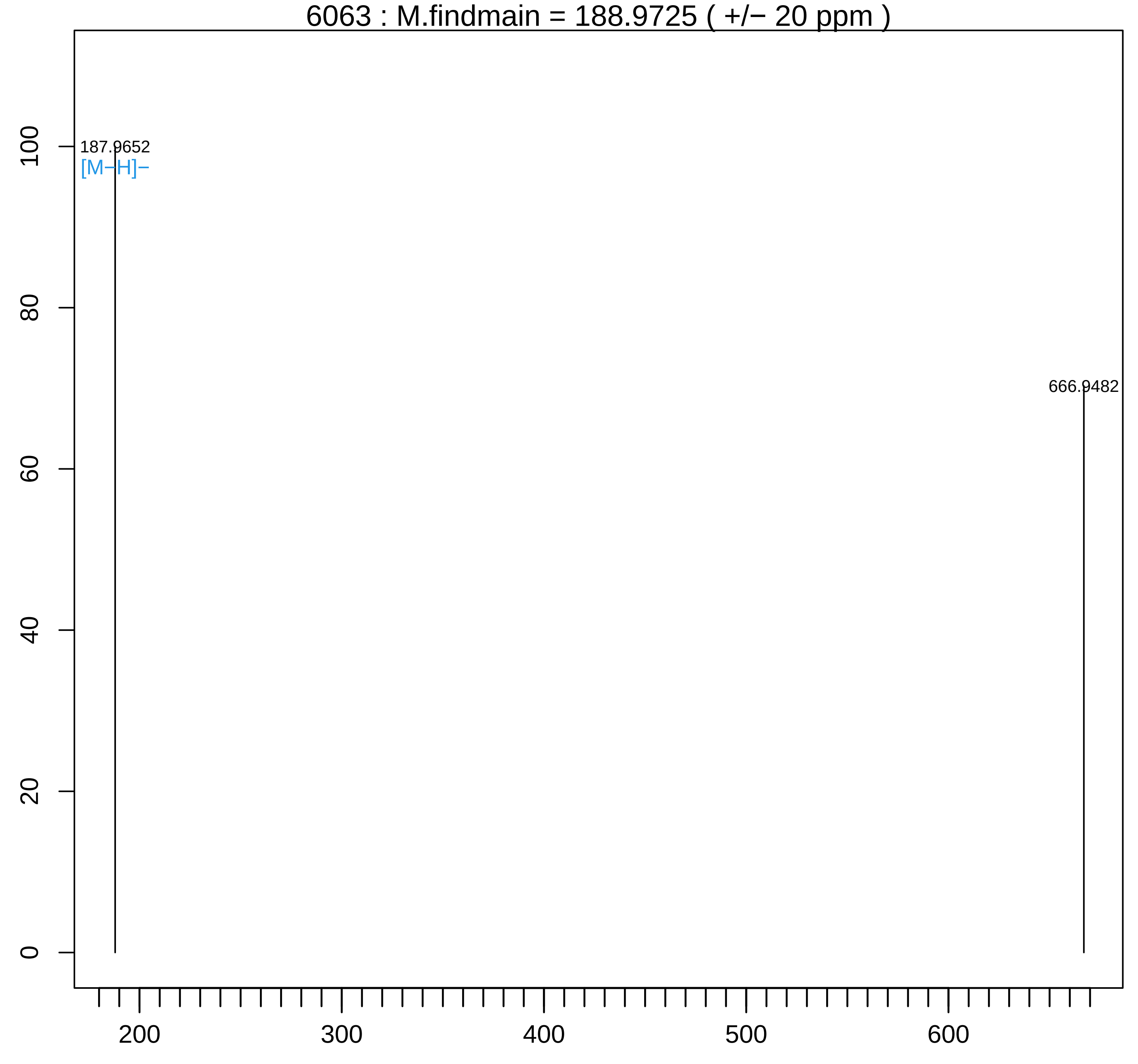
6063 : M.findmain = 188.9725 (+/− 20 ppm)
187.9652
[M−H]−
666.9482
0
20
40
60
80
100
200
300
400
500
600

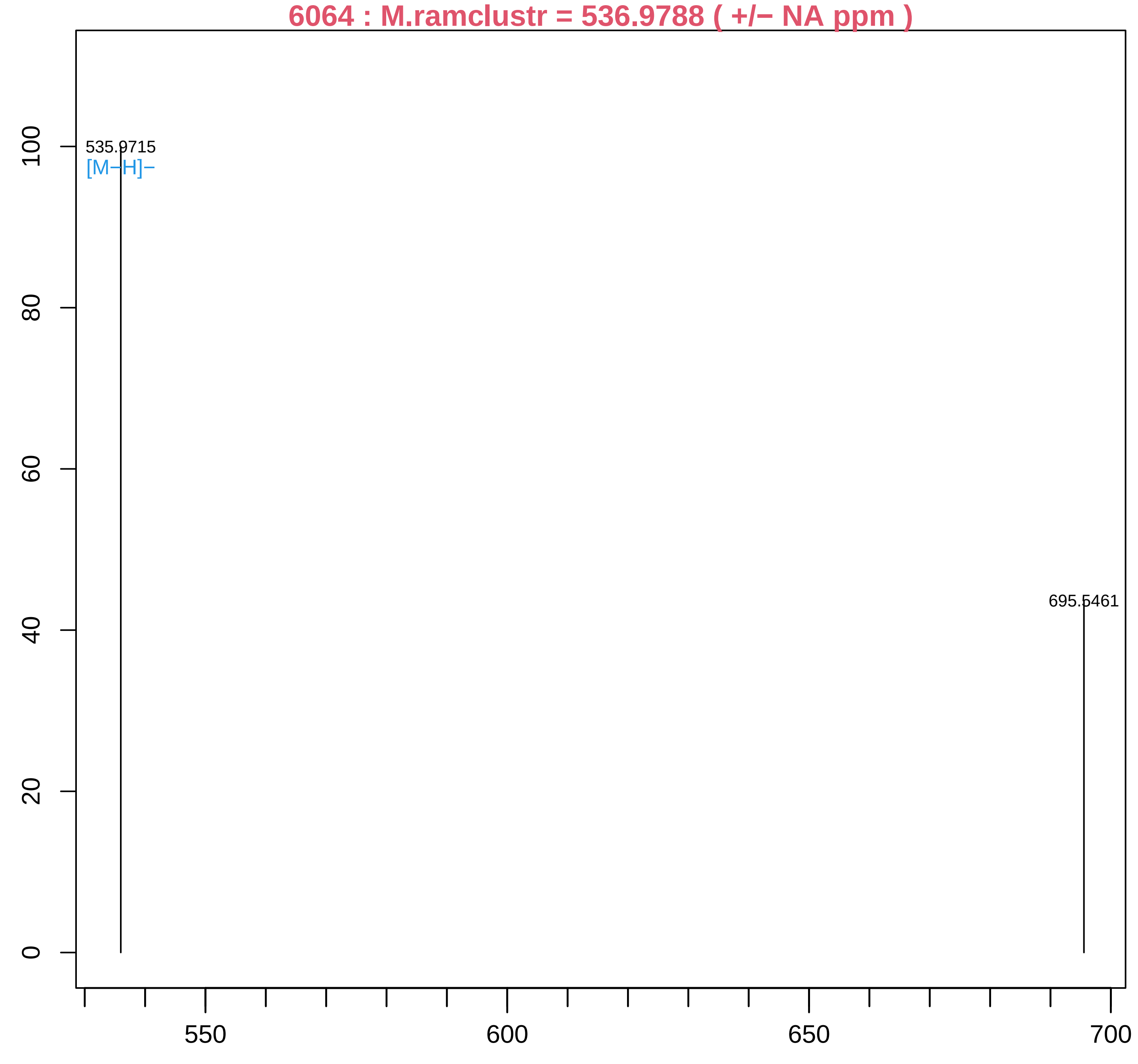
6064 : M.ramclustr = 536.9788 (+/− NA ppm)
535.9715
[M−H]−
695.5461
0
20
40
60
80
100
550
600
650
700

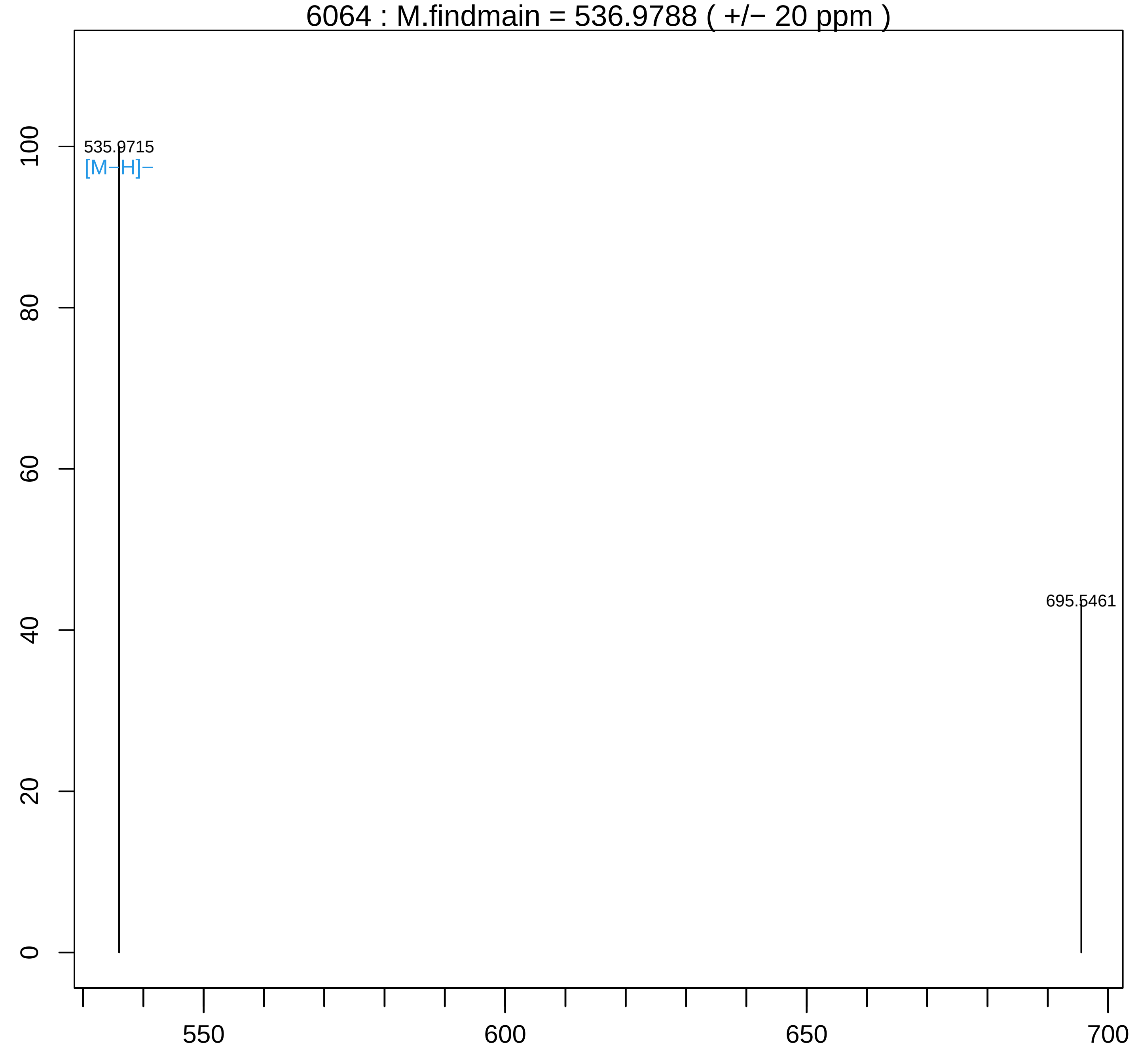
6064 : M.findmain = 536.9788 (+/− 20 ppm)
535.9715
[M−H]−
695.5461
0
20
40
60
80
100
550
600
650
700

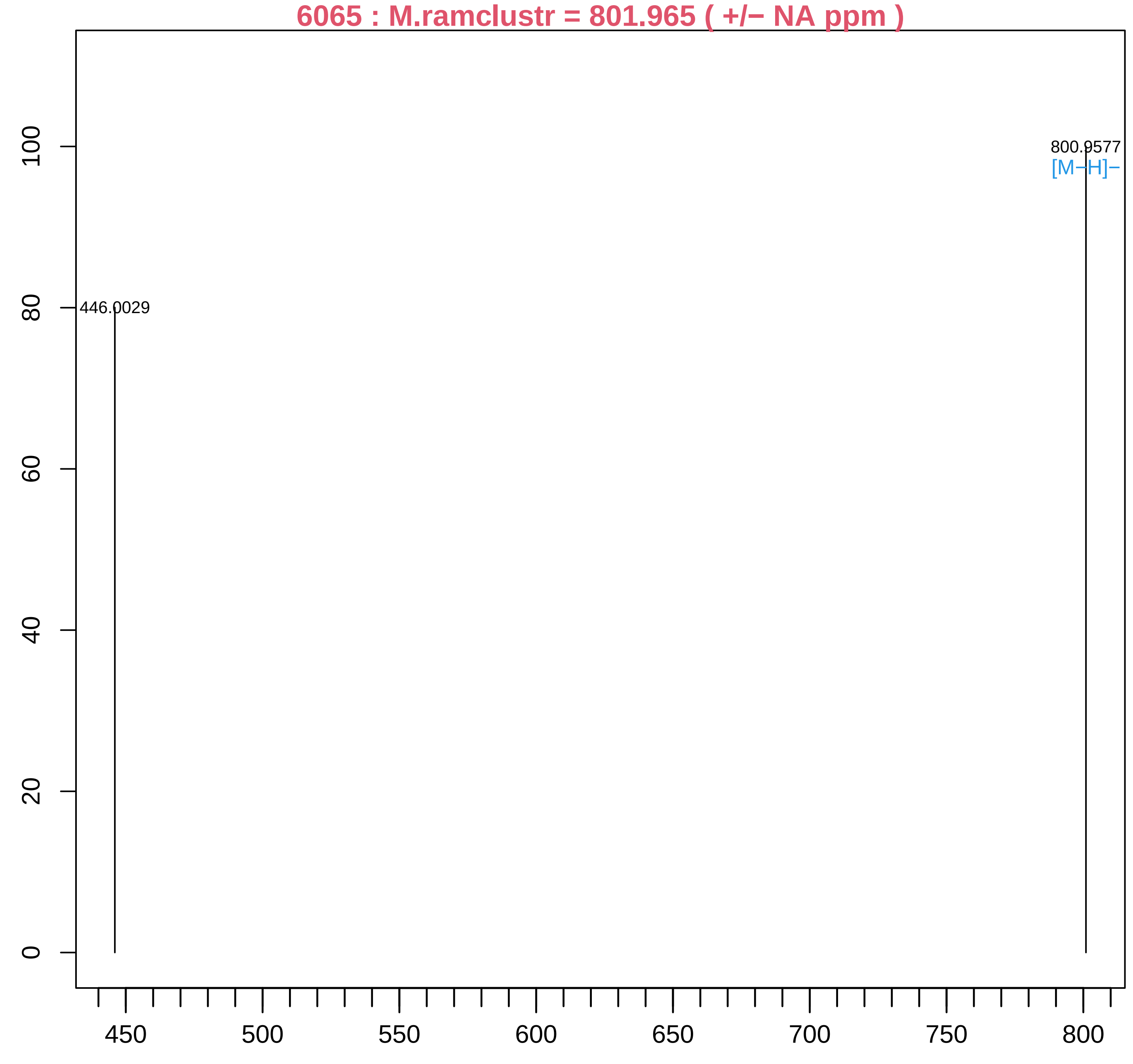
6065 : M.ramclustr = 801.965 (+/− NA ppm)
800.9577
[M−H]−
446.0029
0
20
40
60
80
100
450
500
550
600
650
700
750
800

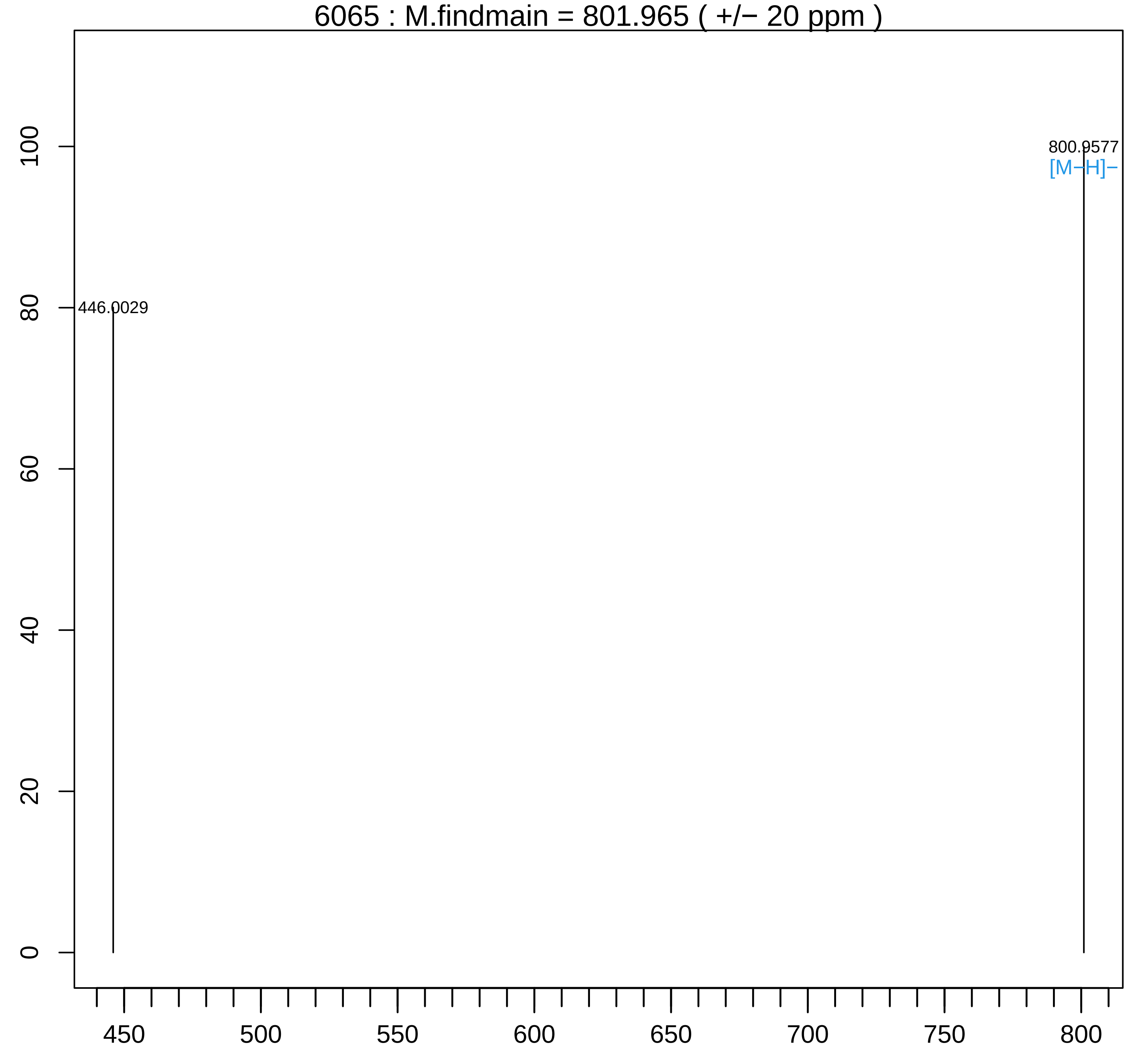
6065 : M.findmain = 801.965 (+/− 20 ppm)
800.9577
[M−H]−
446.0029
0
20
40
60
80
100
450
500
550
600
650
700
750
800

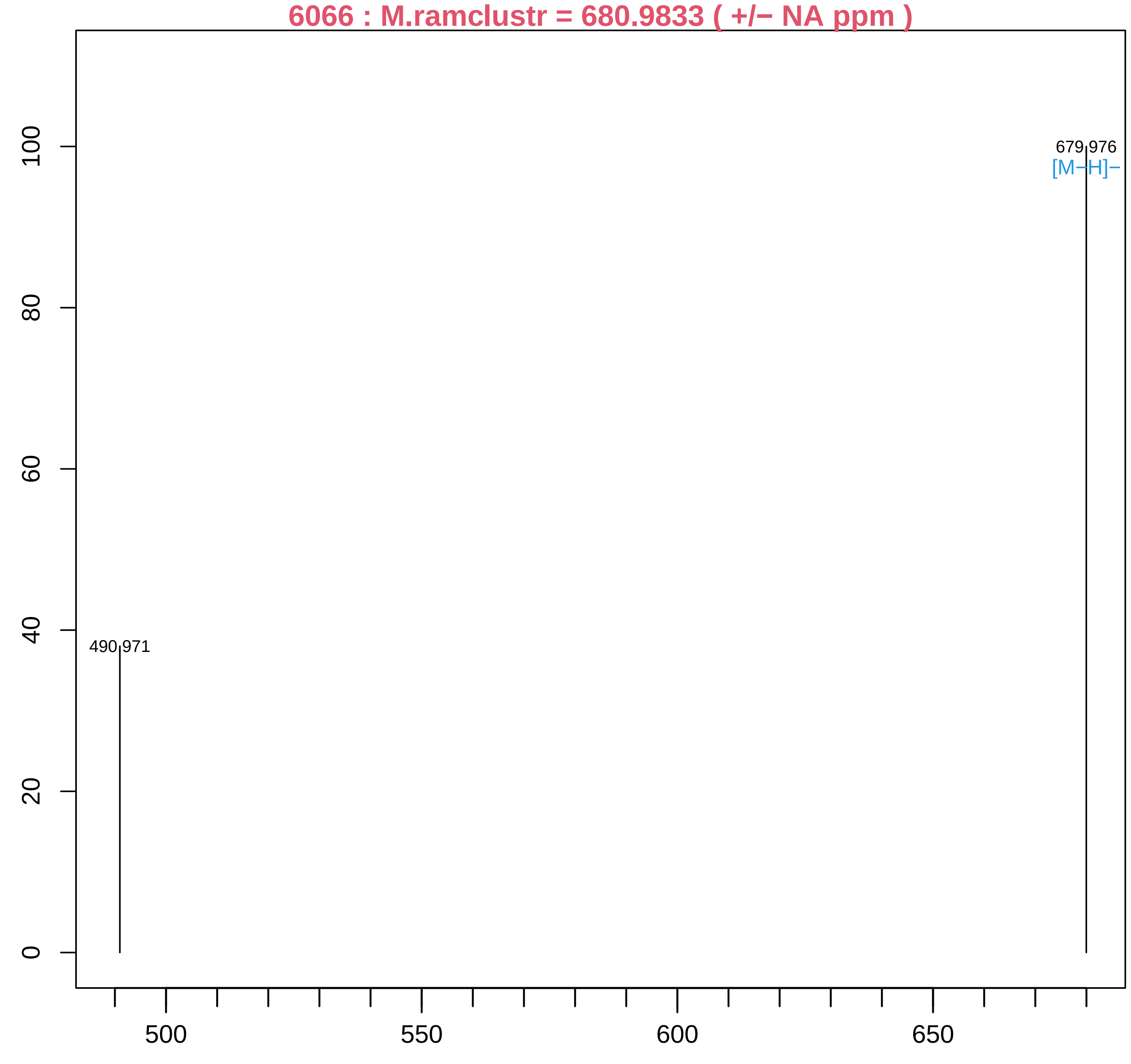
6066 : M.ramclustr = 680.9833 (+/− NA ppm)
679.976
[M−H]−
490.971
0
20
40
60
80
100
500
550
600
650

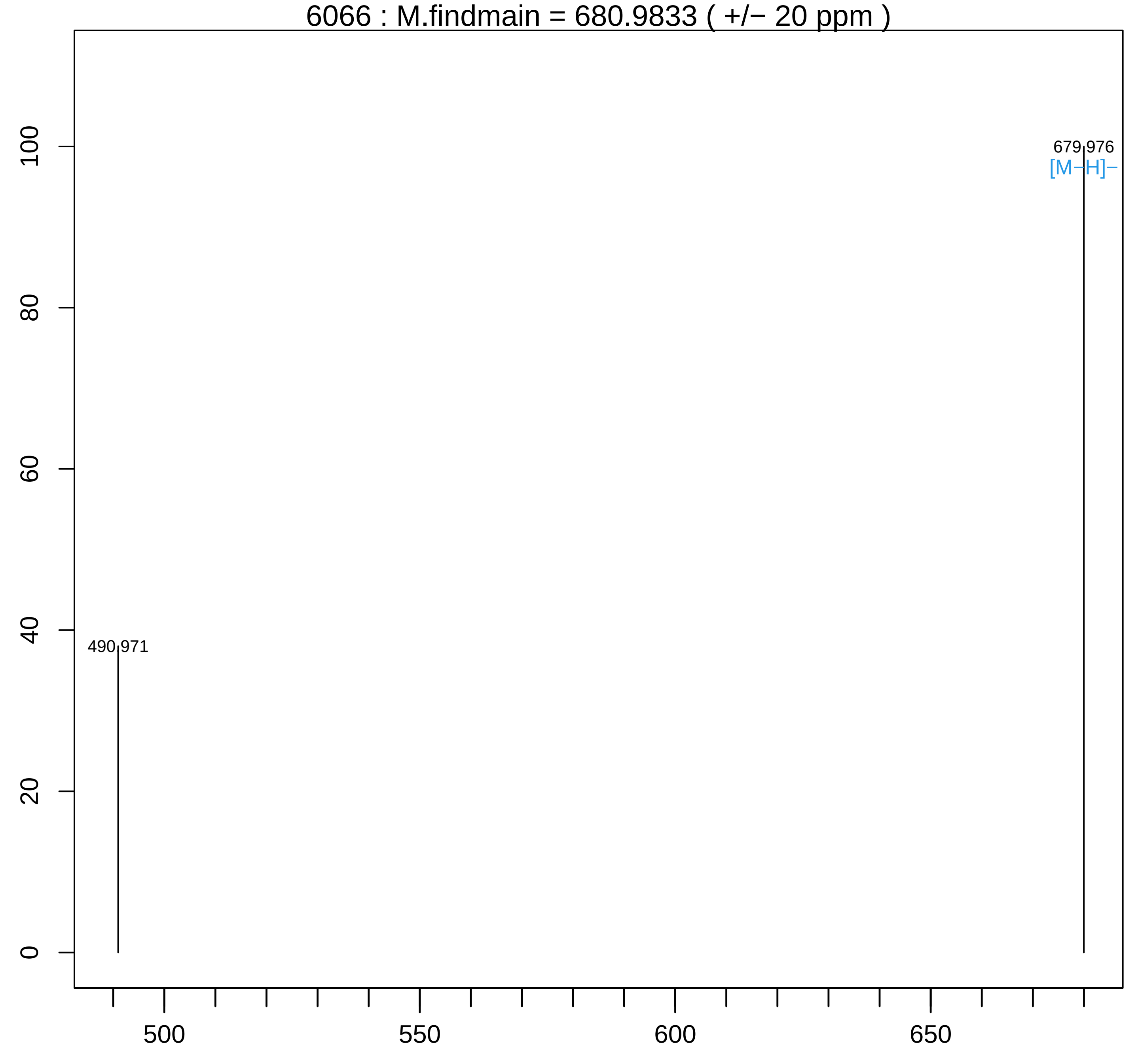
6066 : M.findmain = 680.9833 (+/− 20 ppm)
679.976
[M−H]−
490.971
0
20
40
60
80
100
500
550
600
650

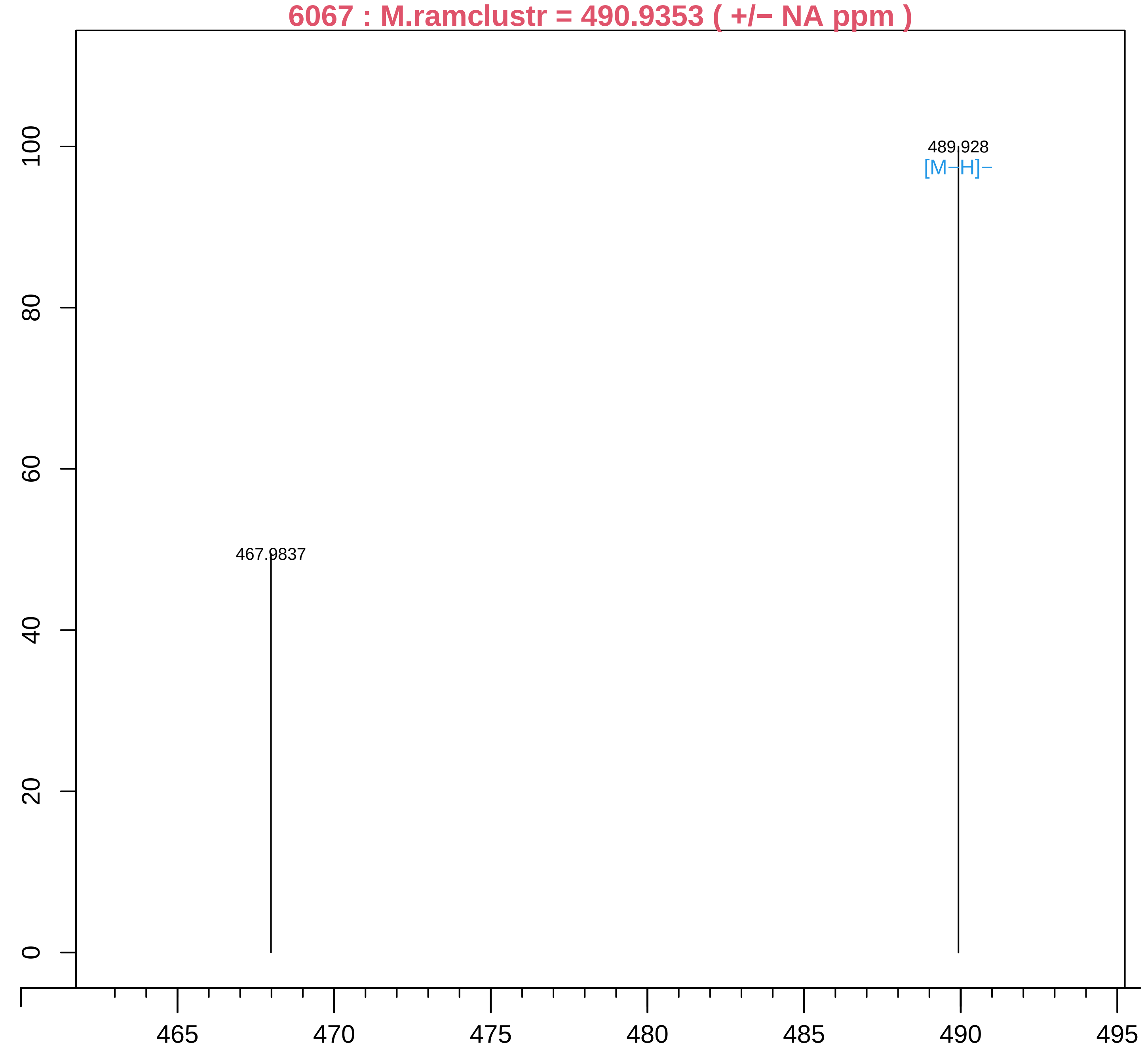
6067 : M.ramclustr = 490.9353 (+/− NA ppm)
489.928
[M−H]−
467.9837
0
20
40
60
80
100
465
470
475
480
485
490
495

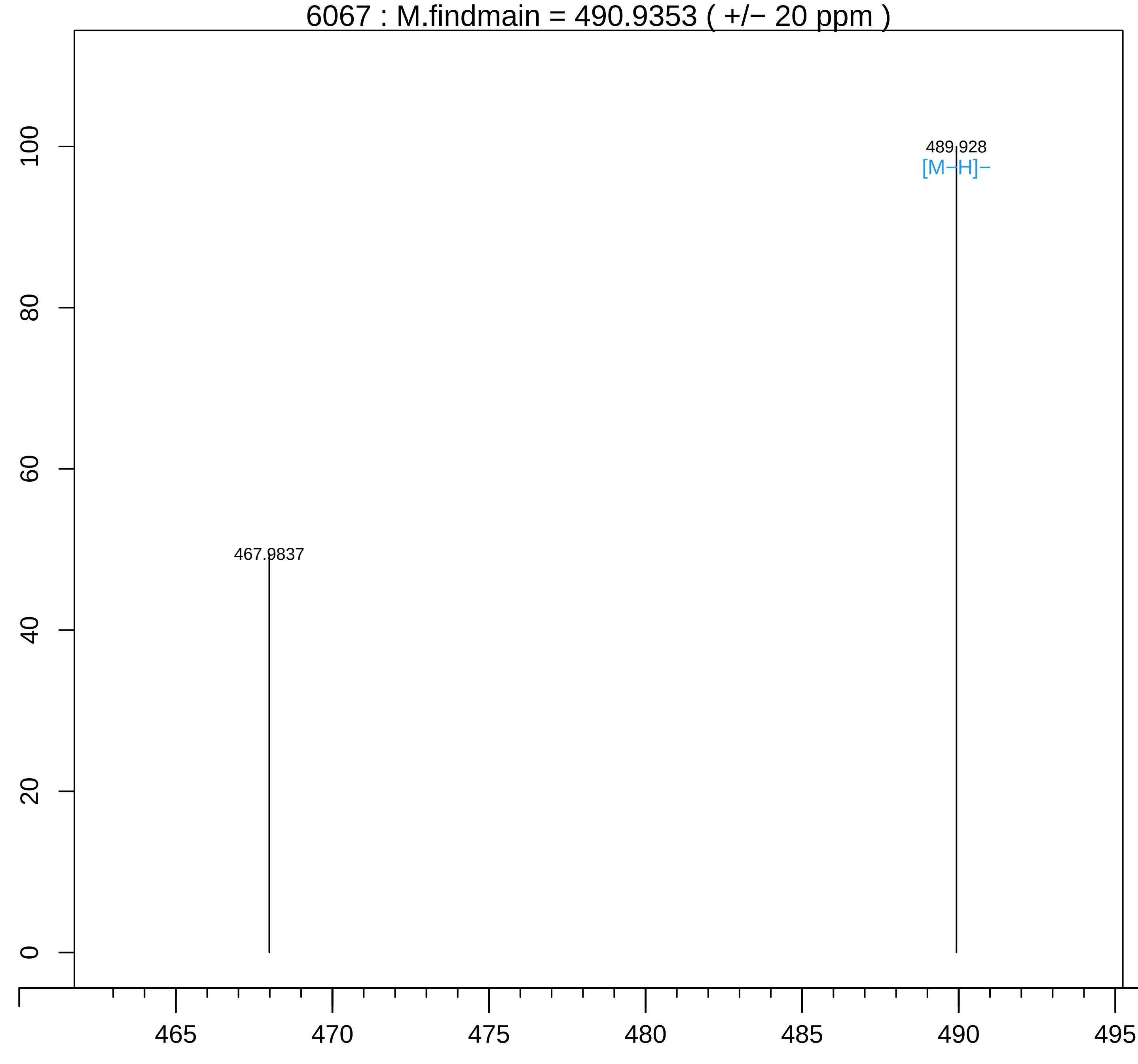
6067 : M.findmain = 490.9353 (+/− 20 ppm)
489.928
[M−H]−
467.9837
0
20
40
60
80
100
465
470
475
480
485
490
495

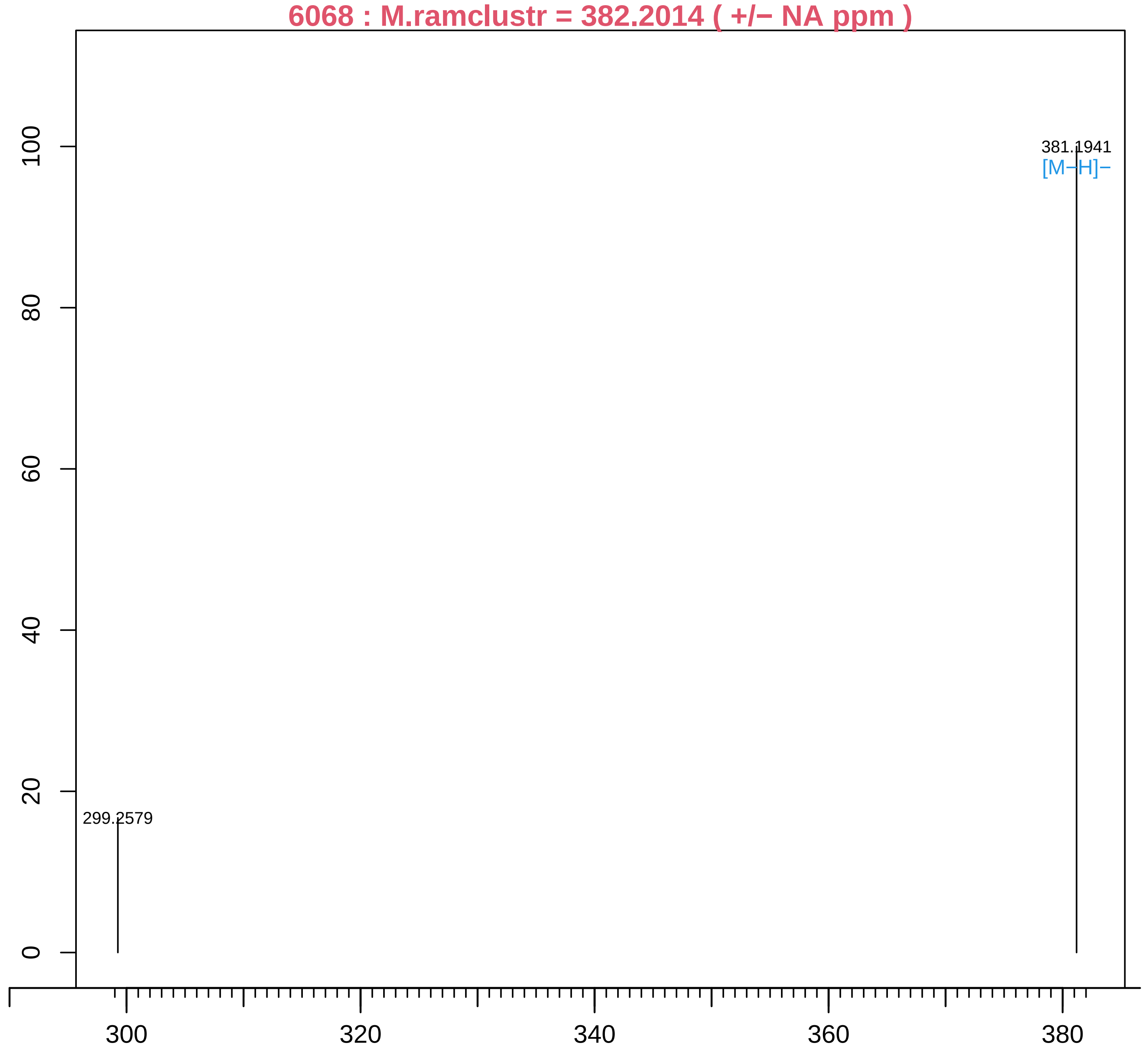
6068 : M.ramclustr = 382.2014 (+/− NA ppm)
381.1941
[M−H]−
299.2579
0
20
40
60
80
100
300
320
340
360
380

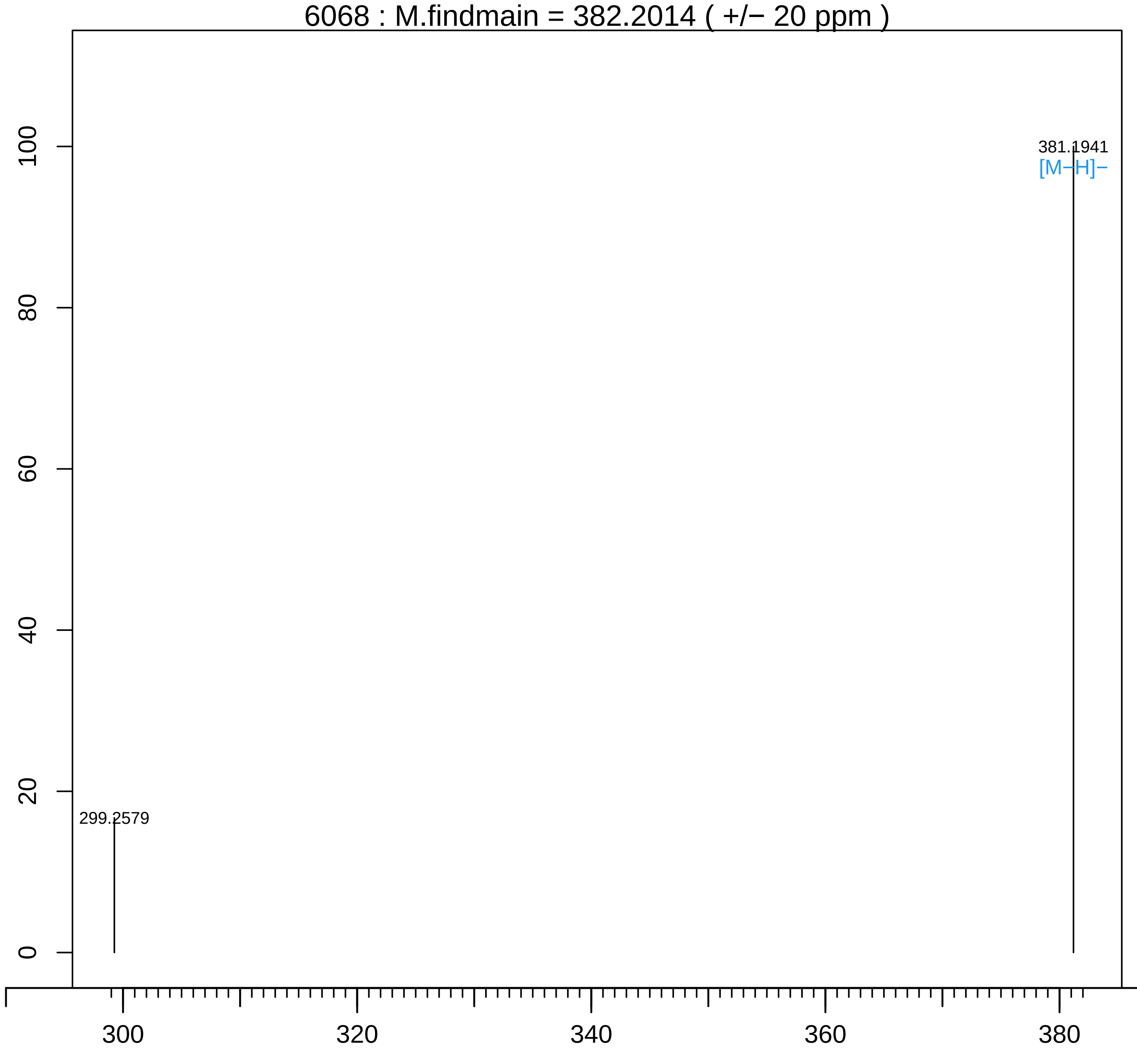
6068 : M.findmain = 382.2014 (+/− 20 ppm)
381.1941
[M−H]−
299.2579
0
20
40
60
80
100
300
320
340
360
380

6069 : M.ramclustr = 452.0462 (+/− NA ppm)

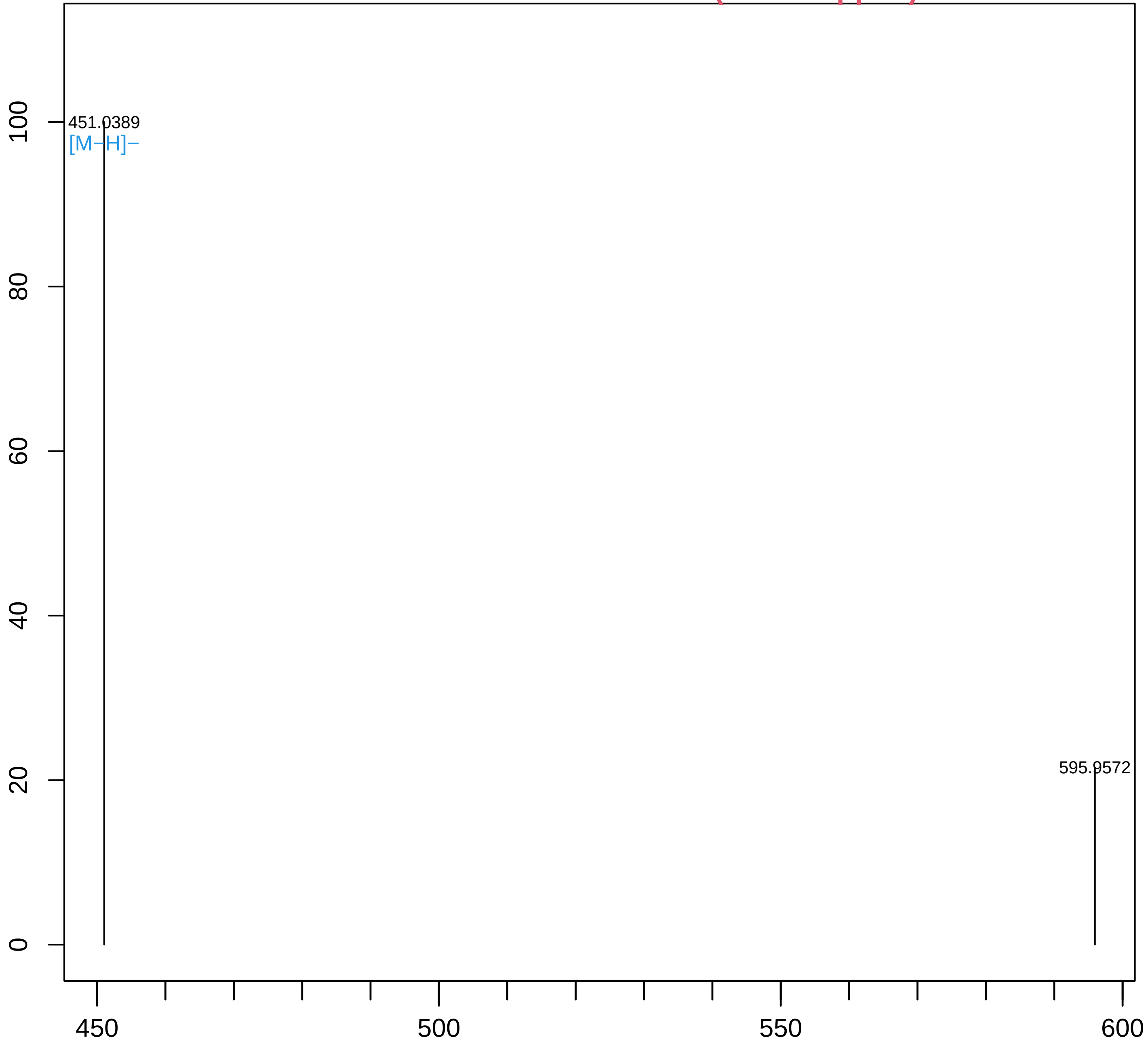

6069 : M.findmain = 452.0462 (+/− 20 ppm)

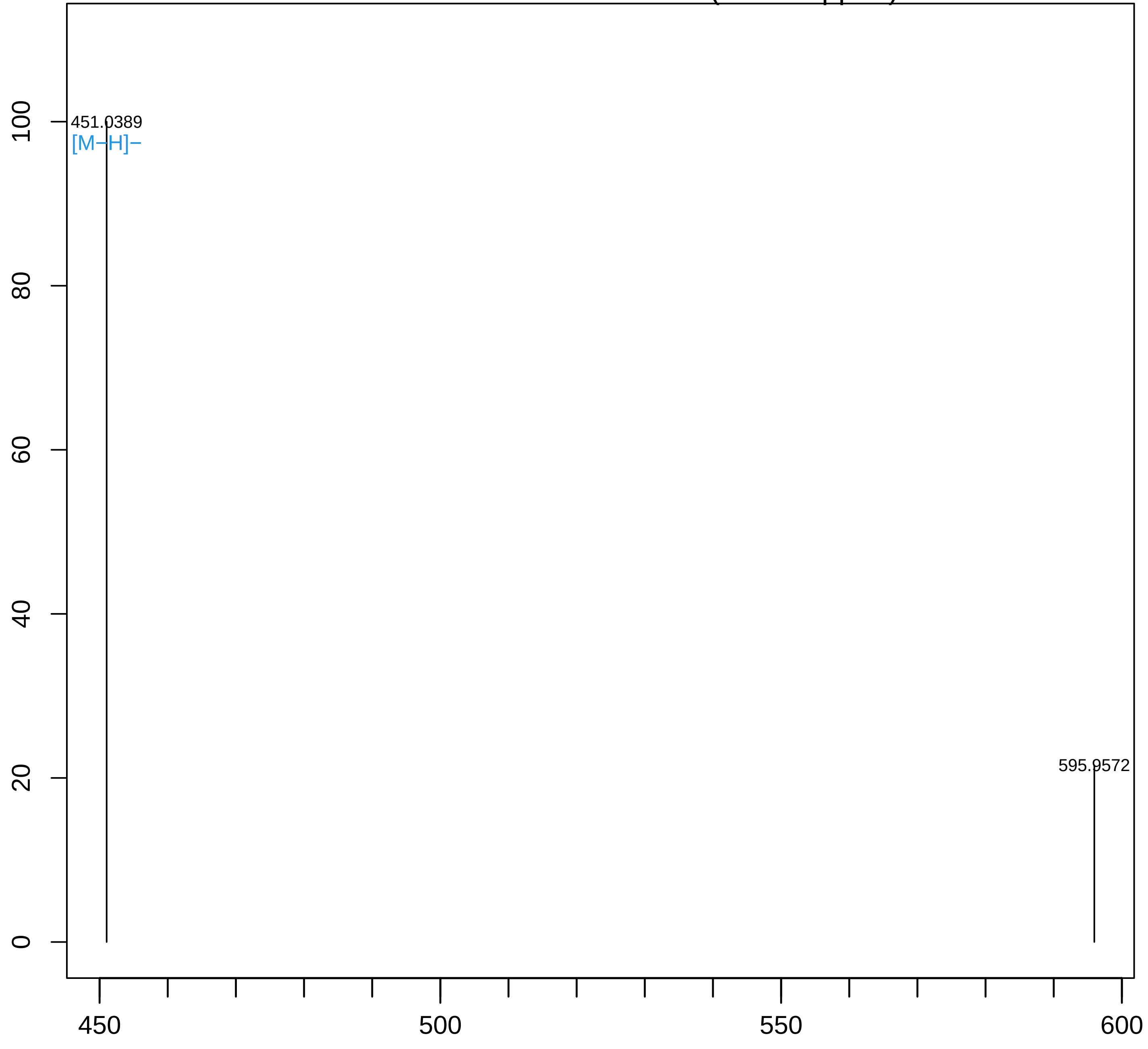

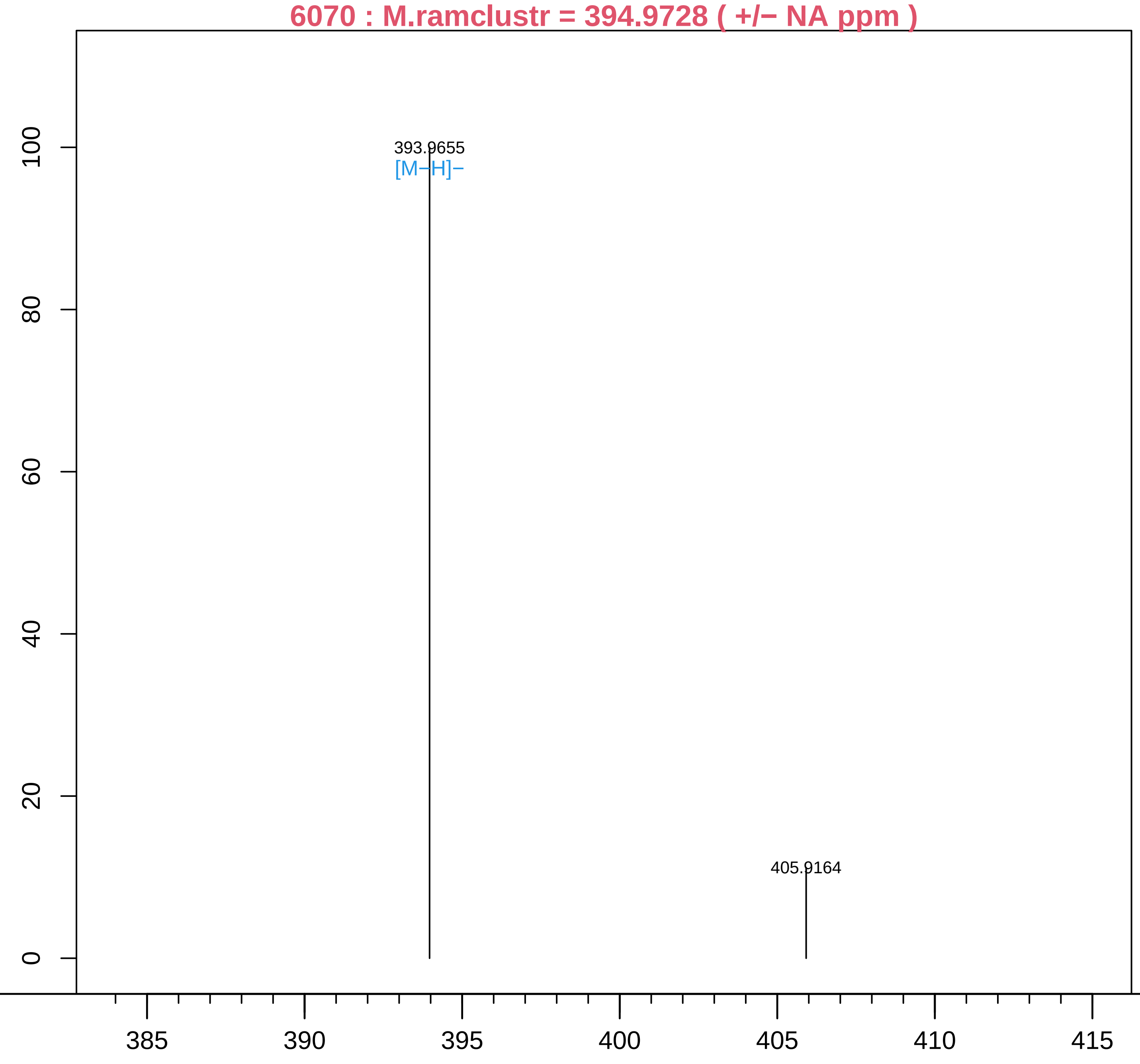
6070 : M.ramclustr = 394.9728 (+/− NA ppm)
393.9655
[M−H]−
405.9164
0
20
40
60
80
100
385
390
395
400
405
410
415

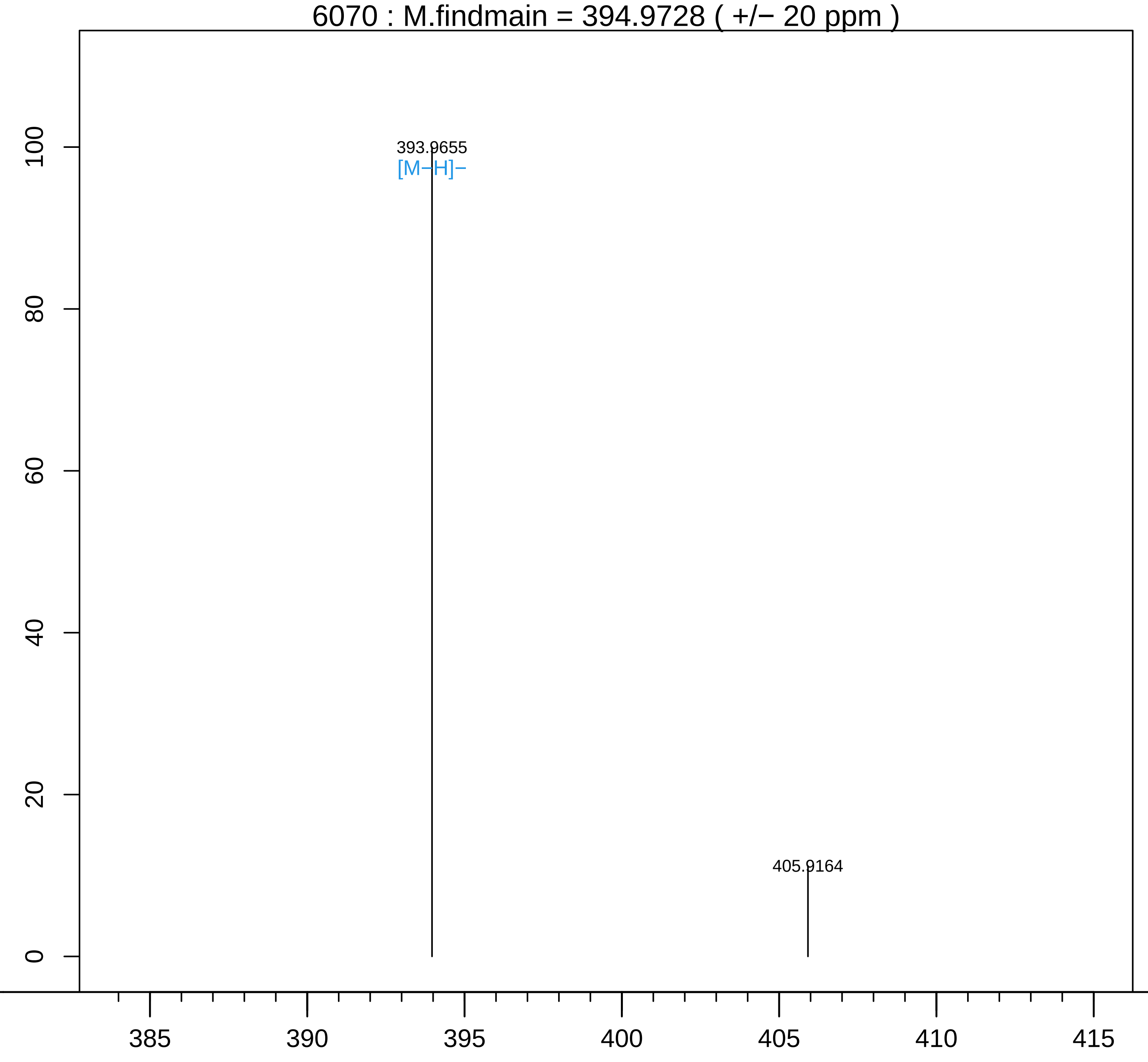
6070 : M.findmain = 394.9728 (+/− 20 ppm)
393.9655
[M−H]−
405.9164
0
20
40
60
80
100
385
390
395
400
405
410
415

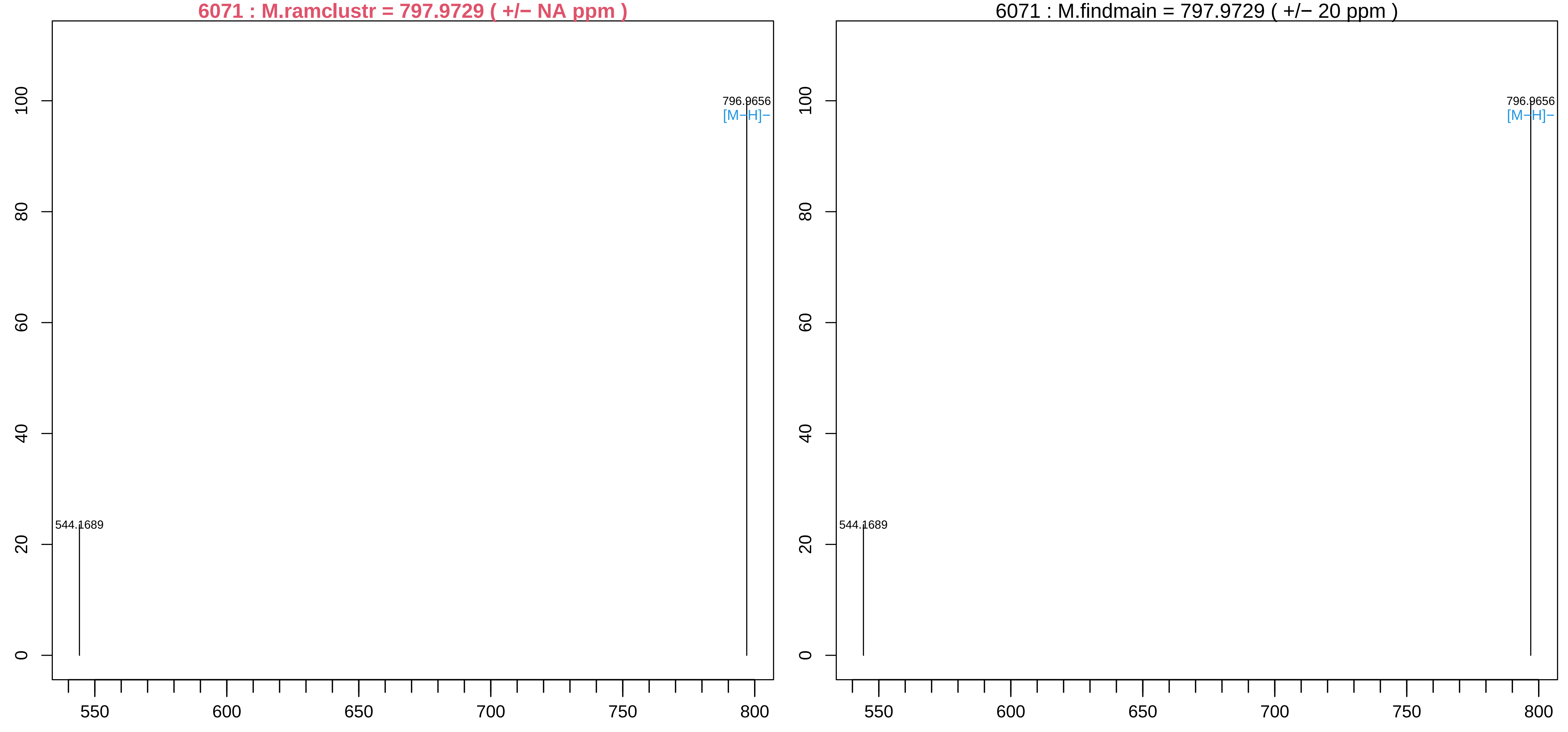

6071 : M.ramclustr = 797.9729 (+/− NA ppm)
796.9656
[M−H]−
544.1689
0
20
40
60
80
100
550
600
650
700
750
800
6071 : M.findmain = 797.9729 (+/− 20 ppm)
796.9656
[M−H]−
544.1689
0
20
40
60
80
100
550
600
650
700
750
800

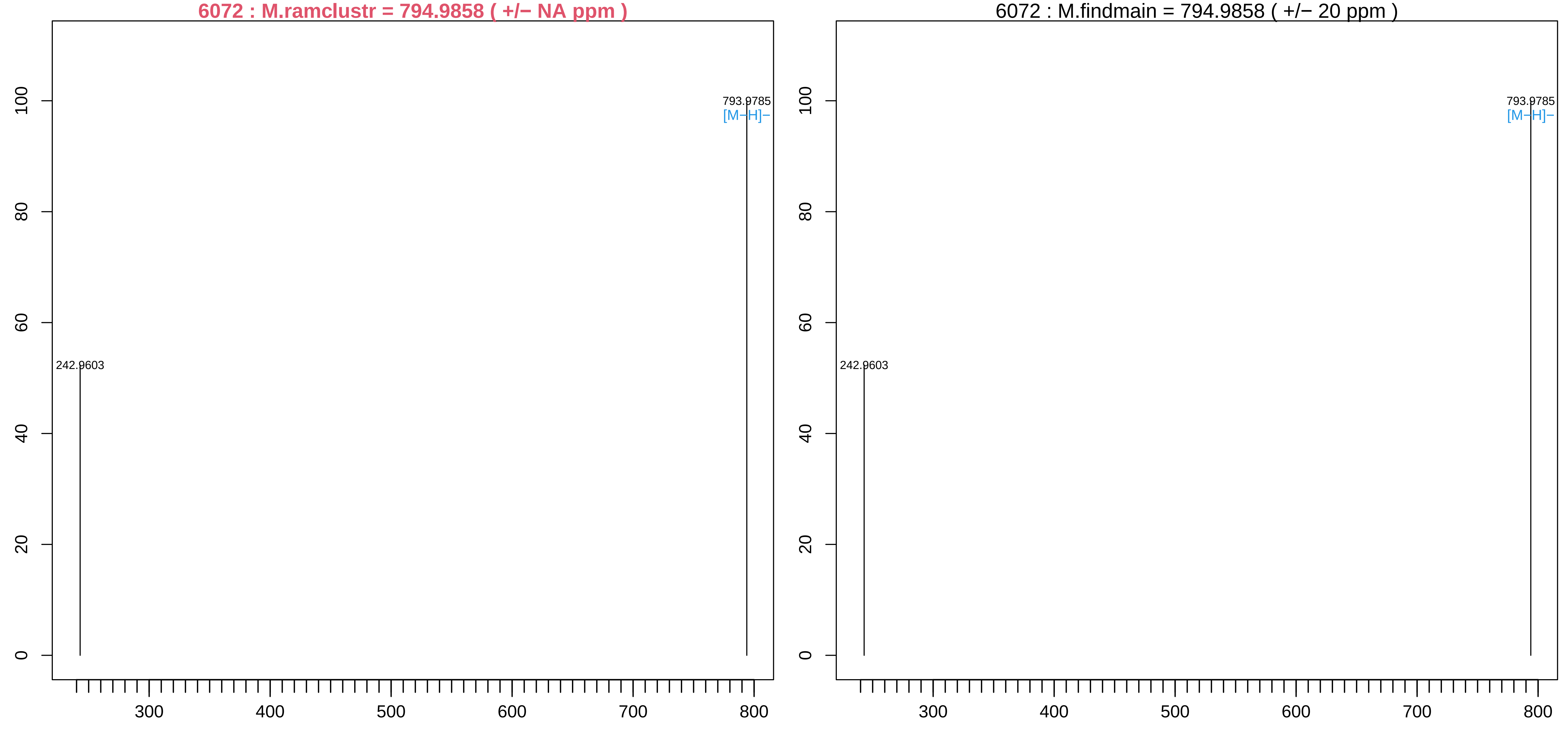

6072 : M.ramclustr = 794.9858 (+/− NA ppm)
793.9785
[M−H]−
242.9603
0
20
40
60
80
100
300
400
500
600
700
800
6072 : M.findmain = 794.9858 (+/− 20 ppm)
793.9785
[M−H]−
242.9603
0
20
40
60
80
100
300
400
500
600
700
800

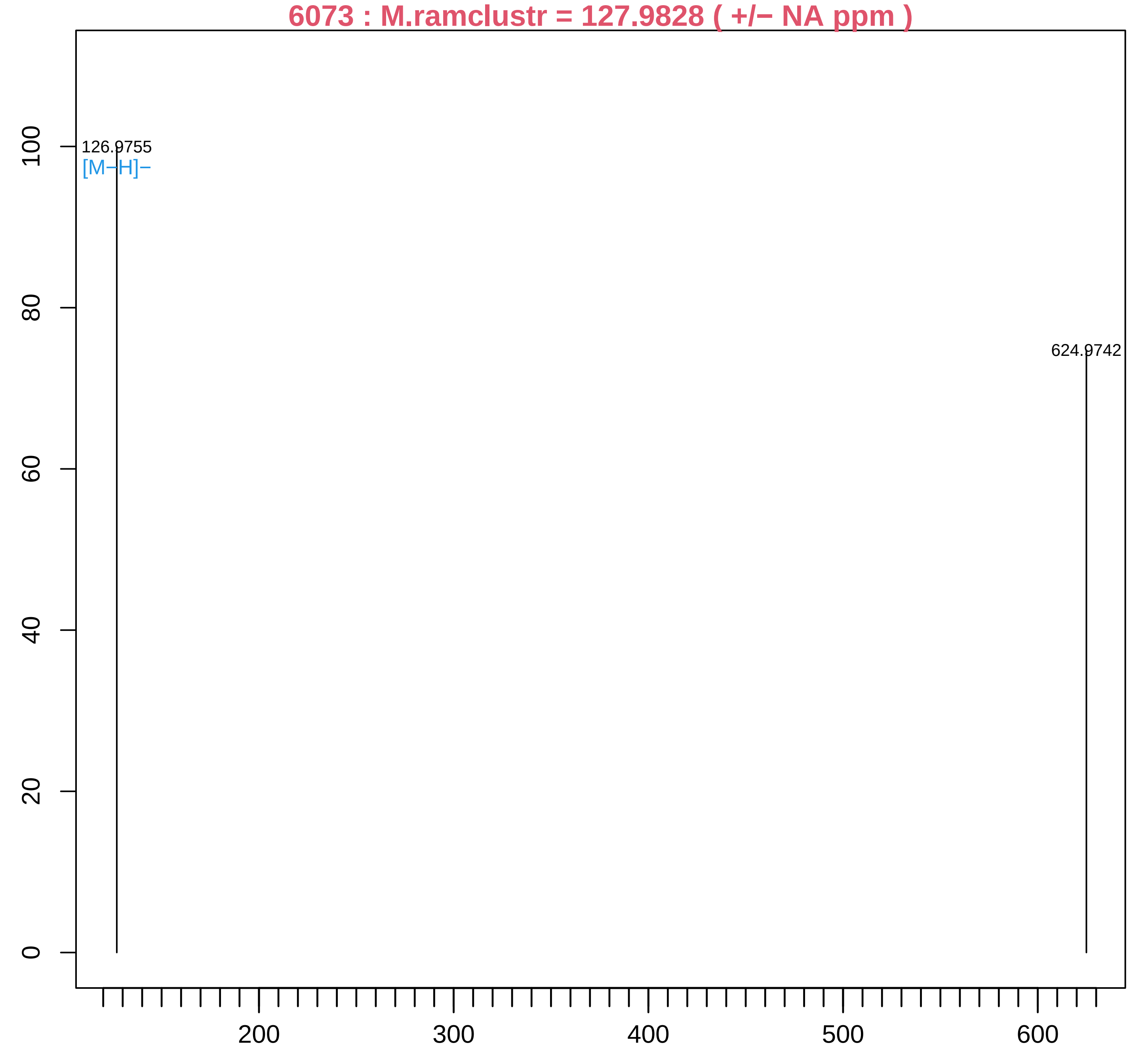
6073 : M.ramclustr = 127.9828 (+/− NA ppm)
126.9755
[M−H]−
624.9742
0
20
40
60
80
100
200
300
400
500
600
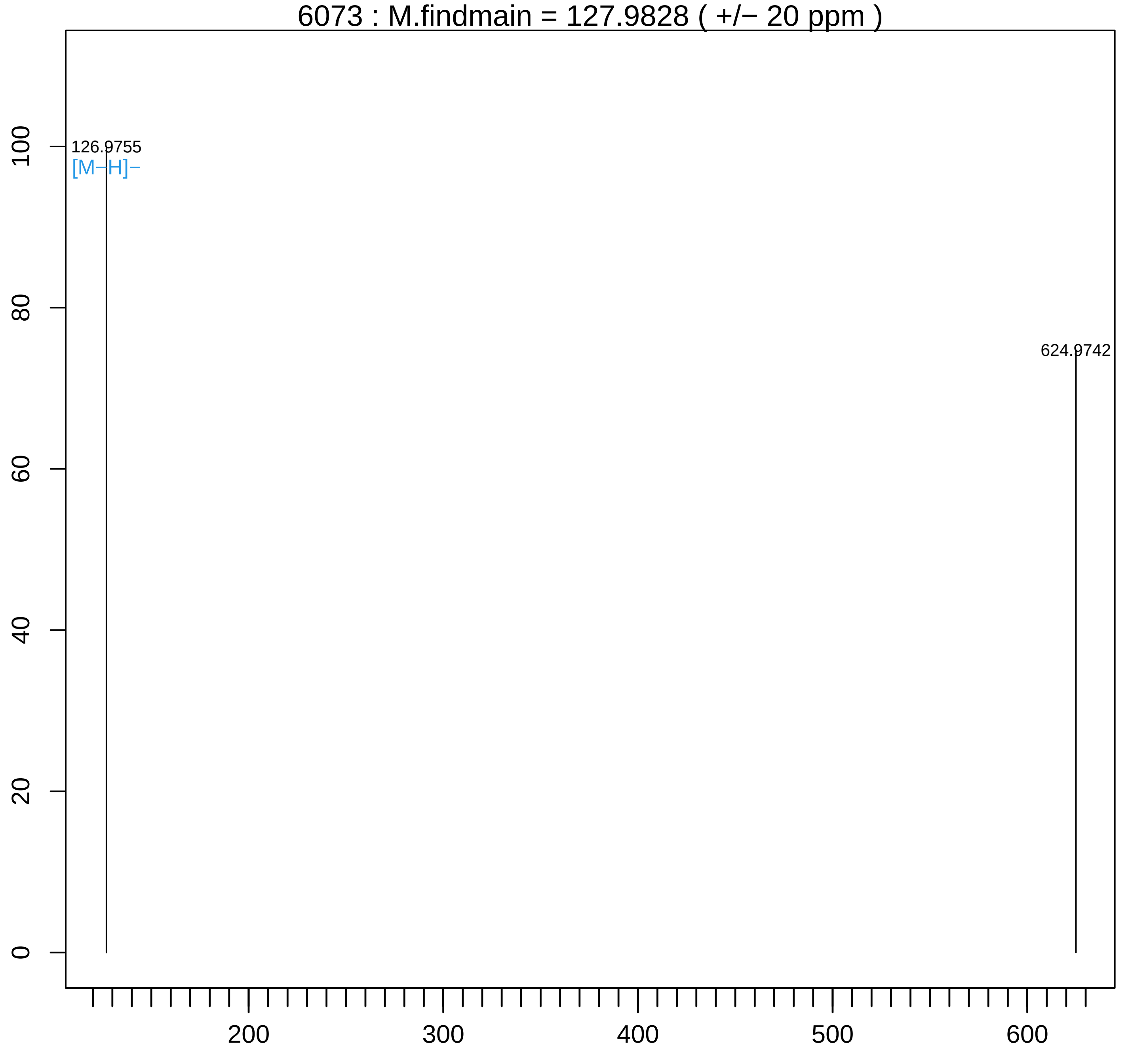
6073 : M.findmain = 127.9828 (+/− 20 ppm)
126.9755
[M−H]−
624.9742
0
20
40
60
80
100
200
300
400
500
600

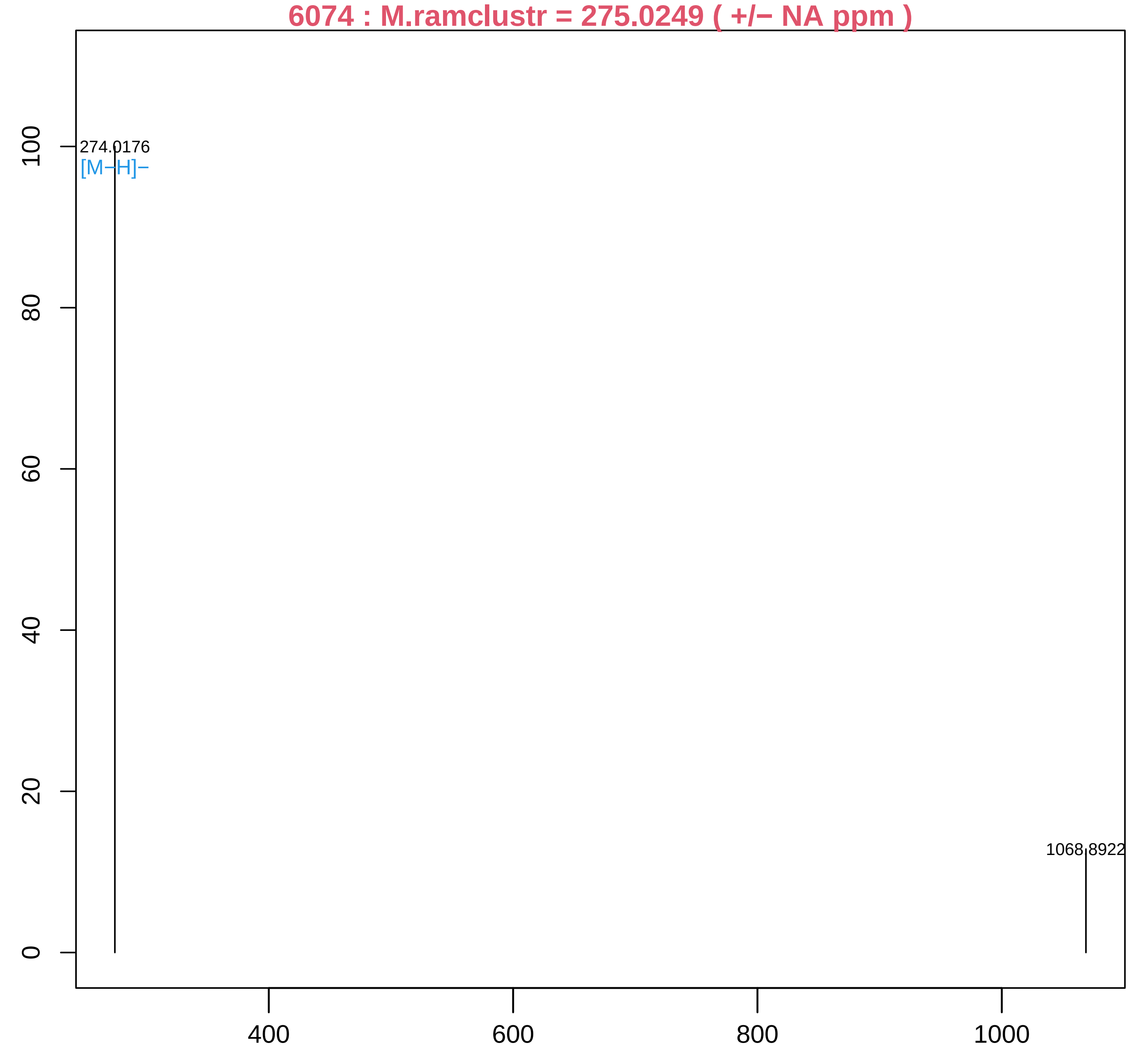

6074 : M.ramclustr = 275.0249 (+/− NA ppm)
274.0176
[M−H]−
1068.8922
0
20
40
60
80
100
400
600
800
1000

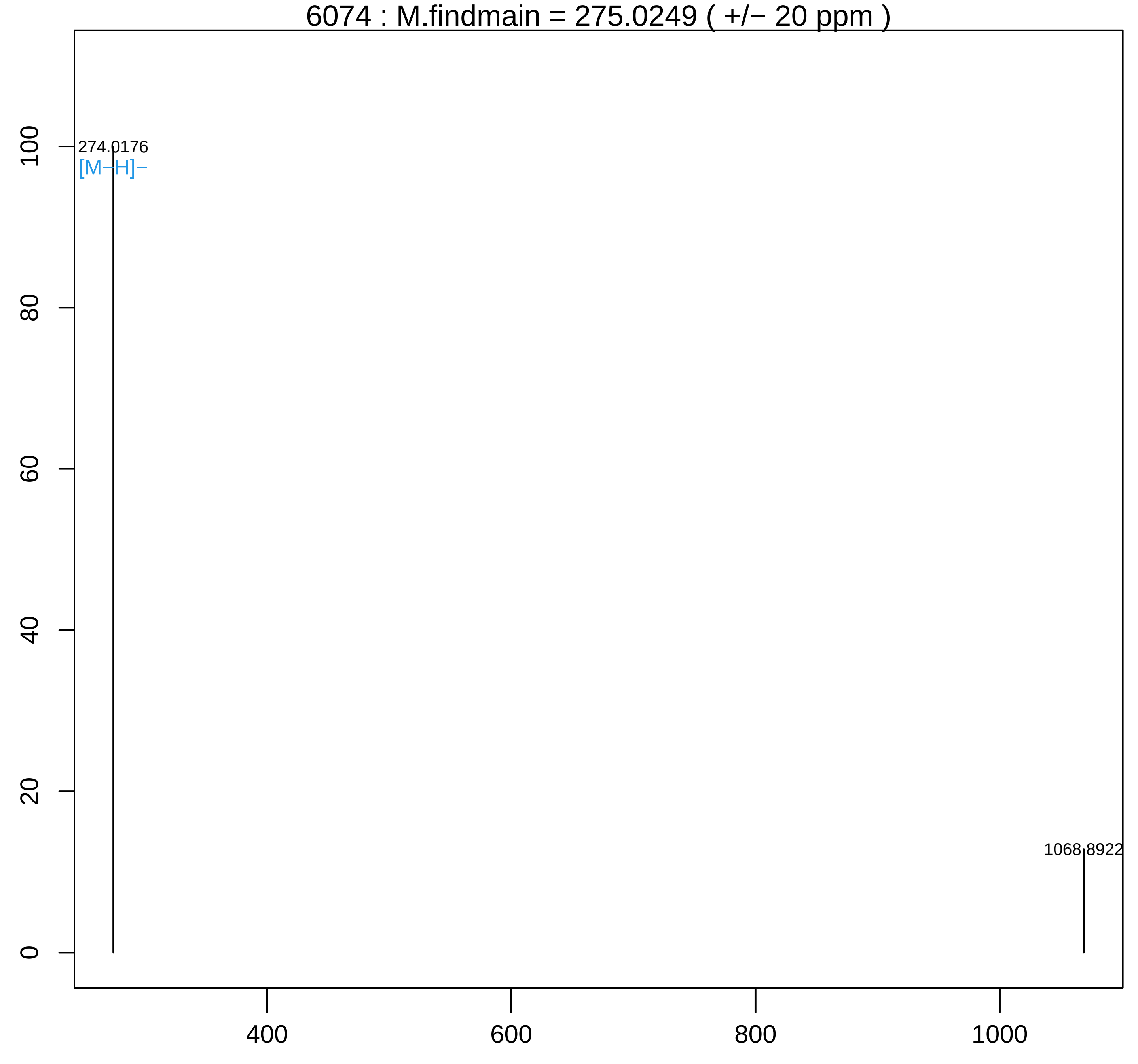

6074 : M.findmain = 275.0249 (+/− 20 ppm)
274.0176
[M−H]−
1068.8922
0
20
40
60
80
100
400
600
800
1000

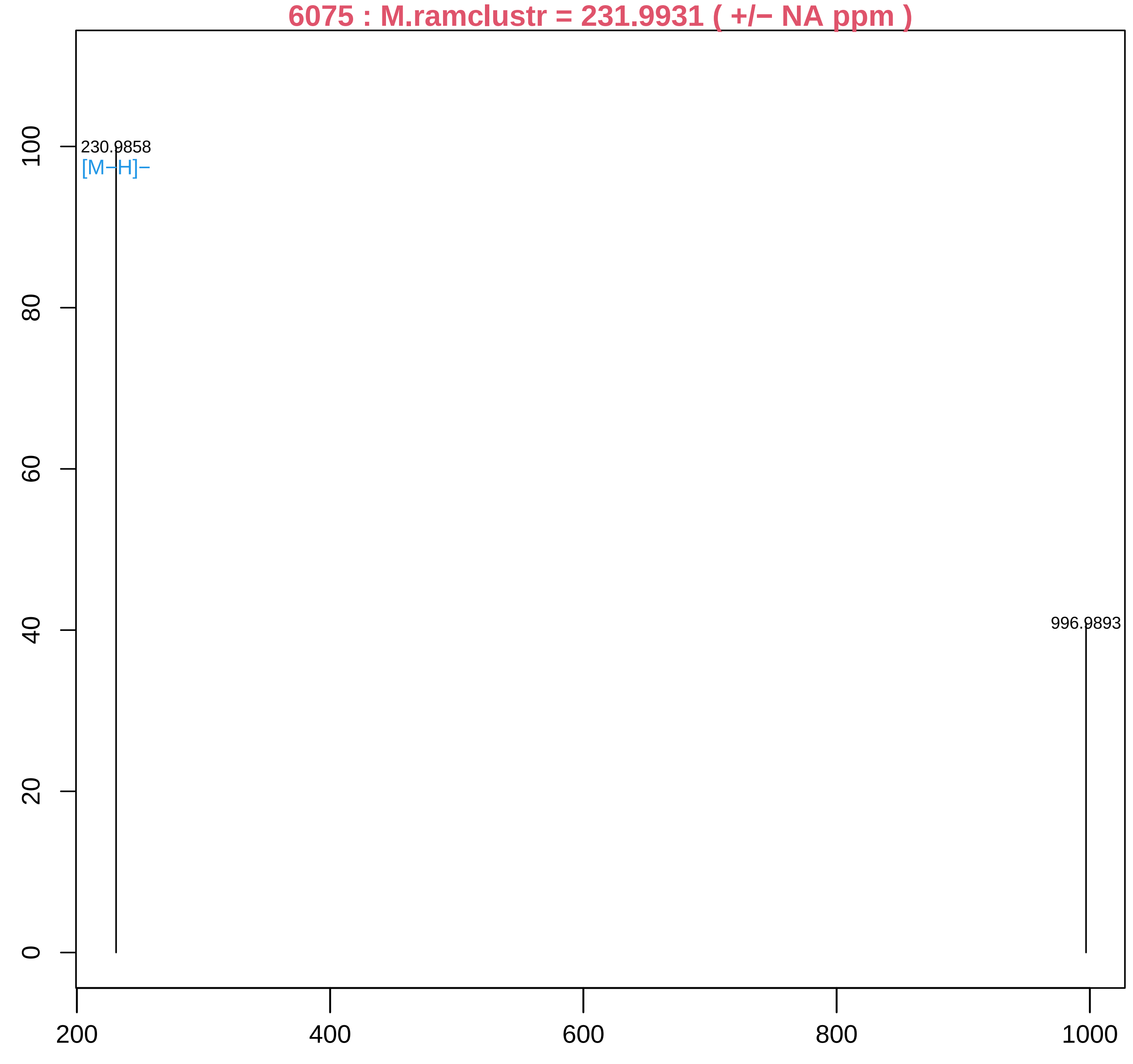
6075 : M.ramclustr = 231.9931 (+/− NA ppm)
230.9858
[M−H]−
996.9893
0
20
40
60
80
100
200
400
600
800
1000

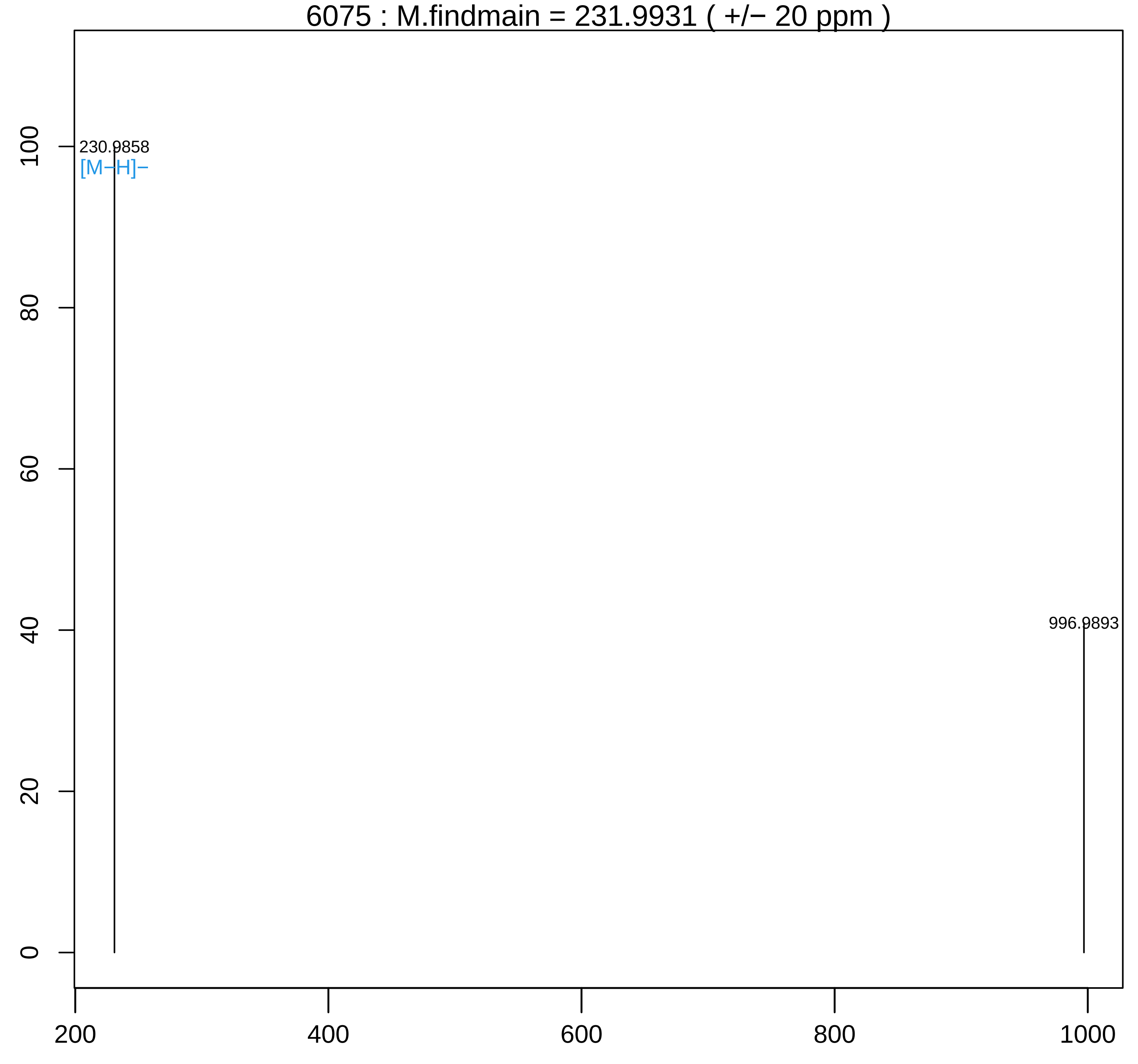
6075 : M.findmain = 231.9931 (+/− 20 ppm)
230.9858
[M−H]−
996.9893
0
20
40
60
80
100
200
400
600
800
1000

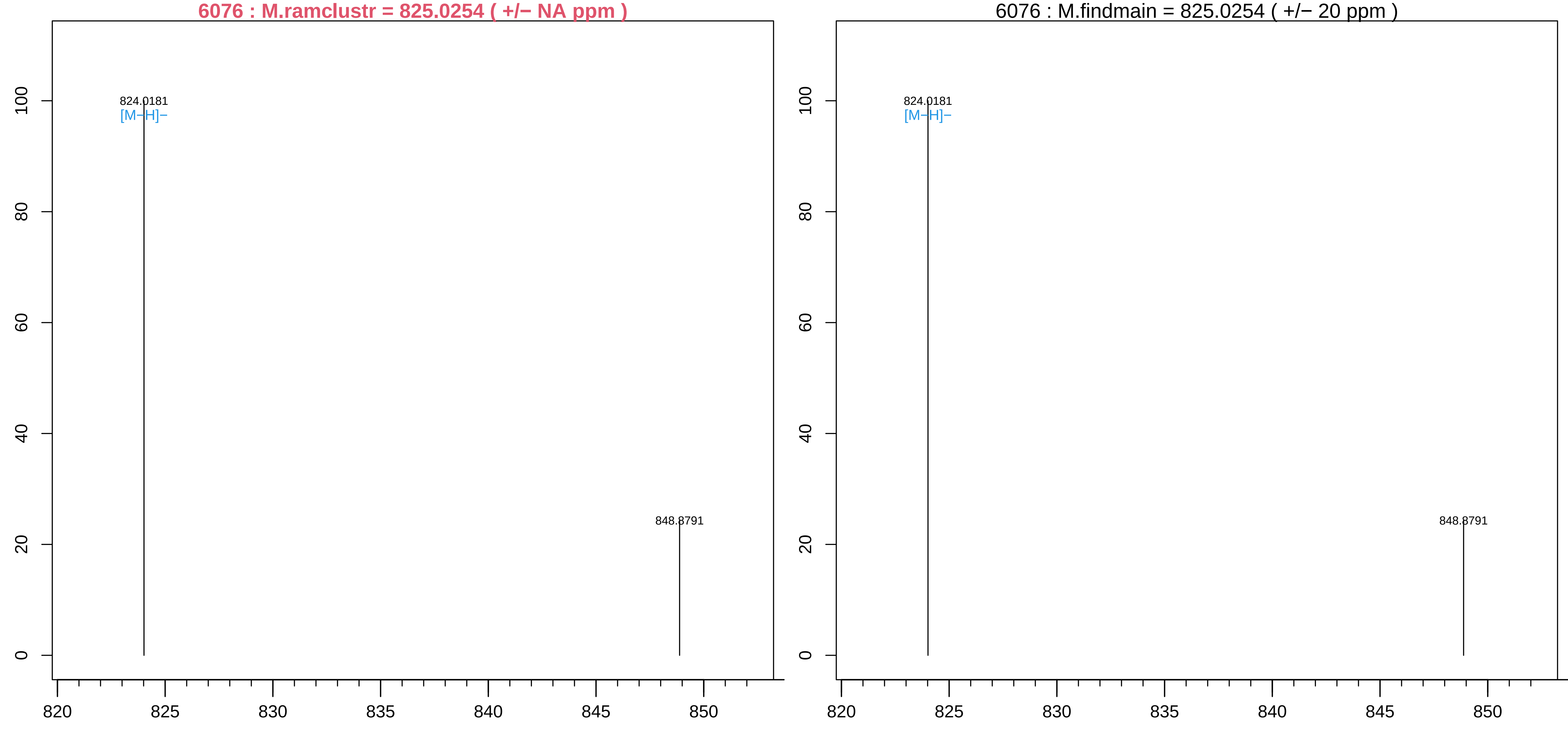
6076 : M.ramclustr = 825.0254 (+/− NA ppm)
6076 : M.findmain = 825.0254 (+/− 20 ppm)
824.0181
[M−H]−
848.8791
0
20
40
60
80
100
820
825
830
835
840
845
850

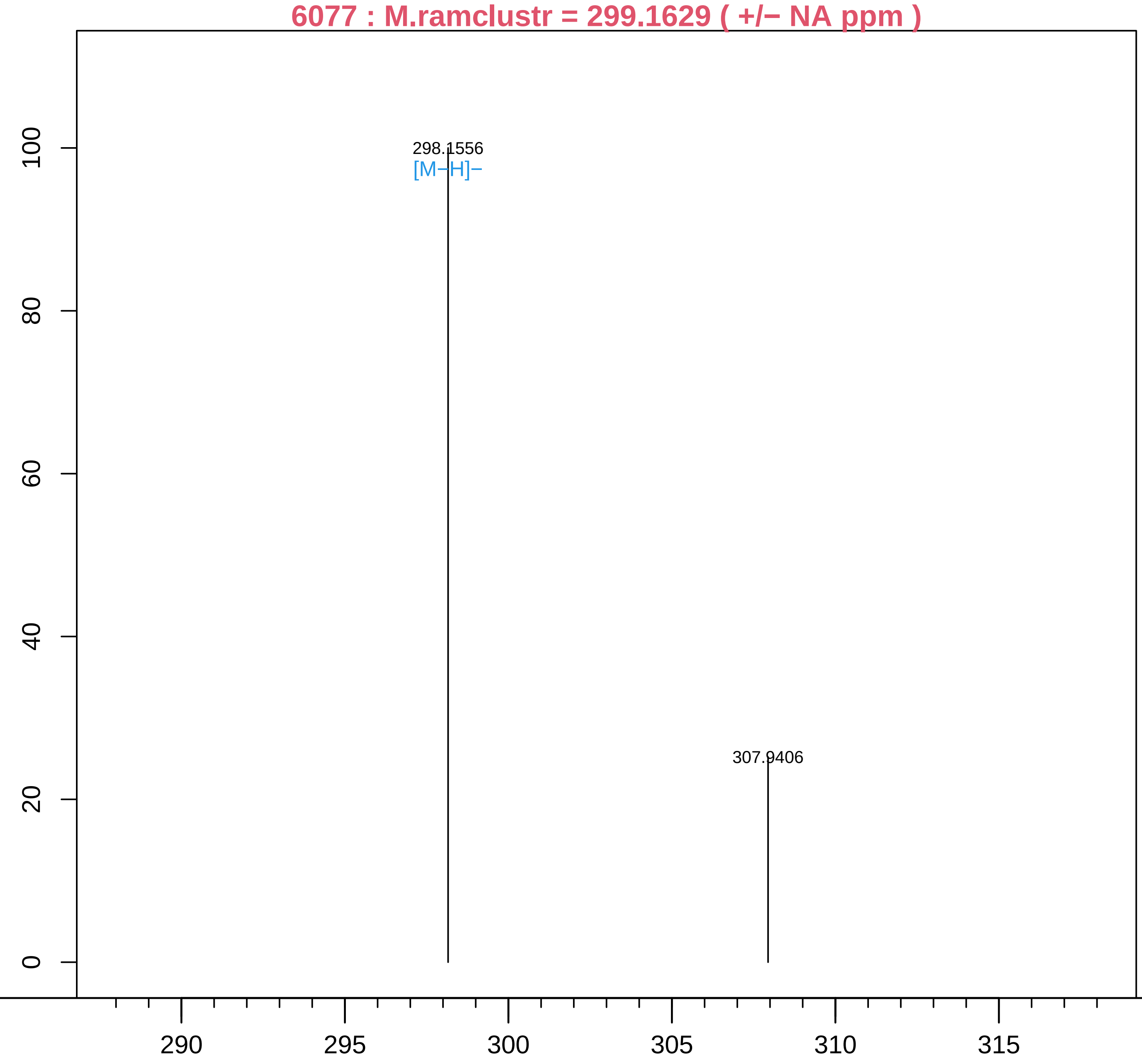

6077 : M.ramclustr = 299.1629 (+/− NA ppm)
298.1556
[M−H]−
307.9406
0
20
40
60
80
100
290
295
300
305
310
315

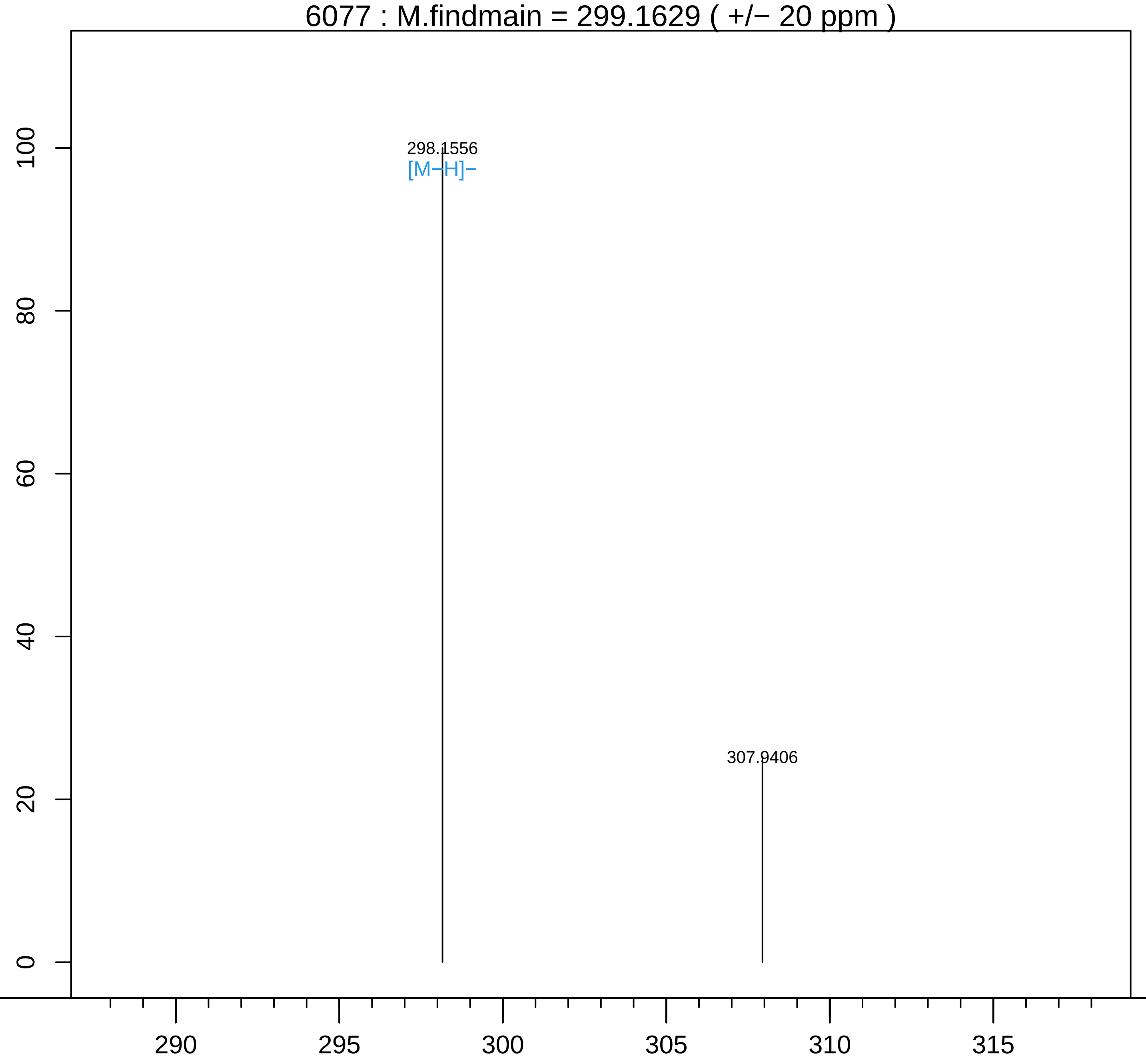

6077 : M.findmain = 299.1629 (+/− 20 ppm)
298.1556
[M−H]−
307.9406
0
20
40
60
80
100
290
295
300
305
310
315

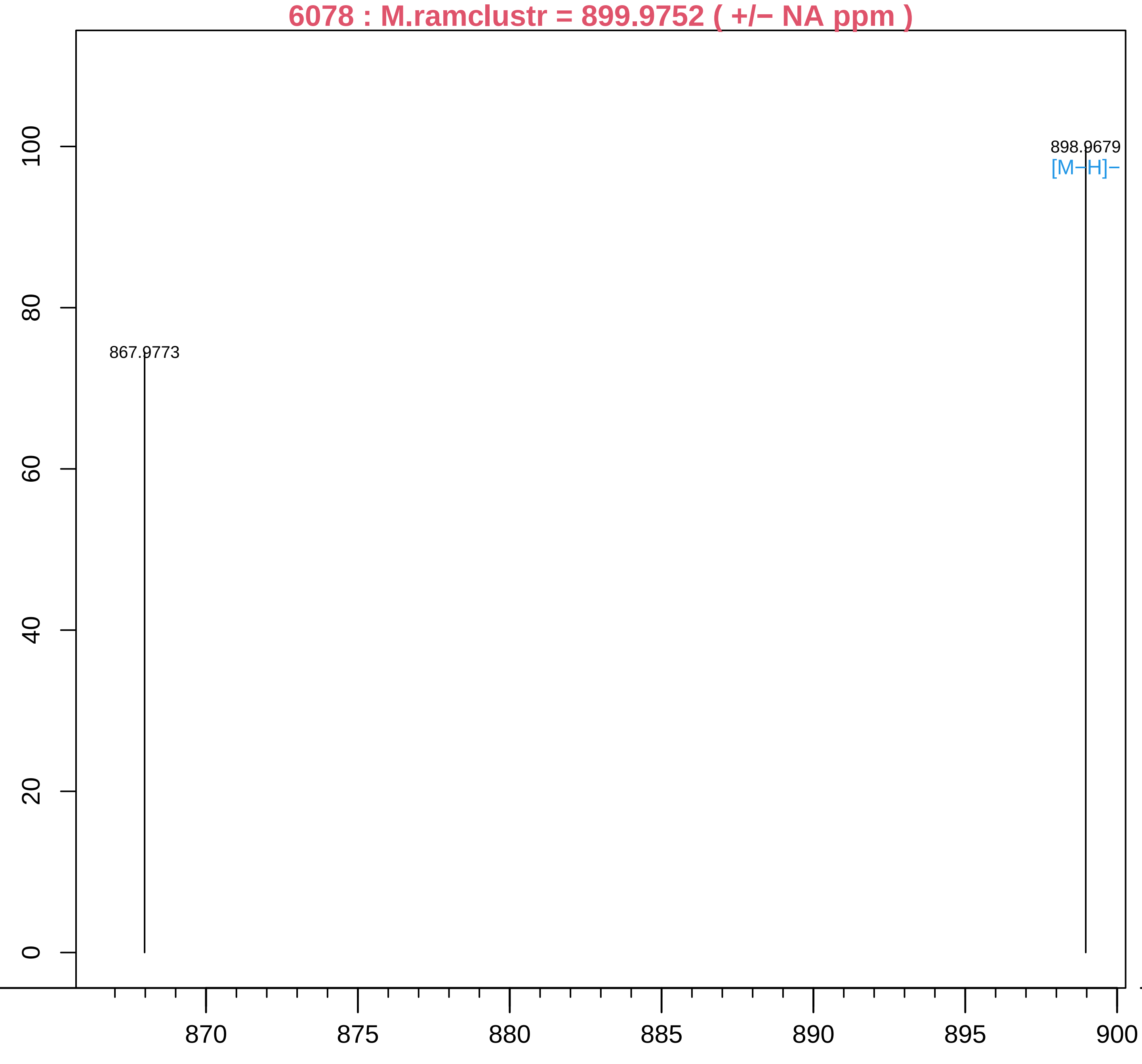
6078 : M.ramclustr = 899.9752 (+/− NA ppm)
898.9679
[M−H]−
867.9773
0
20
40
60
80
100
870
875
880
885
890
895
900

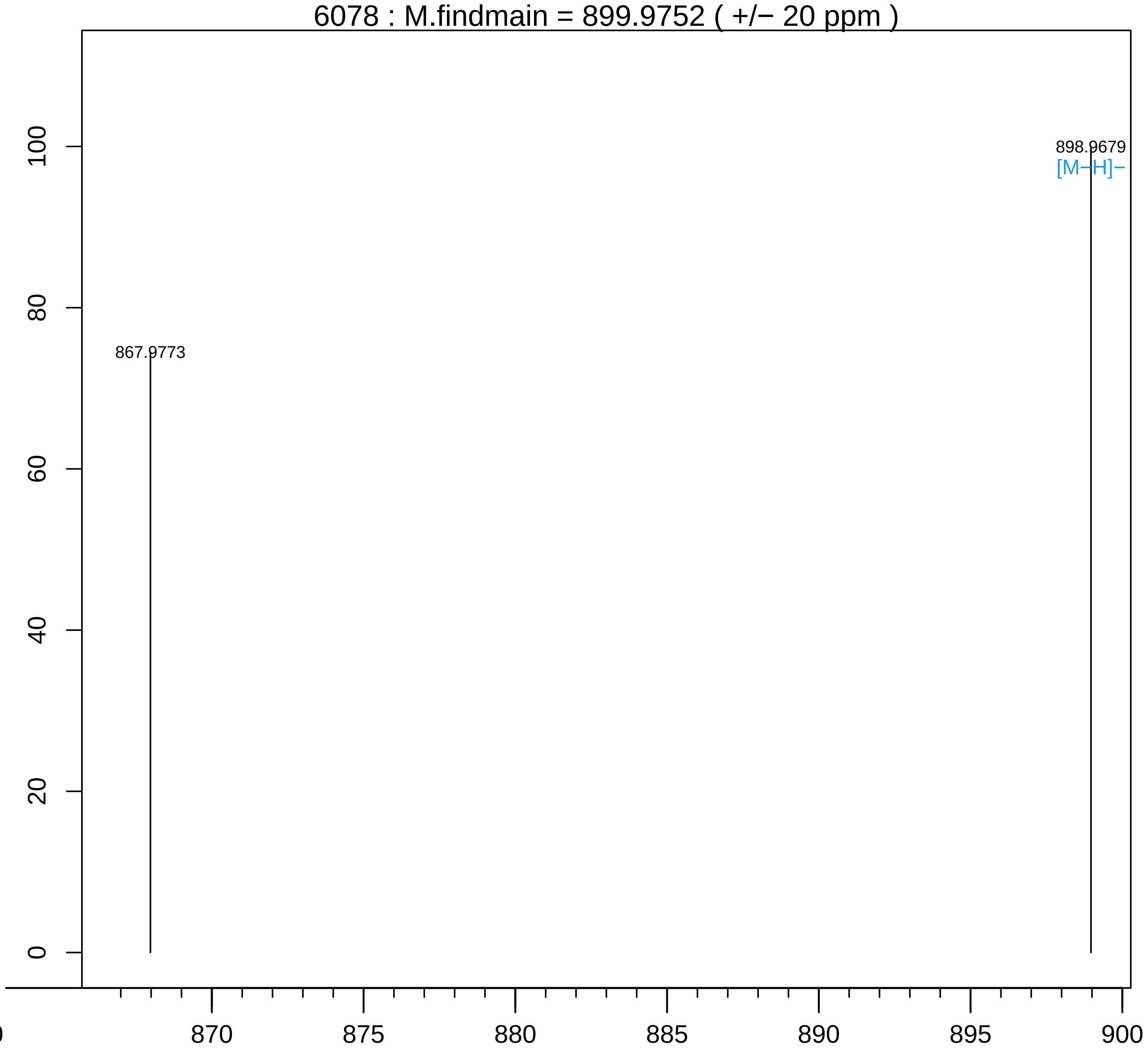
6078 : M.findmain = 899.9752 (+/− 20 ppm)
898.9679
[M−H]−
867.9773
0
20
40
60
80
100
870
875
880
885
890
895
900

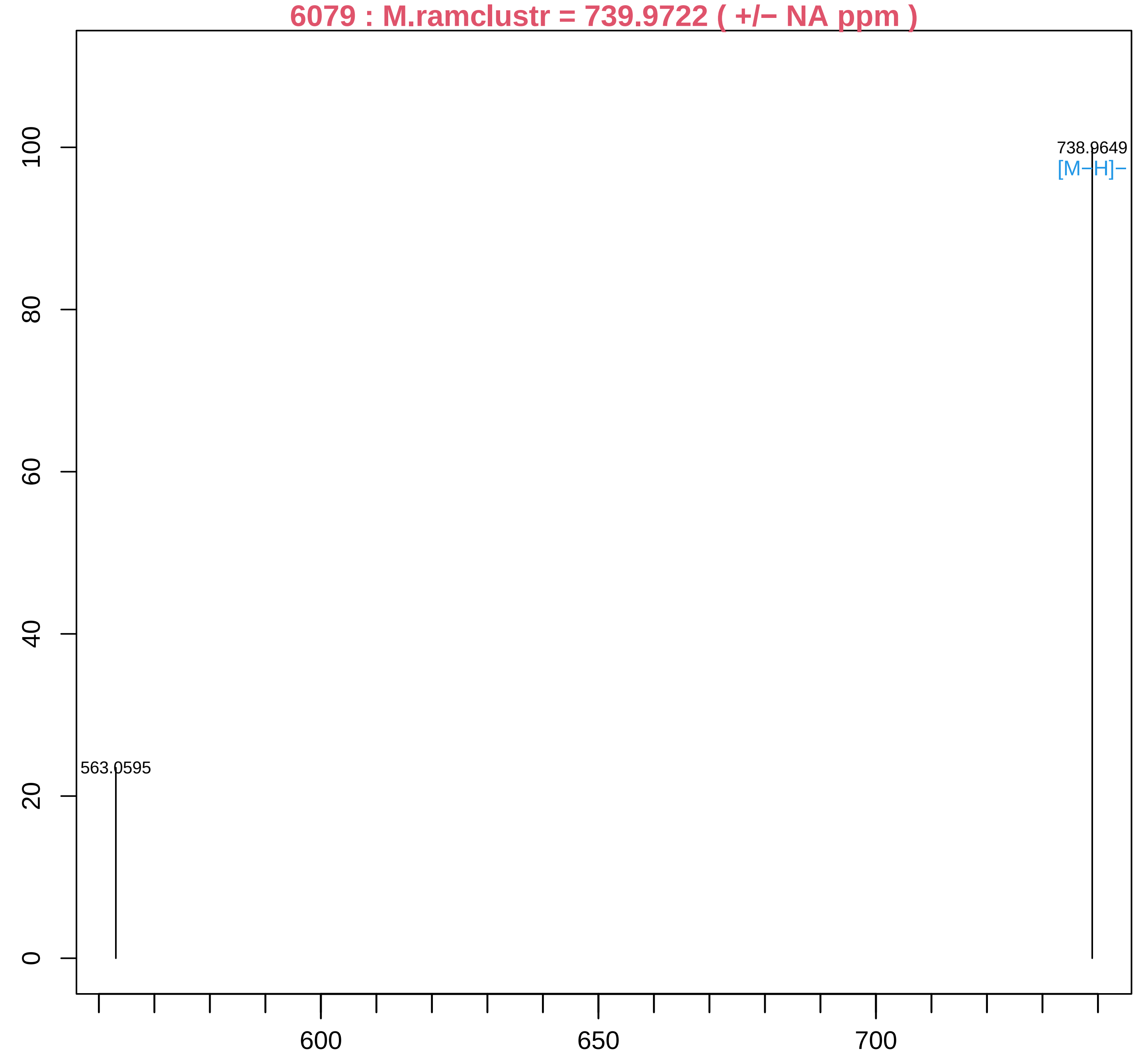
6079 : M.ramclustr = 739.9722 (+/− NA ppm)
738.9649
[M−H]−
563.0595
0
20
40
60
80
100
600
650
700

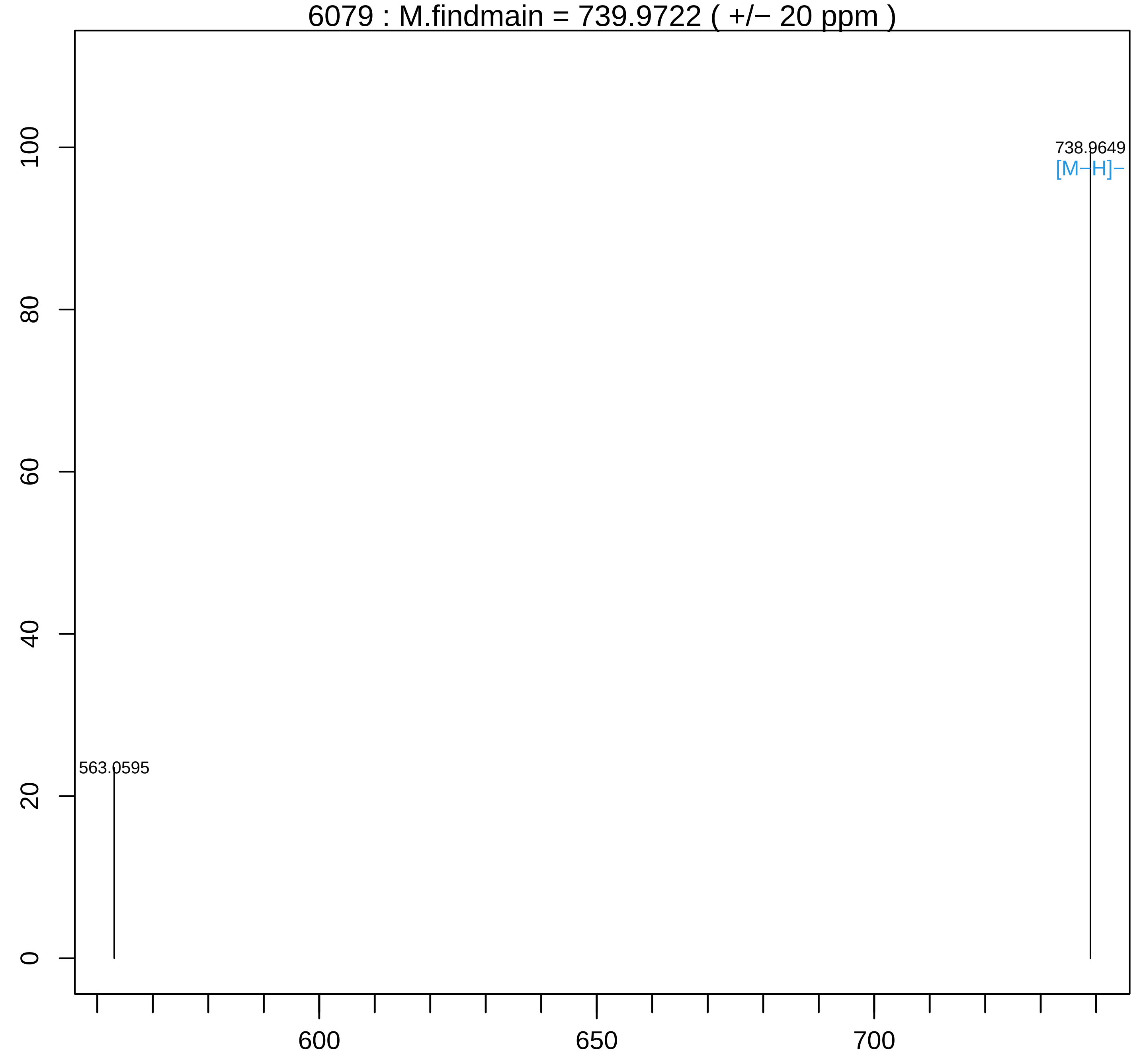
6079 : M.findmain = 739.9722 (+/− 20 ppm)
738.9649
[M−H]−
563.0595
0
20
40
60
80
100
600
650
700

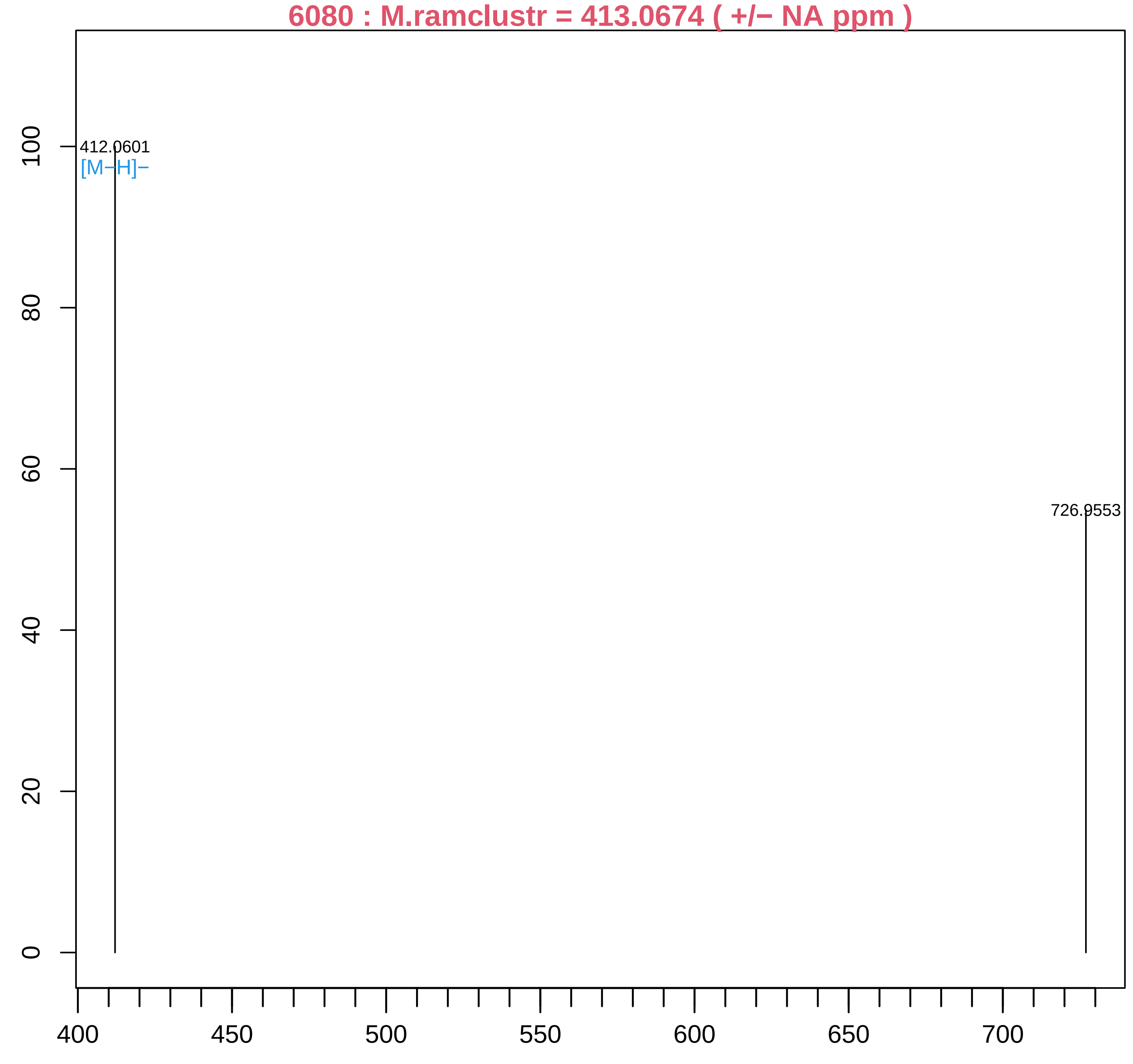
6080 : M.ramclustr = 413.0674 (+/− NA ppm)
412.0601
[M−H]−
726.9553
0
20
40
60
80
100
400
450
500
550
600
650
700

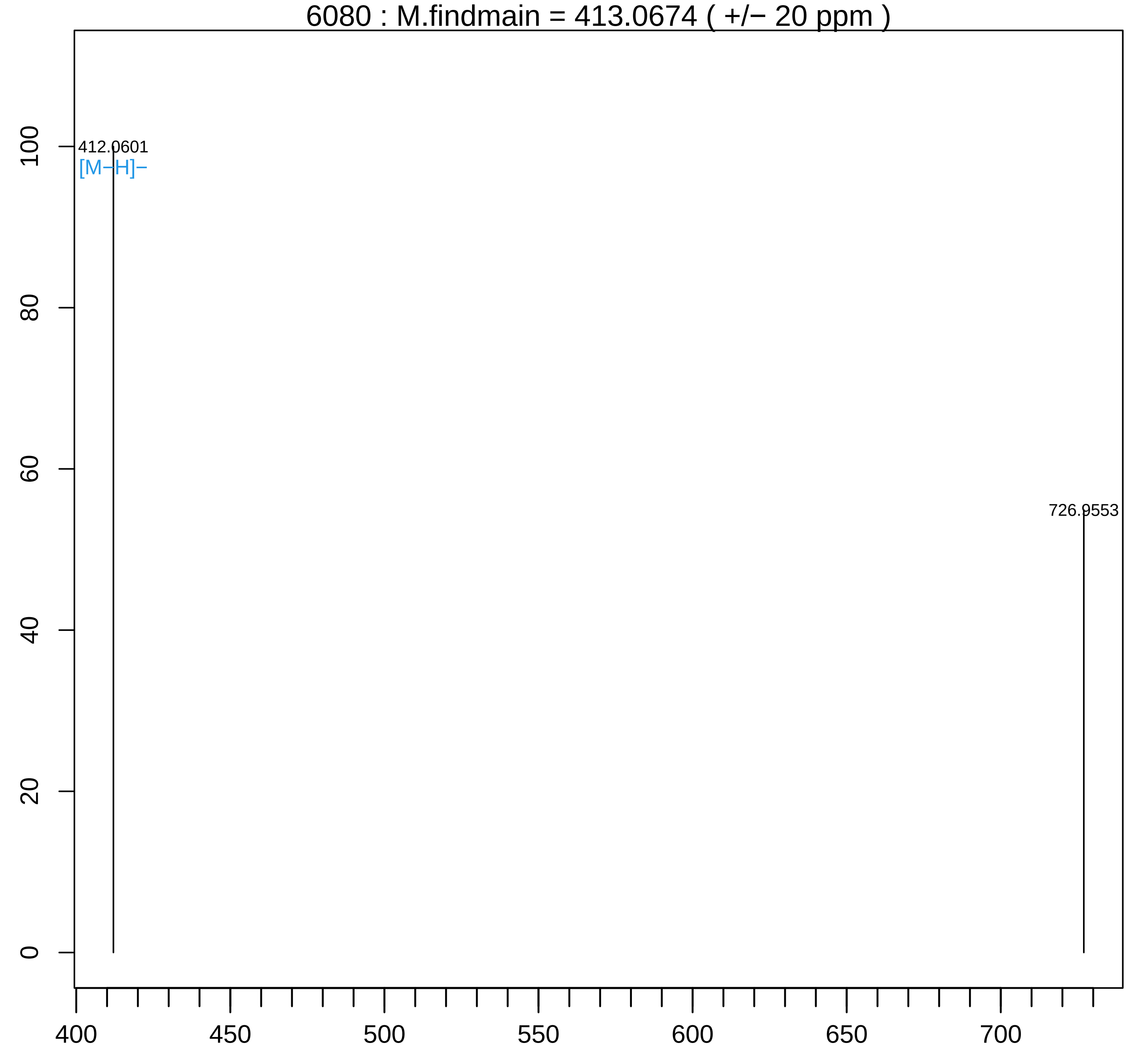
6080 : M.findmain = 413.0674 (+/− 20 ppm)
412.0601
[M−H]−
726.9553
0
20
40
60
80
100
400
450
500
550
600
650
700

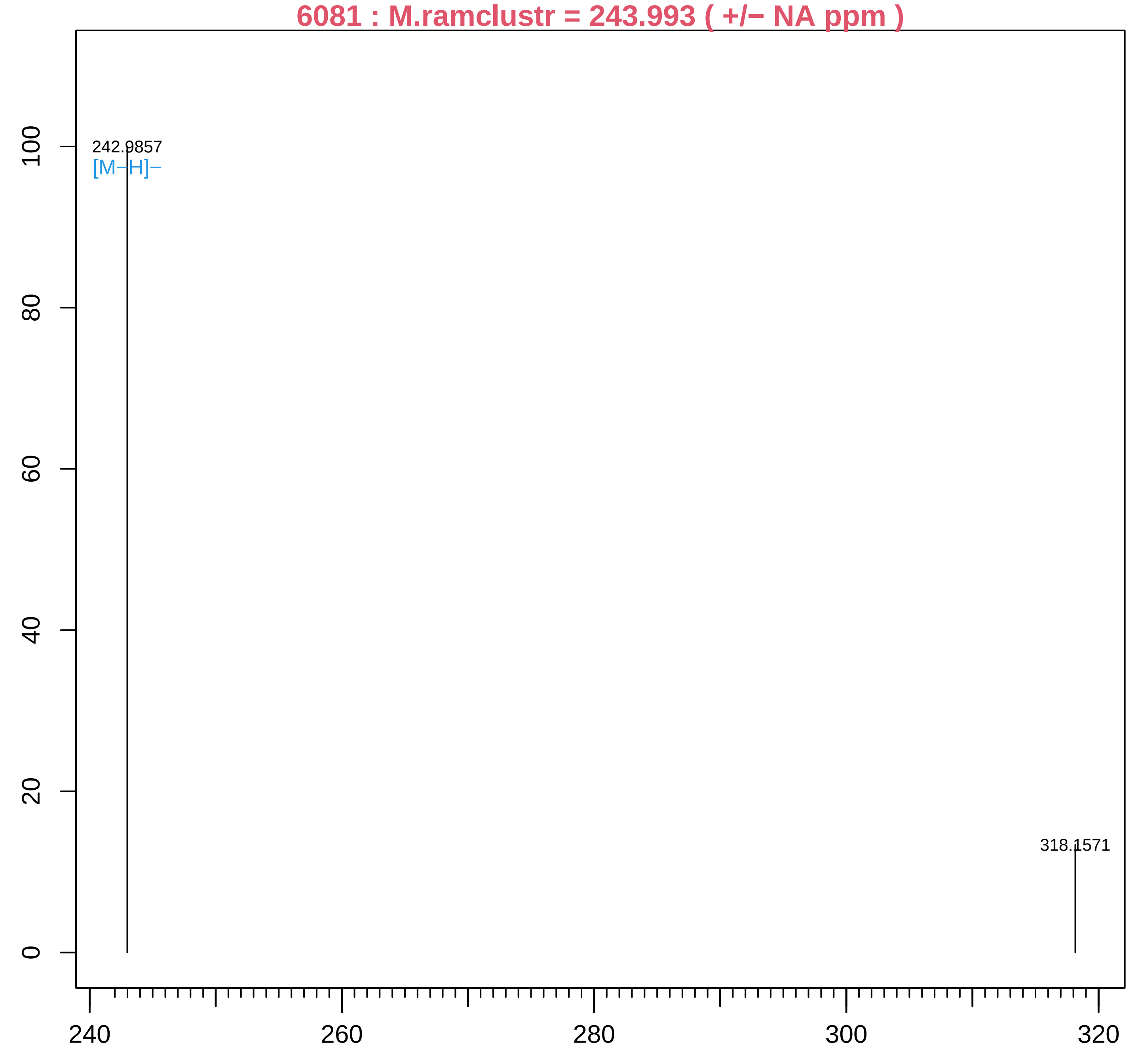
6081 : M.ramclustr = 243.993 (+/− NA ppm)
242.9857
[M−H]−
318.1571
0
20
40
60
80
100
240
260
280
300
320

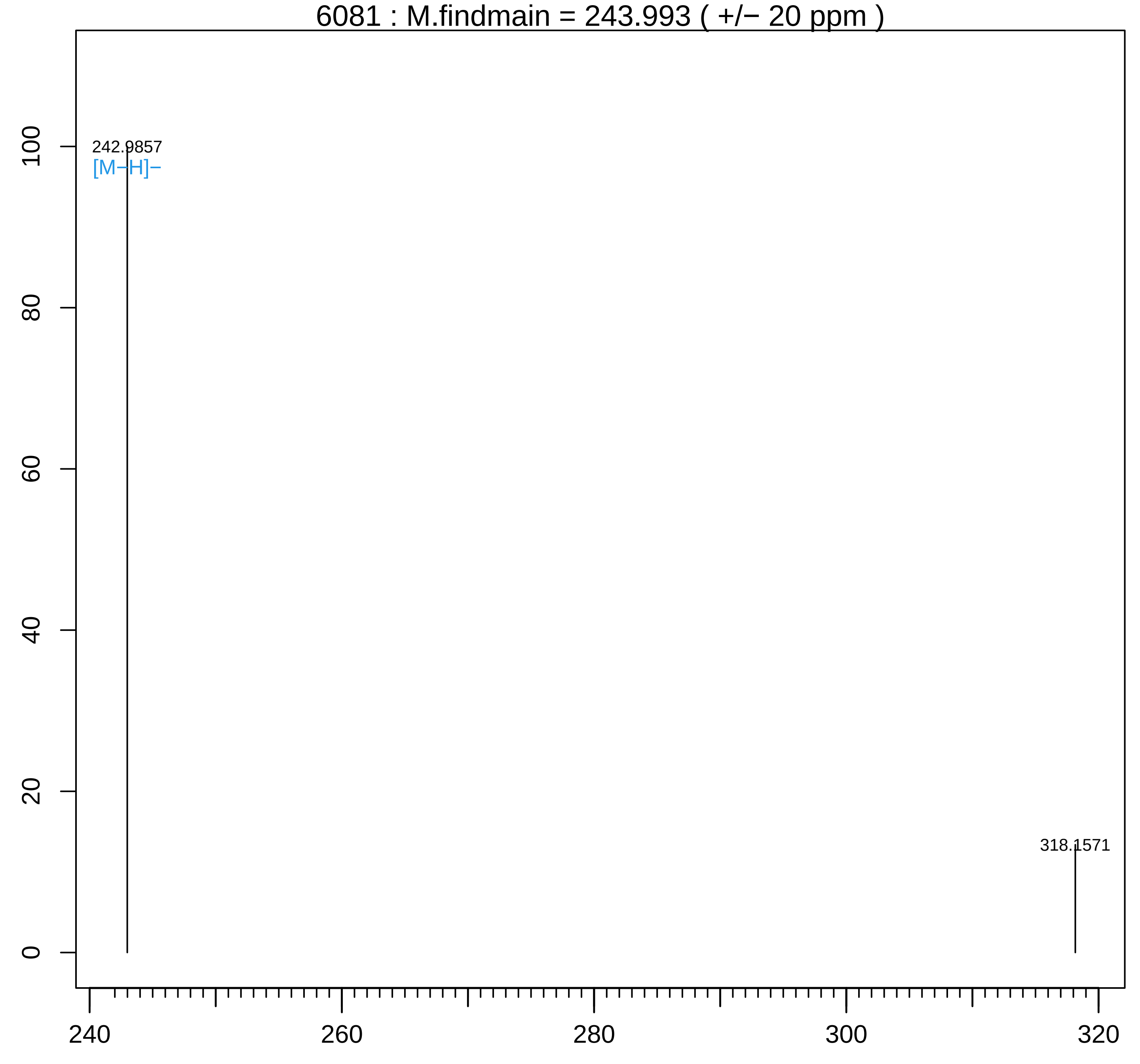
6081 : M.findmain = 243.993 (+/− 20 ppm)
242.9857
[M−H]−
318.1571
0
20
40
60
80
100
240
260
280
300
320

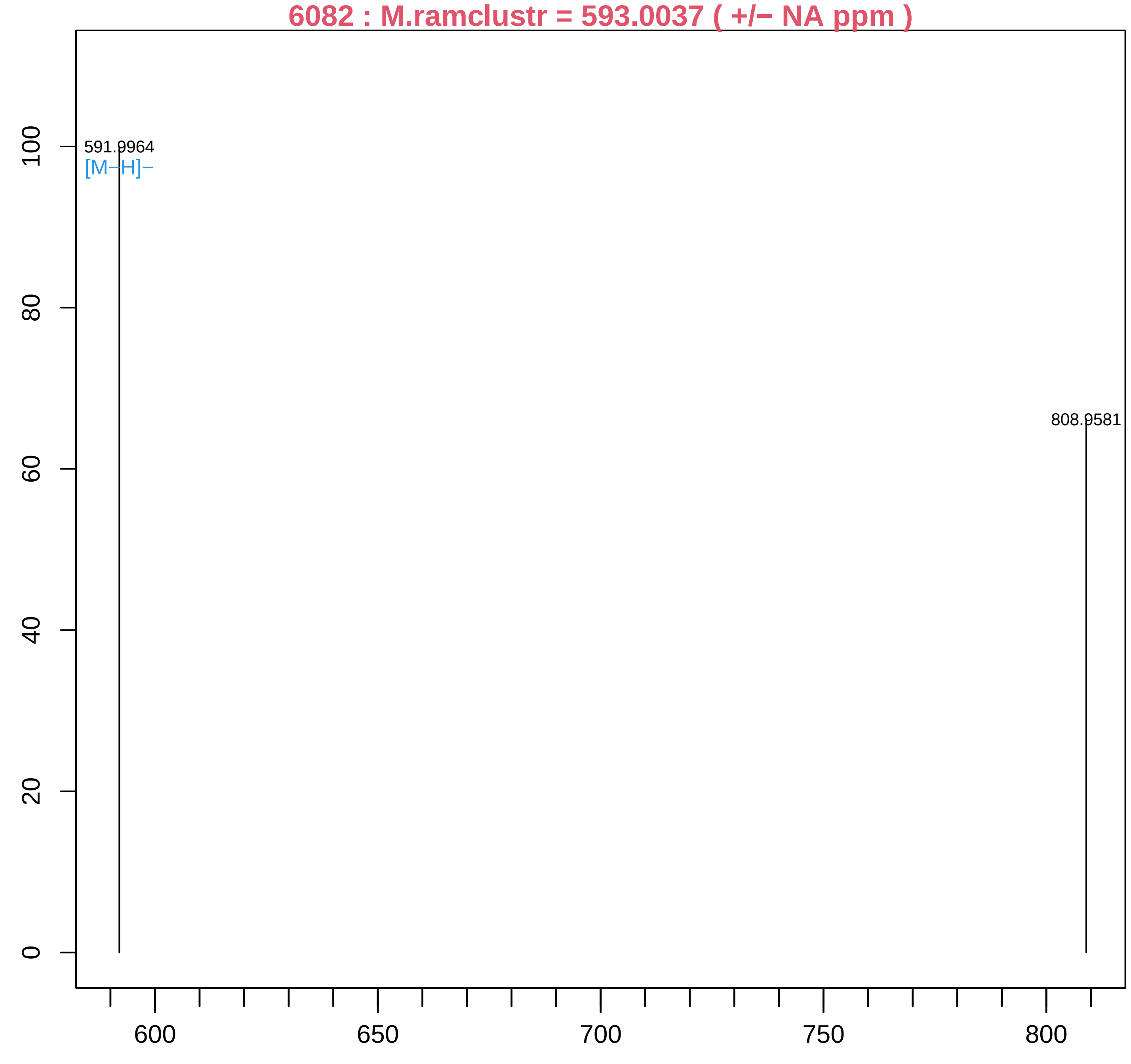

6082 : M.ramclustr = 593.0037 (+/− NA ppm)
591.9964
[M−H]−
808.9581
0
20
40
60
80
100
600
650
700
750
800

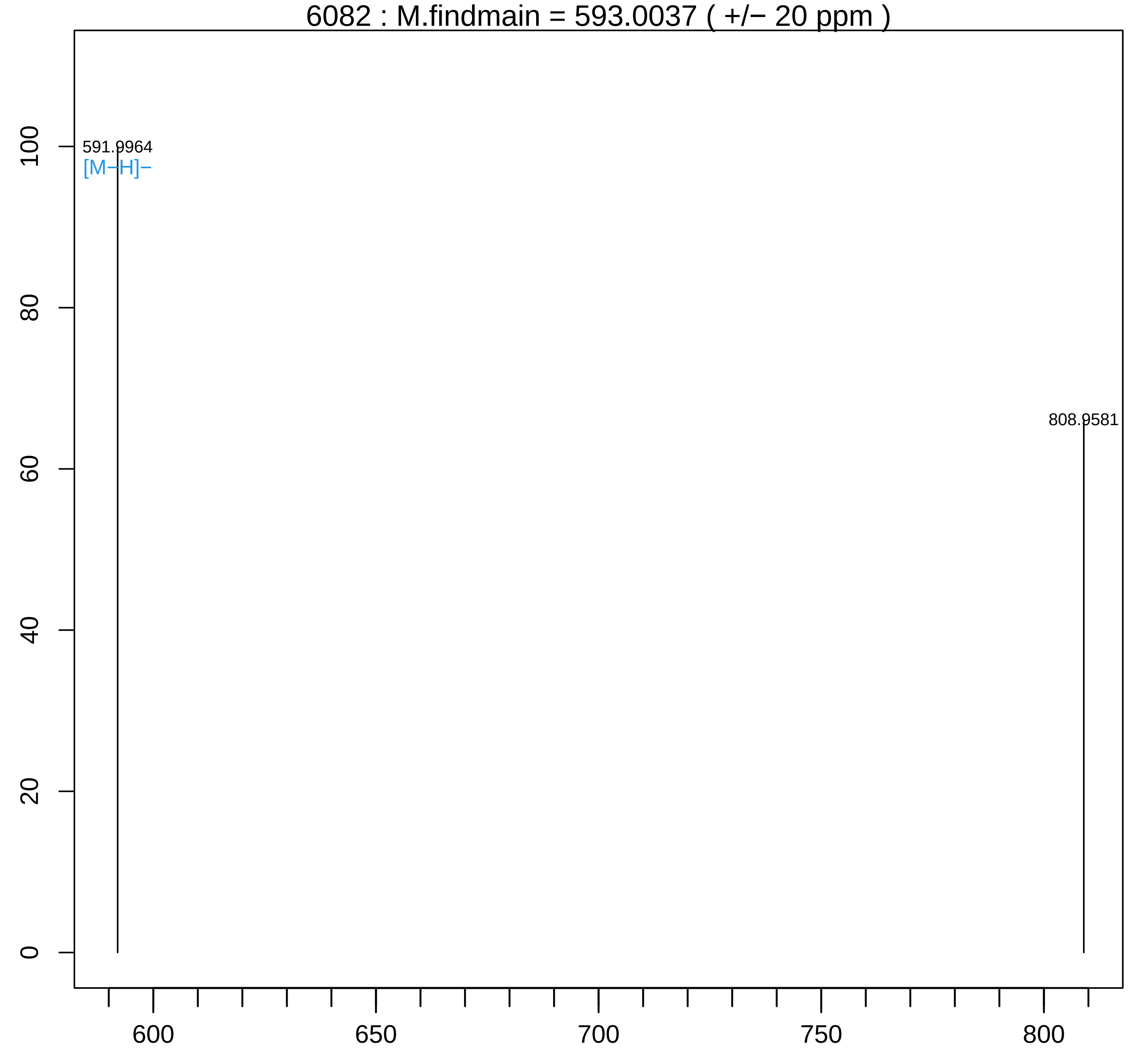

6082 : M.findmain = 593.0037 (+/− 20 ppm)
591.9964
[M−H]−
808.9581
0
20
40
60
80
100
600
650
700
750
800

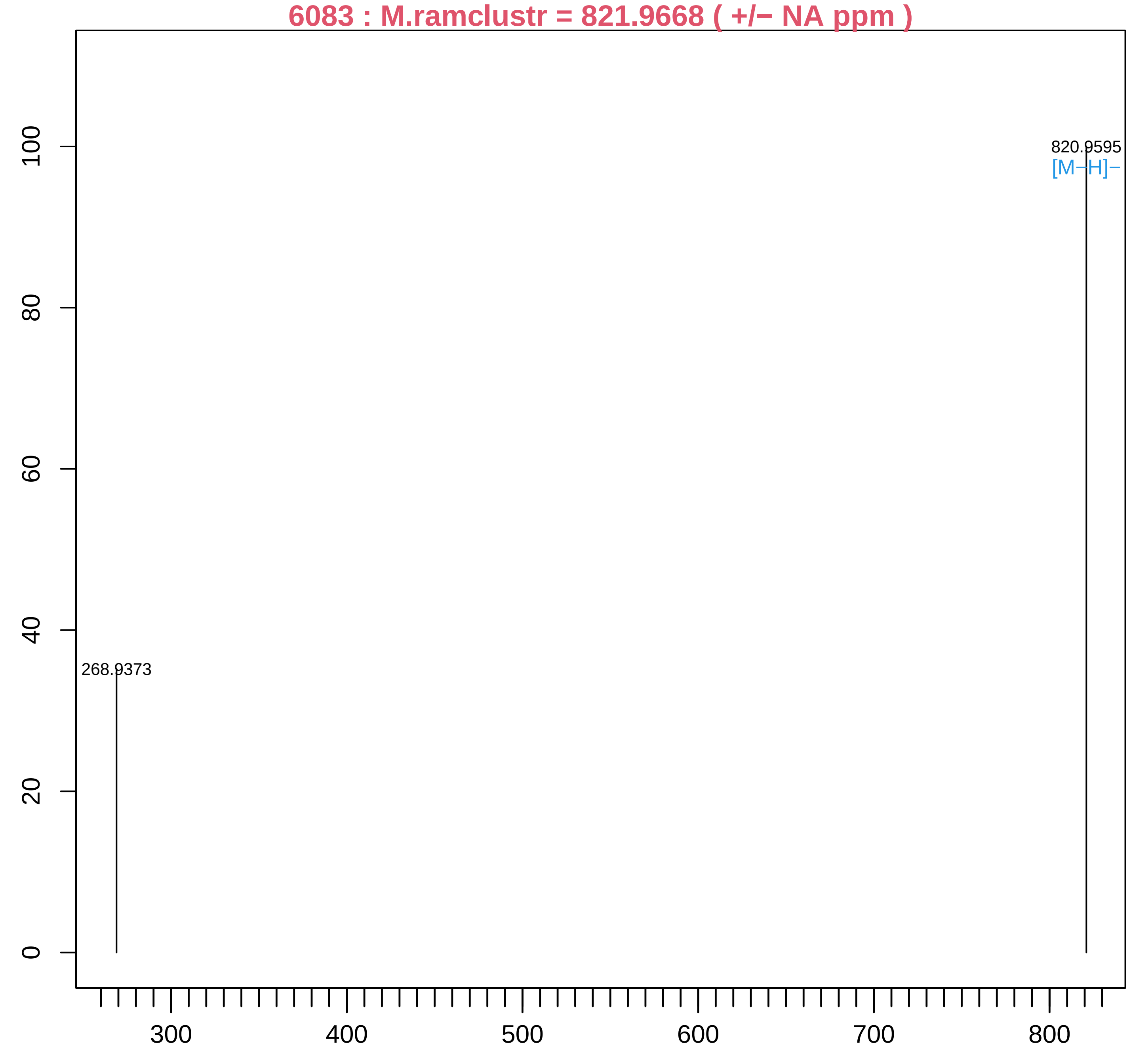

6083 : M.ramclustr = 821.9668 (+/− NA ppm)
820.9595
[M−H]−
268.9373
0
20
40
60
80
100
300
400
500
600
700
800

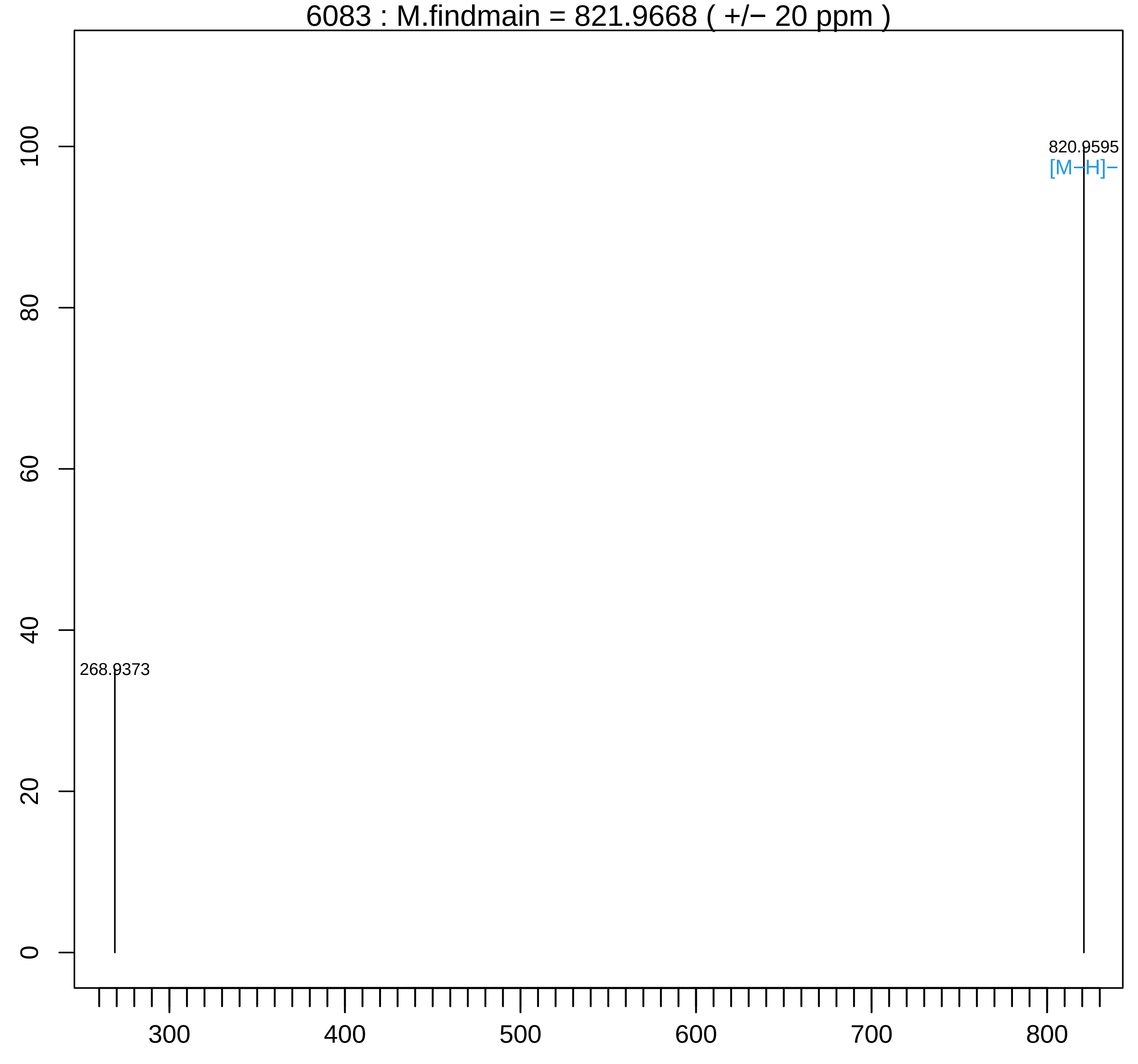

6083 : M.findmain = 821.9668 (+/− 20 ppm)
820.9595
[M−H]−
268.9373
0
20
40
60
80
100
300
400
500
600
700
800

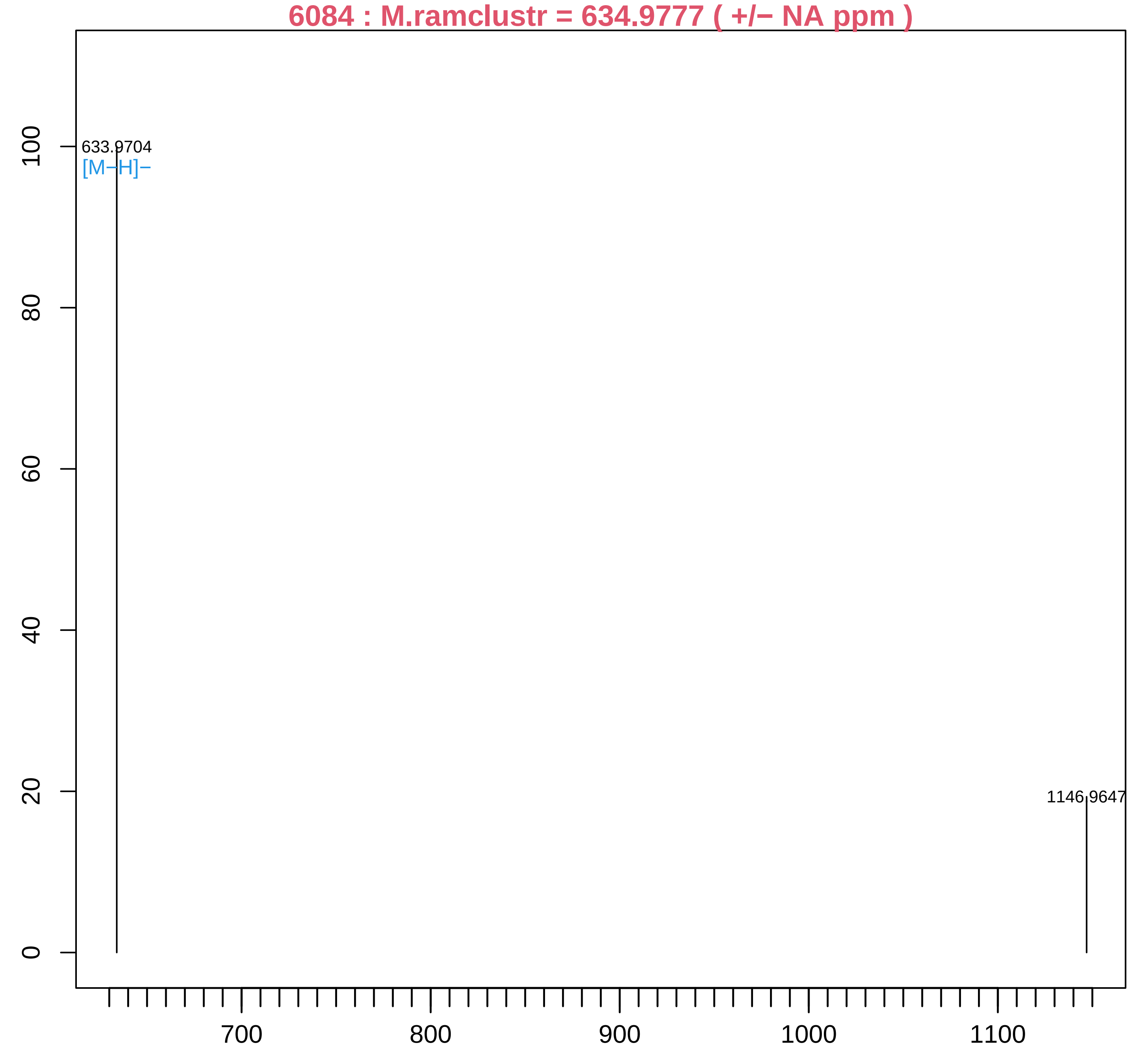

6084 : M.ramclustr = 634.9777 (+/− NA ppm)
633.9704
[M−H]−
1146.9647
0
20
40
60
80
100
700
800
900
1000
1100

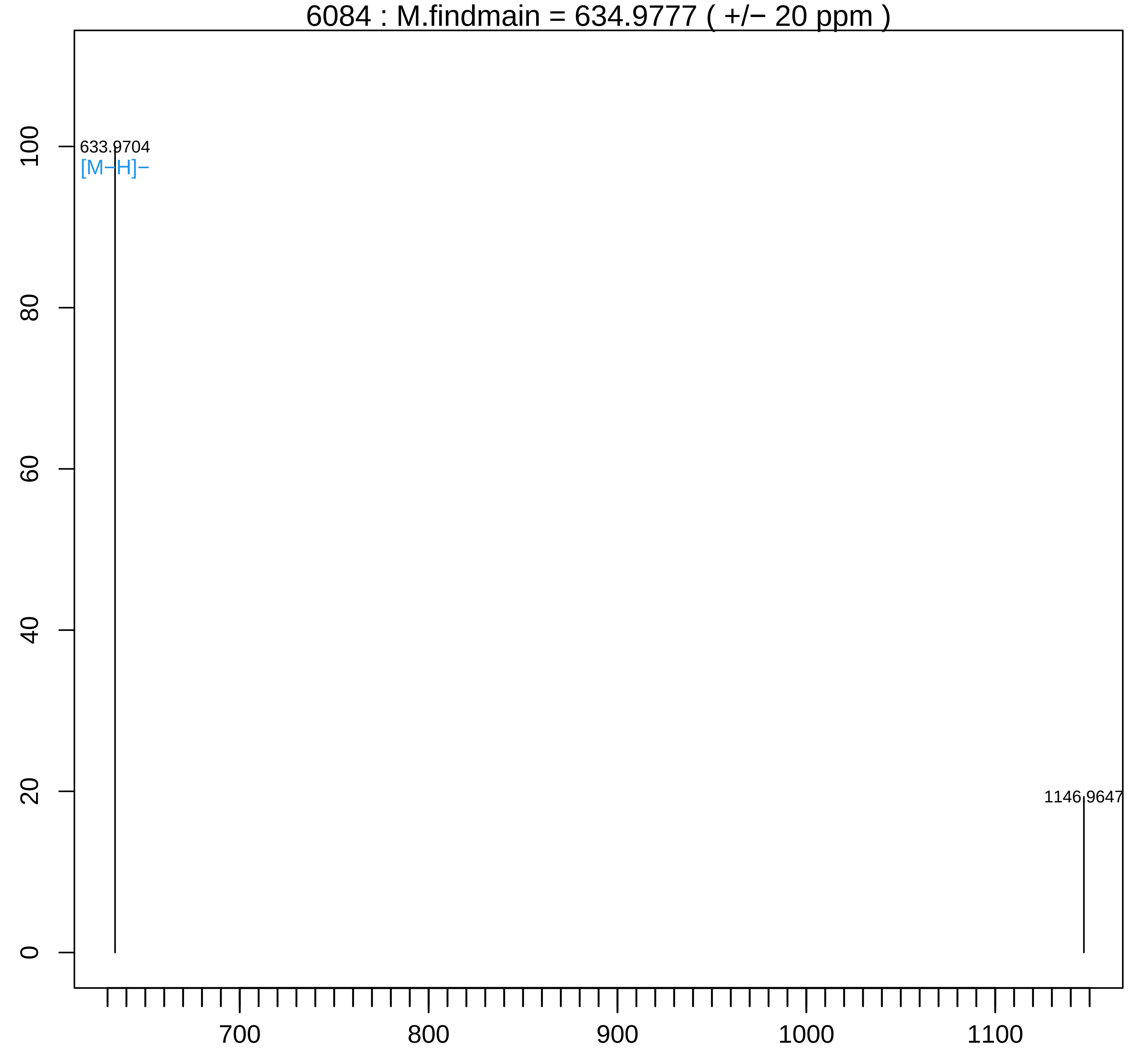

6084 : M.findmain = 634.9777 (+/− 20 ppm)
633.9704
[M−H]−
1146.9647
0
20
40
60
80
100
700
800
900
1000
1100

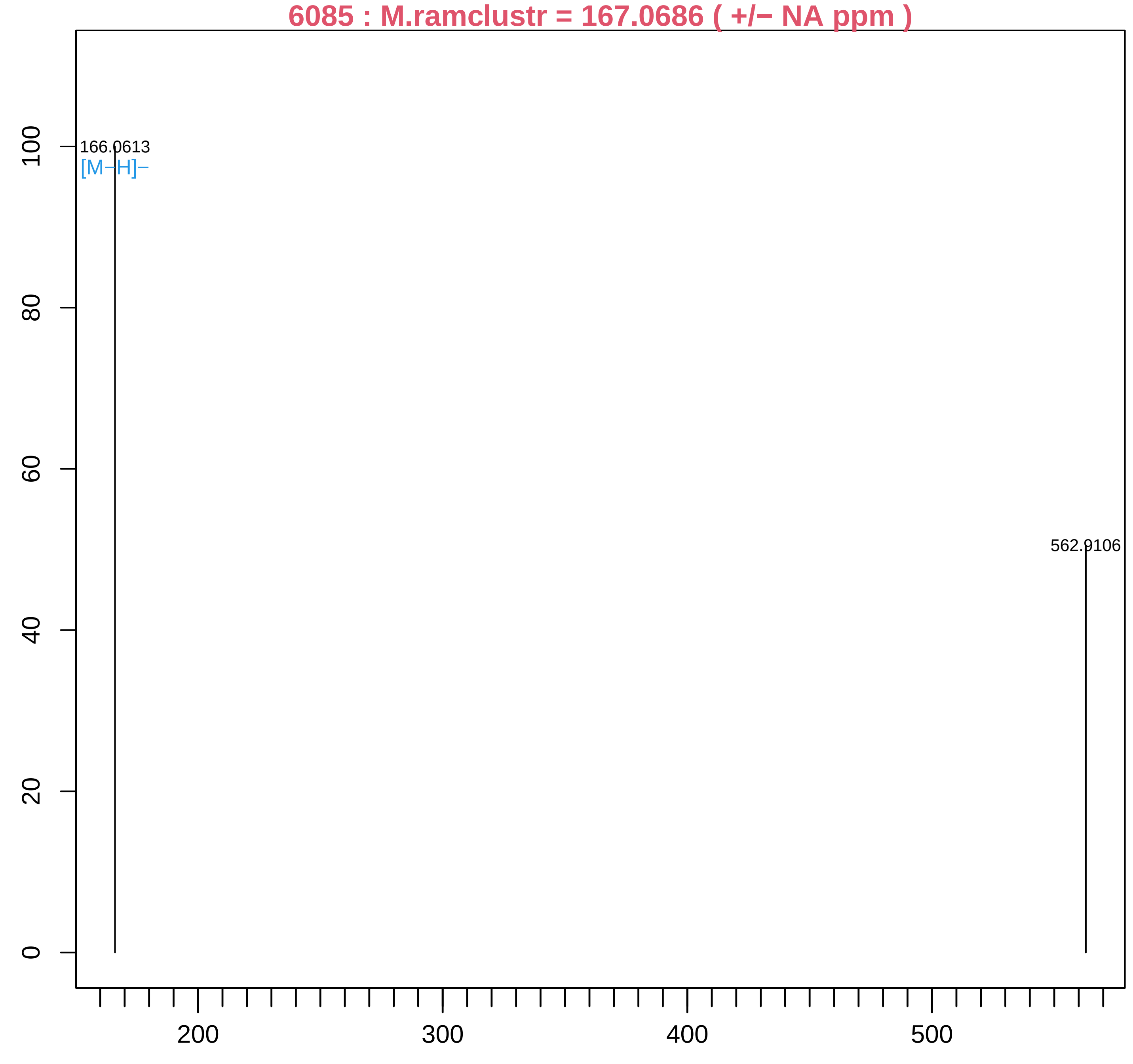
6085 : M.ramclustr = 167.0686 (+/− NA ppm)
166.0613
[M−H]−
562.9106
0
20
40
60
80
100
200
300
400
500

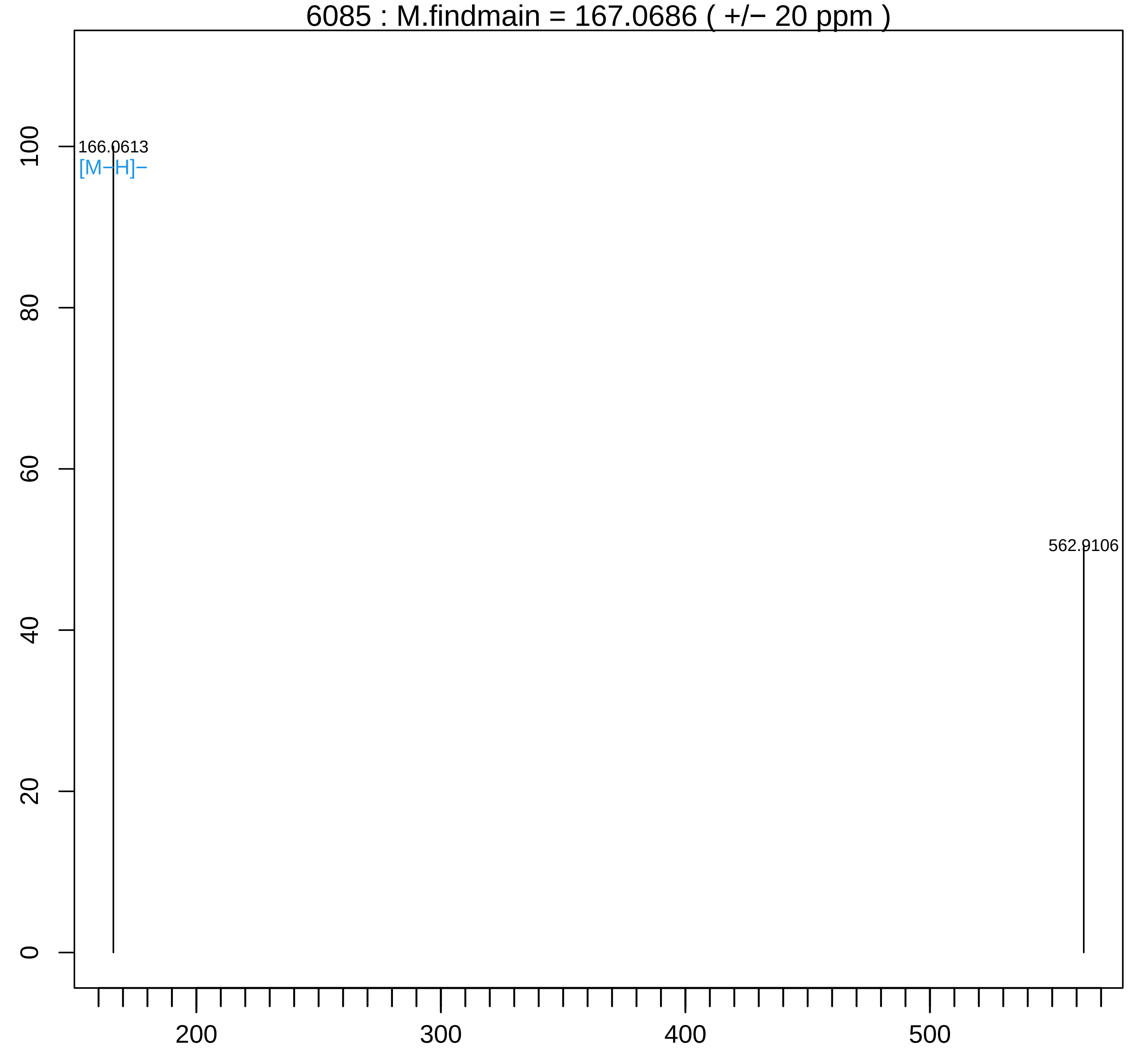
6085 : M.findmain = 167.0686 (+/− 20 ppm)
166.0613
[M−H]−
562.9106
0
20
40
60
80
100
200
300
400
500

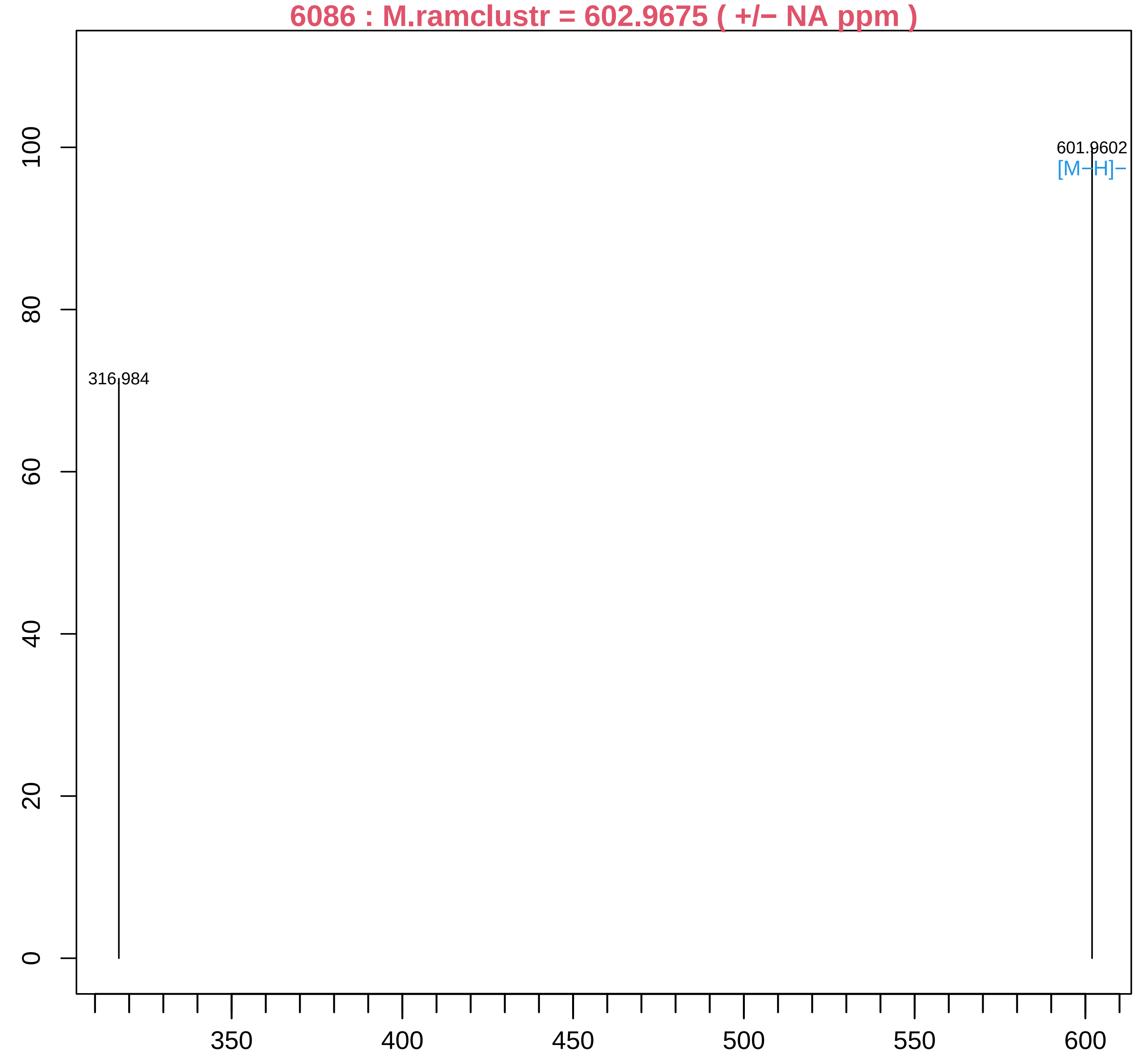

6086 : M.ramclustr = 602.9675 (+/− NA ppm)
601.9602
[M−H]−
316.984
0
20
40
60
80
100
350
400
450
500
550
600

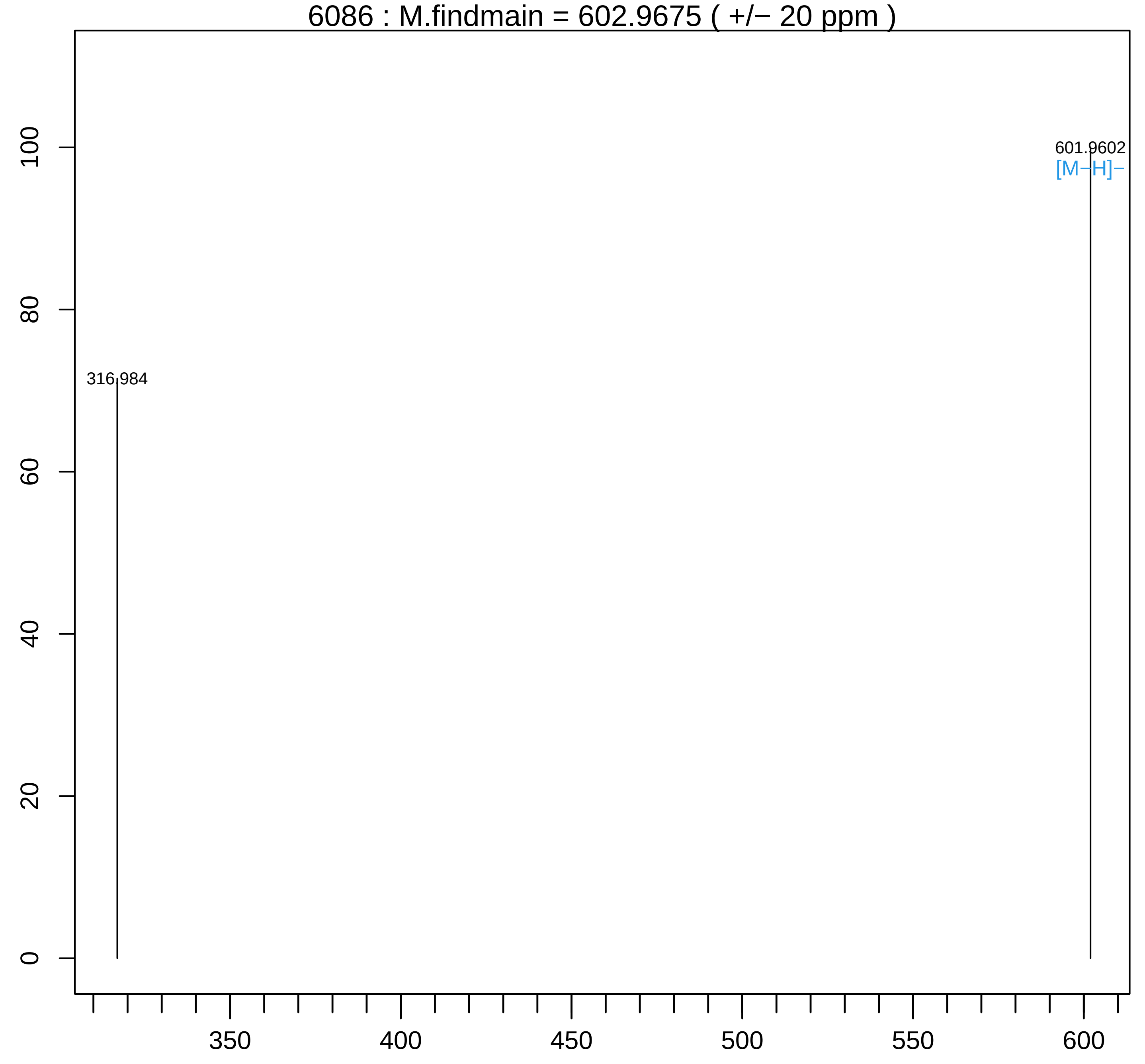

6086 : M.findmain = 602.9675 (+/− 20 ppm)
601.9602
[M−H]−
316.984
0
20
40
60
80
100
350
400
450
500
550
600

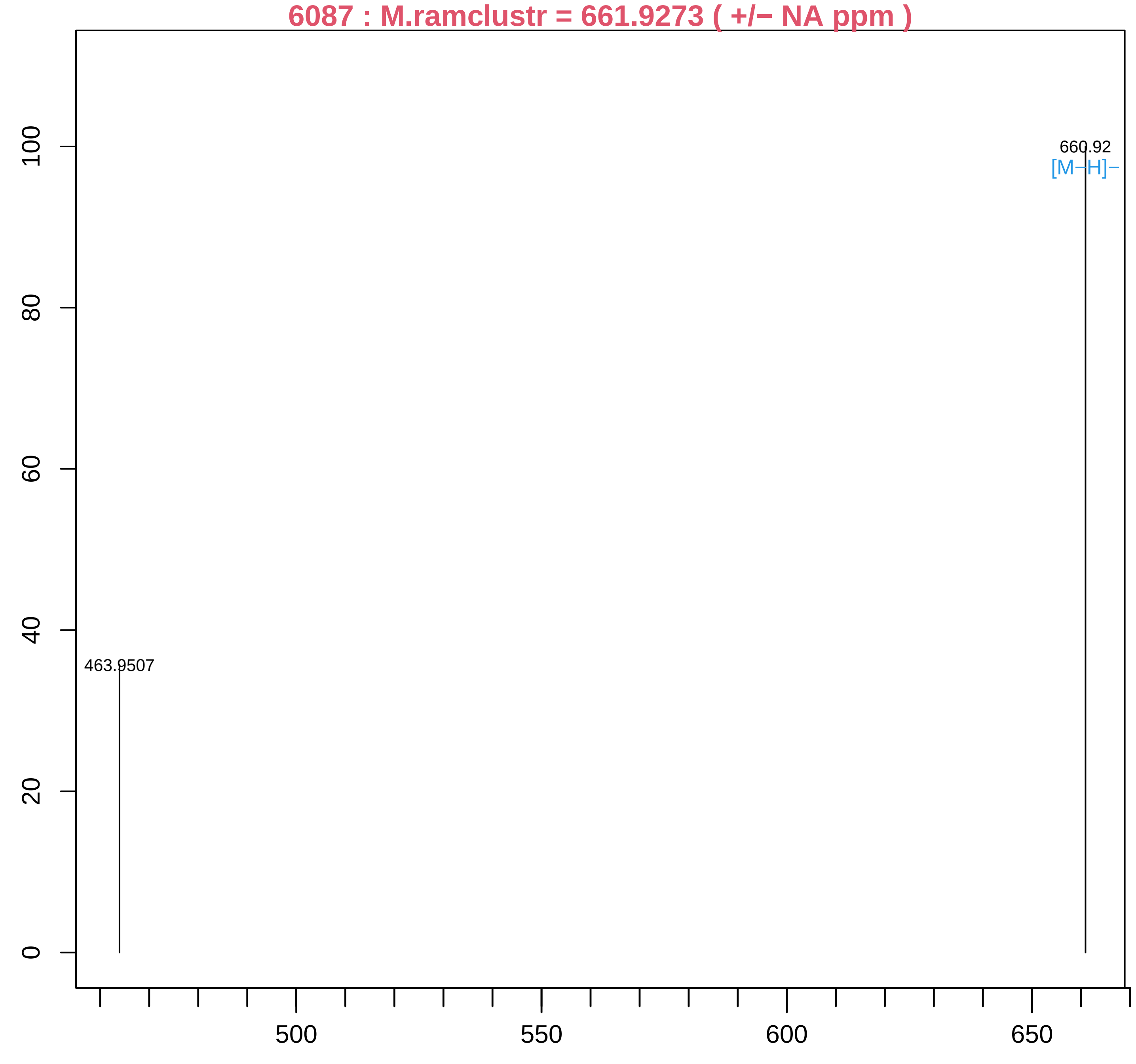

6087 : M.ramclustr = 661.9273 (+/− NA ppm)
660.92
[M−H]−
463.9507
0
20
40
60
80
100
500
550
600
650

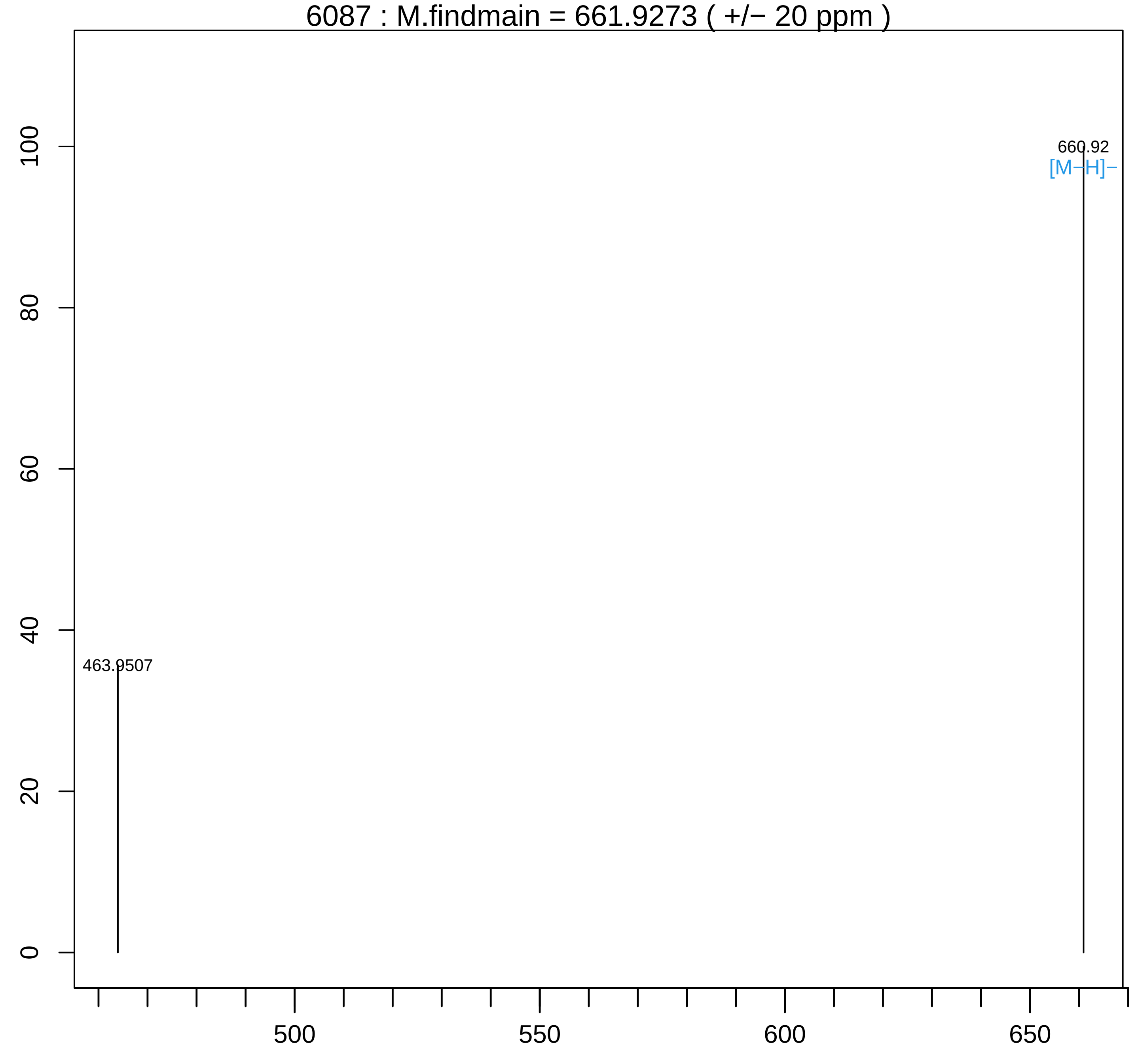

6087 : M.findmain = 661.9273 (+/− 20 ppm)
660.92
[M−H]−
463.9507
0
20
40
60
80
100
500
550
600
650

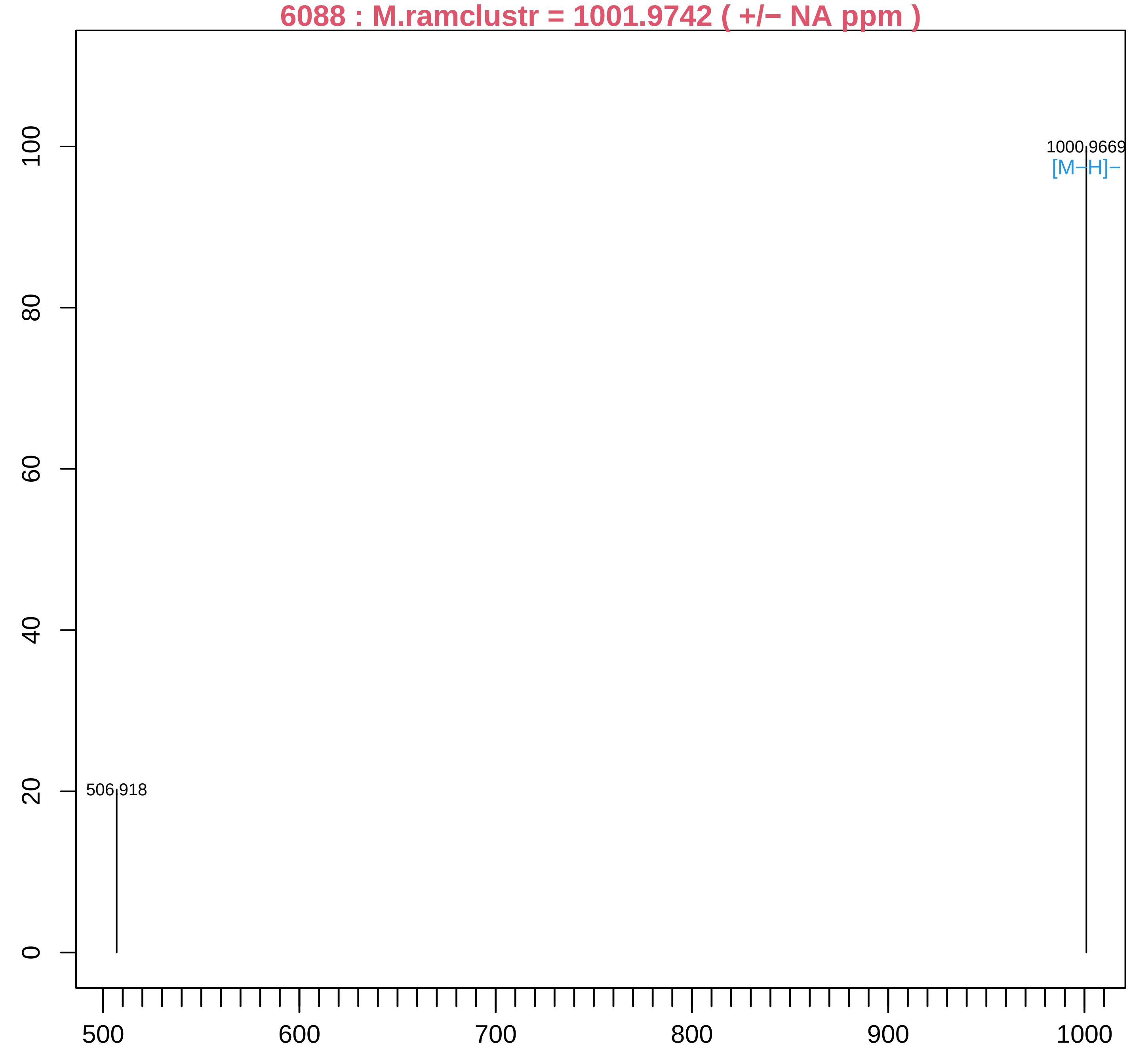
6088 : M.ramclustr = 1001.9742 (+/− NA ppm)
1000.9669
[M−H]−
506.918
0
20
40
60
80
100
500
600
700
800
900
1000

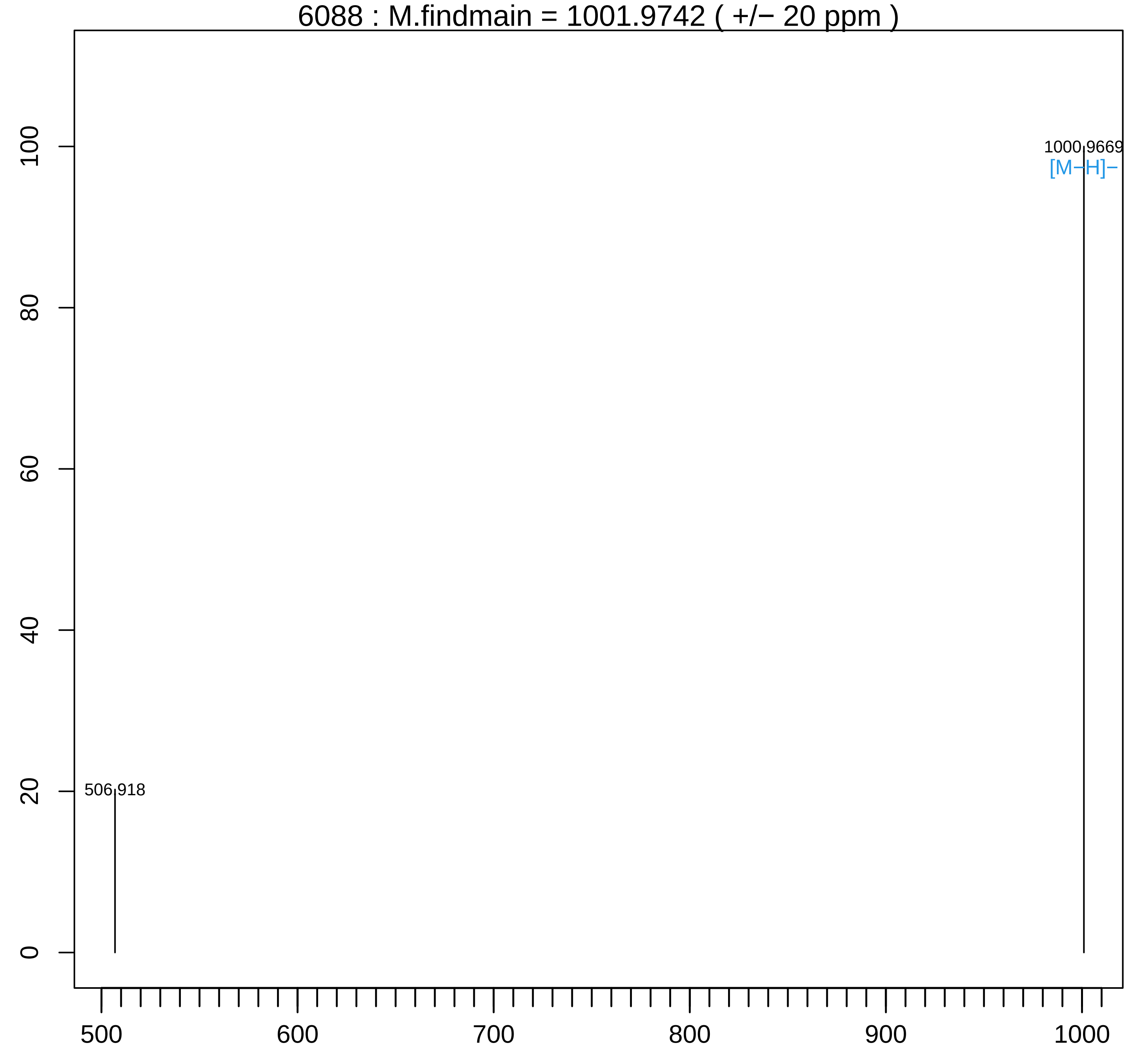
6088 : M.findmain = 1001.9742 (+/− 20 ppm)
1000.9669
[M−H]−
506.918
0
20
40
60
80
100
500
600
700
800
900
1000

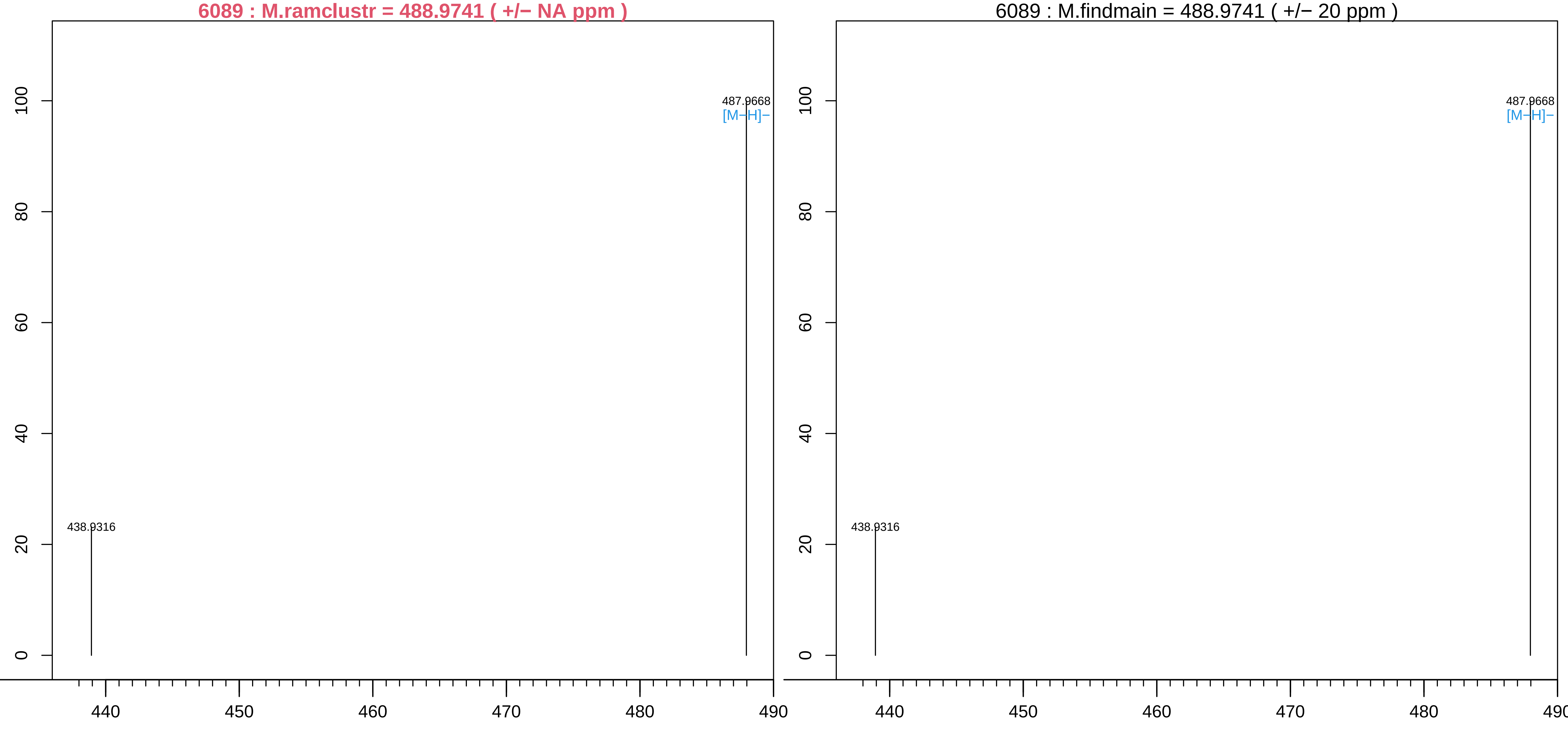
6089 : M.ramclustr = 488.9741 (+/− NA ppm)
487.9668
[M−H]−
438.9316
0
20
40
60
80
100
440
450
460
470
480
490
6089 : M.findmain = 488.9741 (+/− 20 ppm)
487.9668
[M−H]−
438.9316
0
20
40
60
80
100
440
450
460
470
480
490

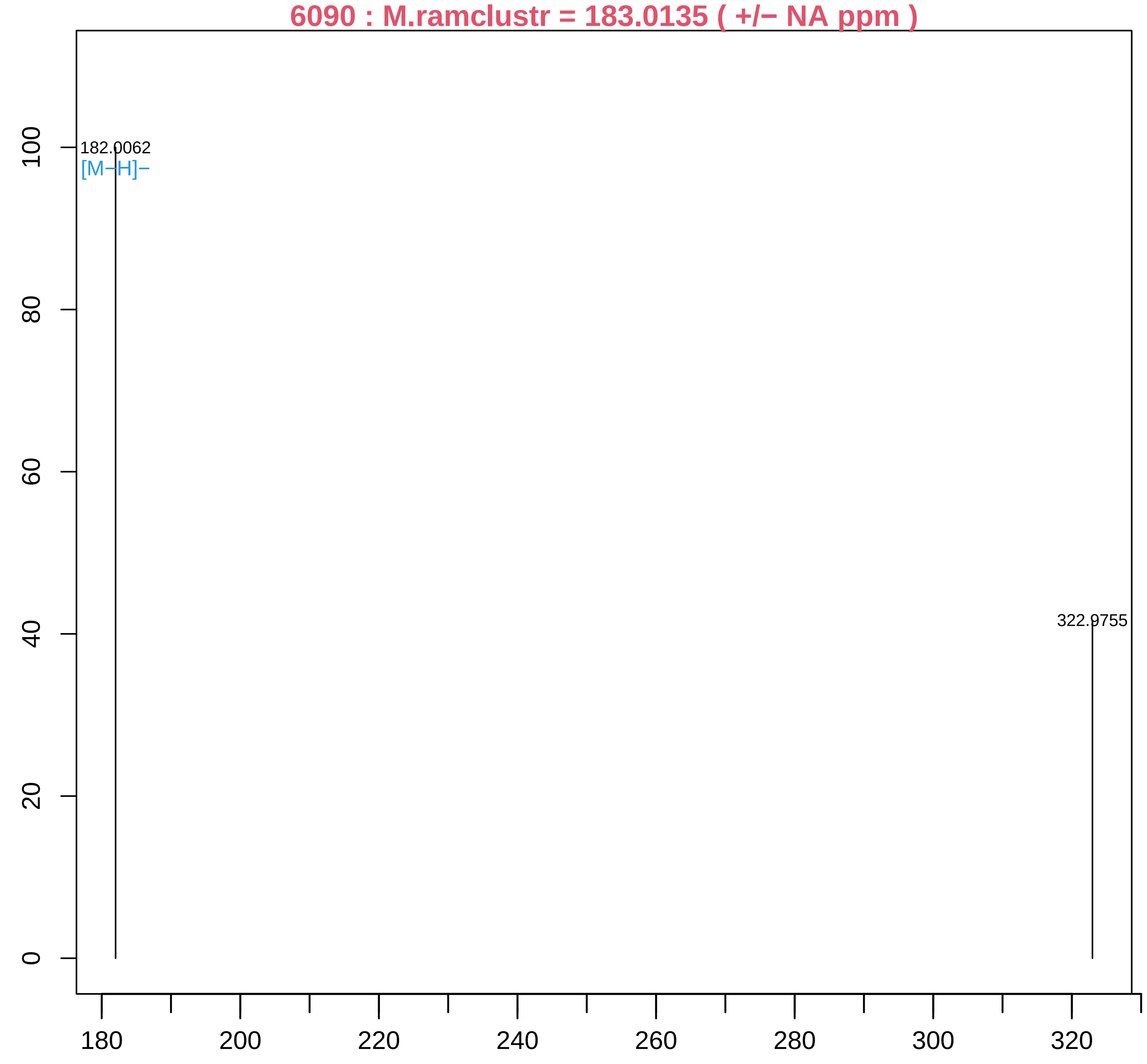
6090 : M.ramclustr = 183.0135 (+/− NA ppm)
182.0062
[M−H]−
322.9755
0
20
40
60
80
100
180
200
220
240
260
280
300
320

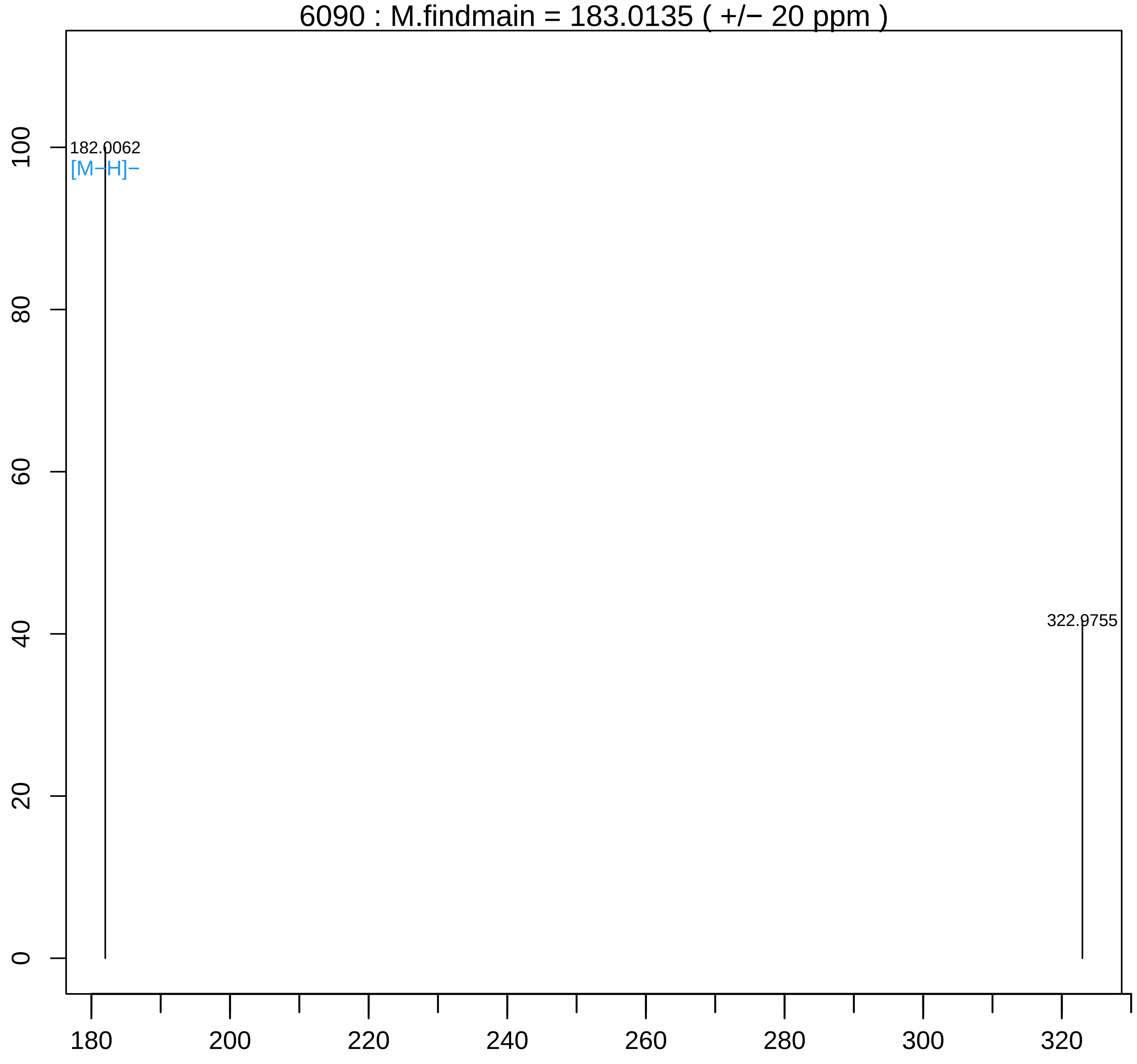
6090 : M.findmain = 183.0135 (+/− 20 ppm)
182.0062
[M−H]−
322.9755
0
20
40
60
80
100
180
200
220
240
260
280
300
320

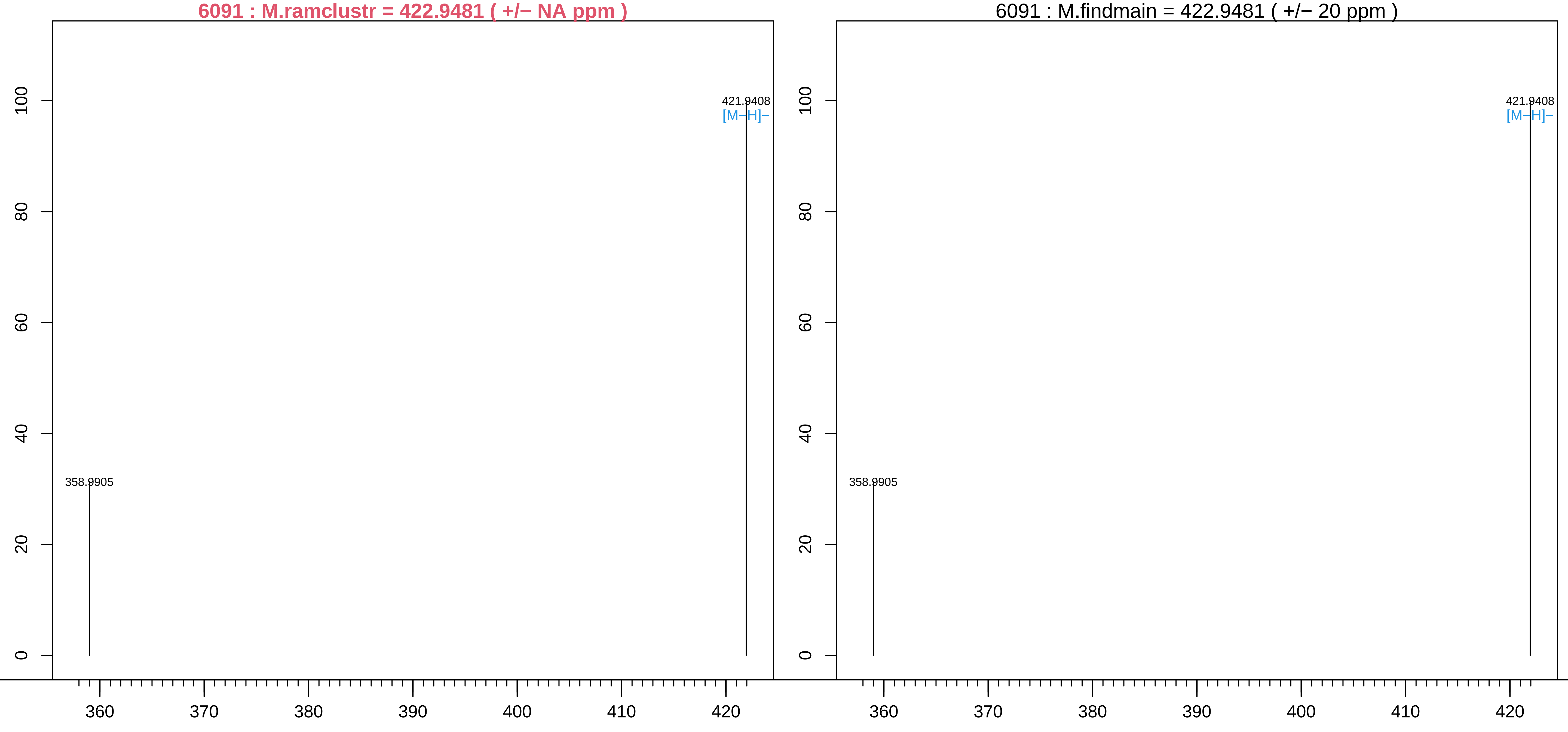

6091 : M.ramclustr = 422.9481 (+/− NA ppm)
6091 : M.findmain = 422.9481 (+/− 20 ppm)
421.9408
[M−H]−
358.9905
0
20
40
60
80
100
360
370
380
390
400
410
420

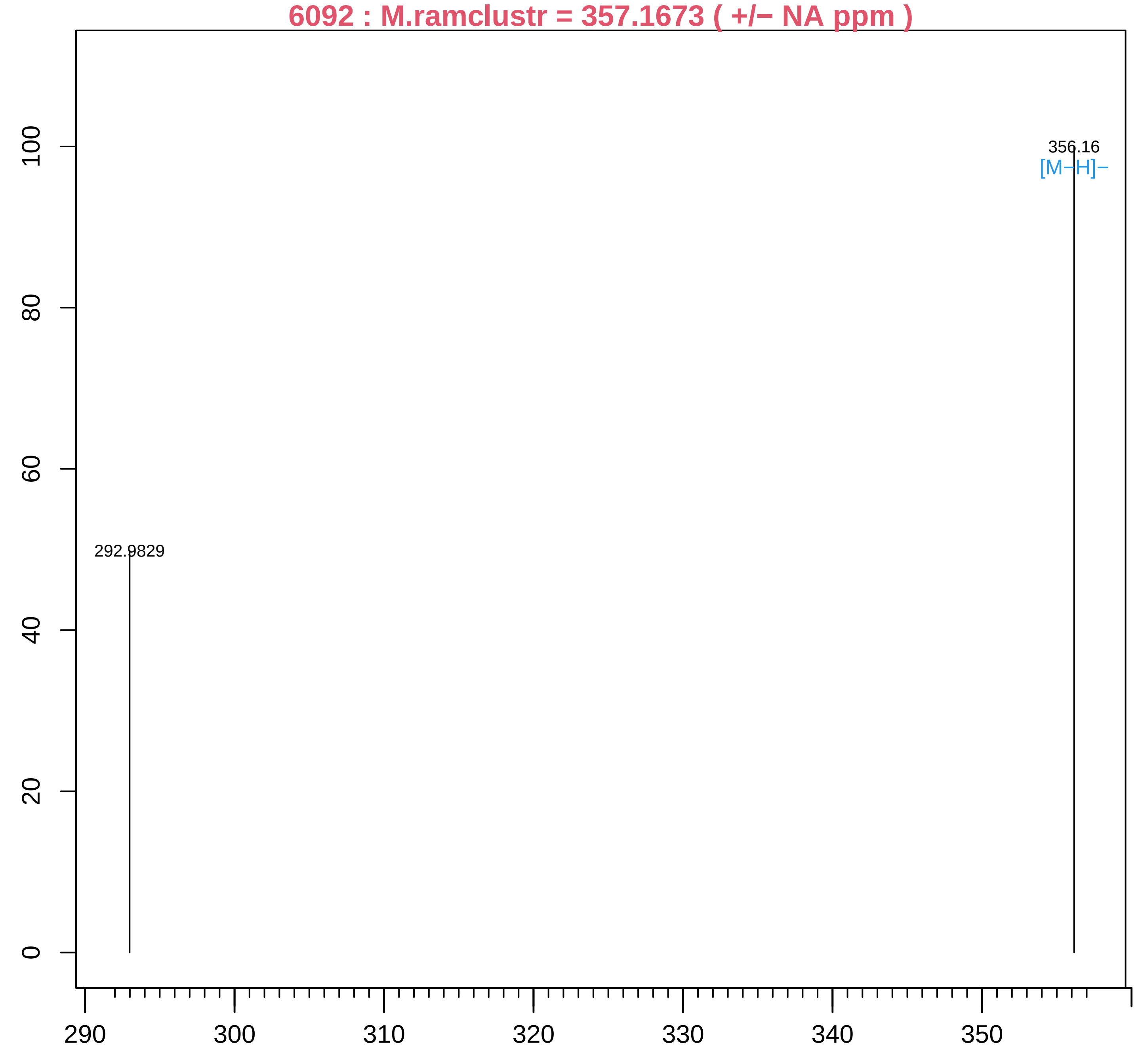
6092 : M.ramclustr = 357.1673 (+/− NA ppm)
356.16
[M−H]−
292.9829
0
20
40
60
80
100
290
300
310
320
330
340
350

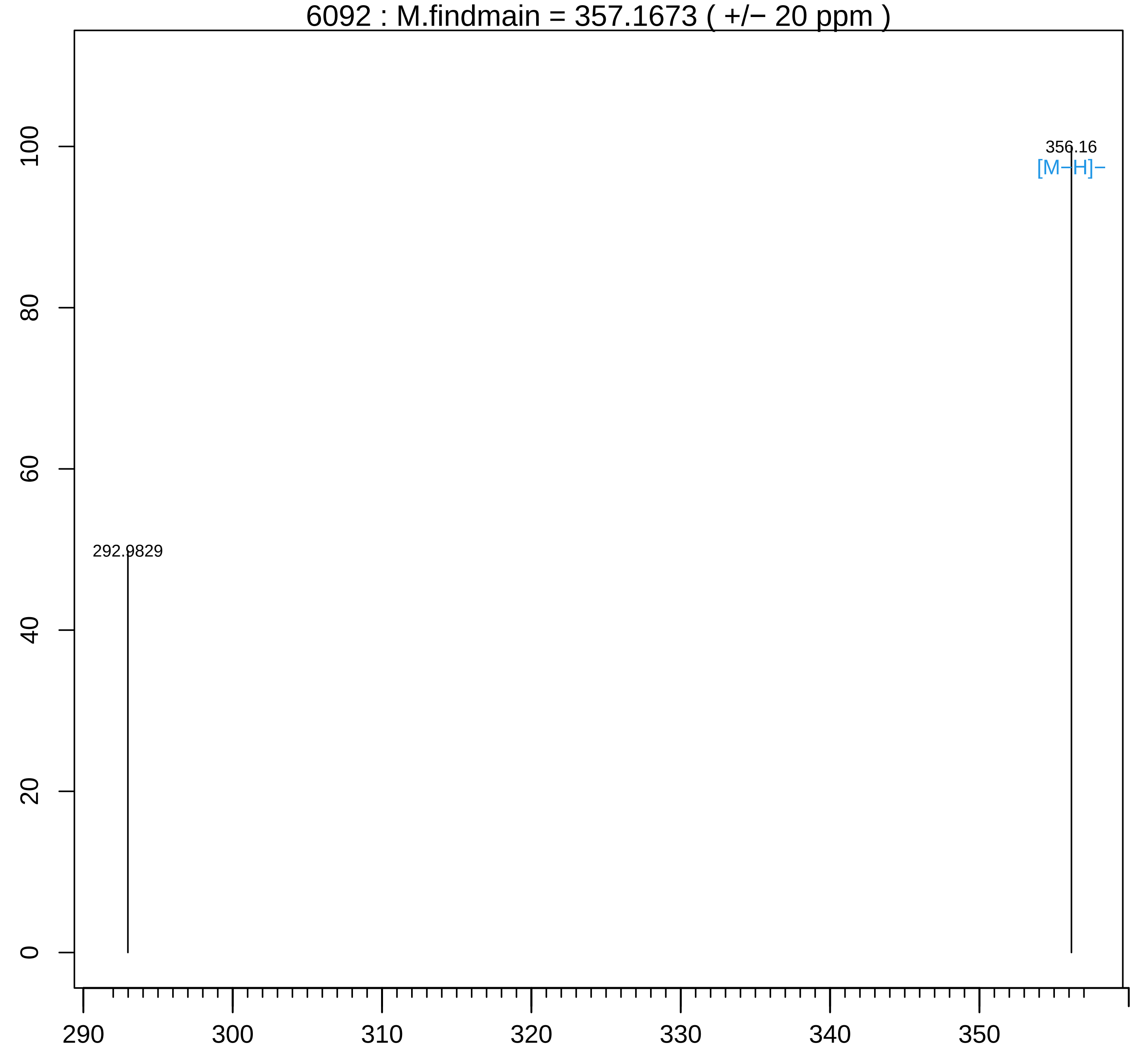
6092 : M.findmain = 357.1673 (+/− 20 ppm)
356.16
[M−H]−
292.9829
0
20
40
60
80
100
290
300
310
320
330
340
350

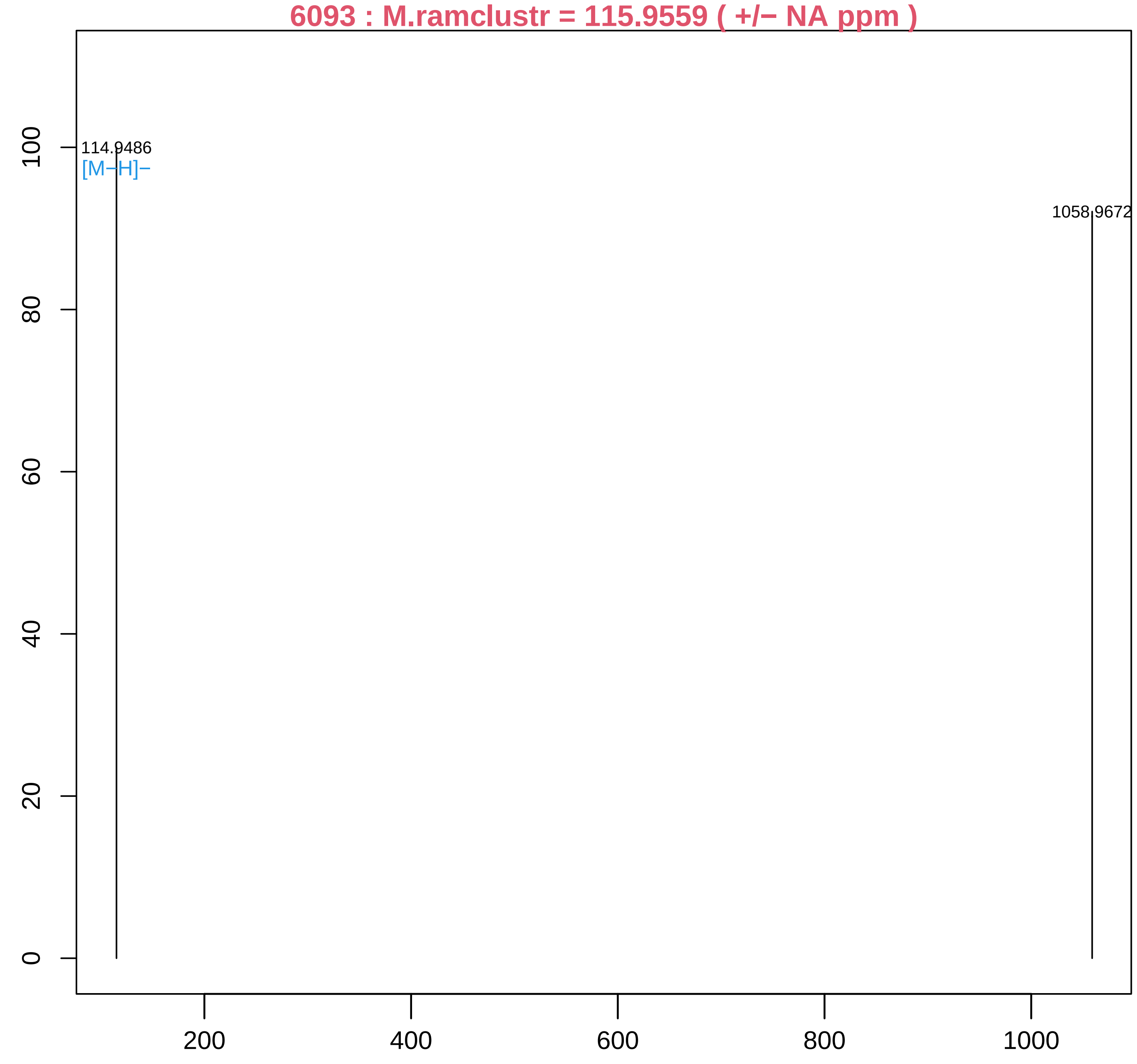

6093 : M.ramclustr = 115.9559 (+/− NA ppm)
114.9486
[M−H]−
1058.9672
0
20
40
60
80
100
200
400
600
800
1000

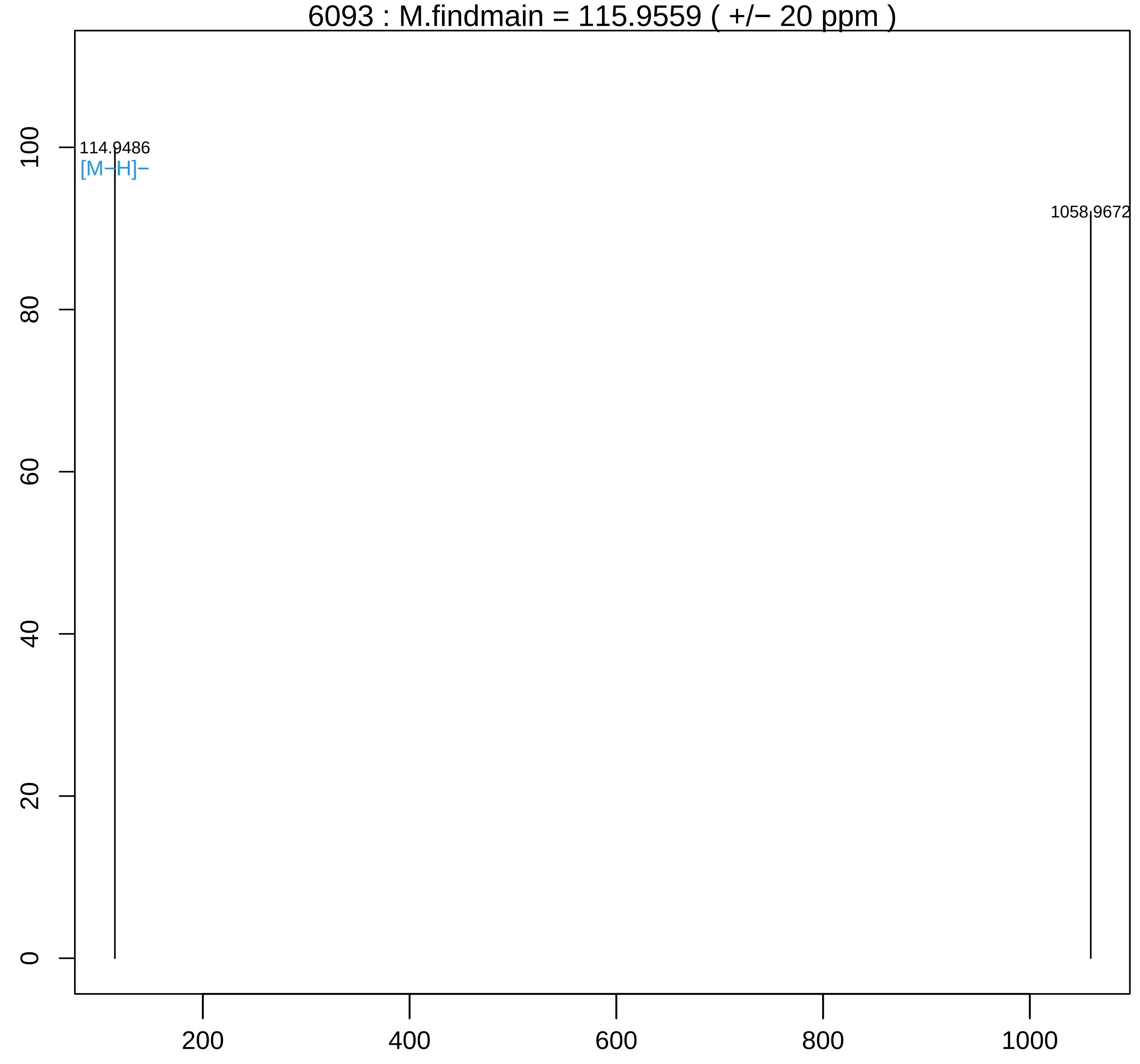

6093 : M.findmain = 115.9559 (+/− 20 ppm)
114.9486
[M−H]−
1058.9672
0
20
40
60
80
100
200
400
600
800
1000

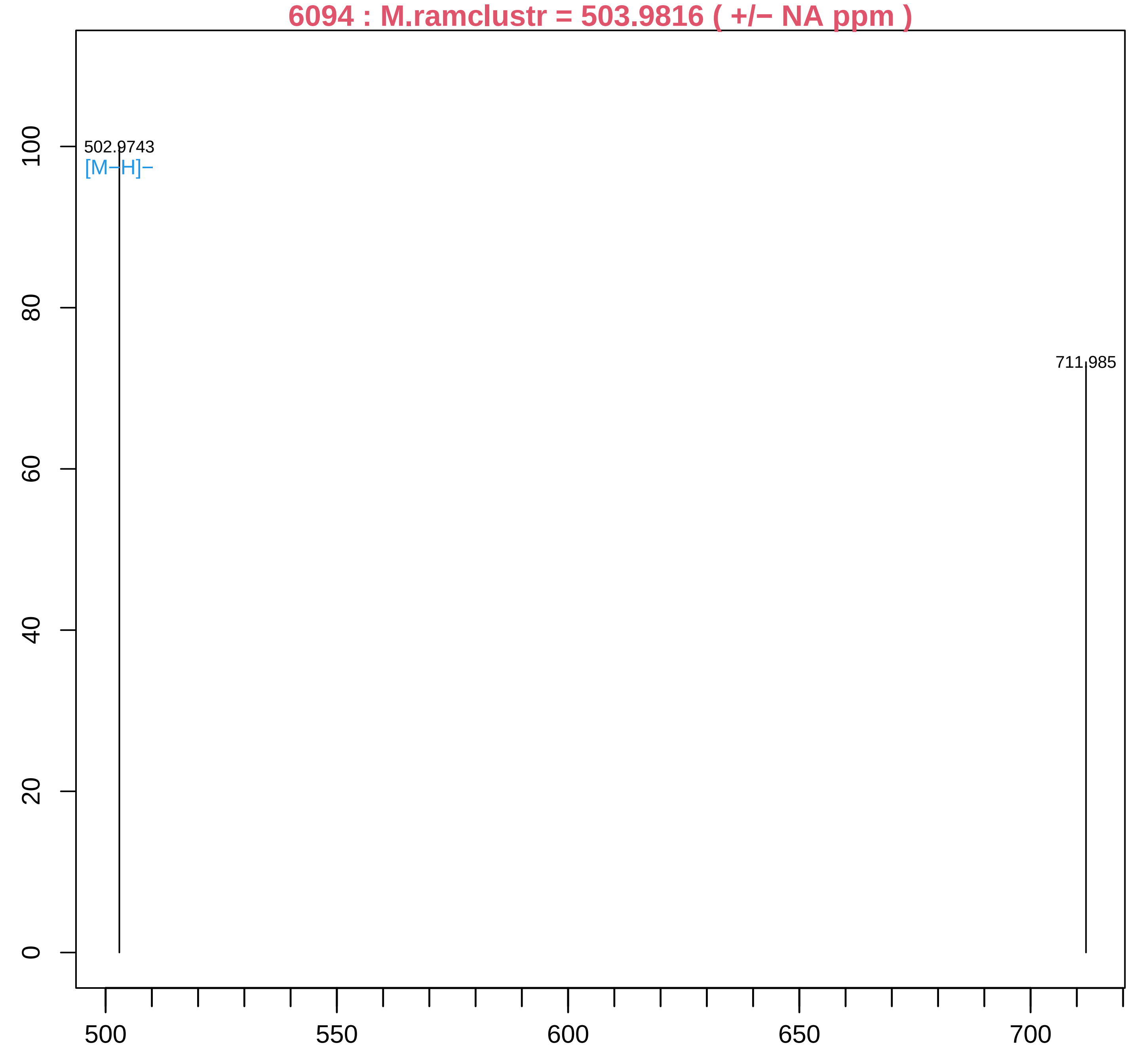

6094 : M.ramclustr = 503.9816 (+/− NA ppm)
502.9743
[M−H]−
711.985
0
20
40
60
80
100
500
550
600
650
700

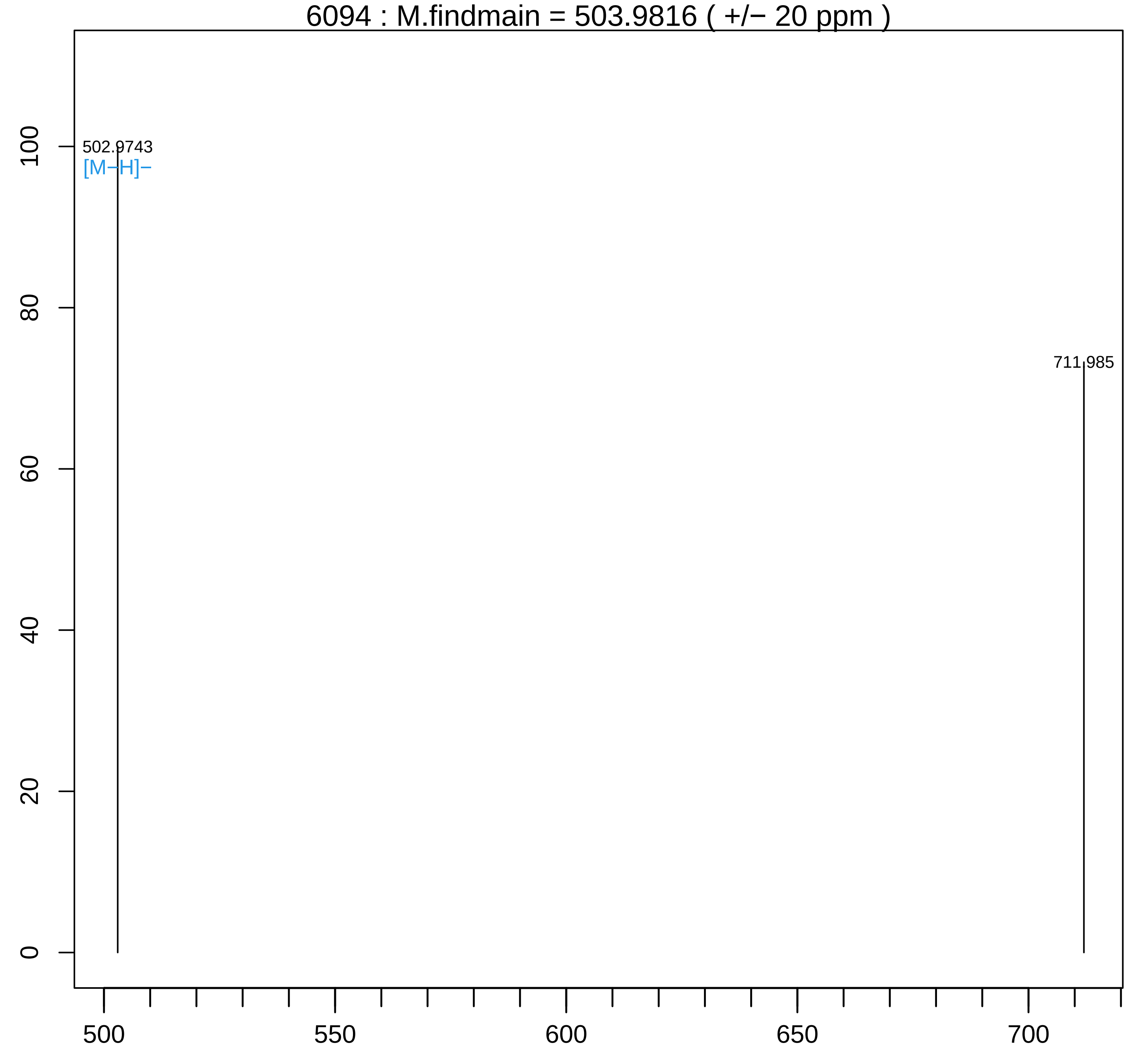

6094 : M.findmain = 503.9816 (+/− 20 ppm)
502.9743
[M−H]−
711.985
0
20
40
60
80
100
500
550
600
650
700

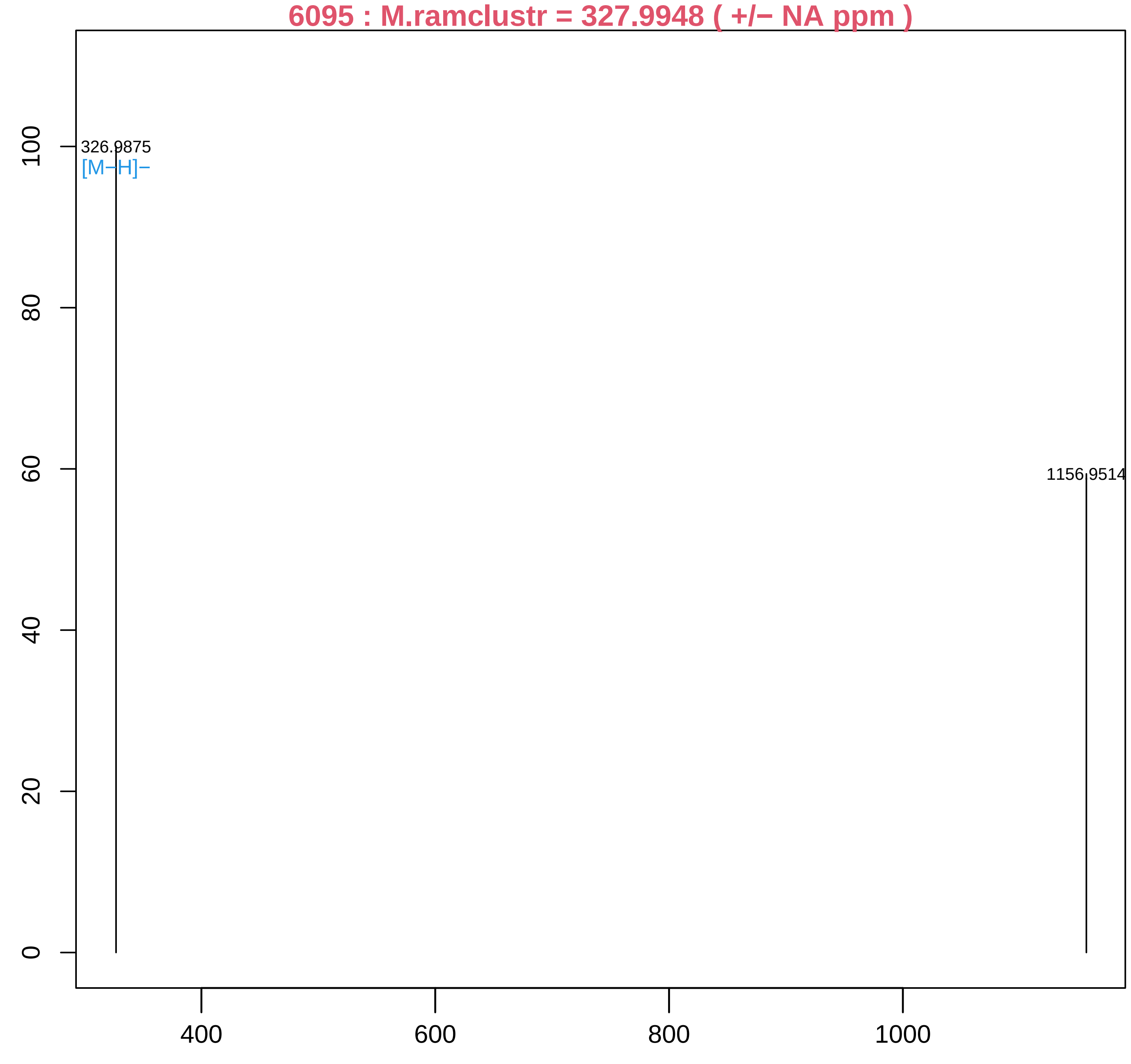
6095 : M.ramclustr = 327.9948 (+/− NA ppm)
326.9875
[M−H]−
1156.9514
0
20
40
60
80
100
400
600
800
1000

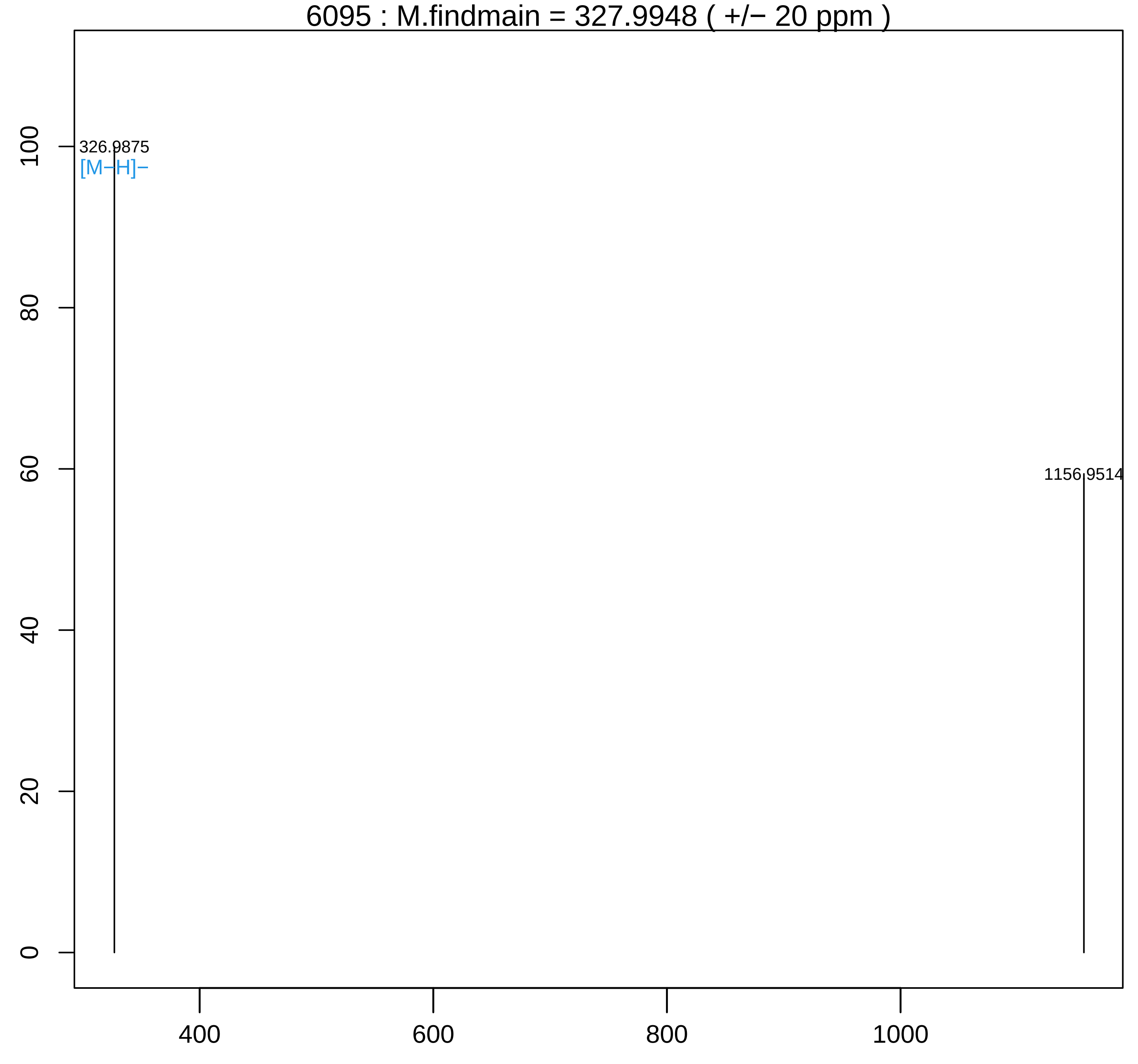
6095 : M.findmain = 327.9948 (+/− 20 ppm)
326.9875
[M−H]−
1156.9514
0
20
40
60
80
100
400
600
800
1000

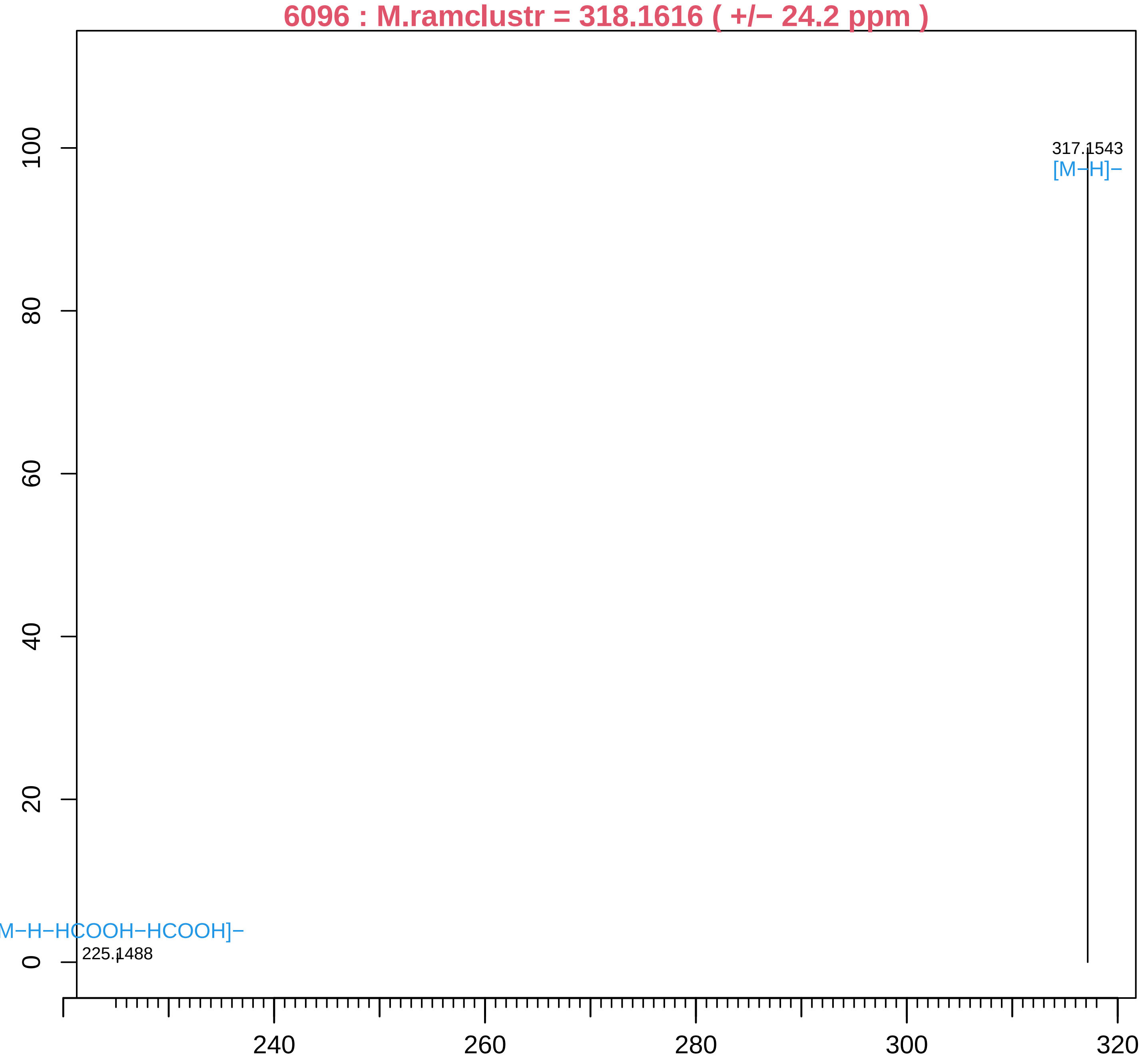
6096 : M.ramclustr = 318.1616 (+/− 24.2 ppm)
317.1543
[M−H]−
225.1488
[M−H−HCOOH−HCOOH]−
0
20
40
60
80
100
240
260
280
300
320

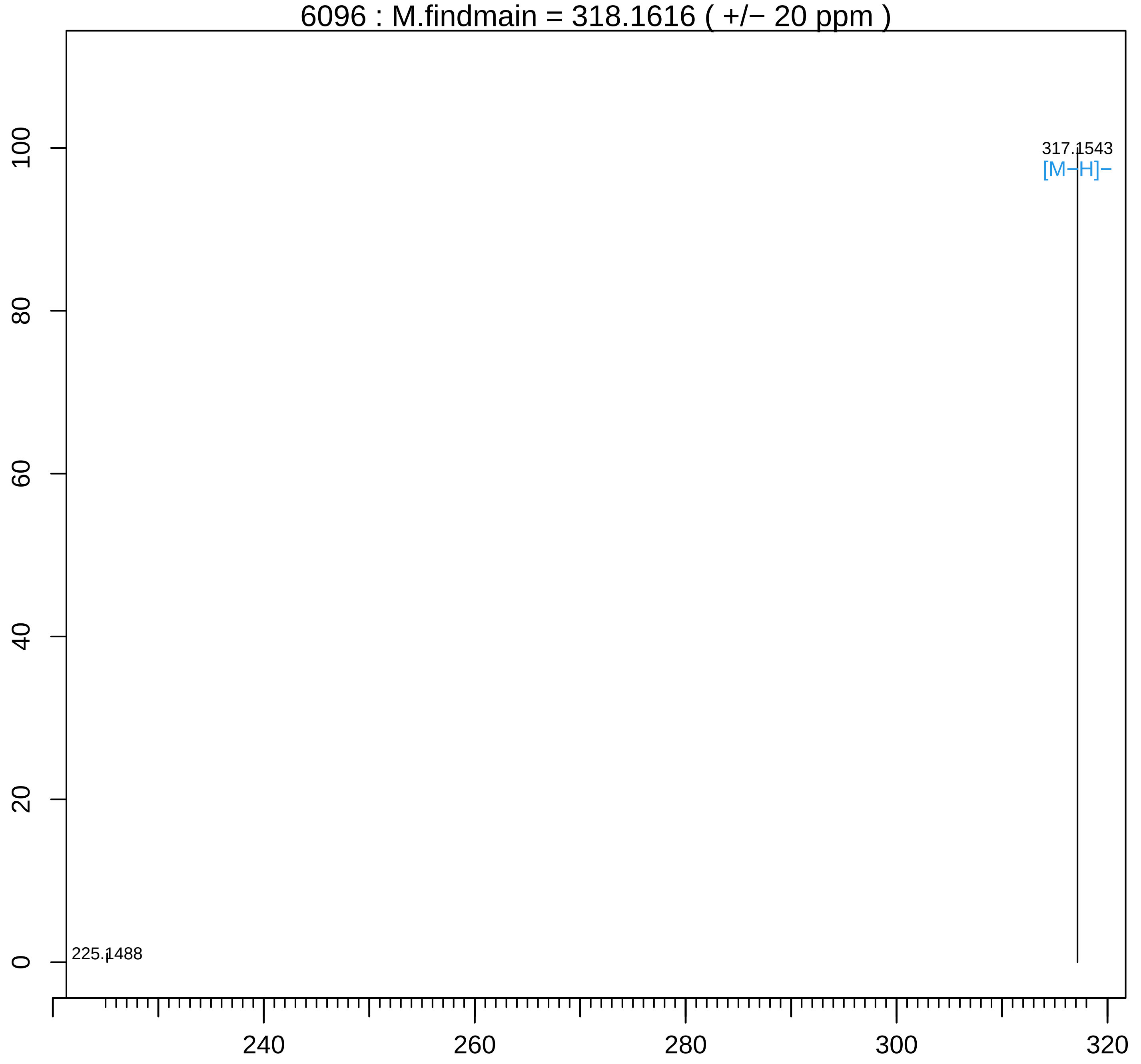
6096 : M.findmain = 318.1616 (+/− 20 ppm)
317.1543
[M−H]−
225.1488
0
20
40
60
80
100
240
260
280
300
320

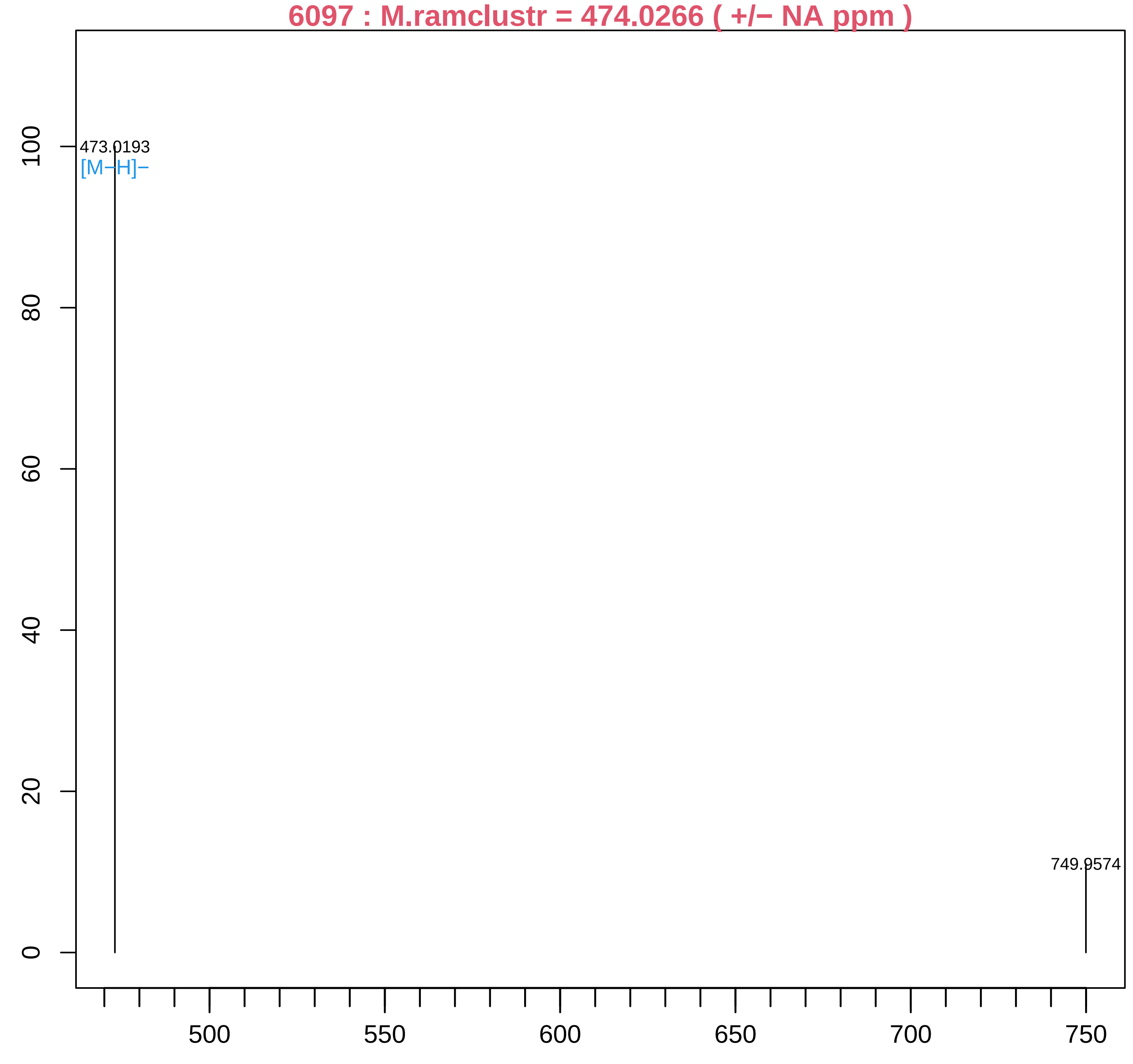
6097 : M.ramclustr = 474.0266 (+/− NA ppm)
473.0193
[M−H]−
749.9574
0
20
40
60
80
100
500
550
600
650
700
750

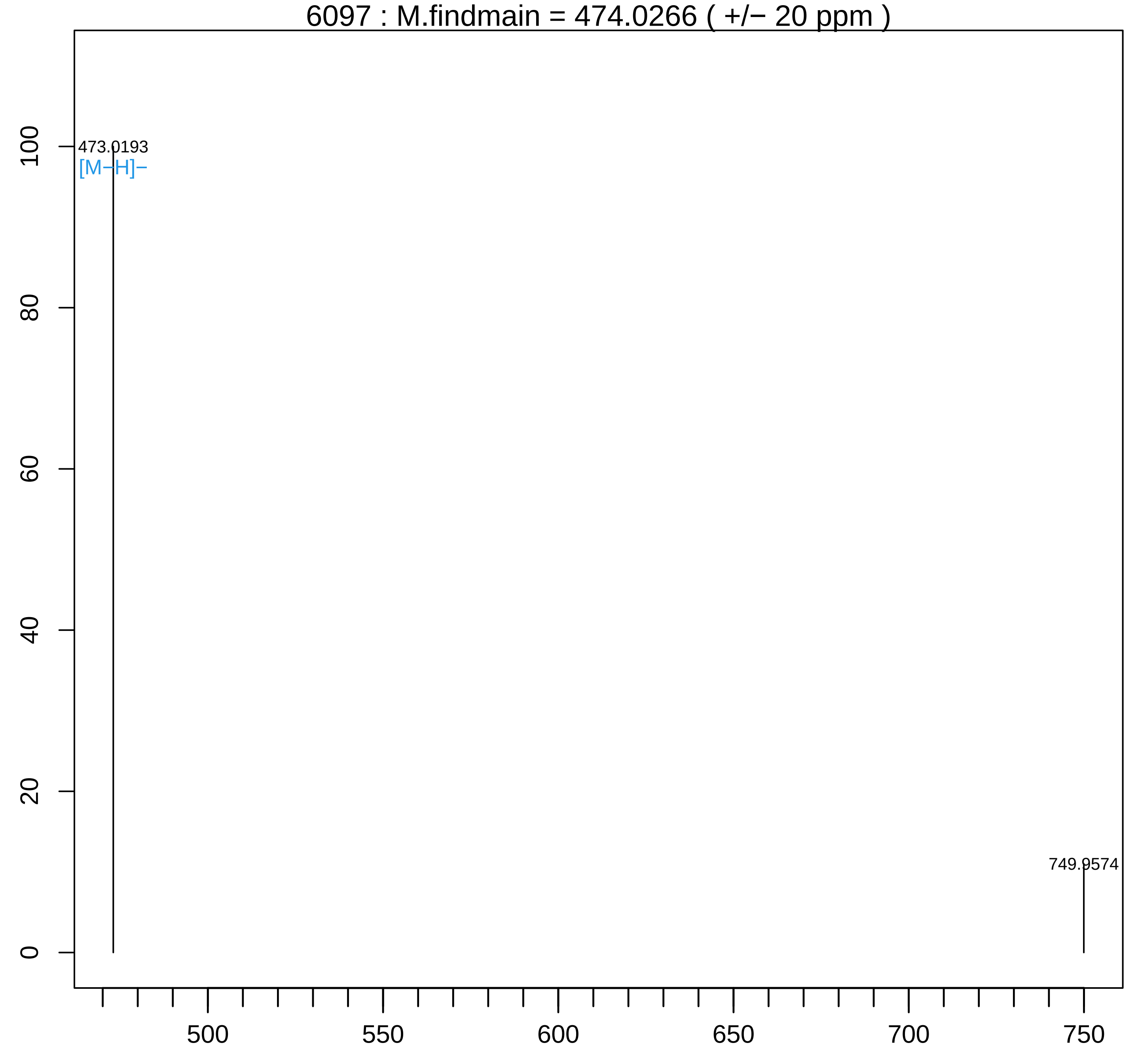
6097 : M.findmain = 474.0266 (+/− 20 ppm)
473.0193
[M−H]−
749.9574
0
20
40
60
80
100
500
550
600
650
700
750

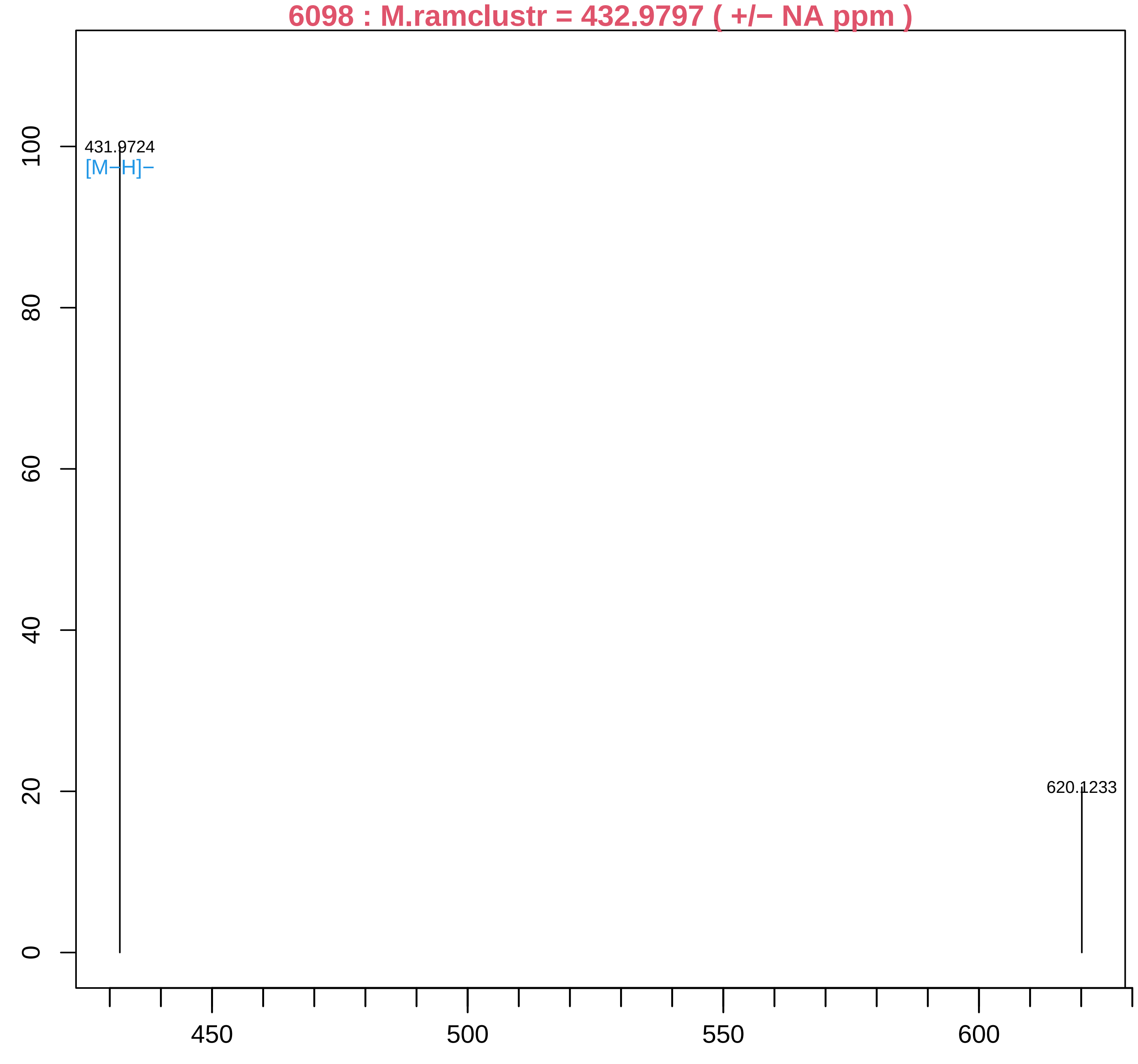
6098 : M.ramclustr = 432.9797 (+/− NA ppm)
431.9724
[M−H]−
620.1233
0
20
40
60
80
100
450
500
550
600

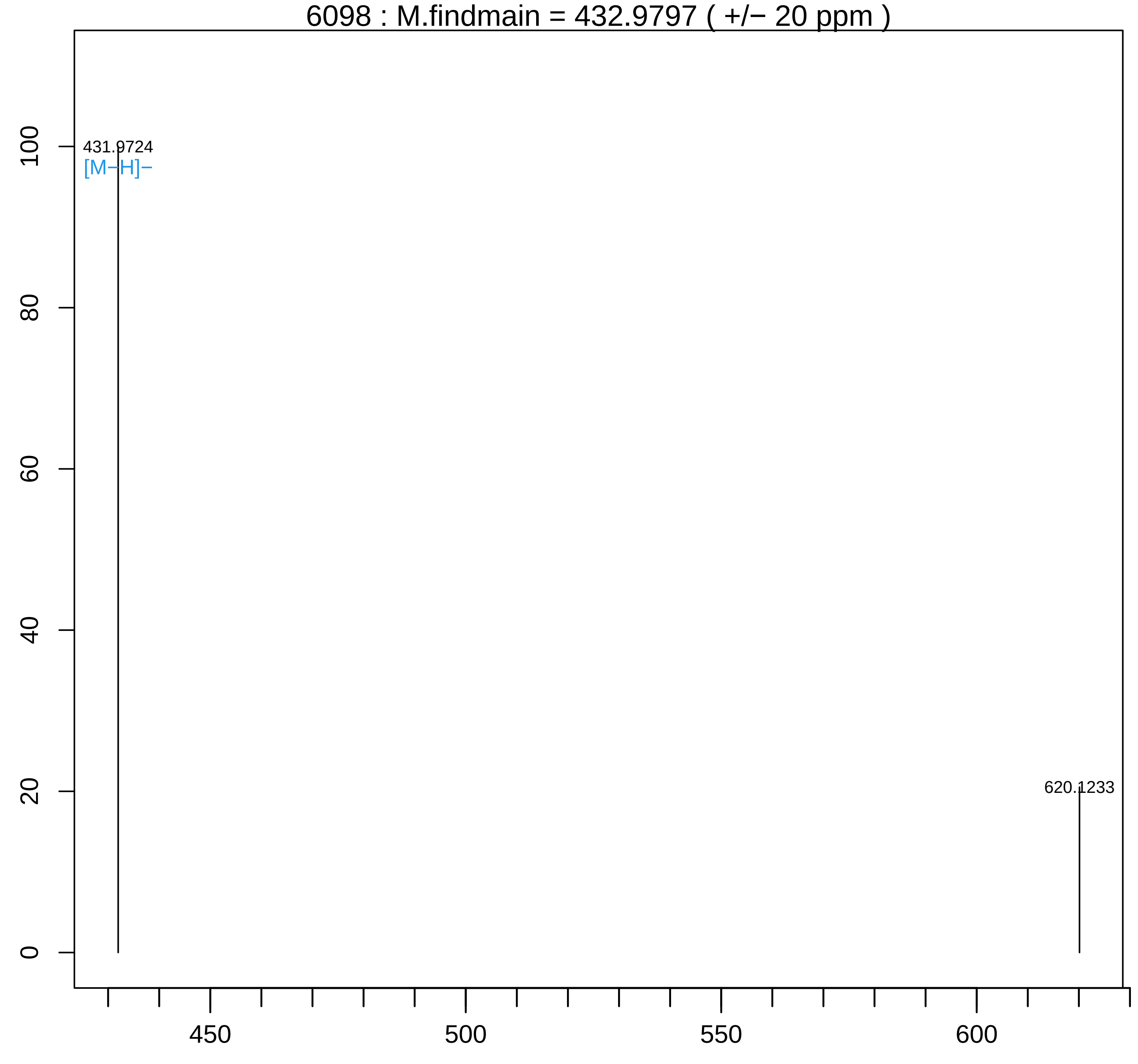
6098 : M.findmain = 432.9797 (+/− 20 ppm)
431.9724
[M−H]−
620.1233
0
20
40
60
80
100
450
500
550
600

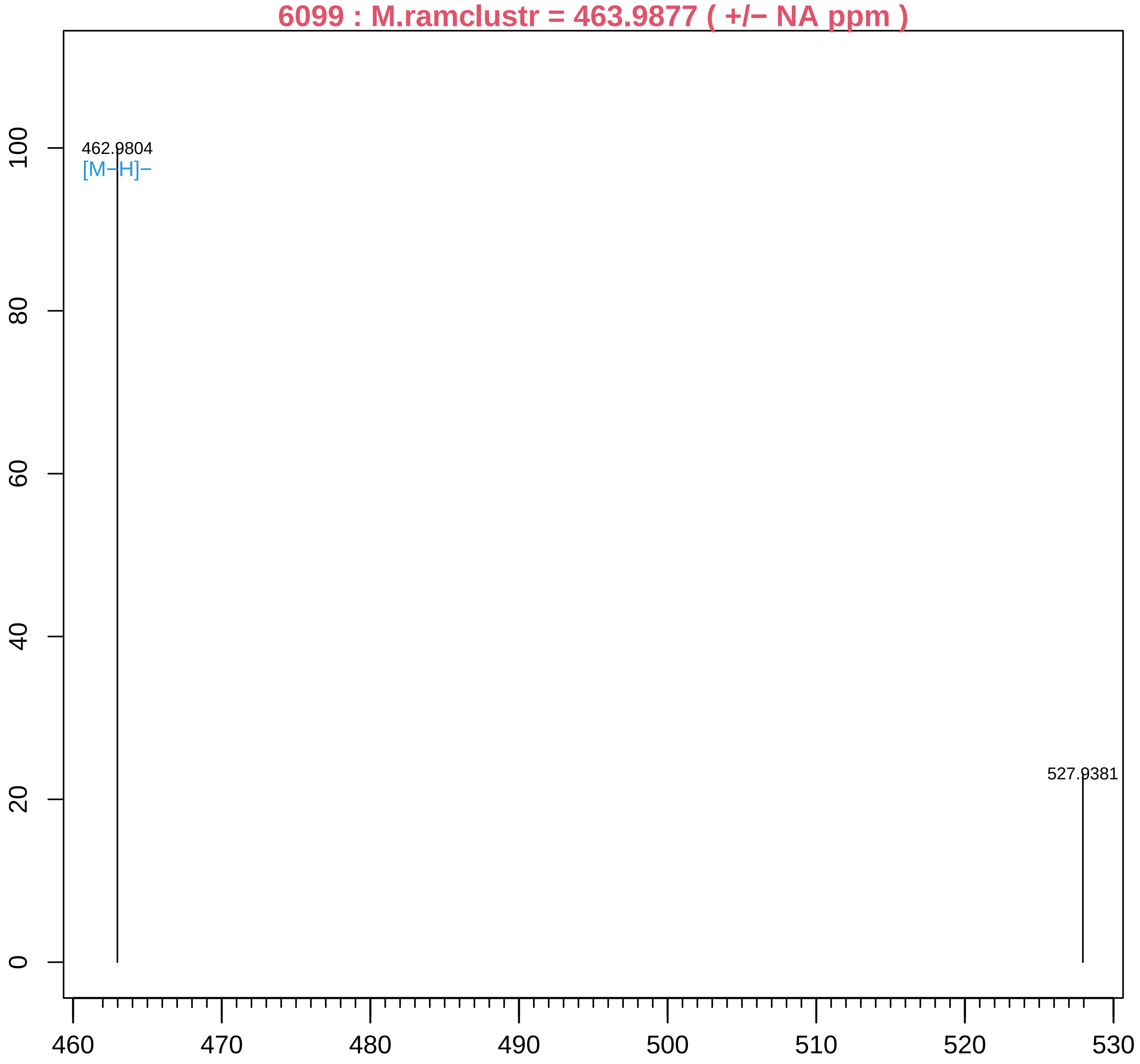
6099 : M.ramclustr = 463.9877 (+/− NA ppm)
462.9804
[M−H]−
527.9381
0
20
40
60
80
100
460
470
480
490
500
510
520
530

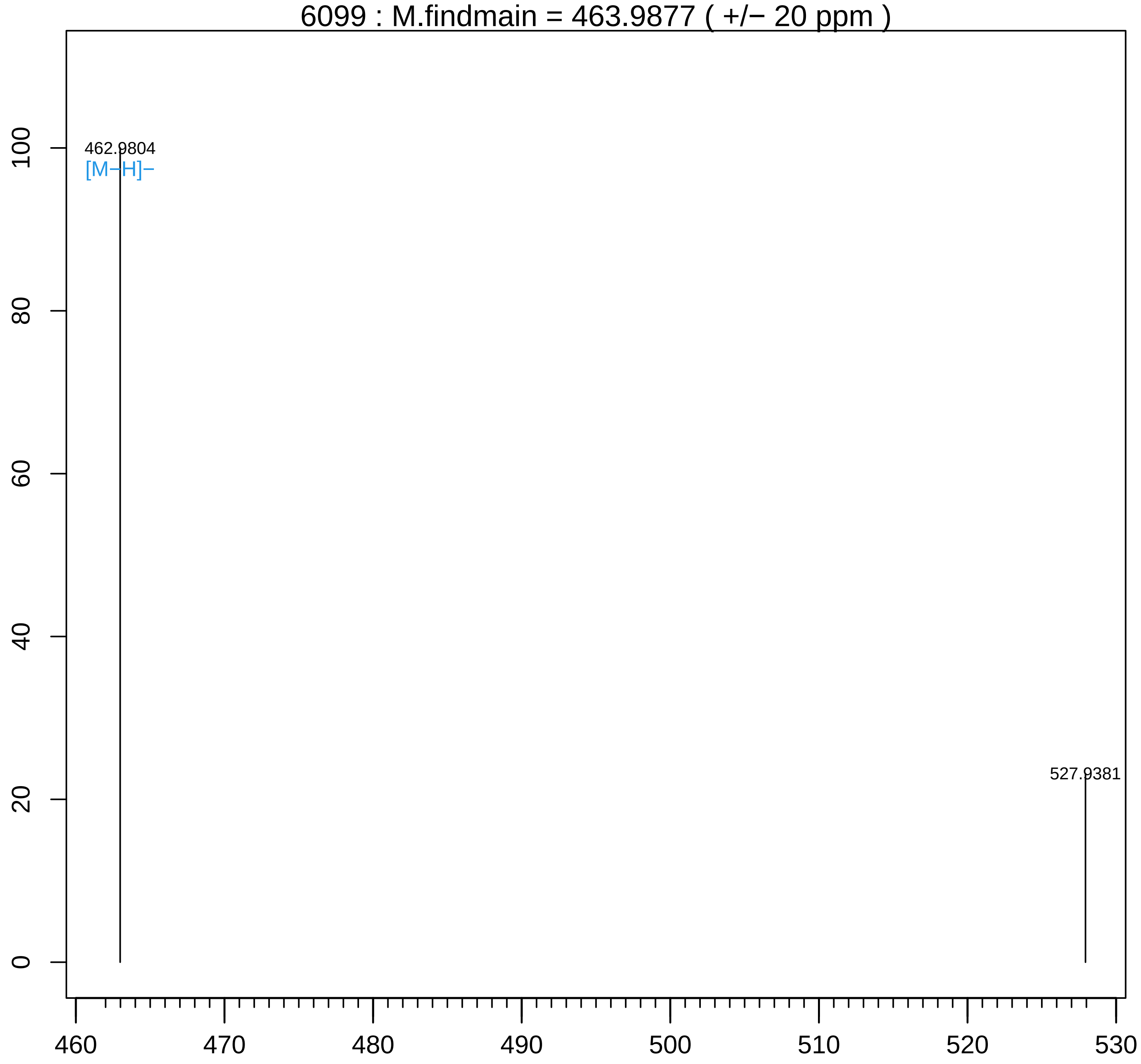
6099 : M.findmain = 463.9877 (+/− 20 ppm)
462.9804
[M−H]−
527.9381
0
20
40
60
80
100
460
470
480
490
500
510
520
530

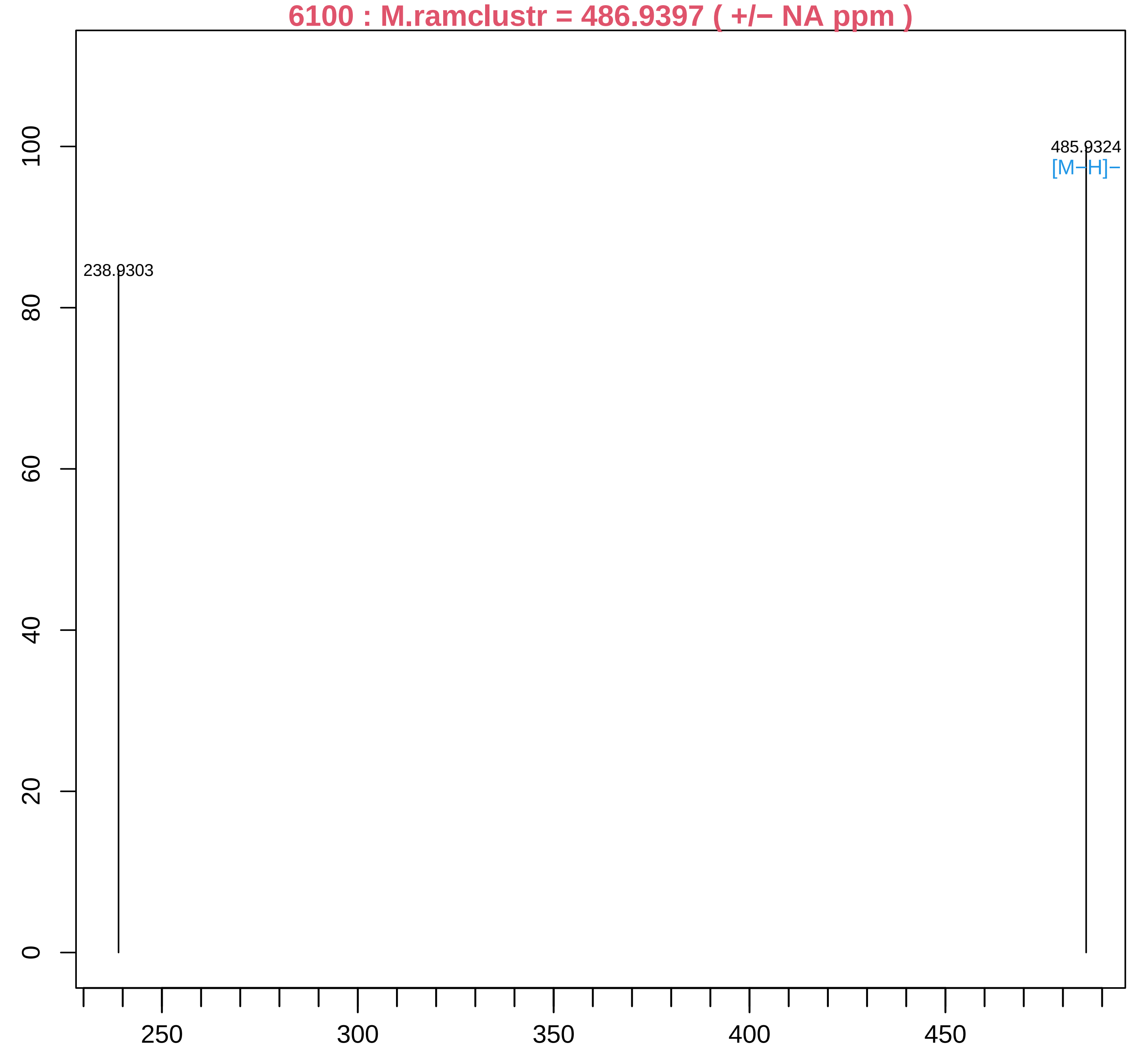

6100 : M.ramclustr = 486.9397 (+/− NA ppm)
485.9324
[M−H]−
238.9303
0
20
40
60
80
100
250
300
350
400
450

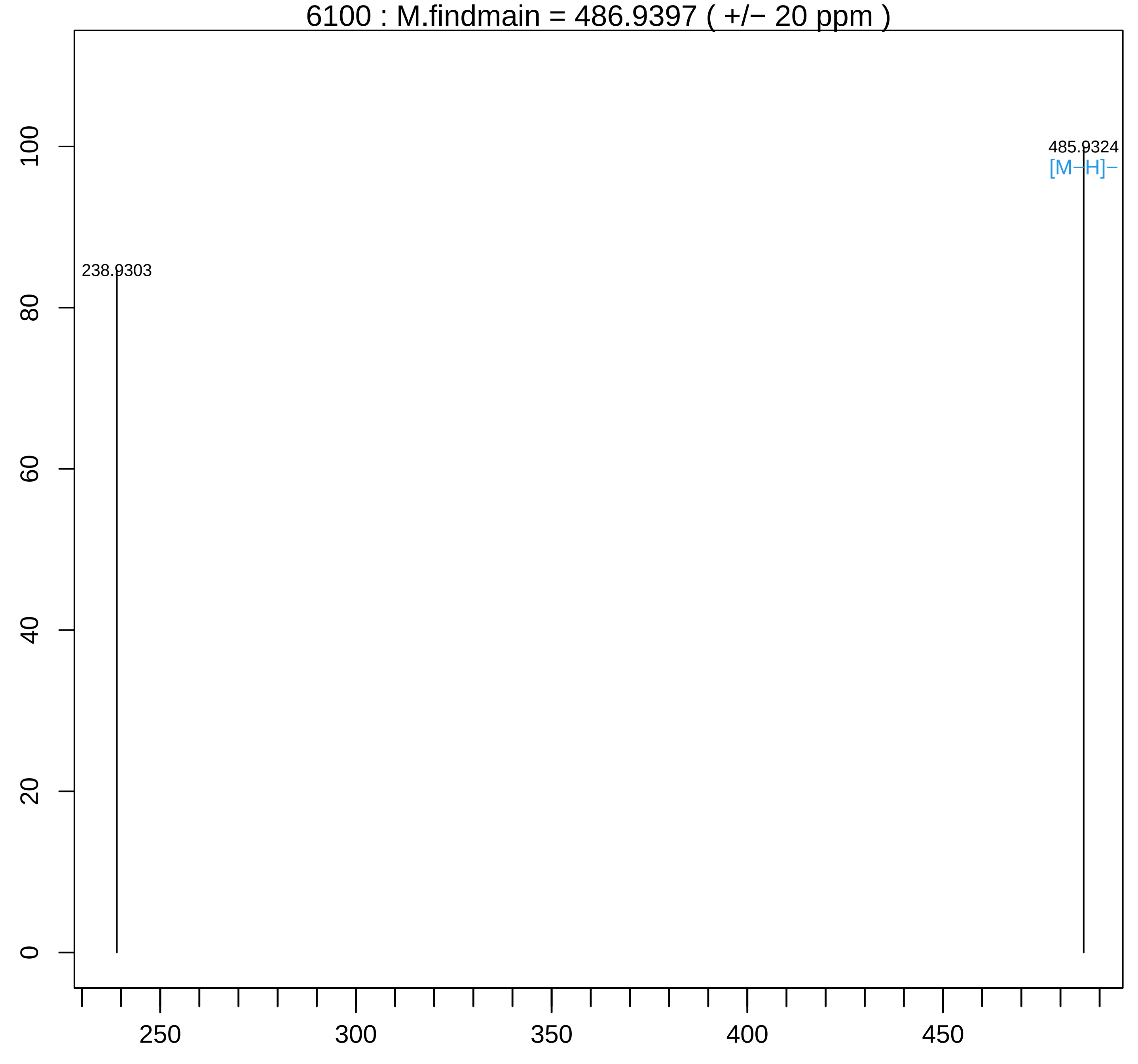

6100 : M.findmain = 486.9397 (+/− 20 ppm)
485.9324
[M−H]−
238.9303
0
20
40
60
80
100
250
300
350
400
450

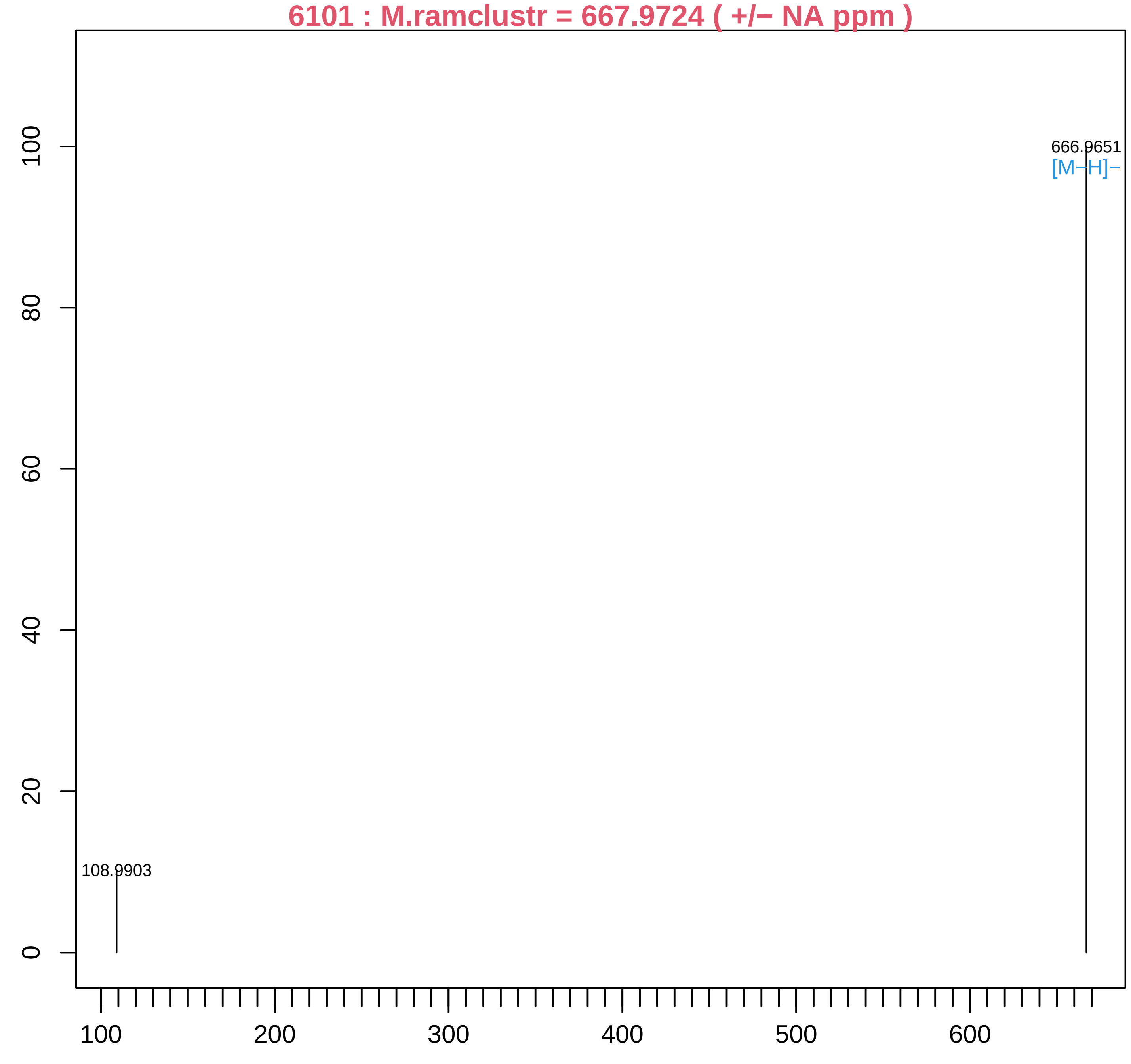
6101 : M.ramclustr = 667.9724 (+/− NA ppm)
666.9651
[M−H]−
108.9903
0
20
40
60
80
100
100
200
300
400
500
600

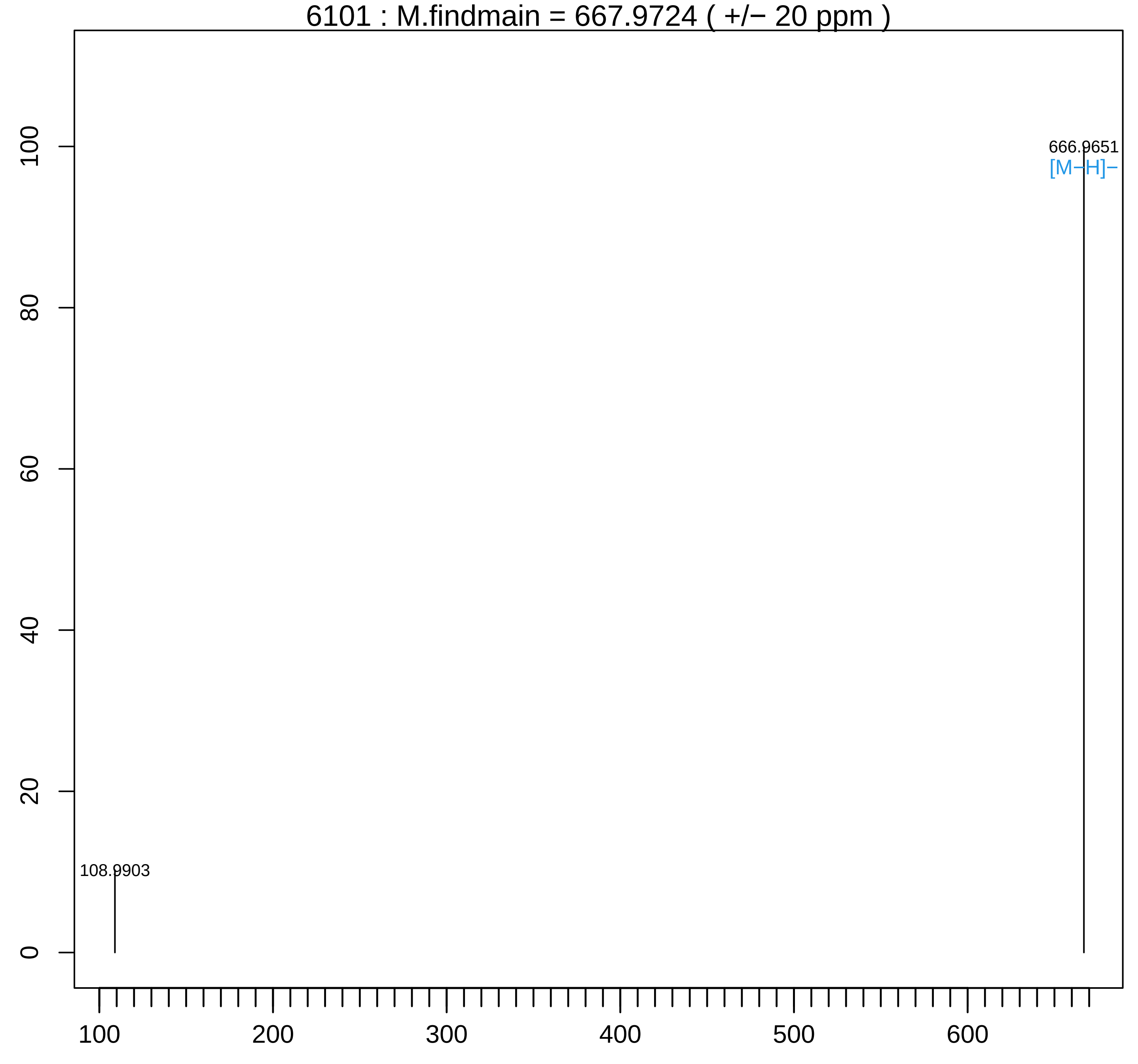
6101 : M.findmain = 667.9724 (+/− 20 ppm)
666.9651
[M−H]−
108.9903
0
20
40
60
80
100
100
200
300
400
500
600

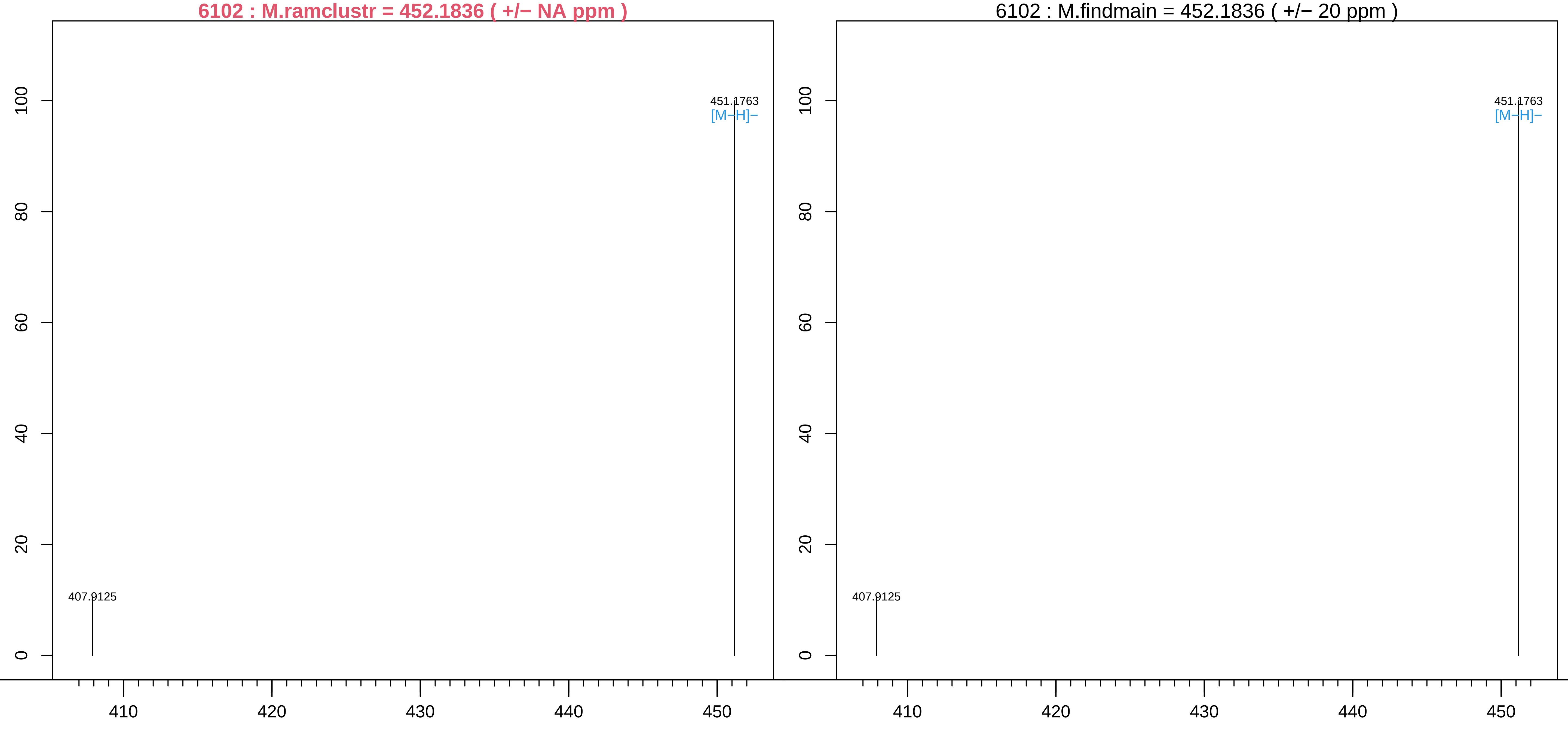
6102 : M.ramclustr = 452.1836 (+/− NA ppm)
451.1763
[M−H]−
407.9125
6102 : M.findmain = 452.1836 (+/− 20 ppm)
451.1763
[M−H]−
407.9125
0
20
40
60
80
100
410
420
430
440
450

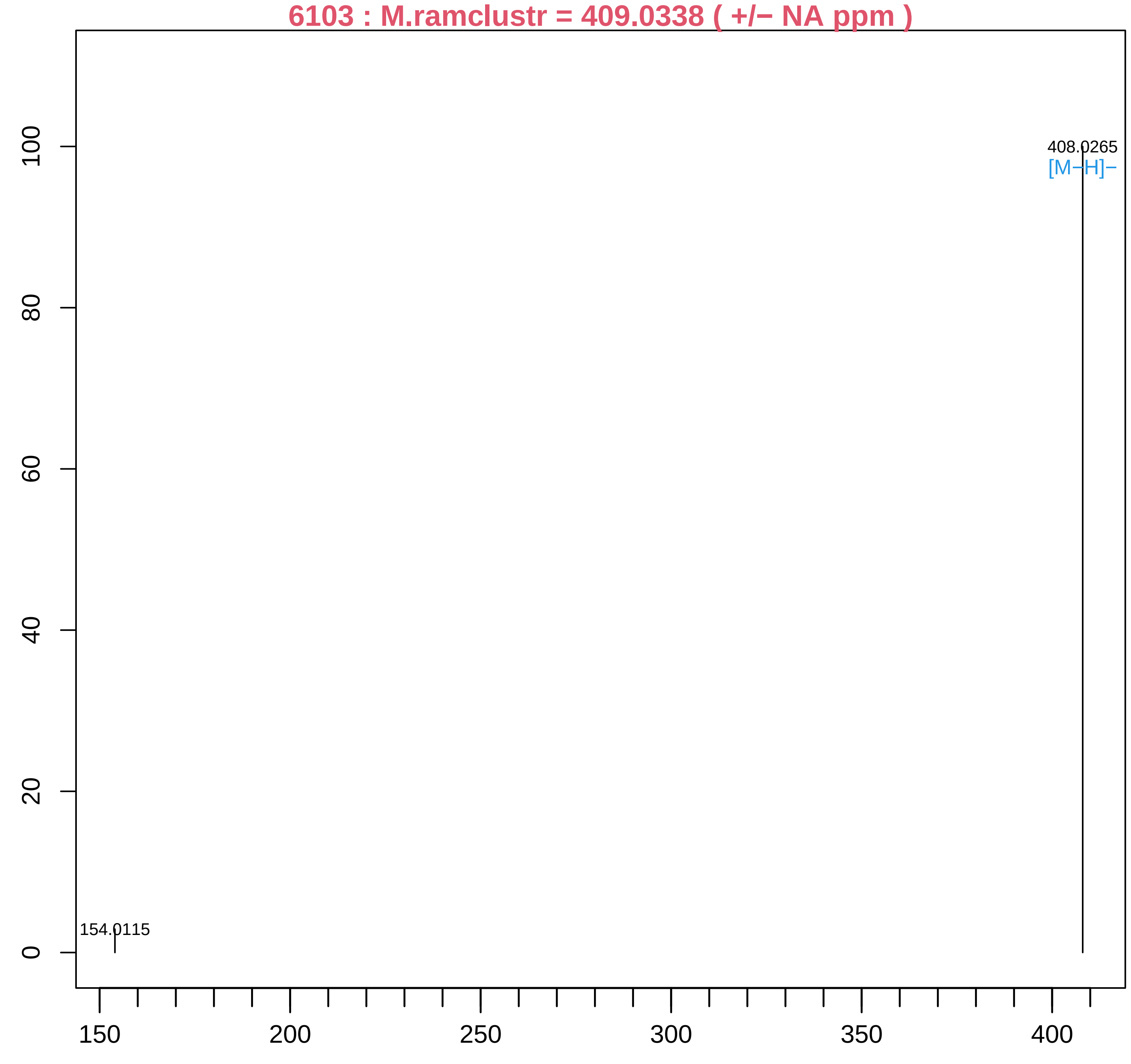
6103 : M.ramclustr = 409.0338 (+/− NA ppm)
408.0265
[M−H]−
154.0115
0
20
40
60
80
100
150
200
250
300
350
400

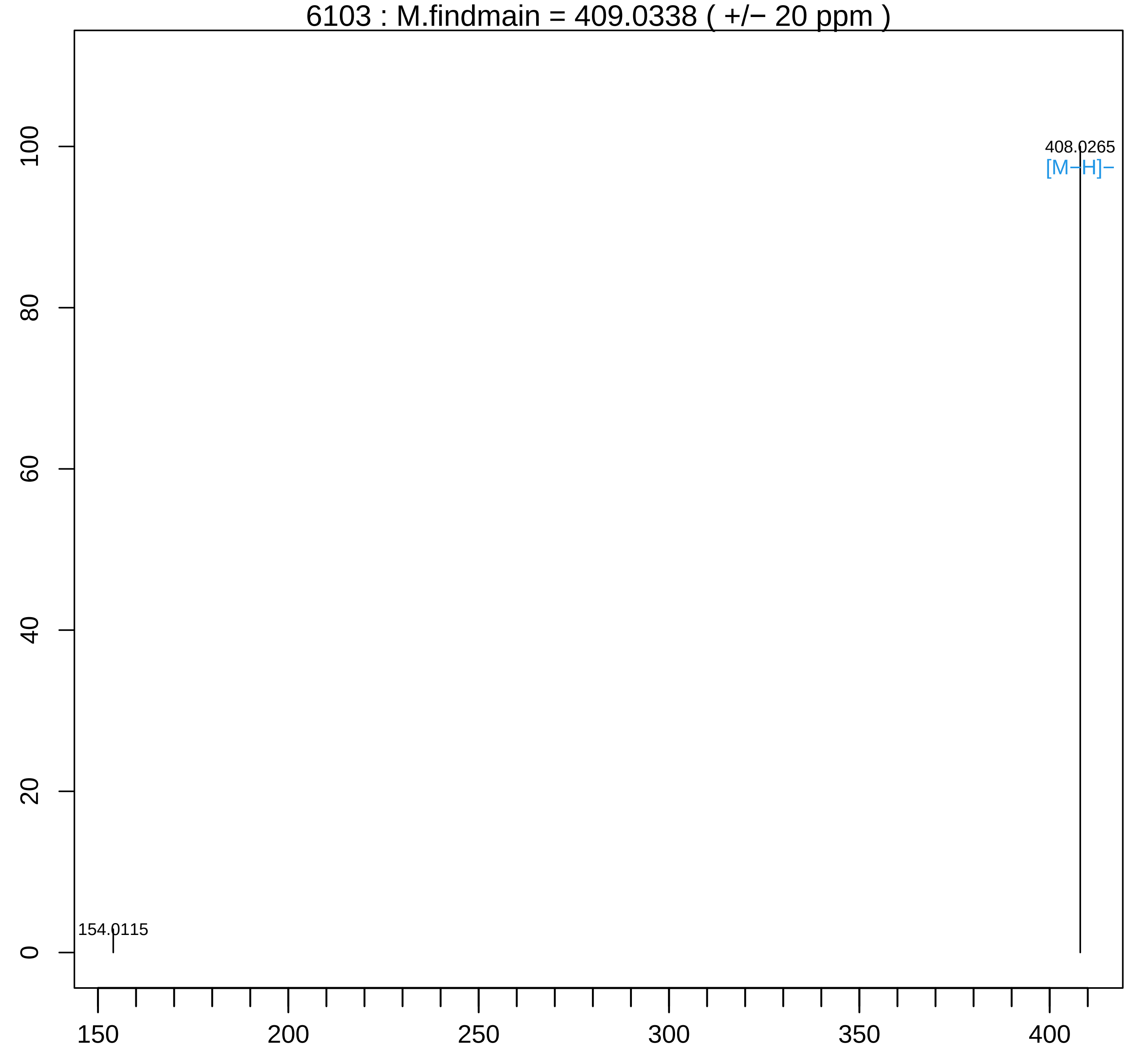
6103 : M.findmain = 409.0338 (+/− 20 ppm)
408.0265
[M−H]−
154.0115
0
20
40
60
80
100
150
200
250
300
350
400

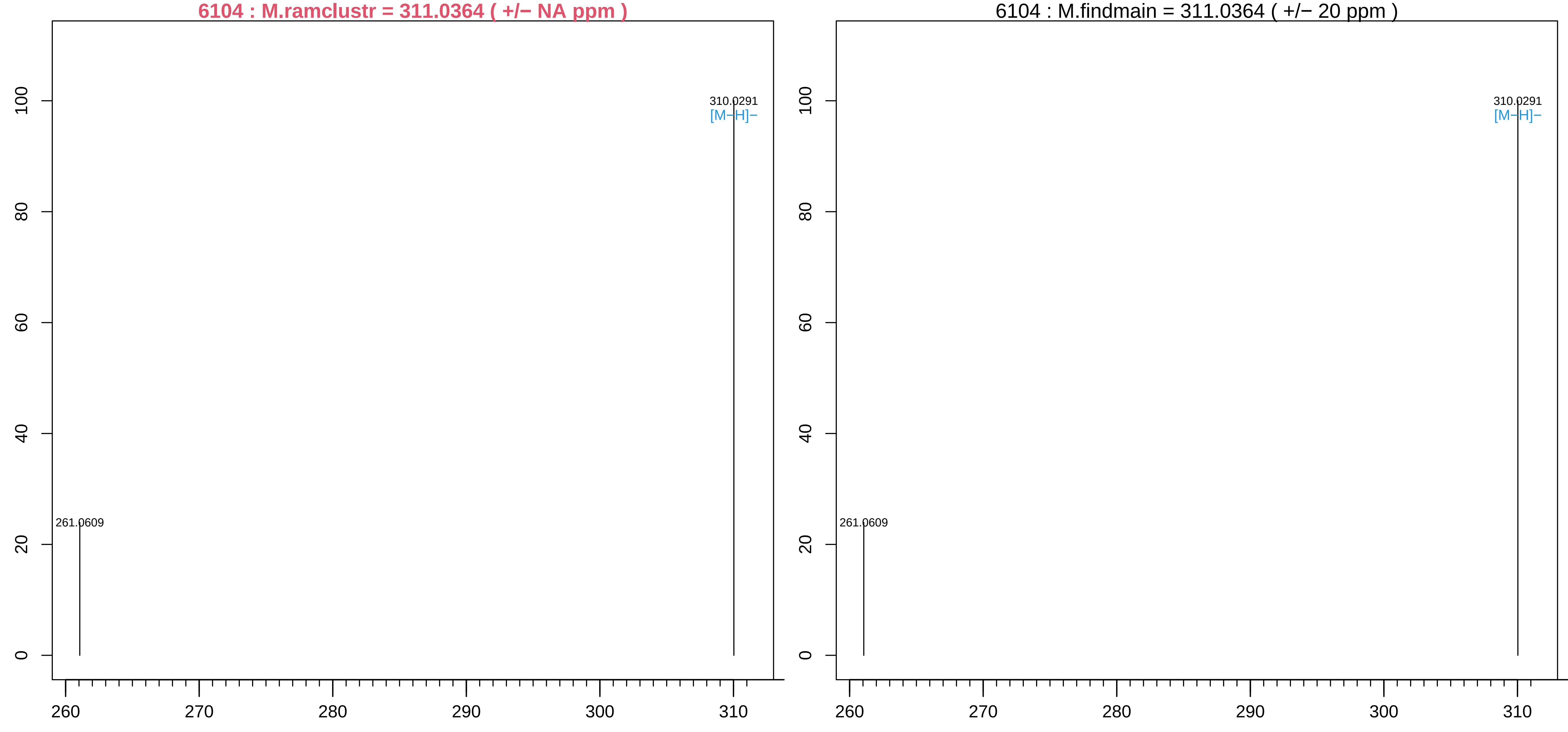
6104 : M.ramclustr = 311.0364 (+/− NA ppm)
6104 : M.findmain = 311.0364 (+/− 20 ppm)
310.0291
[M−H]−
261.0609
0
20
40
60
80
100
260
270
280
290
300
310

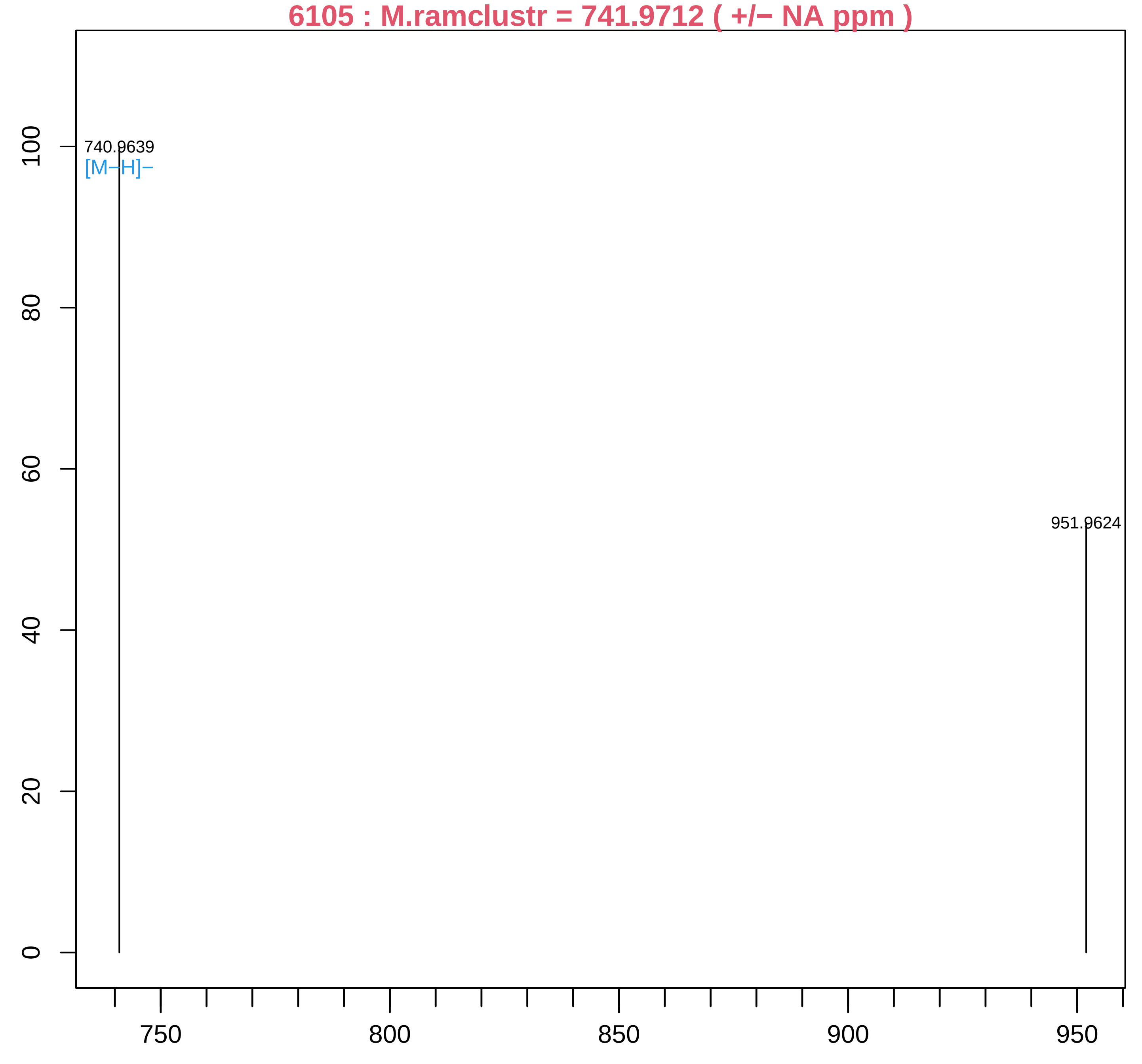
6105 : M.ramclustr = 741.9712 (+/− NA ppm)
740.9639
[M−H]−
951.9624
0
20
40
60
80
100
750
800
850
900
950

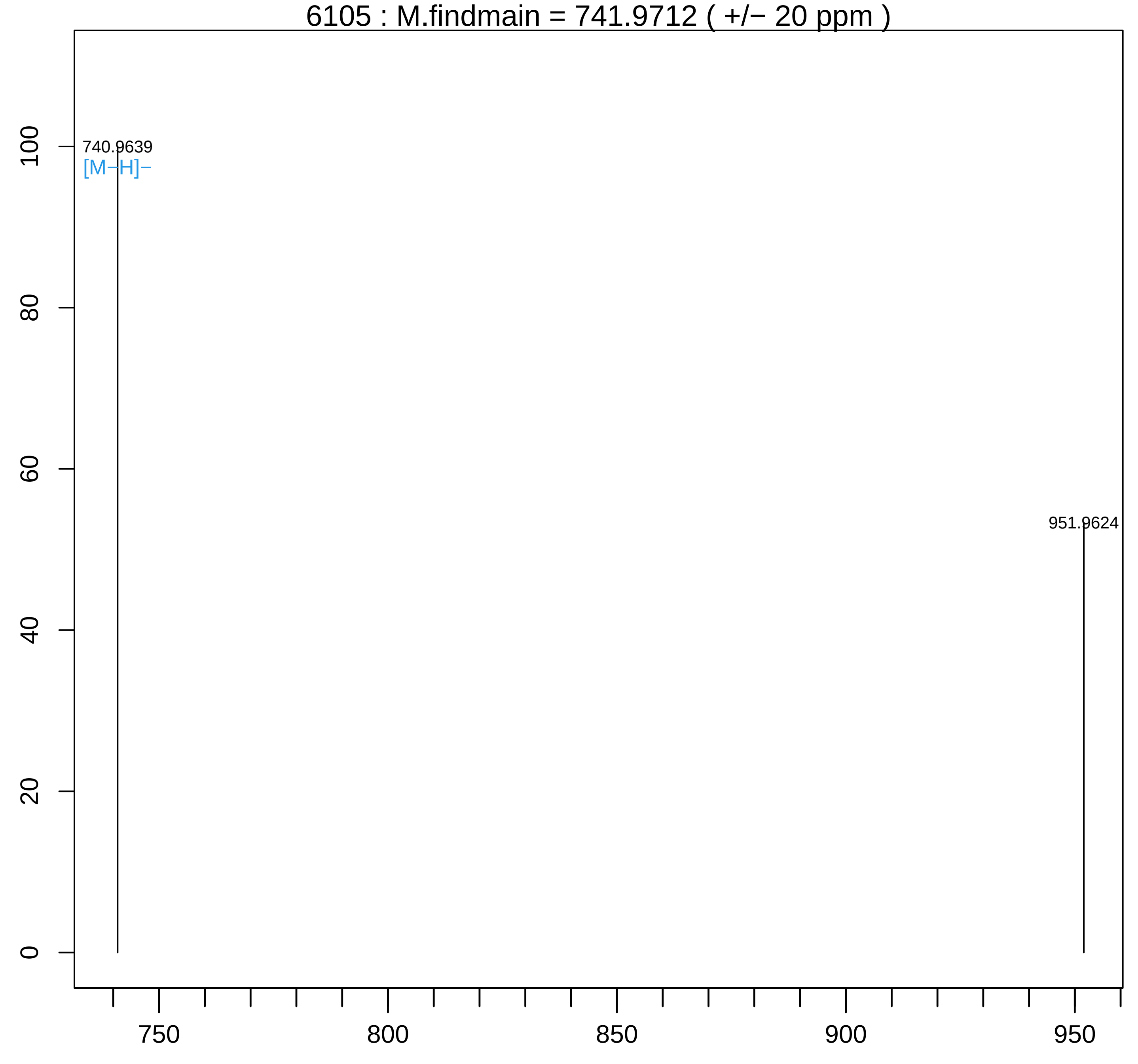
6105 : M.findmain = 741.9712 (+/− 20 ppm)
740.9639
[M−H]−
951.9624
0
20
40
60
80
100
750
800
850
900
950

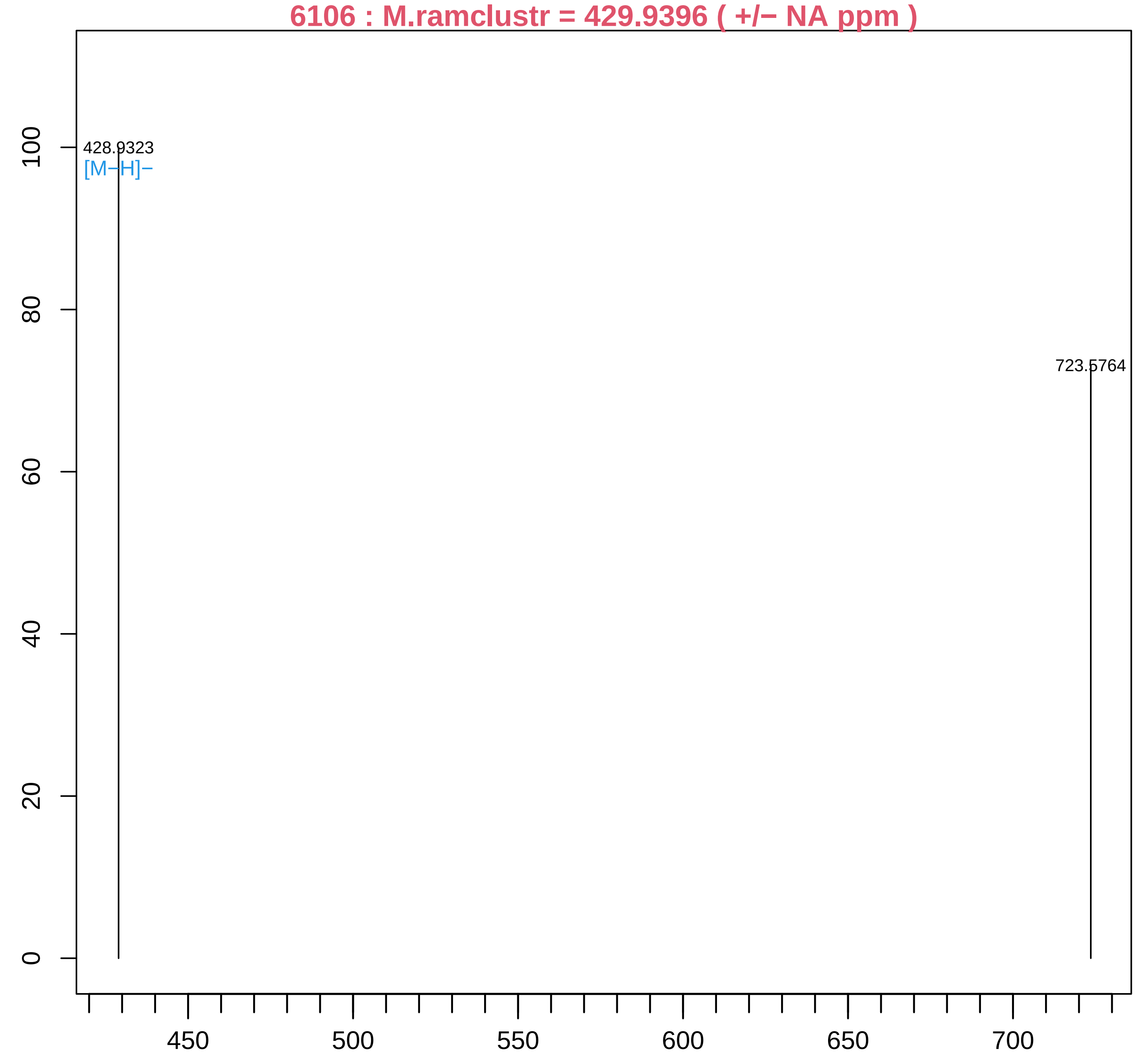
6106 : M.ramclustr = 429.9396 (+/− NA ppm)
428.9323
[M−H]−
723.5764
0
20
40
60
80
100
450
500
550
600
650
700

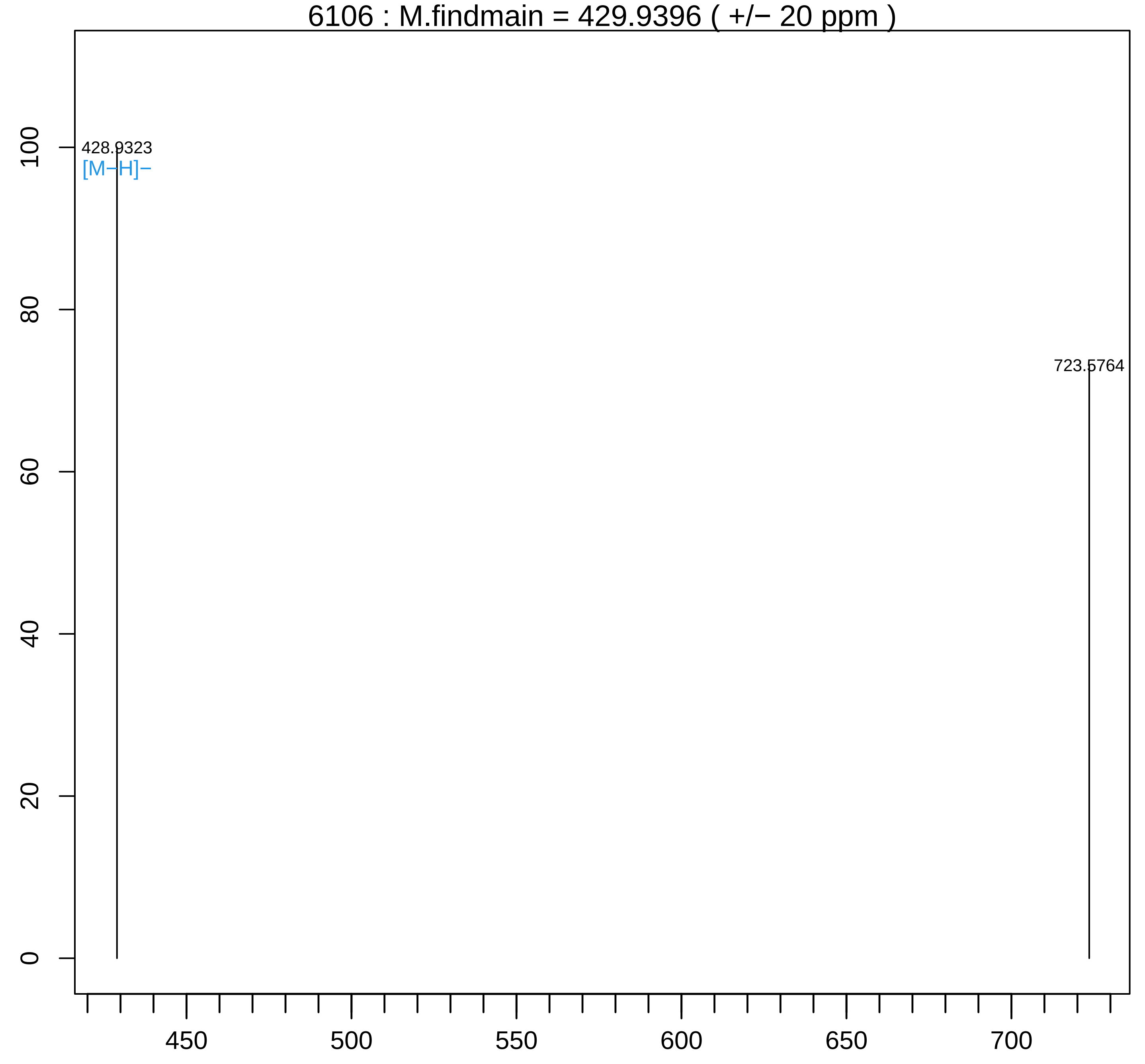
6106 : M.findmain = 429.9396 (+/− 20 ppm)
428.9323
[M−H]−
723.5764
0
20
40
60
80
100
450
500
550
600
650
700

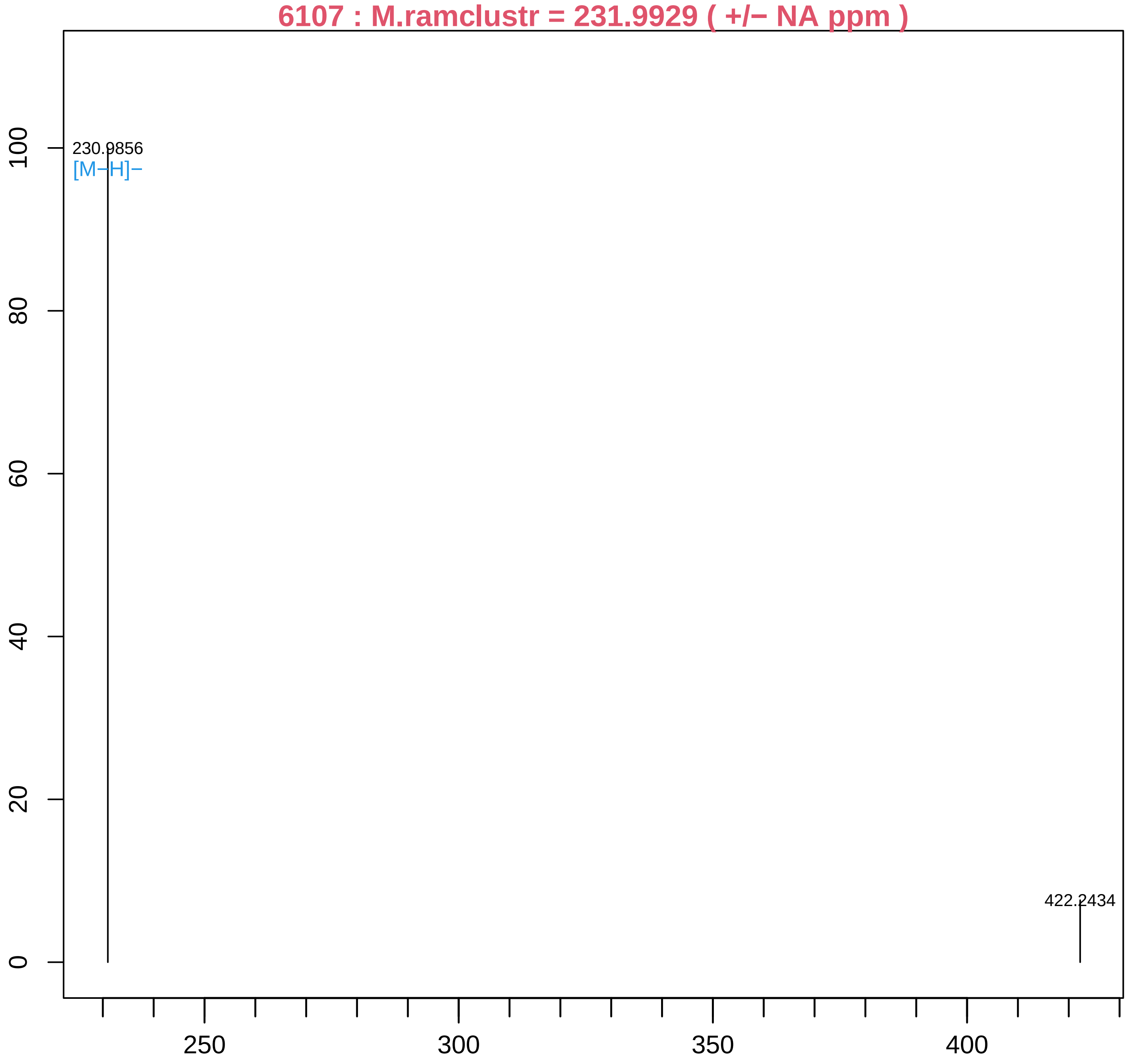
6107 : M.ramclustr = 231.9929 (+/− NA ppm)
230.9856
[M−H]−
422.2434
0
20
40
60
80
100
250
300
350
400

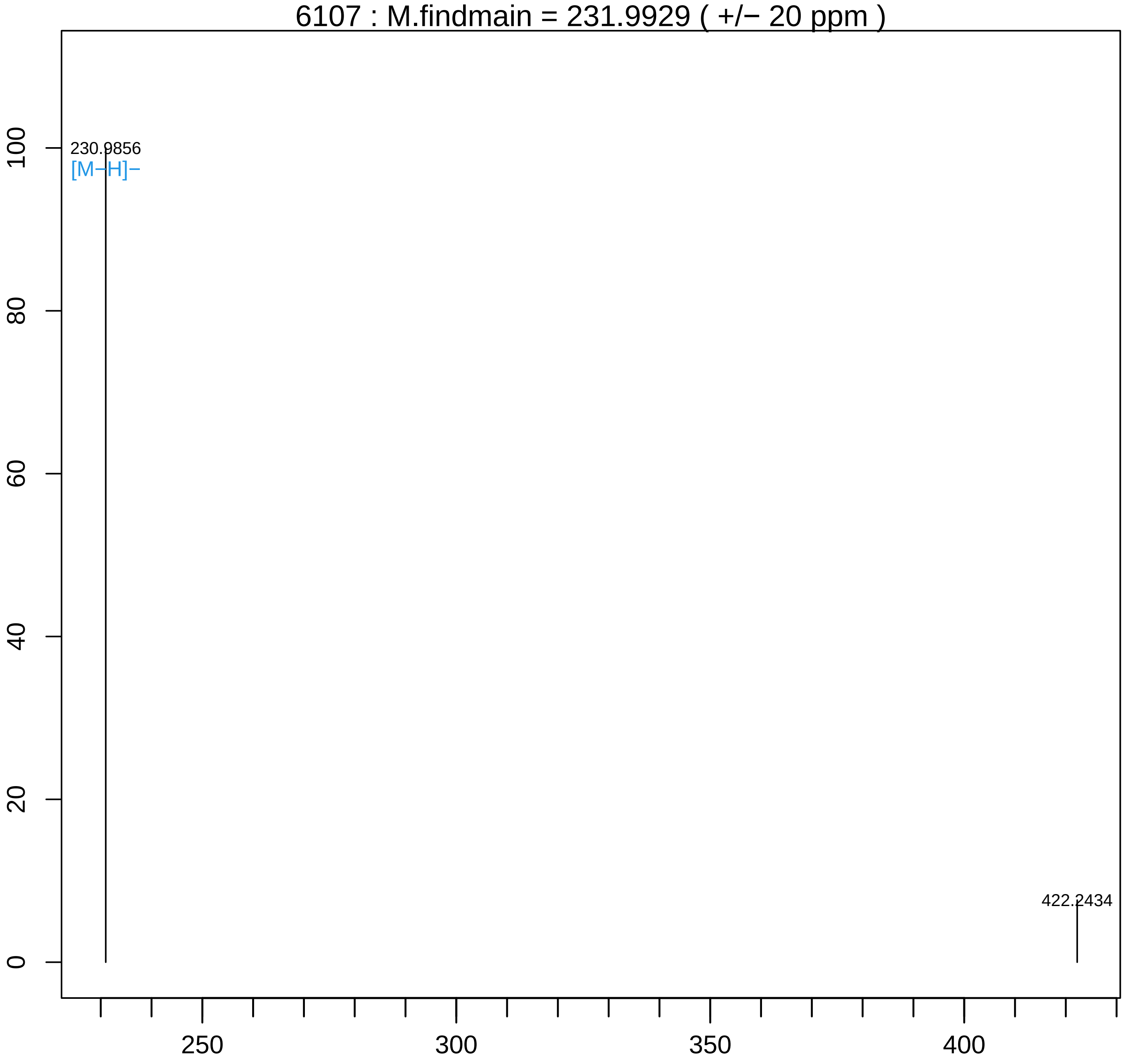
6107 : M.findmain = 231.9929 (+/− 20 ppm)
230.9856
[M−H]−
422.2434
0
20
40
60
80
100
250
300
350
400

6108 : M.ramclustr = 276.9445 (+/− NA ppm)

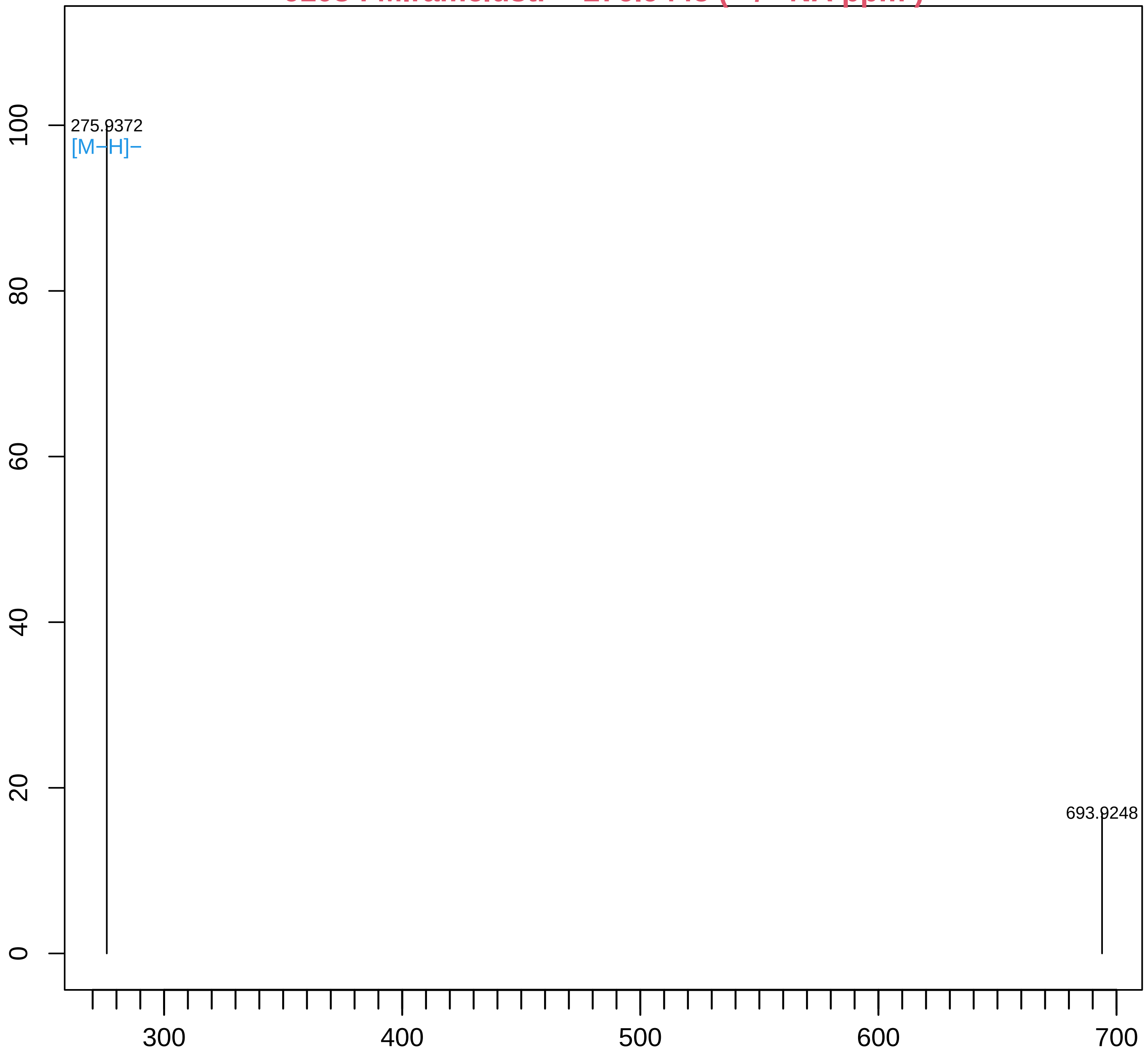

6108 : M.findmain = 276.9445 (+/− 20 ppm)

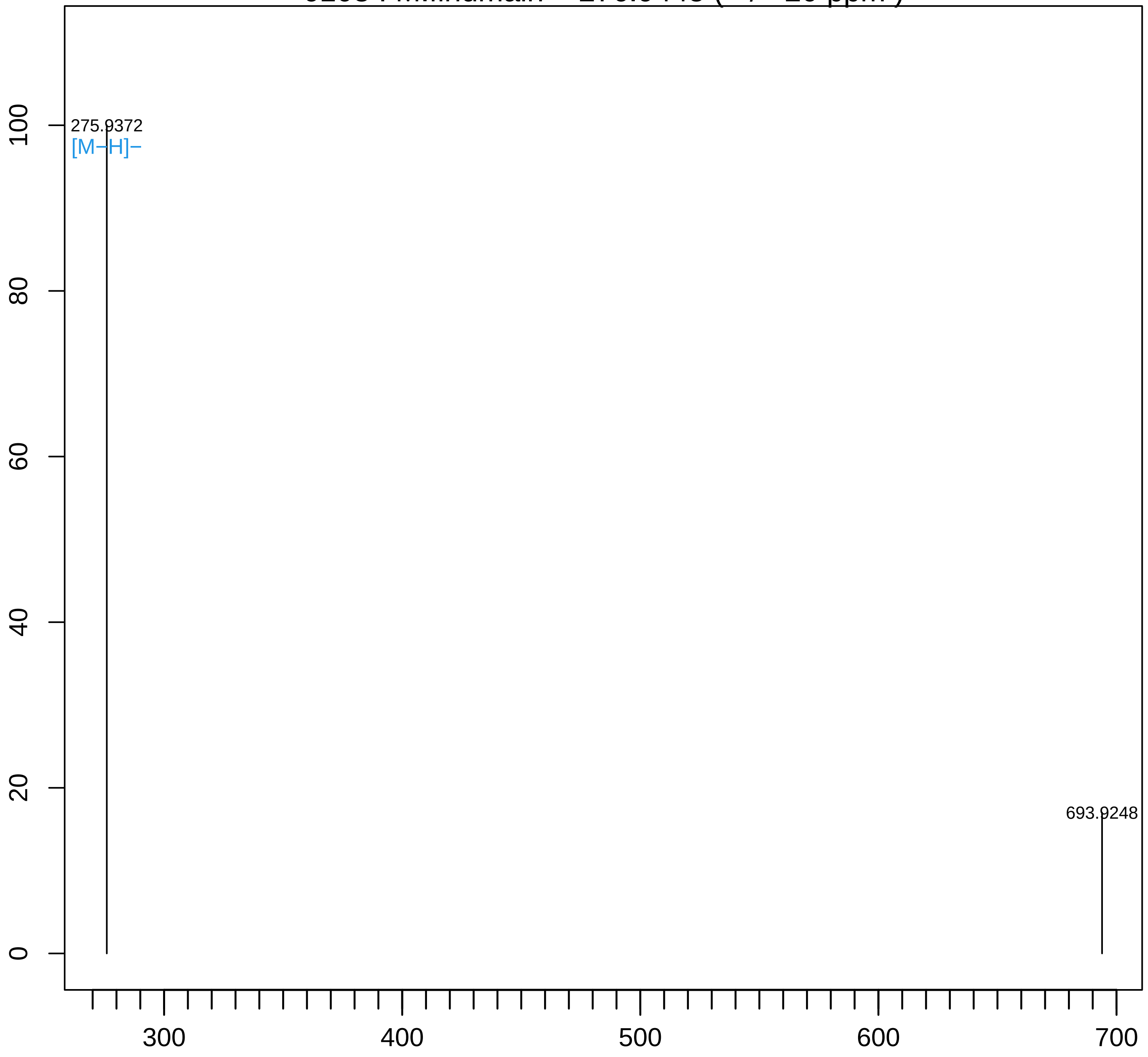

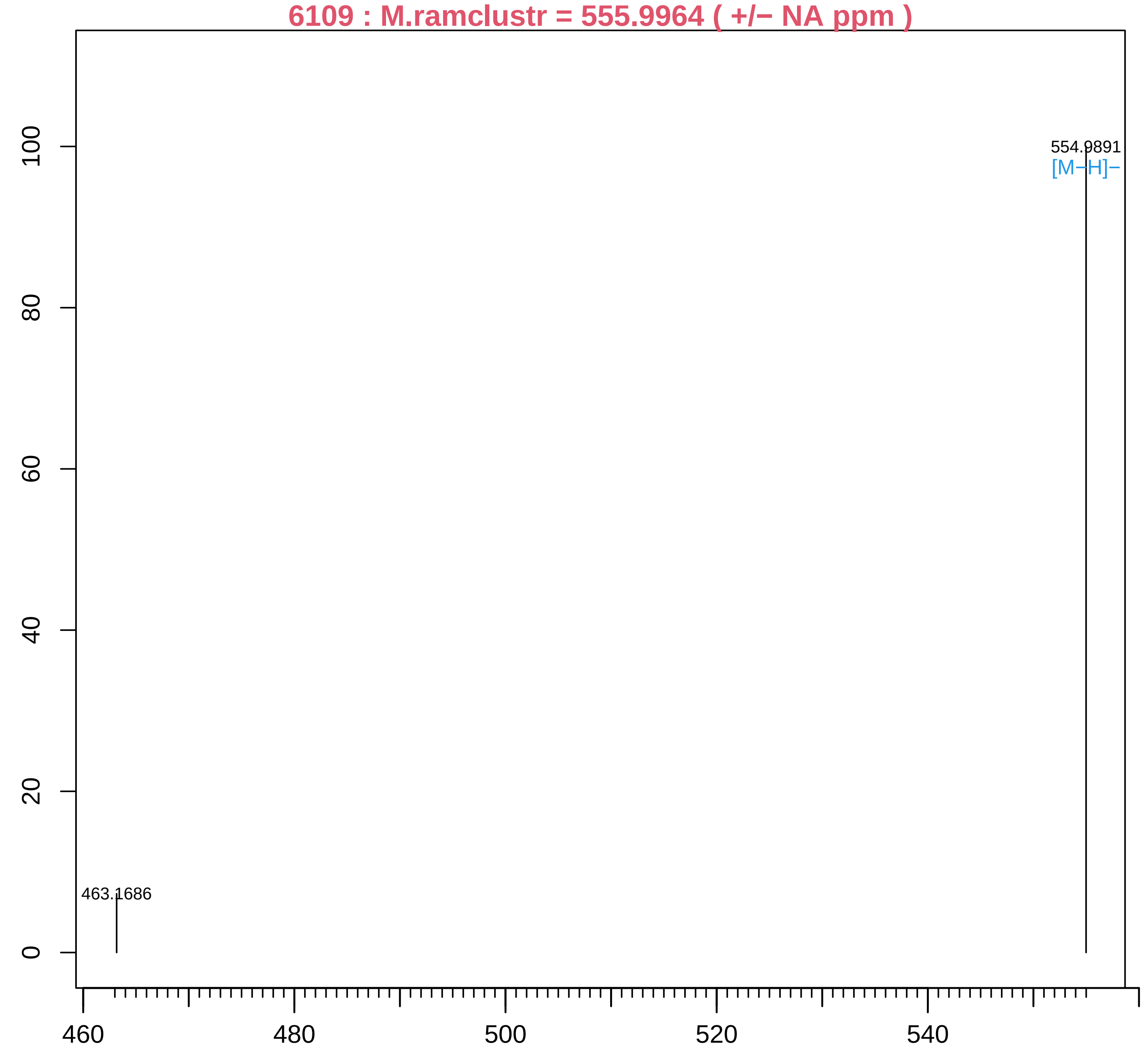
6109 : M.ramclustr = 555.9964 (+/− NA ppm)
554.9891
[M−H]−
463.1686
0
20
40
60
80
100
460
480
500
520
540

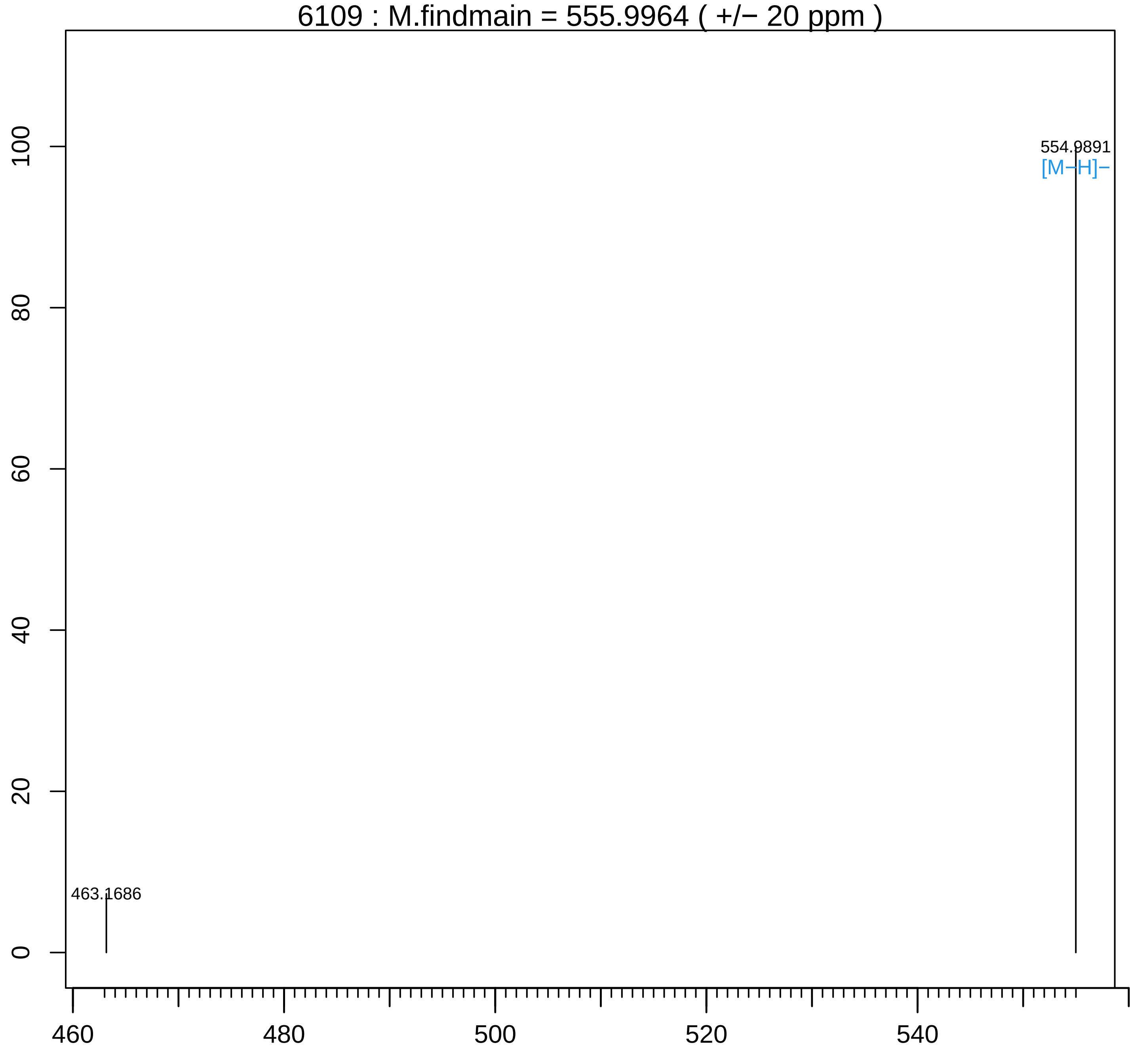
6109 : M.findmain = 555.9964 (+/− 20 ppm)
554.9891
[M−H]−
463.1686
0
20
40
60
80
100
460
480
500
520
540

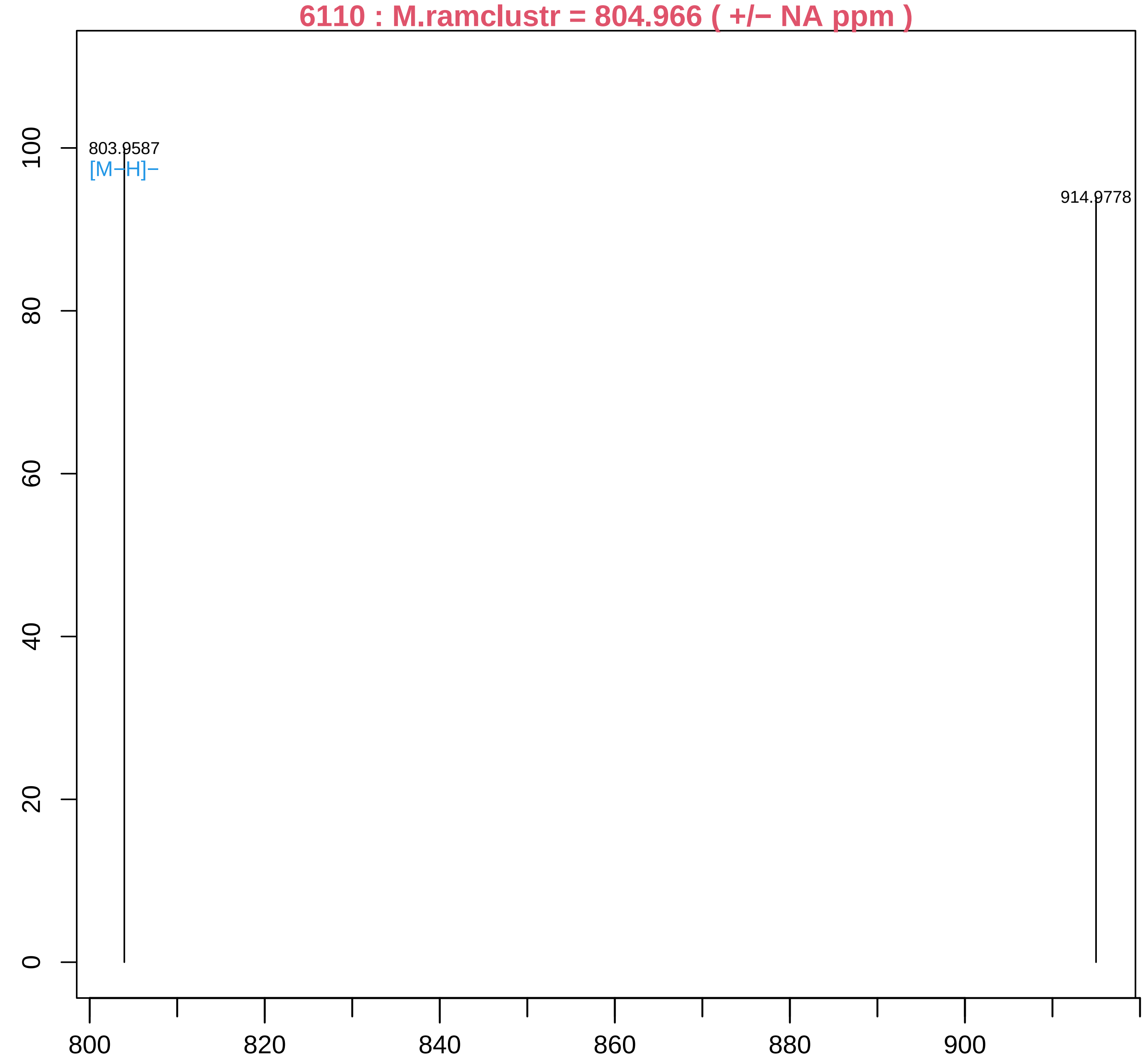

6110 : M.ramclustr = 804.966 (+/− NA ppm)
803.9587
[M−H]−
914.9778
0
20
40
60
80
100
800
820
840
860
880
900

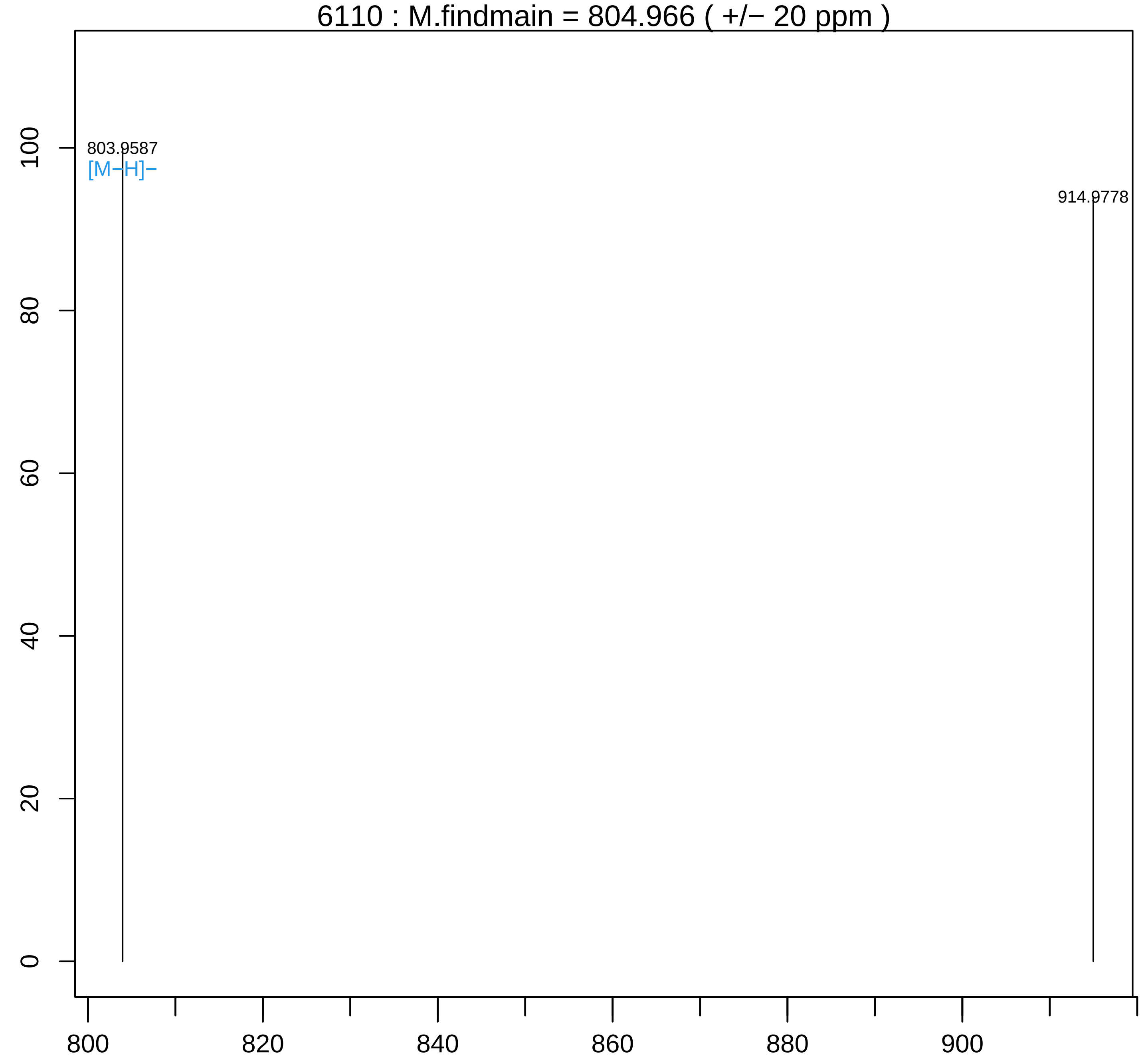

6110 : M.findmain = 804.966 (+/− 20 ppm)
803.9587
[M−H]−
914.9778
0
20
40
60
80
100
800
820
840
860
880
900

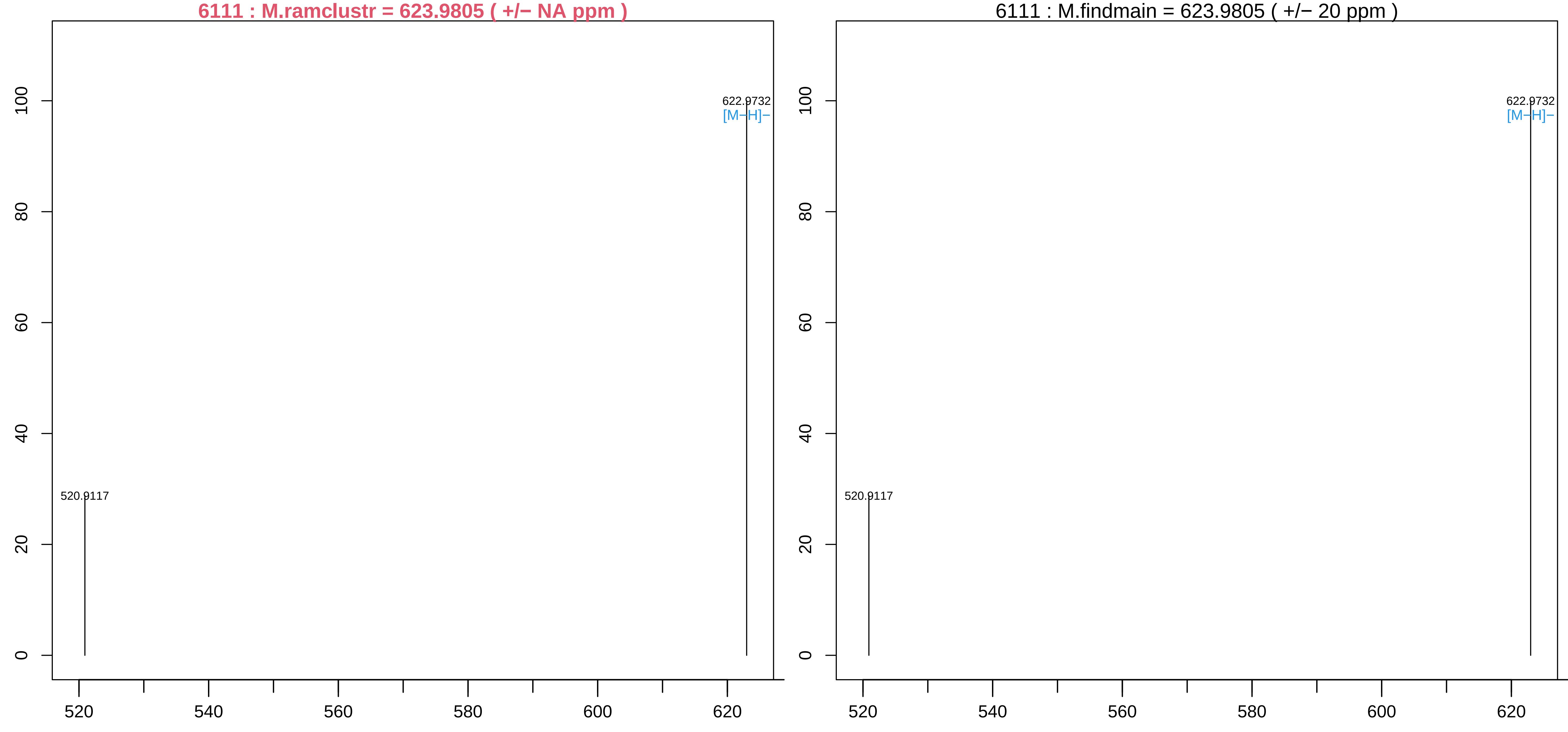

6111 : M.ramclustr = 623.9805 (+/− NA ppm)
622.9732
[M−H]−
520.9117
0
20
40
60
80
100
520
540
560
580
600
620
6111 : M.findmain = 623.9805 (+/− 20 ppm)
622.9732
[M−H]−
520.9117
0
20
40
60
80
100
520
540
560
580
600
620

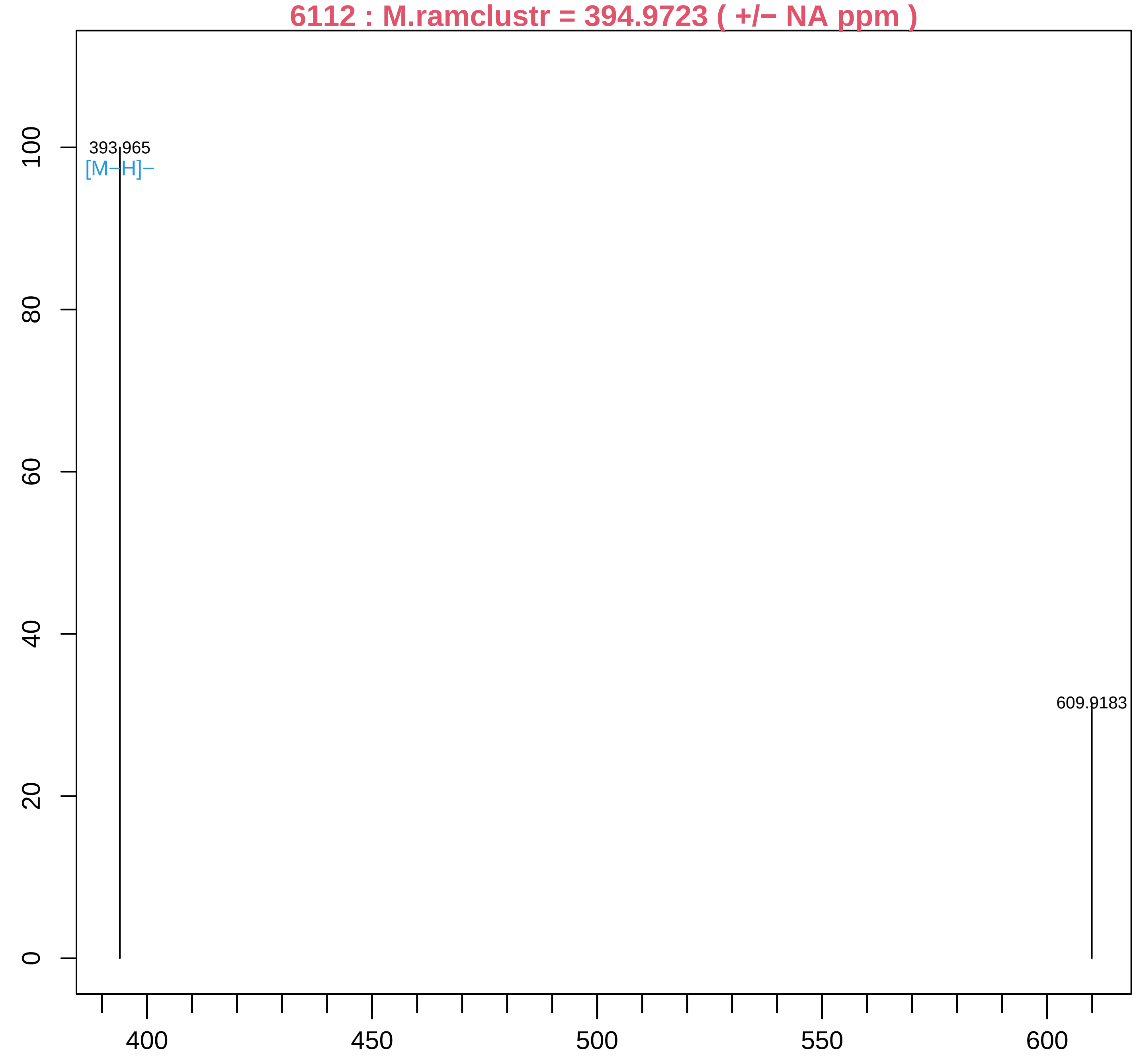

6112 : M.ramclustr = 394.9723 (+/− NA ppm)
393.965
[M−H]−
609.9183
0
20
40
60
80
100
400
450
500
550
600

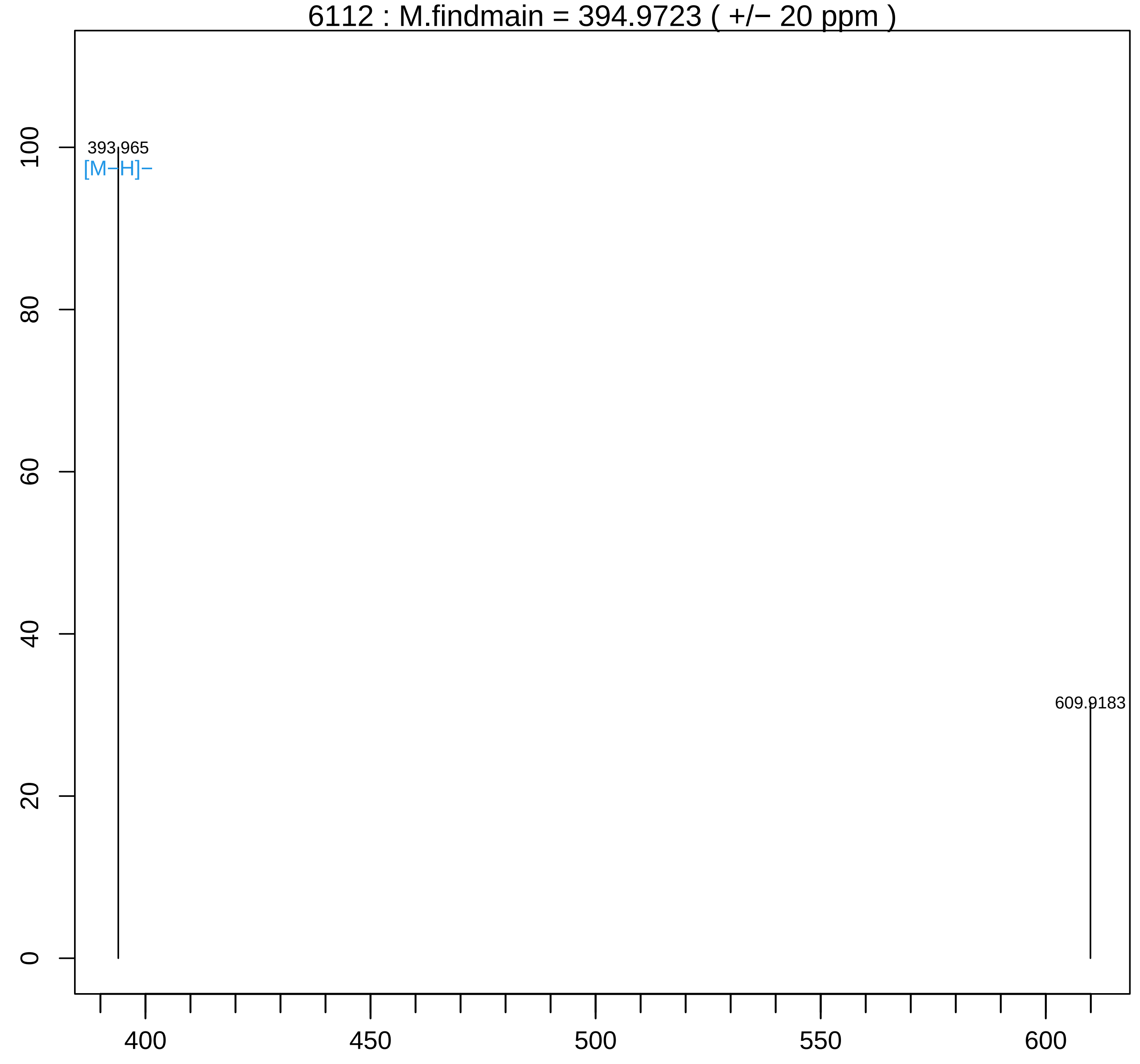

6112 : M.findmain = 394.9723 (+/− 20 ppm)
393.965
[M−H]−
609.9183
0
20
40
60
80
100
400
450
500
550
600

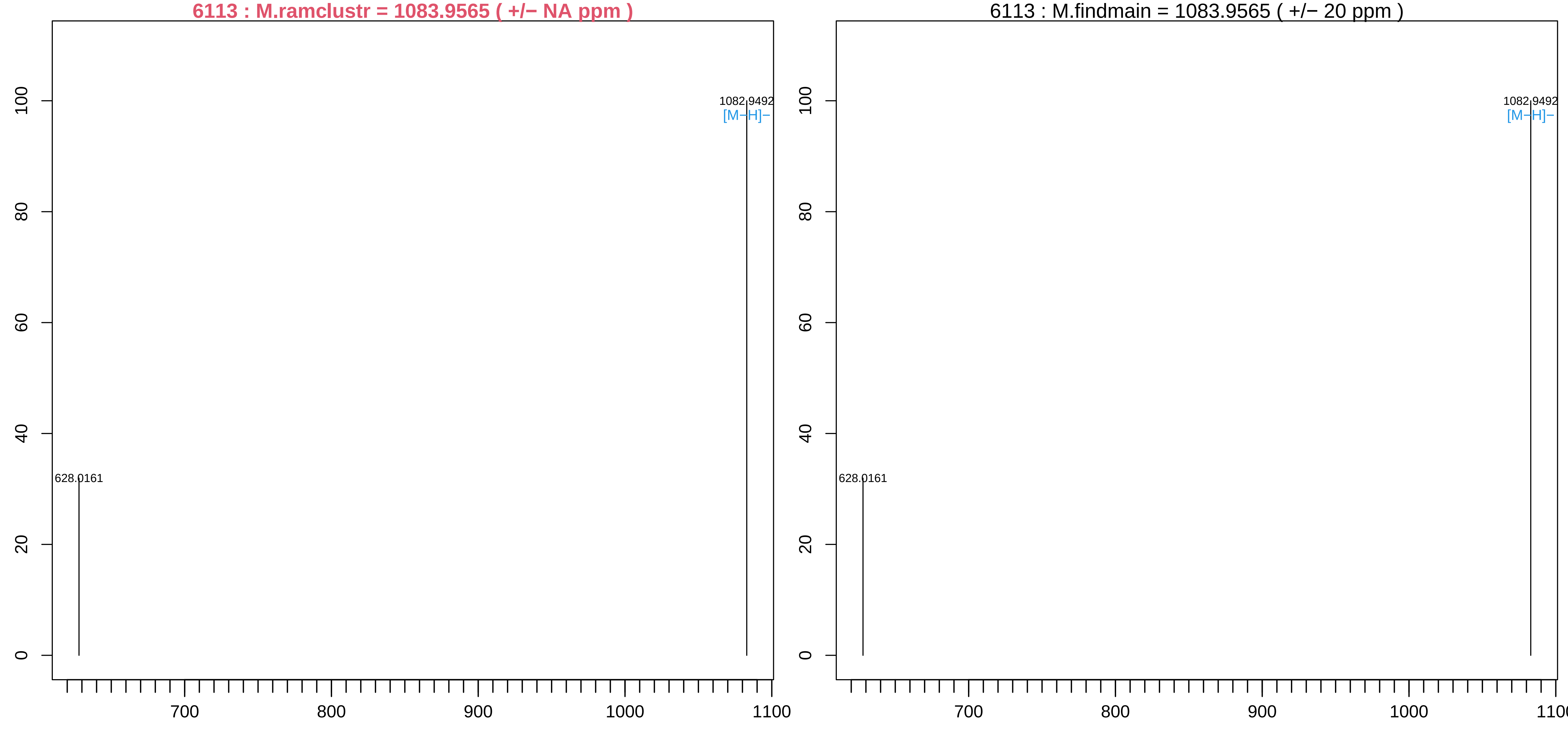
6113 : M.ramclustr = 1083.9565 (+/− NA ppm)
1082.9492
[M−H]−
628.0161
6113 : M.findmain = 1083.9565 (+/− 20 ppm)
1082.9492
[M−H]−
628.0161
0
20
40
60
80
100
700
800
900
1000
1100

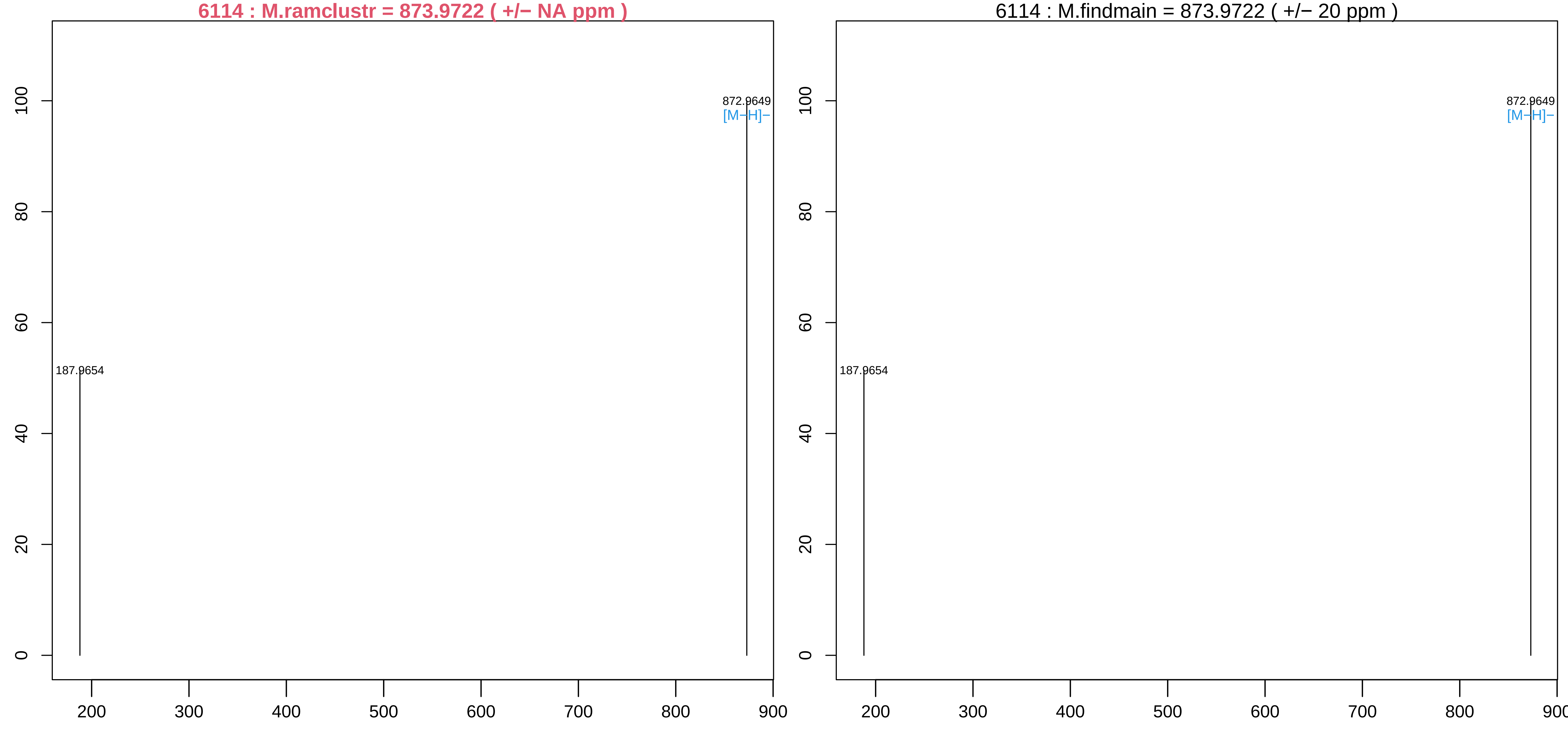
6114 : M.ramclustr = 873.9722 (+/− NA ppm)
872.9649
[M−H]−
187.9654
6114 : M.findmain = 873.9722 (+/− 20 ppm)
872.9649
[M−H]−
187.9654
0
20
40
60
80
100
200
300
400
500
600
700
800
900

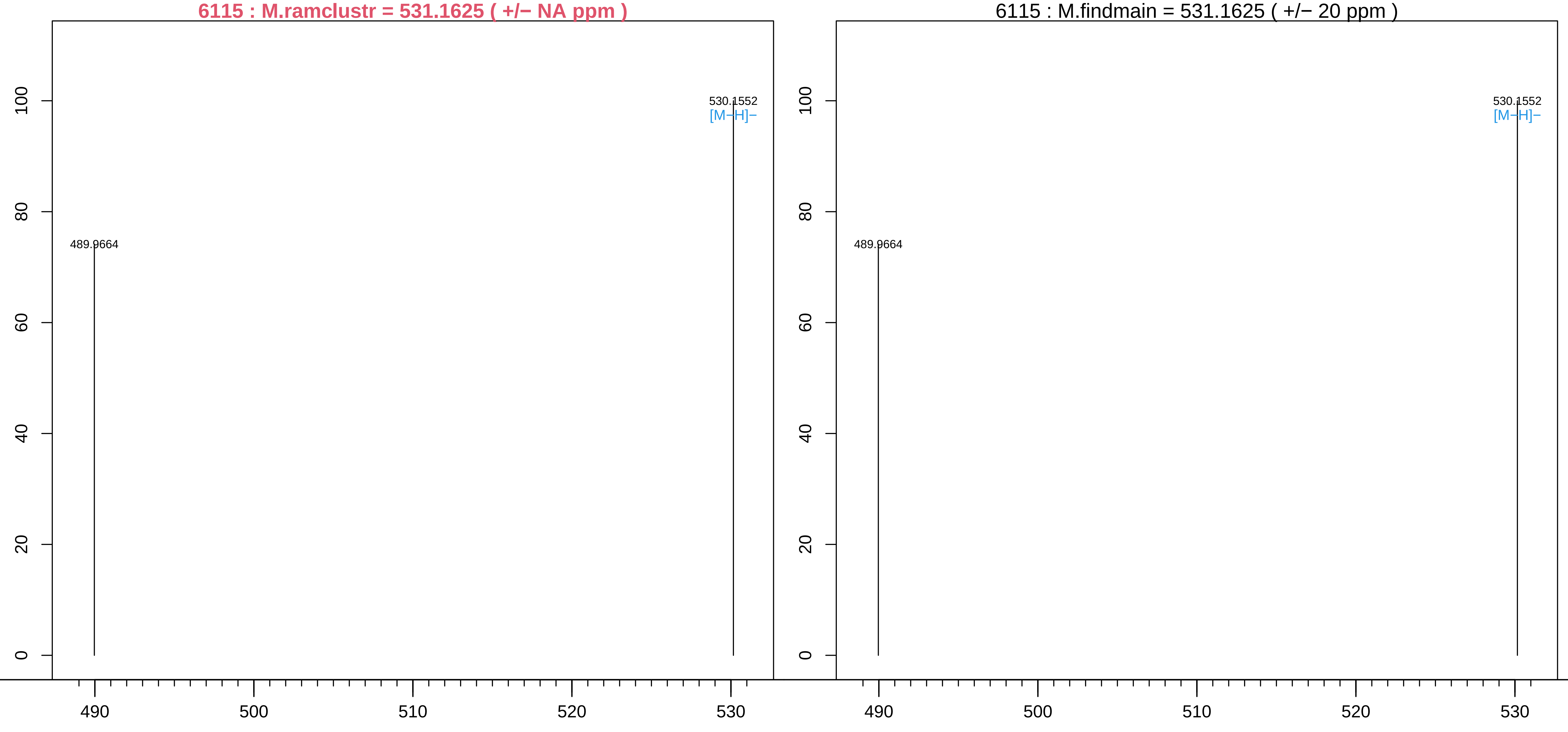
6115 : M.ramclustr = 531.1625 (+/− NA ppm)
6115 : M.findmain = 531.1625 (+/− 20 ppm)
530.1552
[M−H]−
489.9664
0
20
40
60
80
100
490
500
510
520
530

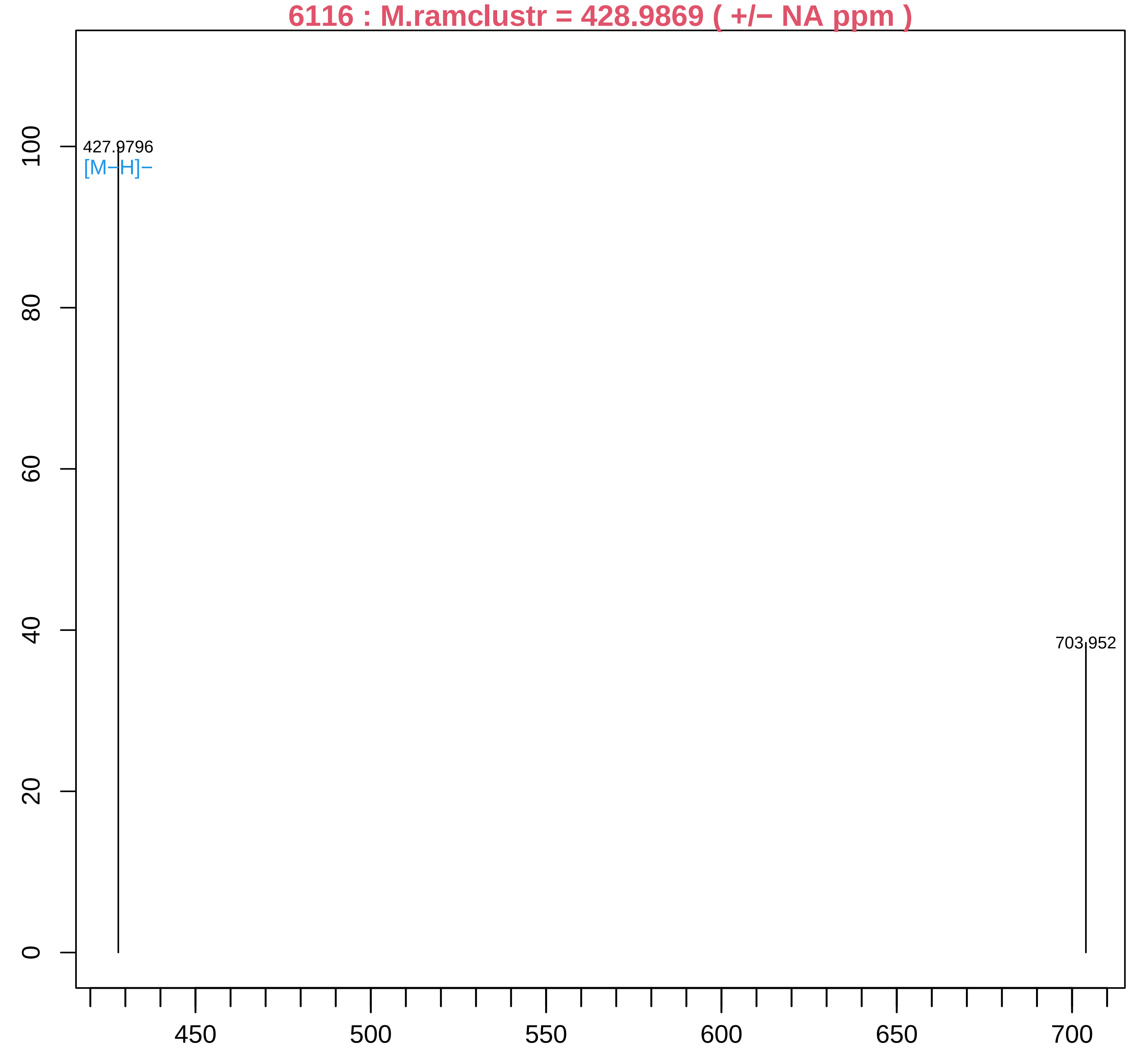
6116 : M.ramclustr = 428.9869 (+/− NA ppm)
427.9796
[M−H]−
703.952
0
20
40
60
80
100
450
500
550
600
650
700

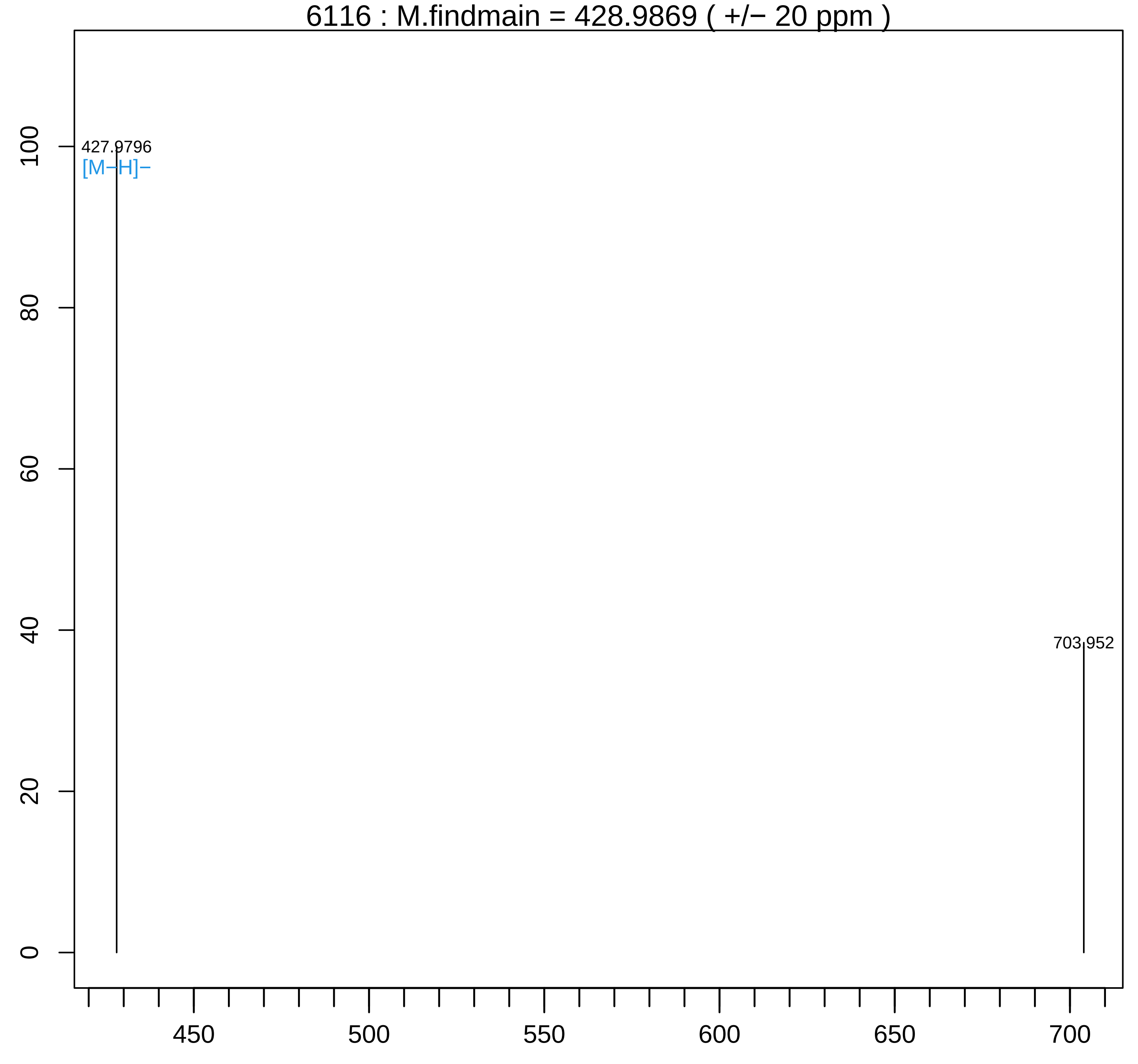
6116 : M.findmain = 428.9869 (+/− 20 ppm)
427.9796
[M−H]−
703.952
0
20
40
60
80
100
450
500
550
600
650
700

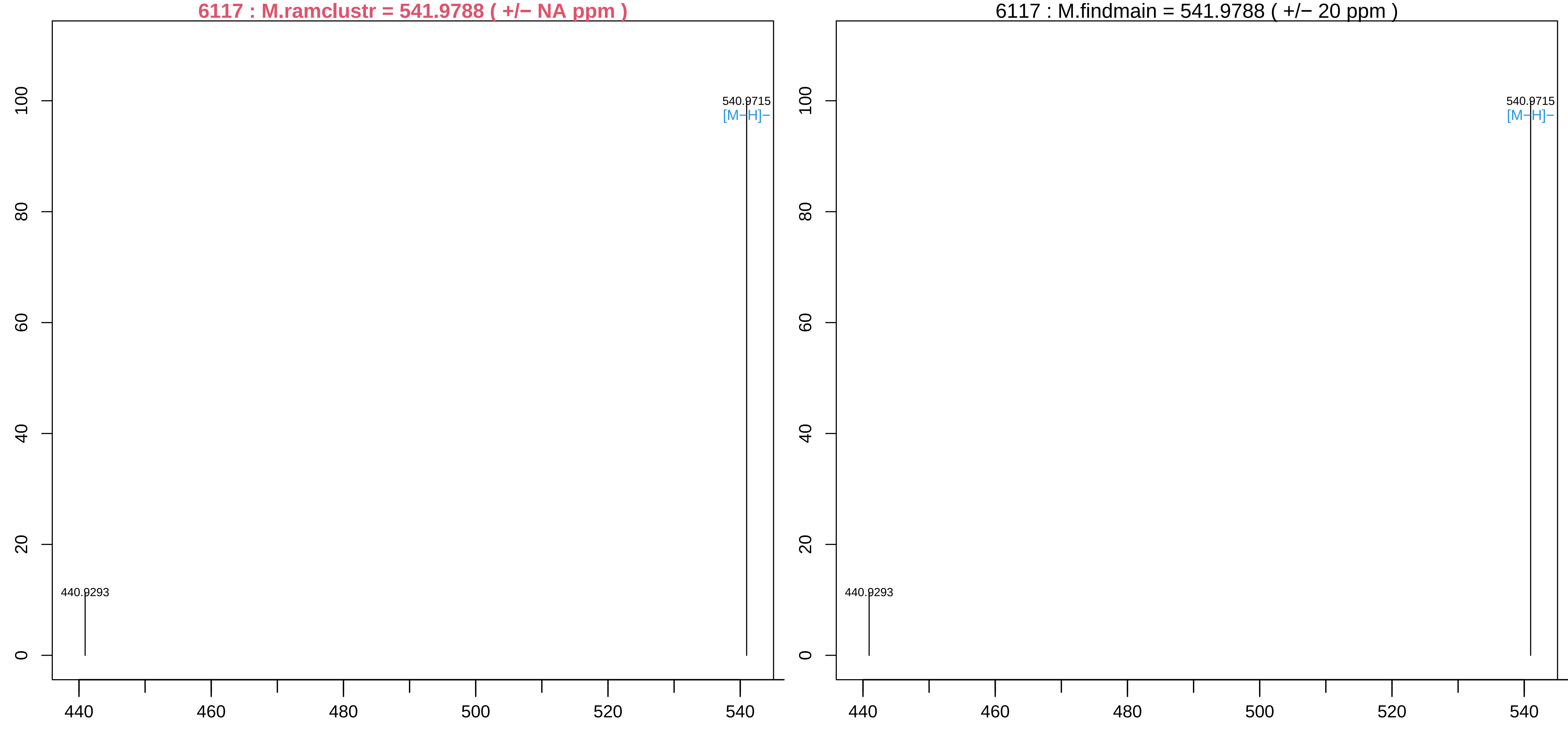
6117 : M.ramclustr = 541.9788 (+/− NA ppm)
540.9715
[M−H]−
440.9293
0
20
40
60
80
100
440
460
480
500
520
540
6117 : M.findmain = 541.9788 (+/− 20 ppm)
540.9715
[M−H]−
440.9293
0
20
40
60
80
100
440
460
480
500
520
540

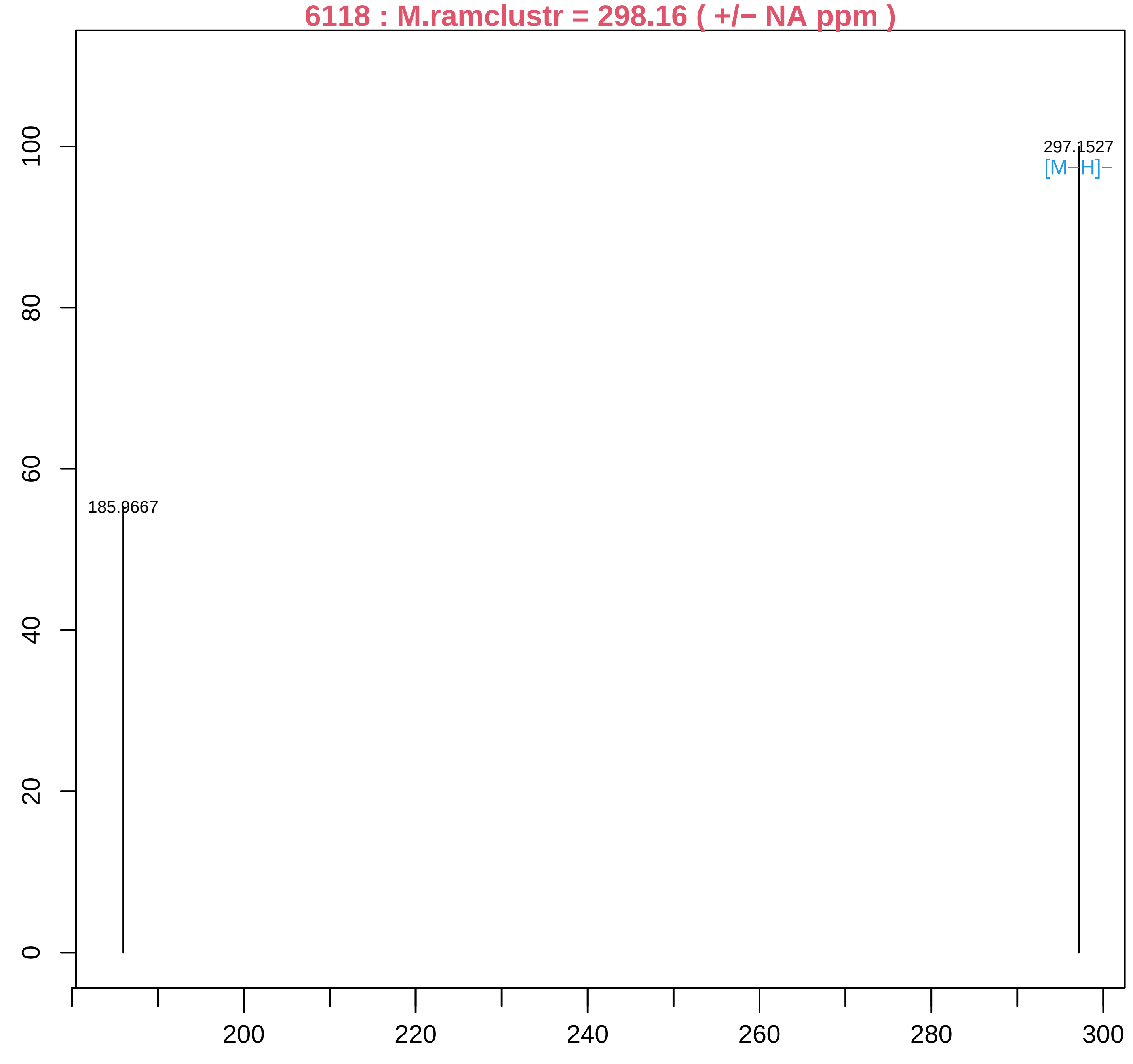

6118 : M.ramclustr = 298.16 (+/− NA ppm)
185.9667
297.1527
[M−H]−

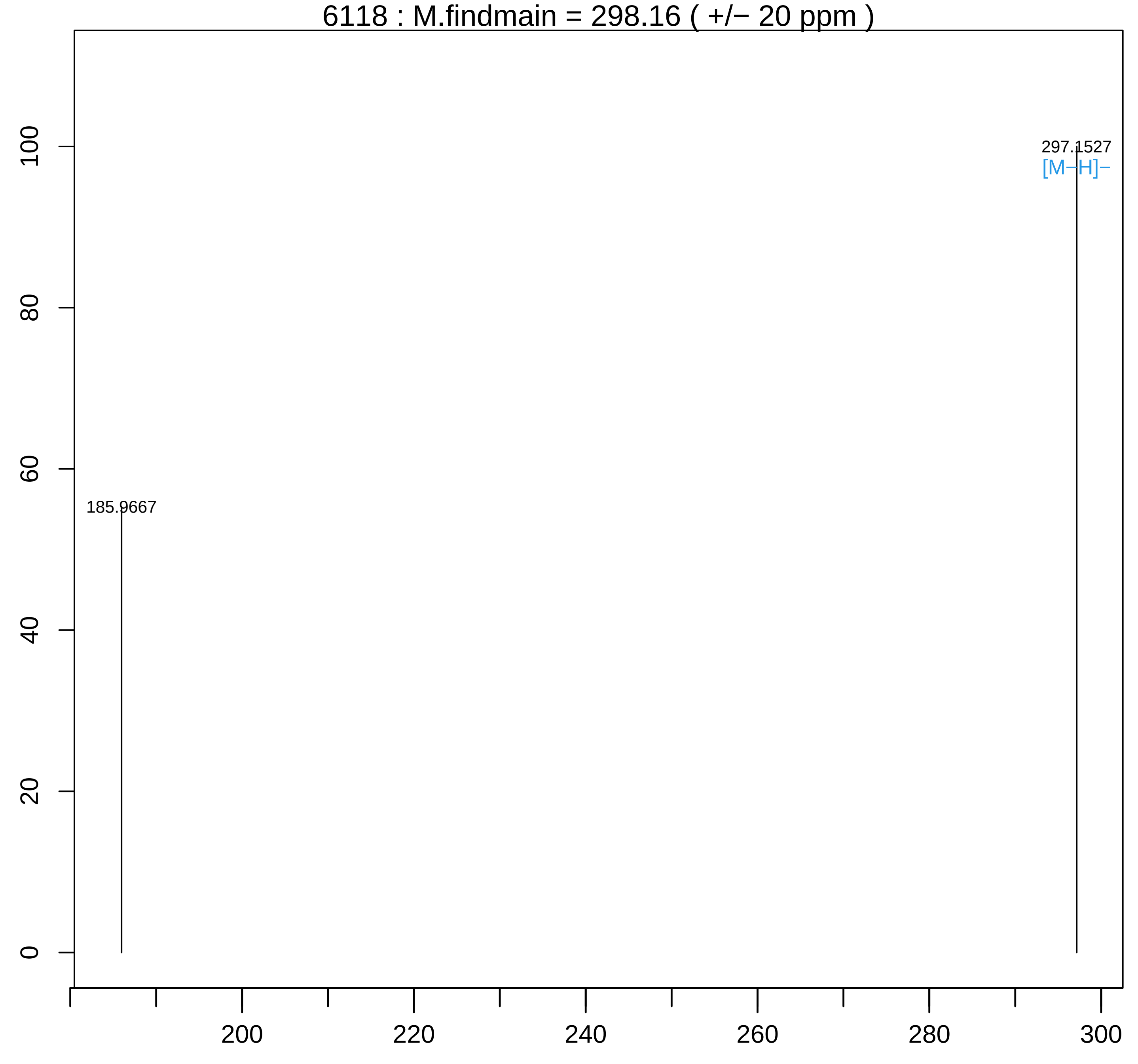

6118 : M.findmain = 298.16 (+/− 20 ppm)
185.9667
297.1527
[M−H]−

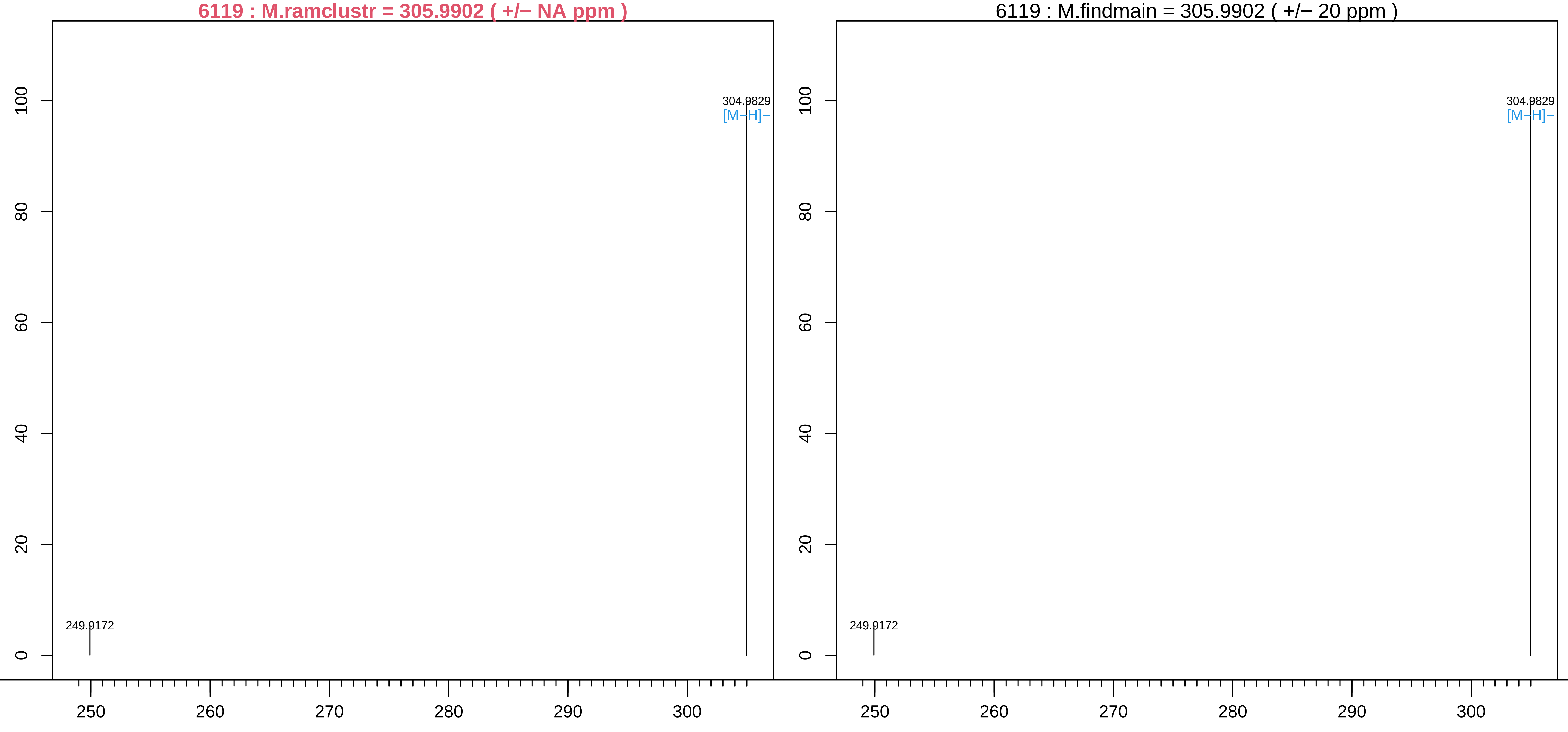

6119 : M.ramclustr = 305.9902 (+/− NA ppm)
304.9829
[M−H]−
249.9172
6119 : M.findmain = 305.9902 (+/− 20 ppm)
304.9829
[M−H]−
249.9172

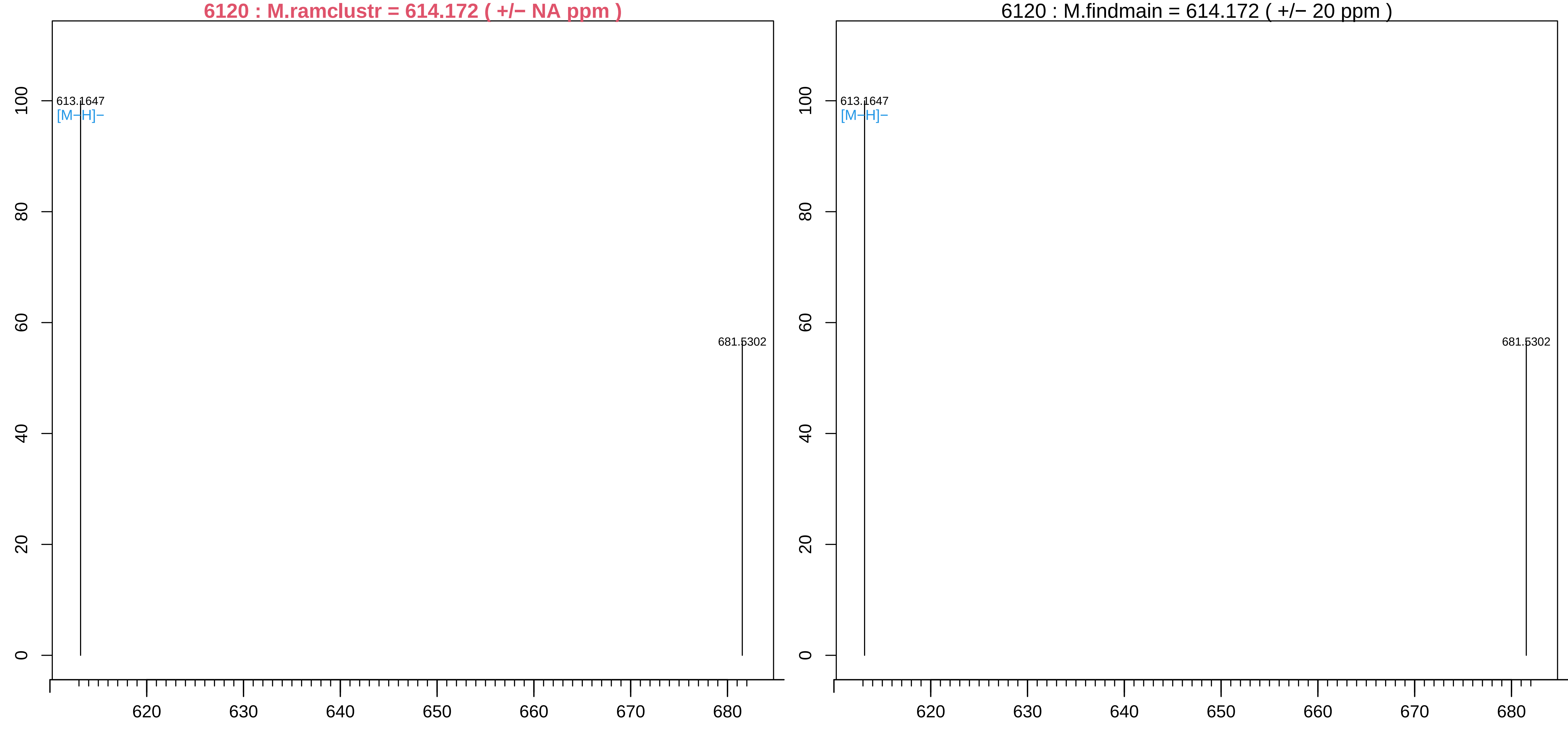
6120 : M.ramclustr = 614.172 (+/− NA ppm)
613.1647
[M−H]−
681.5302
6120 : M.findmain = 614.172 (+/− 20 ppm)
613.1647
[M−H]−
681.5302

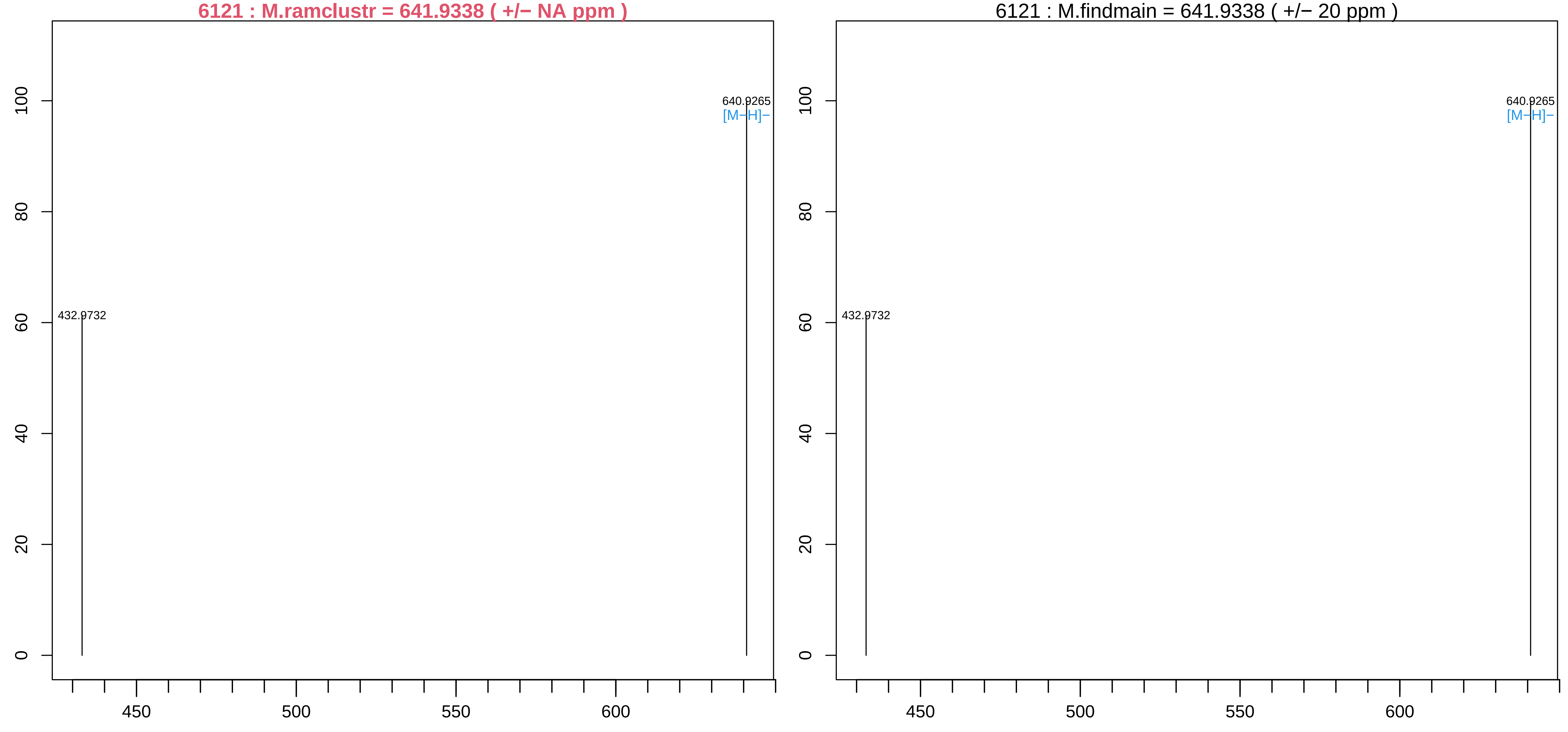
6121 : M.ramclustr = 641.9338 (+/− NA ppm)
640.9265
[M−H]−
432.9732
6121 : M.findmain = 641.9338 (+/− 20 ppm)
640.9265
[M−H]−
432.9732
0
20
40
60
80
100
450
500
550
600

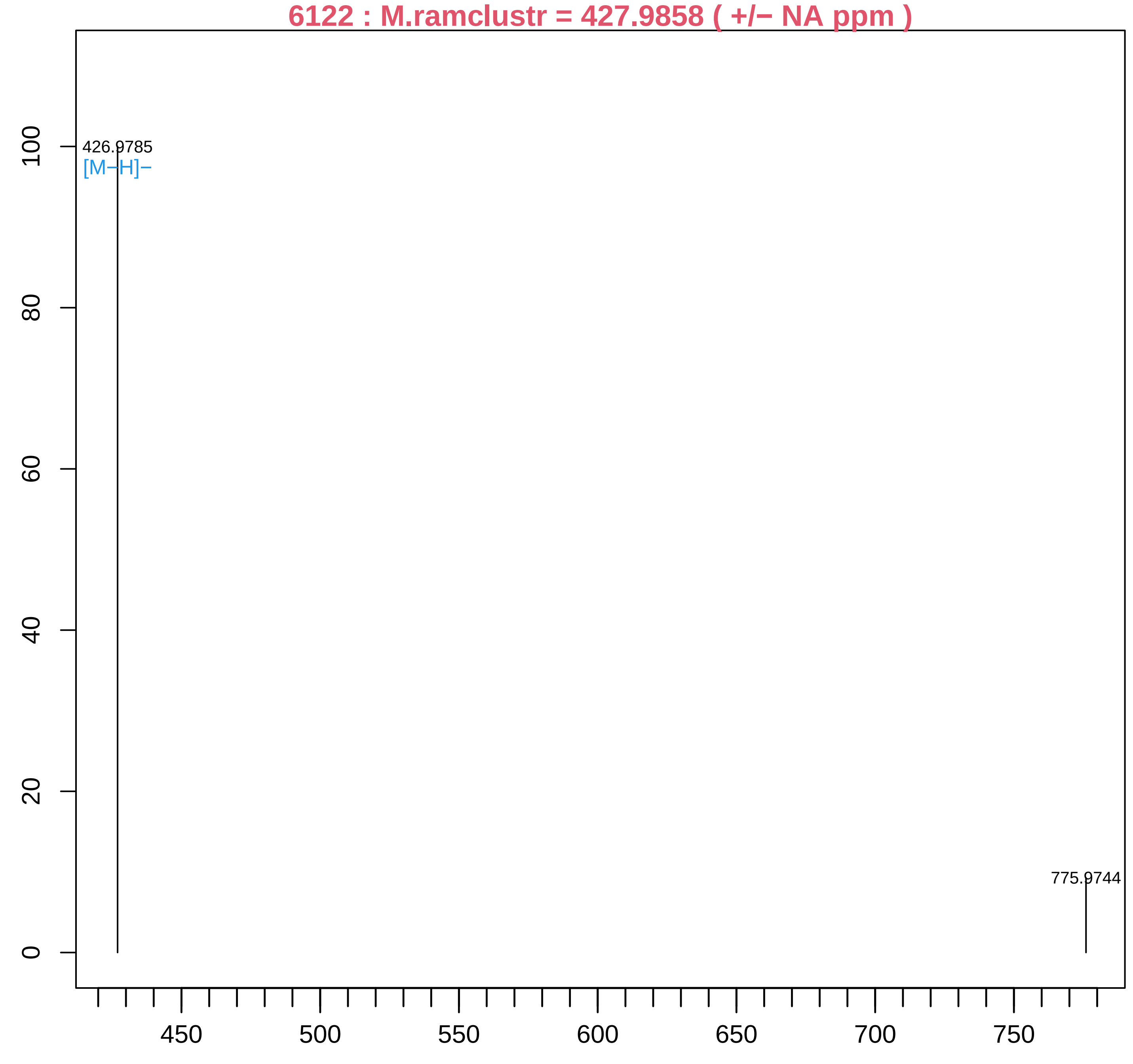
6122 : M.ramclustr = 427.9858 (+/− NA ppm)
426.9785
[M−H]−
775.9744
0
20
40
60
80
100
450
500
550
600
650
700
750

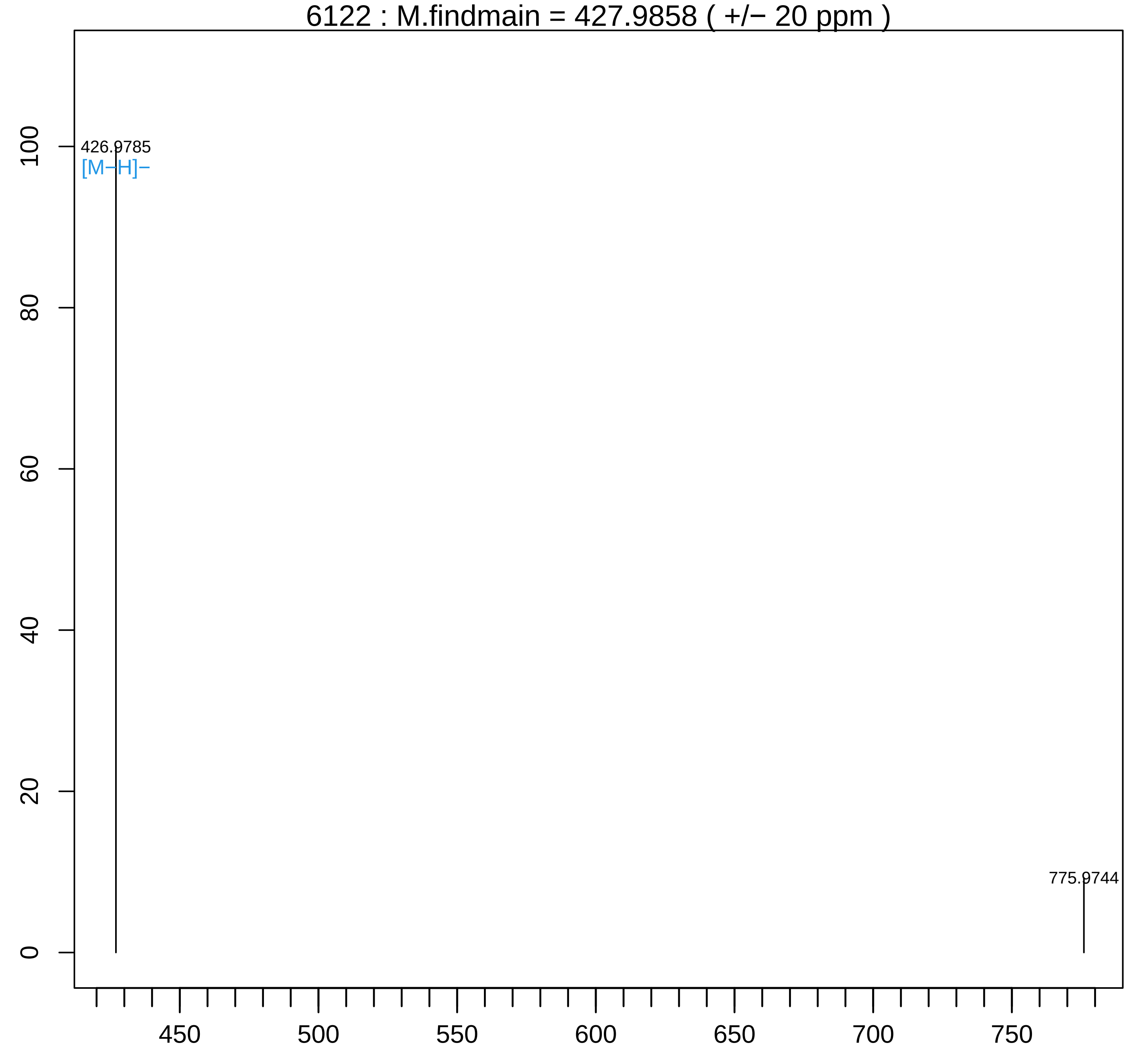
6122 : M.findmain = 427.9858 (+/− 20 ppm)
426.9785
[M−H]−
775.9744
0
20
40
60
80
100
450
500
550
600
650
700
750

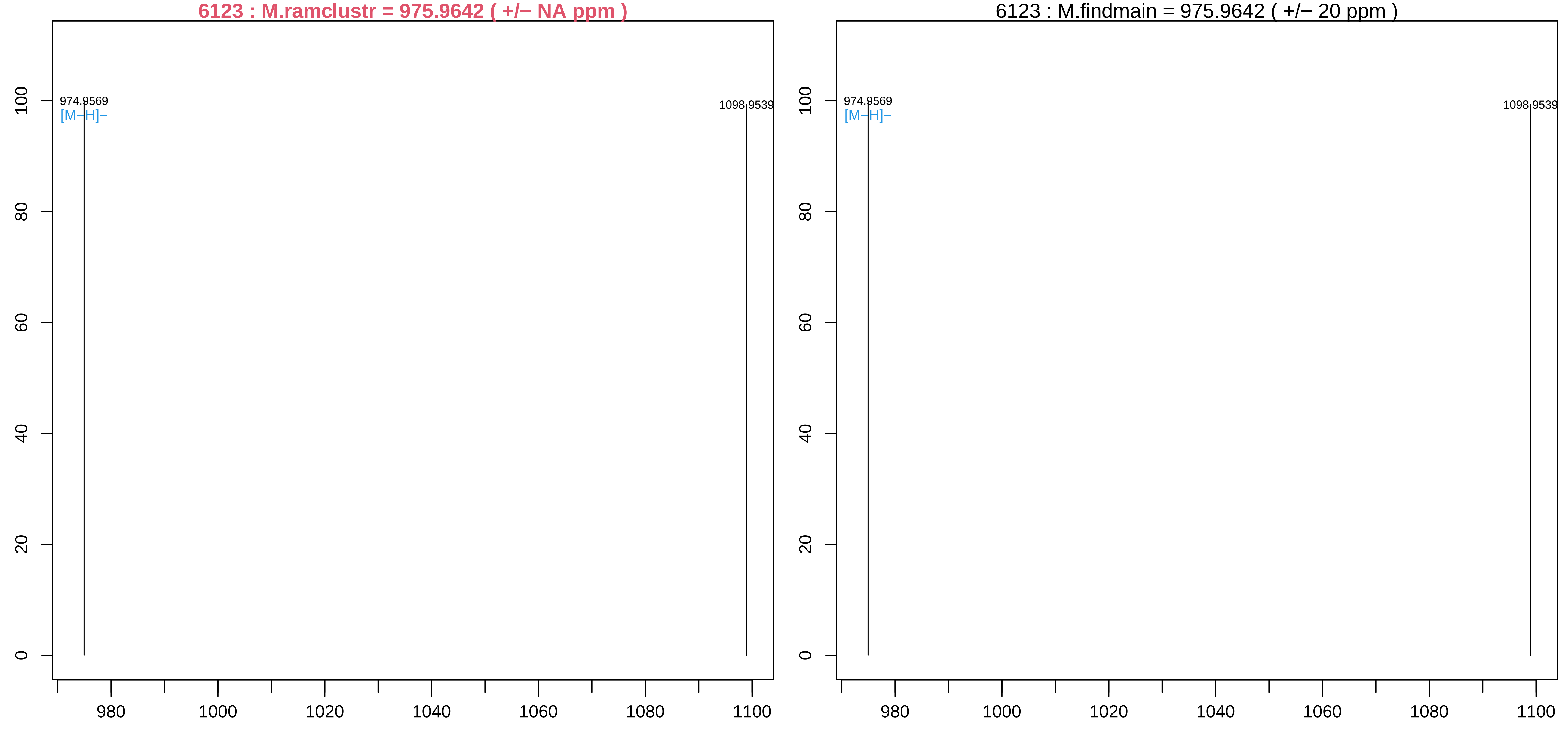
6123 : M.ramclustr = 975.9642 (+/− NA ppm)
974.9569
[M−H]−
1098.9539
6123 : M.findmain = 975.9642 (+/− 20 ppm)
974.9569
[M−H]−
1098.9539
0
20
40
60
80
100
980
1000
1020
1040
1060
1080
1100

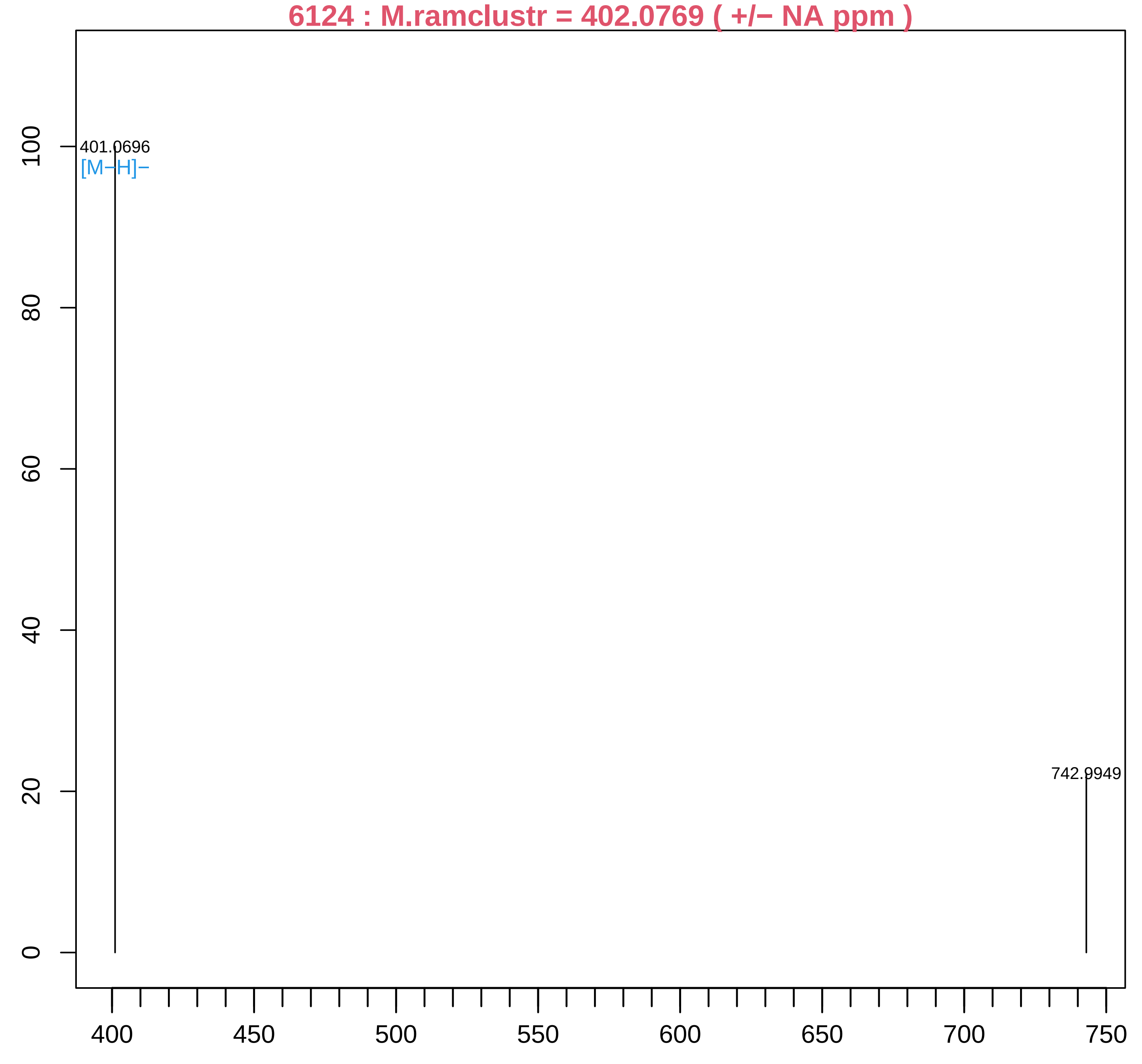
6124 : M.ramclustr = 402.0769 (+/− NA ppm)
401.0696
[M−H]−
742.9949
0
20
40
60
80
100
400
450
500
550
600
650
700
750

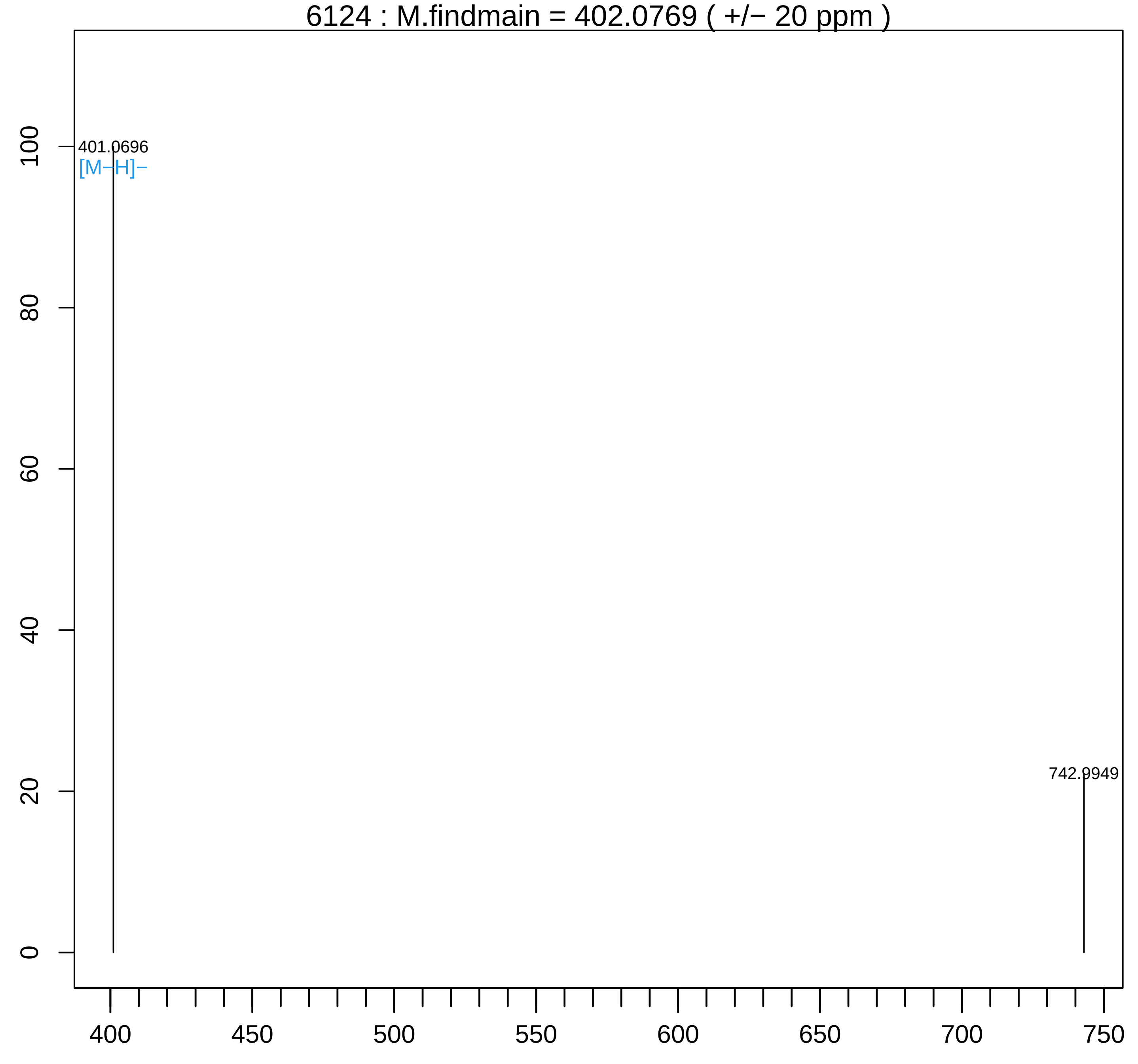
6124 : M.findmain = 402.0769 (+/− 20 ppm)
401.0696
[M−H]−
742.9949
0
20
40
60
80
100
400
450
500
550
600
650
700
750

6125 : M.ramclustr = 546.9741 (+/− NA ppm)

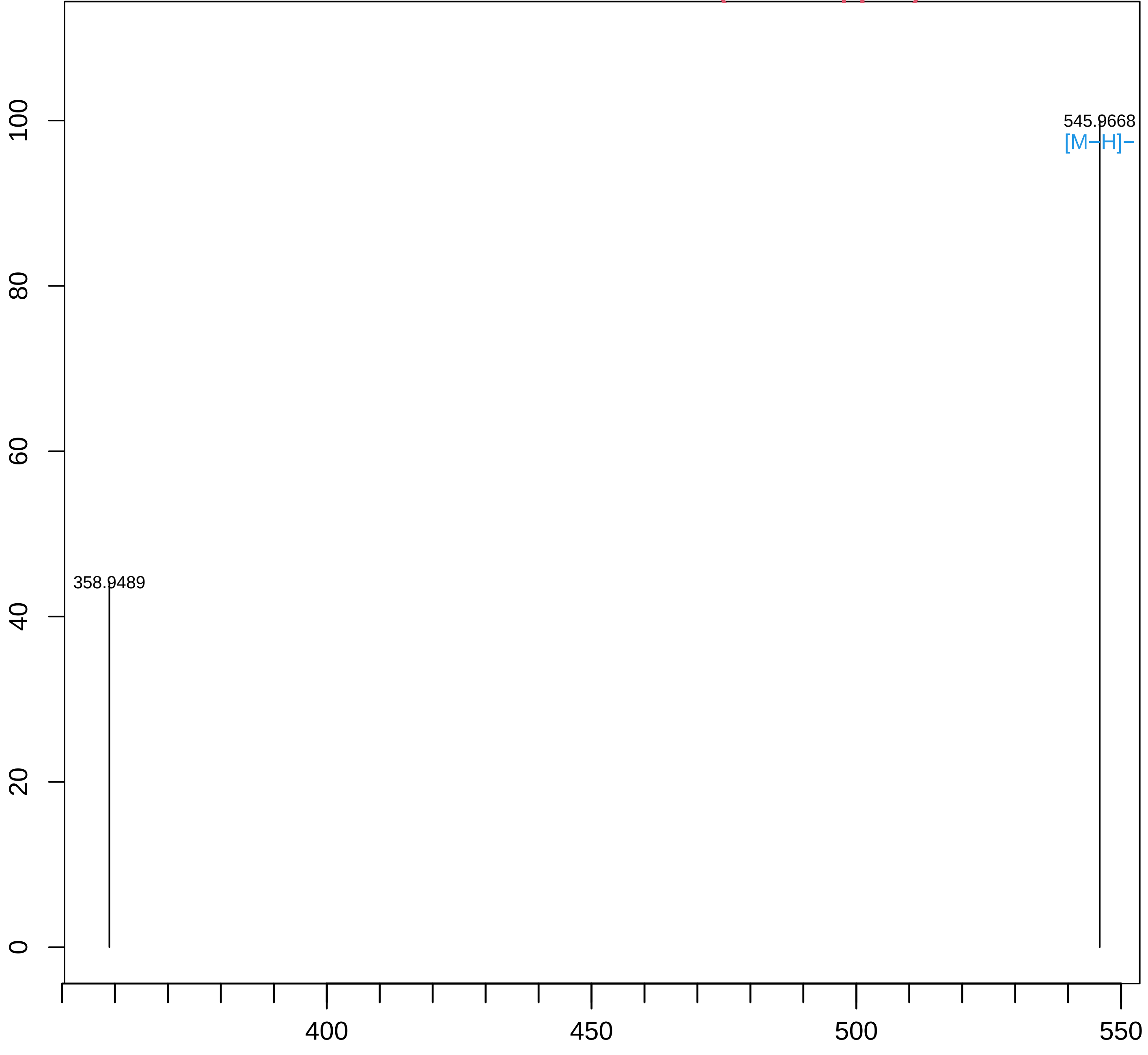

6125 : M.findmain = 546.9741 (+/− 20 ppm)

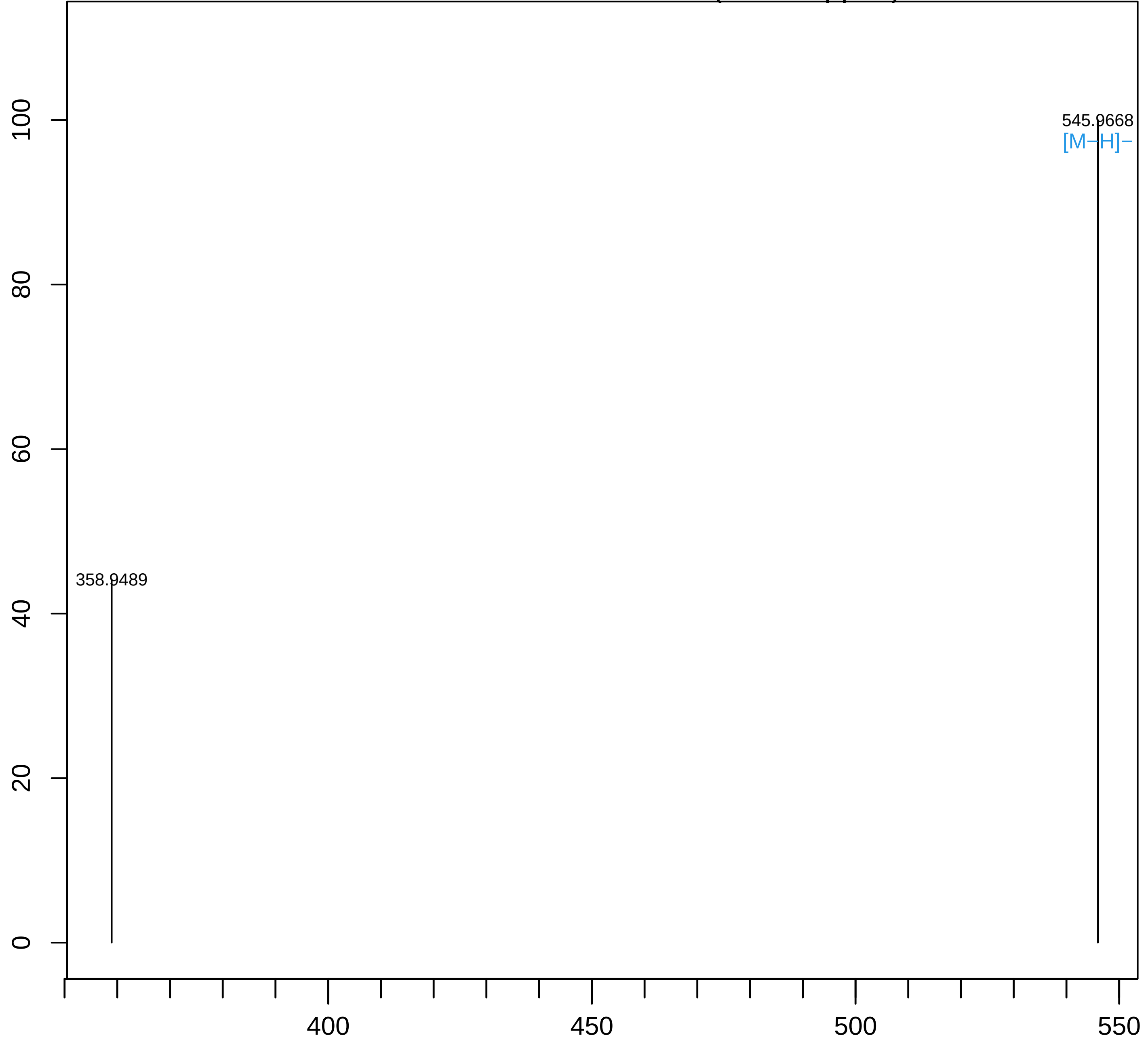

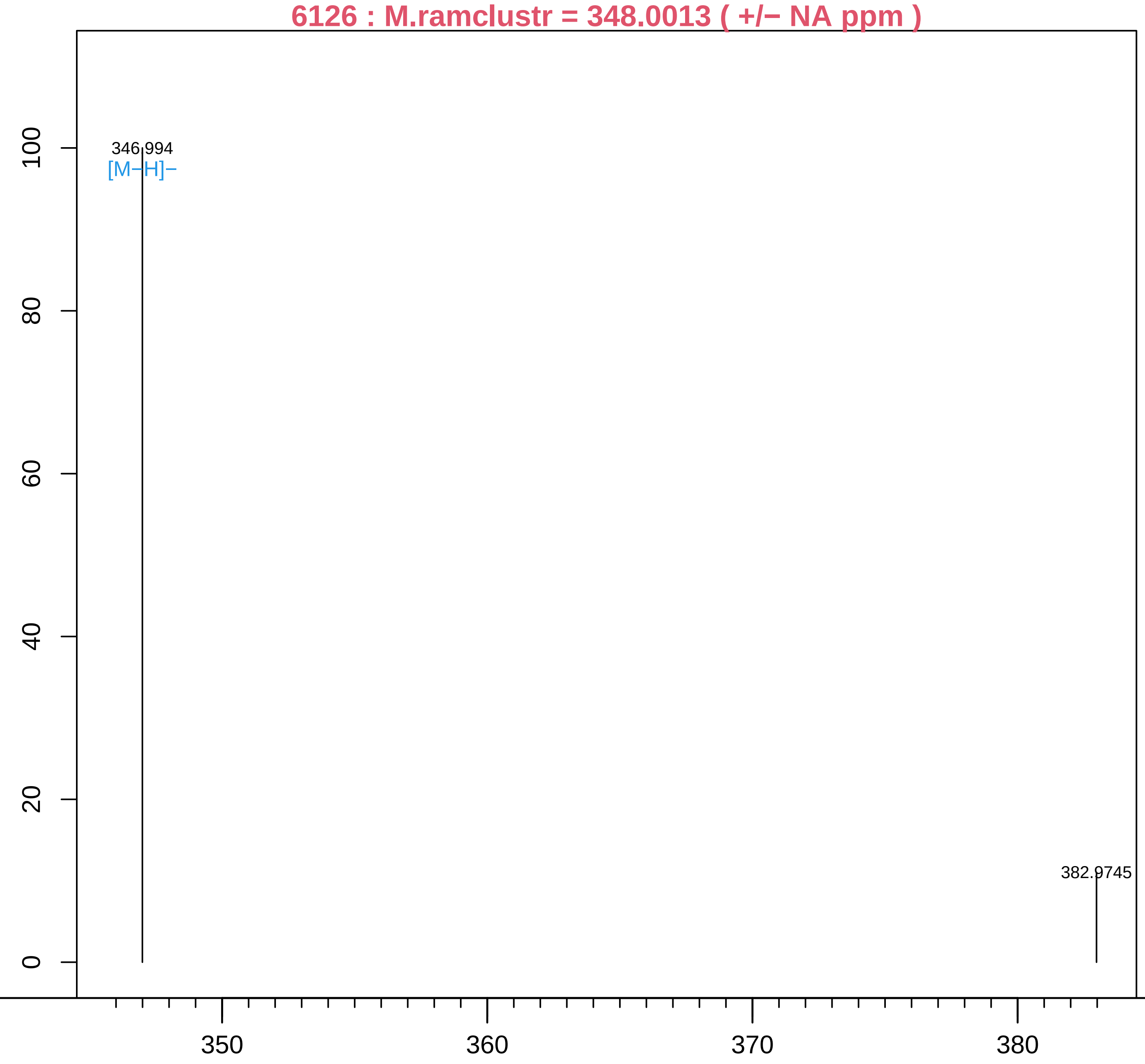

6126 : M.ramclustr = 348.0013 (+/− NA ppm)
346.994
[M−H]−
382.9745
0
20
40
60
80
100
350
360
370
380

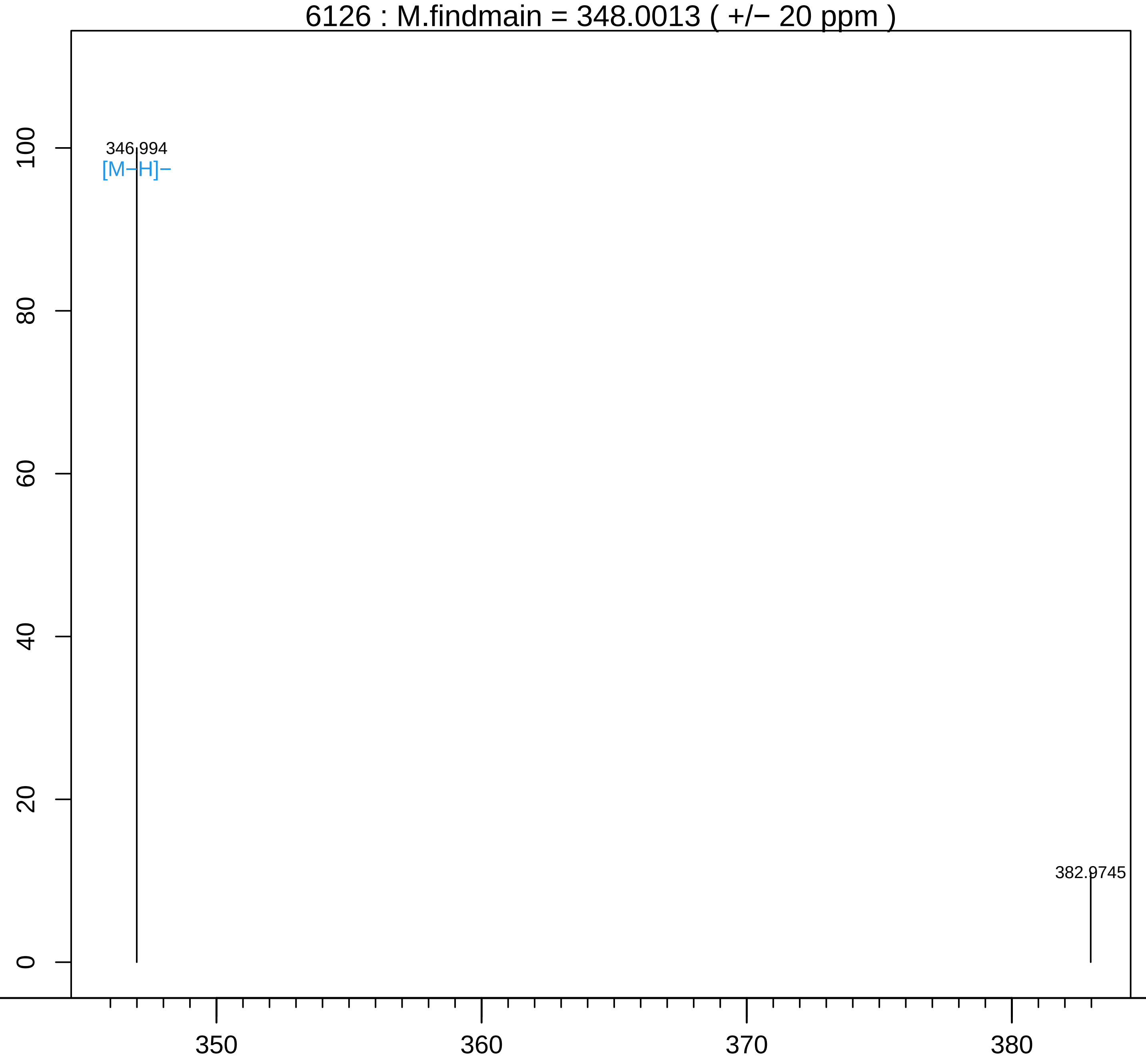

6126 : M.findmain = 348.0013 (+/− 20 ppm)
346.994
[M−H]−
382.9745
0
20
40
60
80
100
350
360
370
380

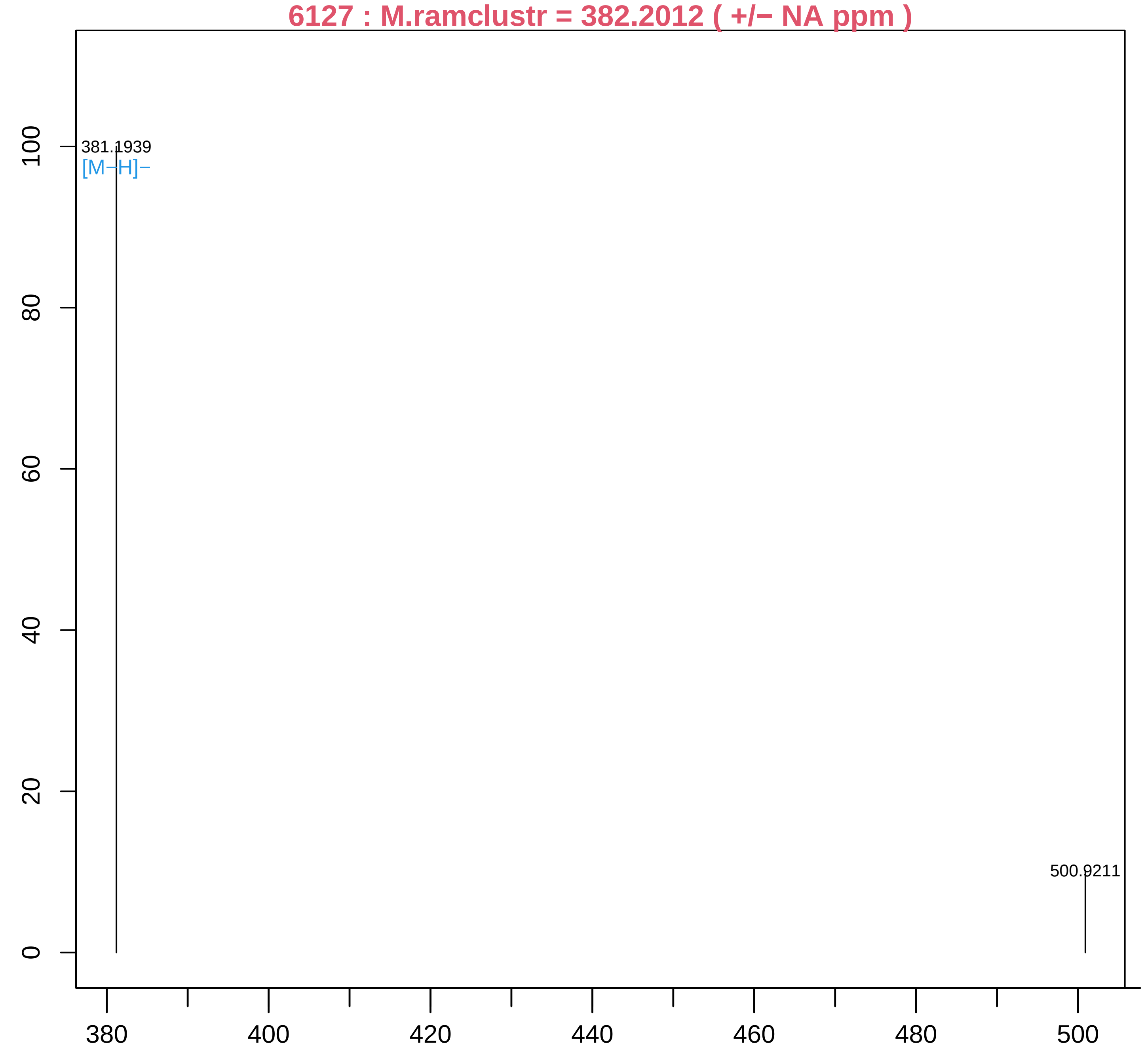
6127 : M.ramclustr = 382.2012 (+/− NA ppm)
381.1939
[M−H]−
500.9211
0
20
40
60
80
100
380
400
420
440
460
480
500

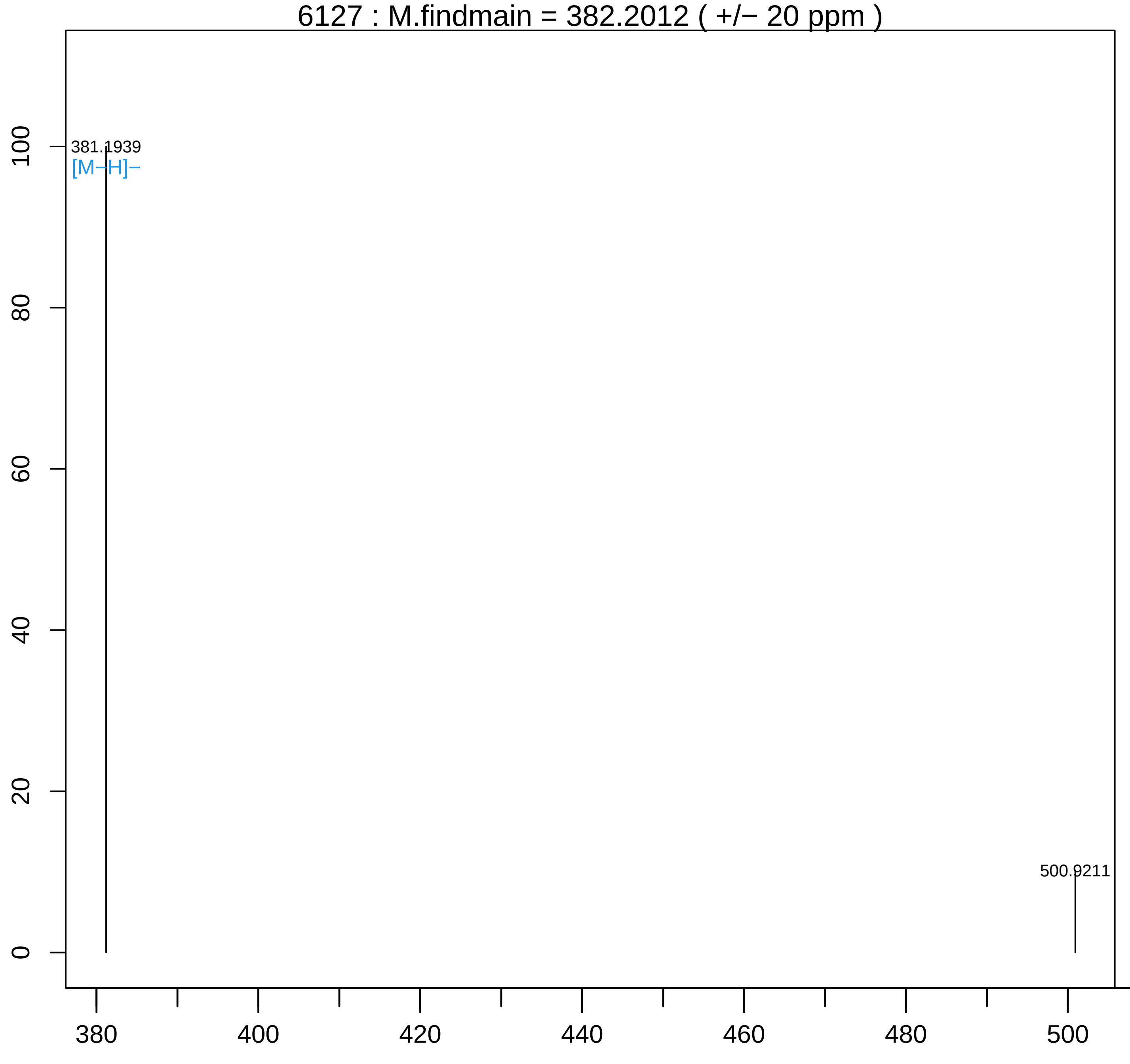
6127 : M.findmain = 382.2012 (+/− 20 ppm)
381.1939
[M−H]−
500.9211
0
20
40
60
80
100
380
400
420
440
460
480
500

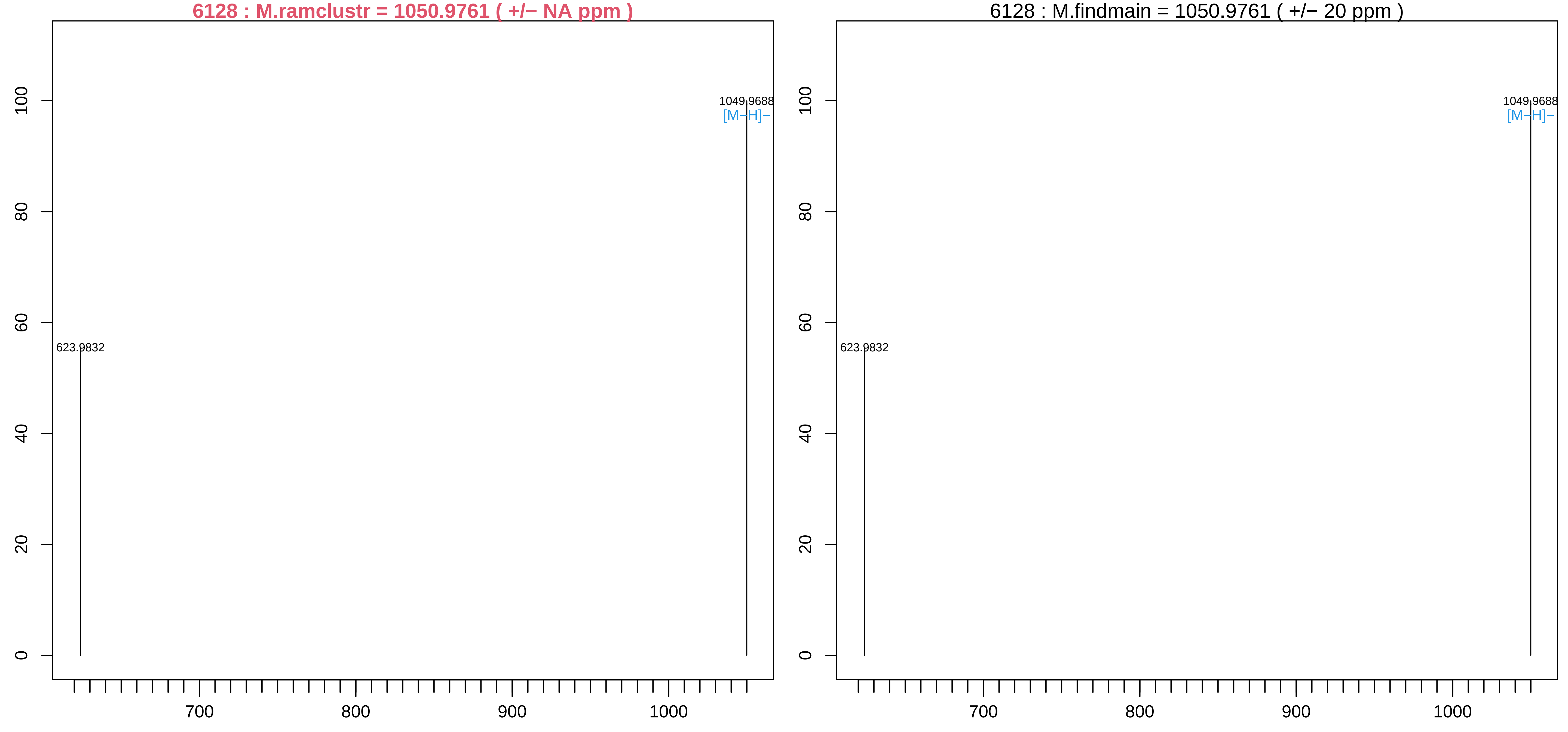
6128 : M.ramclustr = 1050.9761 (+/− NA ppm)
6128 : M.findmain = 1050.9761 (+/− 20 ppm)
1049.9688
[M−H]−
623.9832
1049.9688
[M−H]−
623.9832
0
20
40
60
80
100
700
800
900
1000

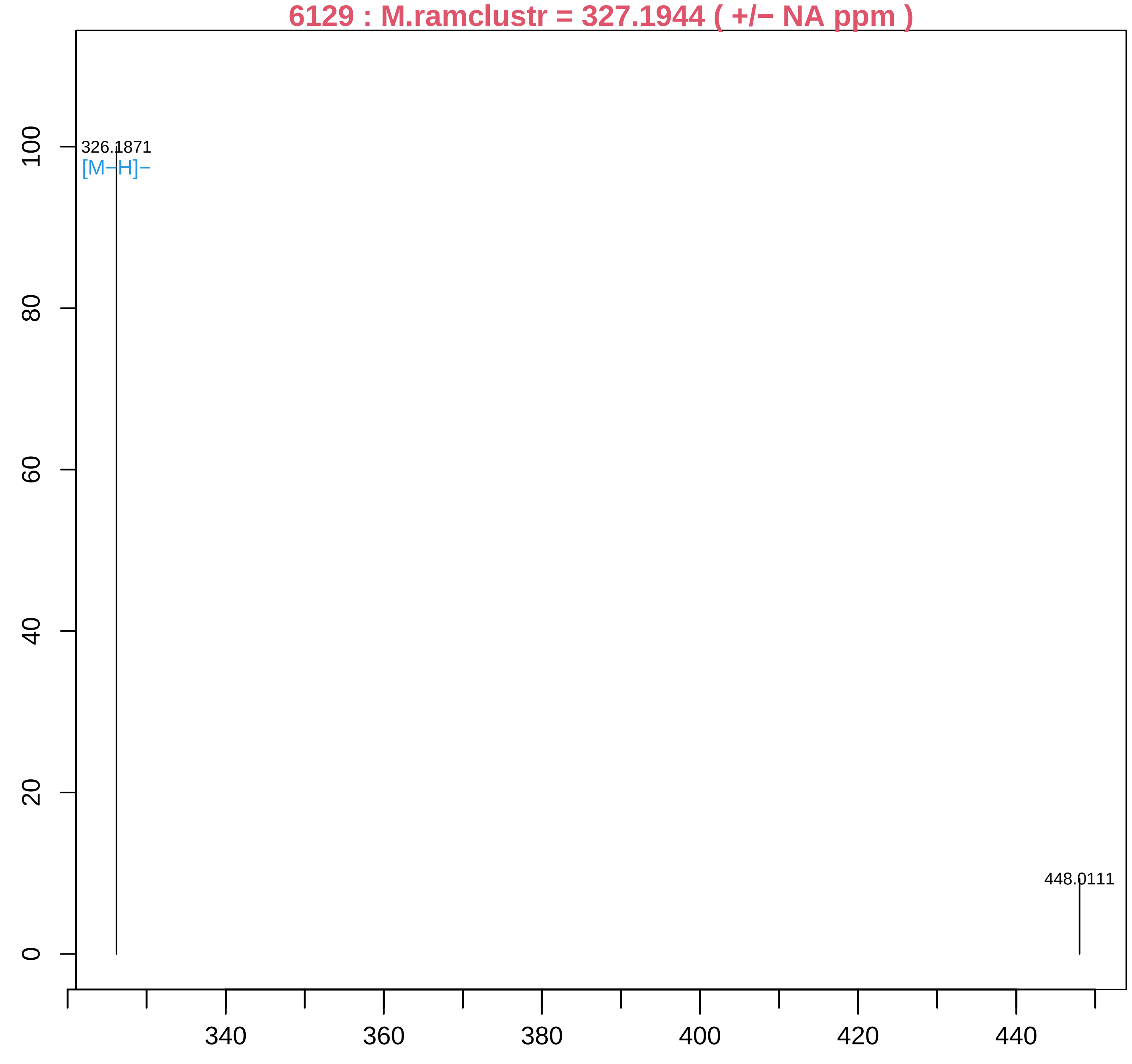
6129 : M.ramclustr = 327.1944 (+/− NA ppm)
326.1871
[M−H]−
448.0111
0
20
40
60
80
100
340
360
380
400
420
440

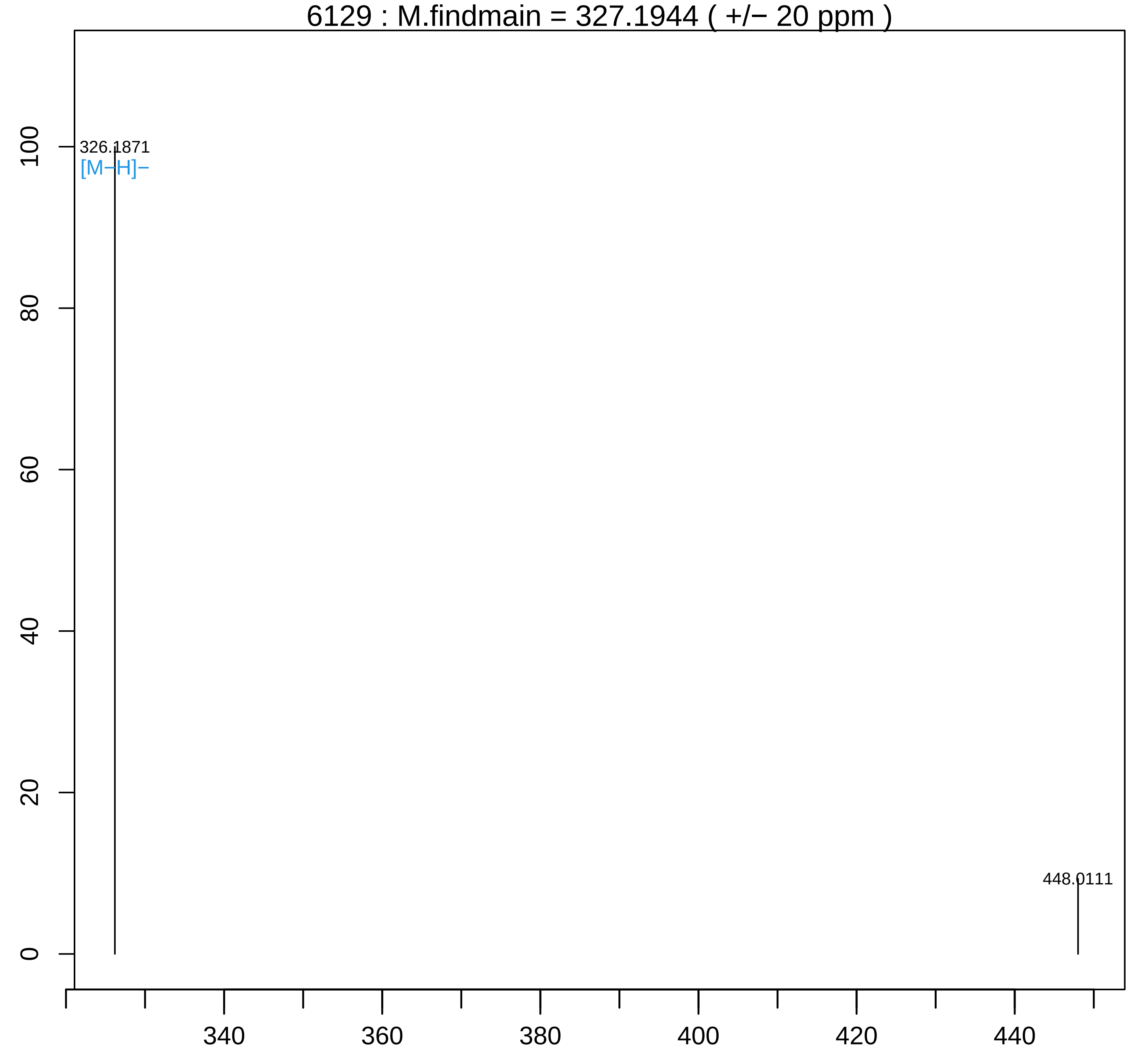
6129 : M.findmain = 327.1944 (+/− 20 ppm)
326.1871
[M−H]−
448.0111
0
20
40
60
80
100
340
360
380
400
420
440

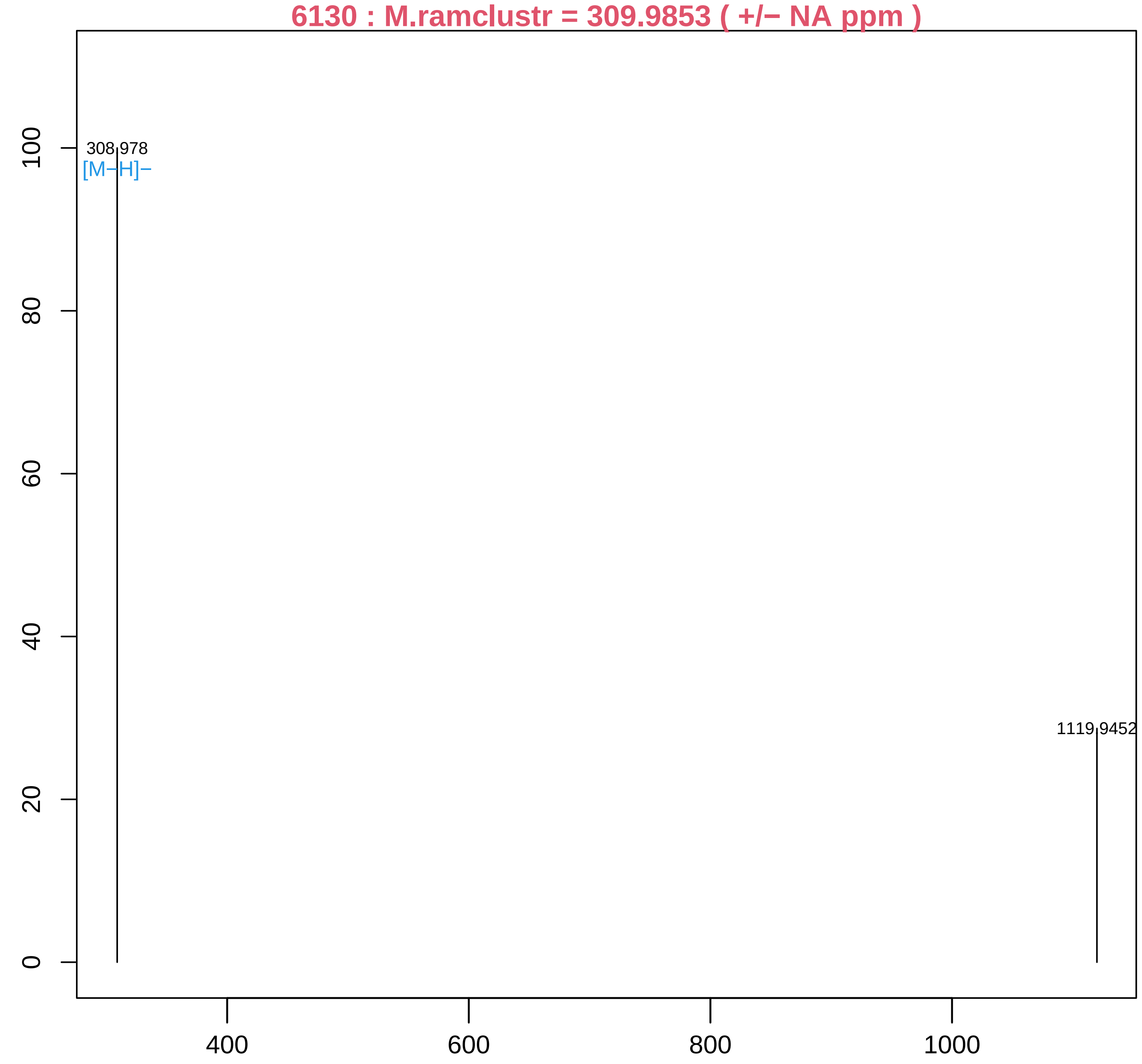

6130 : M.ramclustr = 309.9853 (+/− NA ppm)
308.978
[M−H]−
1119.9452
0
20
40
60
80
100
400
600
800
1000

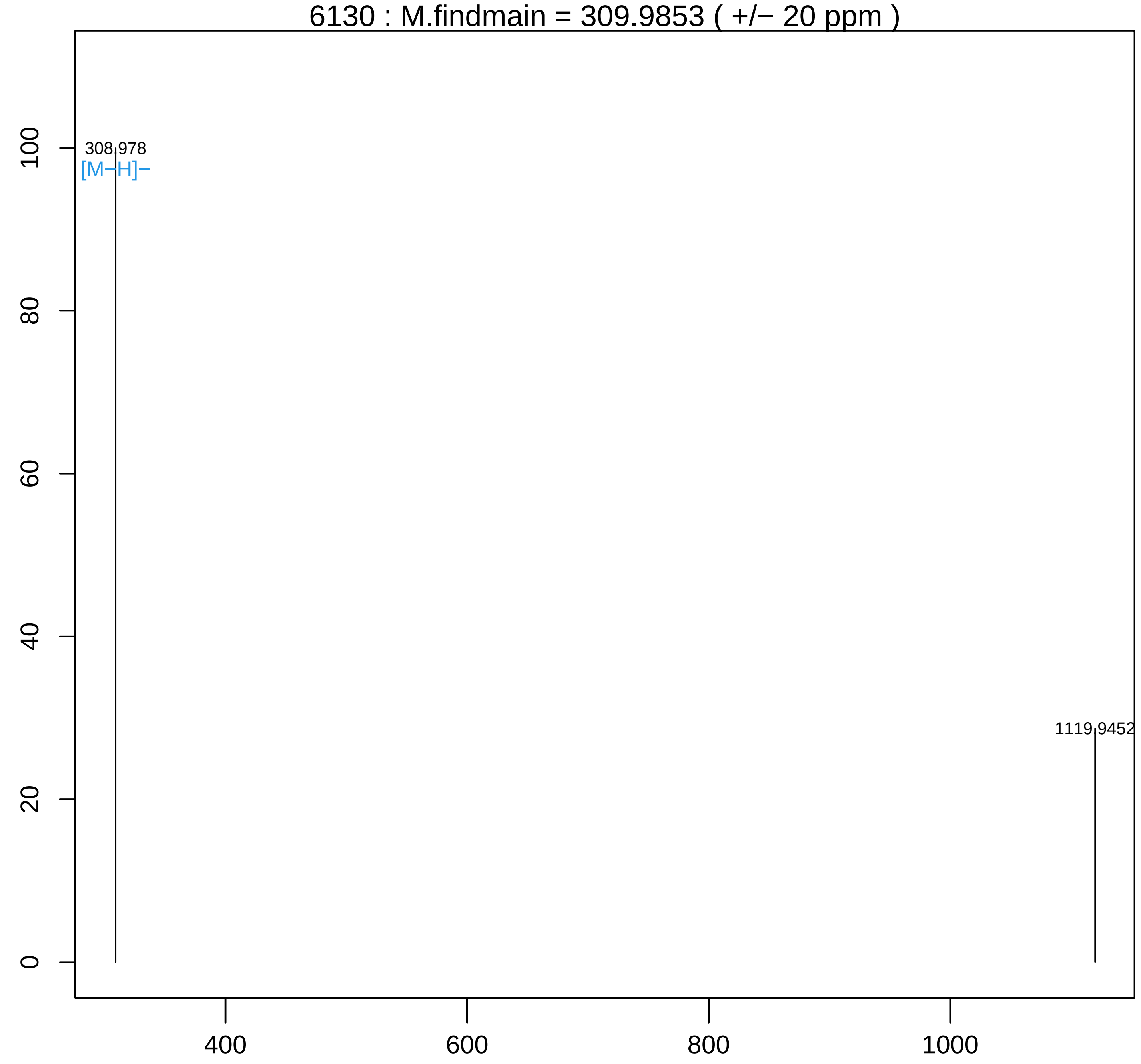

6130 : M.findmain = 309.9853 (+/− 20 ppm)
308.978
[M−H]−
1119.9452
0
20
40
60
80
100
400
600
800
1000

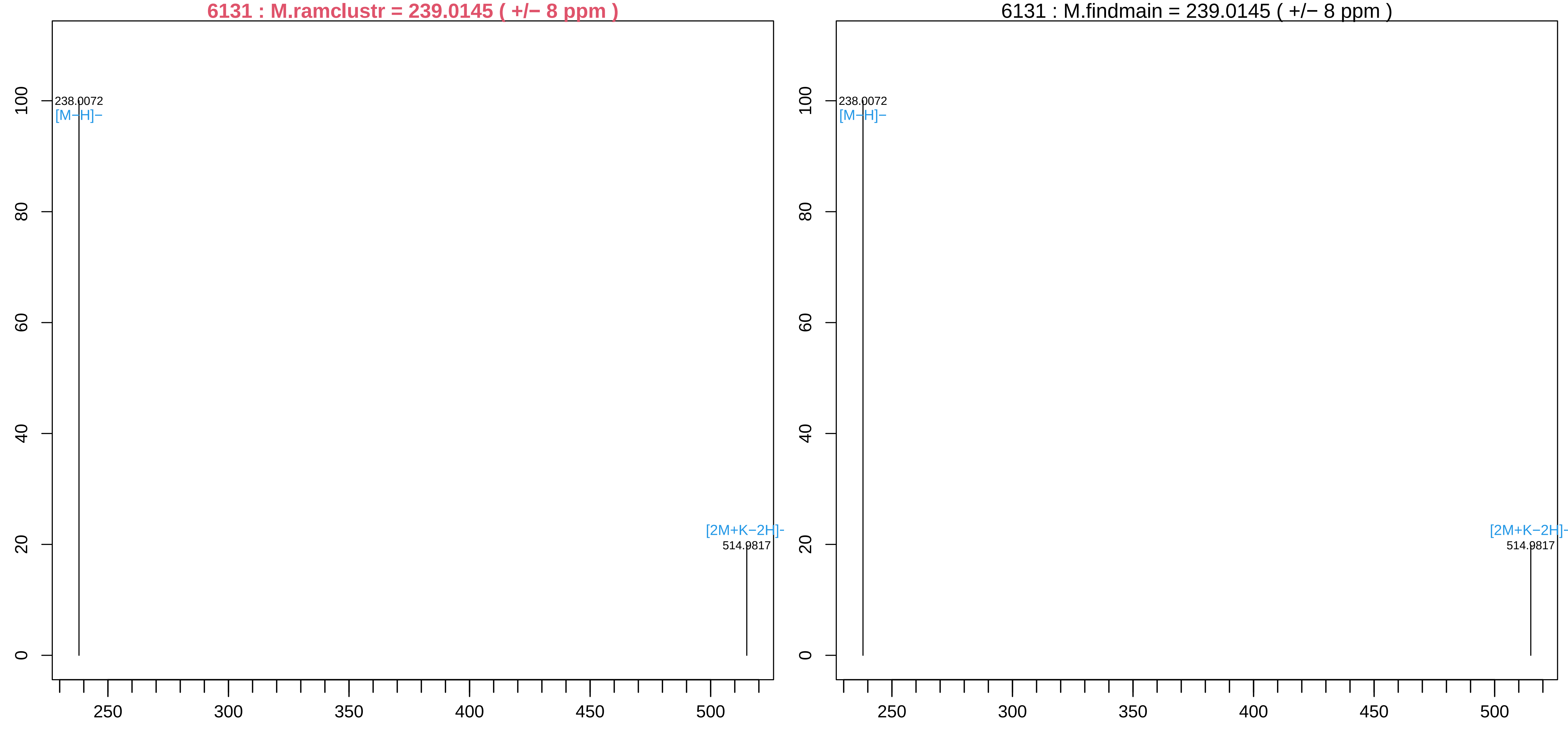
6131 : M.ramclustr = 239.0145 (+/− 8 ppm)
238.0072
[M−H]−
[2M+K−2H]-
514.9817
0
20
40
60
80
100
250
300
350
400
450
500
6131 : M.findmain = 239.0145 (+/− 8 ppm)
238.0072
[M−H]−
[2M+K−2H]-
514.9817
0
20
40
60
80
100
250
300
350
400
450
500

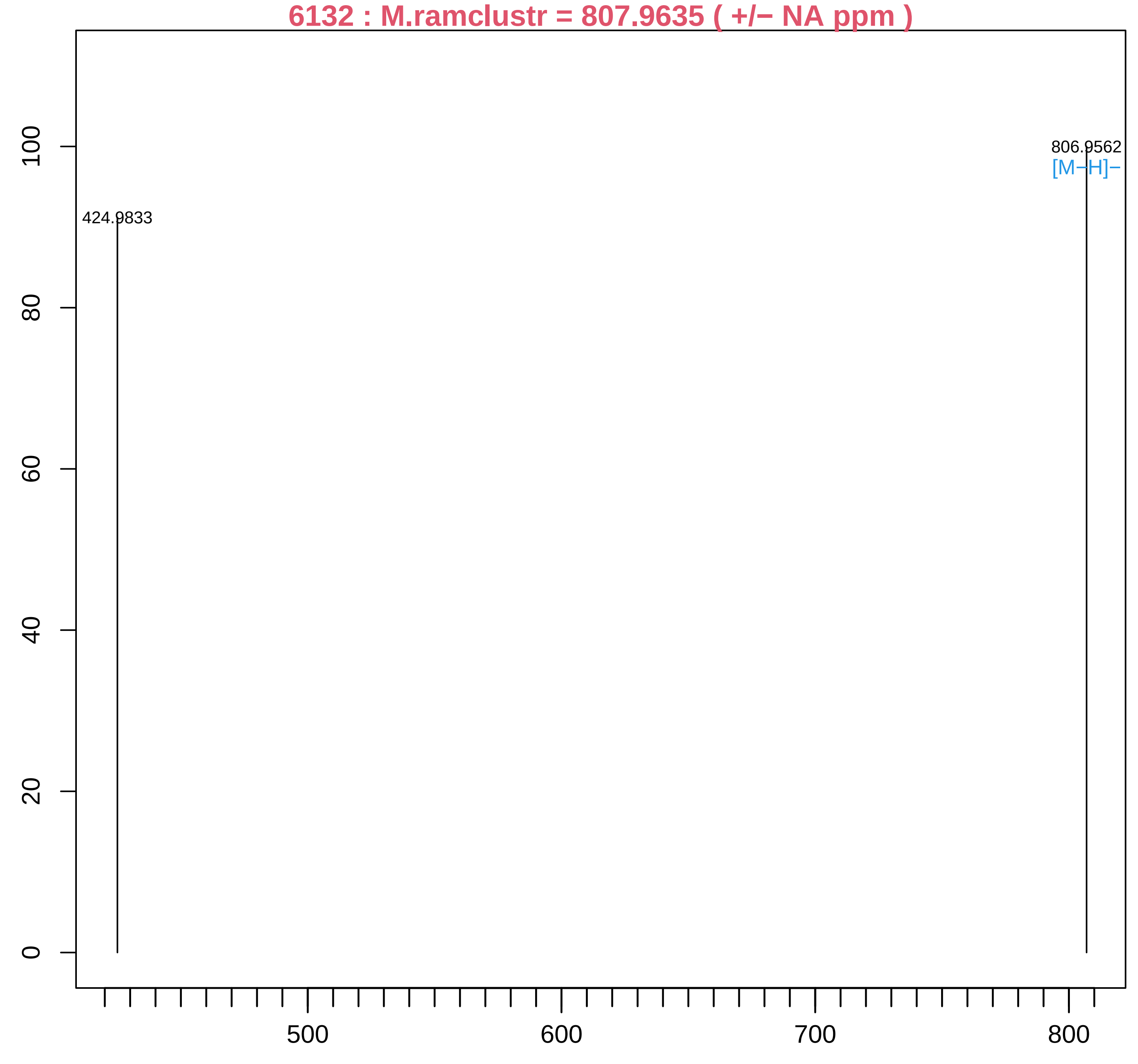
6132 : M.ramclustr = 807.9635 (+/− NA ppm)
806.9562
[M−H]−
424.9833
0
20
40
60
80
100
500
600
700
800

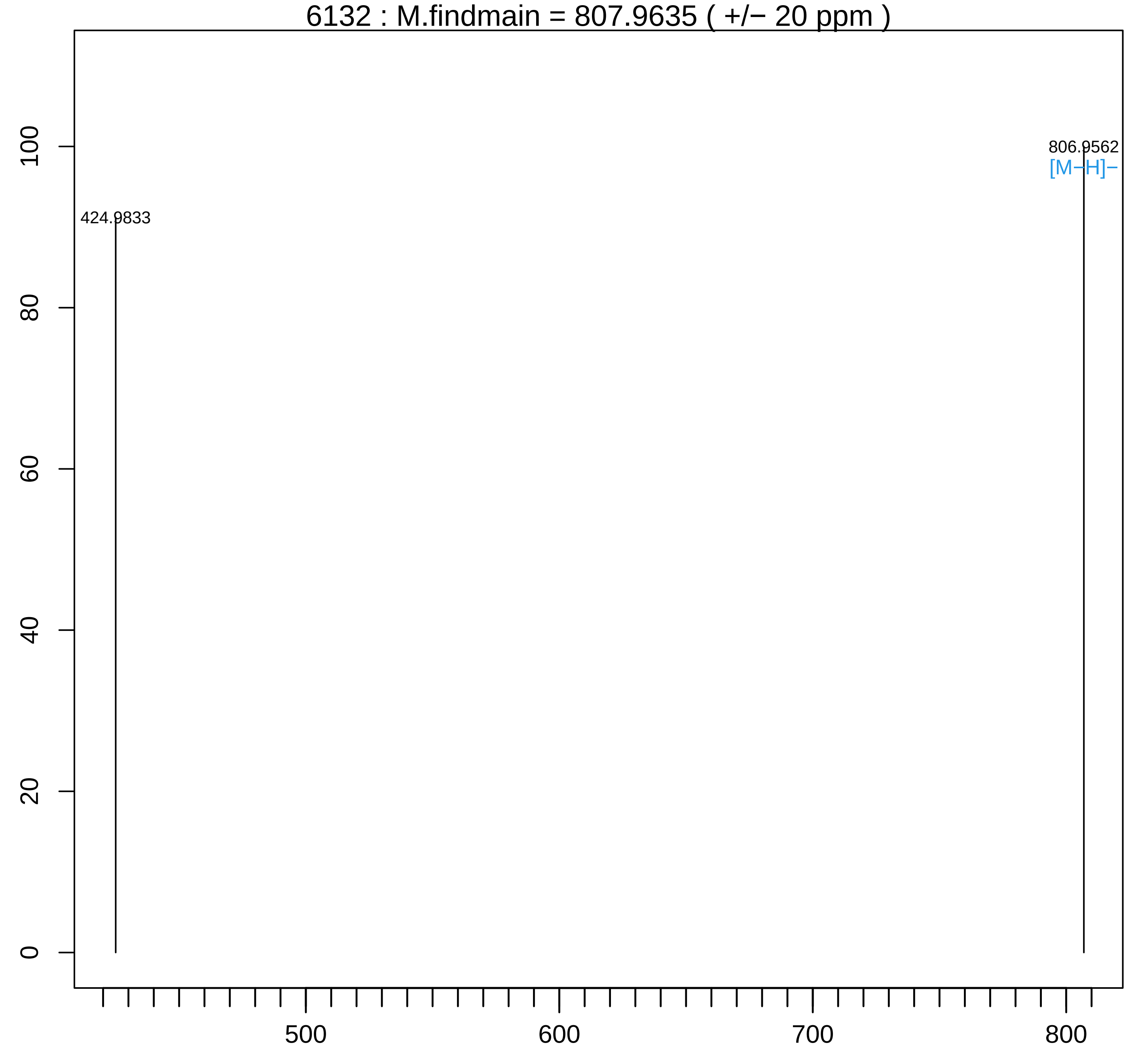
6132 : M.findmain = 807.9635 (+/− 20 ppm)
806.9562
[M−H]−
424.9833
0
20
40
60
80
100
500
600
700
800

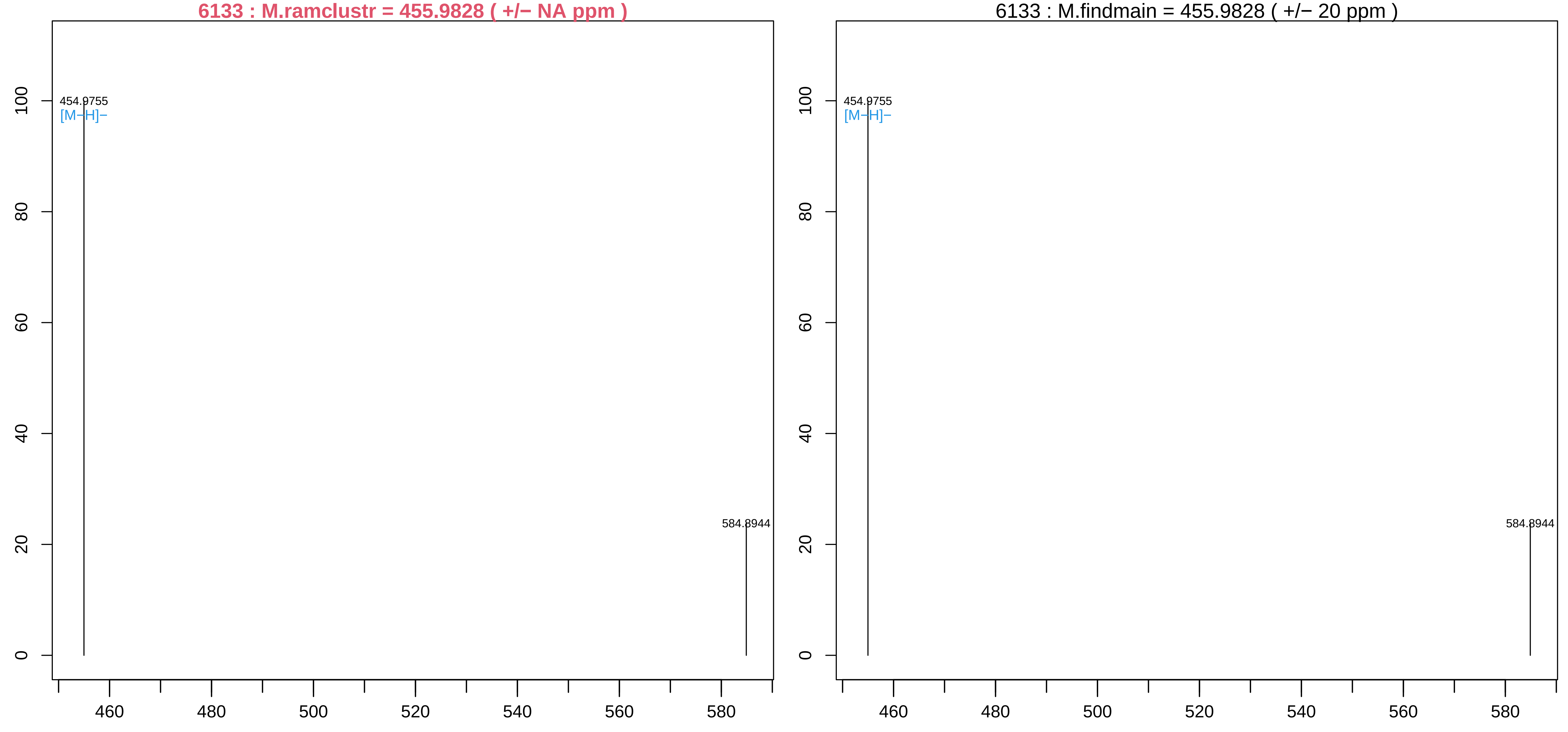

6133 : M.ramclustr = 455.9828 (+/− NA ppm)
454.9755
[M−H]−
584.8944
0
20
40
60
80
100
460
480
500
520
540
560
580
6133 : M.findmain = 455.9828 (+/− 20 ppm)
454.9755
[M−H]−
584.8944
0
20
40
60
80
100
460
480
500
520
540
560
580

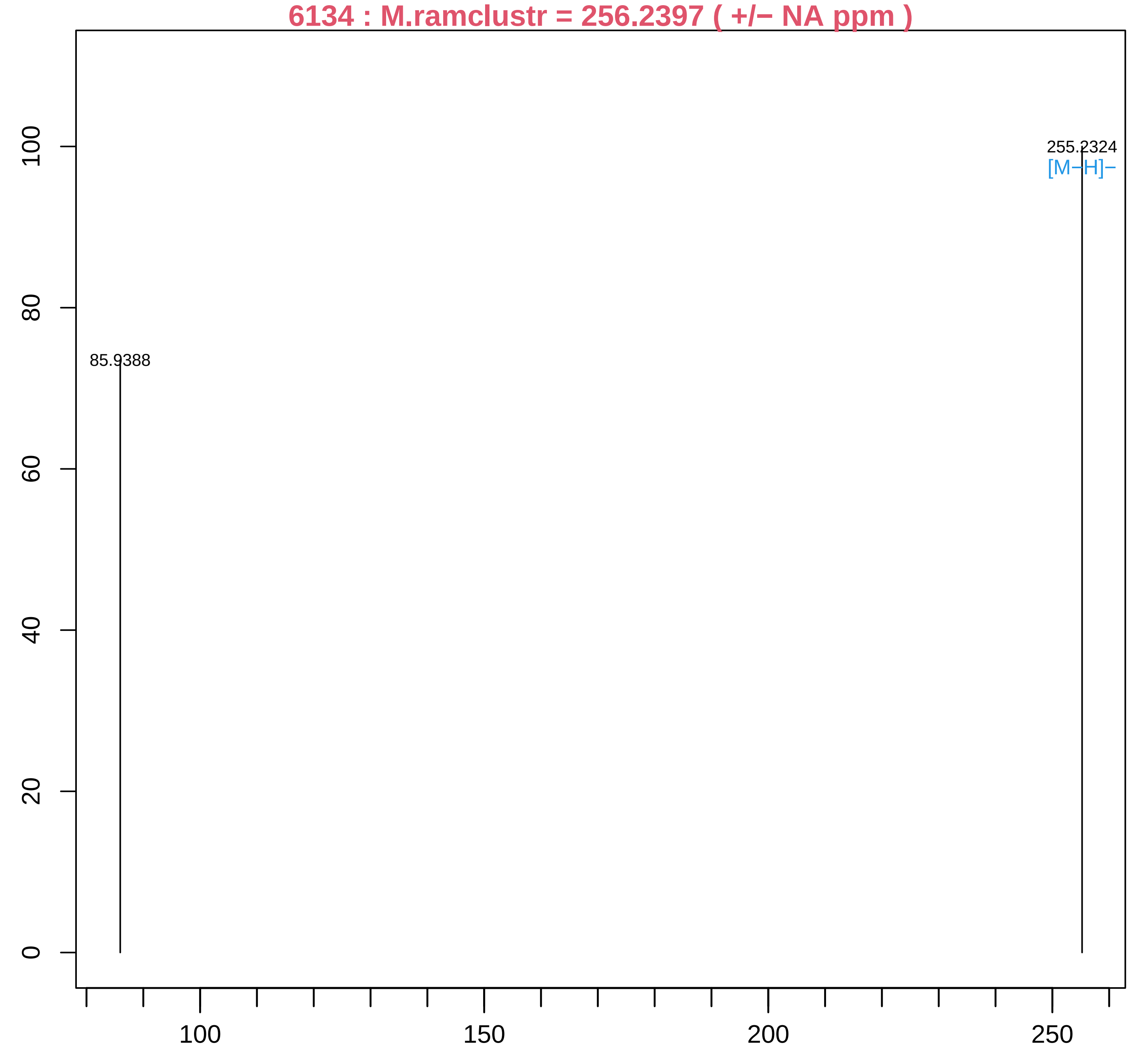
6134 : M.ramclustr = 256.2397 (+/− NA ppm)
255.2324
[M−H]−
85.9388
0
20
40
60
80
100
100
150
200
250

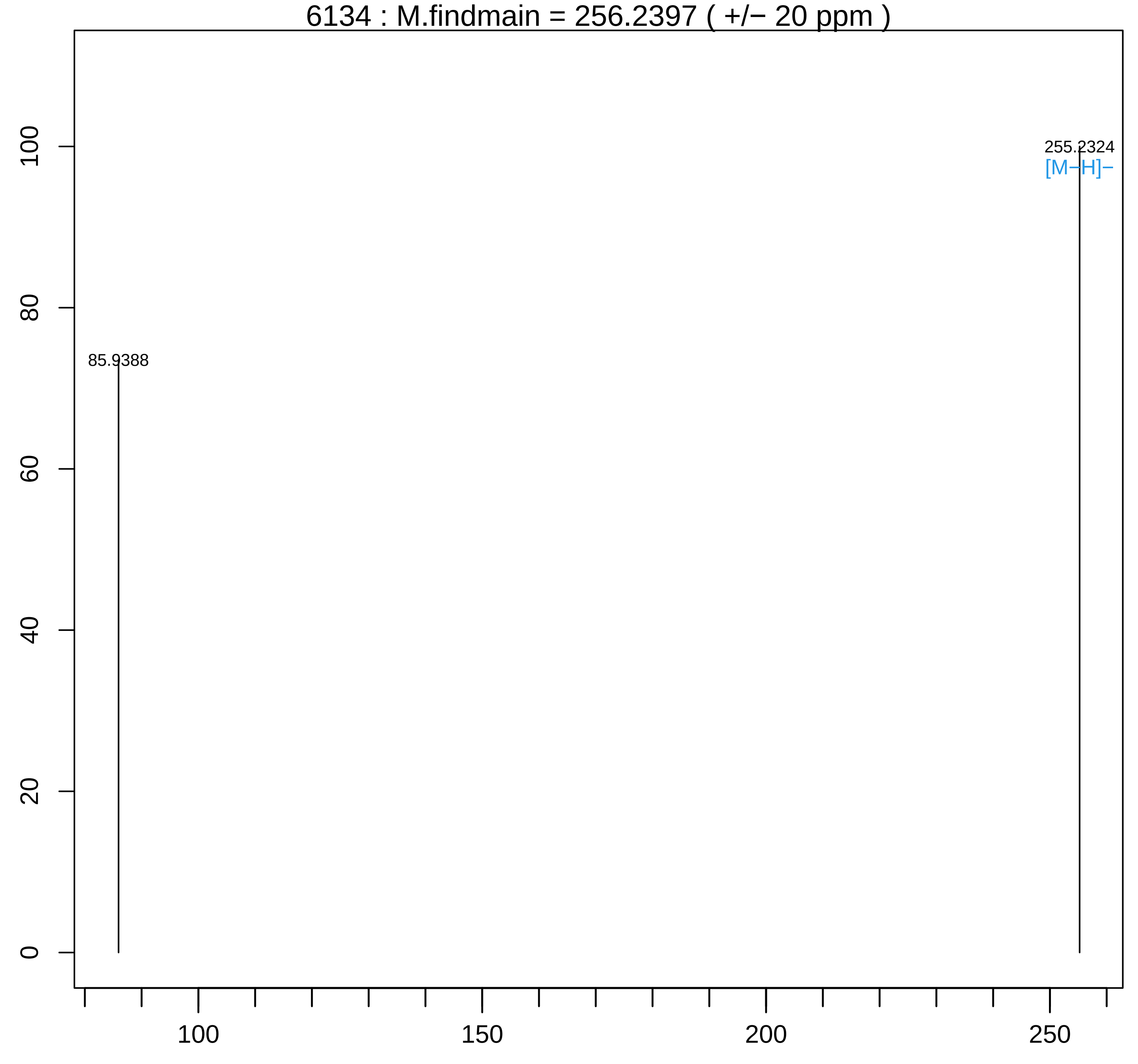
6134 : M.findmain = 256.2397 (+/− 20 ppm)
255.2324
[M−H]−
85.9388
0
20
40
60
80
100
100
150
200
250

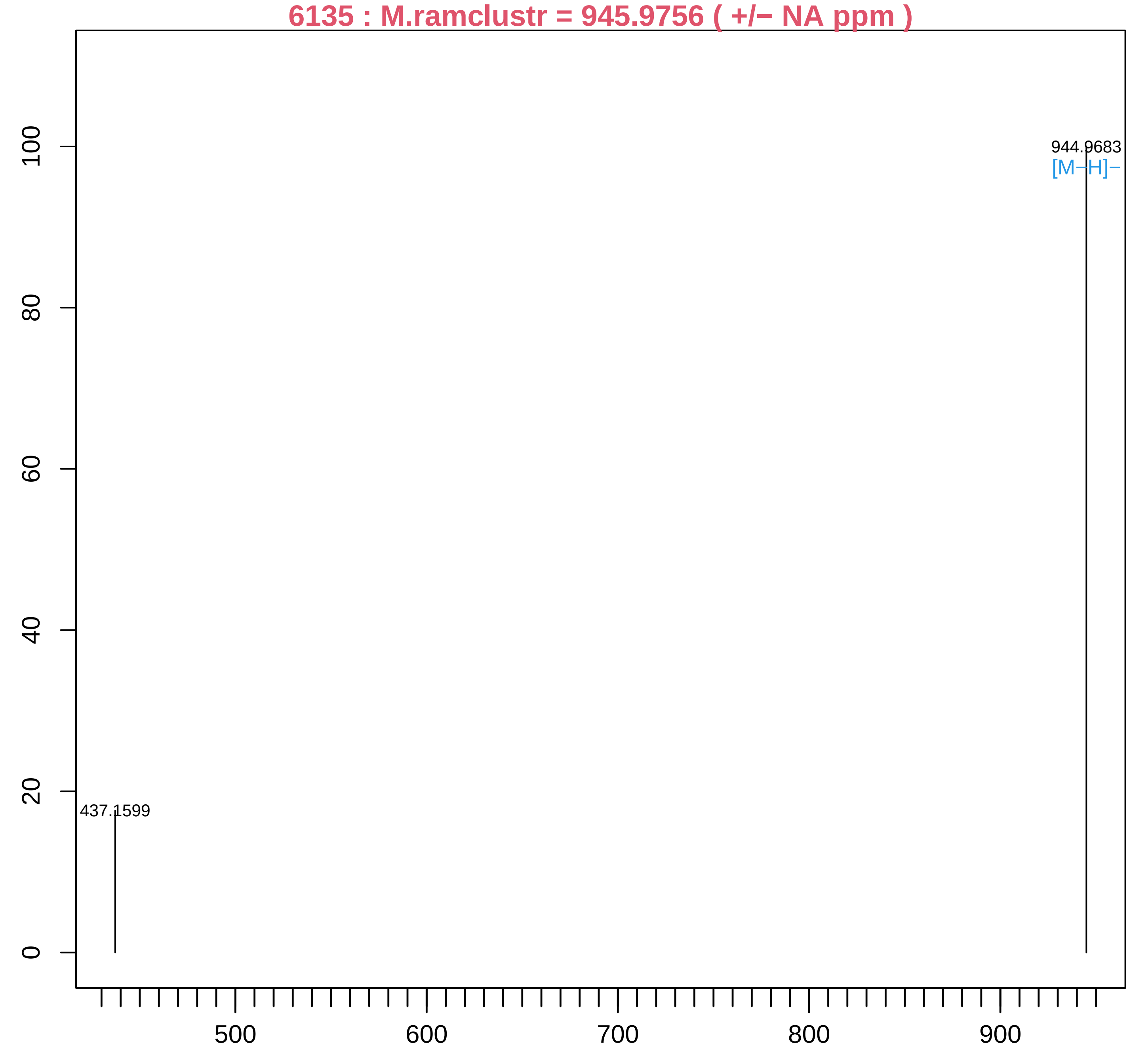
6135 : M.ramclustr = 945.9756 (+/− NA ppm)
944.9683
[M−H]−
437.1599
0
20
40
60
80
100
500
600
700
800
900

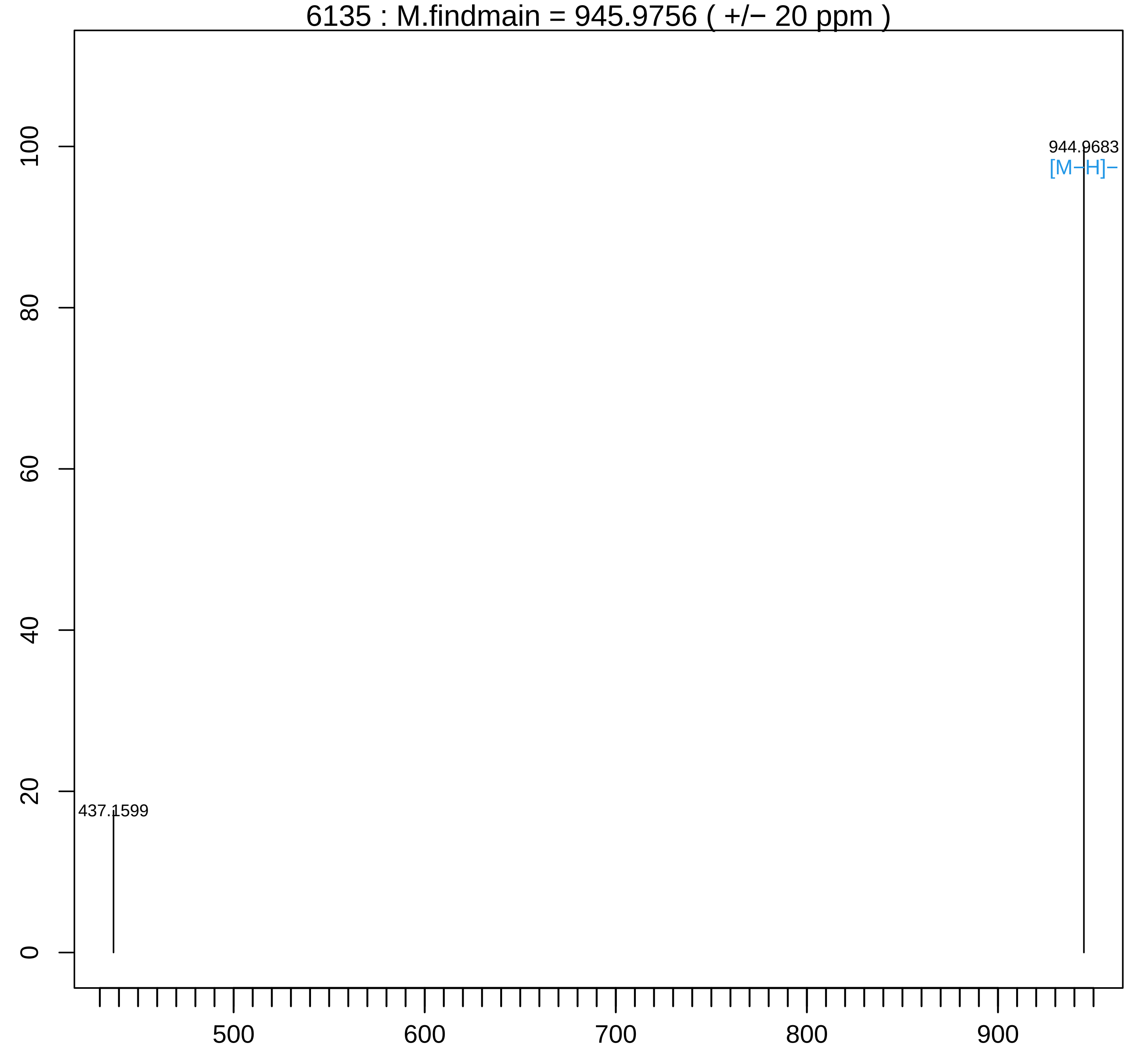
6135 : M.findmain = 945.9756 (+/− 20 ppm)
944.9683
[M−H]−
437.1599
0
20
40
60
80
100
500
600
700
800
900

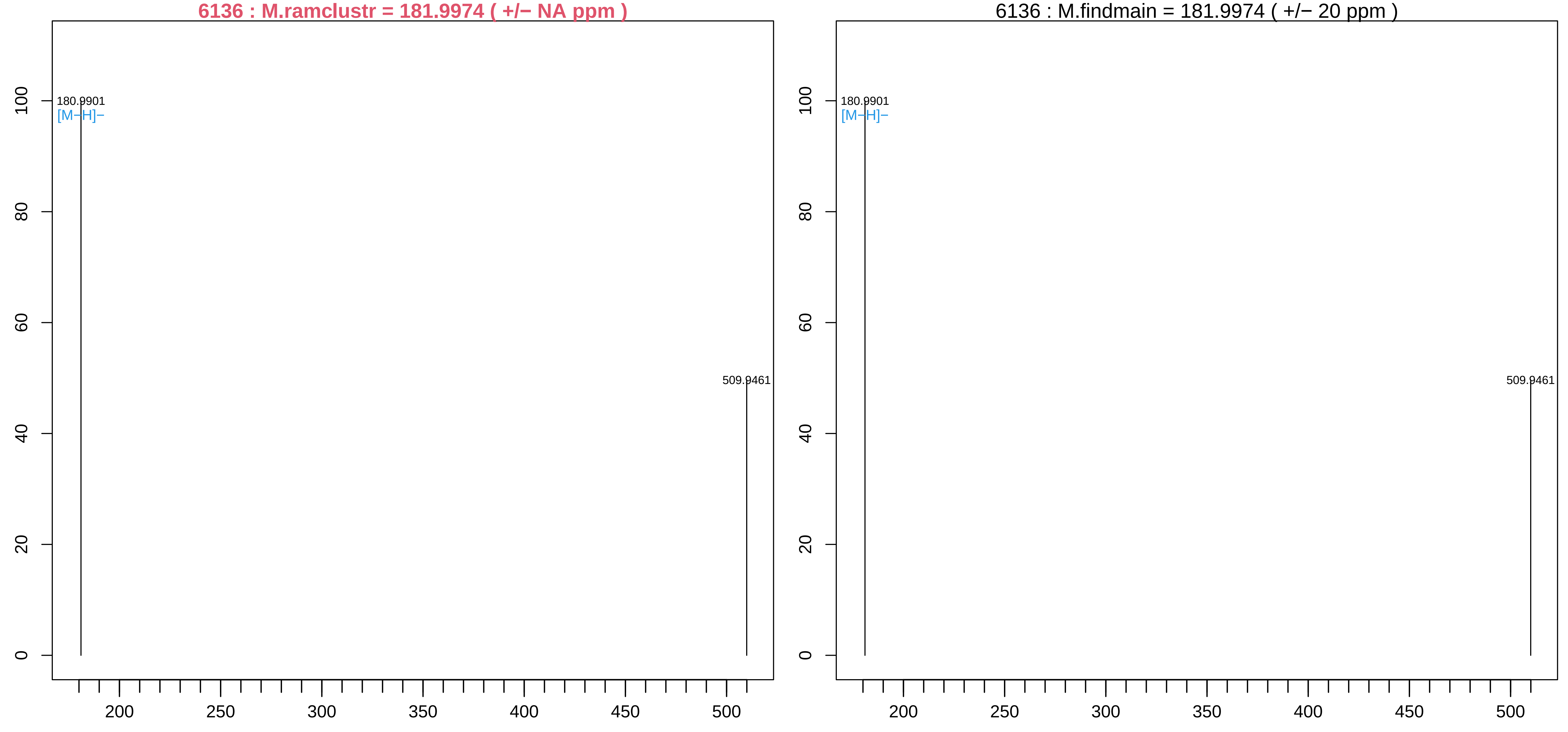
6136 : M.ramclustr = 181.9974 (+/− NA ppm)
6136 : M.findmain = 181.9974 (+/− 20 ppm)
180.9901
[M−H]−
509.9461
0
20
40
60
80
100
200
250
300
350
400
450
500

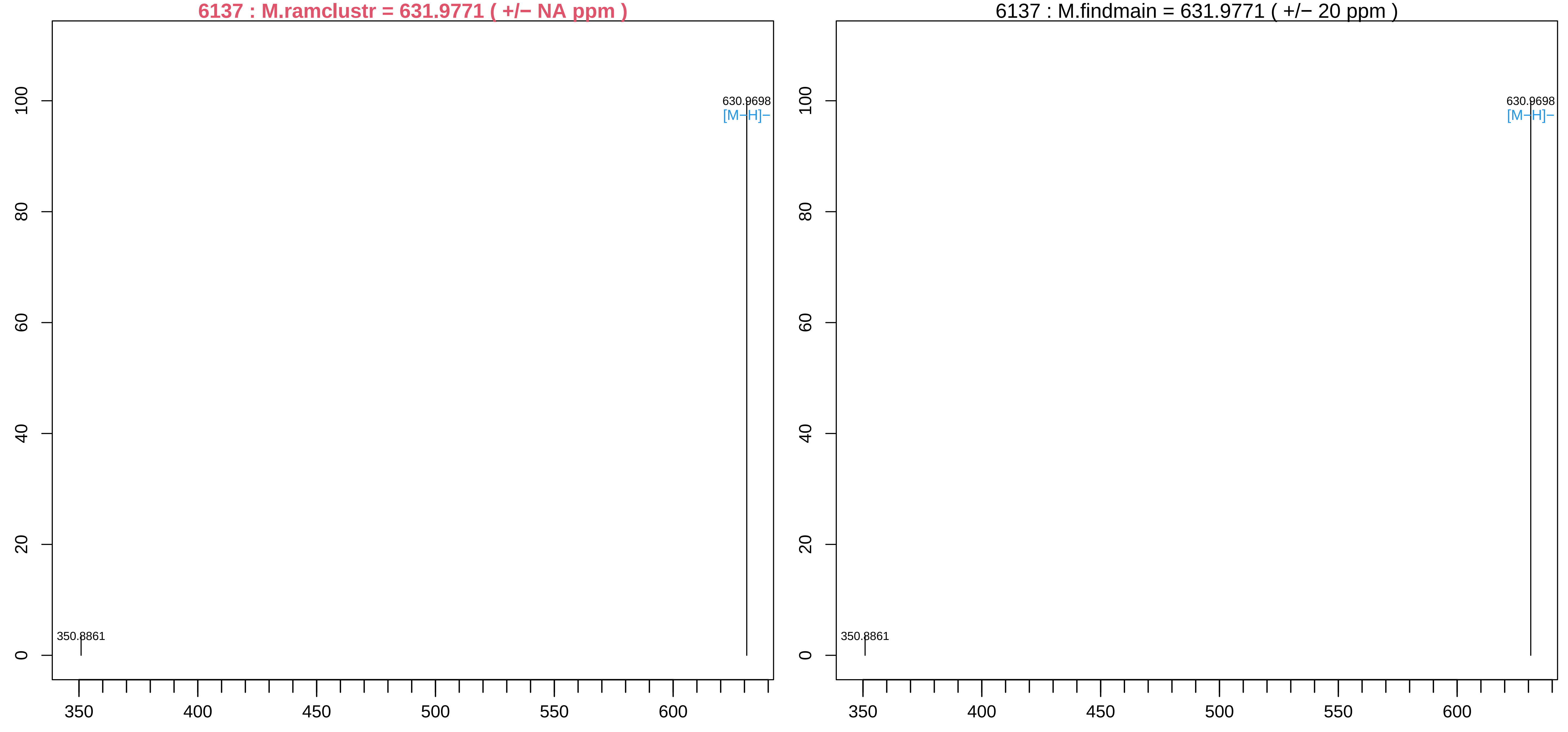
6137 : M.ramclustr = 631.9771 (+/− NA ppm)
6137 : M.findmain = 631.9771 (+/− 20 ppm)
630.9698
[M−H]−
350.8861
630.9698
[M−H]−
350.8861
0
20
40
60
80
100
350
400
450
500
550
600

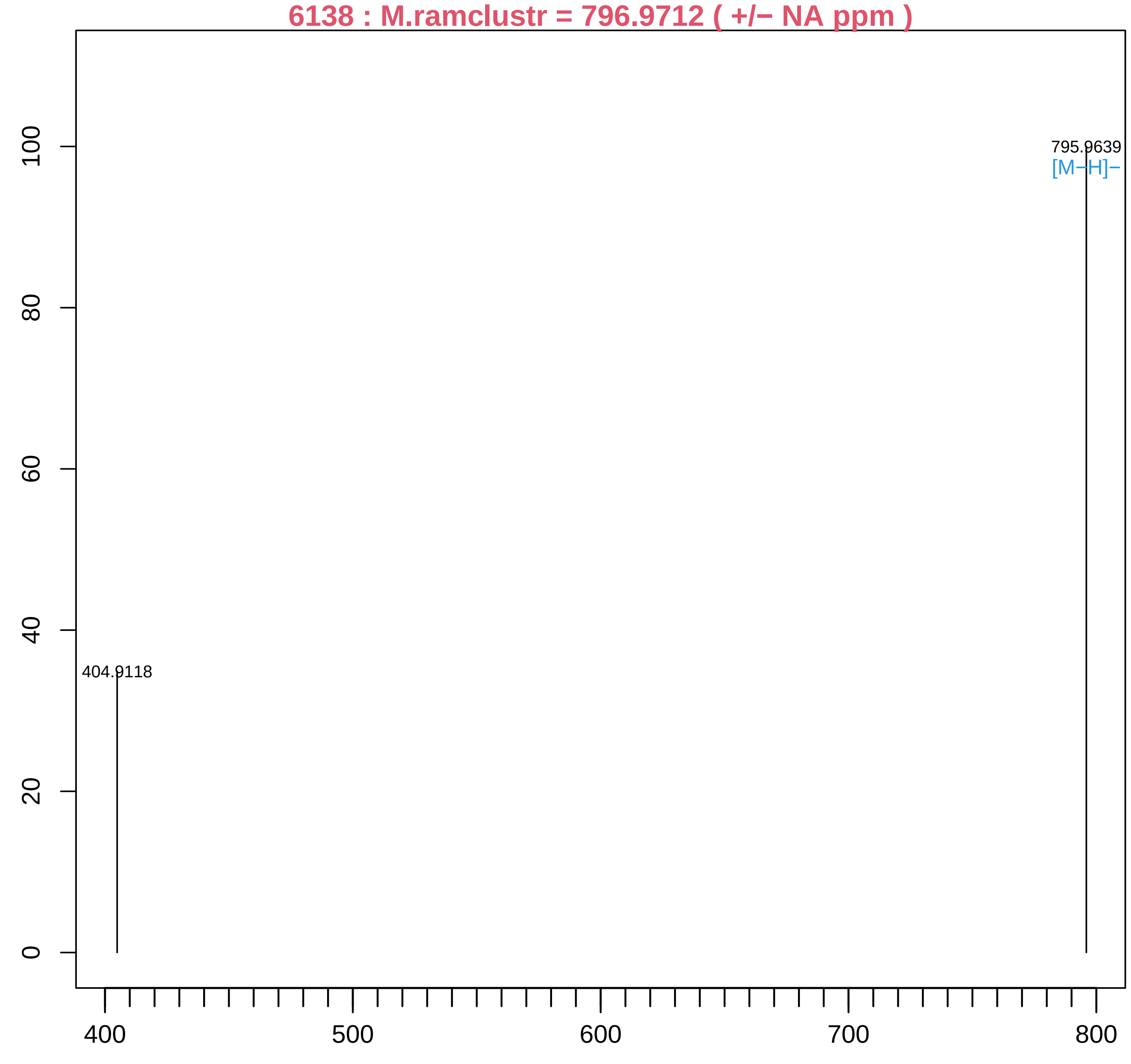
6138 : M.ramclustr = 796.9712 (+/− NA ppm)
795.9639
[M−H]−
404.9118
0
20
40
60
80
100
400
500
600
700
800

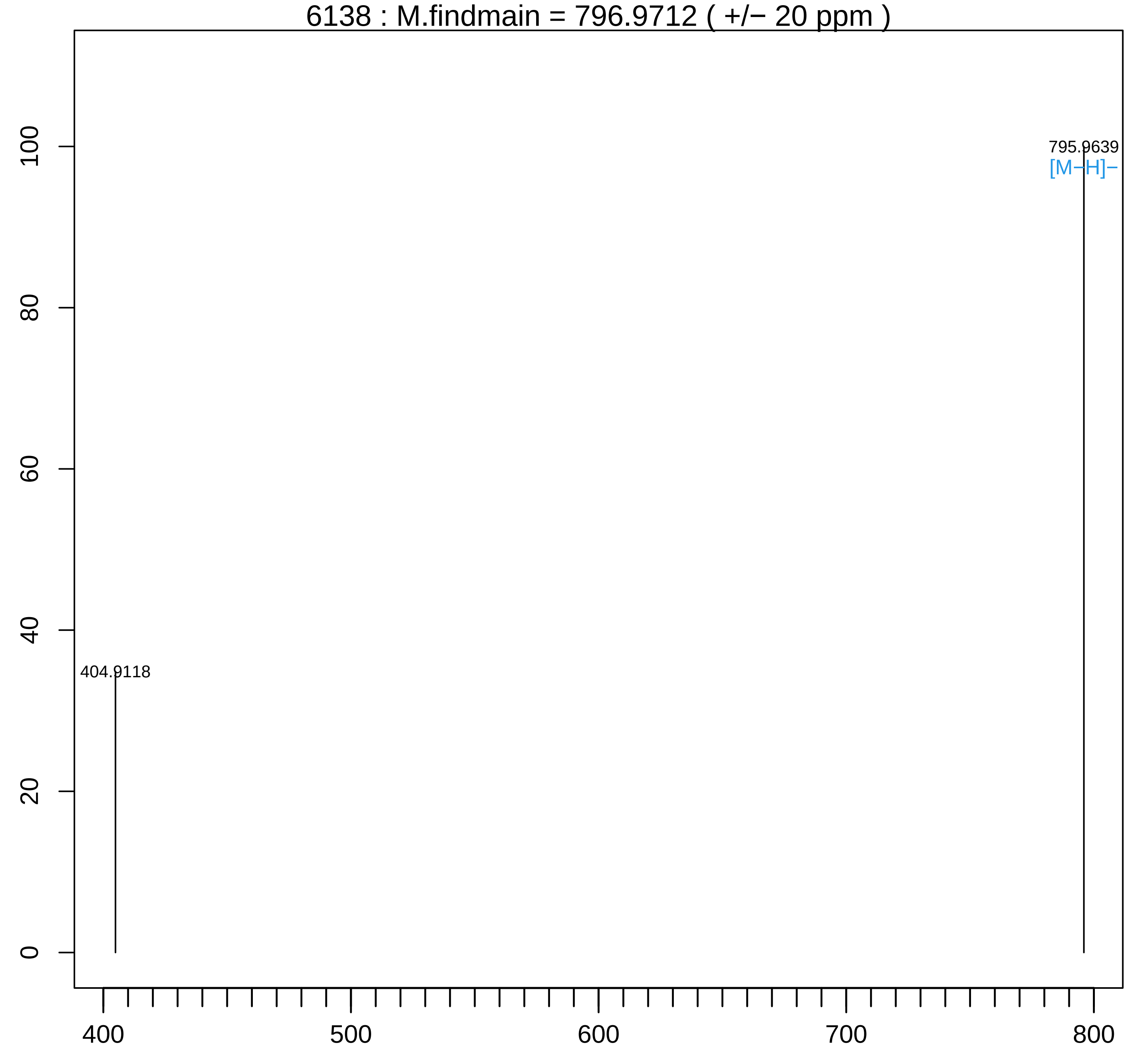
6138 : M.findmain = 796.9712 (+/− 20 ppm)
795.9639
[M−H]−
404.9118
0
20
40
60
80
100
400
500
600
700
800

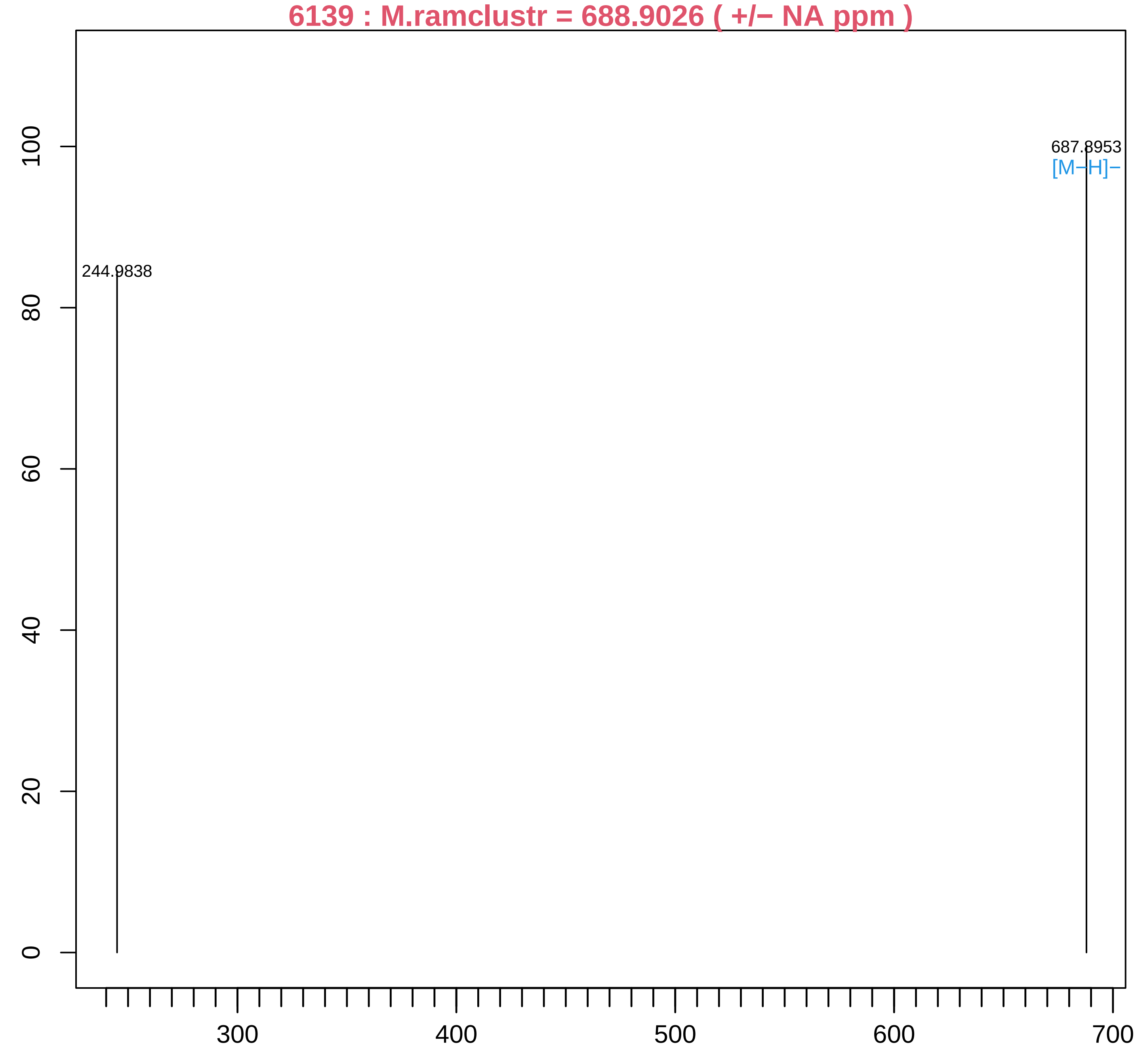
6139 : M.ramclustr = 688.9026 (+/− NA ppm)
687.8953
[M−H]−
244.9838
0
20
40
60
80
100
300
400
500
600
700

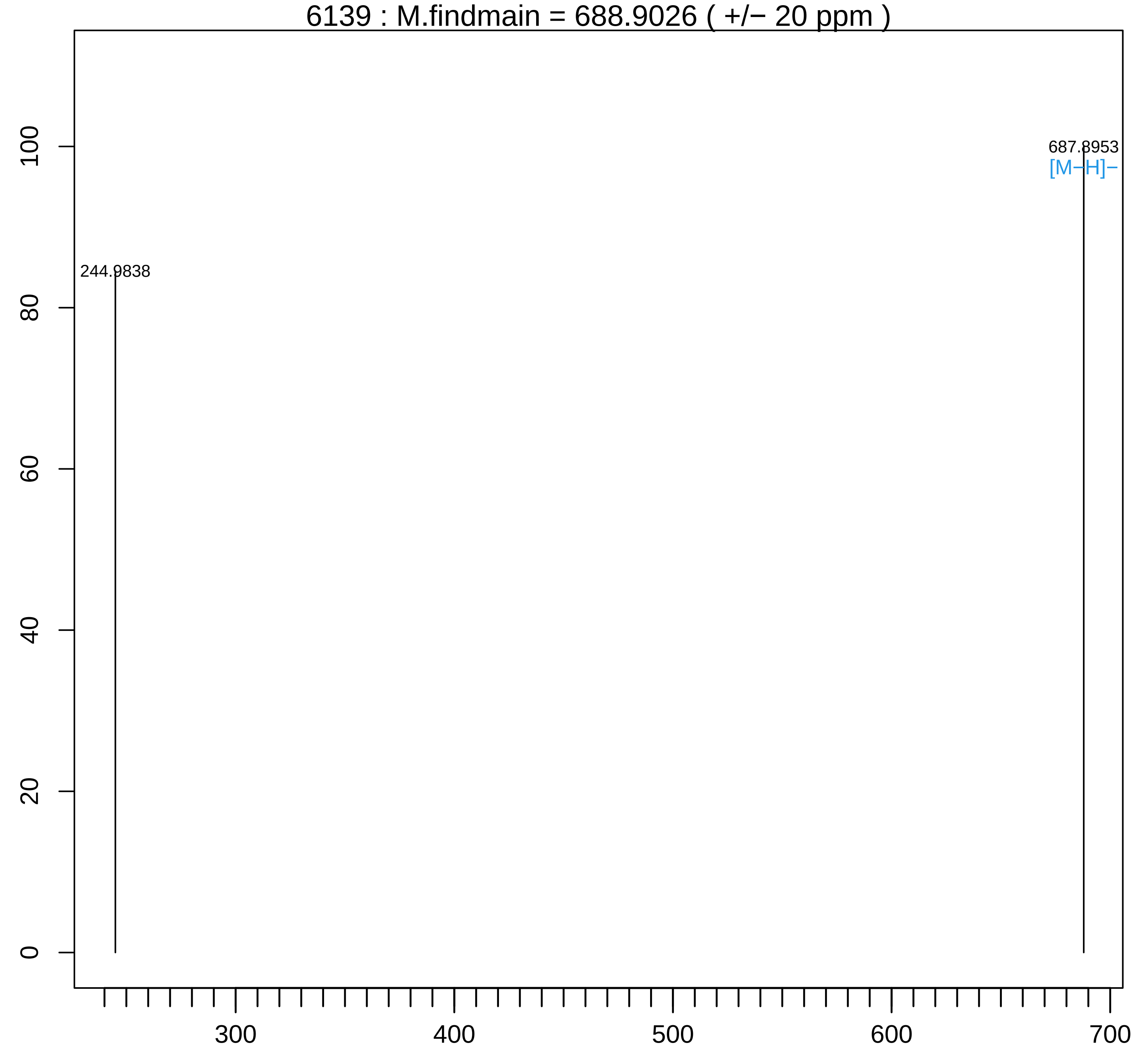
6139 : M.findmain = 688.9026 (+/− 20 ppm)
687.8953
[M−H]−
244.9838
0
20
40
60
80
100
300
400
500
600
700

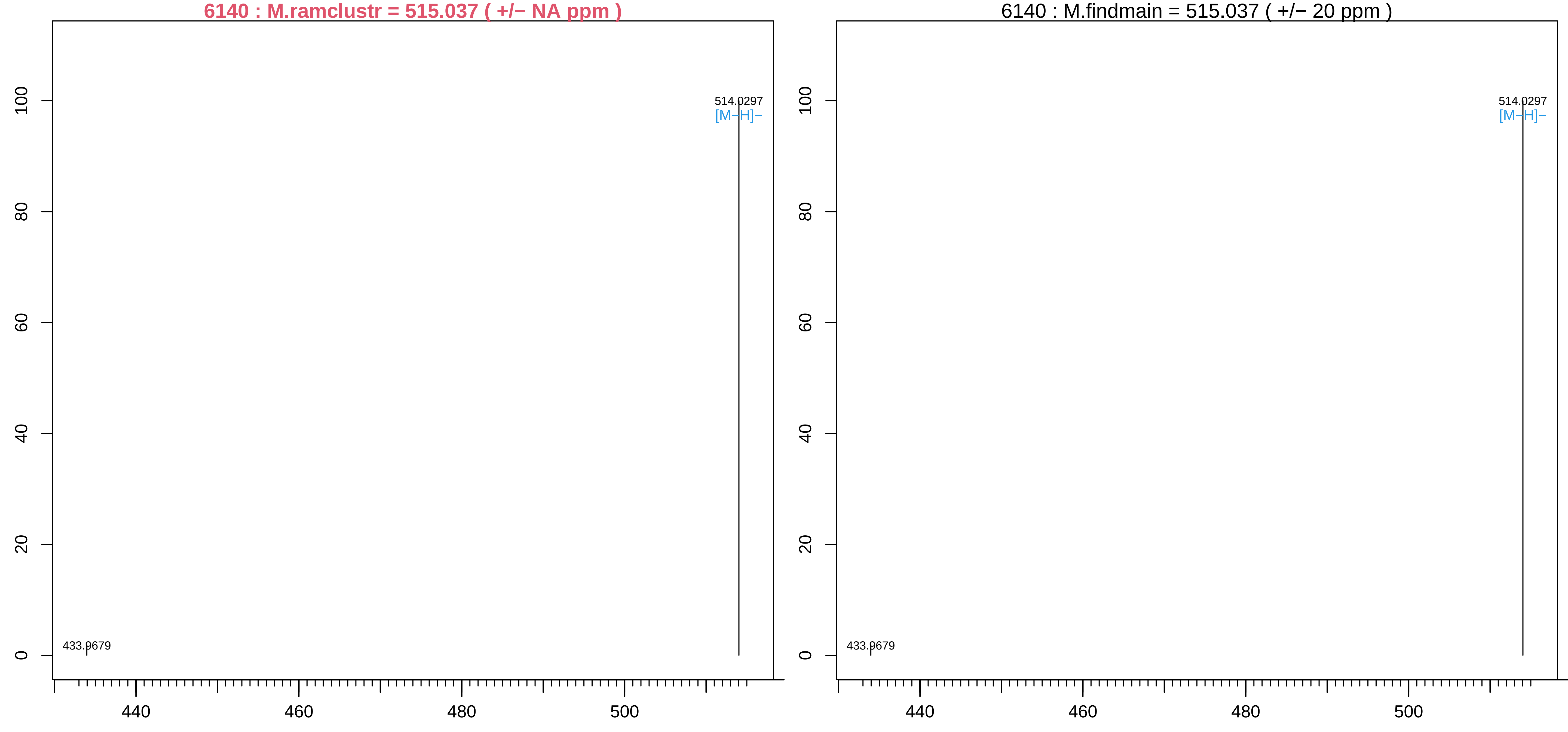
6140 : M.ramclustr = 515.037 (+/− NA ppm)
6140 : M.findmain = 515.037 (+/− 20 ppm)
514.0297
[M−H]−
433.9679
0
20
40
60
80
100
440
460
480
500

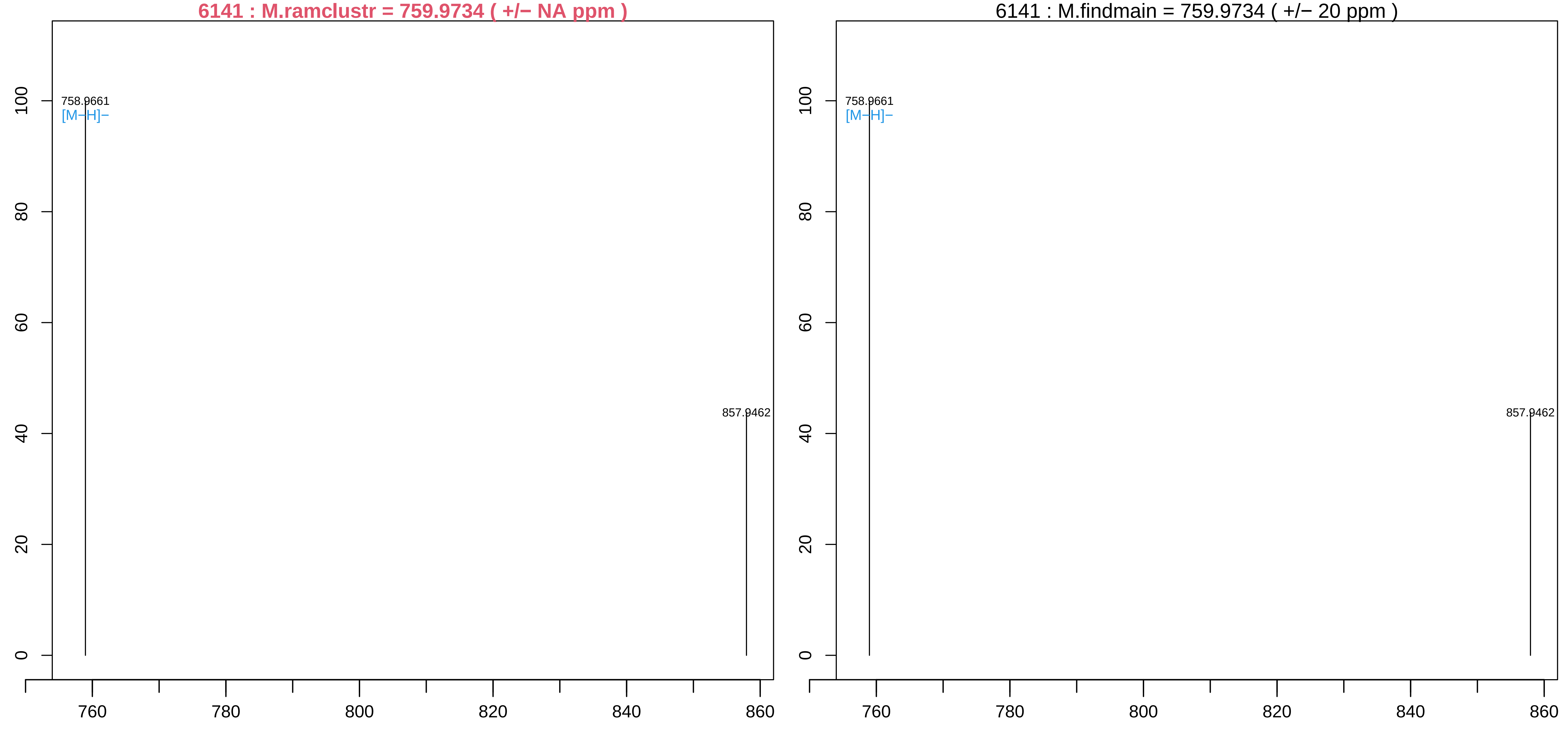
6141 : M.ramclustr = 759.9734 (+/− NA ppm)
758.9661
[M−H]−
857.9462
6141 : M.findmain = 759.9734 (+/− 20 ppm)
758.9661
[M−H]−
857.9462
0
20
40
60
80
100
760
780
800
820
840
860

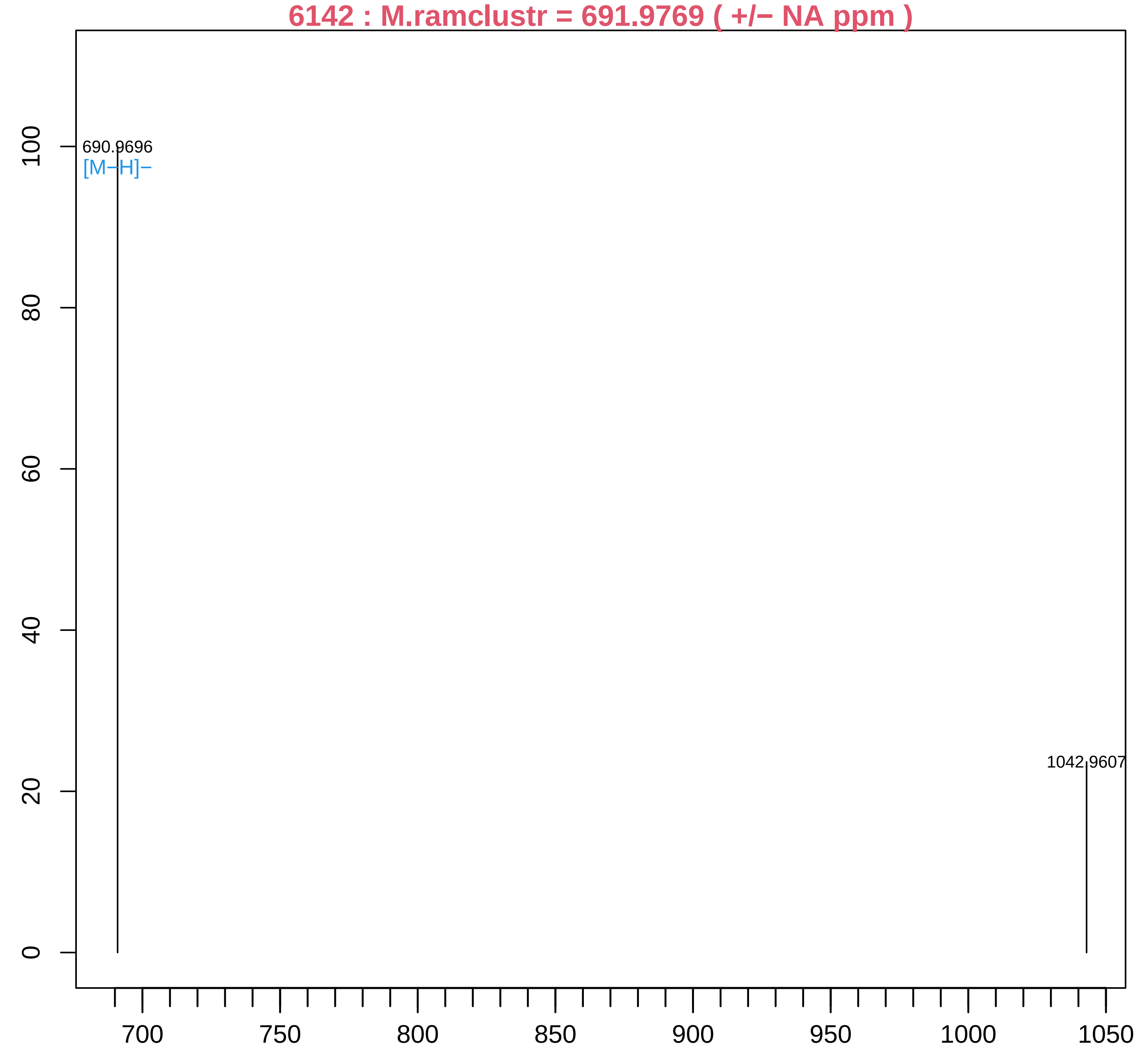
6142 : M.ramclustr = 691.9769 (+/− NA ppm)
690.9696
[M−H]−
1042.9607
0
20
40
60
80
100
700
750
800
850
900
950
1000
1050

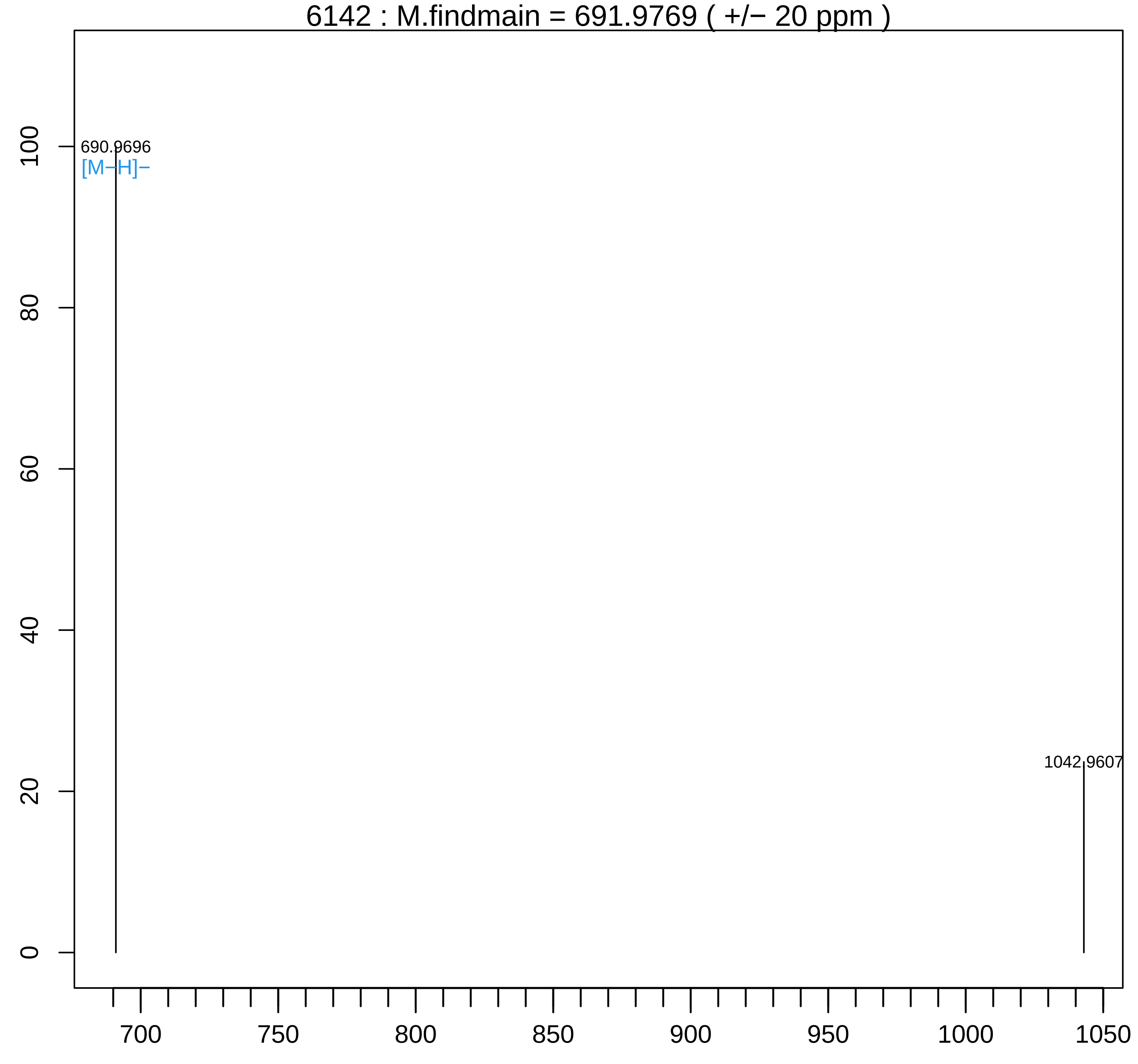
6142 : M.findmain = 691.9769 (+/− 20 ppm)
690.9696
[M−H]−
1042.9607
0
20
40
60
80
100
700
750
800
850
900
950
1000
1050

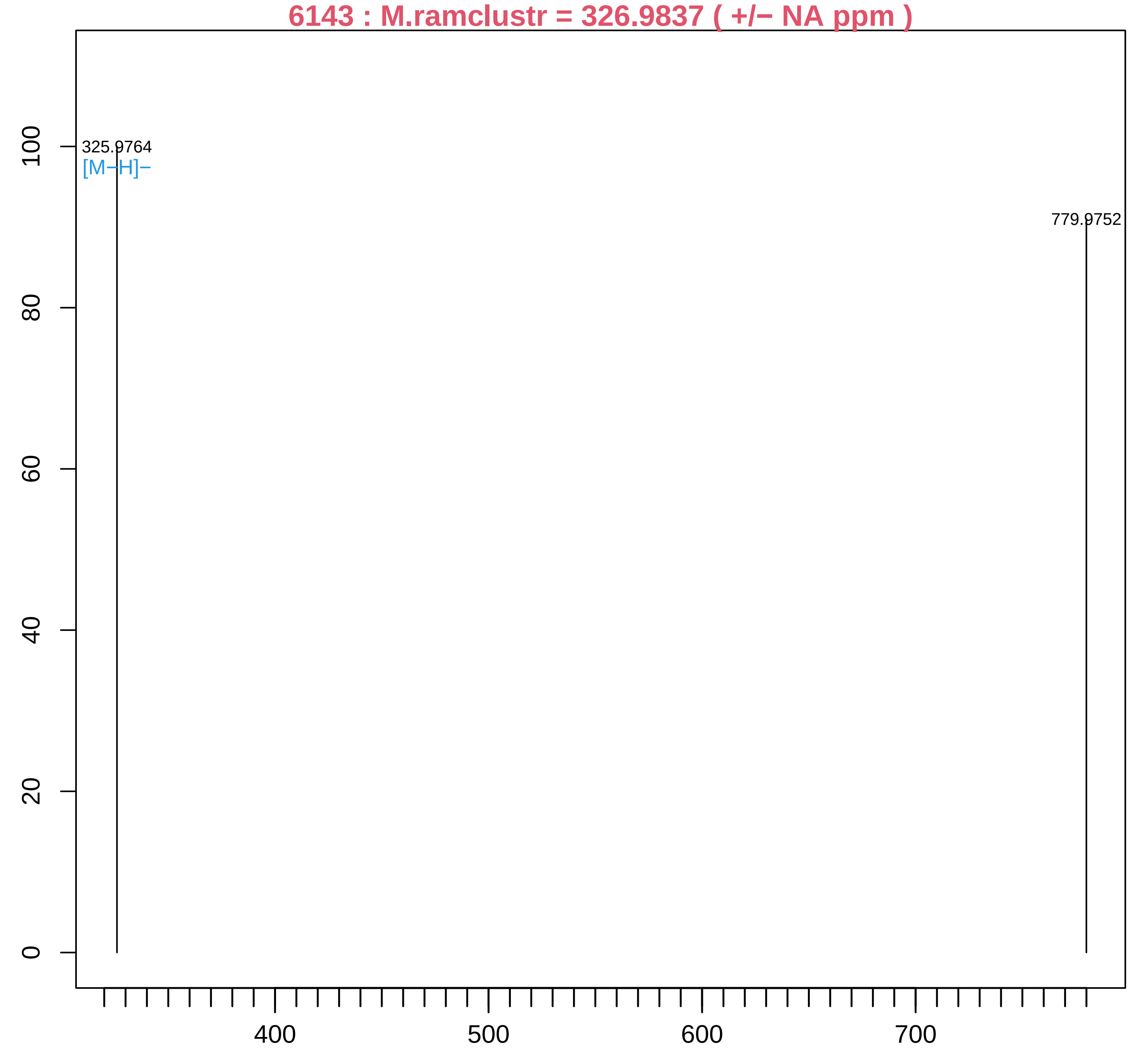
6143 : M.ramclustr = 326.9837 (+/− NA ppm)
325.9764
[M−H]−
779.9752
0
20
40
60
80
100
400
500
600
700

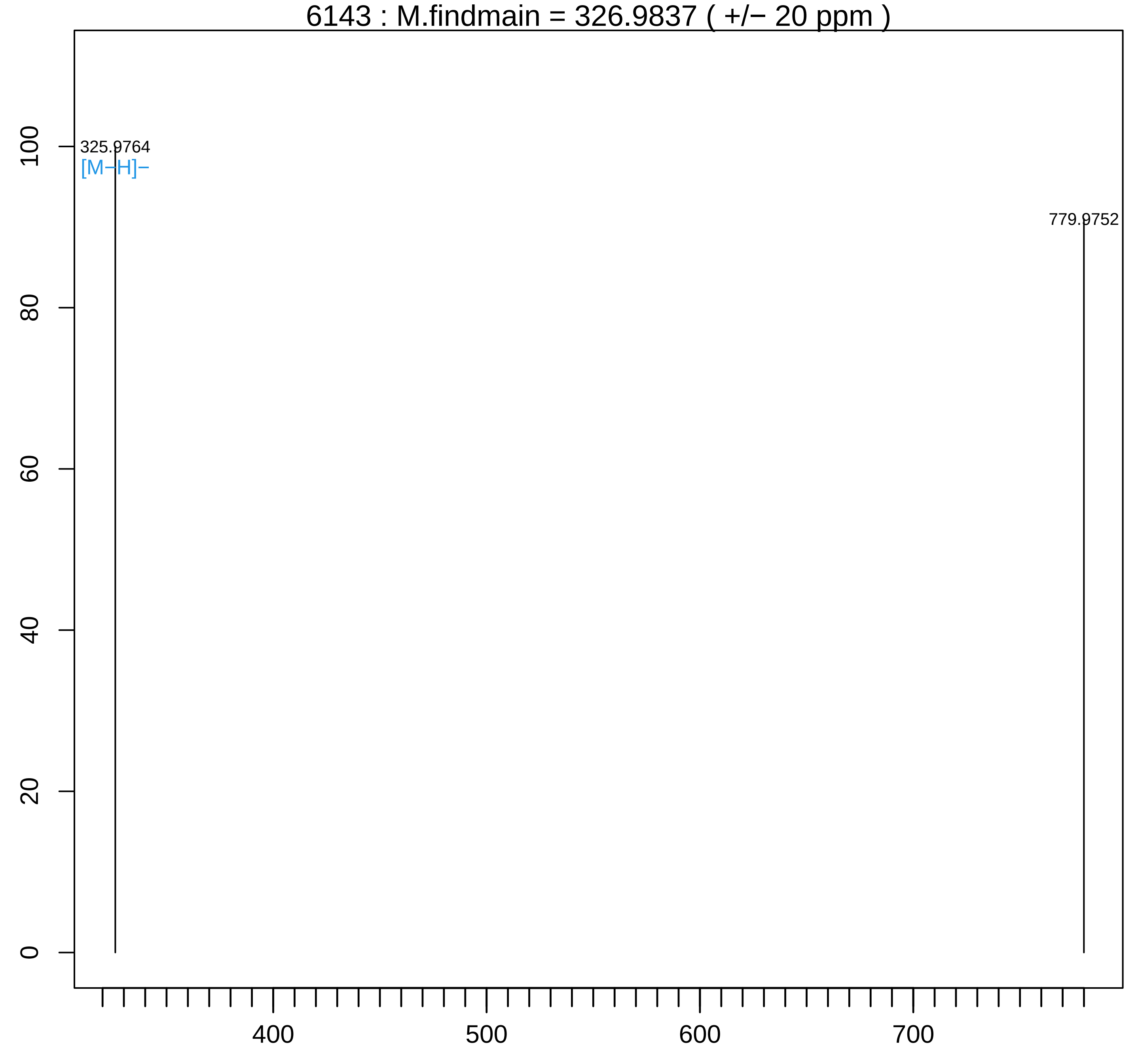
6143 : M.findmain = 326.9837 (+/− 20 ppm)
325.9764
[M−H]−
779.9752
0
20
40
60
80
100
400
500
600
700

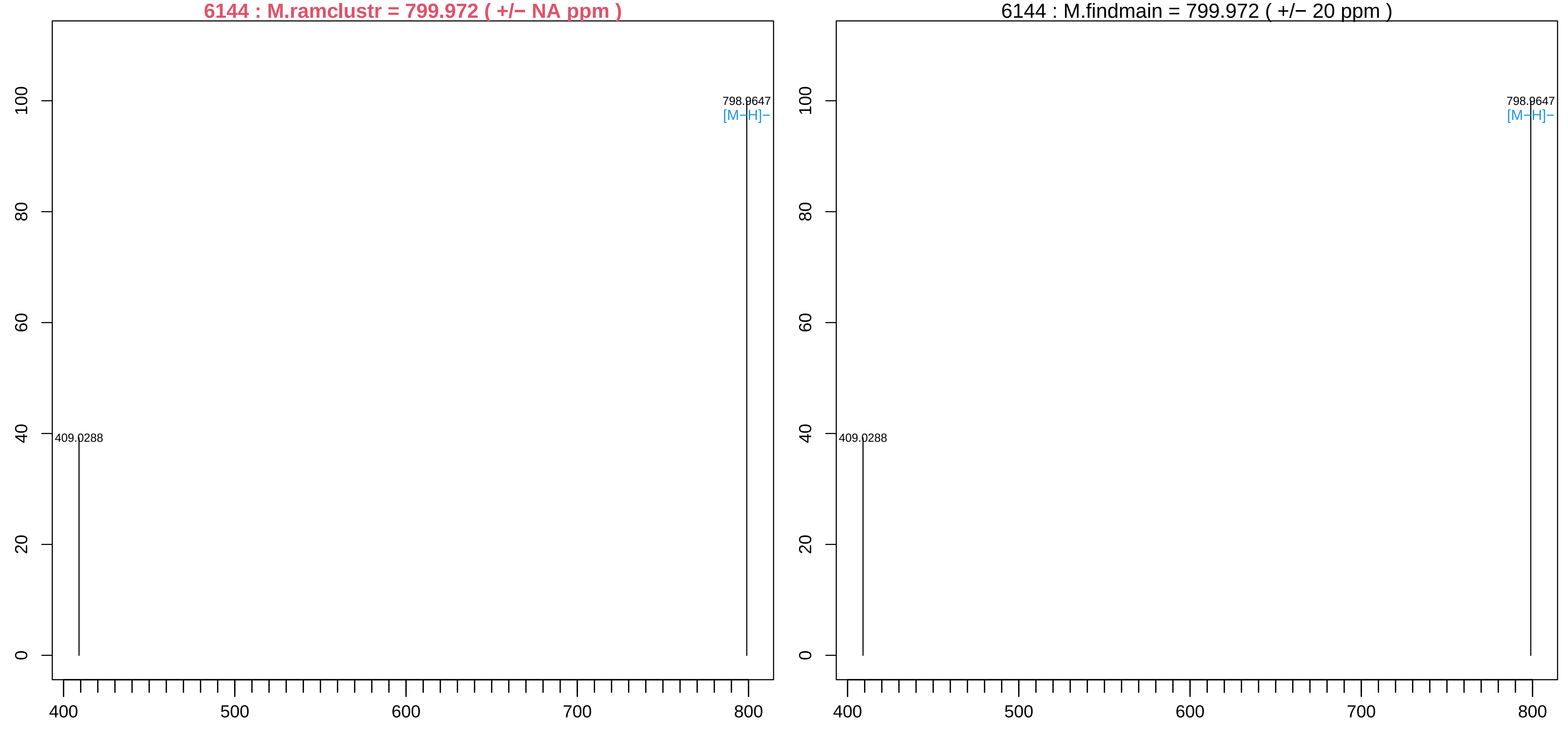
6144 : M.ramclustr = 799.972 (+/− NA ppm)
798.9647
[M−H]−
409.0288
6144 : M.findmain = 799.972 (+/− 20 ppm)
798.9647
[M−H]−
409.0288
0
20
40
60
80
100
400
500
600
700
800

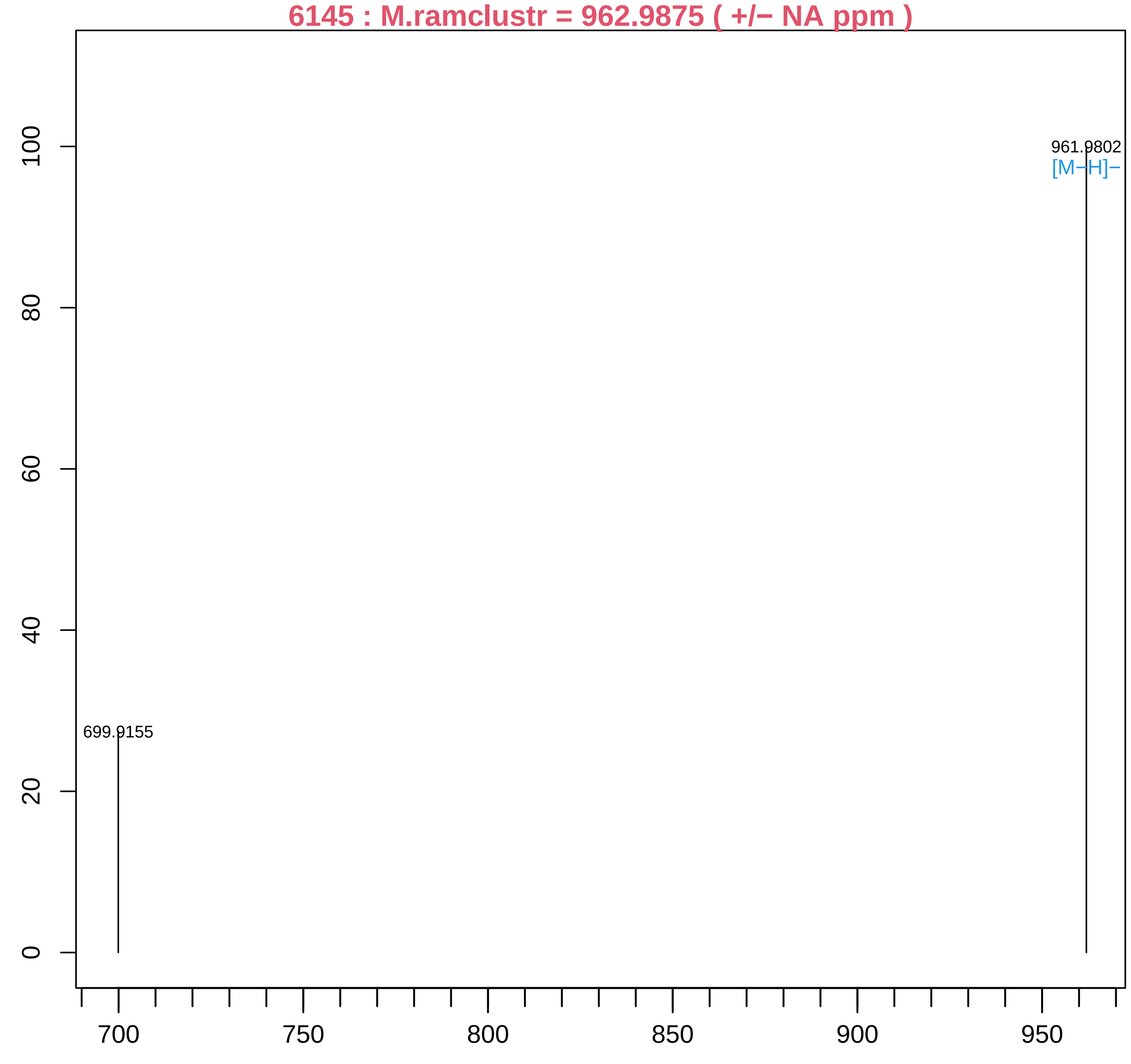
6145 : M.ramclustr = 962.9875 (+/− NA ppm)
961.9802
[M−H]−
699.9155
0
20
40
60
80
100
700
750
800
850
900
950

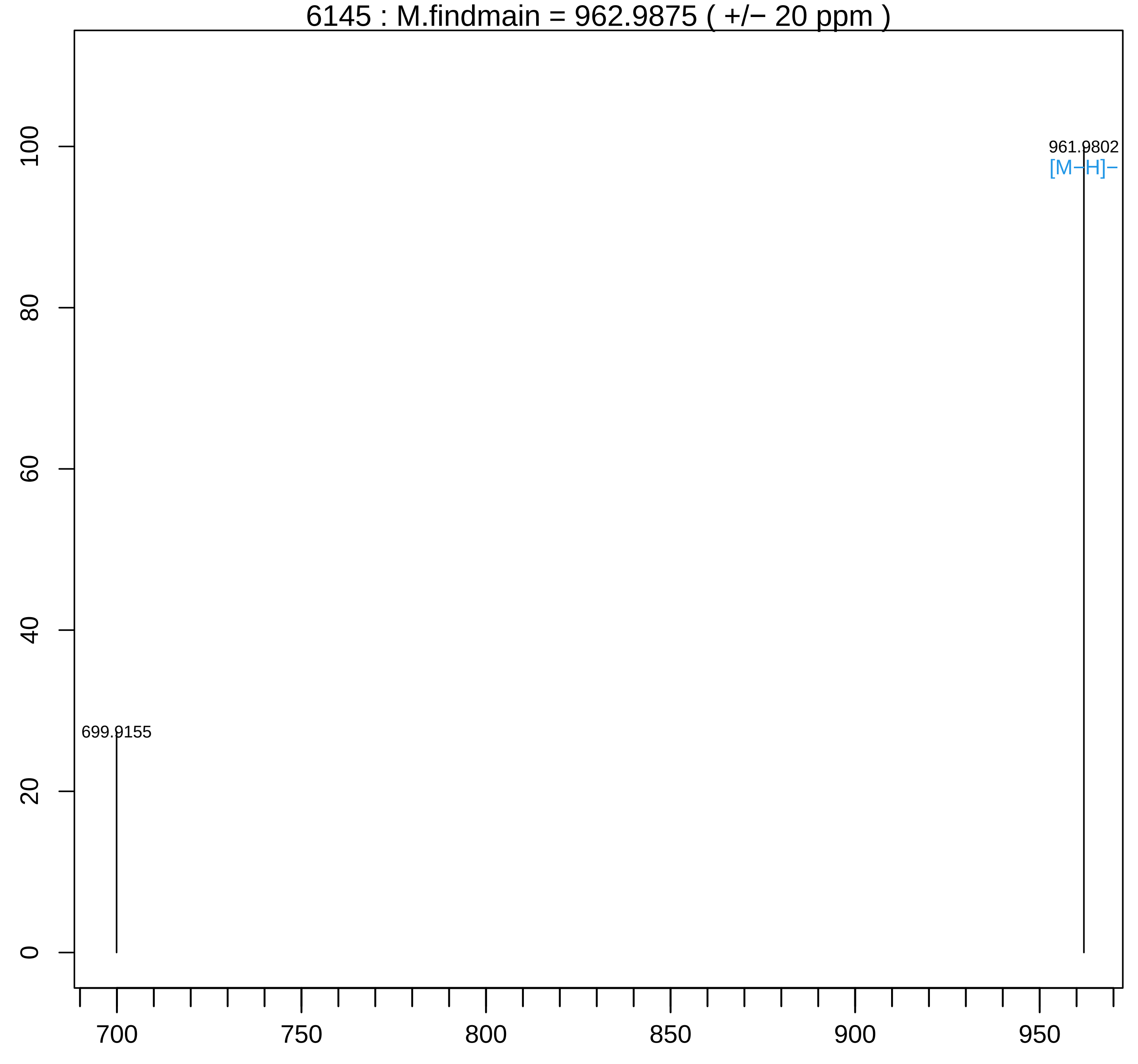
6145 : M.findmain = 962.9875 (+/− 20 ppm)
961.9802
[M−H]−
699.9155
0
20
40
60
80
100
700
750
800
850
900
950

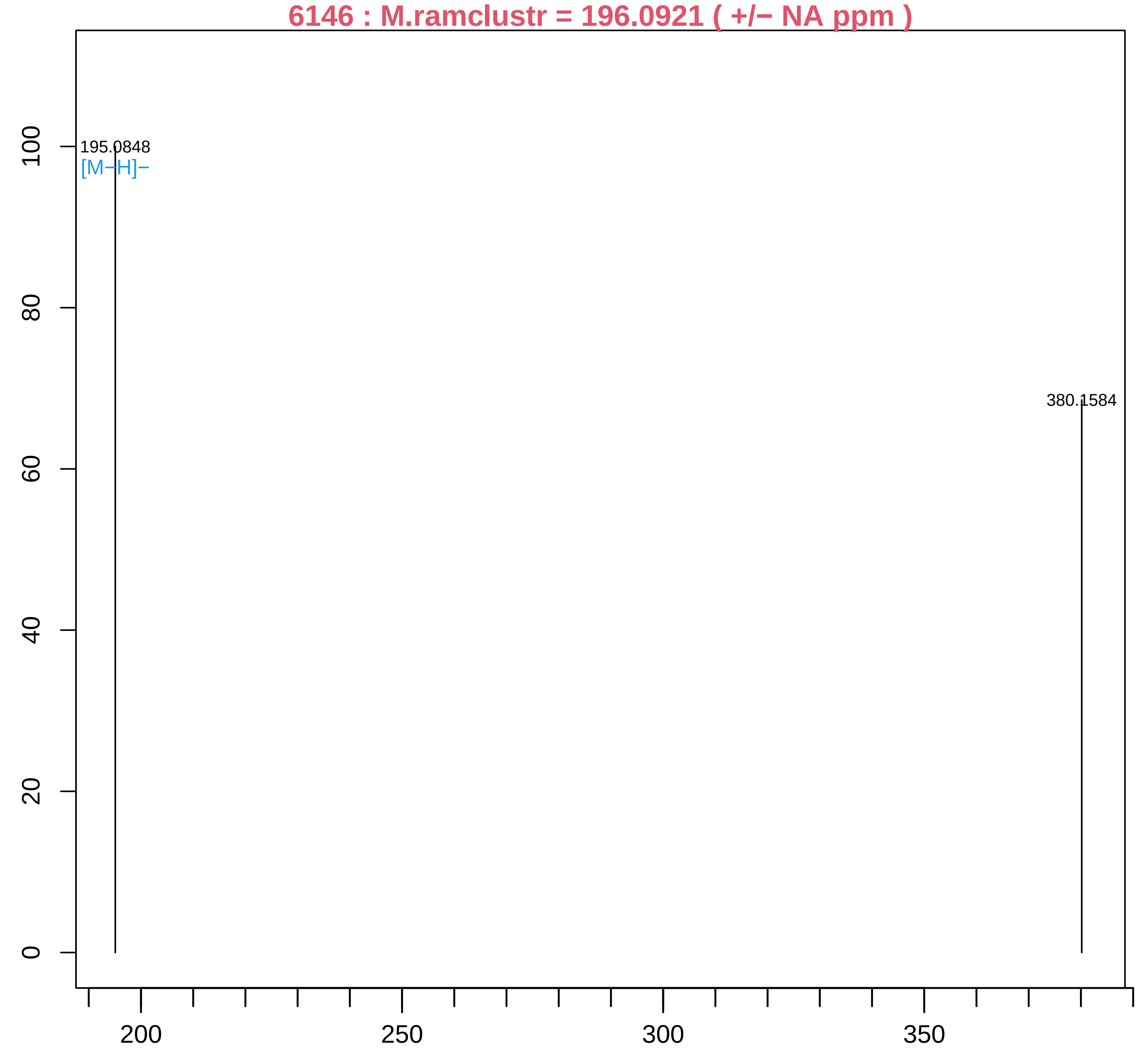
6146 : M.ramclustr = 196.0921 (+/− NA ppm)
195.0848
[M−H]−
380.1584
100
80
60
40
20
0
200
250
300
350

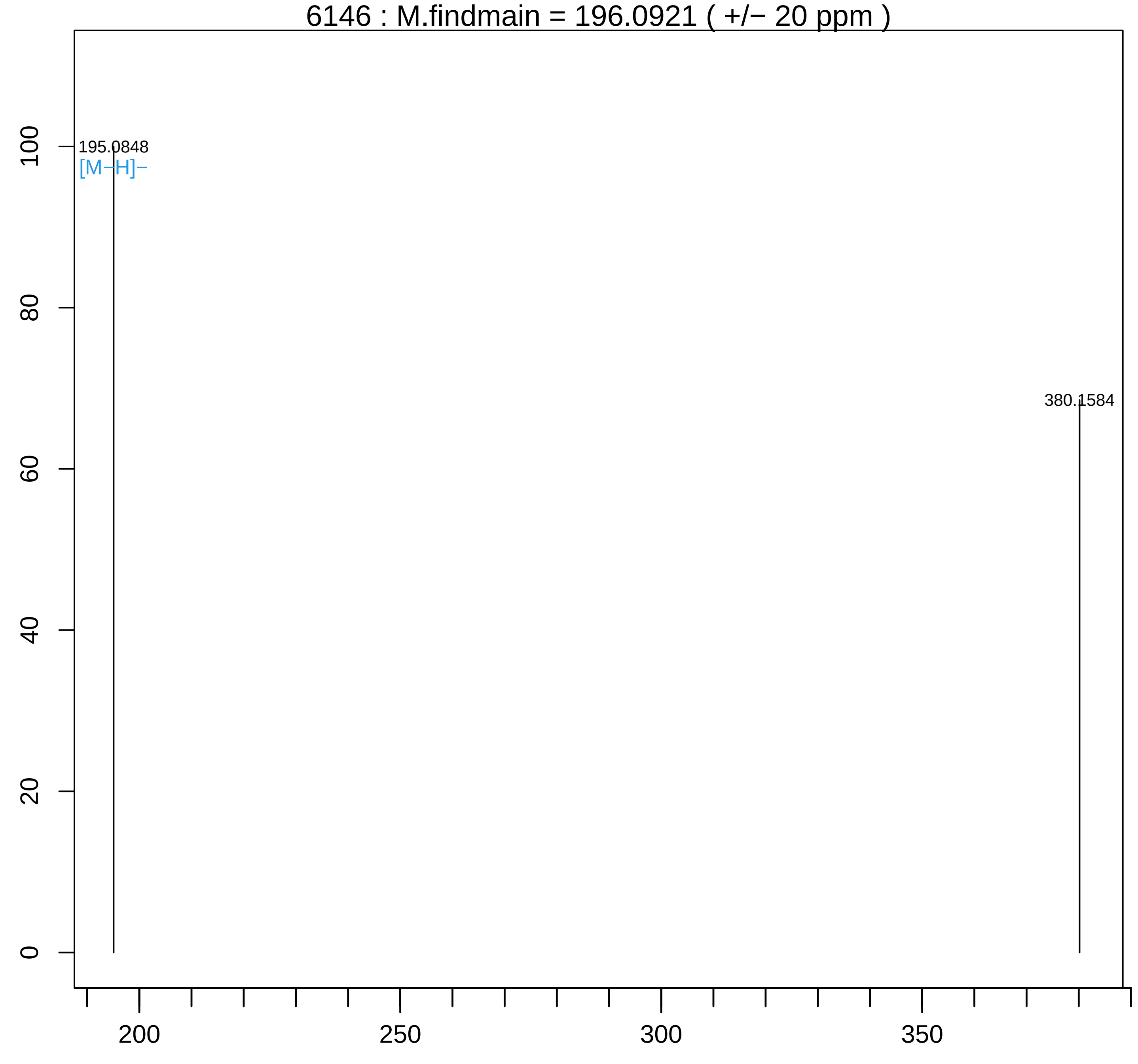
6146 : M.findmain = 196.0921 (+/− 20 ppm)
195.0848
[M−H]−
380.1584
100
80
60
40
20
0
200
250
300
350

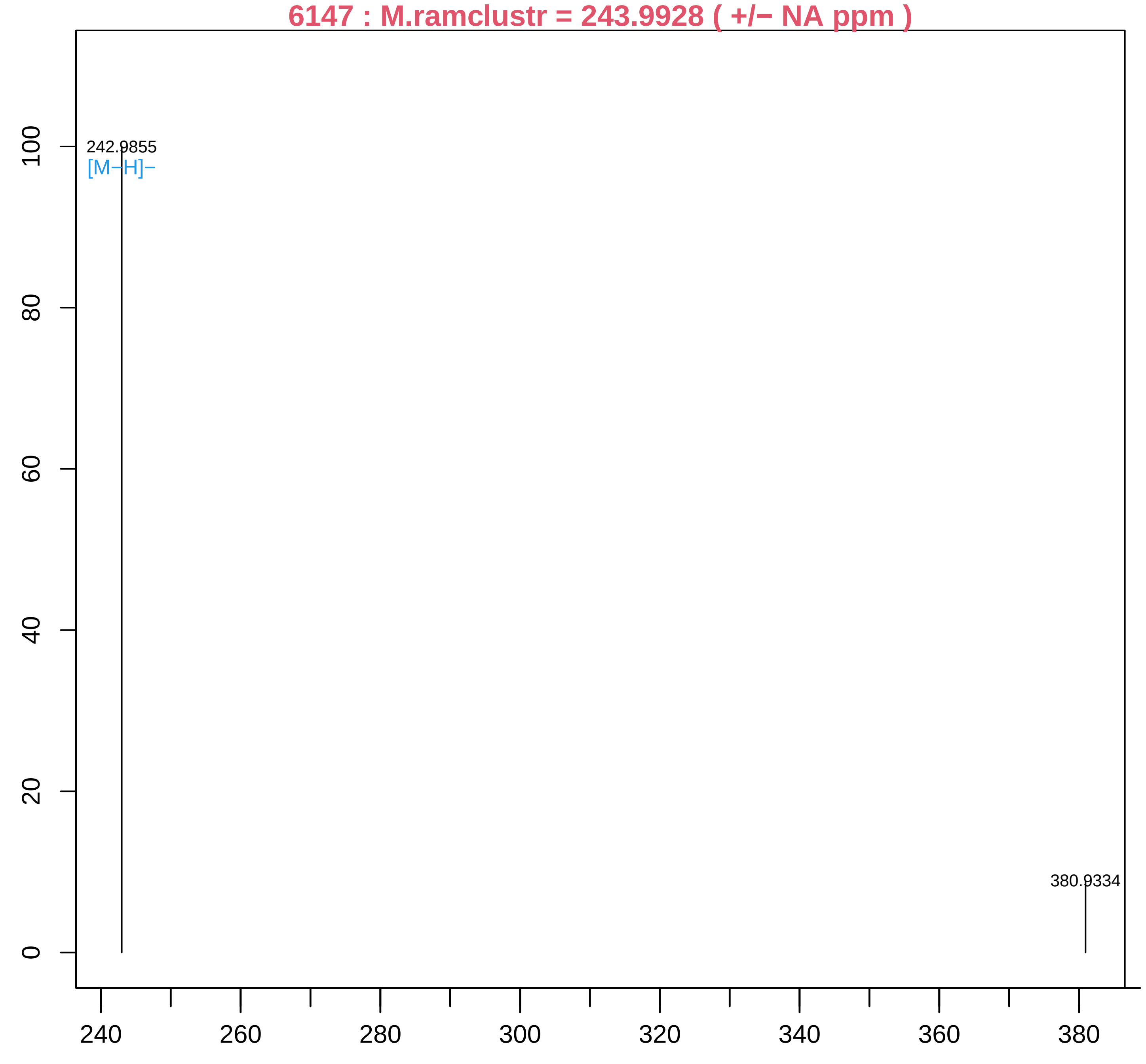
6147 : M.ramclustr = 243.9928 (+/− NA ppm)
242.9855
[M−H]−
380.9334
0
20
40
60
80
100
240
260
280
300
320
340
360
380

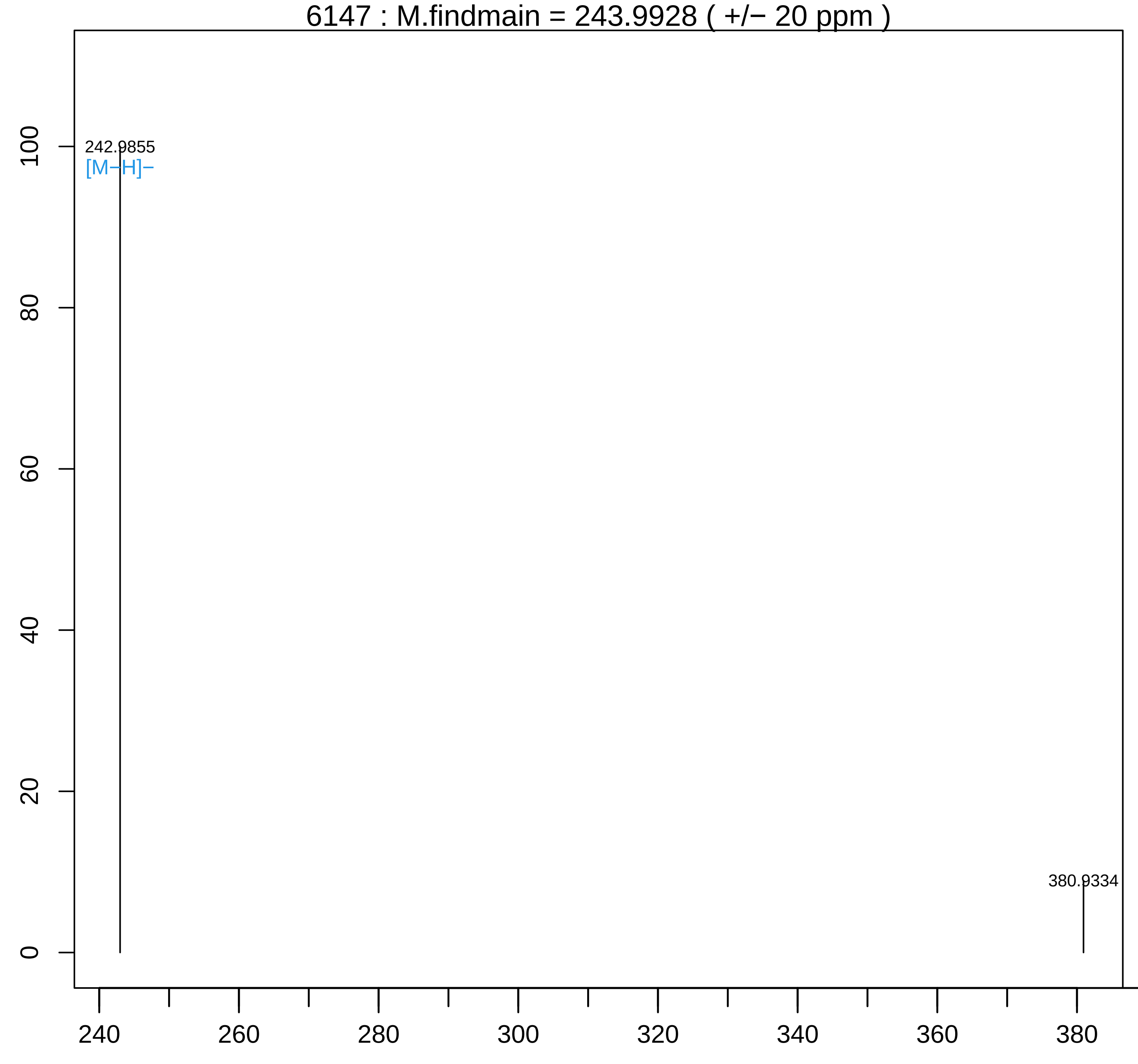
6147 : M.findmain = 243.9928 (+/− 20 ppm)
242.9855
[M−H]−
380.9334
0
20
40
60
80
100
240
260
280
300
320
340
360
380

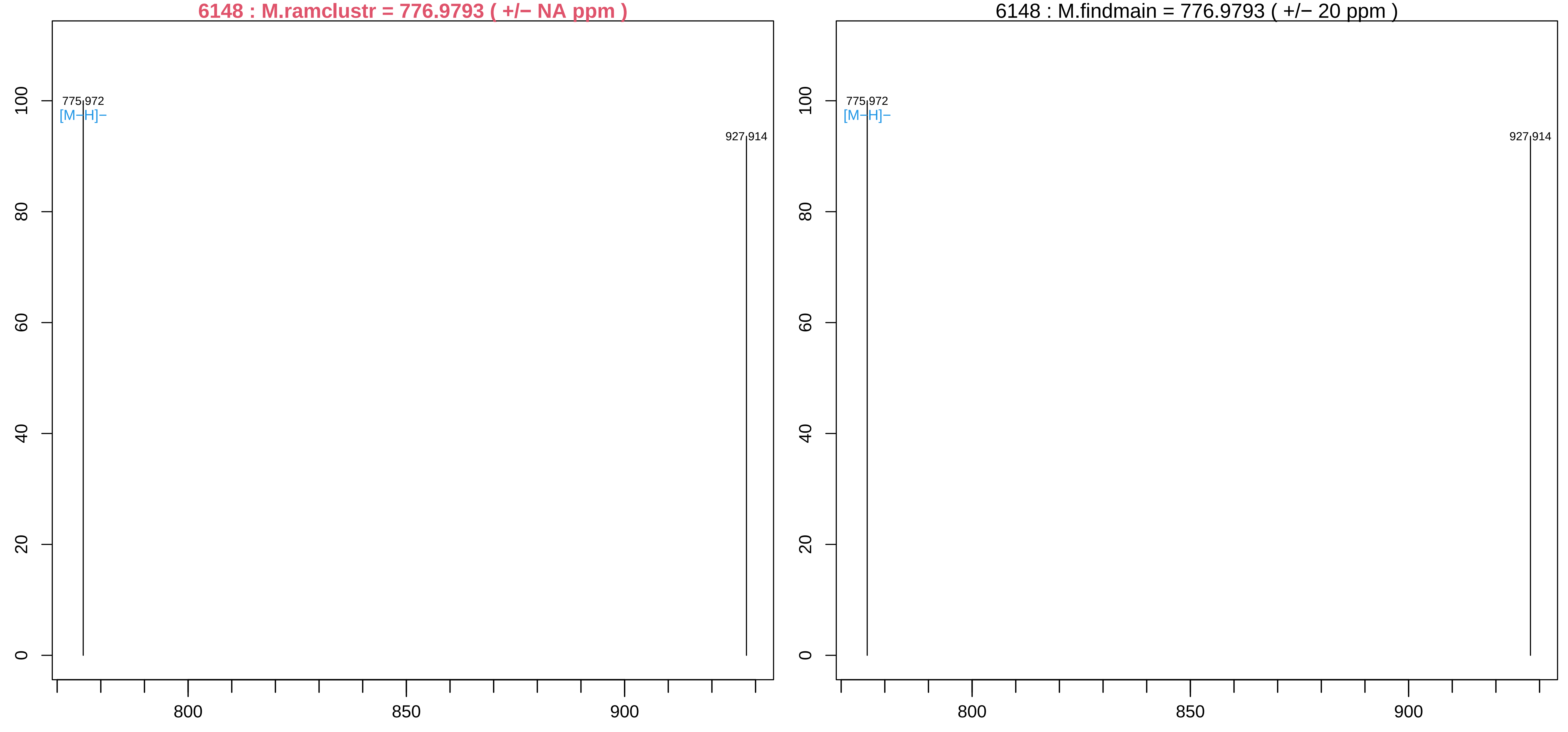
6148 : M.ramclustr = 776.9793 (+/− NA ppm)
775.972
[M−H]−
927.914
0
20
40
60
80
100
800
850
900
6148 : M.findmain = 776.9793 (+/− 20 ppm)
775.972
[M−H]−
927.914
0
20
40
60
80
100
800
850
900

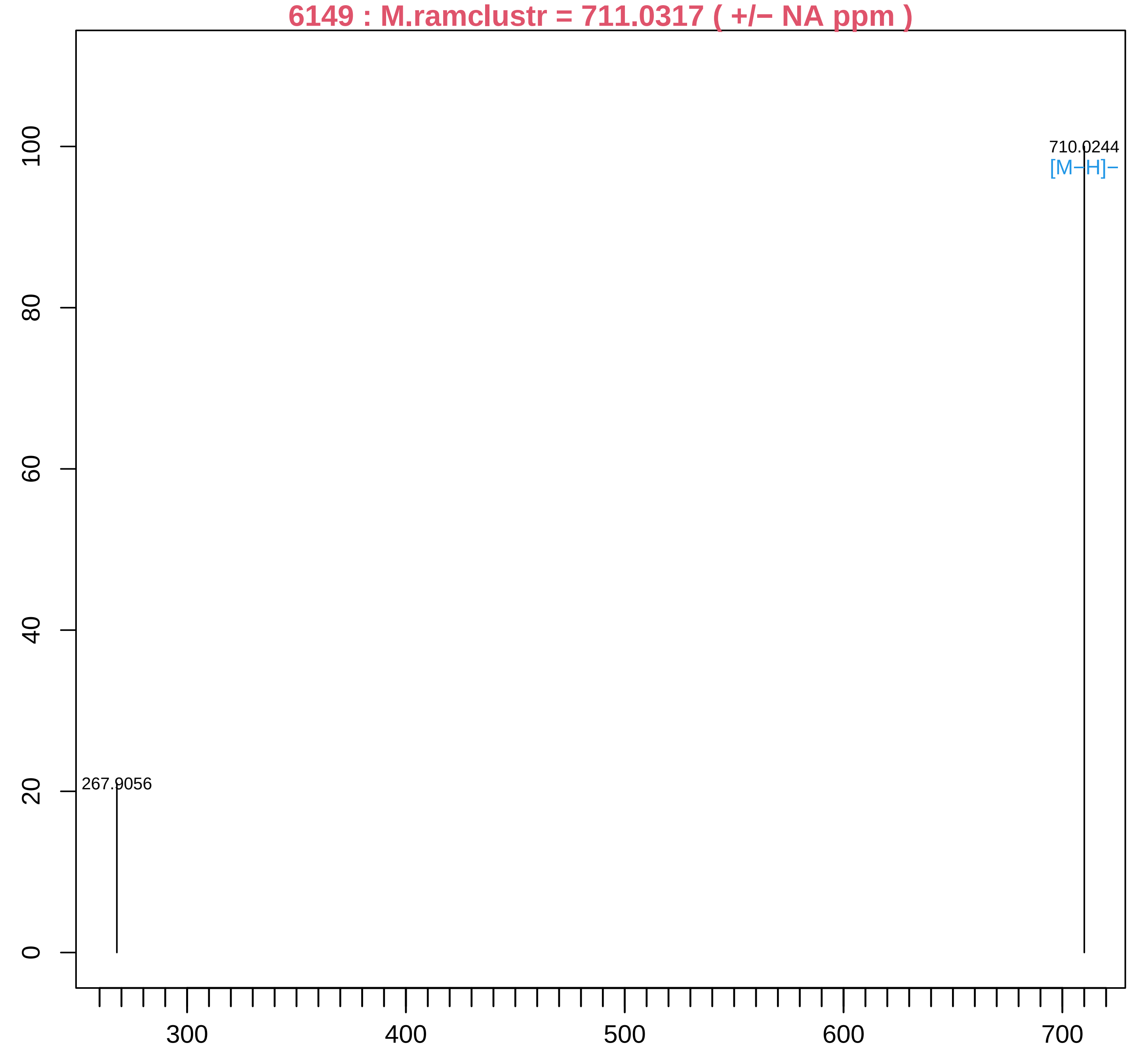
6149 : M.ramclustr = 711.0317 (+/− NA ppm)
710.0244
[M−H]−
267.9056
0
20
40
60
80
100
300
400
500
600
700

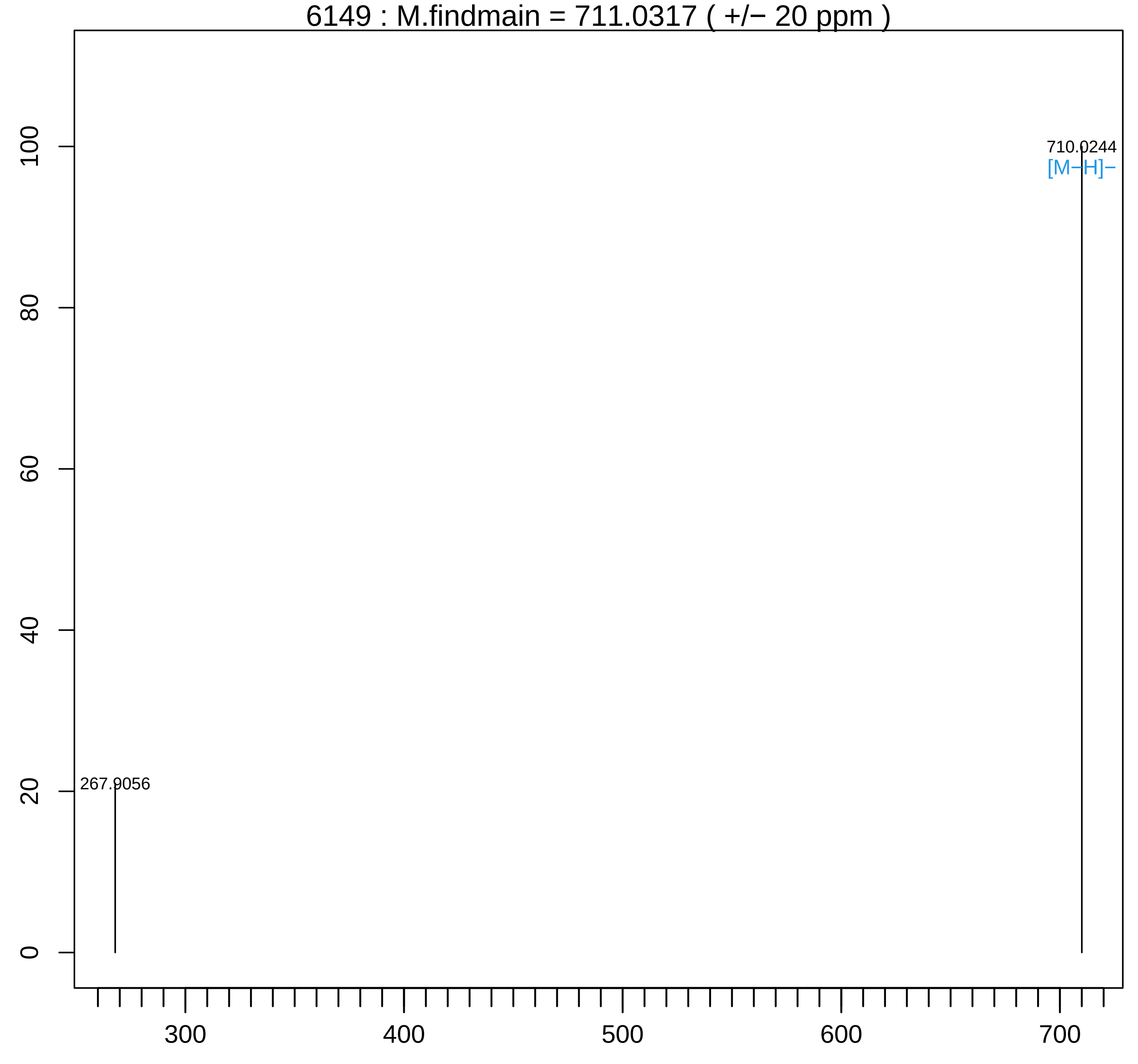
6149 : M.findmain = 711.0317 (+/− 20 ppm)
710.0244
[M−H]−
267.9056
0
20
40
60
80
100
300
400
500
600
700

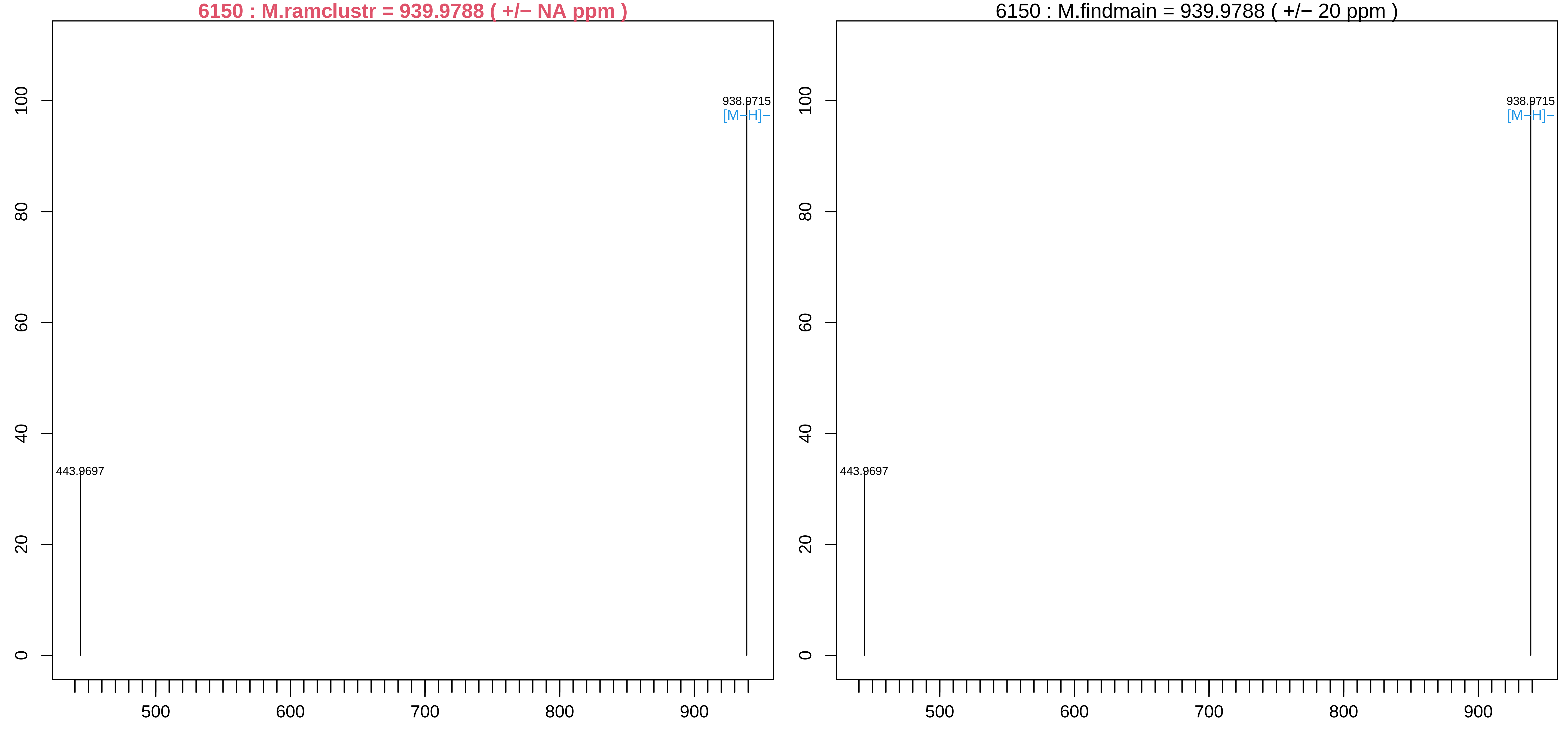
6150 : M.ramclustr = 939.9788 (+/− NA ppm)
938.9715
[M−H]−
443.9697
6150 : M.findmain = 939.9788 (+/− 20 ppm)
938.9715
[M−H]−
443.9697
0
20
40
60
80
100
500
600
700
800
900

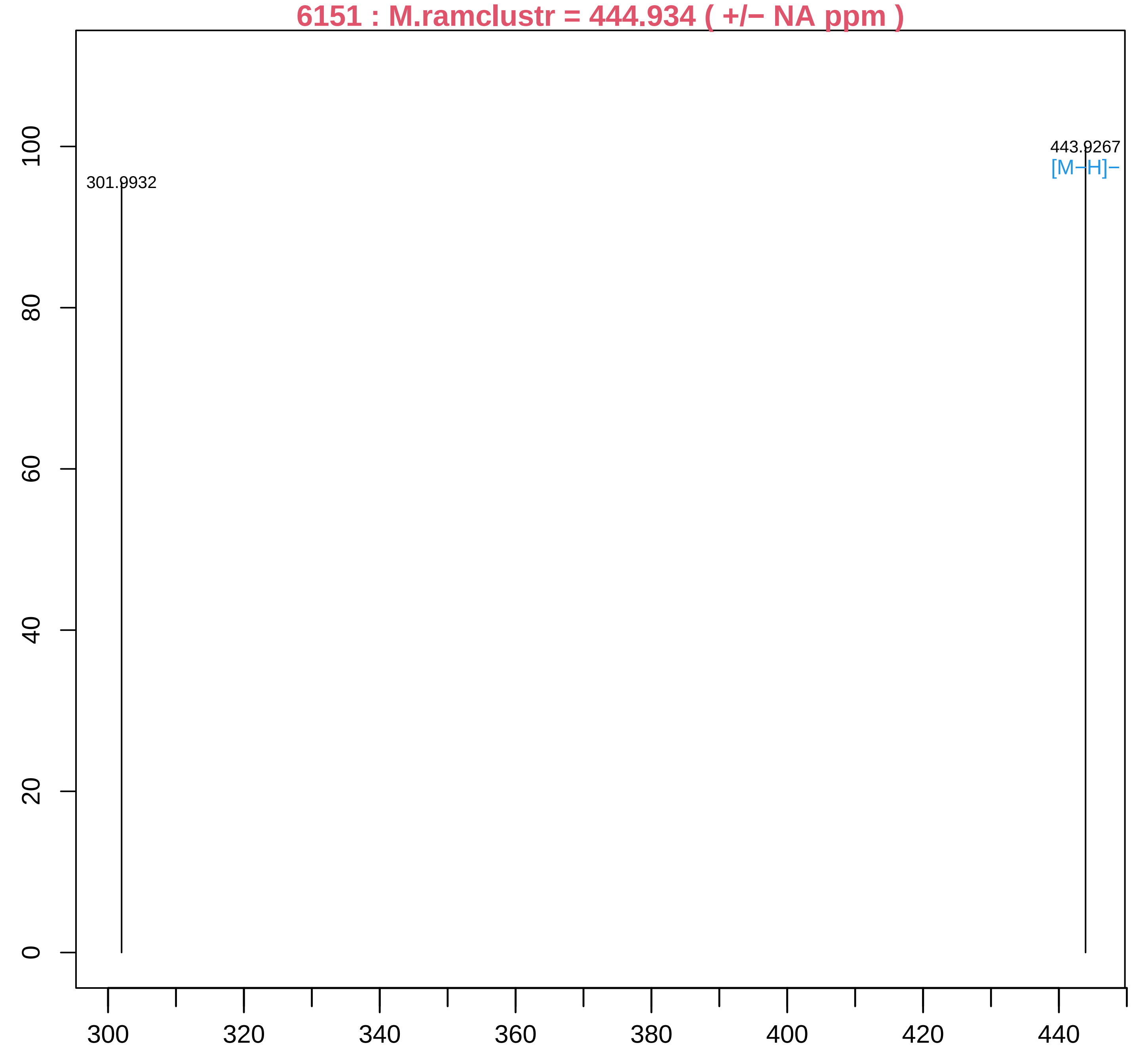
6151 : M.ramclustr = 444.934 (+/− NA ppm)
301.9932
443.9267
[M−H]−
0
20
40
60
80
100
300
320
340
360
380
400
420
440

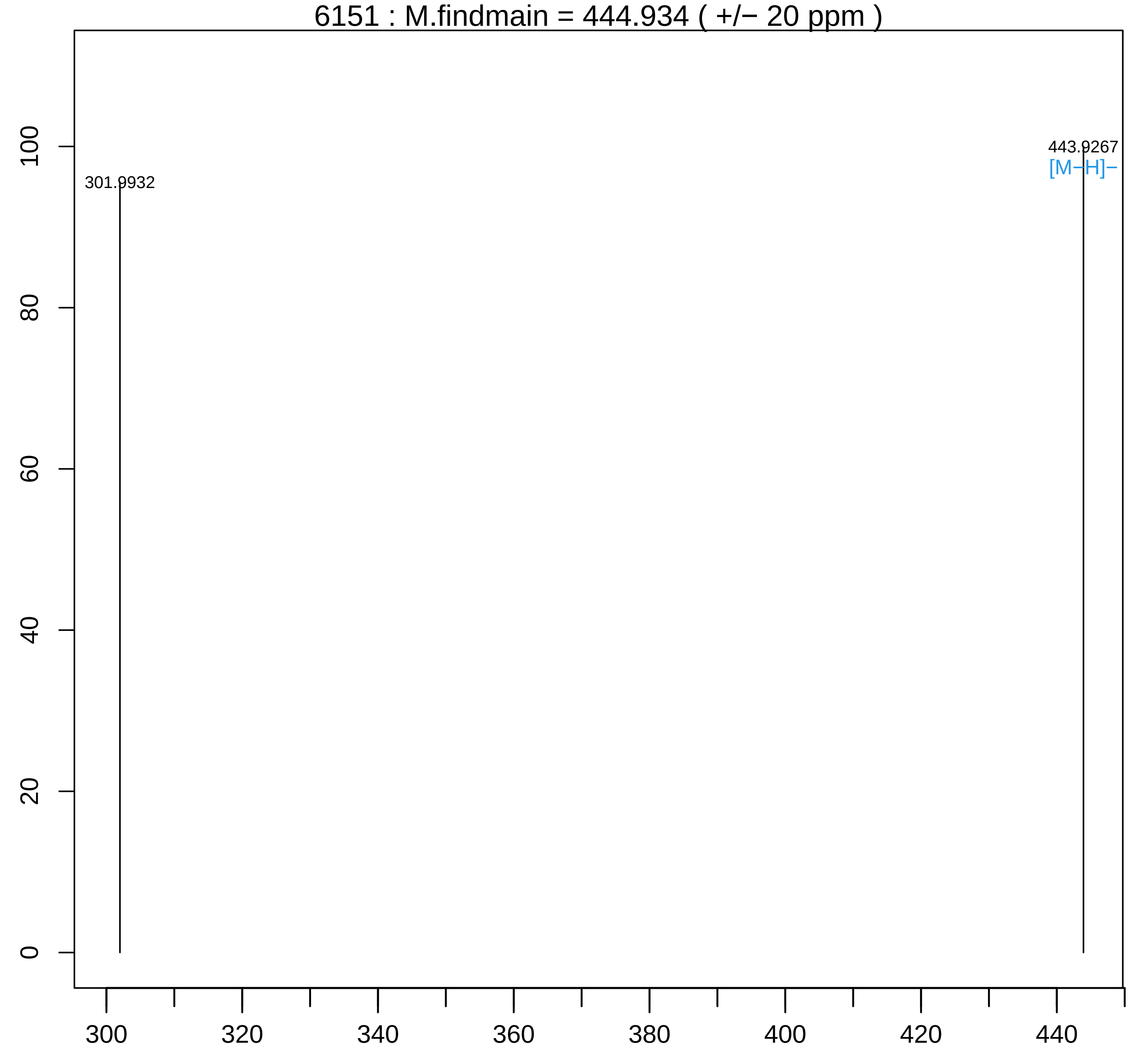
6151 : M.findmain = 444.934 (+/− 20 ppm)
301.9932
443.9267
[M−H]−
0
20
40
60
80
100
300
320
340
360
380
400
420
440

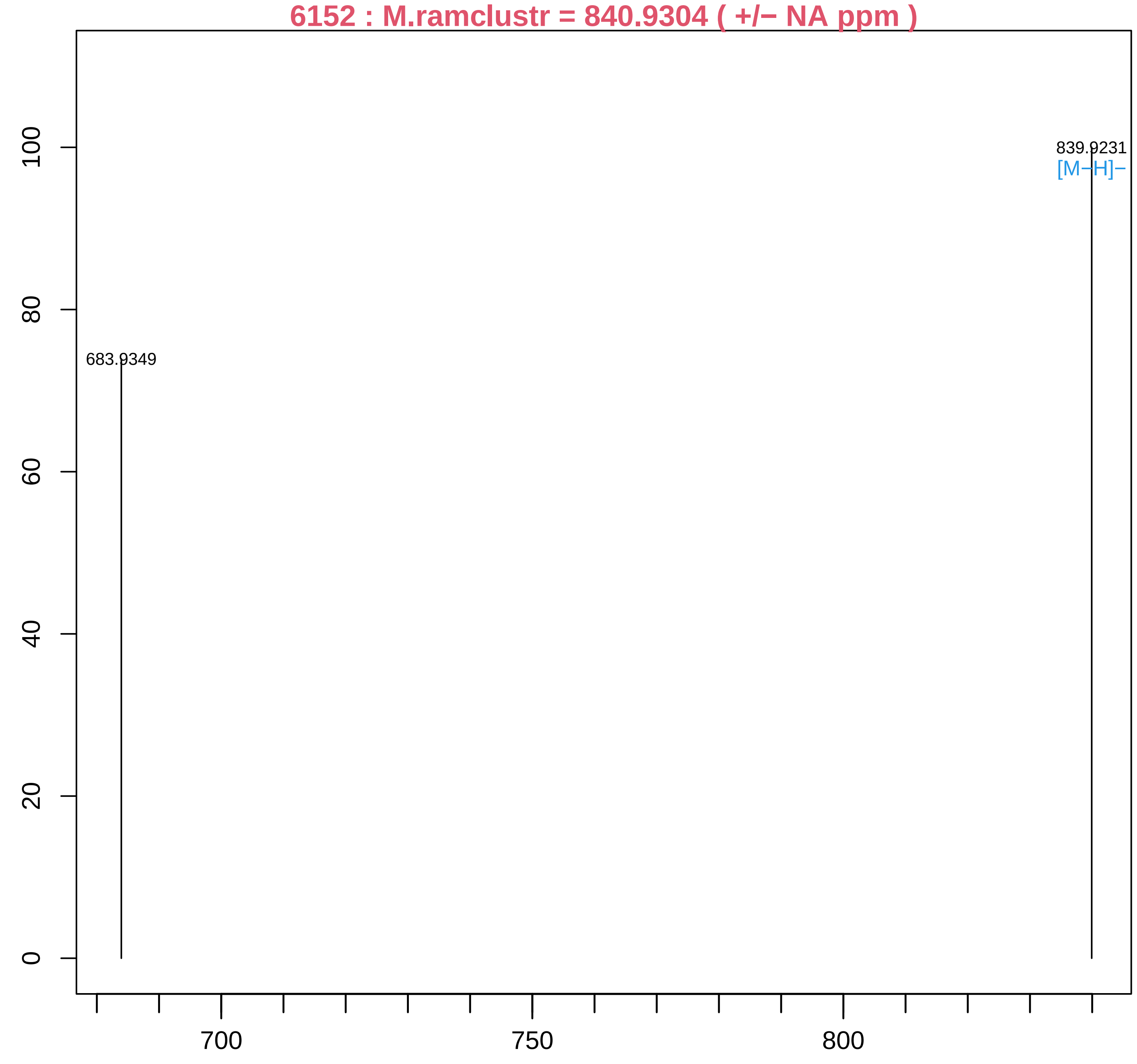

6152 : M.ramclustr = 840.9304 (+/− NA ppm)
839.9231
[M−H]−
683.9349
0
20
40
60
80
100
700
750
800

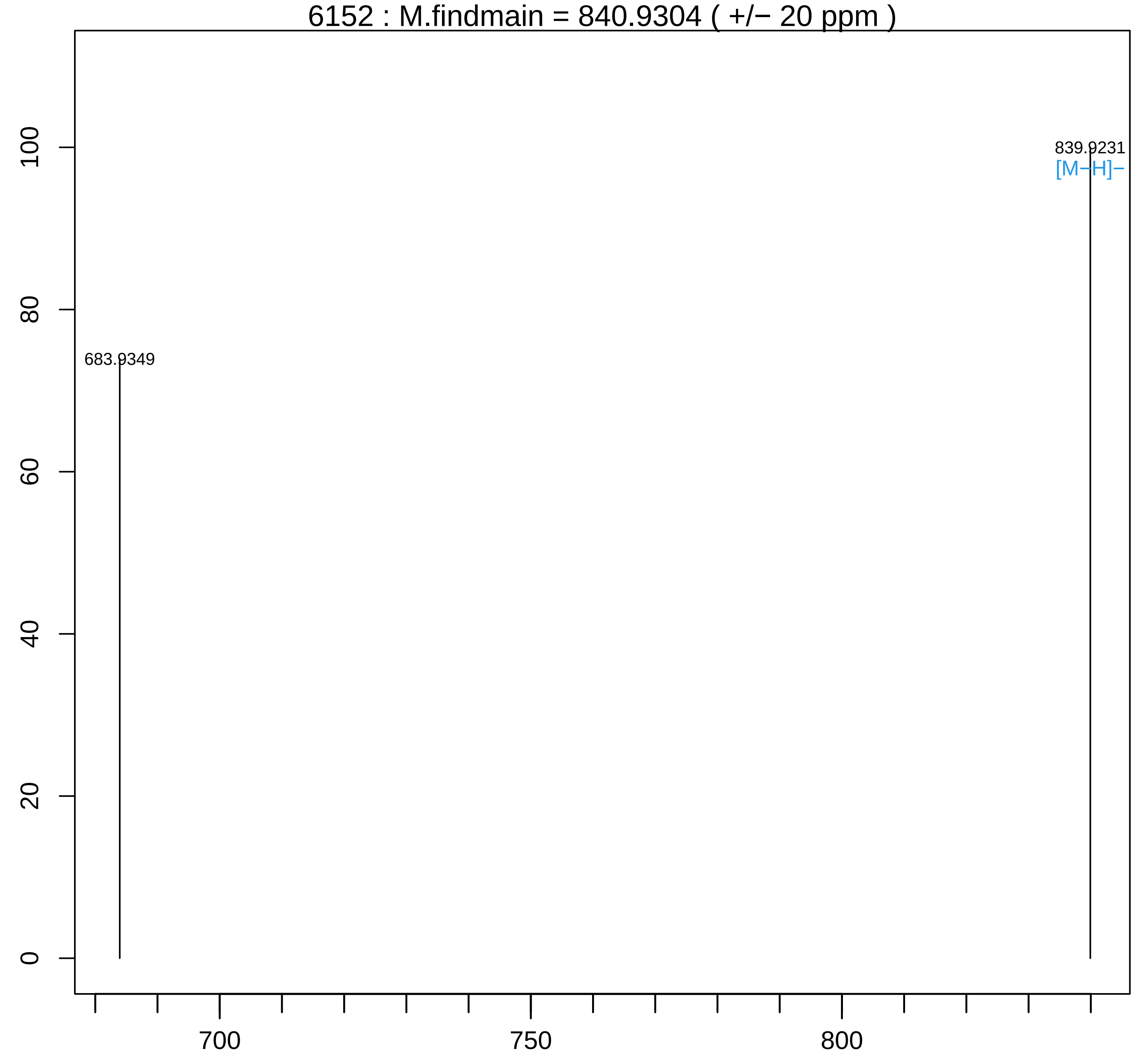

6152 : M.findmain = 840.9304 (+/− 20 ppm)
839.9231
[M−H]−
683.9349
0
20
40
60
80
100
700
750
800

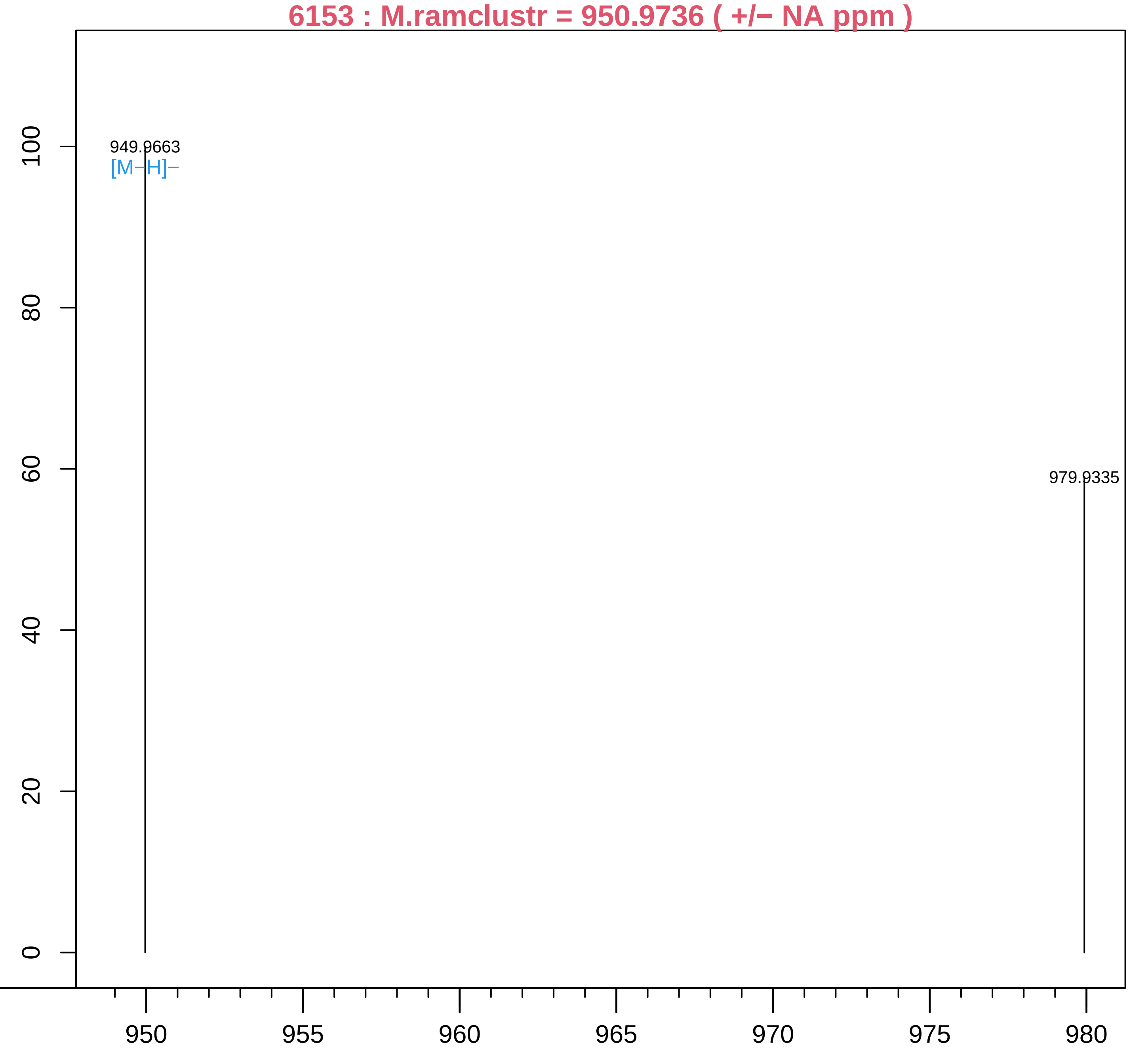
6153 : M.ramclustr = 950.9736 (+/− NA ppm)
949.9663
[M−H]−
979.9335
0
20
40
60
80
100
950
955
960
965
970
975
980

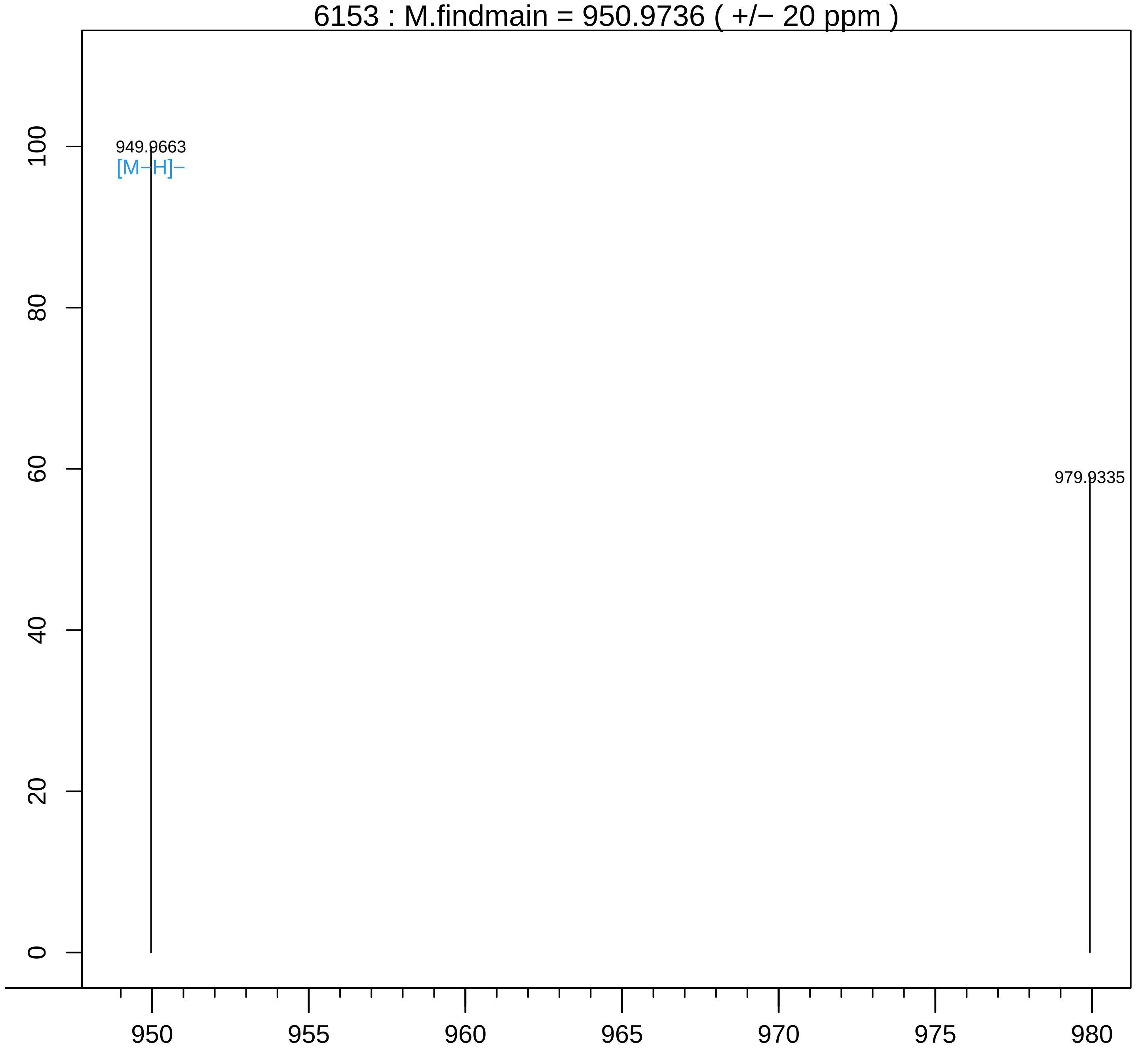
6153 : M.findmain = 950.9736 (+/− 20 ppm)
949.9663
[M−H]−
979.9335
0
20
40
60
80
100
950
955
960
965
970
975
980

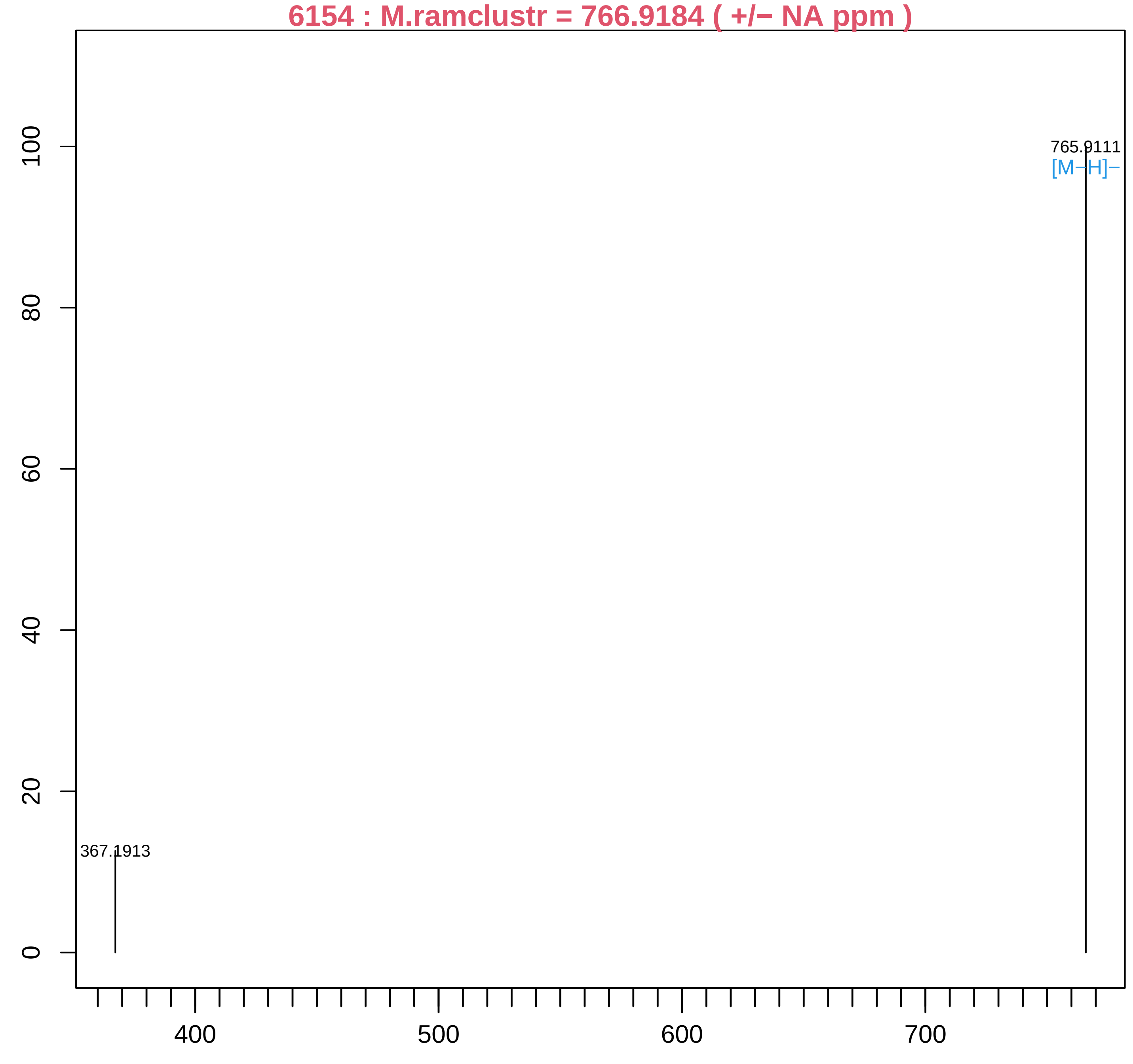
6154 : M.ramclustr = 766.9184 (+/− NA ppm)
765.9111
[M−H]−
367.1913
0
20
40
60
80
100
400
500
600
700

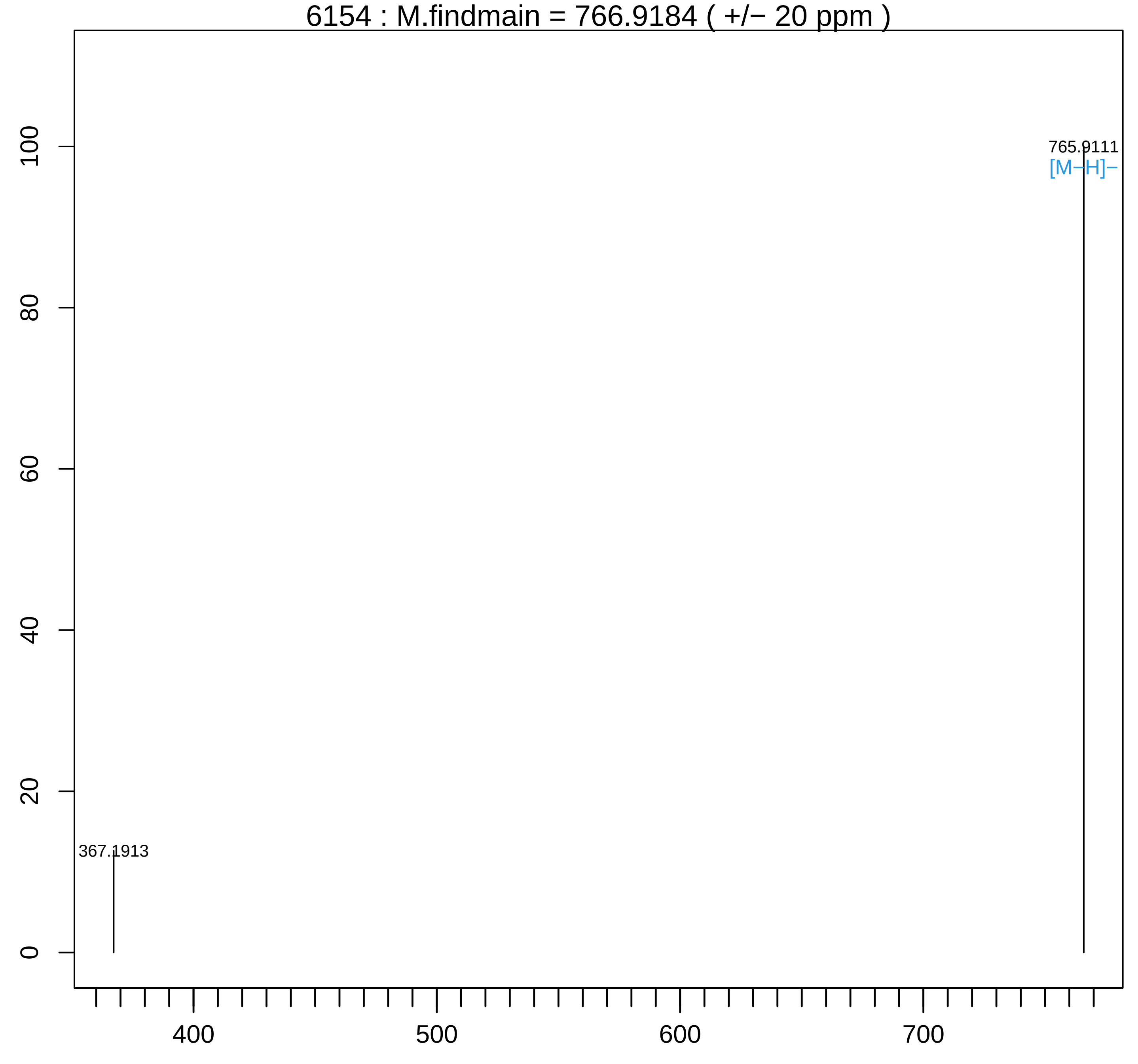
6154 : M.findmain = 766.9184 (+/− 20 ppm)
765.9111
[M−H]−
367.1913
0
20
40
60
80
100
400
500
600
700

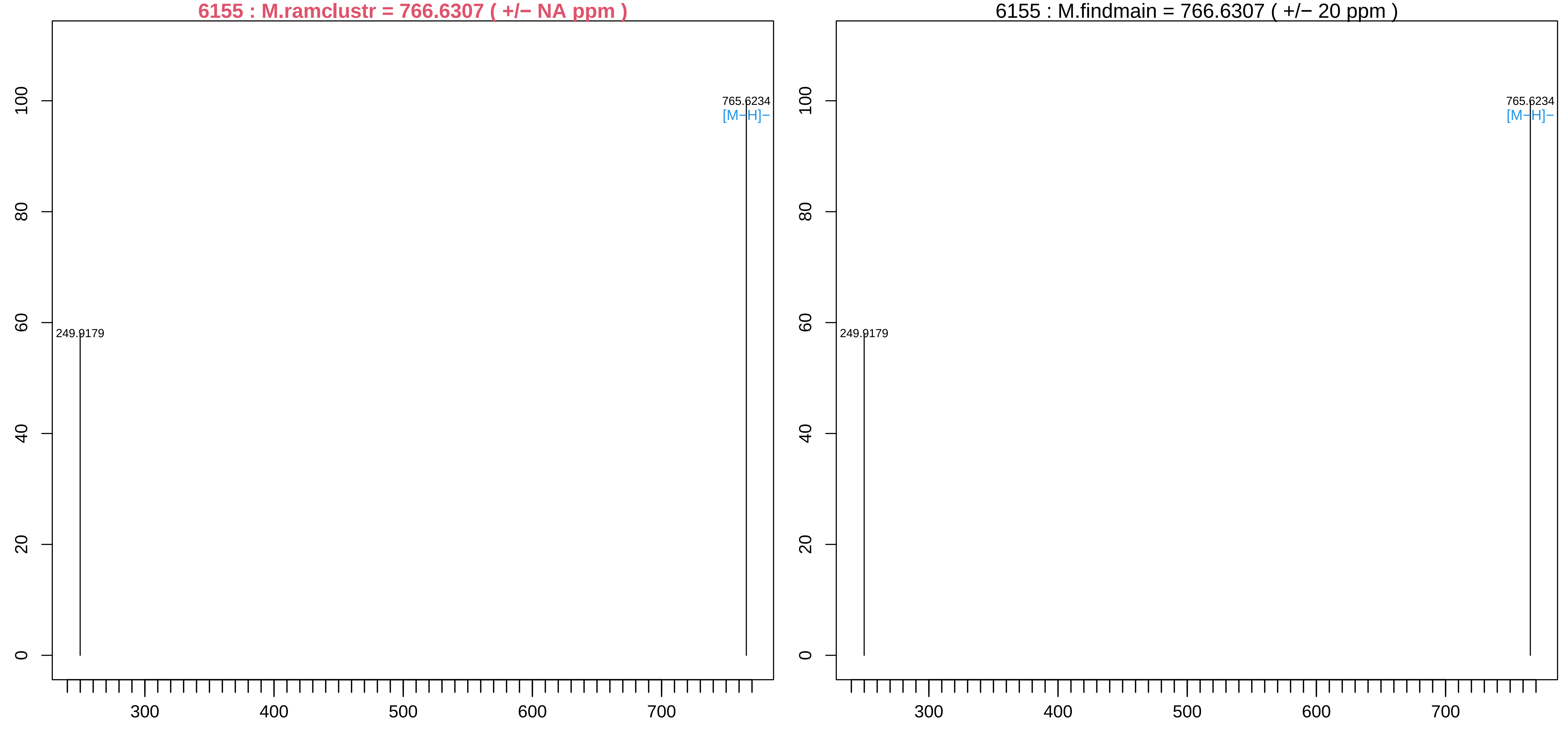

6155 : M.ramclustr = 766.6307 (+/− NA ppm)
765.6234
[M−H]−
249.9179
0
20
40
60
80
100
300
400
500
600
700
6155 : M.findmain = 766.6307 (+/− 20 ppm)
765.6234
[M−H]−
249.9179
0
20
40
60
80
100
300
400
500
600
700

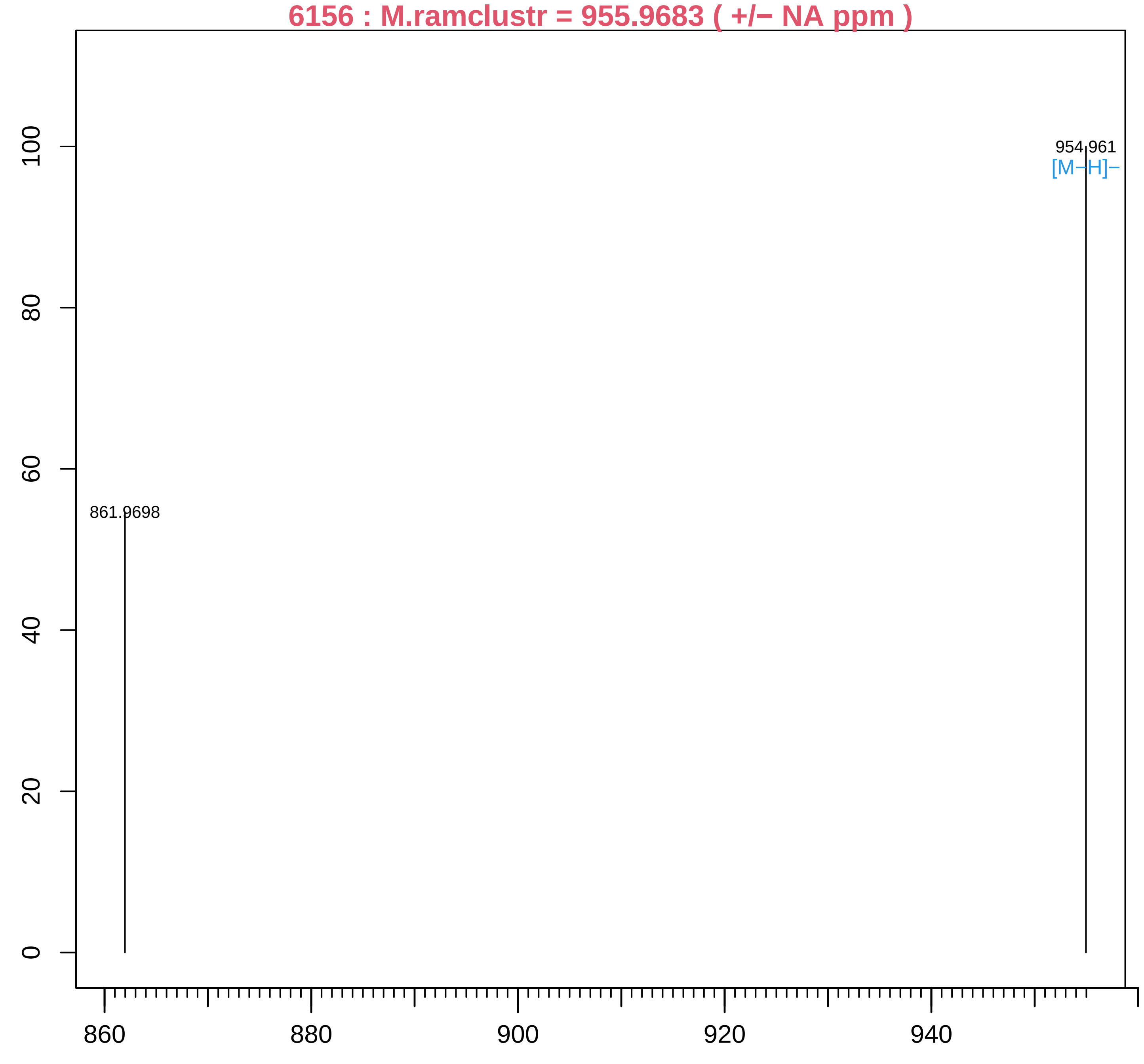
6156 : M.ramclustr = 955.9683 (+/− NA ppm)
954.961
[M−H]−
861.9698
0
20
40
60
80
100
860
880
900
920
940

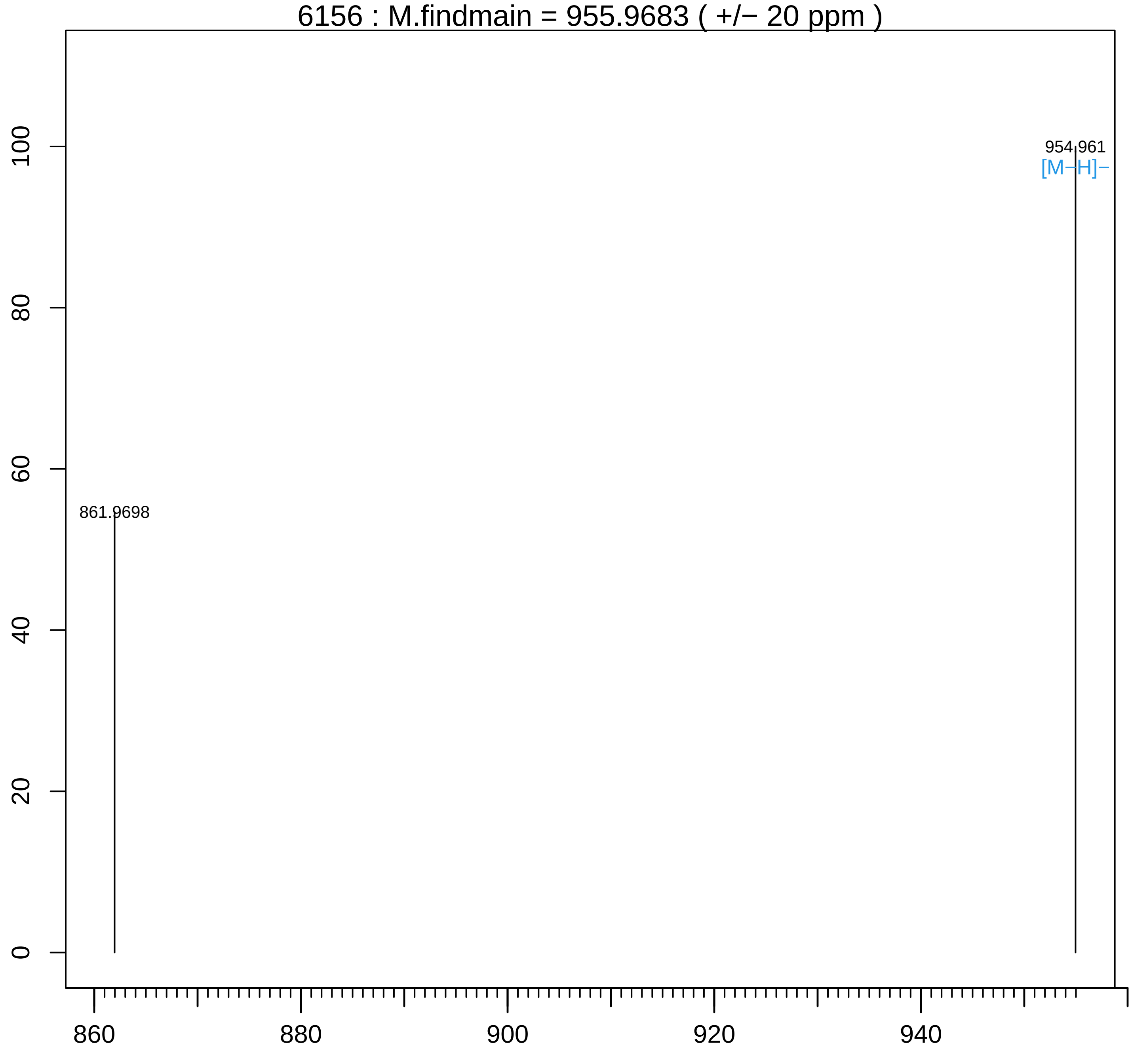
6156 : M.findmain = 955.9683 (+/− 20 ppm)
954.961
[M−H]−
861.9698
0
20
40
60
80
100
860
880
900
920
940

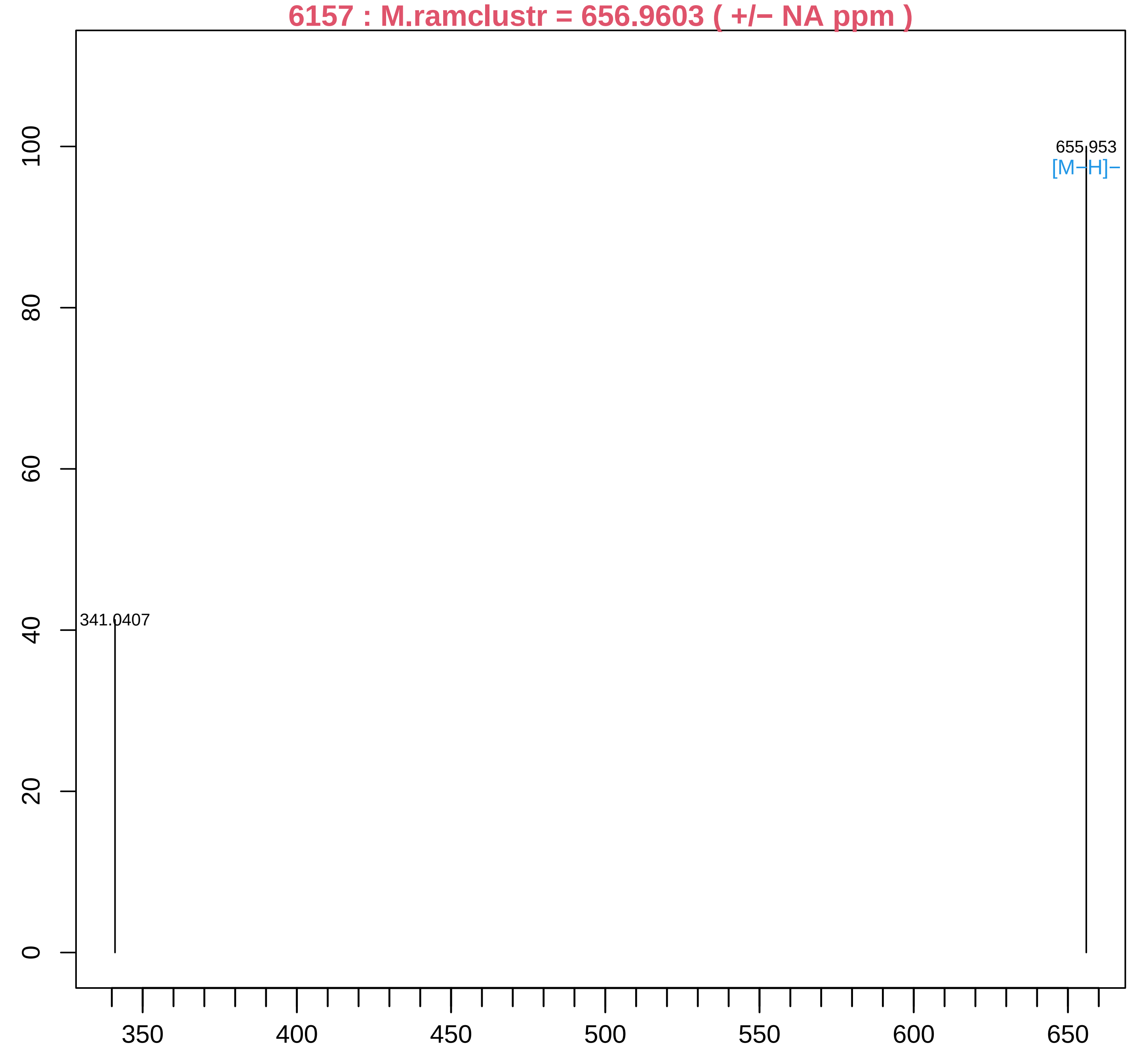
6157 : M.ramclustr = 656.9603 (+/− NA ppm)
655.953
[M−H]−
341.0407
0
20
40
60
80
100
350
400
450
500
550
600
650

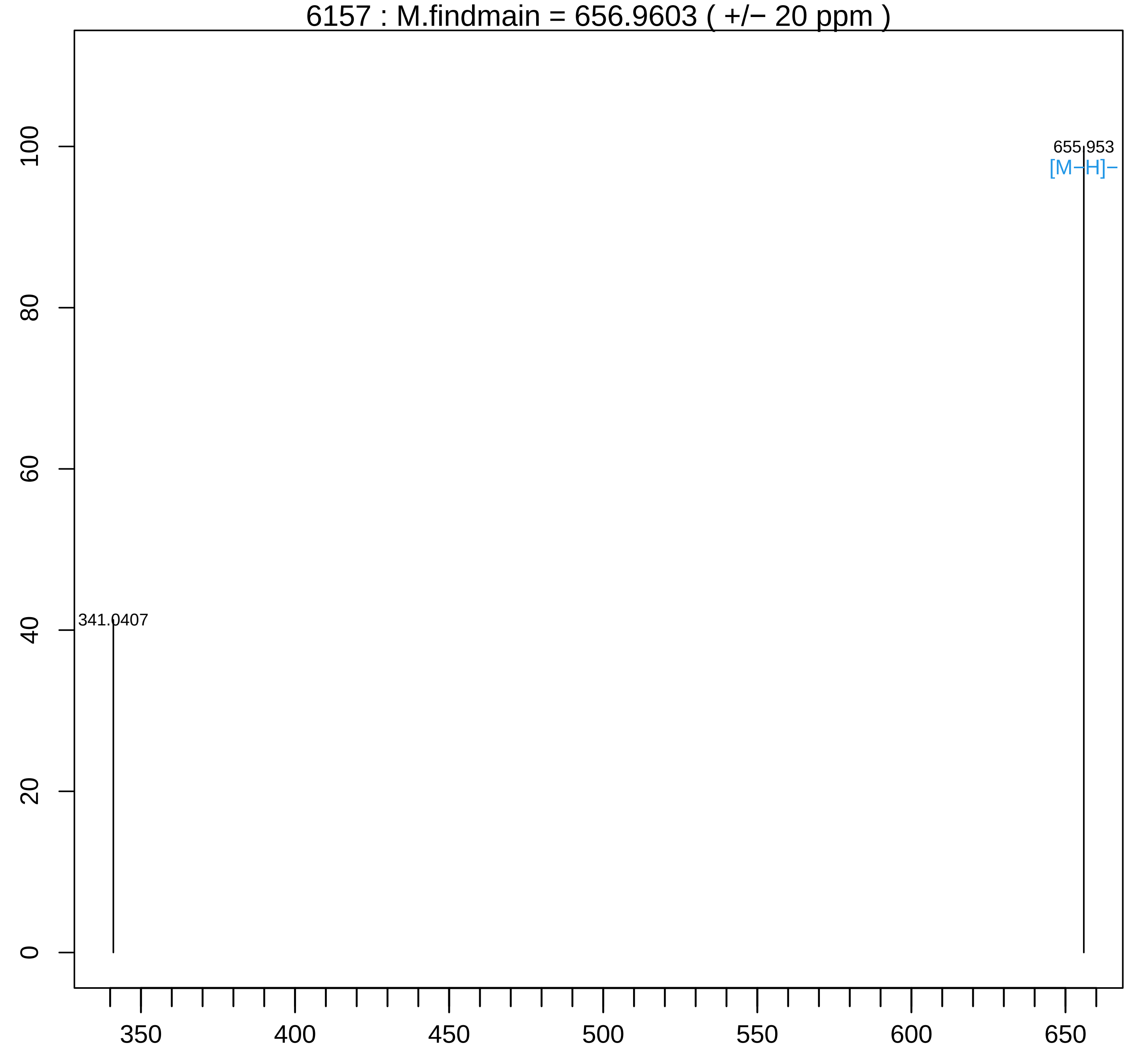
6157 : M.findmain = 656.9603 (+/− 20 ppm)
655.953
[M−H]−
341.0407
0
20
40
60
80
100
350
400
450
500
550
600
650

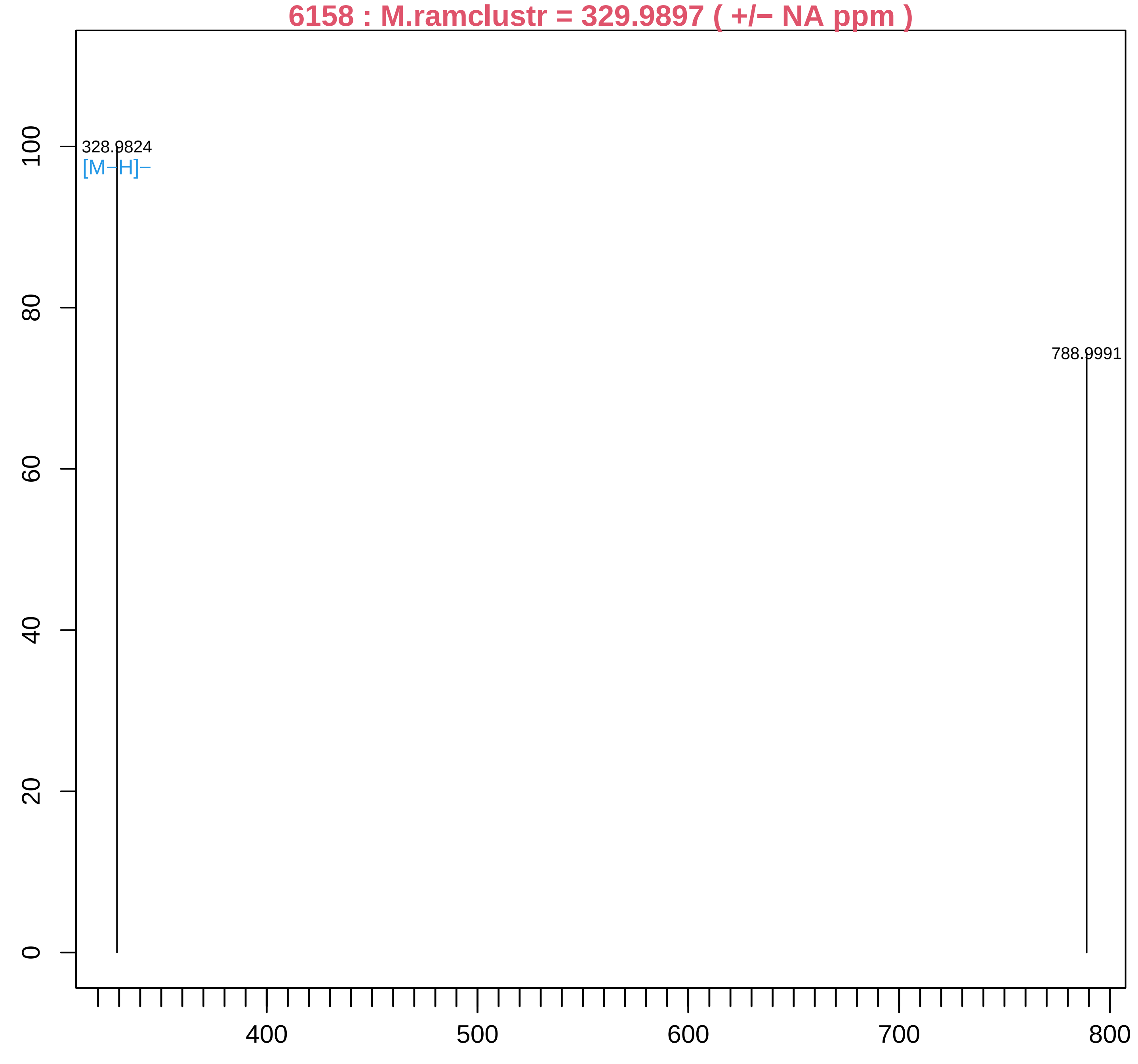
6158 : M.ramclustr = 329.9897 (+/− NA ppm)
328.9824
[M−H]−
788.9991
0
20
40
60
80
100
400
500
600
700
800

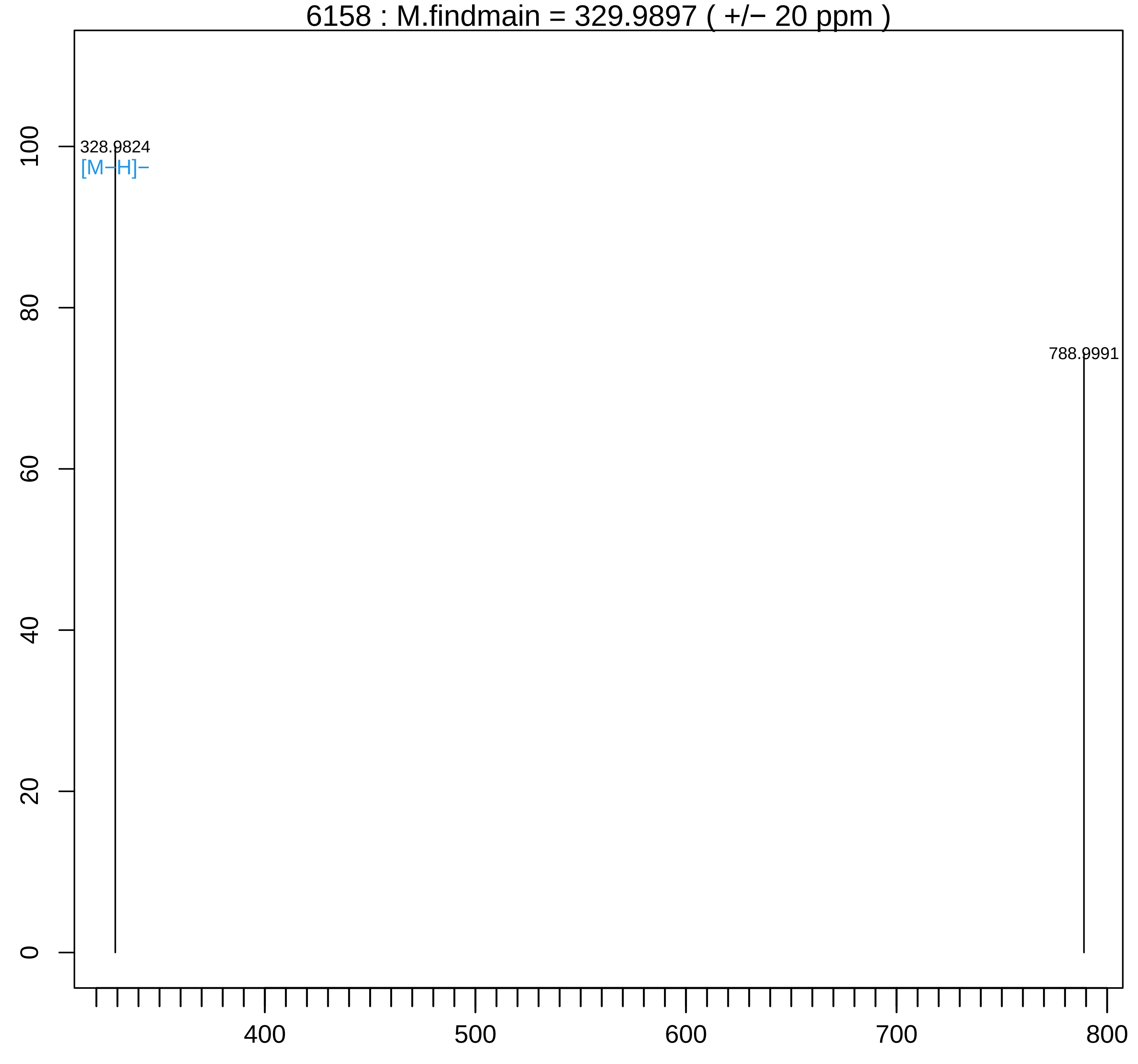
6158 : M.findmain = 329.9897 (+/− 20 ppm)
328.9824
[M−H]−
788.9991
0
20
40
60
80
100
400
500
600
700
800

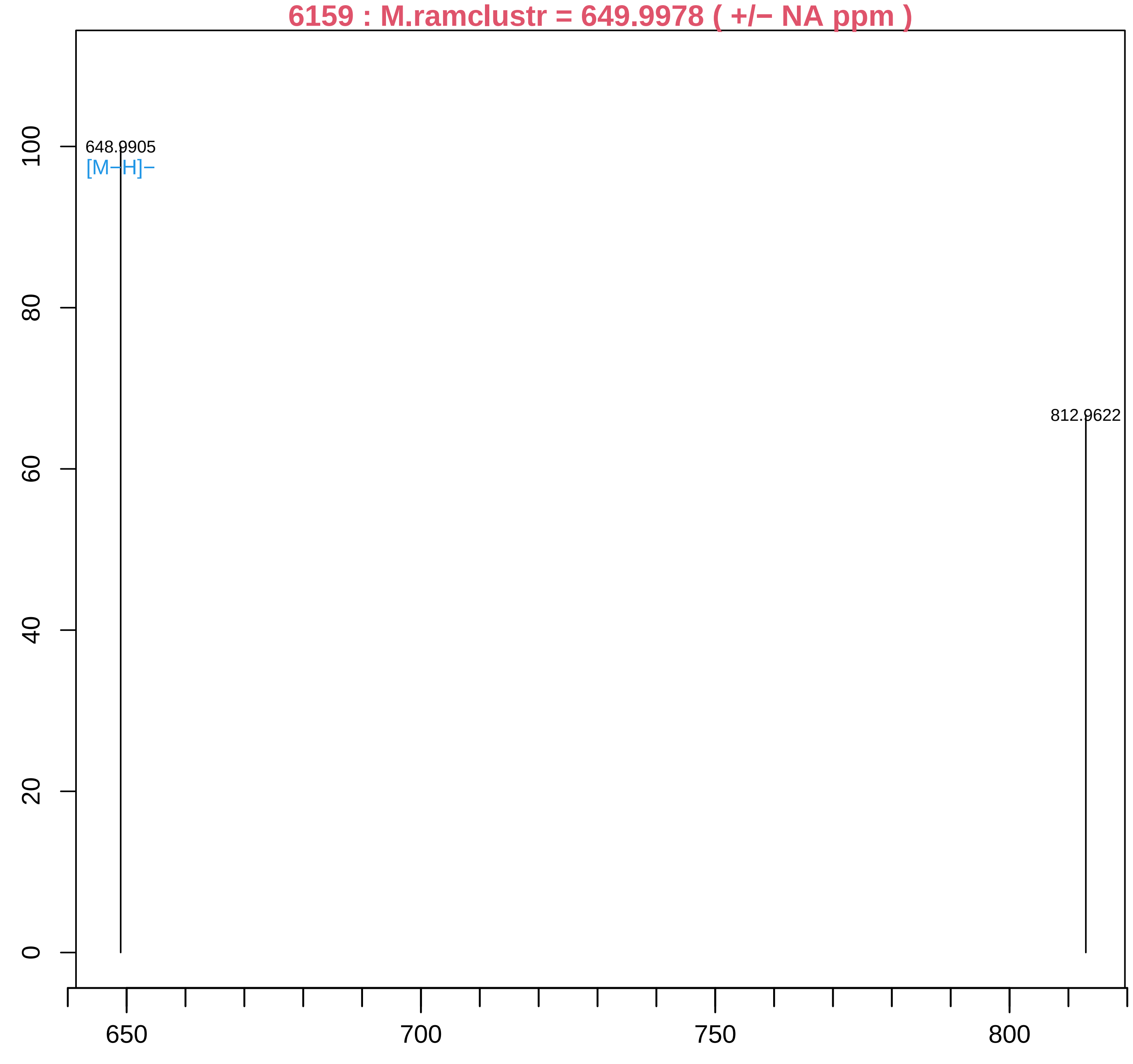
6159 : M.ramclustr = 649.9978 (+/− NA ppm)
648.9905
[M−H]−
812.9622
0
20
40
60
80
100
650
700
750
800

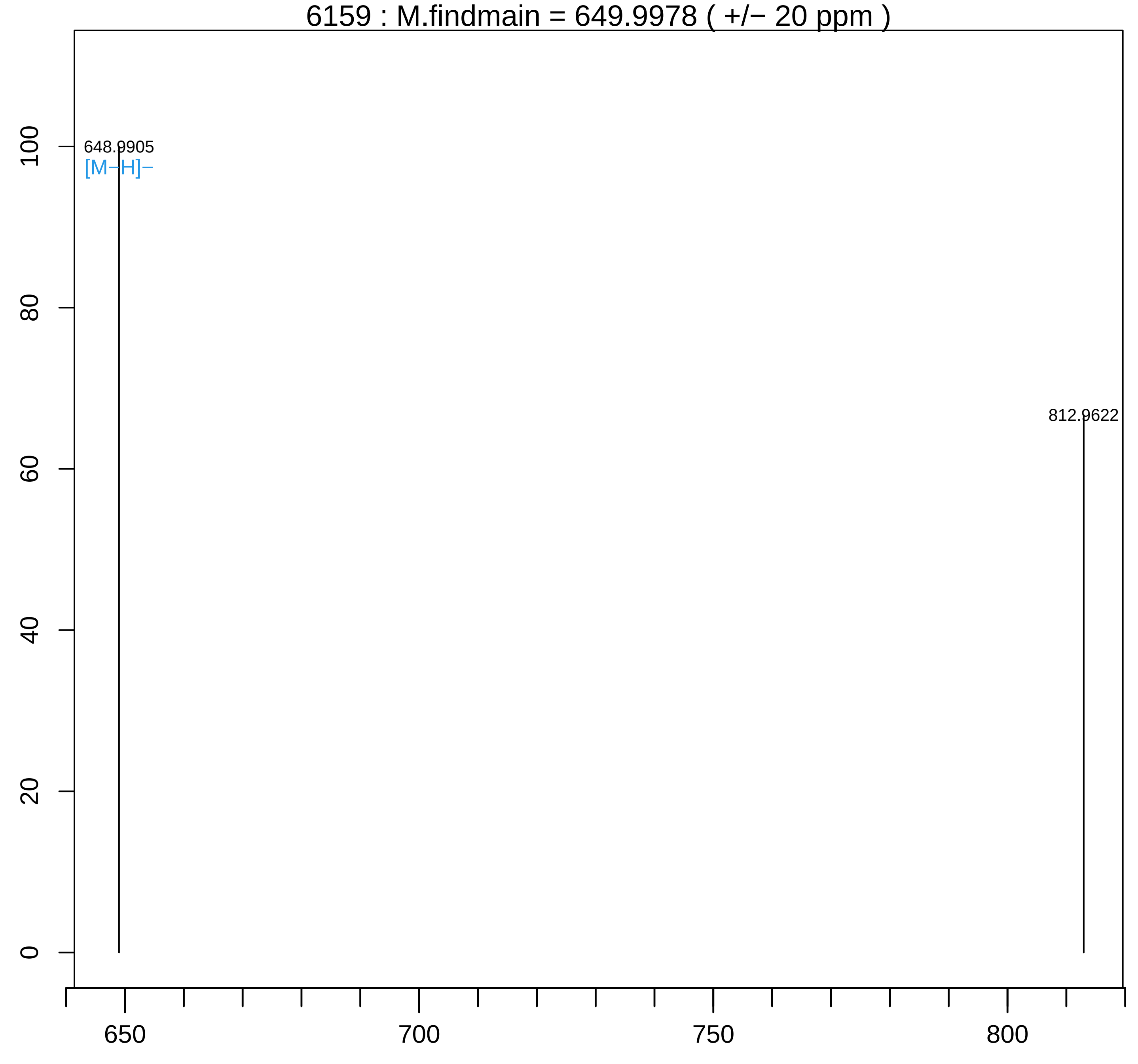
6159 : M.findmain = 649.9978 (+/− 20 ppm)
648.9905
[M−H]−
812.9622
0
20
40
60
80
100
650
700
750
800

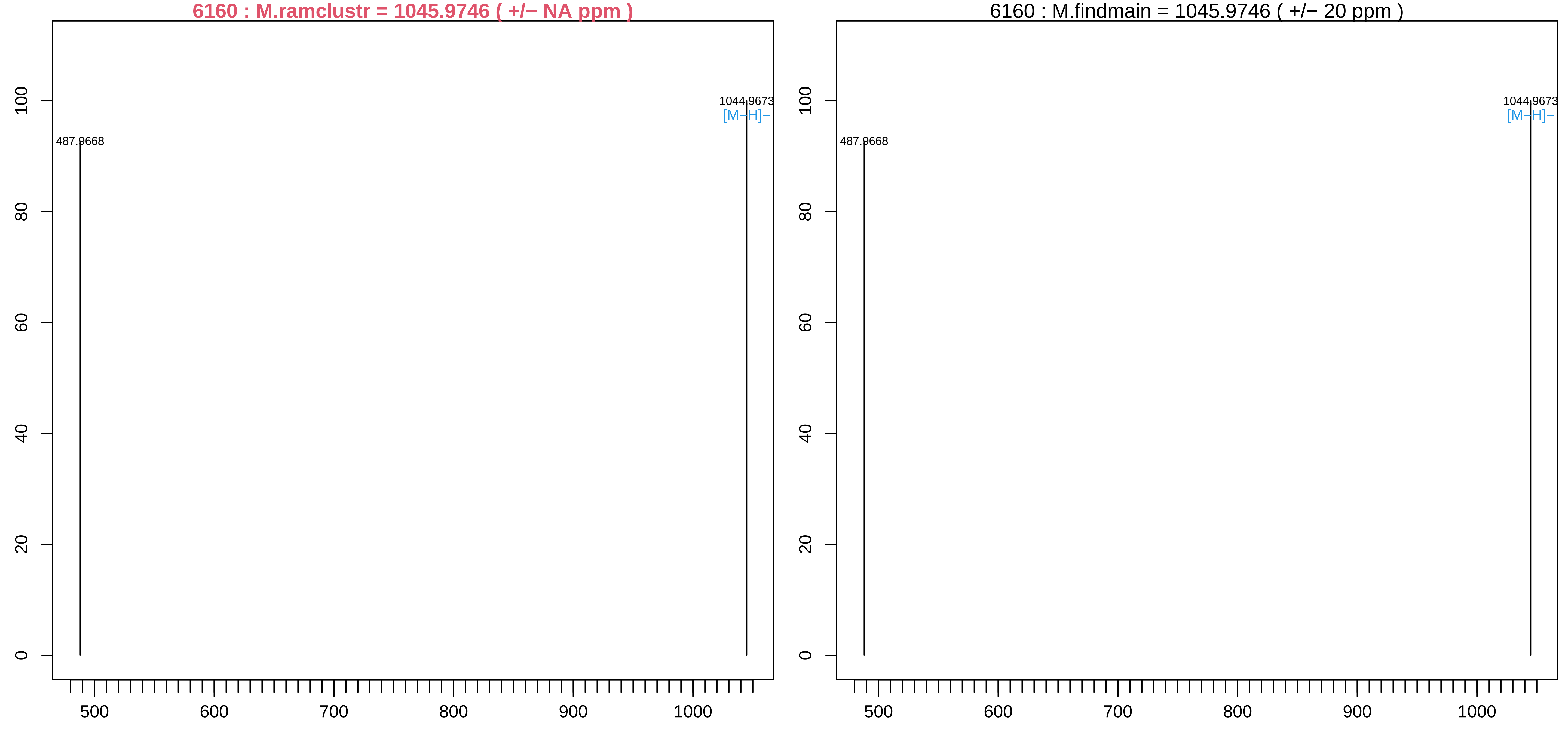
6160 : M.ramclustr = 1045.9746 (+/− NA ppm)
1044.9673
[M−H]−
487.9668
0
20
40
60
80
100
500
600
700
800
900
1000
6160 : M.findmain = 1045.9746 (+/− 20 ppm)
1044.9673
[M−H]−
487.9668
0
20
40
60
80
100
500
600
700
800
900
1000

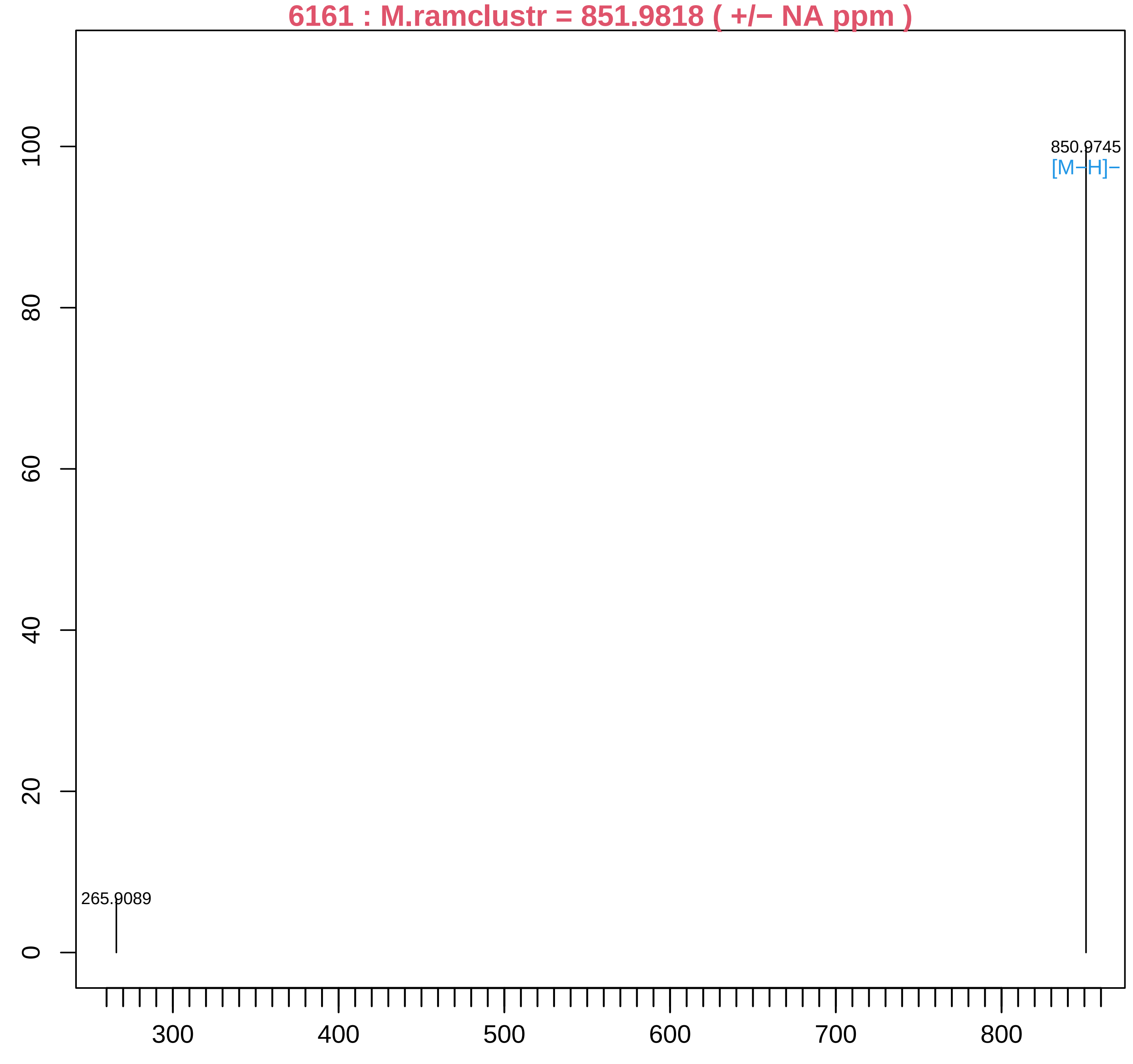
6161 : M.ramclustr = 851.9818 (+/− NA ppm)
850.9745
[M−H]−
265.9089
0
20
40
60
80
100
300
400
500
600
700
800

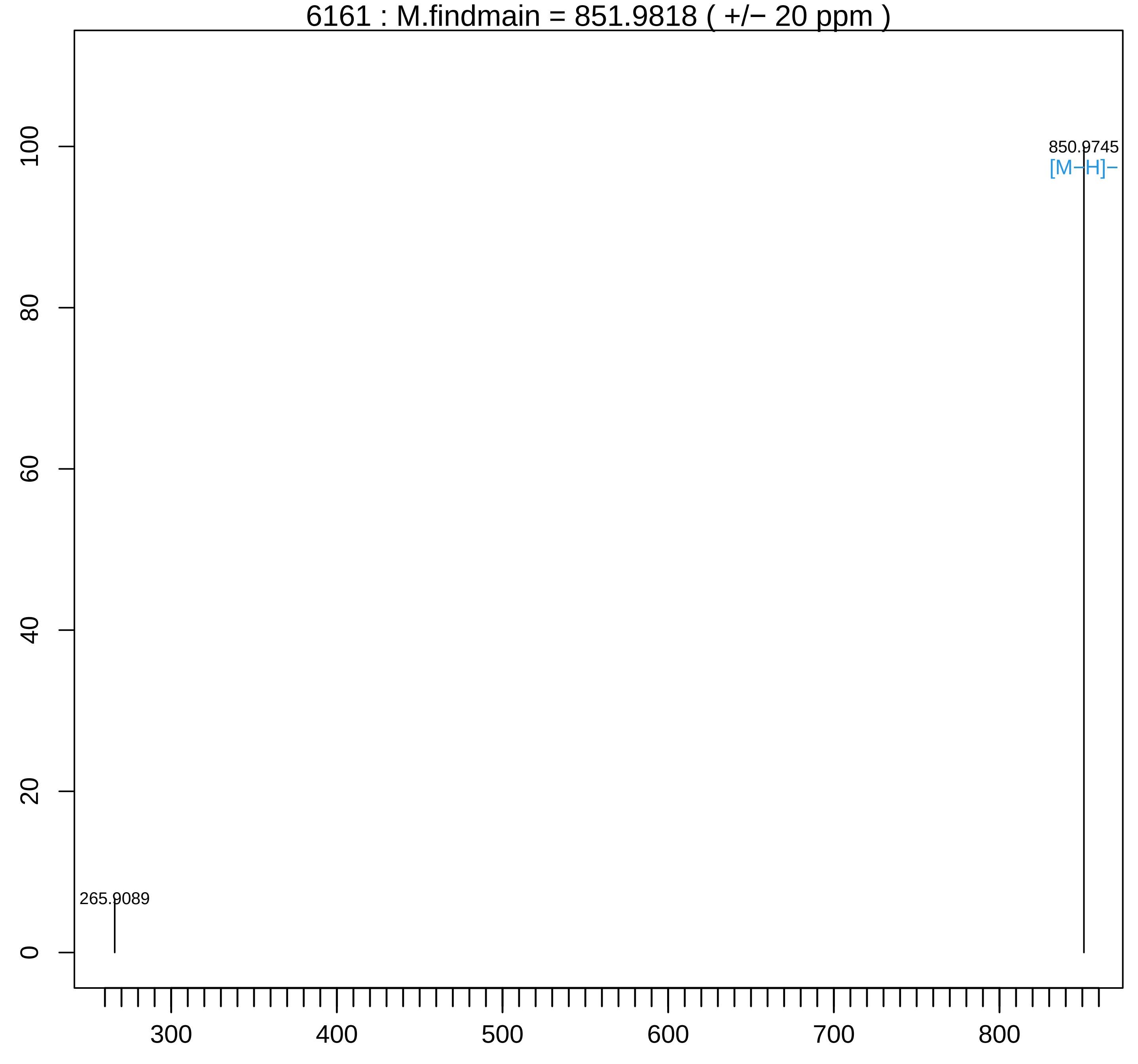
6161 : M.findmain = 851.9818 (+/− 20 ppm)
850.9745
[M−H]−
265.9089
0
20
40
60
80
100
300
400
500
600
700
800

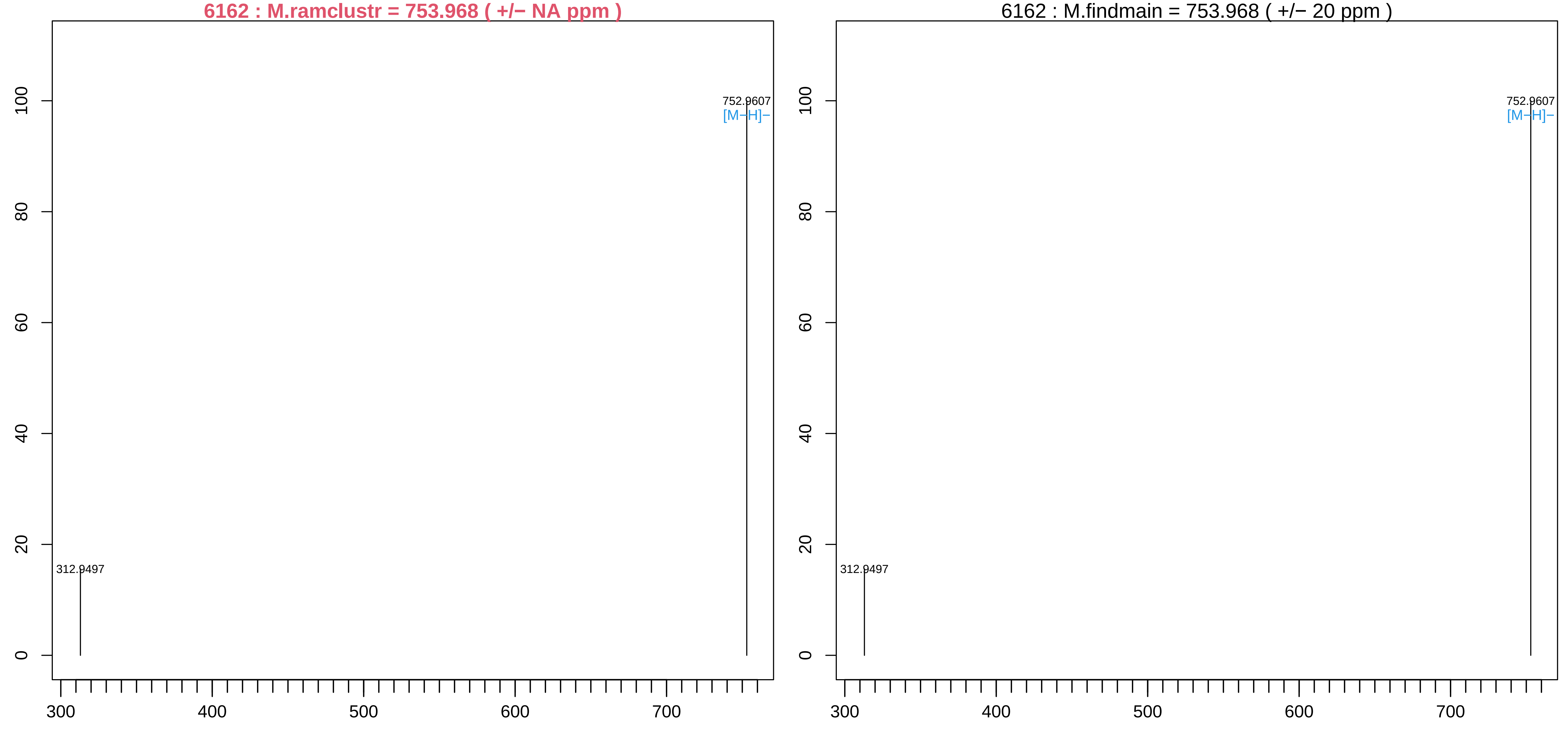

6162 : M.ramclustr = 753.968 (+/− NA ppm)
752.9607
[M−H]−
312.9497
0
20
40
60
80
100
300
400
500
600
700
6162 : M.findmain = 753.968 (+/− 20 ppm)
752.9607
[M−H]−
312.9497
0
20
40
60
80
100
300
400
500
600
700

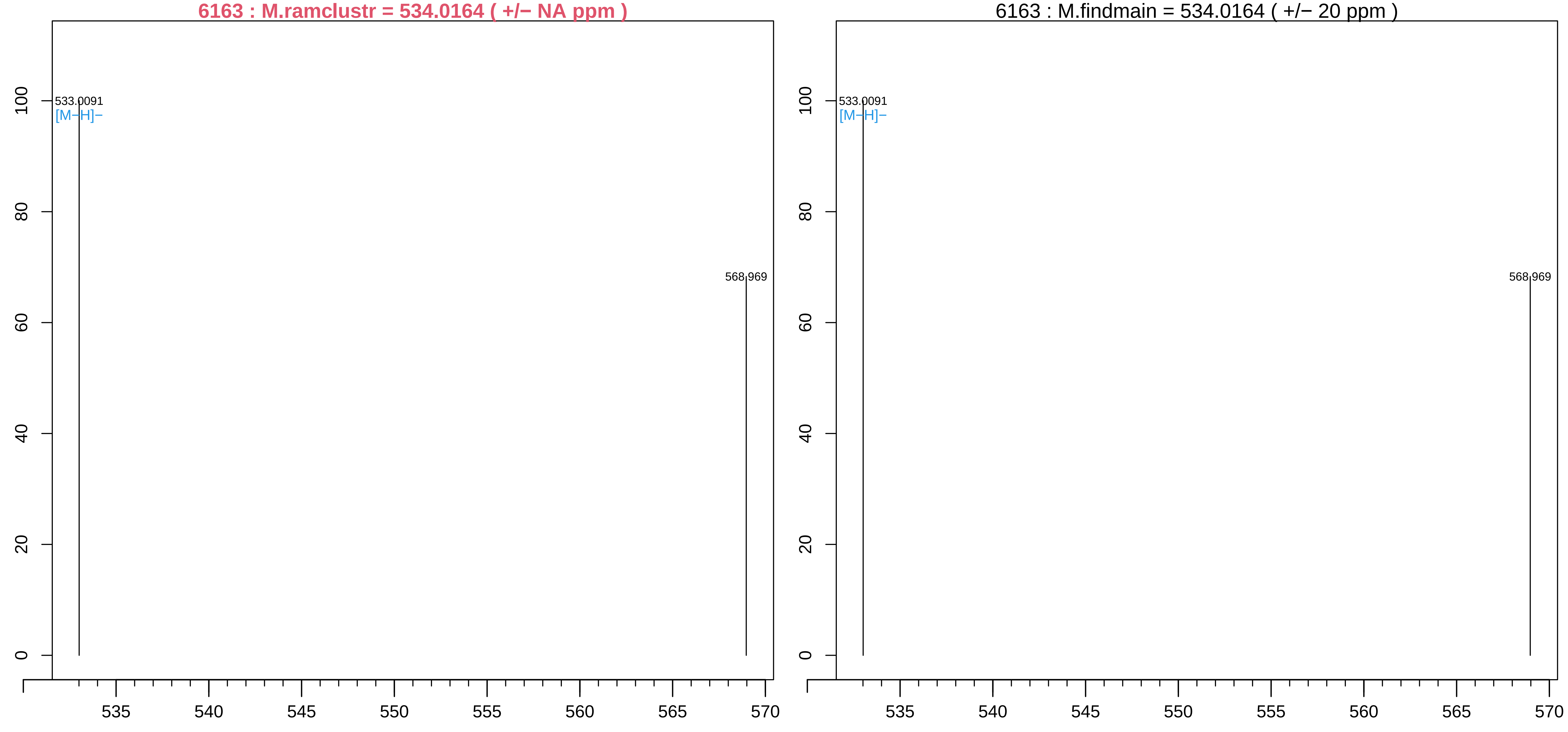

6163 : M.ramclustr = 534.0164 (+/− NA ppm)
533.0091
[M−H]−
568.969
6163 : M.findmain = 534.0164 (+/− 20 ppm)
533.0091
[M−H]−
568.969
0
20
40
60
80
100
535
540
545
550
555
560
565
570

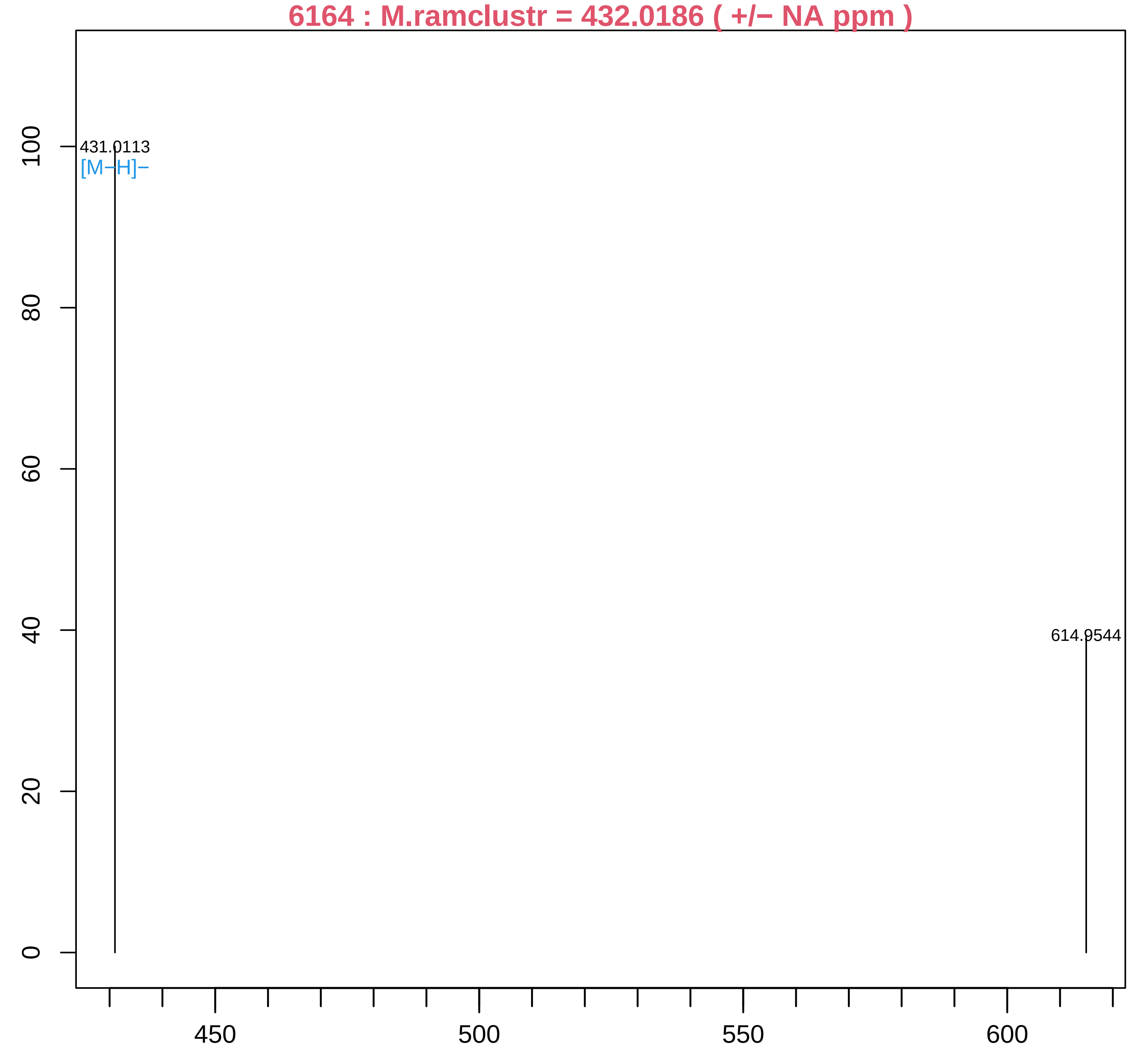
6164 : M.ramclustr = 432.0186 (+/− NA ppm)
431.0113
[M−H]−
614.9544
0
20
40
60
80
100
450
500
550
600

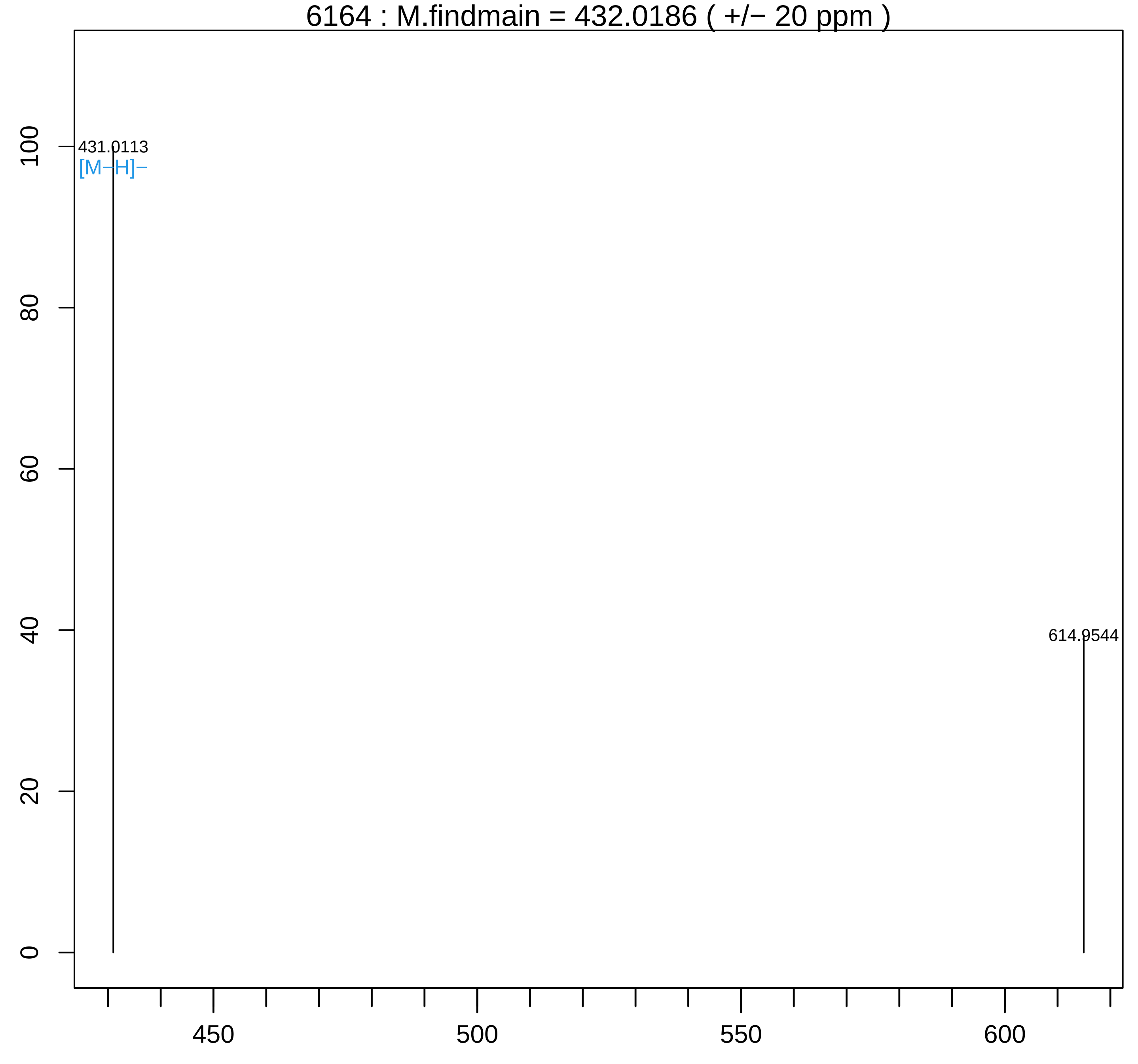
6164 : M.findmain = 432.0186 (+/− 20 ppm)
431.0113
[M−H]−
614.9544
0
20
40
60
80
100
450
500
550
600

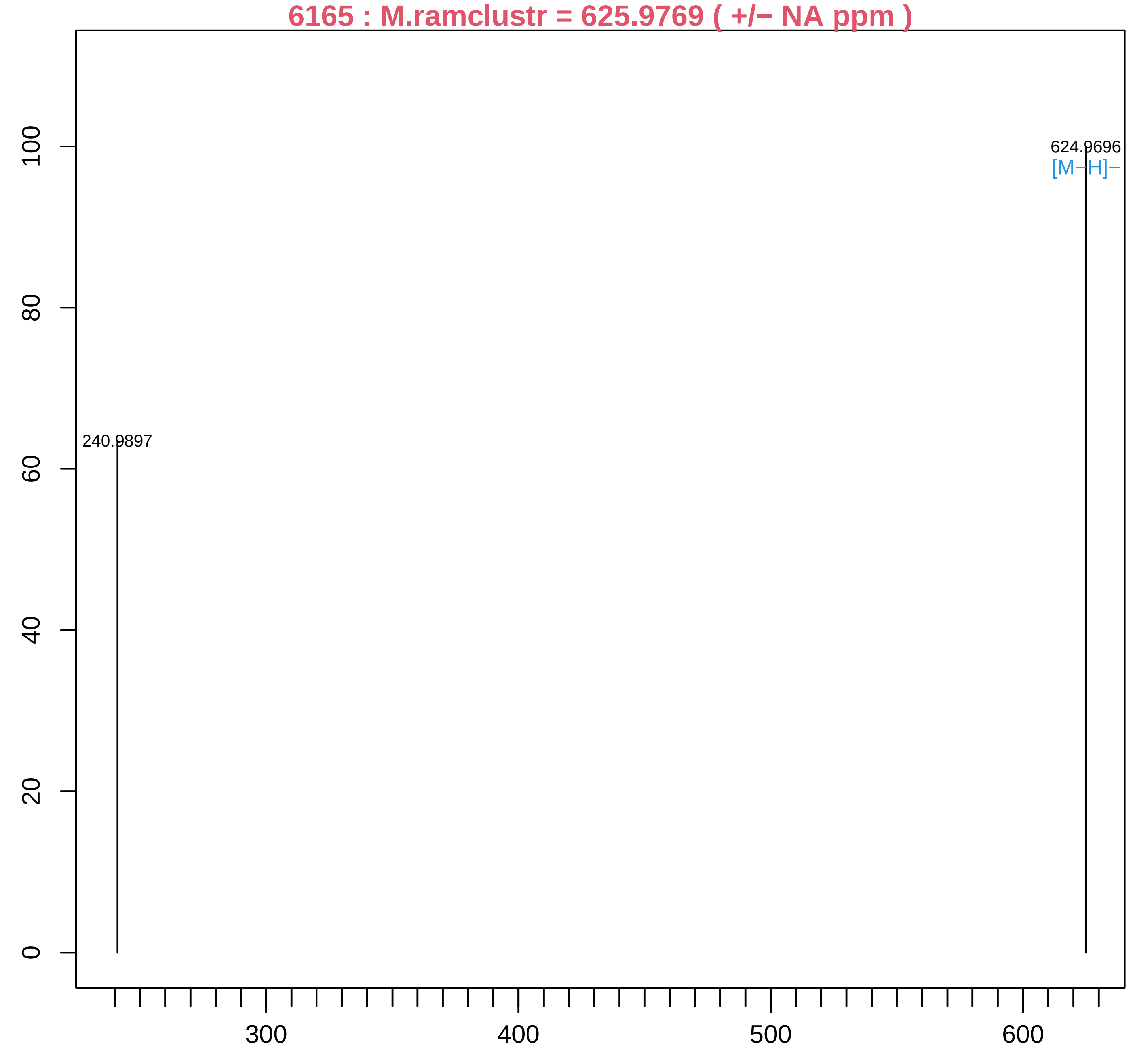

6165 : M.ramclustr = 625.9769 (+/− NA ppm)
624.9696
[M−H]−
240.9897
0
20
40
60
80
100
300
400
500
600

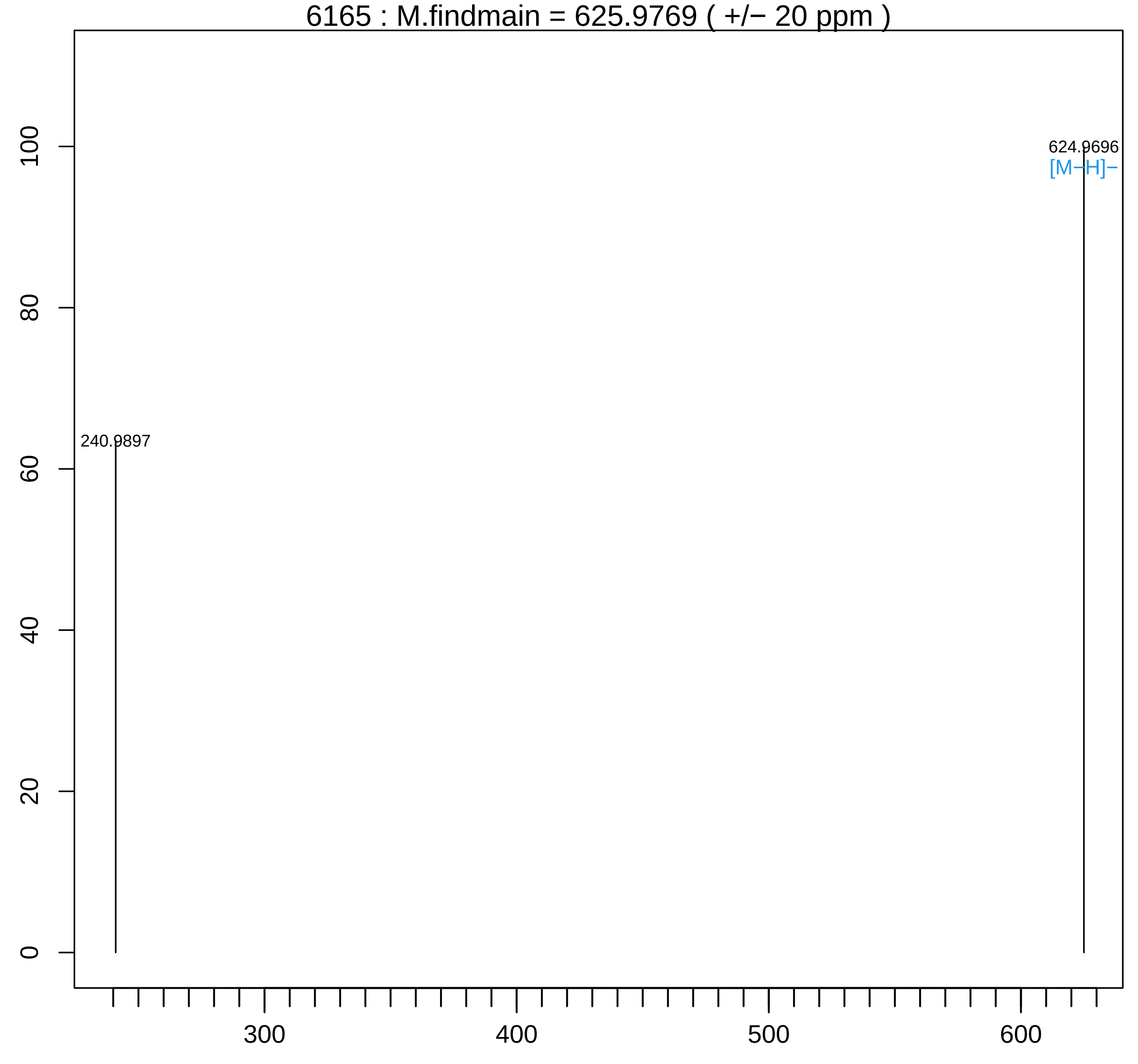

6165 : M.findmain = 625.9769 (+/− 20 ppm)
624.9696
[M−H]−
240.9897
0
20
40
60
80
100
300
400
500
600

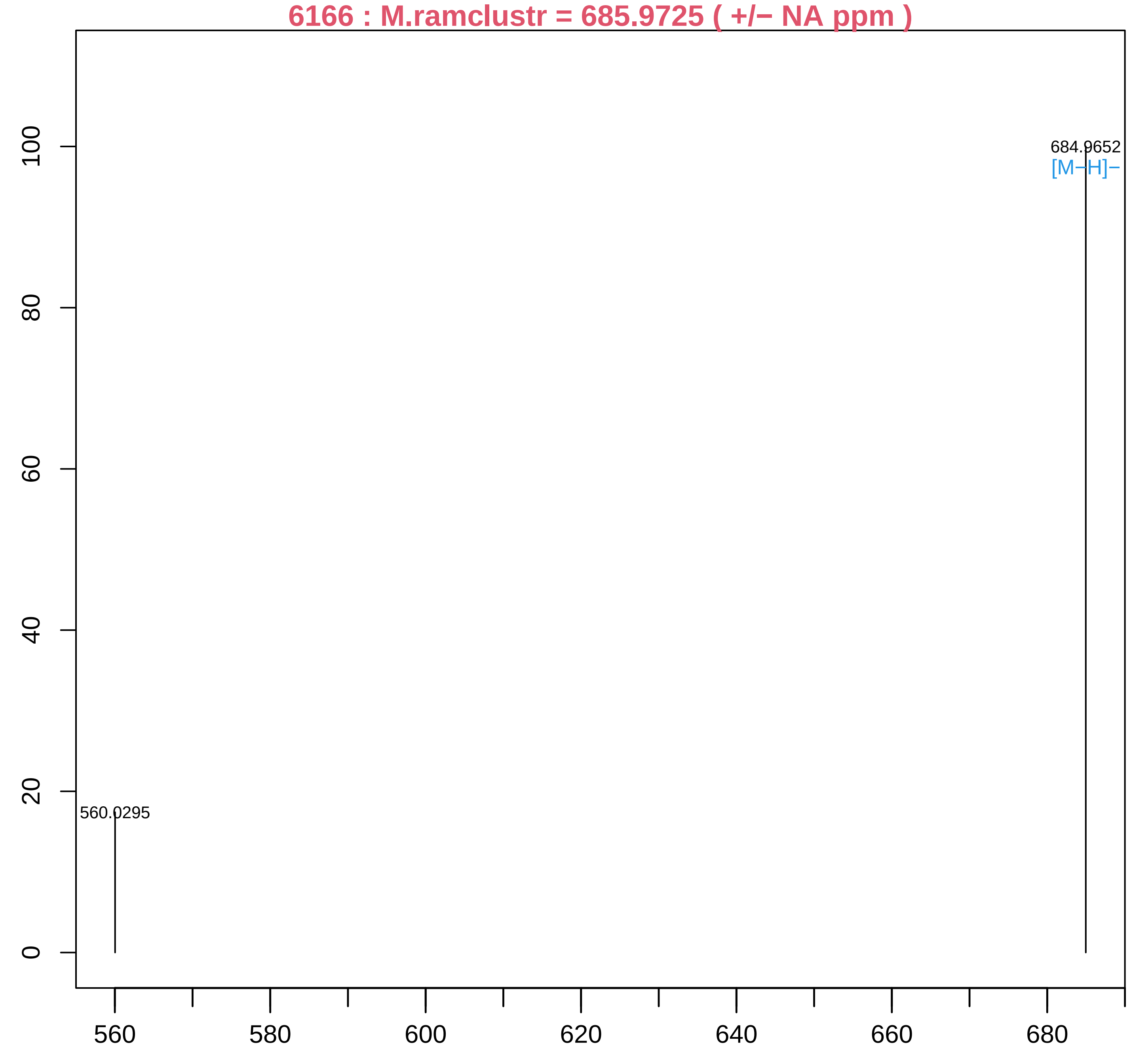
6166 : M.ramclustr = 685.9725 (+/− NA ppm)
684.9652
[M−H]−
560.0295
0
20
40
60
80
100
560
580
600
620
640
660
680

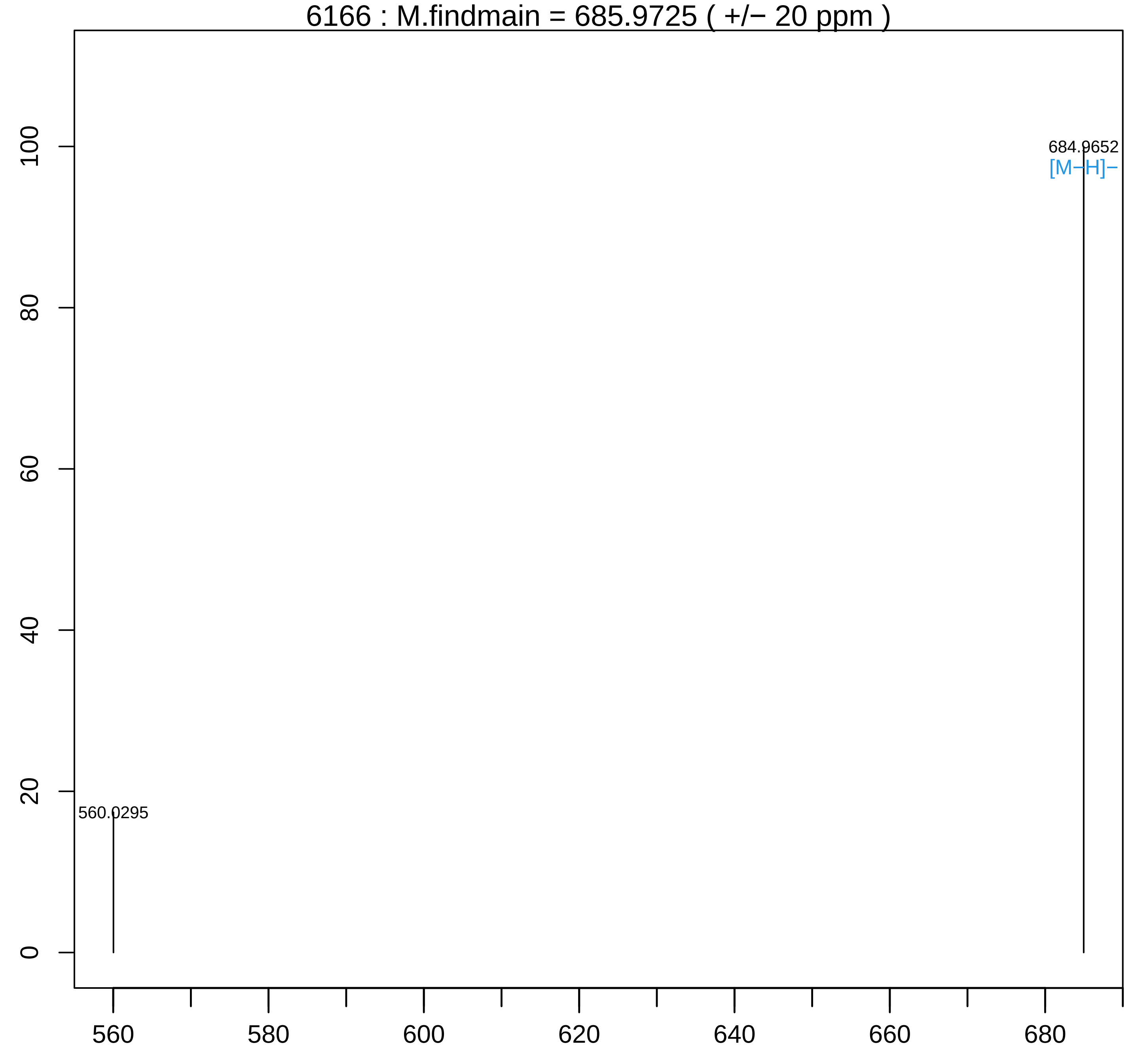
6166 : M.findmain = 685.9725 (+/− 20 ppm)
684.9652
[M−H]−
560.0295
0
20
40
60
80
100
560
580
600
620
640
660
680

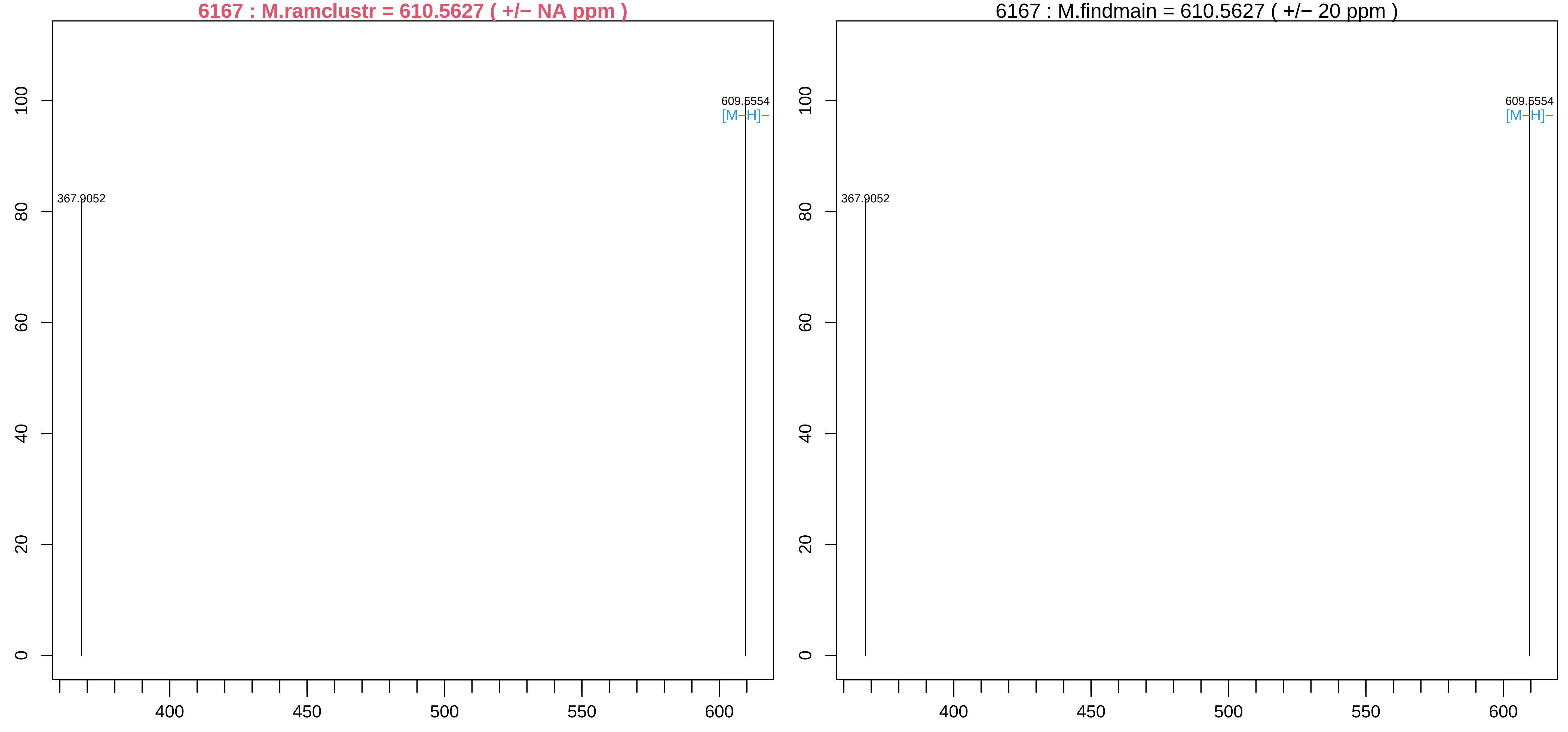

6167 : M.ramclustr = 610.5627 (+/− NA ppm)
367.9052
609.5554
[M−H]−
6167 : M.findmain = 610.5627 (+/− 20 ppm)
367.9052
609.5554
[M−H]−

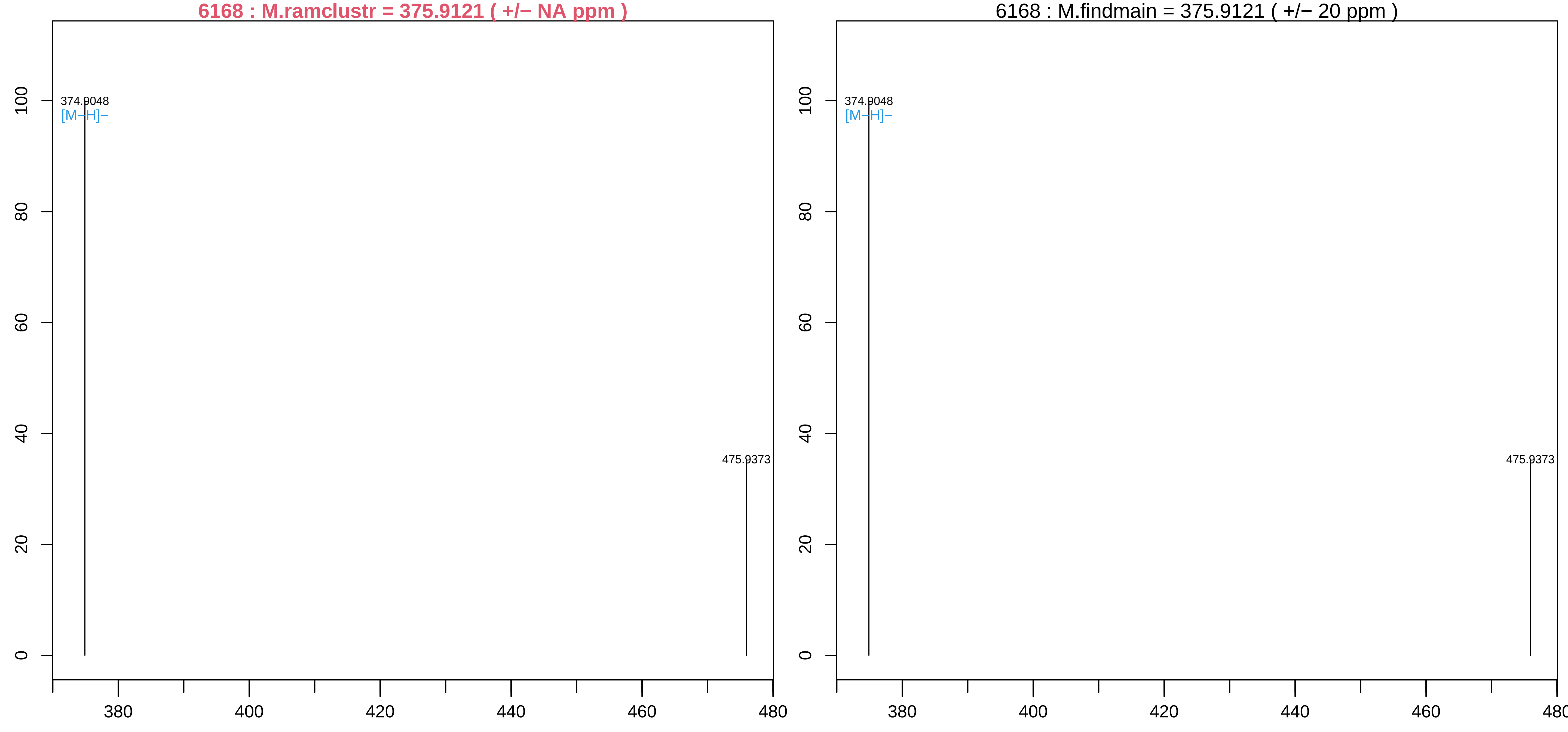
6168 : M.ramclustr = 375.9121 (+/− NA ppm)
374.9048
[M−H]−
475.9373
6168 : M.findmain = 375.9121 (+/− 20 ppm)
374.9048
[M−H]−
475.9373

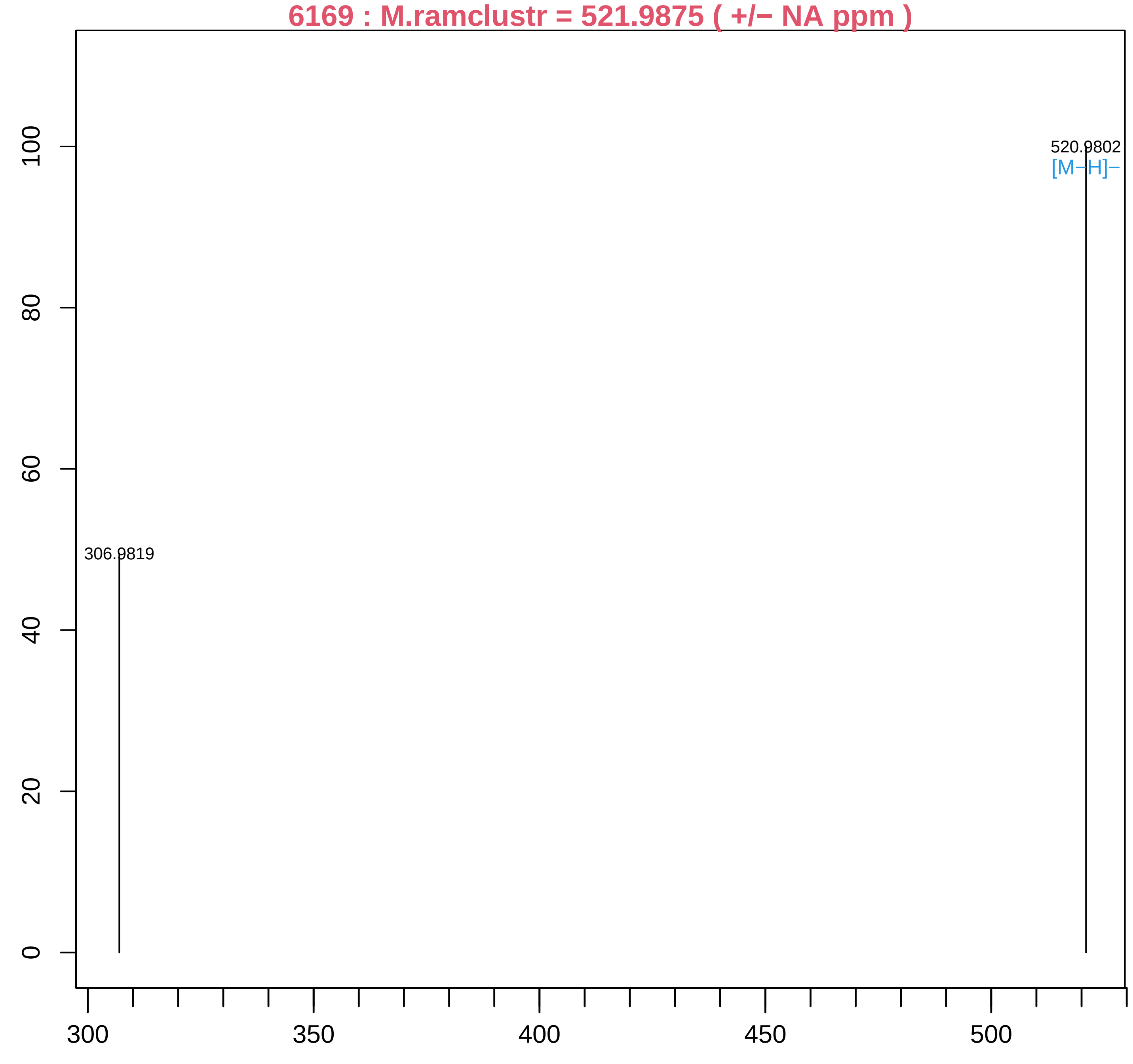

6169 : M.ramclustr = 521.9875 (+/− NA ppm)
520.9802
[M−H]−
306.9819
0
20
40
60
80
100
300
350
400
450
500

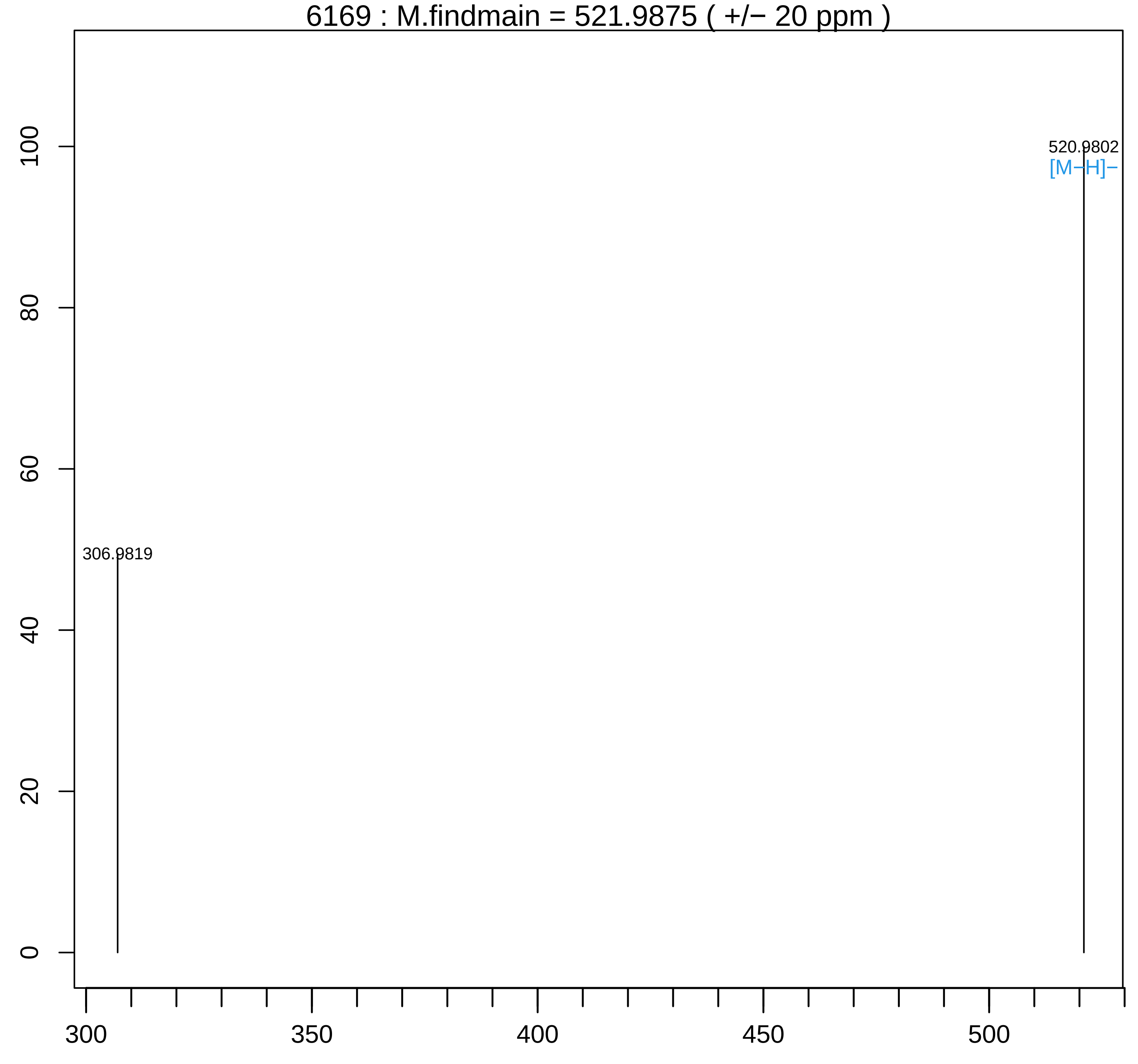

6169 : M.findmain = 521.9875 (+/− 20 ppm)
520.9802
[M−H]−
306.9819
0
20
40
60
80
100
300
350
400
450
500

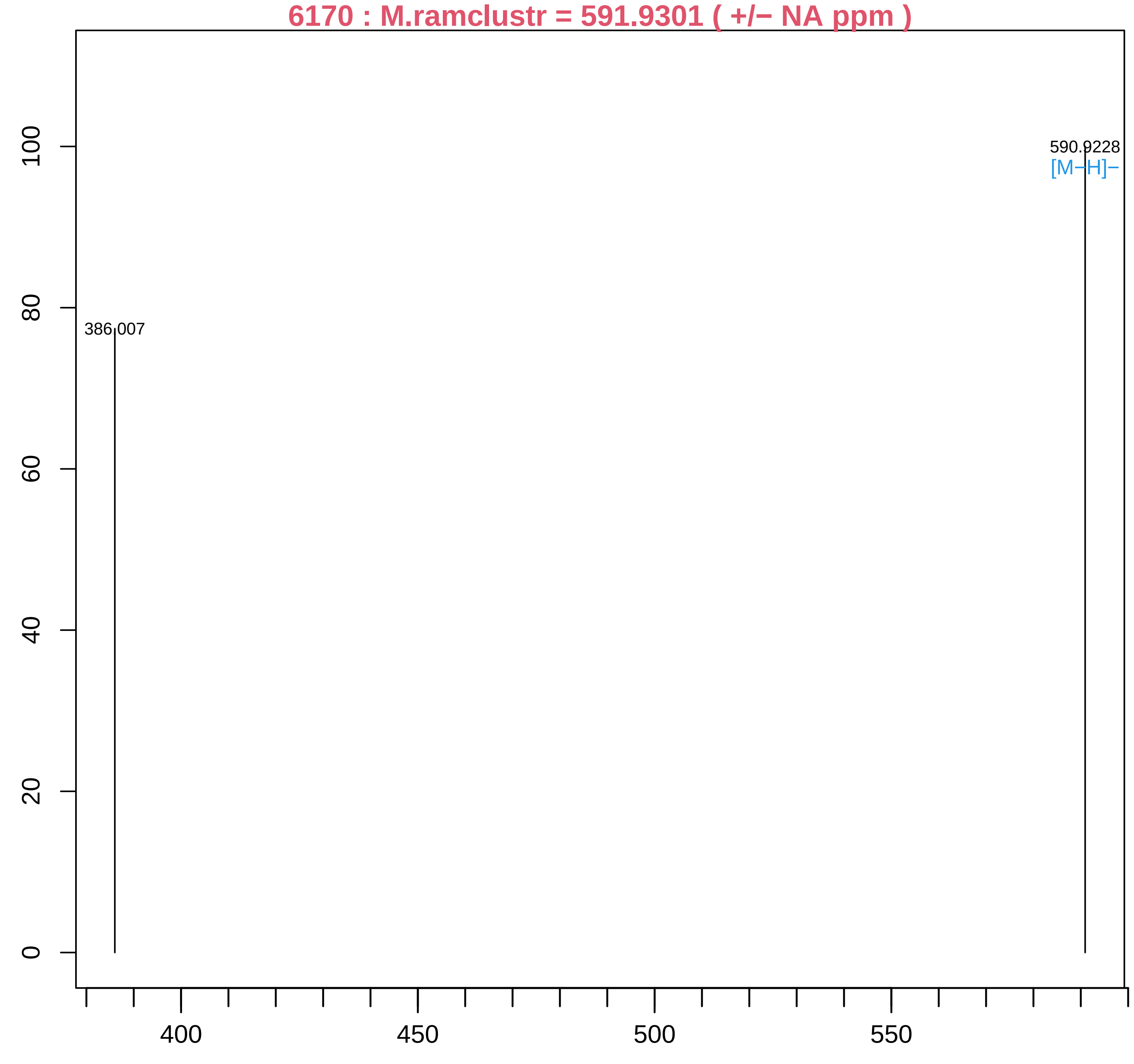
6170 : M.ramclustr = 591.9301 (+/− NA ppm)
590.9228
[M−H]−
386.007
0
20
40
60
80
100
400
450
500
550

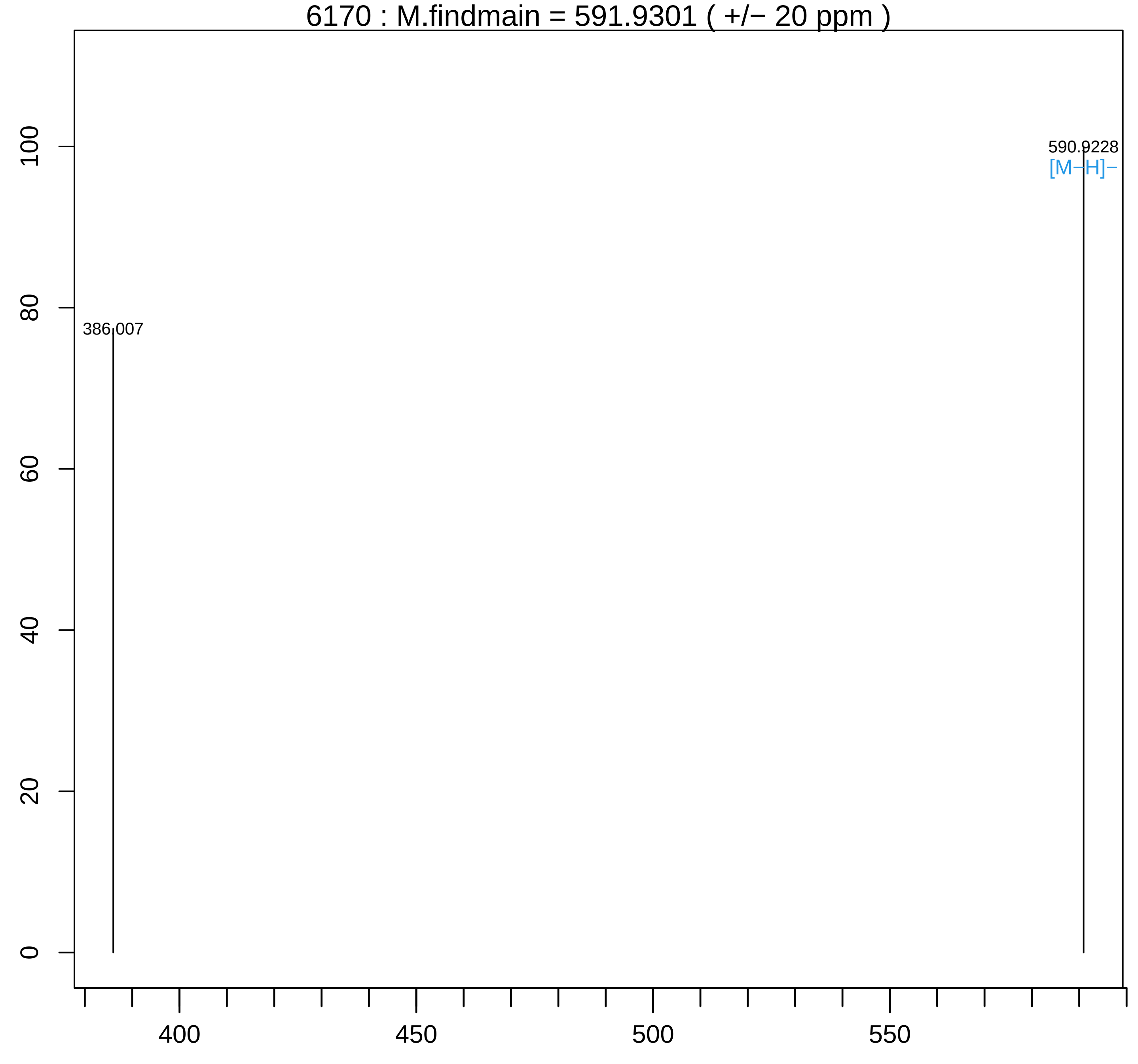
6170 : M.findmain = 591.9301 (+/− 20 ppm)
590.9228
[M−H]−
386.007
0
20
40
60
80
100
400
450
500
550

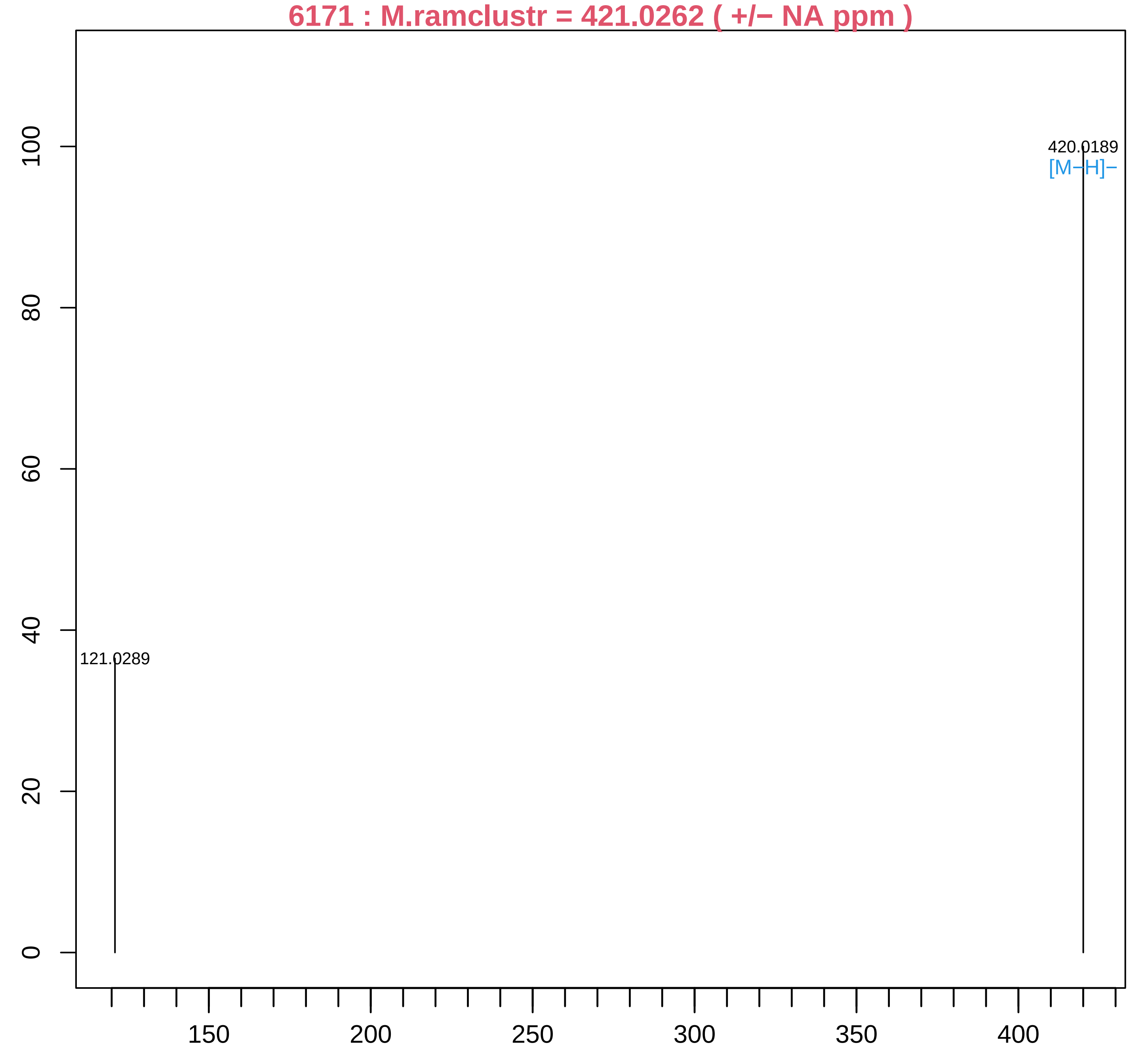
6171 : M.ramclustr = 421.0262 (+/− NA ppm)
420.0189
[M−H]−
121.0289
0
20
40
60
80
100
150
200
250
300
350
400
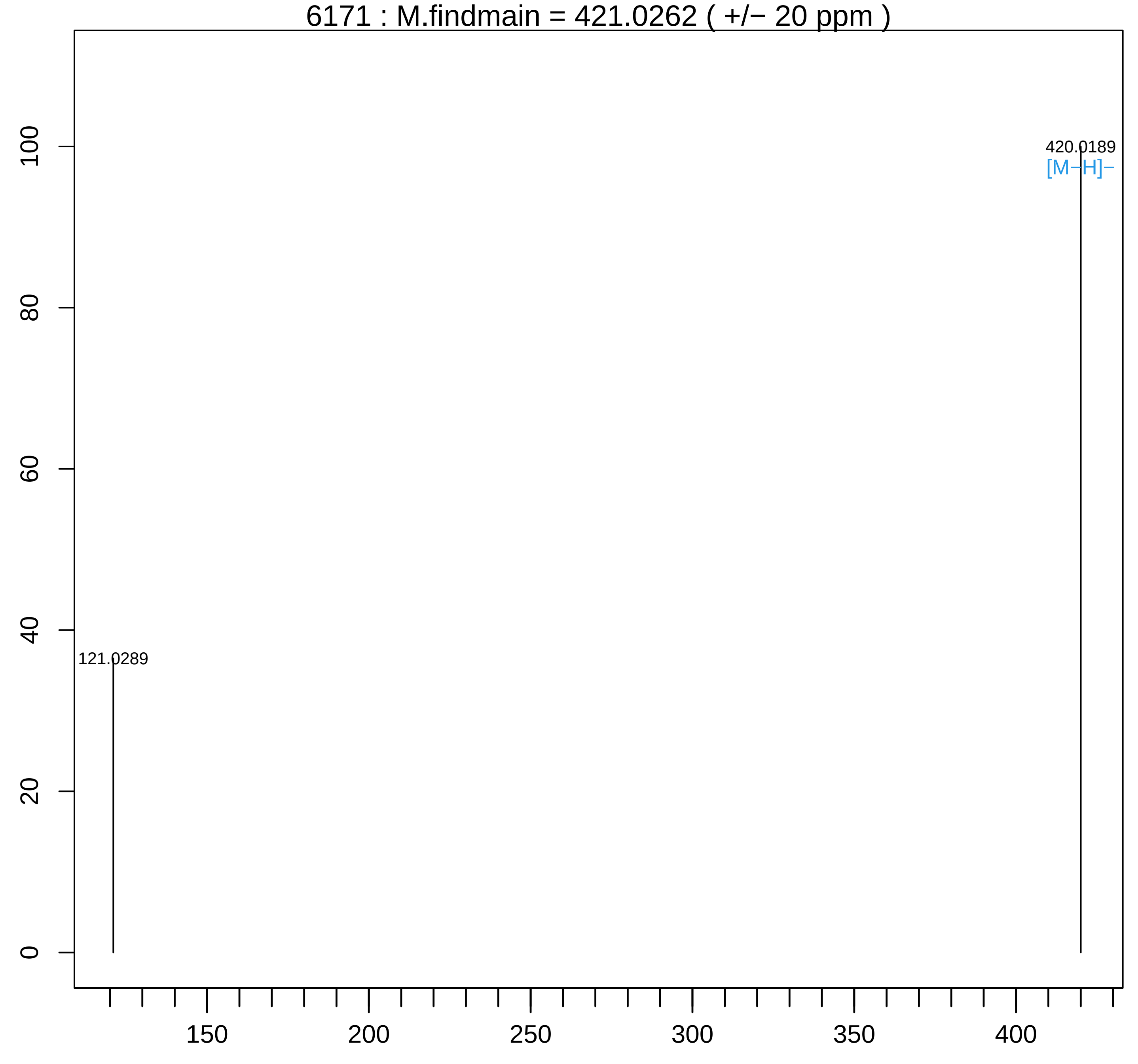
6171 : M.findmain = 421.0262 (+/− 20 ppm)
420.0189
[M−H]−
121.0289
0
20
40
60
80
100
150
200
250
300
350
400

6172 : M.ramclustr = 624.4349 (+/− NA ppm)

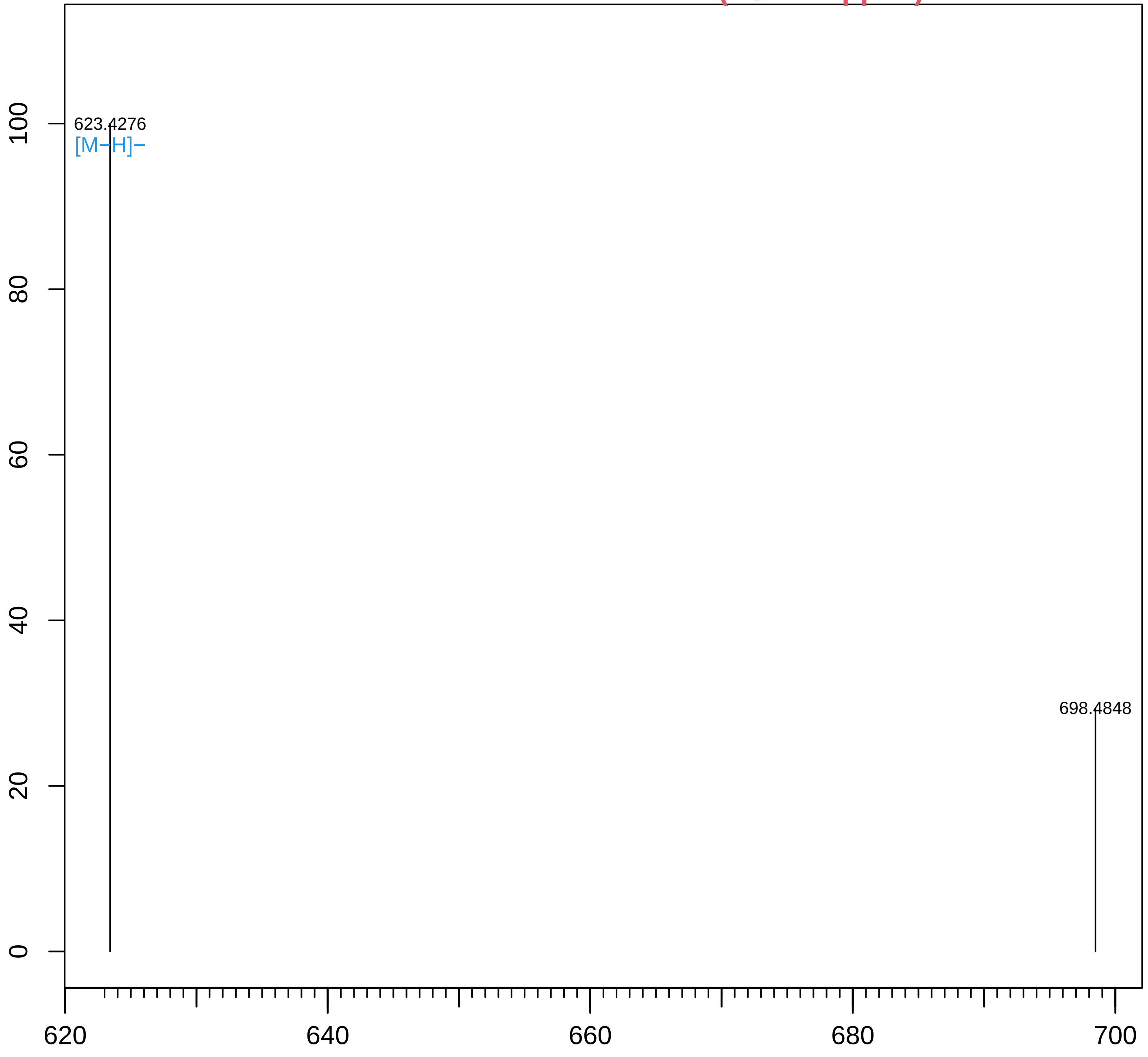

6172 : M.findmain = 624.4349 (+/− 20 ppm)

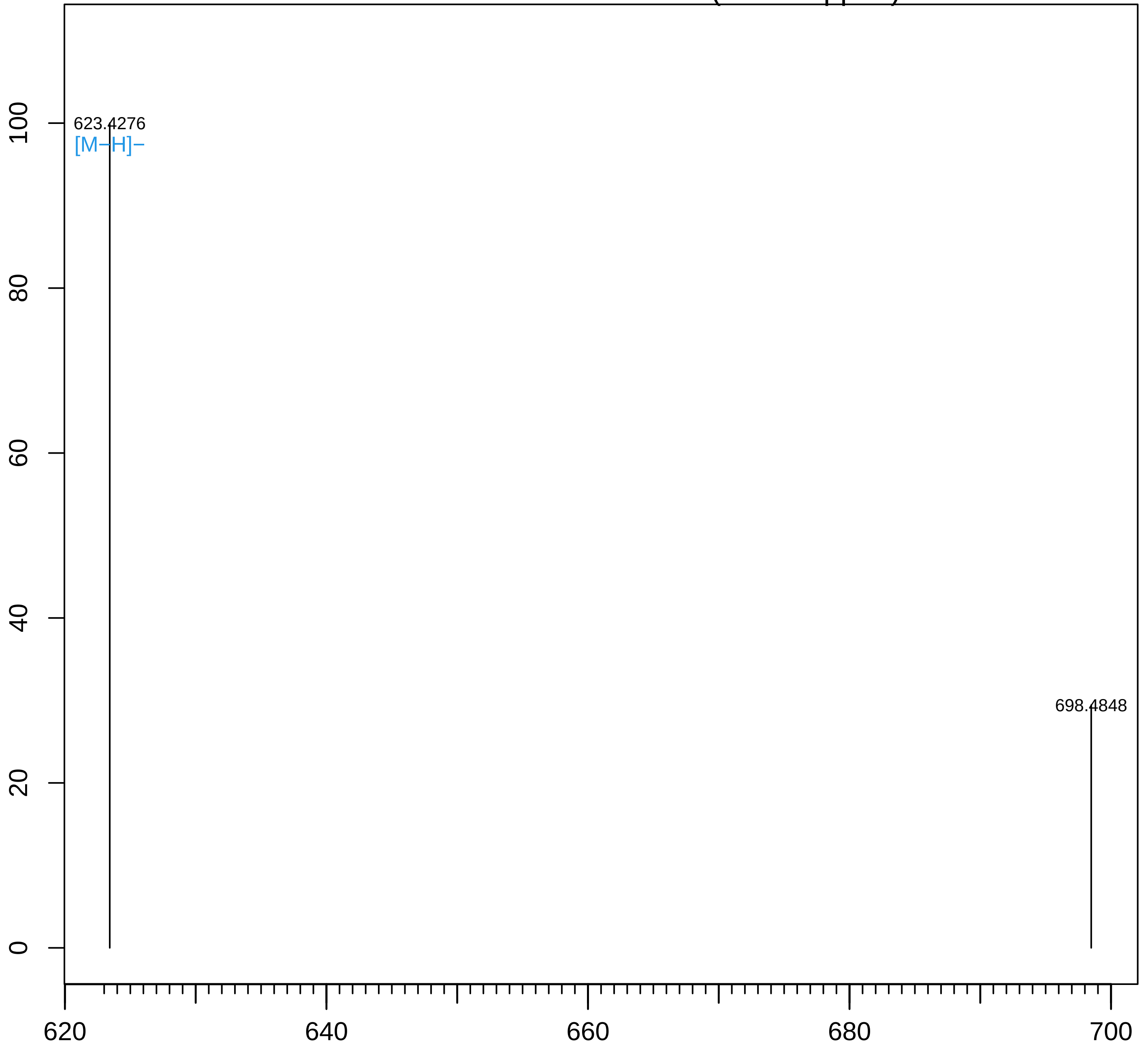

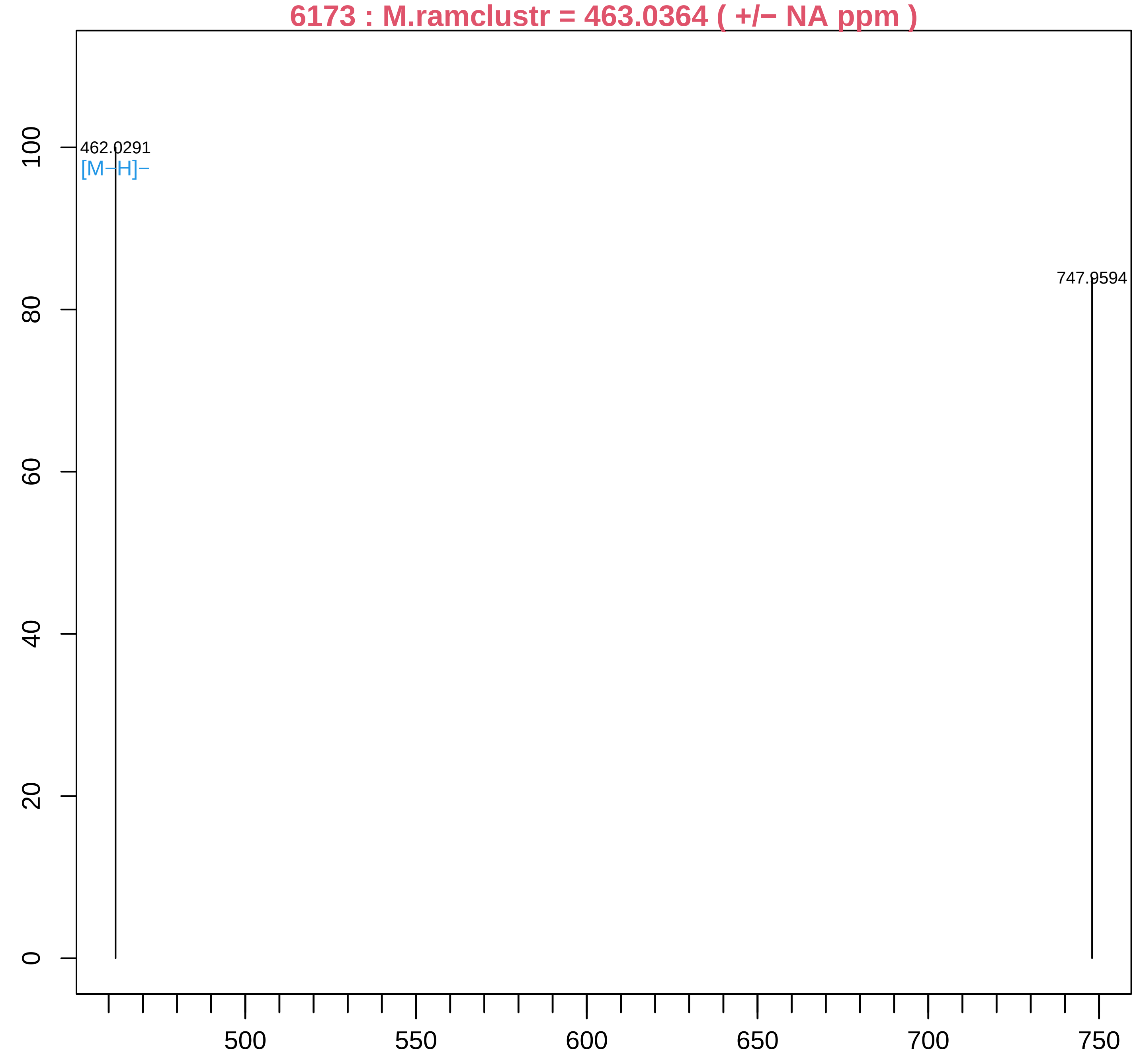
6173 : M.ramclustr = 463.0364 (+/− NA ppm)
462.0291
[M−H]−
747.9594
0
20
40
60
80
100
500
550
600
650
700
750

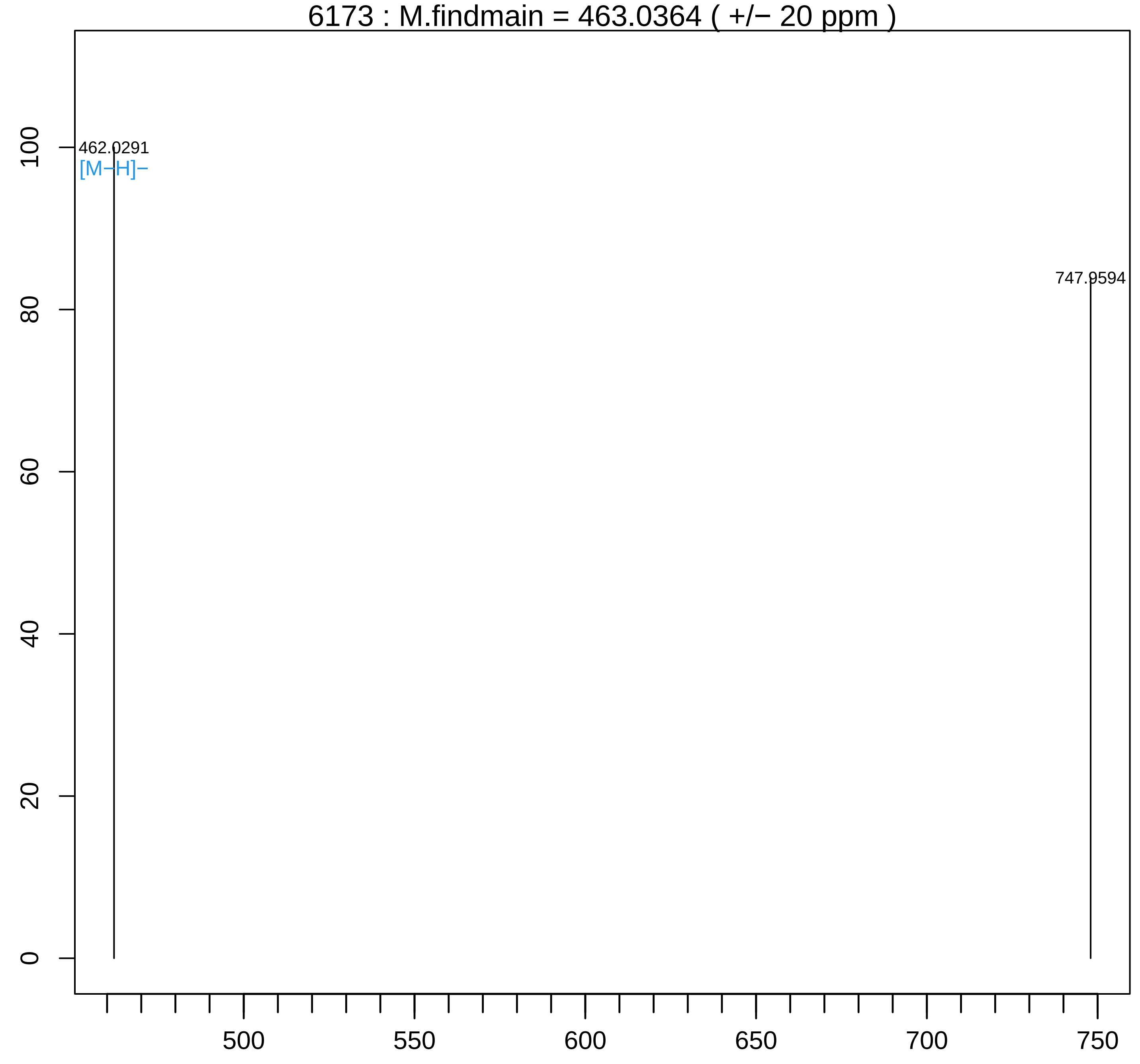
6173 : M.findmain = 463.0364 (+/− 20 ppm)
462.0291
[M−H]−
747.9594
0
20
40
60
80
100
500
550
600
650
700
750

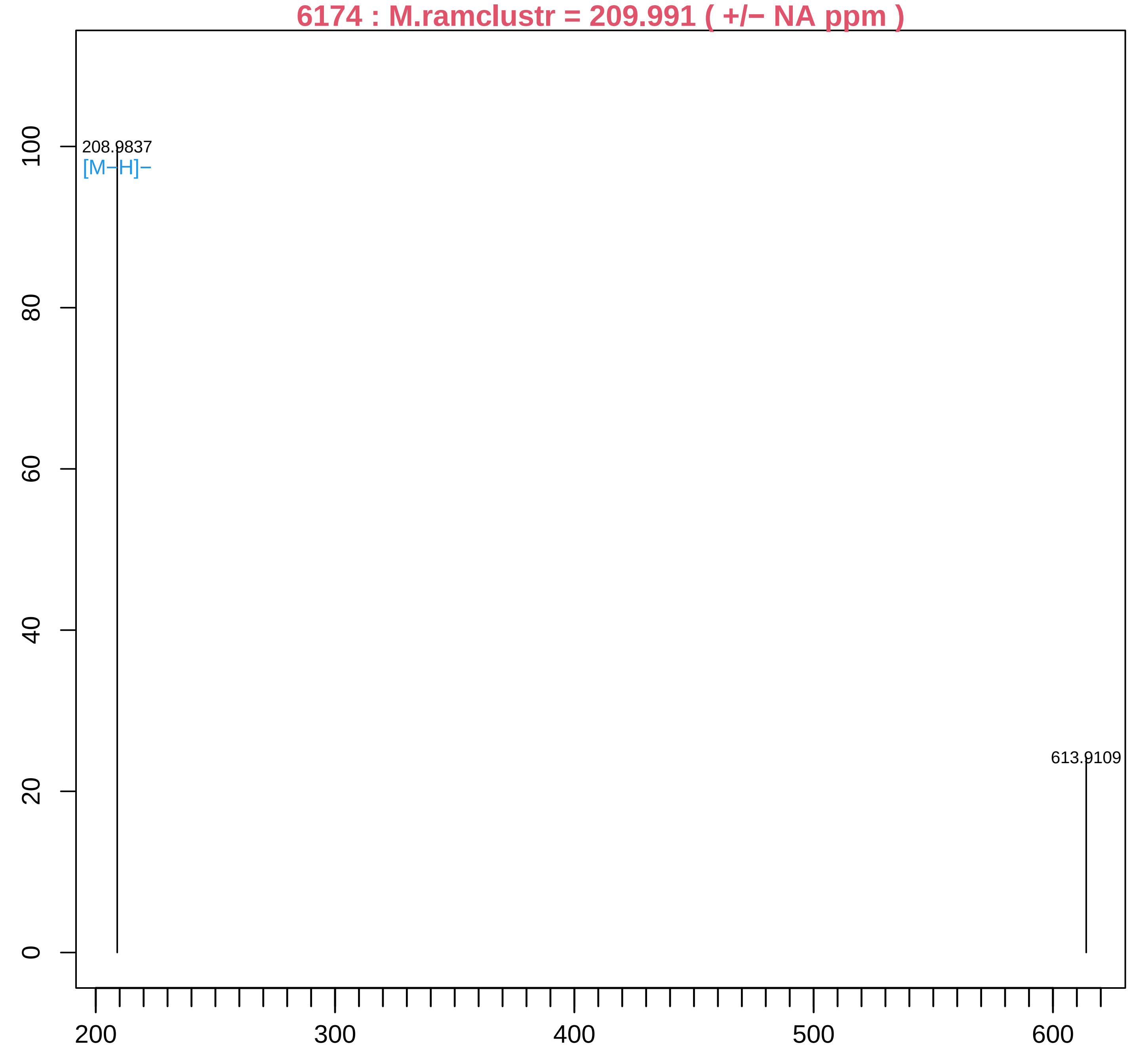
6174 : M.ramclustr = 209.991 (+/− NA ppm)
208.9837
[M−H]−
613.9109
0
20
40
60
80
100
200
300
400
500
600

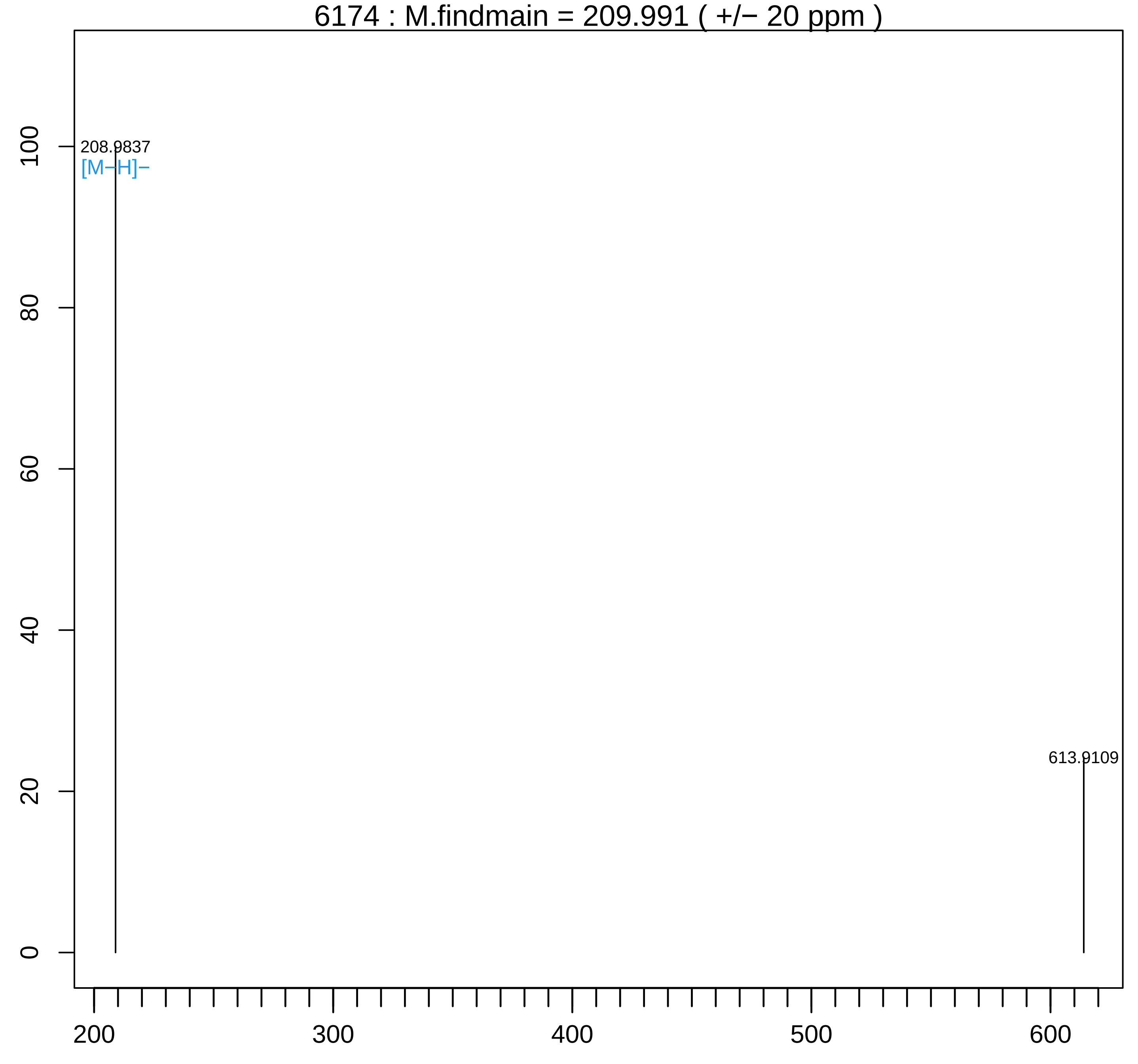
6174 : M.findmain = 209.991 (+/− 20 ppm)
208.9837
[M−H]−
613.9109
0
20
40
60
80
100
200
300
400
500
600

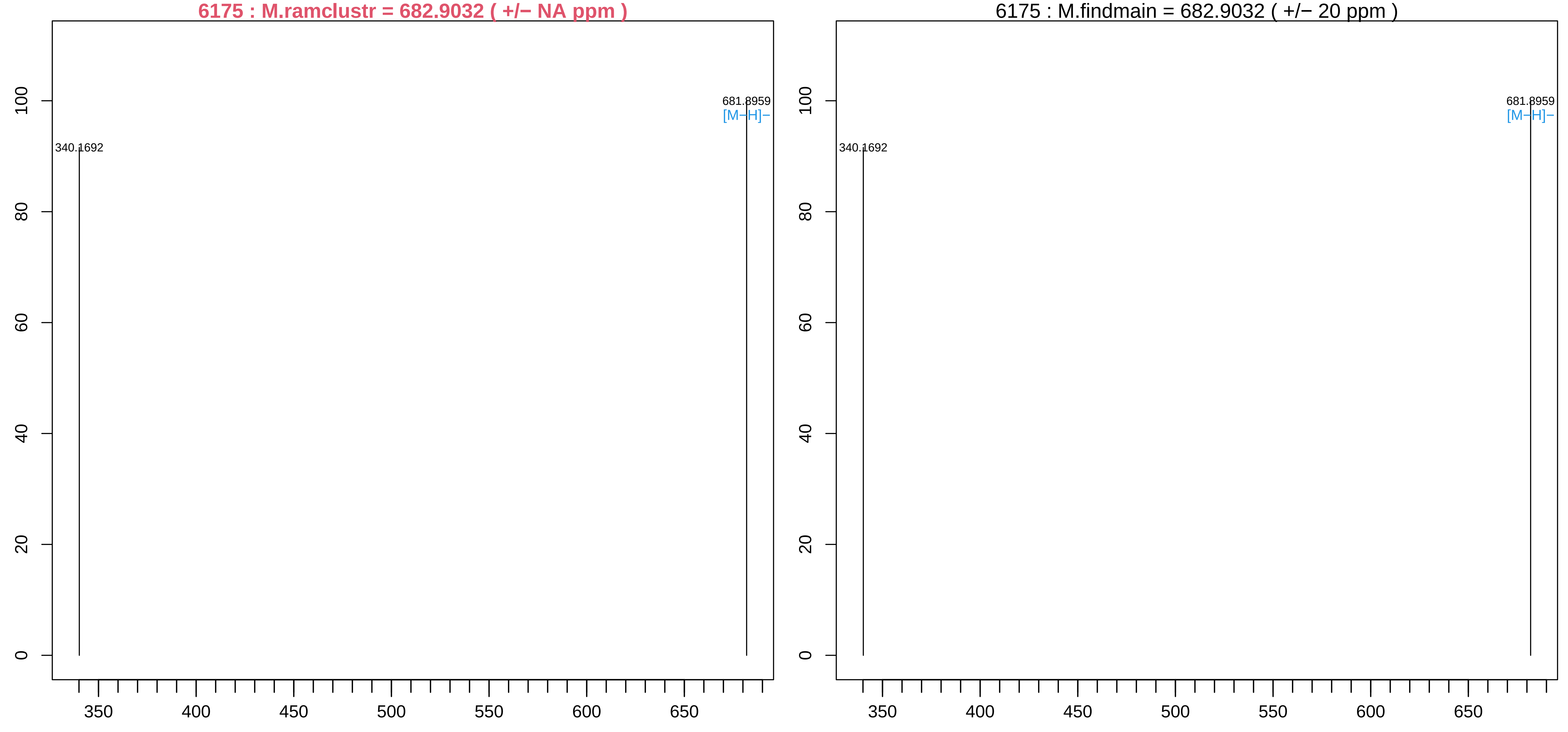
6175 : M.ramclustr = 682.9032 (+/− NA ppm)
681.8959
[M−H]−
340.1692
6175 : M.findmain = 682.9032 (+/− 20 ppm)
681.8959
[M−H]−
340.1692
0
20
40
60
80
100
350
400
450
500
550
600
650

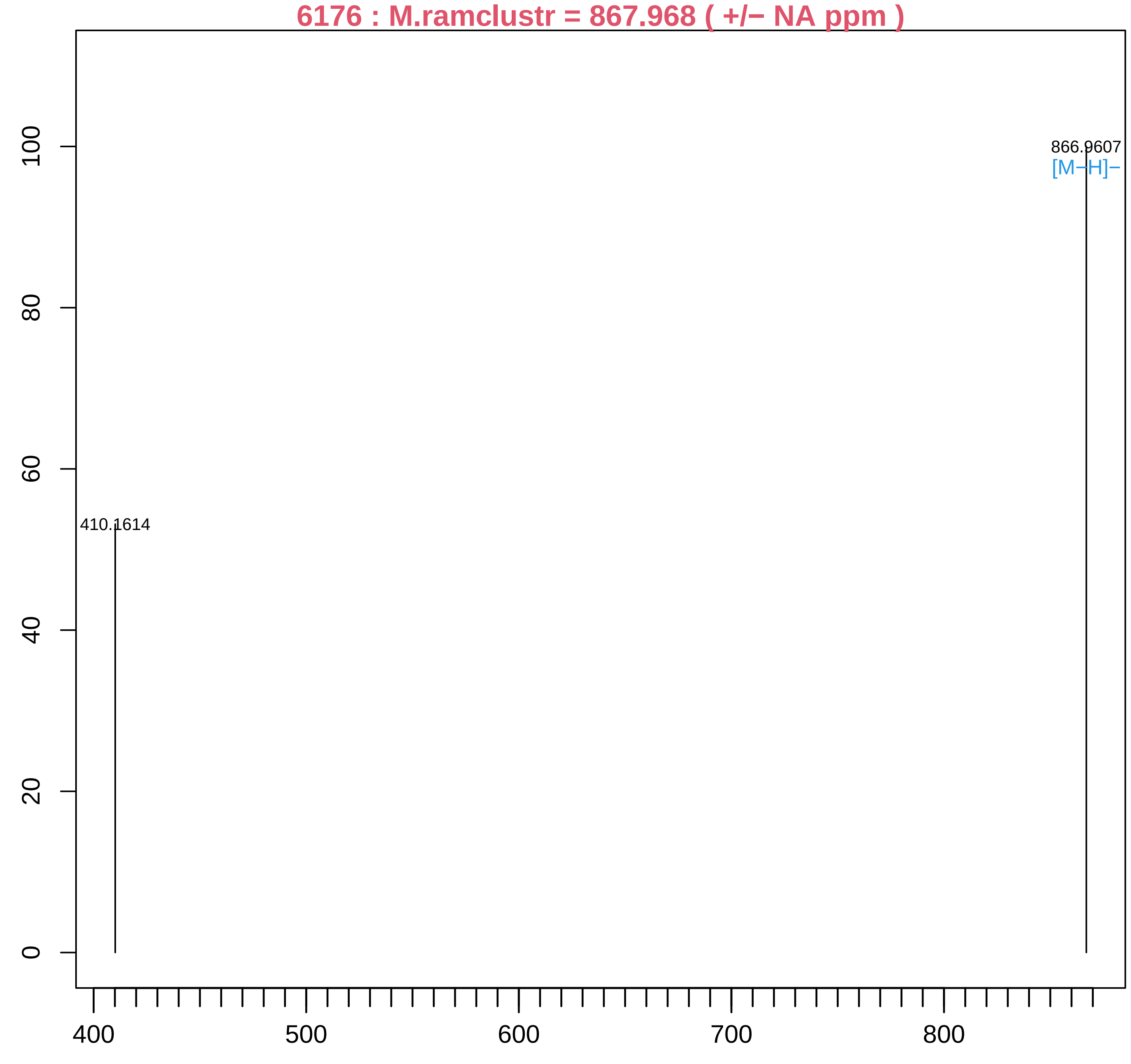
6176 : M.ramclustr = 867.968 (+/− NA ppm)
866.9607
[M−H]−
410.1614
0
20
40
60
80
100
400
500
600
700
800

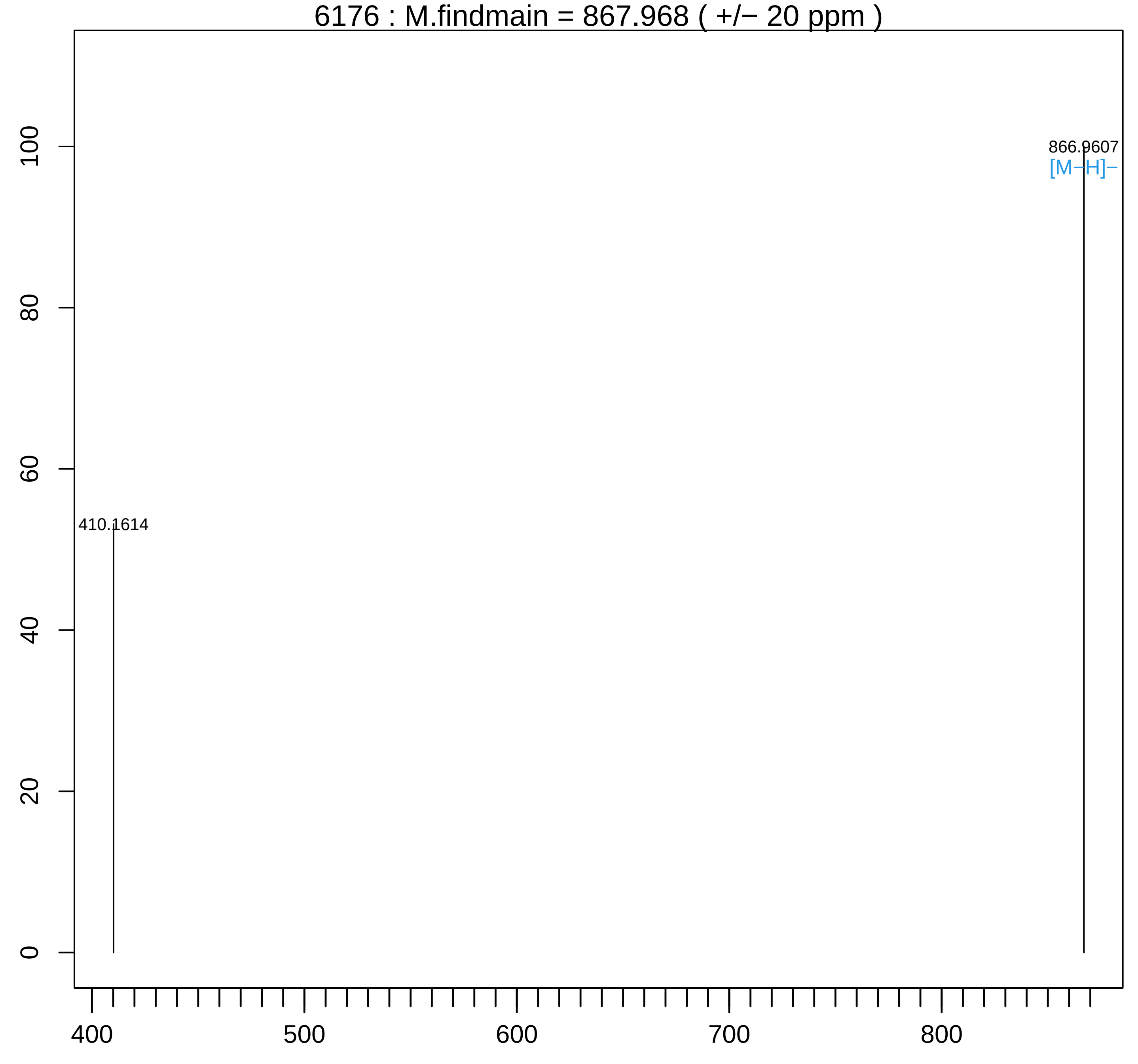
6176 : M.findmain = 867.968 (+/− 20 ppm)
866.9607
[M−H]−
410.1614
0
20
40
60
80
100
400
500
600
700
800

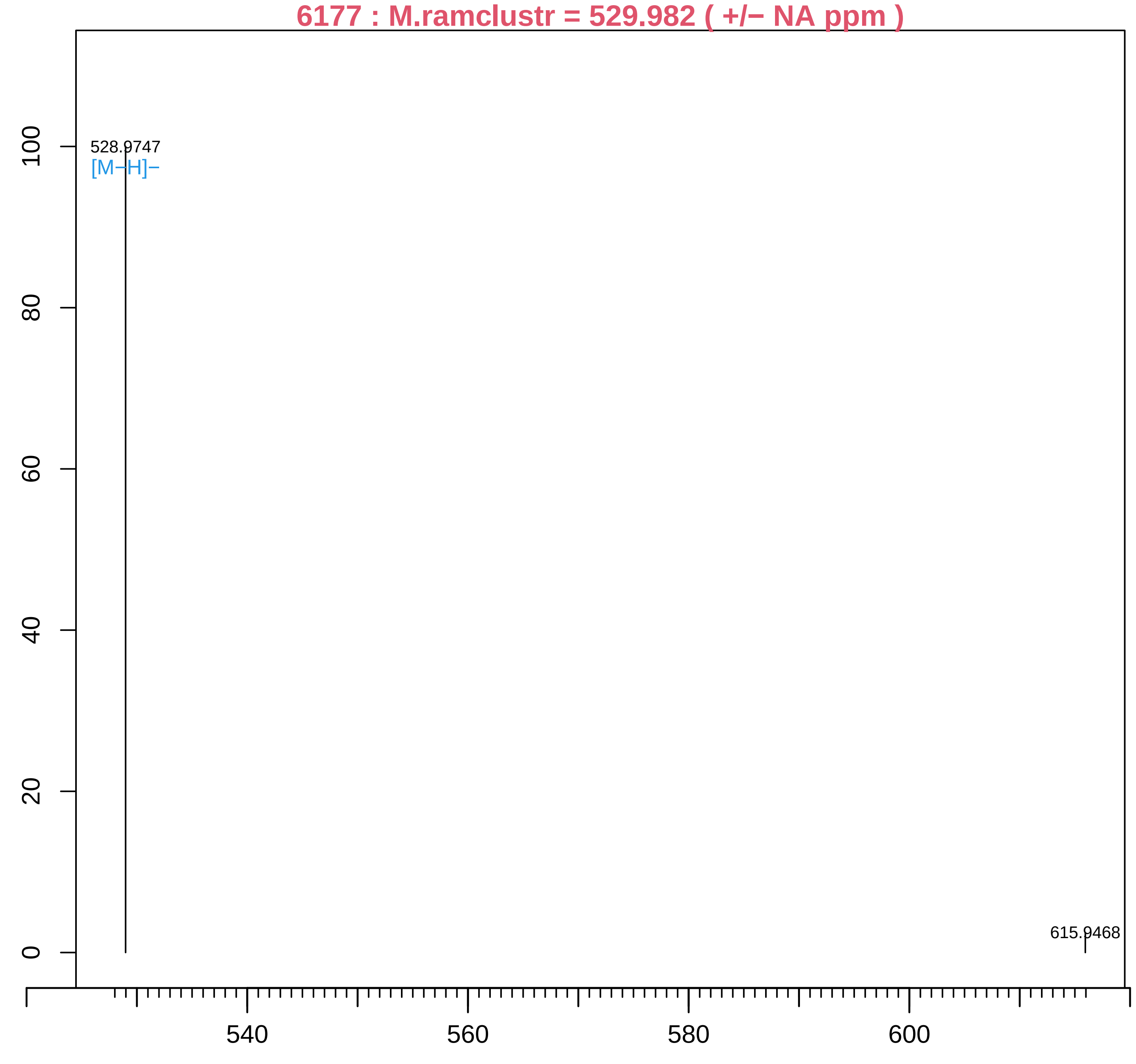

6177 : M.ramclustr = 529.982 (+/− NA ppm)
528.9747
[M−H]−
615.9468
0
20
40
60
80
100
540
560
580
600

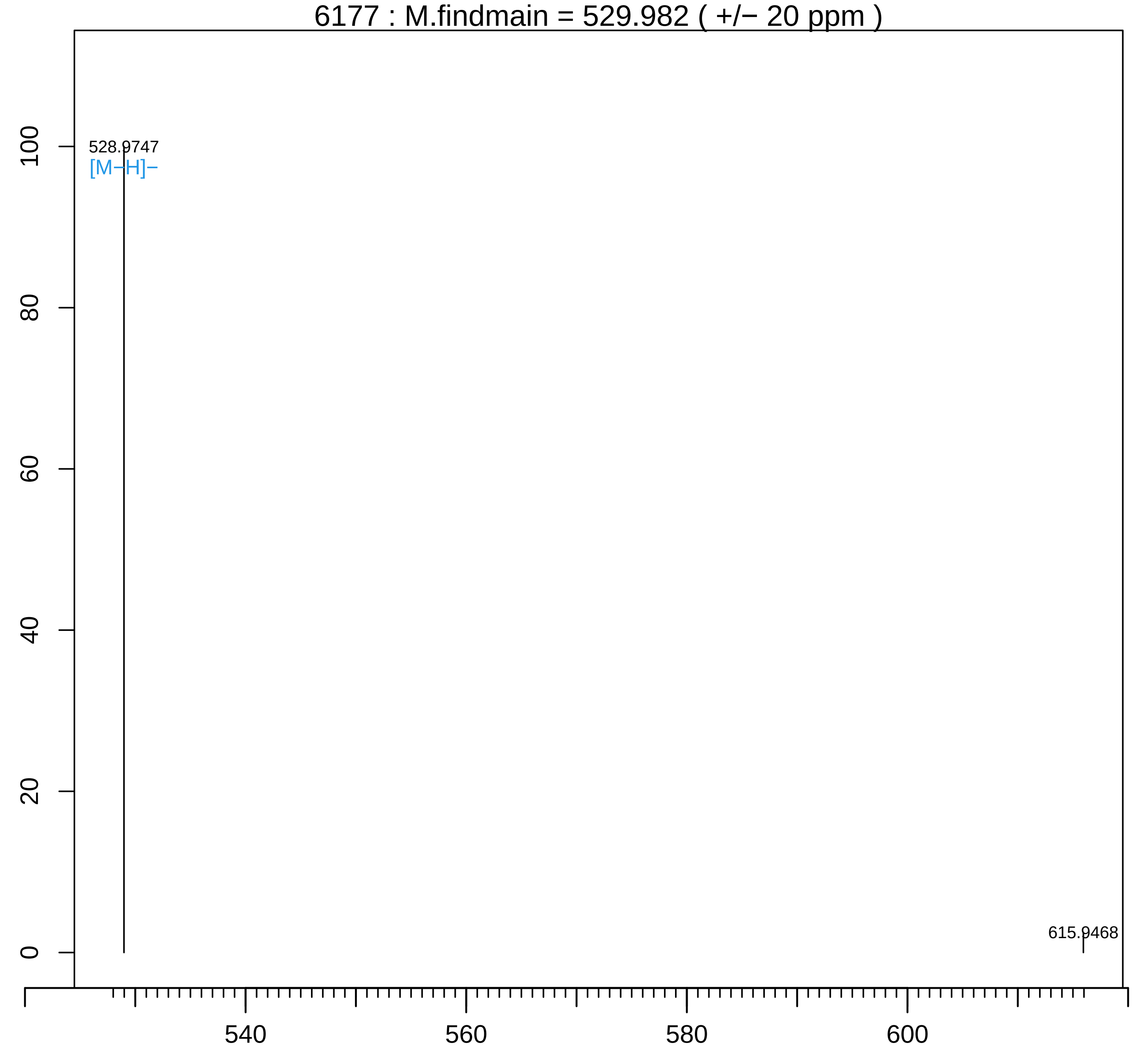

6177 : M.findmain = 529.982 (+/− 20 ppm)
528.9747
[M−H]−
615.9468
0
20
40
60
80
100
540
560
580
600

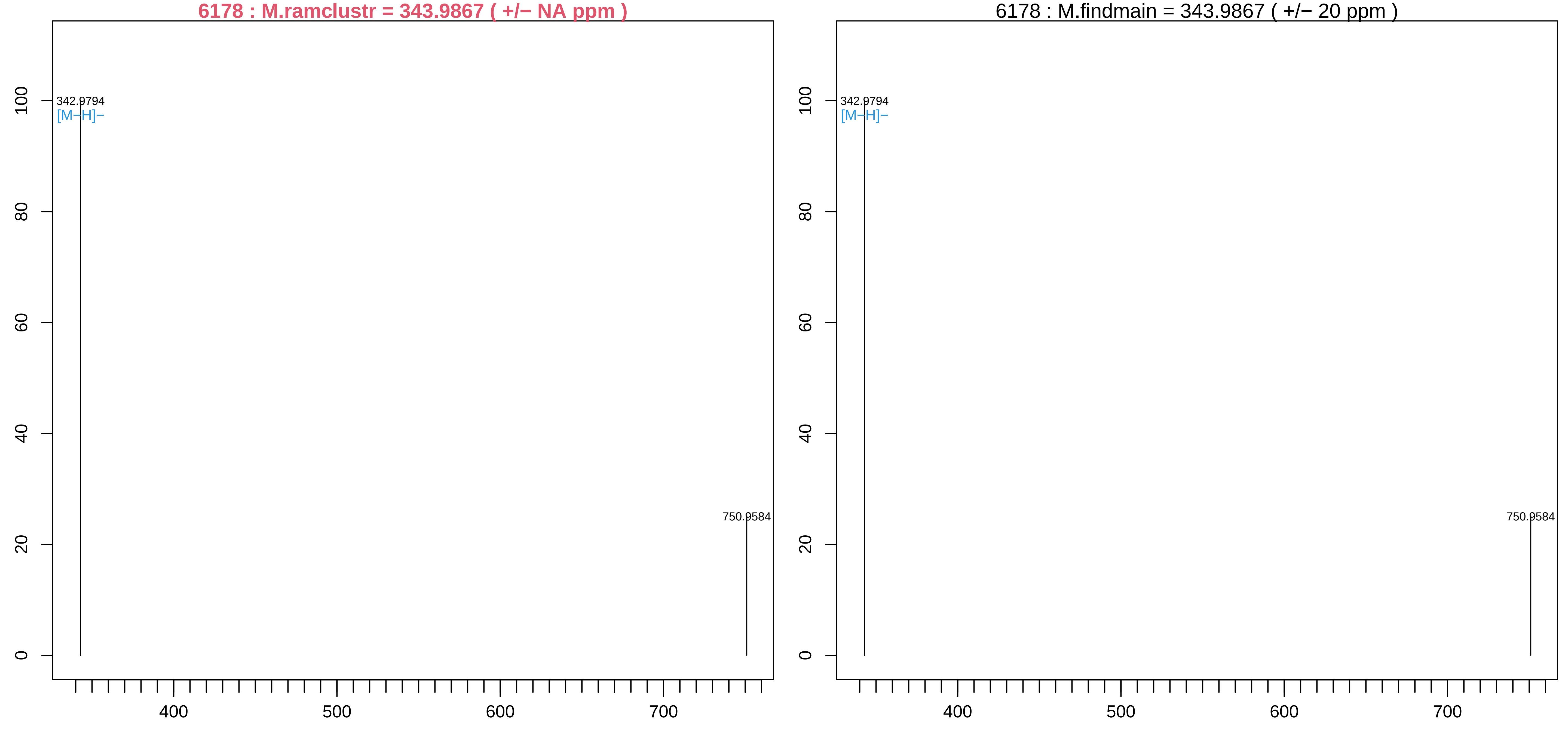

6178 : M.ramclustr = 343.9867 (+/− NA ppm)
342.9794
[M−H]−
750.9584
6178 : M.findmain = 343.9867 (+/− 20 ppm)
342.9794
[M−H]−
750.9584

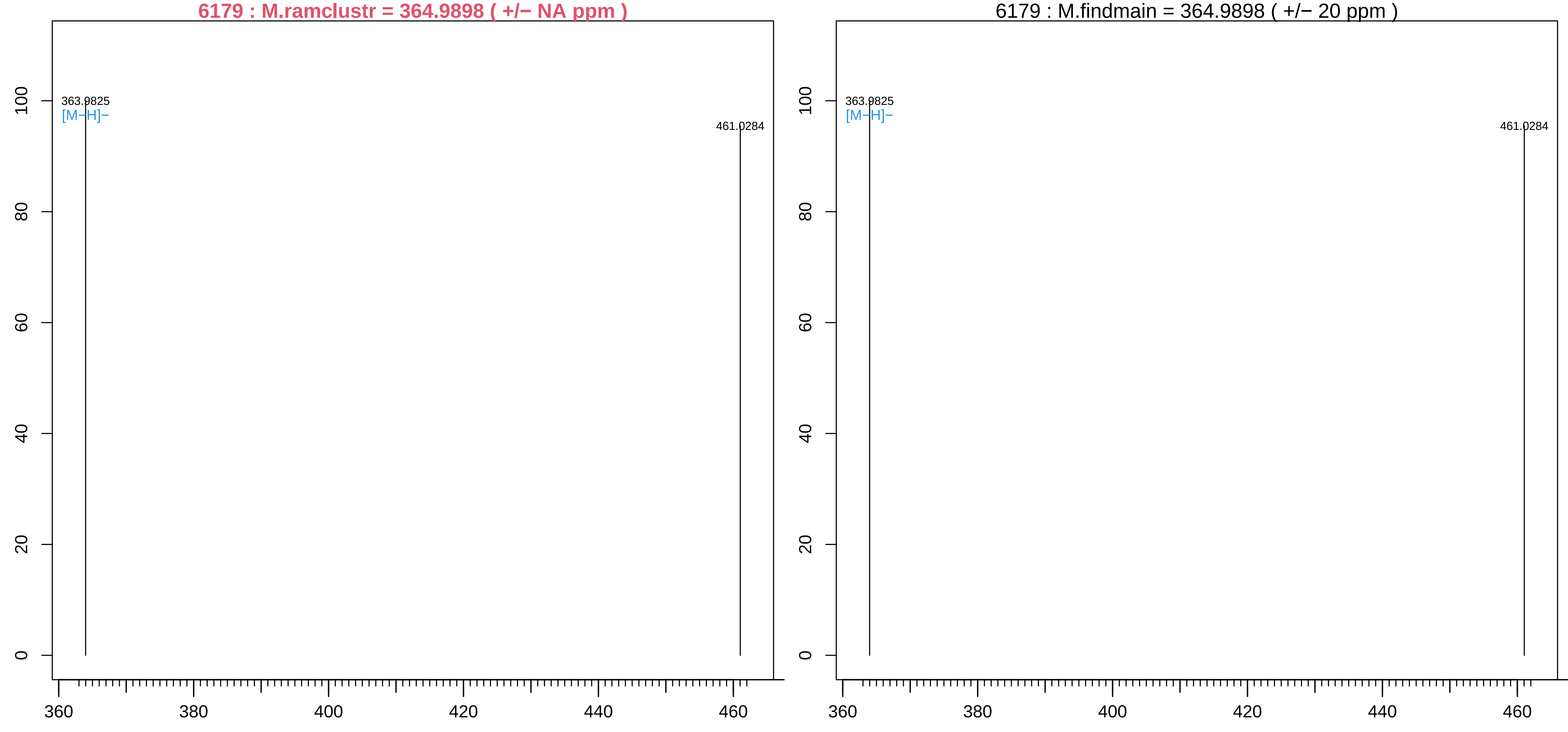

6179 : M.ramclustr = 364.9898 (+/− NA ppm)
363.9825
[M−H]−
461.0284
6179 : M.findmain = 364.9898 (+/− 20 ppm)
363.9825
[M−H]−
461.0284
0
20
40
60
80
100
360
380
400
420
440
460

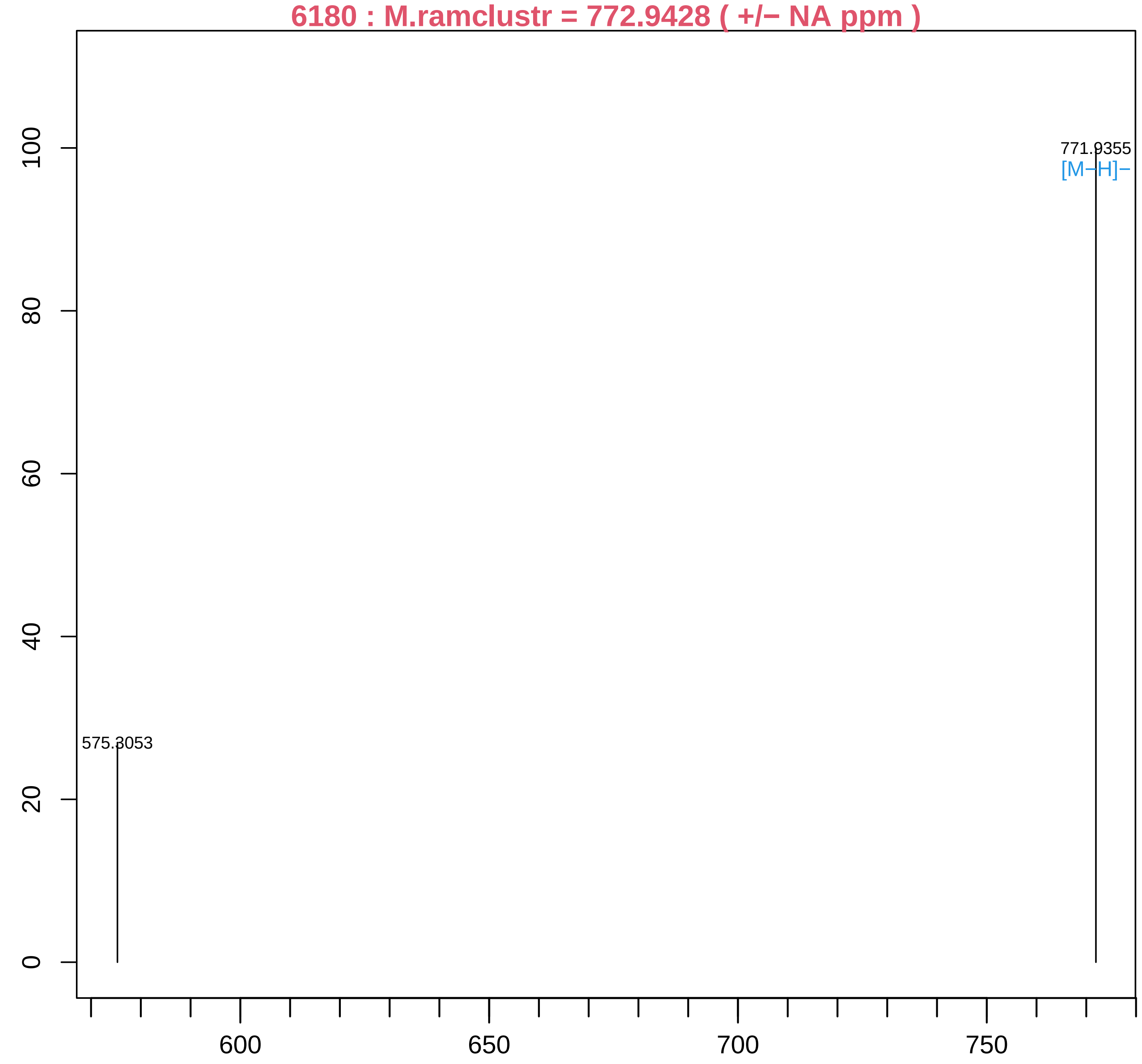

6180 : M.ramclustr = 772.9428 (+/− NA ppm)
771.9355
[M−H]−
575.3053
0
20
40
60
80
100
600
650
700
750

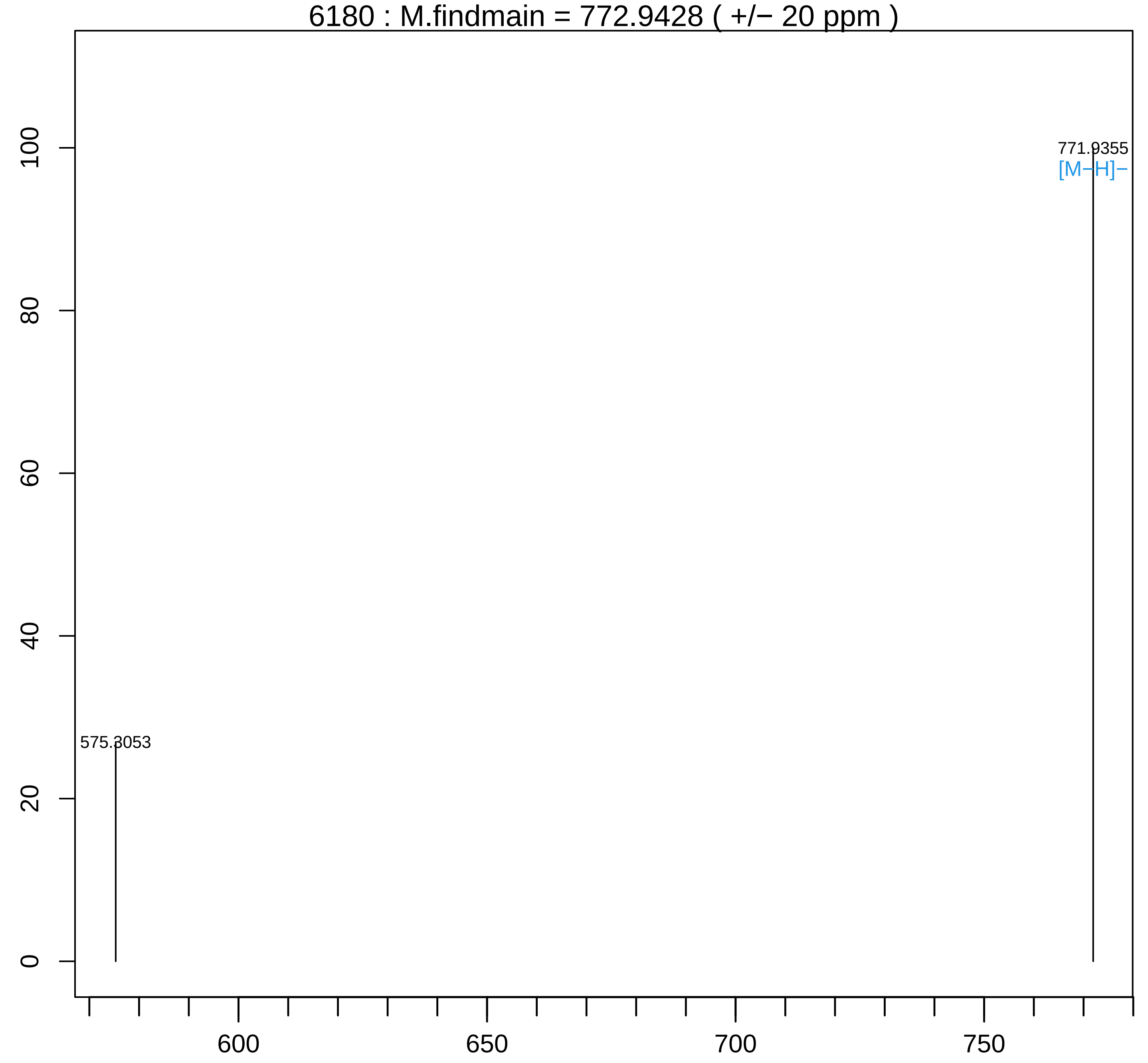

6180 : M.findmain = 772.9428 (+/− 20 ppm)
771.9355
[M−H]−
575.3053
0
20
40
60
80
100
600
650
700
750

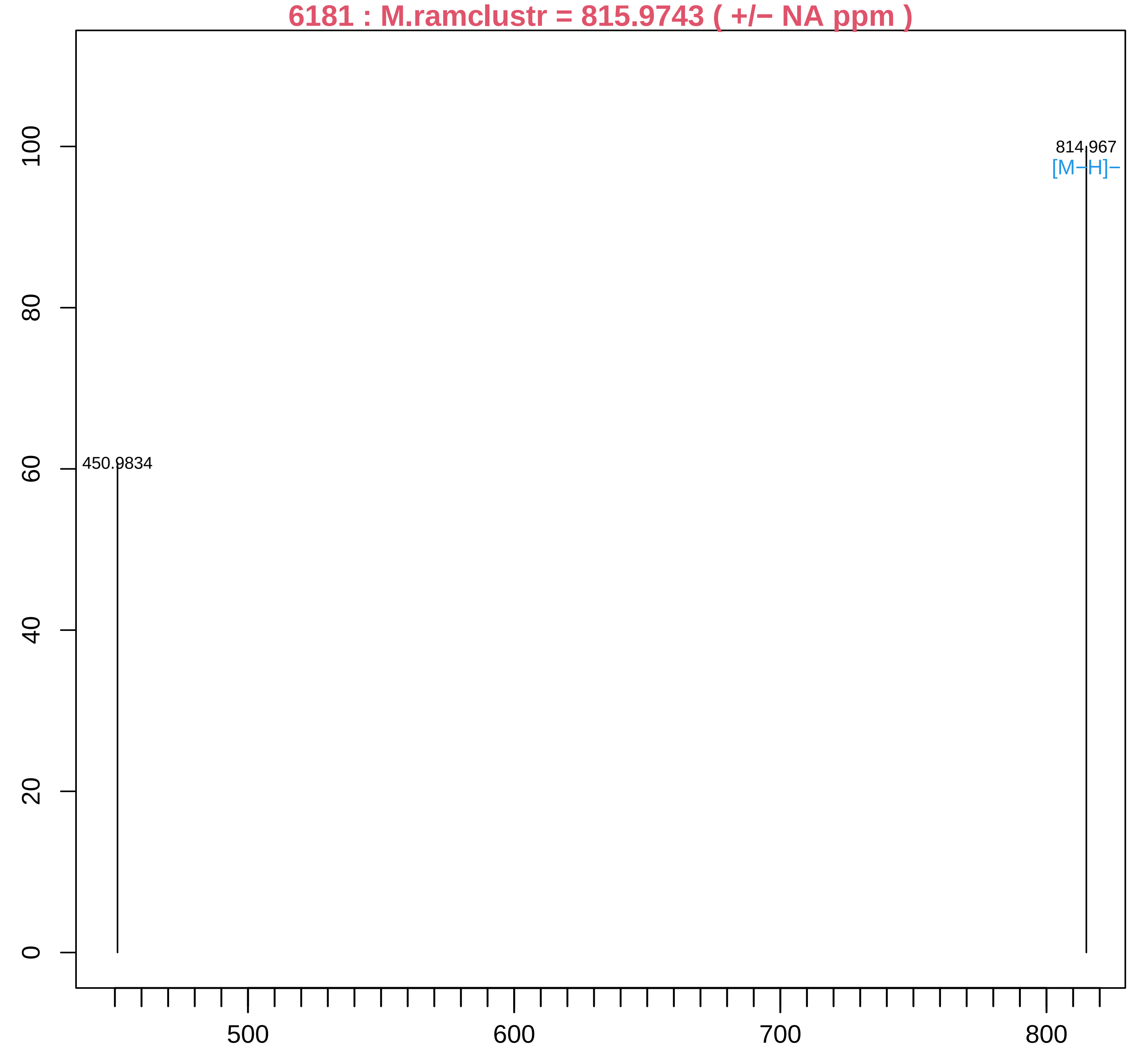
6181 : M.ramclustr = 815.9743 (+/− NA ppm)
814.967
[M−H]−
450.9834
0
20
40
60
80
100
500
600
700
800

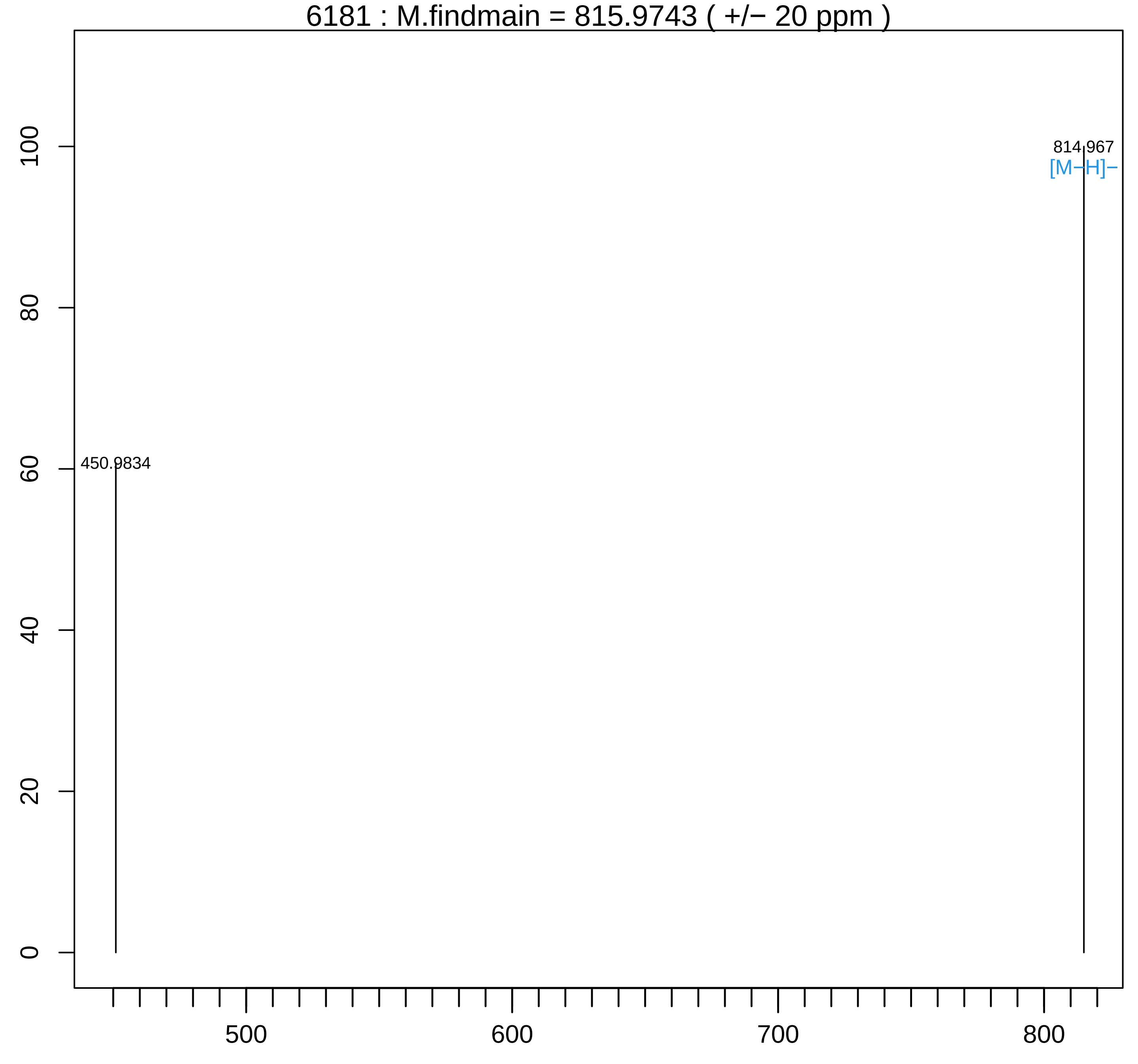
6181 : M.findmain = 815.9743 (+/− 20 ppm)
814.967
[M−H]−
450.9834
0
20
40
60
80
100
500
600
700
800

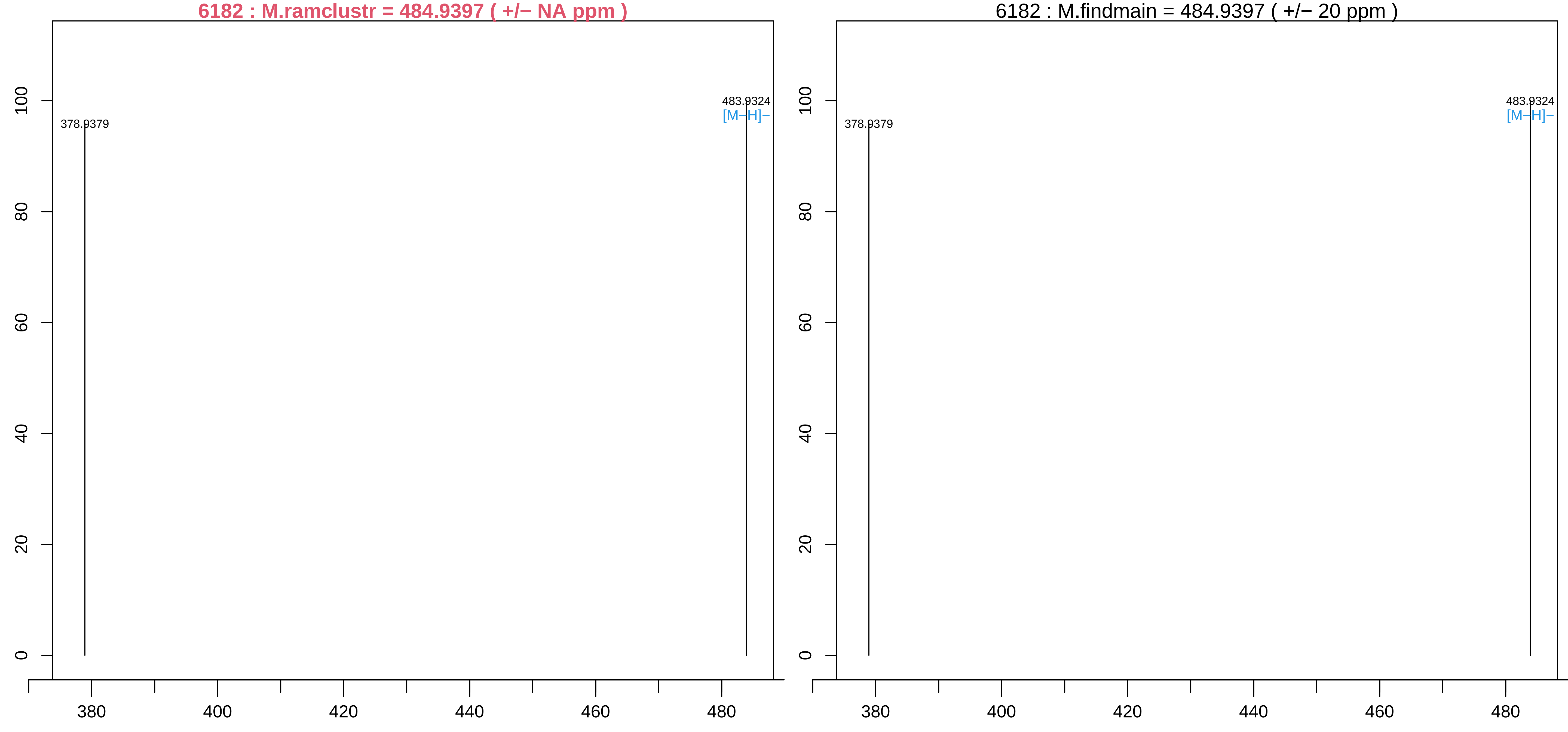

6182 : M.ramclustr = 484.9397 (+/− NA ppm)
378.9379
483.9324
[M−H]−
0
20
40
60
80
100
380
400
420
440
460
480
6182 : M.findmain = 484.9397 (+/− 20 ppm)
378.9379
483.9324
[M−H]−
0
20
40
60
80
100
380
400
420
440
460
480

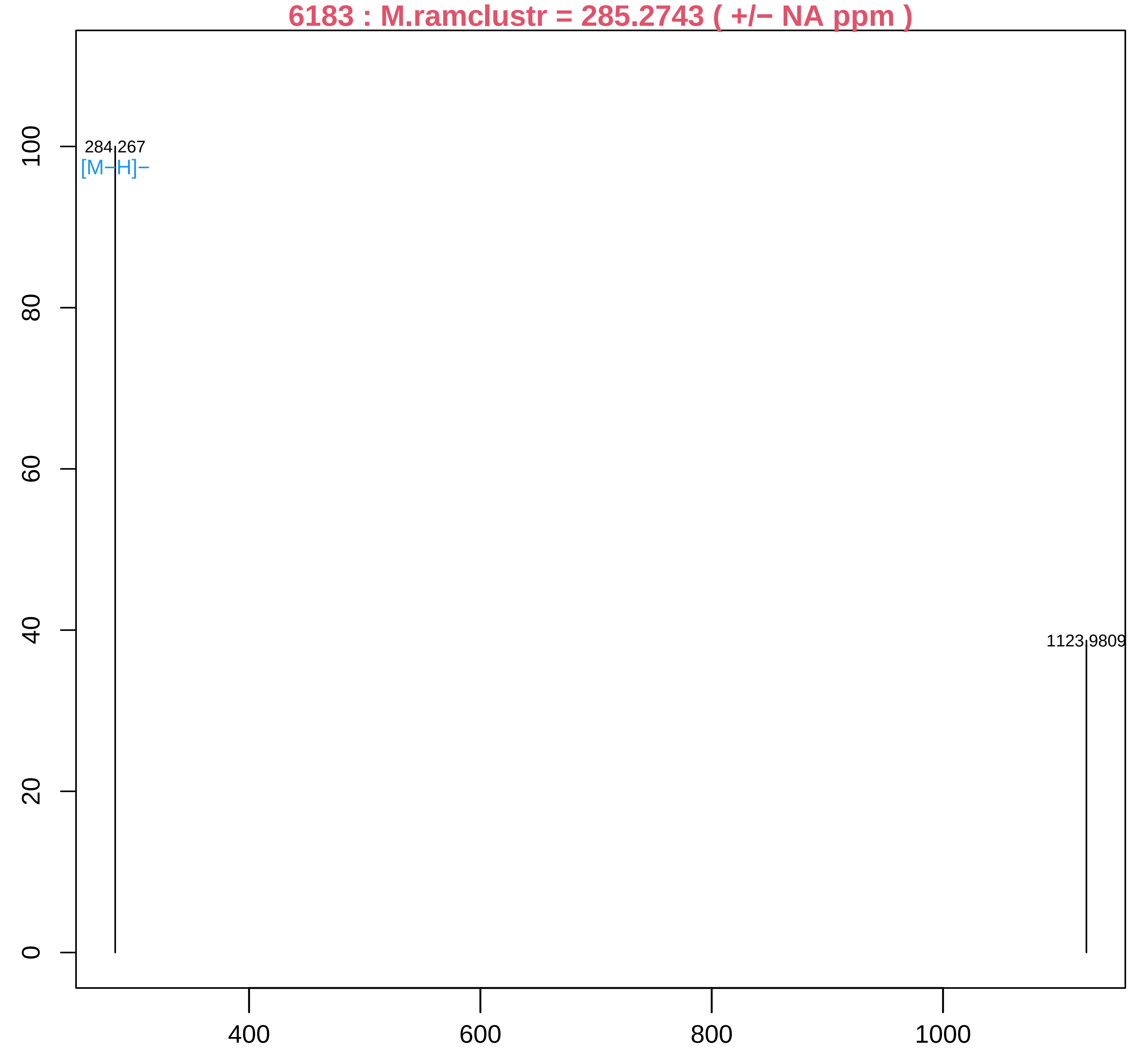

6183 : M.ramclustr = 285.2743 (+/− NA ppm)
284.267
[M−H]−
1123.9809
0
20
40
60
80
100
400
600
800
1000

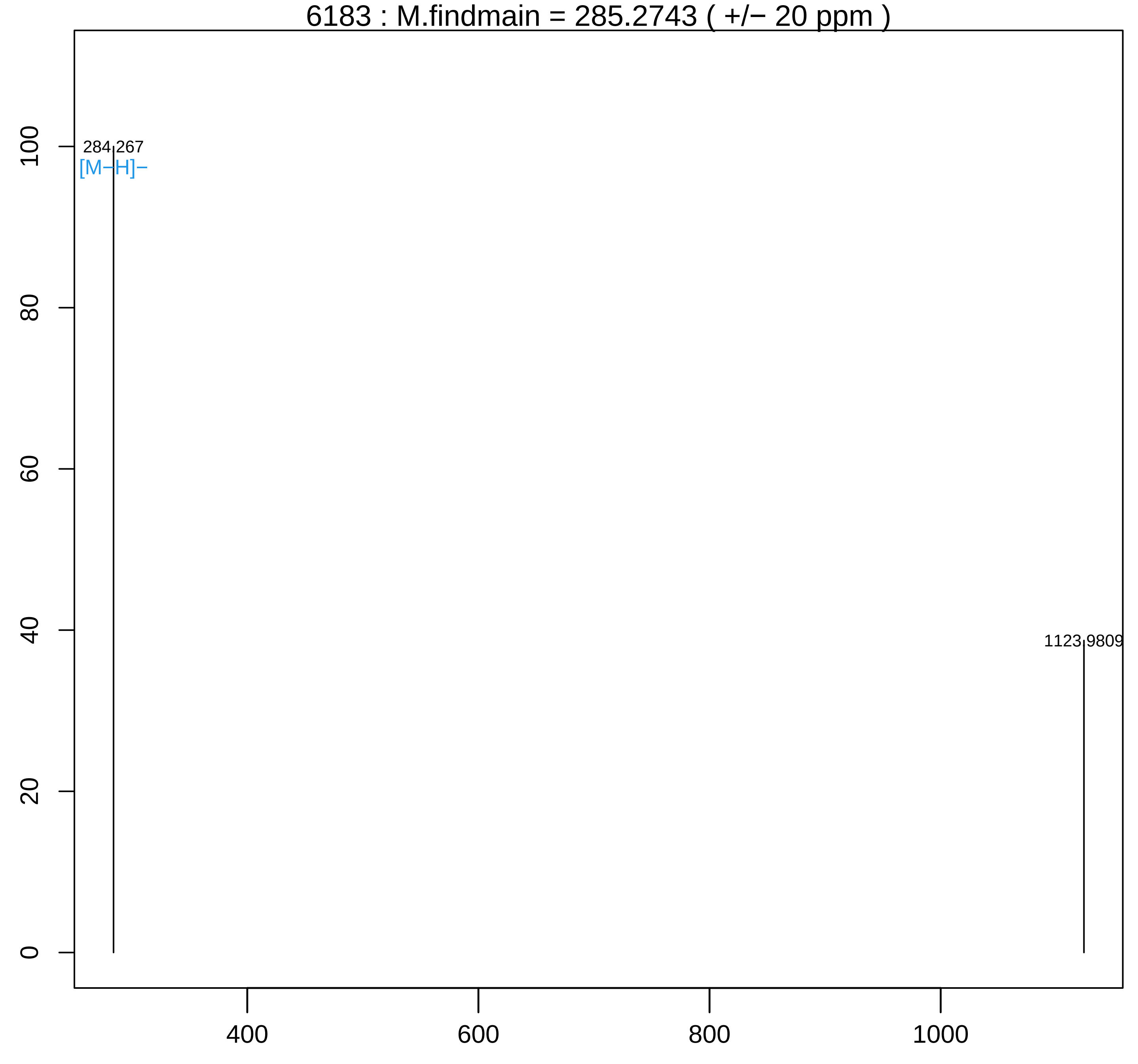

6183 : M.findmain = 285.2743 (+/− 20 ppm)
284.267
[M−H]−
1123.9809
0
20
40
60
80
100
400
600
800
1000

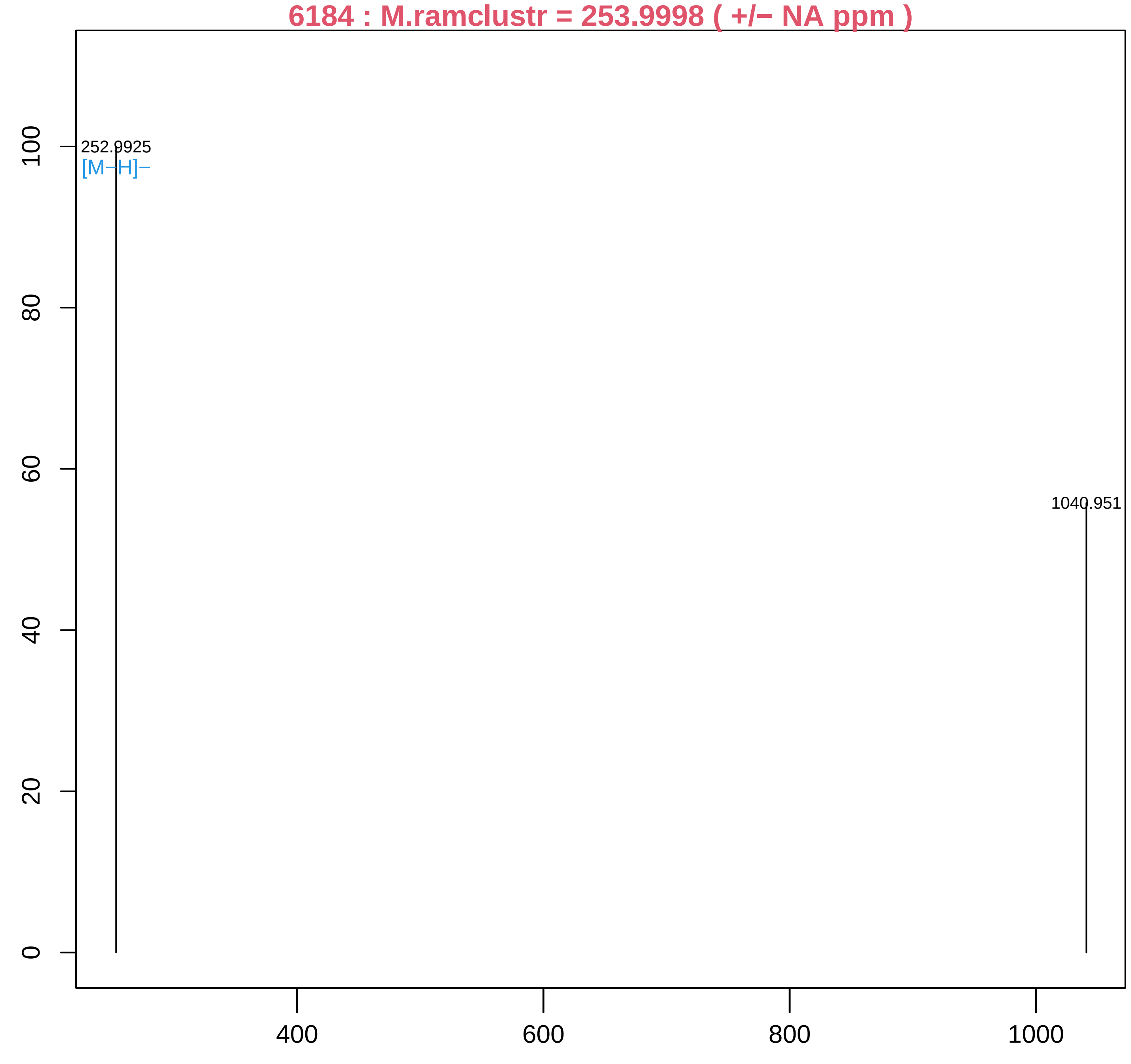

6184 : M.ramclustr = 253.9998 (+/− NA ppm)
252.9925
[M−H]−
1040.951
0
20
40
60
80
100
400
600
800
1000

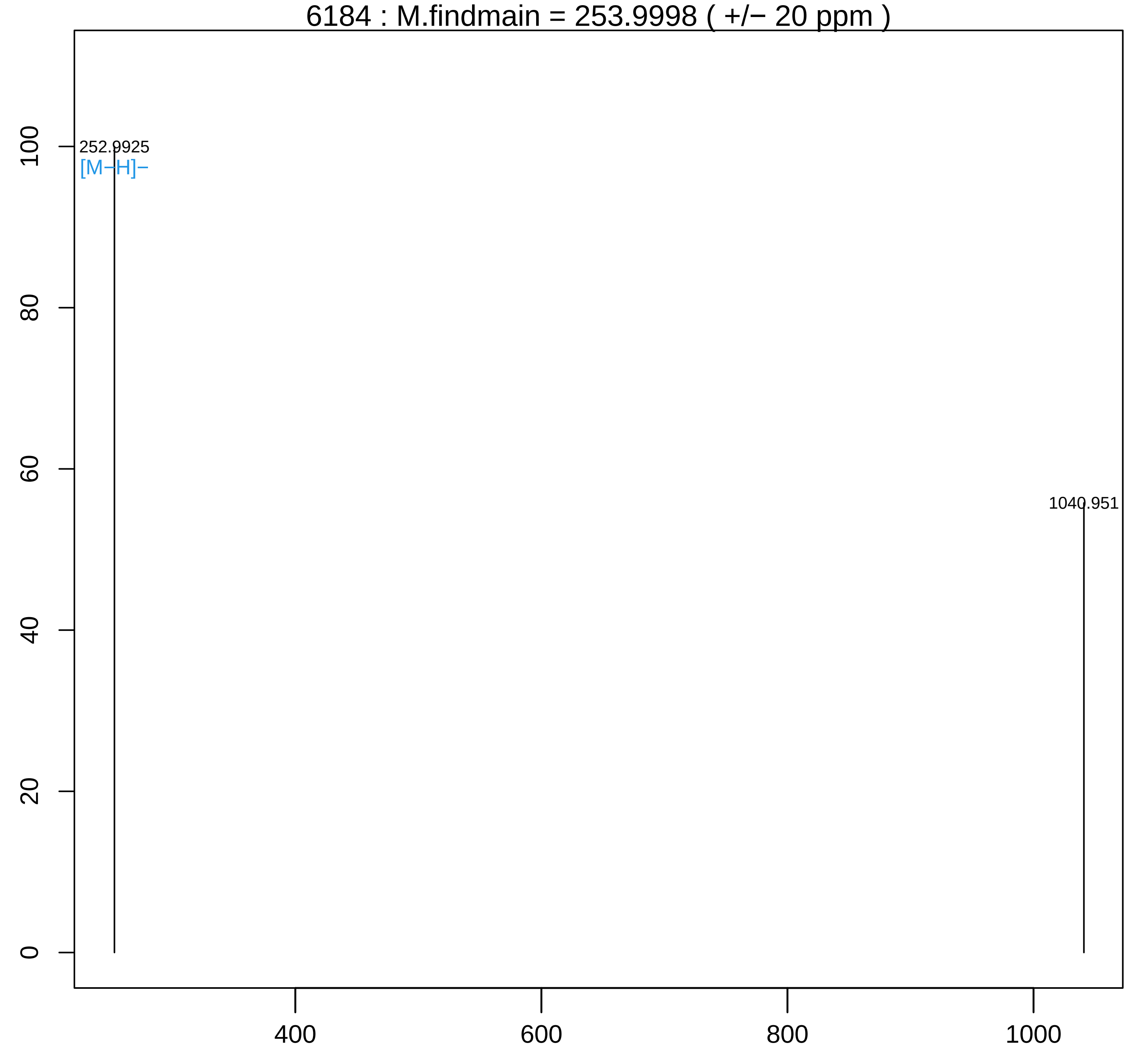

6184 : M.findmain = 253.9998 (+/− 20 ppm)
252.9925
[M−H]−
1040.951
0
20
40
60
80
100
400
600
800
1000

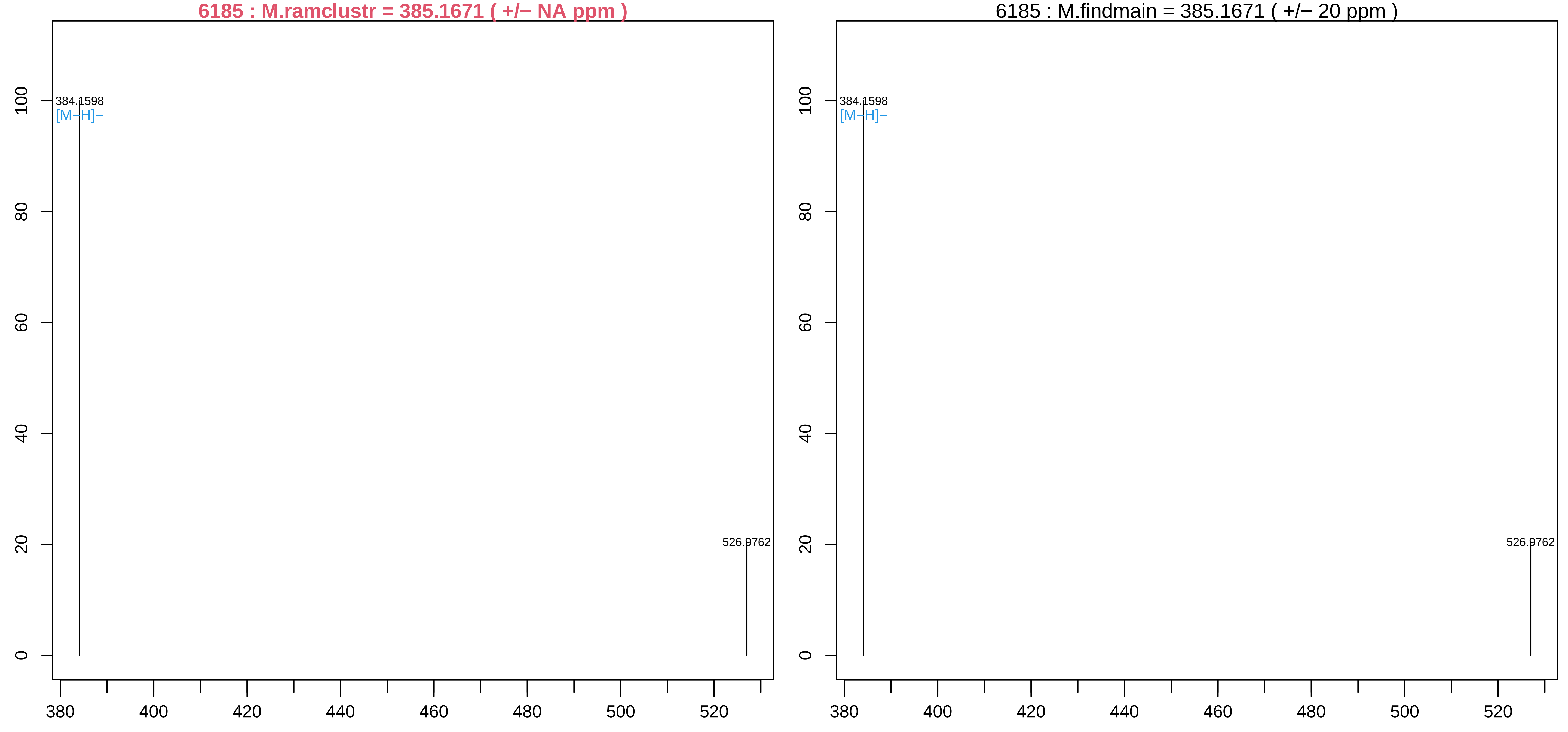

6185 : M.ramclustr = 385.1671 (+/− NA ppm)
384.1598
[M−H]−
526.9762
6185 : M.findmain = 385.1671 (+/− 20 ppm)
384.1598
[M−H]−
526.9762
0
20
40
60
80
100
380
400
420
440
460
480
500
520

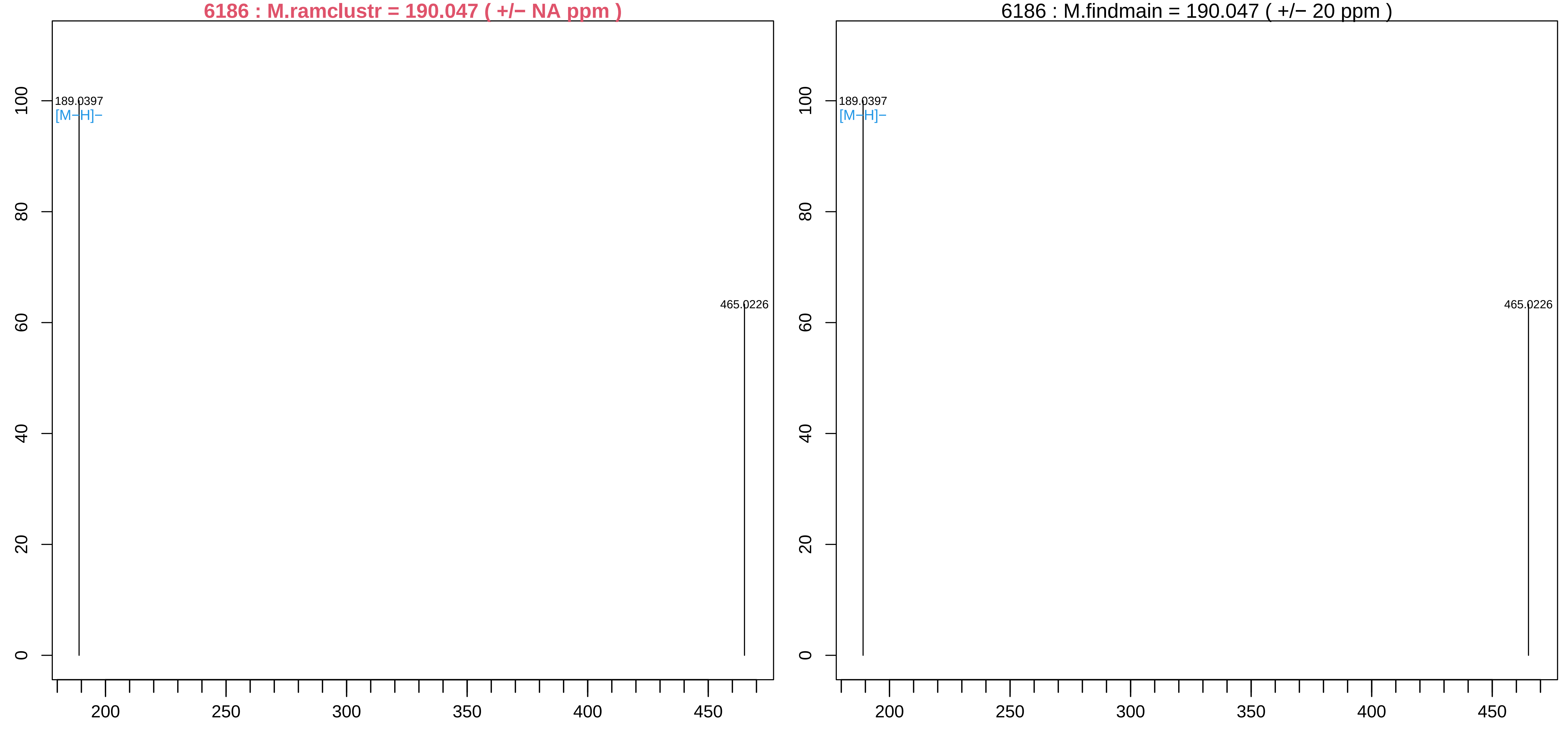
6186 : M.ramclustr = 190.047 (+/− NA ppm)
189.0397
[M−H]−
465.0226
6186 : M.findmain = 190.047 (+/− 20 ppm)
189.0397
[M−H]−
465.0226
0
20
40
60
80
100
200
250
300
350
400
450

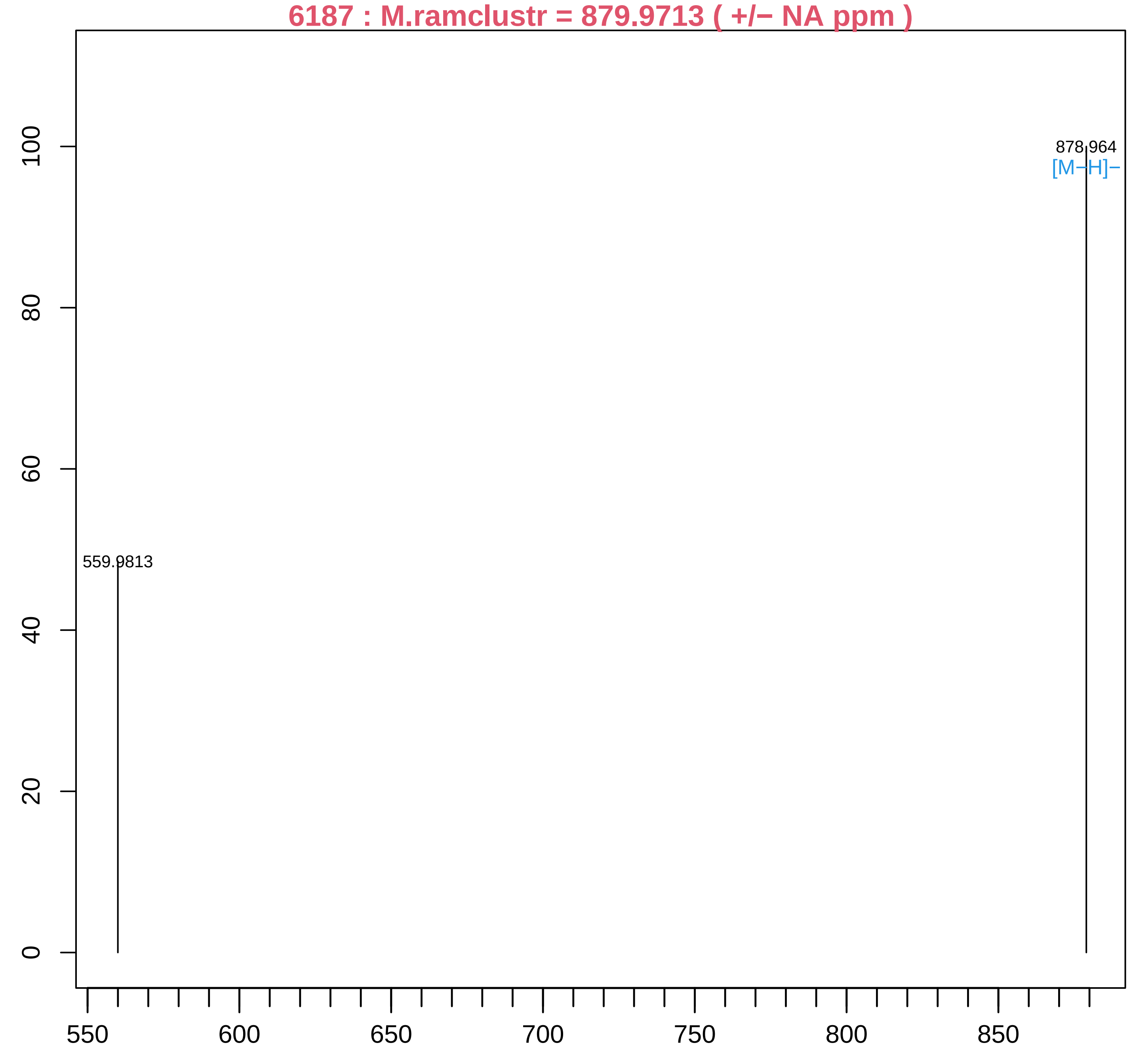
6187 : M.ramclustr = 879.9713 (+/− NA ppm)
878.964
[M−H]−
559.9813
0
20
40
60
80
100
550
600
650
700
750
800
850

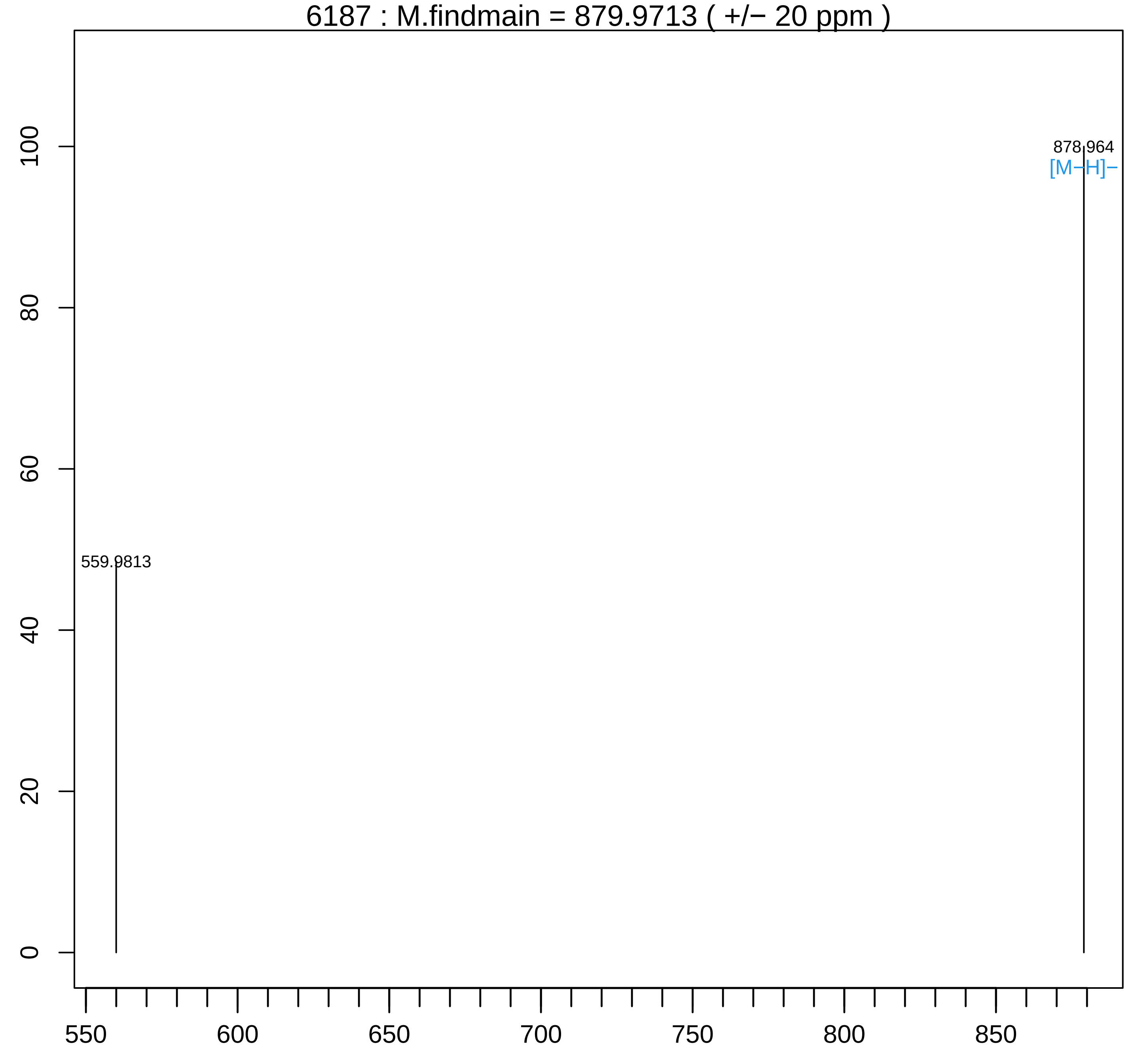
6187 : M.findmain = 879.9713 (+/− 20 ppm)
878.964
[M−H]−
559.9813
0
20
40
60
80
100
550
600
650
700
750
800
850

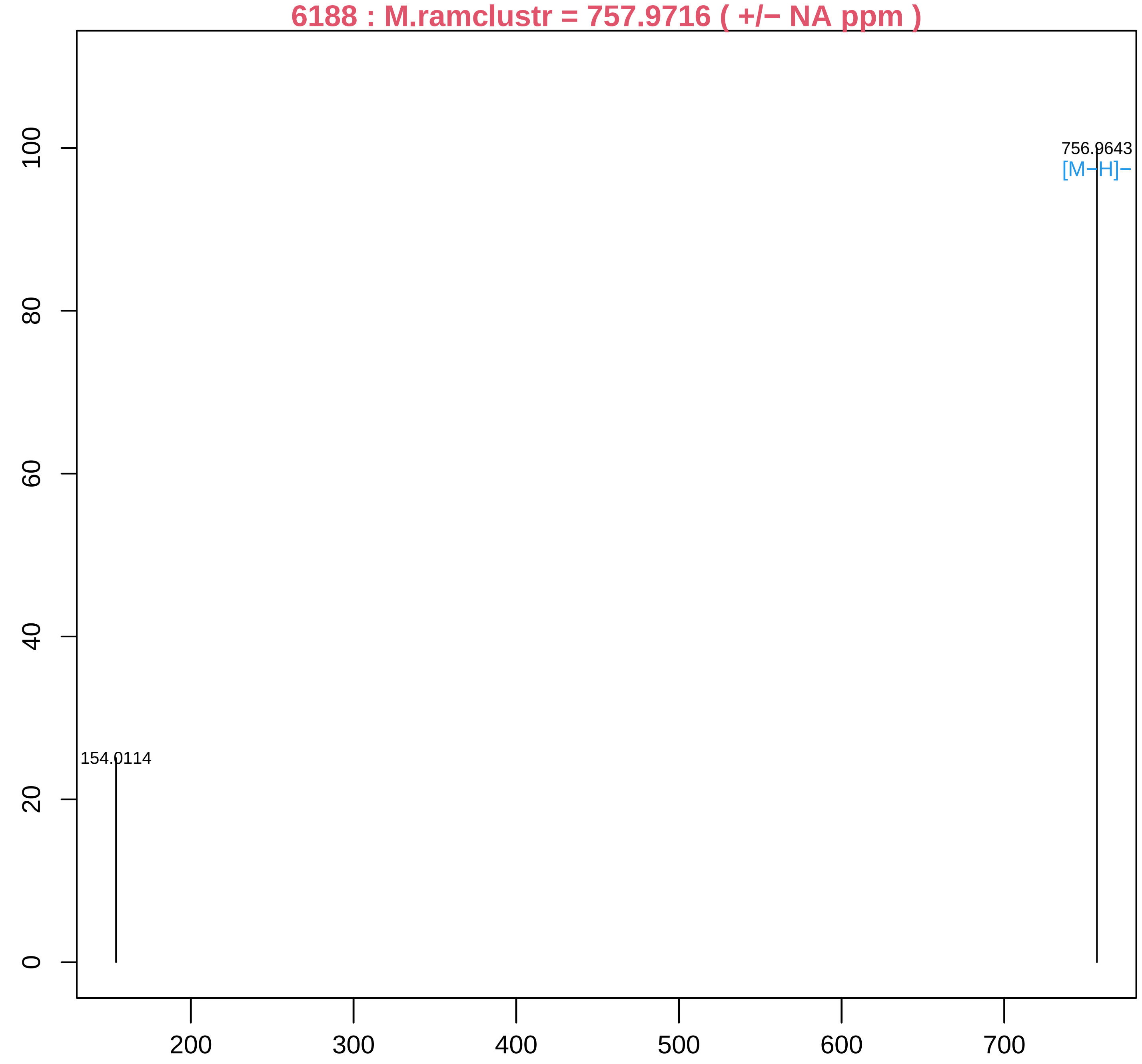

6188 : M.ramclustr = 757.9716 (+/− NA ppm)
756.9643
[M−H]−
154.0114
0
20
40
60
80
100
200
300
400
500
600
700

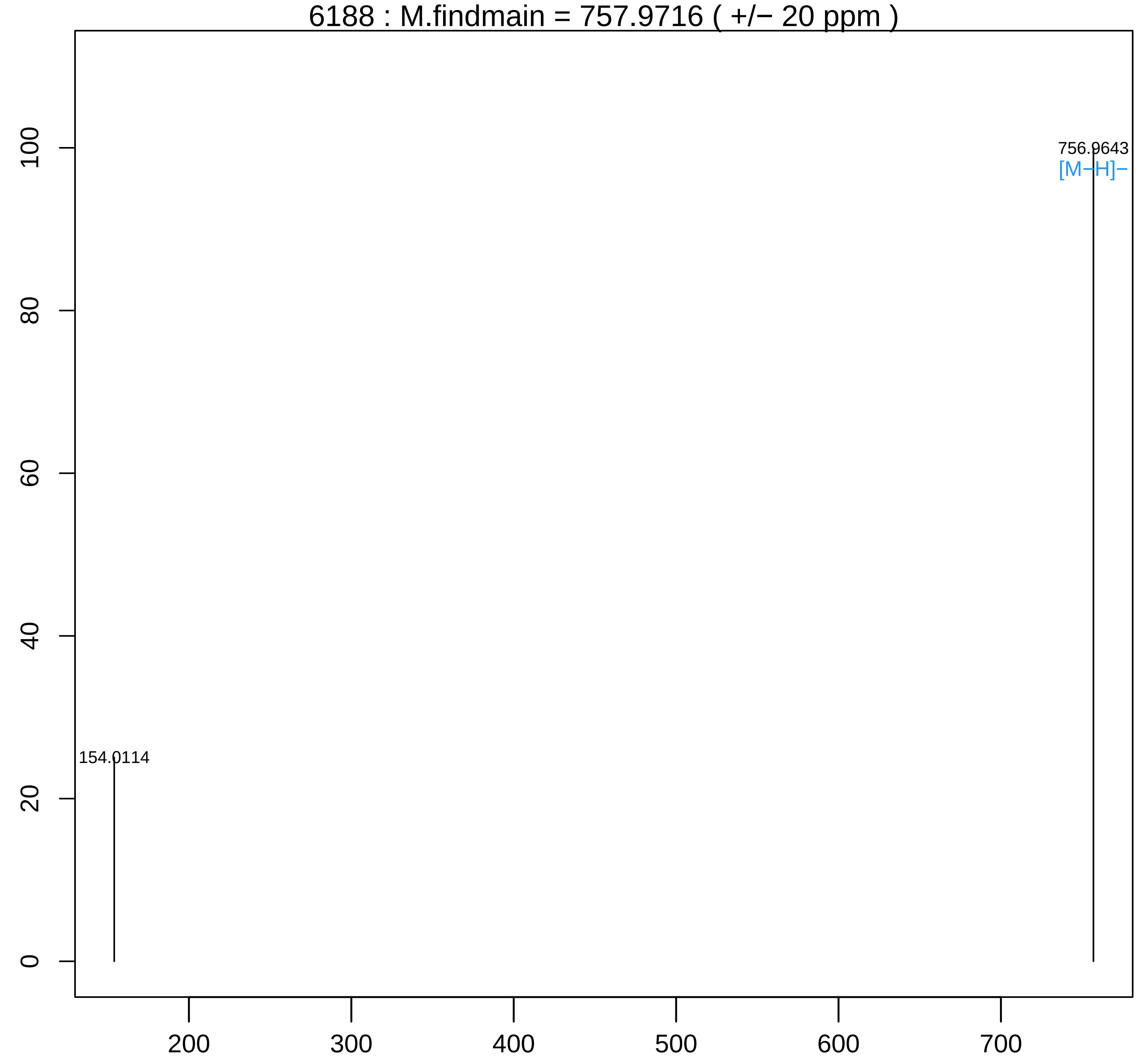

6188 : M.findmain = 757.9716 (+/− 20 ppm)
756.9643
[M−H]−
154.0114
0
20
40
60
80
100
200
300
400
500
600
700

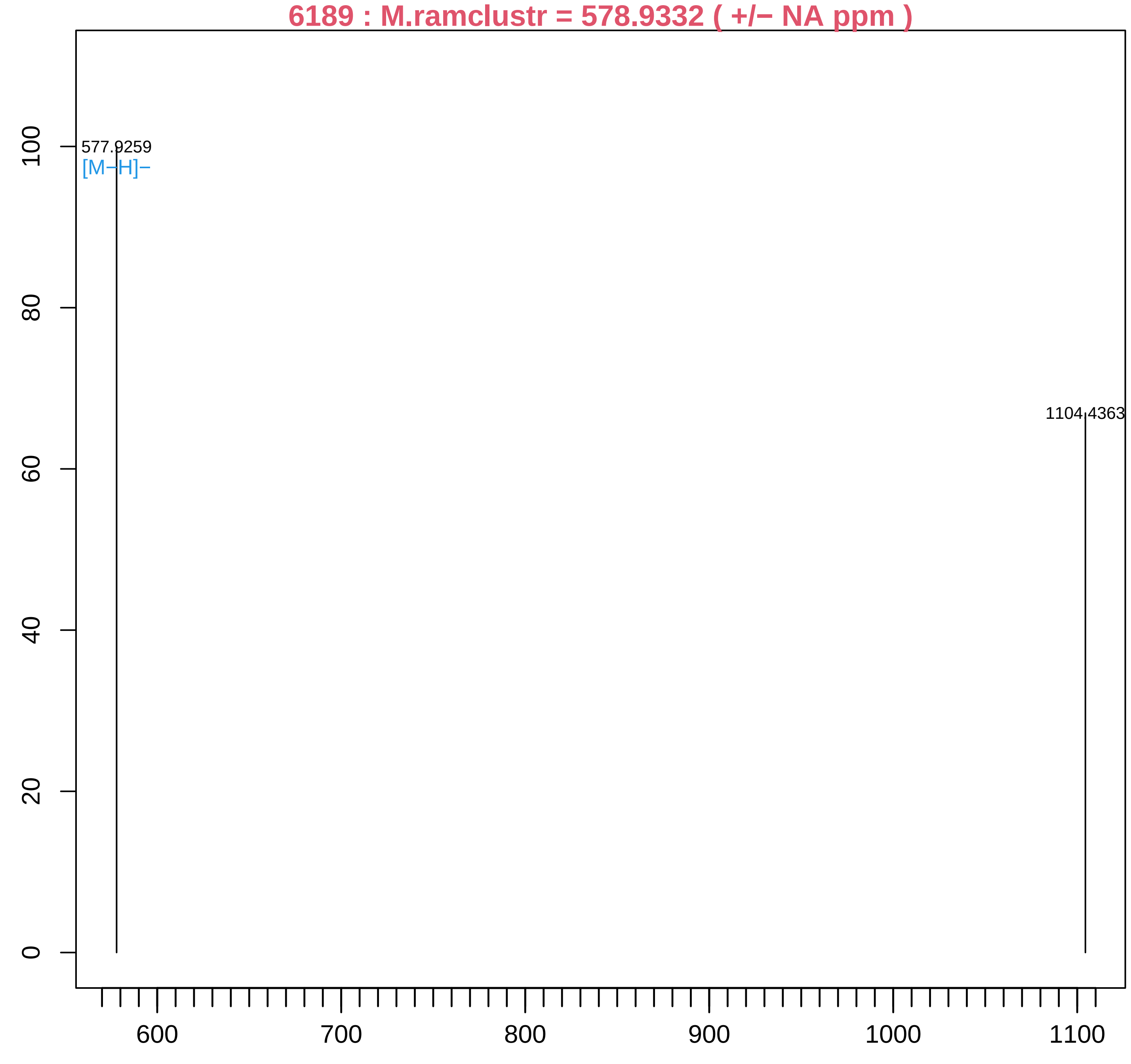
6189 : M.ramclustr = 578.9332 (+/− NA ppm)
577.9259
[M−H]−
1104.4363
0
20
40
60
80
100
600
700
800
900
1000
1100

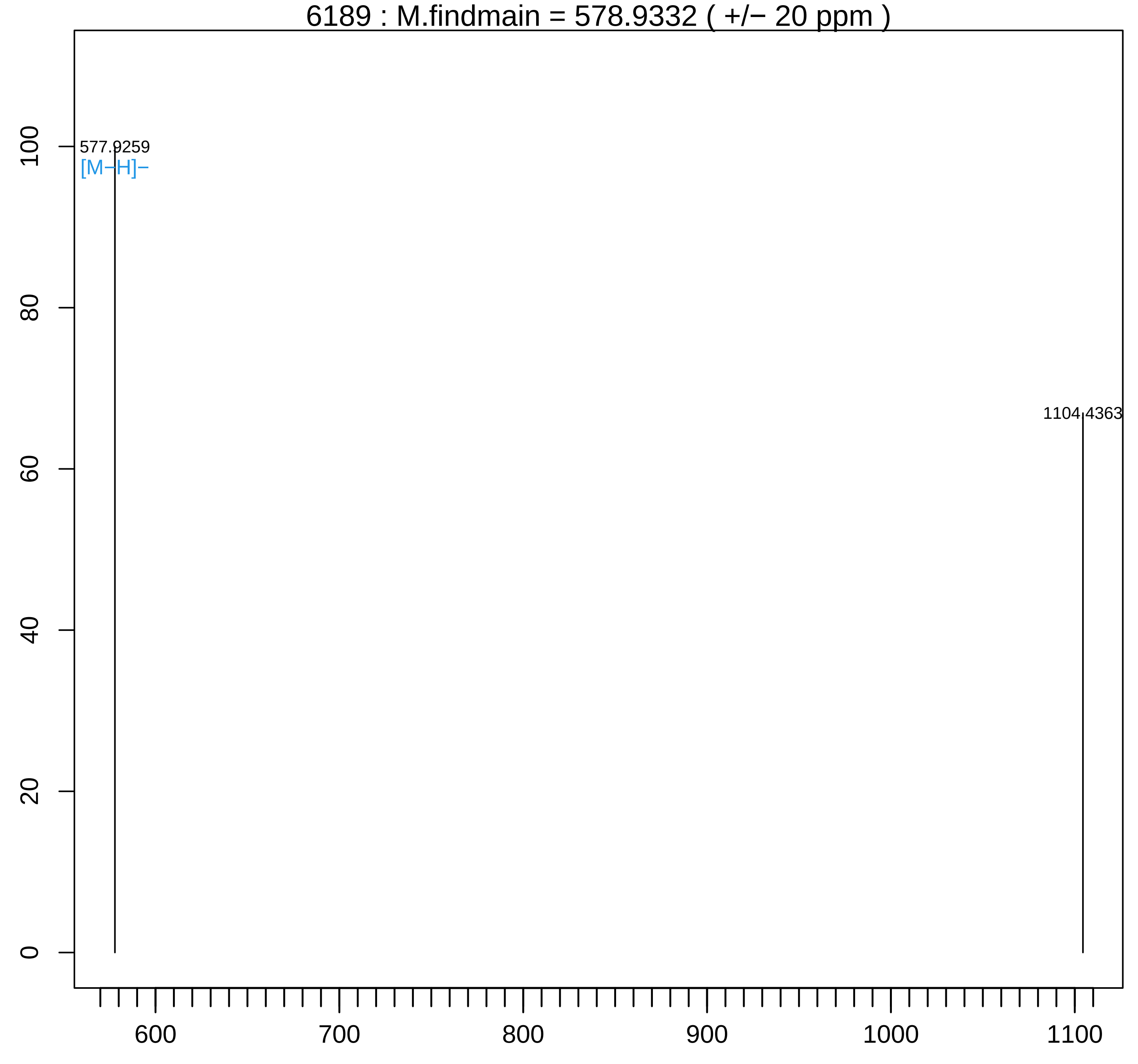
6189 : M.findmain = 578.9332 (+/− 20 ppm)
577.9259
[M−H]−
1104.4363
0
20
40
60
80
100
600
700
800
900
1000
1100

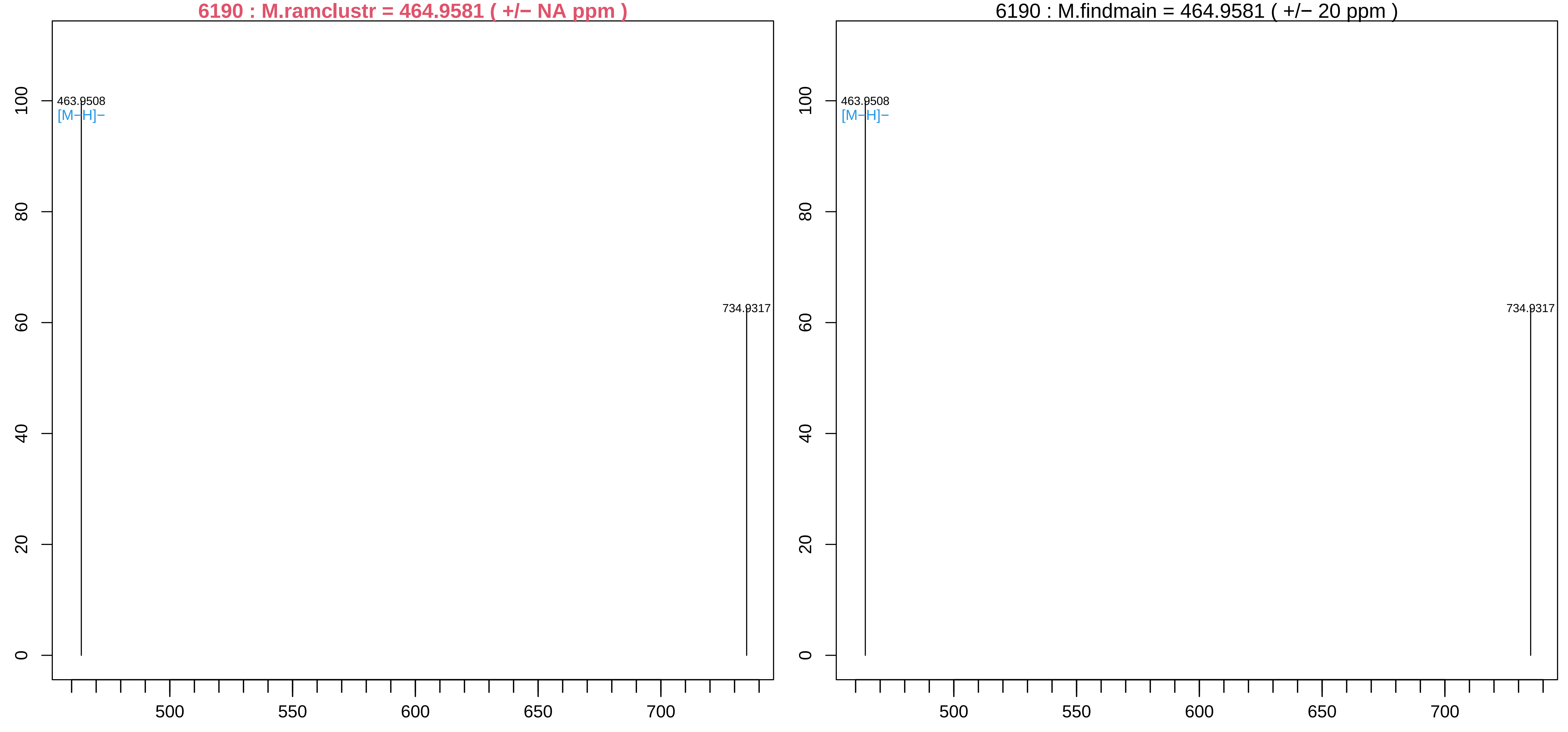
6190 : M.ramclustr = 464.9581 (+/− NA ppm)
463.9508
[M−H]−
734.9317
6190 : M.findmain = 464.9581 (+/− 20 ppm)
463.9508
[M−H]−
734.9317
0
20
40
60
80
100
500
550
600
650
700

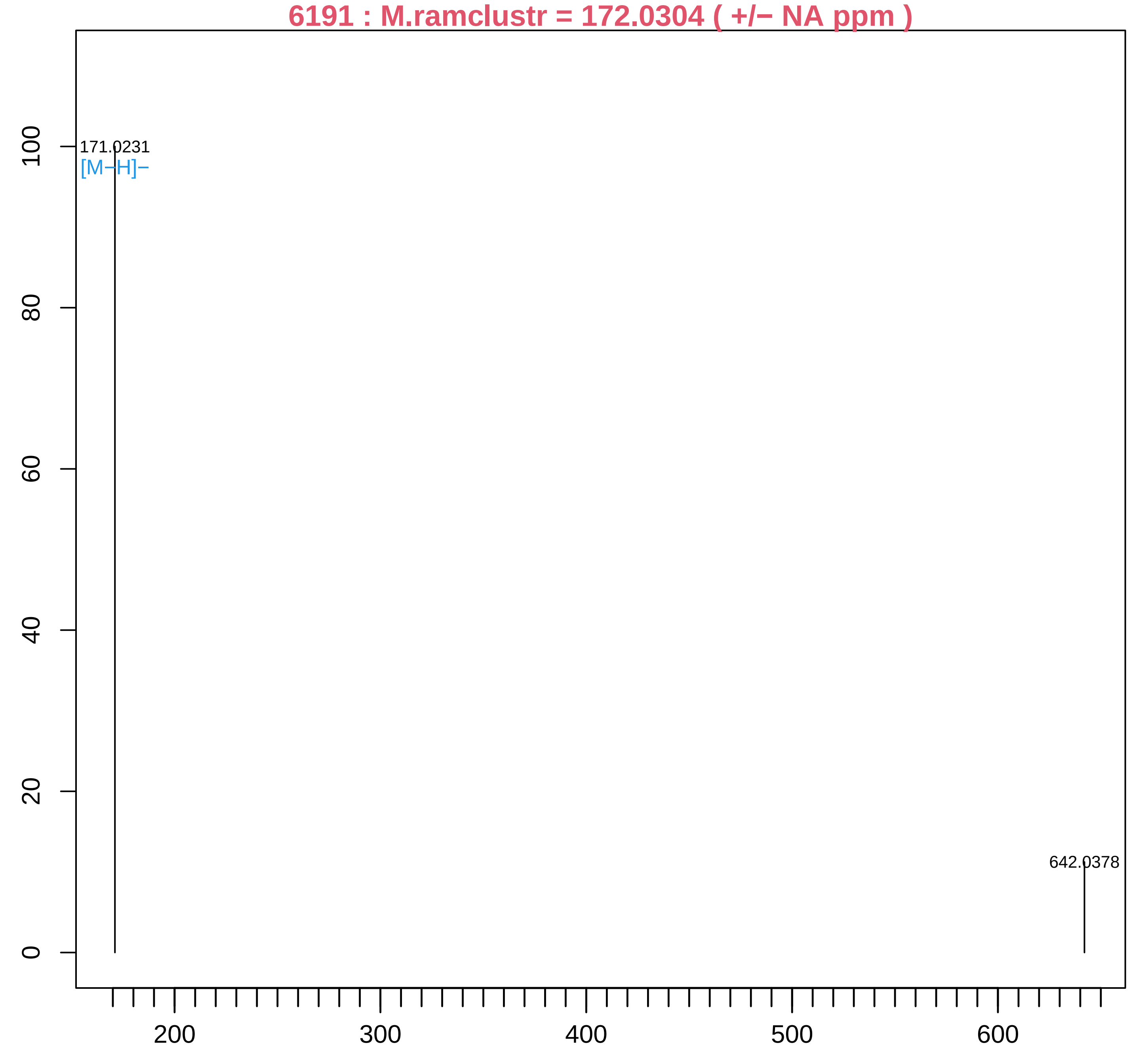

6191 : M.ramclustr = 172.0304 (+/− NA ppm)
171.0231
[M−H]−
642.0378
0
20
40
60
80
100
200
300
400
500
600

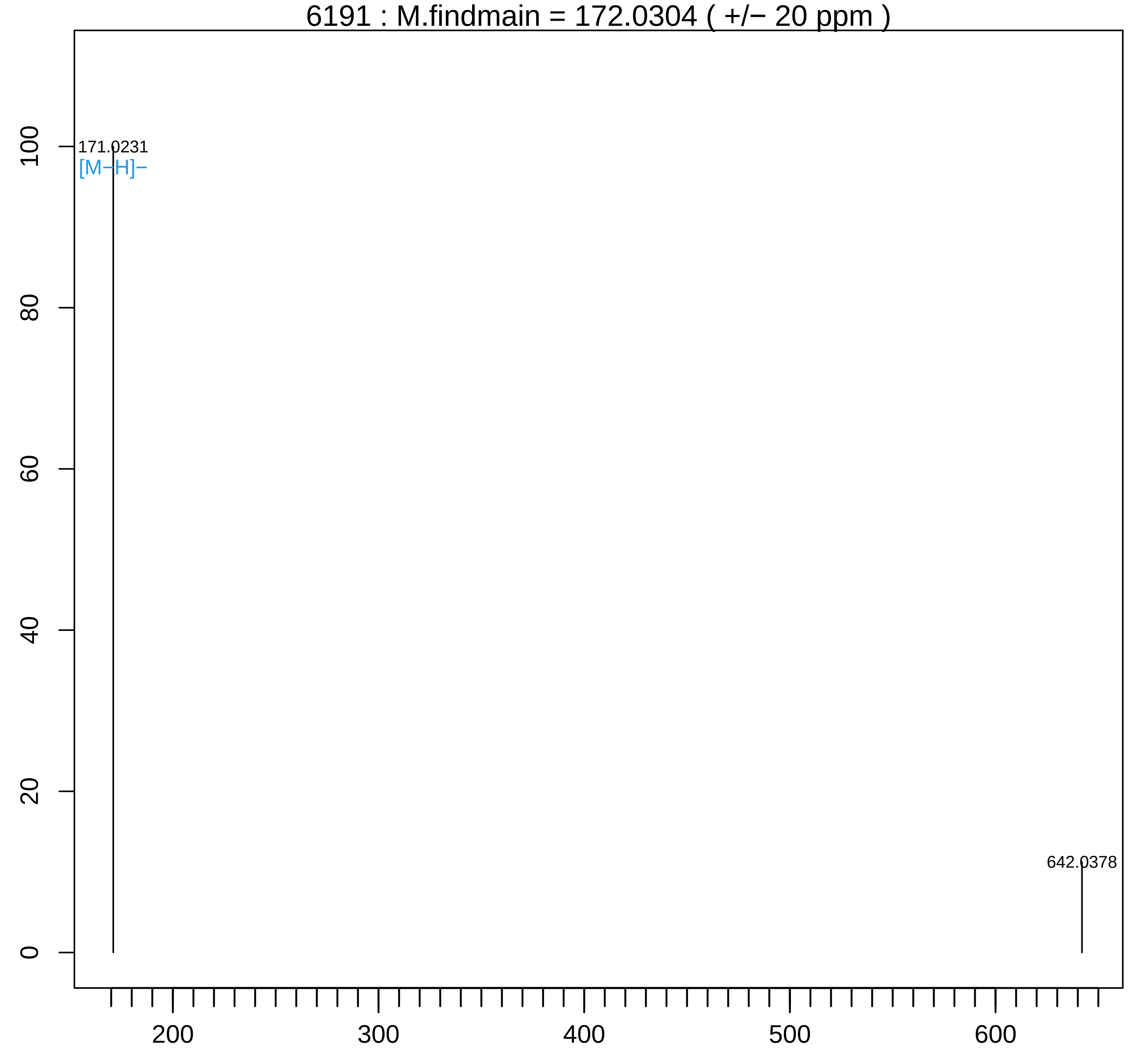

6191 : M.findmain = 172.0304 (+/− 20 ppm)
171.0231
[M−H]−
642.0378
0
20
40
60
80
100
200
300
400
500
600

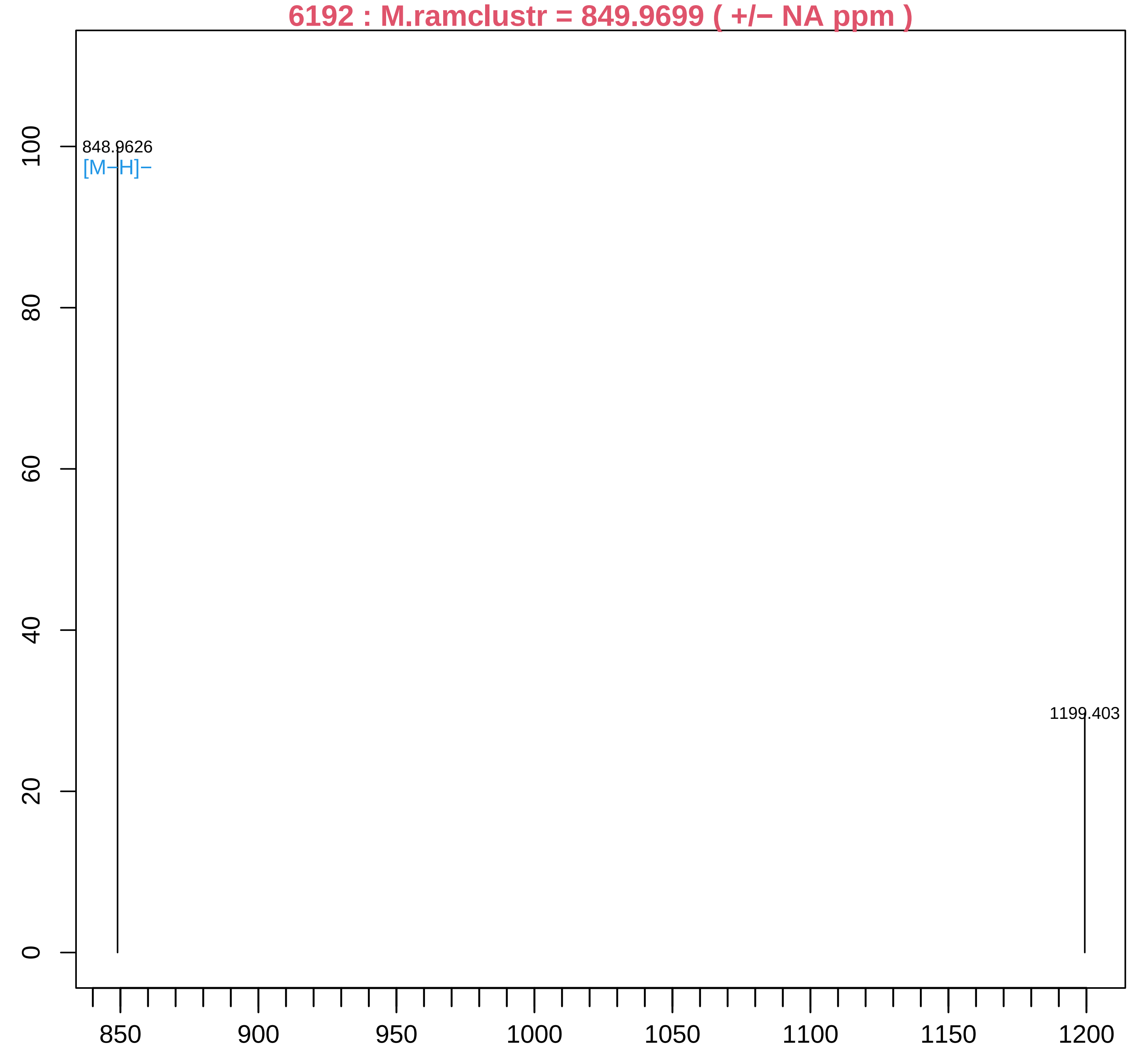
6192 : M.ramclustr = 849.9699 (+/− NA ppm)
848.9626
[M−H]−
1199.403
0
20
40
60
80
100
850
900
950
1000
1050
1100
1150
1200

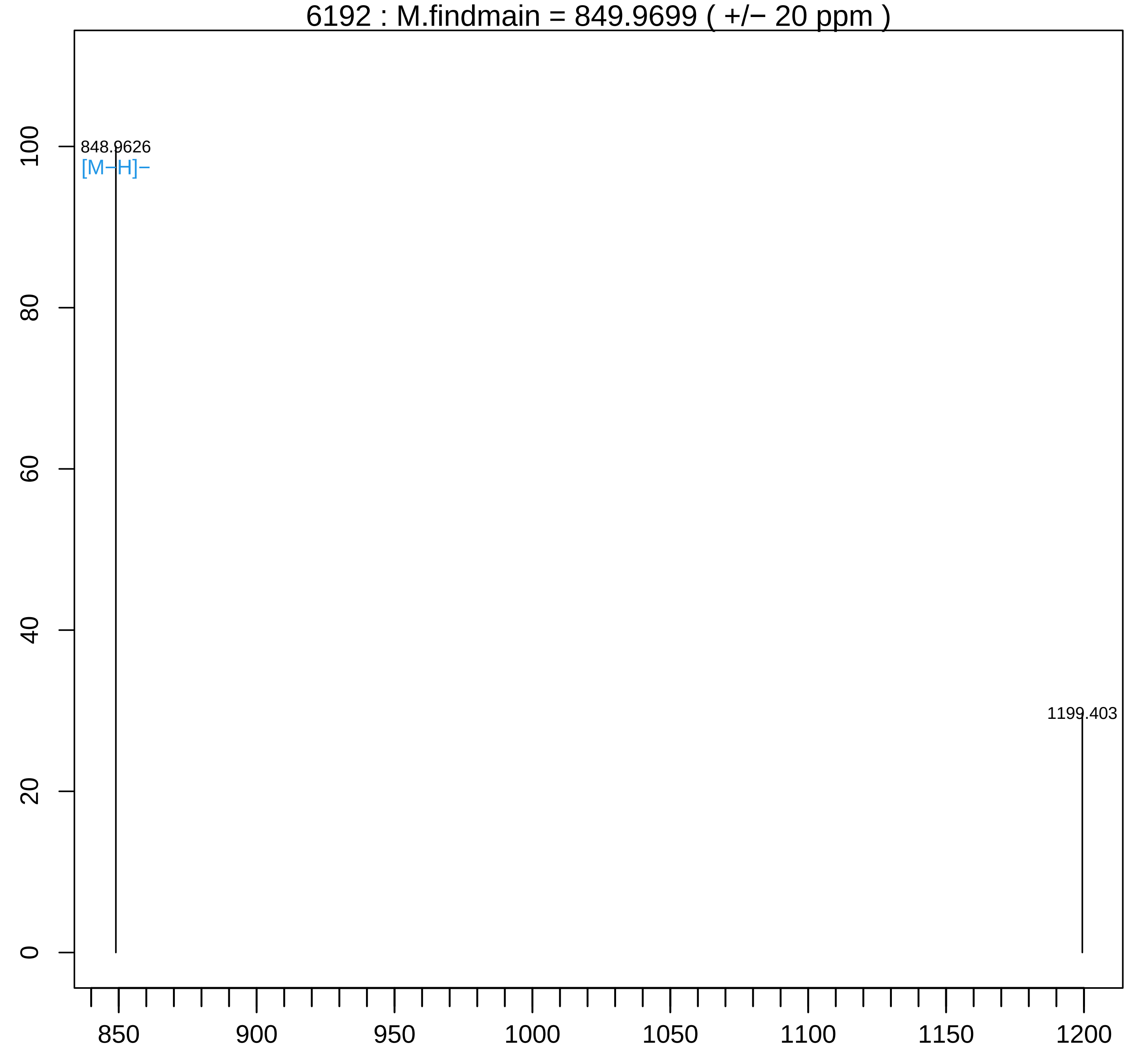
6192 : M.findmain = 849.9699 (+/− 20 ppm)
848.9626
[M−H]−
1199.403
0
20
40
60
80
100
850
900
950
1000
1050
1100
1150
1200

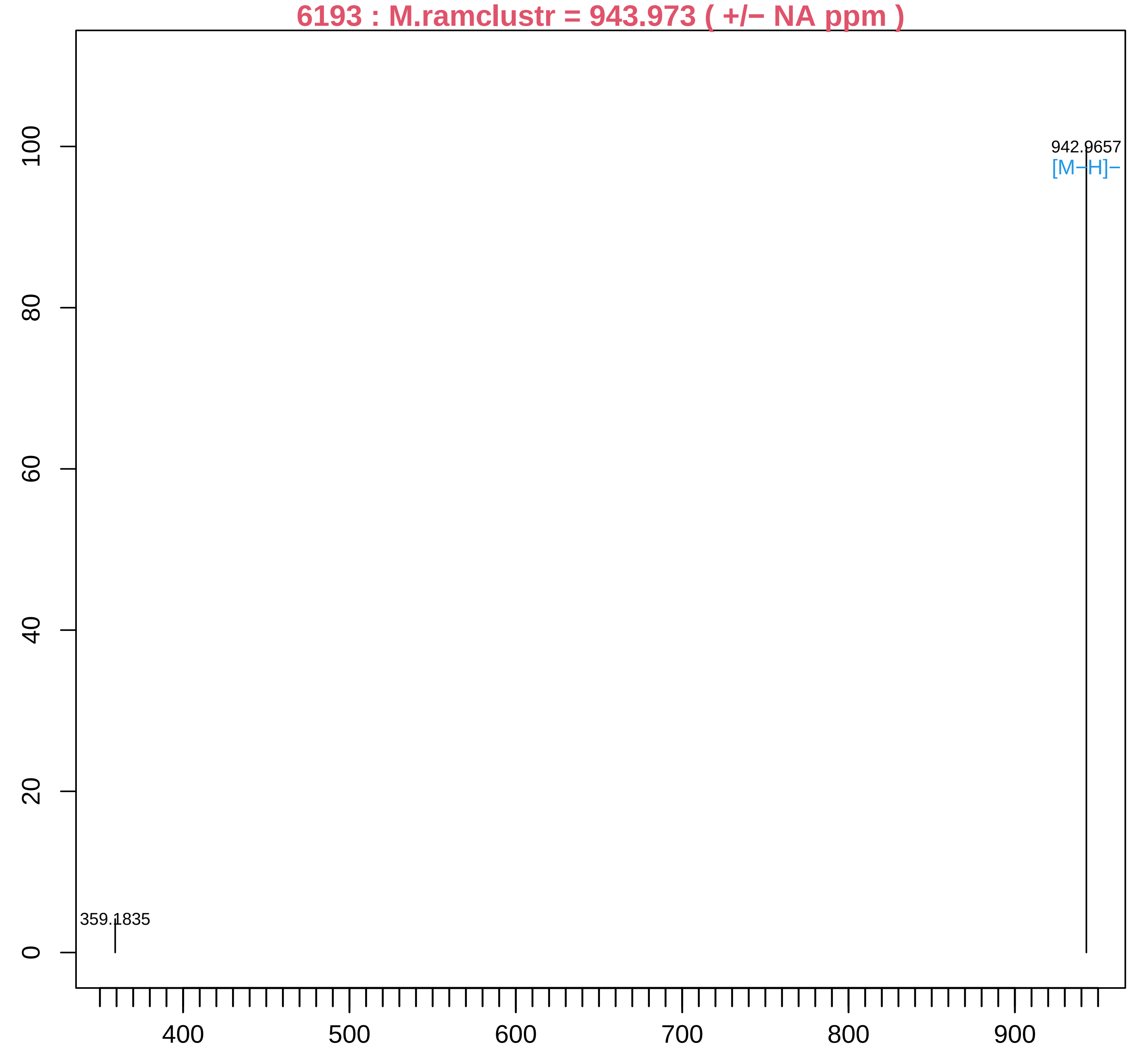
6193 : M.ramclustr = 943.973 (+/− NA ppm)
942.9657
[M−H]−
359.1835
0
20
40
60
80
100
400
500
600
700
800
900

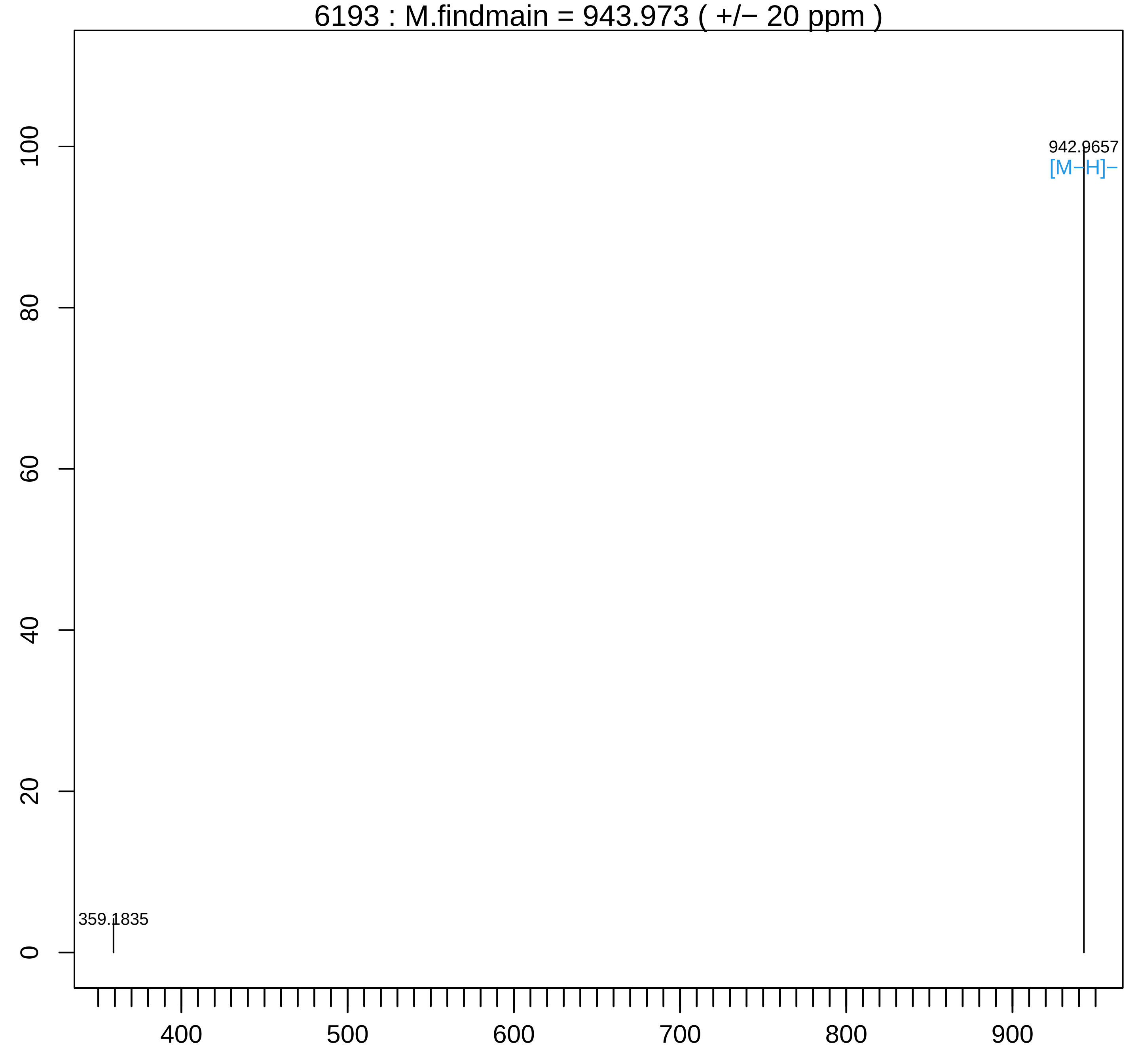
6193 : M.findmain = 943.973 (+/− 20 ppm)
942.9657
[M−H]−
359.1835
0
20
40
60
80
100
400
500
600
700
800
900

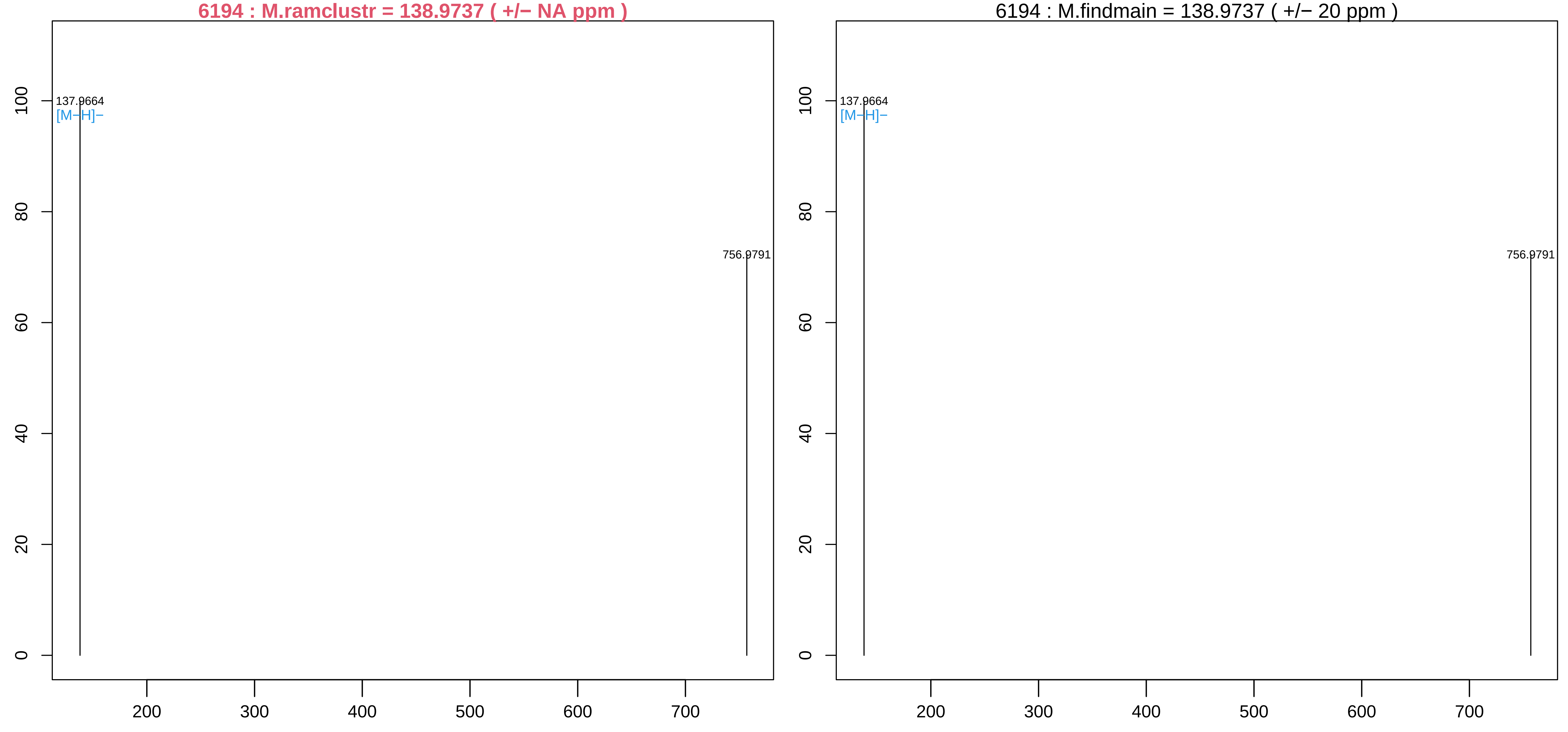
6194 : M.ramclustr = 138.9737 (+/− NA ppm)
137.9664
[M−H]−
756.9791
0
20
40
60
80
100
200
300
400
500
600
700
6194 : M.findmain = 138.9737 (+/− 20 ppm)
137.9664
[M−H]−
756.9791
0
20
40
60
80
100
200
300
400
500
600
700

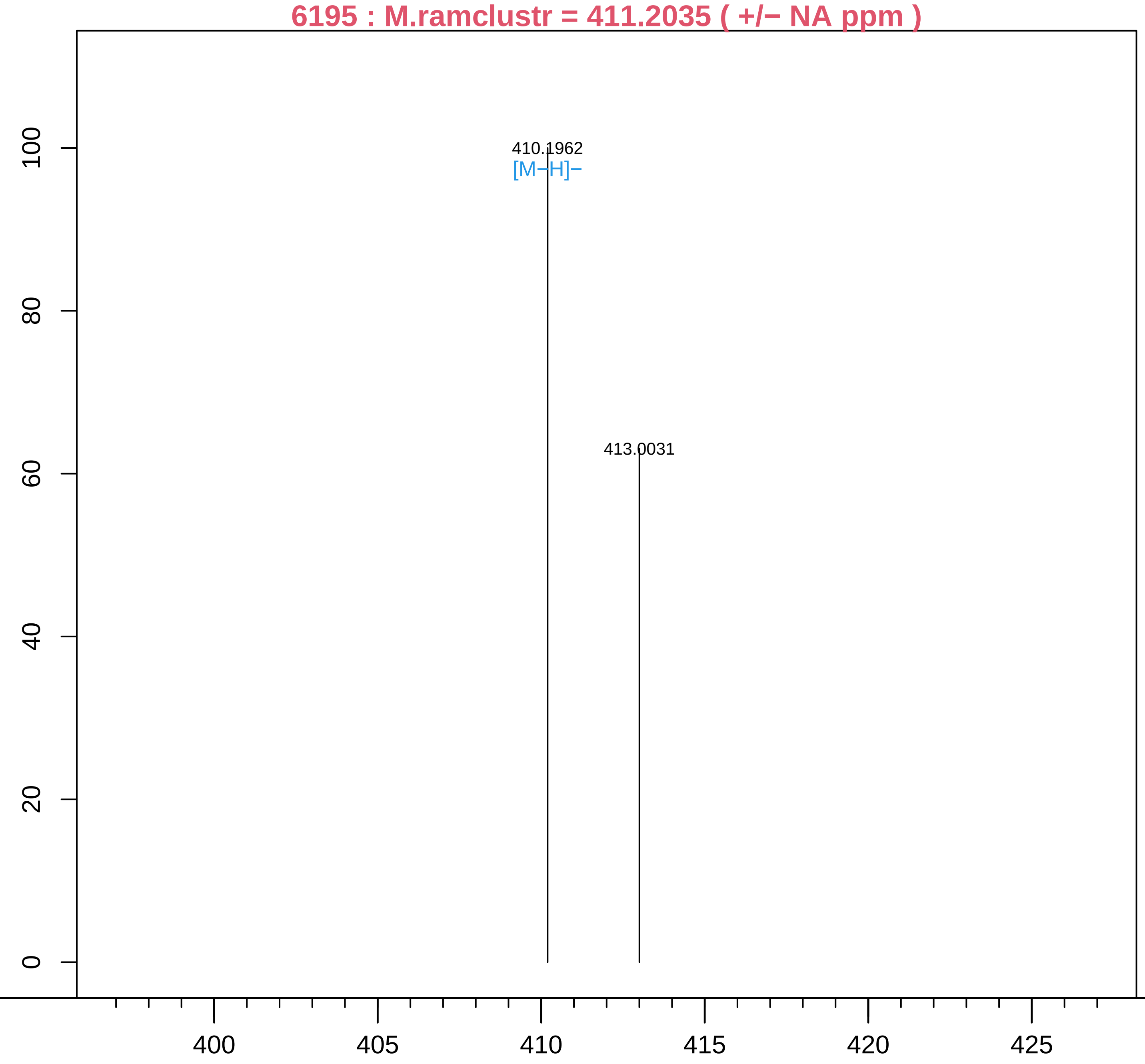
6195 : M.ramclustr = 411.2035 (+/− NA ppm)
410.1962
[M−H]−
413.0031
0
20
40
60
80
100
400
405
410
415
420
425

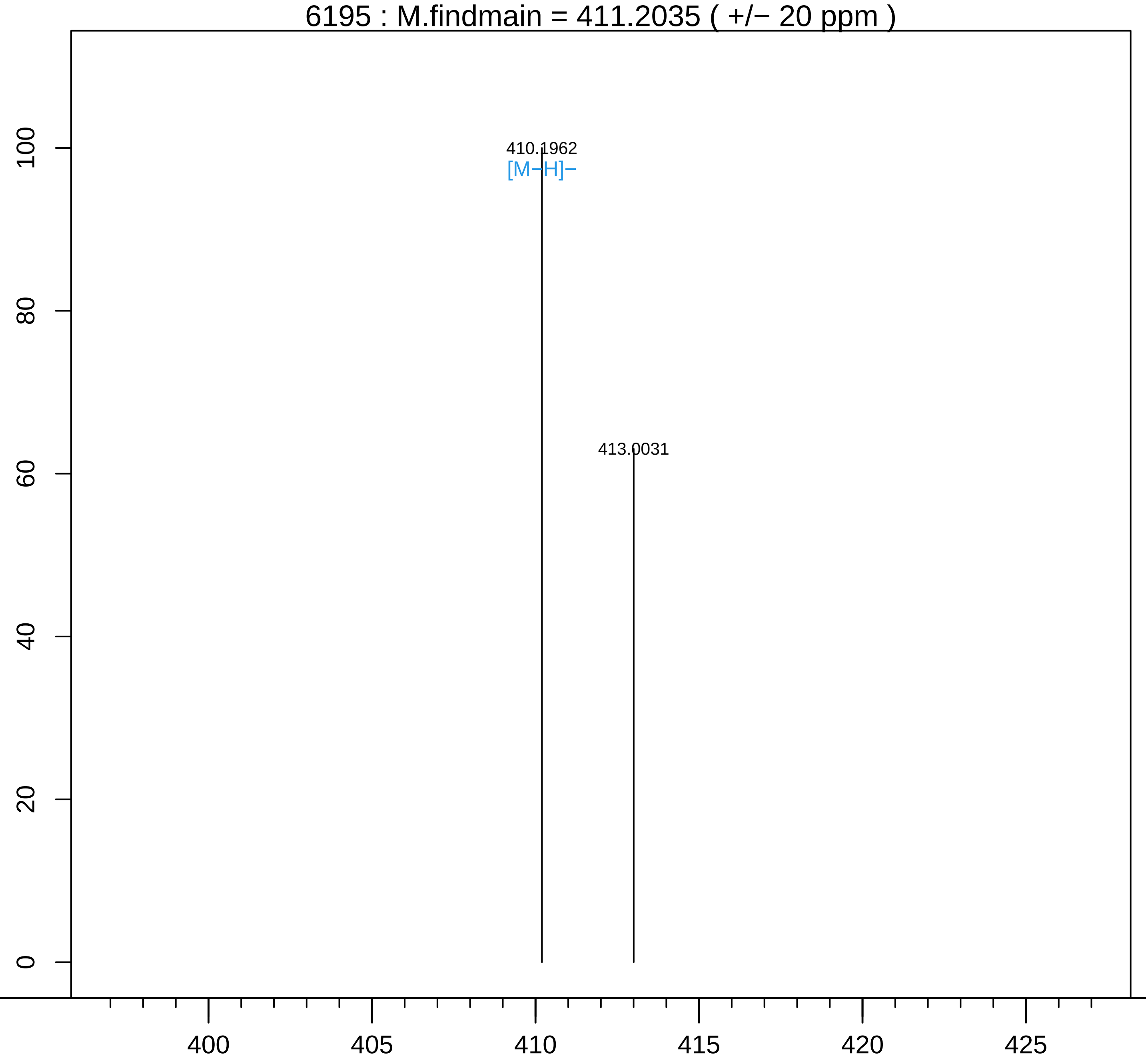
6195 : M.findmain = 411.2035 (+/− 20 ppm)
410.1962
[M−H]−
413.0031
0
20
40
60
80
100
400
405
410
415
420
425

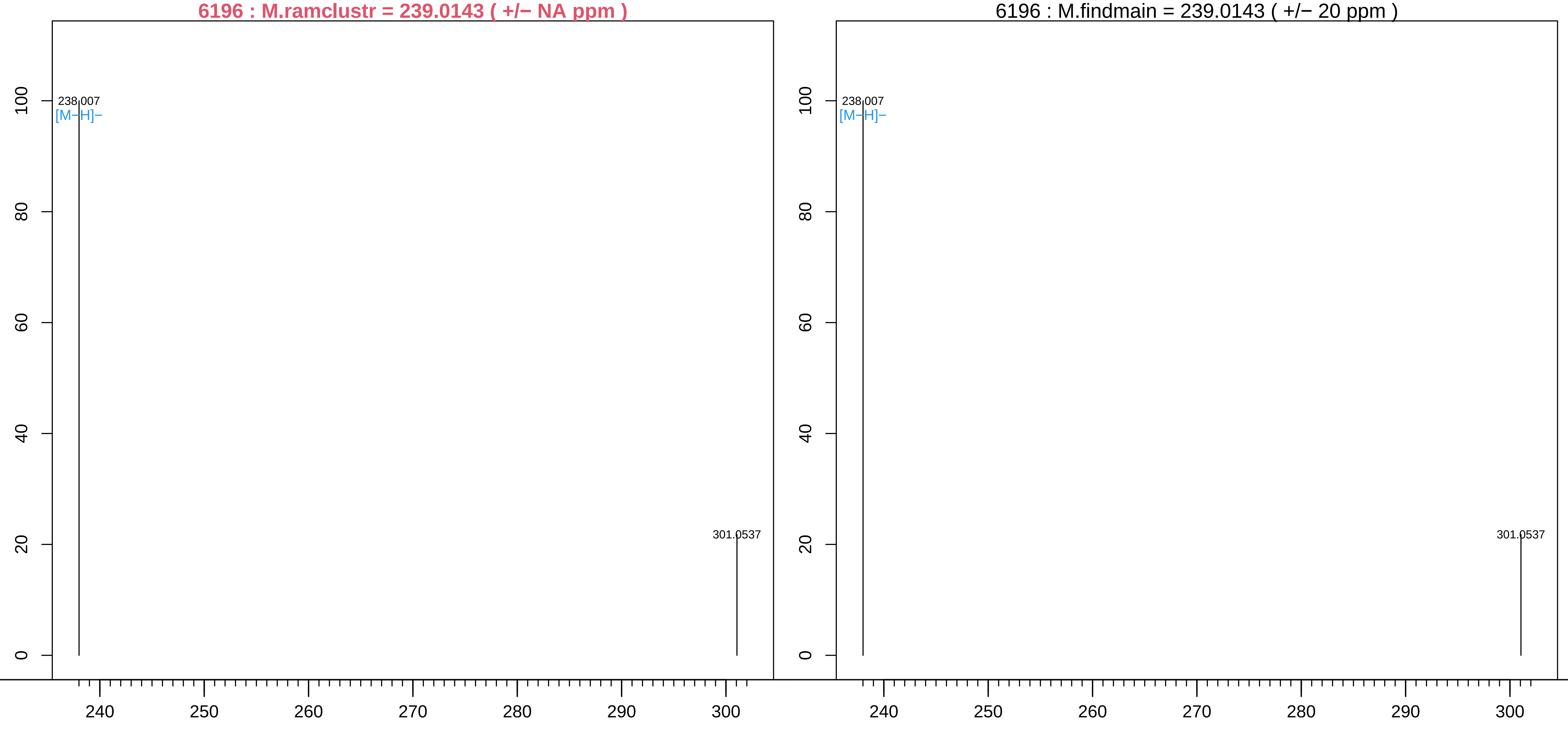
6196 : M.ramclustr = 239.0143 (+/− NA ppm)
238.007
[M−H]−
301.0537
6196 : M.findmain = 239.0143 (+/− 20 ppm)
238.007
[M−H]−
301.0537

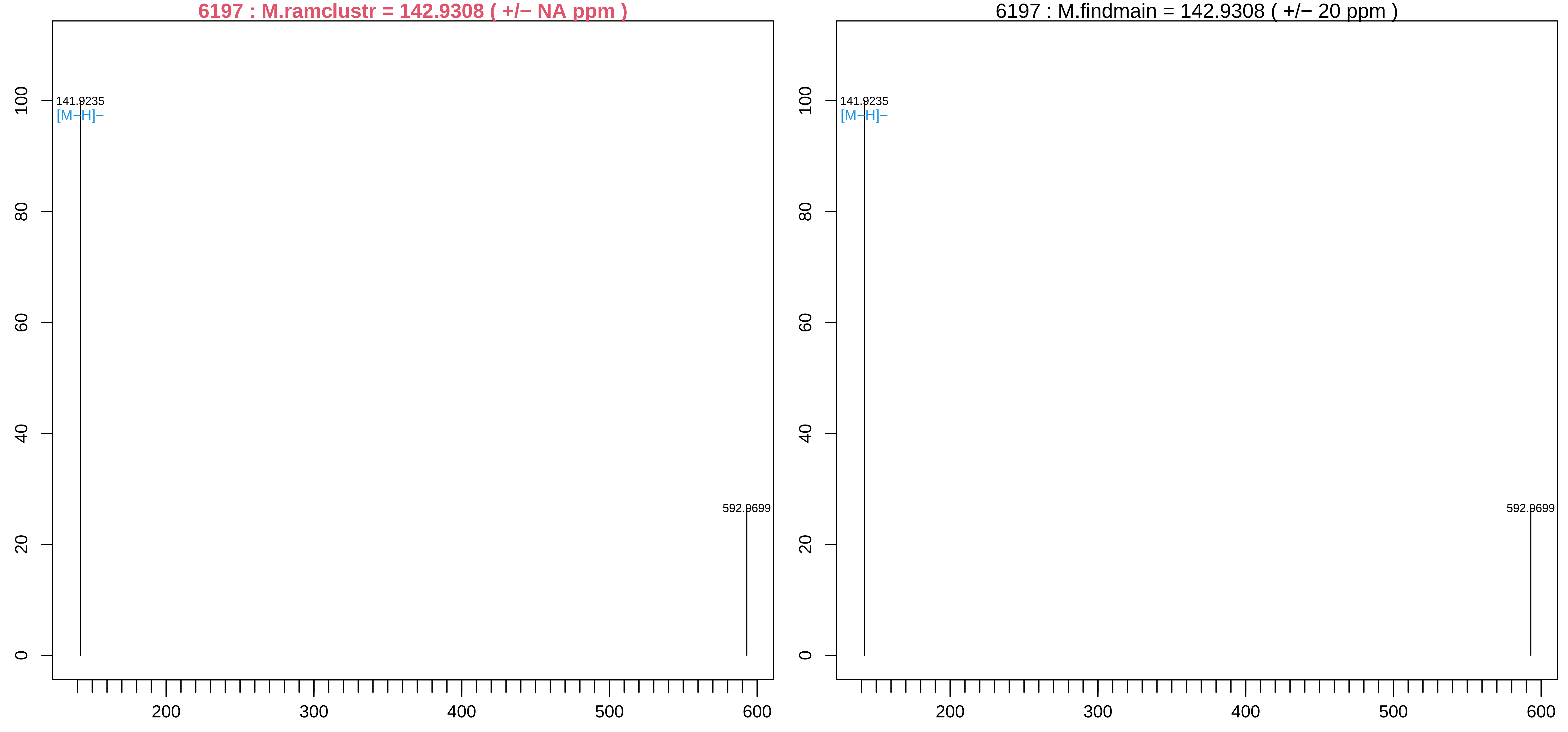
6197 : M.ramclustr = 142.9308 (+/− NA ppm)
141.9235
[M−H]−
592.9699
6197 : M.findmain = 142.9308 (+/− 20 ppm)
141.9235
[M−H]−
592.9699
0
20
40
60
80
100
200
300
400
500
600

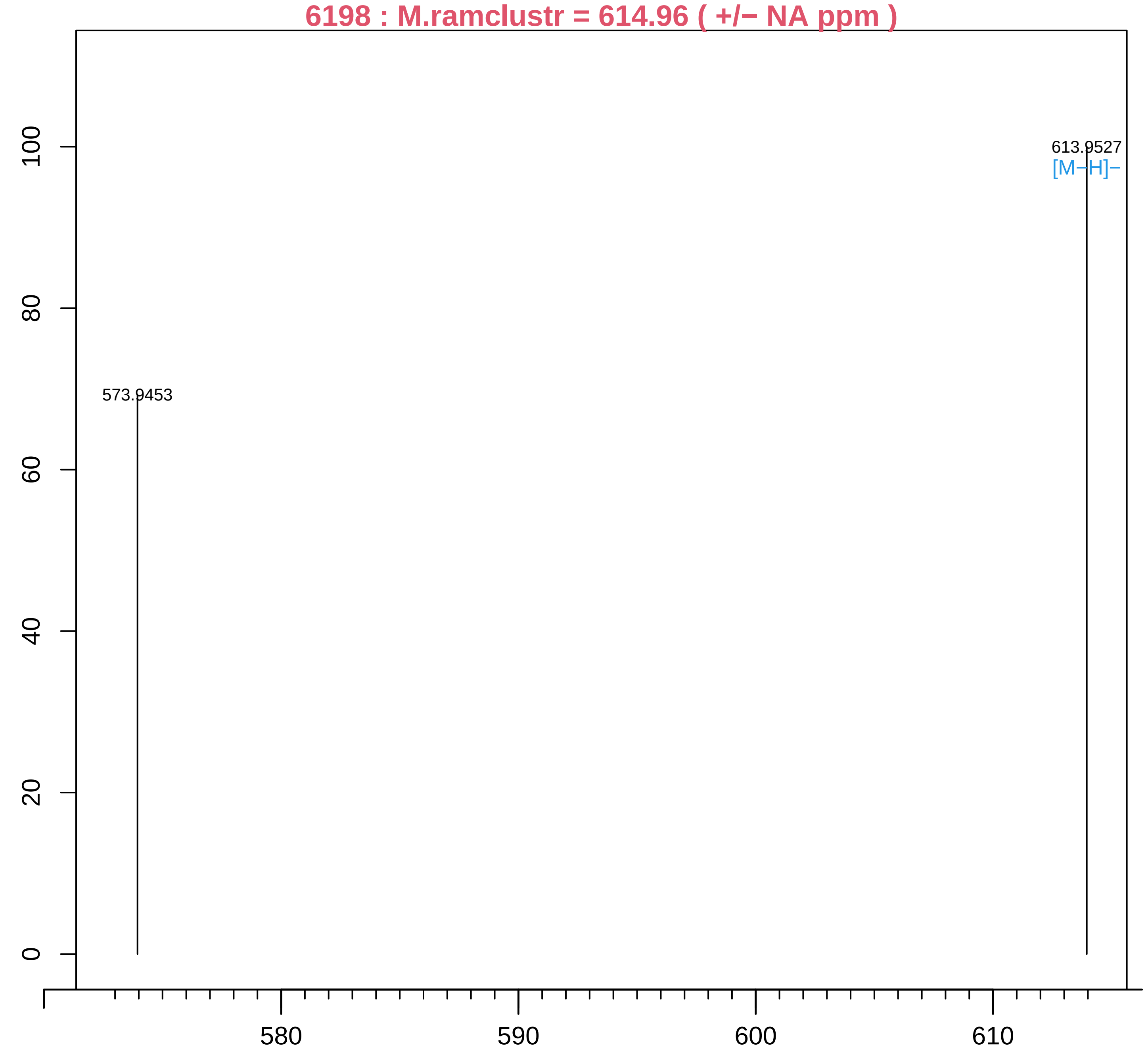
6198 : M.ramclustr = 614.96 (+/− NA ppm)
613.9527
[M−H]−
573.9453
0
20
40
60
80
100
580
590
600
610

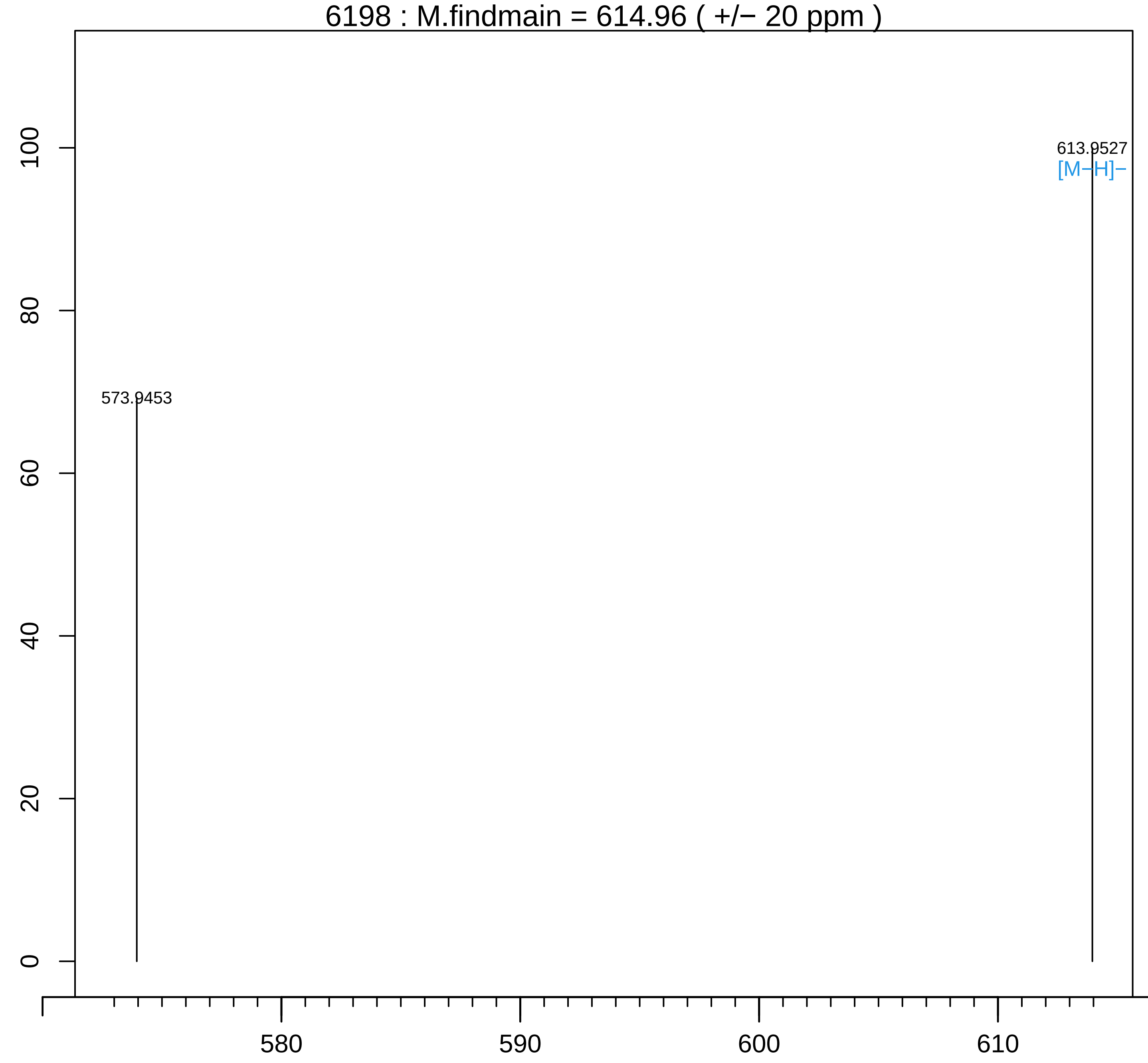
6198 : M.findmain = 614.96 (+/− 20 ppm)
613.9527
[M−H]−
573.9453
0
20
40
60
80
100
580
590
600
610

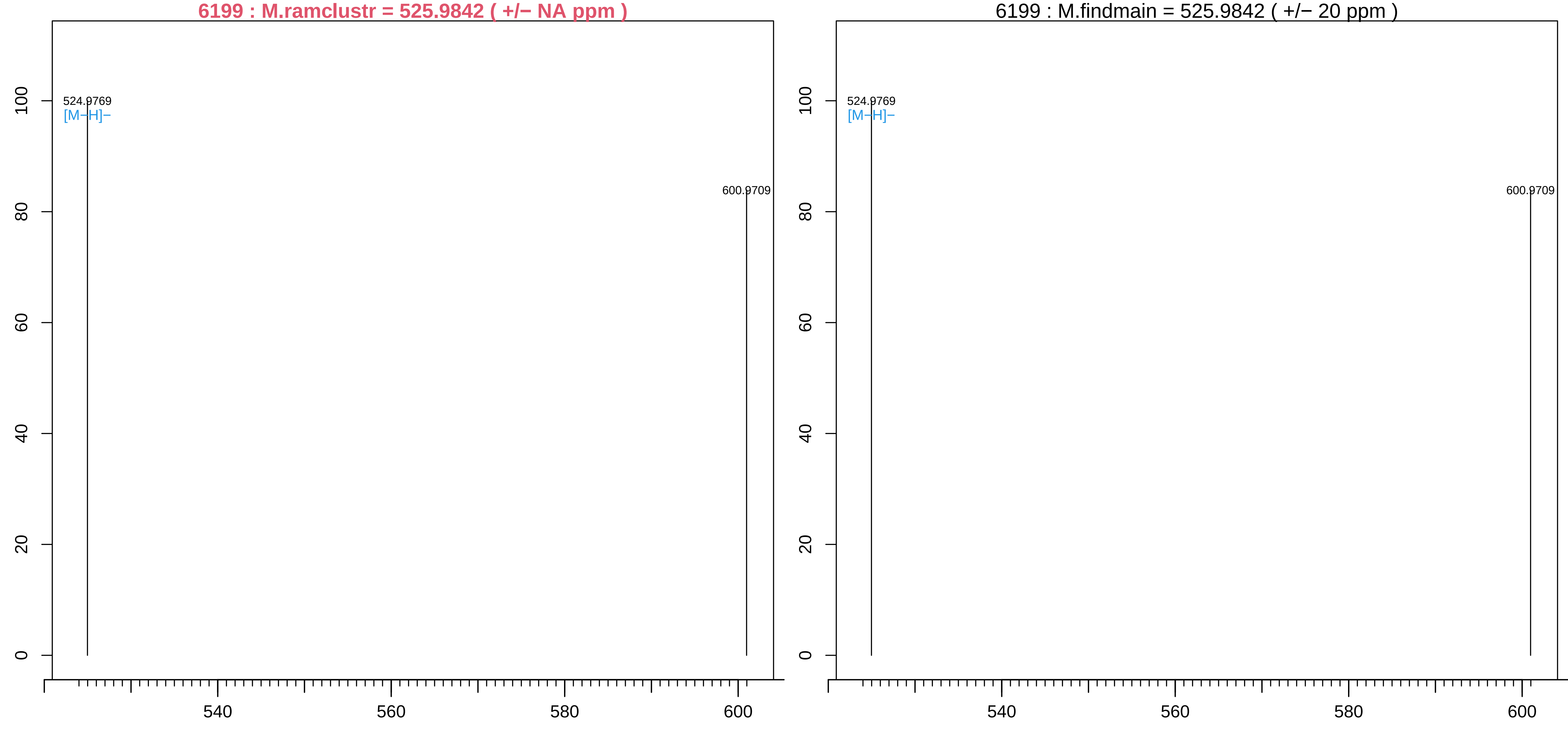

6199 : M.ramclustr = 525.9842 (+/− NA ppm)
524.9769
[M−H]−
600.9709
0
20
40
60
80
100
540
560
580
600
6199 : M.findmain = 525.9842 (+/− 20 ppm)
524.9769
[M−H]−
600.9709
0
20
40
60
80
100
540
560
580
600

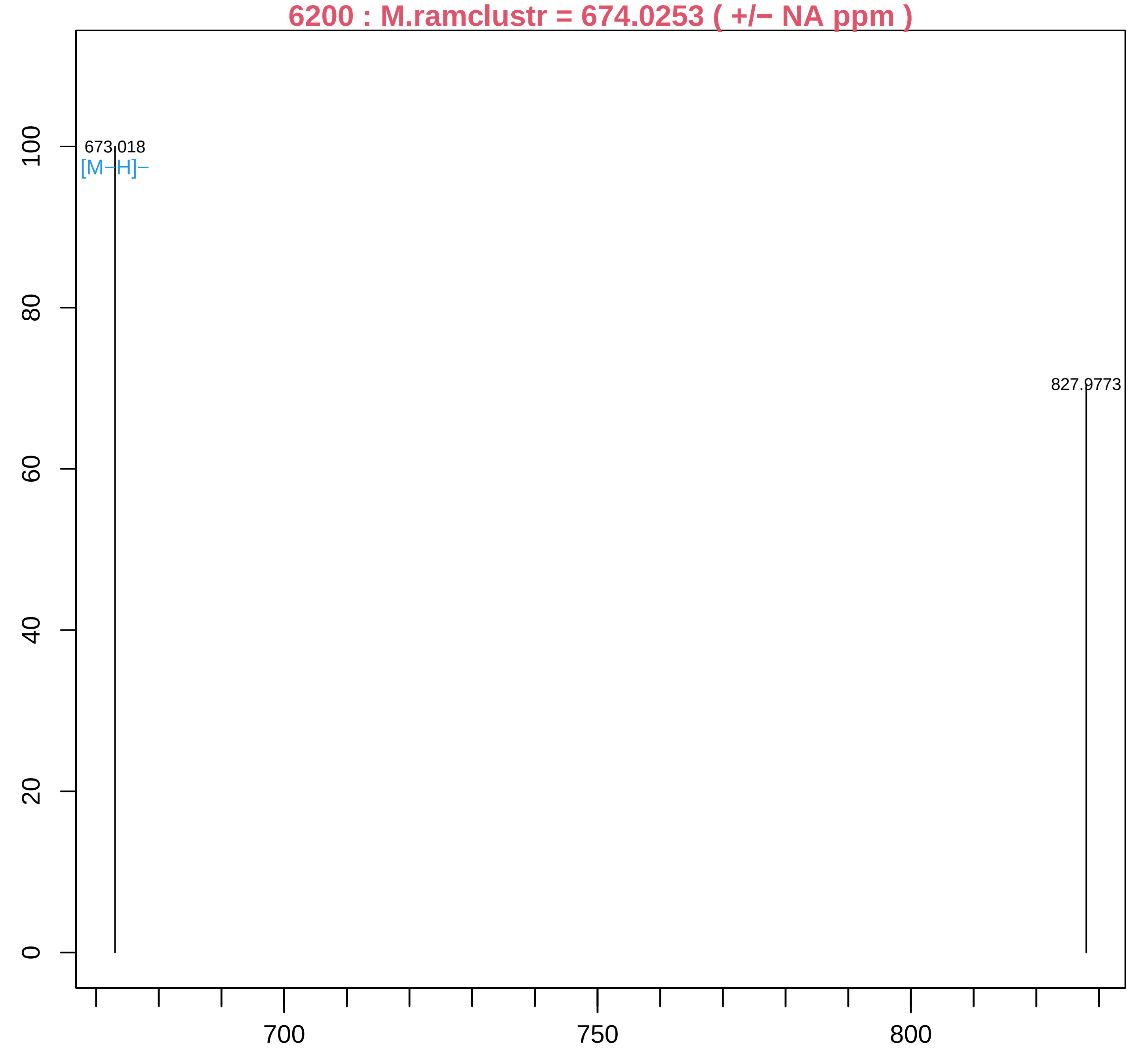
6200 : M.ramclustr = 674.0253 (+/− NA ppm)
673.018
[M−H]−
827.9773
0
20
40
60
80
100
700
750
800
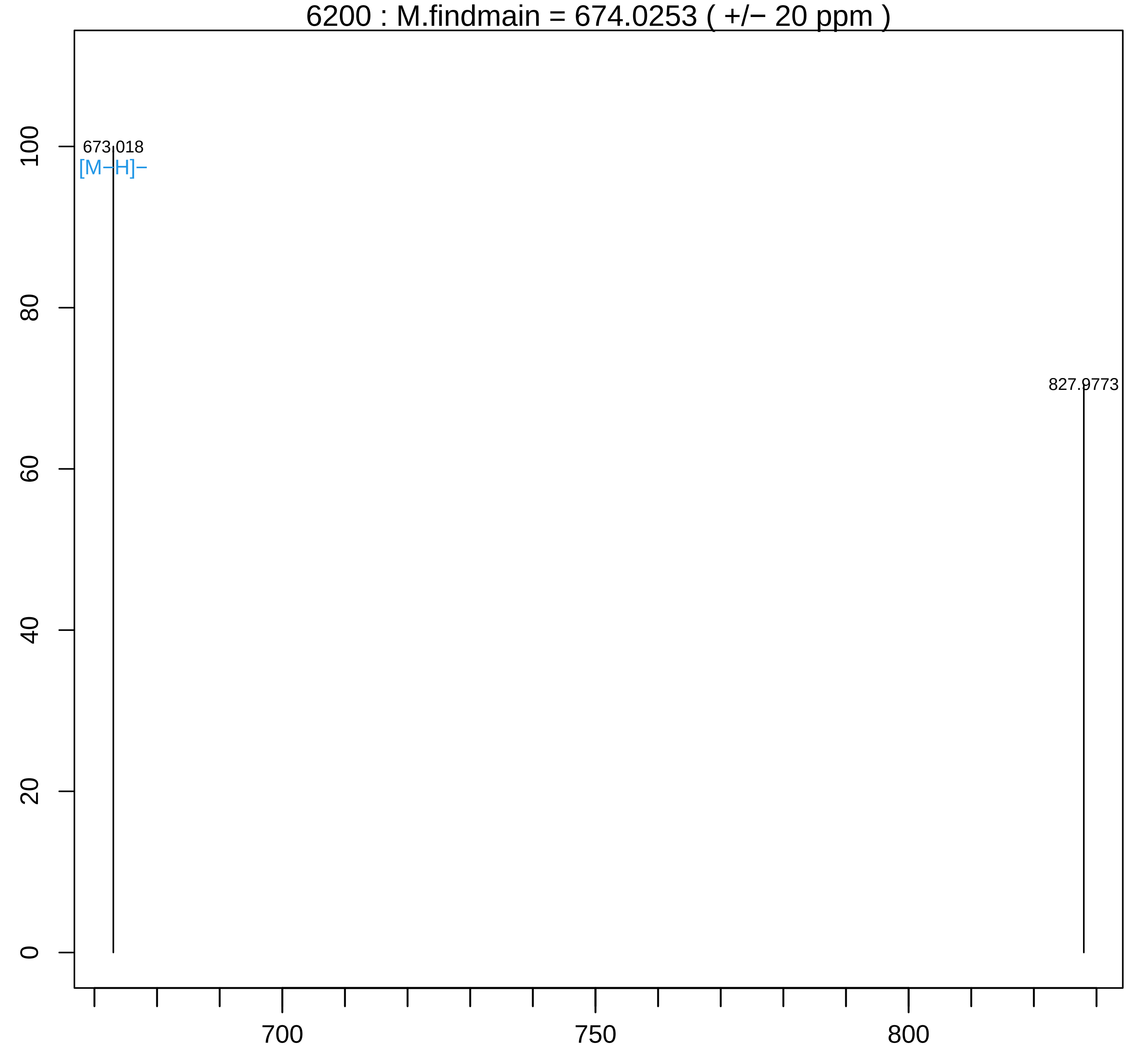
6200 : M.findmain = 674.0253 (+/− 20 ppm)
673.018
[M−H]−
827.9773
0
20
40
60
80
100
700
750
800

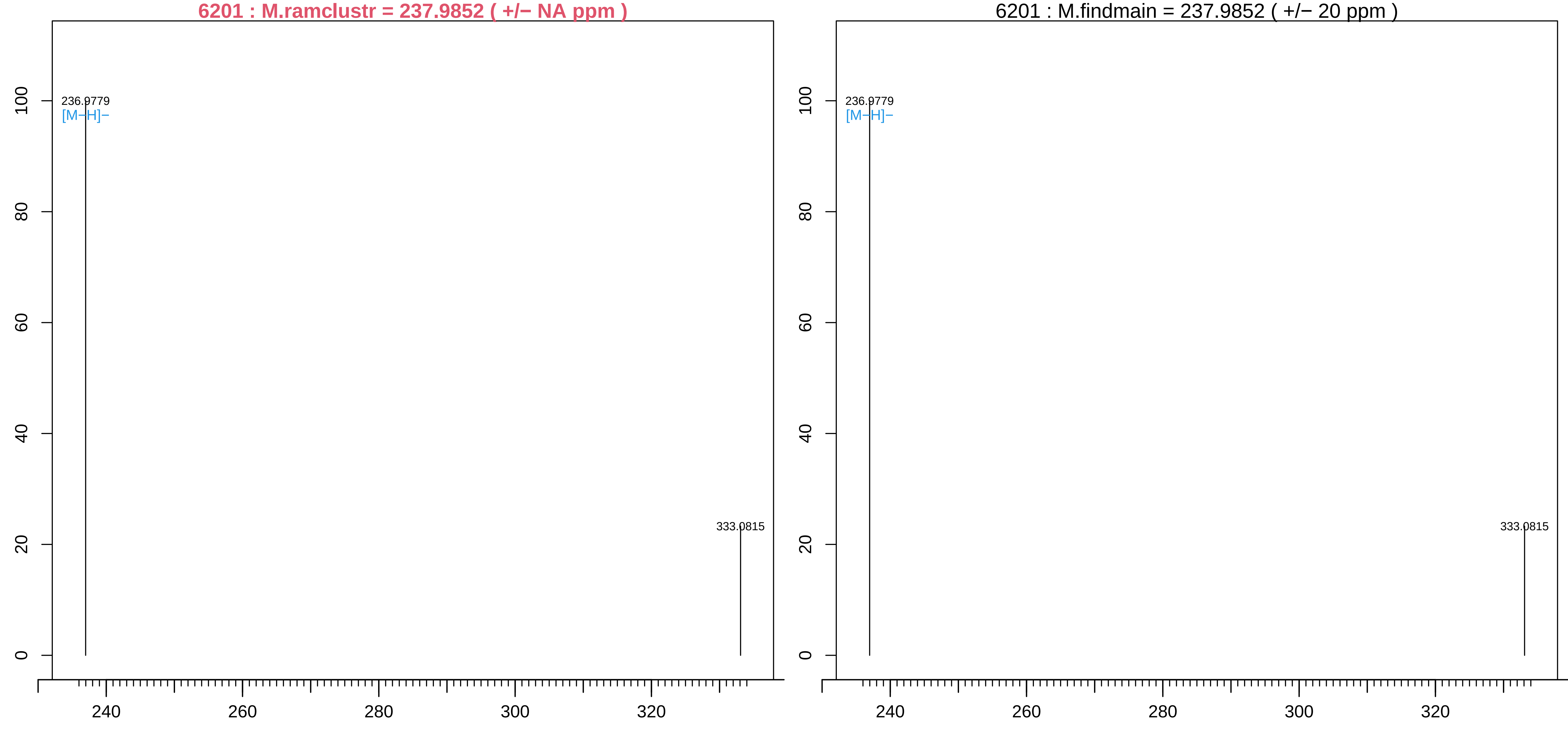
6201 : M.ramclustr = 237.9852 (+/− NA ppm)
236.9779
[M−H]−
333.0815
6201 : M.findmain = 237.9852 (+/− 20 ppm)
236.9779
[M−H]−
333.0815
0
20
40
60
80
100
240
260
280
300
320

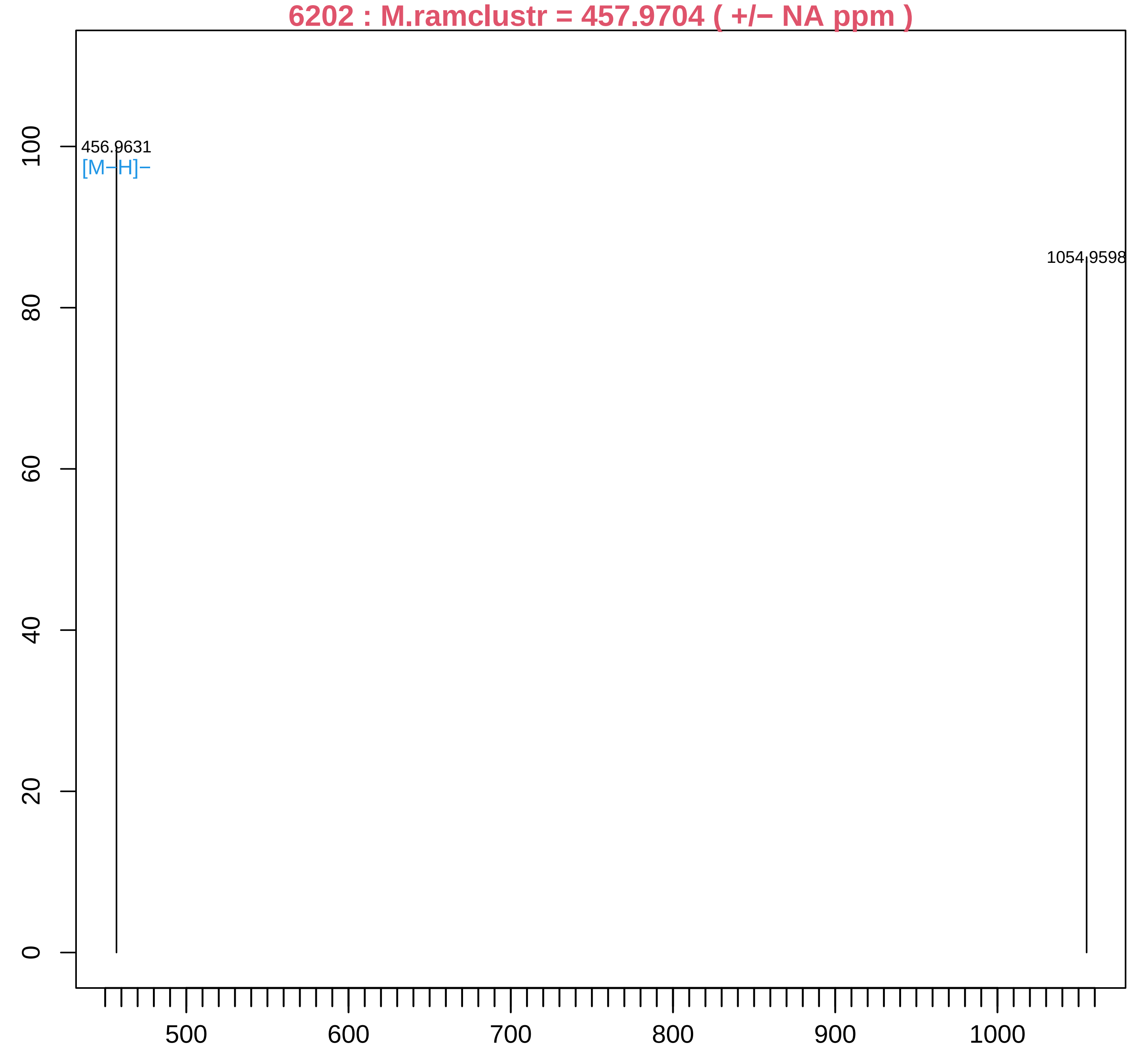
6202 : M.ramclustr = 457.9704 (+/− NA ppm)
456.9631
[M−H]−
1054.9598
0
20
40
60
80
100
500
600
700
800
900
1000

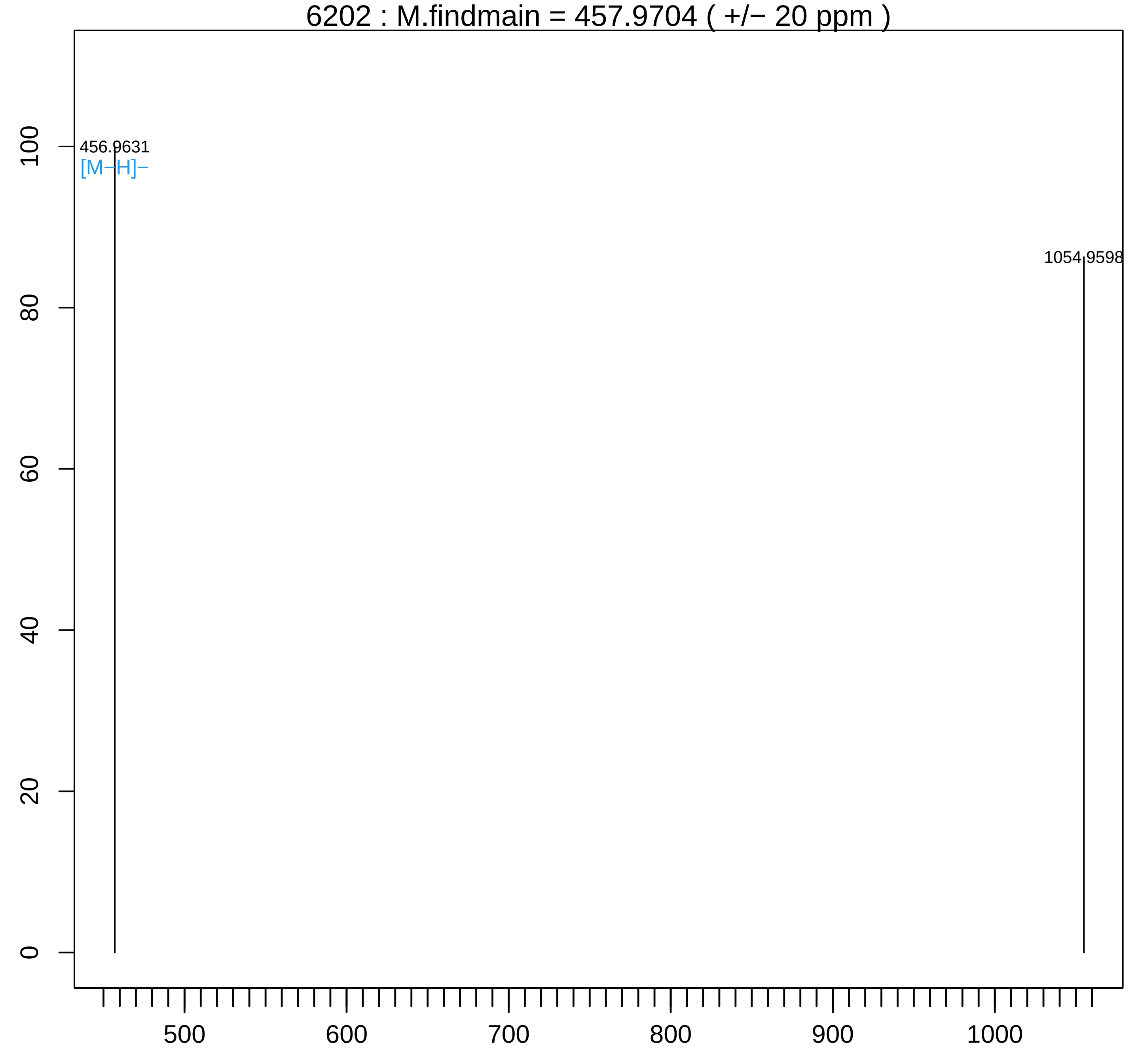
6202 : M.findmain = 457.9704 (+/− 20 ppm)
456.9631
[M−H]−
1054.9598
0
20
40
60
80
100
500
600
700
800
900
1000

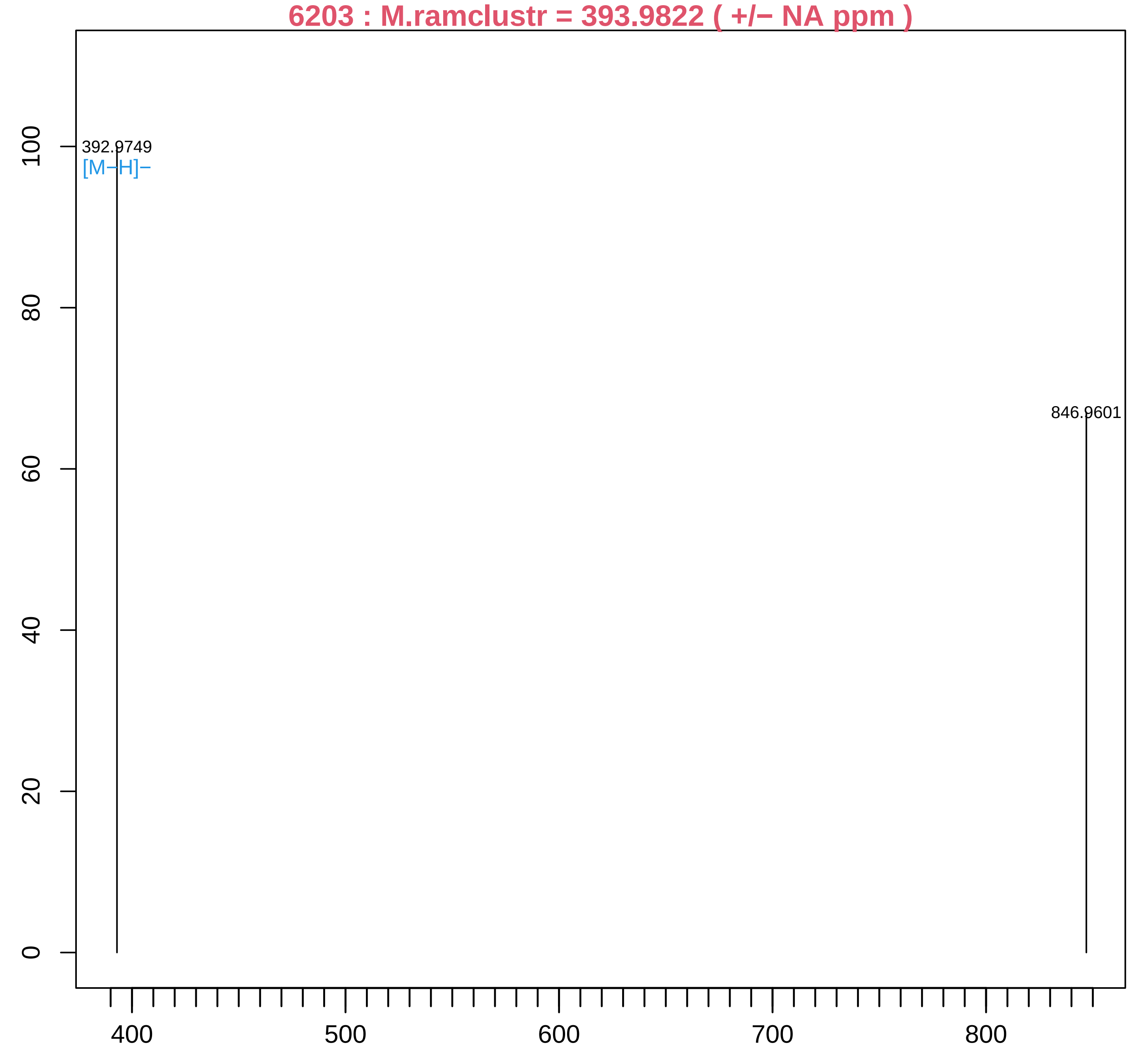

6203 : M.ramclustr = 393.9822 (+/− NA ppm)
392.9749
[M−H]−
846.9601
0
20
40
60
80
100
400
500
600
700
800

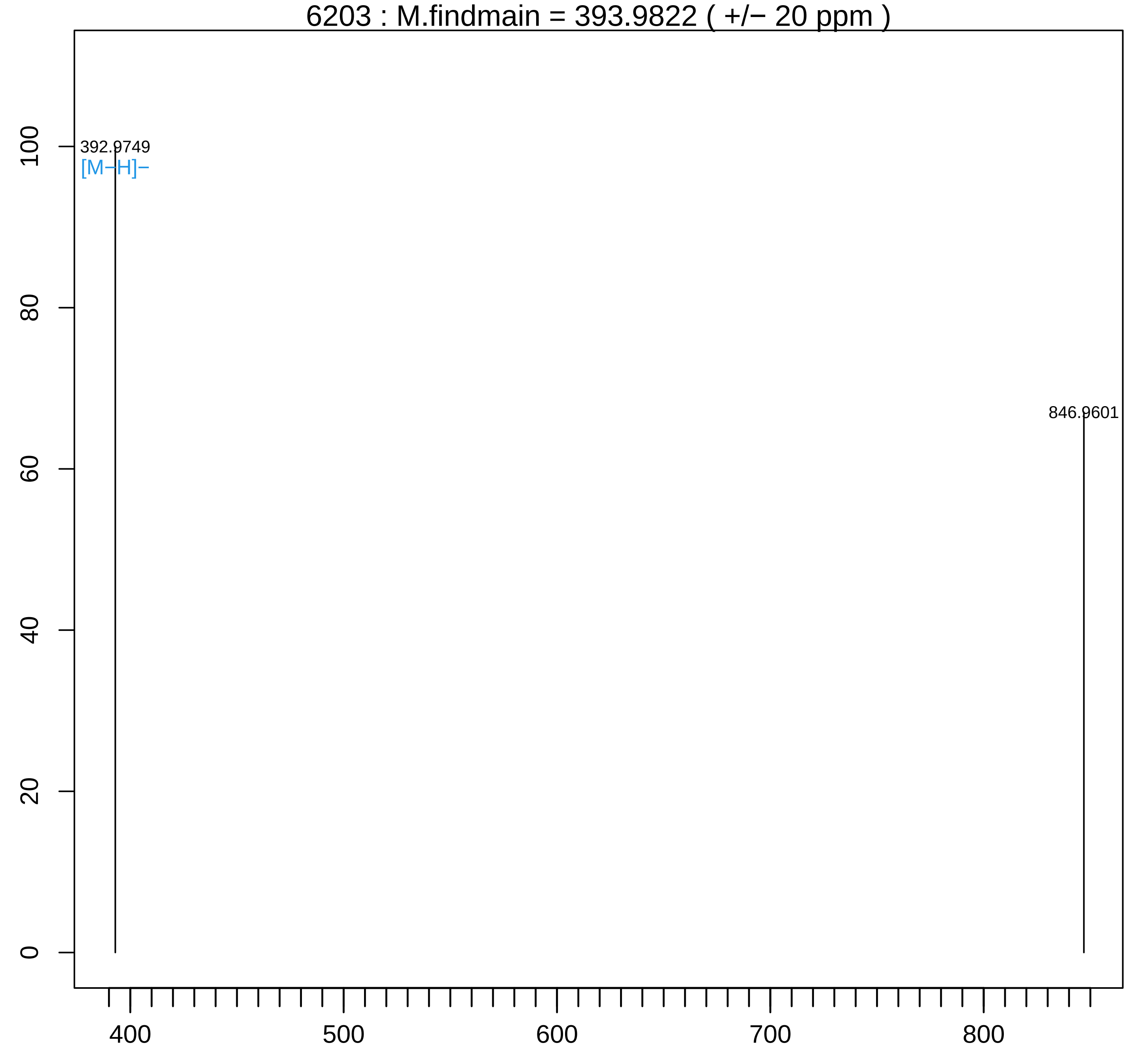

6203 : M.findmain = 393.9822 (+/− 20 ppm)
392.9749
[M−H]−
846.9601
0
20
40
60
80
100
400
500
600
700
800

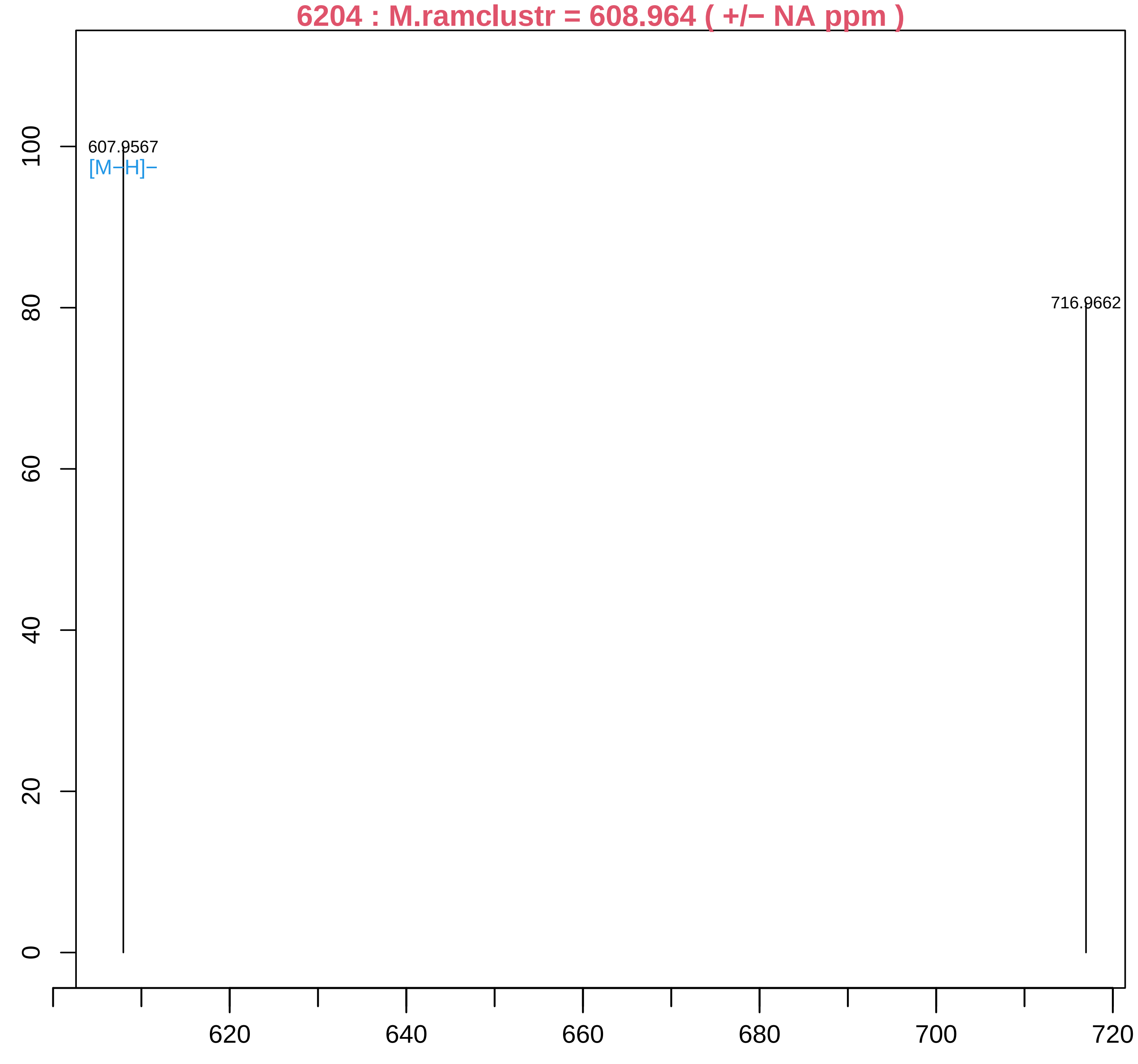
6204 : M.ramclustr = 608.964 (+/− NA ppm)
607.9567
[M−H]−
716.9662
0
20
40
60
80
100
620
640
660
680
700
720

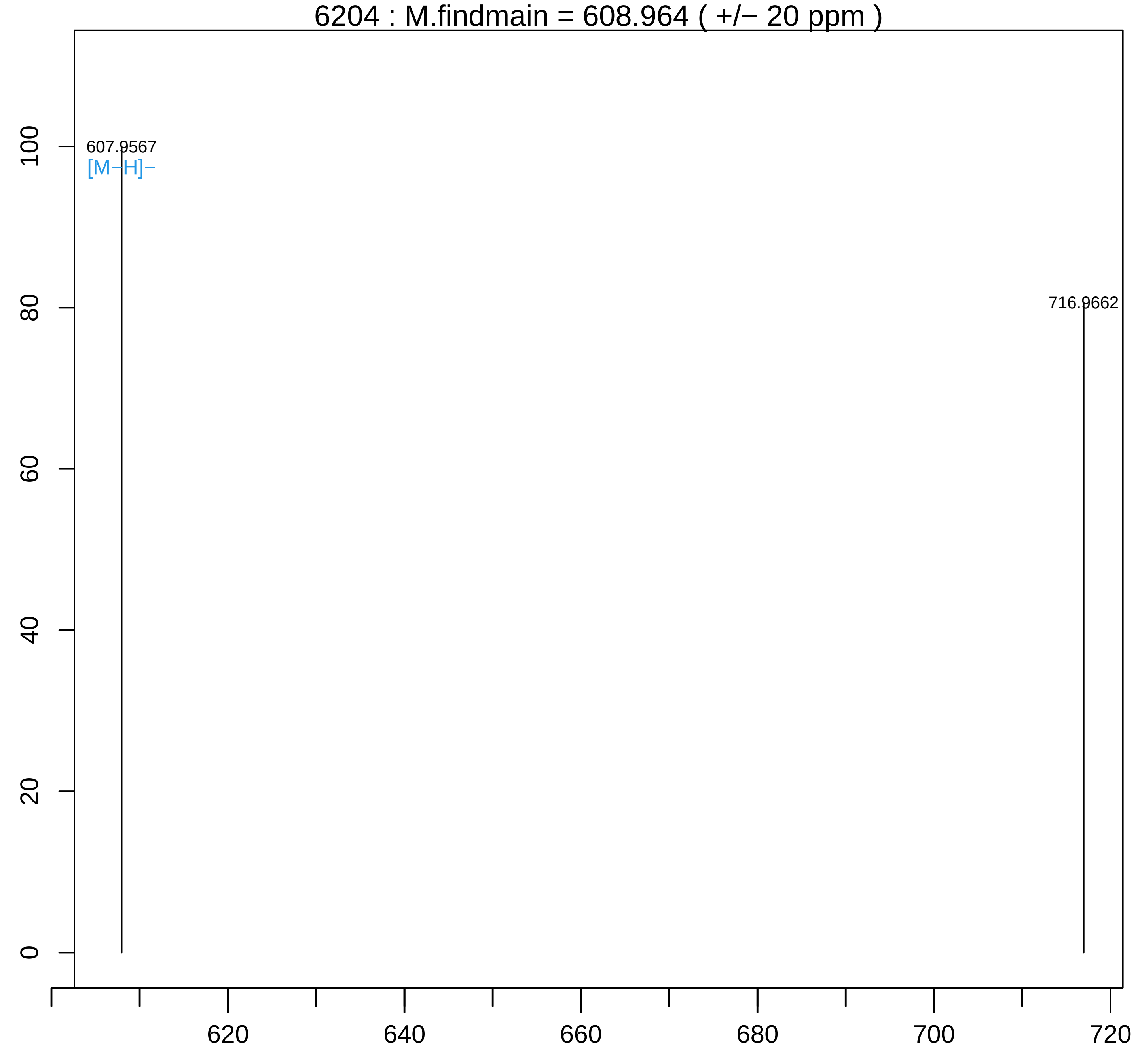
6204 : M.findmain = 608.964 (+/− 20 ppm)
607.9567
[M−H]−
716.9662
0
20
40
60
80
100
620
640
660
680
700
720

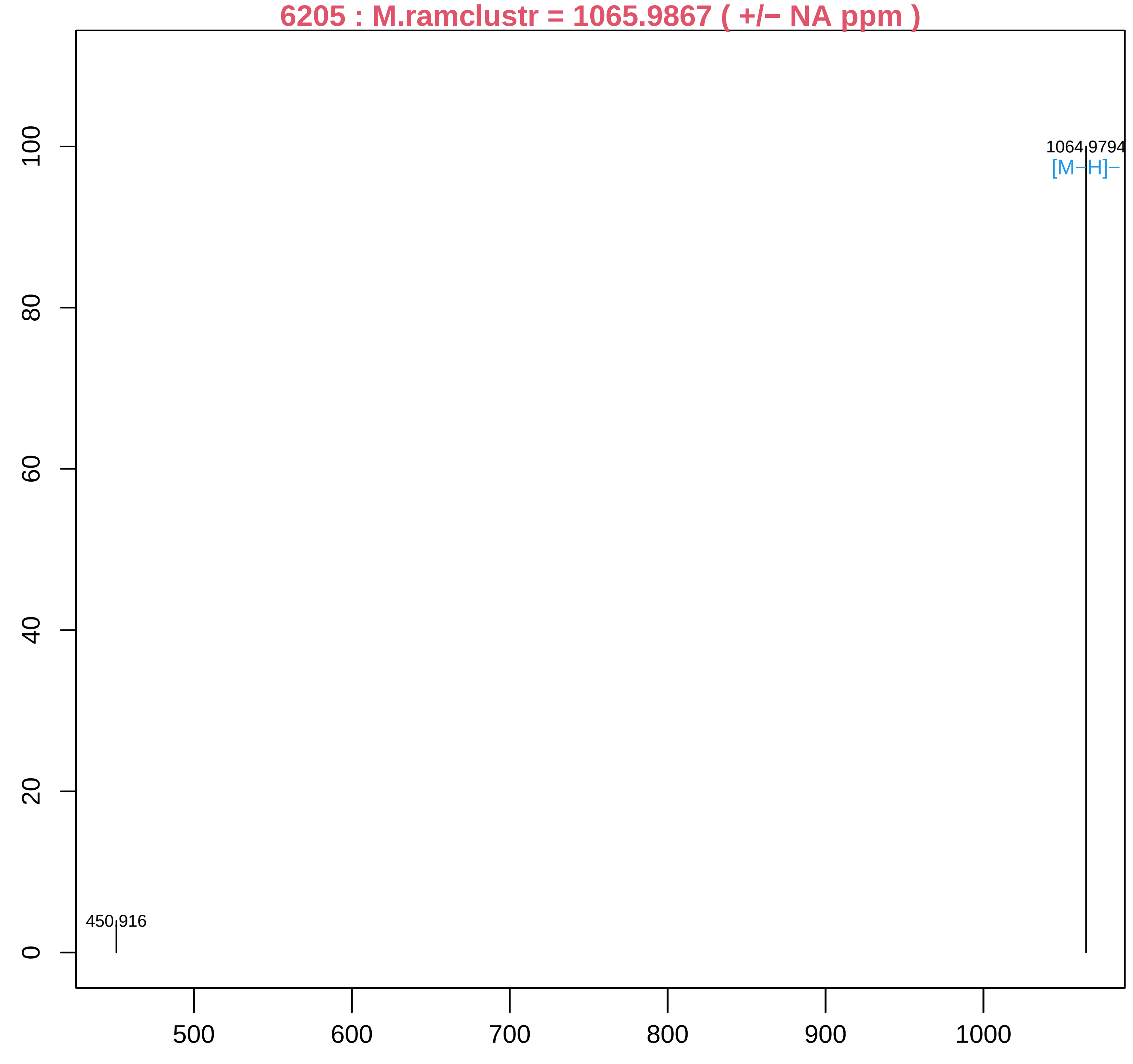

6205 : M.ramclustr = 1065.9867 (+/− NA ppm)
1064.9794
[M−H]−
450.916
0
20
40
60
80
100
500
600
700
800
900
1000

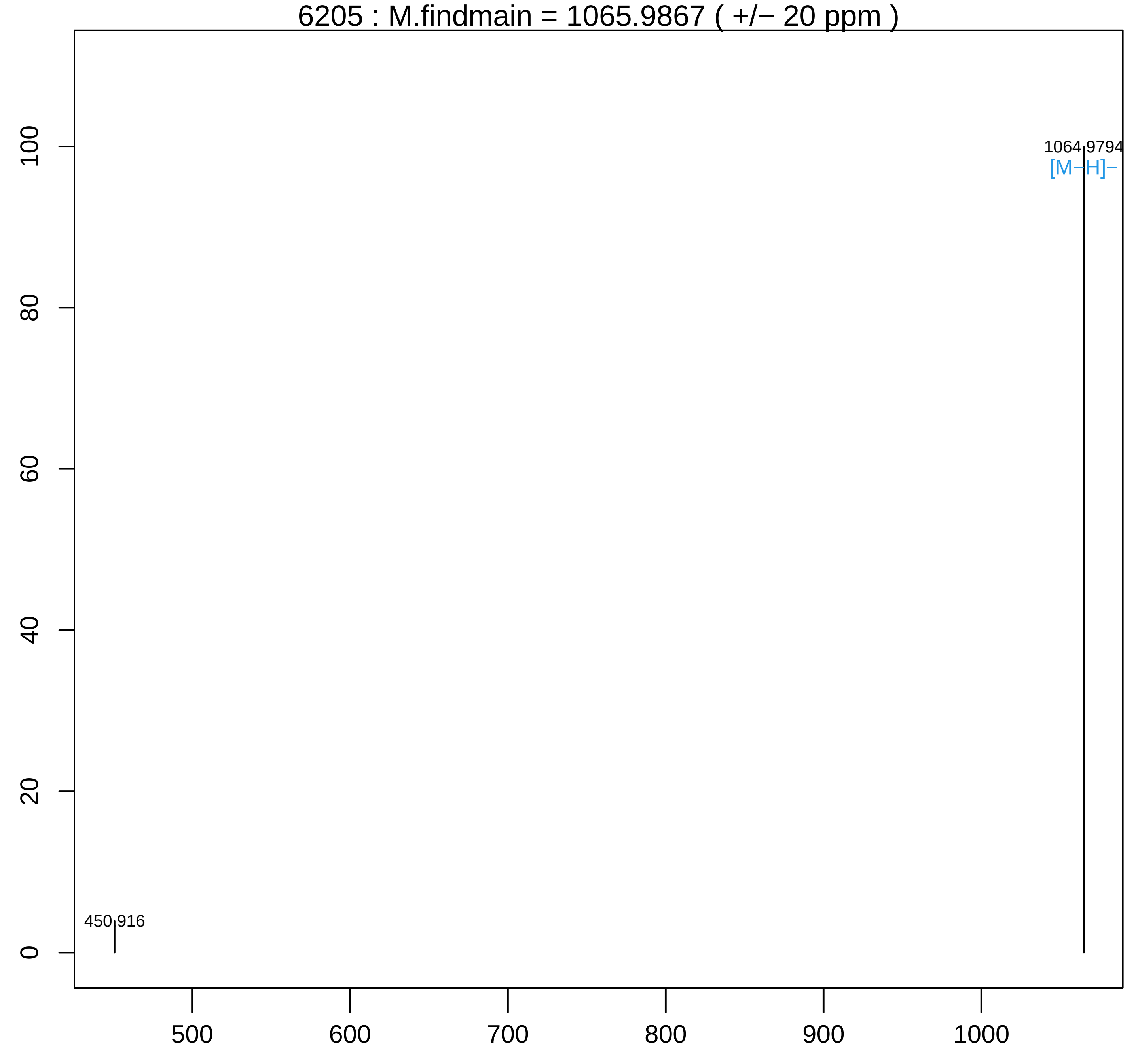

6205 : M.findmain = 1065.9867 (+/− 20 ppm)
1064.9794
[M−H]−
450.916
0
20
40
60
80
100
500
600
700
800
900
1000

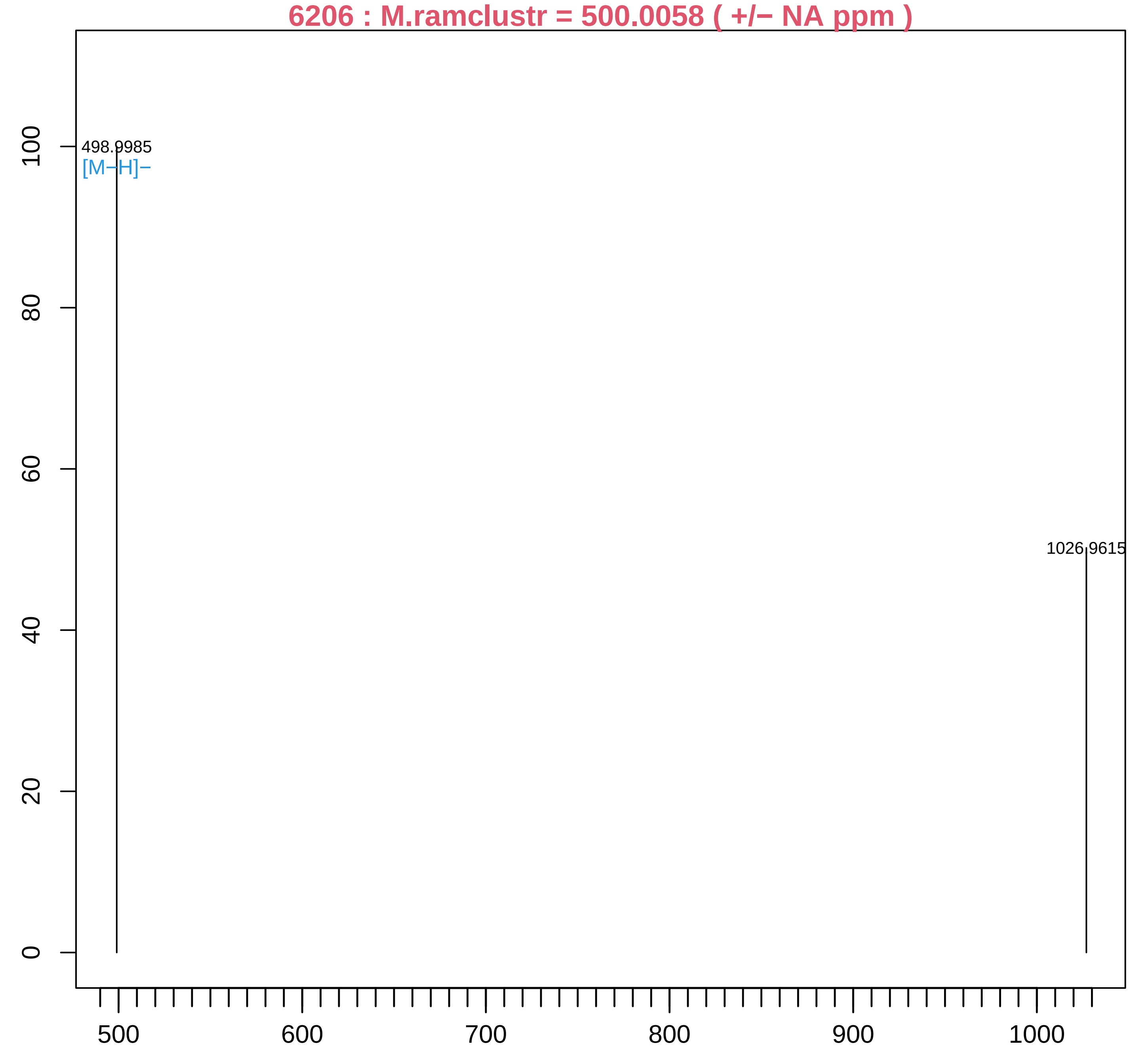
6206 : M.ramclustr = 500.0058 (+/− NA ppm)
498.9985
[M−H]−
1026.9615
0
20
40
60
80
100
500
600
700
800
900
1000

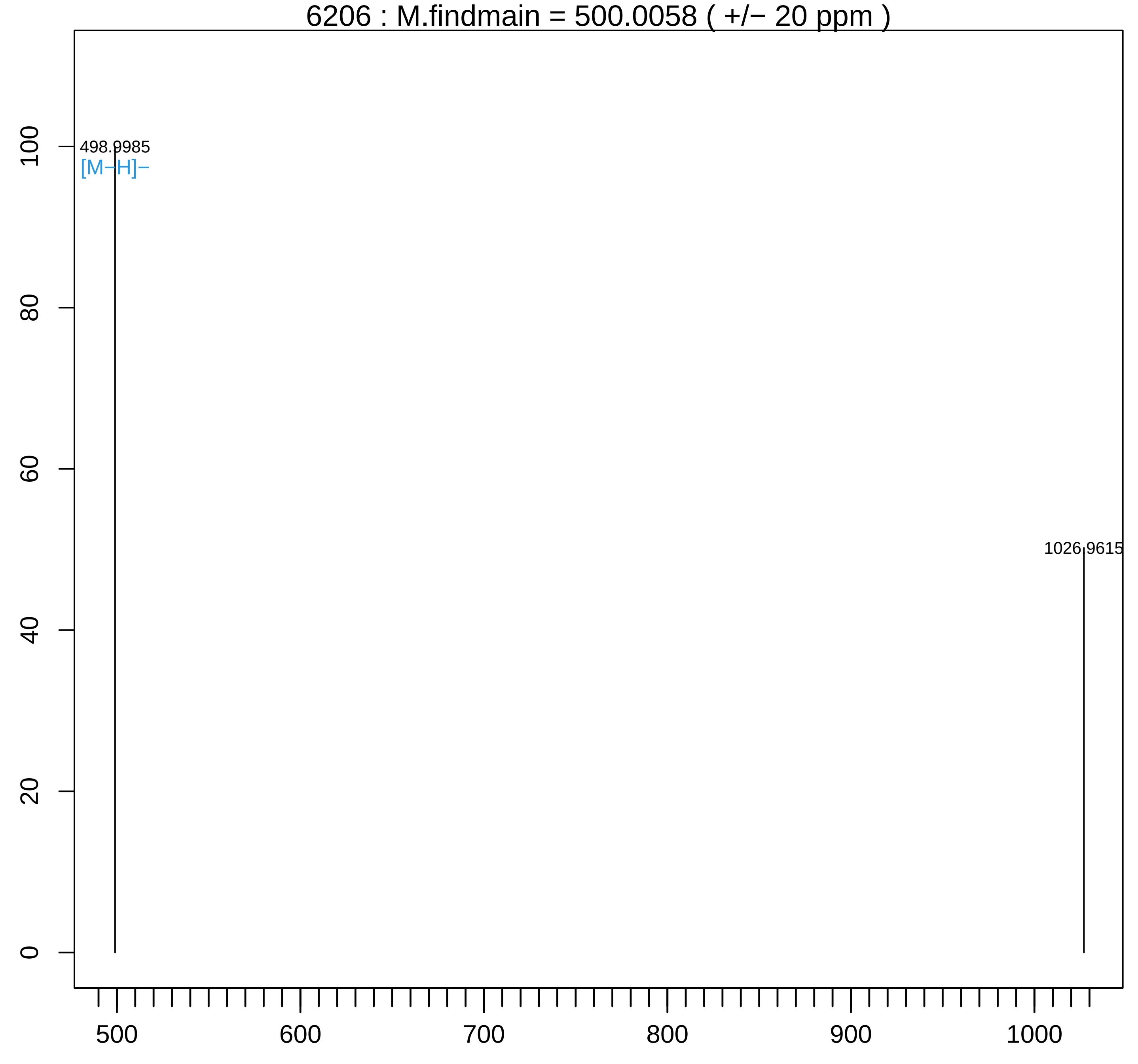
6206 : M.findmain = 500.0058 (+/− 20 ppm)
498.9985
[M−H]−
1026.9615
0
20
40
60
80
100
500
600
700
800
900
1000

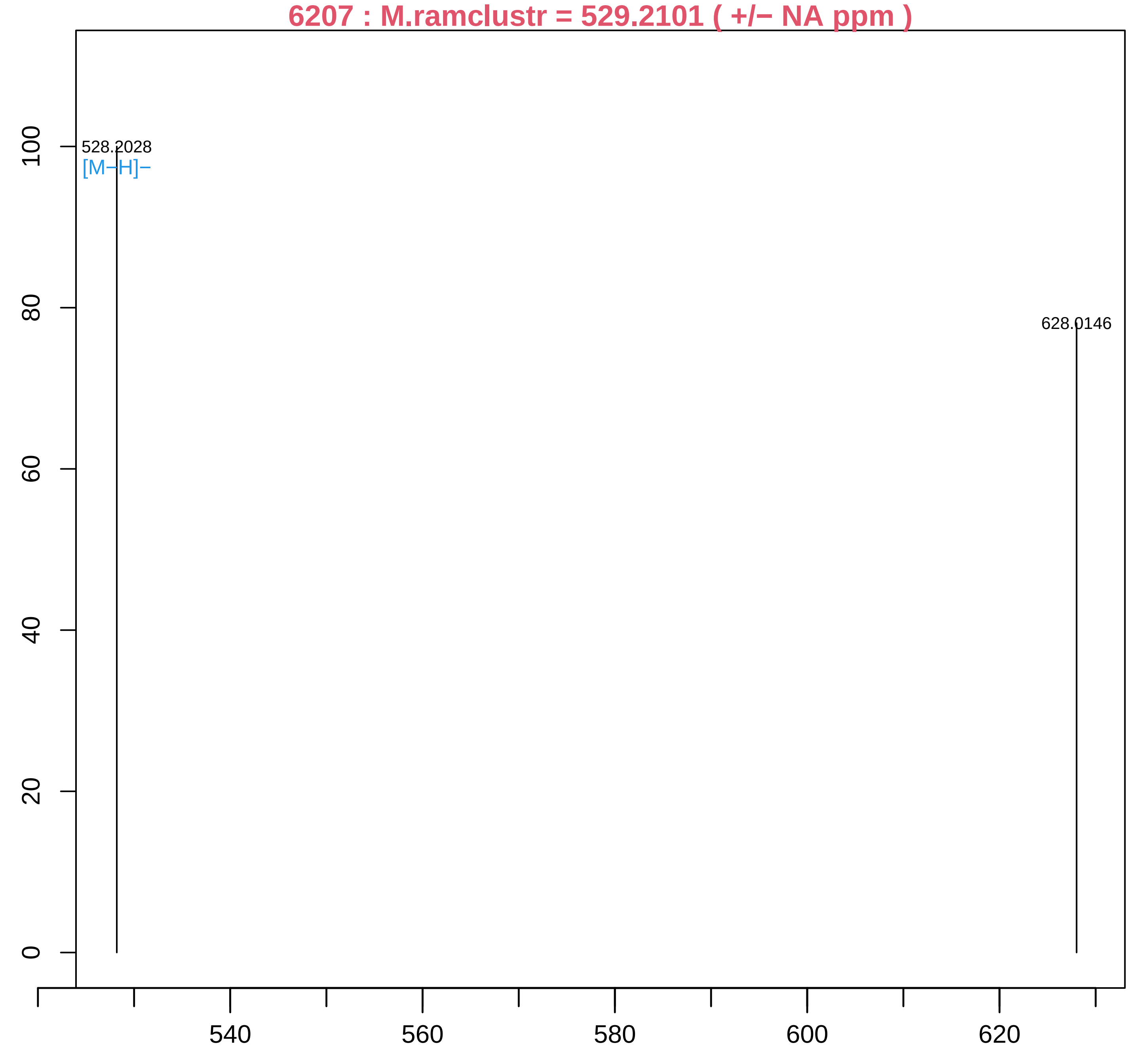
6207 : M.ramclustr = 529.2101 (+/− NA ppm)
528.2028
[M−H]−
628.0146
0
20
40
60
80
100
540
560
580
600
620

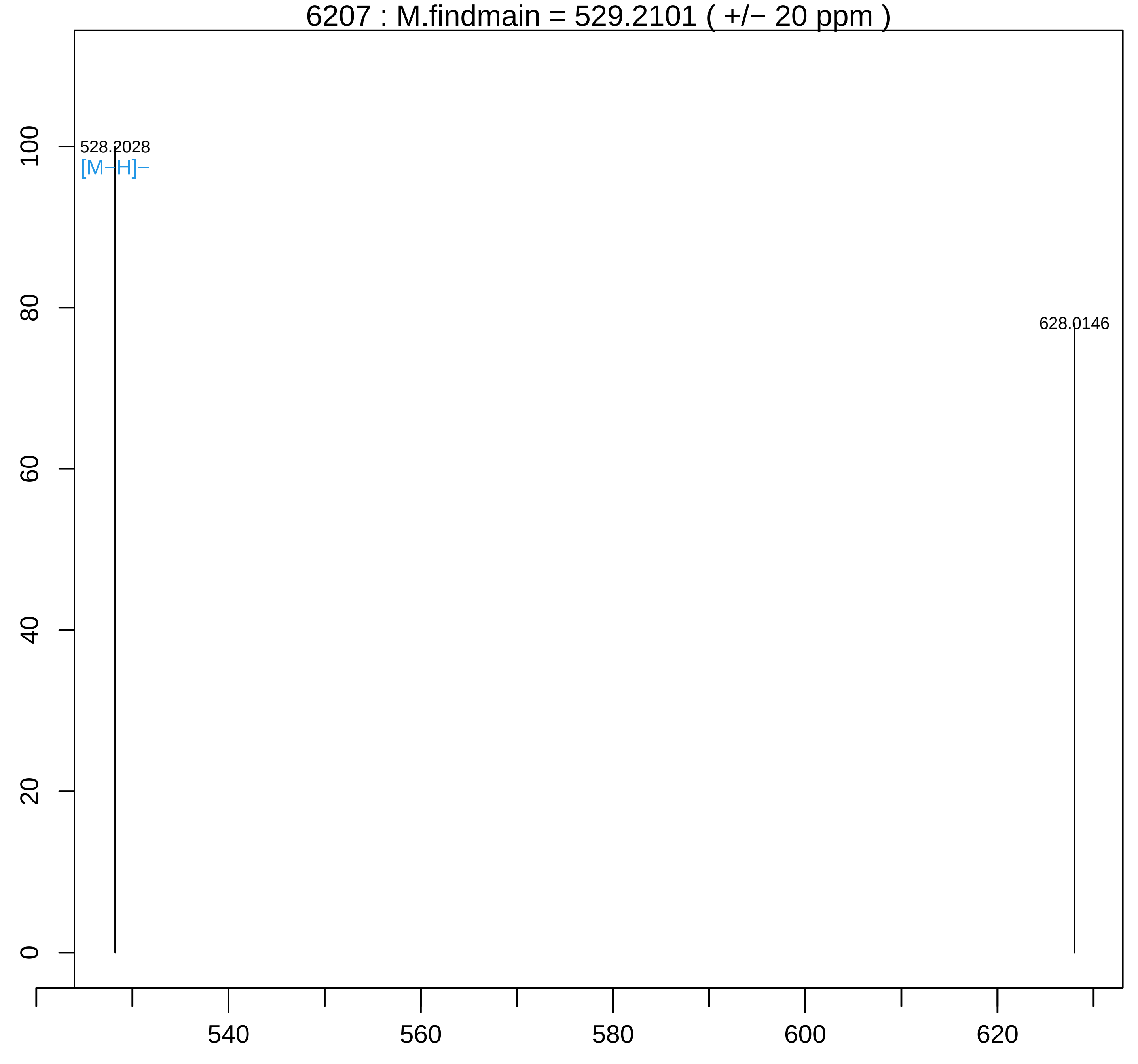
6207 : M.findmain = 529.2101 (+/− 20 ppm)
528.2028
[M−H]−
628.0146
0
20
40
60
80
100
540
560
580
600
620

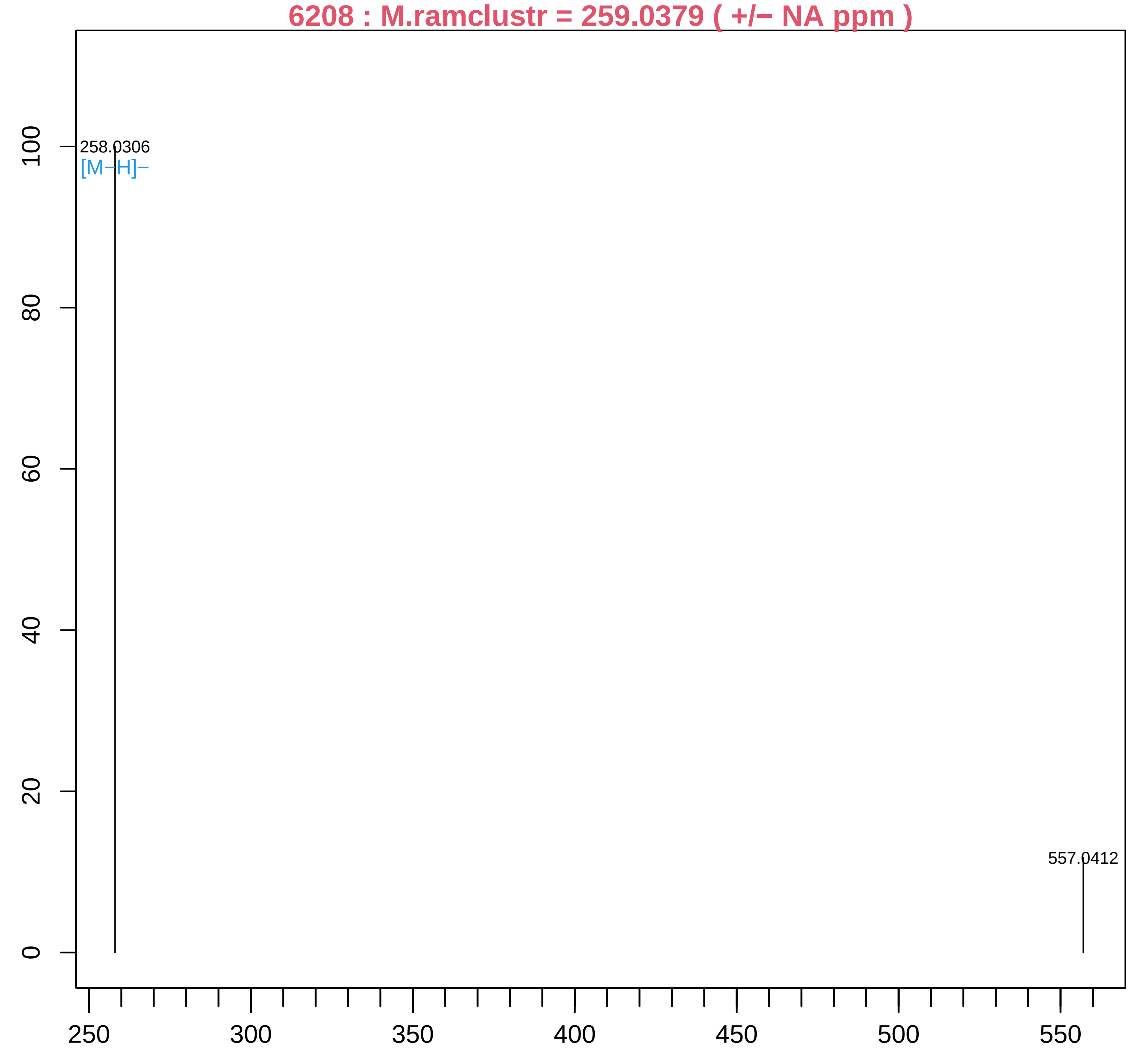
6208 : M.ramclustr = 259.0379 (+/− NA ppm)
258.0306
[M−H]−
557.0412
0
20
40
60
80
100
250
300
350
400
450
500
550

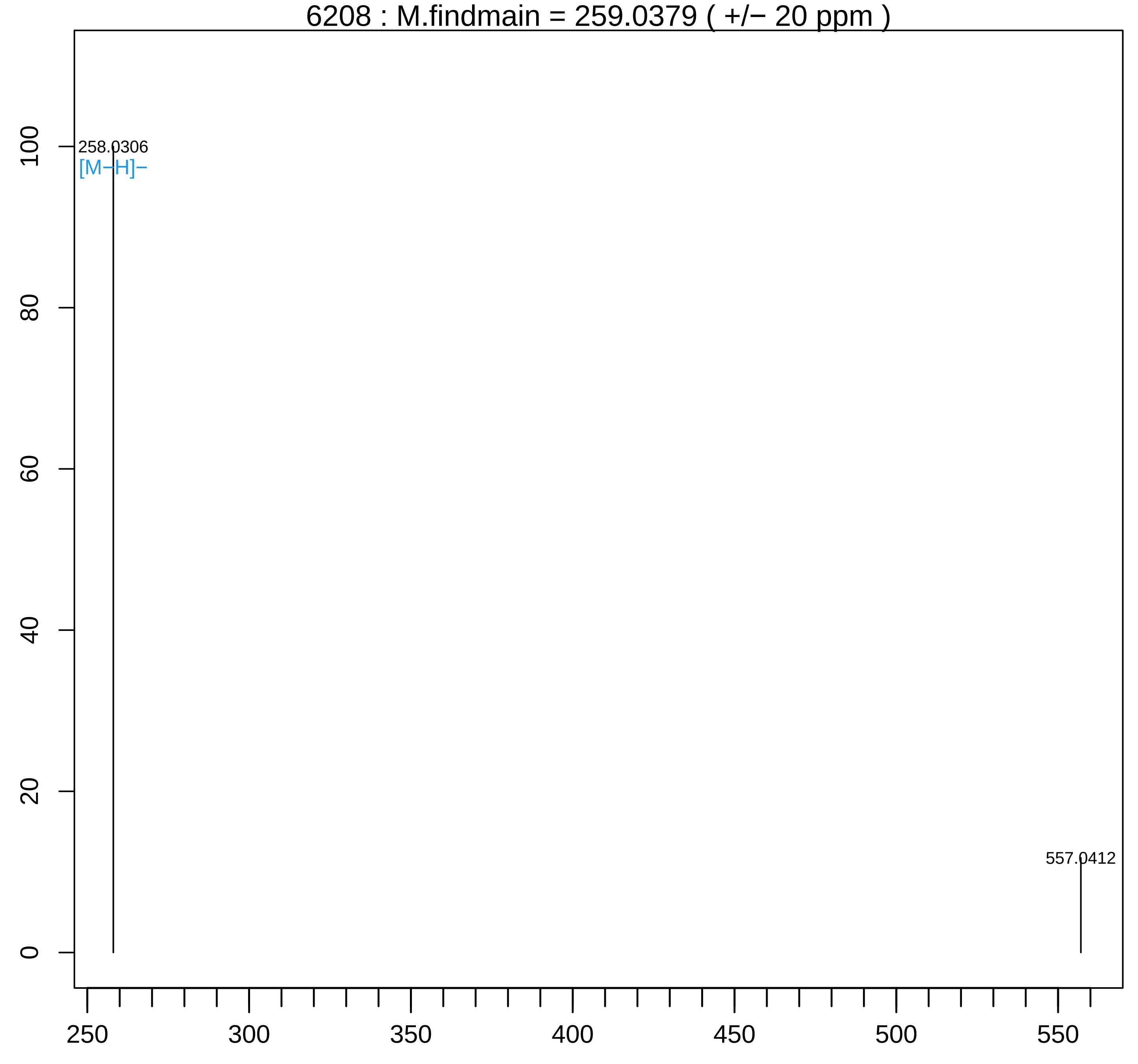
6208 : M.findmain = 259.0379 (+/− 20 ppm)
258.0306
[M−H]−
557.0412
0
20
40
60
80
100
250
300
350
400
450
500
550

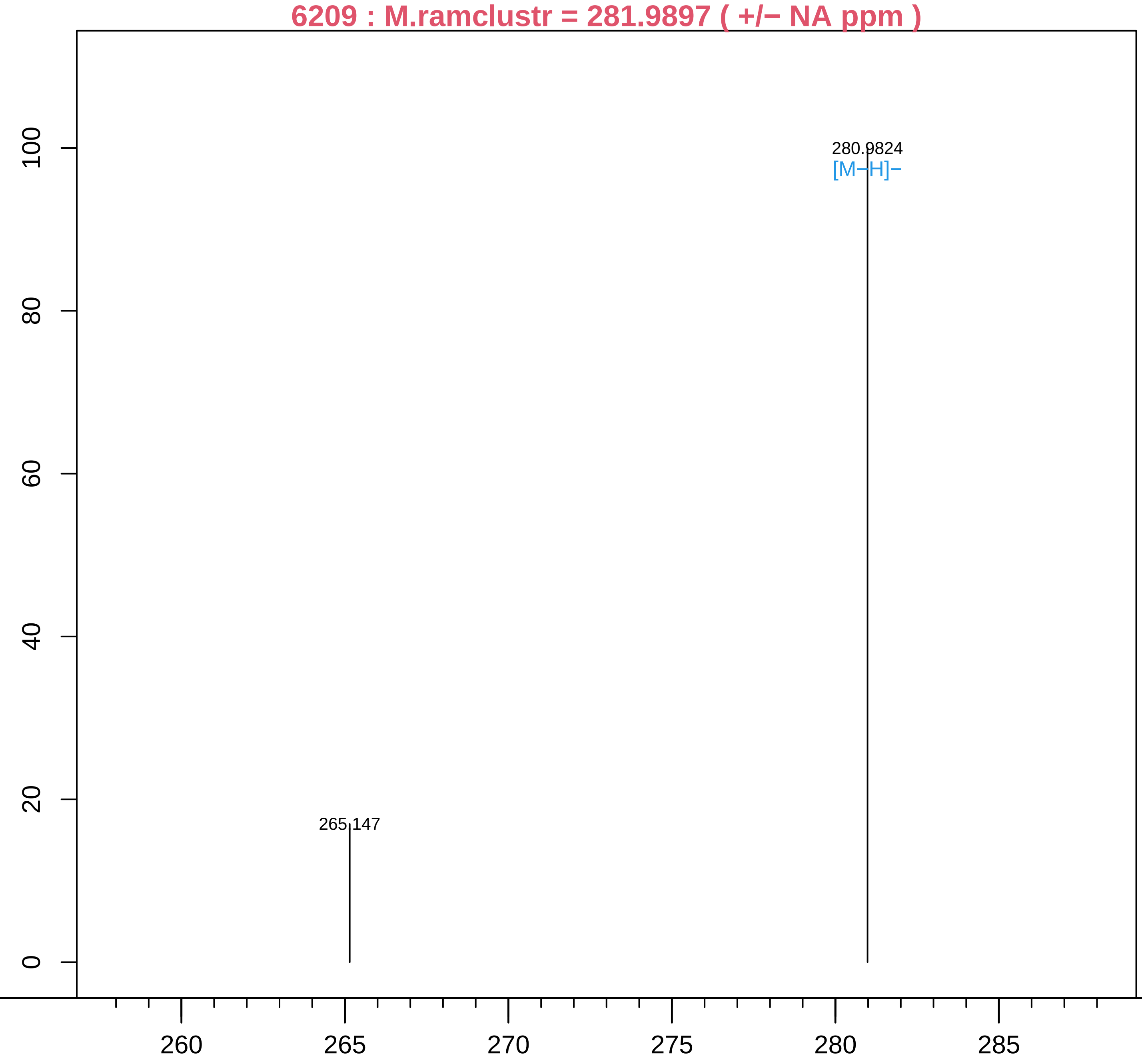

6209 : M.ramclustr = 281.9897 (+/− NA ppm)
280.9824
[M−H]−
265.147
0
20
40
60
80
100
260
265
270
275
280
285

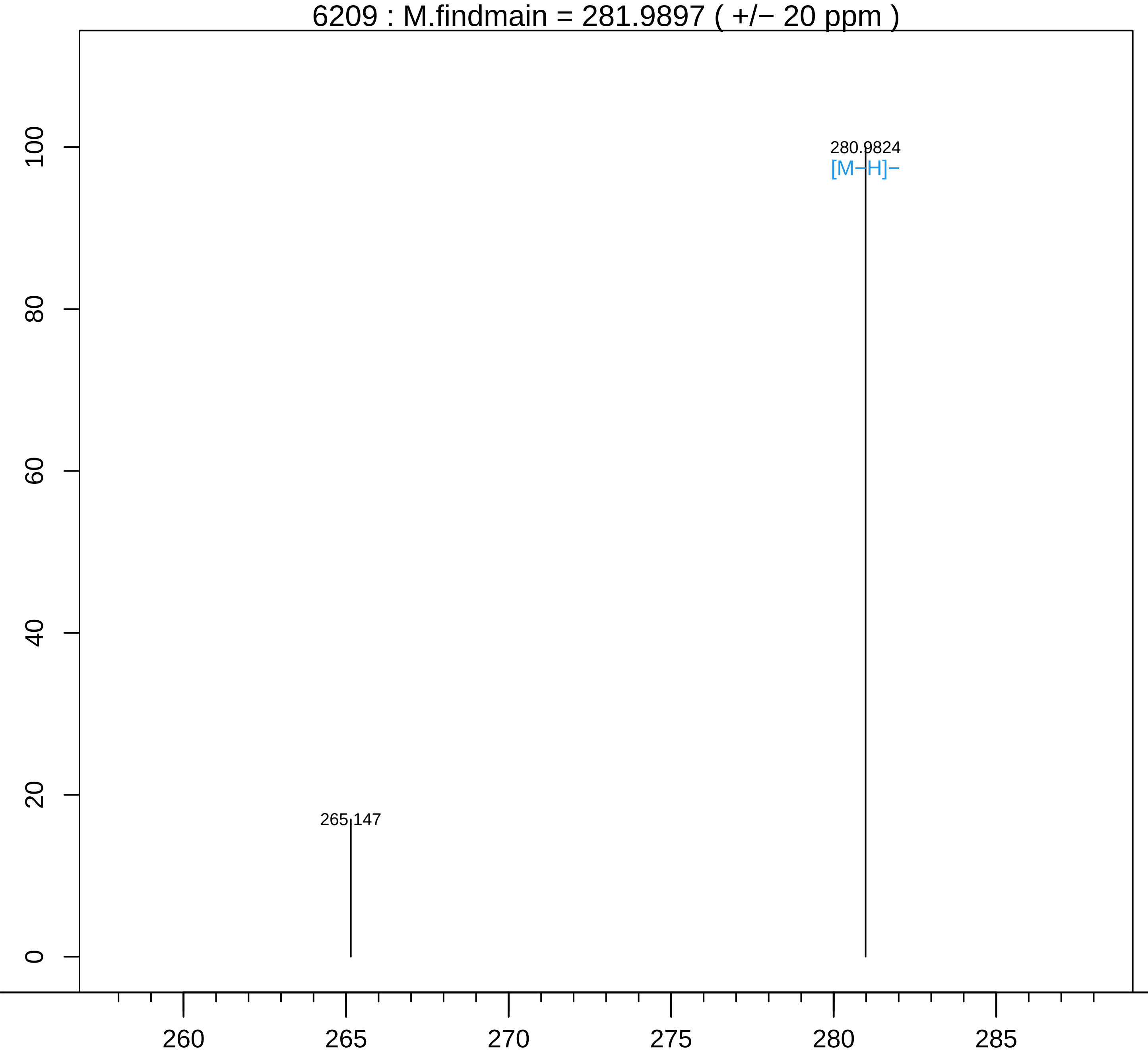

6209 : M.findmain = 281.9897 (+/− 20 ppm)
280.9824
[M−H]−
265.147
0
20
40
60
80
100
260
265
270
275
280
285

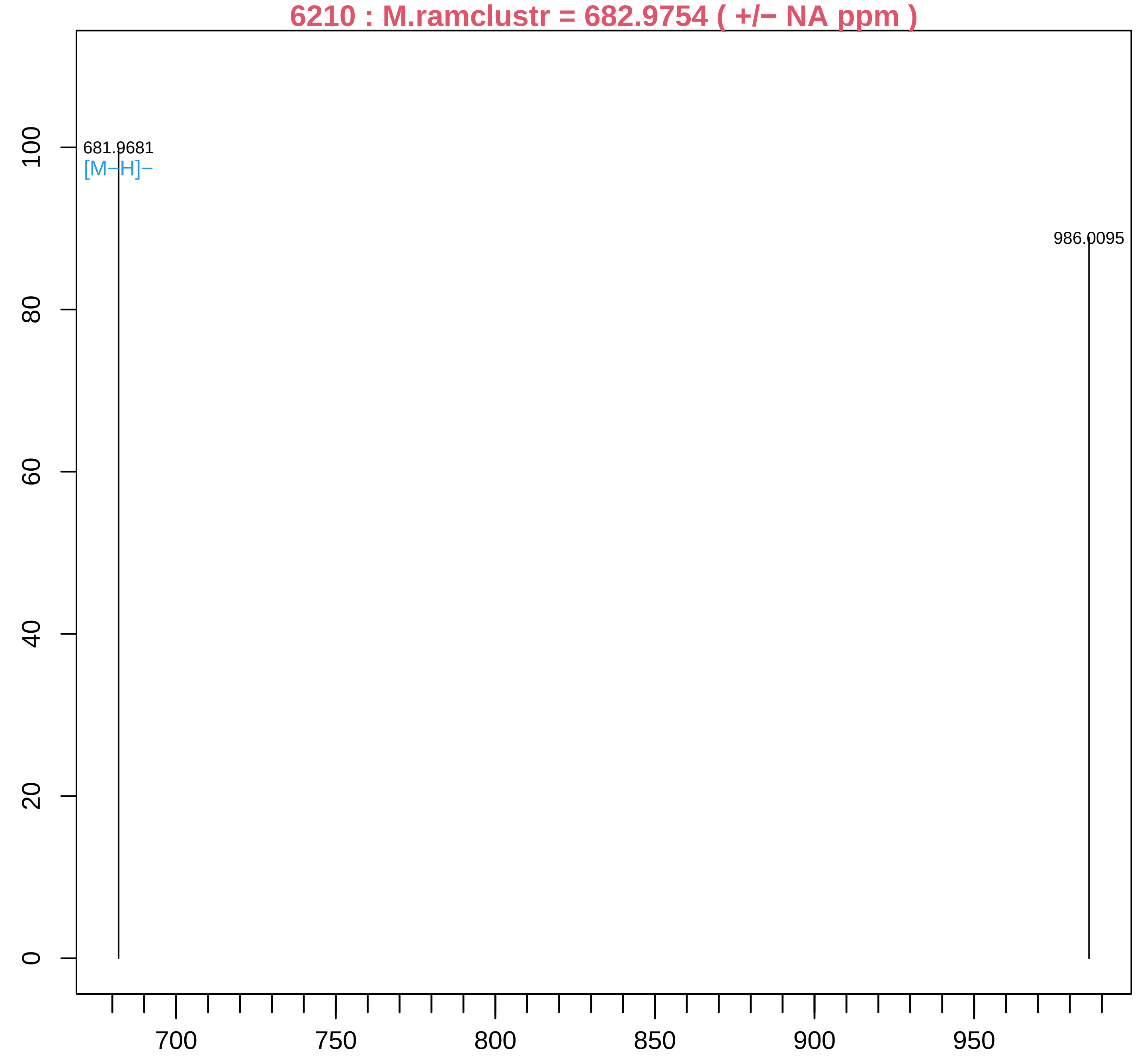
6210 : M.ramclustr = 682.9754 (+/− NA ppm)
681.9681
[M−H]−
986.0095
0
20
40
60
80
100
700
750
800
850
900
950

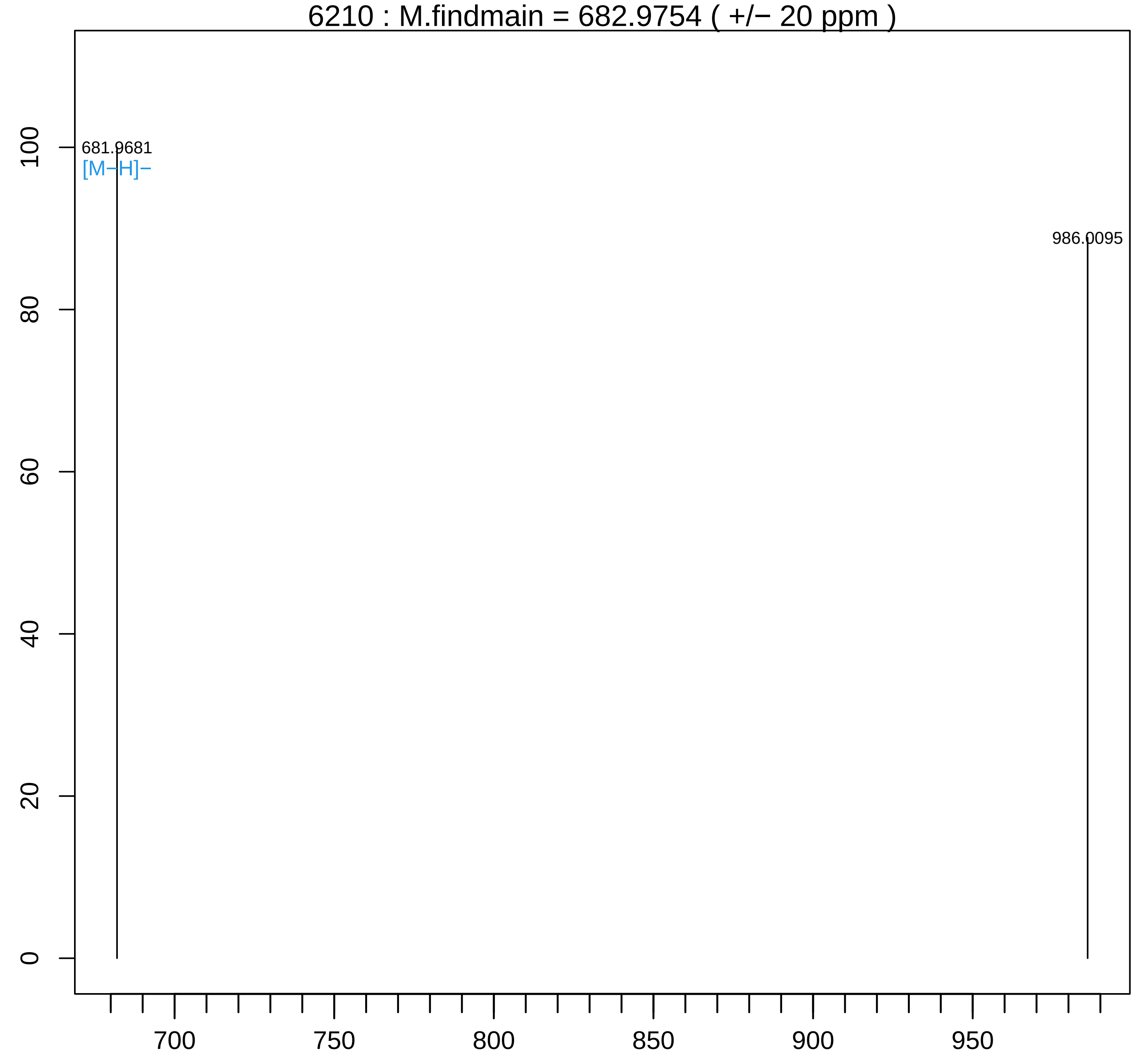
6210 : M.findmain = 682.9754 (+/− 20 ppm)
681.9681
[M−H]−
986.0095
0
20
40
60
80
100
700
750
800
850
900
950

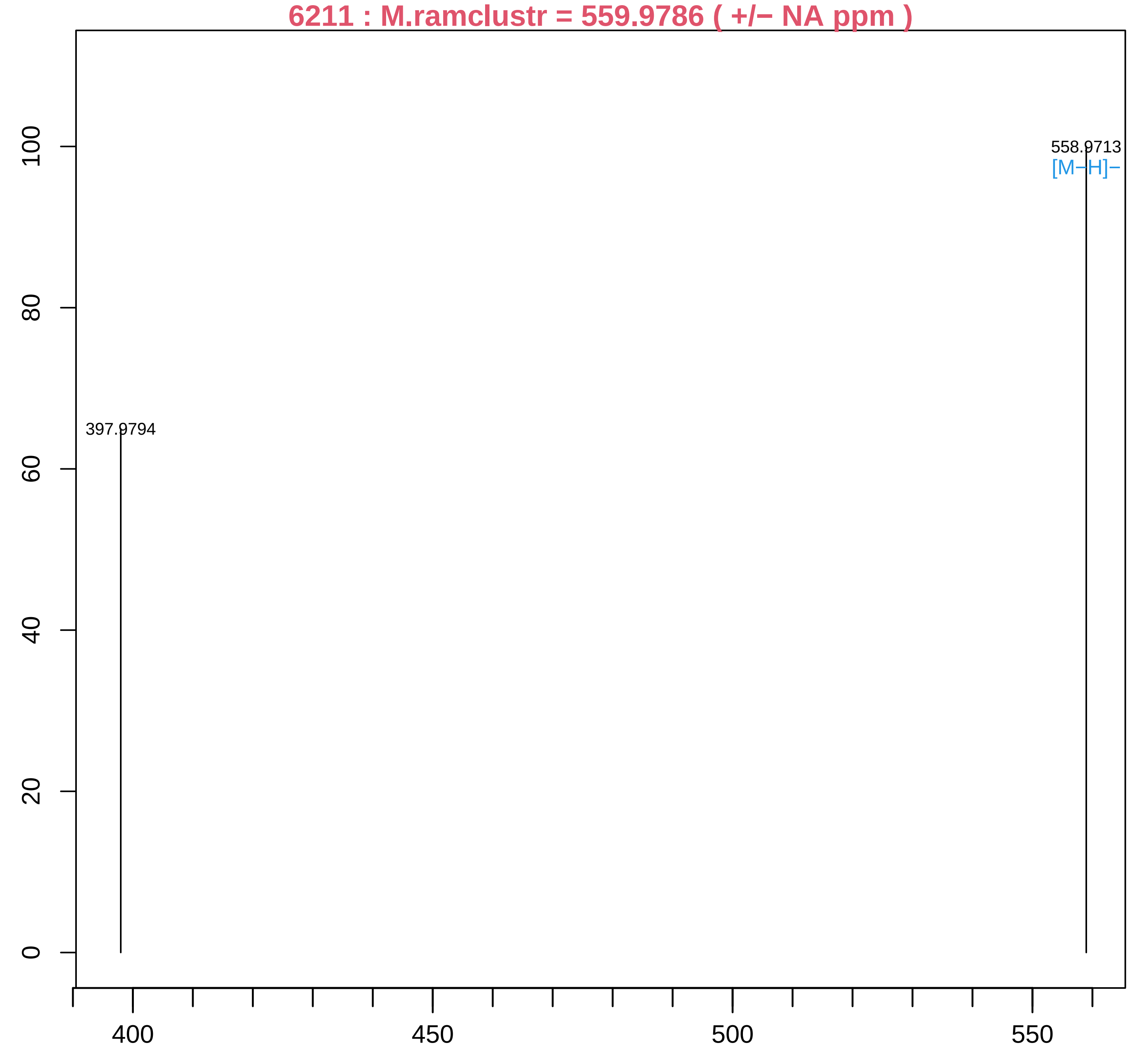
6211 : M.ramclustr = 559.9786 (+/− NA ppm)
558.9713
[M−H]−
397.9794
0
20
40
60
80
100
400
450
500
550

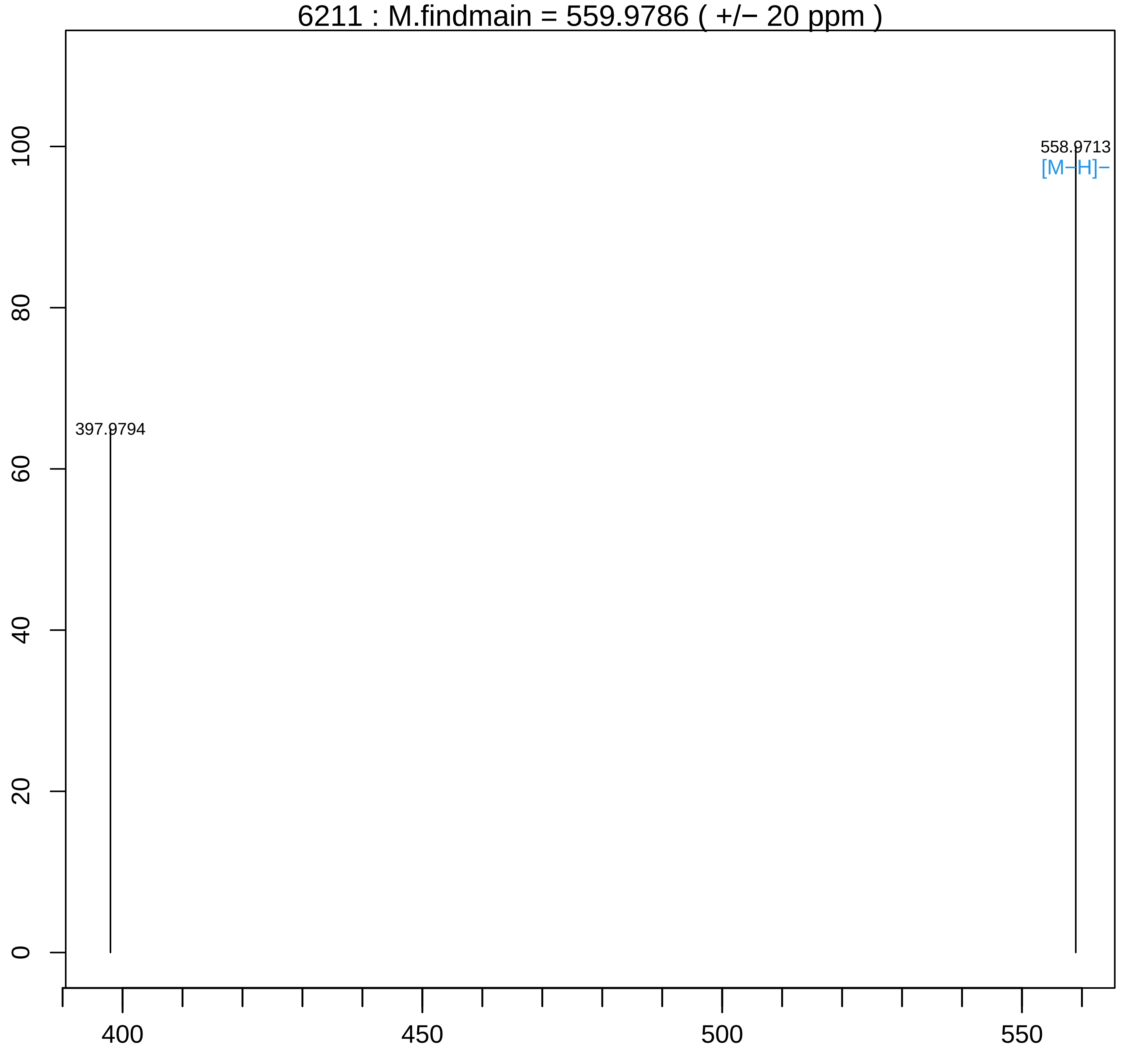
6211 : M.findmain = 559.9786 (+/− 20 ppm)
558.9713
[M−H]−
397.9794
0
20
40
60
80
100
400
450
500
550

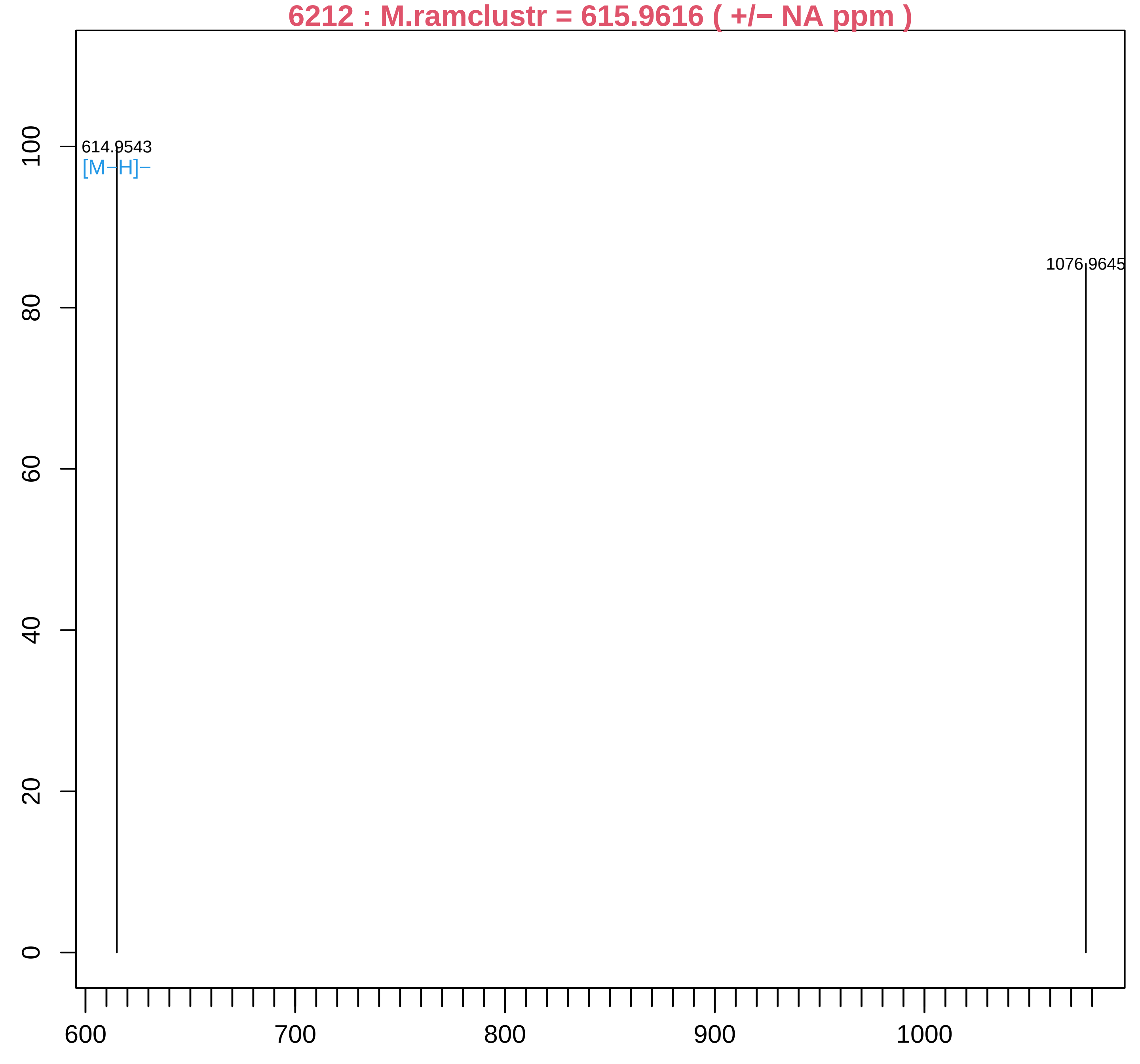
6212 : M.ramclustr = 615.9616 (+/− NA ppm)
614.9543
[M−H]−
1076.9645
0
20
40
60
80
100
600
700
800
900
1000

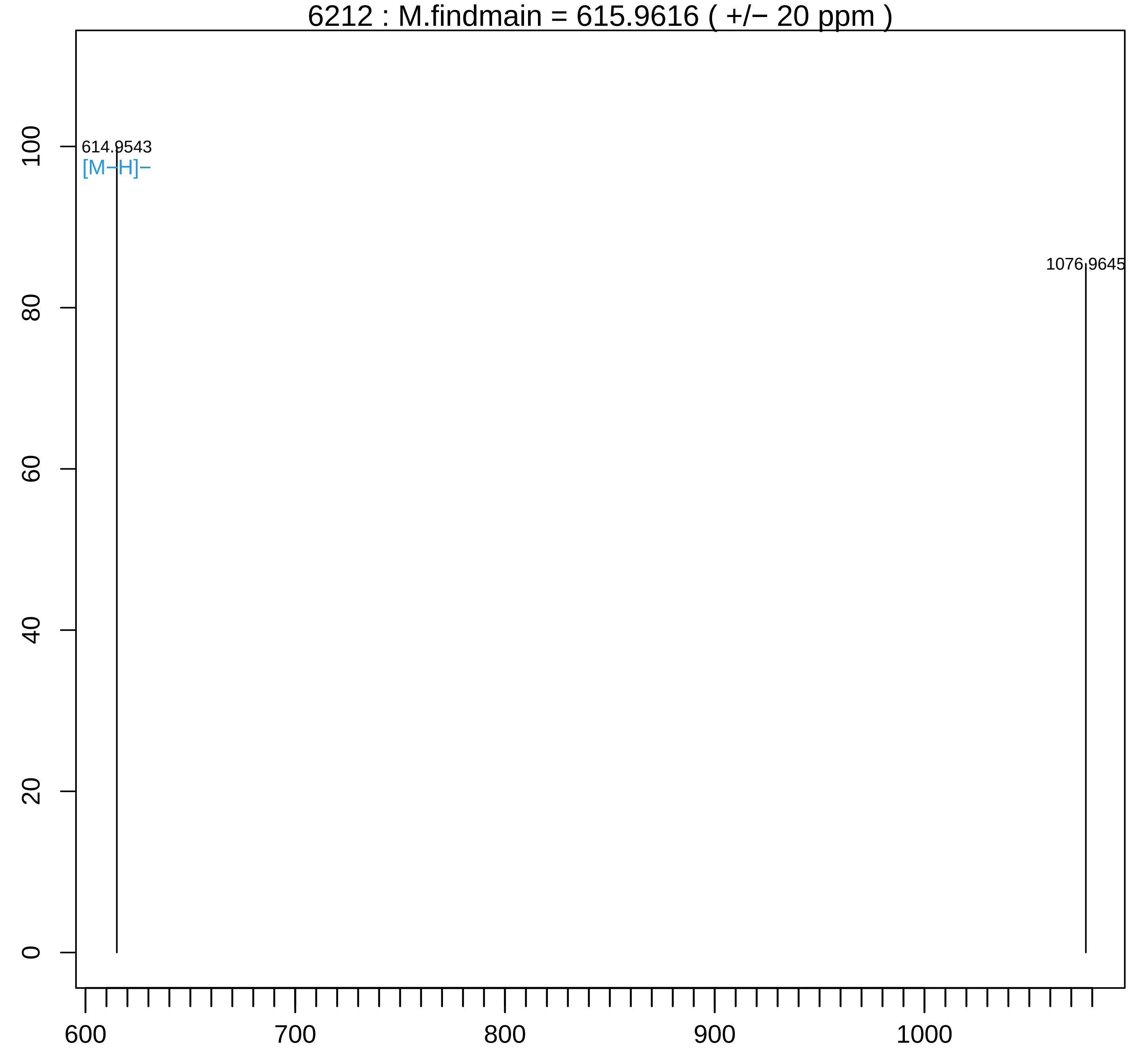
6212 : M.findmain = 615.9616 (+/− 20 ppm)
614.9543
[M−H]−
1076.9645
0
20
40
60
80
100
600
700
800
900
1000

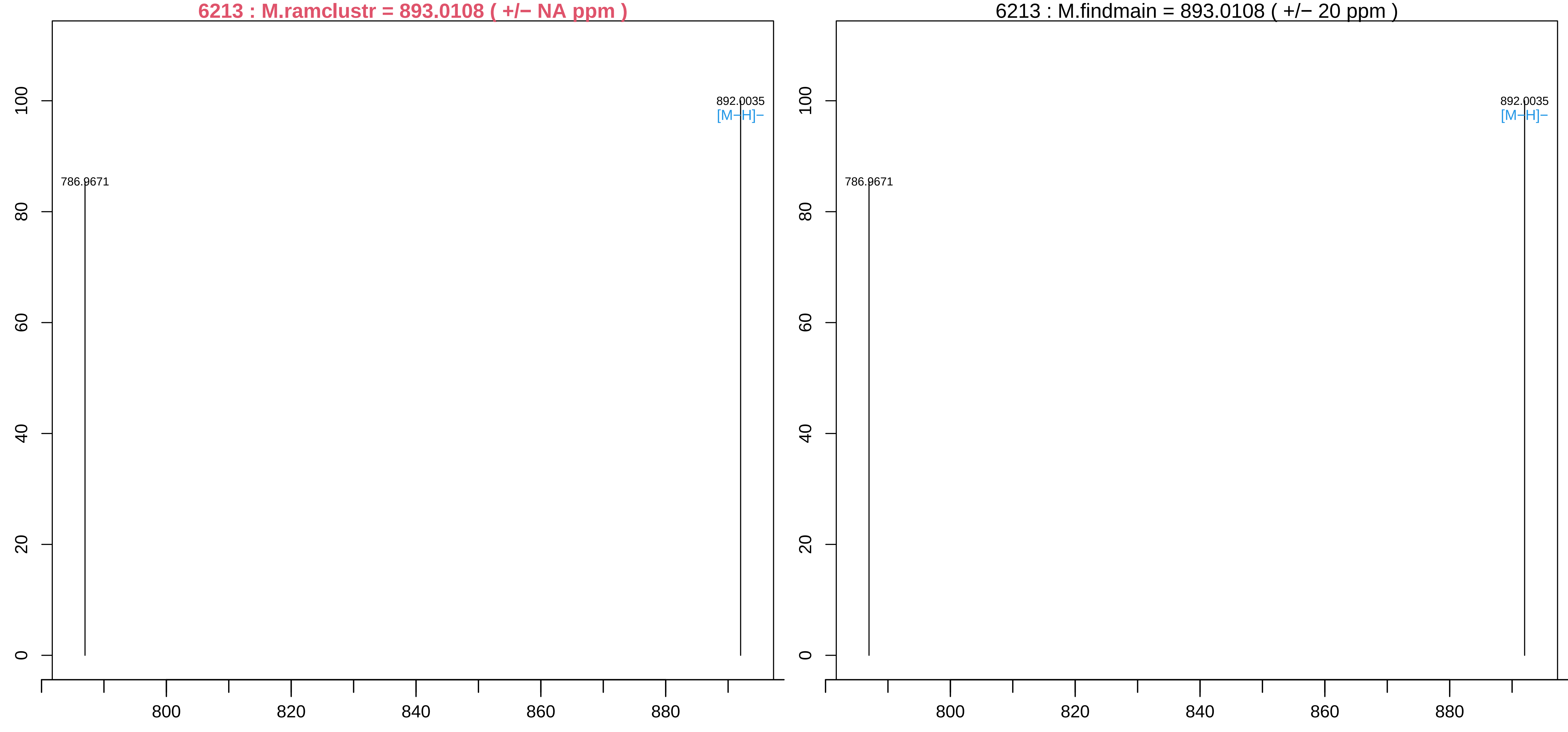
6213 : M.ramclustr = 893.0108 (+/− NA ppm)
6213 : M.findmain = 893.0108 (+/− 20 ppm)
786.9671
892.0035
[M−H]−
0
20
40
60
80
100
800
820
840
860
880

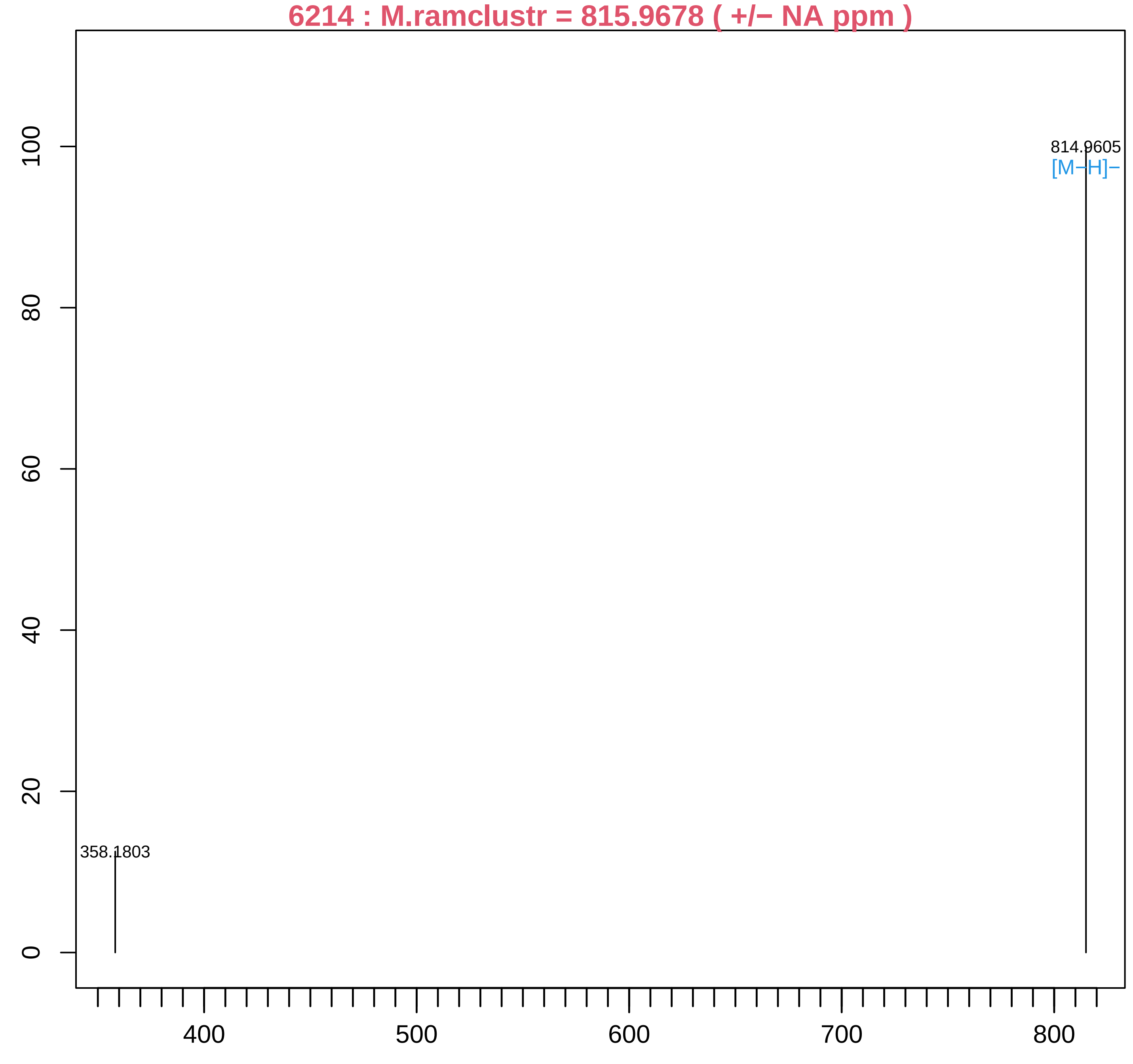
6214 : M.ramclustr = 815.9678 (+/− NA ppm)
814.9605
[M−H]−
358.1803
0
20
40
60
80
100
400
500
600
700
800

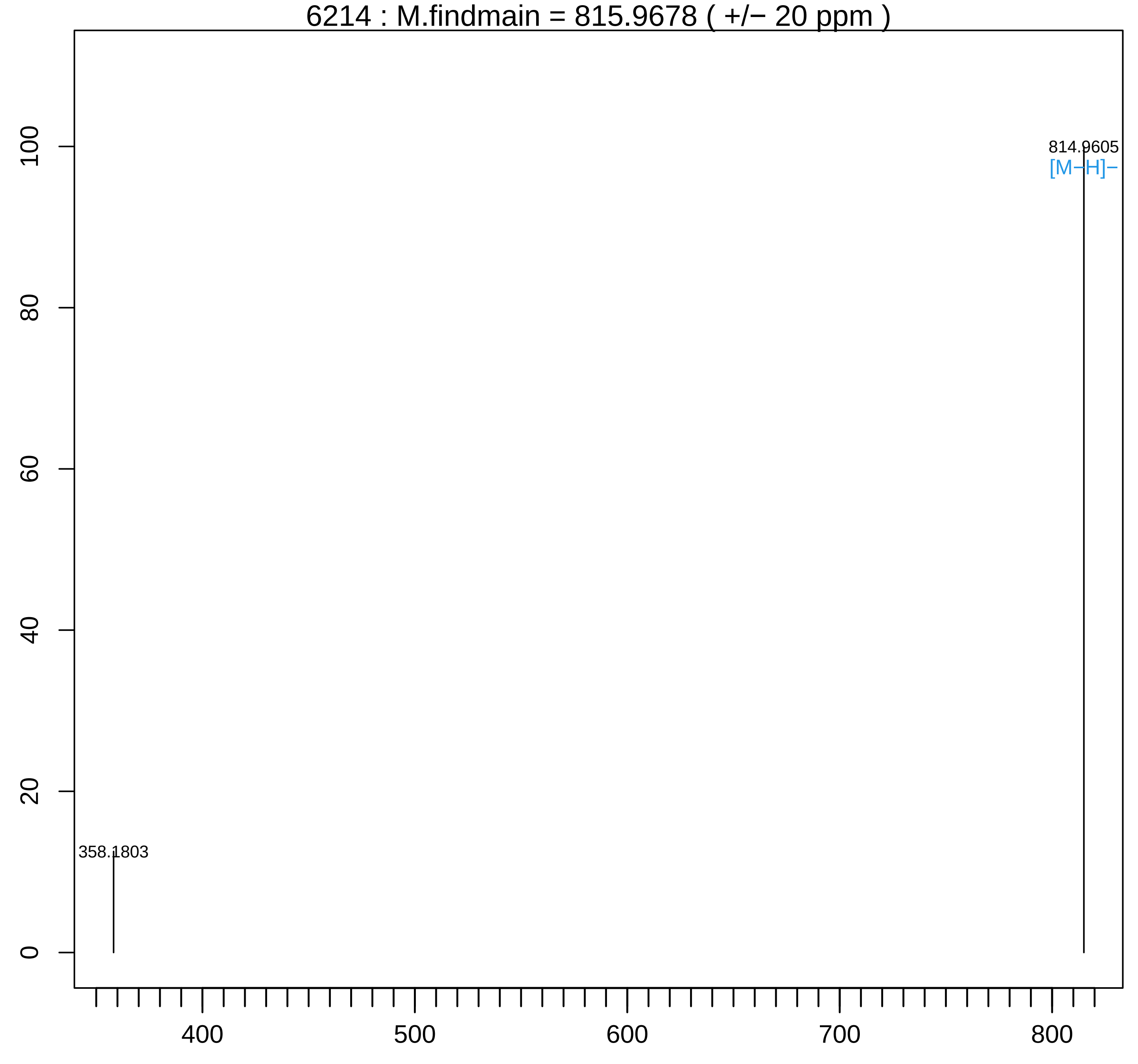
6214 : M.findmain = 815.9678 (+/− 20 ppm)
814.9605
[M−H]−
358.1803
0
20
40
60
80
100
400
500
600
700
800

6215 : M.ramclustr = 578.9856 (+/− NA ppm)

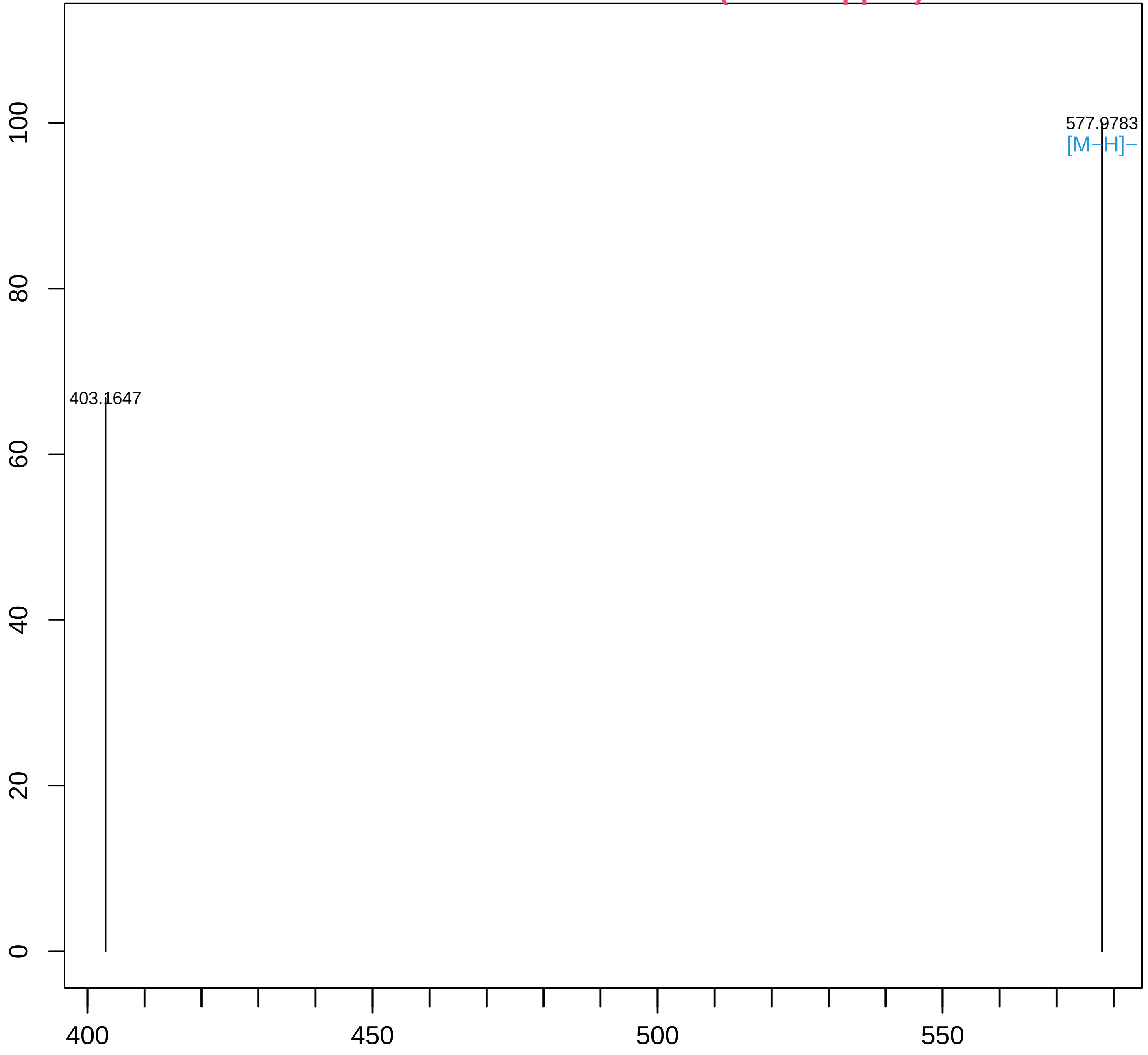

6215 : M.findmain = 578.9856 (+/− 20 ppm)

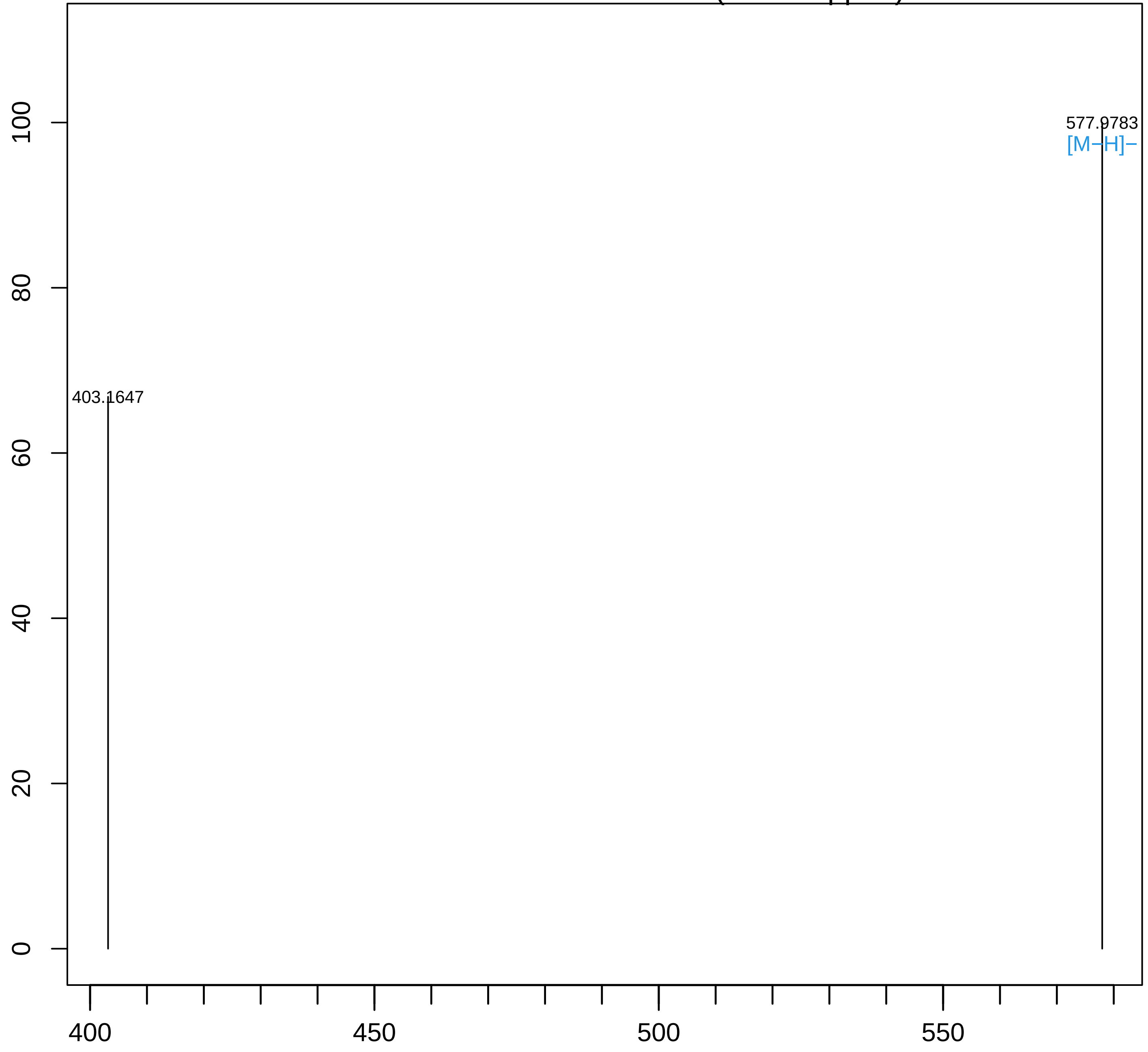

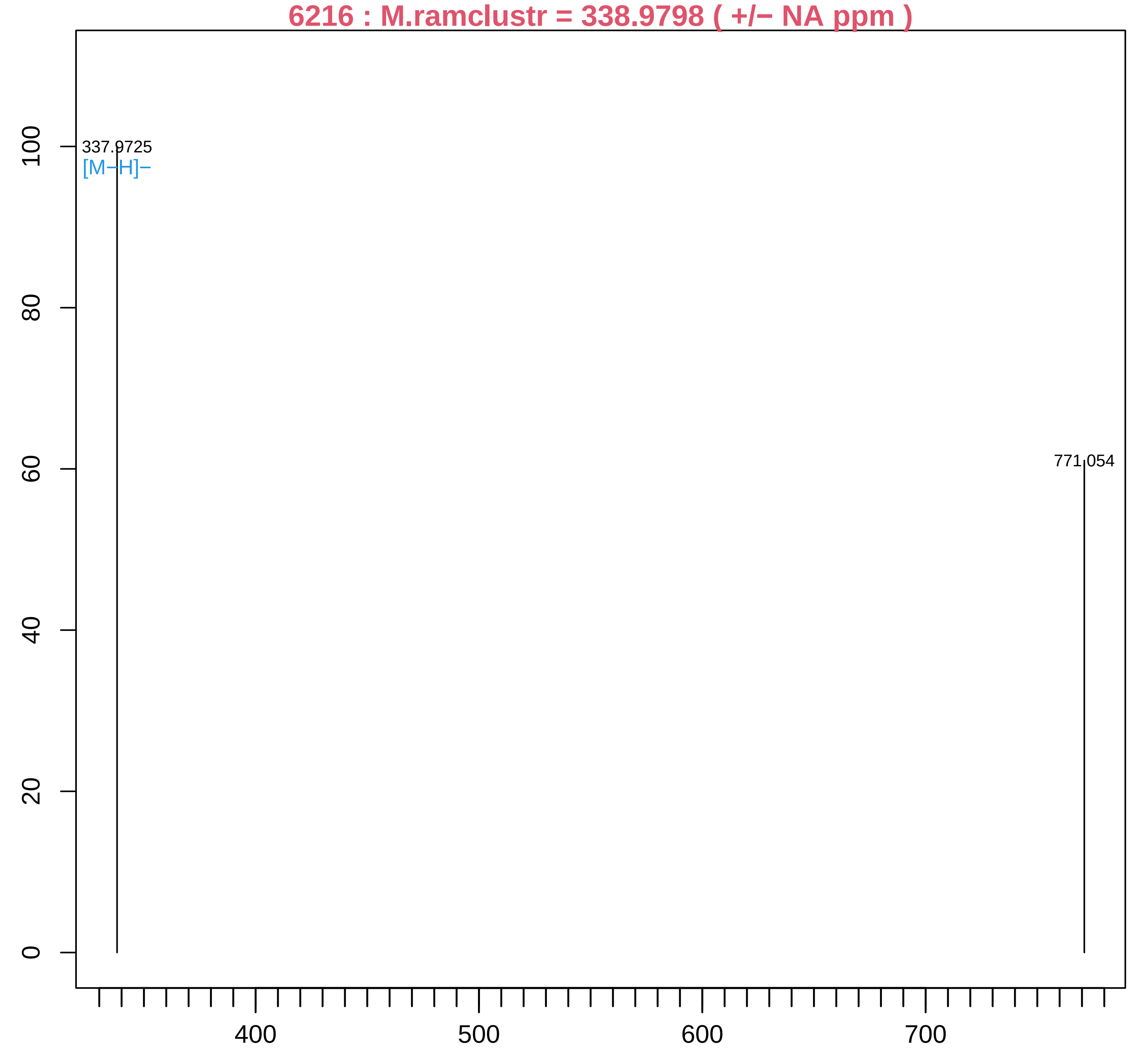
6216 : M.ramclustr = 338.9798 (+/− NA ppm)
337.9725
[M−H]−
771.054
0
20
40
60
80
100
400
500
600
700

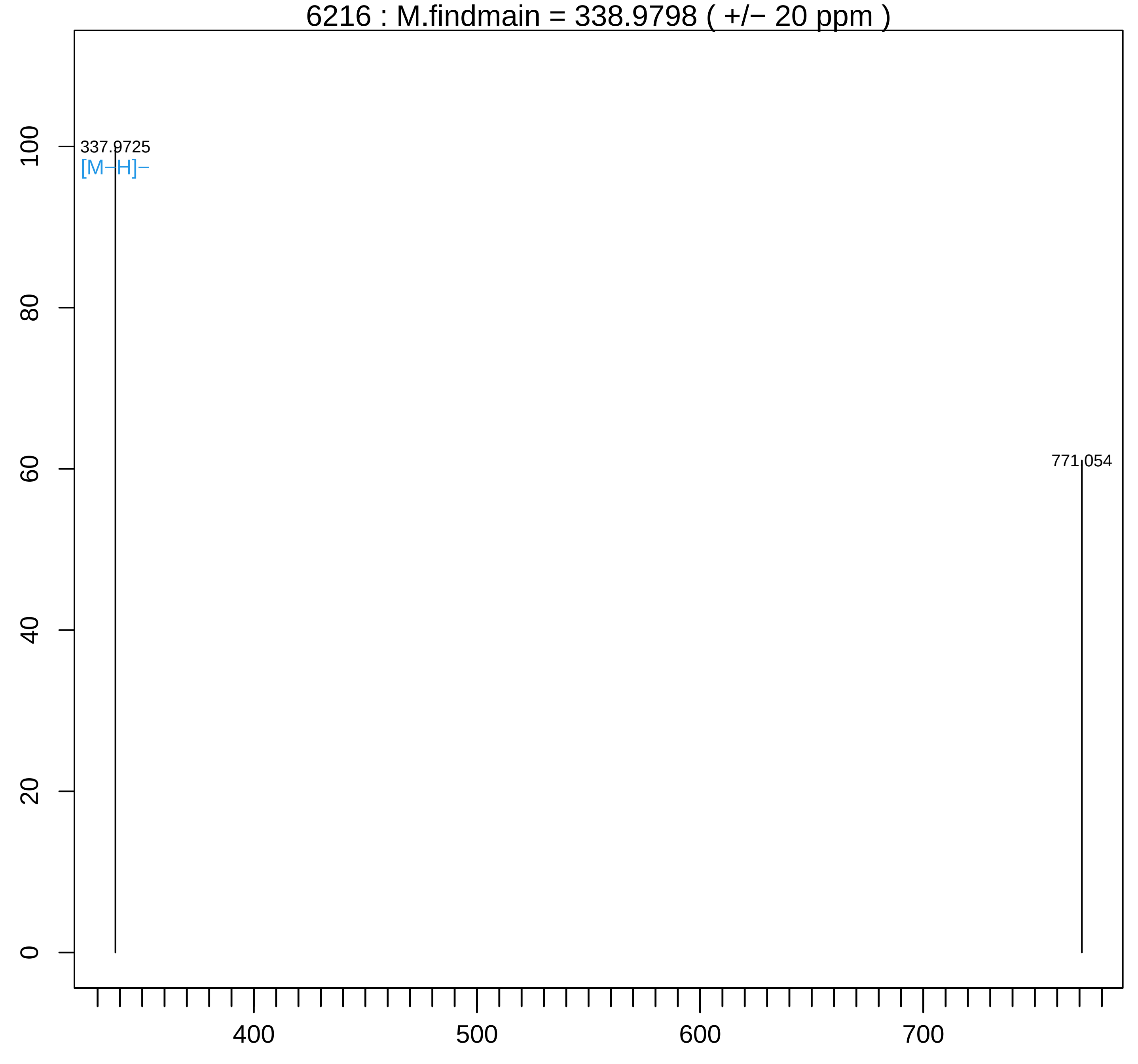
6216 : M.findmain = 338.9798 (+/− 20 ppm)
337.9725
[M−H]−
771.054
0
20
40
60
80
100
400
500
600
700

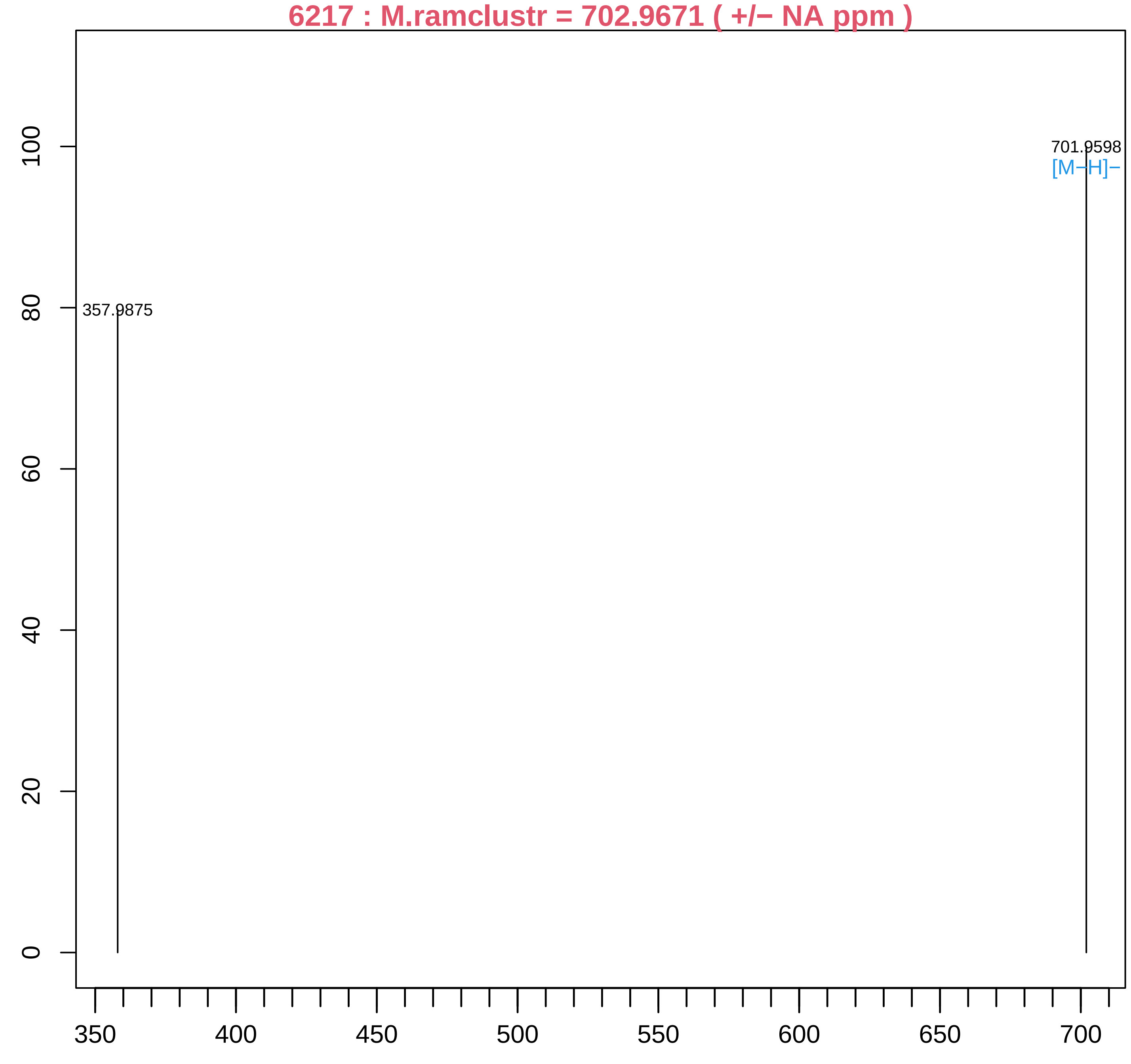
6217 : M.ramclustr = 702.9671 (+/− NA ppm)
701.9598
[M−H]−
357.9875
0
20
40
60
80
100
350
400
450
500
550
600
650
700

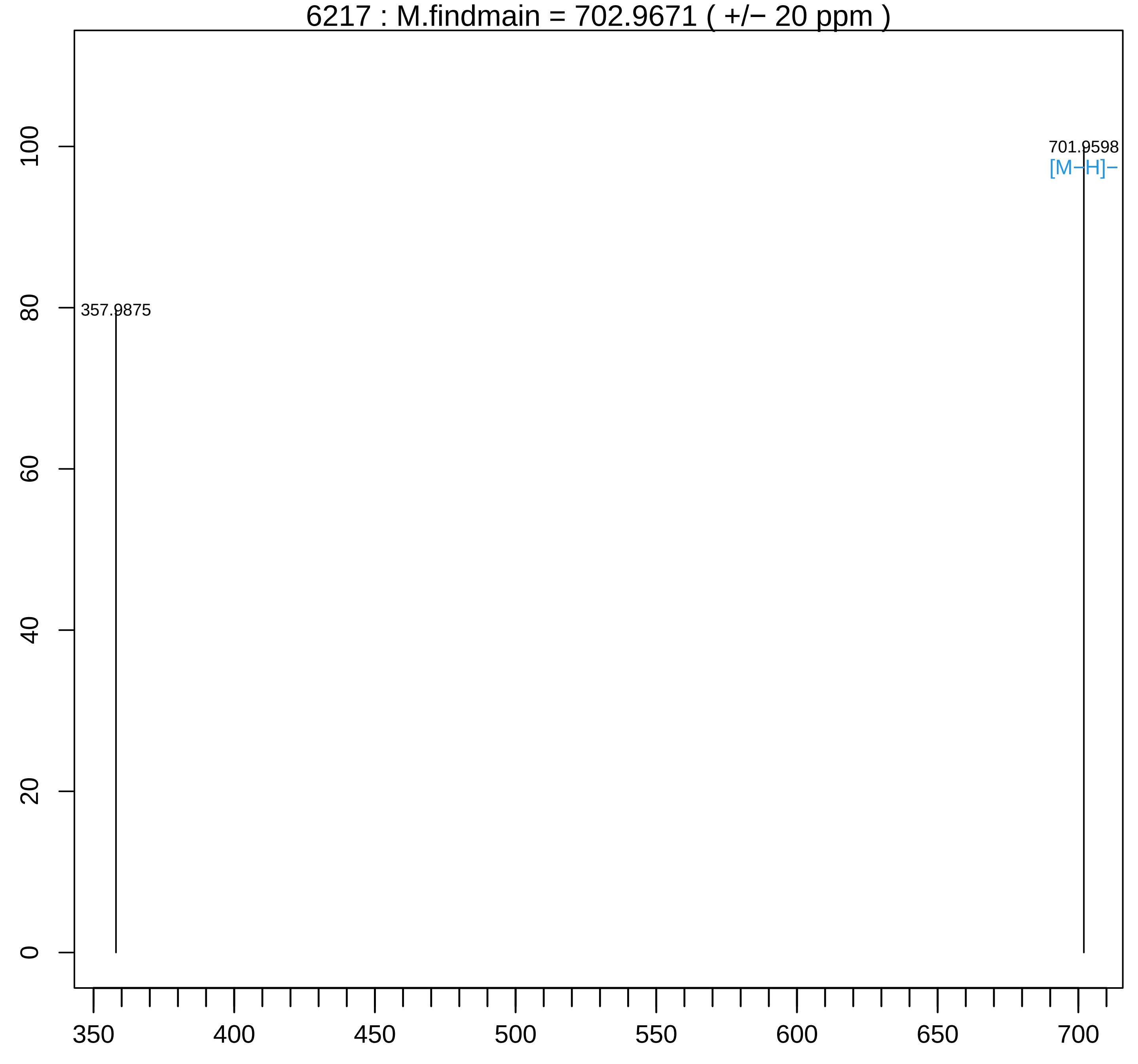
6217 : M.findmain = 702.9671 (+/− 20 ppm)
701.9598
[M−H]−
357.9875
0
20
40
60
80
100
350
400
450
500
550
600
650
700

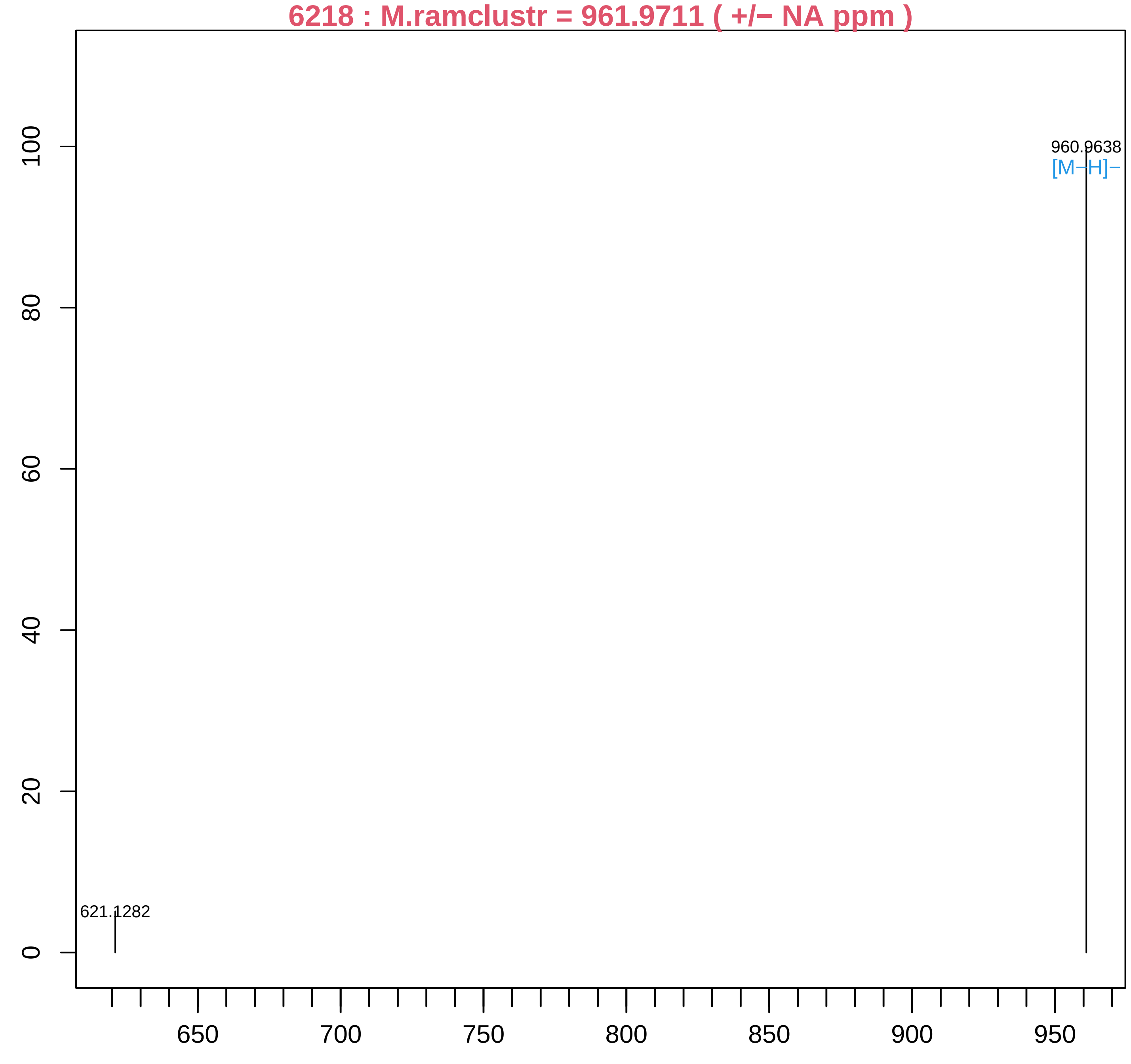
6218 : M.ramclustr = 961.9711 (+/− NA ppm)
960.9638
[M−H]−
621.1282
0
20
40
60
80
100
650
700
750
800
850
900
950

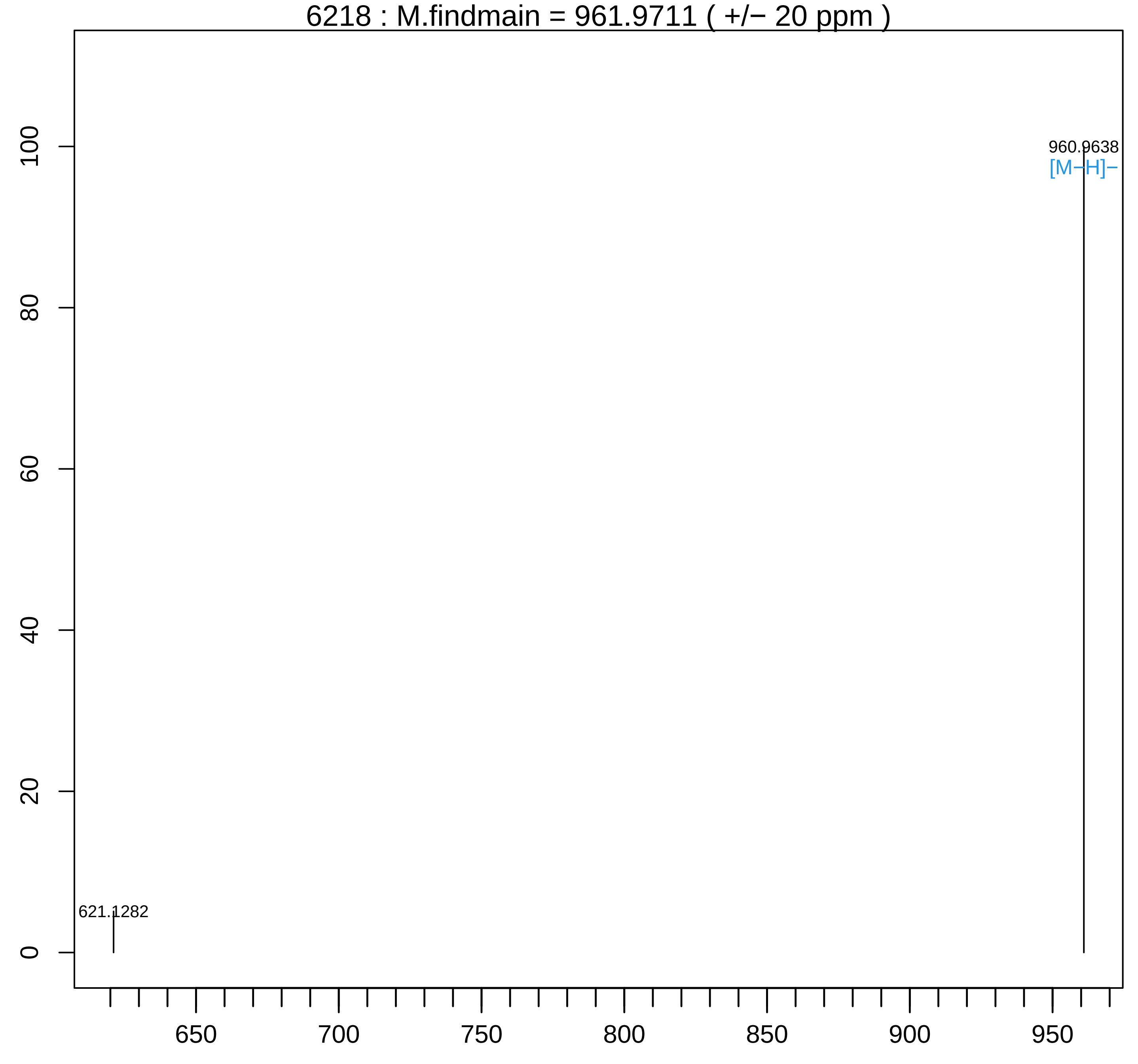
6218 : M.findmain = 961.9711 (+/− 20 ppm)
960.9638
[M−H]−
621.1282
0
20
40
60
80
100
650
700
750
800
850
900
950

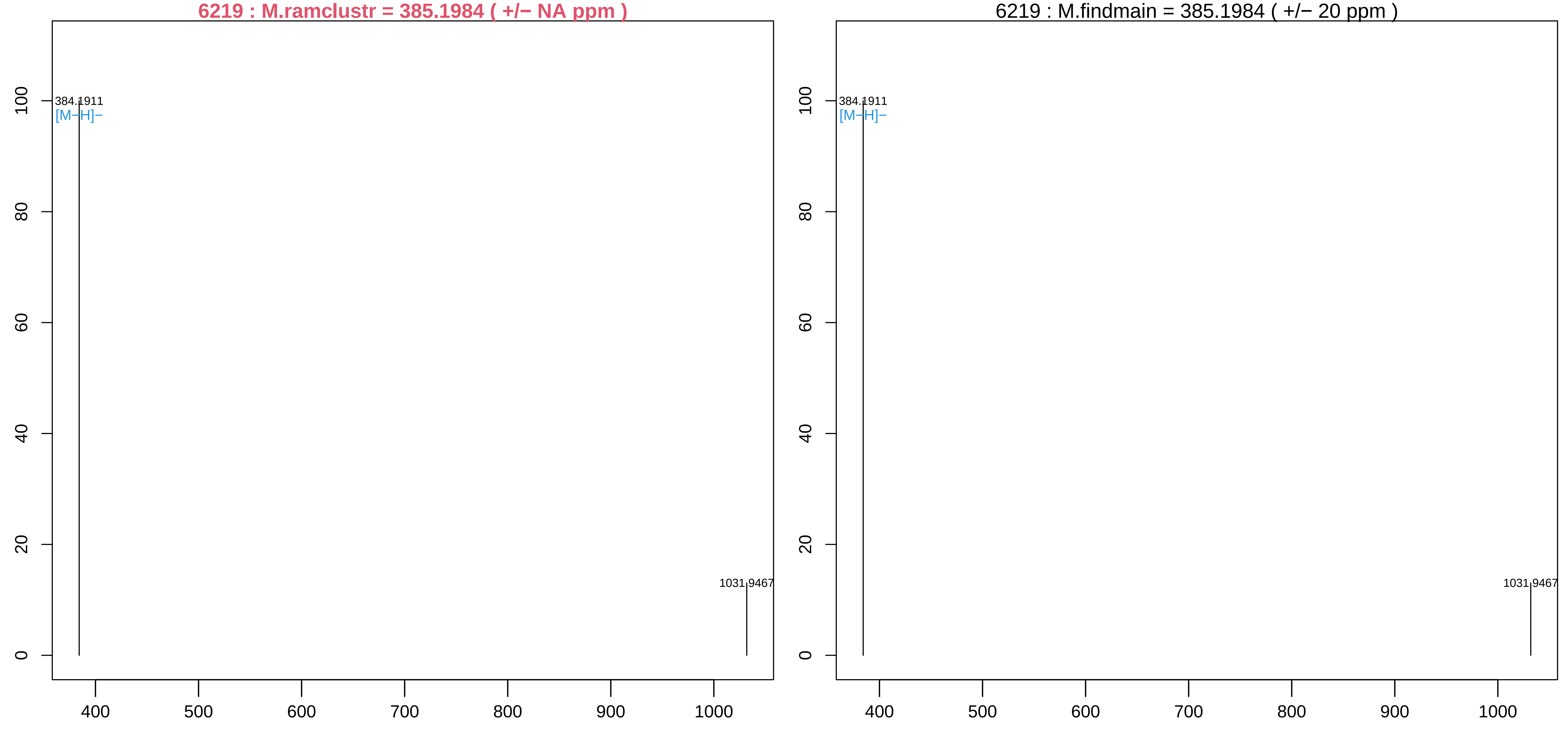
6219 : M.ramclustr = 385.1984 (+/− NA ppm)
6219 : M.findmain = 385.1984 (+/− 20 ppm)
384.1911
[M−H]−
1031.9467
384.1911
[M−H]−
1031.9467
0
20
40
60
80
100
400
500
600
700
800
900
1000

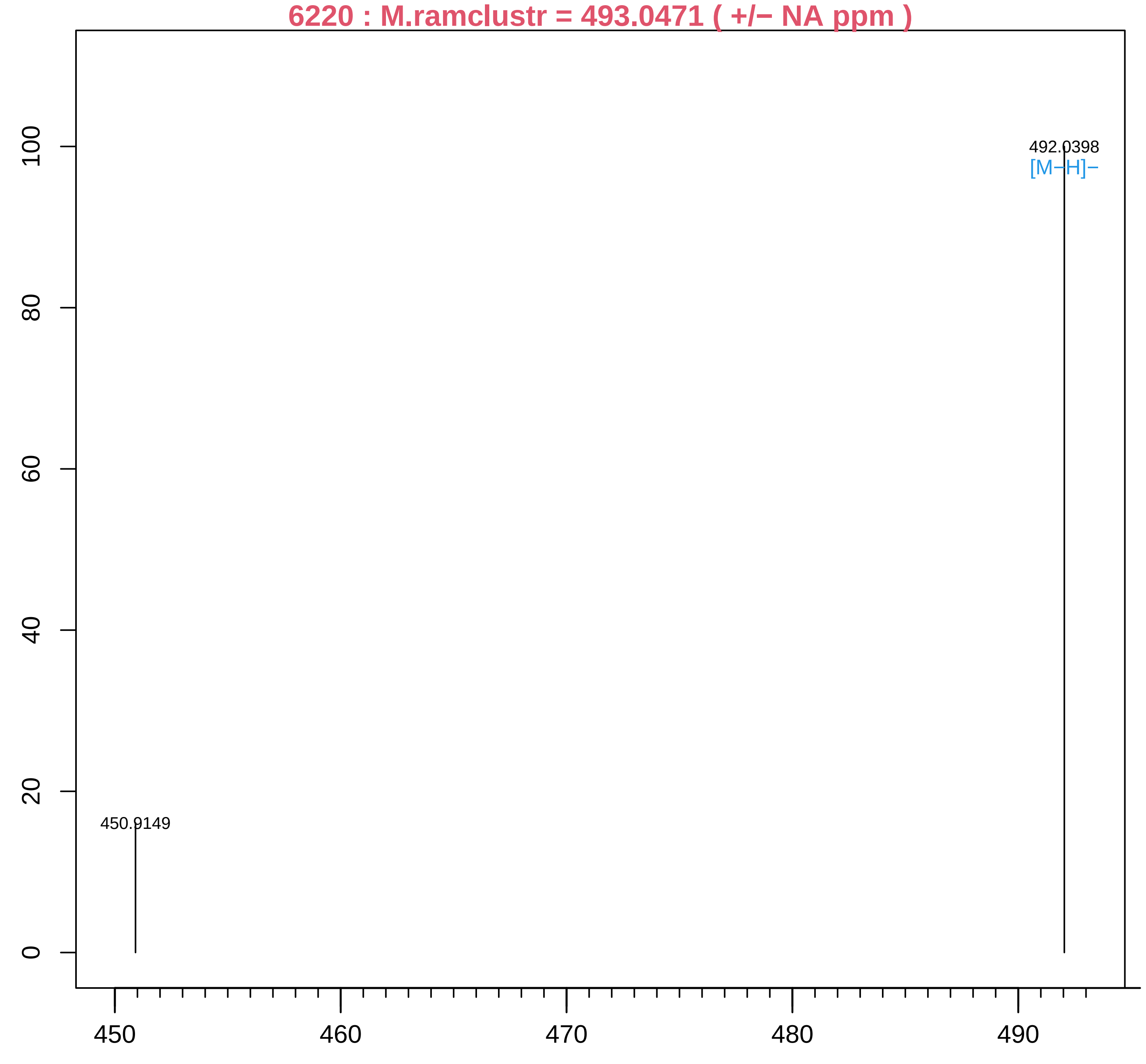
6220 : M.ramclustr = 493.0471 (+/− NA ppm)
492.0398
[M−H]−
450.9149
0
20
40
60
80
100
450
460
470
480
490

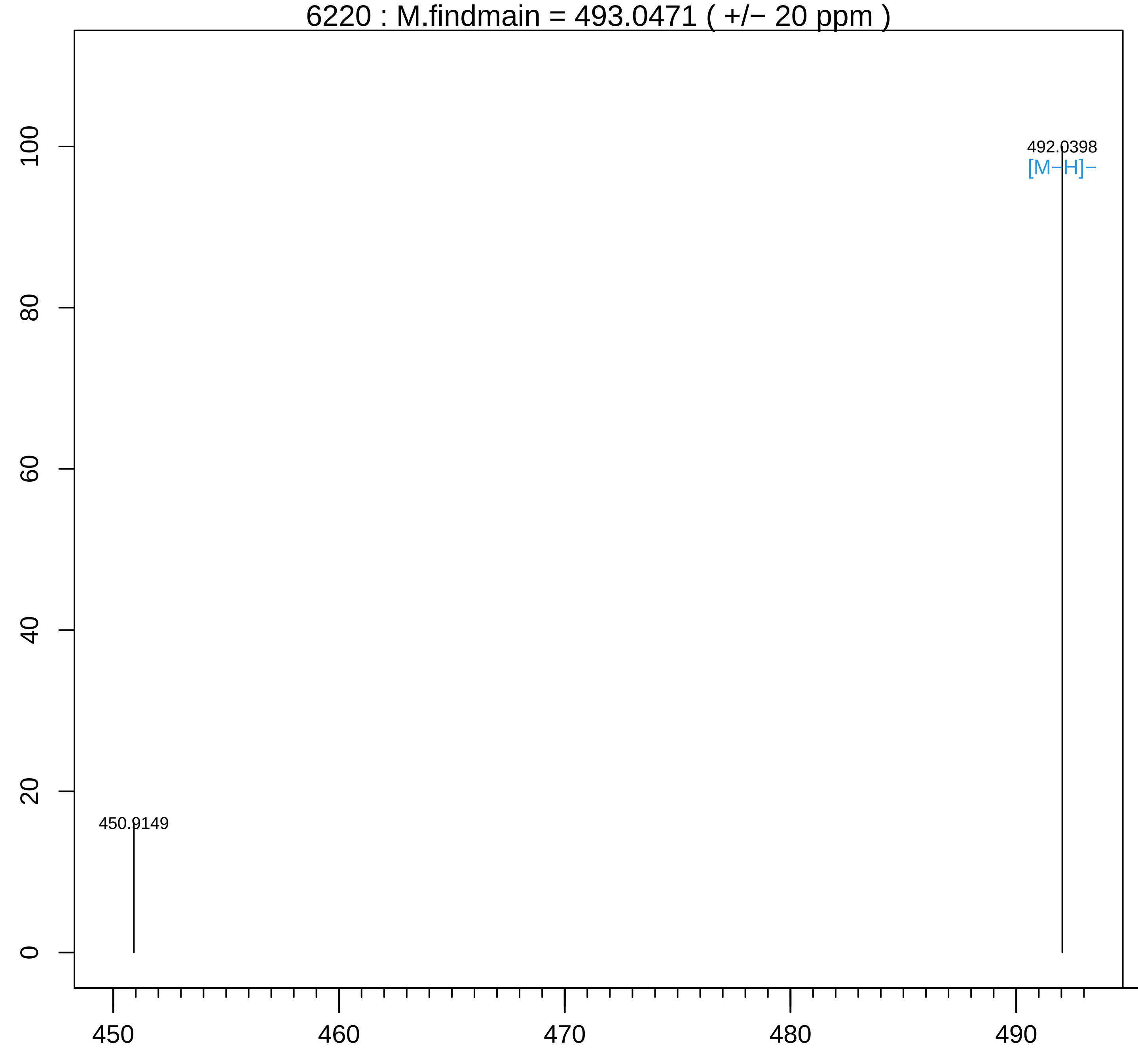
6220 : M.findmain = 493.0471 (+/− 20 ppm)
492.0398
[M−H]−
450.9149
0
20
40
60
80
100
450
460
470
480
490

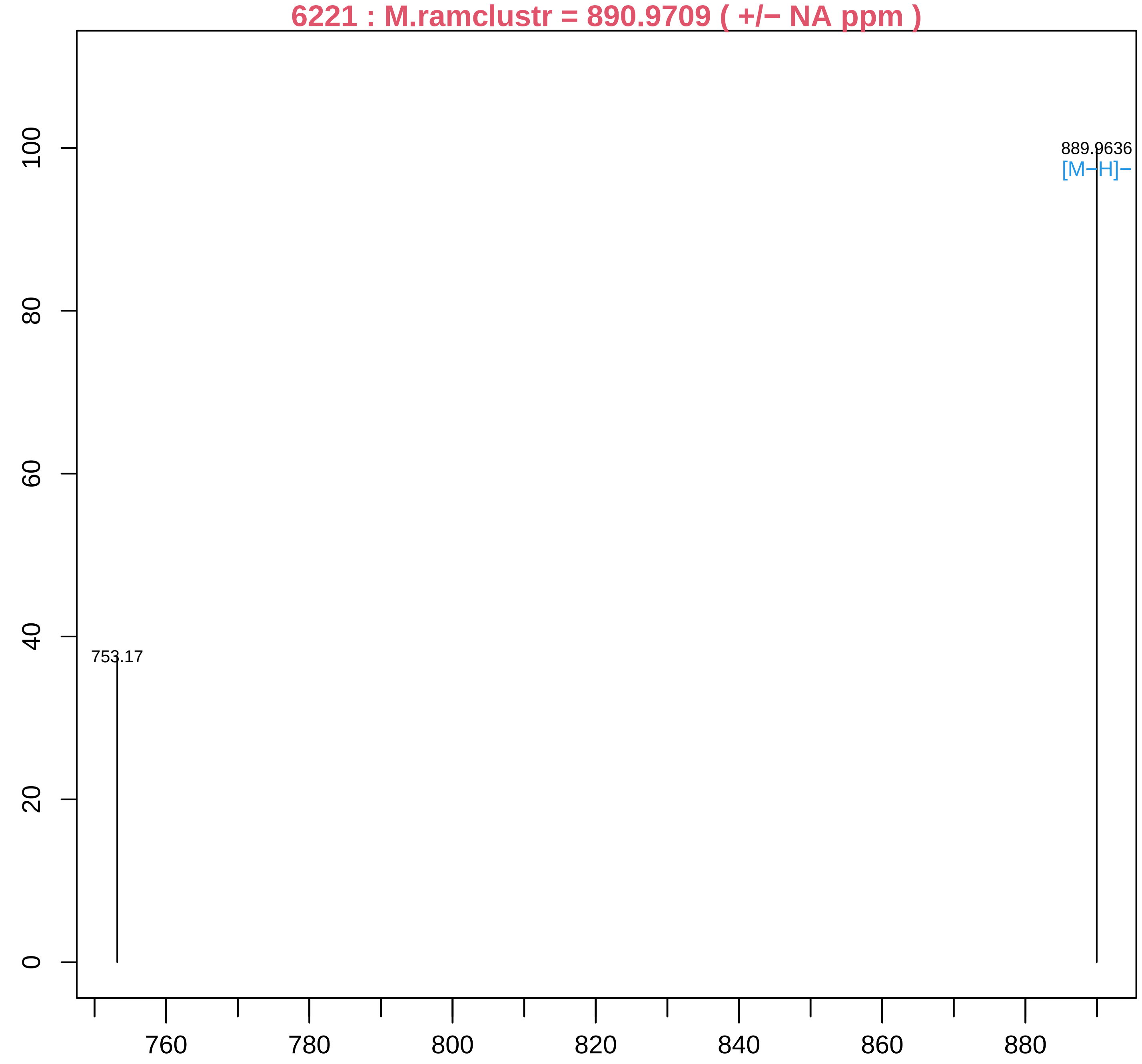
6221 : M.ramclustr = 890.9709 (+/− NA ppm)
889.9636
[M−H]−
753.17
0
20
40
60
80
100
760
780
800
820
840
860
880

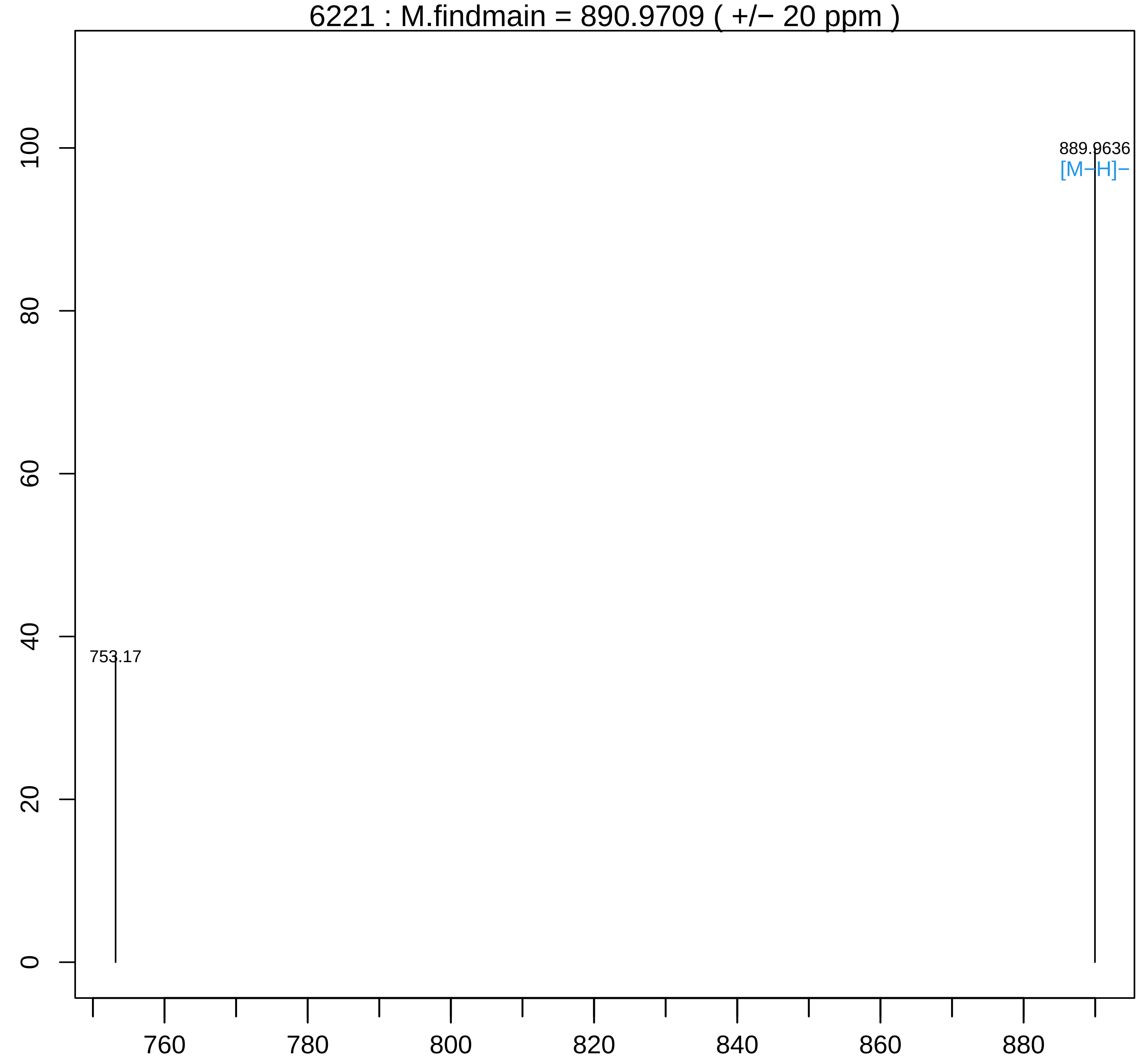
6221 : M.findmain = 890.9709 (+/− 20 ppm)
889.9636
[M−H]−
753.17
0
20
40
60
80
100
760
780
800
820
840
860
880

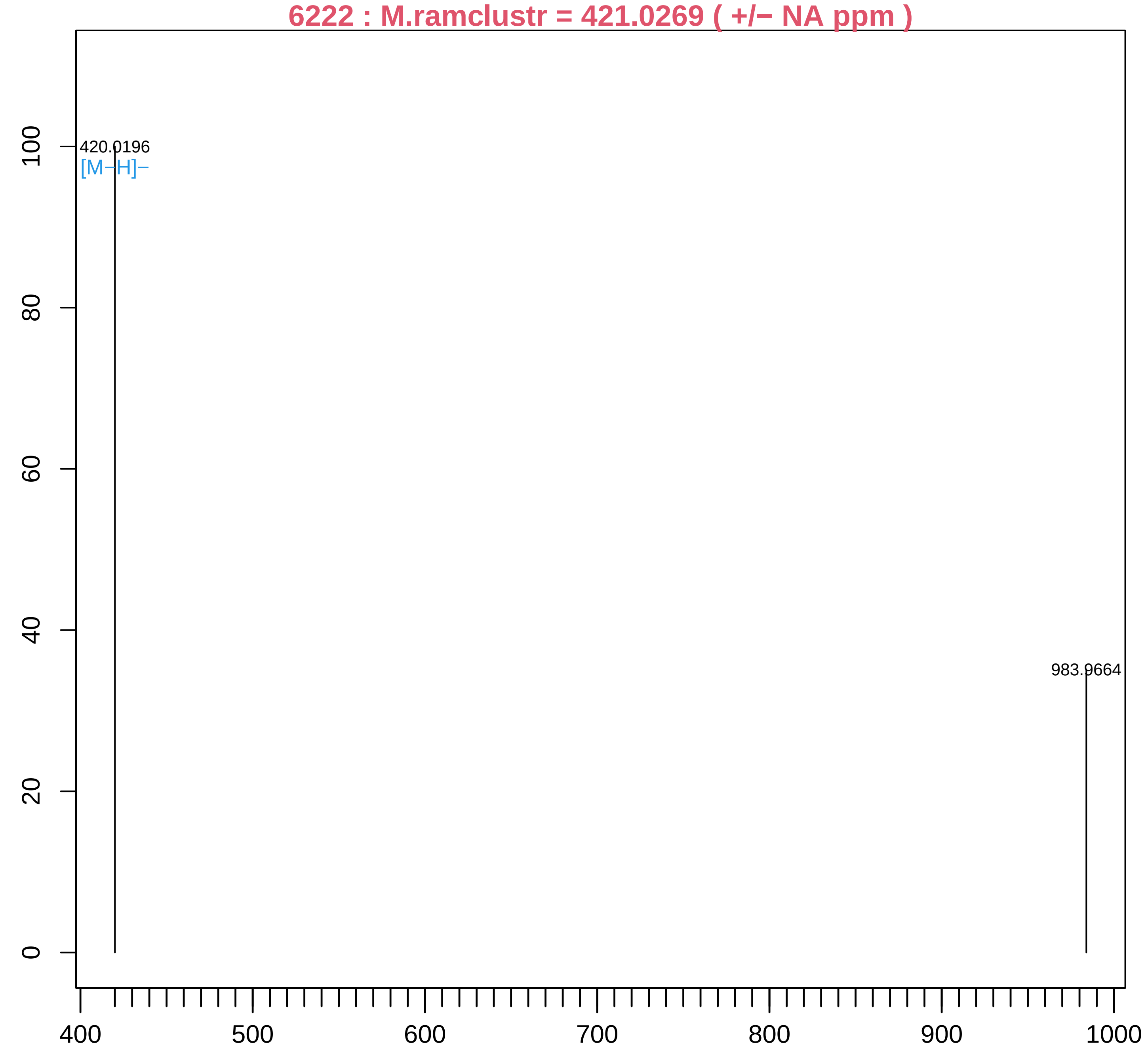
6222 : M.ramclustr = 421.0269 (+/− NA ppm)
420.0196
[M−H]−
983.9664
0
20
40
60
80
100
400
500
600
700
800
900
1000

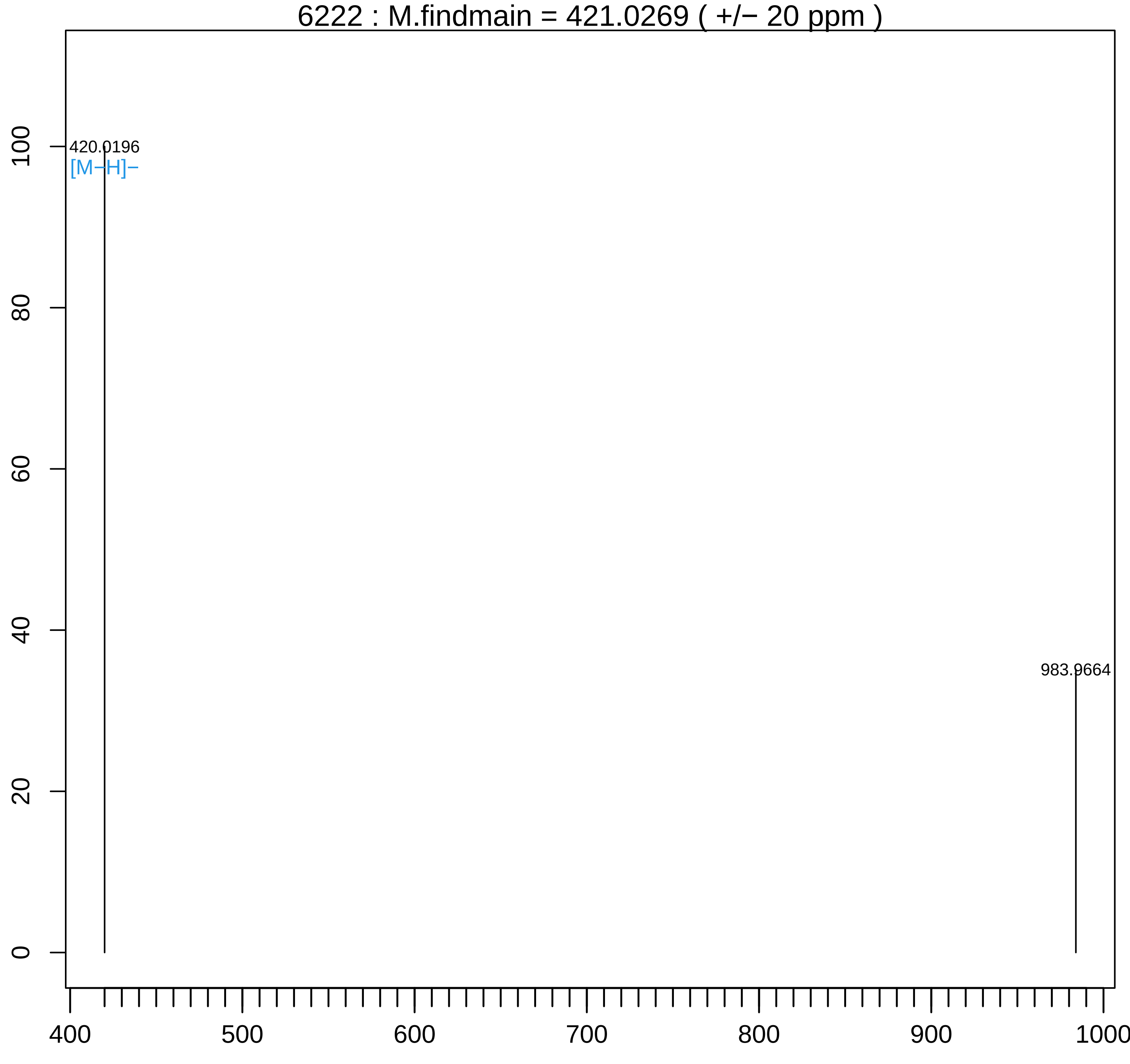
6222 : M.findmain = 421.0269 (+/− 20 ppm)
420.0196
[M−H]−
983.9664
0
20
40
60
80
100
400
500
600
700
800
900
1000

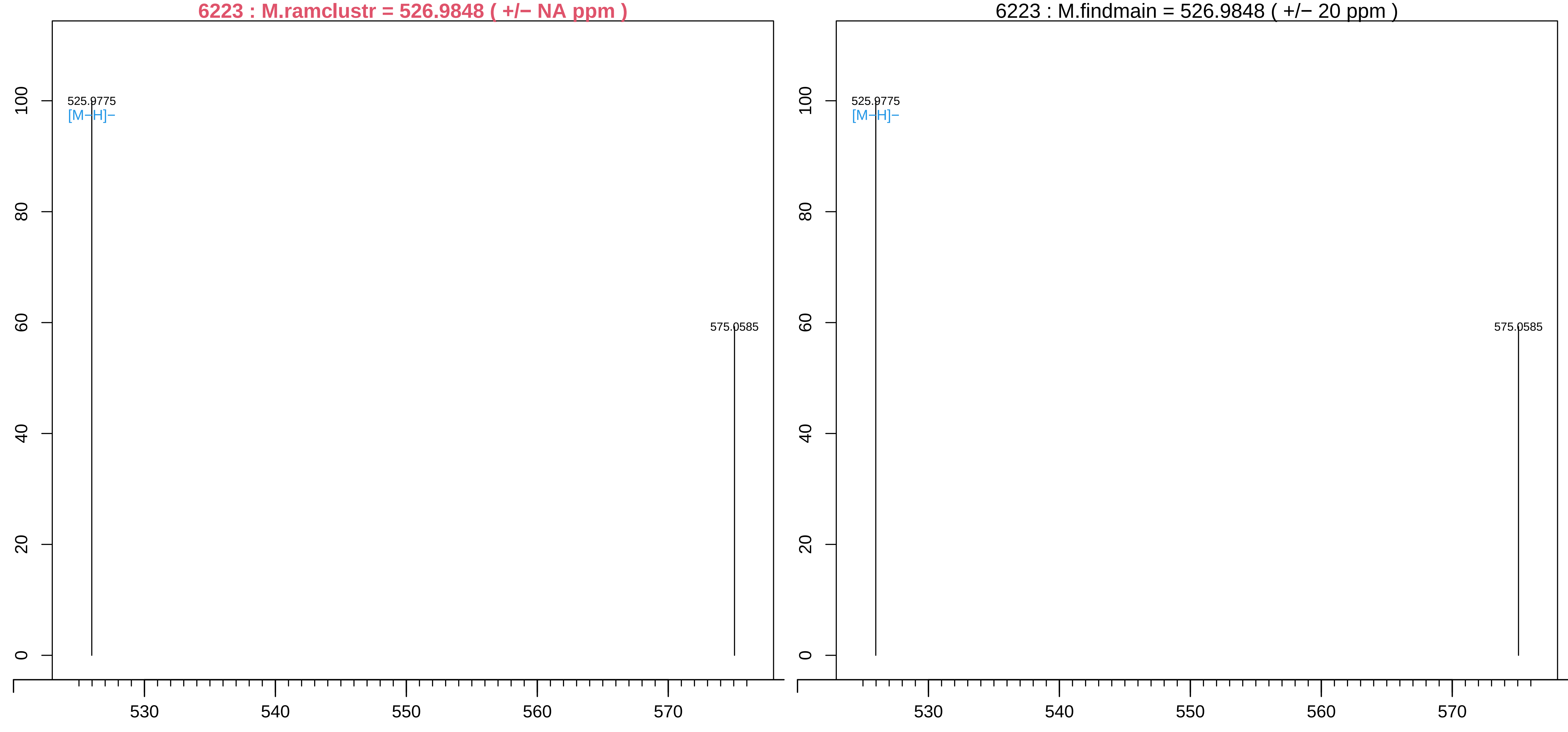

6223 : M.ramclustr = 526.9848 (+/− NA ppm)
525.9775
[M−H]−
575.0585
6223 : M.findmain = 526.9848 (+/− 20 ppm)
525.9775
[M−H]−
575.0585
0
20
40
60
80
100
530
540
550
560
570

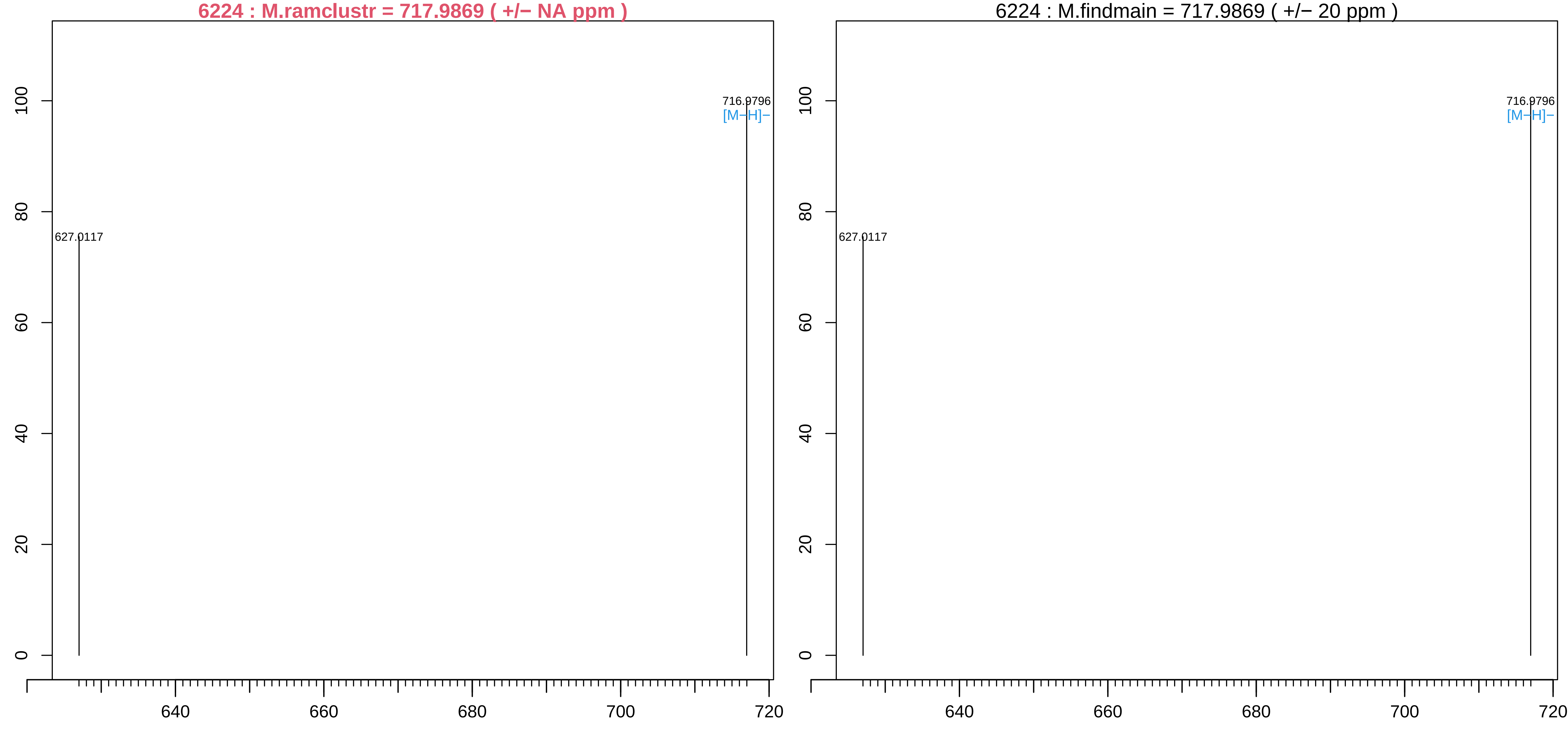
6224 : M.ramclustr = 717.9869 (+/− NA ppm)
627.0117
716.9796
[M−H]−
6224 : M.findmain = 717.9869 (+/− 20 ppm)
627.0117
716.9796
[M−H]−
0
20
40
60
80
100
640
660
680
700
720

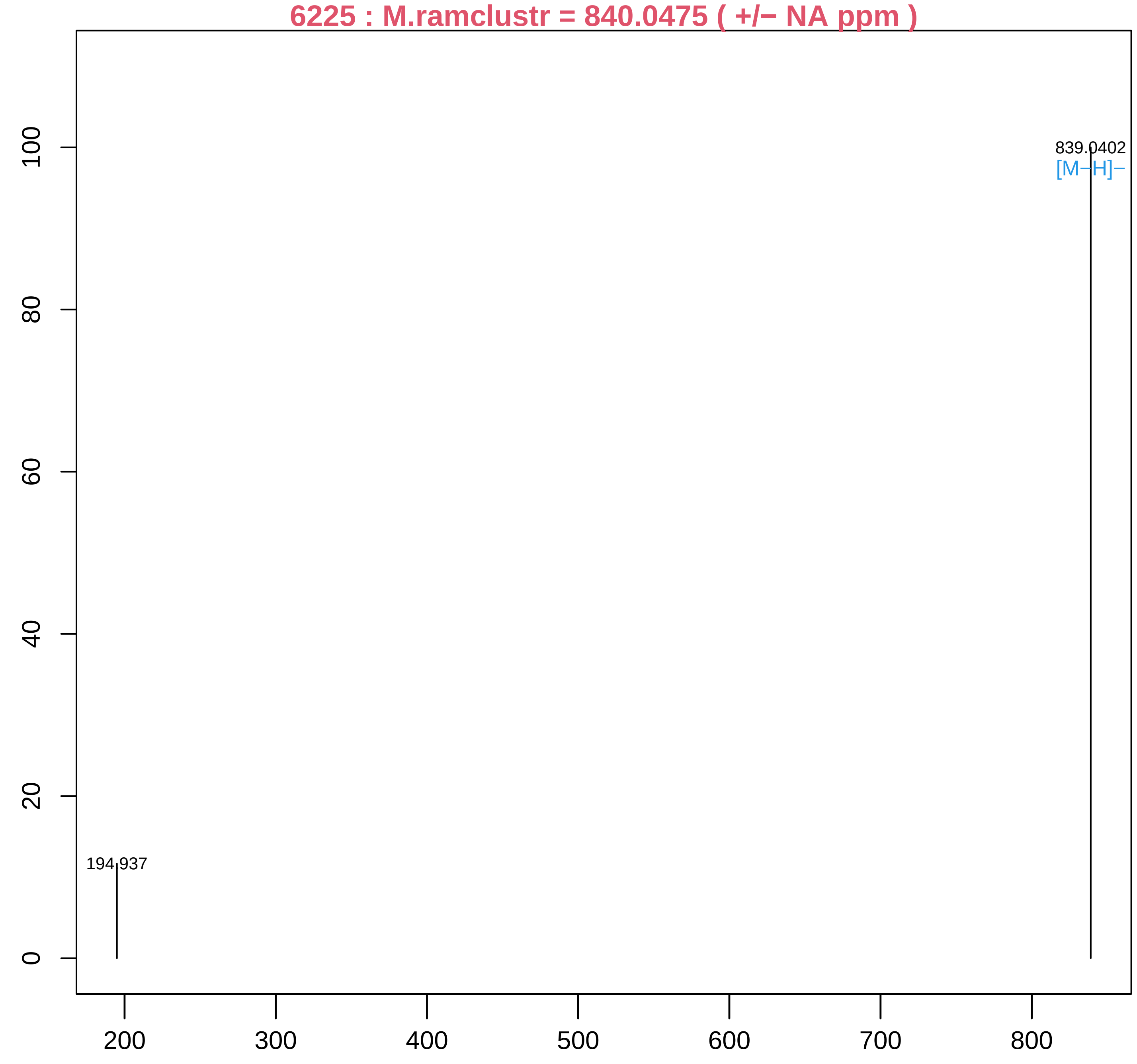
6225 : M.ramclustr = 840.0475 (+/− NA ppm)
839.0402
[M−H]−
194.937
0
20
40
60
80
100
200
300
400
500
600
700
800

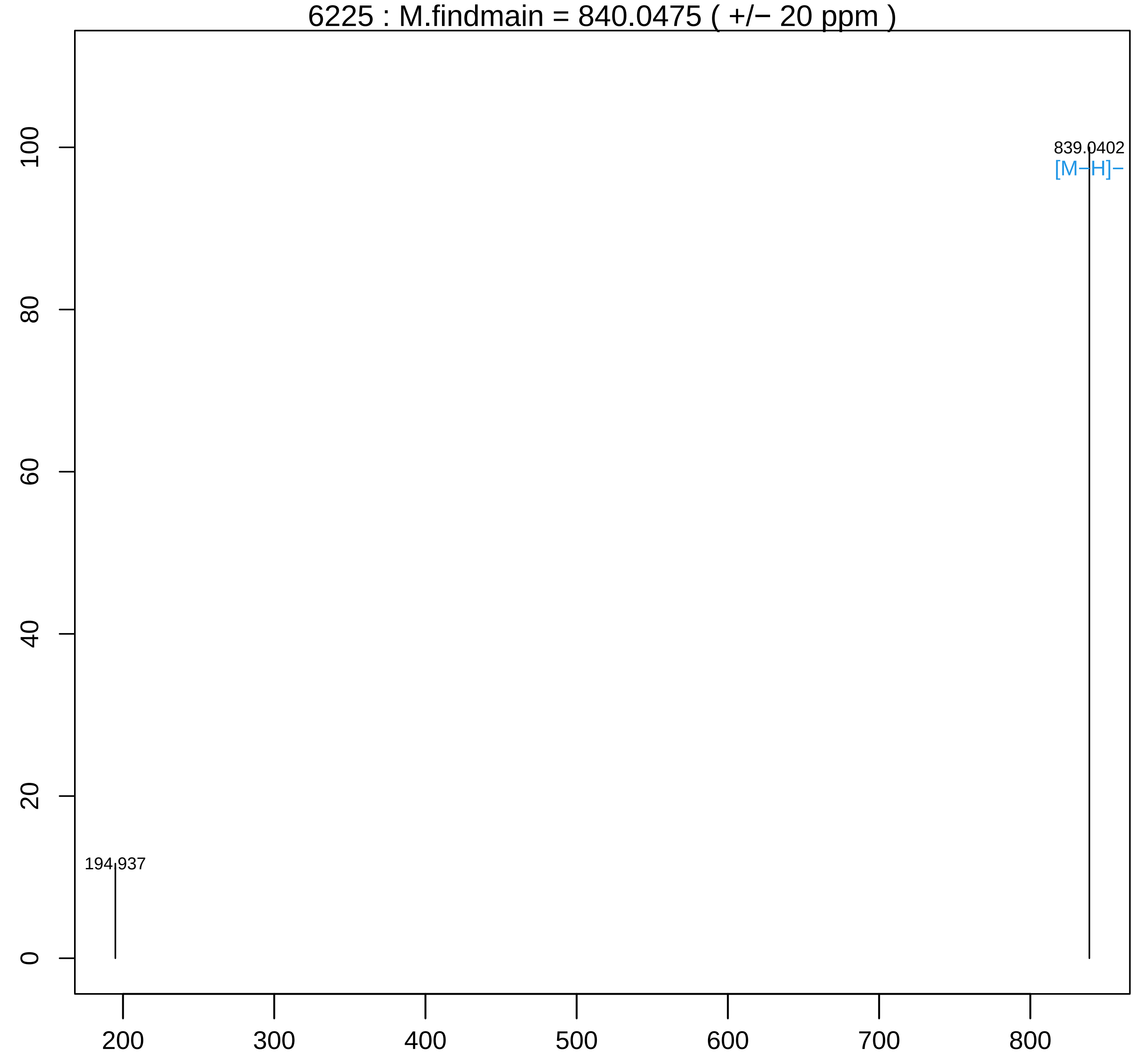
6225 : M.findmain = 840.0475 (+/− 20 ppm)
839.0402
[M−H]−
194.937
0
20
40
60
80
100
200
300
400
500
600
700
800

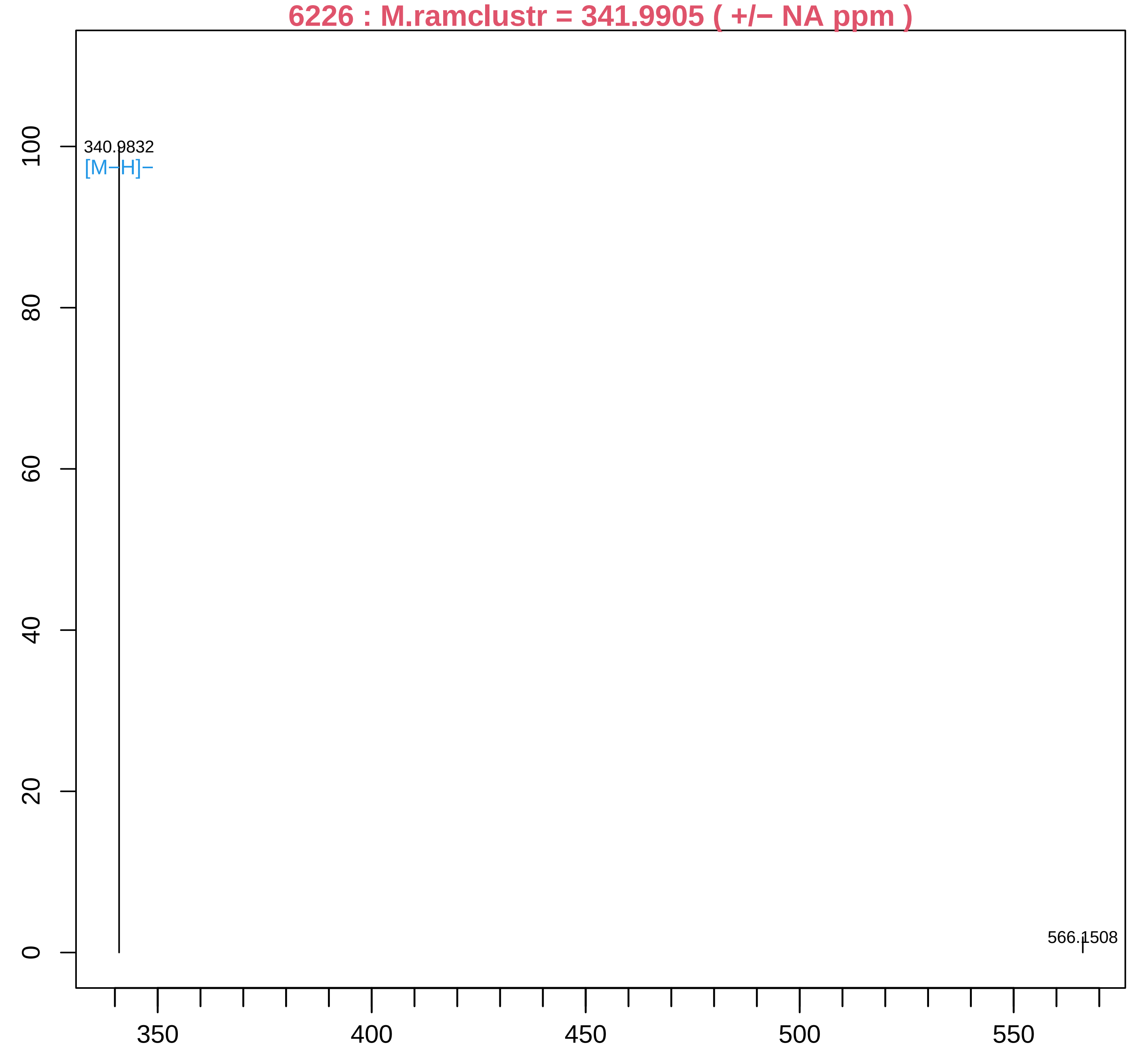
6226 : M.ramclustr = 341.9905 (+/− NA ppm)
340.9832
[M−H]−
566.1508
0
20
40
60
80
100
350
400
450
500
550

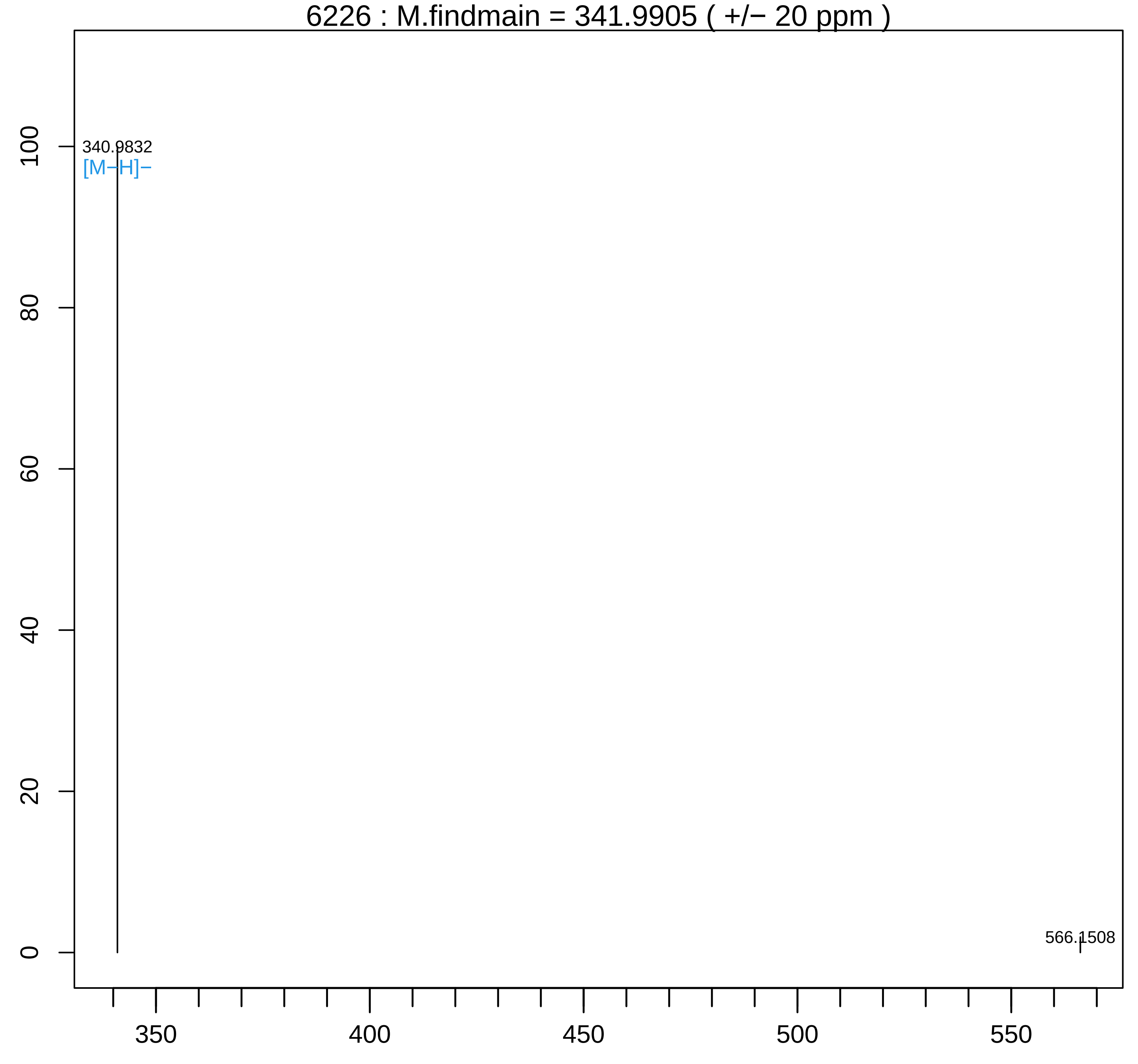
6226 : M.findmain = 341.9905 (+/− 20 ppm)
340.9832
[M−H]−
566.1508
0
20
40
60
80
100
350
400
450
500
550

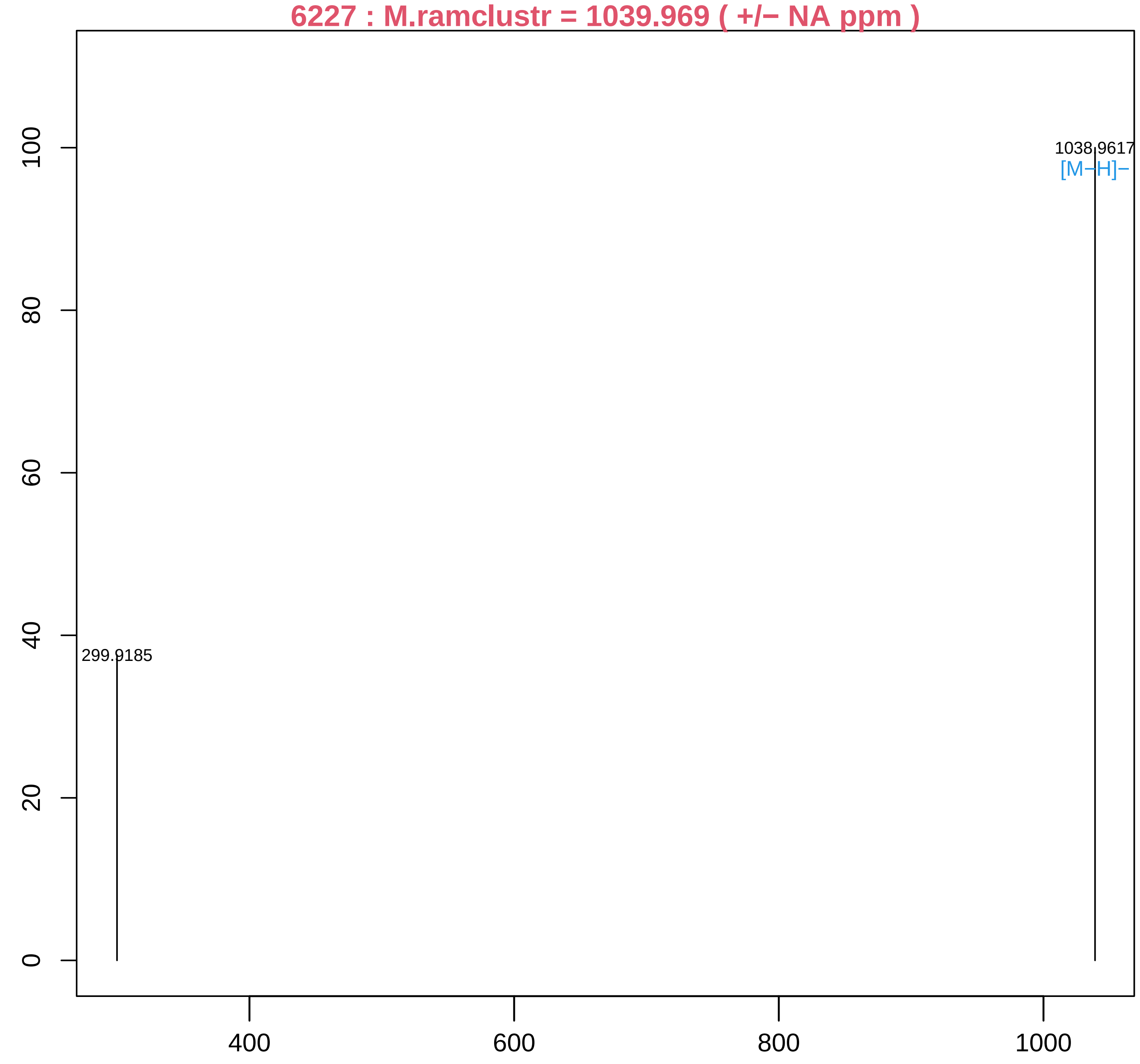
6227 : M.ramclustr = 1039.969 (+/− NA ppm)
1038.9617
[M−H]−
299.9185
0
20
40
60
80
100
400
600
800
1000

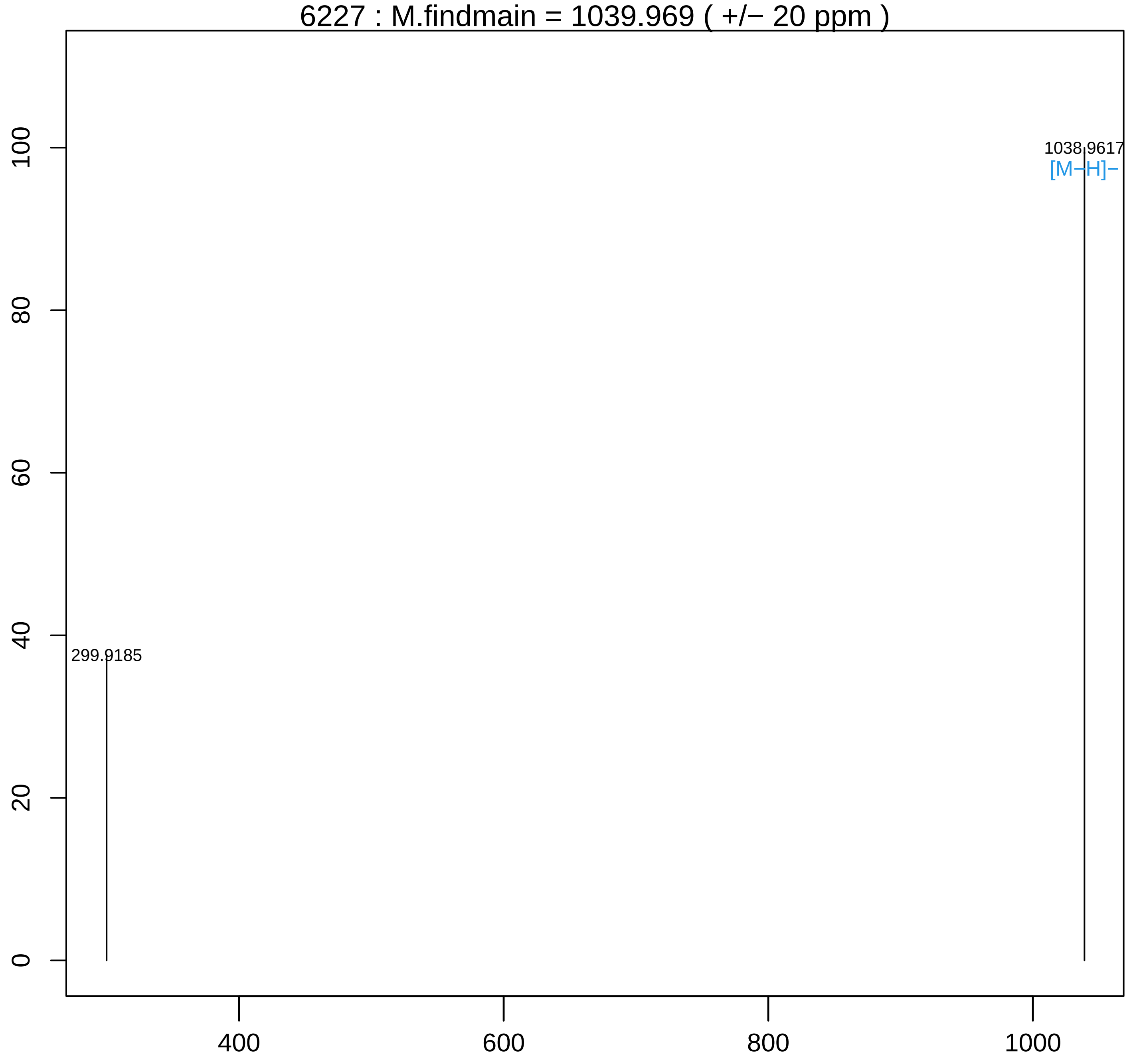
6227 : M.findmain = 1039.969 (+/− 20 ppm)
1038.9617
[M−H]−
299.9185
0
20
40
60
80
100
400
600
800
1000

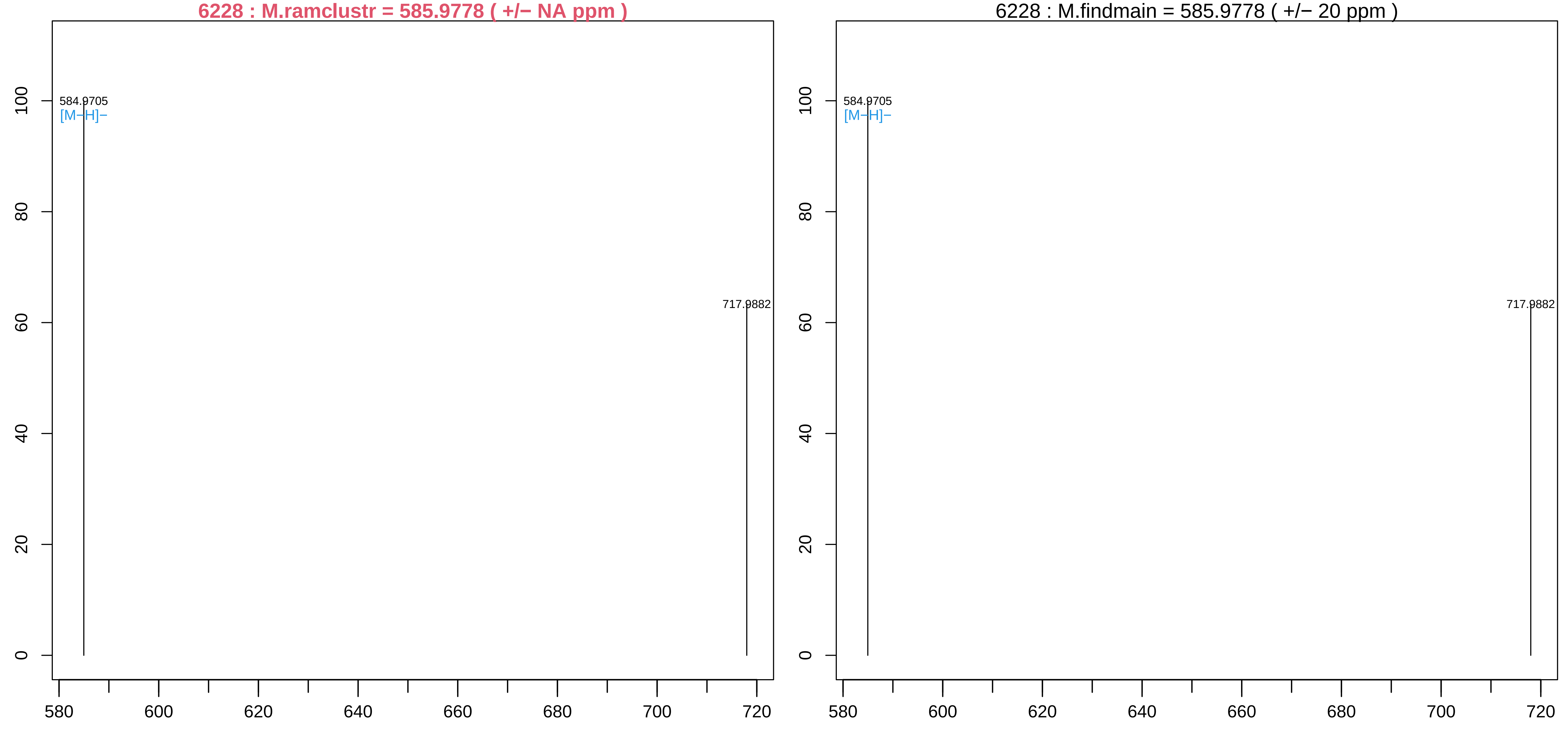
6228 : M.ramclustr = 585.9778 (+/− NA ppm)
584.9705
[M−H]−
717.9882
6228 : M.findmain = 585.9778 (+/− 20 ppm)
584.9705
[M−H]−
717.9882
0
20
40
60
80
100
580
600
620
640
660
680
700
720

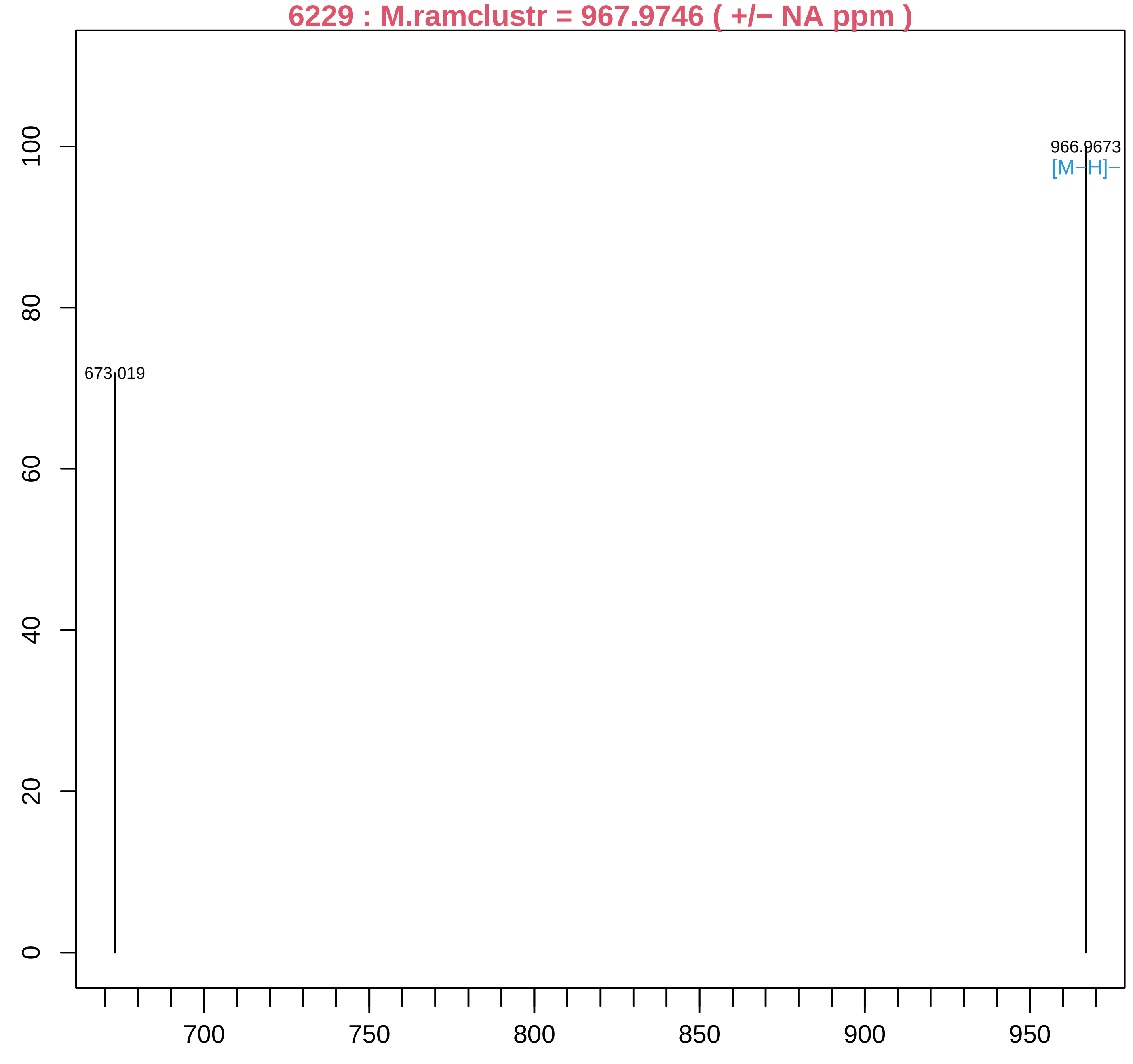

6229 : M.ramclustr = 967.9746 (+/− NA ppm)
966.9673
[M−H]−
673.019
0
20
40
60
80
100
700
750
800
850
900
950

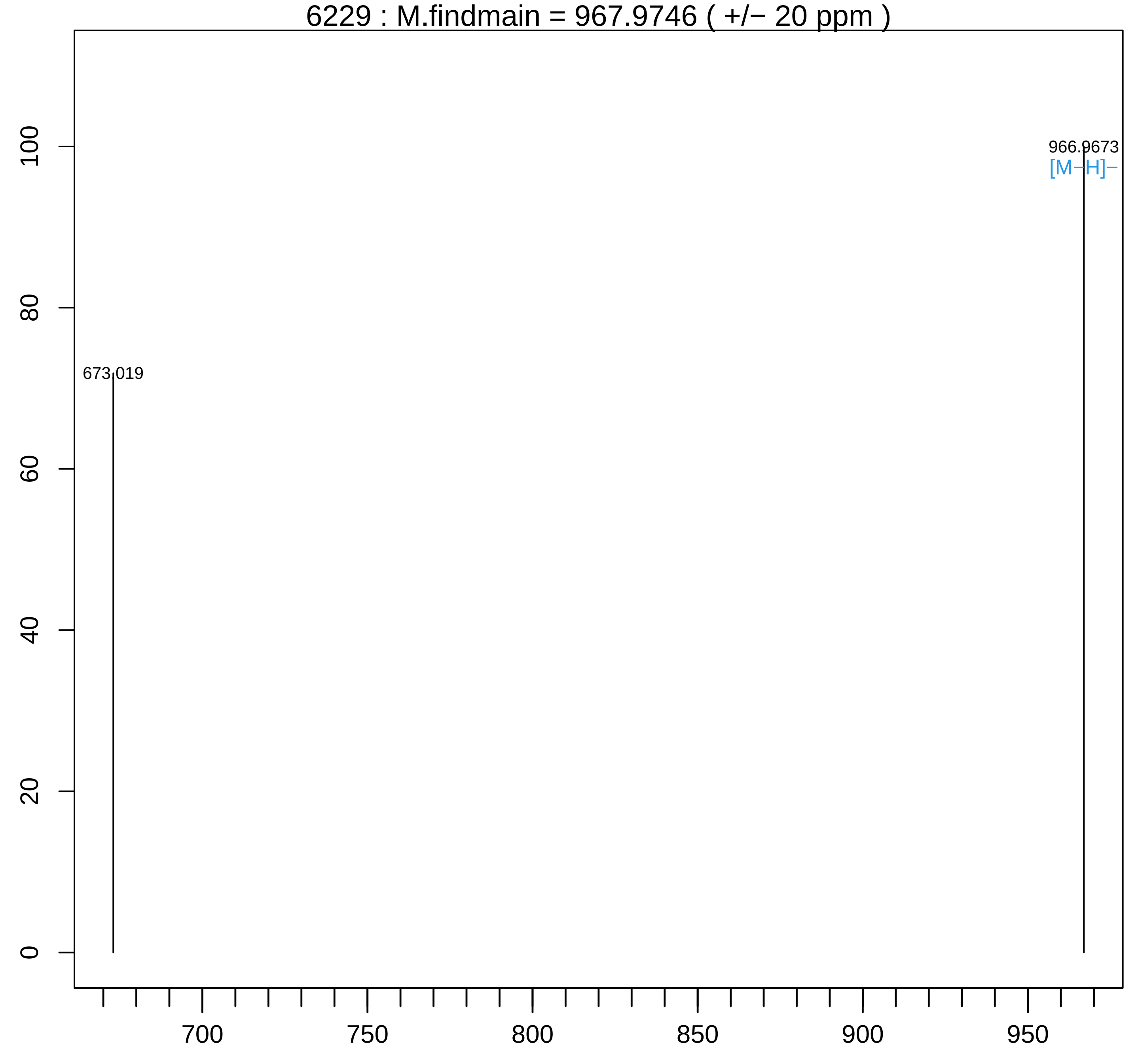

6229 : M.findmain = 967.9746 (+/− 20 ppm)
966.9673
[M−H]−
673.019
0
20
40
60
80
100
700
750
800
850
900
950

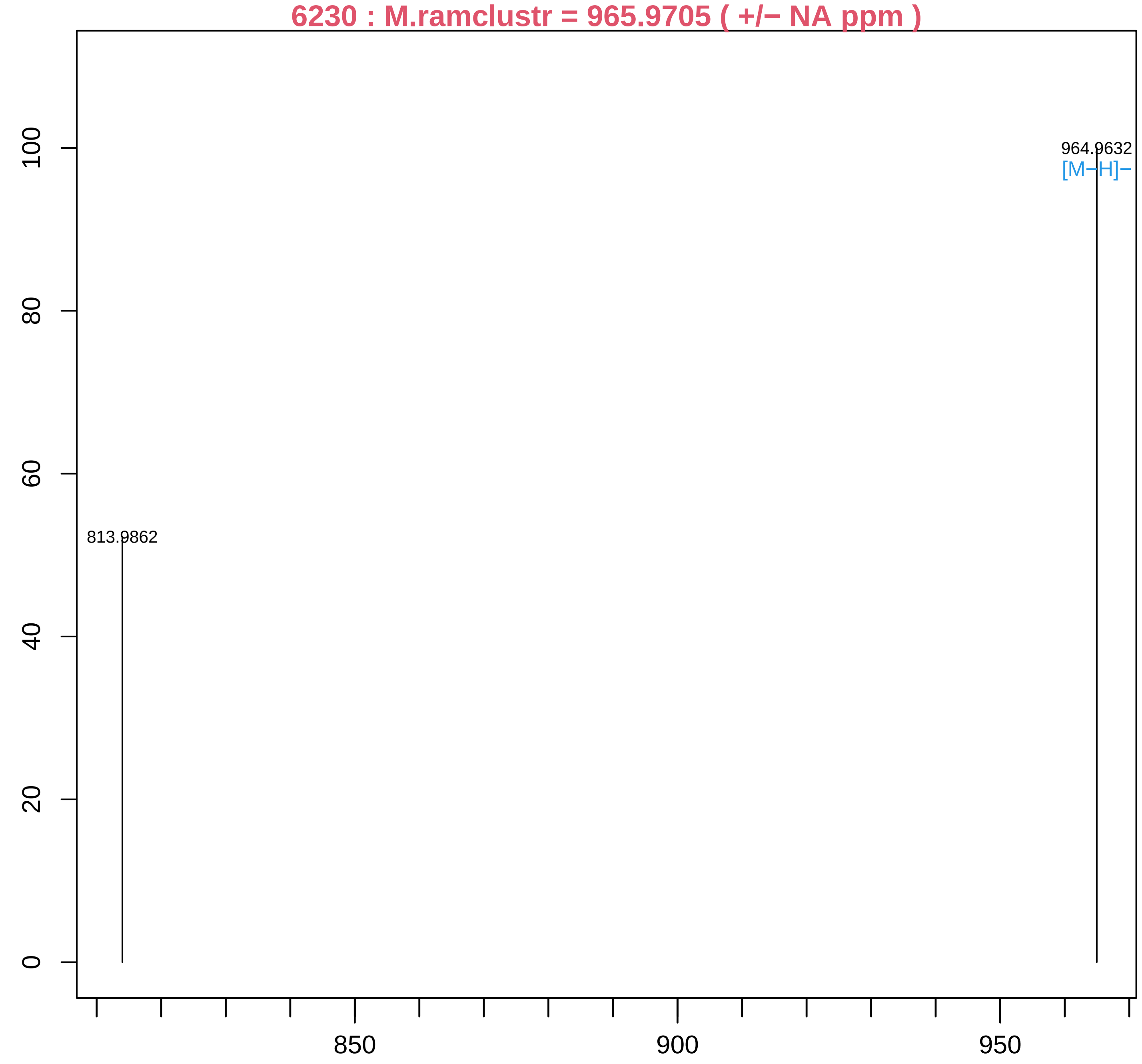
6230 : M.ramclustr = 965.9705 (+/− NA ppm)
964.9632
[M−H]−
813.9862
0
20
40
60
80
100
850
900
950

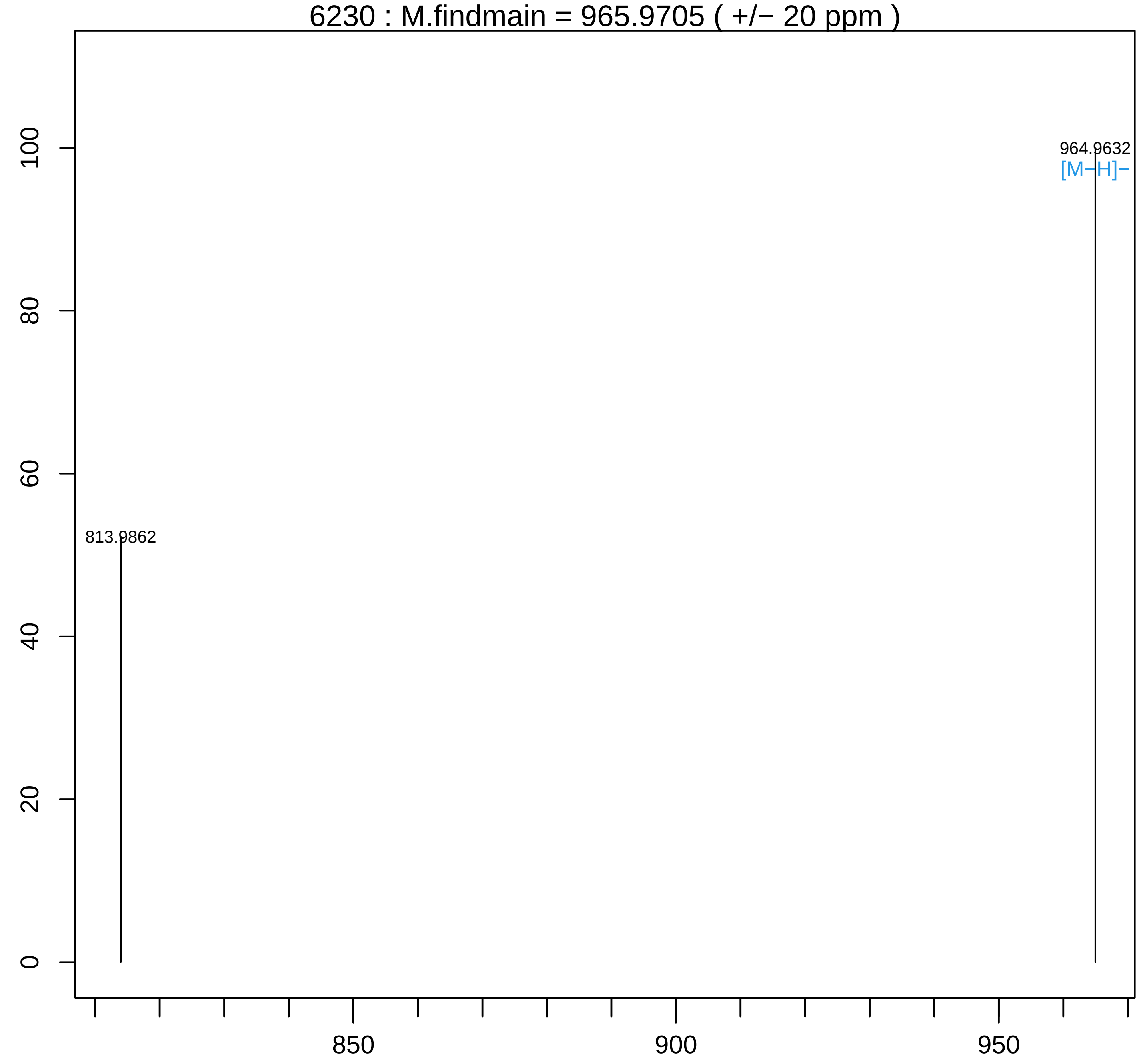
6230 : M.findmain = 965.9705 (+/− 20 ppm)
964.9632
[M−H]−
813.9862
0
20
40
60
80
100
850
900
950

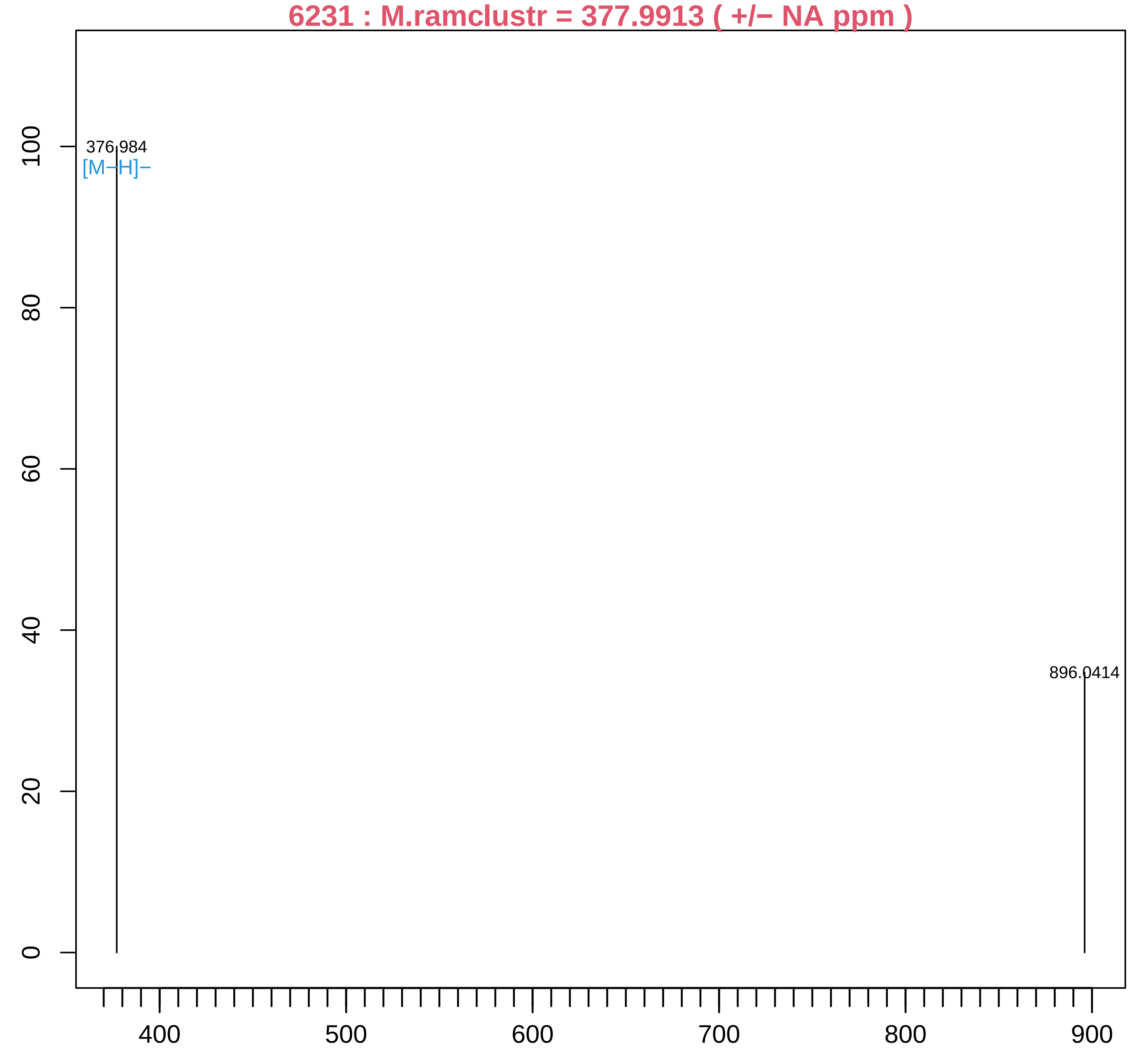
6231 : M.ramclustr = 377.9913 (+/− NA ppm)
376.984
[M−H]−
896.0414
0
20
40
60
80
100
400
500
600
700
800
900

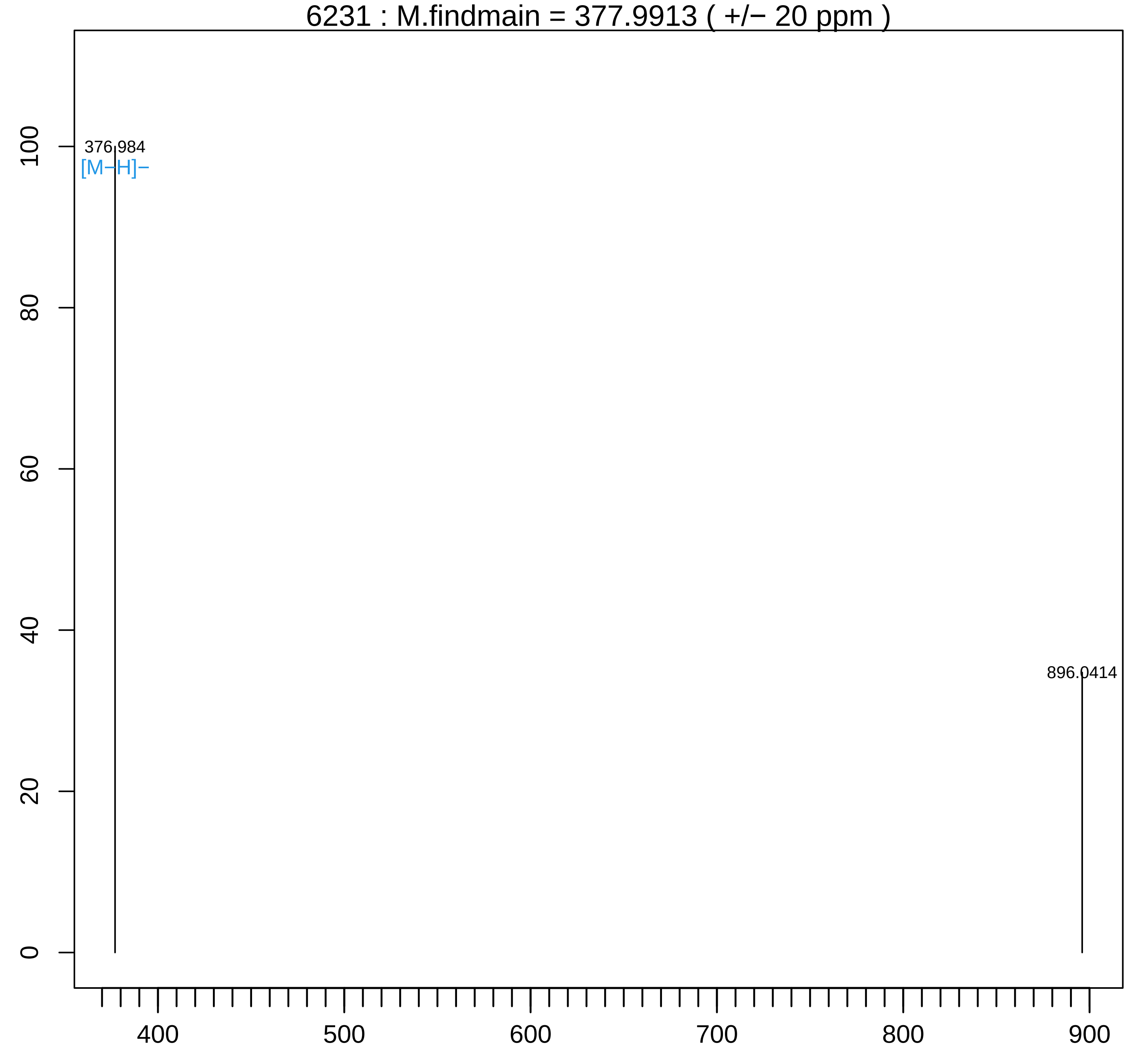
6231 : M.findmain = 377.9913 (+/− 20 ppm)
376.984
[M−H]−
896.0414
0
20
40
60
80
100
400
500
600
700
800
900

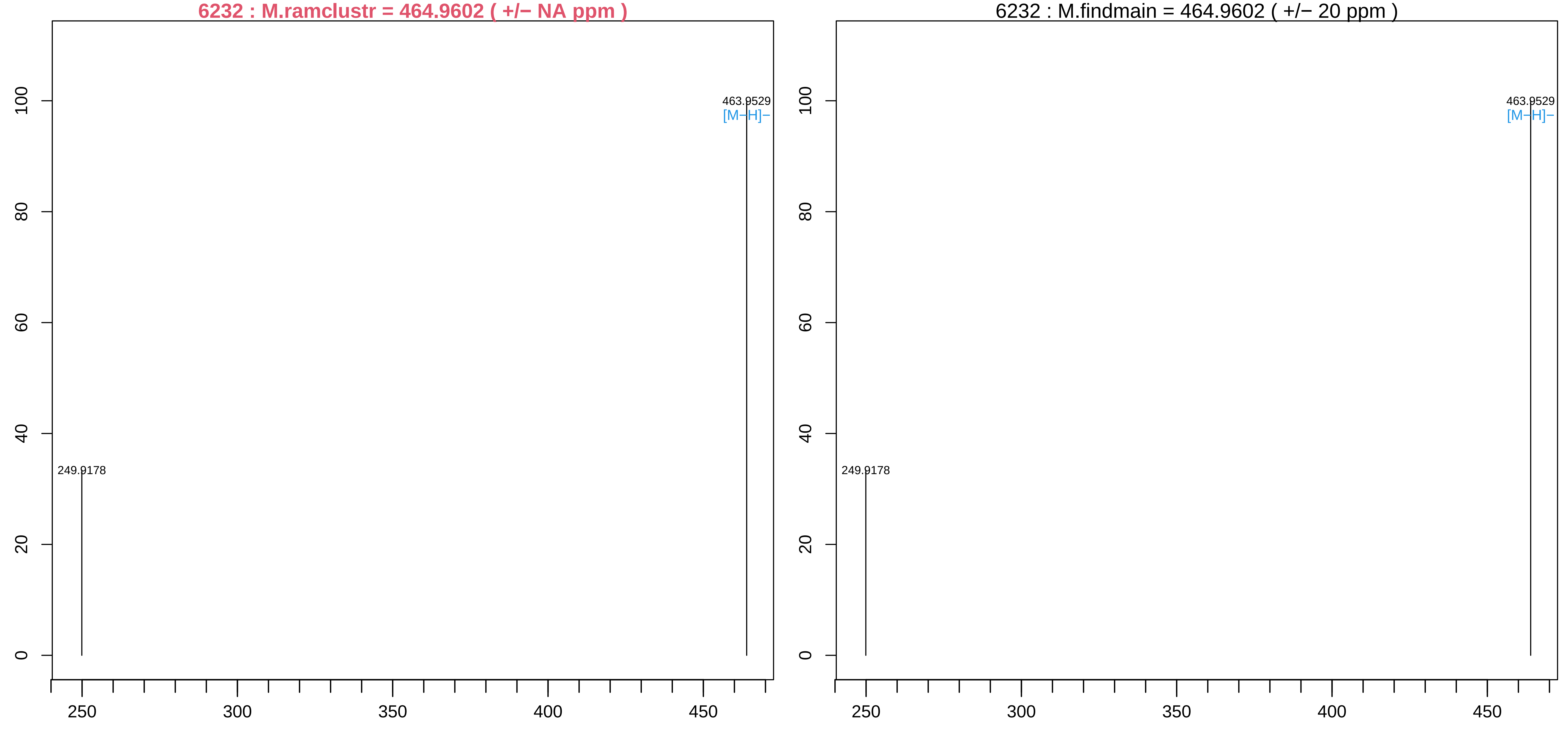

6232 : M.ramclustr = 464.9602 (+/− NA ppm)
463.9529
[M−H]−
249.9178
6232 : M.findmain = 464.9602 (+/− 20 ppm)
463.9529
[M−H]−
249.9178
0
20
40
60
80
100
250
300
350
400
450

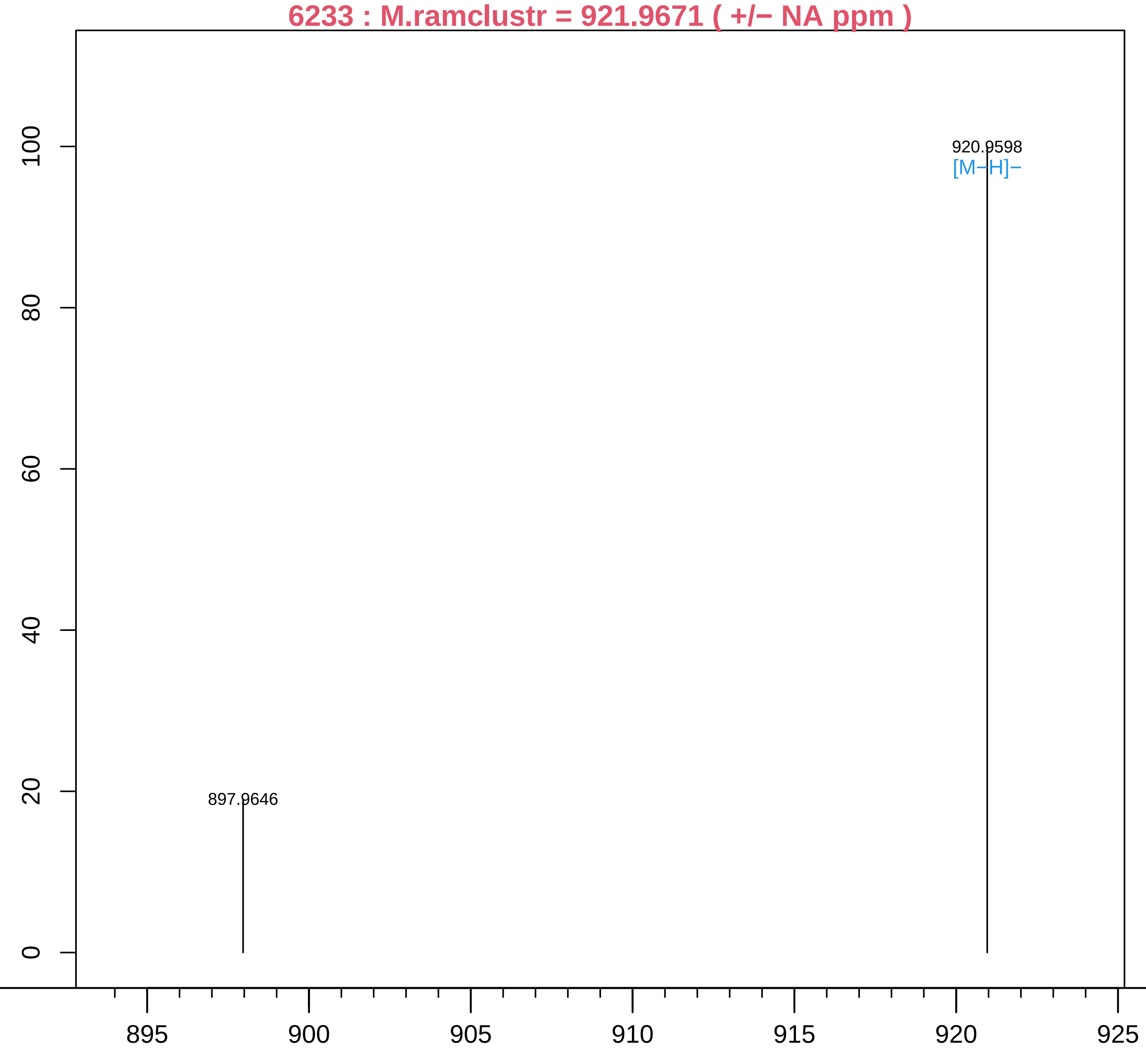

6233 : M.ramclustr = 921.9671 (+/− NA ppm)
920.9598
[M−H]−
897.9646
0
20
40
60
80
100
895
900
905
910
915
920
925

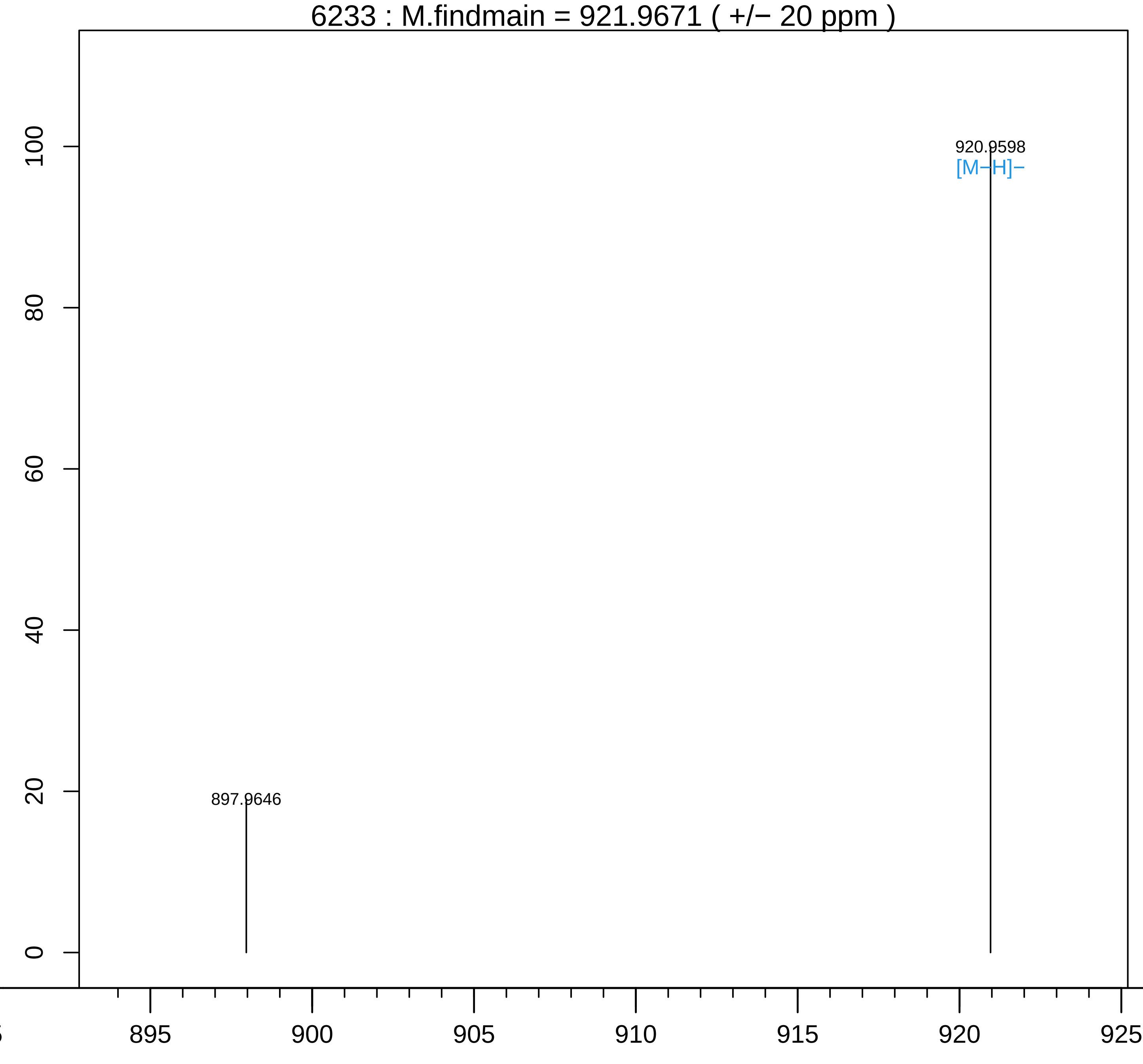

6233 : M.findmain = 921.9671 (+/− 20 ppm)
920.9598
[M−H]−
897.9646
0
20
40
60
80
100
895
900
905
910
915
920
925

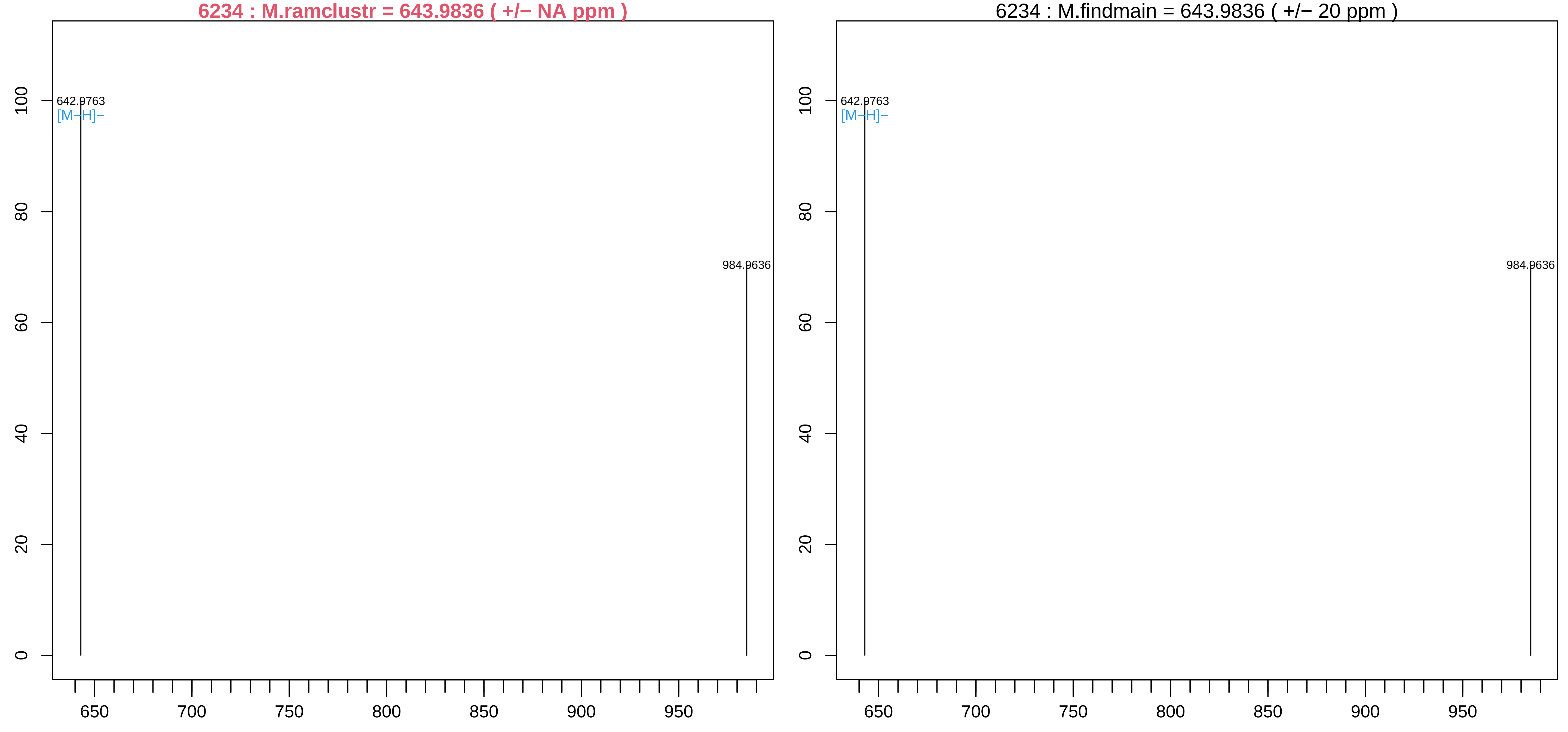
6234 : M.ramclustr = 643.9836 (+/− NA ppm)
642.9763
[M−H]−
984.9636
0
20
40
60
80
100
650
700
750
800
850
900
950
6234 : M.findmain = 643.9836 (+/− 20 ppm)
642.9763
[M−H]−
984.9636
0
20
40
60
80
100
650
700
750
800
850
900
950

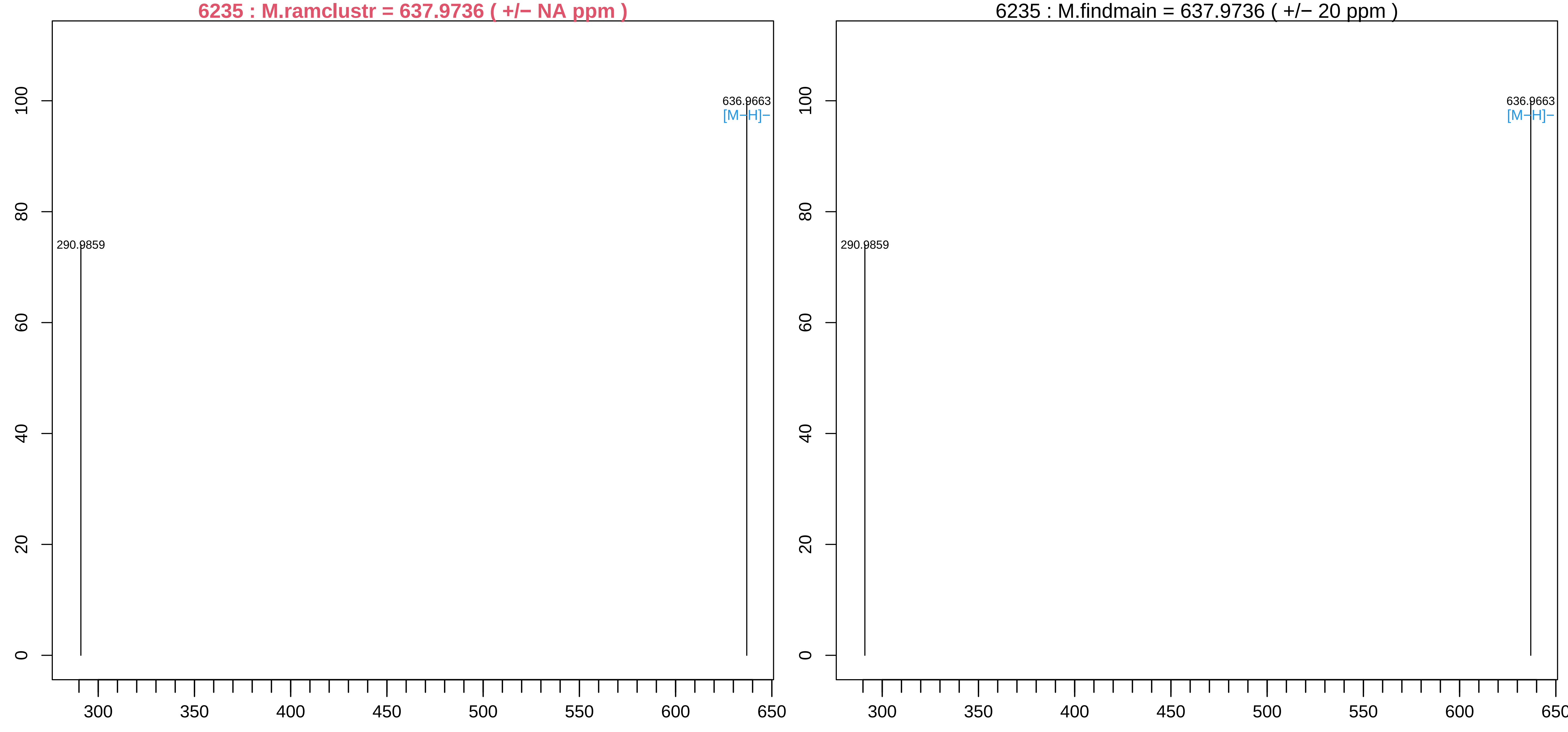
6235 : M.ramclustr = 637.9736 (+/− NA ppm)
636.9663
[M−H]−
290.9859
6235 : M.findmain = 637.9736 (+/− 20 ppm)
636.9663
[M−H]−
290.9859
0
20
40
60
80
100
300
350
400
450
500
550
600
650

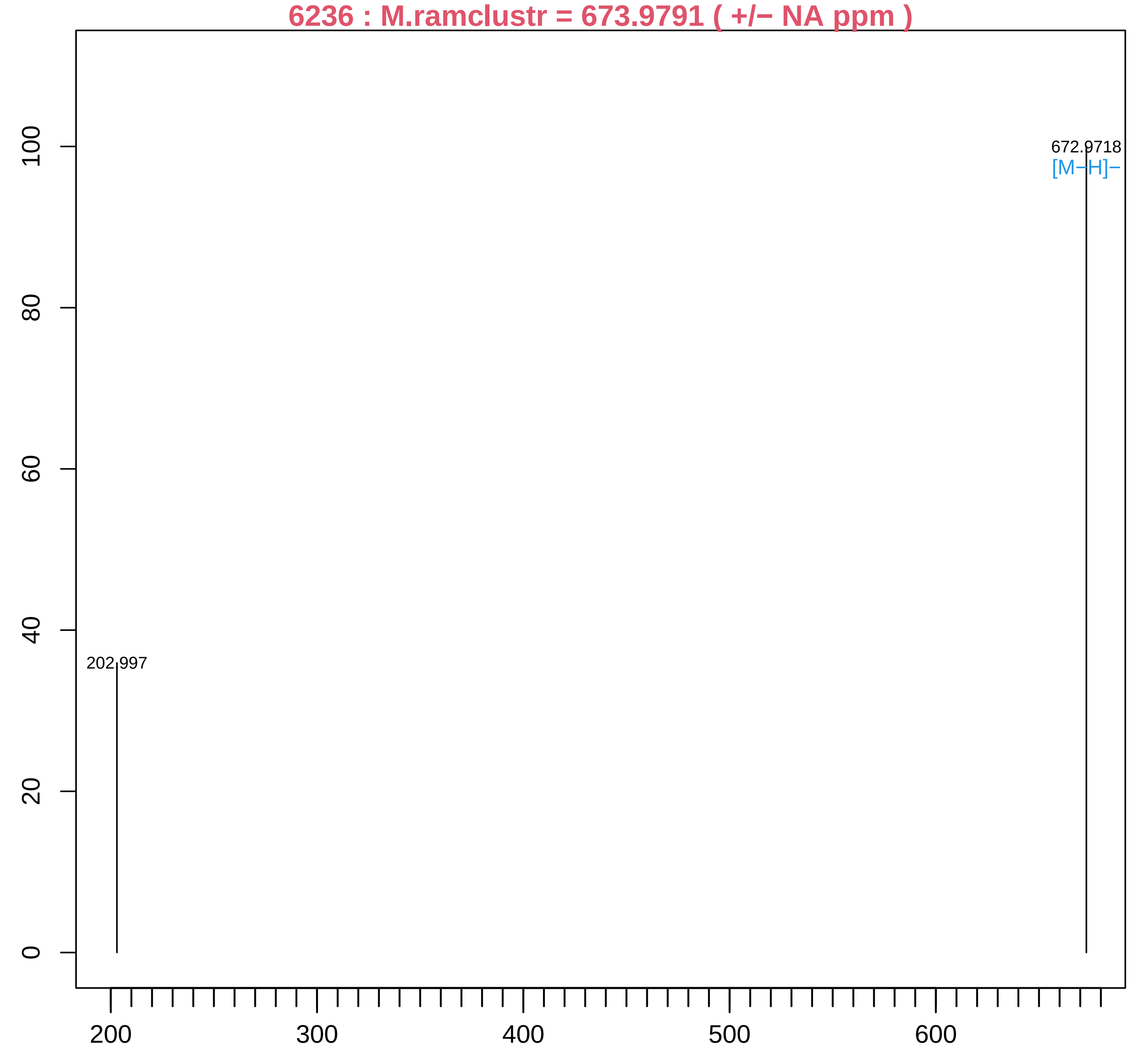
6236 : M.ramclustr = 673.9791 (+/− NA ppm)
672.9718
[M−H]−
202.997
0
20
40
60
80
100
200
300
400
500
600

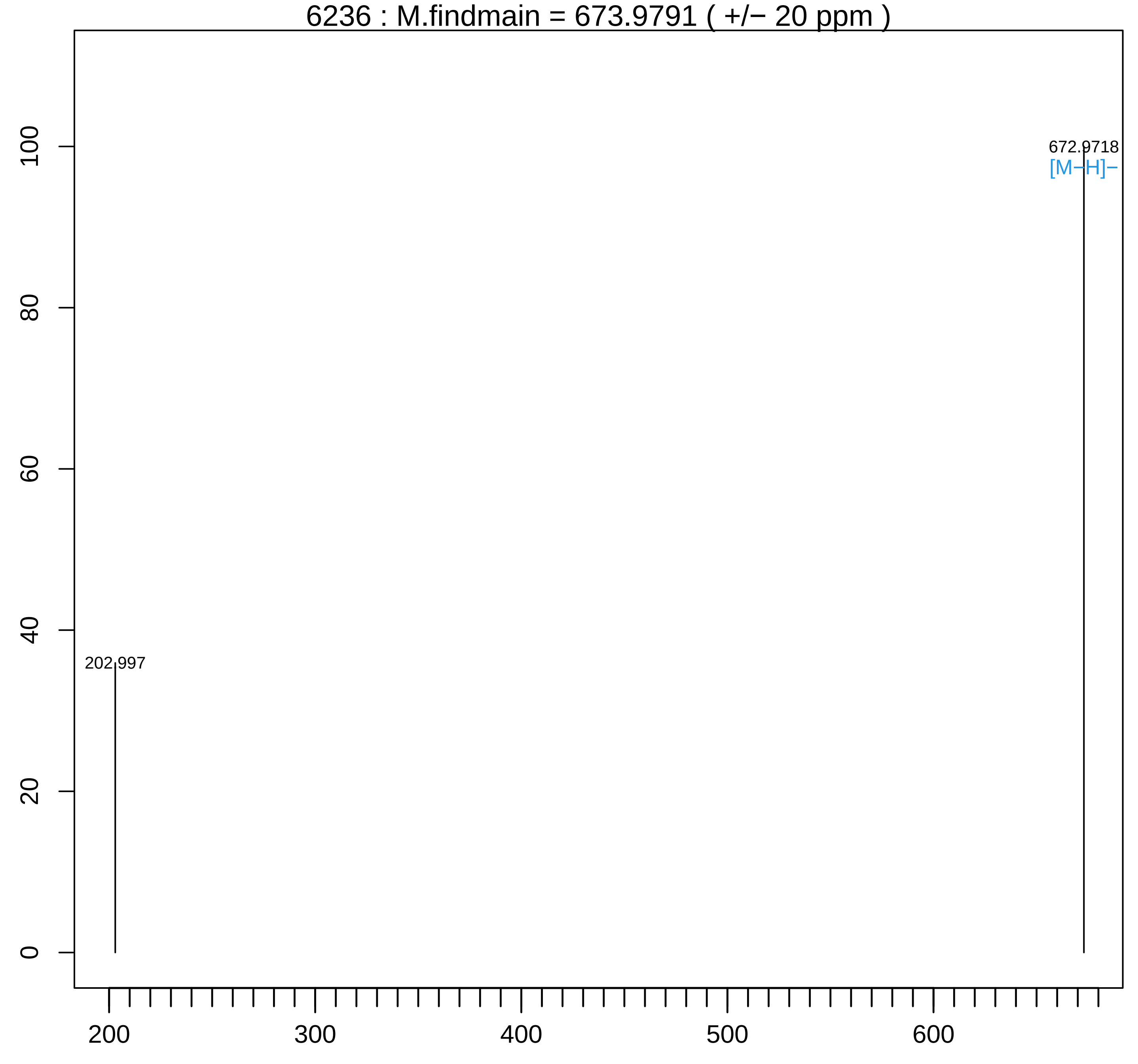
6236 : M.findmain = 673.9791 (+/− 20 ppm)
672.9718
[M−H]−
202.997
0
20
40
60
80
100
200
300
400
500
600

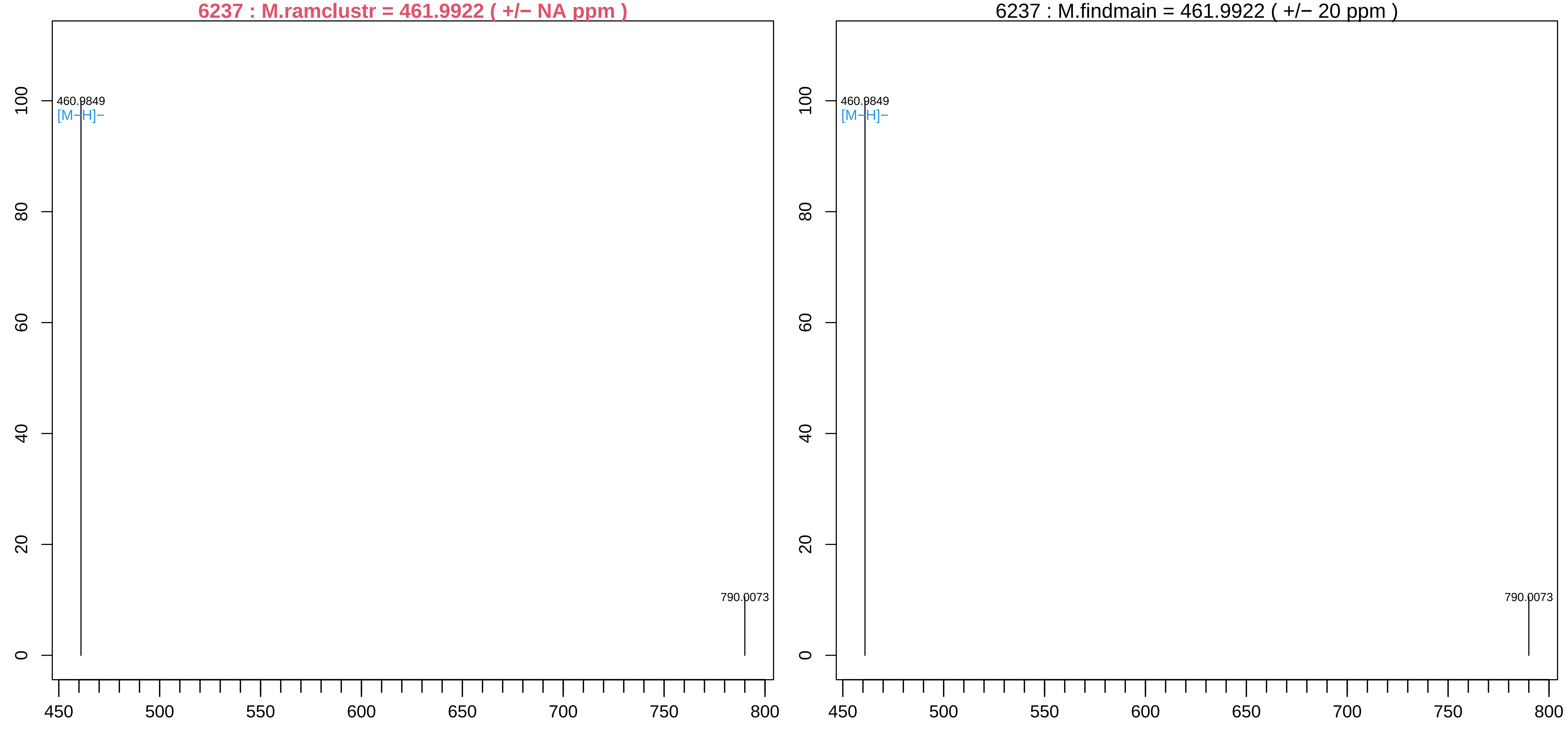
6237 : M.ramclustr = 461.9922 (+/− NA ppm)
460.9849
[M−H]−
790.0073
0
20
40
60
80
100
450
500
550
600
650
700
750
800
6237 : M.findmain = 461.9922 (+/− 20 ppm)
460.9849
[M−H]−
790.0073
0
20
40
60
80
100
450
500
550
600
650
700
750
800

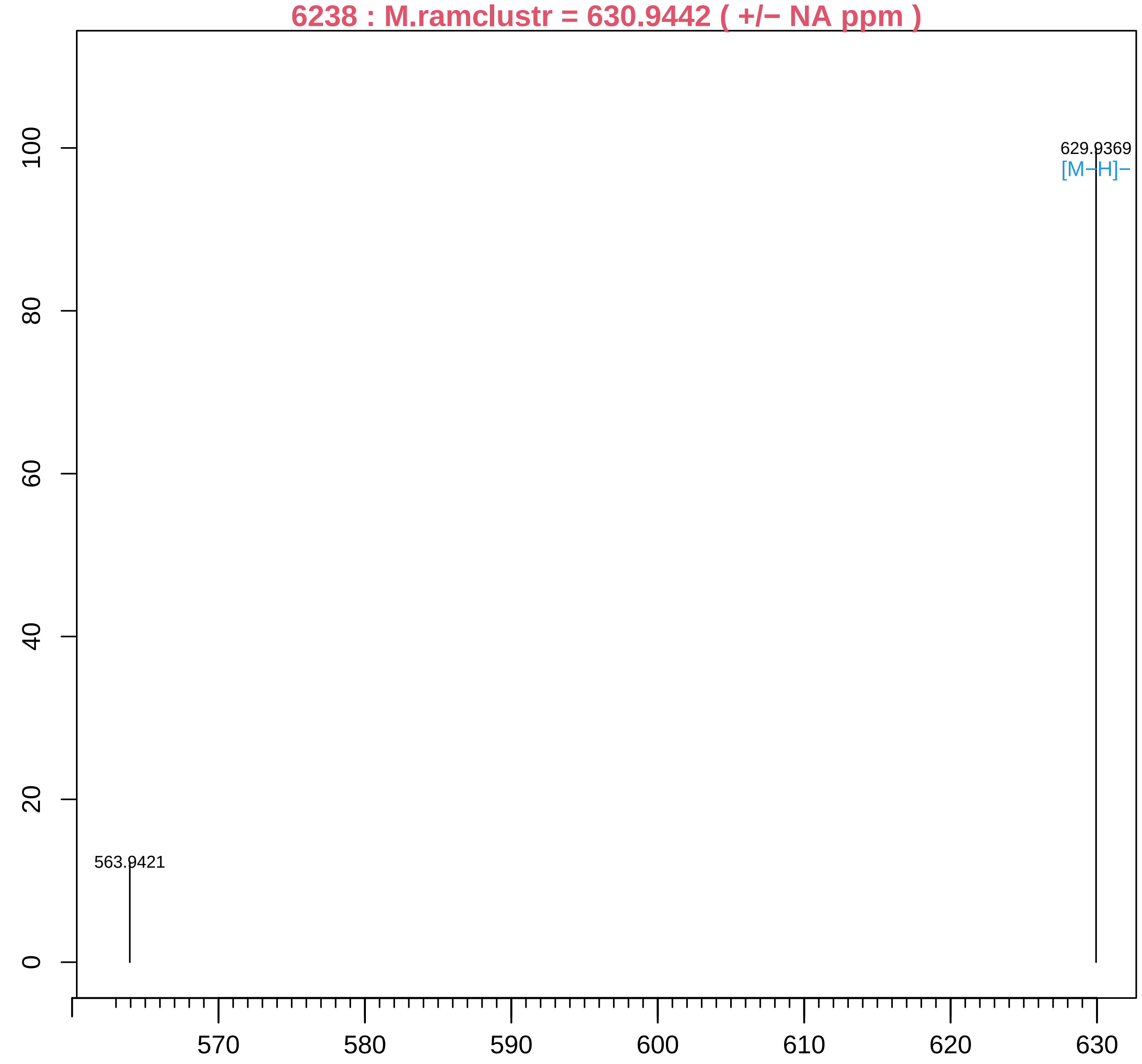
6238 : M.ramclustr = 630.9442 (+/− NA ppm)
629.9369
[M−H]−
563.9421
0
20
40
60
80
100
570
580
590
600
610
620
630

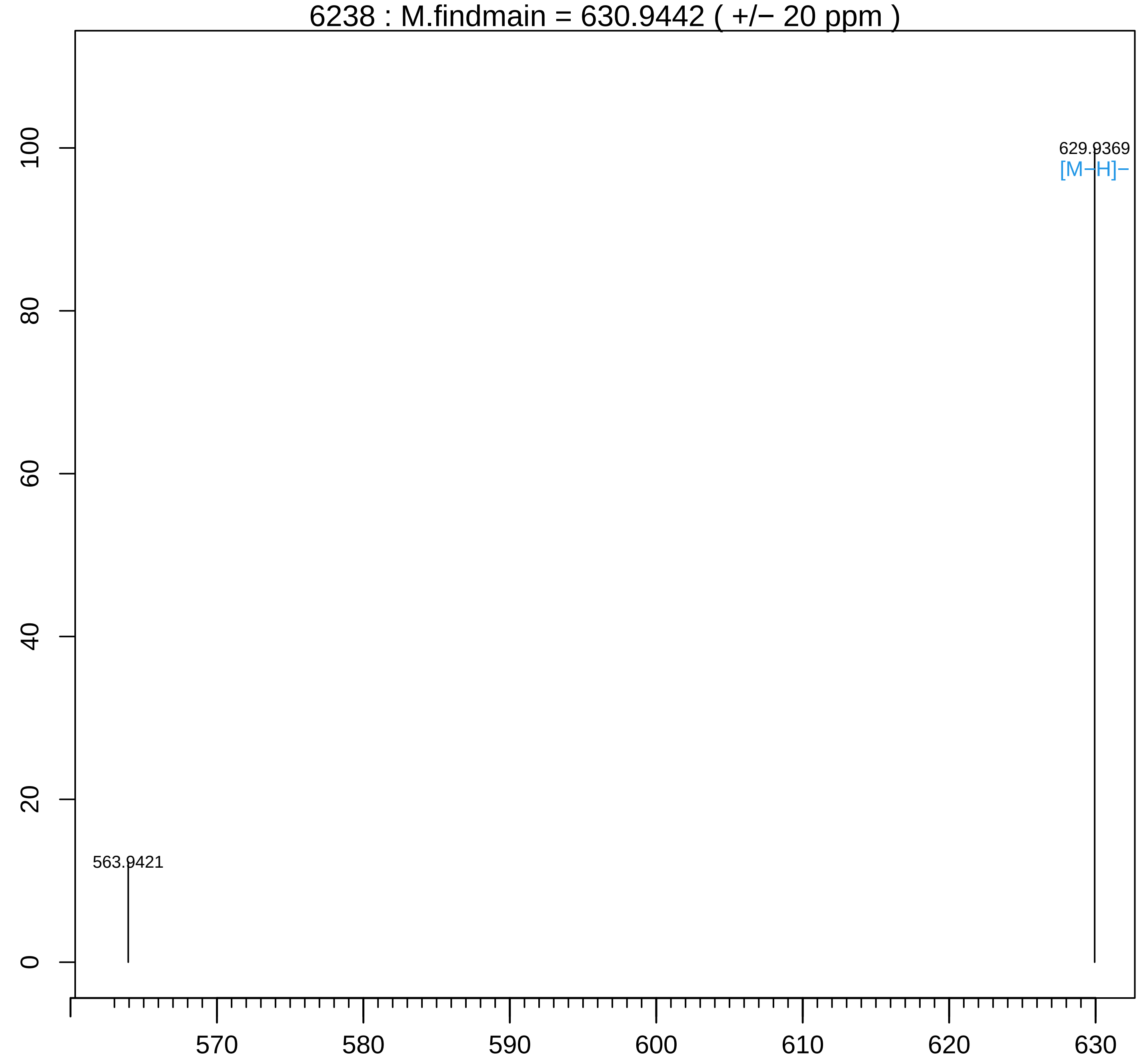
6238 : M.findmain = 630.9442 (+/− 20 ppm)
629.9369
[M−H]−
563.9421
0
20
40
60
80
100
570
580
590
600
610
620
630

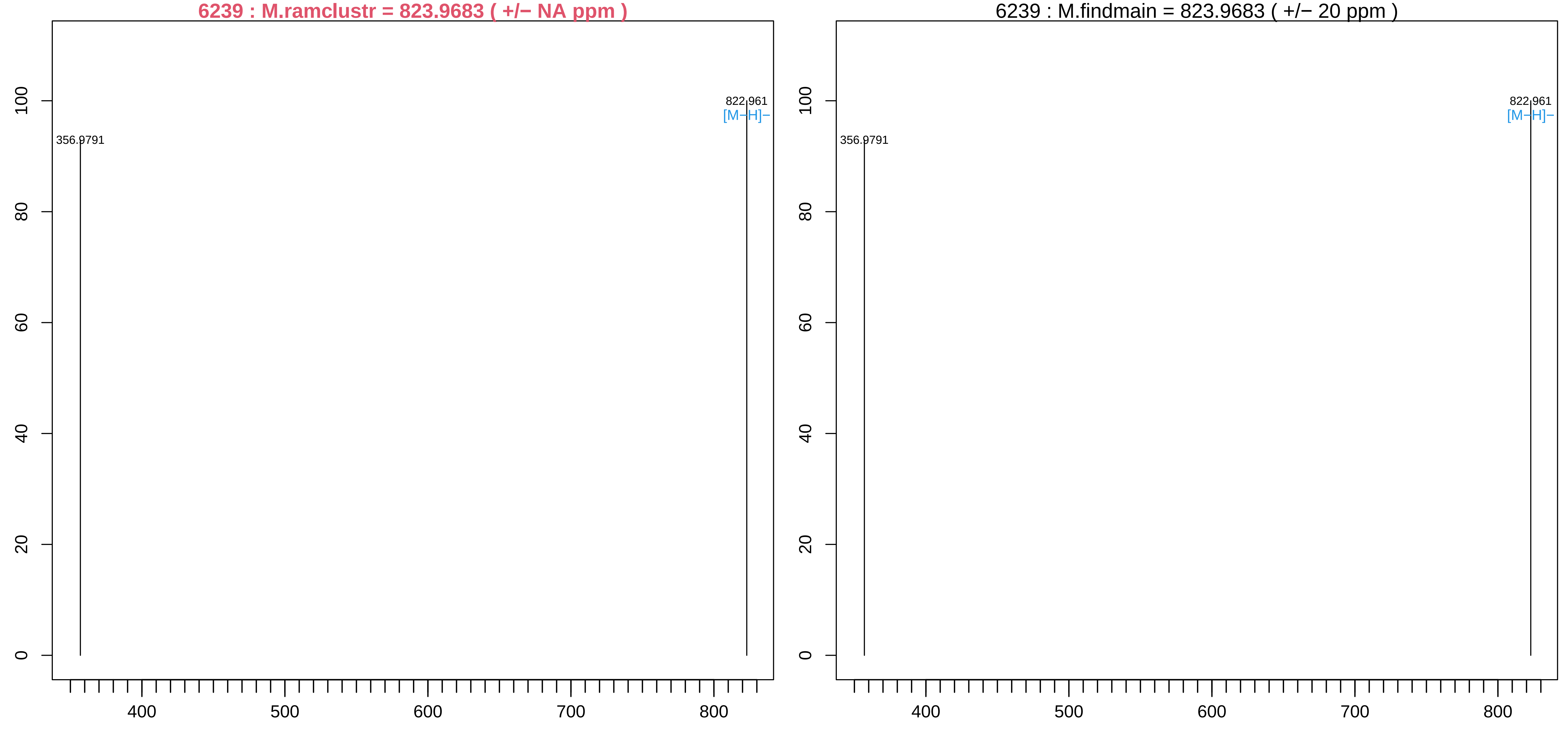
6239 : M.ramclustr = 823.9683 (+/− NA ppm)
356.9791
822.961
[M−H]−
6239 : M.findmain = 823.9683 (+/− 20 ppm)
356.9791
822.961
[M−H]−
0
20
40
60
80
100
400
500
600
700
800

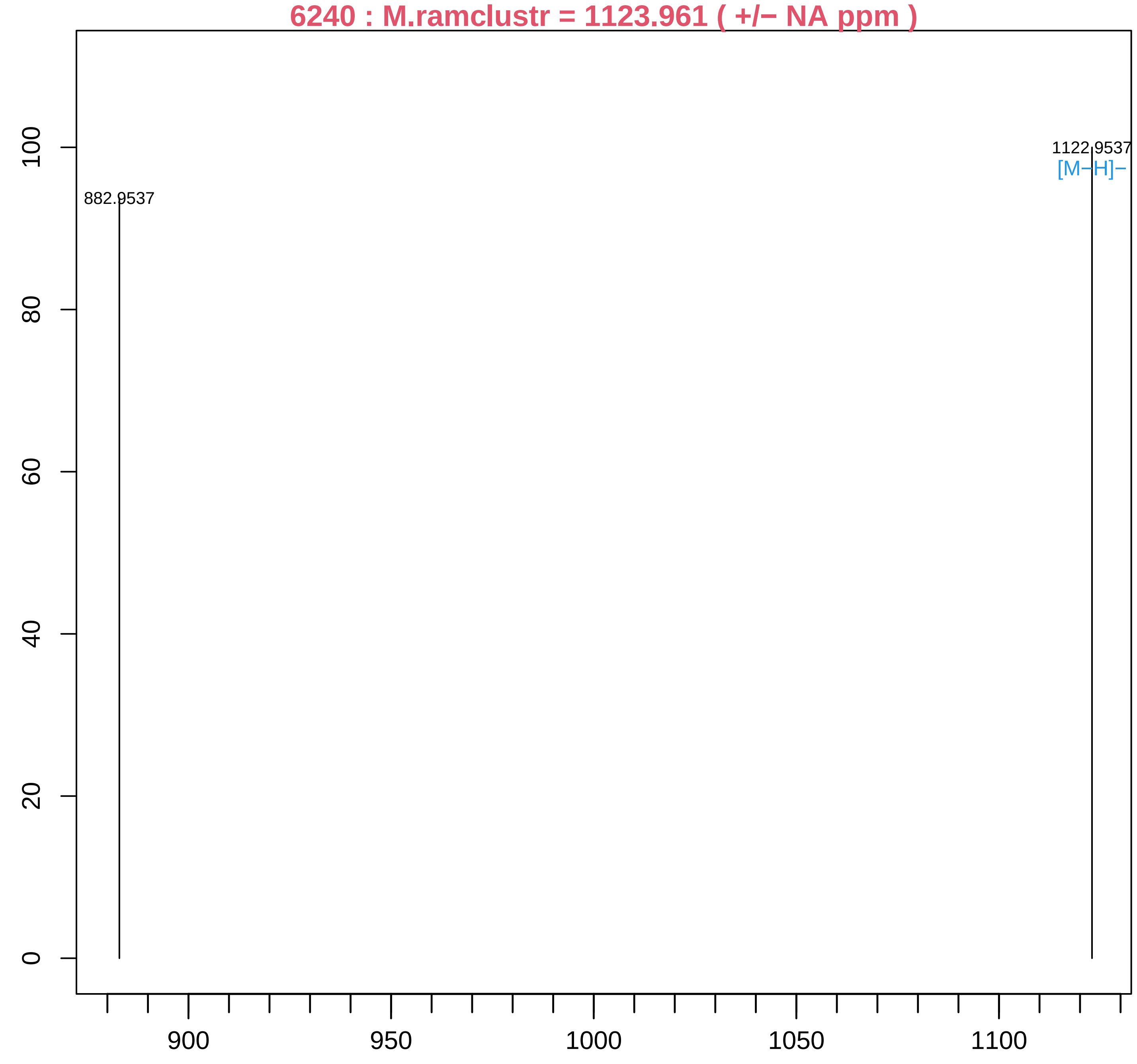
6240 : M.ramclustr = 1123.961 (+/− NA ppm)
1122.9537
[M−H]−
882.9537
0
20
40
60
80
100
900
950
1000
1050
1100

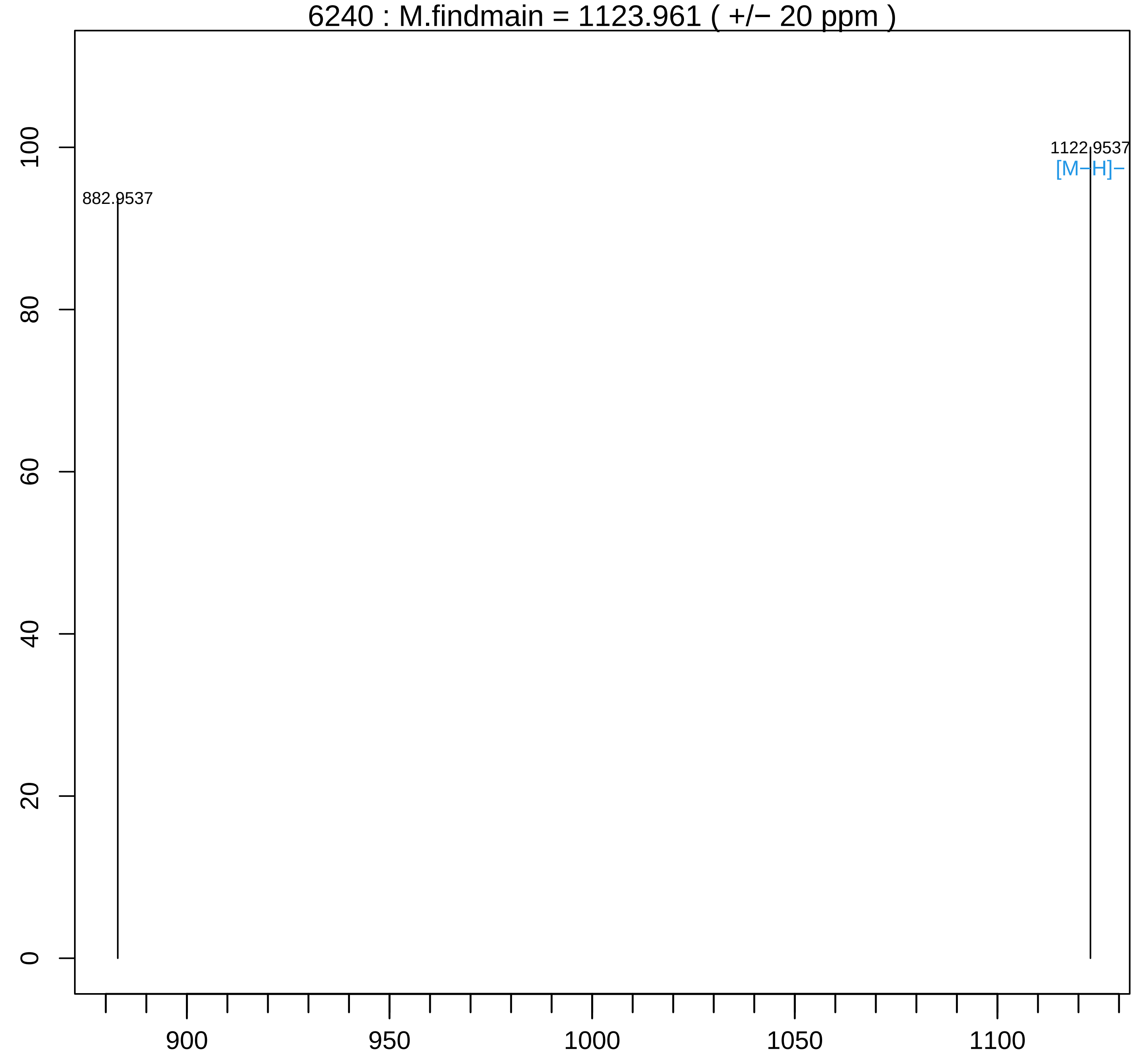
6240 : M.findmain = 1123.961 (+/− 20 ppm)
1122.9537
[M−H]−
882.9537
0
20
40
60
80
100
900
950
1000
1050
1100

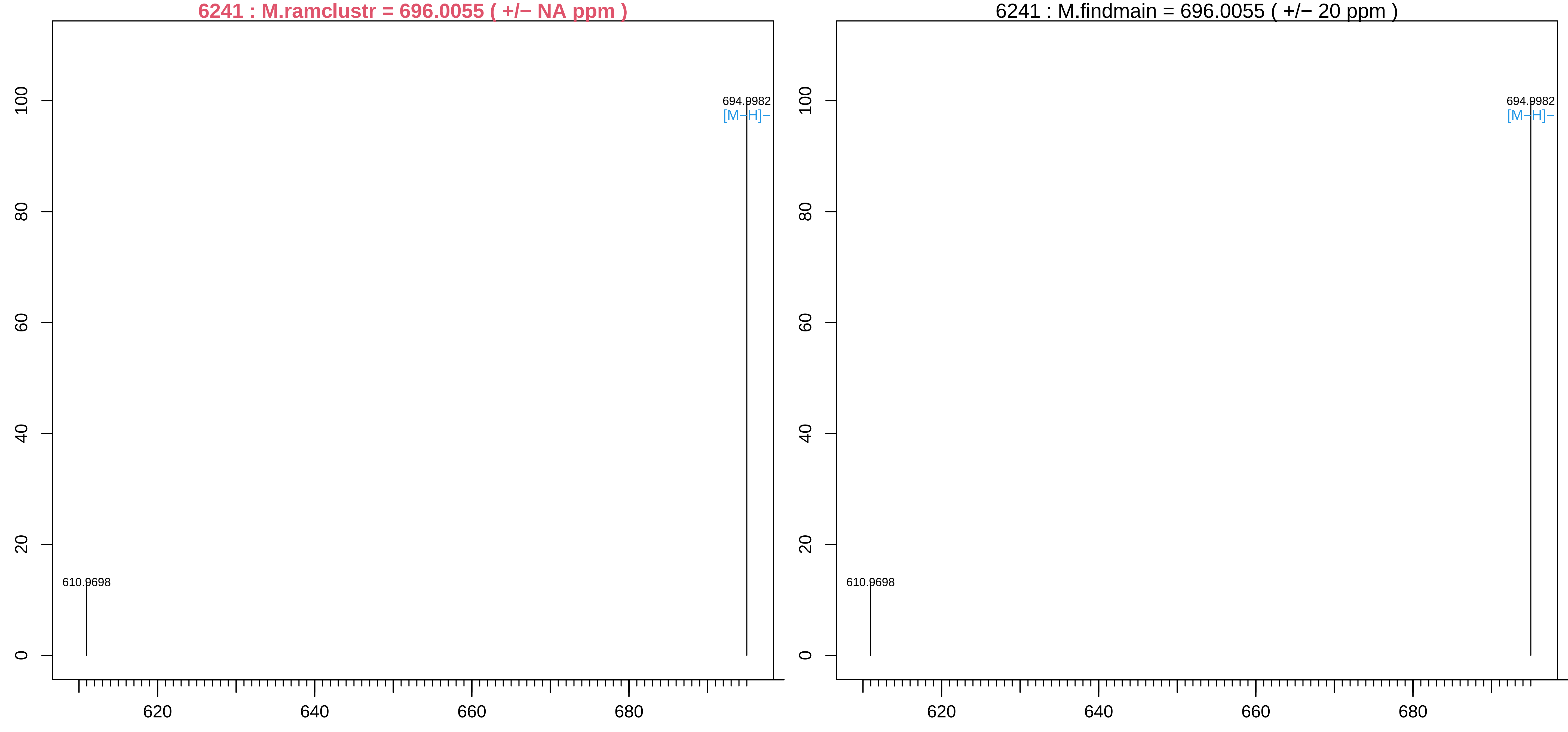

6241 : M.ramclustr = 696.0055 (+/− NA ppm)
694.9982
[M−H]−
610.9698
0
20
40
60
80
100
620
640
660
680
6241 : M.findmain = 696.0055 (+/− 20 ppm)
694.9982
[M−H]−
610.9698
0
20
40
60
80
100
620
640
660
680

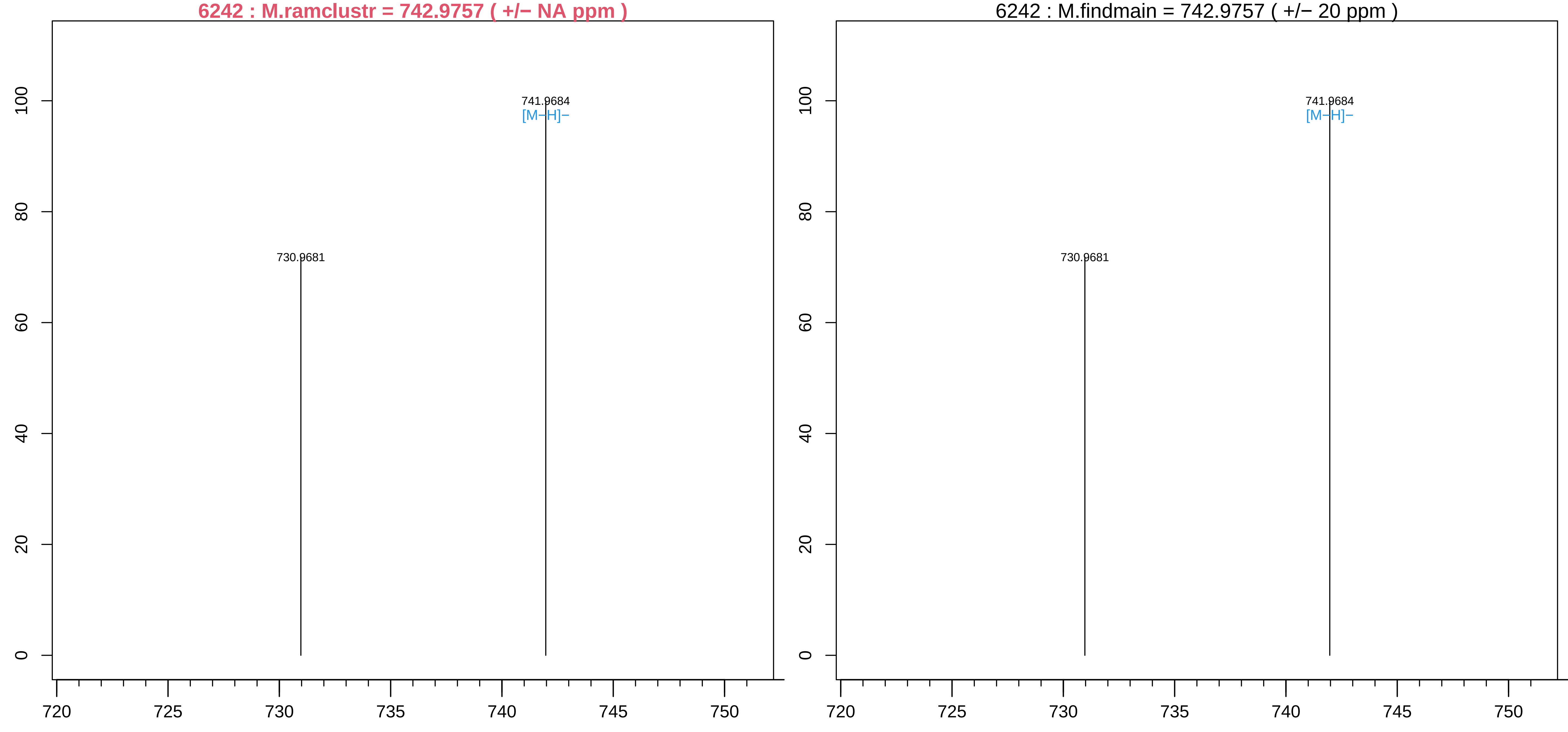
6242 : M.ramclustr = 742.9757 (+/− NA ppm)
741.9684
[M−H]−
730.9681
6242 : M.findmain = 742.9757 (+/− 20 ppm)
741.9684
[M−H]−
730.9681
0
20
40
60
80
100
720
725
730
735
740
745
750

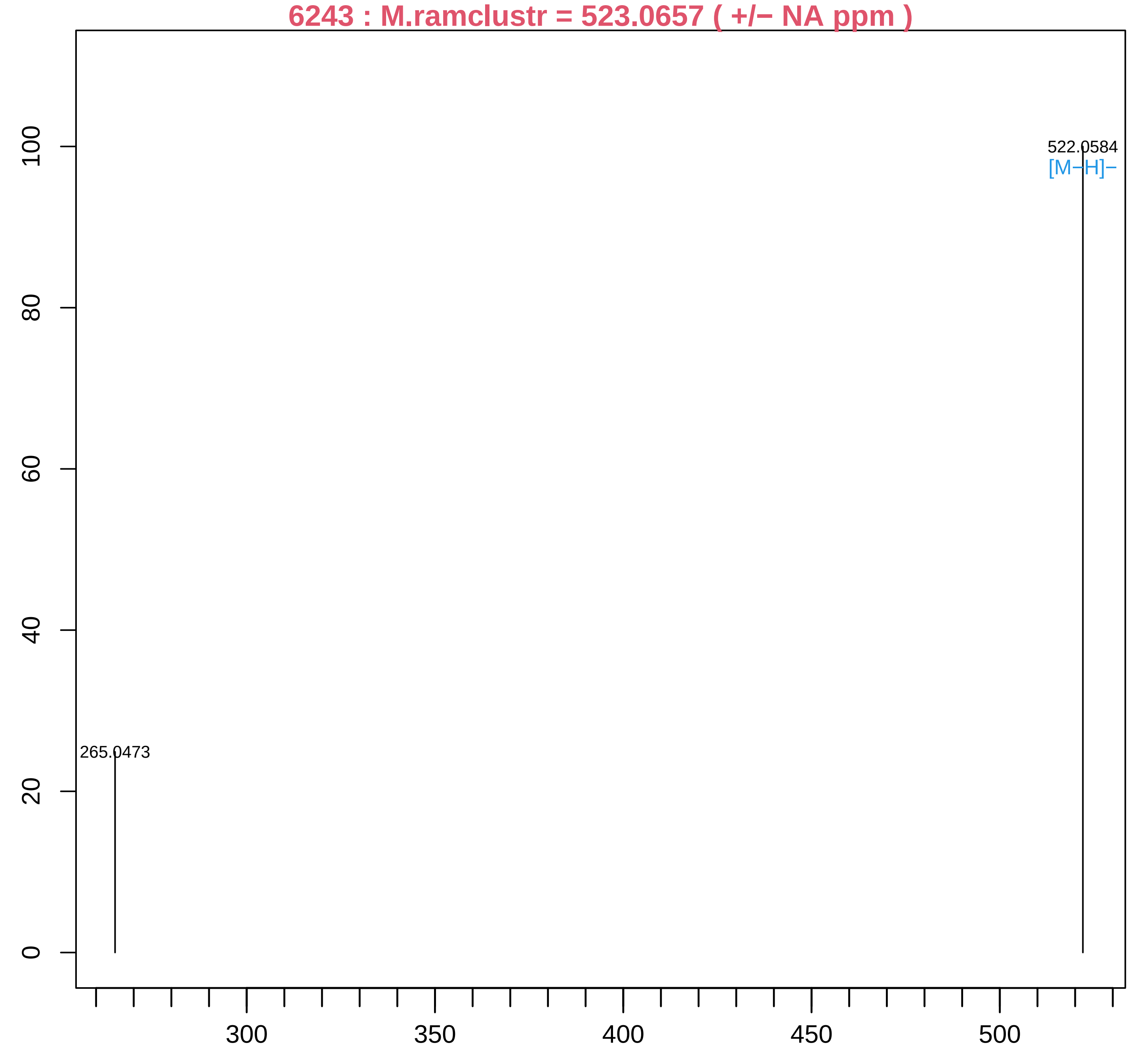
6243 : M.ramclustr = 523.0657 (+/− NA ppm)
522.0584
[M−H]−
265.0473
0
20
40
60
80
100
300
350
400
450
500

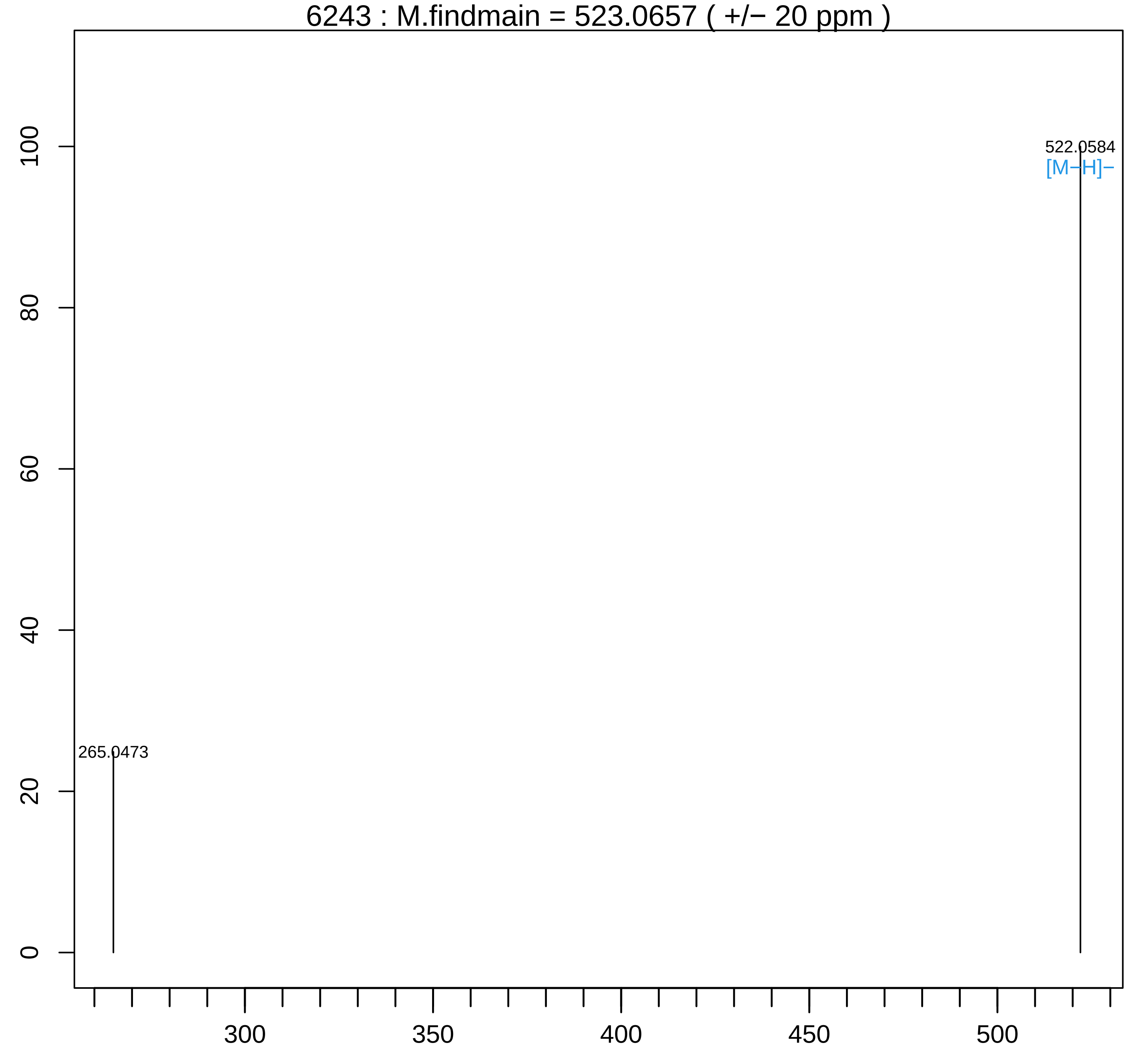
6243 : M.findmain = 523.0657 (+/− 20 ppm)
522.0584
[M−H]−
265.0473
0
20
40
60
80
100
300
350
400
450
500

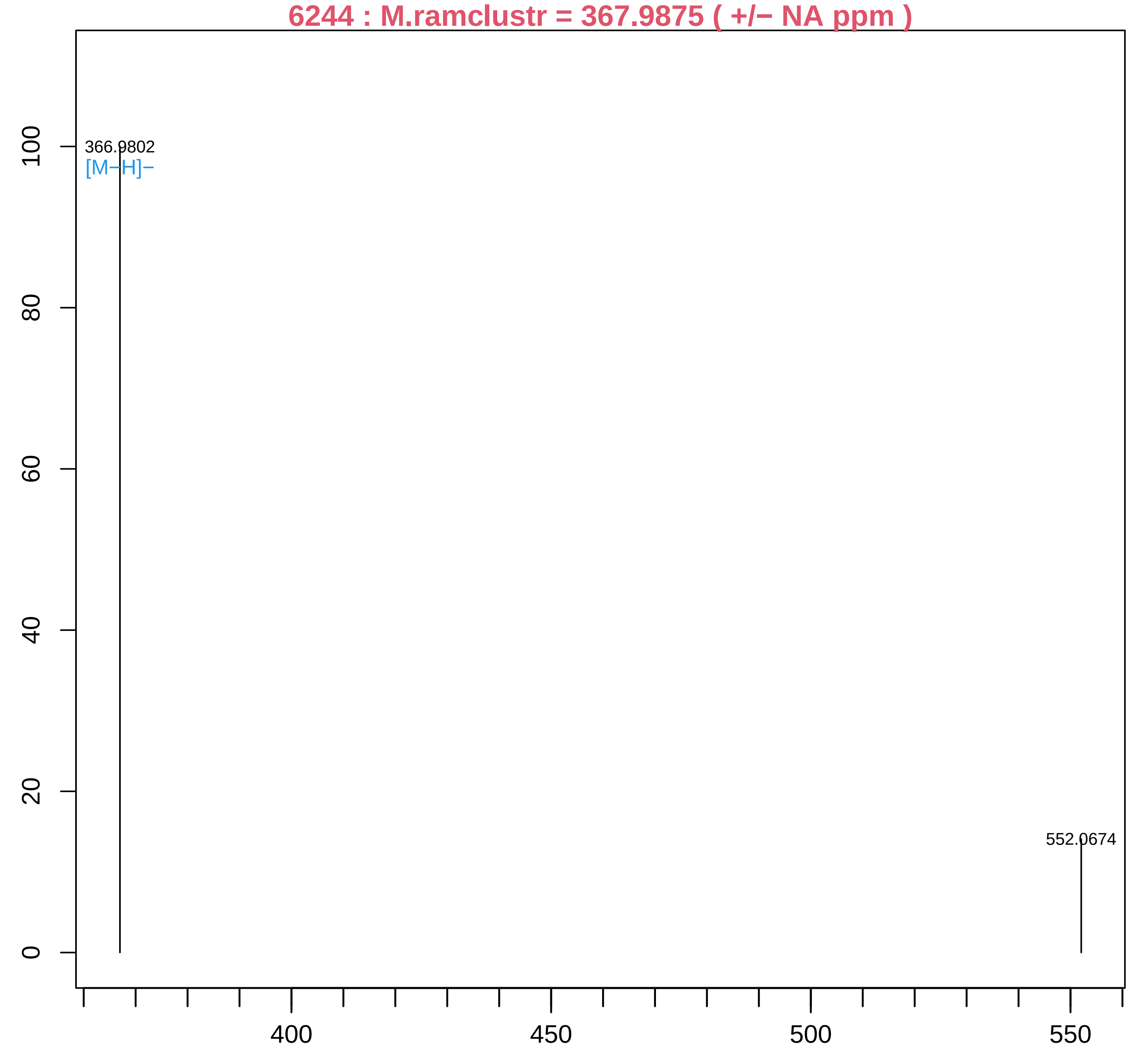
6244 : M.ramclustr = 367.9875 (+/− NA ppm)
366.9802
[M−H]−
552.0674
0
20
40
60
80
100
400
450
500
550

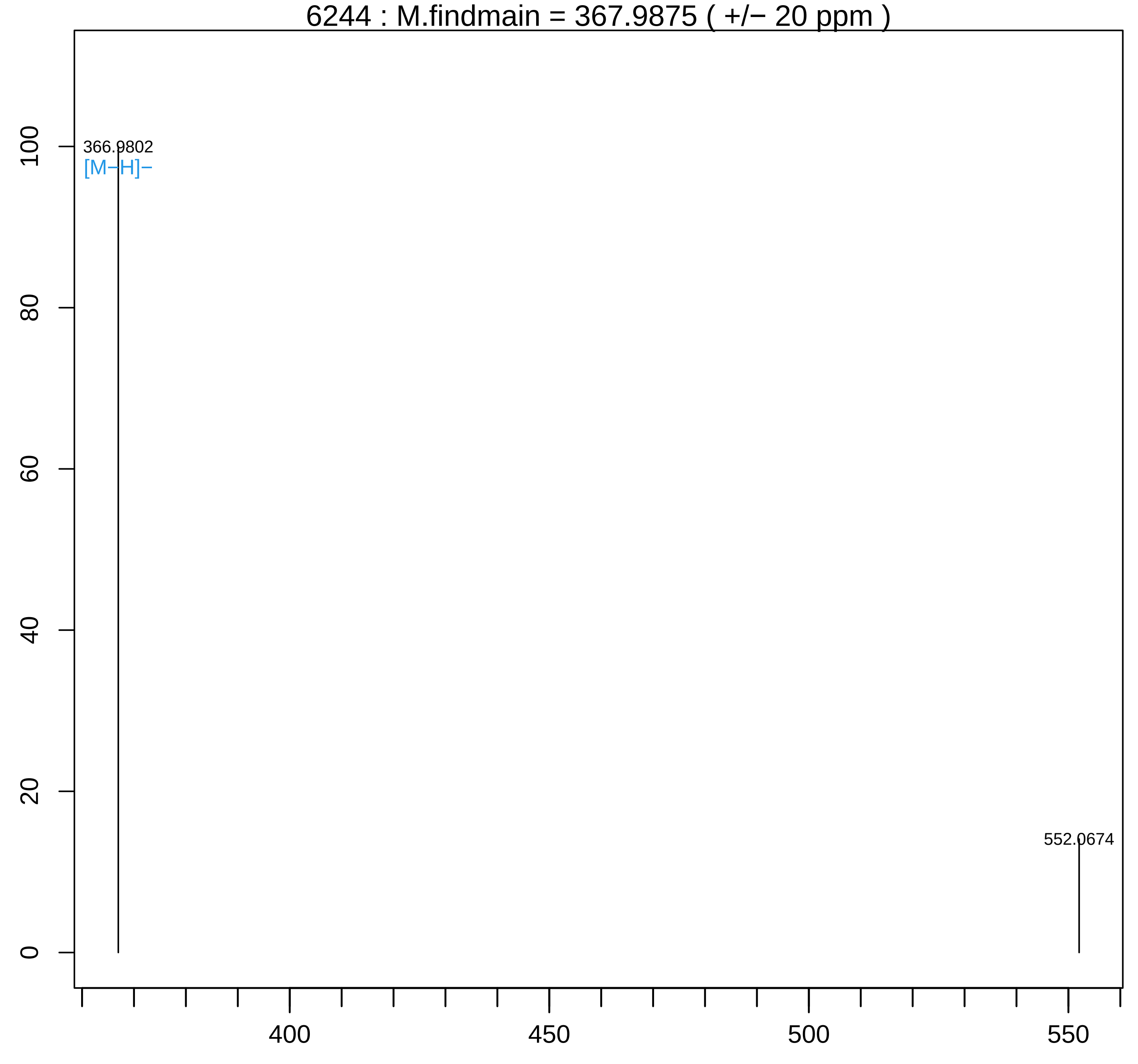
6244 : M.findmain = 367.9875 (+/− 20 ppm)
366.9802
[M−H]−
552.0674
0
20
40
60
80
100
400
450
500
550

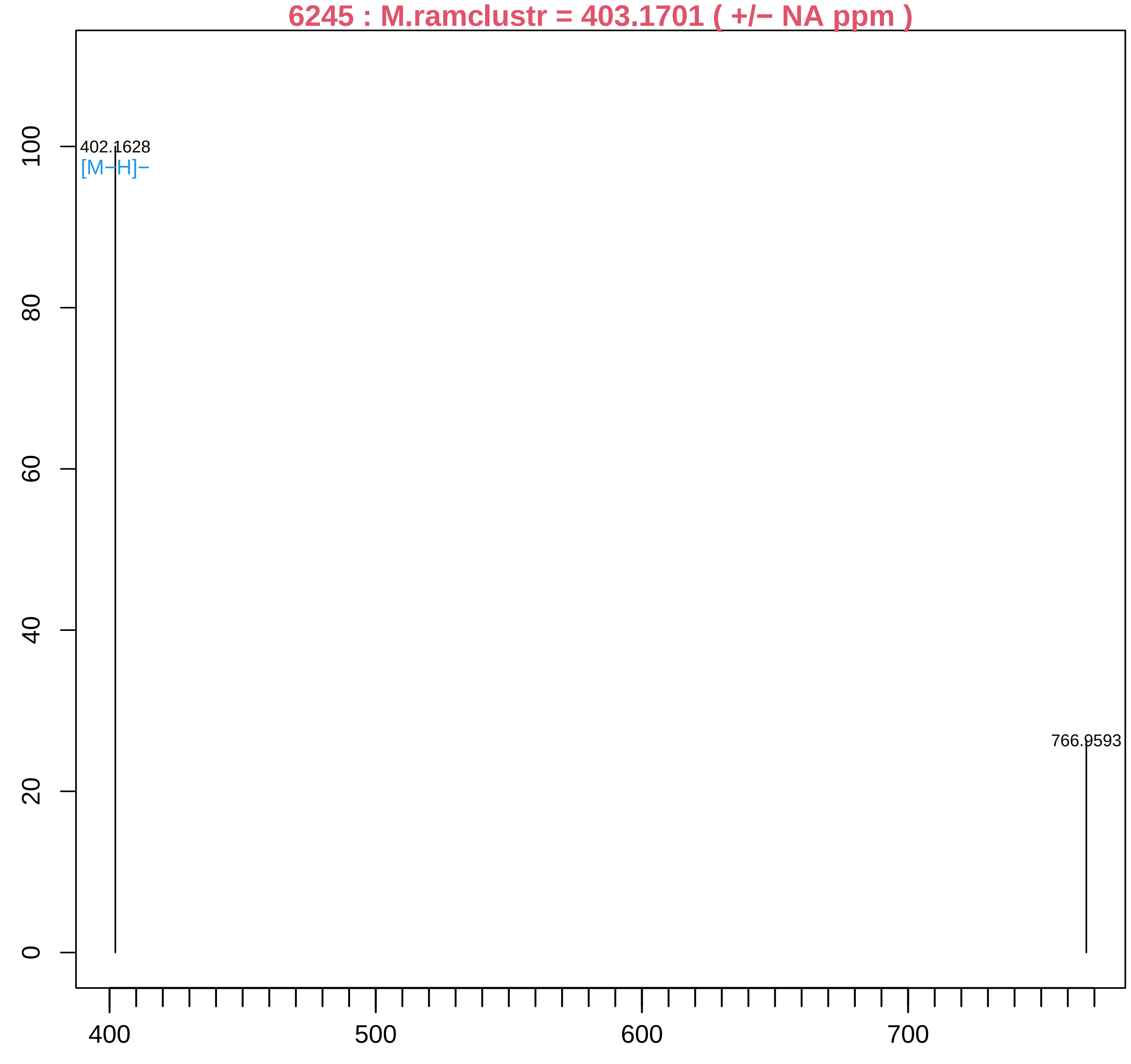
6245 : M.ramclustr = 403.1701 (+/− NA ppm)
402.1628
[M−H]−
766.9593
0
20
40
60
80
100
400
500
600
700

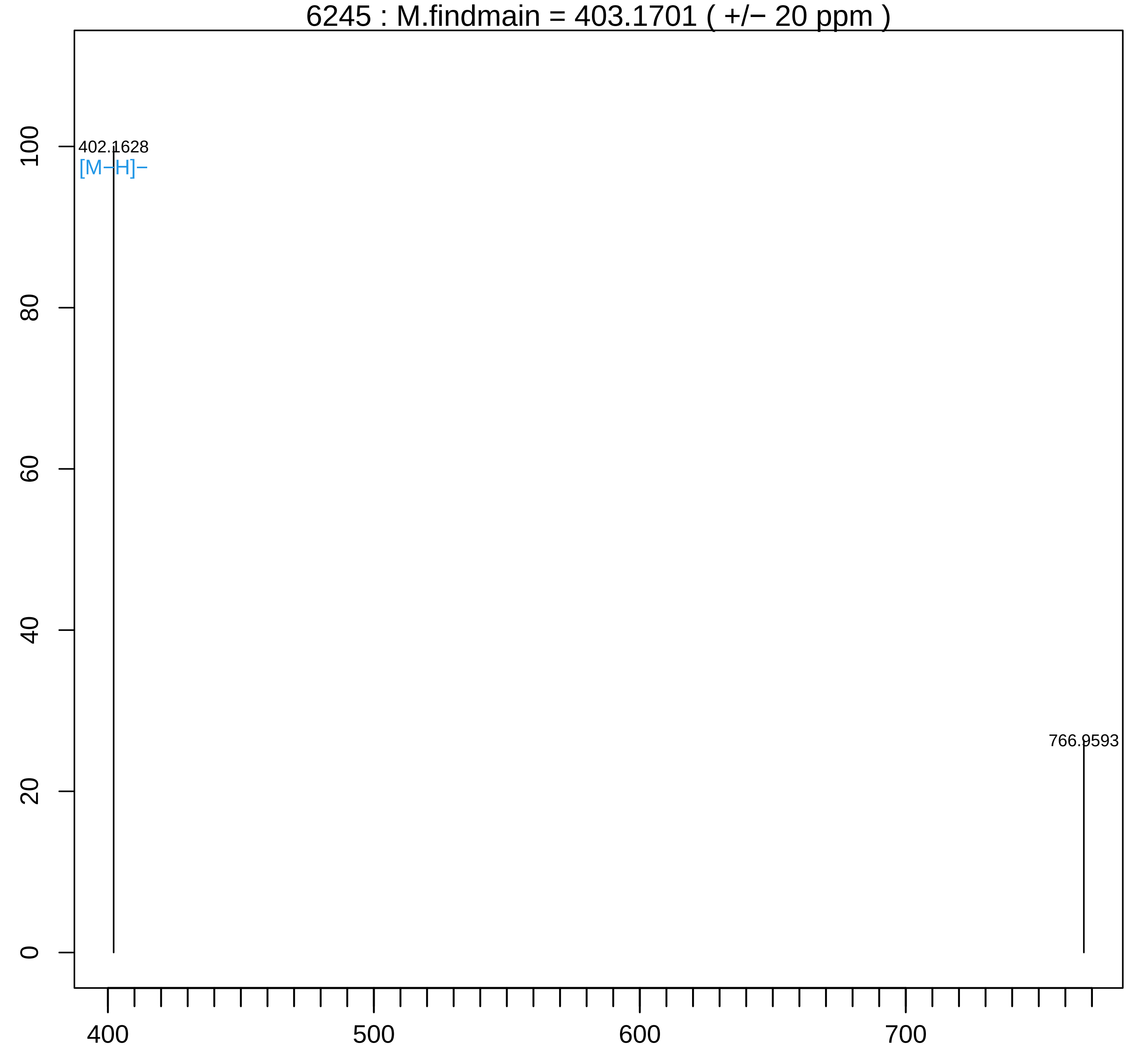
6245 : M.findmain = 403.1701 (+/− 20 ppm)
402.1628
[M−H]−
766.9593
0
20
40
60
80
100
400
500
600
700

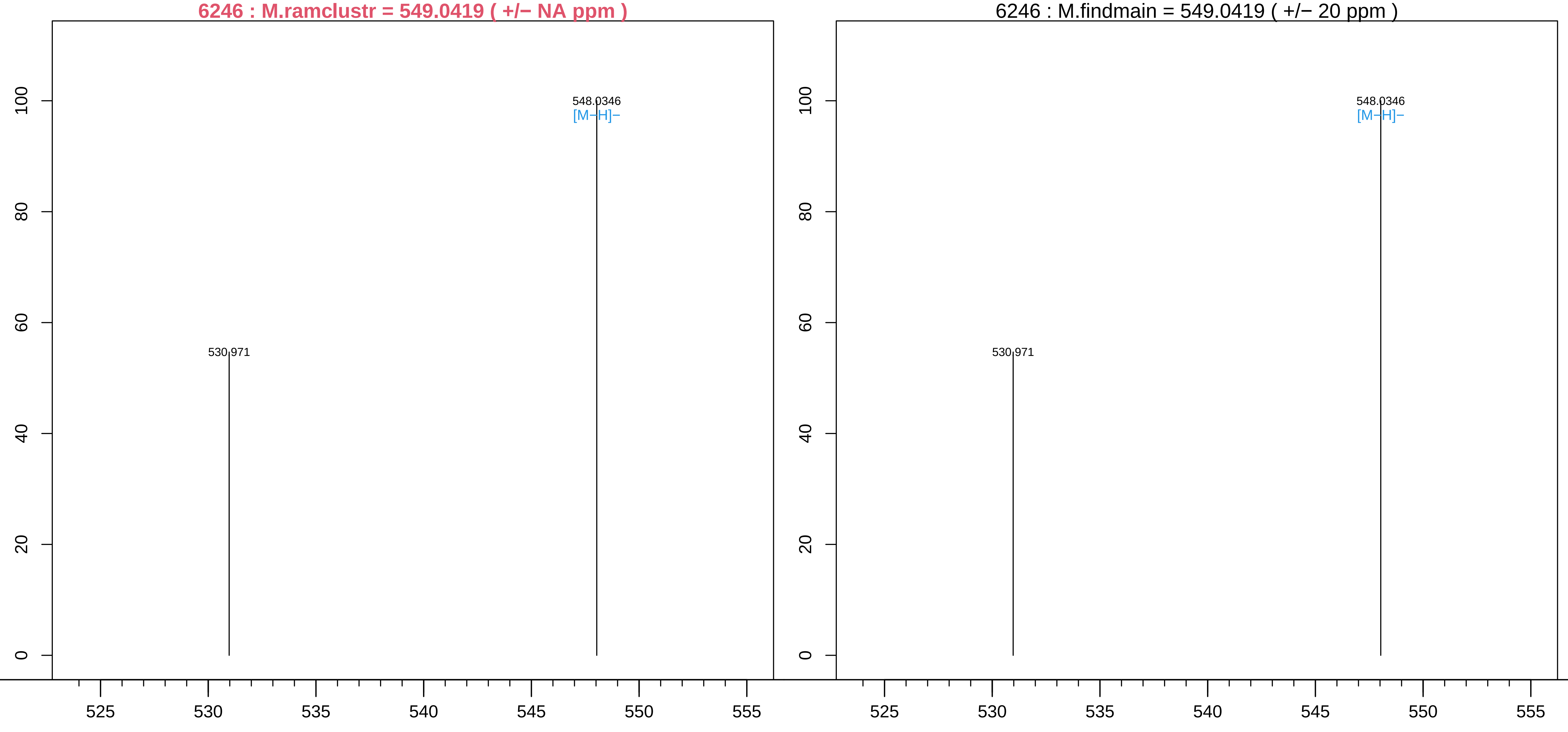
6246 : M.ramclustr = 549.0419 (+/− NA ppm)
6246 : M.findmain = 549.0419 (+/− 20 ppm)
548.0346
[M−H]−
530.971
548.0346
[M−H]−
530.971
0
20
40
60
80
100
525
530
535
540
545
550
555

6247 : M.ramclustr = 777.9755 (+/− NA ppm)

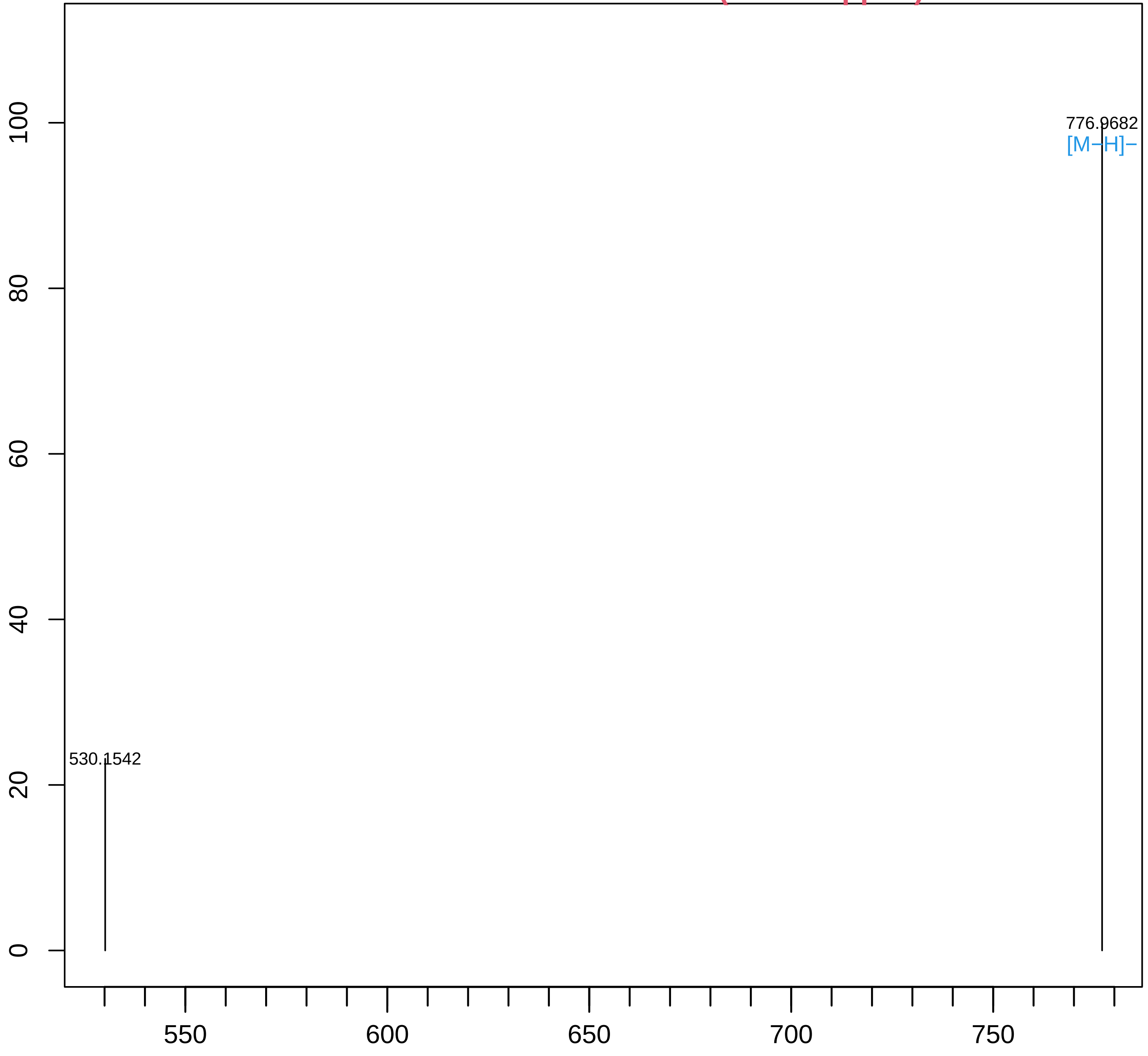

6247 : M.findmain = 777.9755 (+/− 20 ppm)

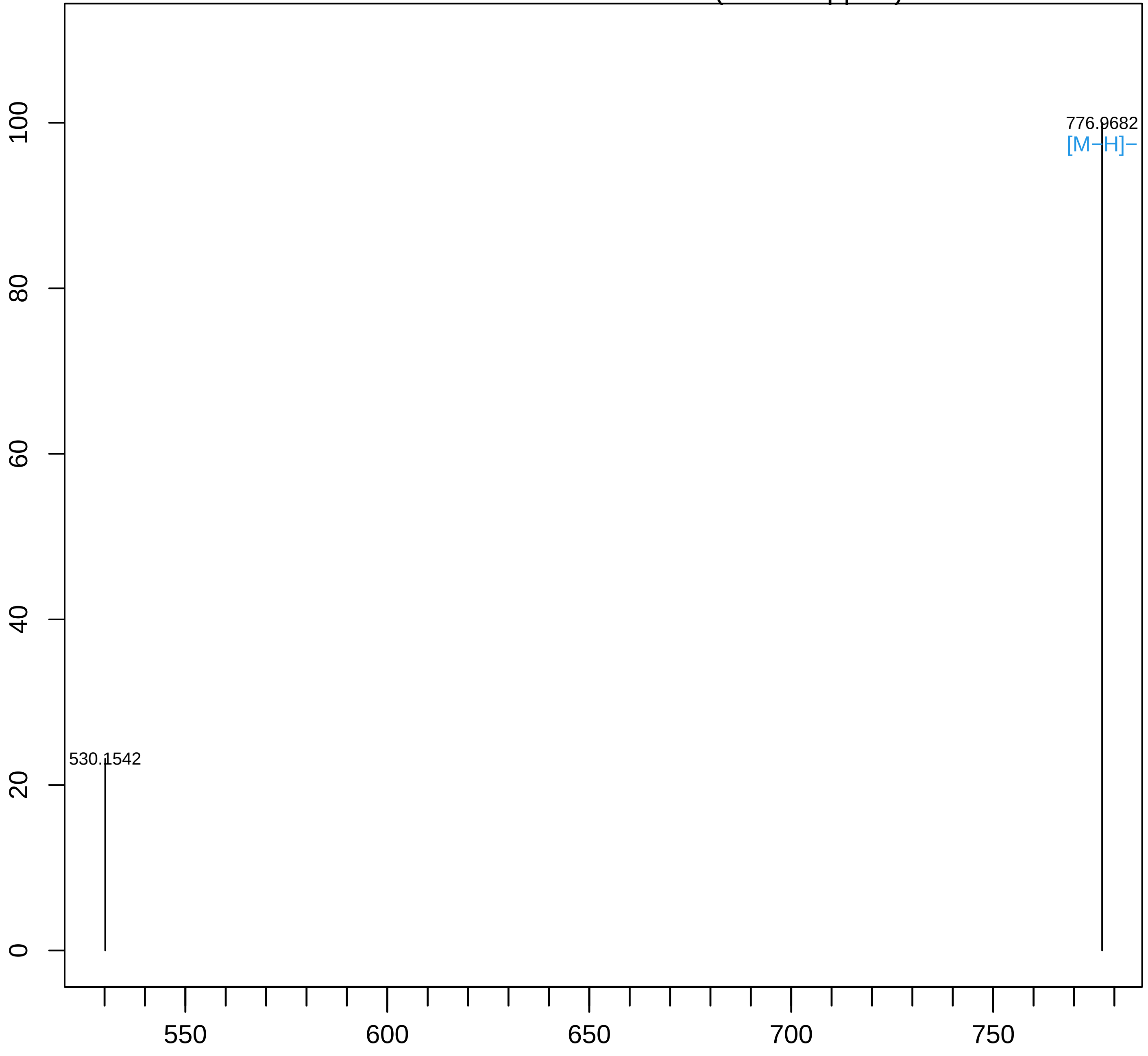

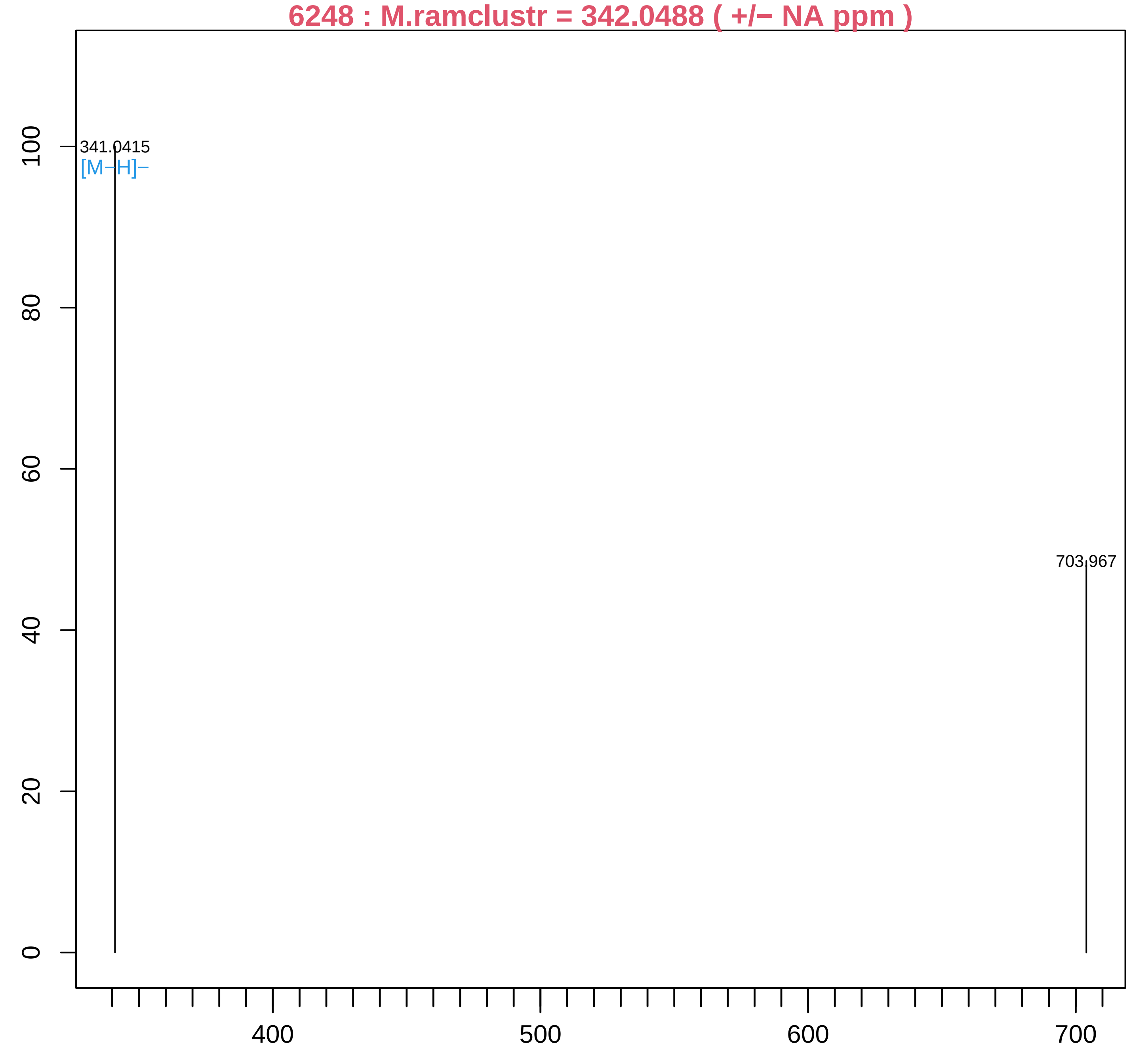
6248 : M.ramclustr = 342.0488 (+/− NA ppm)
341.0415
[M−H]−
703.967
0
20
40
60
80
100
400
500
600
700

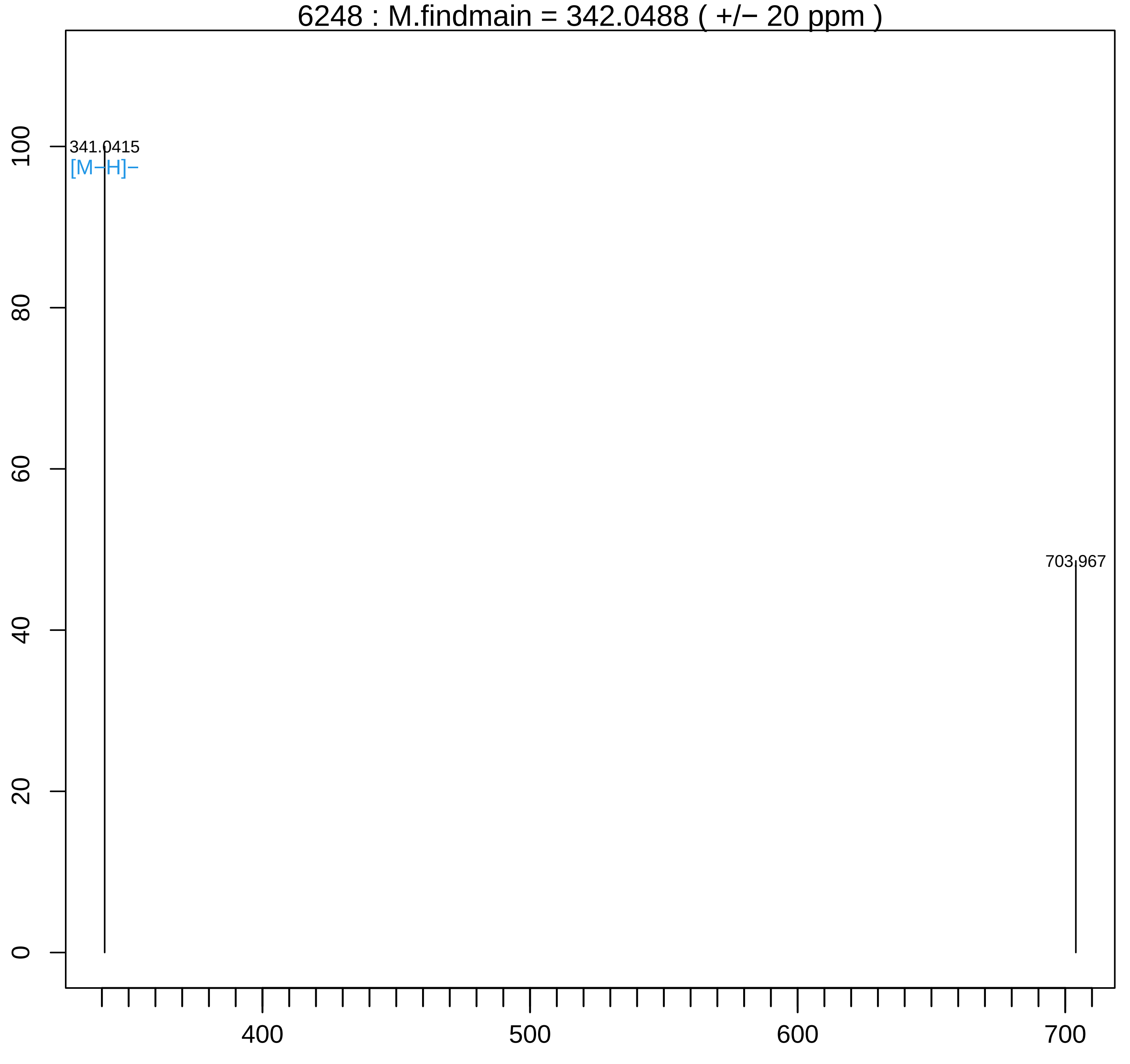
6248 : M.findmain = 342.0488 (+/− 20 ppm)
341.0415
[M−H]−
703.967
0
20
40
60
80
100
400
500
600
700

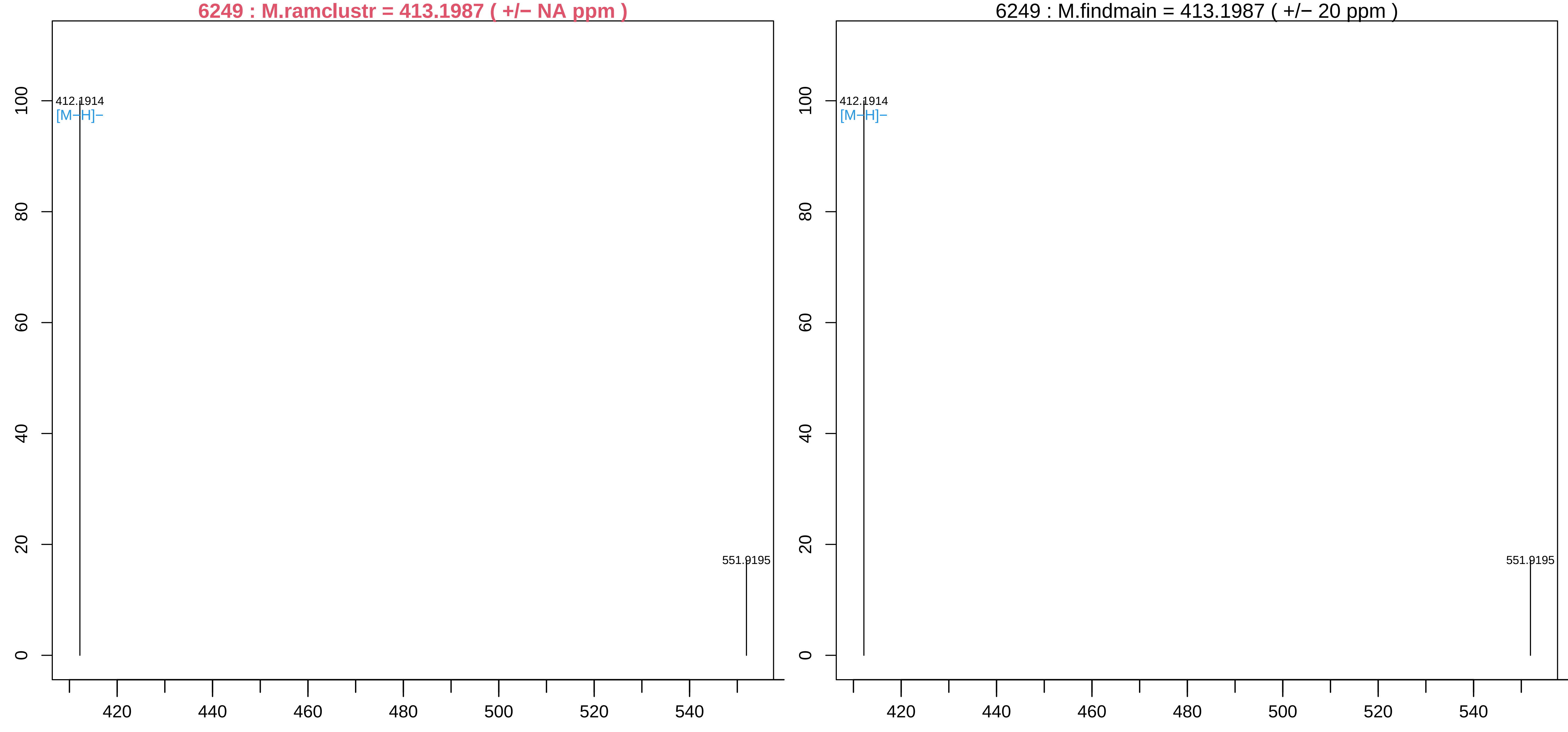
6249 : M.ramclustr = 413.1987 (+/− NA ppm)
412.1914
[M−H]−
551.9195
6249 : M.findmain = 413.1987 (+/− 20 ppm)
412.1914
[M−H]−
551.9195
0
20
40
60
80
100
420
440
460
480
500
520
540

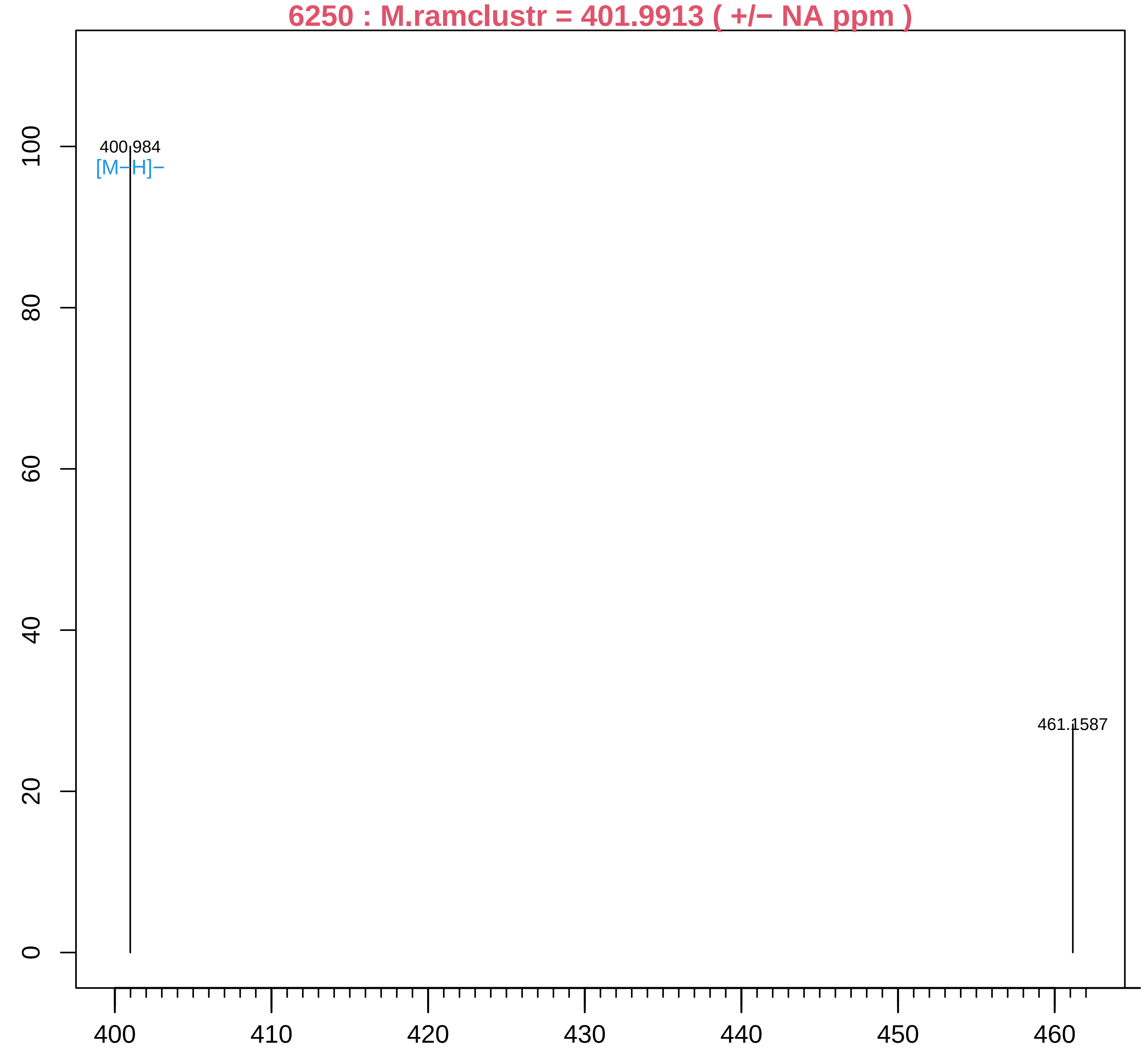
6250 : M.ramclustr = 401.9913 (+/− NA ppm)
400.984
[M−H]−
461.1587
0
20
40
60
80
100
400
410
420
430
440
450
460

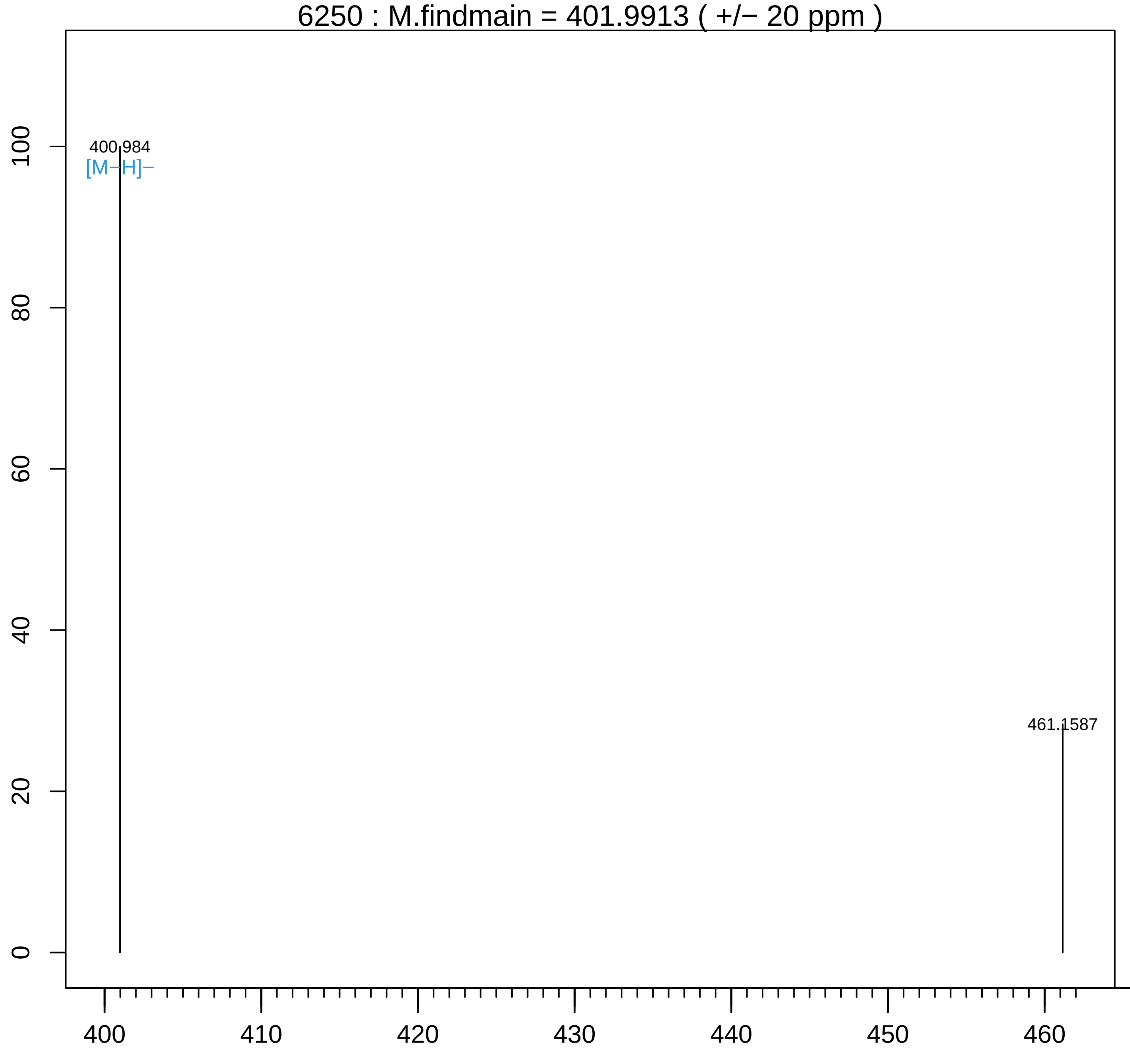
6250 : M.findmain = 401.9913 (+/− 20 ppm)
400.984
[M−H]−
461.1587
0
20
40
60
80
100
400
410
420
430
440
450
460

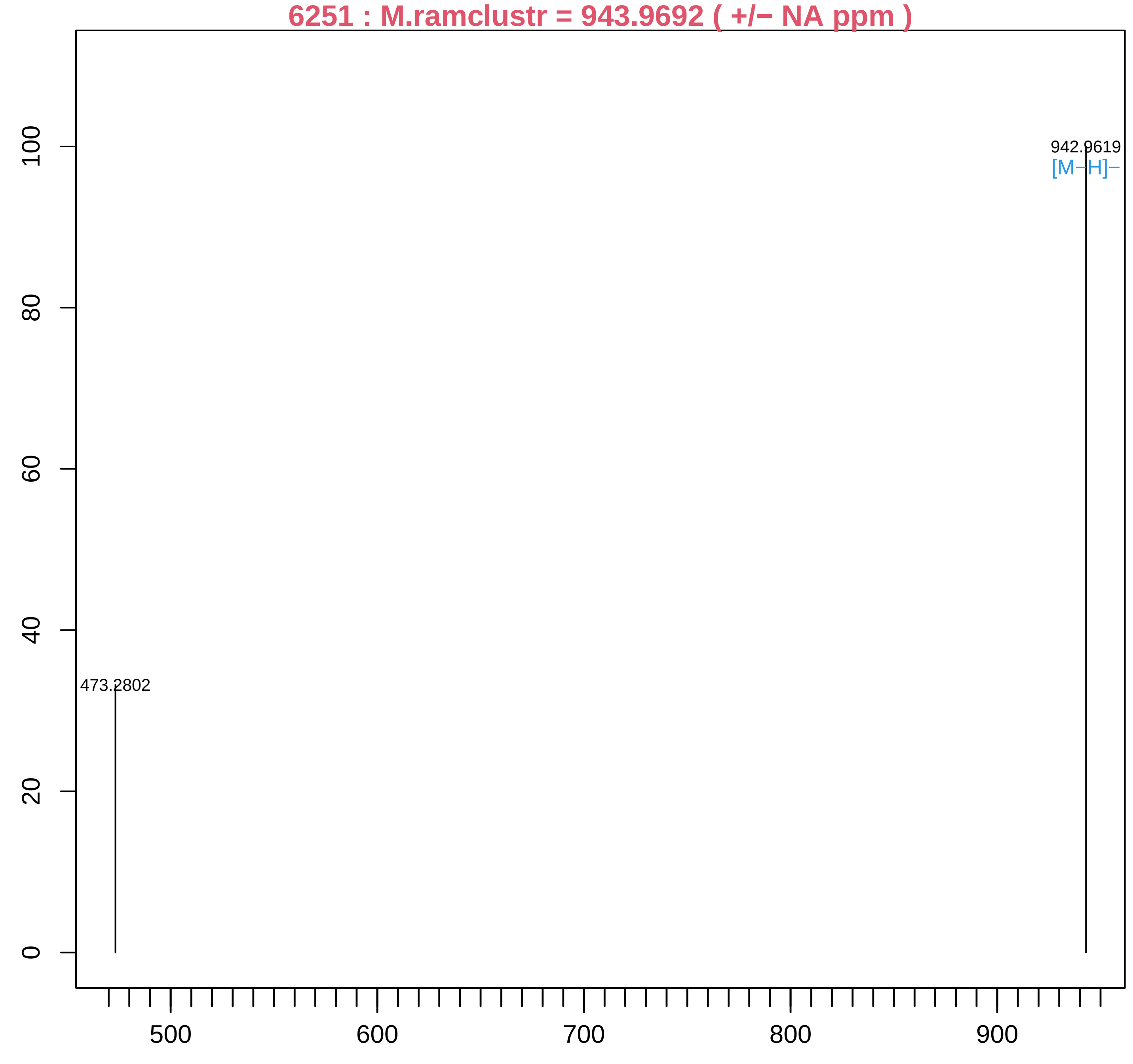

6251 : M.ramclustr = 943.9692 (+/− NA ppm)
942.9619
[M−H]−
473.2802
0
20
40
60
80
100
500
600
700
800
900

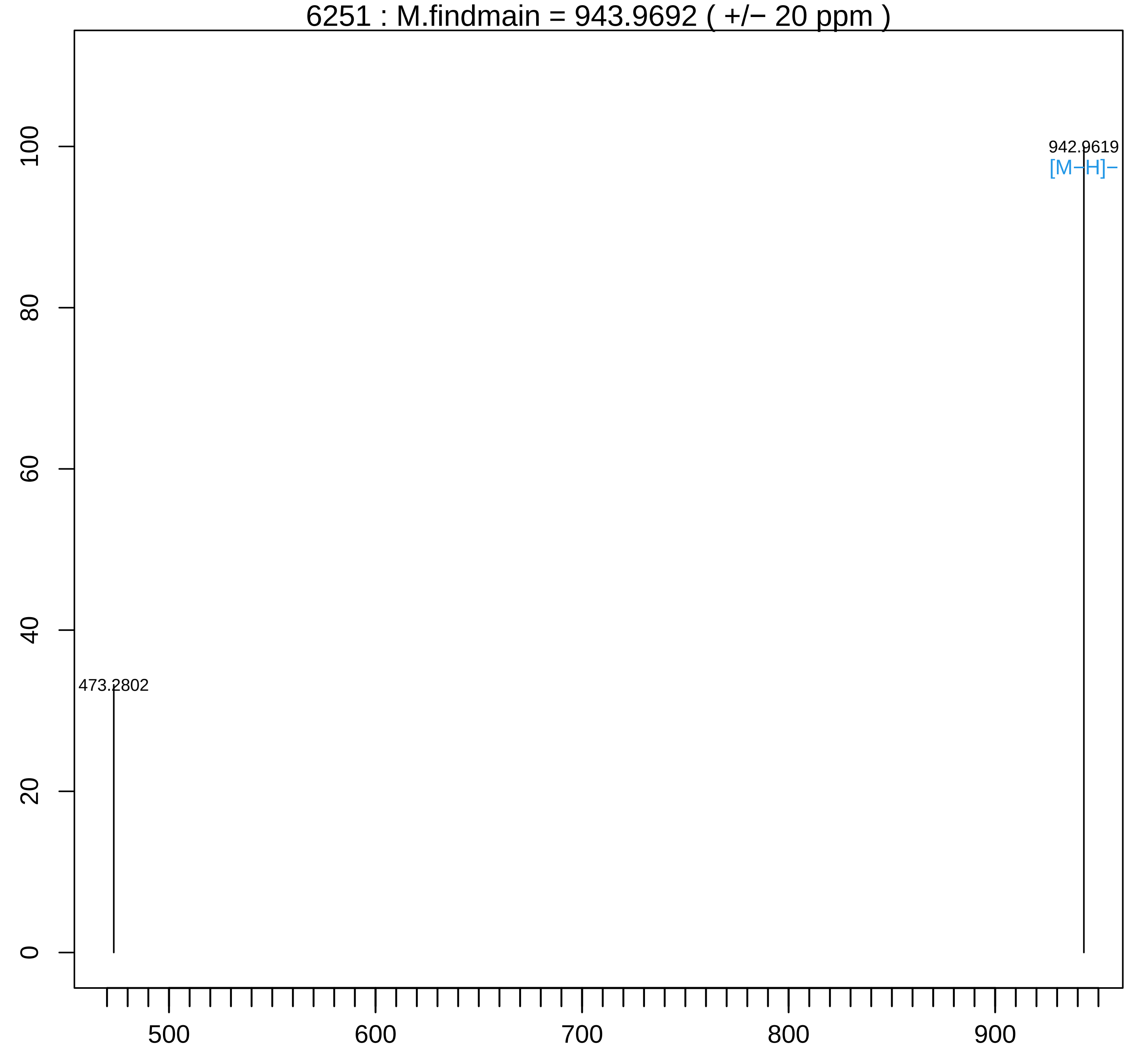

6251 : M.findmain = 943.9692 (+/− 20 ppm)
942.9619
[M−H]−
473.2802
0
20
40
60
80
100
500
600
700
800
900

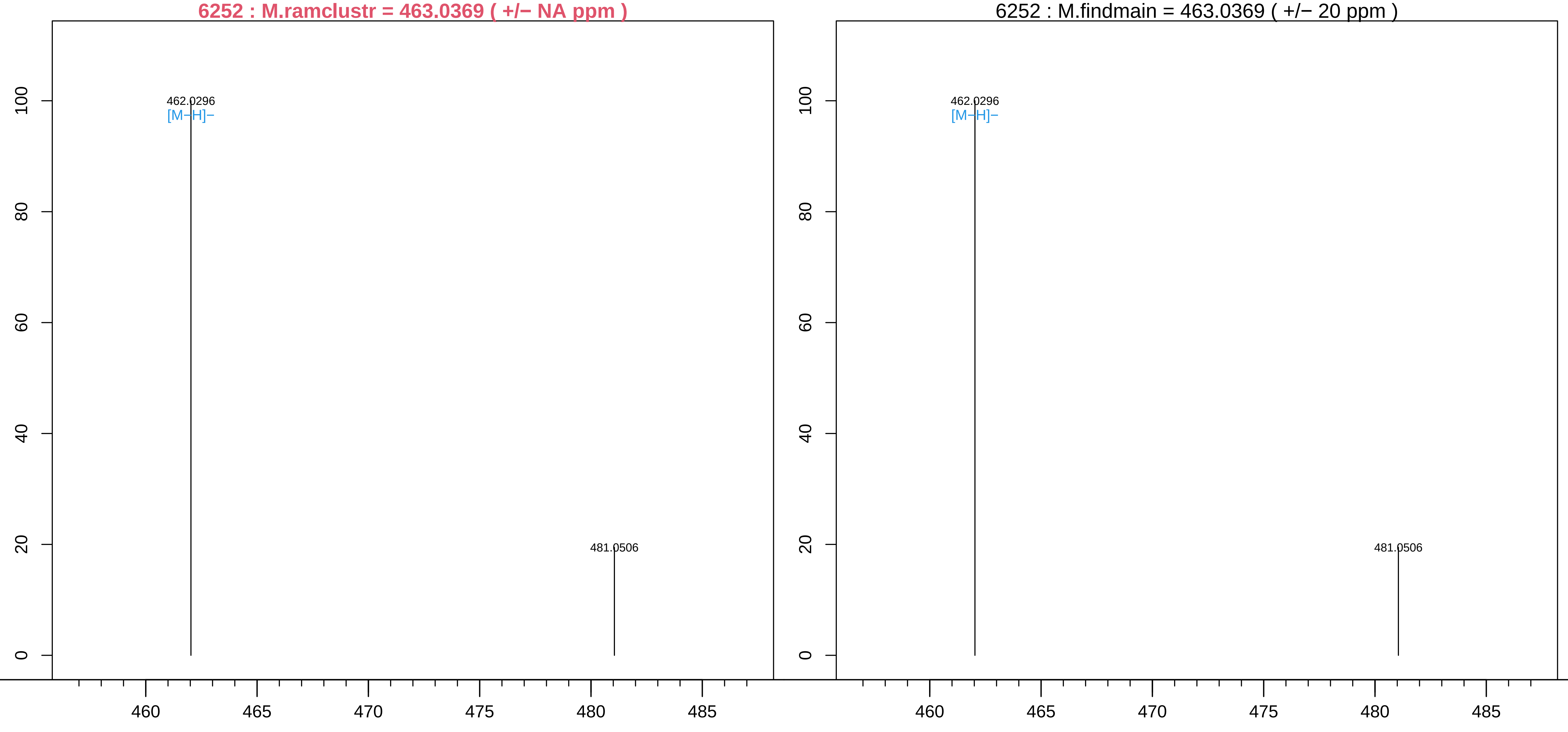
6252 : M.ramclustr = 463.0369 (+/− NA ppm)
462.0296
[M−H]−
481.0506
6252 : M.findmain = 463.0369 (+/− 20 ppm)
462.0296
[M−H]−
481.0506
0
20
40
60
80
100
460
465
470
475
480
485

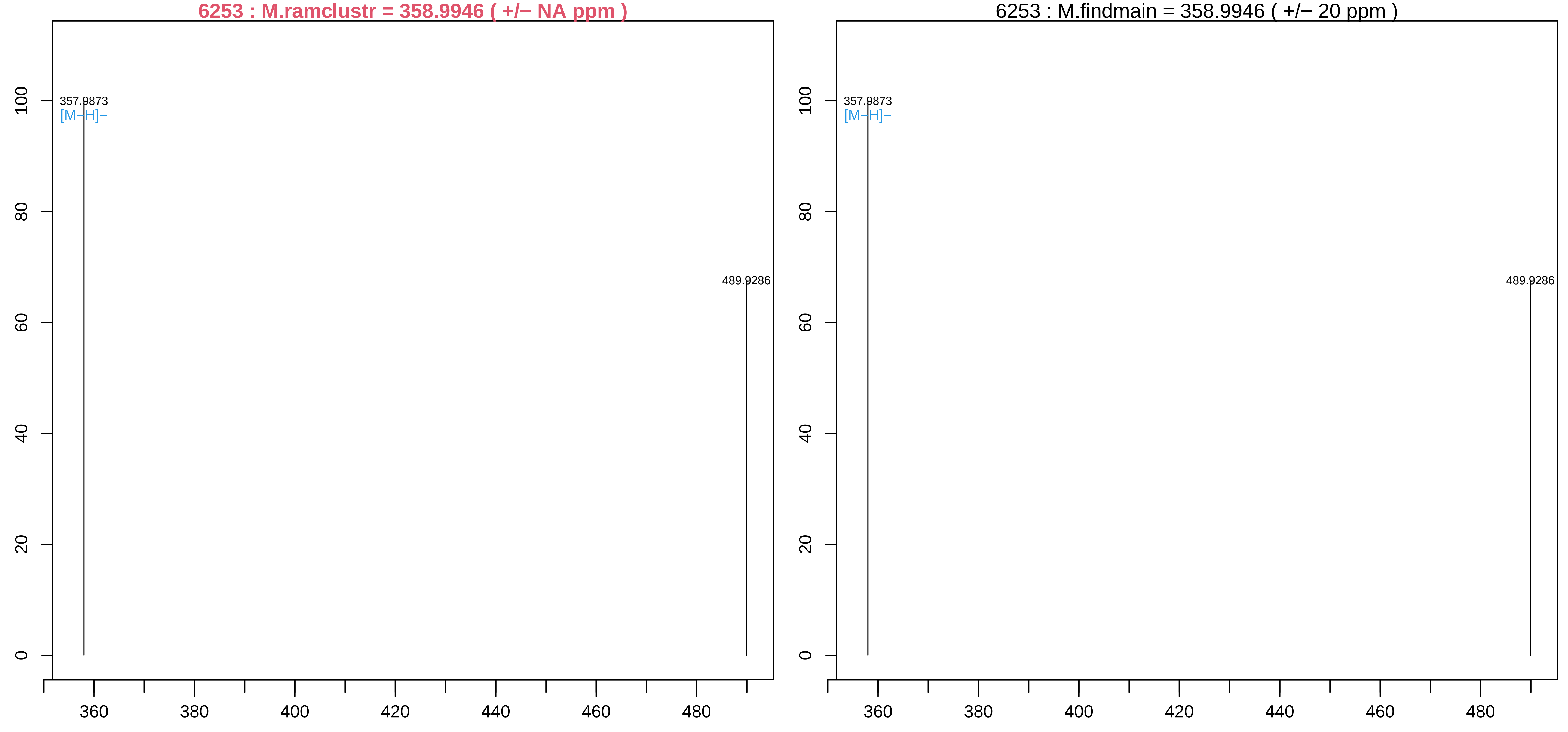
6253 : M.ramclustr = 358.9946 (+/− NA ppm)
357.9873
[M−H]−
489.9286
6253 : M.findmain = 358.9946 (+/− 20 ppm)
357.9873
[M−H]−
489.9286
0
20
40
60
80
100
360
380
400
420
440
460
480

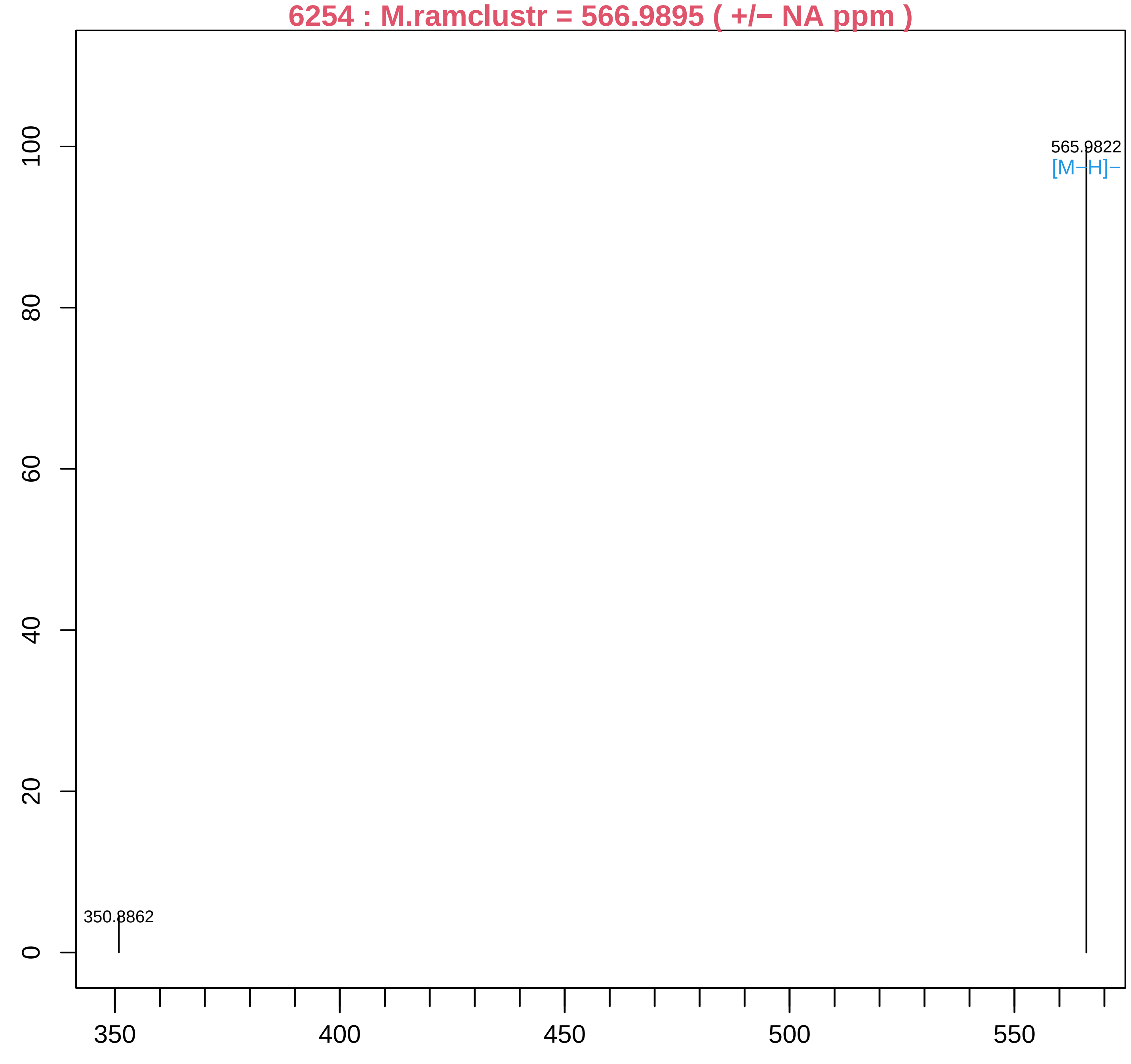
6254 : M.ramclustr = 566.9895 (+/− NA ppm)
565.9822
[M−H]−
350.8862
0
20
40
60
80
100
350
400
450
500
550

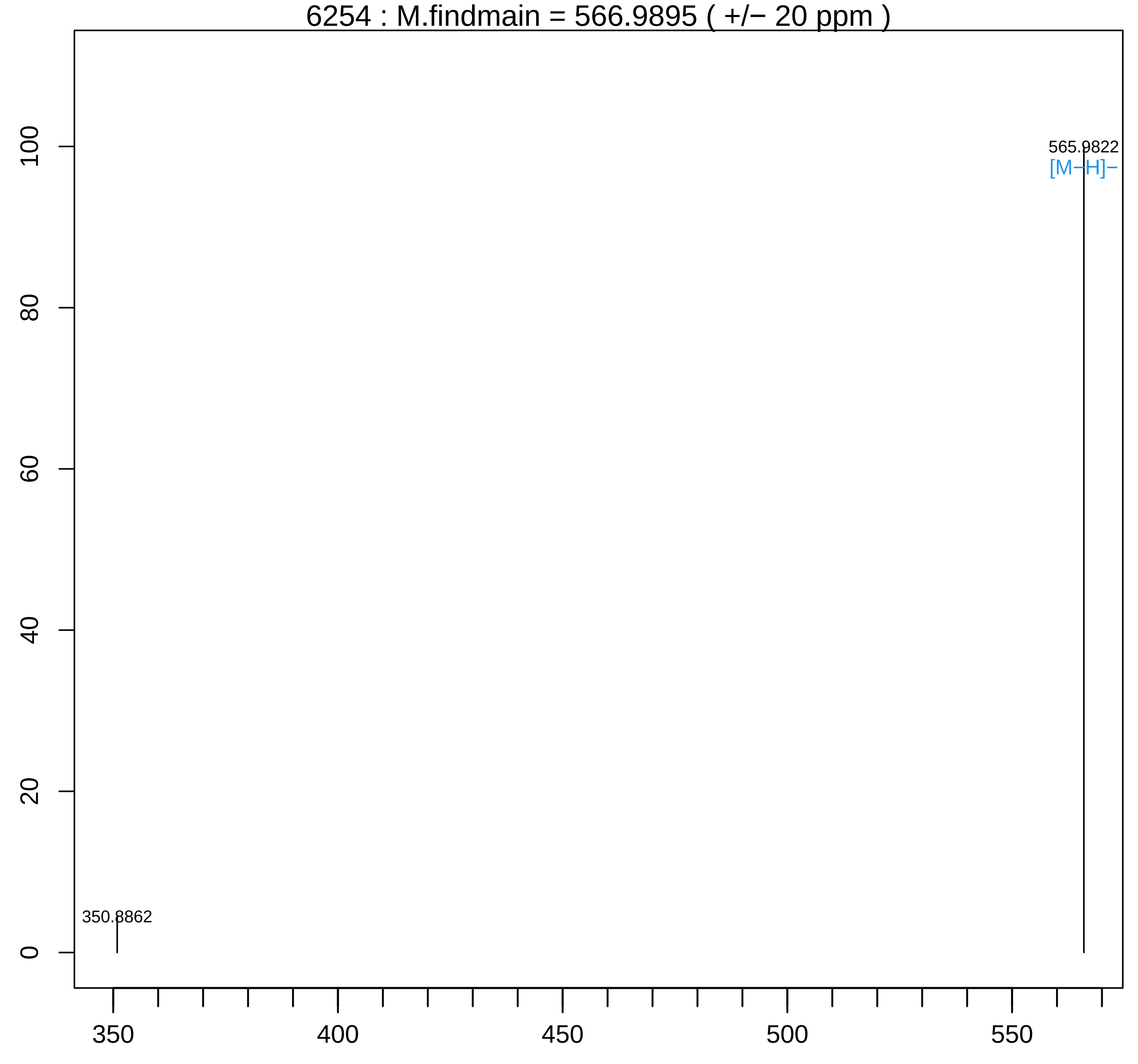
6254 : M.findmain = 566.9895 (+/− 20 ppm)
565.9822
[M−H]−
350.8862
0
20
40
60
80
100
350
400
450
500
550

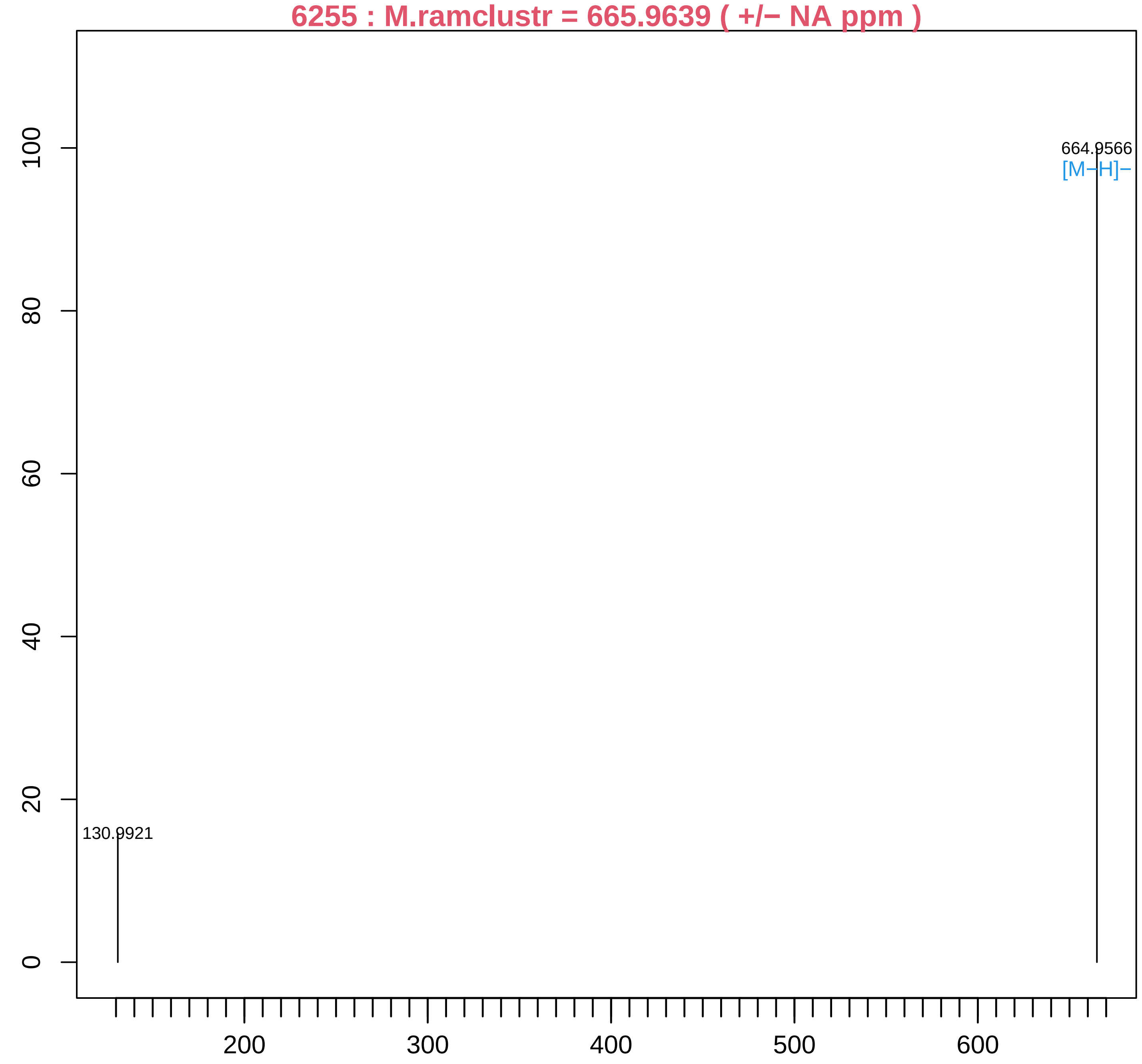
6255 : M.ramclustr = 665.9639 (+/− NA ppm)
664.9566
[M−H]−
130.9921
0
20
40
60
80
100
200
300
400
500
600

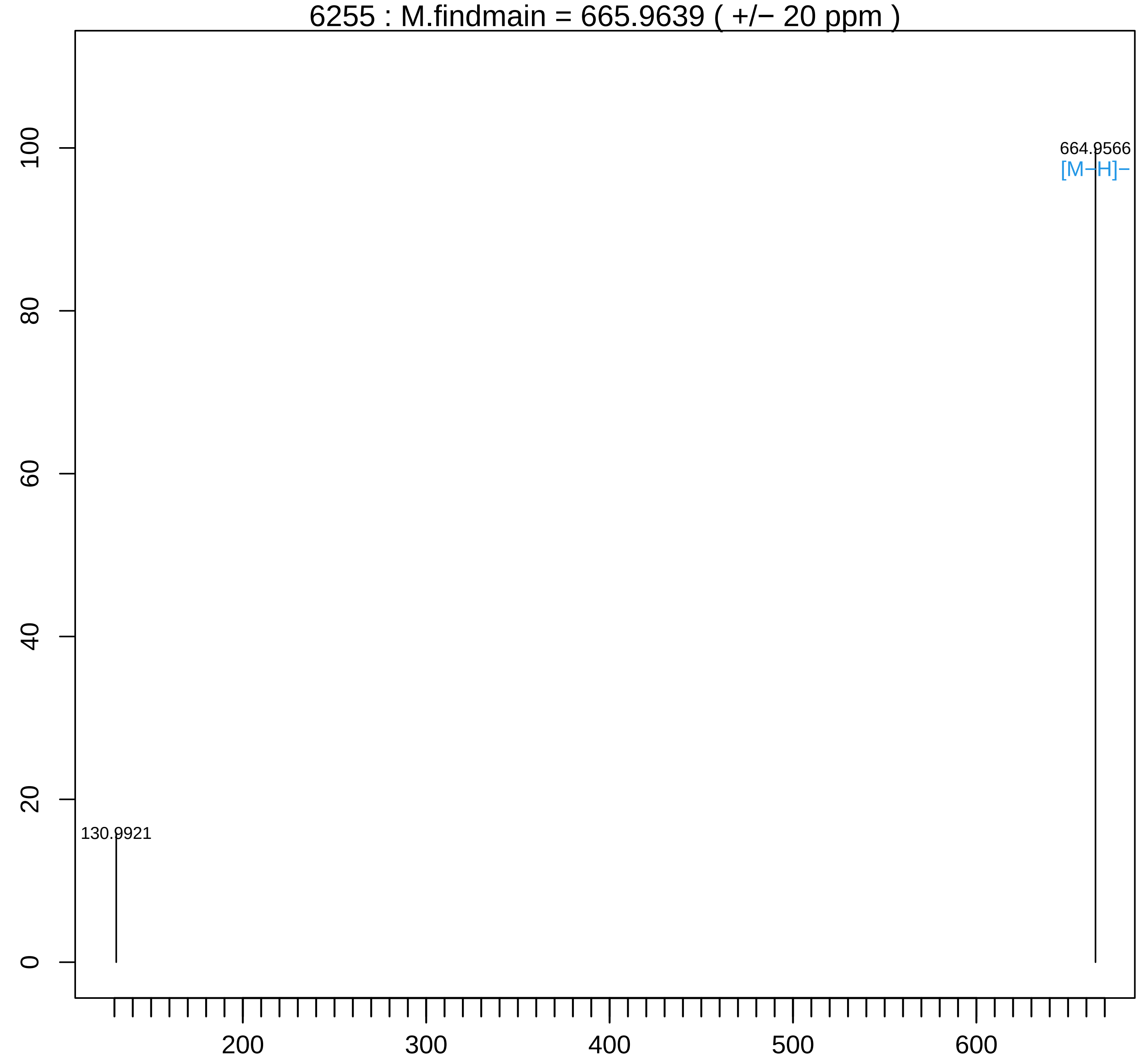
6255 : M.findmain = 665.9639 (+/− 20 ppm)
664.9566
[M−H]−
130.9921
0
20
40
60
80
100
200
300
400
500
600

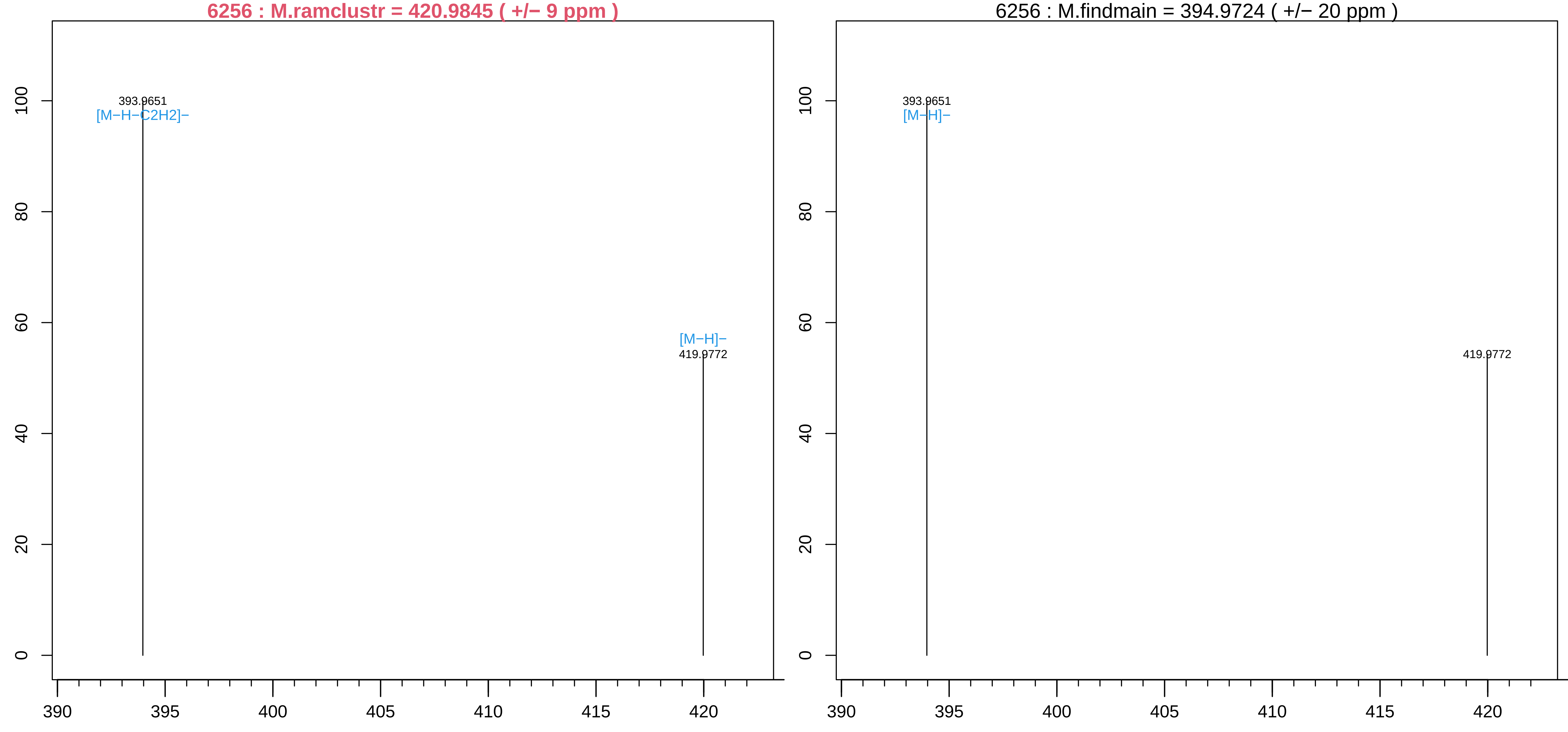
6256 : M.ramclustr = 420.9845 (+/− 9 ppm)
393.9651
[M−H−C2H2]−
[M−H]−
419.9772
6256 : M.findmain = 394.9724 (+/− 20 ppm)
393.9651
[M−H]−
419.9772

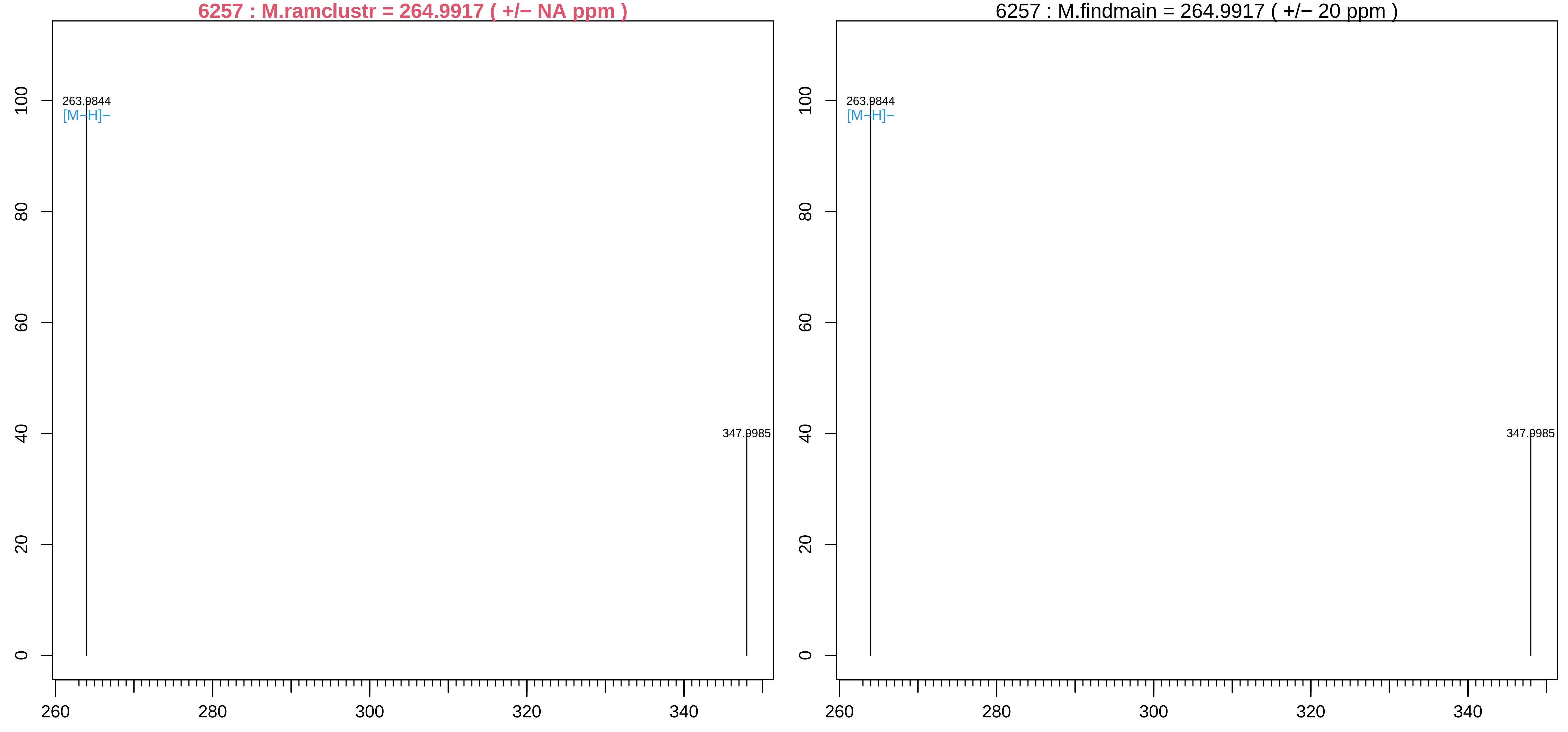
6257 : M.ramclustr = 264.9917 (+/− NA ppm)
263.9844
[M−H]−
347.9985
6257 : M.findmain = 264.9917 (+/− 20 ppm)
263.9844
[M−H]−
347.9985
0
20
40
60
80
100
260
280
300
320
340

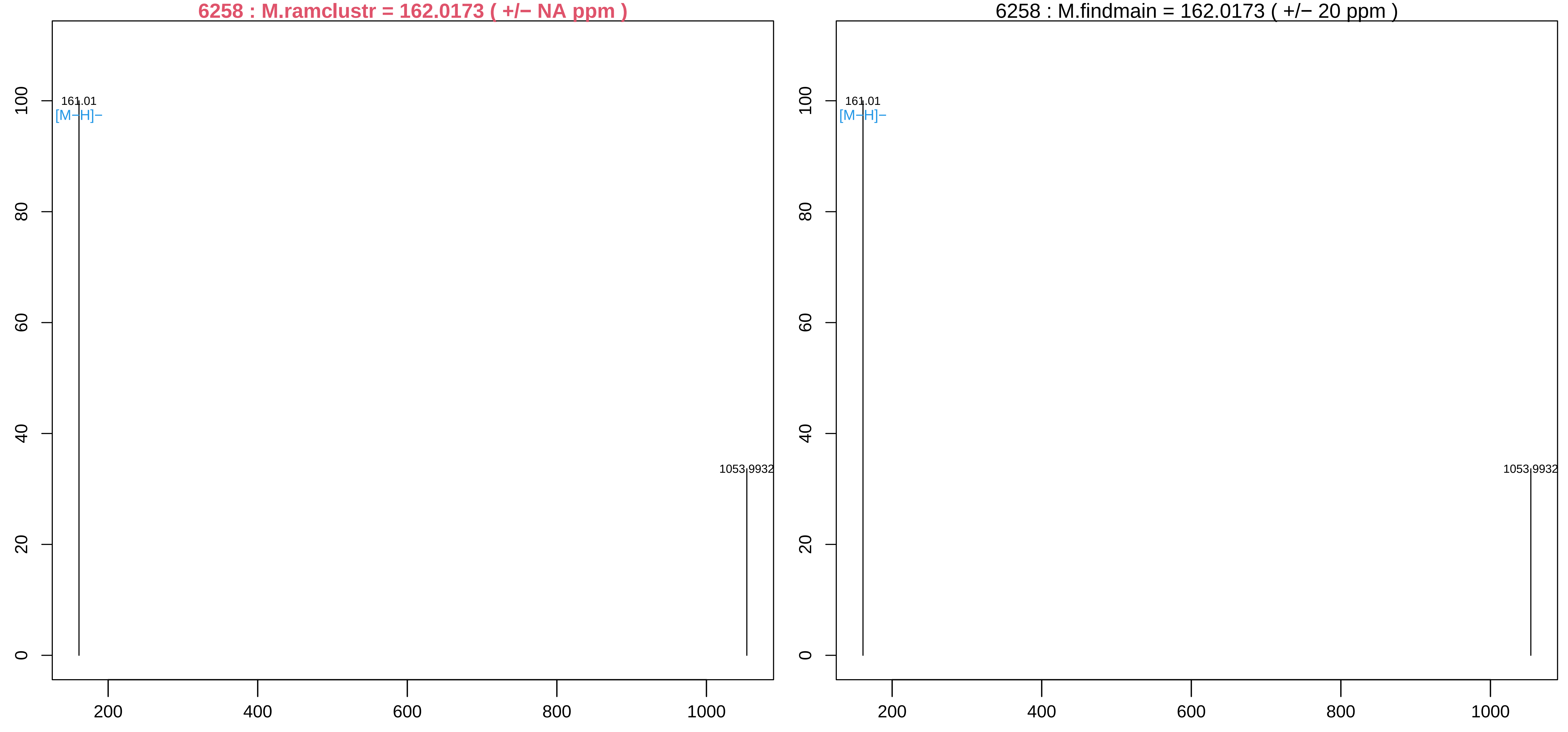
6258 : M.ramclustr = 162.0173 (+/− NA ppm)
161.01
[M−H]−
1053.9932
6258 : M.findmain = 162.0173 (+/− 20 ppm)
161.01
[M−H]−
1053.9932
0
20
40
60
80
100
200
400
600
800
1000

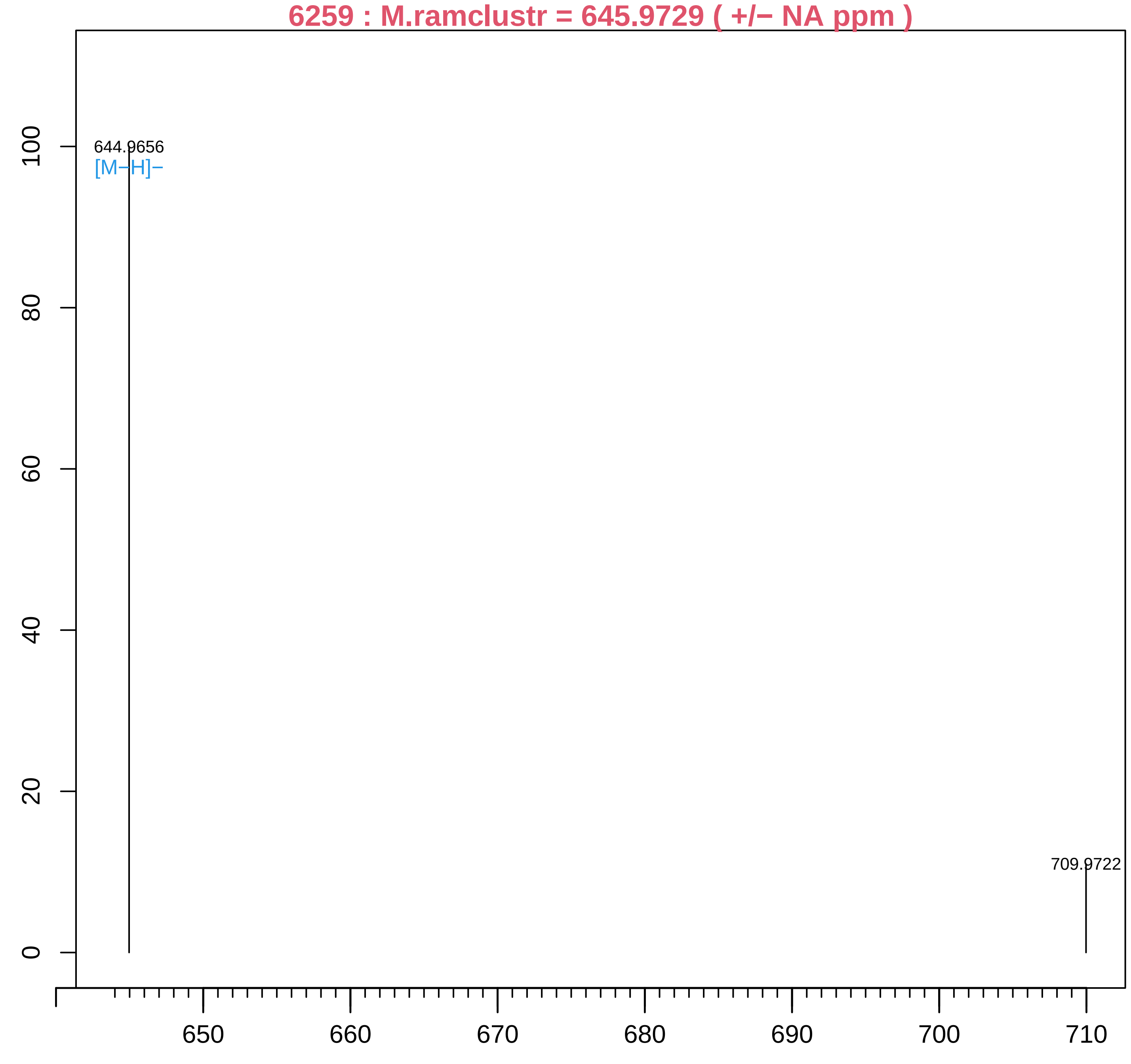

6259 : M.ramclustr = 645.9729 (+/− NA ppm)
644.9656
[M−H]−
709.9722

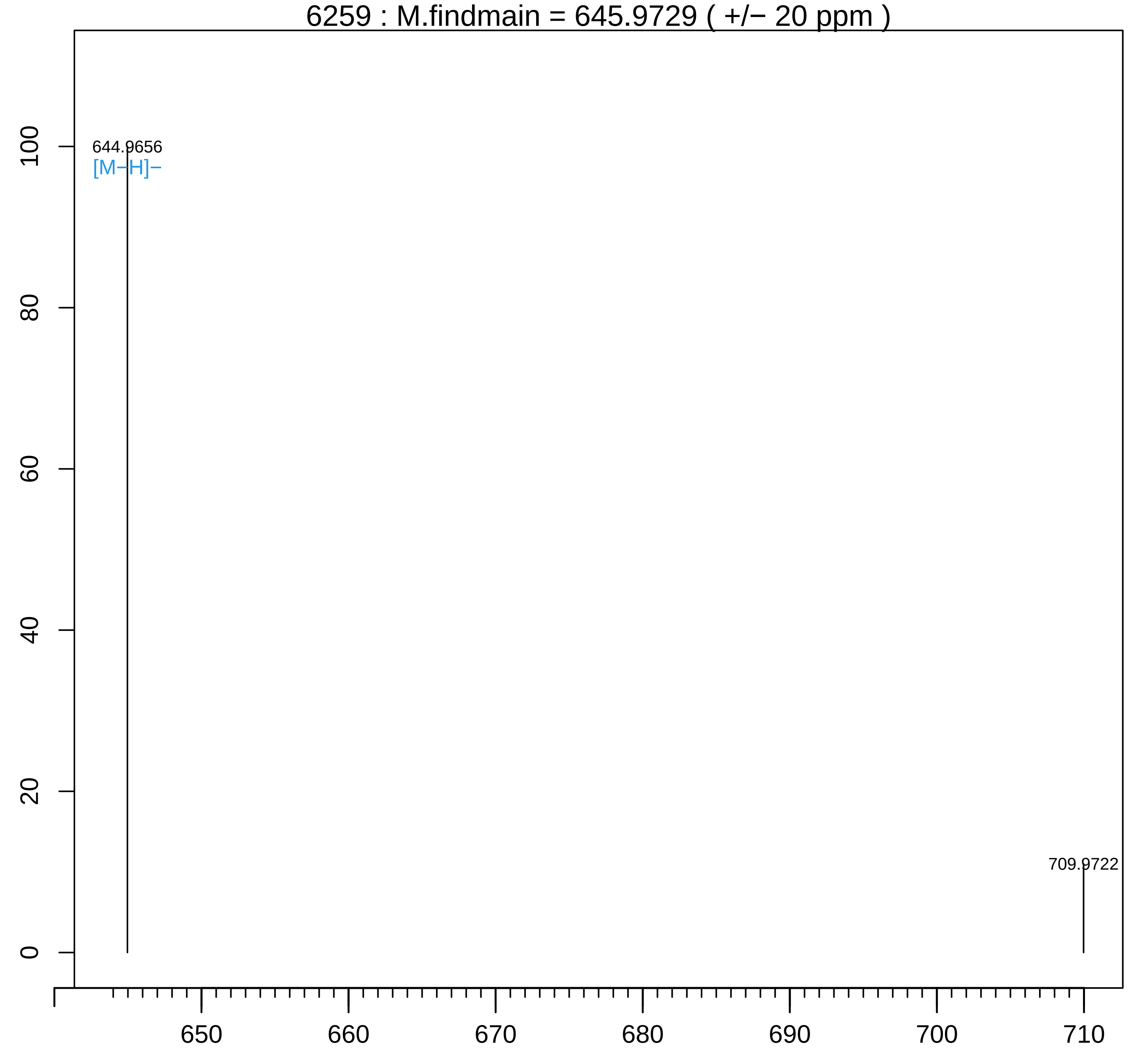

6259 : M.findmain = 645.9729 (+/− 20 ppm)
644.9656
[M−H]−
709.9722

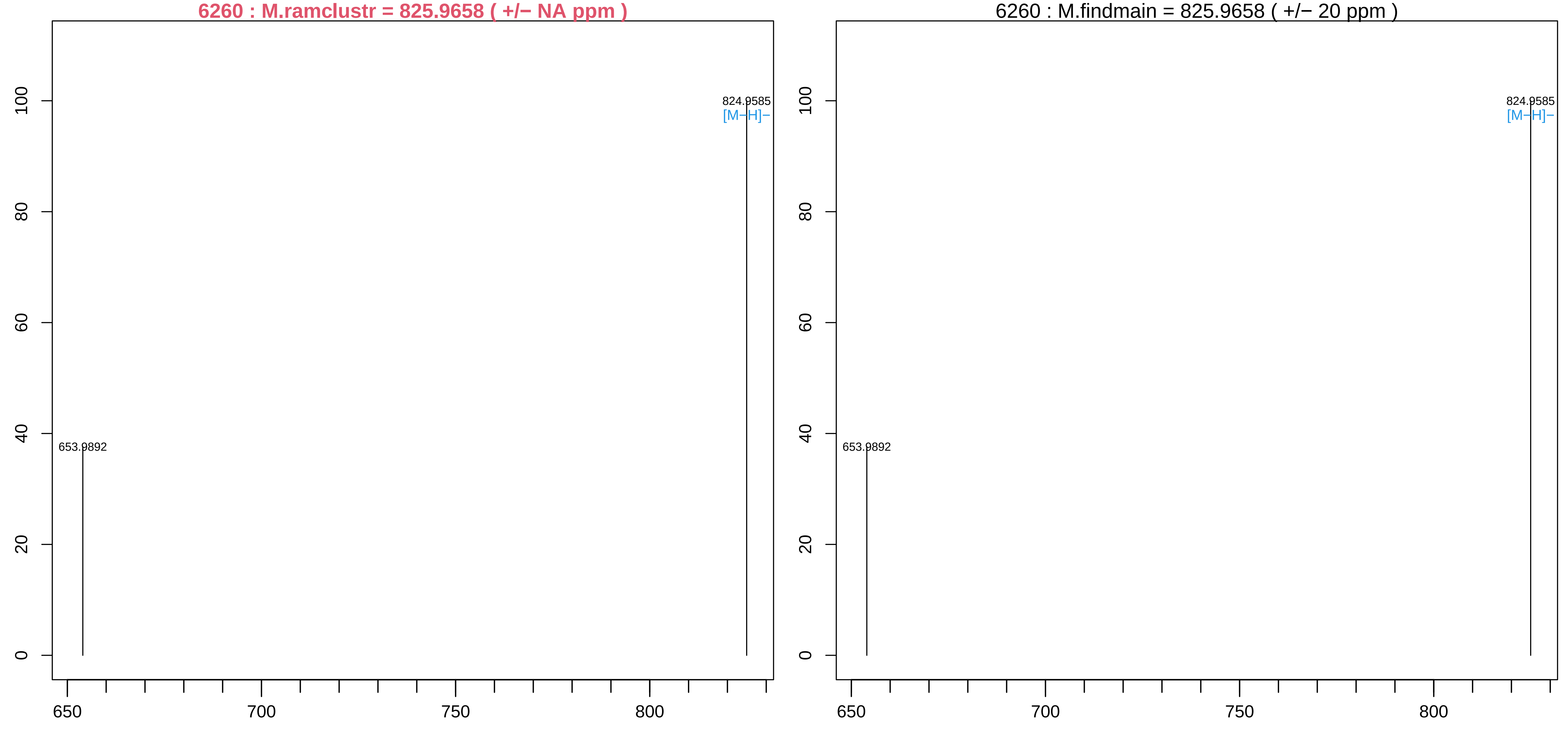
6260 : M.ramclustr = 825.9658 (+/− NA ppm)
824.9585
[M−H]−
653.9892
0
20
40
60
80
100
650
700
750
800
6260 : M.findmain = 825.9658 (+/− 20 ppm)
824.9585
[M−H]−
653.9892
0
20
40
60
80
100
650
700
750
800

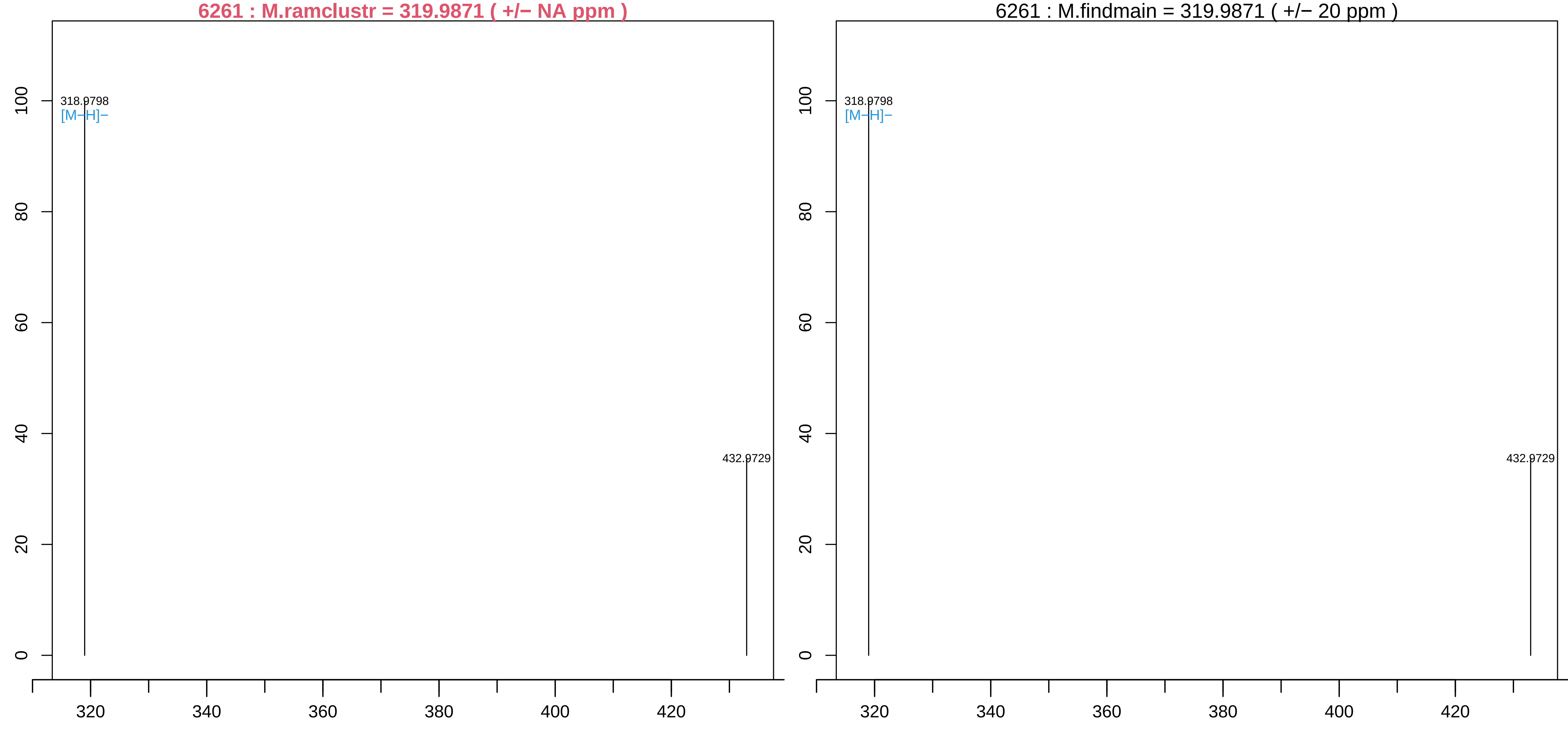

6261 : M.ramclustr = 319.9871 (+/− NA ppm)
318.9798
[M−H]−
432.9729
6261 : M.findmain = 319.9871 (+/− 20 ppm)
318.9798
[M−H]−
432.9729

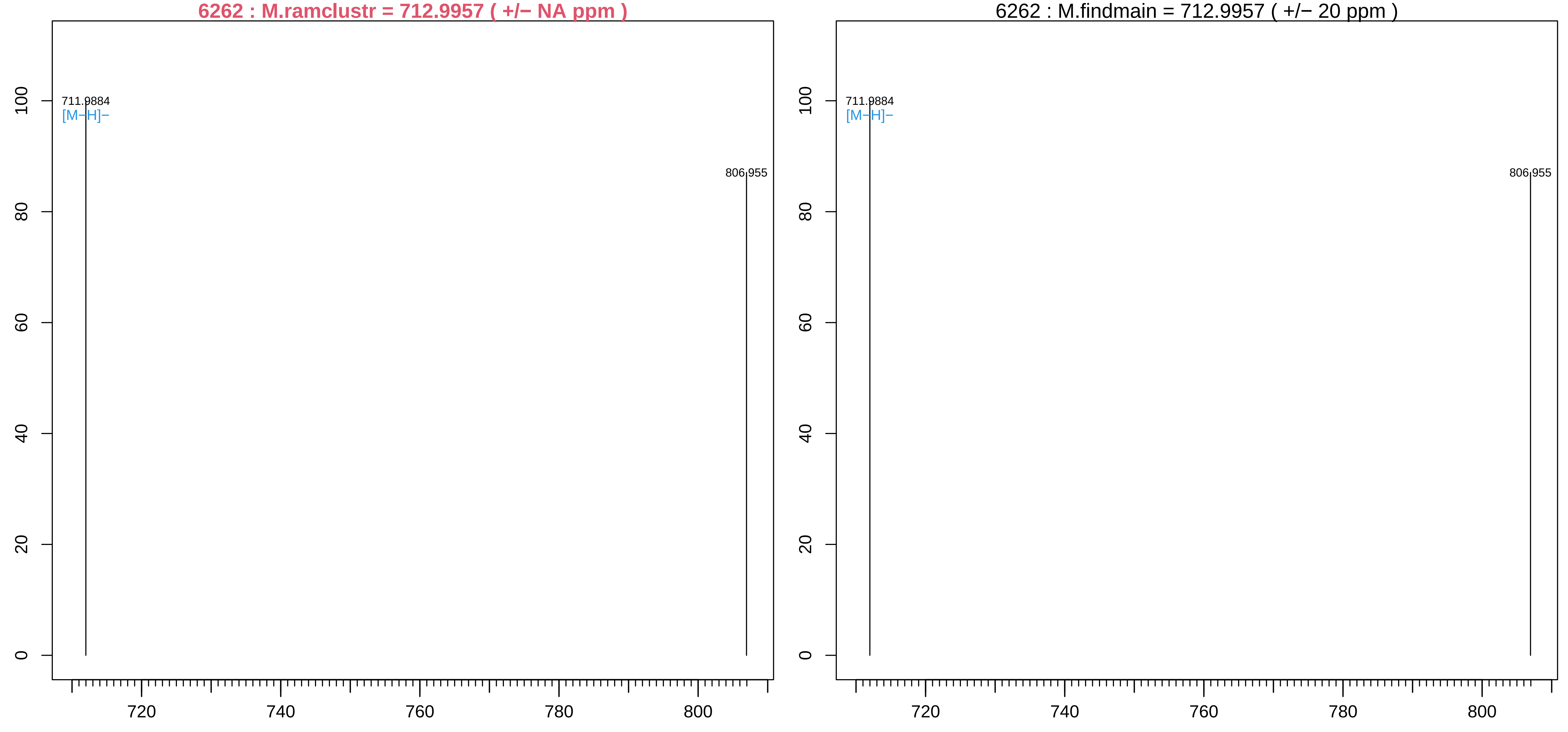

6262 : M.ramclustr = 712.9957 (+/− NA ppm)
711.9884
[M−H]−
806.955
0
20
40
60
80
100
720
740
760
780
800
6262 : M.findmain = 712.9957 (+/− 20 ppm)
711.9884
[M−H]−
806.955
0
20
40
60
80
100
720
740
760
780
800

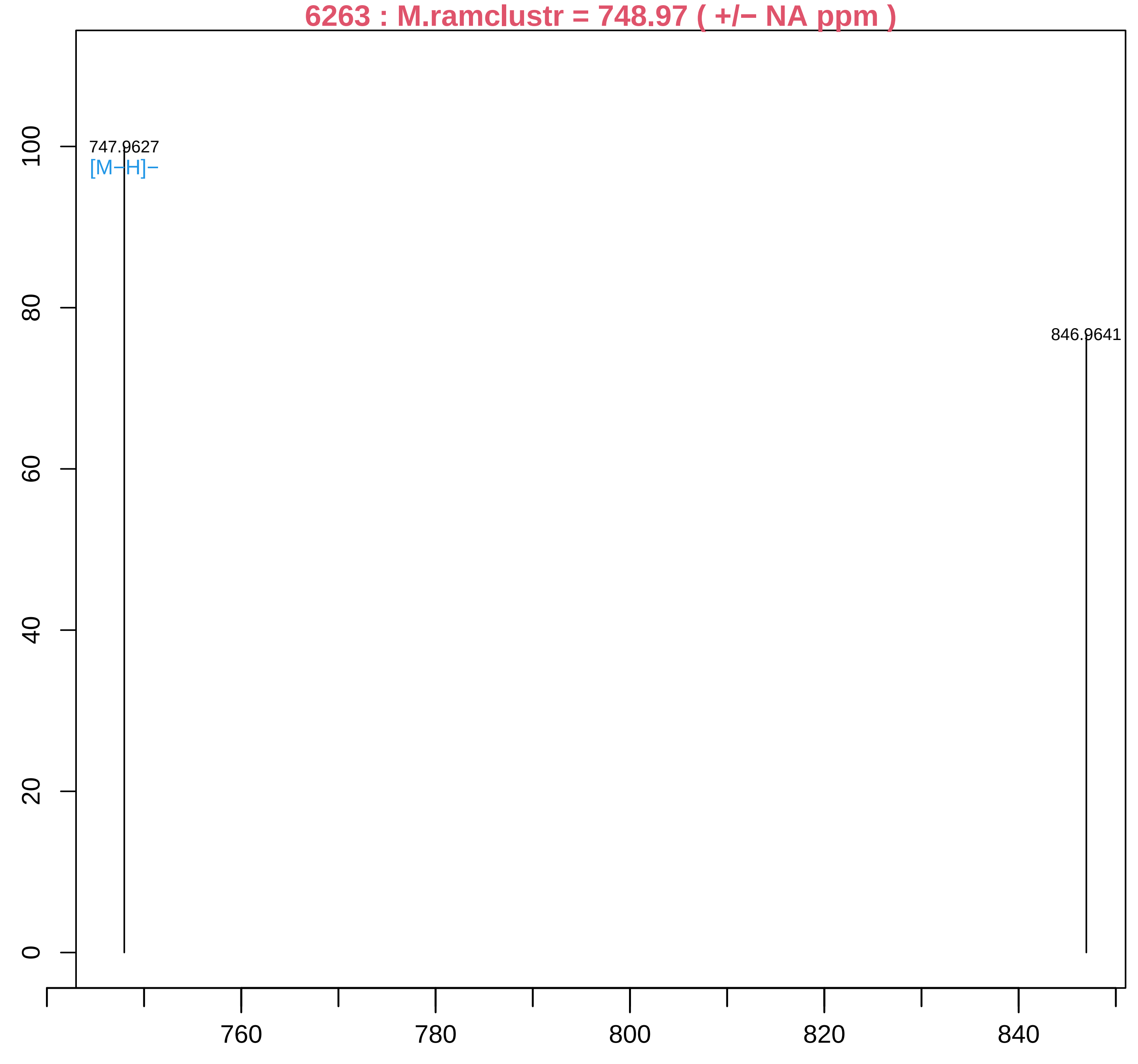
6263 : M.ramclustr = 748.97 (+/− NA ppm)
747.9627
[M−H]−
846.9641
0
20
40
60
80
100
760
780
800
820
840
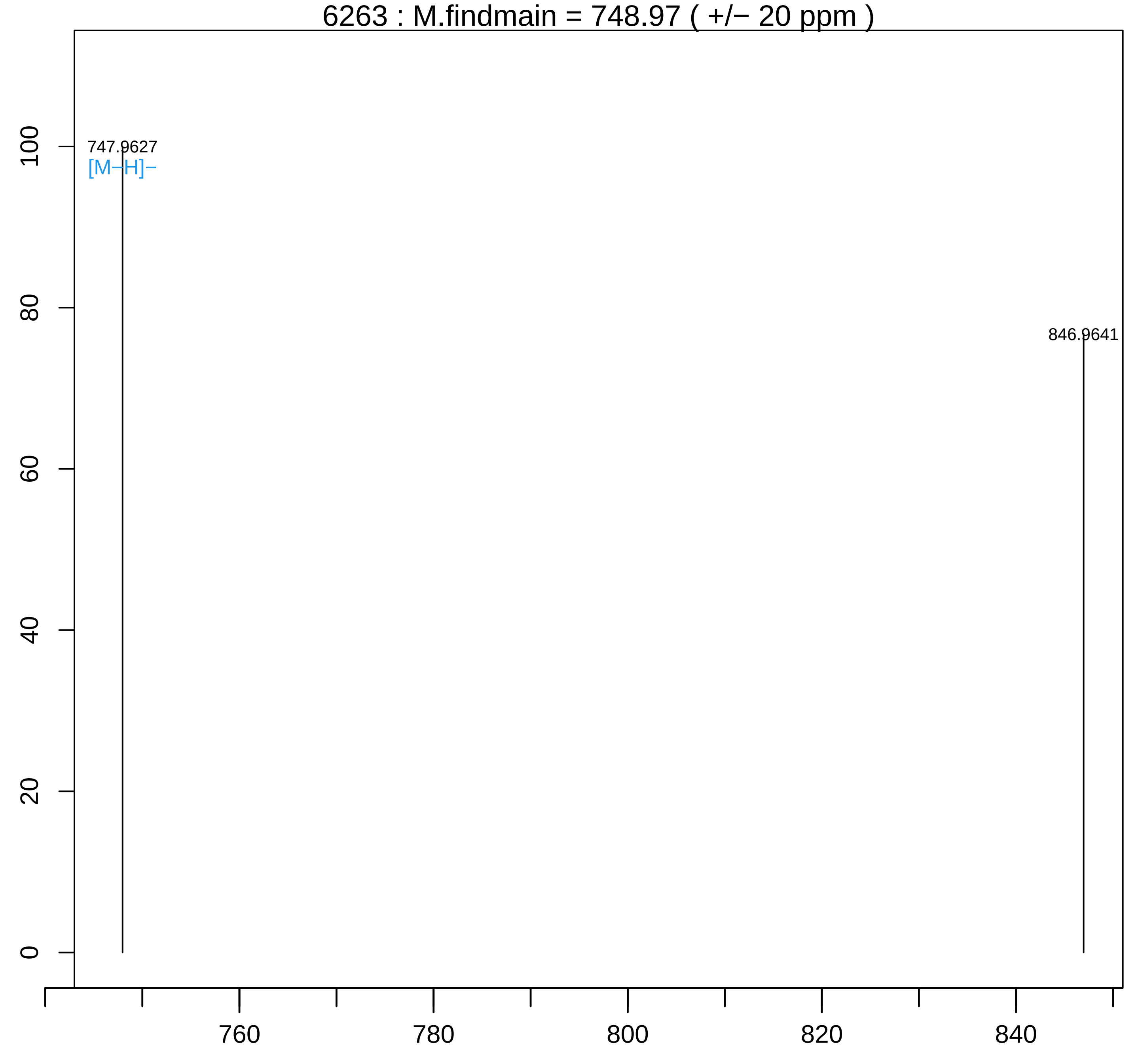
6263 : M.findmain = 748.97 (+/− 20 ppm)
747.9627
[M−H]−
846.9641
0
20
40
60
80
100
760
780
800
820
840

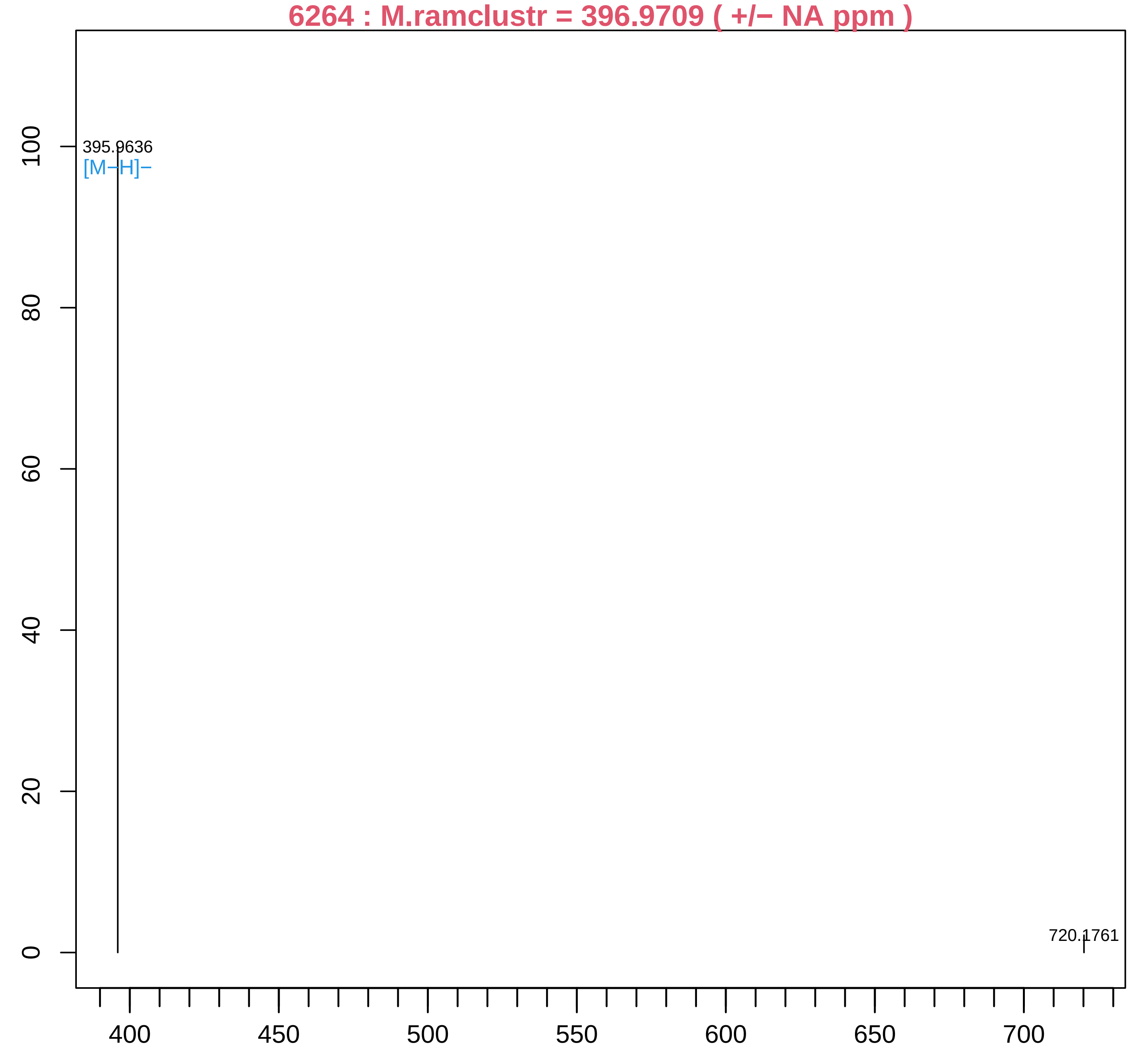

6264 : M.ramclustr = 396.9709 (+/− NA ppm)
395.9636
[M−H]−
720.1761
0
20
40
60
80
100
400
450
500
550
600
650
700

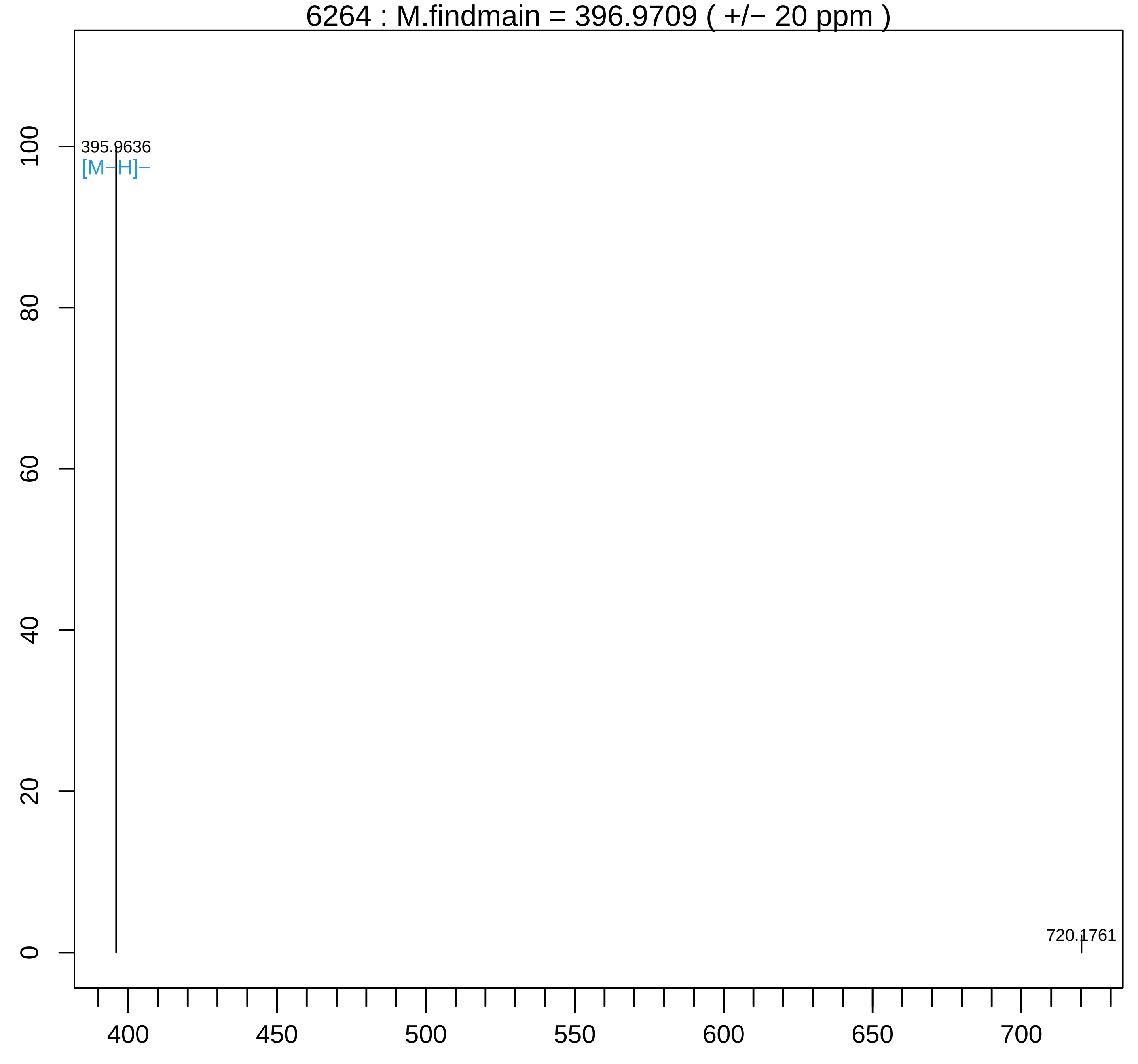

6264 : M.findmain = 396.9709 (+/− 20 ppm)
395.9636
[M−H]−
720.1761
0
20
40
60
80
100
400
450
500
550
600
650
700

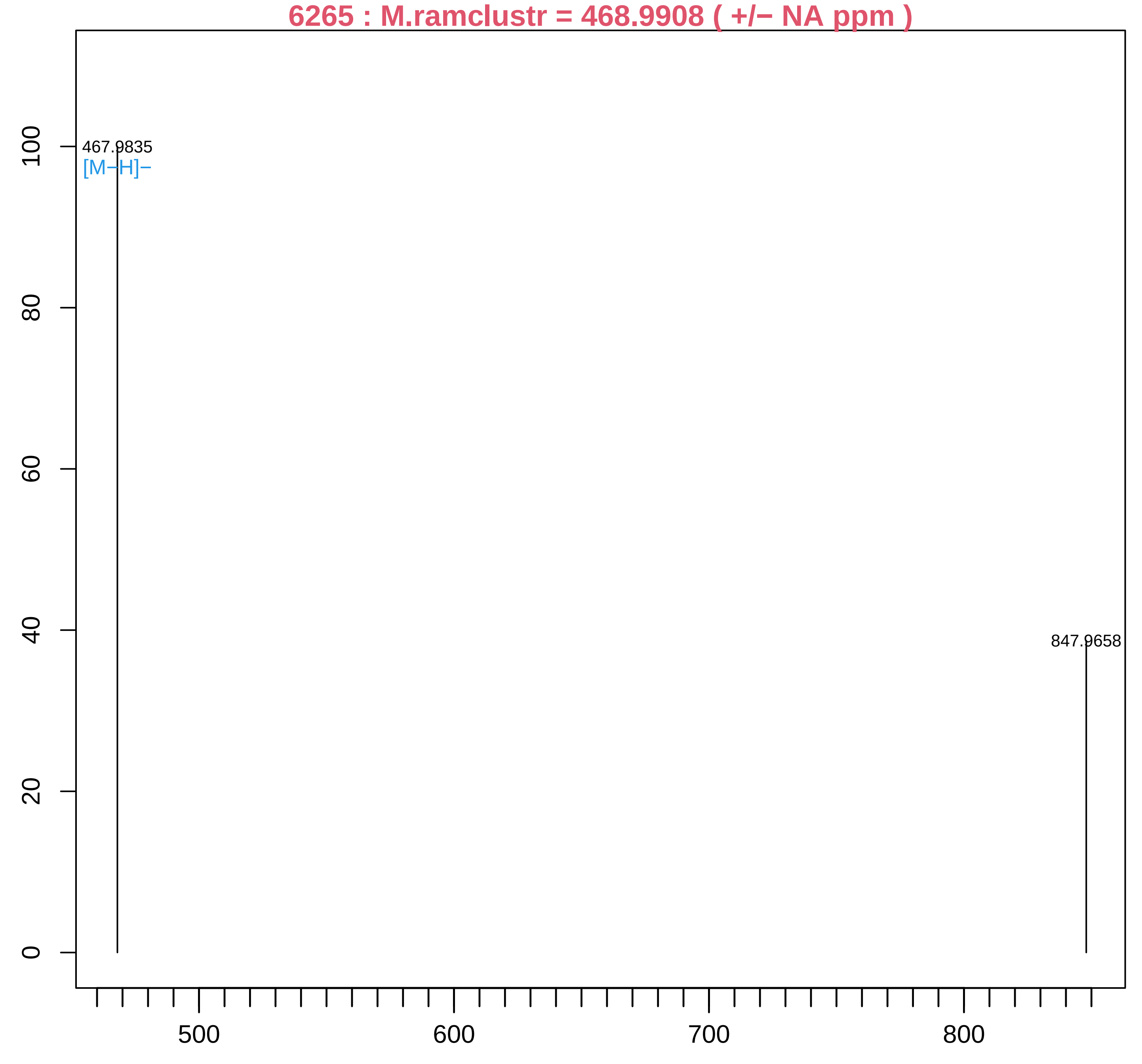
6265 : M.ramclustr = 468.9908 (+/− NA ppm)
467.9835
[M−H]−
847.9658
0
20
40
60
80
100
500
600
700
800

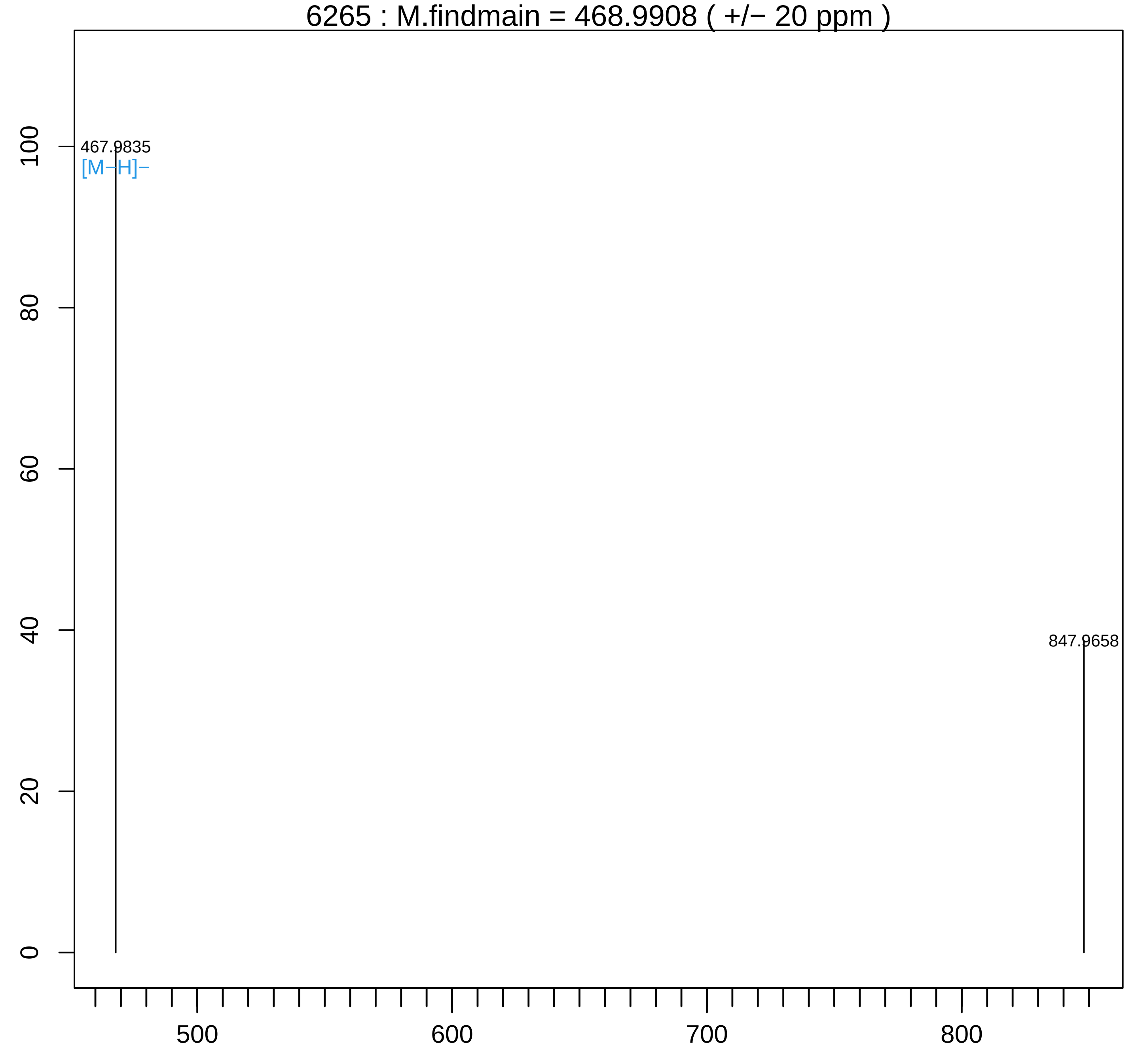
6265 : M.findmain = 468.9908 (+/− 20 ppm)
467.9835
[M−H]−
847.9658
0
20
40
60
80
100
500
600
700
800

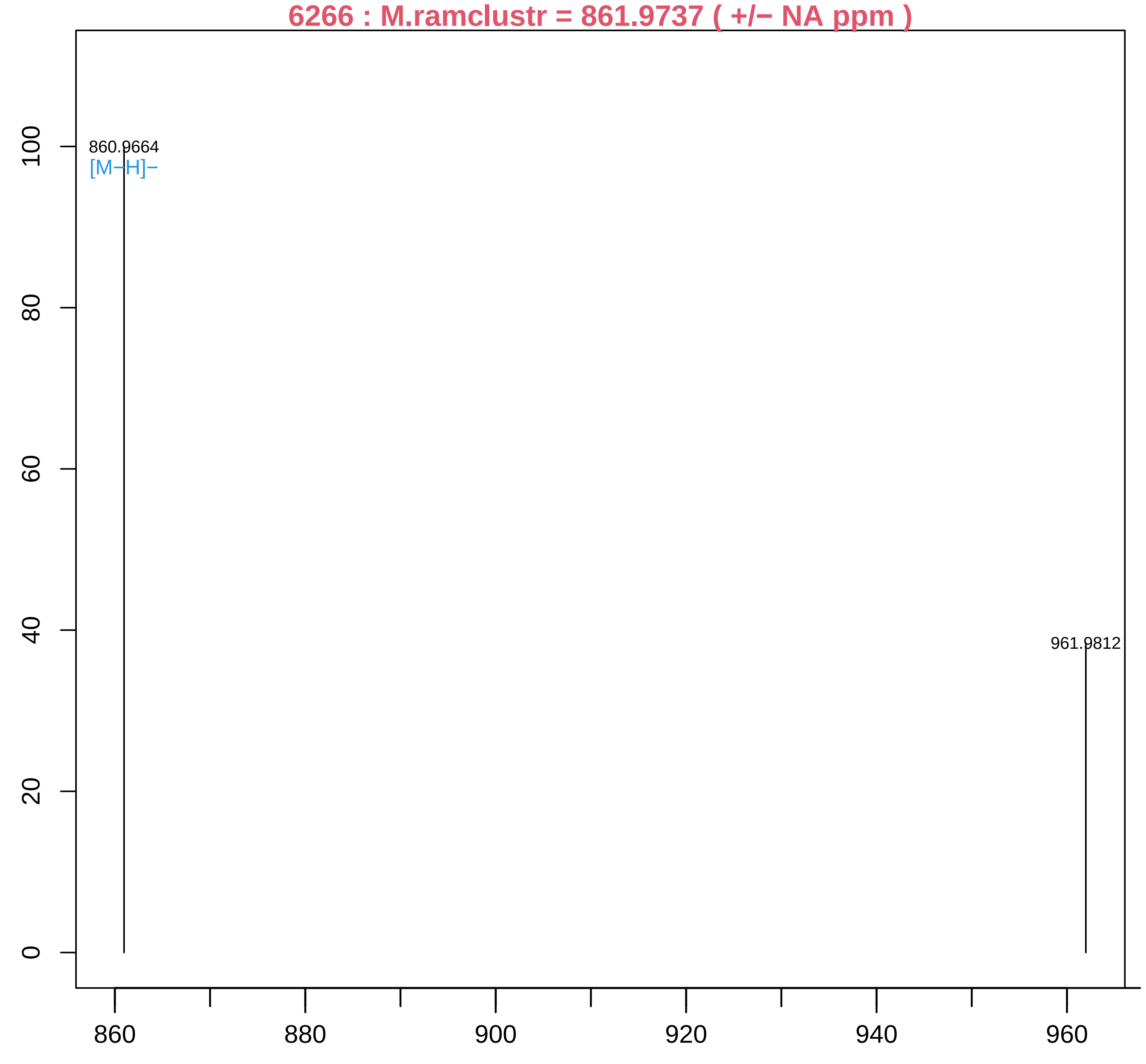
6266 : M.ramclustr = 861.9737 (+/− NA ppm)
860.9664
[M−H]−
961.9812
0
20
40
60
80
100
860
880
900
920
940
960

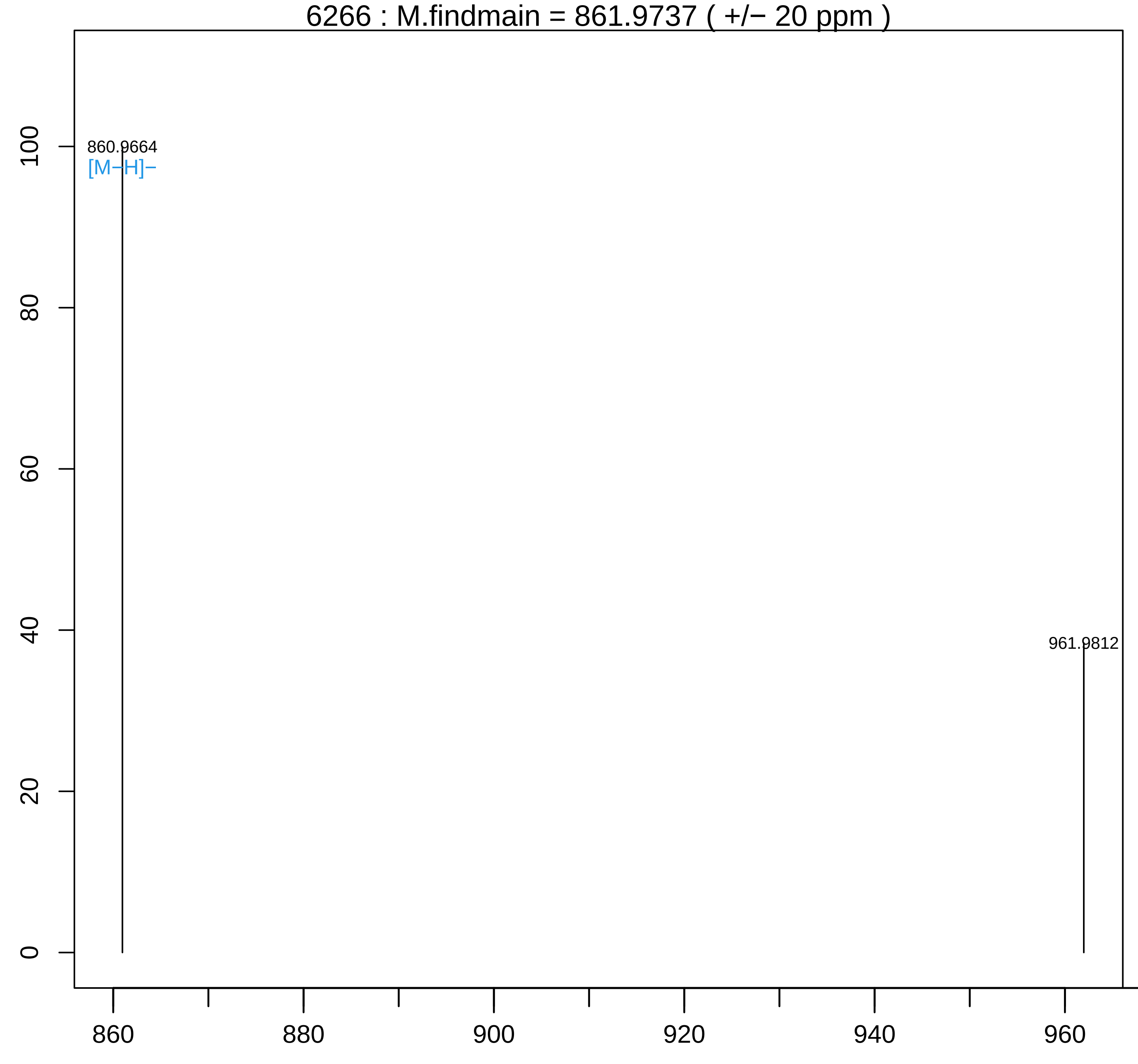
6266 : M.findmain = 861.9737 (+/− 20 ppm)
860.9664
[M−H]−
961.9812
0
20
40
60
80
100
860
880
900
920
940
960

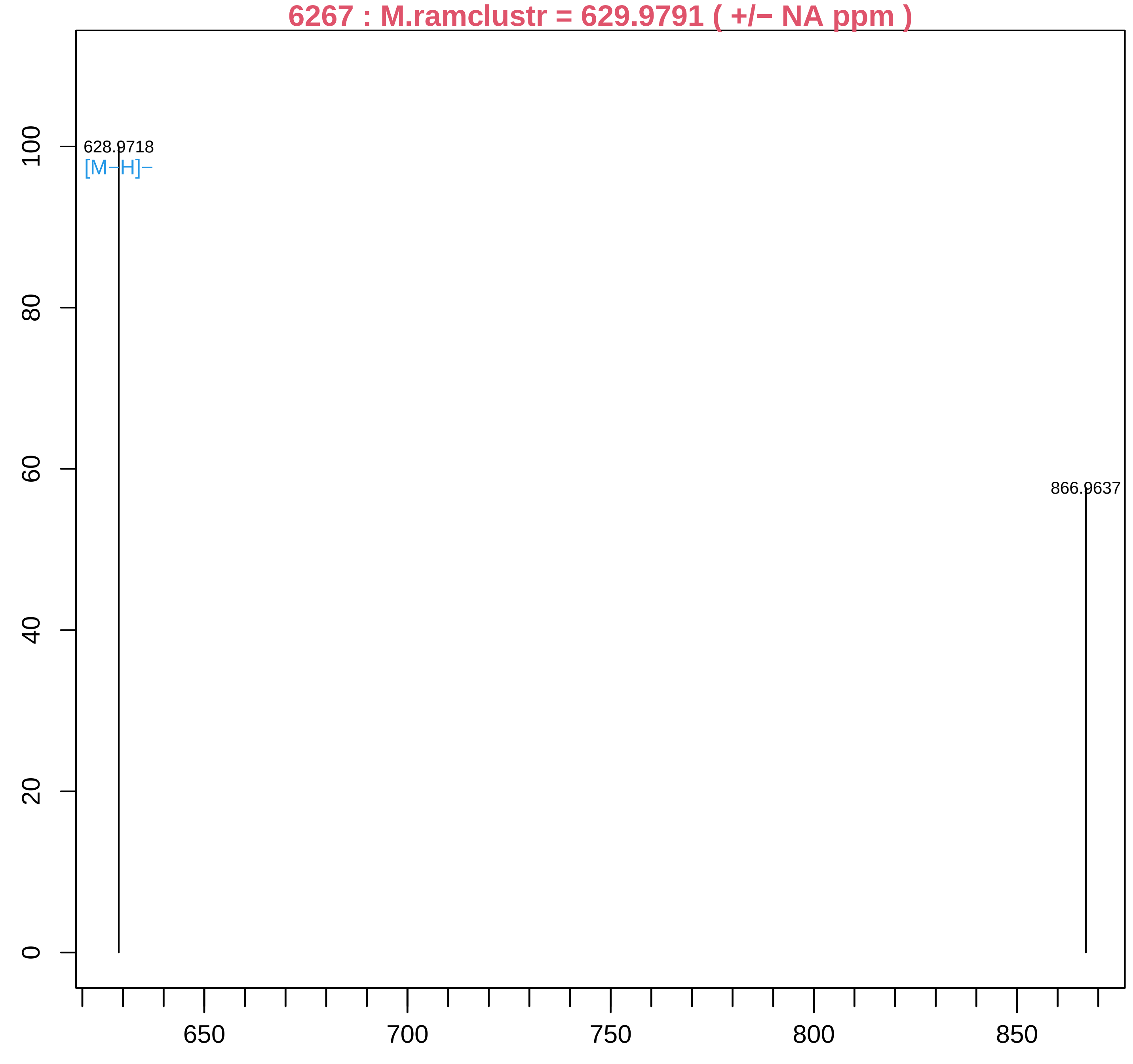

6267 : M.ramclustr = 629.9791 (+/− NA ppm)
628.9718
[M−H]−
866.9637
0
20
40
60
80
100
650
700
750
800
850

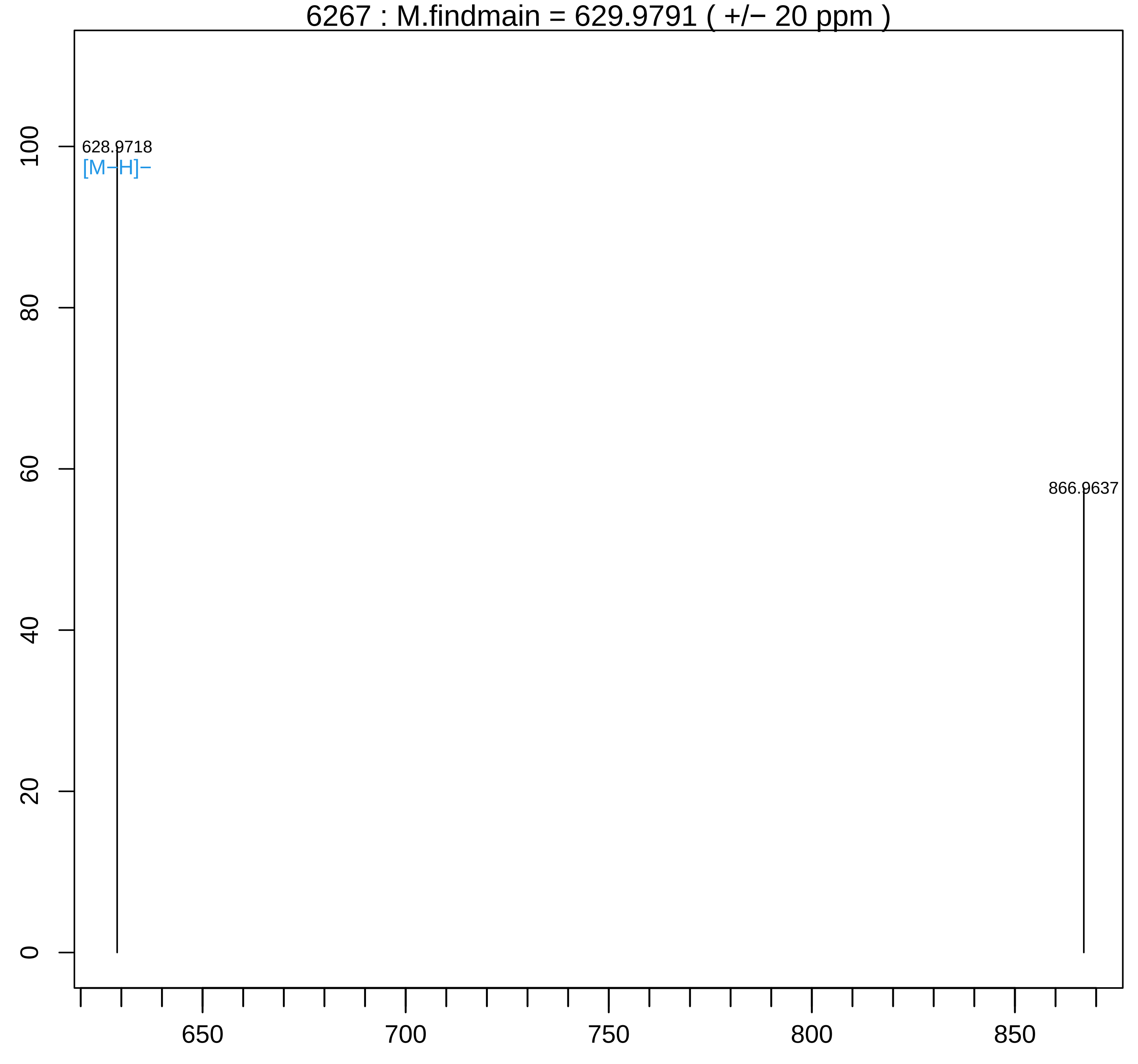

6267 : M.findmain = 629.9791 (+/− 20 ppm)
628.9718
[M−H]−
866.9637
0
20
40
60
80
100
650
700
750
800
850

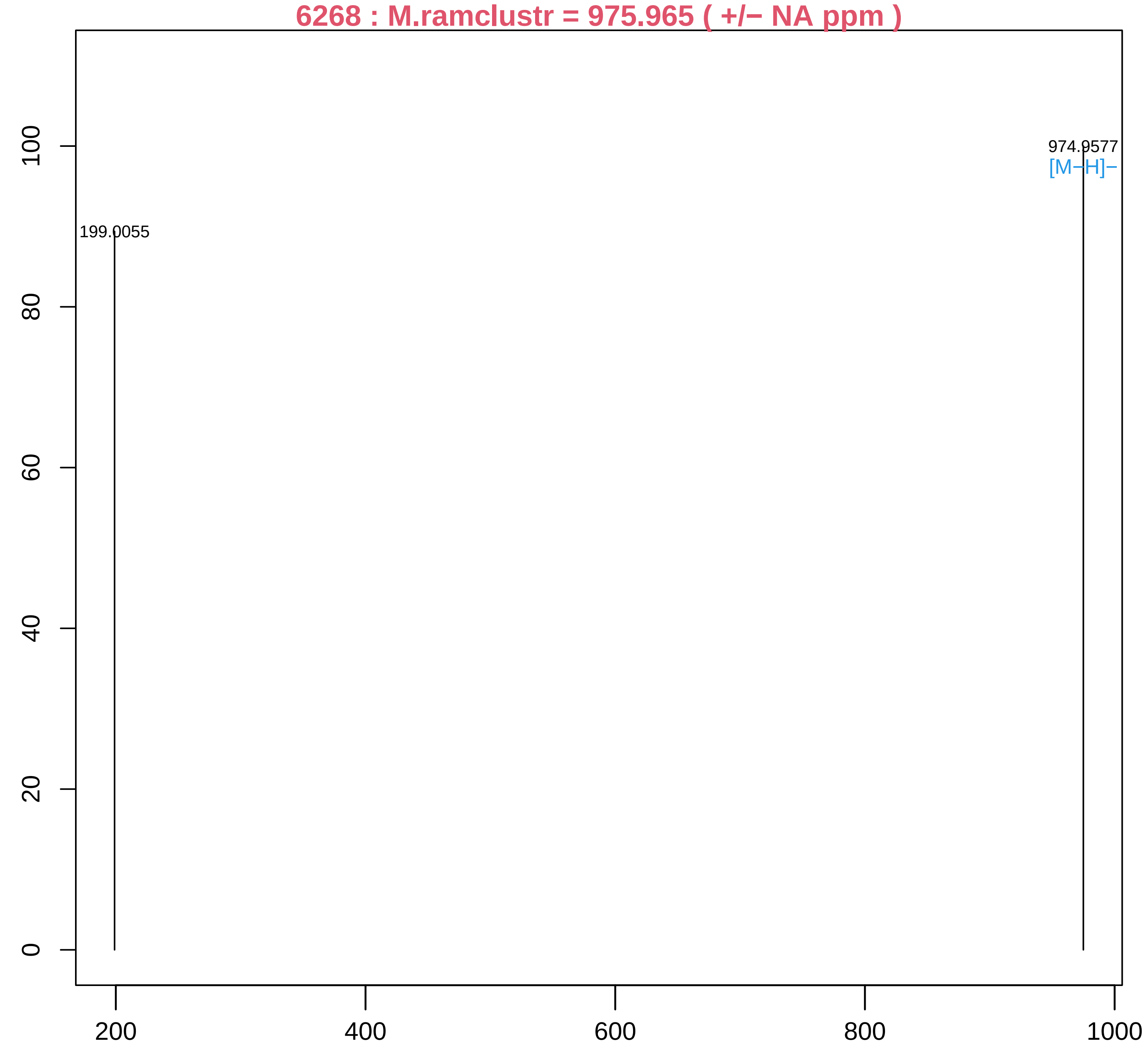
6268 : M.ramclustr = 975.965 (+/− NA ppm)
974.9577
[M−H]−
199.0055
0
20
40
60
80
100
200
400
600
800
1000

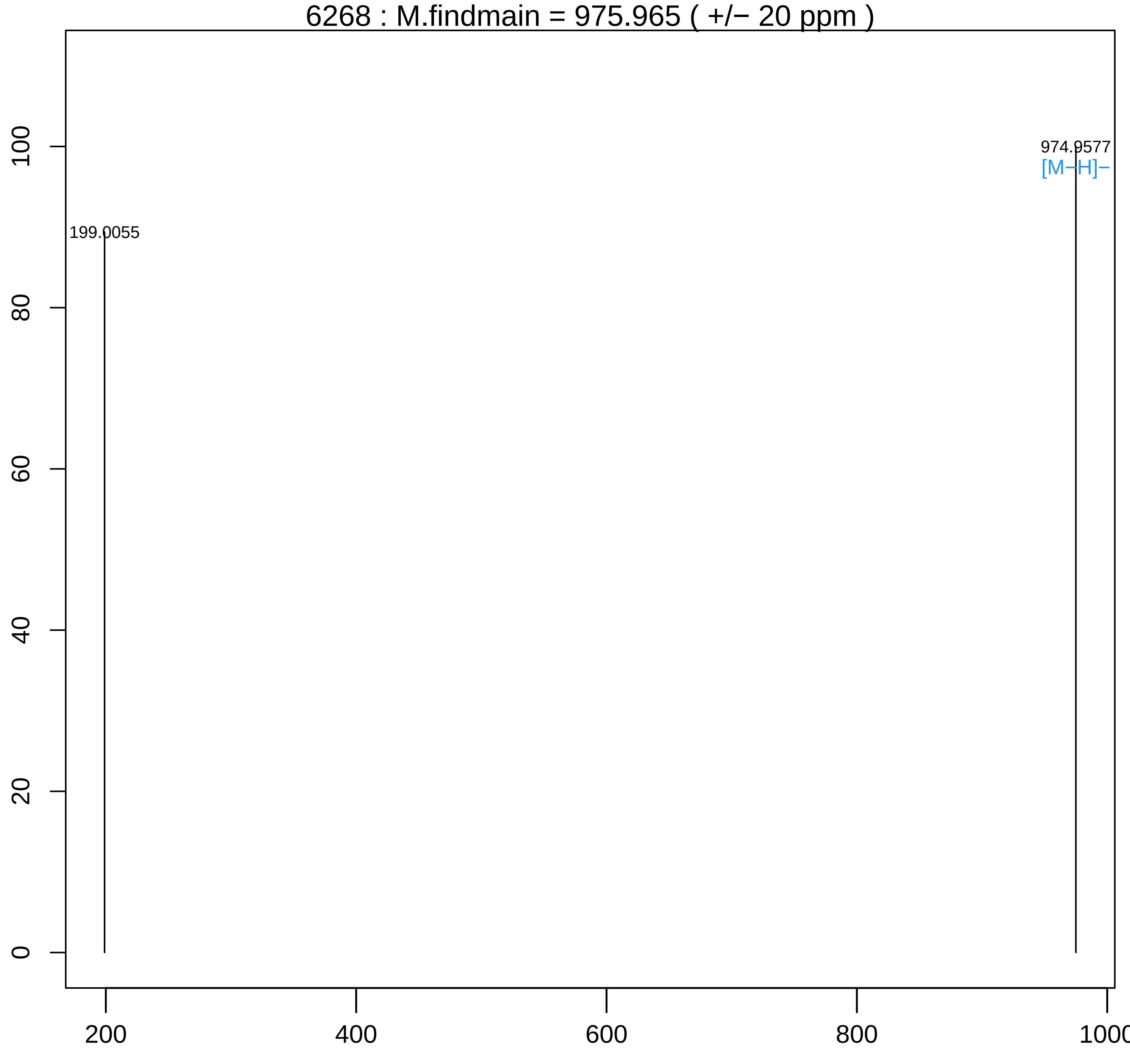
6268 : M.findmain = 975.965 (+/− 20 ppm)
974.9577
[M−H]−
199.0055
0
20
40
60
80
100
200
400
600
800
1000

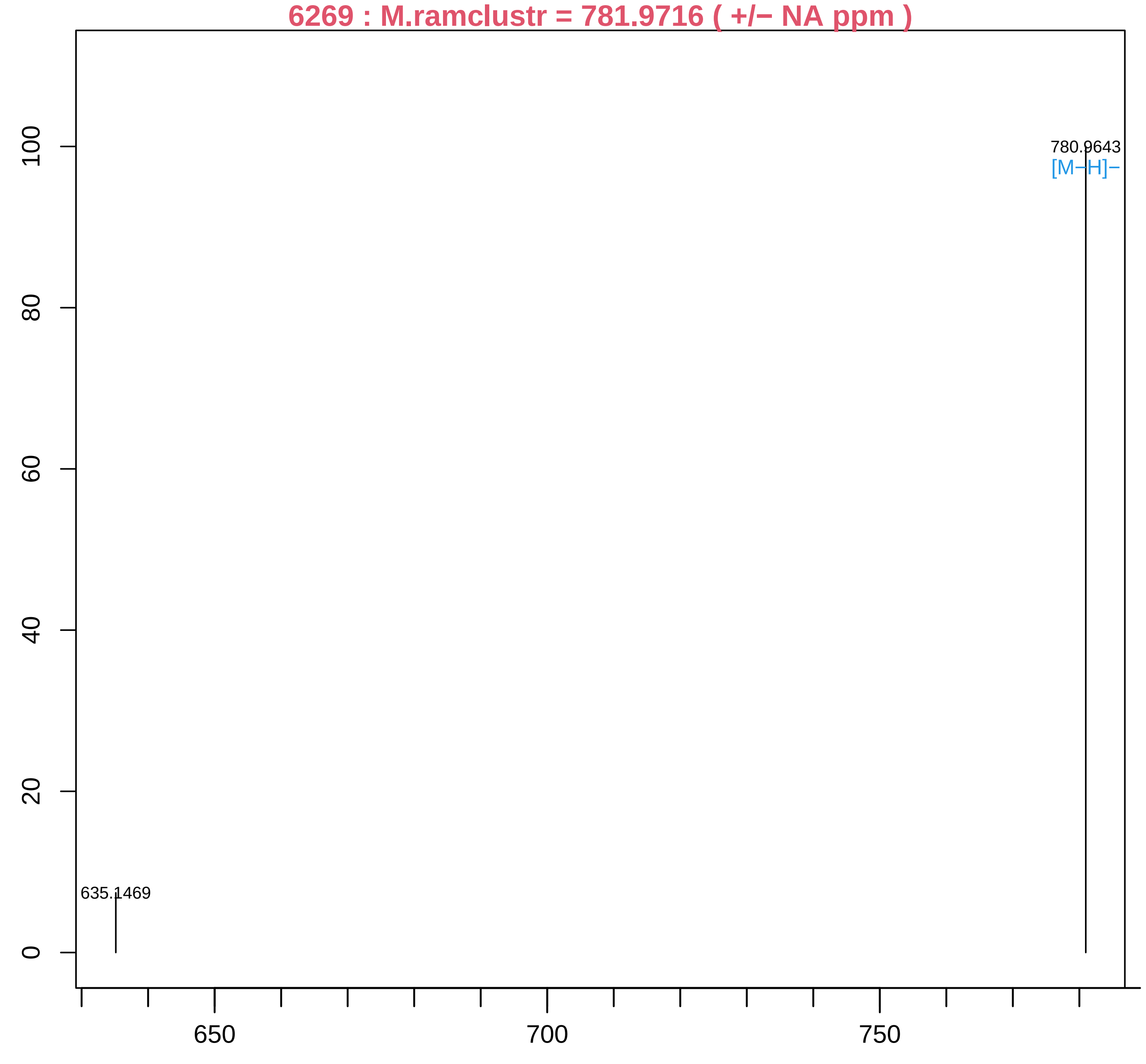
6269 : M.ramclustr = 781.9716 (+/− NA ppm)
780.9643
[M−H]−
635.1469
0
20
40
60
80
100
650
700
750

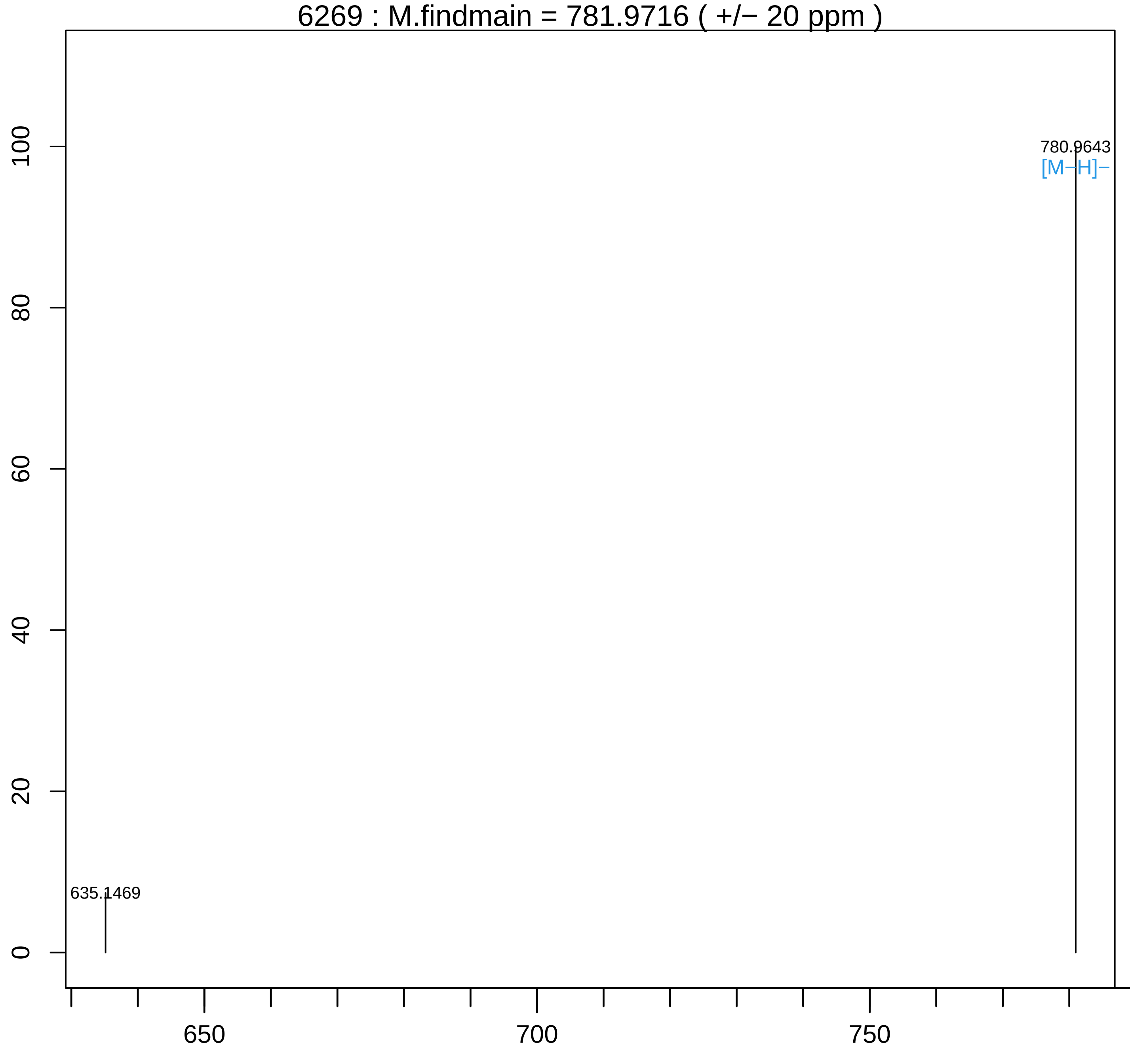
6269 : M.findmain = 781.9716 (+/− 20 ppm)
780.9643
[M−H]−
635.1469
0
20
40
60
80
100
650
700
750

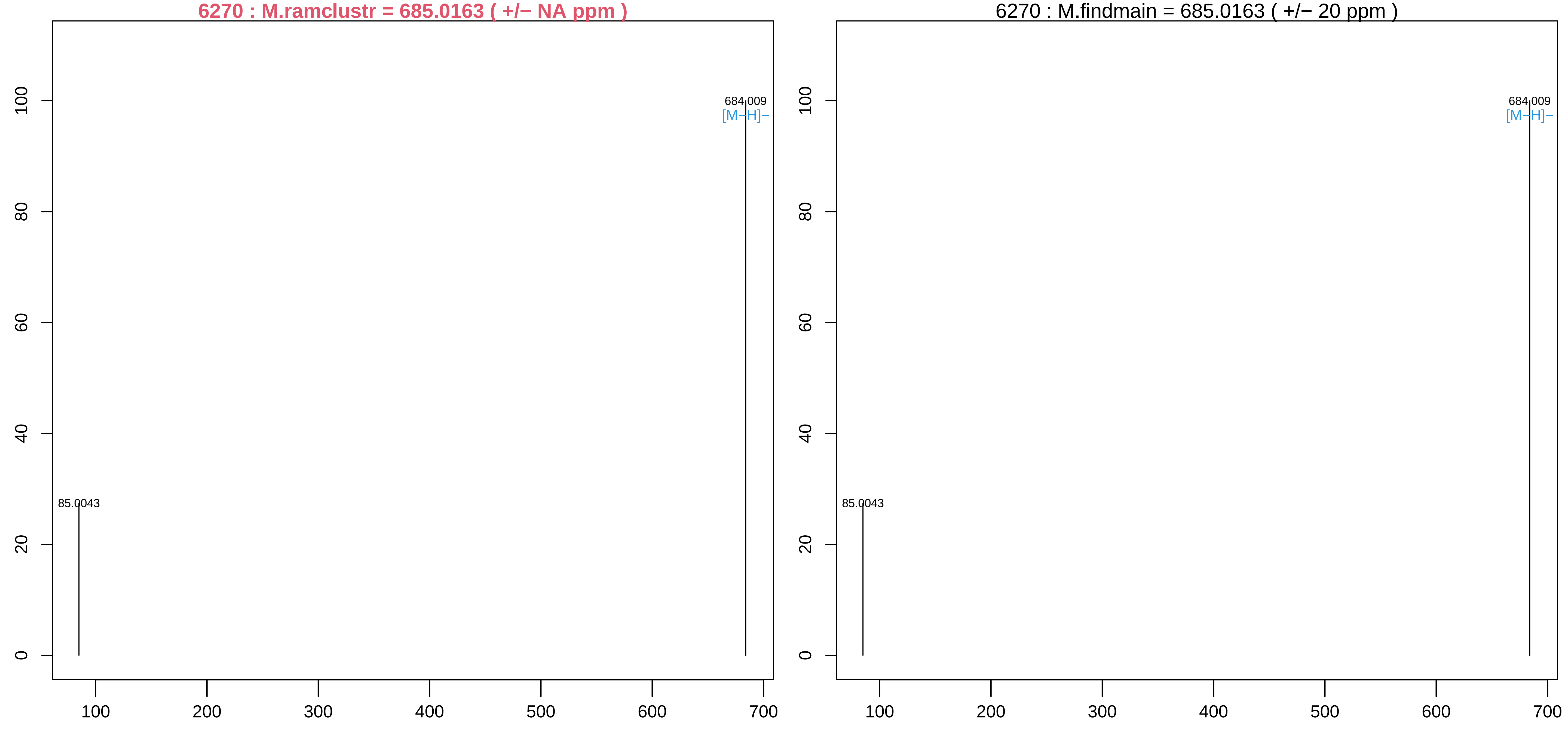

6270 : M.ramclustr = 685.0163 (+/− NA ppm)
684.009
[M−H]−
85.0043
0
20
40
60
80
100
100
200
300
400
500
600
700
6270 : M.findmain = 685.0163 (+/− 20 ppm)
684.009
[M−H]−
85.0043
0
20
40
60
80
100
100
200
300
400
500
600
700

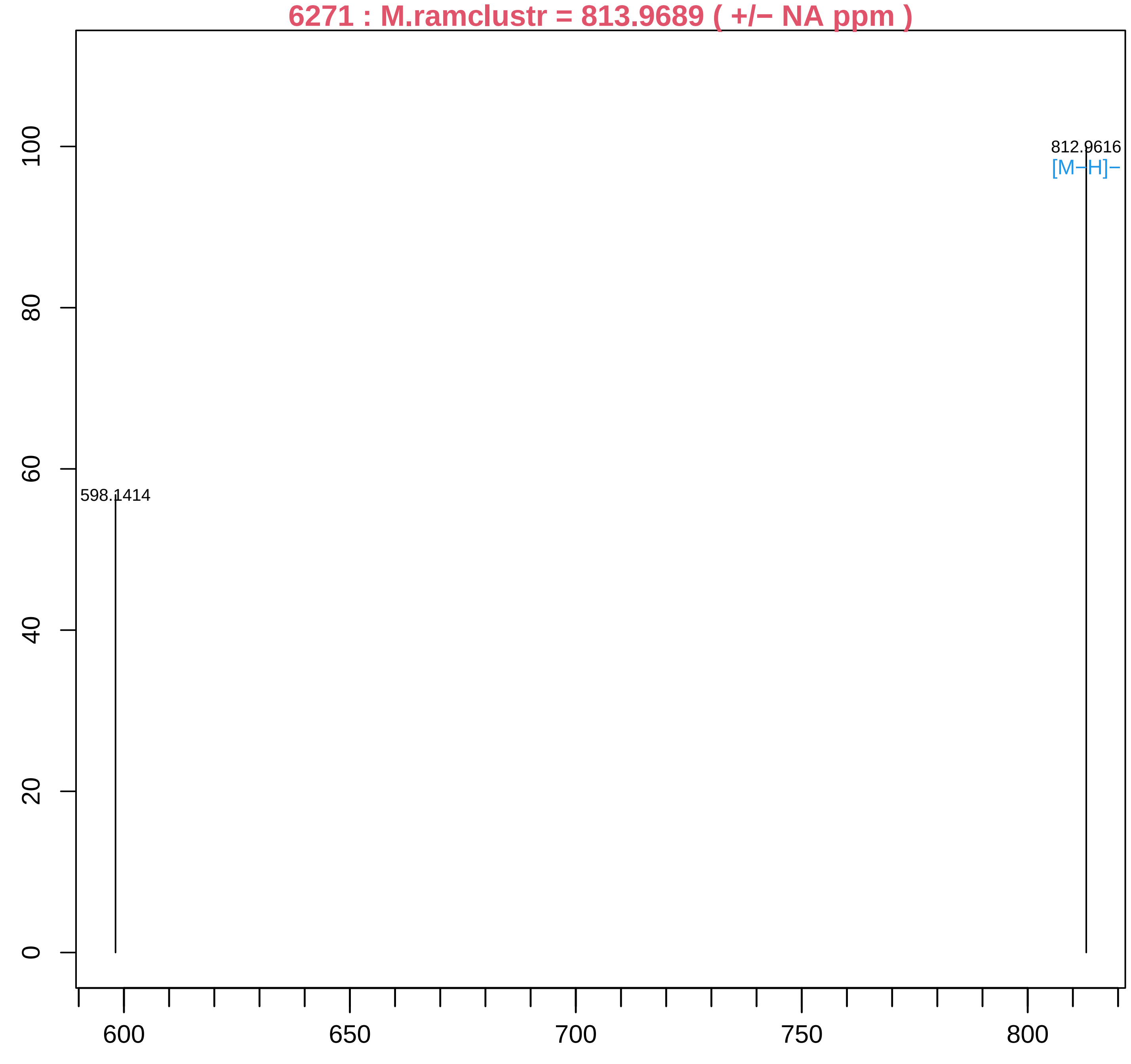
6271 : M.ramclustr = 813.9689 (+/− NA ppm)
812.9616
[M−H]−
598.1414
0
20
40
60
80
100
600
650
700
750
800

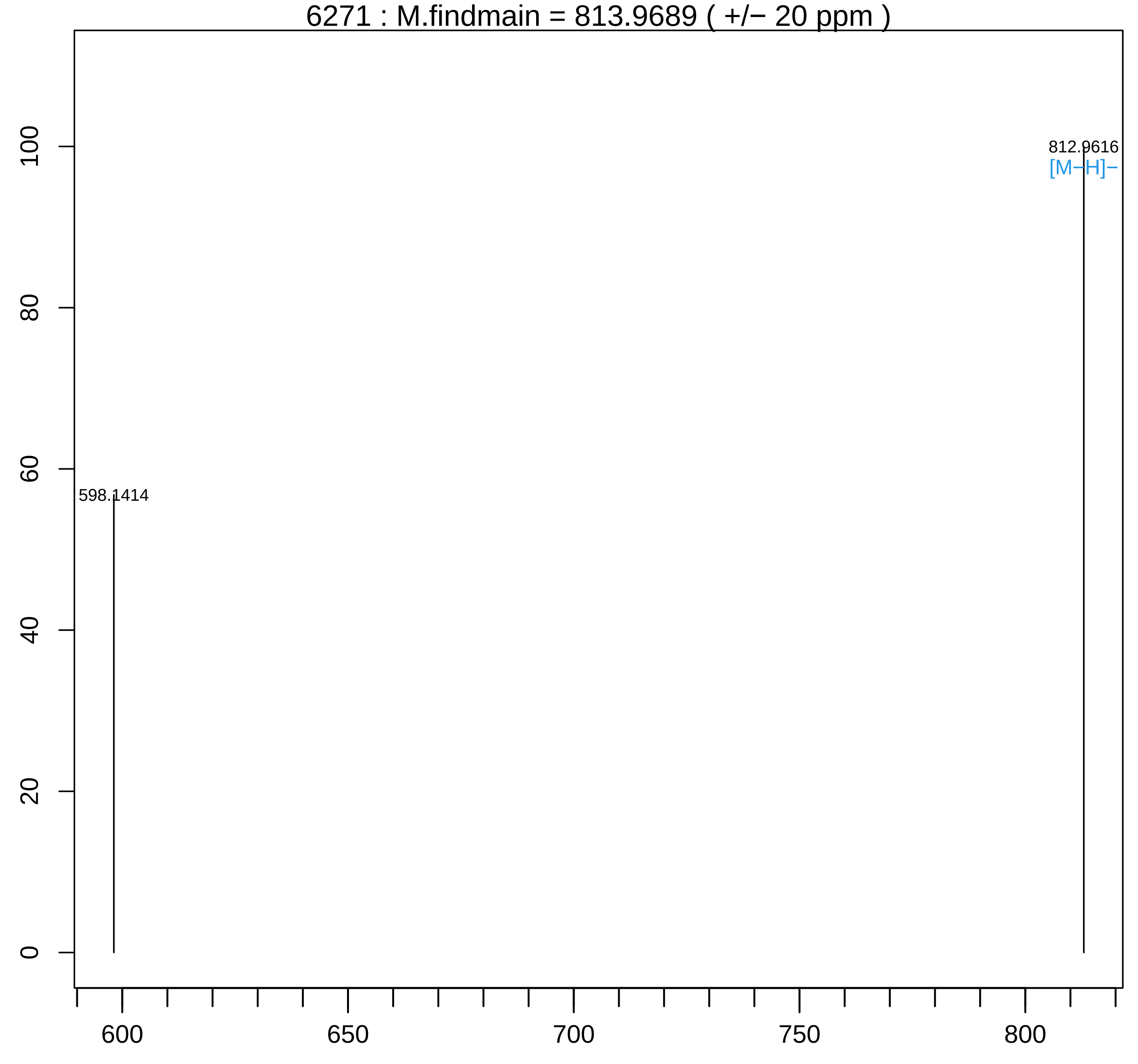
6271 : M.findmain = 813.9689 (+/− 20 ppm)
812.9616
[M−H]−
598.1414
0
20
40
60
80
100
600
650
700
750
800

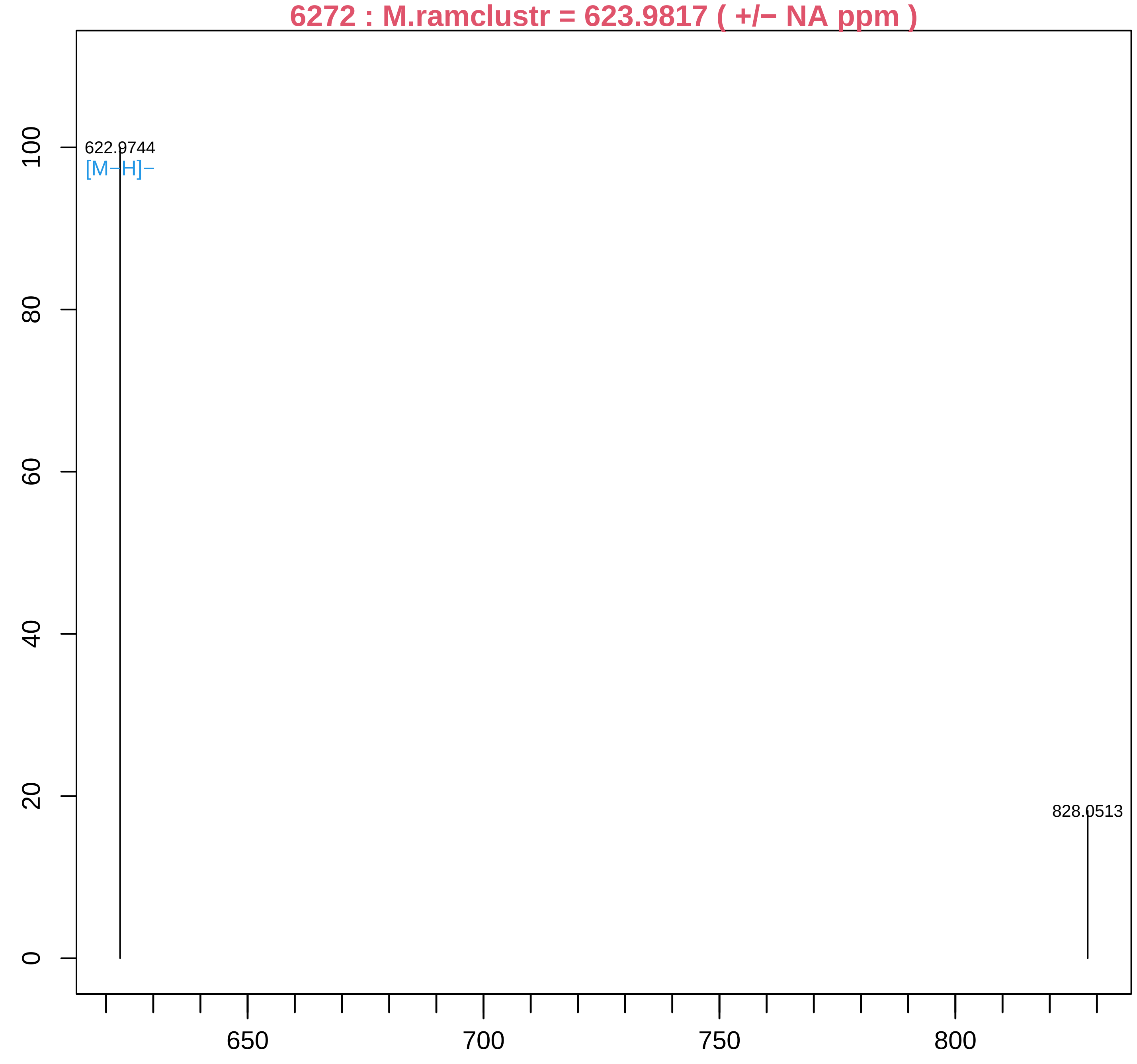
6272 : M.ramclustr = 623.9817 (+/− NA ppm)
622.9744
[M−H]−
828.0513
0
20
40
60
80
100
650
700
750
800

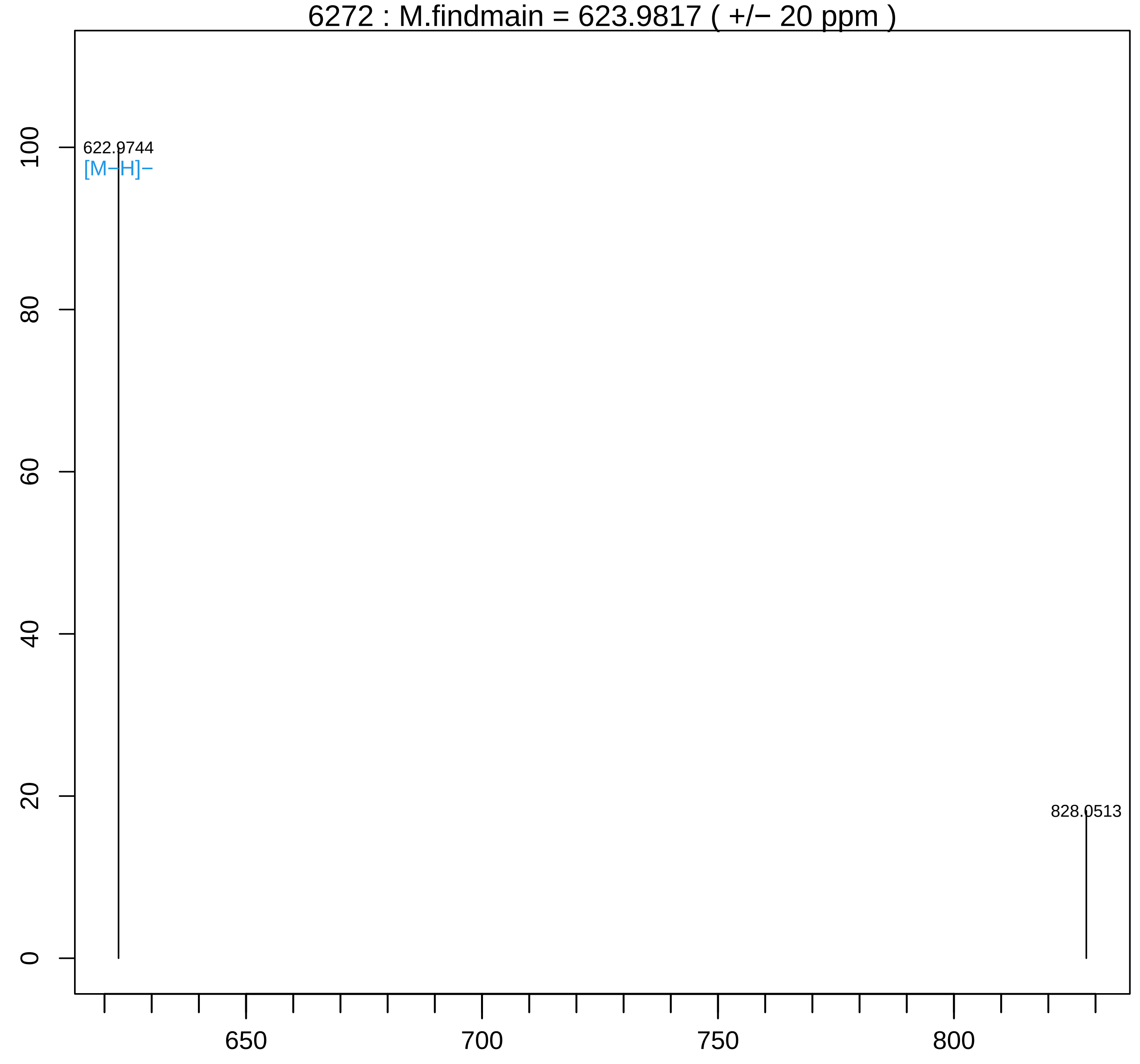
6272 : M.findmain = 623.9817 (+/− 20 ppm)
622.9744
[M−H]−
828.0513
0
20
40
60
80
100
650
700
750
800

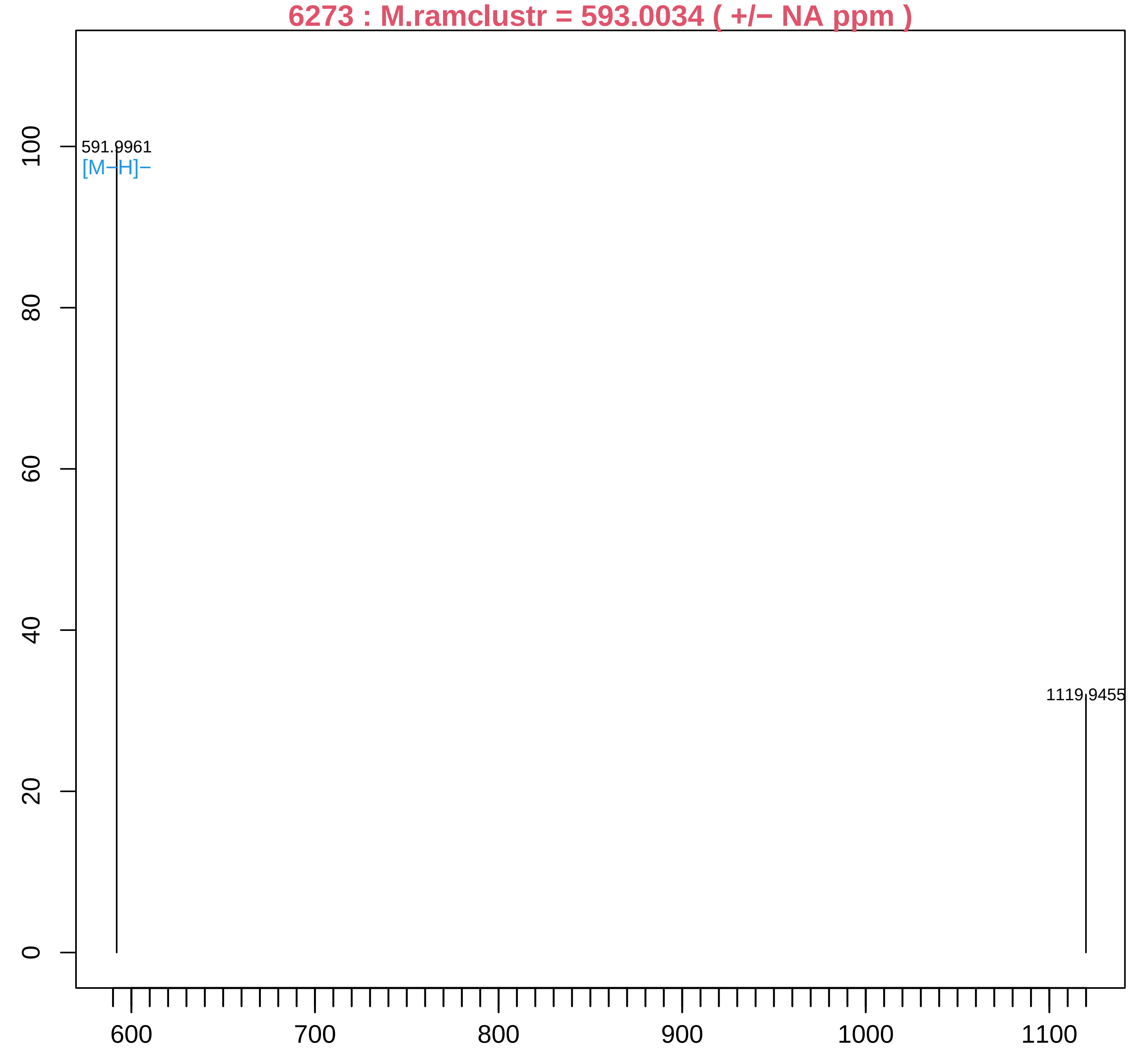
6273 : M.ramclustr = 593.0034 (+/− NA ppm)
591.9961
[M−H]−
1119.9455
0
20
40
60
80
100
600
700
800
900
1000
1100

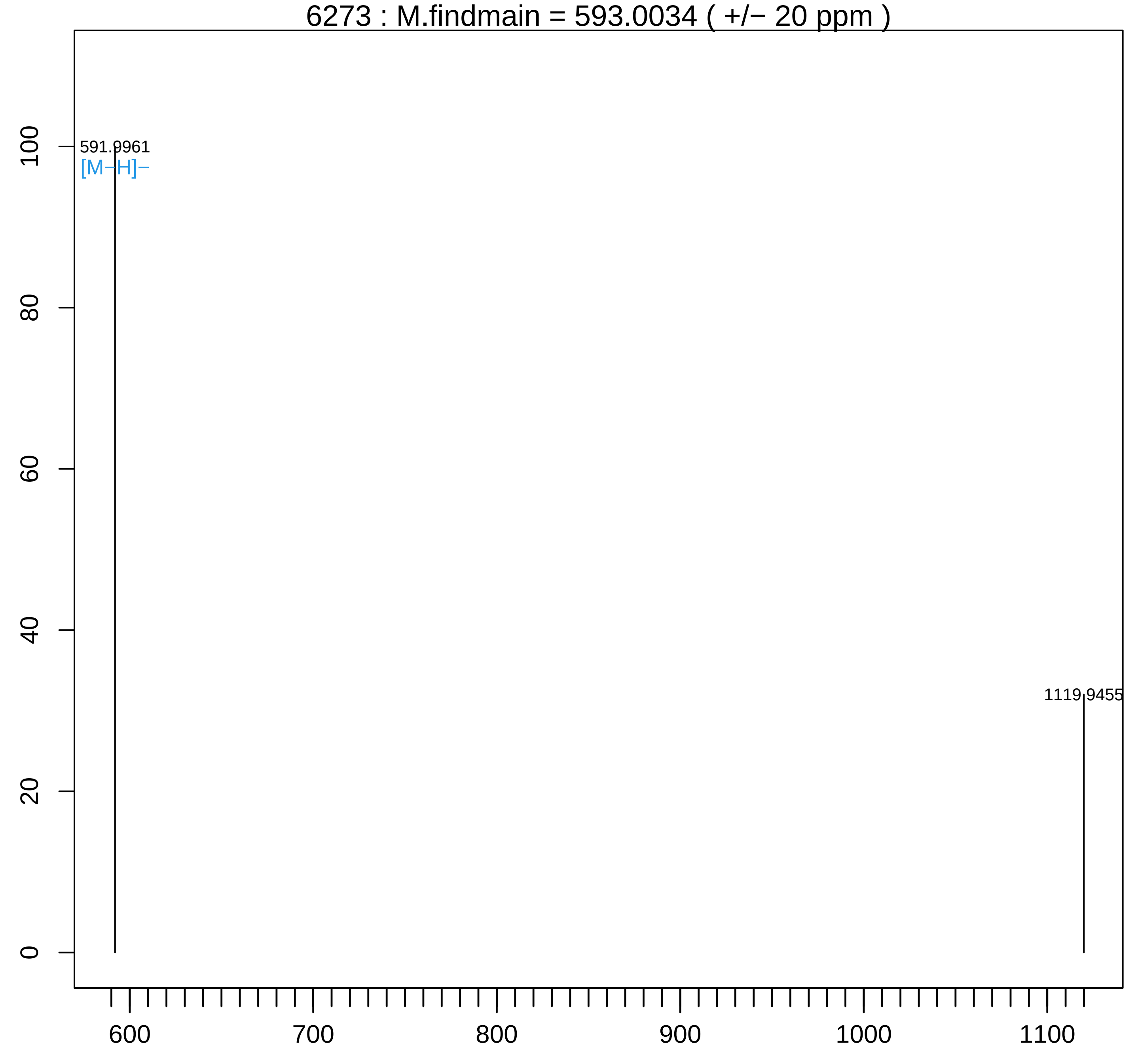
6273 : M.findmain = 593.0034 (+/− 20 ppm)
591.9961
[M−H]−
1119.9455
0
20
40
60
80
100
600
700
800
900
1000
1100

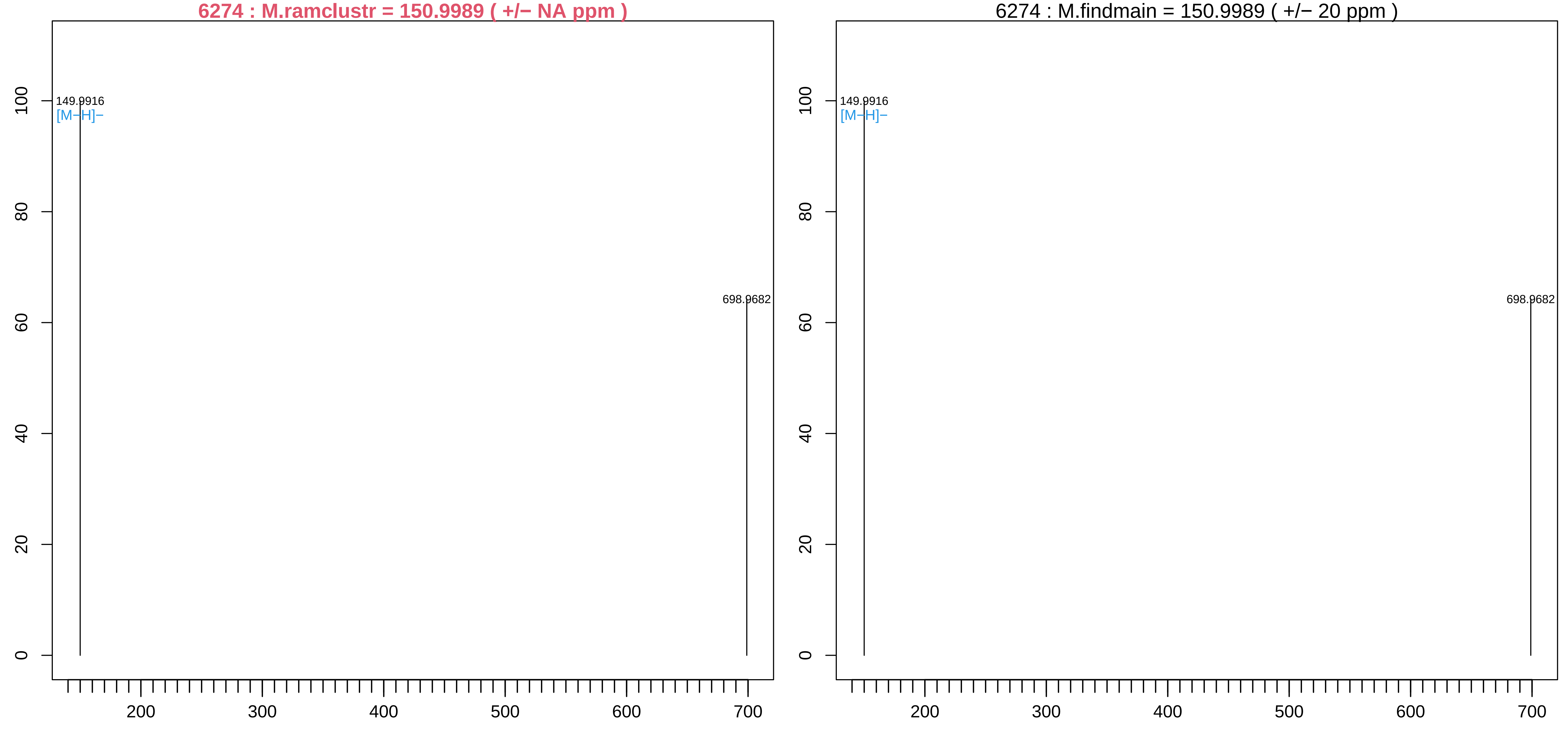
6274 : M.ramclustr = 150.9989 (+/− NA ppm)
149.9916
[M−H]−
698.9682
6274 : M.findmain = 150.9989 (+/− 20 ppm)
149.9916
[M−H]−
698.9682
0
20
40
60
80
100
200
300
400
500
600
700

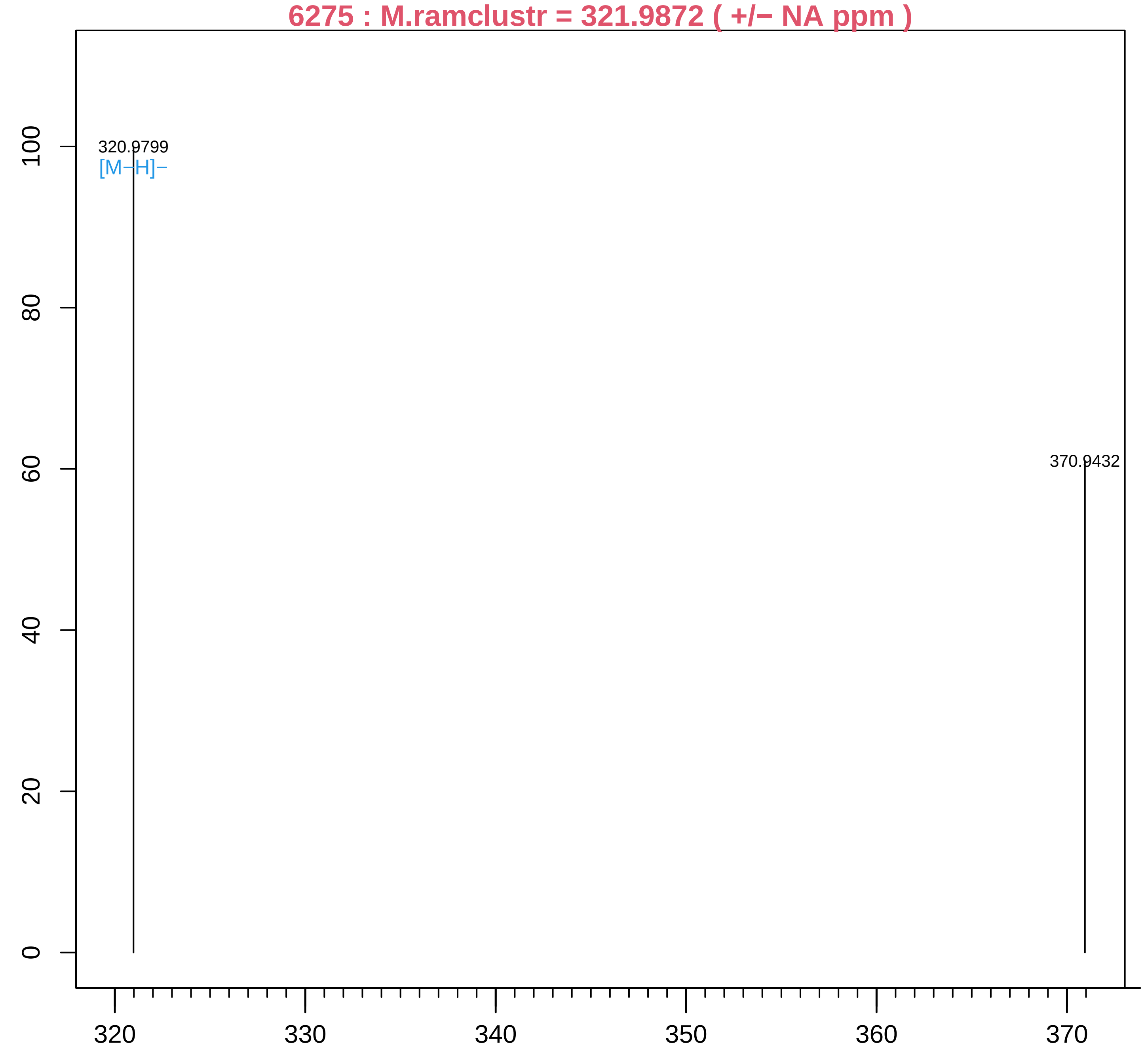
6275 : M.ramclustr = 321.9872 (+/− NA ppm)
320.9799
[M−H]−
370.9432
0
20
40
60
80
100
320
330
340
350
360
370

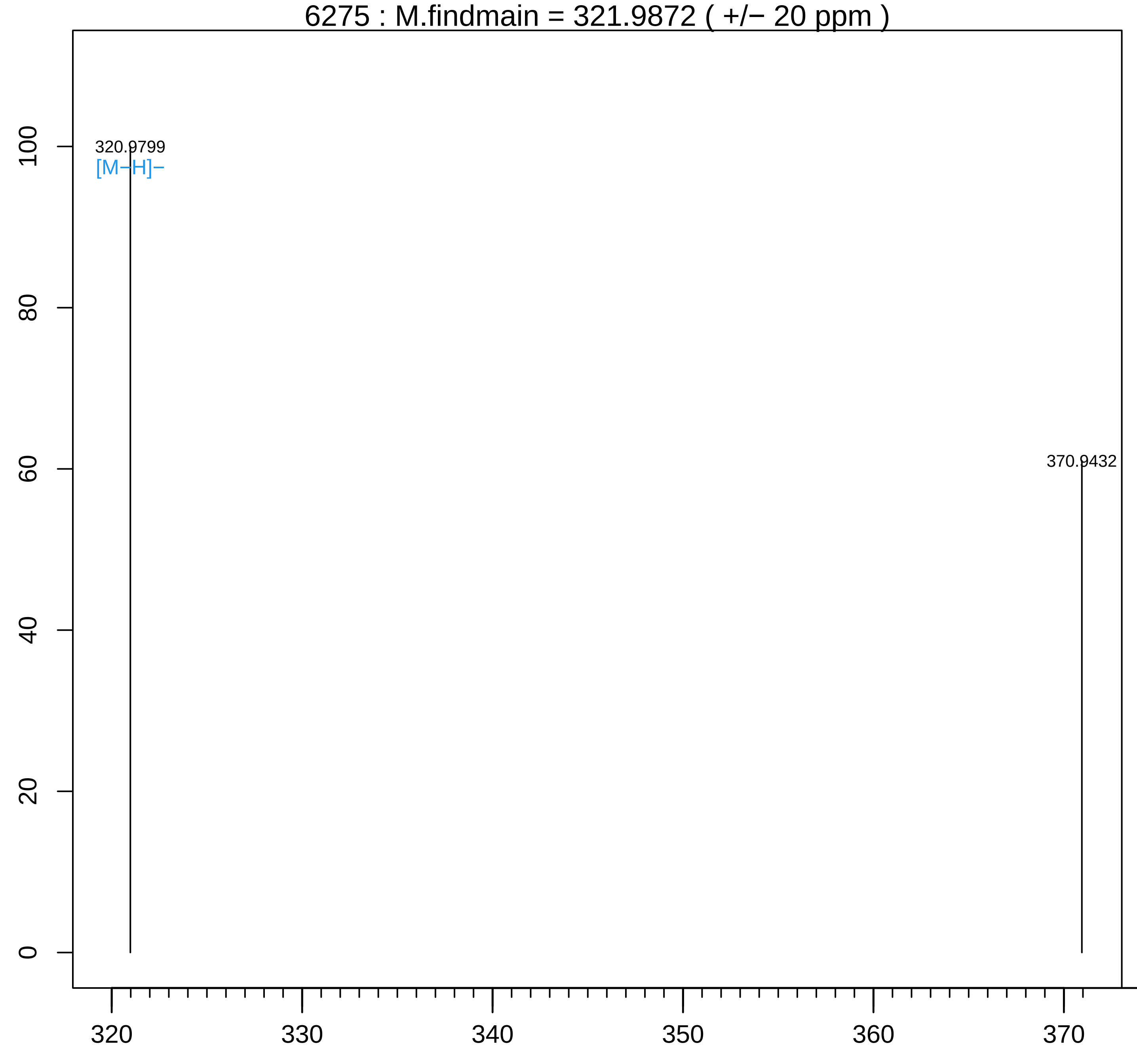
6275 : M.findmain = 321.9872 (+/− 20 ppm)
320.9799
[M−H]−
370.9432
0
20
40
60
80
100
320
330
340
350
360
370

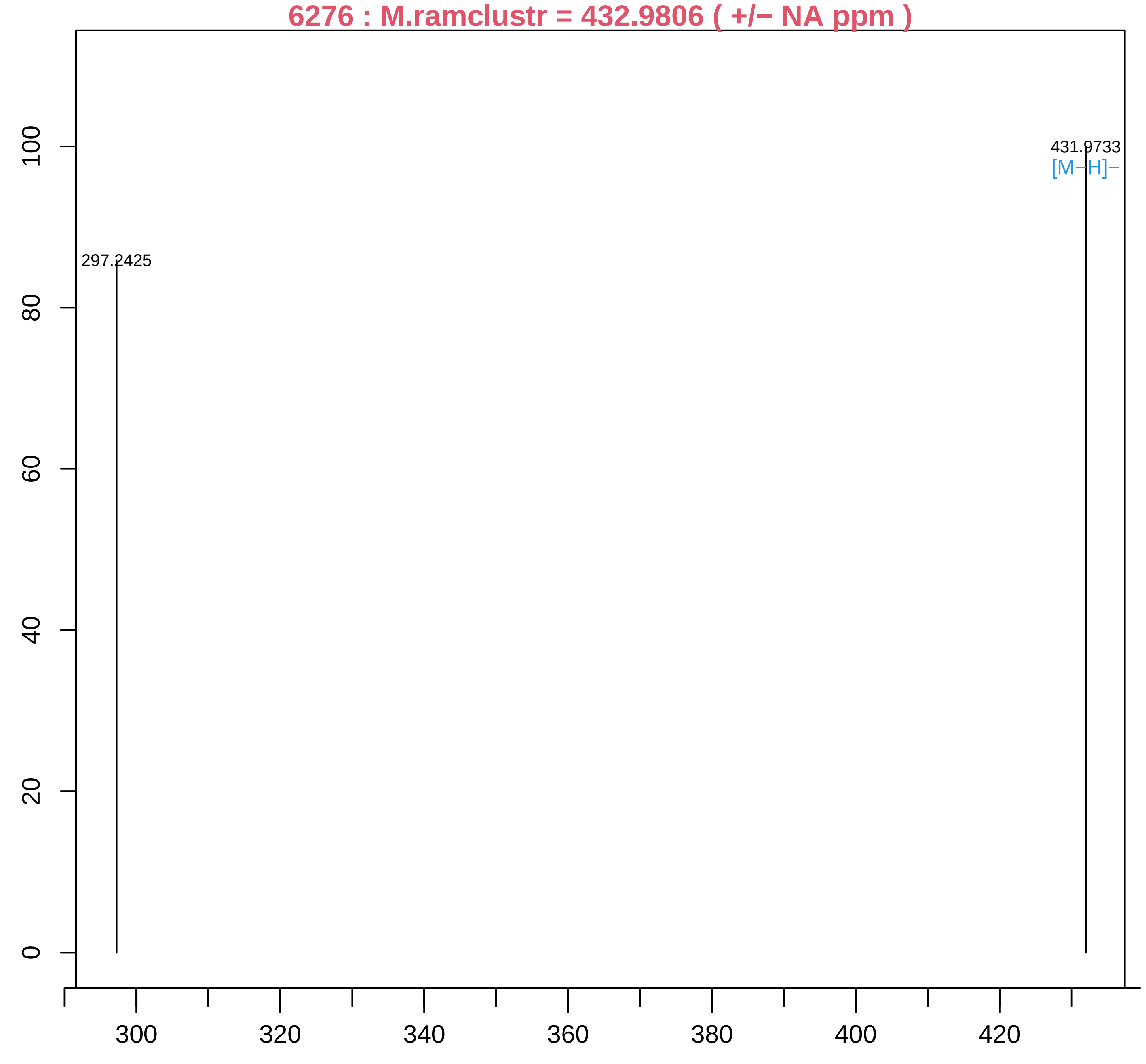
6276 : M.ramclustr = 432.9806 (+/− NA ppm)
431.9733
[M−H]−
297.2425

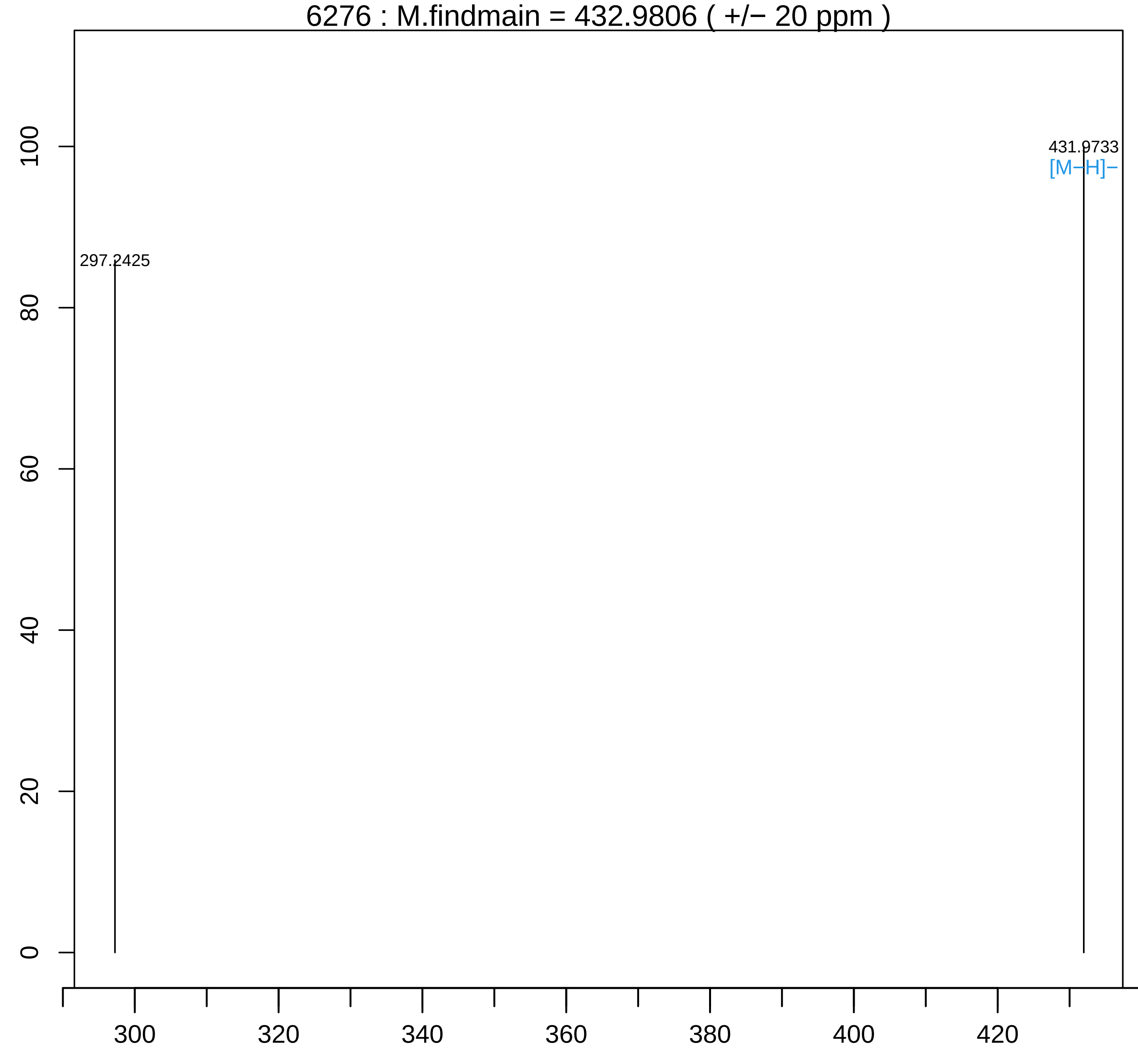
6276 : M.findmain = 432.9806 (+/− 20 ppm)
431.9733
[M−H]−
297.2425

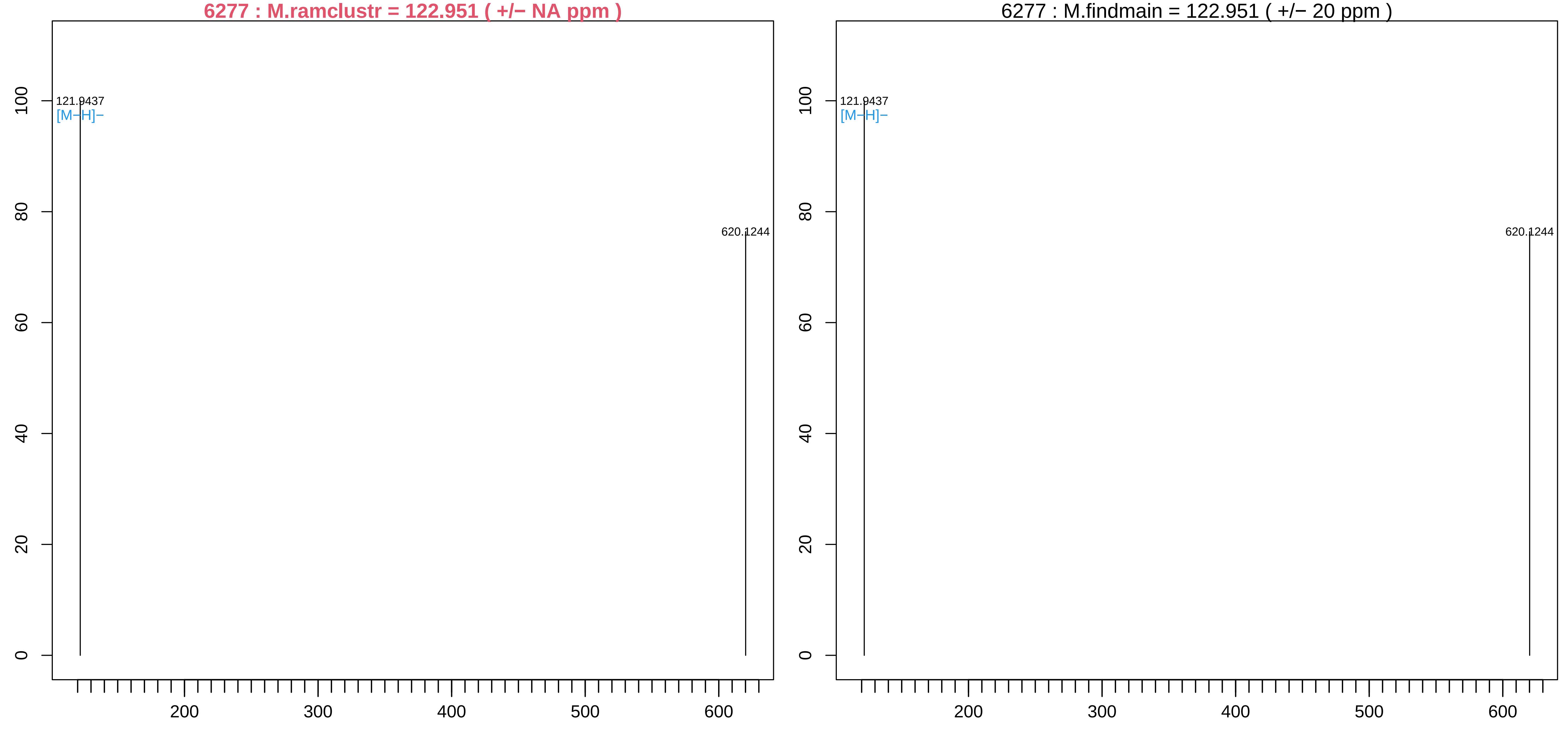
6277 : M.ramclustr = 122.951 (+/− NA ppm)
6277 : M.findmain = 122.951 (+/− 20 ppm)
121.9437
[M−H]−
620.1244
0
20
40
60
80
100
200
300
400
500
600

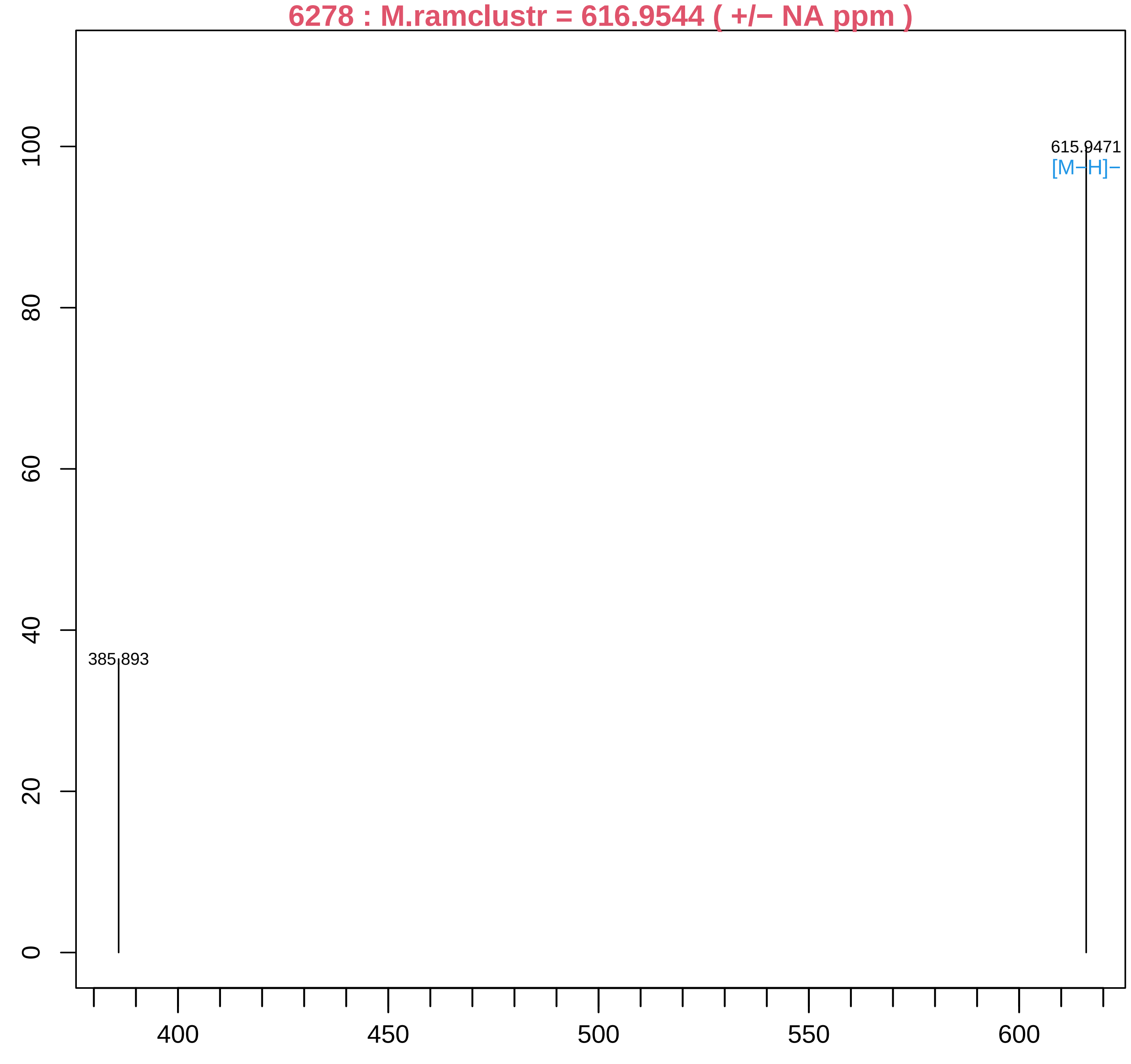

6278 : M.ramclustr = 616.9544 (+/− NA ppm)
615.9471
[M−H]−
385.893
0
20
40
60
80
100
400
450
500
550
600

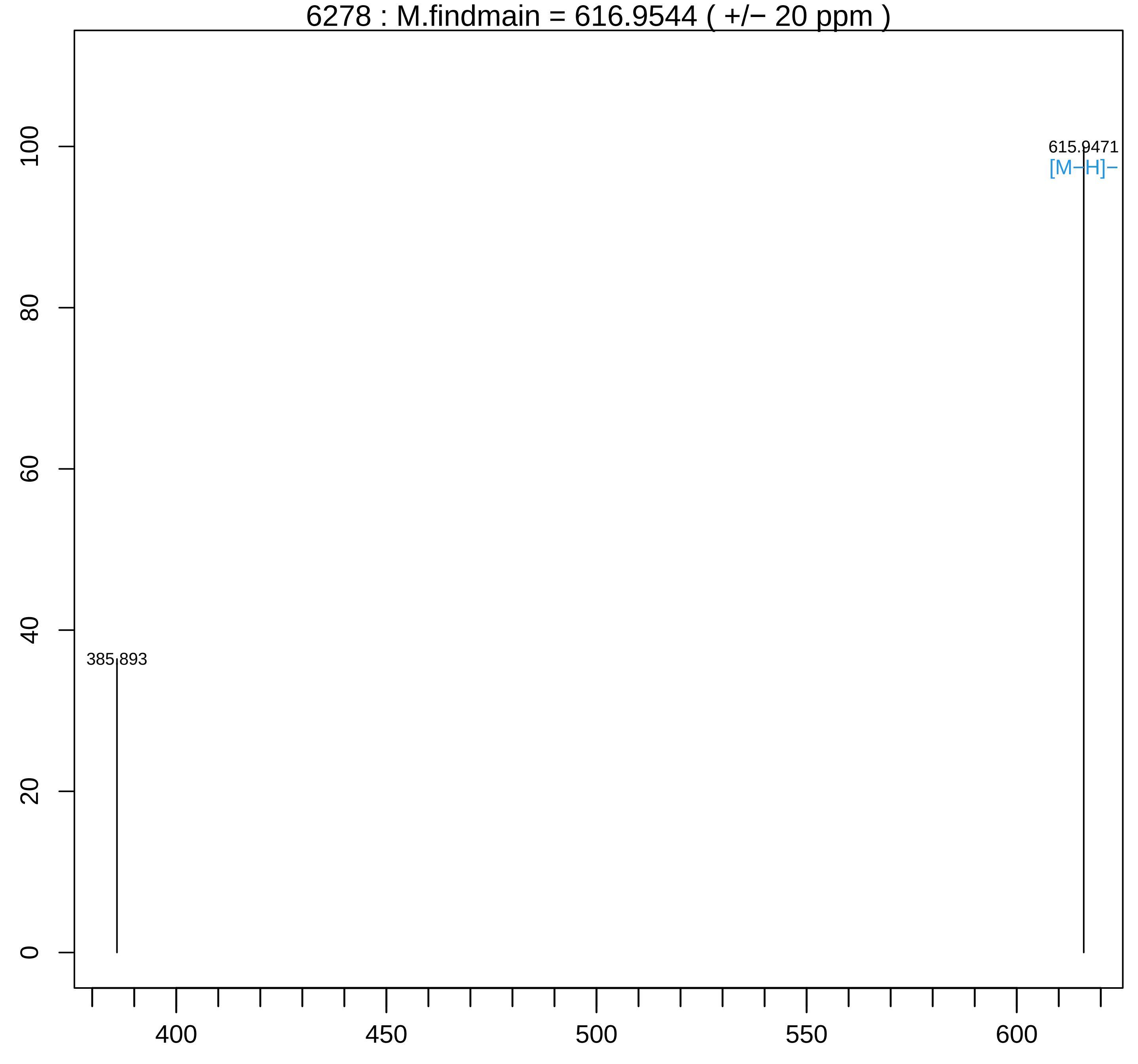

6278 : M.findmain = 616.9544 (+/− 20 ppm)
615.9471
[M−H]−
385.893
0
20
40
60
80
100
400
450
500
550
600

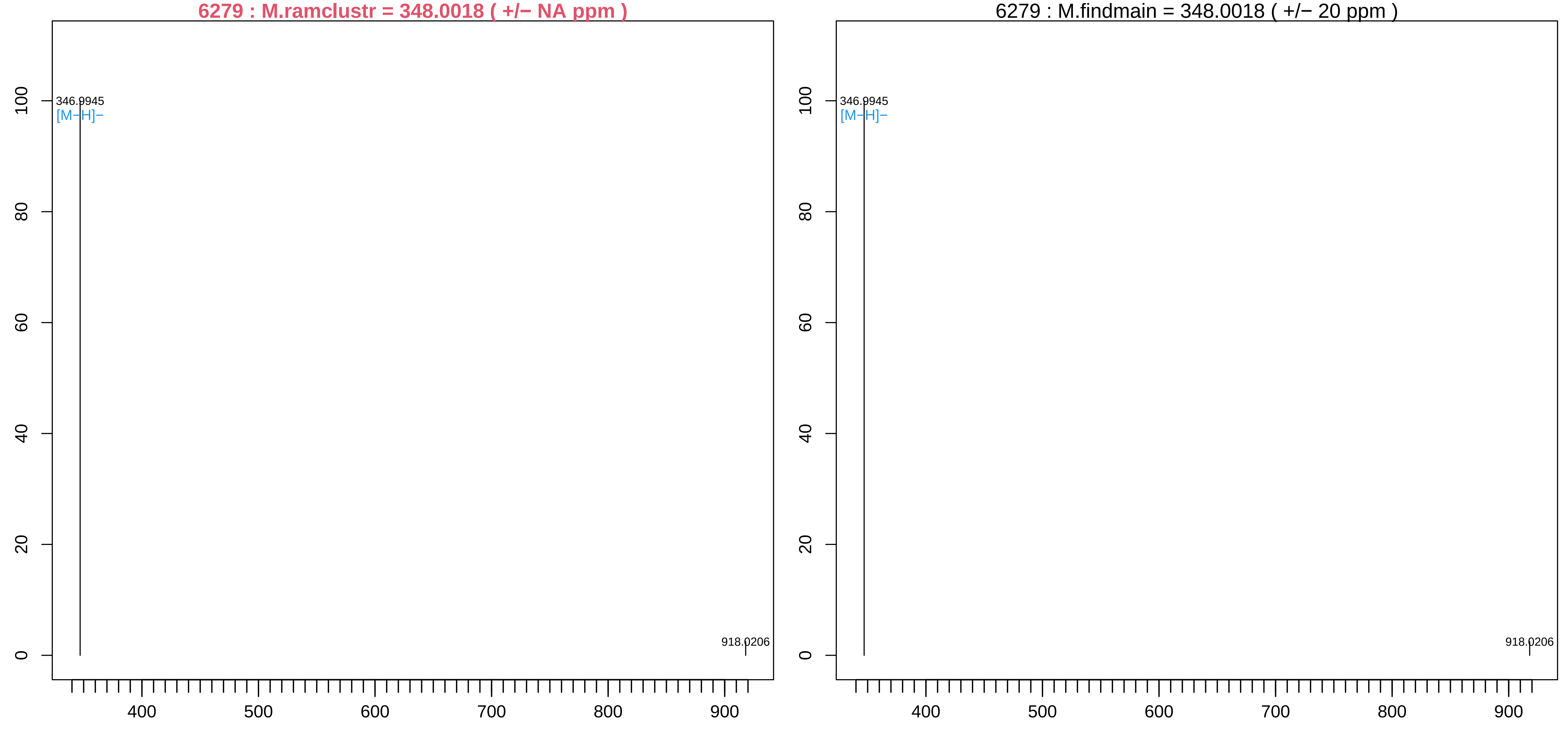
6279 : M.ramclustr = 348.0018 (+/− NA ppm)
6279 : M.findmain = 348.0018 (+/− 20 ppm)
346.9945
[M−H]−
918.0206
0
20
40
60
80
100
400
500
600
700
800
900

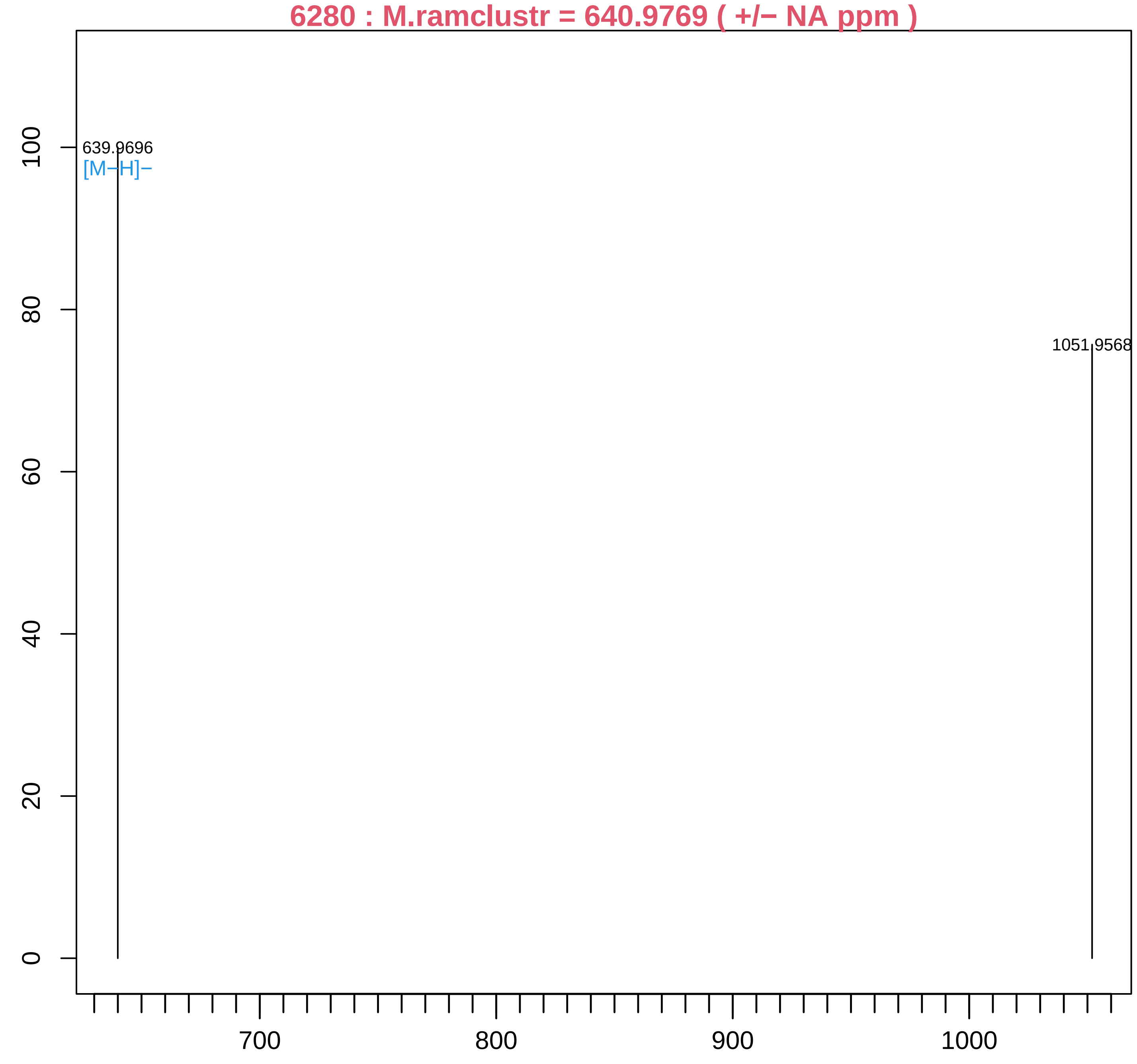
6280 : M.ramclustr = 640.9769 (+/− NA ppm)
639.9696
[M−H]−
1051.9568
0
20
40
60
80
100
700
800
900
1000

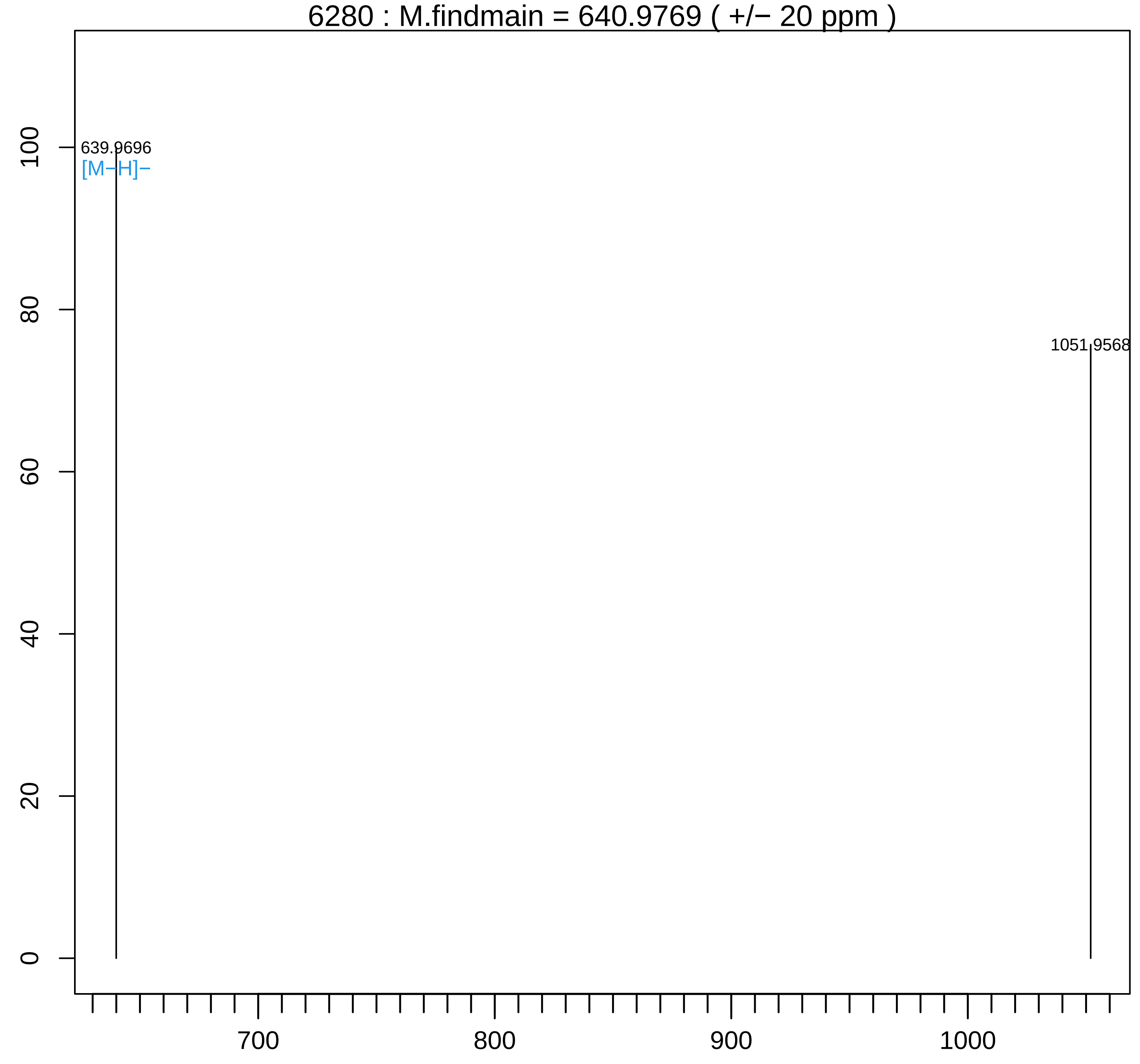
6280 : M.findmain = 640.9769 (+/− 20 ppm)
639.9696
[M−H]−
1051.9568
0
20
40
60
80
100
700
800
900
1000

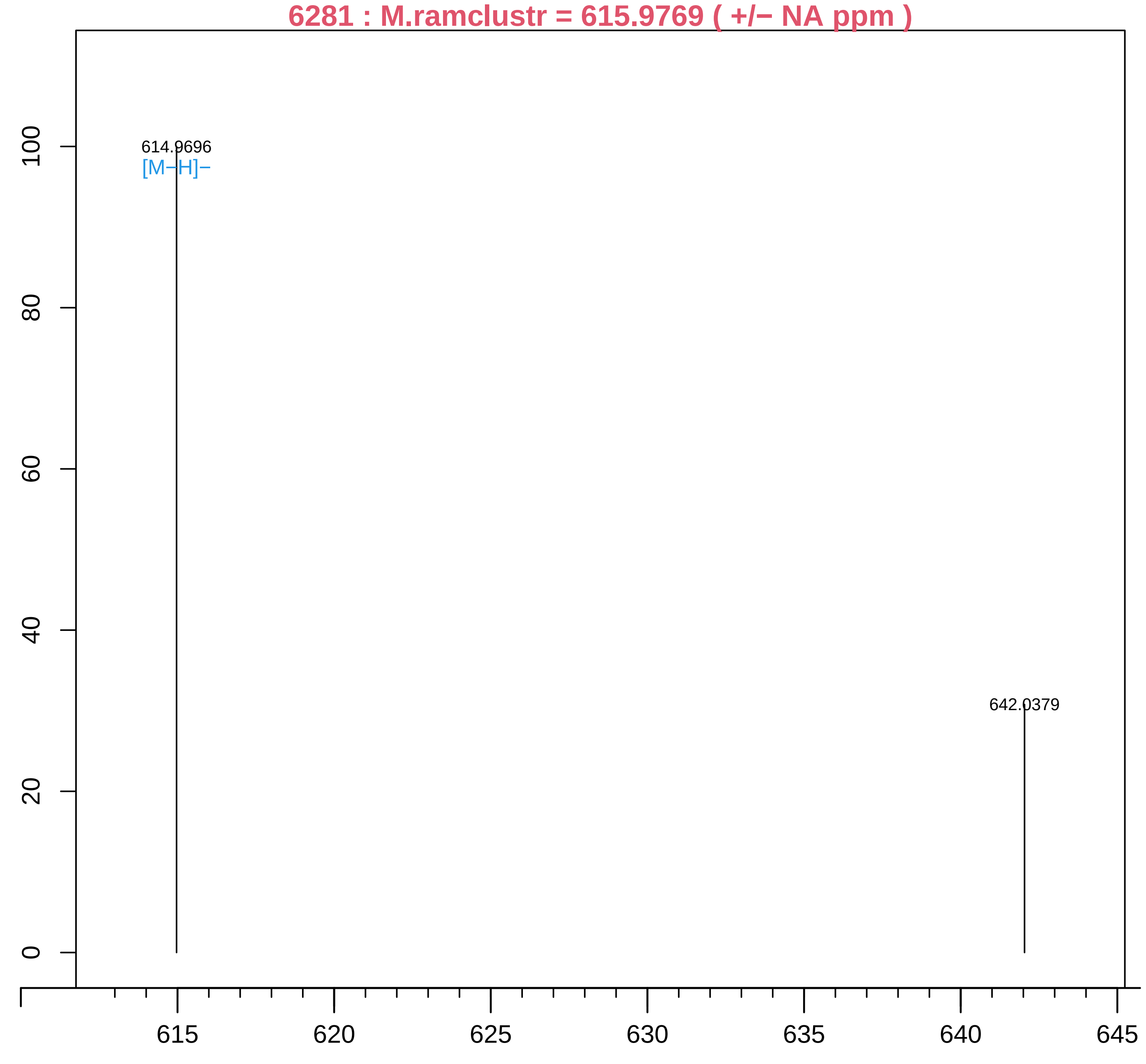
6281 : M.ramclustr = 615.9769 (+/− NA ppm)
614.9696
[M−H]−
642.0379
0
20
40
60
80
100
615
620
625
630
635
640
645

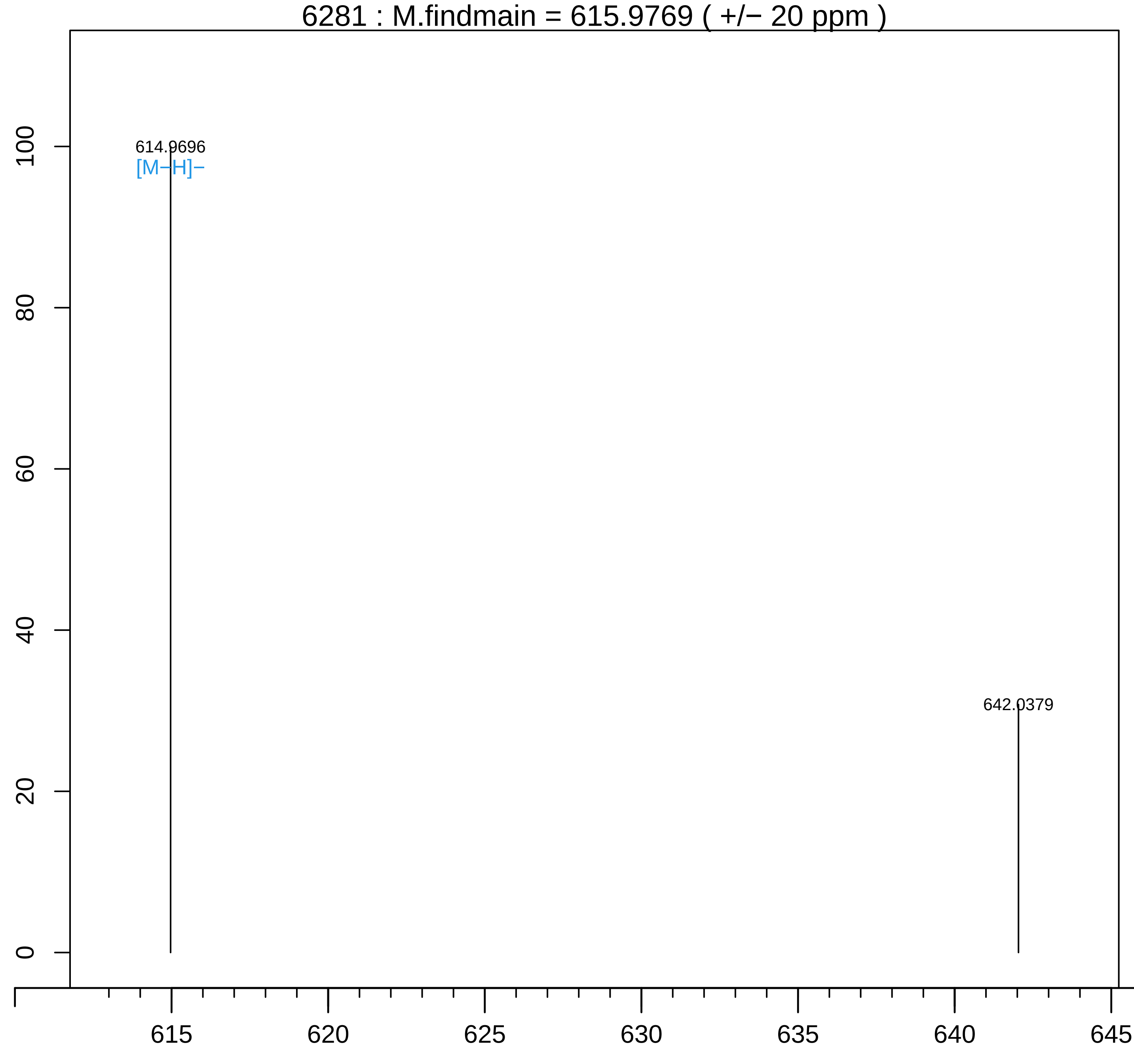
6281 : M.findmain = 615.9769 (+/− 20 ppm)
614.9696
[M−H]−
642.0379
0
20
40
60
80
100
615
620
625
630
635
640
645

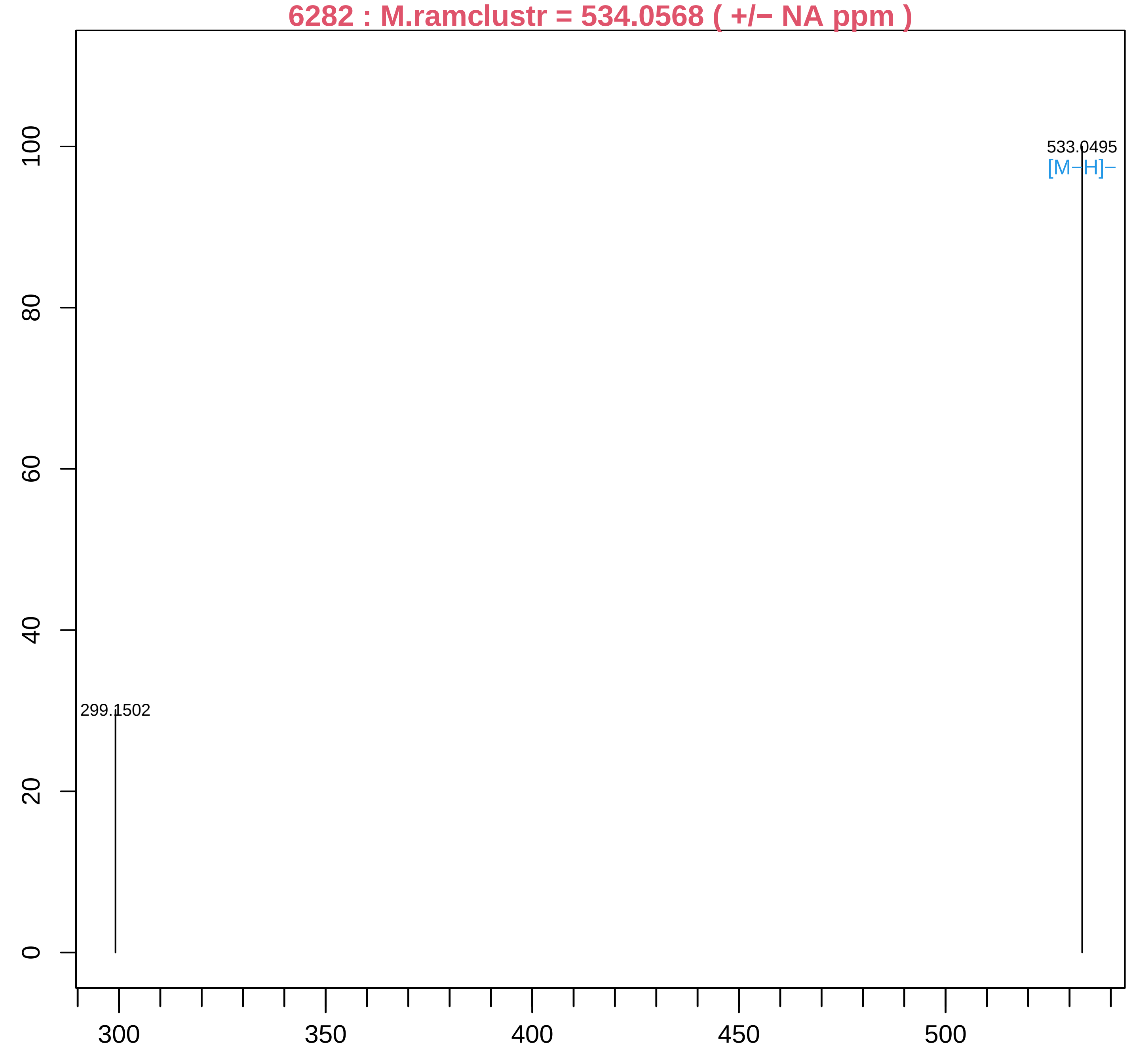
6282 : M.ramclustr = 534.0568 (+/− NA ppm)
533.0495
[M−H]−
299.1502
0
20
40
60
80
100
300
350
400
450
500

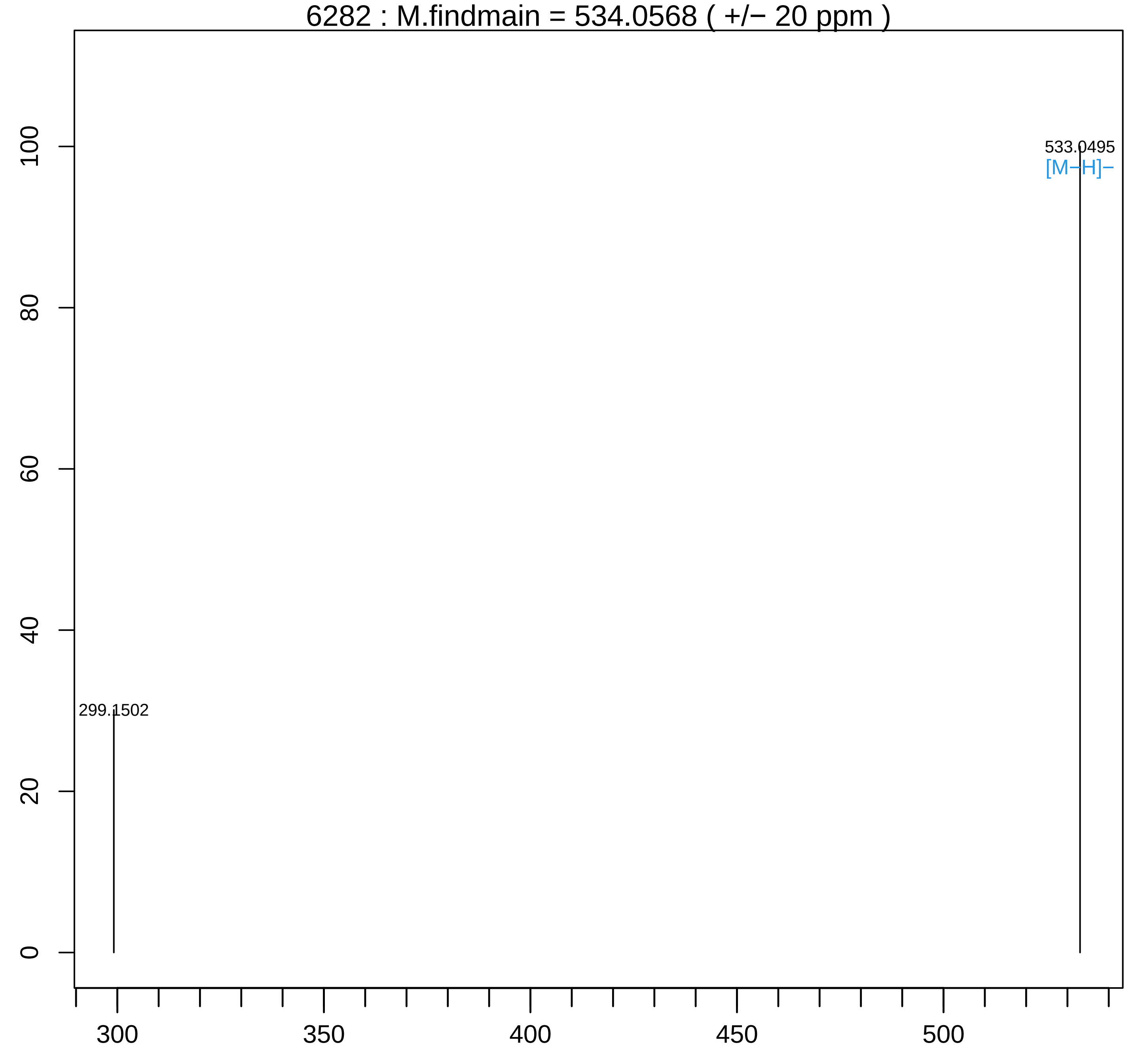
6282 : M.findmain = 534.0568 (+/− 20 ppm)
533.0495
[M−H]−
299.1502
0
20
40
60
80
100
300
350
400
450
500

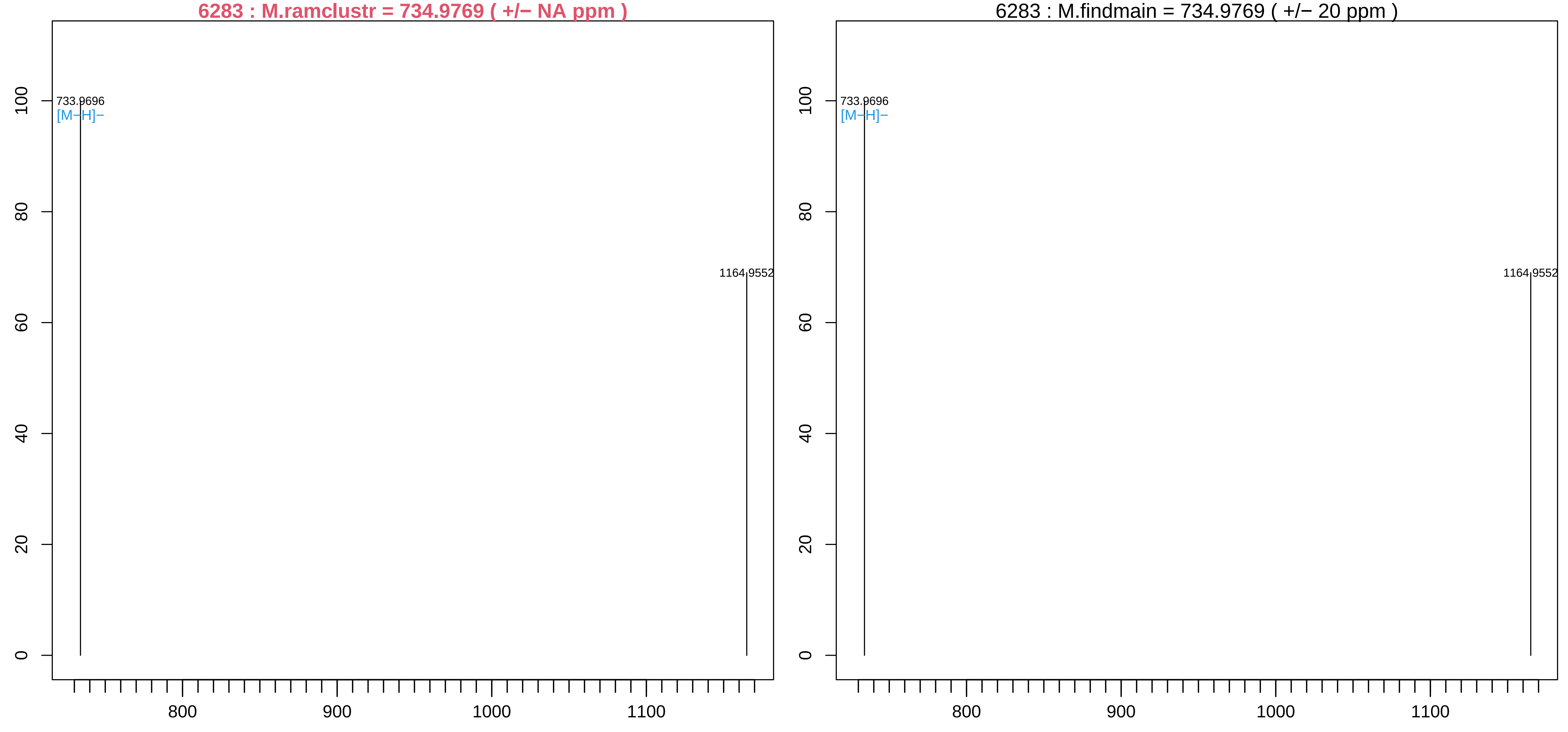

6283 : M.ramclustr = 734.9769 (+/− NA ppm)
733.9696
[M−H]−
1164.9552
0
20
40
60
80
100
800
900
1000
1100
6283 : M.findmain = 734.9769 (+/− 20 ppm)
733.9696
[M−H]−
1164.9552
0
20
40
60
80
100
800
900
1000
1100

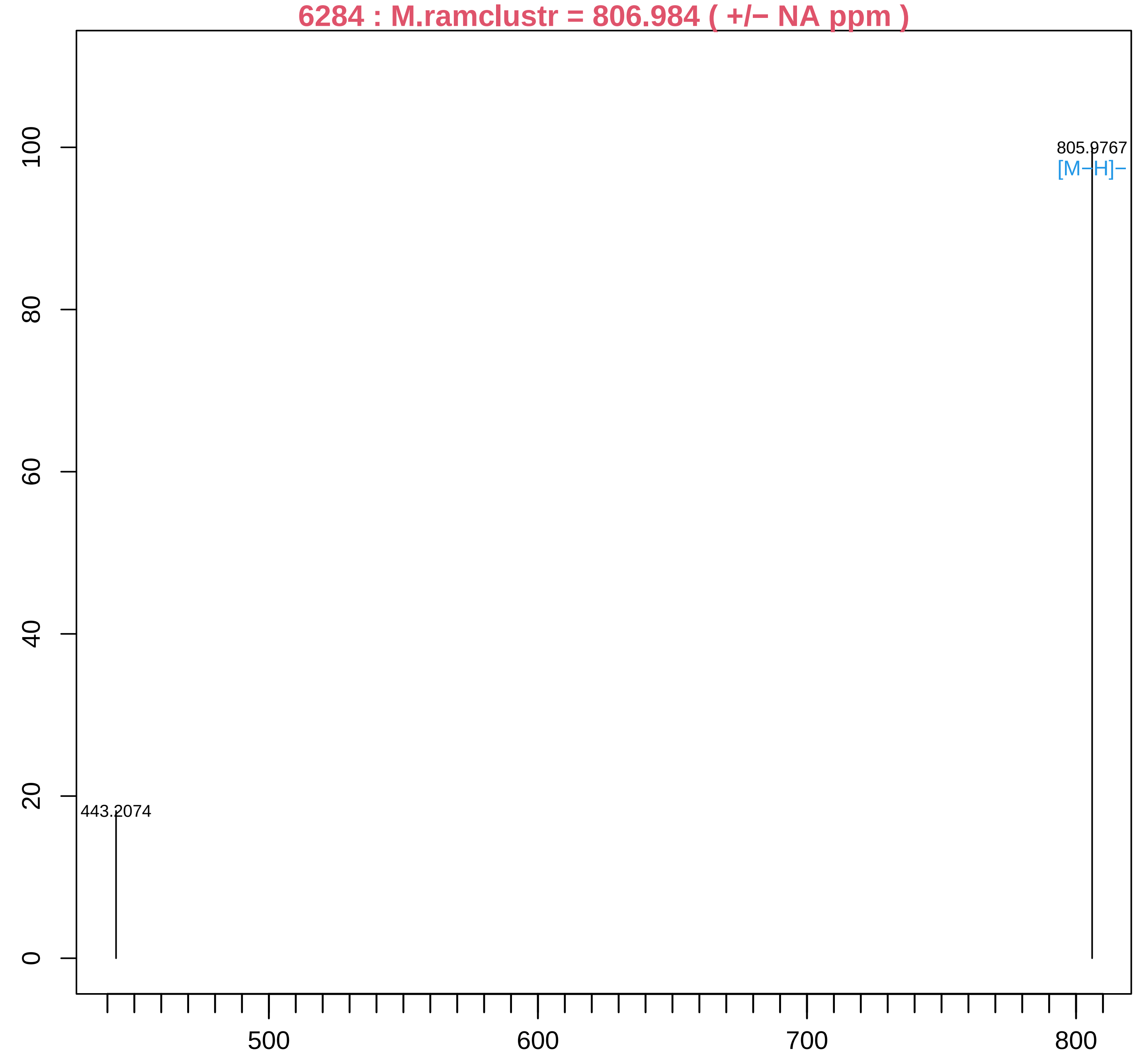
6284 : M.ramclustr = 806.984 (+/− NA ppm)
805.9767
[M−H]−
443.2074
0
20
40
60
80
100
500
600
700
800

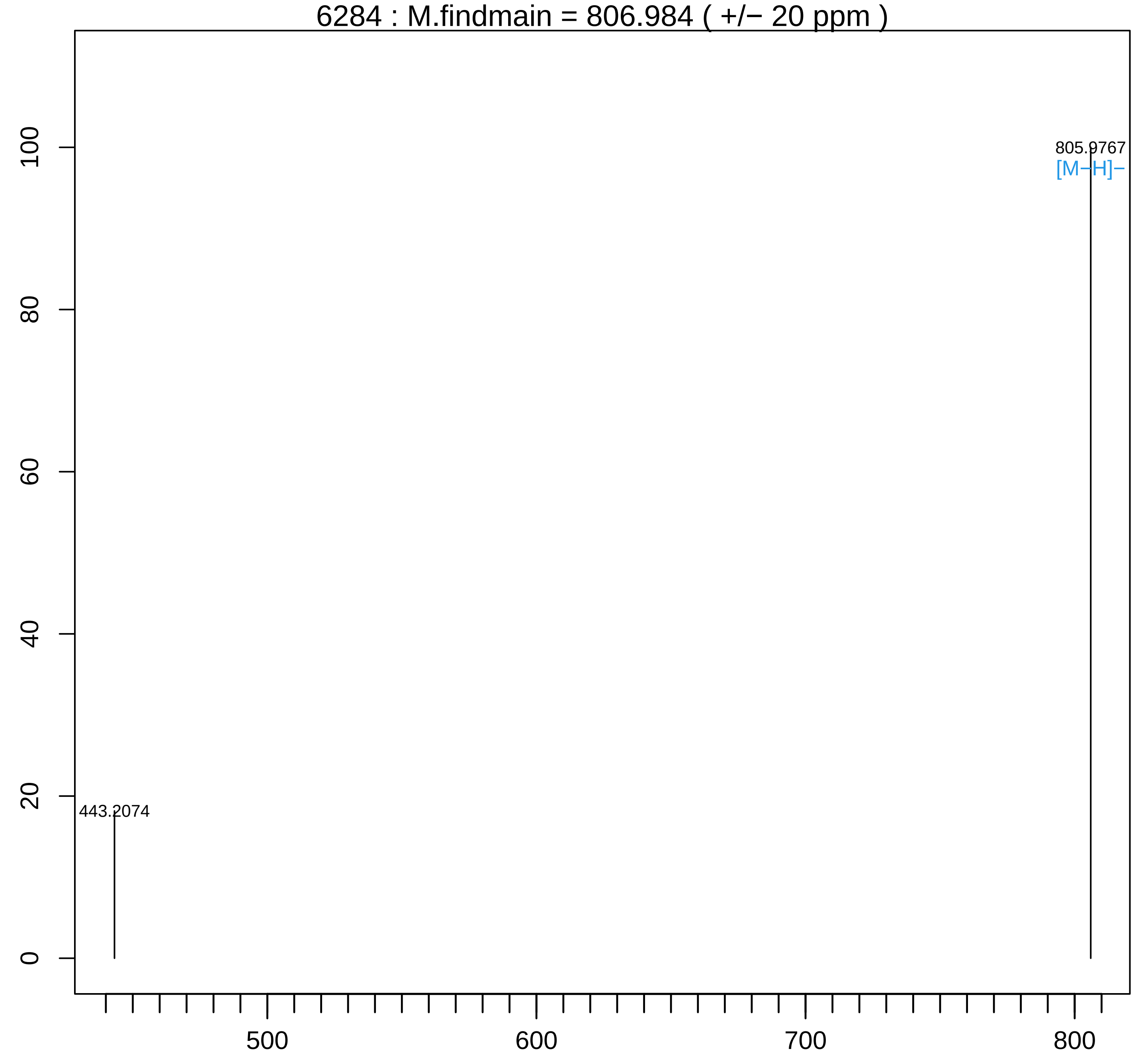
6284 : M.findmain = 806.984 (+/− 20 ppm)
805.9767
[M−H]−
443.2074
0
20
40
60
80
100
500
600
700
800

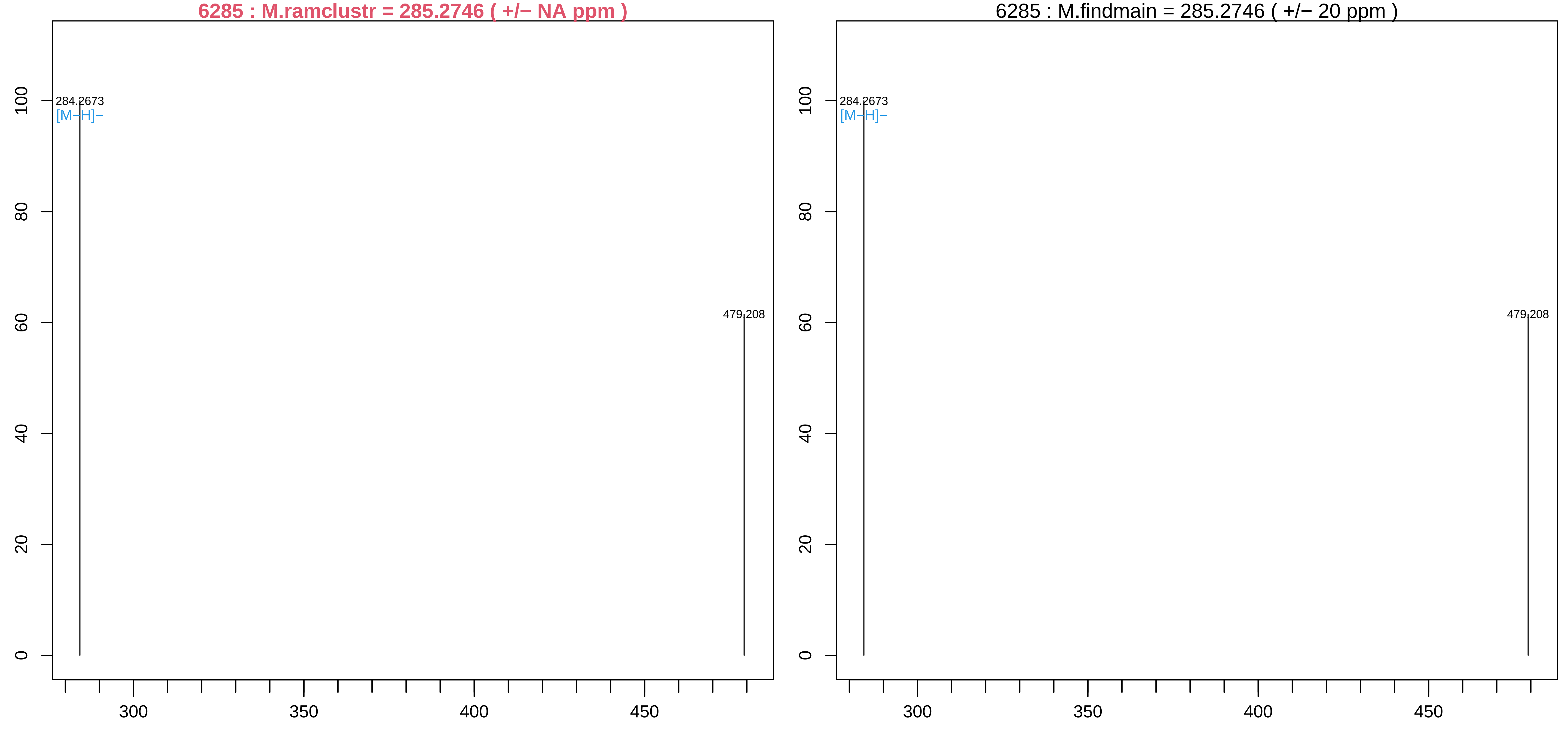
6285 : M.ramclustr = 285.2746 (+/− NA ppm)
284.2673
[M−H]−
479.208
6285 : M.findmain = 285.2746 (+/− 20 ppm)
284.2673
[M−H]−
479.208
0
20
40
60
80
100
300
350
400
450

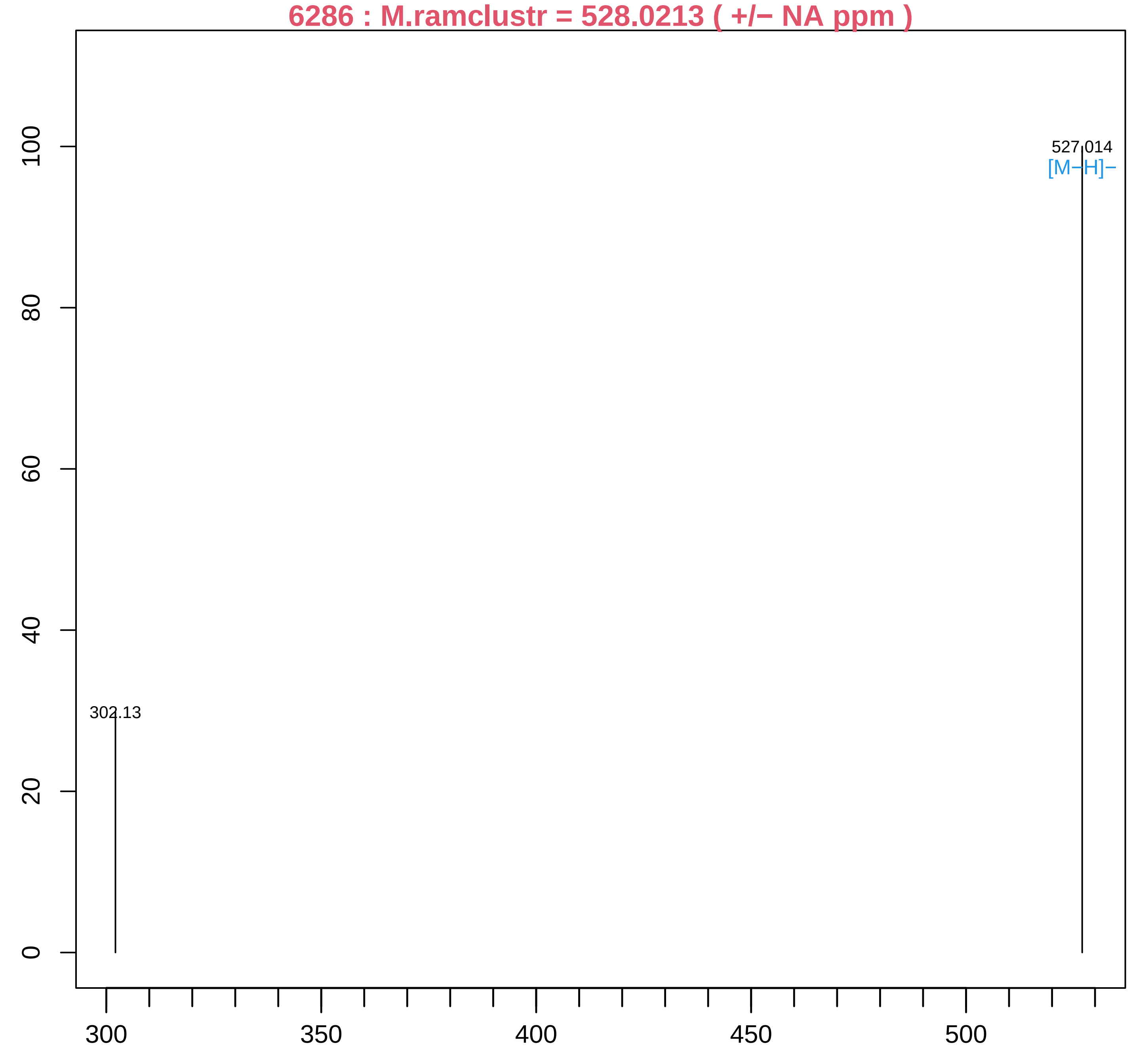
6286 : M.ramclustr = 528.0213 (+/− NA ppm)
527.014
[M−H]−
302.13
0
20
40
60
80
100
300
350
400
450
500

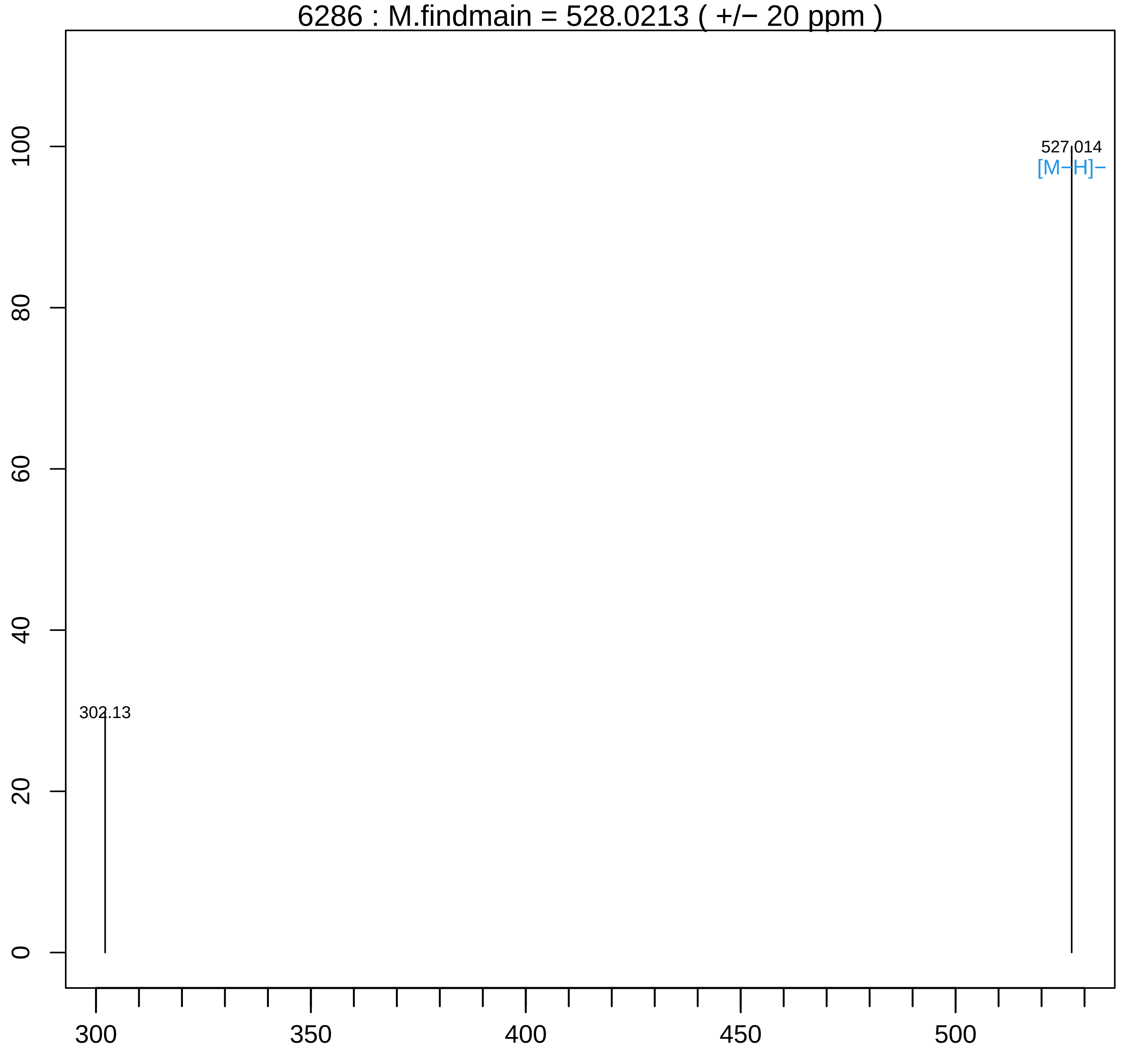
6286 : M.findmain = 528.0213 (+/− 20 ppm)
527.014
[M−H]−
302.13
0
20
40
60
80
100
300
350
400
450
500

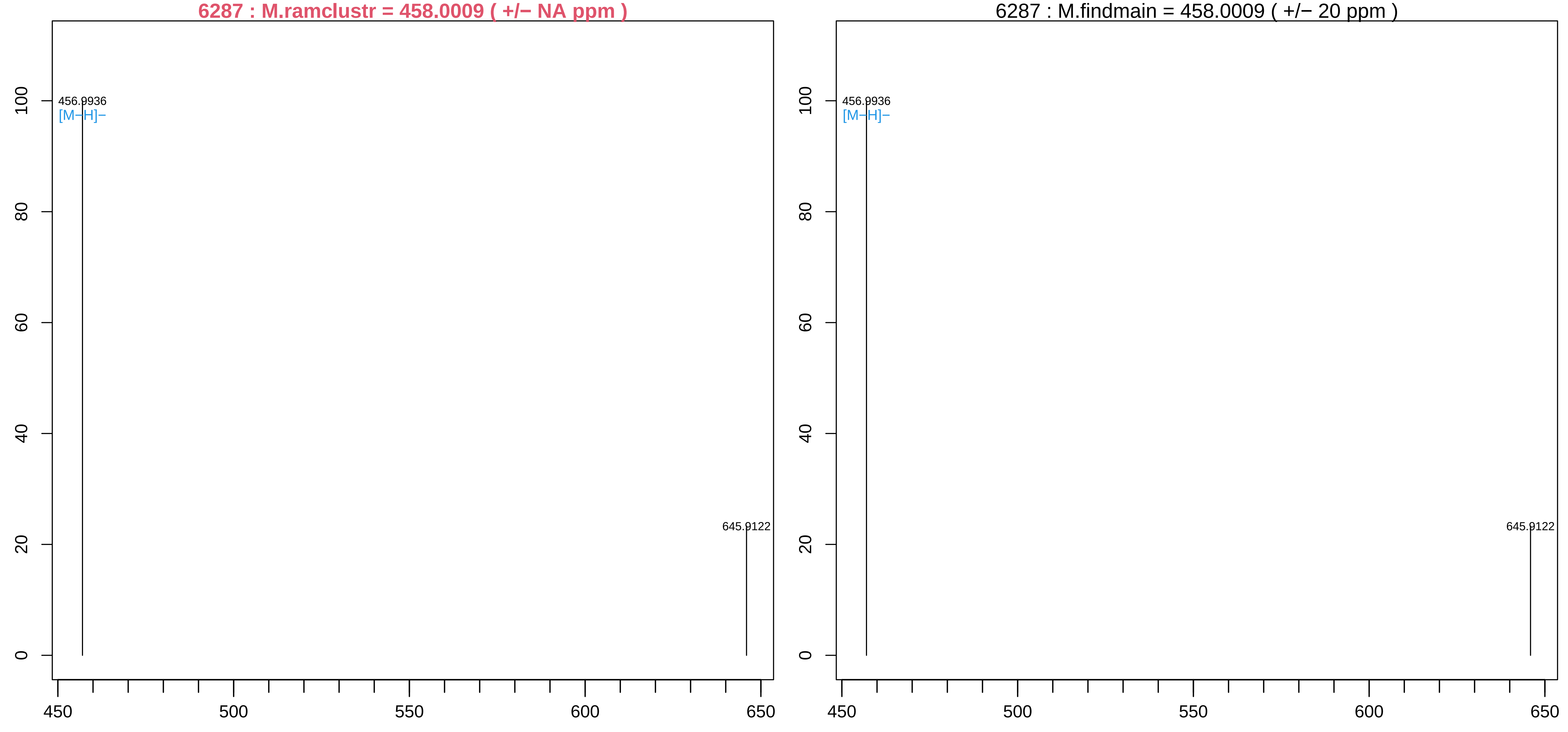

6287 : M.ramclustr = 458.0009 (+/− NA ppm)
456.9936
[M−H]−
645.9122
0
20
40
60
80
100
450
500
550
600
650
6287 : M.findmain = 458.0009 (+/− 20 ppm)
456.9936
[M−H]−
645.9122
0
20
40
60
80
100
450
500
550
600
650

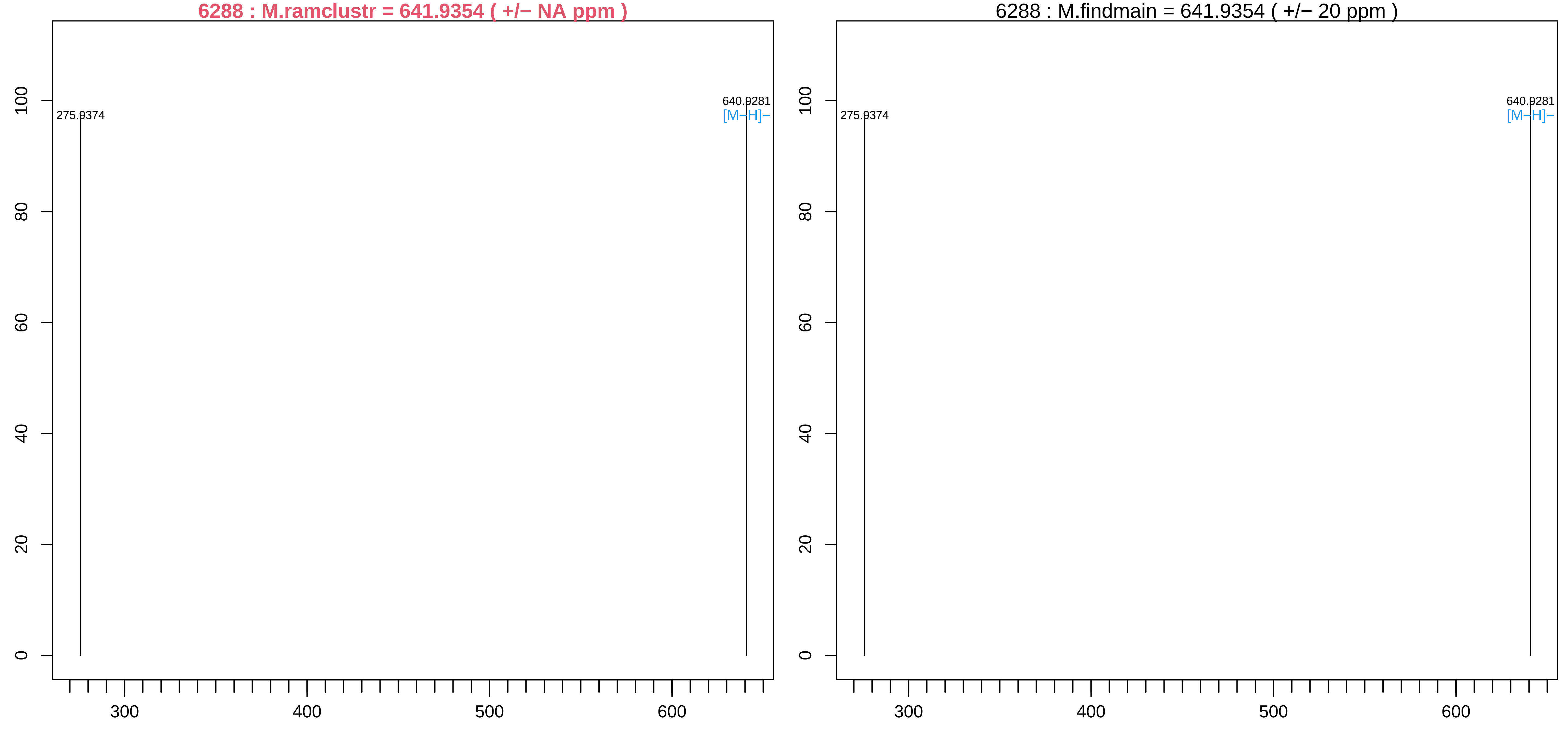
6288 : M.ramclustr = 641.9354 (+/− NA ppm)
275.9374
640.9281
[M−H]−
6288 : M.findmain = 641.9354 (+/− 20 ppm)
275.9374
640.9281
[M−H]−
0
20
40
60
80
100
300
400
500
600

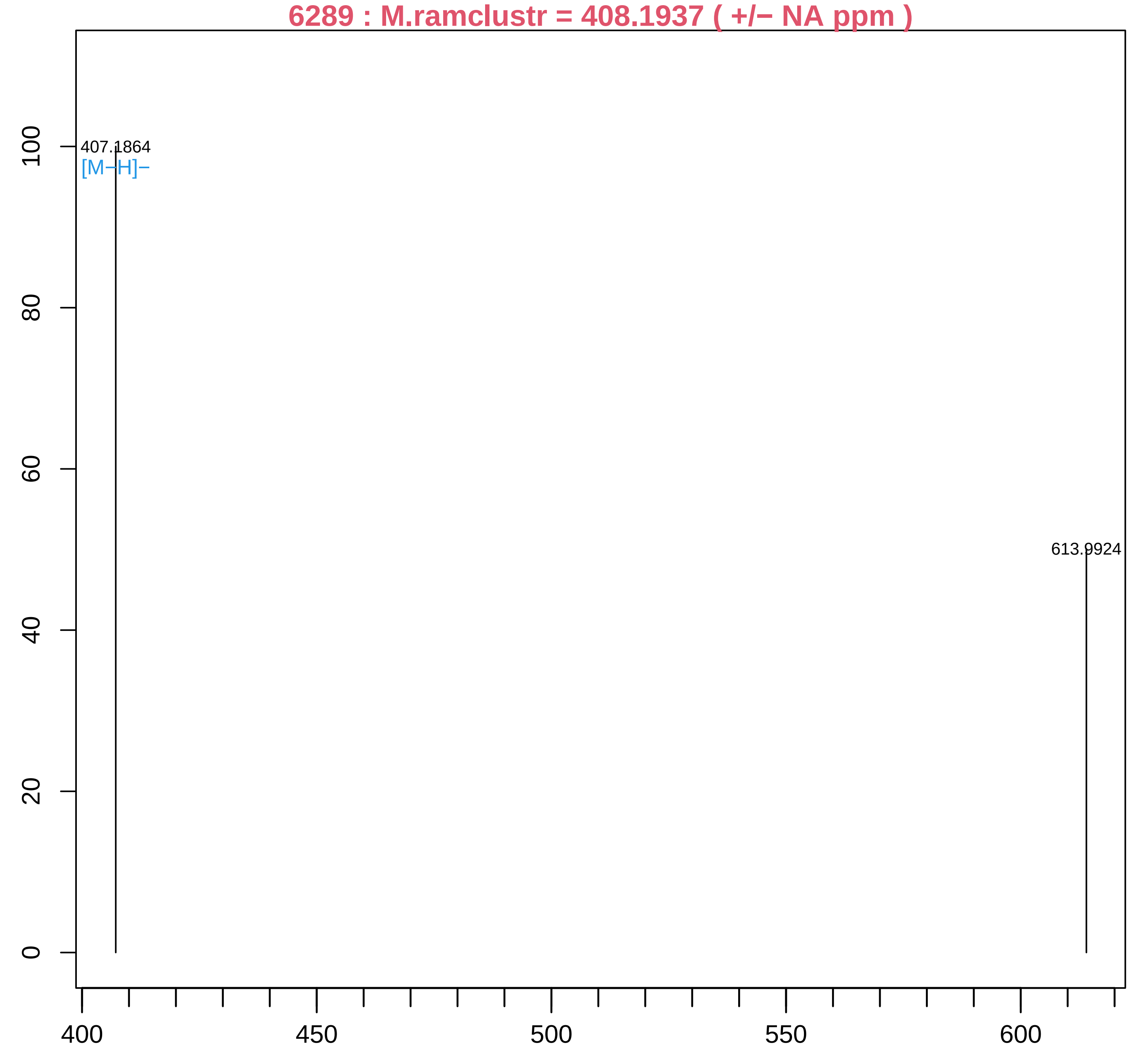
6289 : M.ramclustr = 408.1937 (+/− NA ppm)
407.1864
[M−H]−
613.9924
0
20
40
60
80
100
400
450
500
550
600

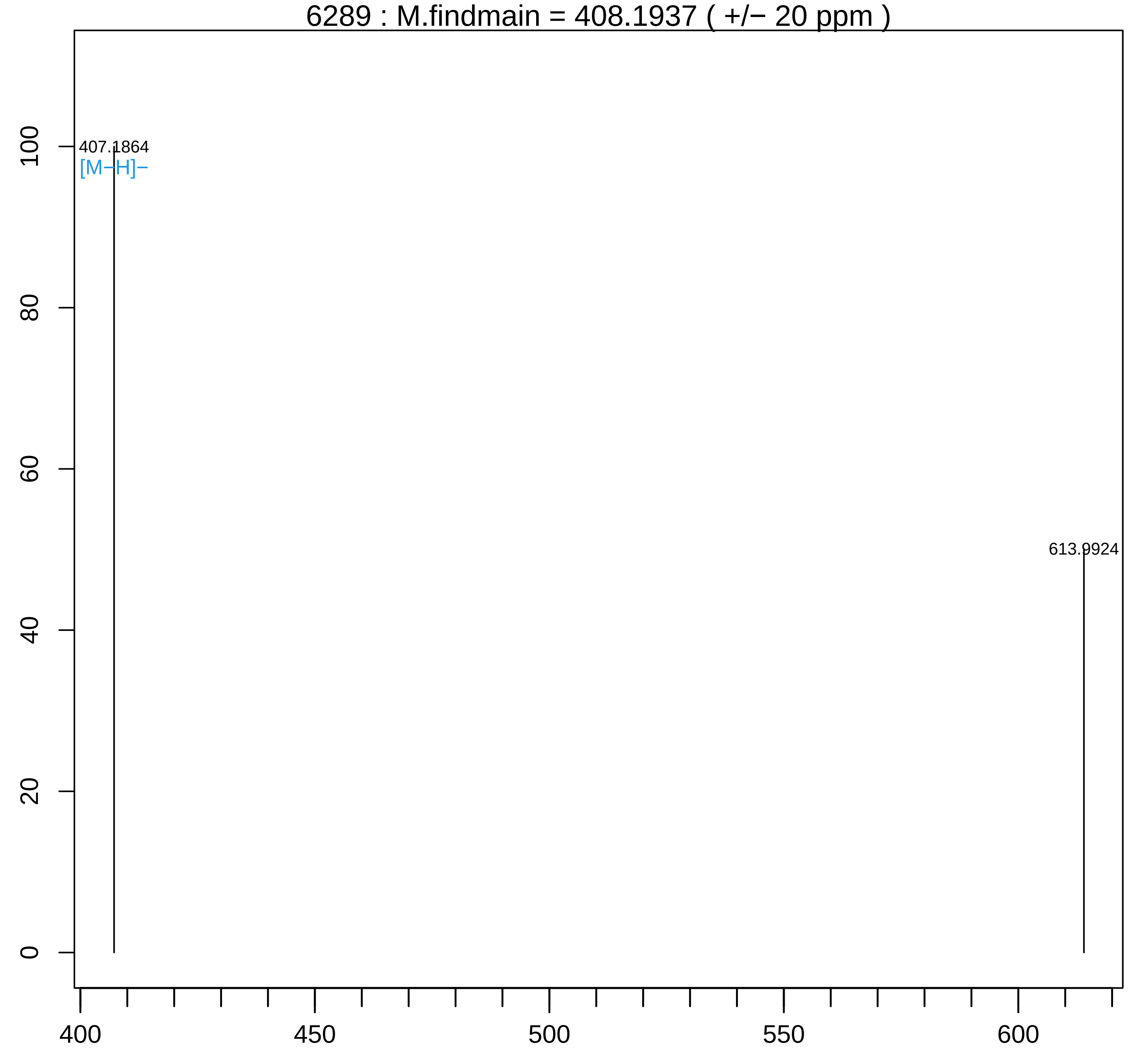
6289 : M.findmain = 408.1937 (+/− 20 ppm)
407.1864
[M−H]−
613.9924
0
20
40
60
80
100
400
450
500
550
600

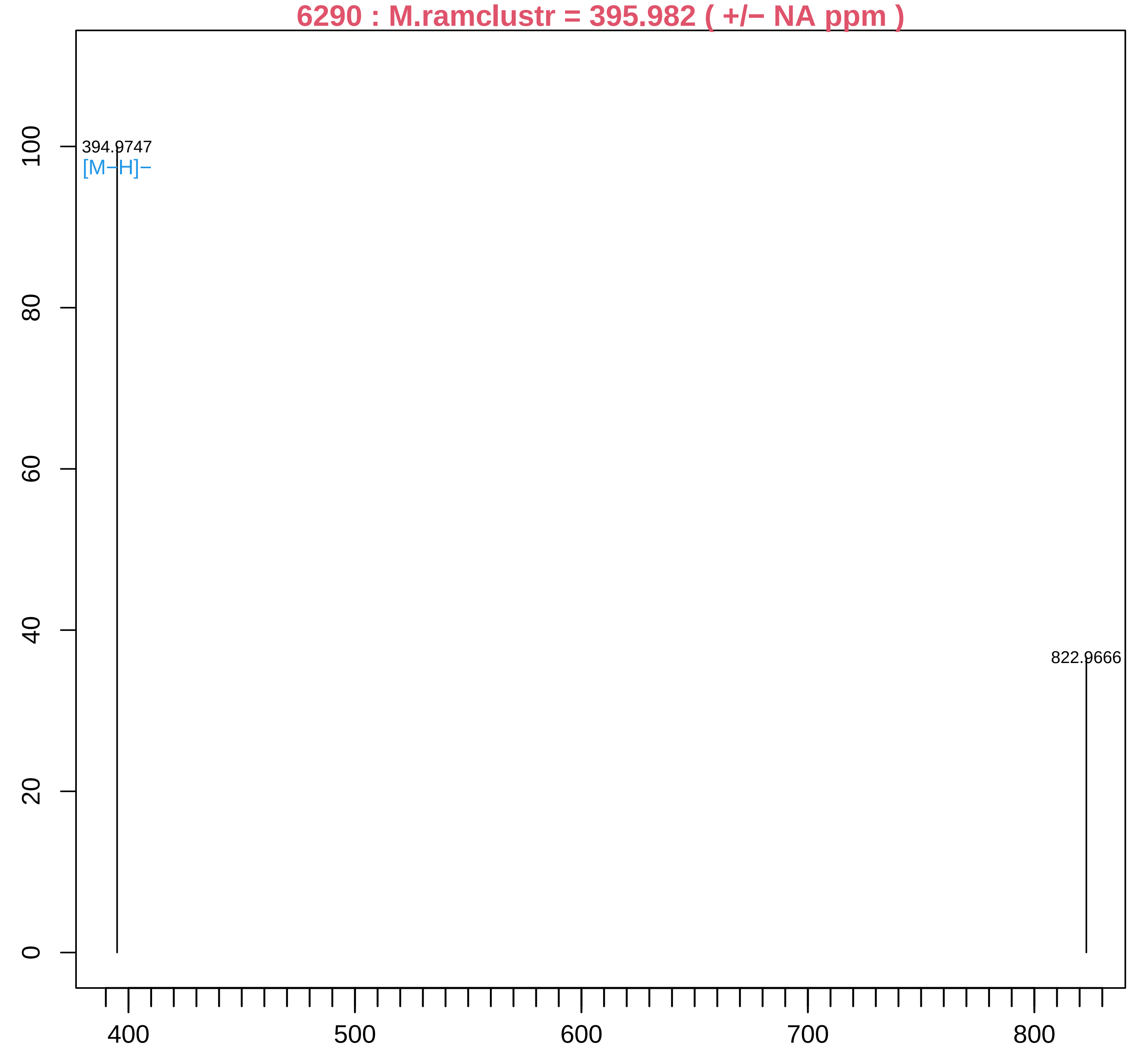
6290 : M.ramclustr = 395.982 (+/− NA ppm)
394.9747
[M−H]−
822.9666
0
20
40
60
80
100
400
500
600
700
800

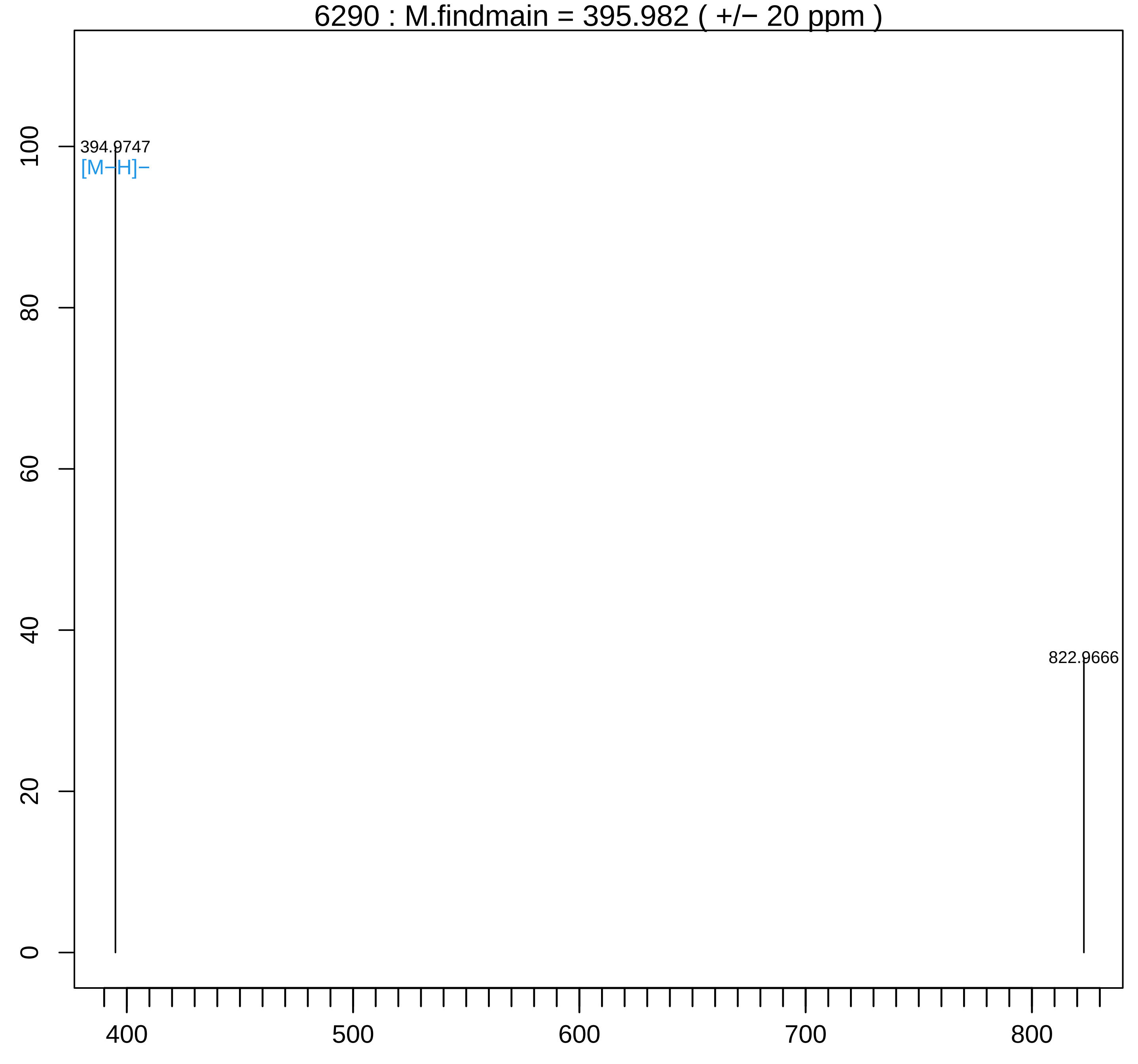
6290 : M.findmain = 395.982 (+/− 20 ppm)
394.9747
[M−H]−
822.9666
0
20
40
60
80
100
400
500
600
700
800

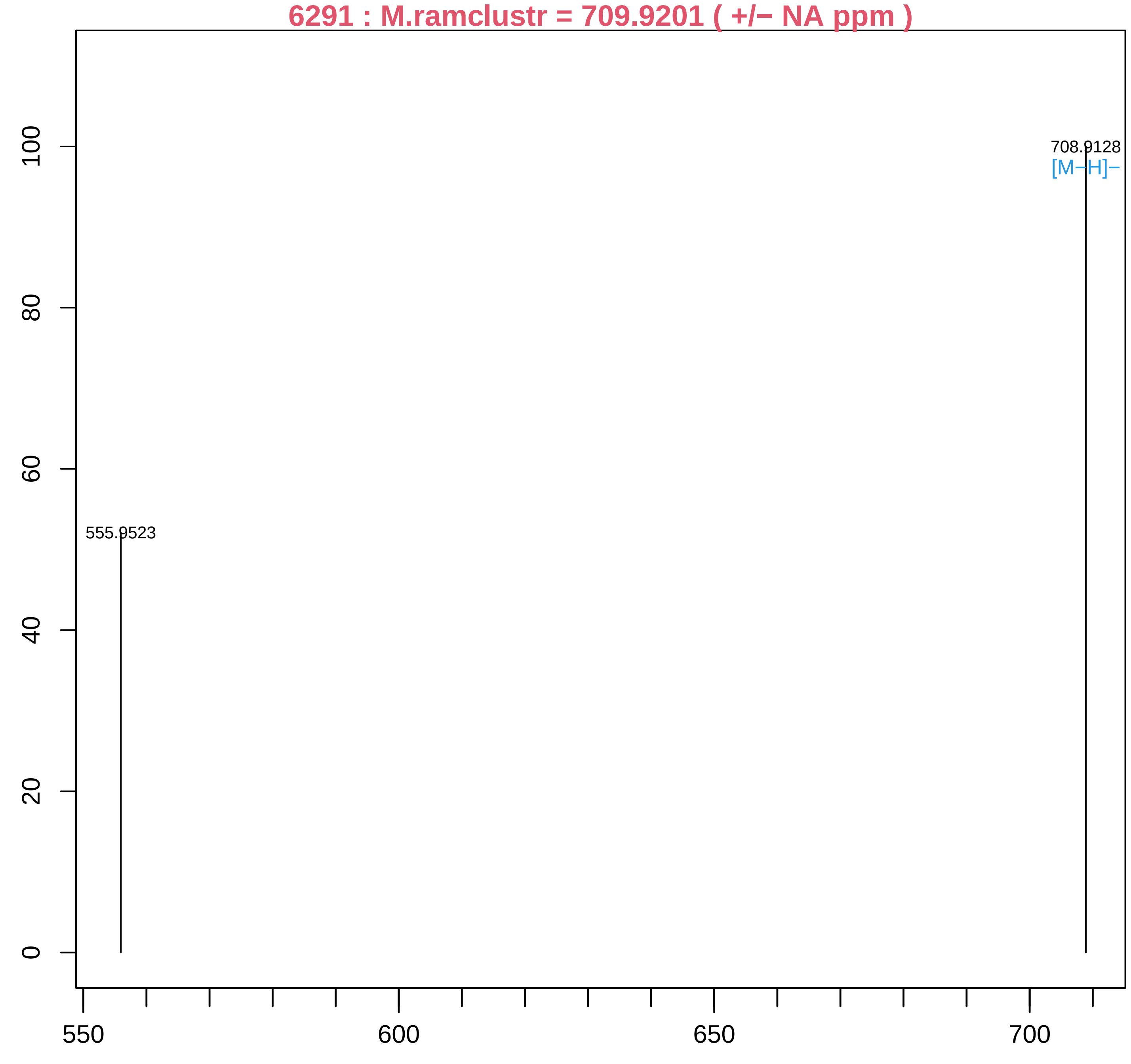
6291 : M.ramclustr = 709.9201 (+/− NA ppm)
708.9128
[M−H]−
555.9523
0
20
40
60
80
100
550
600
650
700

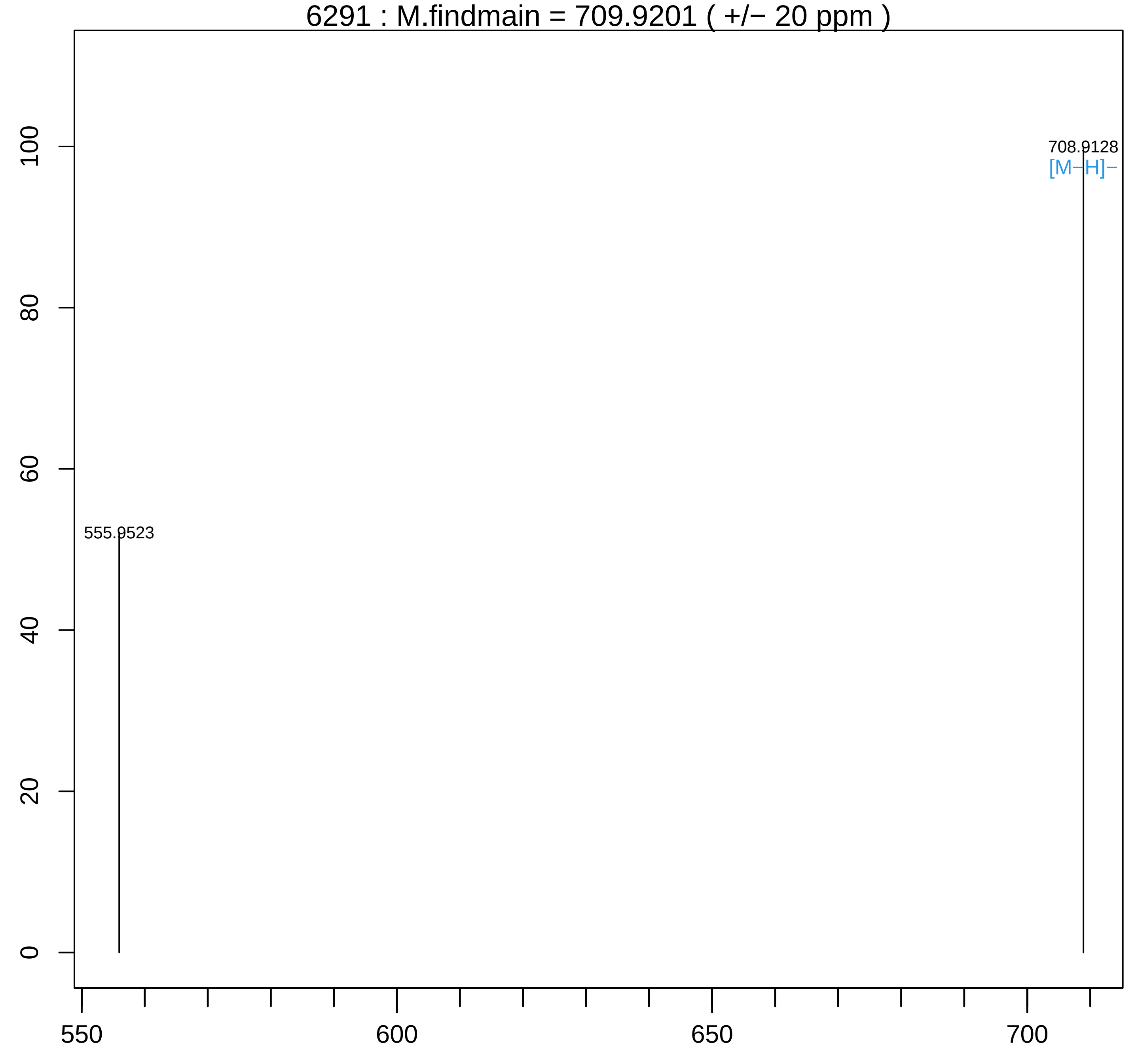
6291 : M.findmain = 709.9201 (+/− 20 ppm)
708.9128
[M−H]−
555.9523
0
20
40
60
80
100
550
600
650
700

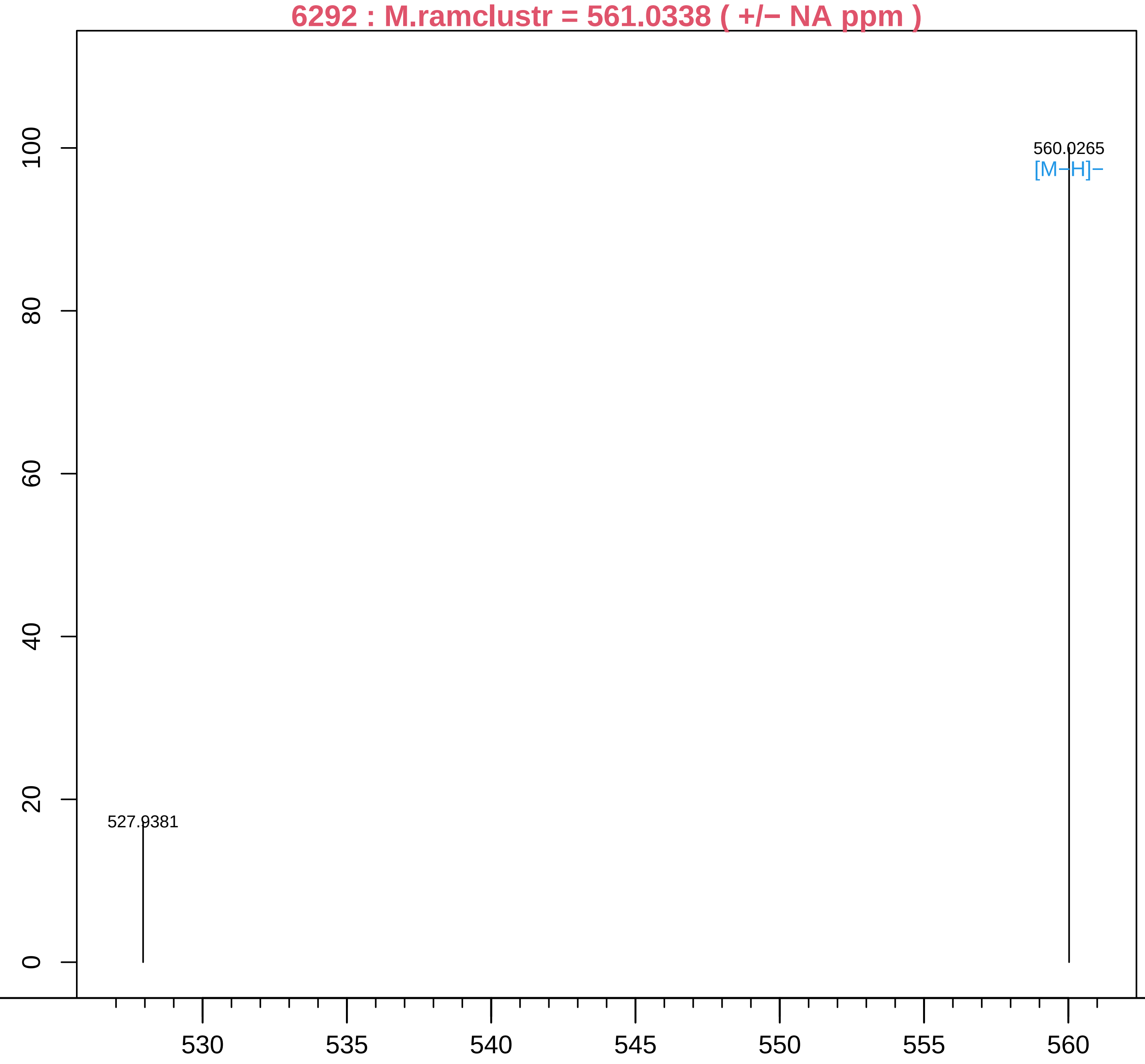
6292 : M.ramclustr = 561.0338 (+/− NA ppm)
560.0265
[M−H]−
527.9381
0
20
40
60
80
100
530
535
540
545
550
555
560

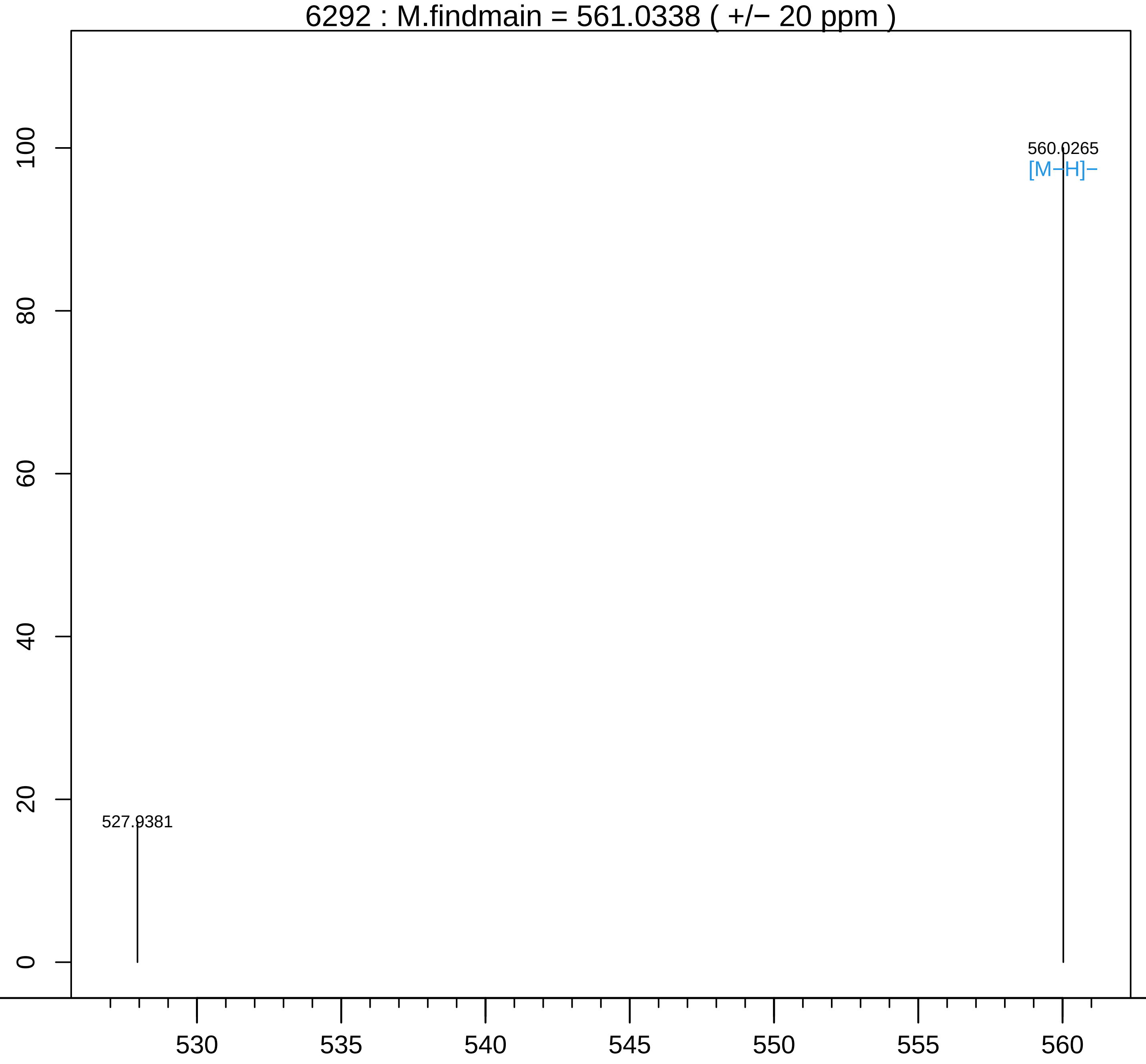
6292 : M.findmain = 561.0338 (+/− 20 ppm)
560.0265
[M−H]−
527.9381
0
20
40
60
80
100
530
535
540
545
550
555
560

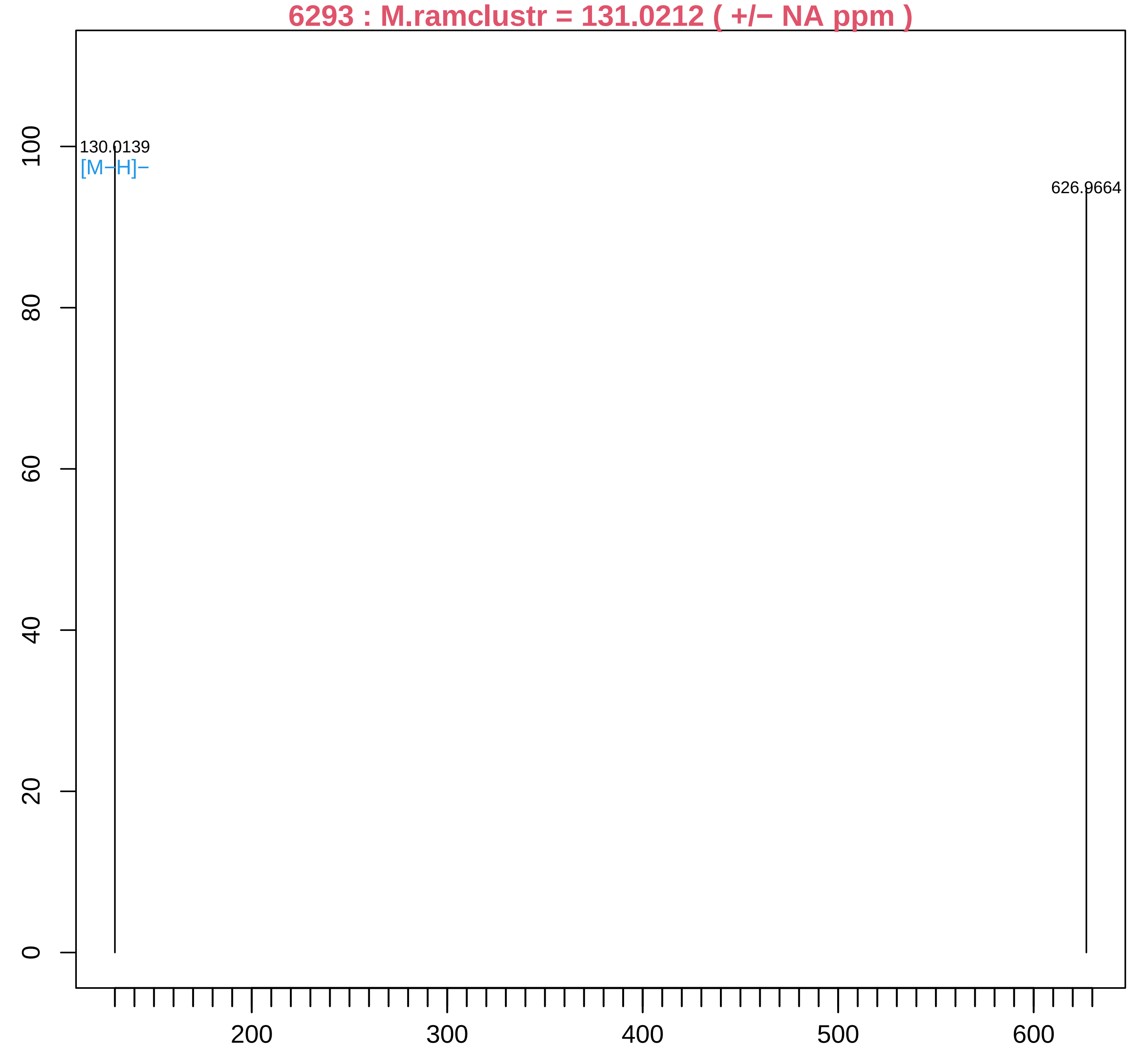
6293 : M.ramclustr = 131.0212 (+/− NA ppm)
130.0139
[M−H]−
626.9664
0
20
40
60
80
100
200
300
400
500
600
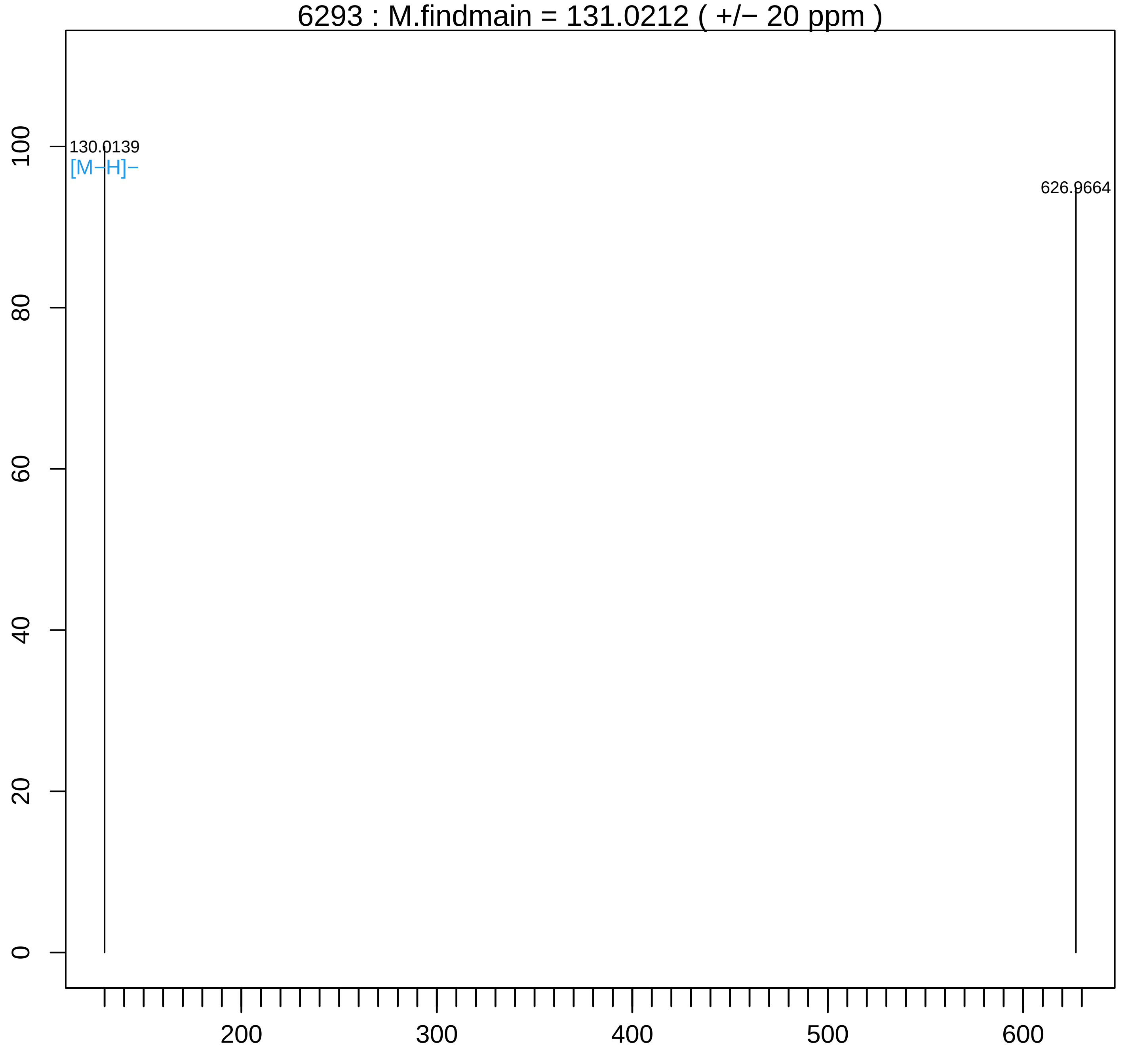
6293 : M.findmain = 131.0212 (+/− 20 ppm)
130.0139
[M−H]−
626.9664
0
20
40
60
80
100
200
300
400
500
600

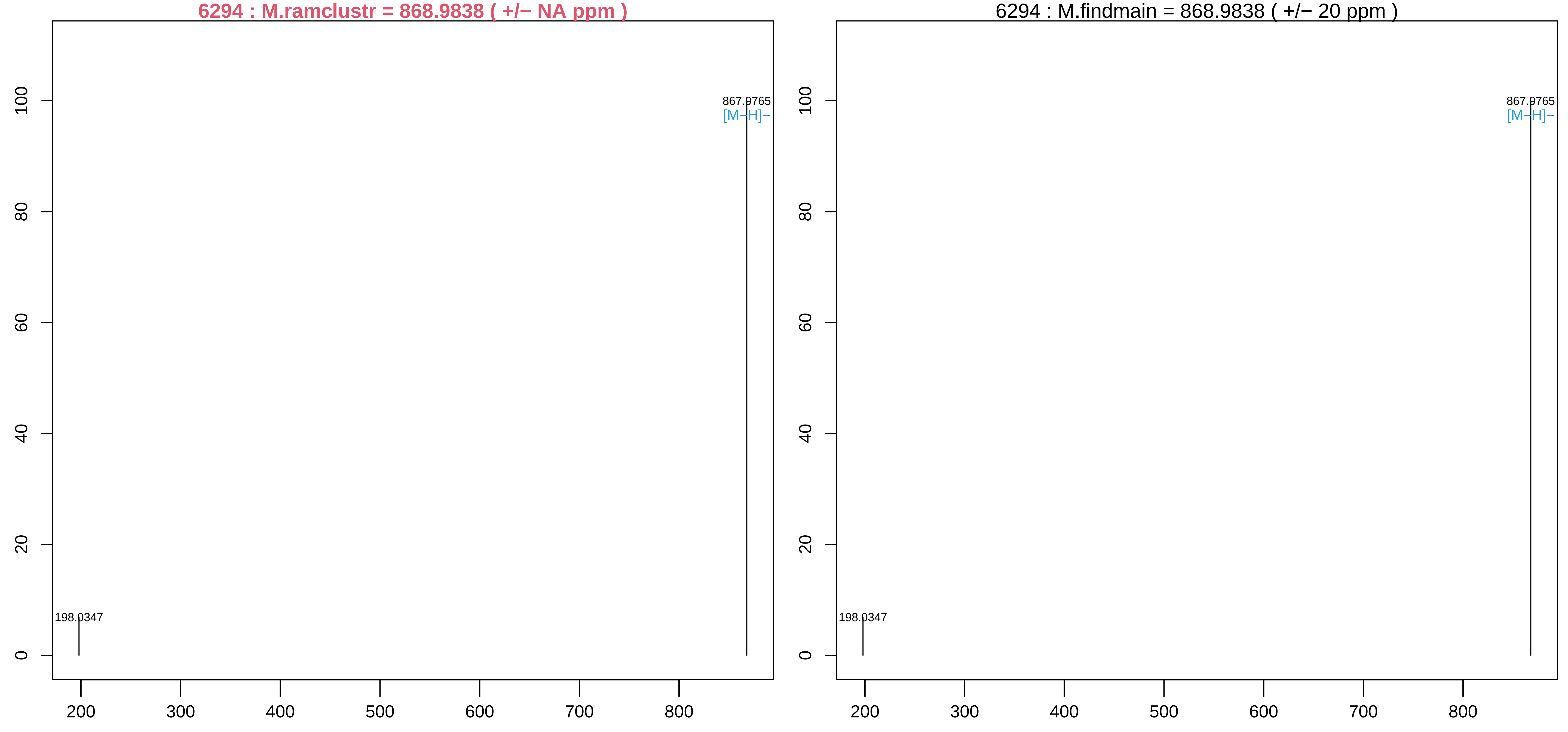
6294 : M.ramclustr = 868.9838 (+/− NA ppm)
6294 : M.findmain = 868.9838 (+/− 20 ppm)
867.9765
[M−H]−
198.0347
867.9765
[M−H]−
198.0347
0
20
40
60
80
100
200
300
400
500
600
700
800

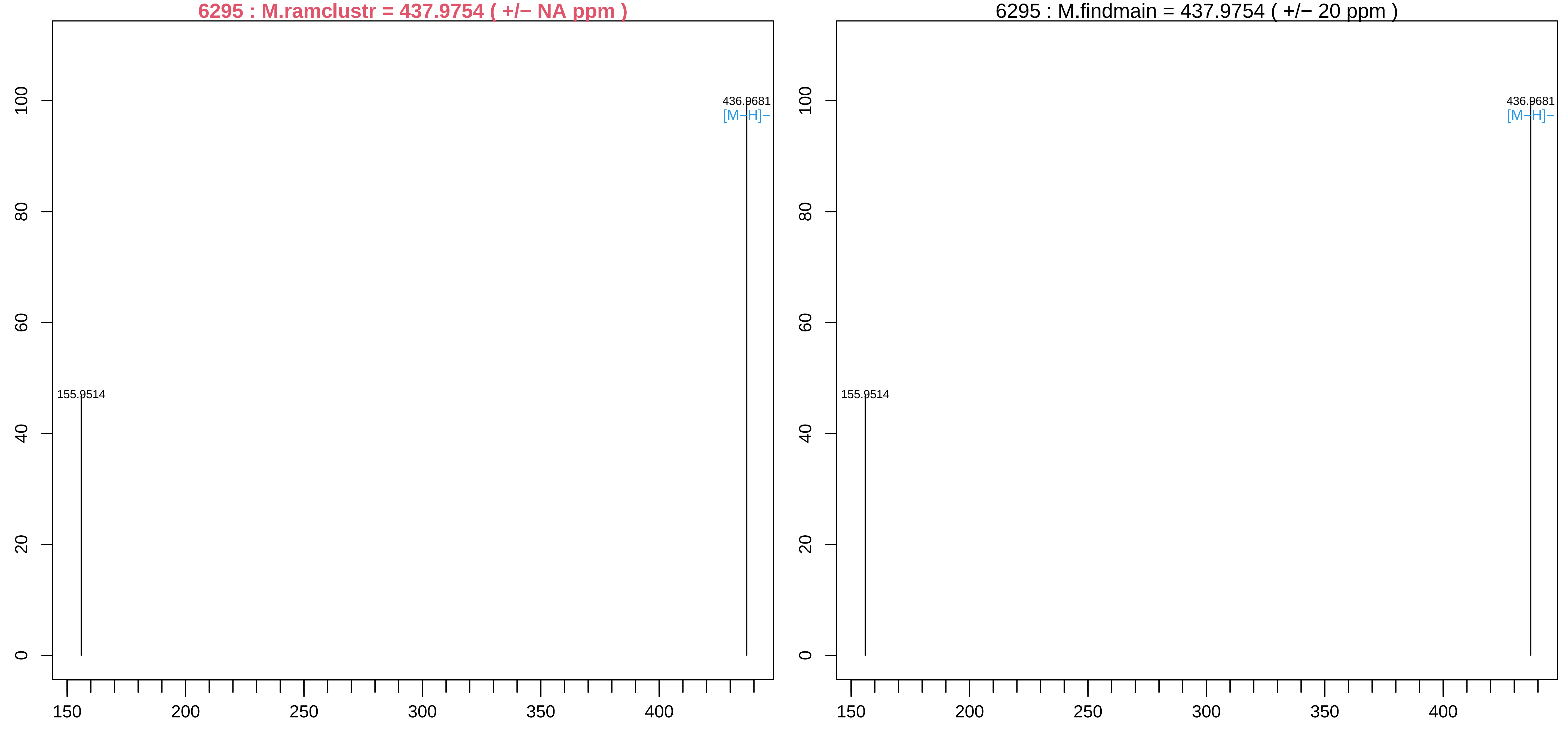
6295 : M.ramclustr = 437.9754 (+/− NA ppm)
6295 : M.findmain = 437.9754 (+/− 20 ppm)
436.9681
[M−H]−
155.9514
436.9681
[M−H]−
155.9514
0
20
40
60
80
100
150
200
250
300
350
400

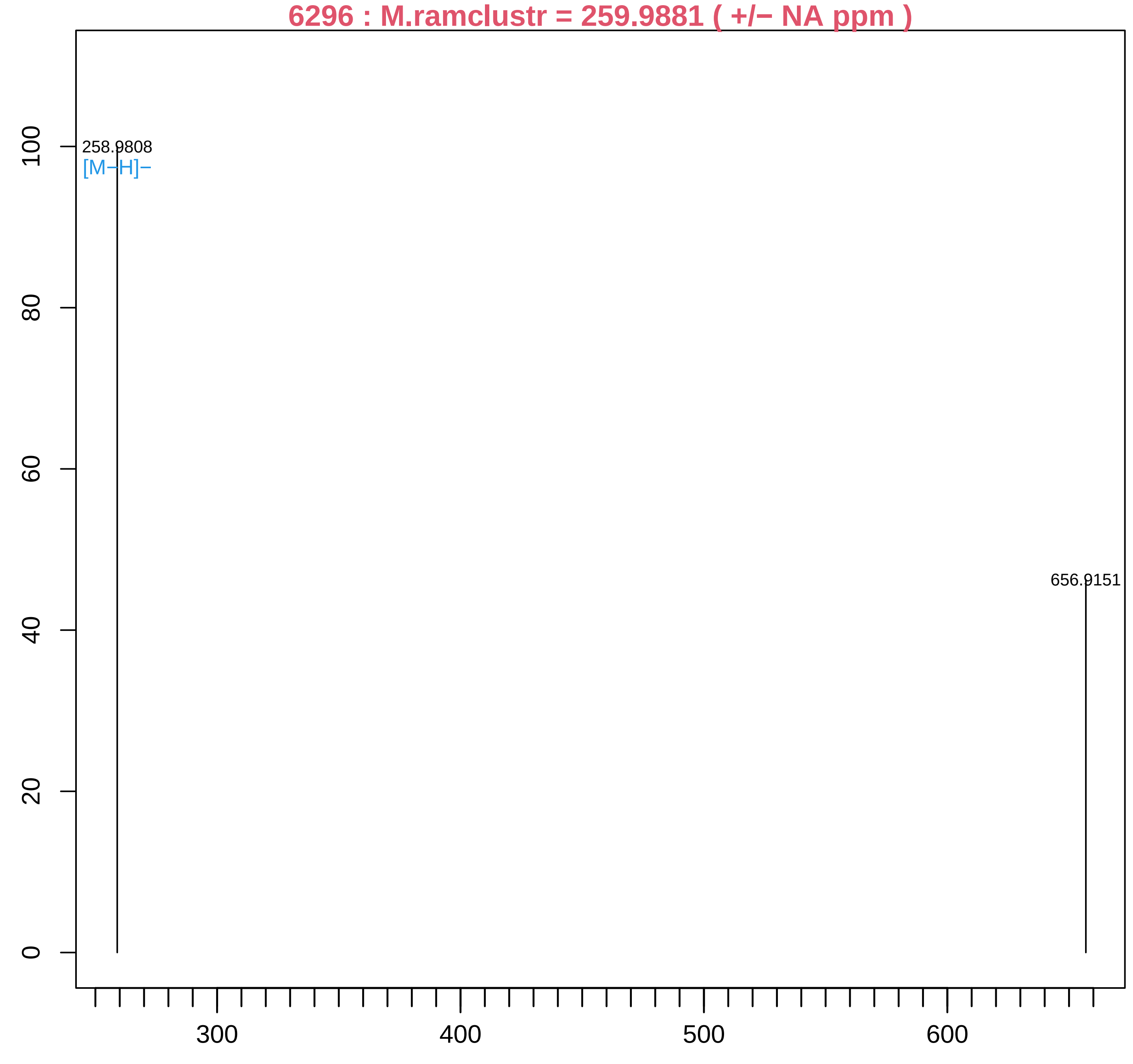
6296 : M.ramclustr = 259.9881 (+/− NA ppm)
258.9808
[M−H]−
656.9151
0
20
40
60
80
100
300
400
500
600

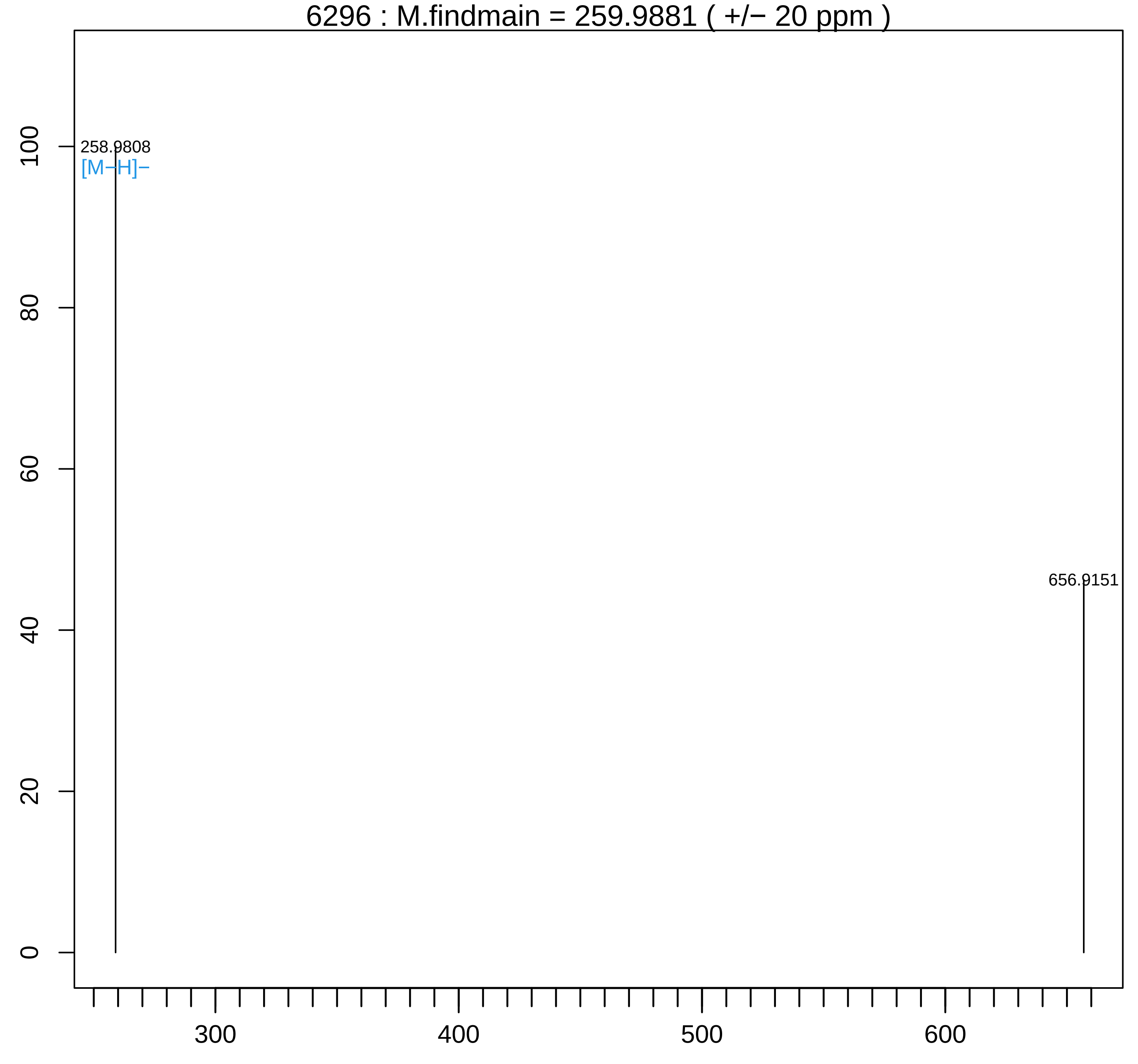
6296 : M.findmain = 259.9881 (+/− 20 ppm)
258.9808
[M−H]−
656.9151
0
20
40
60
80
100
300
400
500
600

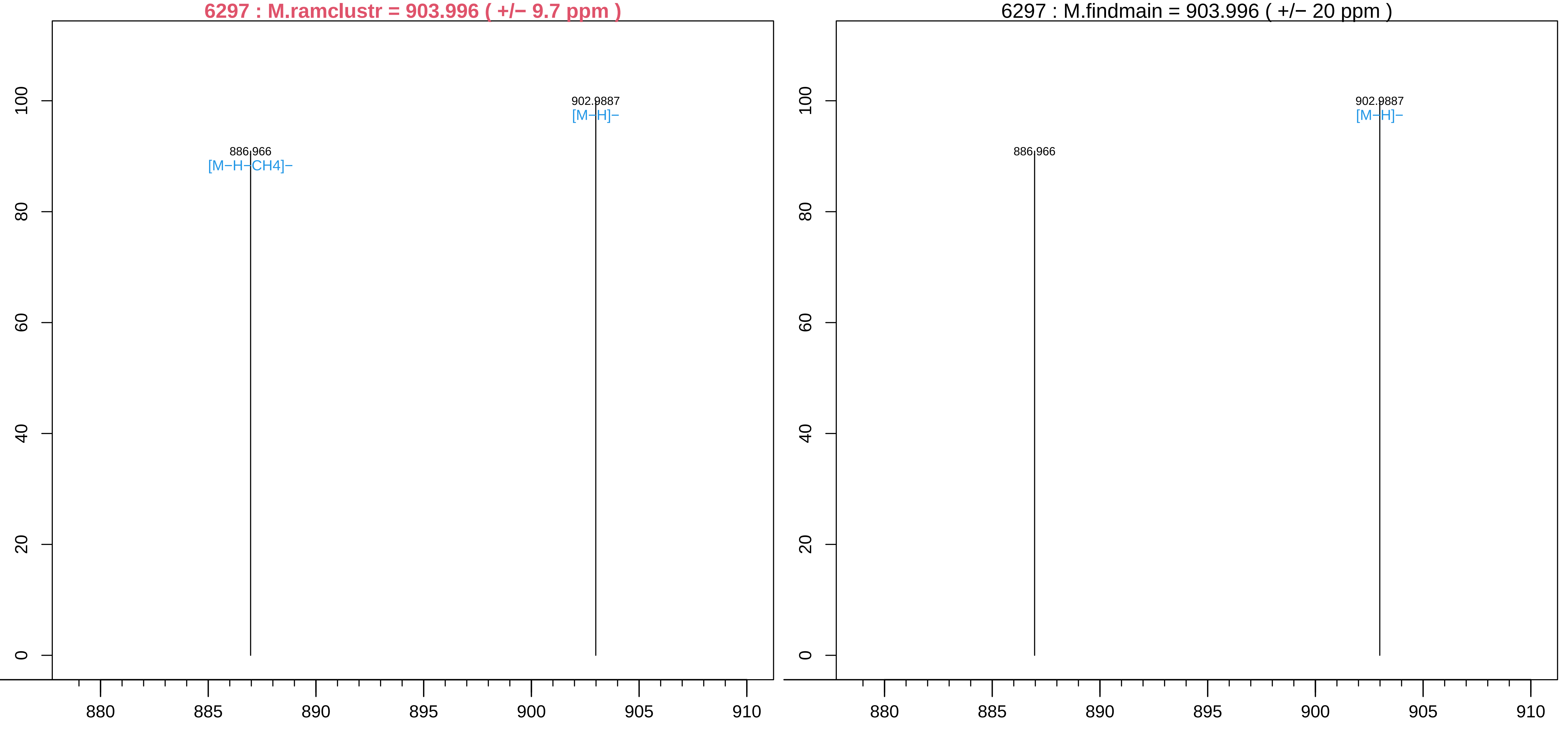

6297 : M.ramclustr = 903.996 (+/− 9.7 ppm)
902.9887
[M−H]−
886.966
[M−H−CH4]−
0
20
40
60
80
100
880
885
890
895
900
905
910
6297 : M.findmain = 903.996 (+/− 20 ppm)
902.9887
[M−H]−
886.966
0
20
40
60
80
100
880
885
890
895
900
905
910

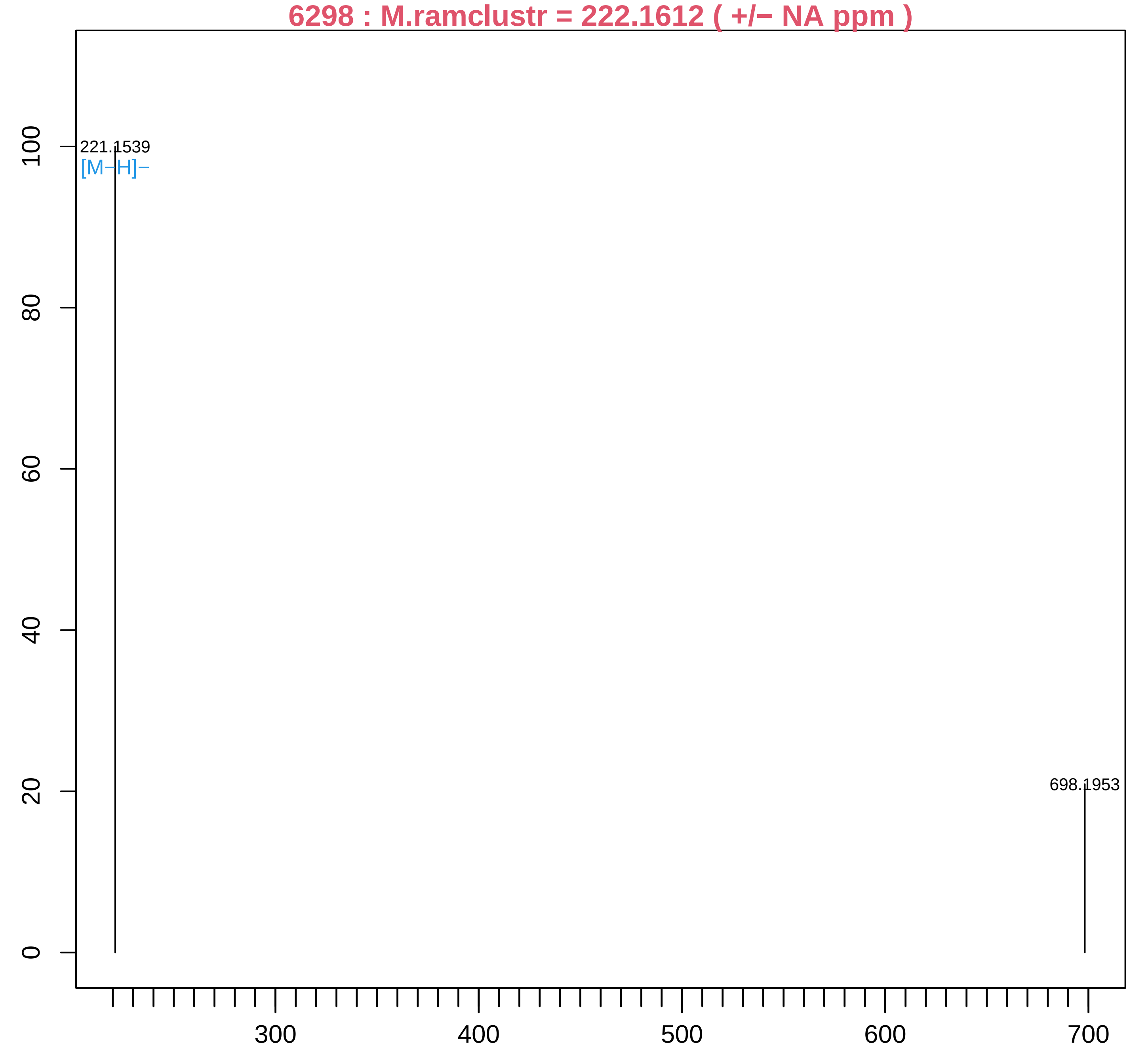
6298 : M.ramclustr = 222.1612 (+/− NA ppm)
221.1539
[M−H]−
698.1953
0
20
40
60
80
100
300
400
500
600
700

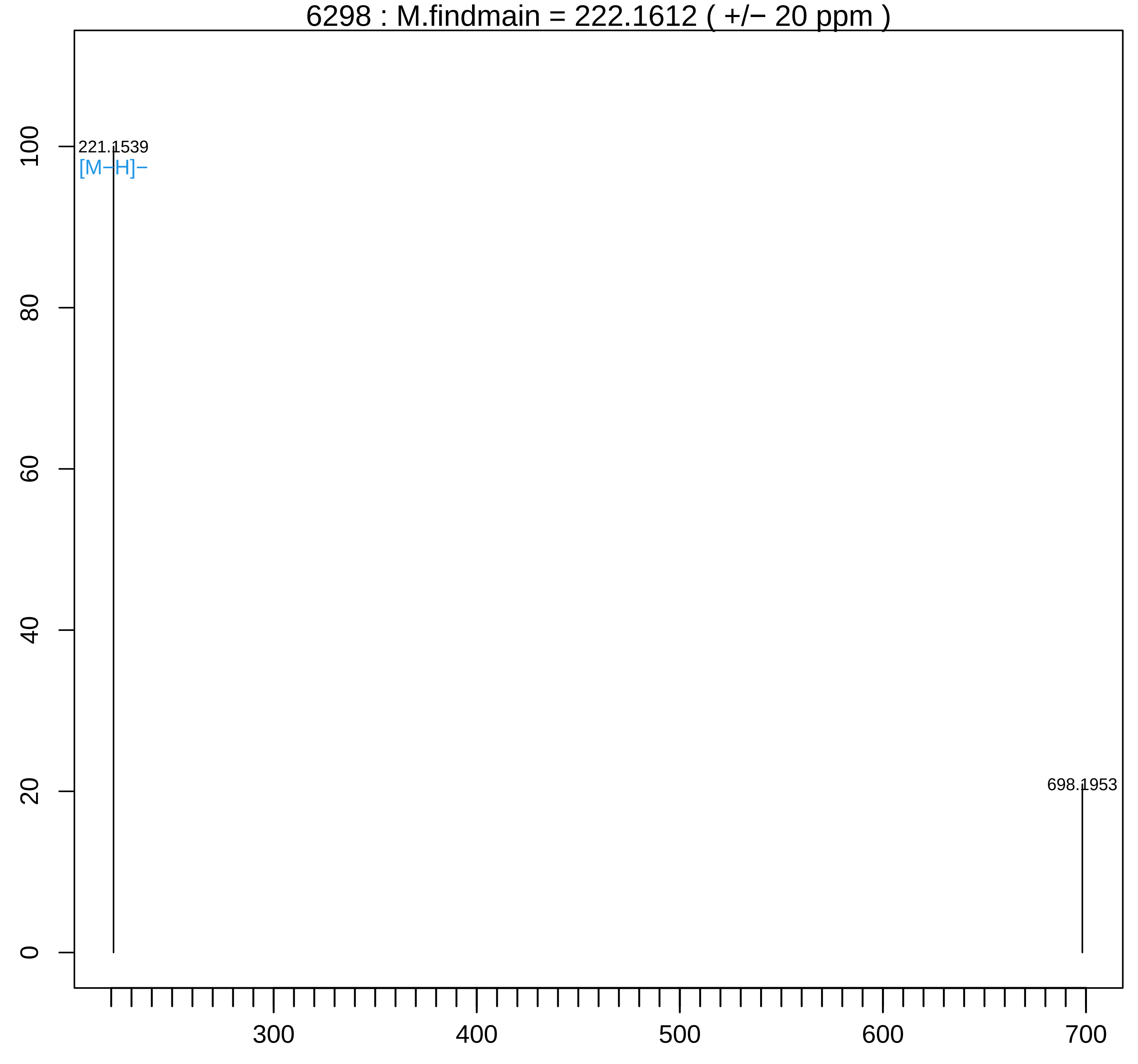
6298 : M.findmain = 222.1612 (+/− 20 ppm)
221.1539
[M−H]−
698.1953
0
20
40
60
80
100
300
400
500
600
700

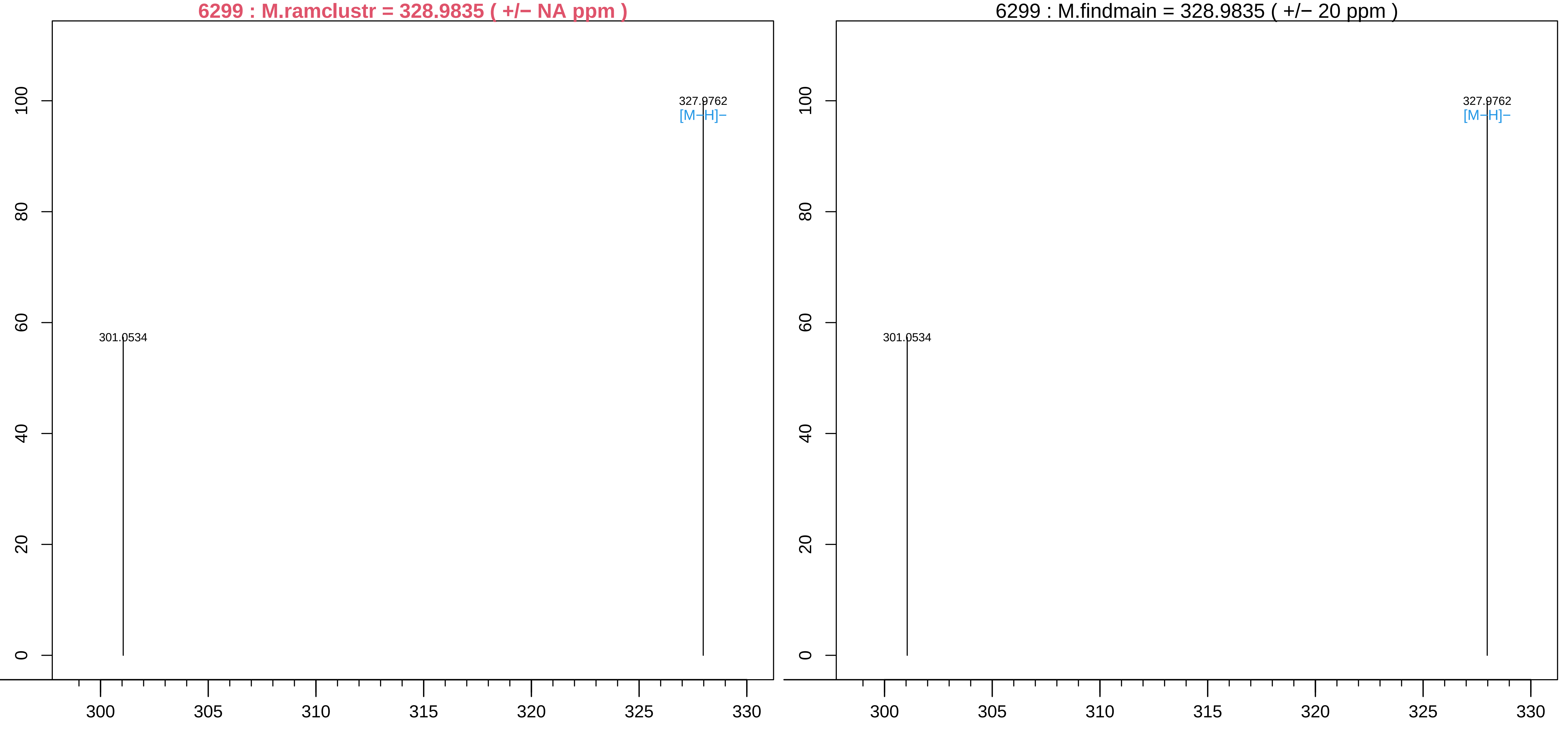
6299 : M.ramclustr = 328.9835 (+/− NA ppm)
6299 : M.findmain = 328.9835 (+/− 20 ppm)
327.9762
[M−H]−
301.0534
327.9762
[M−H]−
301.0534
0
20
40
60
80
100
300
305
310
315
320
325
330

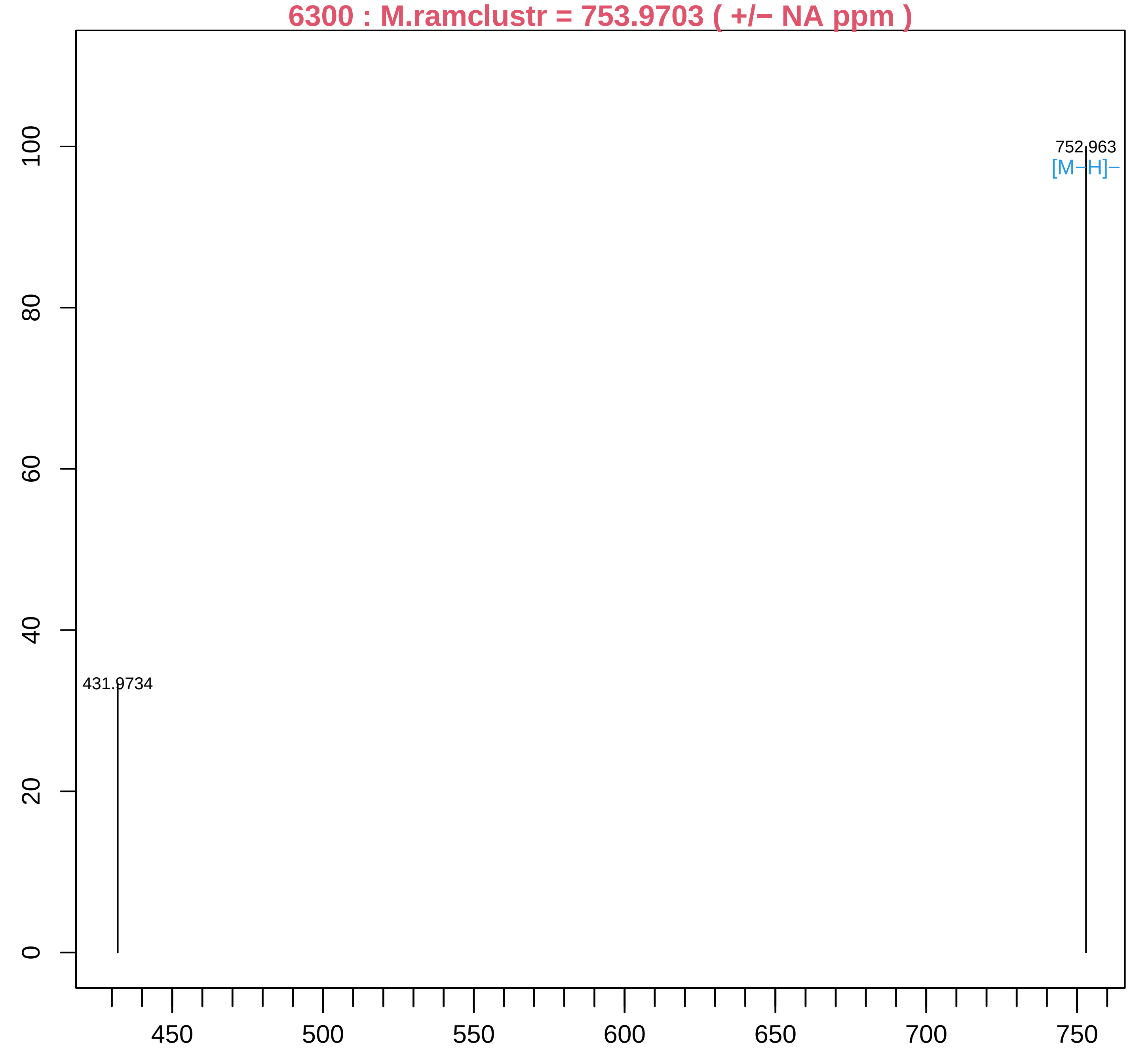
6300 : M.ramclustr = 753.9703 (+/− NA ppm)
752.963
[M−H]−
431.9734
0
20
40
60
80
100
450
500
550
600
650
700
750

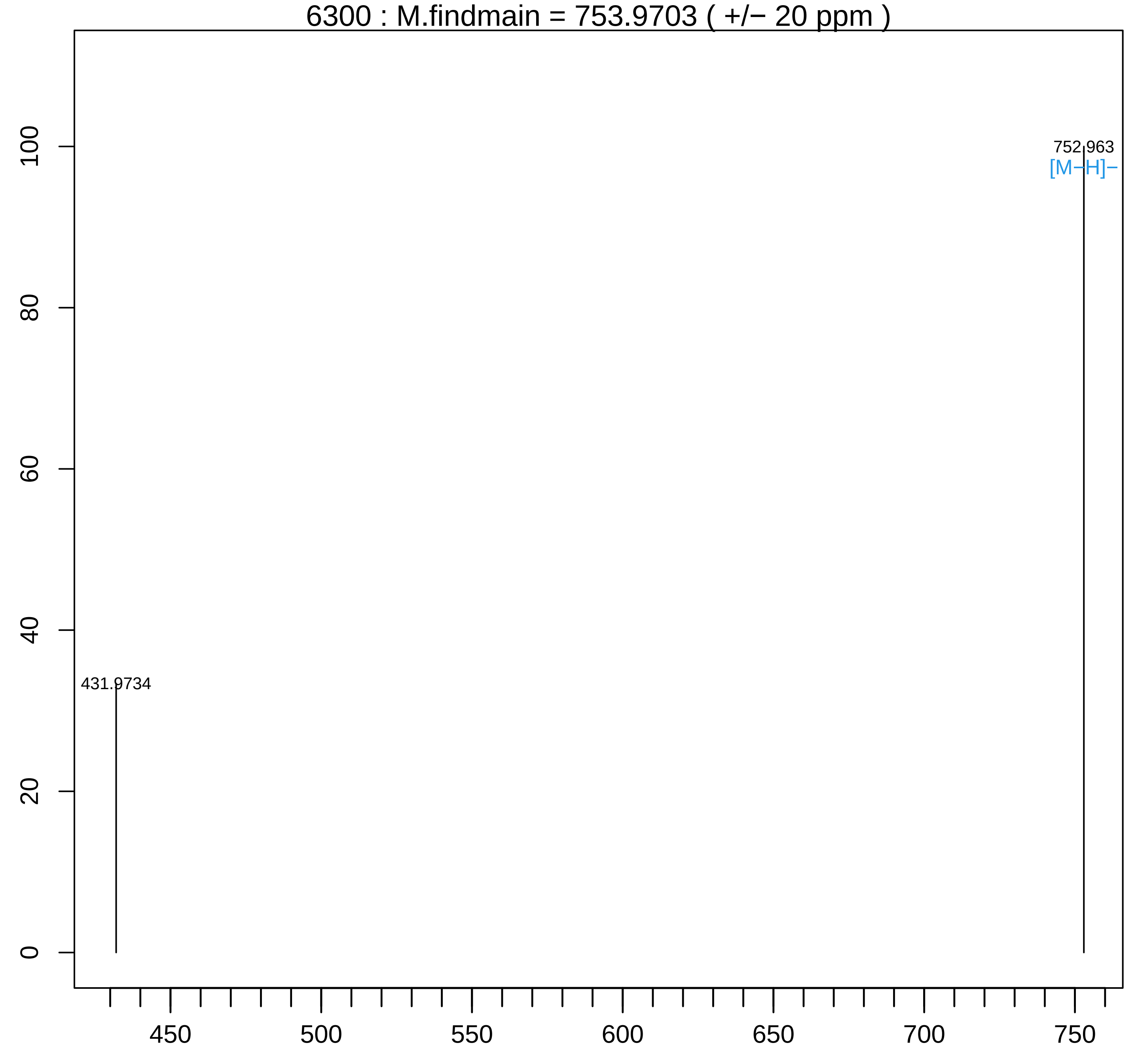
6300 : M.findmain = 753.9703 (+/− 20 ppm)
752.963
[M−H]−
431.9734
0
20
40
60
80
100
450
500
550
600
650
700
750

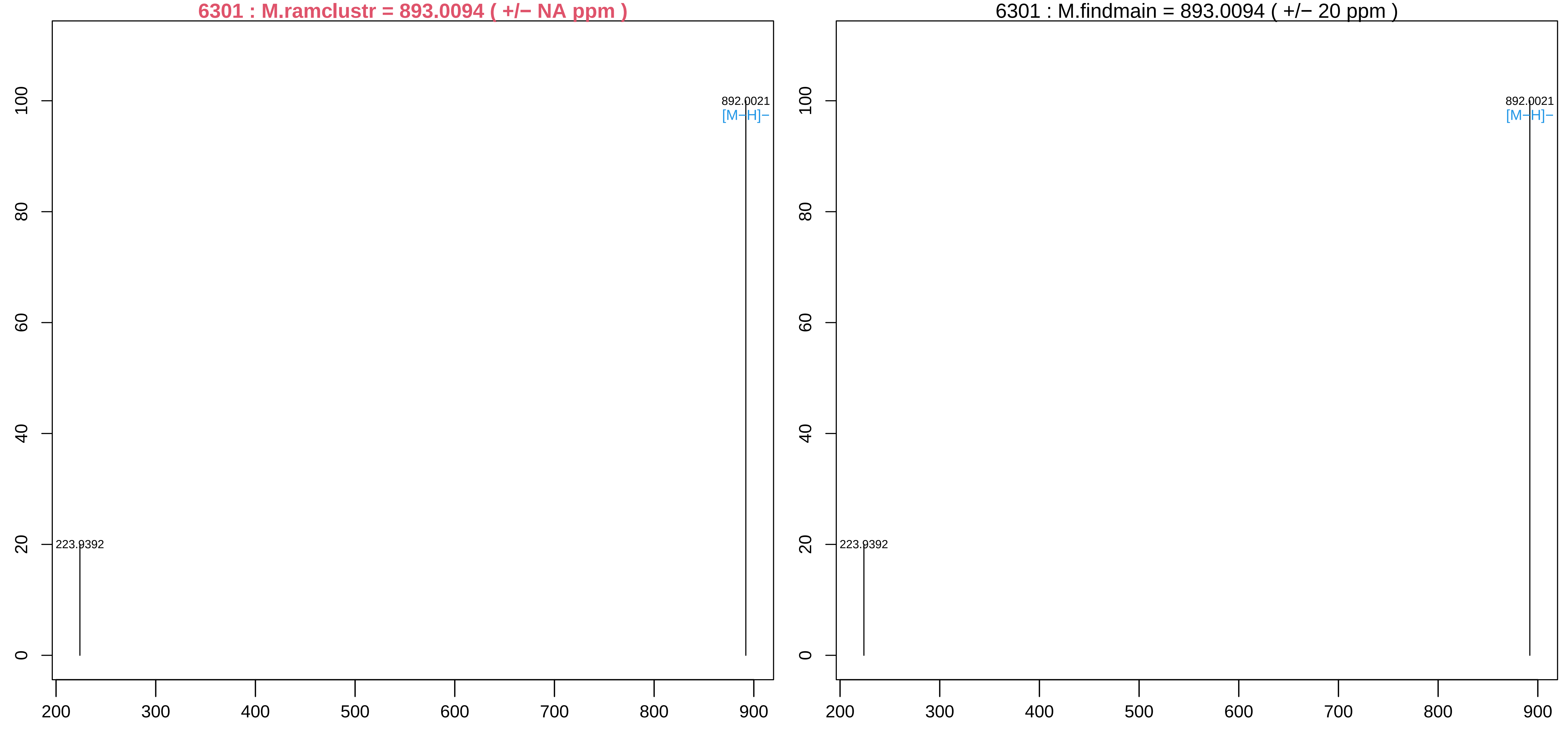
6301 : M.ramclustr = 893.0094 (+/− NA ppm)
892.0021
[M−H]−
223.9392
6301 : M.findmain = 893.0094 (+/− 20 ppm)
892.0021
[M−H]−
223.9392
0
20
40
60
80
100
200
300
400
500
600
700
800
900

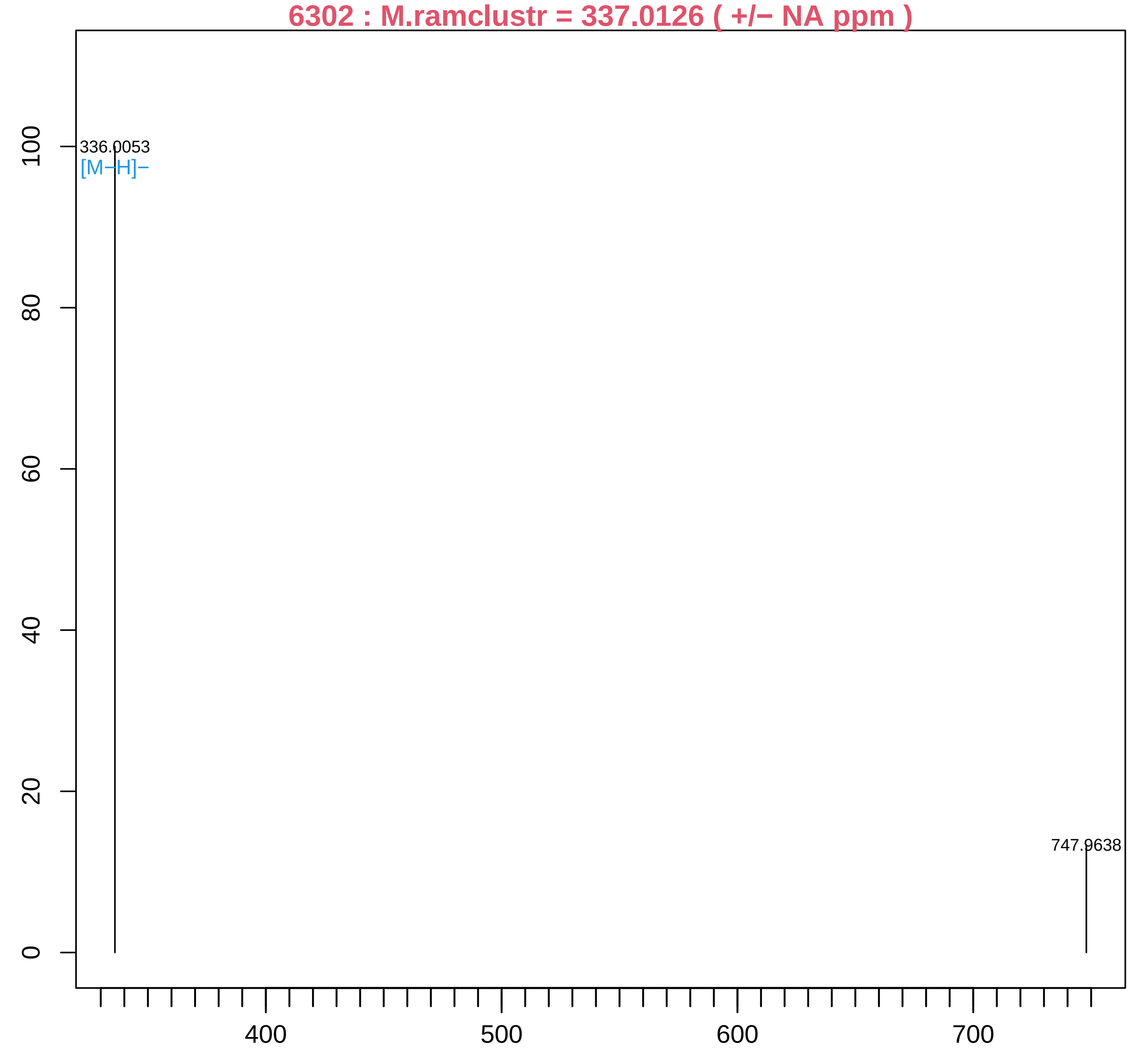
6302 : M.ramclustr = 337.0126 (+/− NA ppm)
336.0053
[M−H]−
747.9638
0
20
40
60
80
100
400
500
600
700

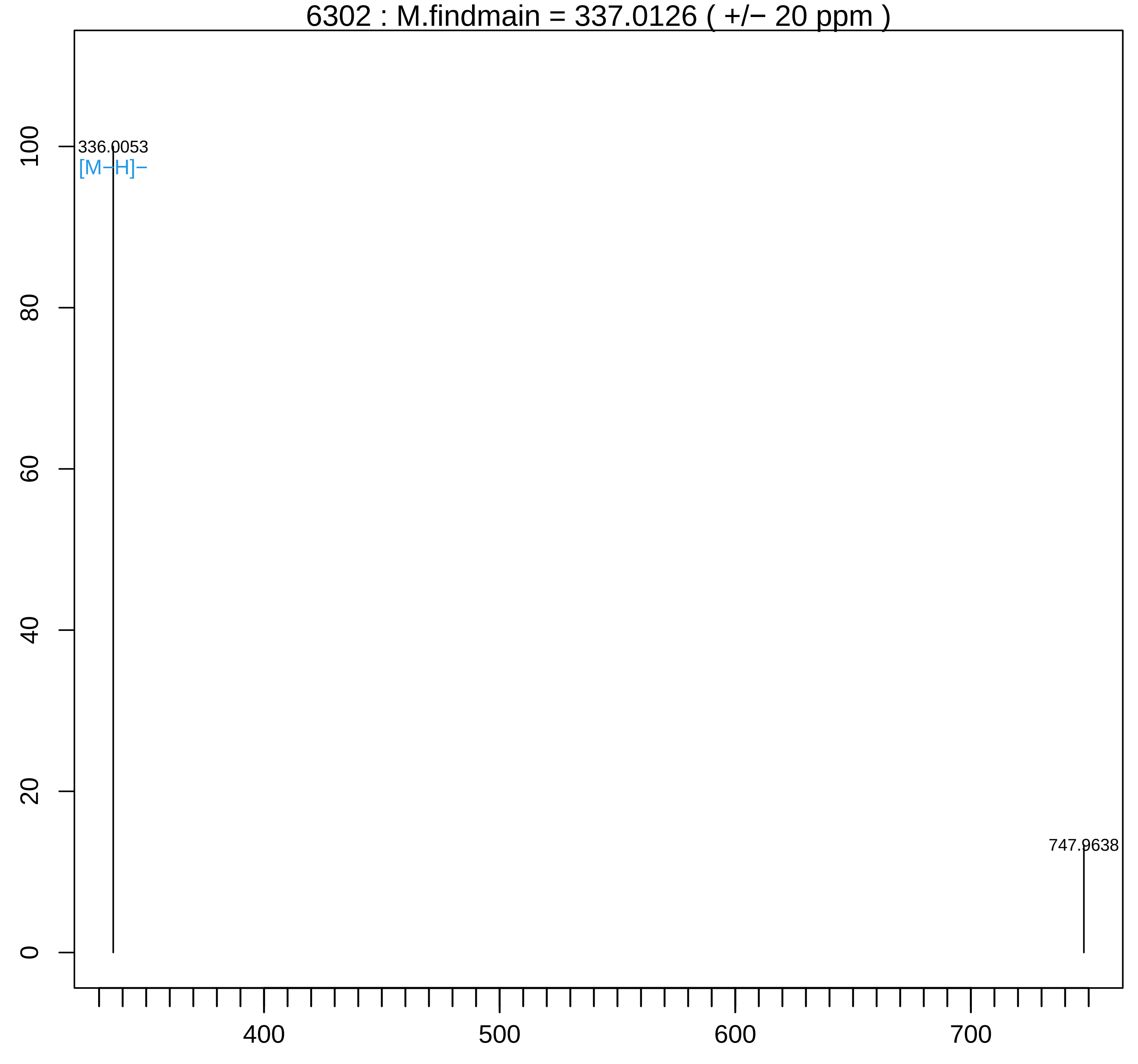
6302 : M.findmain = 337.0126 (+/− 20 ppm)
336.0053
[M−H]−
747.9638
0
20
40
60
80
100
400
500
600
700

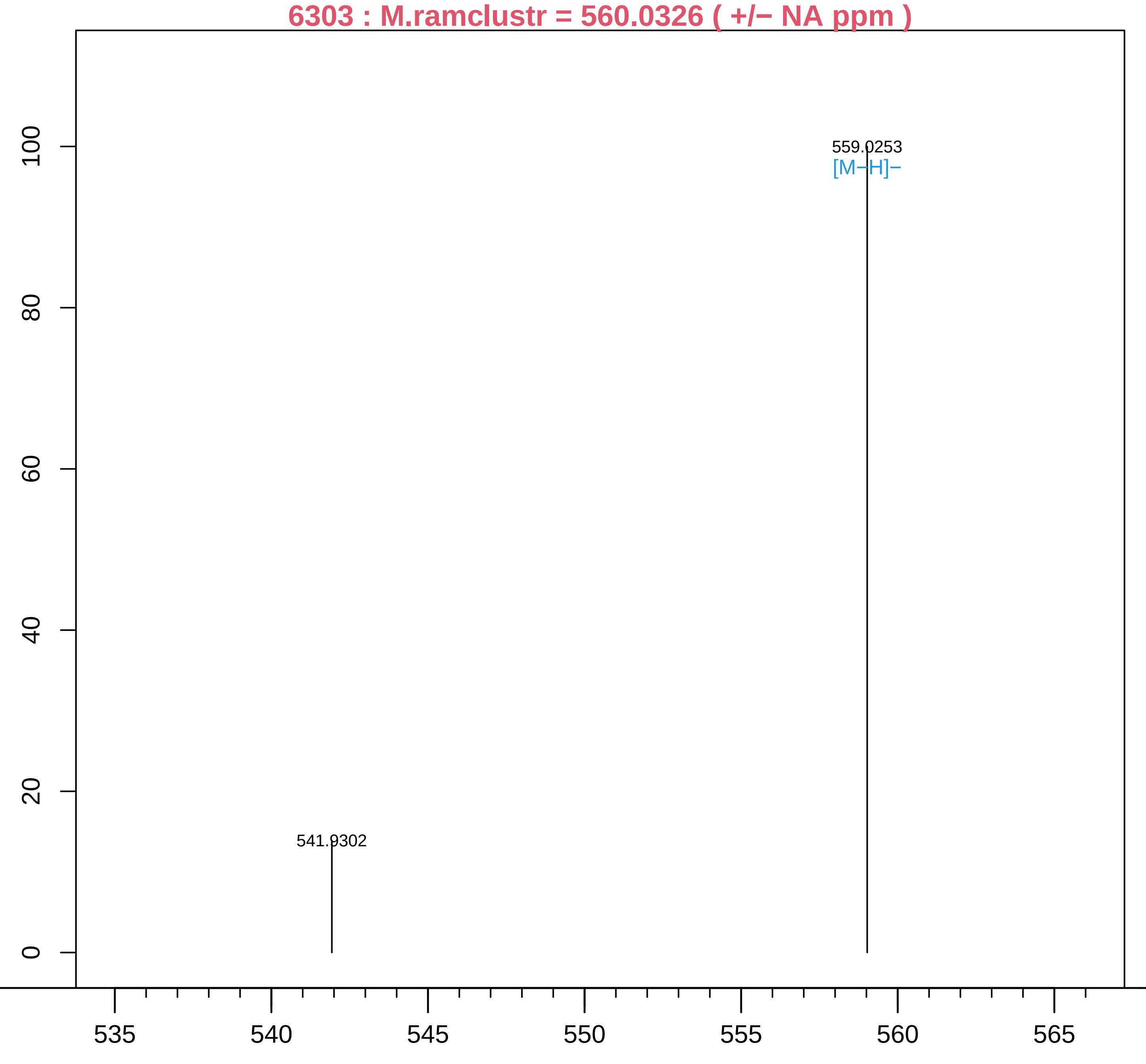
6303 : M.ramclustr = 560.0326 (+/− NA ppm)
559.0253
[M−H]−
541.9302
0
20
40
60
80
100
535
540
545
550
555
560
565

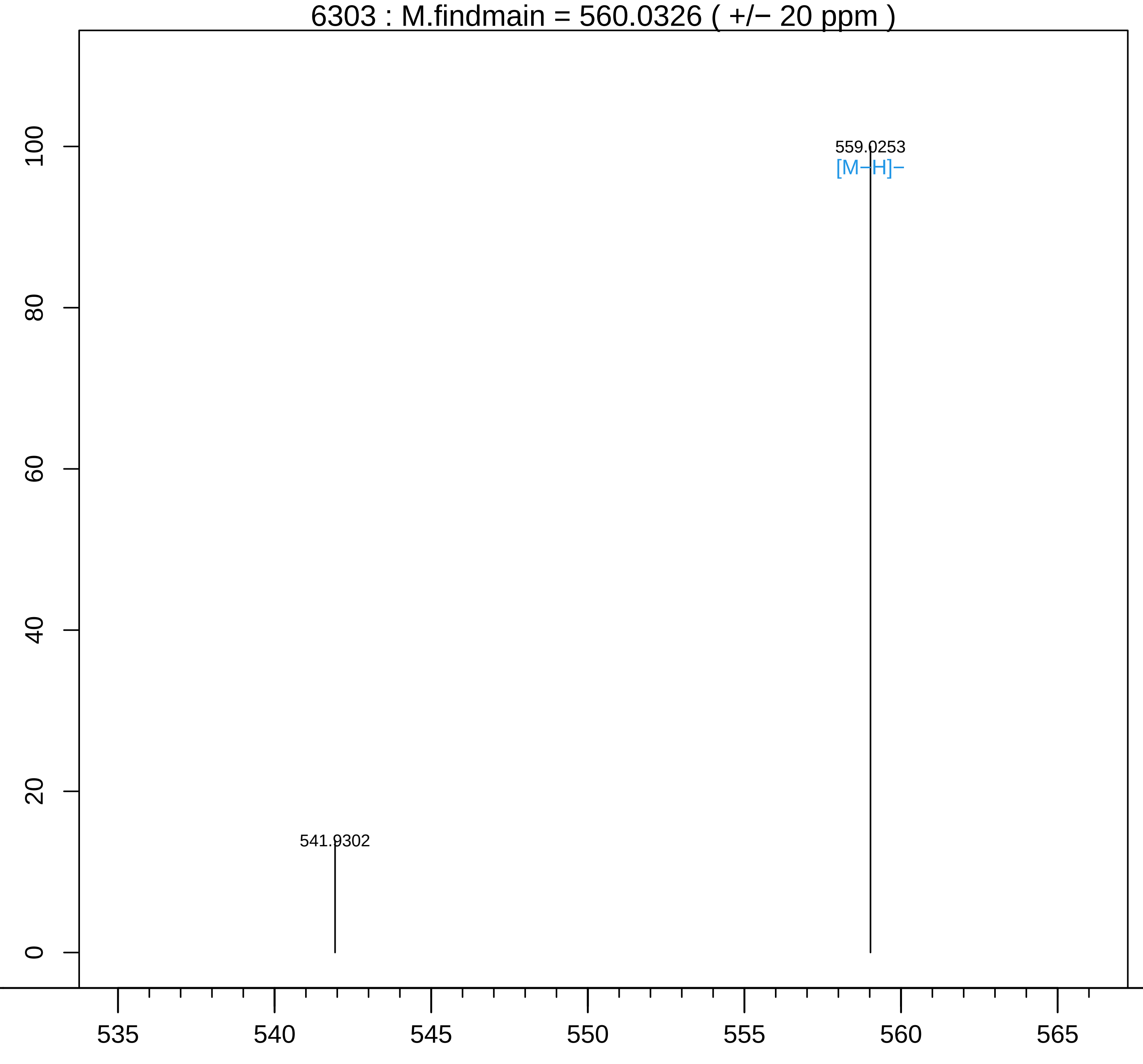
6303 : M.findmain = 560.0326 (+/− 20 ppm)
559.0253
[M−H]−
541.9302
0
20
40
60
80
100
535
540
545
550
555
560
565

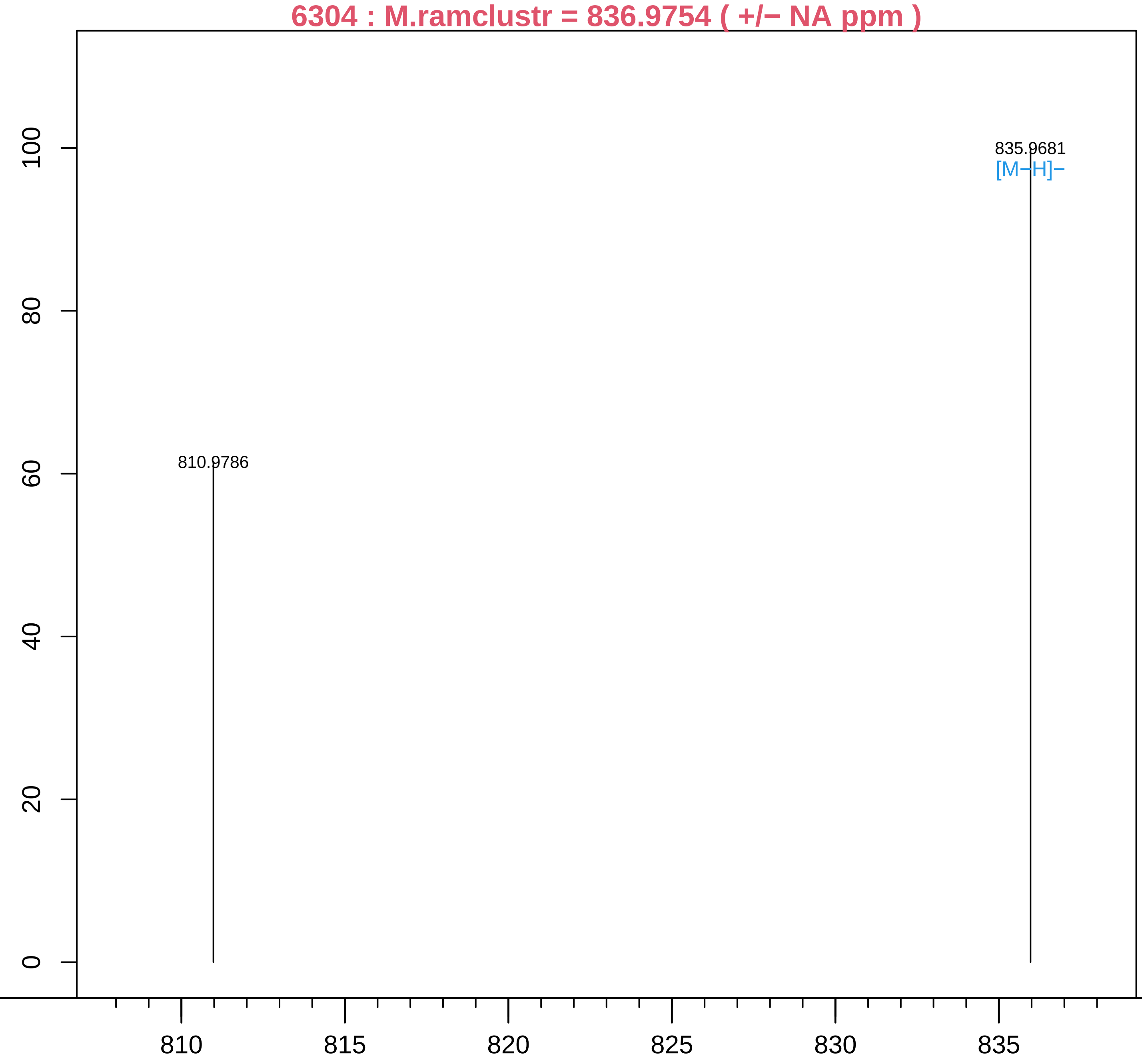
6304 : M.ramclustr = 836.9754 (+/− NA ppm)
835.9681
[M−H]−
810.9786
0
20
40
60
80
100
810
815
820
825
830
835

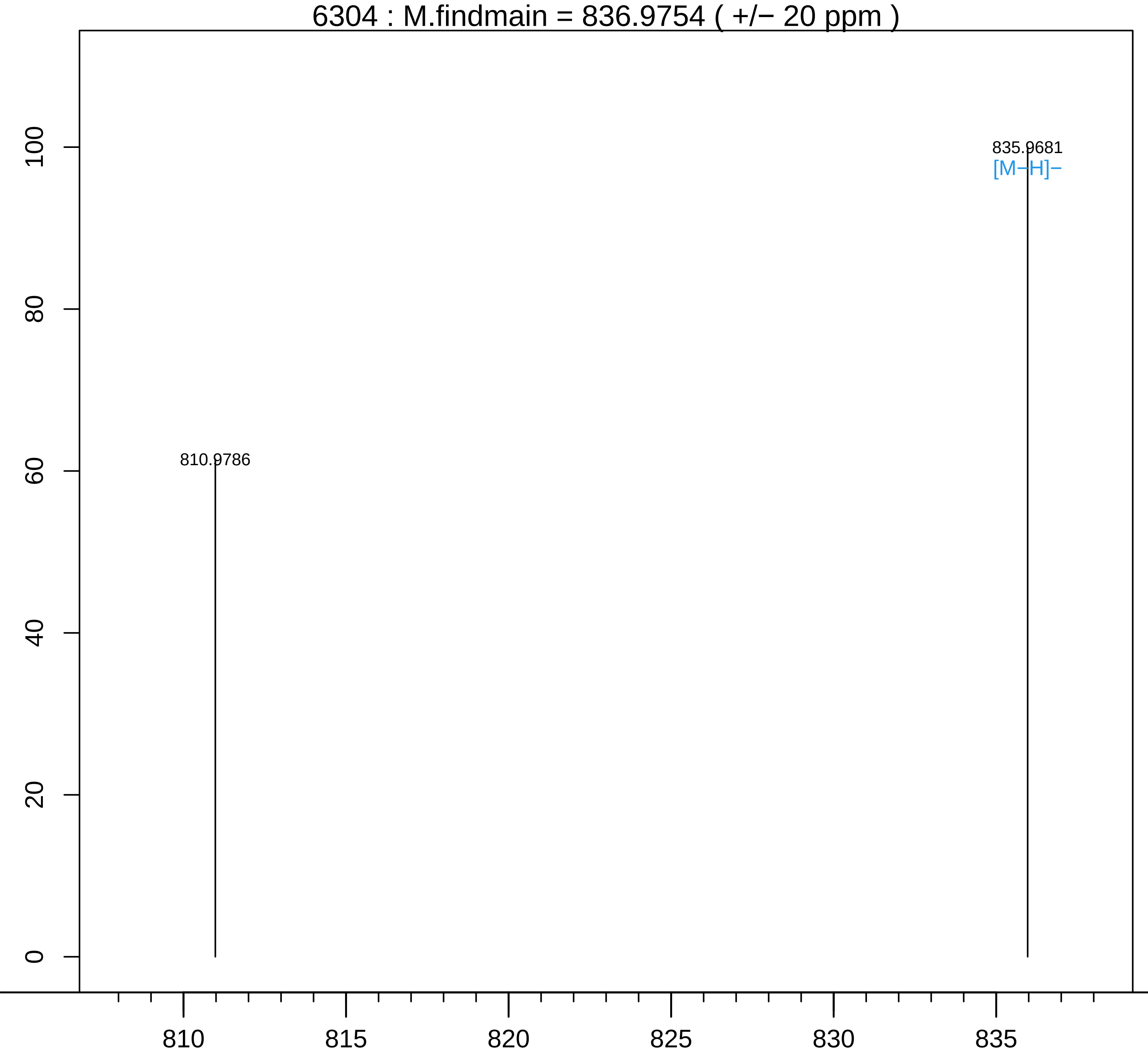
6304 : M.findmain = 836.9754 (+/− 20 ppm)
835.9681
[M−H]−
810.9786
0
20
40
60
80
100
810
815
820
825
830
835

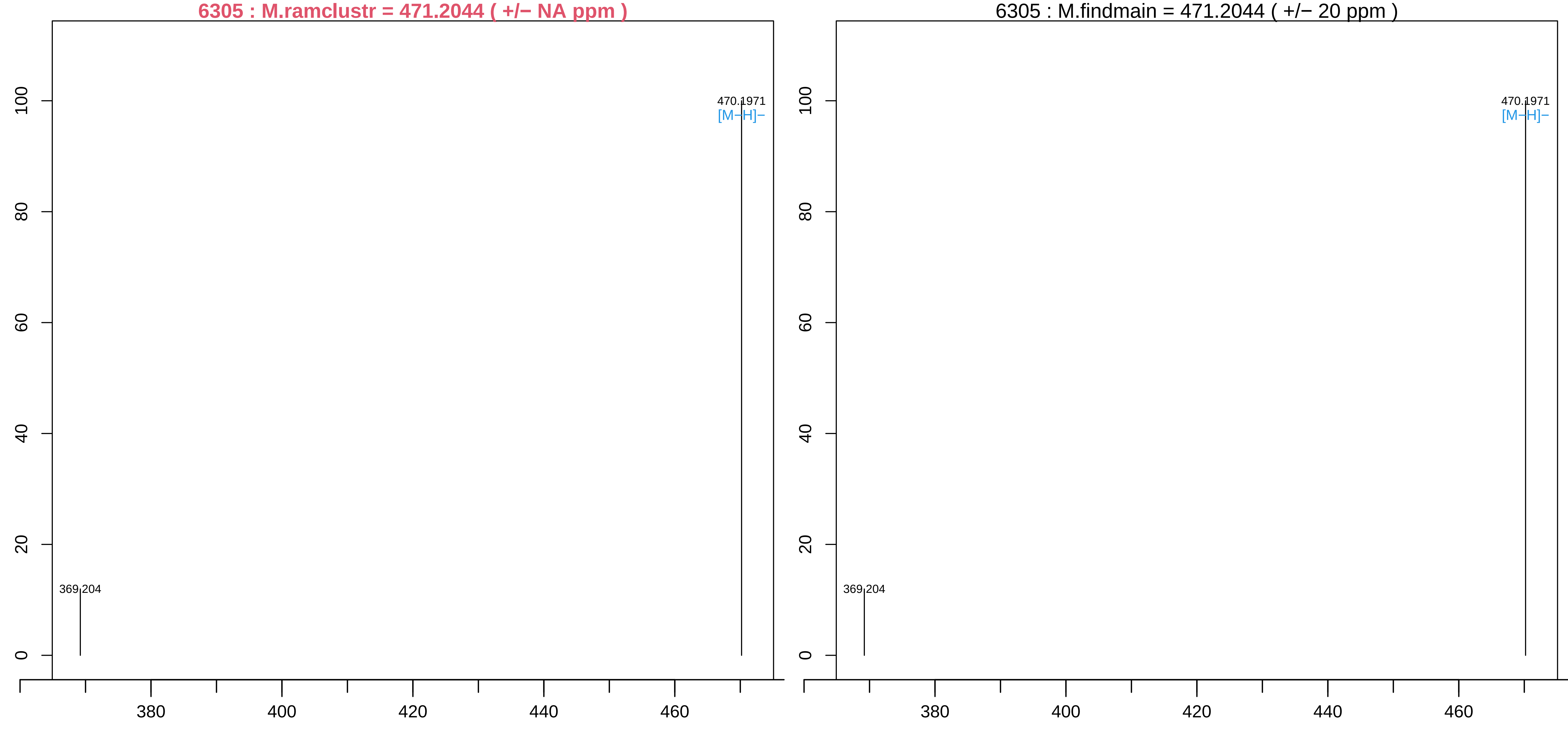
6305 : M.ramclustr = 471.2044 (+/− NA ppm)
470.1971
[M−H]−
369.204
6305 : M.findmain = 471.2044 (+/− 20 ppm)
470.1971
[M−H]−
369.204
0
20
40
60
80
100
380
400
420
440
460

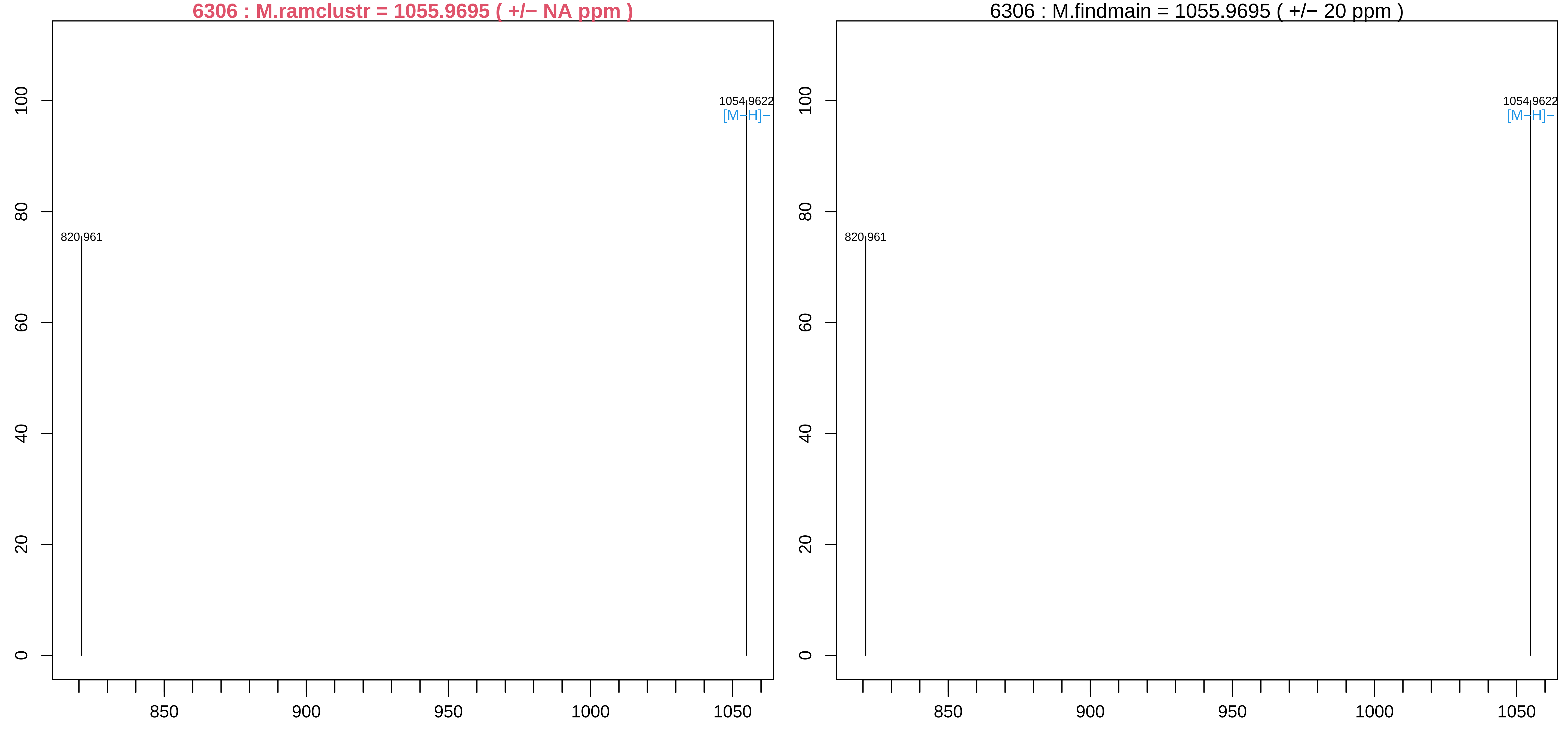
6306 : M.ramclustr = 1055.9695 (+/− NA ppm)
820.961
1054.9622
[M−H]−
6306 : M.findmain = 1055.9695 (+/− 20 ppm)
820.961
1054.9622
[M−H]−
0
20
40
60
80
100
850
900
950
1000
1050

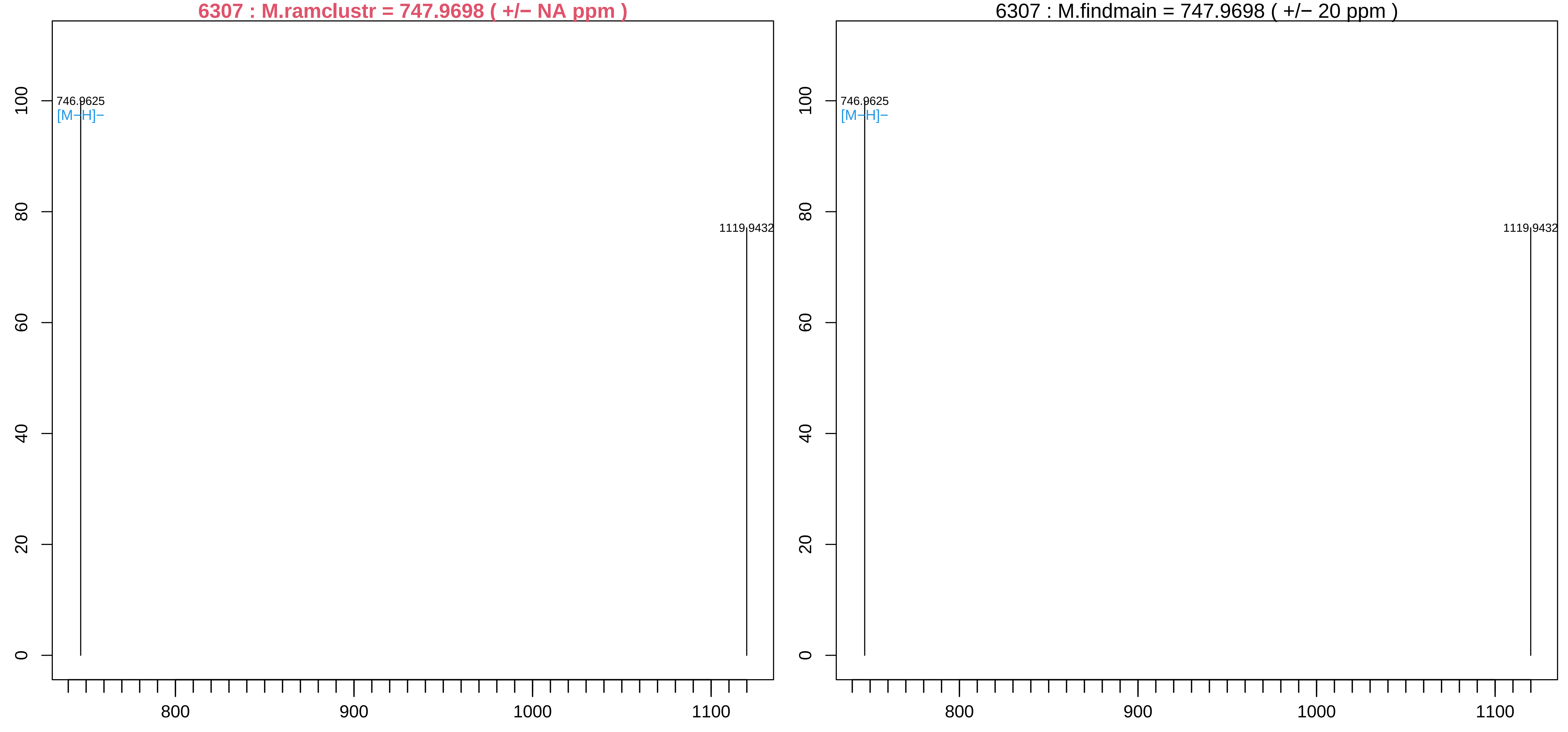
6307 : M.ramclustr = 747.9698 (+/− NA ppm)
746.9625
[M−H]−
1119.9432
6307 : M.findmain = 747.9698 (+/− 20 ppm)
746.9625
[M−H]−
1119.9432
0
20
40
60
80
100
800
900
1000
1100

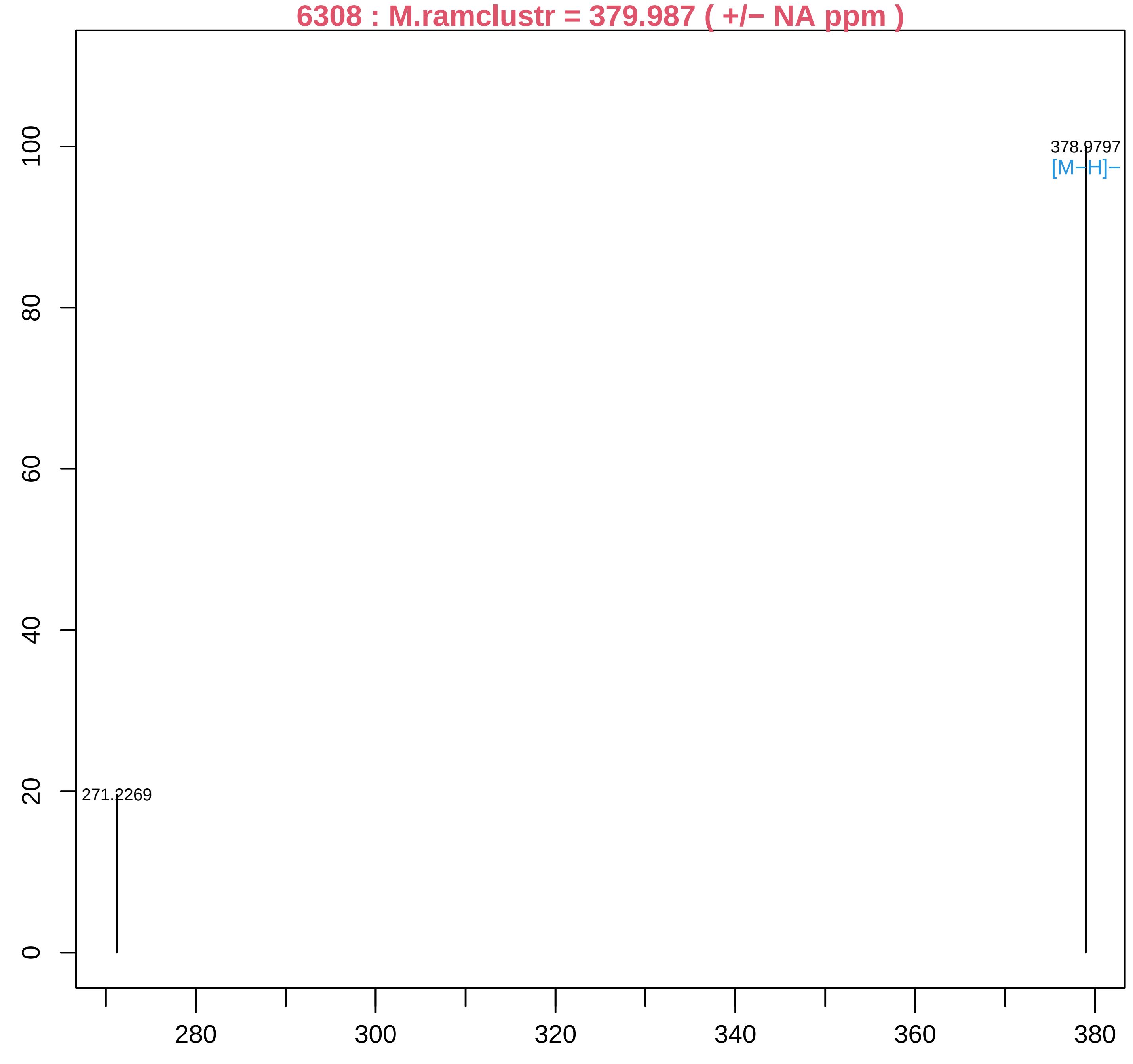
6308 : M.ramclustr = 379.987 (+/− NA ppm)
378.9797
[M−H]−
271.2269
0
20
40
60
80
100
280
300
320
340
360
380

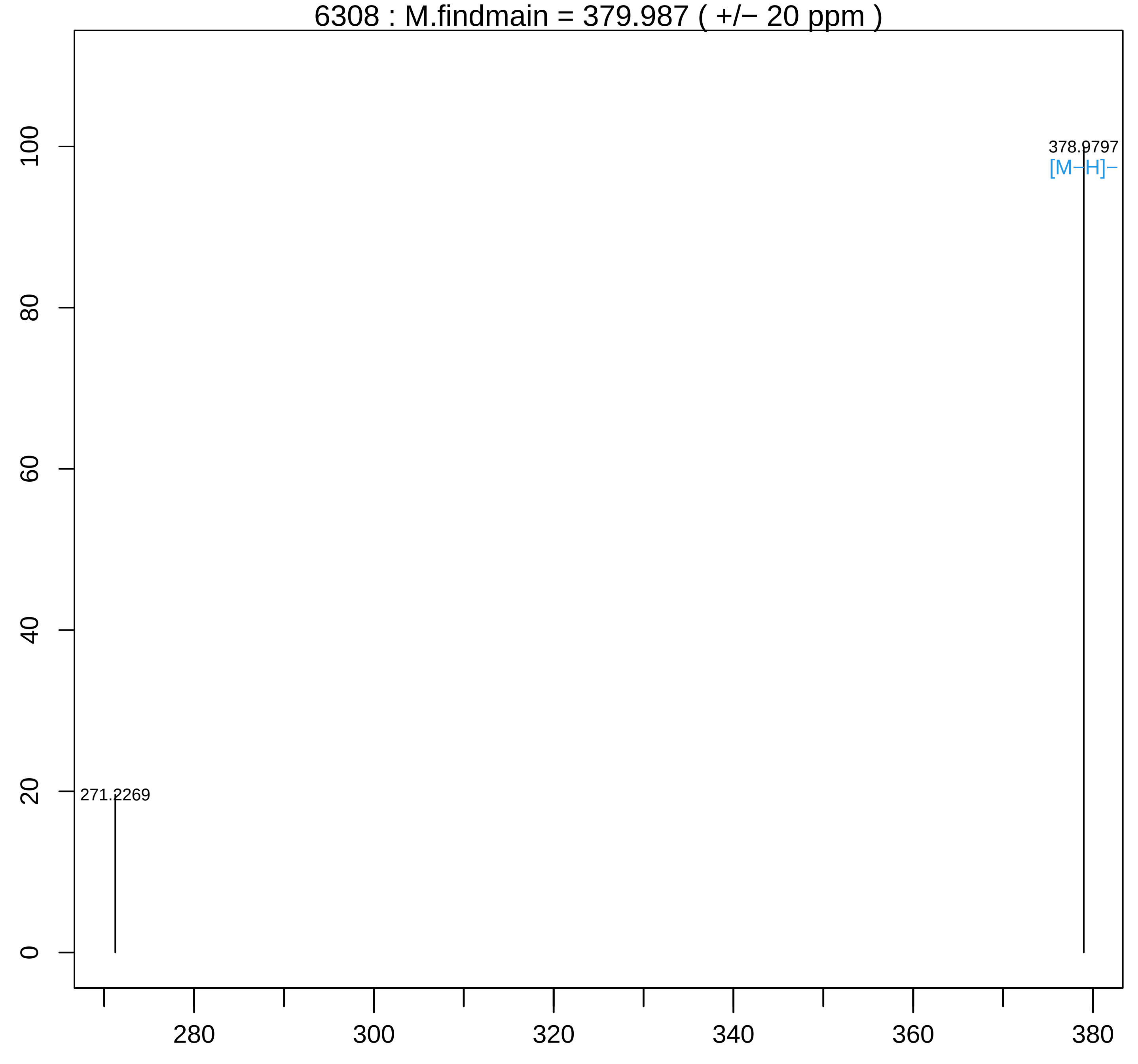
6308 : M.findmain = 379.987 (+/− 20 ppm)
378.9797
[M−H]−
271.2269
0
20
40
60
80
100
280
300
320
340
360
380

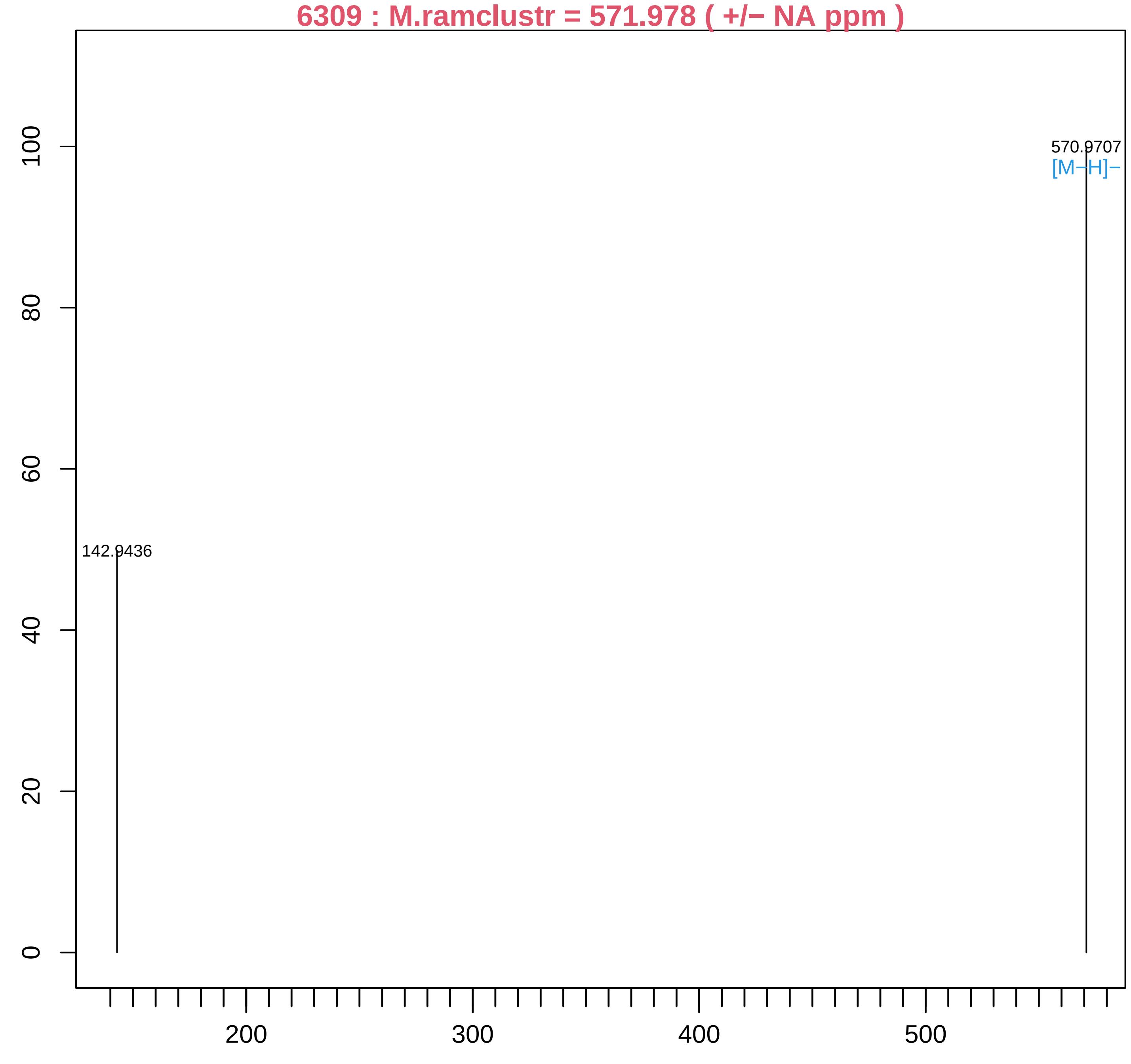
6309 : M.ramclustr = 571.978 (+/− NA ppm)
570.9707
[M−H]−
142.9436
0
20
40
60
80
100
200
300
400
500

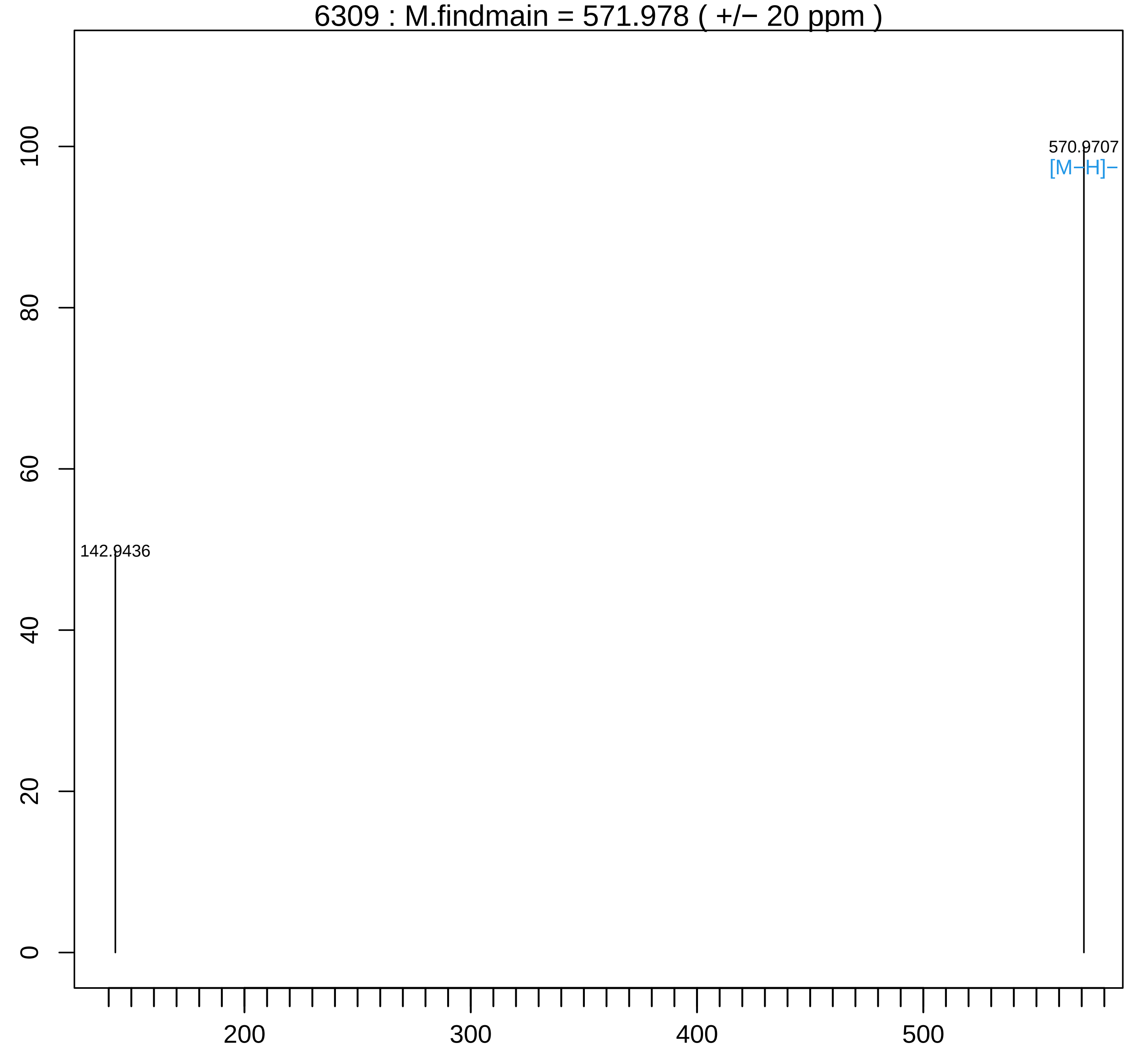
6309 : M.findmain = 571.978 (+/− 20 ppm)
570.9707
[M−H]−
142.9436
0
20
40
60
80
100
200
300
400
500

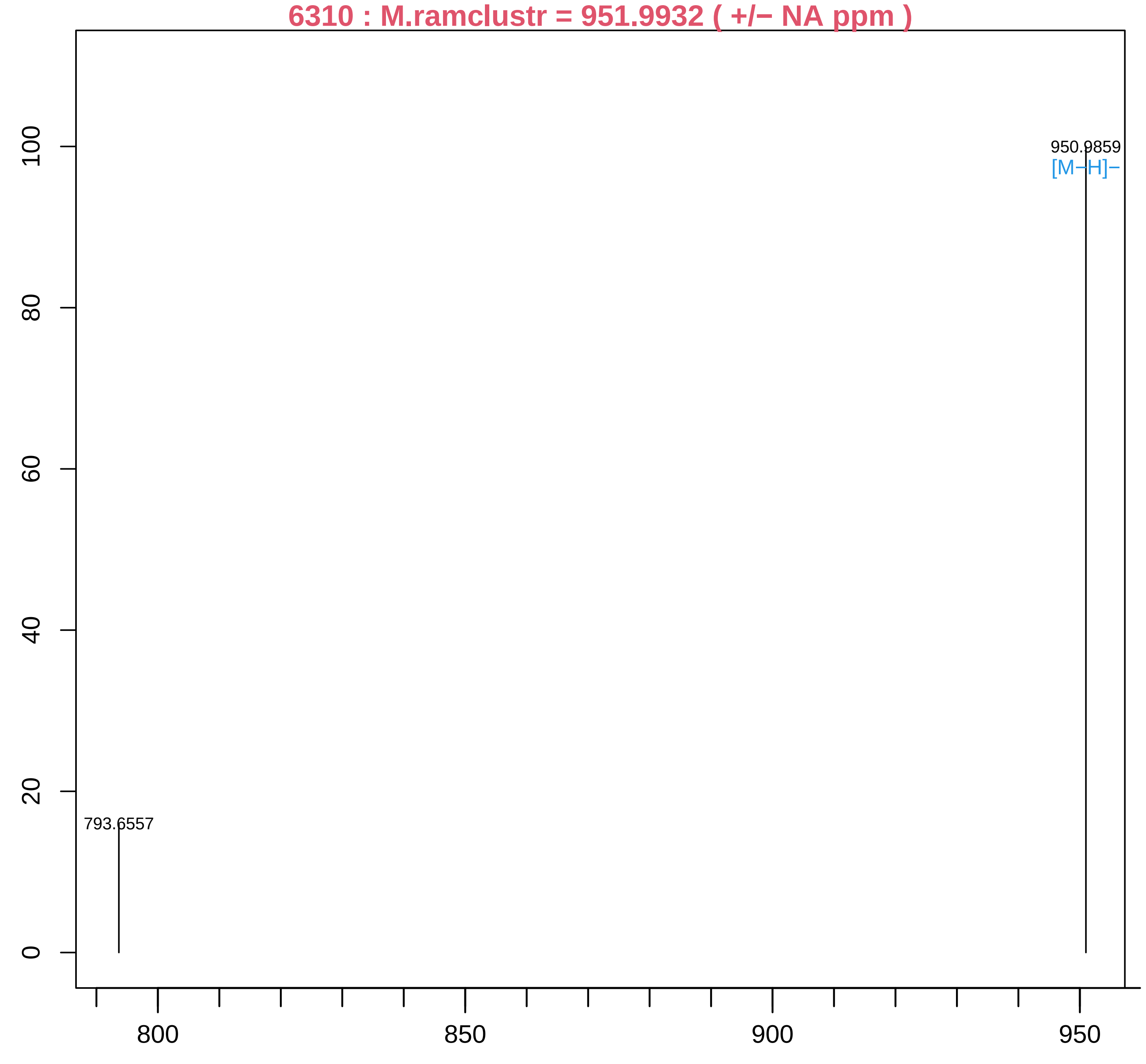
6310 : M.ramclustr = 951.9932 (+/− NA ppm)
950.9859
[M−H]−
793.6557
0
20
40
60
80
100
800
850
900
950

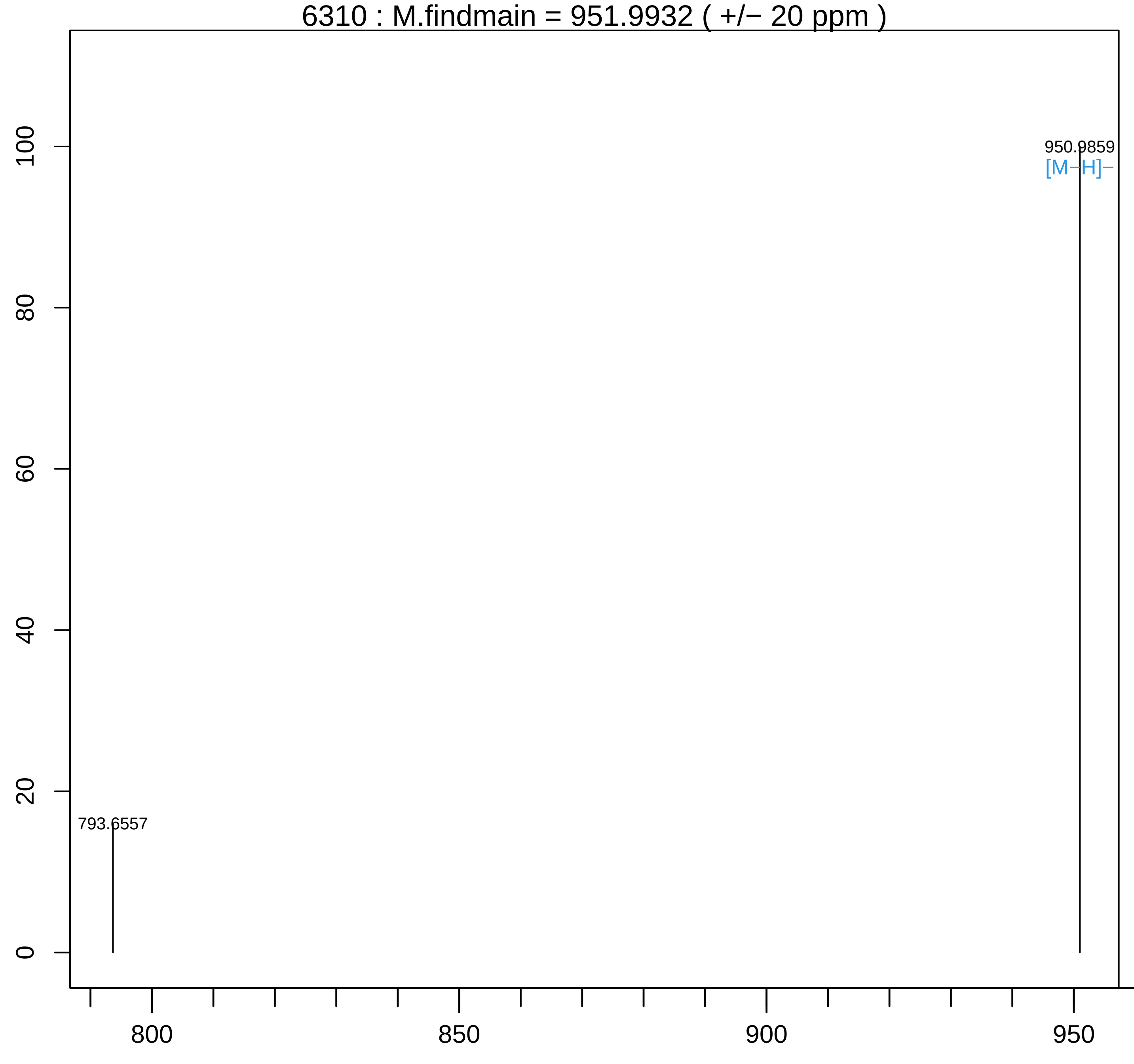
6310 : M.findmain = 951.9932 (+/− 20 ppm)
950.9859
[M−H]−
793.6557
0
20
40
60
80
100
800
850
900
950

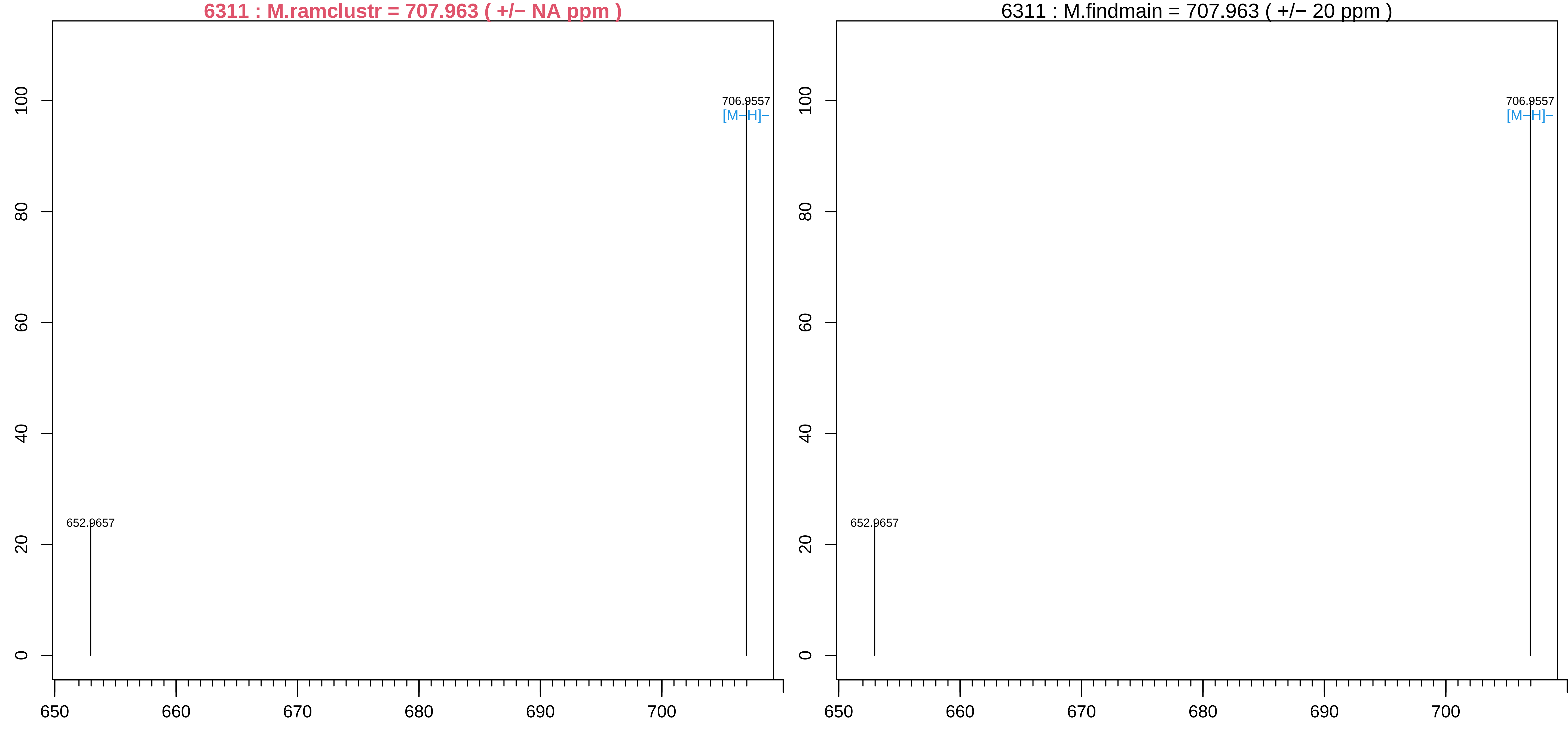
6311 : M.ramclustr = 707.963 (+/− NA ppm)
706.9557
[M−H]−
652.9657
6311 : M.findmain = 707.963 (+/− 20 ppm)
706.9557
[M−H]−
652.9657

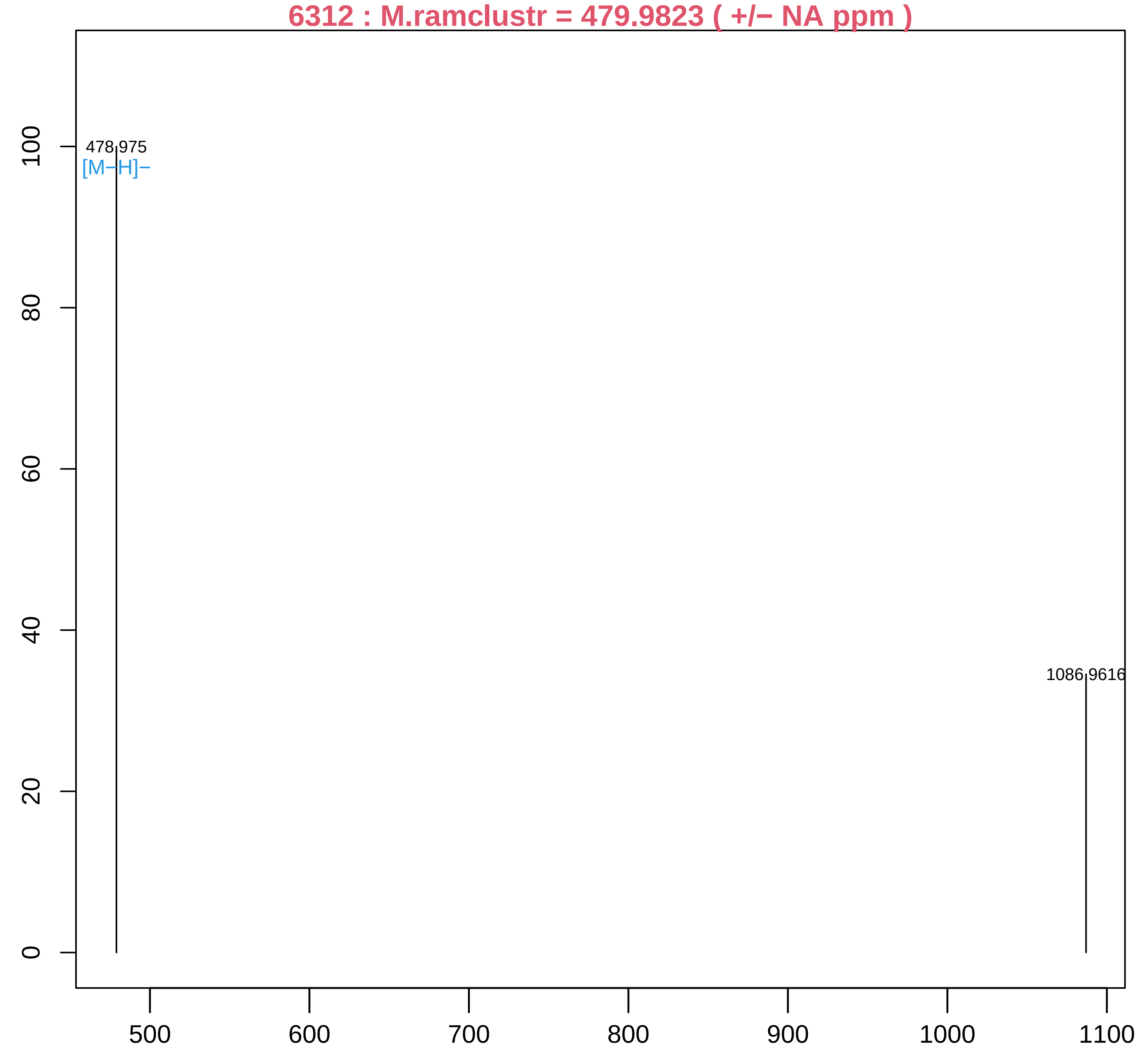
6312 : M.ramclustr = 479.9823 (+/− NA ppm)
478.975
[M−H]−
1086.9616
0
20
40
60
80
100
500
600
700
800
900
1000
1100
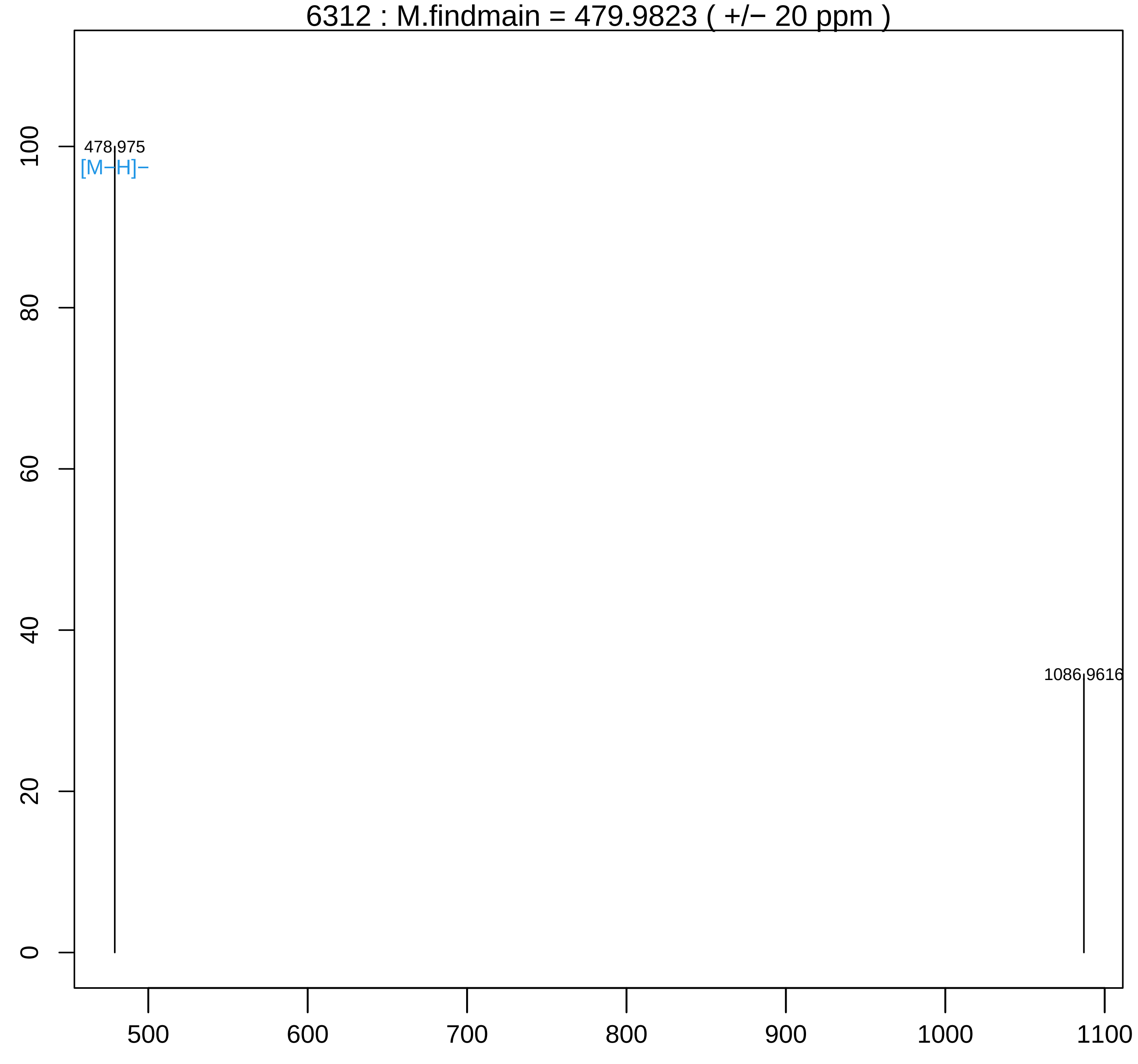
6312 : M.findmain = 479.9823 (+/− 20 ppm)
478.975
[M−H]−
1086.9616
0
20
40
60
80
100
500
600
700
800
900
1000
1100

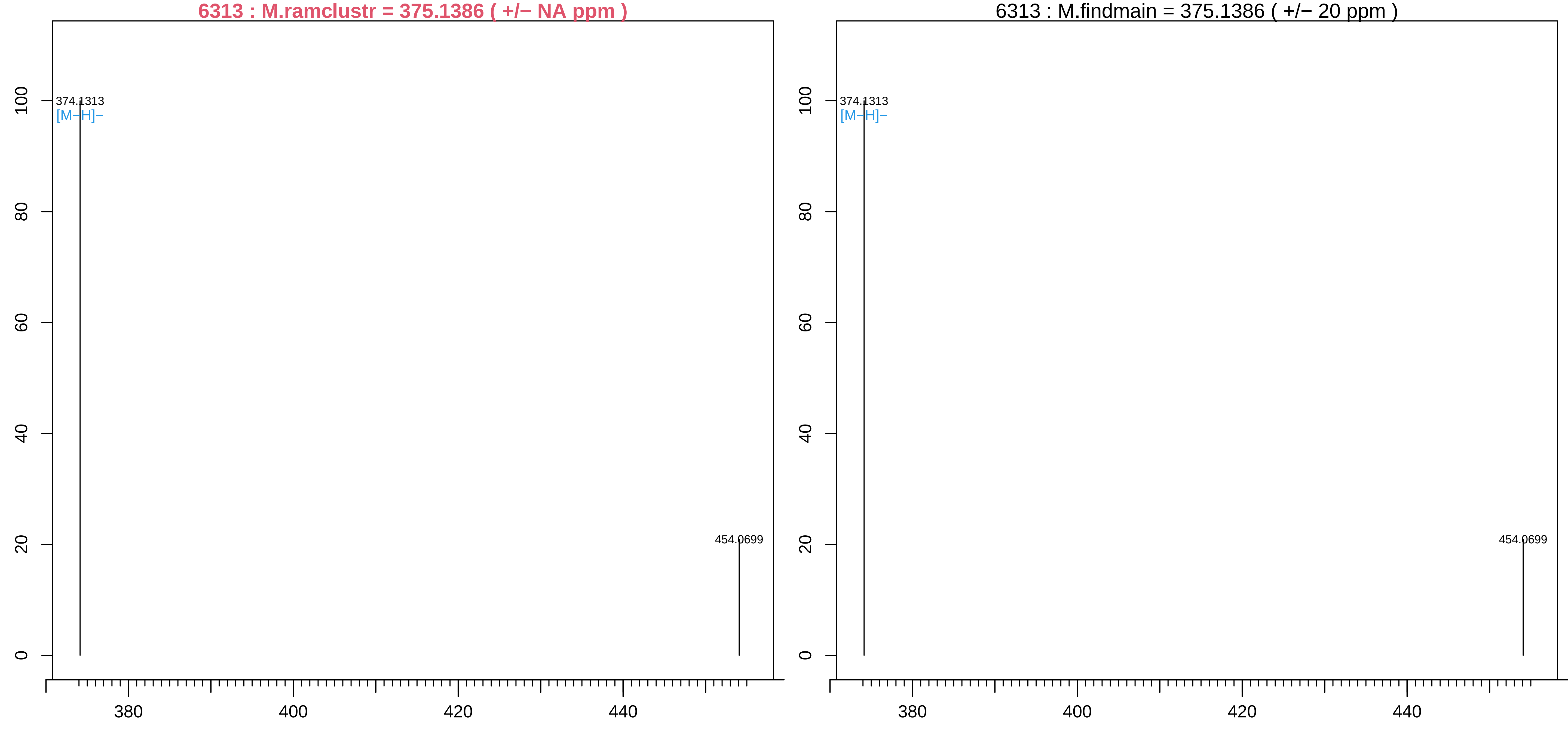
6313 : M.ramclustr = 375.1386 (+/− NA ppm)
374.1313
[M−H]−
454.0699
6313 : M.findmain = 375.1386 (+/− 20 ppm)
374.1313
[M−H]−
454.0699
0
20
40
60
80
100
380
400
420
440

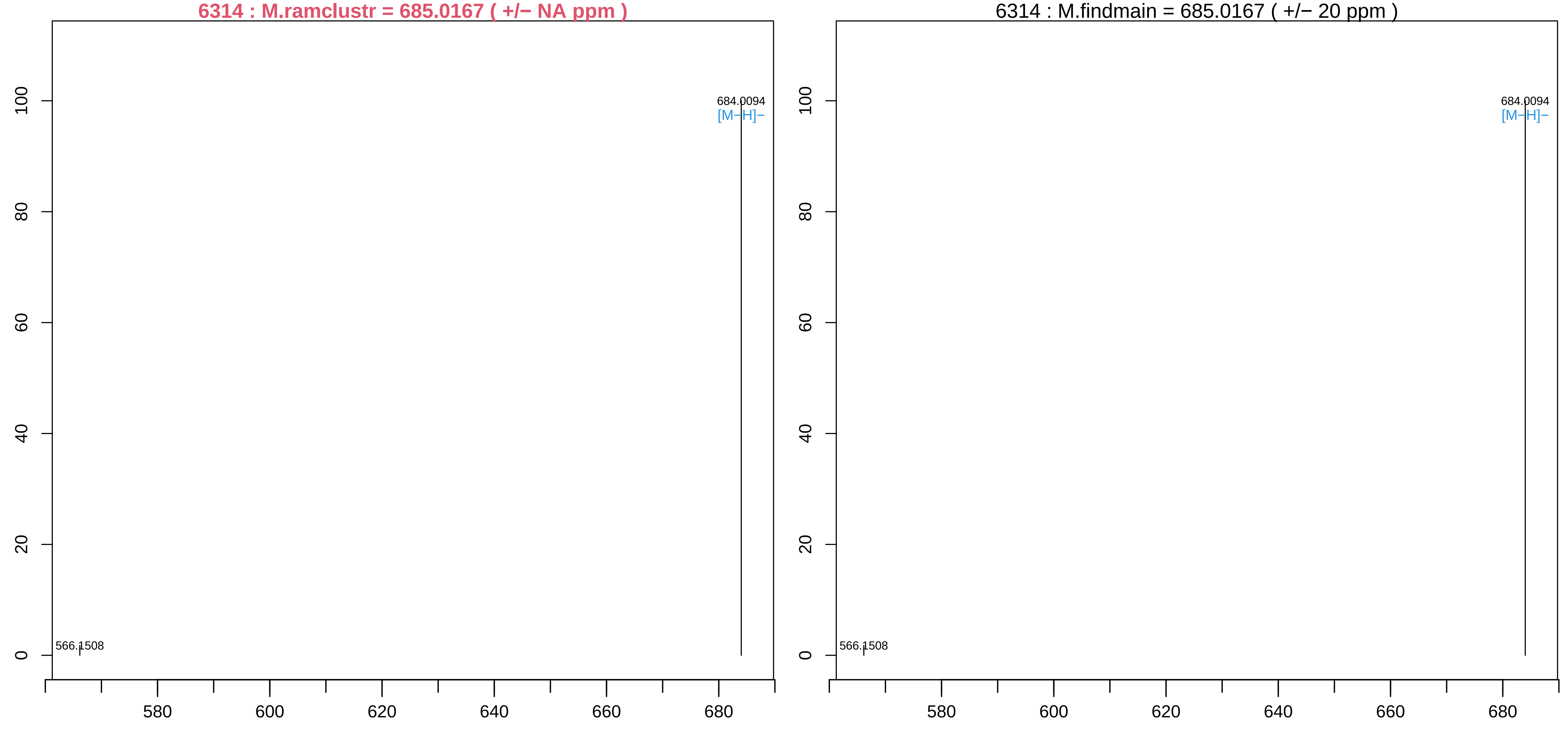
6314 : M.ramclustr = 685.0167 (+/− NA ppm)
6314 : M.findmain = 685.0167 (+/− 20 ppm)
684.0094
[M−H]−
566.1508
684.0094
[M−H]−
566.1508
0
20
40
60
80
100
580
600
620
640
660
680

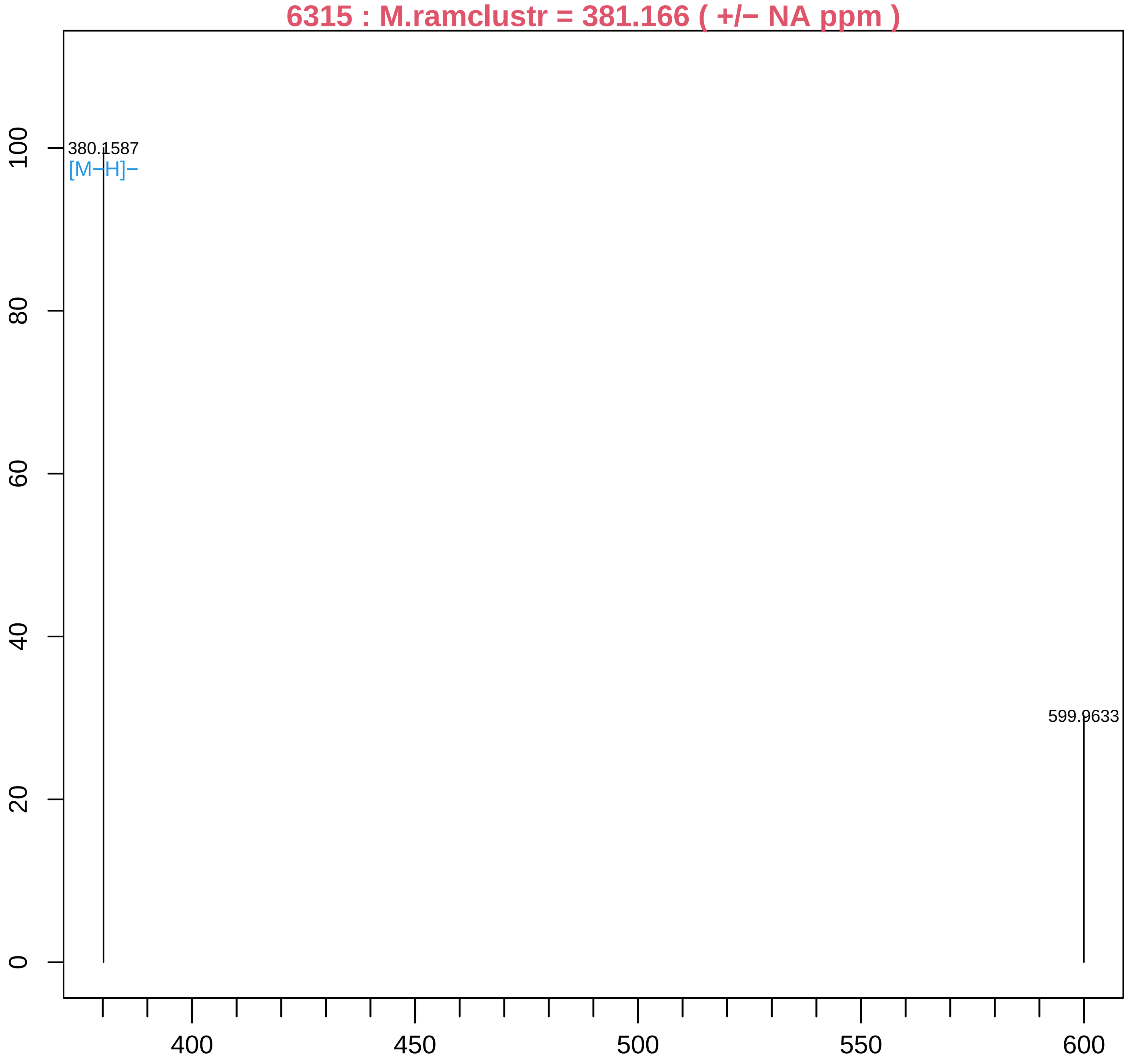
6315 : M.ramclustr = 381.166 (+/− NA ppm)
380.1587
[M−H]−
599.9633
0
20
40
60
80
100
400
450
500
550
600

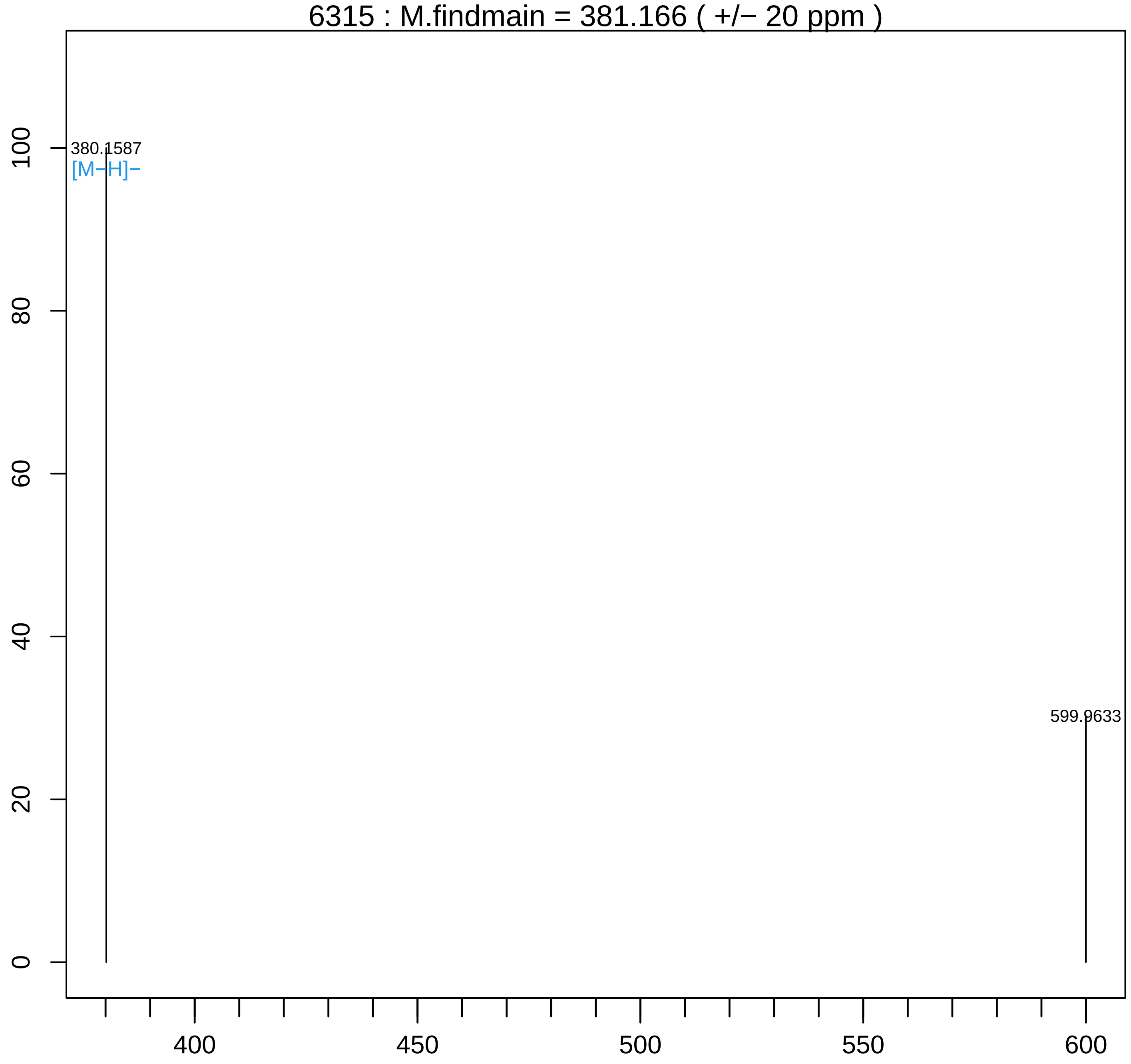
6315 : M.findmain = 381.166 (+/− 20 ppm)
380.1587
[M−H]−
599.9633
0
20
40
60
80
100
400
450
500
550
600

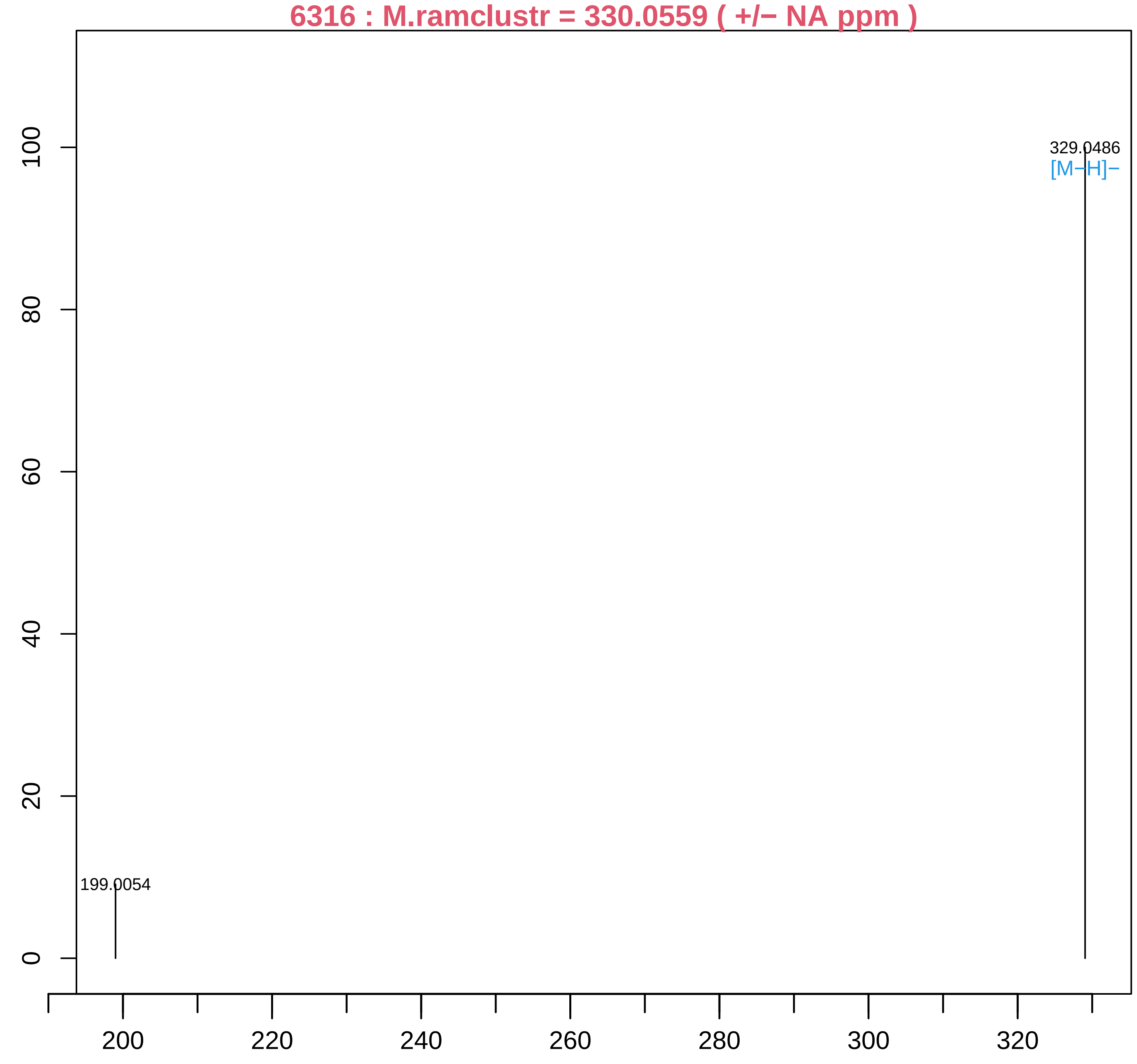
6316 : M.ramclustr = 330.0559 (+/− NA ppm)
329.0486
[M−H]−
199.0054
0
20
40
60
80
100
200
220
240
260
280
300
320

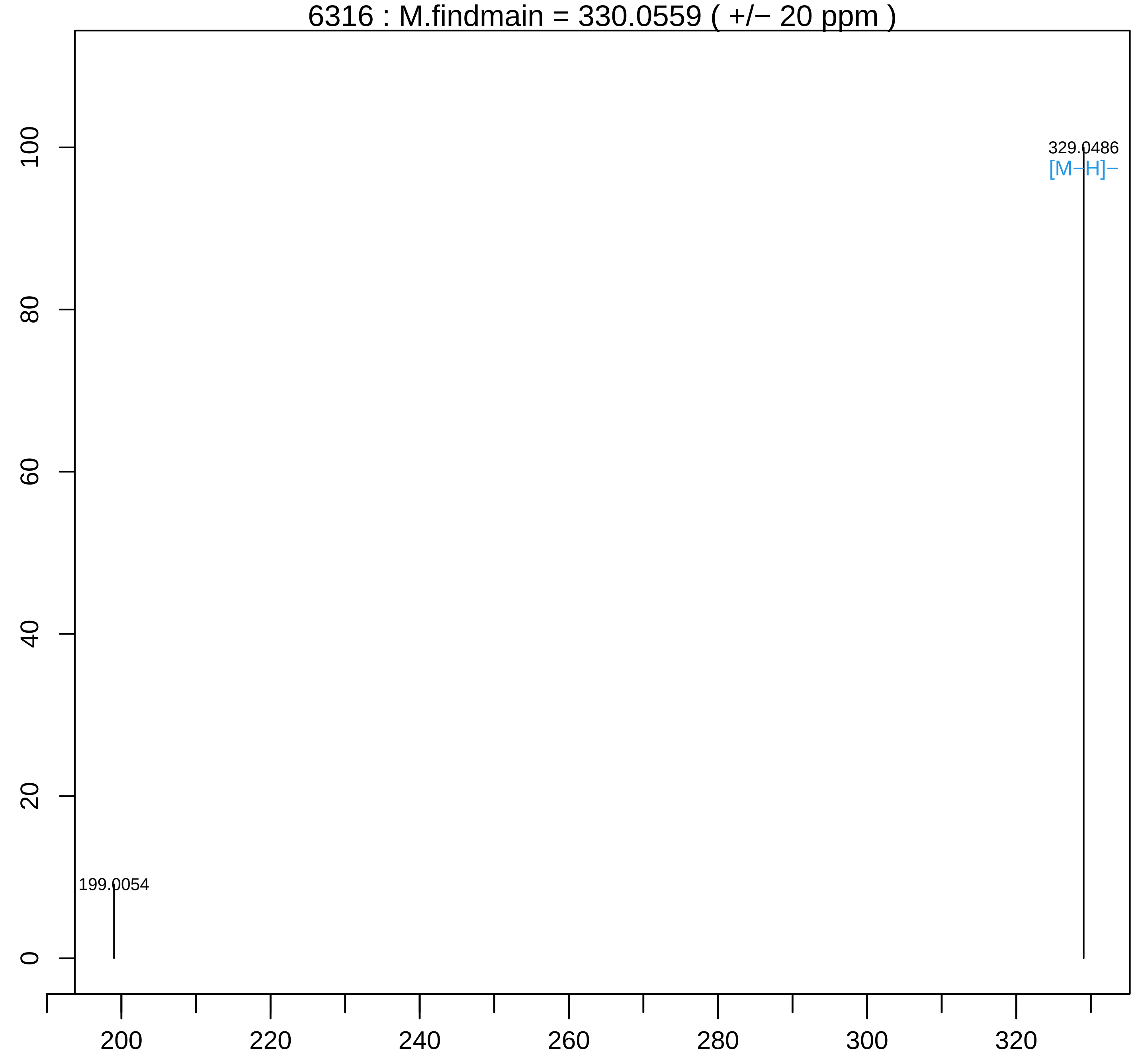
6316 : M.findmain = 330.0559 (+/− 20 ppm)
329.0486
[M−H]−
199.0054
0
20
40
60
80
100
200
220
240
260
280
300
320

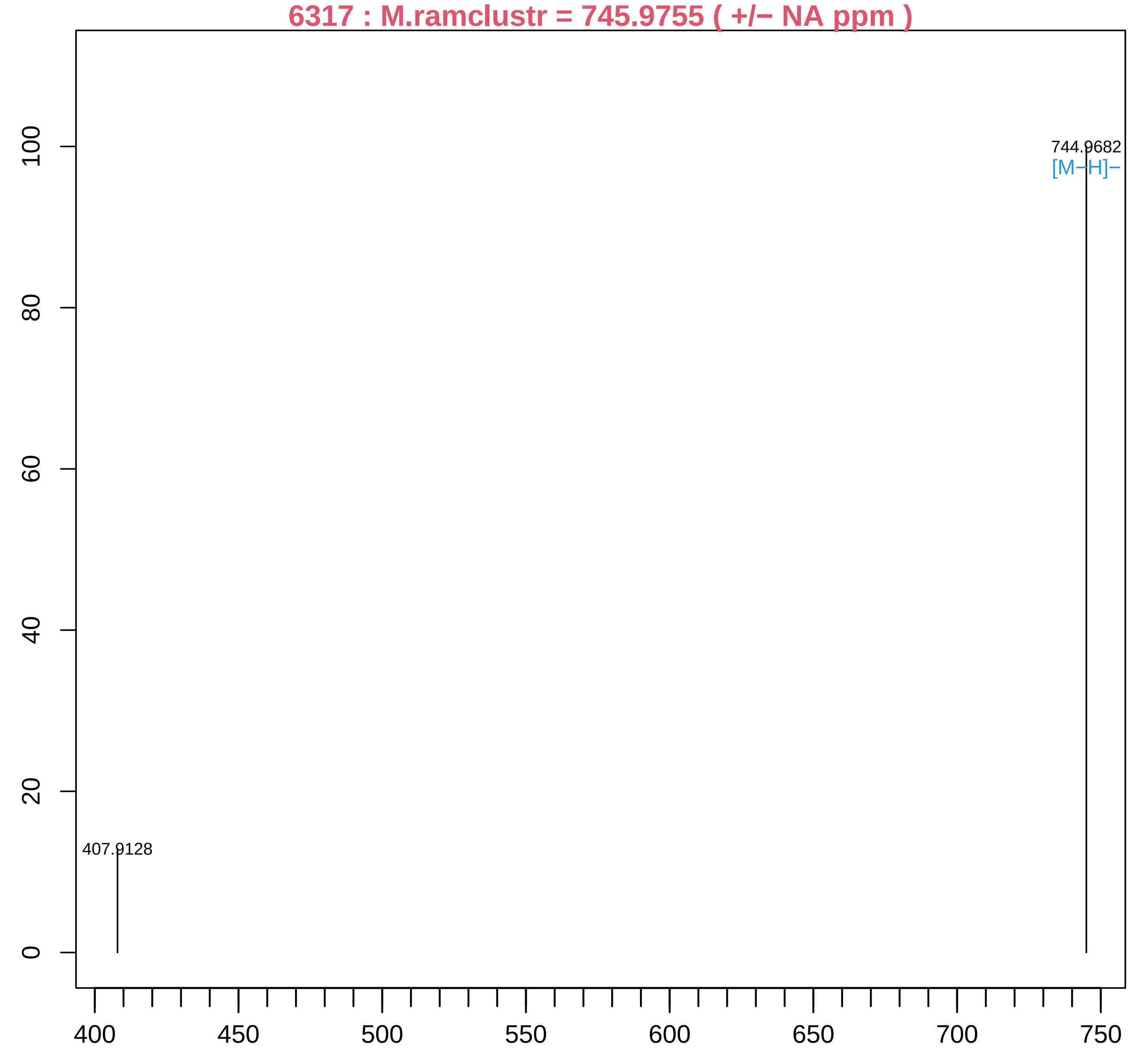
6317 : M.ramclustr = 745.9755 (+/− NA ppm)
744.9682
[M−H]−
407.9128
0
20
40
60
80
100
400
450
500
550
600
650
700
750

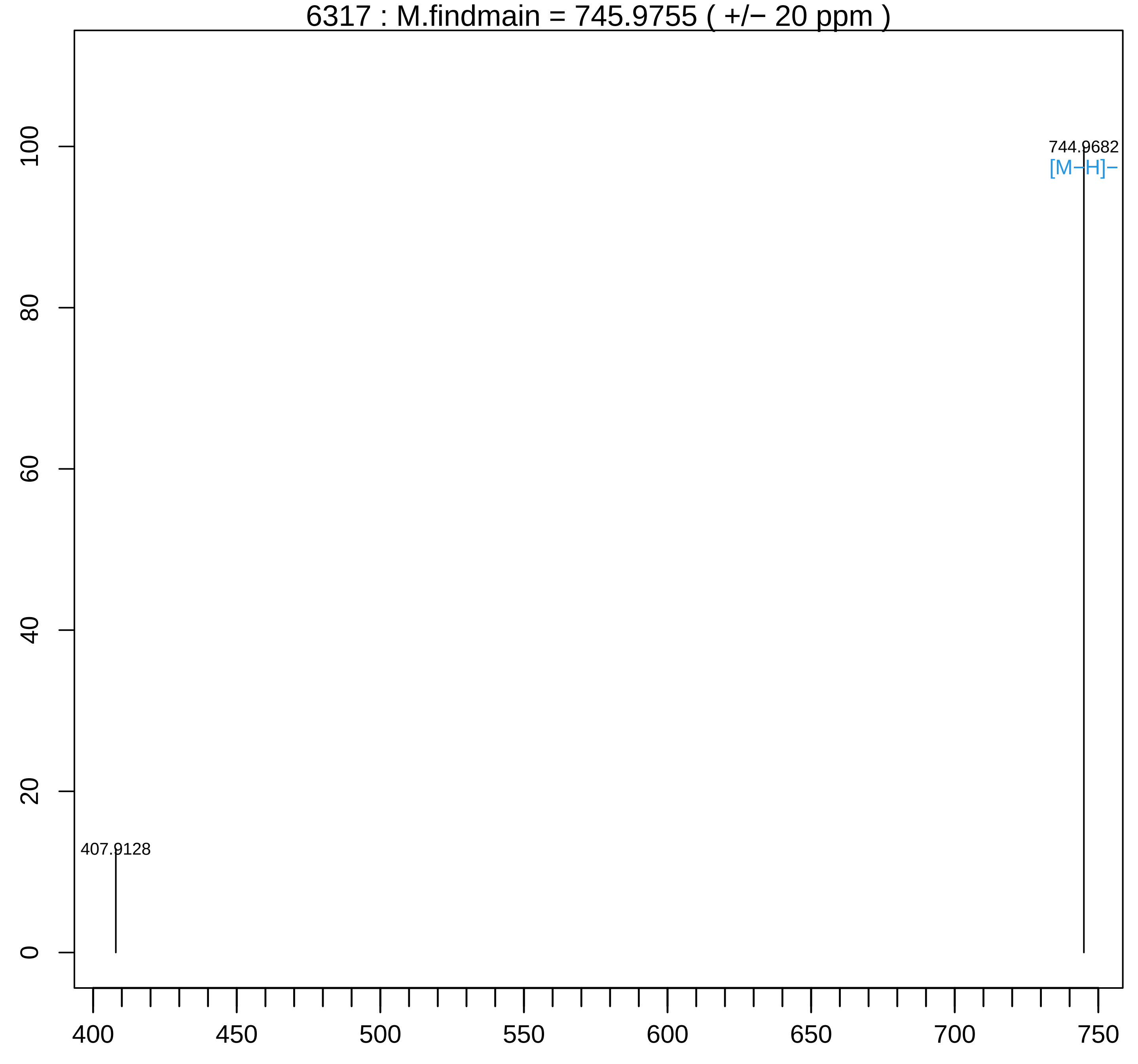
6317 : M.findmain = 745.9755 (+/− 20 ppm)
744.9682
[M−H]−
407.9128
0
20
40
60
80
100
400
450
500
550
600
650
700
750

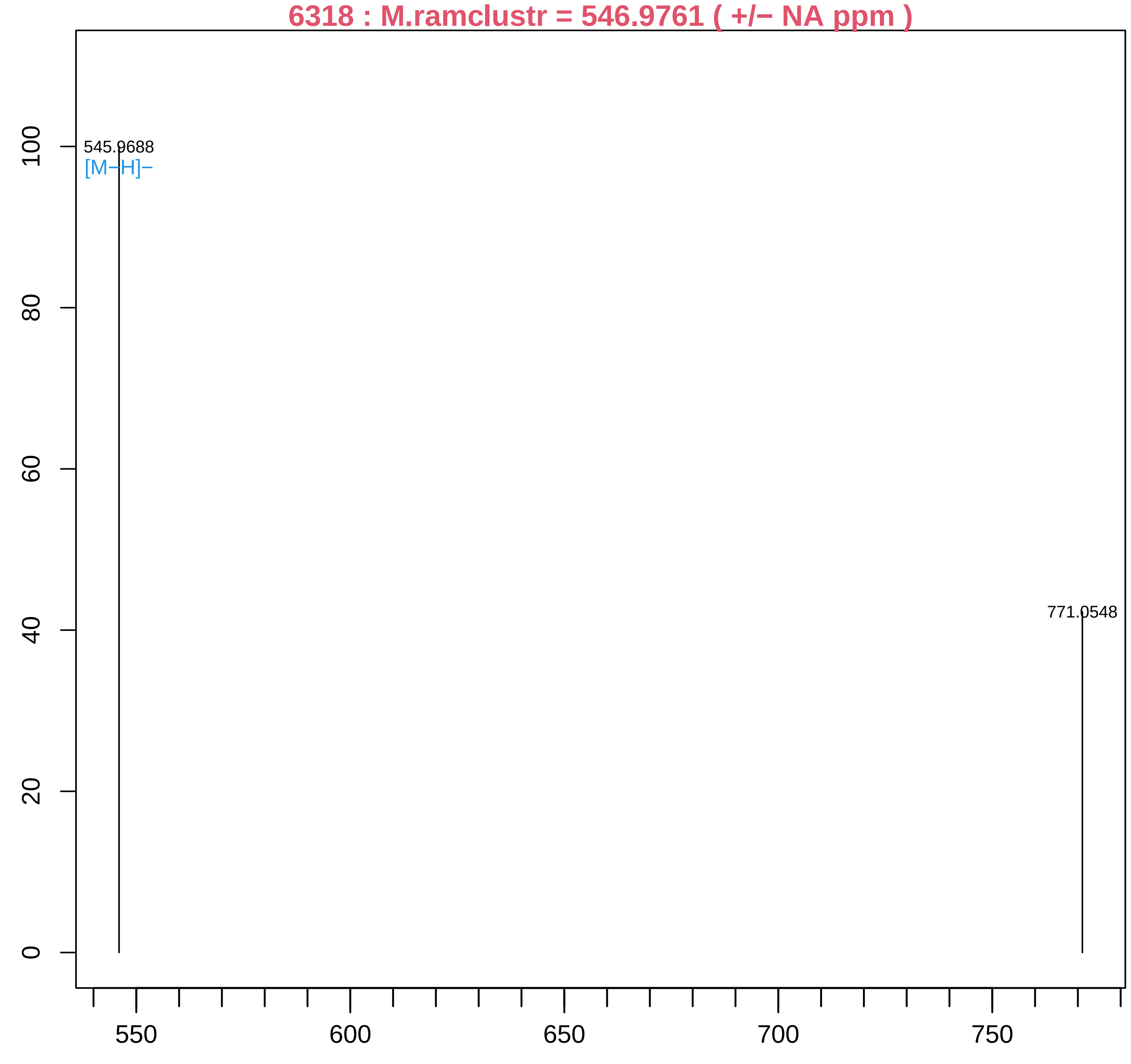
6318 : M.ramclustr = 546.9761 (+/− NA ppm)
545.9688
[M−H]−
771.0548
0
20
40
60
80
100
550
600
650
700
750

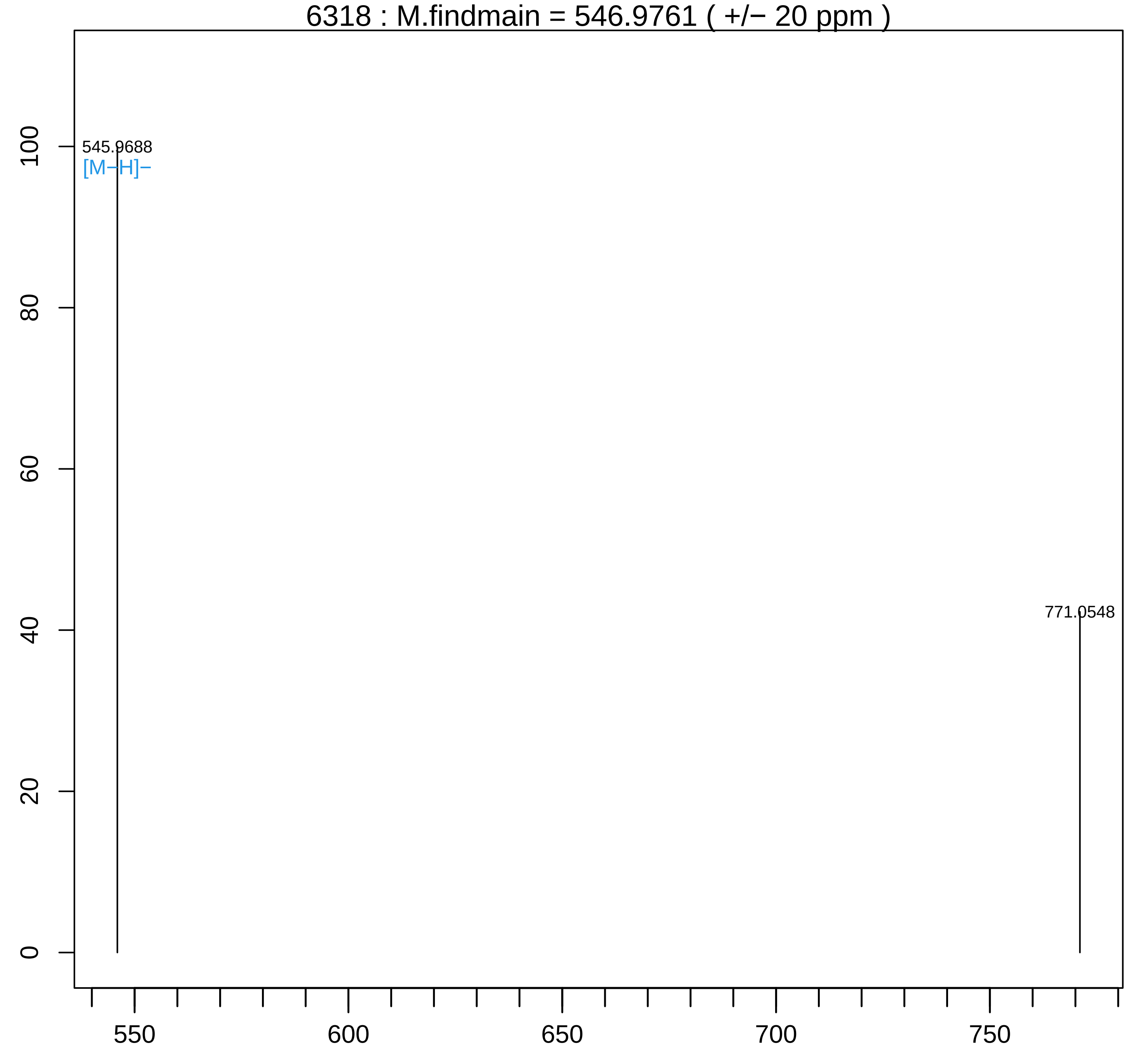
6318 : M.findmain = 546.9761 (+/− 20 ppm)
545.9688
[M−H]−
771.0548
0
20
40
60
80
100
550
600
650
700
750

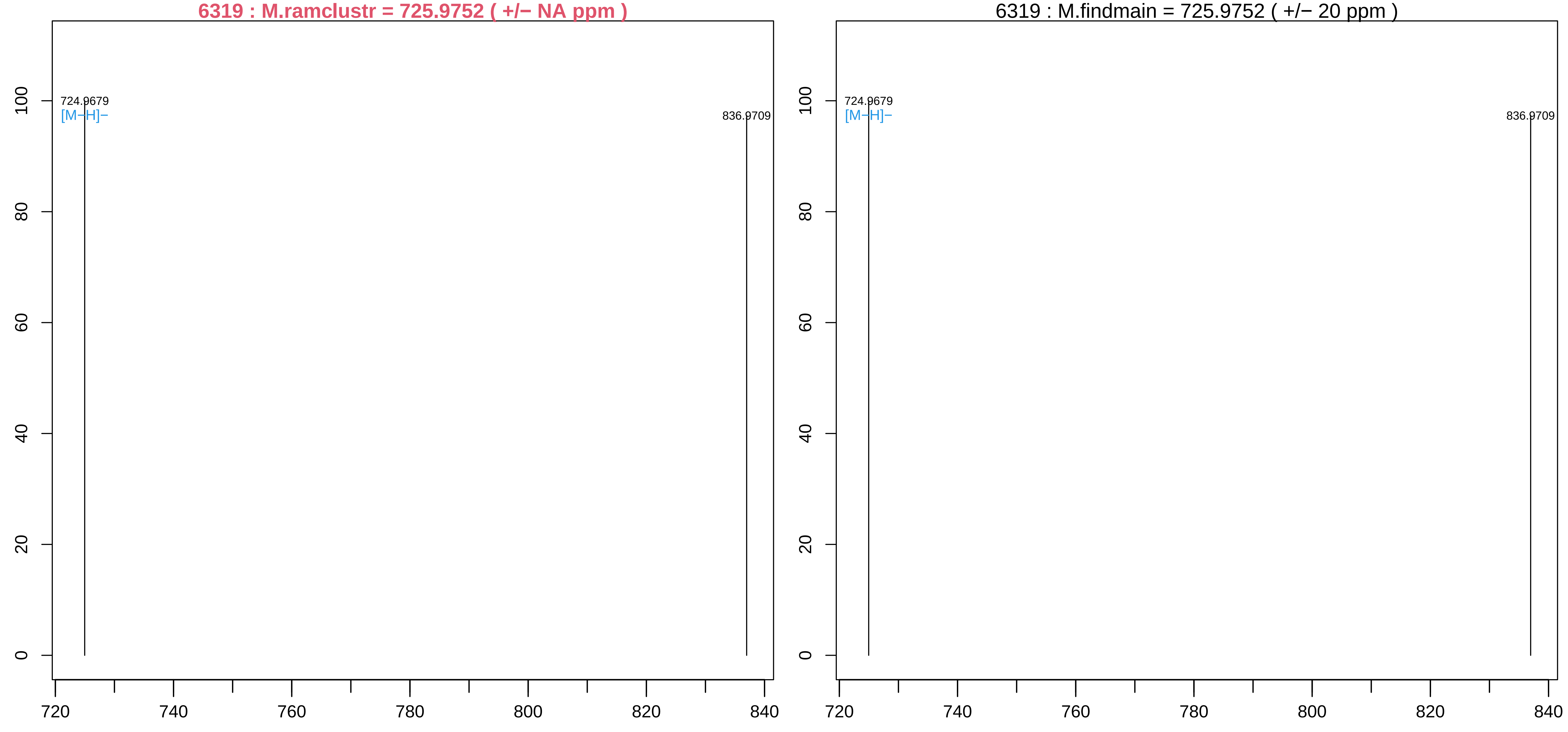
6319 : M.ramclustr = 725.9752 (+/− NA ppm)
724.9679
[M−H]−
836.9709
0
20
40
60
80
100
720
740
760
780
800
820
840
6319 : M.findmain = 725.9752 (+/− 20 ppm)
724.9679
[M−H]−
836.9709
0
20
40
60
80
100
720
740
760
780
800
820
840

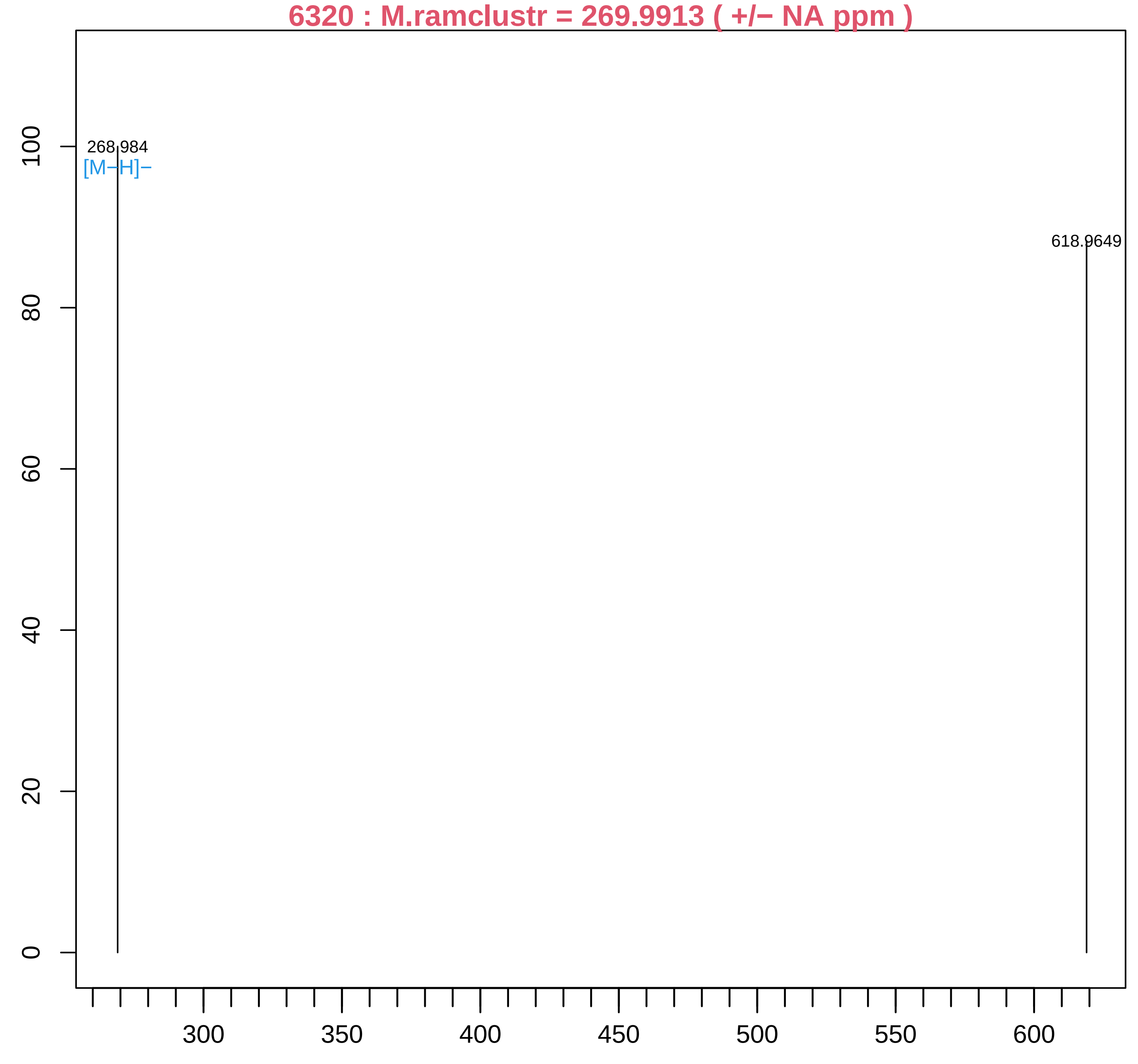
6320 : M.ramclustr = 269.9913 (+/− NA ppm)
268.984
[M−H]−
618.9649
0
20
40
60
80
100
300
350
400
450
500
550
600

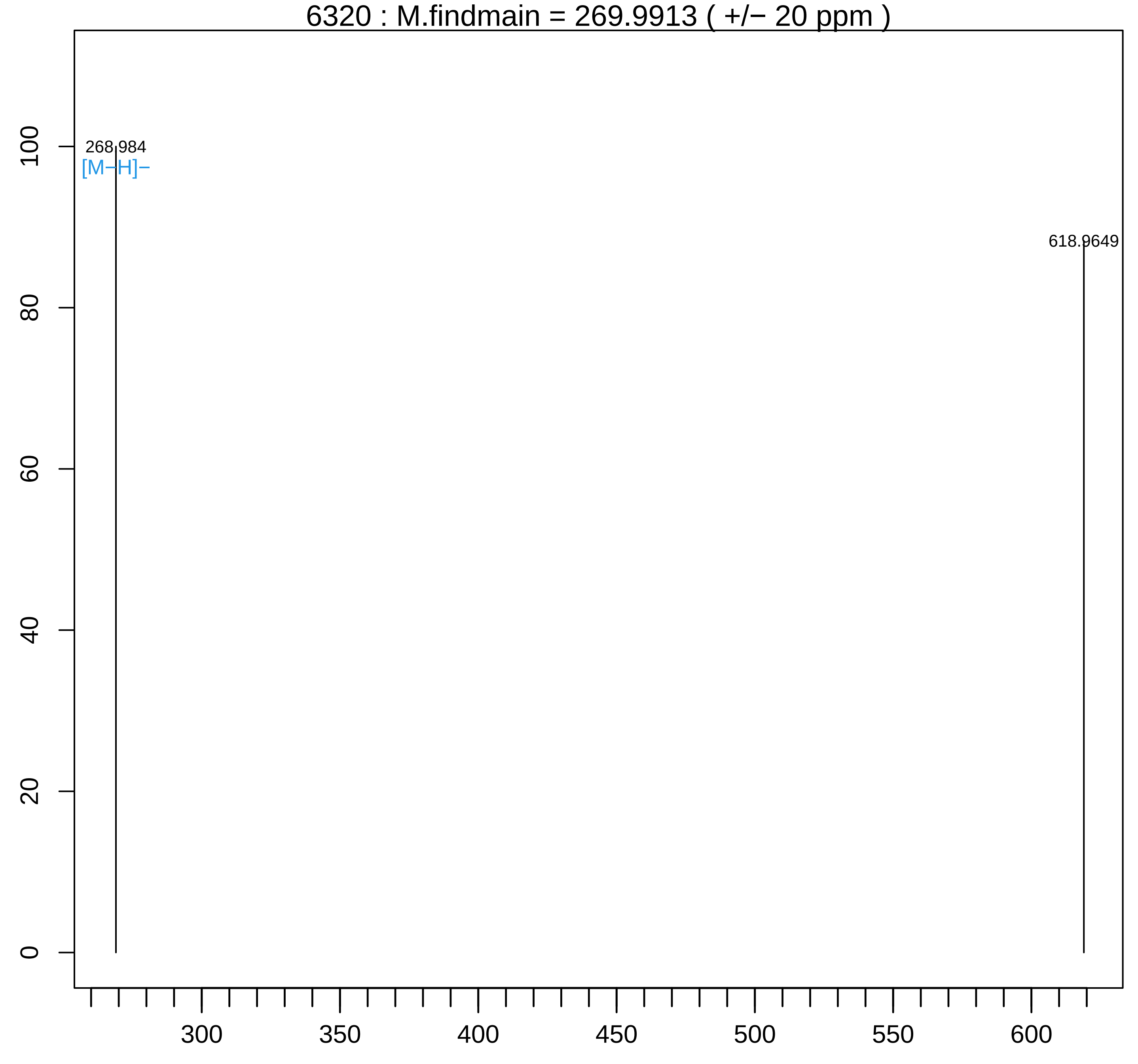
6320 : M.findmain = 269.9913 (+/− 20 ppm)
268.984
[M−H]−
618.9649
0
20
40
60
80
100
300
350
400
450
500
550
600

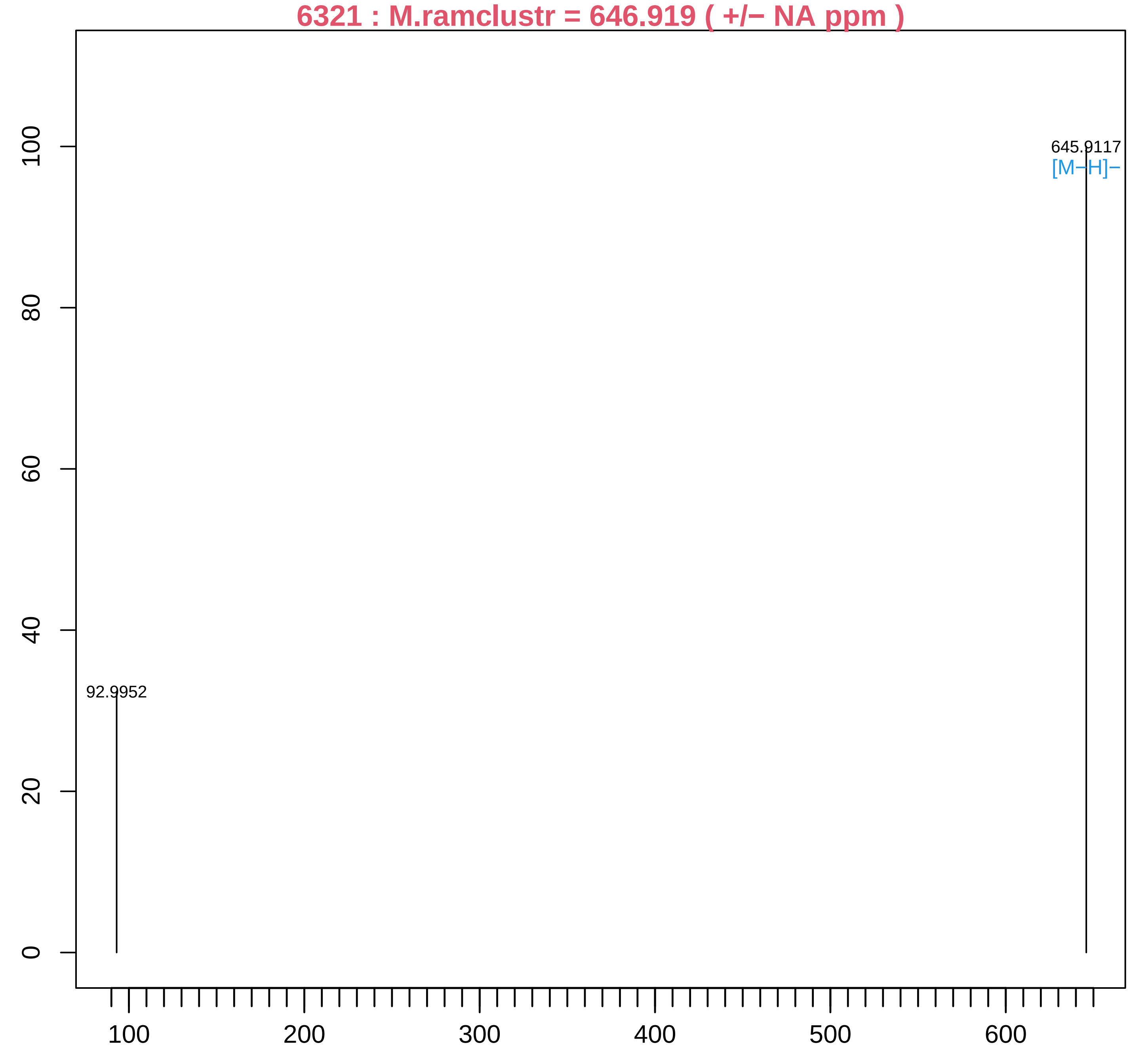
6321 : M.ramclustr = 646.919 (+/− NA ppm)
645.9117
[M−H]−
92.9952
0
20
40
60
80
100
100
200
300
400
500
600

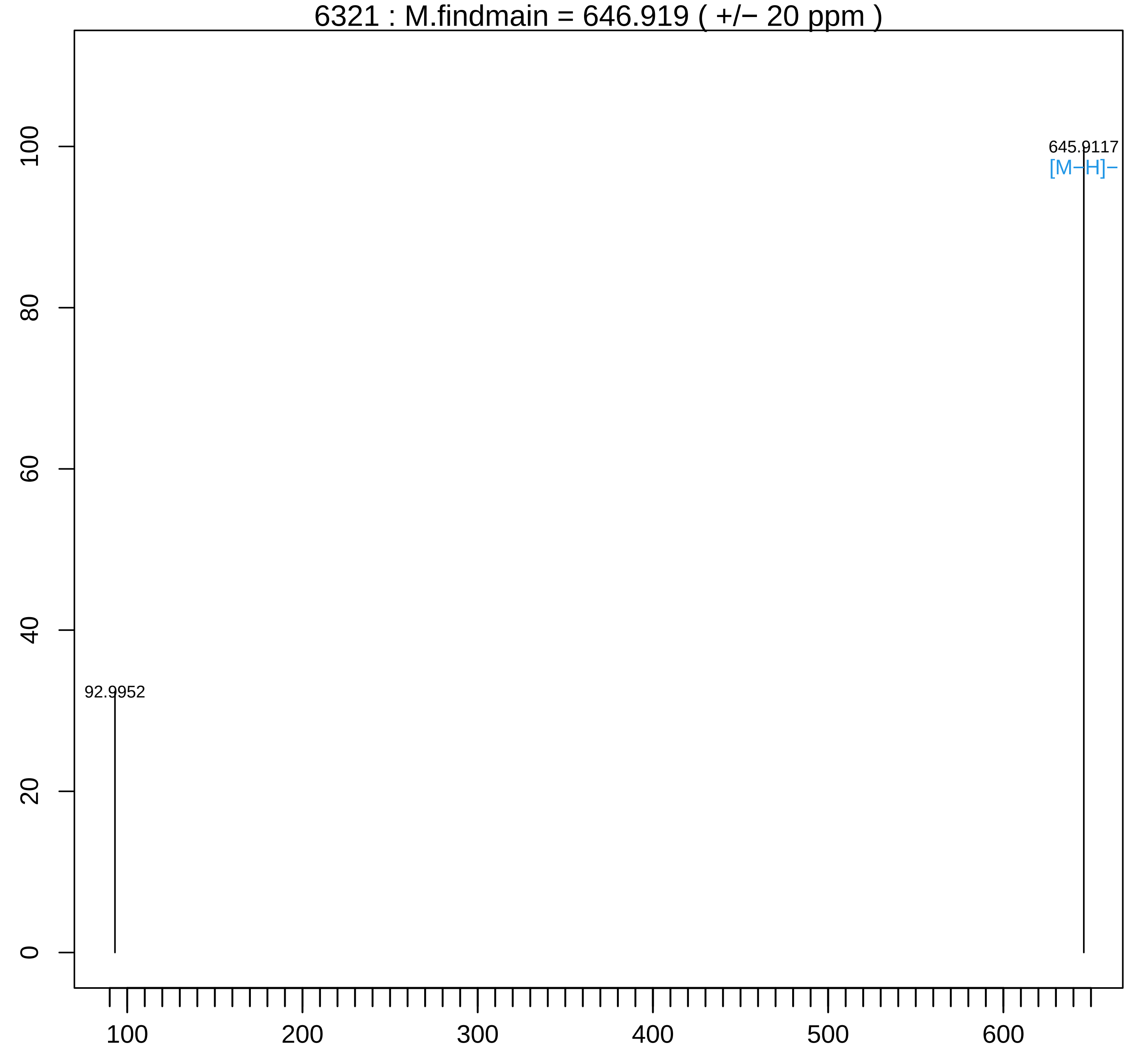
6321 : M.findmain = 646.919 (+/− 20 ppm)
645.9117
[M−H]−
92.9952
0
20
40
60
80
100
100
200
300
400
500
600

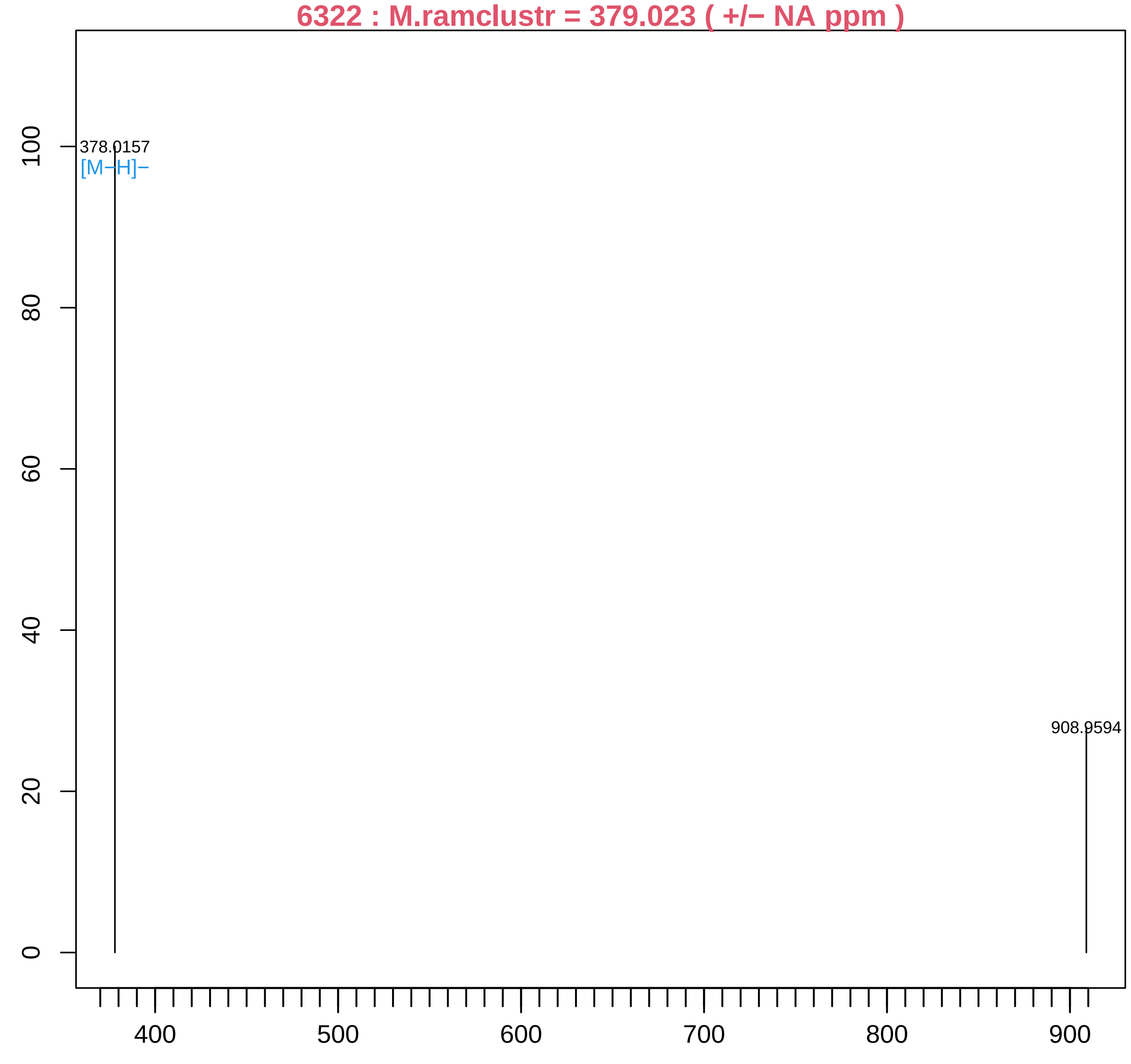
6322 : M.ramclustr = 379.023 (+/− NA ppm)
378.0157
[M−H]−
908.9594
0
20
40
60
80
100
400
500
600
700
800
900

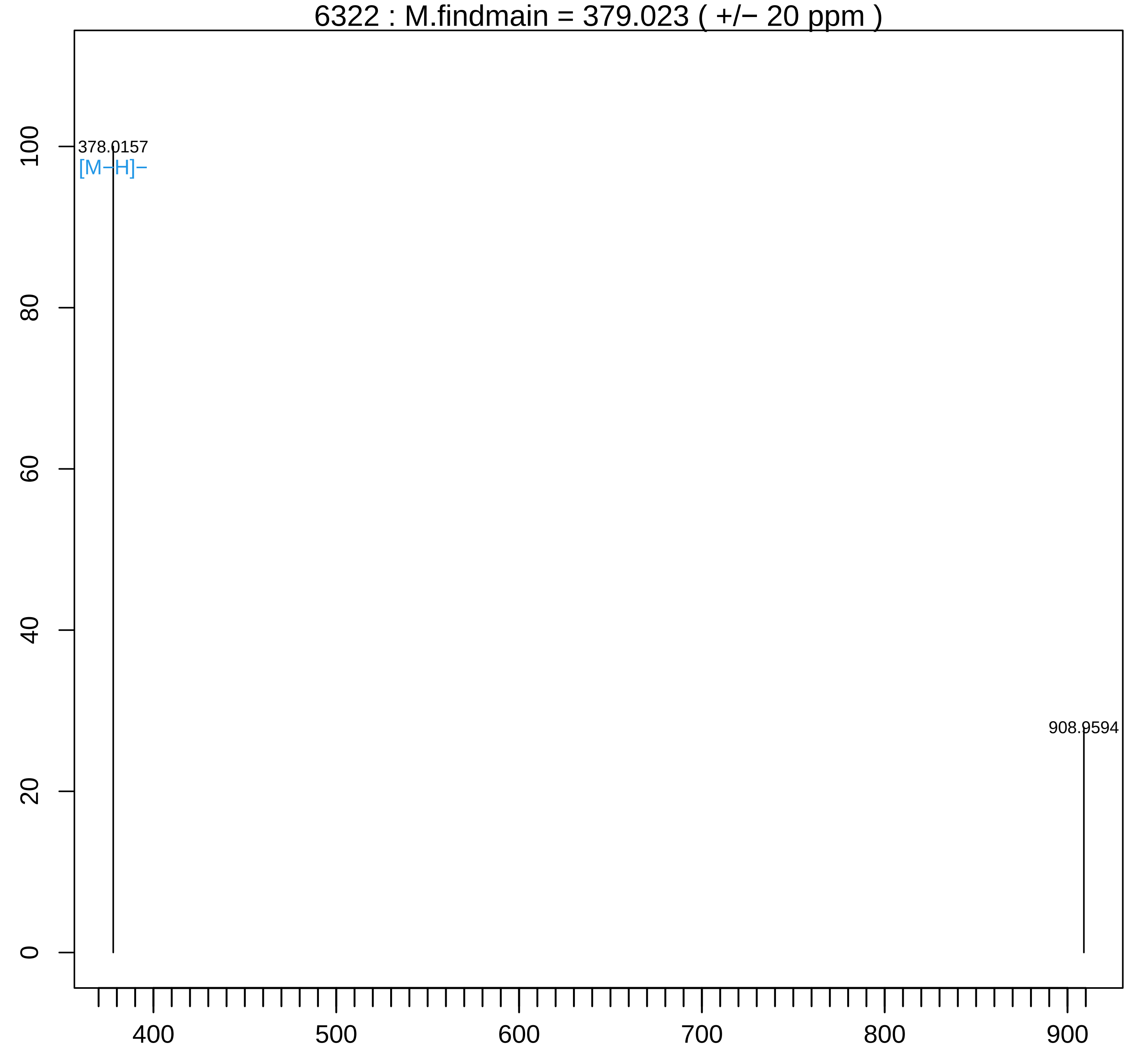
6322 : M.findmain = 379.023 (+/− 20 ppm)
378.0157
[M−H]−
908.9594
0
20
40
60
80
100
400
500
600
700
800
900

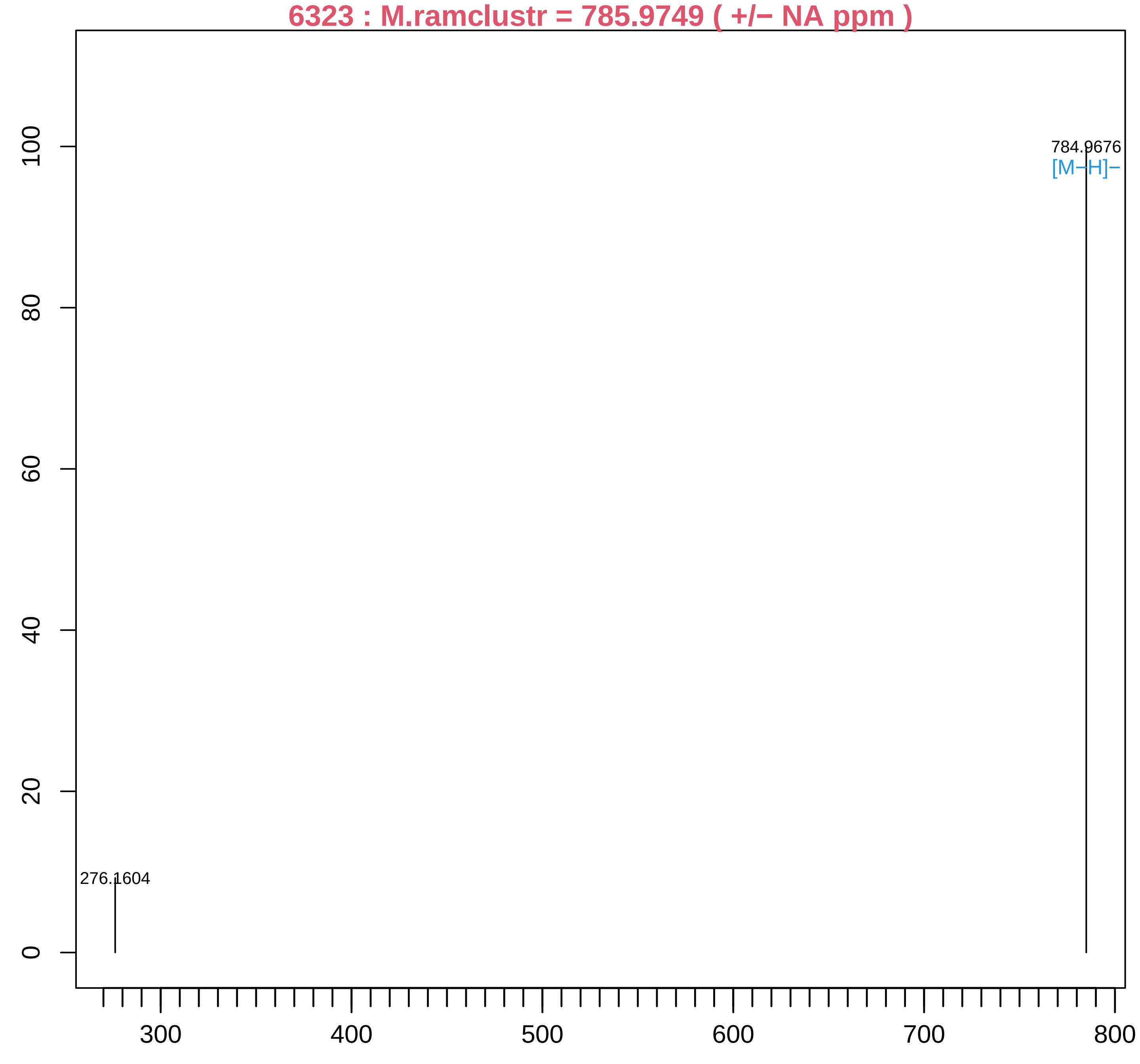
6323 : M.ramclustr = 785.9749 (+/− NA ppm)
784.9676
[M−H]−
276.1604
0
20
40
60
80
100
300
400
500
600
700
800

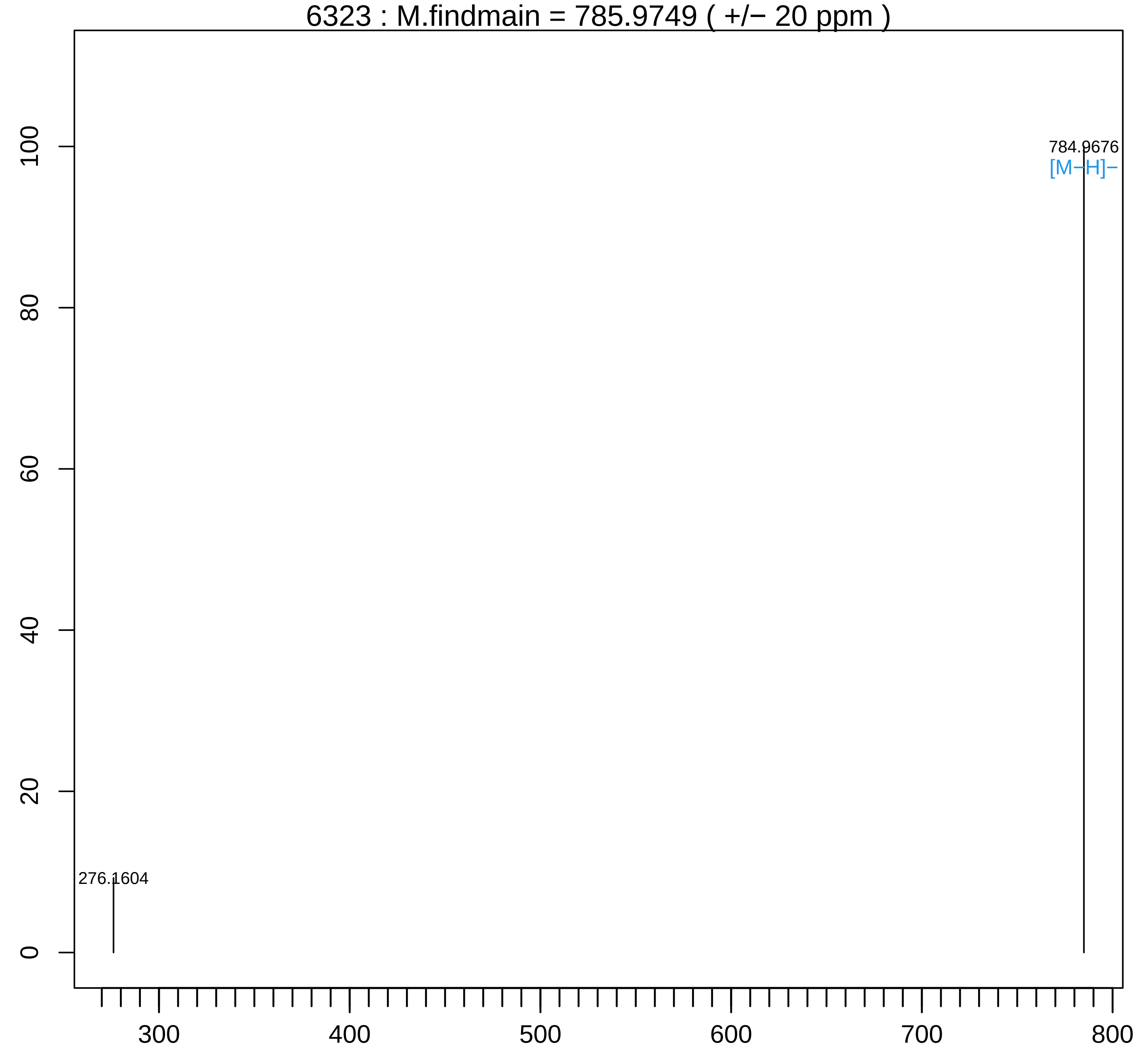
6323 : M.findmain = 785.9749 (+/− 20 ppm)
784.9676
[M−H]−
276.1604
0
20
40
60
80
100
300
400
500
600
700
800

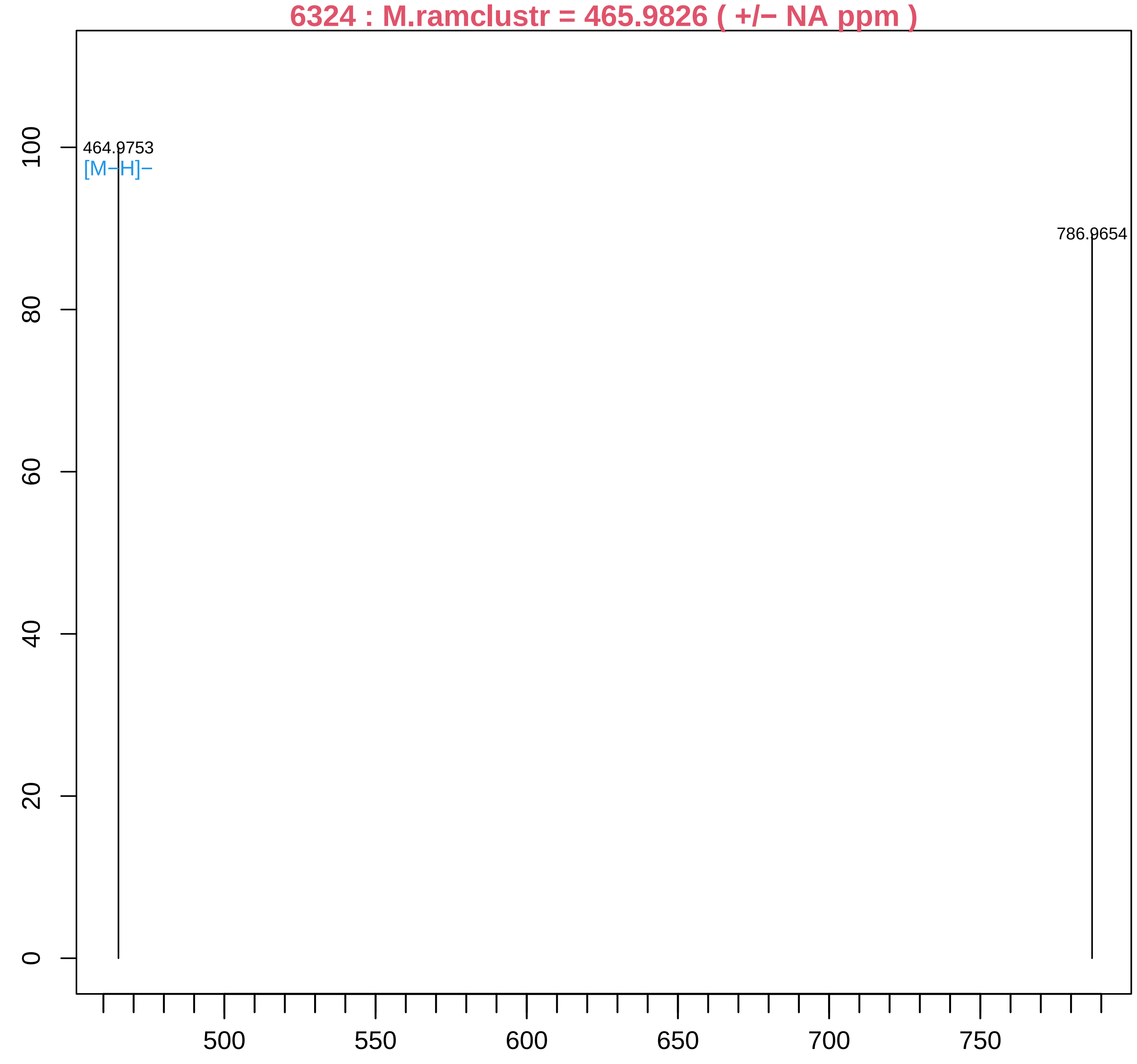
6324 : M.ramclustr = 465.9826 (+/− NA ppm)
464.9753
[M−H]−
786.9654
0
20
40
60
80
100
500
550
600
650
700
750

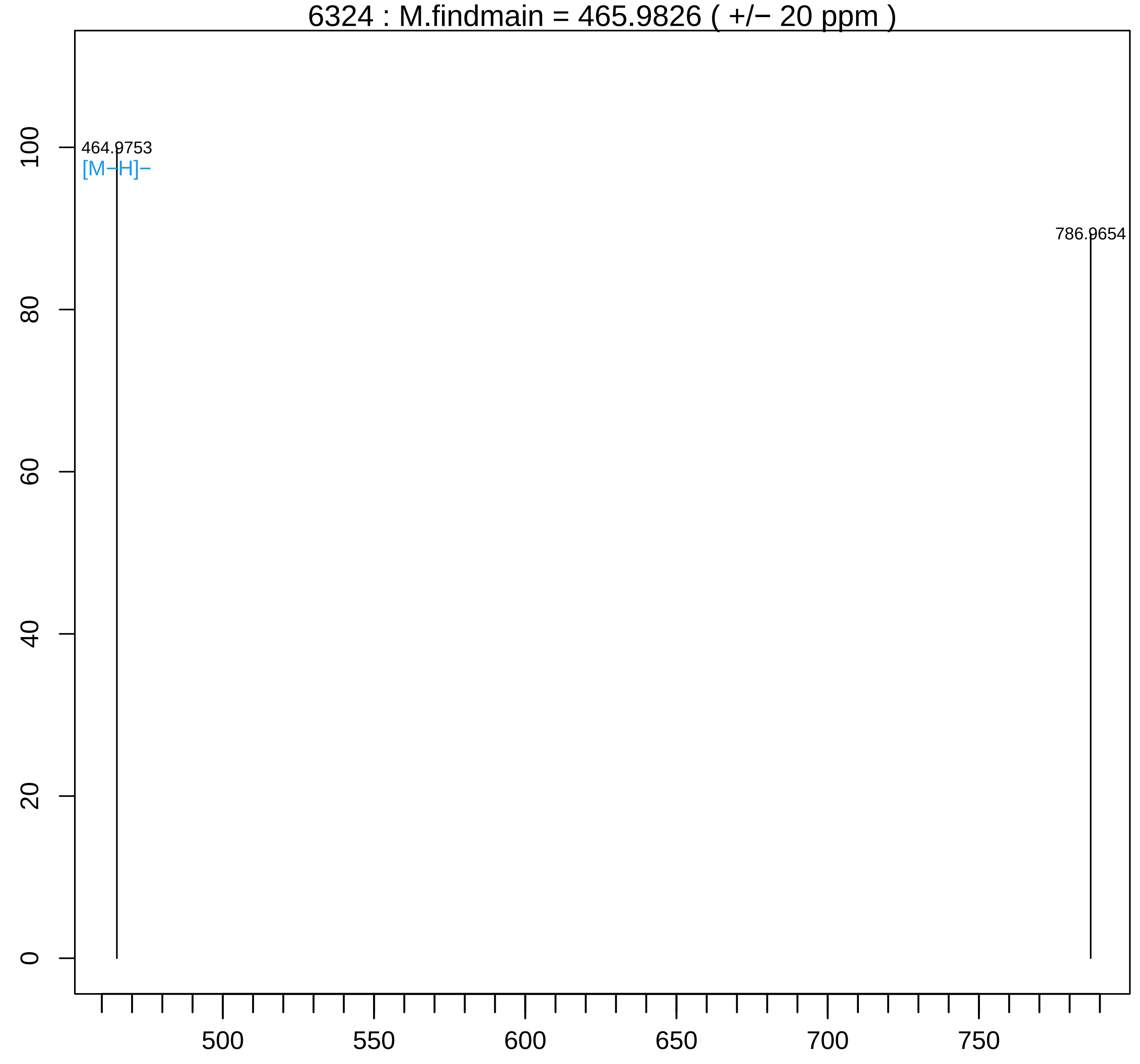
6324 : M.findmain = 465.9826 (+/− 20 ppm)
464.9753
[M−H]−
786.9654
0
20
40
60
80
100
500
550
600
650
700
750

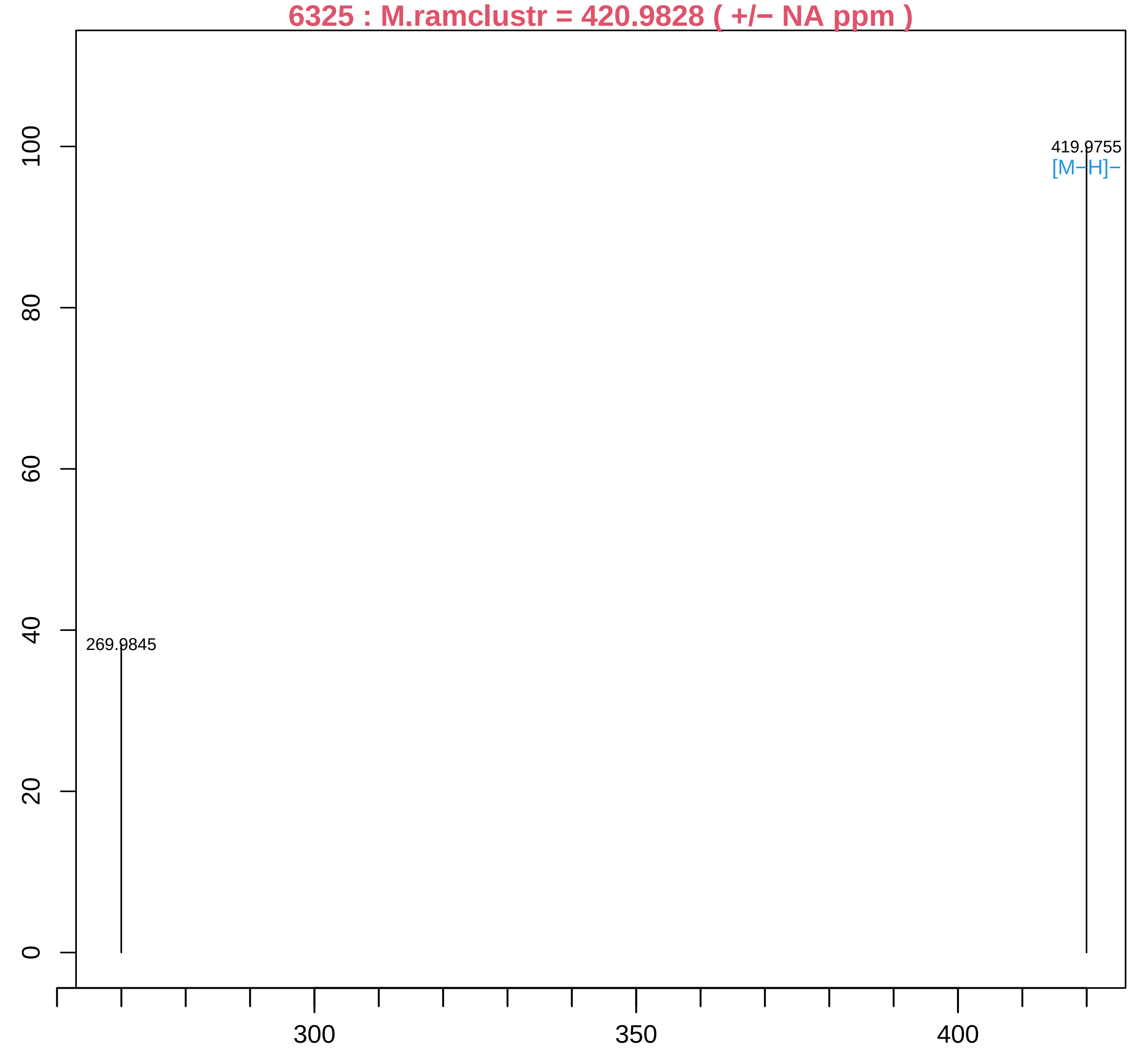
6325 : M.ramclustr = 420.9828 (+/− NA ppm)
419.9755
[M−H]−
269.9845
0
20
40
60
80
100
300
350
400

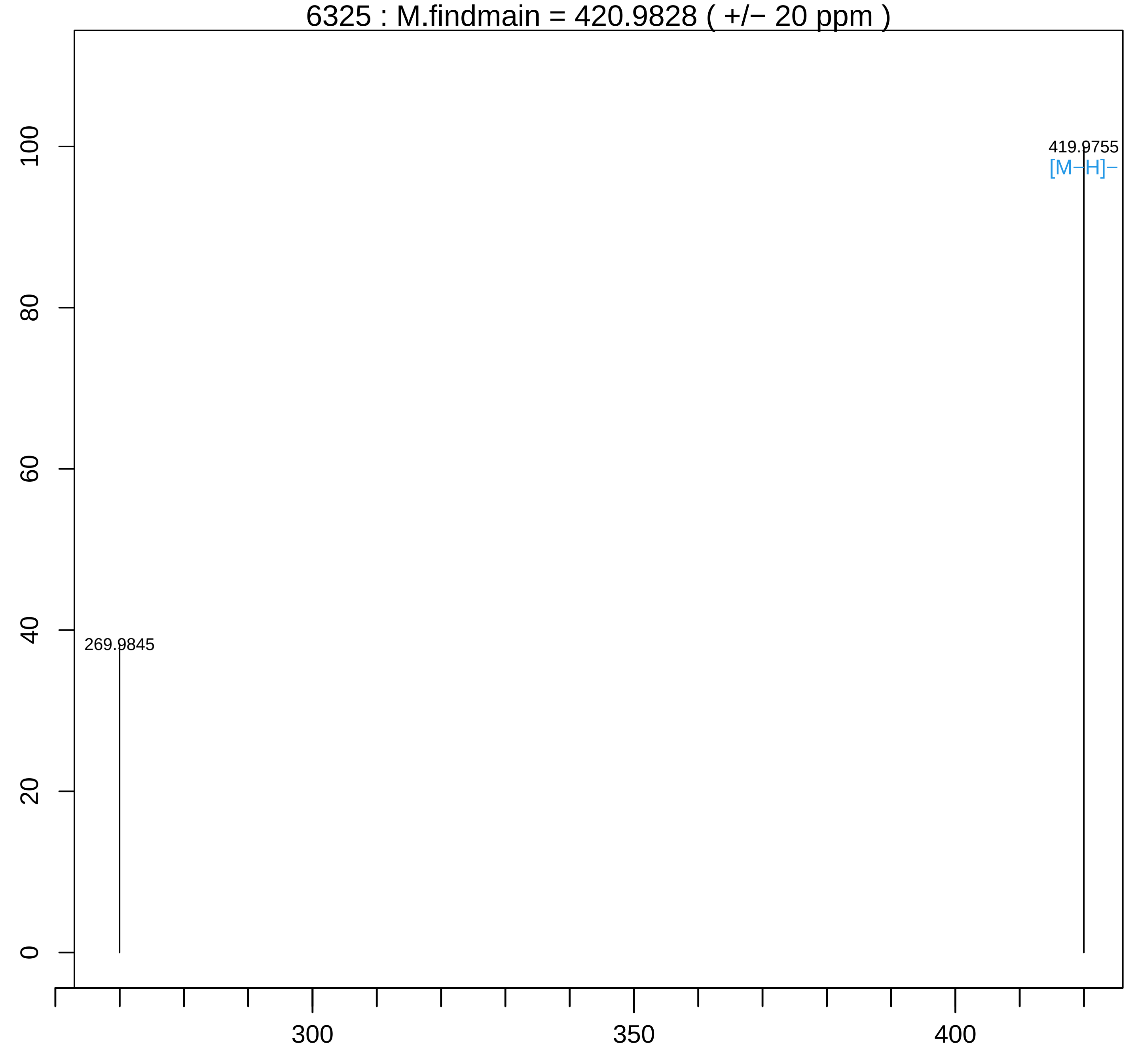
6325 : M.findmain = 420.9828 (+/− 20 ppm)
419.9755
[M−H]−
269.9845
0
20
40
60
80
100
300
350
400

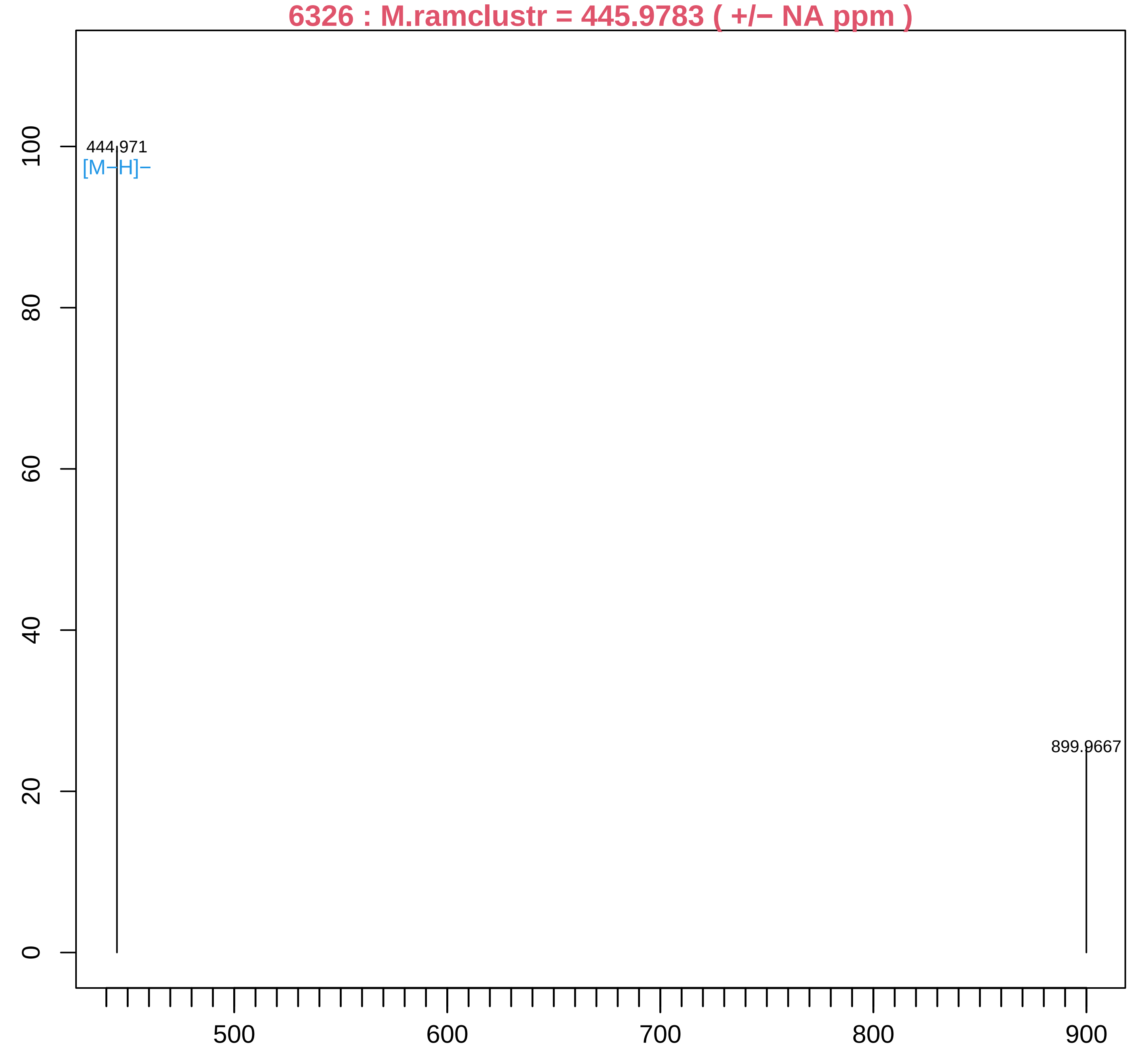
6326 : M.ramclustr = 445.9783 (+/− NA ppm)
444.971
[M−H]−
899.9667
0
20
40
60
80
100
500
600
700
800
900

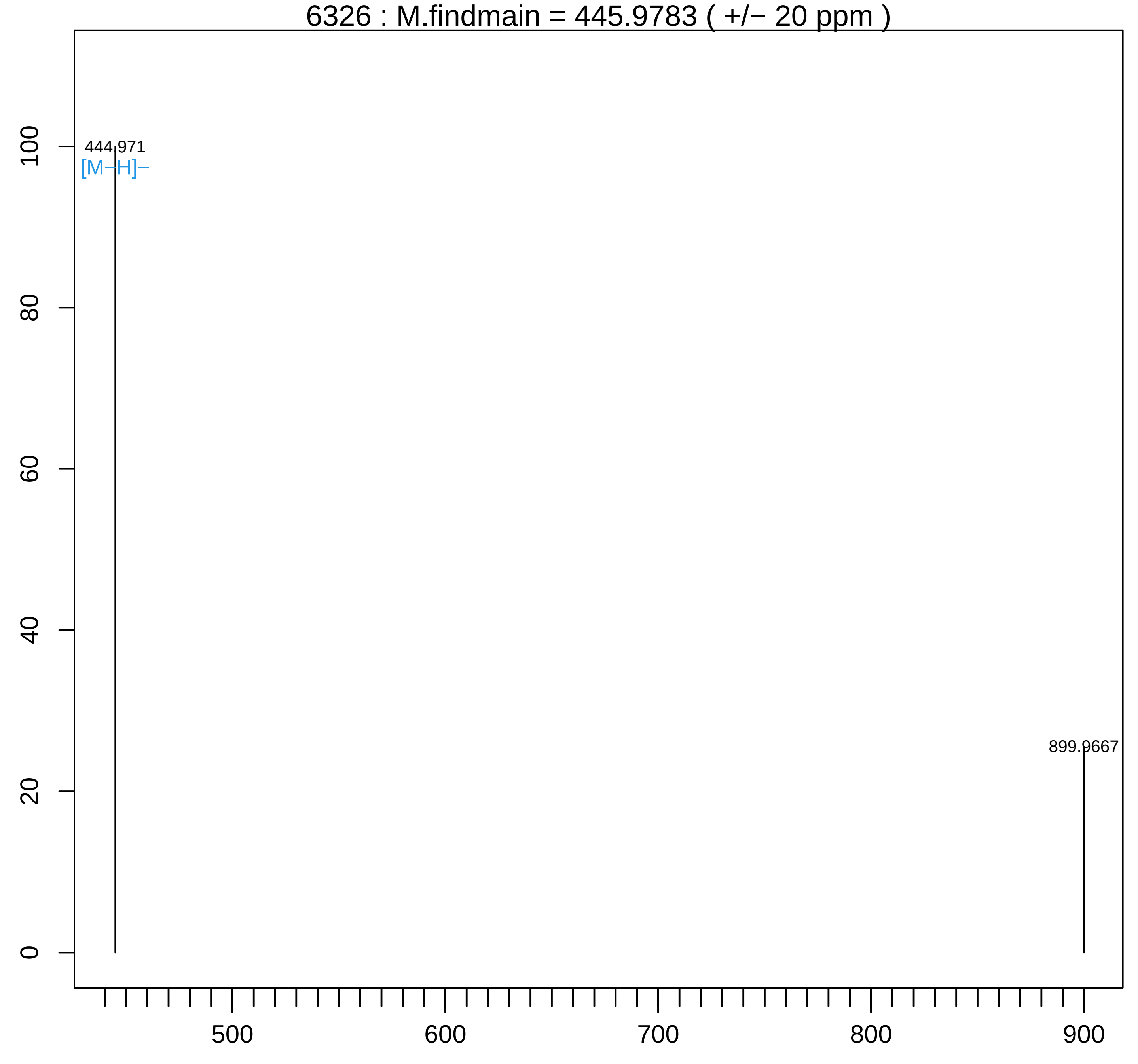
6326 : M.findmain = 445.9783 (+/− 20 ppm)
444.971
[M−H]−
899.9667
0
20
40
60
80
100
500
600
700
800
900

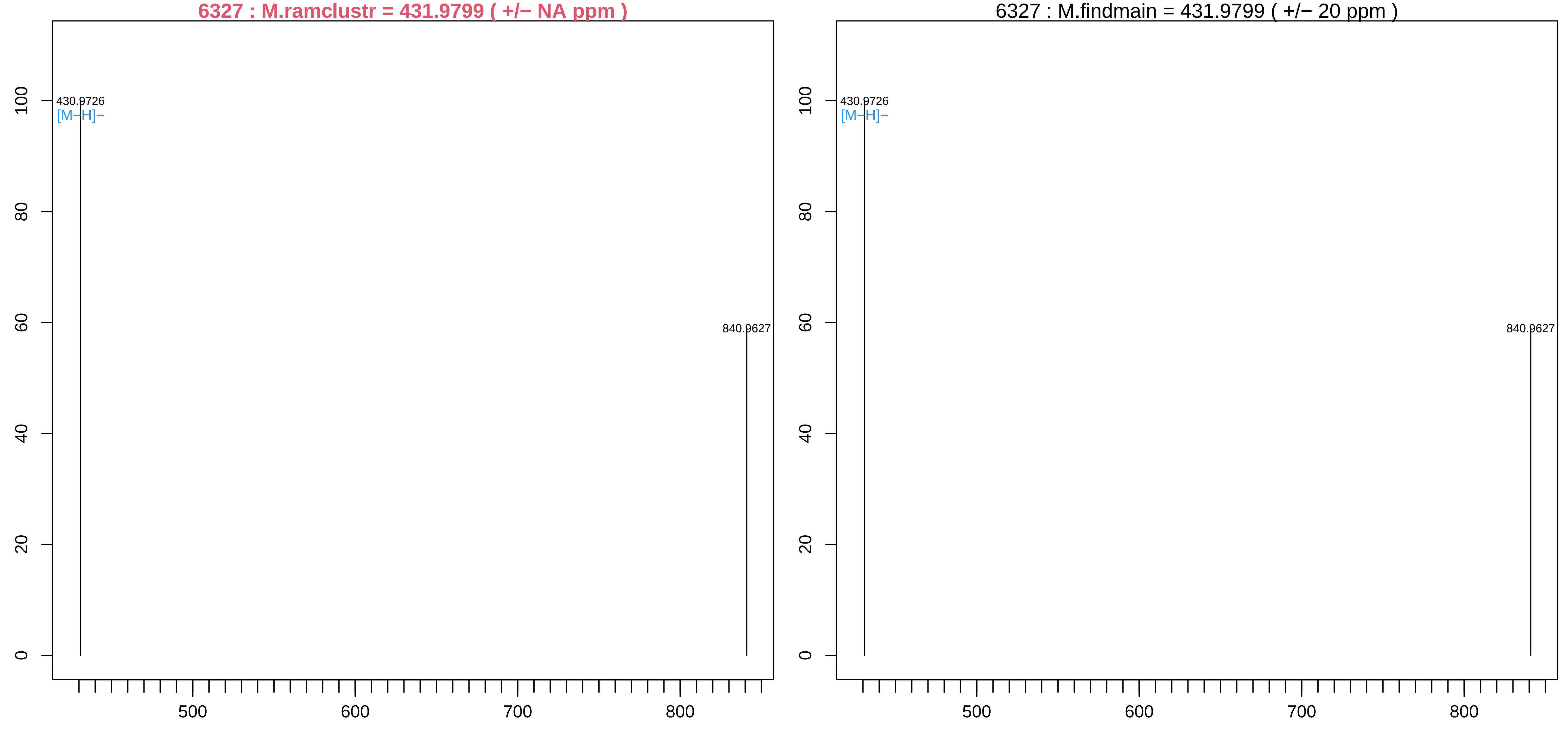
6327 : M.ramclustr = 431.9799 (+/− NA ppm)
430.9726
[M−H]−
840.9627
100
80
60
40
20
0
500
600
700
800
6327 : M.findmain = 431.9799 (+/− 20 ppm)
430.9726
[M−H]−
840.9627
100
80
60
40
20
0
500
600
700
800

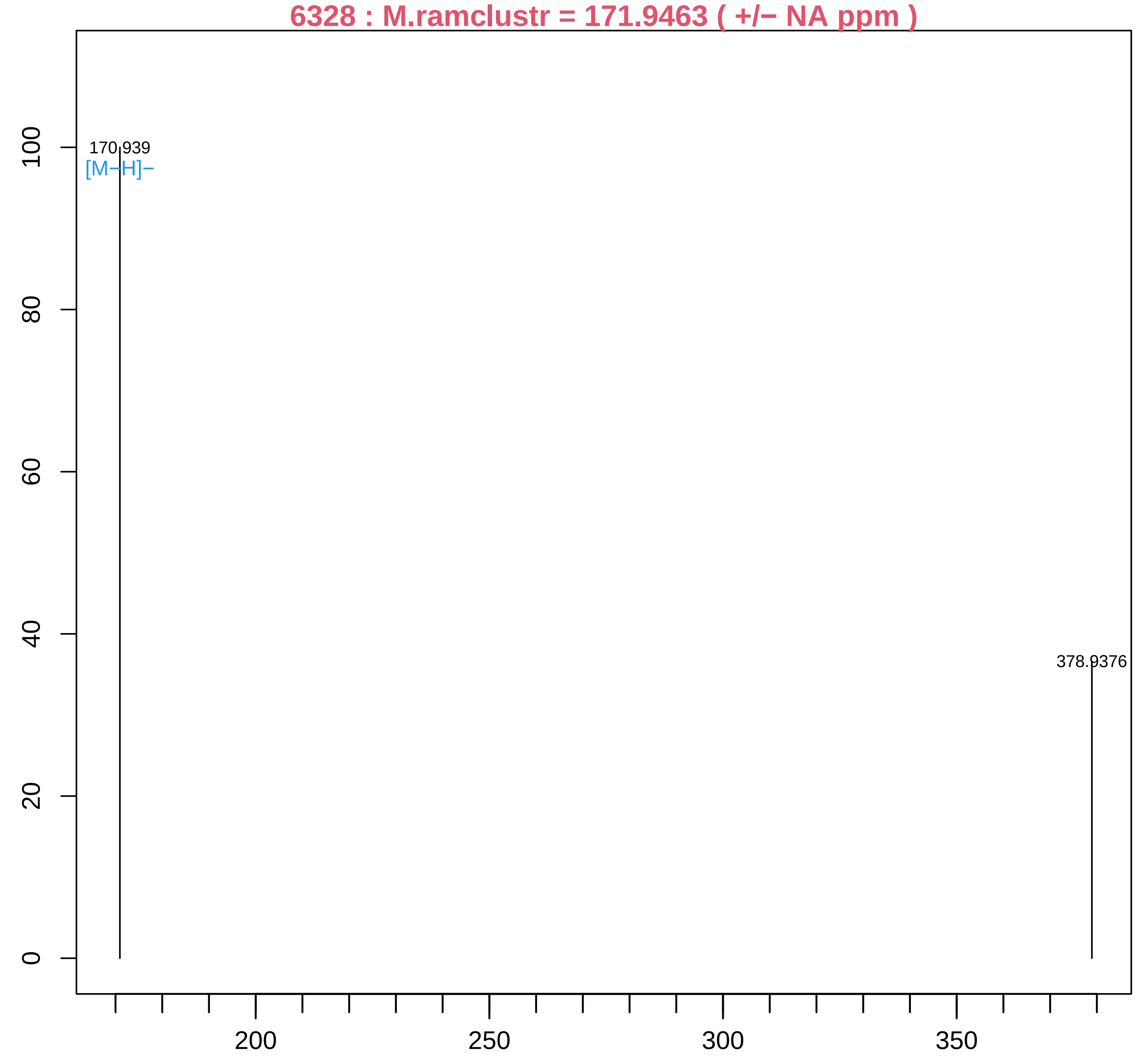
6328 : M.ramclustr = 171.9463 (+/− NA ppm)
170.939
[M−H]−
378.9376
0
20
40
60
80
100
200
250
300
350

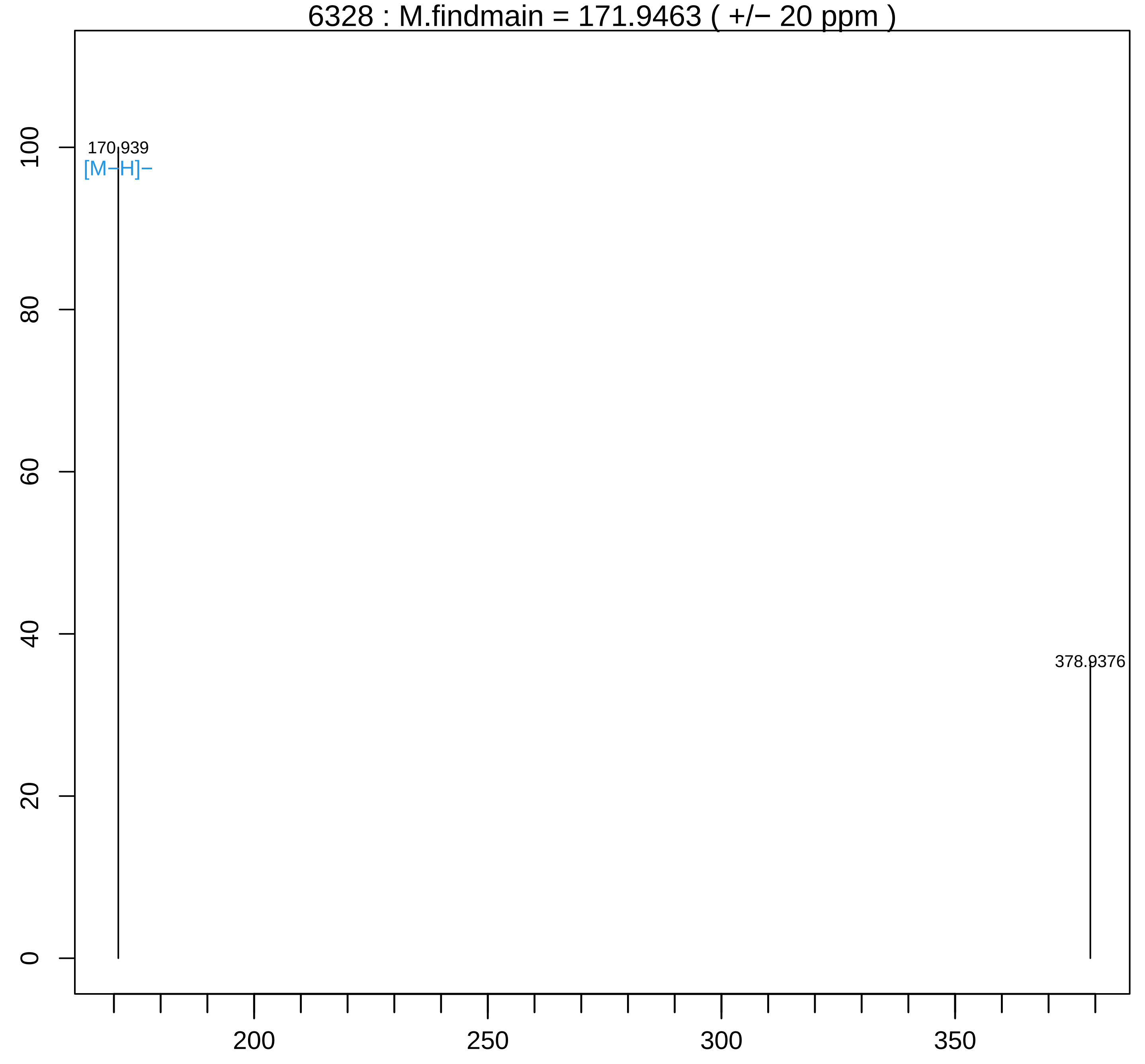
6328 : M.findmain = 171.9463 (+/− 20 ppm)
170.939
[M−H]−
378.9376
0
20
40
60
80
100
200
250
300
350

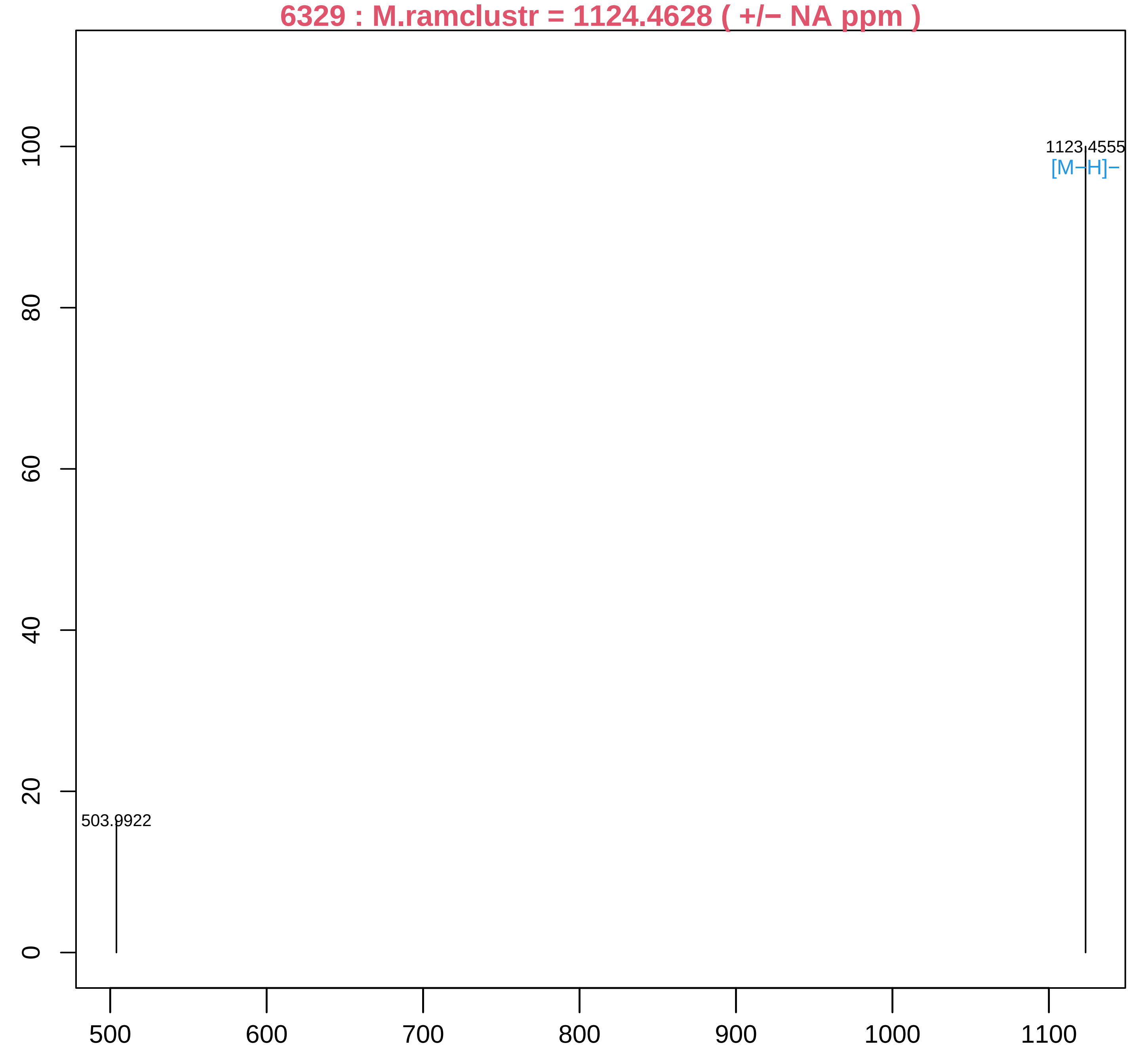

6329 : M.ramclustr = 1124.4628 (+/− NA ppm)
1123.4555
[M−H]−
503.9922
0
20
40
60
80
100
500
600
700
800
900
1000
1100

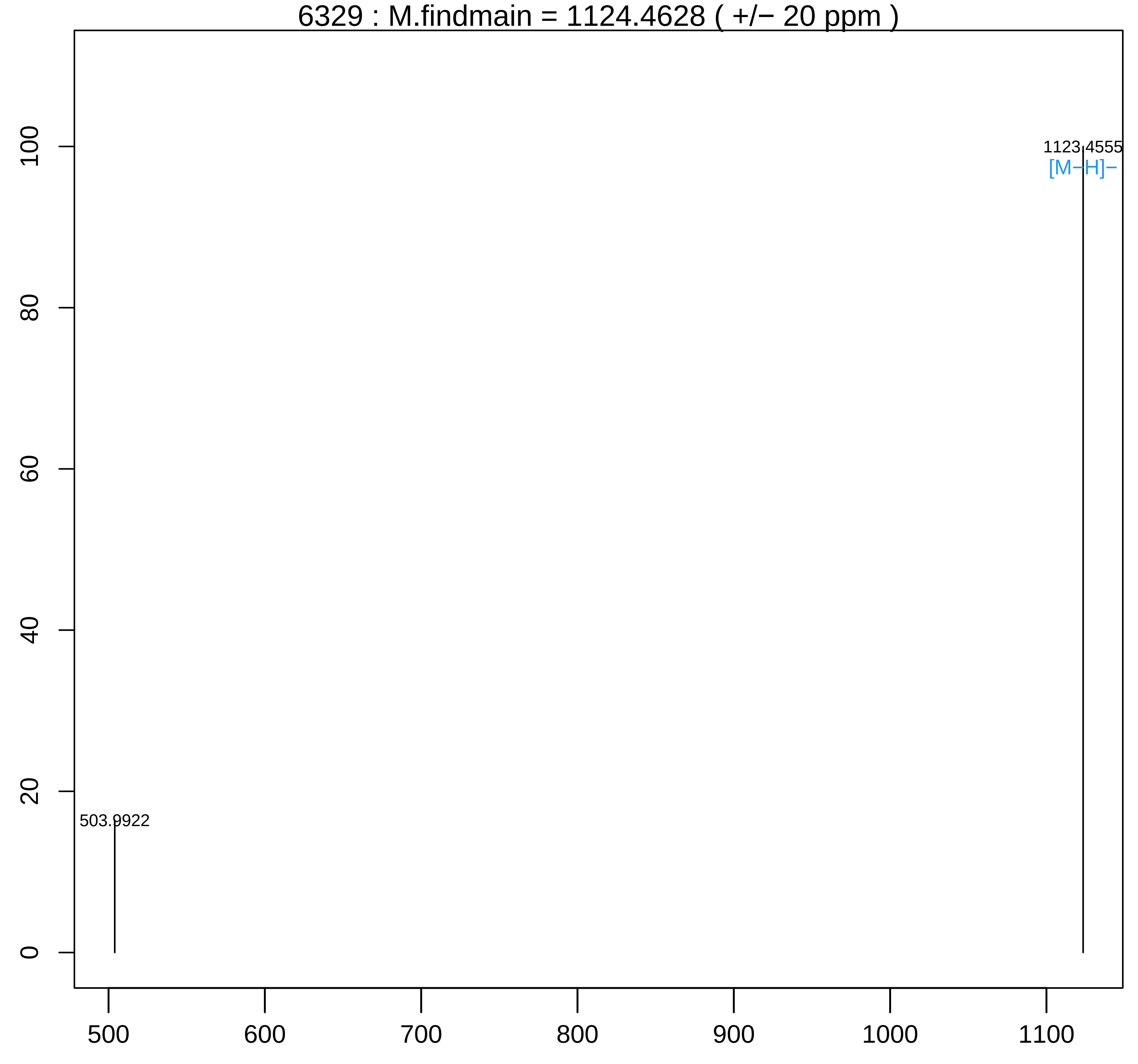

6329 : M.findmain = 1124.4628 (+/− 20 ppm)
1123.4555
[M−H]−
503.9922
0
20
40
60
80
100
500
600
700
800
900
1000
1100

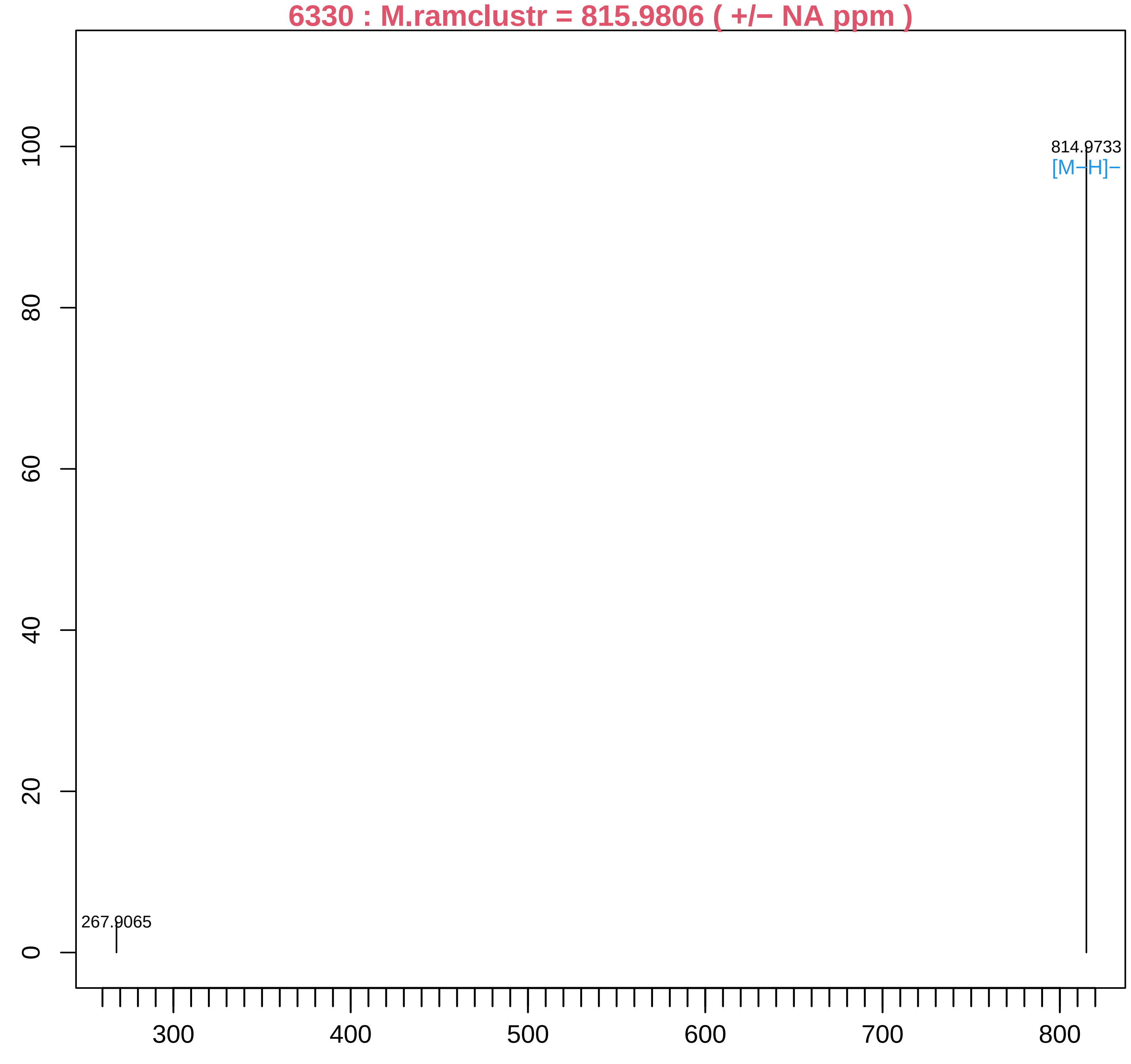

6330 : M.ramclustr = 815.9806 (+/− NA ppm)
814.9733
[M−H]−
267.9065
0
20
40
60
80
100
300
400
500
600
700
800

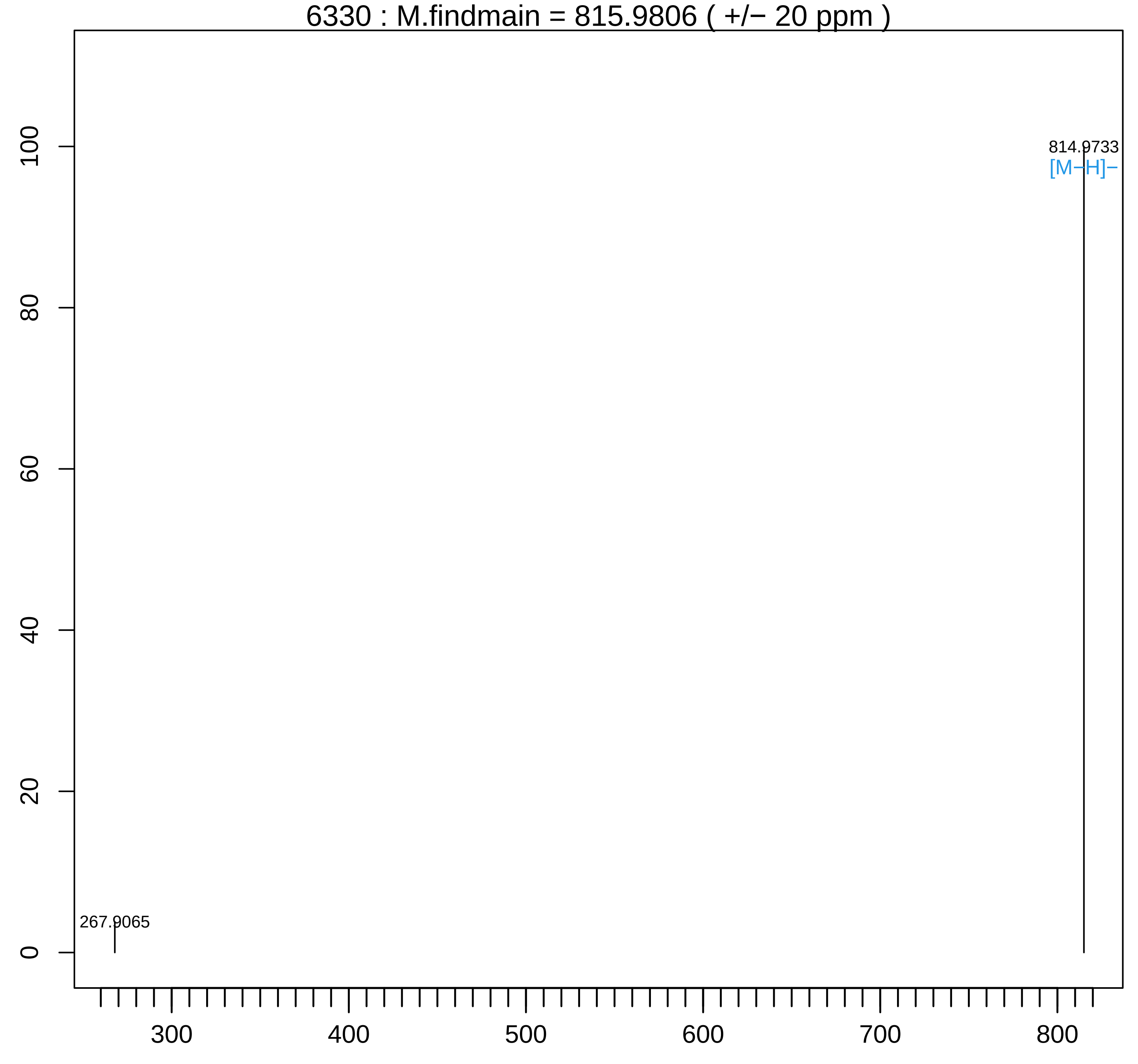

6330 : M.findmain = 815.9806 (+/− 20 ppm)
814.9733
[M−H]−
267.9065
0
20
40
60
80
100
300
400
500
600
700
800

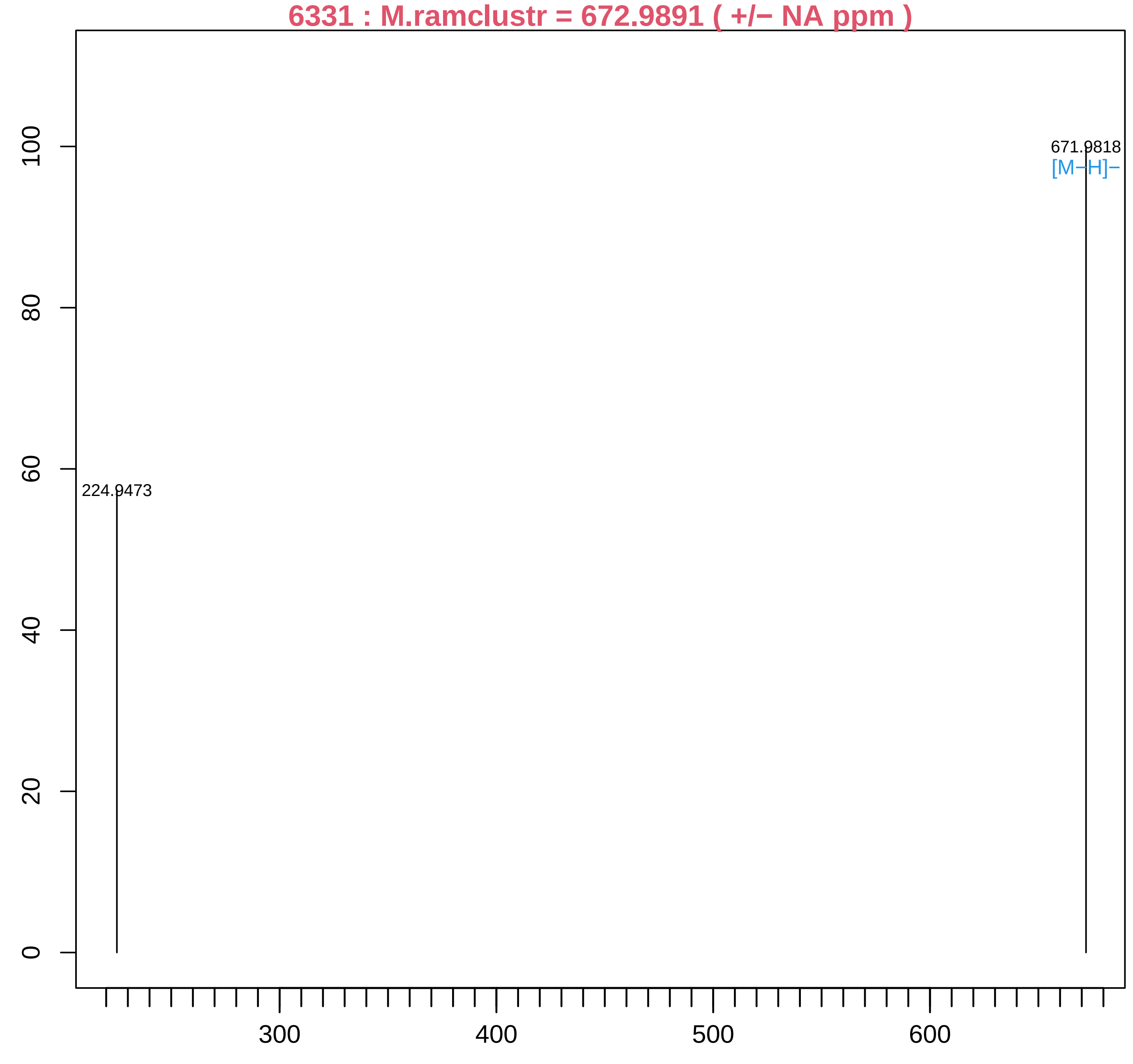
6331 : M.ramclustr = 672.9891 (+/− NA ppm)
671.9818
[M−H]−
224.9473
0
20
40
60
80
100
300
400
500
600

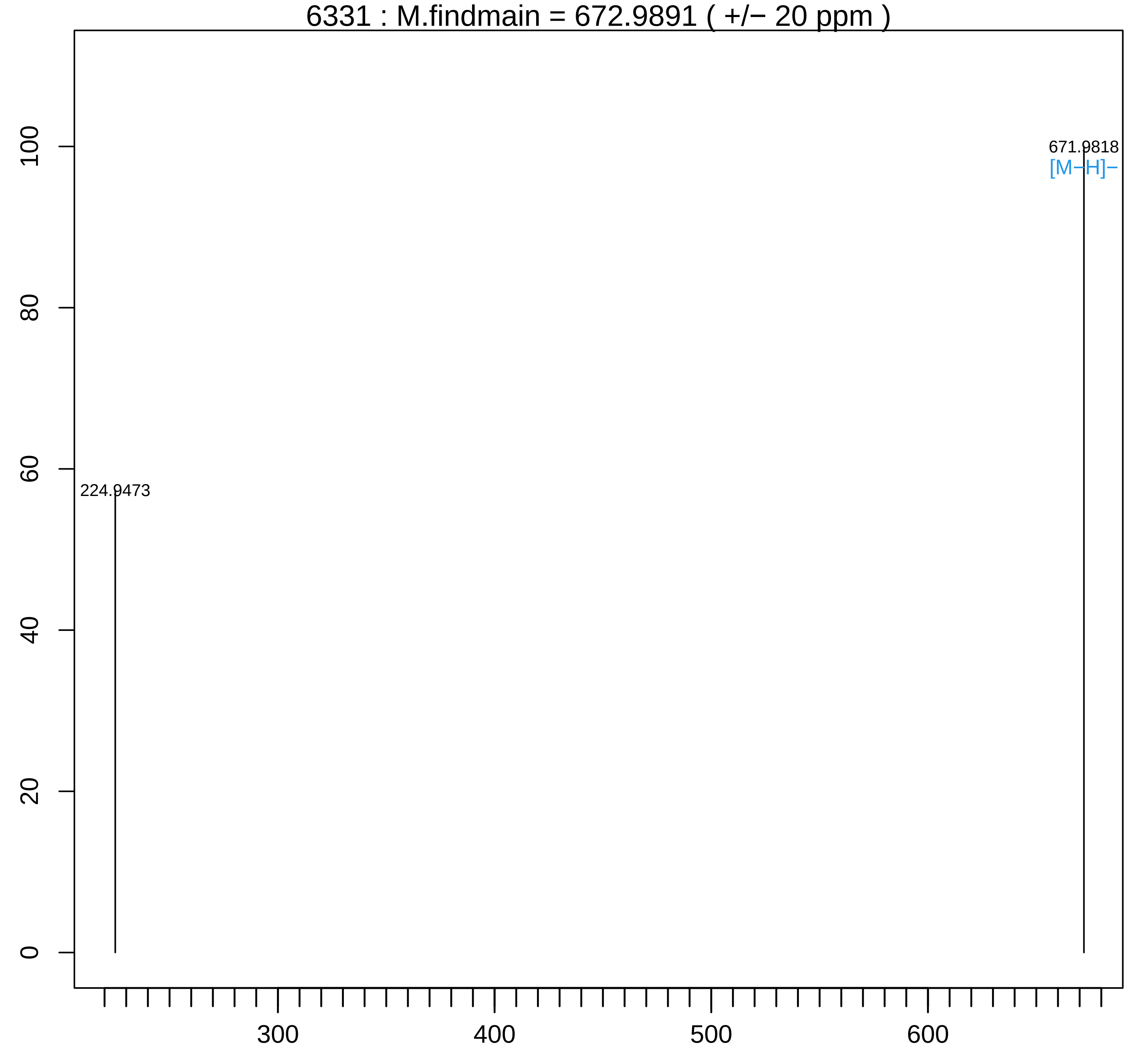
6331 : M.findmain = 672.9891 (+/− 20 ppm)
671.9818
[M−H]−
224.9473
0
20
40
60
80
100
300
400
500
600

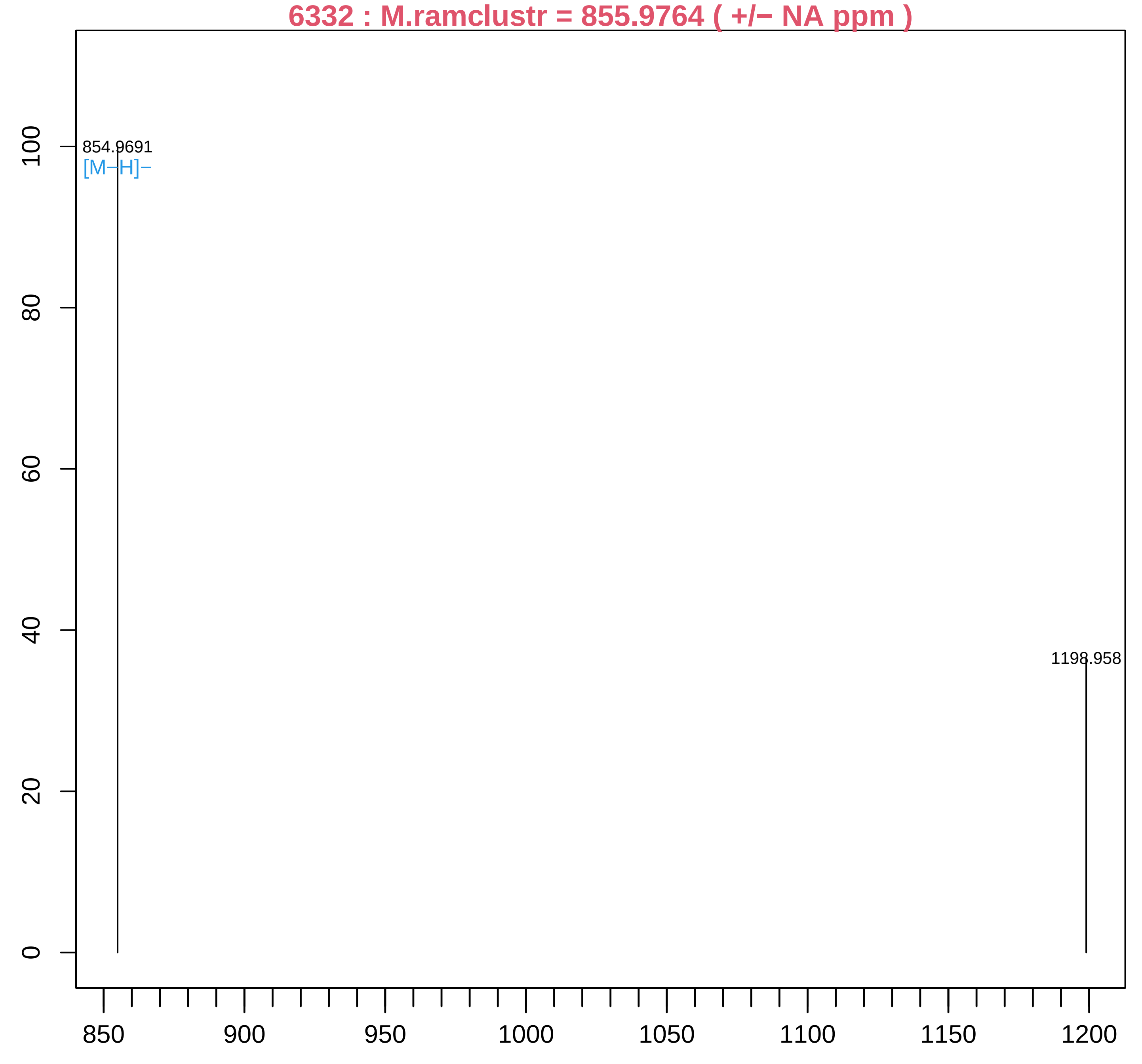
6332 : M.ramclustr = 855.9764 (+/− NA ppm)
854.9691
[M−H]−
1198.958
0
20
40
60
80
100
850
900
950
1000
1050
1100
1150
1200

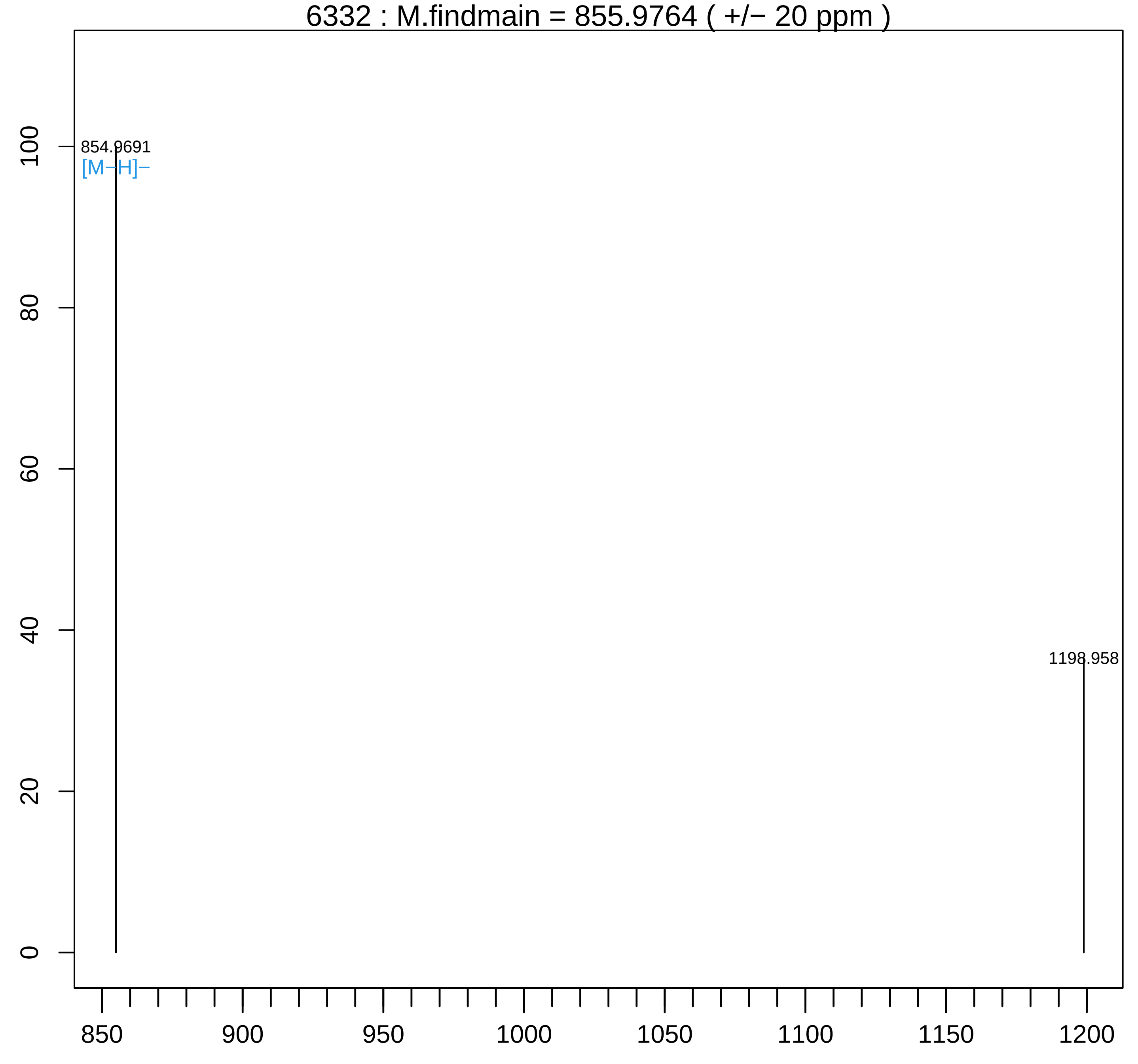
6332 : M.findmain = 855.9764 (+/− 20 ppm)
854.9691
[M−H]−
1198.958
0
20
40
60
80
100
850
900
950
1000
1050
1100
1150
1200

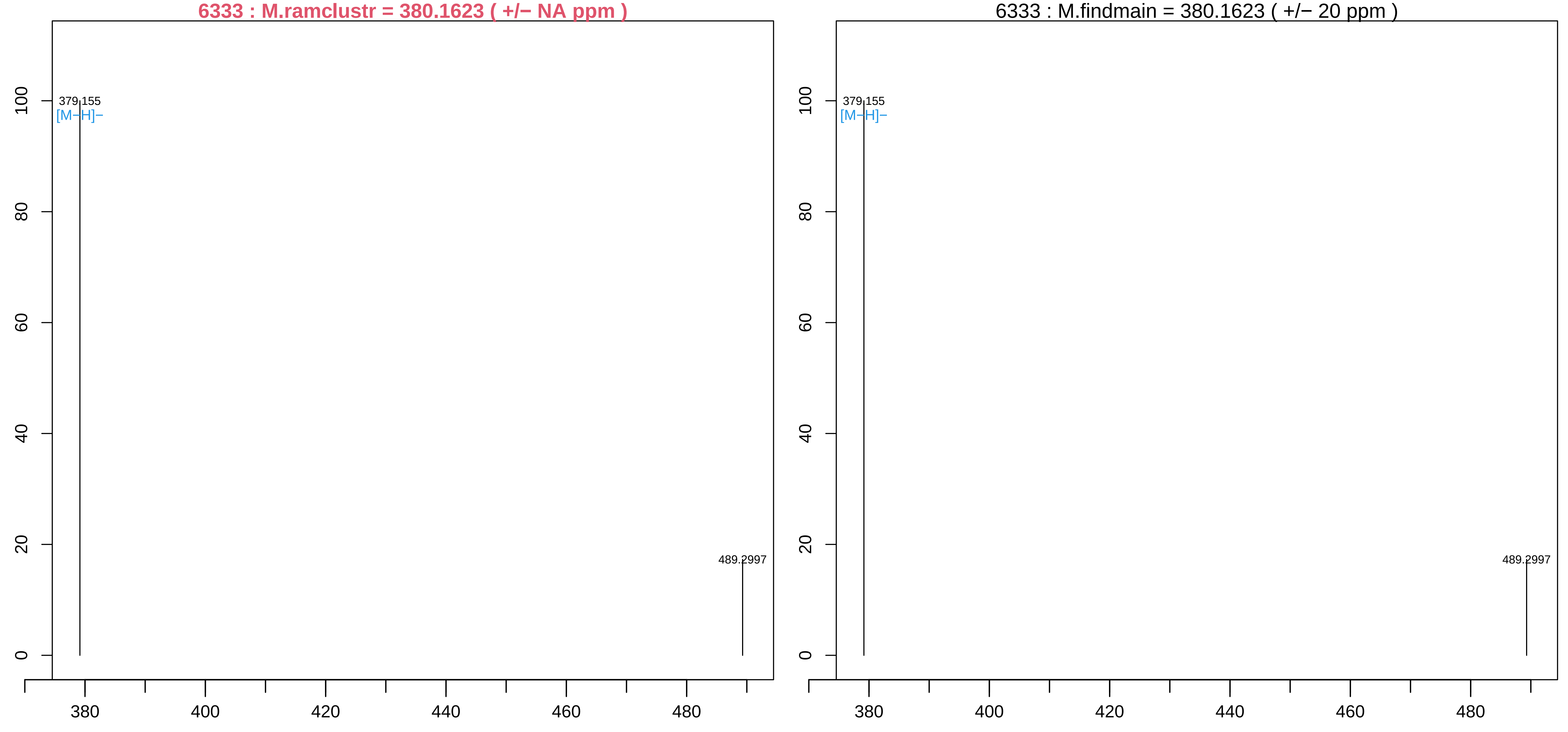
6333 : M.ramclustr = 380.1623 (+/− NA ppm)
6333 : M.findmain = 380.1623 (+/− 20 ppm)
379.155
[M−H]−
489.2997
0
20
40
60
80
100
380
400
420
440
460
480

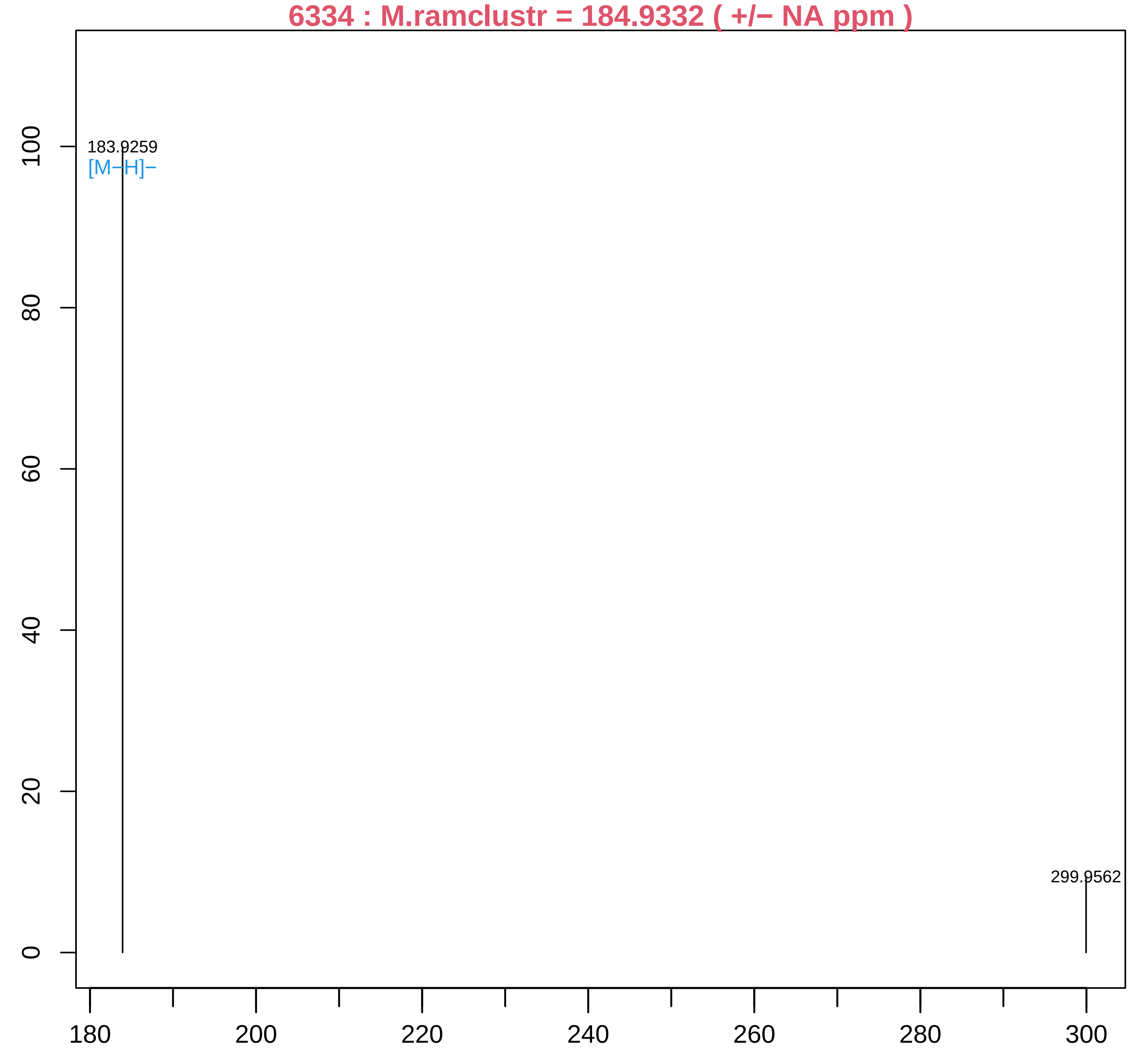
6334 : M.ramclustr = 184.9332 (+/− NA ppm)
183.9259
[M−H]−
299.9562
0
20
40
60
80
100
180
200
220
240
260
280
300

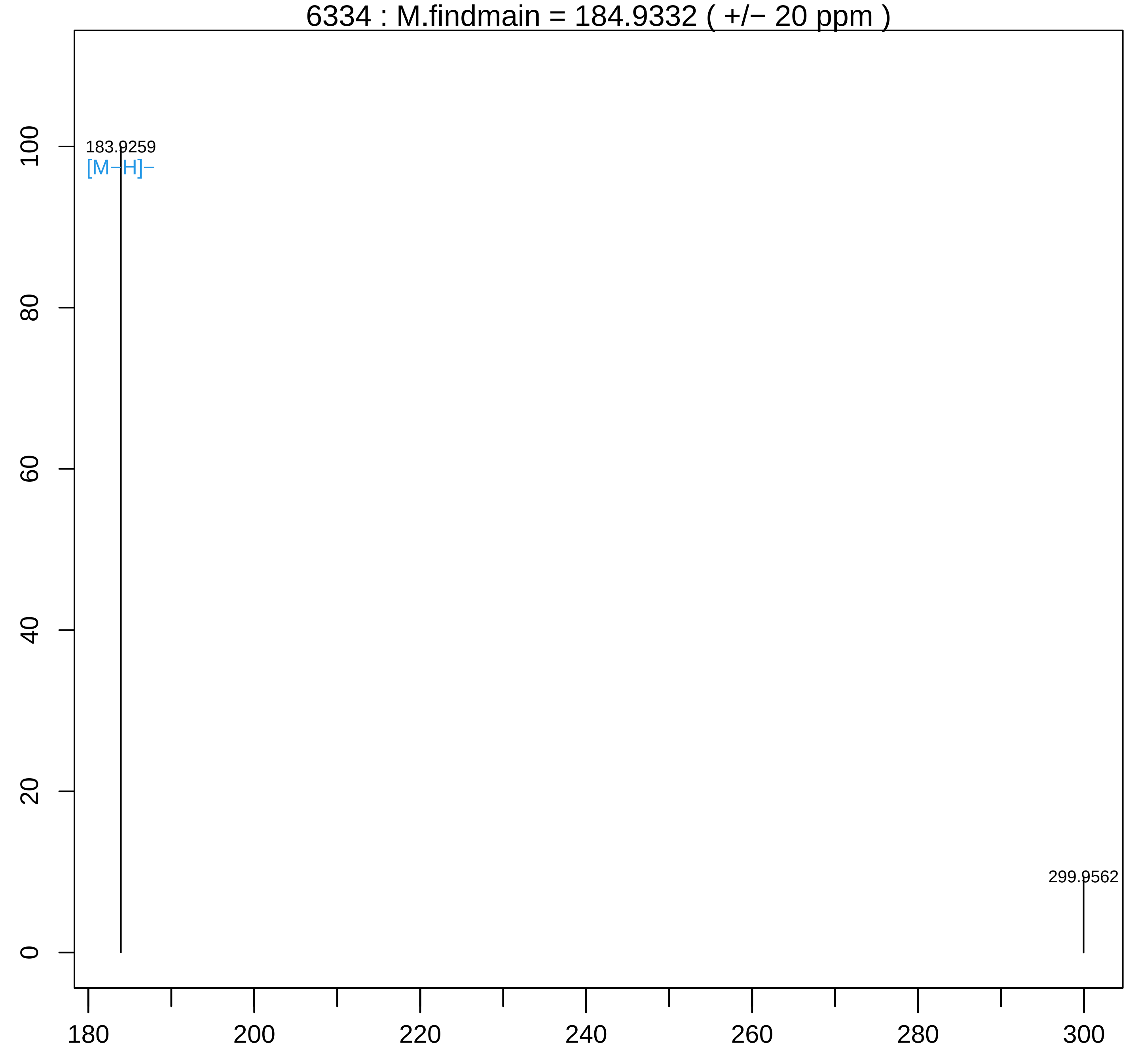
6334 : M.findmain = 184.9332 (+/− 20 ppm)
183.9259
[M−H]−
299.9562
0
20
40
60
80
100
180
200
220
240
260
280
300

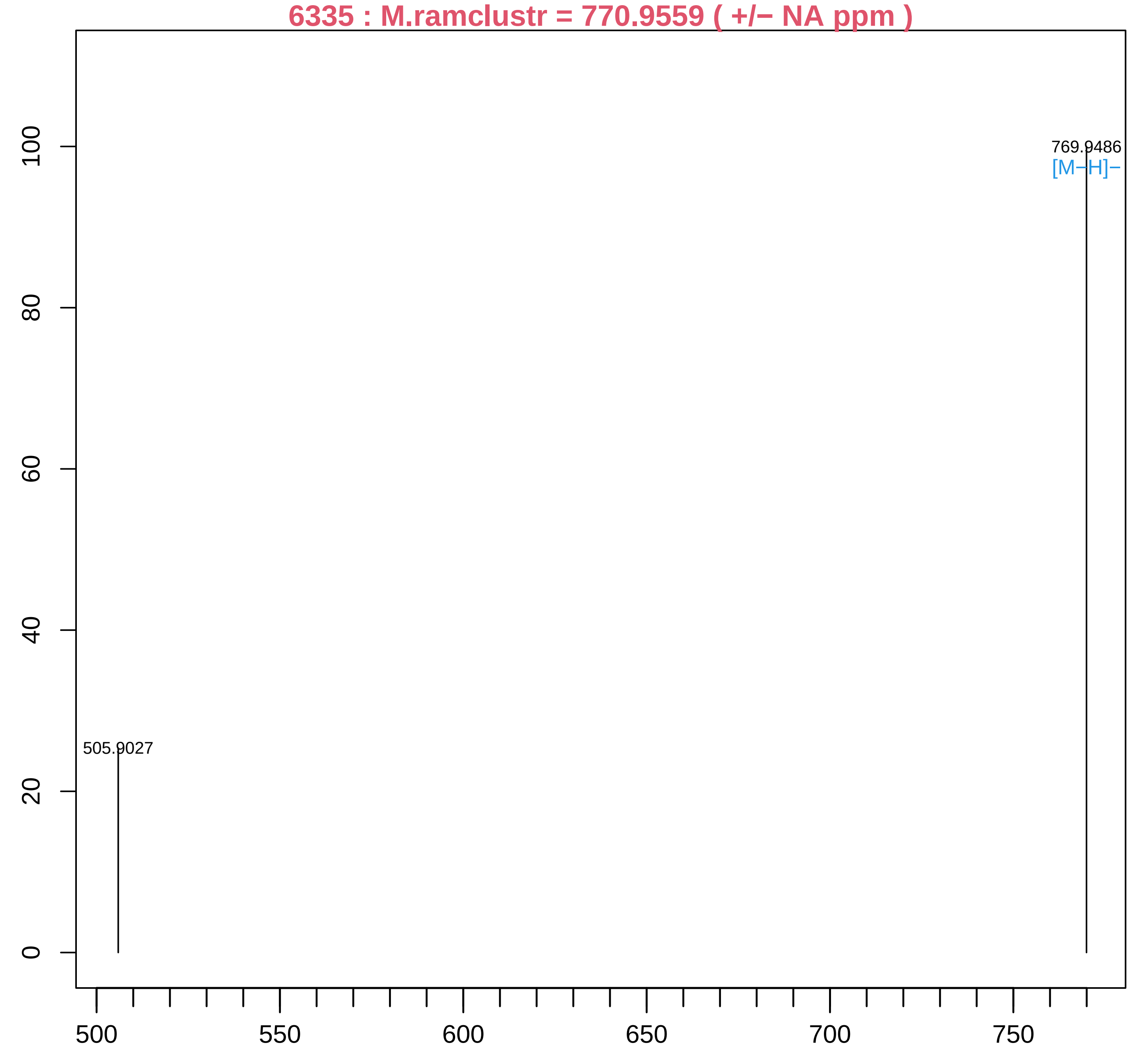
6335 : M.ramclustr = 770.9559 (+/− NA ppm)
769.9486
[M−H]−
505.9027
0
20
40
60
80
100
500
550
600
650
700
750

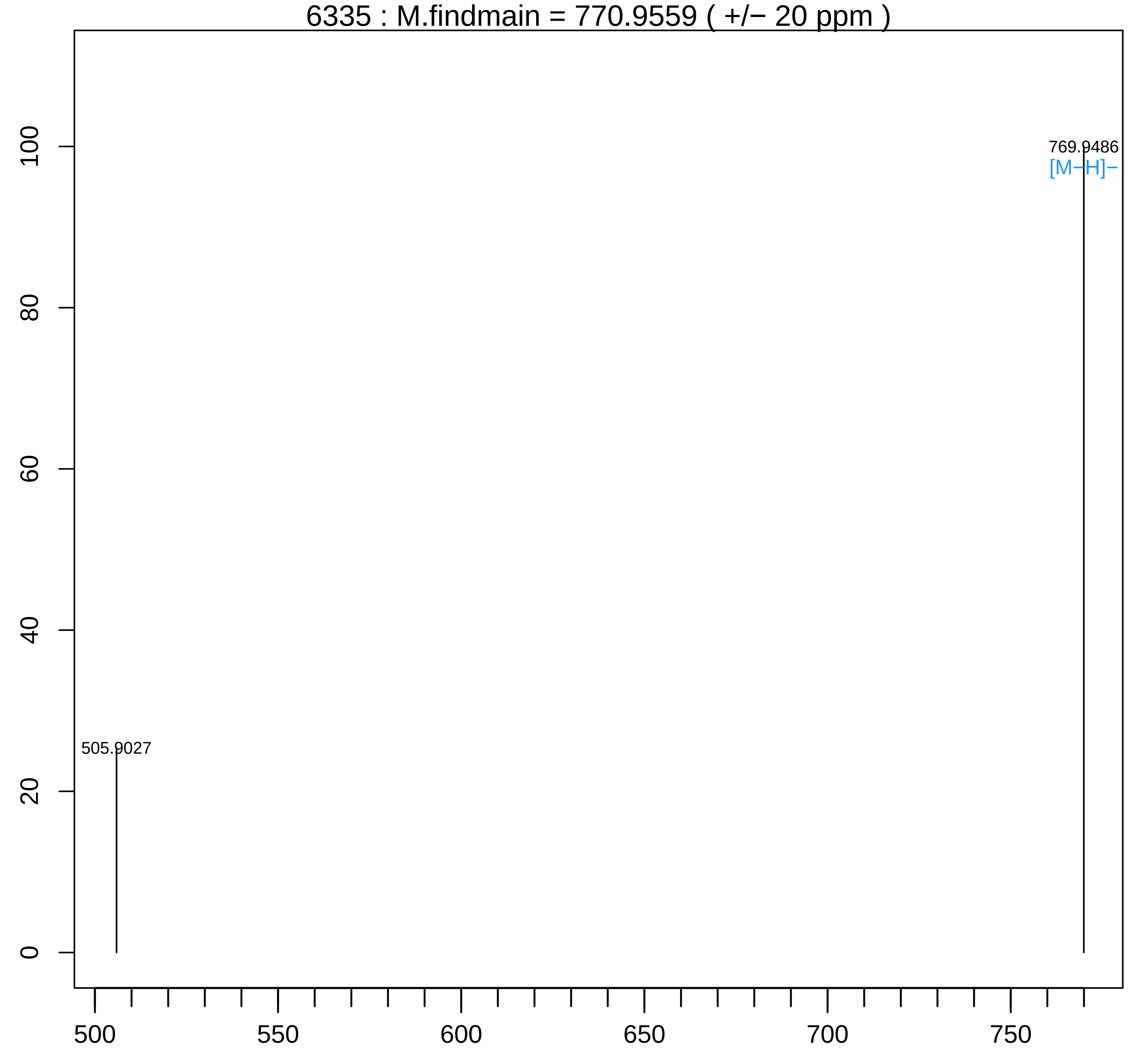
6335 : M.findmain = 770.9559 (+/− 20 ppm)
769.9486
[M−H]−
505.9027
0
20
40
60
80
100
500
550
600
650
700
750

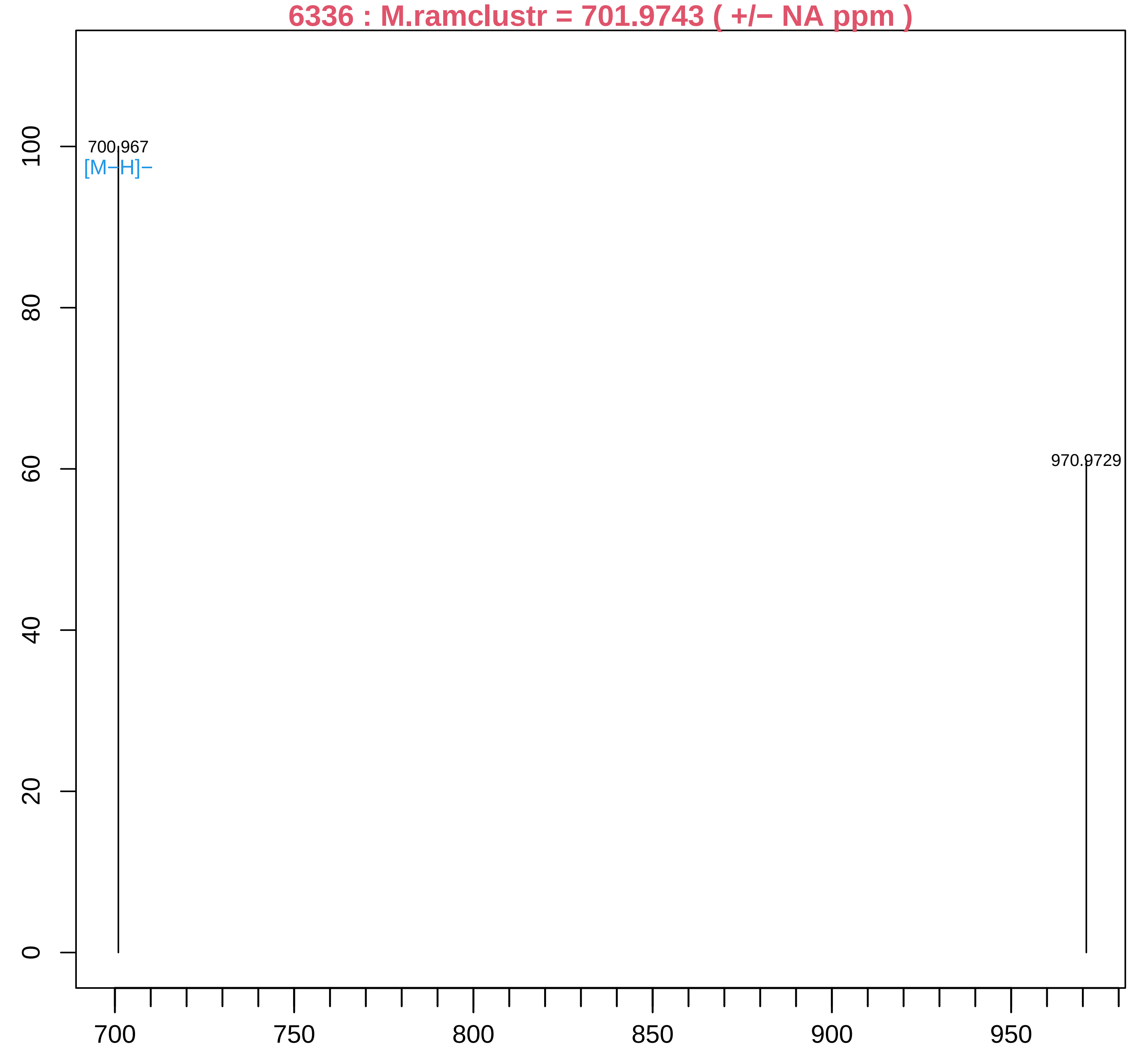
6336 : M.ramclustr = 701.9743 (+/− NA ppm)
700.967
[M−H]−
970.9729
0
20
40
60
80
100
700
750
800
850
900
950

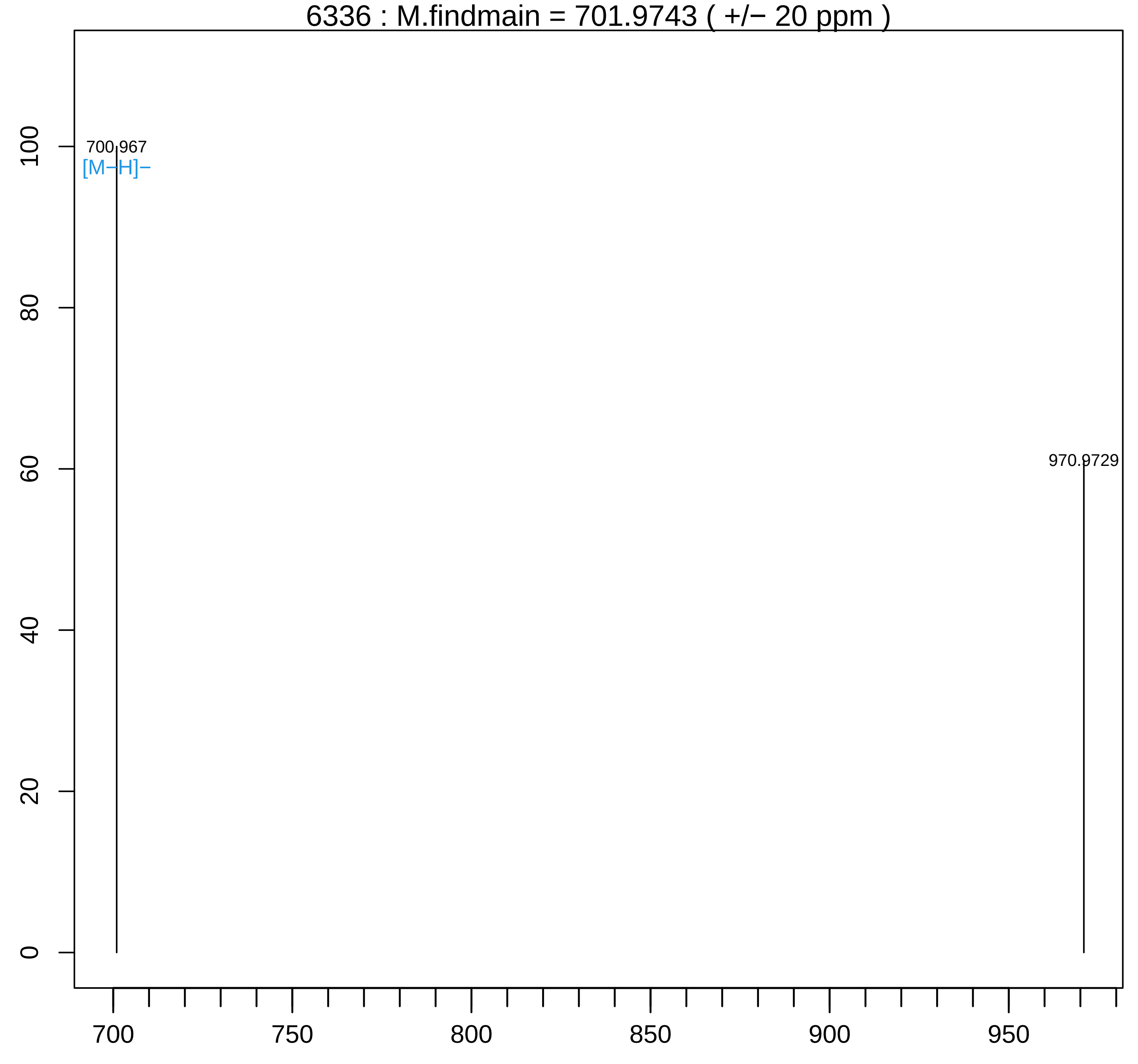
6336 : M.findmain = 701.9743 (+/− 20 ppm)
700.967
[M−H]−
970.9729
0
20
40
60
80
100
700
750
800
850
900
950

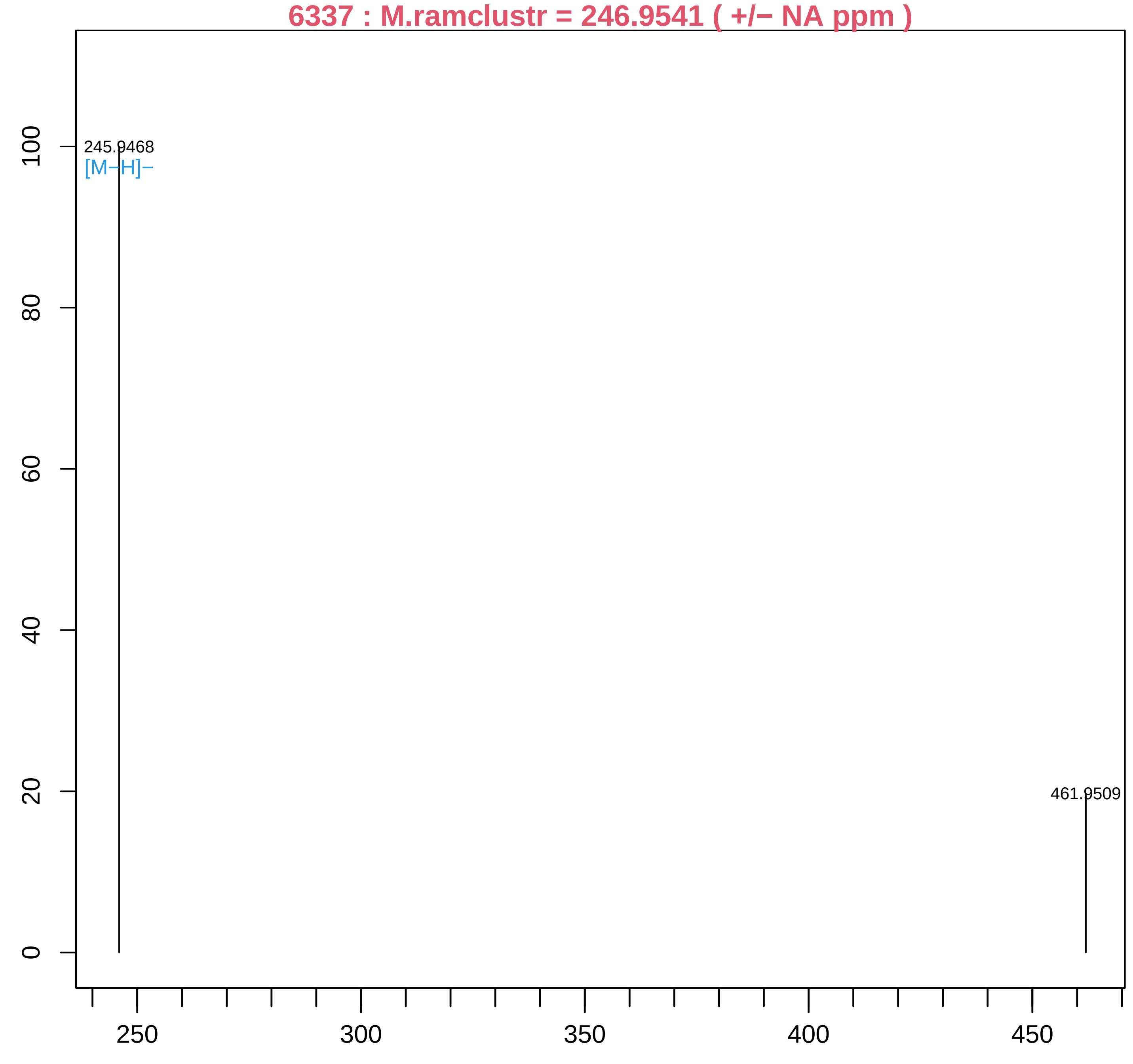
6337 : M.ramclustr = 246.9541 (+/− NA ppm)
245.9468
[M−H]−
461.9509
0
20
40
60
80
100
250
300
350
400
450

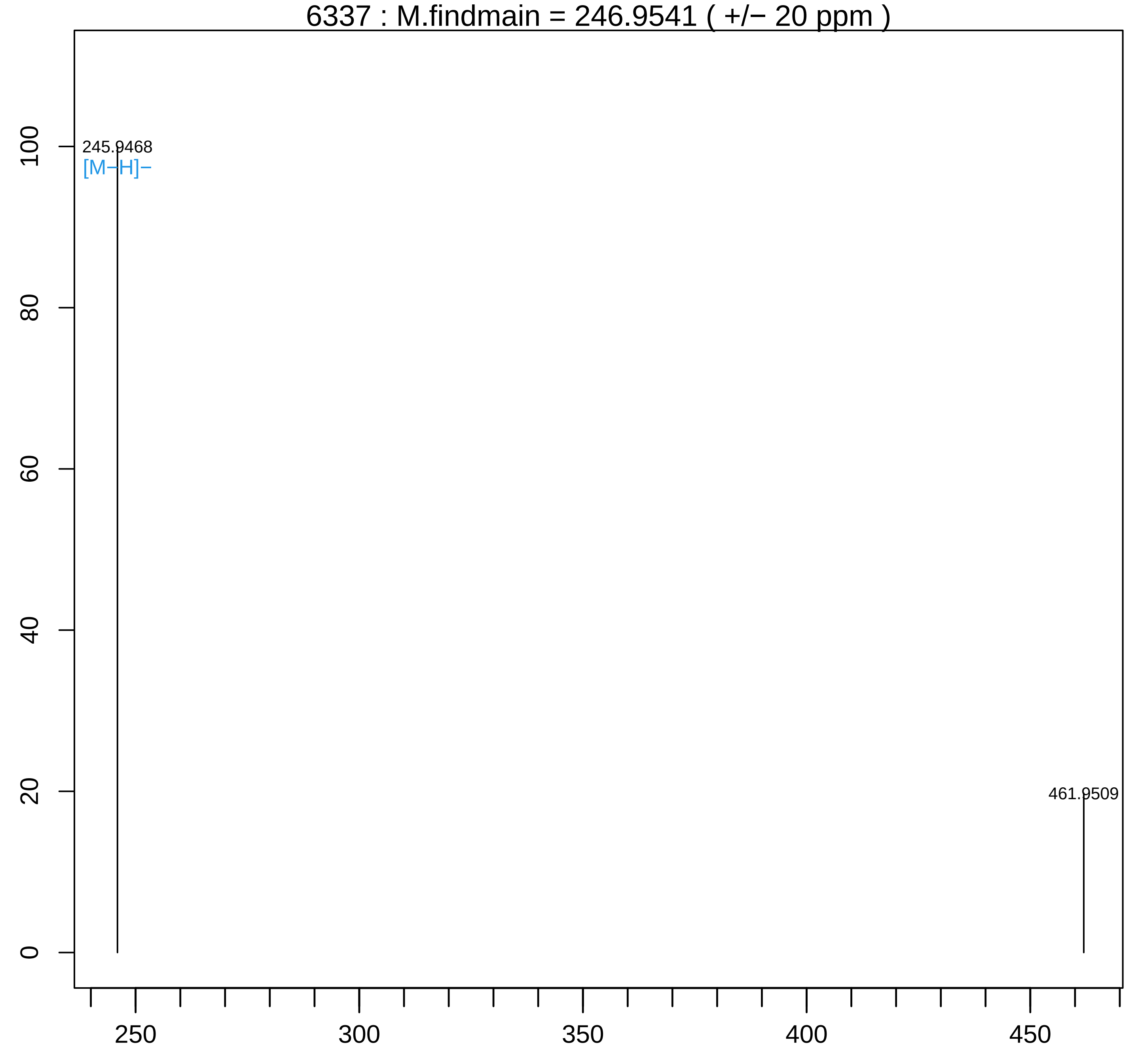
6337 : M.findmain = 246.9541 (+/− 20 ppm)
245.9468
[M−H]−
461.9509
0
20
40
60
80
100
250
300
350
400
450

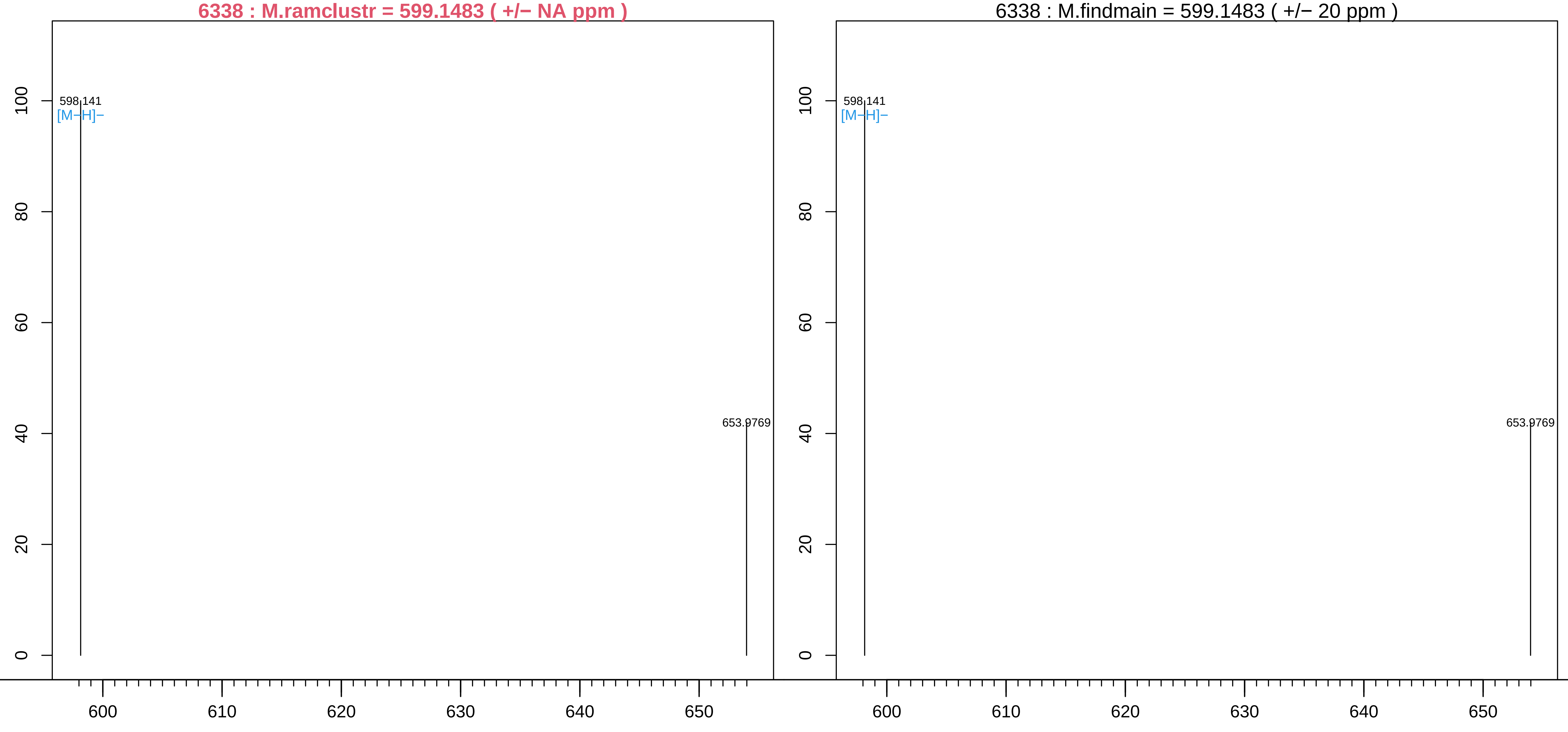
6338 : M.ramclustr = 599.1483 (+/− NA ppm)
598.141
[M−H]−
653.9769
6338 : M.findmain = 599.1483 (+/− 20 ppm)
598.141
[M−H]−
653.9769
0
20
40
60
80
100
600
610
620
630
640
650

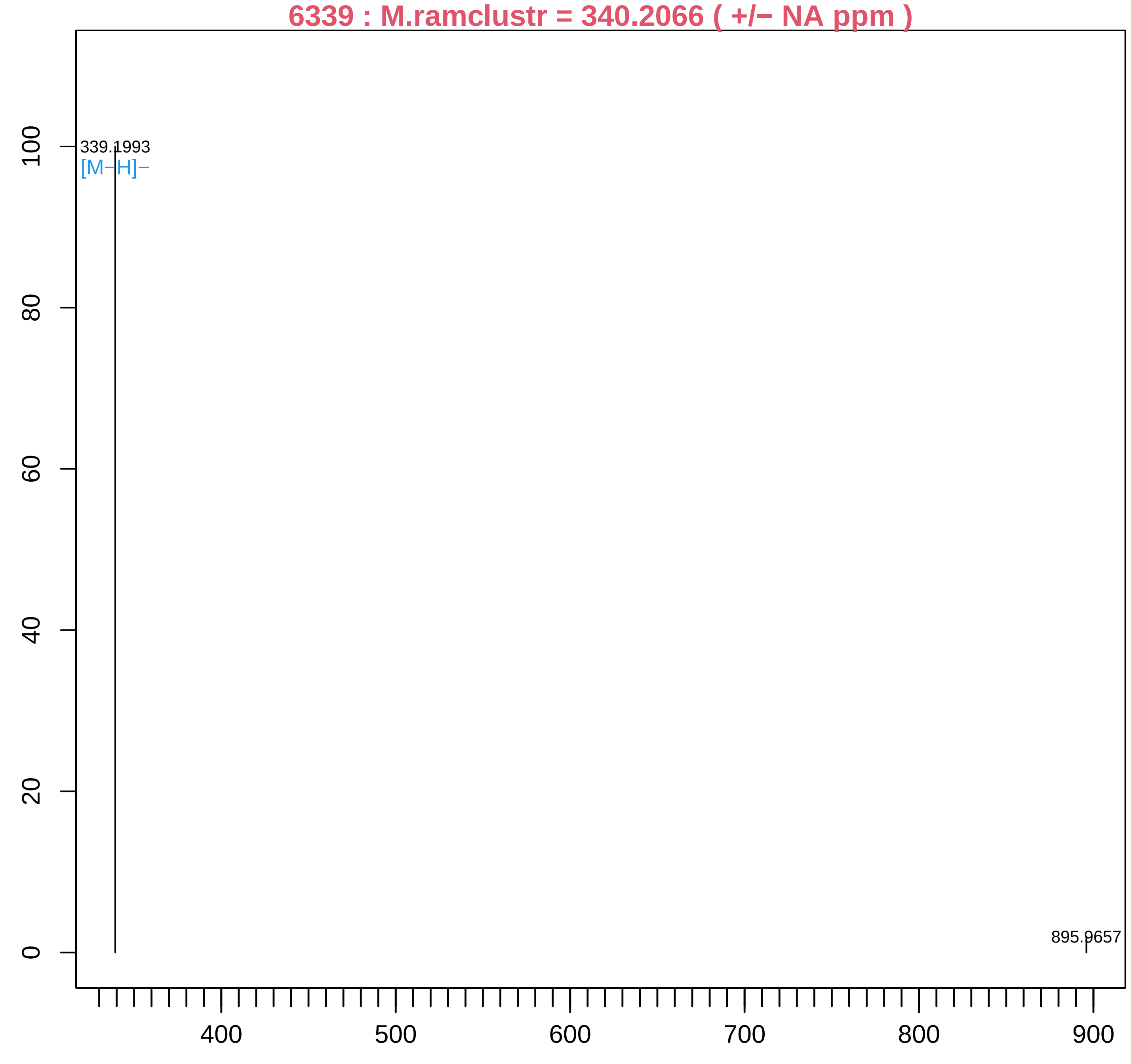
6339 : M.ramclustr = 340.2066 (+/− NA ppm)
339.1993
[M−H]−
895.9657
0
20
40
60
80
100
400
500
600
700
800
900

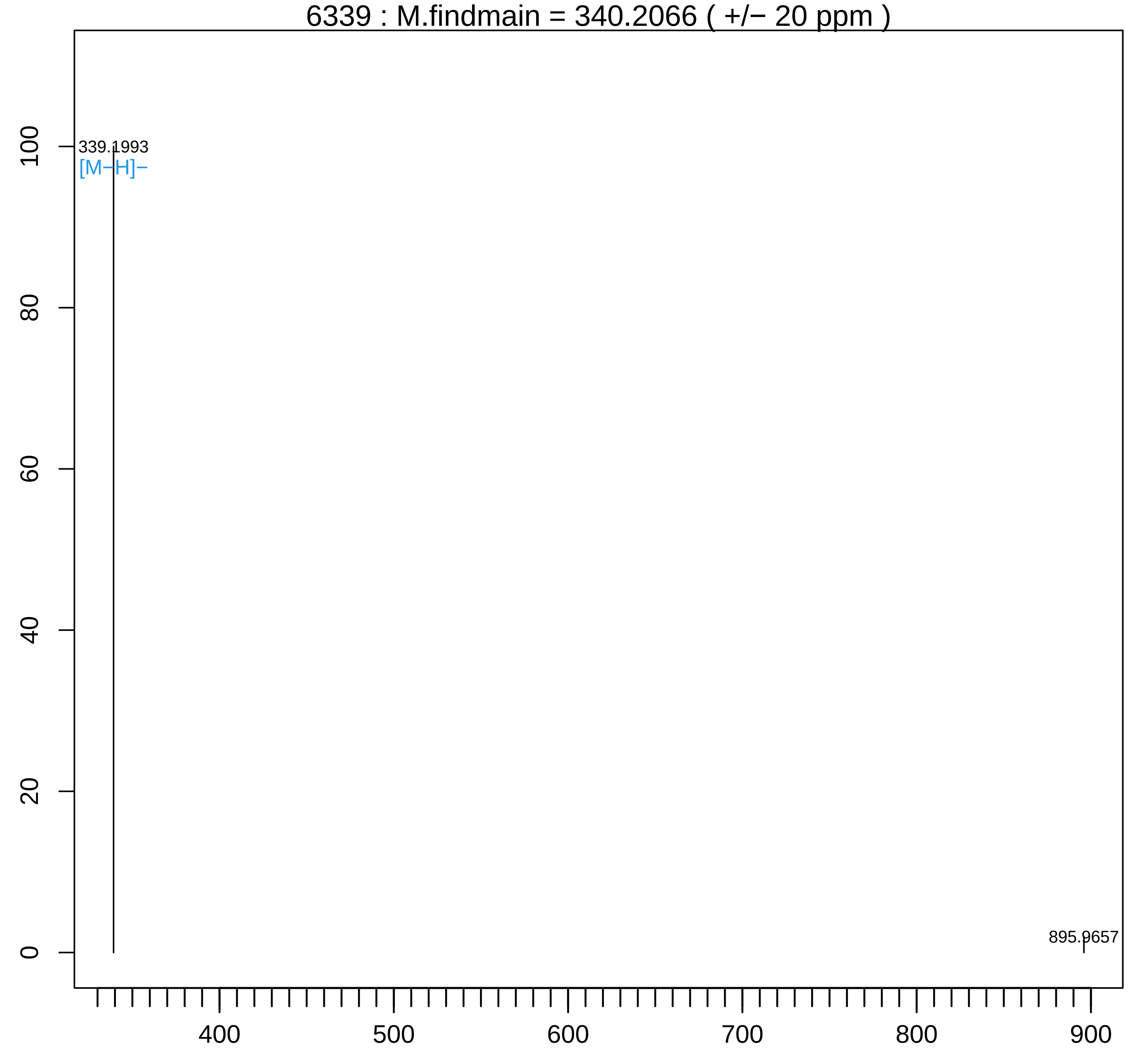
6339 : M.findmain = 340.2066 (+/− 20 ppm)
339.1993
[M−H]−
895.9657
0
20
40
60
80
100
400
500
600
700
800
900

6340 : M.ramclustr = 577.9795 (+/− NA ppm)

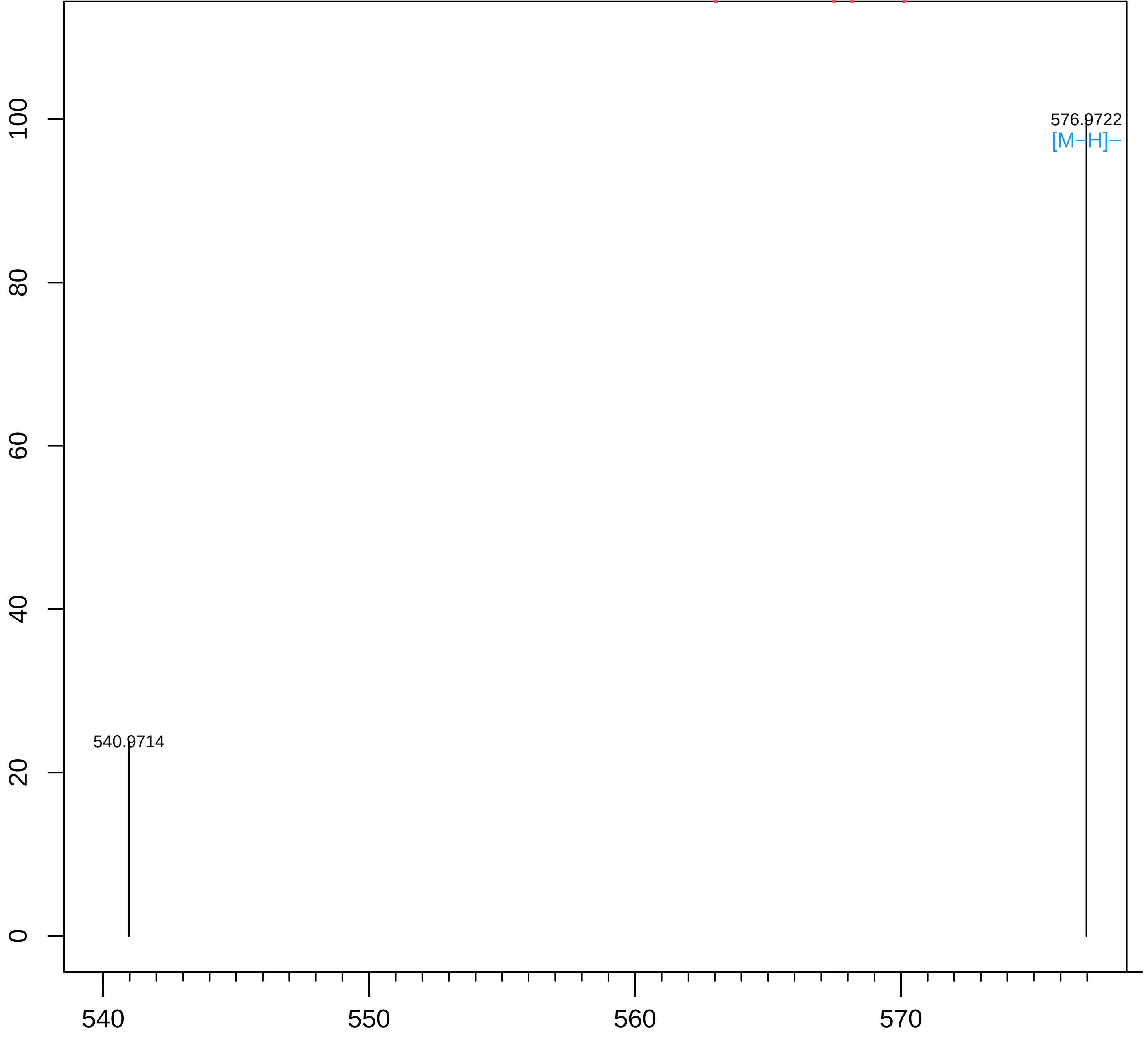

6340 : M.findmain = 577.9795 (+/− 20 ppm)

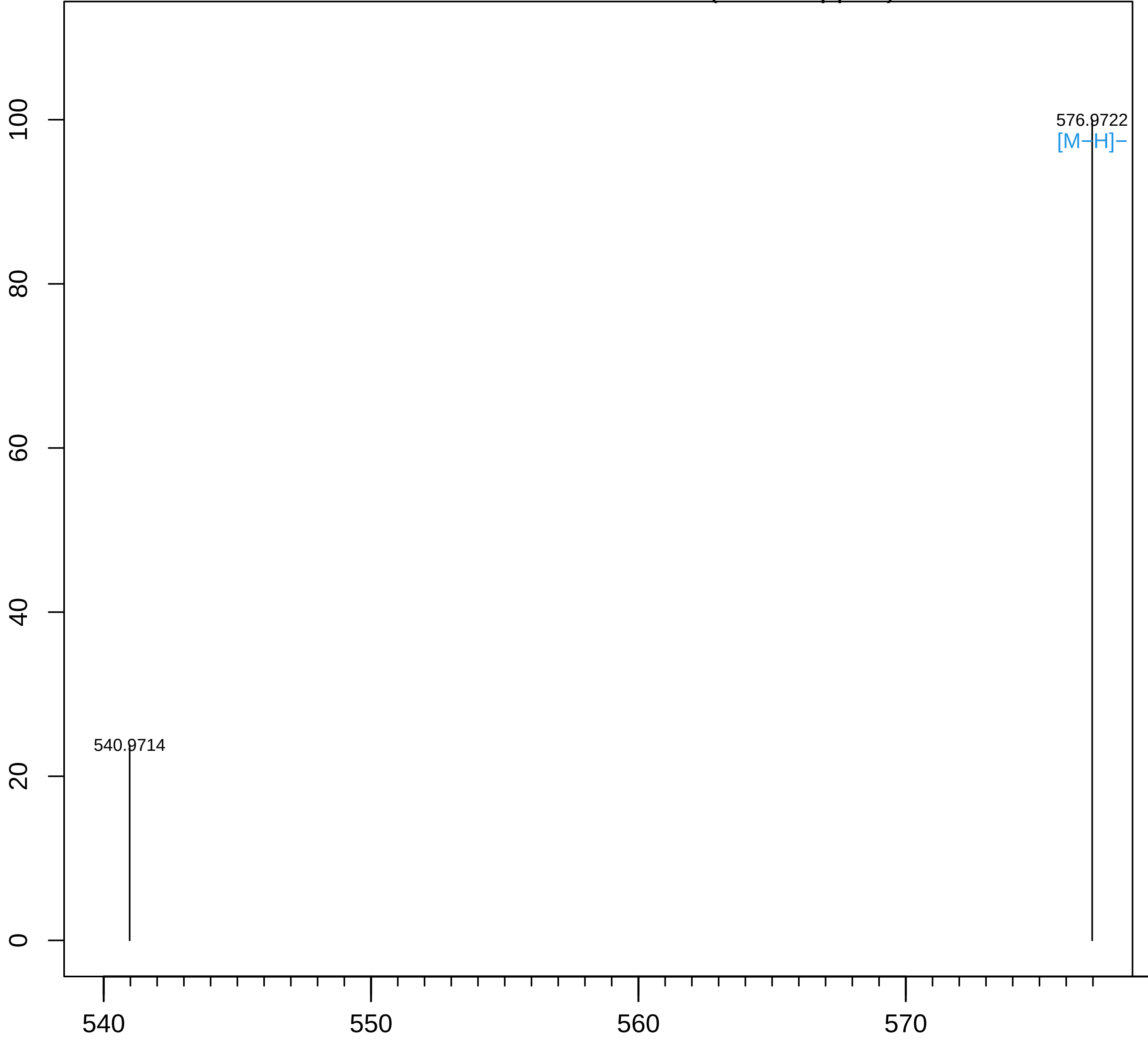

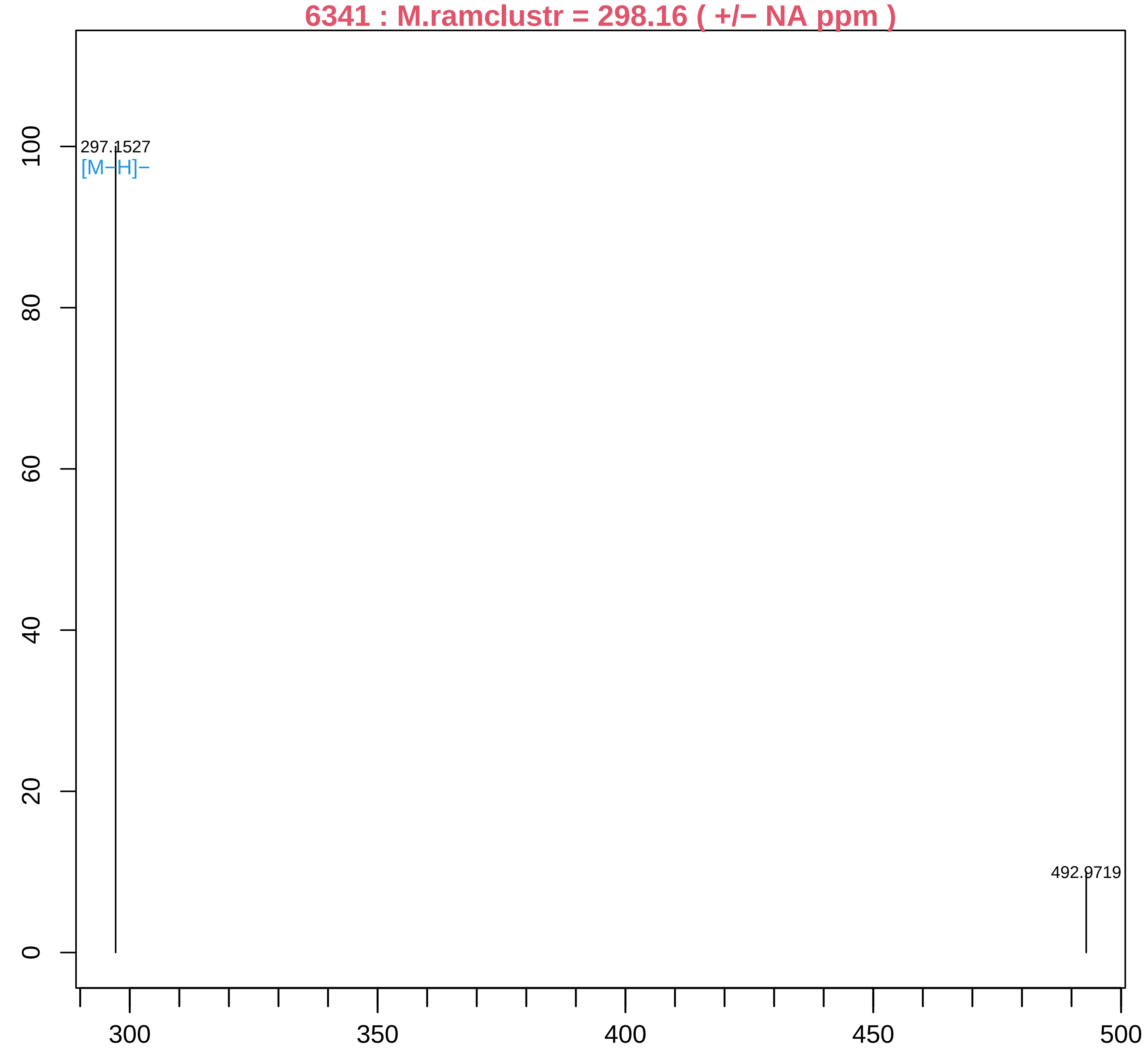
6341 : M.ramclustr = 298.16 (+/− NA ppm)
297.1527
[M−H]−
492.9719
0
20
40
60
80
100
300
350
400
450
500

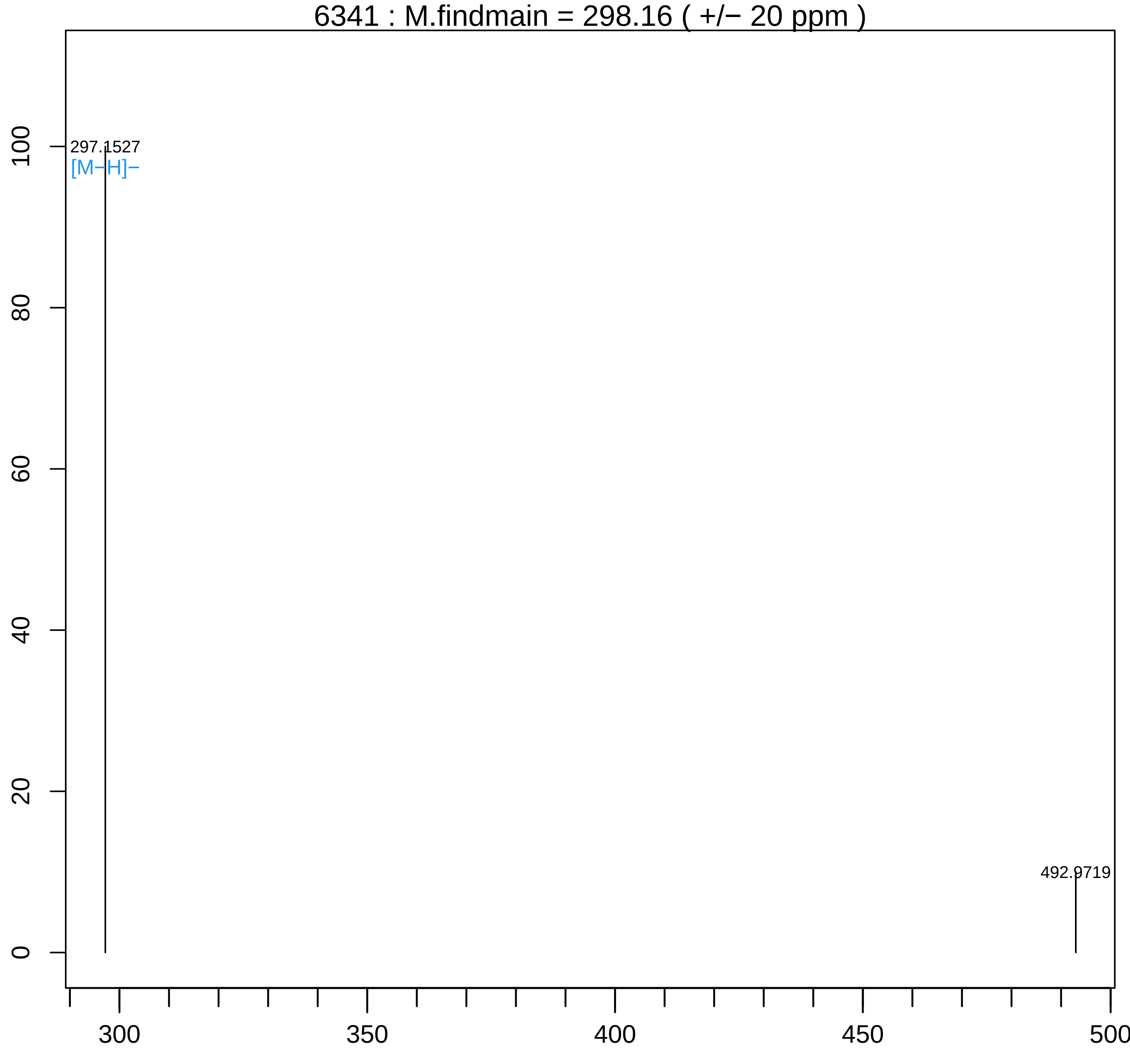
6341 : M.findmain = 298.16 (+/− 20 ppm)
297.1527
[M−H]−
492.9719
0
20
40
60
80
100
300
350
400
450
500

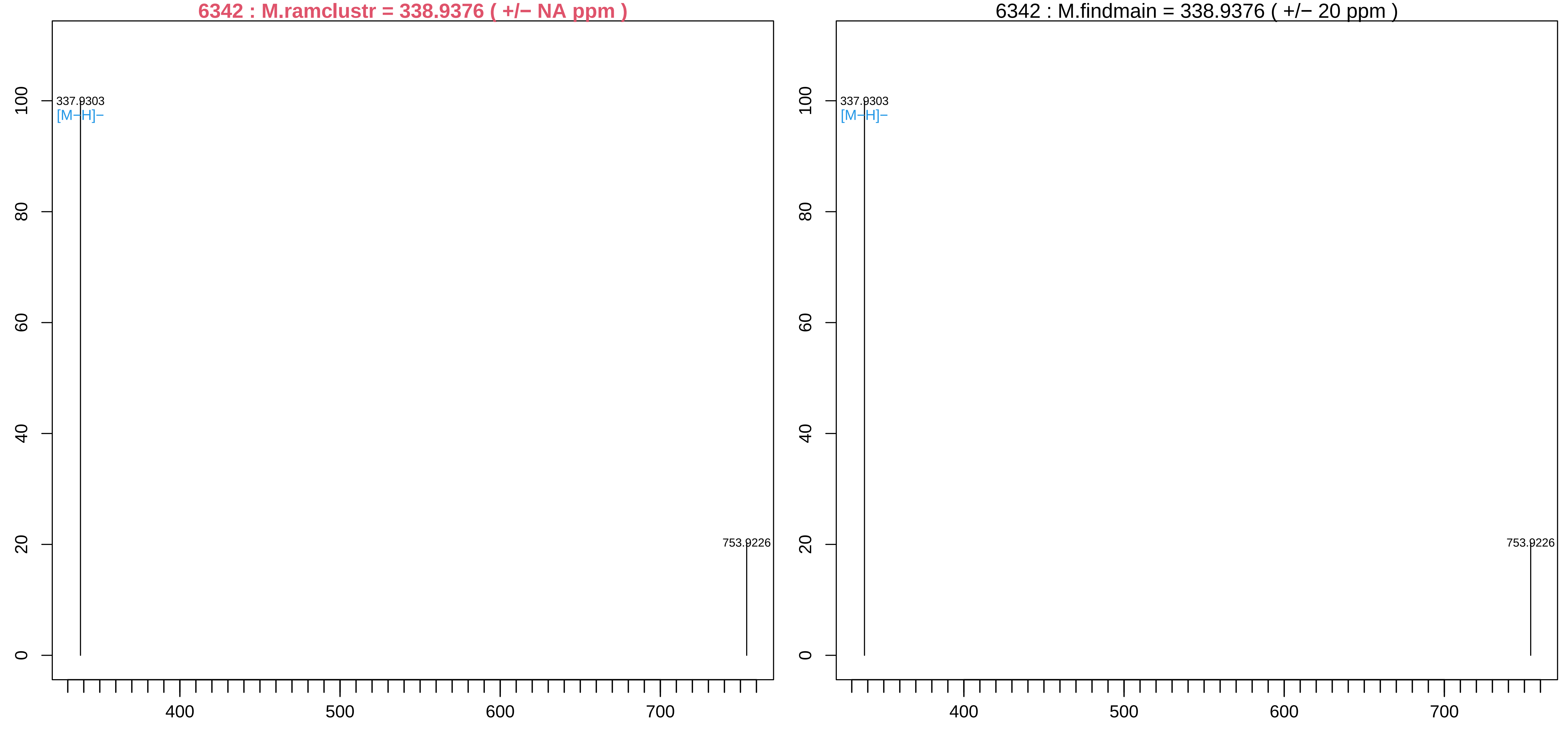
6342 : M.ramclustr = 338.9376 (+/− NA ppm)
6342 : M.findmain = 338.9376 (+/− 20 ppm)
337.9303
[M−H]−
753.9226
0
20
40
60
80
100
400
500
600
700

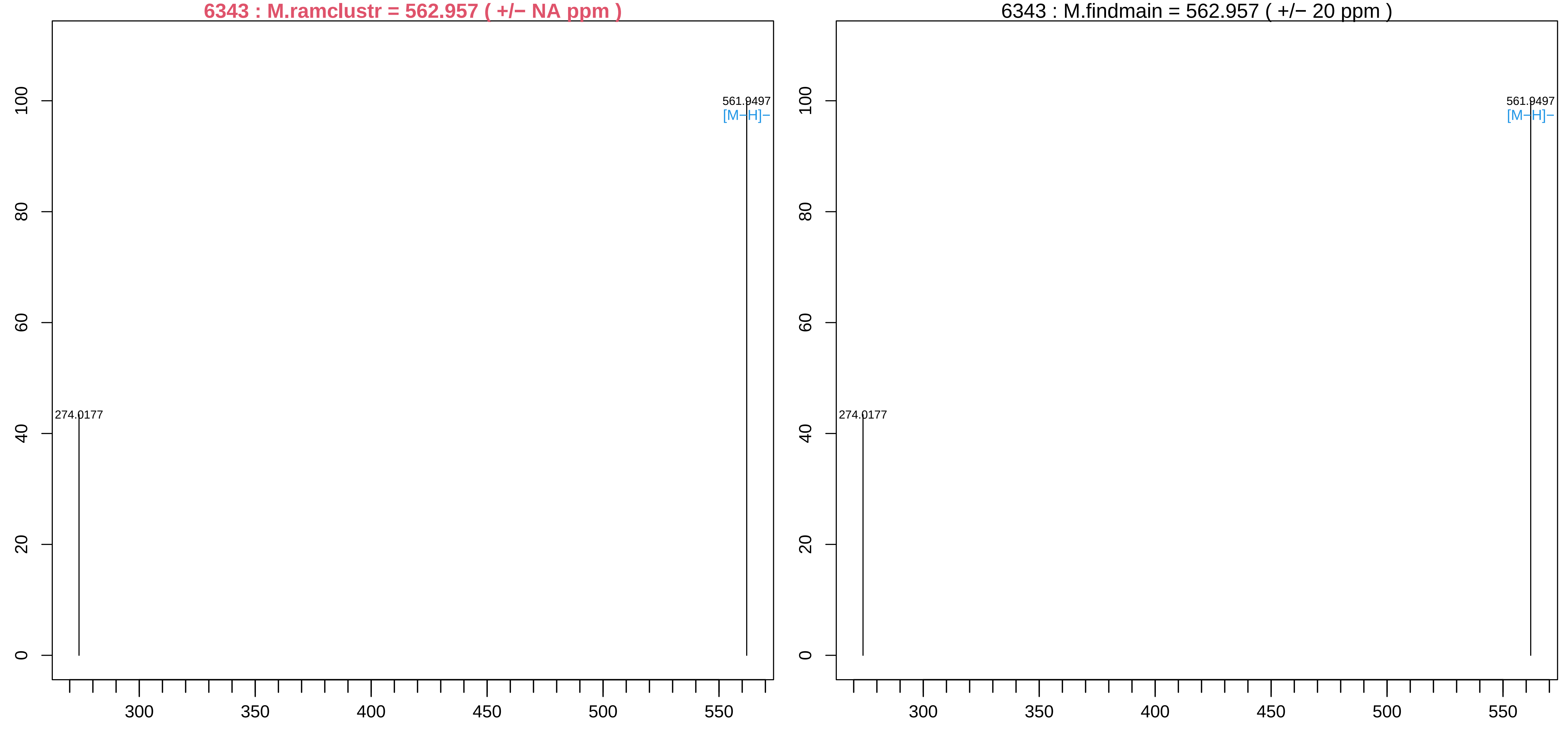

6343 : M.ramclustr = 562.957 (+/− NA ppm)
6343 : M.findmain = 562.957 (+/− 20 ppm)
561.9497
[M−H]−
274.0177
0
20
40
60
80
100
300
350
400
450
500
550

6344 : M.ramclustr = 447.9319 (+/− NA ppm)

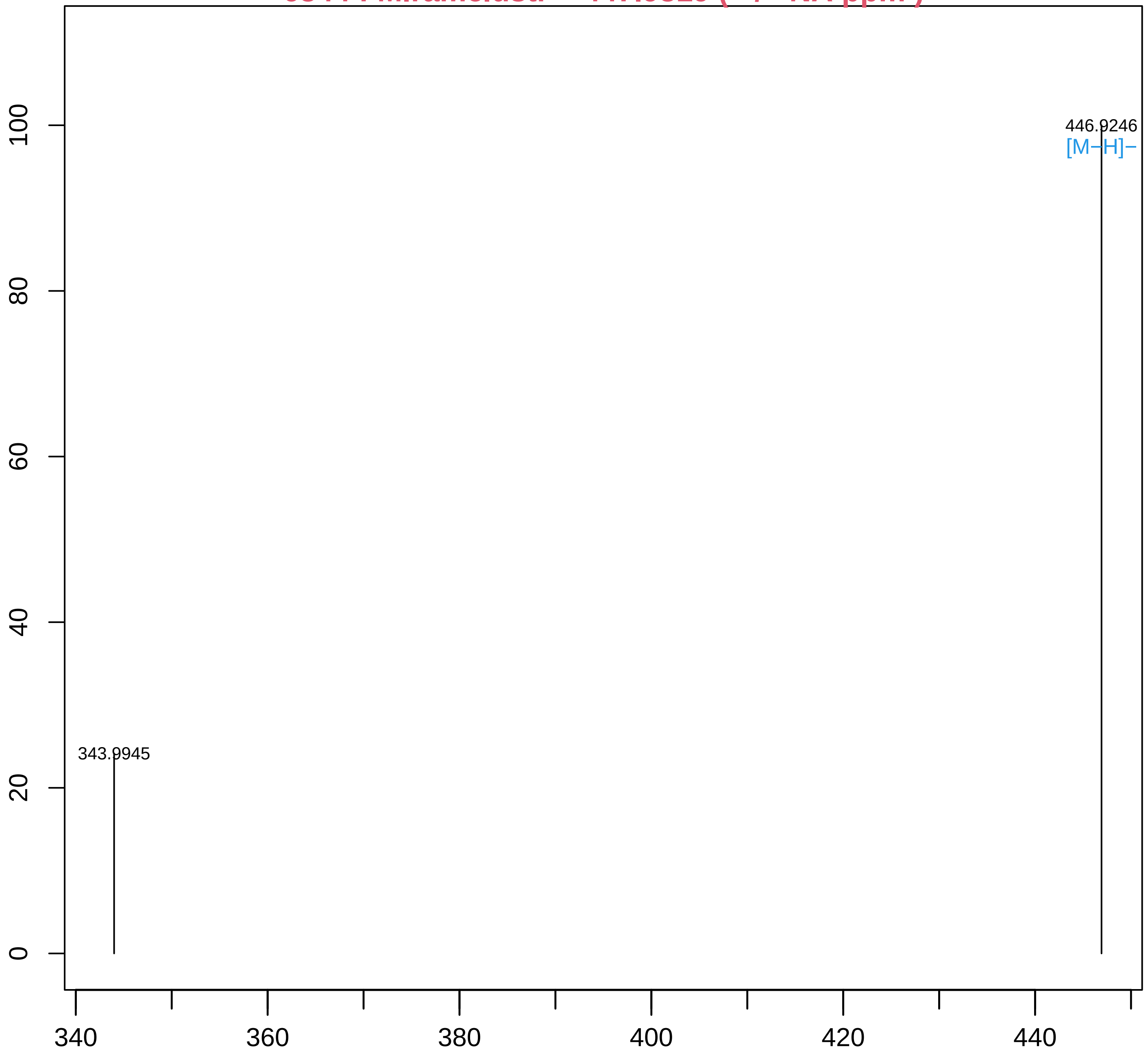

6344 : M.findmain = 447.9319 (+/− 20 ppm)

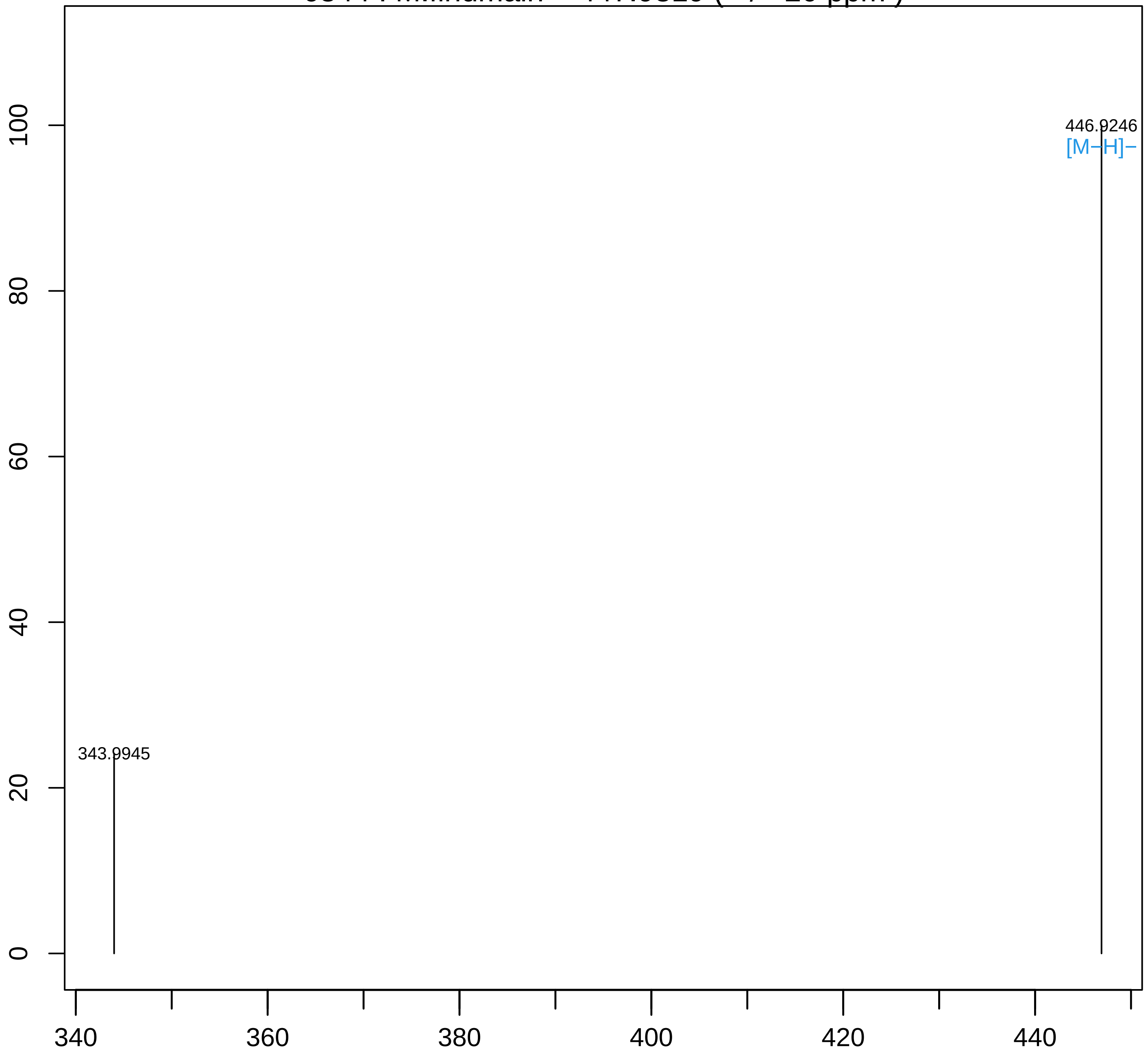

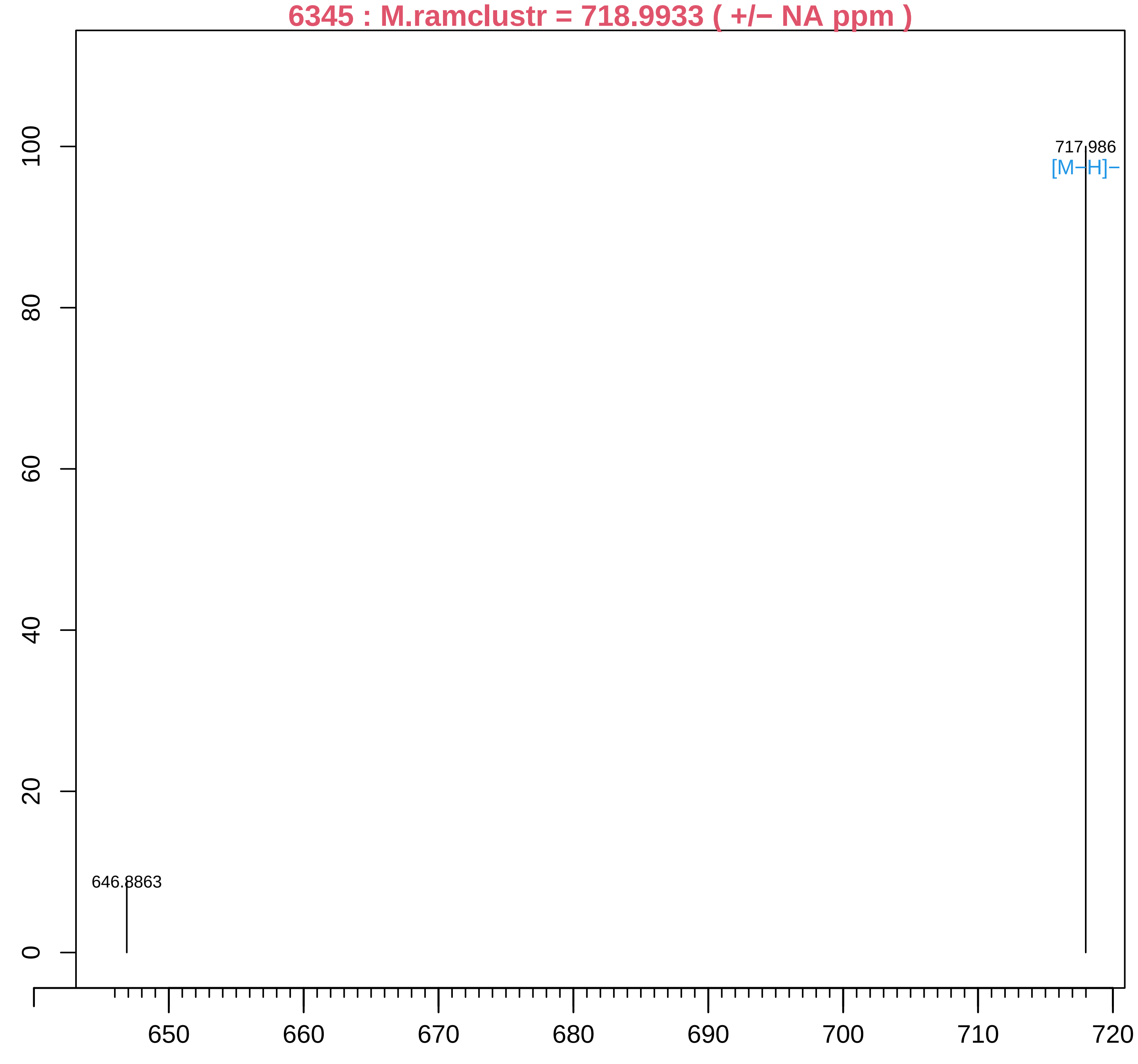
6345 : M.ramclustr = 718.9933 (+/− NA ppm)
717.986
[M−H]−
646.8863
0
20
40
60
80
100
650
660
670
680
690
700
710
720

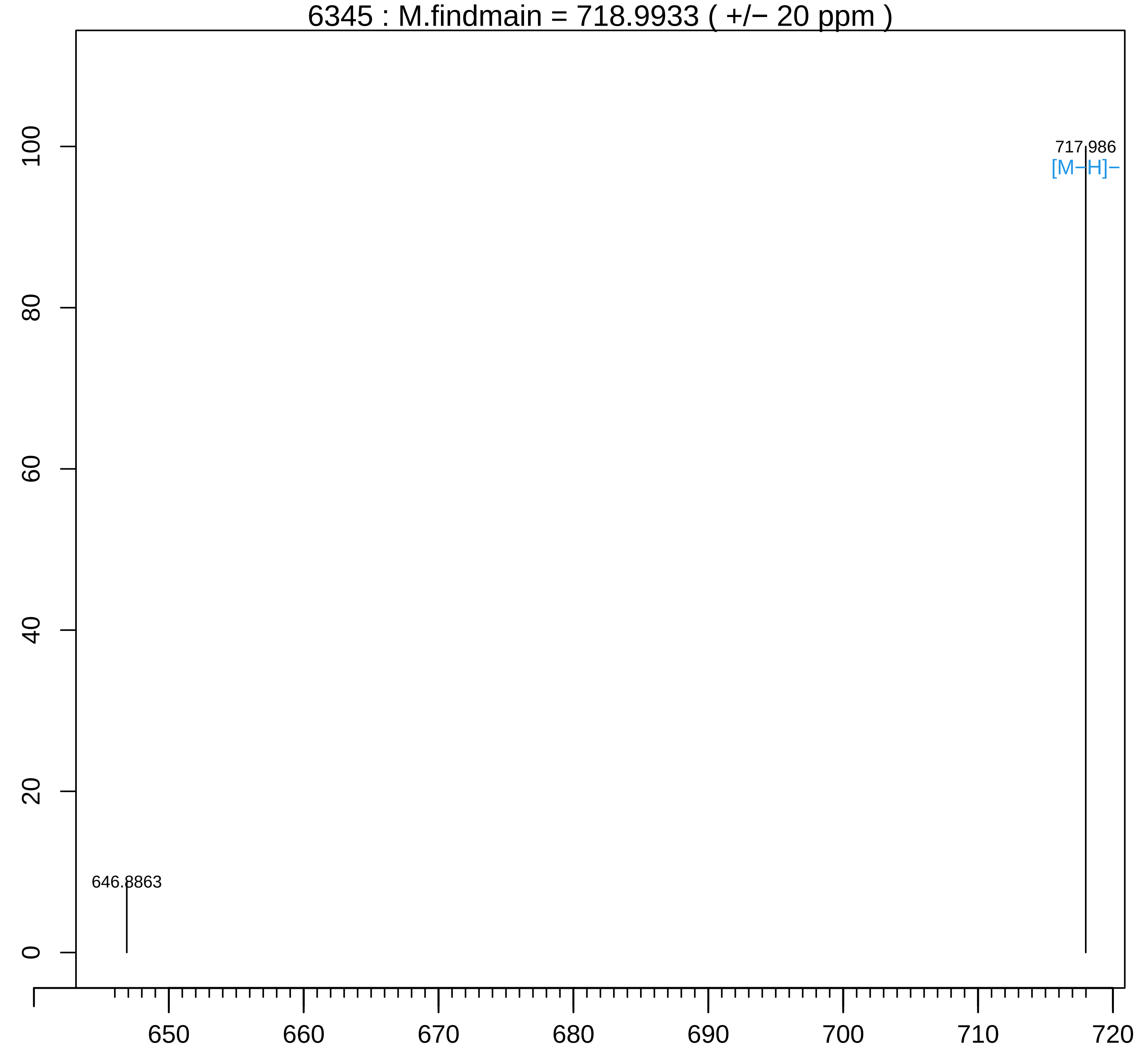
6345 : M.findmain = 718.9933 (+/− 20 ppm)
717.986
[M−H]−
646.8863
0
20
40
60
80
100
650
660
670
680
690
700
710
720

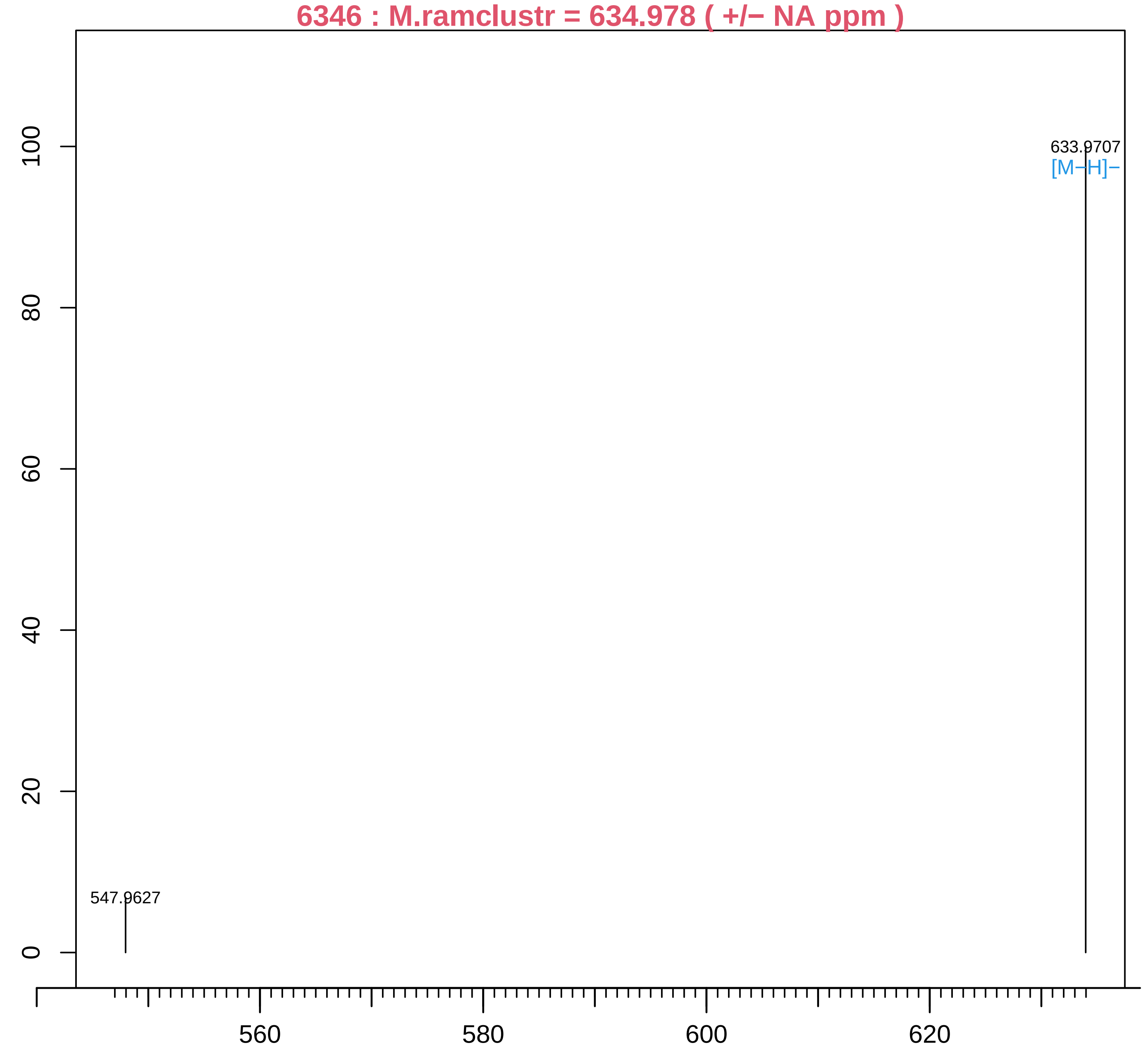

6346 : M.ramclustr = 634.978 (+/− NA ppm)
633.9707
[M−H]−
547.9627
0
20
40
60
80
100
560
580
600
620

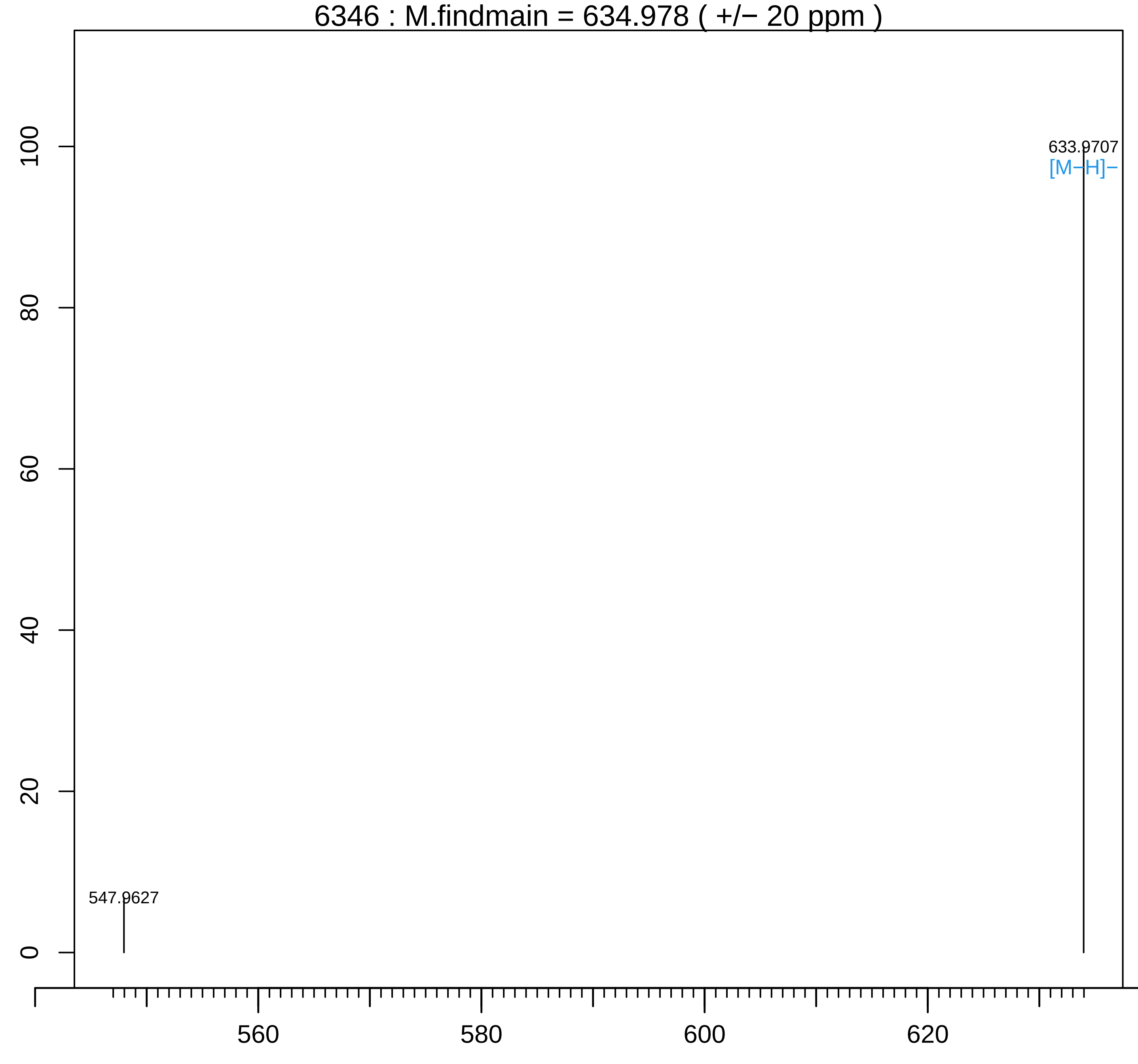

6346 : M.findmain = 634.978 (+/− 20 ppm)
633.9707
[M−H]−
547.9627
0
20
40
60
80
100
560
580
600
620

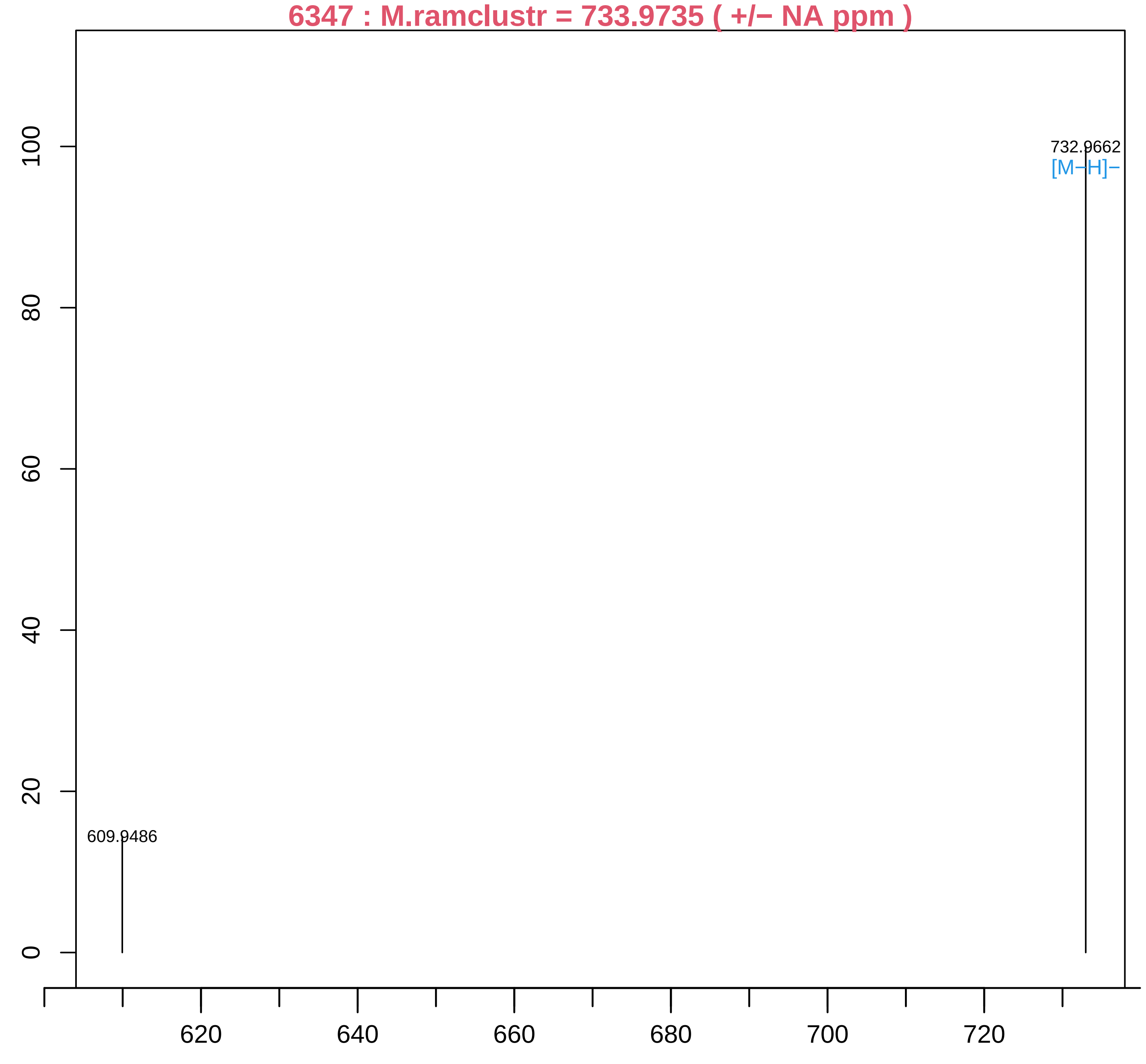
6347 : M.ramclustr = 733.9735 (+/− NA ppm)
732.9662
[M−H]−
609.9486
0
20
40
60
80
100
620
640
660
680
700
720

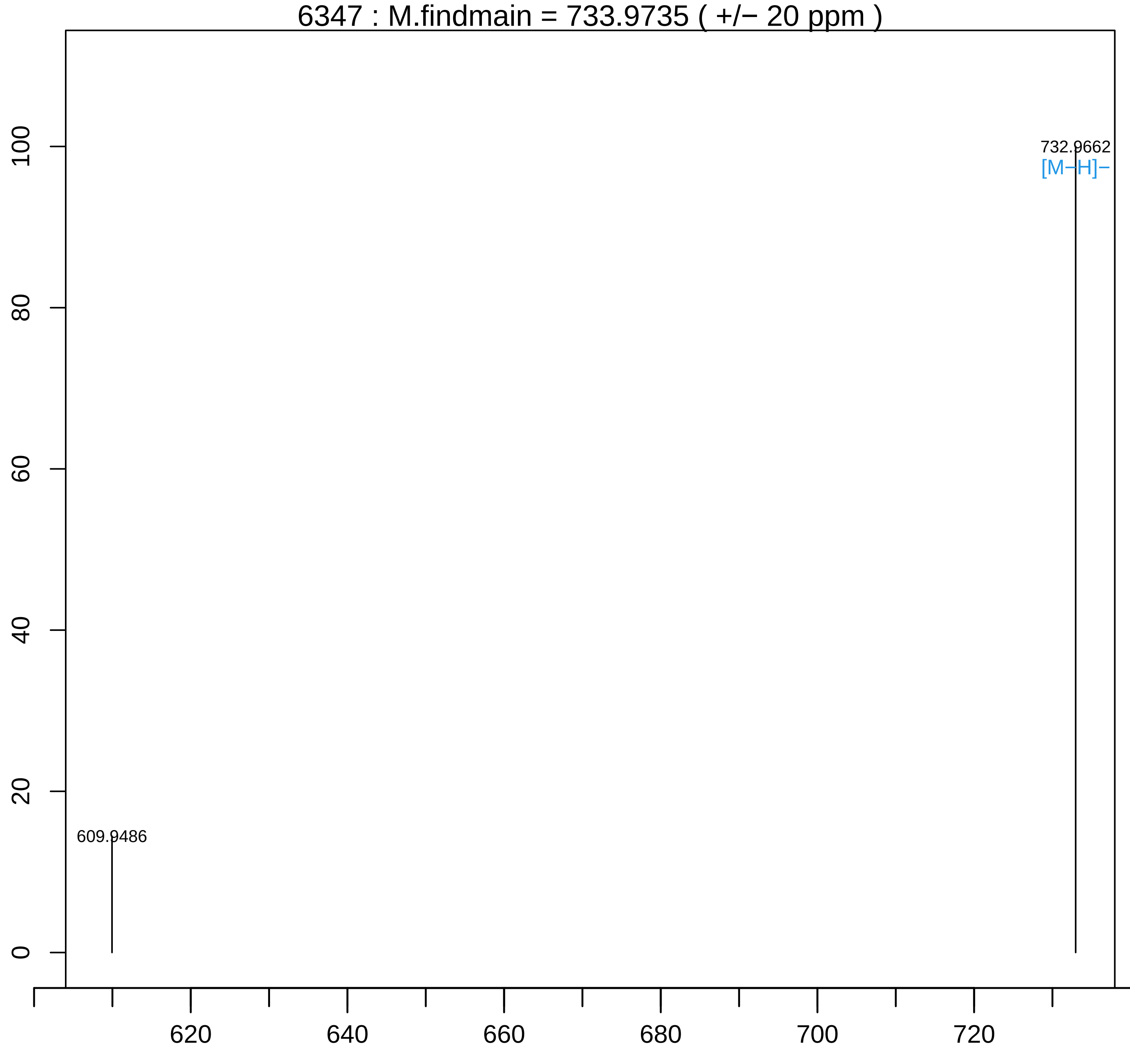
6347 : M.findmain = 733.9735 (+/− 20 ppm)
732.9662
[M−H]−
609.9486
0
20
40
60
80
100
620
640
660
680
700
720

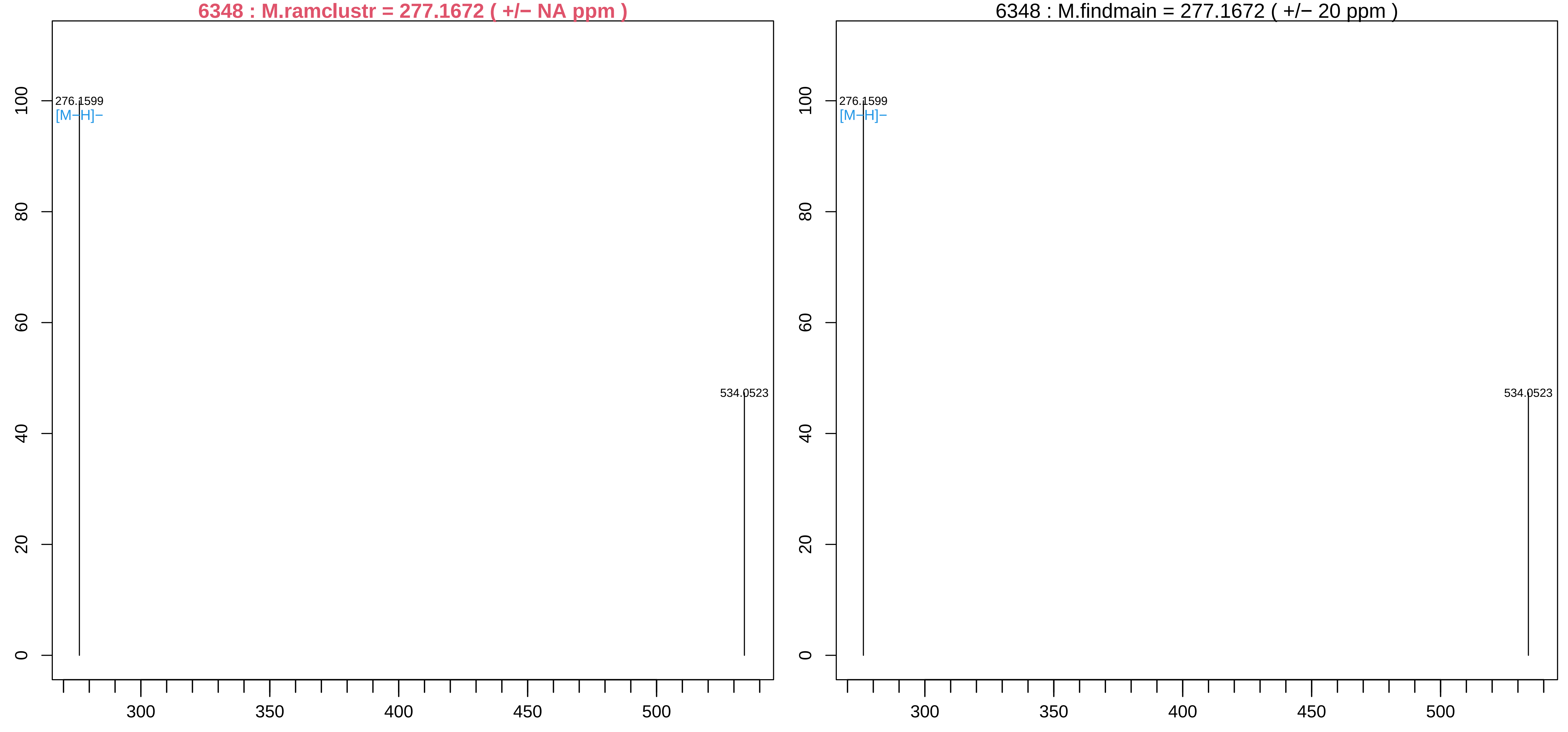
6348 : M.ramclustr = 277.1672 (+/− NA ppm)
276.1599
[M−H]−
534.0523
6348 : M.findmain = 277.1672 (+/− 20 ppm)
276.1599
[M−H]−
534.0523
0
20
40
60
80
100
300
350
400
450
500

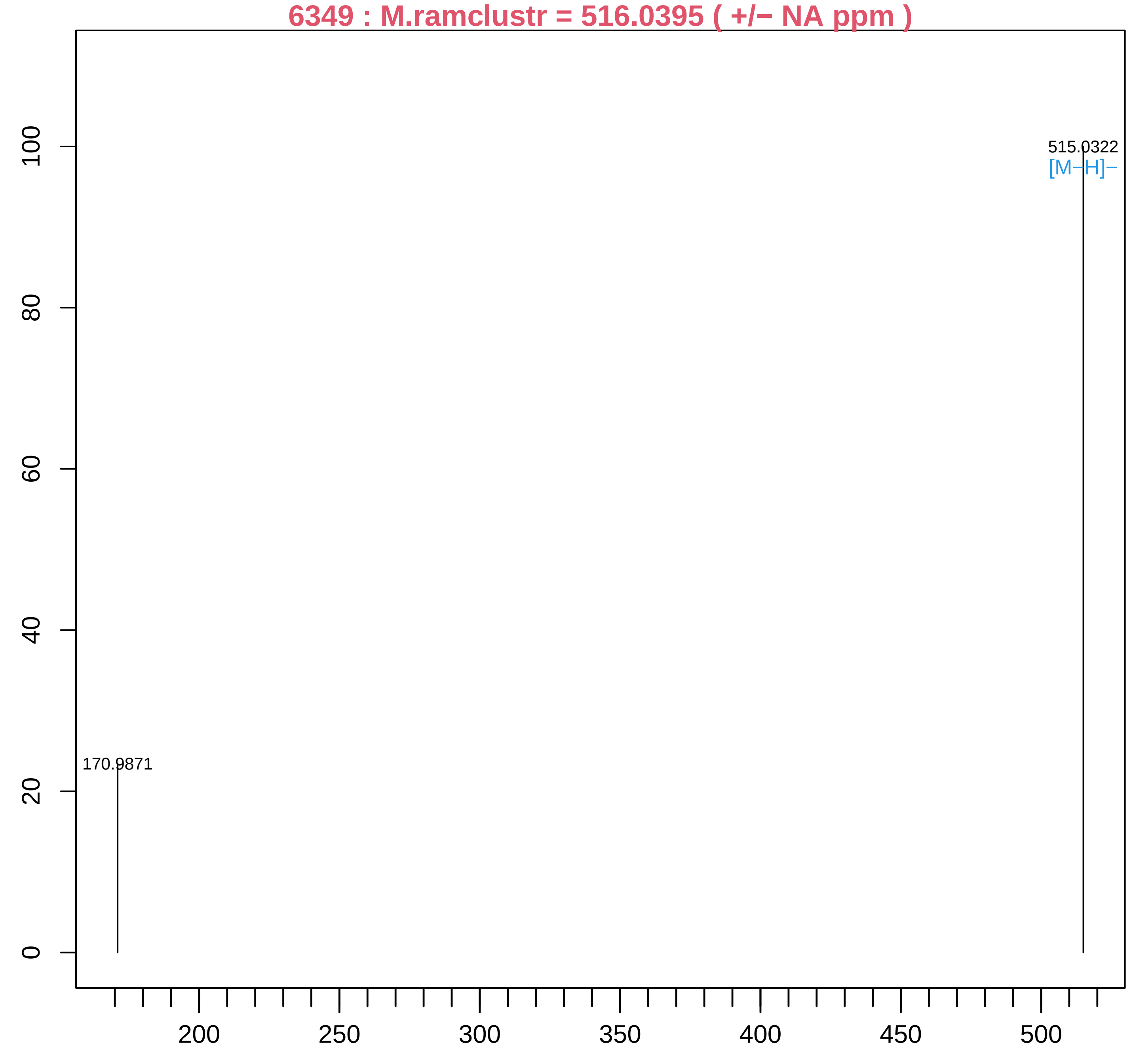
6349 : M.ramclustr = 516.0395 (+/− NA ppm)
515.0322
[M−H]−
170.9871
0
20
40
60
80
100
200
250
300
350
400
450
500

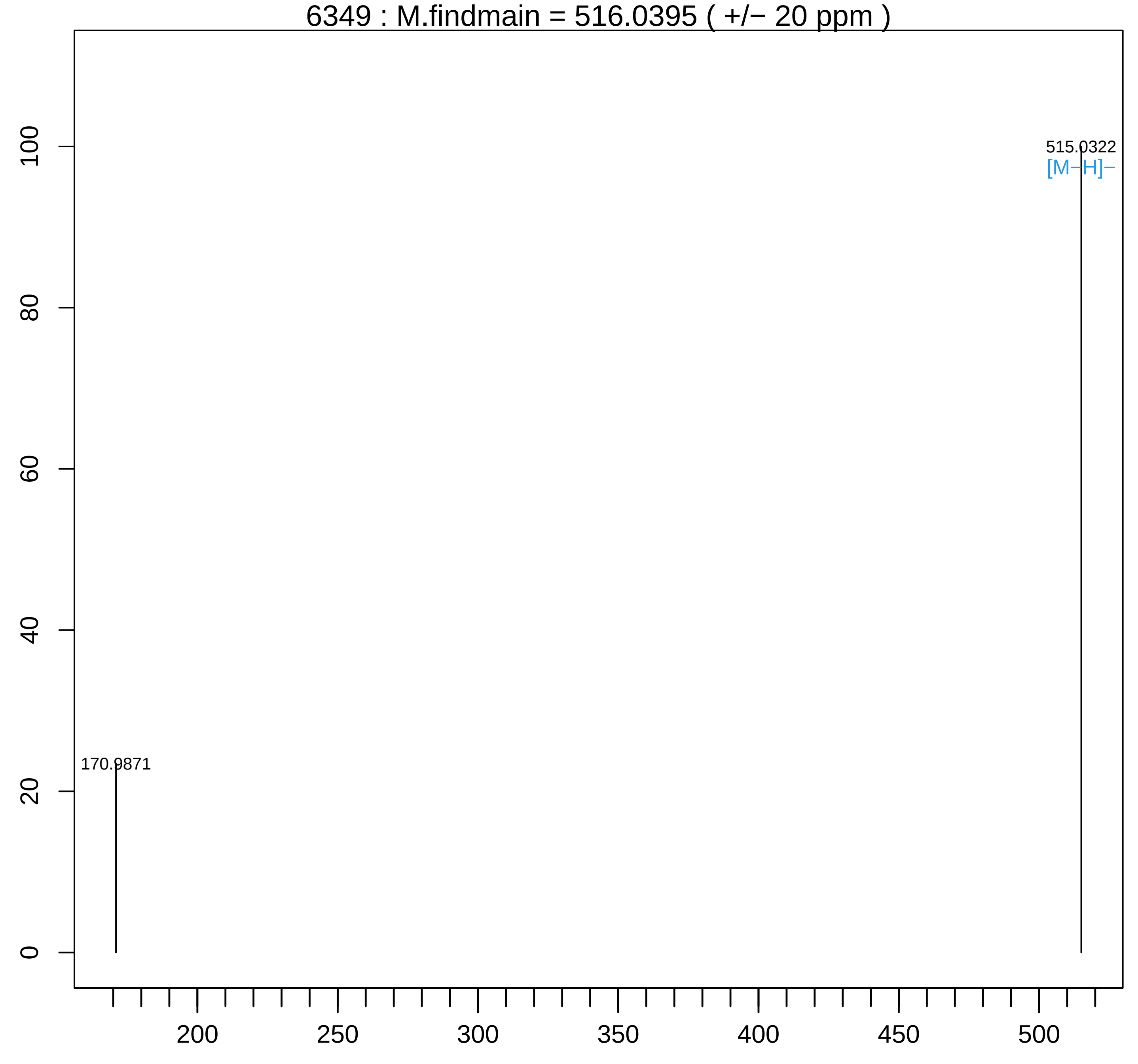
6349 : M.findmain = 516.0395 (+/− 20 ppm)
515.0322
[M−H]−
170.9871
0
20
40
60
80
100
200
250
300
350
400
450
500

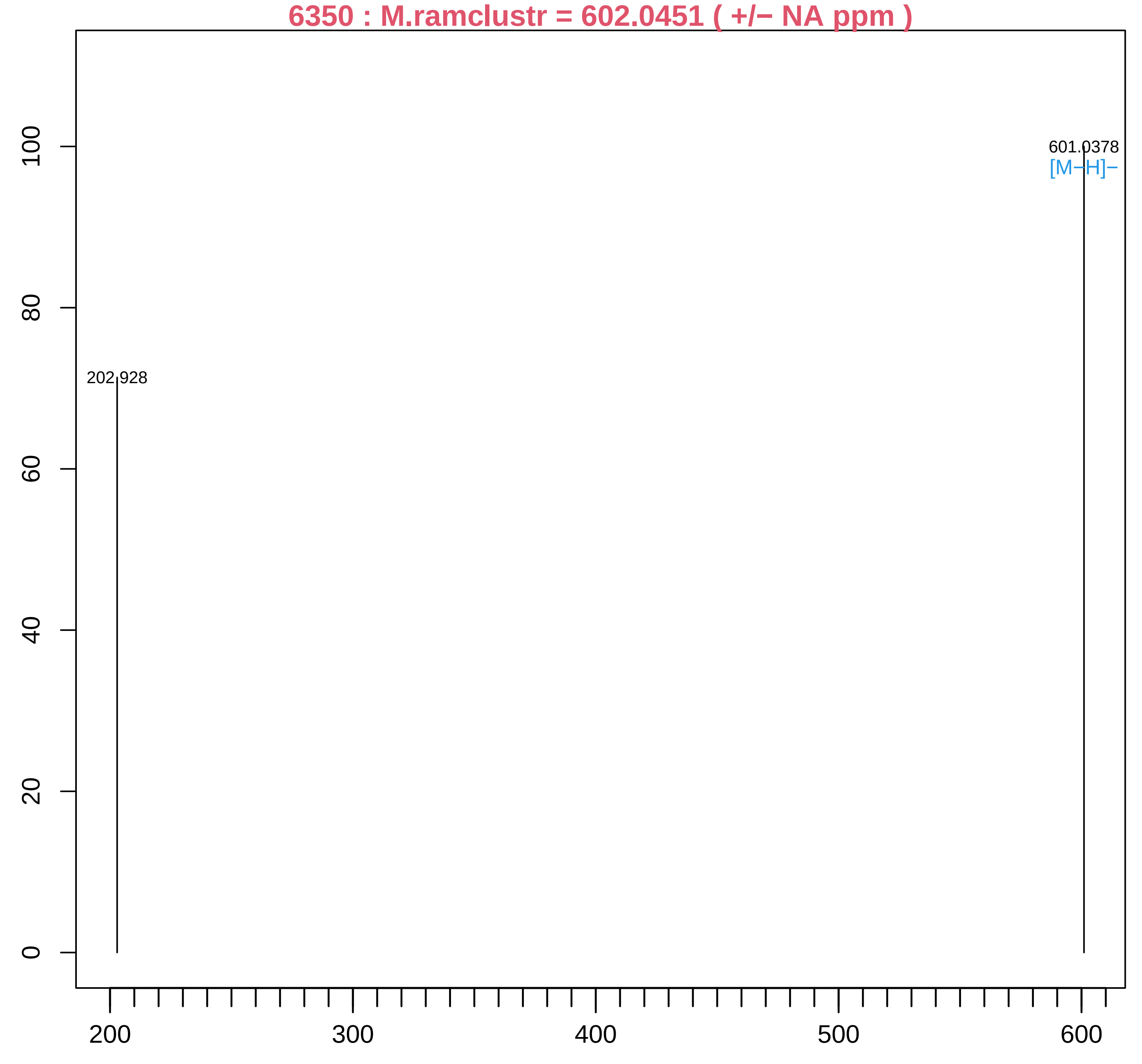
6350 : M.ramclustr = 602.0451 (+/− NA ppm)
601.0378
[M−H]−
202.928
0
20
40
60
80
100
200
300
400
500
600

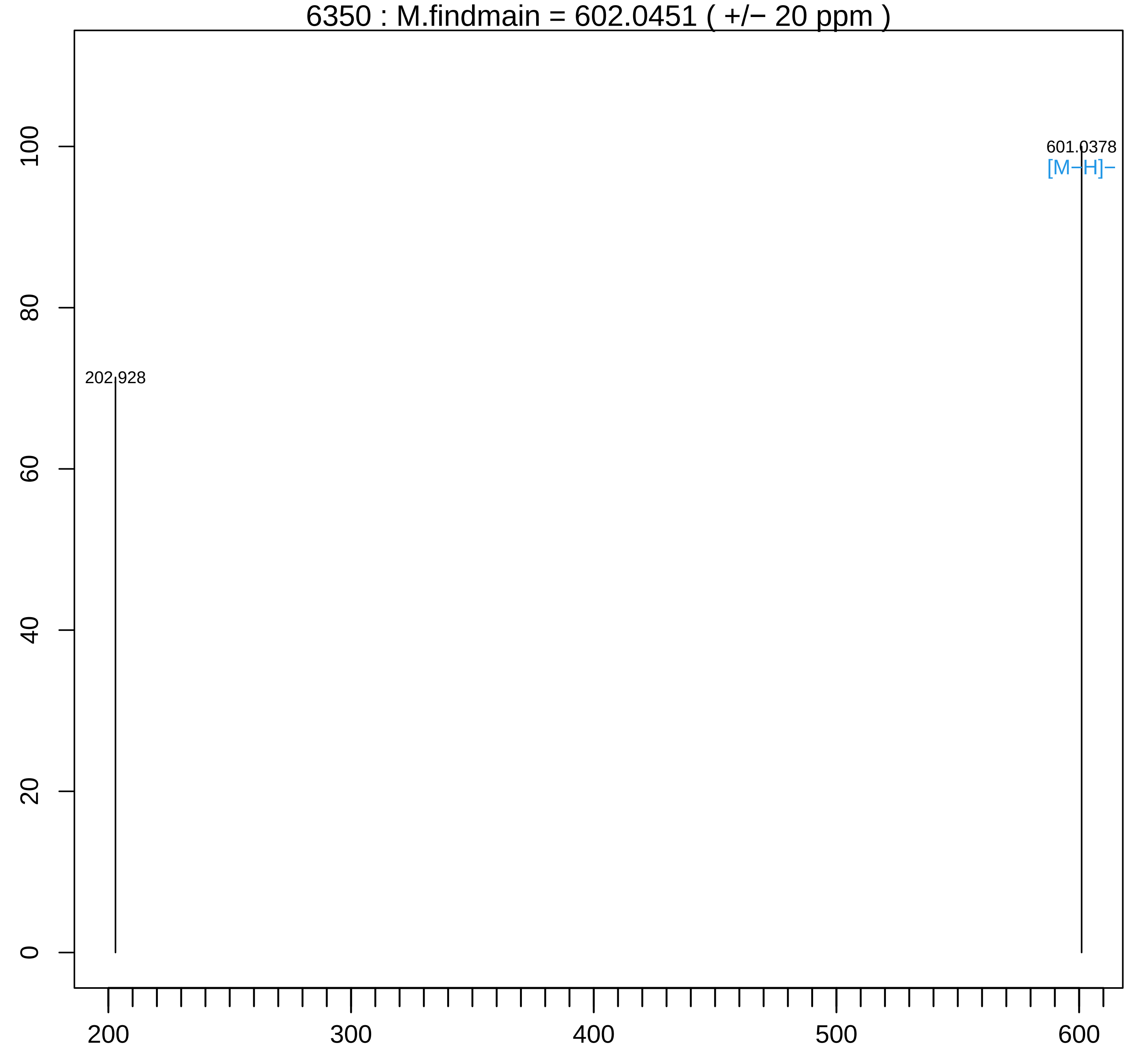
6350 : M.findmain = 602.0451 (+/− 20 ppm)
601.0378
[M−H]−
202.928
0
20
40
60
80
100
200
300
400
500
600

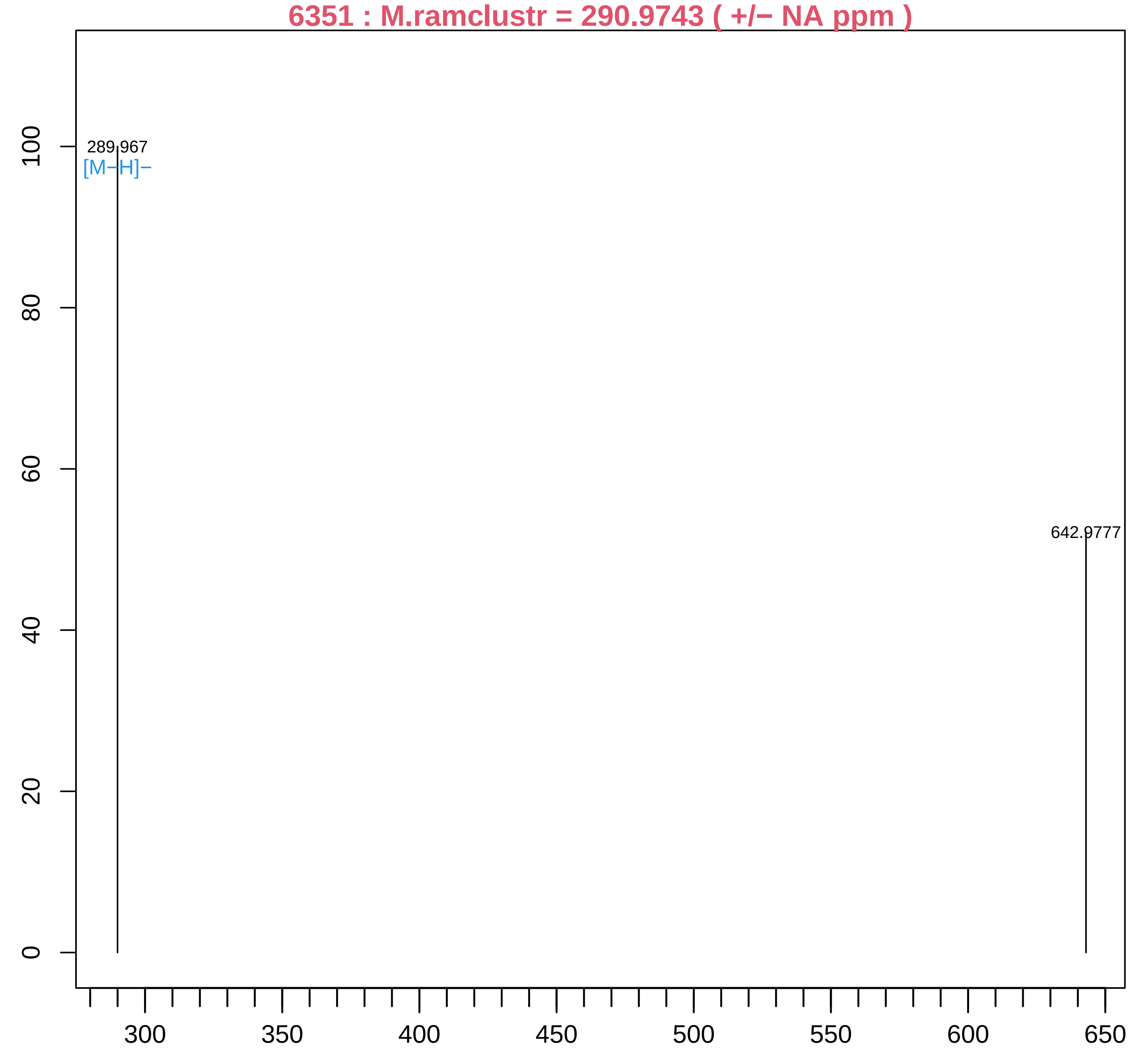
6351 : M.ramclustr = 290.9743 (+/− NA ppm)
289.967
[M−H]−
642.9777
0
20
40
60
80
100
300
350
400
450
500
550
600
650

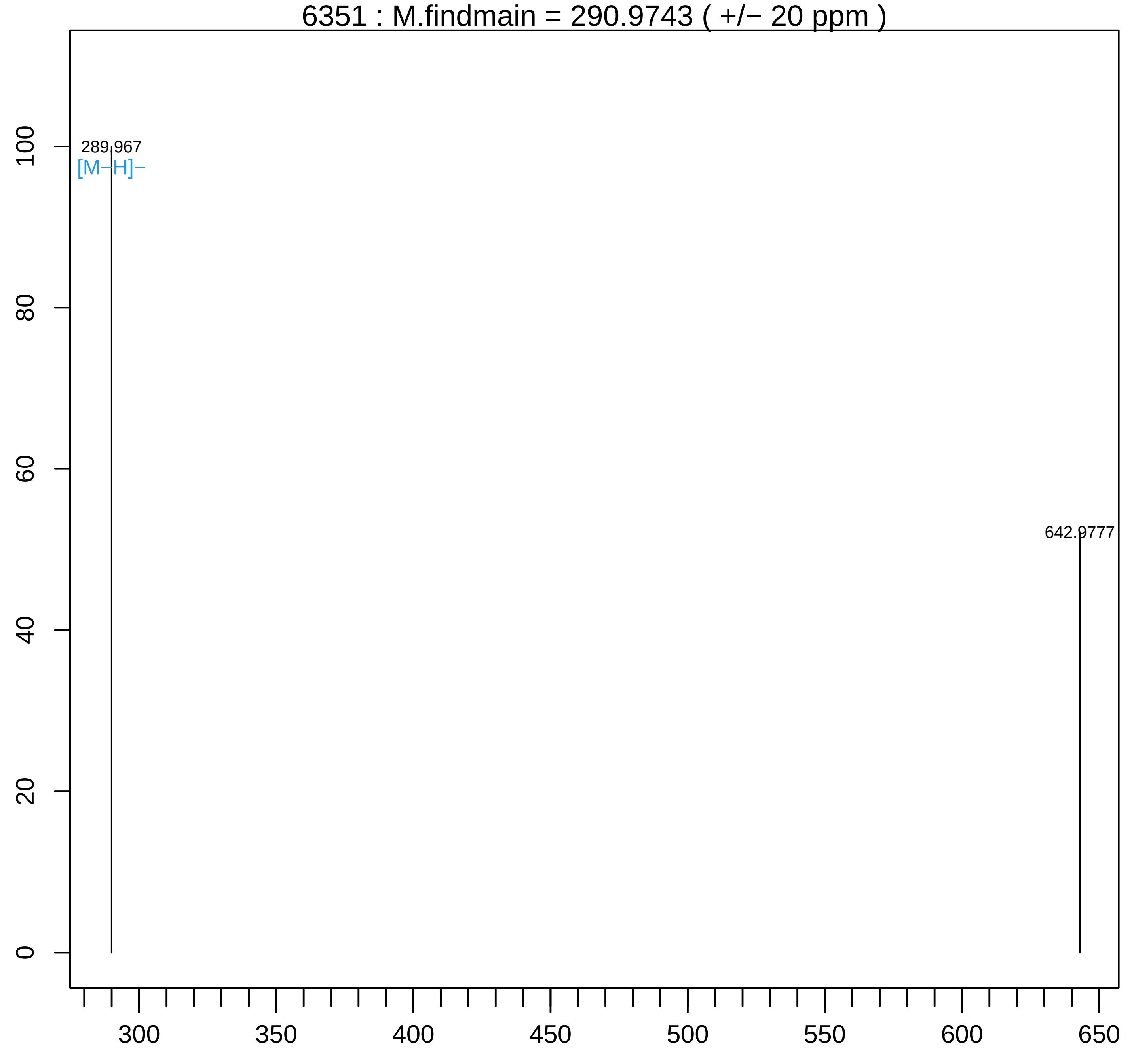
6351 : M.findmain = 290.9743 (+/− 20 ppm)
289.967
[M−H]−
642.9777
0
20
40
60
80
100
300
350
400
450
500
550
600
650

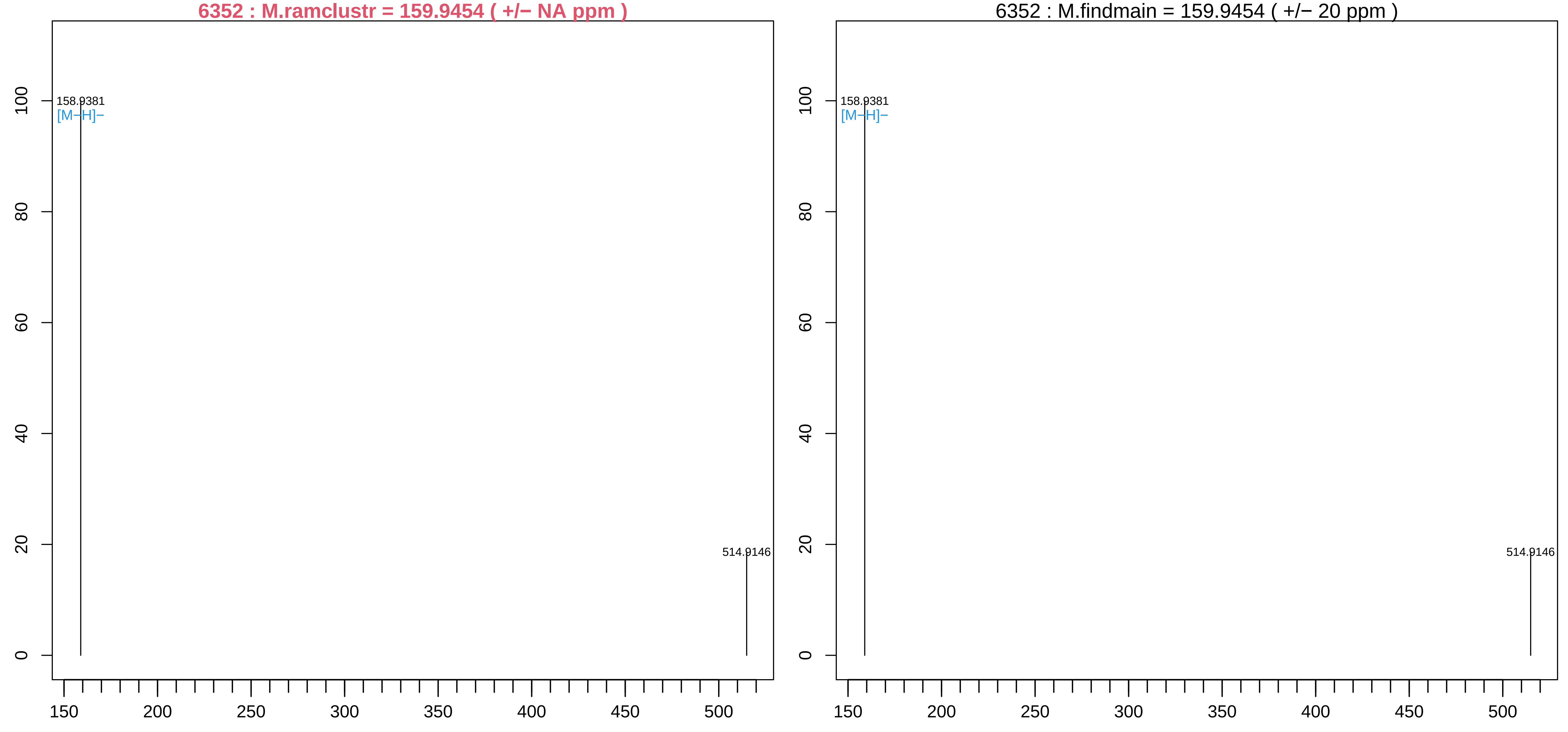
6352 : M.ramclustr = 159.9454 (+/− NA ppm)
158.9381
[M−H]−
514.9146
6352 : M.findmain = 159.9454 (+/− 20 ppm)
158.9381
[M−H]−
514.9146
0
20
40
60
80
100
150
200
250
300
350
400
450
500

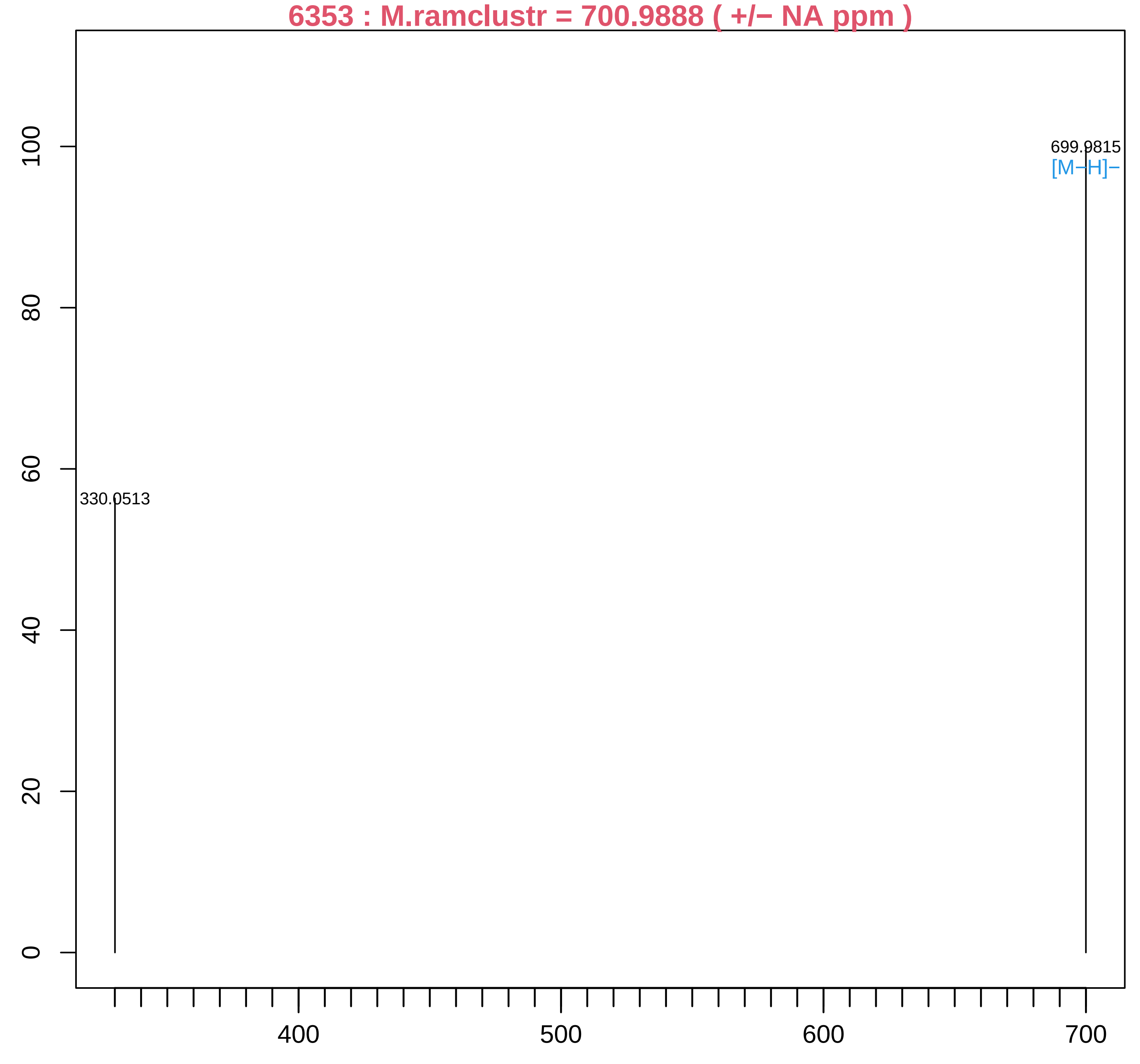
6353 : M.ramclustr = 700.9888 (+/− NA ppm)
330.0513
699.9815
[M−H]−
0
20
40
60
80
100
400
500
600
700

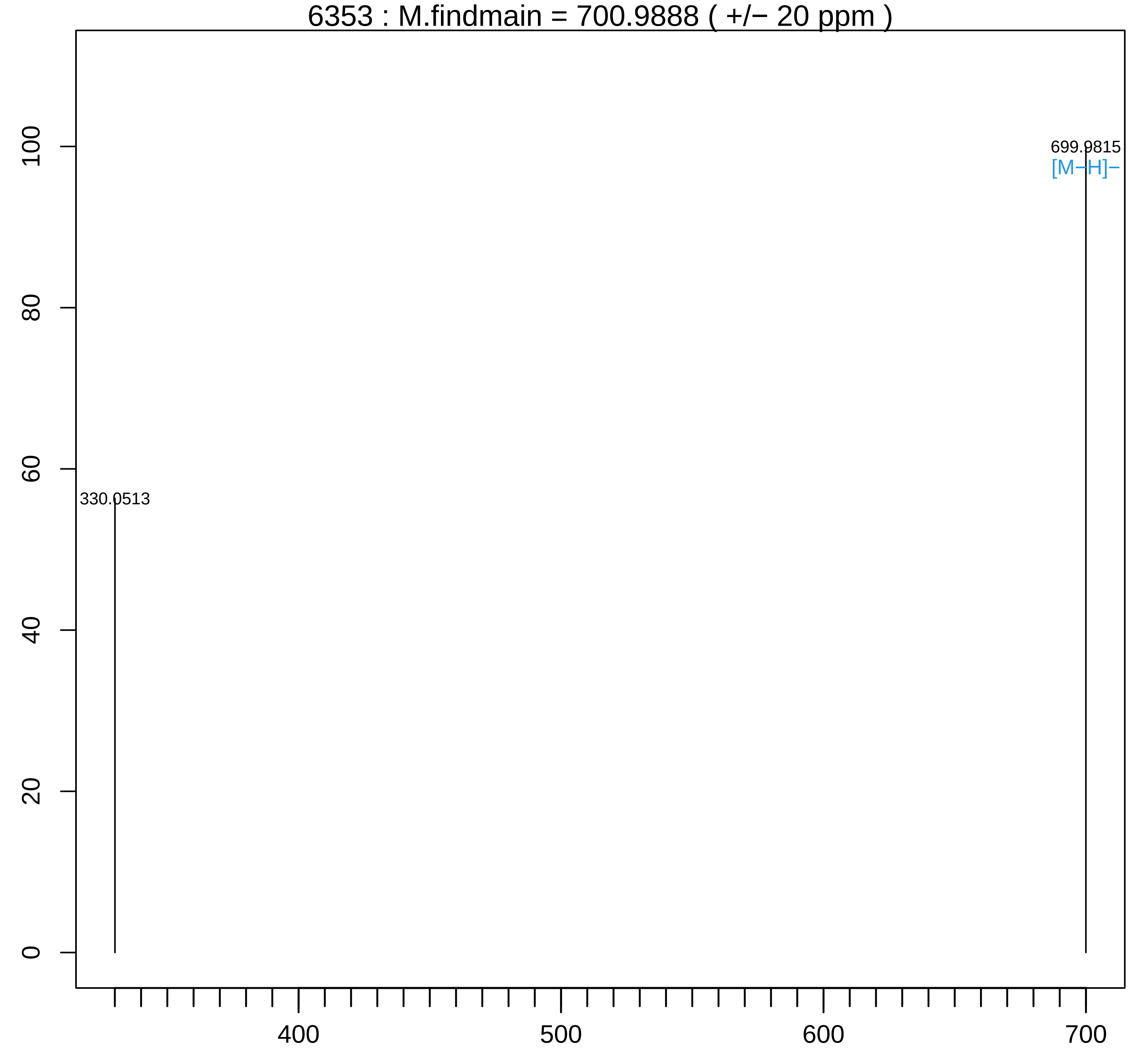
6353 : M.findmain = 700.9888 (+/− 20 ppm)
330.0513
699.9815
[M−H]−
0
20
40
60
80
100
400
500
600
700

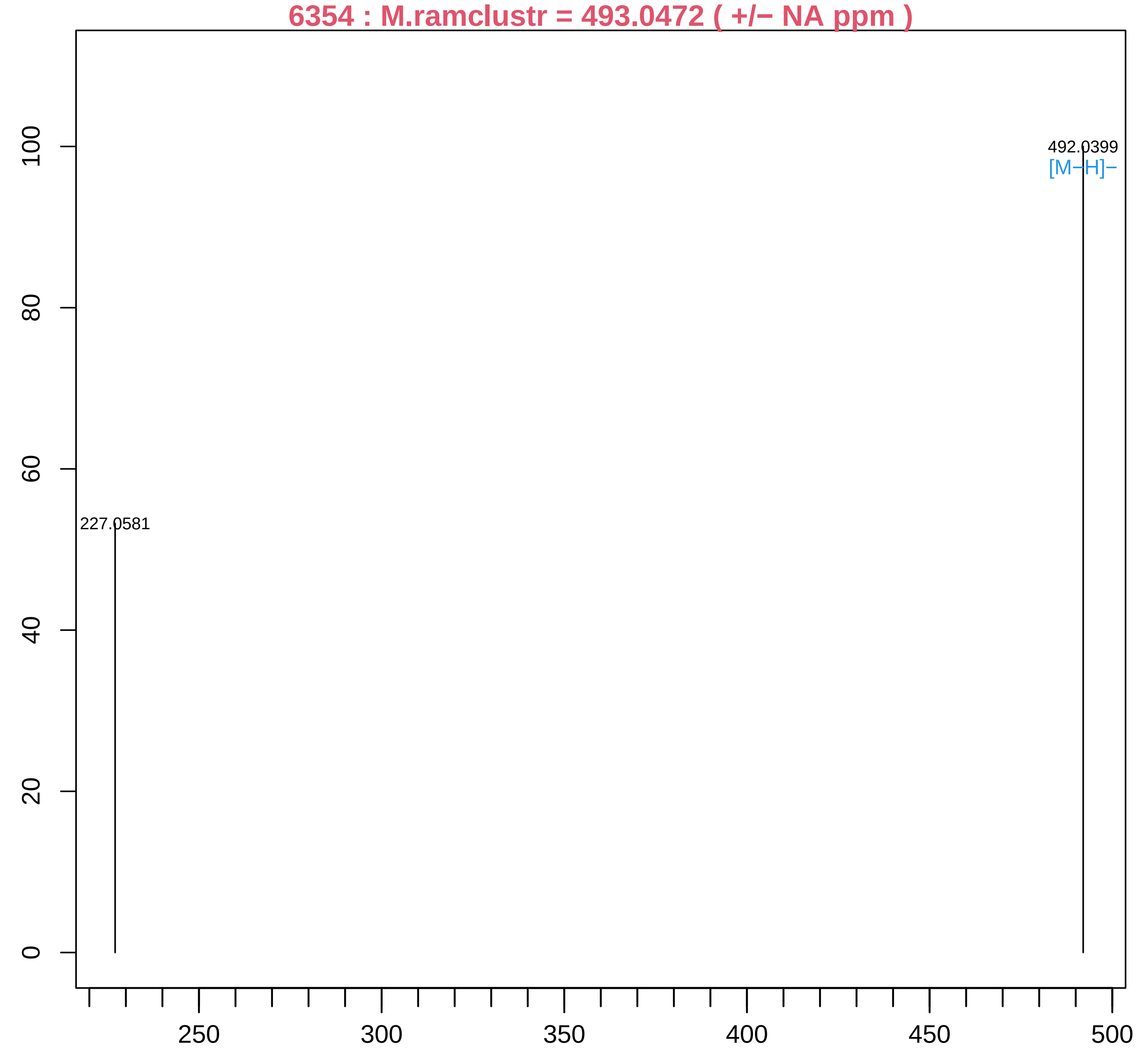
6354 : M.ramclustr = 493.0472 (+/− NA ppm)
492.0399
[M−H]−
227.0581
0
20
40
60
80
100
250
300
350
400
450
500

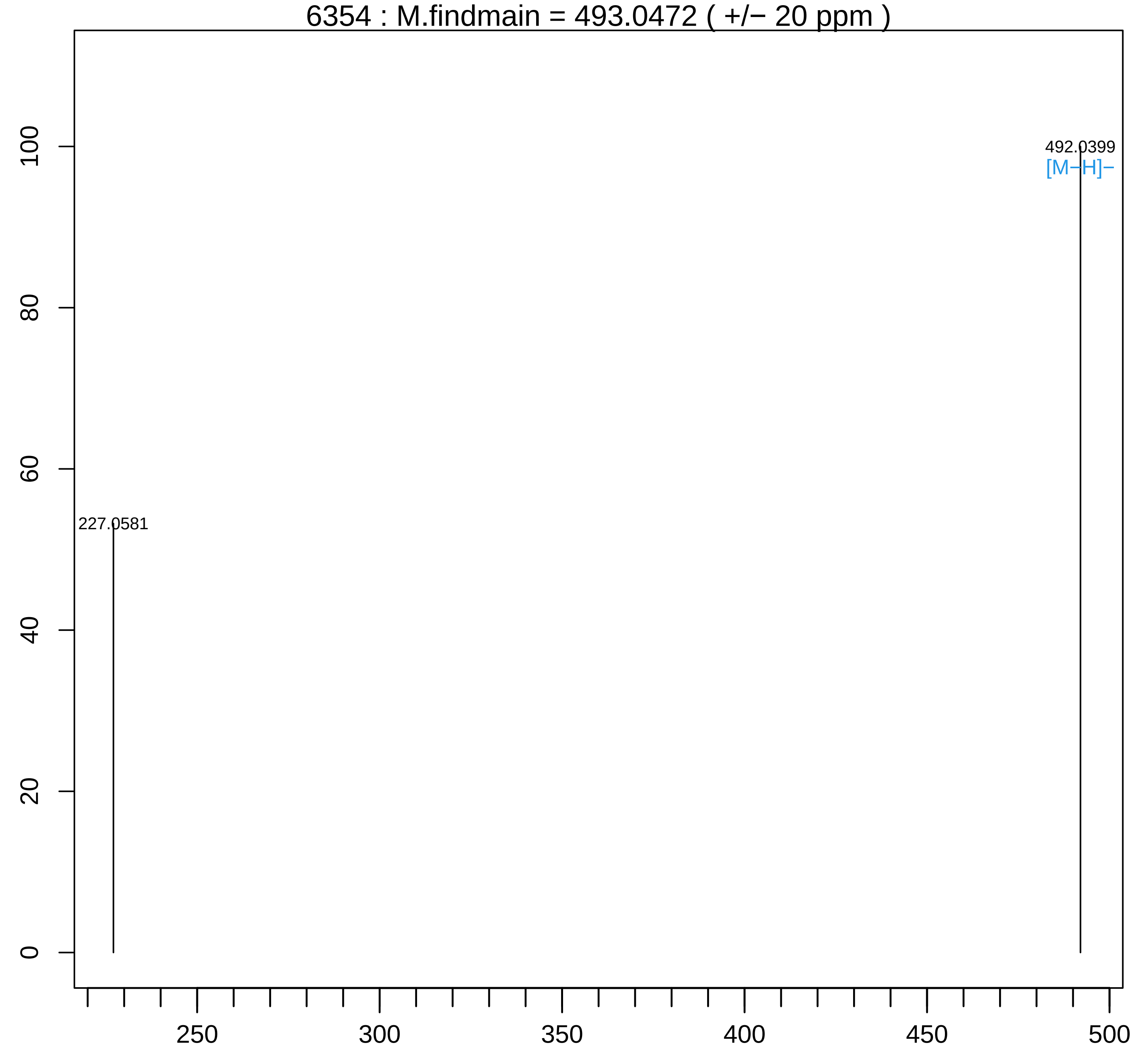
6354 : M.findmain = 493.0472 (+/− 20 ppm)
492.0399
[M−H]−
227.0581
0
20
40
60
80
100
250
300
350
400
450
500

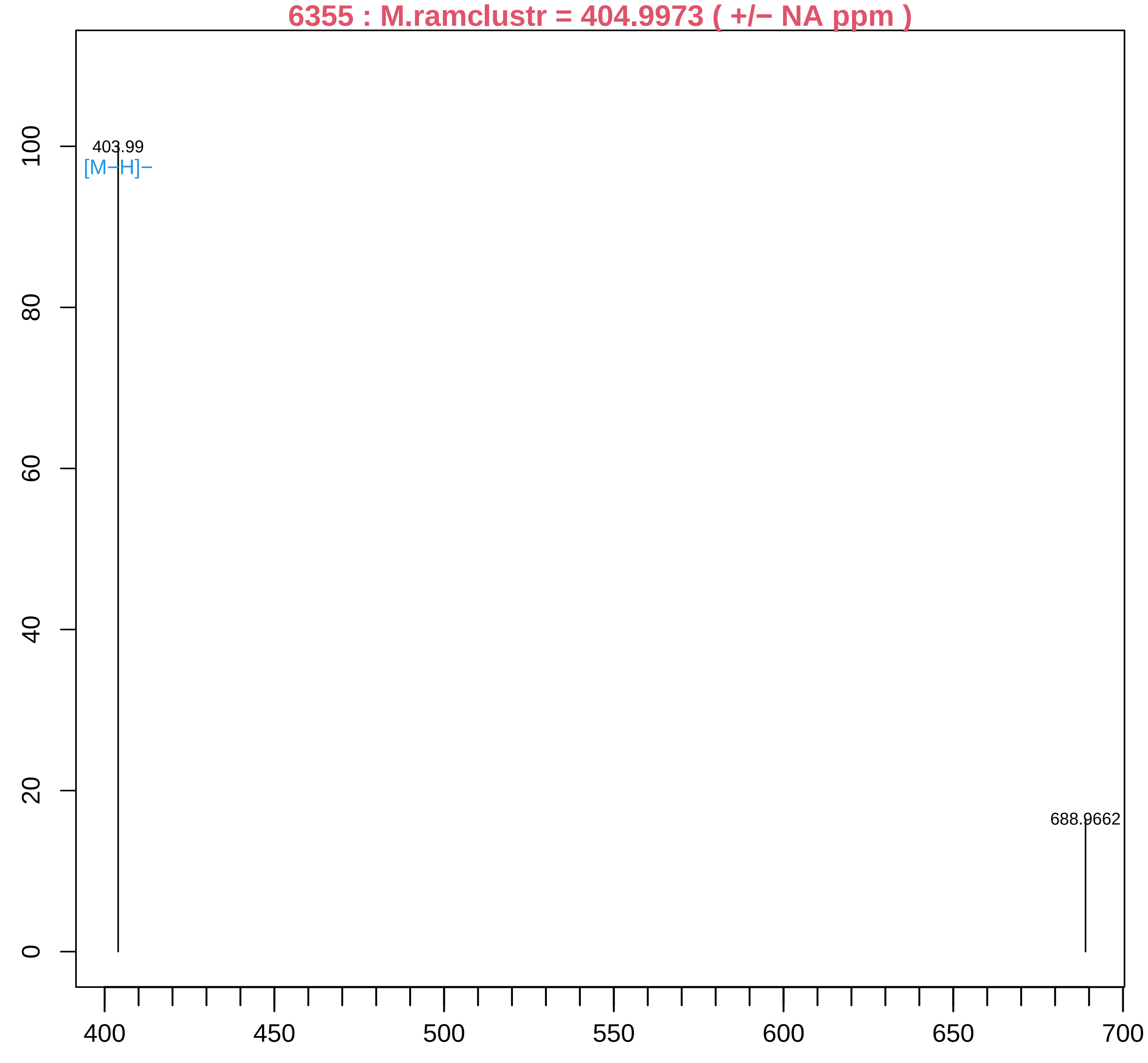
6355 : M.ramclustr = 404.9973 (+/− NA ppm)
403.99
[M−H]−
688.9662
0
20
40
60
80
100
400
450
500
550
600
650
700

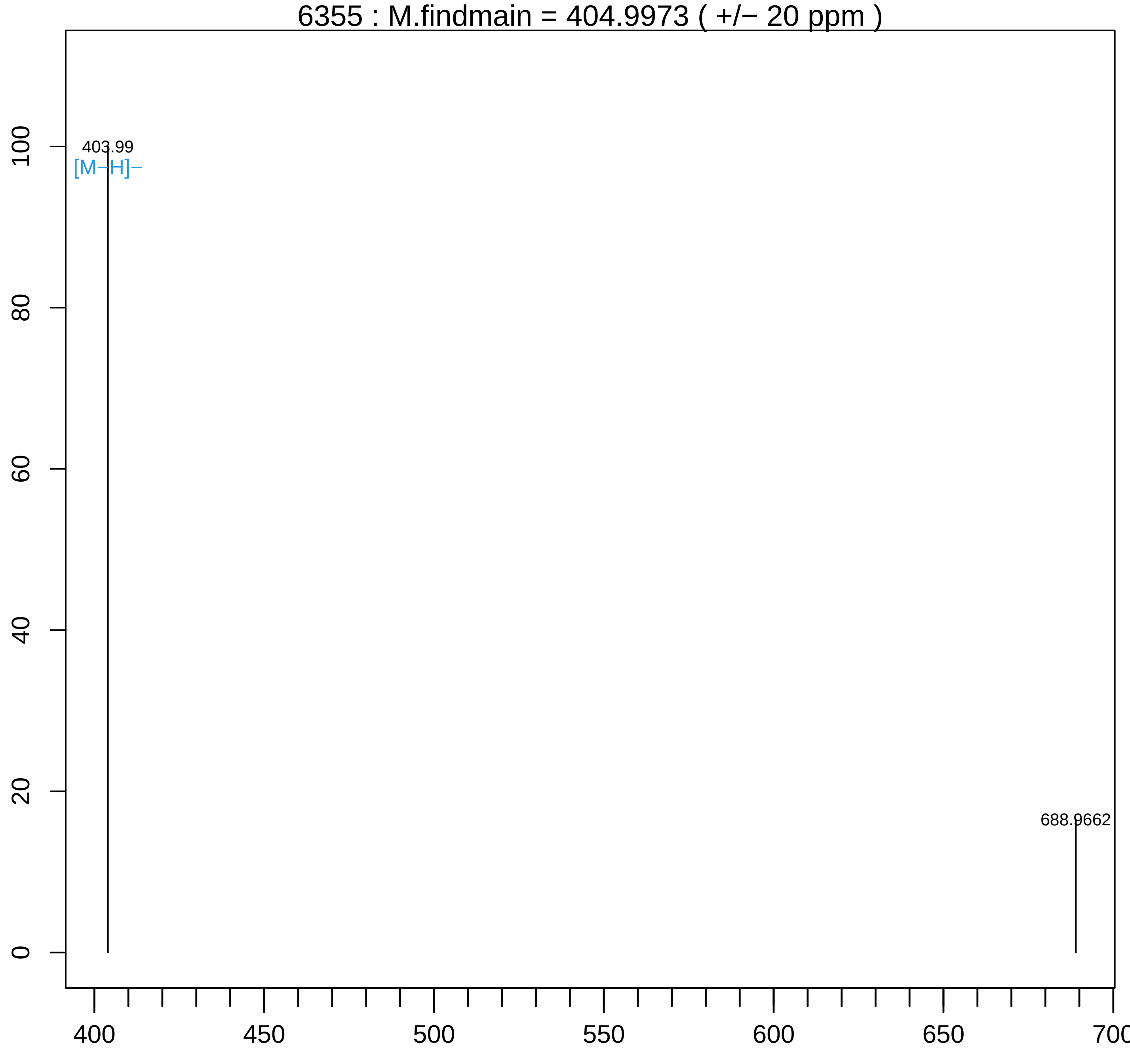
6355 : M.findmain = 404.9973 (+/− 20 ppm)
403.99
[M−H]−
688.9662
0
20
40
60
80
100
400
450
500
550
600
650
700

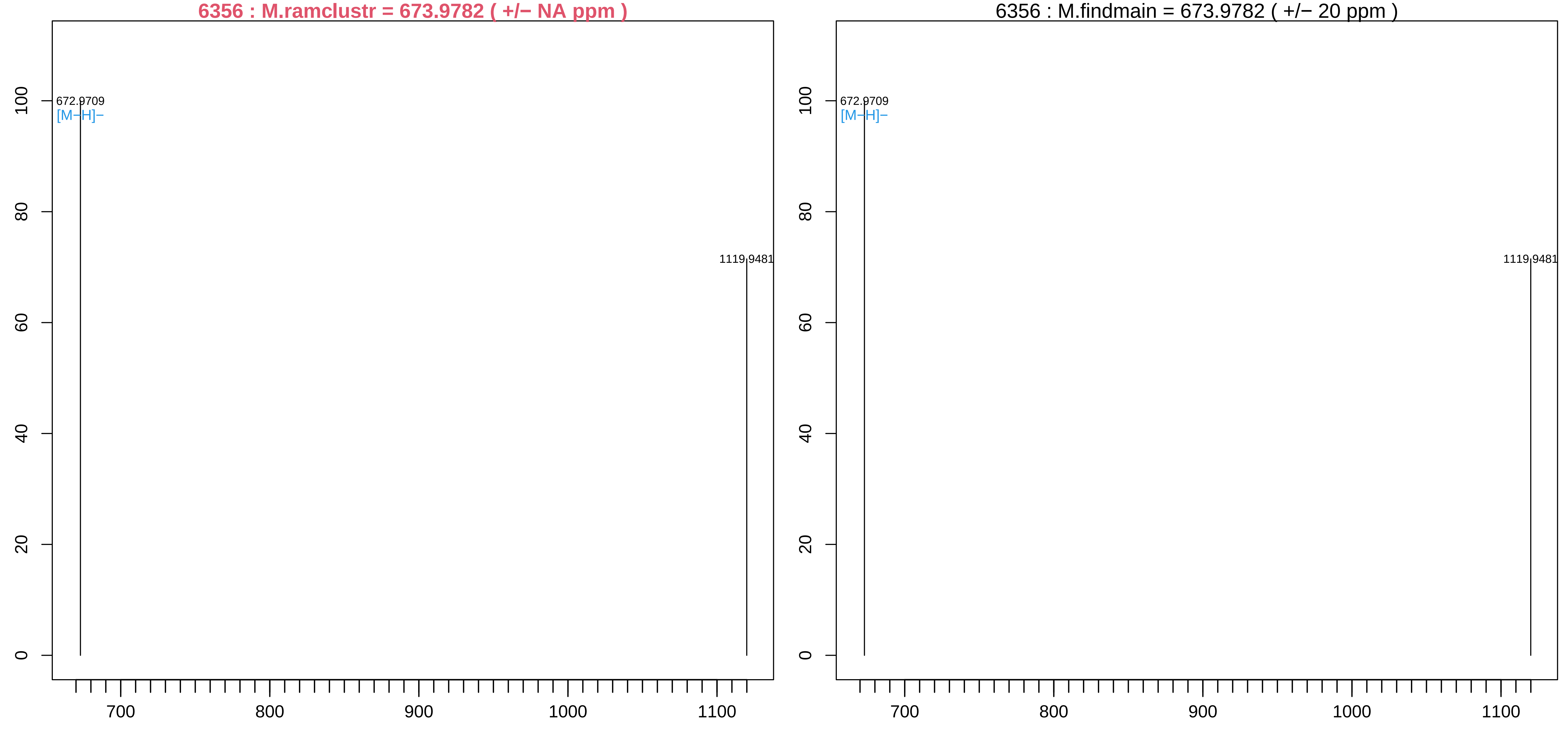

6356 : M.ramclustr = 673.9782 (+/− NA ppm)
672.9709
[M−H]−
1119.9481
0
20
40
60
80
100
700
800
900
1000
1100
6356 : M.findmain = 673.9782 (+/− 20 ppm)
672.9709
[M−H]−
1119.9481
0
20
40
60
80
100
700
800
900
1000
1100

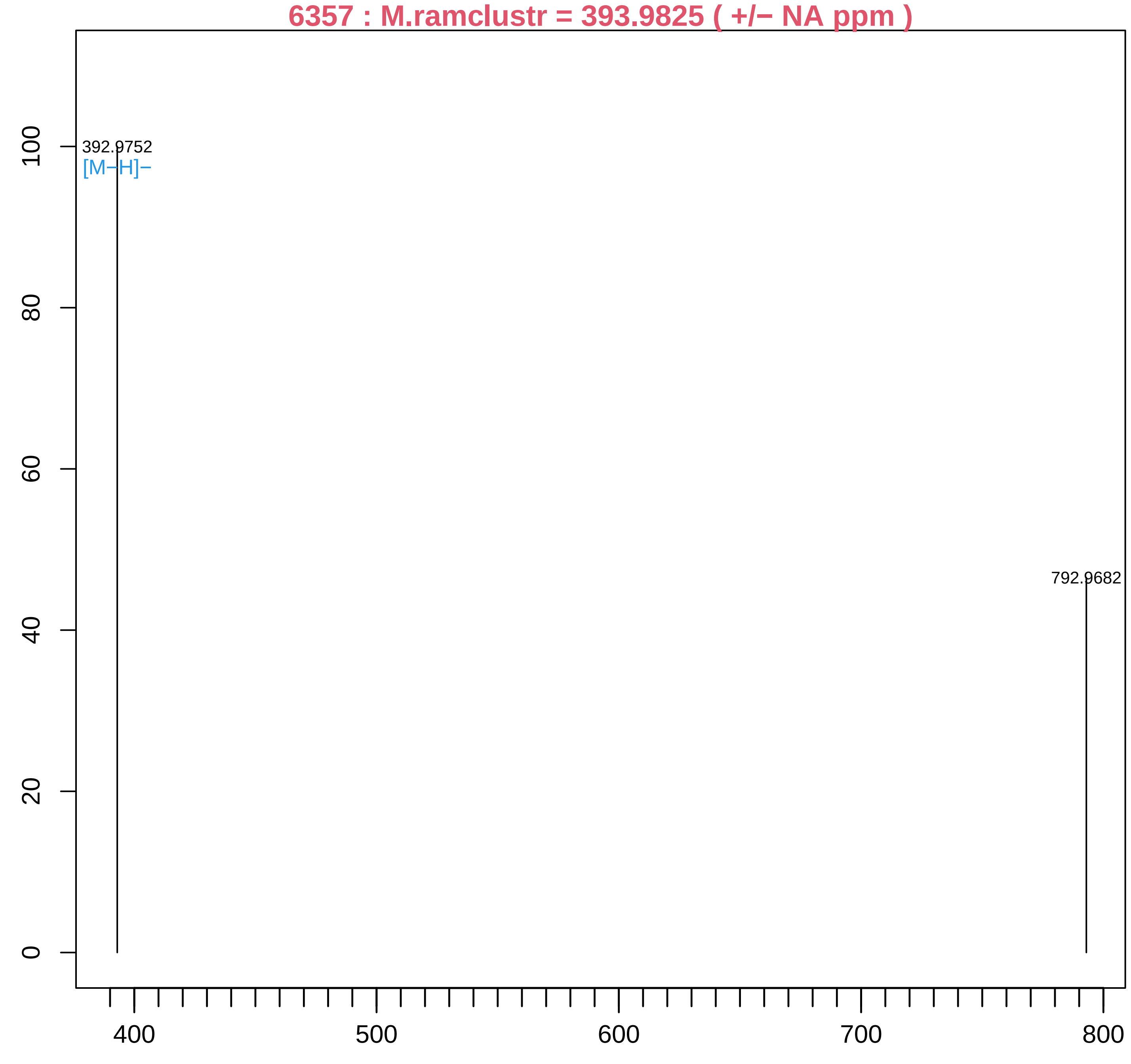
6357 : M.ramclustr = 393.9825 (+/− NA ppm)
392.9752
[M−H]−
792.9682
0
20
40
60
80
100
400
500
600
700
800

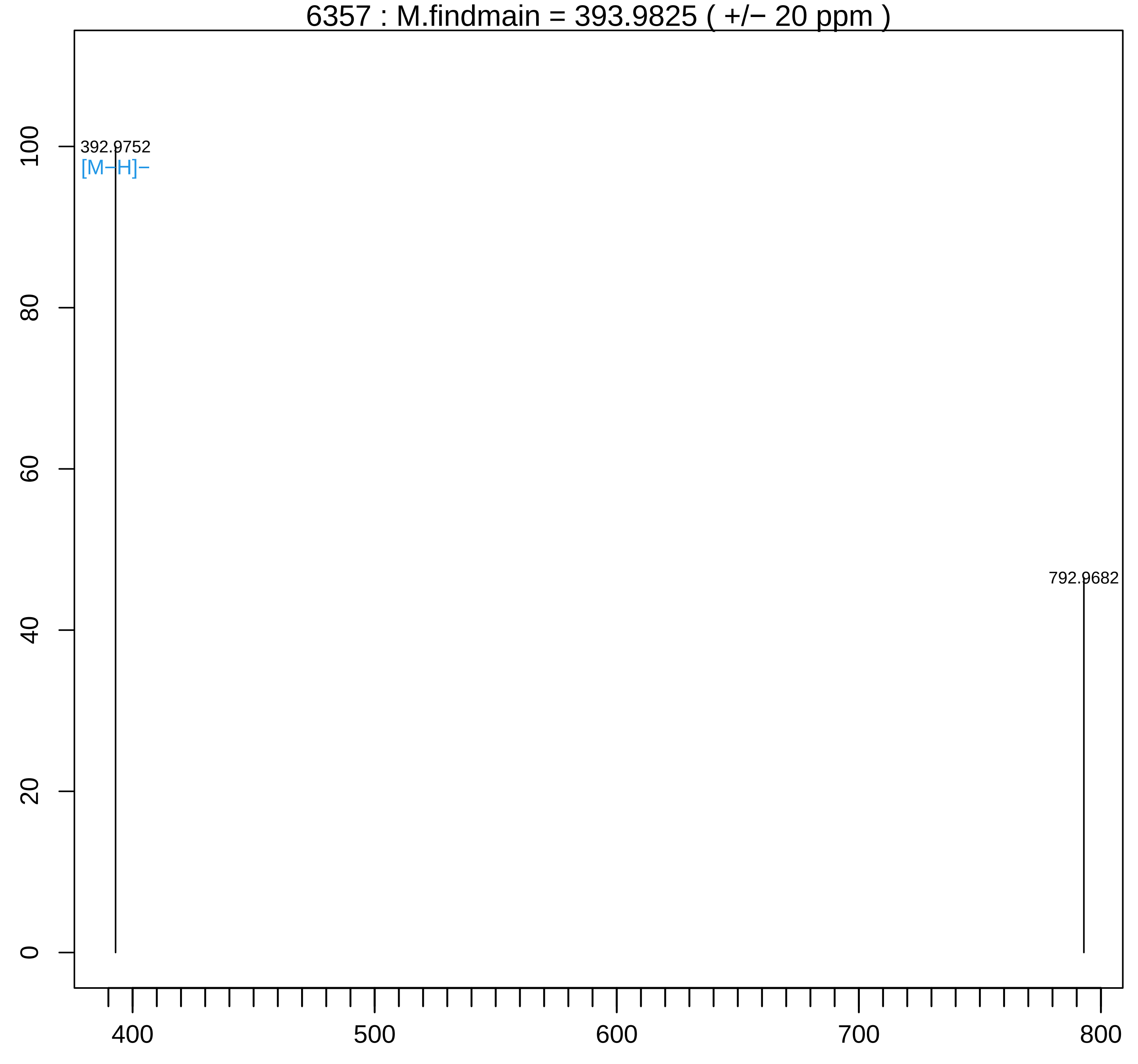
6357 : M.findmain = 393.9825 (+/− 20 ppm)
392.9752
[M−H]−
792.9682
0
20
40
60
80
100
400
500
600
700
800

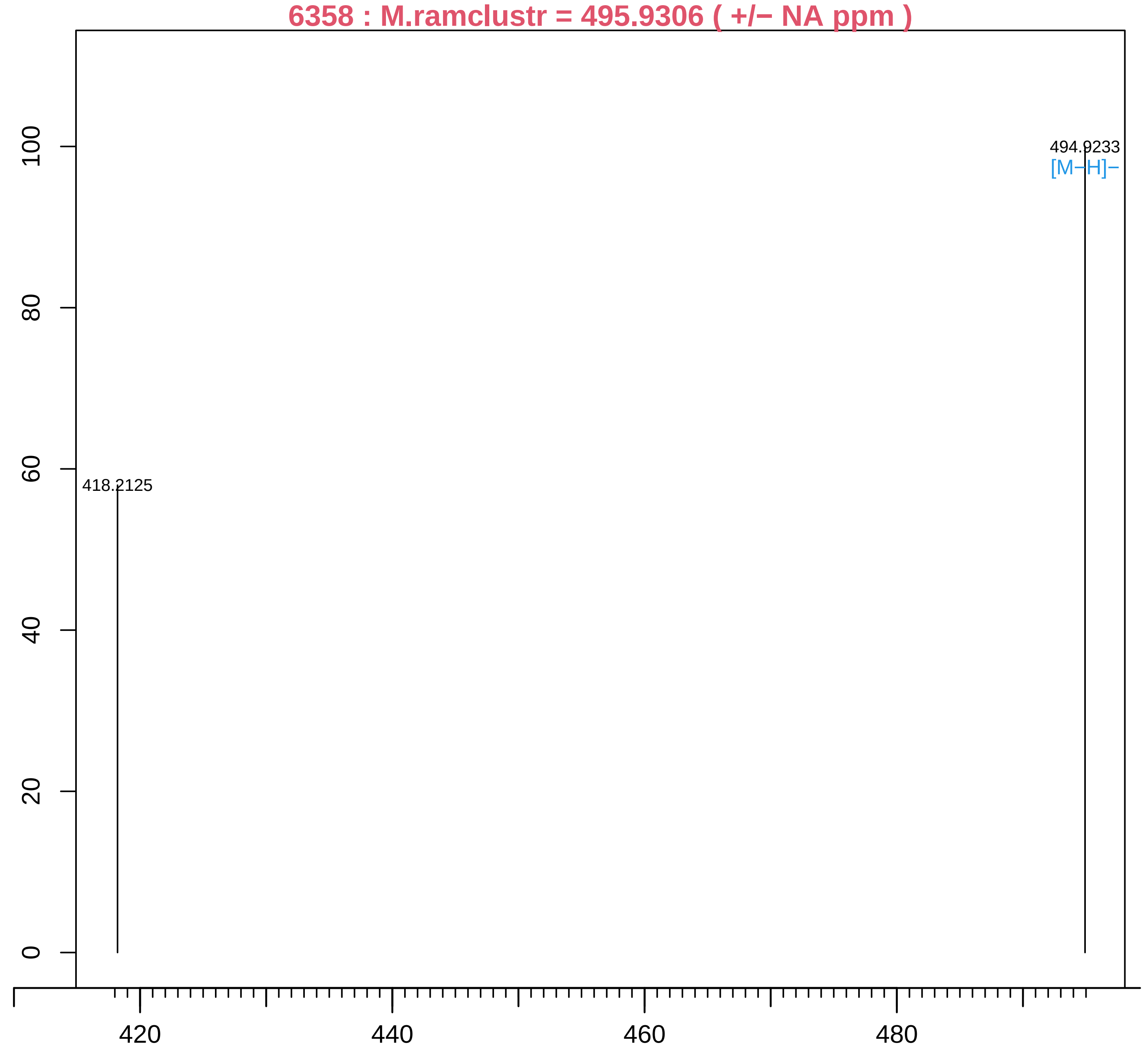
6358 : M.ramclustr = 495.9306 (+/− NA ppm)
494.9233
[M−H]−
418.2125
0
20
40
60
80
100
420
440
460
480

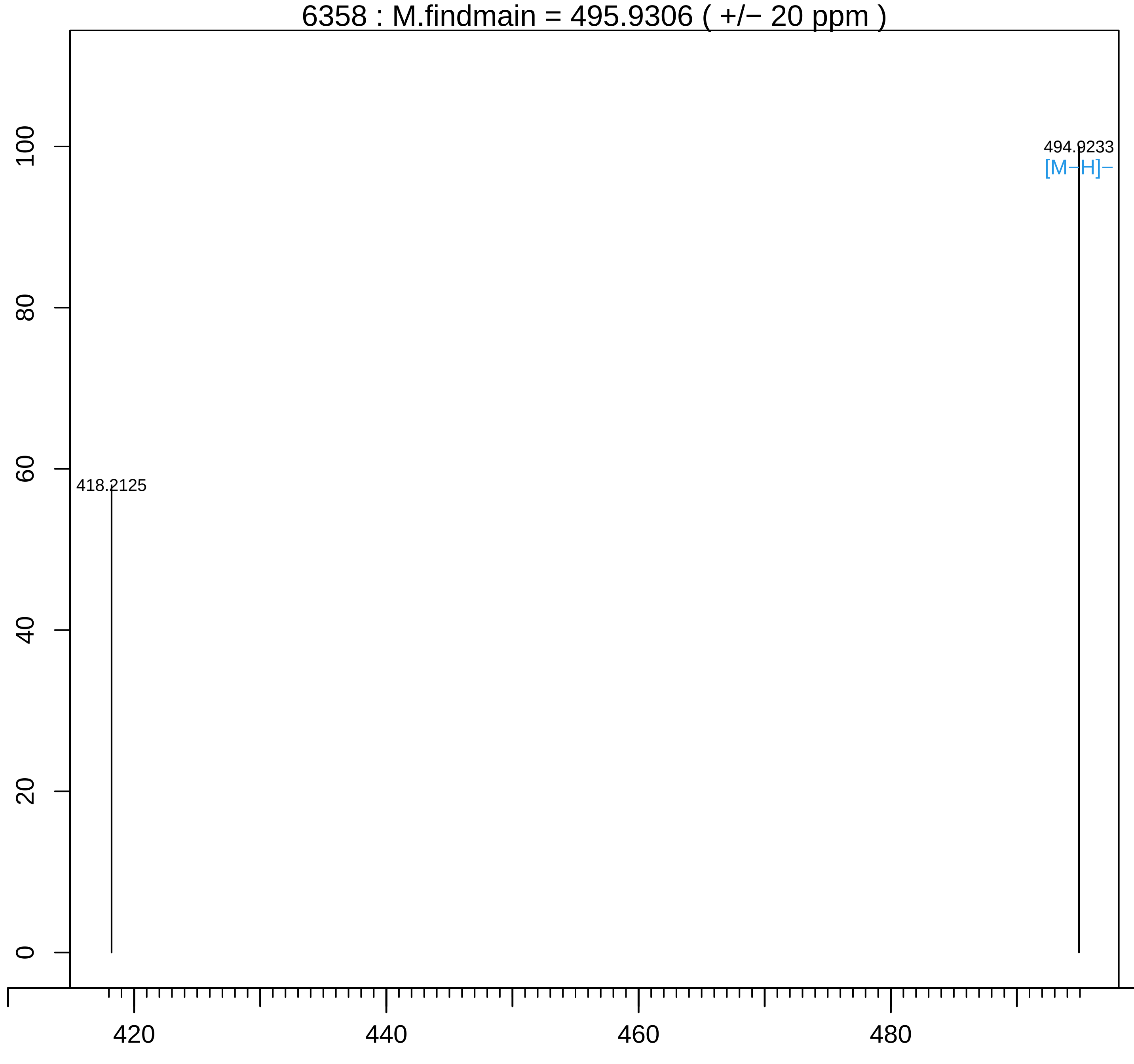
6358 : M.findmain = 495.9306 (+/− 20 ppm)
494.9233
[M−H]−
418.2125
0
20
40
60
80
100
420
440
460
480

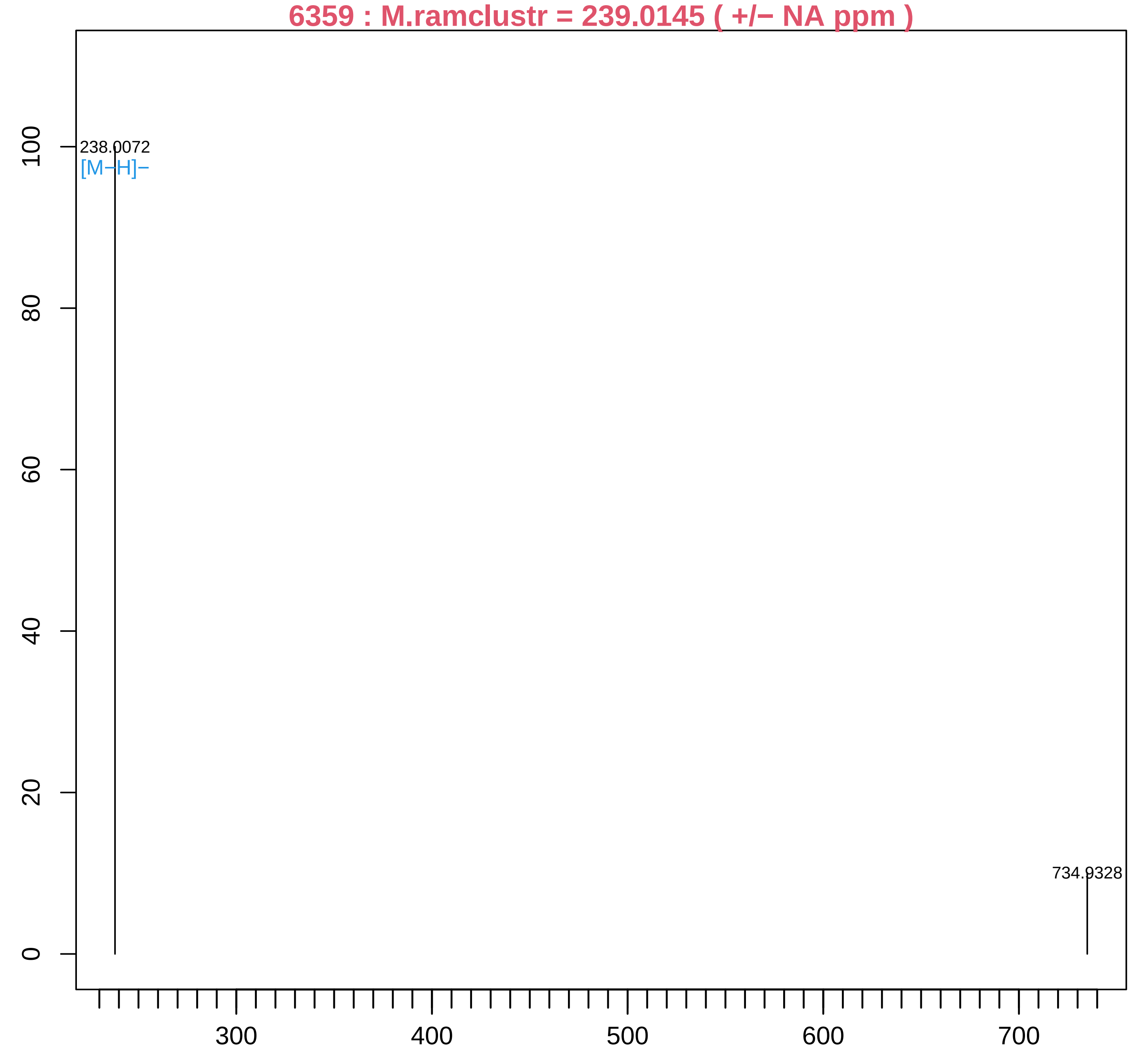
6359 : M.ramclustr = 239.0145 (+/− NA ppm)
238.0072
[M−H]−
734.9328
0
20
40
60
80
100
300
400
500
600
700

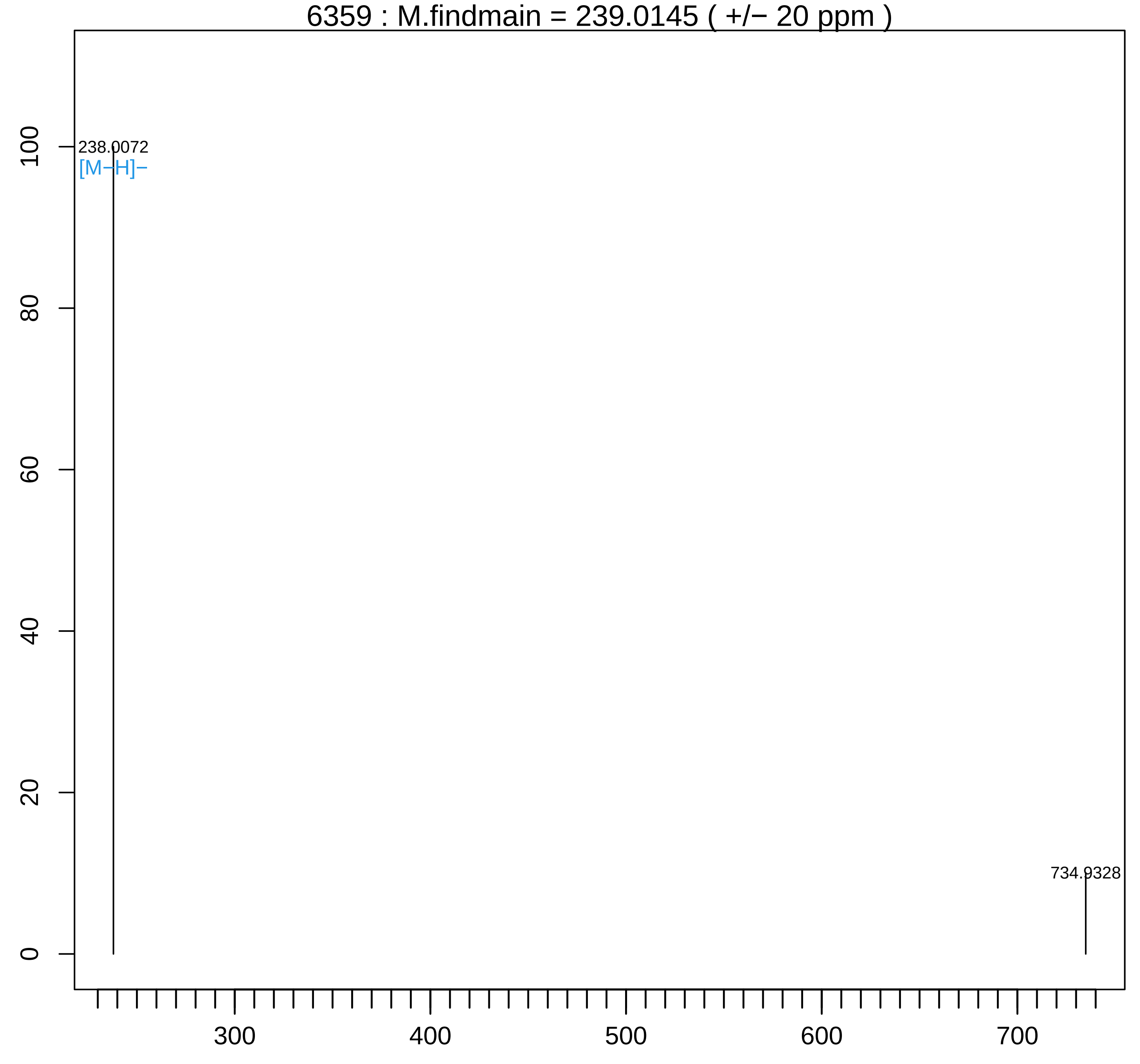
6359 : M.findmain = 239.0145 (+/− 20 ppm)
238.0072
[M−H]−
734.9328
0
20
40
60
80
100
300
400
500
600
700

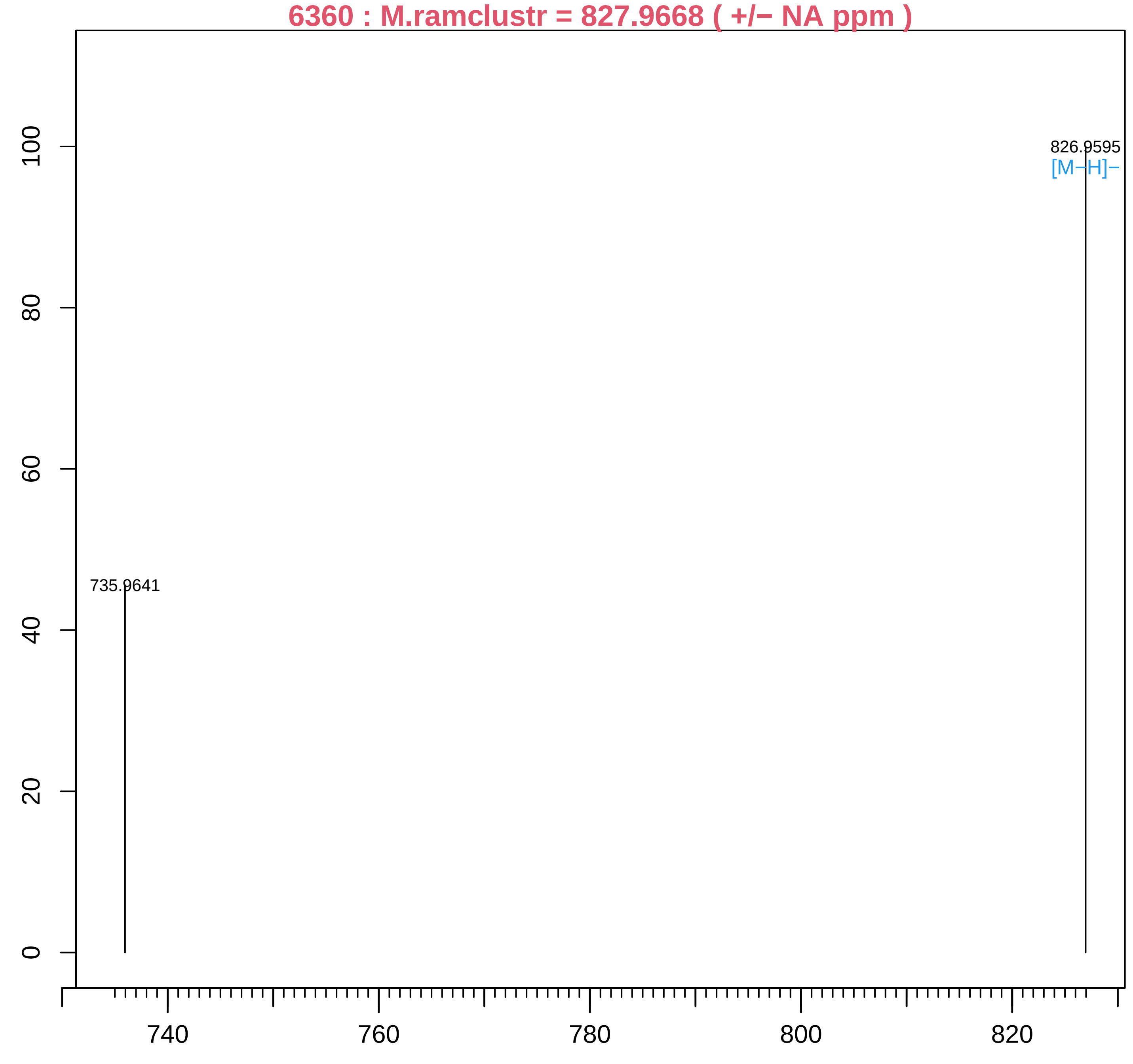
6360 : M.ramclustr = 827.9668 (+/− NA ppm)
826.9595
[M−H]−
735.9641
0
20
40
60
80
100
740
760
780
800
820

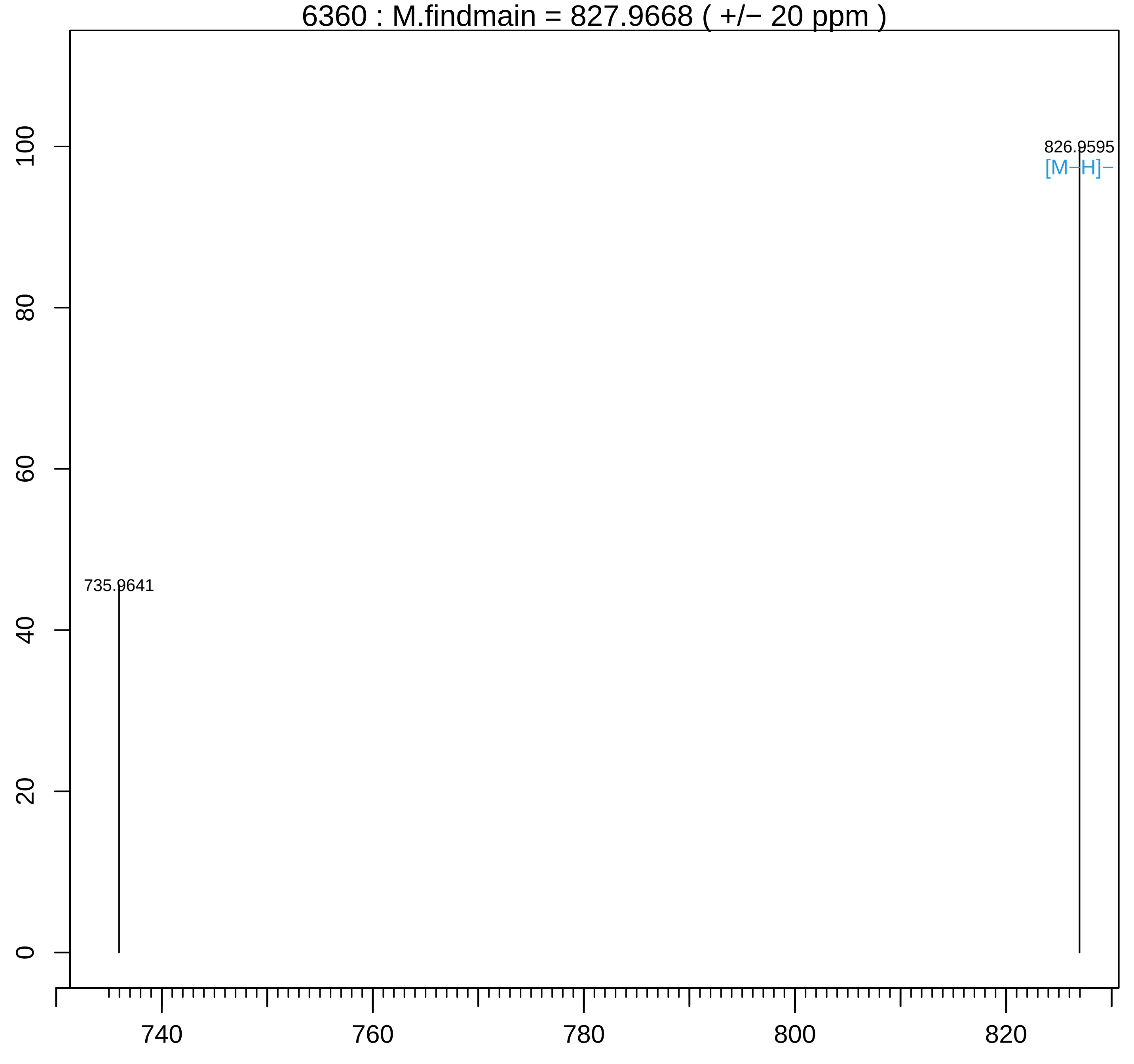
6360 : M.findmain = 827.9668 (+/− 20 ppm)
826.9595
[M−H]−
735.9641
0
20
40
60
80
100
740
760
780
800
820

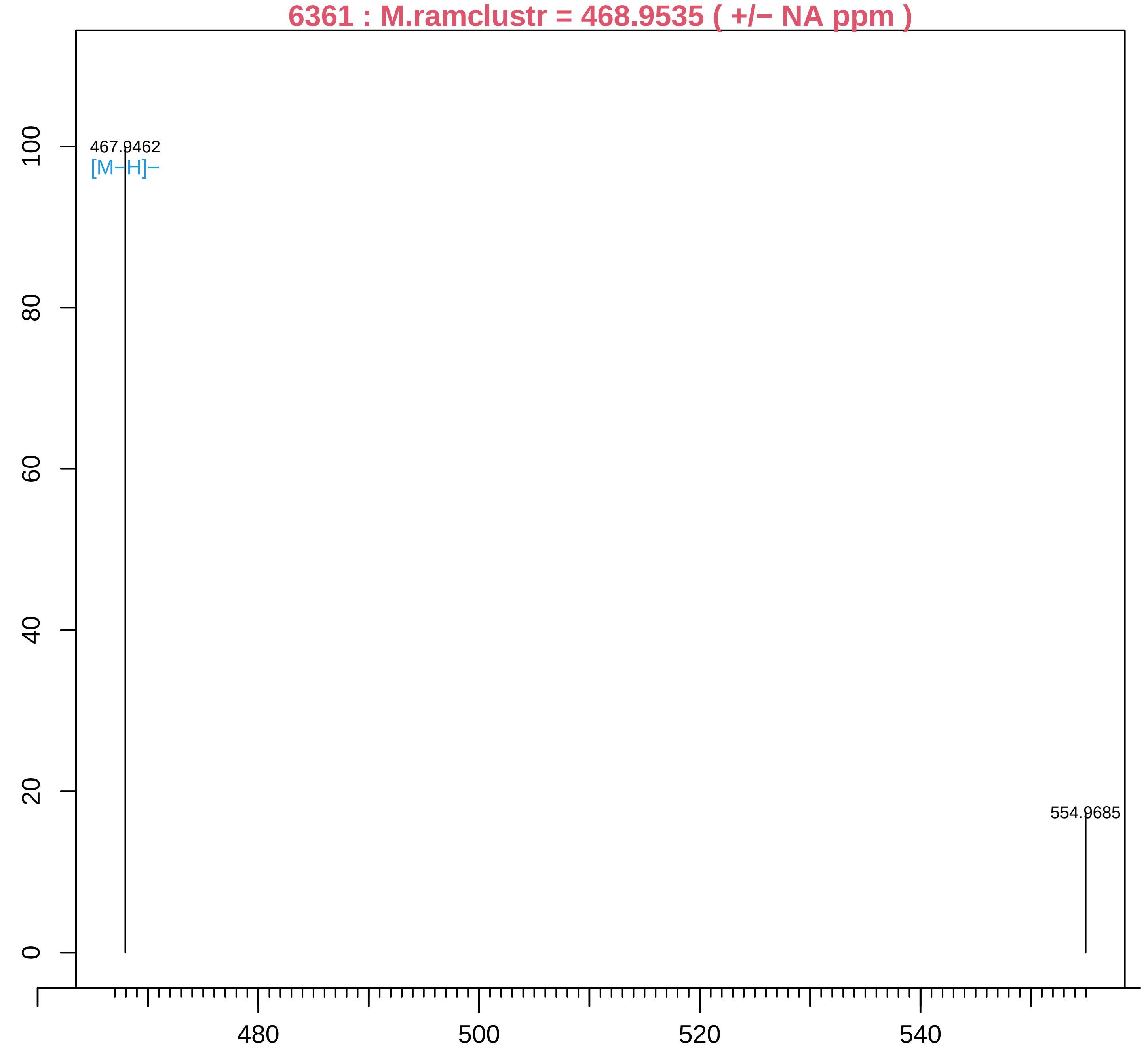
6361 : M.ramclustr = 468.9535 (+/− NA ppm)
467.9462
[M−H]−
554.9685
0
20
40
60
80
100
480
500
520
540

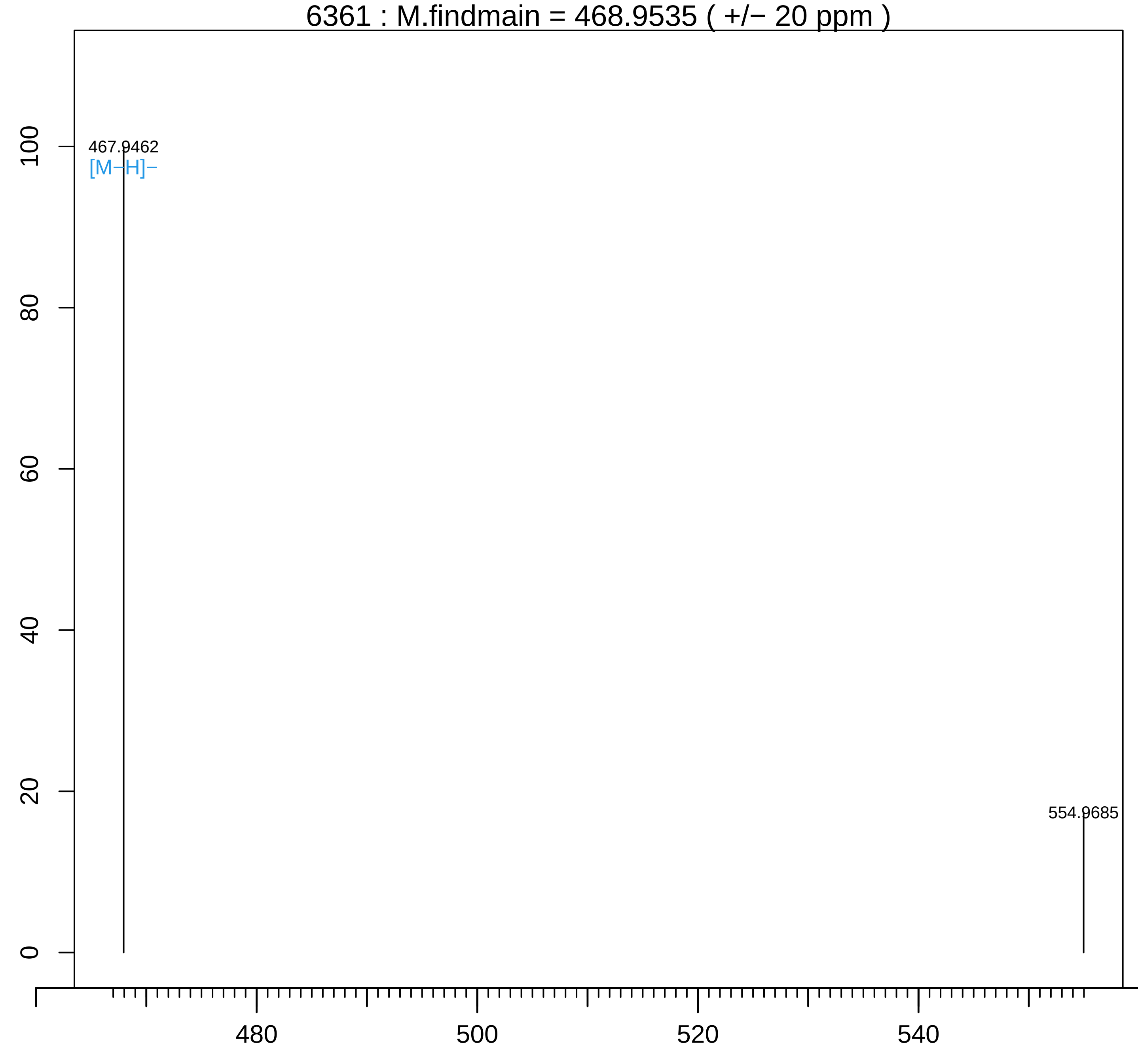
6361 : M.findmain = 468.9535 (+/− 20 ppm)
467.9462
[M−H]−
554.9685
0
20
40
60
80
100
480
500
520
540

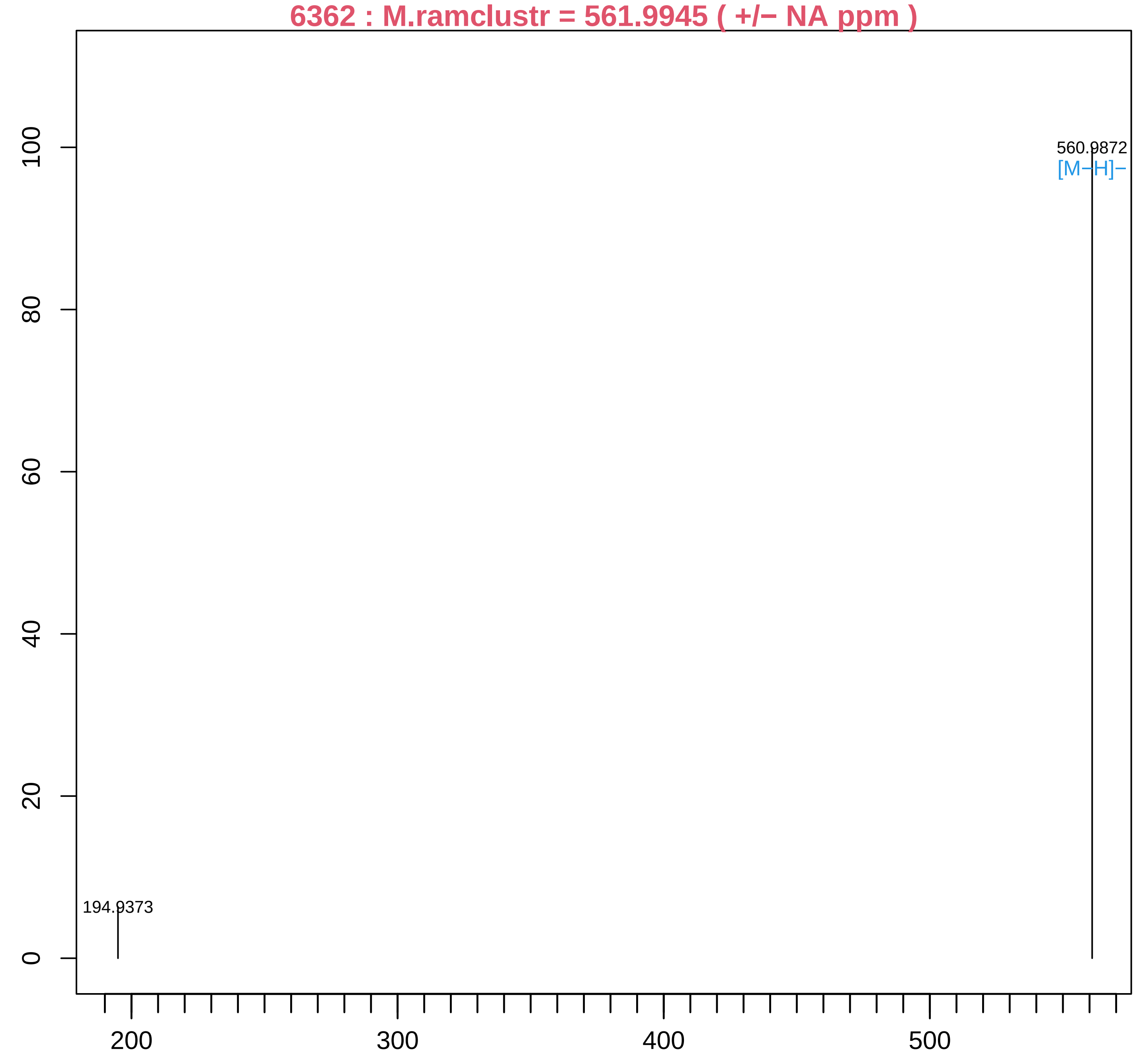
6362 : M.ramclustr = 561.9945 (+/− NA ppm)
560.9872
[M−H]−
194.9373
0
20
40
60
80
100
200
300
400
500

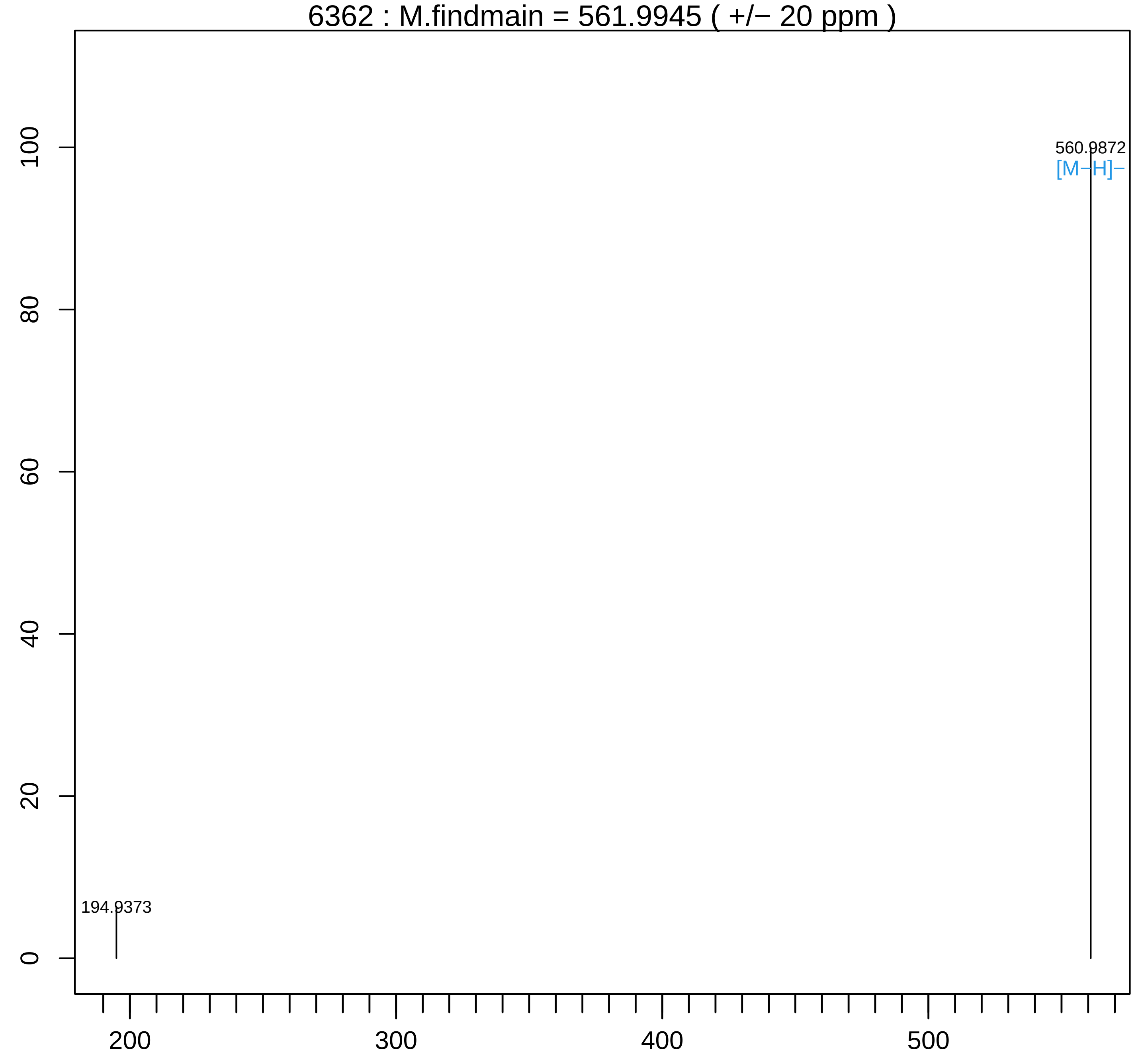
6362 : M.findmain = 561.9945 (+/− 20 ppm)
560.9872
[M−H]−
194.9373
0
20
40
60
80
100
200
300
400
500

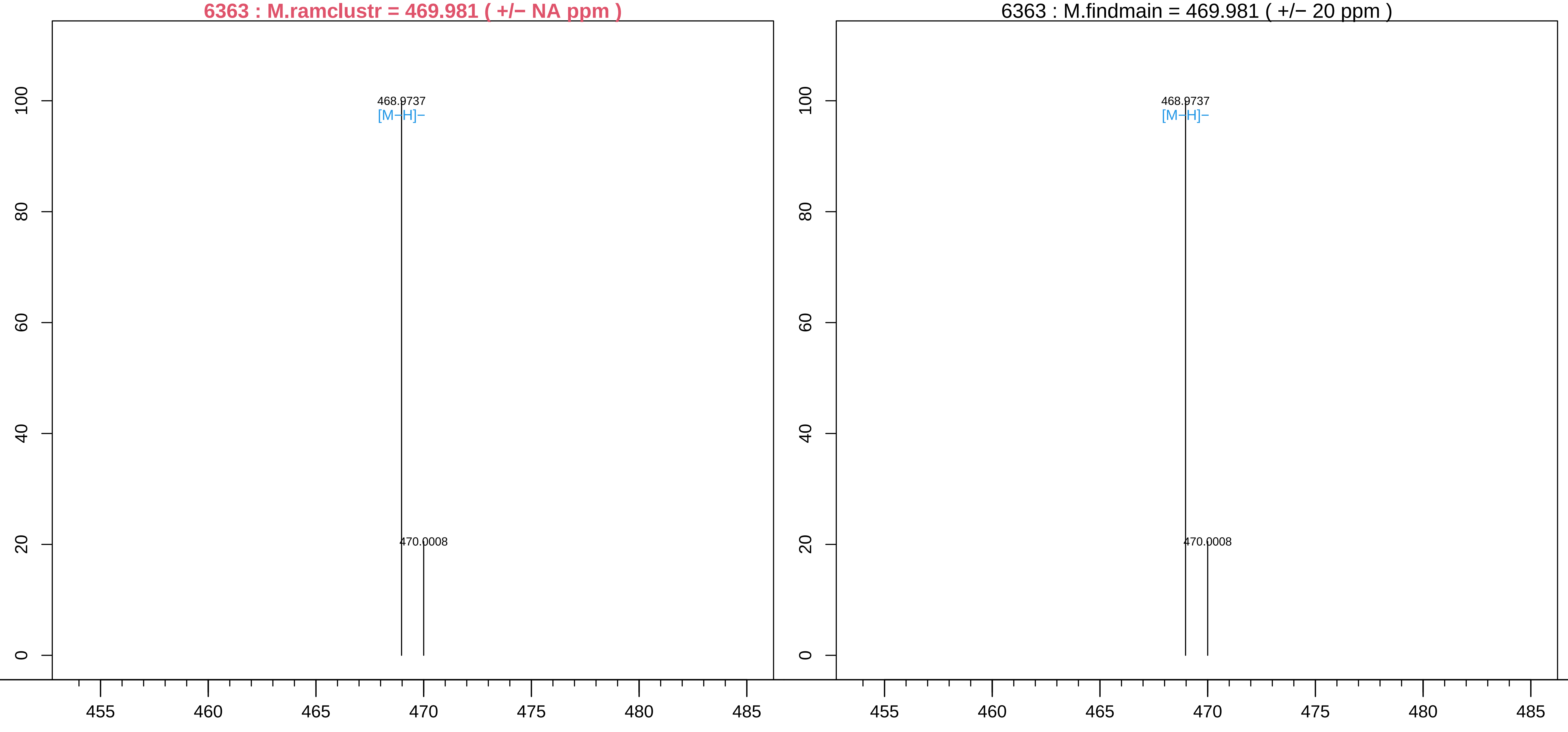
6363 : M.ramclustr = 469.981 (+/− NA ppm)
6363 : M.findmain = 469.981 (+/− 20 ppm)
468.9737
[M−H]−
470.0008
0
20
40
60
80
100
455
460
465
470
475
480
485

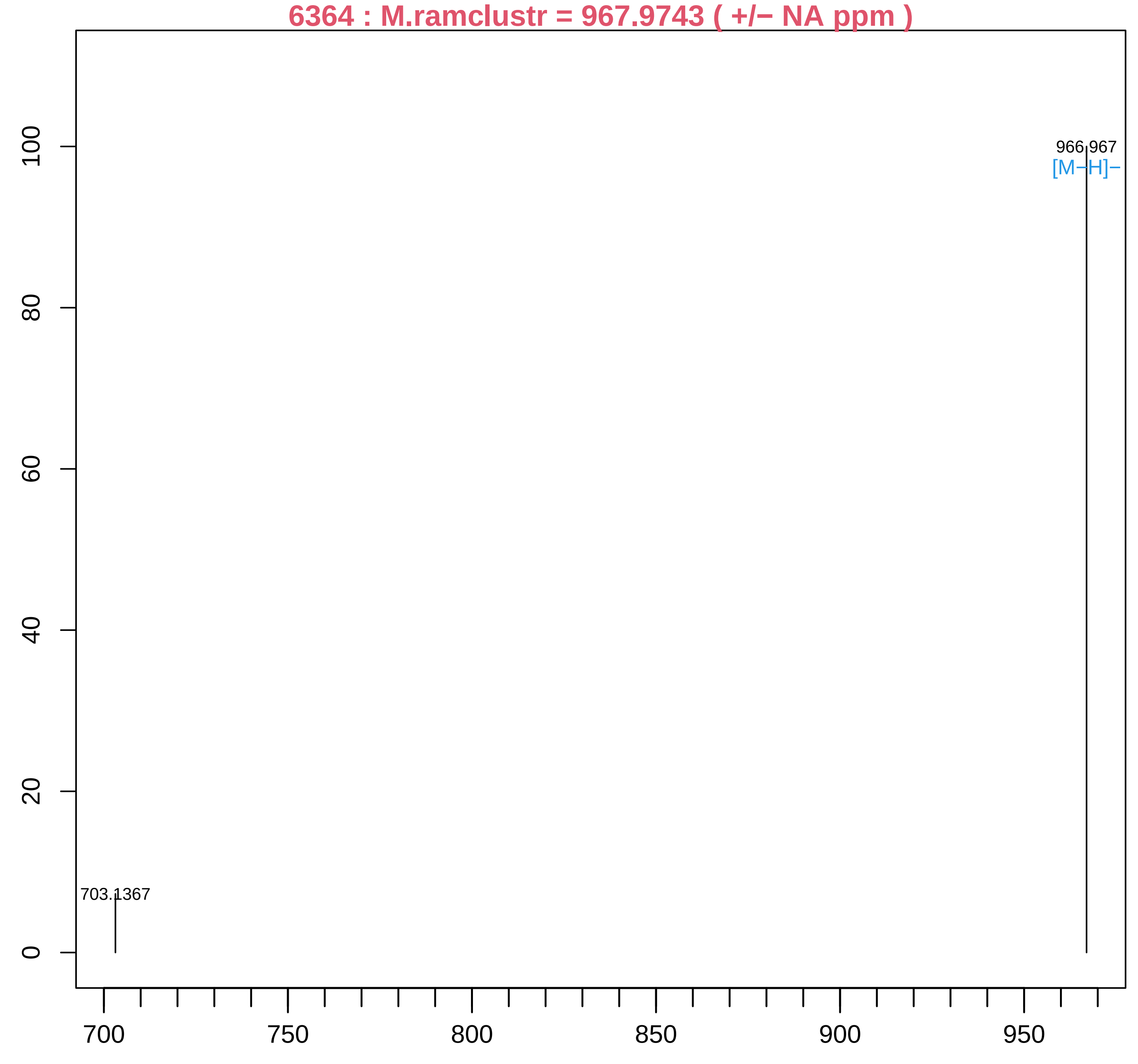
6364 : M.ramclustr = 967.9743 (+/− NA ppm)
966.967
[M−H]−
703.1367
0
20
40
60
80
100
700
750
800
850
900
950

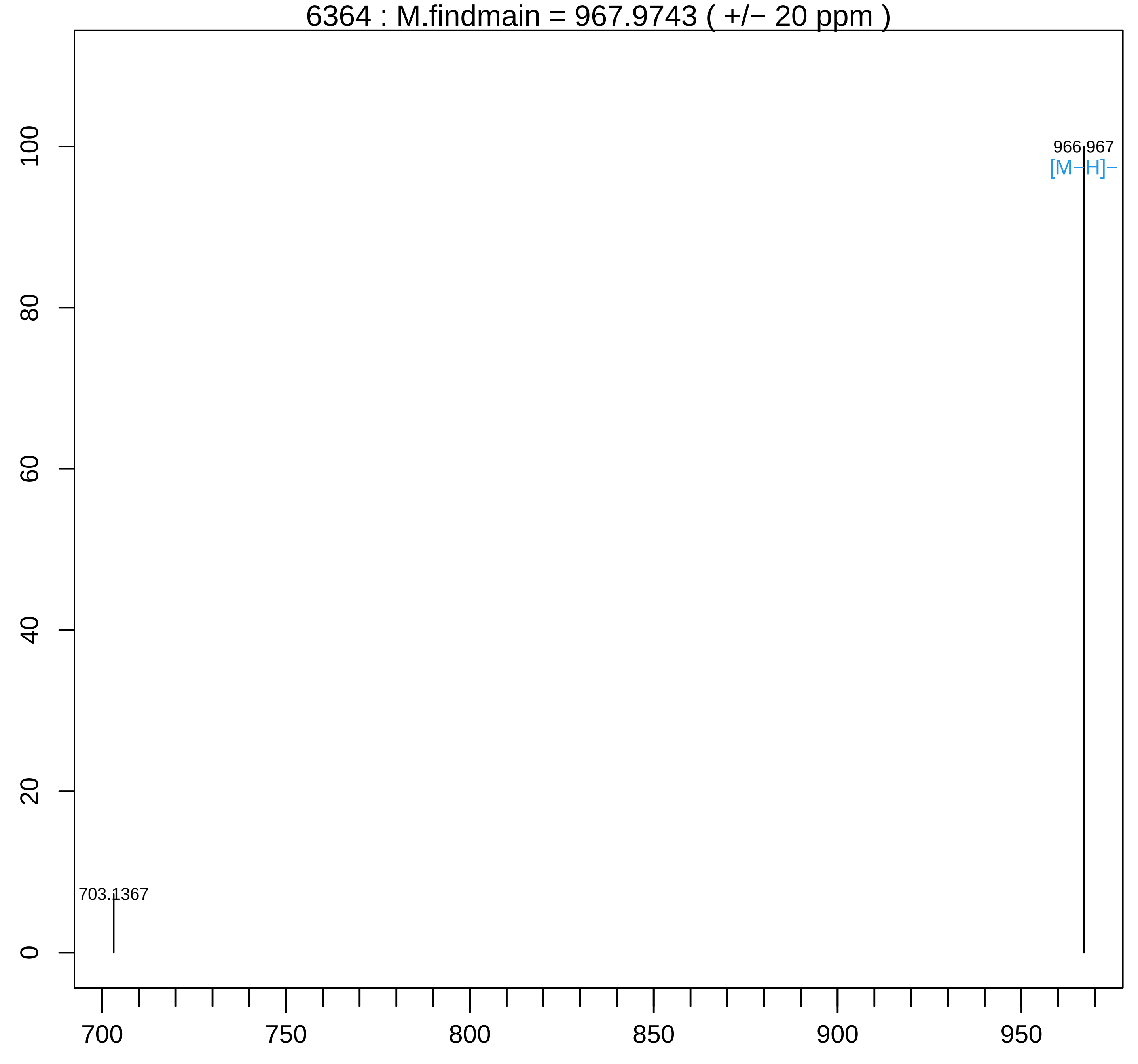
6364 : M.findmain = 967.9743 (+/− 20 ppm)
966.967
[M−H]−
703.1367
0
20
40
60
80
100
700
750
800
850
900
950

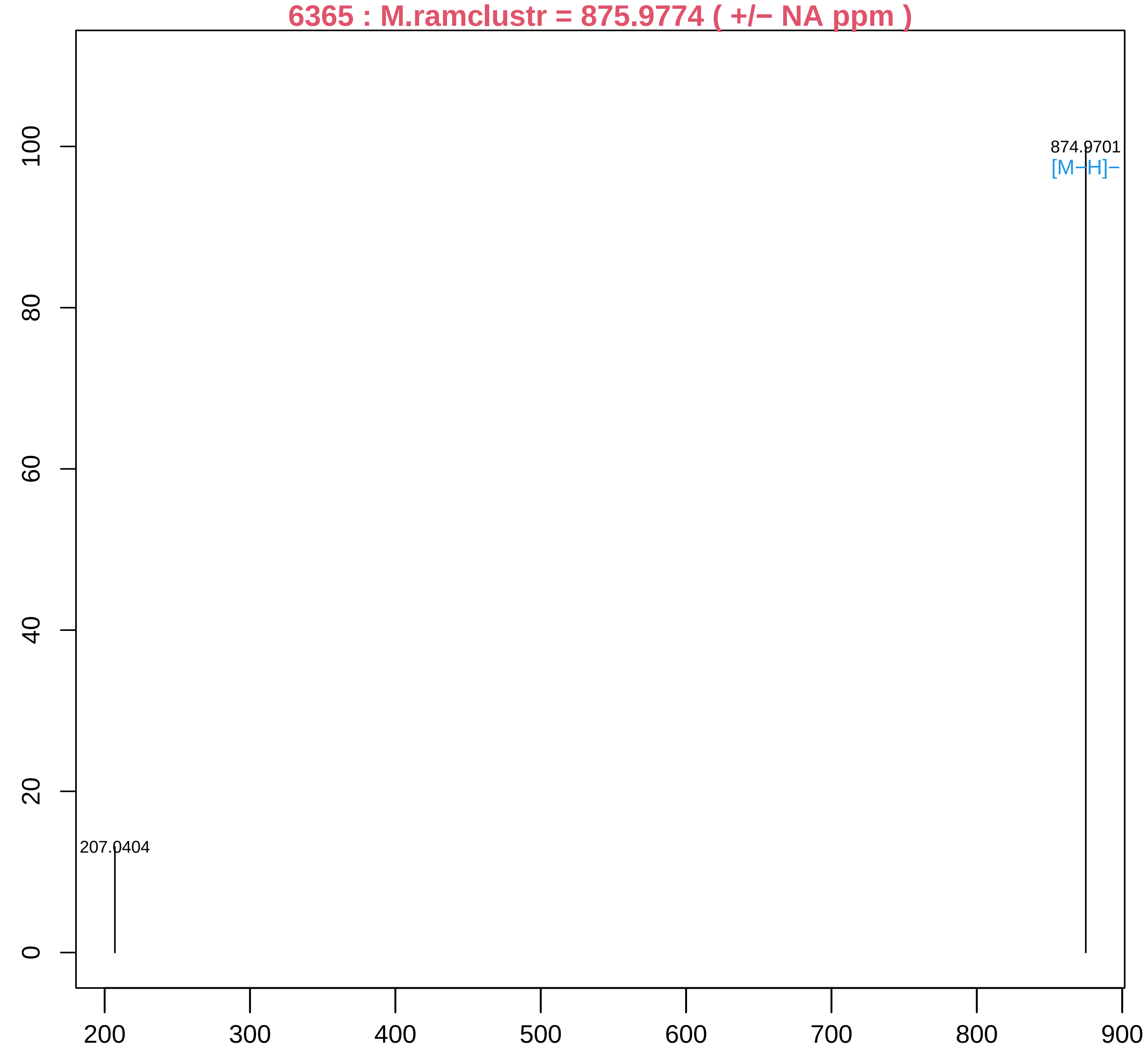
6365 : M.ramclustr = 875.9774 (+/− NA ppm)
874.9701
[M−H]−
207.0404

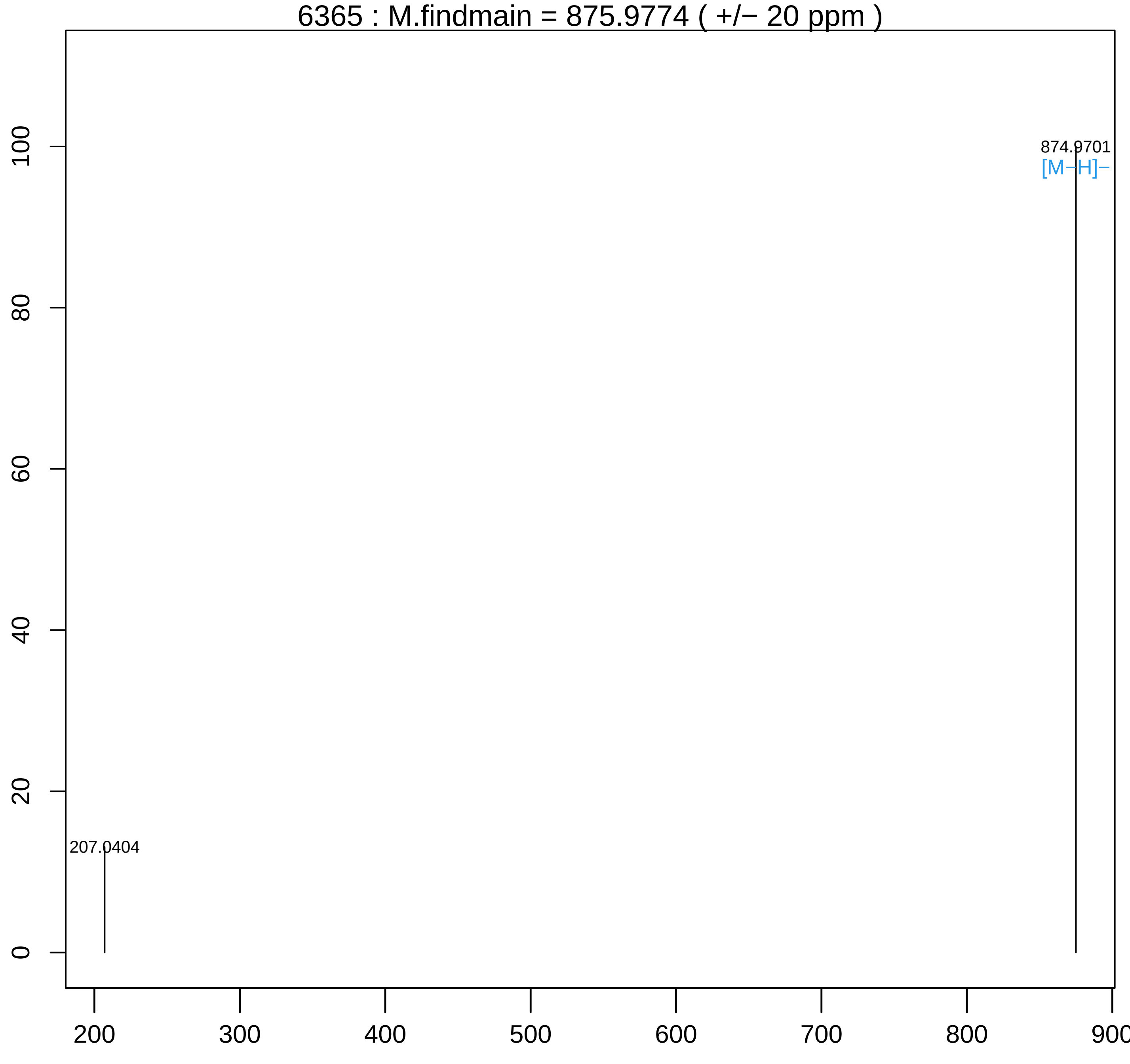
6365 : M.findmain = 875.9774 (+/− 20 ppm)
874.9701
[M−H]−
207.0404

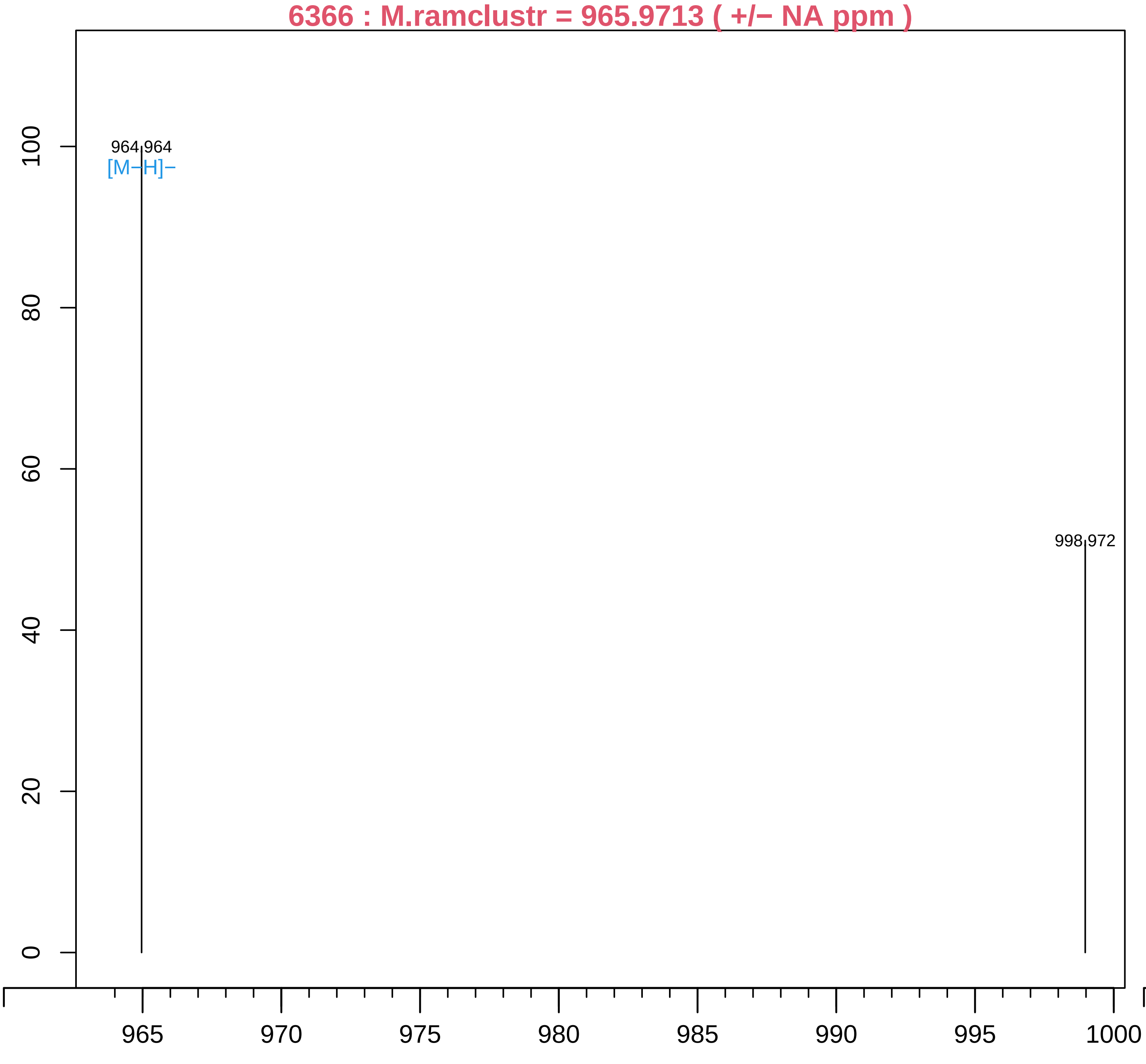
6366 : M.ramclustr = 965.9713 (+/− NA ppm)
964.964
[M−H]−
998.972
0
20
40
60
80
100
965
970
975
980
985
990
995
1000

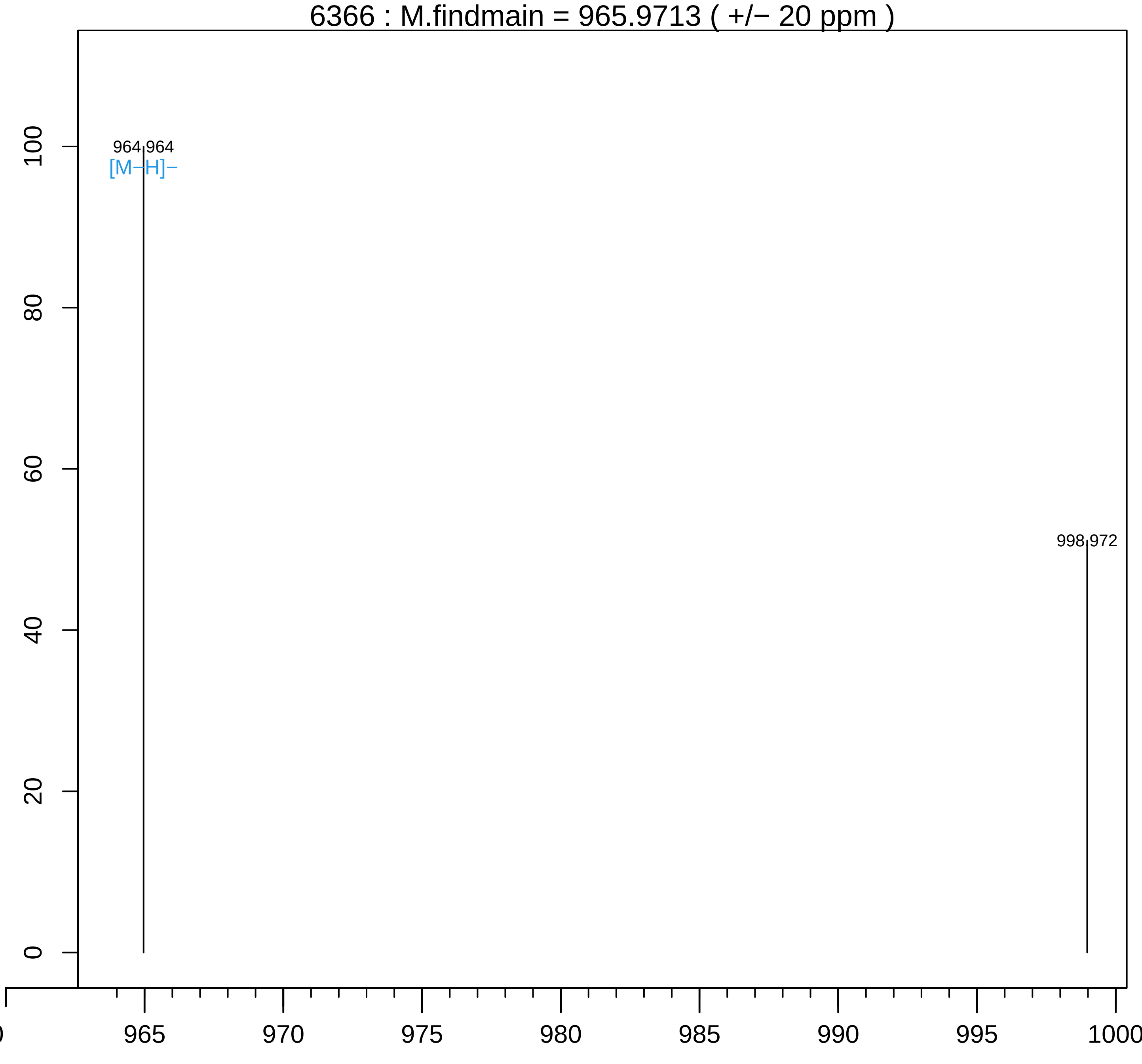
6366 : M.findmain = 965.9713 (+/− 20 ppm)
964.964
[M−H]−
998.972
0
20
40
60
80
100
965
970
975
980
985
990
995
1000

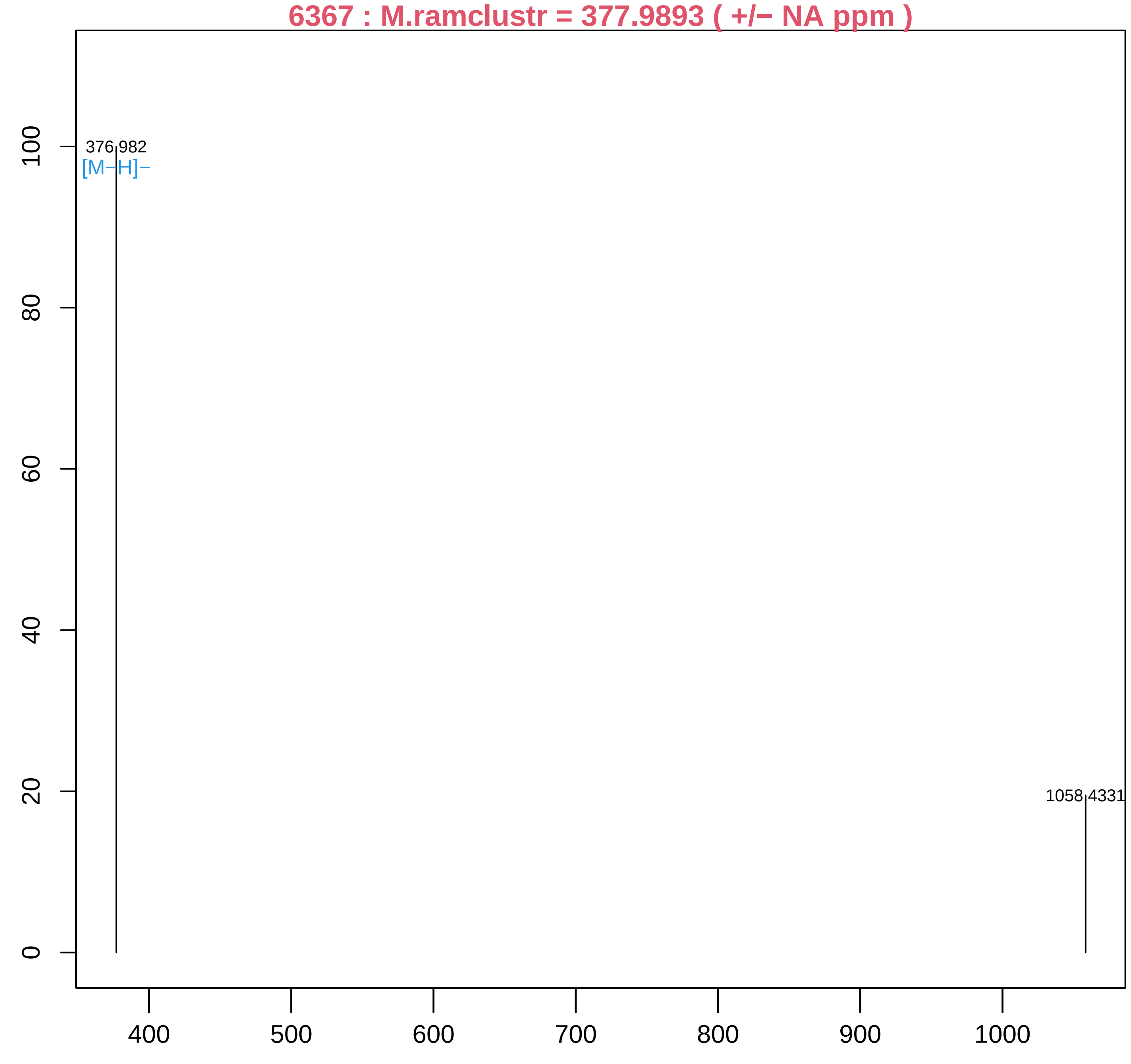
6367 : M.ramclustr = 377.9893 (+/− NA ppm)
376.982
[M−H]−
1058.4331
0
20
40
60
80
100
400
500
600
700
800
900
1000

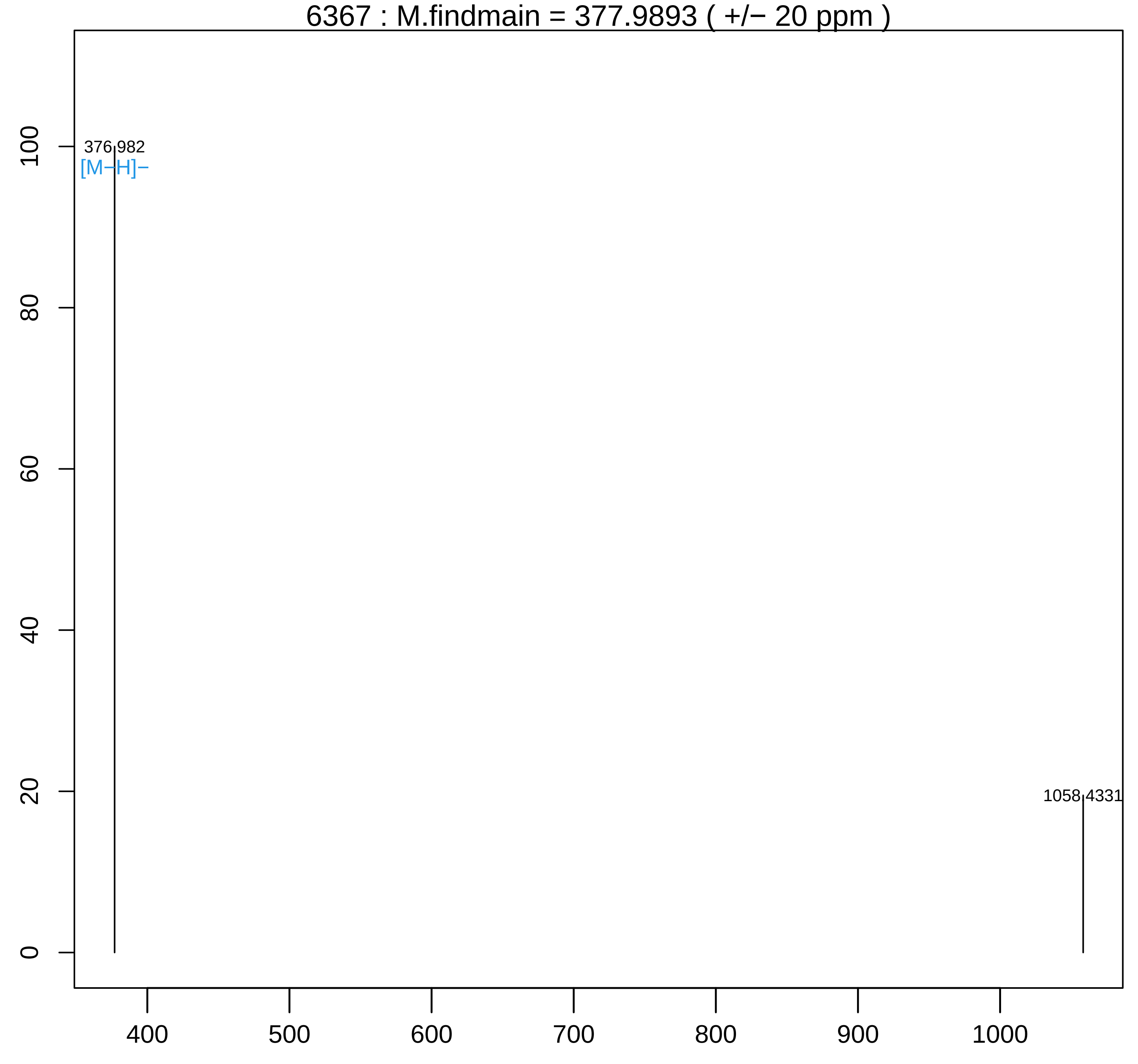
6367 : M.findmain = 377.9893 (+/− 20 ppm)
376.982
[M−H]−
1058.4331
0
20
40
60
80
100
400
500
600
700
800
900
1000

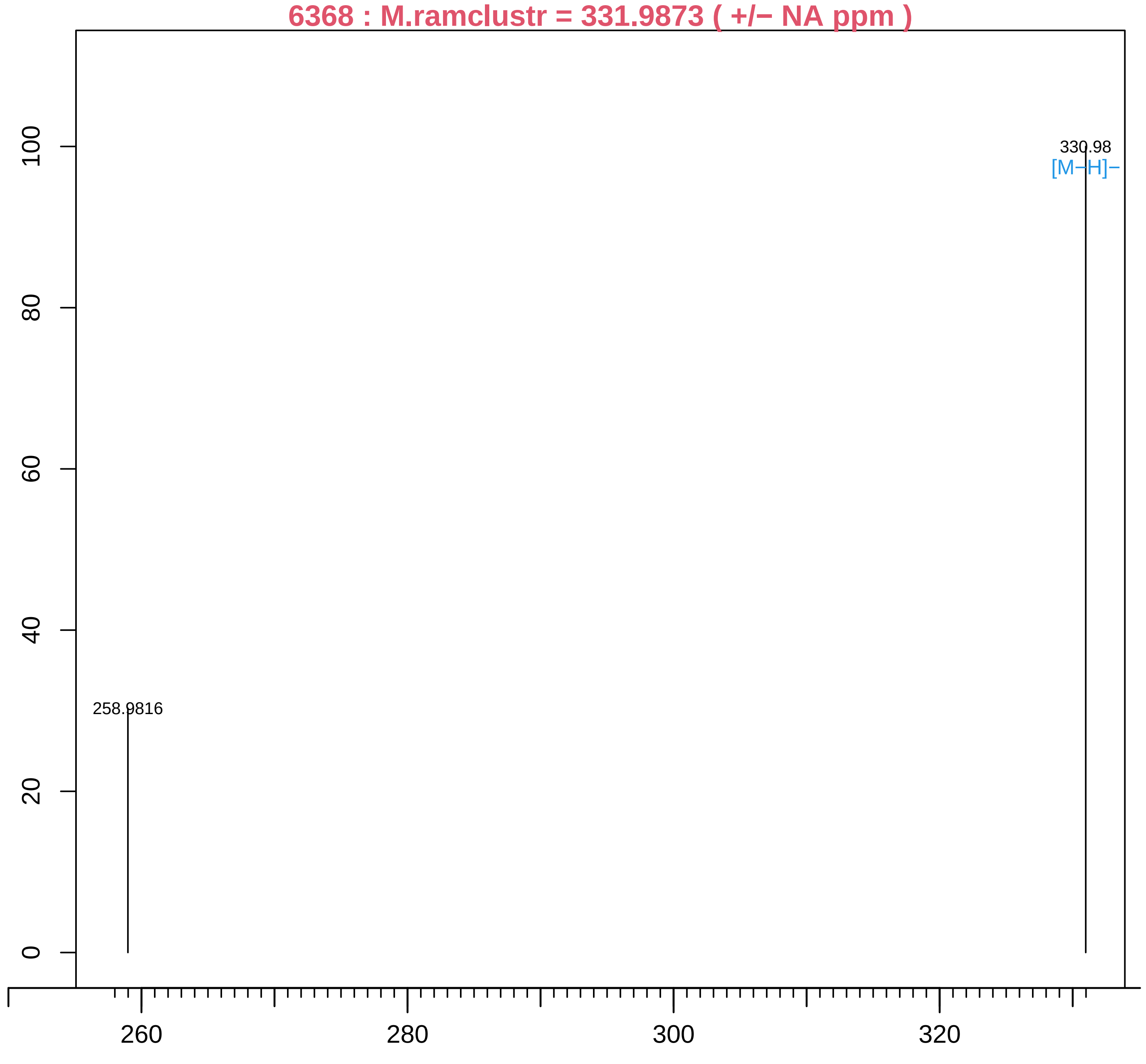

6368 : M.ramclustr = 331.9873 (+/− NA ppm)
330.98
[M−H]−
258.9816
0
20
40
60
80
100
260
280
300
320

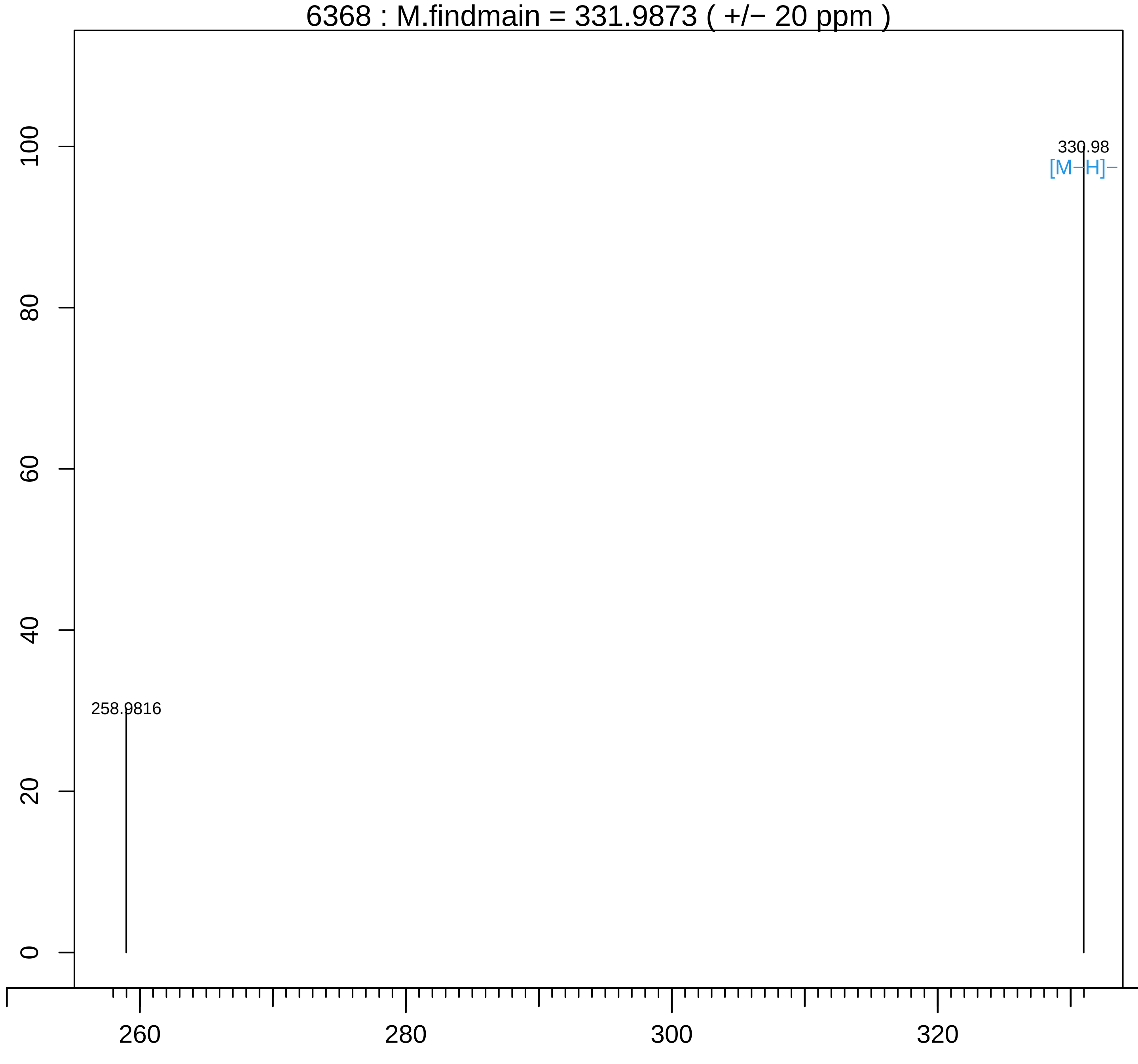

6368 : M.findmain = 331.9873 (+/− 20 ppm)
330.98
[M−H]−
258.9816
0
20
40
60
80
100
260
280
300
320

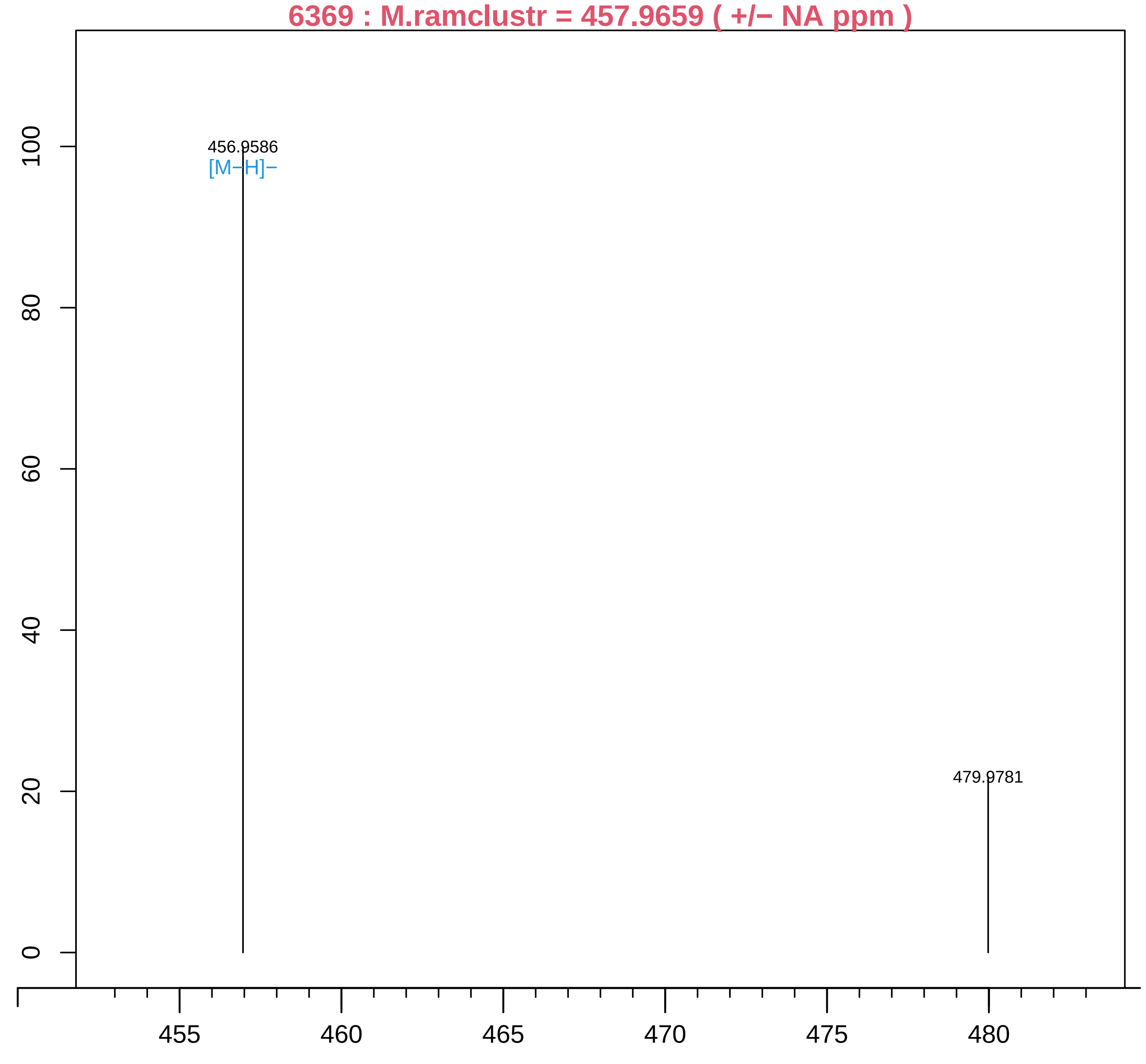
6369 : M.ramclustr = 457.9659 (+/− NA ppm)
456.9586
[M−H]−
479.9781
0
20
40
60
80
100
455
460
465
470
475
480

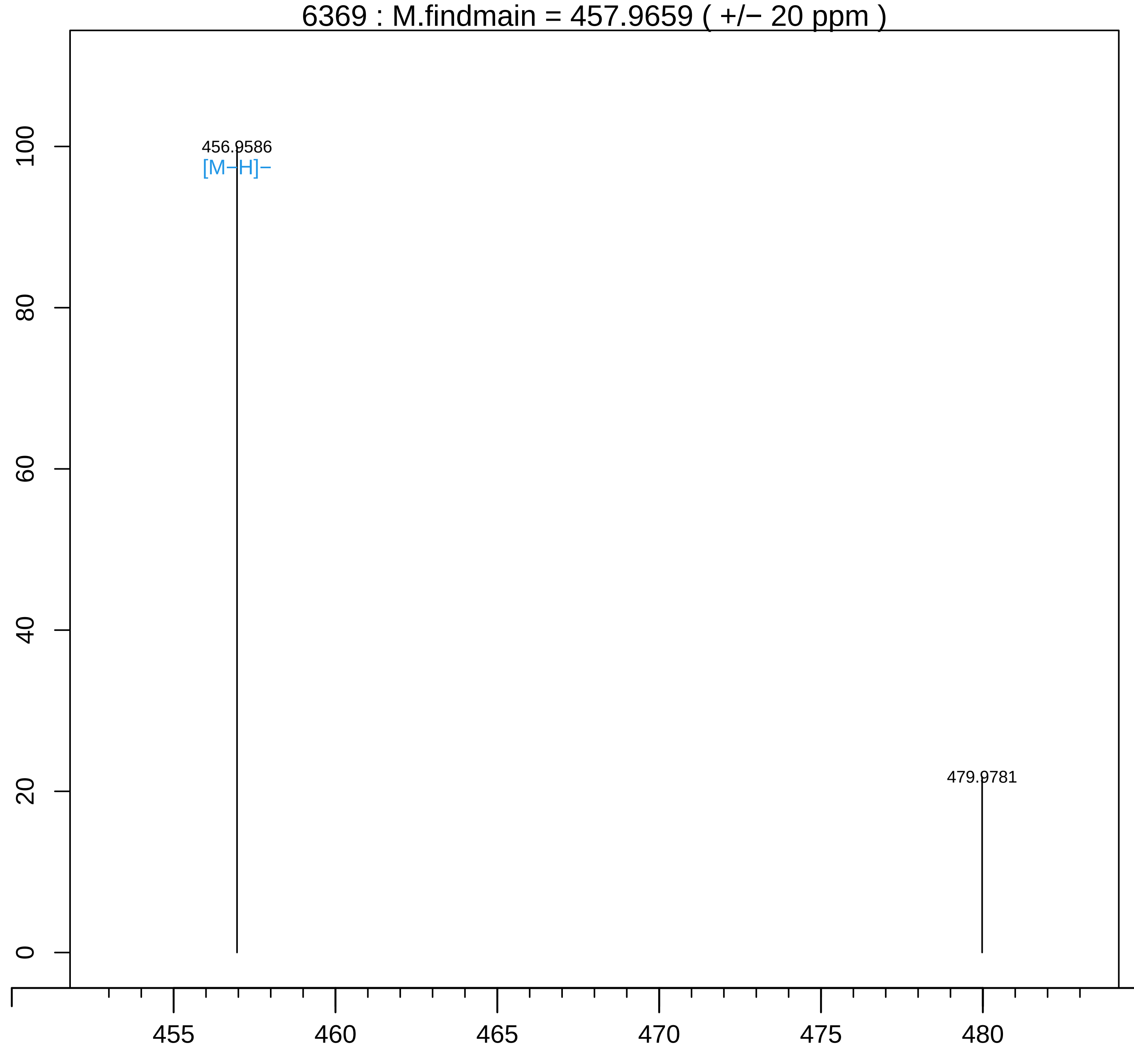
6369 : M.findmain = 457.9659 (+/− 20 ppm)
456.9586
[M−H]−
479.9781
0
20
40
60
80
100
455
460
465
470
475
480

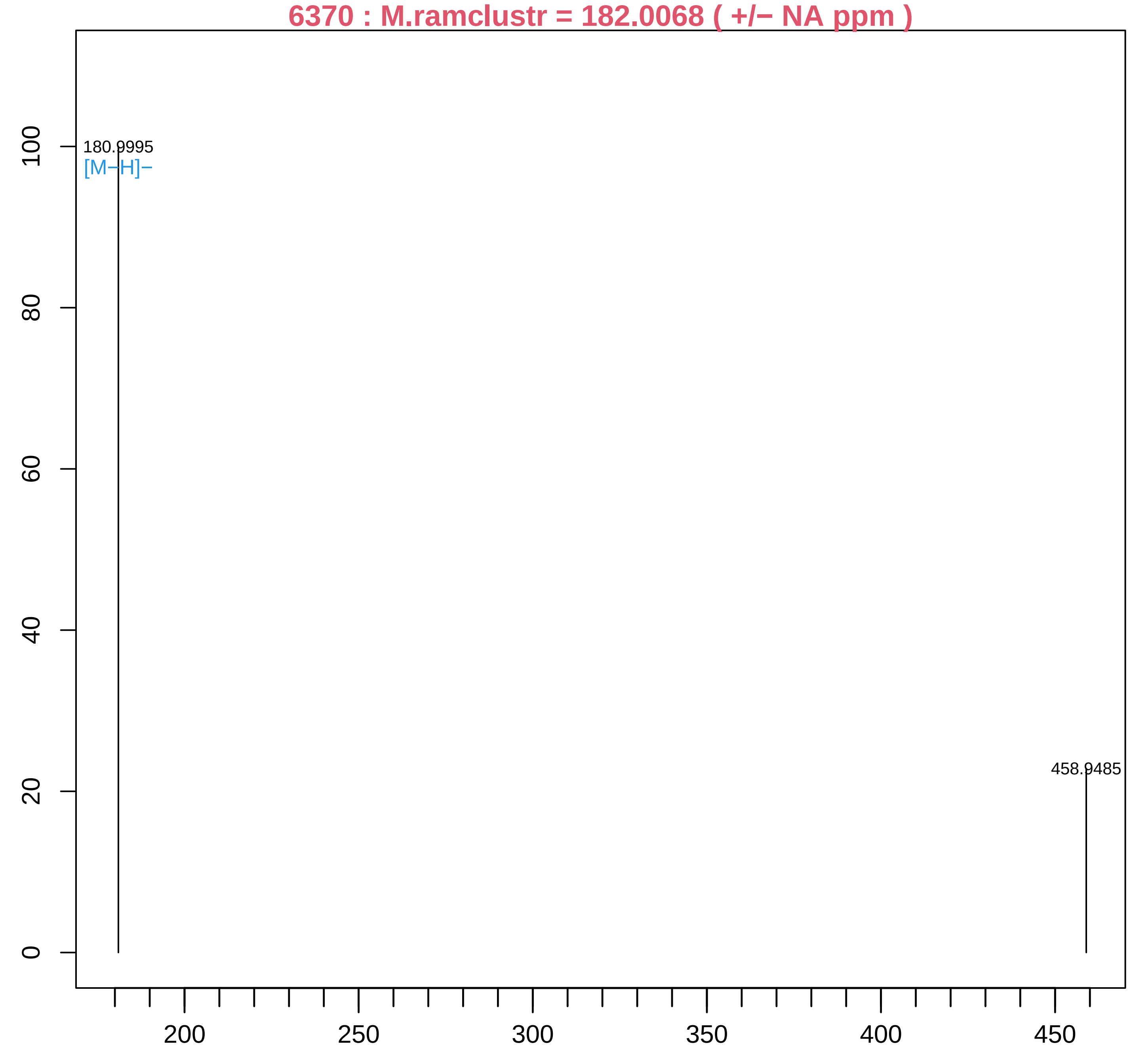
6370 : M.ramclustr = 182.0068 (+/− NA ppm)
180.9995
[M−H]−
458.9485
0
20
40
60
80
100
200
250
300
350
400
450

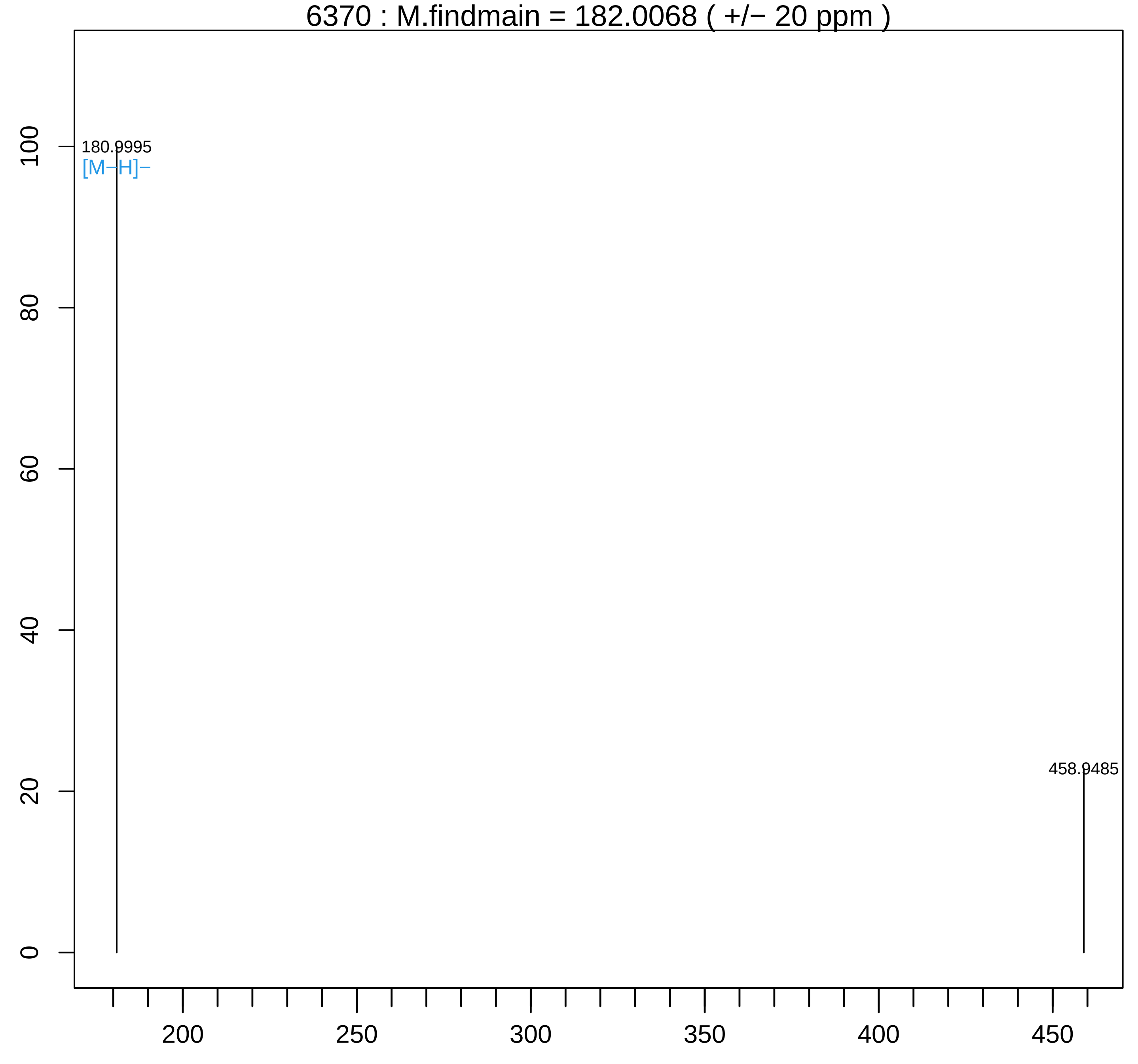
6370 : M.findmain = 182.0068 (+/− 20 ppm)
180.9995
[M−H]−
458.9485
0
20
40
60
80
100
200
250
300
350
400
450

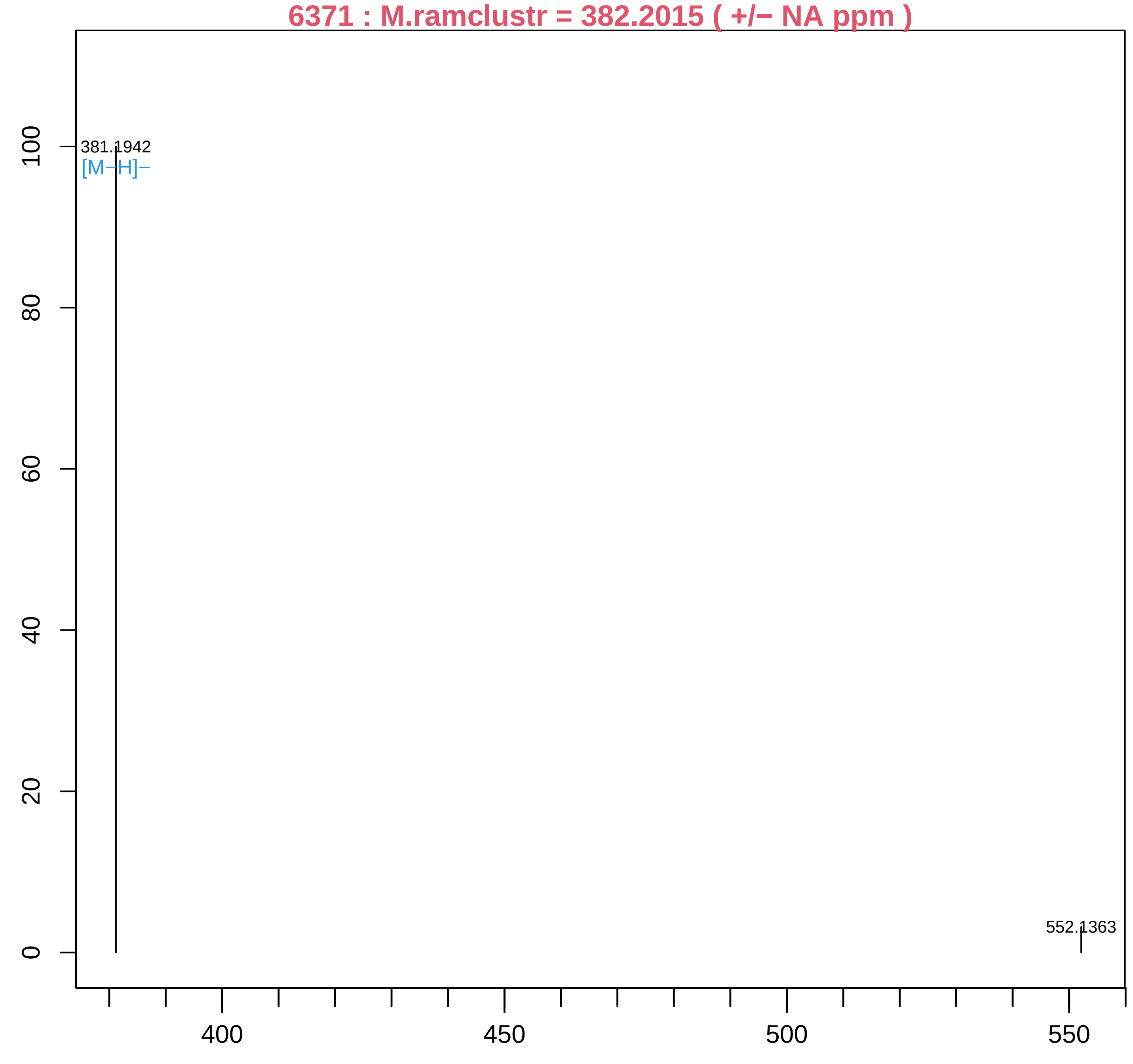
6371 : M.ramclustr = 382.2015 (+/− NA ppm)
381.1942
[M−H]−
552.1363
0
20
40
60
80
100
400
450
500
550

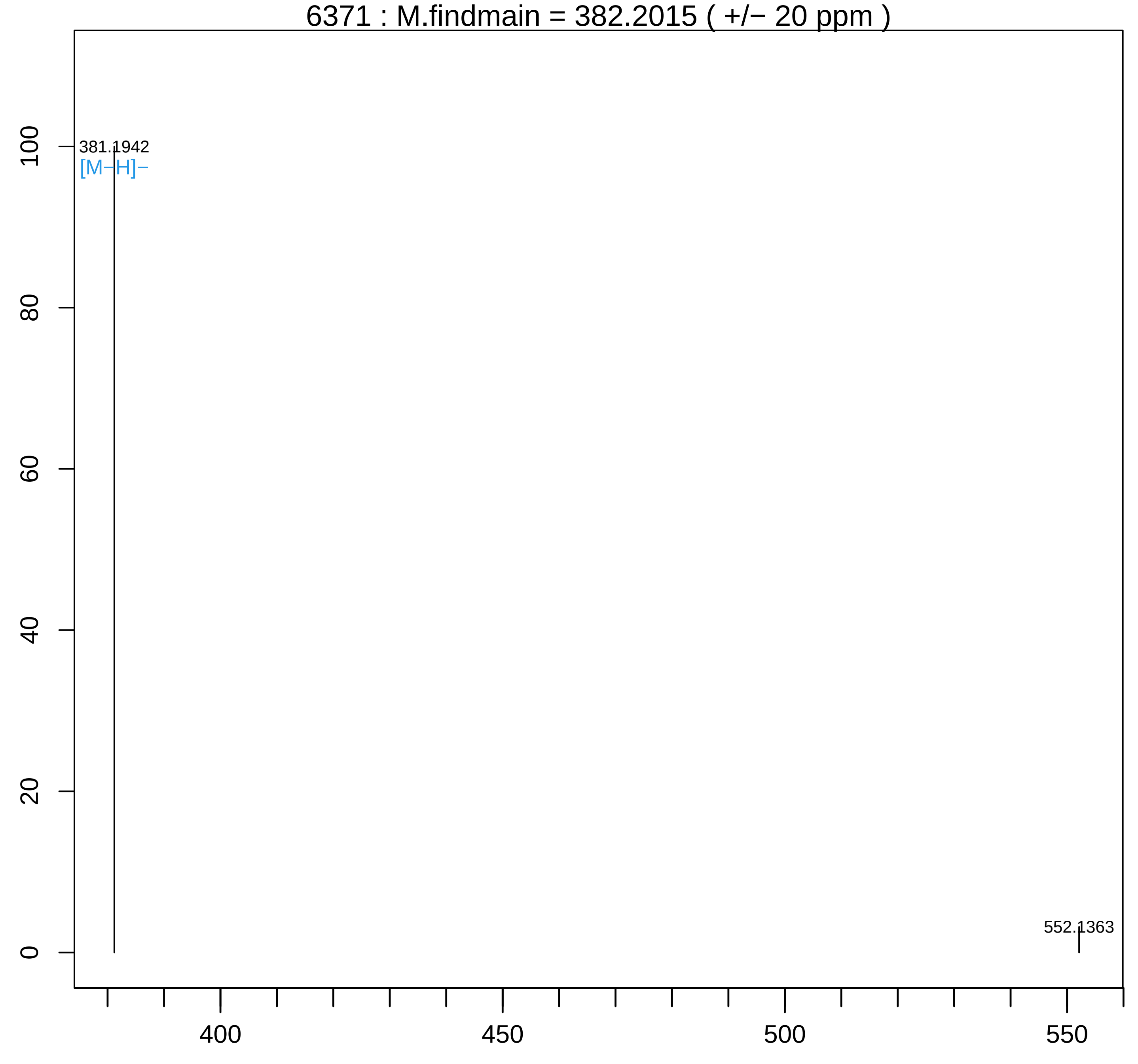
6371 : M.findmain = 382.2015 (+/− 20 ppm)
381.1942
[M−H]−
552.1363
0
20
40
60
80
100
400
450
500
550

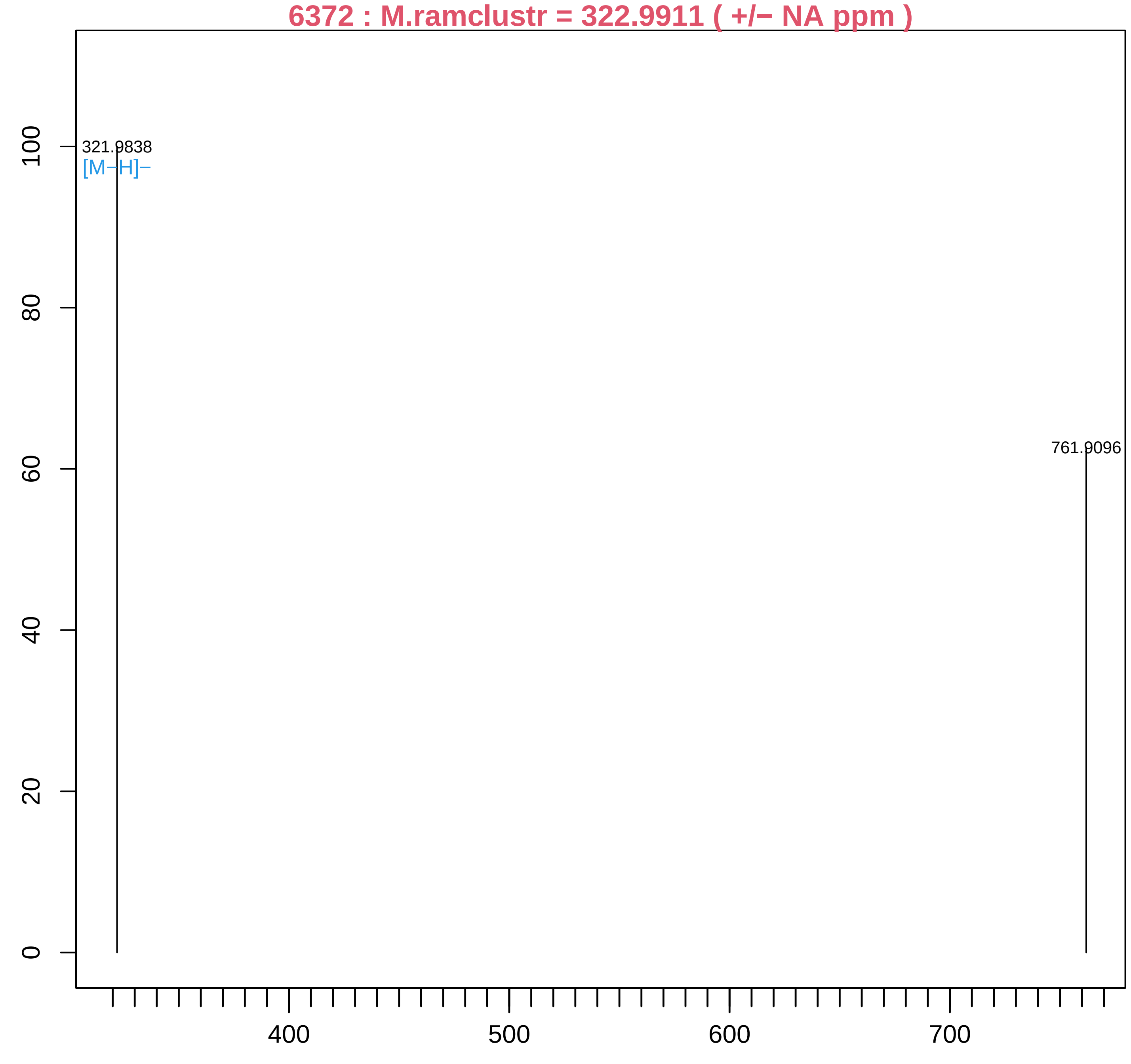
6372 : M.ramclustr = 322.9911 (+/− NA ppm)
321.9838
[M−H]−
761.9096
0
20
40
60
80
100
400
500
600
700

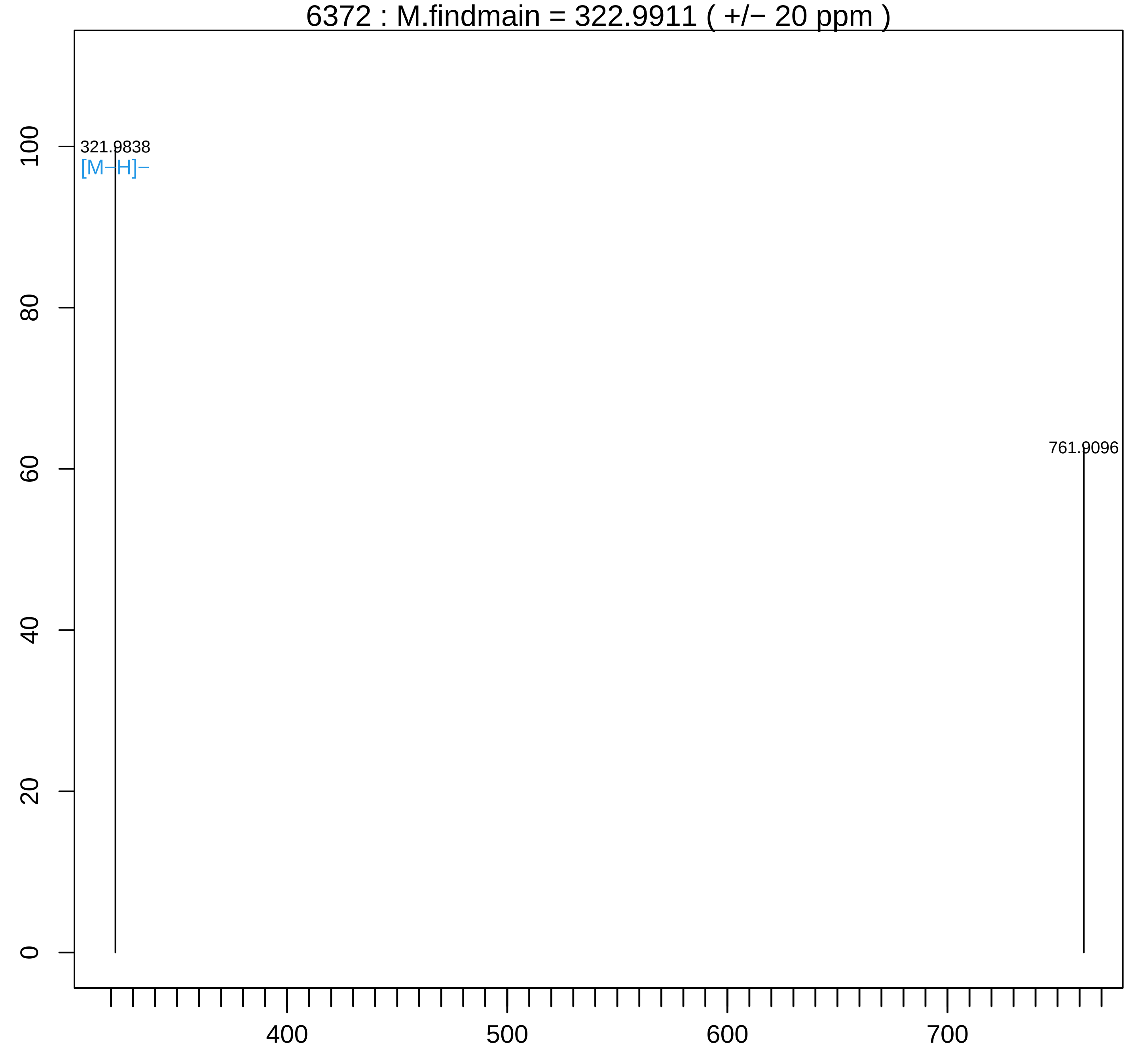
6372 : M.findmain = 322.9911 (+/− 20 ppm)
321.9838
[M−H]−
761.9096
0
20
40
60
80
100
400
500
600
700

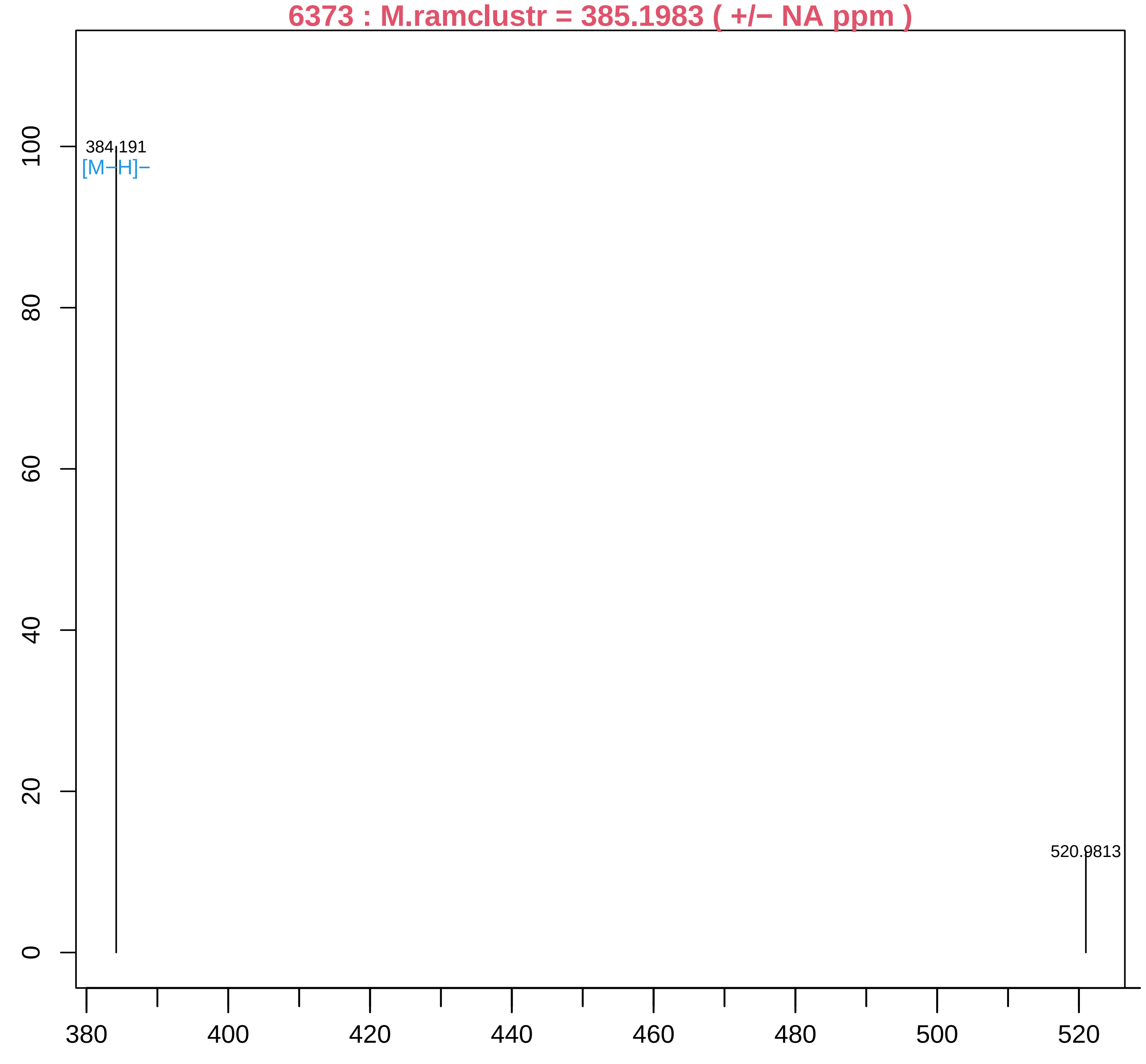
6373 : M.ramclustr = 385.1983 (+/− NA ppm)
384.191
[M−H]−
520.9813
0
20
40
60
80
100
380
400
420
440
460
480
500
520

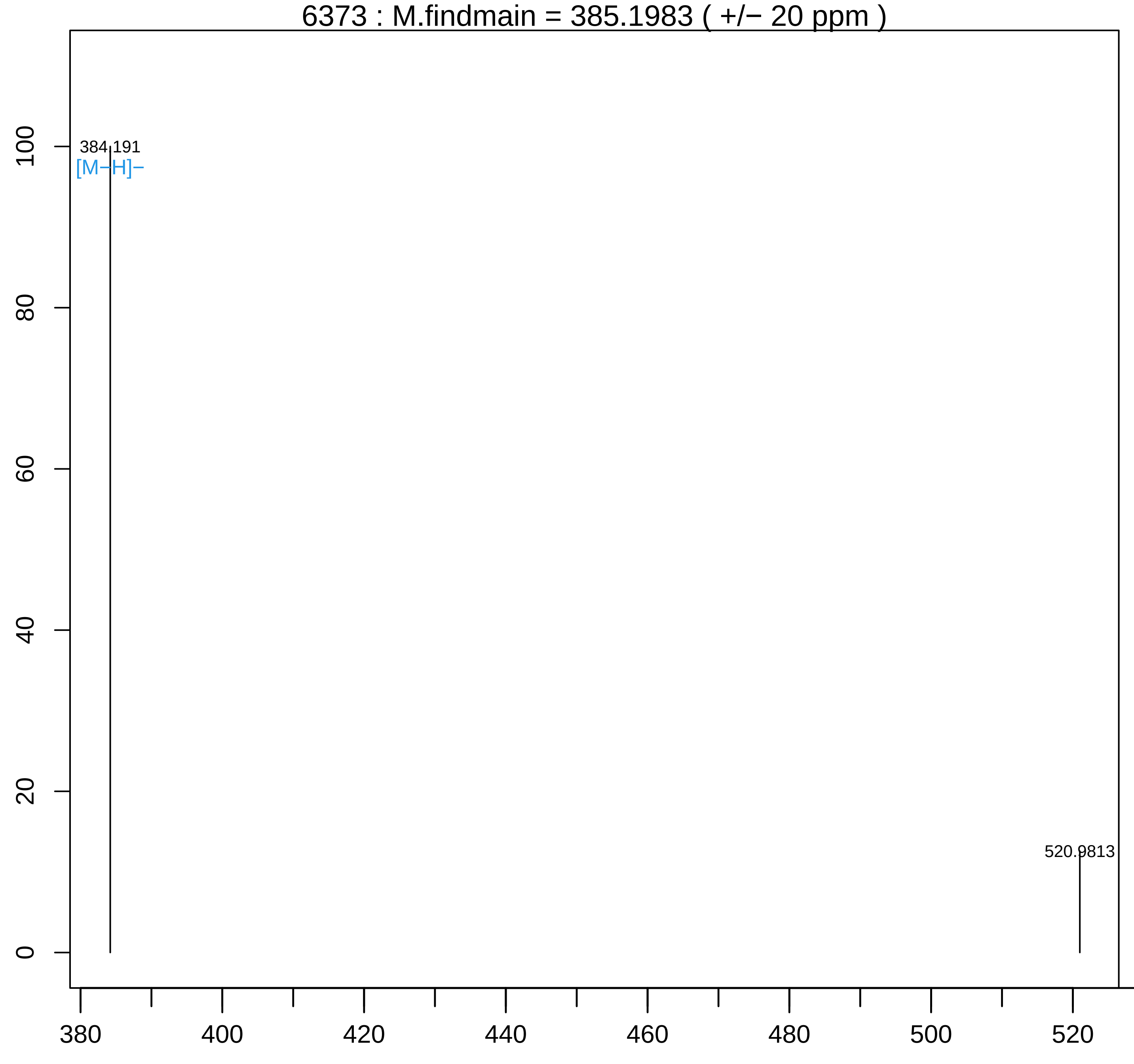
6373 : M.findmain = 385.1983 (+/− 20 ppm)
384.191
[M−H]−
520.9813
0
20
40
60
80
100
380
400
420
440
460
480
500
520

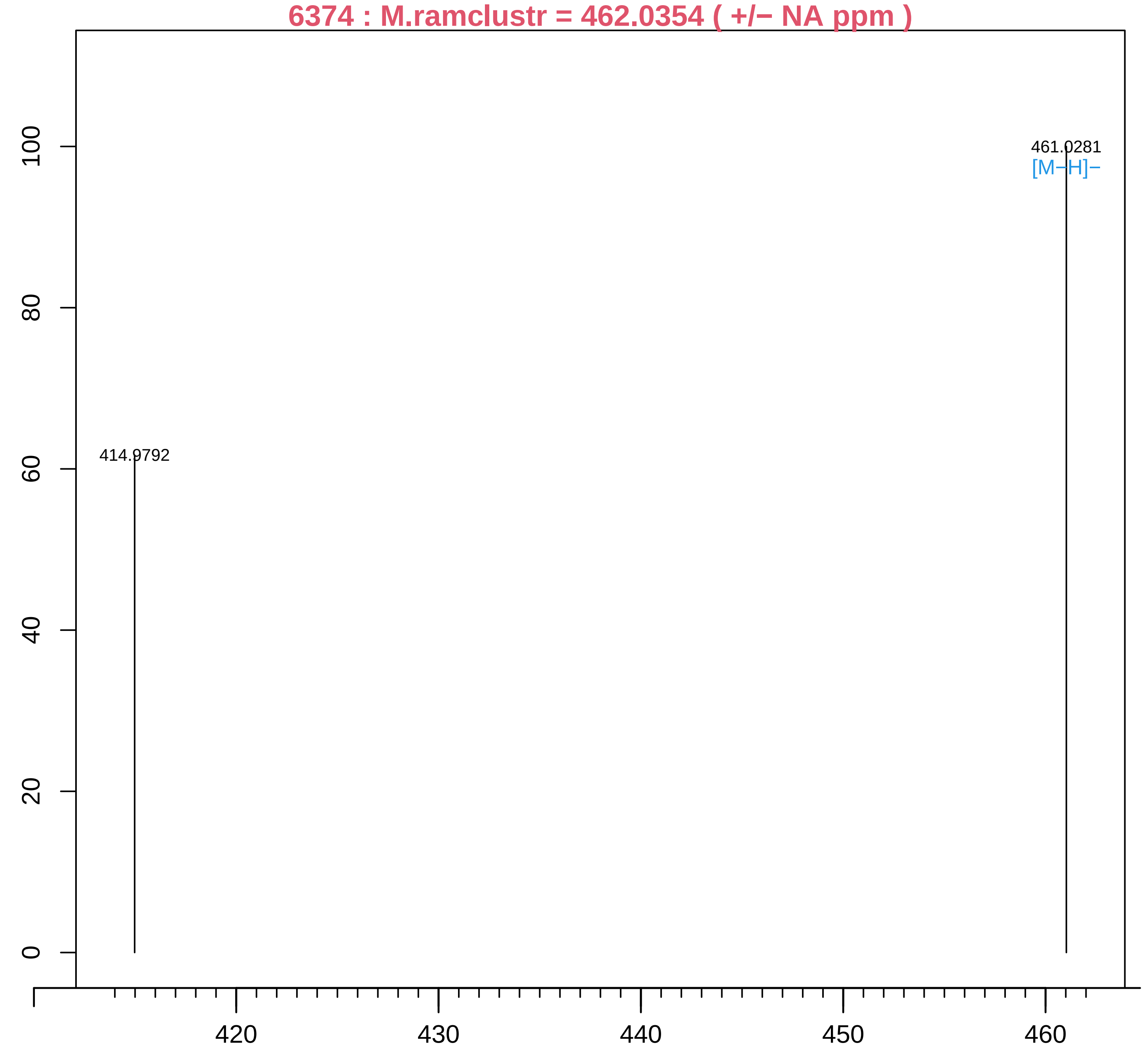
6374 : M.ramclustr = 462.0354 (+/− NA ppm)
414.9792
461.0281
[M−H]−
0
20
40
60
80
100
420
430
440
450
460

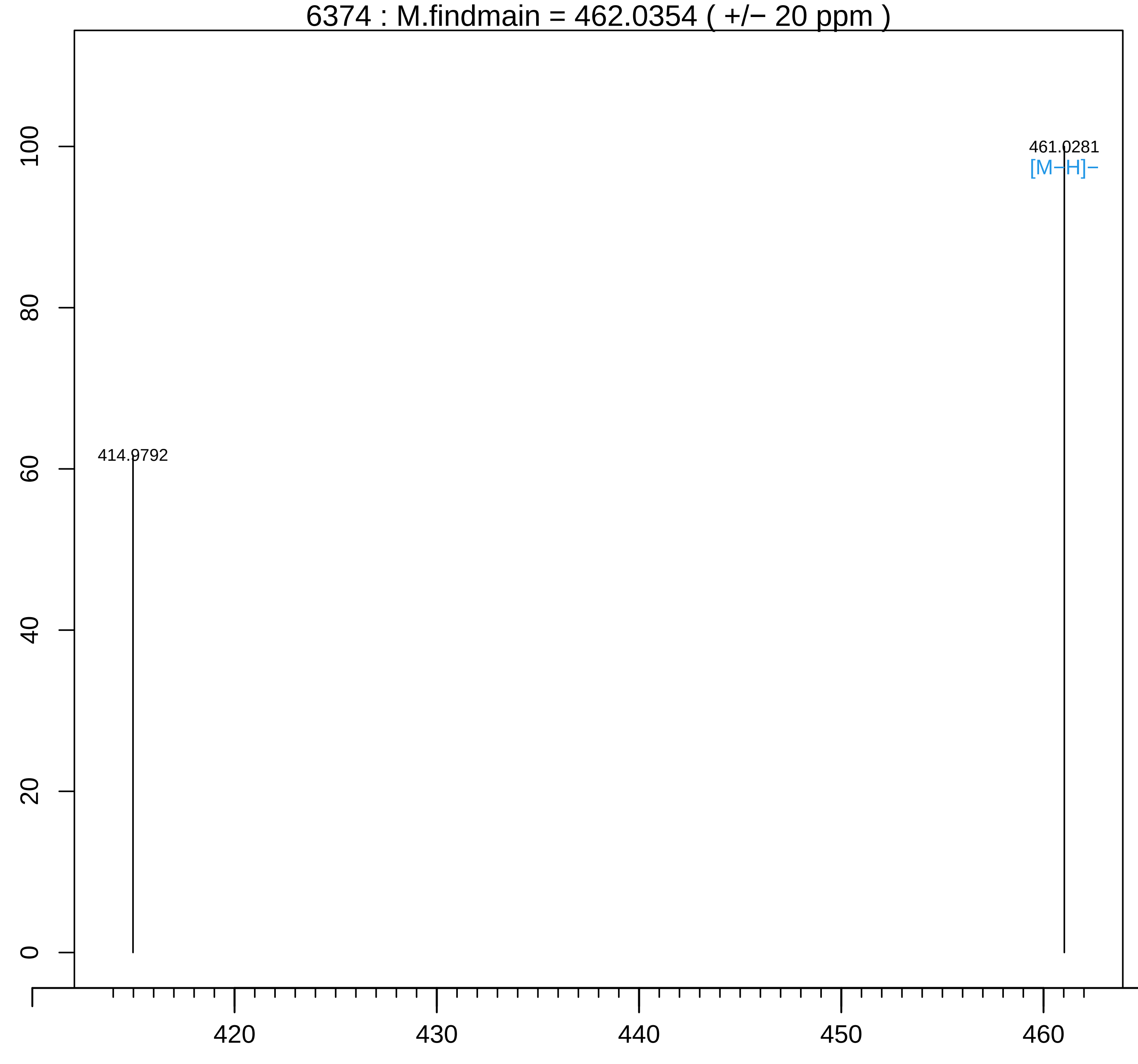
6374 : M.findmain = 462.0354 (+/− 20 ppm)
414.9792
461.0281
[M−H]−
0
20
40
60
80
100
420
430
440
450
460

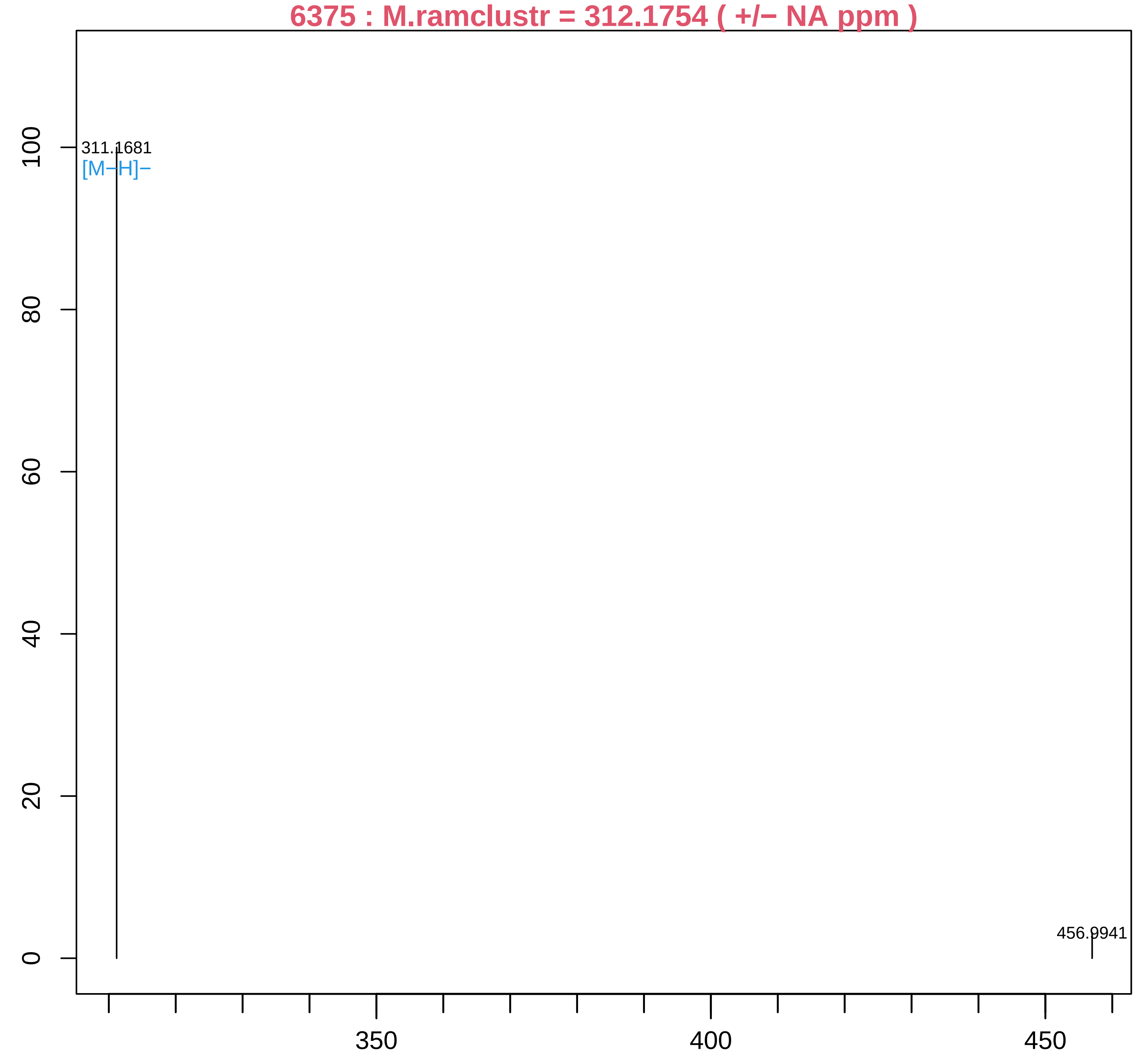

6375 : M.ramclustr = 312.1754 (+/− NA ppm)
311.1681
[M−H]−
456.9941
0
20
40
60
80
100
350
400
450

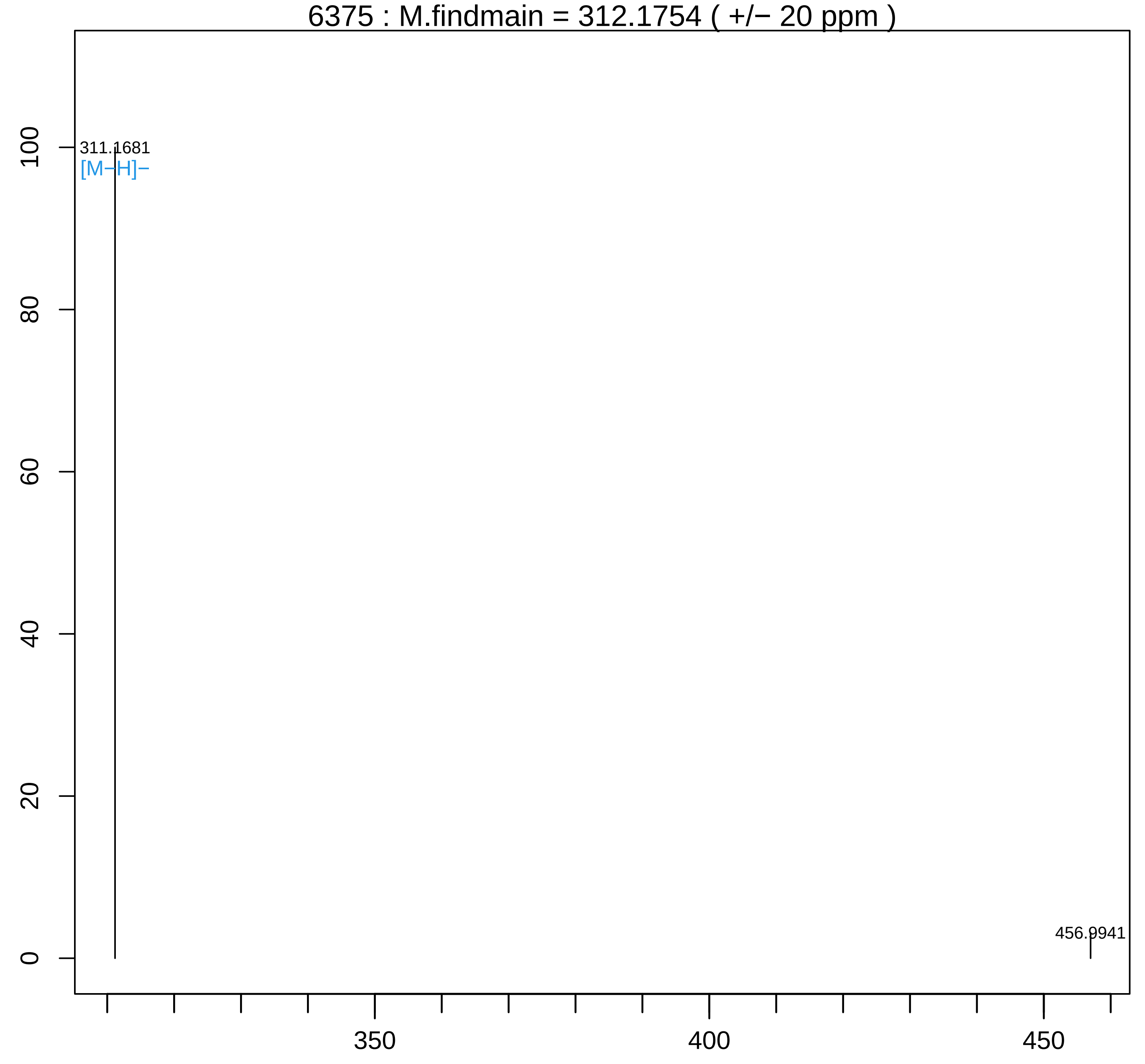

6375 : M.findmain = 312.1754 (+/− 20 ppm)
311.1681
[M−H]−
456.9941
0
20
40
60
80
100
350
400
450

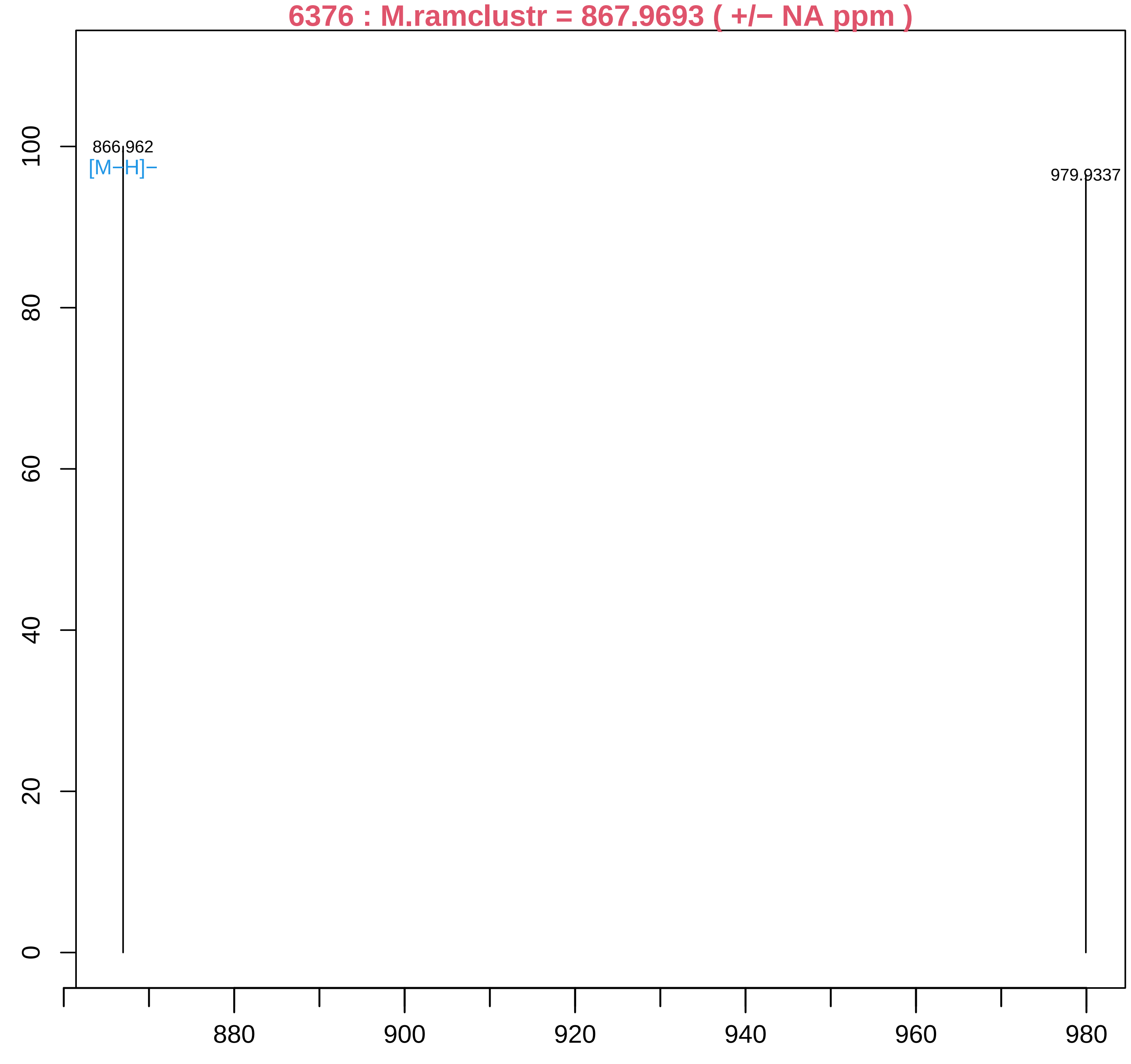
6376 : M.ramclustr = 867.9693 (+/− NA ppm)
866.962
[M−H]−
979.9337
0
20
40
60
80
100
880
900
920
940
960
980

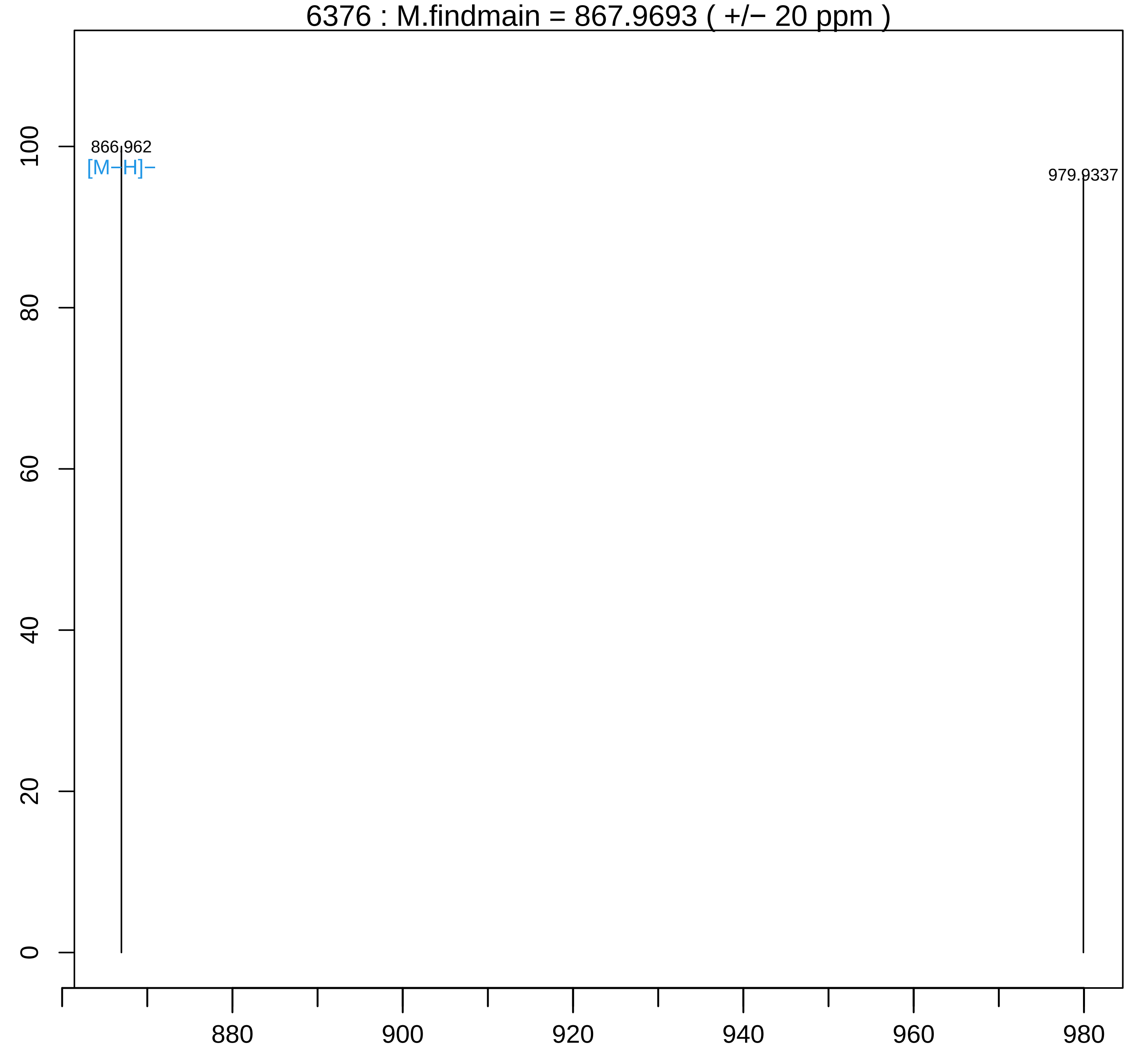
6376 : M.findmain = 867.9693 (+/− 20 ppm)
866.962
[M−H]−
979.9337
0
20
40
60
80
100
880
900
920
940
960
980

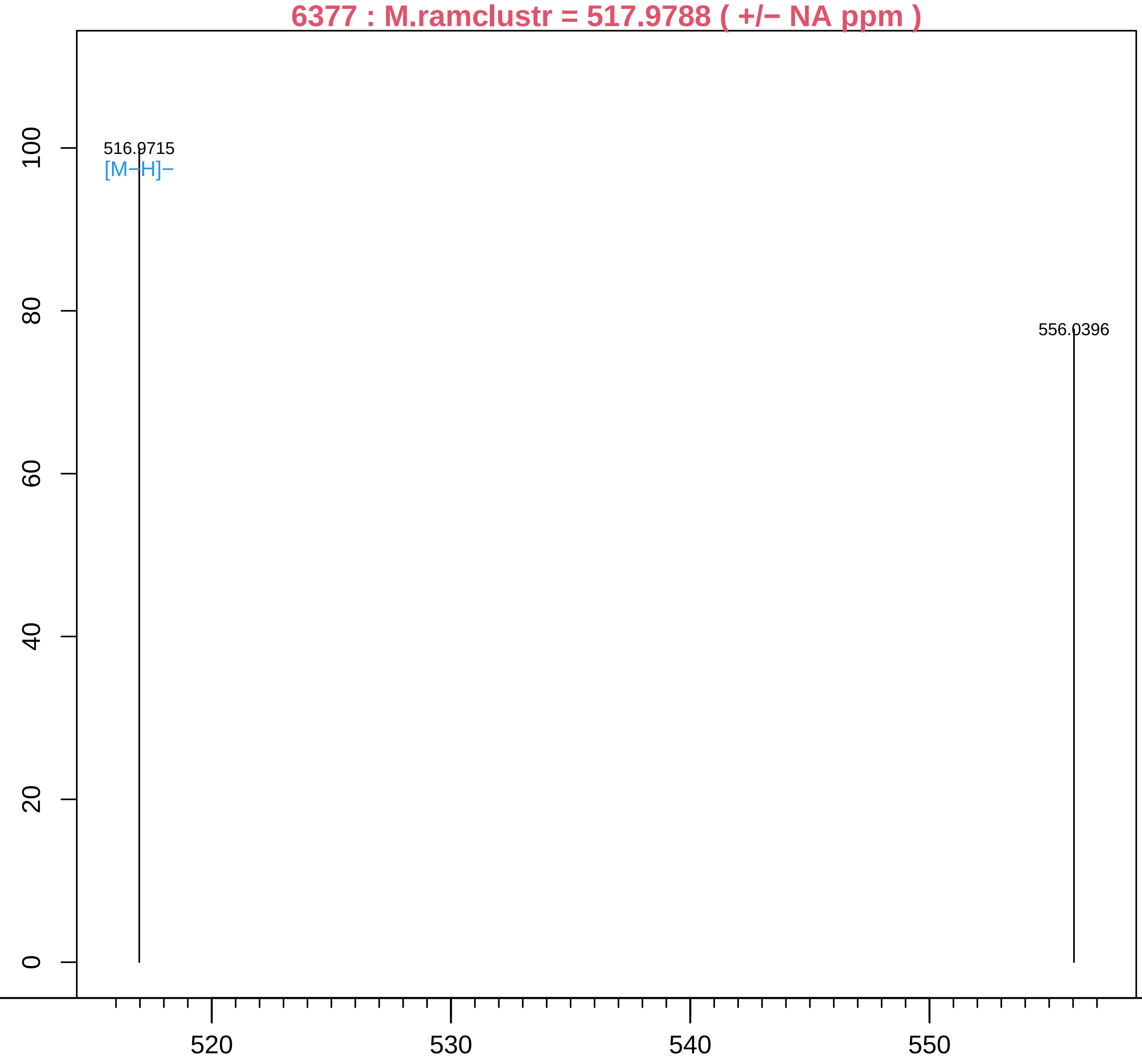

6377 : M.ramclustr = 517.9788 (+/− NA ppm)
516.9715
[M−H]−
556.0396
0
20
40
60
80
100
520
530
540
550

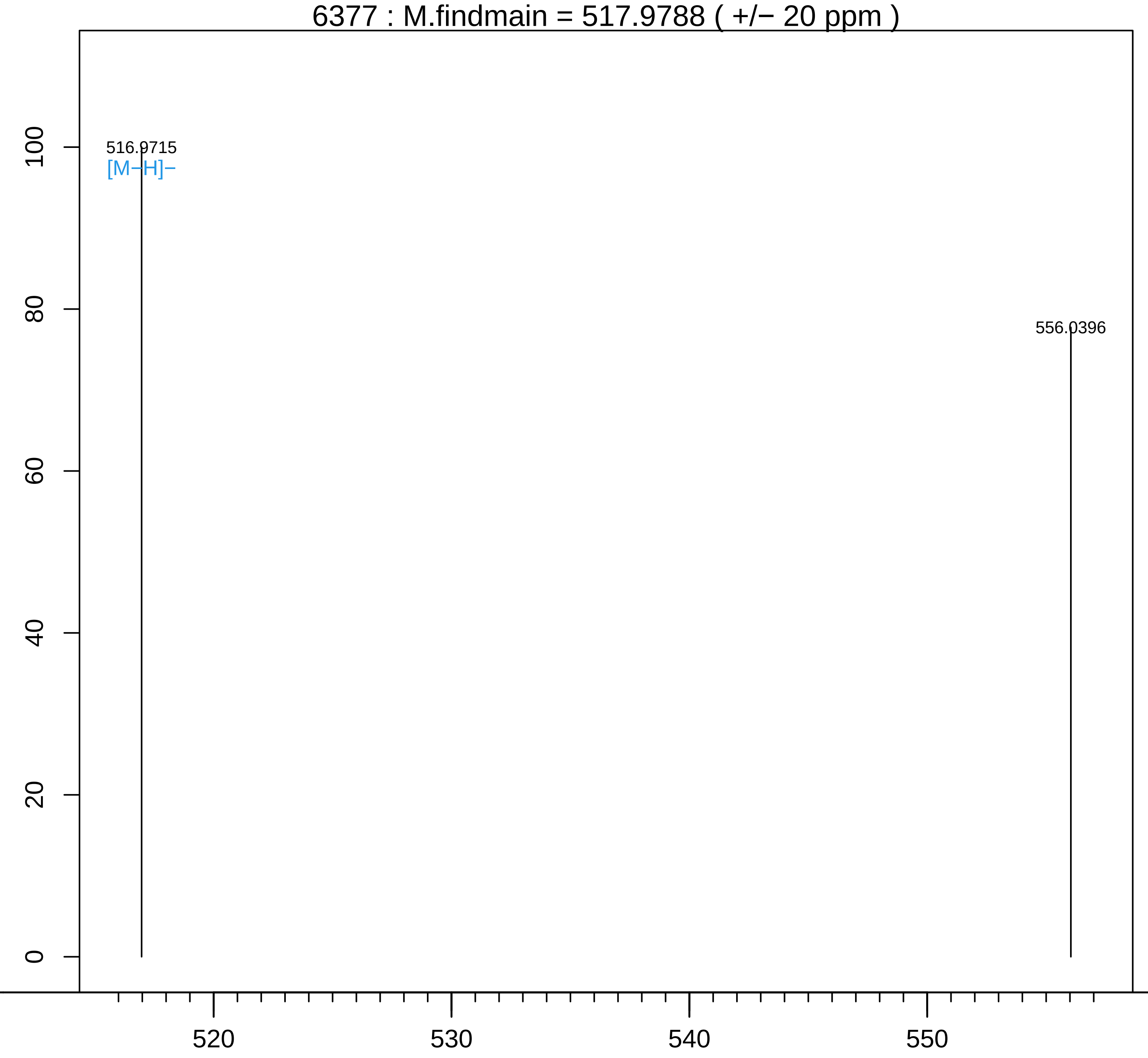

6377 : M.findmain = 517.9788 (+/− 20 ppm)
516.9715
[M−H]−
556.0396
0
20
40
60
80
100
520
530
540
550

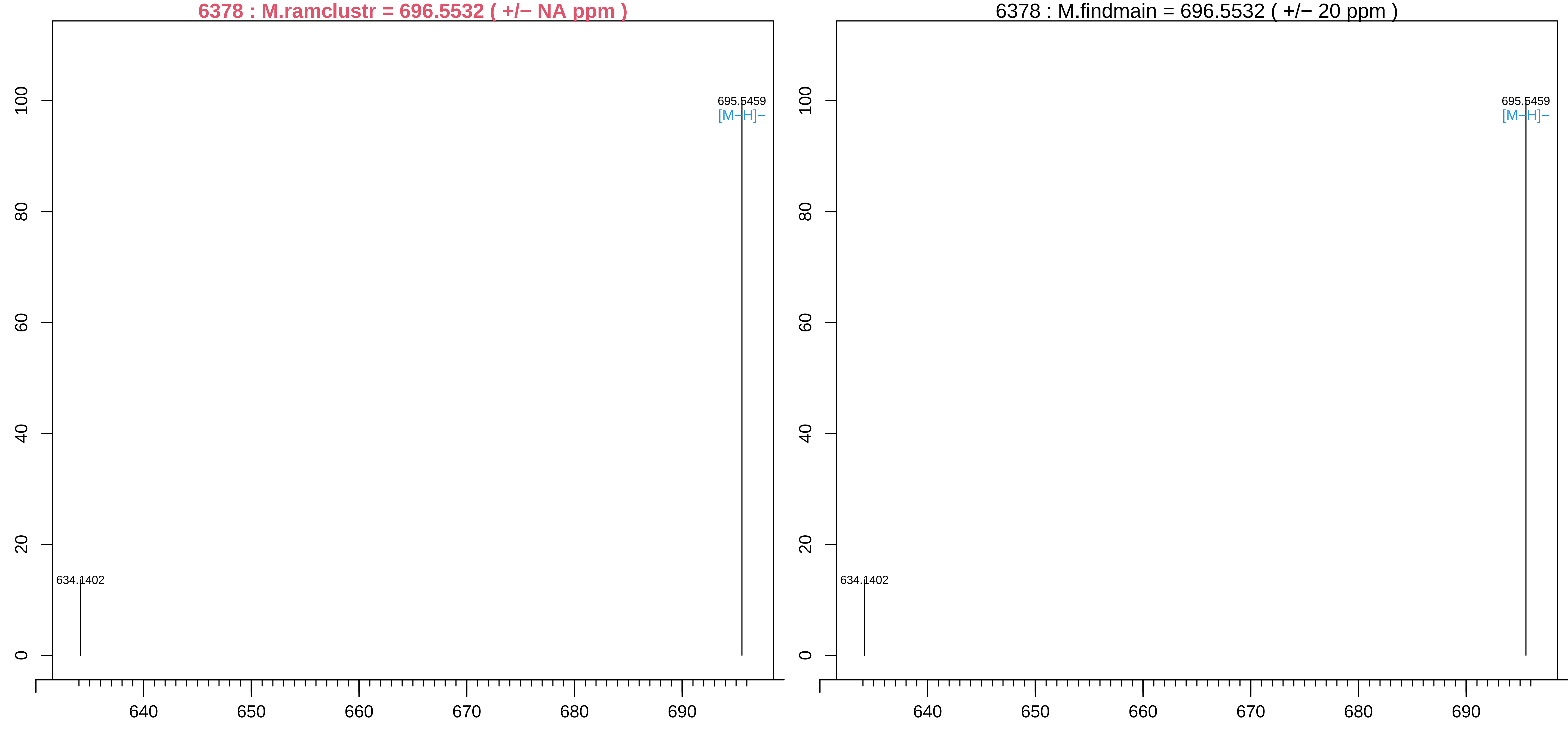
6378 : M.ramclustr = 696.5532 (+/− NA ppm)
6378 : M.findmain = 696.5532 (+/− 20 ppm)
695.5459
[M−H]−
634.1402
695.5459
[M−H]−
634.1402
0
20
40
60
80
100
640
650
660
670
680
690

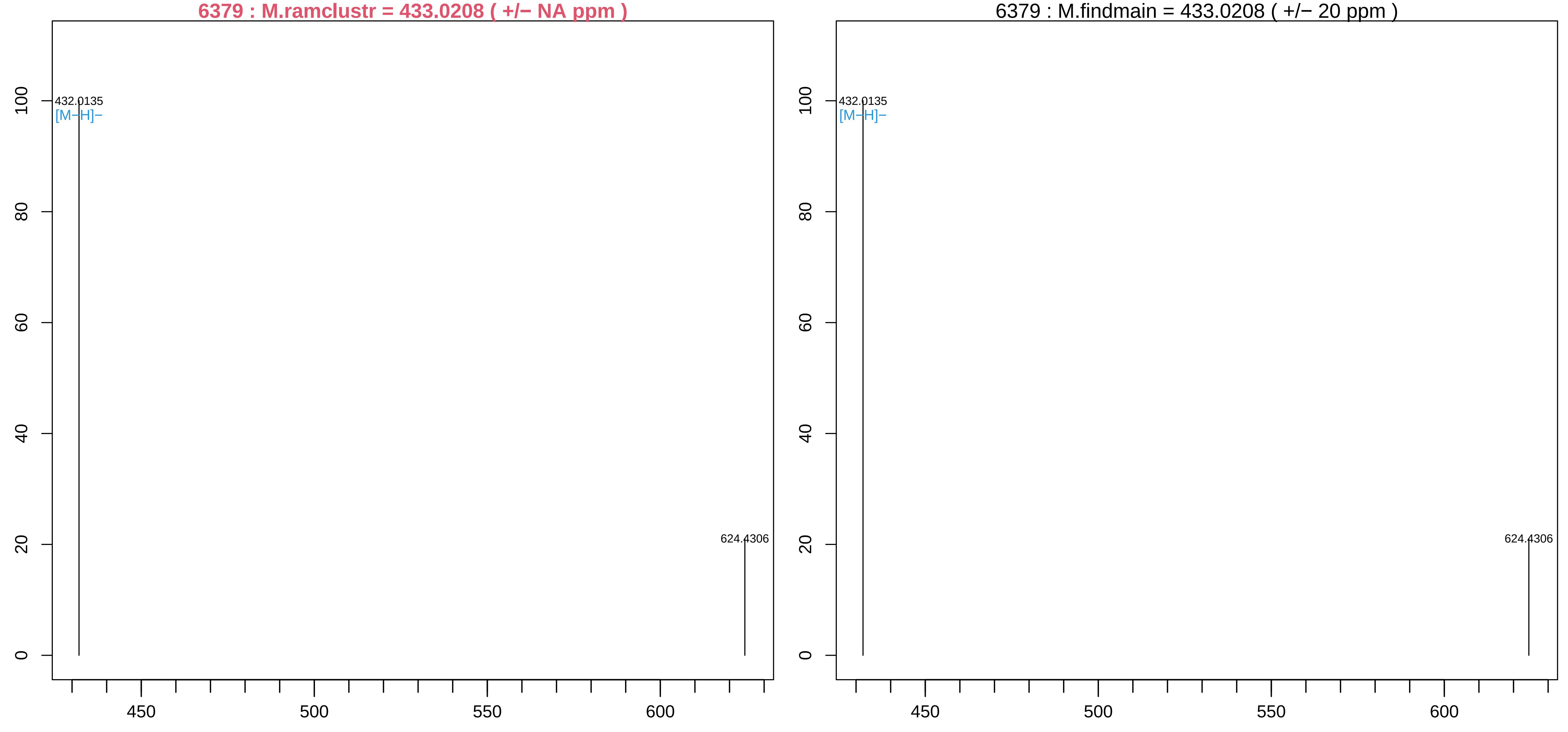

6379 : M.ramclustr = 433.0208 (+/− NA ppm)
432.0135
[M−H]−
624.4306
6379 : M.findmain = 433.0208 (+/− 20 ppm)
432.0135
[M−H]−
624.4306
0
20
40
60
80
100
450
500
550
600

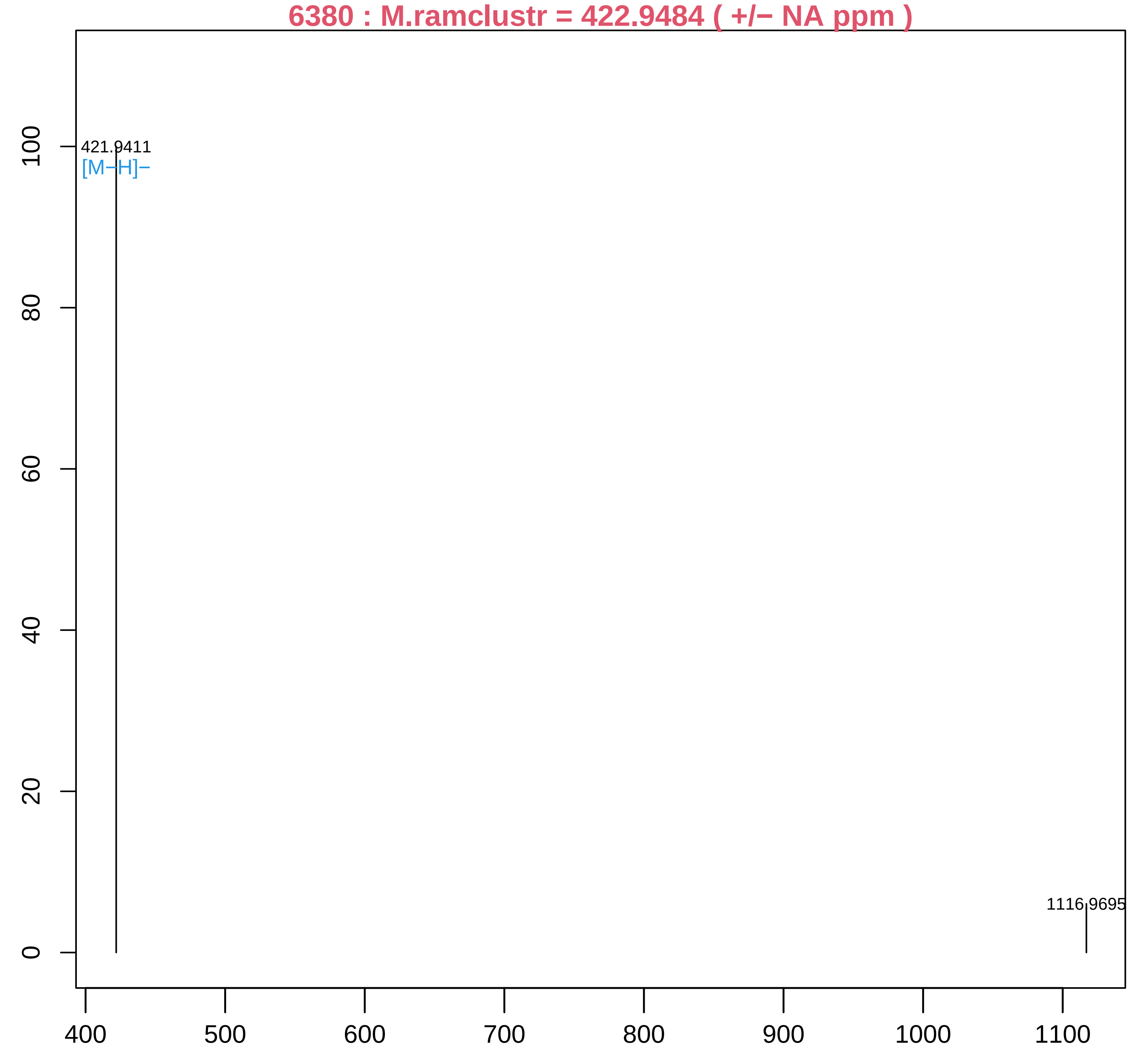
6380 : M.ramclustr = 422.9484 (+/− NA ppm)
421.9411
[M−H]−
1116.9695
0
20
40
60
80
100
400
500
600
700
800
900
1000
1100

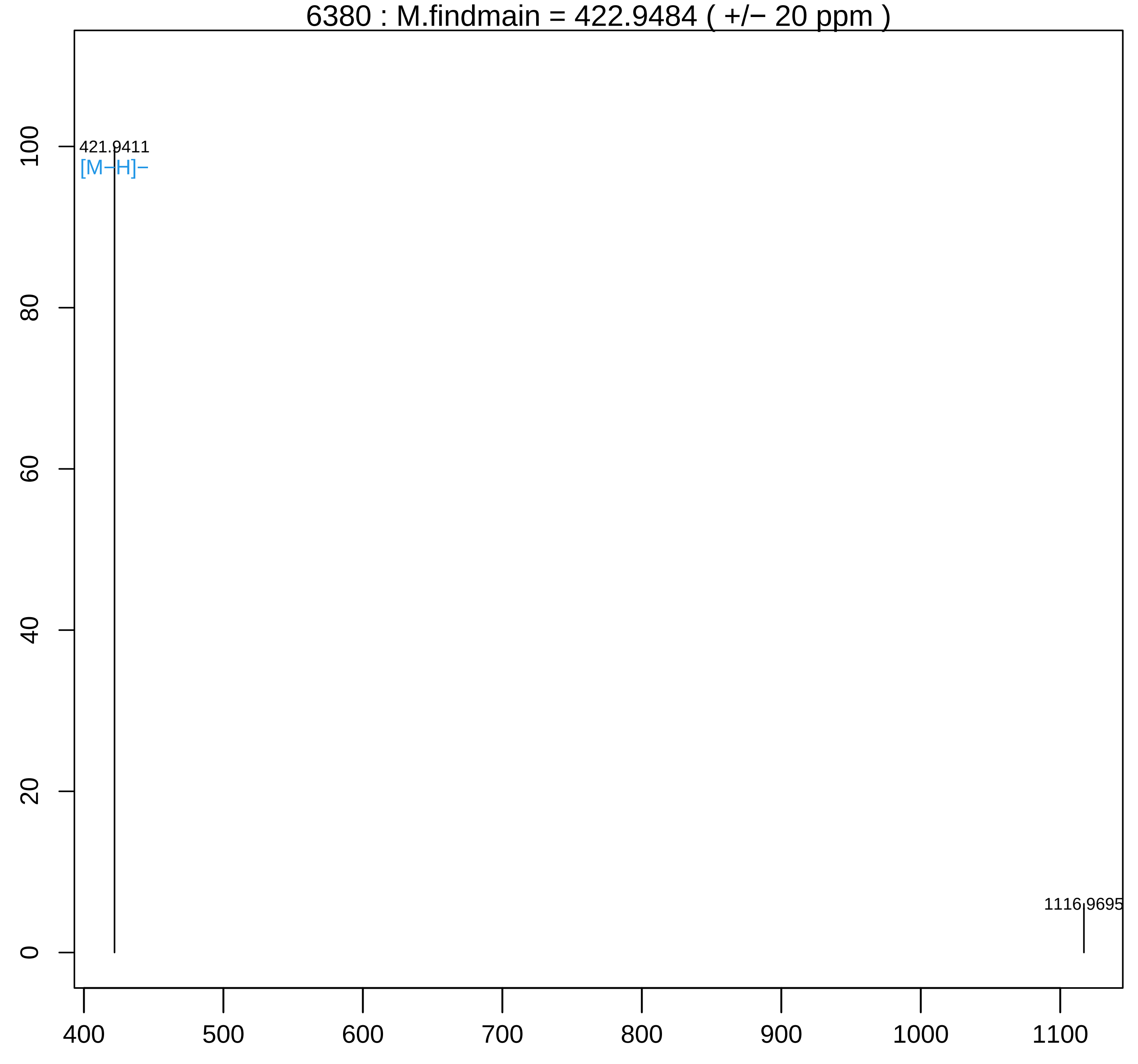
6380 : M.findmain = 422.9484 (+/− 20 ppm)
421.9411
[M−H]−
1116.9695
0
20
40
60
80
100
400
500
600
700
800
900
1000
1100

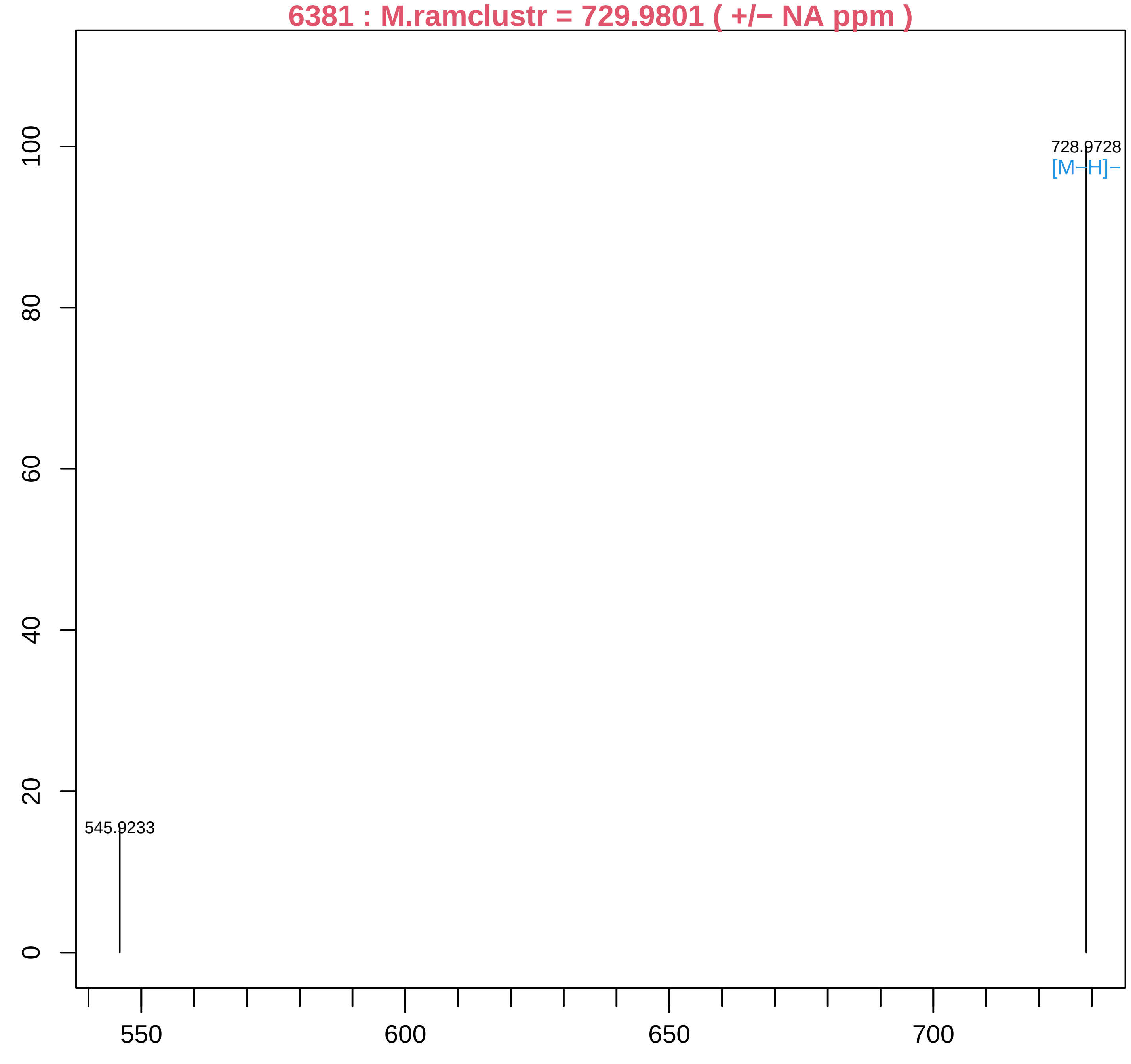

6381 : M.ramclustr = 729.9801 (+/− NA ppm)
728.9728
[M−H]−
545.9233
0
20
40
60
80
100
550
600
650
700

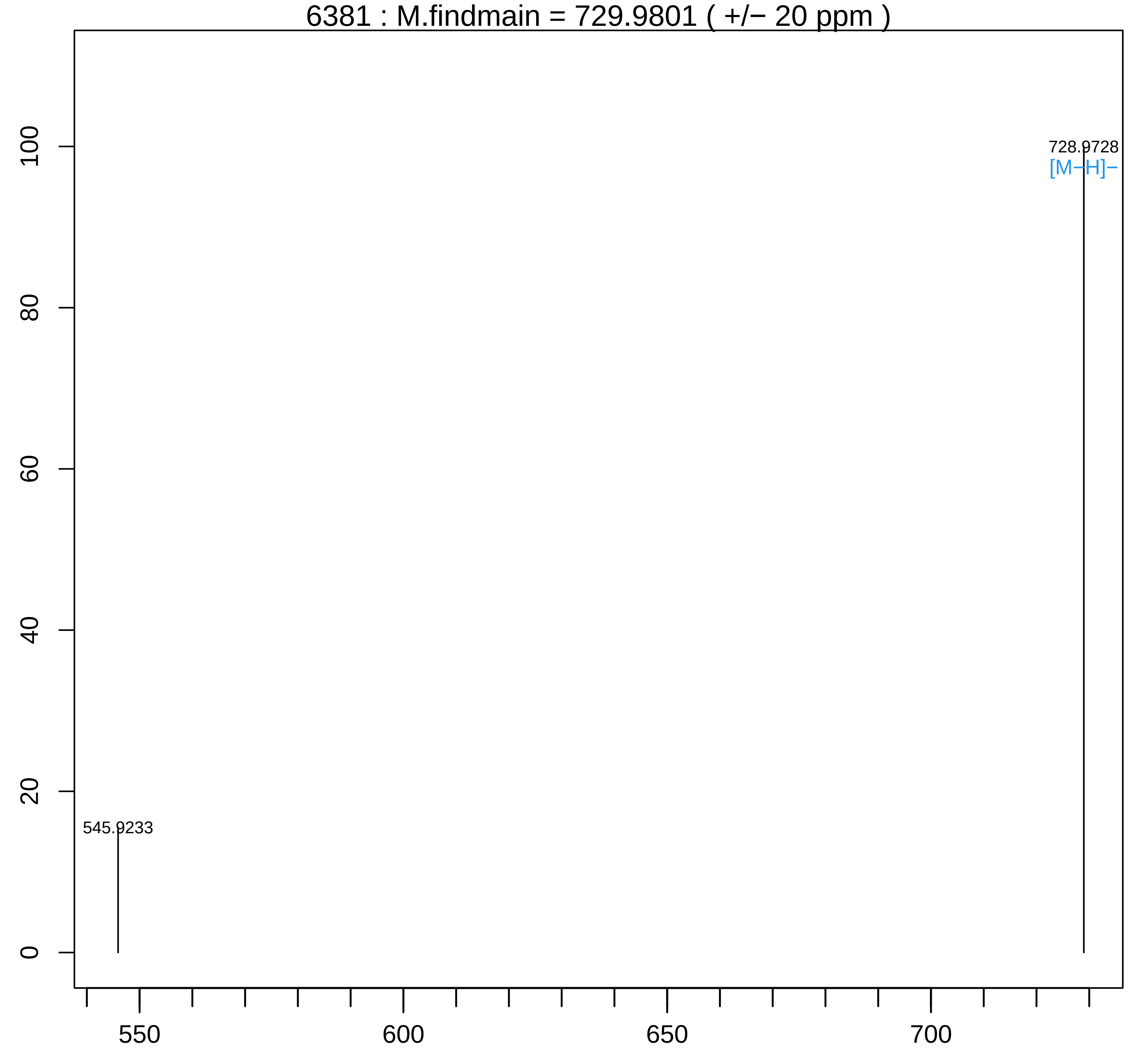

6381 : M.findmain = 729.9801 (+/− 20 ppm)
728.9728
[M−H]−
545.9233
0
20
40
60
80
100
550
600
650
700

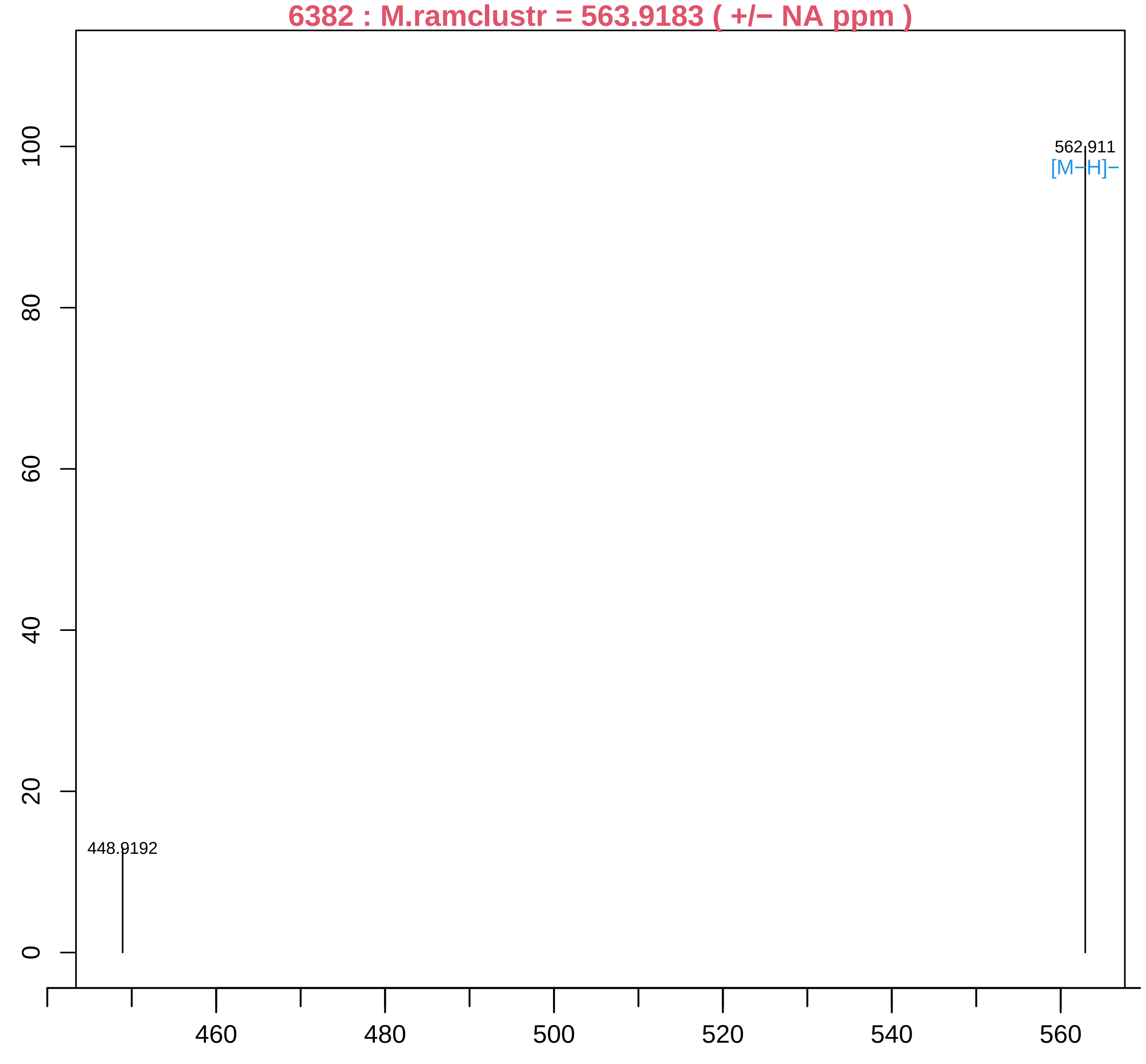
6382 : M.ramclustr = 563.9183 (+/− NA ppm)
562.911
[M−H]−
448.9192
0
20
40
60
80
100
460
480
500
520
540
560

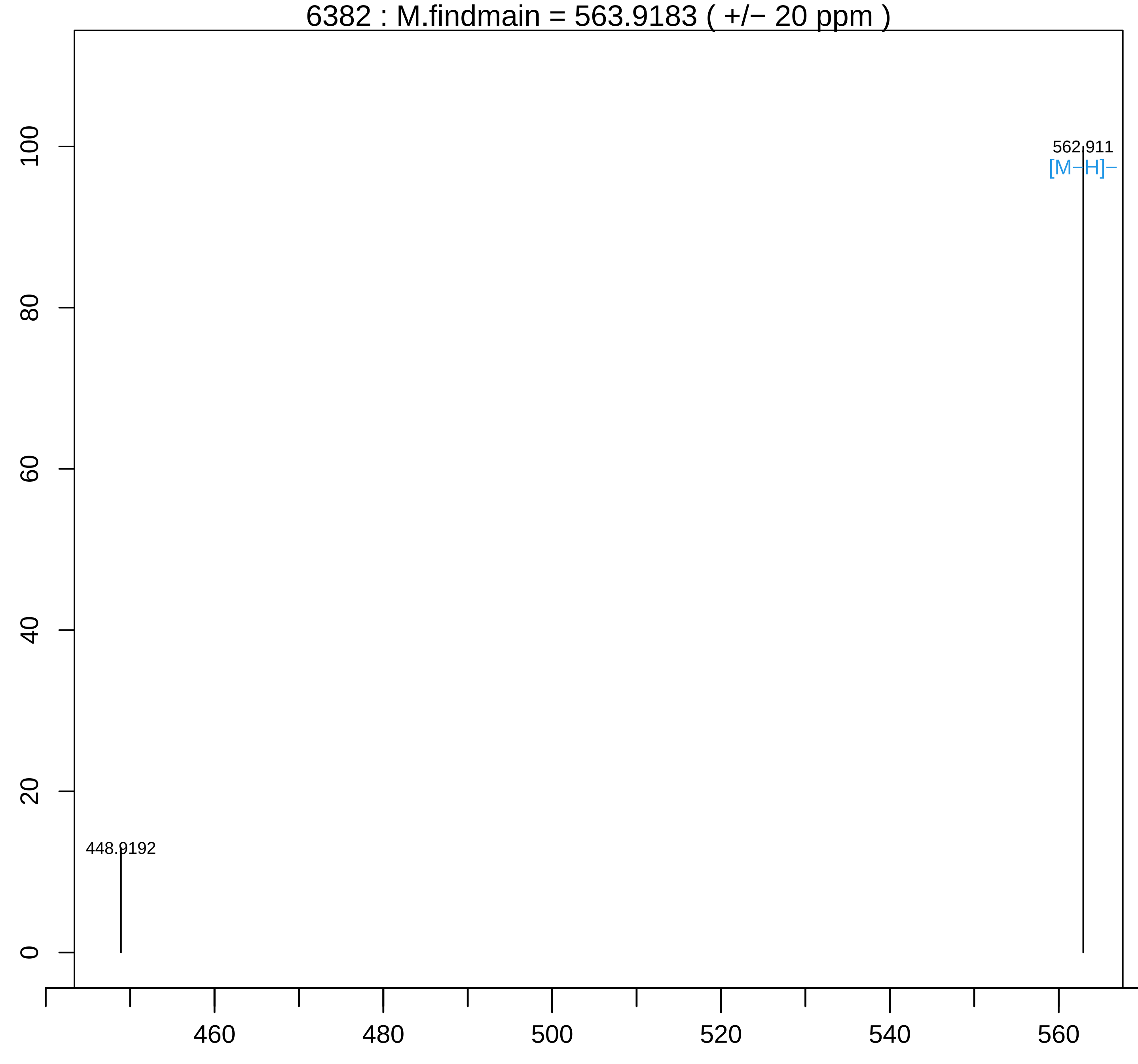
6382 : M.findmain = 563.9183 (+/− 20 ppm)
562.911
[M−H]−
448.9192
0
20
40
60
80
100
460
480
500
520
540
560

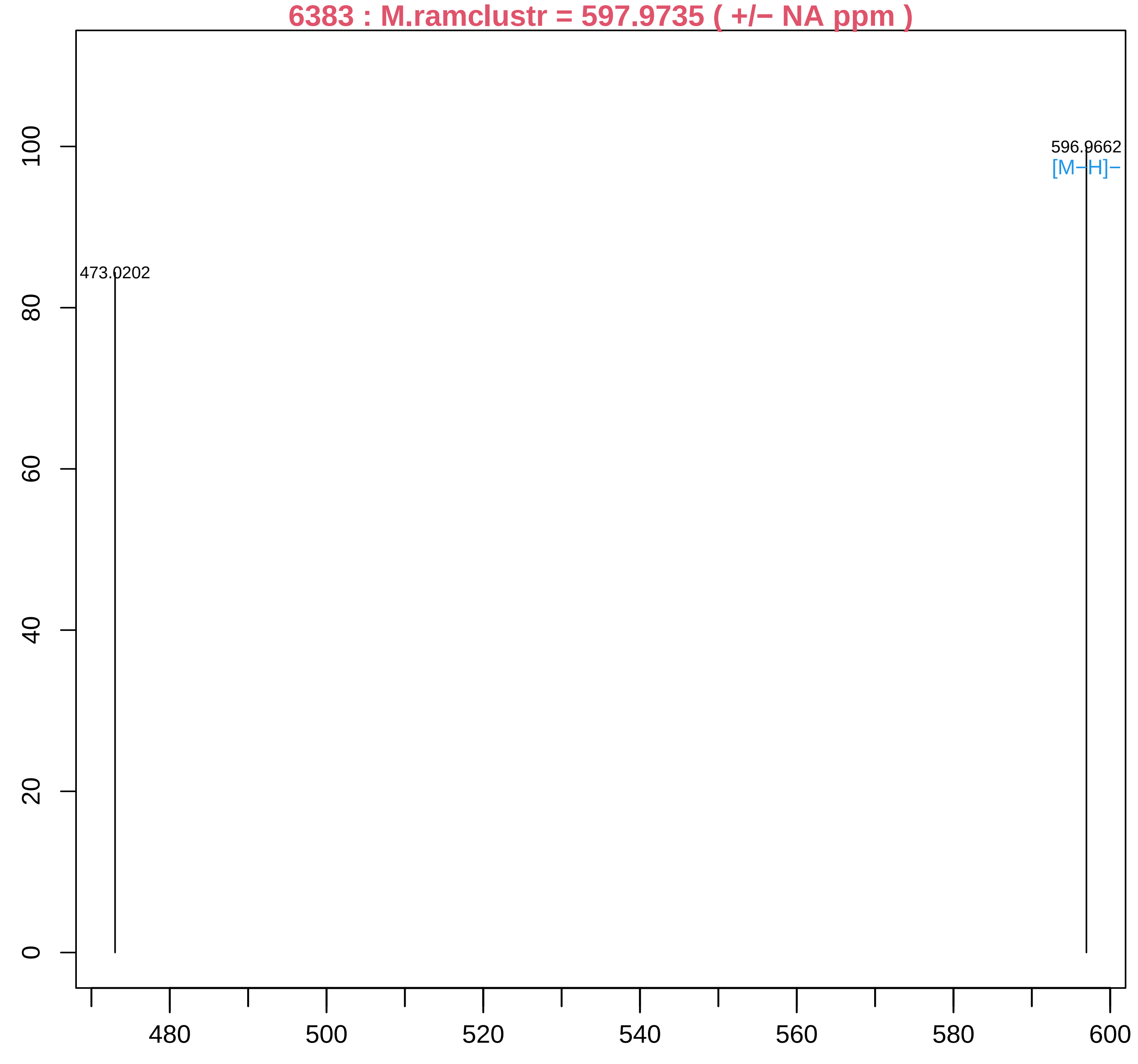
6383 : M.ramclustr = 597.9735 (+/− NA ppm)
596.9662
[M−H]−
473.0202
0
20
40
60
80
100
480
500
520
540
560
580
600

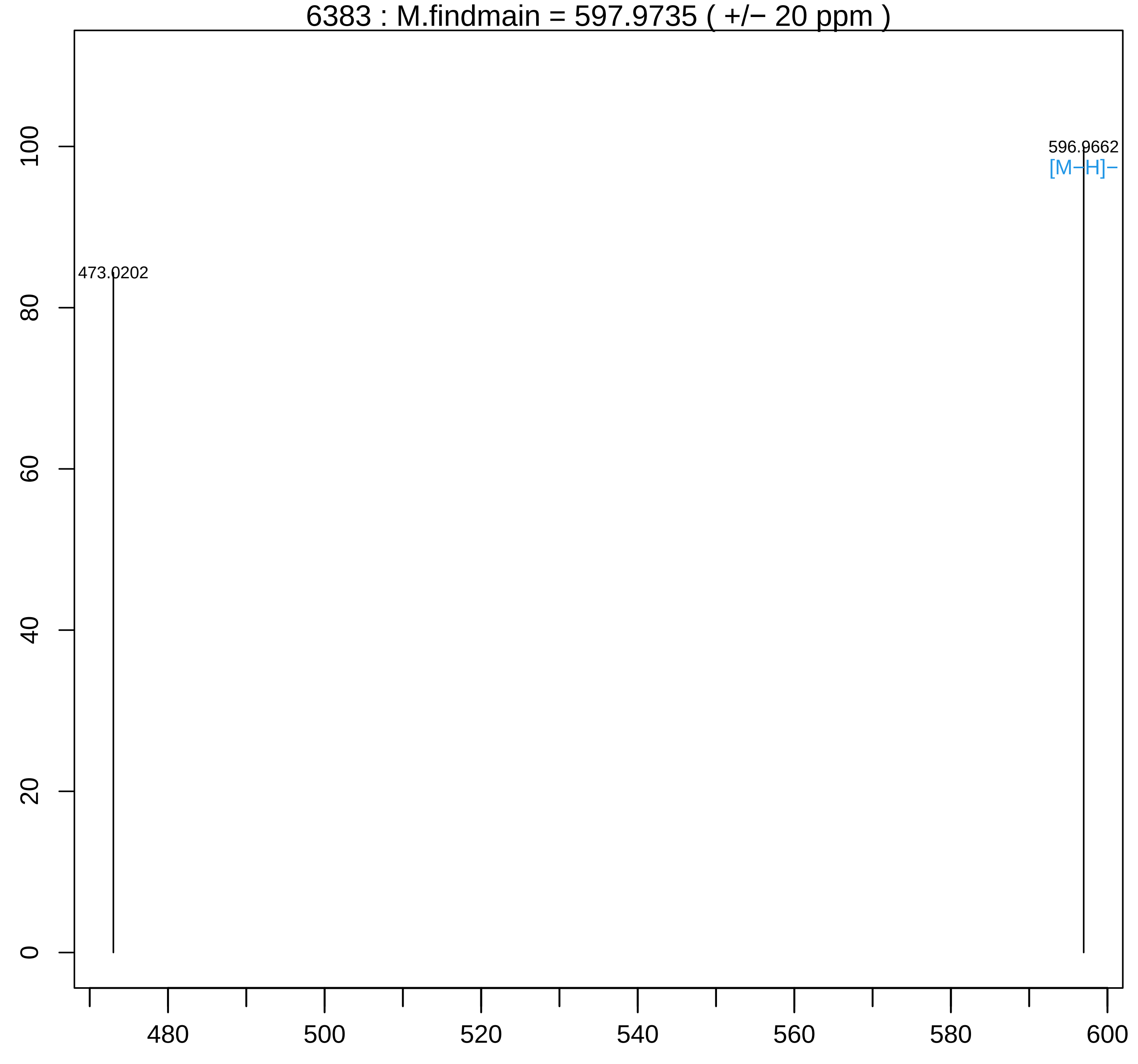
6383 : M.findmain = 597.9735 (+/− 20 ppm)
596.9662
[M−H]−
473.0202
0
20
40
60
80
100
480
500
520
540
560
580
600

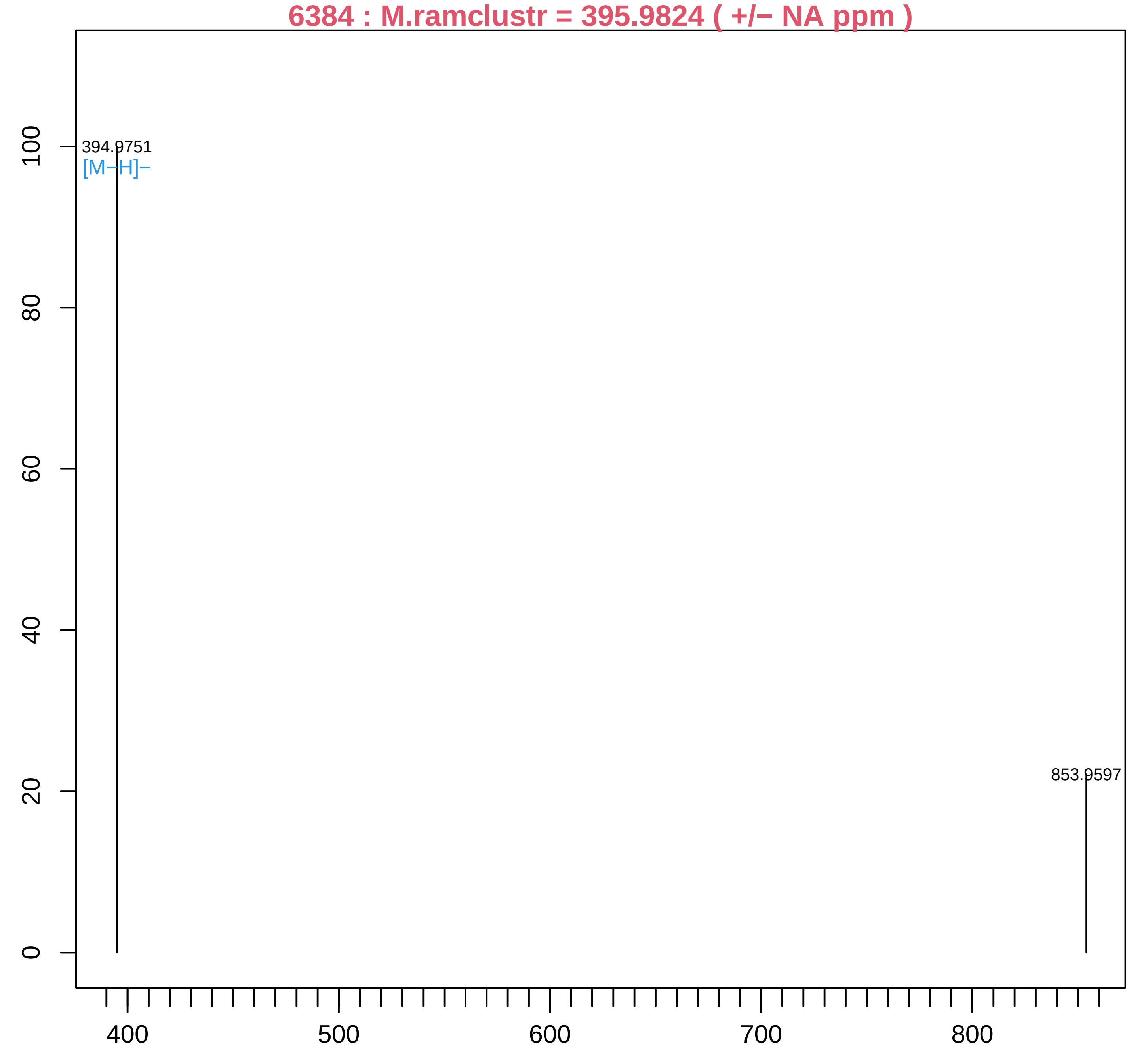
6384 : M.ramclustr = 395.9824 (+/− NA ppm)
394.9751
[M−H]−
853.9597
0
20
40
60
80
100
400
500
600
700
800

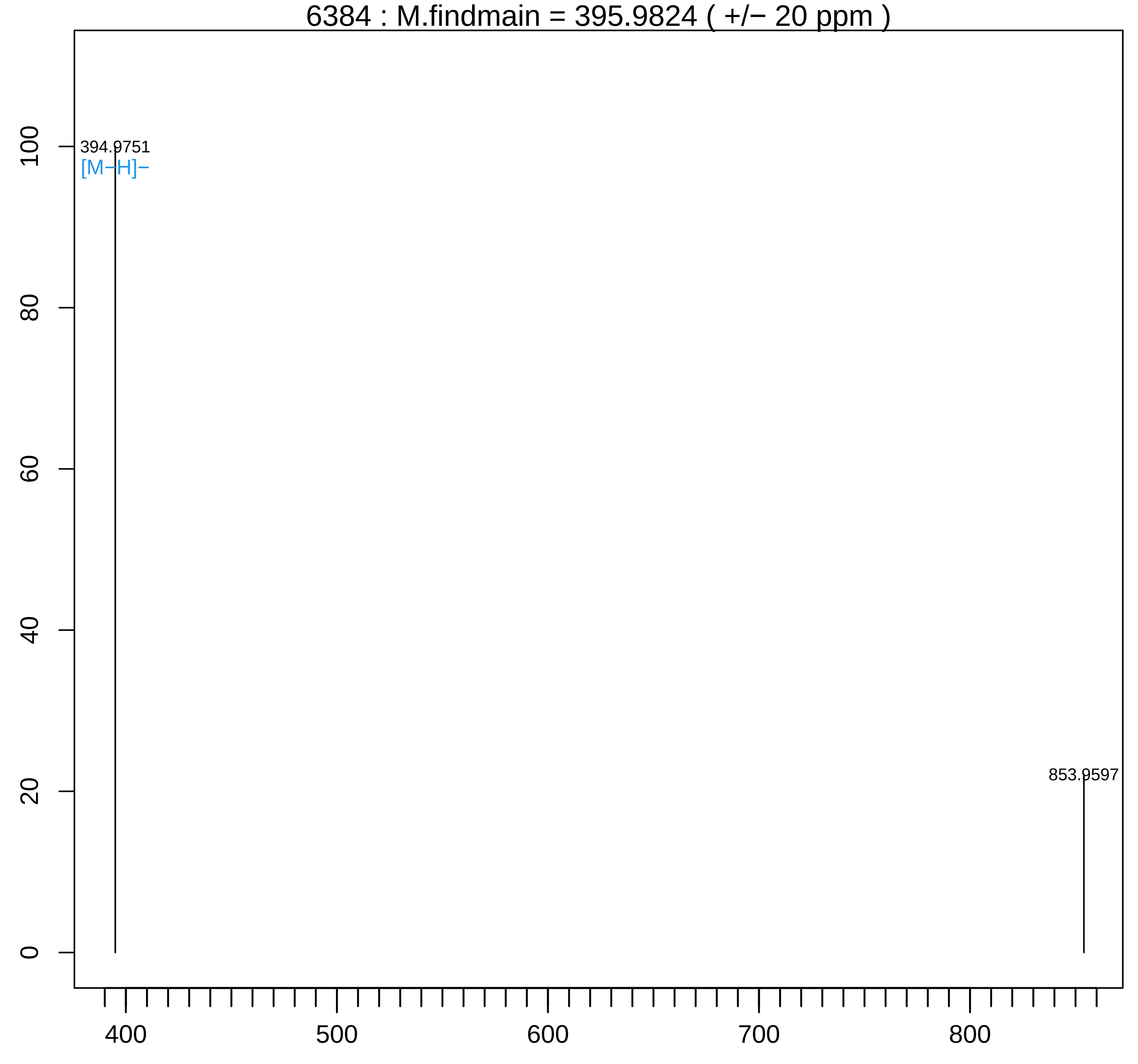
6384 : M.findmain = 395.9824 (+/− 20 ppm)
394.9751
[M−H]−
853.9597
0
20
40
60
80
100
400
500
600
700
800

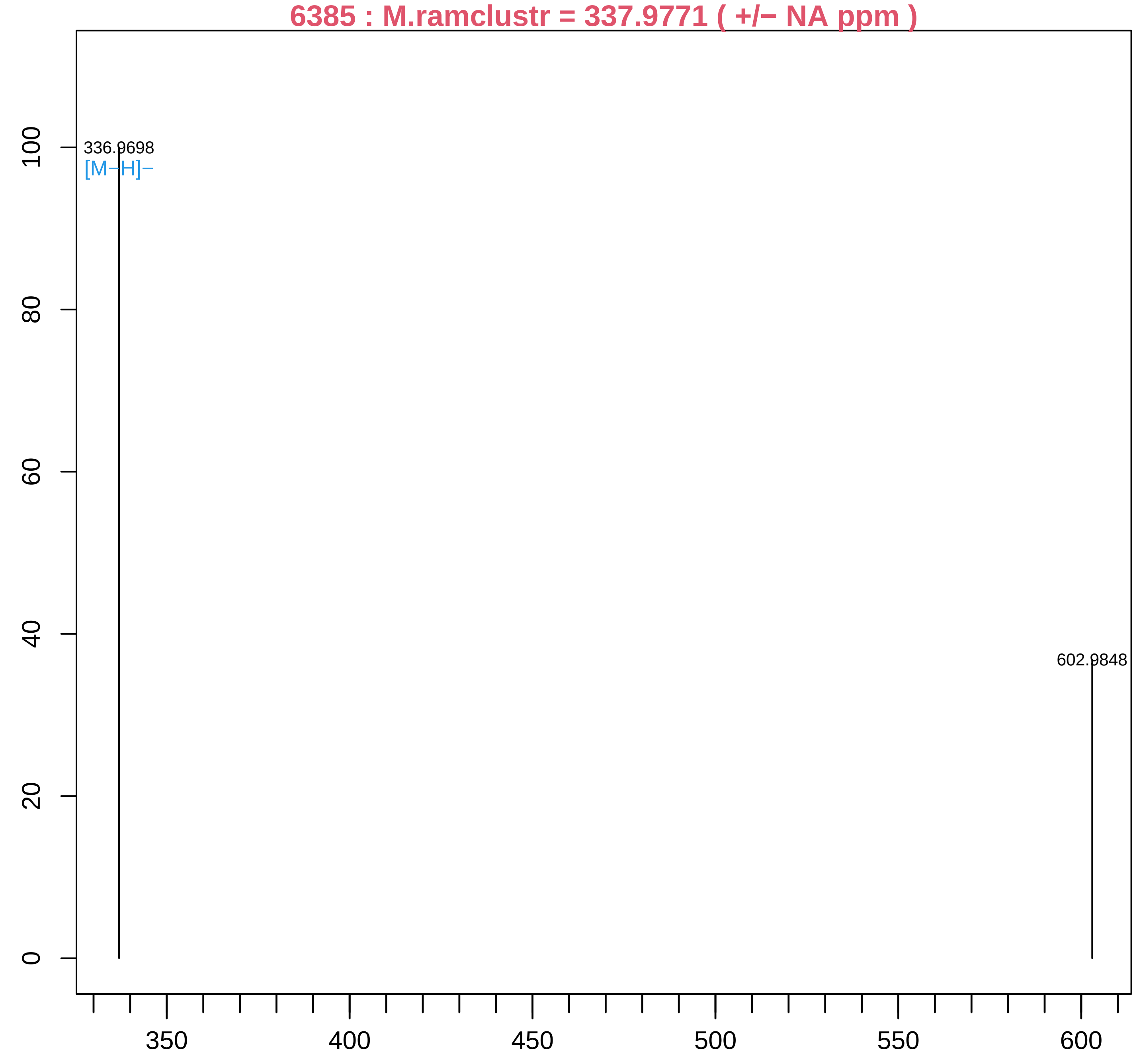
6385 : M.ramclustr = 337.9771 (+/− NA ppm)
336.9698
[M−H]−
602.9848
0
20
40
60
80
100
350
400
450
500
550
600

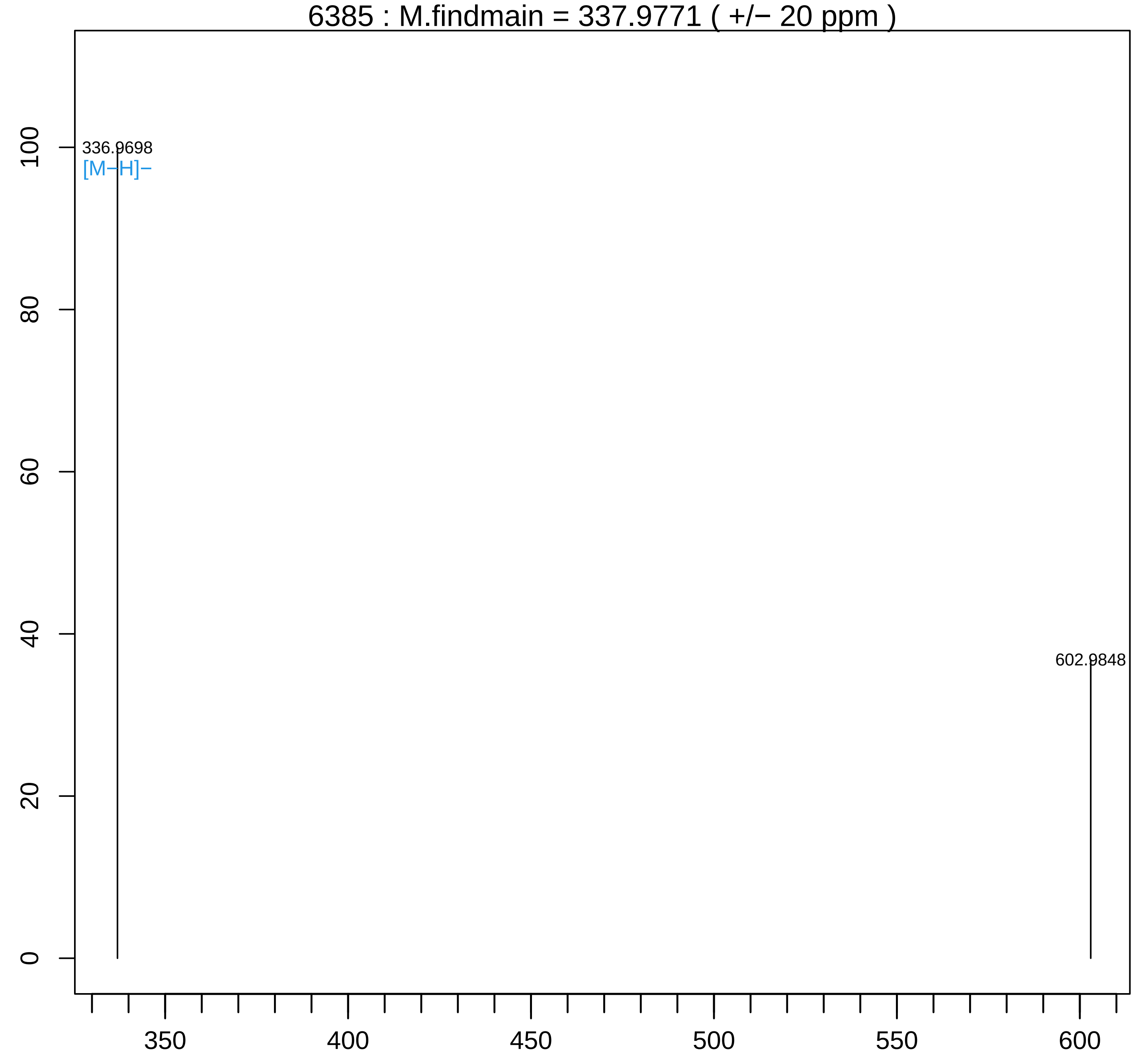
6385 : M.findmain = 337.9771 (+/− 20 ppm)
336.9698
[M−H]−
602.9848
0
20
40
60
80
100
350
400
450
500
550
600

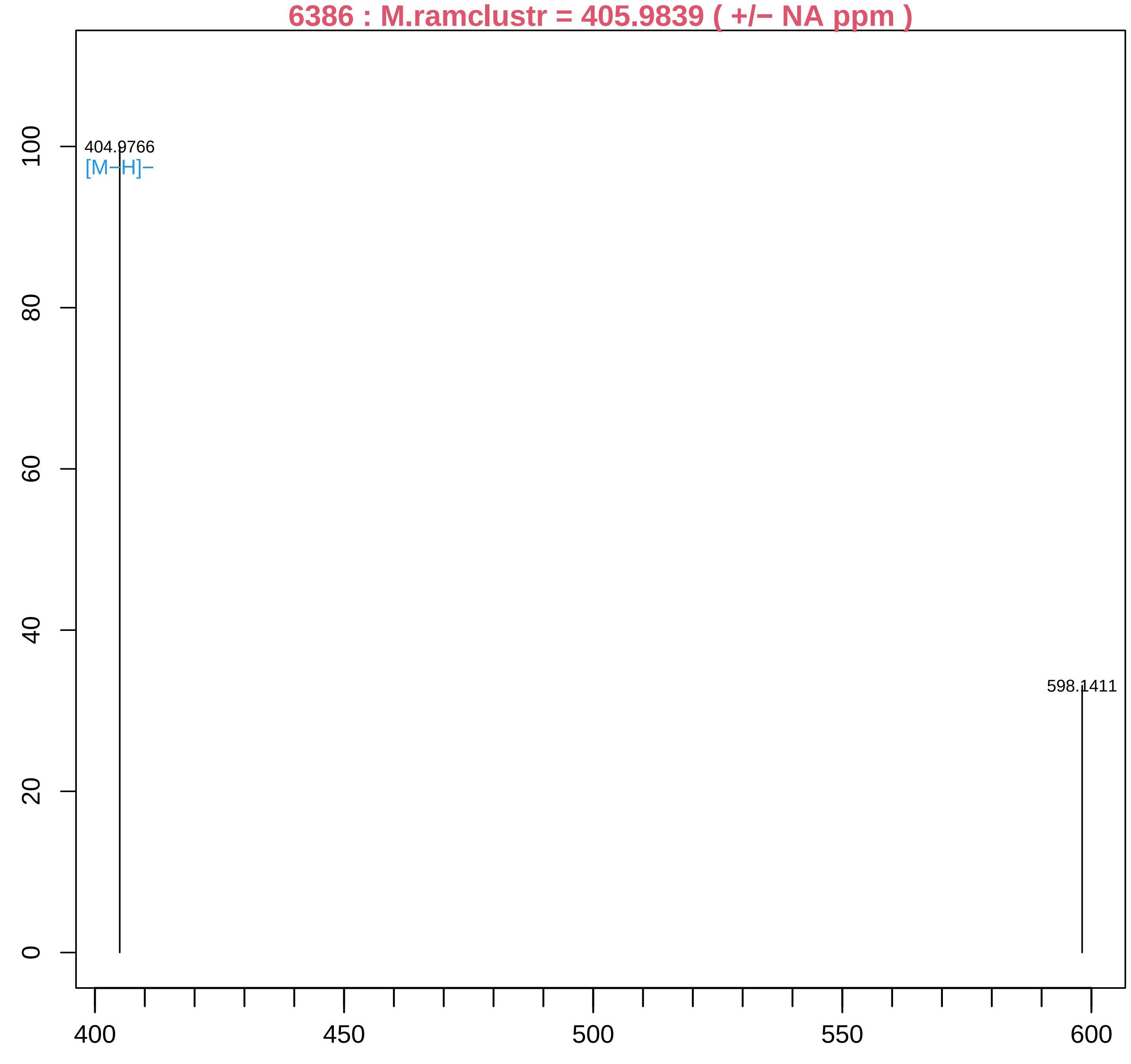
6386 : M.ramclustr = 405.9839 (+/− NA ppm)
404.9766
[M−H]−
598.1411
0
20
40
60
80
100
400
450
500
550
600

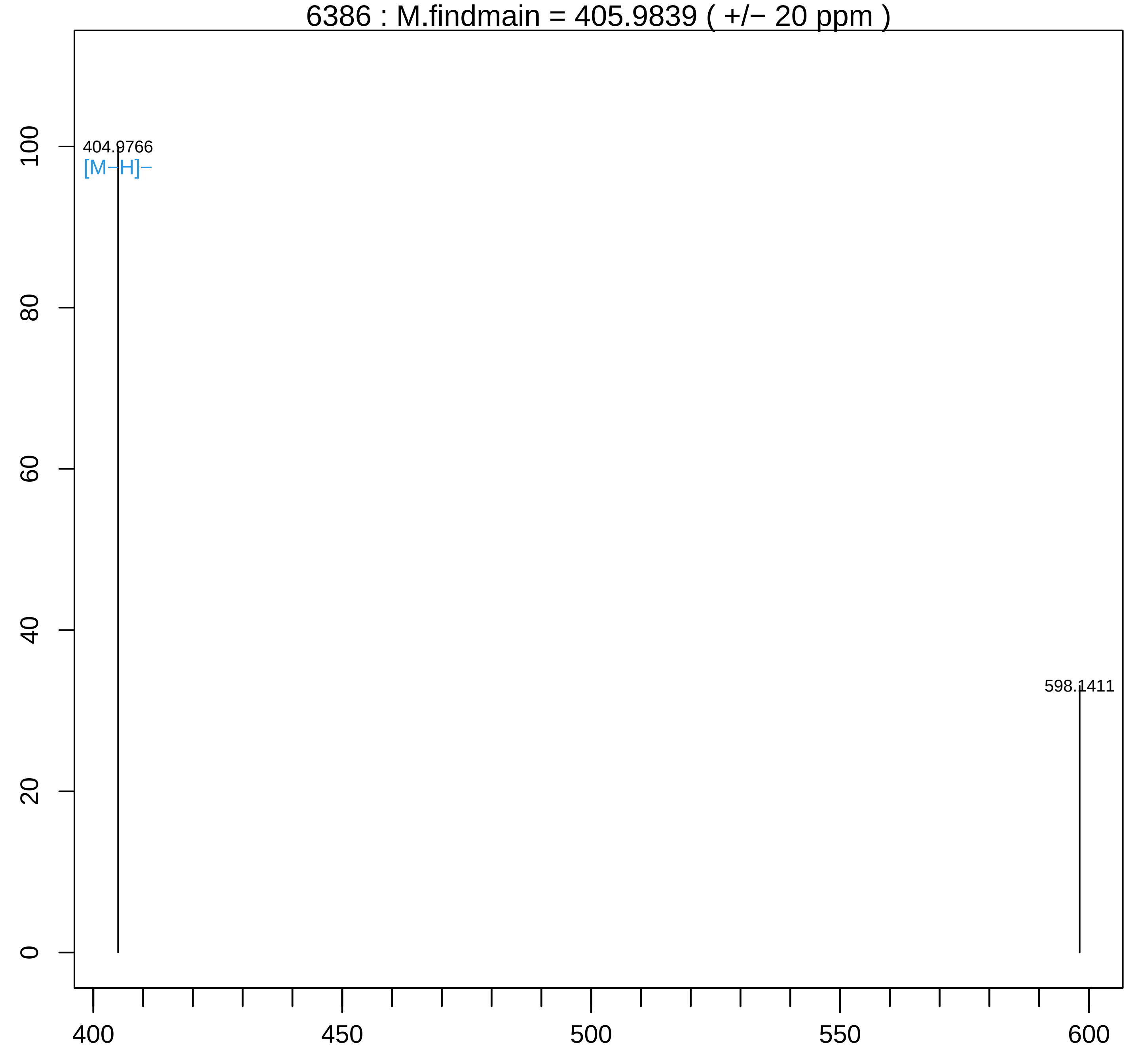
6386 : M.findmain = 405.9839 (+/− 20 ppm)
404.9766
[M−H]−
598.1411
0
20
40
60
80
100
400
450
500
550
600

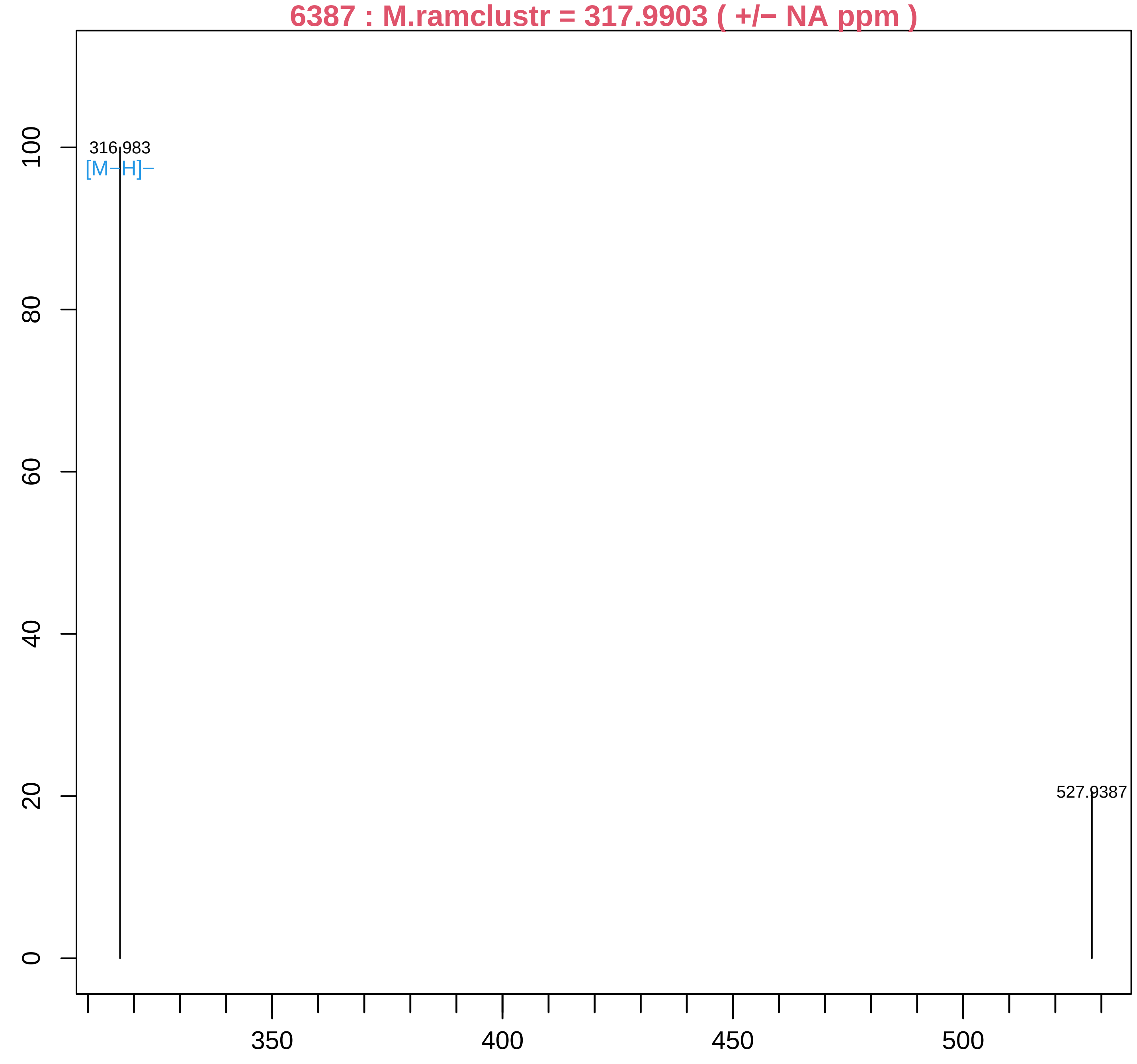

6387 : M.ramclustr = 317.9903 (+/− NA ppm)
316.983
[M−H]−
527.9387
0
20
40
60
80
100
350
400
450
500

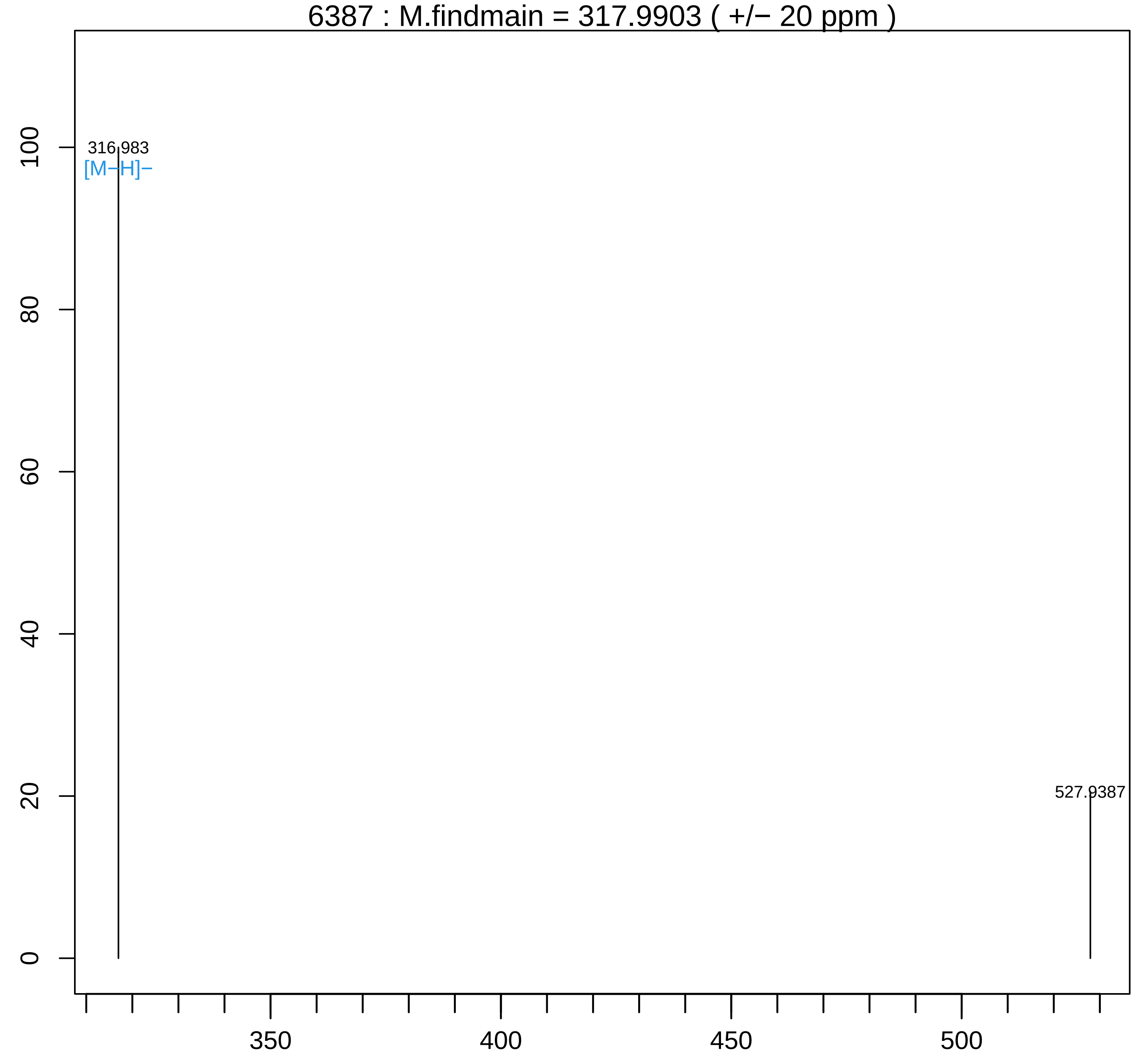

6387 : M.findmain = 317.9903 (+/− 20 ppm)
316.983
[M−H]−
527.9387
0
20
40
60
80
100
350
400
450
500

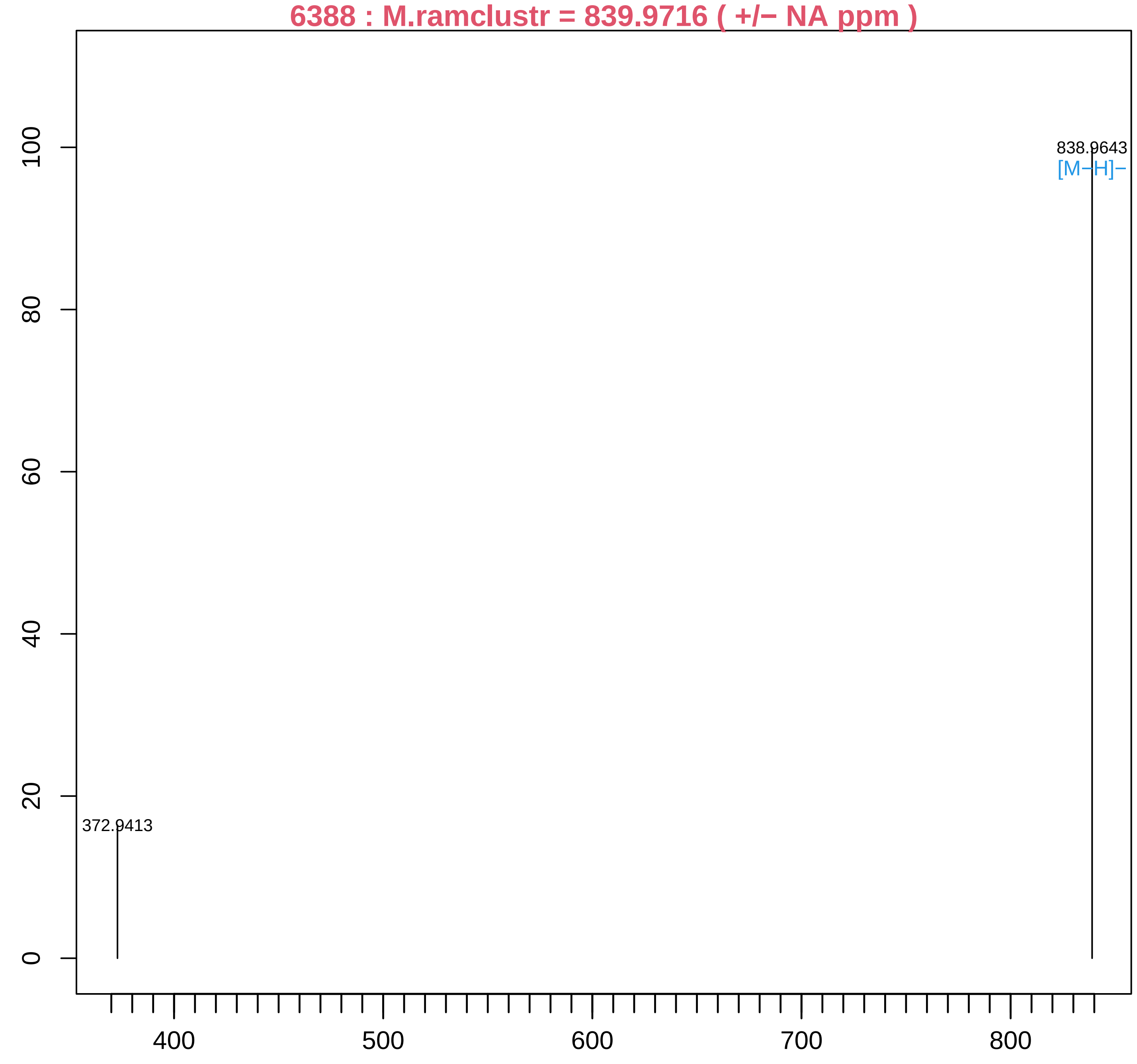
6388 : M.ramclustr = 839.9716 (+/− NA ppm)
838.9643
[M−H]−
372.9413
0
20
40
60
80
100
400
500
600
700
800

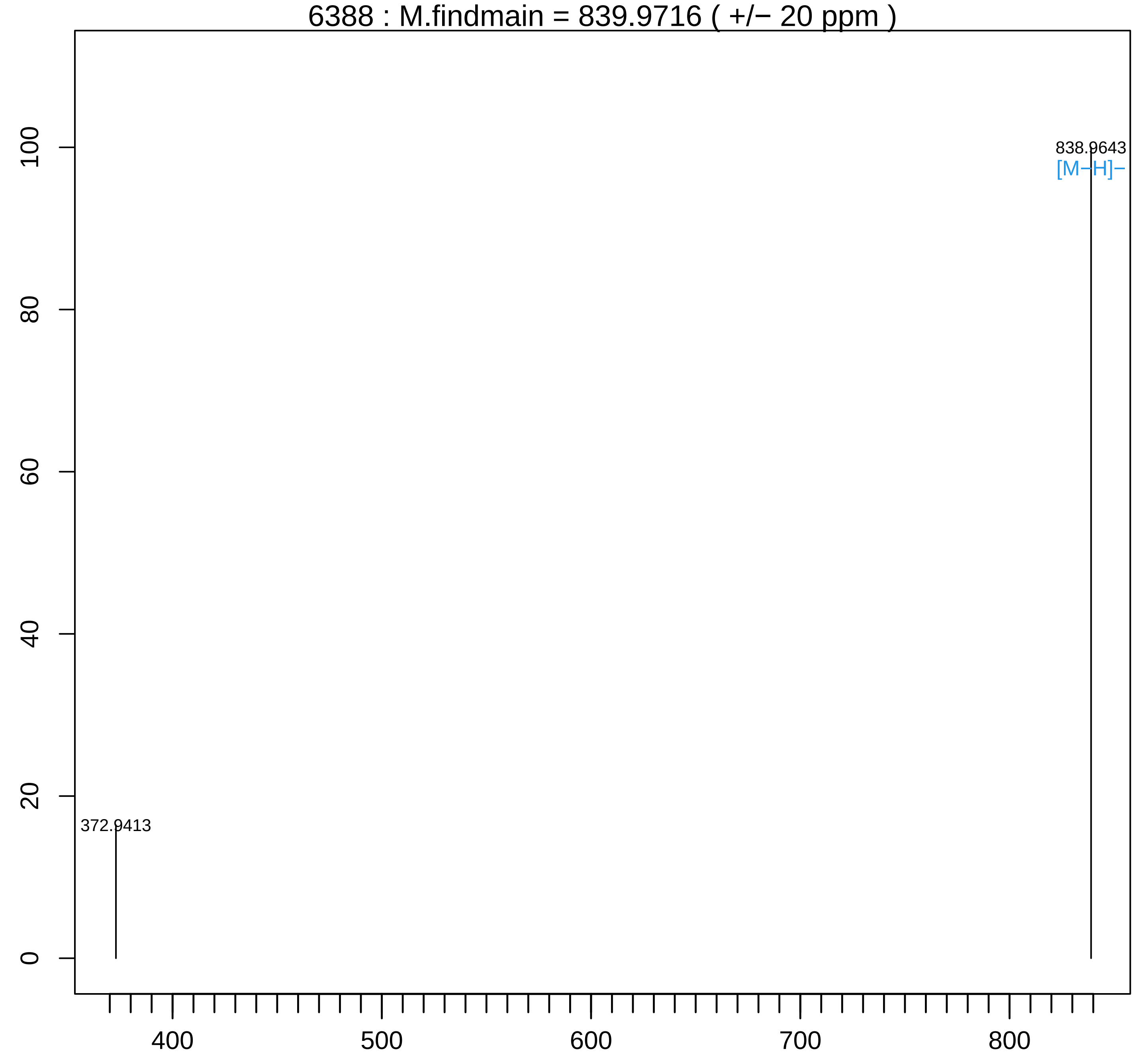
6388 : M.findmain = 839.9716 (+/− 20 ppm)
838.9643
[M−H]−
372.9413
0
20
40
60
80
100
400
500
600
700
800

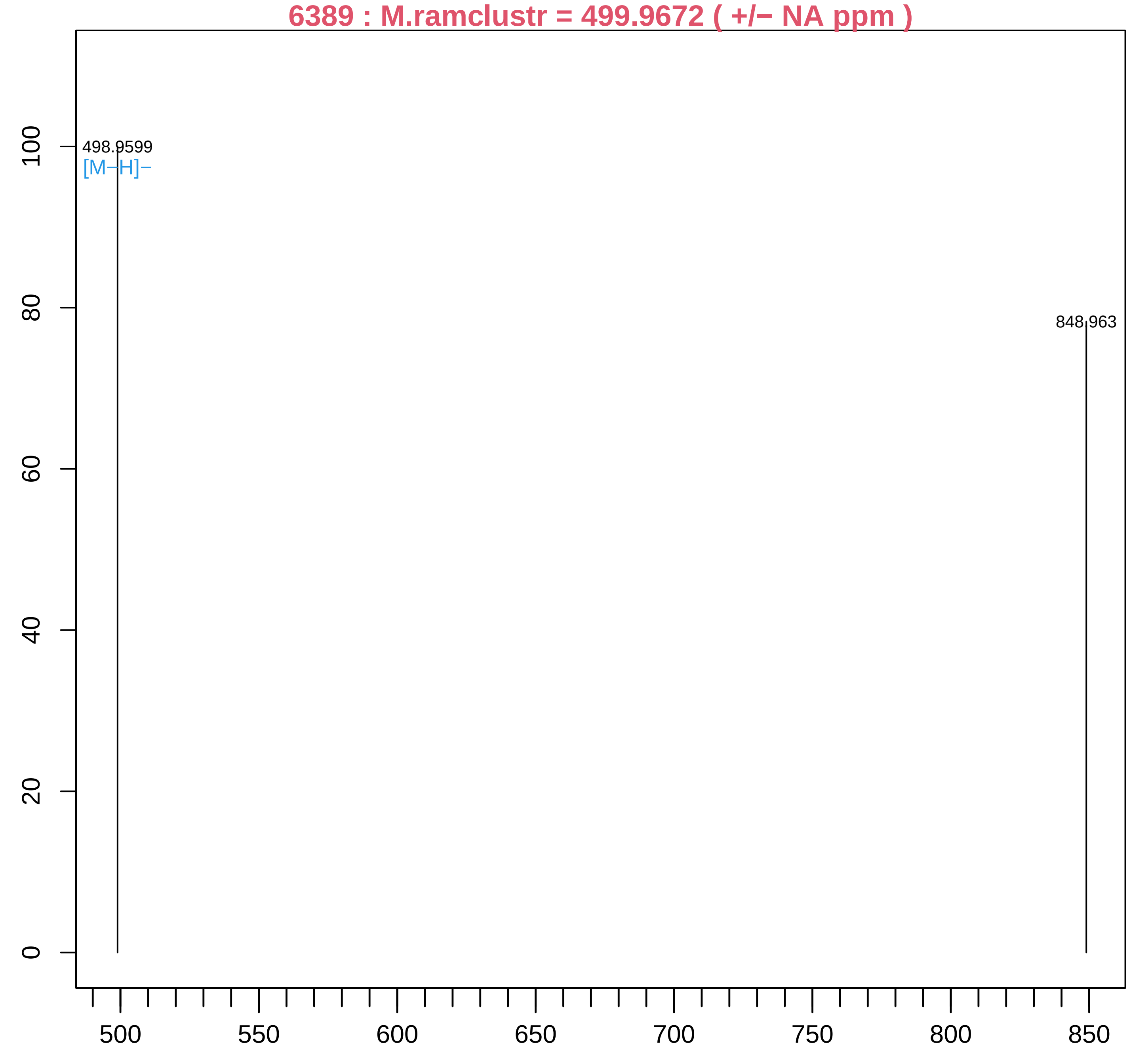
6389 : M.ramclustr = 499.9672 (+/− NA ppm)
498.9599
[M−H]−
848.963
0
20
40
60
80
100
500
550
600
650
700
750
800
850

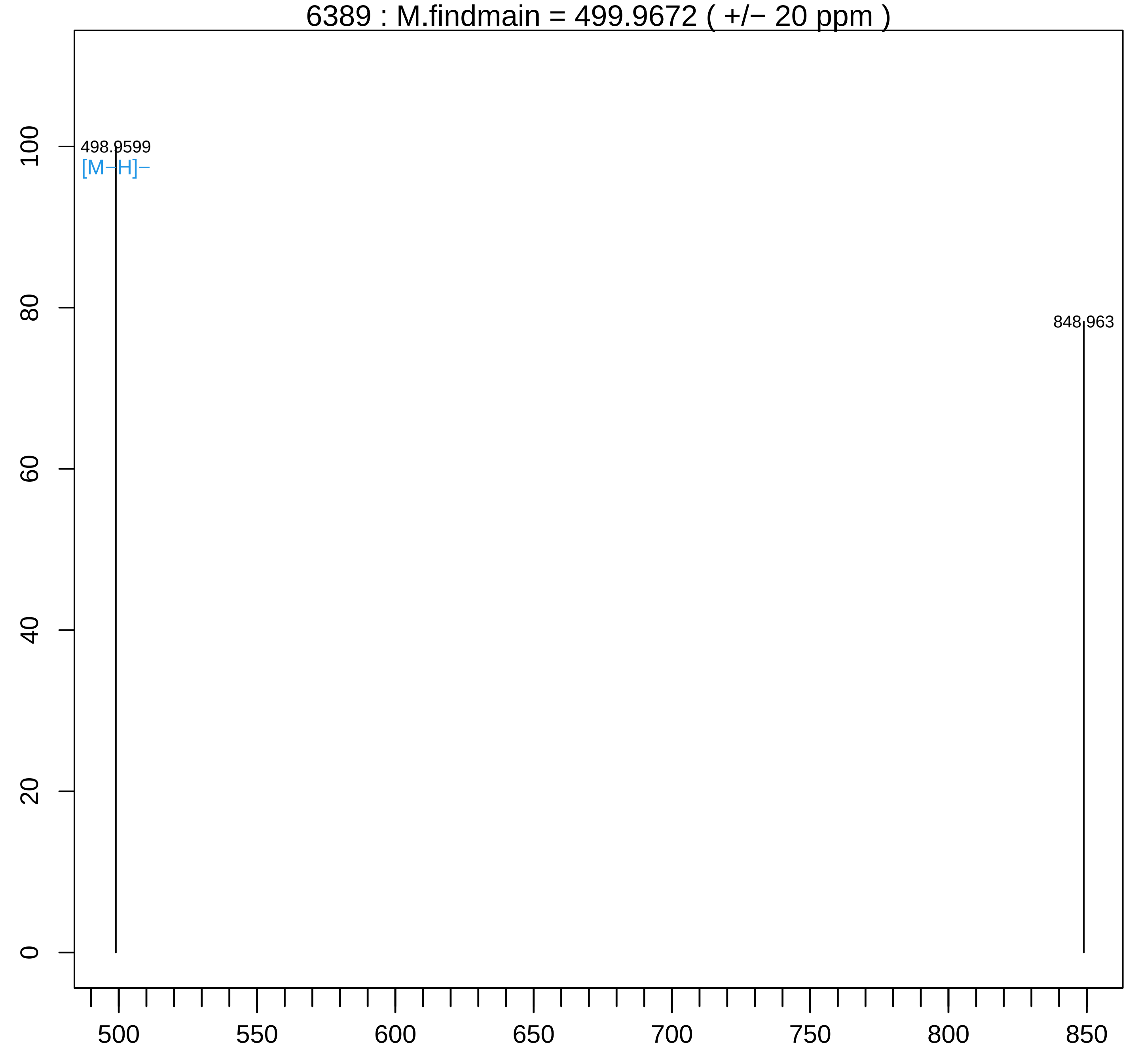
6389 : M.findmain = 499.9672 (+/− 20 ppm)
498.9599
[M−H]−
848.963
0
20
40
60
80
100
500
550
600
650
700
750
800
850

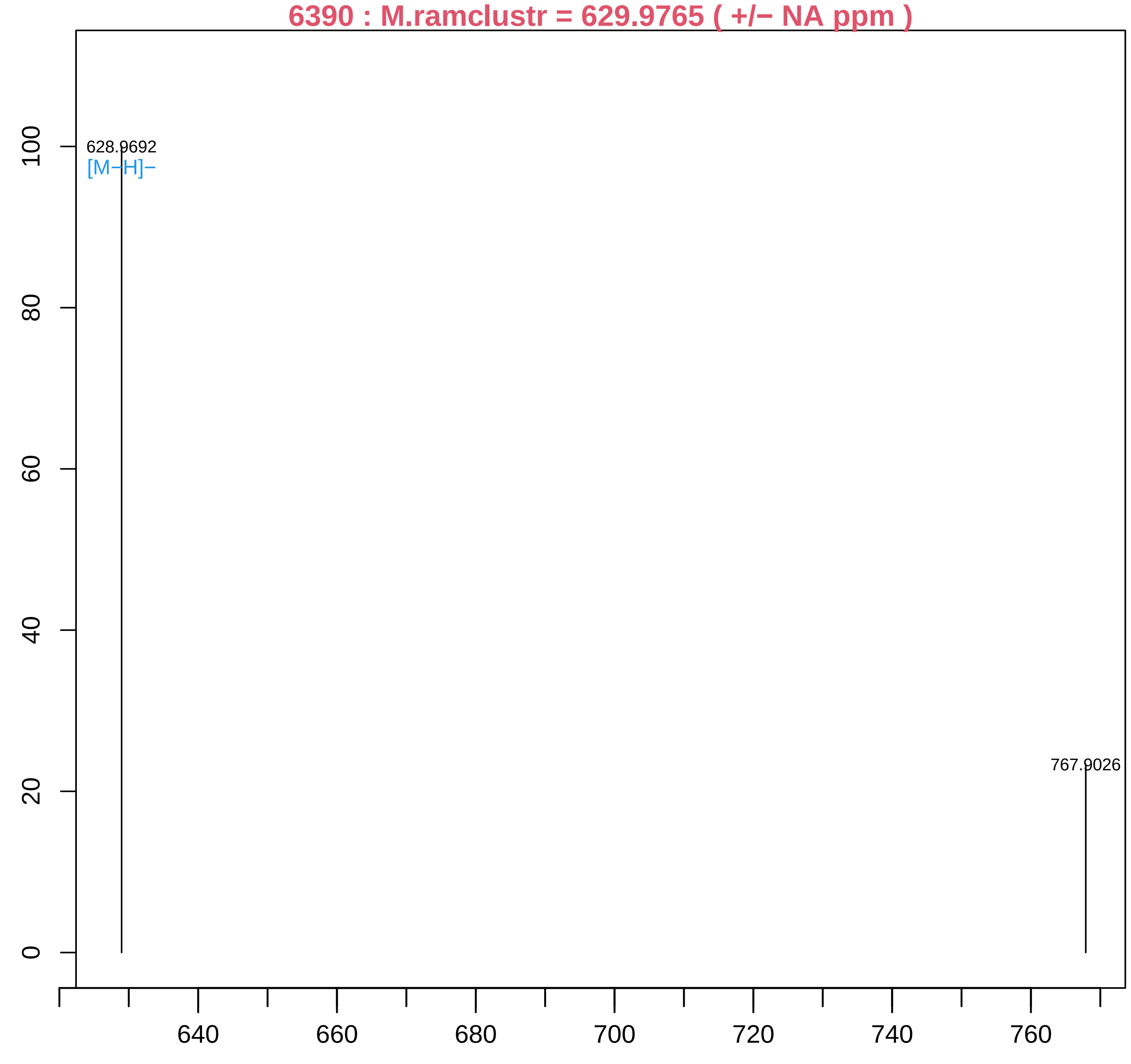
6390 : M.ramclustr = 629.9765 (+/− NA ppm)
628.9692
[M−H]−
767.9026
0
20
40
60
80
100
640
660
680
700
720
740
760

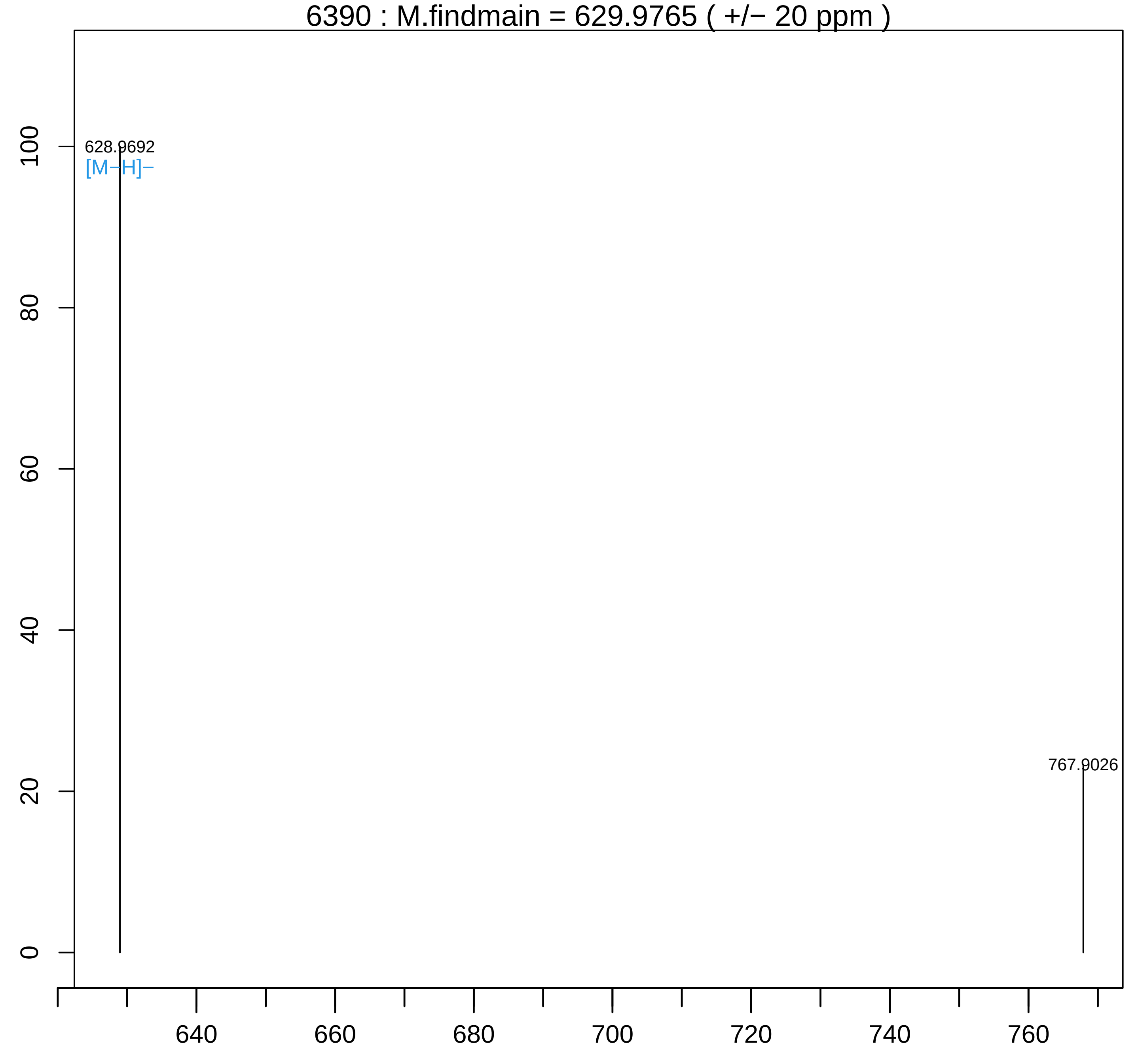
6390 : M.findmain = 629.9765 (+/− 20 ppm)
628.9692
[M−H]−
767.9026
0
20
40
60
80
100
640
660
680
700
720
740
760

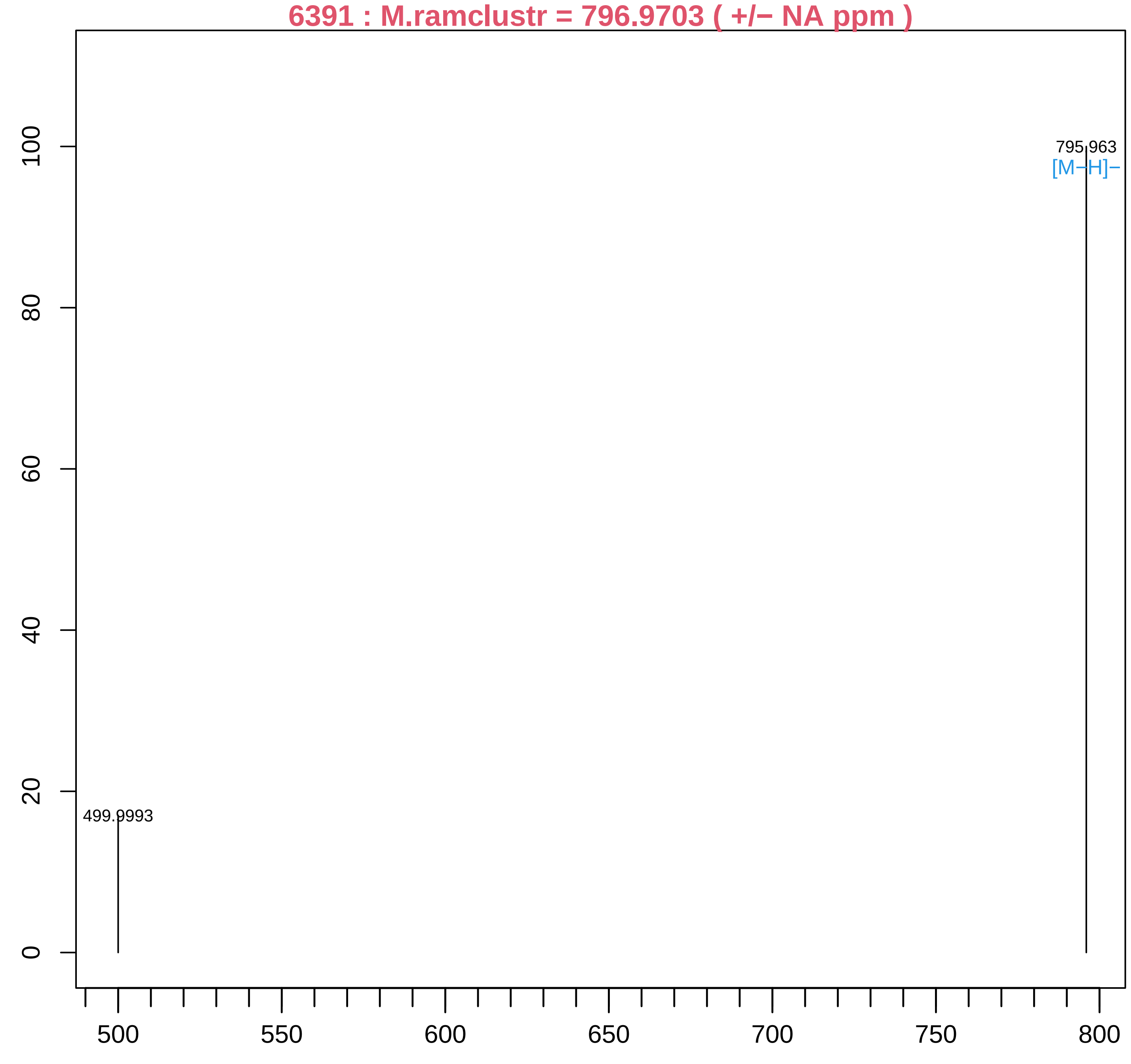
6391 : M.ramclustr = 796.9703 (+/− NA ppm)
795.963
[M−H]−
499.9993
0
20
40
60
80
100
500
550
600
650
700
750
800

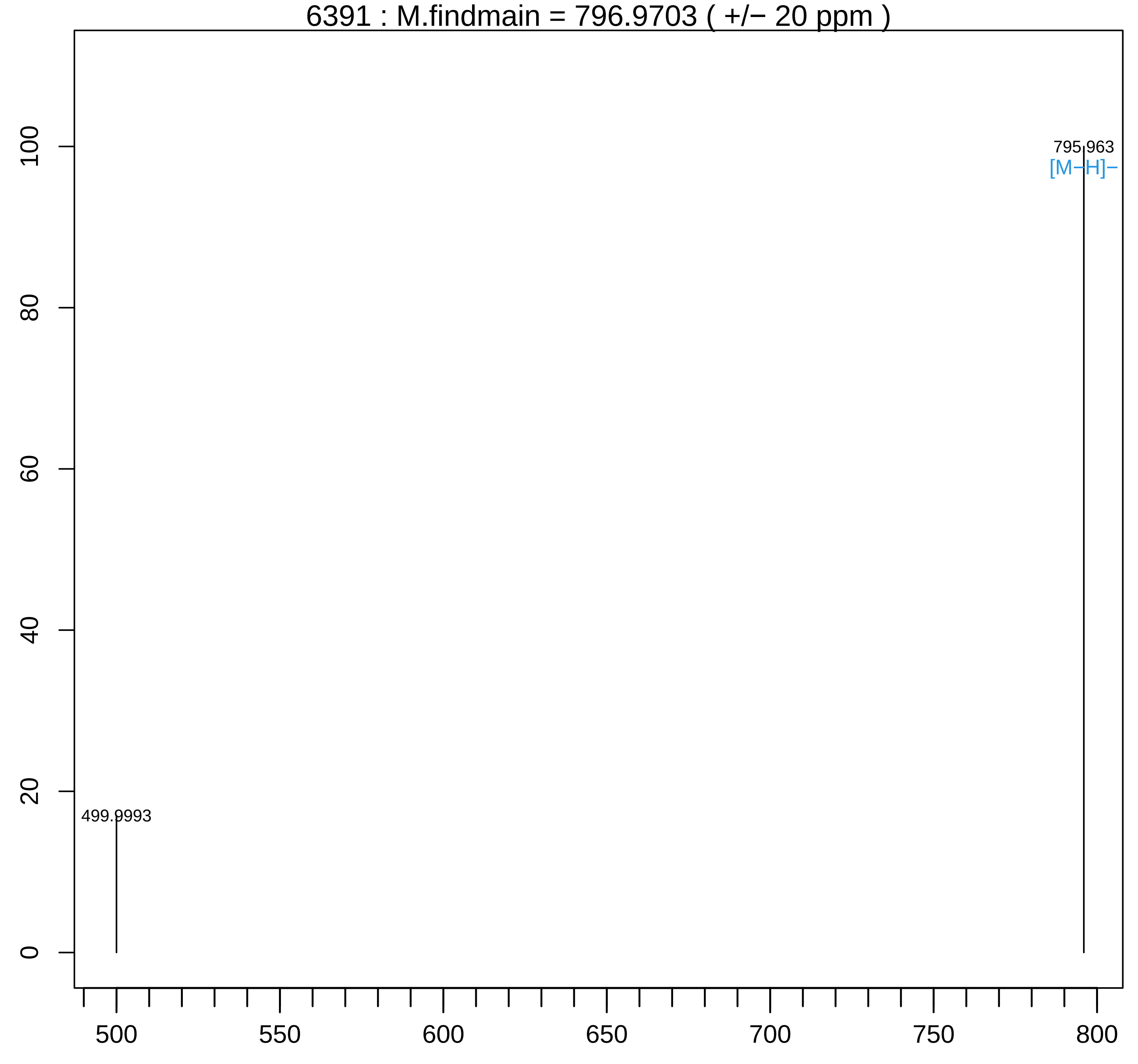
6391 : M.findmain = 796.9703 (+/− 20 ppm)
795.963
[M−H]−
499.9993
0
20
40
60
80
100
500
550
600
650
700
750
800

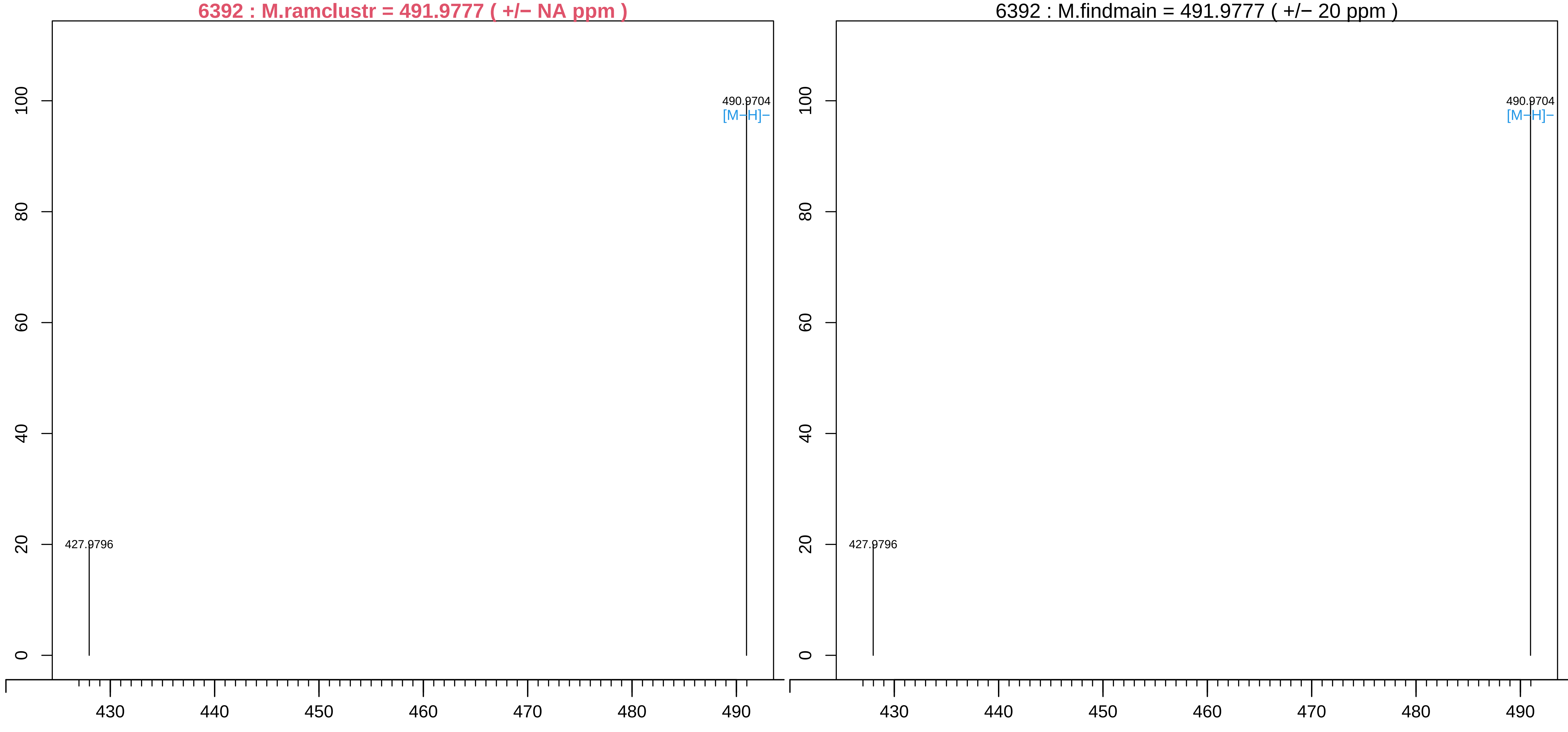
6392 : M.ramclustr = 491.9777 (+/− NA ppm)
490.9704
[M−H]−
427.9796
0
20
40
60
80
100
430
440
450
460
470
480
490
6392 : M.findmain = 491.9777 (+/− 20 ppm)
490.9704
[M−H]−
427.9796
0
20
40
60
80
100
430
440
450
460
470
480
490

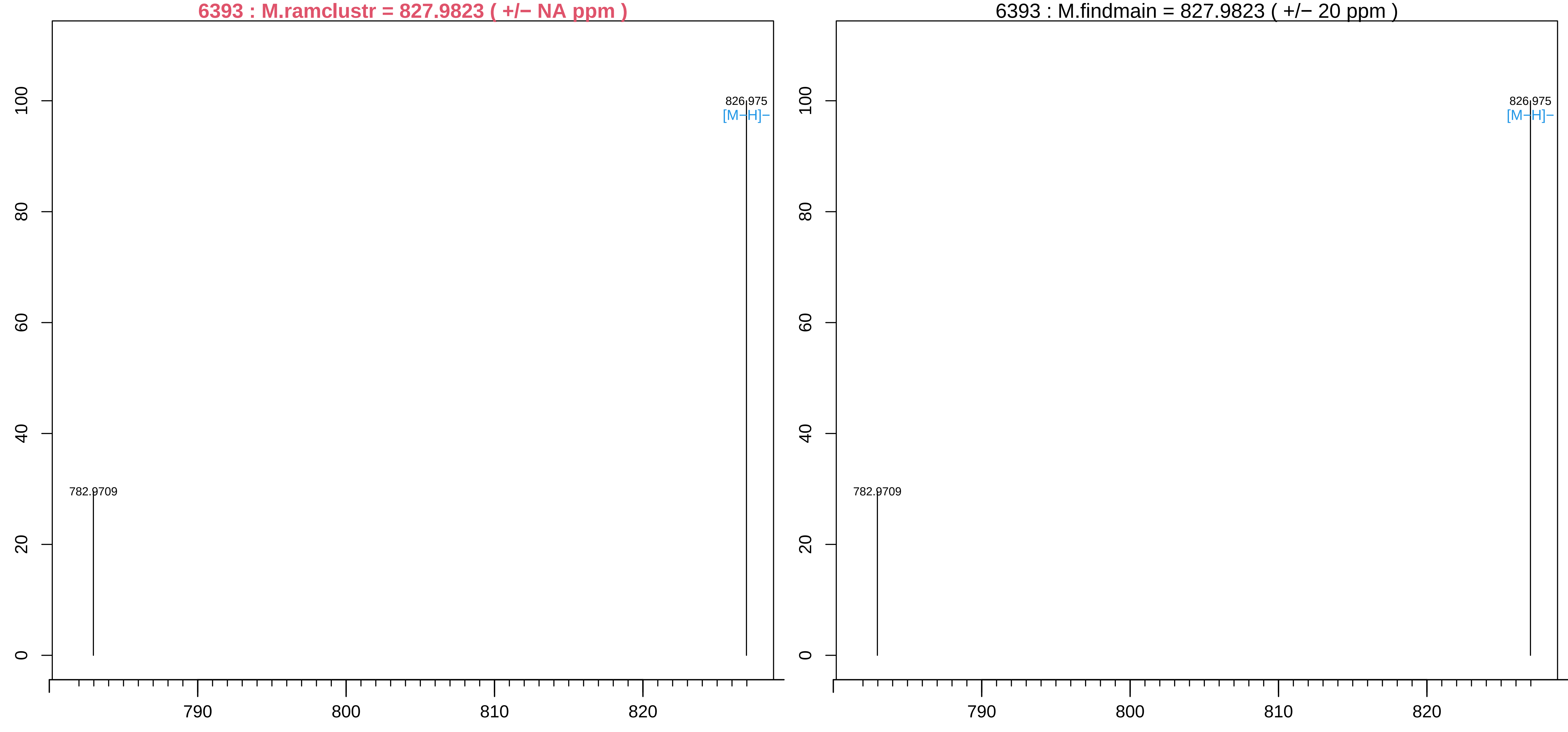
6393 : M.ramclustr = 827.9823 (+/− NA ppm)
6393 : M.findmain = 827.9823 (+/− 20 ppm)
826.975
[M−H]−
782.9709
0
20
40
60
80
100
790
800
810
820

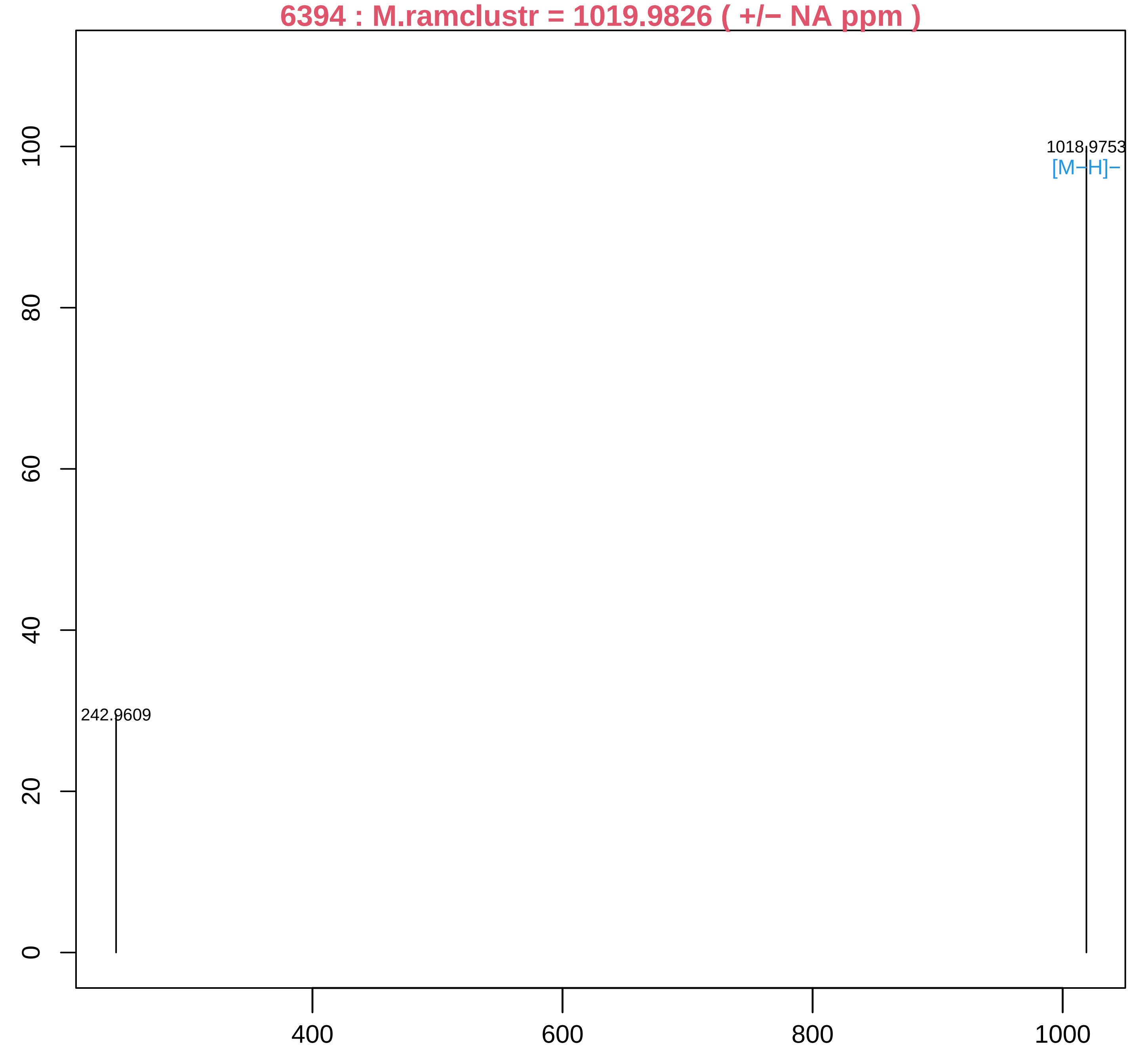

6394 : M.ramclustr = 1019.9826 (+/− NA ppm)
1018.9753
[M−H]−
242.9609
0
20
40
60
80
100
400
600
800
1000

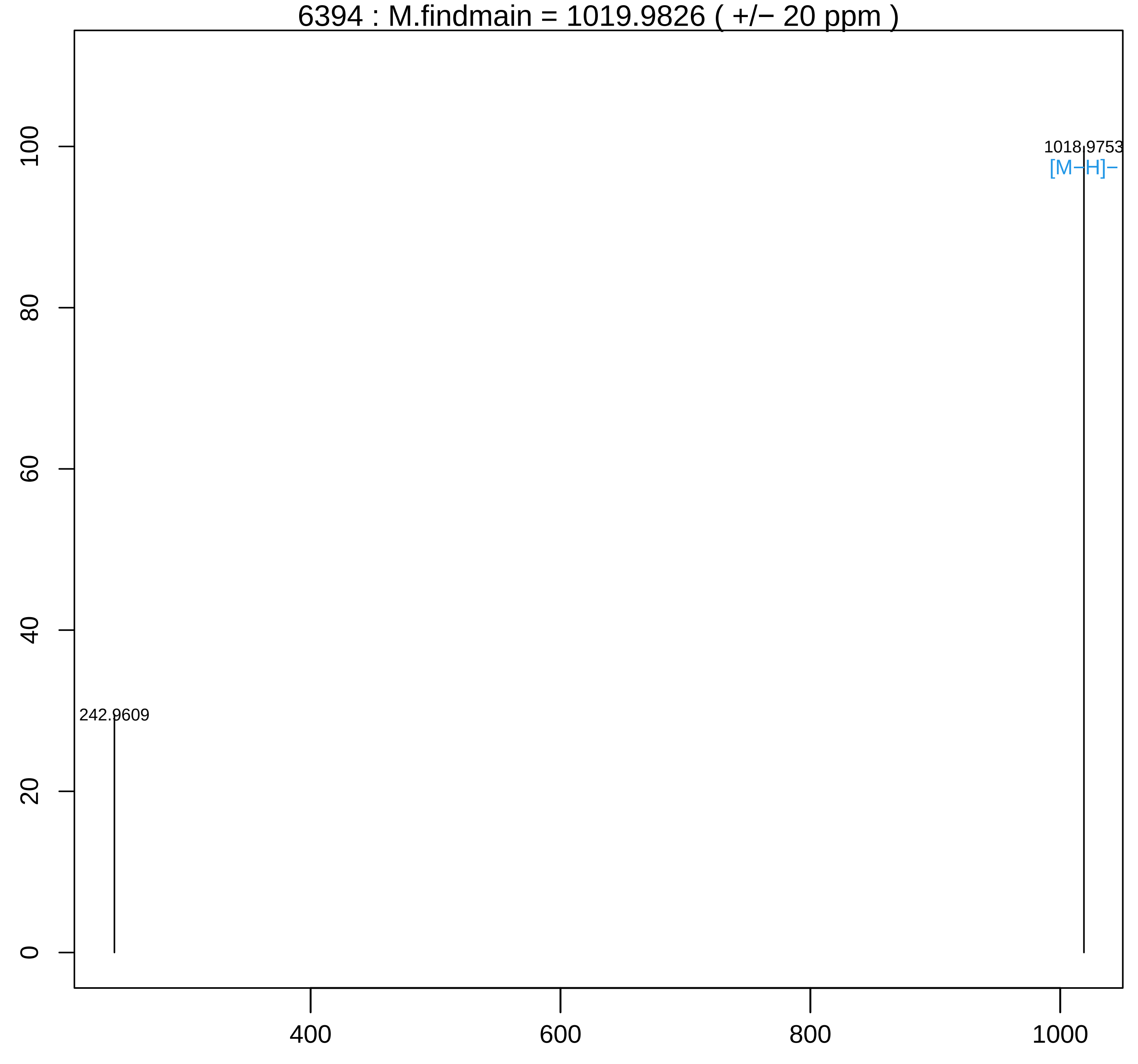

6394 : M.findmain = 1019.9826 (+/− 20 ppm)
1018.9753
[M−H]−
242.9609
0
20
40
60
80
100
400
600
800
1000

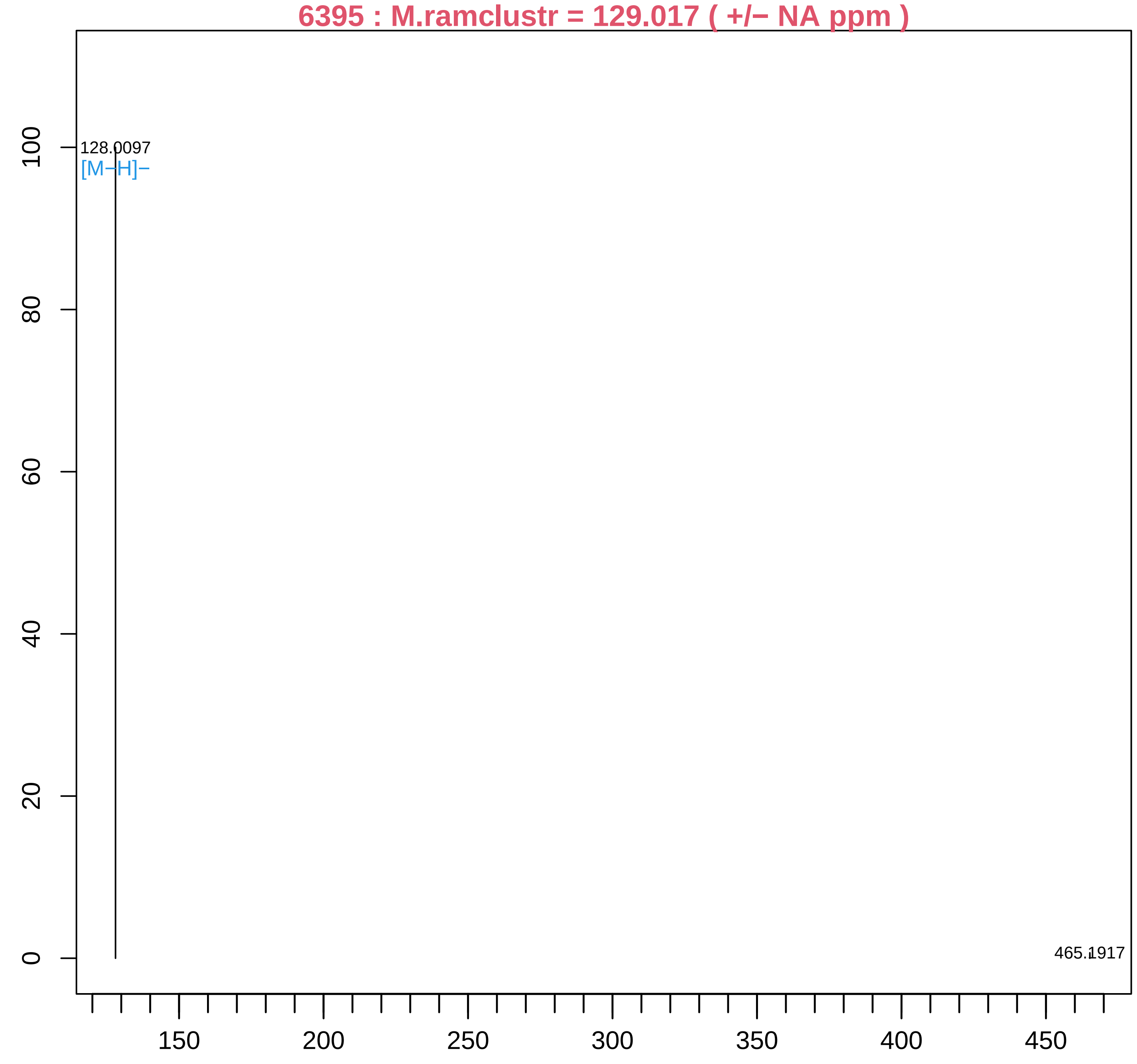
6395 : M.ramclustr = 129.017 (+/− NA ppm)
128.0097
[M−H]−
465.1917
0
20
40
60
80
100
150
200
250
300
350
400
450

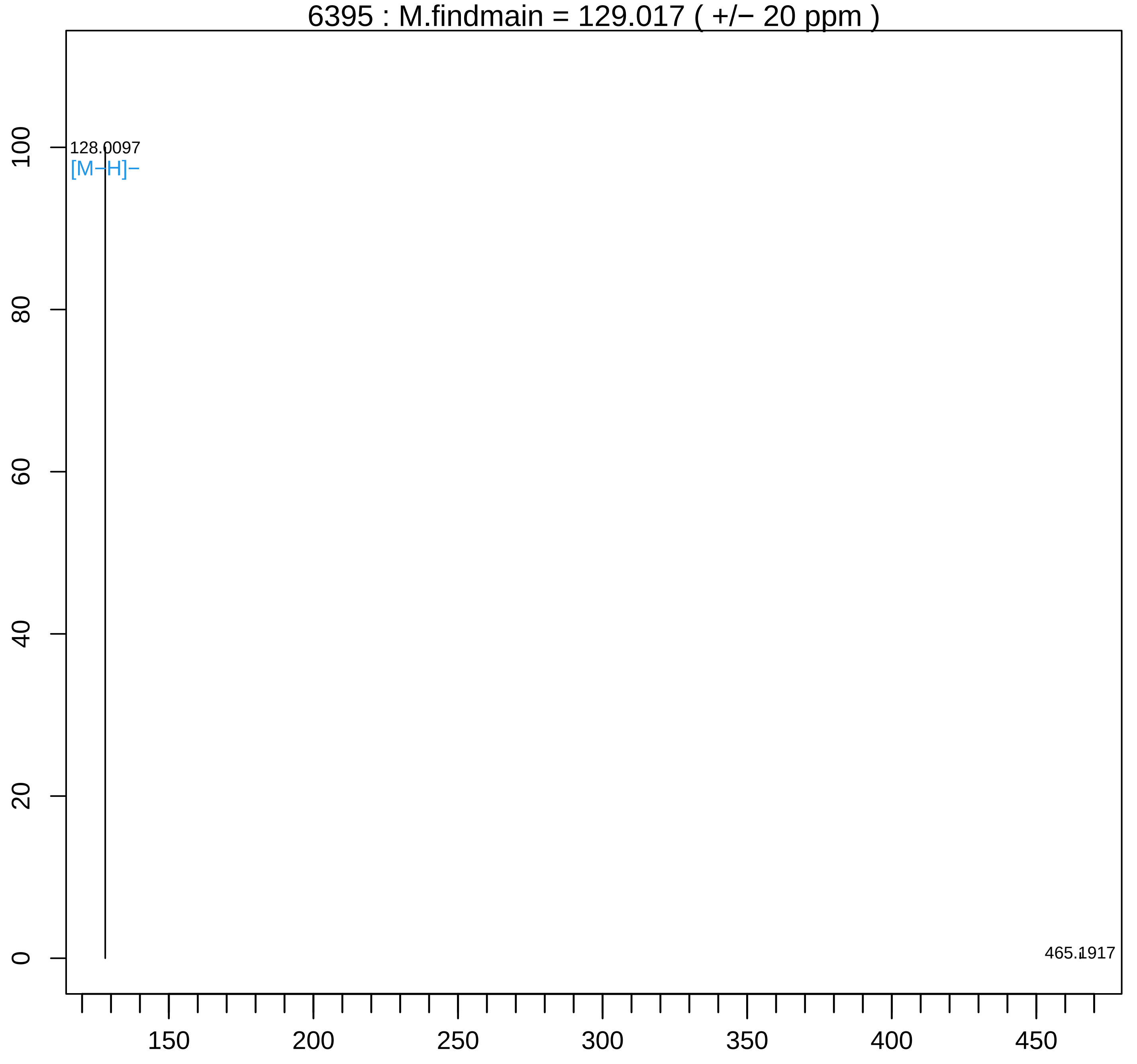
6395 : M.findmain = 129.017 (+/− 20 ppm)
128.0097
[M−H]−
465.1917
0
20
40
60
80
100
150
200
250
300
350
400
450

6396 : M.ramclustr = 331.9876 (+/− NA ppm)

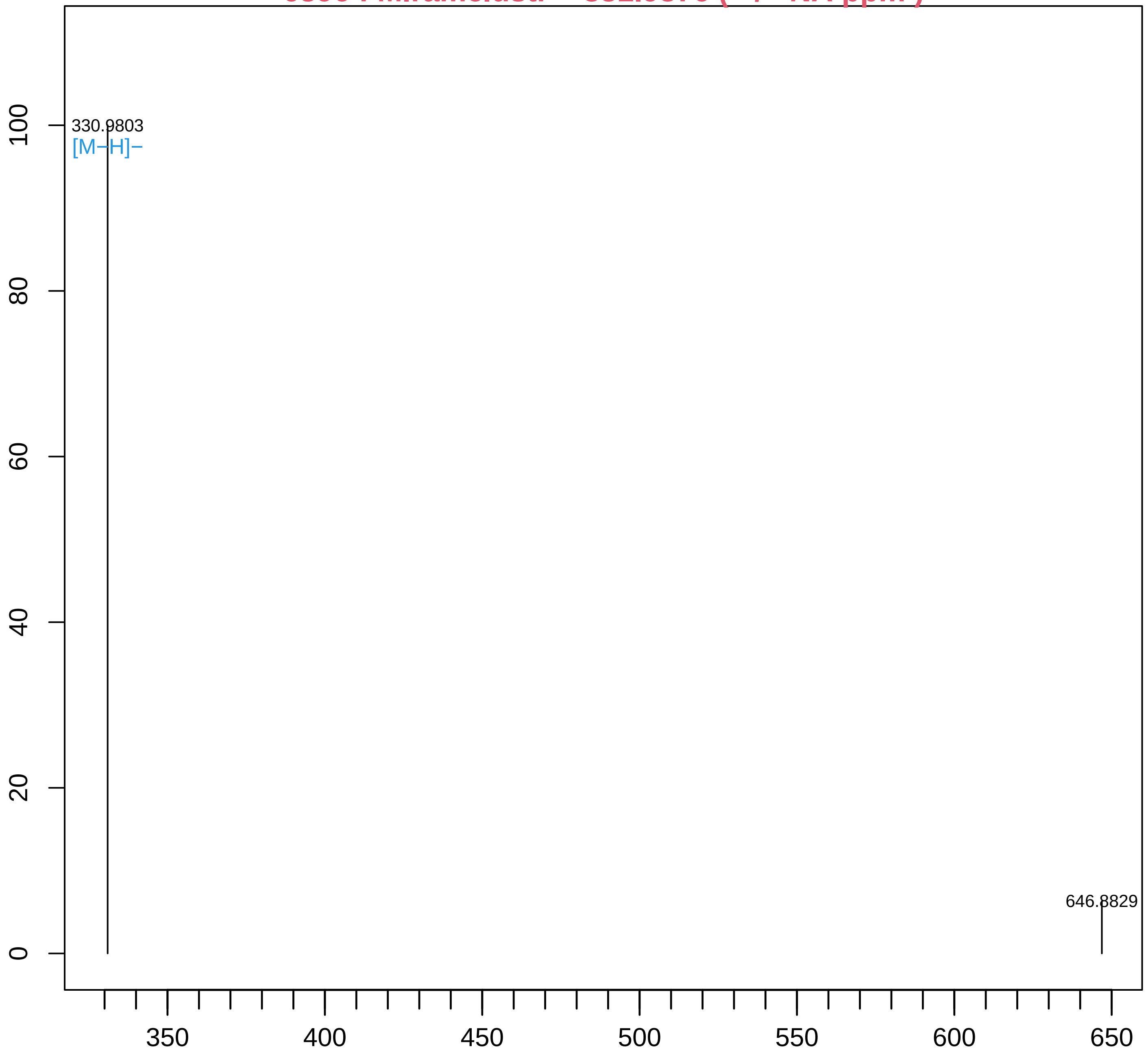

6396 : M.findmain = 331.9876 (+/− 20 ppm)

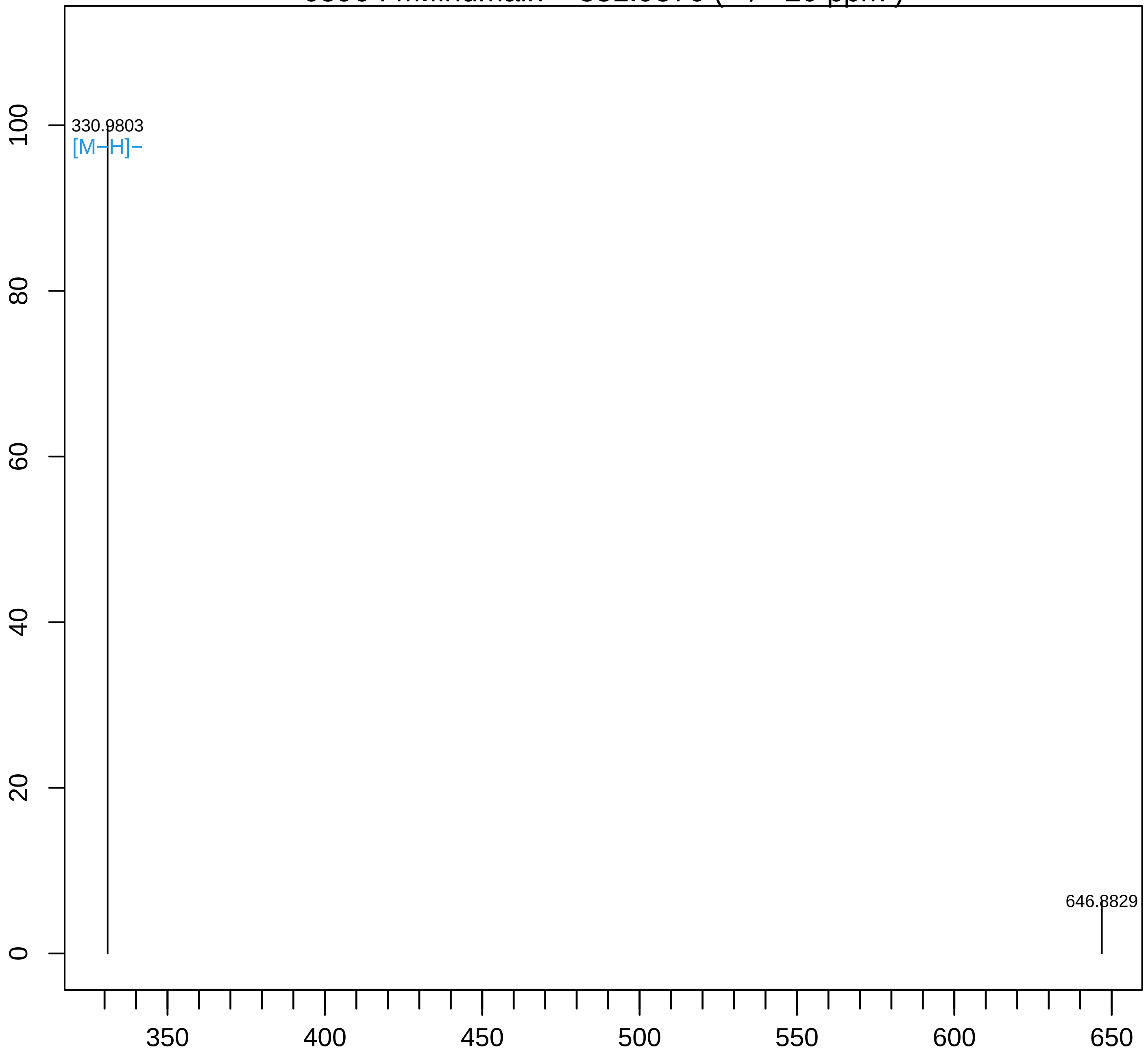

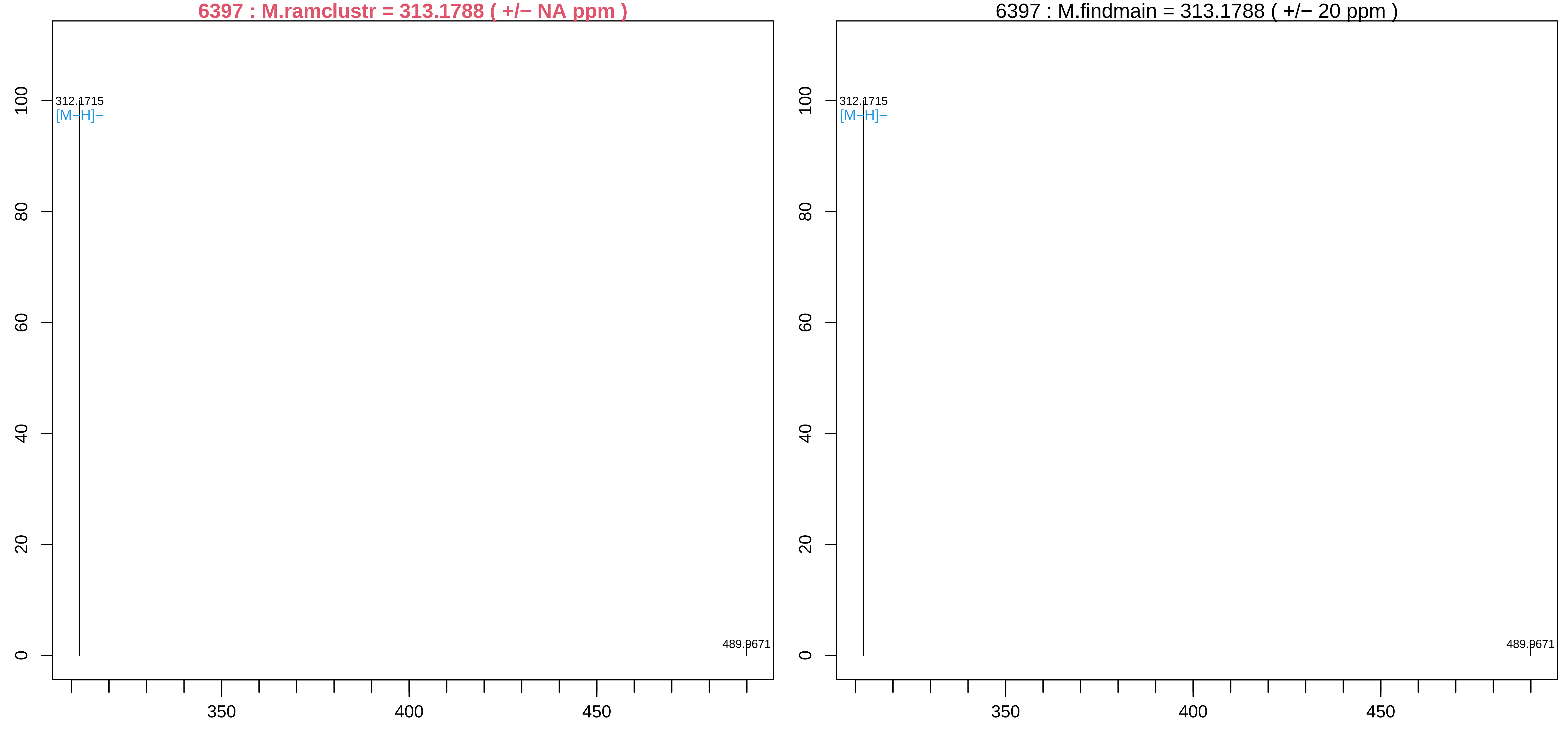
6397 : M.ramclustr = 313.1788 (+/− NA ppm)
312.1715
[M−H]−
489.9671
6397 : M.findmain = 313.1788 (+/− 20 ppm)
312.1715
[M−H]−
489.9671
0
20
40
60
80
100
350
400
450

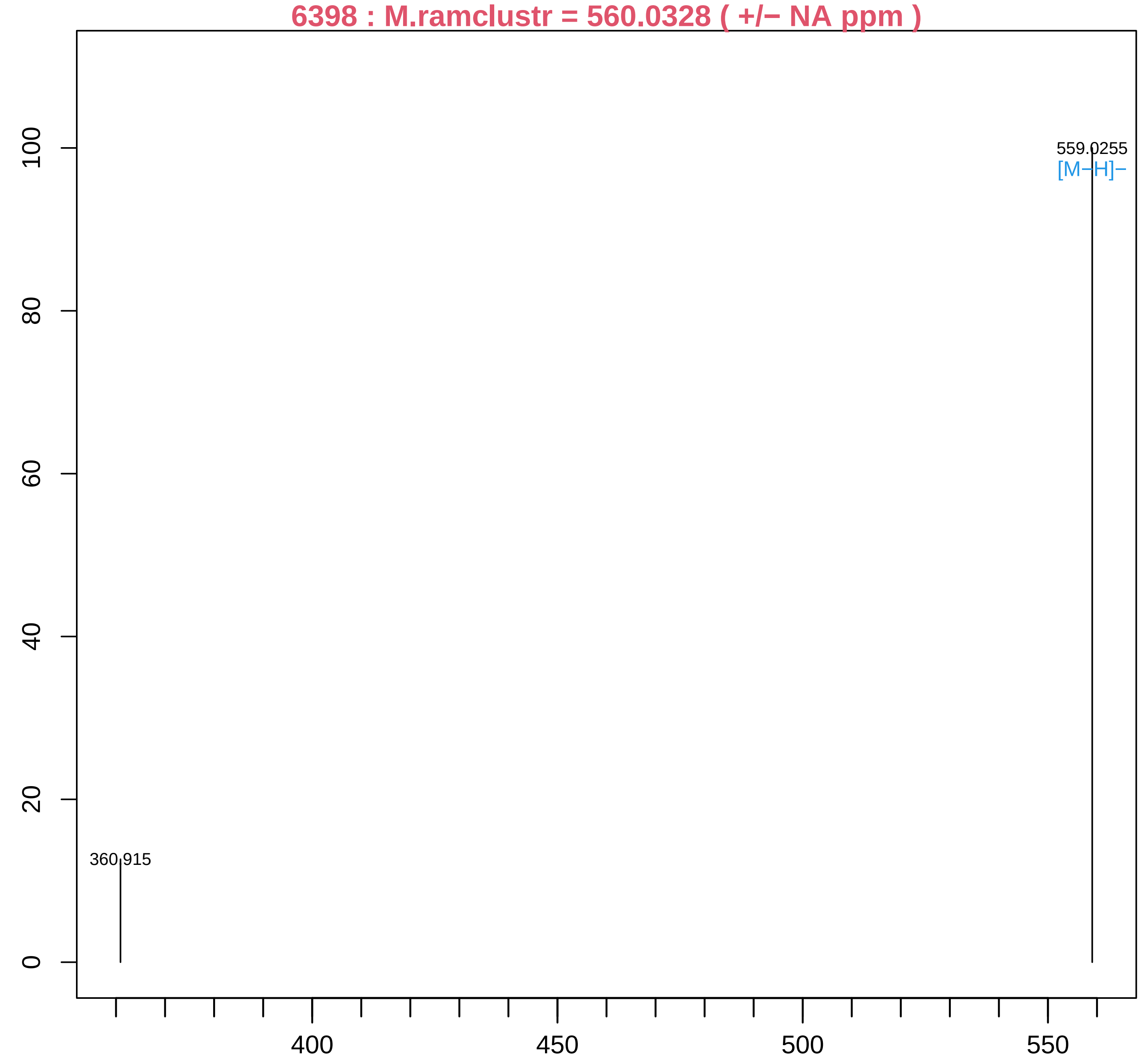
6398 : M.ramclustr = 560.0328 (+/− NA ppm)
559.0255
[M−H]−
360.915
0
20
40
60
80
100
400
450
500
550

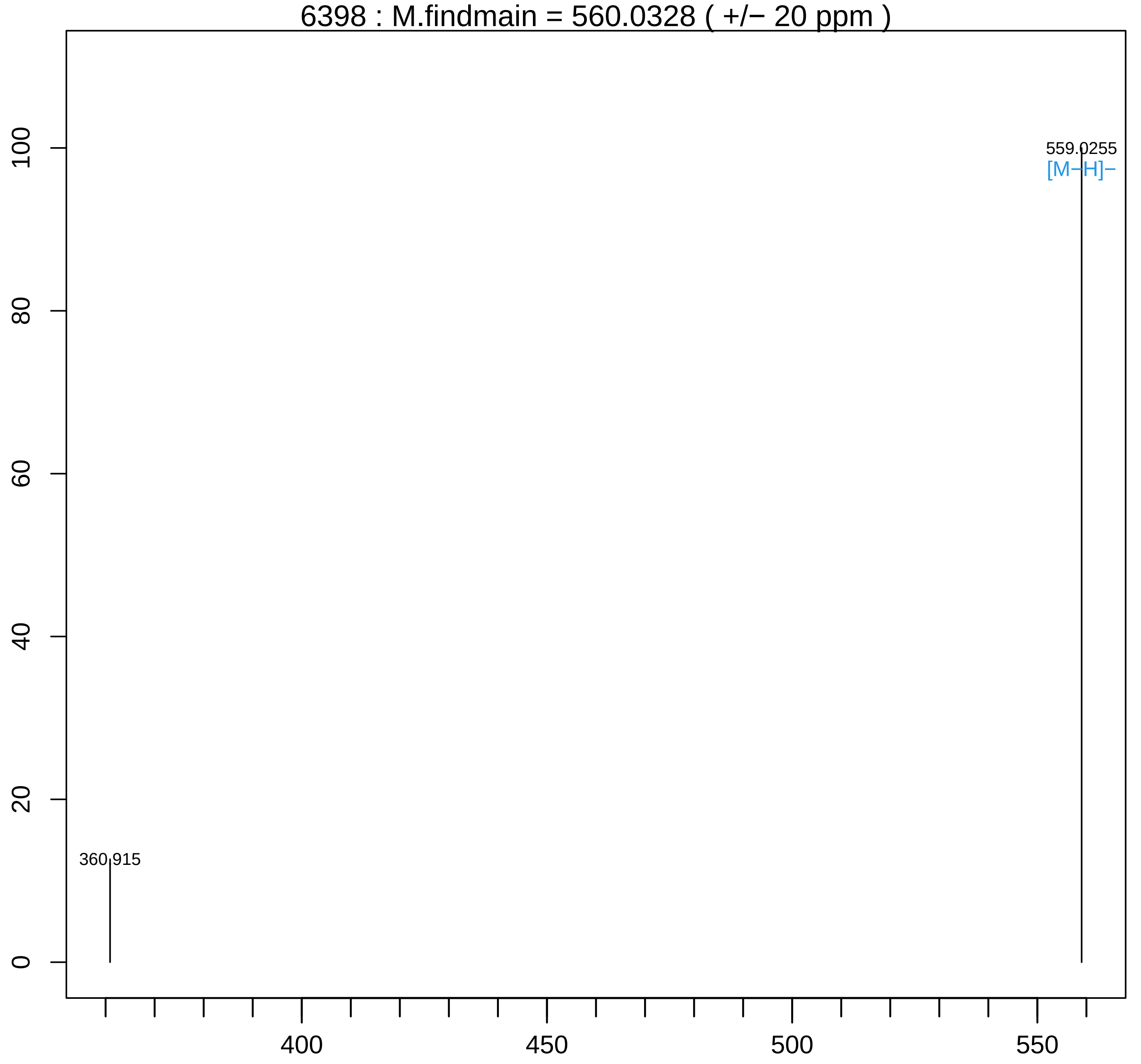
6398 : M.findmain = 560.0328 (+/− 20 ppm)
559.0255
[M−H]−
360.915
0
20
40
60
80
100
400
450
500
550

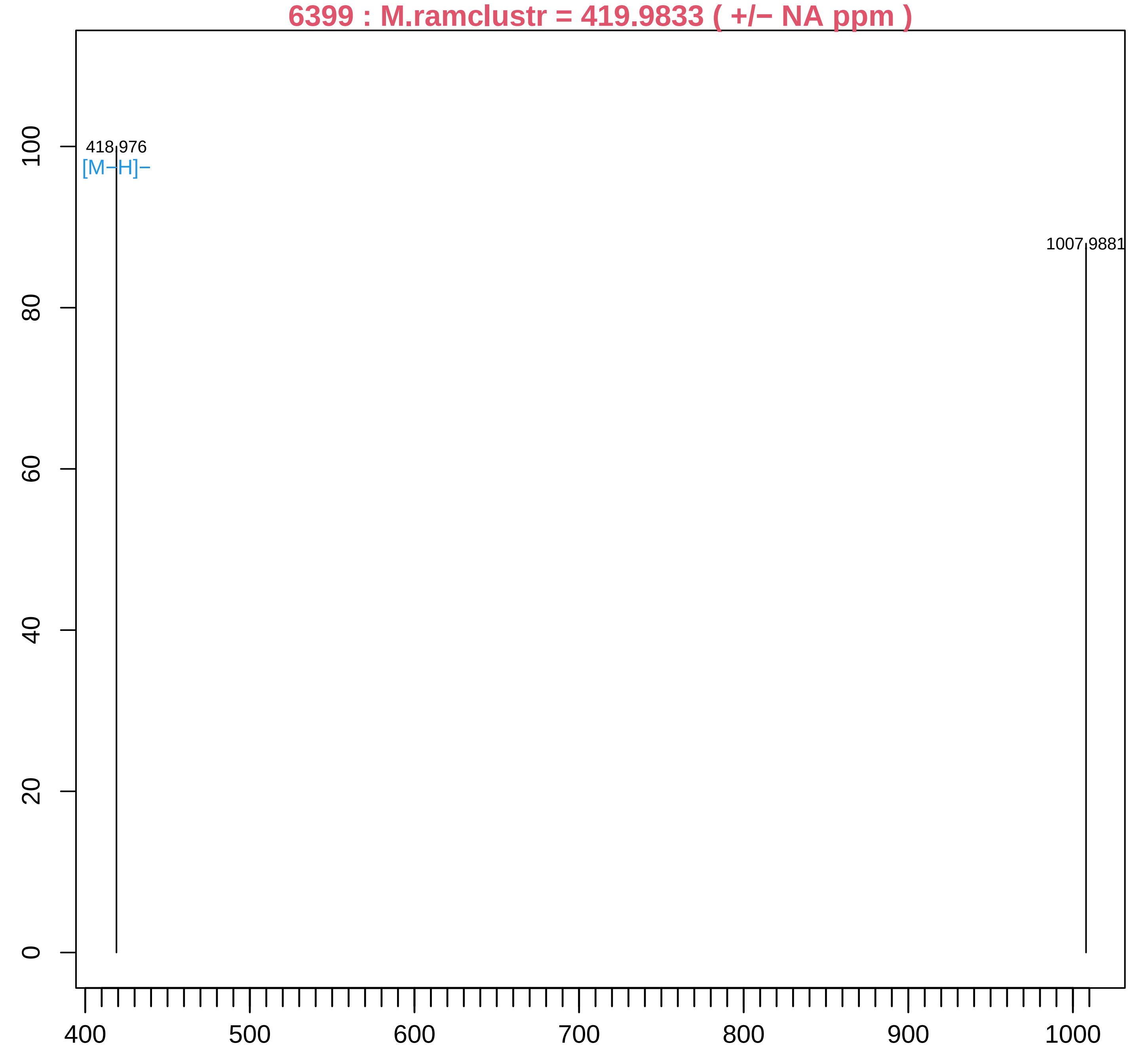
6399 : M.ramclustr = 419.9833 (+/− NA ppm)
418.976
[M−H]−
1007.9881

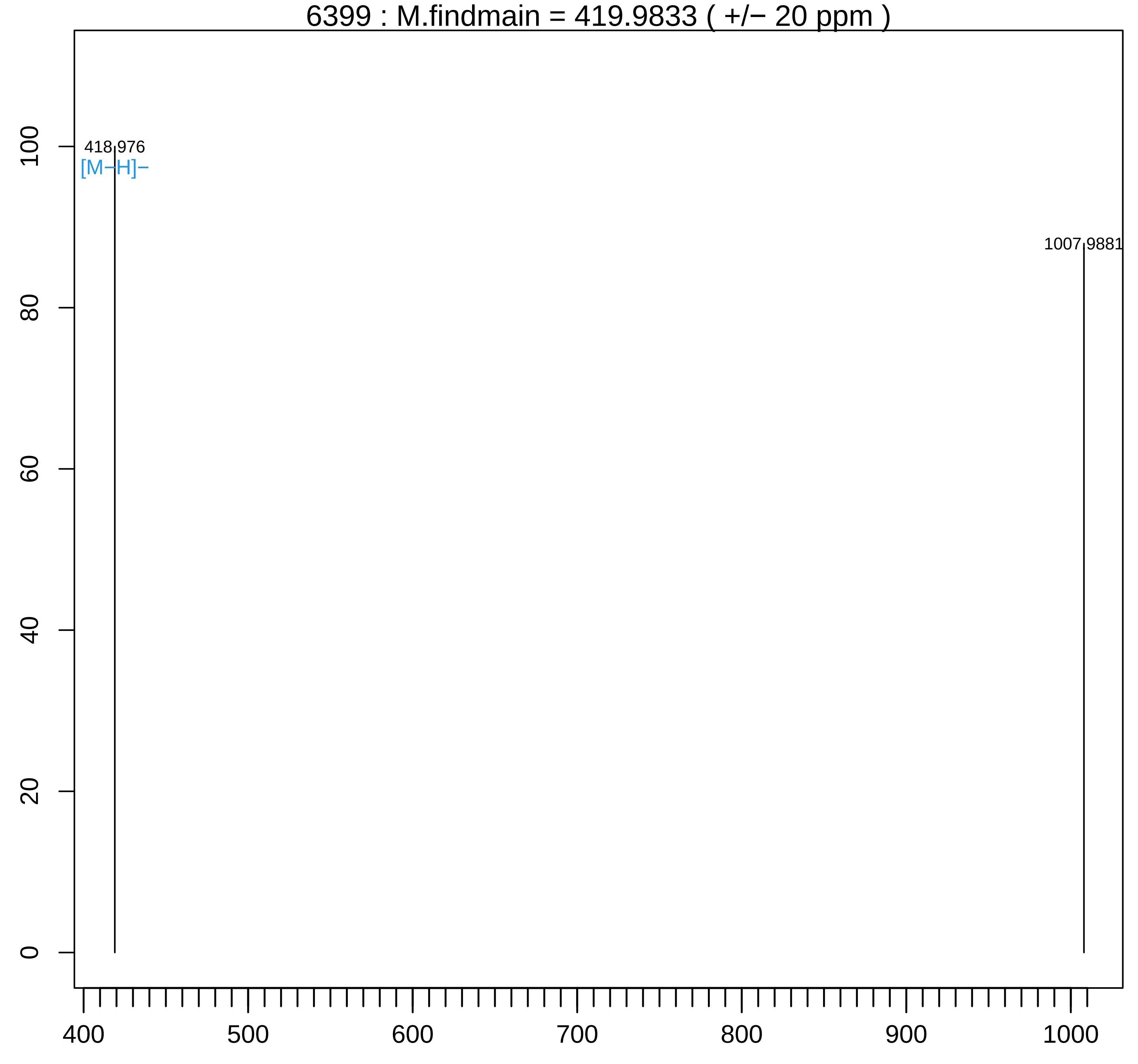
6399 : M.findmain = 419.9833 (+/− 20 ppm)
418.976
[M−H]−
1007.9881

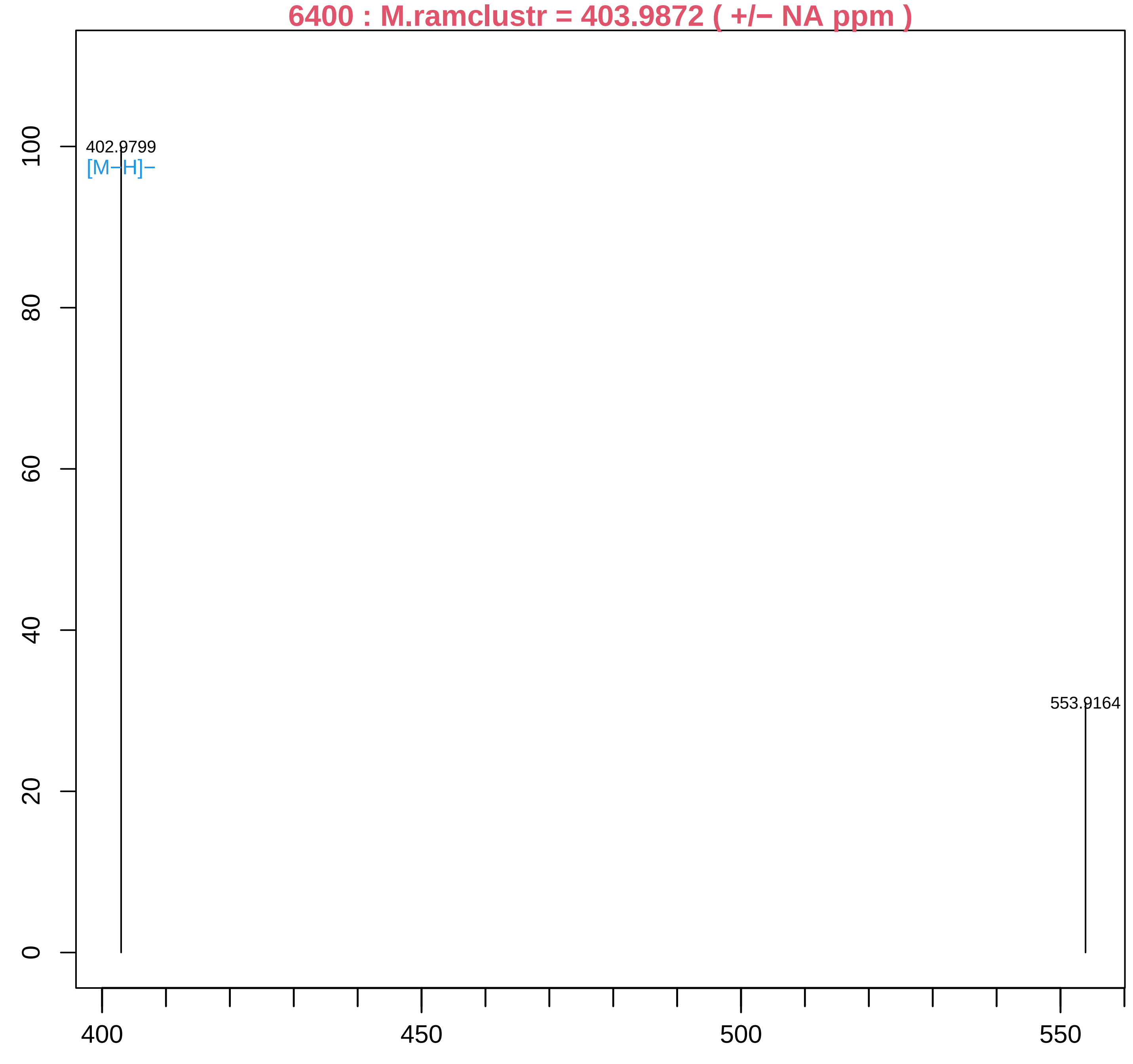
6400 : M.ramclustr = 403.9872 (+/− NA ppm)
402.9799
[M−H]−
553.9164
0
20
40
60
80
100
400
450
500
550

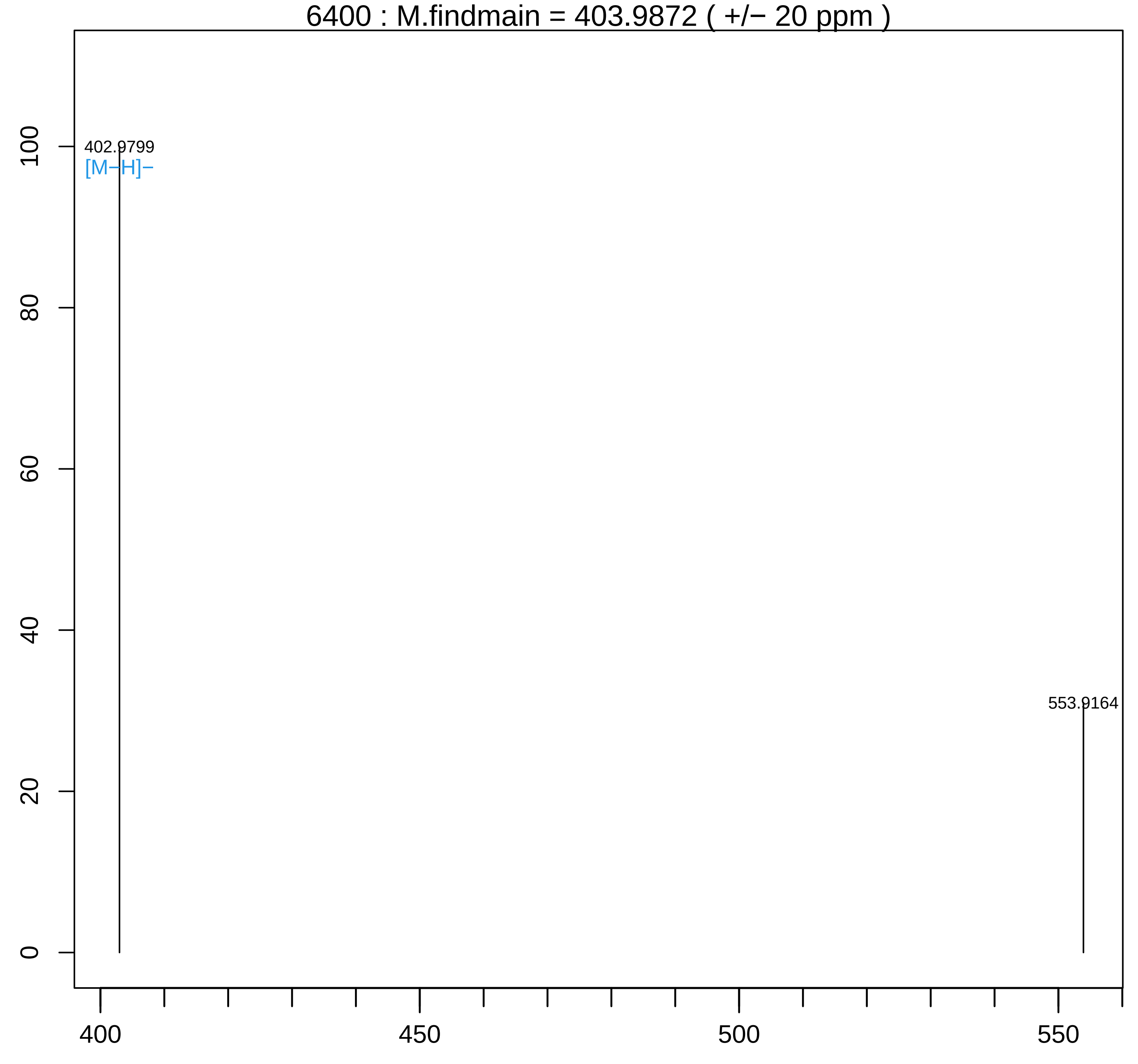
6400 : M.findmain = 403.9872 (+/− 20 ppm)
402.9799
[M−H]−
553.9164
0
20
40
60
80
100
400
450
500
550

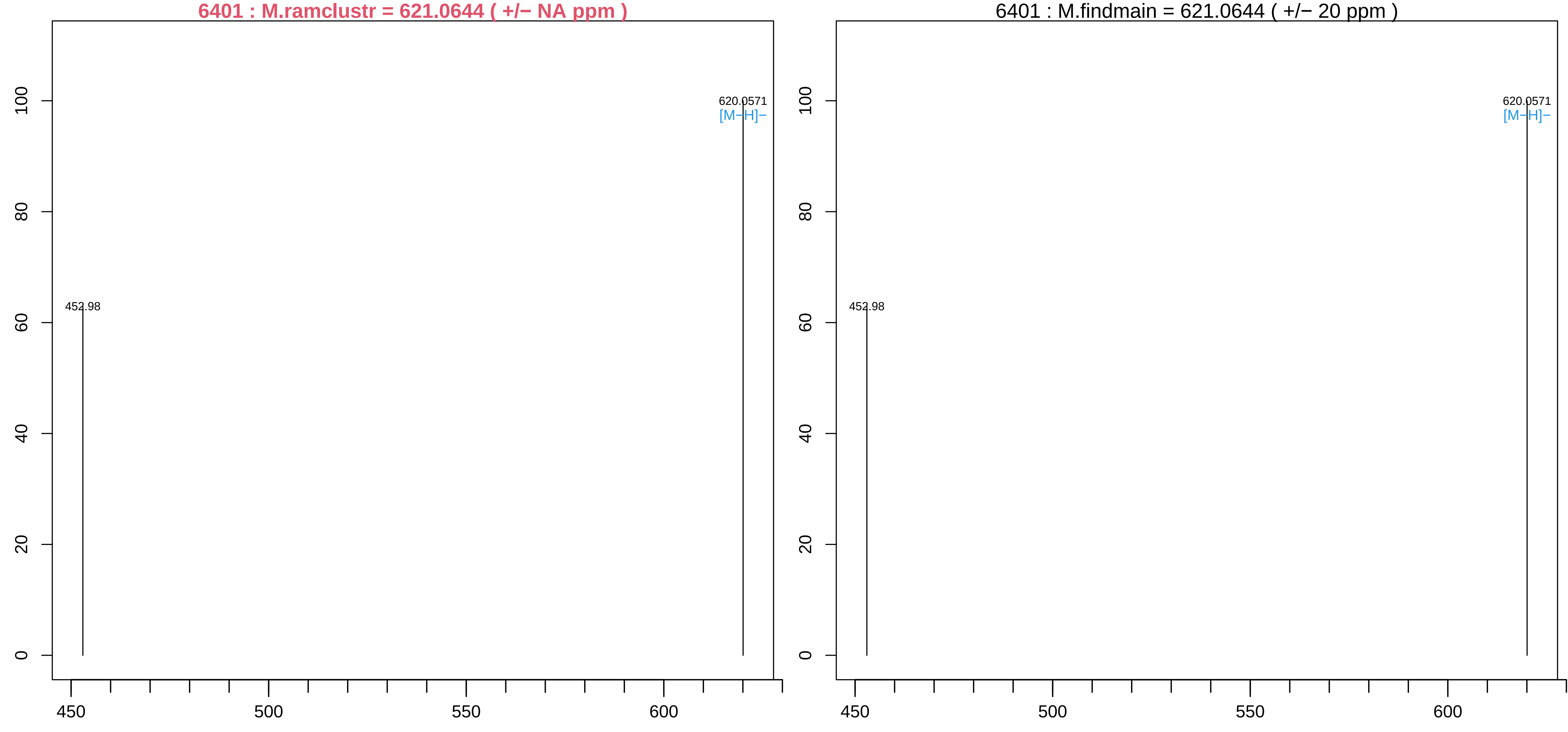

6401 : M.ramclustr = 621.0644 (+/− NA ppm)
620.0571
[M−H]−
452.98
6401 : M.findmain = 621.0644 (+/− 20 ppm)
620.0571
[M−H]−
452.98

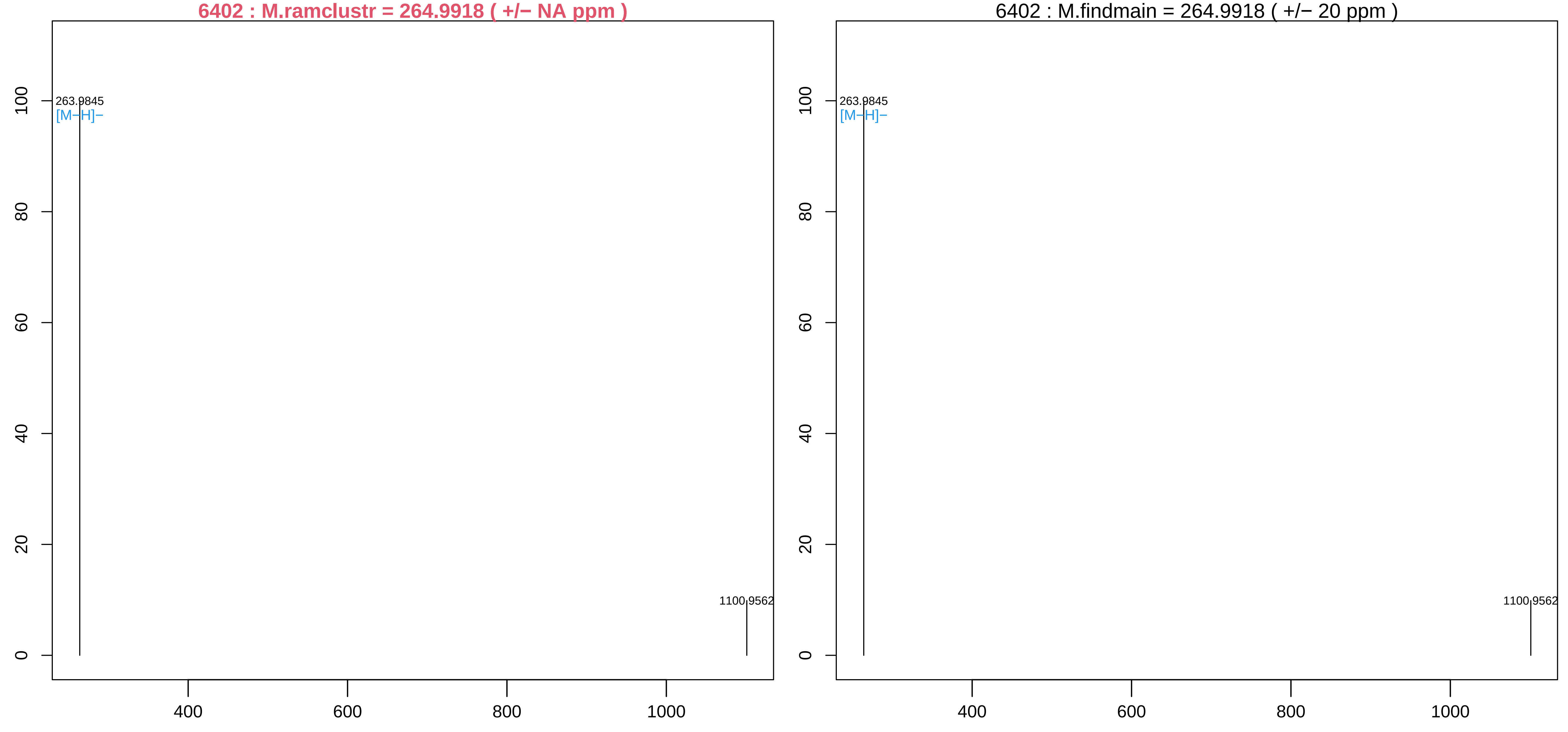
6402 : M.ramclustr = 264.9918 (+/− NA ppm)
263.9845
[M−H]−
1100.9562
0
20
40
60
80
100
400
600
800
1000
6402 : M.findmain = 264.9918 (+/− 20 ppm)
263.9845
[M−H]−
1100.9562
0
20
40
60
80
100
400
600
800
1000

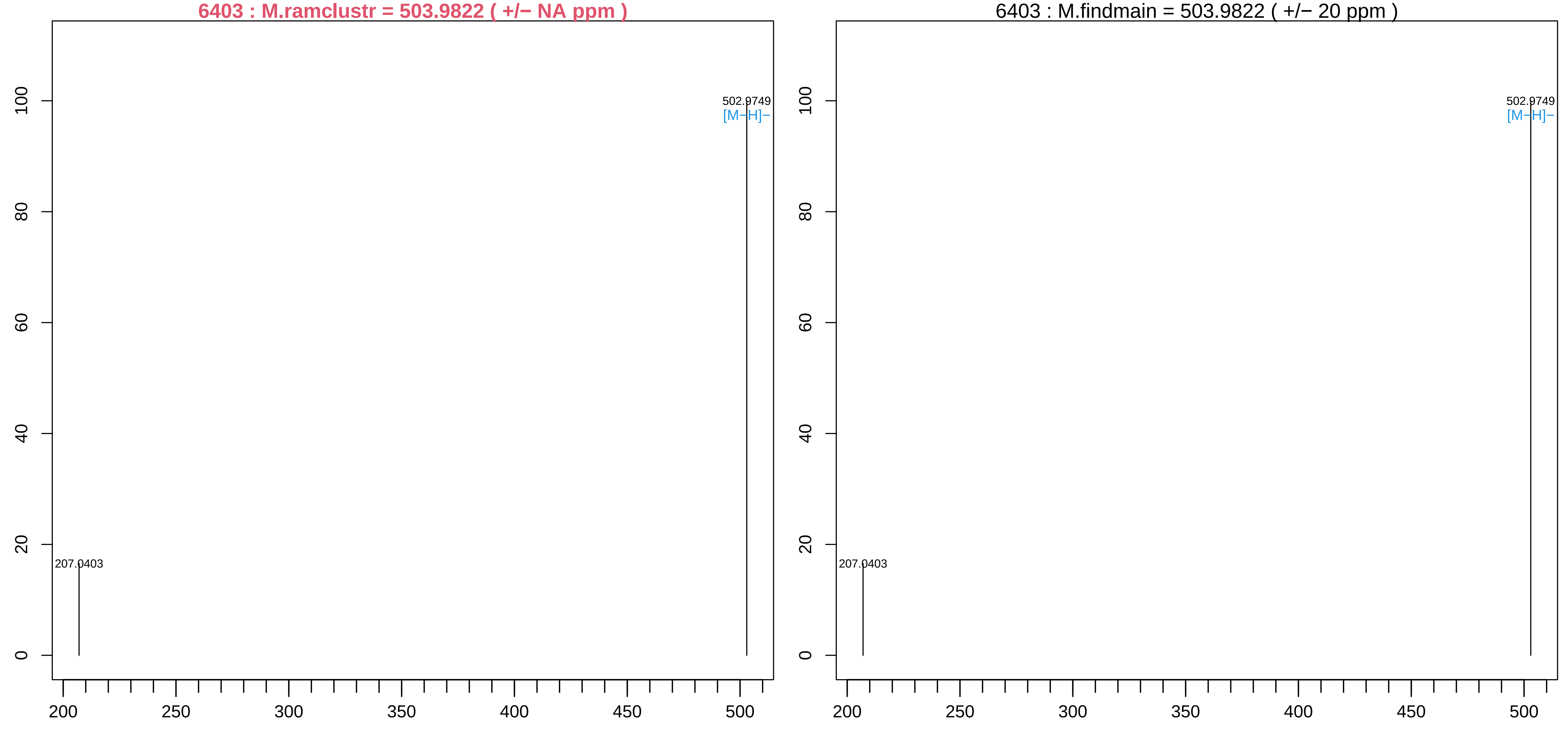

6403 : M.ramclustr = 503.9822 (+/− NA ppm)
502.9749
[M−H]−
207.0403
6403 : M.findmain = 503.9822 (+/− 20 ppm)
502.9749
[M−H]−
207.0403
0
20
40
60
80
100
200
250
300
350
400
450
500

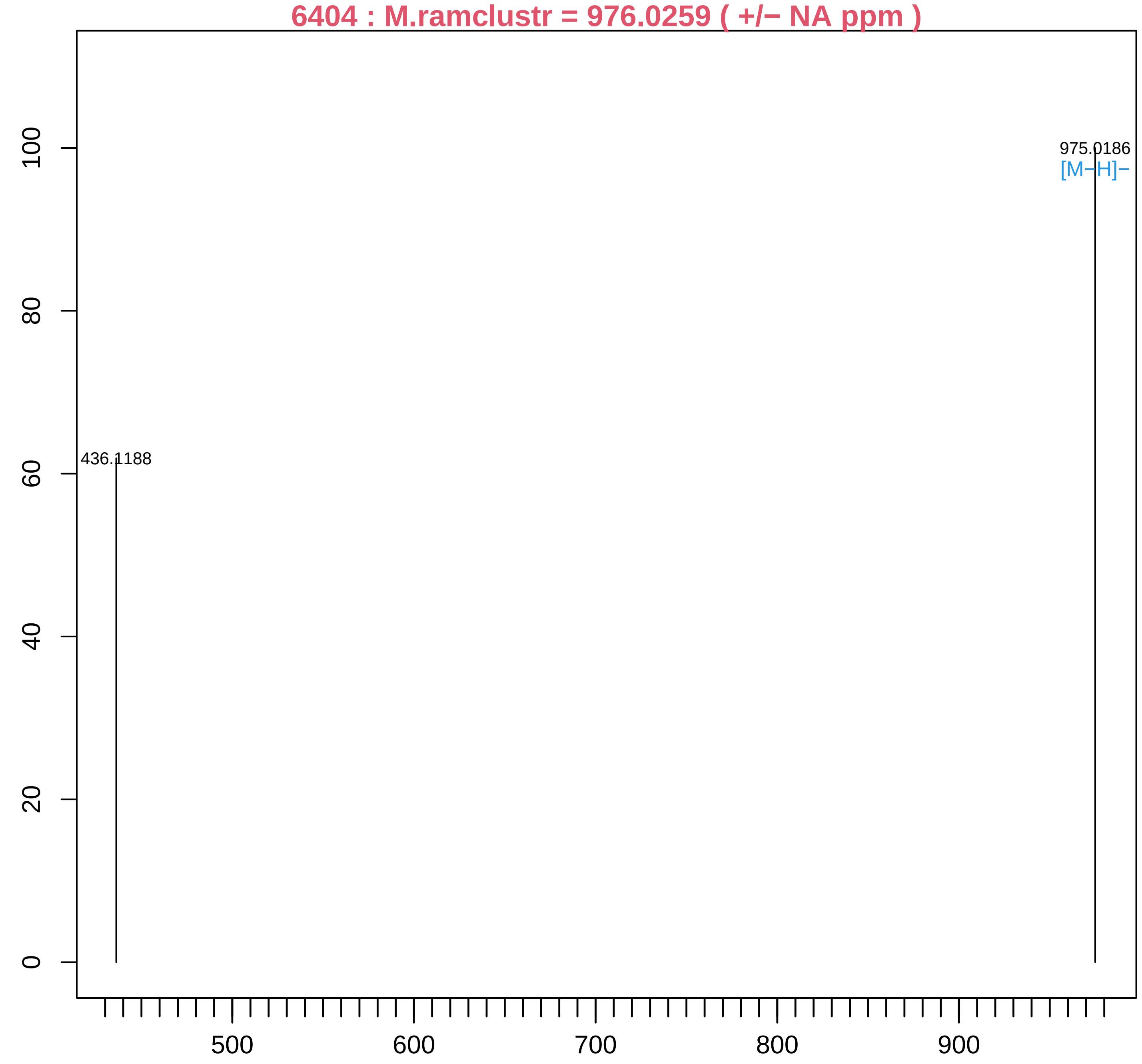

6404 : M.ramclustr = 976.0259 (+/− NA ppm)
975.0186
[M−H]−
436.1188
0
20
40
60
80
100
500
600
700
800
900

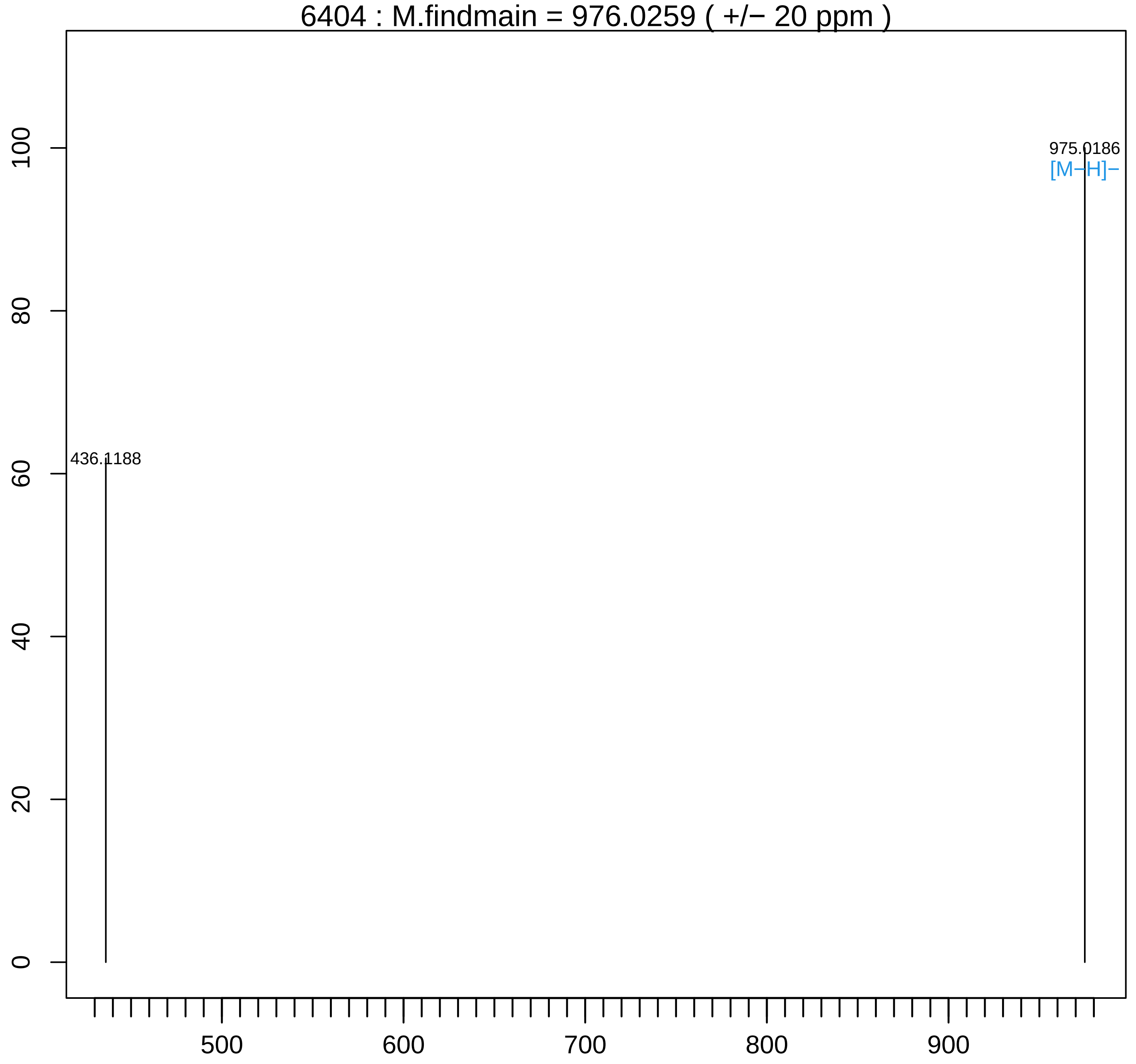

6404 : M.findmain = 976.0259 (+/− 20 ppm)
975.0186
[M−H]−
436.1188
0
20
40
60
80
100
500
600
700
800
900

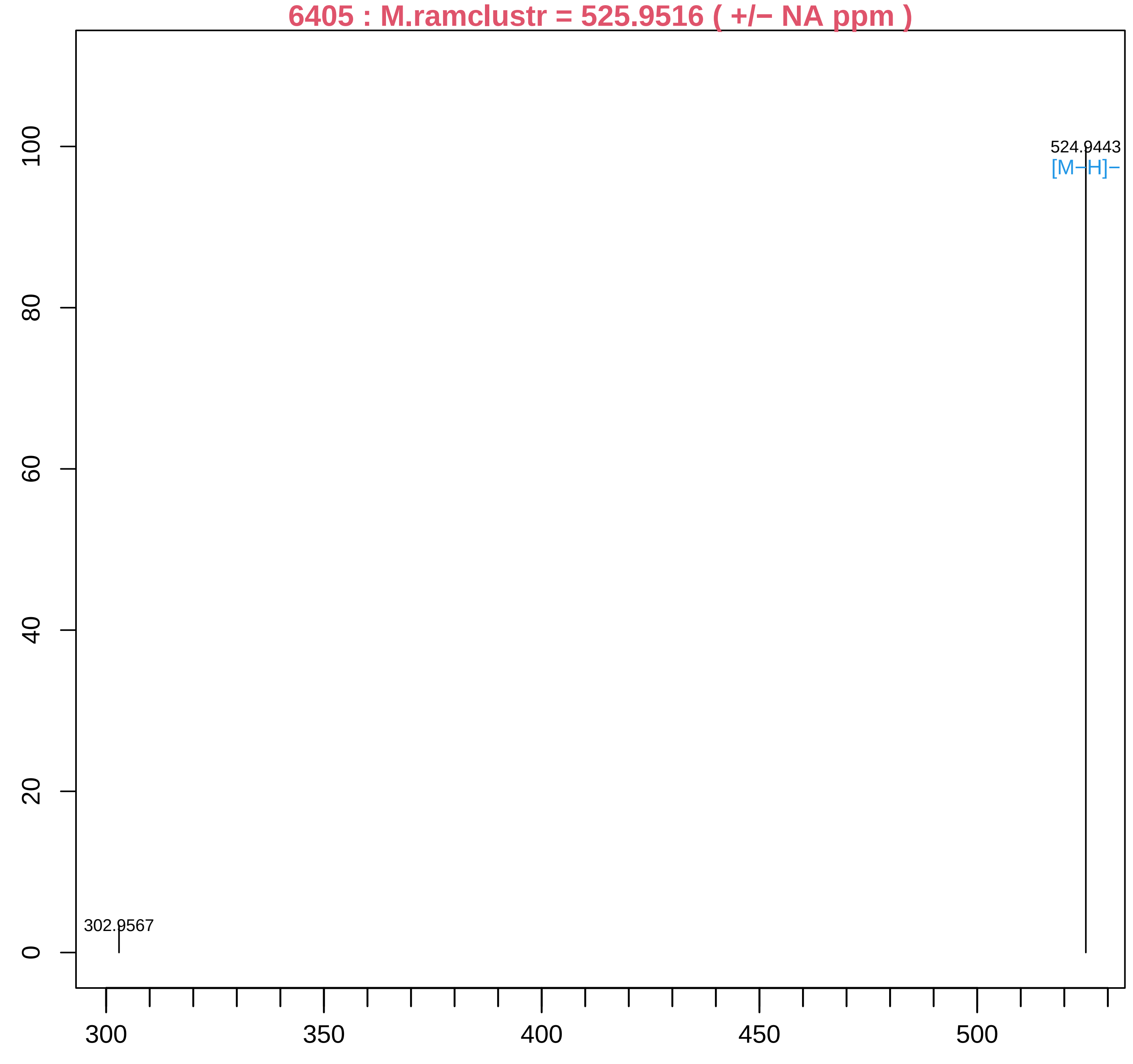
6405 : M.ramclustr = 525.9516 (+/− NA ppm)
524.9443
[M−H]−
302.9567
0
20
40
60
80
100
300
350
400
450
500

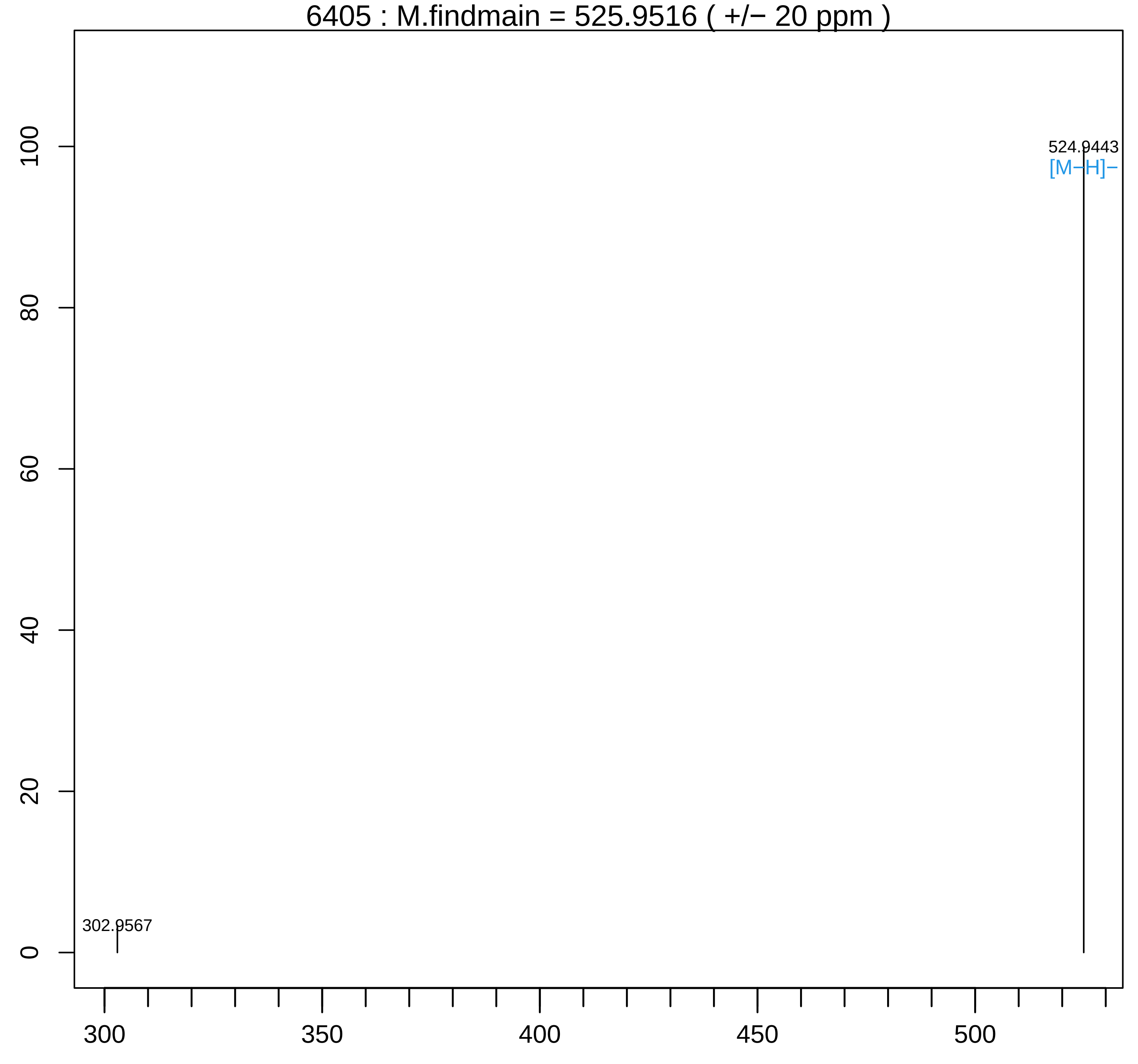
6405 : M.findmain = 525.9516 (+/− 20 ppm)
524.9443
[M−H]−
302.9567
0
20
40
60
80
100
300
350
400
450
500

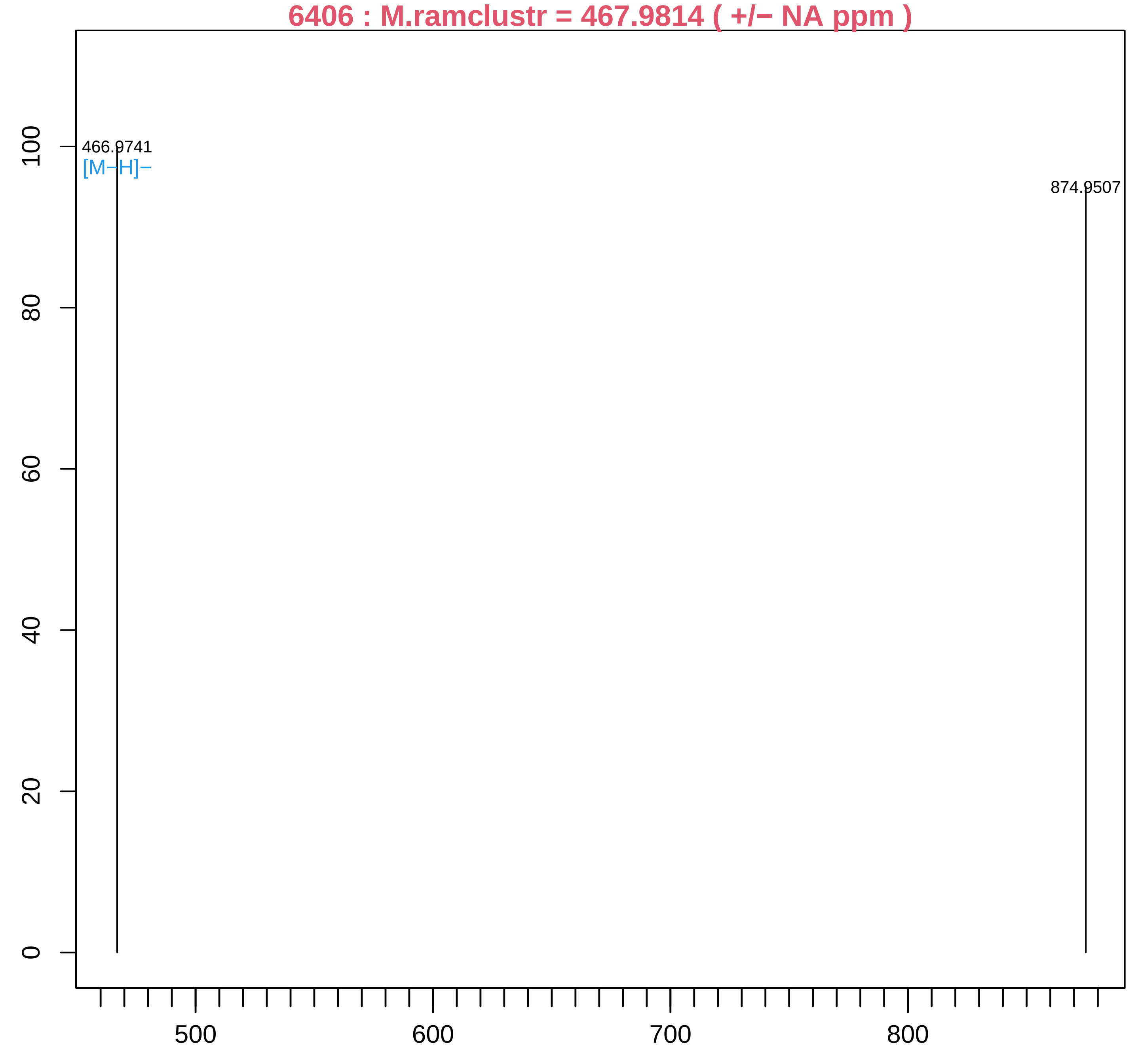
6406 : M.ramclustr = 467.9814 (+/− NA ppm)
466.9741
[M−H]−
874.9507
0
20
40
60
80
100
500
600
700
800

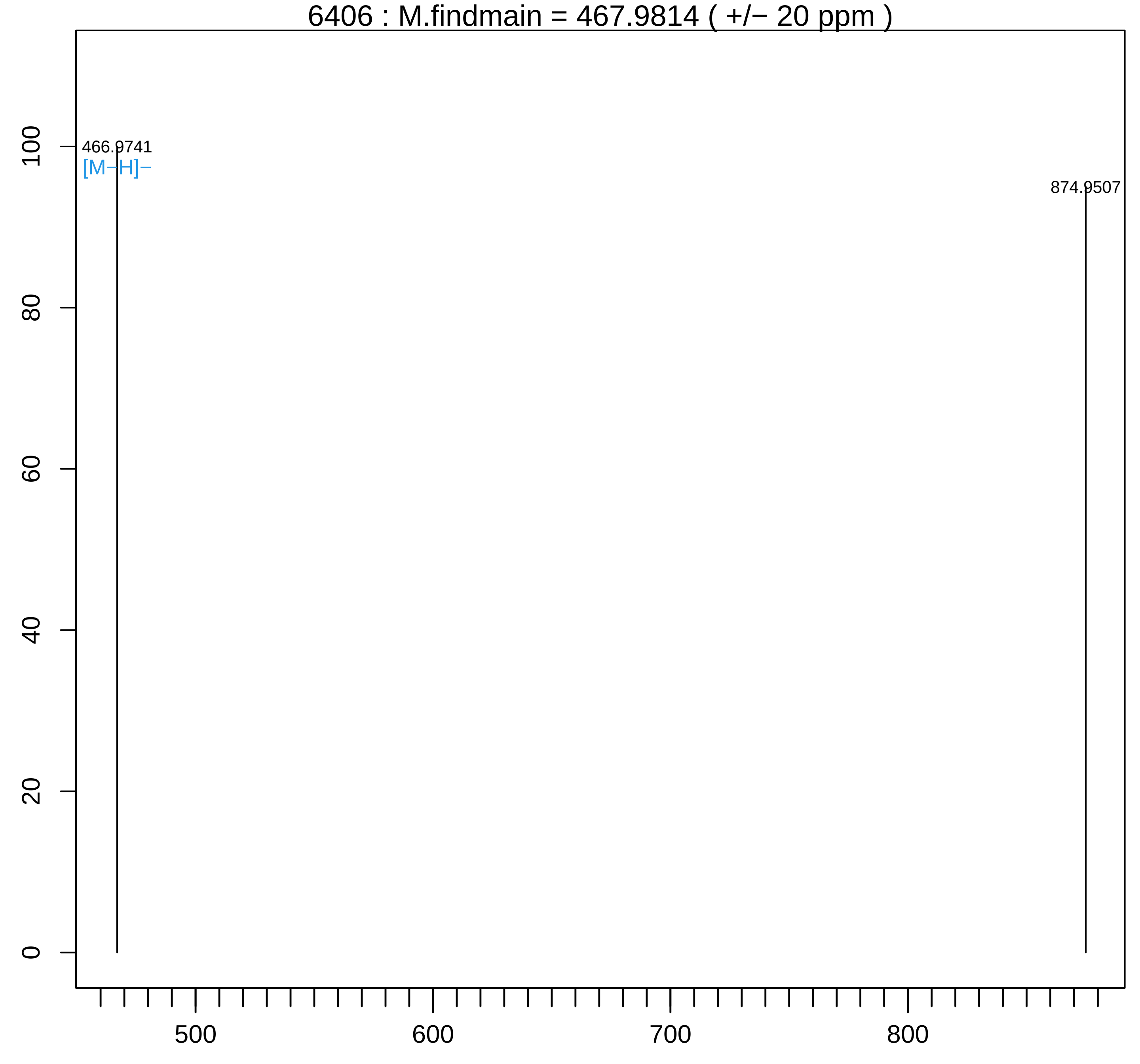
6406 : M.findmain = 467.9814 (+/− 20 ppm)
466.9741
[M−H]−
874.9507
0
20
40
60
80
100
500
600
700
800

6407 : M.ramclustr = 280.0161 (+/− NA ppm)

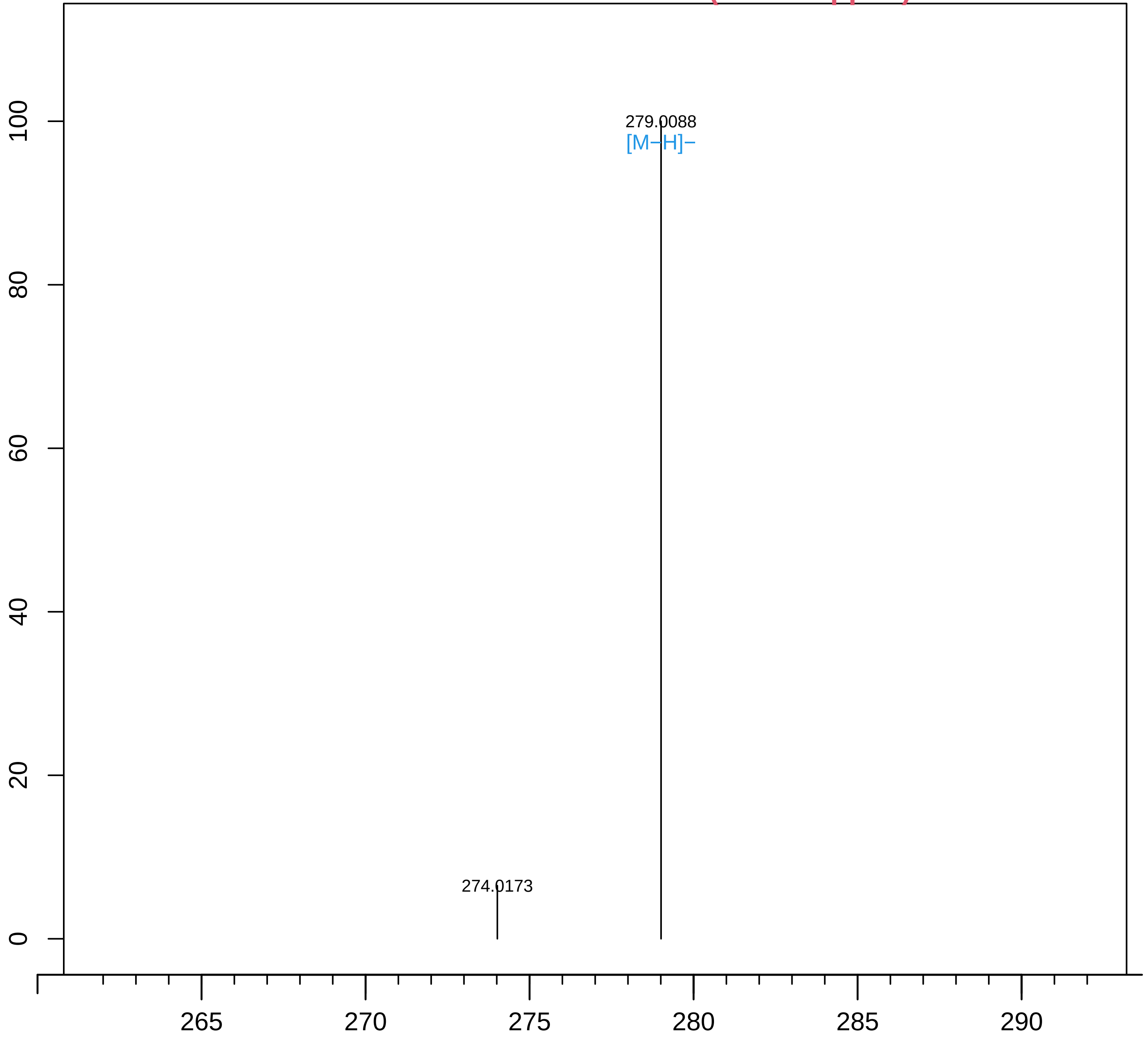

6407 : M.findmain = 280.0161 (+/− 20 ppm)

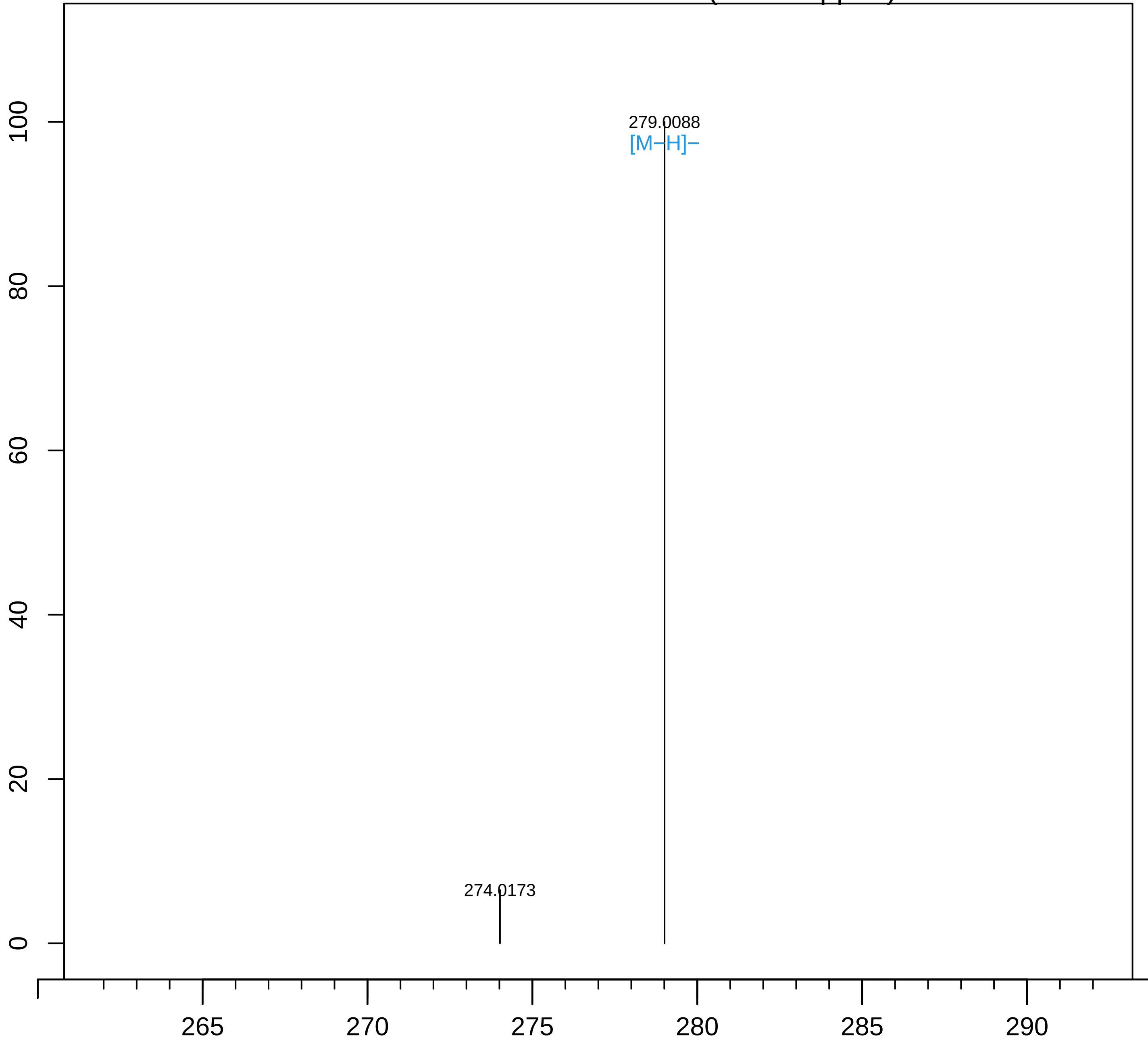

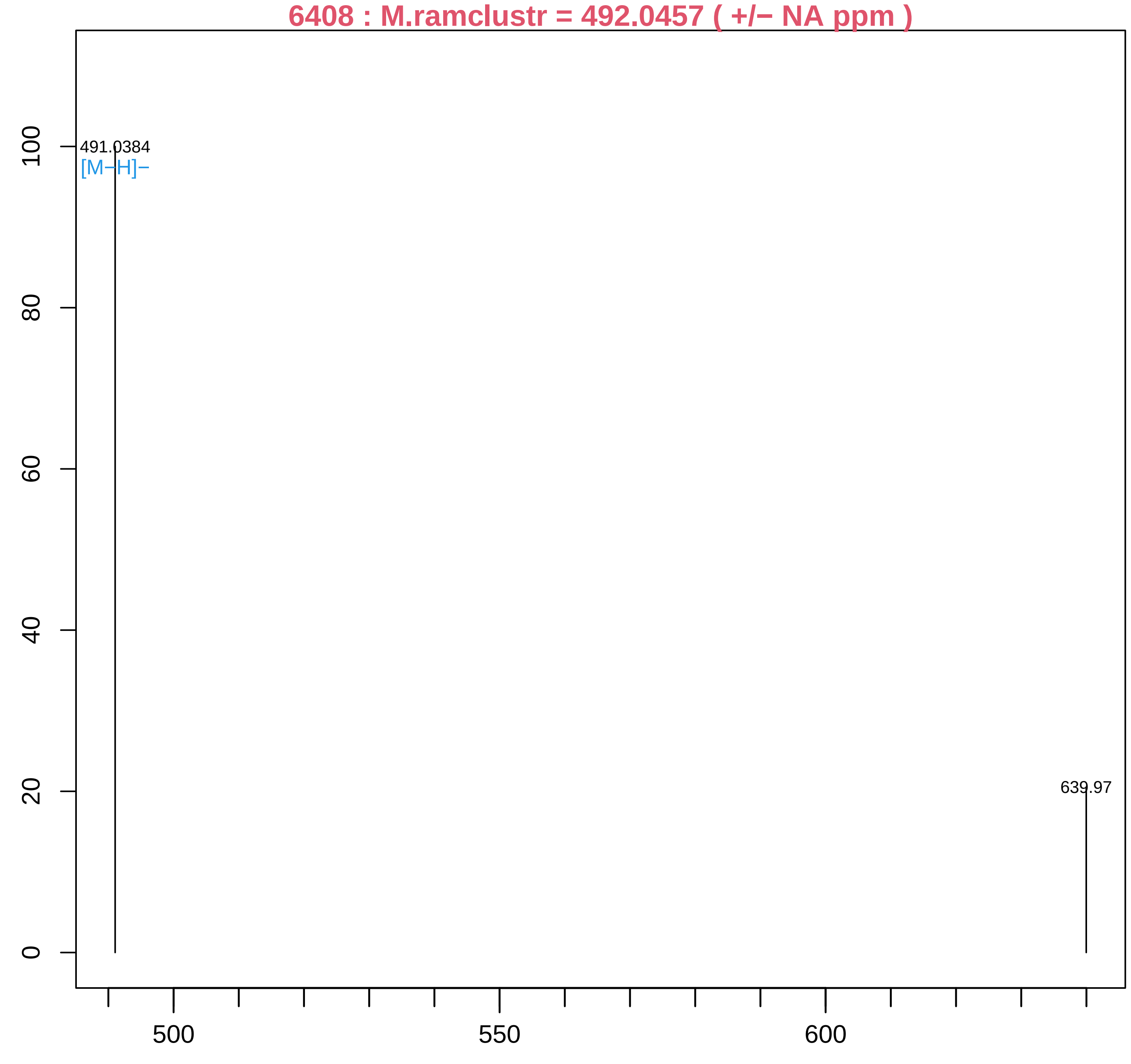
6408 : M.ramclustr = 492.0457 (+/− NA ppm)
491.0384
[M−H]−
639.97
0
20
40
60
80
100
500
550
600

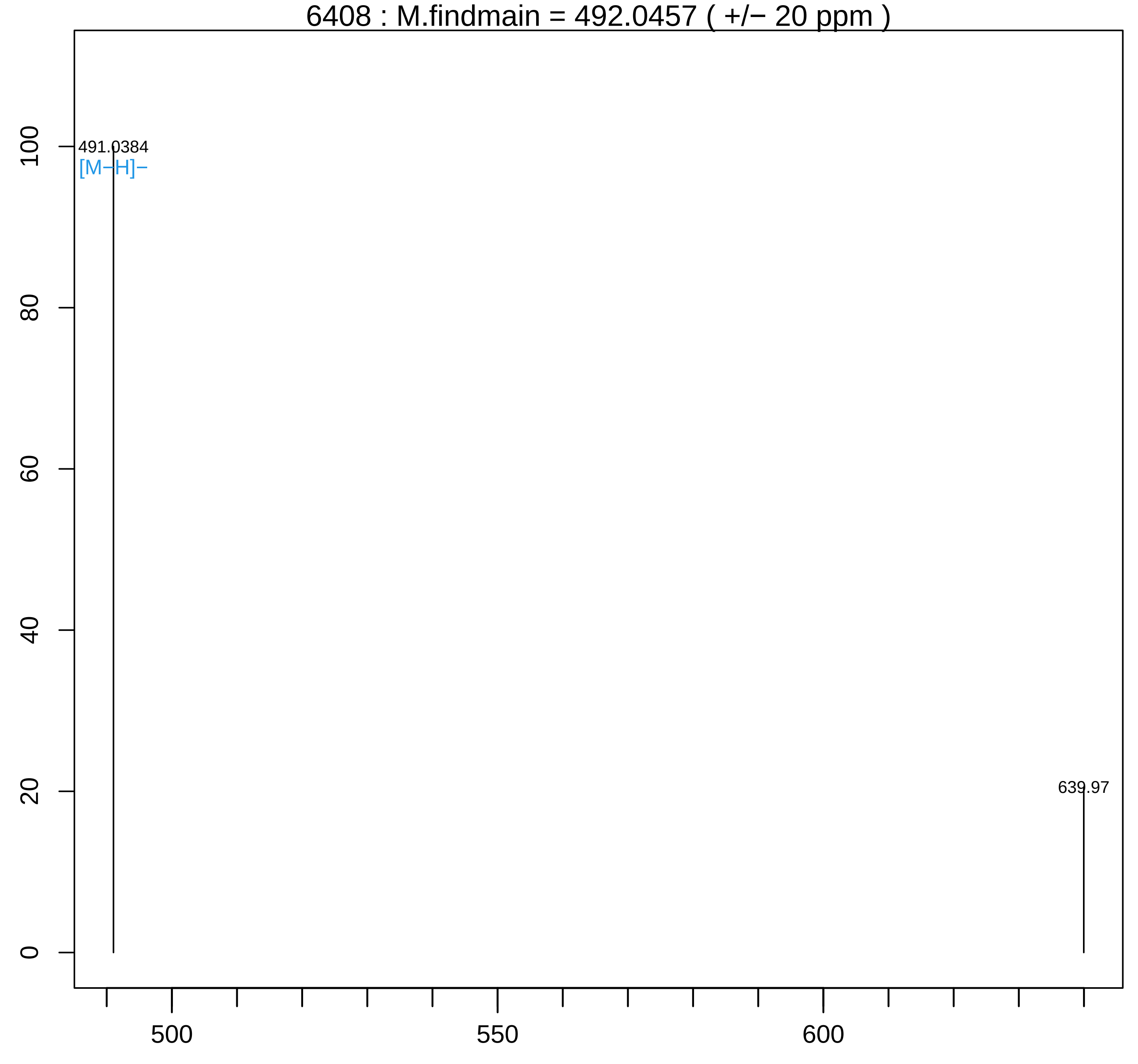
6408 : M.findmain = 492.0457 (+/− 20 ppm)
491.0384
[M−H]−
639.97
0
20
40
60
80
100
500
550
600

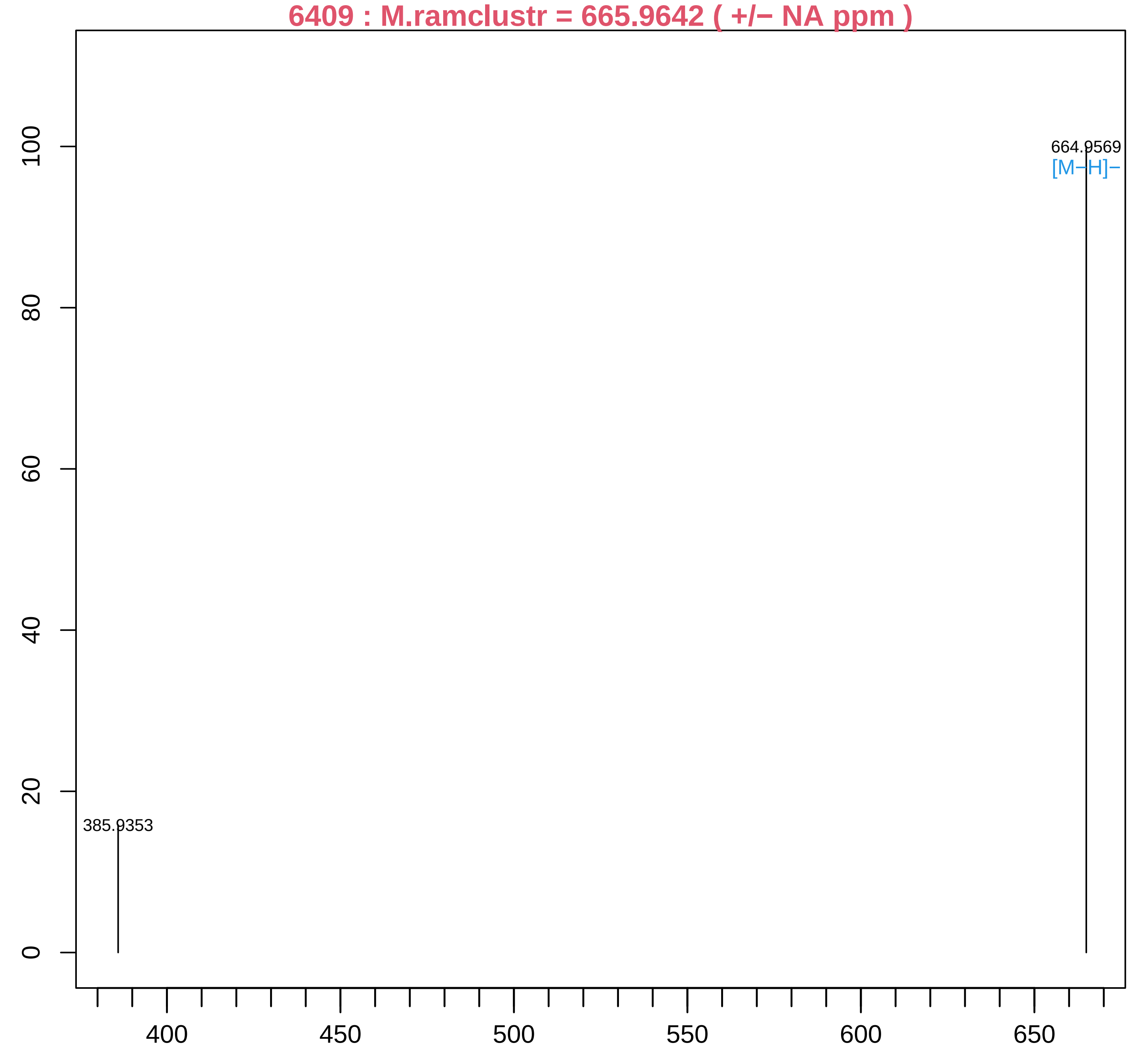
6409 : M.ramclustr = 665.9642 (+/− NA ppm)
664.9569
[M−H]−
385.9353
0
20
40
60
80
100
400
450
500
550
600
650

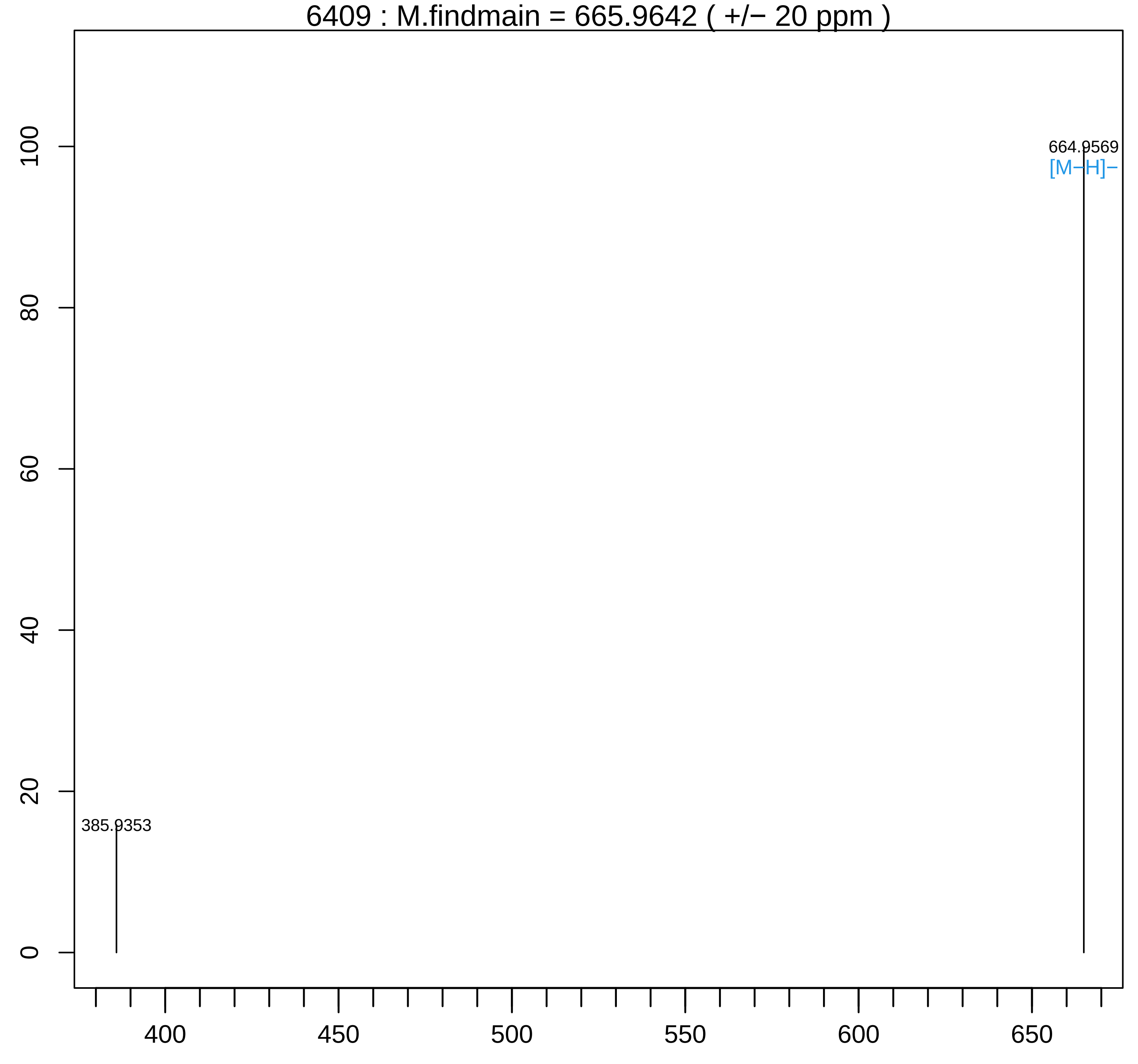
6409 : M.findmain = 665.9642 (+/− 20 ppm)
664.9569
[M−H]−
385.9353
0
20
40
60
80
100
400
450
500
550
600
650

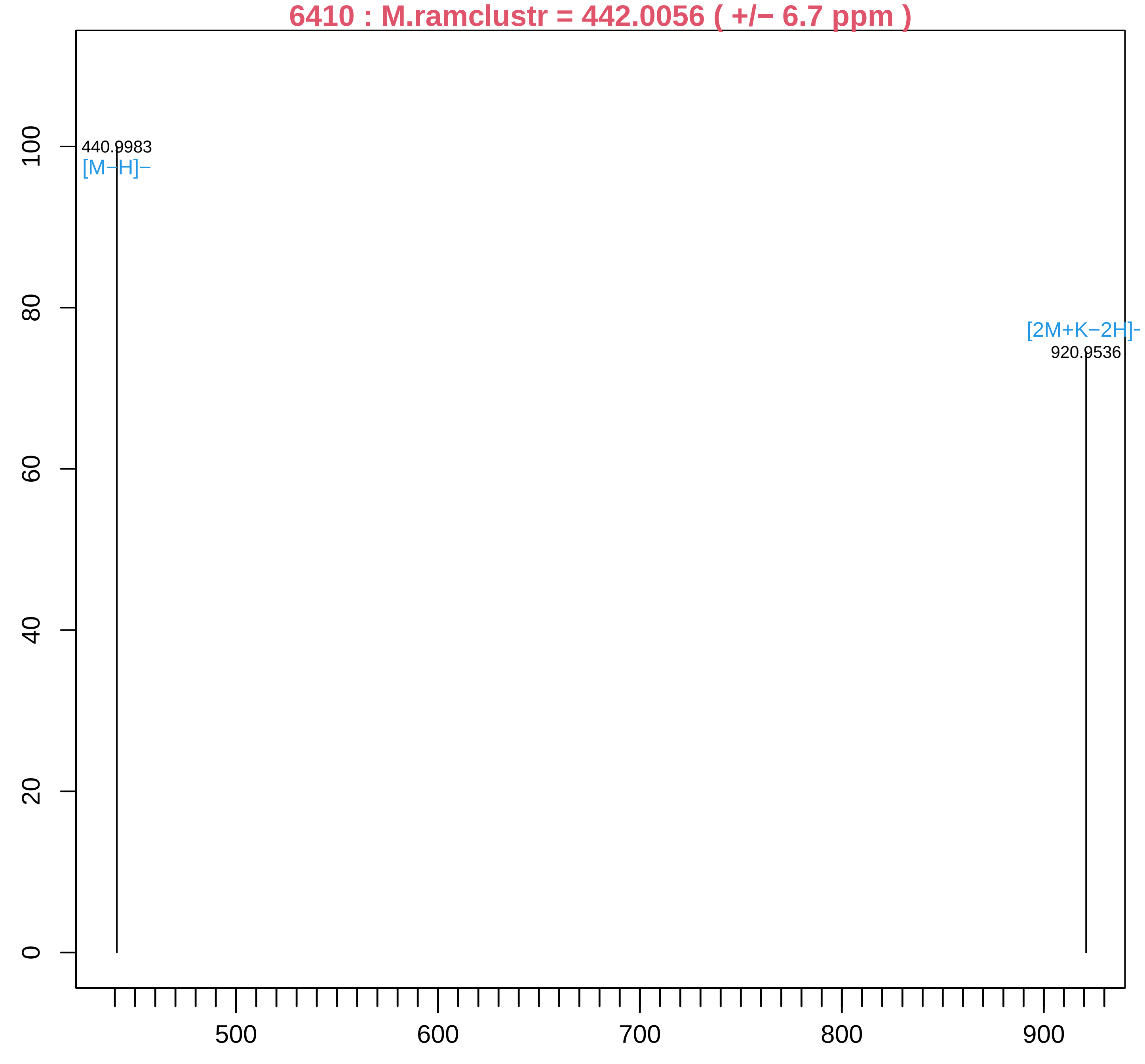
6410 : M.ramclustr = 442.0056 (+/− 6.7 ppm)
440.9983
[M−H]−
[2M+K−2H]-
920.9536
0
20
40
60
80
100
500
600
700
800
900

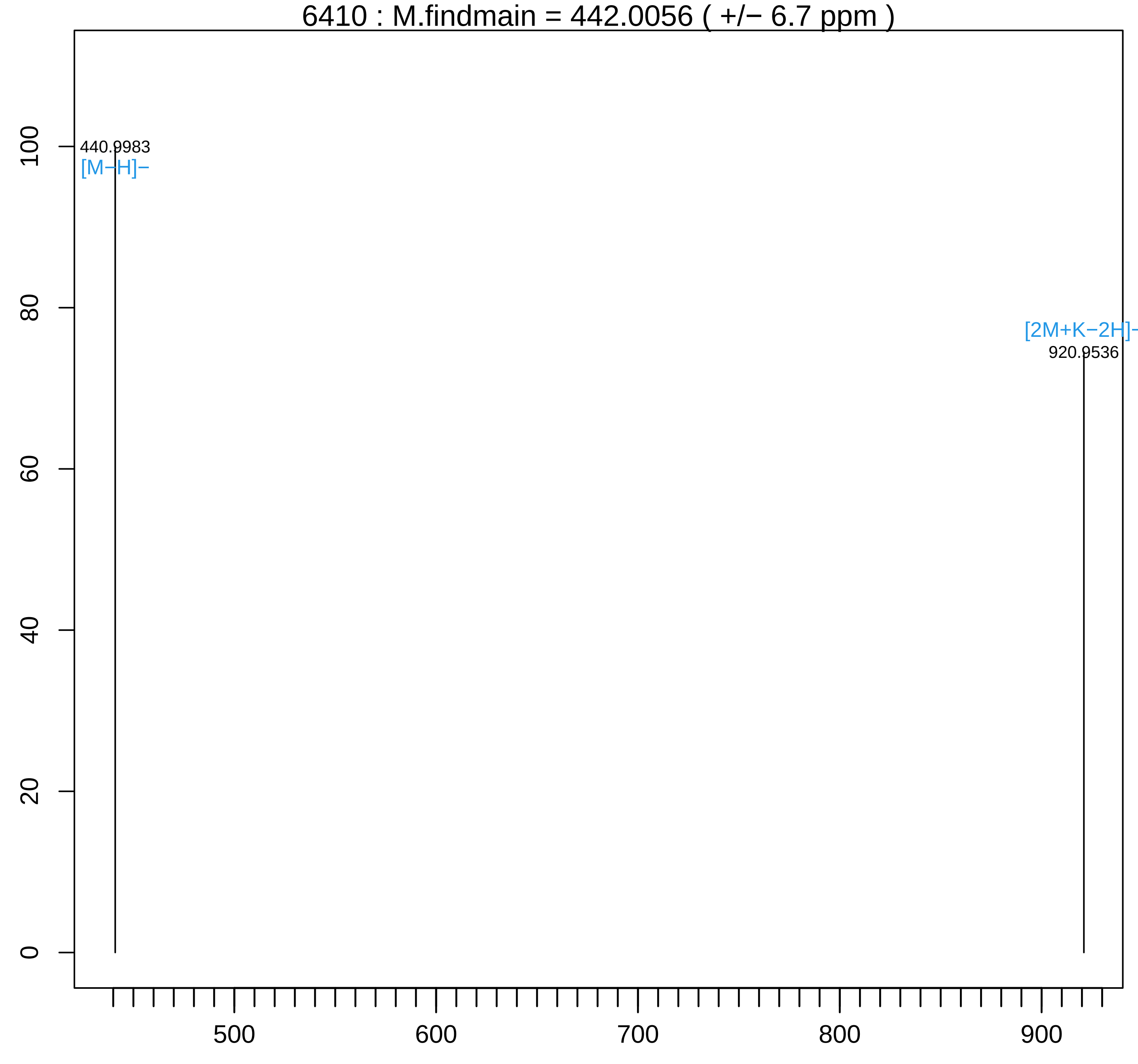
6410 : M.findmain = 442.0056 (+/− 6.7 ppm)
440.9983
[M−H]−
[2M+K−2H]-
920.9536
0
20
40
60
80
100
500
600
700
800
900

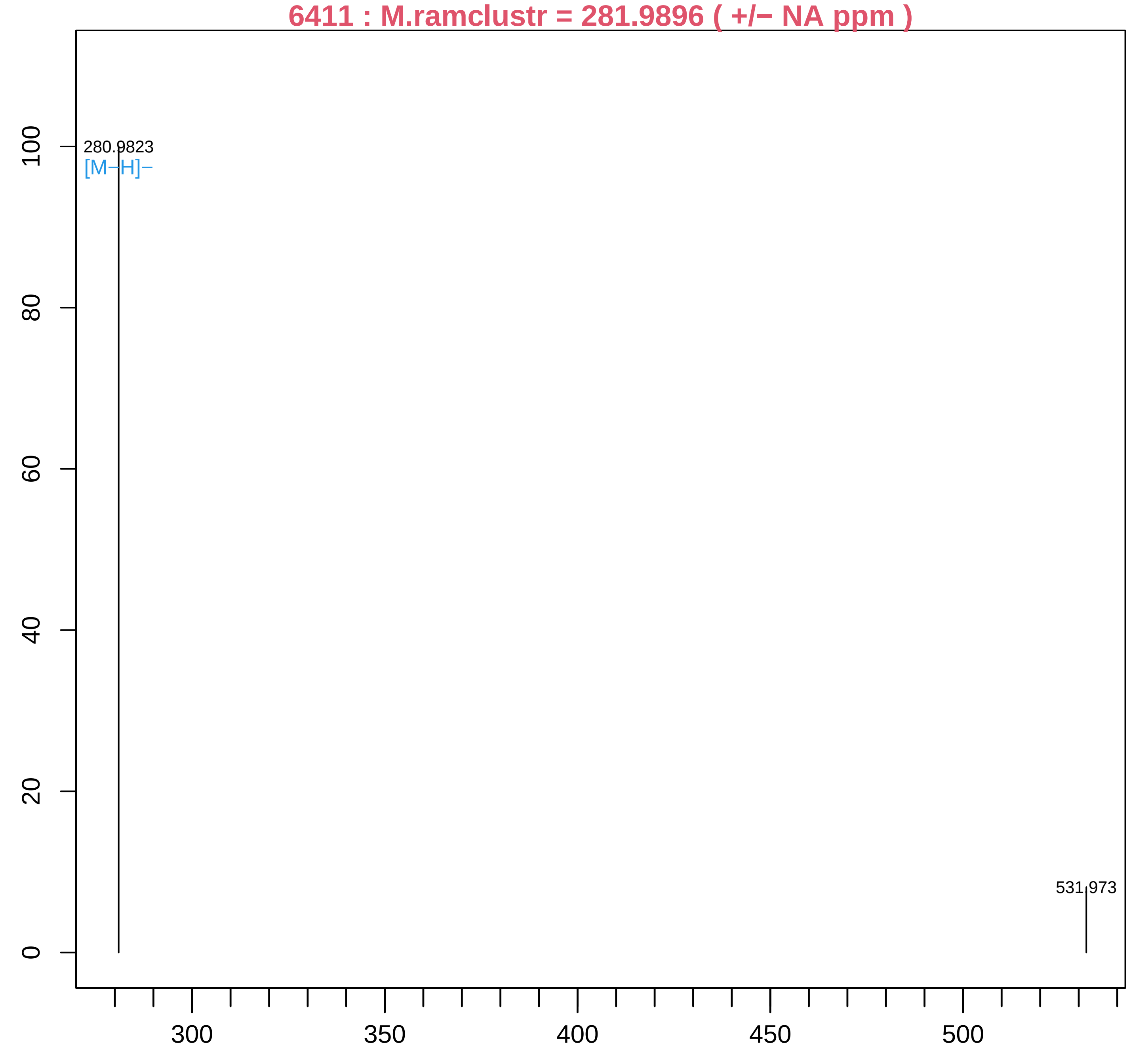
6411 : M.ramclustr = 281.9896 (+/− NA ppm)
280.9823
[M−H]−
531.973
0
20
40
60
80
100
300
350
400
450
500
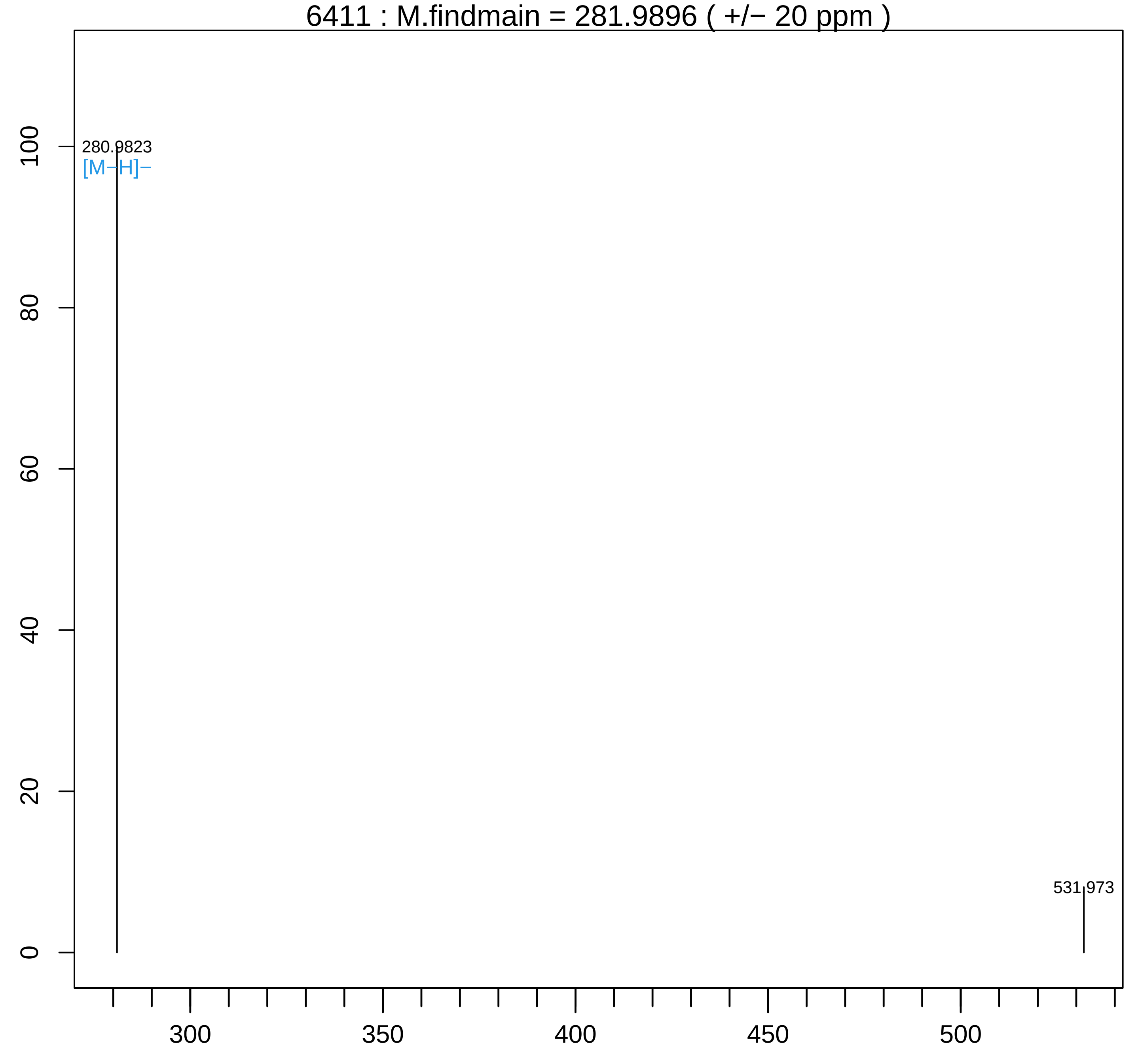
6411 : M.findmain = 281.9896 (+/− 20 ppm)
280.9823
[M−H]−
531.973
0
20
40
60
80
100
300
350
400
450
500

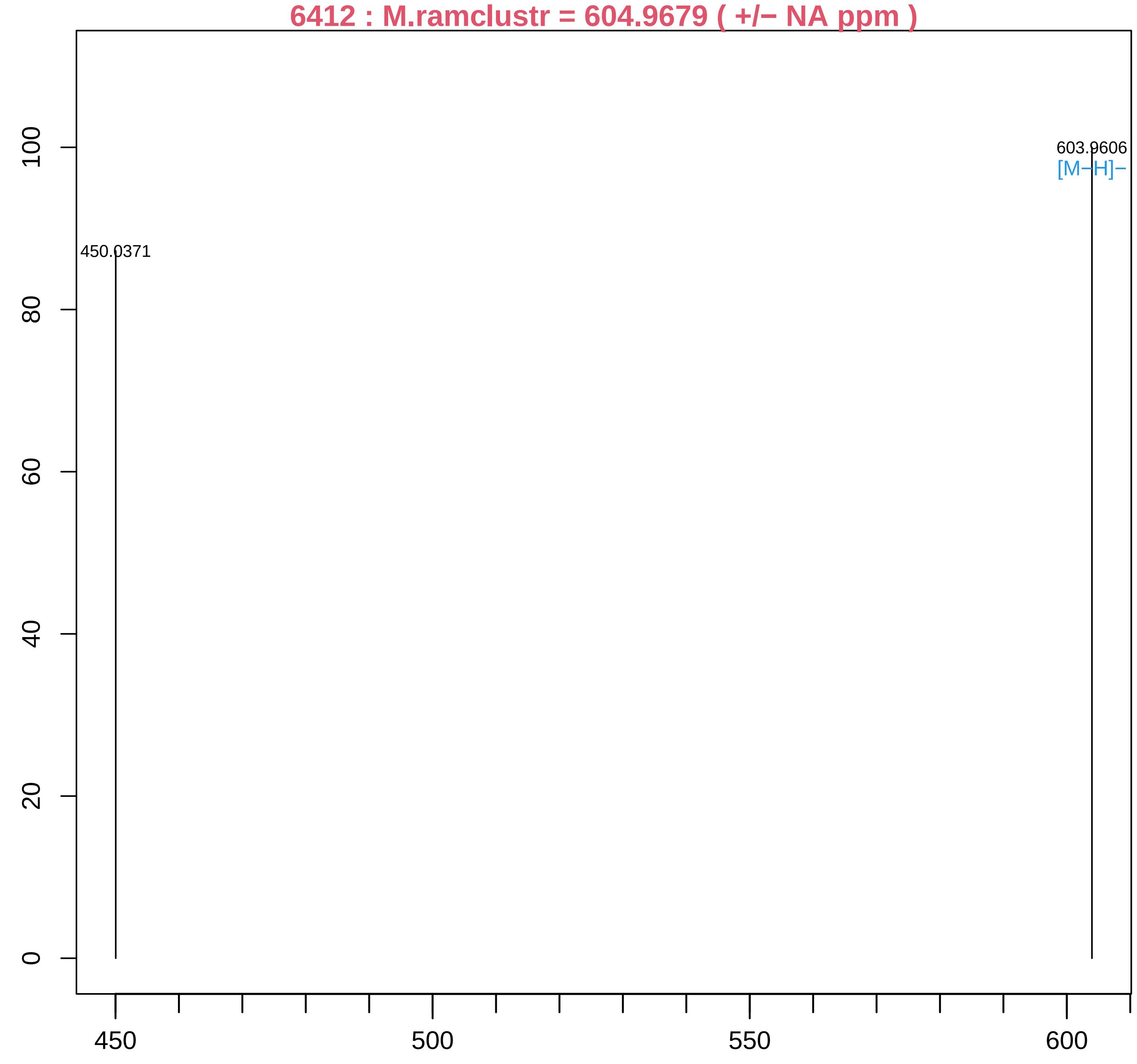
6412 : M.ramclustr = 604.9679 (+/− NA ppm)
603.9606
[M−H]−
450.0371
0
20
40
60
80
100
450
500
550
600

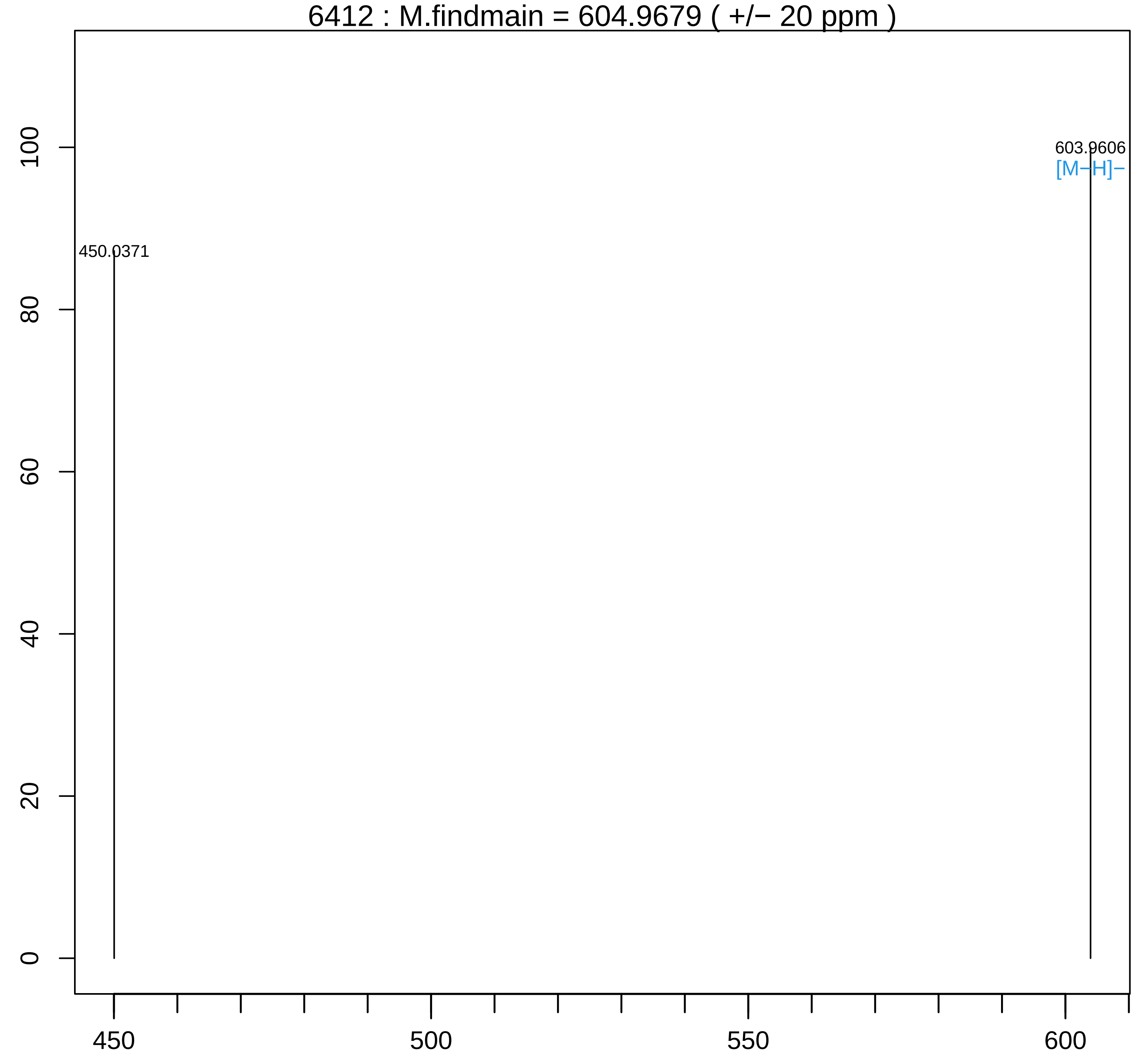
6412 : M.findmain = 604.9679 (+/− 20 ppm)
603.9606
[M−H]−
450.0371
0
20
40
60
80
100
450
500
550
600

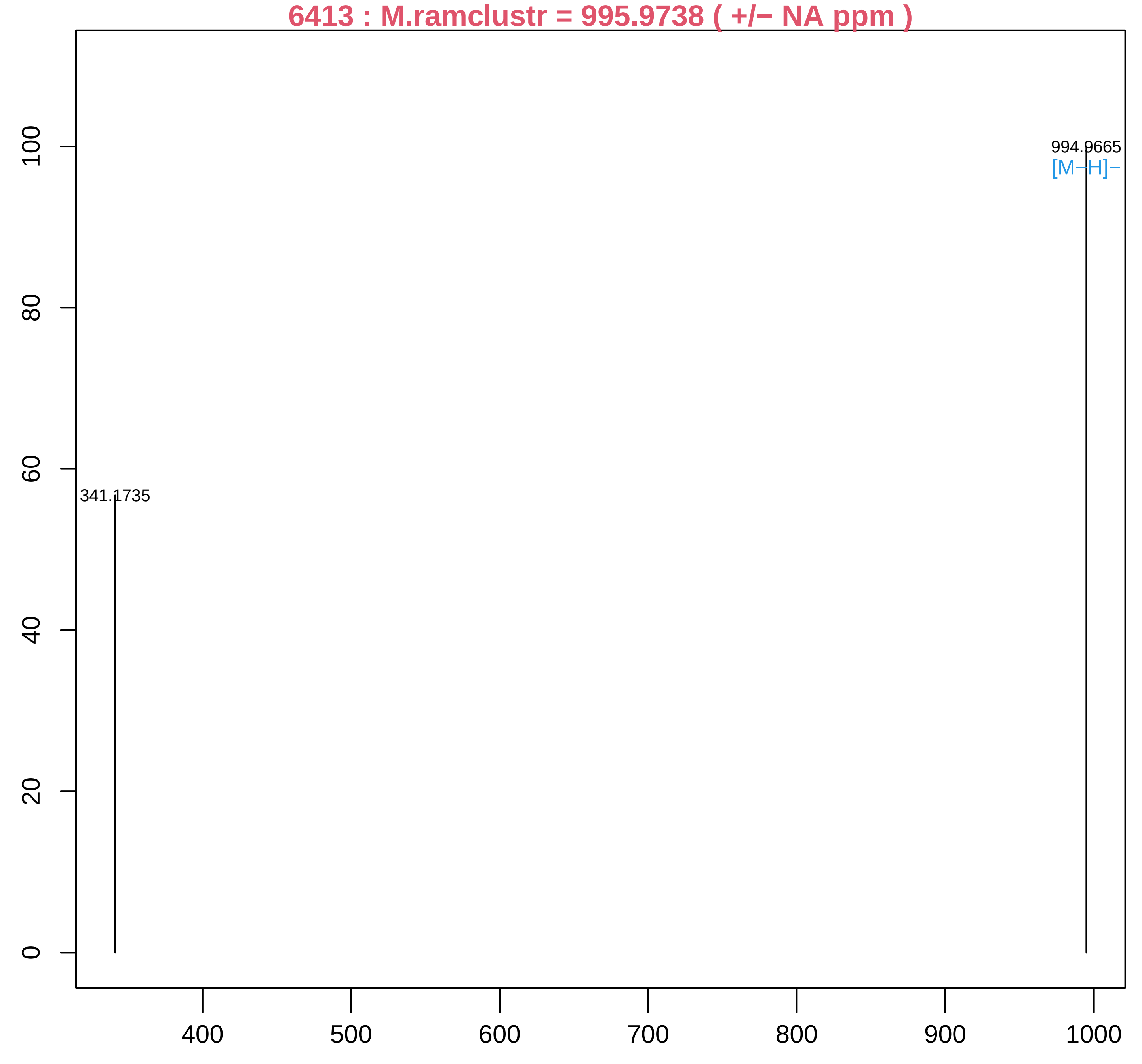

6413 : M.ramclustr = 995.9738 (+/− NA ppm)
994.9665
[M−H]−
341.1735
0
20
40
60
80
100
400
500
600
700
800
900
1000

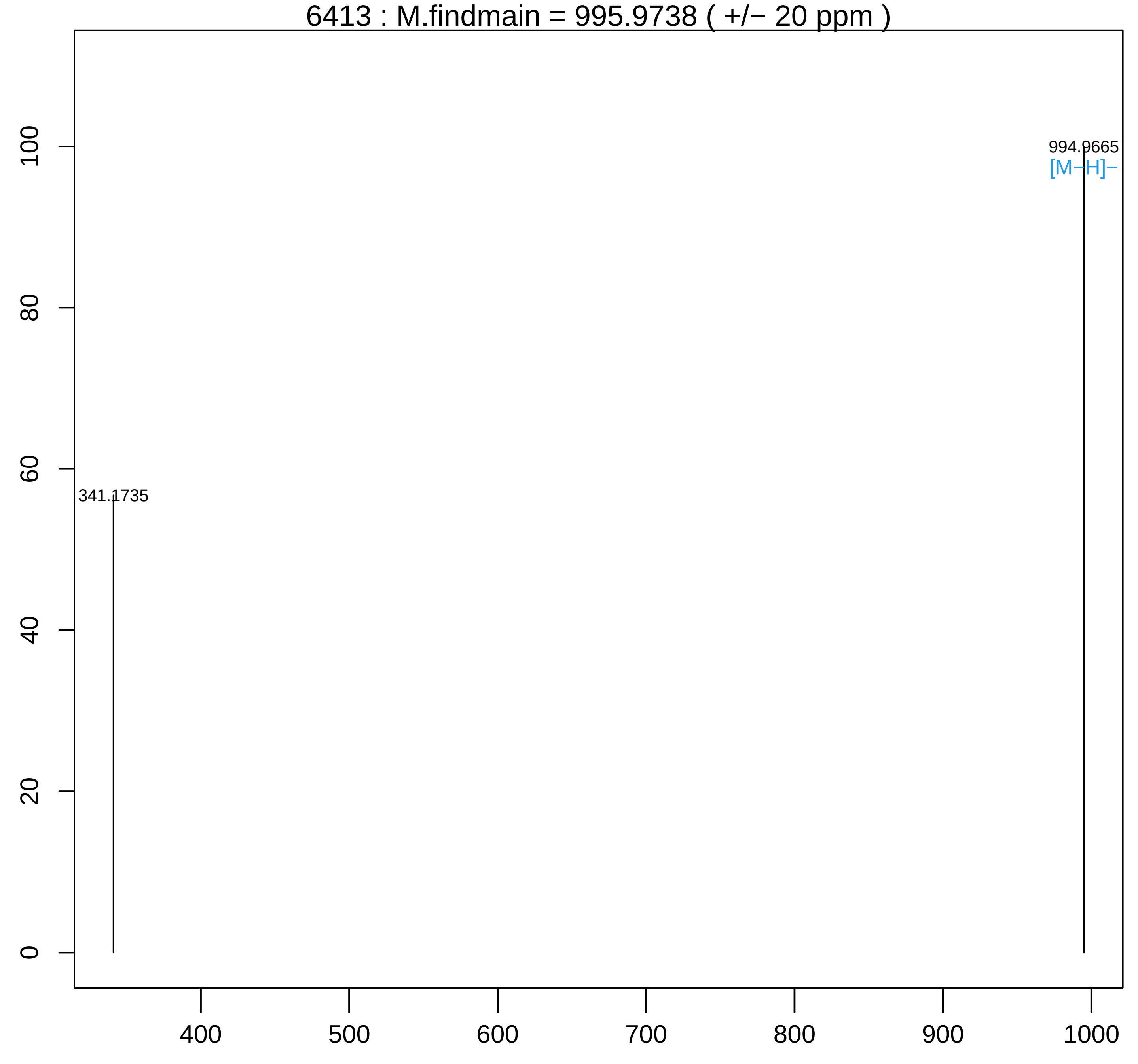

6413 : M.findmain = 995.9738 (+/− 20 ppm)
994.9665
[M−H]−
341.1735
0
20
40
60
80
100
400
500
600
700
800
900
1000

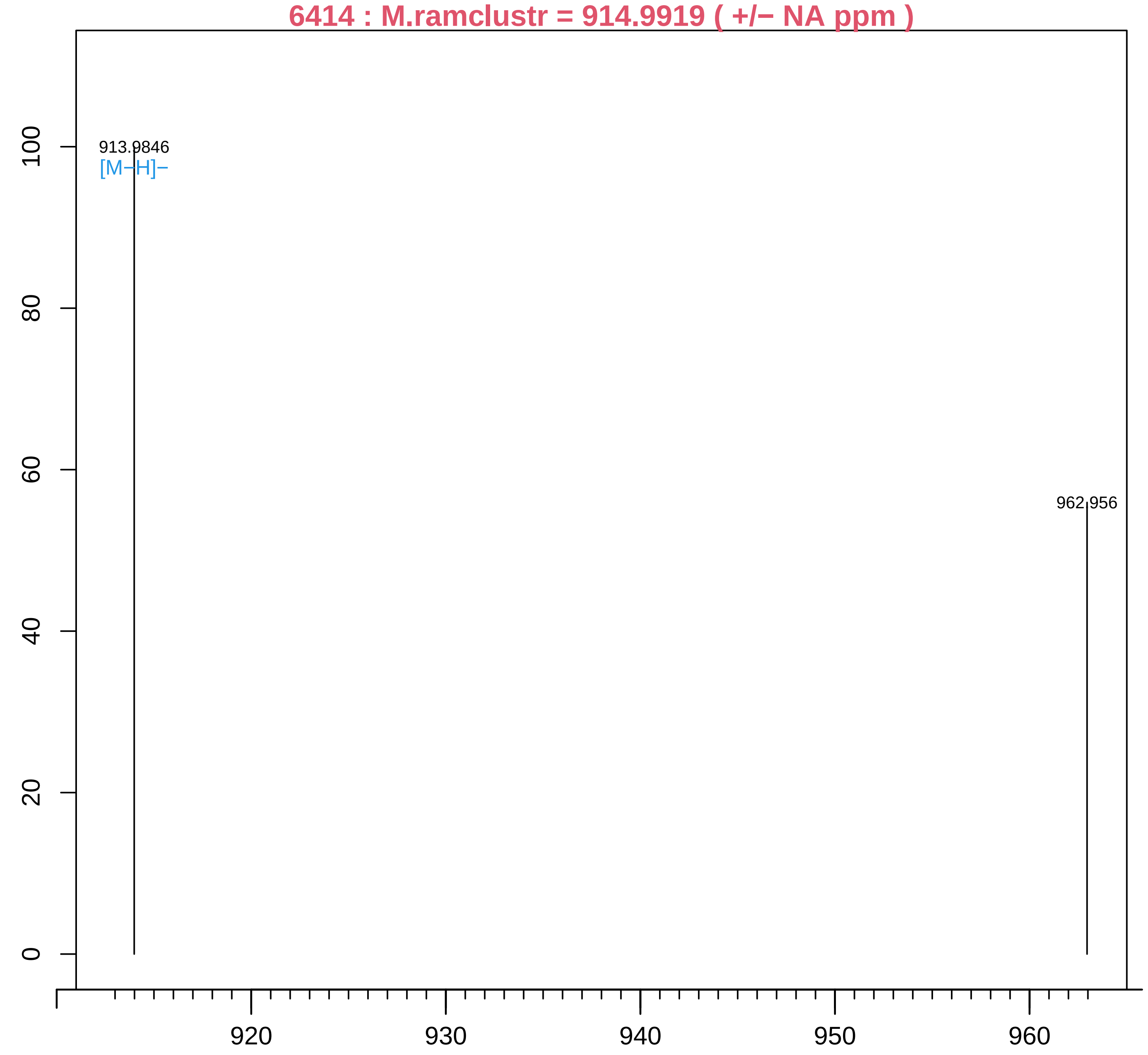
6414 : M.ramclustr = 914.9919 (+/− NA ppm)
913.9846
[M−H]−
962.956
0
20
40
60
80
100
920
930
940
950
960

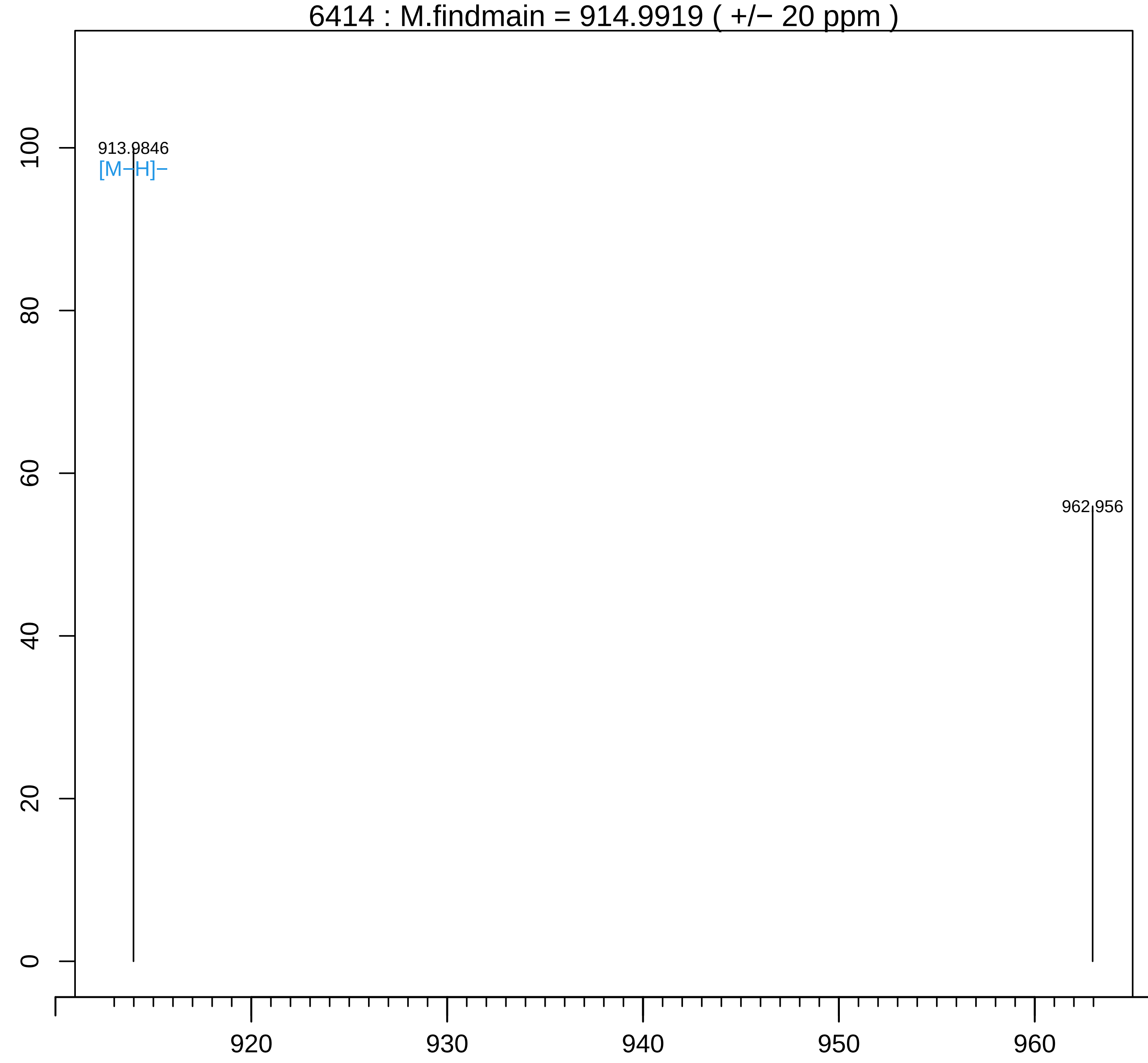
6414 : M.findmain = 914.9919 (+/− 20 ppm)
913.9846
[M−H]−
962.956
0
20
40
60
80
100
920
930
940
950
960

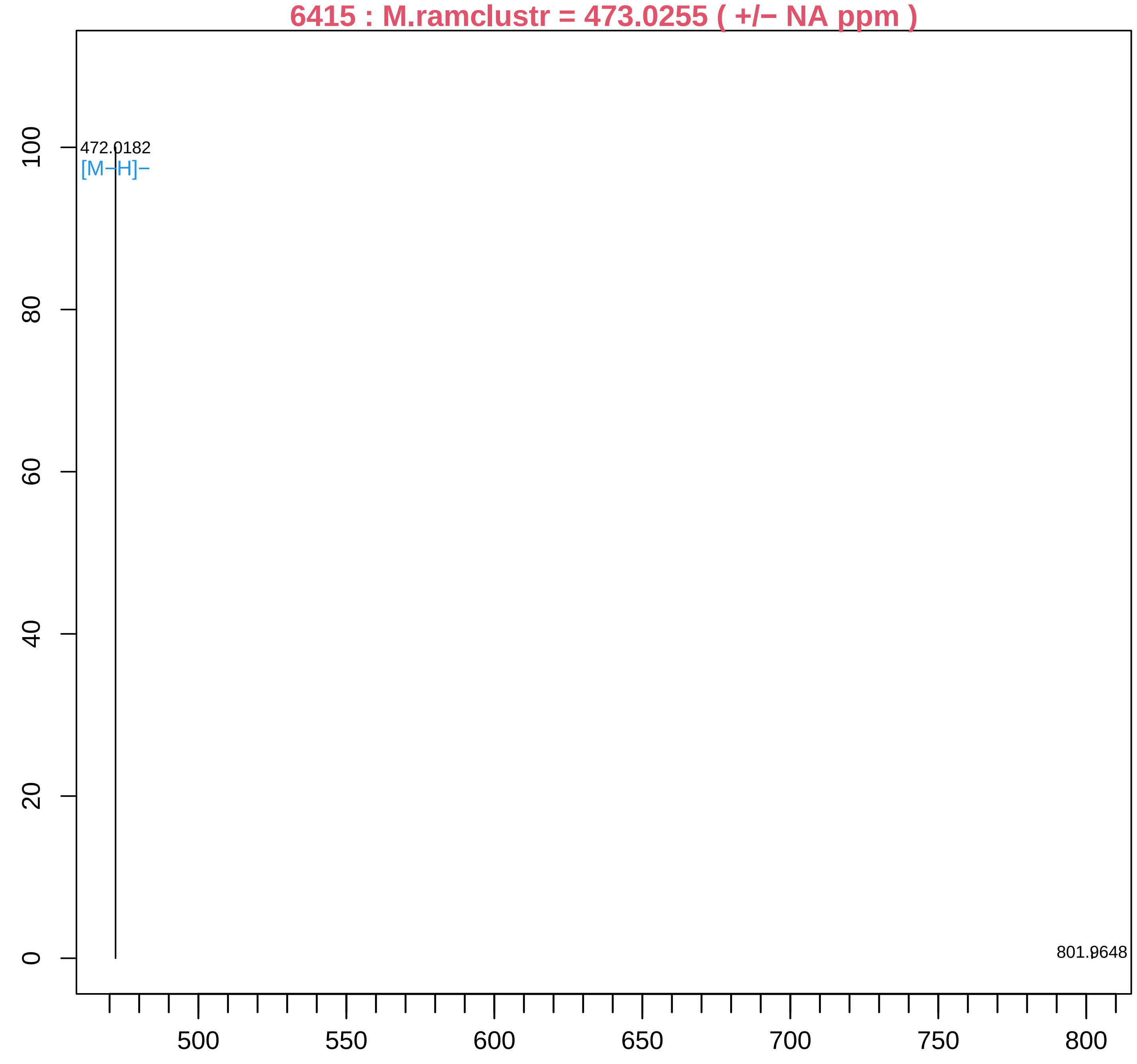
6415 : M.ramclustr = 473.0255 (+/− NA ppm)
472.0182
[M−H]−
801.9648
0
20
40
60
80
100
500
550
600
650
700
750
800

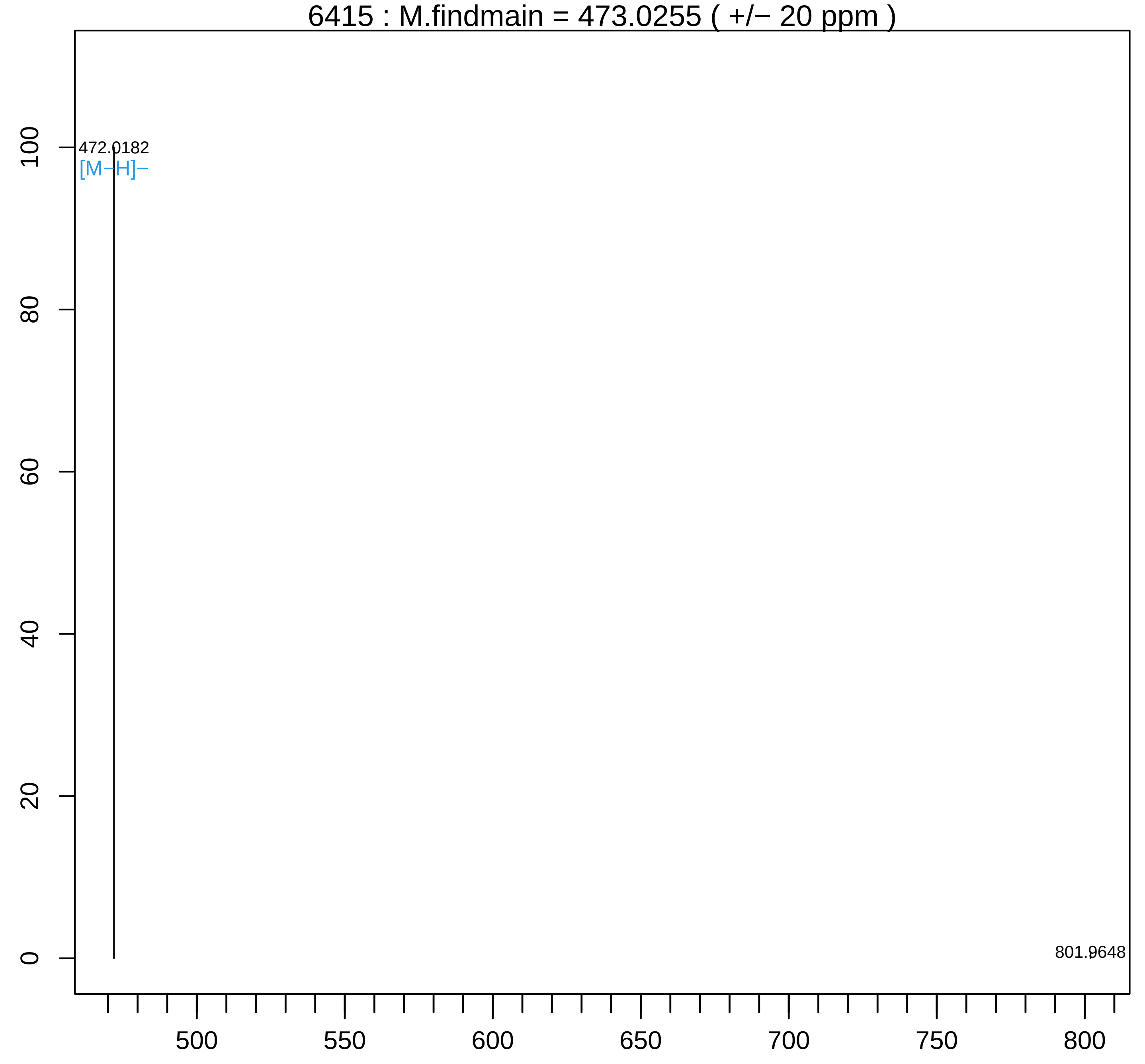
6415 : M.findmain = 473.0255 (+/− 20 ppm)
472.0182
[M−H]−
801.9648
0
20
40
60
80
100
500
550
600
650
700
750
800

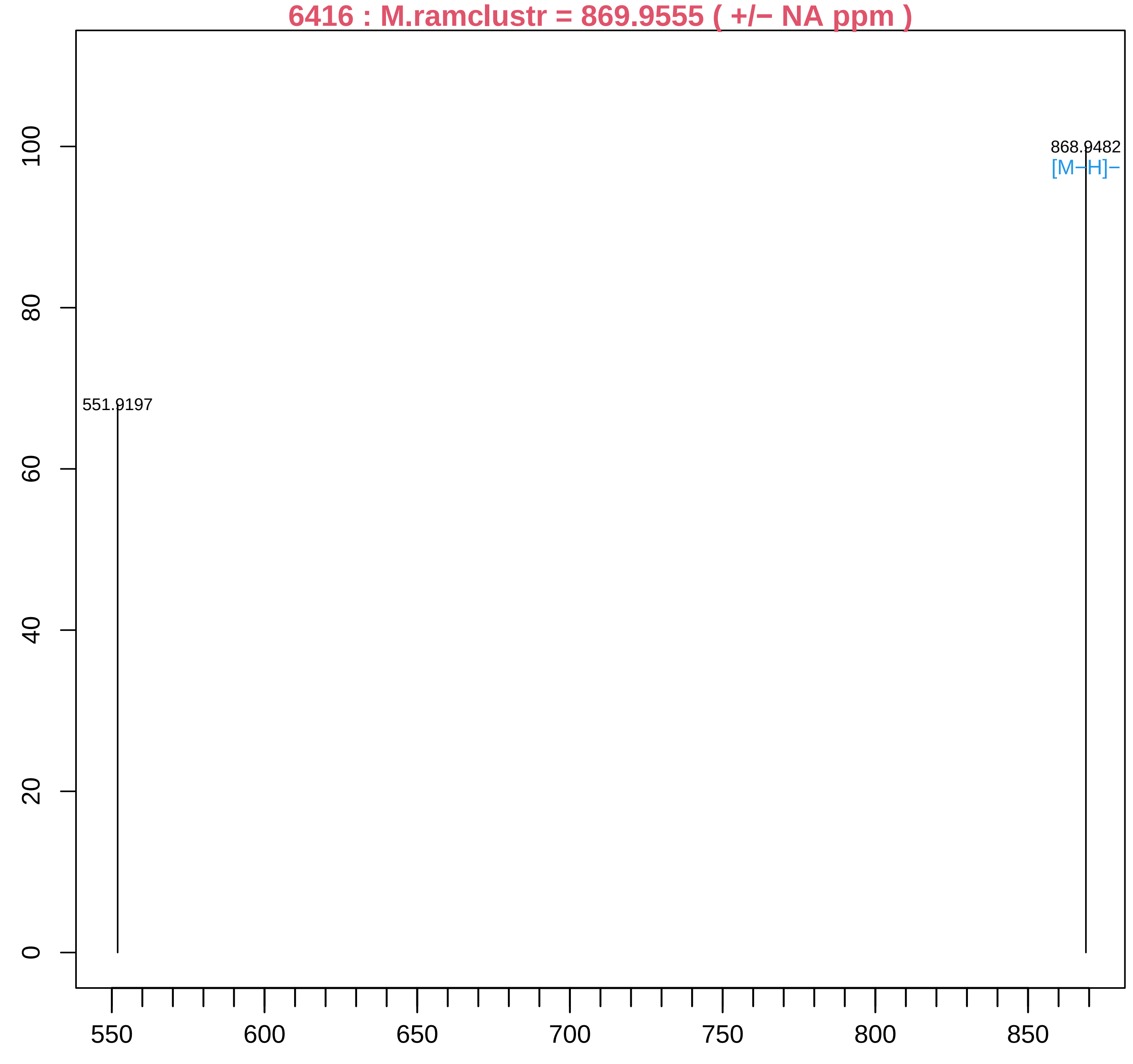
6416 : M.ramclustr = 869.9555 (+/− NA ppm)
868.9482
[M−H]−
551.9197
0
20
40
60
80
100
550
600
650
700
750
800
850

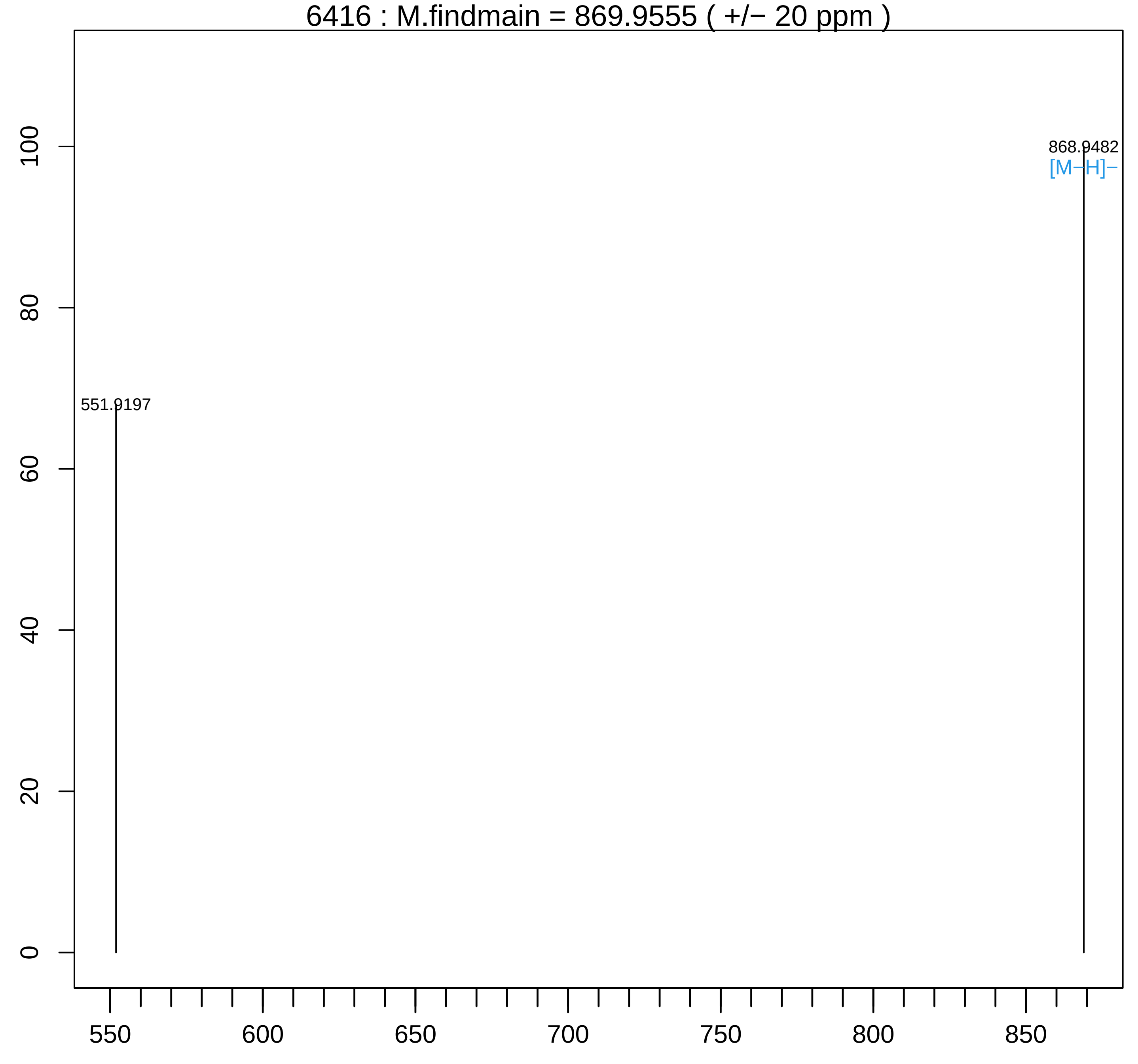
6416 : M.findmain = 869.9555 (+/− 20 ppm)
868.9482
[M−H]−
551.9197
0
20
40
60
80
100
550
600
650
700
750
800
850

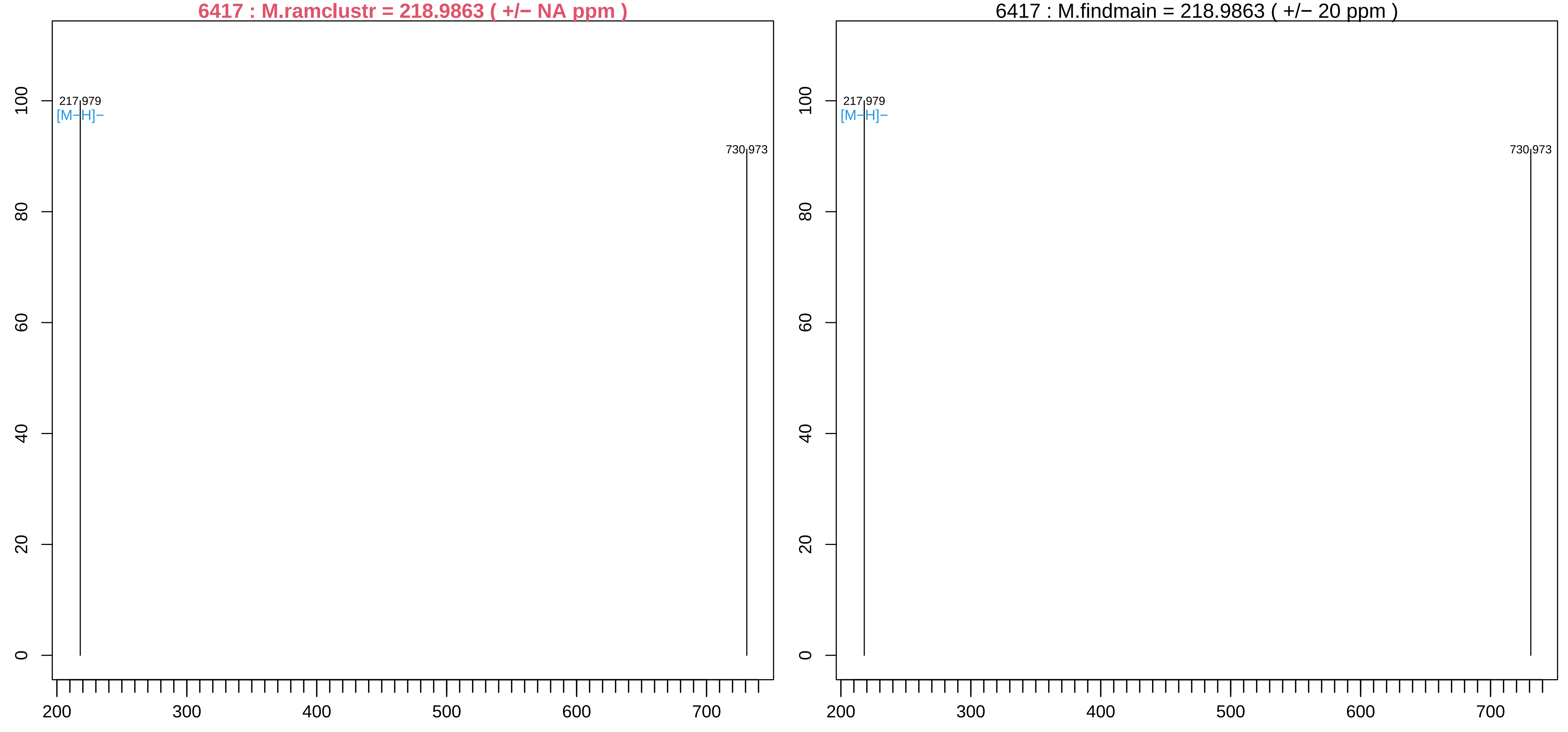
6417 : M.ramclustr = 218.9863 (+/− NA ppm)
217.979
[M−H]−
730.973
6417 : M.findmain = 218.9863 (+/− 20 ppm)
217.979
[M−H]−
730.973
0
20
40
60
80
100
200
300
400
500
600
700

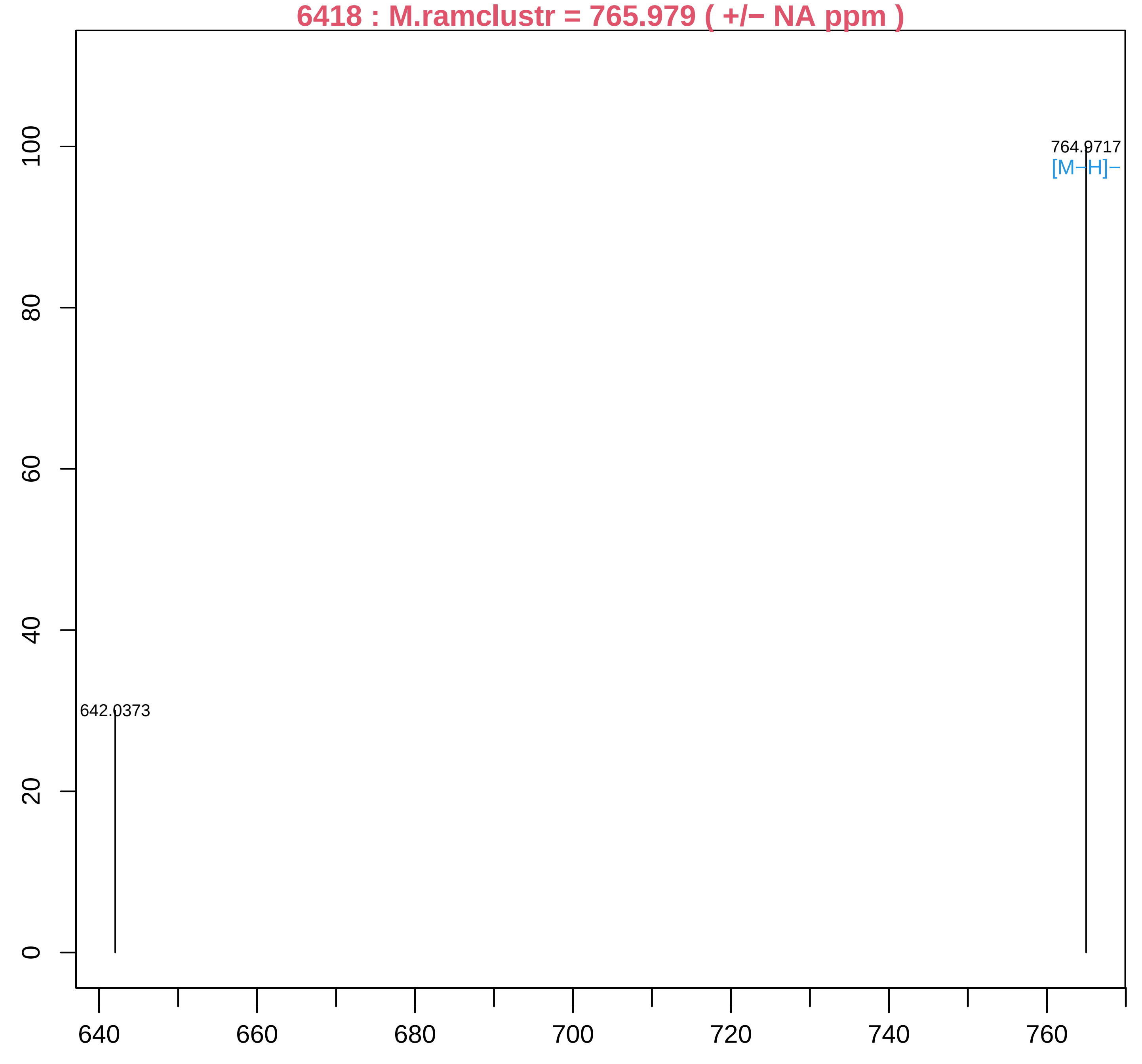
6418 : M.ramclustr = 765.979 (+/− NA ppm)
764.9717
[M−H]−
642.0373
0
20
40
60
80
100
640
660
680
700
720
740
760

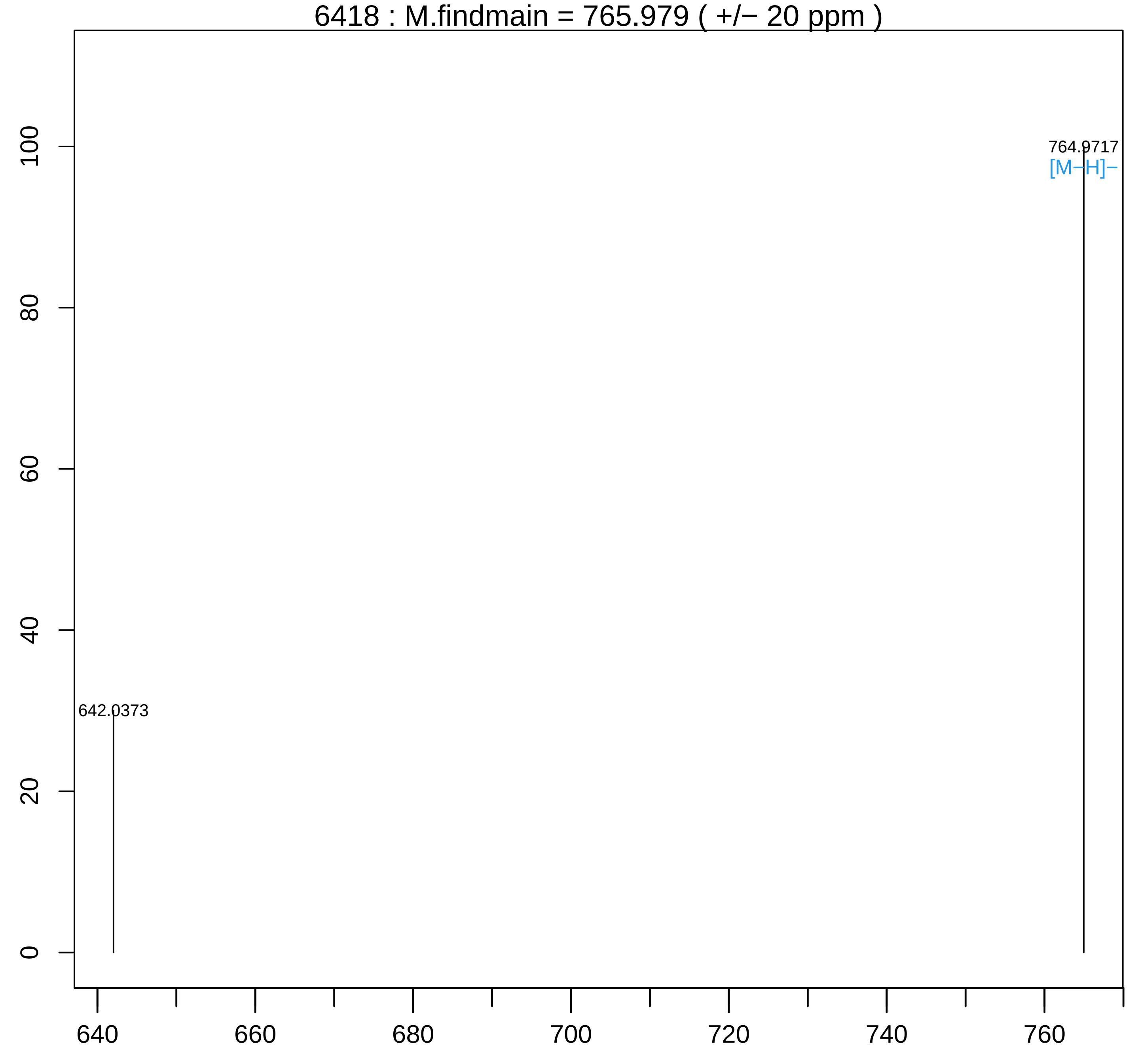
6418 : M.findmain = 765.979 (+/− 20 ppm)
764.9717
[M−H]−
642.0373
0
20
40
60
80
100
640
660
680
700
720
740
760

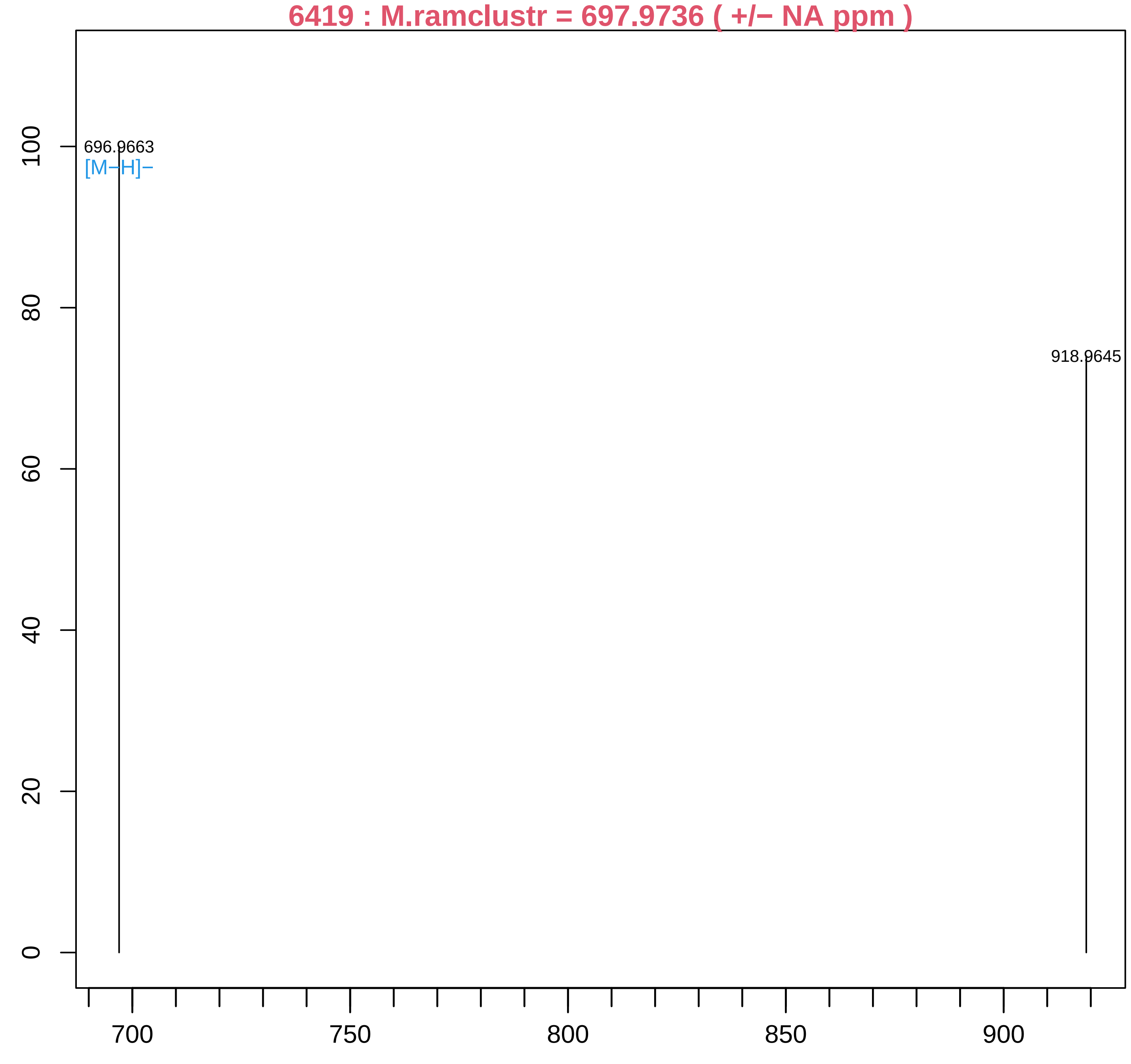
6419 : M.ramclustr = 697.9736 (+/− NA ppm)
696.9663
[M−H]−
918.9645
0
20
40
60
80
100
700
750
800
850
900

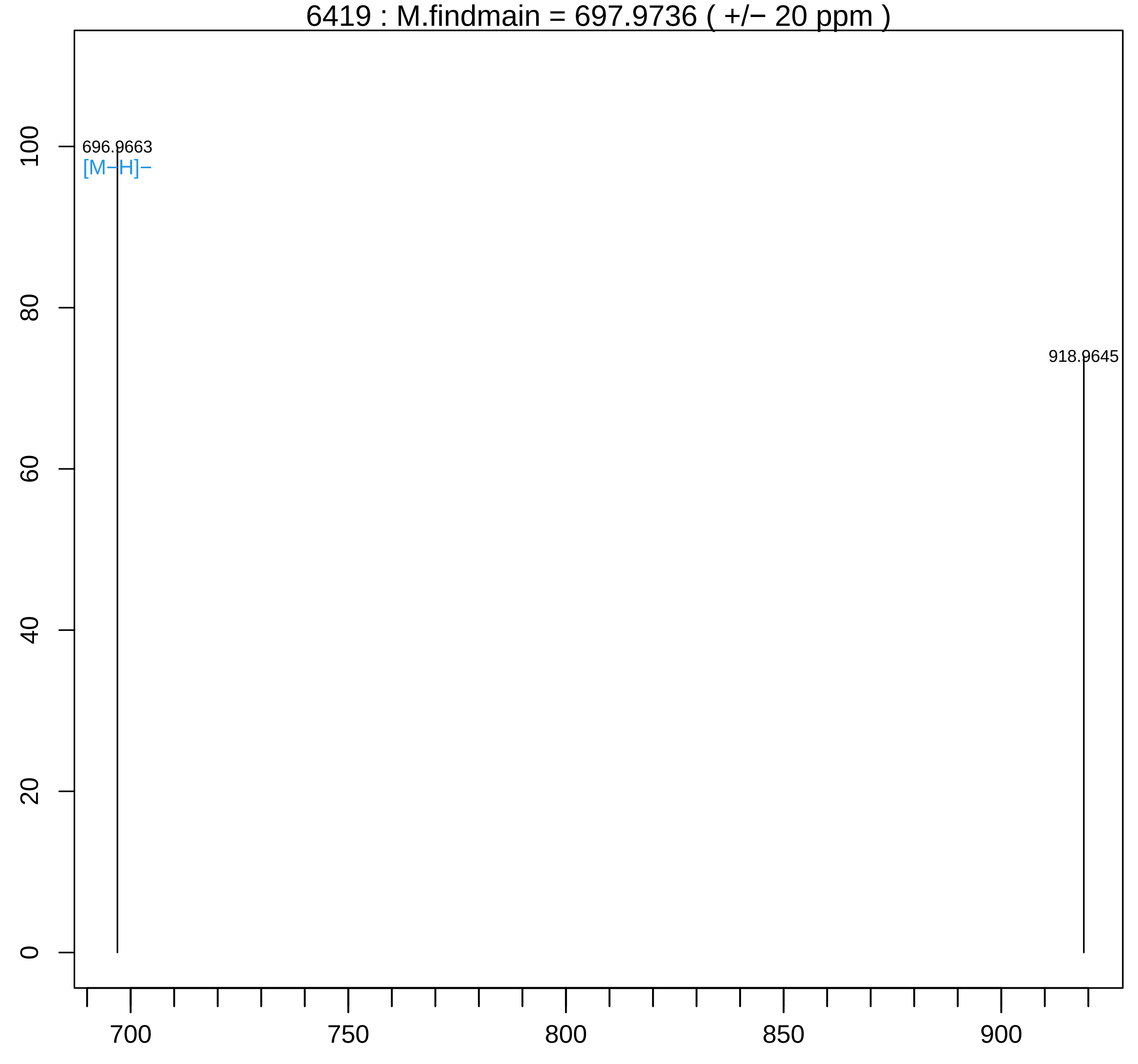
6419 : M.findmain = 697.9736 (+/− 20 ppm)
696.9663
[M−H]−
918.9645
0
20
40
60
80
100
700
750
800
850
900

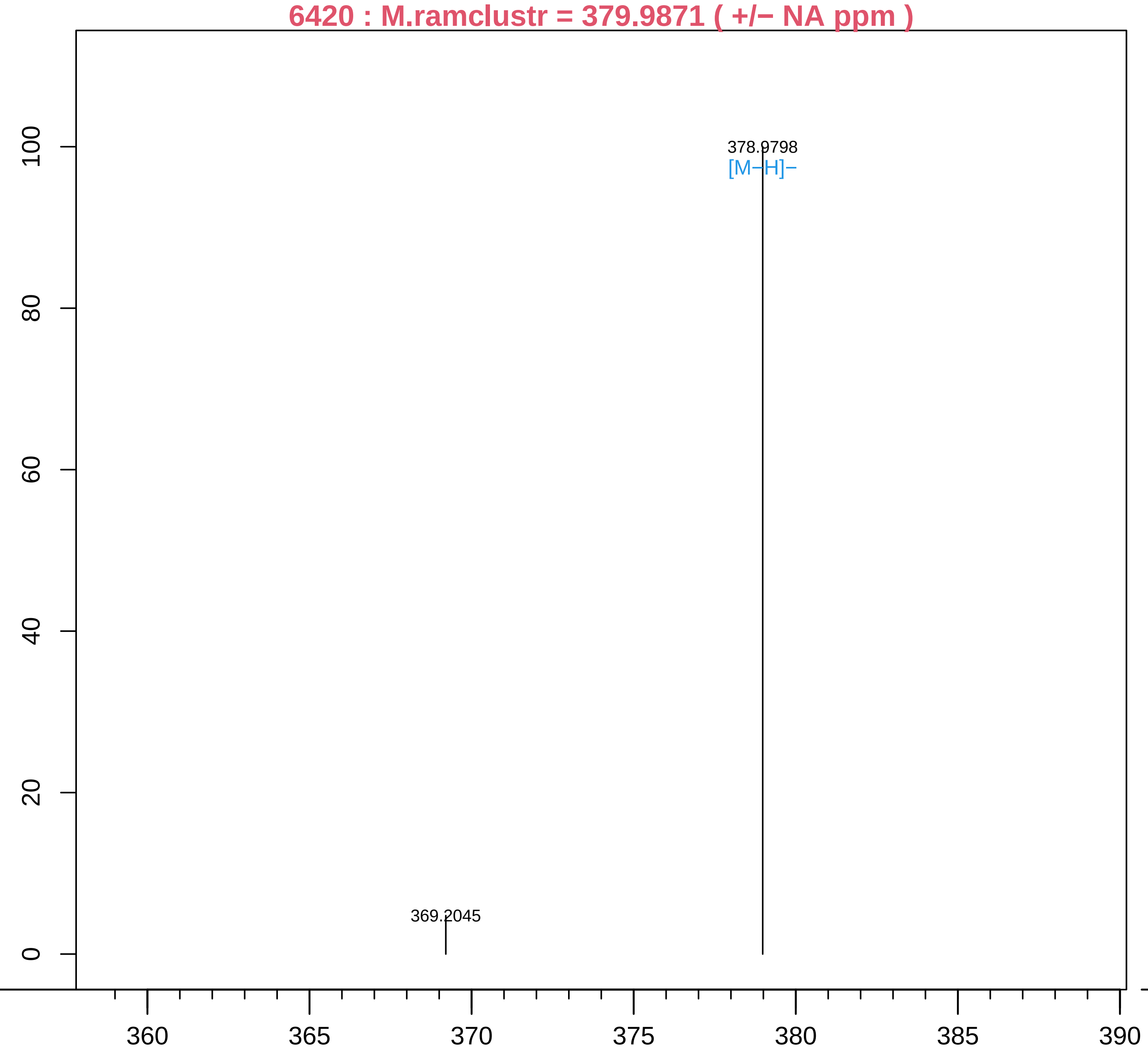
6420 : M.ramclustr = 379.9871 (+/− NA ppm)
378.9798
[M−H]−
369.2045
0
20
40
60
80
100
360
365
370
375
380
385
390

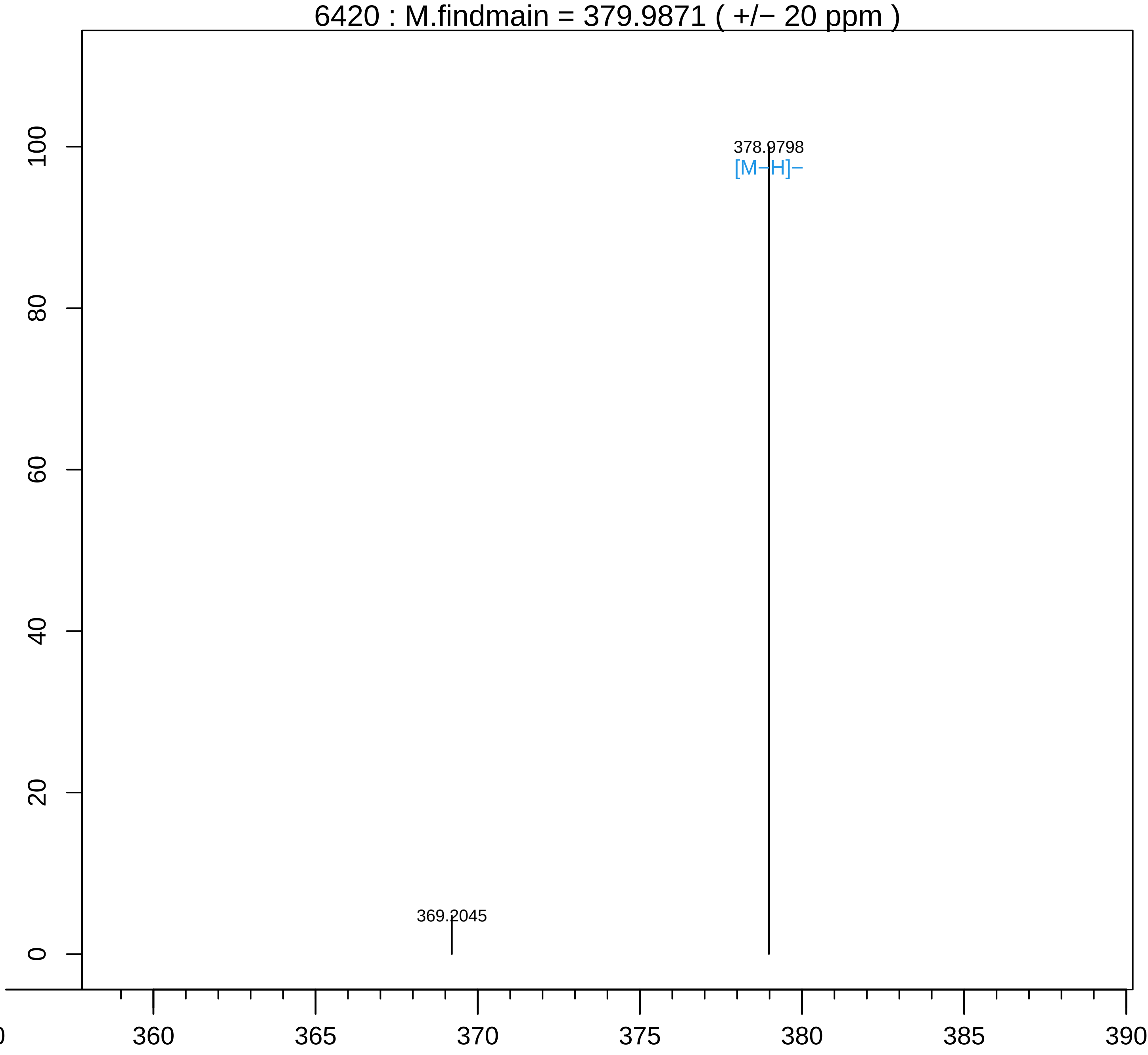
6420 : M.findmain = 379.9871 (+/− 20 ppm)
378.9798
[M−H]−
369.2045
0
20
40
60
80
100
360
365
370
375
380
385
390

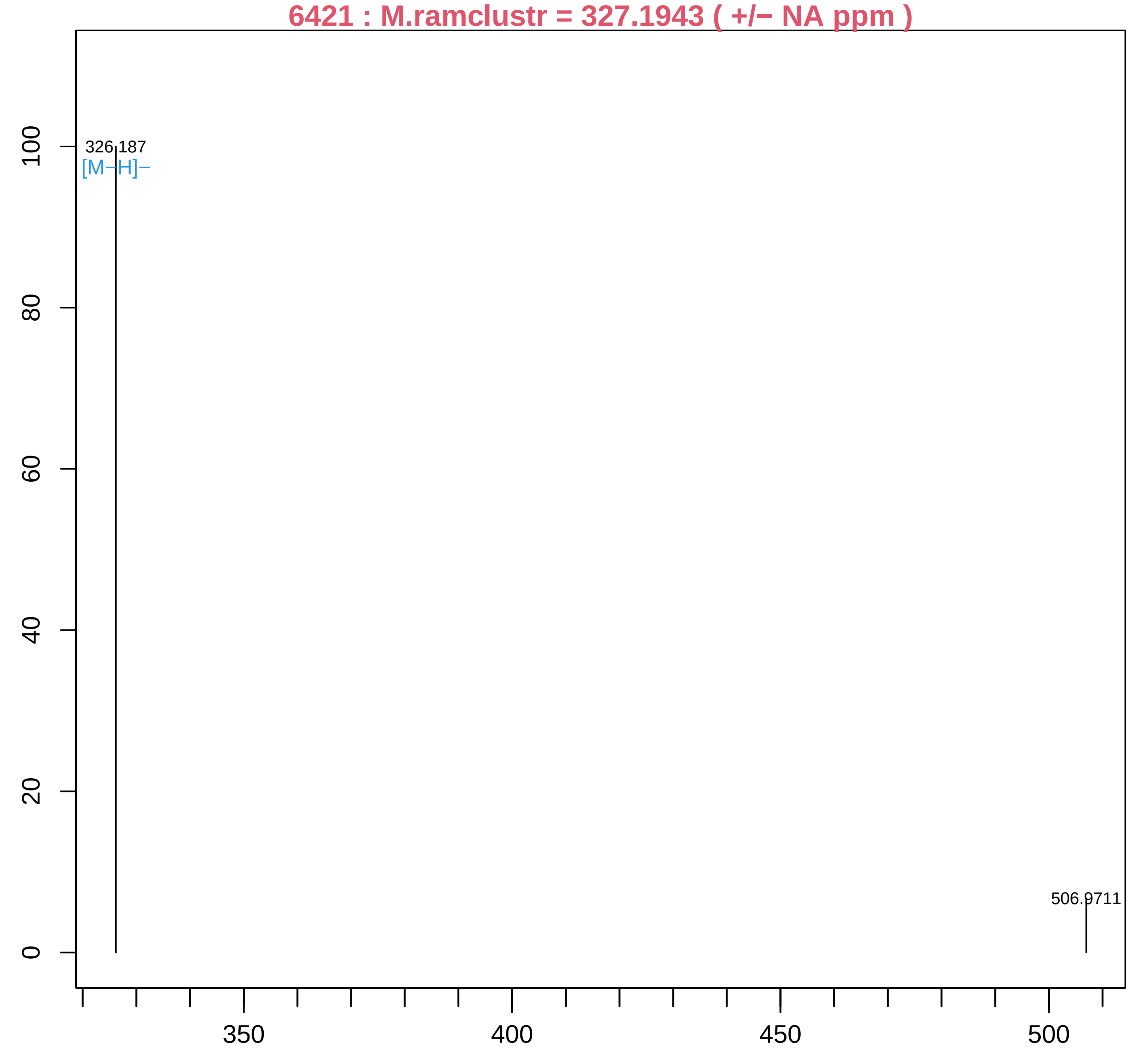
6421 : M.ramclustr = 327.1943 (+/− NA ppm)
326.187
[M−H]−
506.9711
0
20
40
60
80
100
350
400
450
500

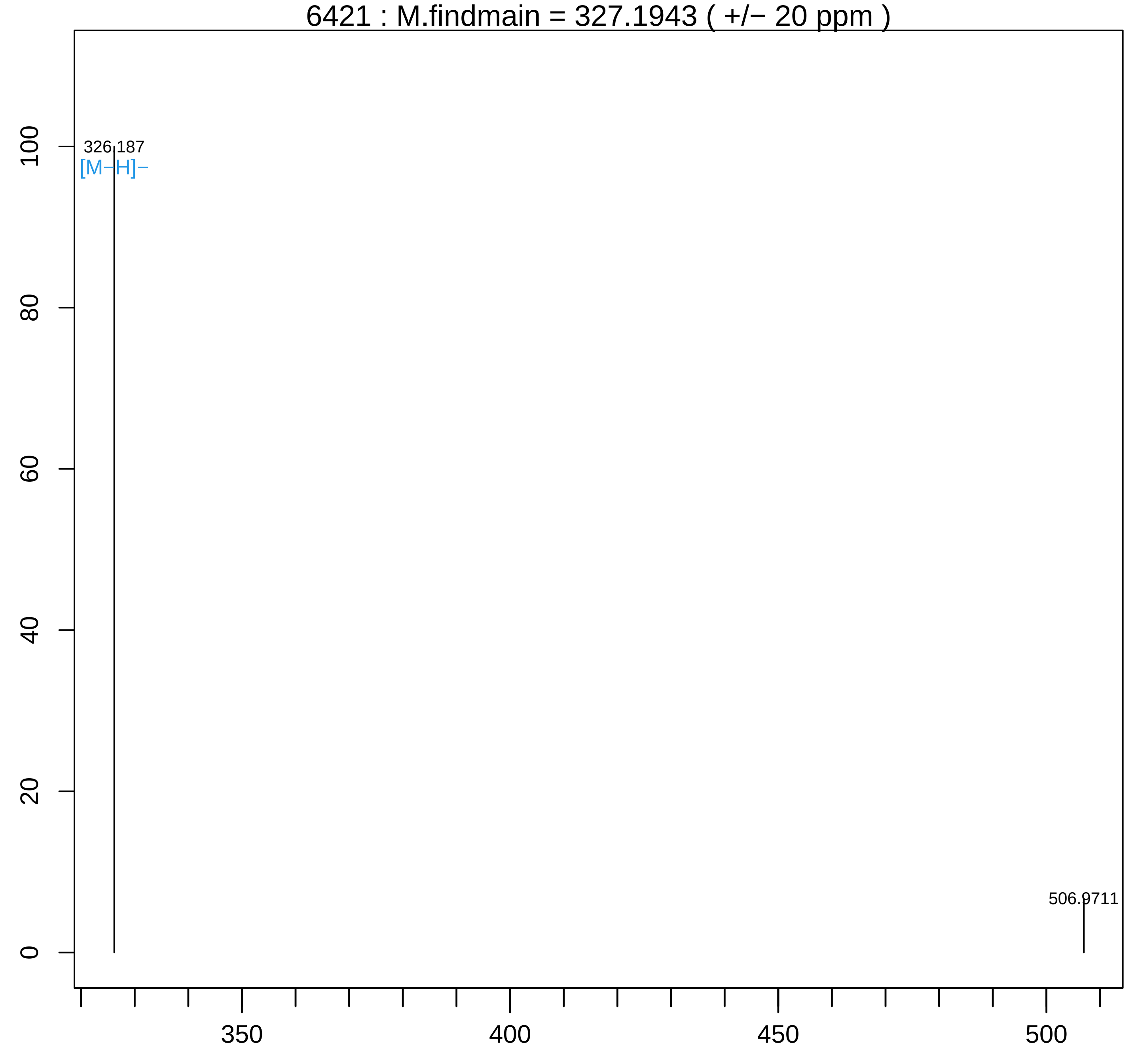
6421 : M.findmain = 327.1943 (+/− 20 ppm)
326.187
[M−H]−
506.9711
0
20
40
60
80
100
350
400
450
500

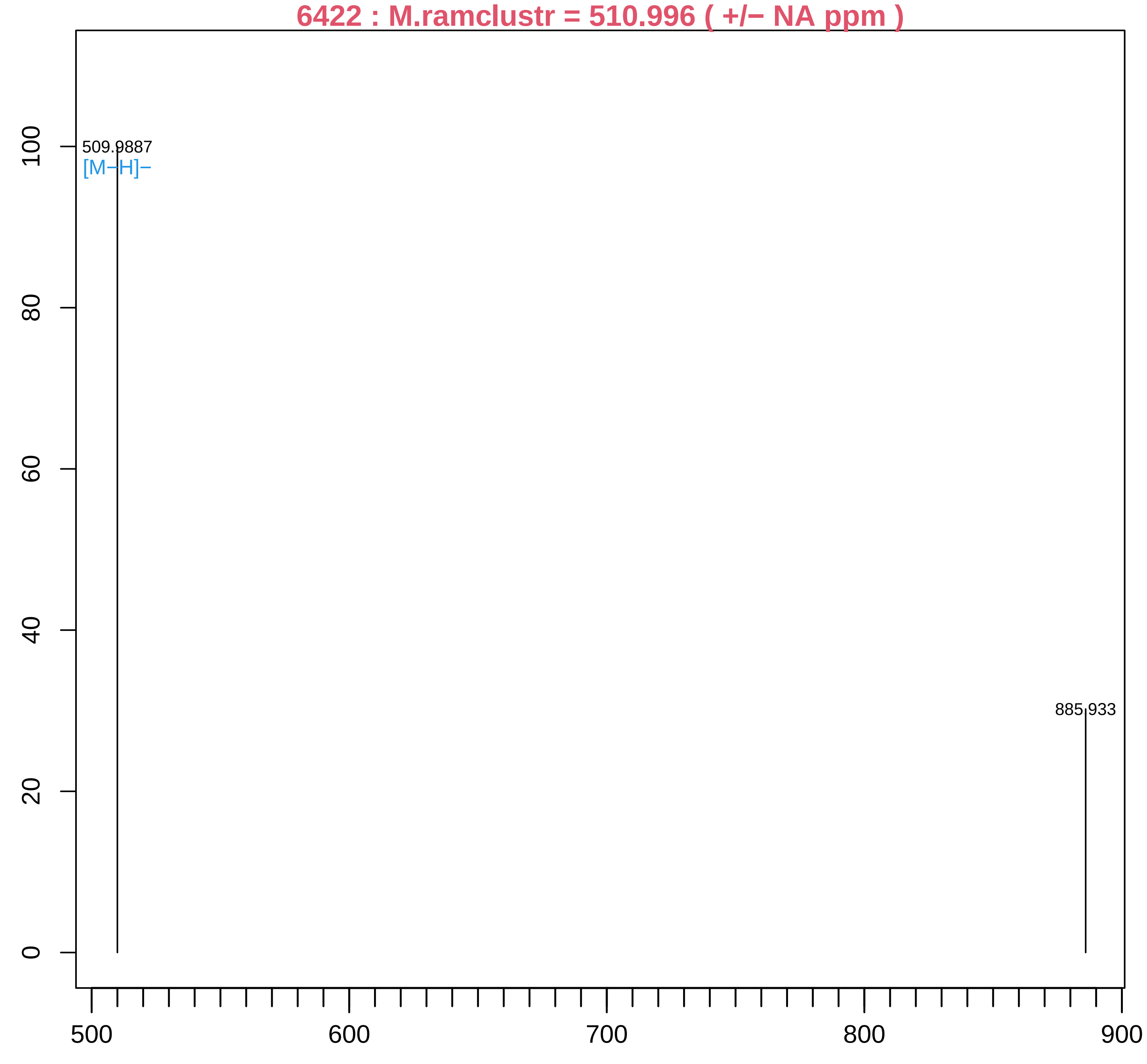
6422 : M.ramclustr = 510.996 (+/− NA ppm)
509.9887
[M−H]−
885.933
0
20
40
60
80
100
500
600
700
800
900

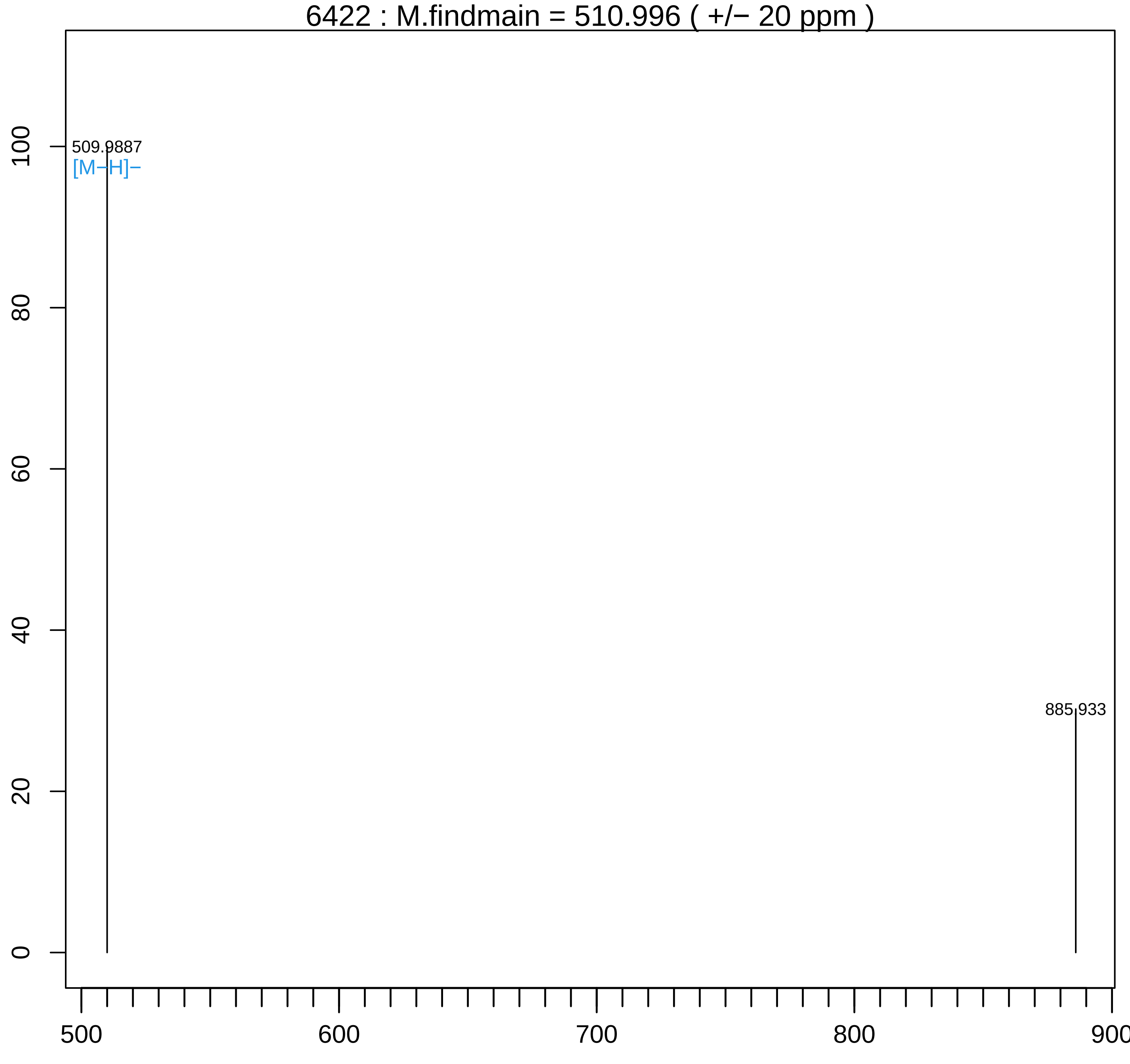
6422 : M.findmain = 510.996 (+/− 20 ppm)
509.9887
[M−H]−
885.933
0
20
40
60
80
100
500
600
700
800
900

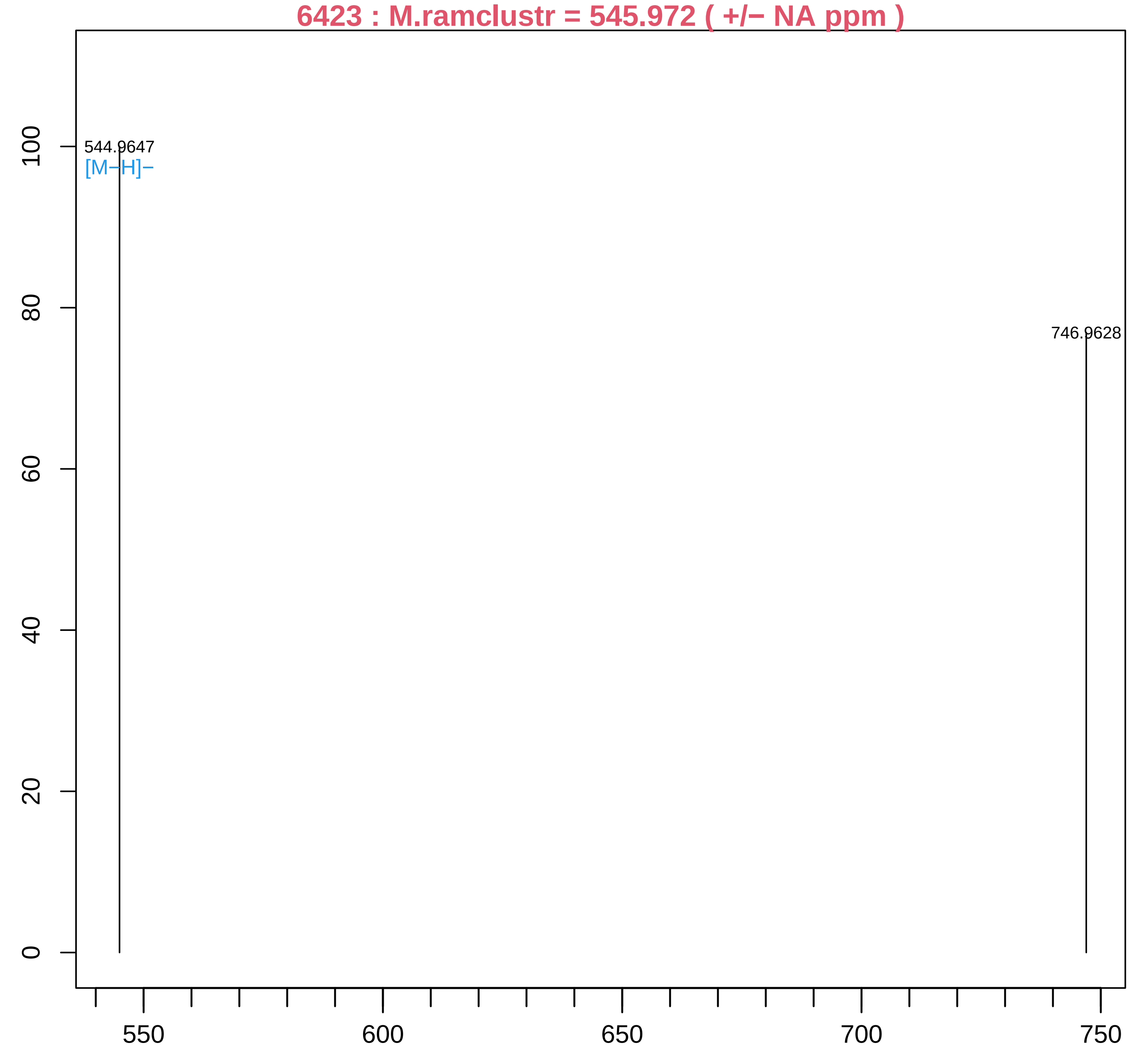
6423 : M.ramclustr = 545.972 (+/− NA ppm)
544.9647
[M−H]−
746.9628
0
20
40
60
80
100
550
600
650
700
750

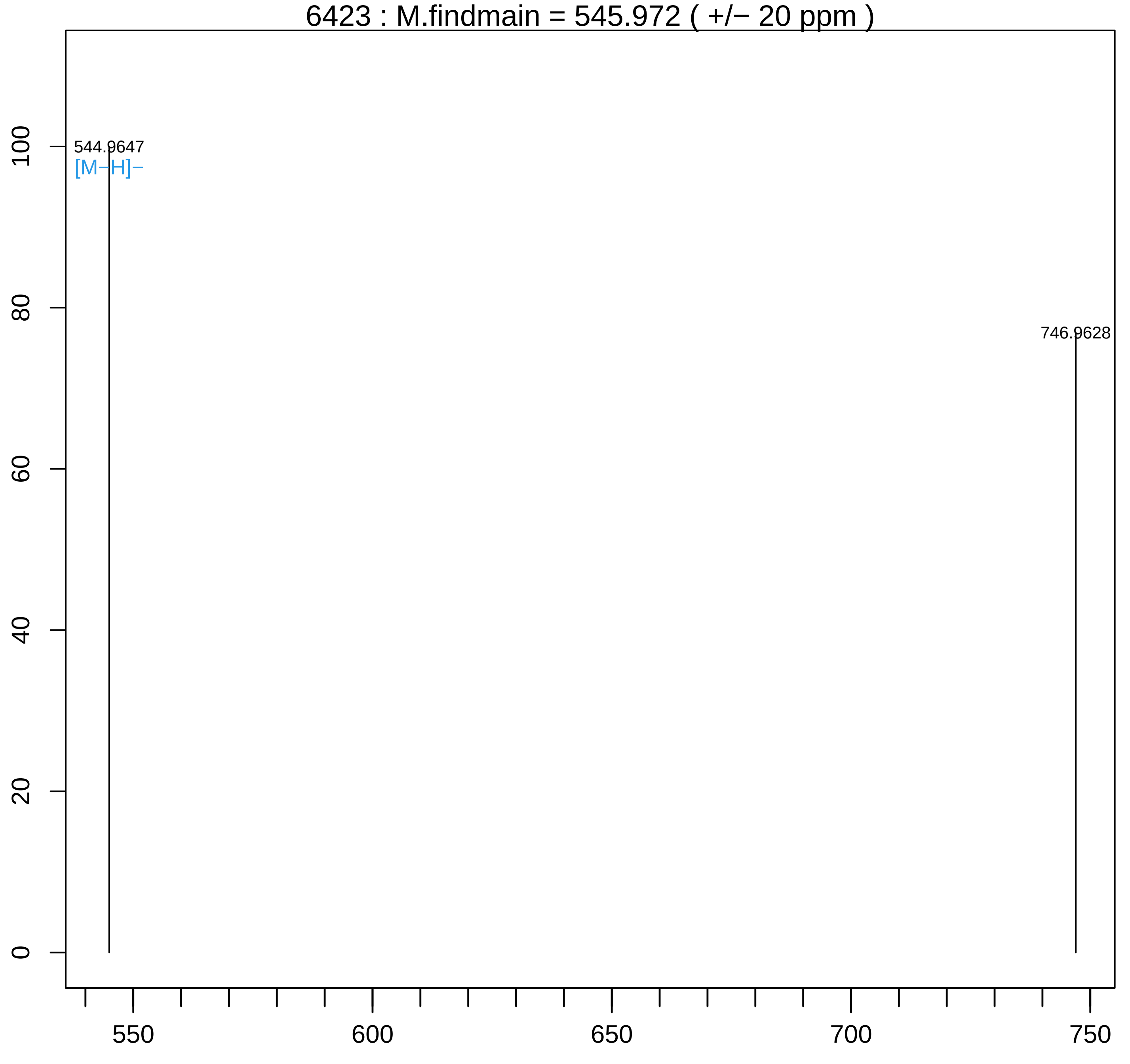
6423 : M.findmain = 545.972 (+/− 20 ppm)
544.9647
[M−H]−
746.9628
0
20
40
60
80
100
550
600
650
700
750

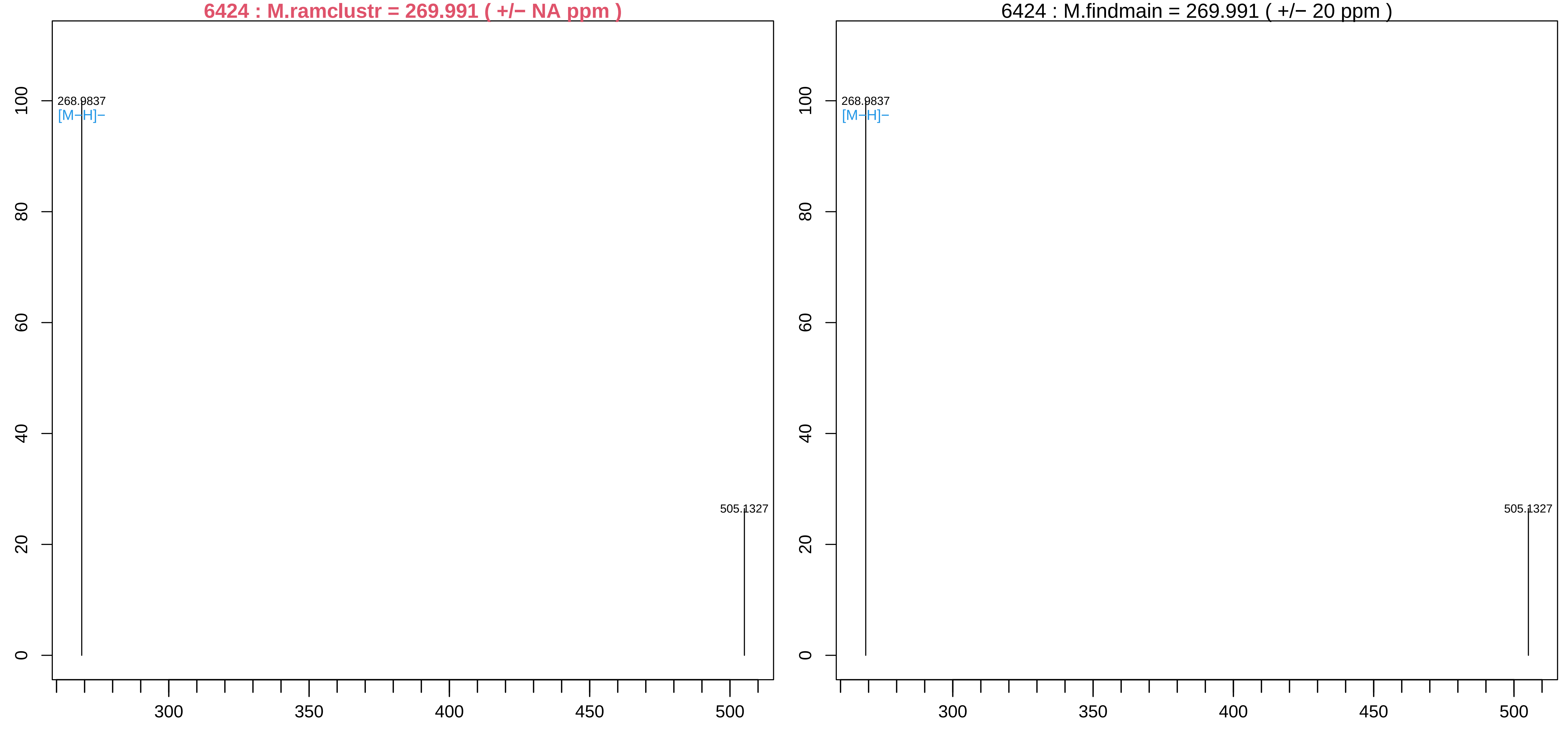
6424 : M.ramclustr = 269.991 (+/− NA ppm)
268.9837
[M−H]−
505.1327
6424 : M.findmain = 269.991 (+/− 20 ppm)
268.9837
[M−H]−
505.1327
0
20
40
60
80
100
300
350
400
450
500

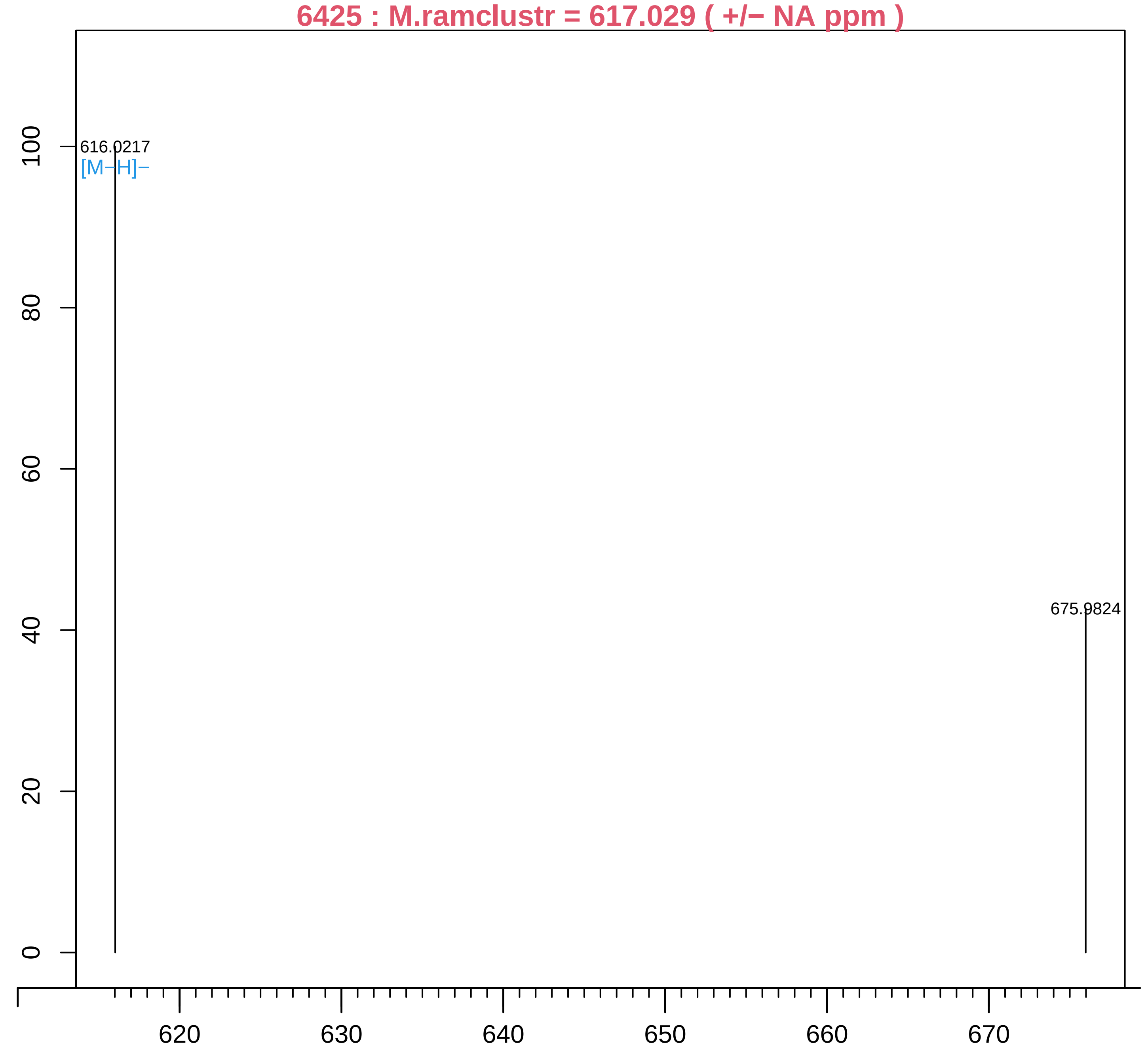
6425 : M.ramclustr = 617.029 (+/− NA ppm)
616.0217
[M−H]−
675.9824
0
20
40
60
80
100
620
630
640
650
660
670

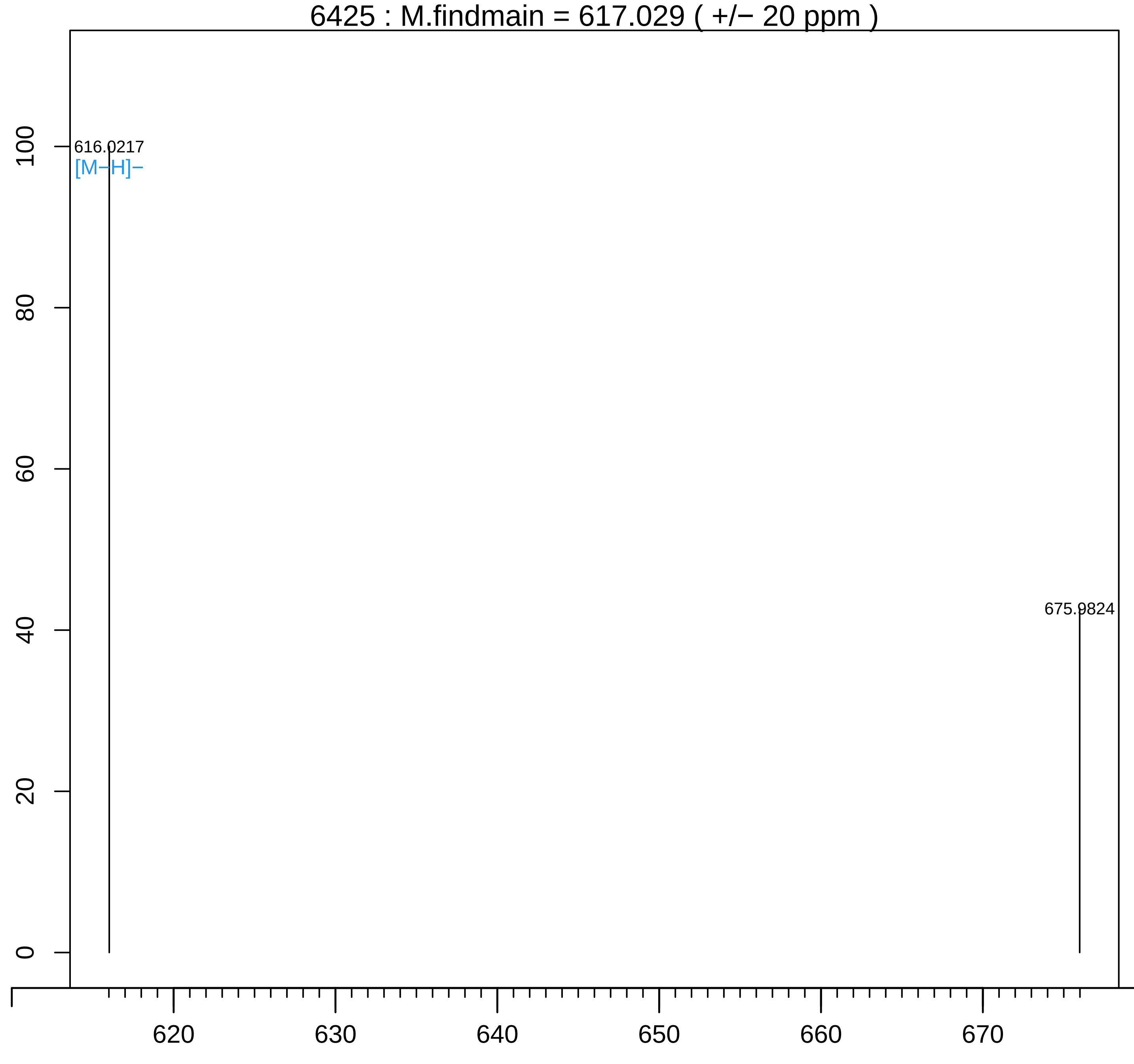
6425 : M.findmain = 617.029 (+/− 20 ppm)
616.0217
[M−H]−
675.9824
0
20
40
60
80
100
620
630
640
650
660
670

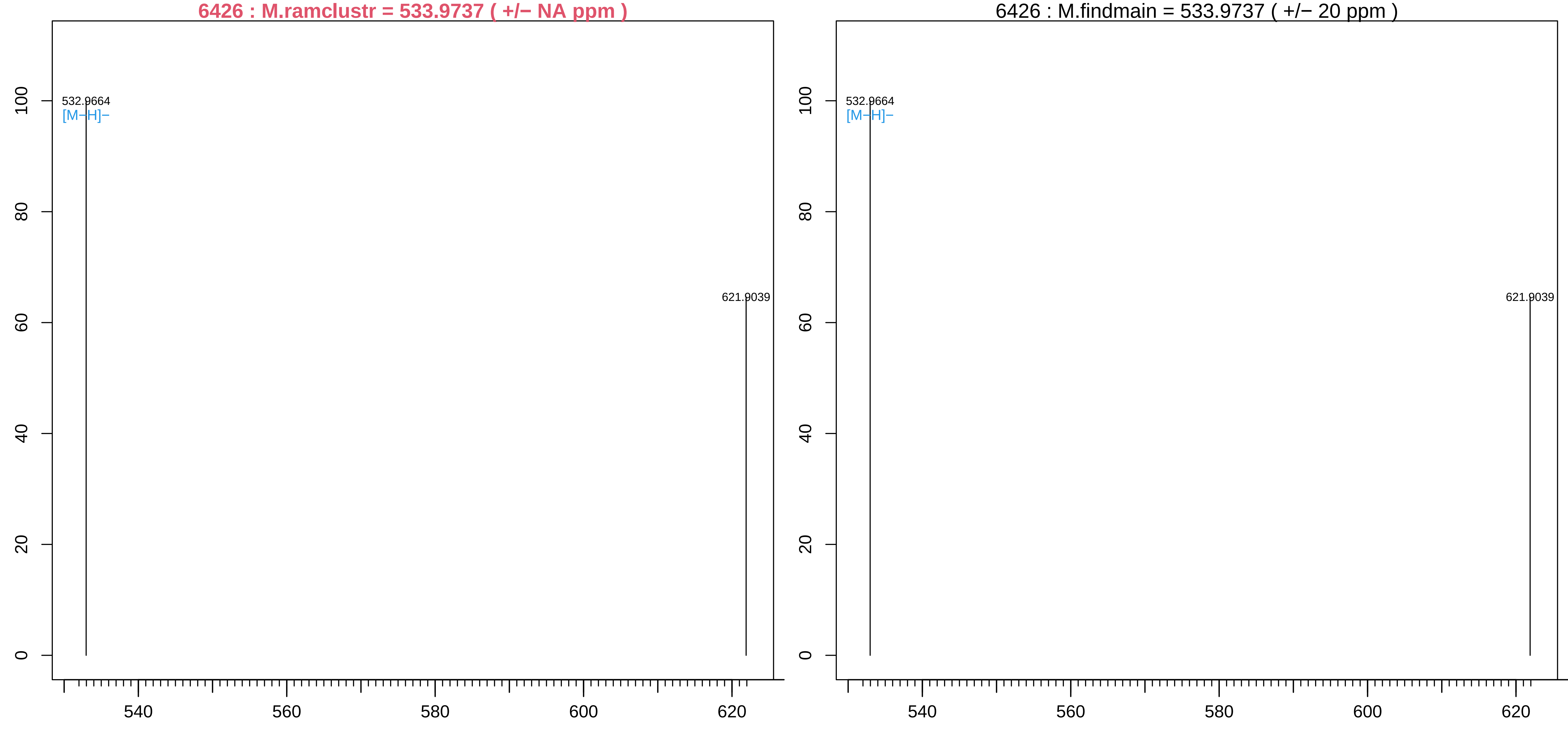

6426 : M.ramclustr = 533.9737 (+/− NA ppm)
532.9664
[M−H]−
621.9039
0
20
40
60
80
100
540
560
580
600
620
6426 : M.findmain = 533.9737 (+/− 20 ppm)
532.9664
[M−H]−
621.9039
0
20
40
60
80
100
540
560
580
600
620

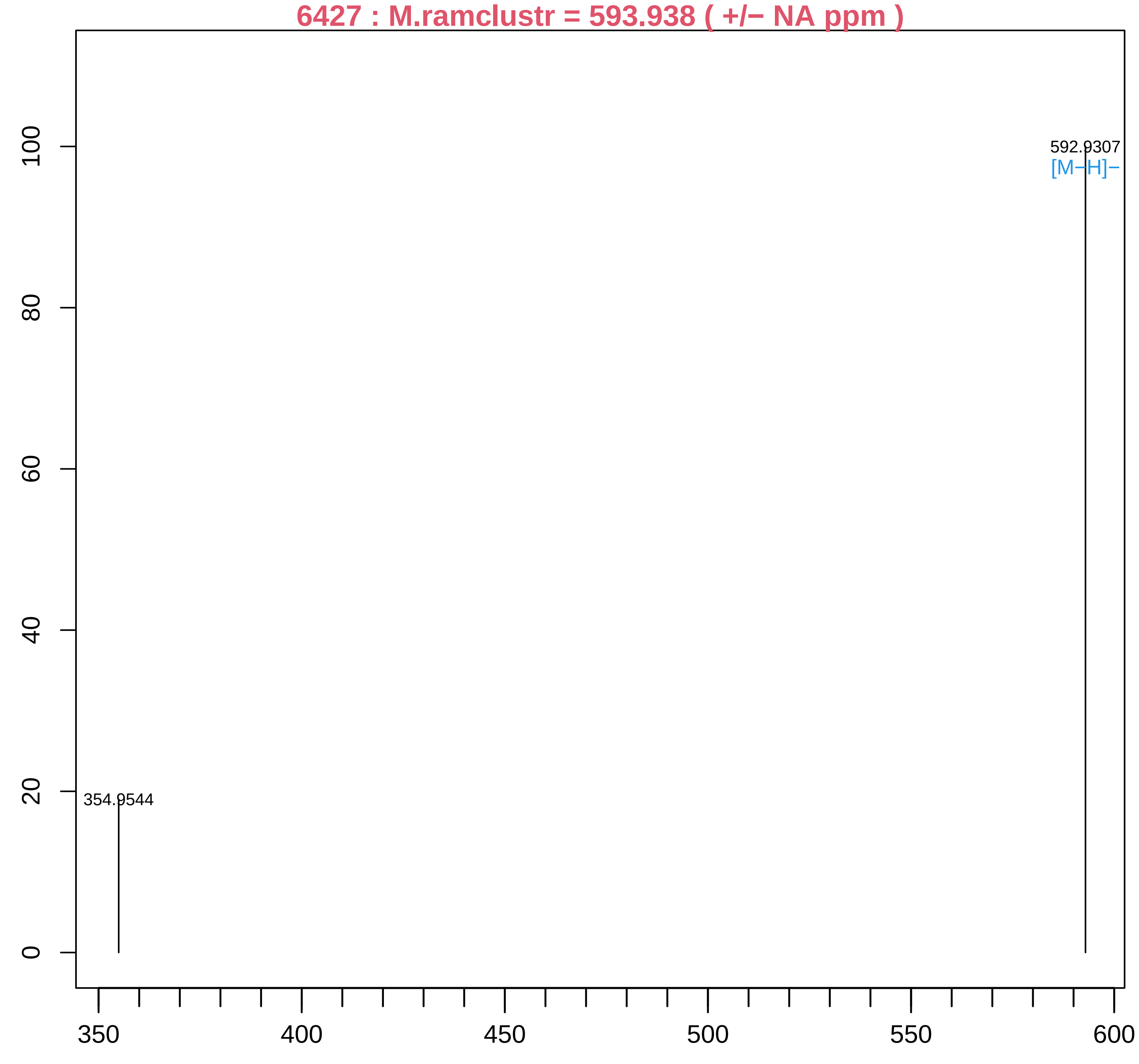
6427 : M.ramclustr = 593.938 (+/− NA ppm)
592.9307
[M−H]−
354.9544
0
20
40
60
80
100
350
400
450
500
550
600

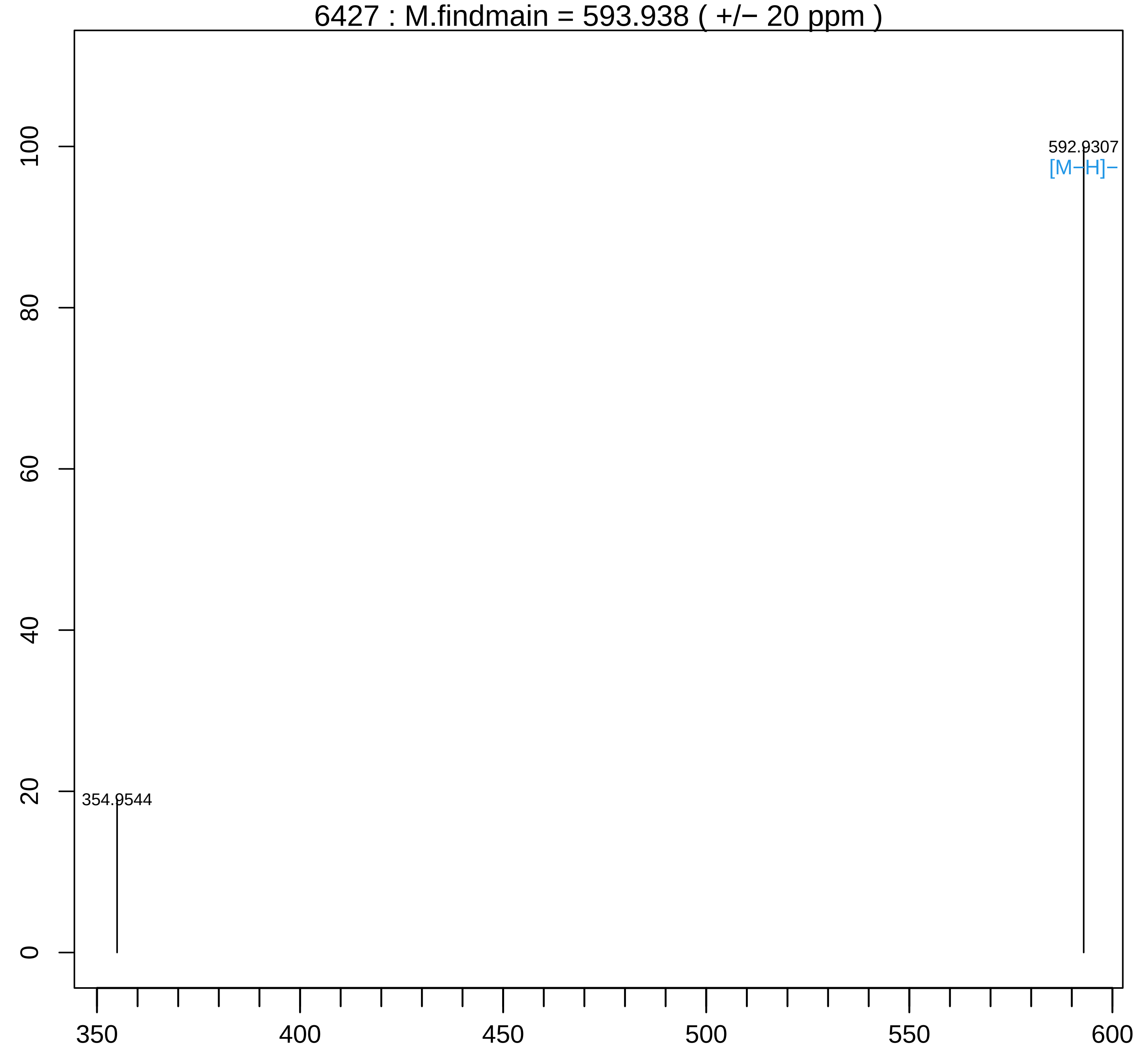
6427 : M.findmain = 593.938 (+/− 20 ppm)
592.9307
[M−H]−
354.9544
0
20
40
60
80
100
350
400
450
500
550
600

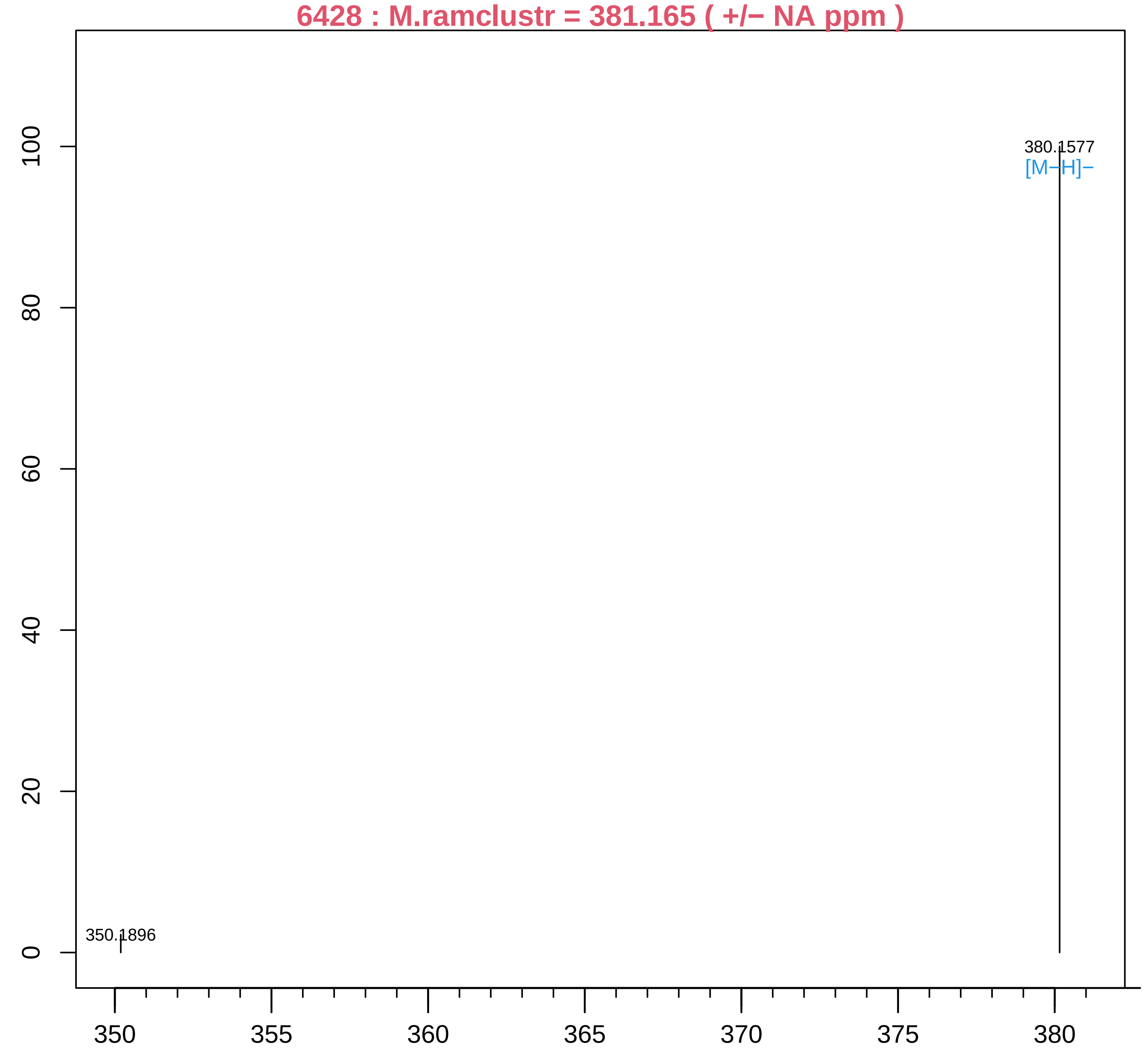
6428 : M.ramclustr = 381.165 (+/− NA ppm)
380.1577
[M−H]−
350.1896
0
20
40
60
80
100
350
355
360
365
370
375
380

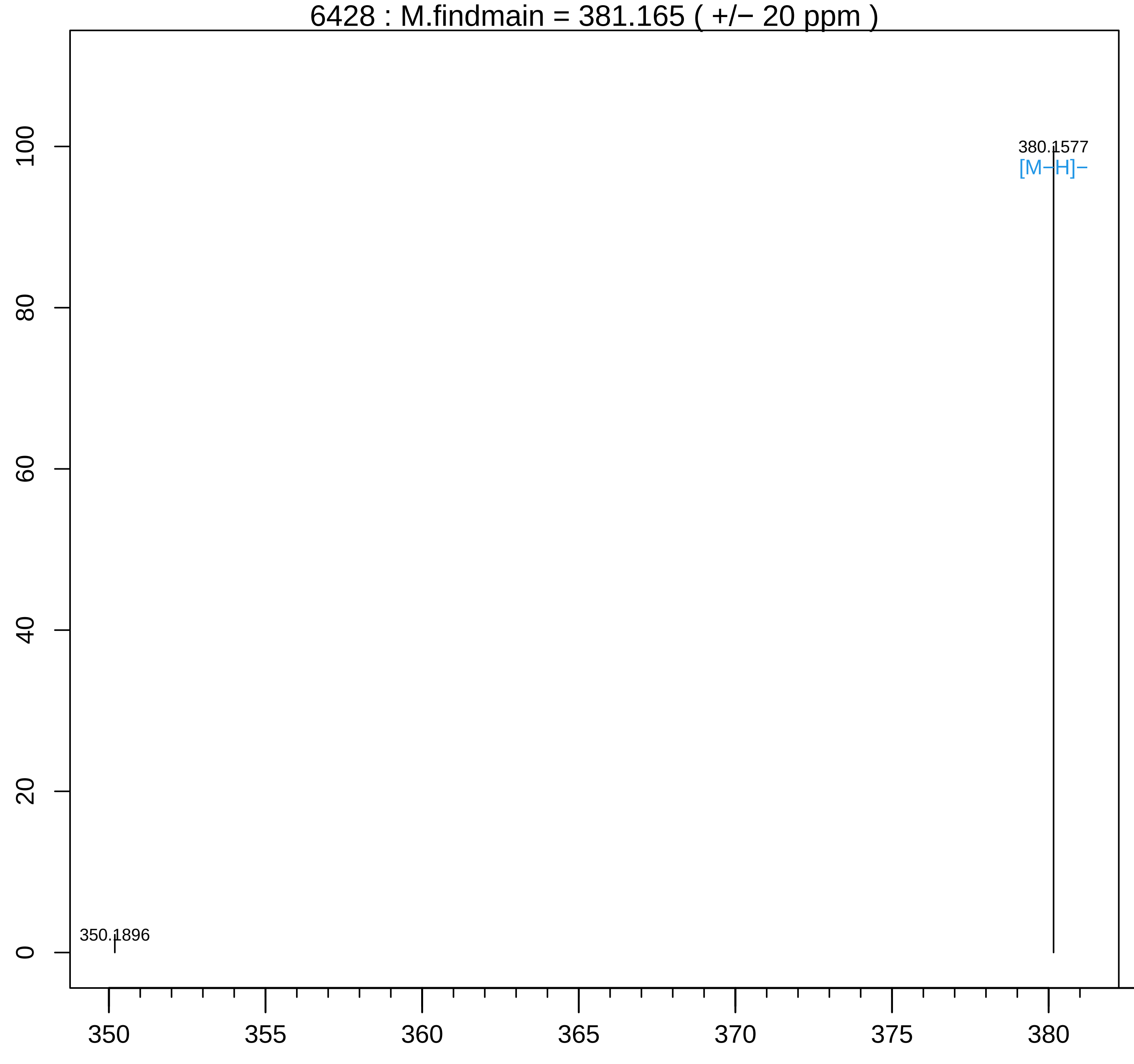
6428 : M.findmain = 381.165 (+/− 20 ppm)
380.1577
[M−H]−
350.1896
0
20
40
60
80
100
350
355
360
365
370
375
380

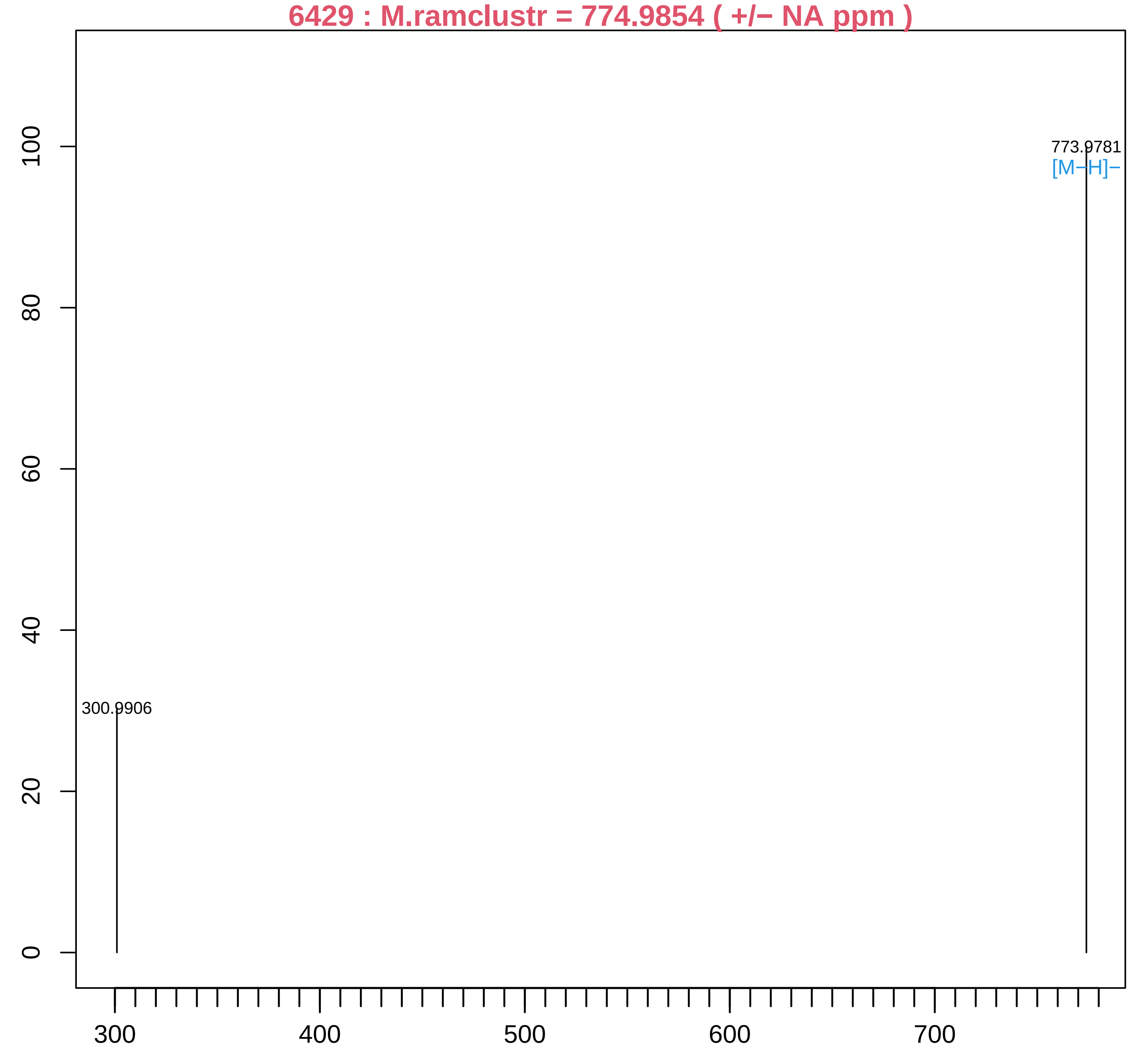
6429 : M.ramclustr = 774.9854 (+/− NA ppm)
773.9781
[M−H]−
300.9906
0
20
40
60
80
100
300
400
500
600
700
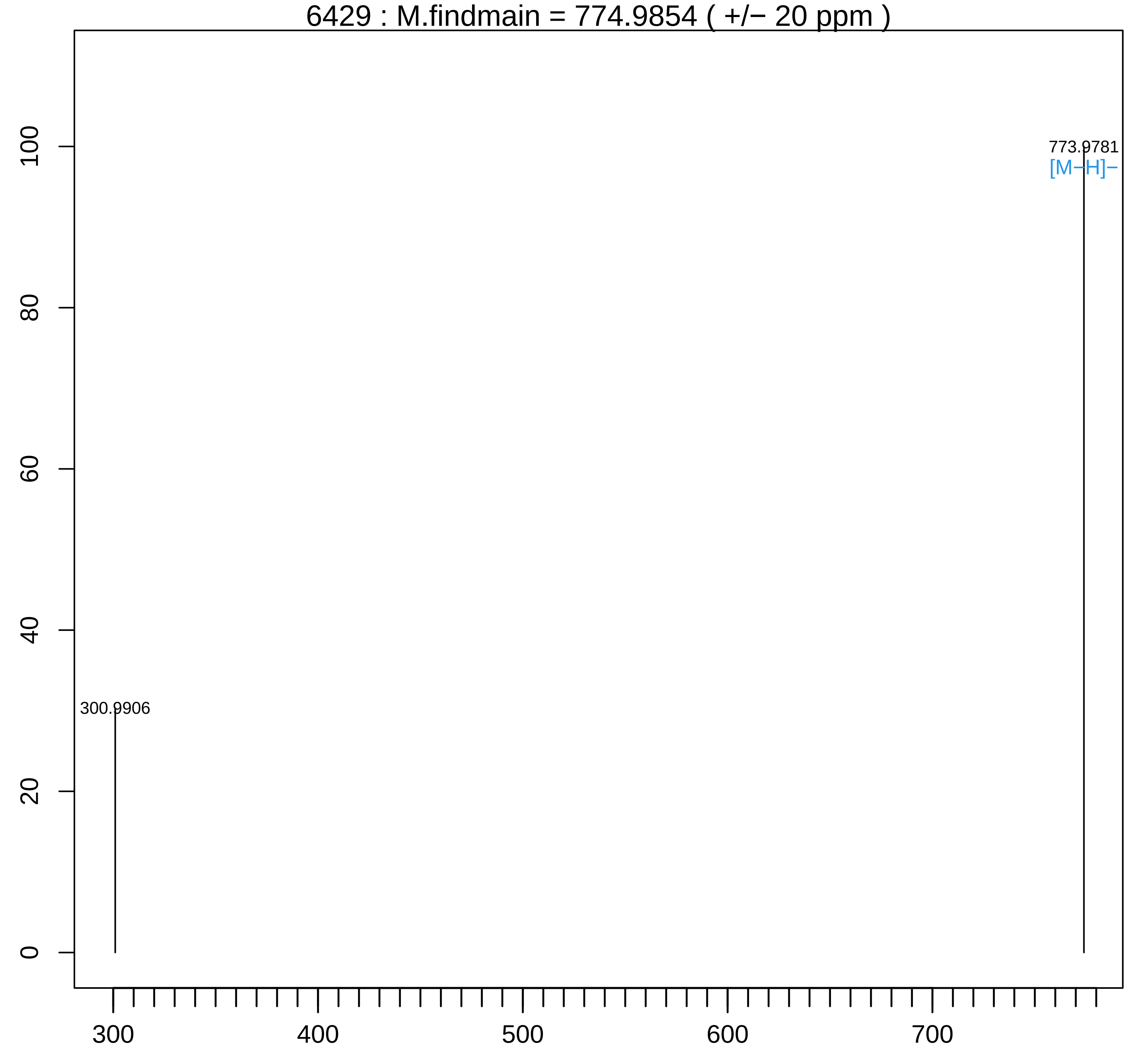
6429 : M.findmain = 774.9854 (+/− 20 ppm)
773.9781
[M−H]−
300.9906
0
20
40
60
80
100
300
400
500
600
700

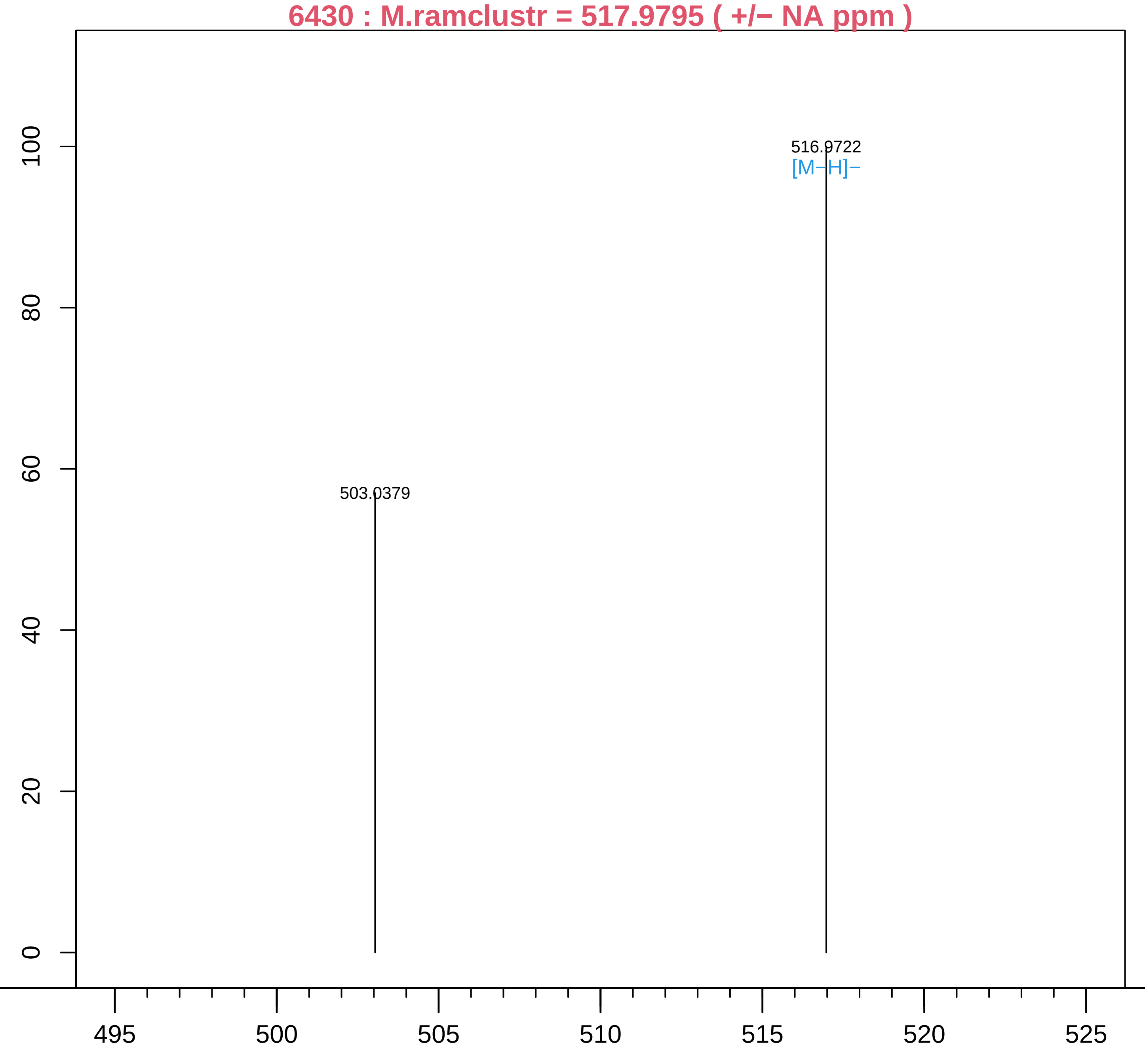
6430 : M.ramclustr = 517.9795 (+/− NA ppm)
516.9722
[M−H]−
503.0379
0
20
40
60
80
100
495
500
505
510
515
520
525

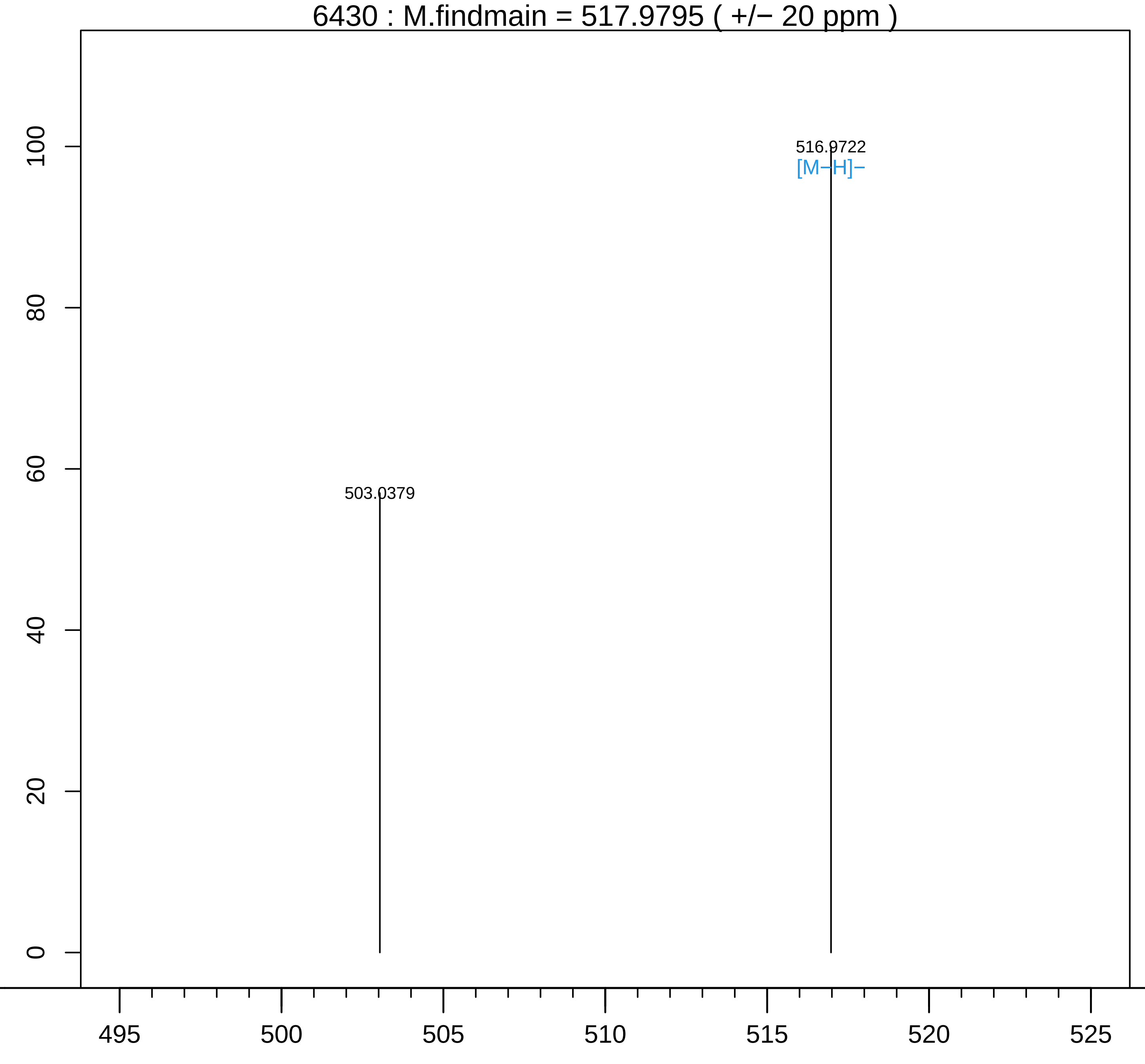
6430 : M.findmain = 517.9795 (+/− 20 ppm)
516.9722
[M−H]−
503.0379
0
20
40
60
80
100
495
500
505
510
515
520
525

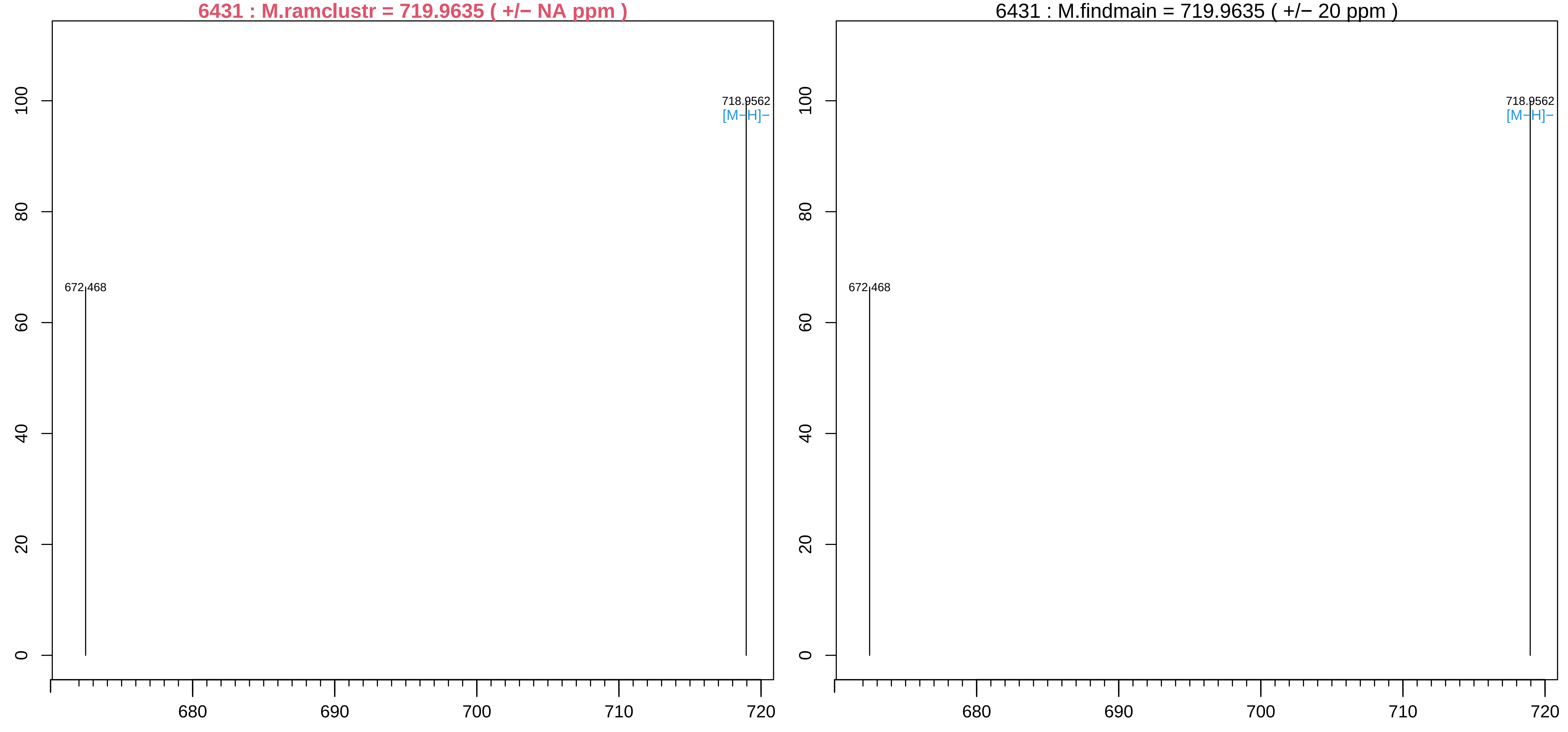
6431 : M.ramclustr = 719.9635 (+/− NA ppm)
718.9562
[M−H]−
672.468
0
20
40
60
80
100
680
690
700
710
720
6431 : M.findmain = 719.9635 (+/− 20 ppm)
718.9562
[M−H]−
672.468
0
20
40
60
80
100
680
690
700
710
720

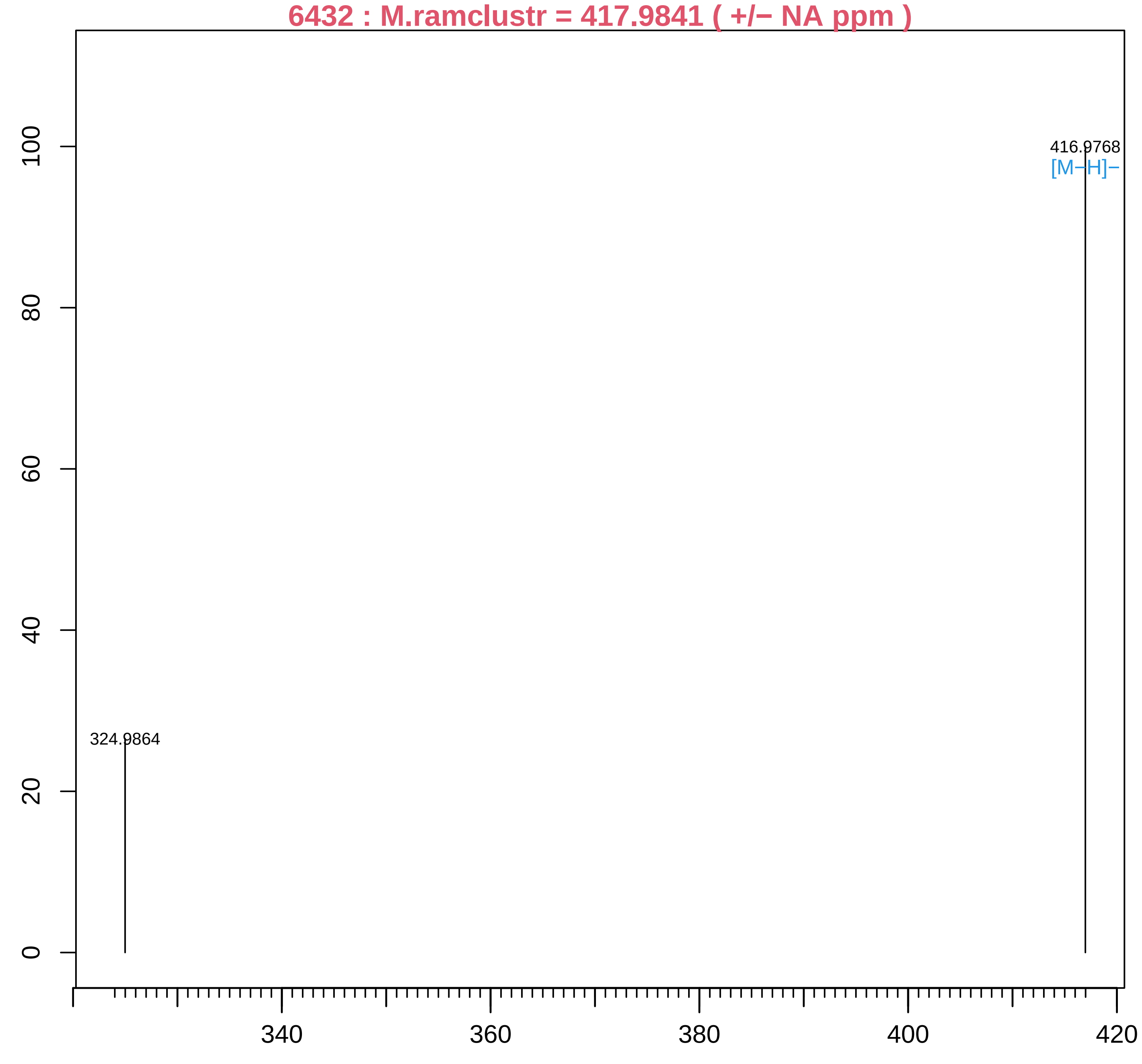
6432 : M.ramclustr = 417.9841 (+/− NA ppm)
416.9768
[M−H]−
324.9864
0
20
40
60
80
100
340
360
380
400
420

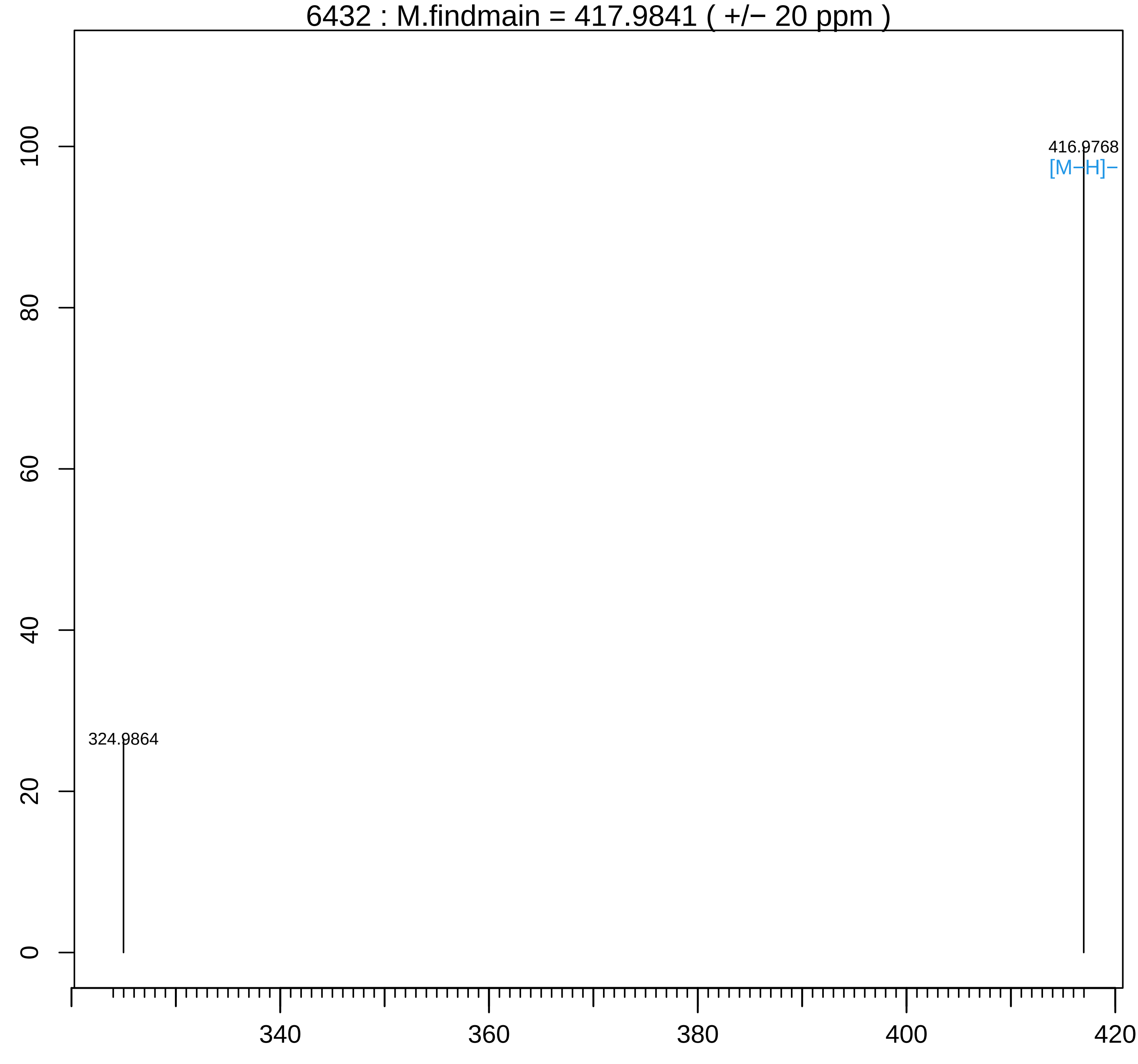
6432 : M.findmain = 417.9841 (+/− 20 ppm)
416.9768
[M−H]−
324.9864
0
20
40
60
80
100
340
360
380
400
420

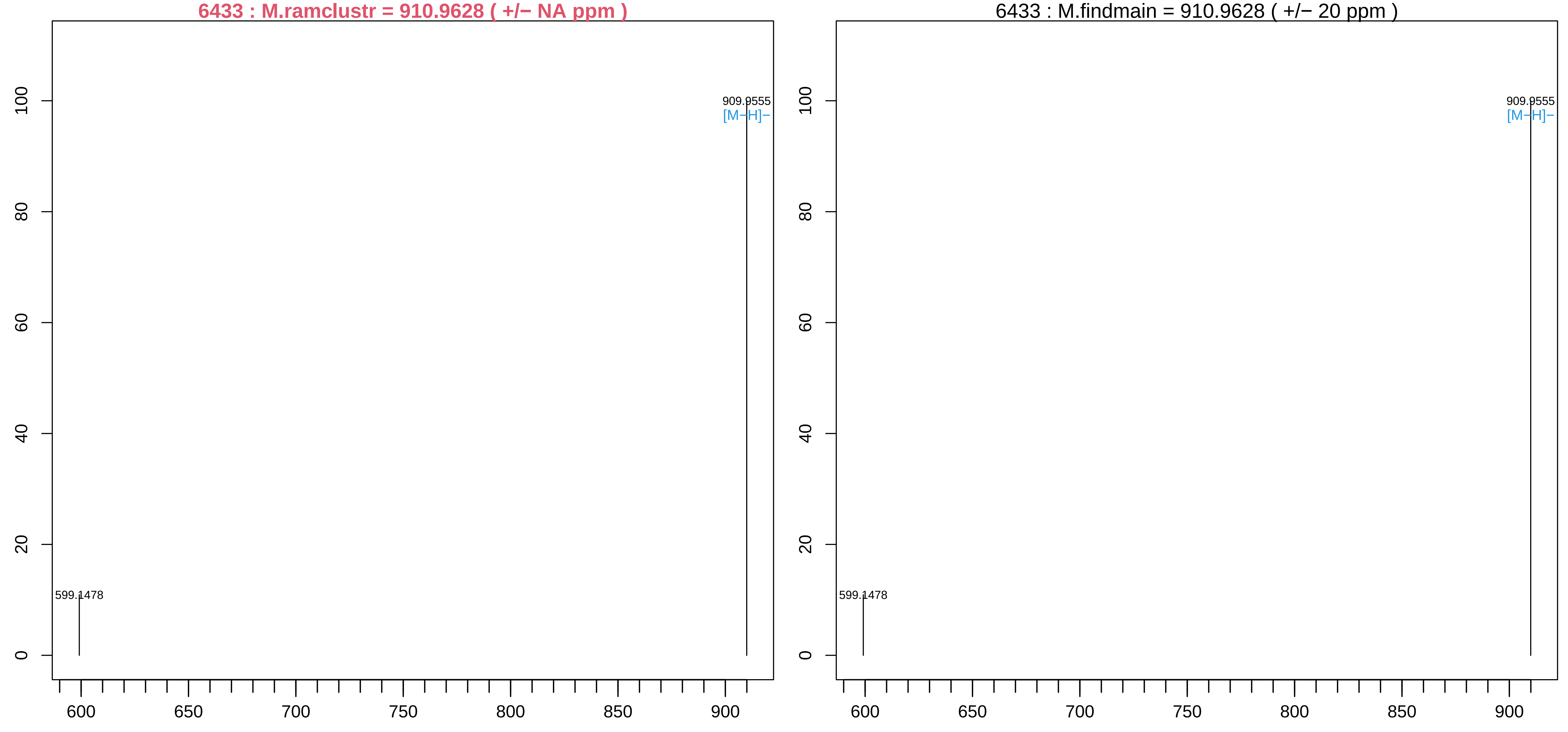
6433 : M.ramclustr = 910.9628 (+/− NA ppm)
909.9555
[M−H]−
599.1478
6433 : M.findmain = 910.9628 (+/− 20 ppm)
909.9555
[M−H]−
599.1478

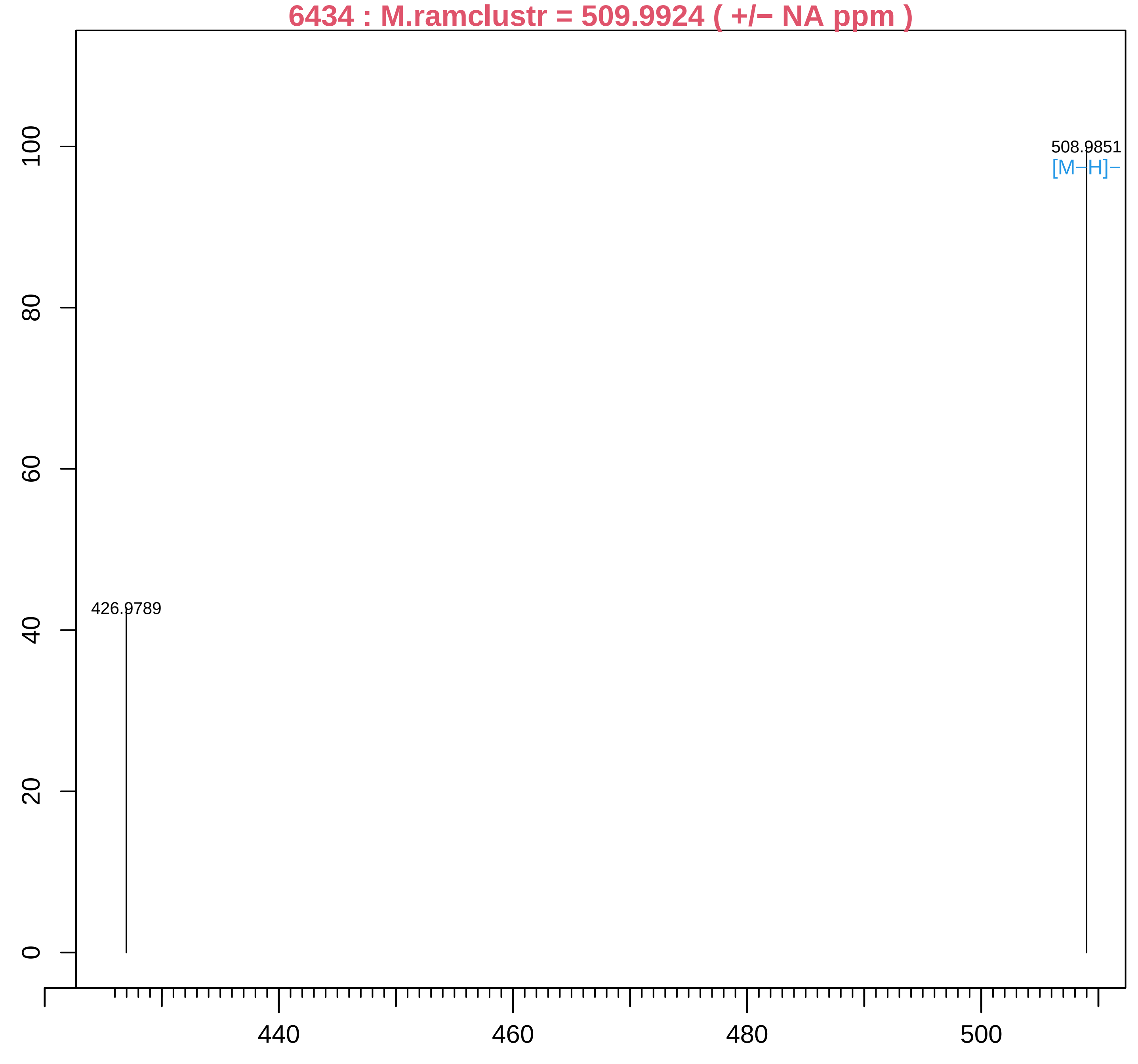

6434 : M.ramclustr = 509.9924 (+/− NA ppm)
508.9851
[M−H]−
426.9789
0
20
40
60
80
100
440
460
480
500

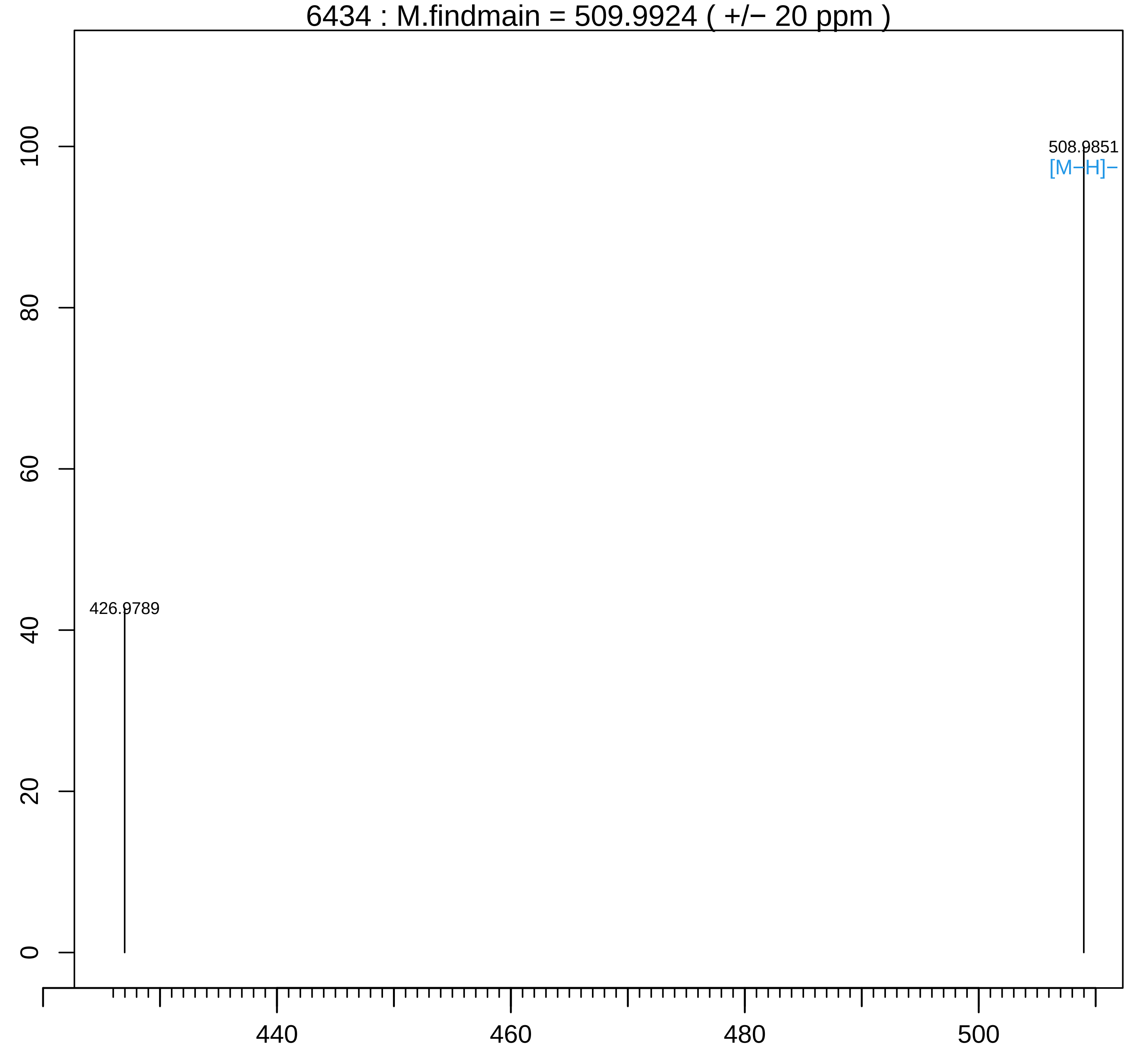

6434 : M.findmain = 509.9924 (+/− 20 ppm)
508.9851
[M−H]−
426.9789
0
20
40
60
80
100
440
460
480
500

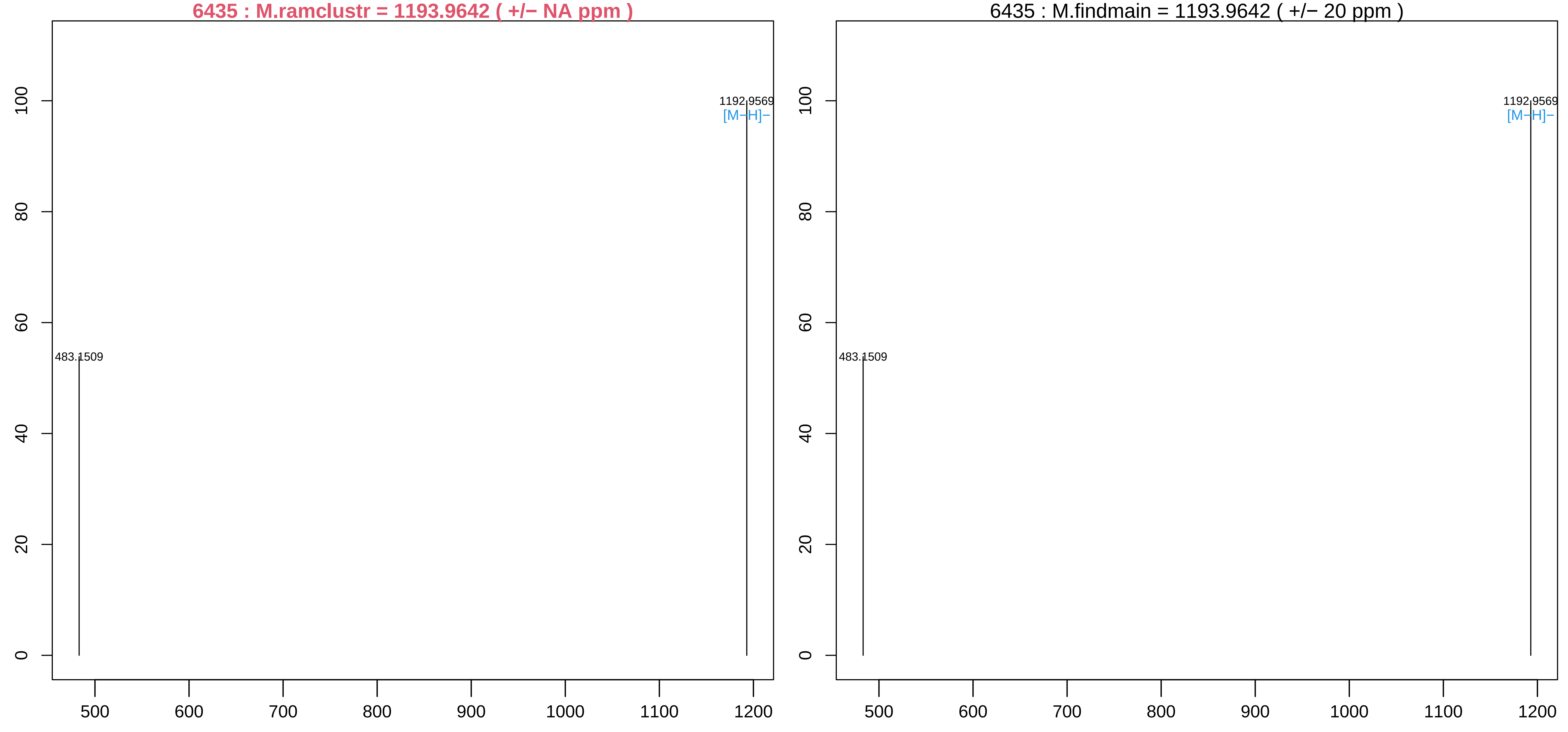
6435 : M.ramclustr = 1193.9642 (+/− NA ppm)
6435 : M.findmain = 1193.9642 (+/− 20 ppm)
1192.9569
[M−H]−
483.1509
0
20
40
60
80
100
500
600
700
800
900
1000
1100
1200

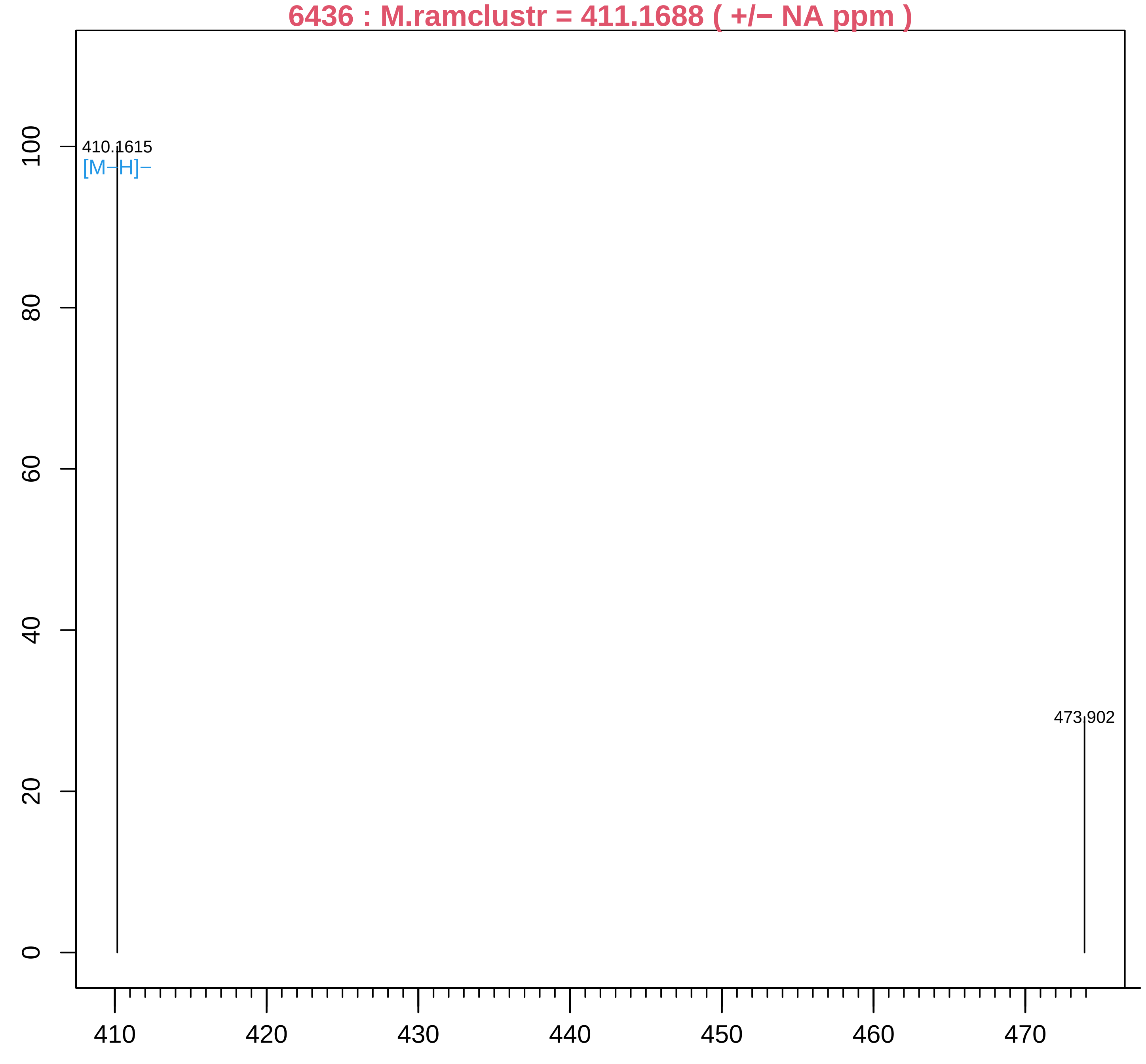
6436 : M.ramclustr = 411.1688 (+/− NA ppm)
410.1615
[M−H]−
473.902
0
20
40
60
80
100
410
420
430
440
450
460
470

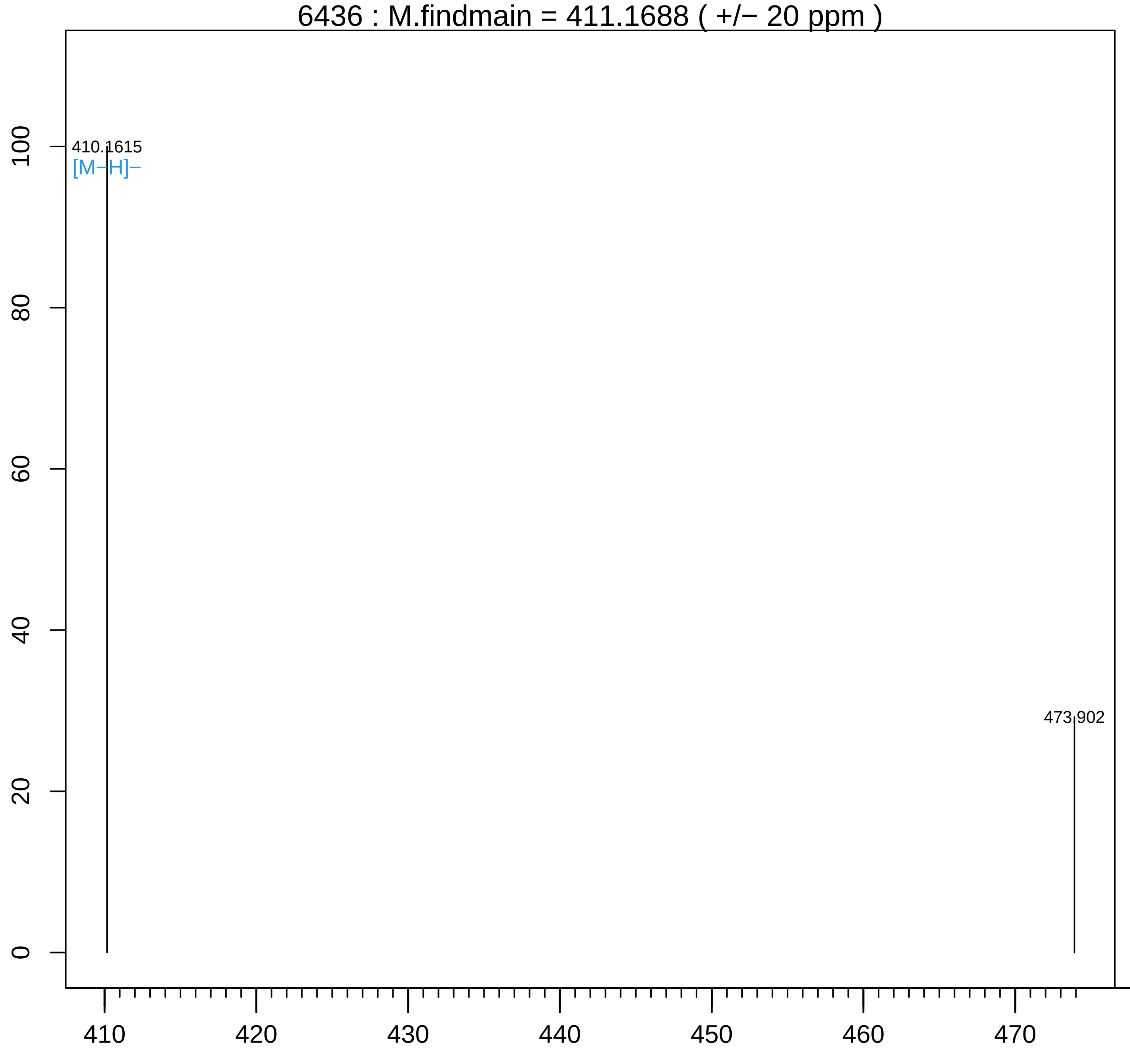
6436 : M.findmain = 411.1688 (+/− 20 ppm)
410.1615
[M−H]−
473.902
0
20
40
60
80
100
410
420
430
440
450
460
470

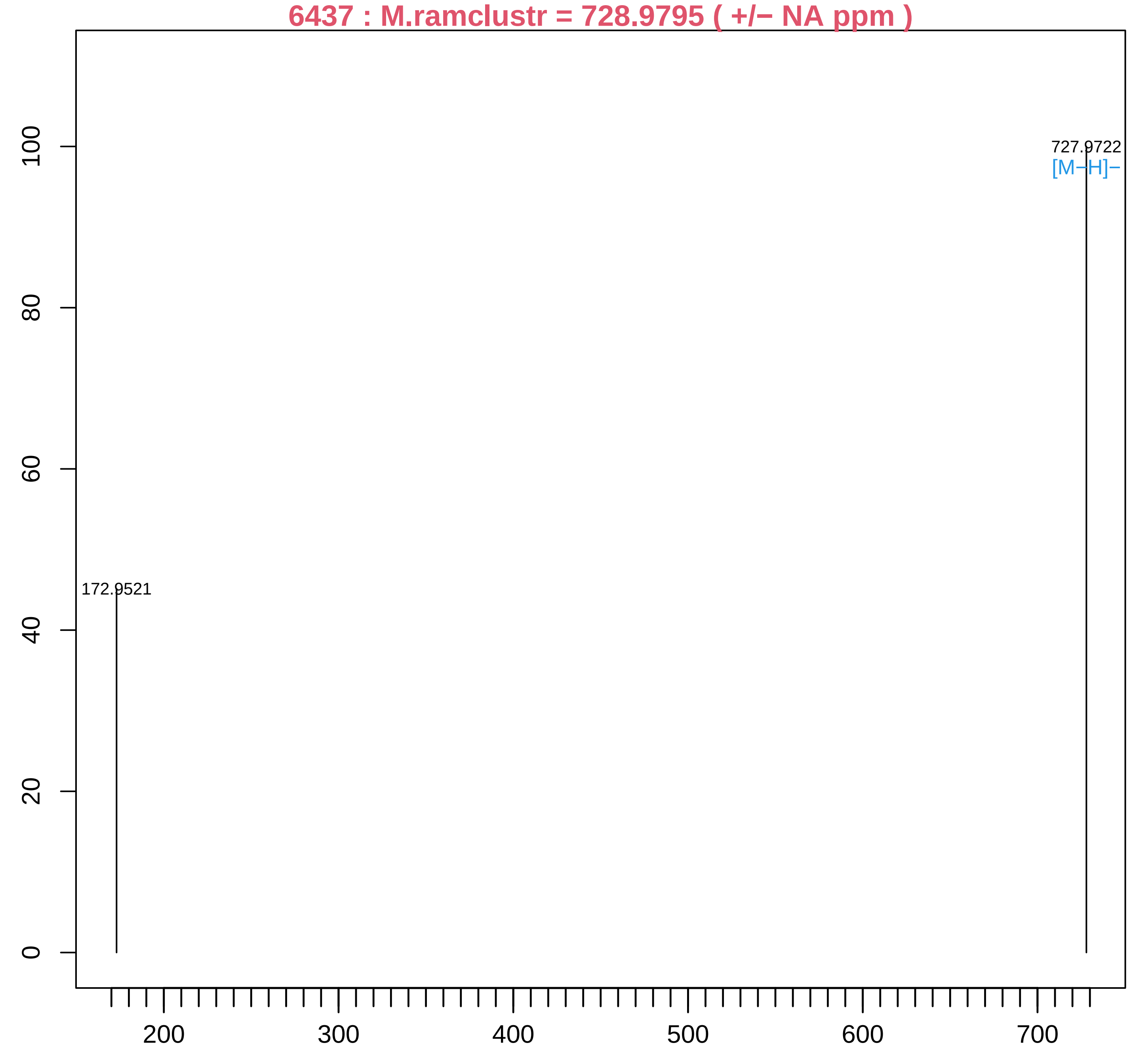
6437 : M.ramclustr = 728.9795 (+/− NA ppm)
727.9722
[M−H]−
172.9521
0
20
40
60
80
100
200
300
400
500
600
700

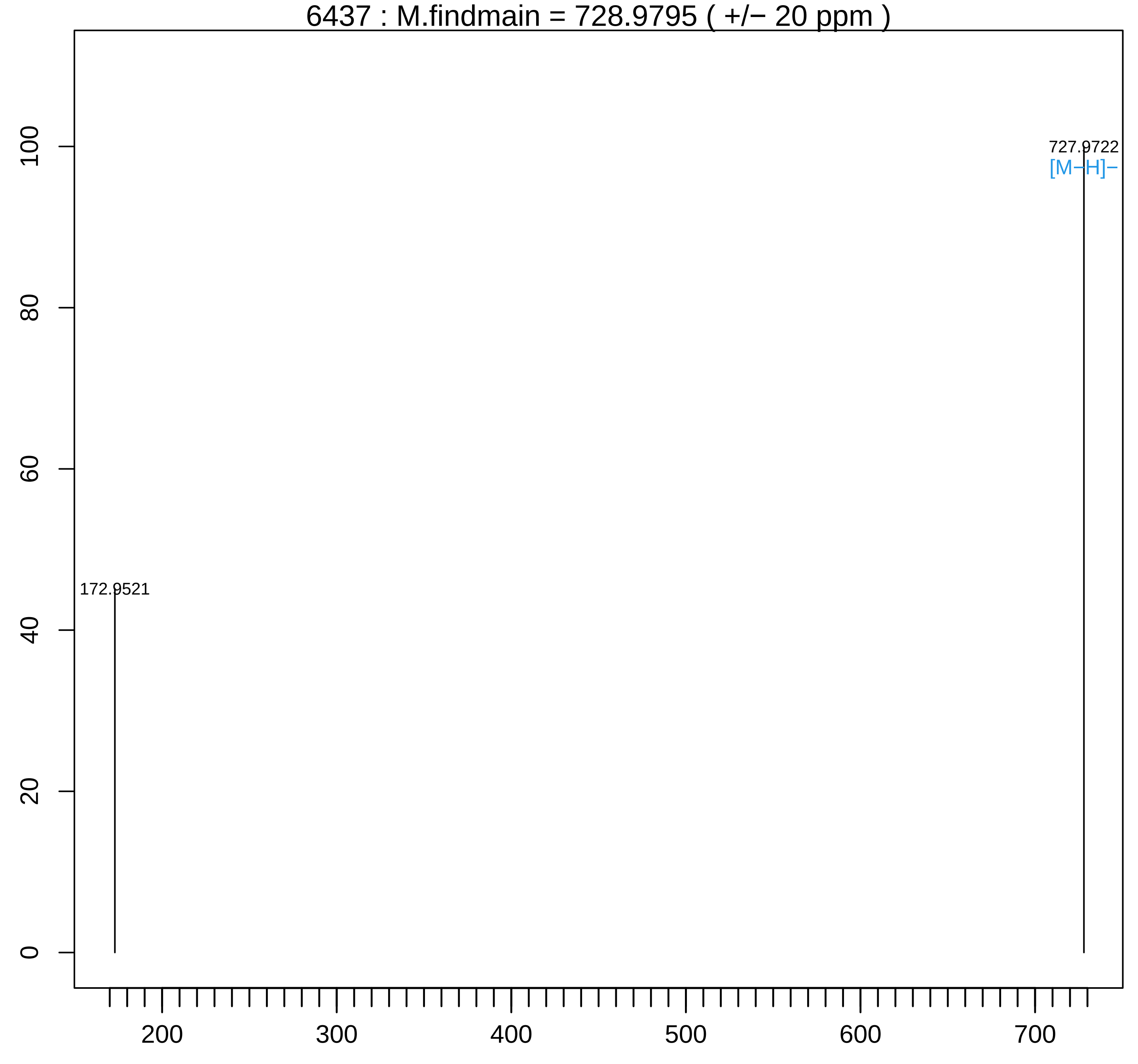
6437 : M.findmain = 728.9795 (+/− 20 ppm)
727.9722
[M−H]−
172.9521
0
20
40
60
80
100
200
300
400
500
600
700

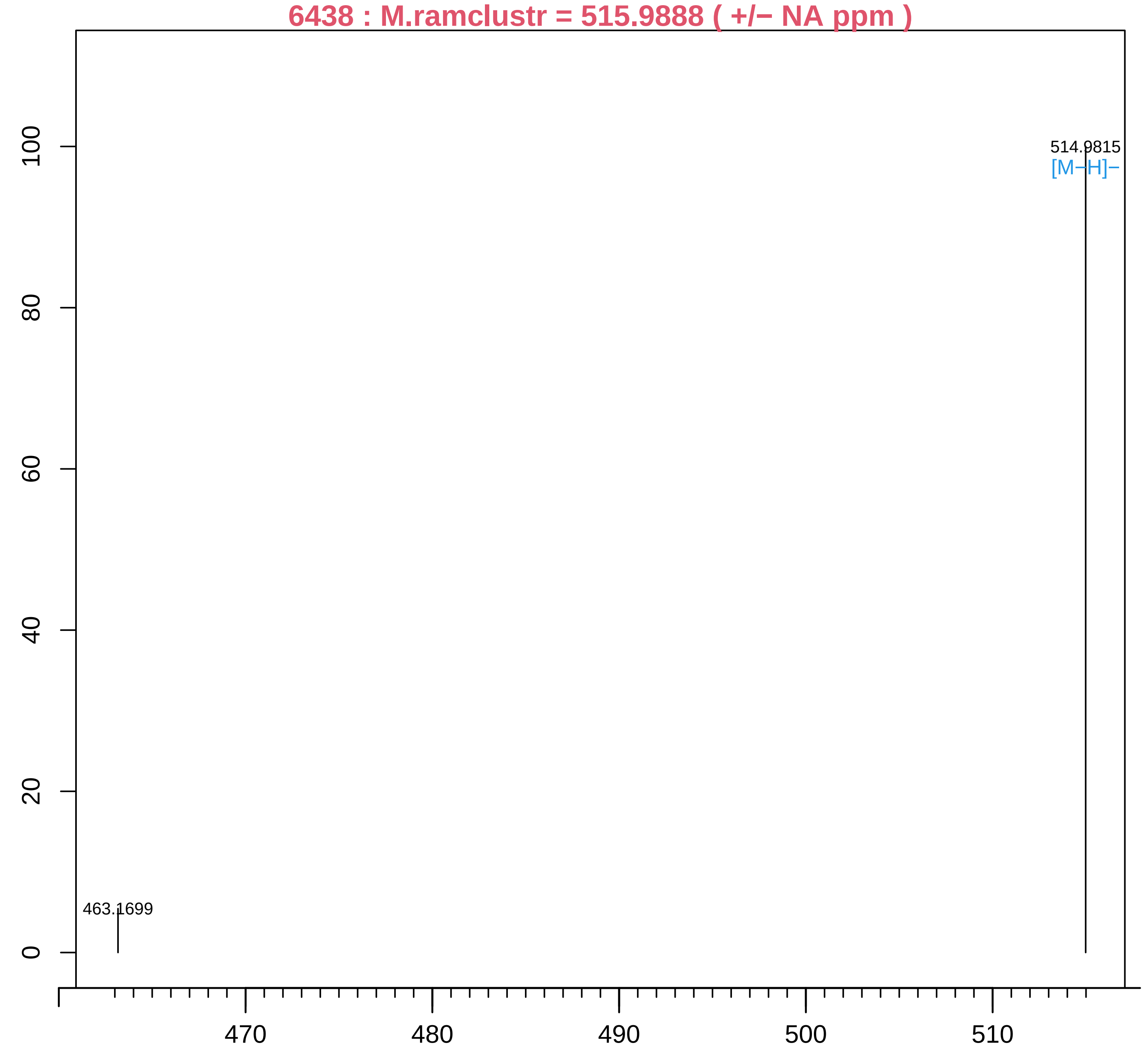
6438 : M.ramclustr = 515.9888 (+/− NA ppm)
514.9815
[M−H]−
463.1699
0
20
40
60
80
100
470
480
490
500
510

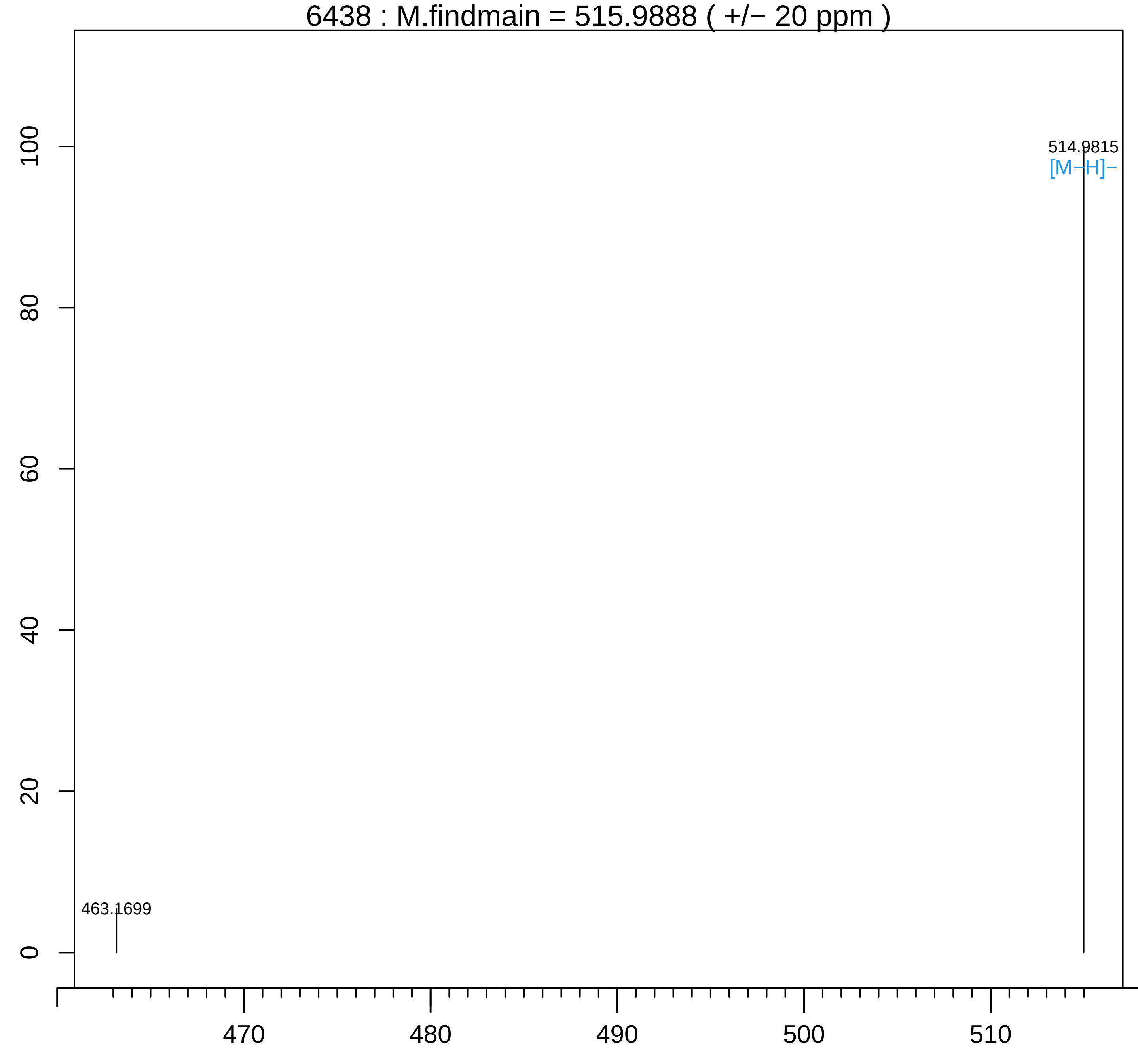
6438 : M.findmain = 515.9888 (+/− 20 ppm)
514.9815
[M−H]−
463.1699
0
20
40
60
80
100
470
480
490
500
510

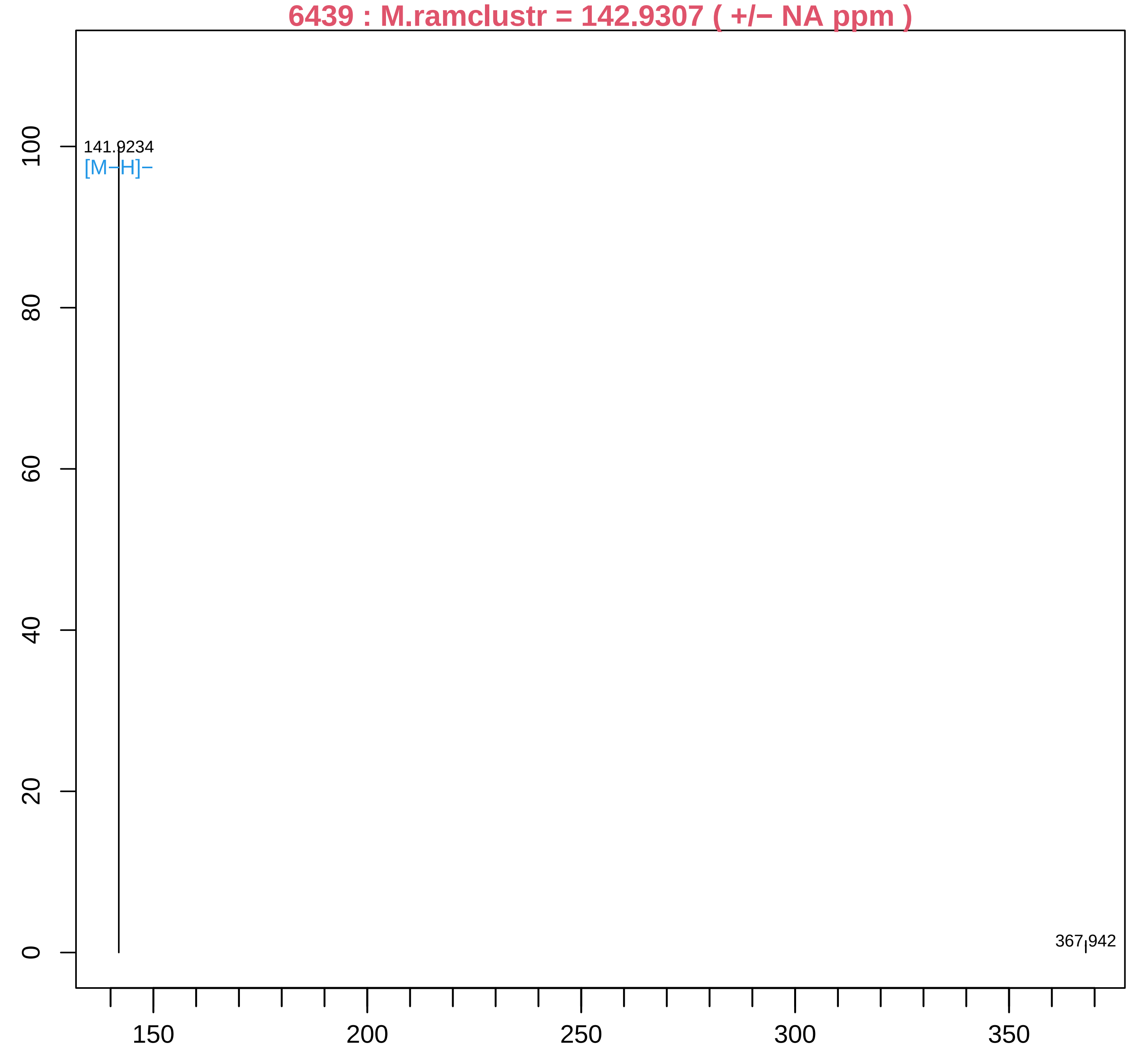
6439 : M.ramclustr = 142.9307 (+/− NA ppm)
141.9234
[M−H]−
367.942
0
20
40
60
80
100
150
200
250
300
350

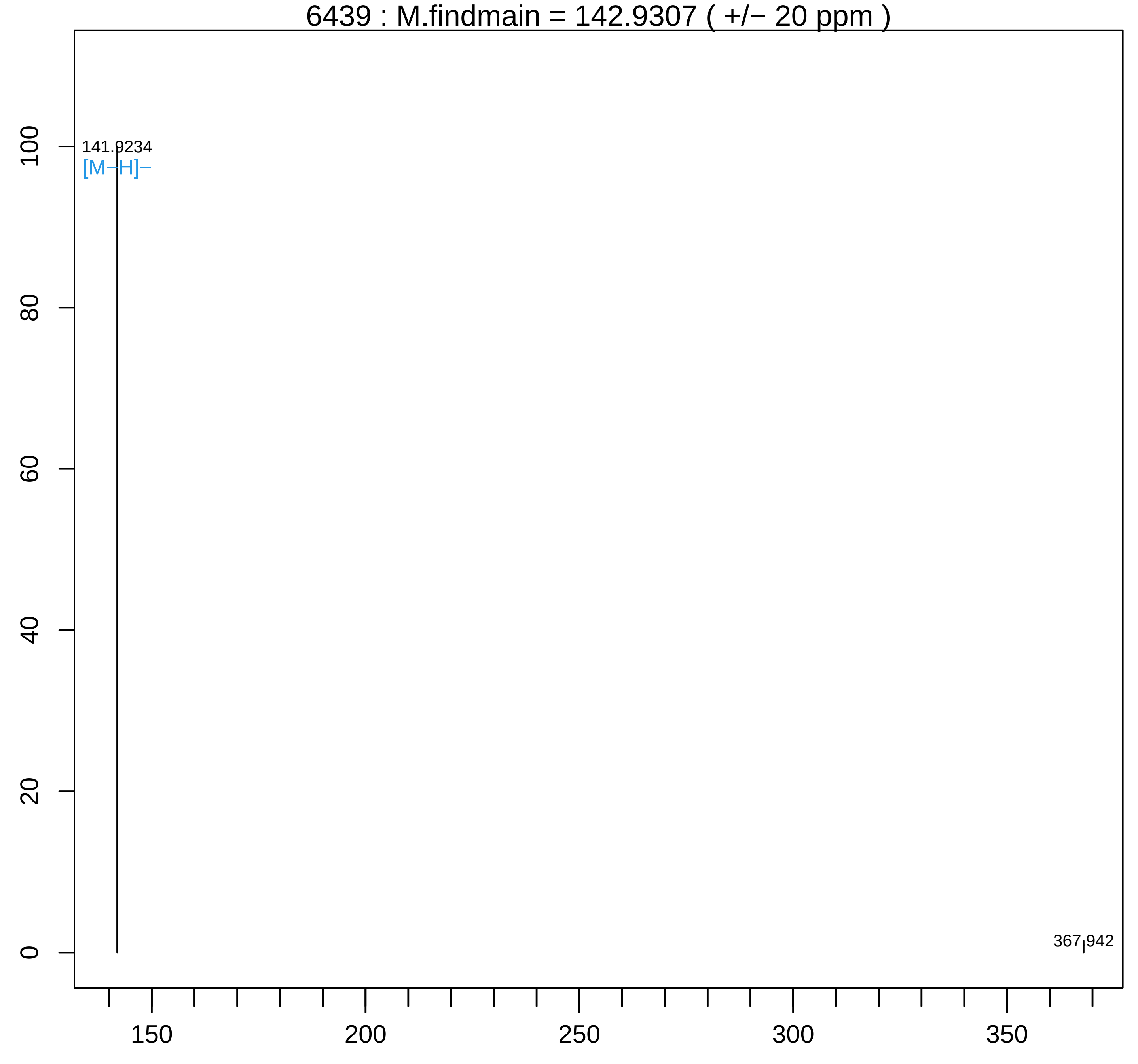
6439 : M.findmain = 142.9307 (+/− 20 ppm)
141.9234
[M−H]−
367.942
0
20
40
60
80
100
150
200
250
300
350

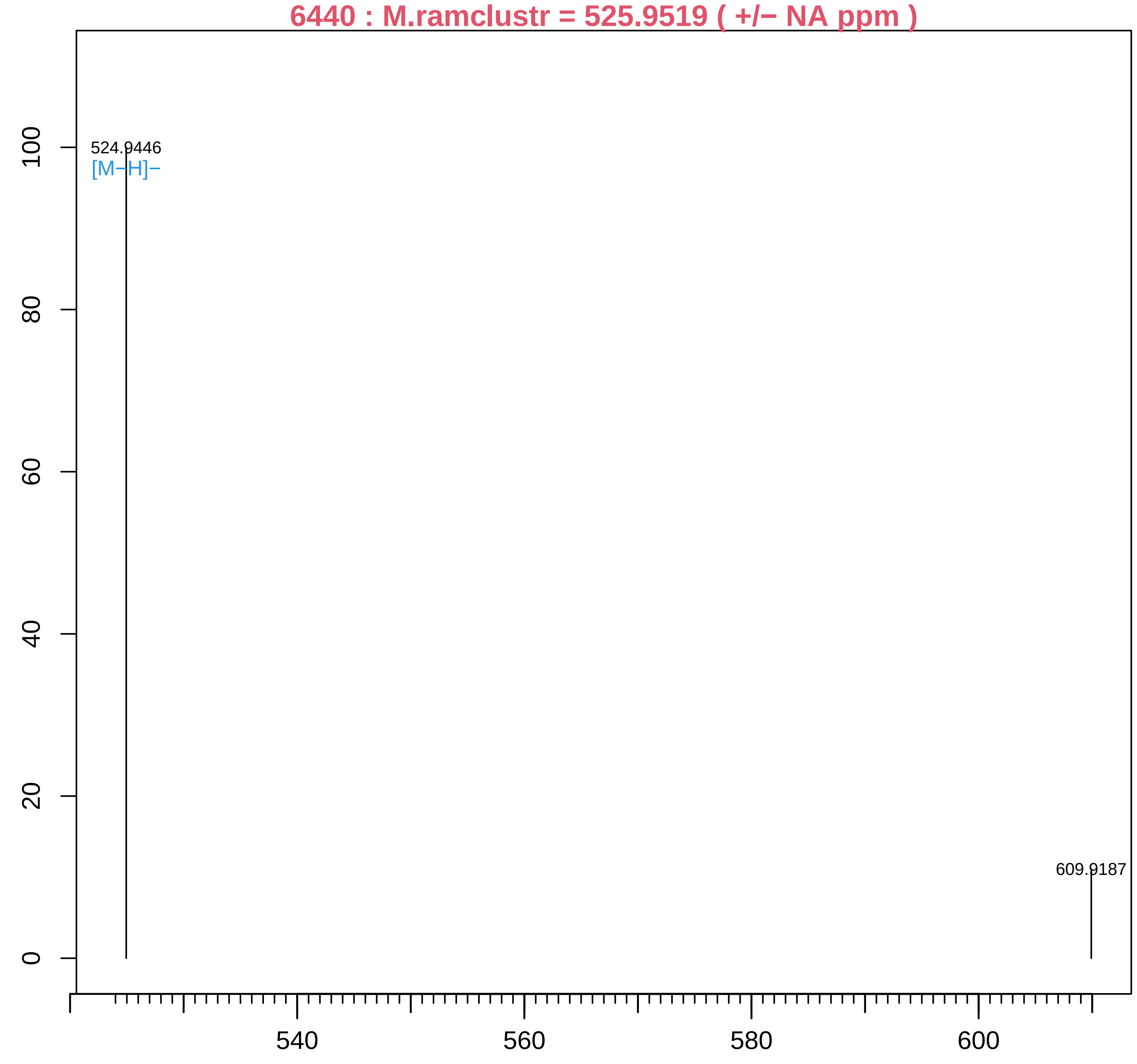

6440 : M.ramclustr = 525.9519 (+/− NA ppm)
524.9446
[M−H]−
609.9187
0
20
40
60
80
100
540
560
580
600

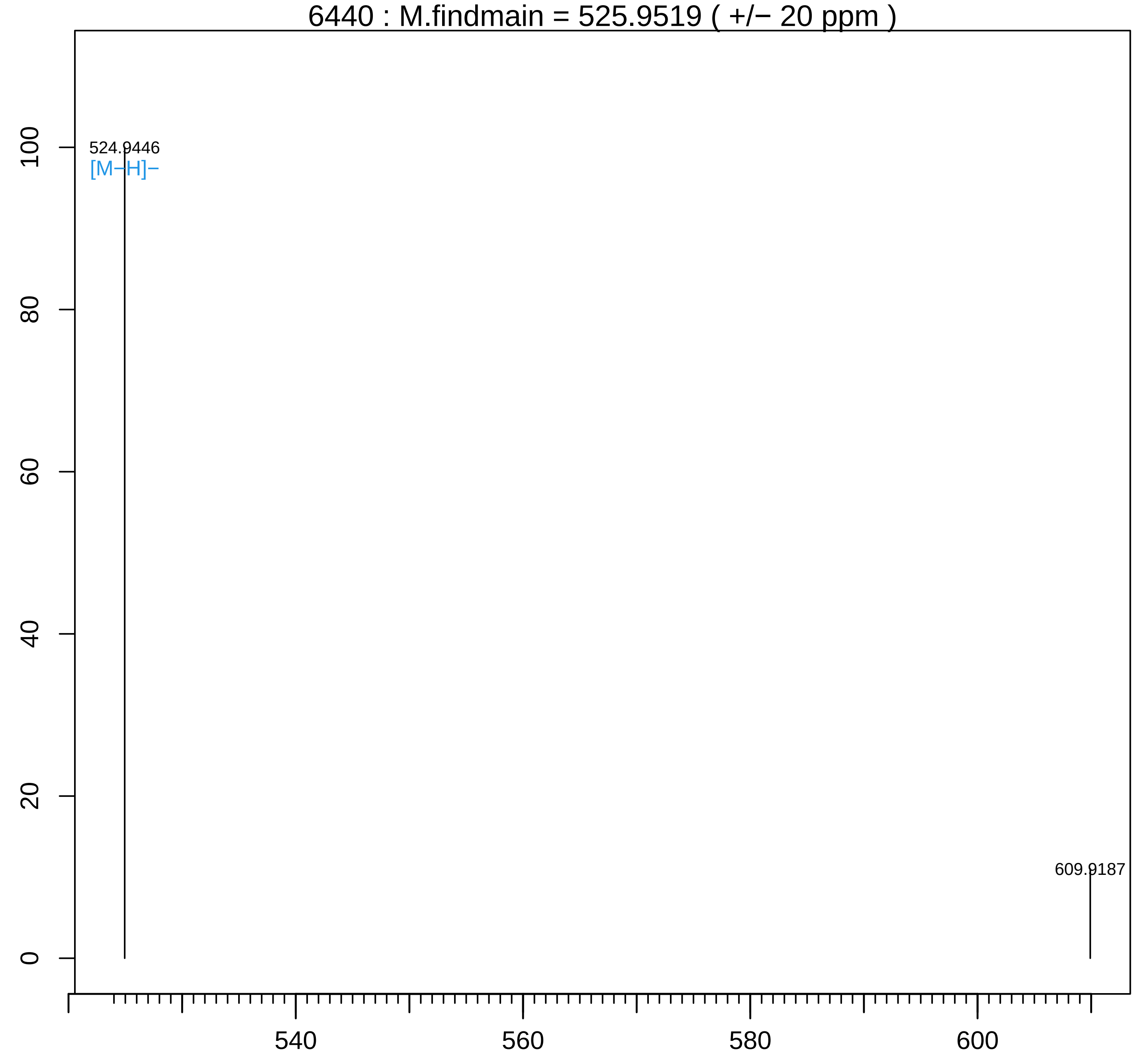

6440 : M.findmain = 525.9519 (+/− 20 ppm)
524.9446
[M−H]−
609.9187
0
20
40
60
80
100
540
560
580
600

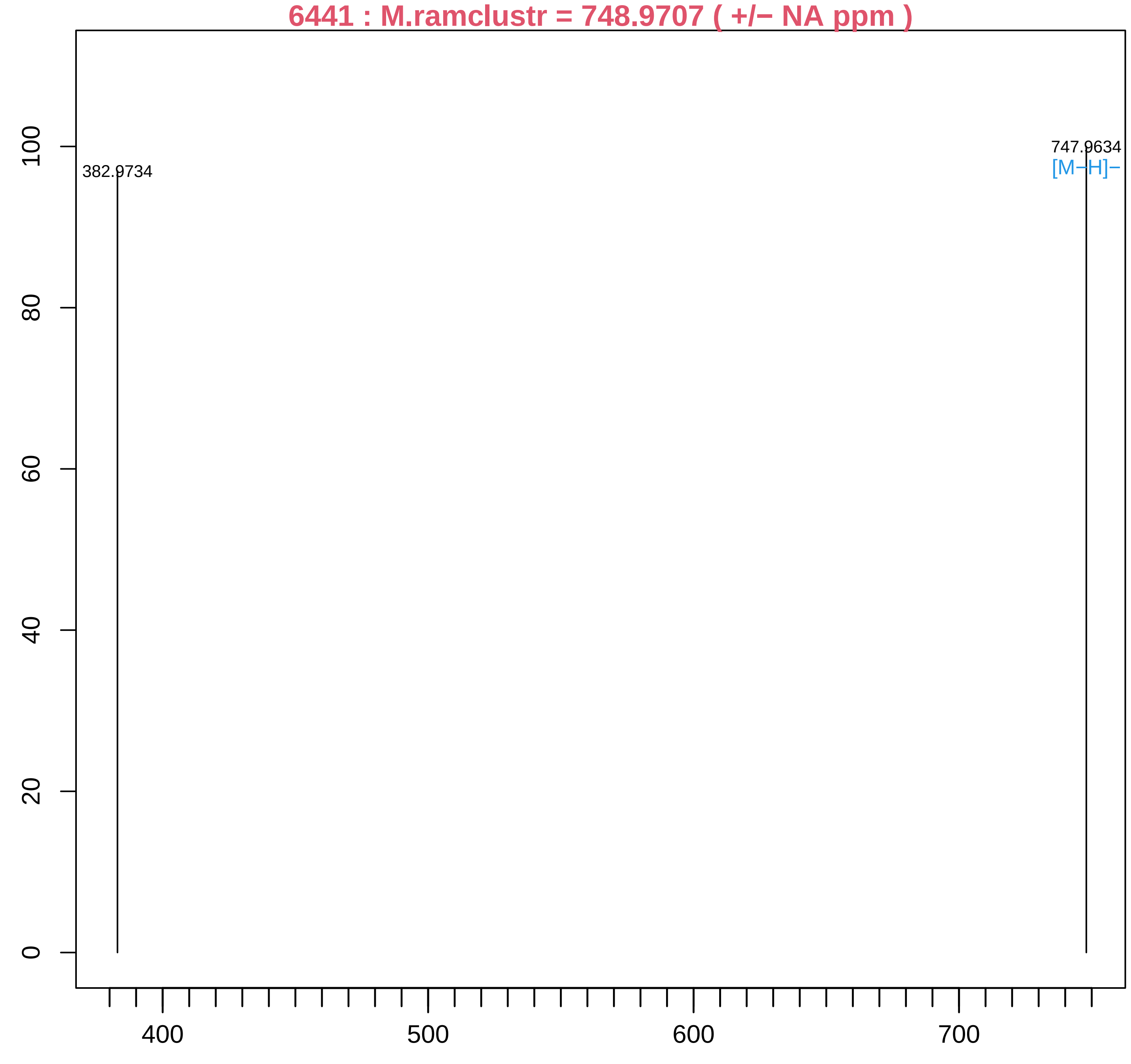
6441 : M.ramclustr = 748.9707 (+/− NA ppm)
382.9734
747.9634
[M−H]−
0
20
40
60
80
100
400
500
600
700

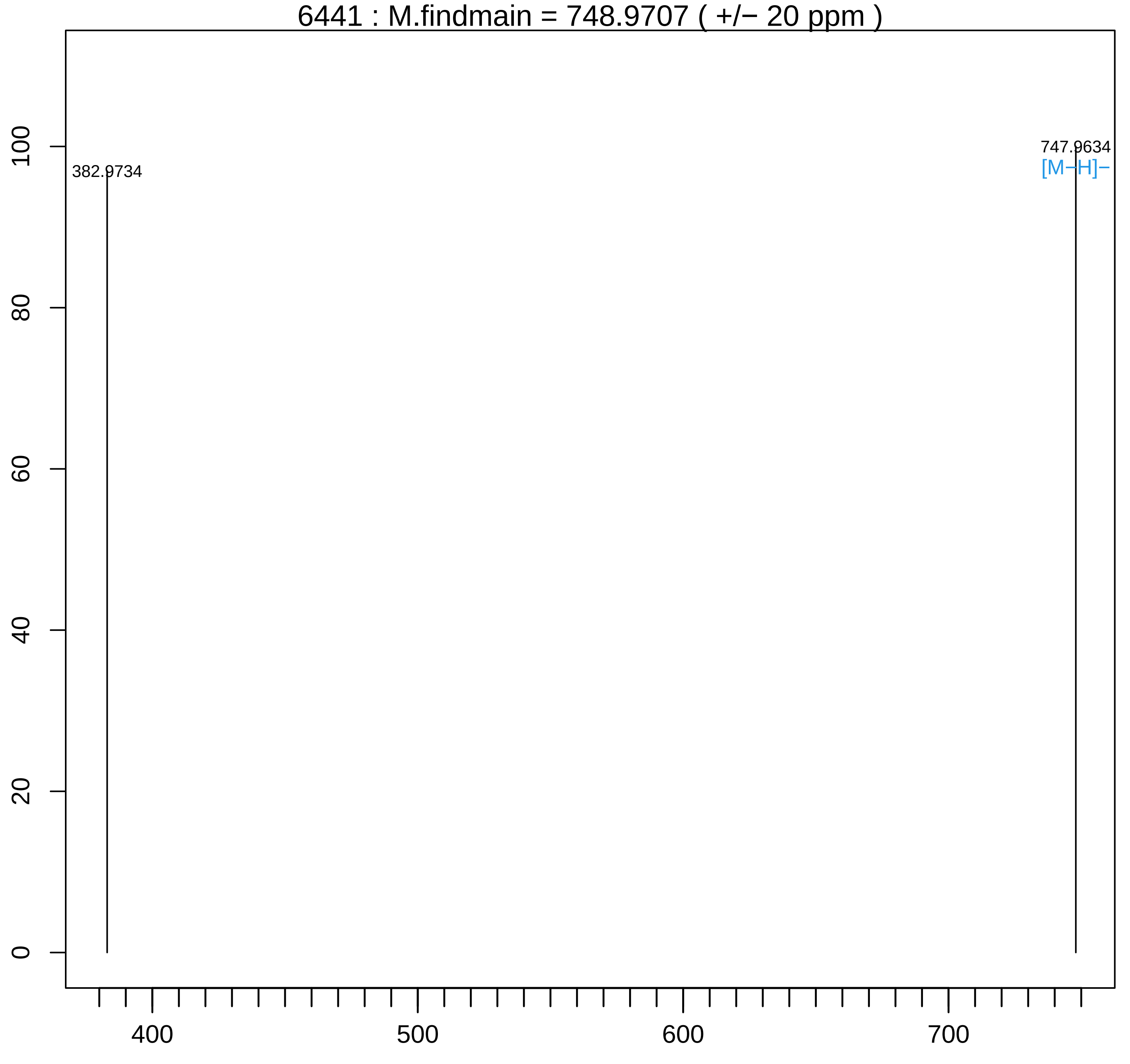
6441 : M.findmain = 748.9707 (+/− 20 ppm)
382.9734
747.9634
[M−H]−
0
20
40
60
80
100
400
500
600
700

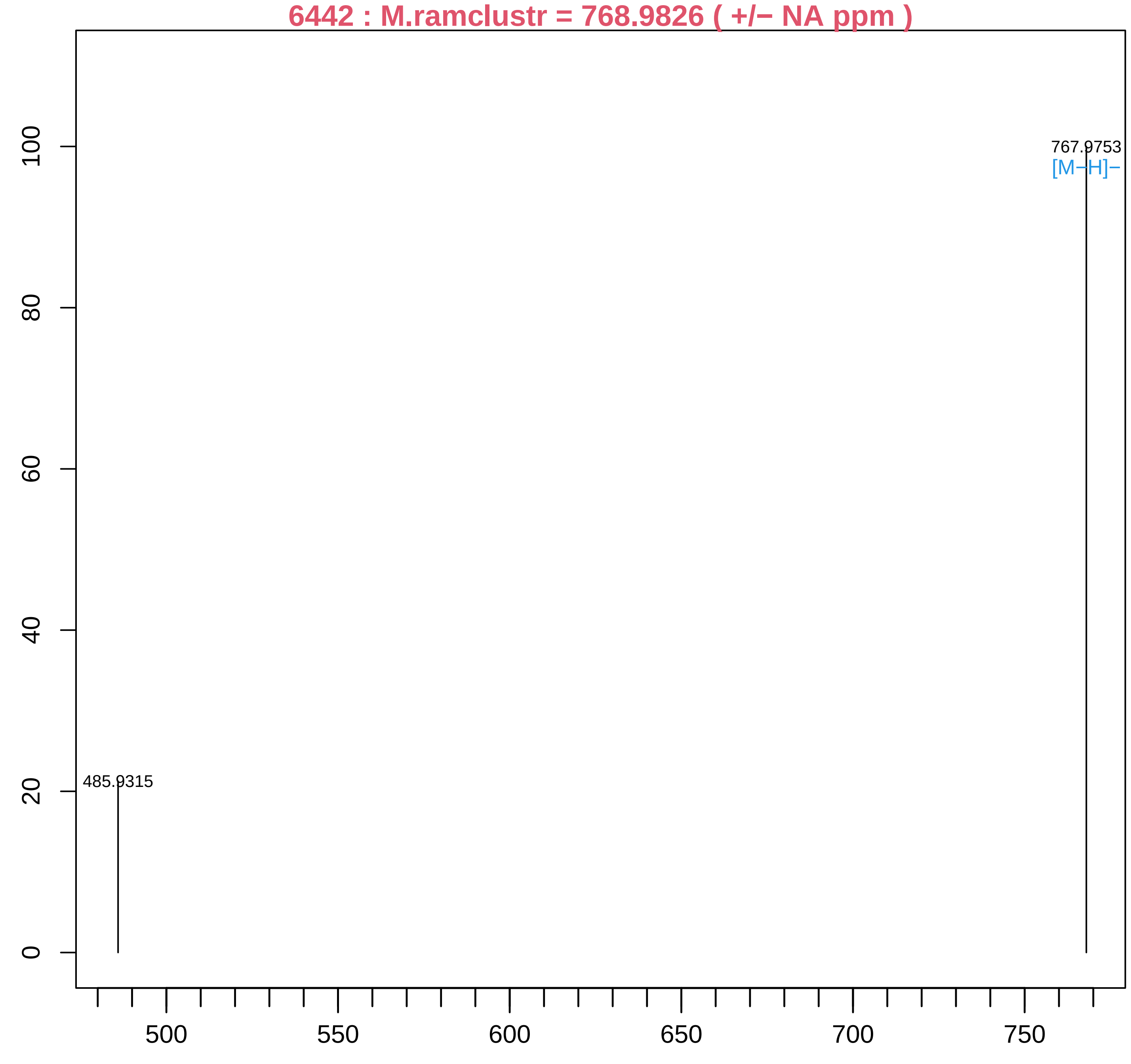
6442 : M.ramclustr = 768.9826 (+/− NA ppm)
767.9753
[M−H]−
485.9315
0
20
40
60
80
100
500
550
600
650
700
750

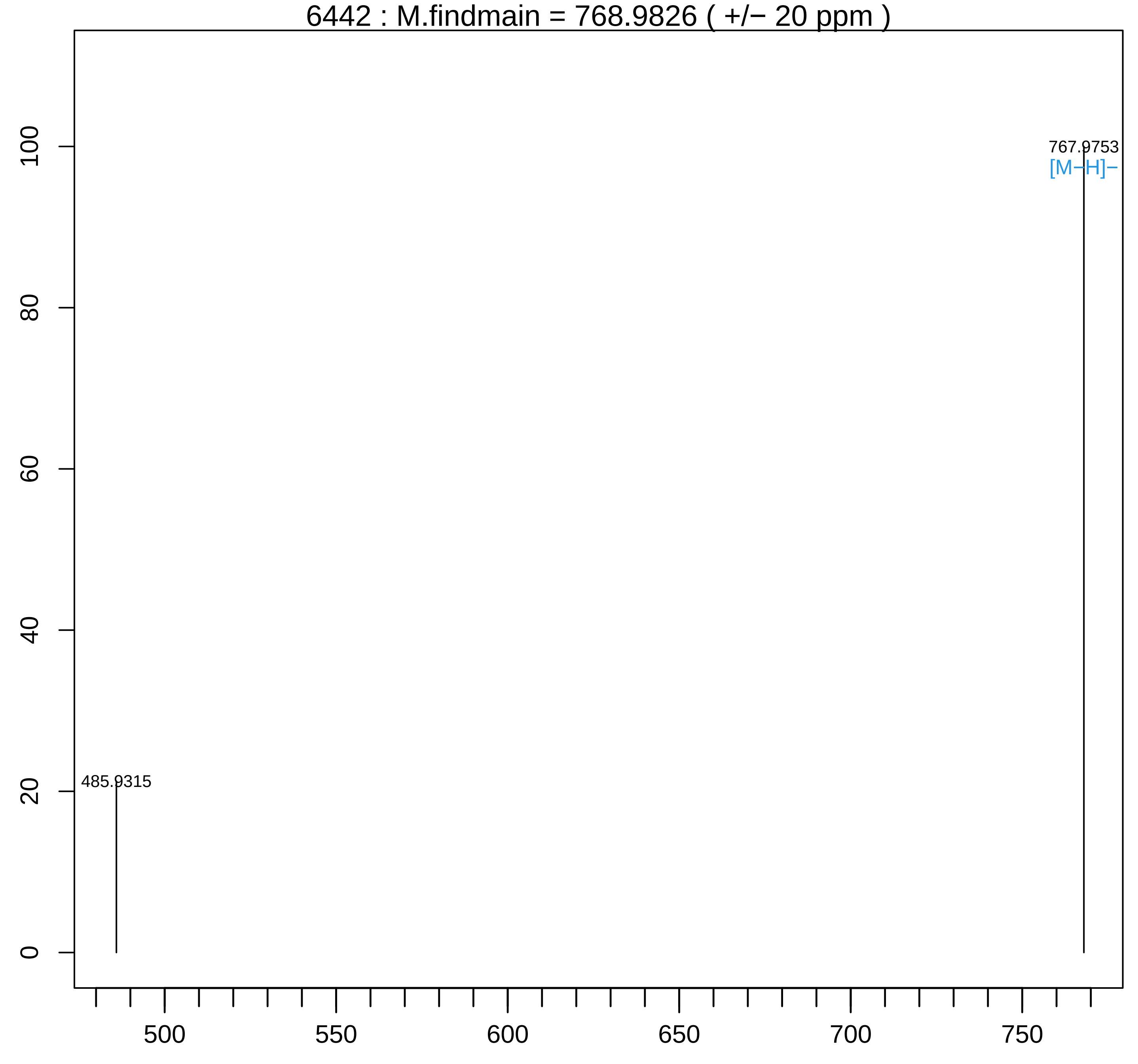
6442 : M.findmain = 768.9826 (+/− 20 ppm)
767.9753
[M−H]−
485.9315
0
20
40
60
80
100
500
550
600
650
700
750

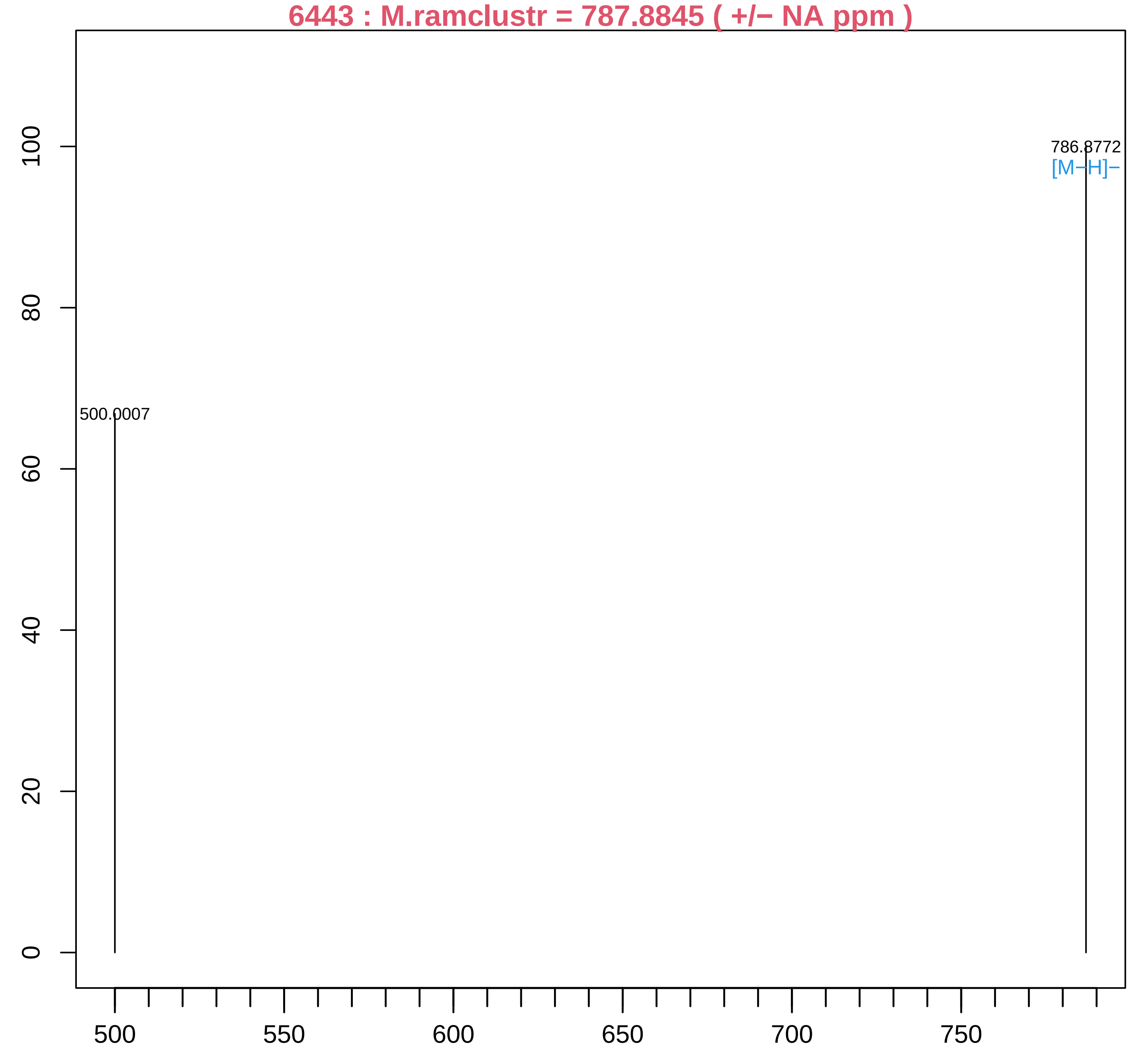
6443 : M.ramclustr = 787.8845 (+/− NA ppm)
786.8772
[M−H]−
500.0007
0
20
40
60
80
100
500
550
600
650
700
750

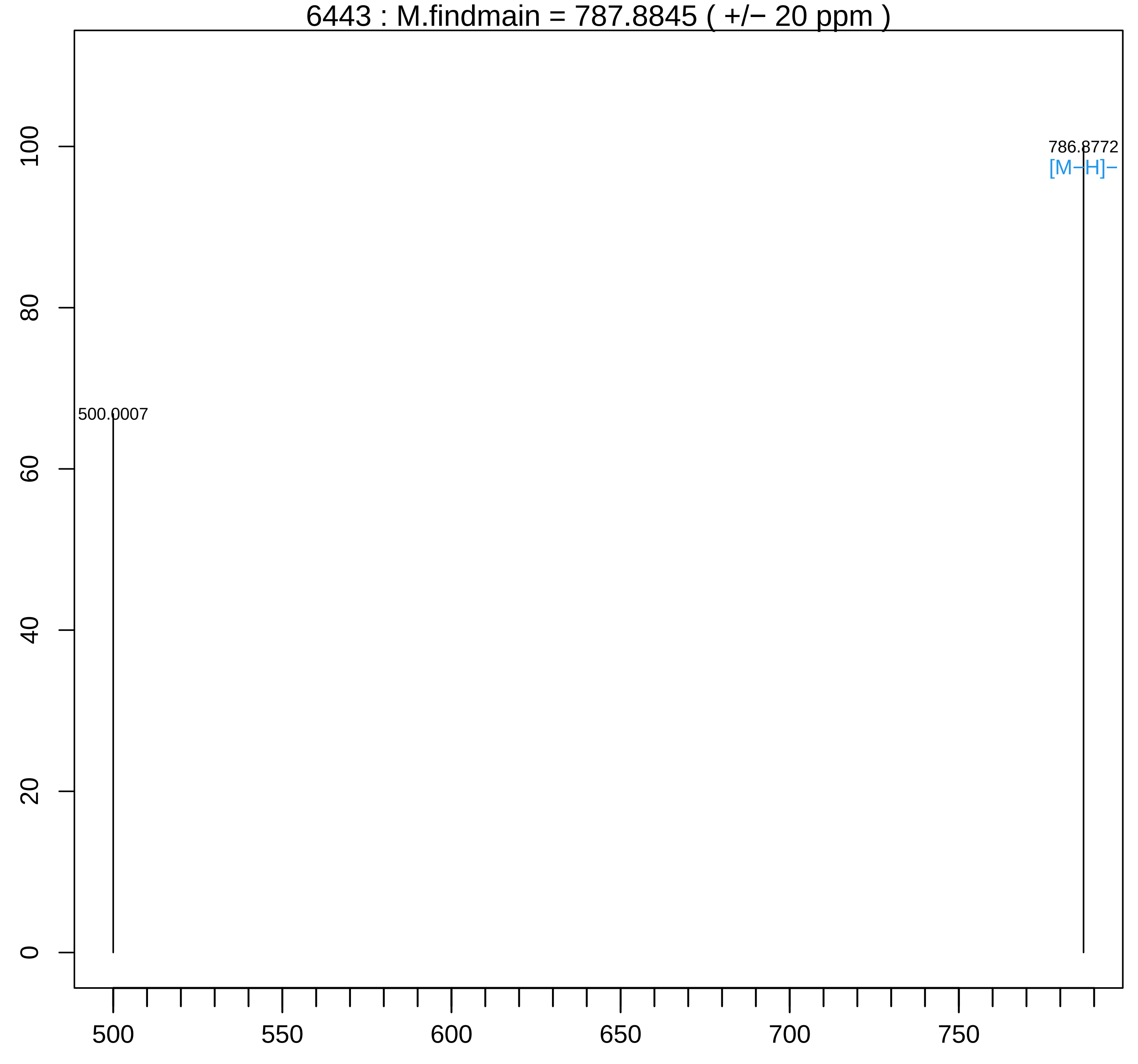
6443 : M.findmain = 787.8845 (+/− 20 ppm)
786.8772
[M−H]−
500.0007
0
20
40
60
80
100
500
550
600
650
700
750

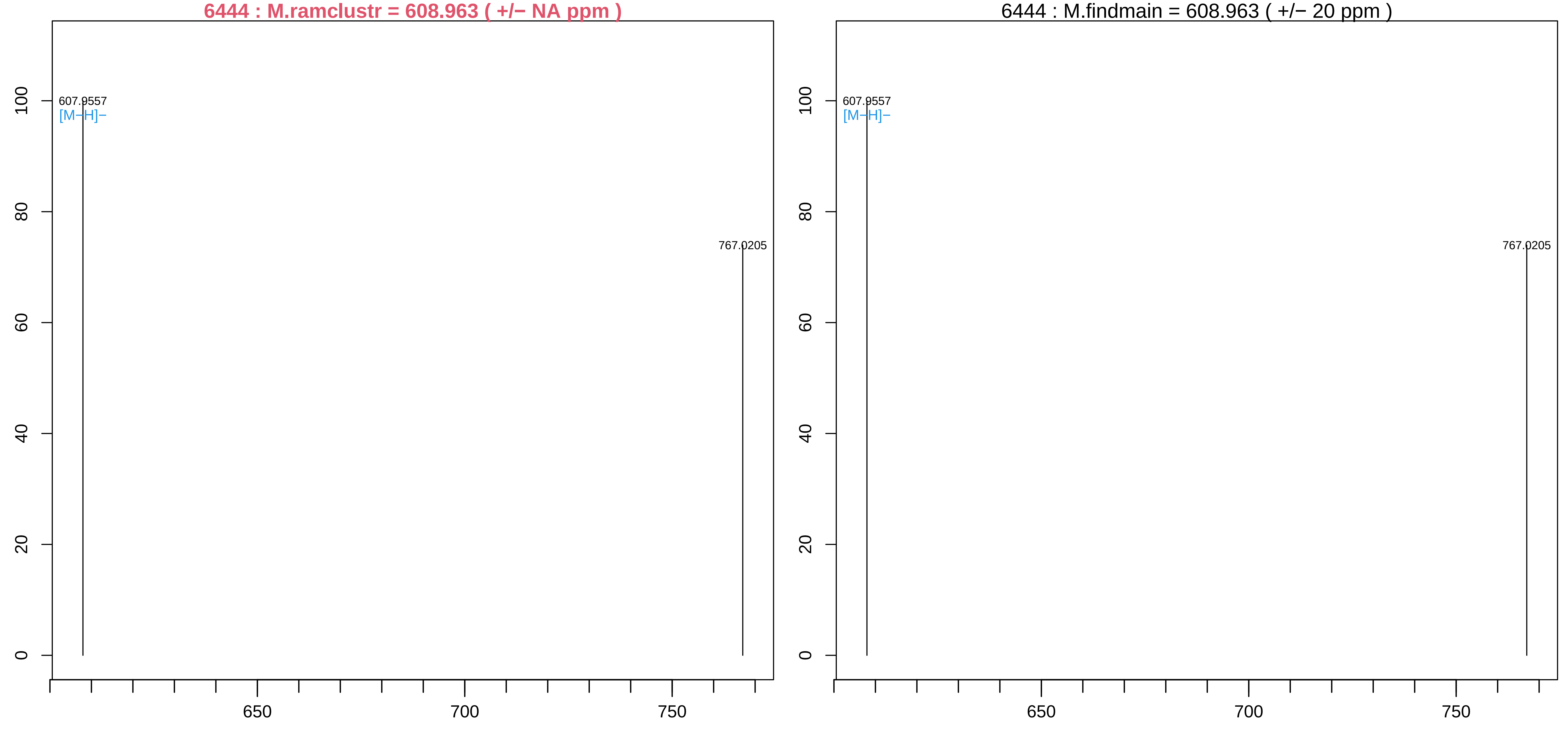

6444 : M.ramclustr = 608.963 (+/− NA ppm)
607.9557
[M−H]−
767.0205
6444 : M.findmain = 608.963 (+/− 20 ppm)
607.9557
[M−H]−
767.0205
0
20
40
60
80
100
650
700
750

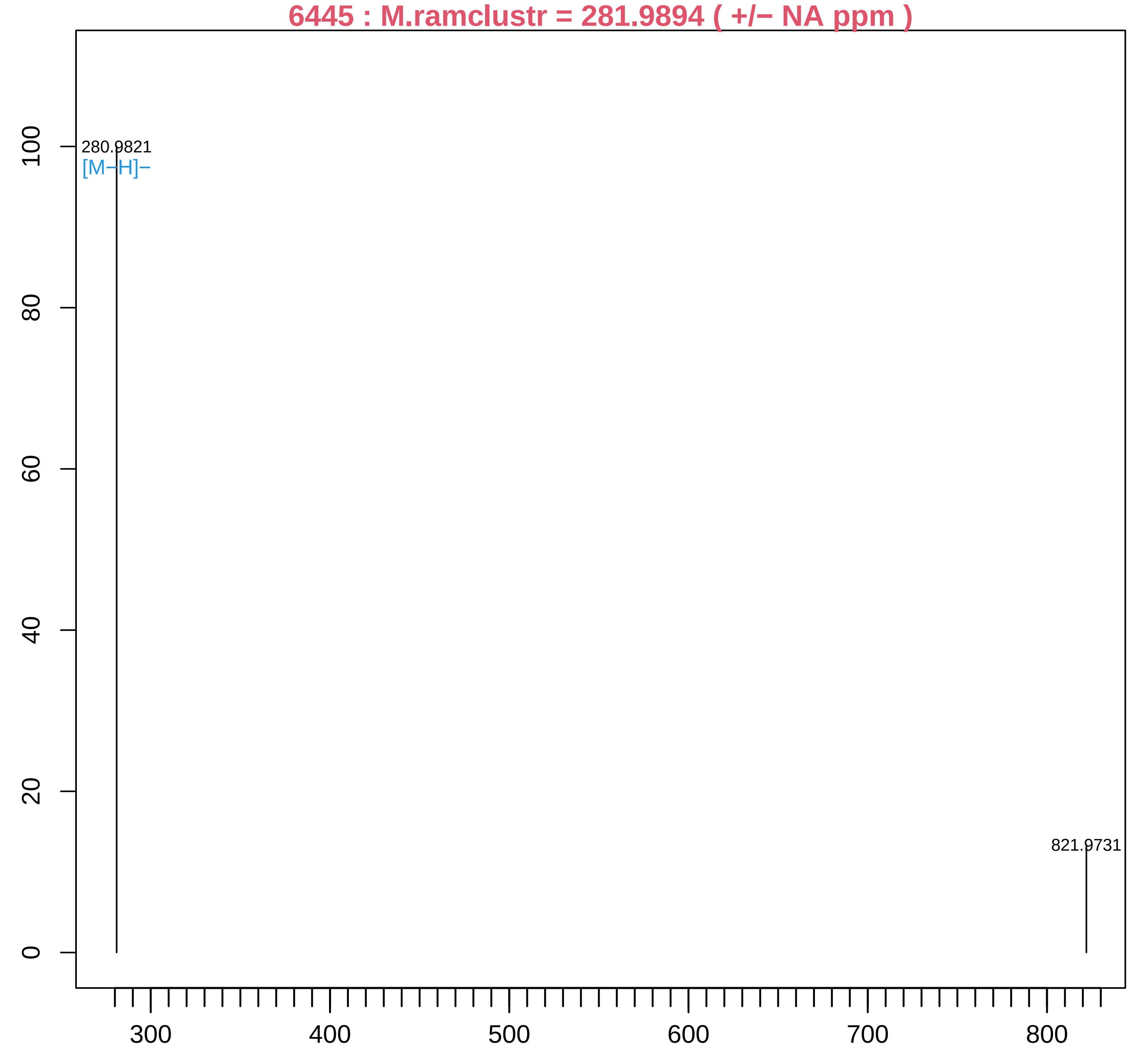
6445 : M.ramclustr = 281.9894 (+/− NA ppm)
280.9821
[M−H]−
821.9731
0
20
40
60
80
100
300
400
500
600
700
800

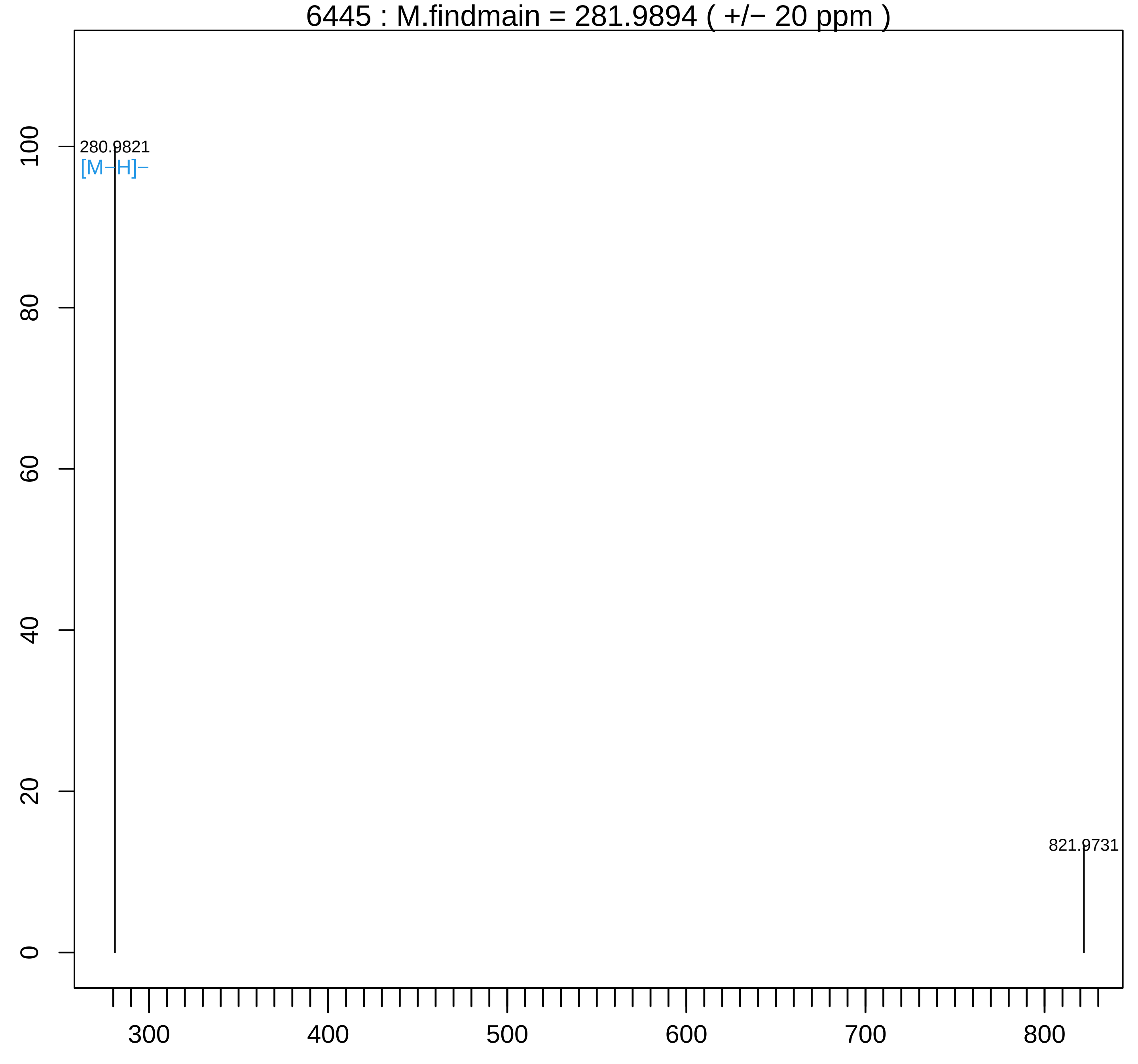
6445 : M.findmain = 281.9894 (+/− 20 ppm)
280.9821
[M−H]−
821.9731
0
20
40
60
80
100
300
400
500
600
700
800

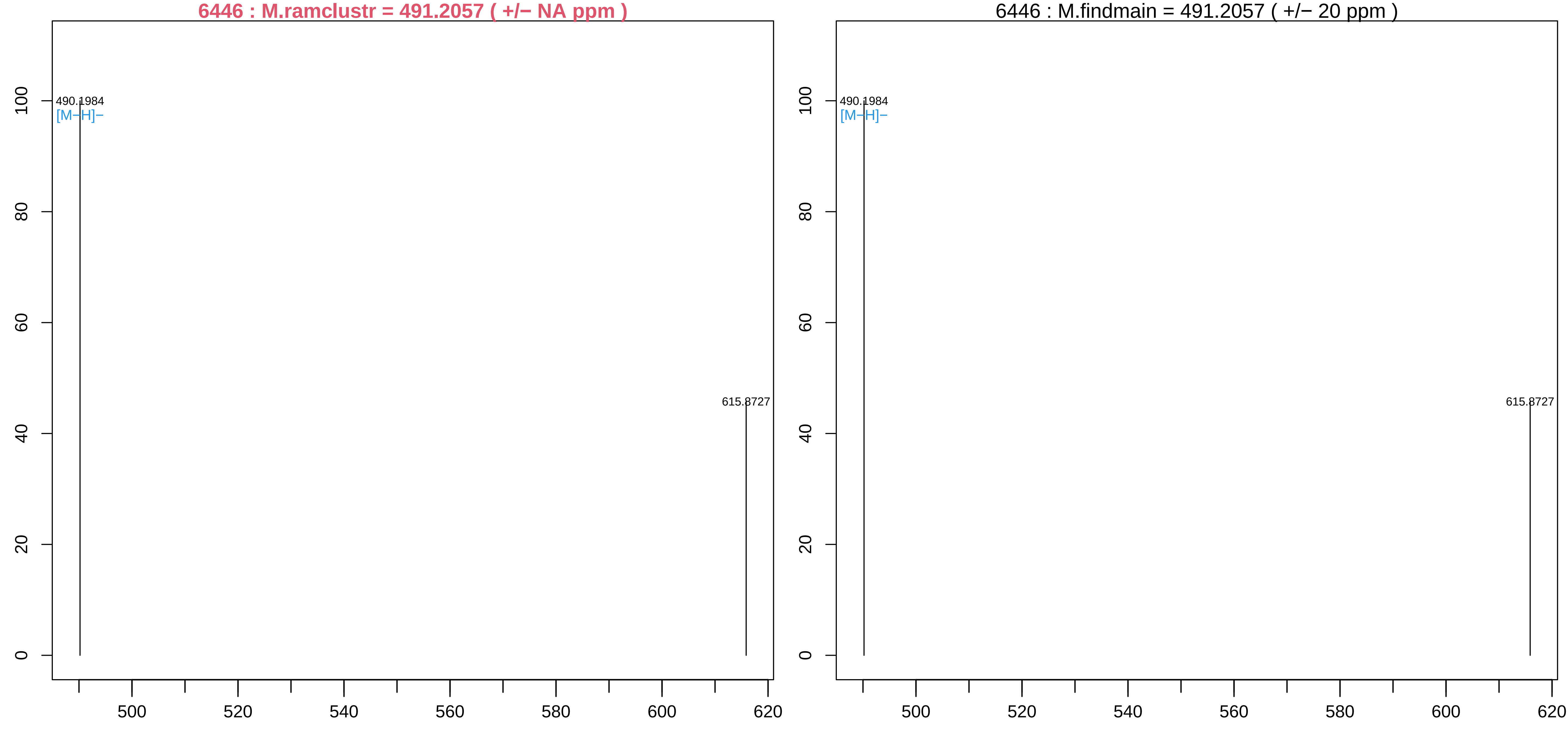
6446 : M.ramclustr = 491.2057 (+/− NA ppm)
490.1984
[M−H]−
615.8727
0
20
40
60
80
100
500
520
540
560
580
600
620
6446 : M.findmain = 491.2057 (+/− 20 ppm)
490.1984
[M−H]−
615.8727
0
20
40
60
80
100
500
520
540
560
580
600
620

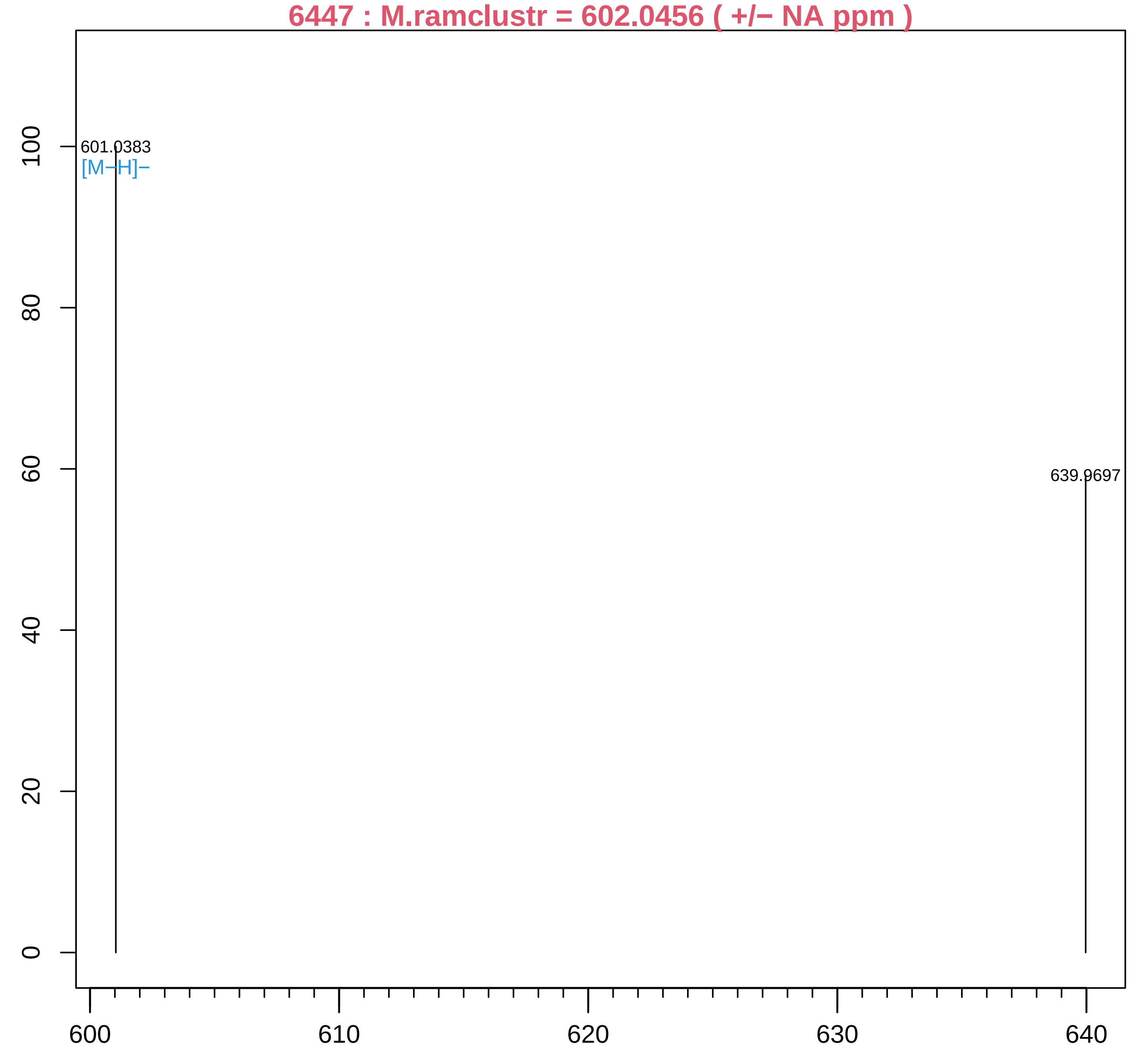

6447 : M.ramclustr = 602.0456 (+/− NA ppm)
601.0383
[M−H]−
639.9697
0
20
40
60
80
100
600
610
620
630
640

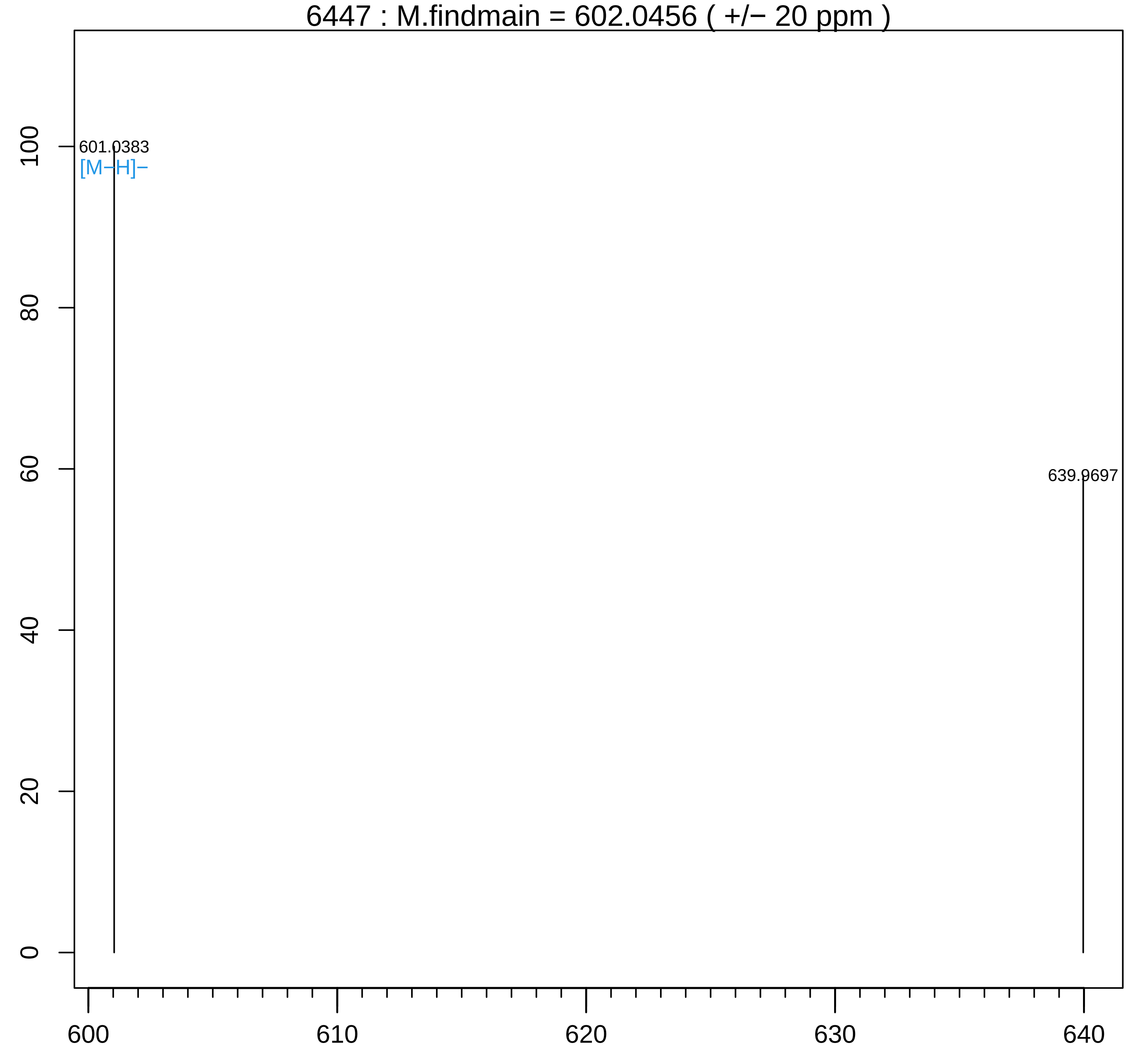

6447 : M.findmain = 602.0456 (+/− 20 ppm)
601.0383
[M−H]−
639.9697
0
20
40
60
80
100
600
610
620
630
640

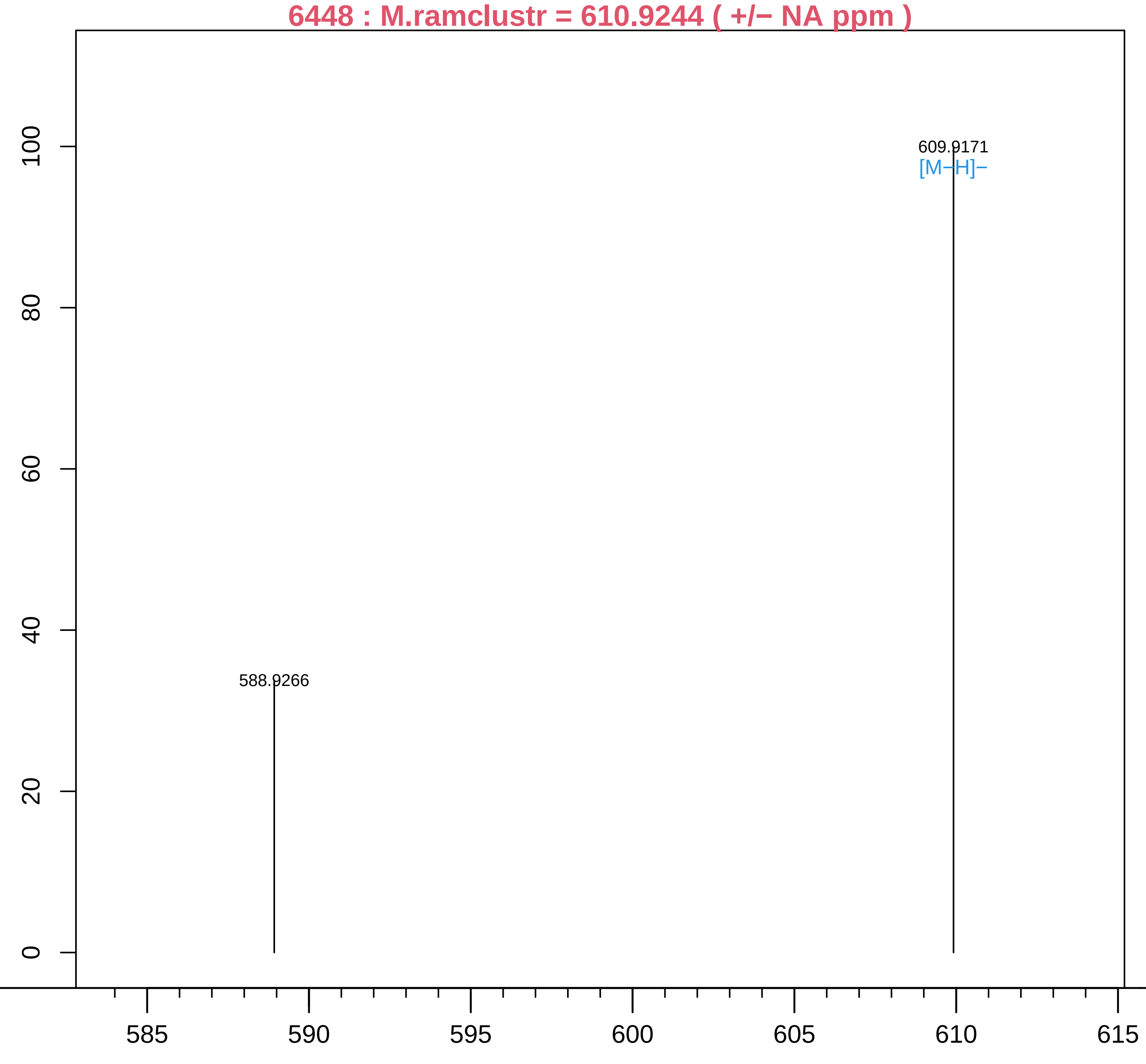
6448 : M.ramclustr = 610.9244 (+/− NA ppm)
609.9171
[M−H]−
588.9266
0
20
40
60
80
100
585
590
595
600
605
610
615

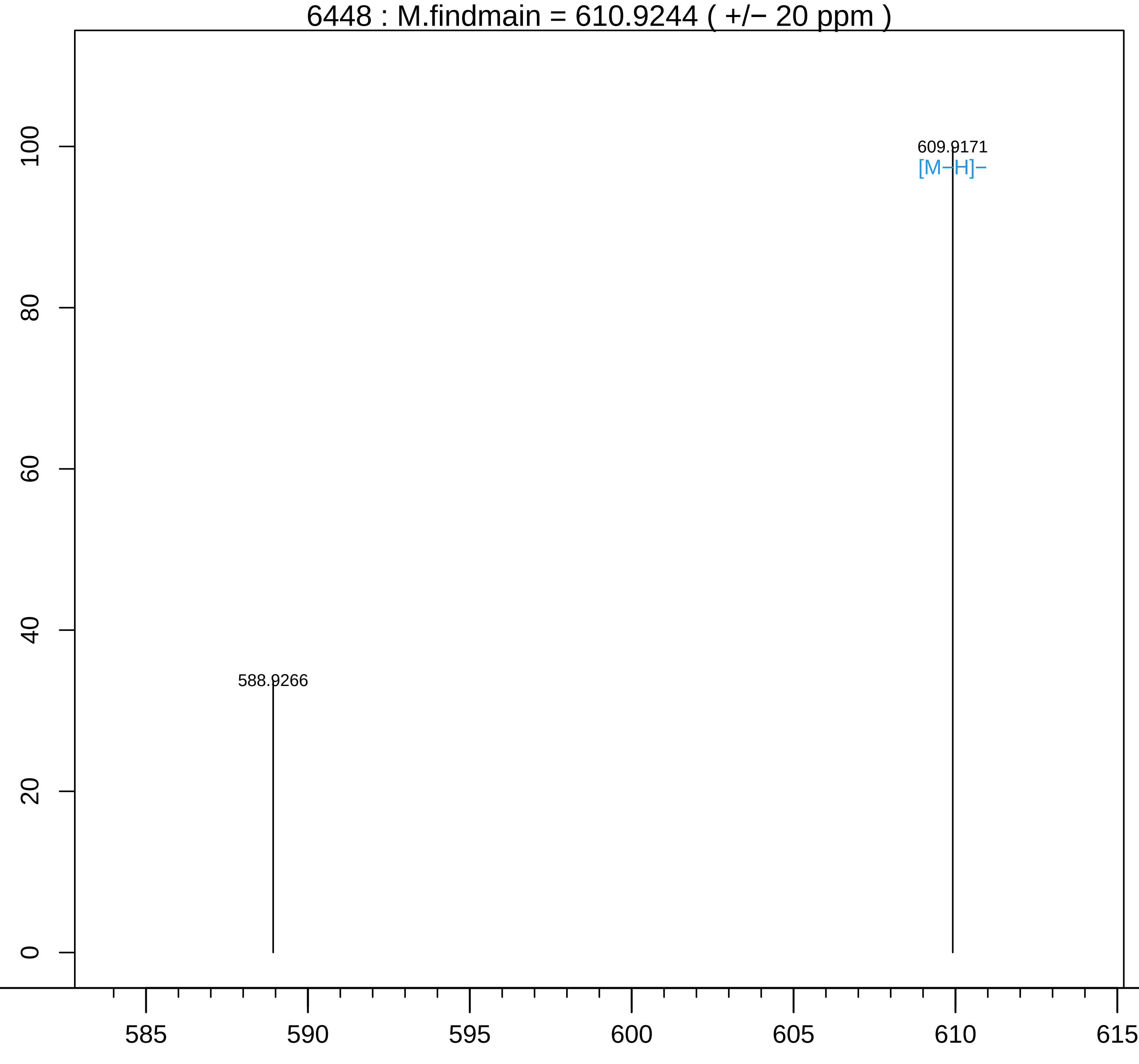
6448 : M.findmain = 610.9244 (+/− 20 ppm)
609.9171
[M−H]−
588.9266
0
20
40
60
80
100
585
590
595
600
605
610
615

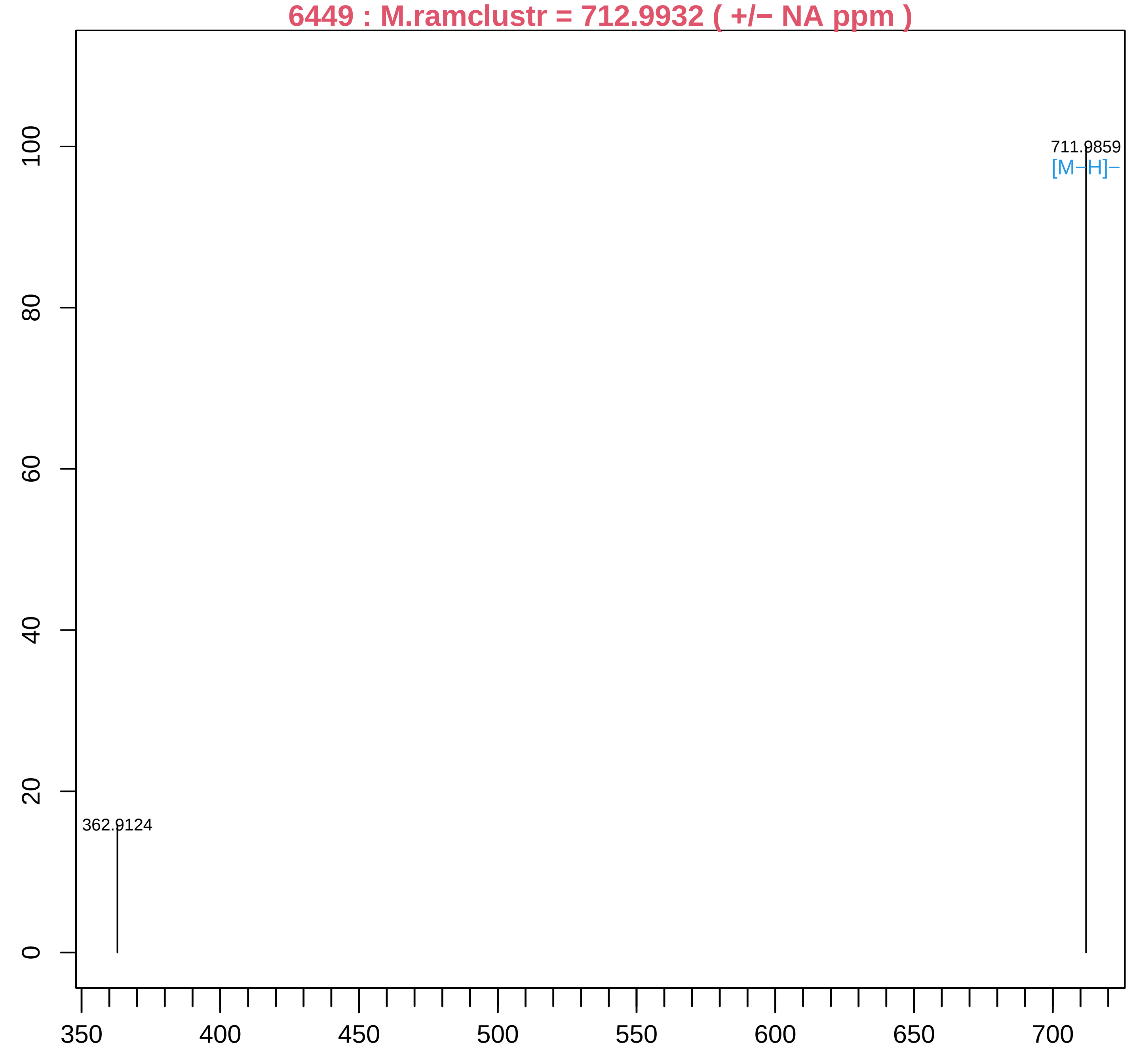
6449 : M.ramclustr = 712.9932 (+/− NA ppm)
711.9859
[M−H]−
362.9124
0
20
40
60
80
100
350
400
450
500
550
600
650
700
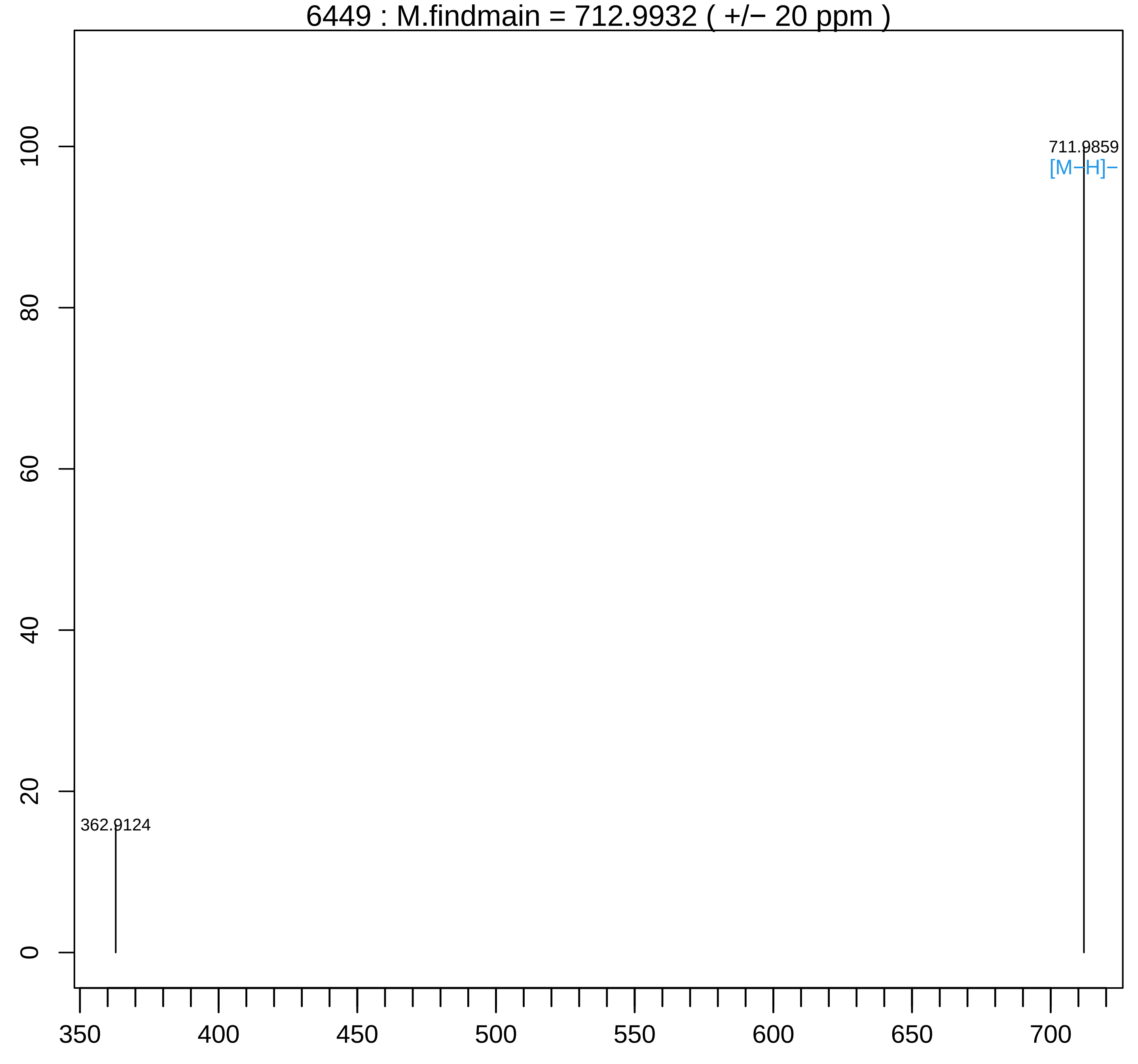
6449 : M.findmain = 712.9932 (+/− 20 ppm)
711.9859
[M−H]−
362.9124
0
20
40
60
80
100
350
400
450
500
550
600
650
700

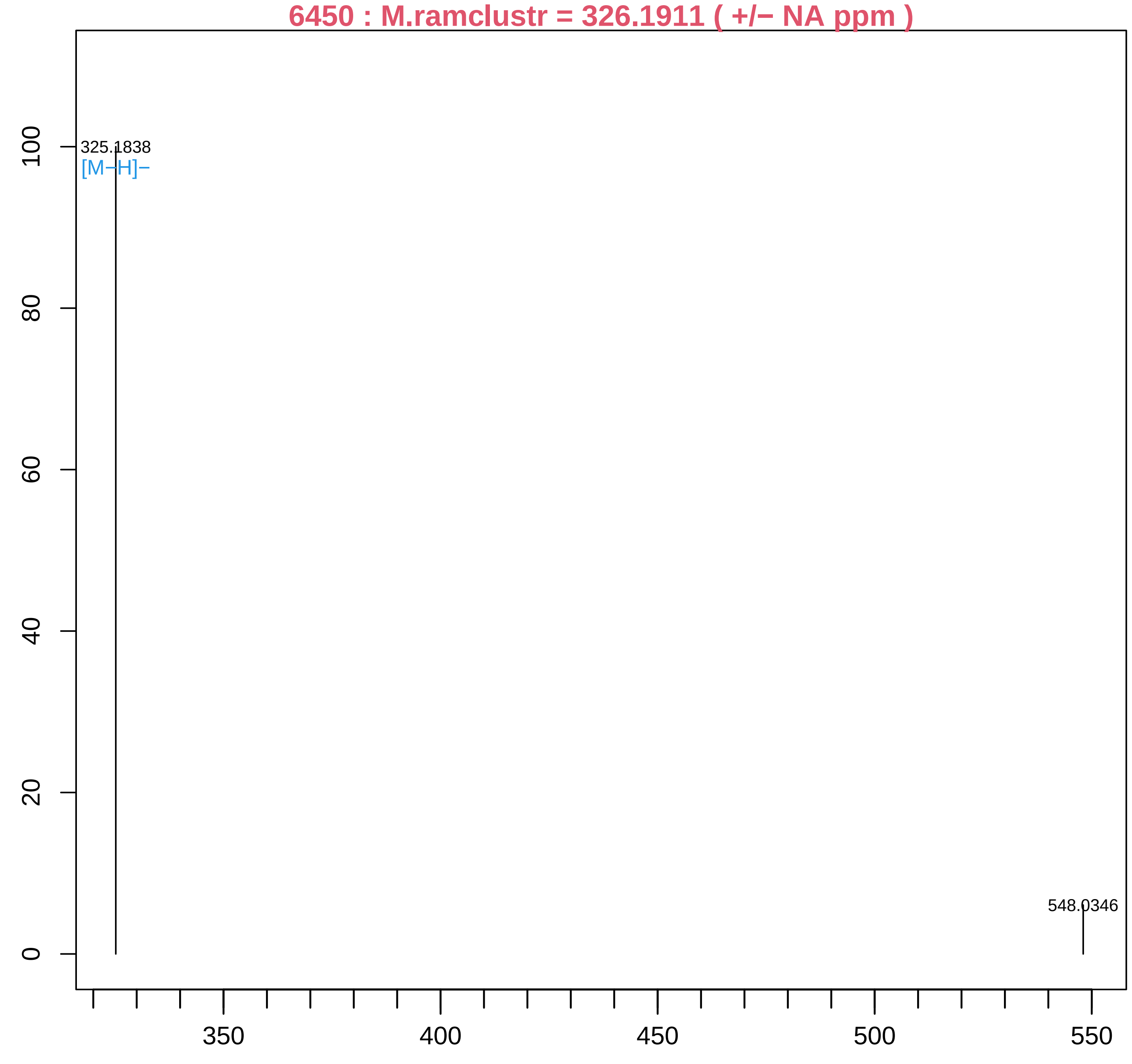
6450 : M.ramclustr = 326.1911 (+/− NA ppm)
325.1838
[M−H]−
548.0346
0
20
40
60
80
100
350
400
450
500
550

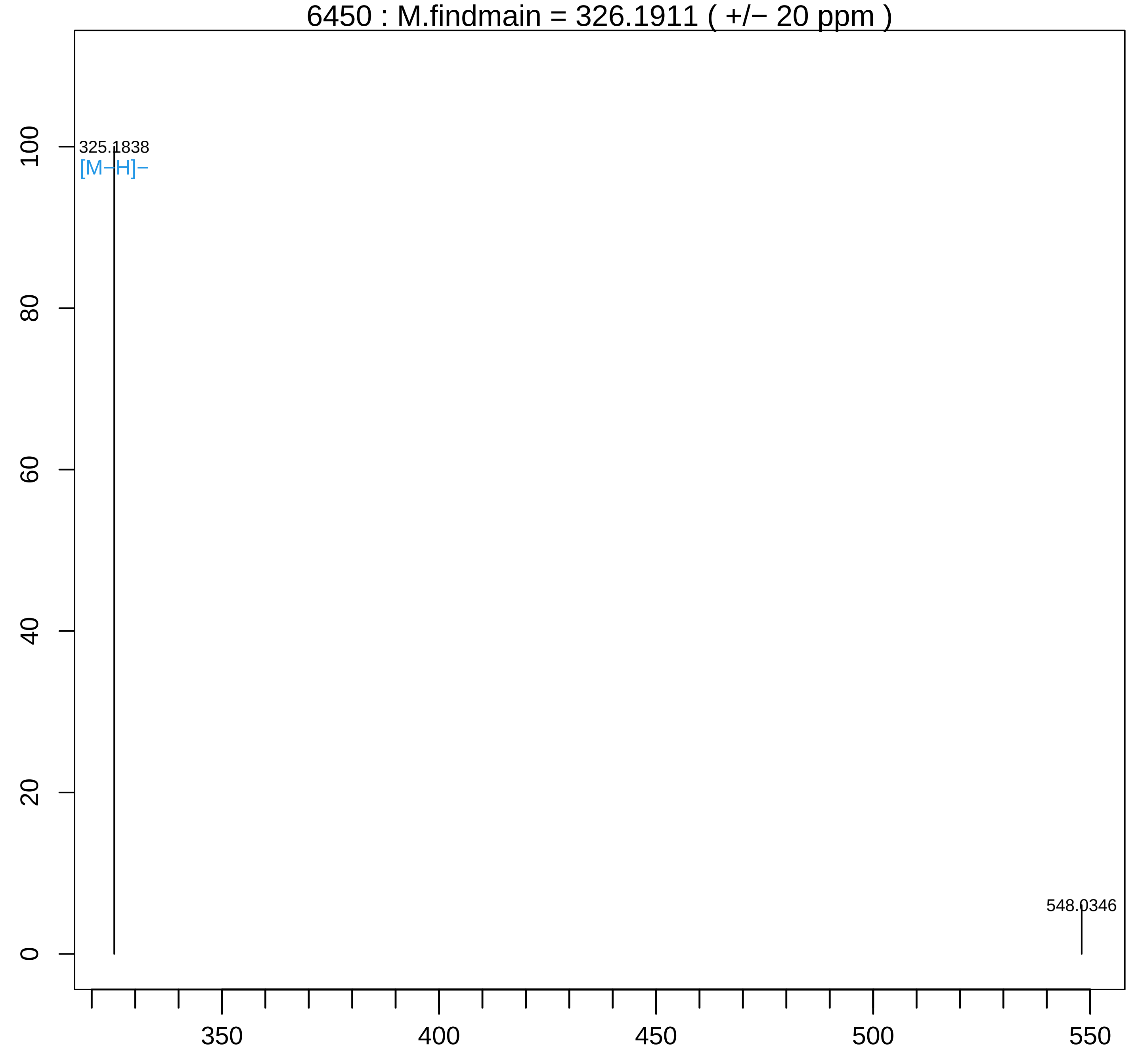
6450 : M.findmain = 326.1911 (+/− 20 ppm)
325.1838
[M−H]−
548.0346
0
20
40
60
80
100
350
400
450
500
550

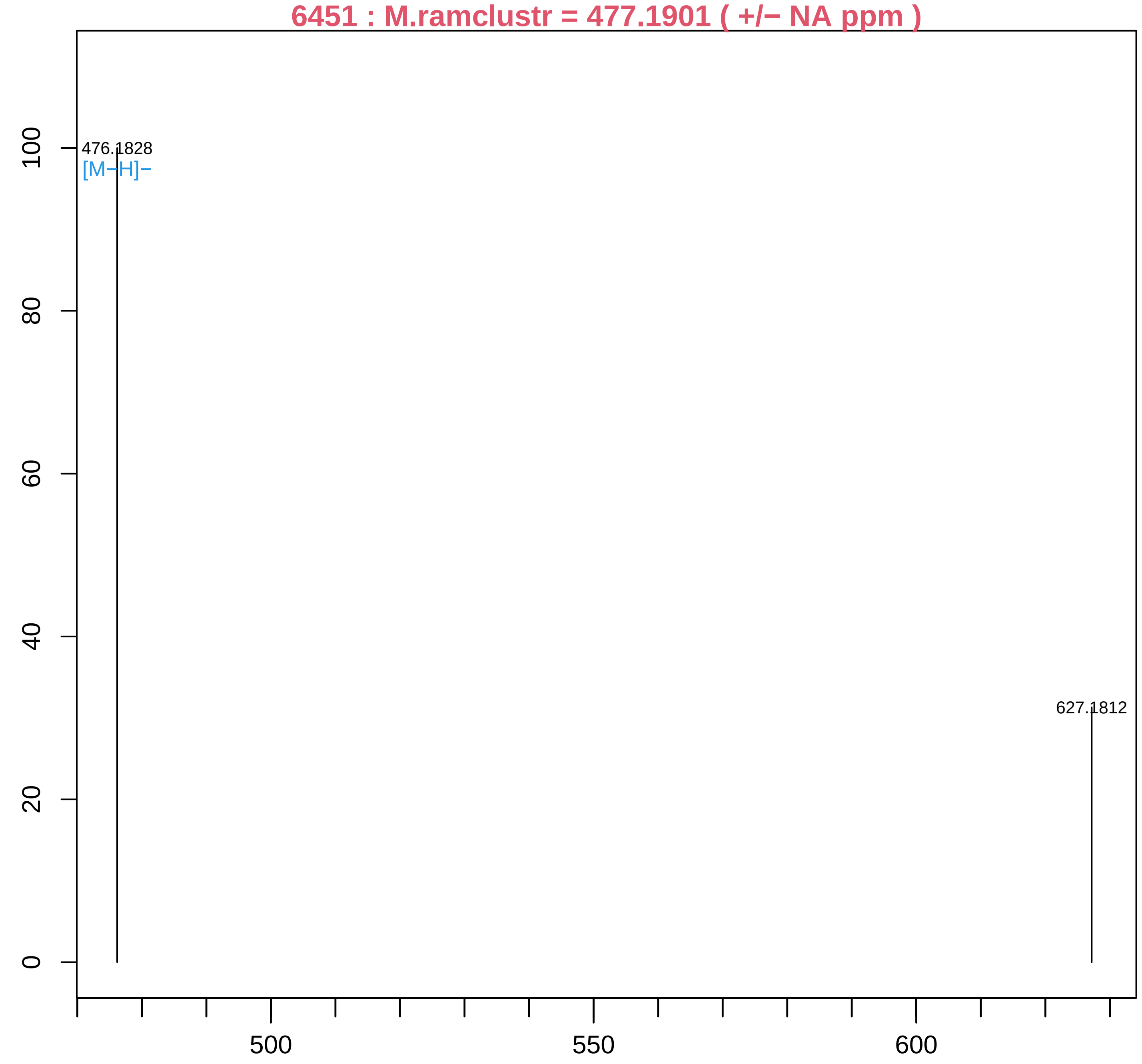
6451 : M.ramclustr = 477.1901 (+/− NA ppm)
476.1828
[M−H]−
627.1812
0
20
40
60
80
100
500
550
600

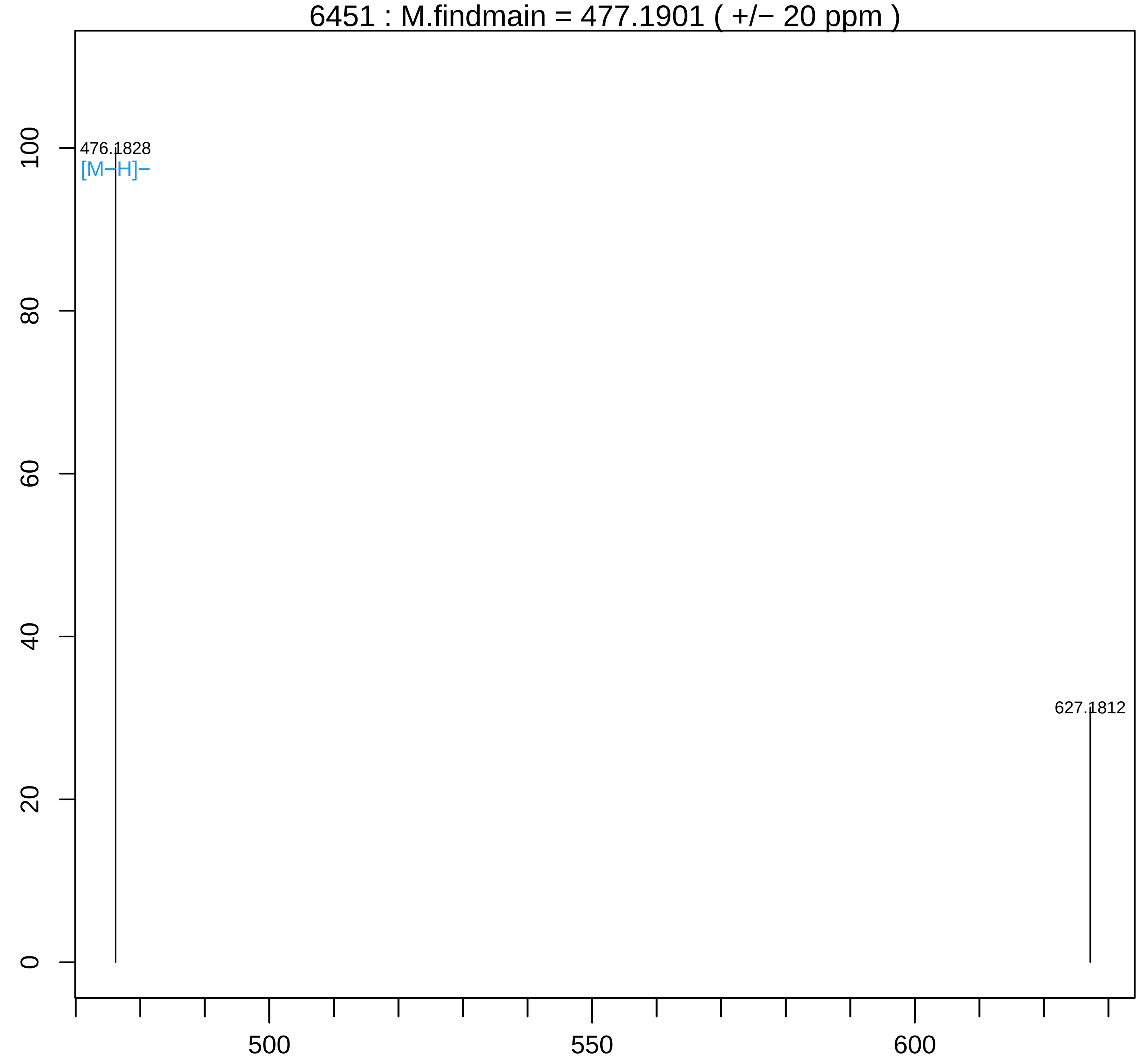
6451 : M.findmain = 477.1901 (+/− 20 ppm)
476.1828
[M−H]−
627.1812
0
20
40
60
80
100
500
550
600

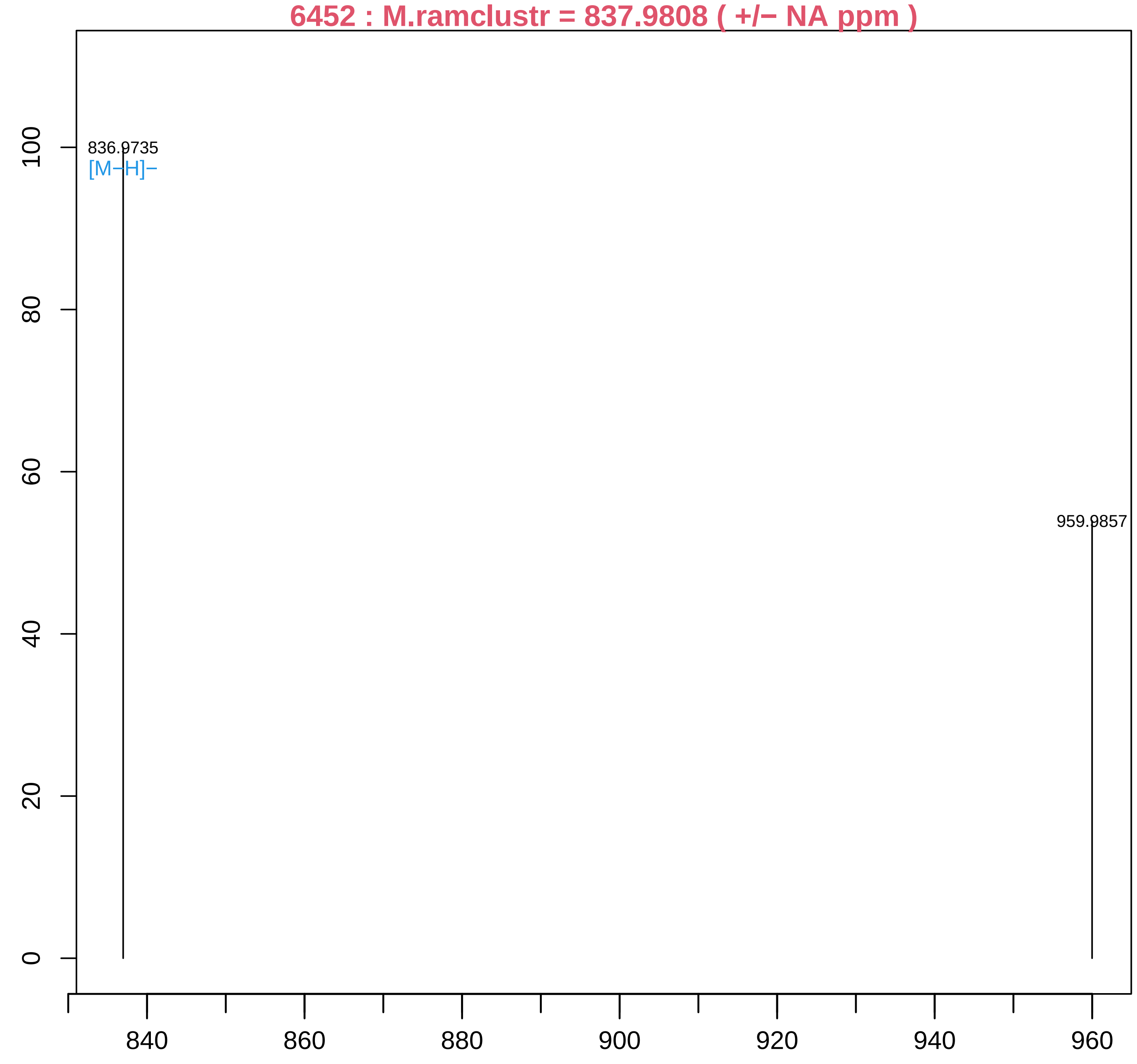
6452 : M.ramclustr = 837.9808 (+/− NA ppm)
836.9735
[M−H]−
959.9857
0
20
40
60
80
100
840
860
880
900
920
940
960

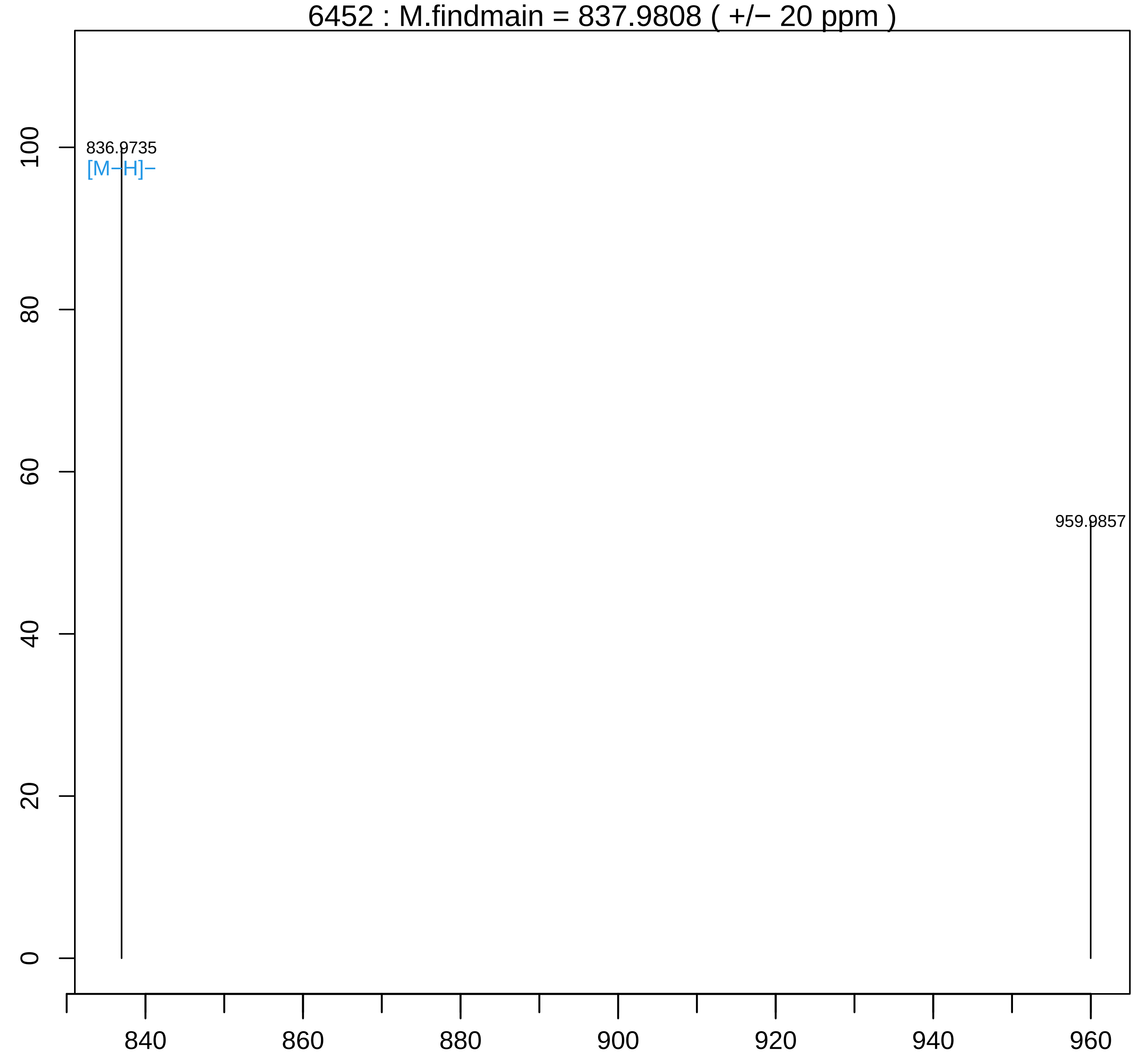
6452 : M.findmain = 837.9808 (+/− 20 ppm)
836.9735
[M−H]−
959.9857
0
20
40
60
80
100
840
860
880
900
920
940
960

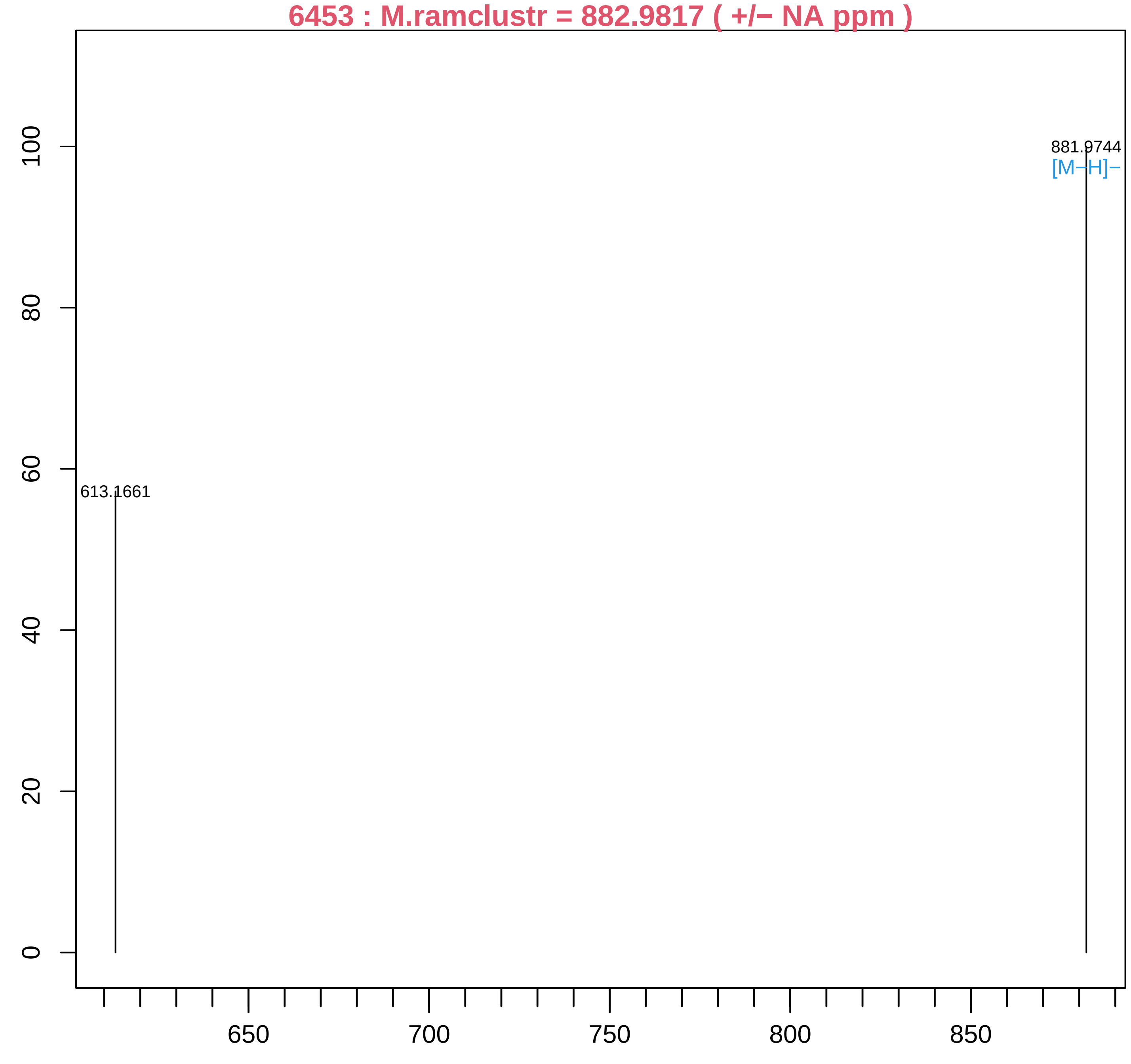
6453 : M.ramclustr = 882.9817 (+/− NA ppm)
881.9744
[M−H]−
613.1661
0
20
40
60
80
100
650
700
750
800
850

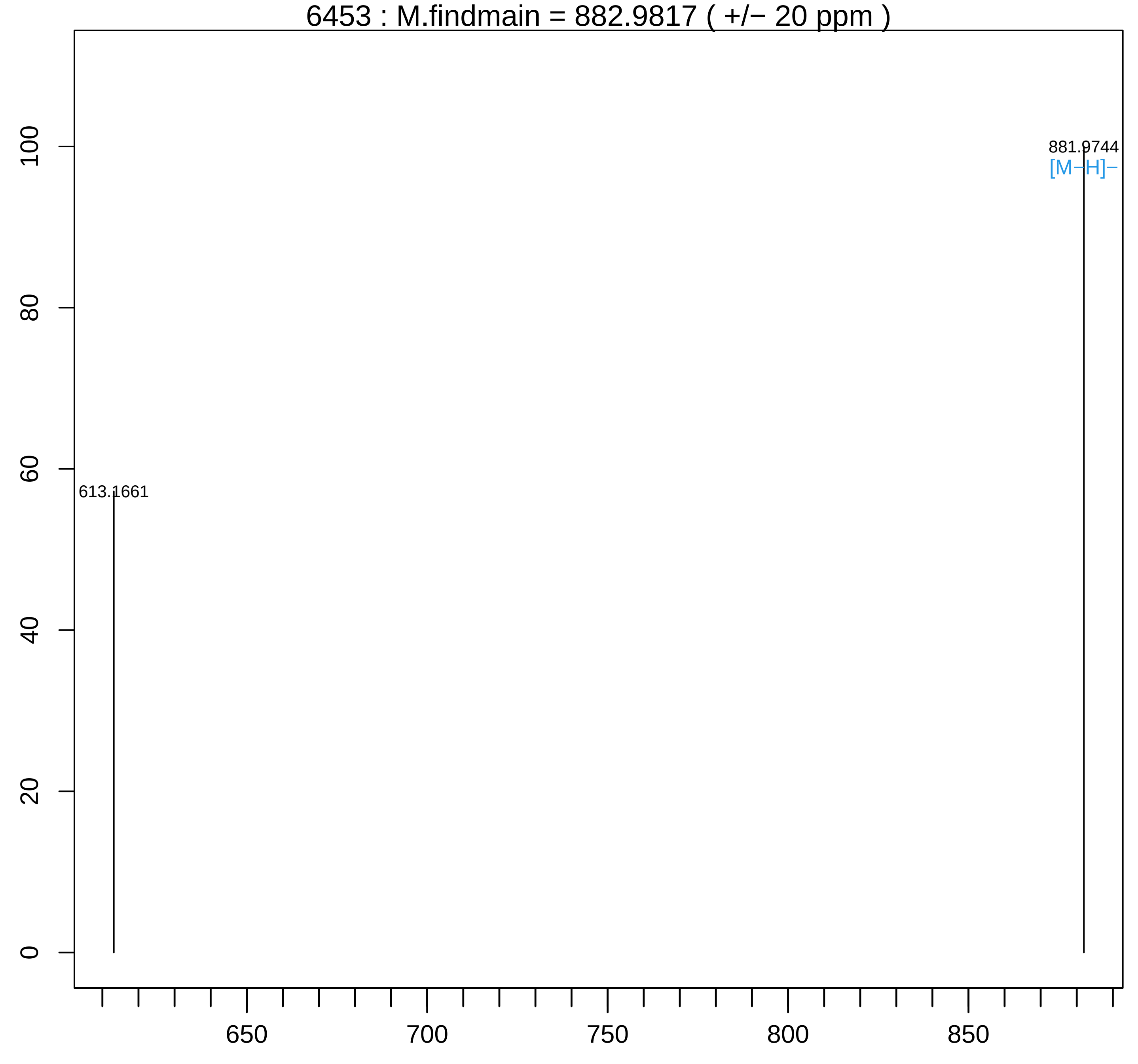
6453 : M.findmain = 882.9817 (+/− 20 ppm)
881.9744
[M−H]−
613.1661
0
20
40
60
80
100
650
700
750
800
850

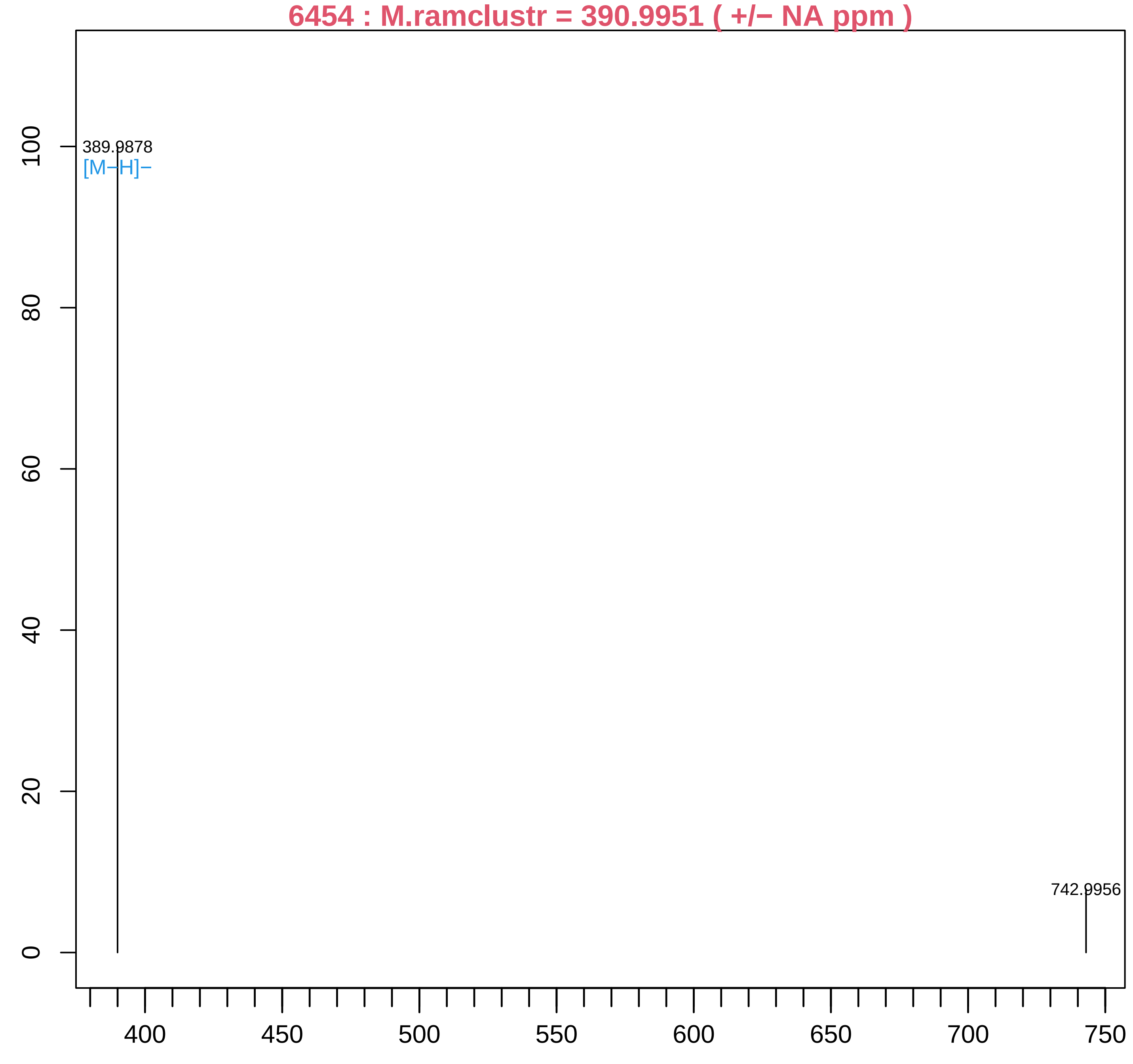

6454 : M.ramclustr = 390.9951 (+/− NA ppm)
389.9878
[M−H]−
742.9956
0
20
40
60
80
100
400
450
500
550
600
650
700
750

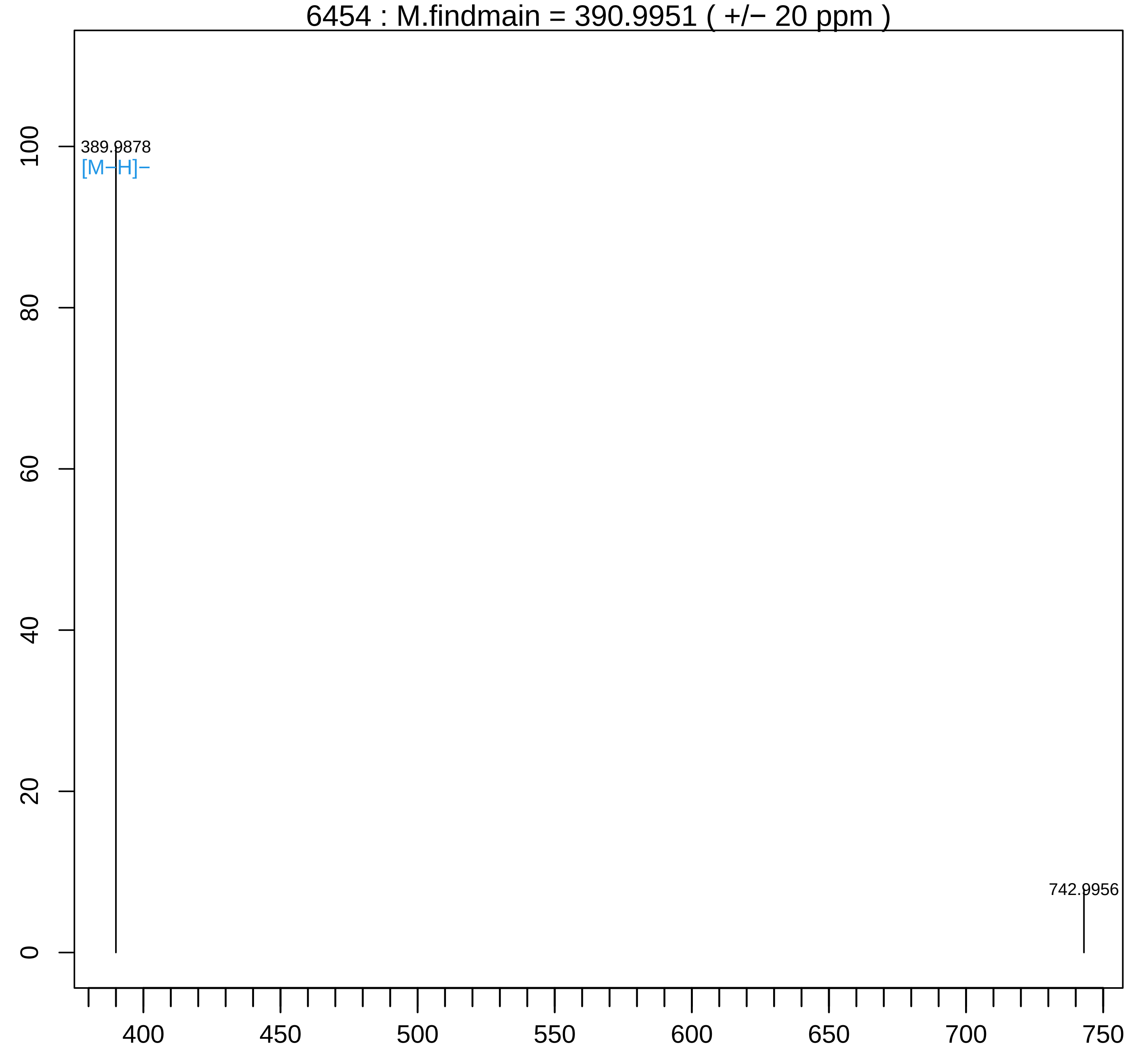

6454 : M.findmain = 390.9951 (+/− 20 ppm)
389.9878
[M−H]−
742.9956
0
20
40
60
80
100
400
450
500
550
600
650
700
750

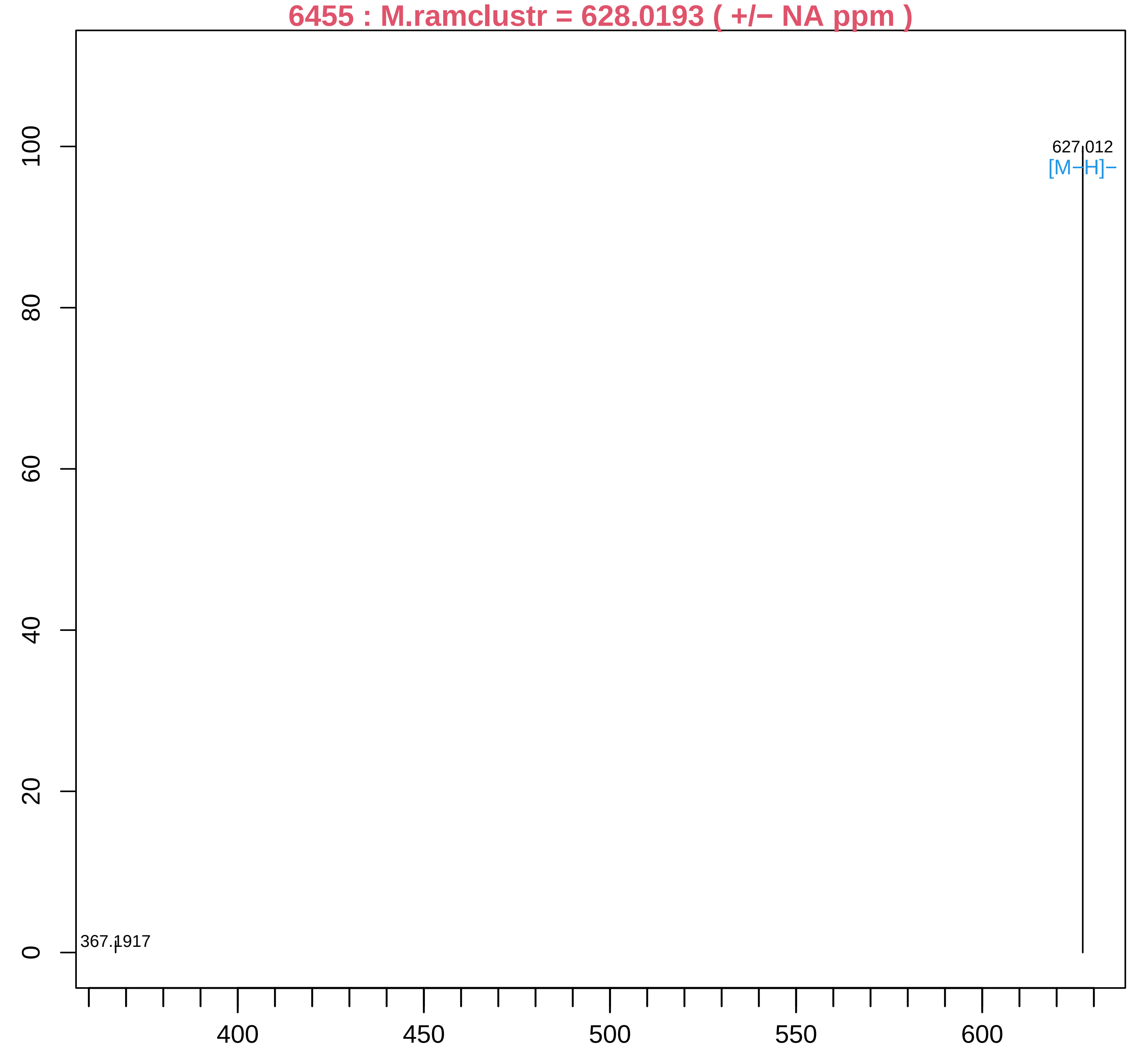

6455 : M.ramclustr = 628.0193 (+/− NA ppm)
627.012
[M−H]−
367.1917
0
20
40
60
80
100
400
450
500
550
600

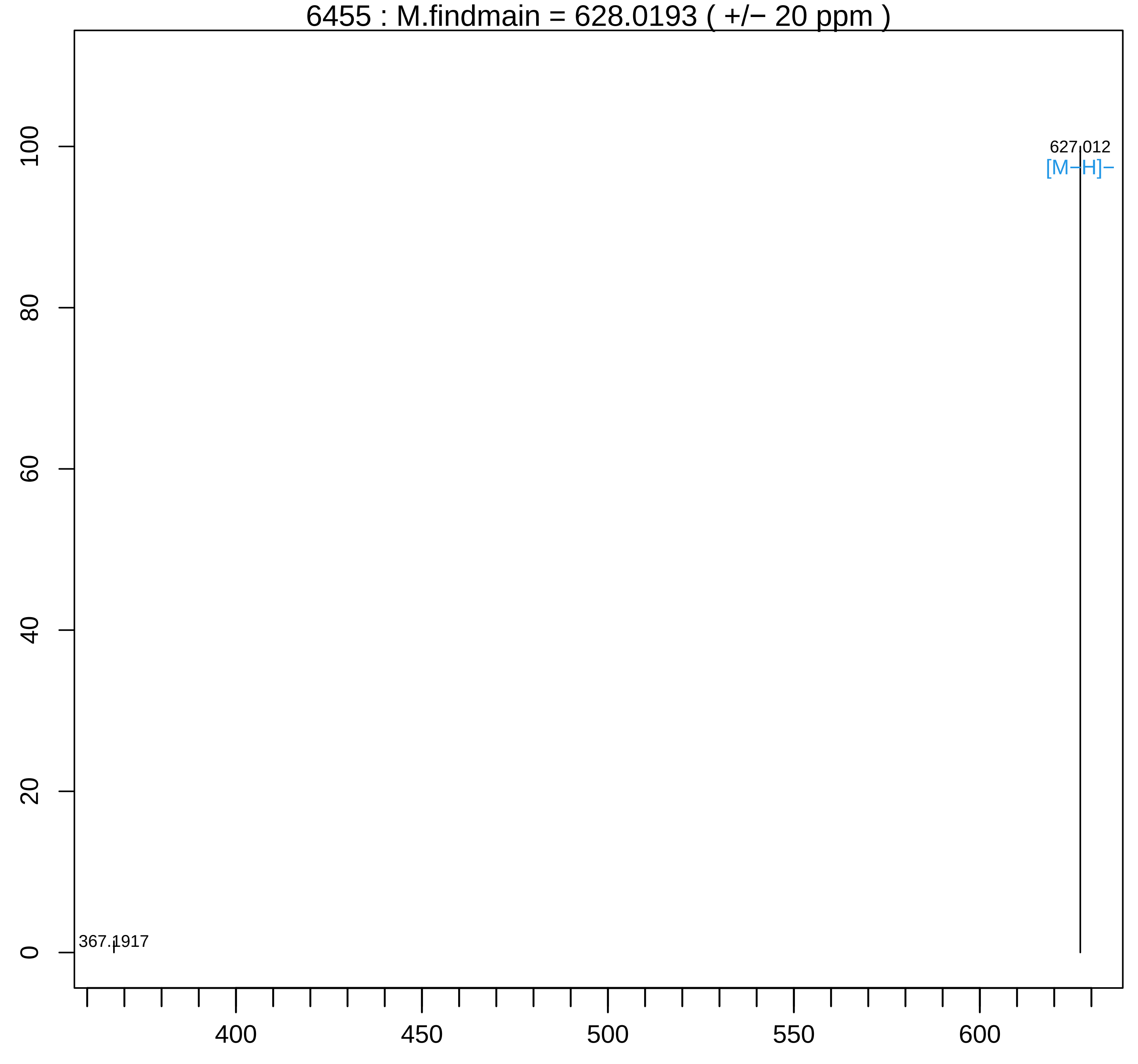

6455 : M.findmain = 628.0193 (+/− 20 ppm)
627.012
[M−H]−
367.1917
0
20
40
60
80
100
400
450
500
550
600

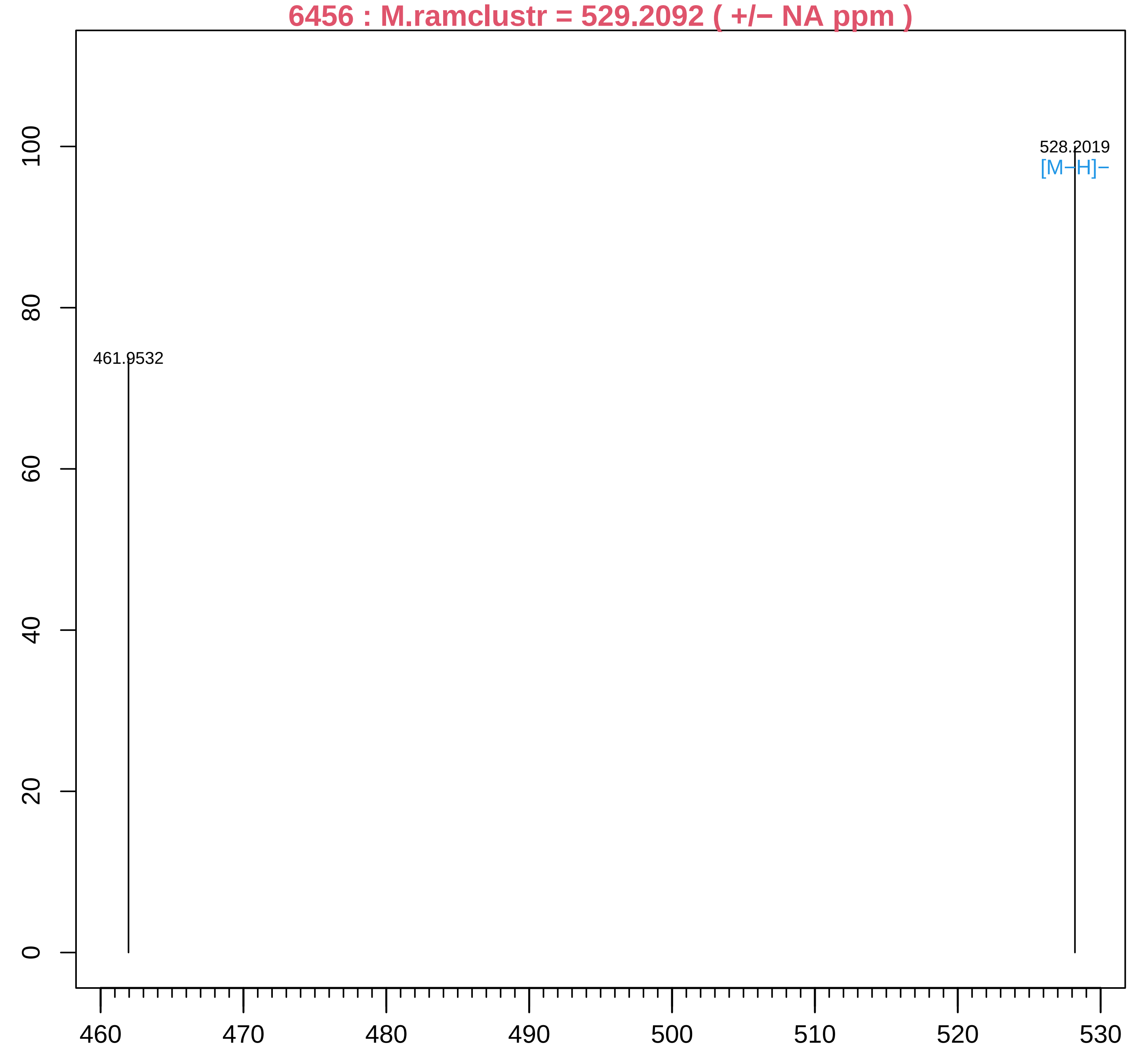
6456 : M.ramclustr = 529.2092 (+/− NA ppm)
461.9532
528.2019
[M−H]−
0
20
40
60
80
100
460
470
480
490
500
510
520
530

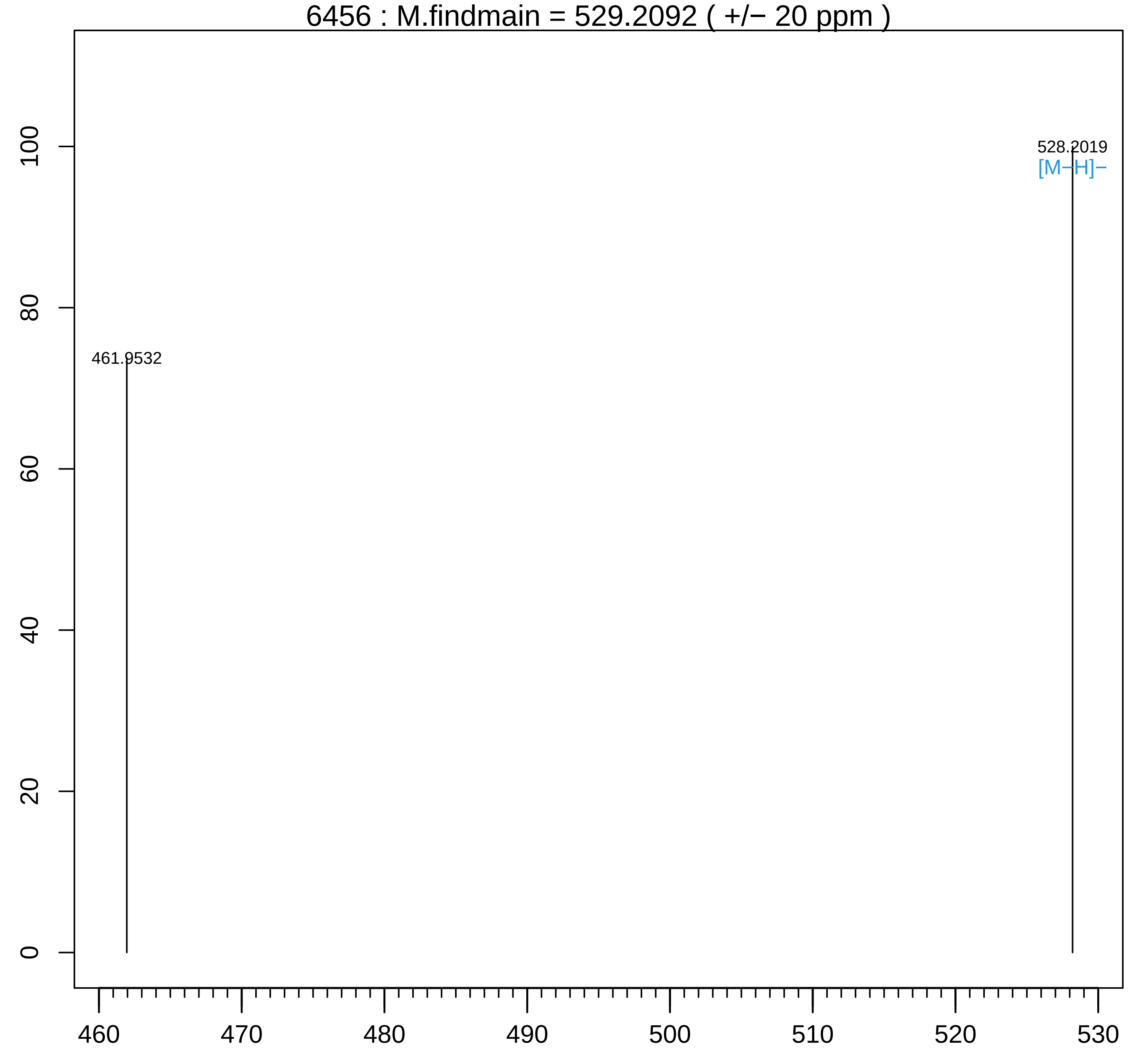
6456 : M.findmain = 529.2092 (+/− 20 ppm)
461.9532
528.2019
[M−H]−
0
20
40
60
80
100
460
470
480
490
500
510
520
530

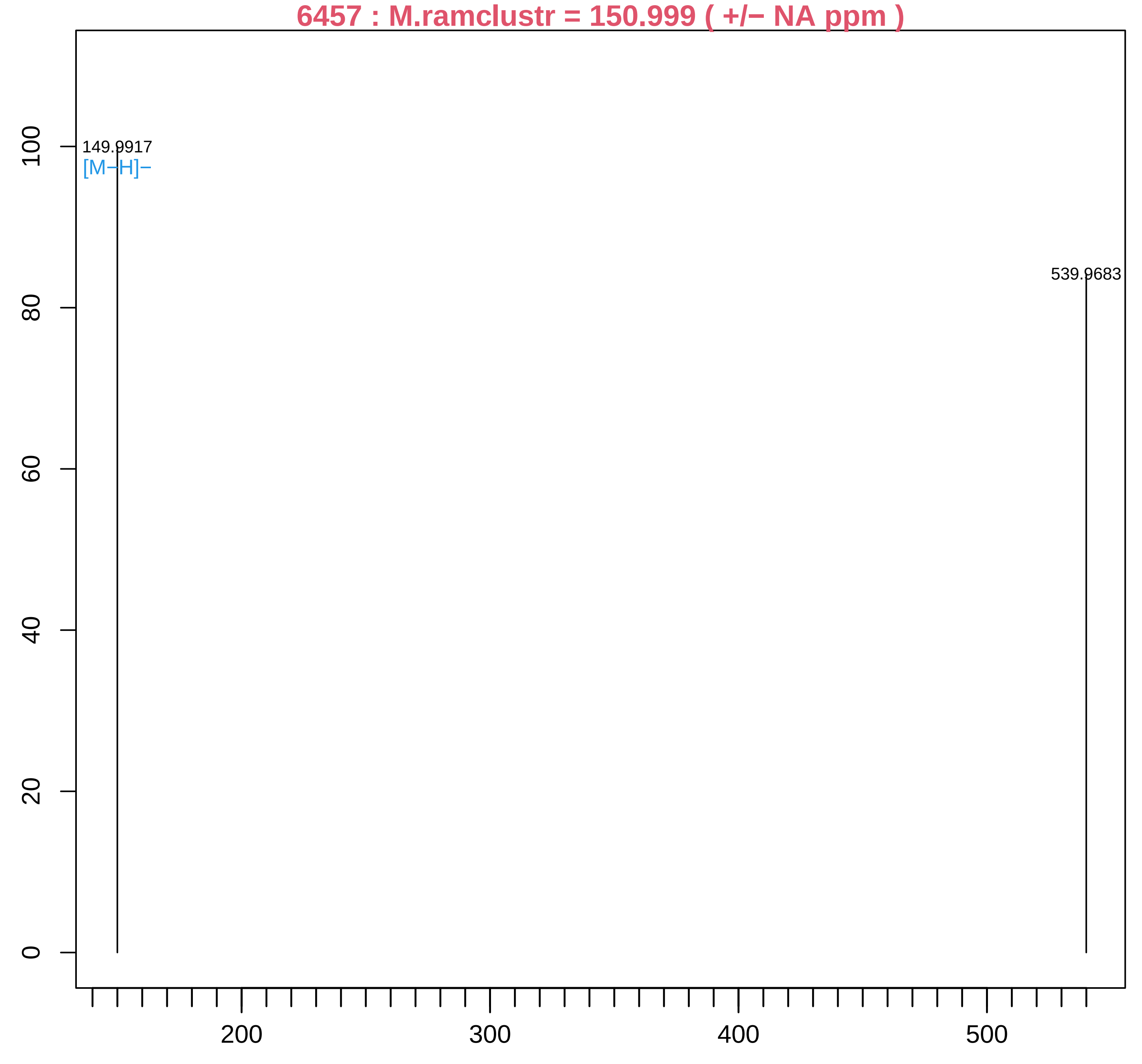
6457 : M.ramclustr = 150.999 (+/− NA ppm)
149.9917
[M−H]−
539.9683
0
20
40
60
80
100
200
300
400
500

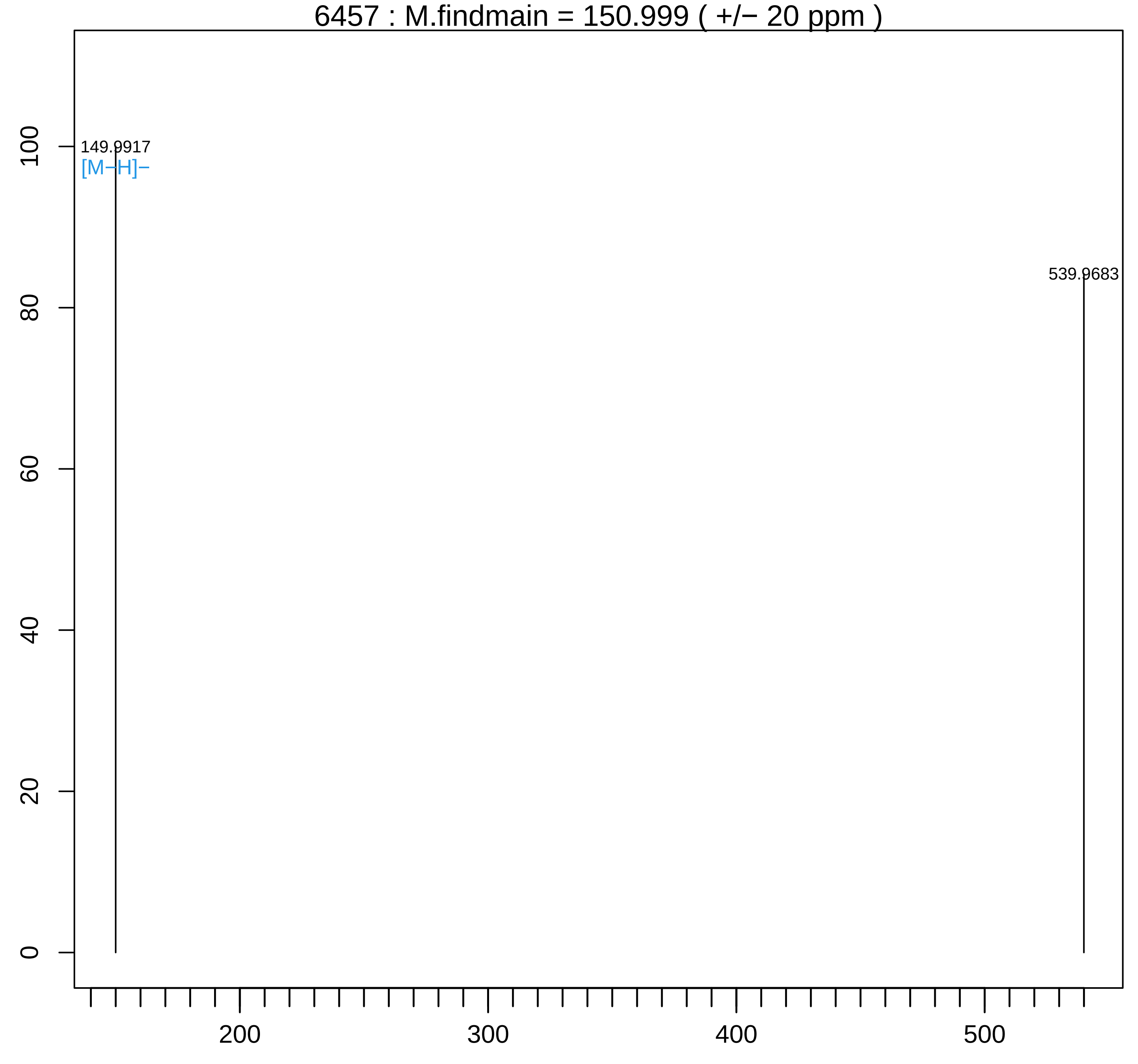
6457 : M.findmain = 150.999 (+/− 20 ppm)
149.9917
[M−H]−
539.9683
0
20
40
60
80
100
200
300
400
500

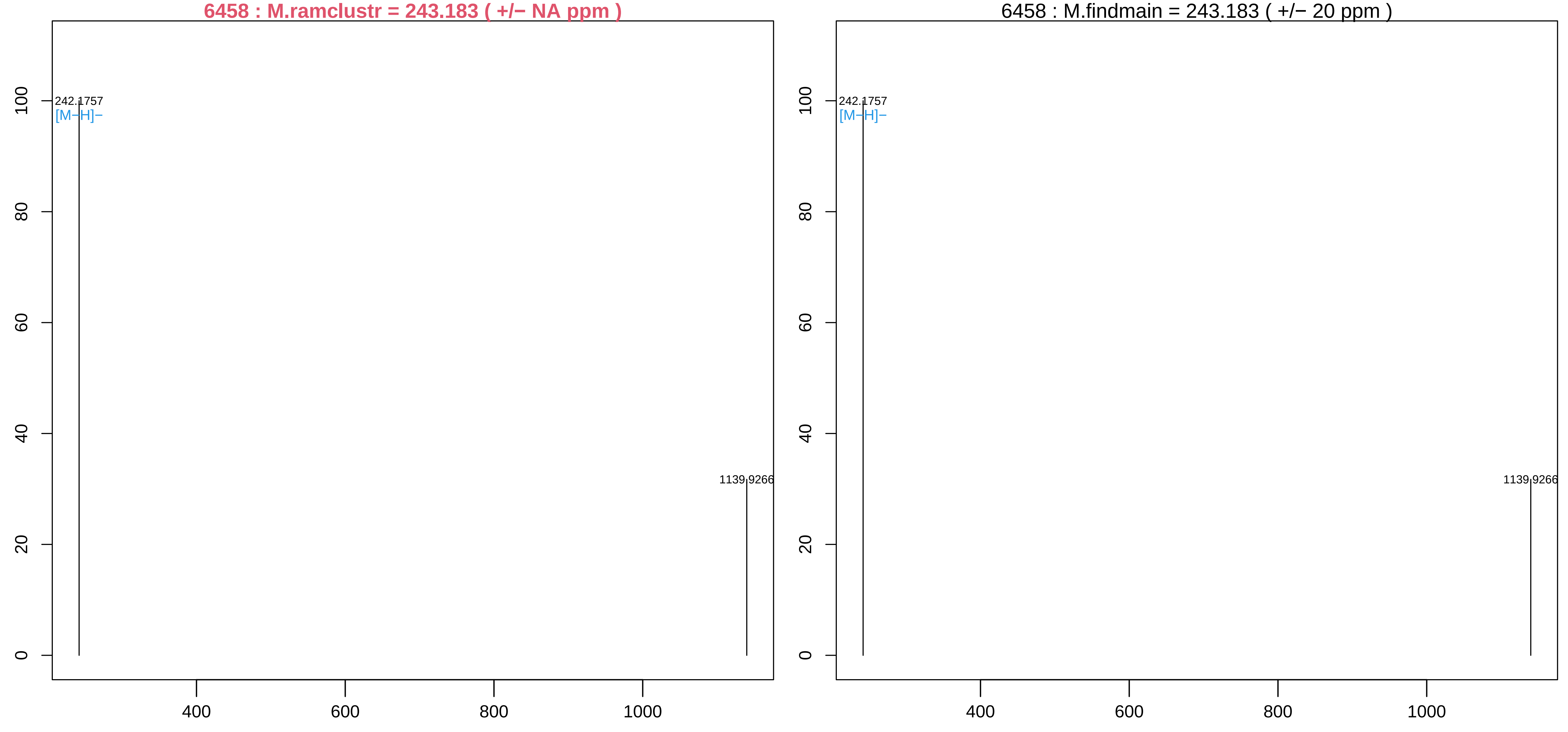

6458 : M.ramclustr = 243.183 (+/− NA ppm)
6458 : M.findmain = 243.183 (+/− 20 ppm)
242.1757
[M−H]−
1139.9266
0
20
40
60
80
100
400
600
800
1000

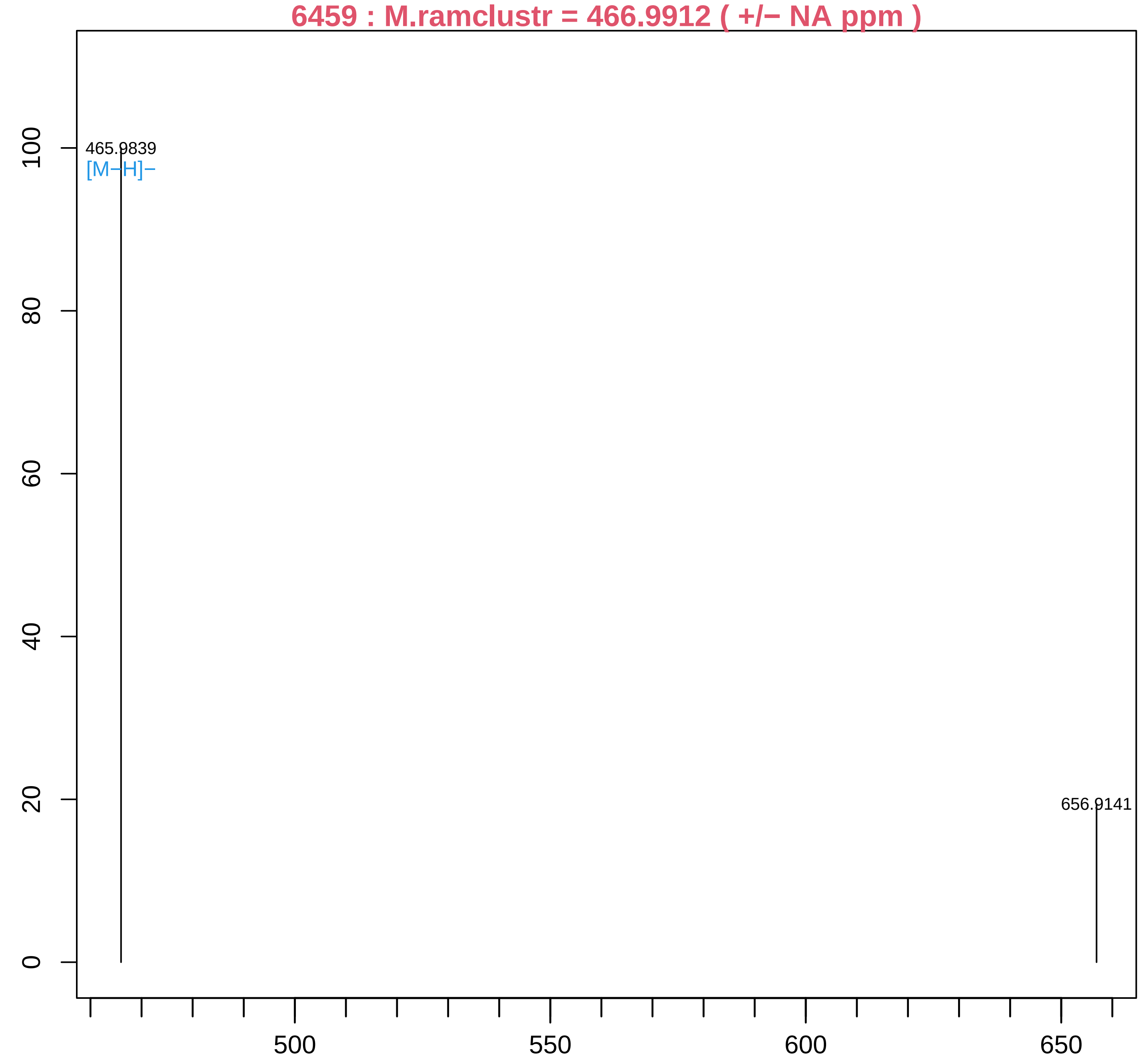

6459 : M.ramclustr = 466.9912 (+/− NA ppm)
465.9839
[M−H]−
656.9141
0
20
40
60
80
100
500
550
600
650

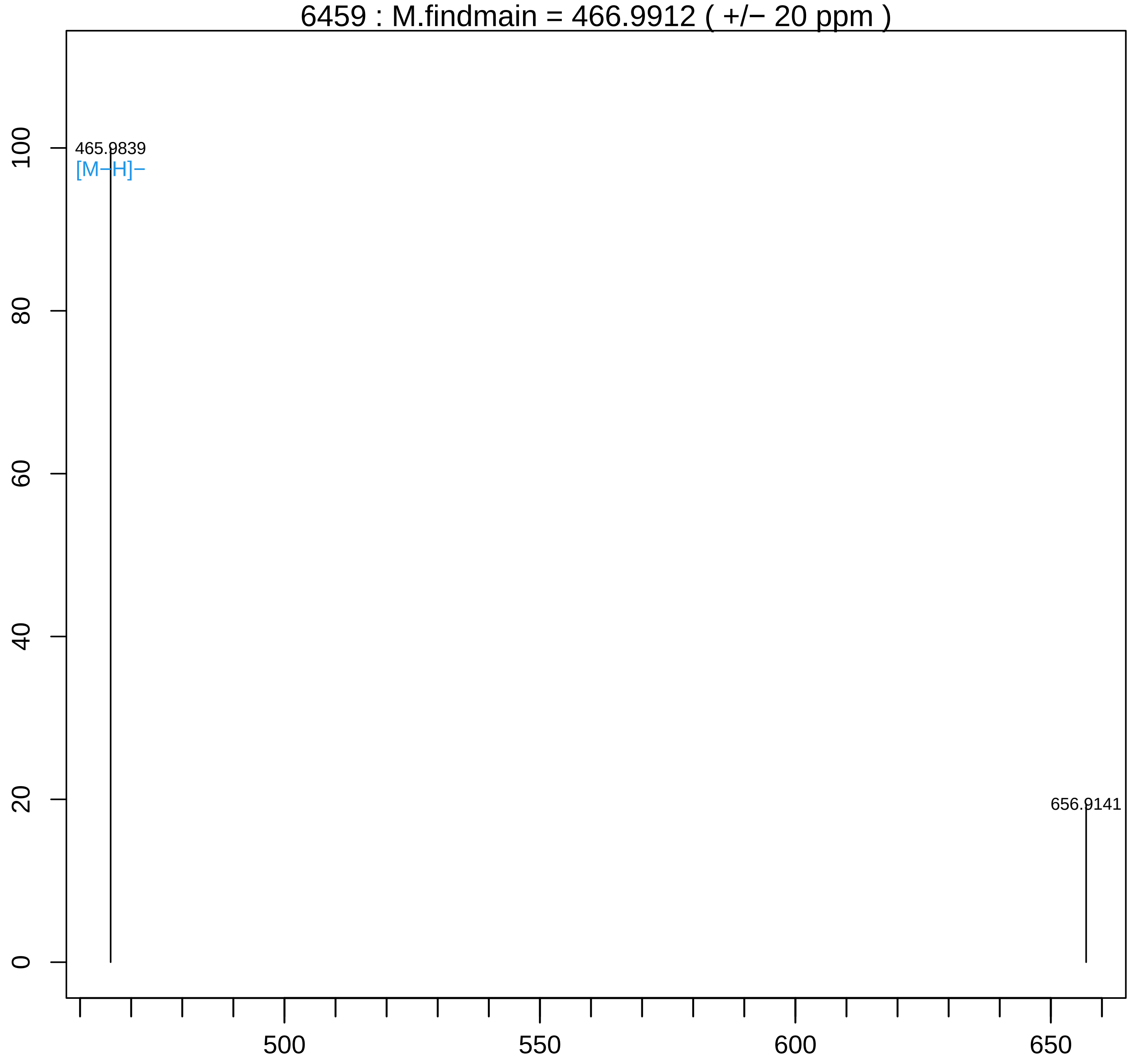

6459 : M.findmain = 466.9912 (+/− 20 ppm)
465.9839
[M−H]−
656.9141
0
20
40
60
80
100
500
550
600
650

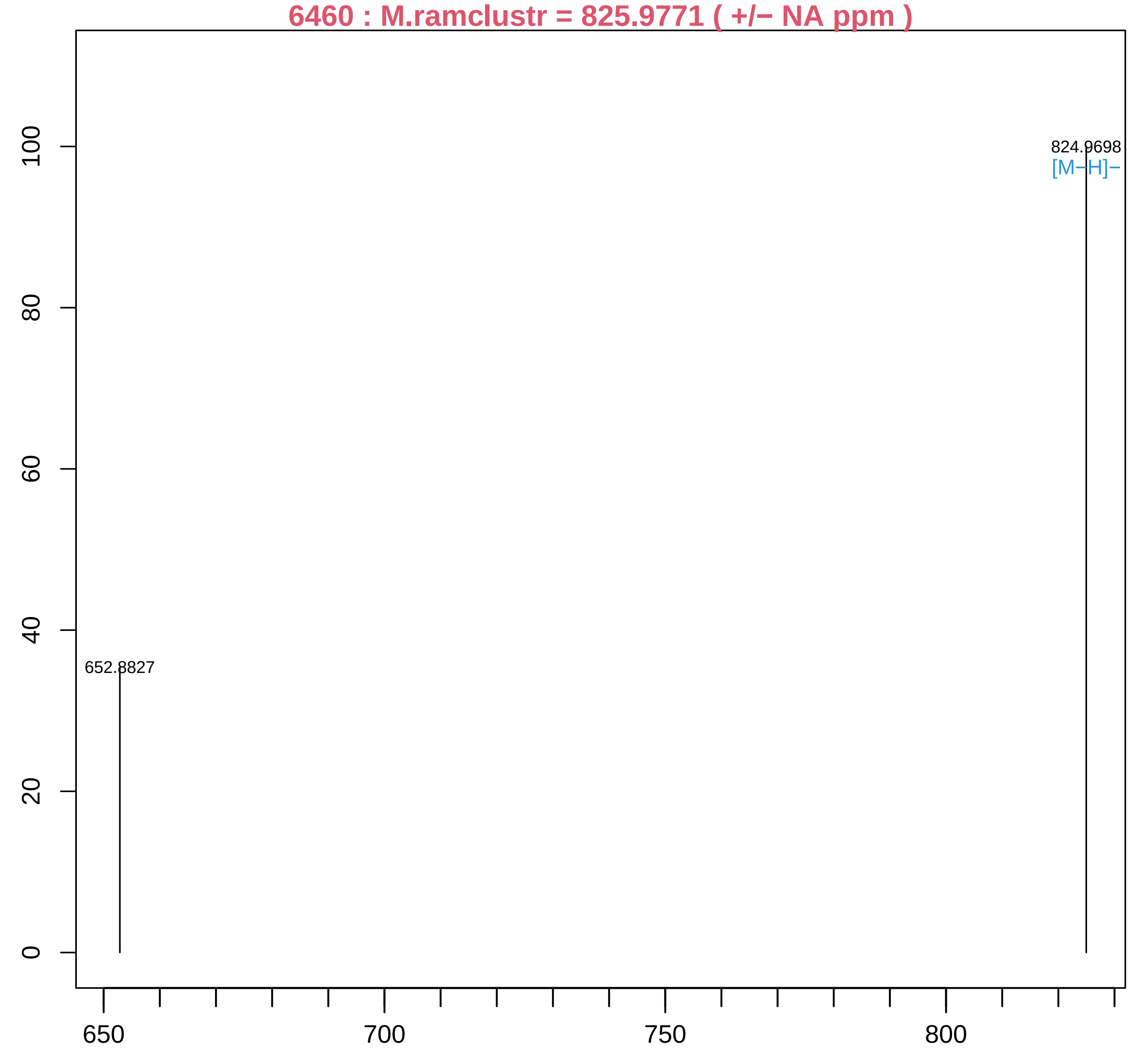
6460 : M.ramclustr = 825.9771 (+/− NA ppm)
824.9698
[M−H]−
652.8827
0
20
40
60
80
100
650
700
750
800

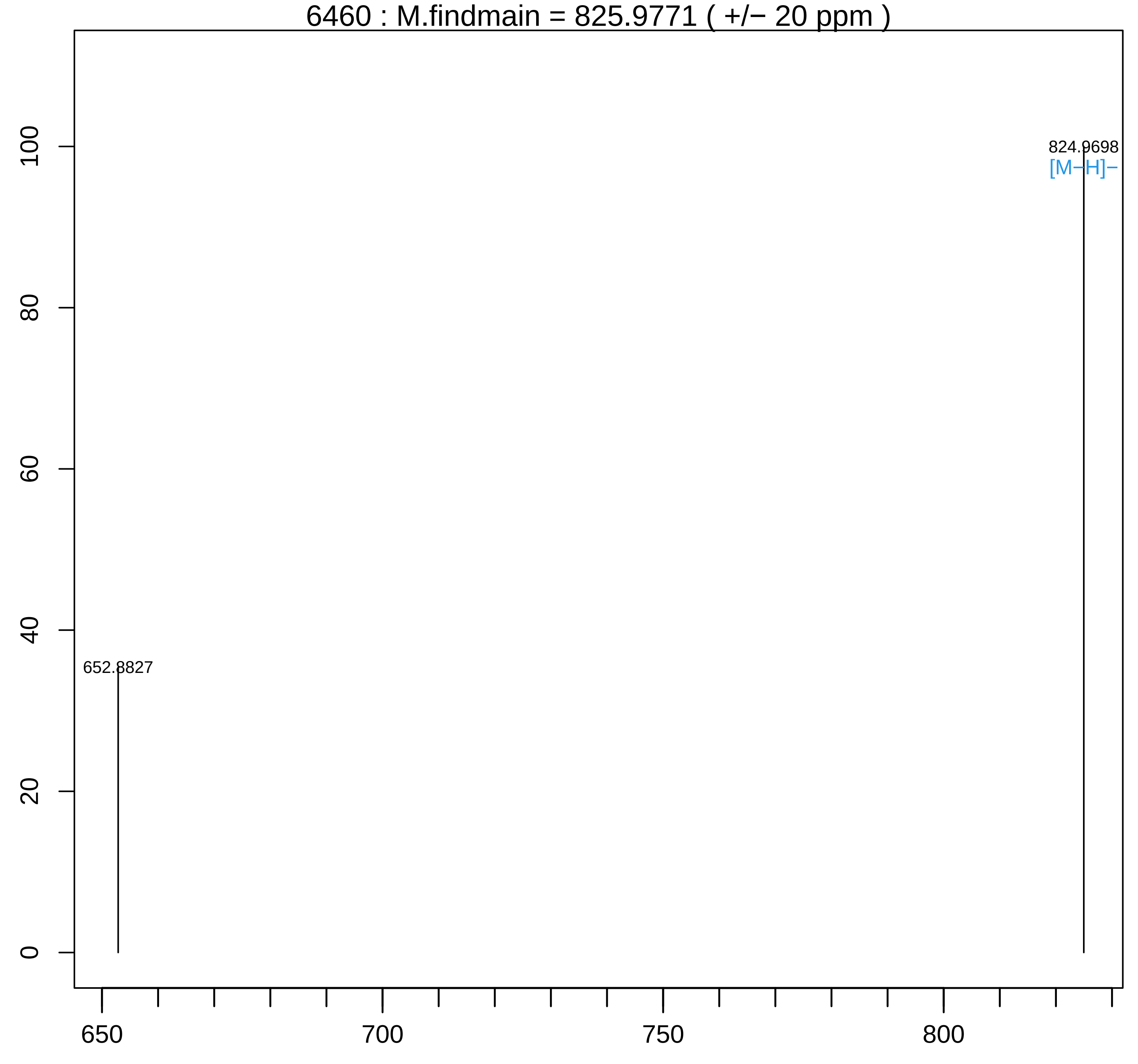
6460 : M.findmain = 825.9771 (+/− 20 ppm)
824.9698
[M−H]−
652.8827
0
20
40
60
80
100
650
700
750
800

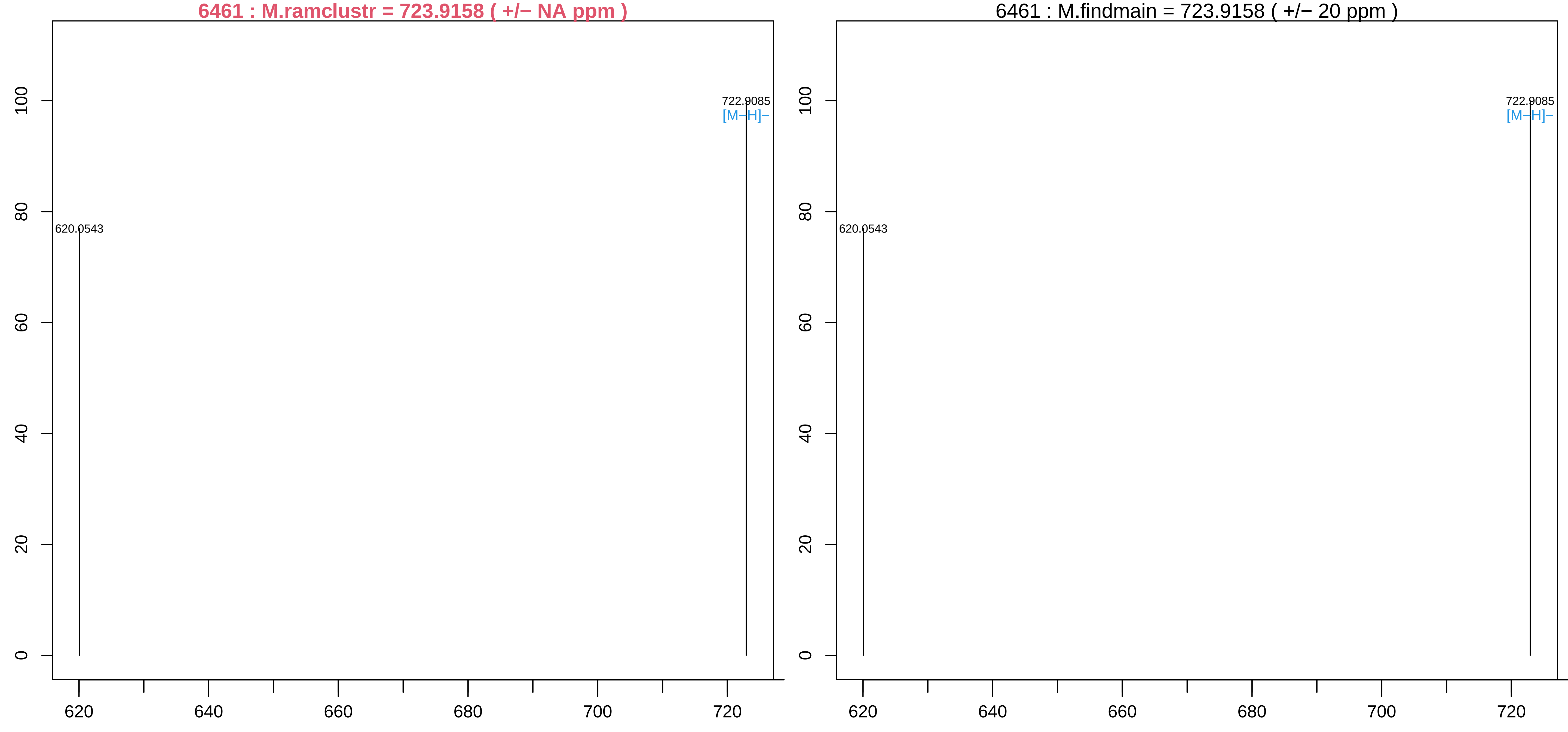
6461 : M.ramclustr = 723.9158 (+/− NA ppm)
722.9085
[M−H]−
620.0543
0
20
40
60
80
100
620
640
660
680
700
720
6461 : M.findmain = 723.9158 (+/− 20 ppm)
722.9085
[M−H]−
620.0543
0
20
40
60
80
100
620
640
660
680
700
720

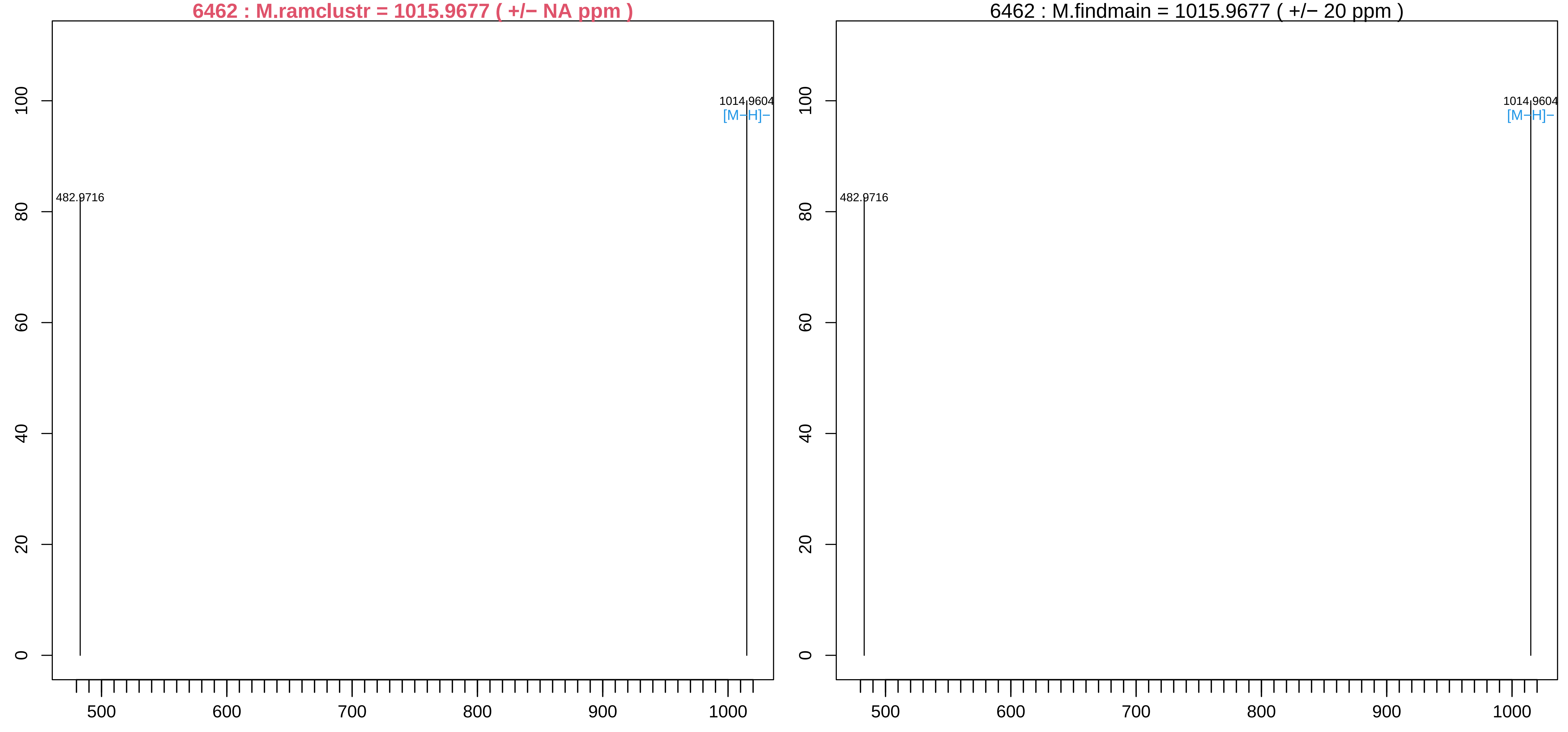
6462 : M.ramclustr = 1015.9677 (+/− NA ppm)
6462 : M.findmain = 1015.9677 (+/− 20 ppm)
482.9716
1014.9604
[M−H]−
482.9716
1014.9604
[M−H]−
0
20
40
60
80
100
500
600
700
800
900
1000

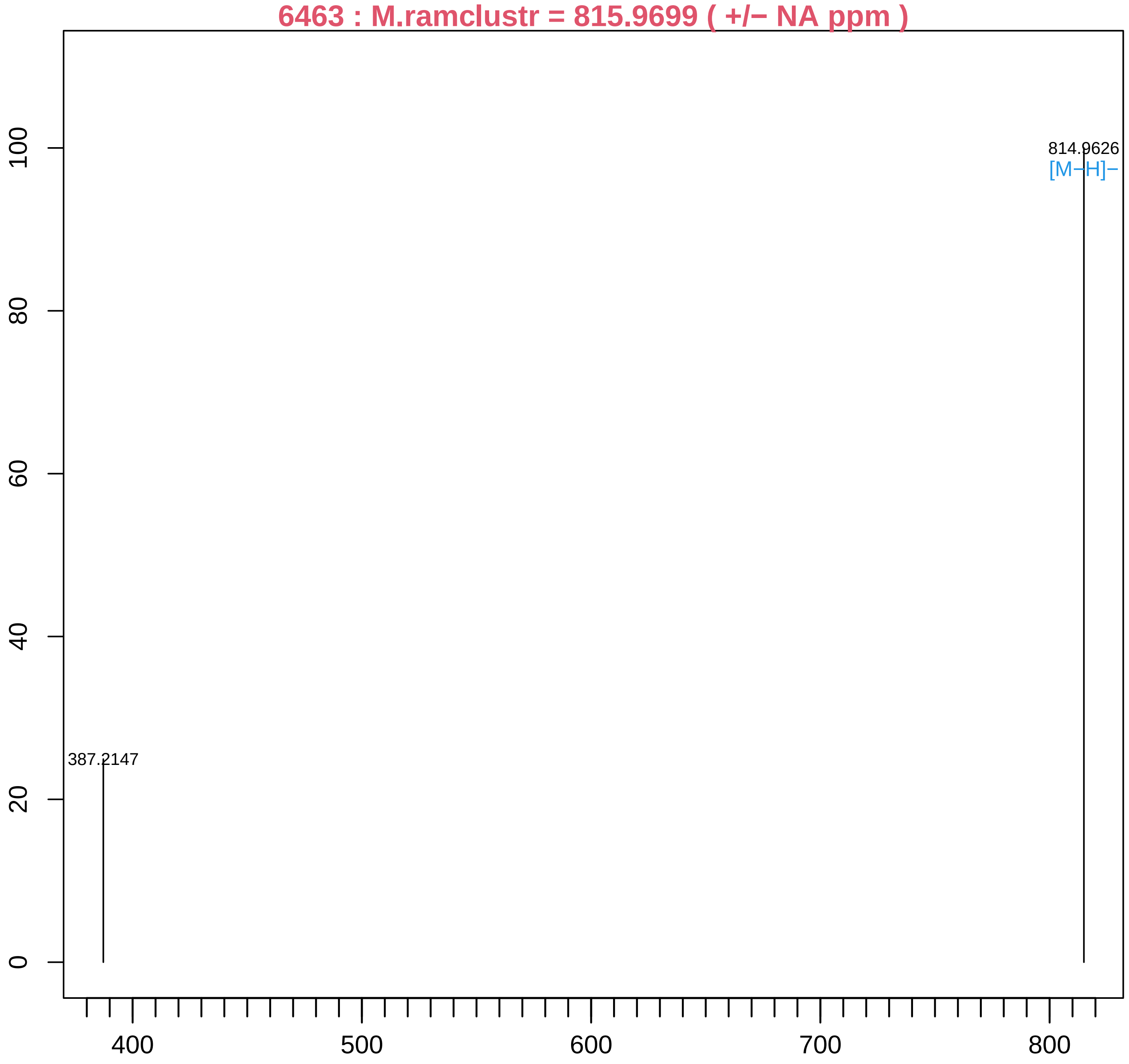
6463 : M.ramclustr = 815.9699 (+/− NA ppm)
814.9626
[M−H]−
387.2147
0
20
40
60
80
100
400
500
600
700
800

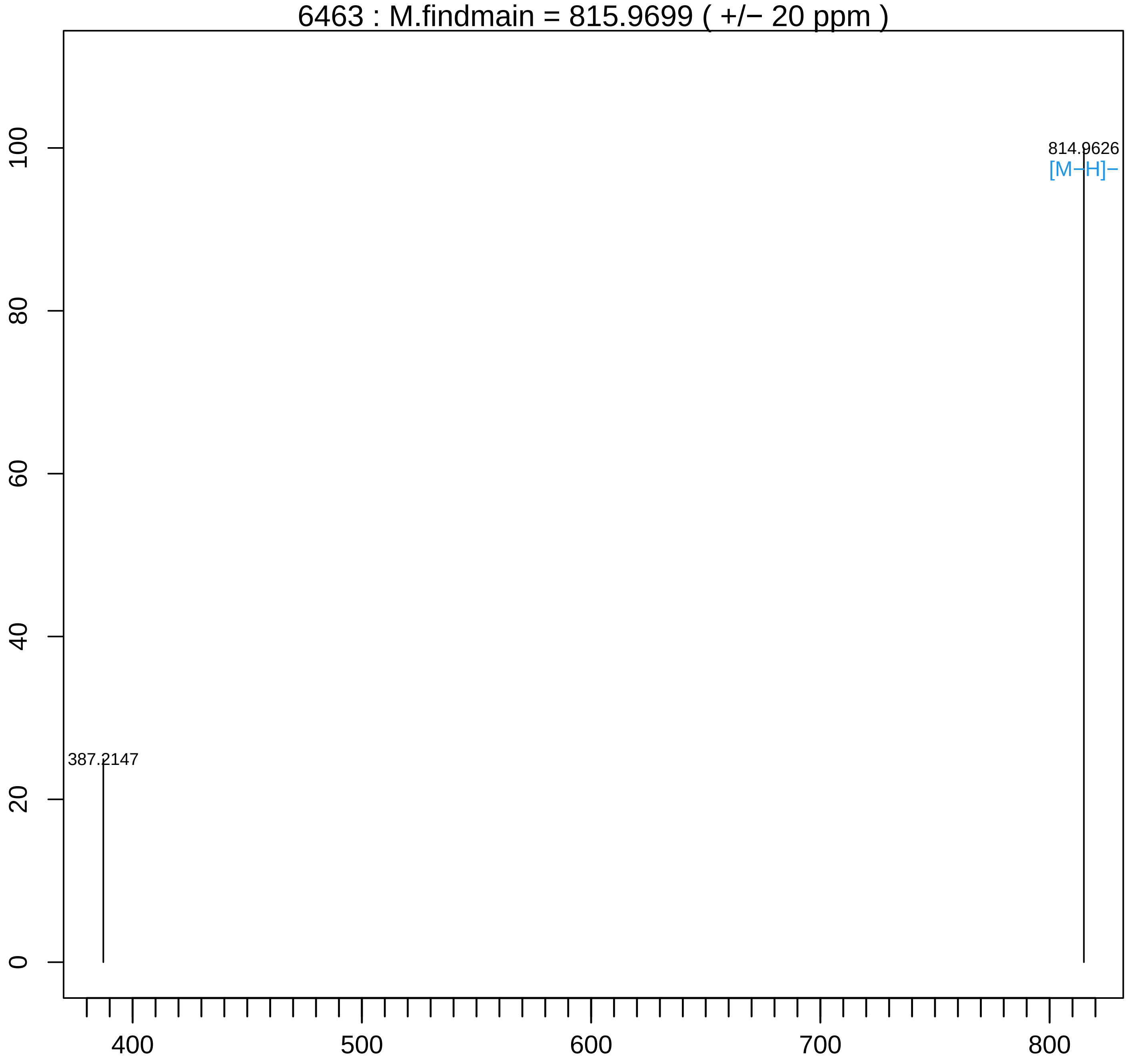
6463 : M.findmain = 815.9699 (+/− 20 ppm)
814.9626
[M−H]−
387.2147
0
20
40
60
80
100
400
500
600
700
800

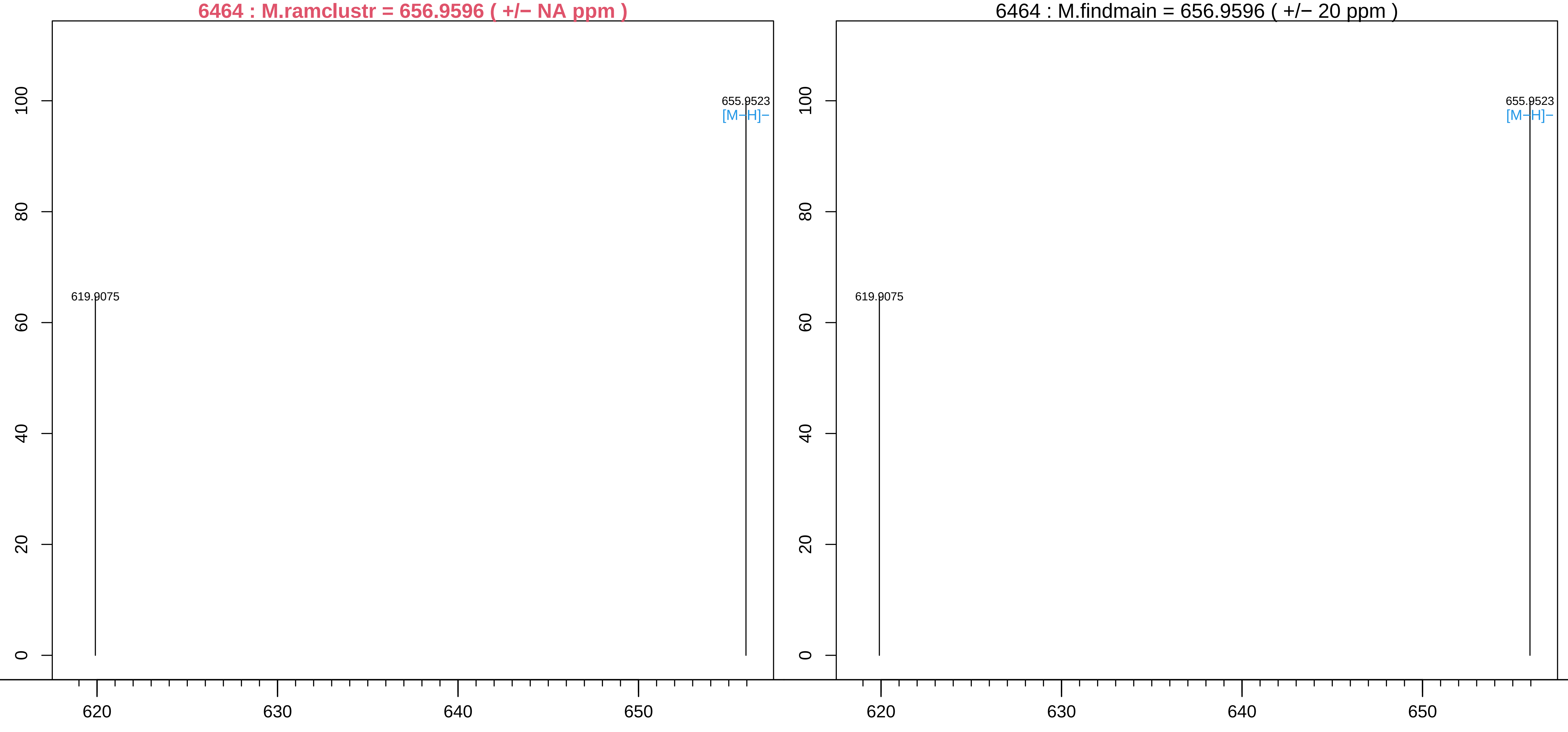
6464 : M.ramclustr = 656.9596 (+/− NA ppm)
6464 : M.findmain = 656.9596 (+/− 20 ppm)
655.9523
[M−H]−
619.9075
0
20
40
60
80
100
620
630
640
650

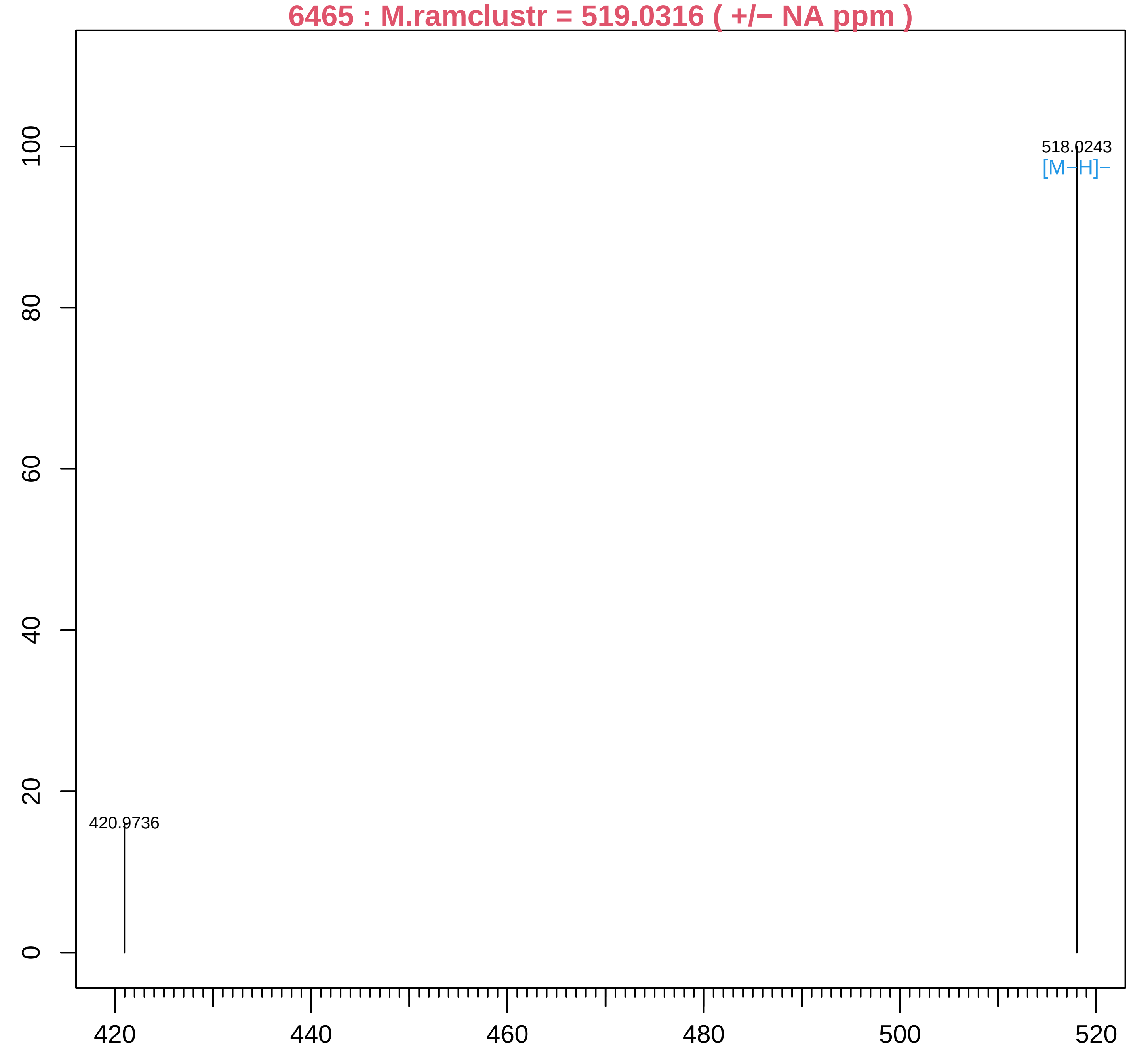

6465 : M.ramclustr = 519.0316 (+/− NA ppm)
518.0243
[M−H]−
420.9736

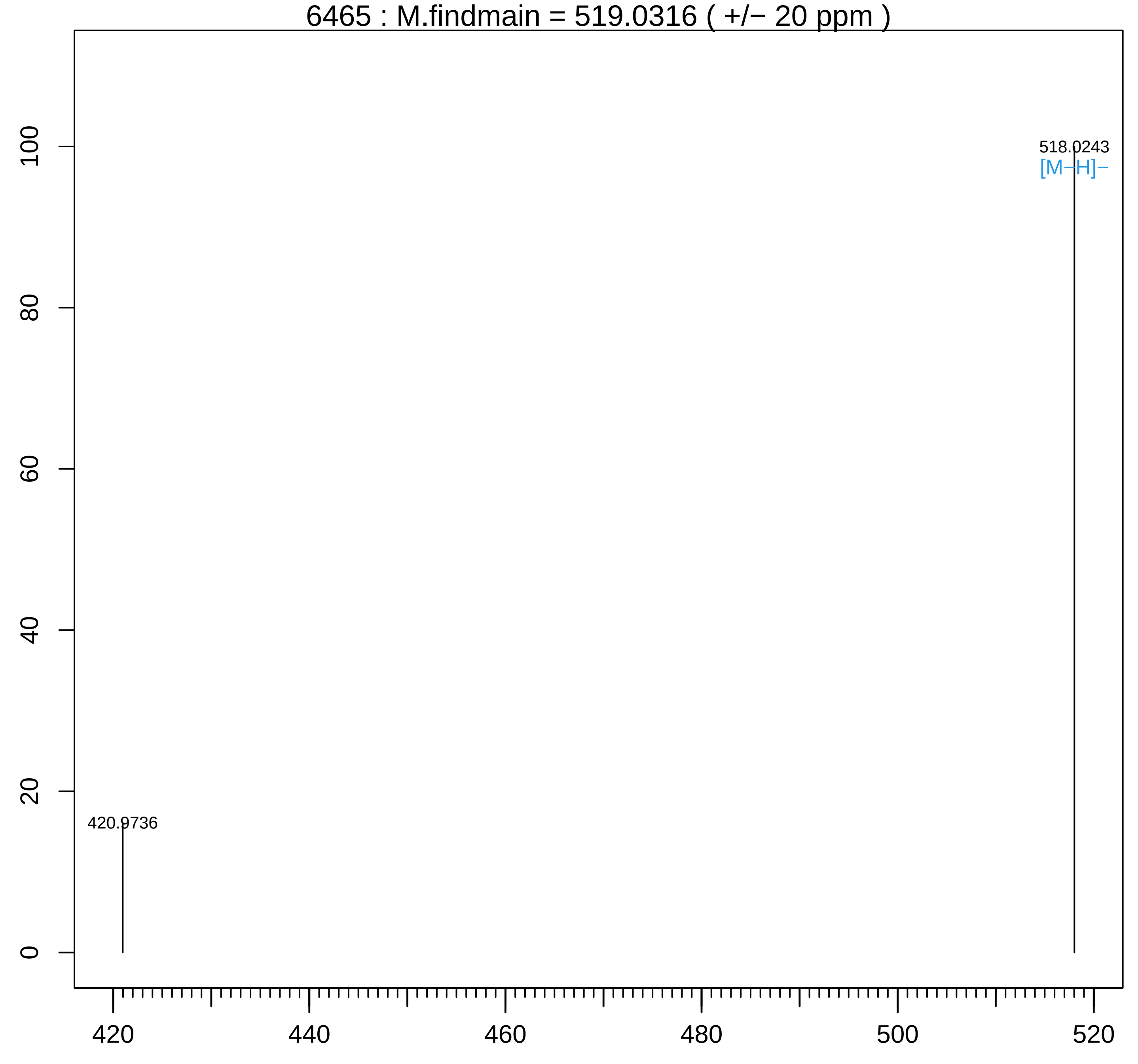

6465 : M.findmain = 519.0316 (+/− 20 ppm)
518.0243
[M−H]−
420.9736

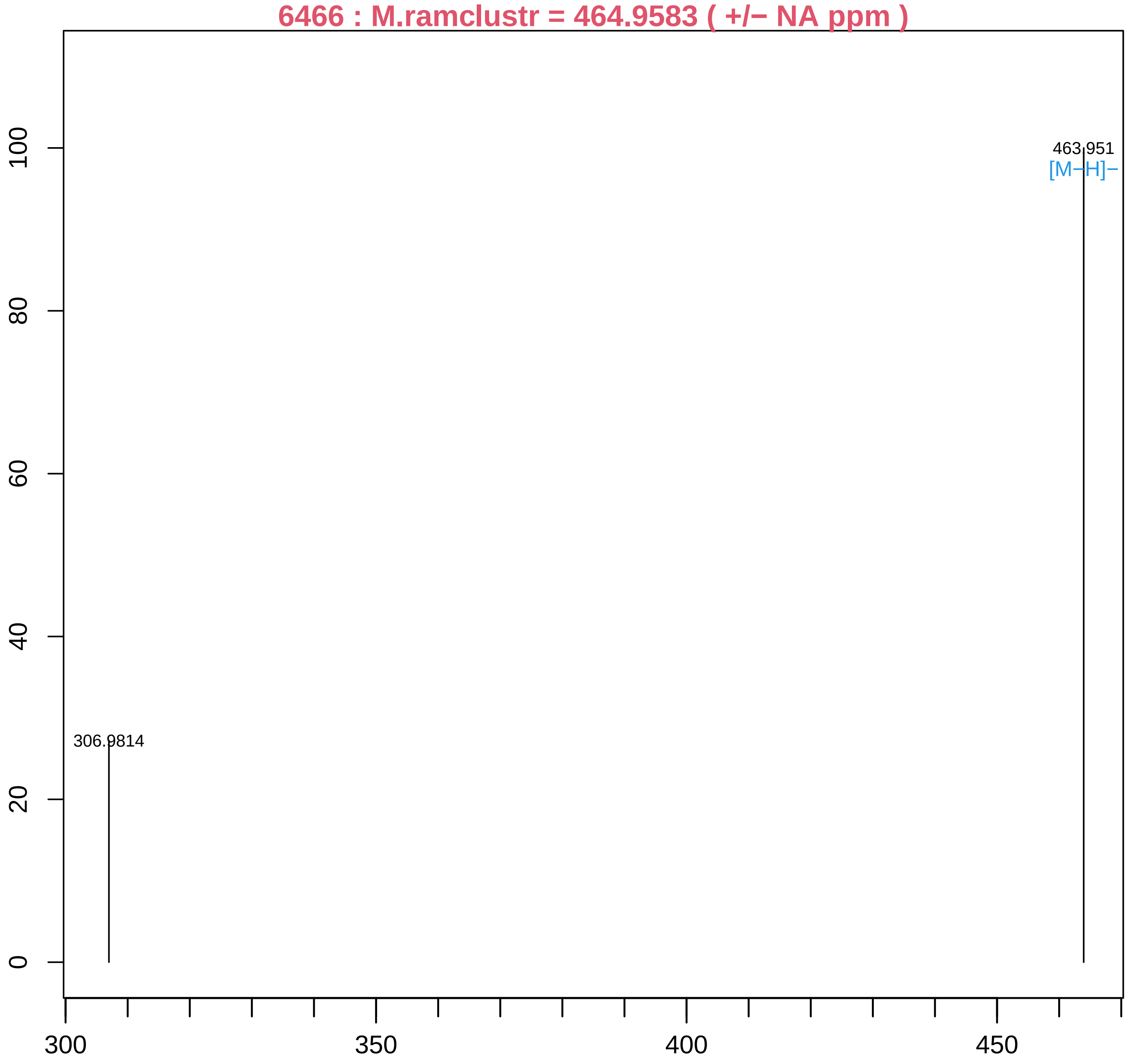
6466 : M.ramclustr = 464.9583 (+/− NA ppm)
463.951
[M−H]−
306.9814
0
20
40
60
80
100
300
350
400
450

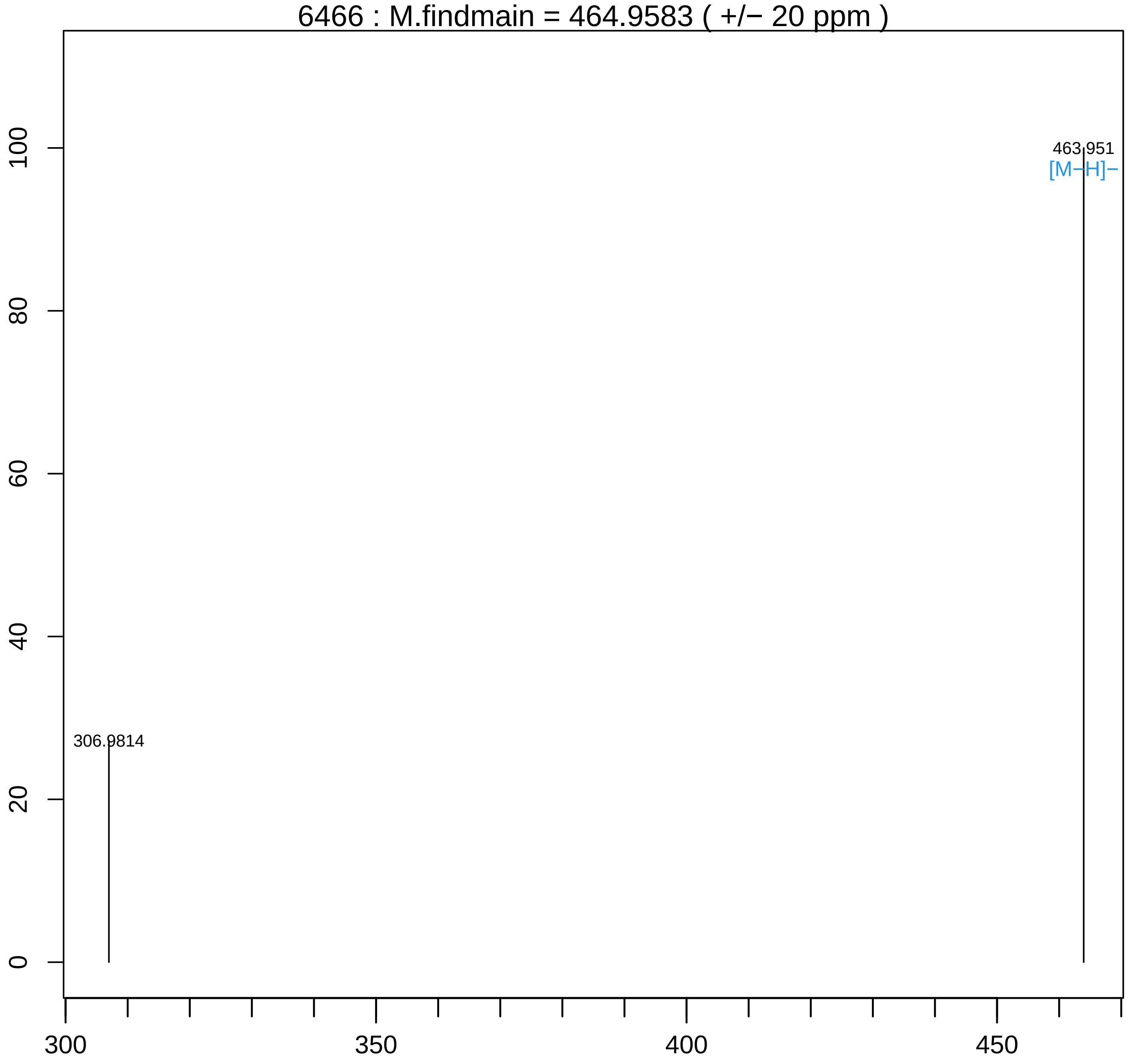
6466 : M.findmain = 464.9583 (+/− 20 ppm)
463.951
[M−H]−
306.9814
0
20
40
60
80
100
300
350
400
450

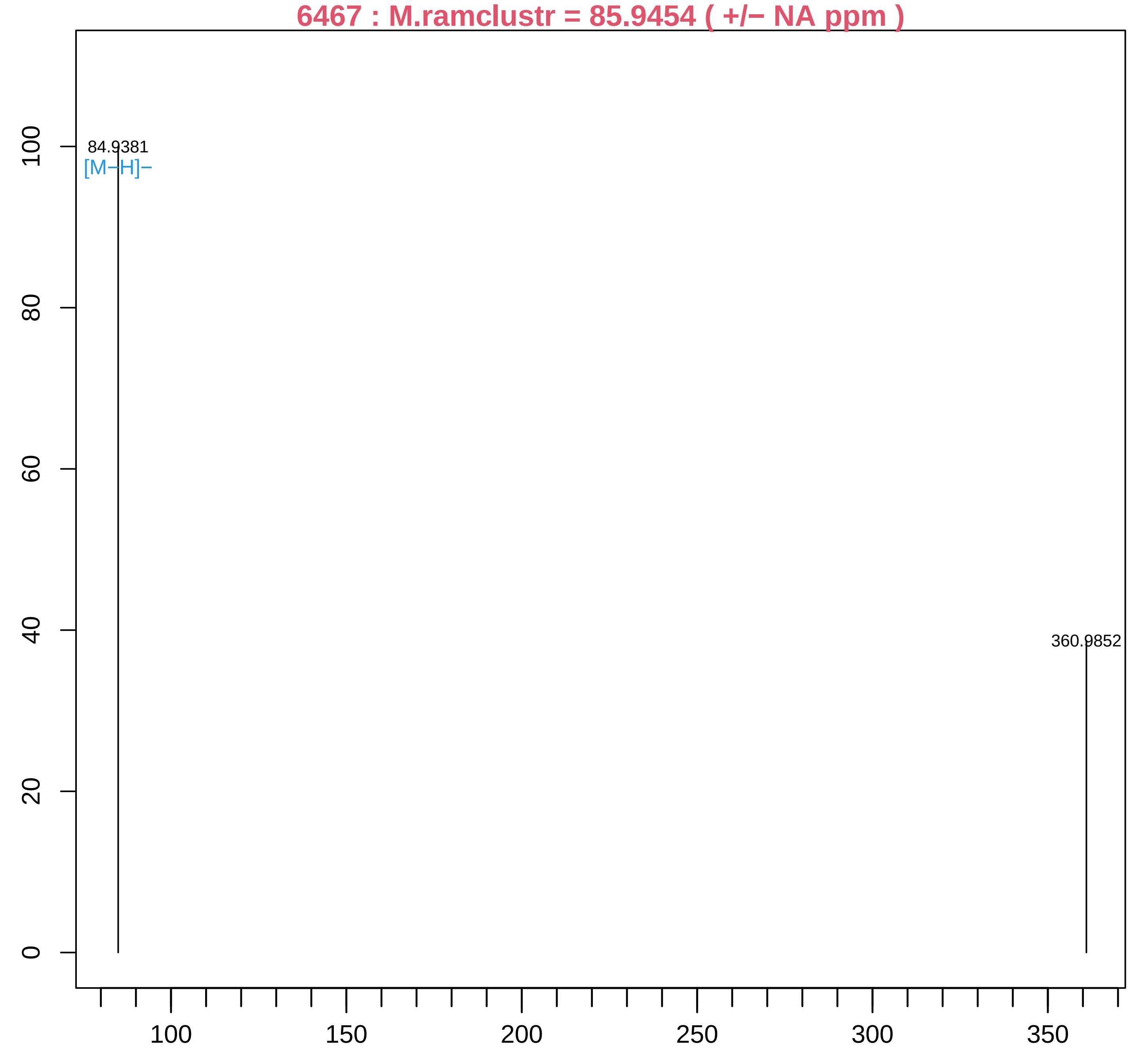
6467 : M.ramclustr = 85.9454 (+/− NA ppm)
84.9381
[M−H]−
360.9852
0
20
40
60
80
100
100
150
200
250
300
350

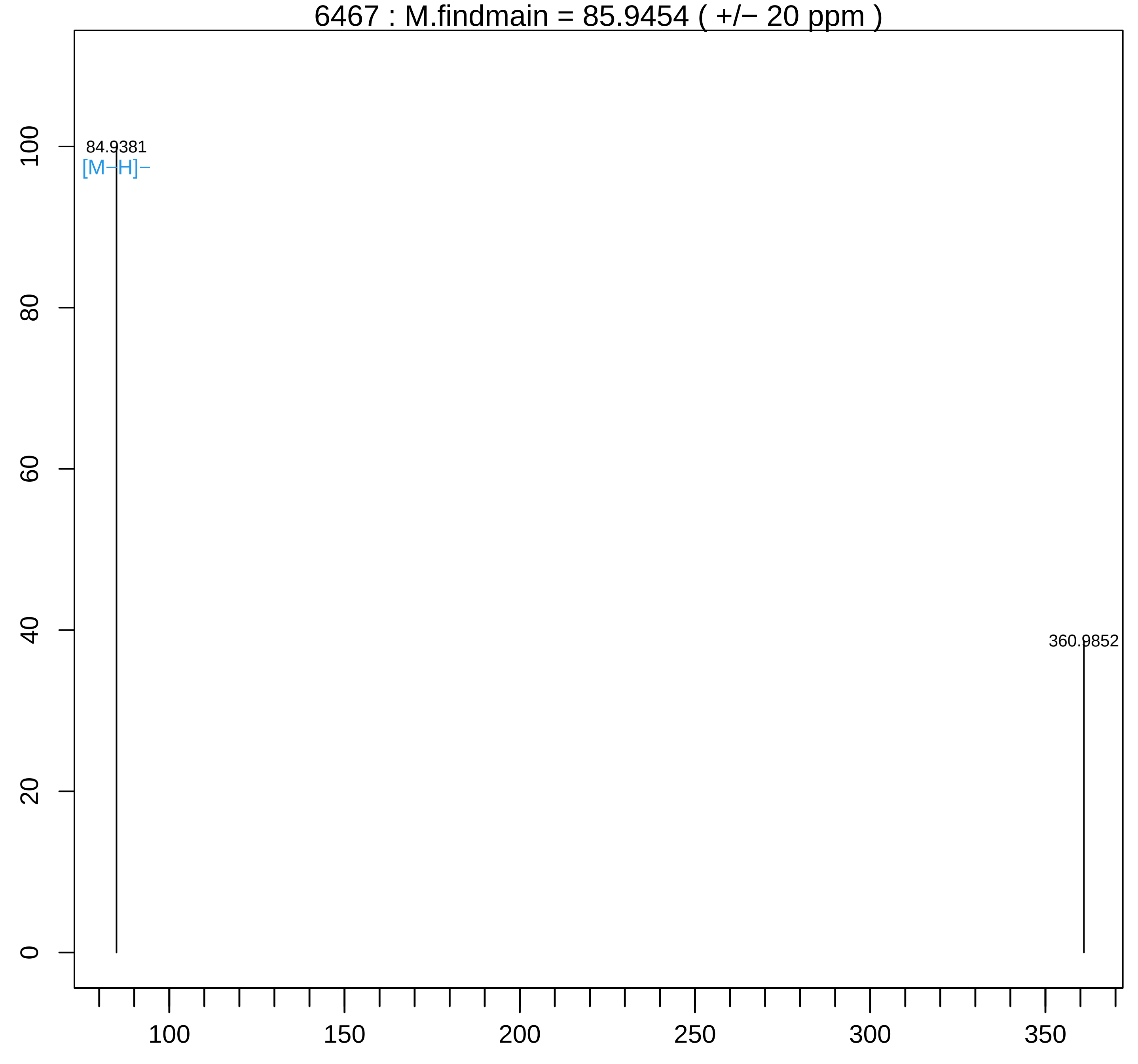
6467 : M.findmain = 85.9454 (+/− 20 ppm)
84.9381
[M−H]−
360.9852
0
20
40
60
80
100
100
150
200
250
300
350

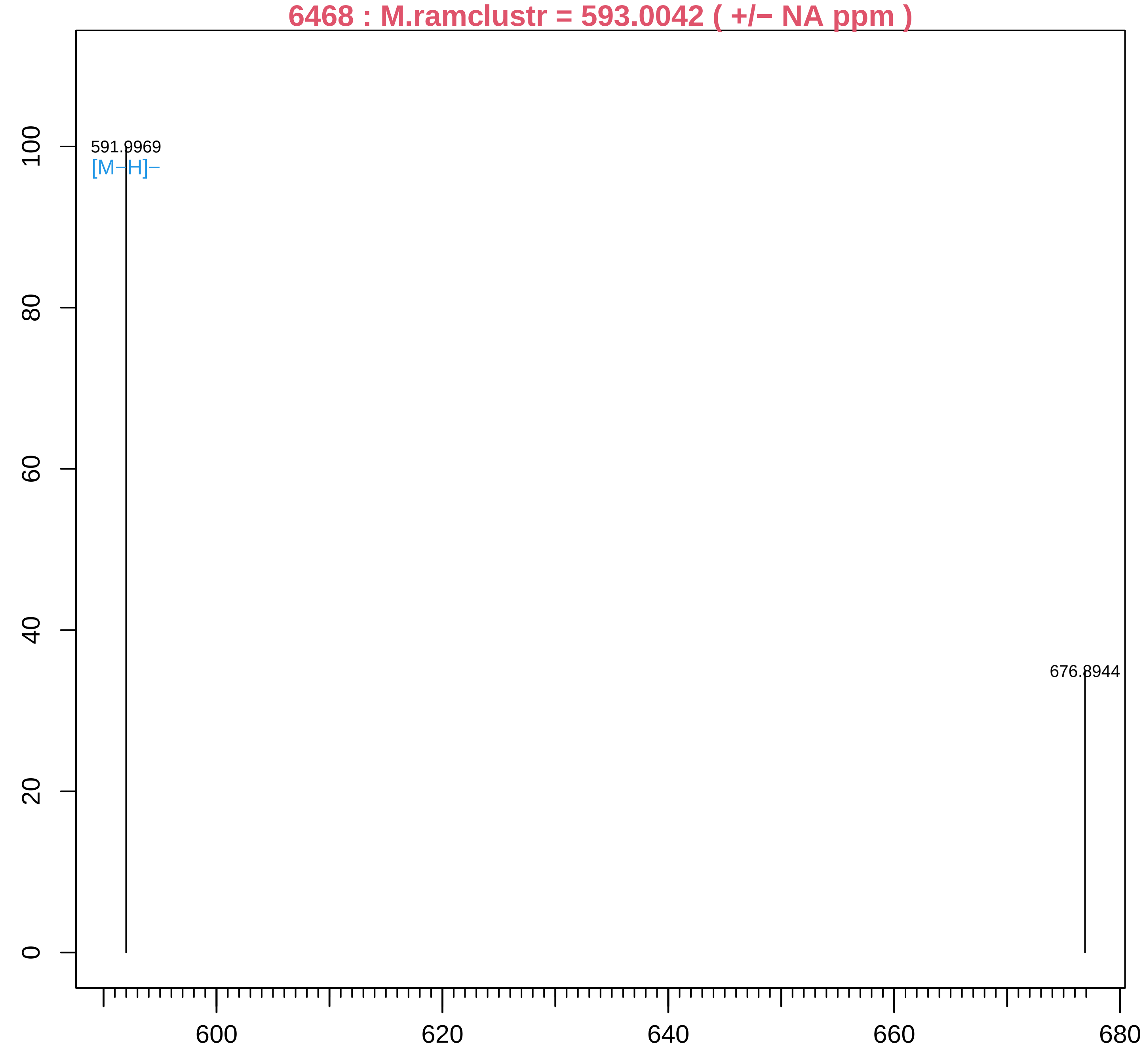

6468 : M.ramclustr = 593.0042 (+/− NA ppm)
591.9969
[M−H]−
676.8944

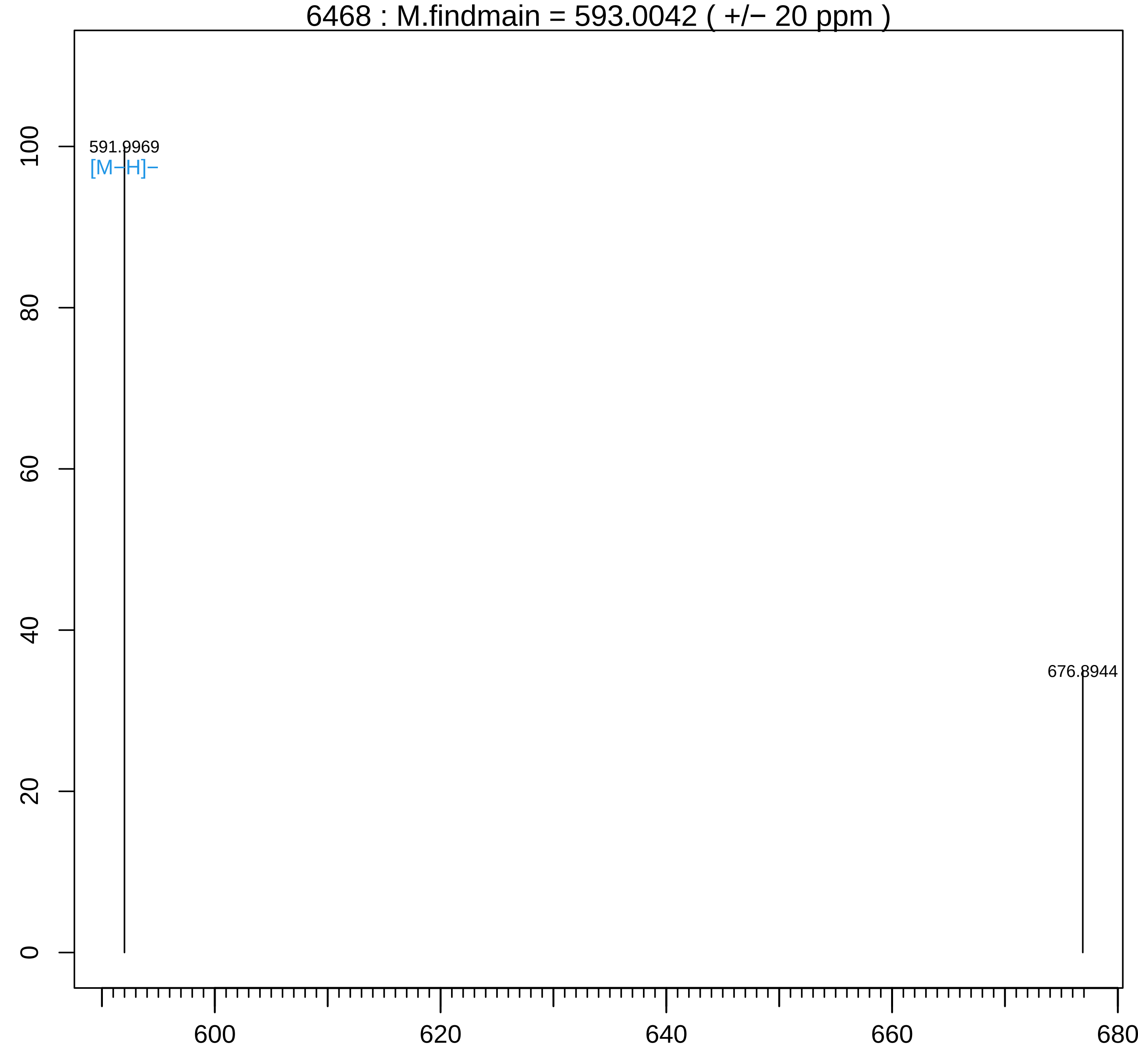

6468 : M.findmain = 593.0042 (+/− 20 ppm)
591.9969
[M−H]−
676.8944

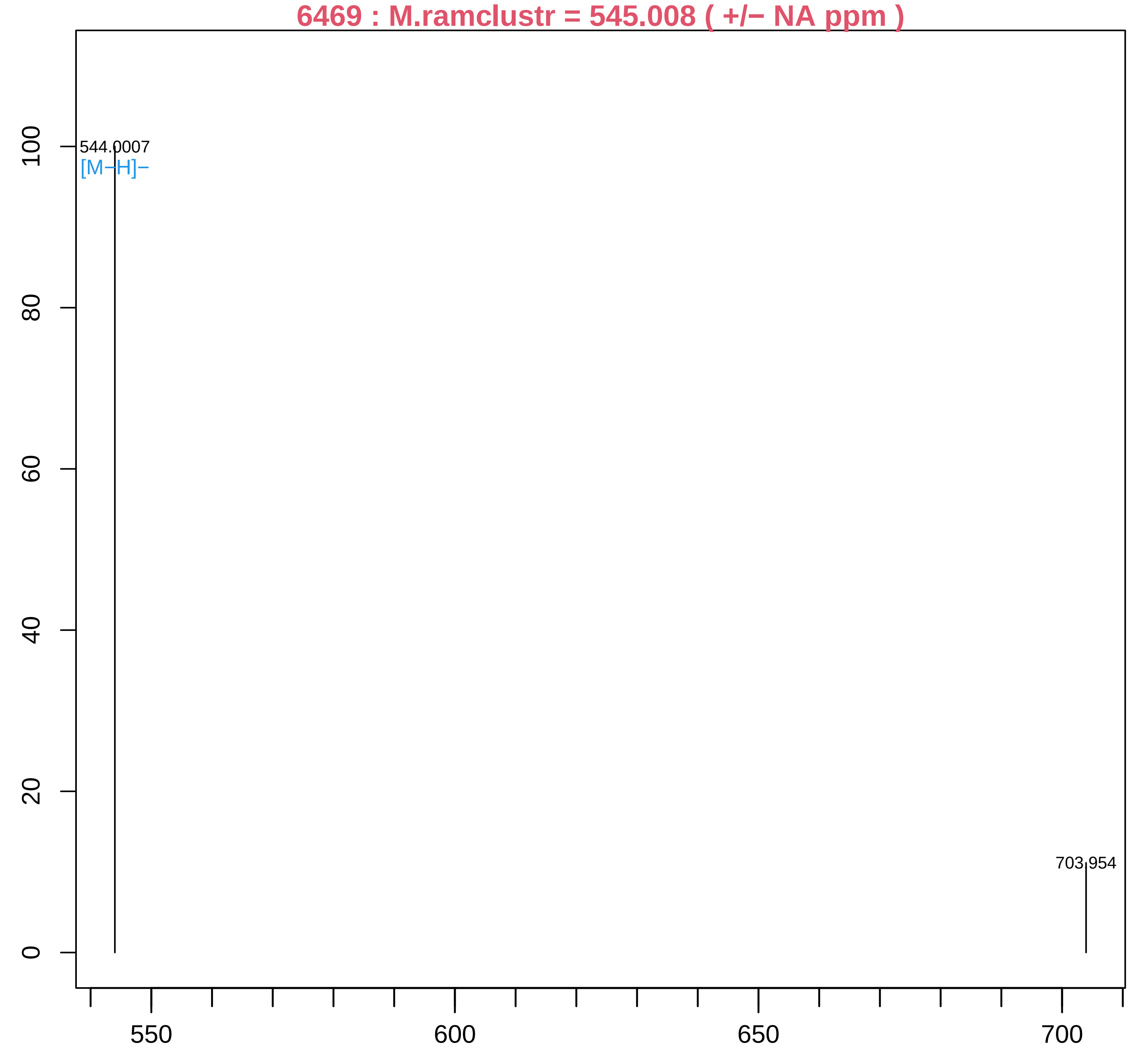
6469 : M.ramclustr = 545.008 (+/− NA ppm)
544.0007
[M−H]−
703.954
0
20
40
60
80
100
550
600
650
700

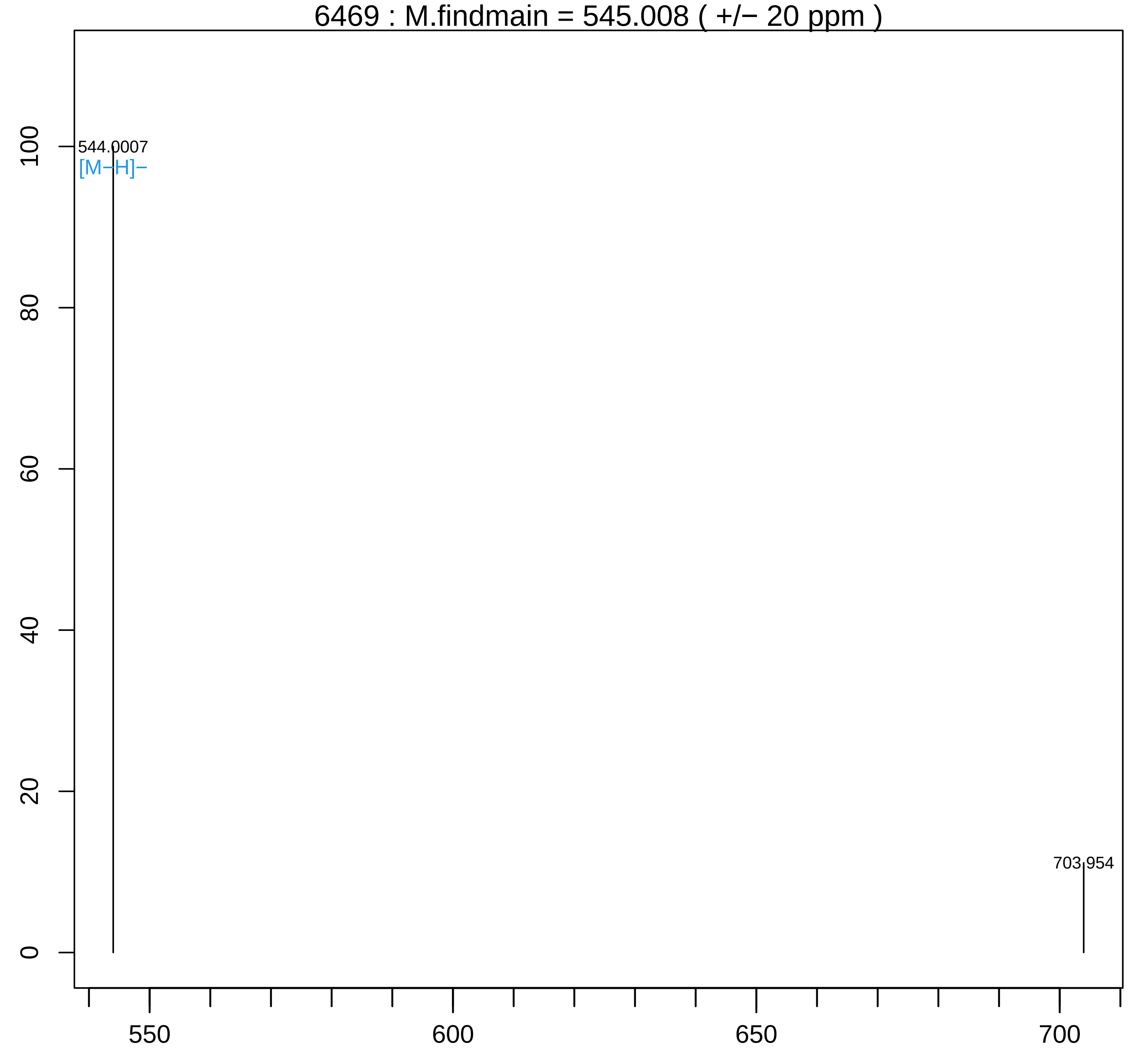
6469 : M.findmain = 545.008 (+/− 20 ppm)
544.0007
[M−H]−
703.954
0
20
40
60
80
100
550
600
650
700

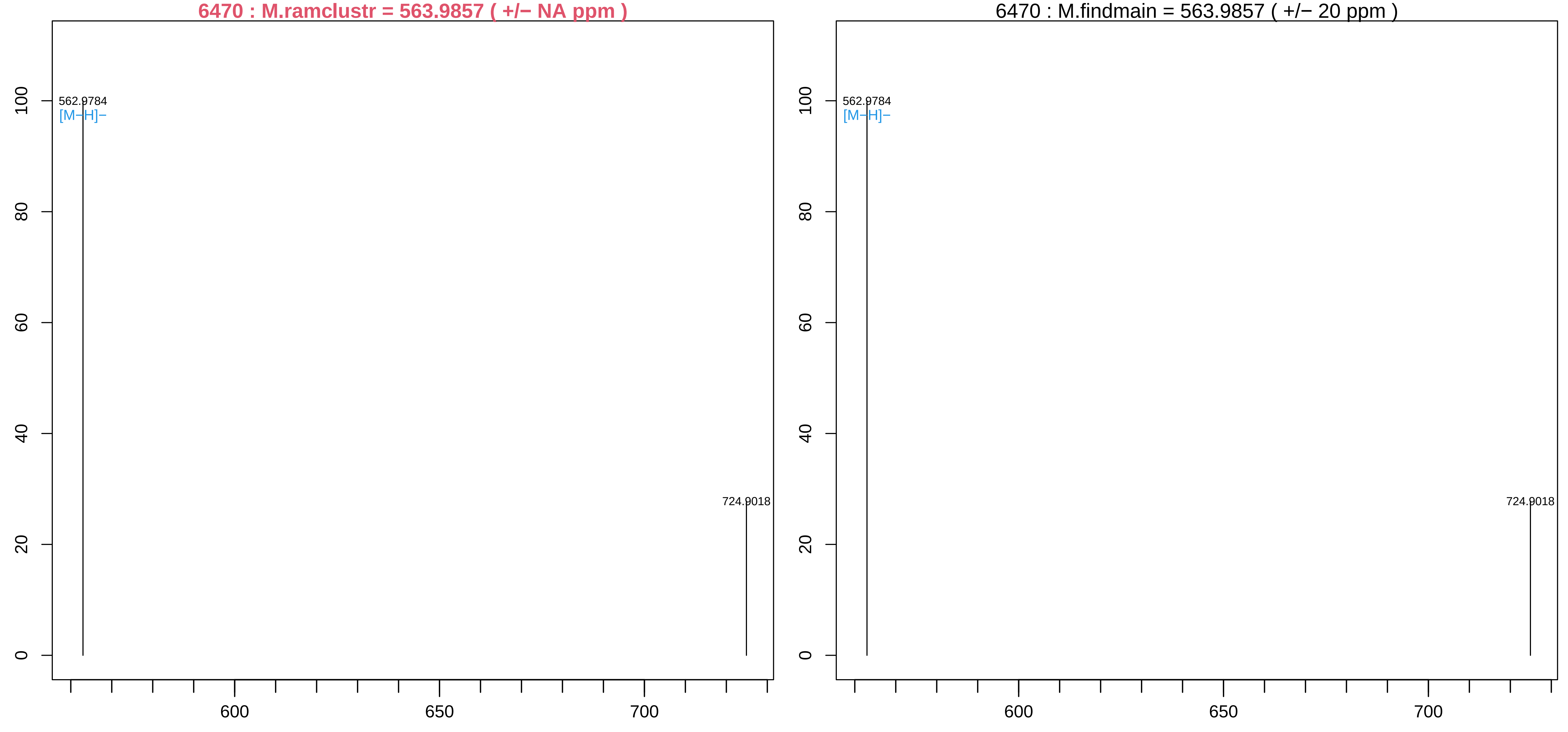
6470 : M.ramclustr = 563.9857 (+/− NA ppm)
562.9784
[M−H]−
724.9018
6470 : M.findmain = 563.9857 (+/− 20 ppm)
562.9784
[M−H]−
724.9018
0
20
40
60
80
100
600
650
700

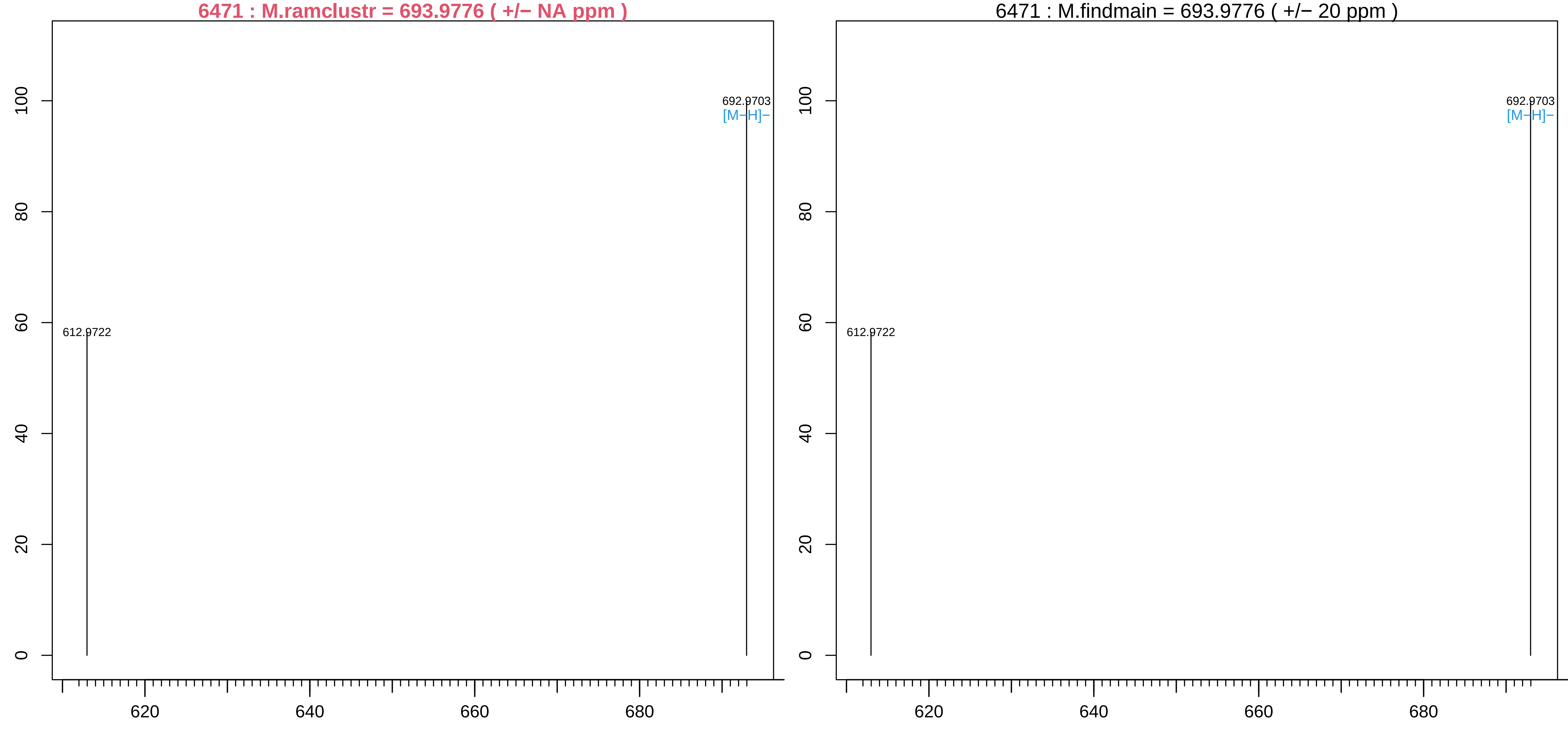
6471 : M.ramclustr = 693.9776 (+/− NA ppm)
612.9722
692.9703
[M−H]−
6471 : M.findmain = 693.9776 (+/− 20 ppm)
612.9722
692.9703
[M−H]−

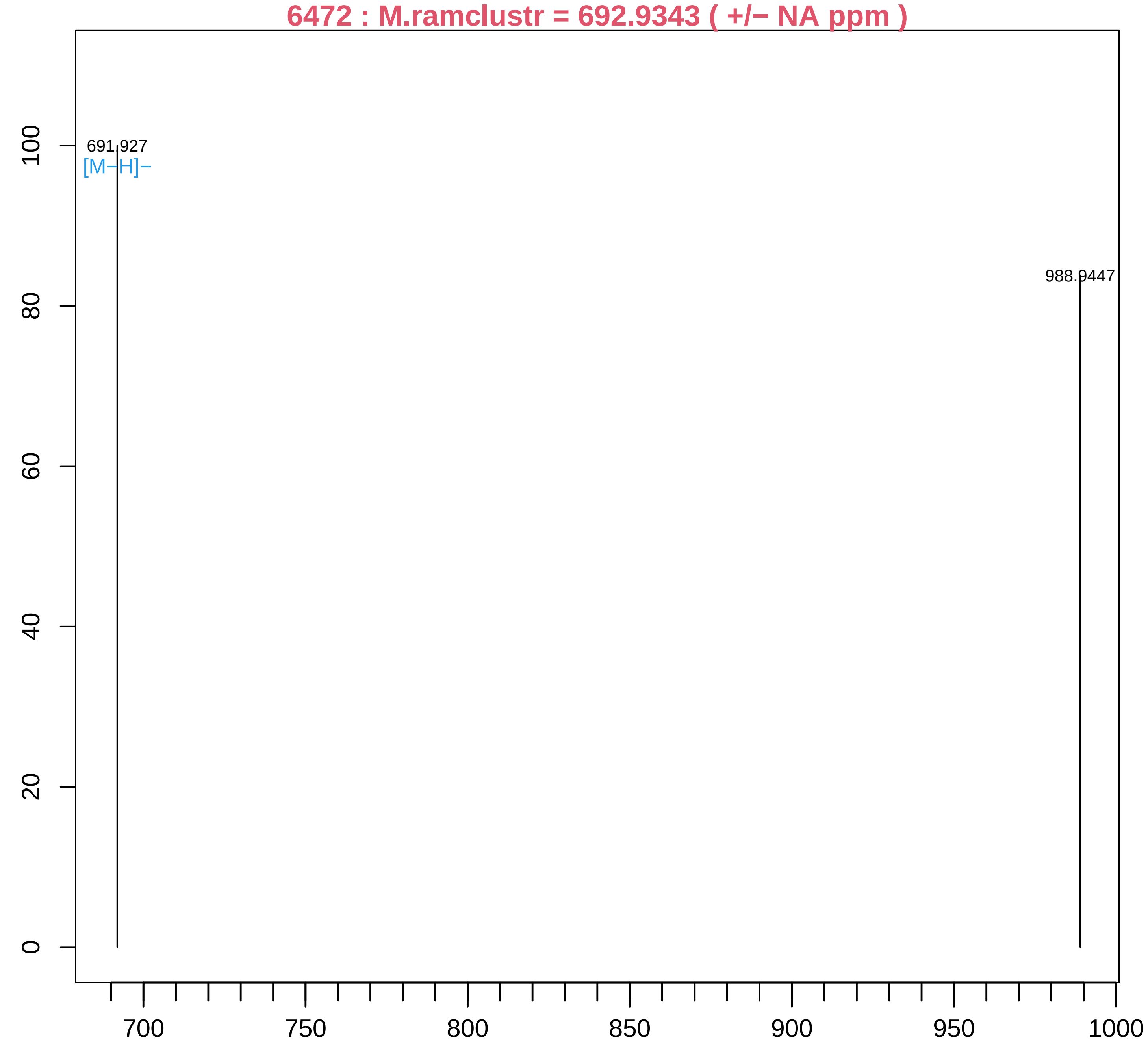
6472 : M.ramclustr = 692.9343 (+/− NA ppm)
691.927
[M−H]−
988.9447
0
20
40
60
80
100
700
750
800
850
900
950
1000

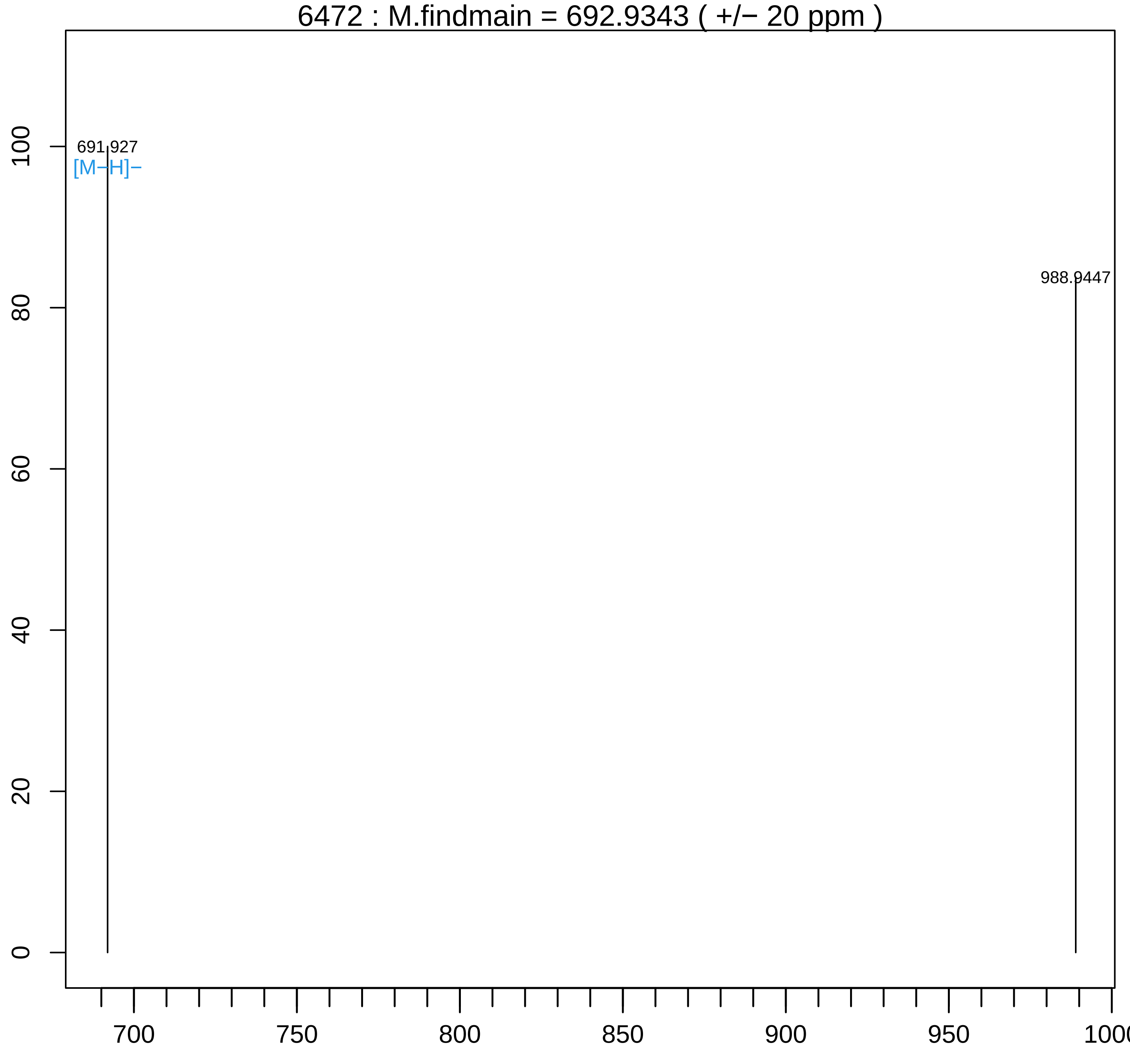
6472 : M.findmain = 692.9343 (+/− 20 ppm)
691.927
[M−H]−
988.9447
0
20
40
60
80
100
700
750
800
850
900
950
1000

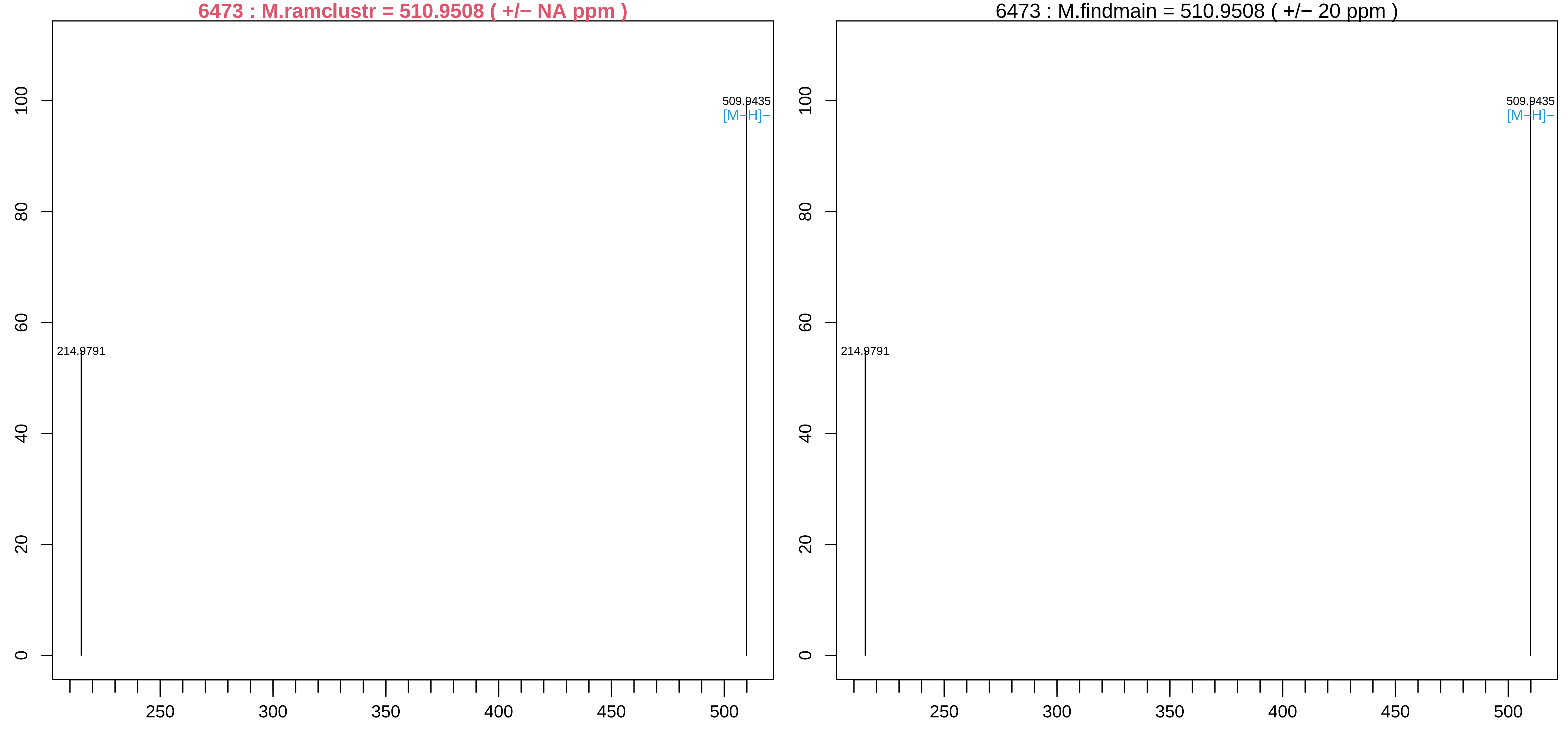
6473 : M.ramclustr = 510.9508 (+/− NA ppm)
6473 : M.findmain = 510.9508 (+/− 20 ppm)
509.9435
[M−H]−
214.9791
509.9435
[M−H]−
214.9791
0
20
40
60
80
100
250
300
350
400
450
500

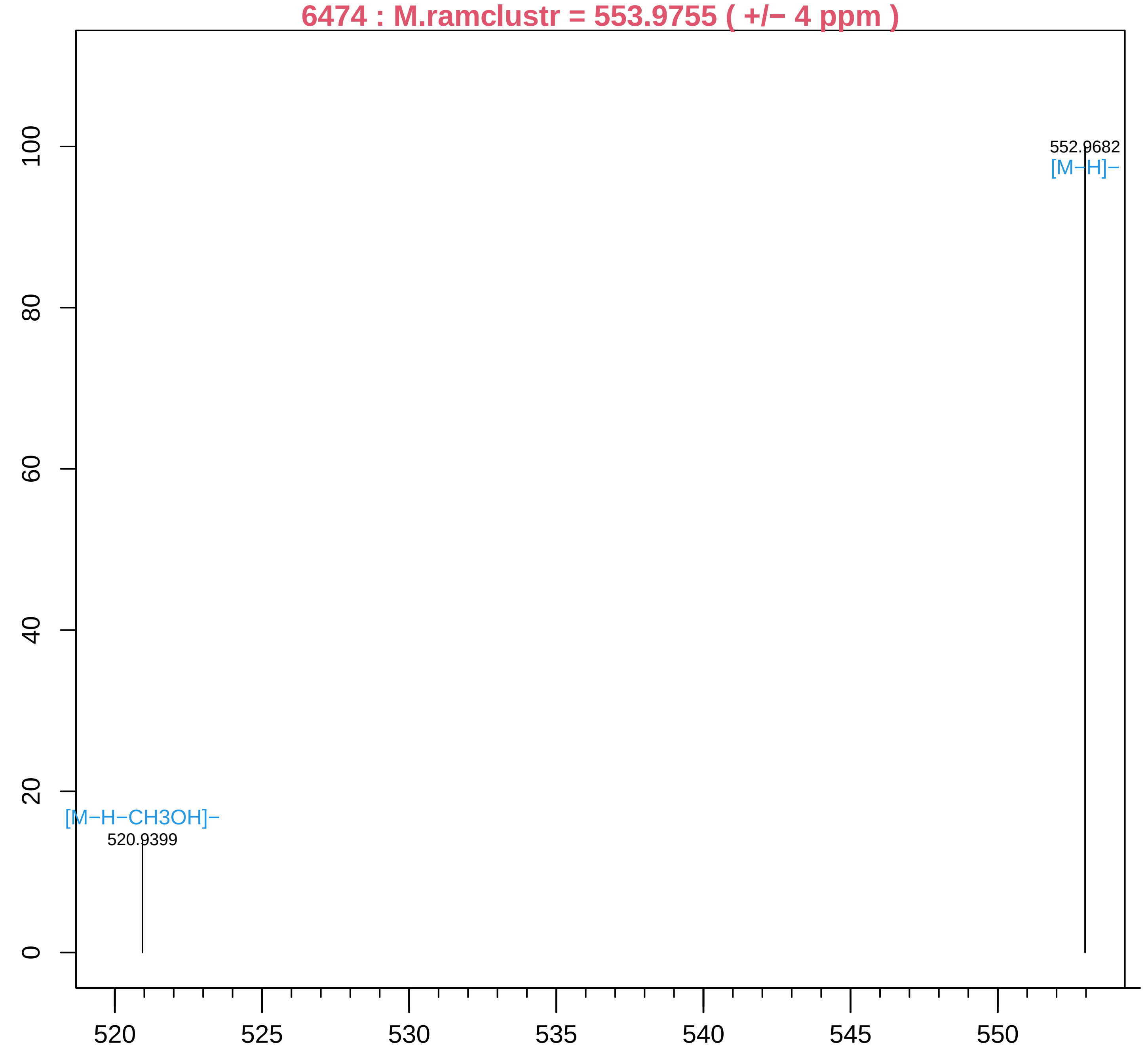
6474 : M.ramclustr = 553.9755 (+/− 4 ppm)
552.9682
[M−H]−
[M−H−CH3OH]−
520.9399
0
20
40
60
80
100
520
525
530
535
540
545
550

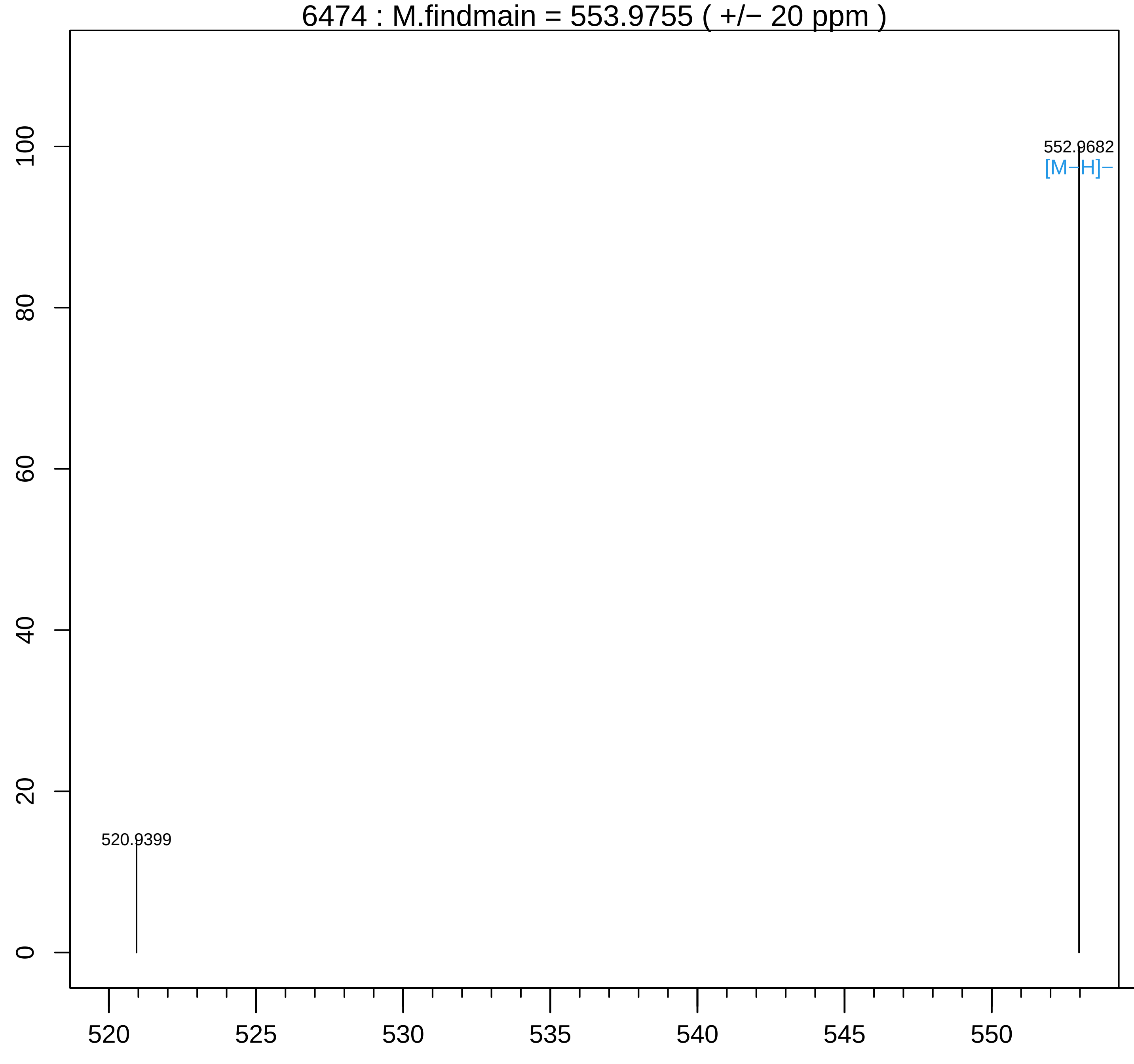
6474 : M.findmain = 553.9755 (+/− 20 ppm)
552.9682
[M−H]−
520.9399
0
20
40
60
80
100
520
525
530
535
540
545
550

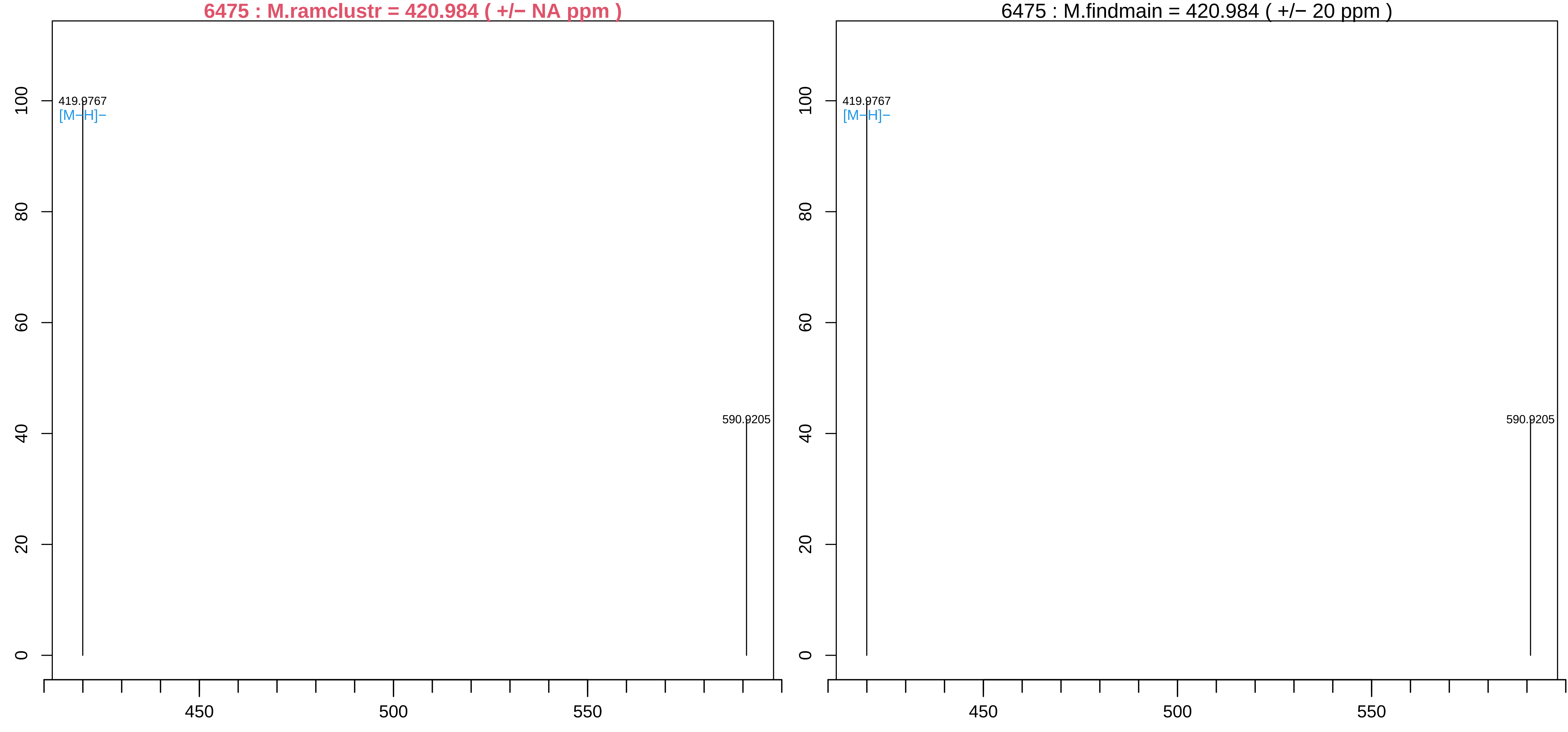
6475 : M.ramclustr = 420.984 (+/− NA ppm)
6475 : M.findmain = 420.984 (+/− 20 ppm)
419.9767
[M−H]−
590.9205
0
20
40
60
80
100
450
500
550

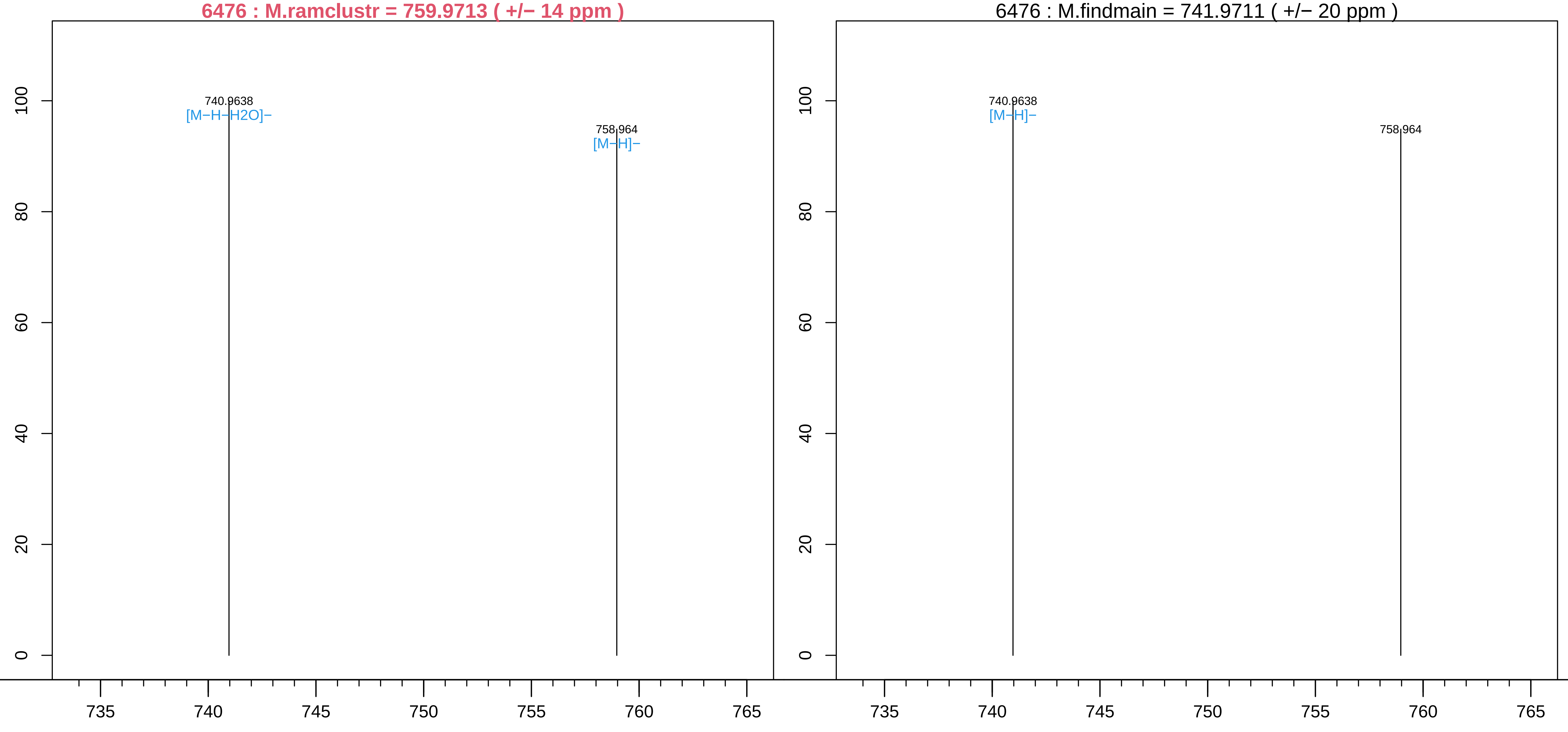
6476 : M.ramclustr = 759.9713 (+/− 14 ppm)
740.9638
[M−H−H2O]−
758.964
[M−H]−
6476 : M.findmain = 741.9711 (+/− 20 ppm)
740.9638
[M−H]−
758.964
0
20
40
60
80
100
735
740
745
750
755
760
765

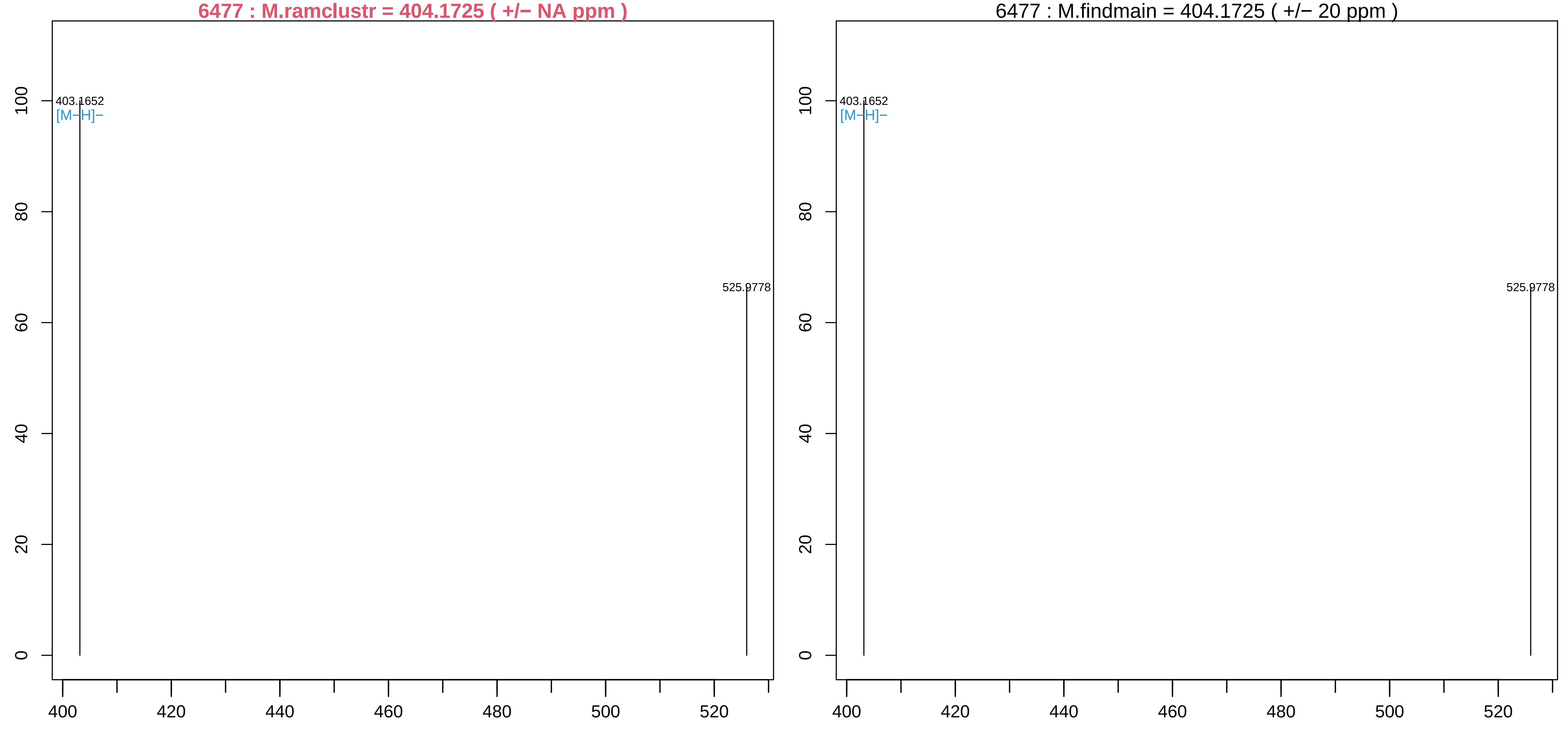

6477 : M.ramclustr = 404.1725 (+/− NA ppm)
403.1652
[M−H]−
525.9778
6477 : M.findmain = 404.1725 (+/− 20 ppm)
403.1652
[M−H]−
525.9778

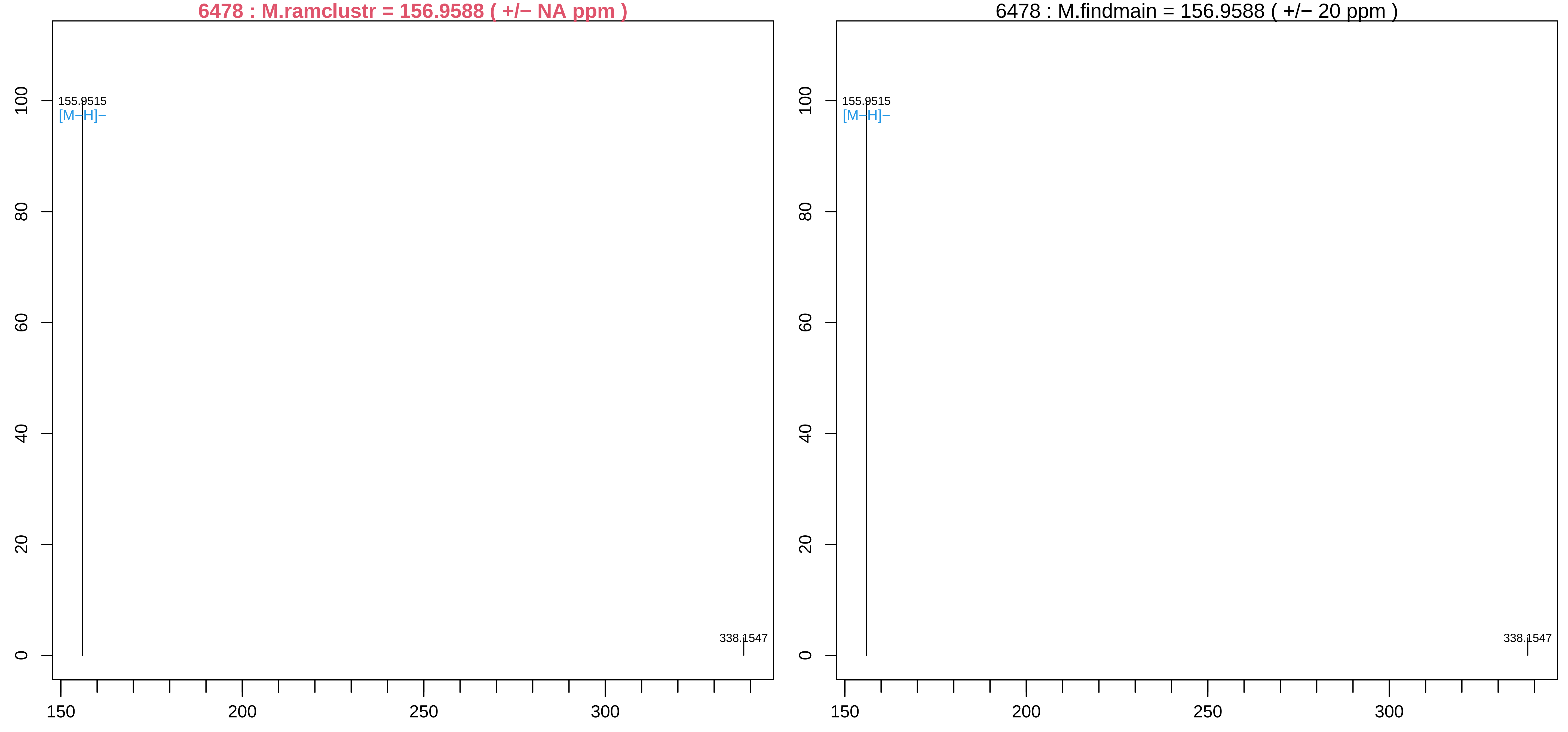
6478 : M.ramclustr = 156.9588 (+/− NA ppm)
155.9515
[M−H]−
338.1547
6478 : M.findmain = 156.9588 (+/− 20 ppm)
155.9515
[M−H]−
338.1547

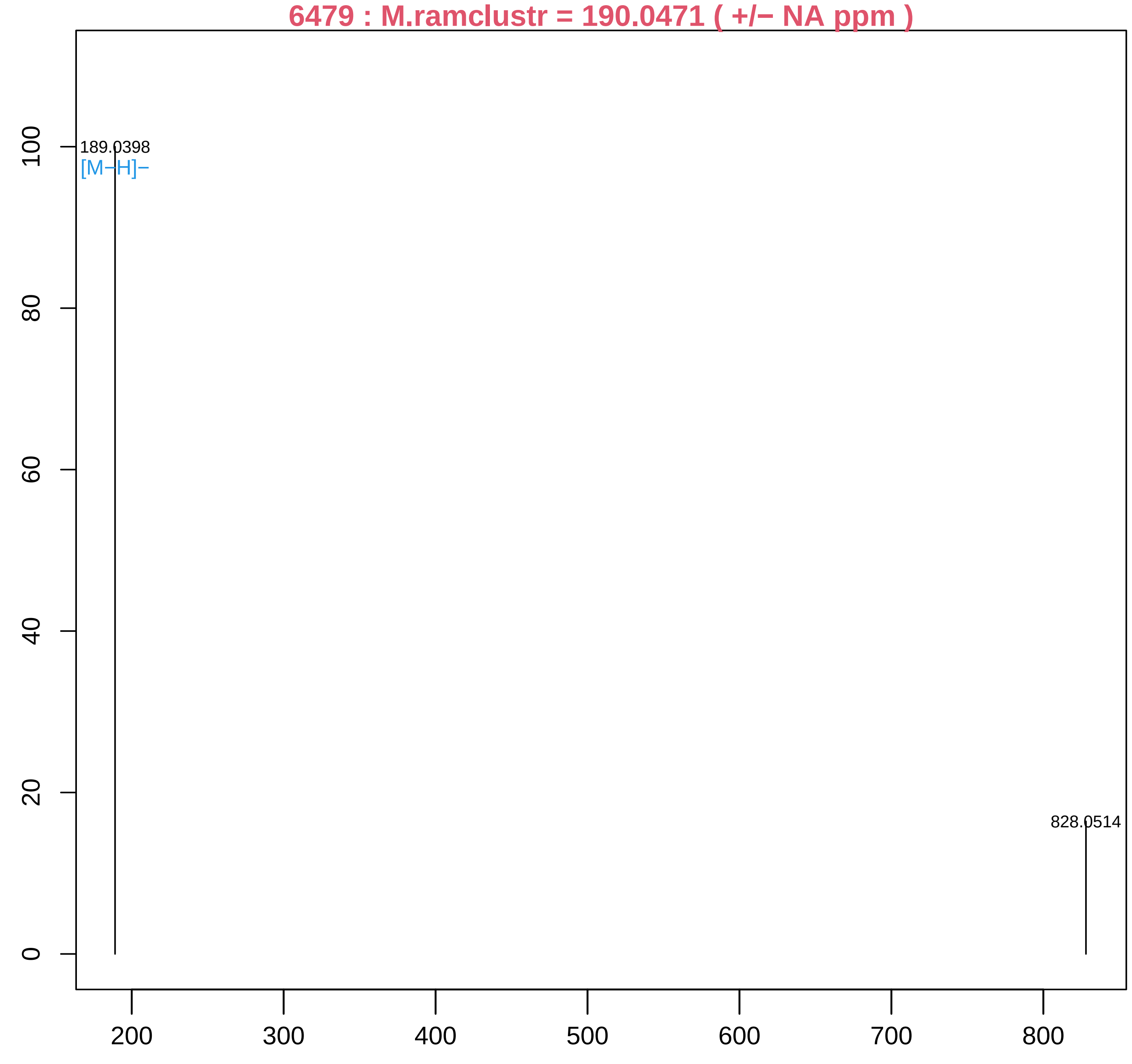
6479 : M.ramclustr = 190.0471 (+/− NA ppm)
189.0398
[M−H]−
828.0514
0
20
40
60
80
100
200
300
400
500
600
700
800

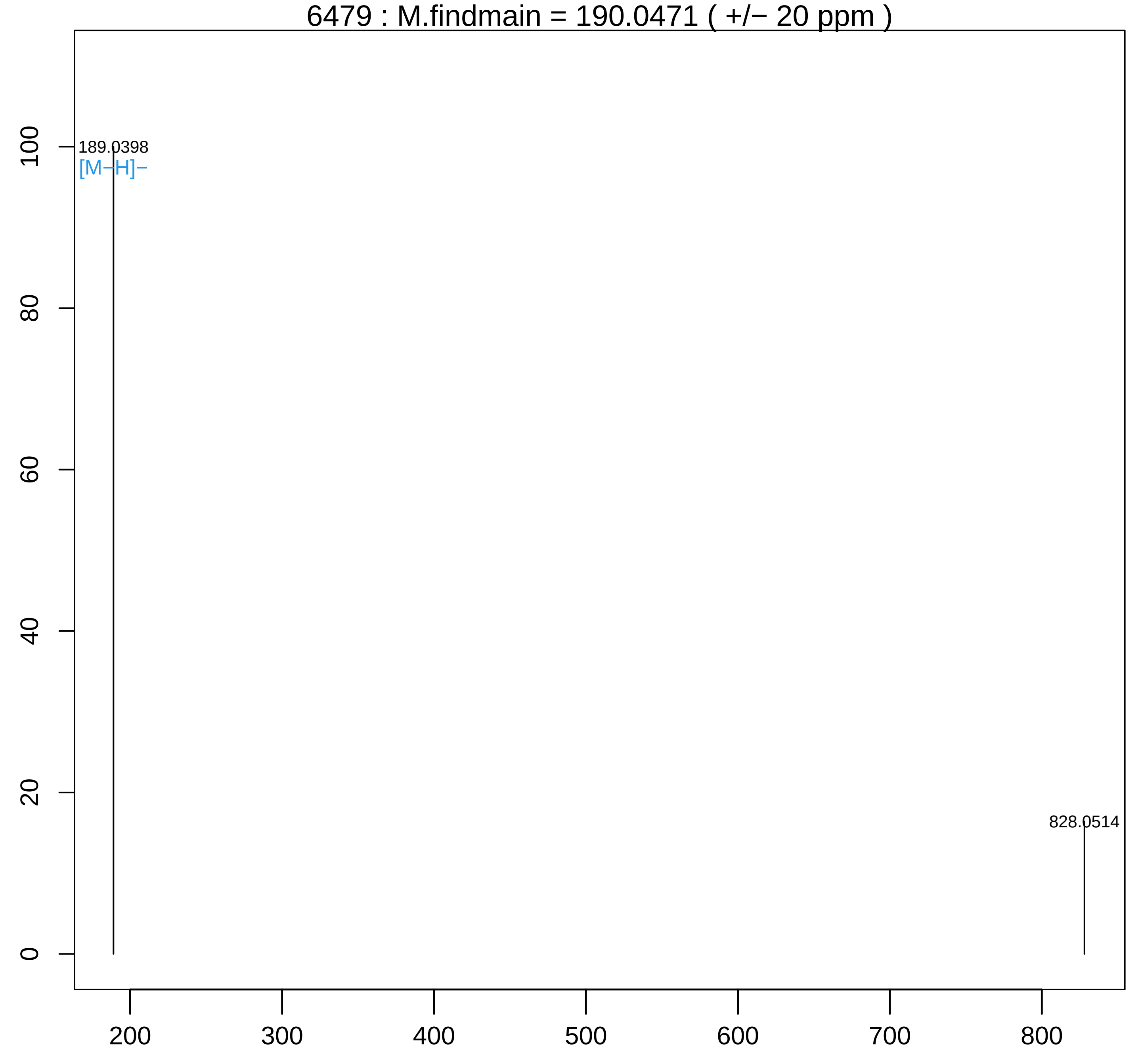
6479 : M.findmain = 190.0471 (+/− 20 ppm)
189.0398
[M−H]−
828.0514
0
20
40
60
80
100
200
300
400
500
600
700
800

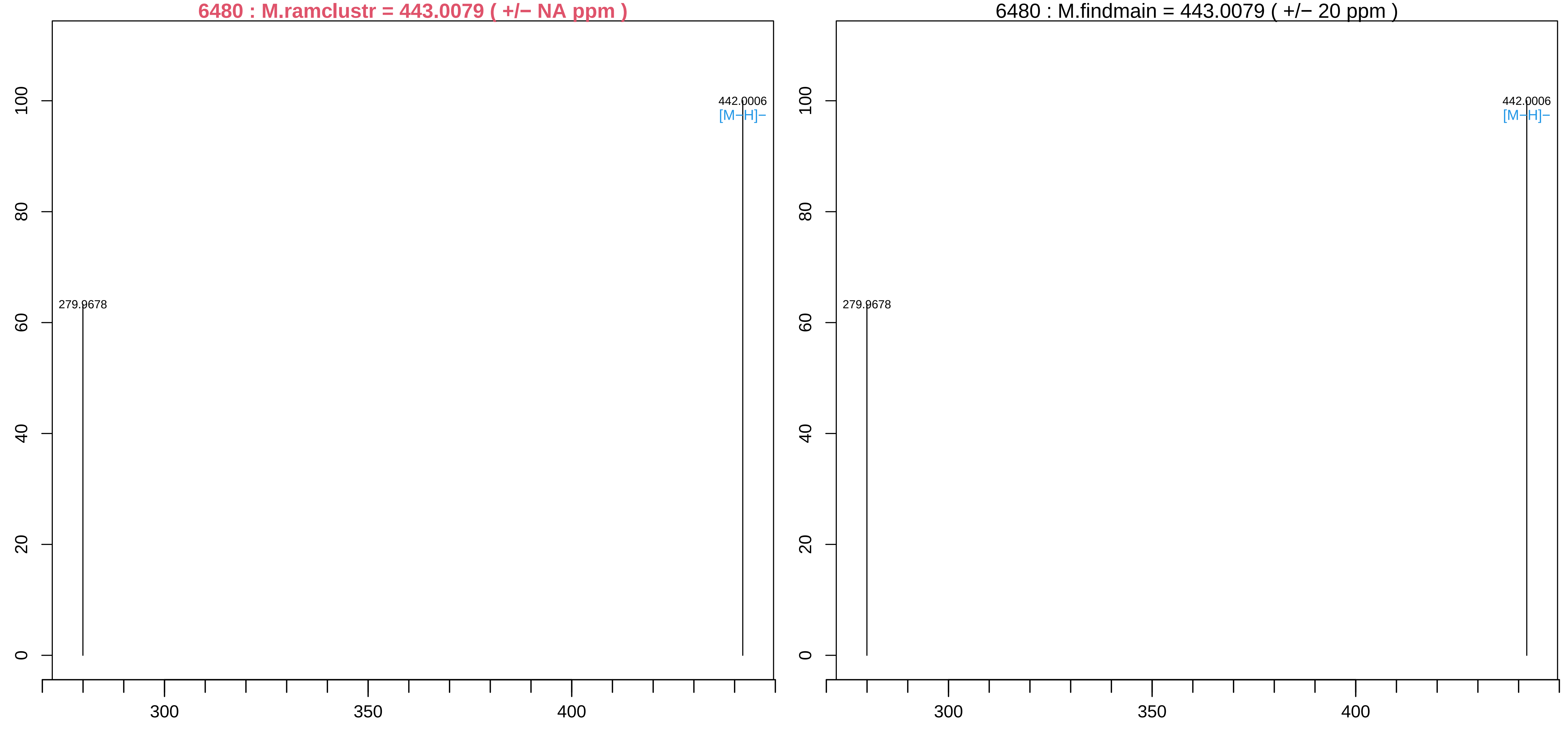
6480 : M.ramclustr = 443.0079 (+/− NA ppm)
279.9678
442.0006
[M−H]−
6480 : M.findmain = 443.0079 (+/− 20 ppm)
279.9678
442.0006
[M−H]−
0
20
40
60
80
100
300
350
400

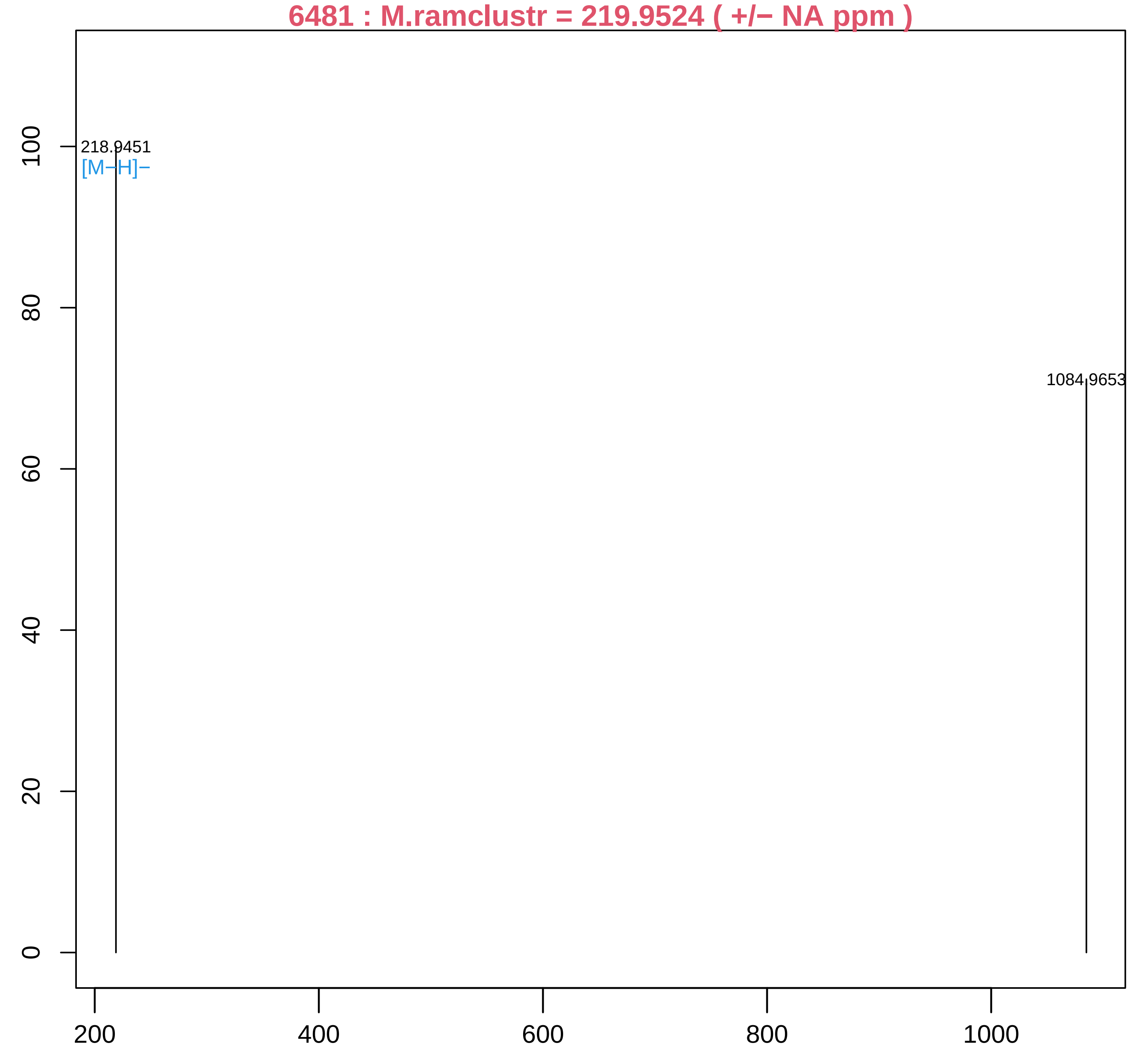
6481 : M.ramclustr = 219.9524 (+/− NA ppm)
218.9451
[M−H]−
1084.9653
0
20
40
60
80
100
200
400
600
800
1000

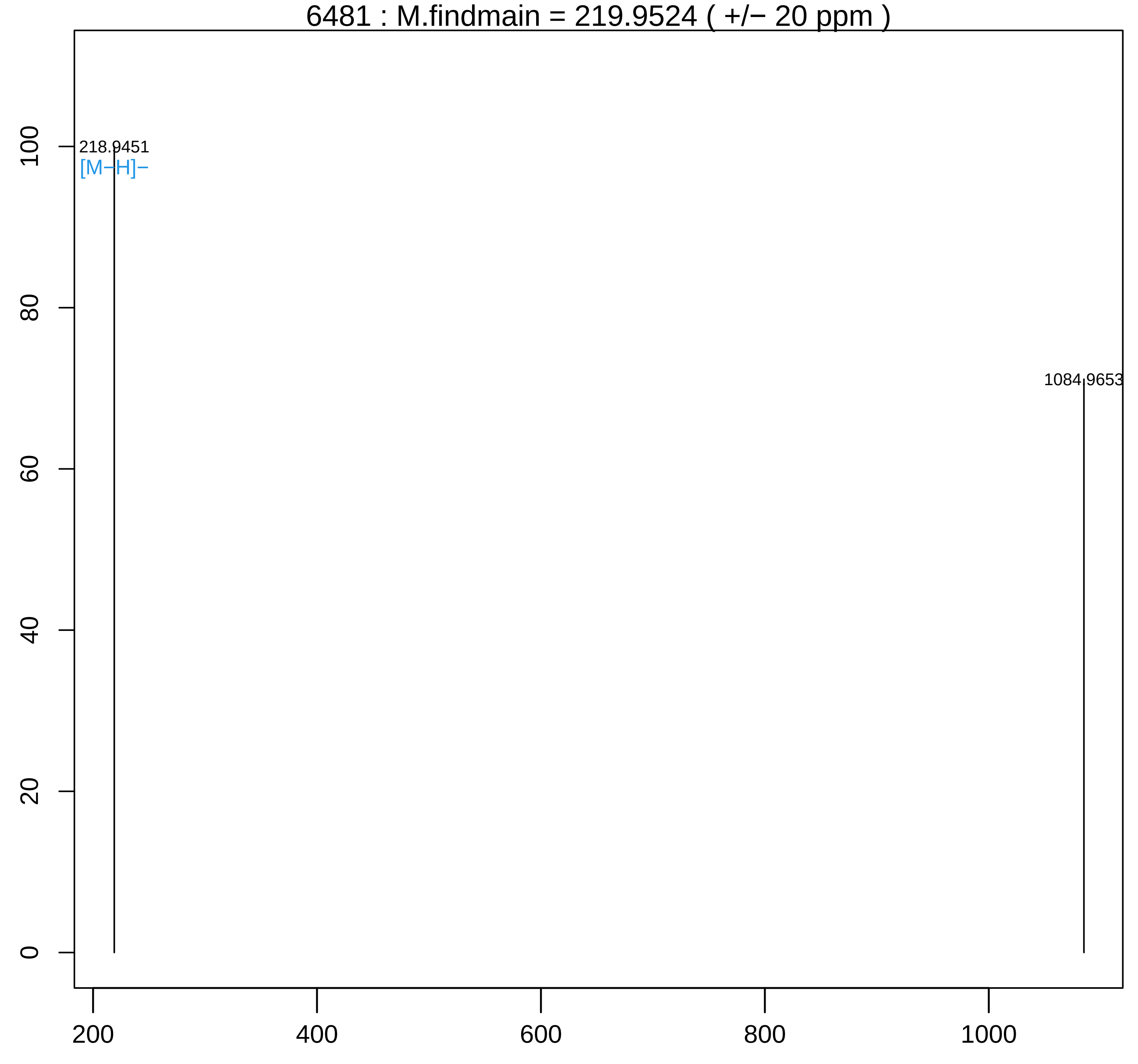
6481 : M.findmain = 219.9524 (+/− 20 ppm)
218.9451
[M−H]−
1084.9653
0
20
40
60
80
100
200
400
600
800
1000

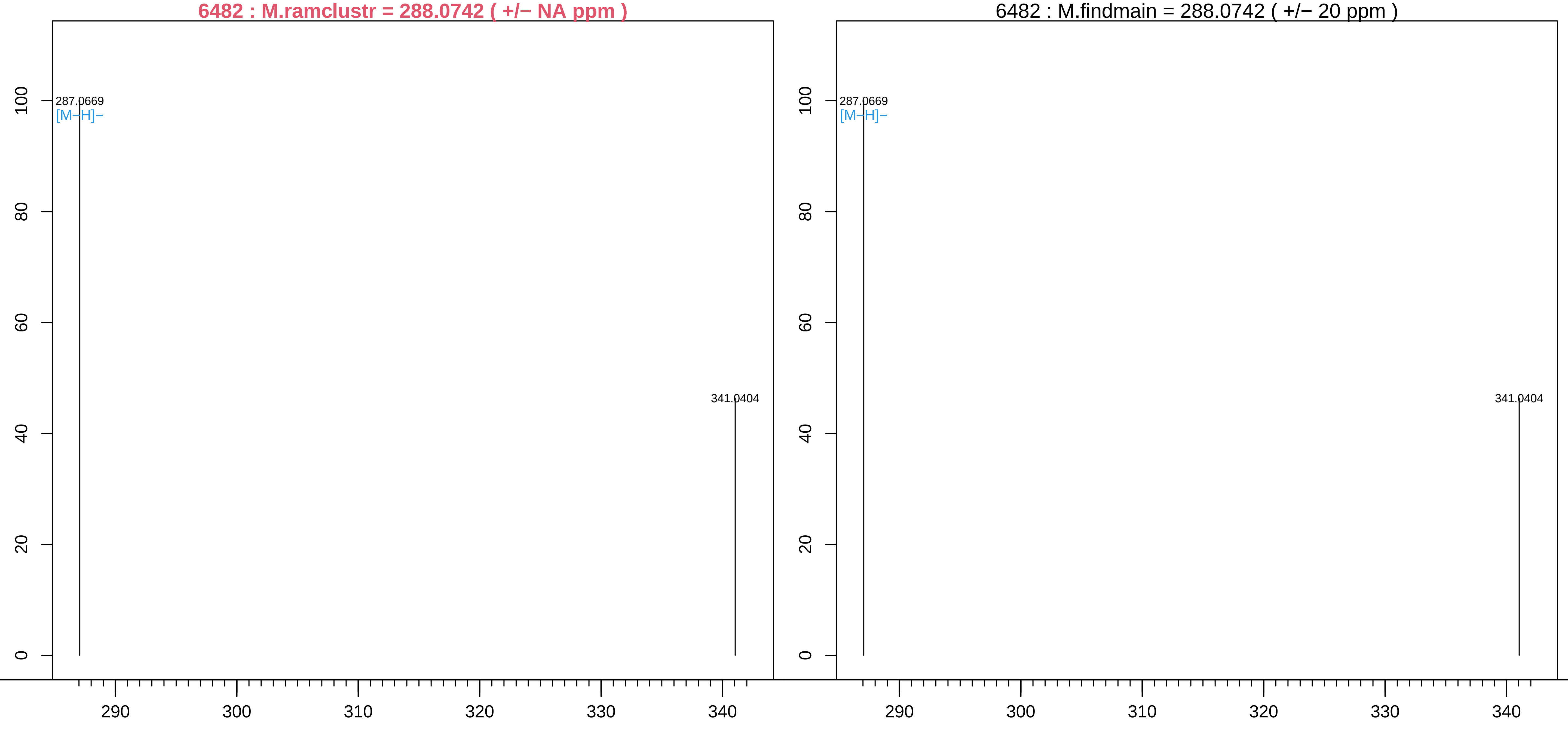

6482 : M.ramclustr = 288.0742 (+/− NA ppm)
287.0669
[M−H]−
341.0404
6482 : M.findmain = 288.0742 (+/− 20 ppm)
287.0669
[M−H]−
341.0404
0
20
40
60
80
100
290
300
310
320
330
340

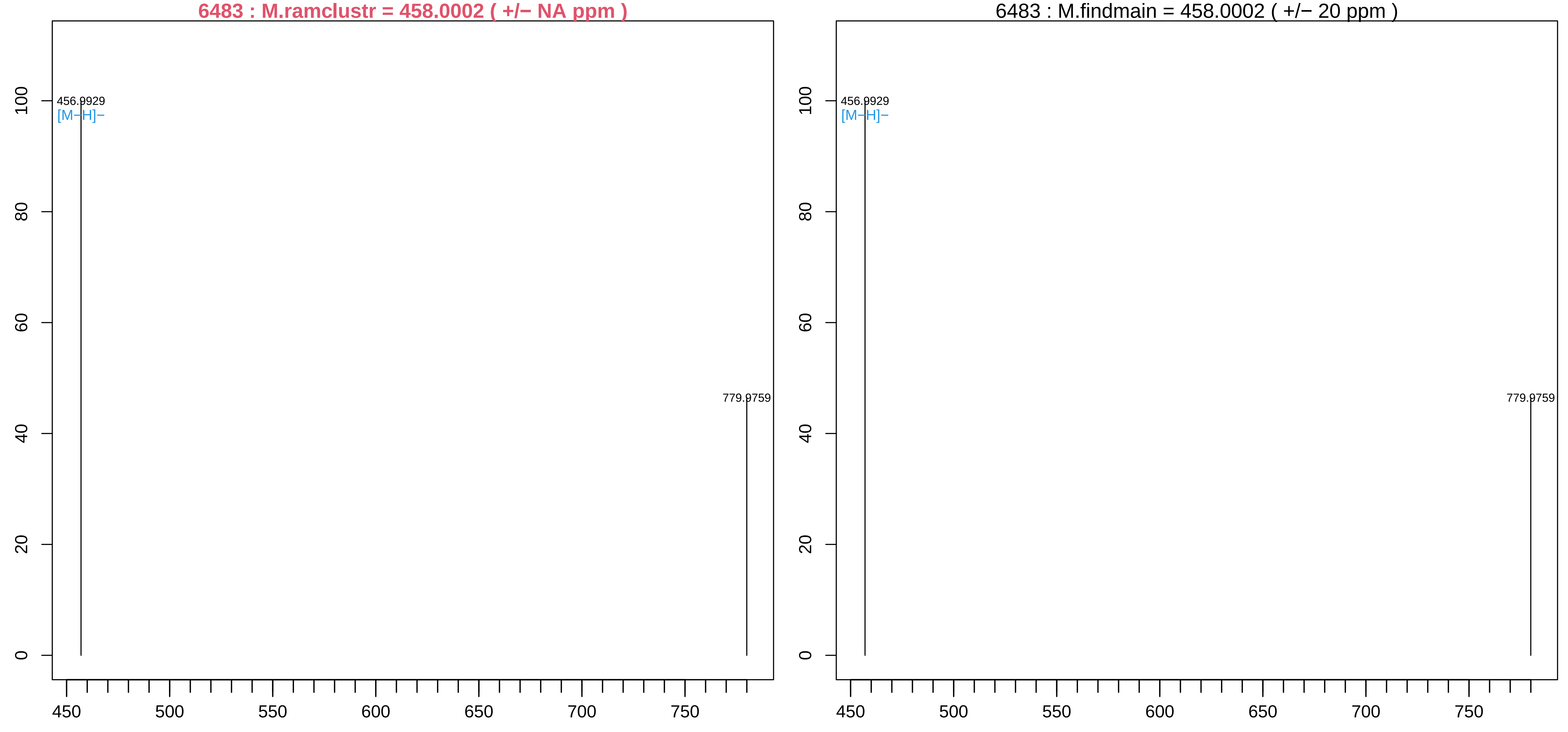
6483 : M.ramclustr = 458.0002 (+/− NA ppm)
456.9929
[M−H]−
779.9759
6483 : M.findmain = 458.0002 (+/− 20 ppm)
456.9929
[M−H]−
779.9759
0
20
40
60
80
100
450
500
550
600
650
700
750

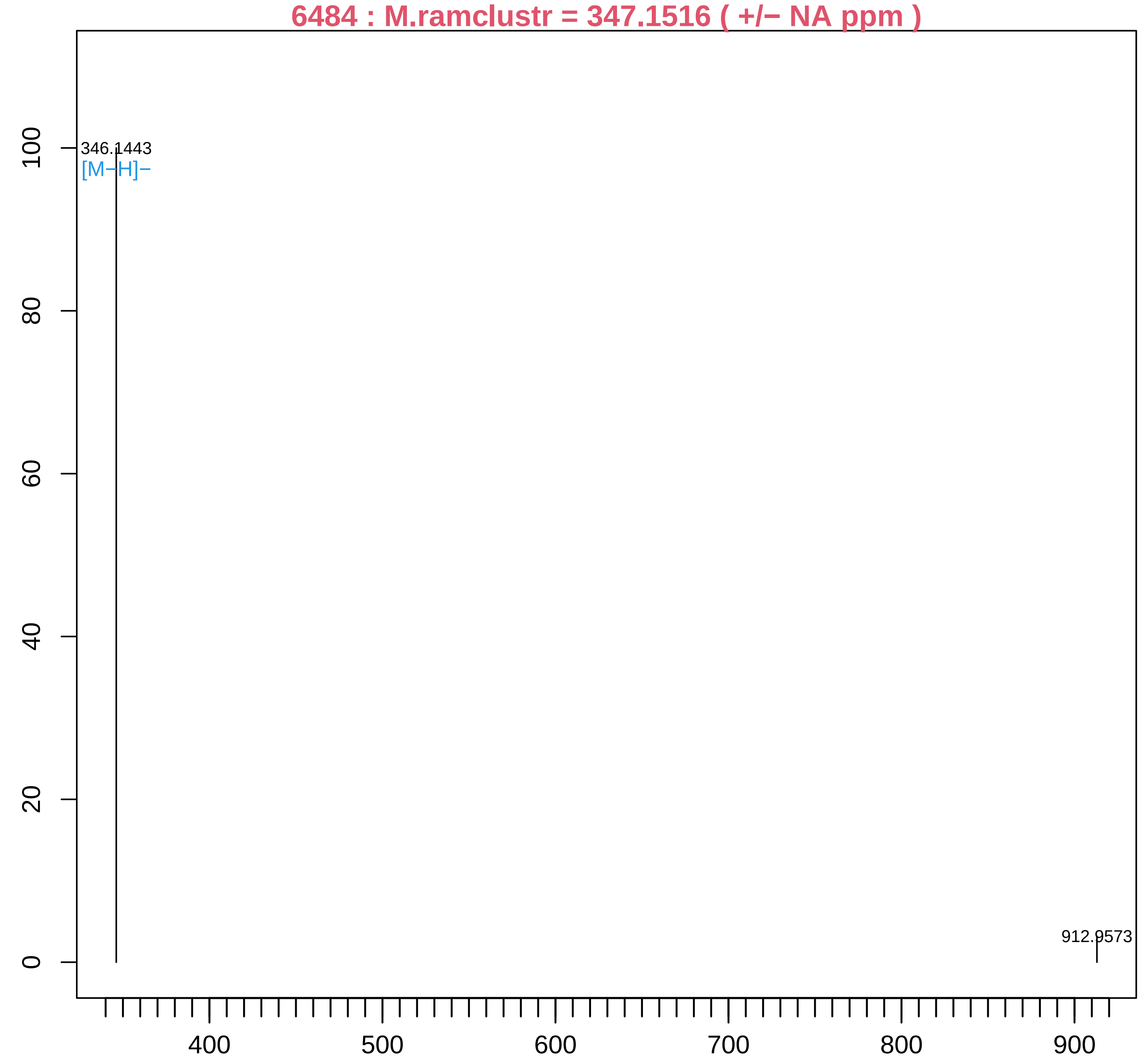
6484 : M.ramclustr = 347.1516 (+/− NA ppm)
346.1443
[M−H]−
912.9573
0
20
40
60
80
100
400
500
600
700
800
900

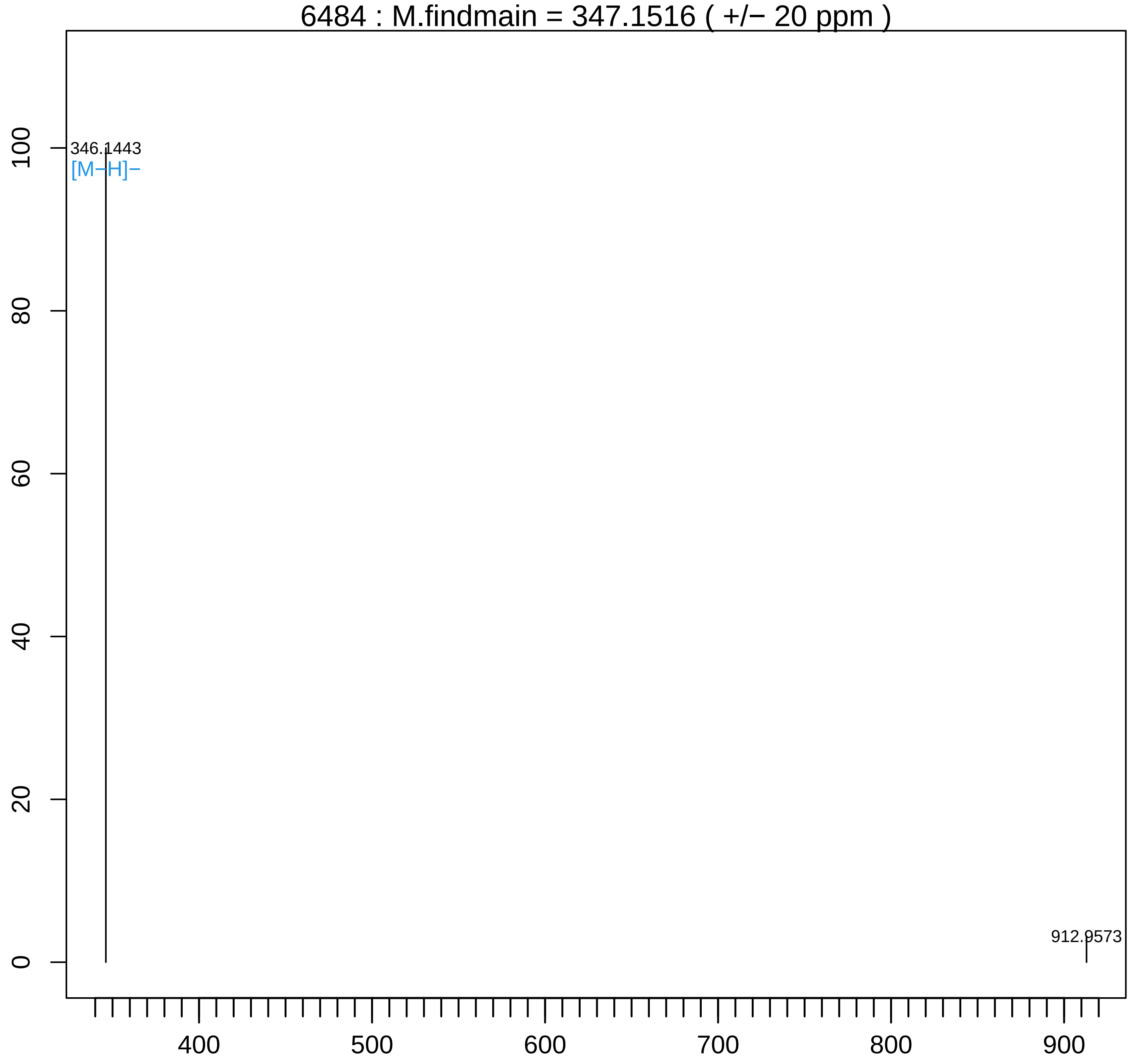
6484 : M.findmain = 347.1516 (+/− 20 ppm)
346.1443
[M−H]−
912.9573
0
20
40
60
80
100
400
500
600
700
800
900

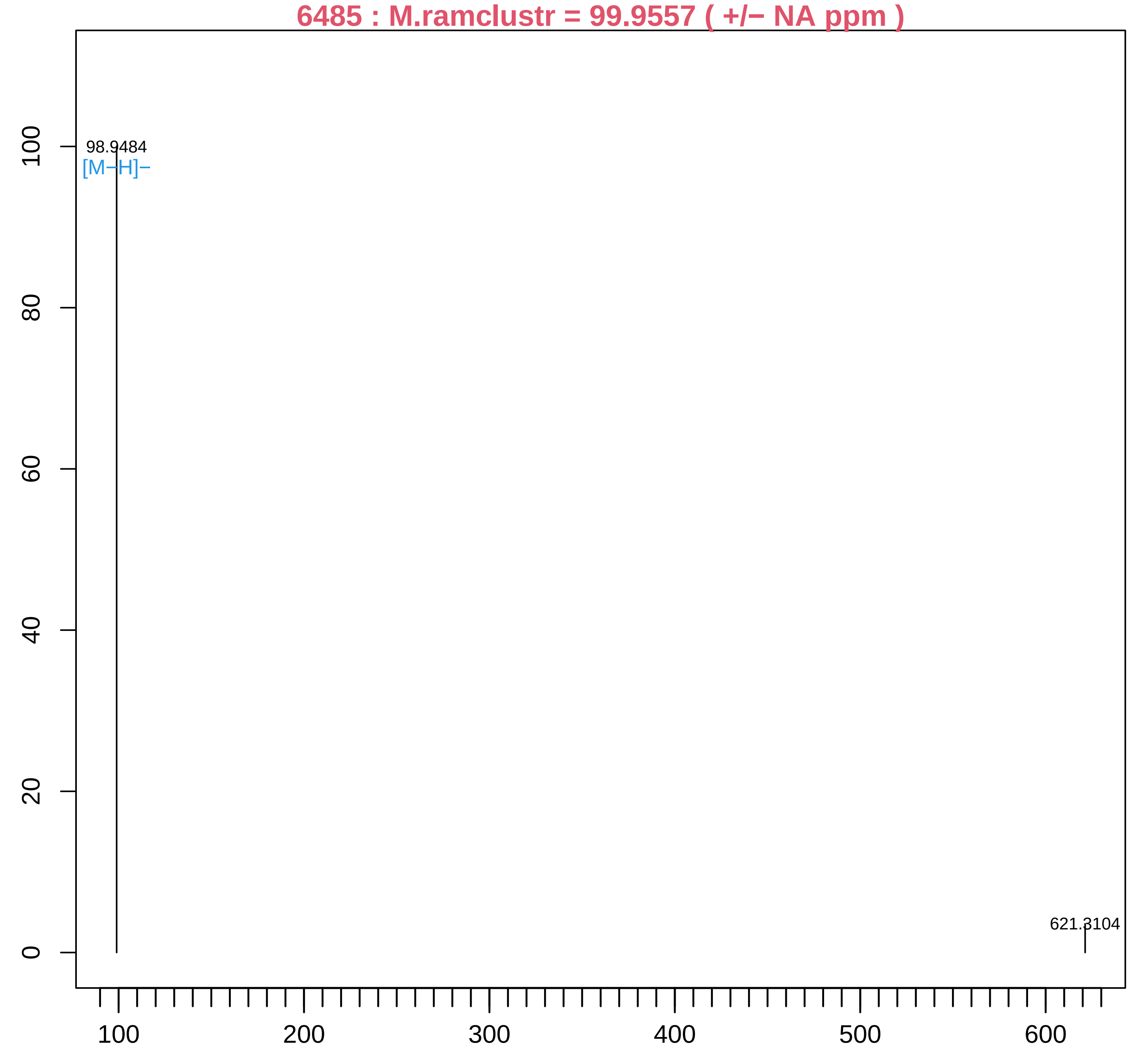

6485 : M.ramclustr = 99.9557 (+/− NA ppm)
98.9484
[M−H]−
621.3104
0
20
40
60
80
100
200
300
400
500
600

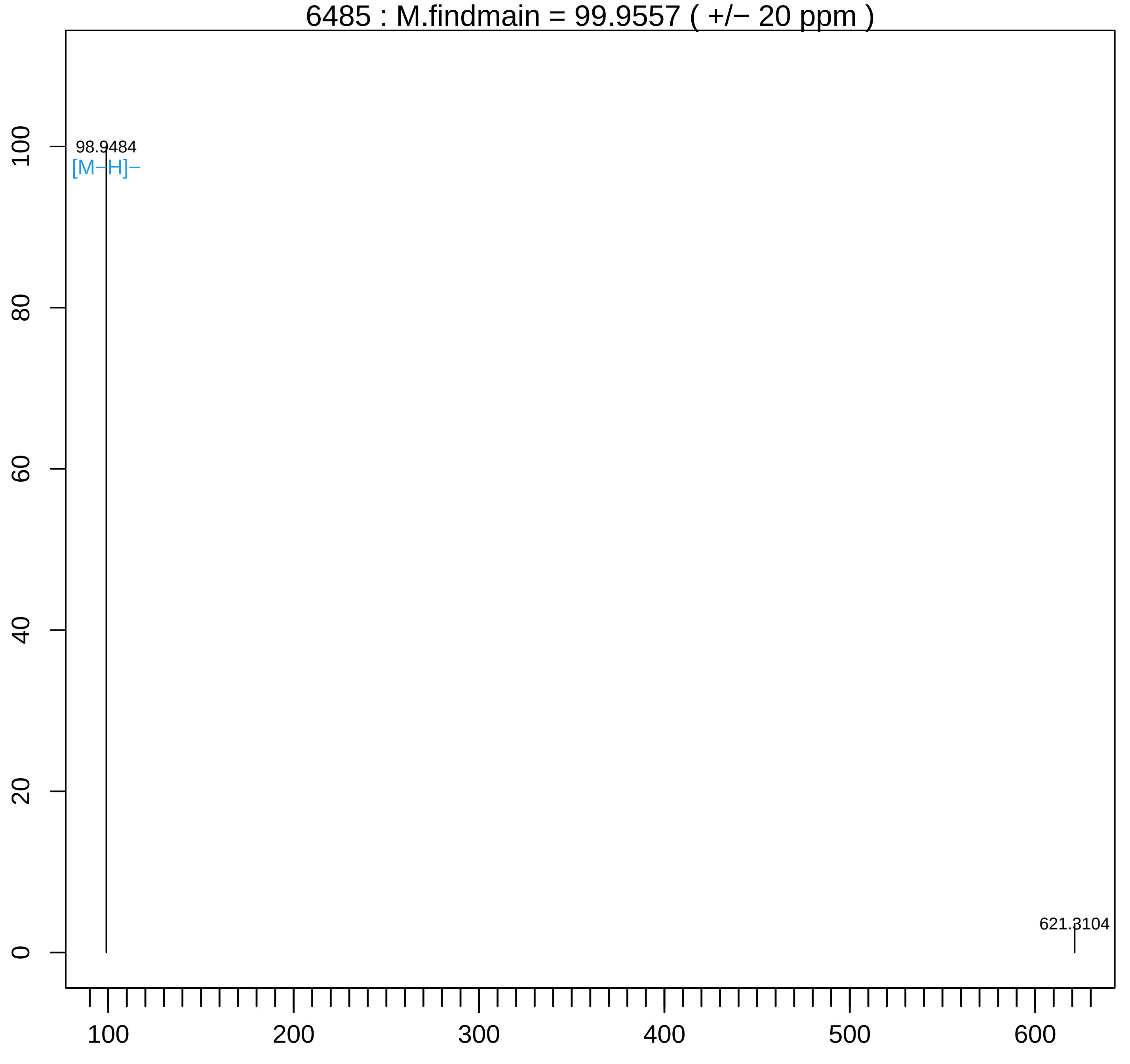

6485 : M.findmain = 99.9557 (+/− 20 ppm)
98.9484
[M−H]−
621.3104
0
20
40
60
80
100
200
300
400
500
600

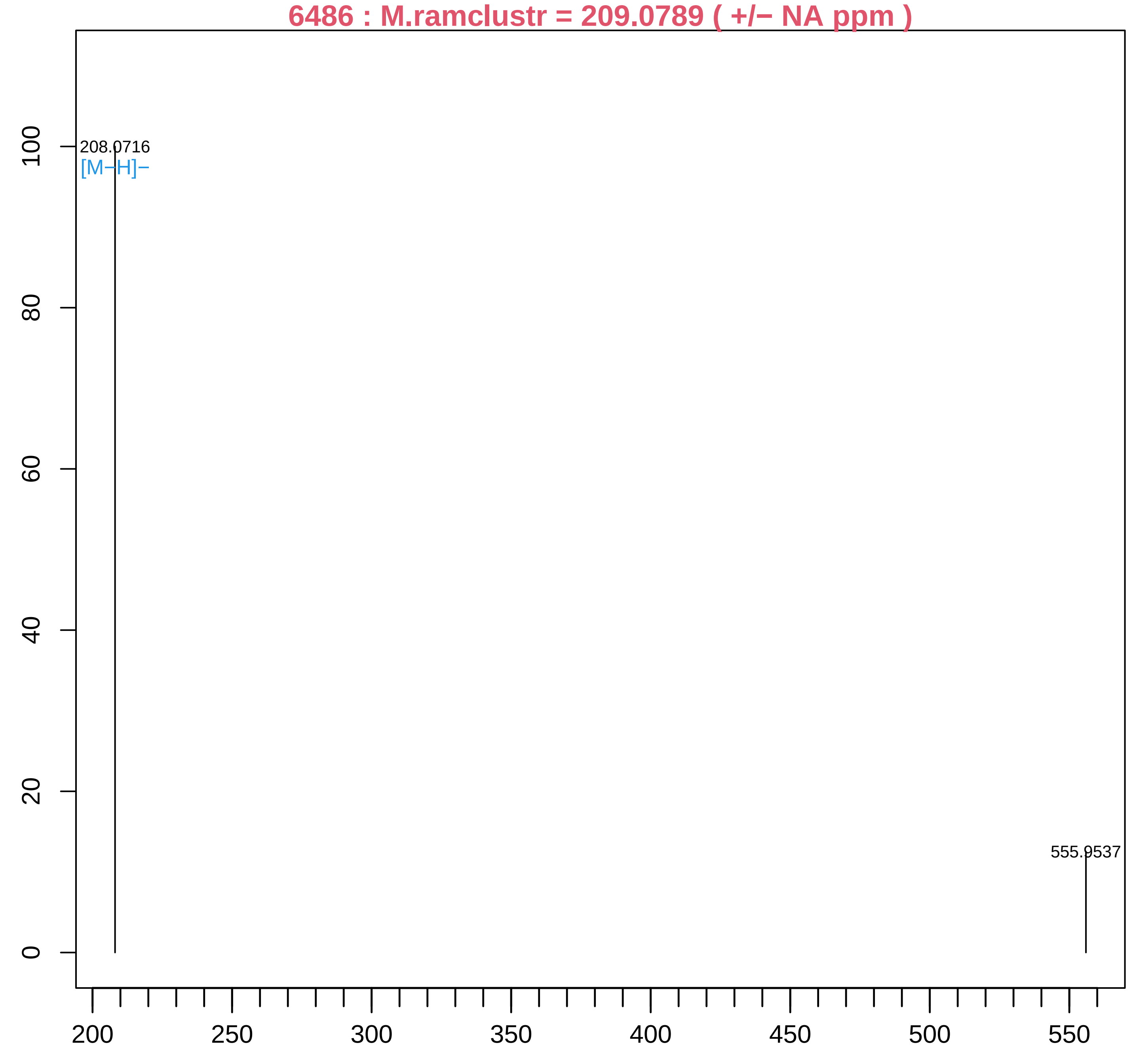
6486 : M.ramclustr = 209.0789 (+/− NA ppm)
208.0716
[M−H]−
555.9537
0
20
40
60
80
100
200
250
300
350
400
450
500
550

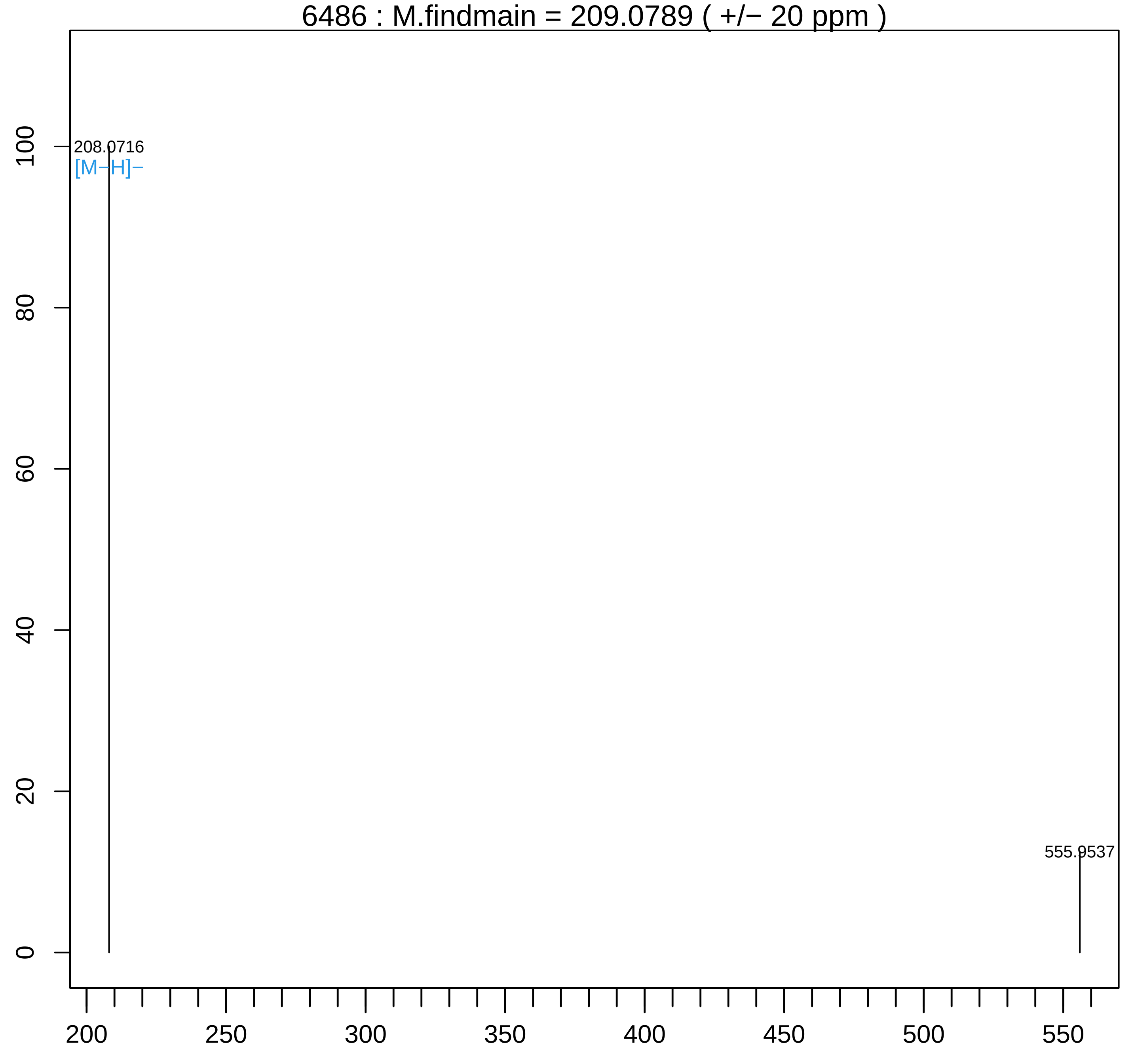
6486 : M.findmain = 209.0789 (+/− 20 ppm)
208.0716
[M−H]−
555.9537
0
20
40
60
80
100
200
250
300
350
400
450
500
550

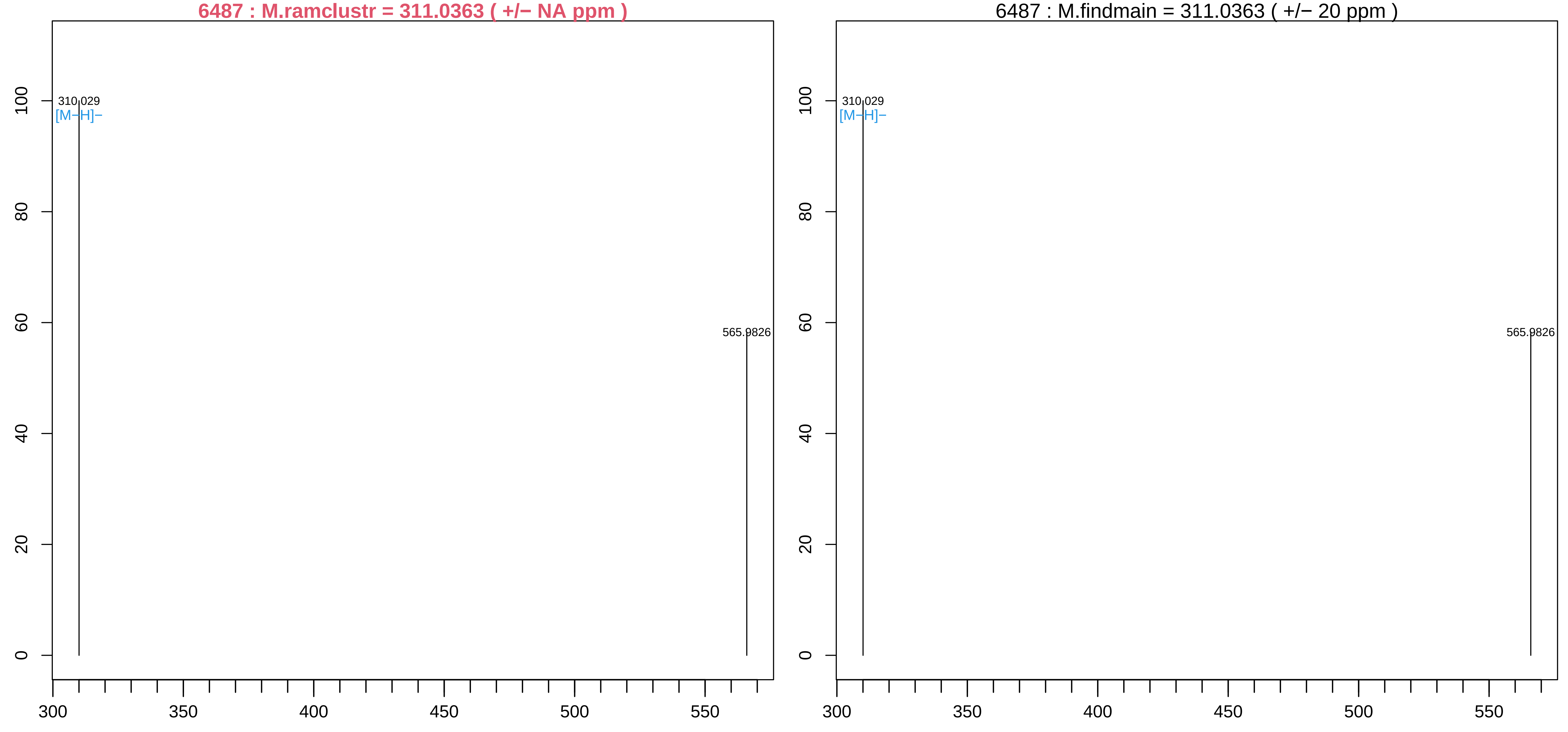
6487 : M.ramclustr = 311.0363 (+/− NA ppm)
6487 : M.findmain = 311.0363 (+/− 20 ppm)
310.029
[M−H]−
565.9826
0
20
40
60
80
100
300
350
400
450
500
550

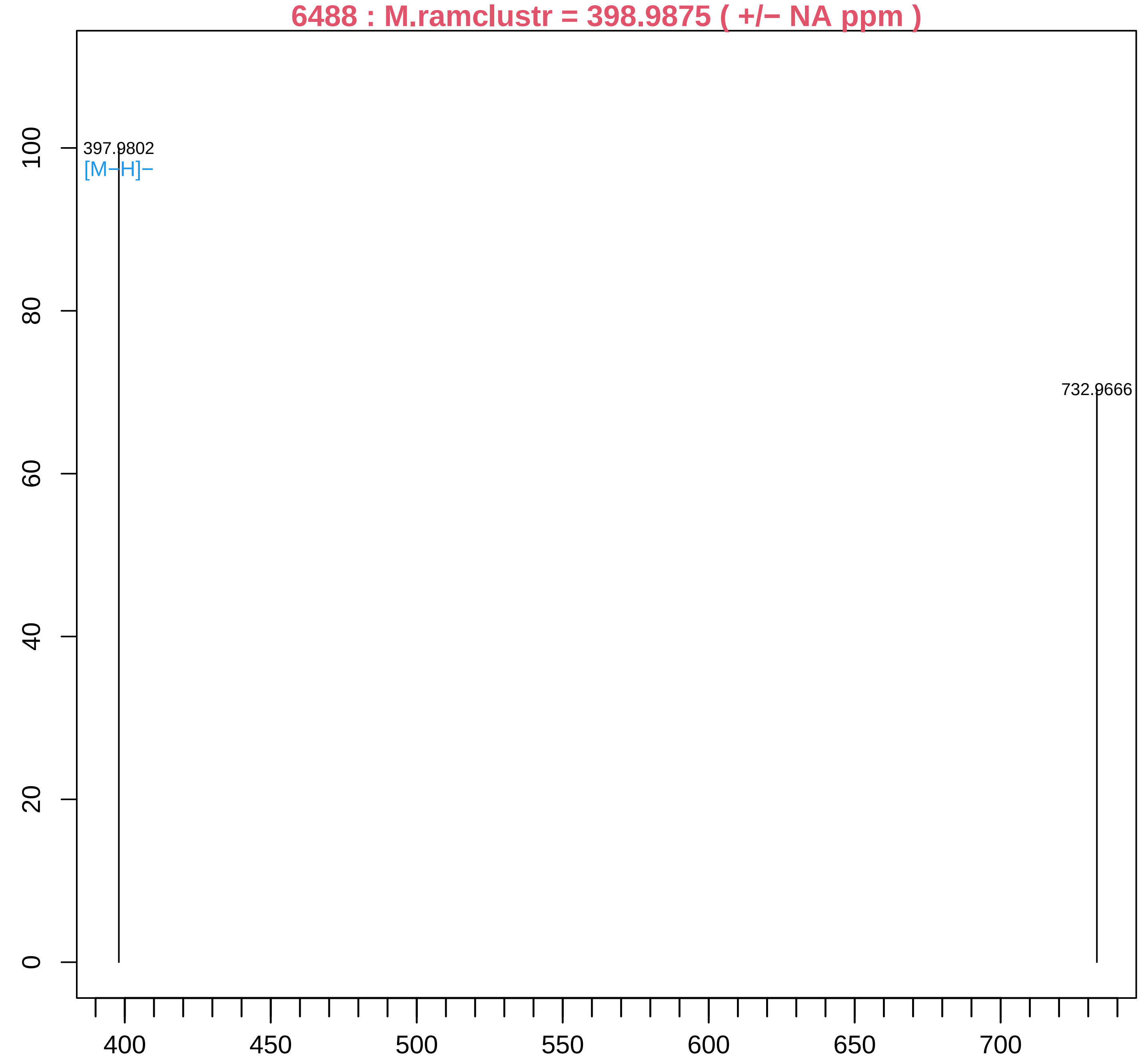
6488 : M.ramclustr = 398.9875 (+/− NA ppm)
397.9802
[M−H]−
732.9666
0
20
40
60
80
100
400
450
500
550
600
650
700

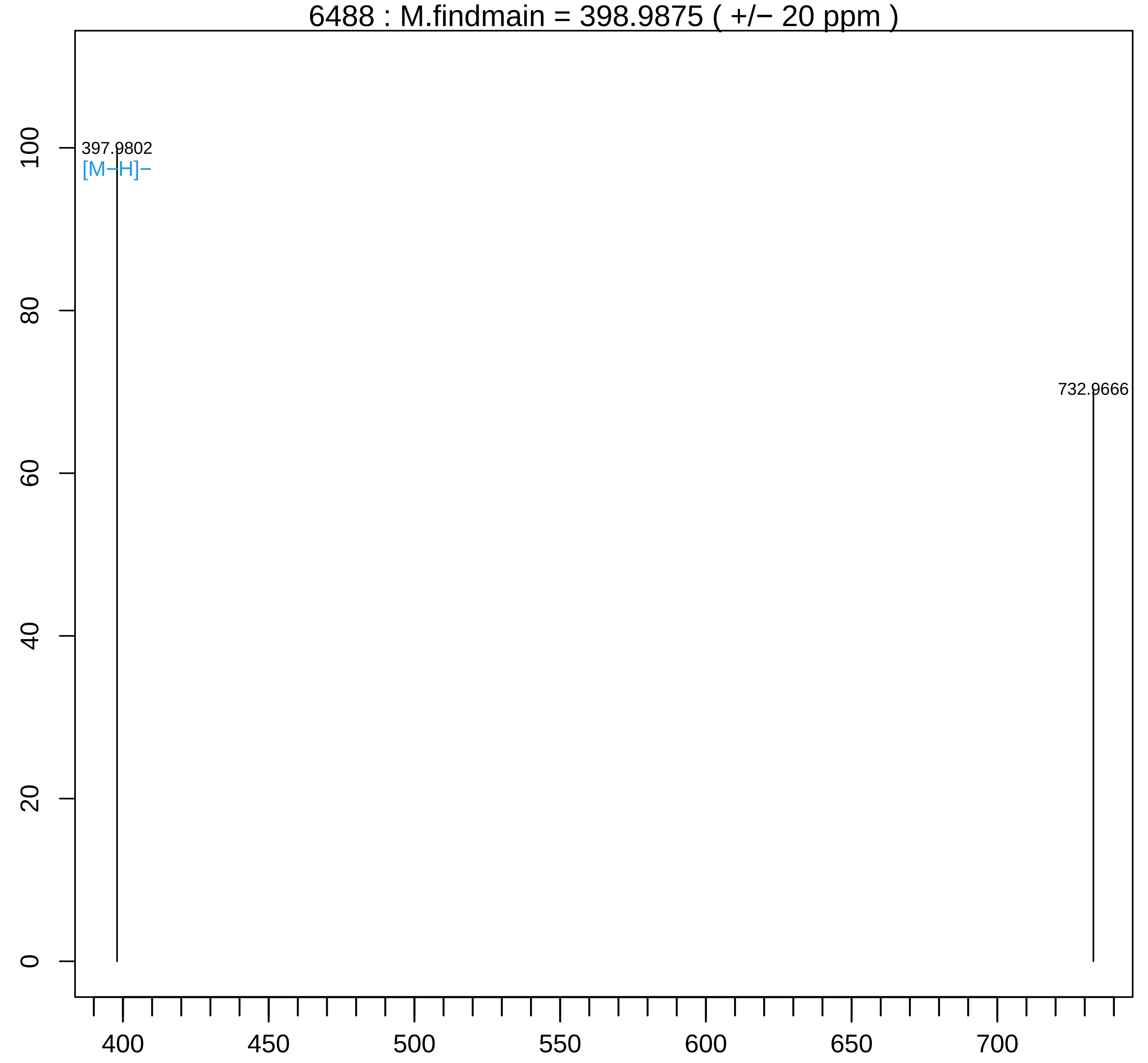
6488 : M.findmain = 398.9875 (+/− 20 ppm)
397.9802
[M−H]−
732.9666
0
20
40
60
80
100
400
450
500
550
600
650
700

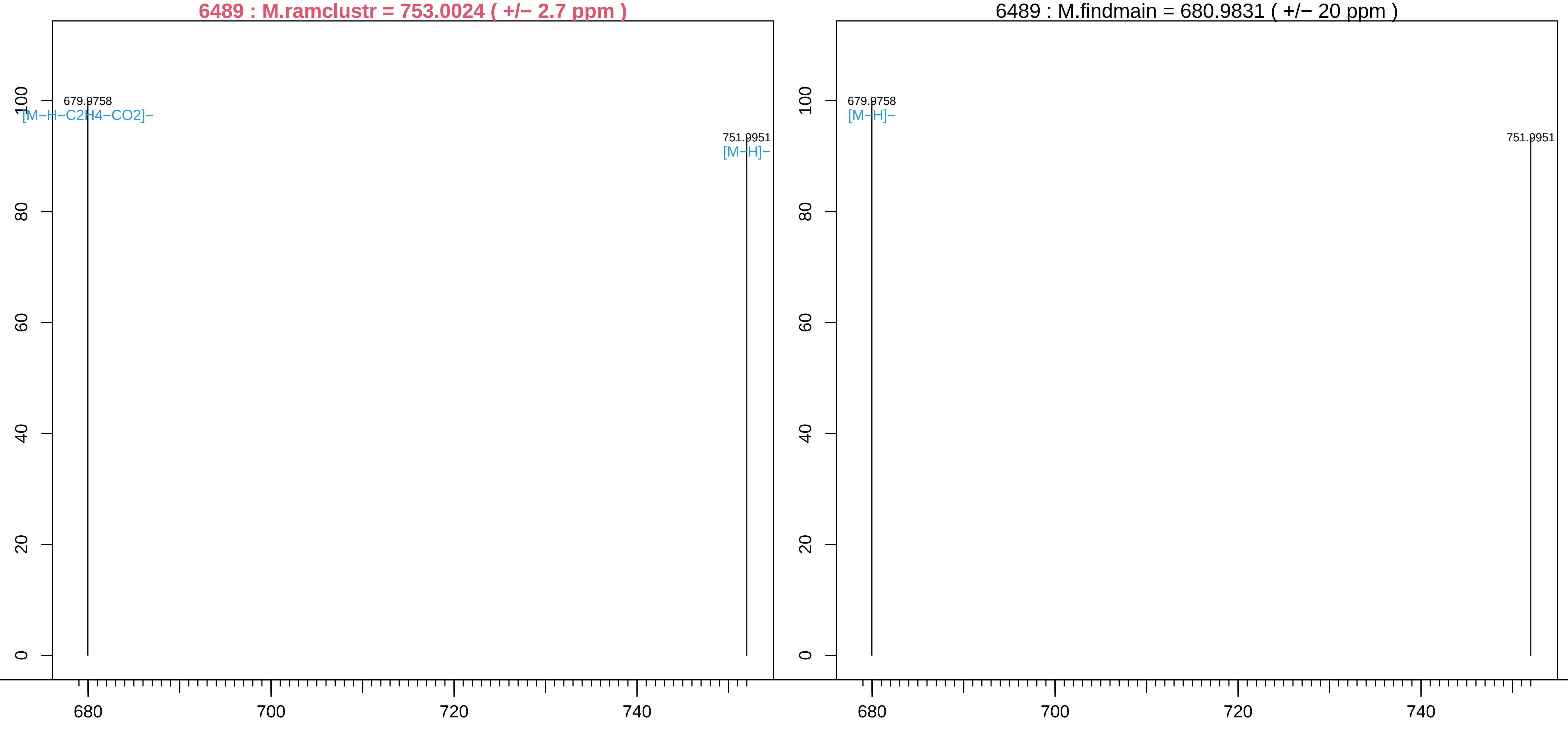
6489 : M.ramclustr = 753.0024 (+/− 2.7 ppm)
679.9758
[M−H−C2H4−CO2]−
751.9951
[M−H]−
6489 : M.findmain = 680.9831 (+/− 20 ppm)
679.9758
[M−H]−
751.9951
0
20
40
60
80
100
680
700
720
740

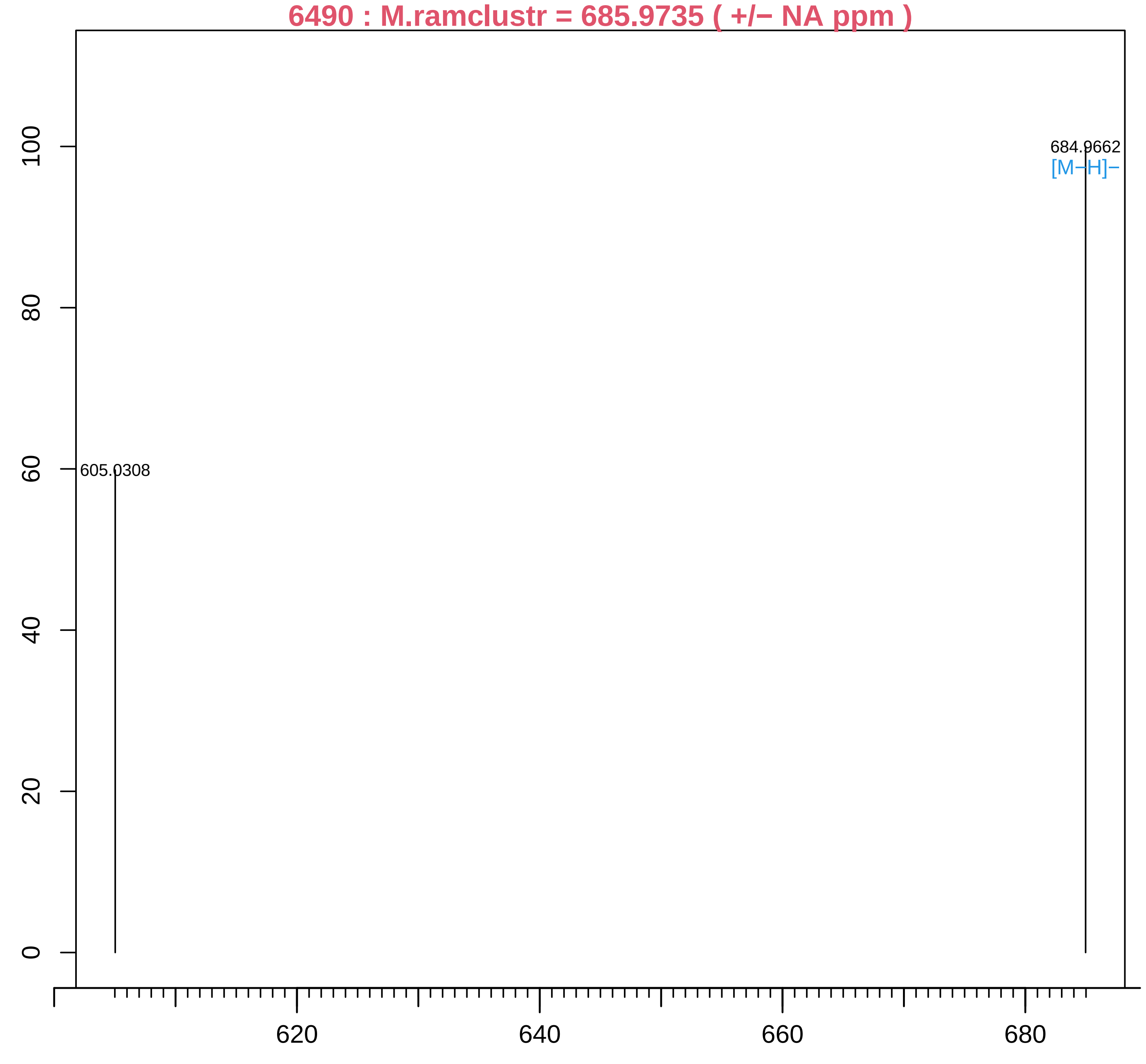
6490 : M.ramclustr = 685.9735 (+/− NA ppm)
684.9662
[M−H]−
605.0308
0
20
40
60
80
100
620
640
660
680

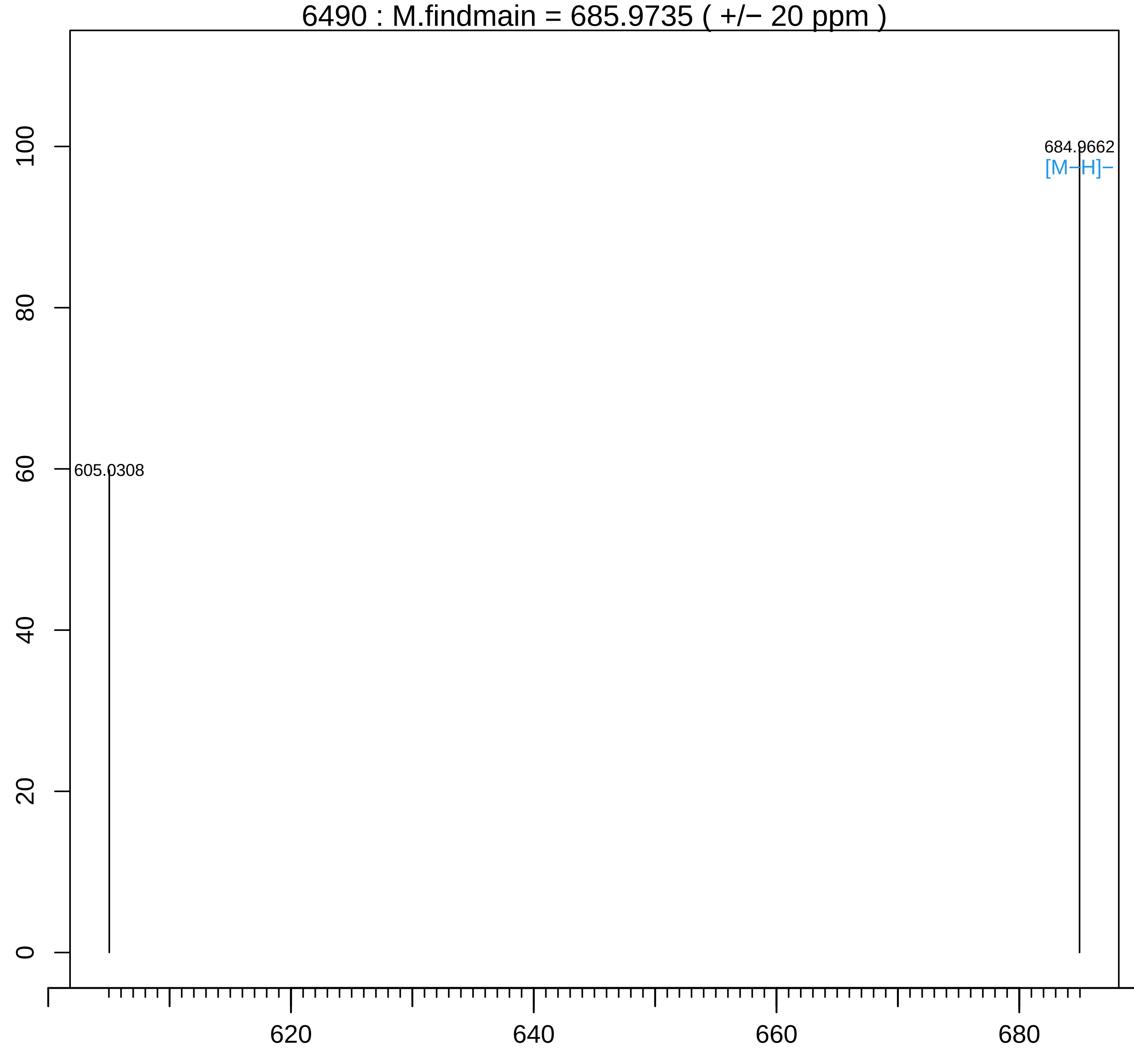
6490 : M.findmain = 685.9735 (+/− 20 ppm)
684.9662
[M−H]−
605.0308
0
20
40
60
80
100
620
640
660
680

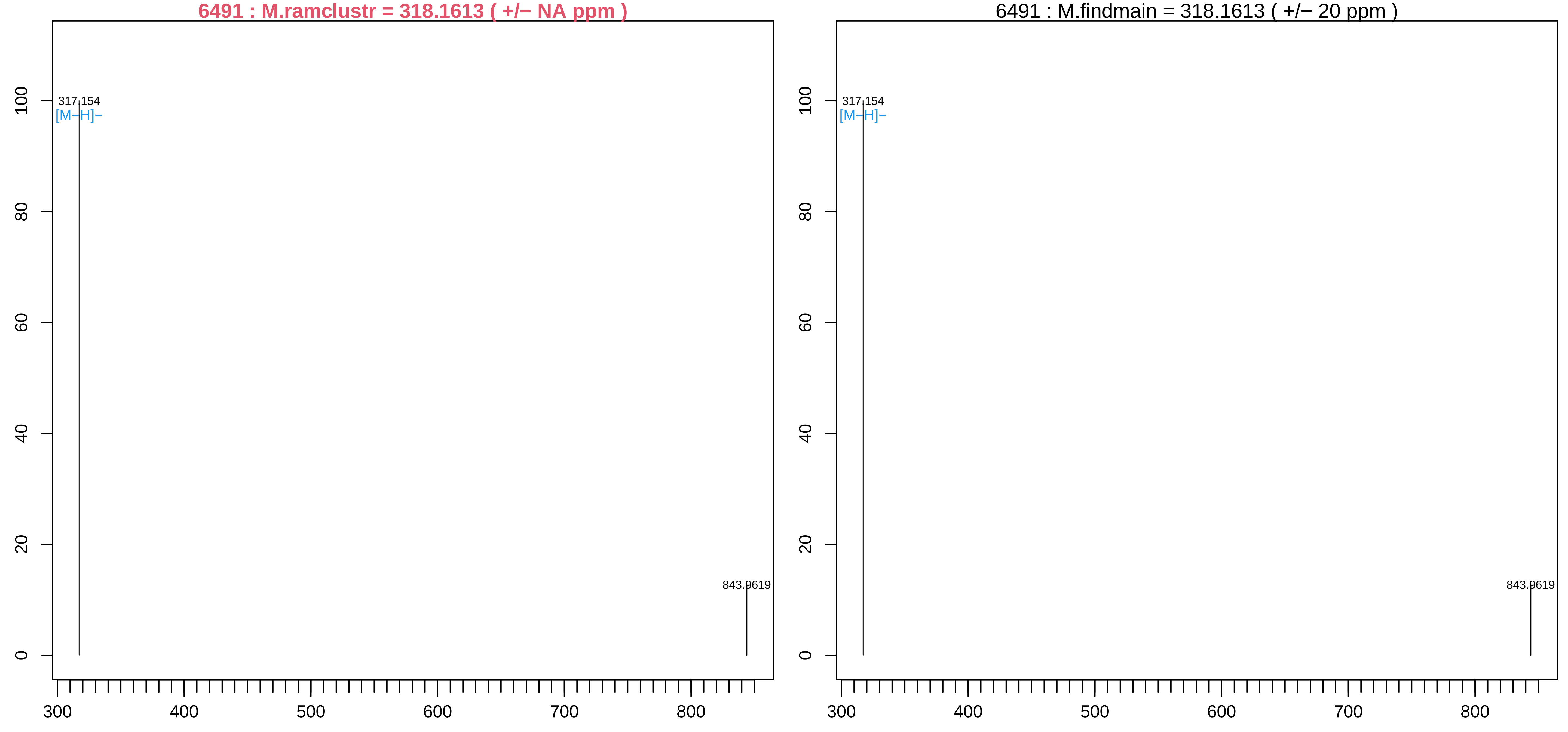

6491 : M.ramclustr = 318.1613 (+/− NA ppm)
317.154
[M−H]−
843.9619
6491 : M.findmain = 318.1613 (+/− 20 ppm)
317.154
[M−H]−
843.9619

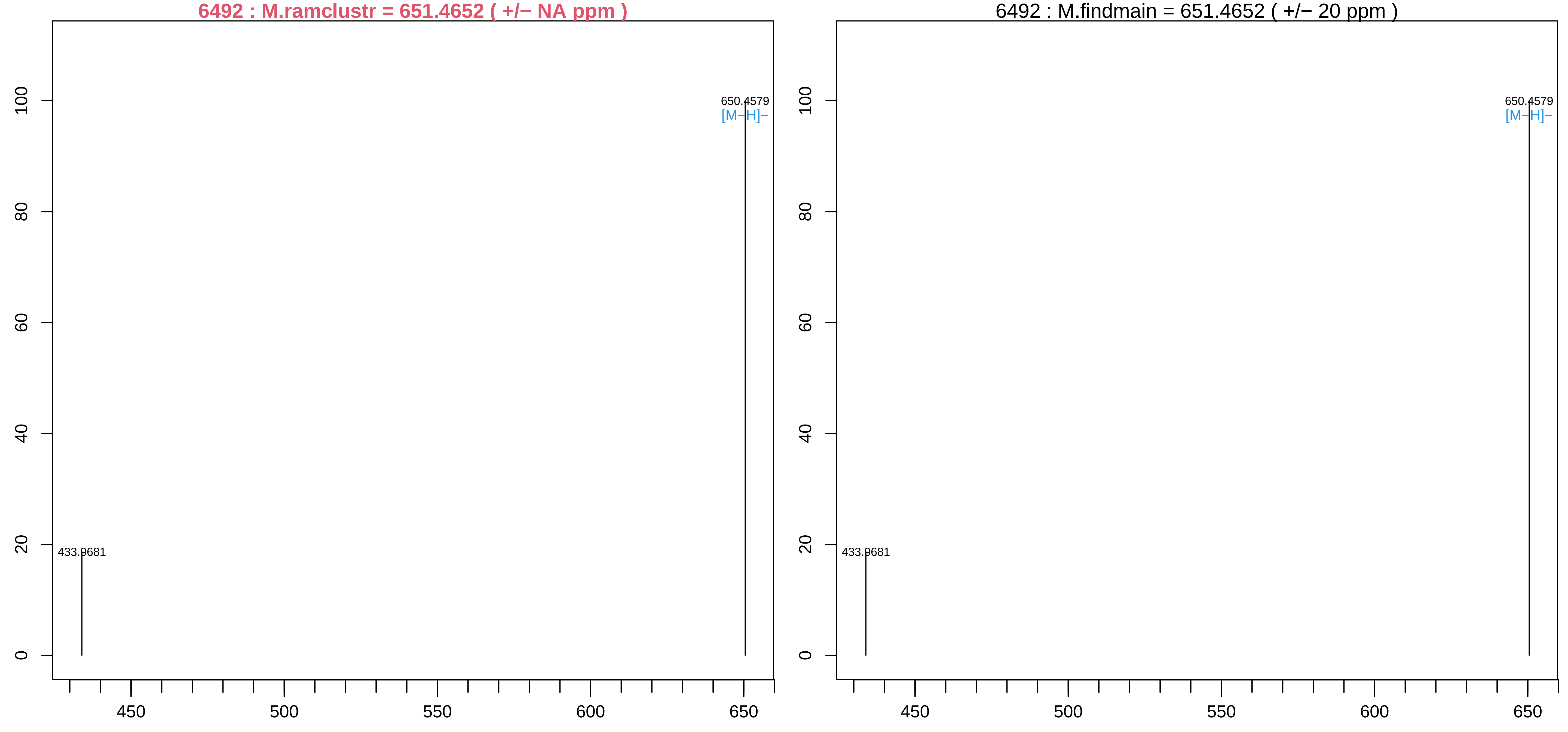
6492 : M.ramclustr = 651.4652 (+/− NA ppm)
650.4579
[M−H]−
433.9681
6492 : M.findmain = 651.4652 (+/− 20 ppm)
650.4579
[M−H]−
433.9681
0
20
40
60
80
100
450
500
550
600
650

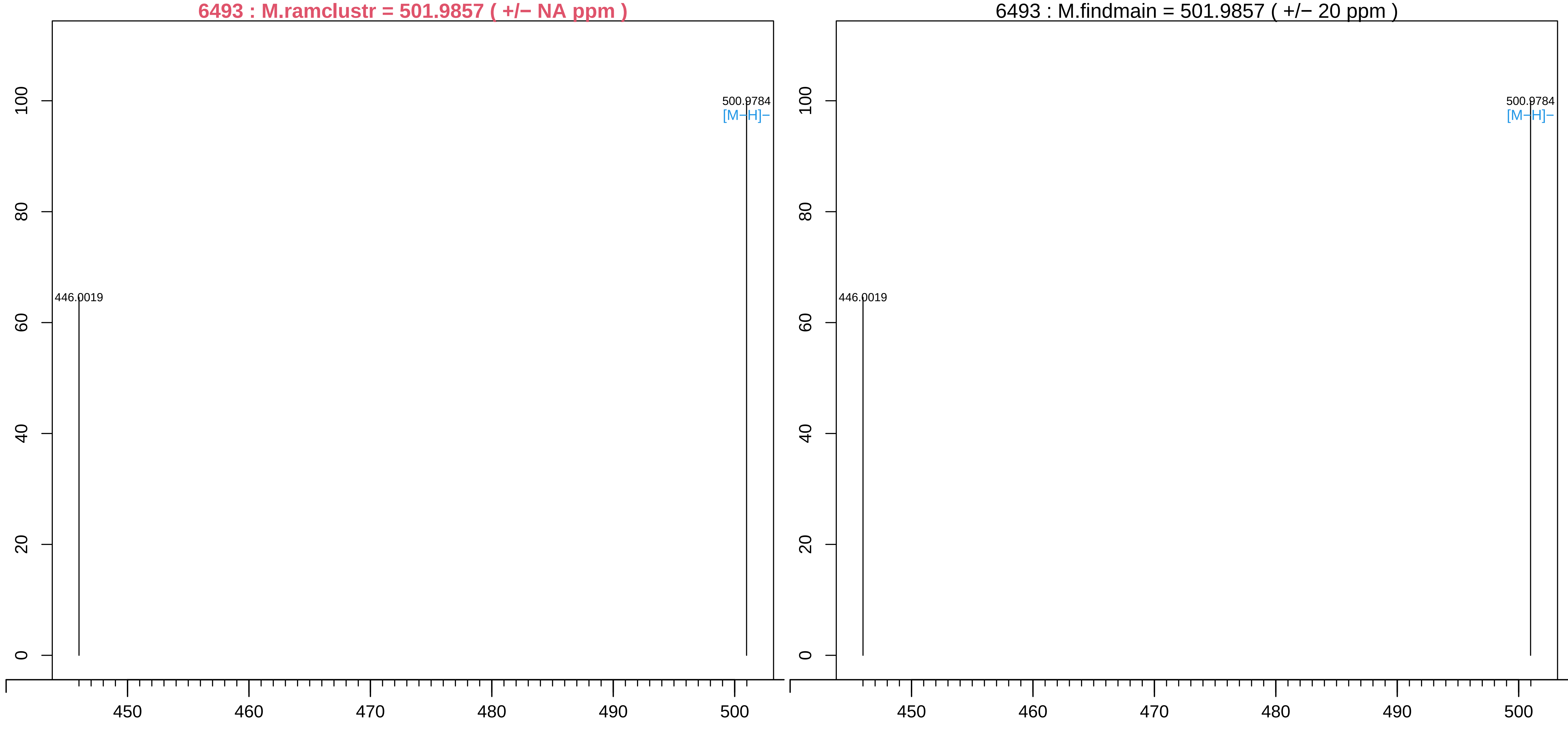

6493 : M.ramclustr = 501.9857 (+/− NA ppm)
6493 : M.findmain = 501.9857 (+/− 20 ppm)
500.9784
[M−H]−
446.0019
0
20
40
60
80
100
450
460
470
480
490
500

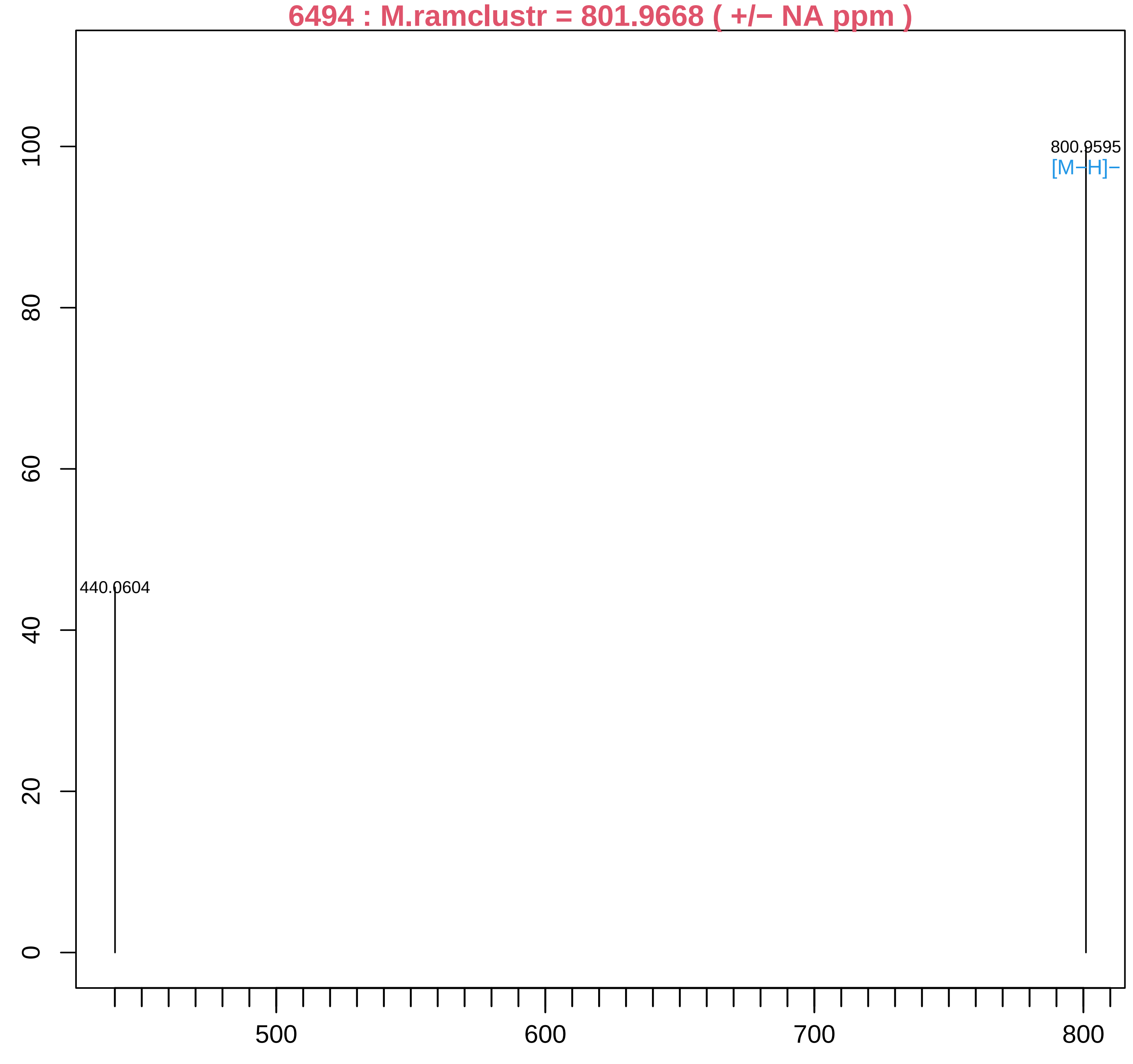
6494 : M.ramclustr = 801.9668 (+/− NA ppm)
800.9595
[M−H]−
440.0604
0
20
40
60
80
100
500
600
700
800

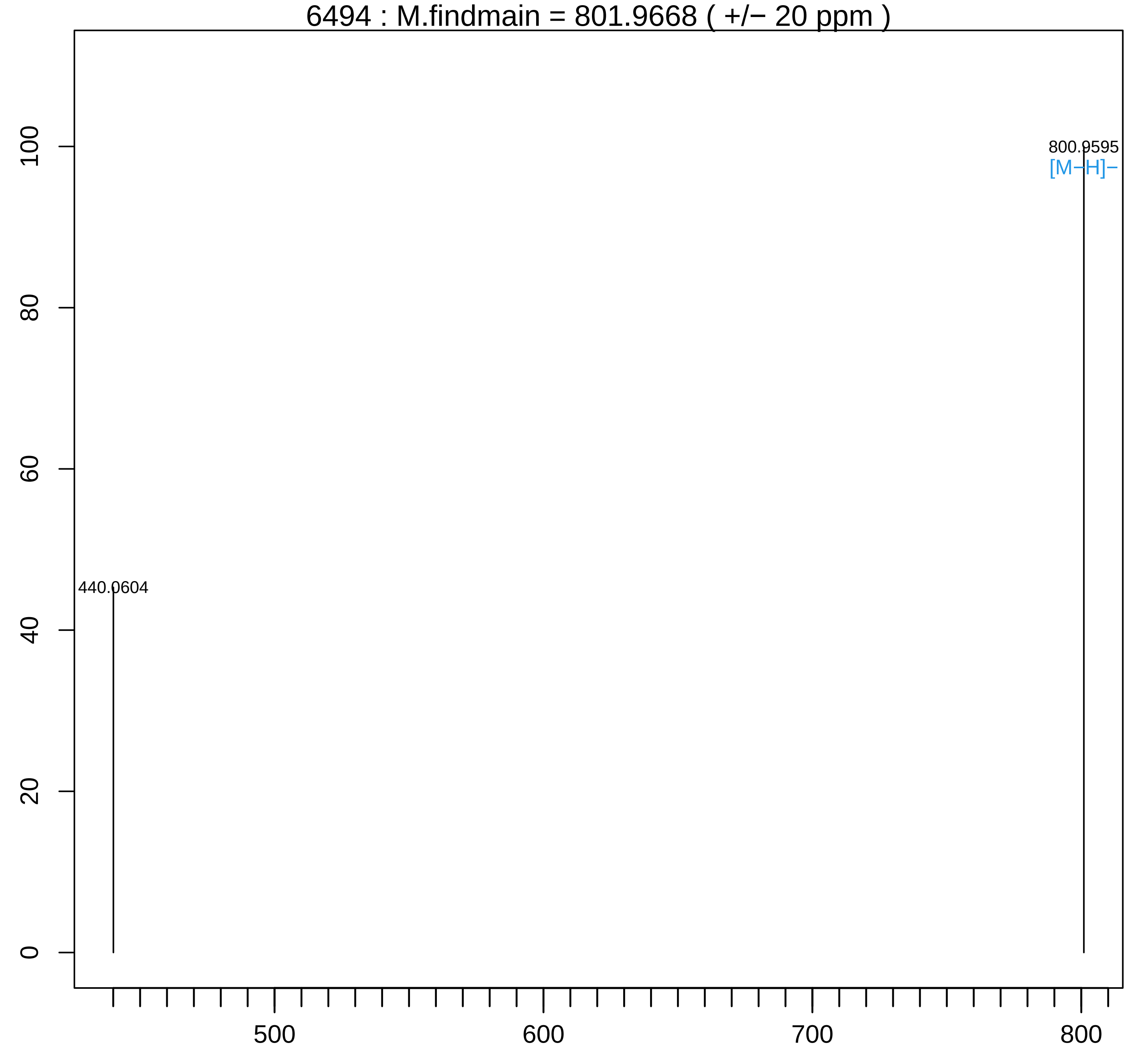
6494 : M.findmain = 801.9668 (+/− 20 ppm)
800.9595
[M−H]−
440.0604
0
20
40
60
80
100
500
600
700
800

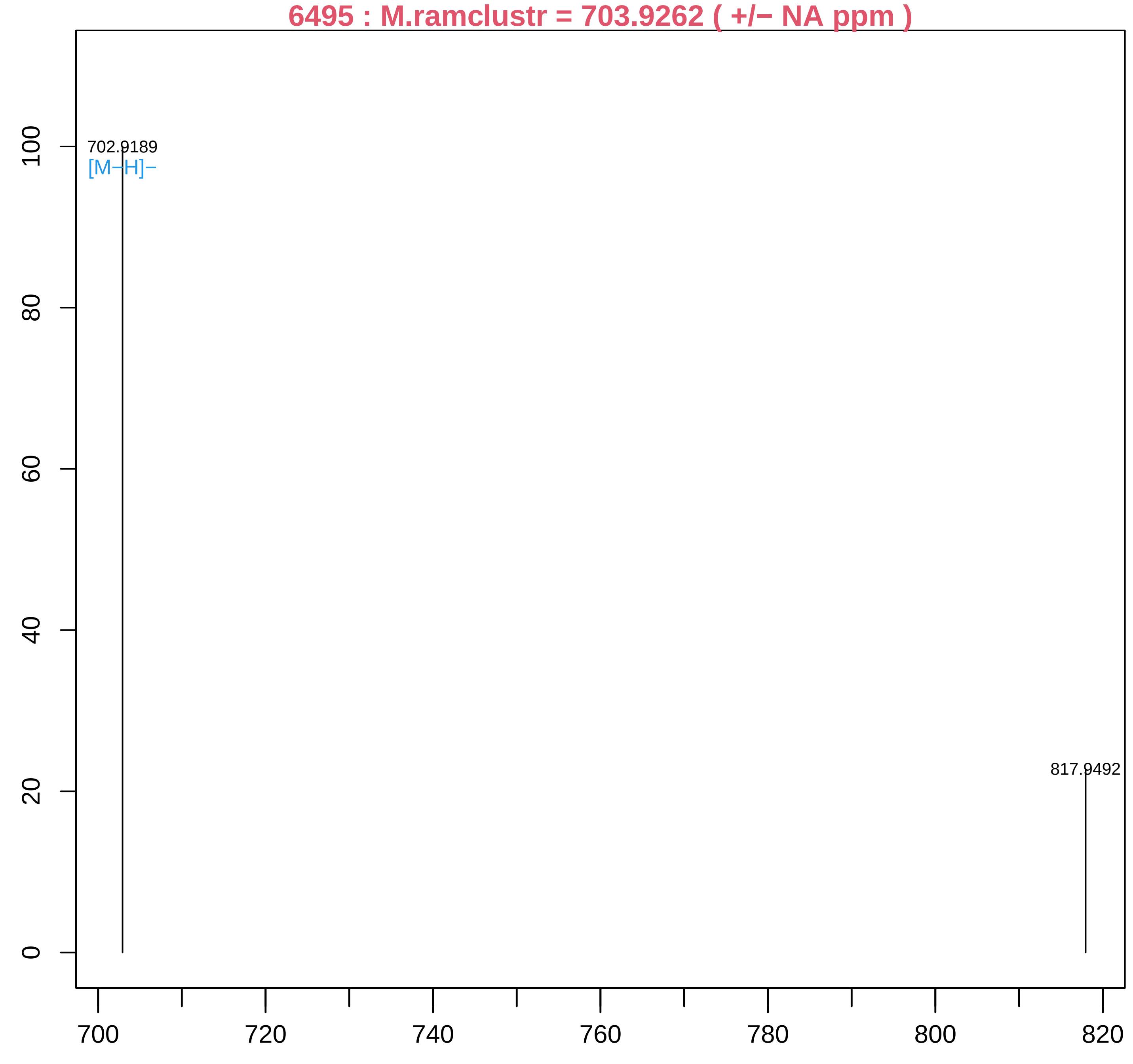

6495 : M.ramclustr = 703.9262 (+/− NA ppm)
702.9189
[M−H]−
817.9492
0
20
40
60
80
100
700
720
740
760
780
800
820

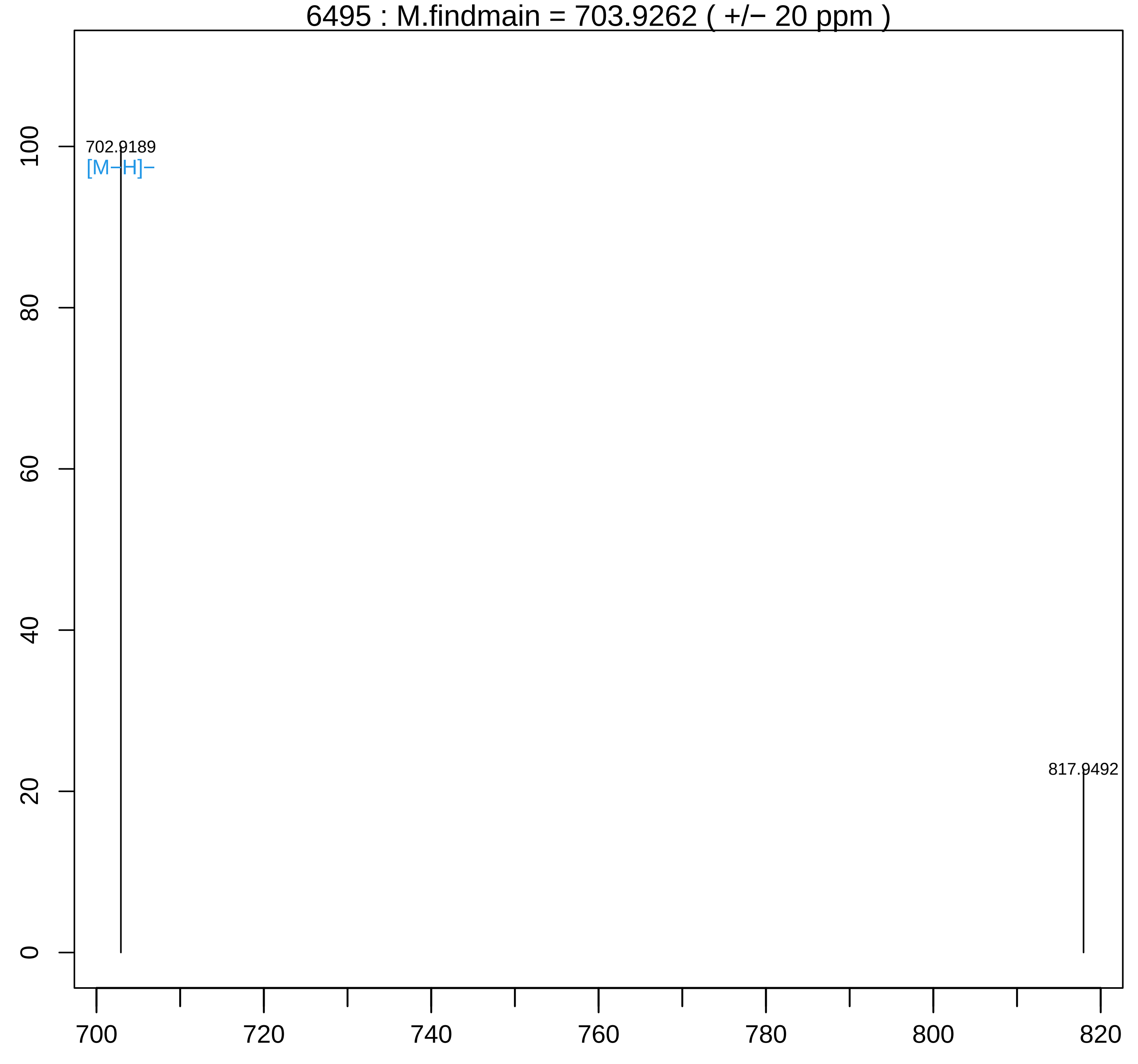

6495 : M.findmain = 703.9262 (+/− 20 ppm)
702.9189
[M−H]−
817.9492
0
20
40
60
80
100
700
720
740
760
780
800
820

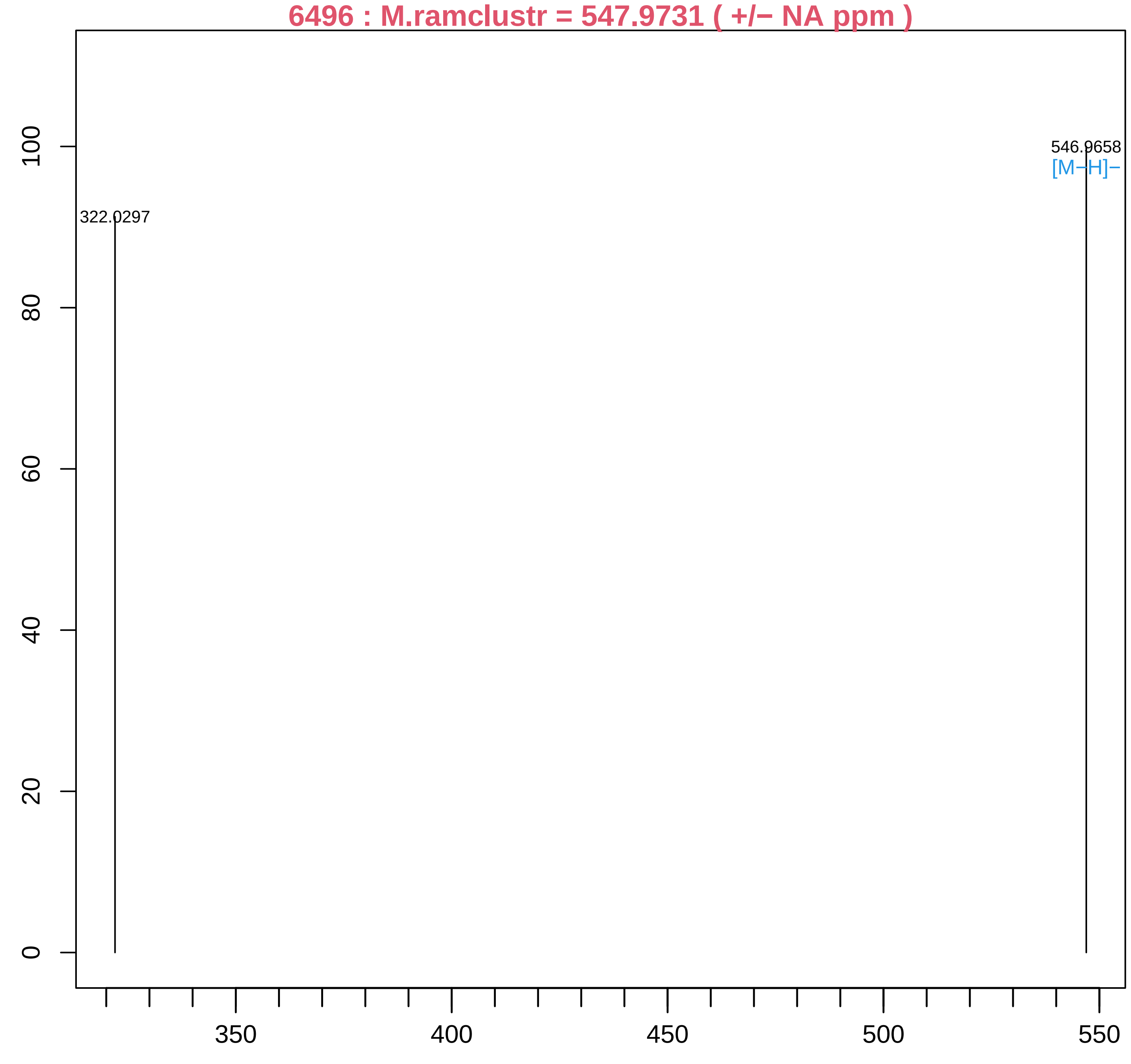
6496 : M.ramclustr = 547.9731 (+/− NA ppm)
546.9658
[M−H]−
322.0297
0
20
40
60
80
100
350
400
450
500
550

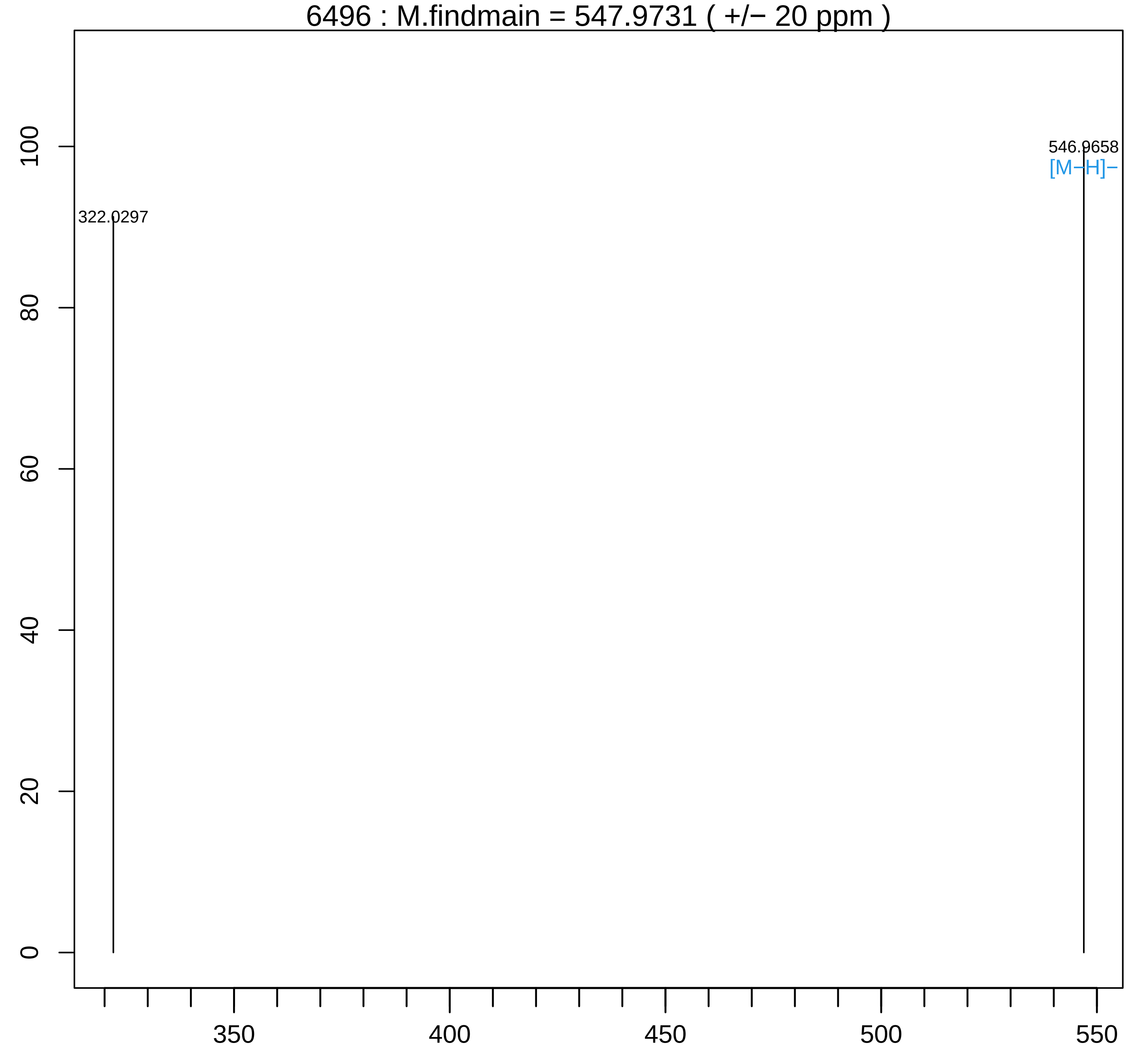
6496 : M.findmain = 547.9731 (+/− 20 ppm)
546.9658
[M−H]−
322.0297
0
20
40
60
80
100
350
400
450
500
550

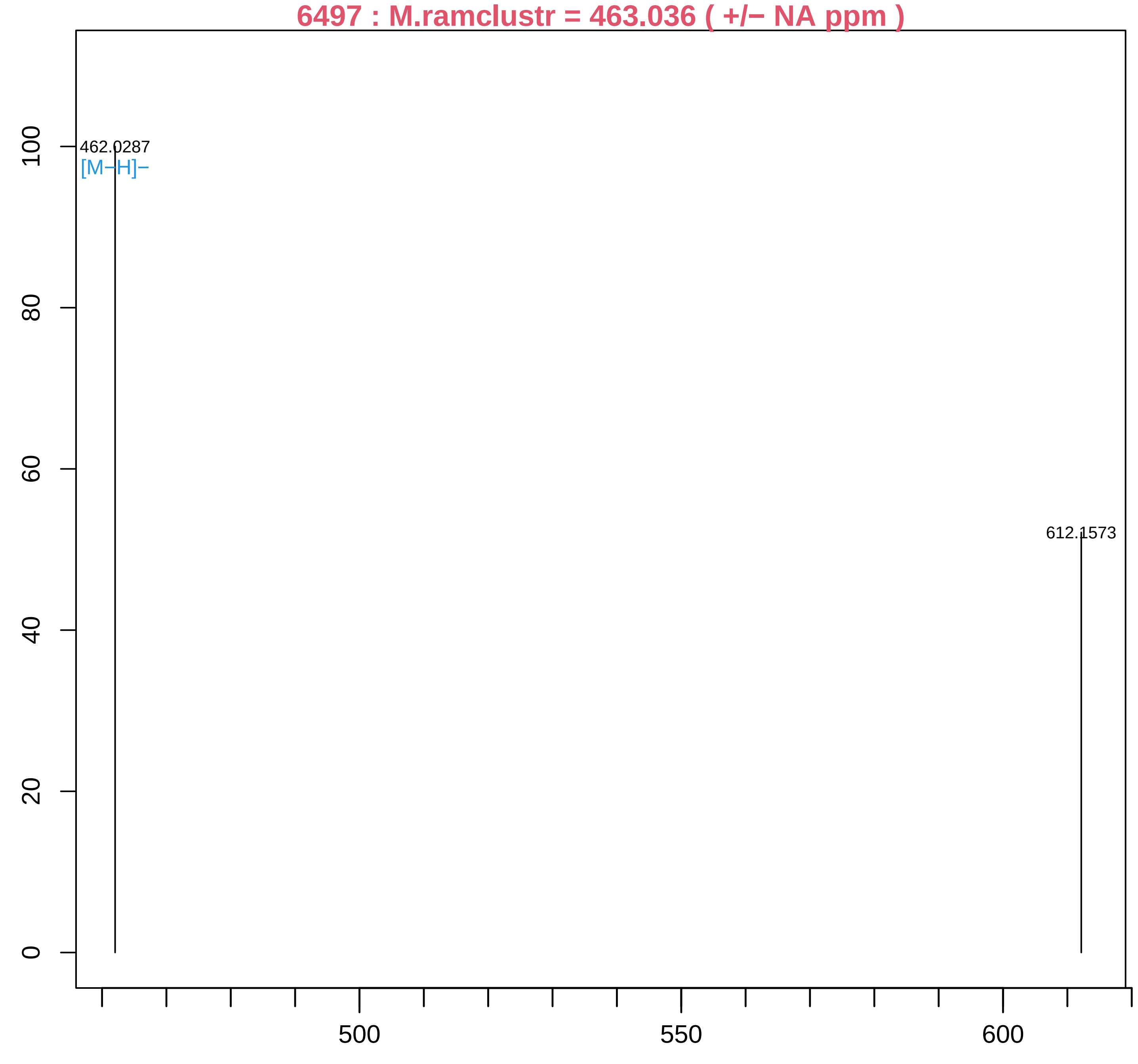
6497 : M.ramclustr = 463.036 (+/− NA ppm)
462.0287
[M−H]−
612.1573
0
20
40
60
80
100
500
550
600

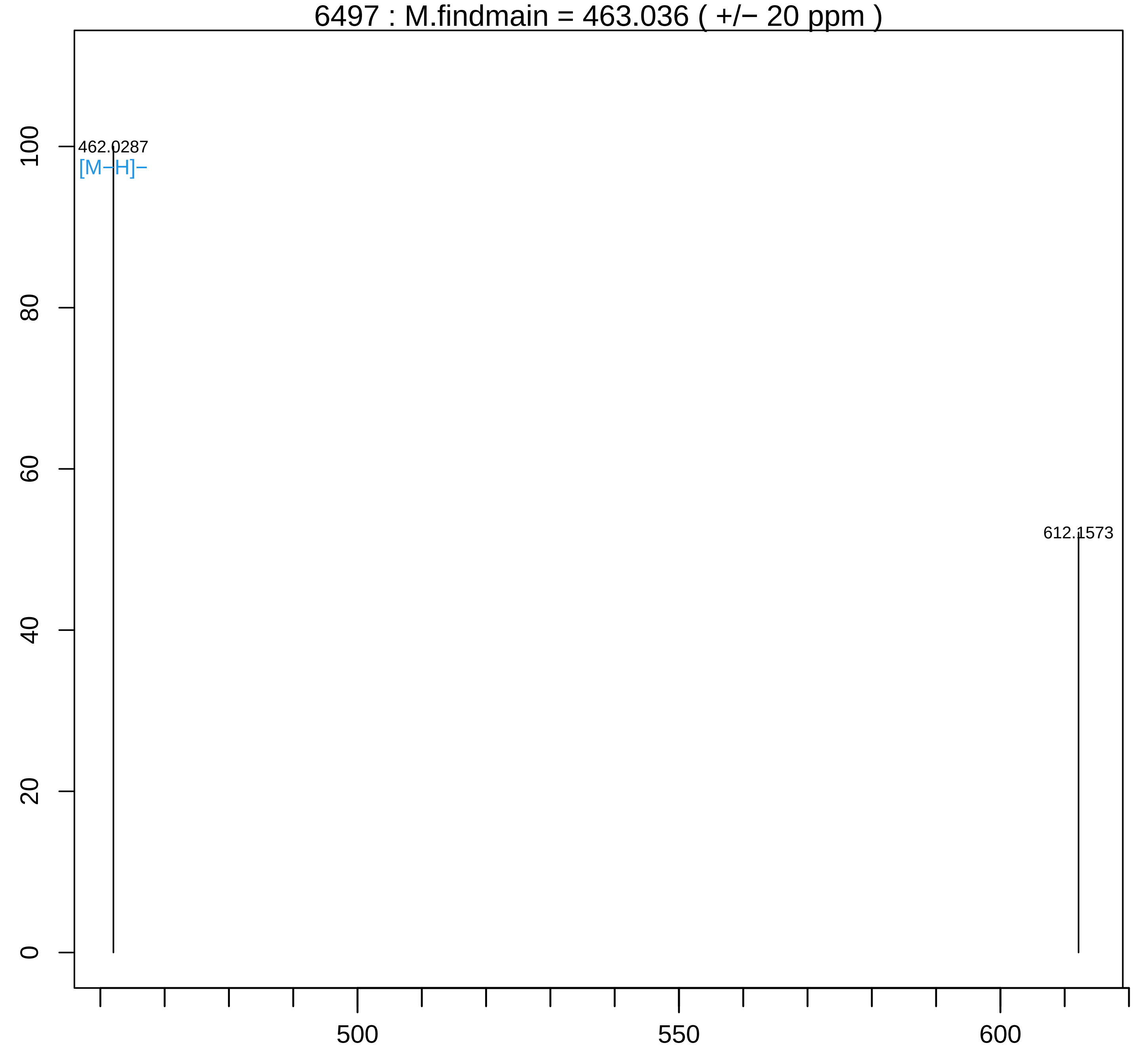
6497 : M.findmain = 463.036 (+/− 20 ppm)
462.0287
[M−H]−
612.1573
0
20
40
60
80
100
500
550
600

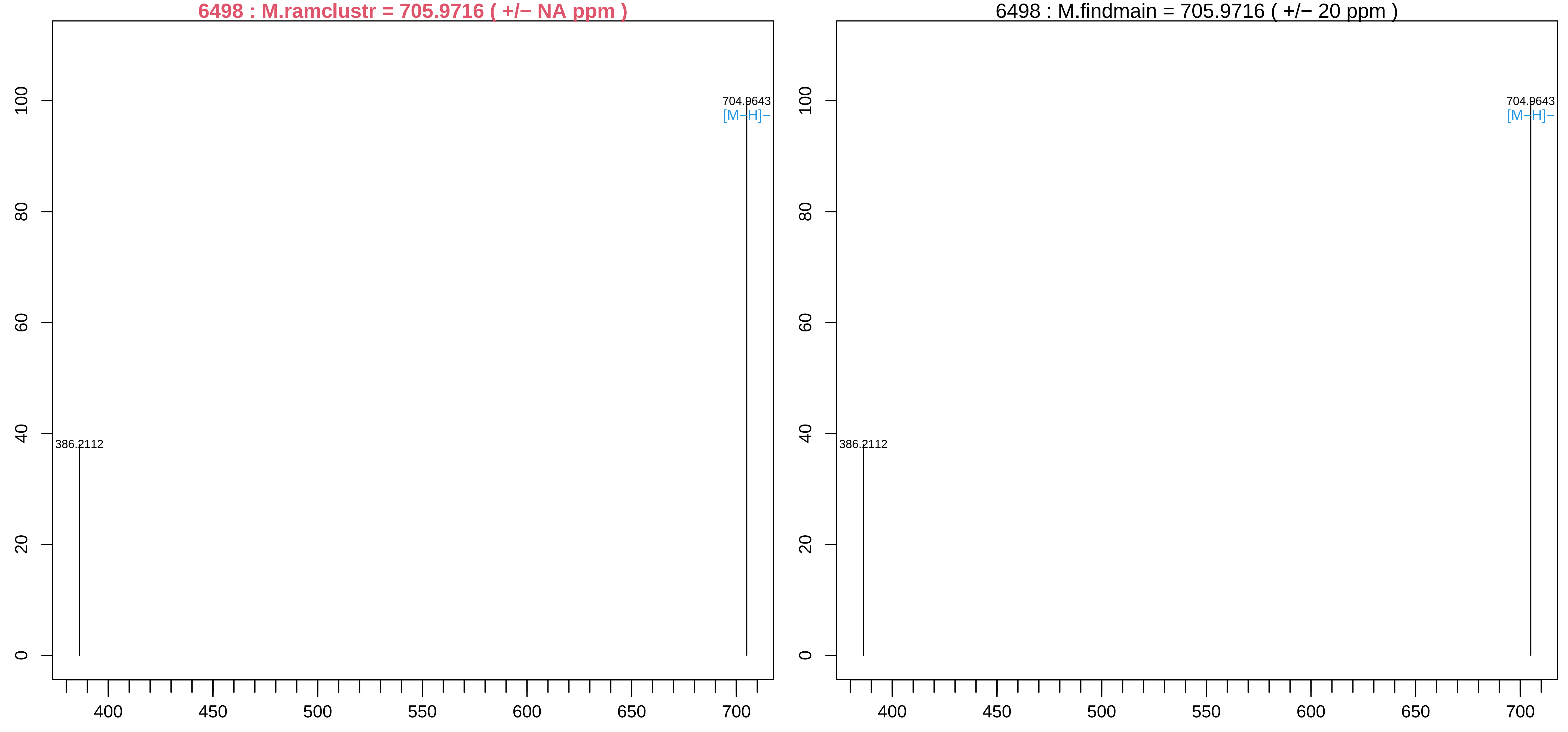
6498 : M.ramclustr = 705.9716 (+/− NA ppm)
704.9643
[M−H]−
386.2112
6498 : M.findmain = 705.9716 (+/− 20 ppm)
704.9643
[M−H]−
386.2112
0
20
40
60
80
100
400
450
500
550
600
650
700

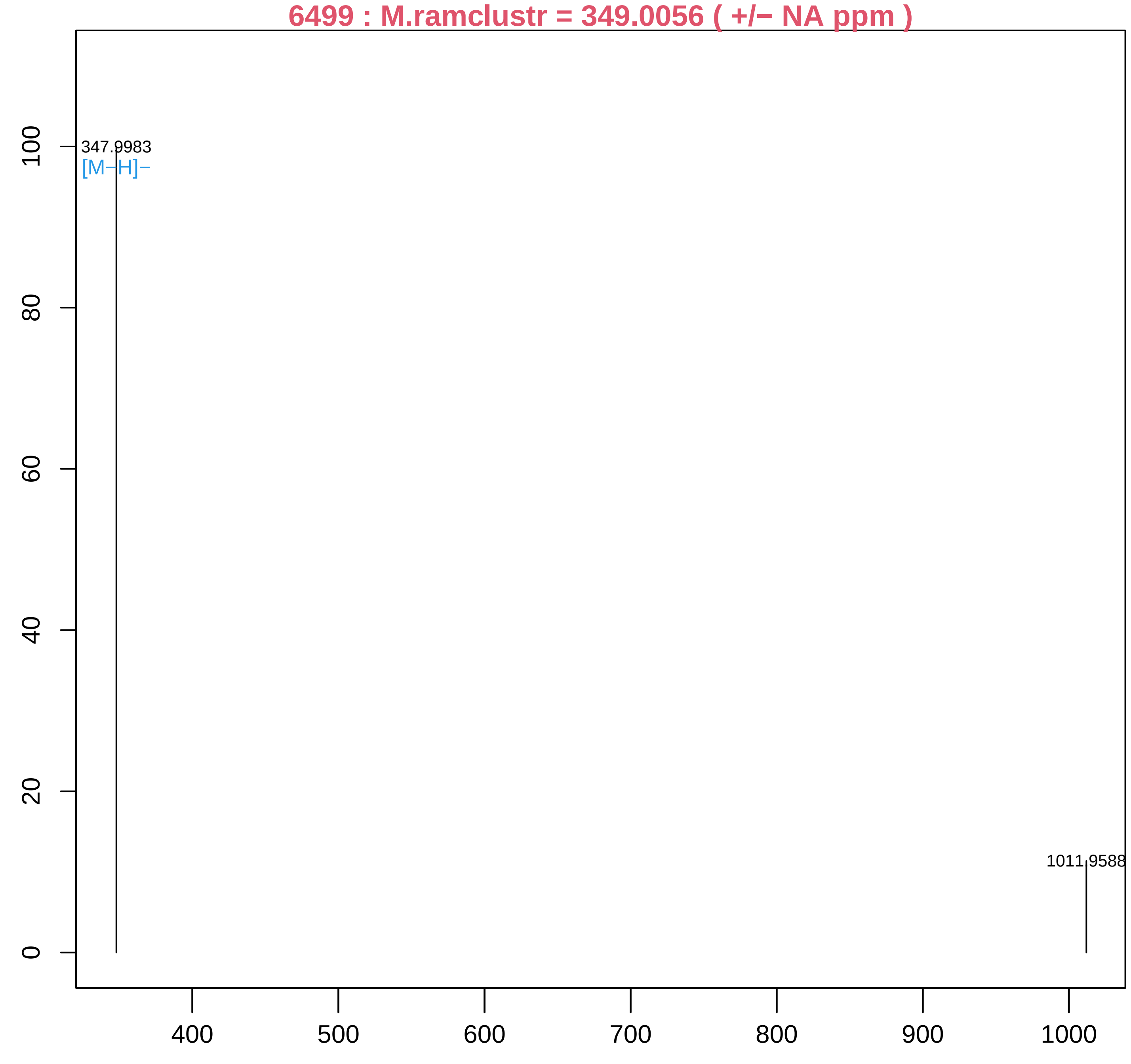
6499 : M.ramclustr = 349.0056 (+/− NA ppm)
347.9983
[M−H]−
1011.9588
0
20
40
60
80
100
400
500
600
700
800
900
1000

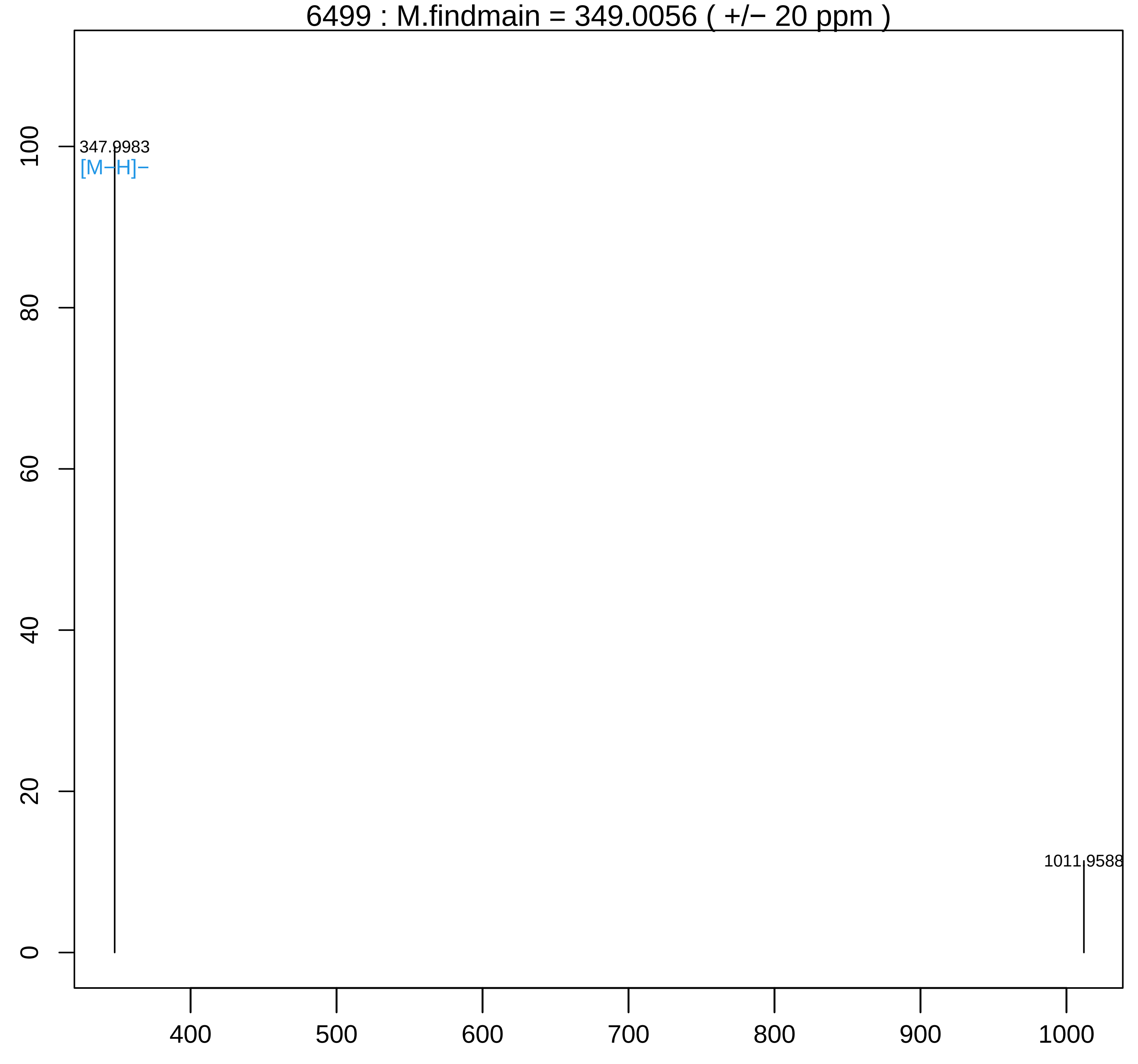
6499 : M.findmain = 349.0056 (+/− 20 ppm)
347.9983
[M−H]−
1011.9588
0
20
40
60
80
100
400
500
600
700
800
900
1000

6500 : M.ramclustr = 1037.9643 (+/− NA ppm)

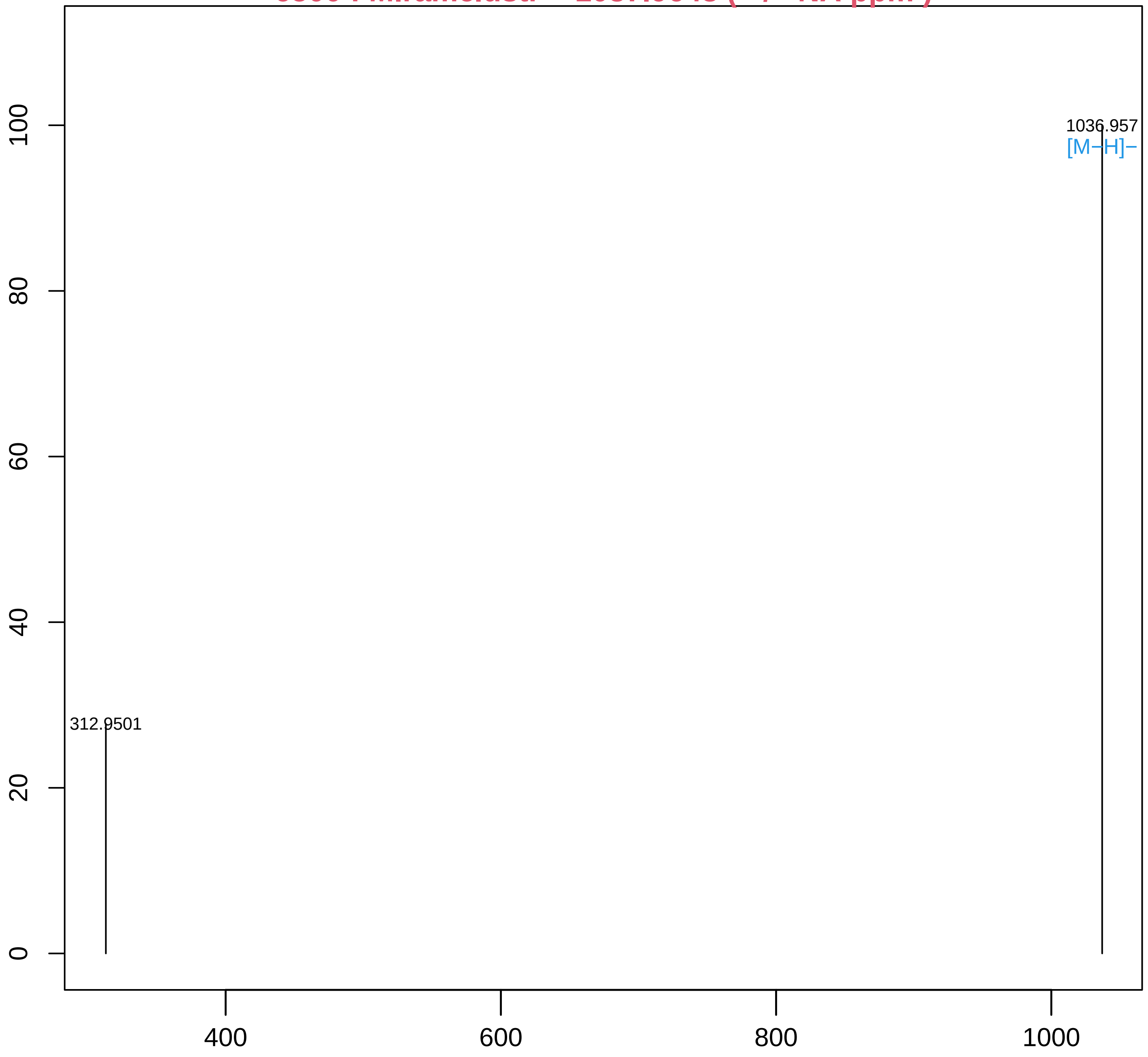

6500 : M.findmain = 1037.9643 (+/− 20 ppm)

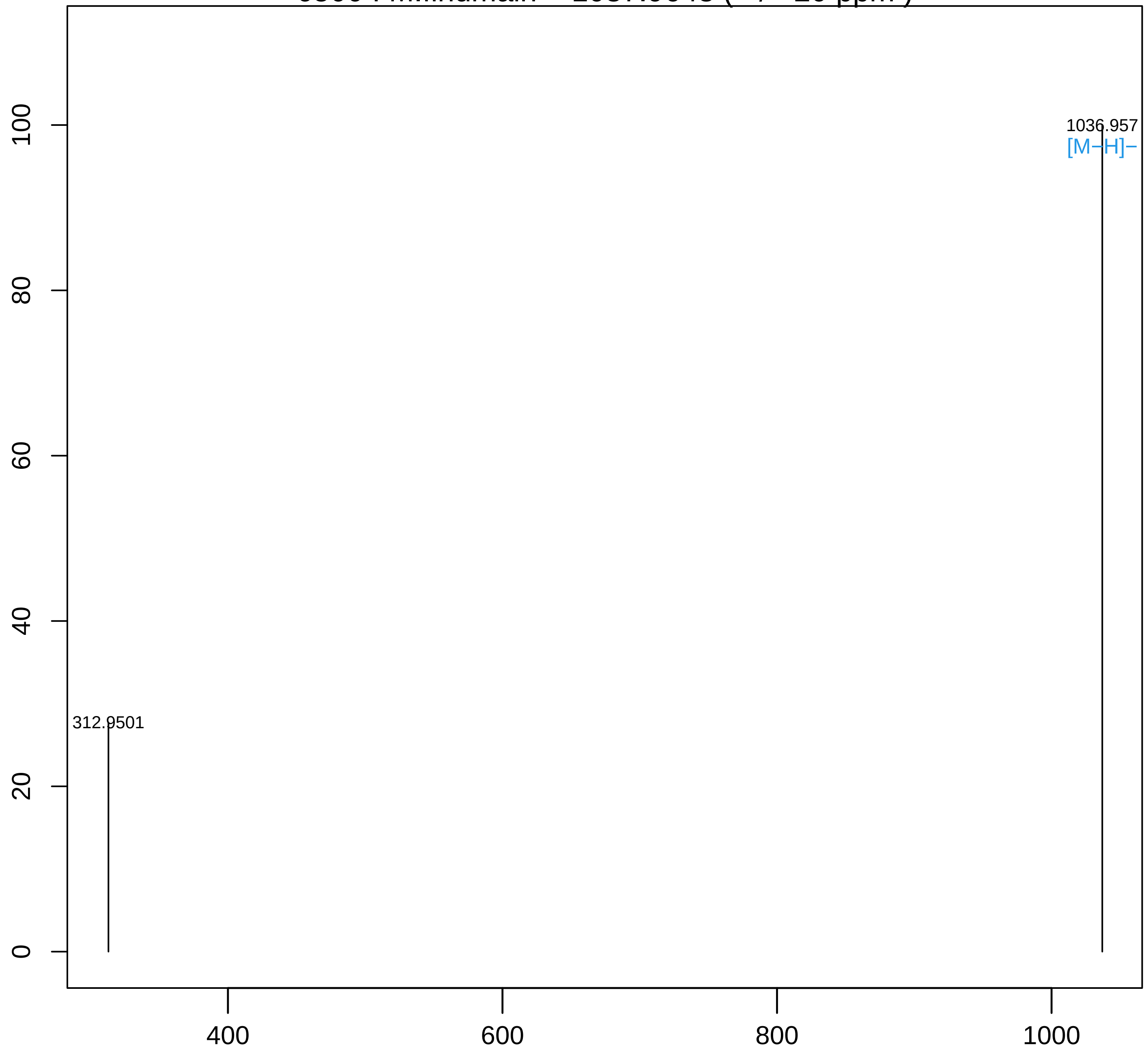

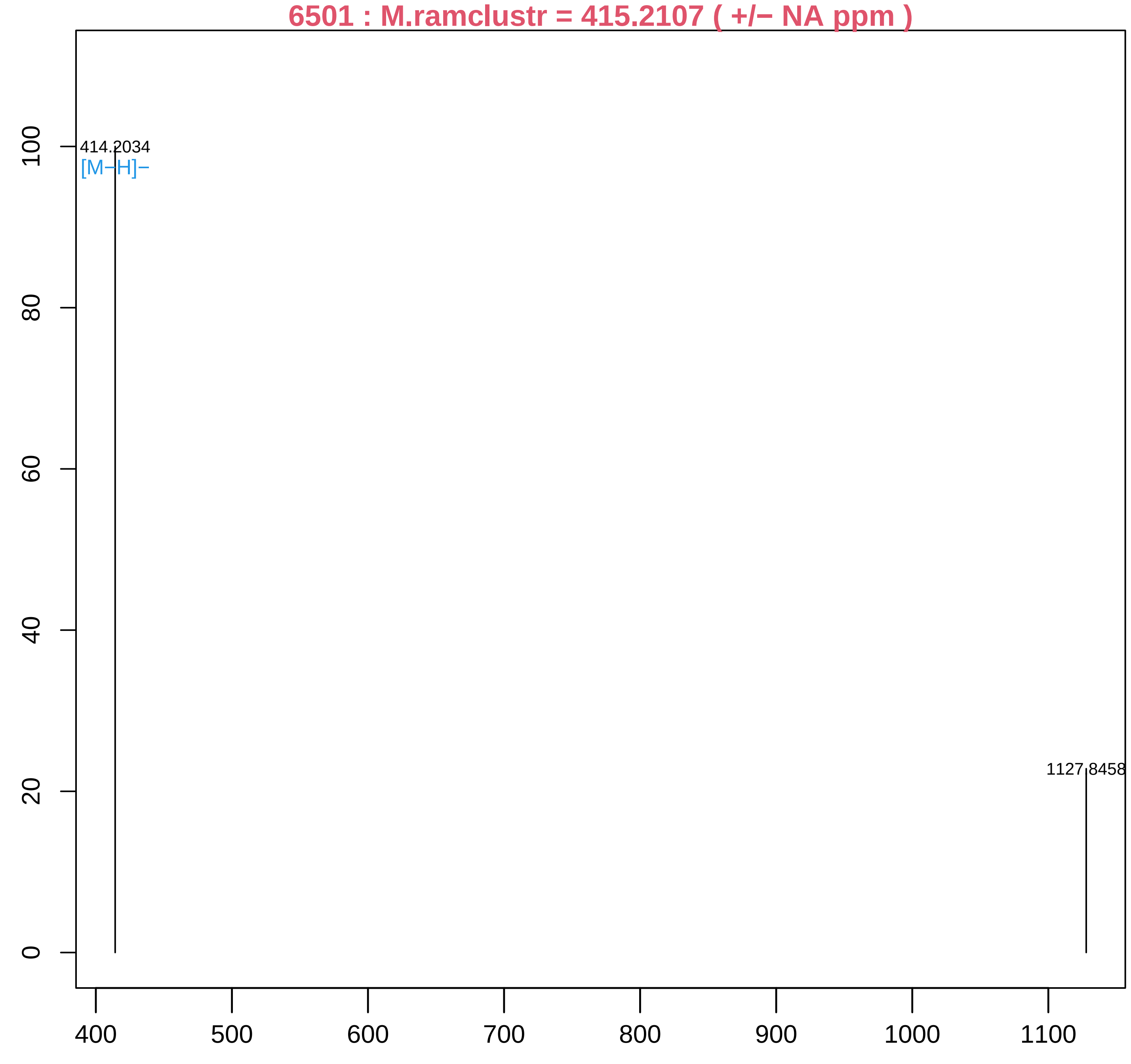
6501 : M.ramclustr = 415.2107 (+/− NA ppm)
414.2034
[M−H]−
1127.8458
0
20
40
60
80
100
400
500
600
700
800
900
1000
1100
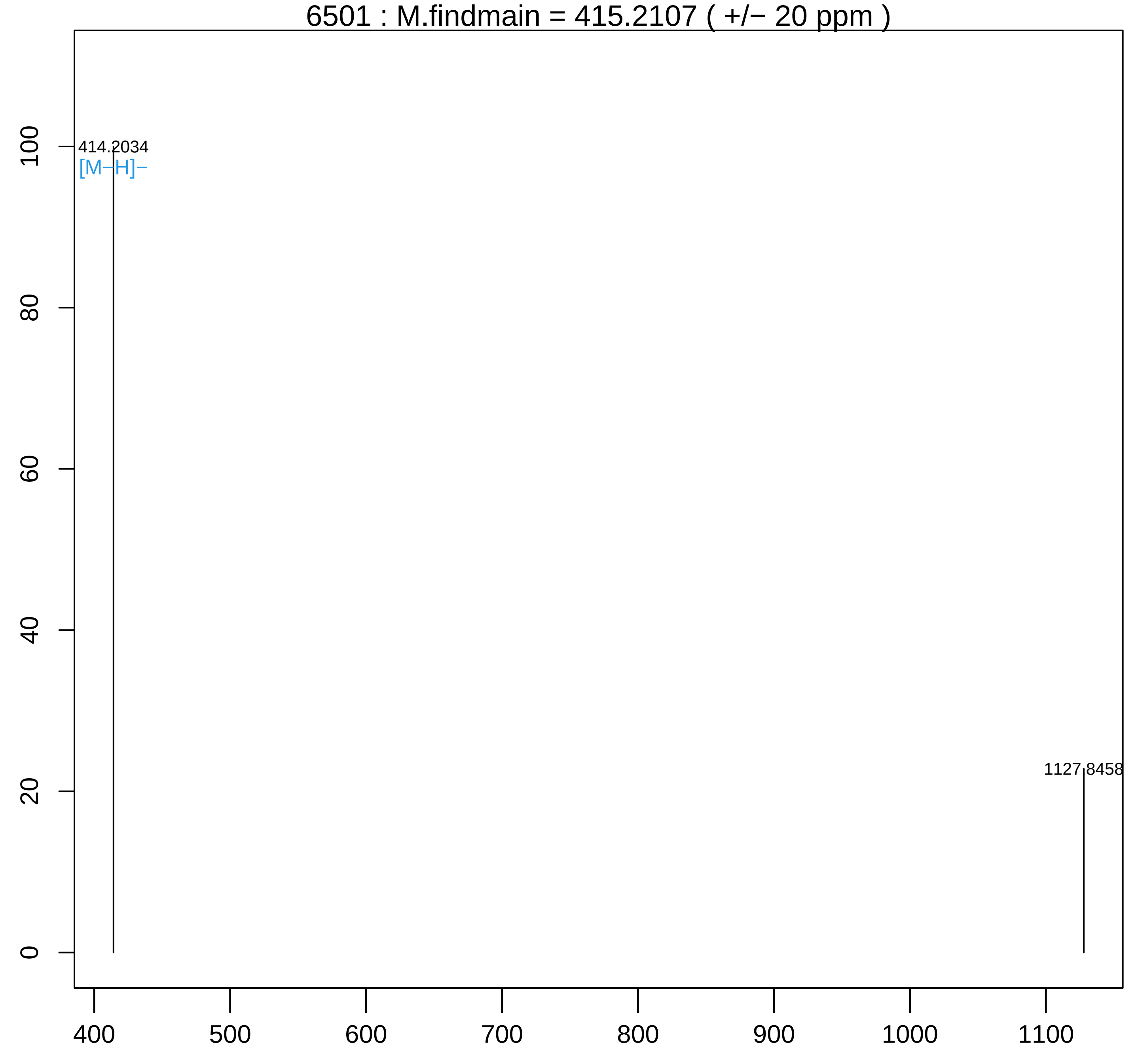
6501 : M.findmain = 415.2107 (+/− 20 ppm)
414.2034
[M−H]−
1127.8458
0
20
40
60
80
100
400
500
600
700
800
900
1000
1100

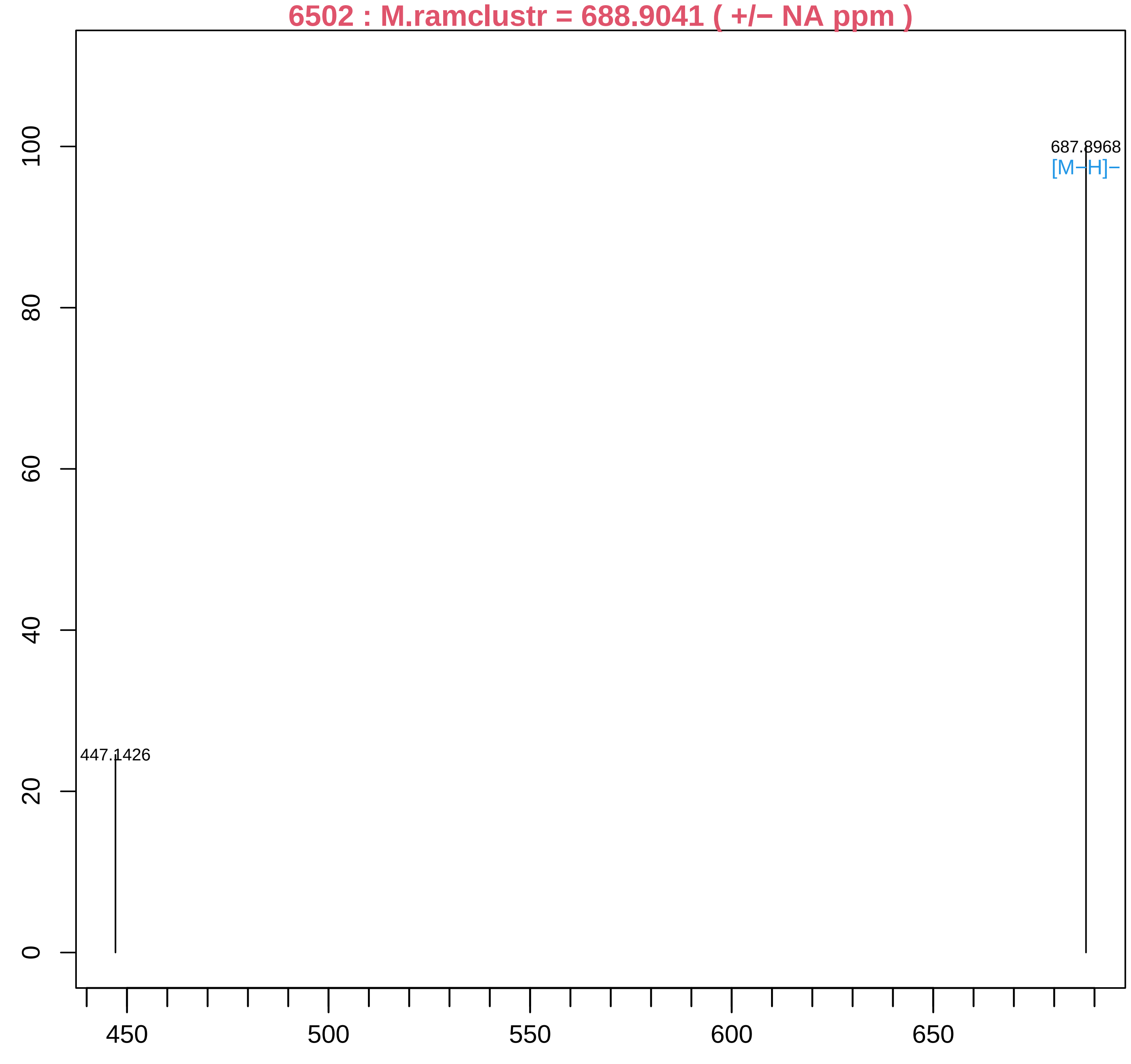
6502 : M.ramclustr = 688.9041 (+/− NA ppm)
687.8968
[M−H]−
447.1426
0
20
40
60
80
100
450
500
550
600
650

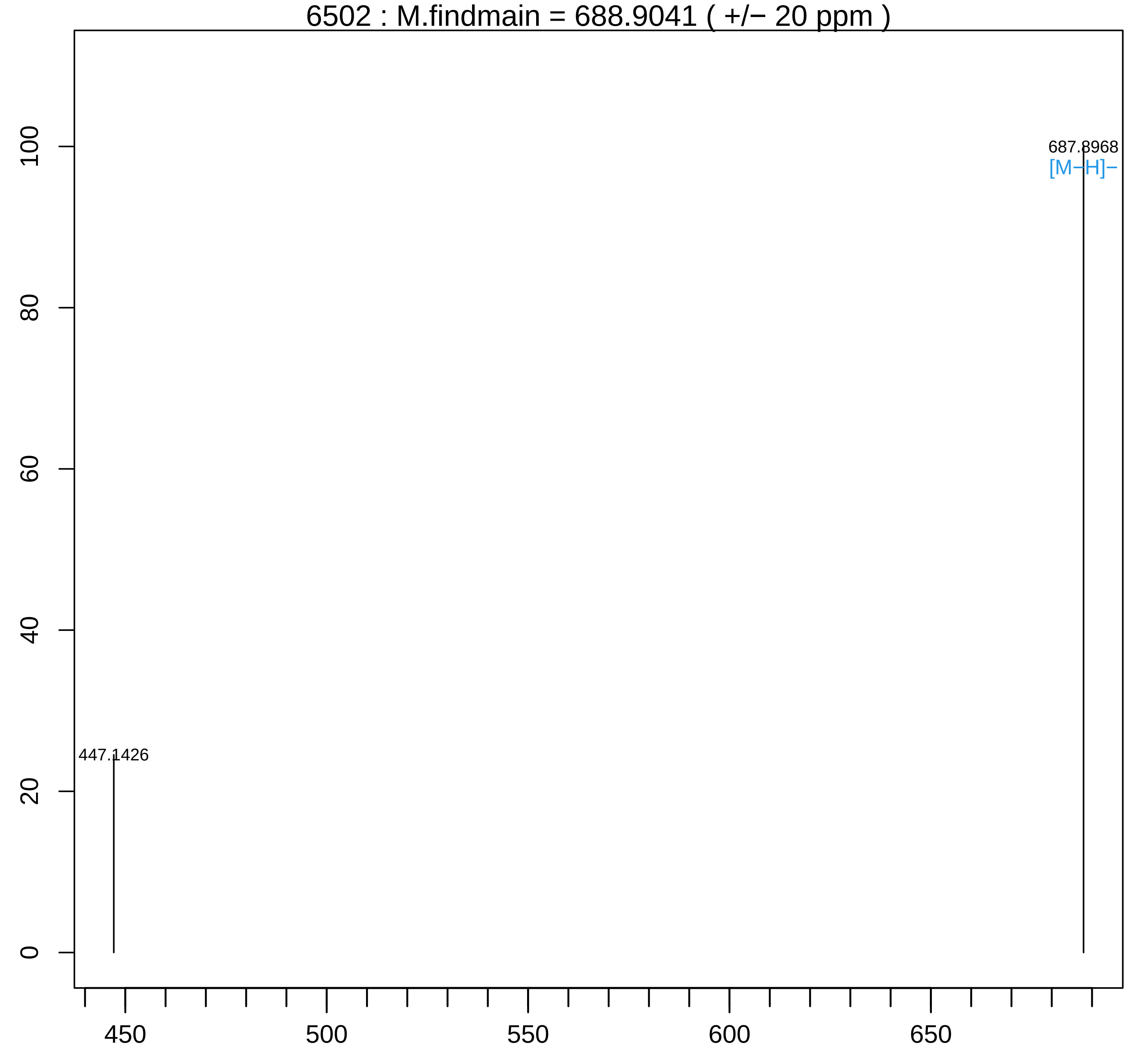
6502 : M.findmain = 688.9041 (+/− 20 ppm)
687.8968
[M−H]−
447.1426
0
20
40
60
80
100
450
500
550
600
650

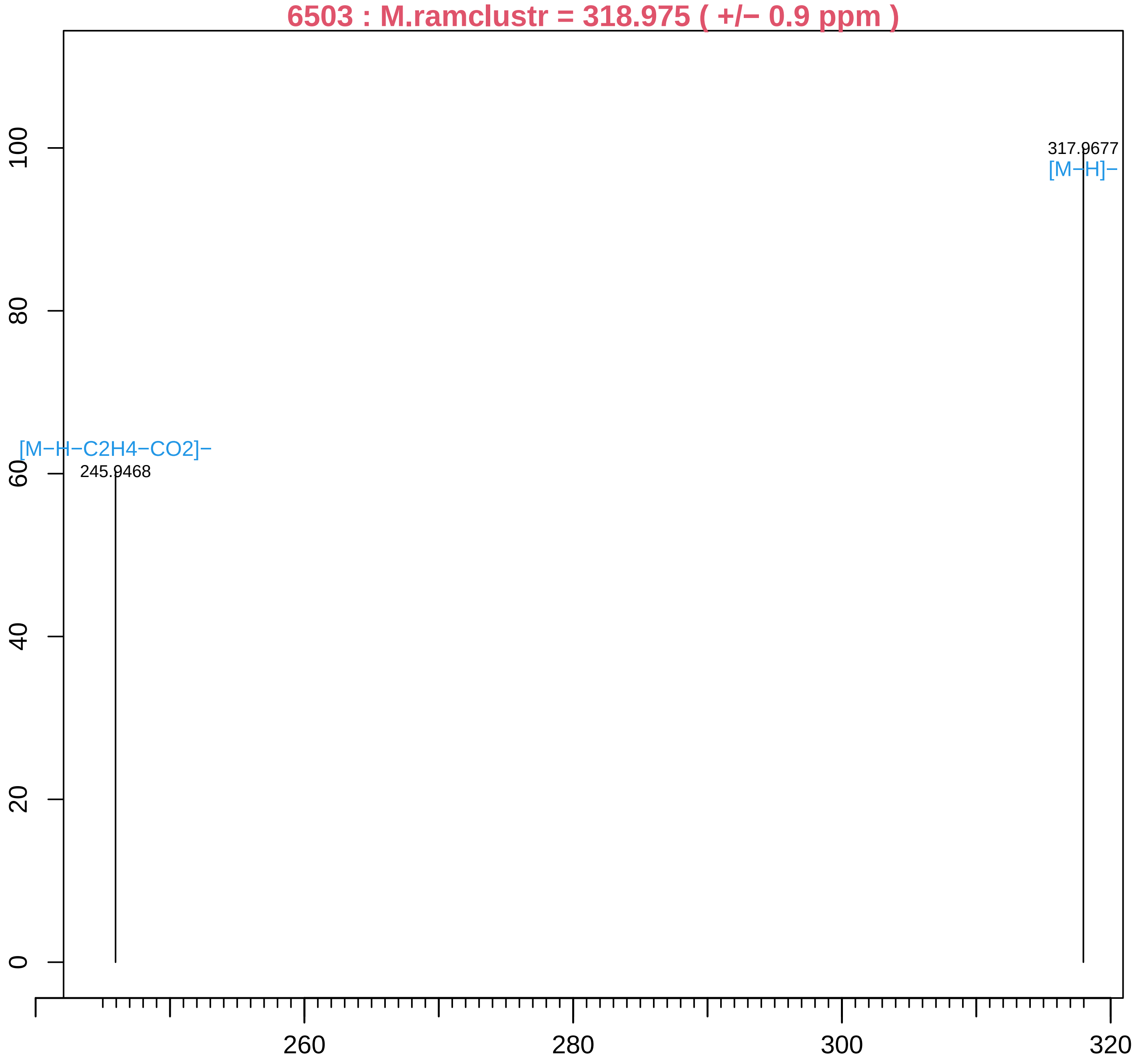
6503 : M.ramclustr = 318.975 (+/− 0.9 ppm)
317.9677
[M−H]−
[M−H−C2H4−CO2]−
245.9468
0
20
40
60
80
100
260
280
300
320

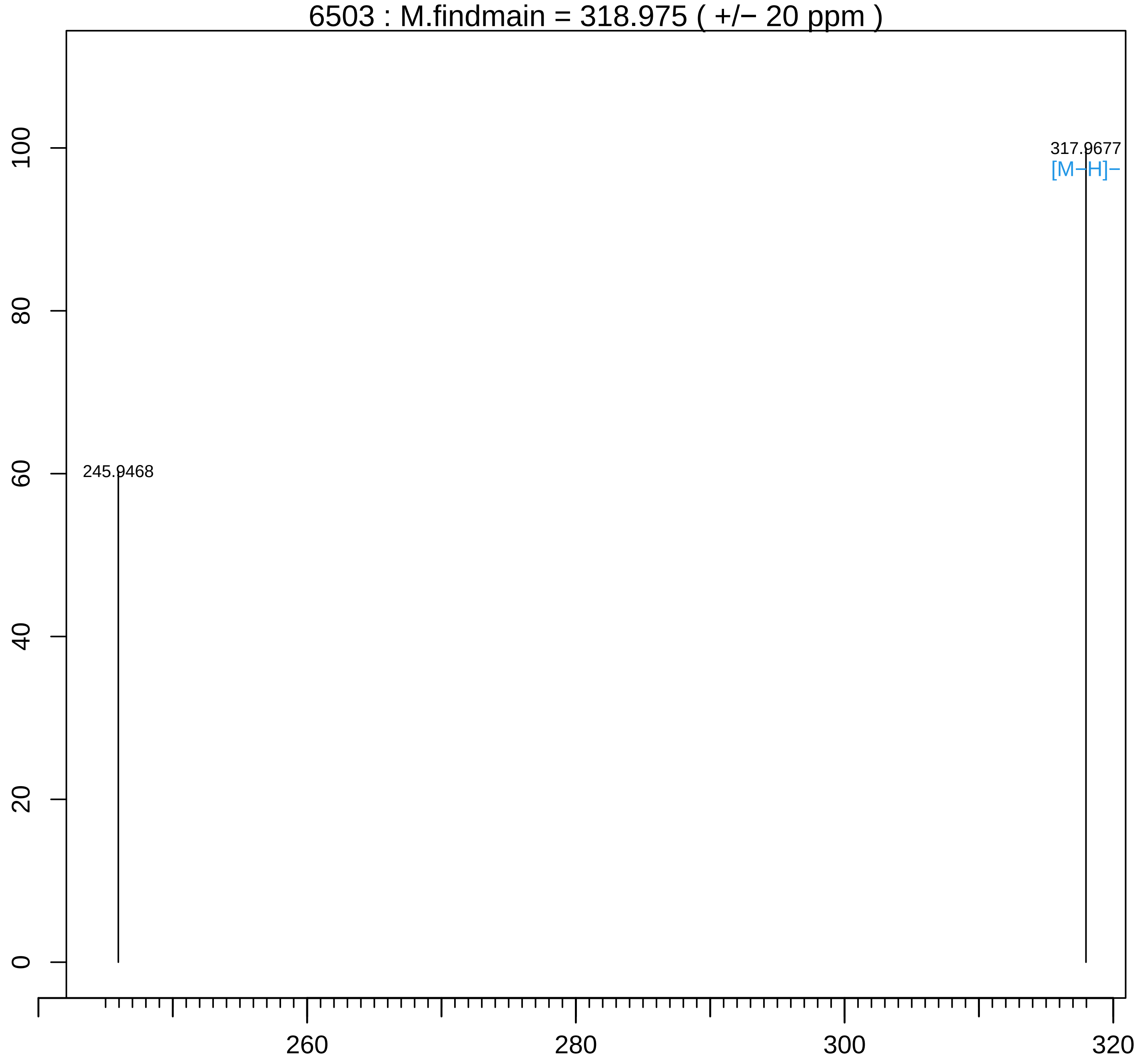
6503 : M.findmain = 318.975 (+/− 20 ppm)
317.9677
[M−H]−
245.9468
0
20
40
60
80
100
260
280
300
320

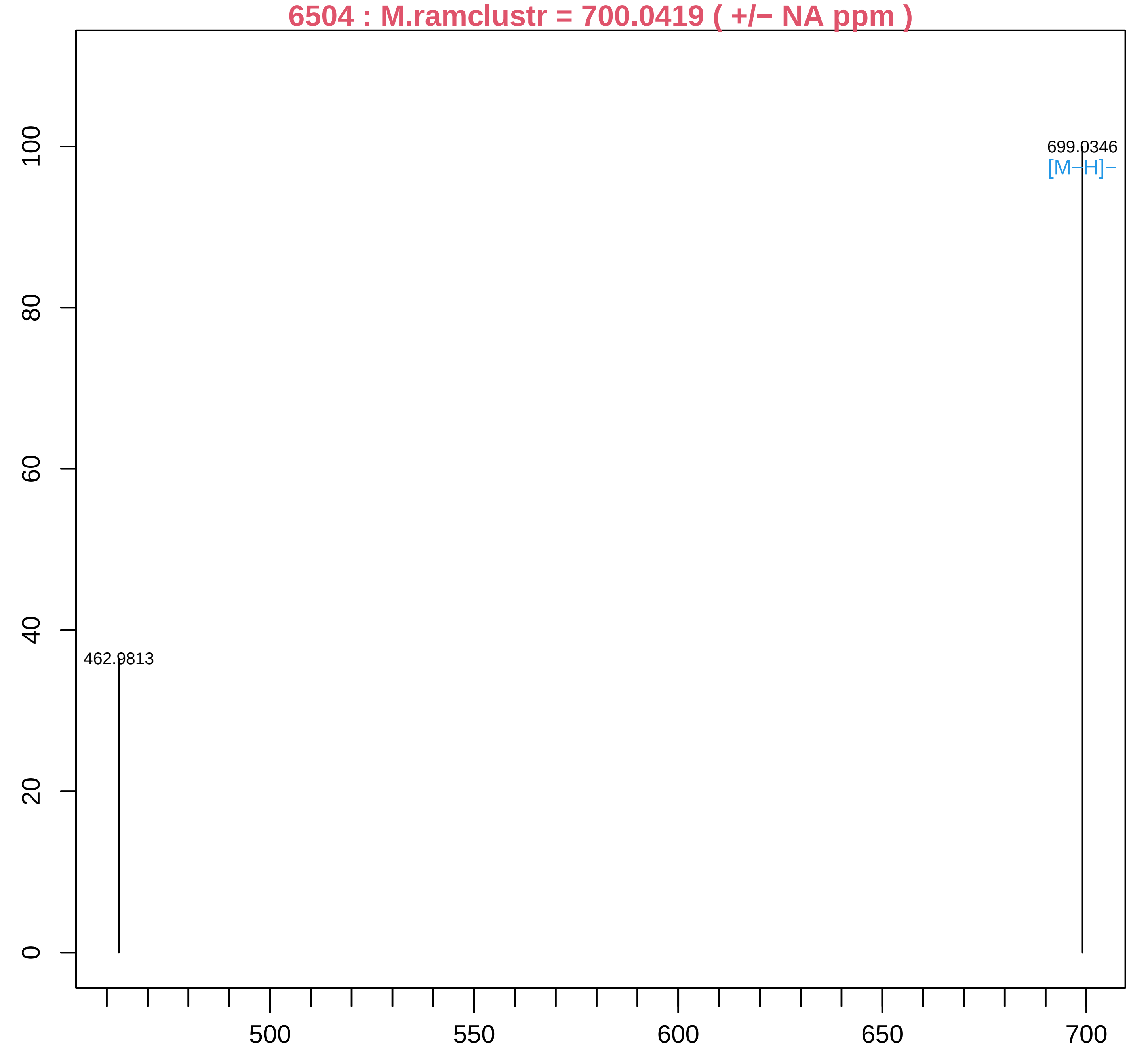

6504 : M.ramclustr = 700.0419 (+/− NA ppm)
699.0346
[M−H]−
462.9813
0
20
40
60
80
100
500
550
600
650
700

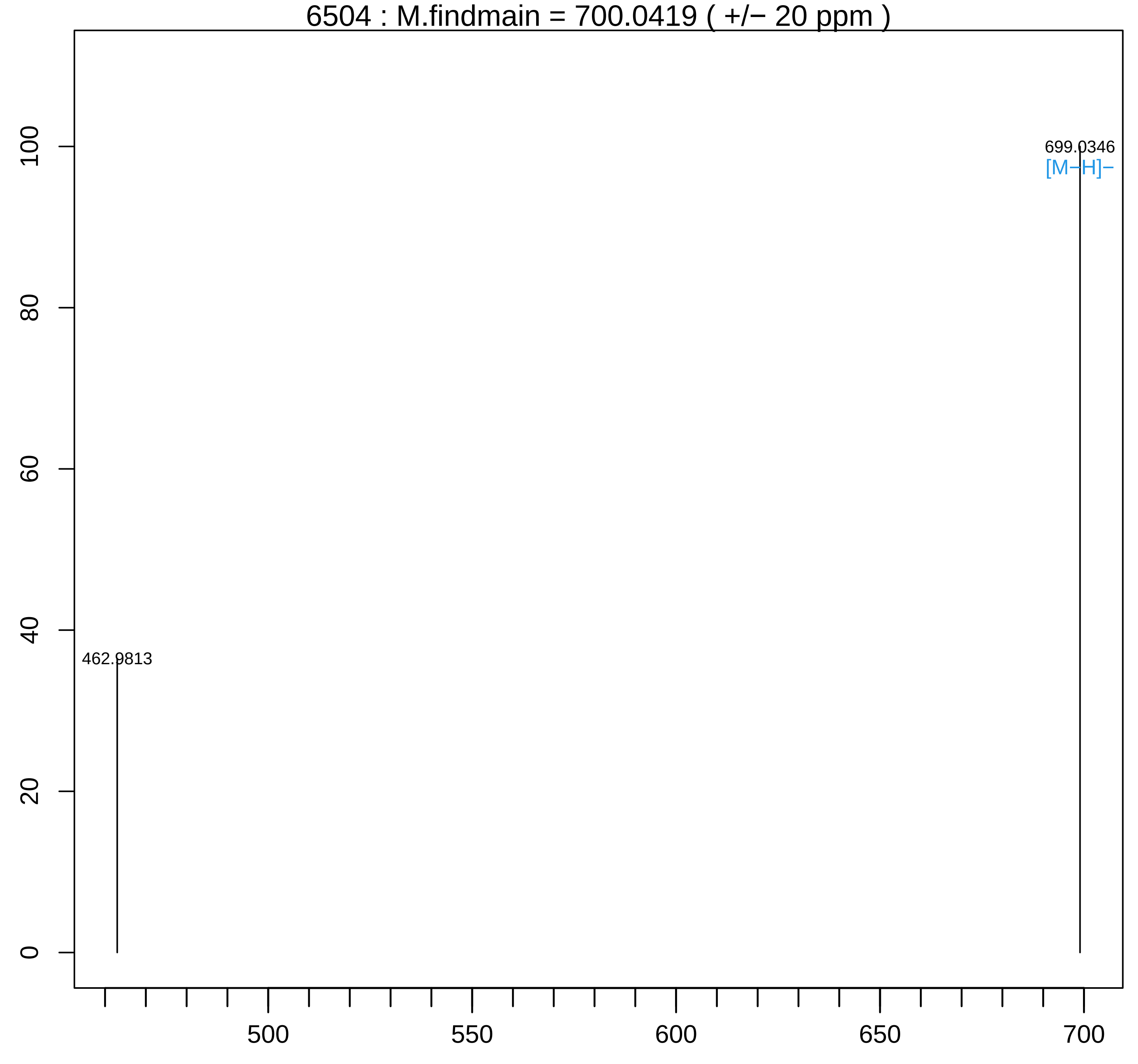

6504 : M.findmain = 700.0419 (+/− 20 ppm)
699.0346
[M−H]−
462.9813
0
20
40
60
80
100
500
550
600
650
700

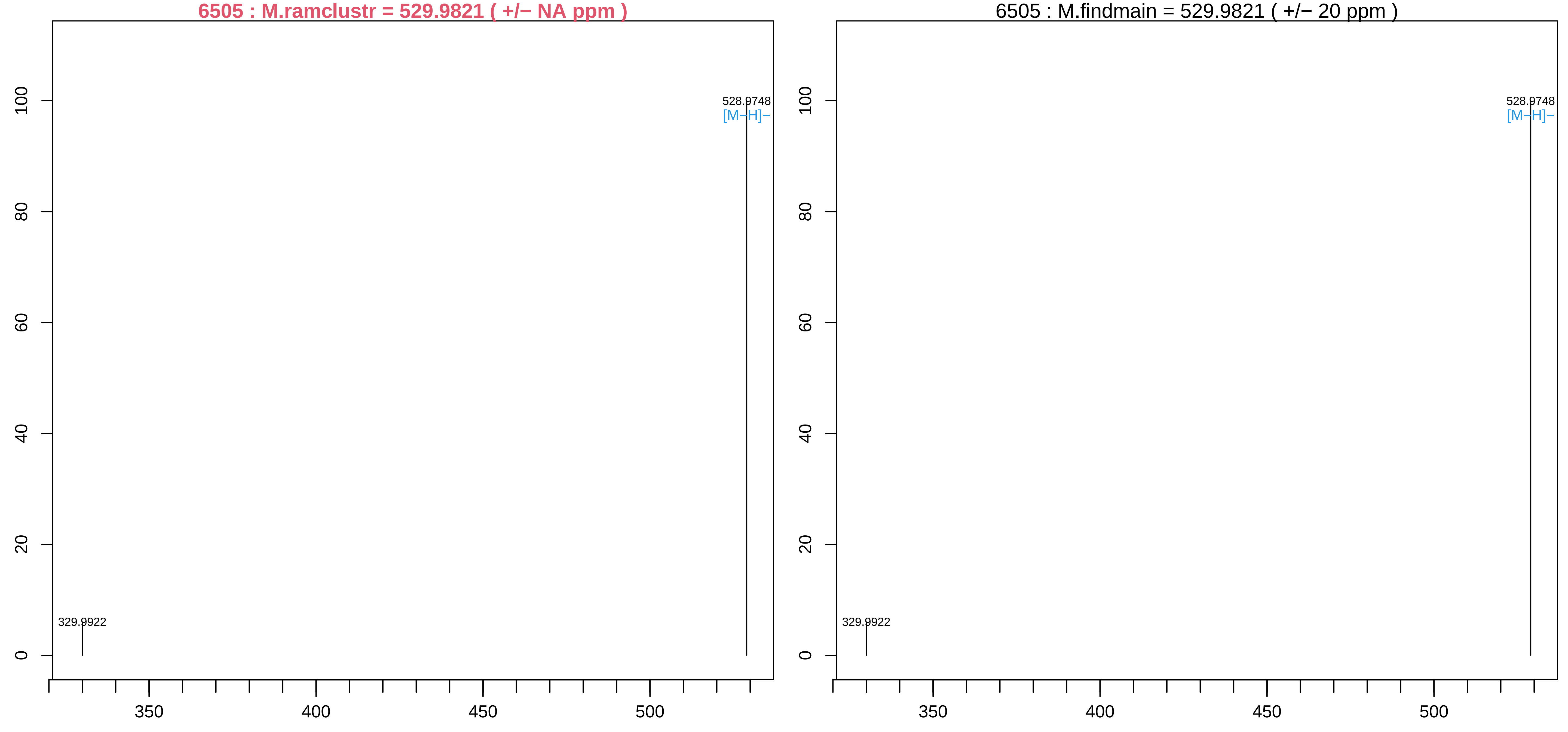

6505 : M.ramclustr = 529.9821 (+/− NA ppm)
528.9748
[M−H]−
329.9922
6505 : M.findmain = 529.9821 (+/− 20 ppm)
528.9748
[M−H]−
329.9922

6506 : M.ramclustr = 696.0051 (+/− NA ppm)

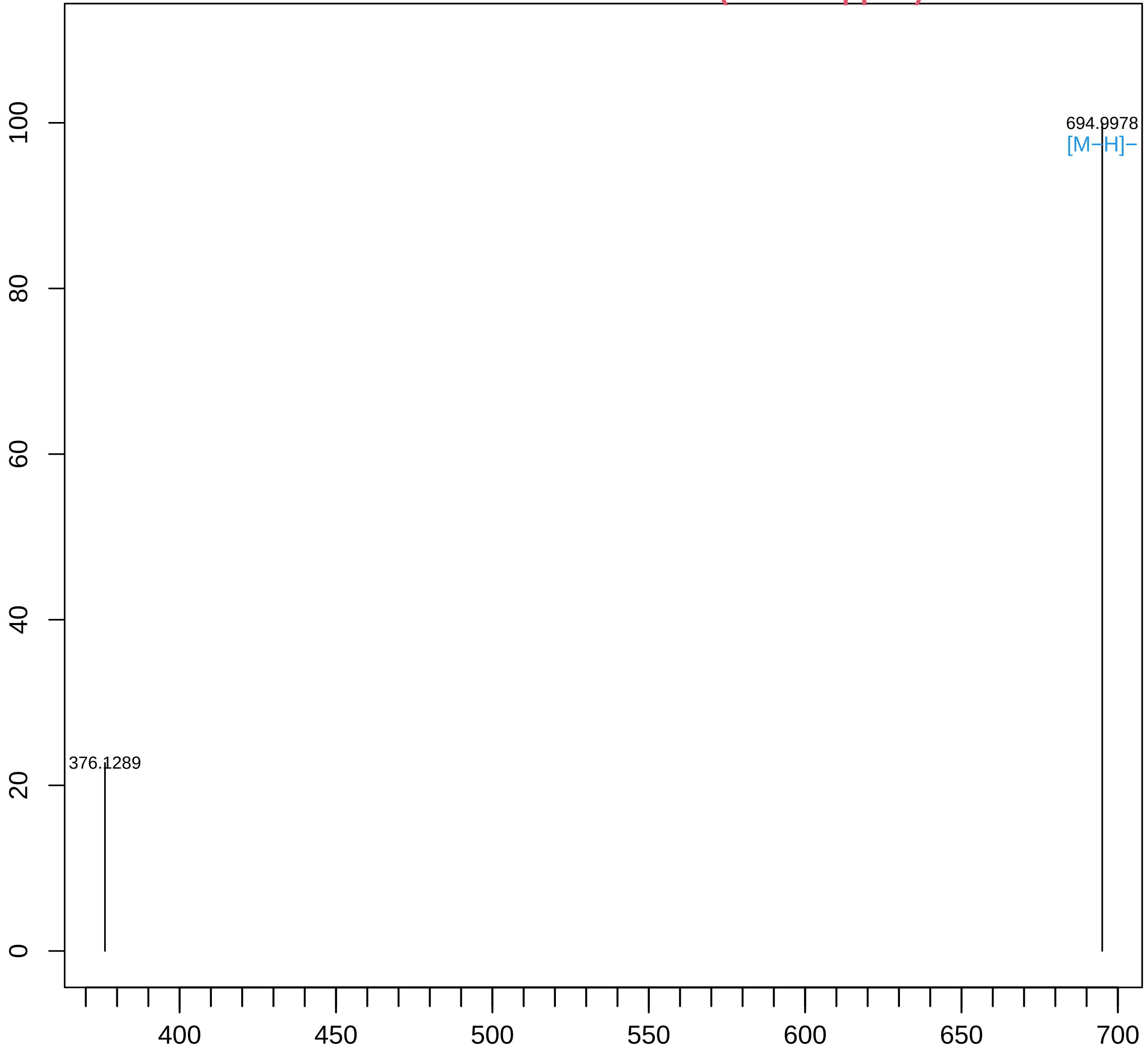

6506 : M.findmain = 696.0051 (+/− 20 ppm)

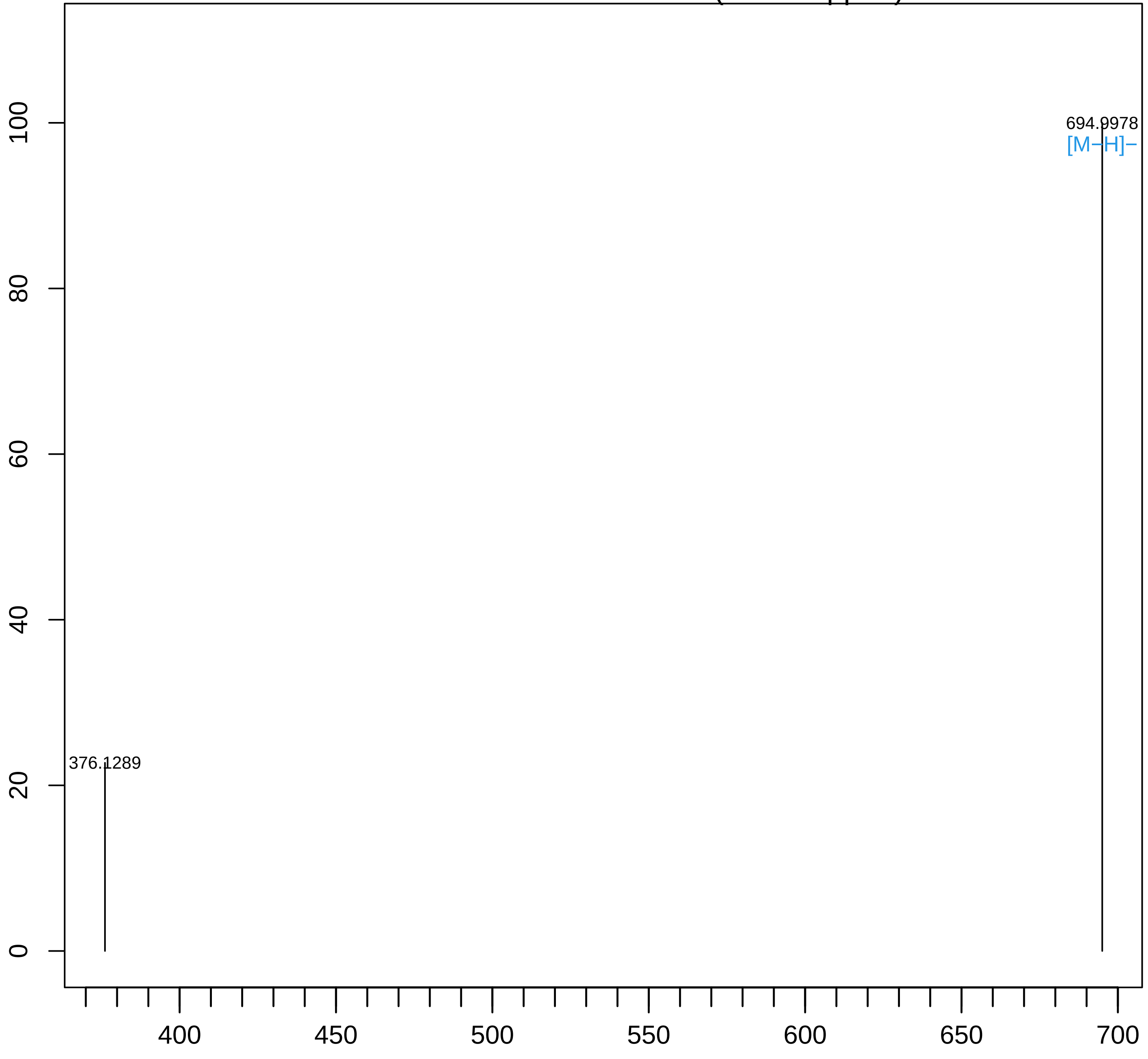

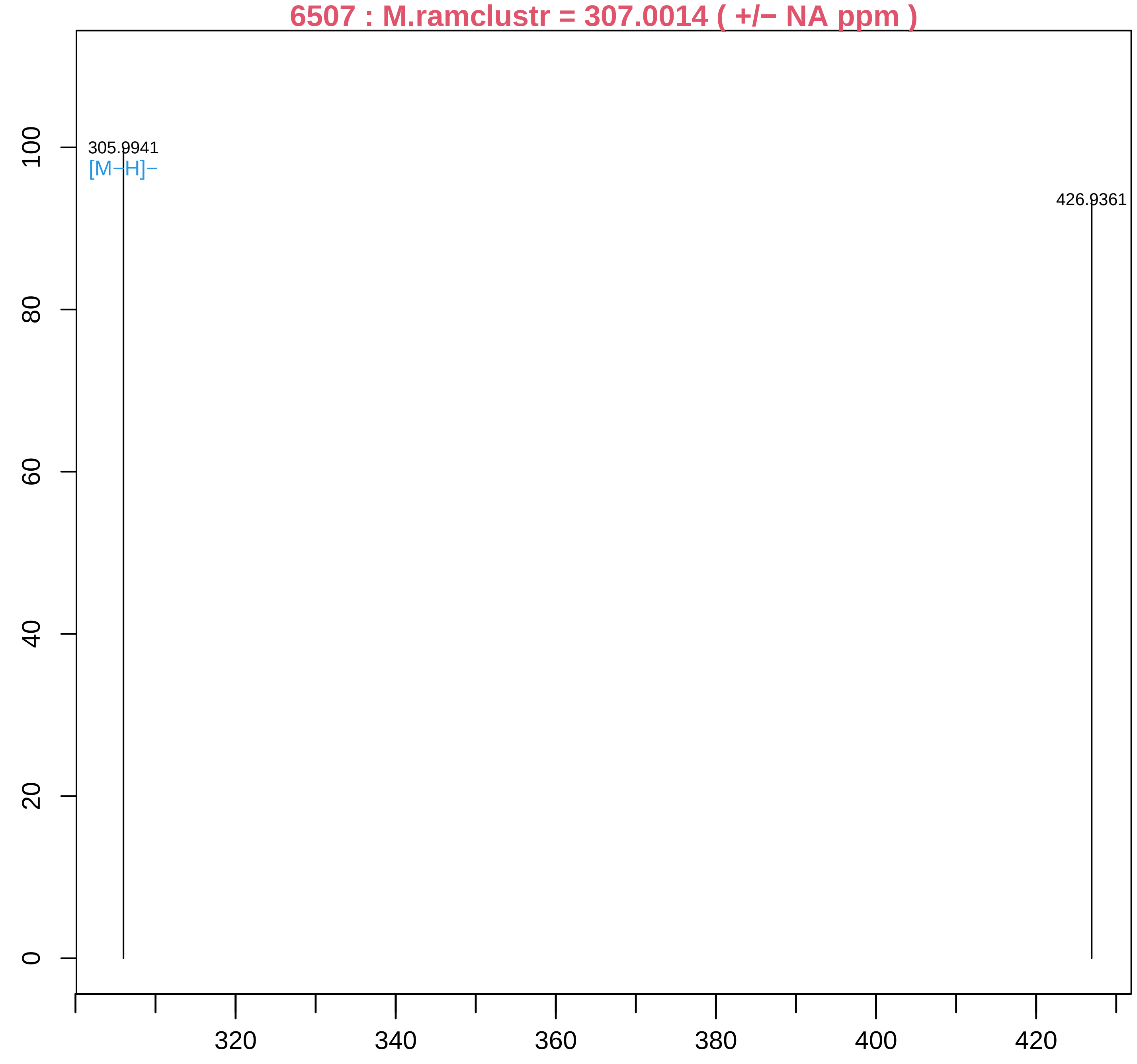
6507 : M.ramclustr = 307.0014 (+/− NA ppm)
305.9941
[M−H]−
426.9361
0
20
40
60
80
100
320
340
360
380
400
420

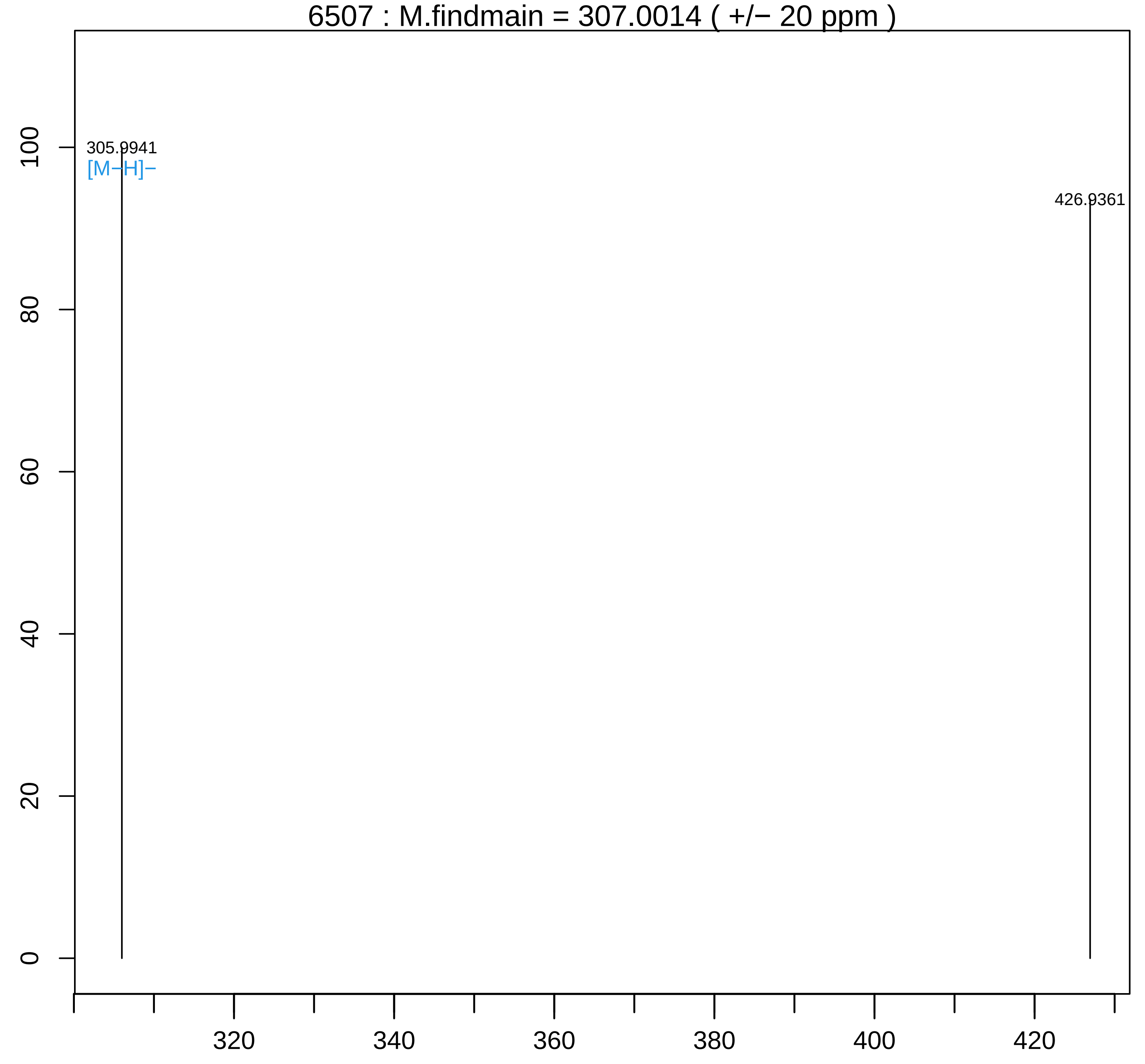
6507 : M.findmain = 307.0014 (+/− 20 ppm)
305.9941
[M−H]−
426.9361
0
20
40
60
80
100
320
340
360
380
400
420

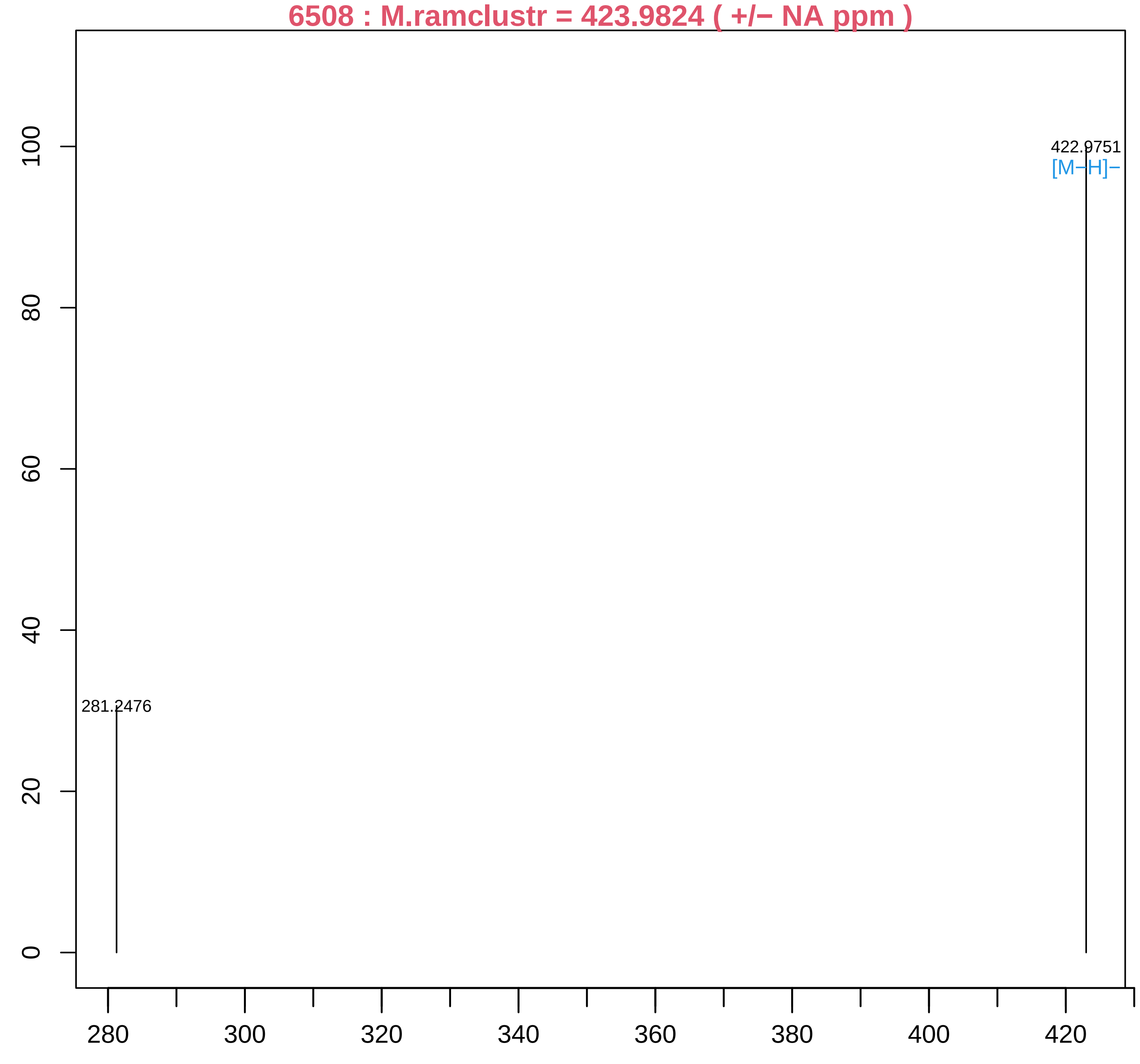

6508 : M.ramclustr = 423.9824 (+/− NA ppm)
422.9751
[M−H]−
281.2476
0
20
40
60
80
100
280
300
320
340
360
380
400
420

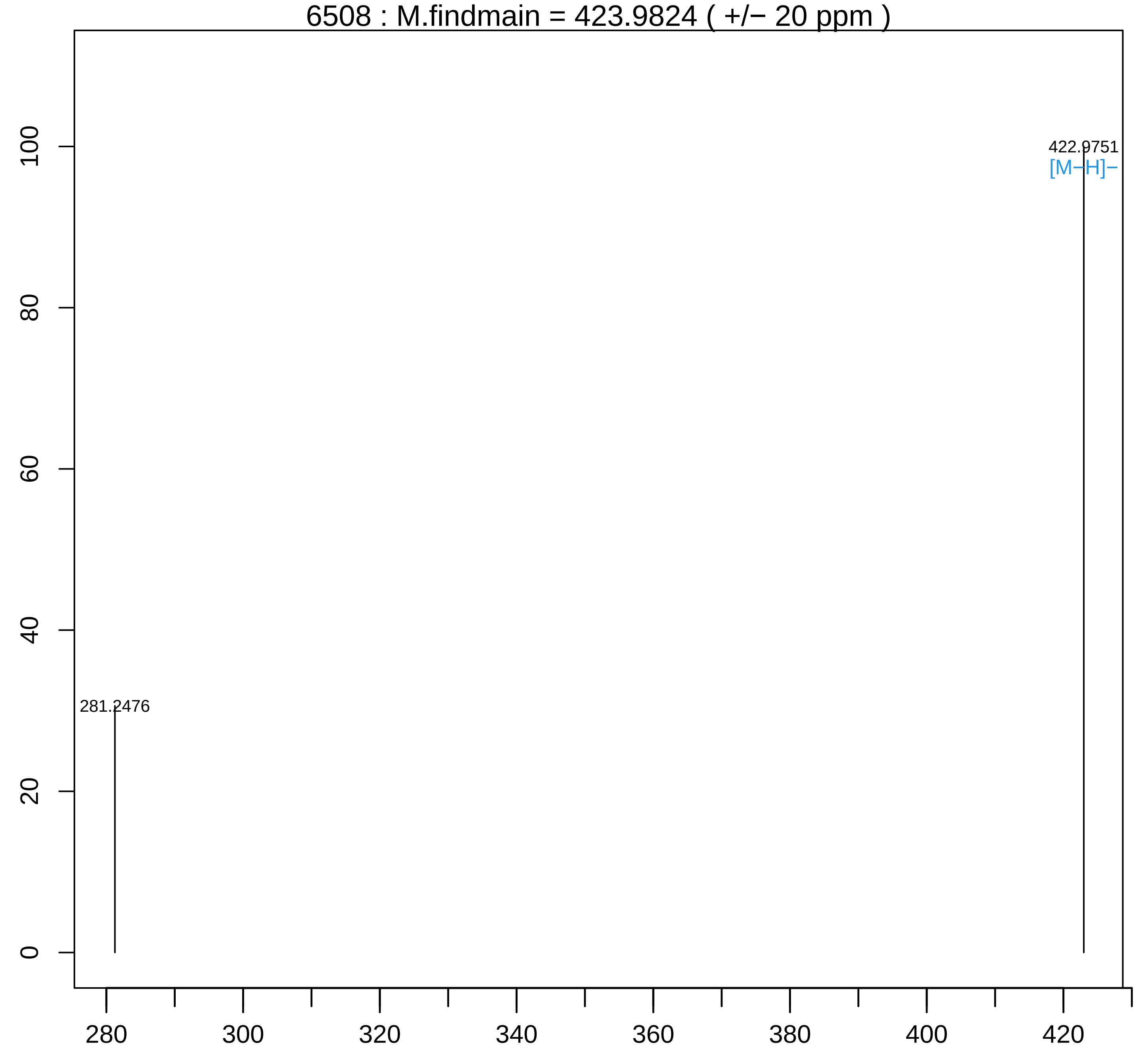

6508 : M.findmain = 423.9824 (+/− 20 ppm)
422.9751
[M−H]−
281.2476
0
20
40
60
80
100
280
300
320
340
360
380
400
420

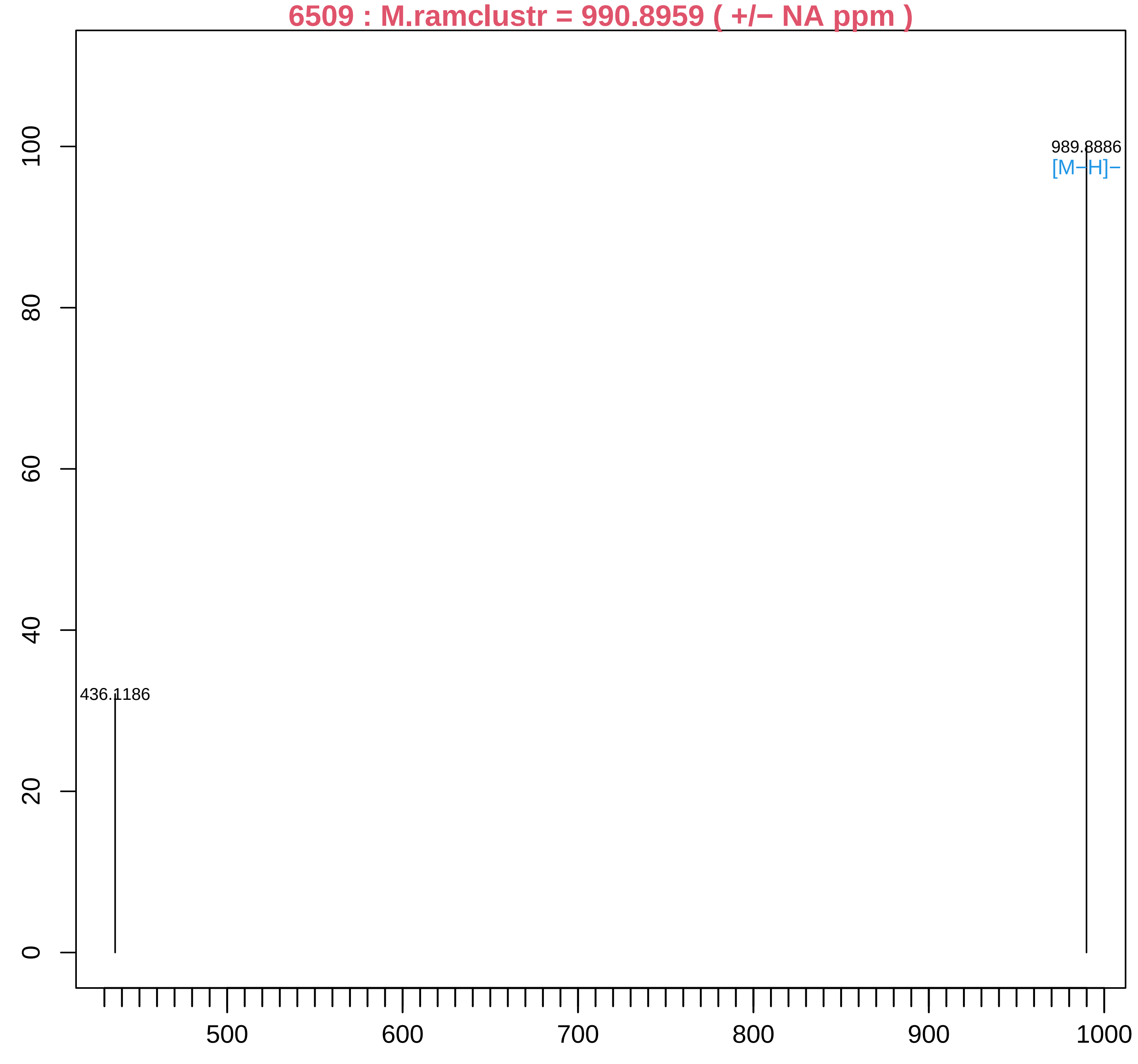
6509 : M.ramclustr = 990.8959 (+/− NA ppm)
989.8886
[M−H]−
436.1186
0
20
40
60
80
100
500
600
700
800
900
1000

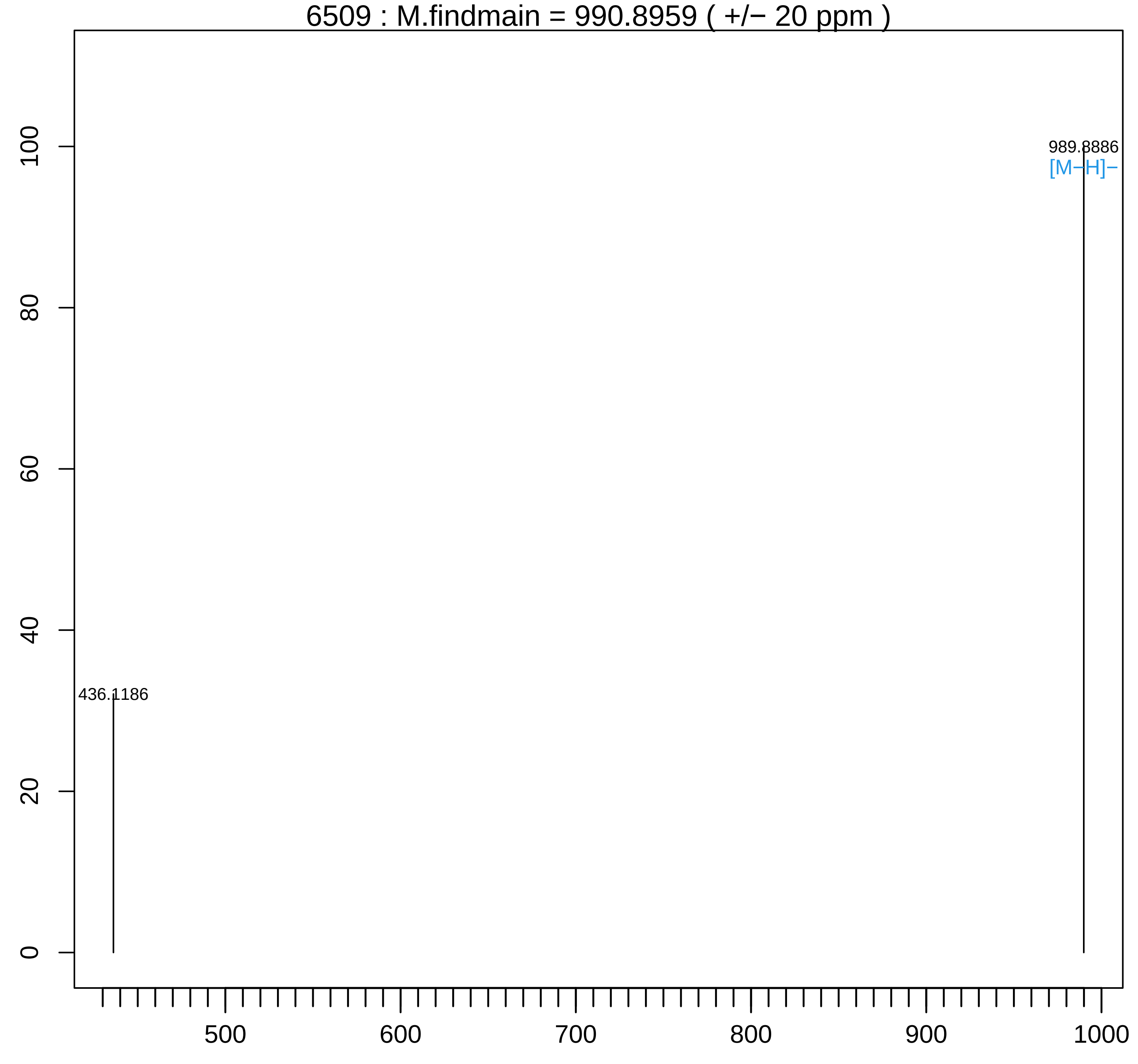
6509 : M.findmain = 990.8959 (+/− 20 ppm)
989.8886
[M−H]−
436.1186
0
20
40
60
80
100
500
600
700
800
900
1000

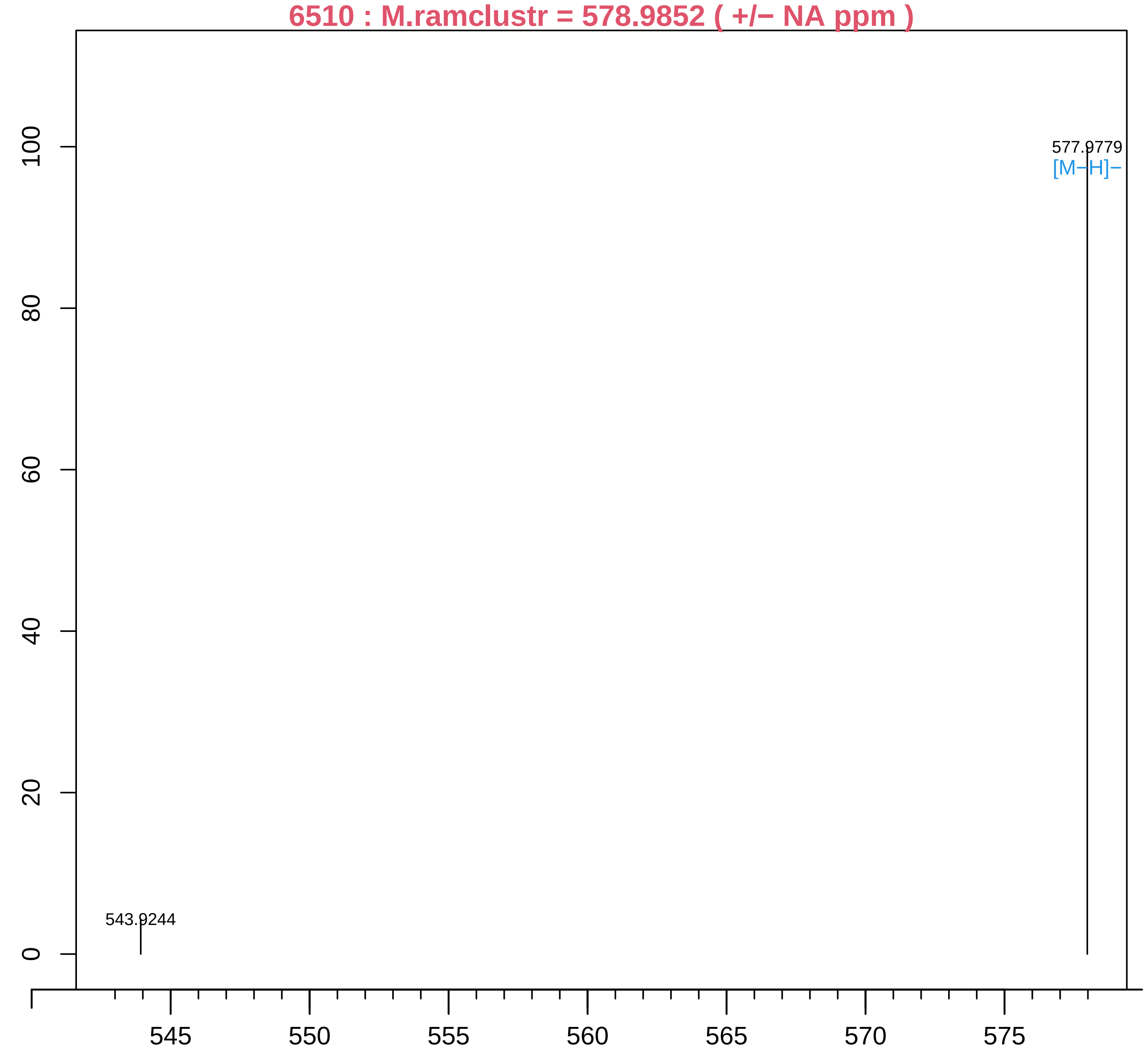
6510 : M.ramclustr = 578.9852 (+/− NA ppm)
577.9779
[M−H]−
543.9244
0
20
40
60
80
100
545
550
555
560
565
570
575

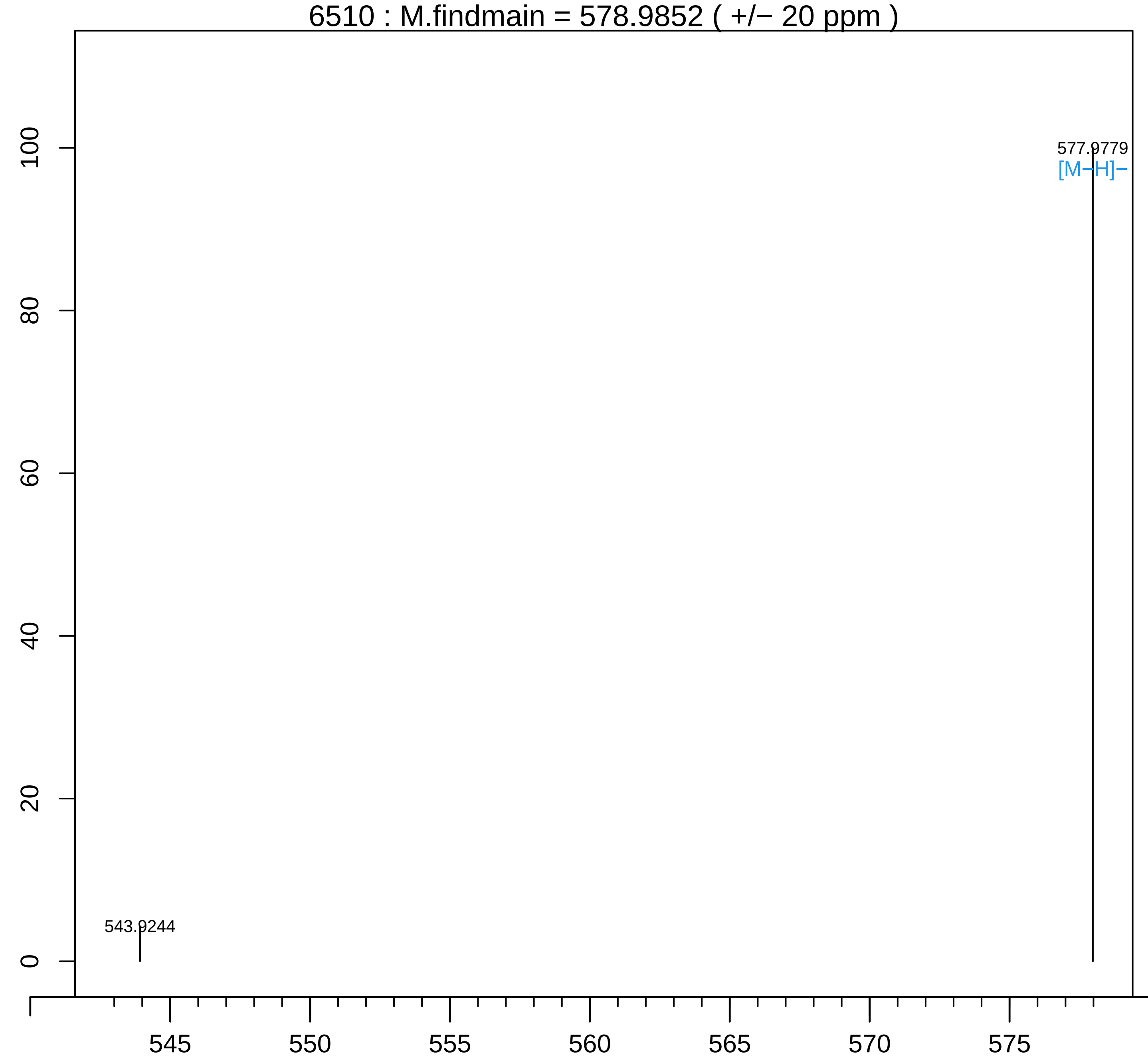
6510 : M.findmain = 578.9852 (+/− 20 ppm)
577.9779
[M−H]−
543.9244
0
20
40
60
80
100
545
550
555
560
565
570
575

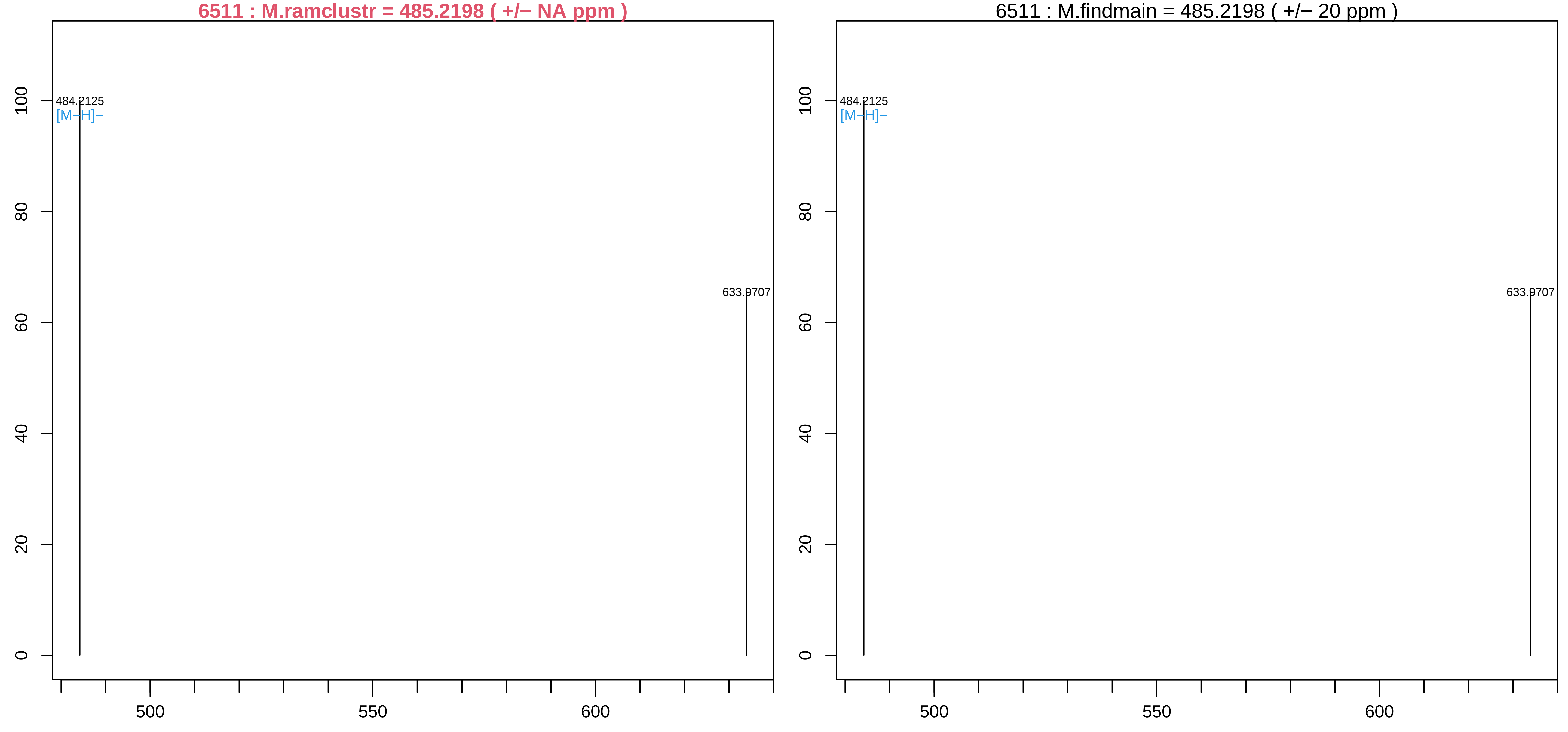
6511 : M.ramclustr = 485.2198 (+/− NA ppm)
484.2125
[M−H]−
633.9707
100
80
60
40
20
0
500
550
600
6511 : M.findmain = 485.2198 (+/− 20 ppm)
484.2125
[M−H]−
633.9707
100
80
60
40
20
0
500
550
600

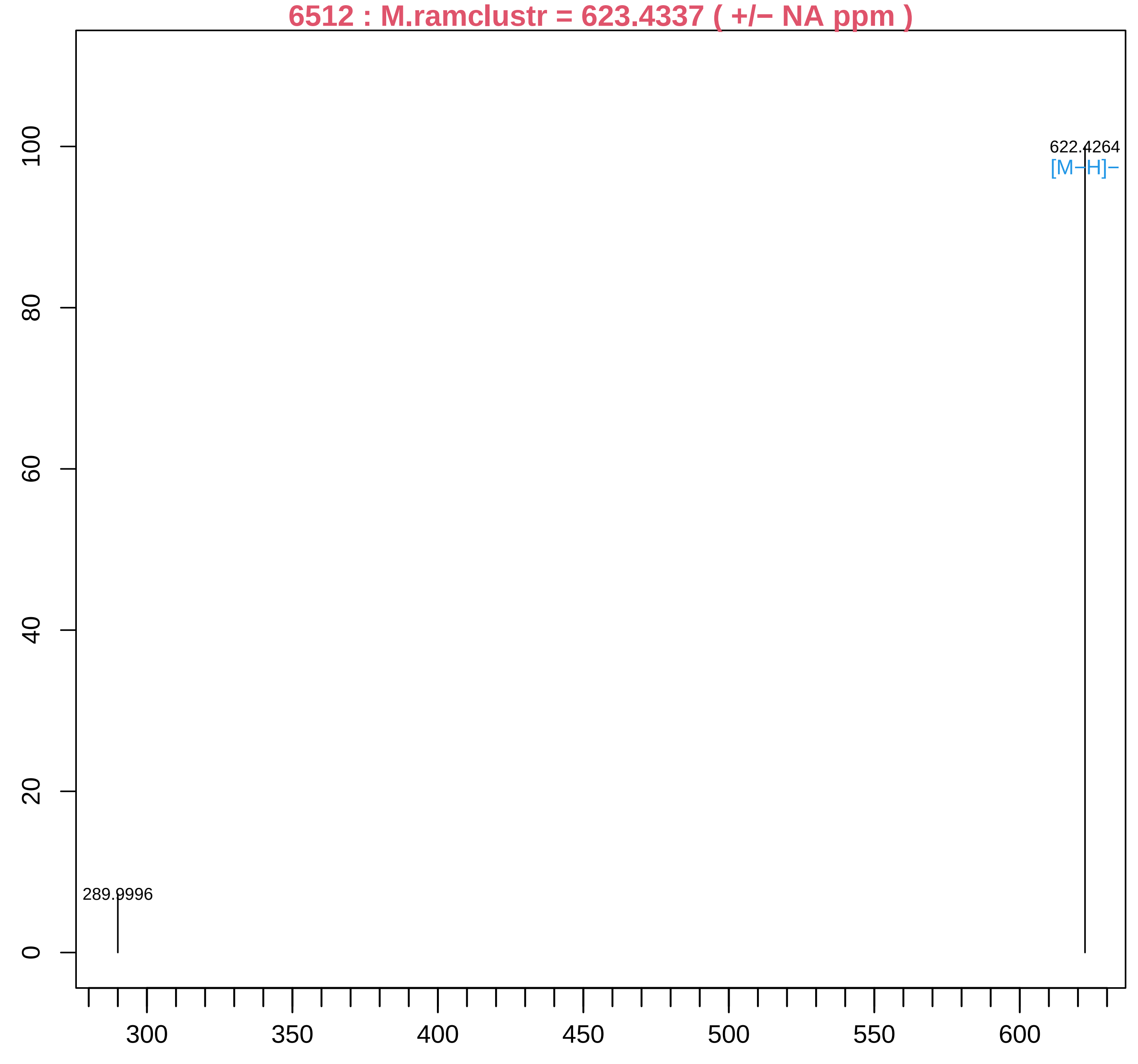
6512 : M.ramclustr = 623.4337 (+/− NA ppm)
622.4264
[M−H]−
289.9996
0
20
40
60
80
100
300
350
400
450
500
550
600

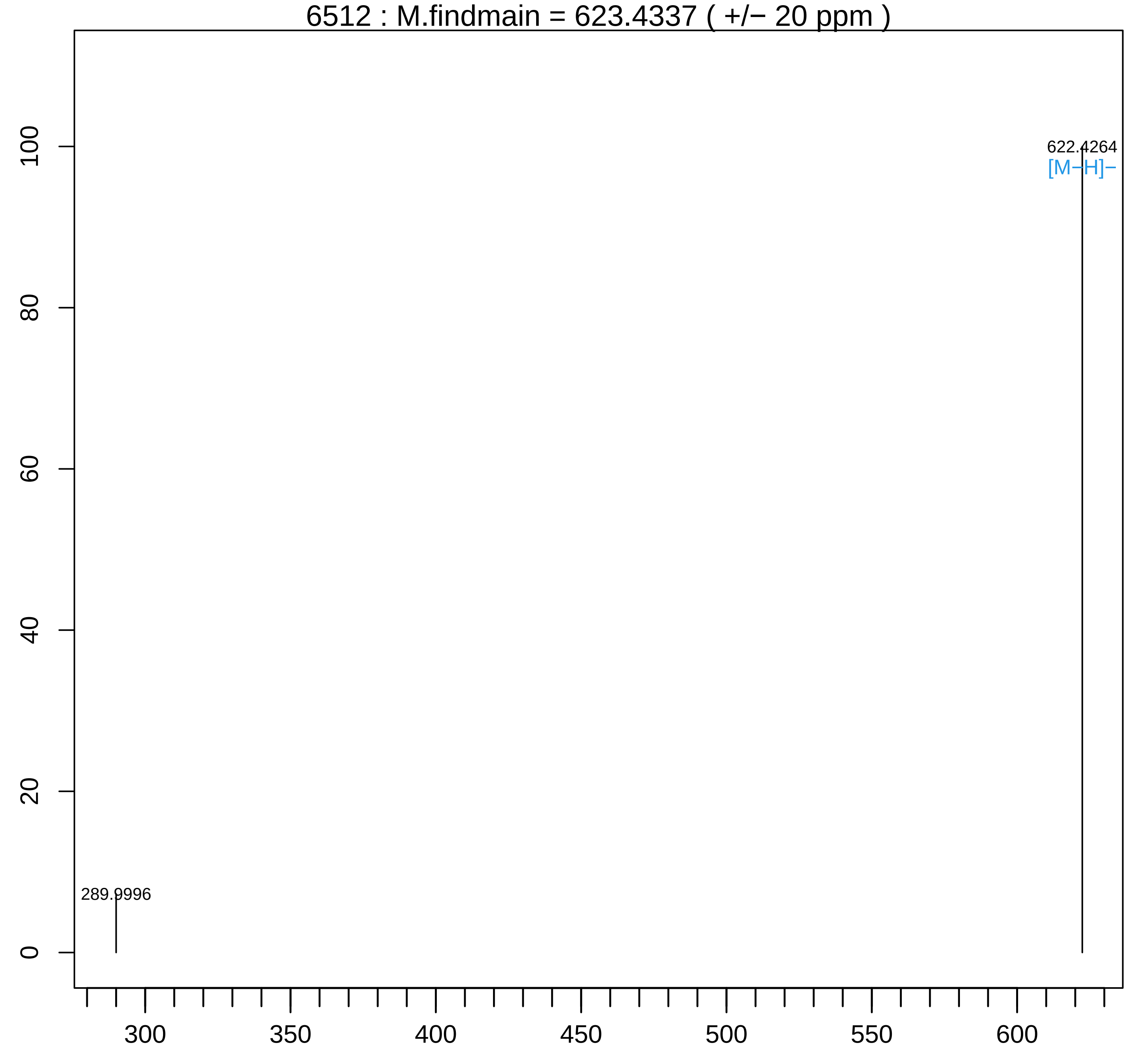
6512 : M.findmain = 623.4337 (+/− 20 ppm)
622.4264
[M−H]−
289.9996
0
20
40
60
80
100
300
350
400
450
500
550
600

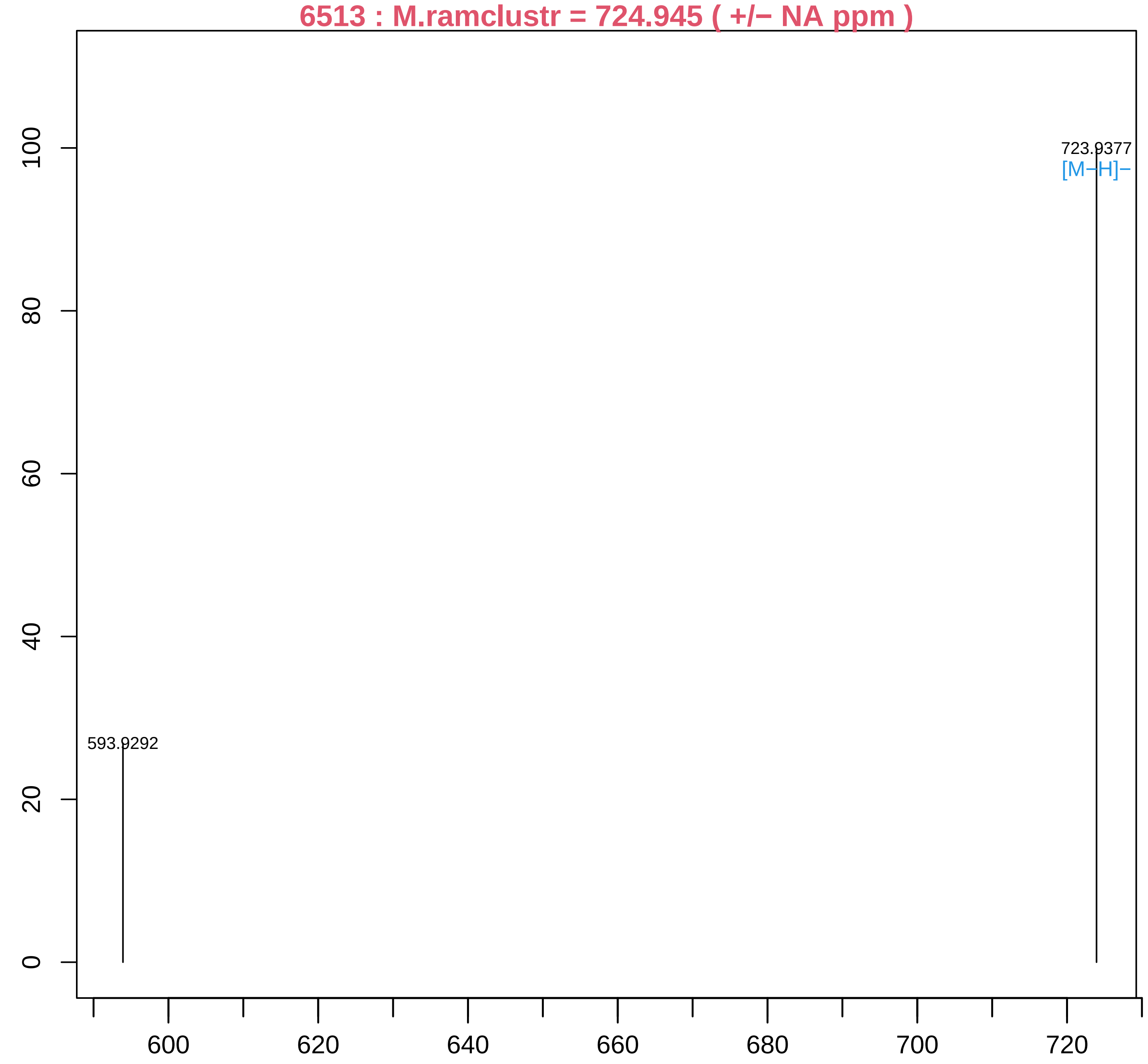
6513 : M.ramclustr = 724.945 (+/− NA ppm)
723.9377
[M−H]−
593.9292
0
20
40
60
80
100
600
620
640
660
680
700
720

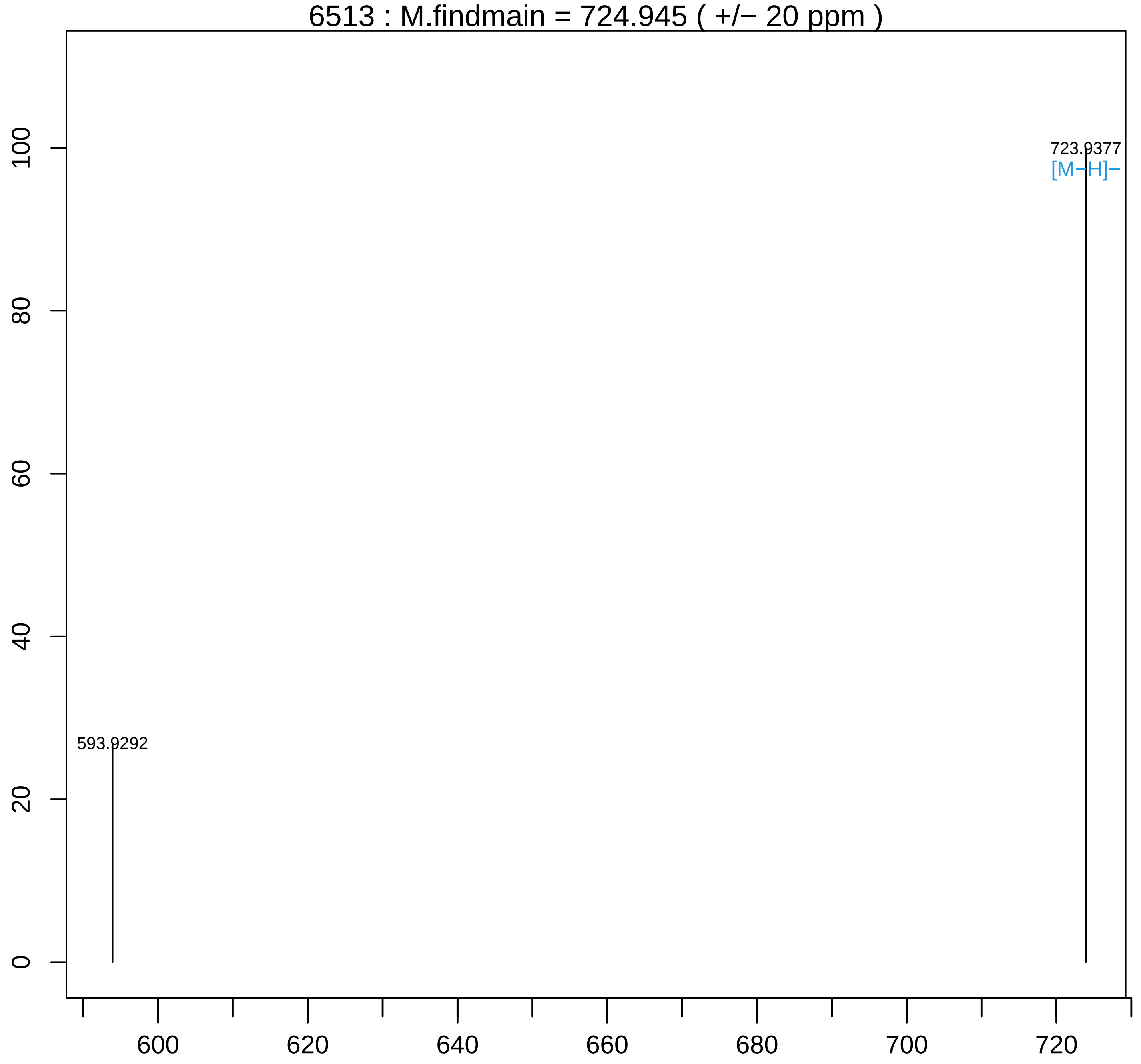
6513 : M.findmain = 724.945 (+/− 20 ppm)
723.9377
[M−H]−
593.9292
0
20
40
60
80
100
600
620
640
660
680
700
720

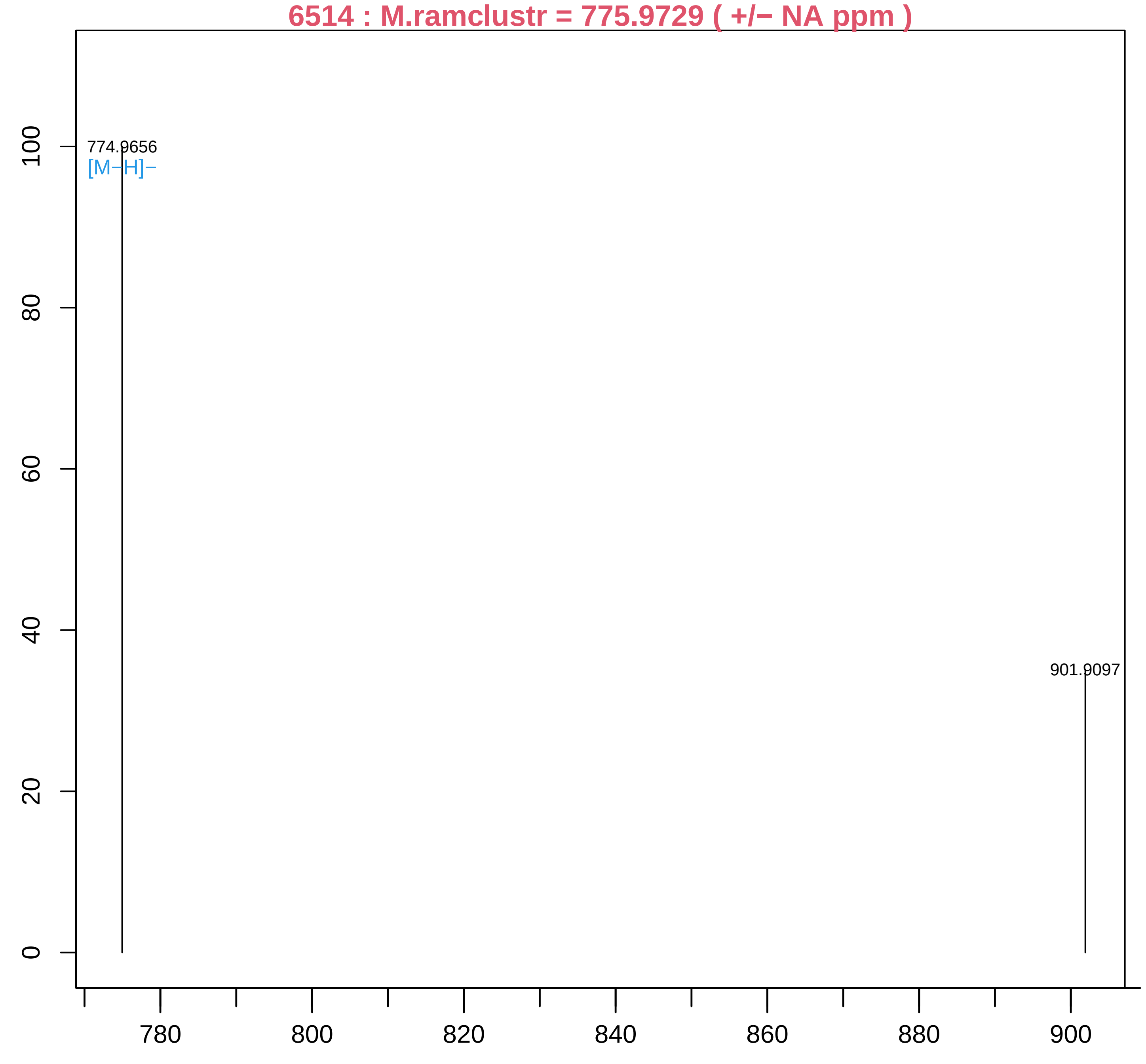
6514 : M.ramclustr = 775.9729 (+/− NA ppm)
774.9656
[M−H]−
901.9097
0
20
40
60
80
100
780
800
820
840
860
880
900

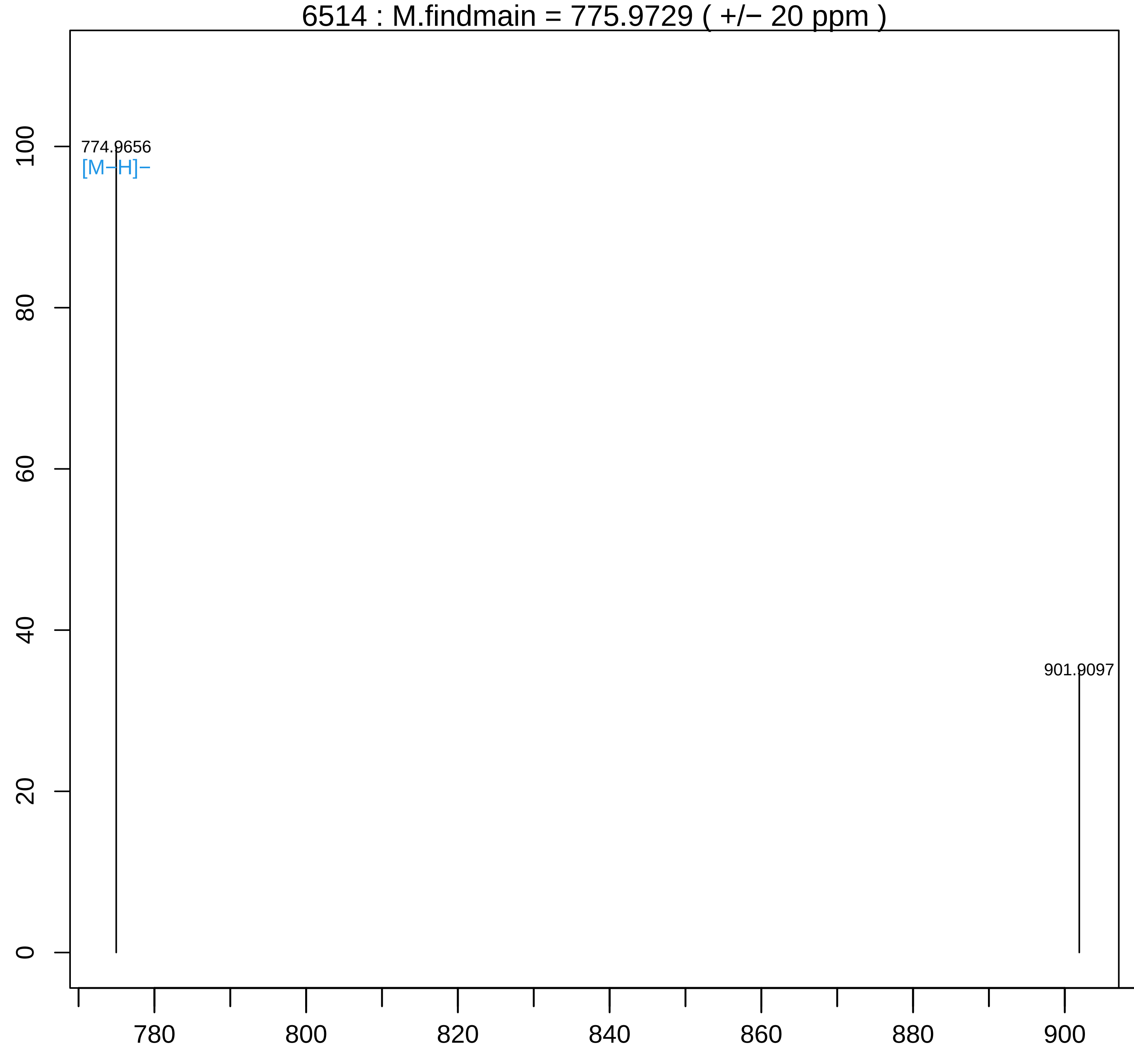
6514 : M.findmain = 775.9729 (+/− 20 ppm)
774.9656
[M−H]−
901.9097
0
20
40
60
80
100
780
800
820
840
860
880
900

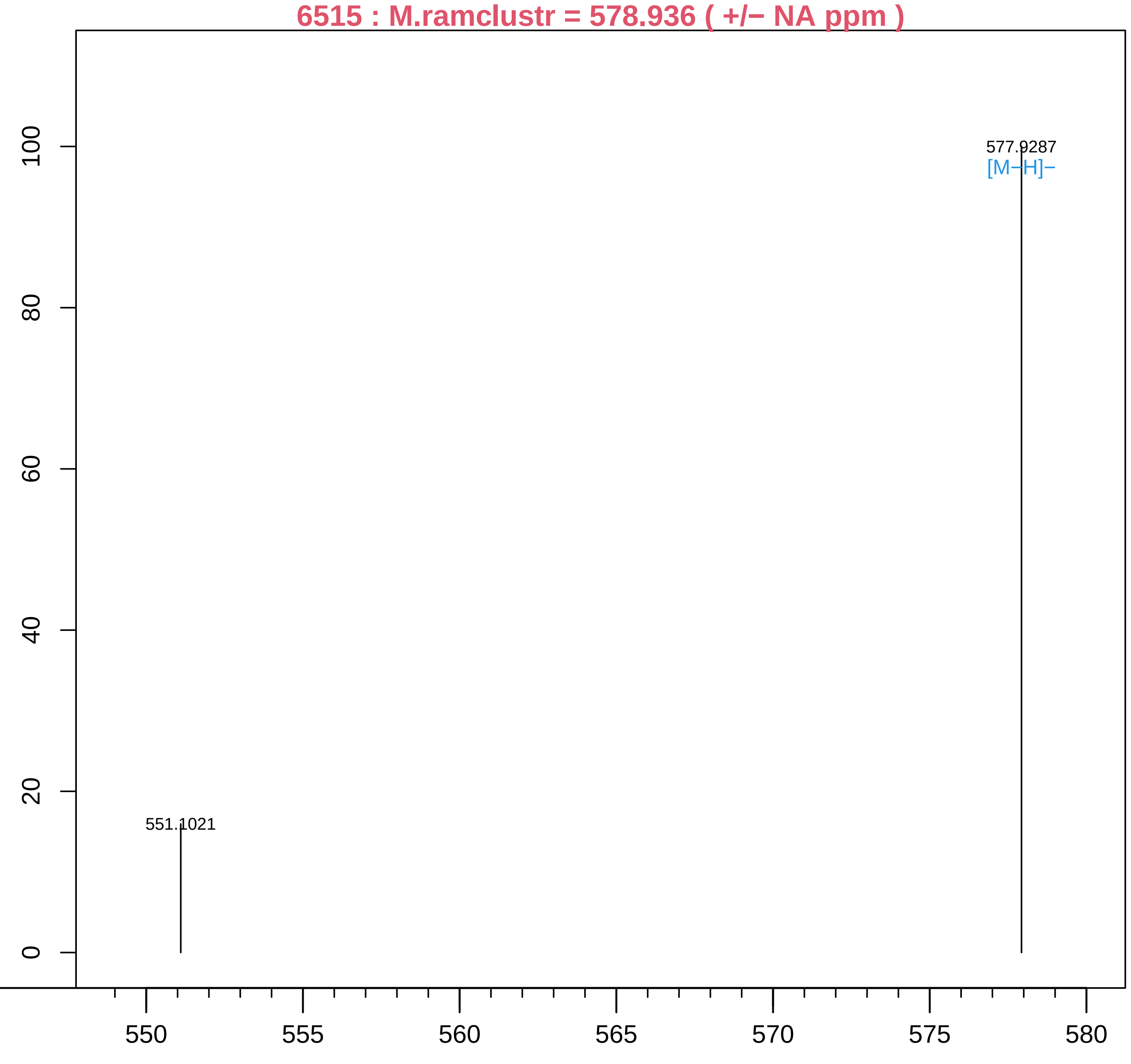
6515 : M.ramclustr = 578.936 (+/− NA ppm)
577.9287
[M−H]−
551.1021
0
20
40
60
80
100
550
555
560
565
570
575
580

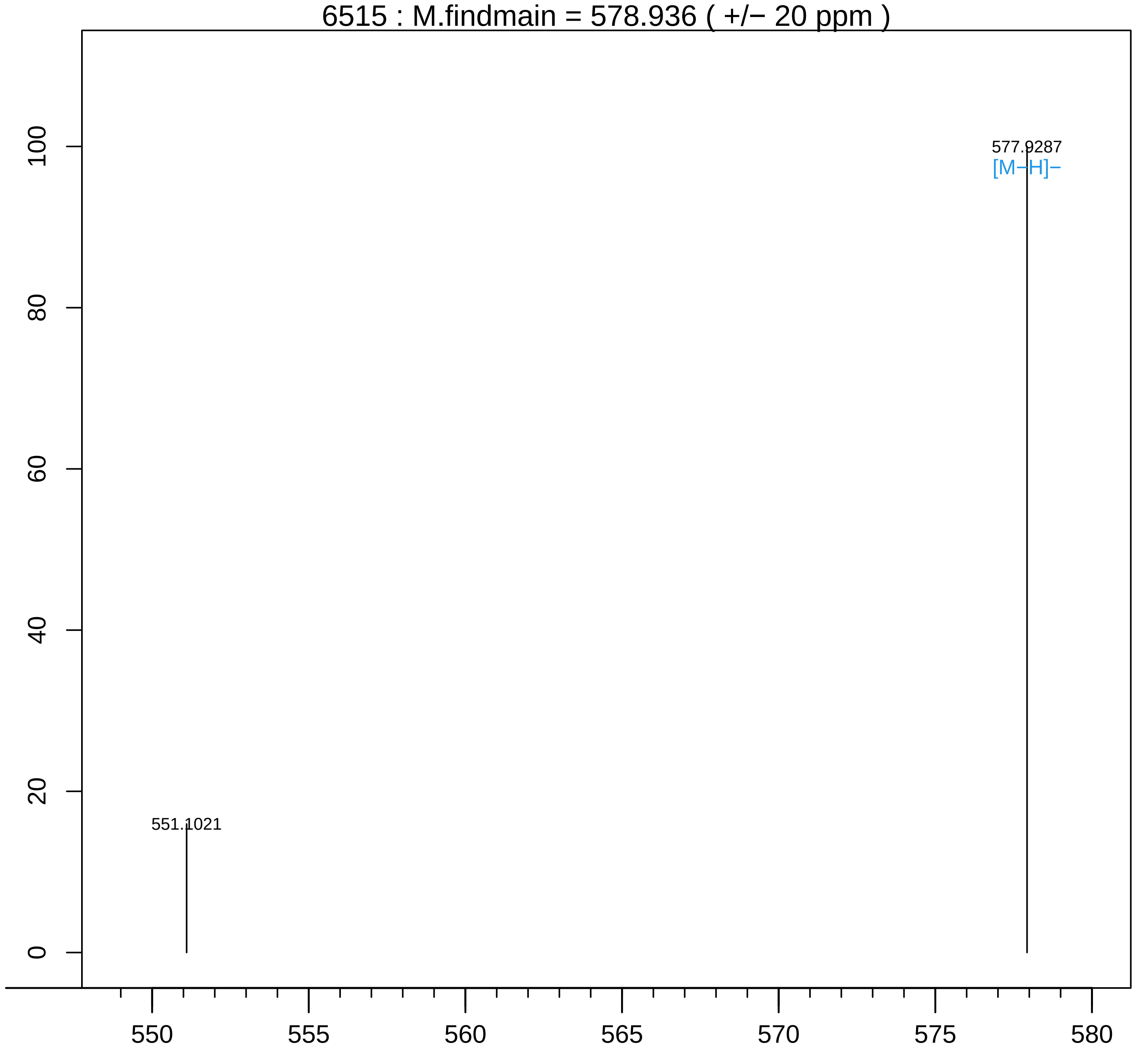
6515 : M.findmain = 578.936 (+/− 20 ppm)
577.9287
[M−H]−
551.1021
0
20
40
60
80
100
550
555
560
565
570
575
580

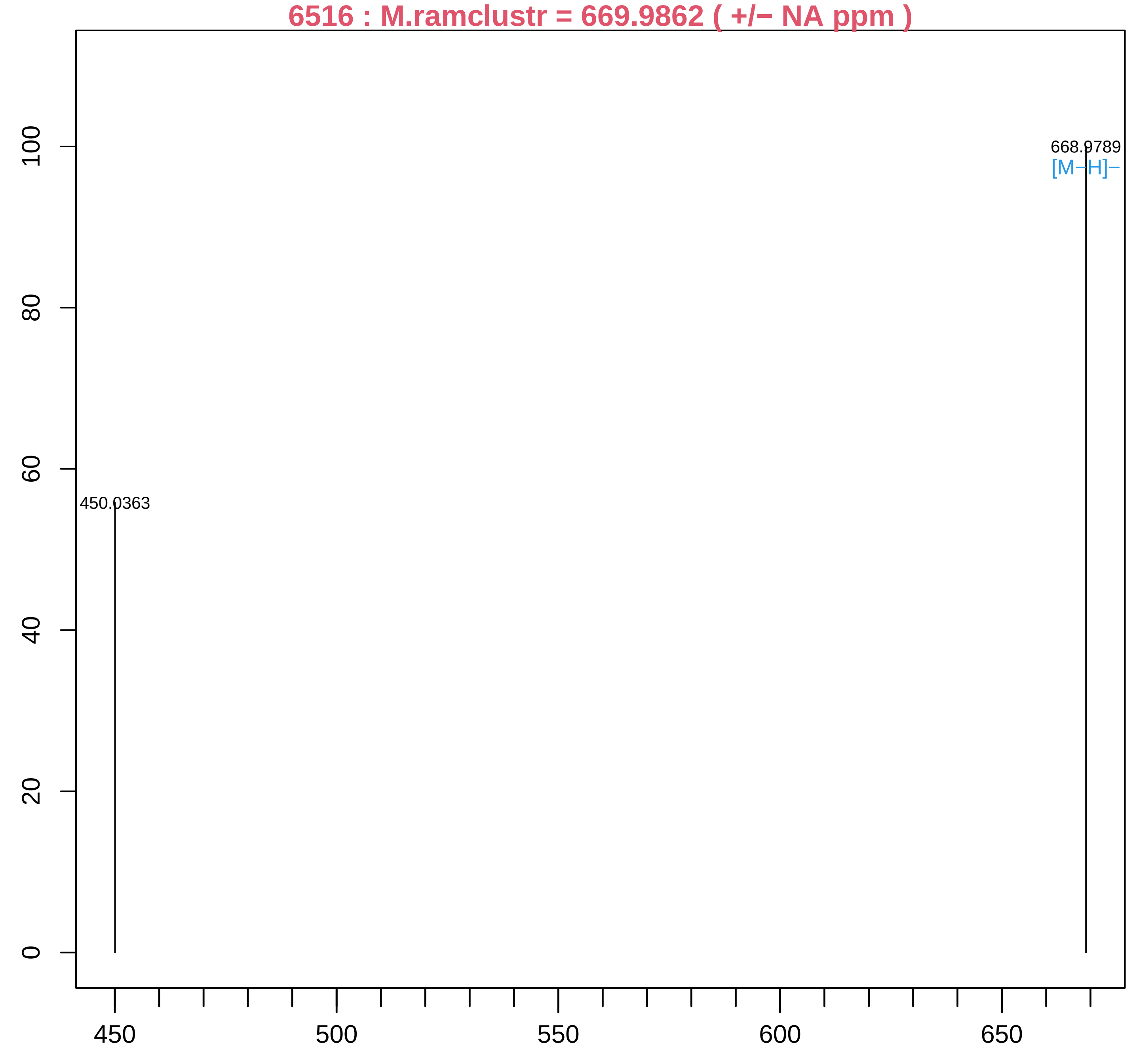

6516 : M.ramclustr = 669.9862 (+/− NA ppm)
668.9789
[M−H]−
450.0363

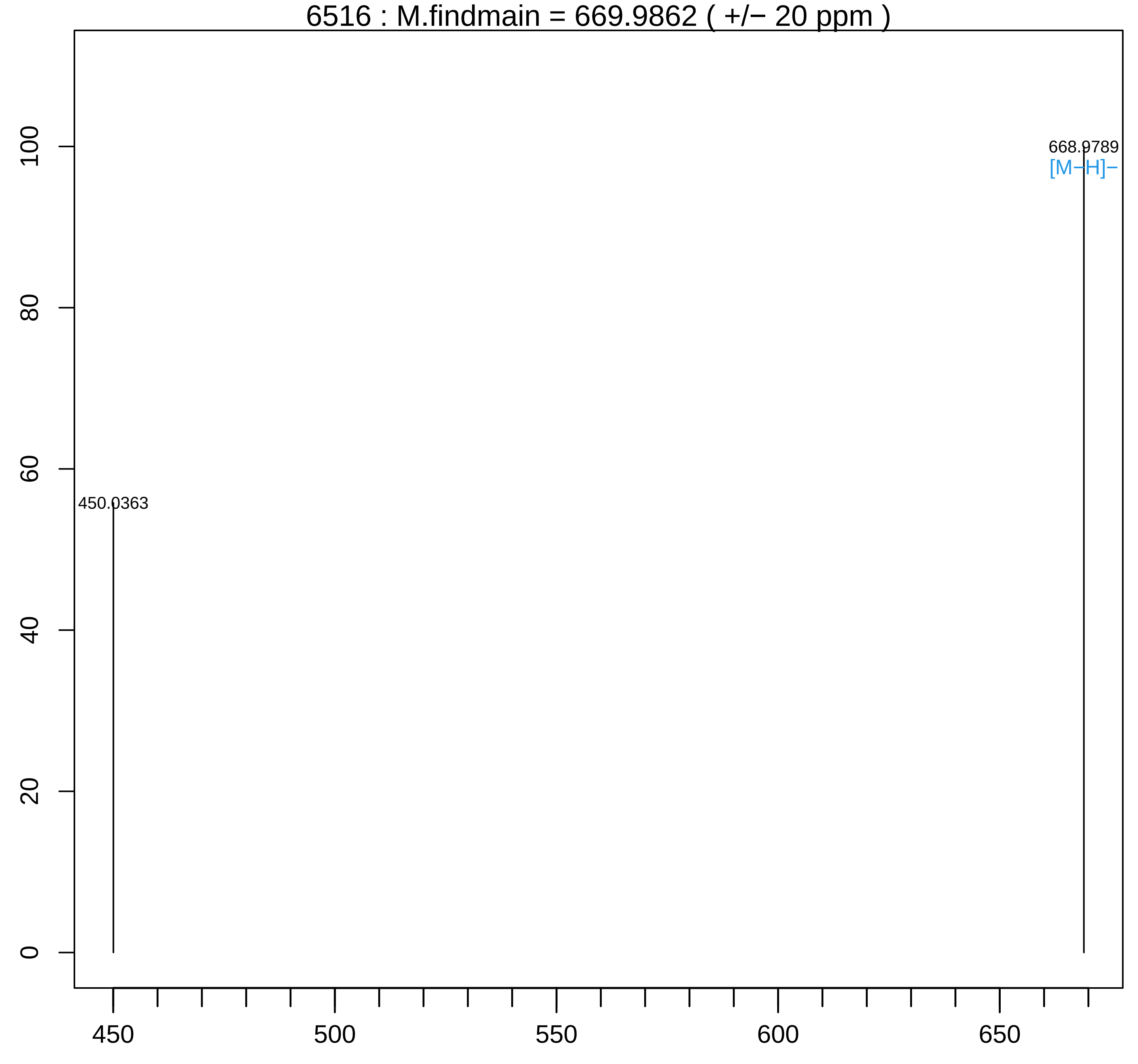

6516 : M.findmain = 669.9862 (+/− 20 ppm)
668.9789
[M−H]−
450.0363

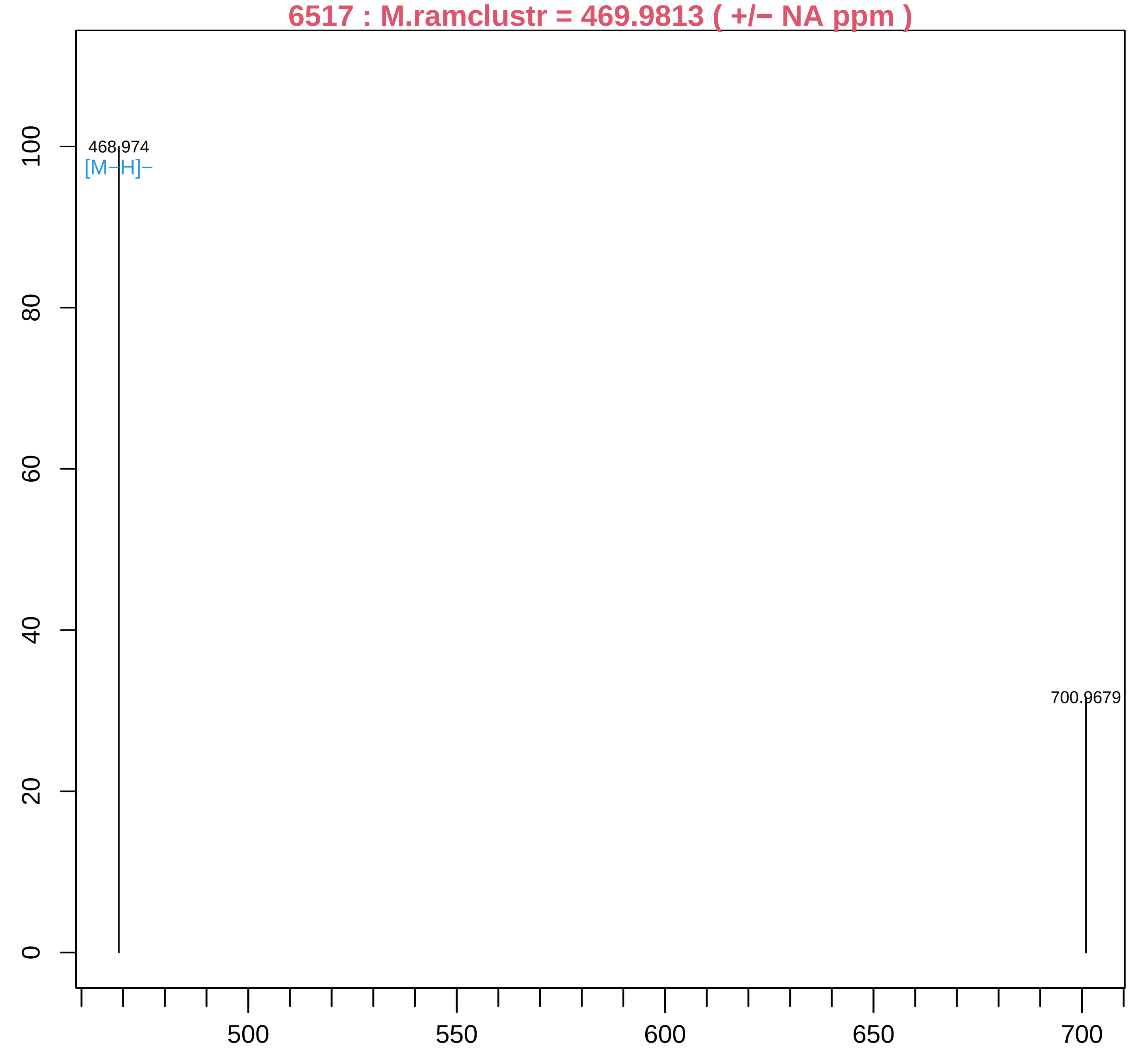
6517 : M.ramclustr = 469.9813 (+/− NA ppm)
468.974
[M−H]−
700.9679
0
20
40
60
80
100
500
550
600
650
700

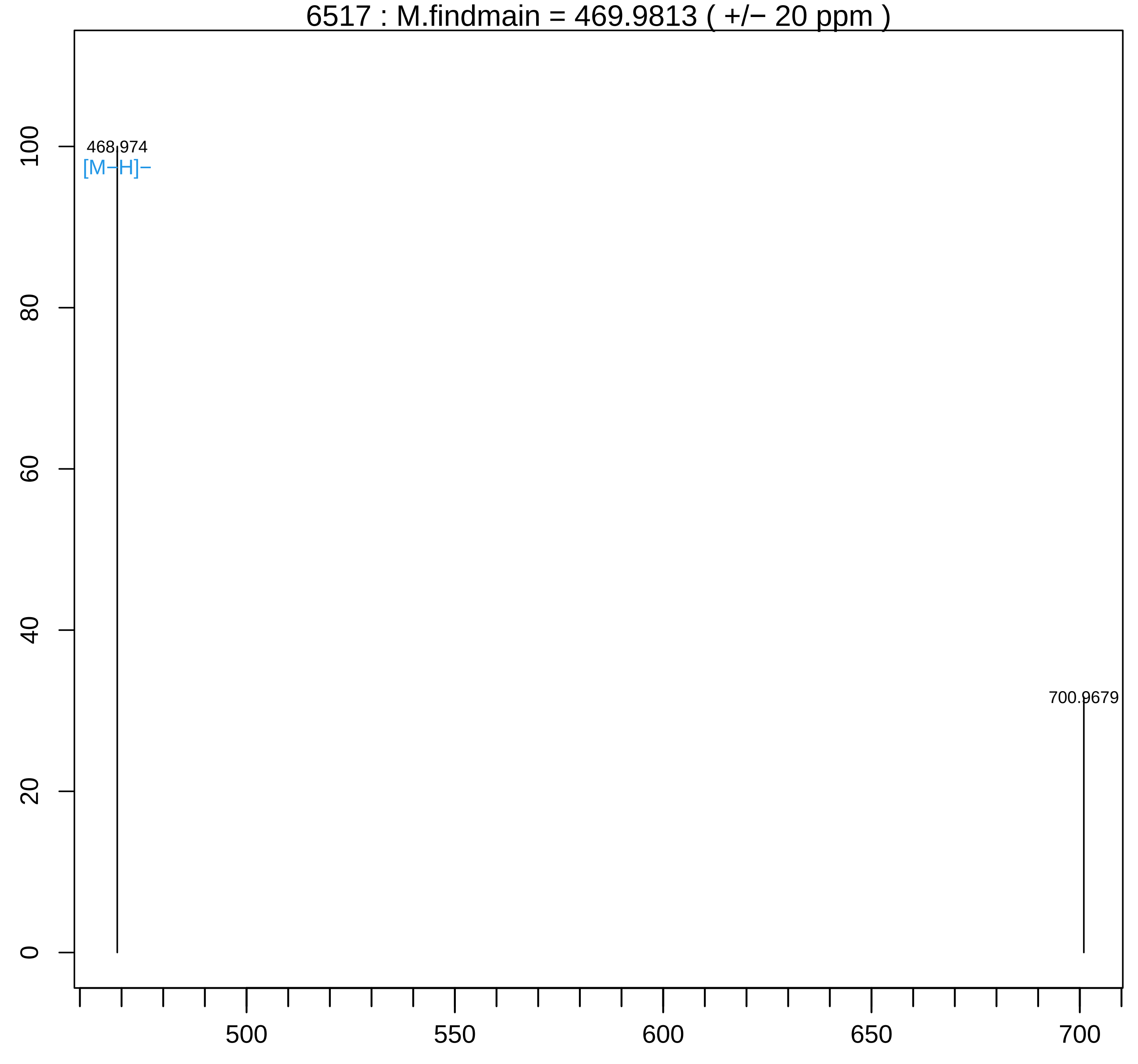
6517 : M.findmain = 469.9813 (+/− 20 ppm)
468.974
[M−H]−
700.9679
0
20
40
60
80
100
500
550
600
650
700

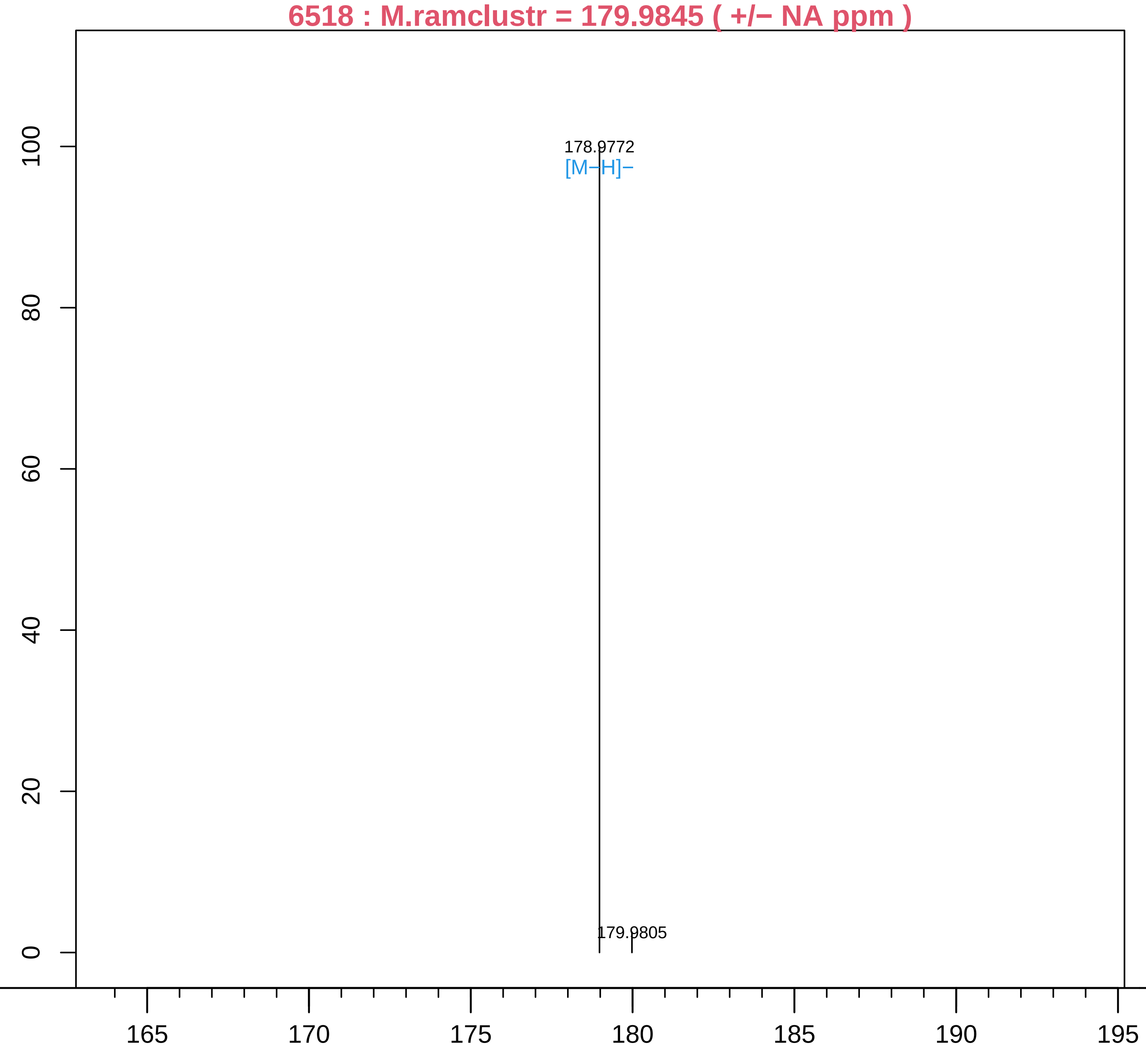
6518 : M.ramclustr = 179.9845 (+/− NA ppm)
178.9772
[M−H]−
179.9805
0
20
40
60
80
100
165
170
175
180
185
190
195

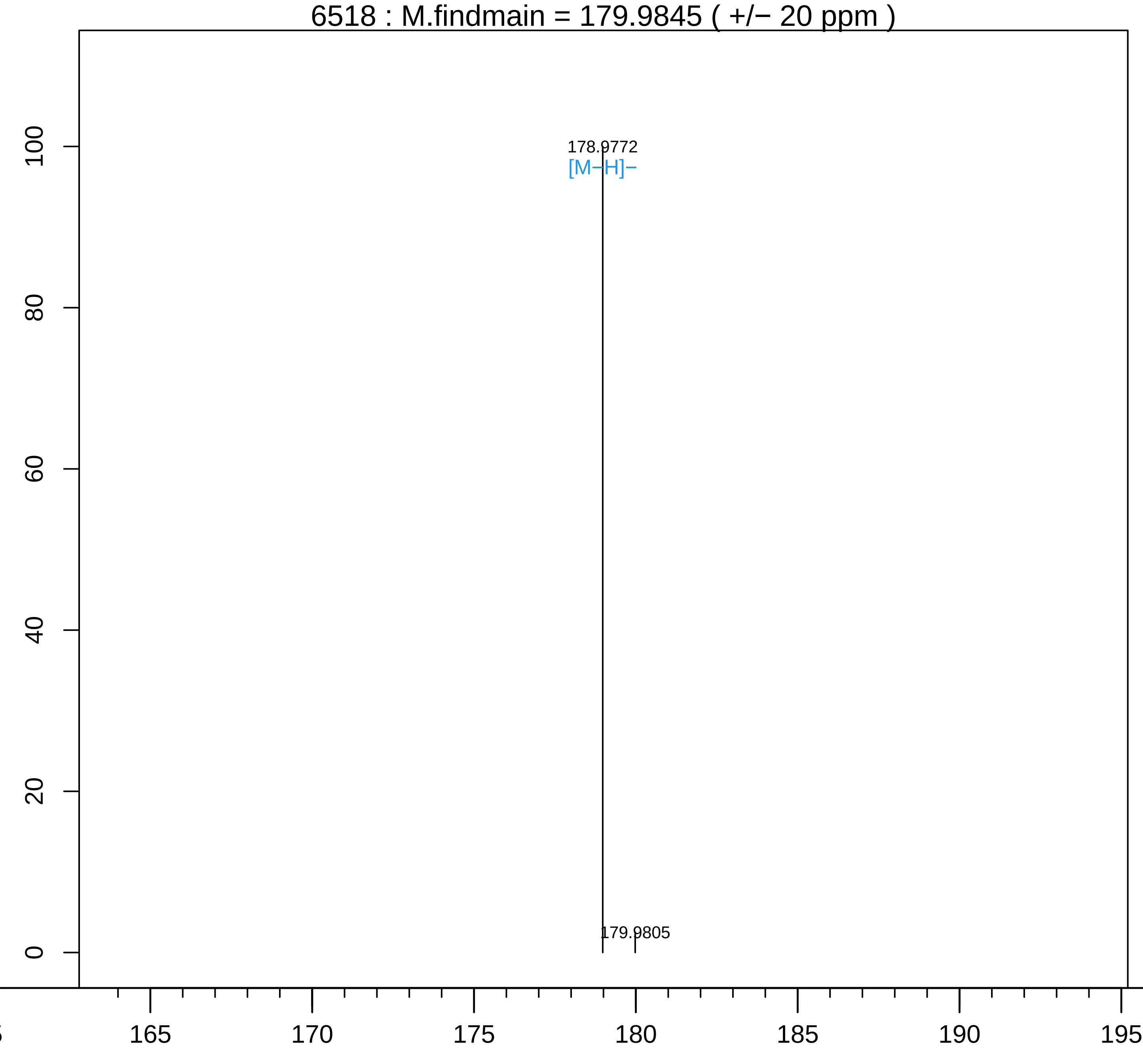
6518 : M.findmain = 179.9845 (+/− 20 ppm)
178.9772
[M−H]−
179.9805
0
20
40
60
80
100
165
170
175
180
185
190
195

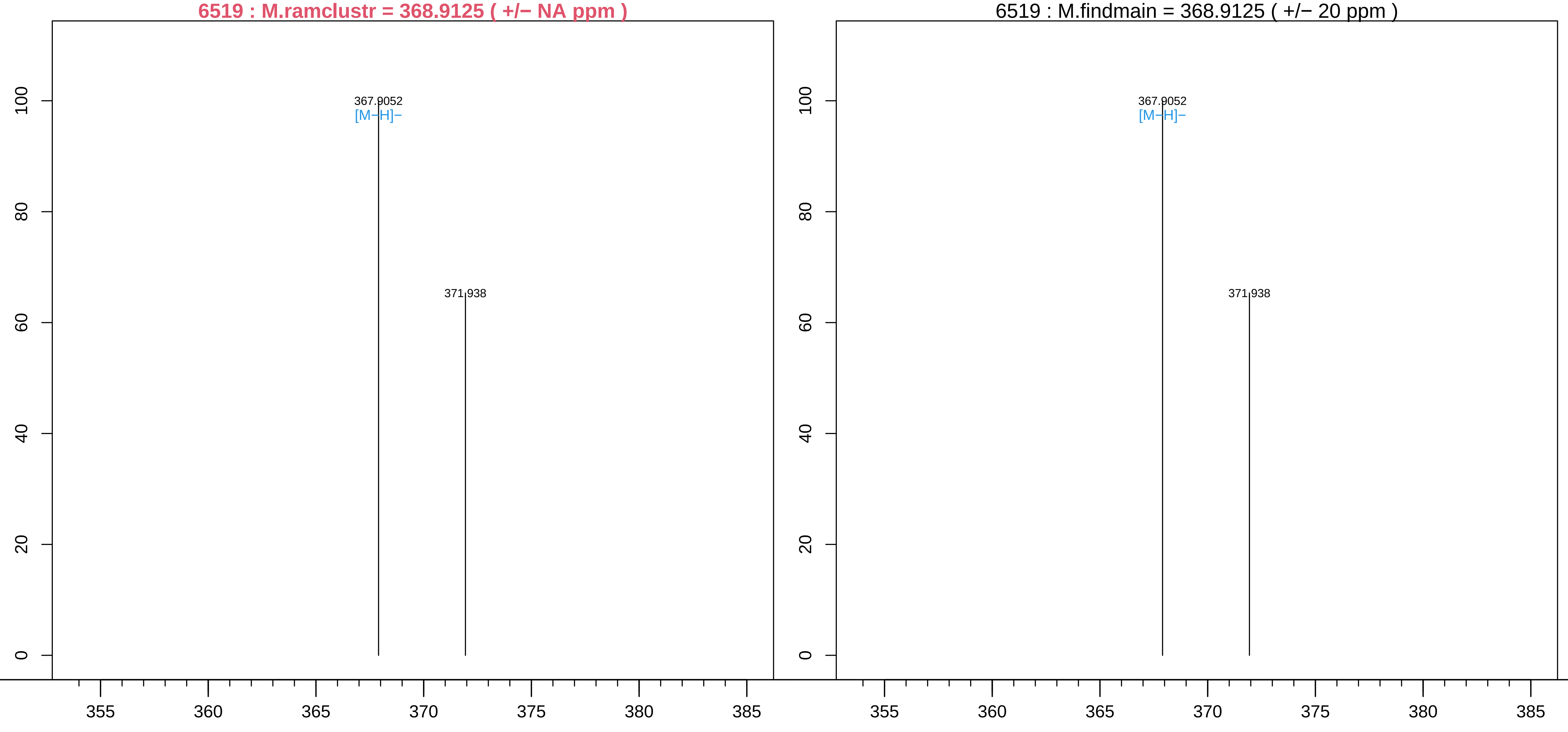

6519 : M.ramclustr = 368.9125 (+/− NA ppm)
367.9052
[M−H]−
371.938
6519 : M.findmain = 368.9125 (+/− 20 ppm)
367.9052
[M−H]−
371.938
0
20
40
60
80
100
355
360
365
370
375
380
385

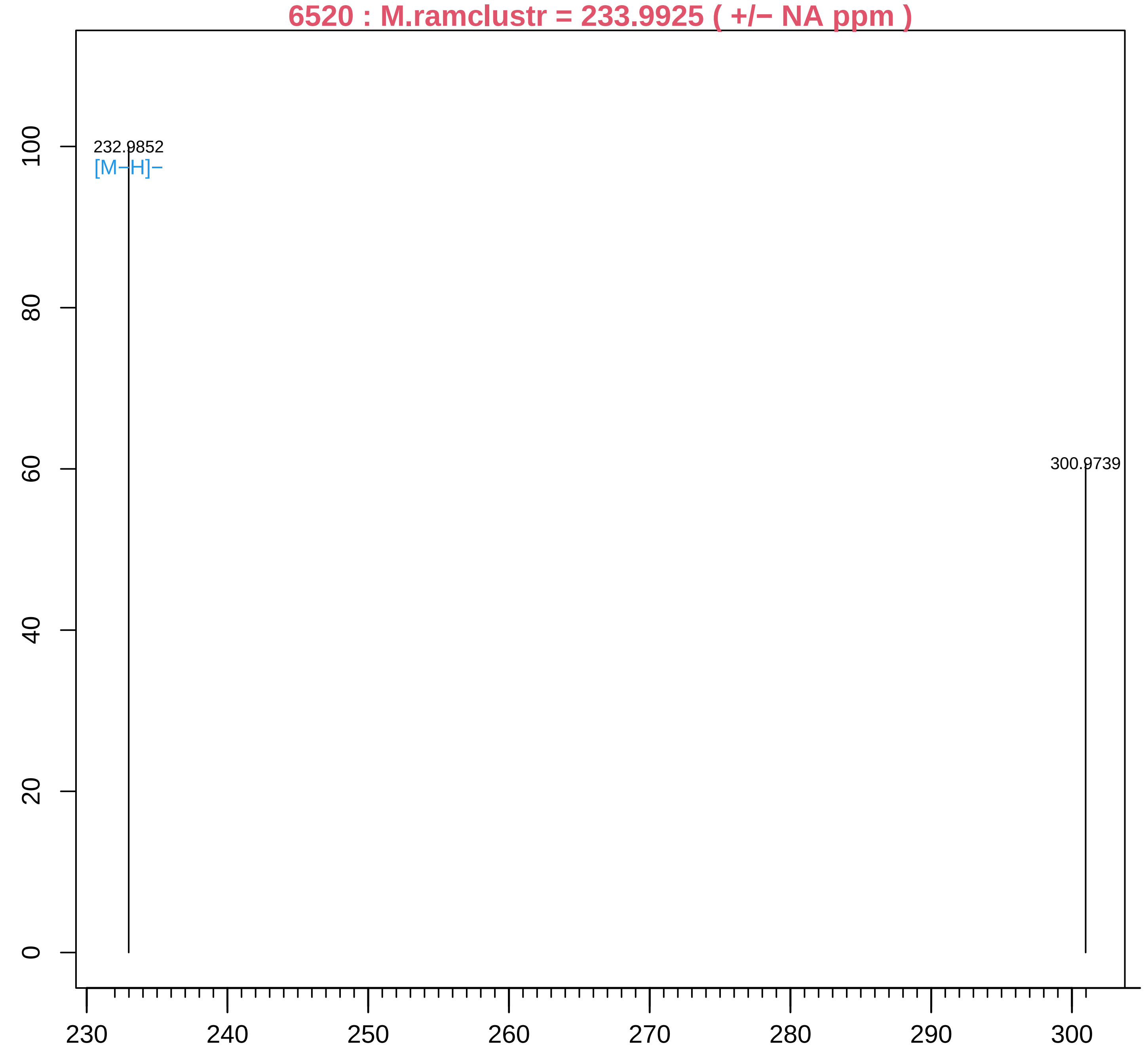

6520 : M.ramclustr = 233.9925 (+/− NA ppm)
232.9852
[M−H]−
300.9739

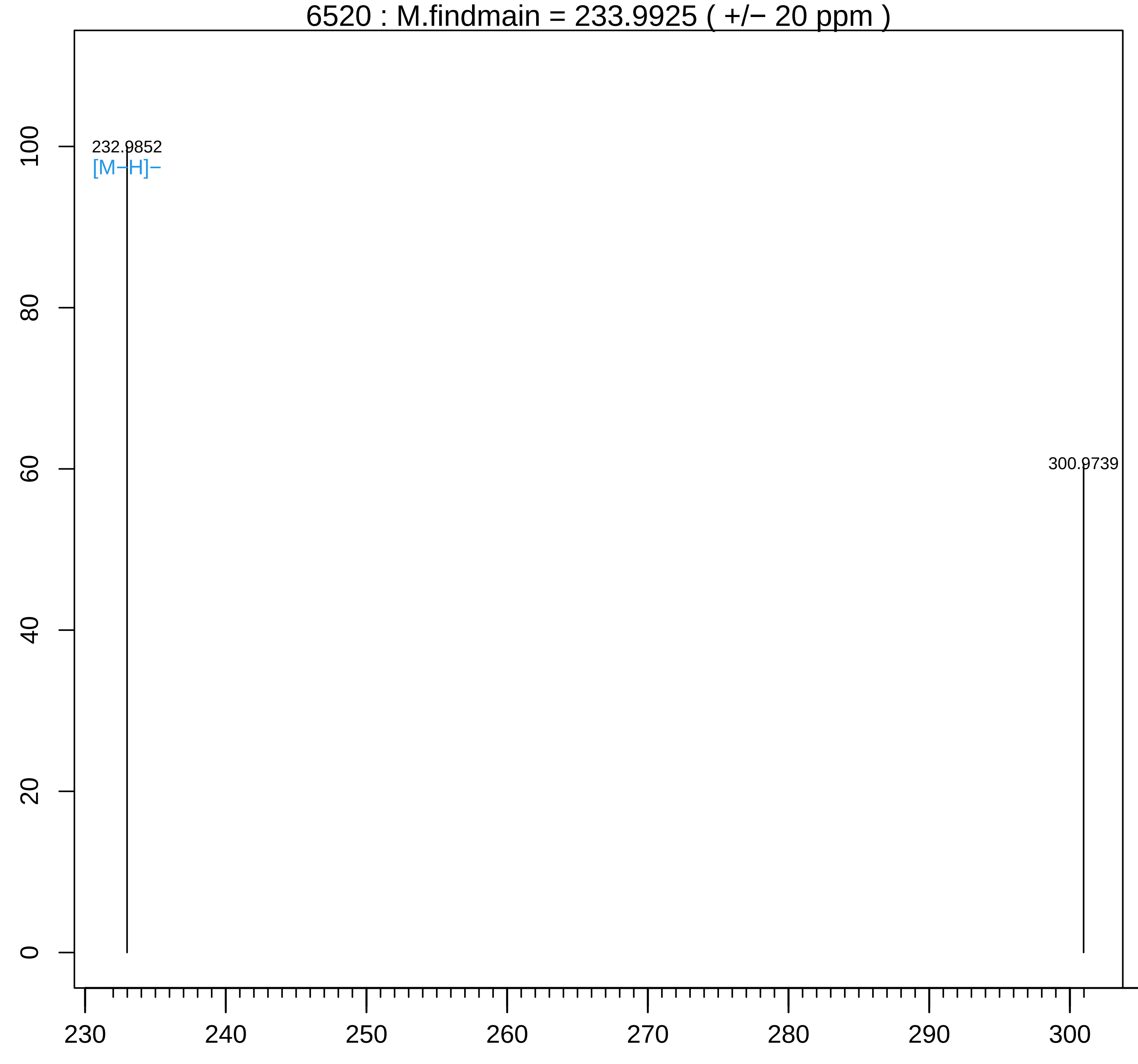

6520 : M.findmain = 233.9925 (+/− 20 ppm)
232.9852
[M−H]−
300.9739

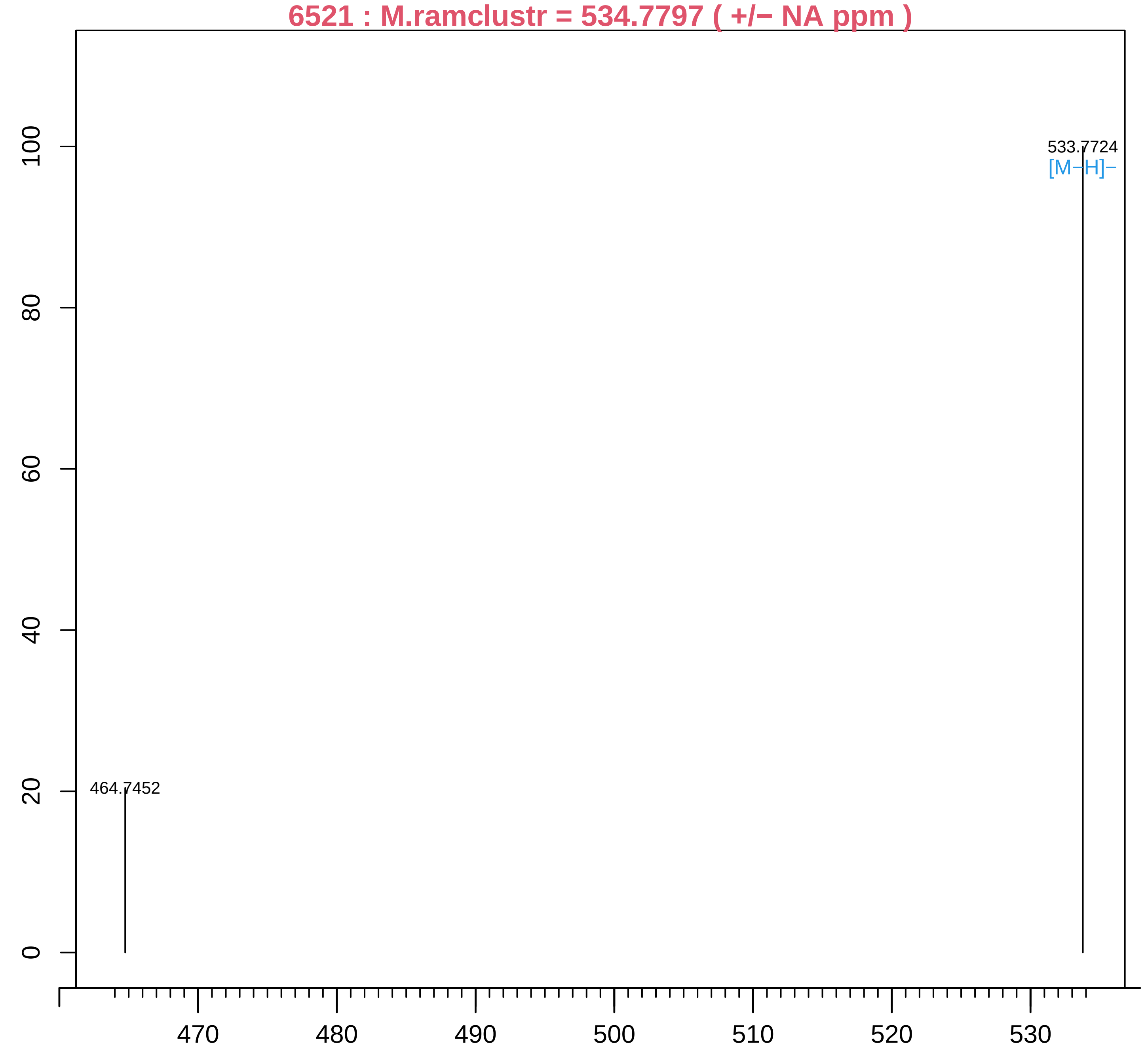
6521 : M.ramclustr = 534.7797 (+/− NA ppm)
533.7724
[M−H]−
464.7452
0
20
40
60
80
100
470
480
490
500
510
520
530

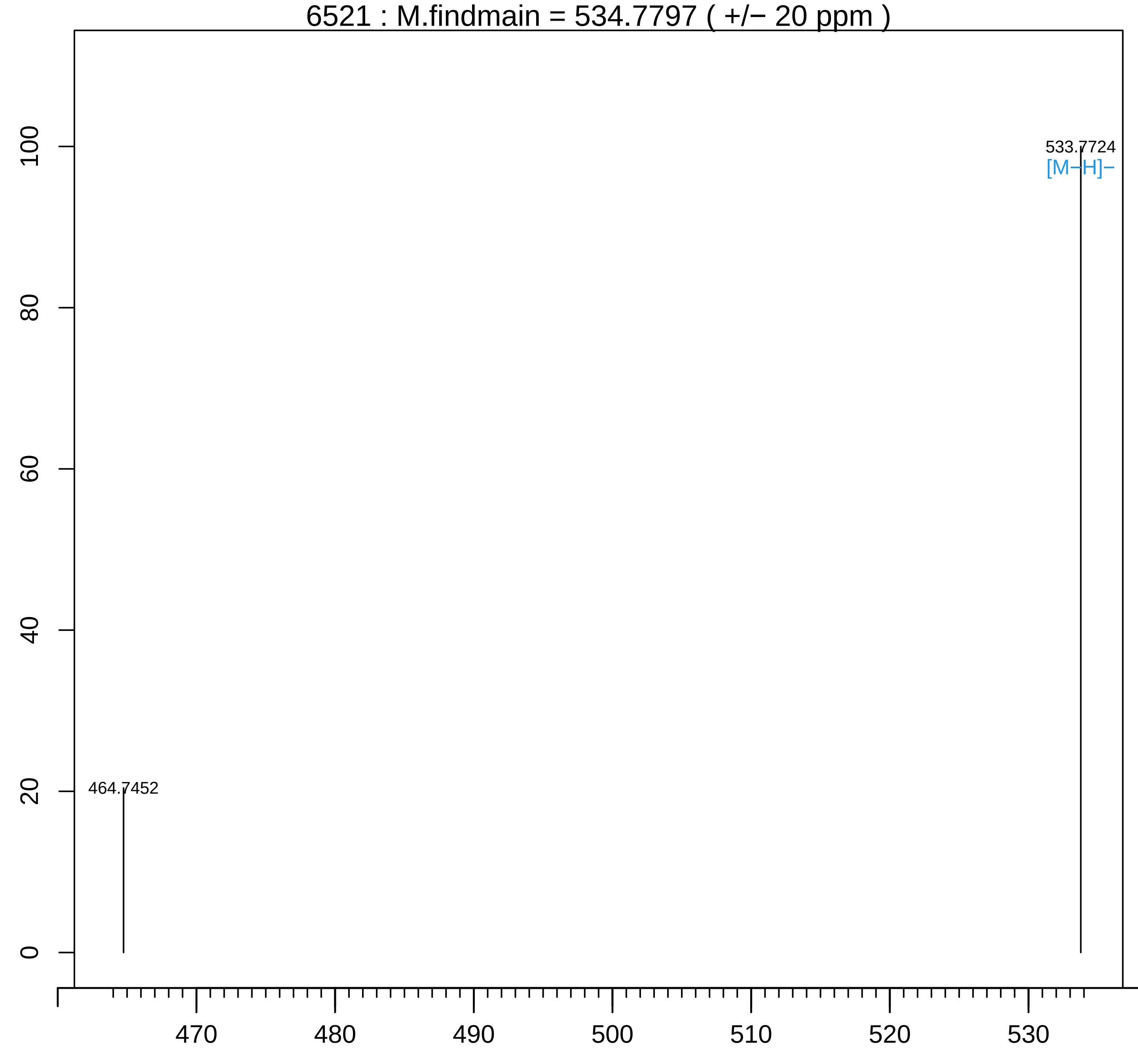
6521 : M.findmain = 534.7797 (+/− 20 ppm)
533.7724
[M−H]−
464.7452
0
20
40
60
80
100
470
480
490
500
510
520
530

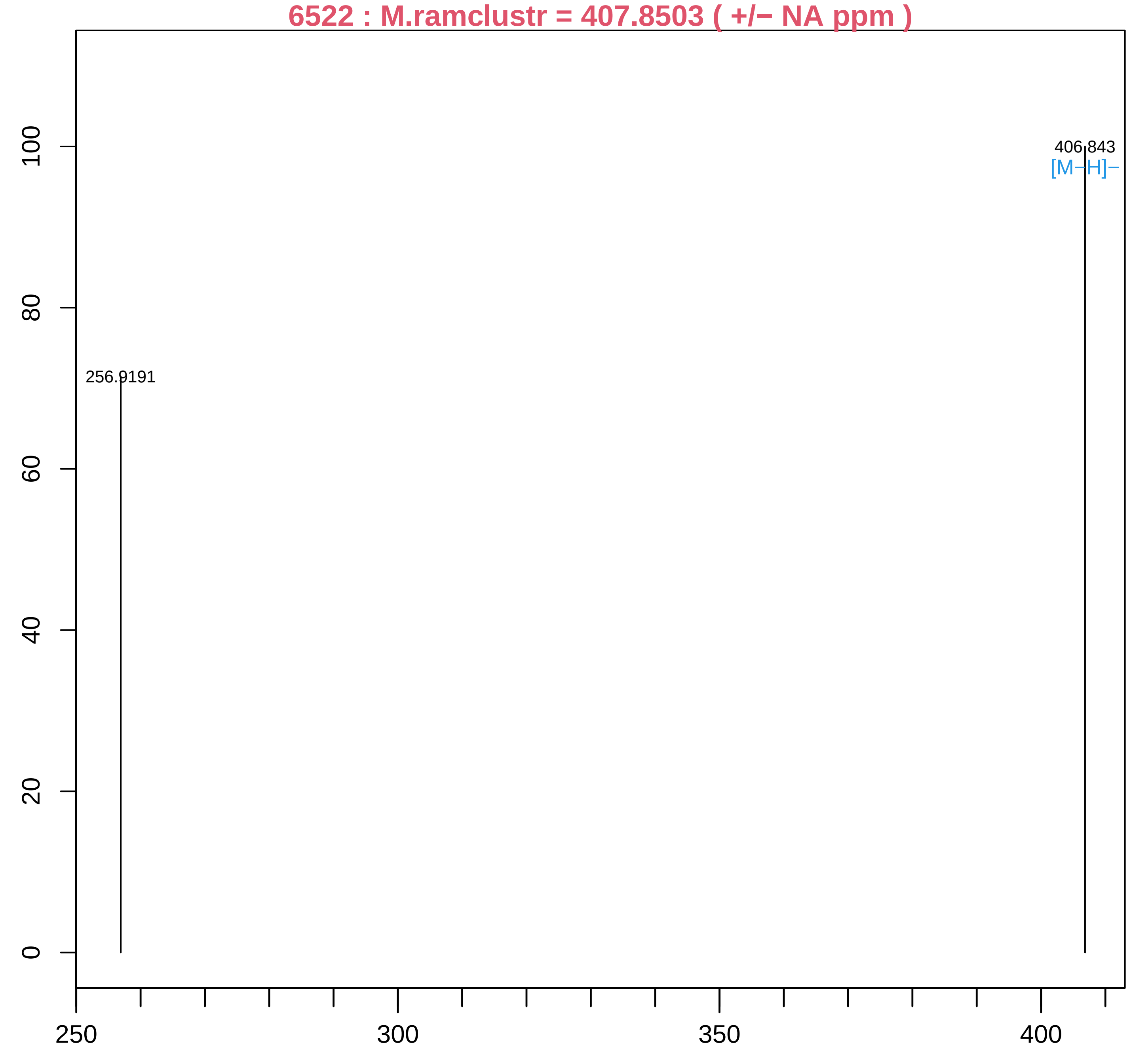
6522 : M.ramclustr = 407.8503 (+/− NA ppm)
406.843
[M−H]−
256.9191
0
20
40
60
80
100
250
300
350
400

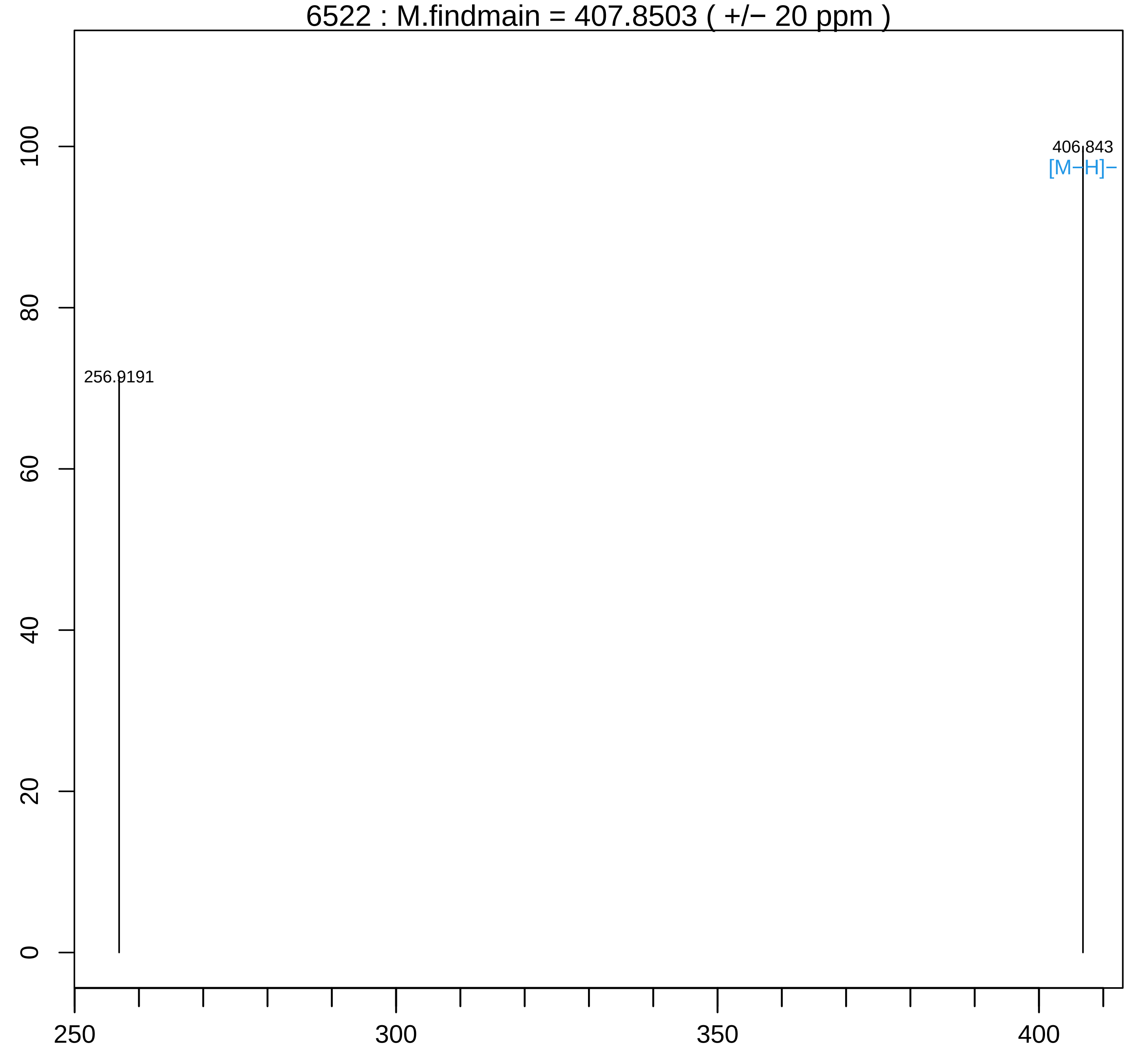
6522 : M.findmain = 407.8503 (+/− 20 ppm)
406.843
[M−H]−
256.9191
0
20
40
60
80
100
250
300
350
400

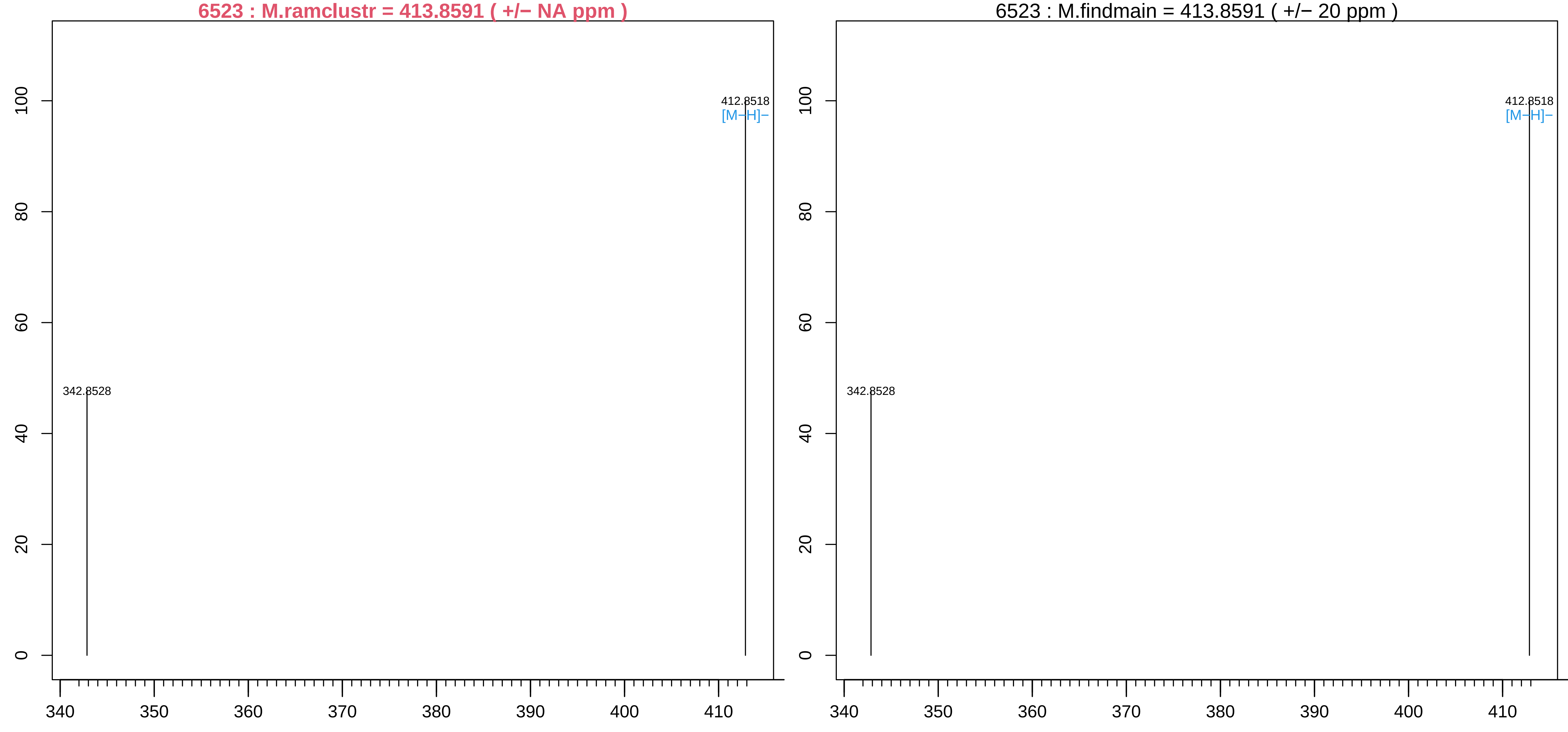
6523 : M.ramclustr = 413.8591 (+/− NA ppm)
412.8518
[M−H]−
342.8528
6523 : M.findmain = 413.8591 (+/− 20 ppm)
412.8518
[M−H]−
342.8528
0
20
40
60
80
100
340
350
360
370
380
390
400
410

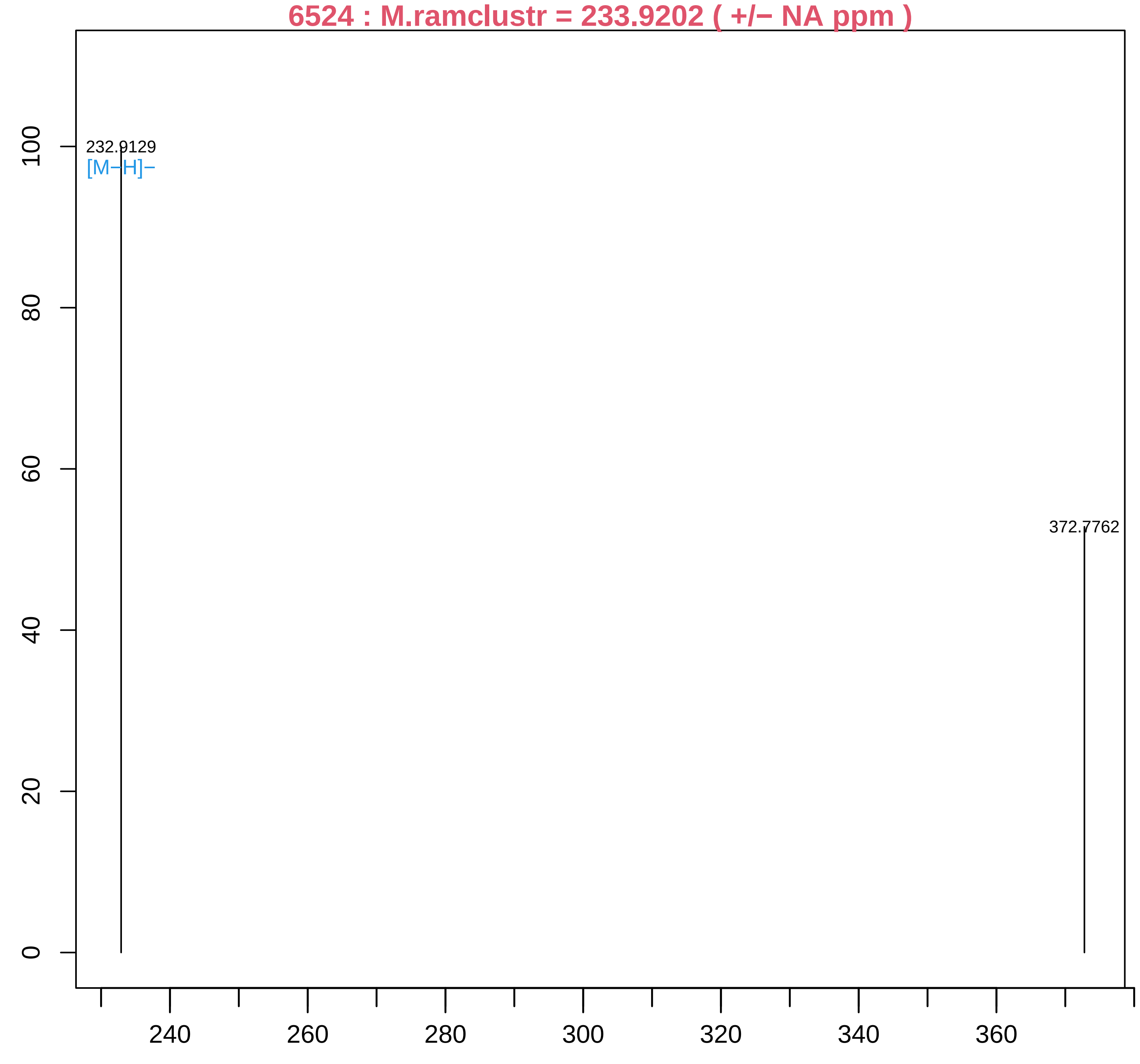
6524 : M.ramclustr = 233.9202 (+/− NA ppm)
232.9129
[M−H]−
372.7762
0
20
40
60
80
100
240
260
280
300
320
340
360
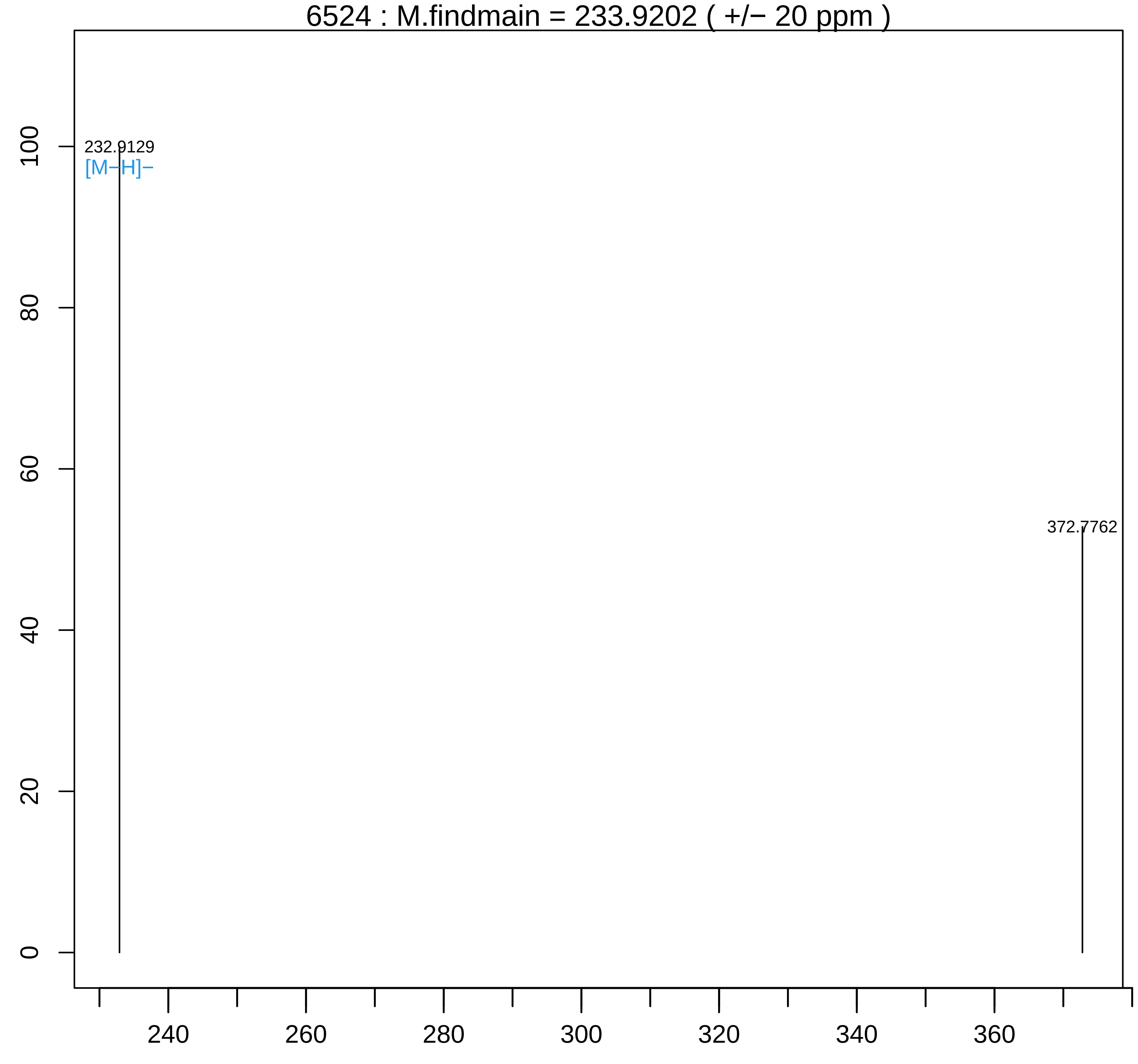
6524 : M.findmain = 233.9202 (+/− 20 ppm)
232.9129
[M−H]−
372.7762
0
20
40
60
80
100
240
260
280
300
320
340
360

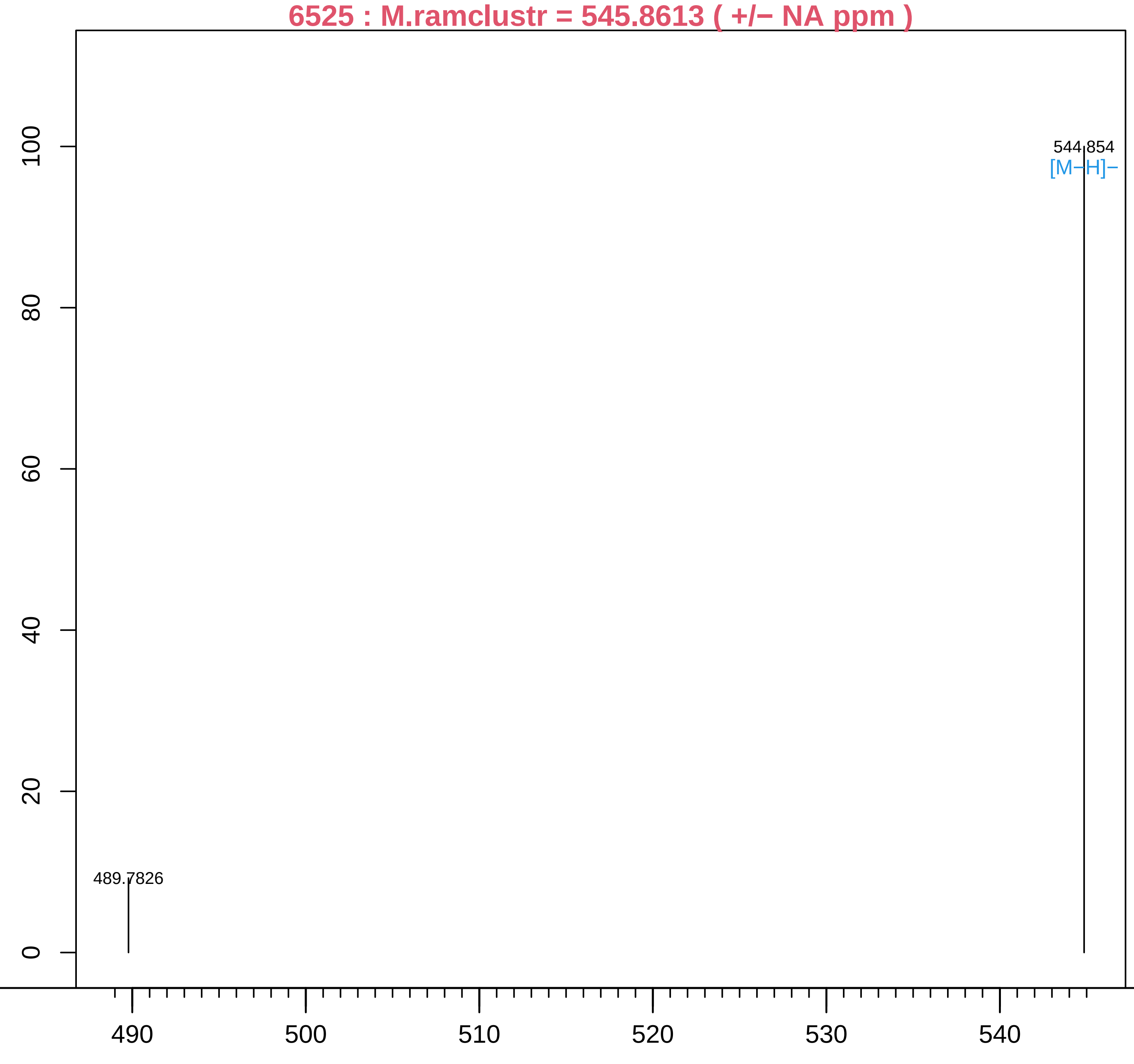
6525 : M.ramclustr = 545.8613 (+/− NA ppm)
544.854
[M−H]−
489.7826
0
20
40
60
80
100
490
500
510
520
530
540

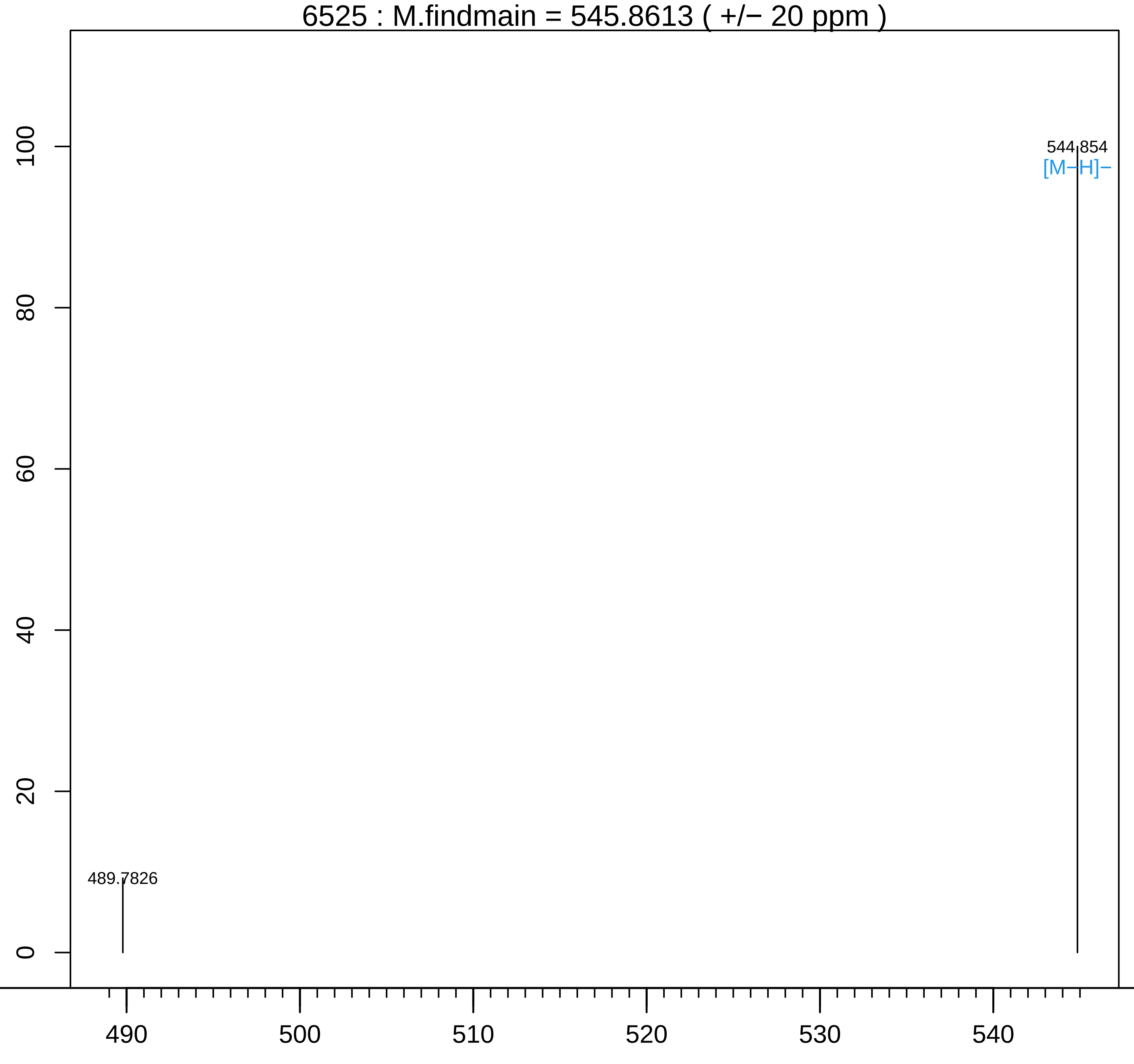
6525 : M.findmain = 545.8613 (+/− 20 ppm)
544.854
[M−H]−
489.7826
0
20
40
60
80
100
490
500
510
520
530
540

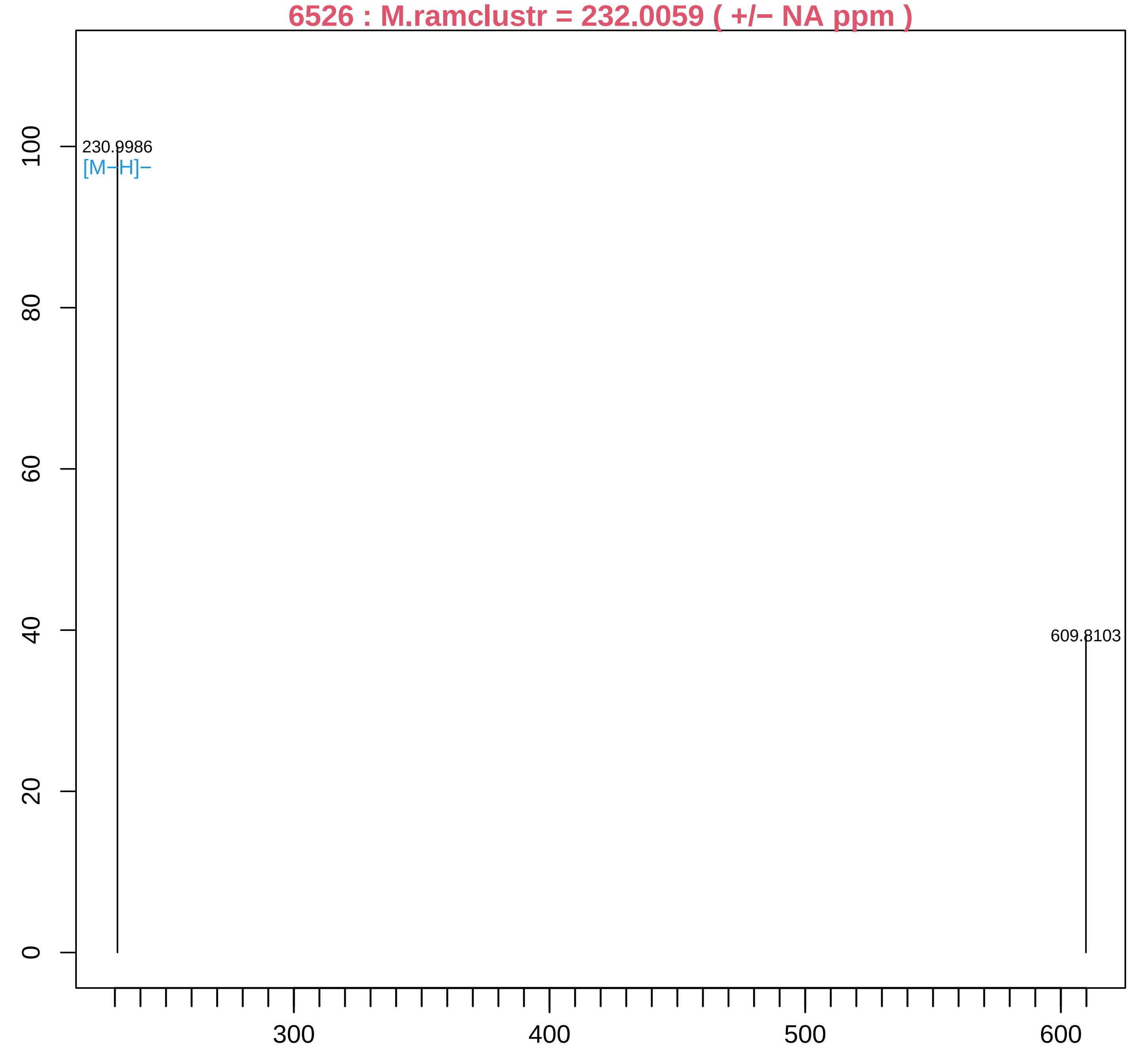
6526 : M.ramclustr = 232.0059 (+/− NA ppm)
230.9986
[M−H]−
609.8103
0
20
40
60
80
100
300
400
500
600

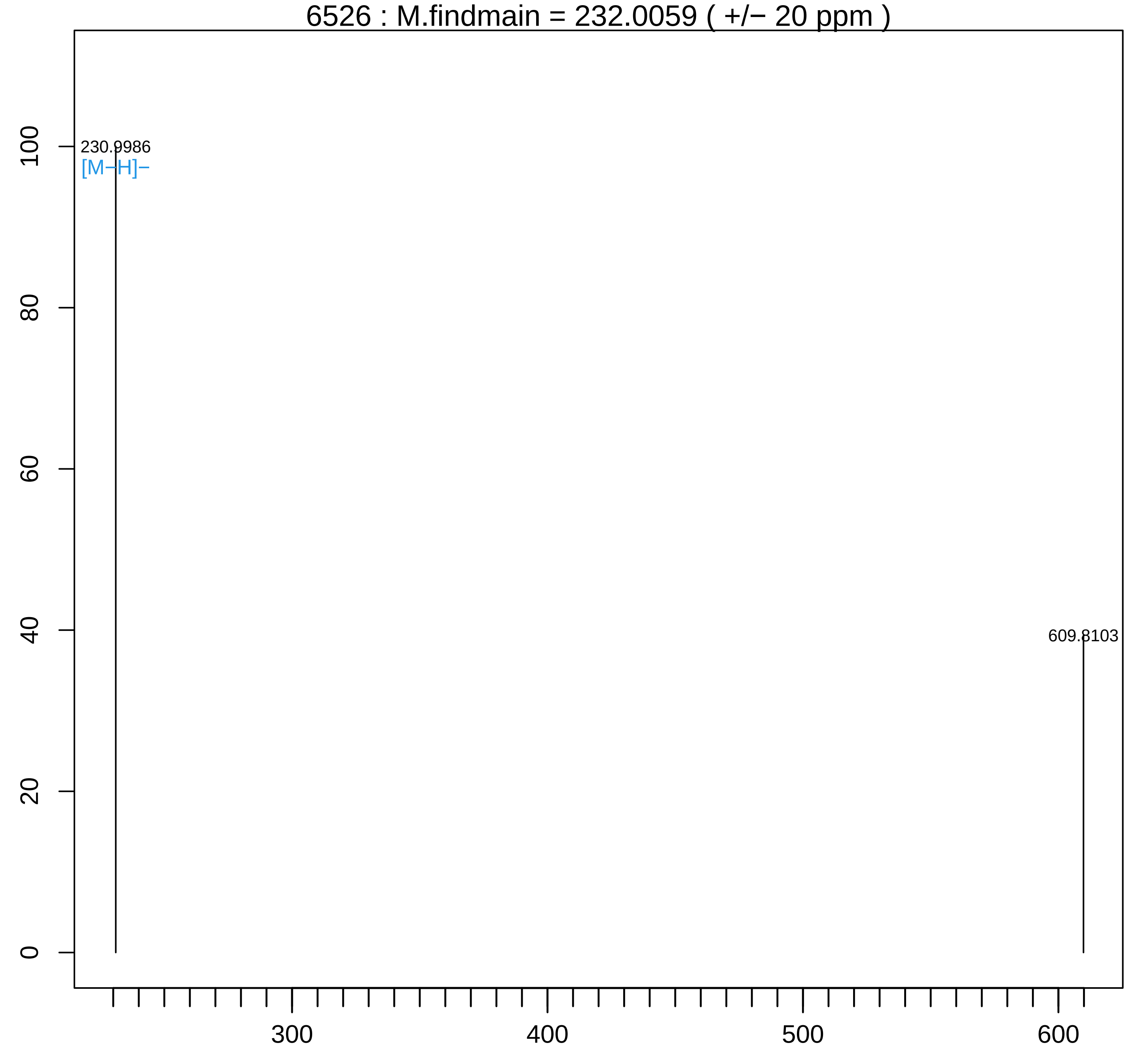
6526 : M.findmain = 232.0059 (+/− 20 ppm)
230.9986
[M−H]−
609.8103
0
20
40
60
80
100
300
400
500
600

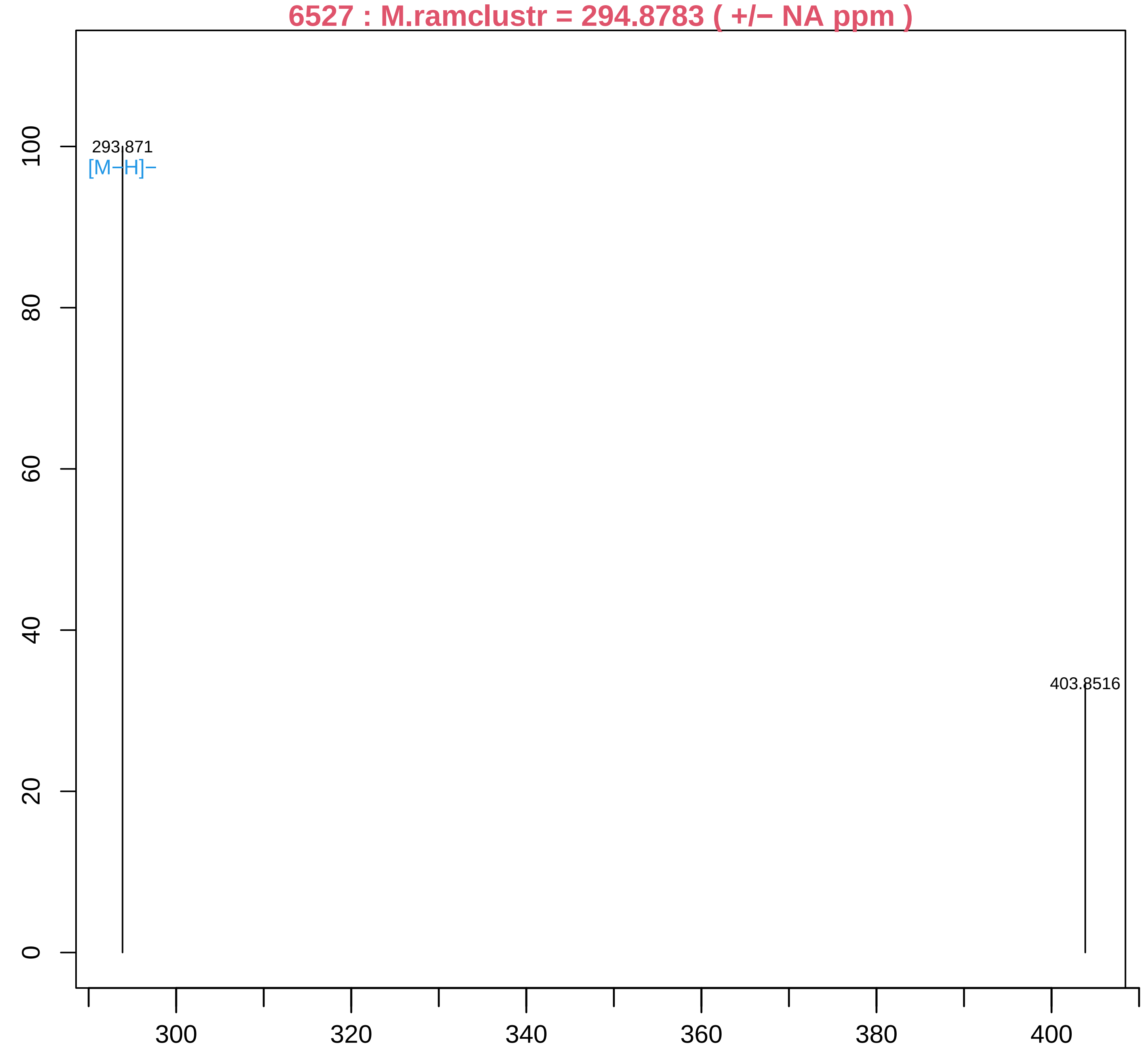
6527 : M.ramclustr = 294.8783 (+/− NA ppm)
293.871
[M−H]−
403.8516
0
20
40
60
80
100
300
320
340
360
380
400

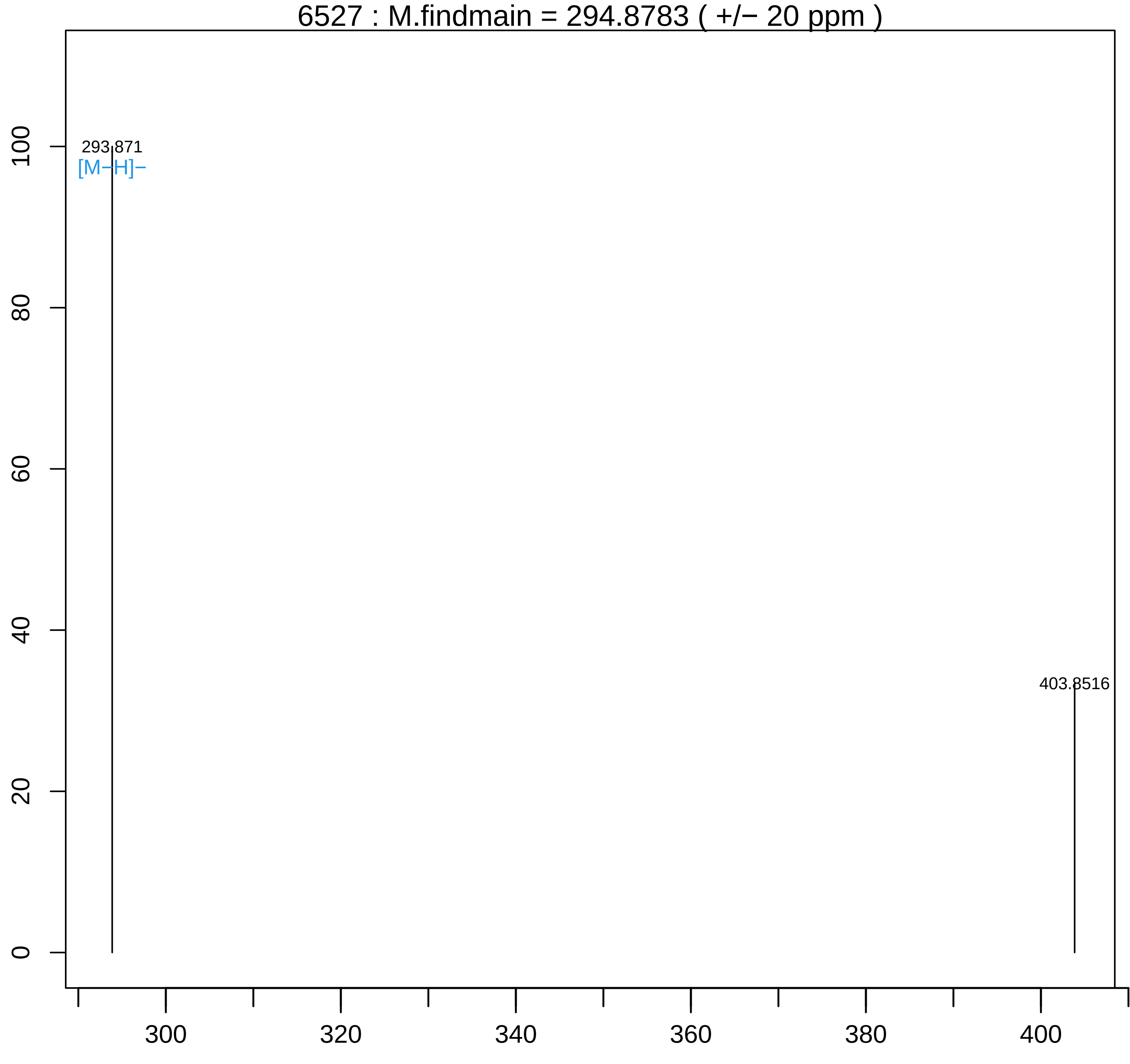
6527 : M.findmain = 294.8783 (+/− 20 ppm)
293.871
[M−H]−
403.8516
0
20
40
60
80
100
300
320
340
360
380
400

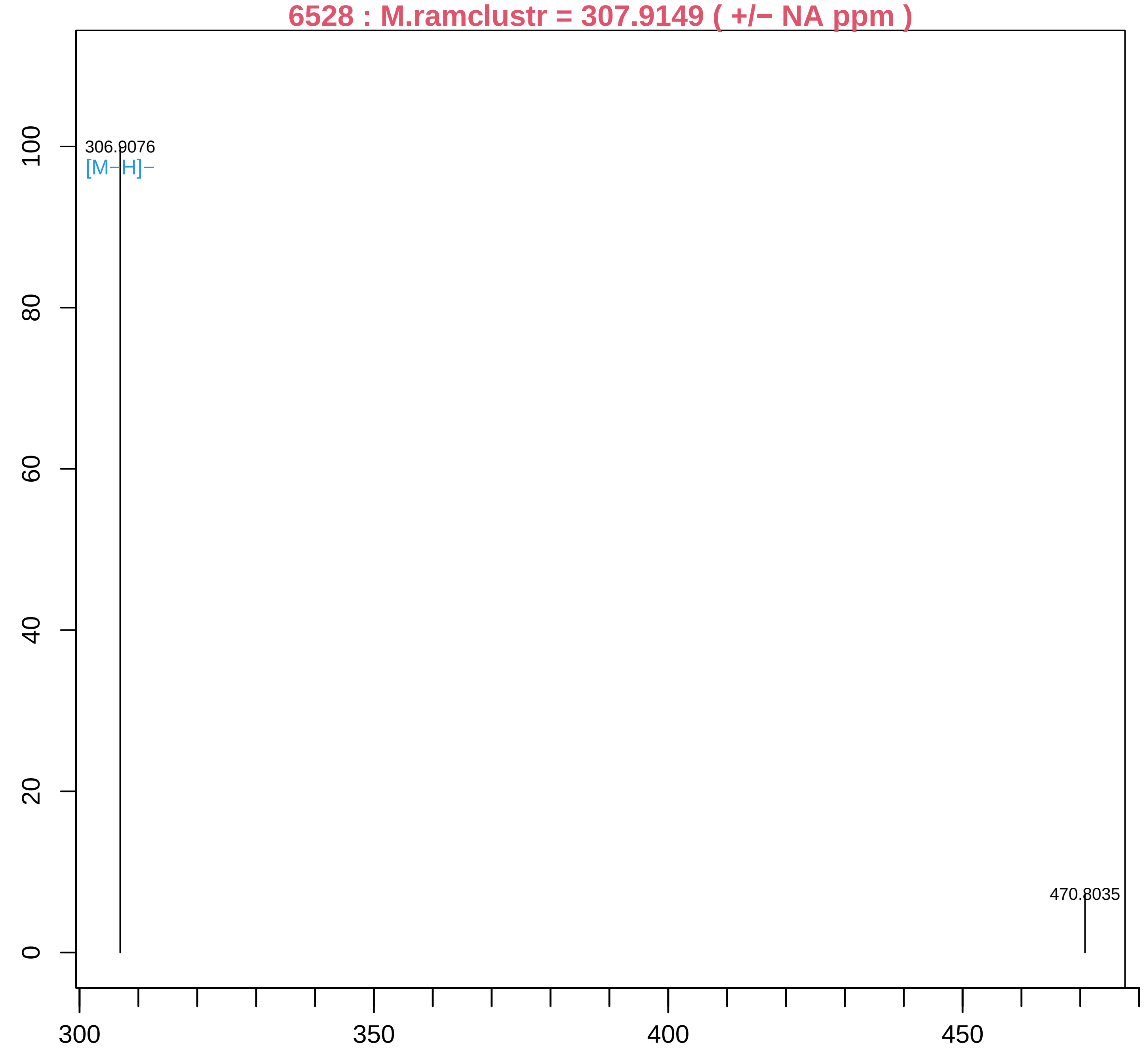
6528 : M.ramclustr = 307.9149 (+/− NA ppm)
306.9076
[M−H]−
470.8035
0
20
40
60
80
100
300
350
400
450

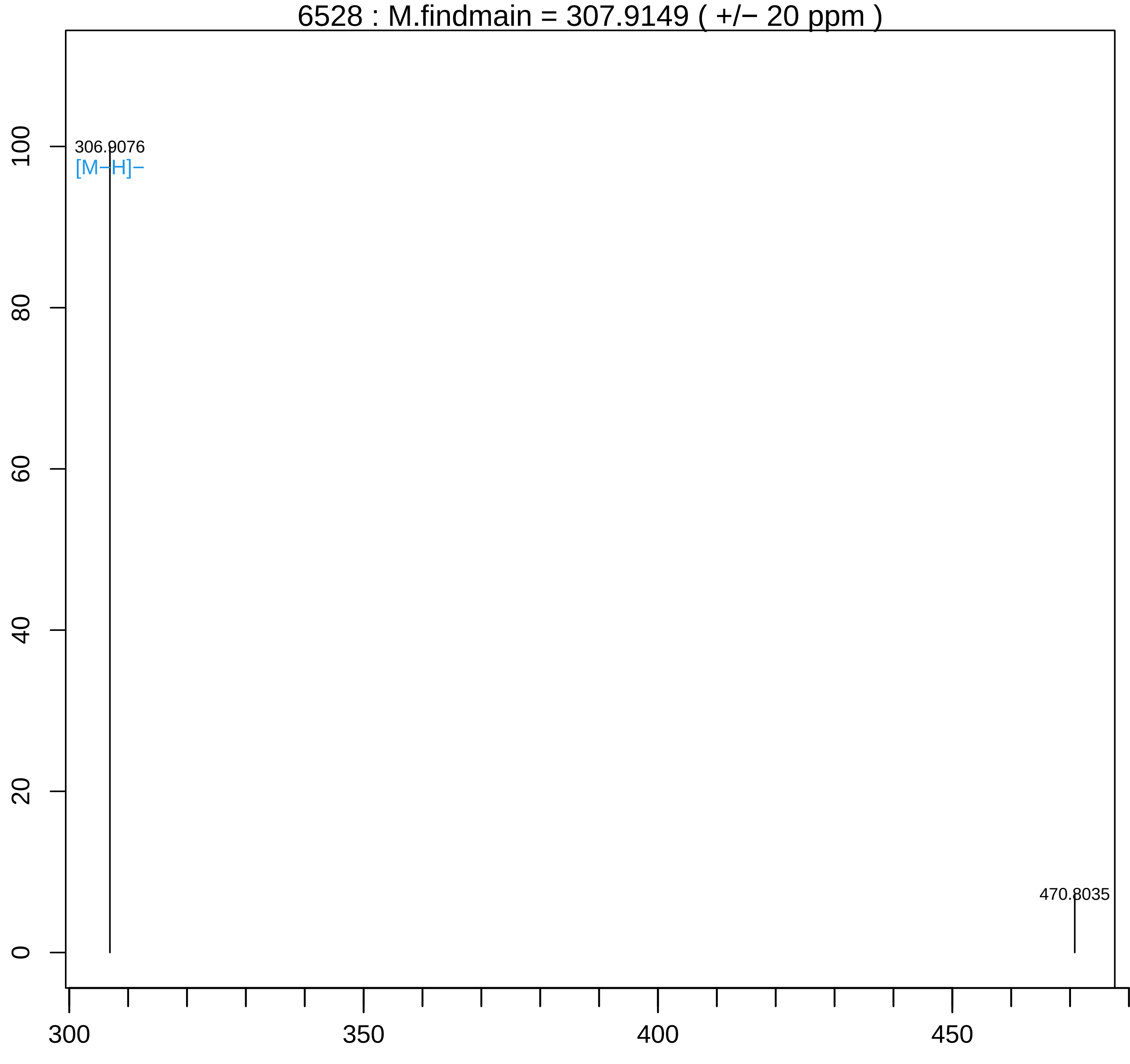
6528 : M.findmain = 307.9149 (+/− 20 ppm)
306.9076
[M−H]−
470.8035
0
20
40
60
80
100
300
350
400
450

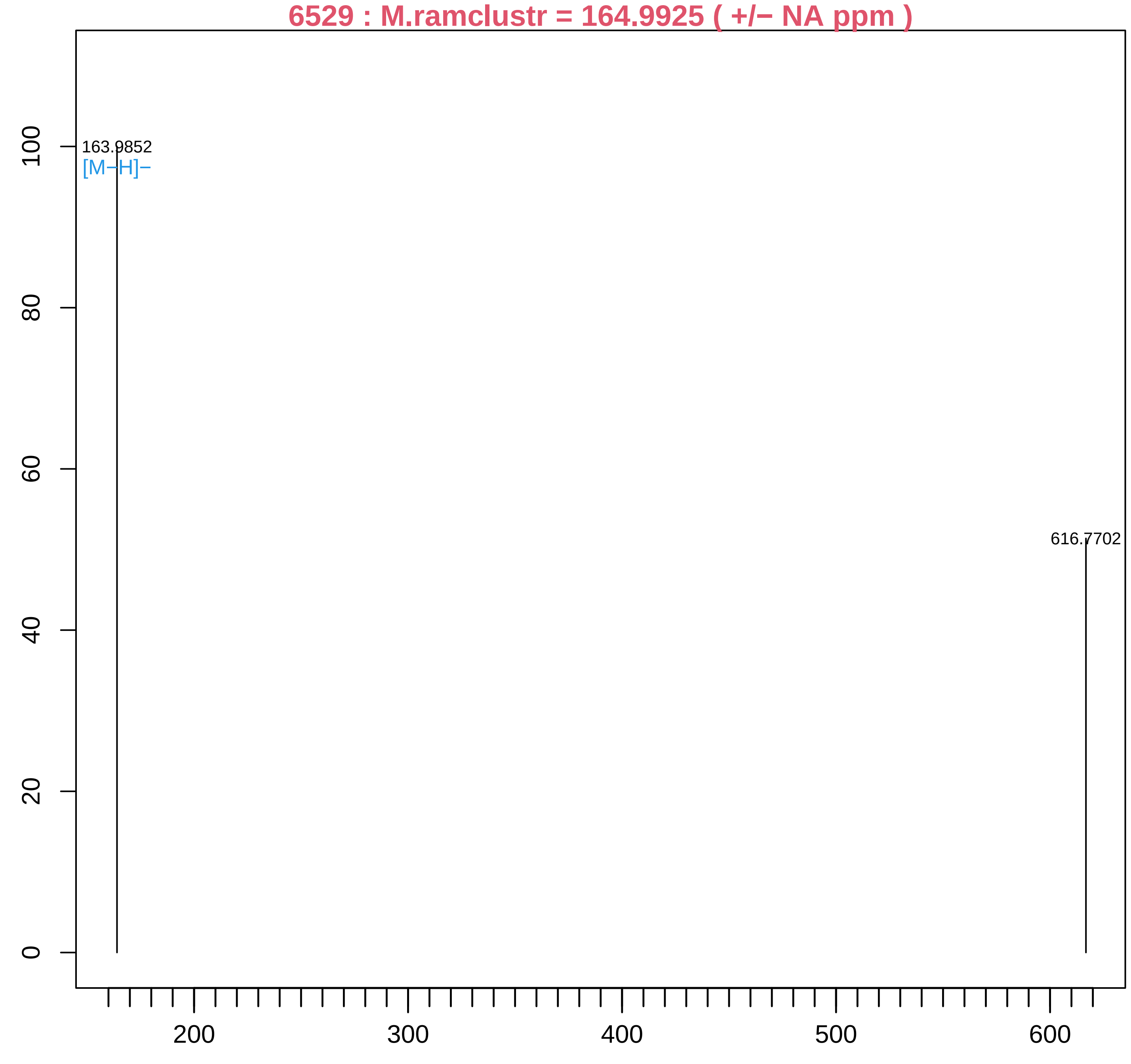
6529 : M.ramclustr = 164.9925 (+/− NA ppm)
163.9852
[M−H]−
616.7702
0
20
40
60
80
100
200
300
400
500
600

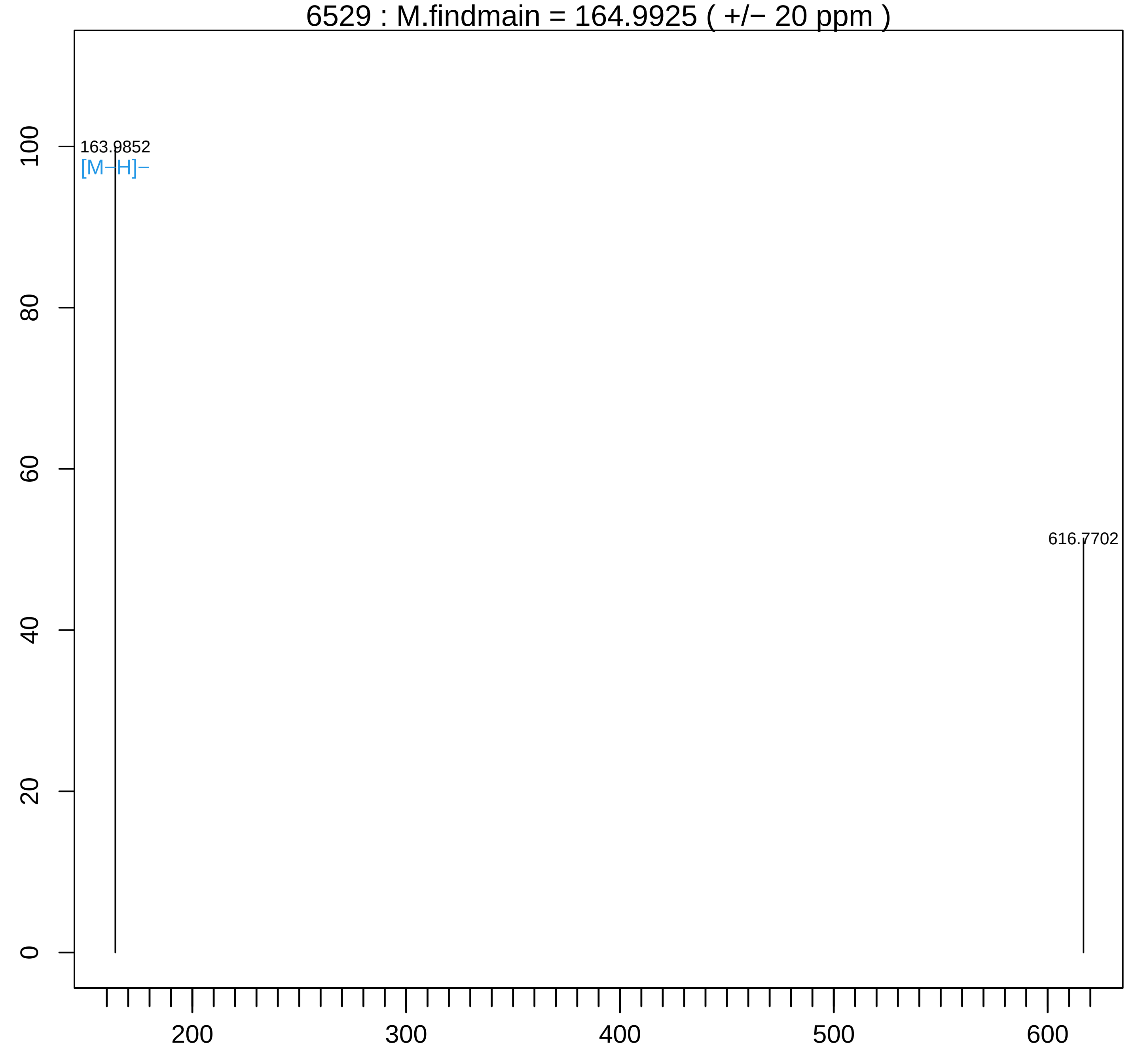
6529 : M.findmain = 164.9925 (+/− 20 ppm)
163.9852
[M−H]−
616.7702
0
20
40
60
80
100
200
300
400
500
600

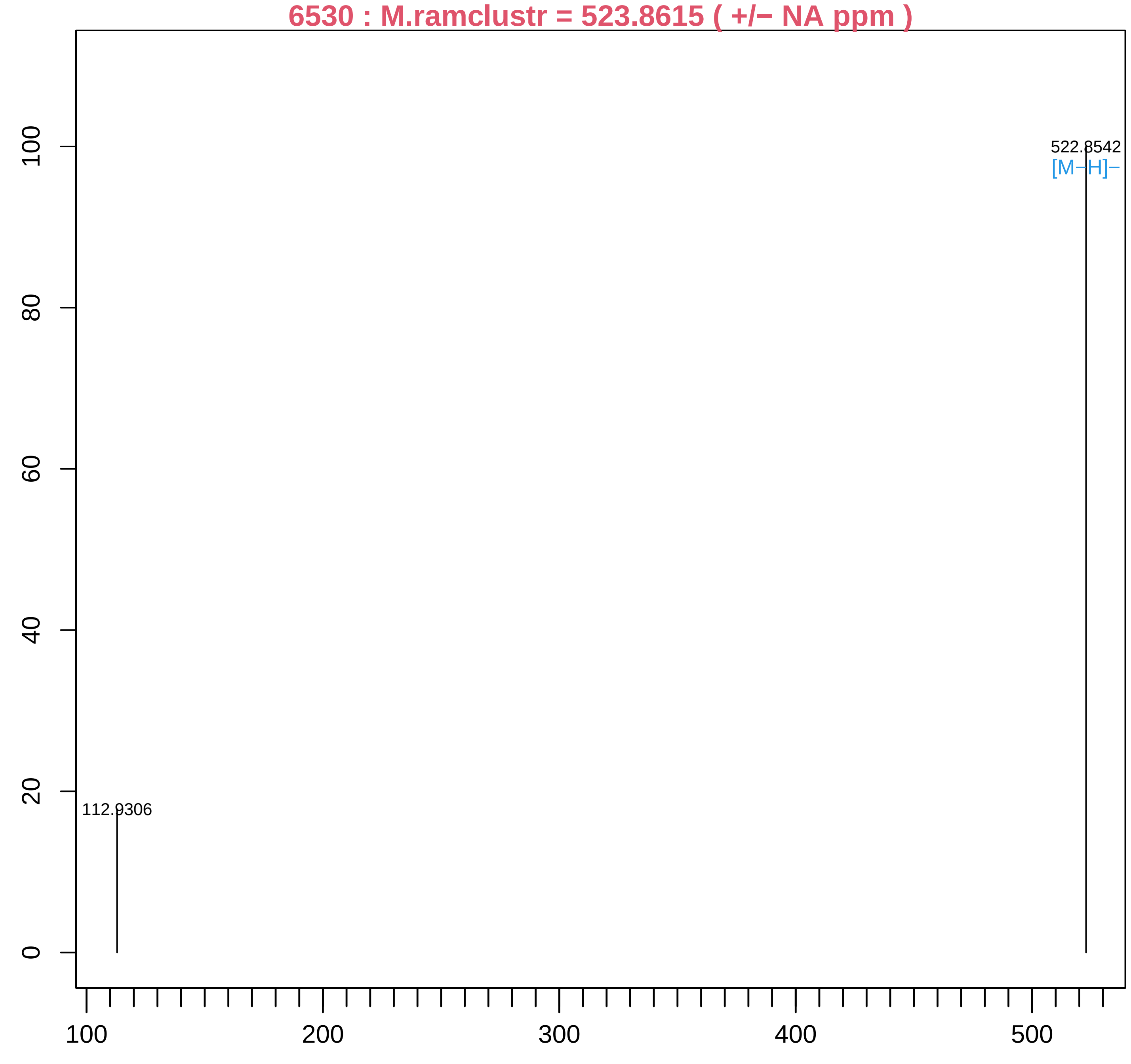
6530 : M.ramclustr = 523.8615 (+/− NA ppm)
522.8542
[M−H]−
112.9306
0
20
40
60
80
100
100
200
300
400
500

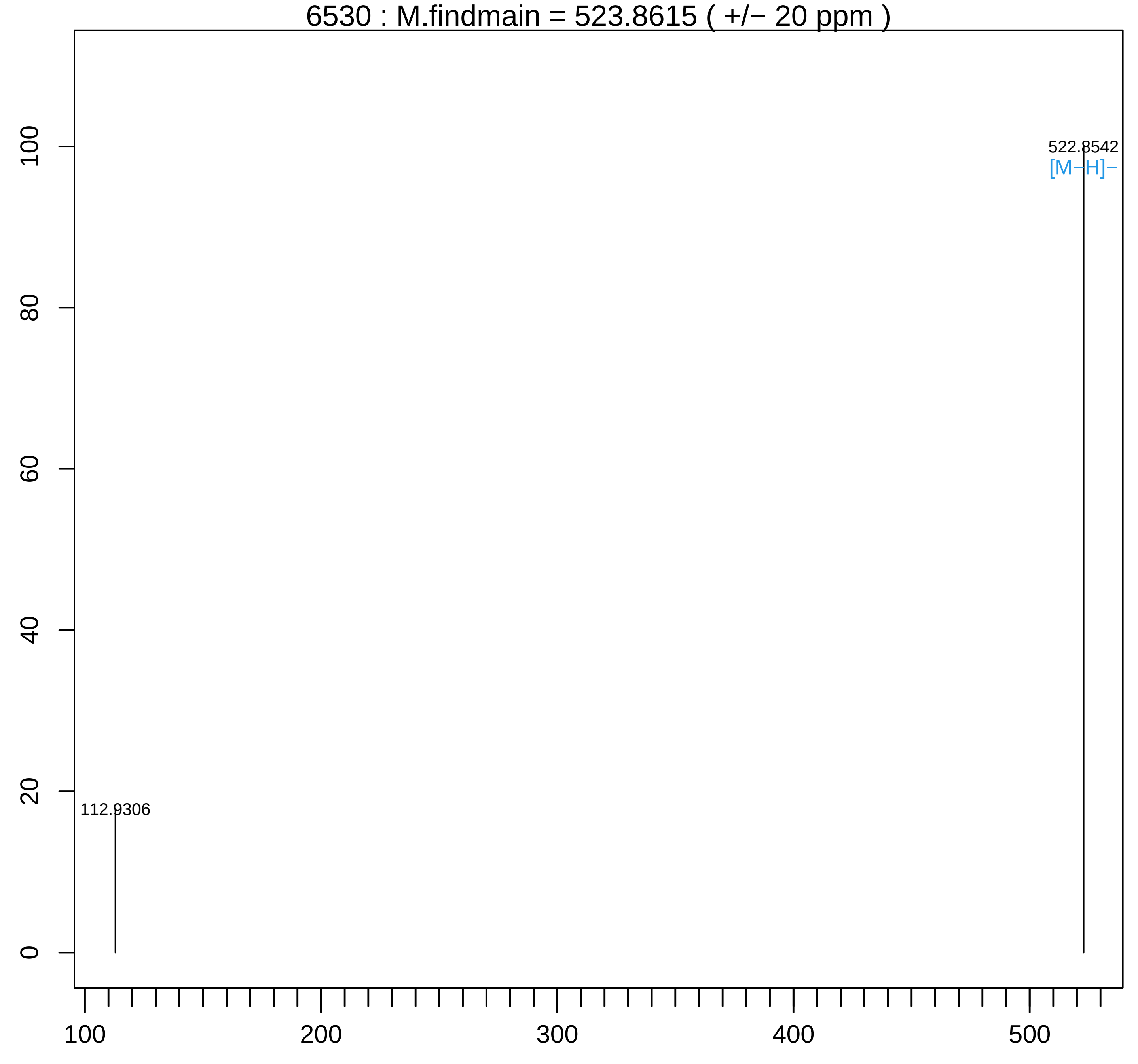
6530 : M.findmain = 523.8615 (+/− 20 ppm)
522.8542
[M−H]−
112.9306
0
20
40
60
80
100
100
200
300
400
500

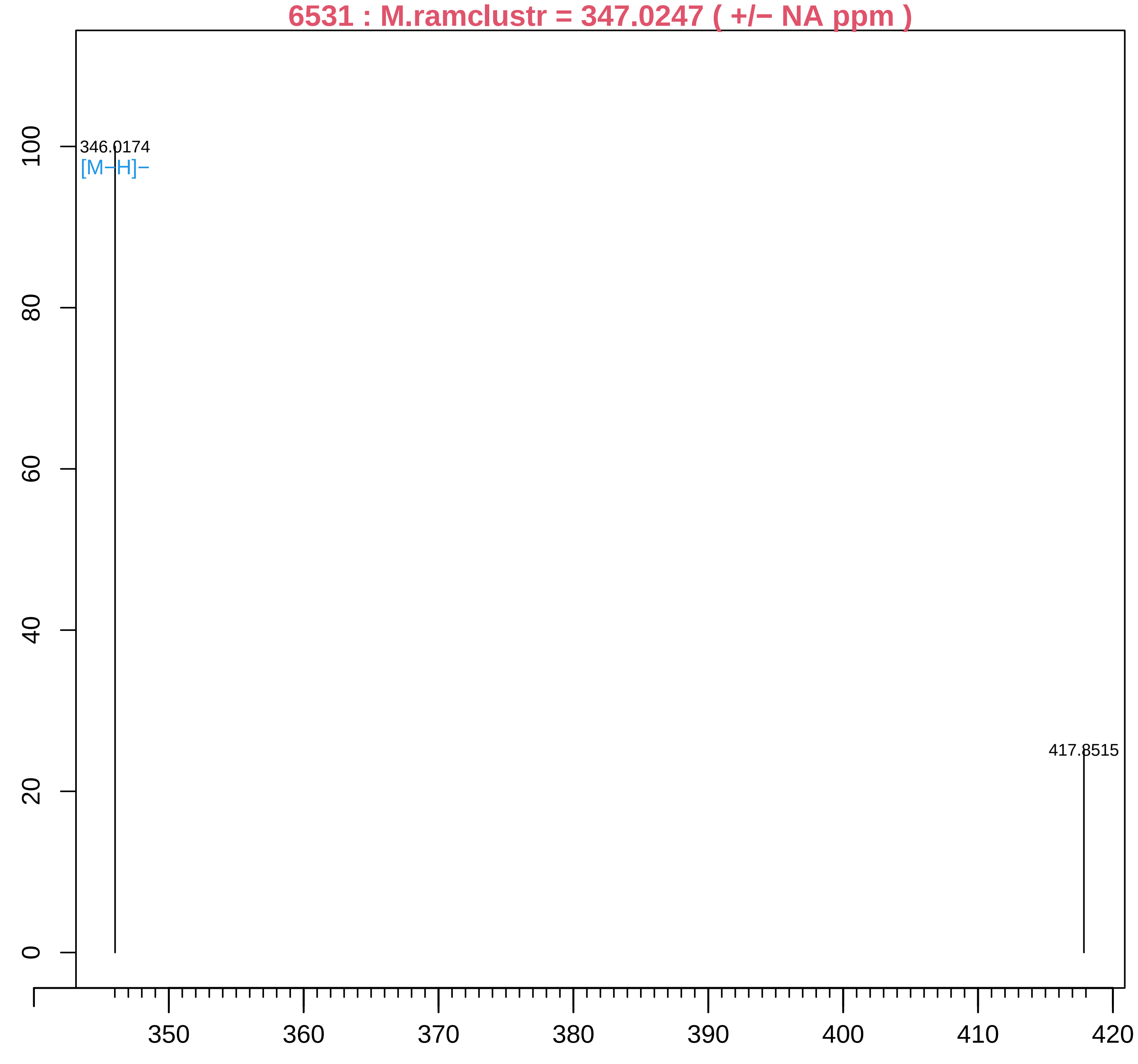
6531 : M.ramclustr = 347.0247 (+/− NA ppm)
346.0174
[M−H]−
417.8515
0
20
40
60
80
100
350
360
370
380
390
400
410
420

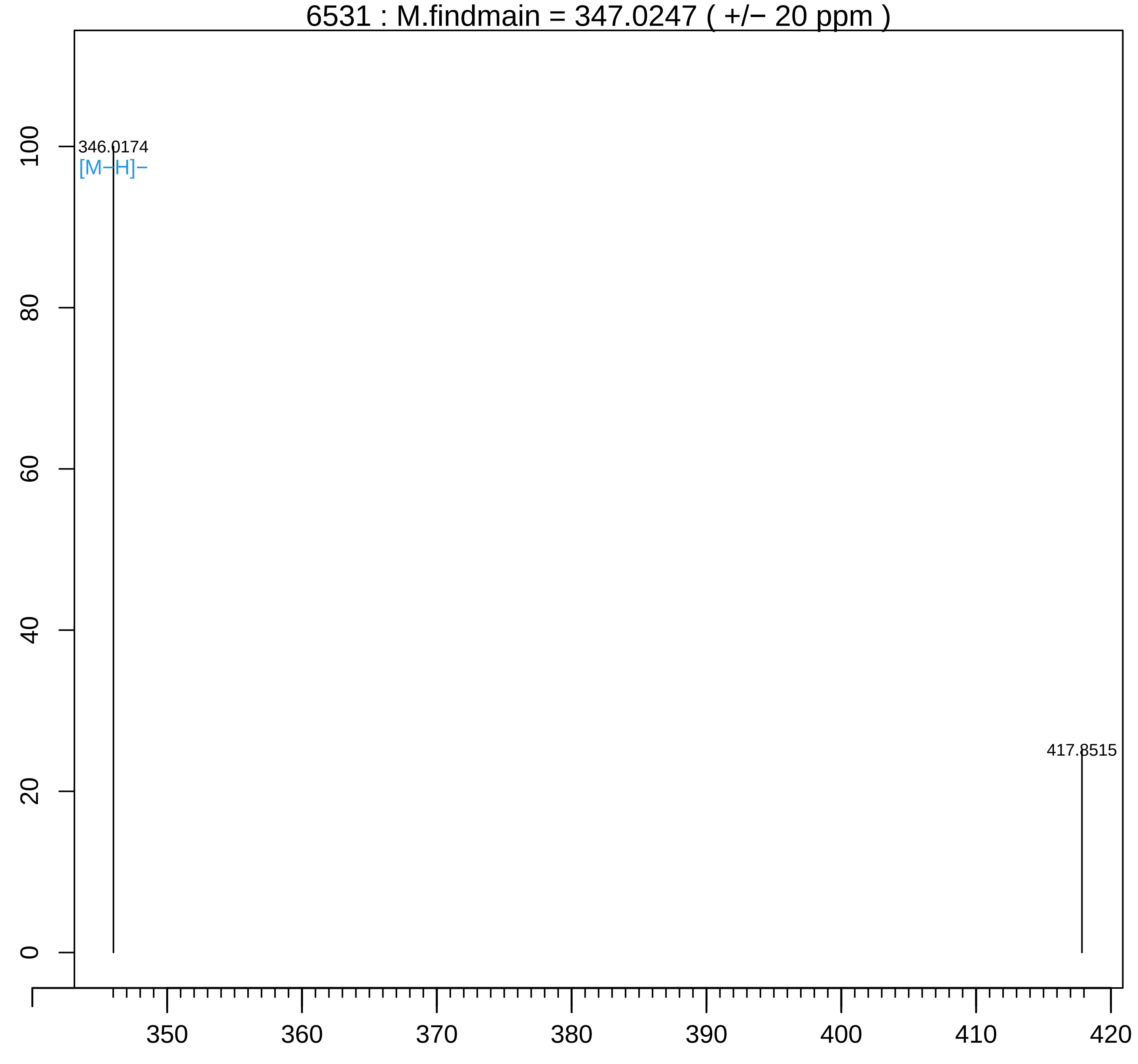
6531 : M.findmain = 347.0247 (+/− 20 ppm)
346.0174
[M−H]−
417.8515
0
20
40
60
80
100
350
360
370
380
390
400
410
420

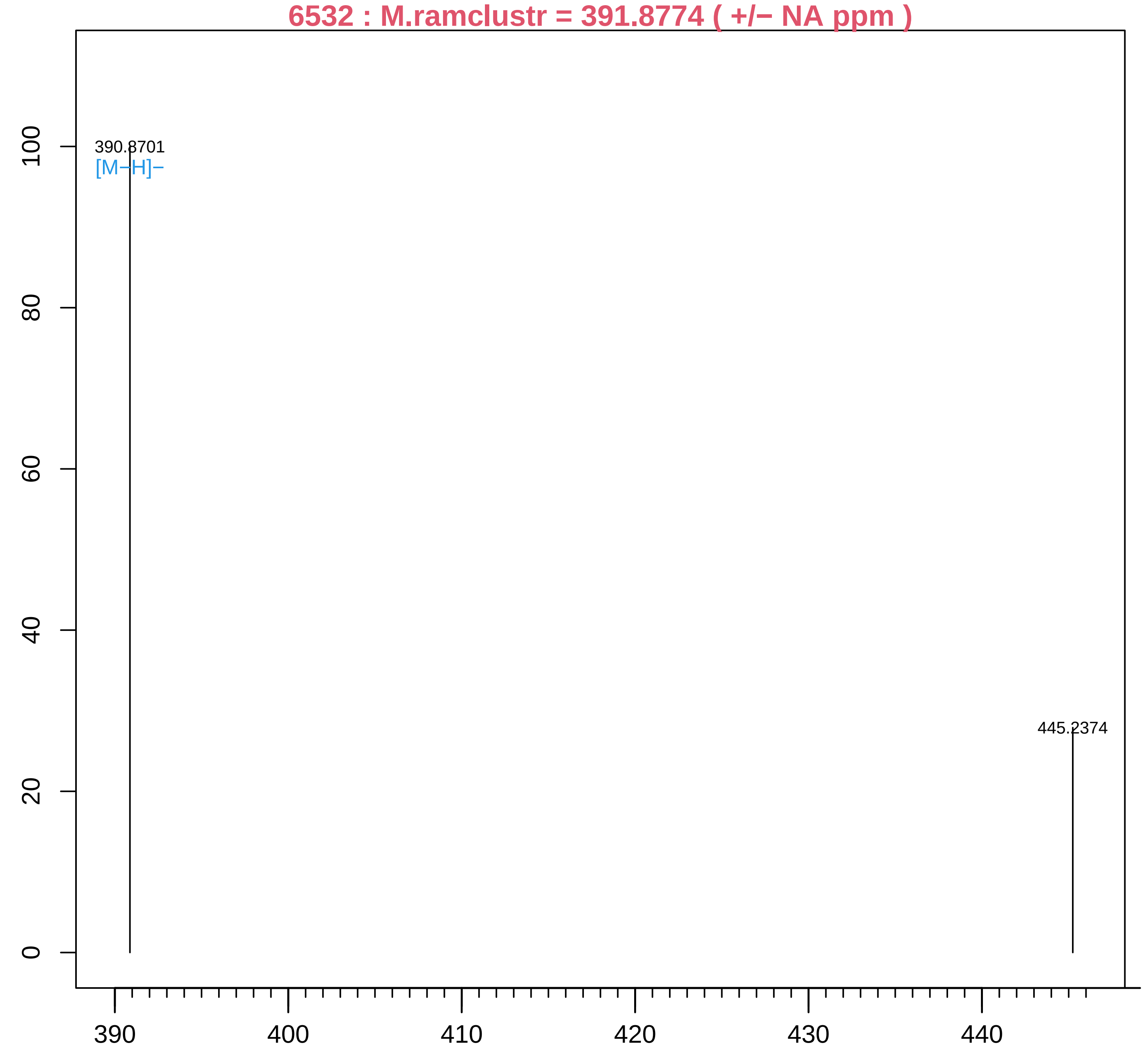
6532 : M.ramclustr = 391.8774 (+/− NA ppm)
390.8701
[M−H]−
445.2374
0
20
40
60
80
100
390
400
410
420
430
440

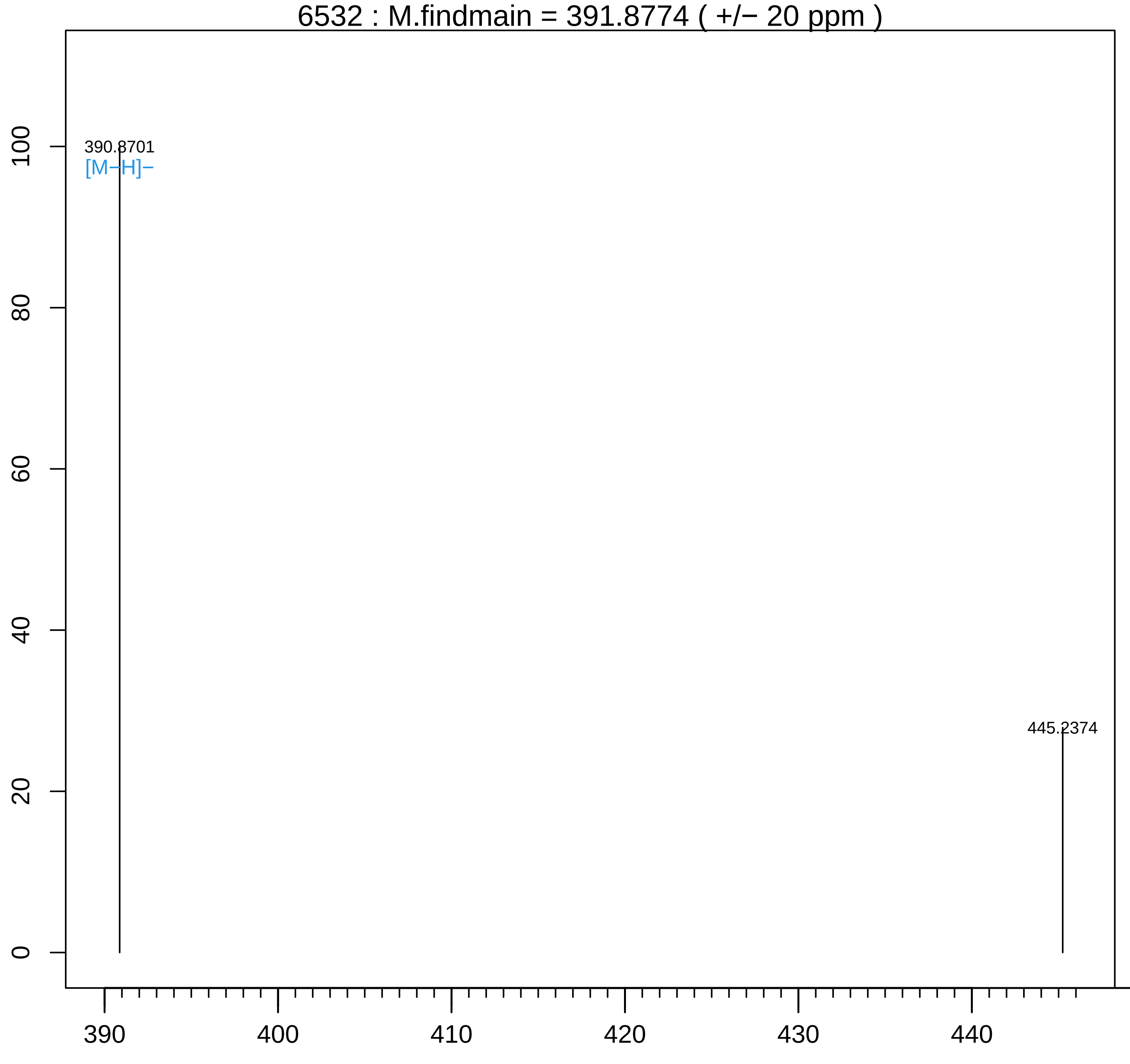
6532 : M.findmain = 391.8774 (+/− 20 ppm)
390.8701
[M−H]−
445.2374
0
20
40
60
80
100
390
400
410
420
430
440

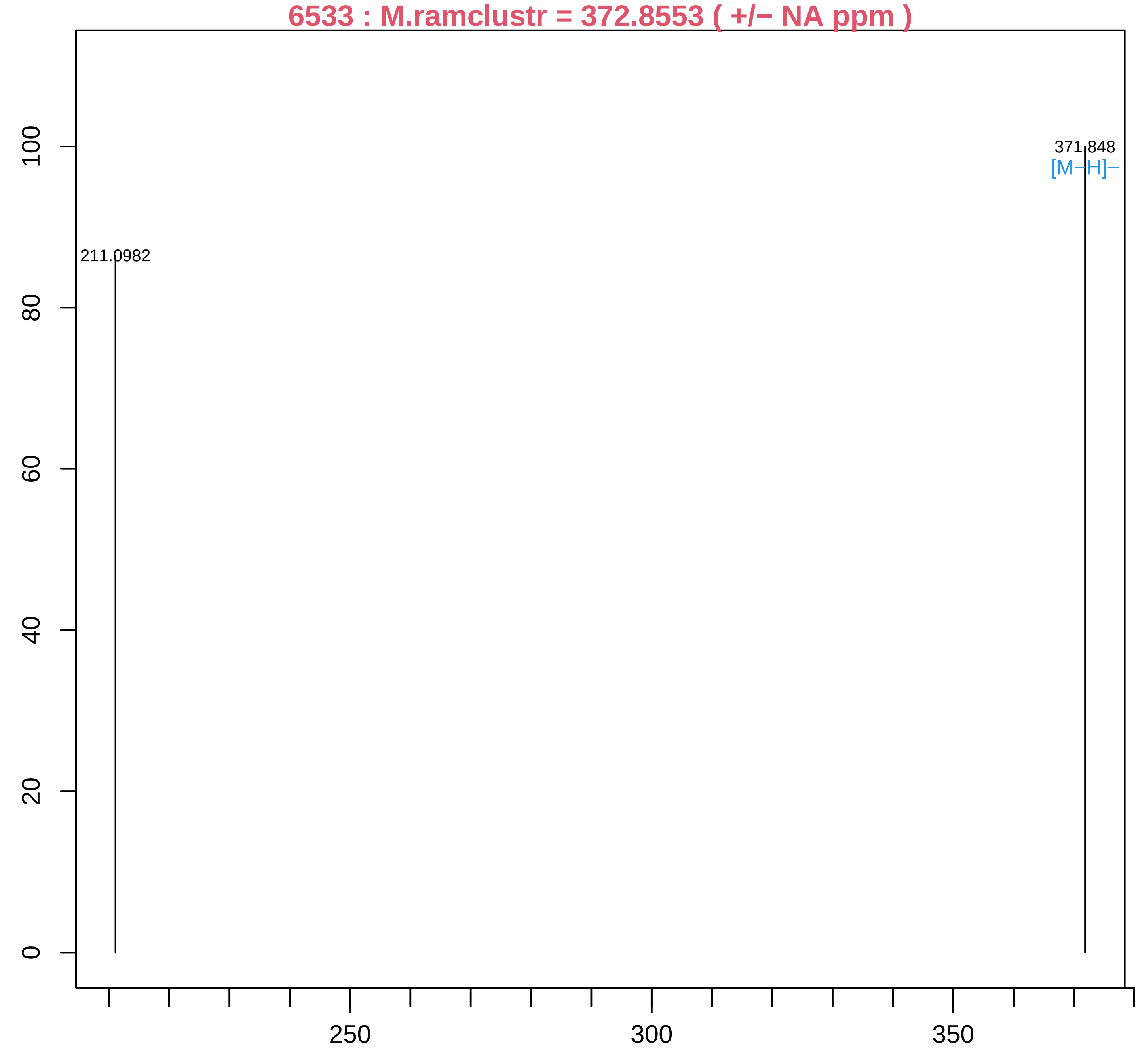
6533 : M.ramclustr = 372.8553 (+/− NA ppm)
211.0982
371.848
[M−H]−
0
20
40
60
80
100
250
300
350

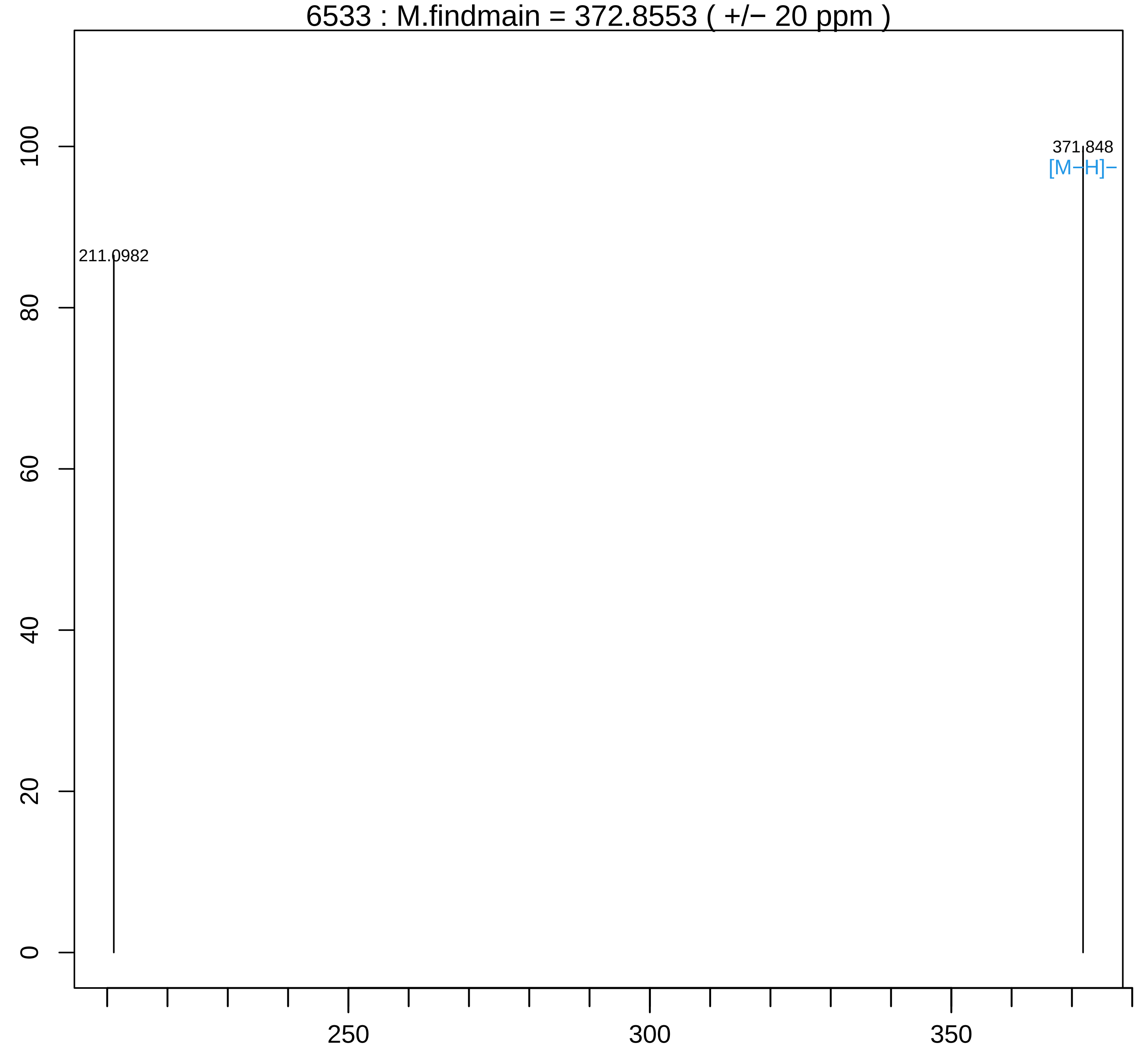
6533 : M.findmain = 372.8553 (+/− 20 ppm)
211.0982
371.848
[M−H]−
0
20
40
60
80
100
250
300
350

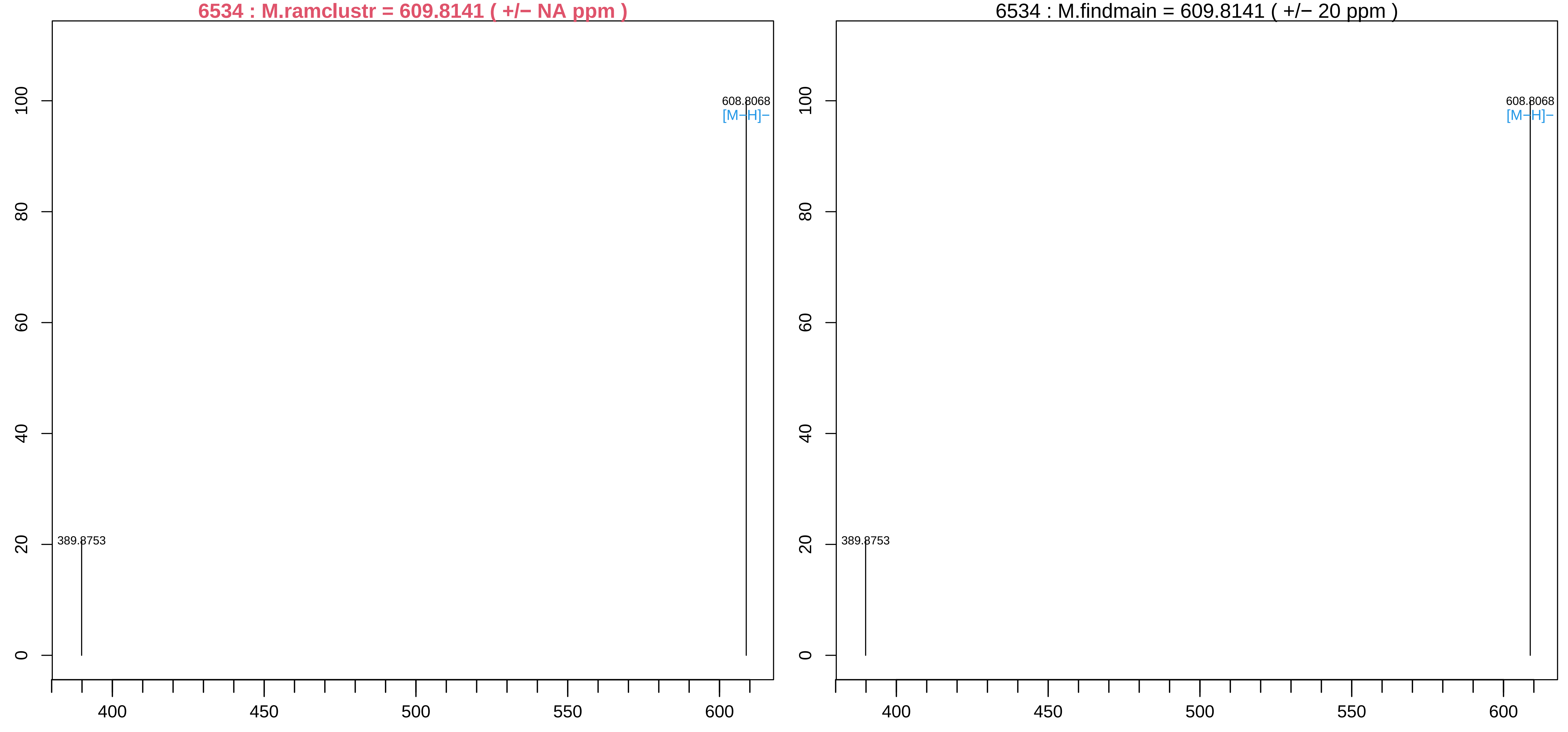
6534 : M.ramclustr = 609.8141 (+/− NA ppm)
6534 : M.findmain = 609.8141 (+/− 20 ppm)
608.8068
[M−H]−
389.8753
0
20
40
60
80
100
400
450
500
550
600

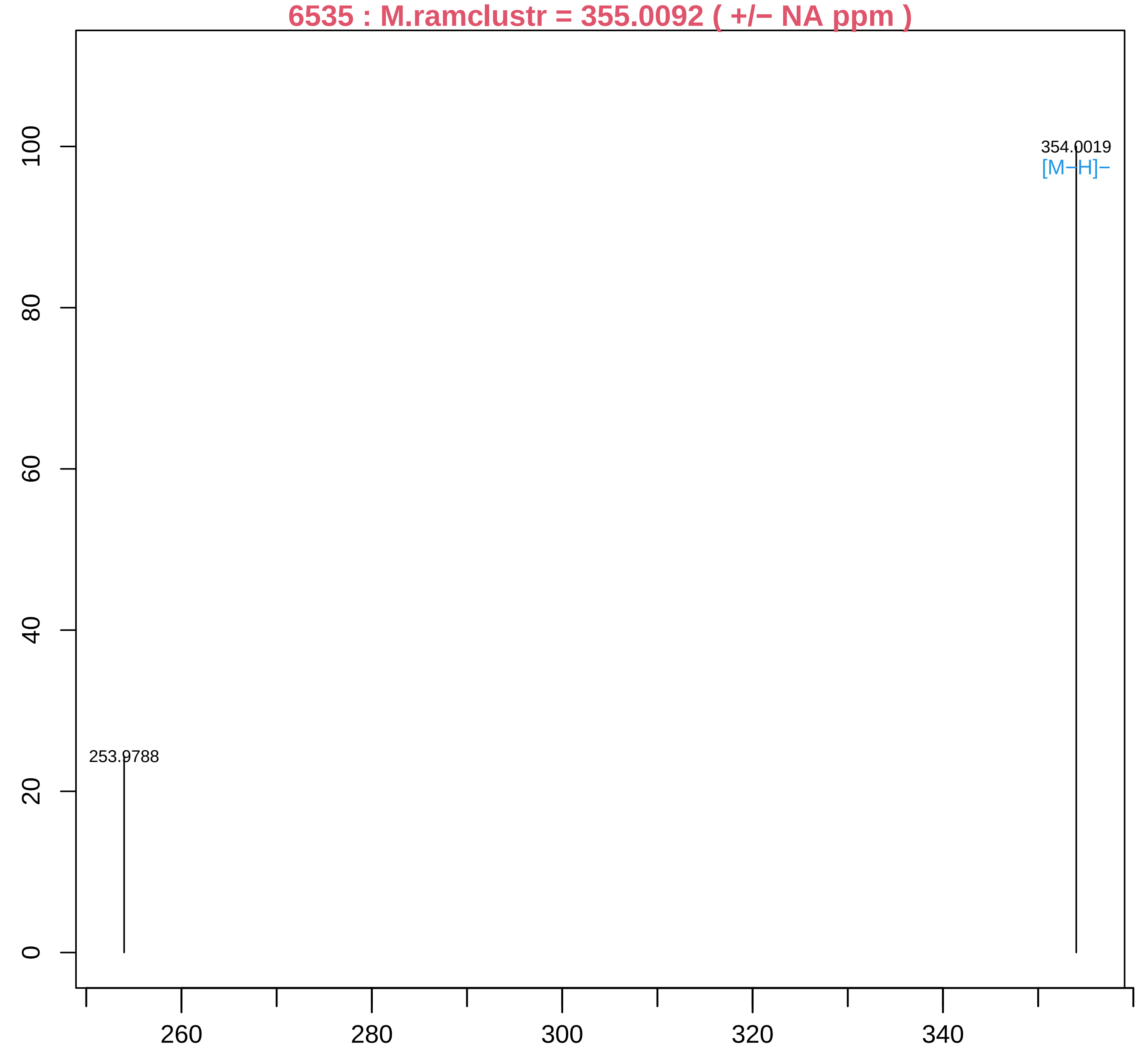
6535 : M.ramclustr = 355.0092 (+/− NA ppm)
354.0019
[M−H]−
253.9788
0
20
40
60
80
100
260
280
300
320
340

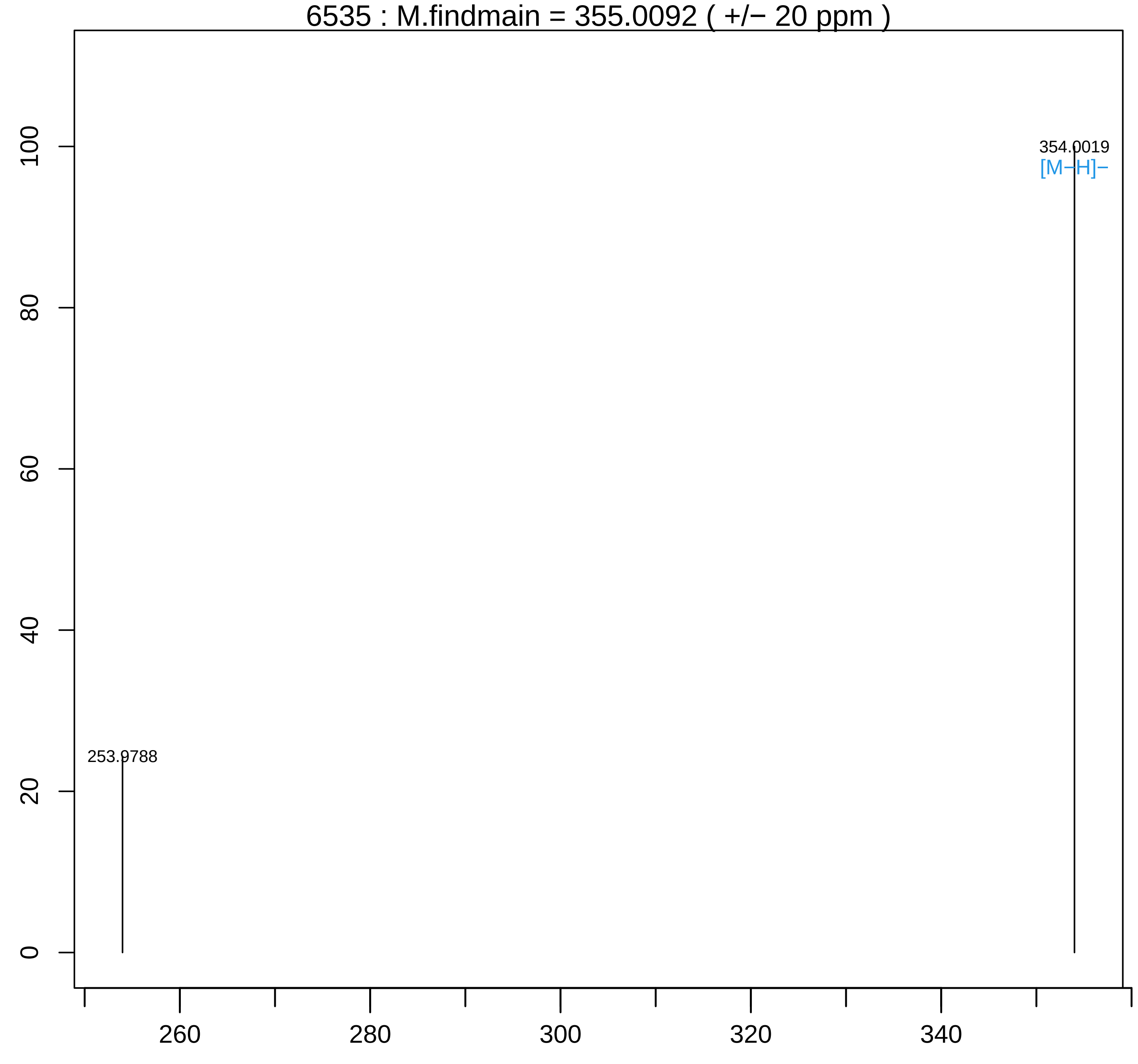
6535 : M.findmain = 355.0092 (+/− 20 ppm)
354.0019
[M−H]−
253.9788
0
20
40
60
80
100
260
280
300
320
340

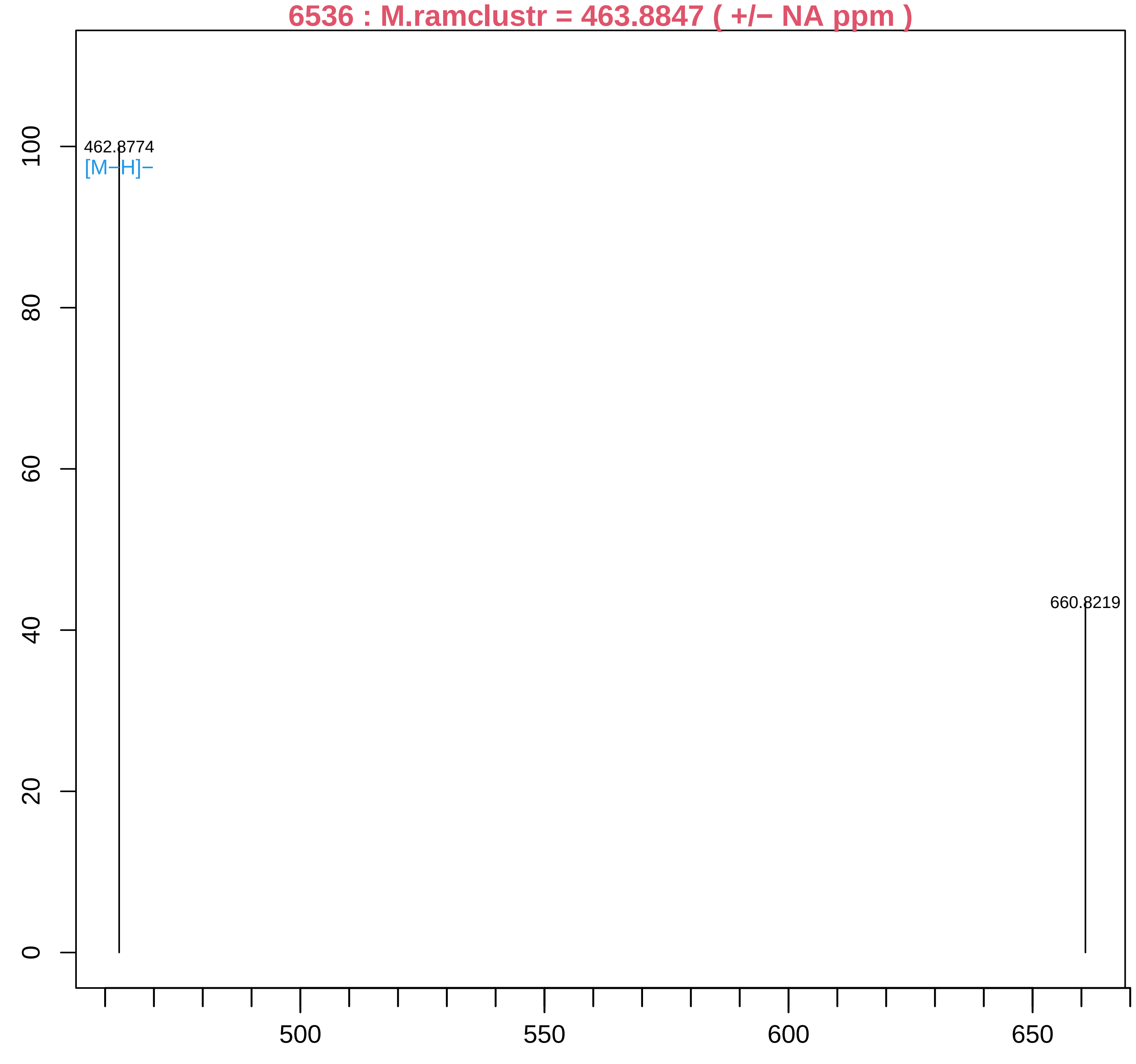
6536 : M.ramclustr = 463.8847 (+/− NA ppm)
462.8774
[M−H]−
660.8219
0
20
40
60
80
100
500
550
600
650

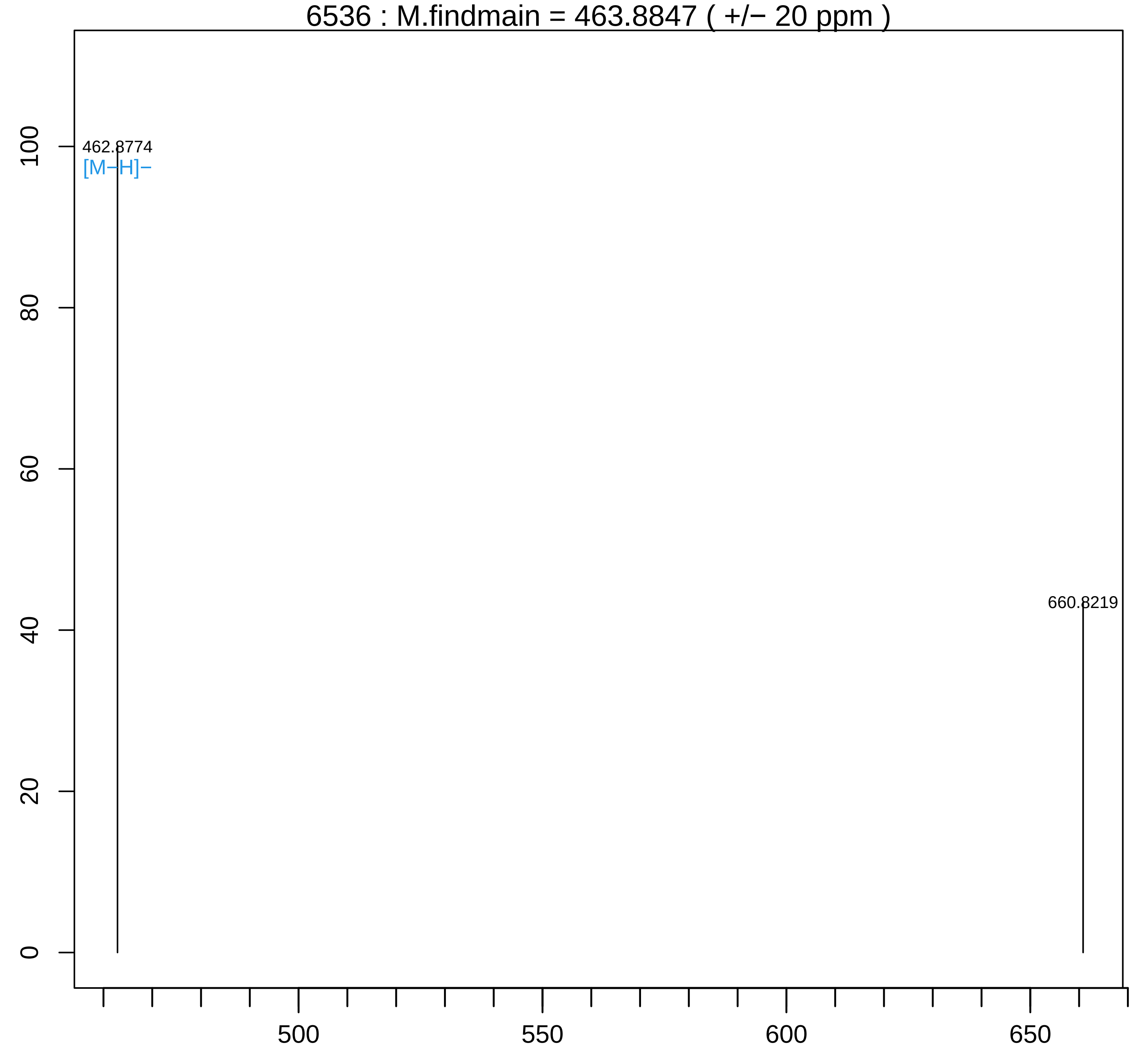
6536 : M.findmain = 463.8847 (+/− 20 ppm)
462.8774
[M−H]−
660.8219
0
20
40
60
80
100
500
550
600
650

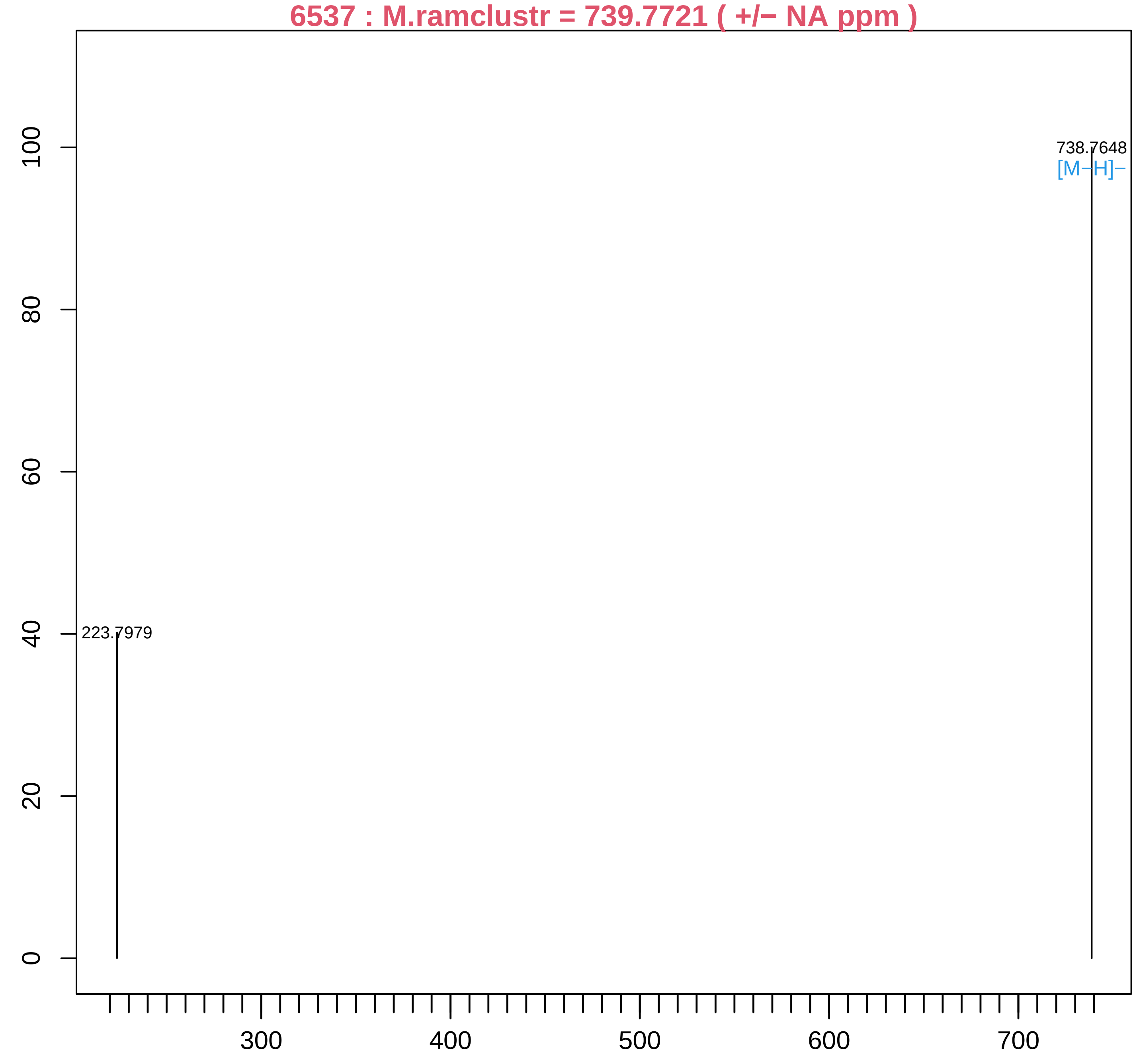
6537 : M.ramclustr = 739.7721 (+/− NA ppm)
738.7648
[M−H]−
223.7979
0
20
40
60
80
100
300
400
500
600
700

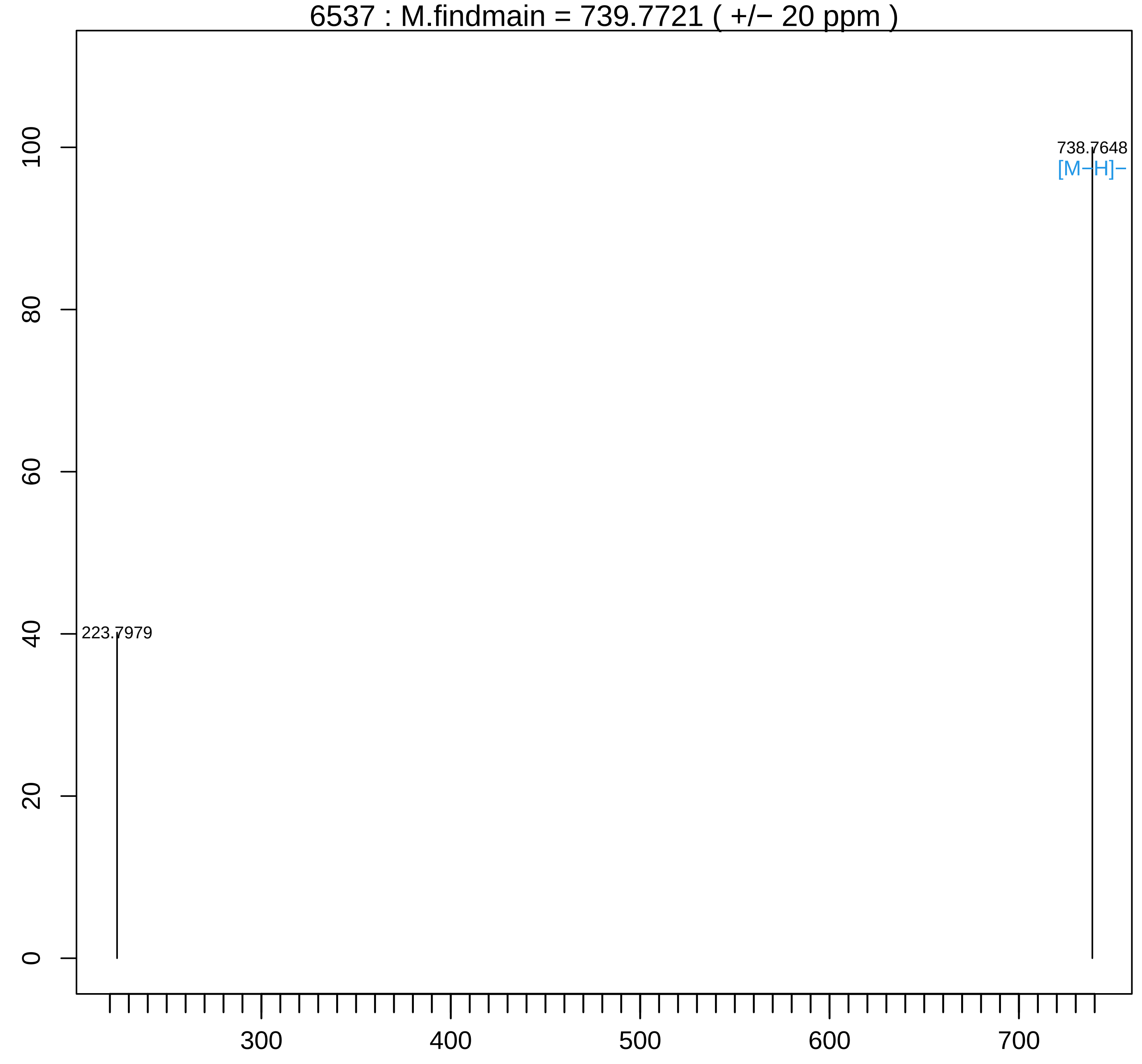
6537 : M.findmain = 739.7721 (+/− 20 ppm)
738.7648
[M−H]−
223.7979
0
20
40
60
80
100
300
400
500
600
700

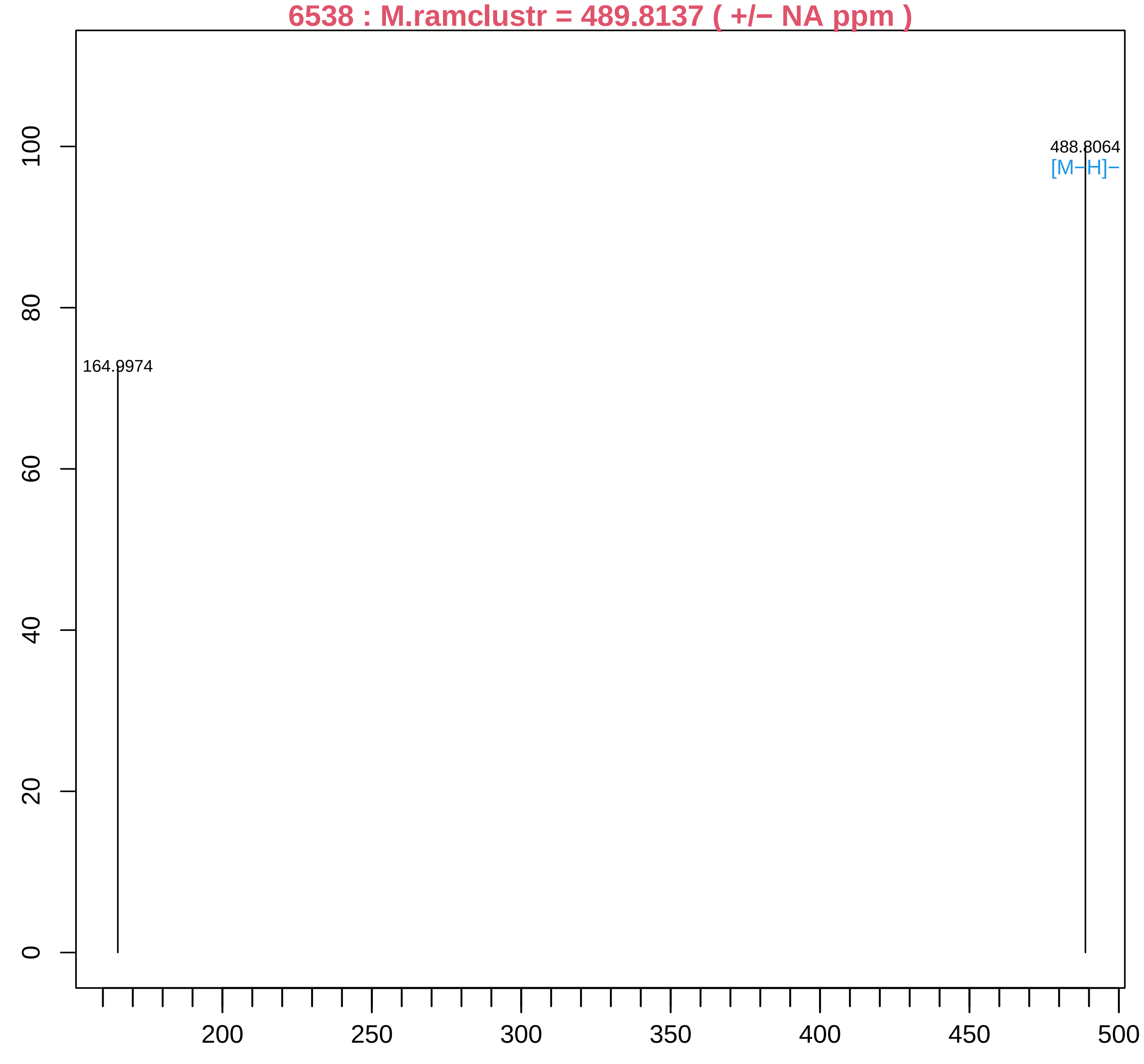
6538 : M.ramclustr = 489.8137 (+/− NA ppm)
488.8064
[M−H]−
164.9974
0
20
40
60
80
100
200
250
300
350
400
450
500

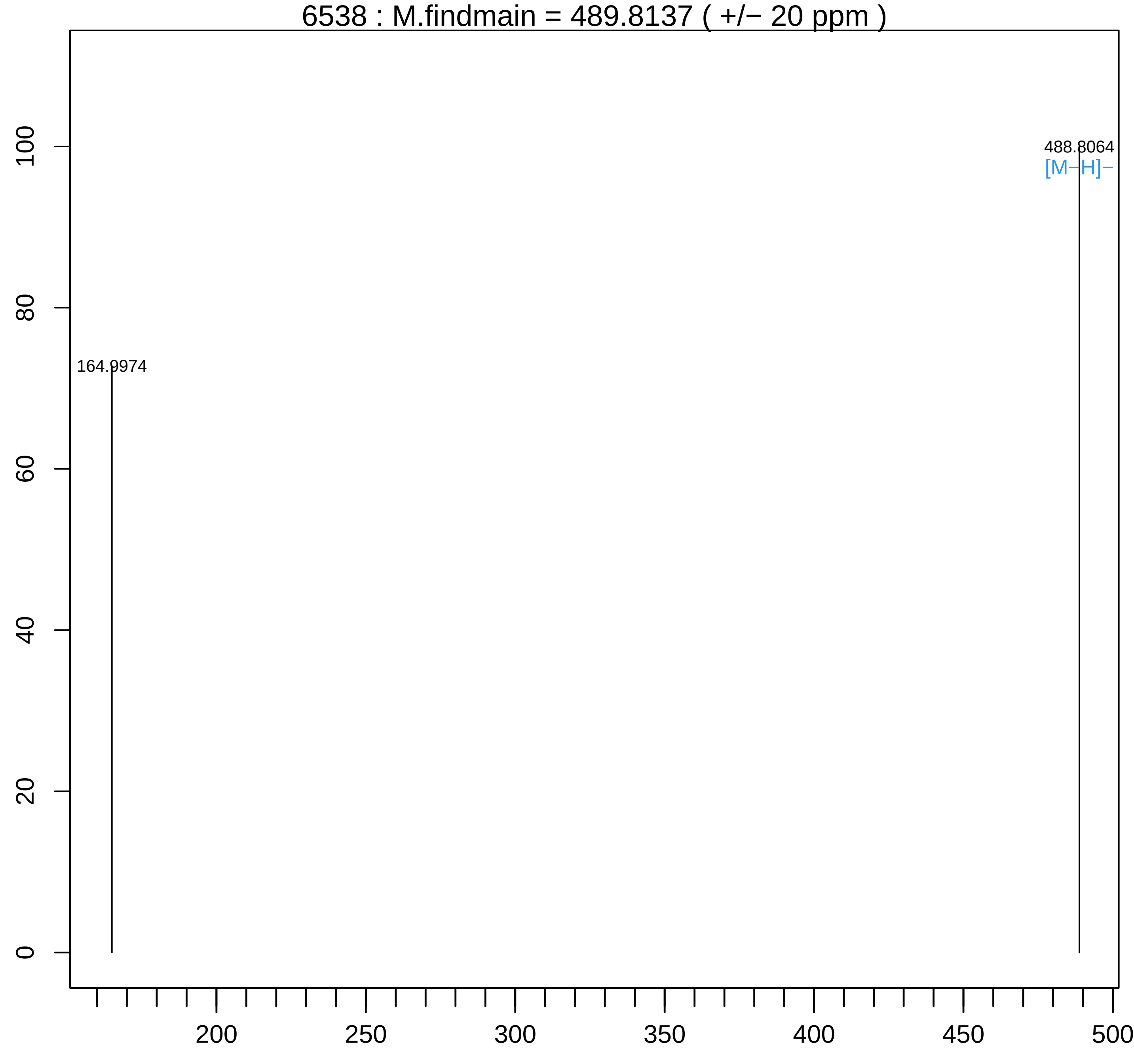
6538 : M.findmain = 489.8137 (+/− 20 ppm)
488.8064
[M−H]−
164.9974
0
20
40
60
80
100
200
250
300
350
400
450
500

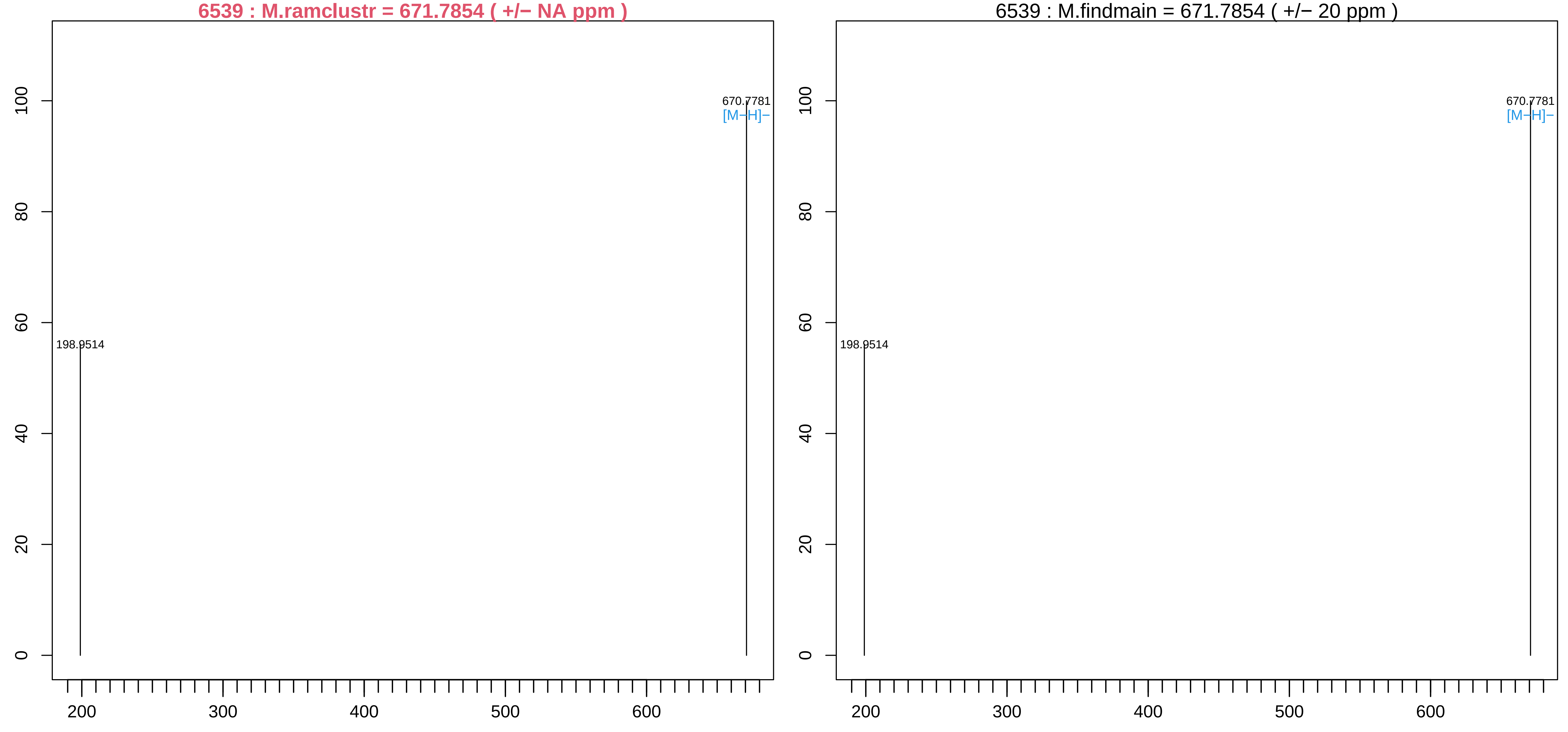
6539 : M.ramclustr = 671.7854 (+/− NA ppm)
670.7781
[M−H]−
198.9514
0
20
40
60
80
100
200
300
400
500
600
6539 : M.findmain = 671.7854 (+/− 20 ppm)
670.7781
[M−H]−
198.9514
0
20
40
60
80
100
200
300
400
500
600

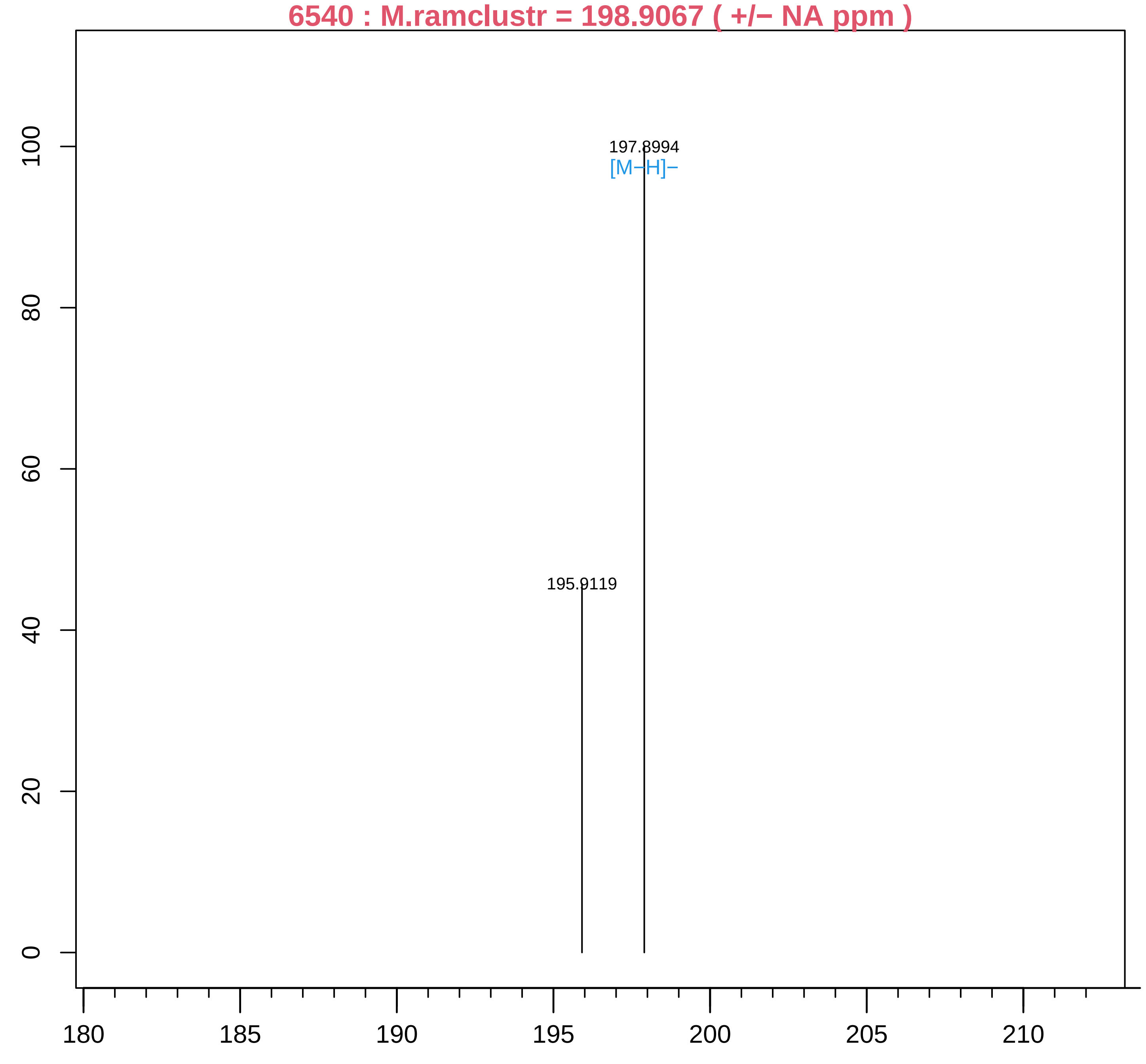
6540 : M.ramclustr = 198.9067 (+/− NA ppm)
197.8994
[M−H]−
195.9119

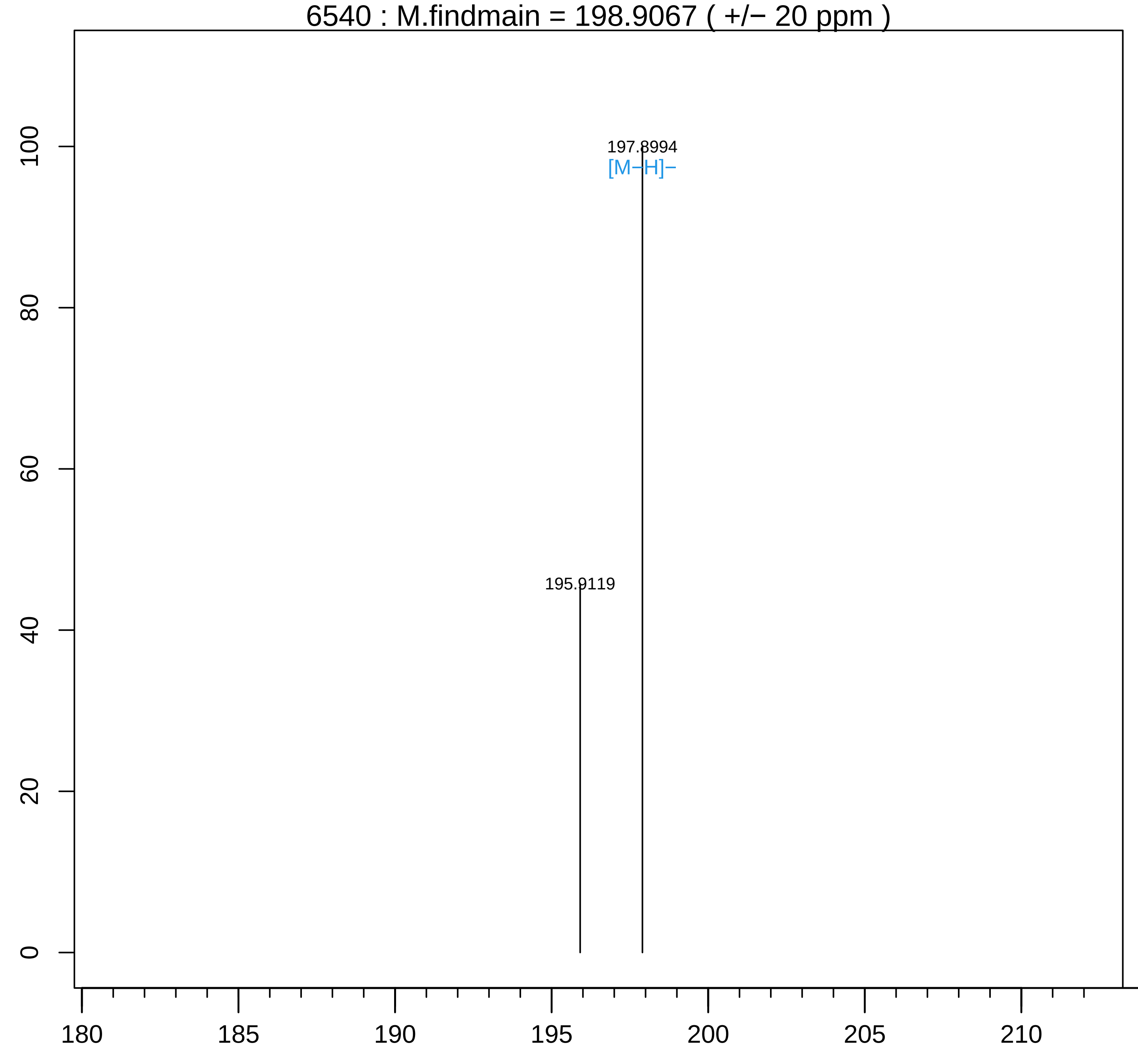
6540 : M.findmain = 198.9067 (+/− 20 ppm)
197.8994
[M−H]−
195.9119

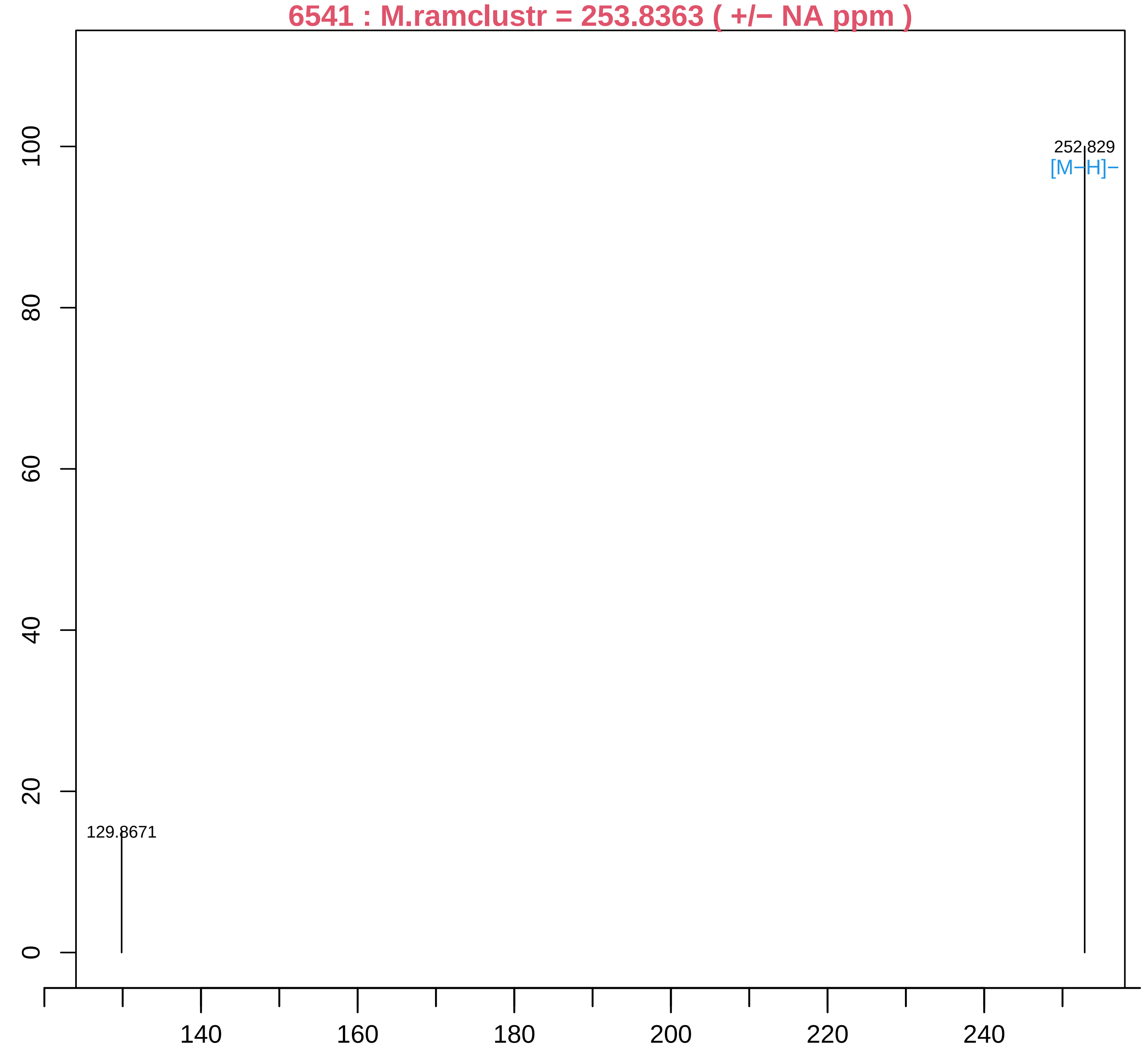

6541 : M.ramclustr = 253.8363 (+/− NA ppm)
252.829
[M−H]−
129.8671
0
20
40
60
80
100
140
160
180
200
220
240

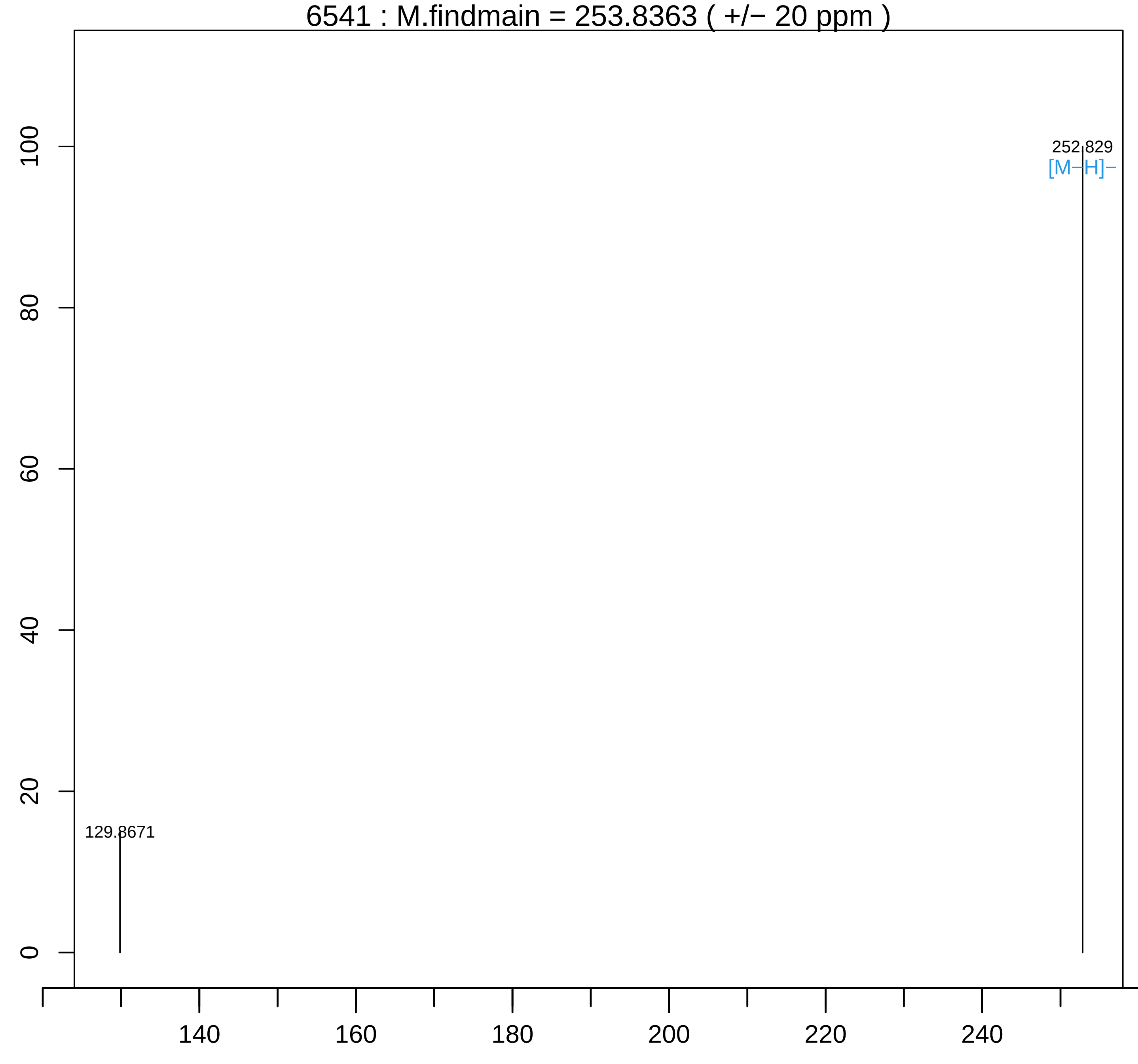

6541 : M.findmain = 253.8363 (+/− 20 ppm)
252.829
[M−H]−
129.8671
0
20
40
60
80
100
140
160
180
200
220
240

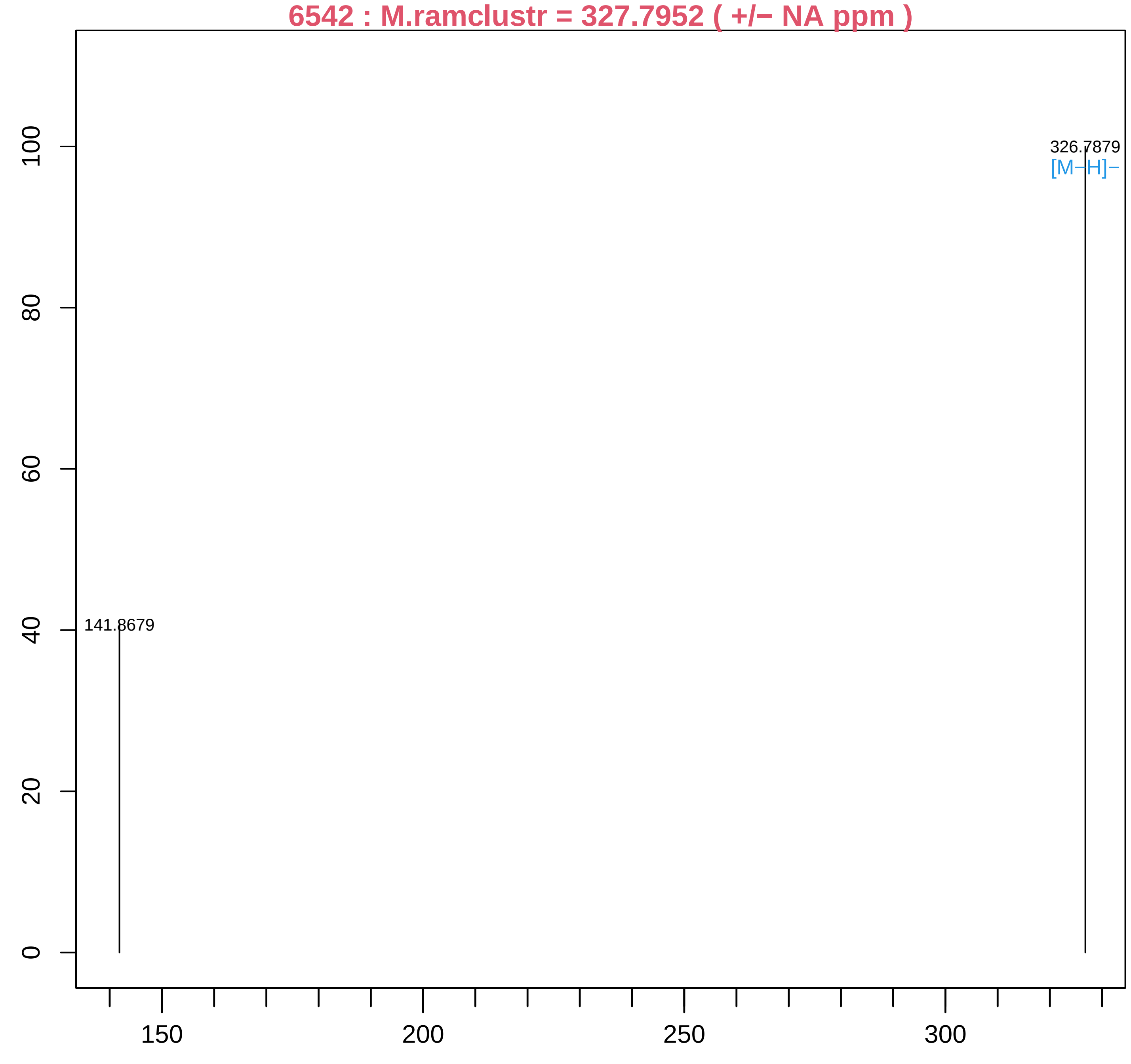
6542 : M.ramclustr = 327.7952 (+/− NA ppm)
326.7879
[M−H]−
141.8679
0
20
40
60
80
100
150
200
250
300

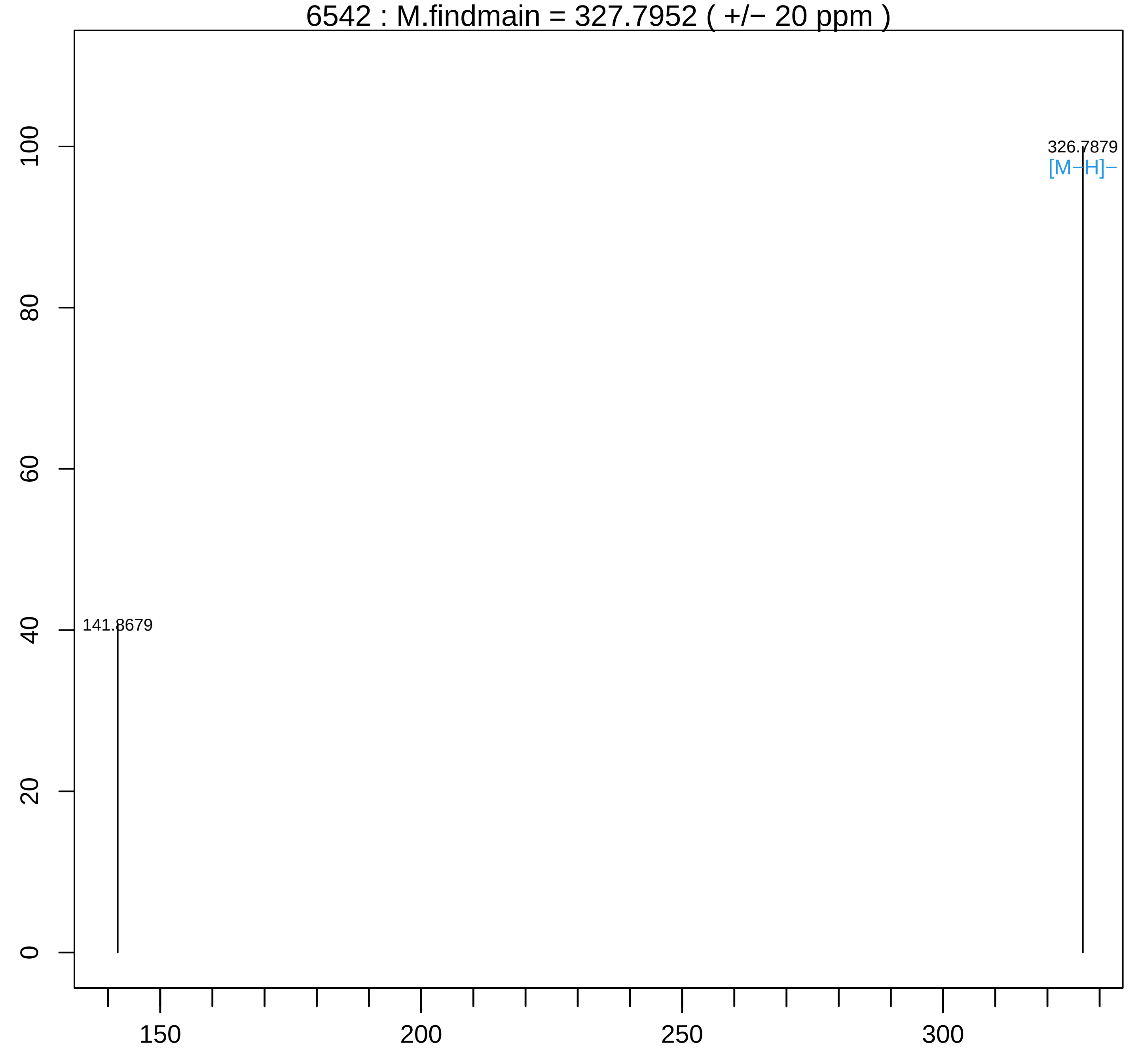
6542 : M.findmain = 327.7952 (+/− 20 ppm)
326.7879
[M−H]−
141.8679
0
20
40
60
80
100
150
200
250
300

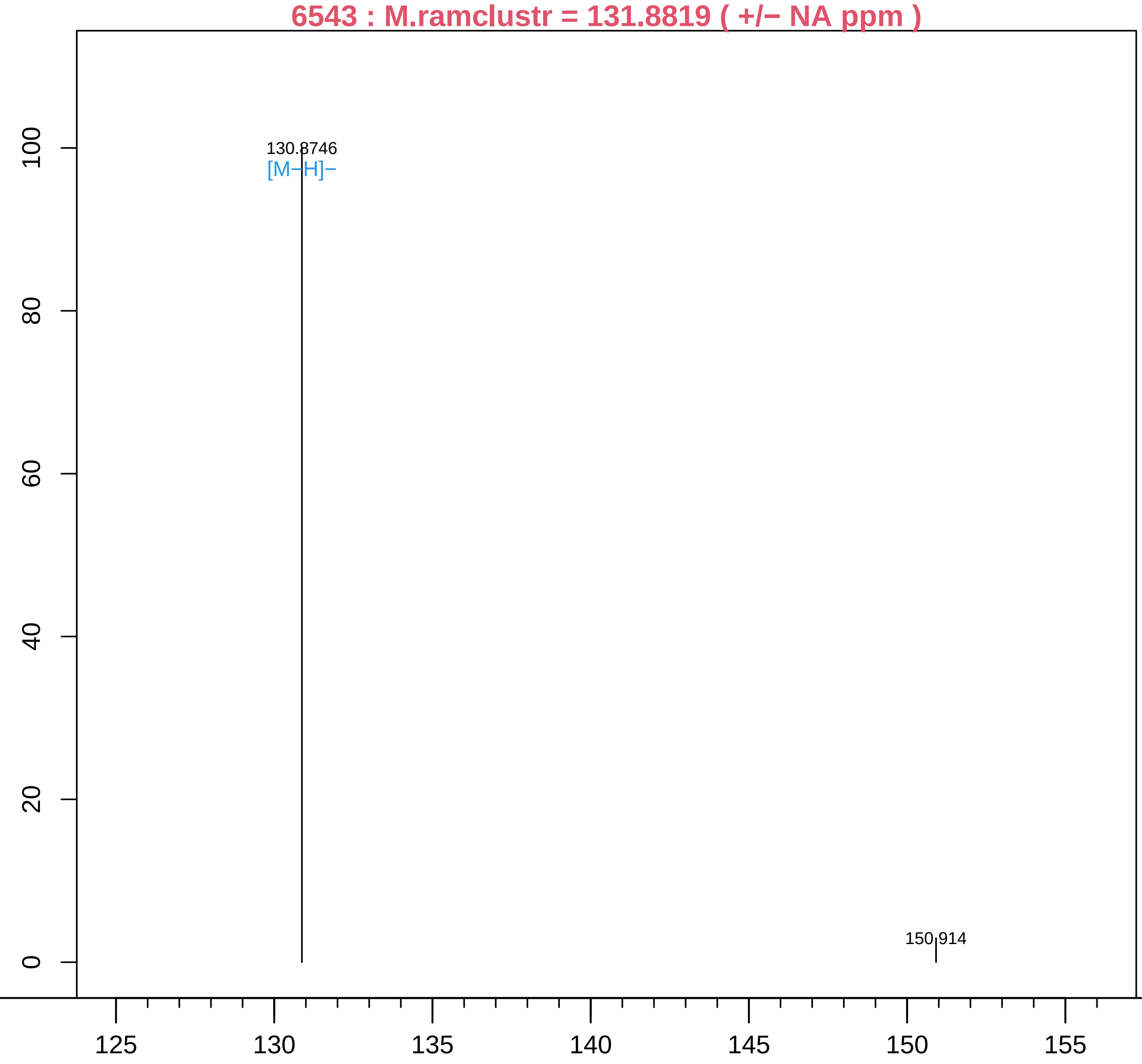

6543 : M.ramclustr = 131.8819 (+/− NA ppm)
130.8746
[M−H]−
150.914
0
20
40
60
80
100
125
130
135
140
145
150
155

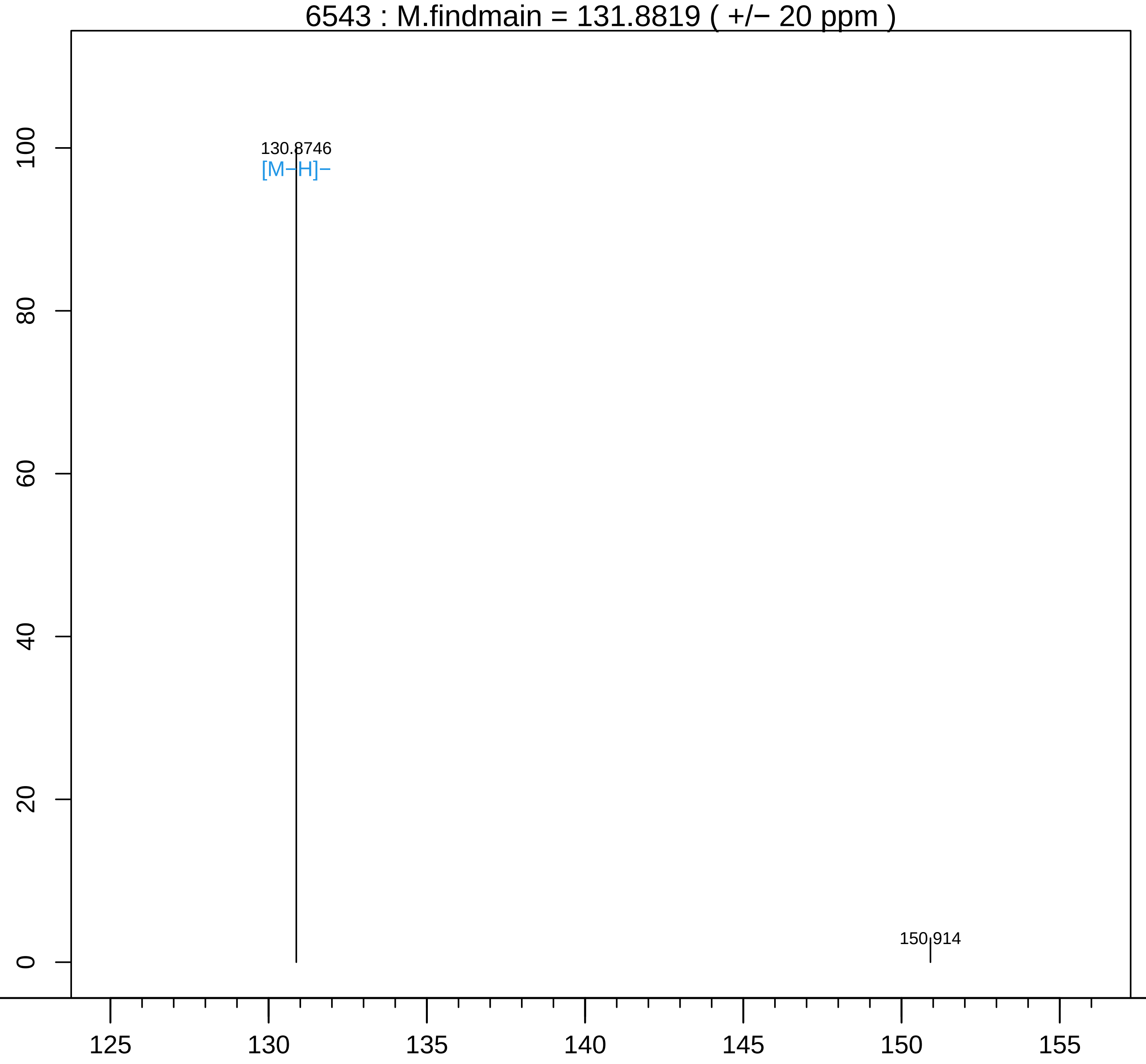

6543 : M.findmain = 131.8819 (+/− 20 ppm)
130.8746
[M−H]−
150.914
0
20
40
60
80
100
125
130
135
140
145
150
155

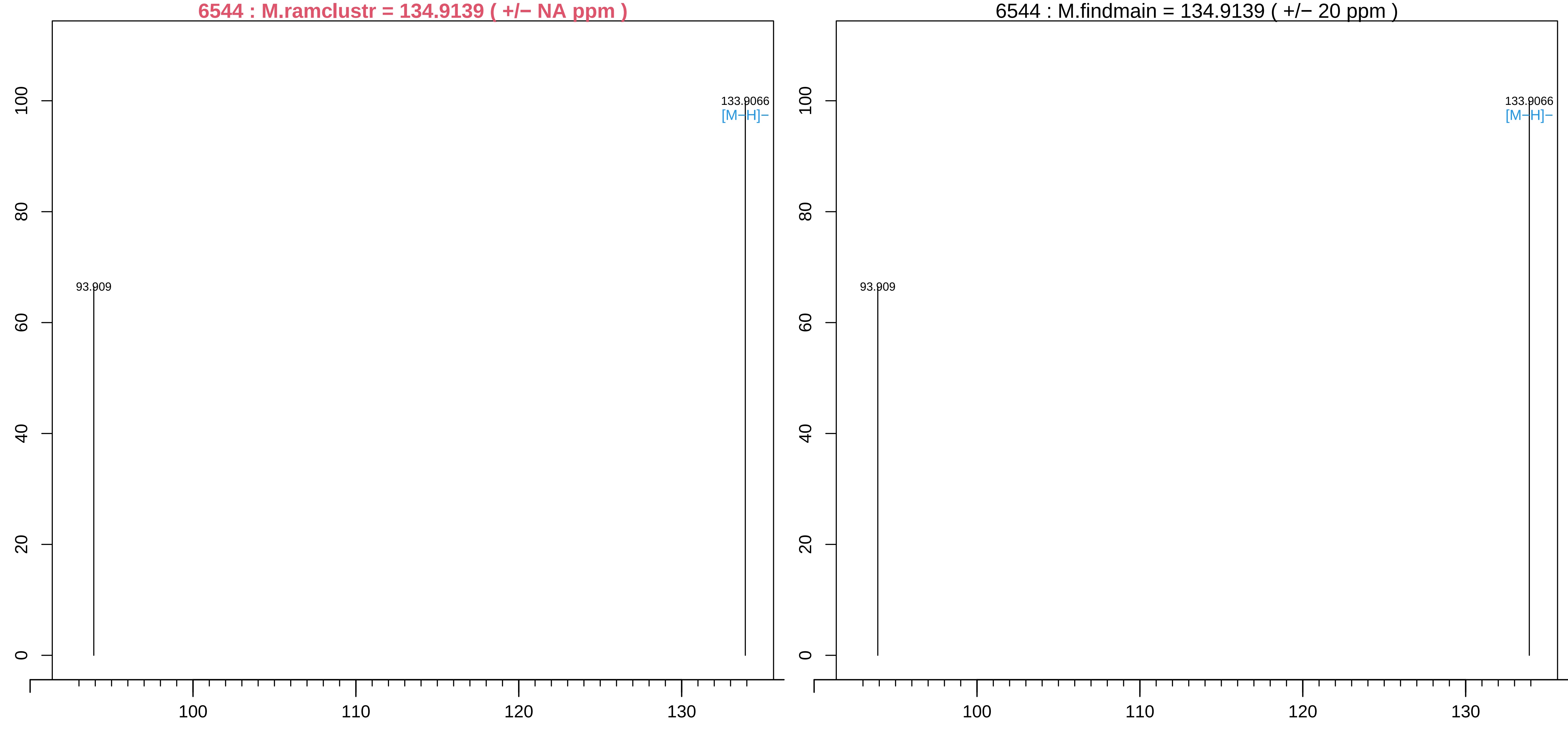

6544 : M.ramclustr = 134.9139 (+/− NA ppm)
133.9066
[M−H]−
93.909
6544 : M.findmain = 134.9139 (+/− 20 ppm)
133.9066
[M−H]−
93.909
0
20
40
60
80
100
100
110
120
130

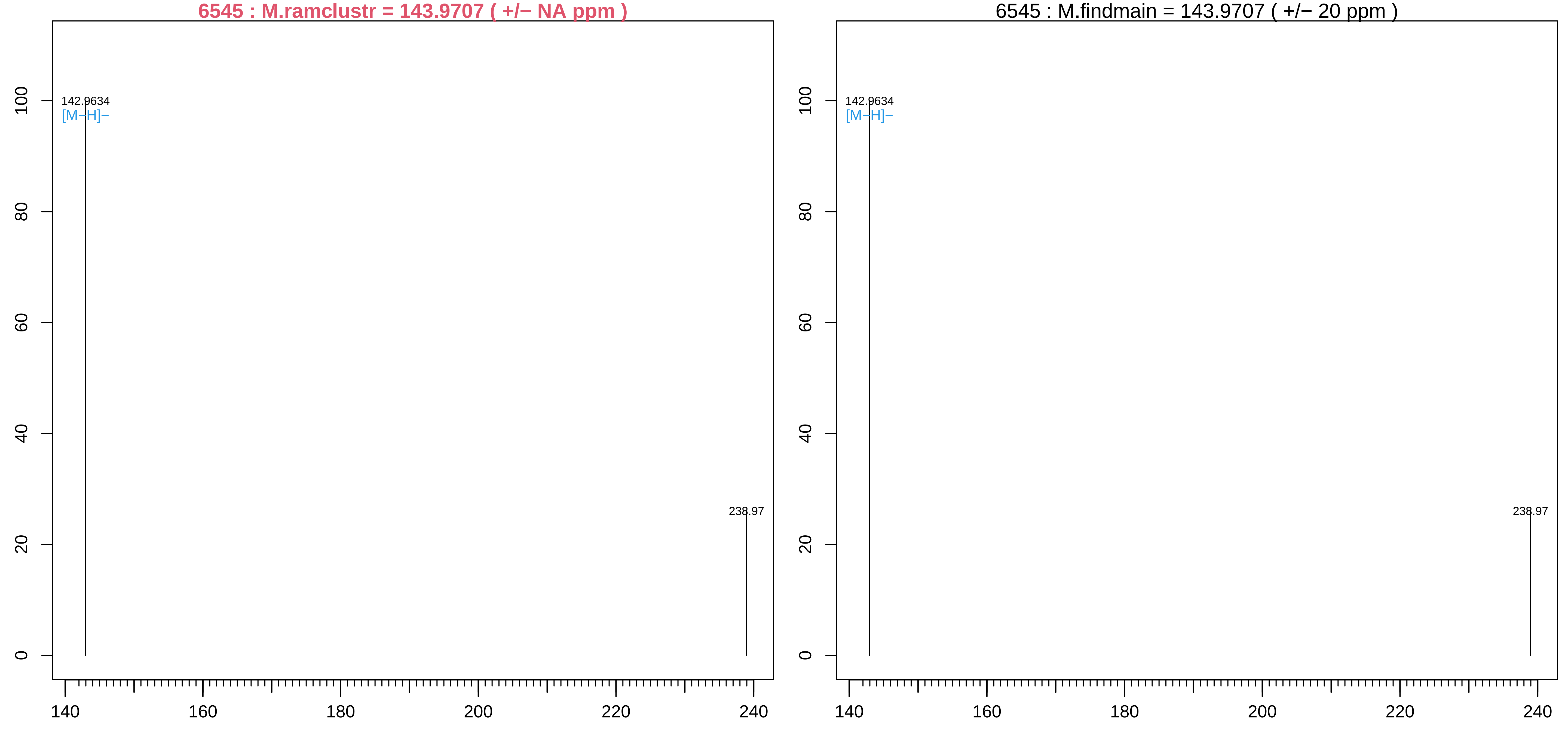

6545 : M.ramclustr = 143.9707 (+/− NA ppm)
142.9634
[M−H]−
238.97
6545 : M.findmain = 143.9707 (+/− 20 ppm)
142.9634
[M−H]−
238.97
0
20
40
60
80
100
140
160
180
200
220
240

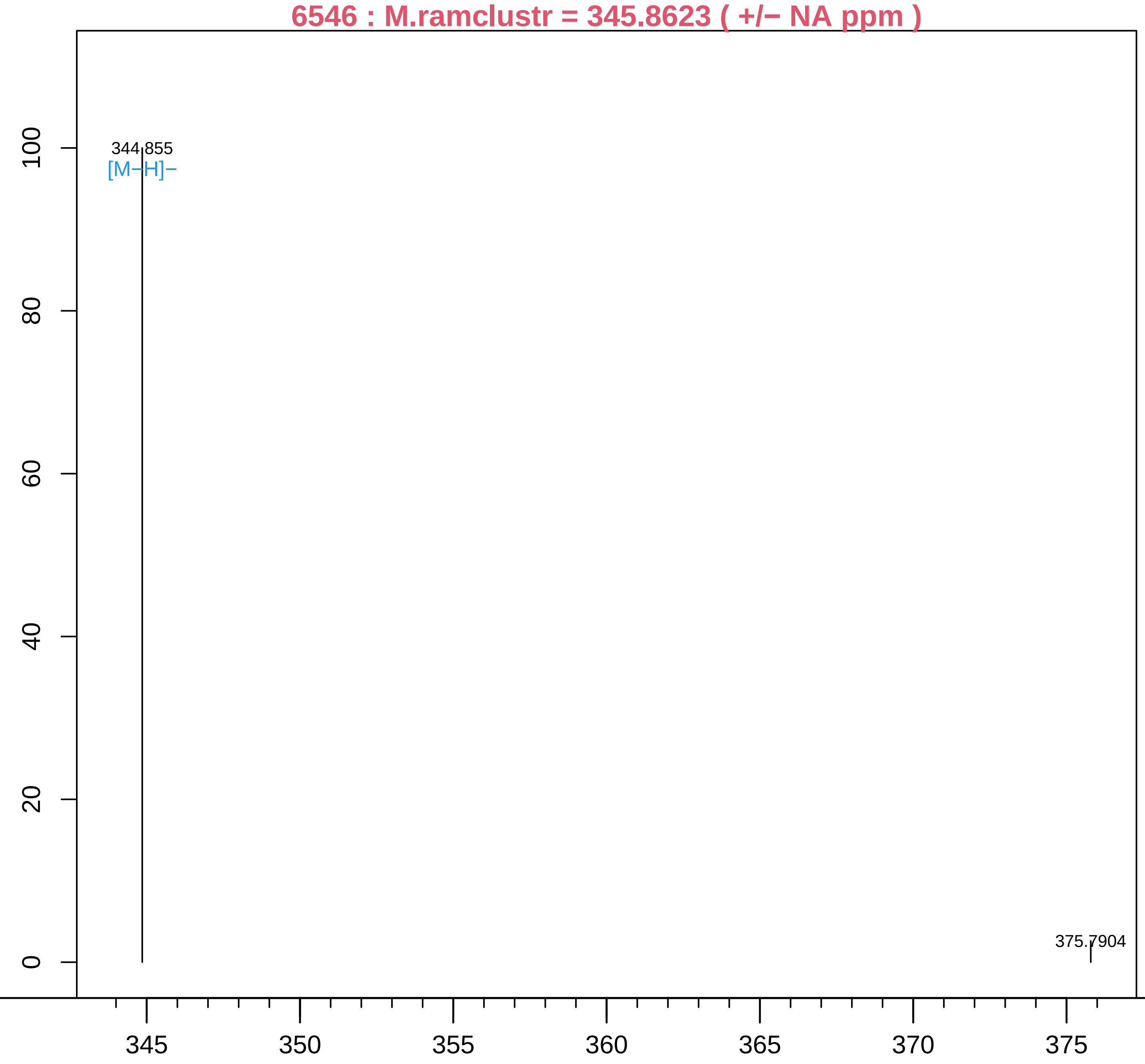

6546 : M.ramclustr = 345.8623 (+/− NA ppm)
344.855
[M−H]−
375.7904
0
20
40
60
80
100
345
350
355
360
365
370
375

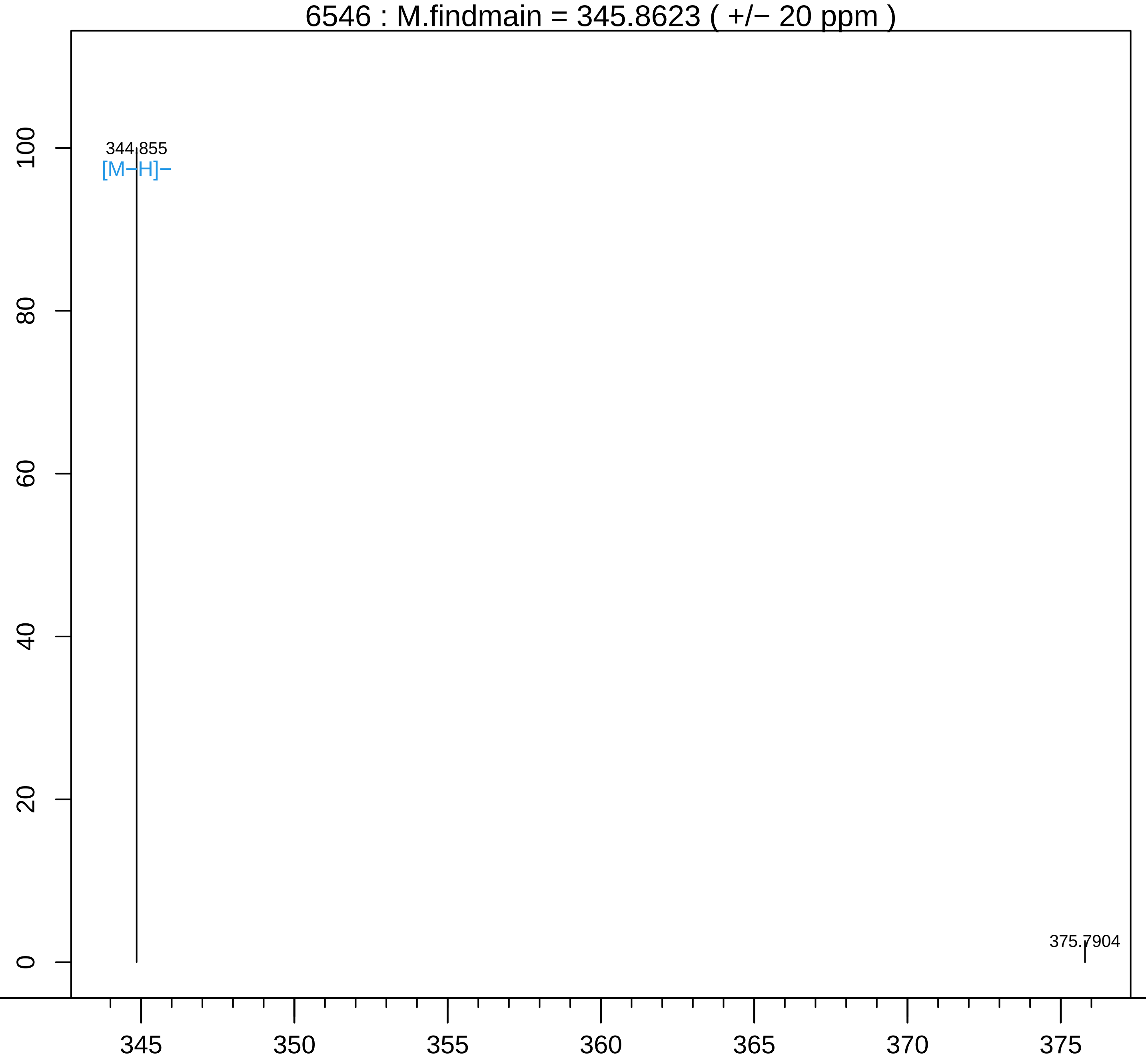

6546 : M.findmain = 345.8623 (+/− 20 ppm)
344.855
[M−H]−
375.7904
0
20
40
60
80
100
345
350
355
360
365
370
375

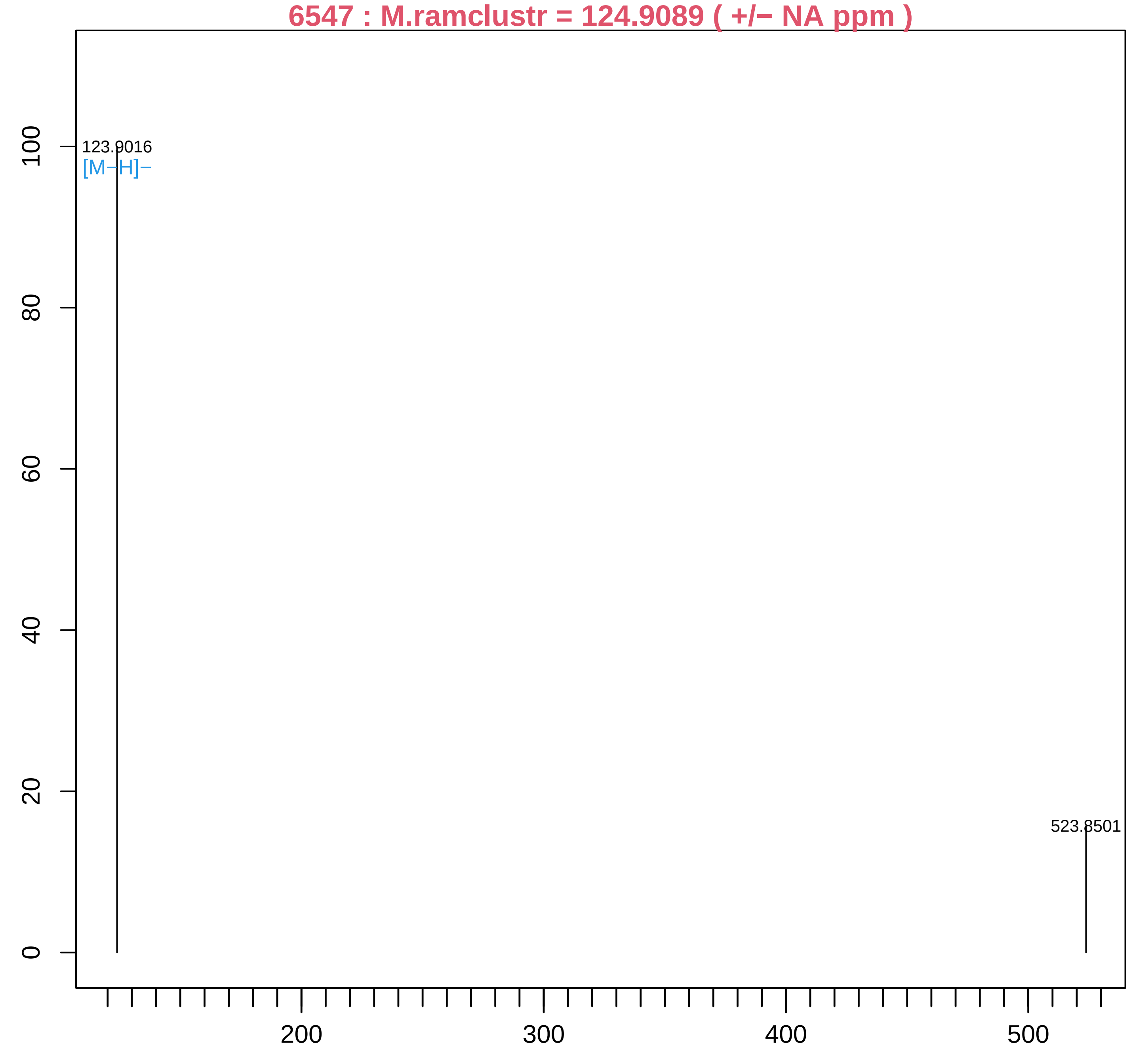
6547 : M.ramclustr = 124.9089 (+/− NA ppm)
123.9016
[M−H]−
523.8501
0
20
40
60
80
100
200
300
400
500

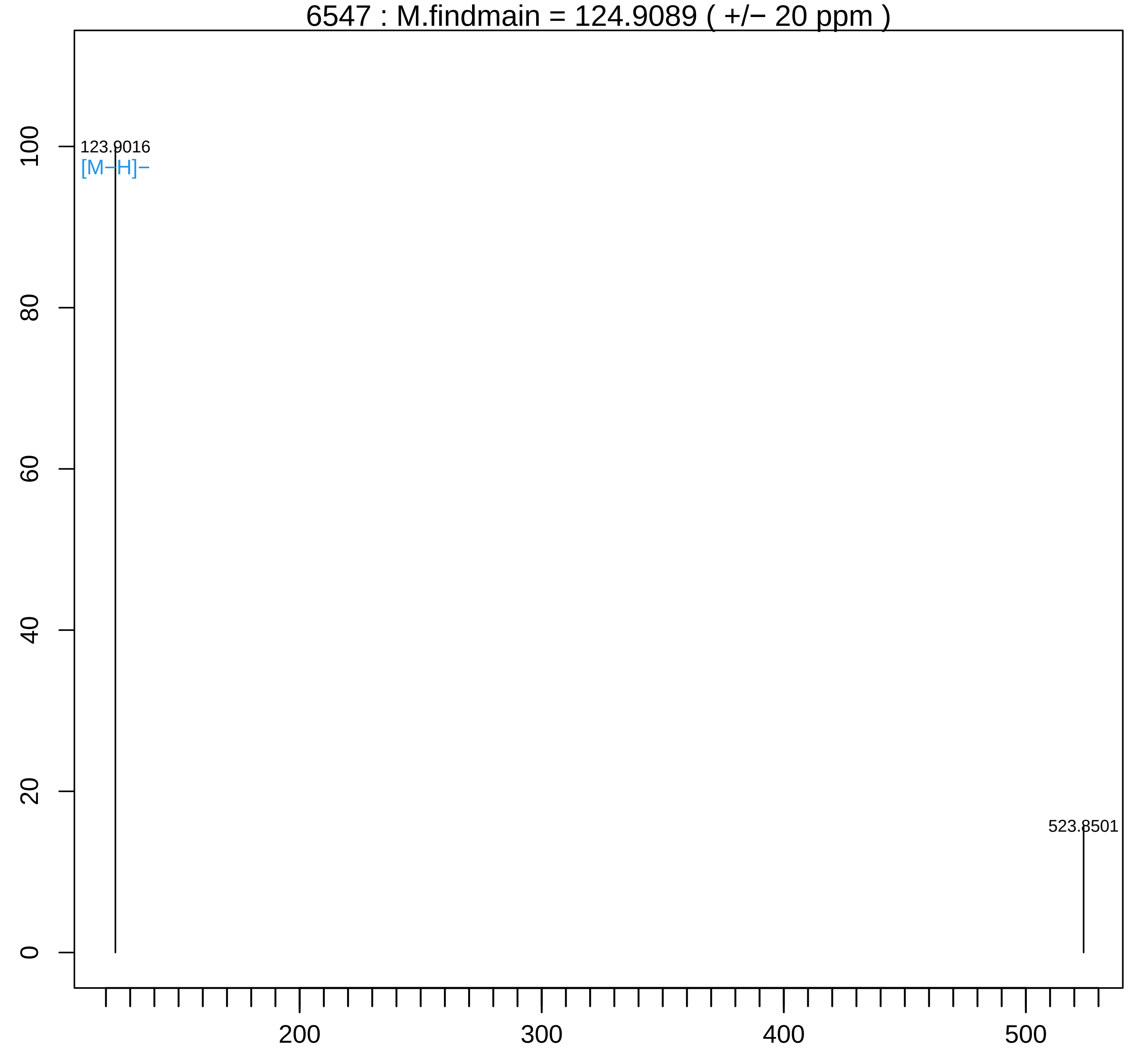
6547 : M.findmain = 124.9089 (+/− 20 ppm)
123.9016
[M−H]−
523.8501
0
20
40
60
80
100
200
300
400
500

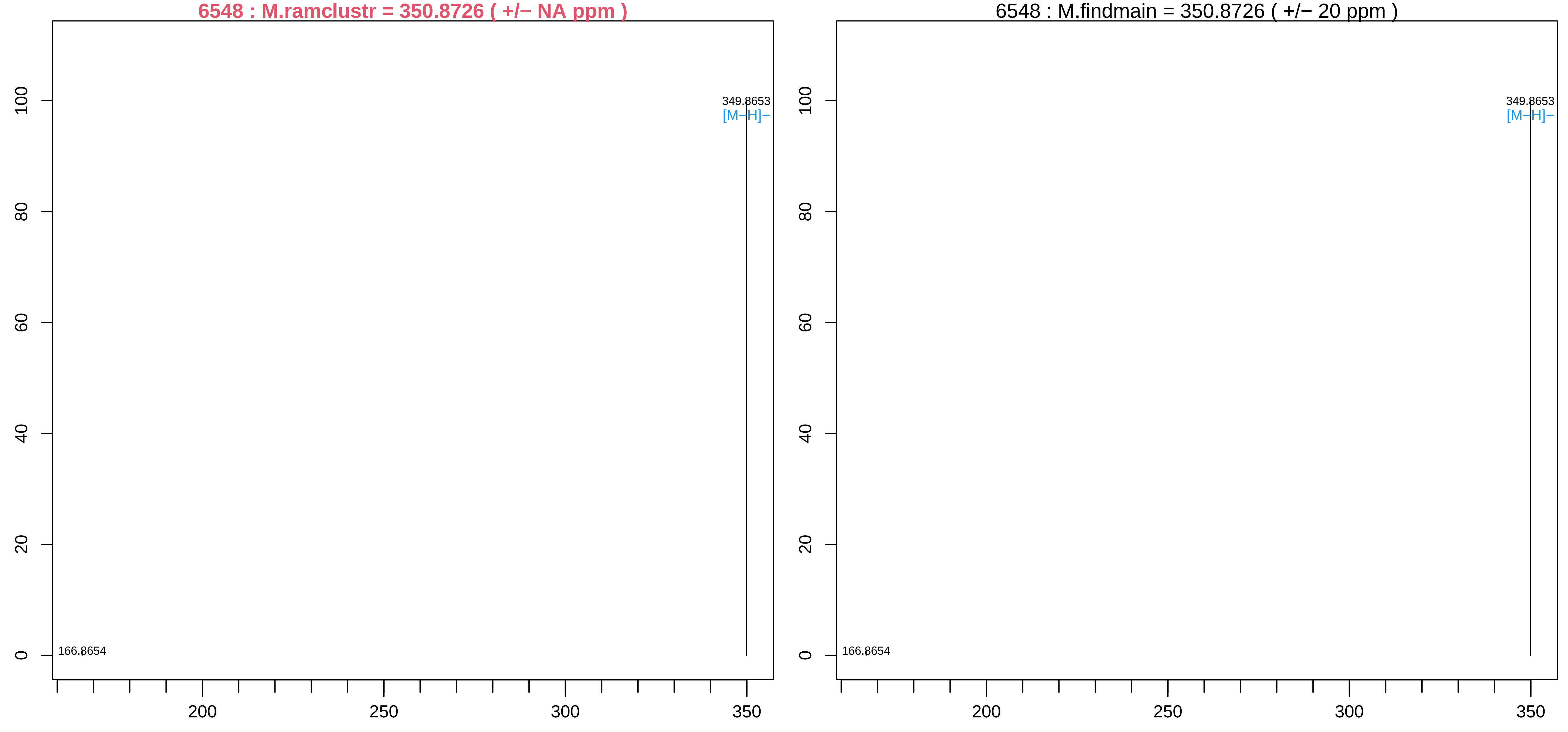
6548 : M.ramclustr = 350.8726 (+/− NA ppm)
349.8653
[M−H]−
166.8654
0
20
40
60
80
100
200
250
300
350
6548 : M.findmain = 350.8726 (+/− 20 ppm)
349.8653
[M−H]−
166.8654
0
20
40
60
80
100
200
250
300
350

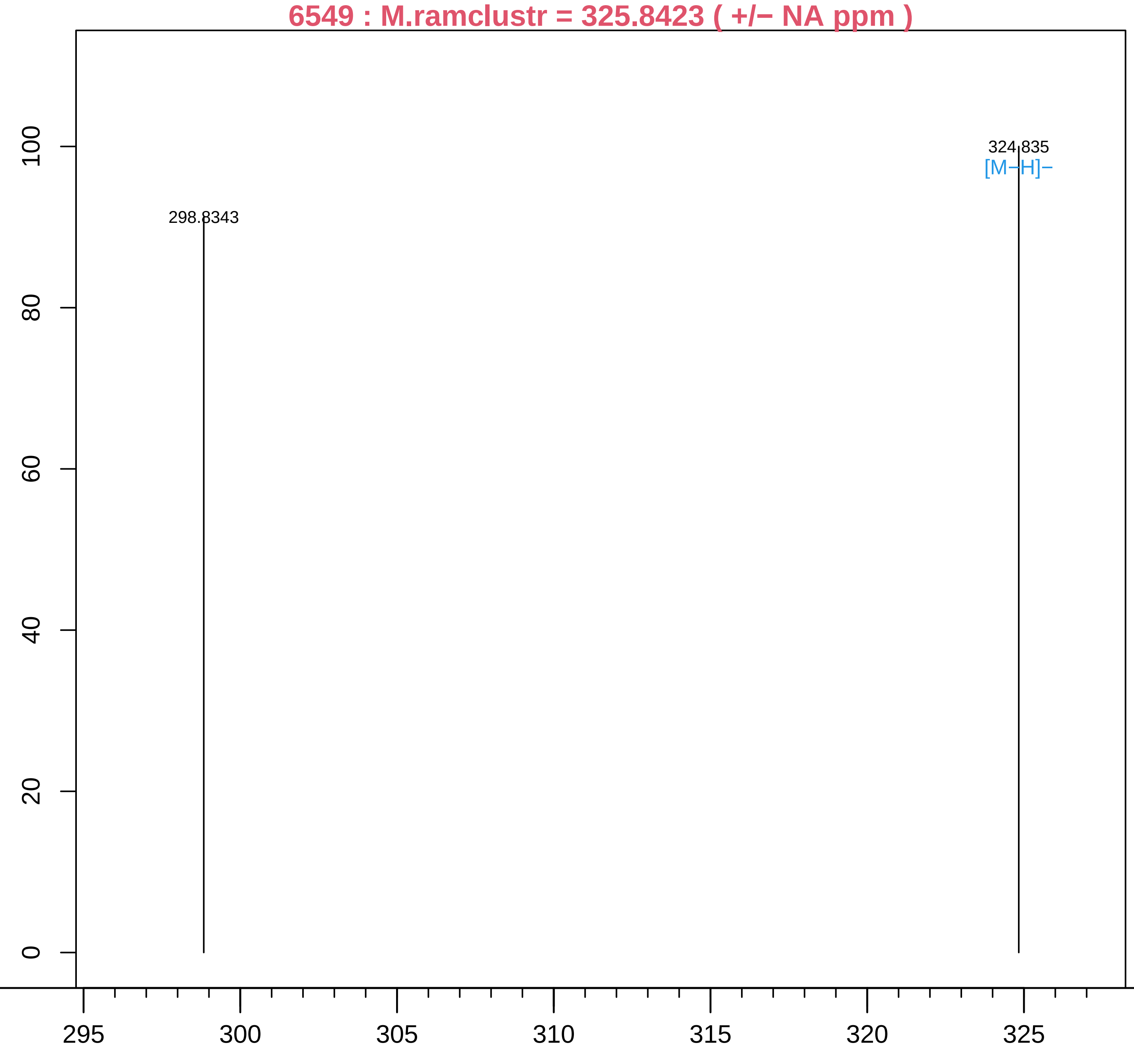
6549 : M.ramclustr = 325.8423 (+/− NA ppm)
298.8343
324.835
[M−H]−
0
20
40
60
80
100
295
300
305
310
315
320
325

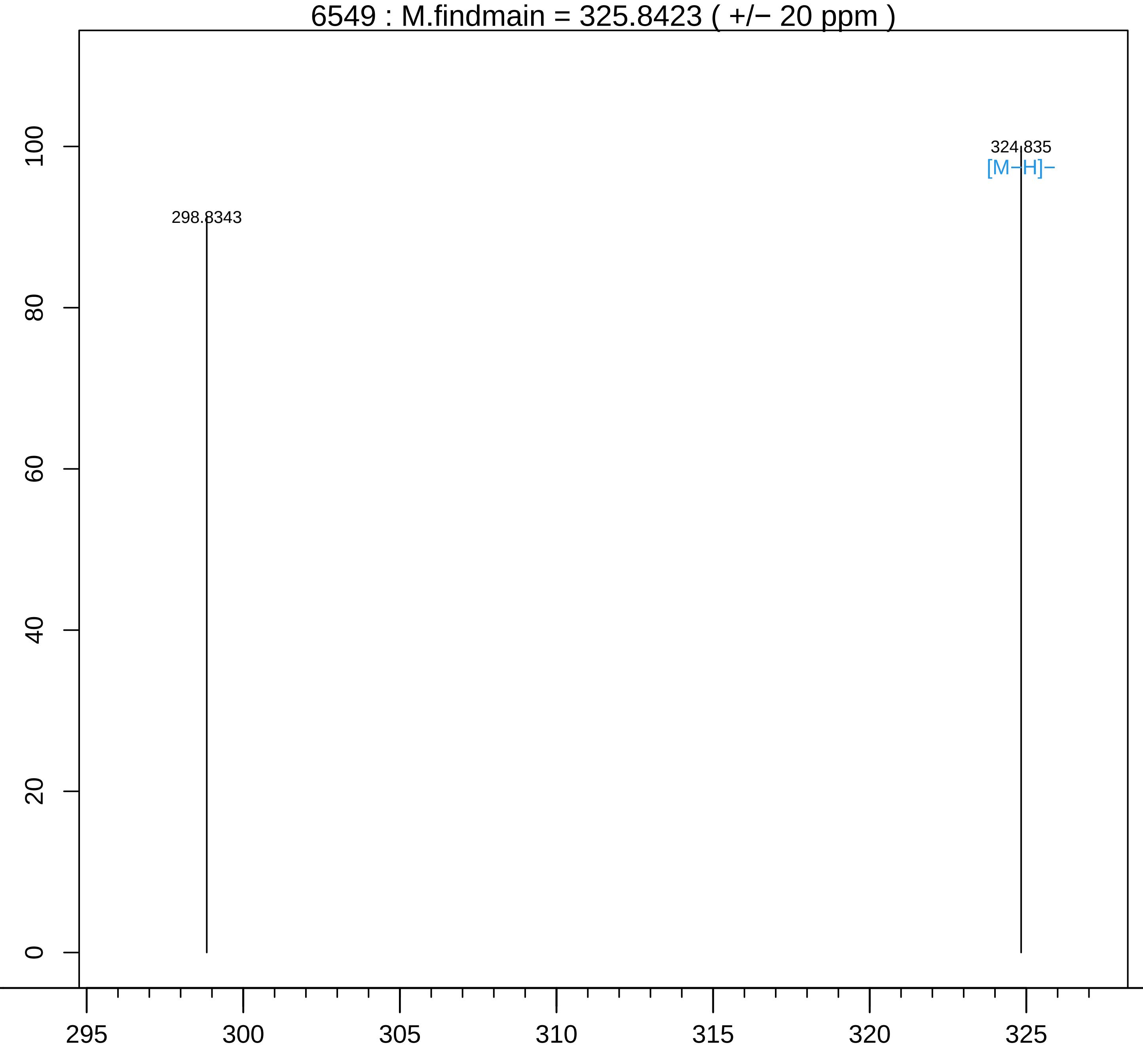
6549 : M.findmain = 325.8423 (+/− 20 ppm)
298.8343
324.835
[M−H]−
0
20
40
60
80
100
295
300
305
310
315
320
325

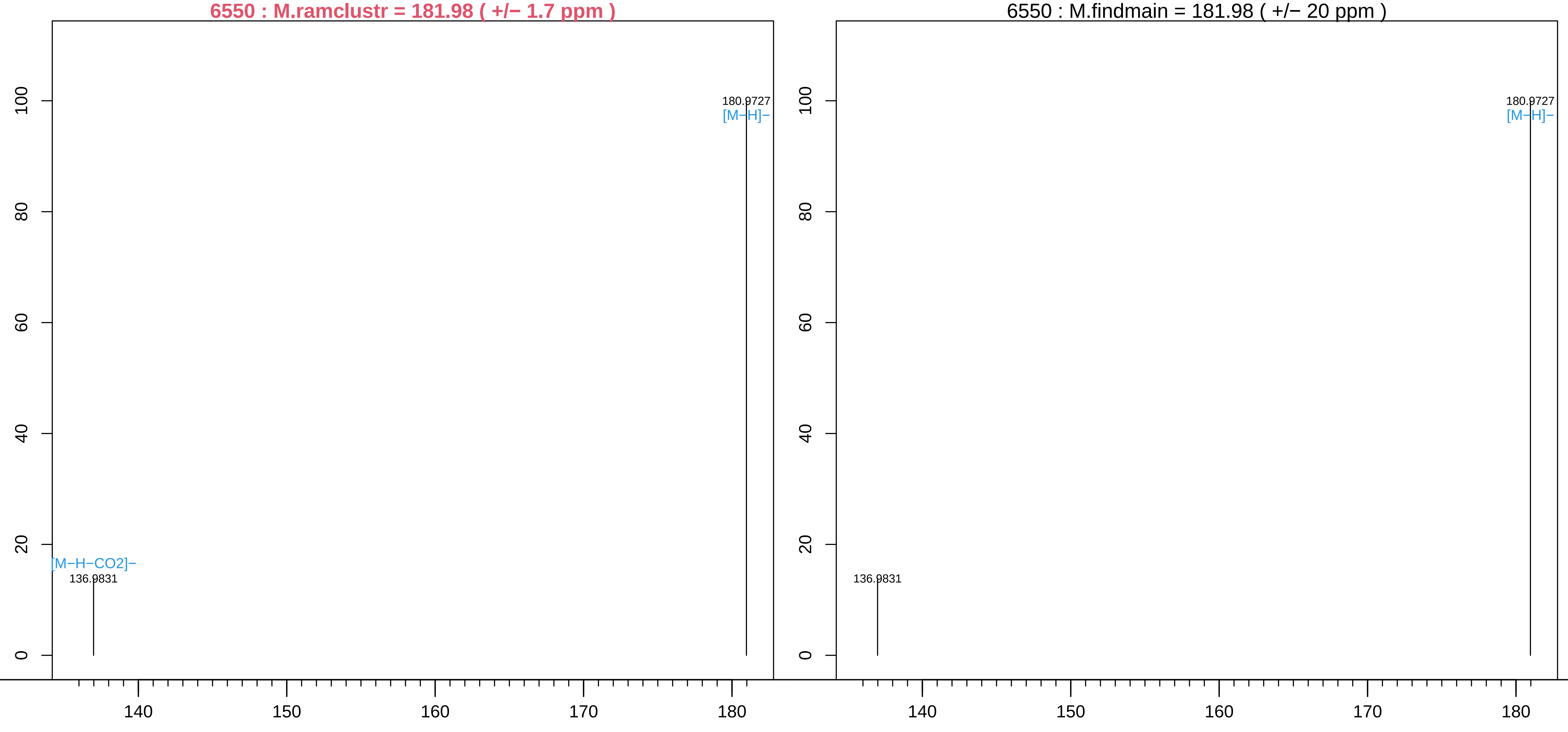

6550 : M.ramclustr = 181.98 (+/− 1.7 ppm)
180.9727
[M−H]−
[M−H−CO2]−
136.9831
0
20
40
60
80
100
140
150
160
170
180
6550 : M.findmain = 181.98 (+/− 20 ppm)
180.9727
[M−H]−
136.9831
0
20
40
60
80
100
140
150
160
170
180

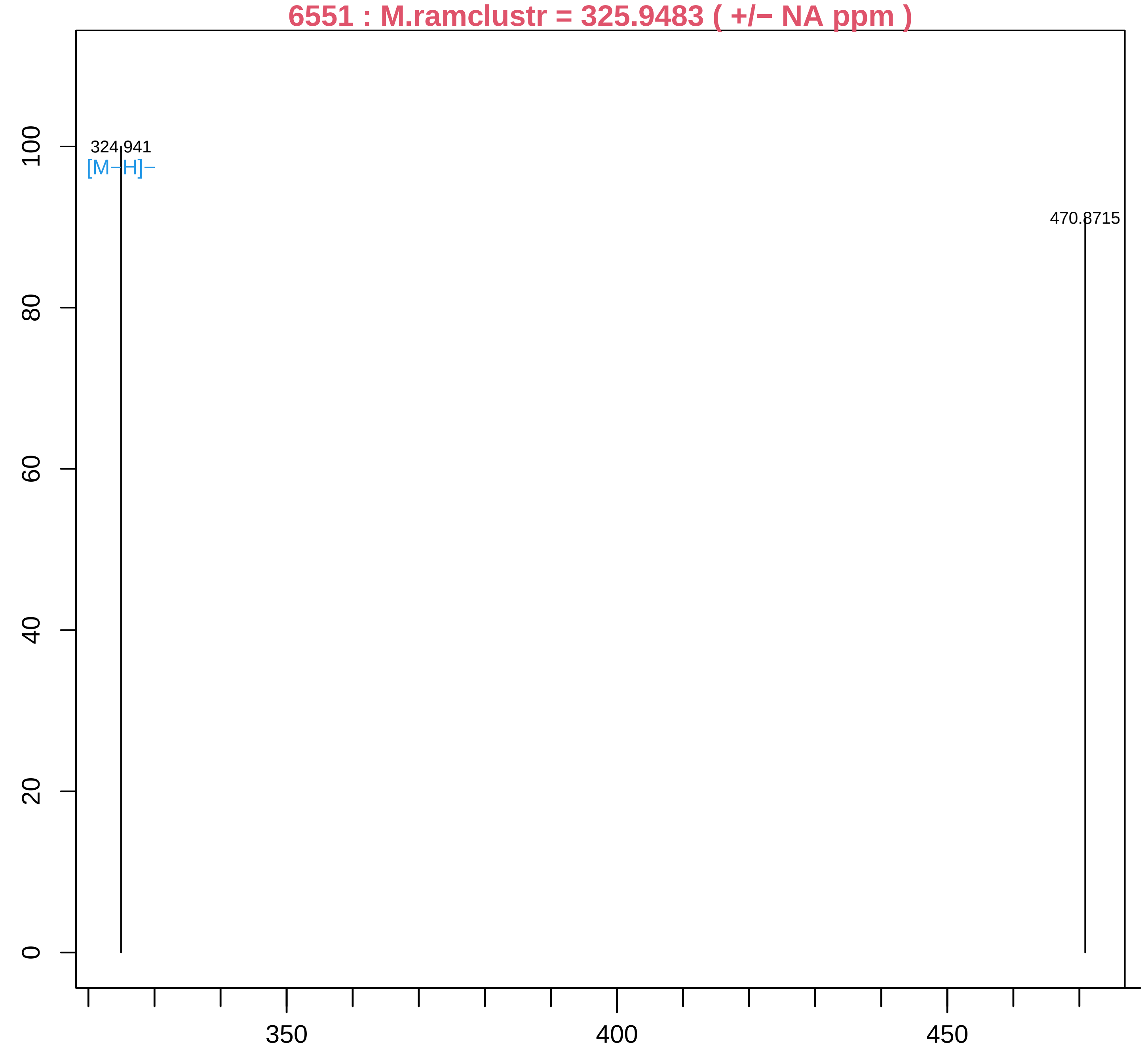
6551 : M.ramclustr = 325.9483 (+/− NA ppm)
324.941
[M−H]−
470.8715
0
20
40
60
80
100
350
400
450

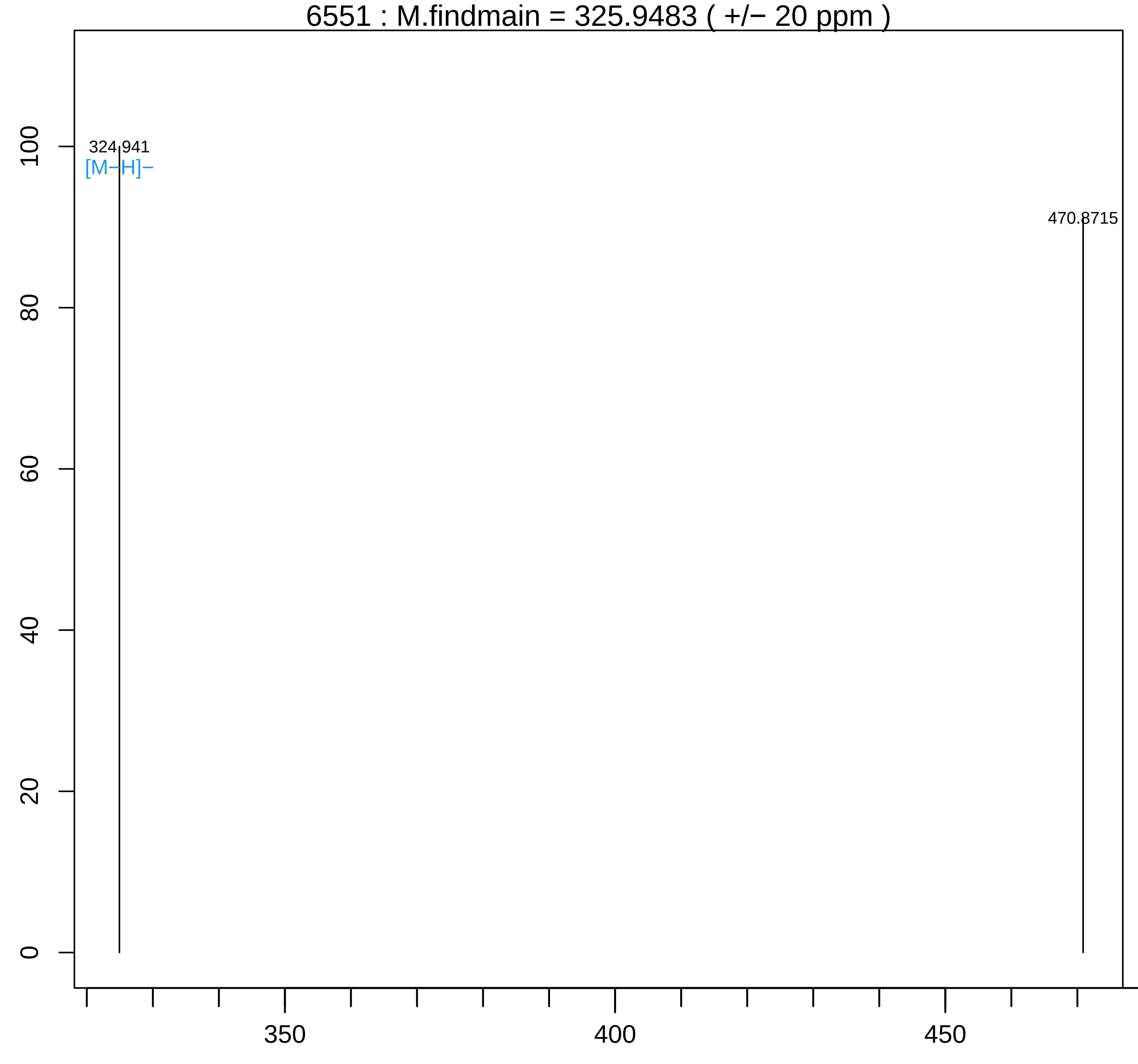
6551 : M.findmain = 325.9483 (+/− 20 ppm)
324.941
[M−H]−
470.8715
0
20
40
60
80
100
350
400
450

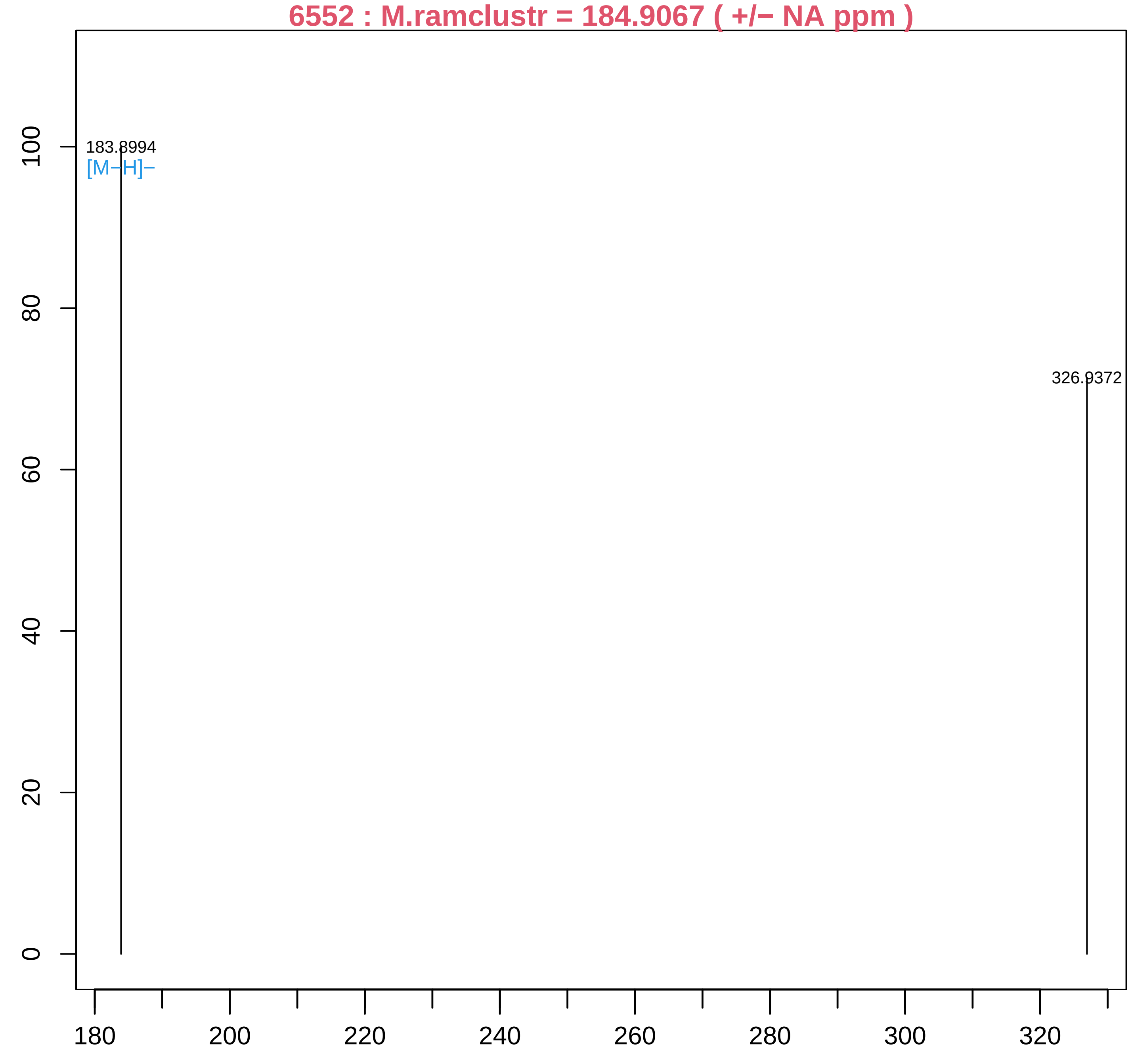
6552 : M.ramclustr = 184.9067 (+/− NA ppm)
183.8994
[M−H]−
326.9372
0
20
40
60
80
100
180
200
220
240
260
280
300
320

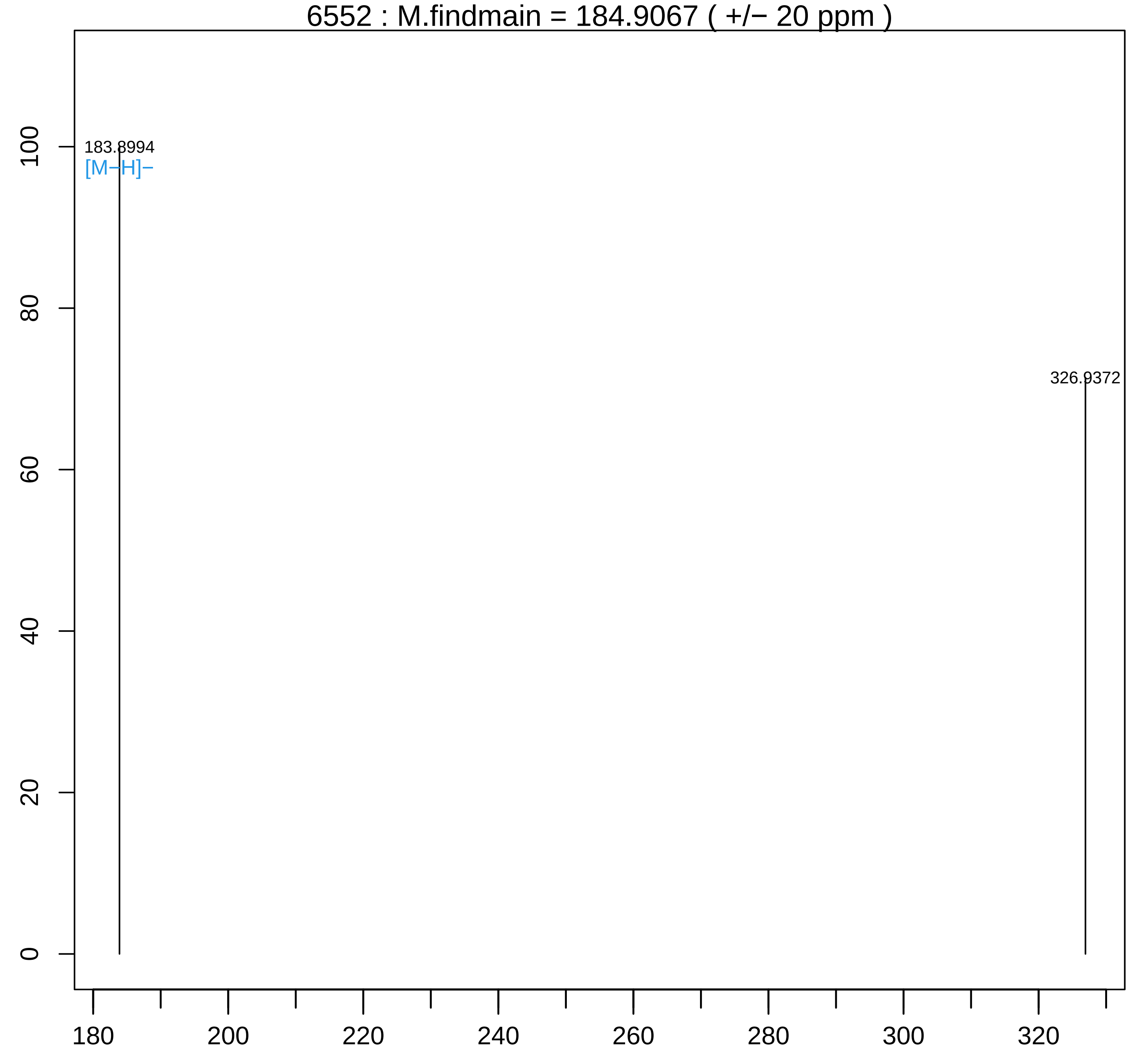
6552 : M.findmain = 184.9067 (+/− 20 ppm)
183.8994
[M−H]−
326.9372
0
20
40
60
80
100
180
200
220
240
260
280
300
320

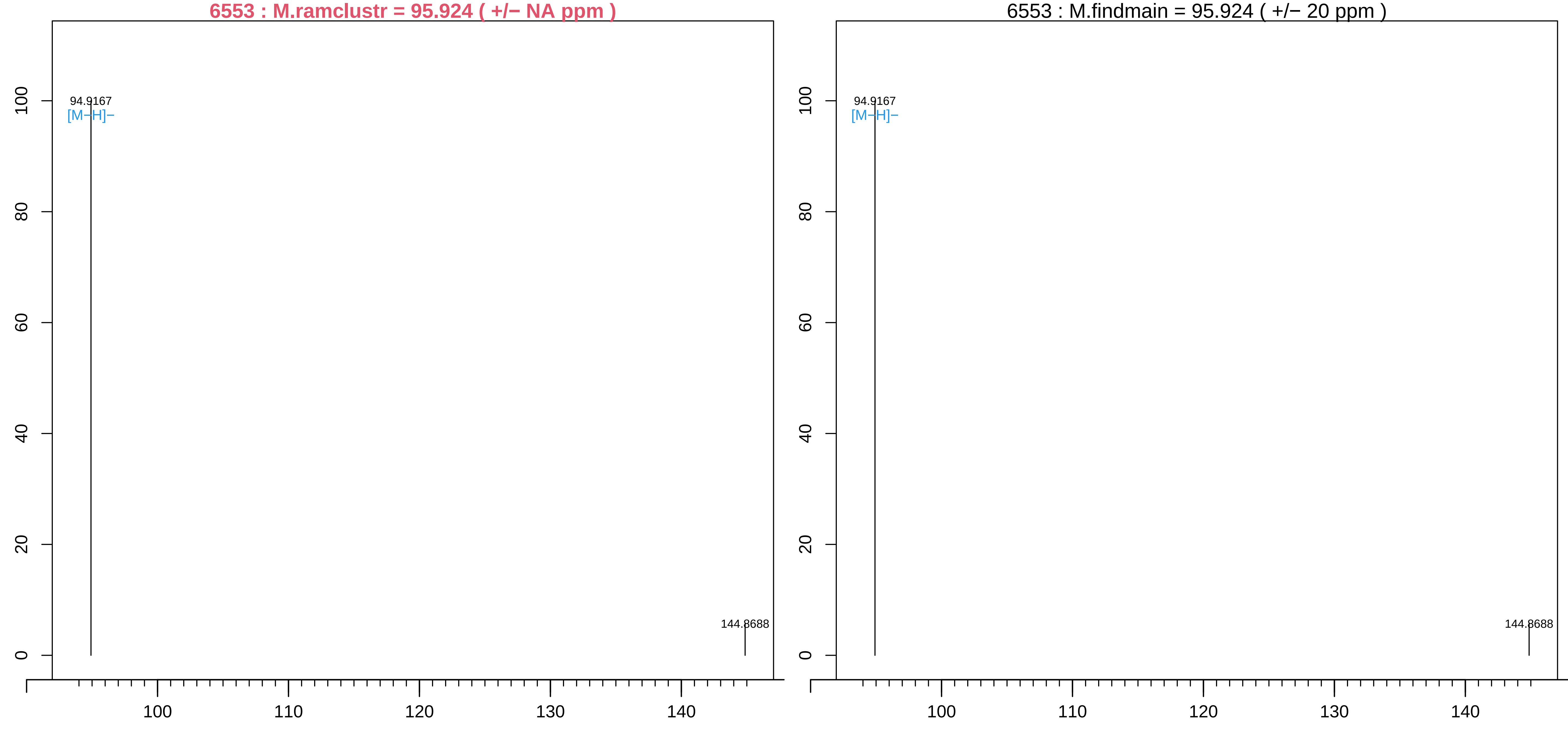
6553 : M.ramclustr = 95.924 (+/− NA ppm)
94.9167
[M−H]−
144.8688
0
20
40
60
80
100
100
110
120
130
140
6553 : M.findmain = 95.924 (+/− 20 ppm)
94.9167
[M−H]−
144.8688
0
20
40
60
80
100
100
110
120
130
140

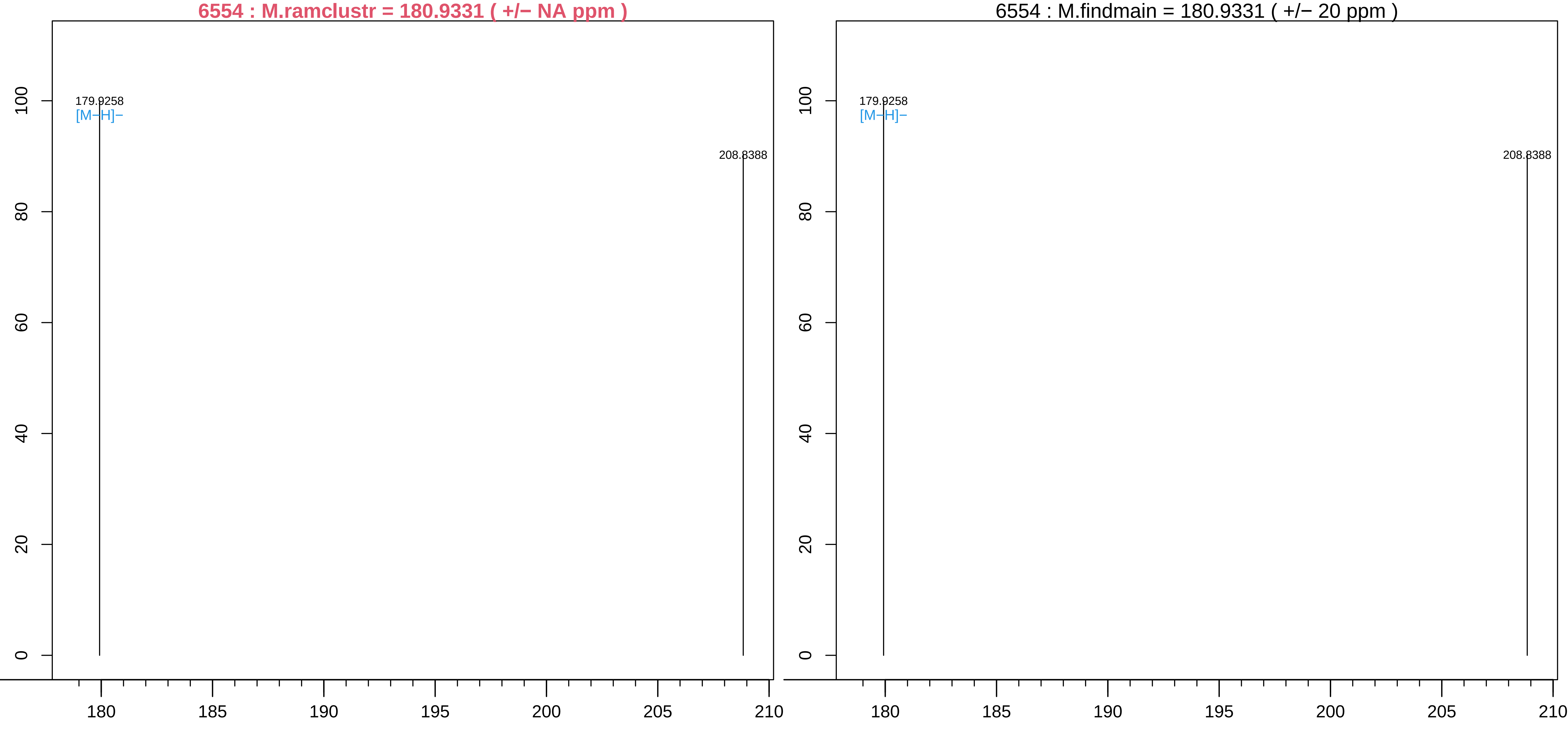

6554 : M.ramclustr = 180.9331 (+/− NA ppm)
179.9258
[M−H]−
208.8388
0
20
40
60
80
100
180
185
190
195
200
205
210
6554 : M.findmain = 180.9331 (+/− 20 ppm)
179.9258
[M−H]−
208.8388
0
20
40
60
80
100
180
185
190
195
200
205
210

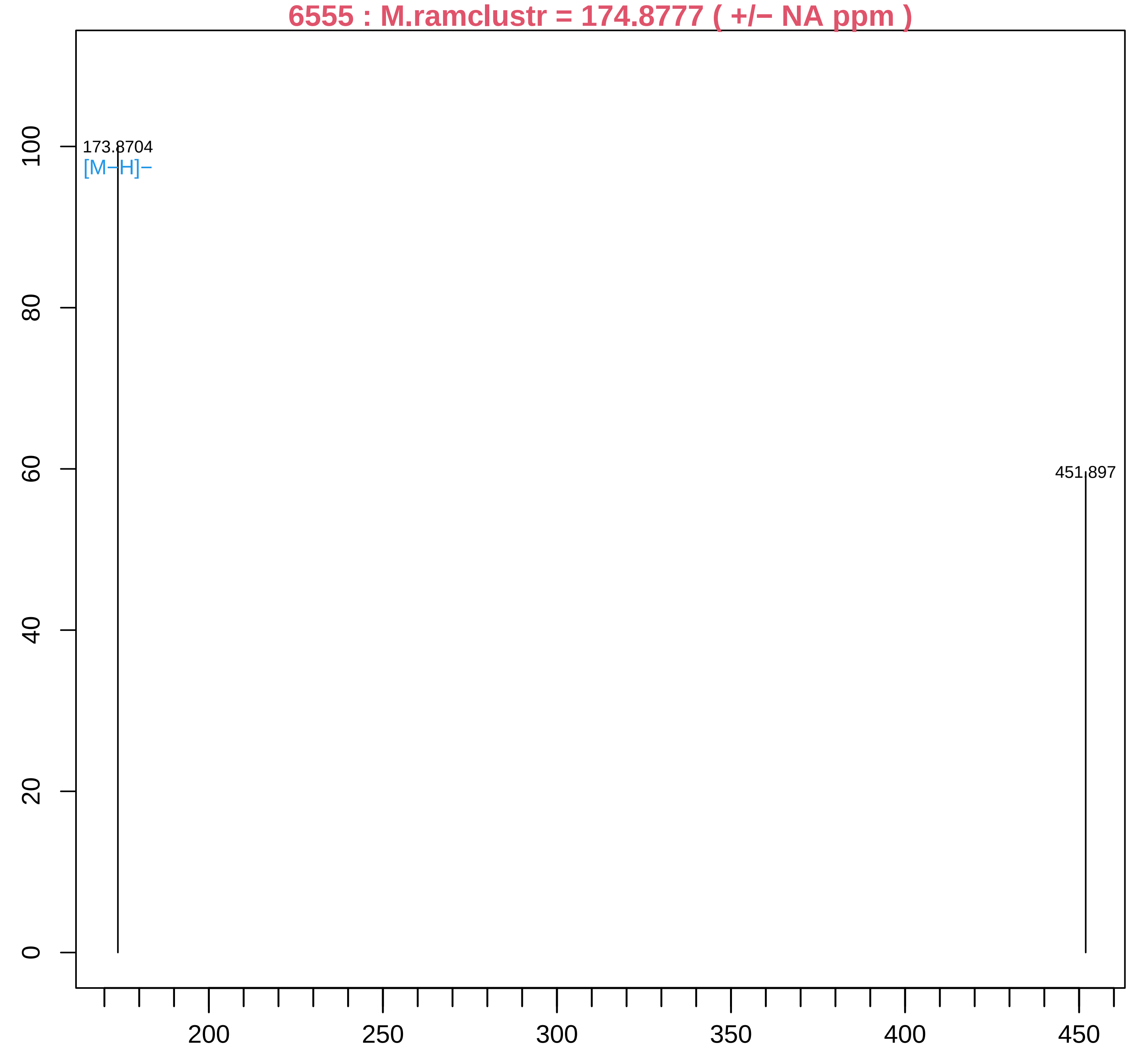
6555 : M.ramclustr = 174.8777 (+/− NA ppm)
173.8704
[M−H]−
451.897
0
20
40
60
80
100
200
250
300
350
400
450

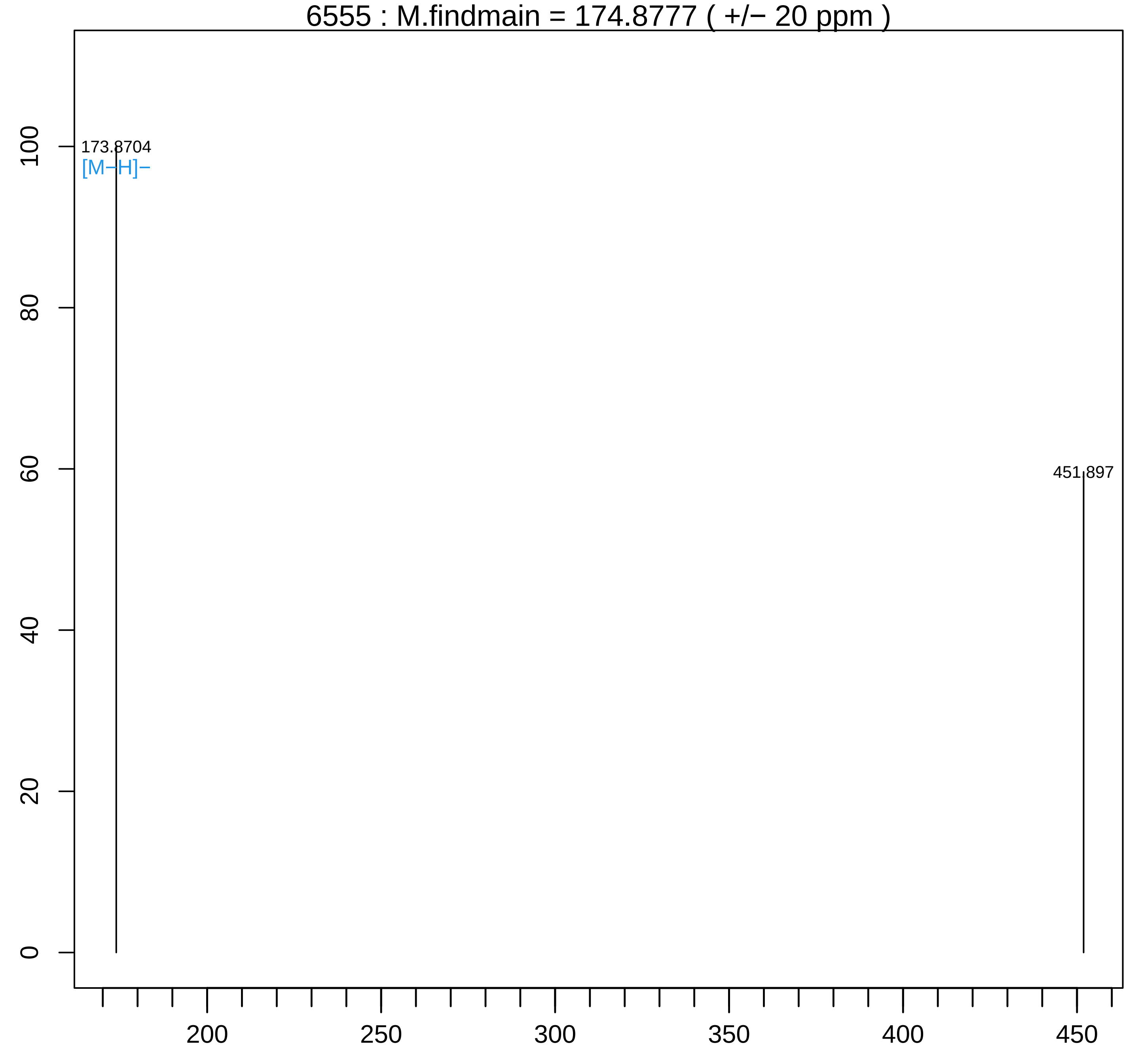
6555 : M.findmain = 174.8777 (+/− 20 ppm)
173.8704
[M−H]−
451.897
0
20
40
60
80
100
200
250
300
350
400
450

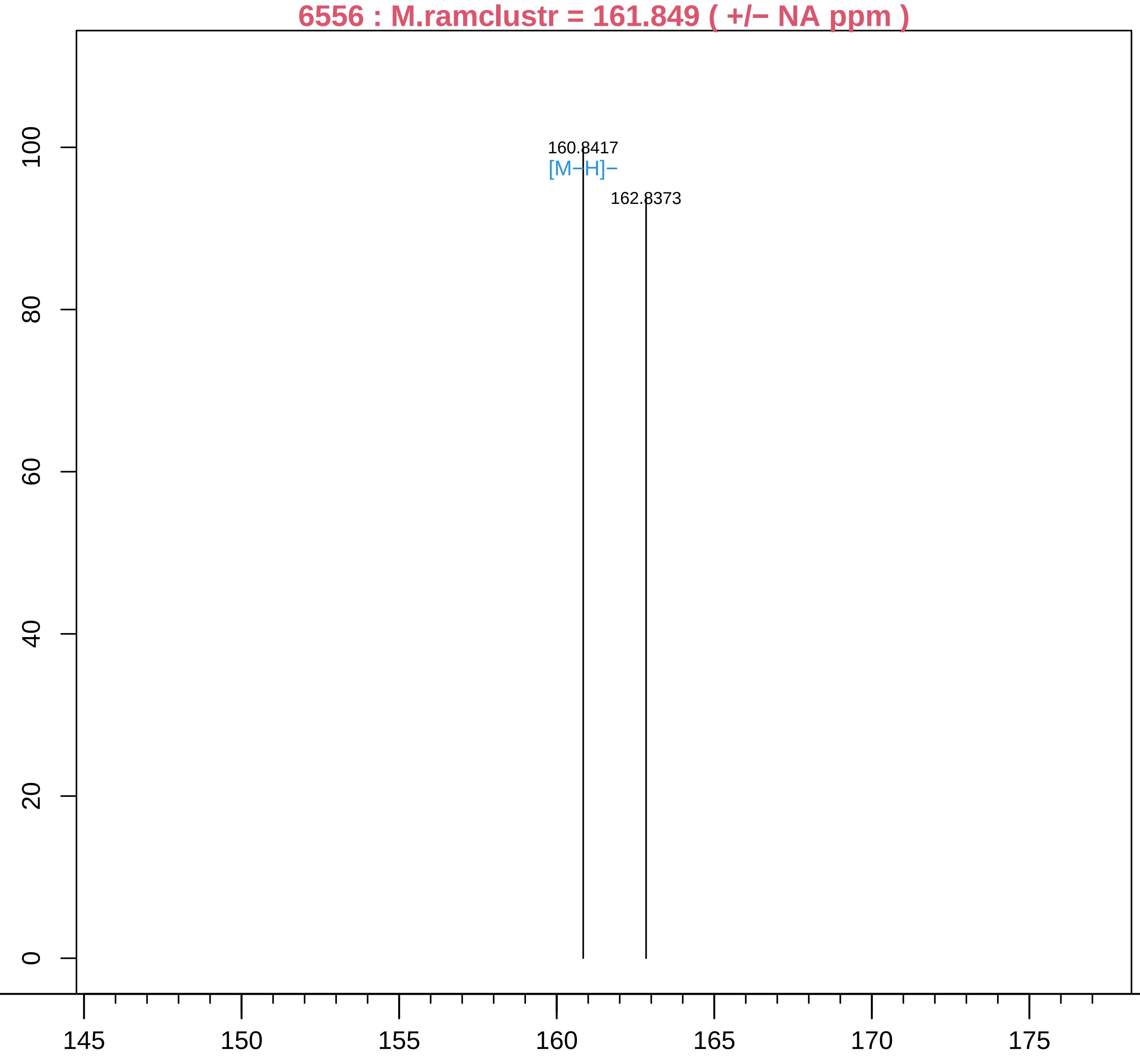
6556 : M.ramclustr = 161.849 (+/− NA ppm)
160.8417
[M−H]−
162.8373
0
20
40
60
80
100
145
150
155
160
165
170
175

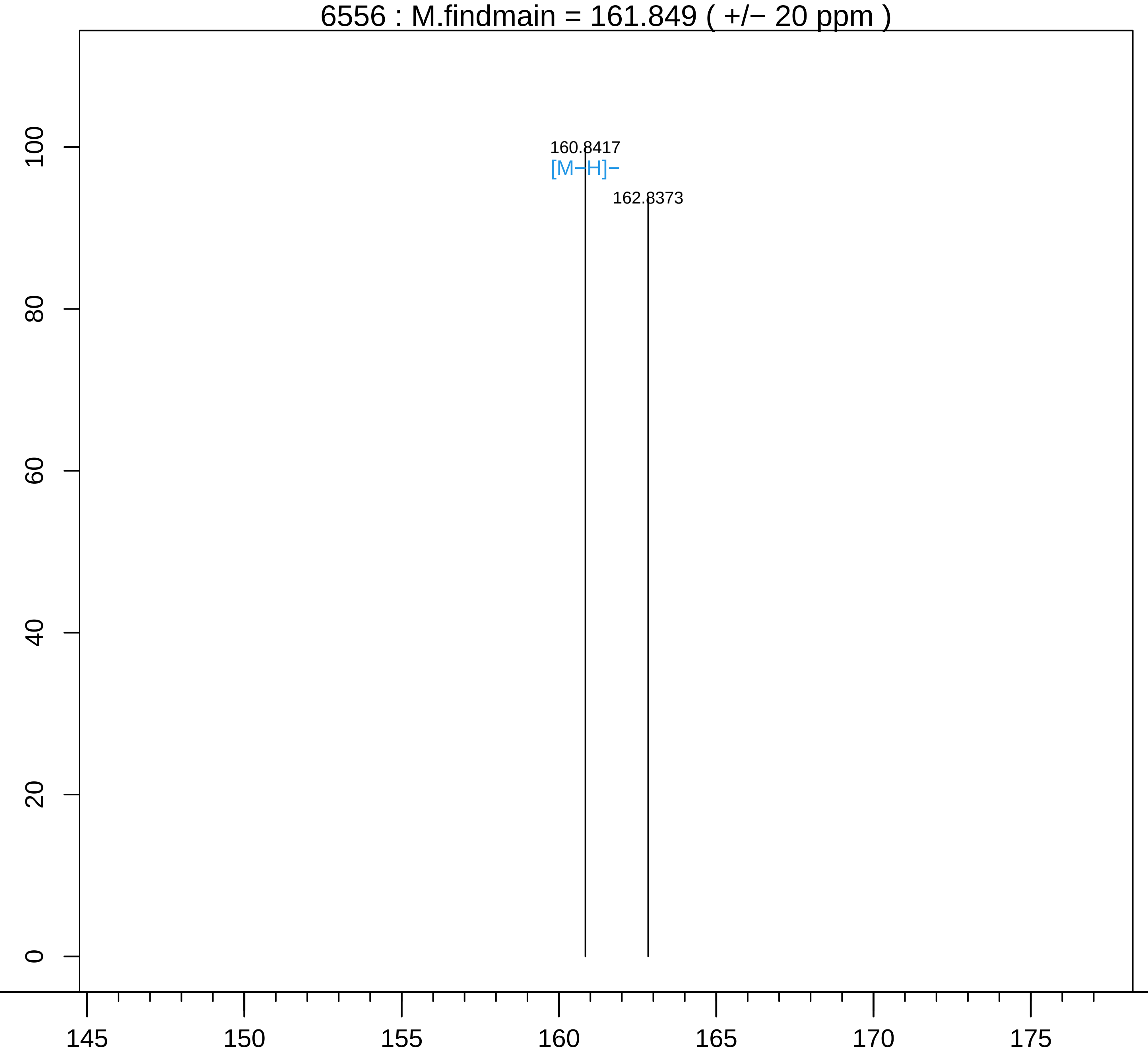
6556 : M.findmain = 161.849 (+/− 20 ppm)
160.8417
[M−H]−
162.8373
0
20
40
60
80
100
145
150
155
160
165
170
175

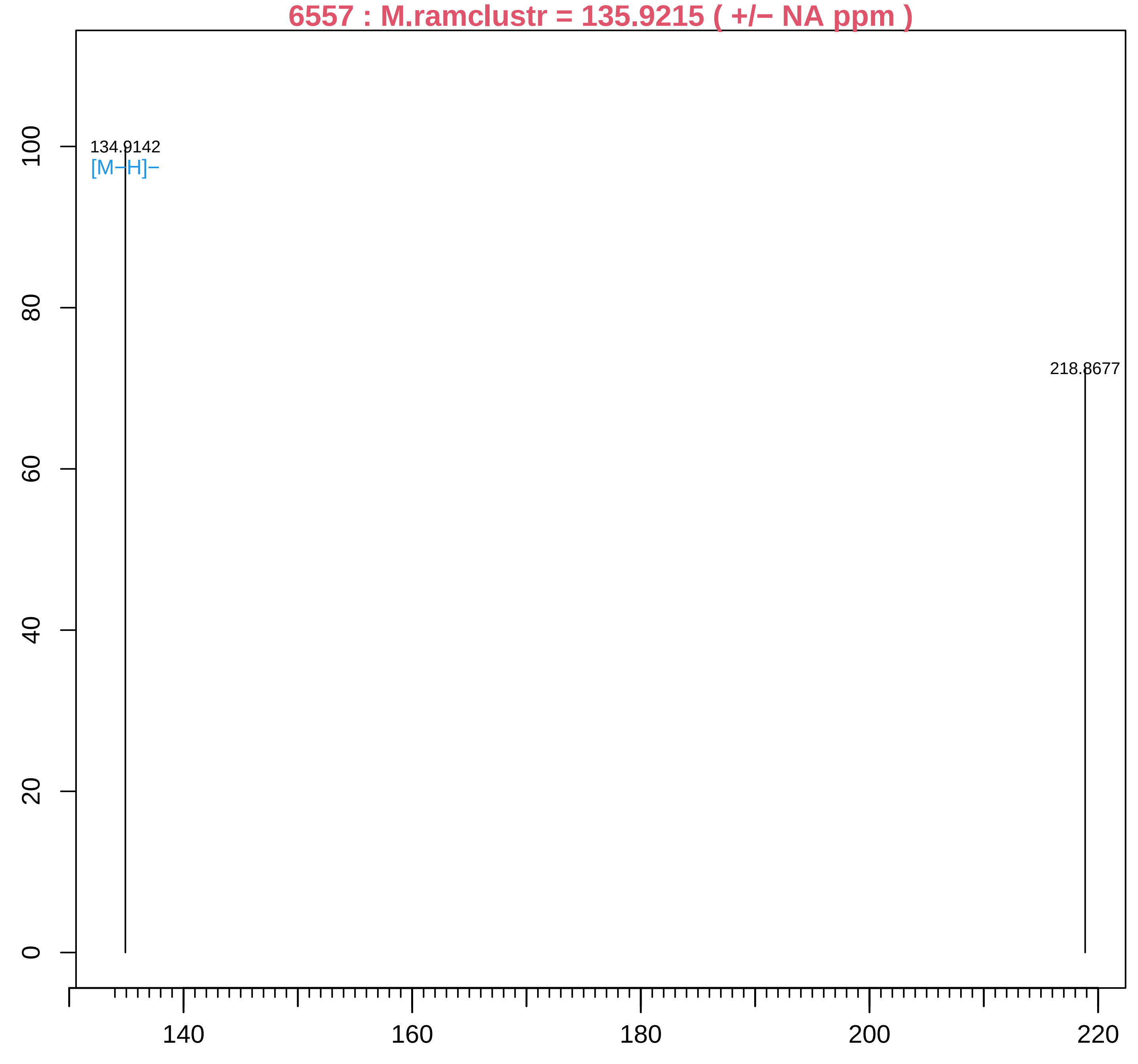
6557 : M.ramclustr = 135.9215 (+/− NA ppm)
134.9142
[M−H]−
218.8677
0
20
40
60
80
100
140
160
180
200
220

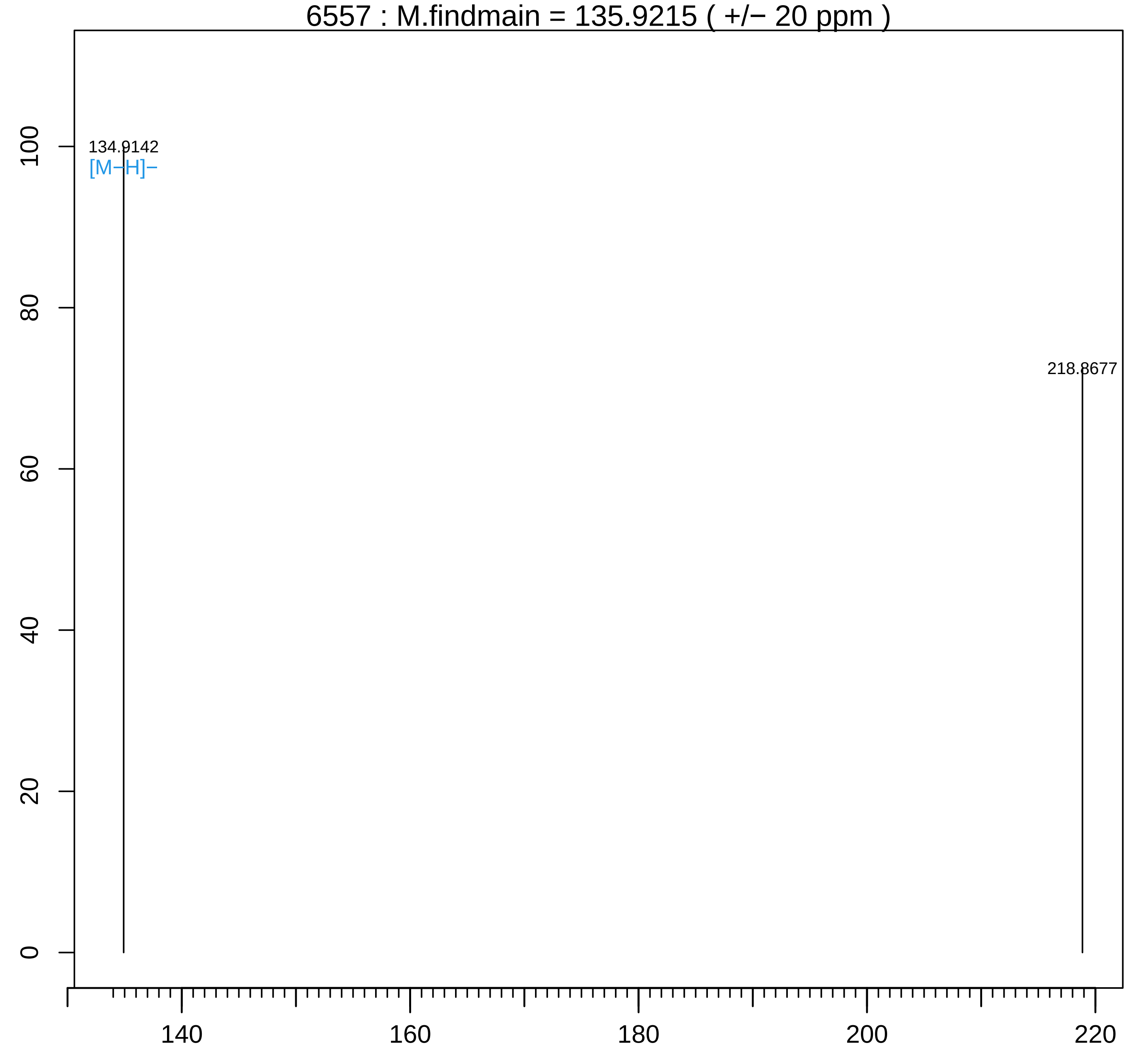
6557 : M.findmain = 135.9215 (+/− 20 ppm)
134.9142
[M−H]−
218.8677
0
20
40
60
80
100
140
160
180
200
220

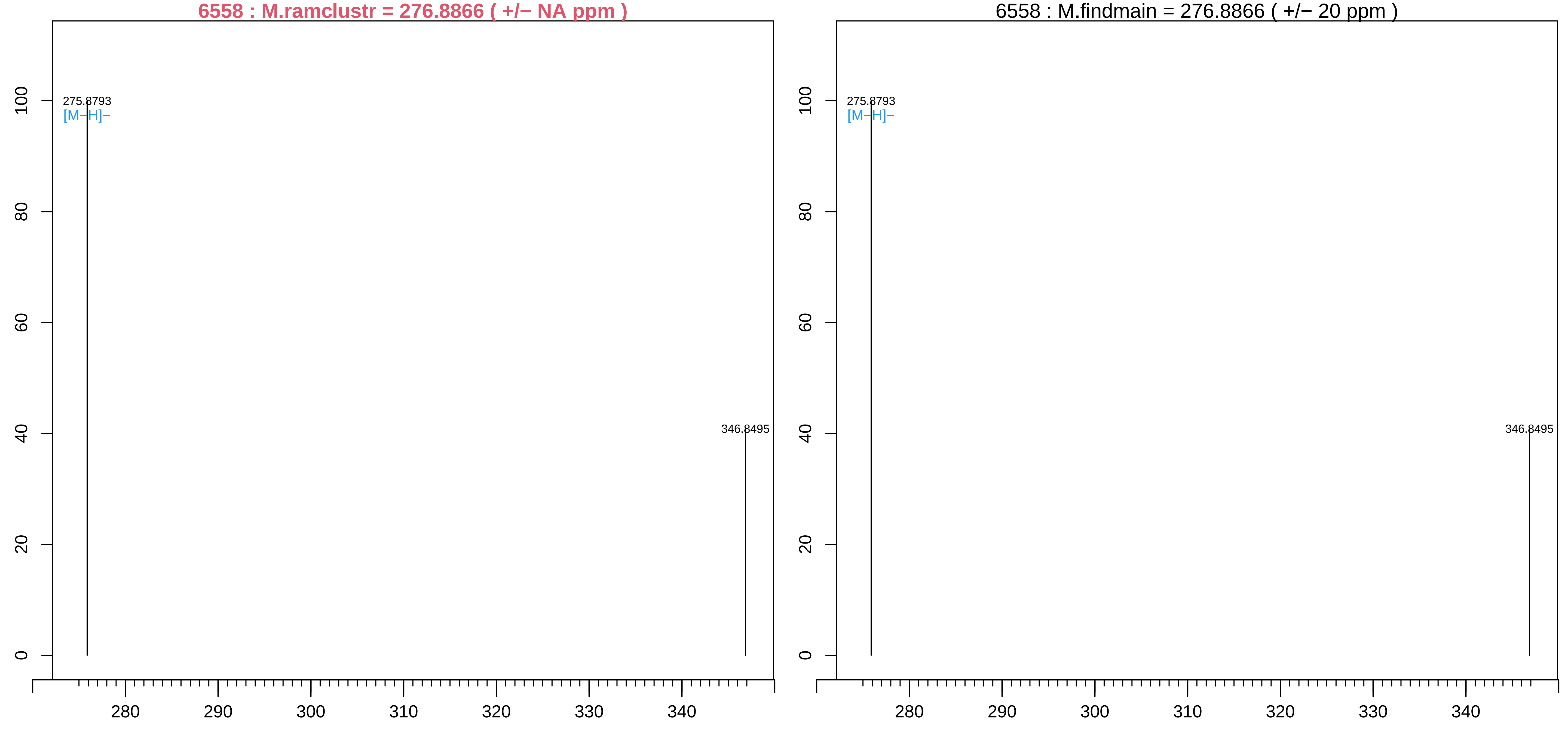
6558 : M.ramclustr = 276.8866 (+/− NA ppm)
275.8793
[M−H]−
346.8495
6558 : M.findmain = 276.8866 (+/− 20 ppm)
275.8793
[M−H]−
346.8495
0
20
40
60
80
100
280
290
300
310
320
330
340

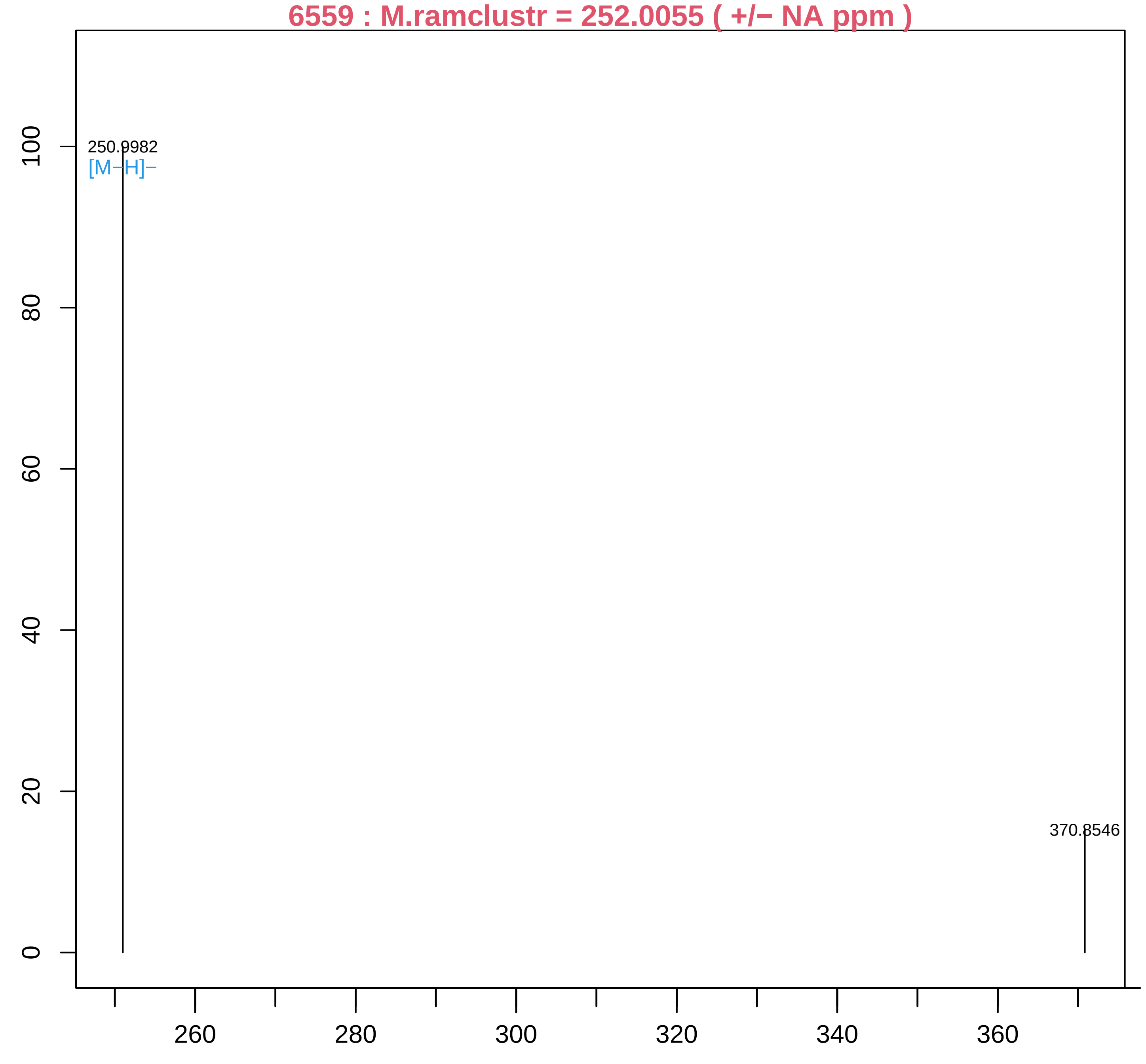

6559 : M.ramclustr = 252.0055 (+/− NA ppm)
250.9982
[M−H]−
370.8546
0
20
40
60
80
100
260
280
300
320
340
360

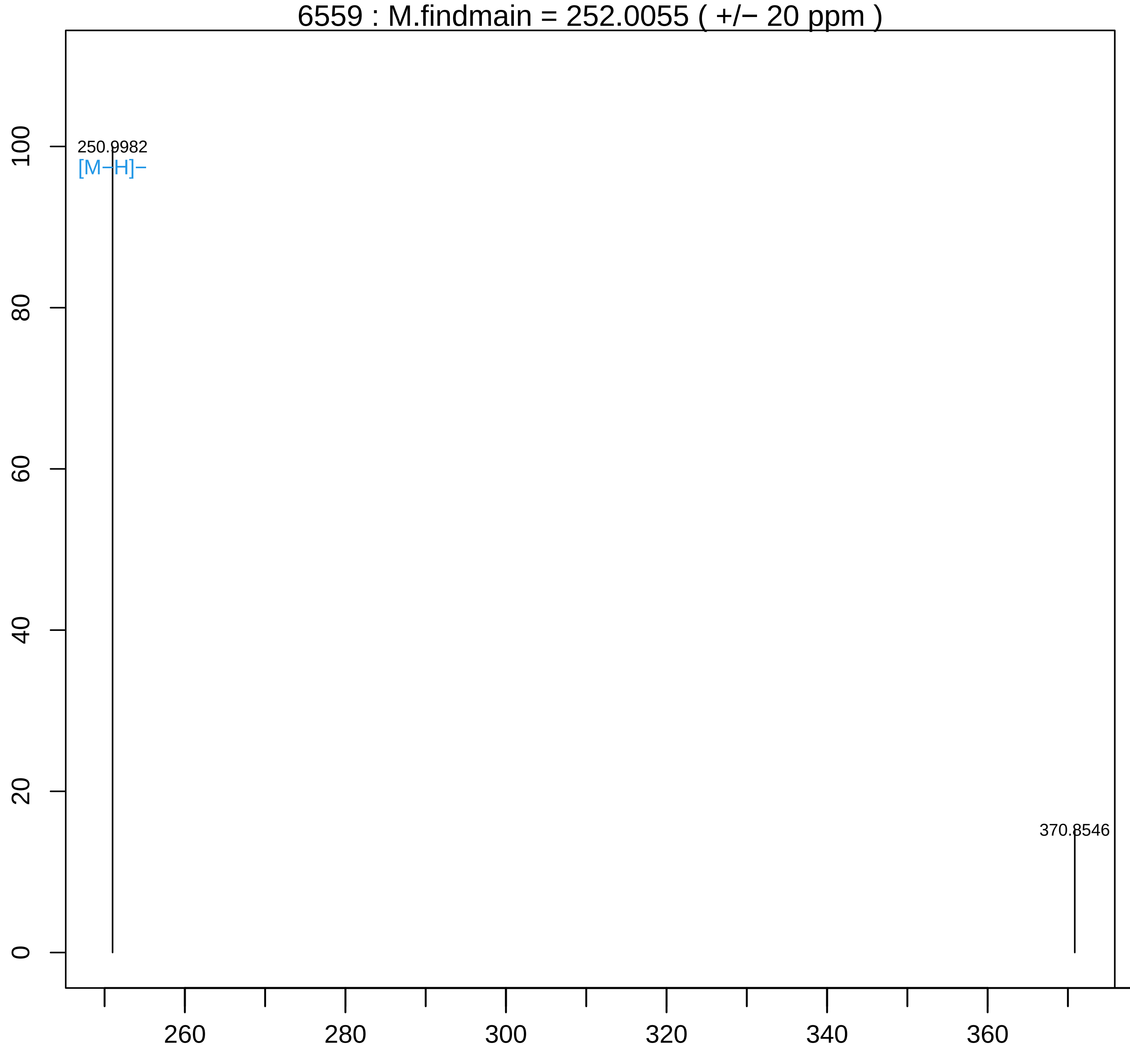

6559 : M.findmain = 252.0055 (+/− 20 ppm)
250.9982
[M−H]−
370.8546
0
20
40
60
80
100
260
280
300
320
340
360

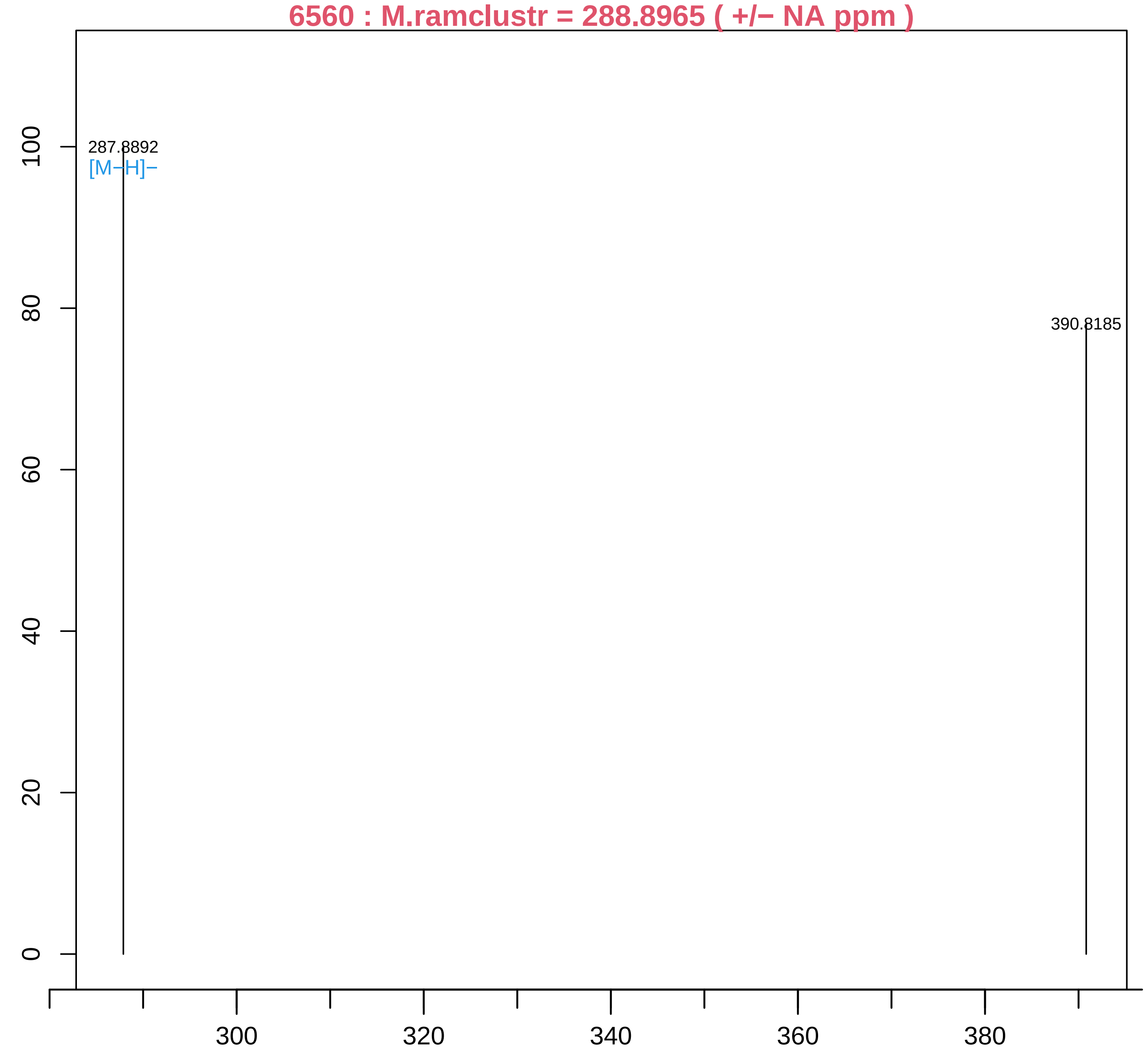
6560 : M.ramclustr = 288.8965 (+/− NA ppm)
287.8892
[M−H]−
390.8185
0
20
40
60
80
100
300
320
340
360
380

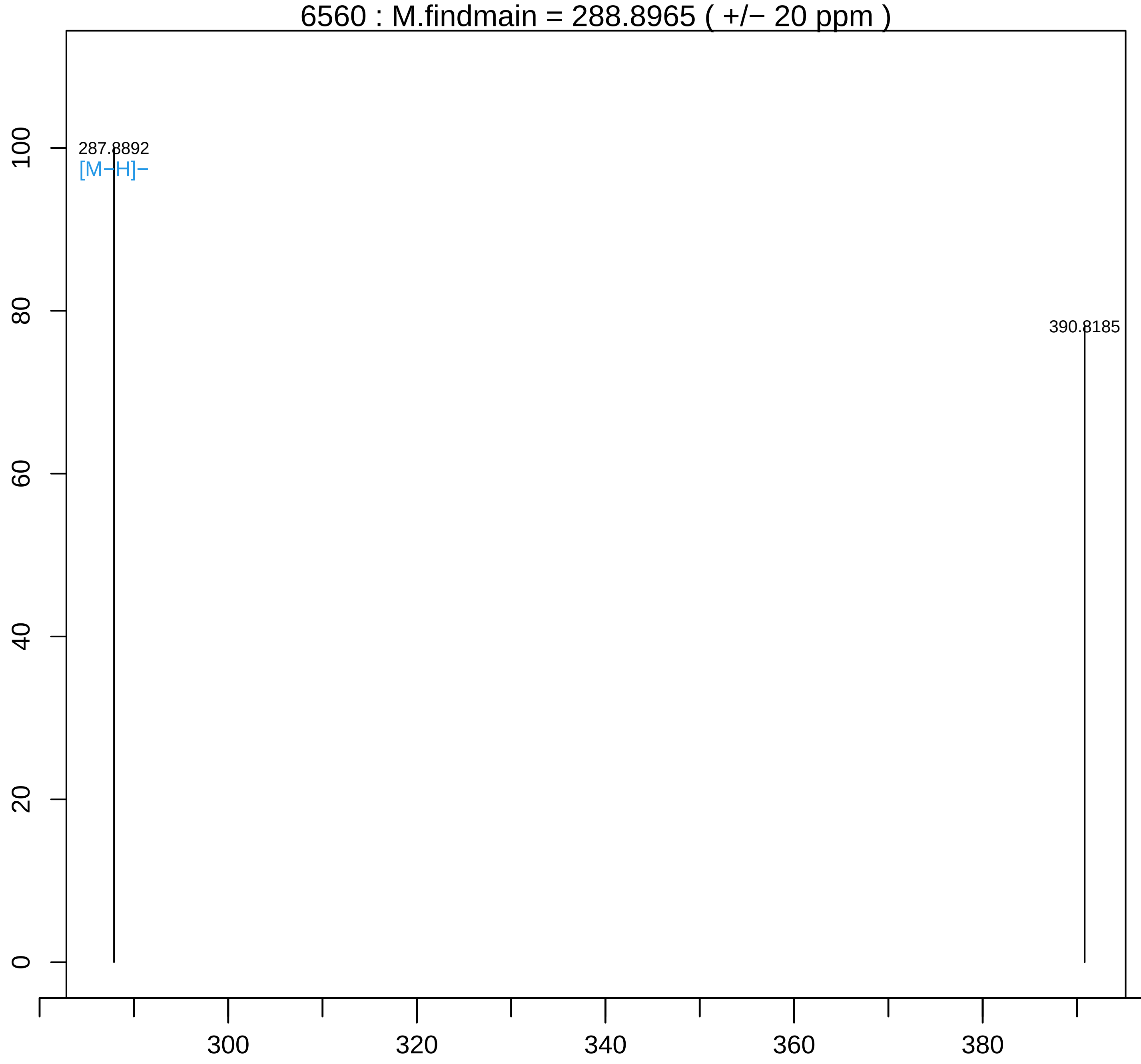
6560 : M.findmain = 288.8965 (+/− 20 ppm)
287.8892
[M−H]−
390.8185
0
20
40
60
80
100
300
320
340
360
380

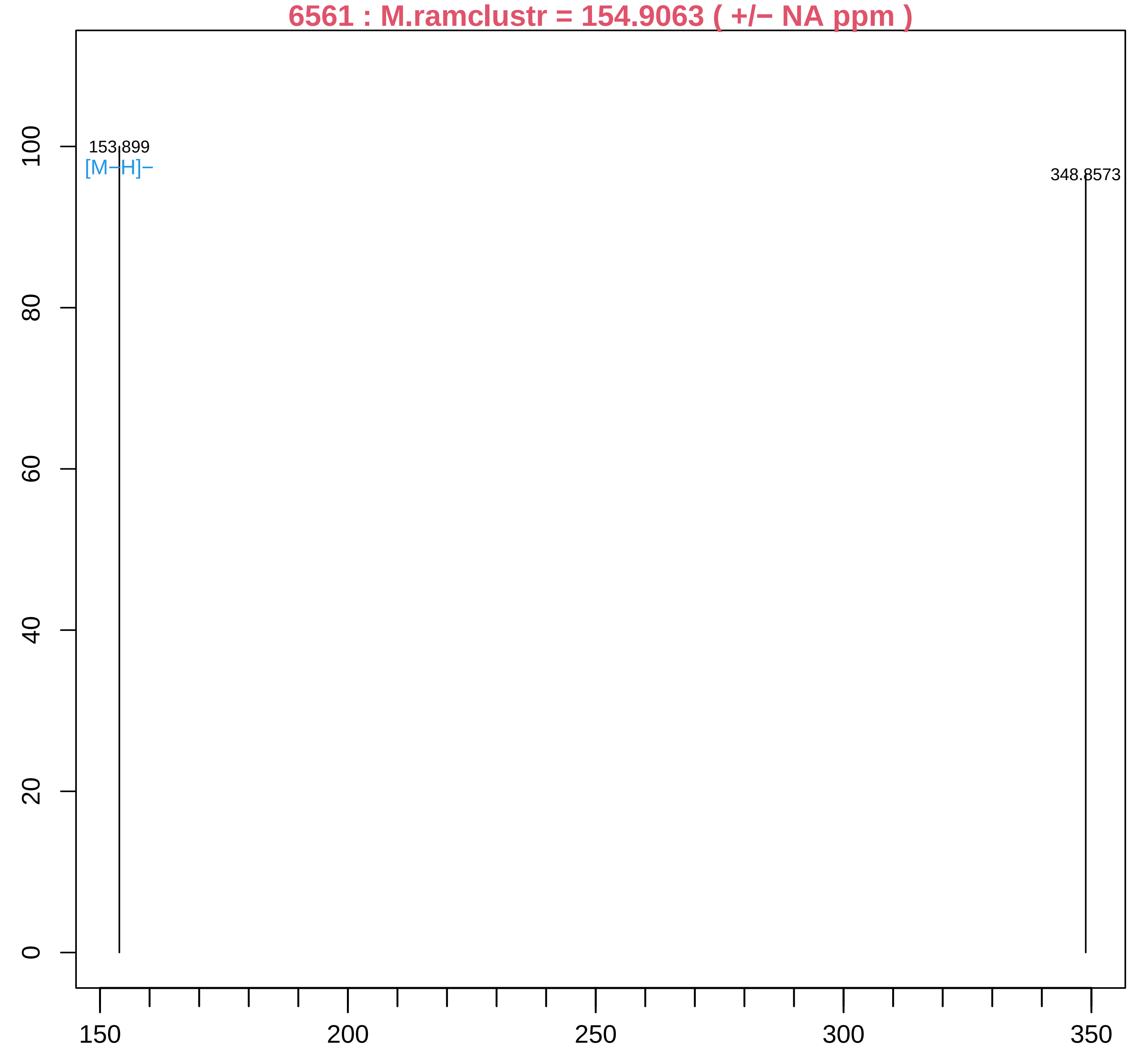

6561 : M.ramclustr = 154.9063 (+/− NA ppm)
153.899
[M−H]−
348.8573
0
20
40
60
80
100
150
200
250
300
350

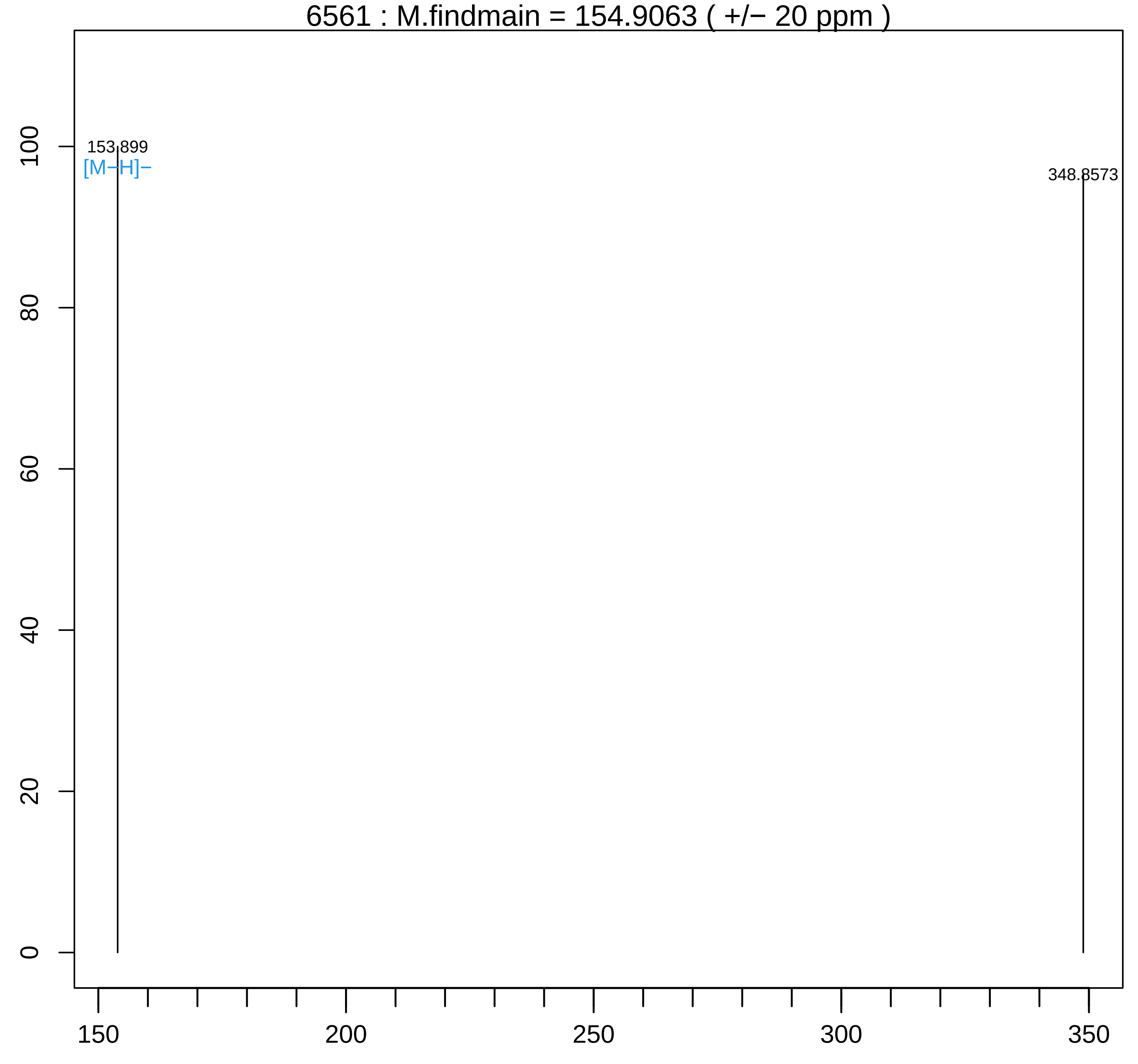

6561 : M.findmain = 154.9063 (+/− 20 ppm)
153.899
[M−H]−
348.8573
0
20
40
60
80
100
150
200
250
300
350

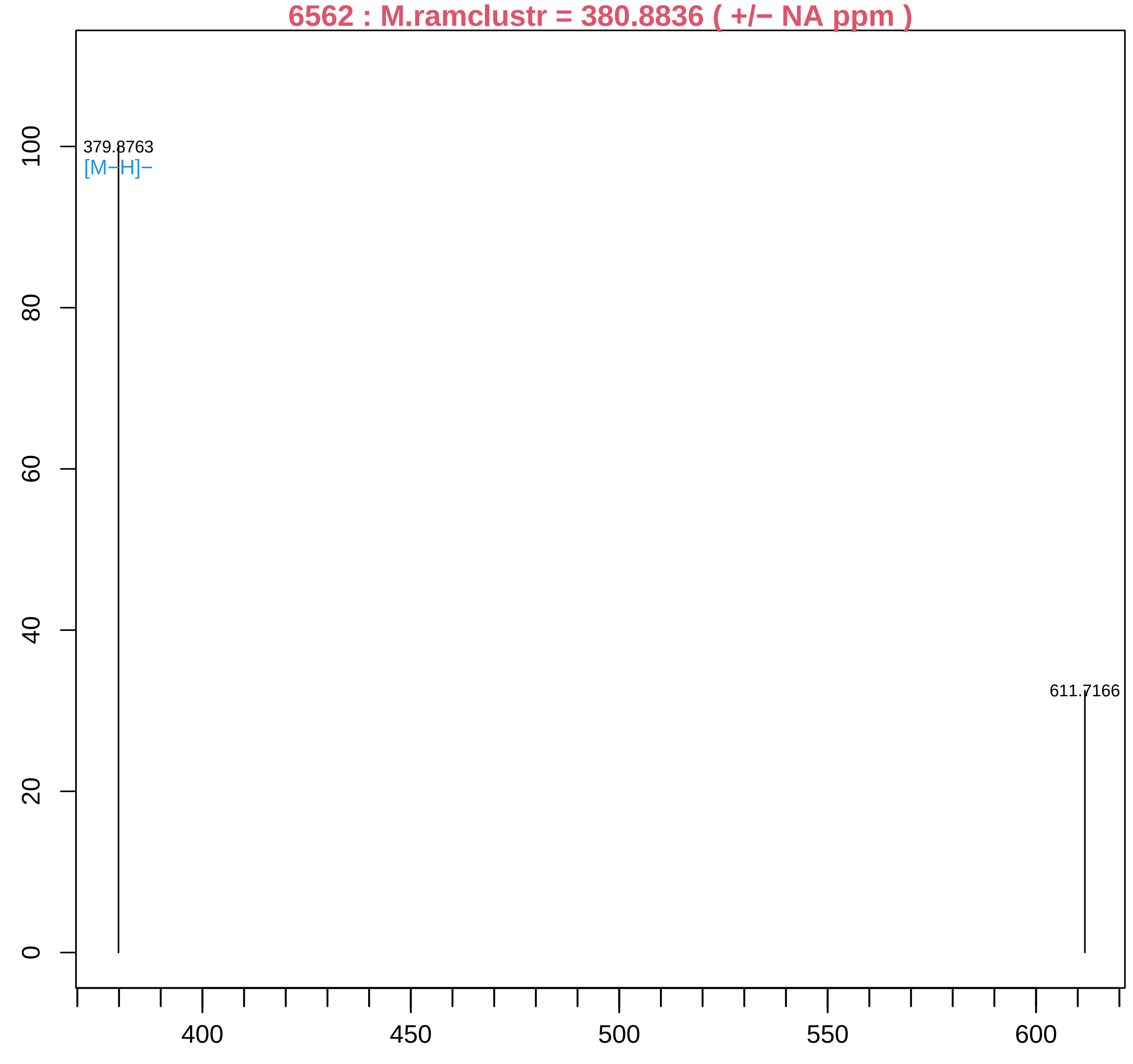
6562 : M.ramclustr = 380.8836 (+/− NA ppm)
379.8763
[M−H]−
611.7166
0
20
40
60
80
100
400
450
500
550
600

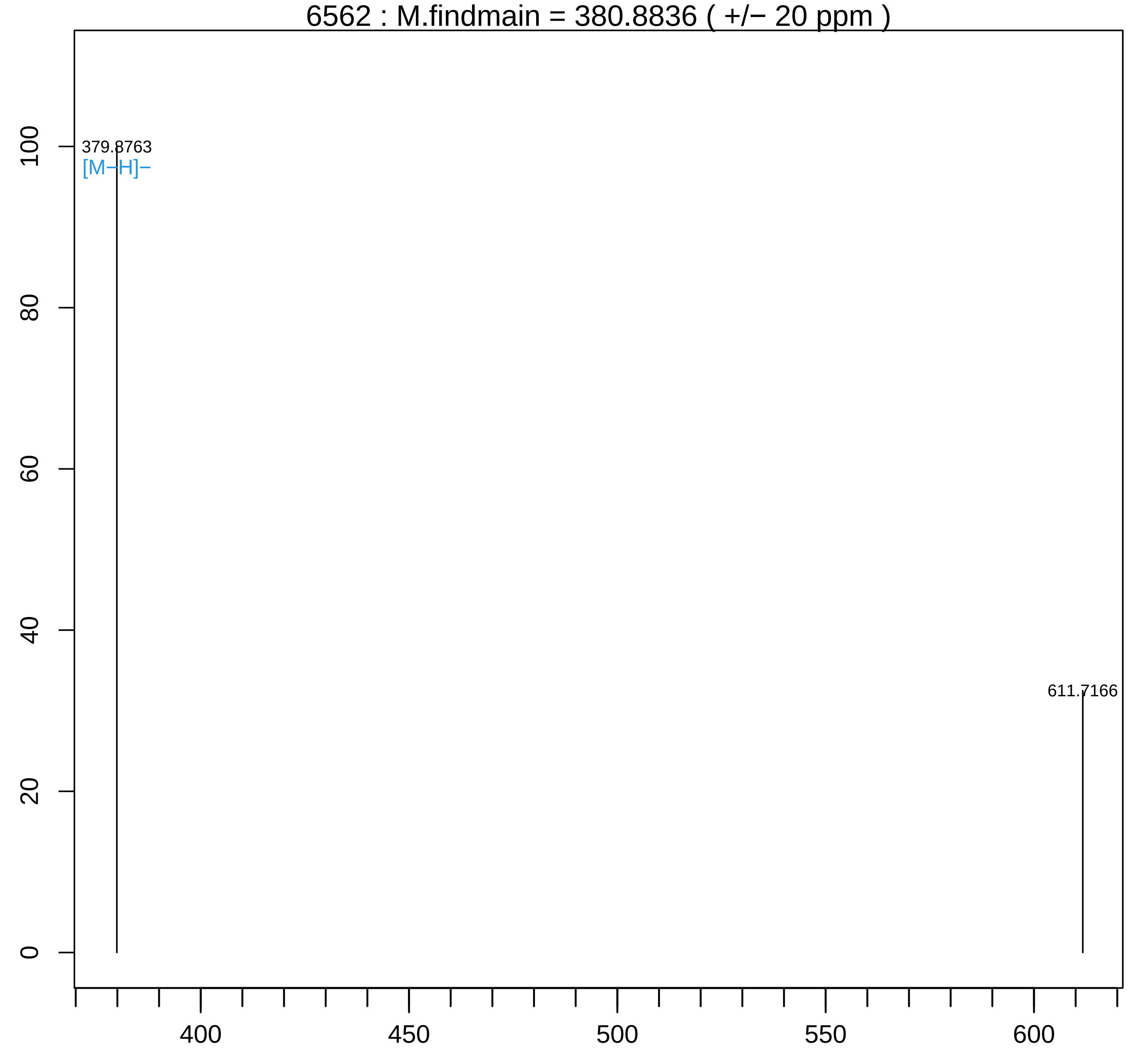
6562 : M.findmain = 380.8836 (+/− 20 ppm)
379.8763
[M−H]−
611.7166
0
20
40
60
80
100
400
450
500
550
600

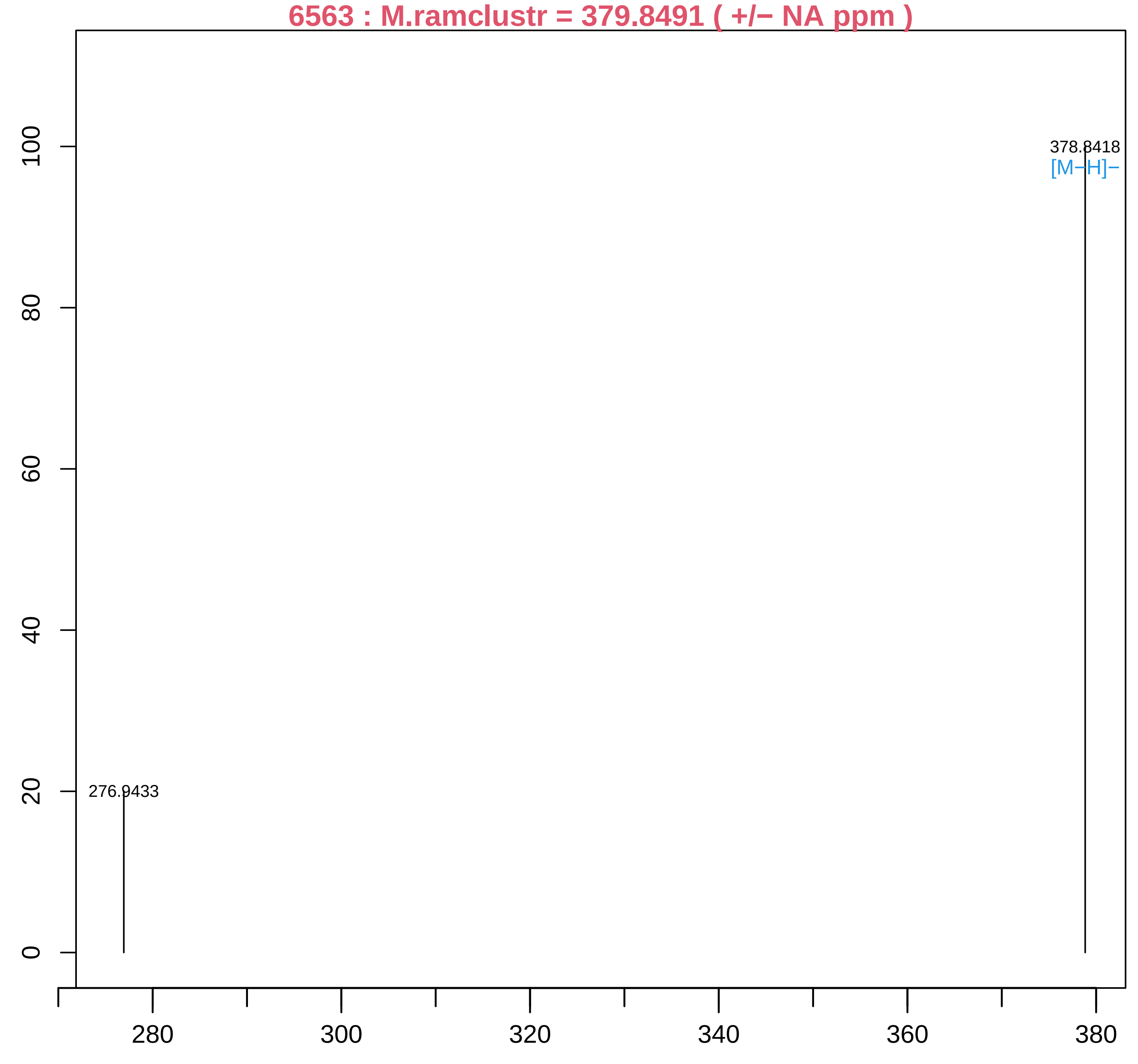

6563 : M.ramclustr = 379.8491 (+/− NA ppm)
378.8418
[M−H]−
276.9433
0
20
40
60
80
100
280
300
320
340
360
380

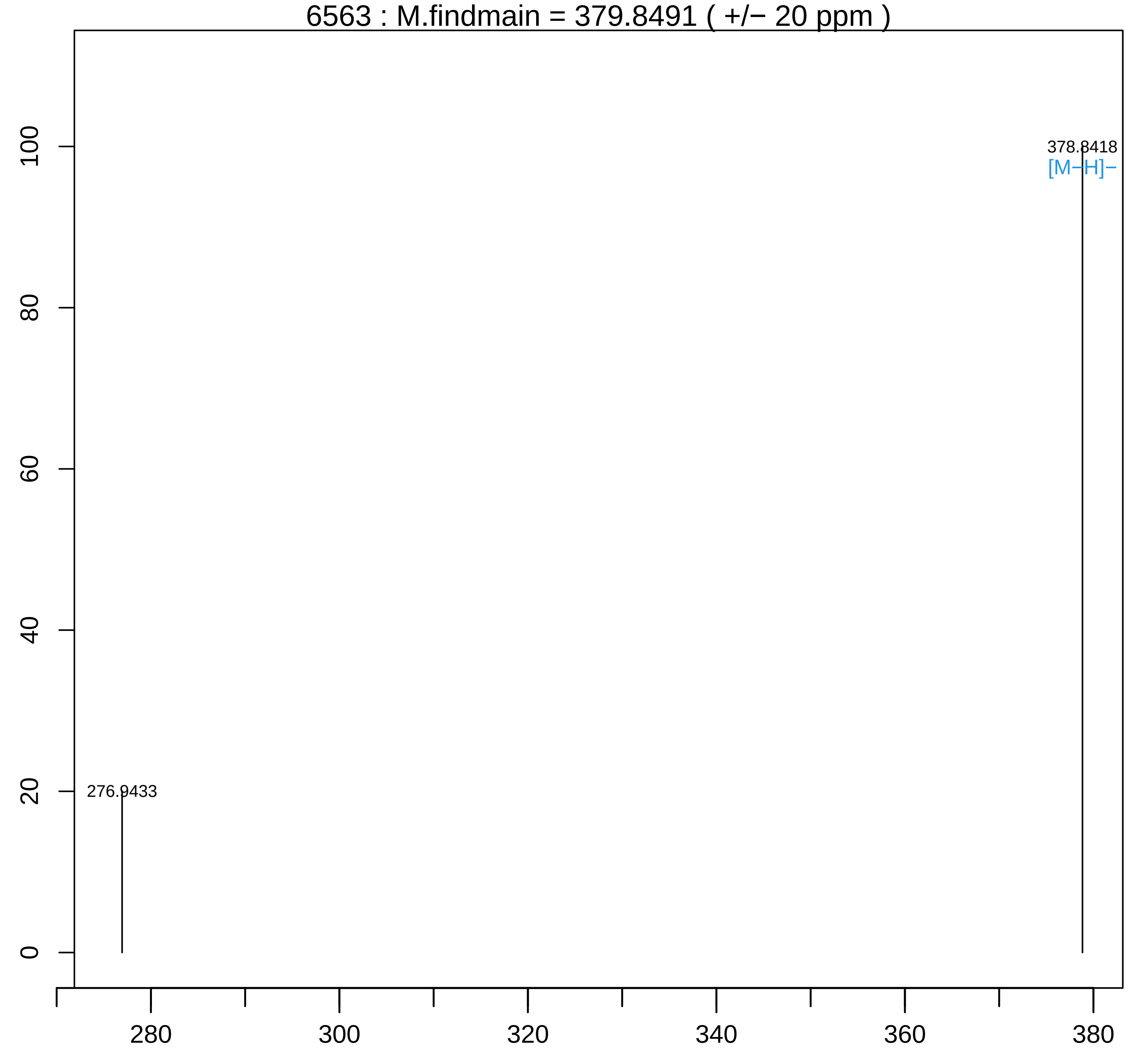

6563 : M.findmain = 379.8491 (+/− 20 ppm)
378.8418
[M−H]−
276.9433
0
20
40
60
80
100
280
300
320
340
360
380

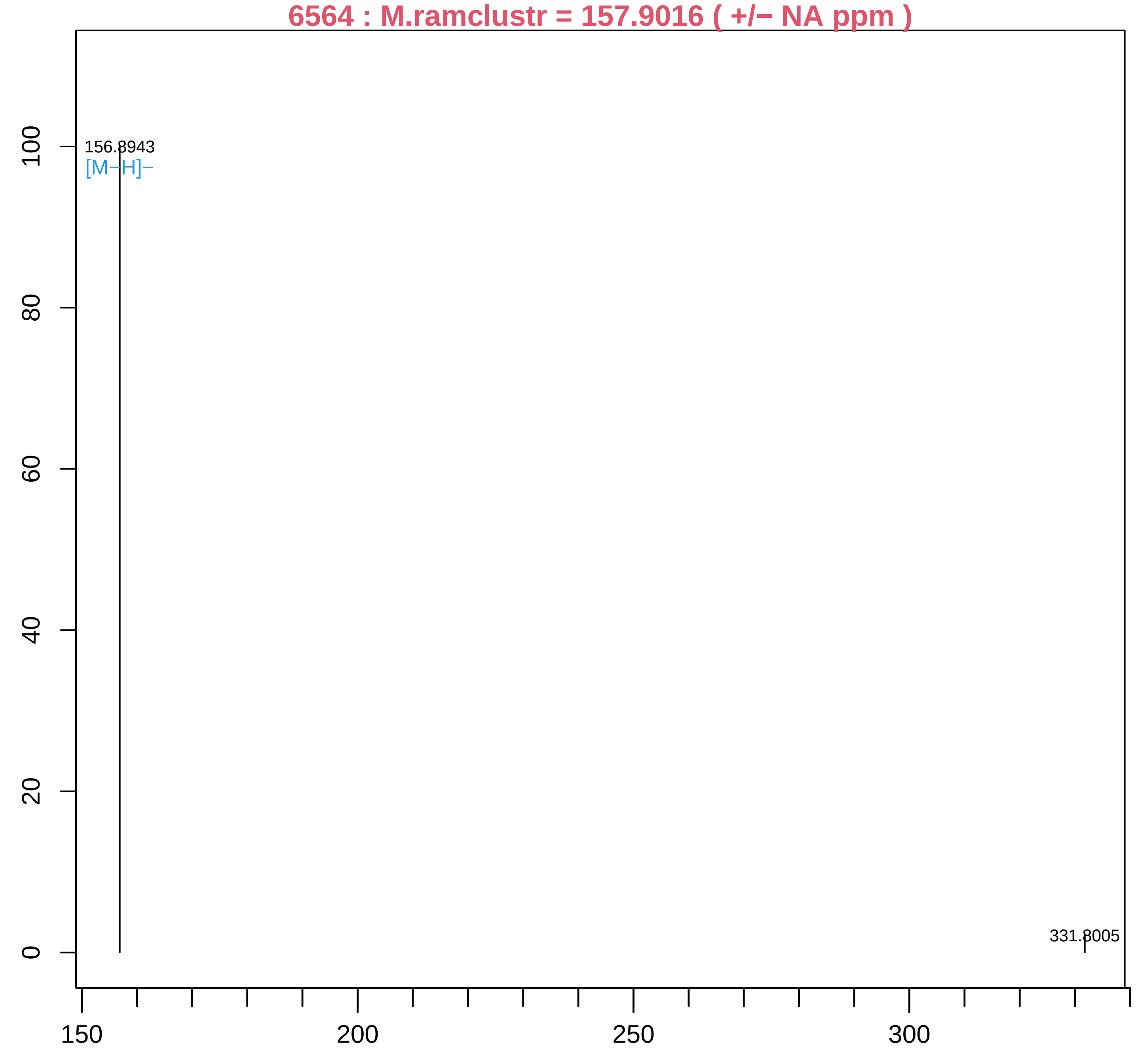
6564 : M.ramclustr = 157.9016 (+/− NA ppm)
156.8943
[M−H]−
331.8005
100
80
60
40
20
0
150
200
250
300

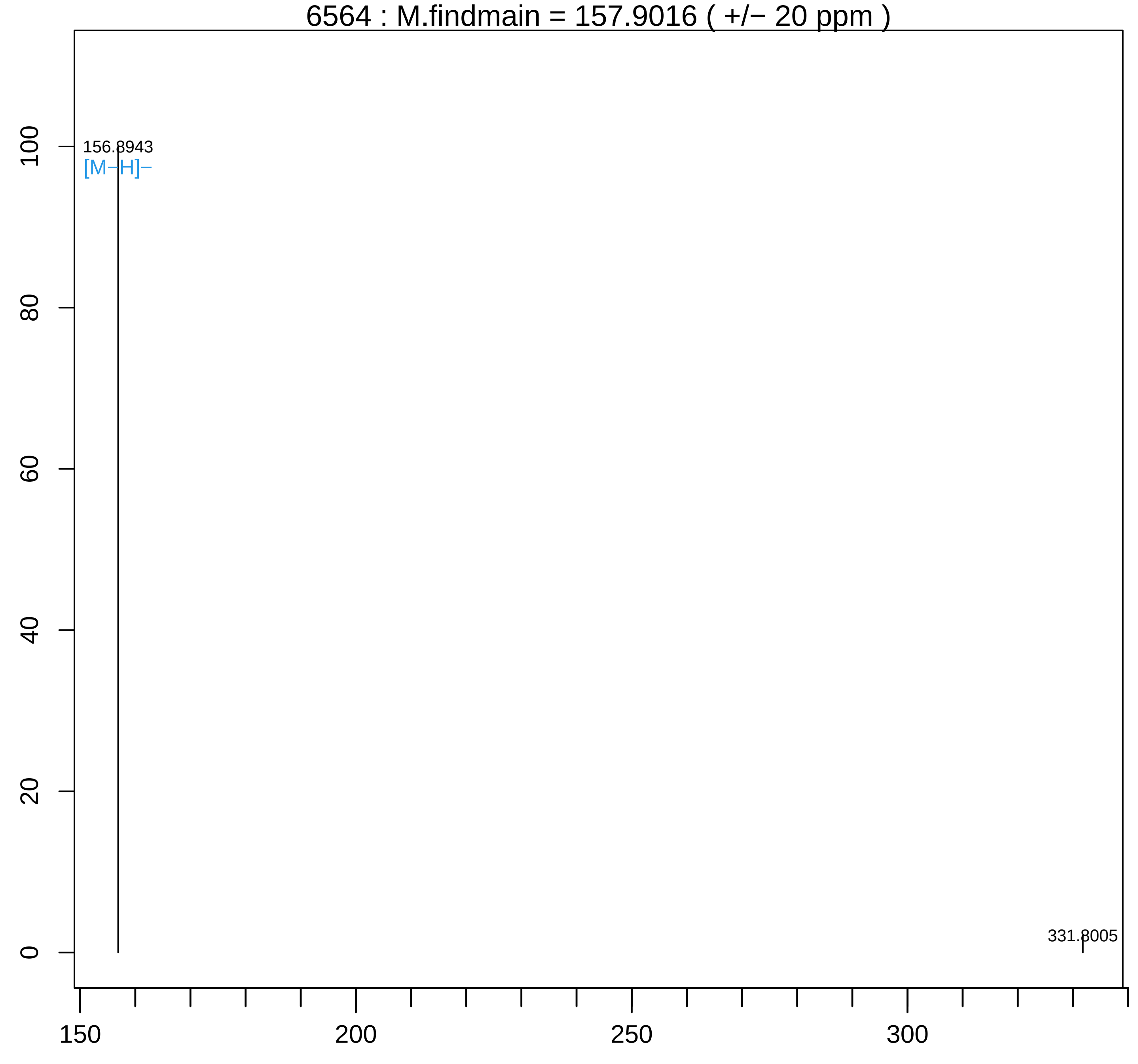
6564 : M.findmain = 157.9016 (+/− 20 ppm)
156.8943
[M−H]−
331.8005
100
80
60
40
20
0
150
200
250
300

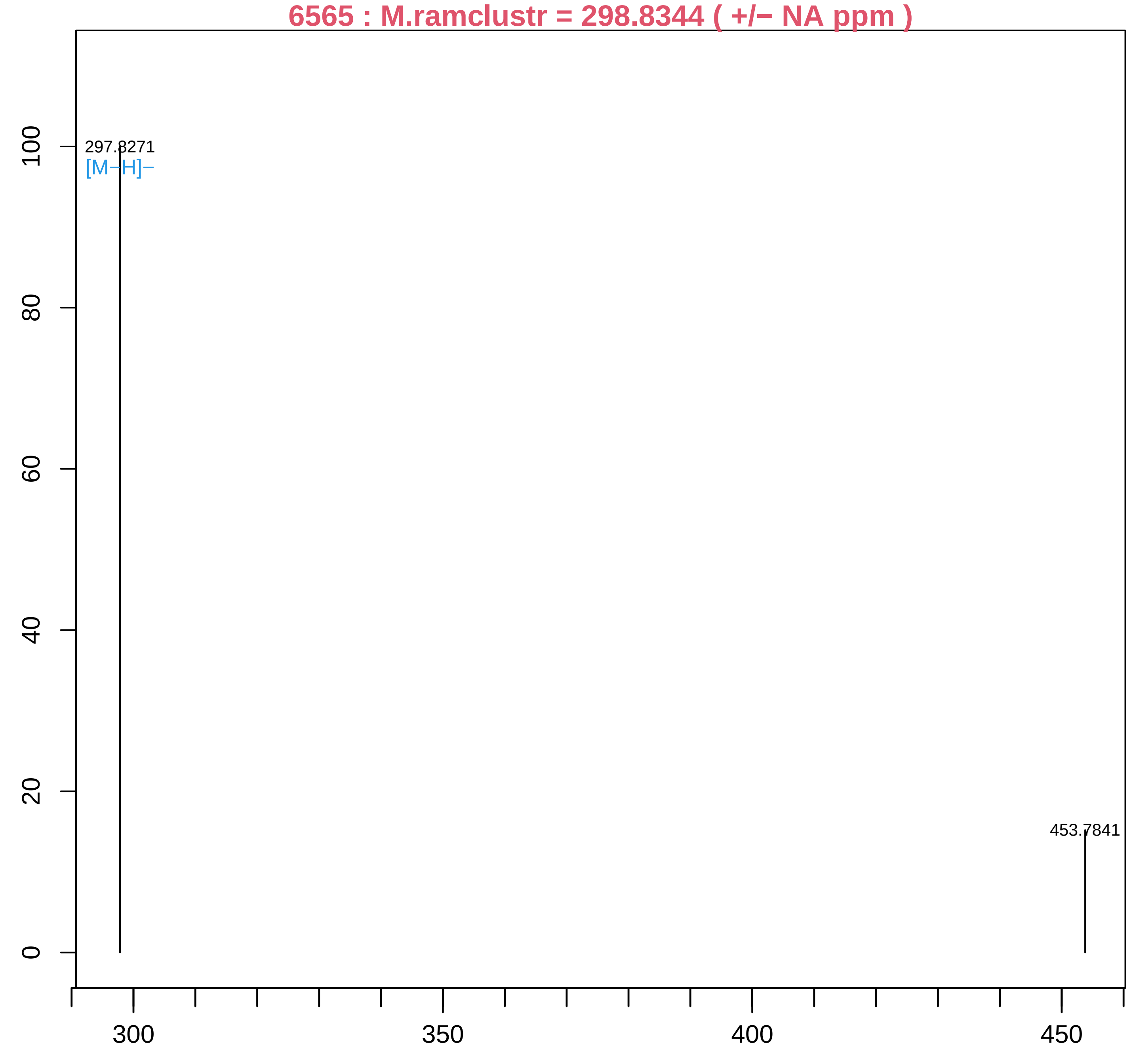
6565 : M.ramclustr = 298.8344 (+/− NA ppm)
297.8271
[M−H]−
453.7841
0
20
40
60
80
100
300
350
400
450

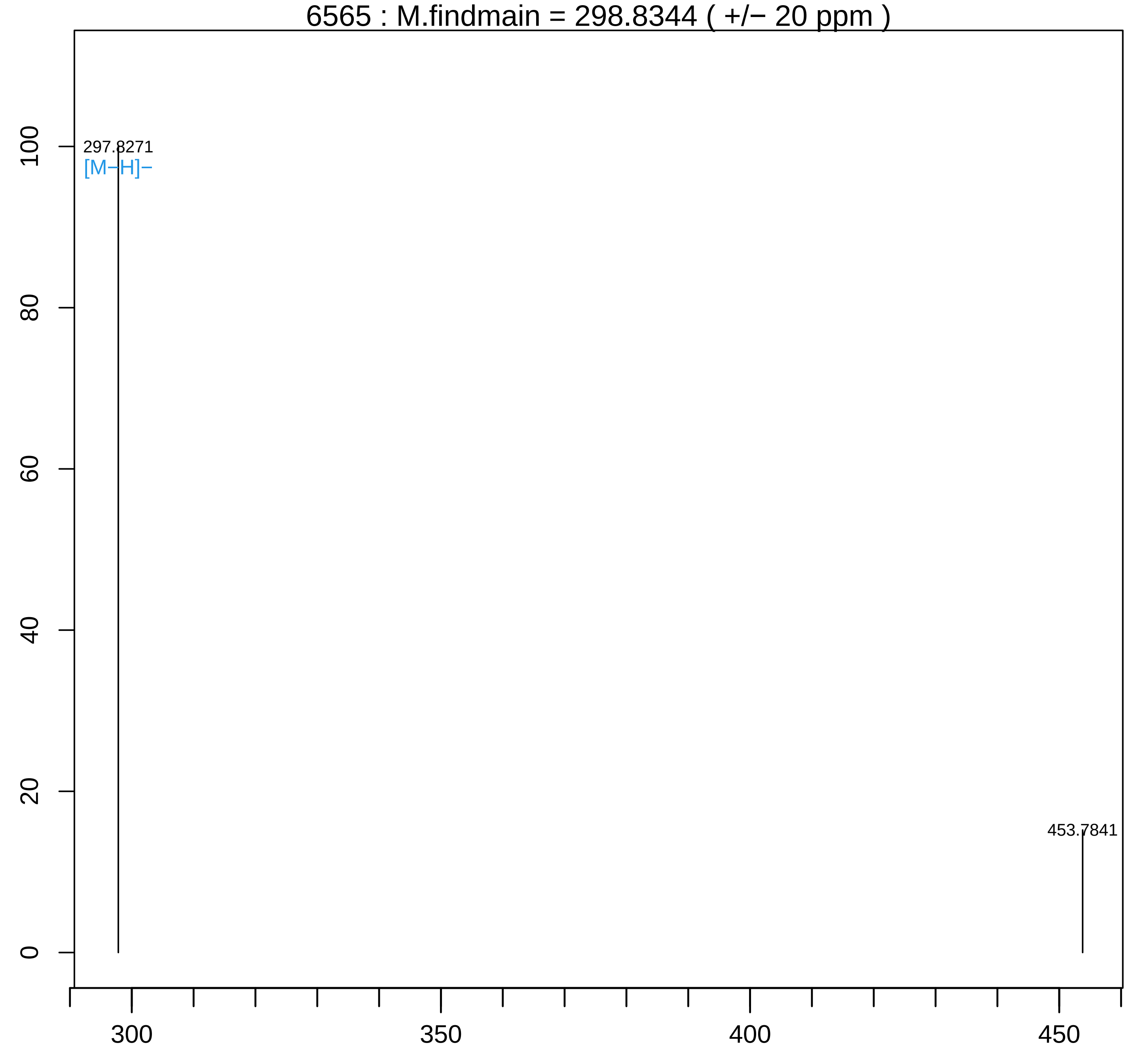
6565 : M.findmain = 298.8344 (+/− 20 ppm)
297.8271
[M−H]−
453.7841
0
20
40
60
80
100
300
350
400
450

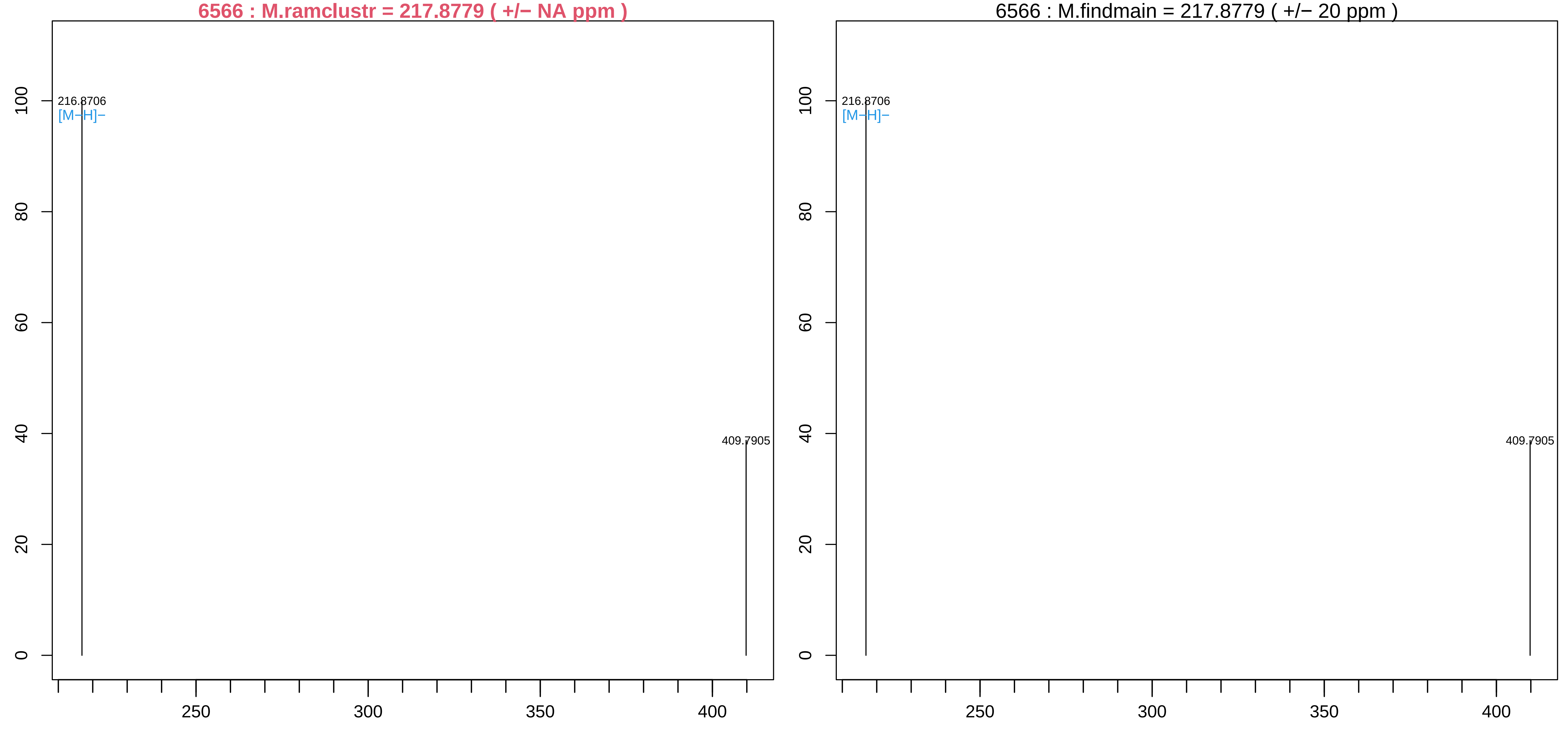
6566 : M.ramclustr = 217.8779 (+/− NA ppm)
216.8706
[M−H]−
409.7905
0
20
40
60
80
100
250
300
350
400
6566 : M.findmain = 217.8779 (+/− 20 ppm)
216.8706
[M−H]−
409.7905
0
20
40
60
80
100
250
300
350
400

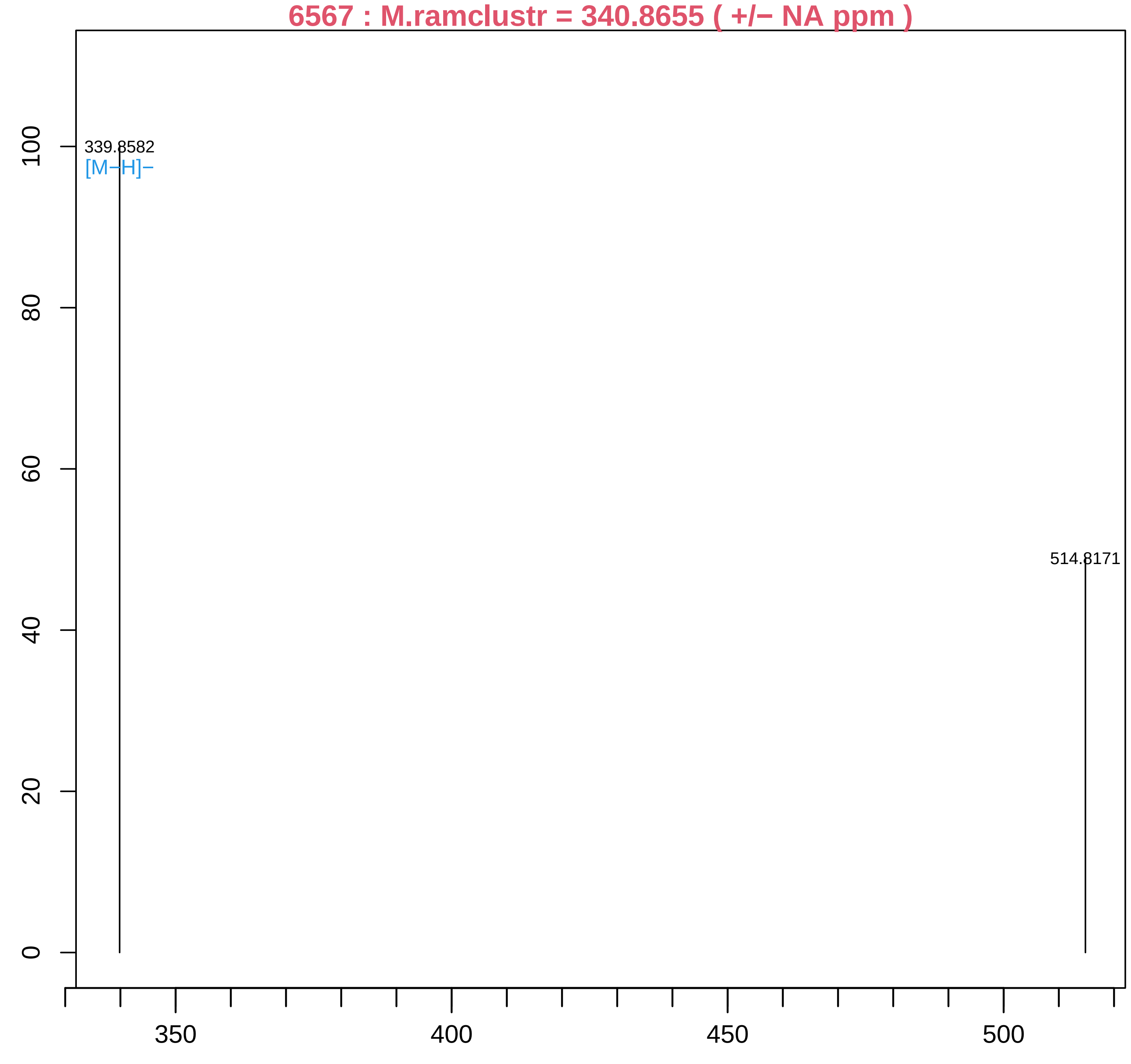
6567 : M.ramclustr = 340.8655 (+/− NA ppm)
339.8582
[M−H]−
514.8171
0
20
40
60
80
100
350
400
450
500

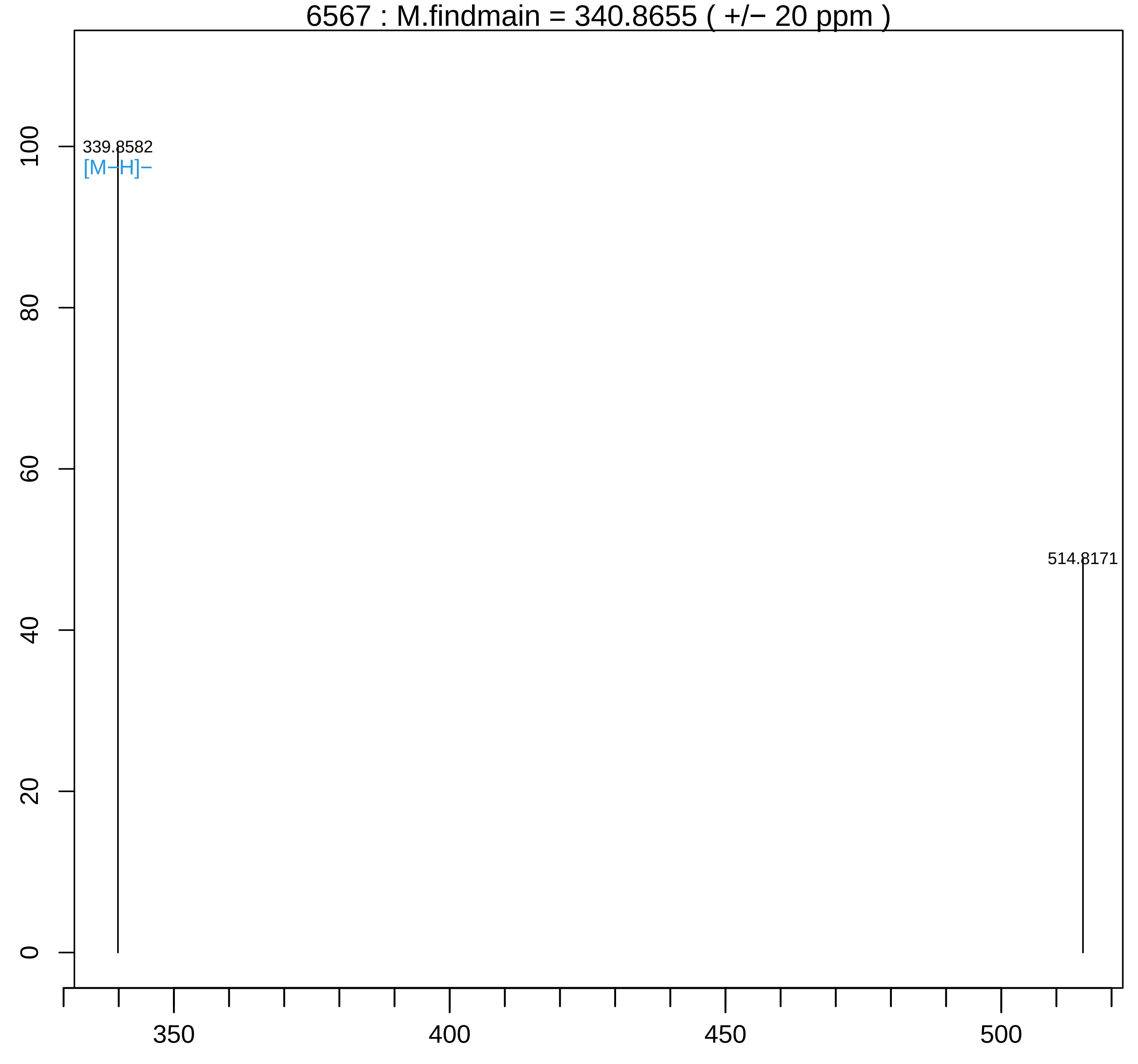
6567 : M.findmain = 340.8655 (+/− 20 ppm)
339.8582
[M−H]−
514.8171
0
20
40
60
80
100
350
400
450
500

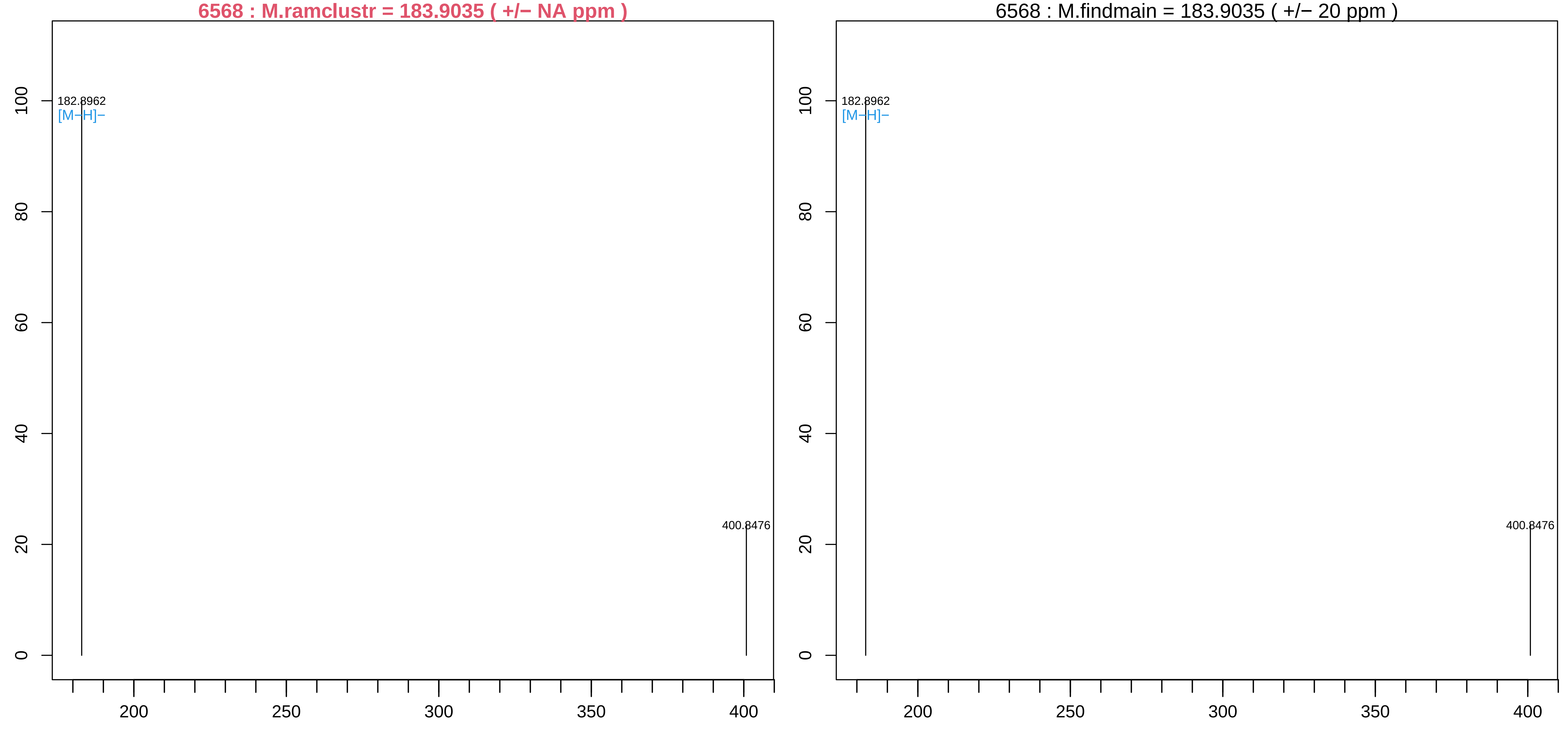

6568 : M.ramclustr = 183.9035 (+/− NA ppm)
182.8962
[M−H]−
400.8476
6568 : M.findmain = 183.9035 (+/− 20 ppm)
182.8962
[M−H]−
400.8476
0
20
40
60
80
100
200
250
300
350
400

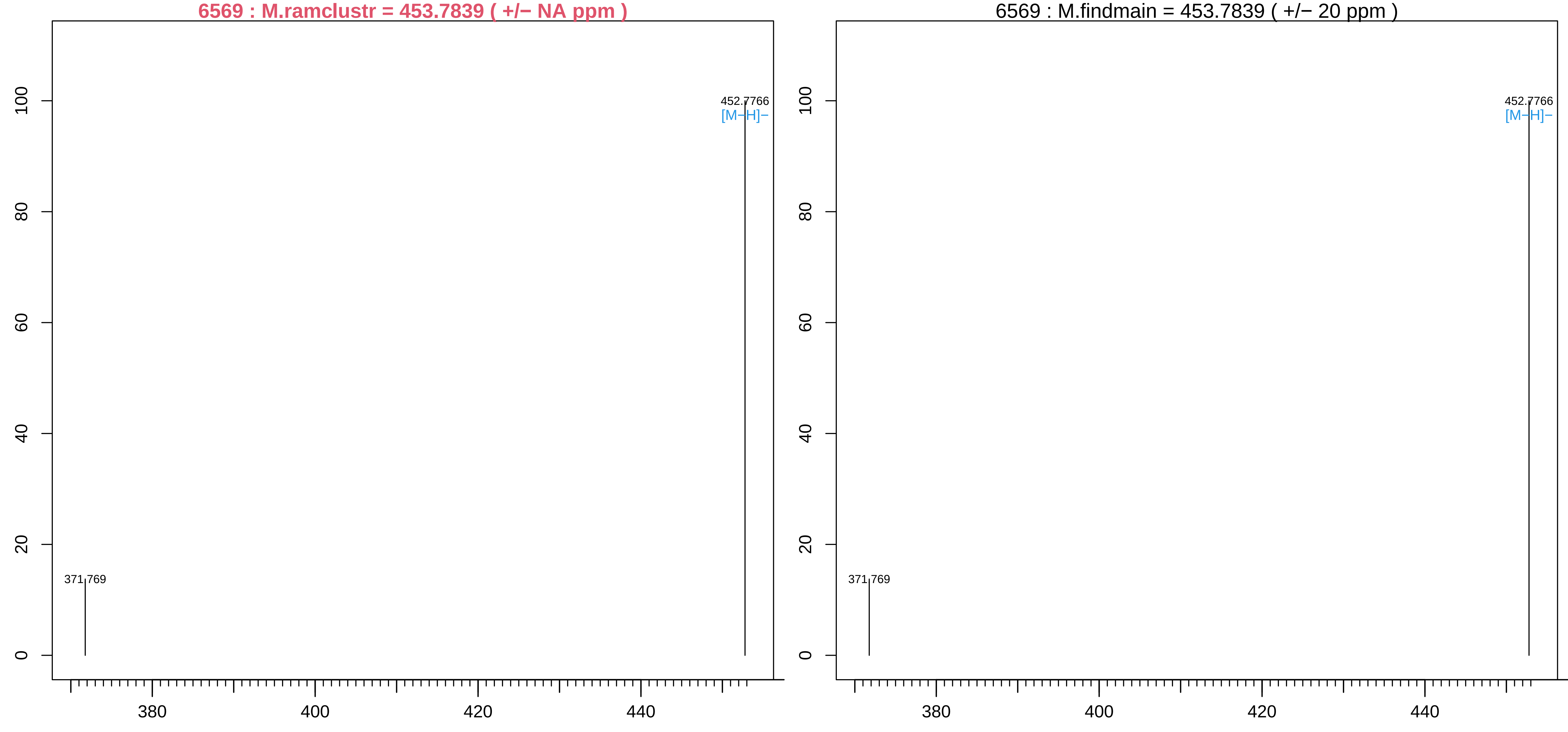

6569 : M.ramclustr = 453.7839 (+/− NA ppm)
452.7766
[M−H]−
371.769
6569 : M.findmain = 453.7839 (+/− 20 ppm)
452.7766
[M−H]−
371.769
0
20
40
60
80
100
380
400
420
440

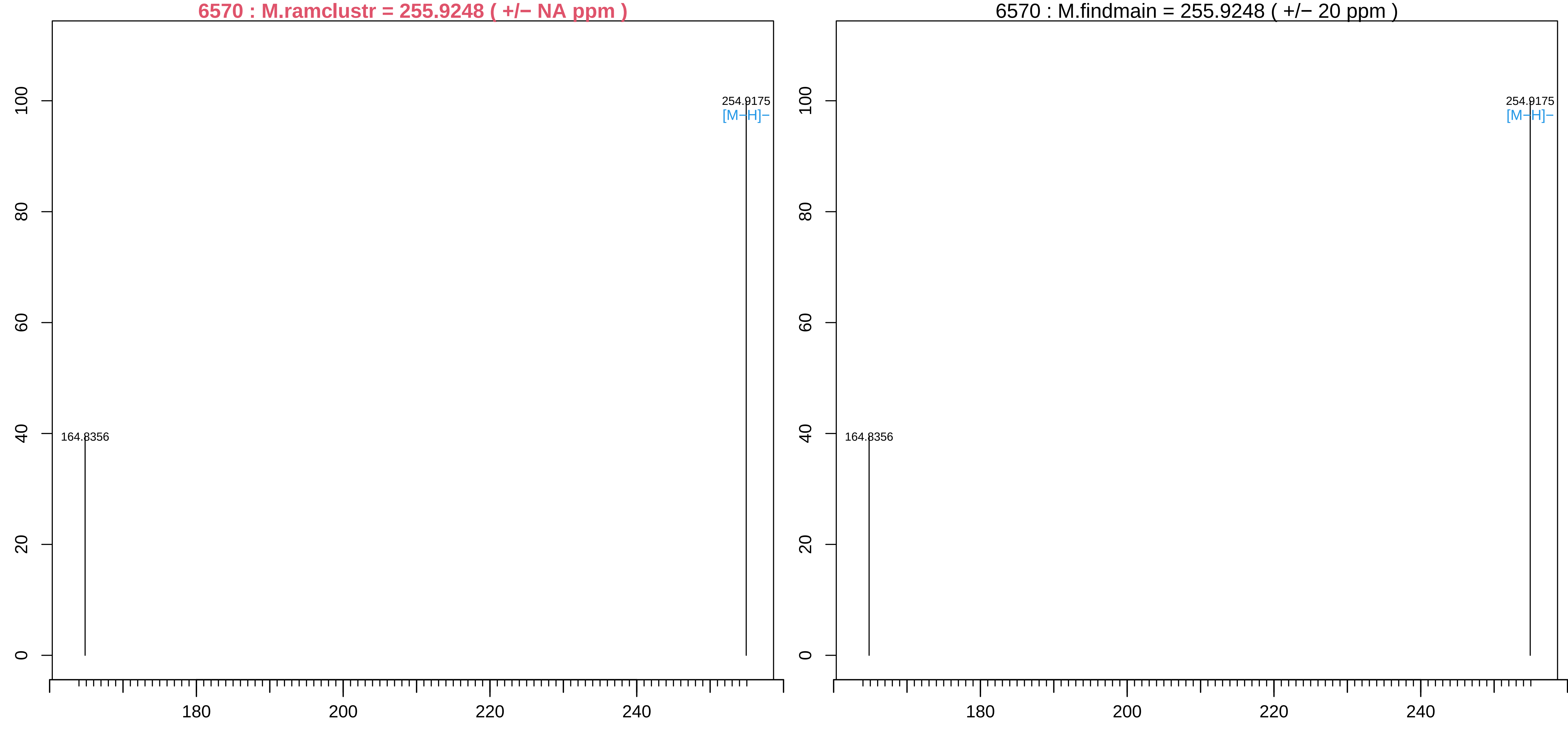

6570 : M.ramclustr = 255.9248 (+/− NA ppm)
254.9175
[M−H]−
164.8356
0
20
40
60
80
100
180
200
220
240
6570 : M.findmain = 255.9248 (+/− 20 ppm)
254.9175
[M−H]−
164.8356
0
20
40
60
80
100
180
200
220
240

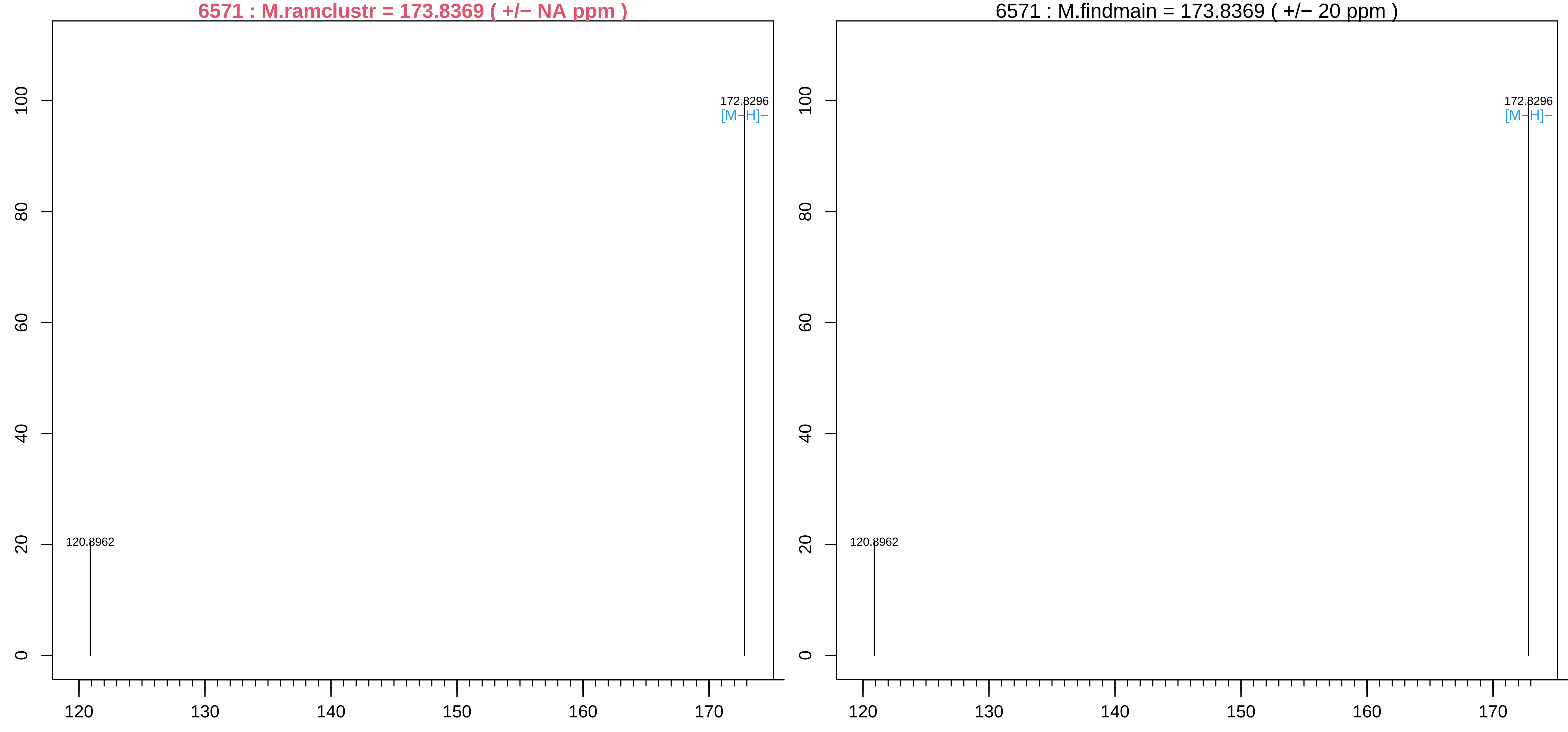
6571 : M.ramclustr = 173.8369 (+/− NA ppm)
172.8296
[M−H]−
120.8962
6571 : M.findmain = 173.8369 (+/− 20 ppm)
172.8296
[M−H]−
120.8962
0
20
40
60
80
100
120
130
140
150
160
170

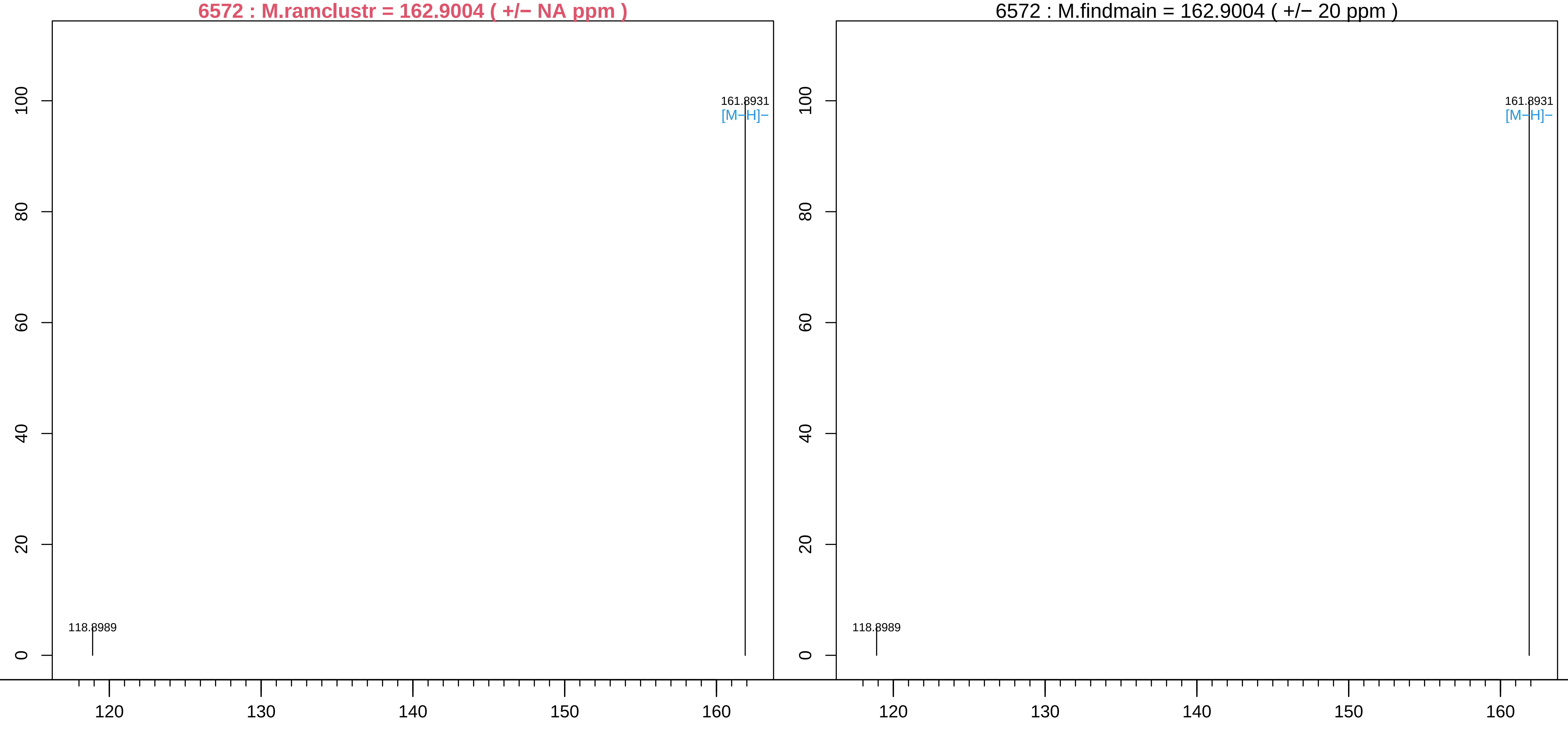

6572 : M.ramclustr = 162.9004 (+/− NA ppm)
161.8931
[M−H]−
118.8989
6572 : M.findmain = 162.9004 (+/− 20 ppm)
161.8931
[M−H]−
118.8989

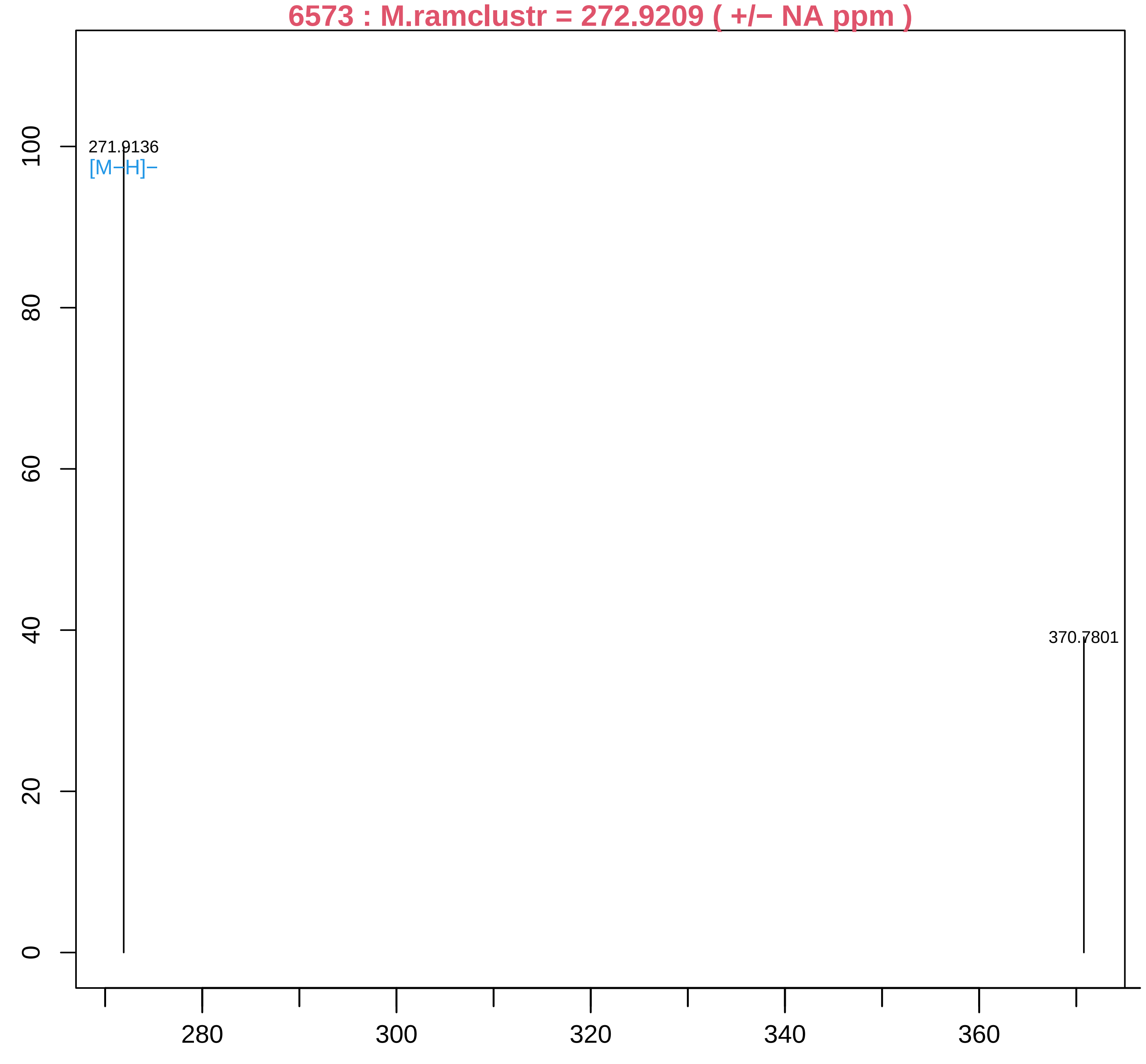
6573 : M.ramclustr = 272.9209 (+/− NA ppm)
271.9136
[M−H]−
370.7801
0
20
40
60
80
100
280
300
320
340
360

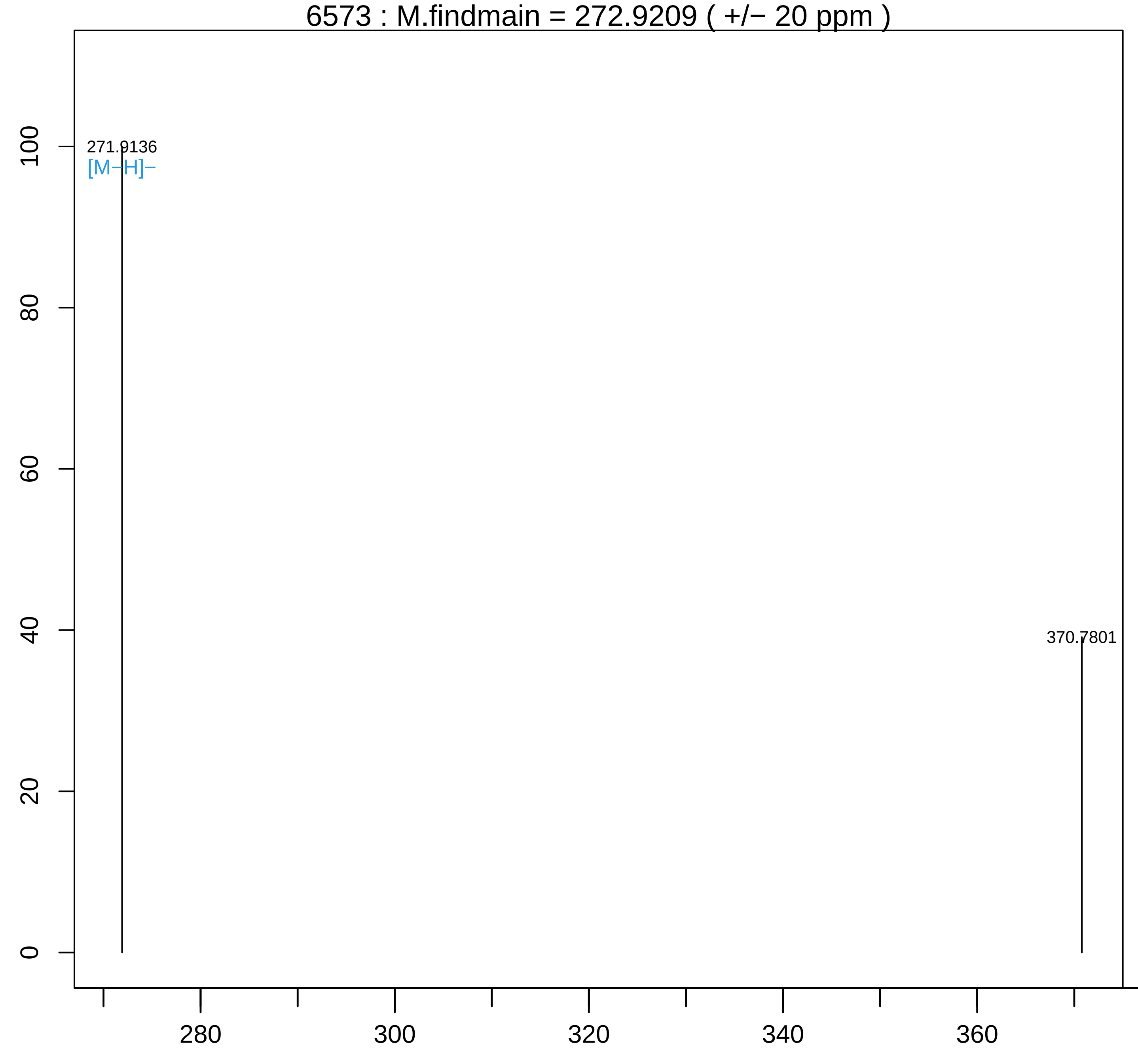
6573 : M.findmain = 272.9209 (+/− 20 ppm)
271.9136
[M−H]−
370.7801
0
20
40
60
80
100
280
300
320
340
360

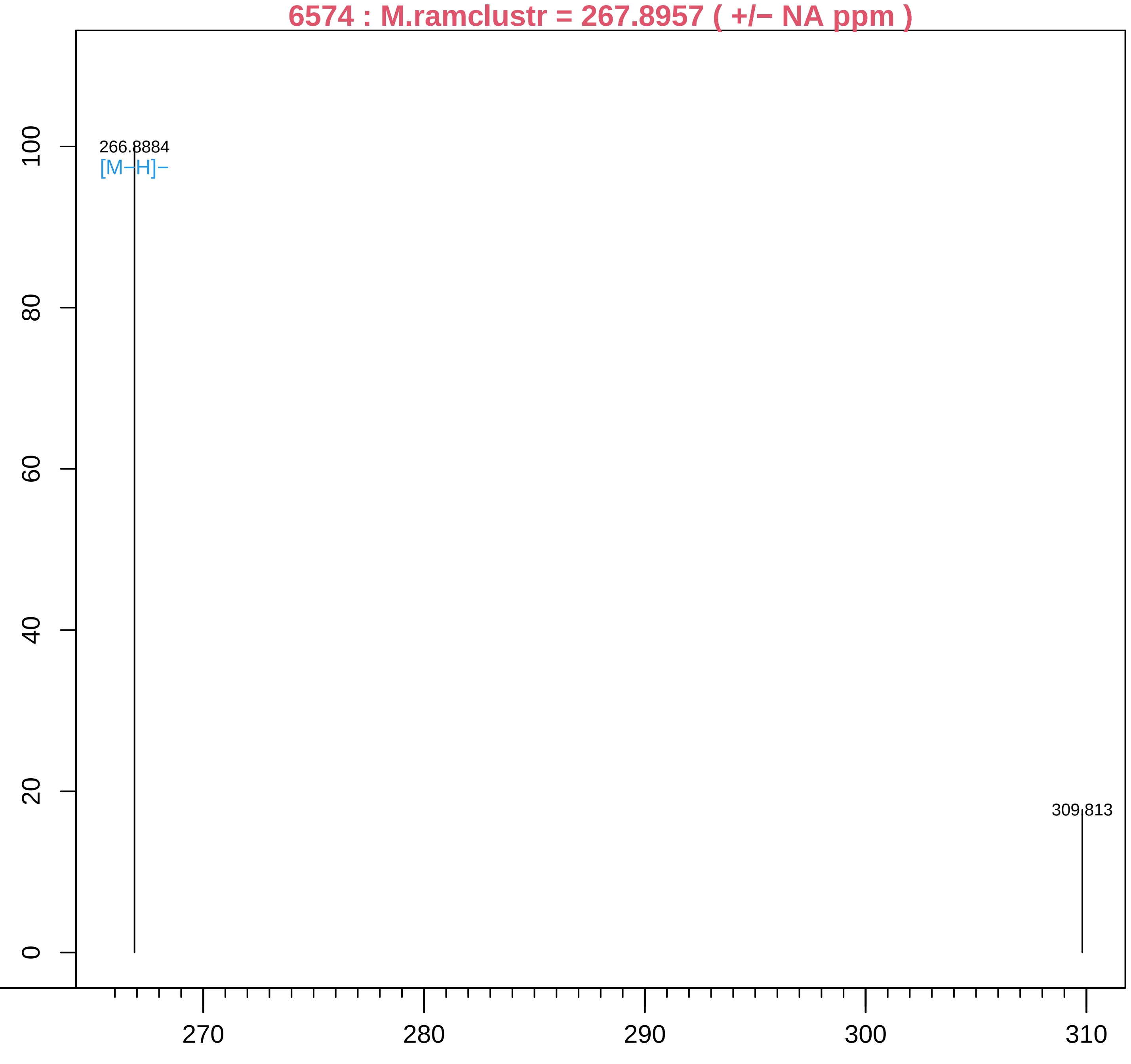
6574 : M.ramclustr = 267.8957 (+/− NA ppm)
266.8884
[M−H]−
309.813
0
20
40
60
80
100
270
280
290
300
310

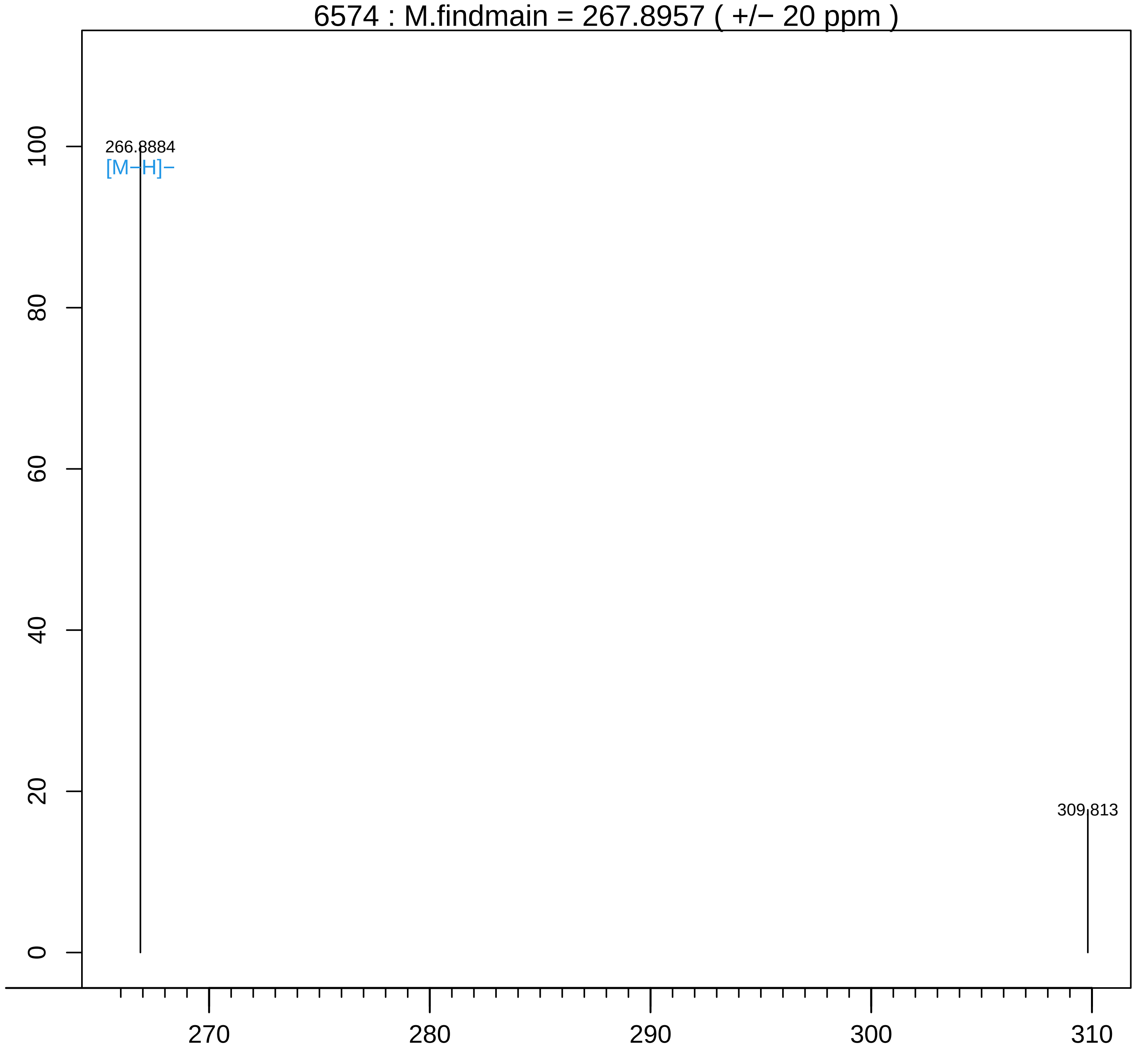
6574 : M.findmain = 267.8957 (+/− 20 ppm)
266.8884
[M−H]−
309.813
0
20
40
60
80
100
270
280
290
300
310

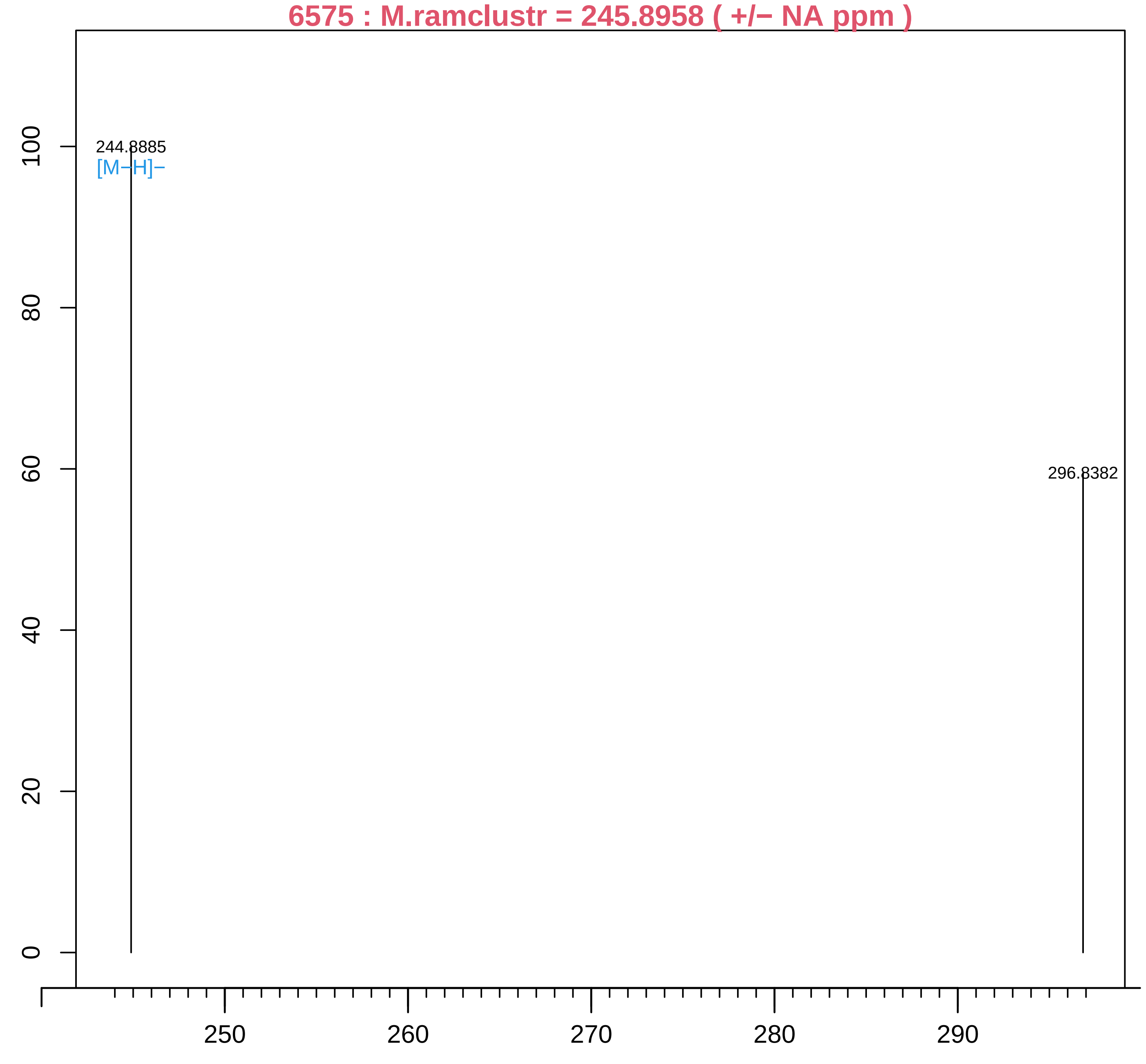
6575 : M.ramclustr = 245.8958 (+/− NA ppm)
244.8885
[M−H]−
296.8382
0
20
40
60
80
100
250
260
270
280
290

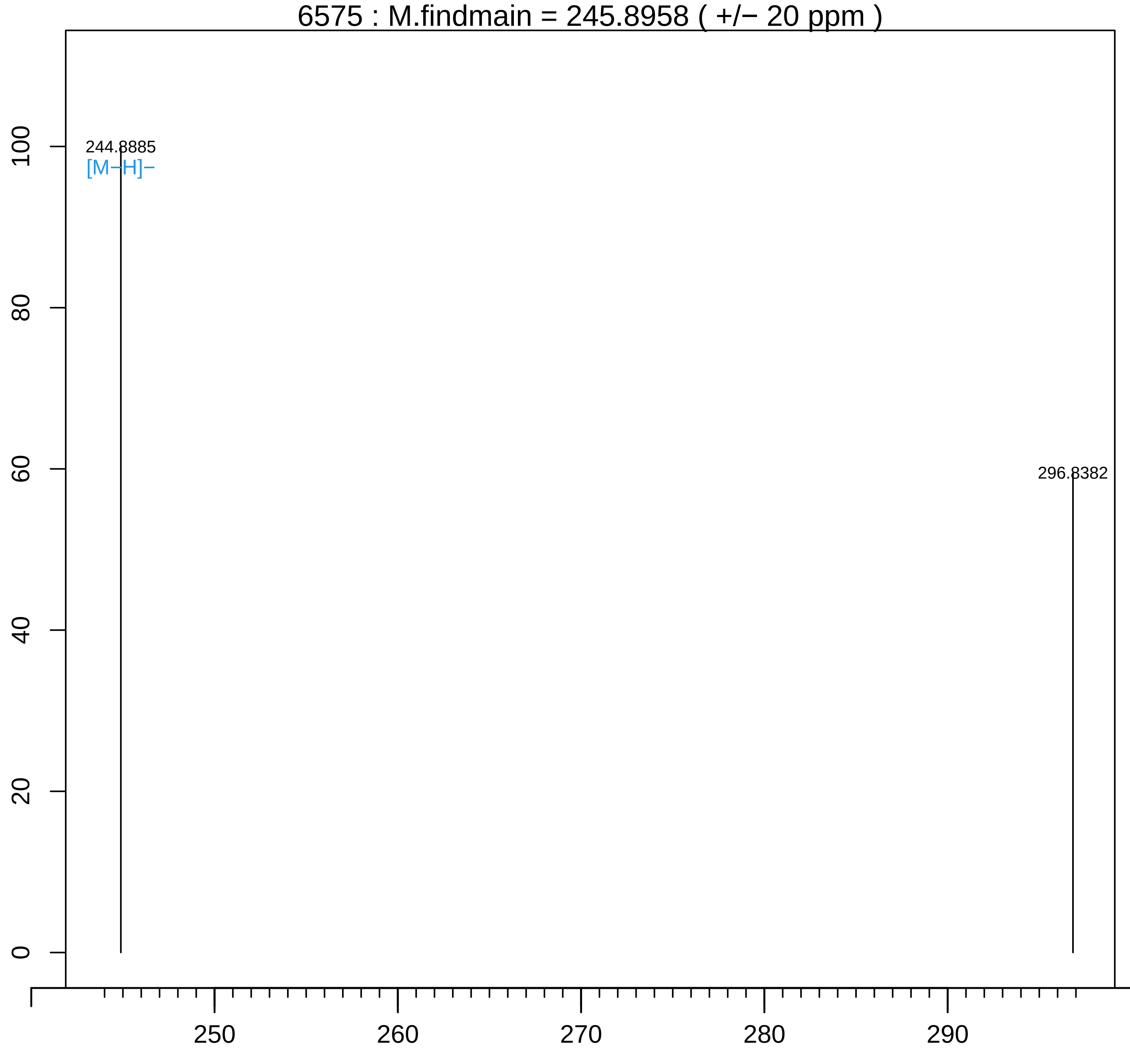
6575 : M.findmain = 245.8958 (+/− 20 ppm)
244.8885
[M−H]−
296.8382
0
20
40
60
80
100
250
260
270
280
290

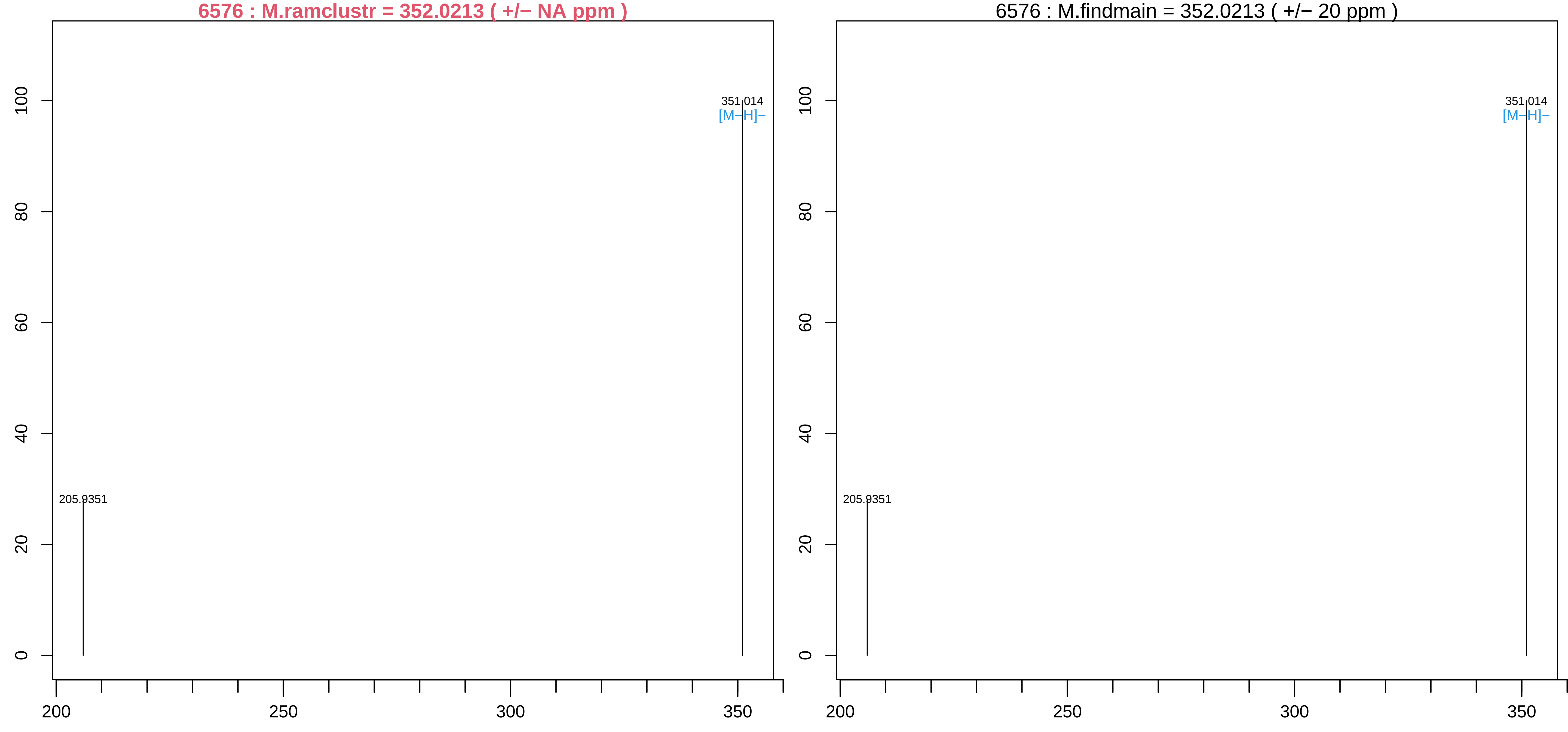

6576 : M.ramclustr = 352.0213 (+/− NA ppm)
351.014
[M−H]−
205.9351
0
20
40
60
80
100
200
250
300
350
6576 : M.findmain = 352.0213 (+/− 20 ppm)
351.014
[M−H]−
205.9351
0
20
40
60
80
100
200
250
300
350

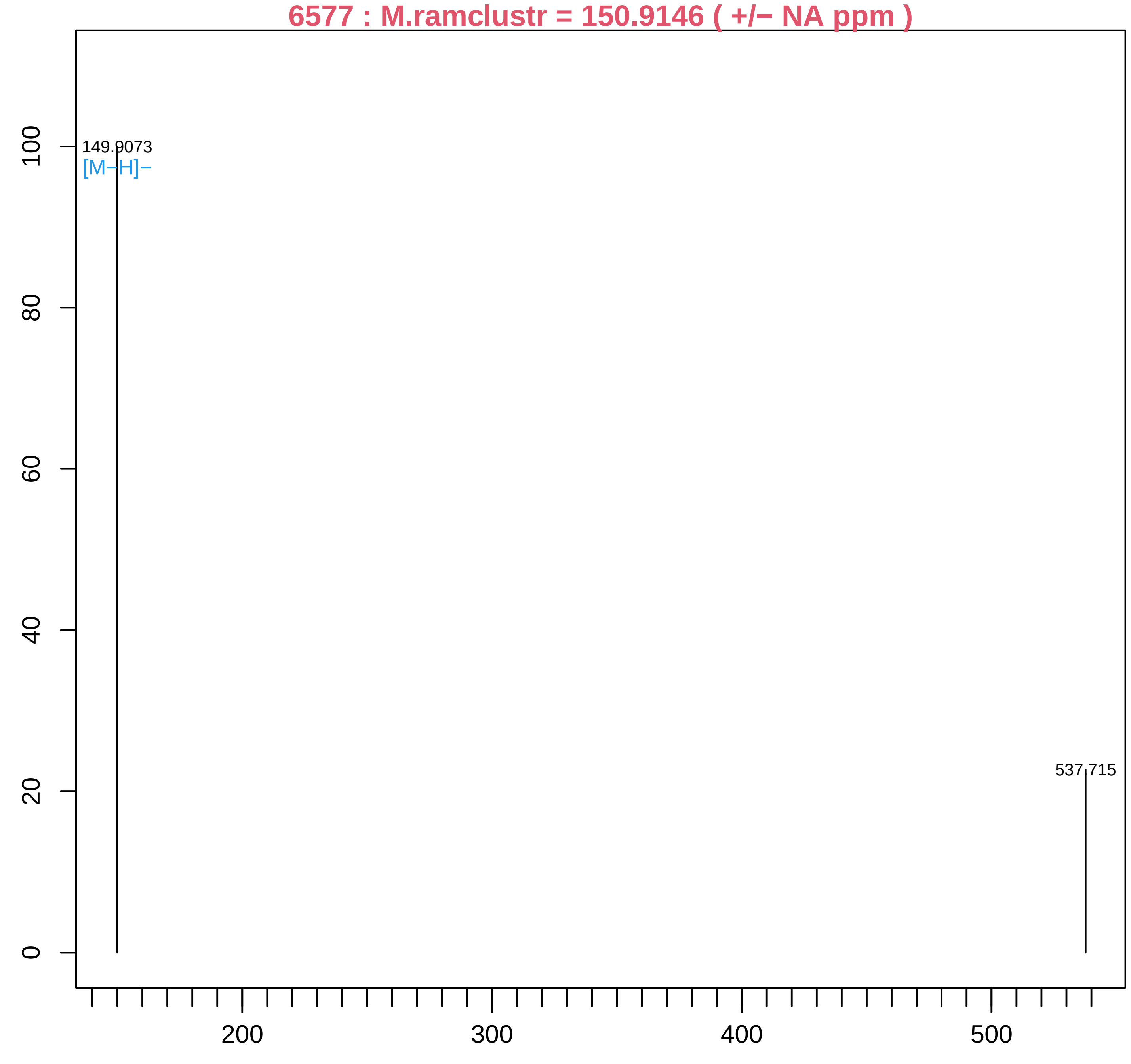
6577 : M.ramclustr = 150.9146 (+/− NA ppm)
149.9073
[M−H]−
537.715
0
20
40
60
80
100
200
300
400
500

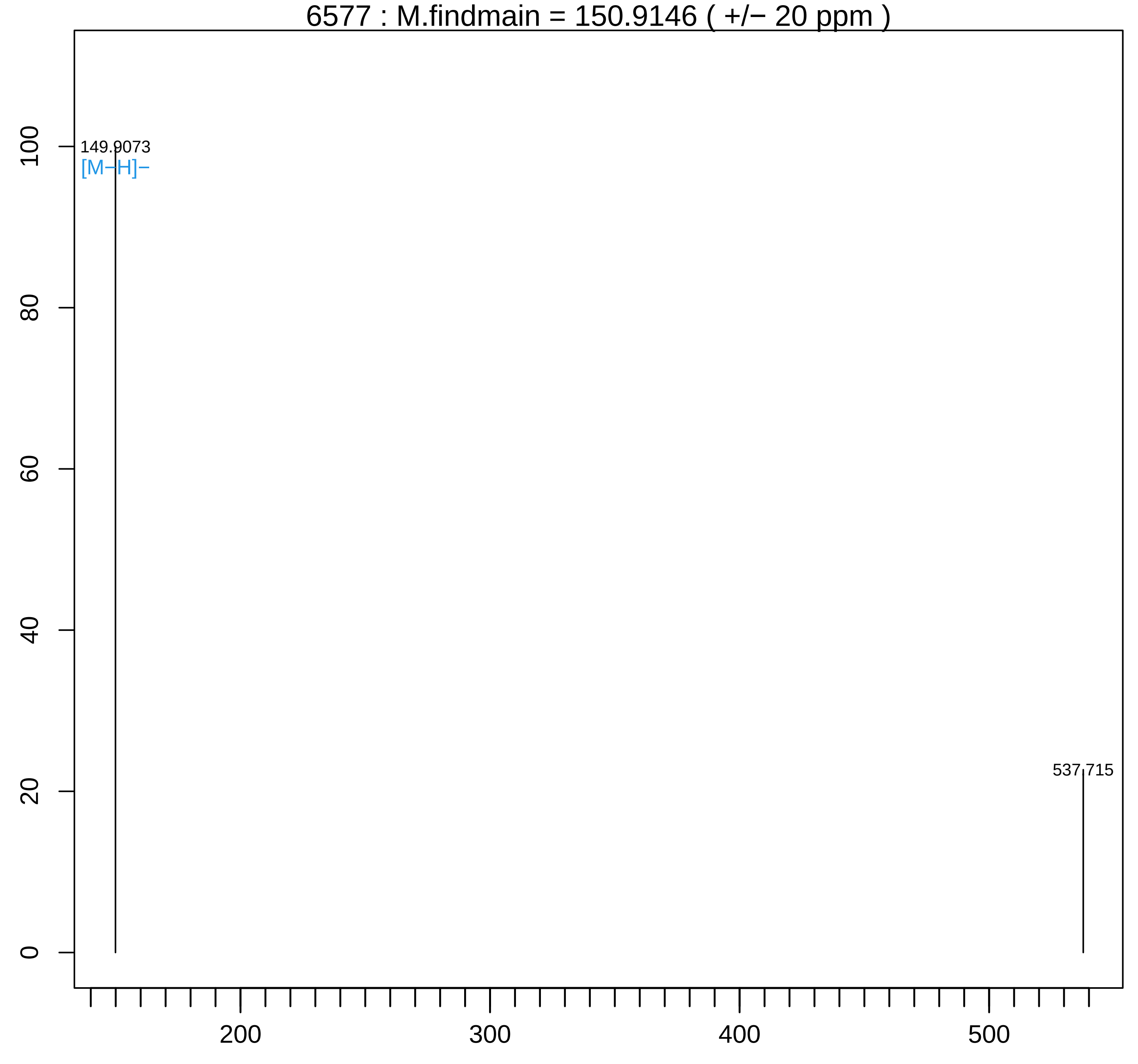
6577 : M.findmain = 150.9146 (+/− 20 ppm)
149.9073
[M−H]−
537.715
0
20
40
60
80
100
200
300
400
500

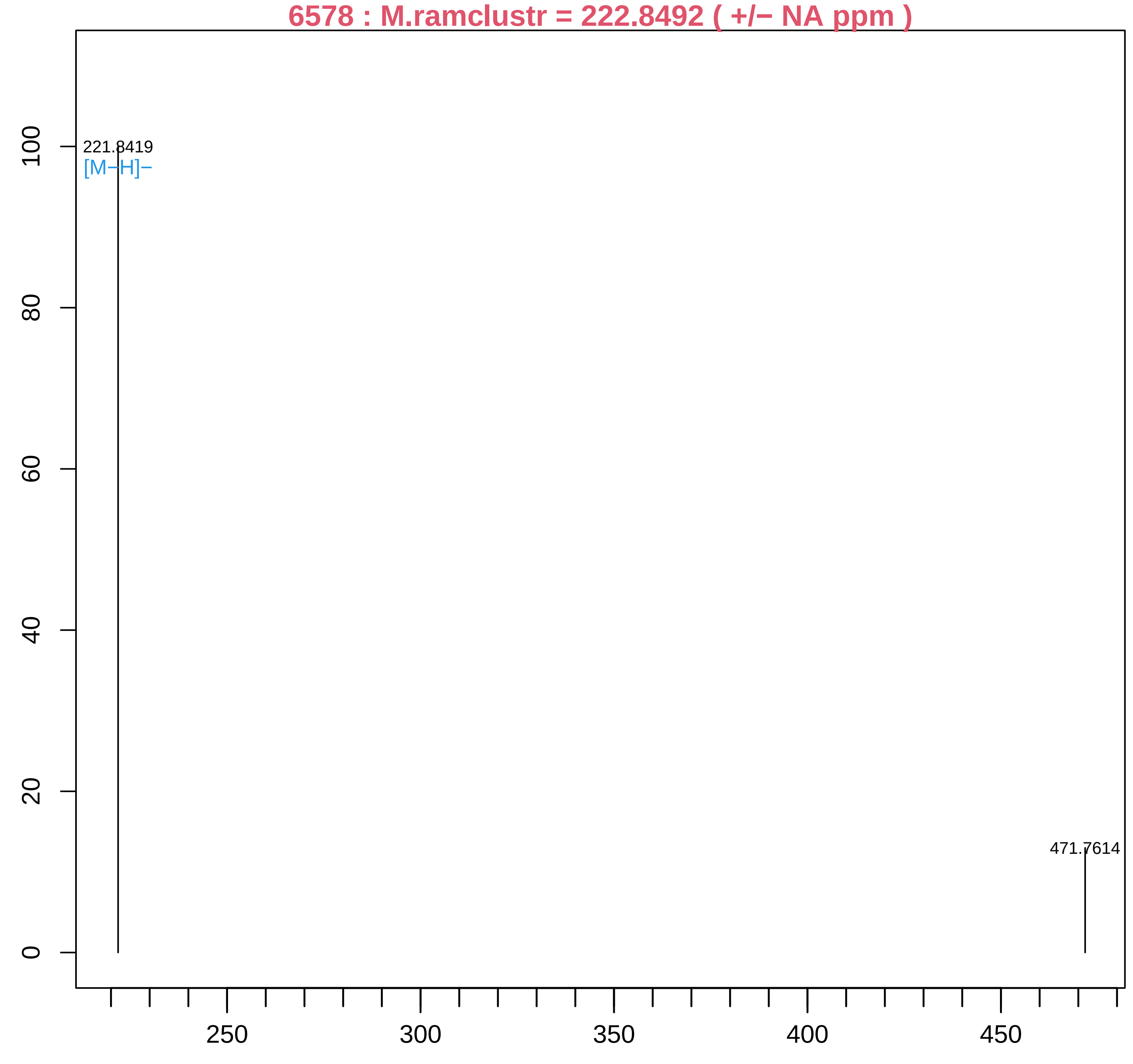
6578 : M.ramclustr = 222.8492 (+/− NA ppm)
221.8419
[M−H]−
471.7614
0
20
40
60
80
100
250
300
350
400
450

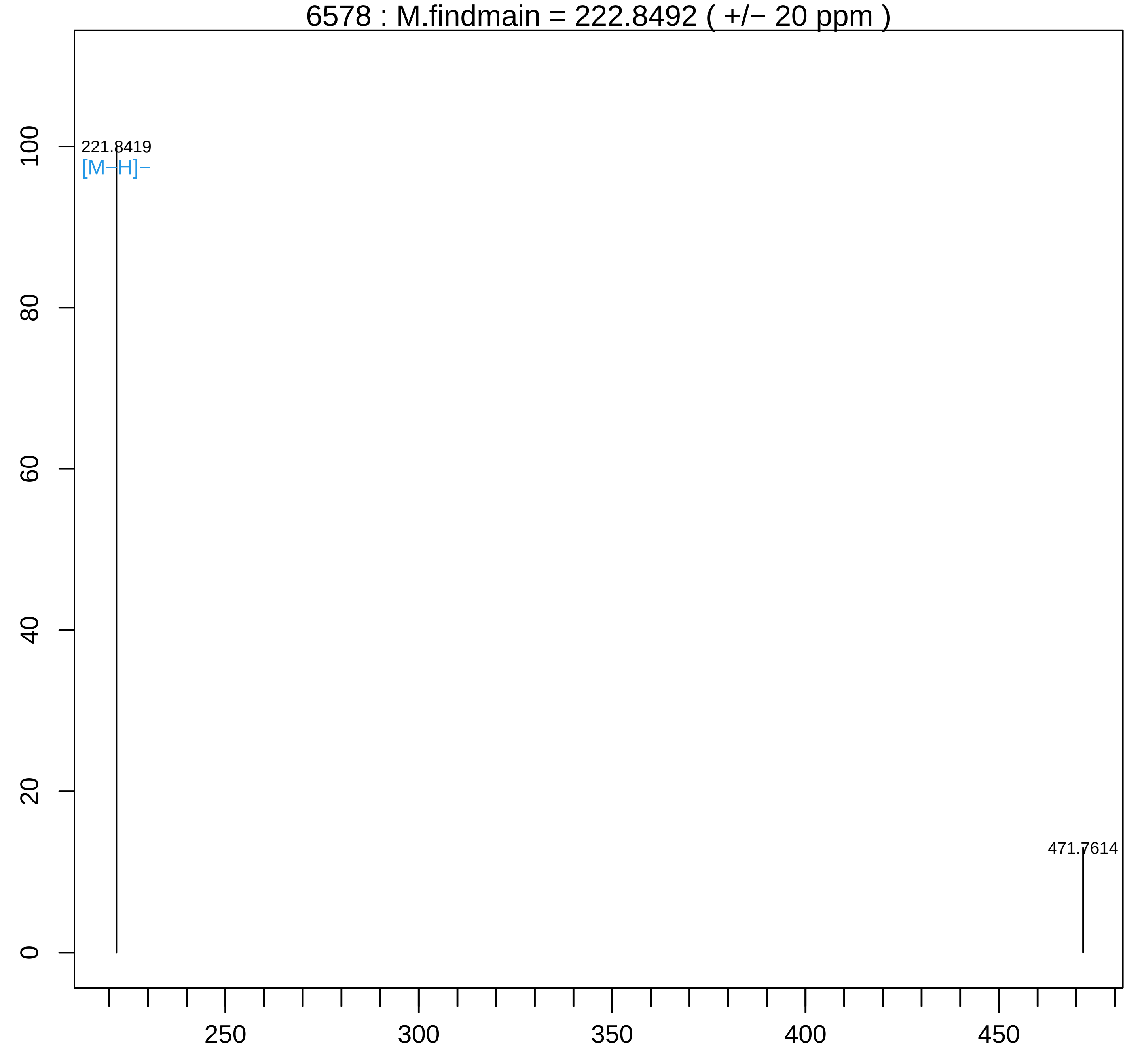
6578 : M.findmain = 222.8492 (+/− 20 ppm)
221.8419
[M−H]−
471.7614
0
20
40
60
80
100
250
300
350
400
450

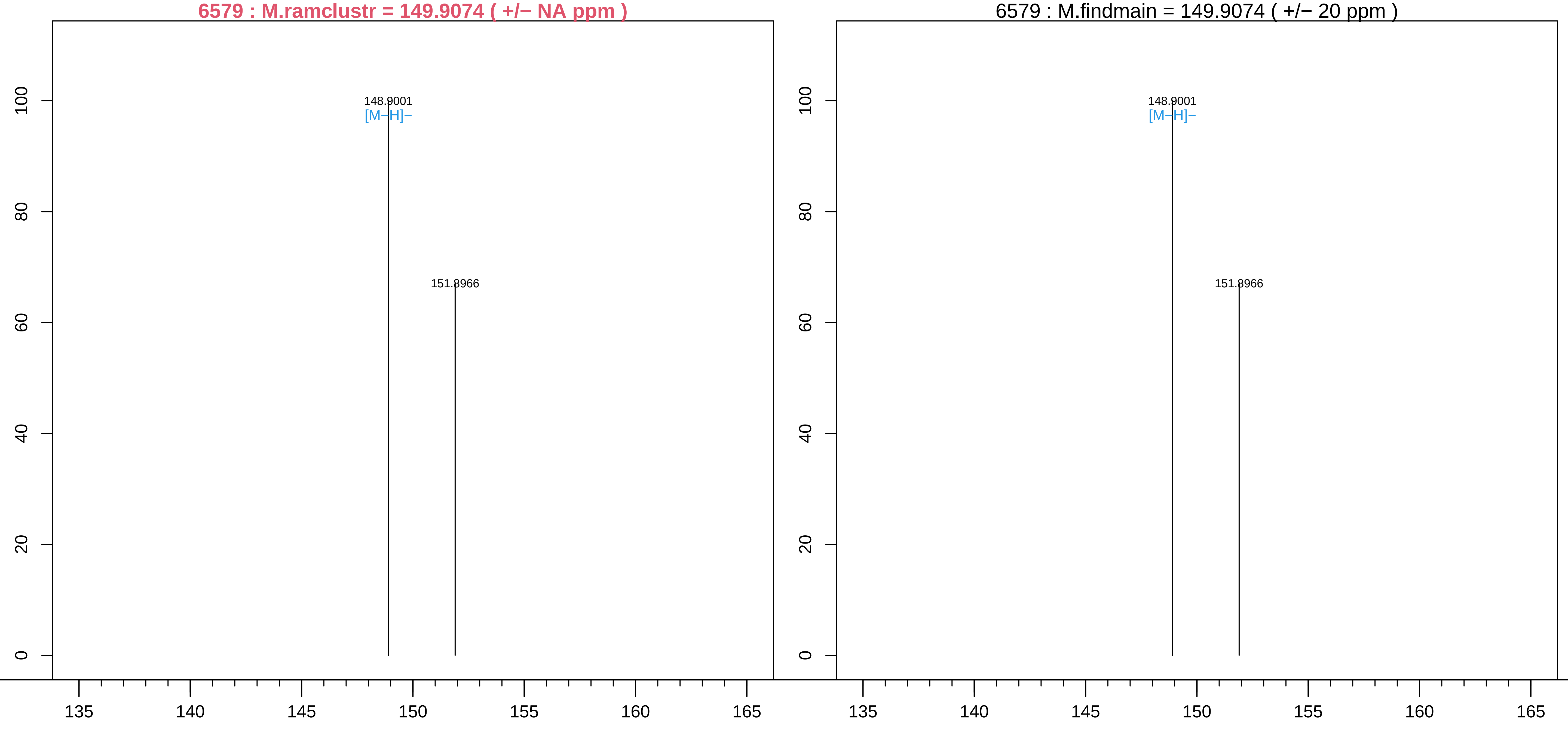
6579 : M.ramclustr = 149.9074 (+/− NA ppm)
148.9001
[M−H]−
151.8966
6579 : M.findmain = 149.9074 (+/− 20 ppm)
148.9001
[M−H]−
151.8966
0
20
40
60
80
100
135
140
145
150
155
160
165

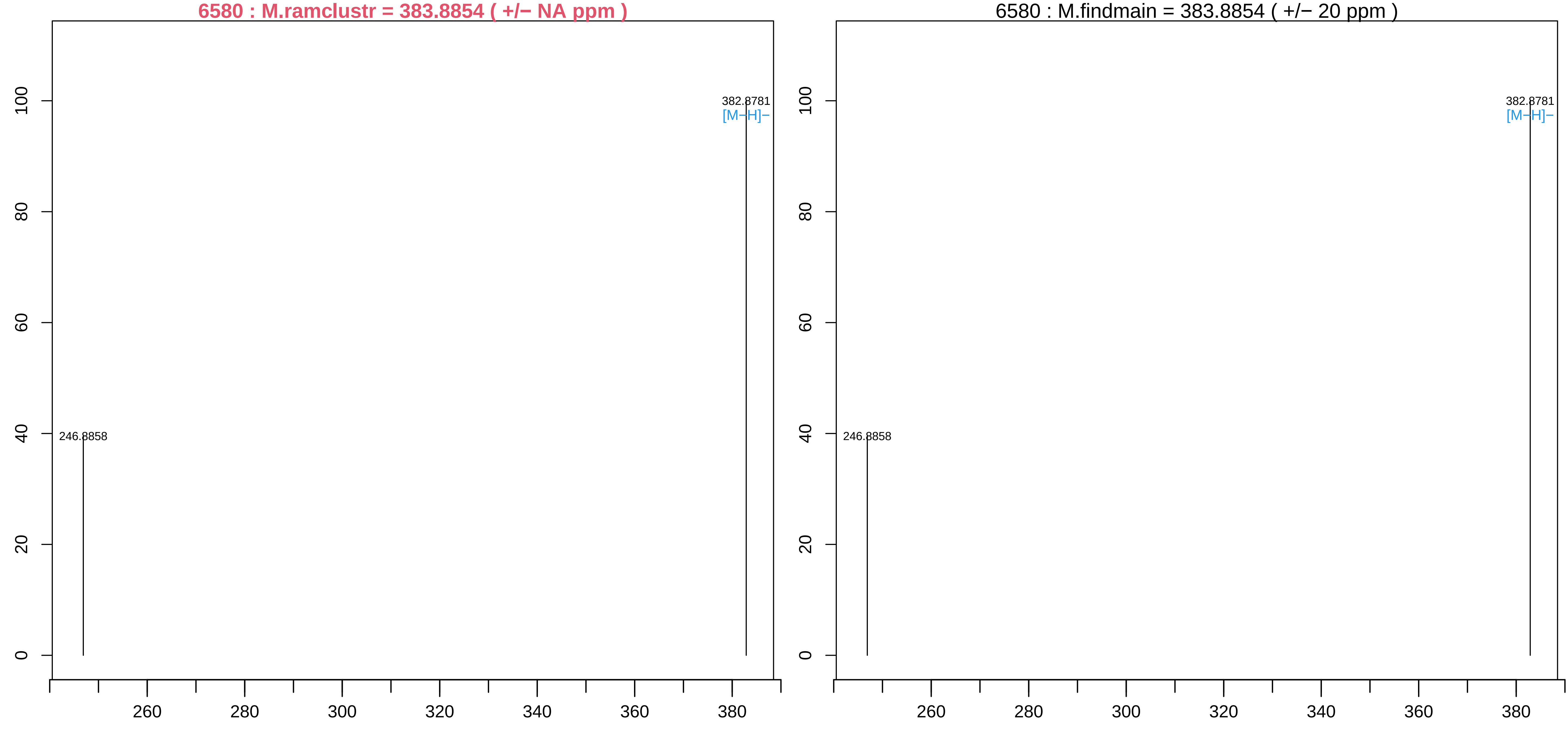
6580 : M.ramclustr = 383.8854 (+/− NA ppm)
382.8781
[M−H]−
246.8858
0
20
40
60
80
100
260
280
300
320
340
360
380
6580 : M.findmain = 383.8854 (+/− 20 ppm)
382.8781
[M−H]−
246.8858
0
20
40
60
80
100
260
280
300
320
340
360
380

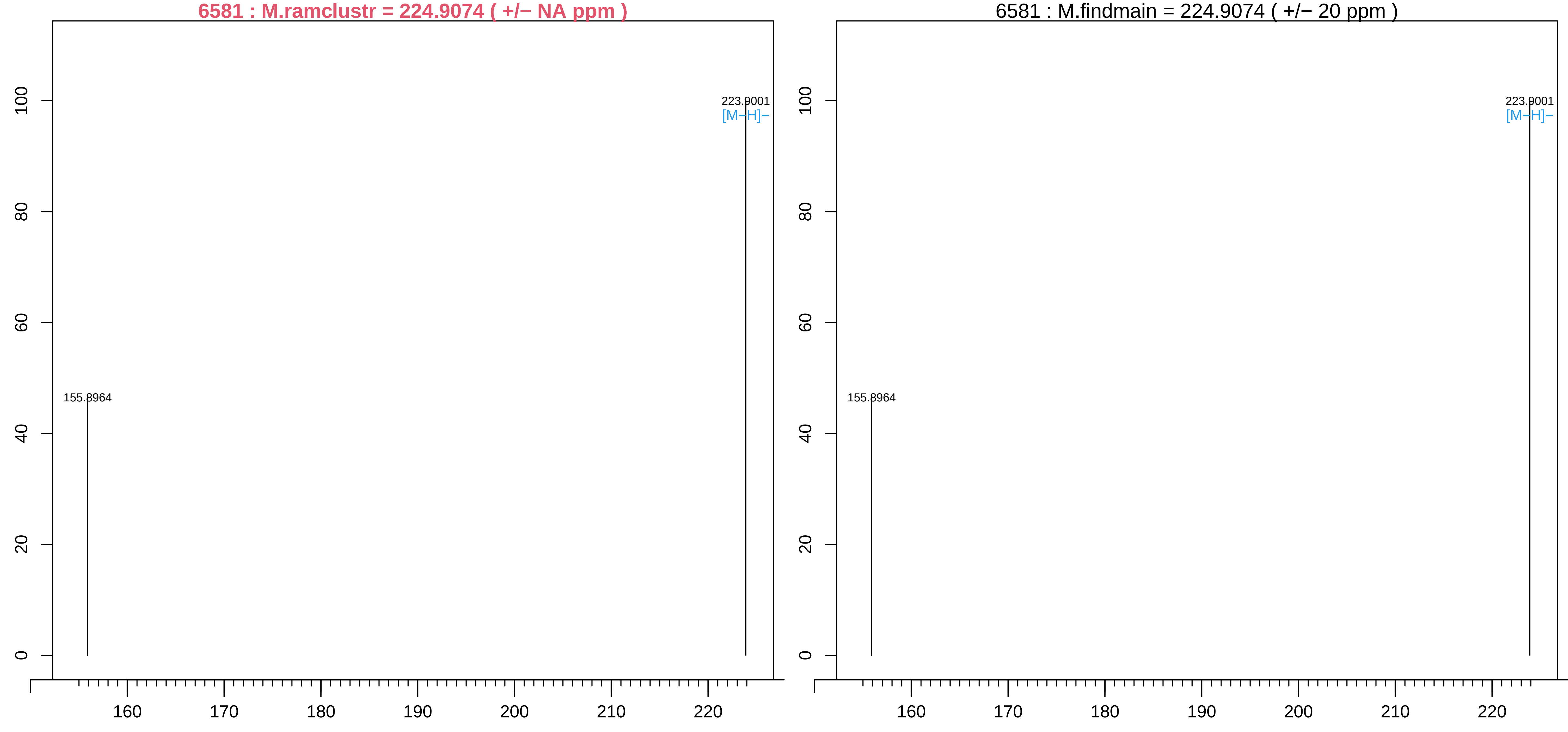

6581 : M.ramclustr = 224.9074 (+/− NA ppm)
6581 : M.findmain = 224.9074 (+/− 20 ppm)
223.9001
[M−H]−
155.8964
0
20
40
60
80
100
160
170
180
190
200
210
220

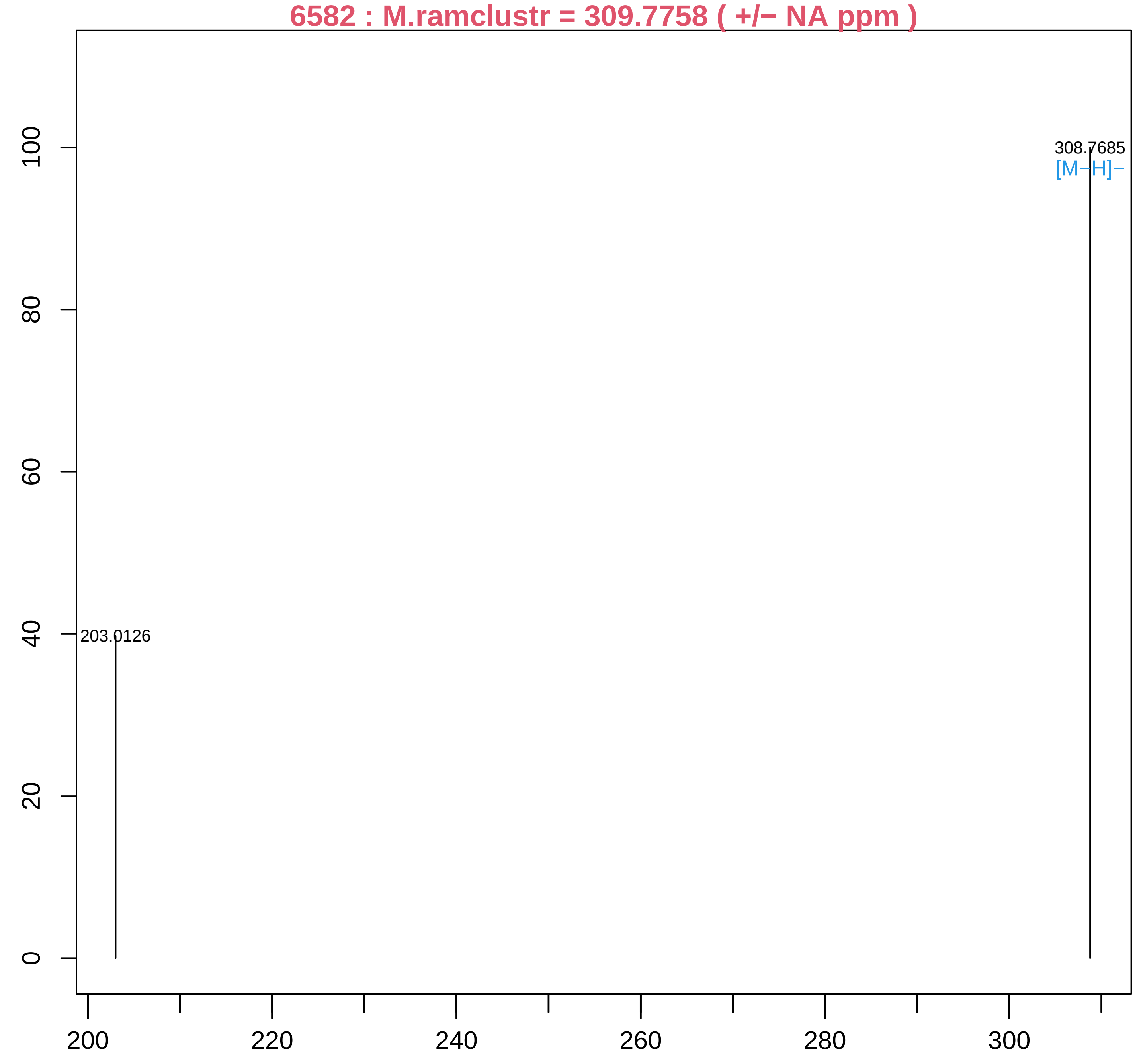
6582 : M.ramclustr = 309.7758 (+/− NA ppm)
308.7685
[M−H]−
203.0126
0
20
40
60
80
100
200
220
240
260
280
300

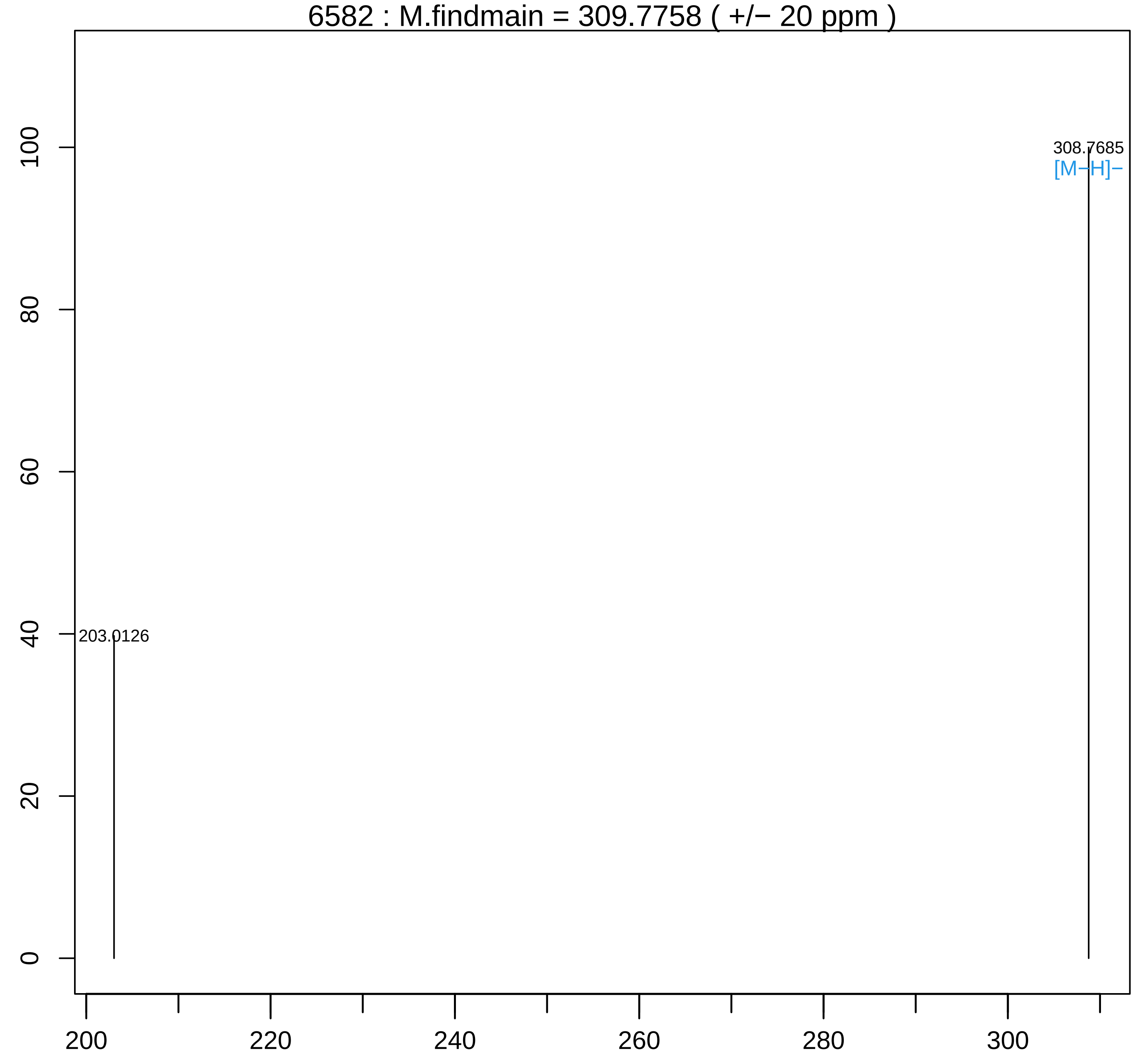
6582 : M.findmain = 309.7758 (+/− 20 ppm)
308.7685
[M−H]−
203.0126
0
20
40
60
80
100
200
220
240
260
280
300

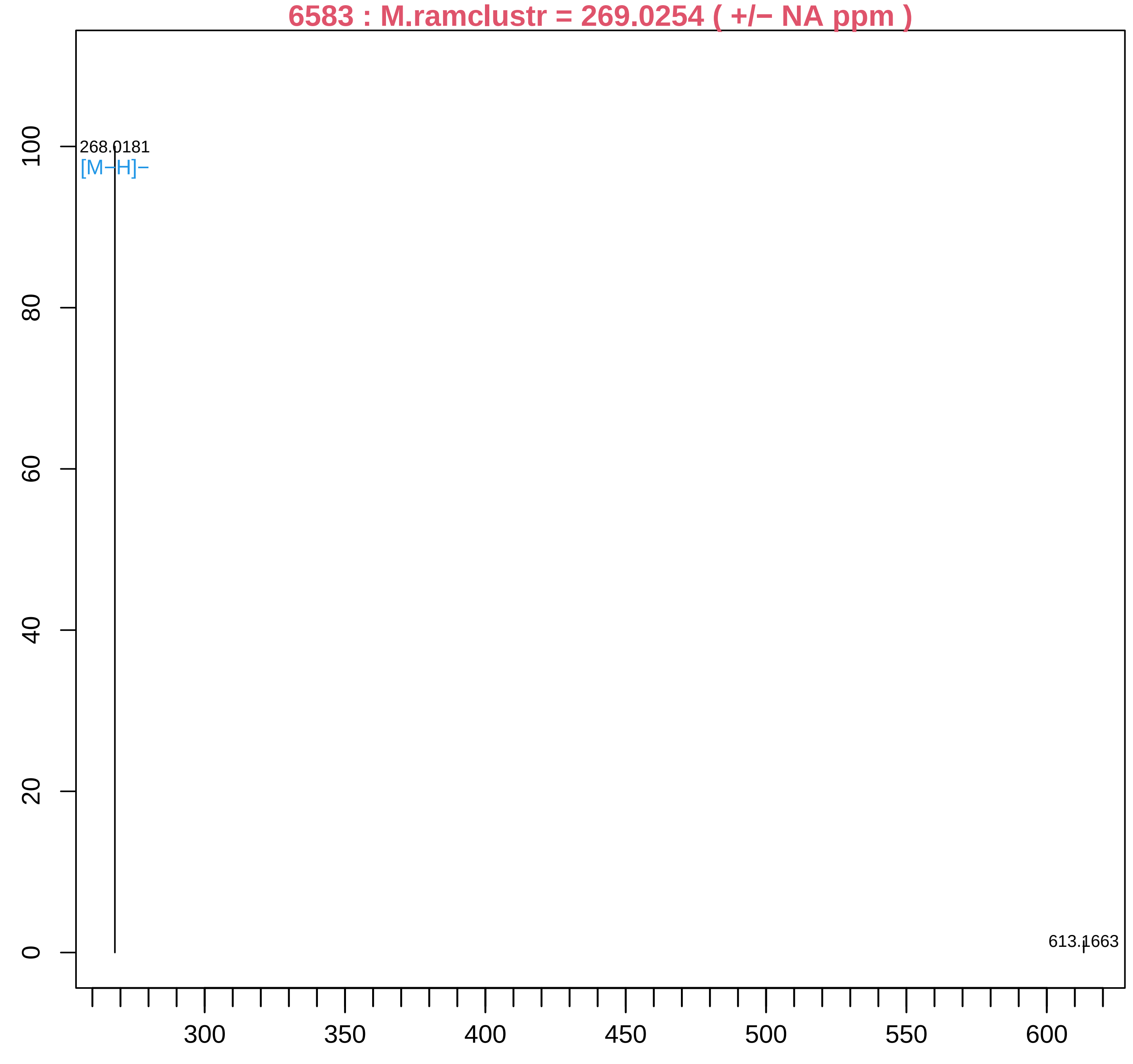
6583 : M.ramclustr = 269.0254 (+/− NA ppm)
268.0181
[M−H]−
613.1663
0
20
40
60
80
100
300
350
400
450
500
550
600

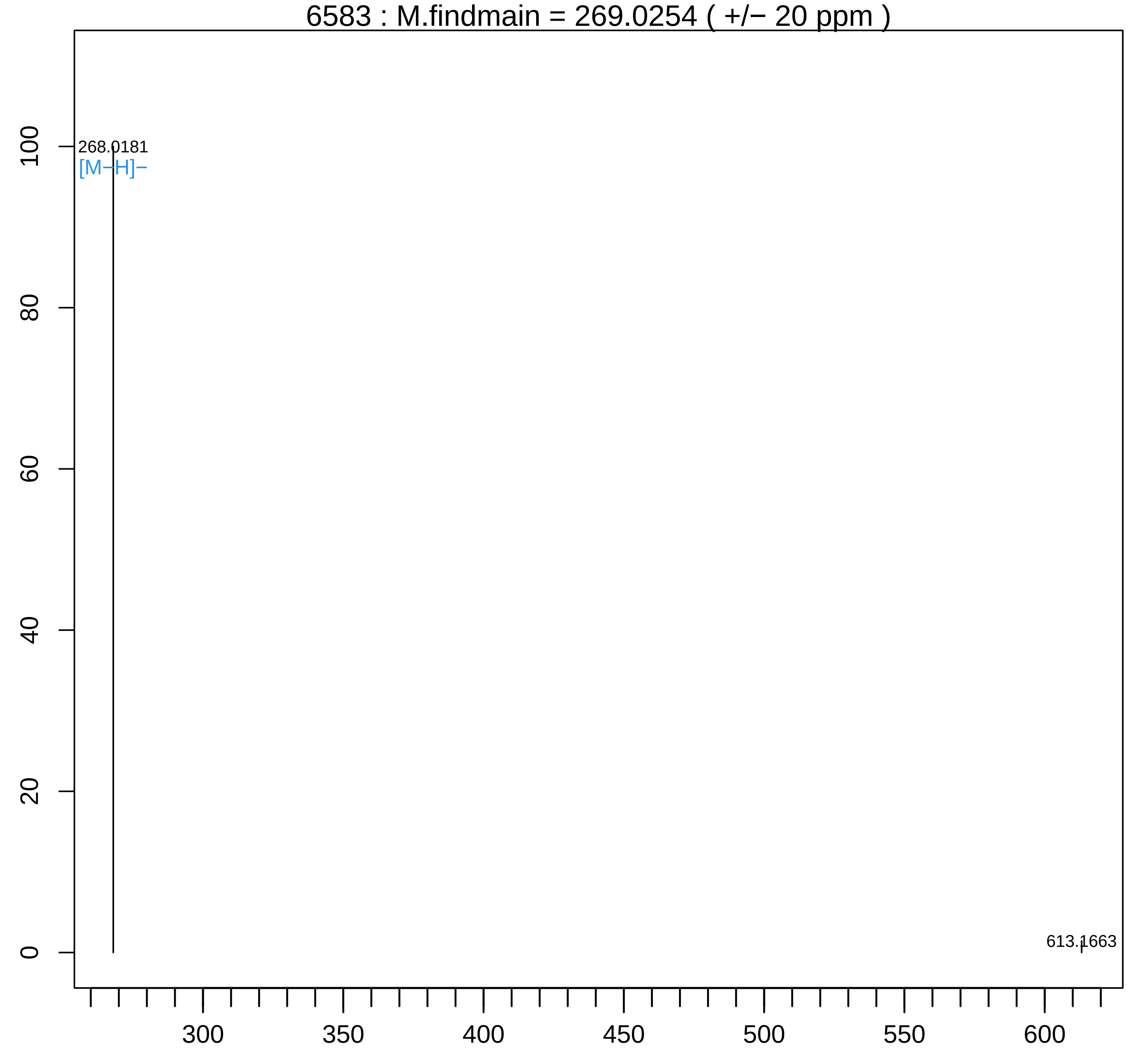
6583 : M.findmain = 269.0254 (+/− 20 ppm)
268.0181
[M−H]−
613.1663
0
20
40
60
80
100
300
350
400
450
500
550
600

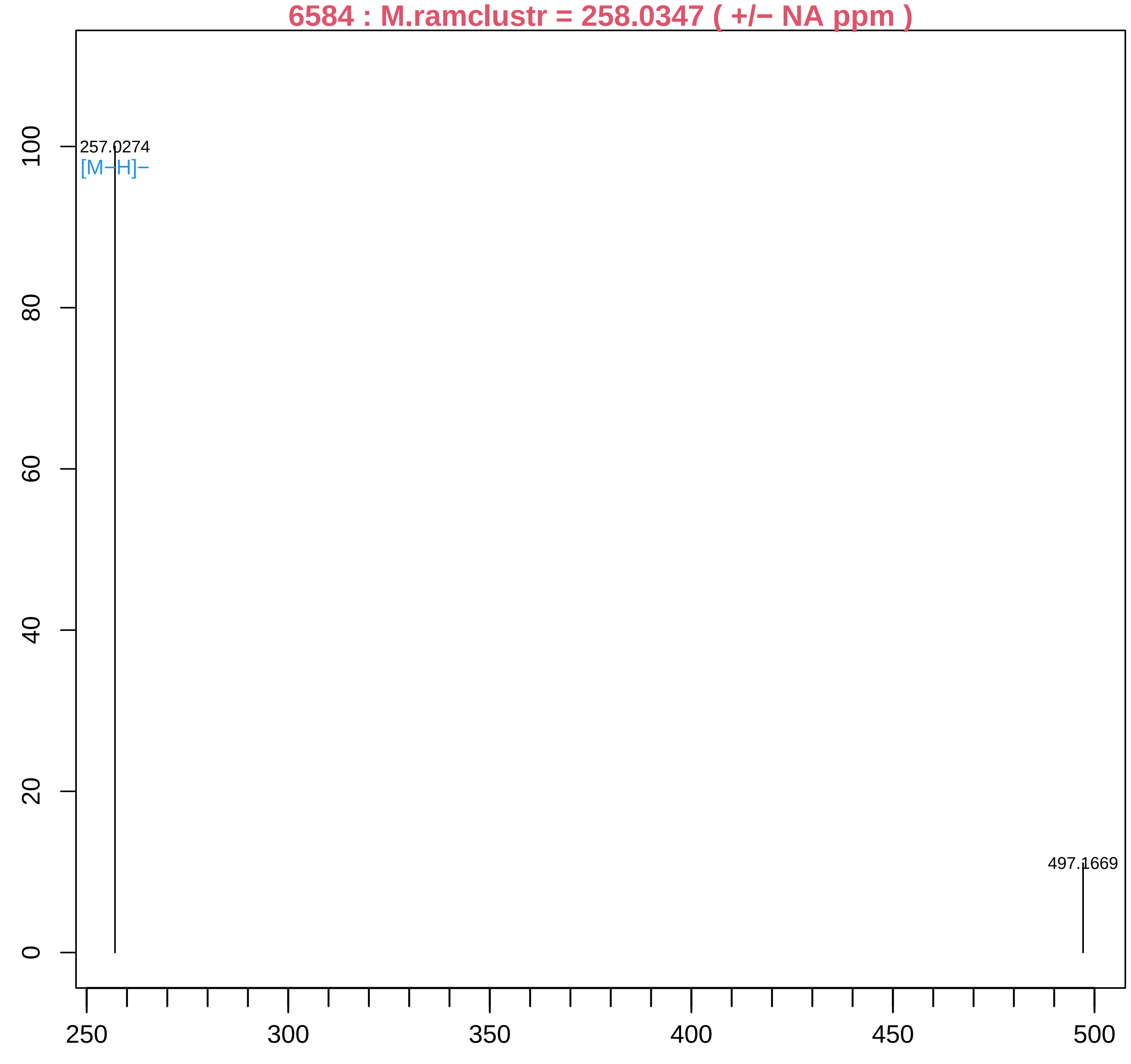
6584 : M.ramclustr = 258.0347 (+/− NA ppm)
257.0274
[M−H]−
497.1669
0
20
40
60
80
100
250
300
350
400
450
500

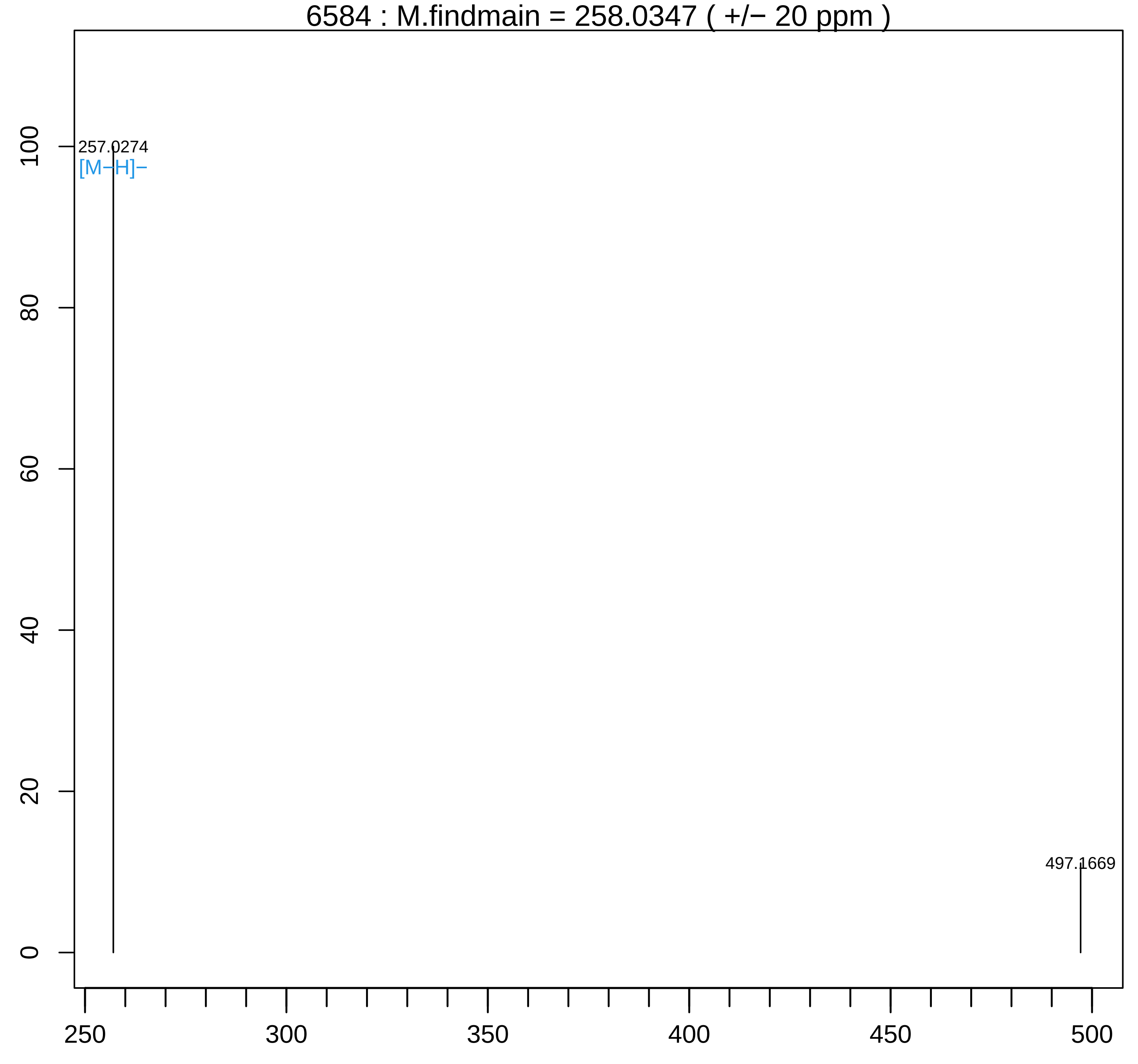
6584 : M.findmain = 258.0347 (+/− 20 ppm)
257.0274
[M−H]−
497.1669
0
20
40
60
80
100
250
300
350
400
450
500

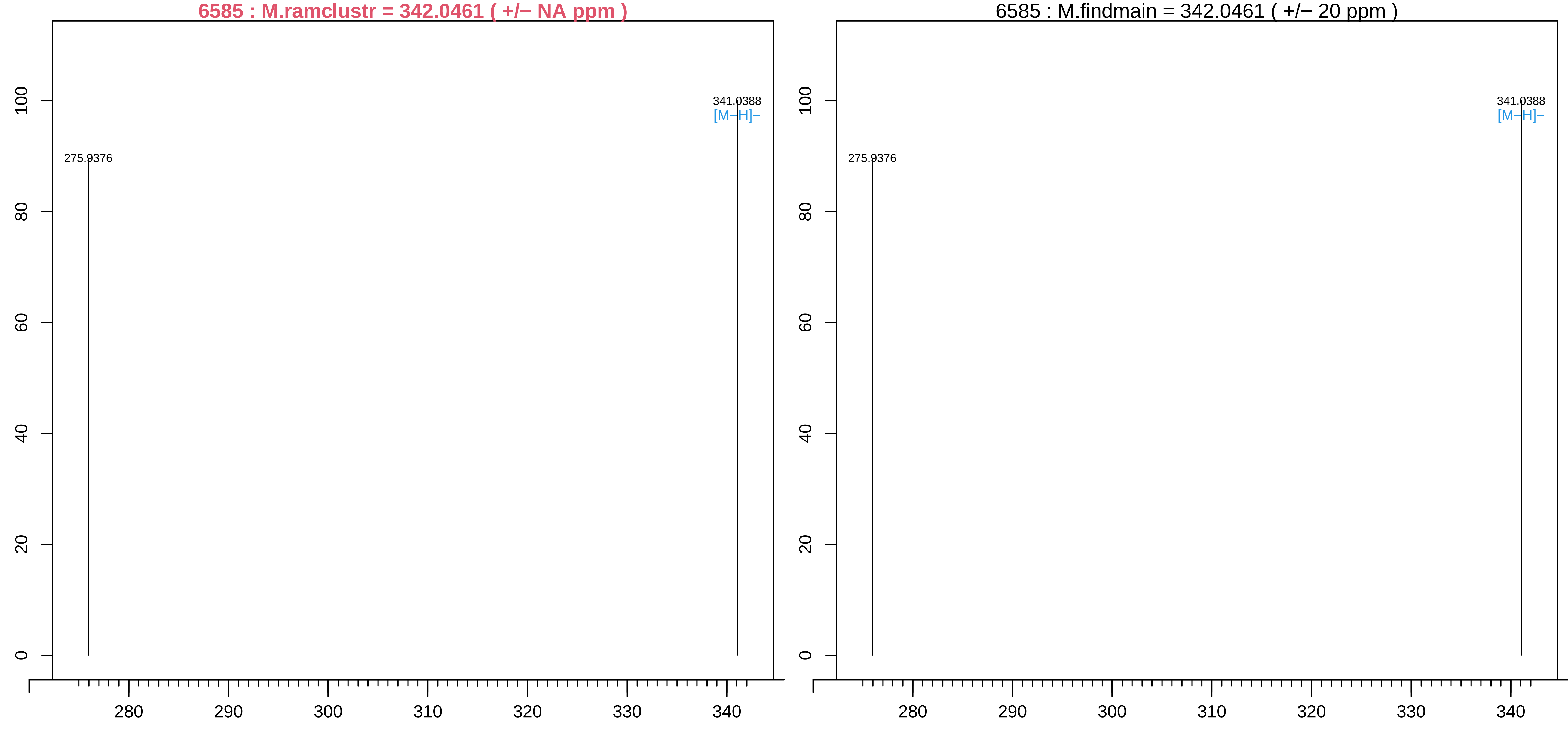
6585 : M.ramclustr = 342.0461 (+/− NA ppm)
341.0388
[M−H]−
275.9376
6585 : M.findmain = 342.0461 (+/− 20 ppm)
341.0388
[M−H]−
275.9376
0
20
40
60
80
100
280
290
300
310
320
330
340

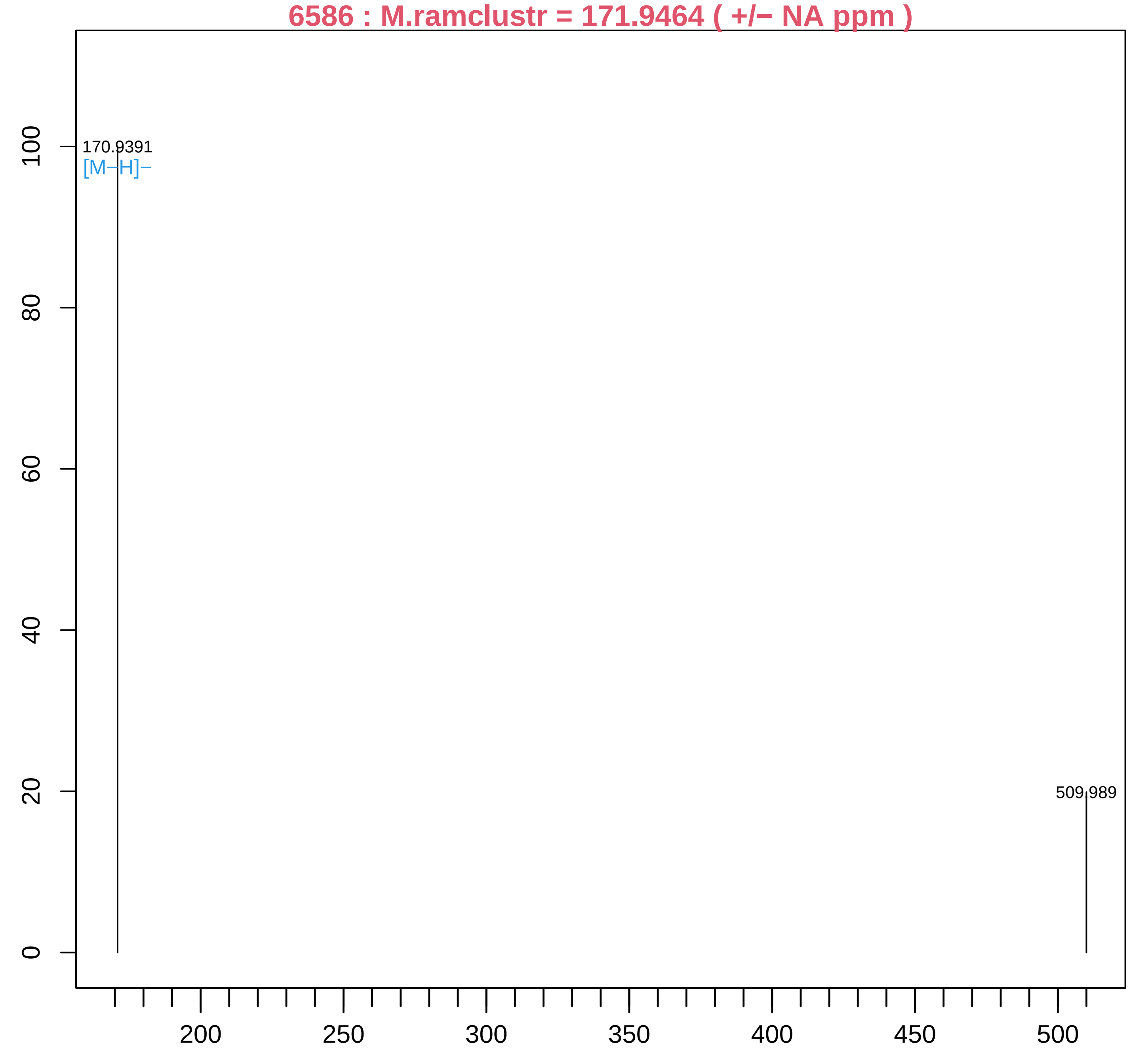

6586 : M.ramclustr = 171.9464 (+/− NA ppm)
170.9391
[M−H]−
509.989
0
20
40
60
80
100
200
250
300
350
400
450
500

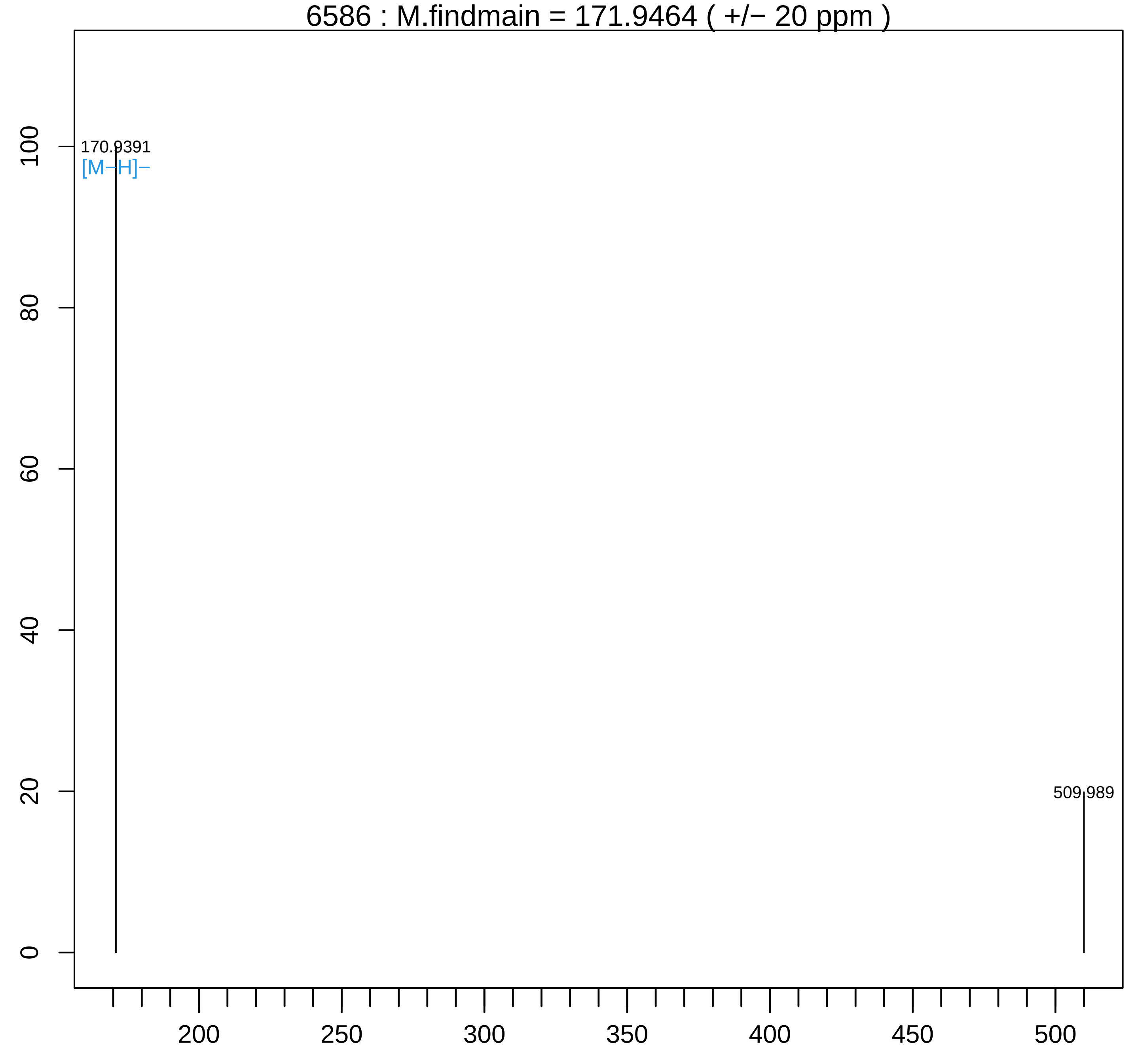

6586 : M.findmain = 171.9464 (+/− 20 ppm)
170.9391
[M−H]−
509.989
0
20
40
60
80
100
200
250
300
350
400
450
500

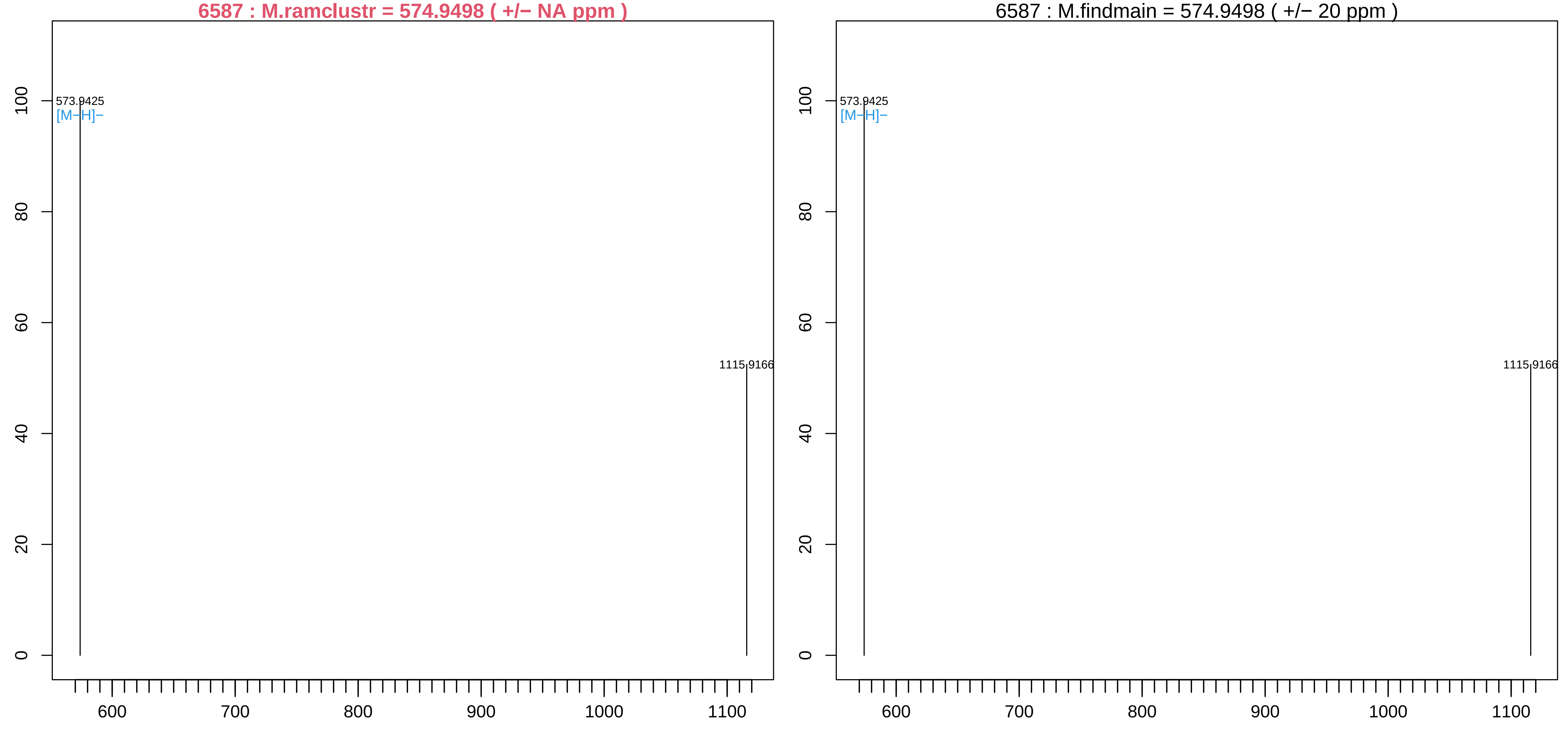
6587 : M.ramclustr = 574.9498 (+/− NA ppm)
573.9425
[M−H]−
1115.9166
0
20
40
60
80
100
600
700
800
900
1000
1100
6587 : M.findmain = 574.9498 (+/− 20 ppm)
573.9425
[M−H]−
1115.9166
0
20
40
60
80
100
600
700
800
900
1000
1100

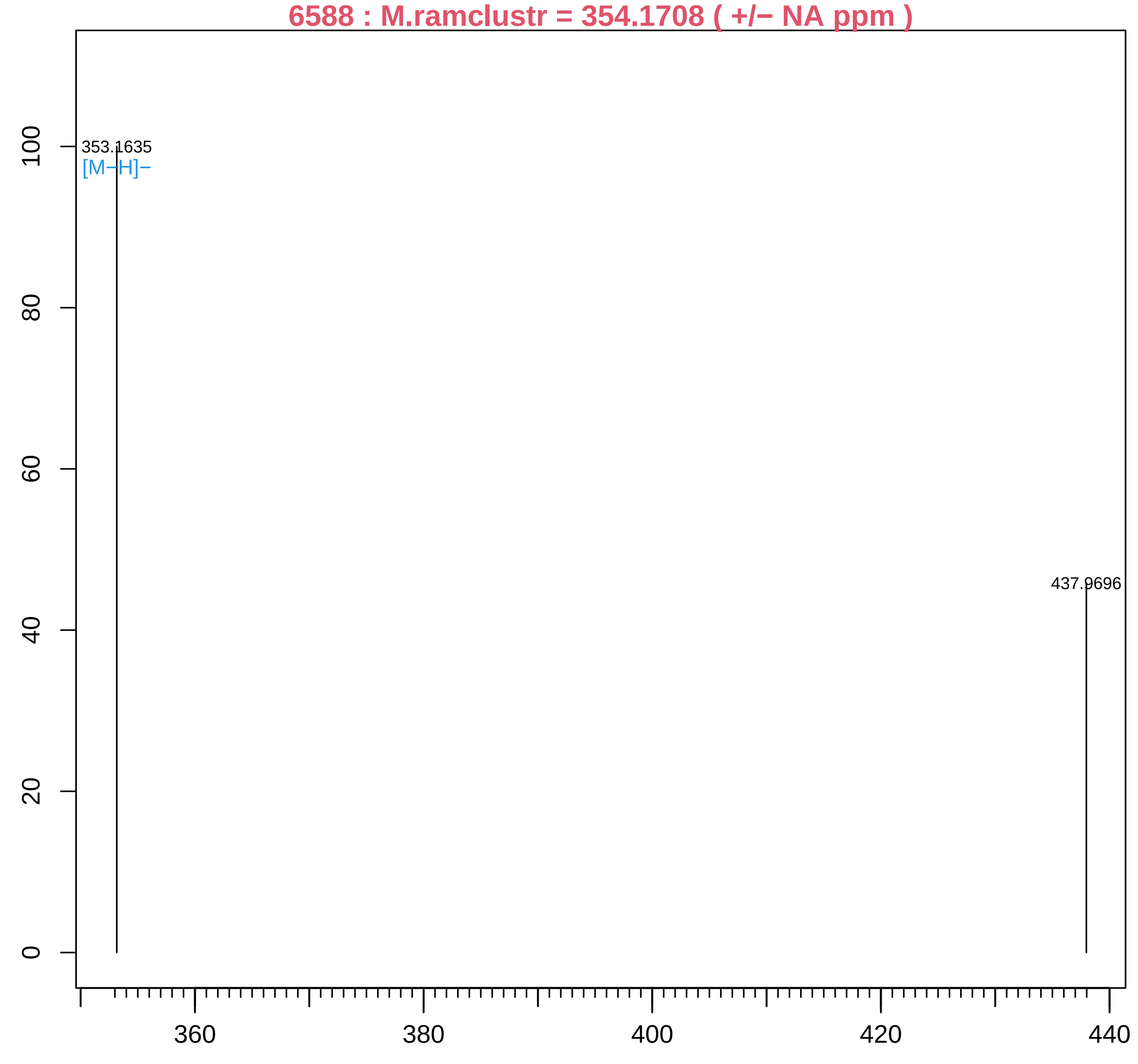
6588 : M.ramclustr = 354.1708 (+/− NA ppm)
353.1635
[M−H]−
437.9696
0
20
40
60
80
100
360
380
400
420
440

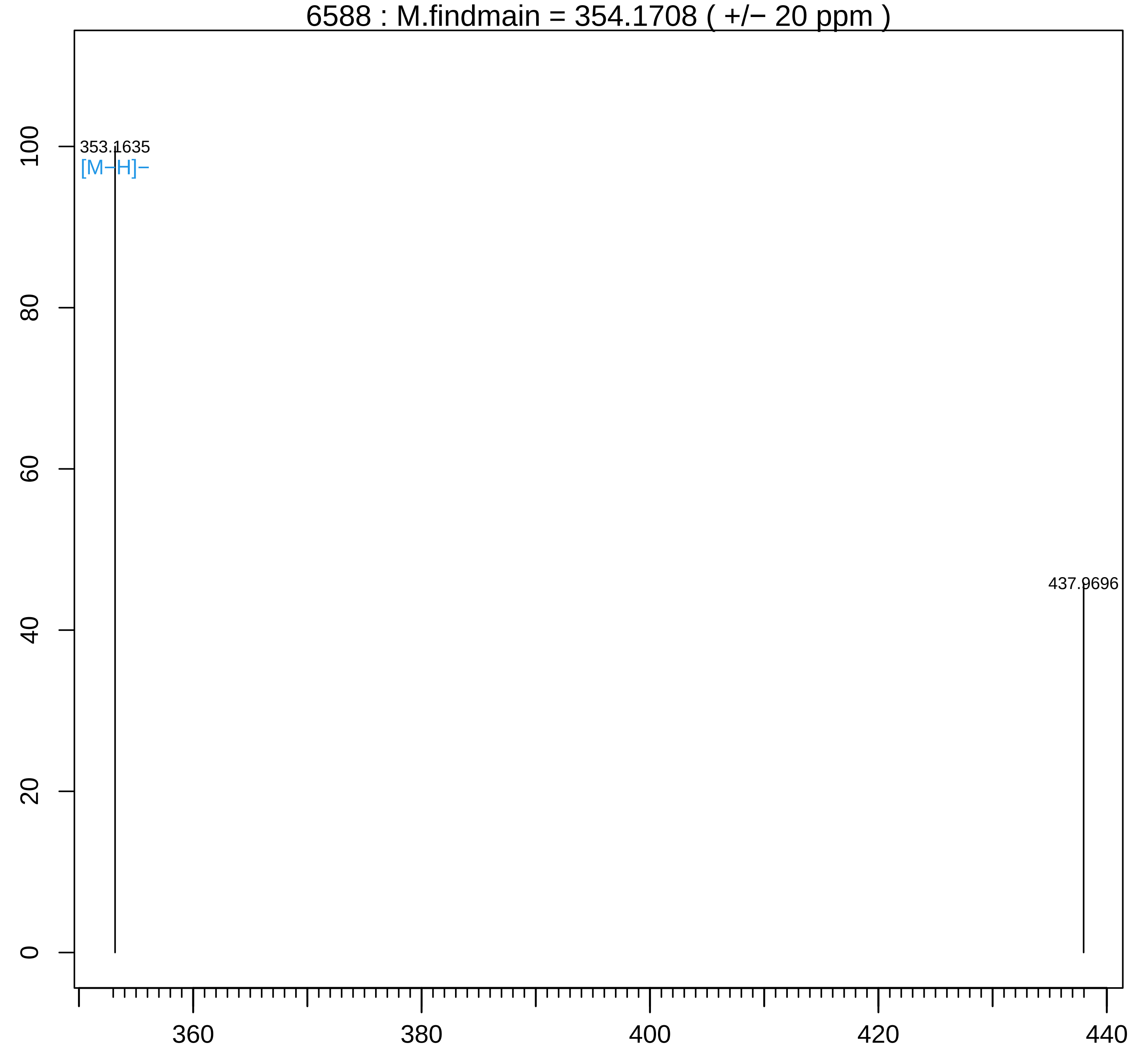
6588 : M.findmain = 354.1708 (+/− 20 ppm)
353.1635
[M−H]−
437.9696
0
20
40
60
80
100
360
380
400
420
440

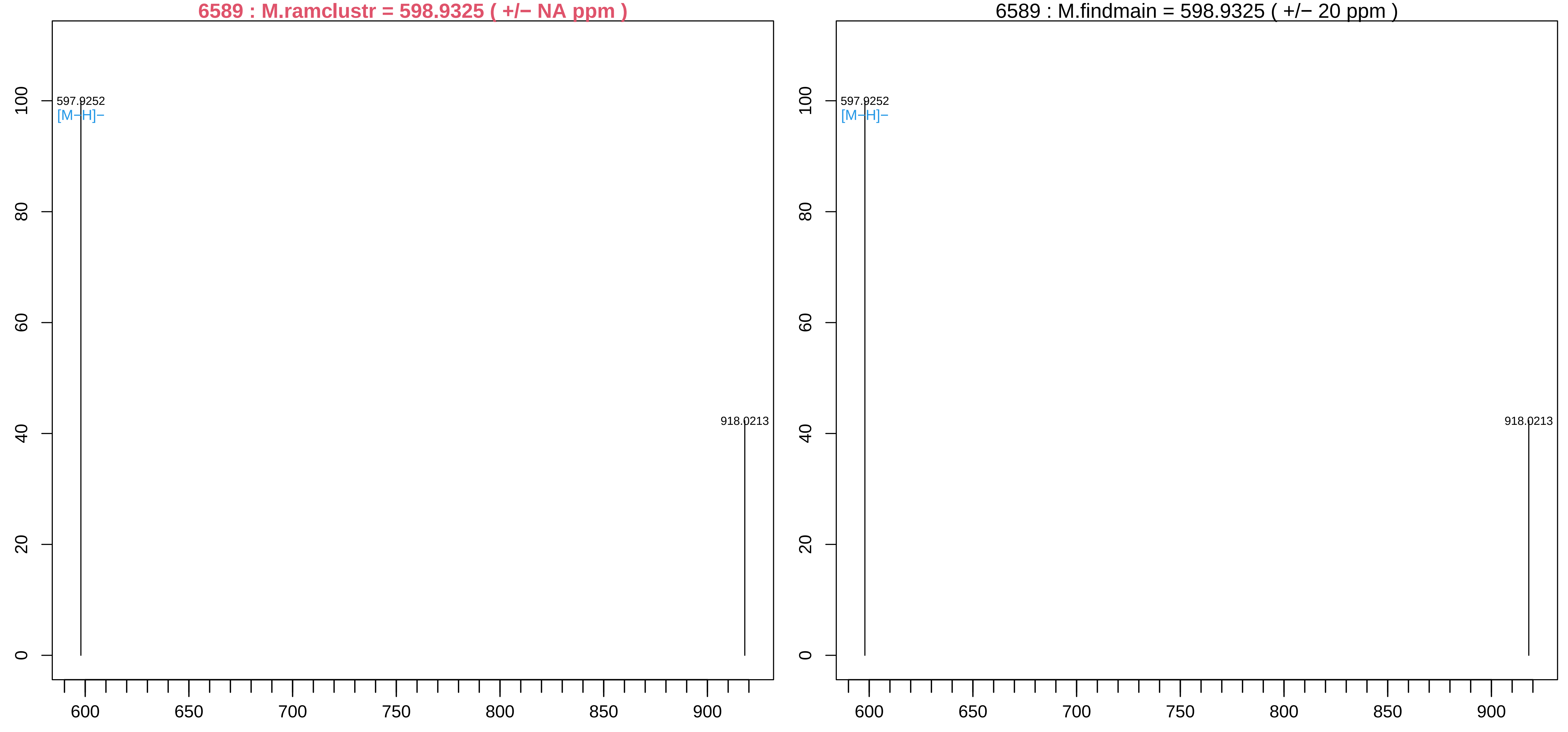
6589 : M.ramclustr = 598.9325 (+/− NA ppm)
597.9252
[M−H]−
918.0213
6589 : M.findmain = 598.9325 (+/− 20 ppm)
597.9252
[M−H]−
918.0213
0
20
40
60
80
100
600
650
700
750
800
850
900

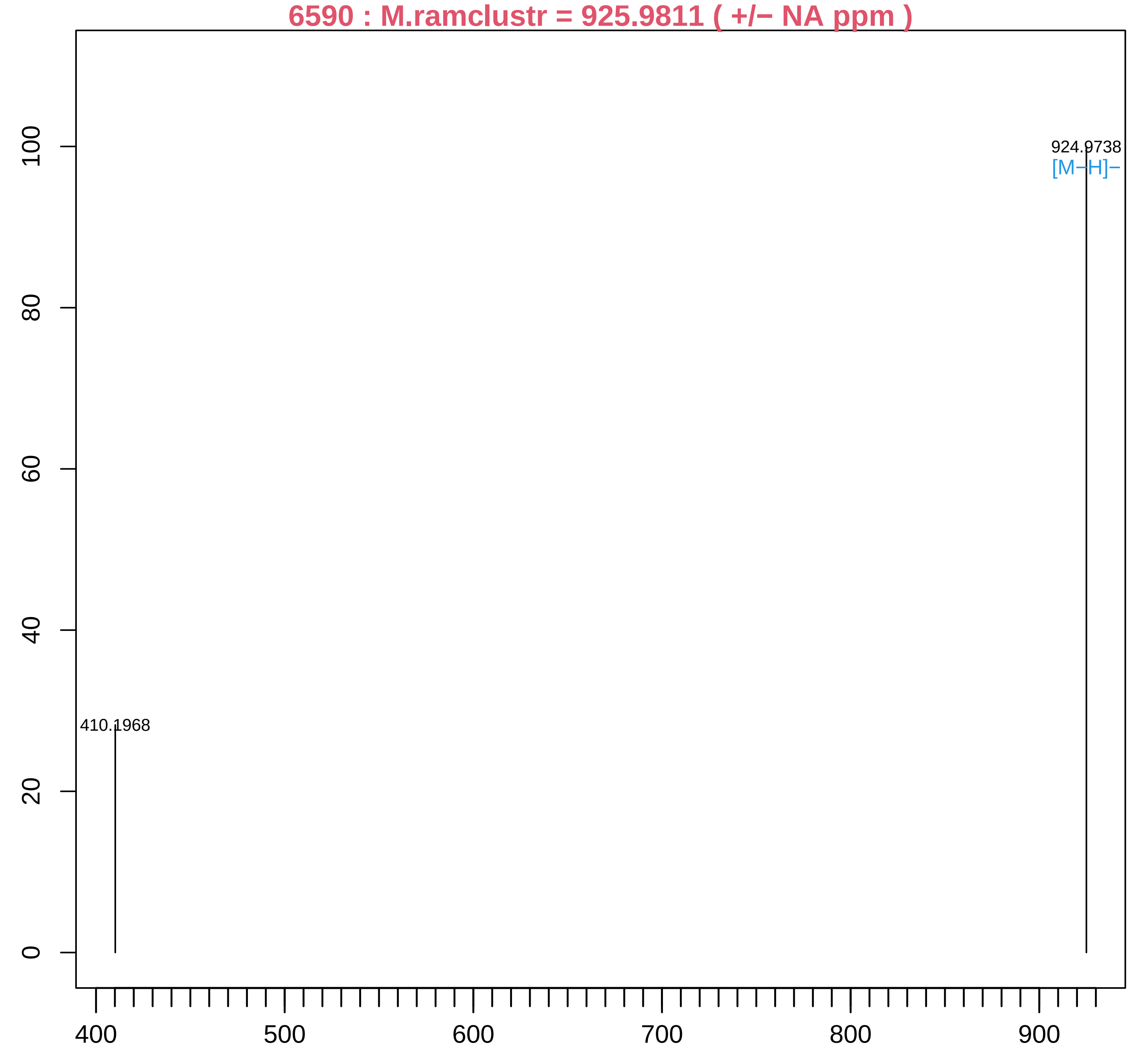
6590 : M.ramclustr = 925.9811 (+/− NA ppm)
924.9738
[M−H]−
410.1968
0
20
40
60
80
100
400
500
600
700
800
900

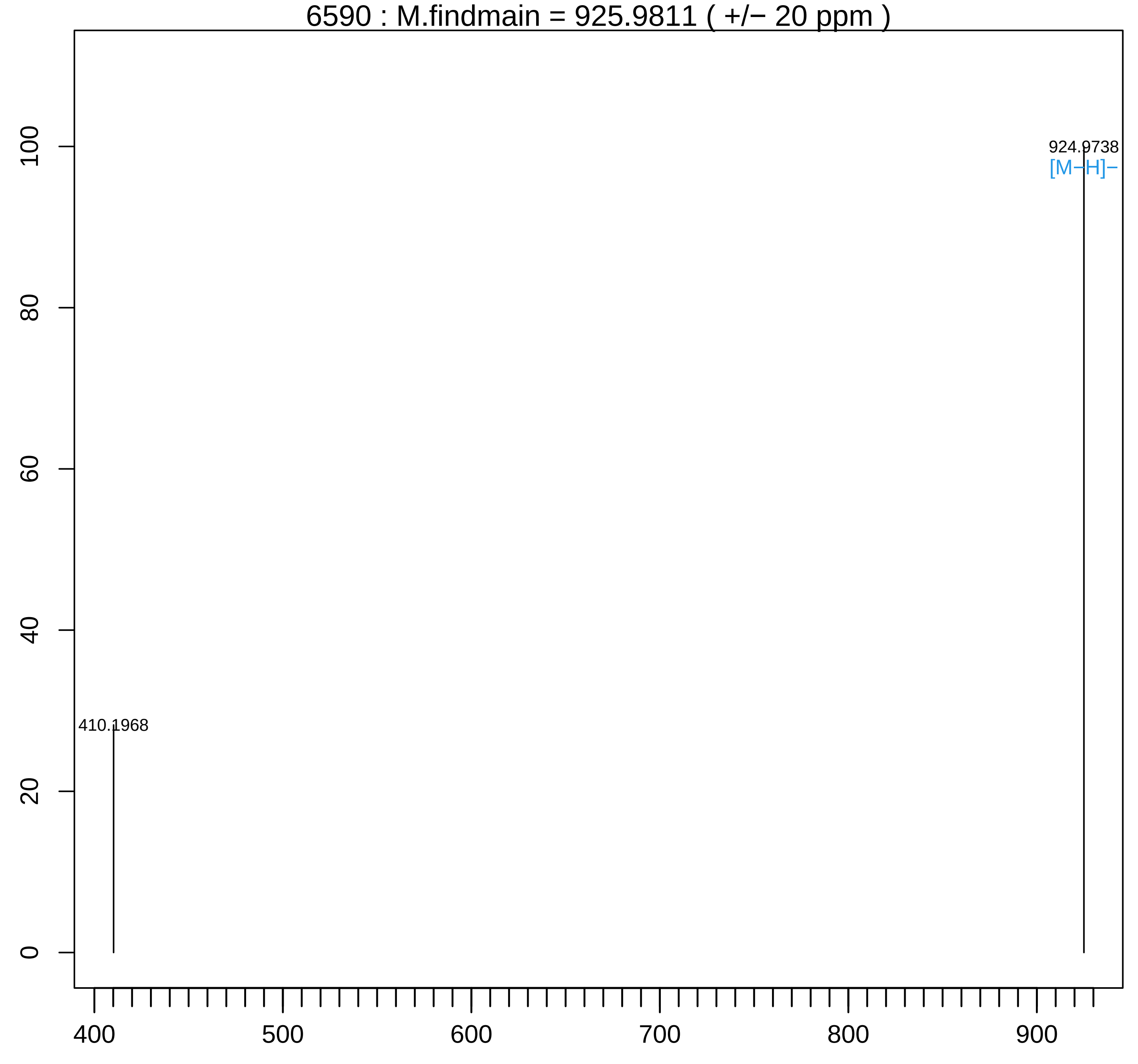
6590 : M.findmain = 925.9811 (+/− 20 ppm)
924.9738
[M−H]−
410.1968
0
20
40
60
80
100
400
500
600
700
800
900

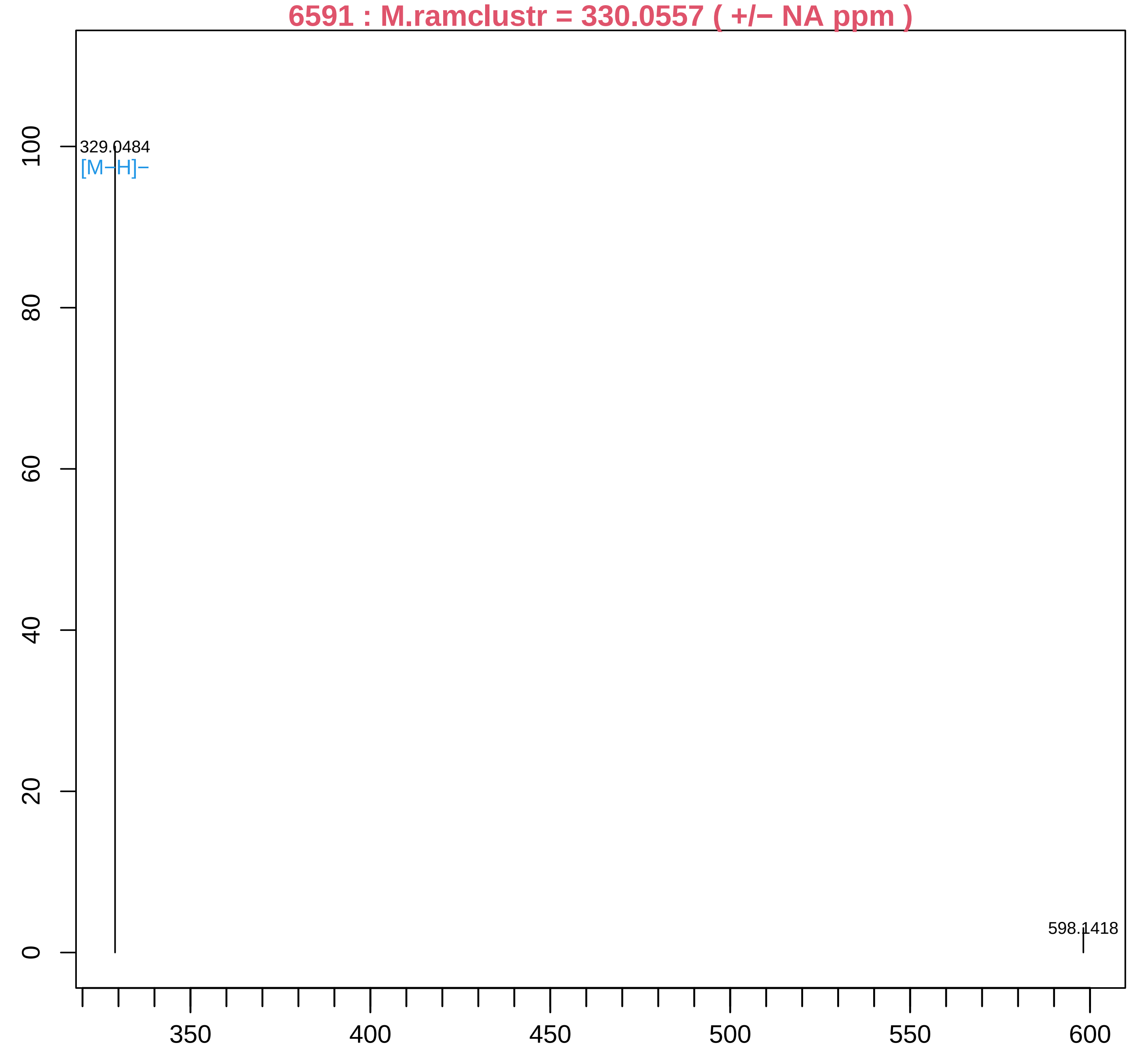
6591 : M.ramclustr = 330.0557 (+/− NA ppm)
329.0484
[M−H]−
598.1418
0
20
40
60
80
100
350
400
450
500
550
600
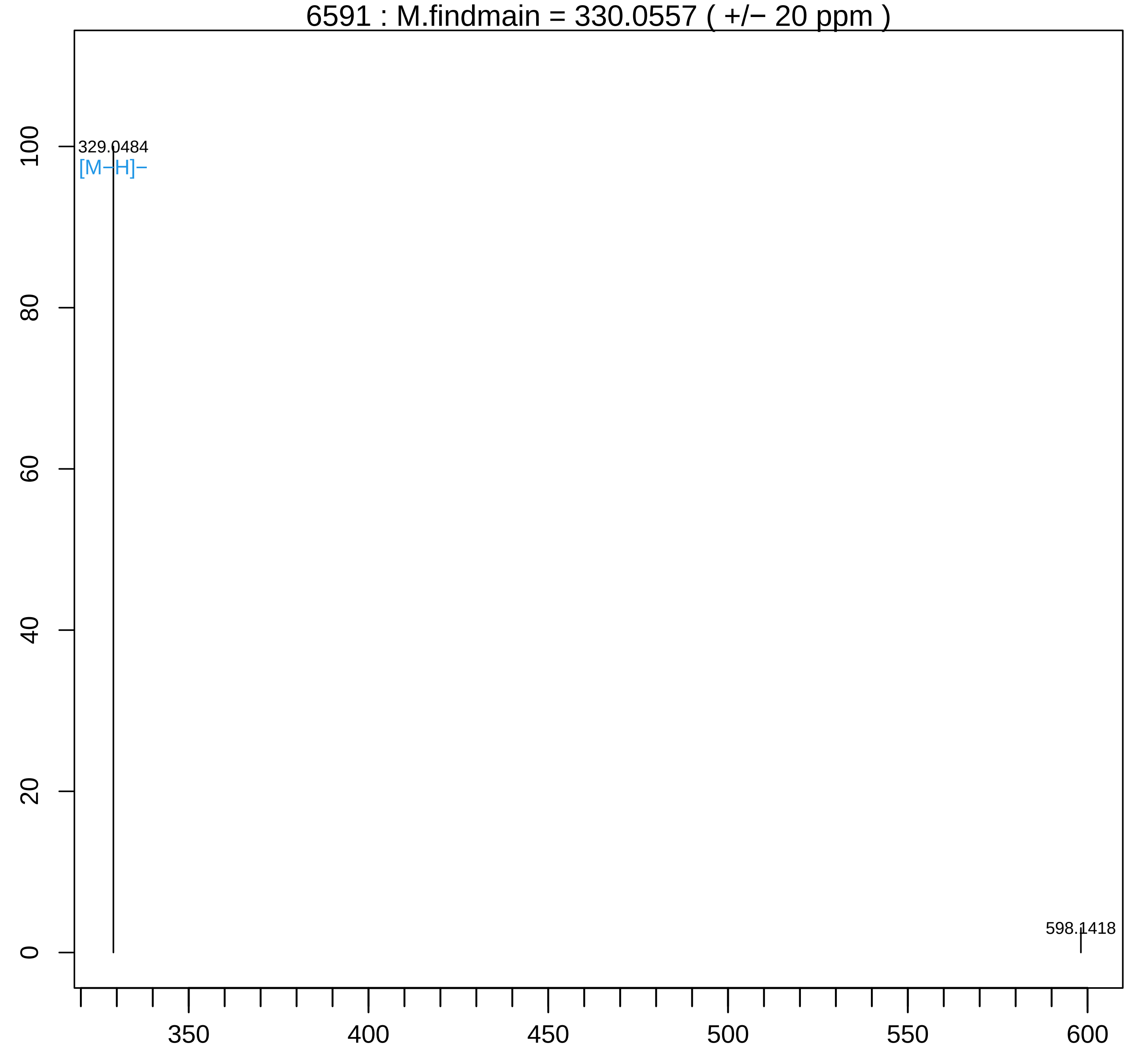
6591 : M.findmain = 330.0557 (+/− 20 ppm)
329.0484
[M−H]−
598.1418
0
20
40
60
80
100
350
400
450
500
550
600

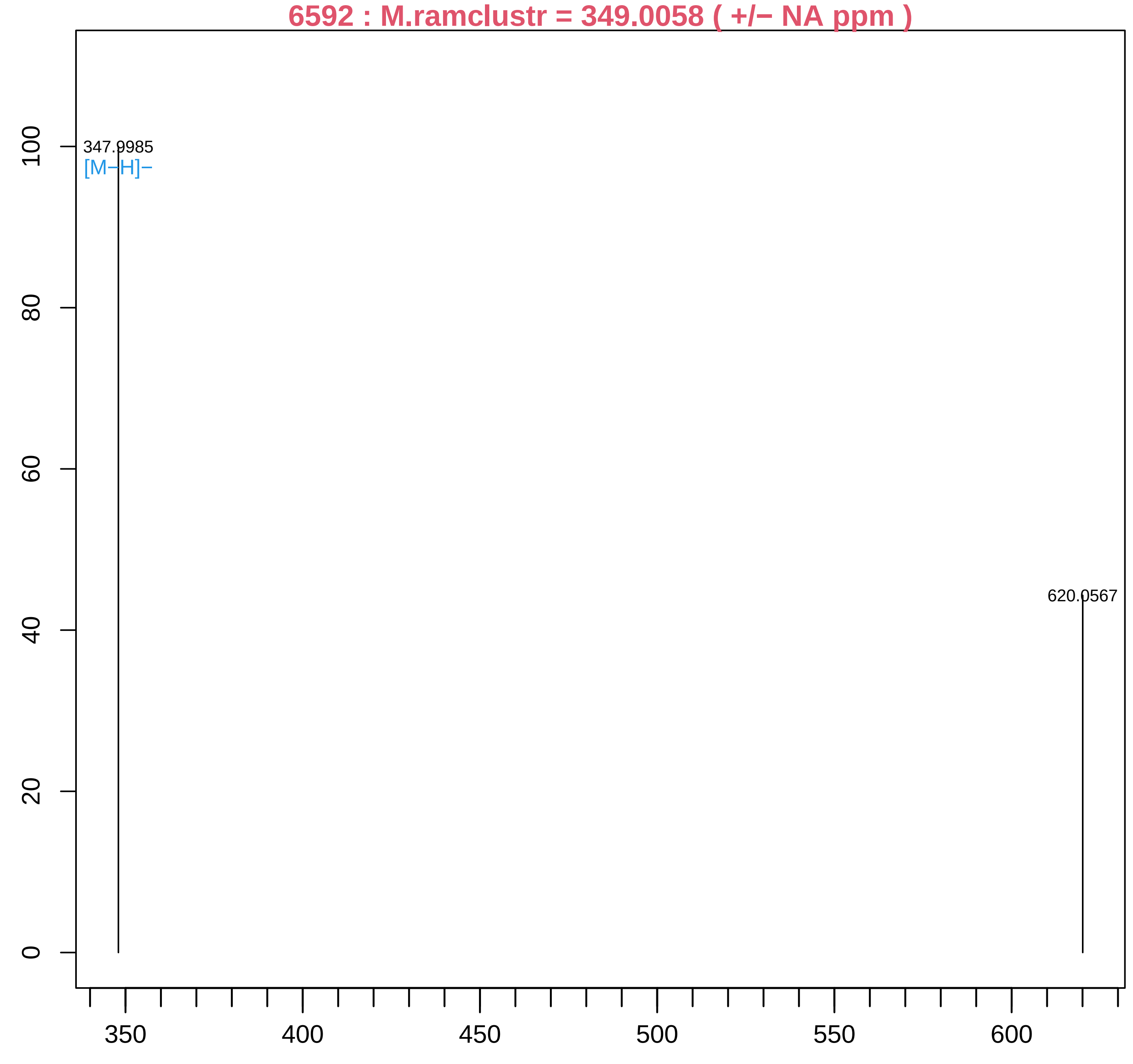
6592 : M.ramclustr = 349.0058 (+/− NA ppm)
347.9985
[M−H]−
620.0567
0
20
40
60
80
100
350
400
450
500
550
600

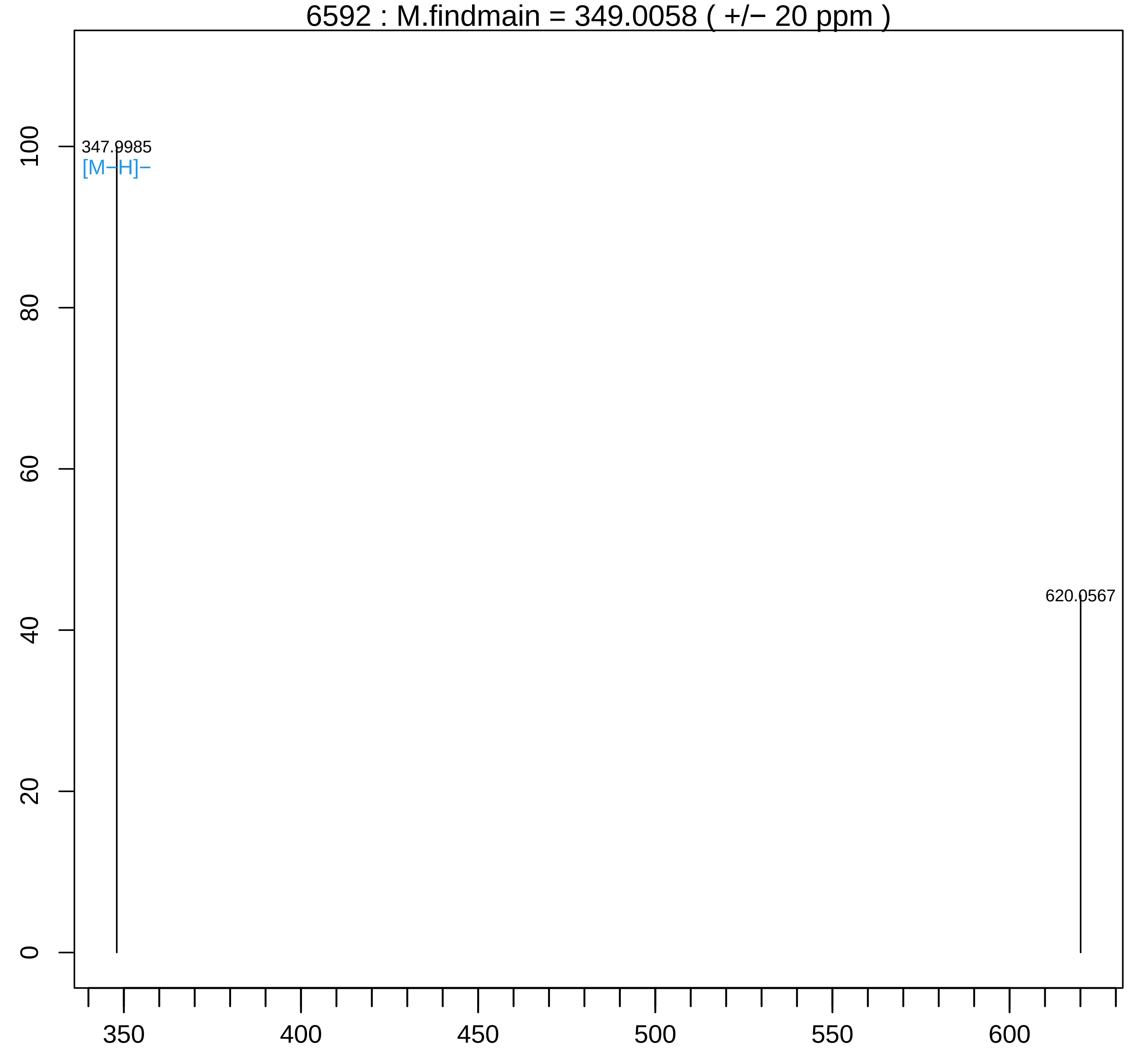
6592 : M.findmain = 349.0058 (+/− 20 ppm)
347.9985
[M−H]−
620.0567
0
20
40
60
80
100
350
400
450
500
550
600

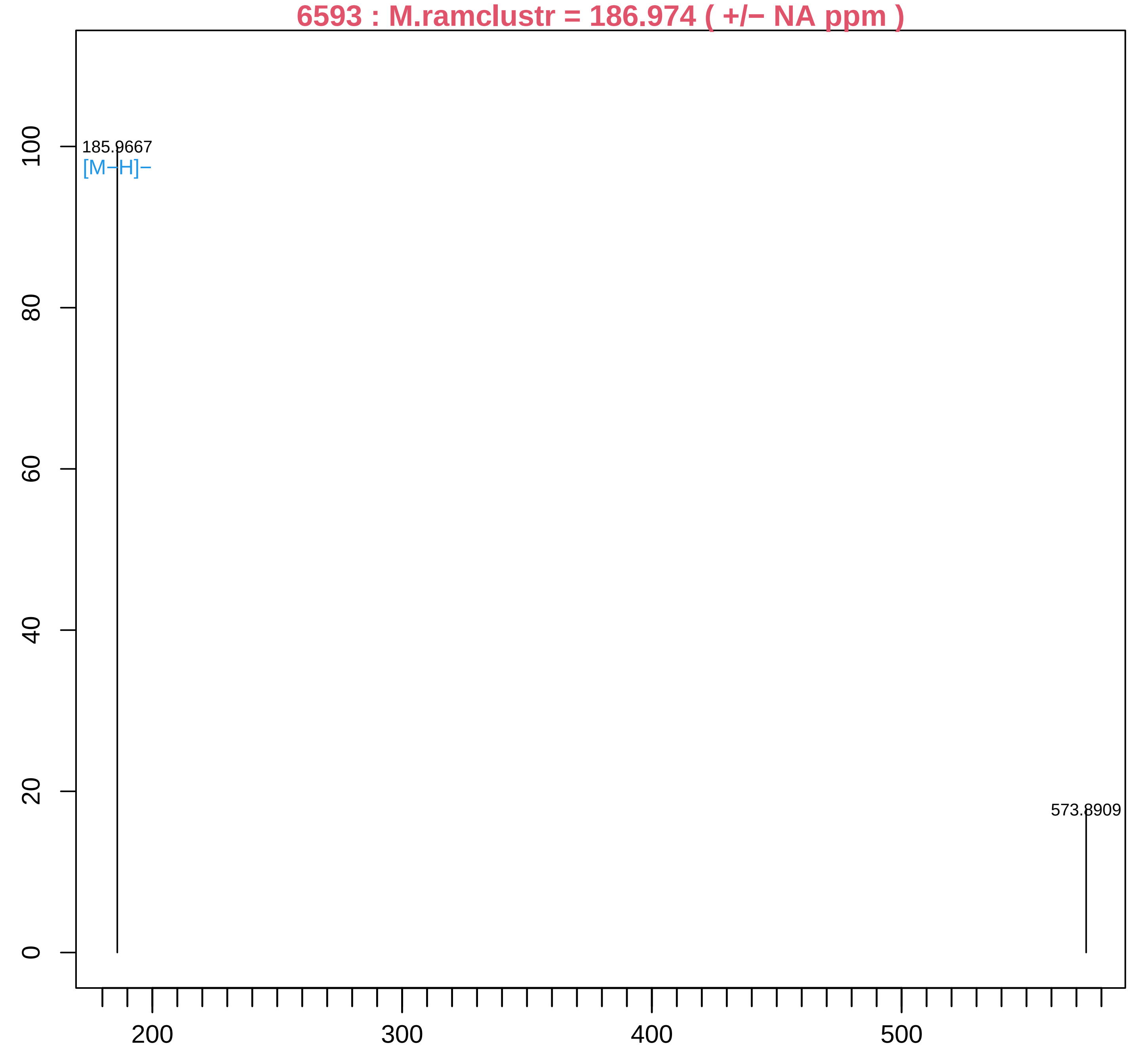
6593 : M.ramclustr = 186.974 (+/− NA ppm)
185.9667
[M−H]−
573.8909
0
20
40
60
80
100
200
300
400
500

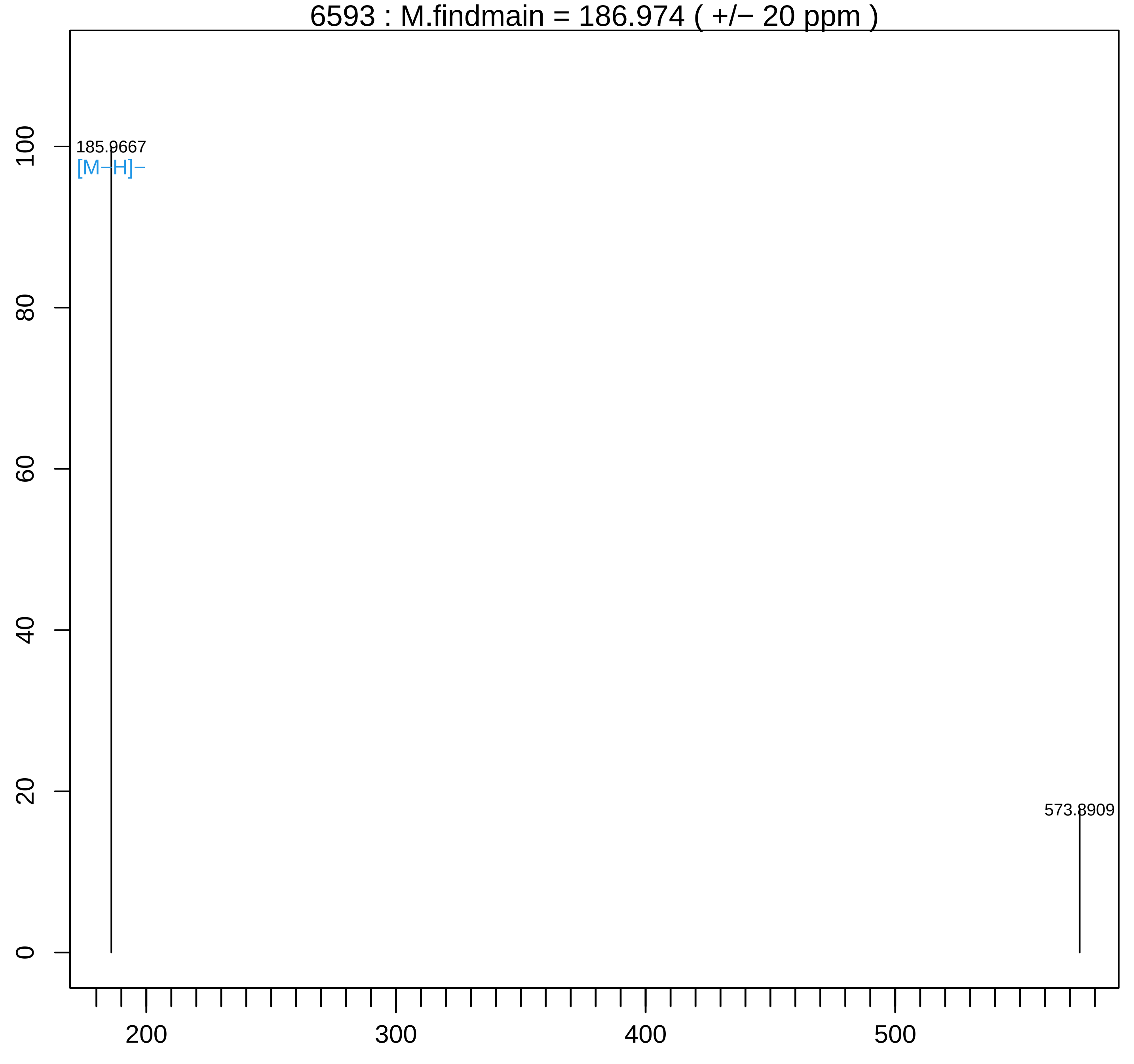
6593 : M.findmain = 186.974 (+/− 20 ppm)
185.9667
[M−H]−
573.8909
0
20
40
60
80
100
200
300
400
500

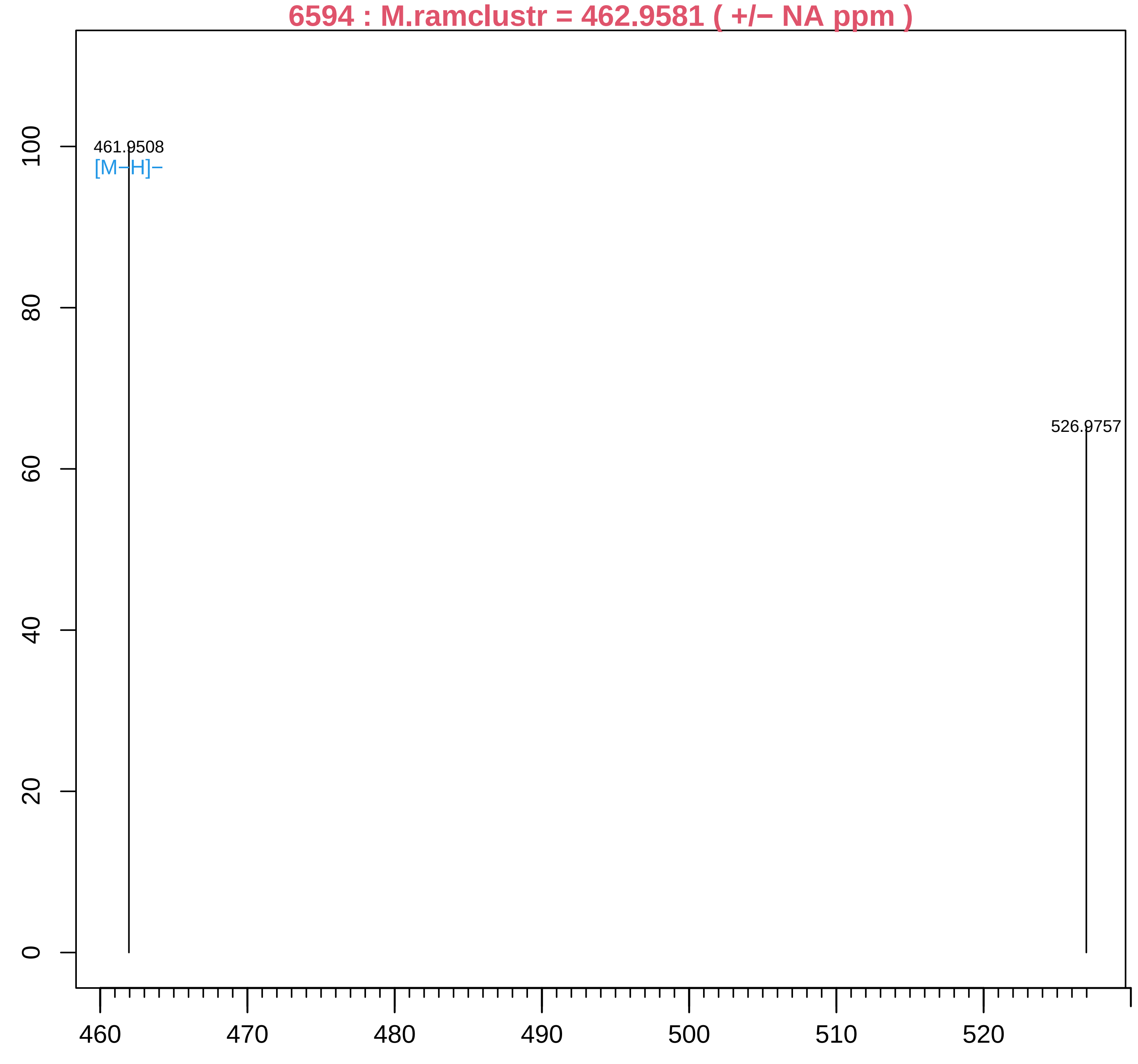
6594 : M.ramclustr = 462.9581 (+/− NA ppm)
461.9508
[M−H]−
526.9757
0
20
40
60
80
100
460
470
480
490
500
510
520

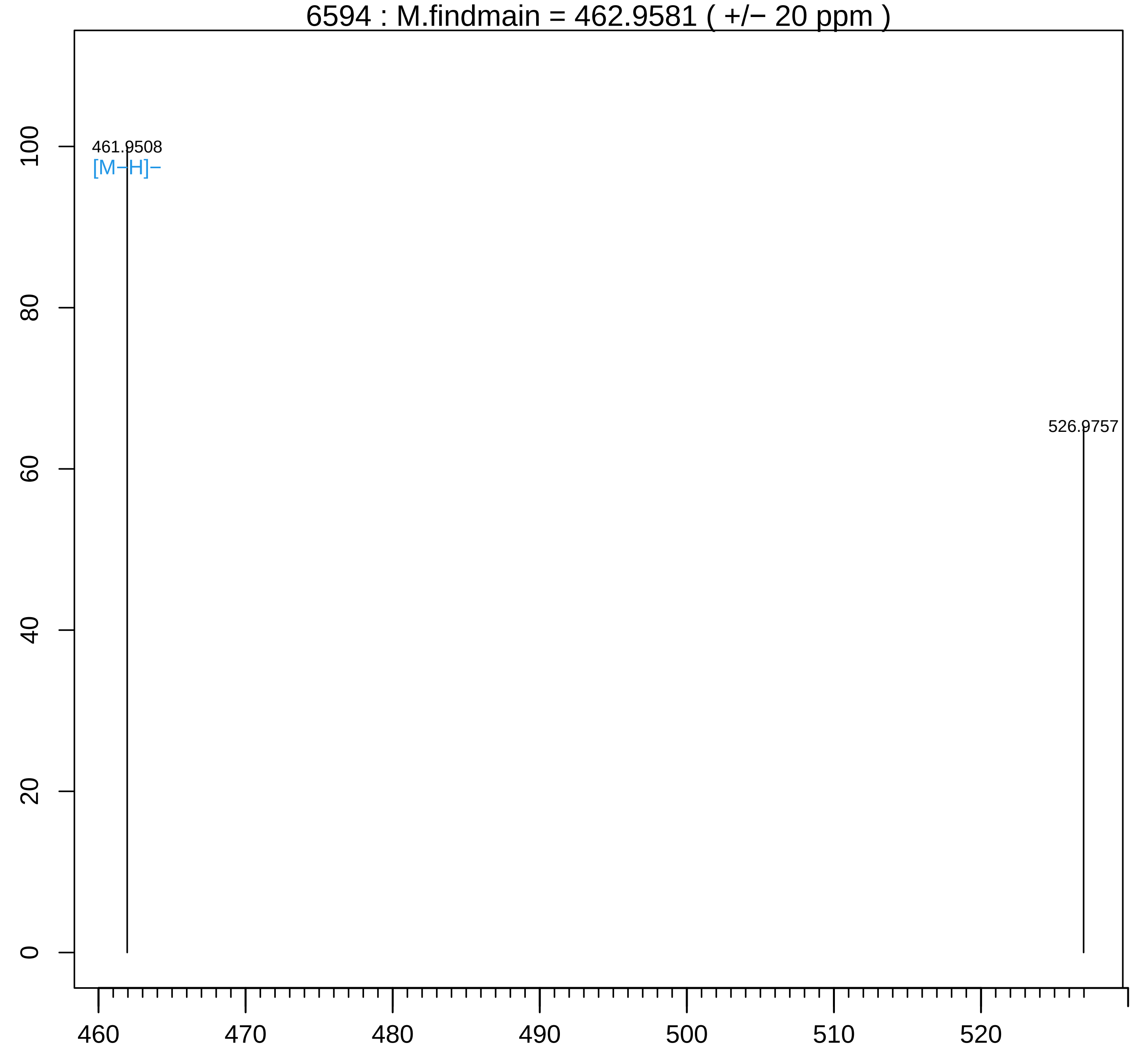
6594 : M.findmain = 462.9581 (+/− 20 ppm)
461.9508
[M−H]−
526.9757
0
20
40
60
80
100
460
470
480
490
500
510
520

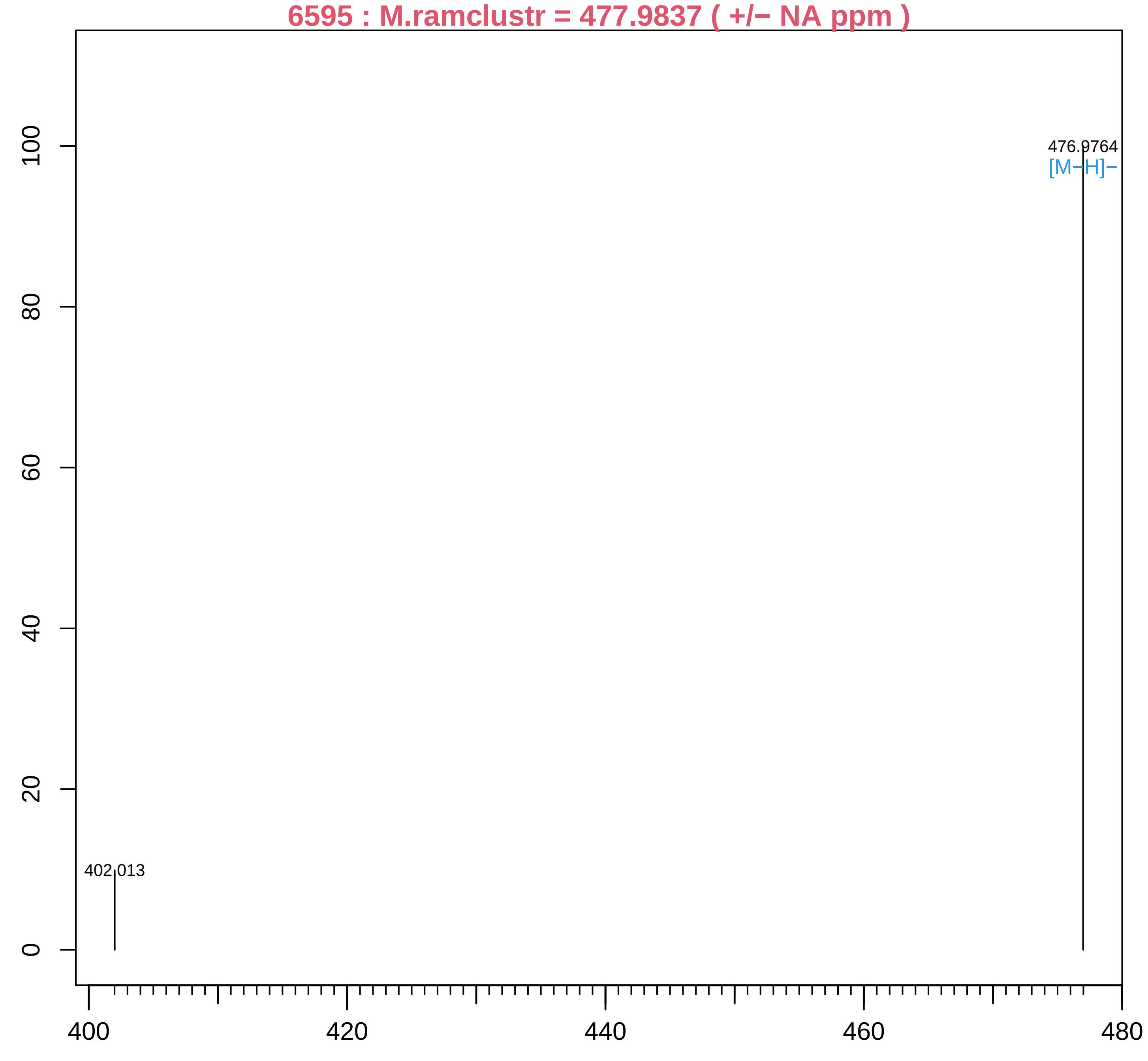
6595 : M.ramclustr = 477.9837 (+/− NA ppm)
476.9764
[M−H]−
402.013
0
20
40
60
80
100
400
420
440
460
480

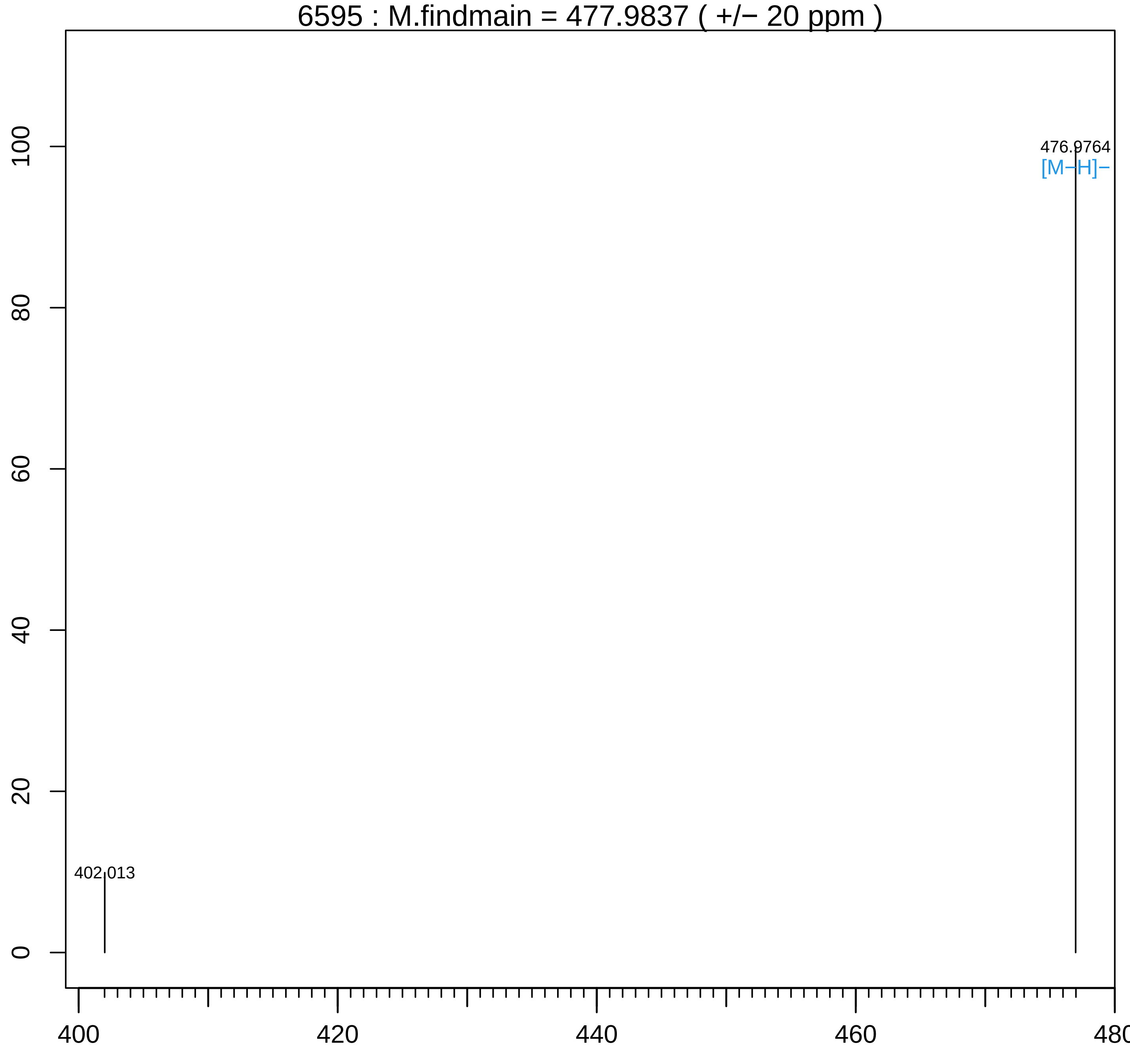
6595 : M.findmain = 477.9837 (+/− 20 ppm)
476.9764
[M−H]−
402.013
0
20
40
60
80
100
400
420
440
460

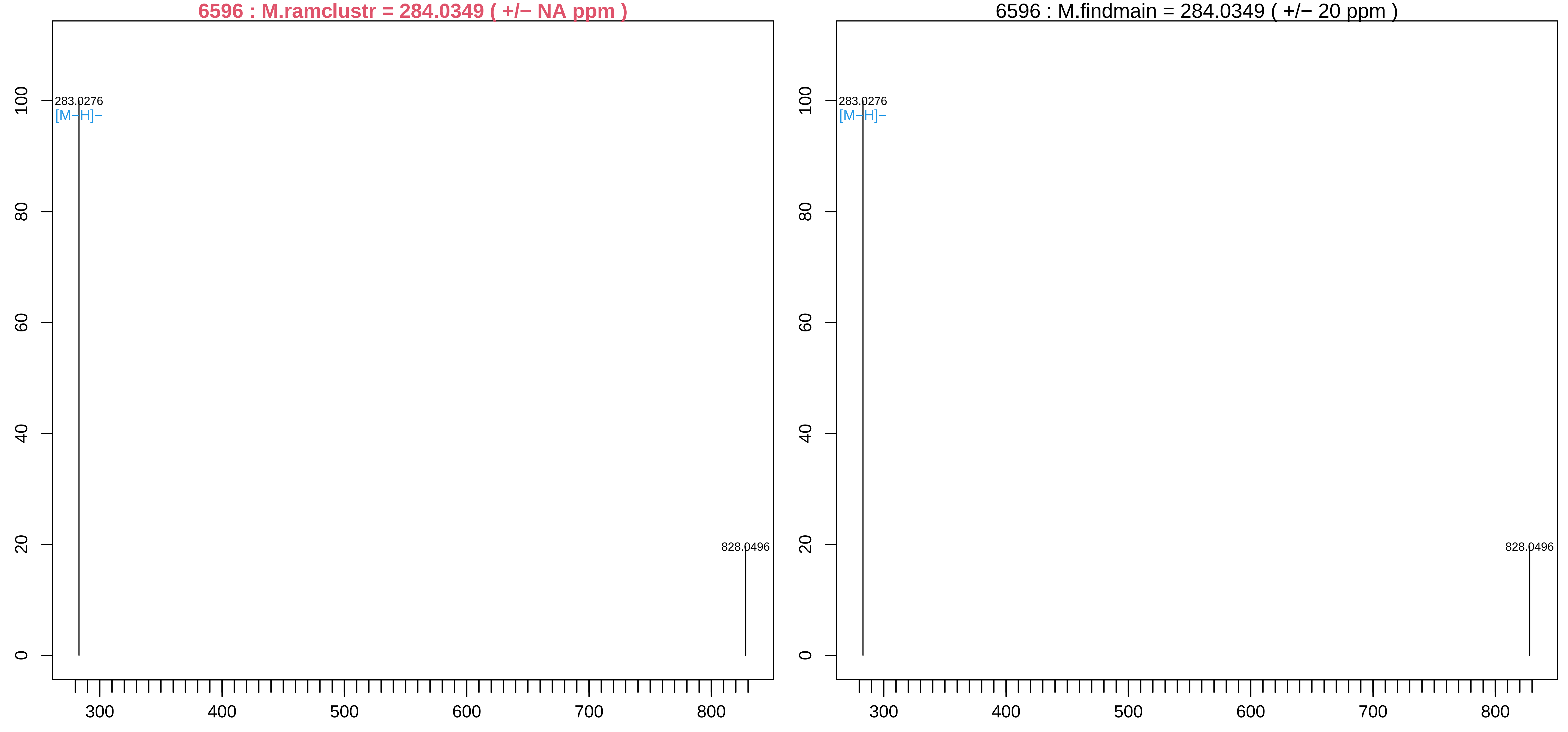
6596 : M.ramclustr = 284.0349 (+/− NA ppm)
283.0276
[M−H]−
828.0496
0
20
40
60
80
100
300
400
500
600
700
800
6596 : M.findmain = 284.0349 (+/− 20 ppm)
283.0276
[M−H]−
828.0496
0
20
40
60
80
100
300
400
500
600
700
800

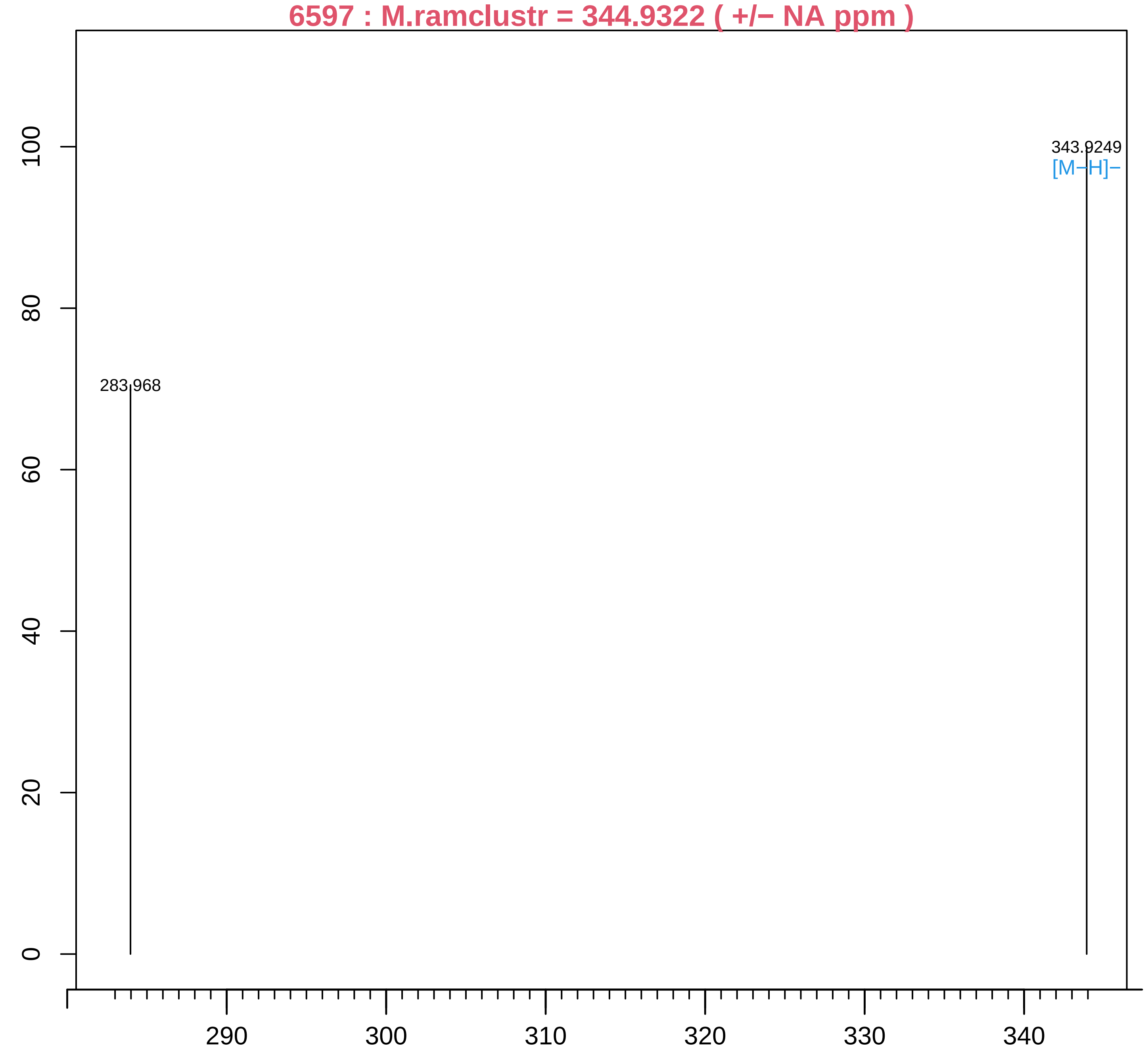
6597 : M.ramclustr = 344.9322 (+/− NA ppm)
343.9249
[M−H]−
283.968
0
20
40
60
80
100
290
300
310
320
330
340

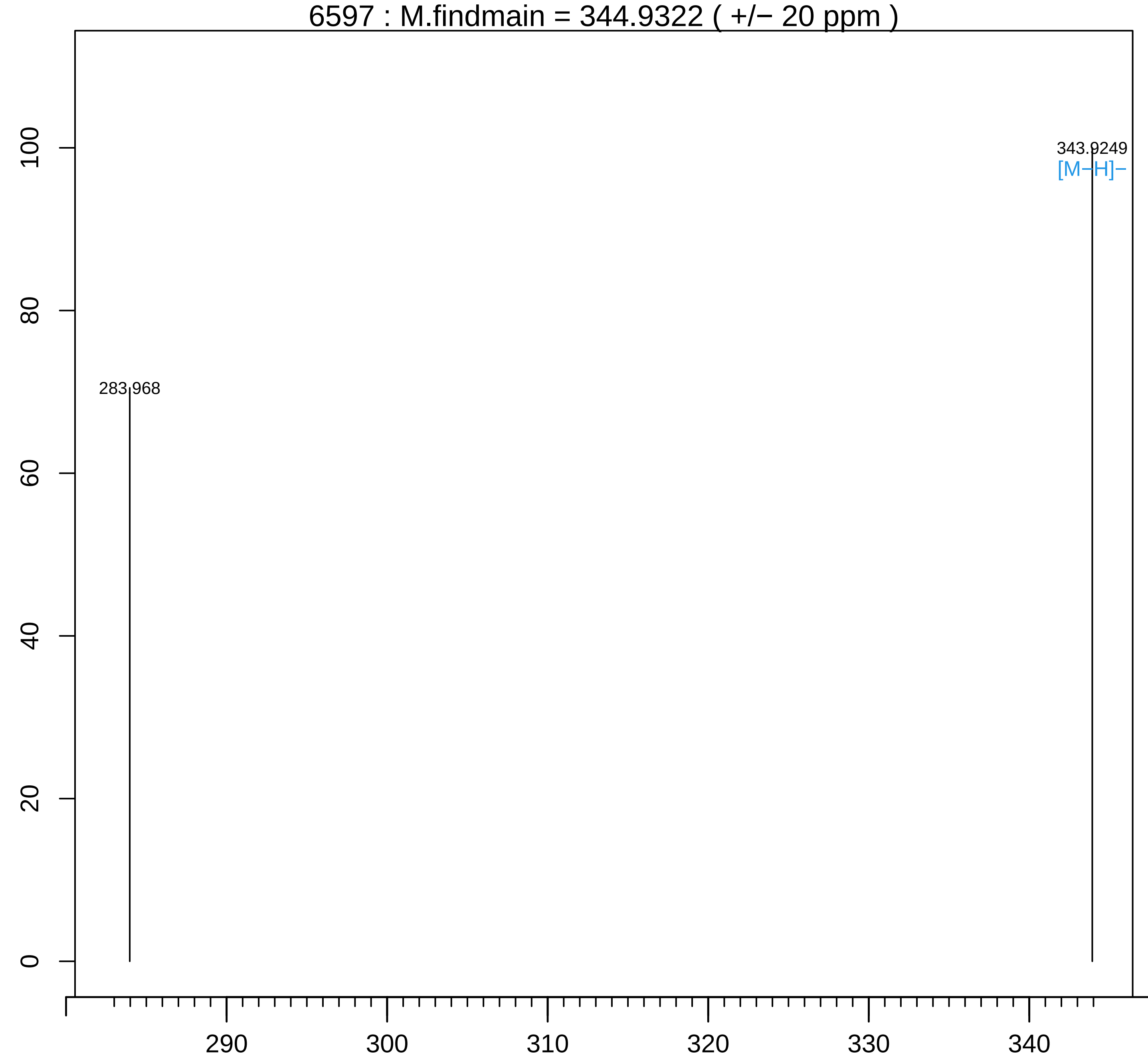
6597 : M.findmain = 344.9322 (+/− 20 ppm)
343.9249
[M−H]−
283.968
0
20
40
60
80
100
290
300
310
320
330
340

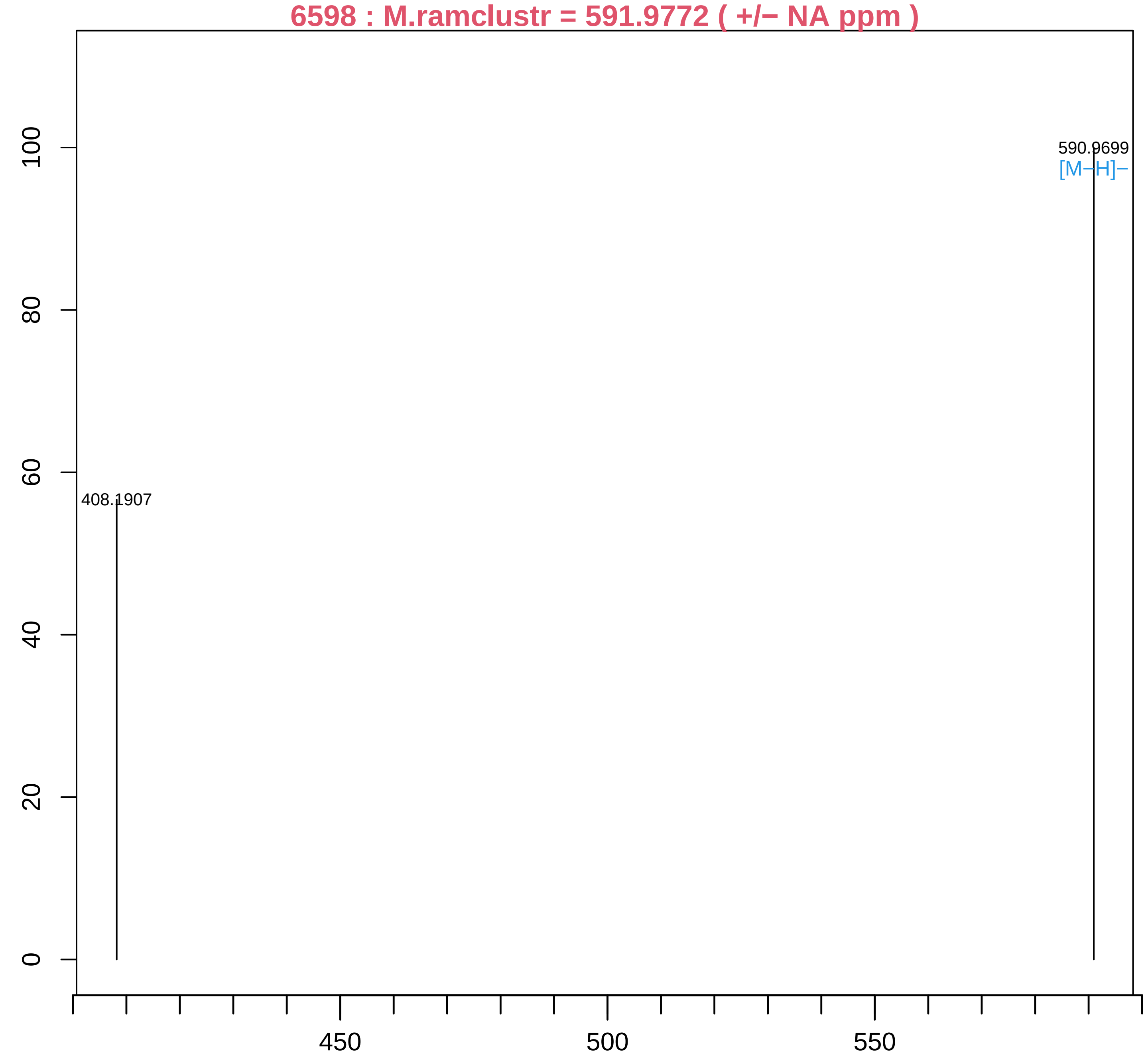
6598 : M.ramclustr = 591.9772 (+/− NA ppm)
590.9699
[M−H]−
408.1907
0
20
40
60
80
100
450
500
550

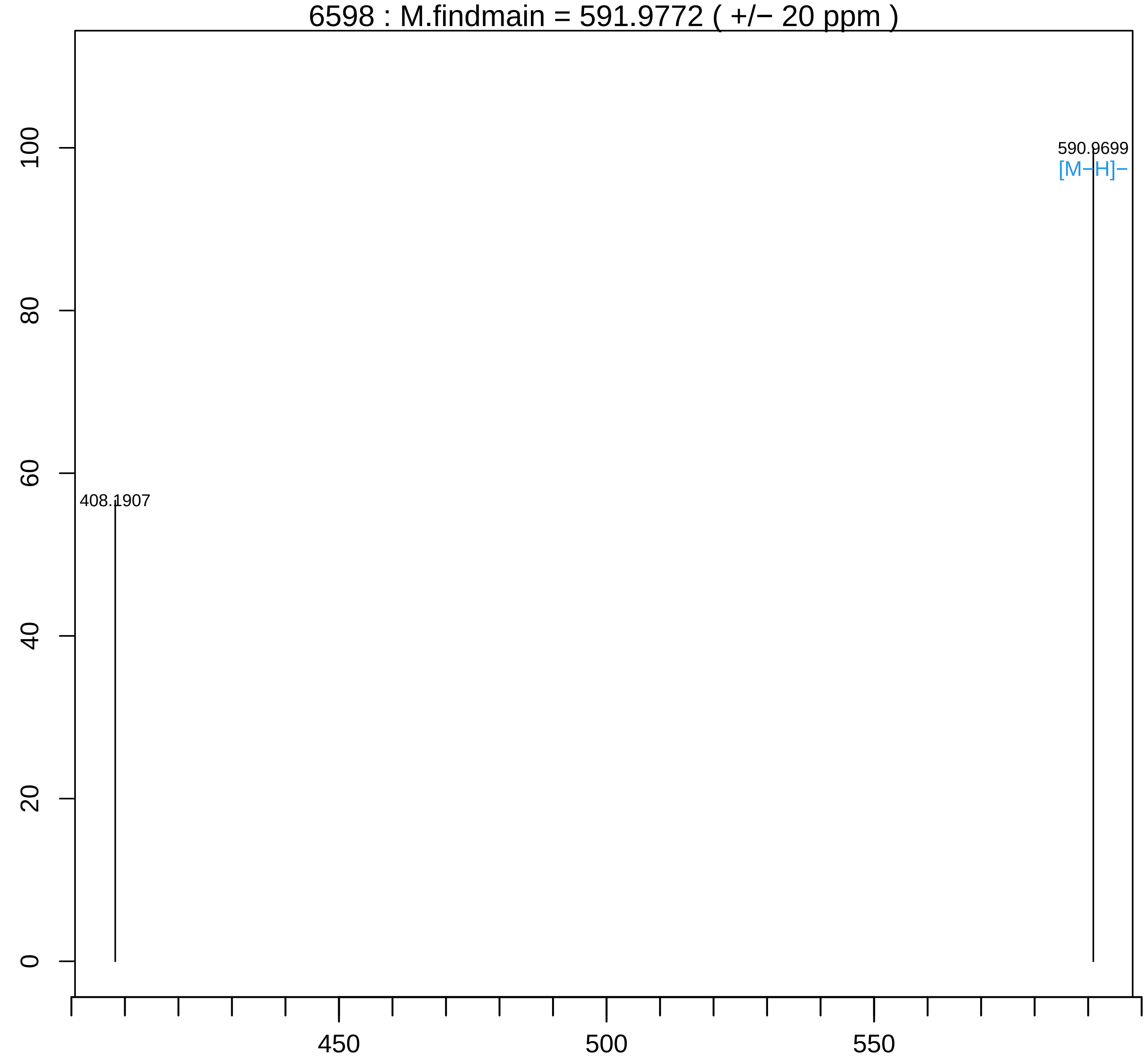
6598 : M.findmain = 591.9772 (+/− 20 ppm)
590.9699
[M−H]−
408.1907
0
20
40
60
80
100
450
500
550

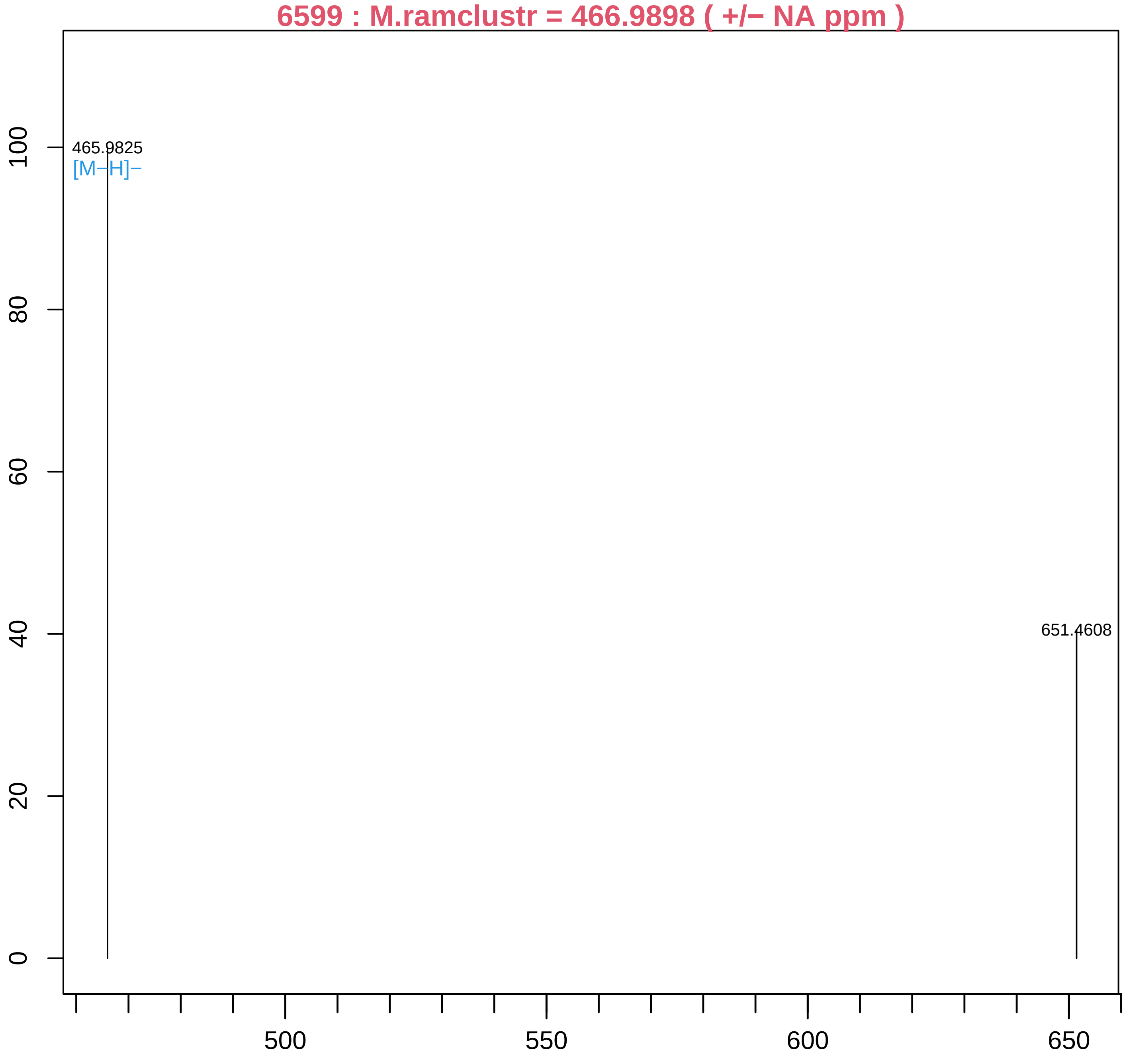

6599 : M.ramclustr = 466.9898 (+/− NA ppm)
465.9825
[M−H]−
651.4608
0
20
40
60
80
100
500
550
600
650

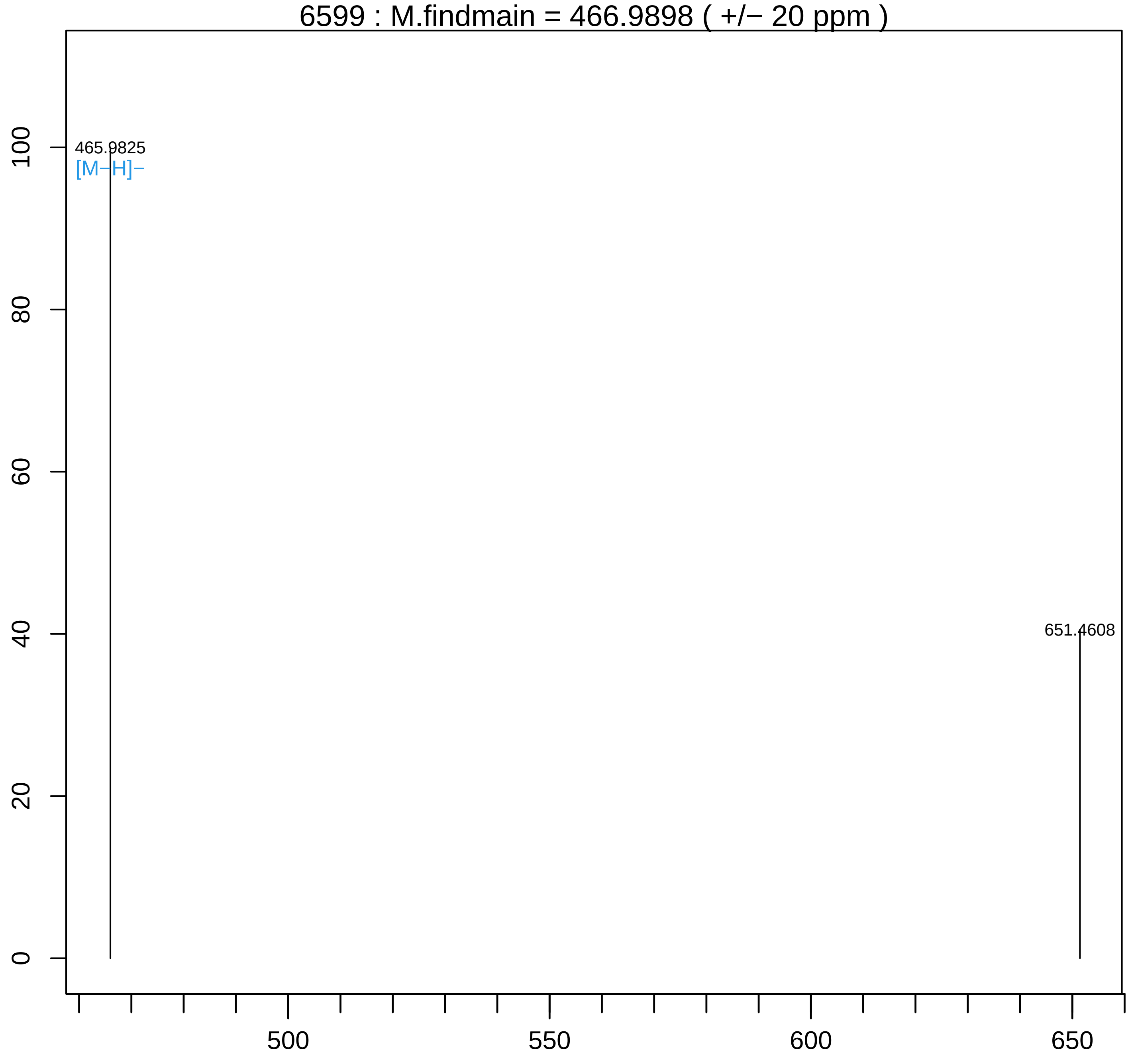

6599 : M.findmain = 466.9898 (+/− 20 ppm)
465.9825
[M−H]−
651.4608
0
20
40
60
80
100
500
550
600
650

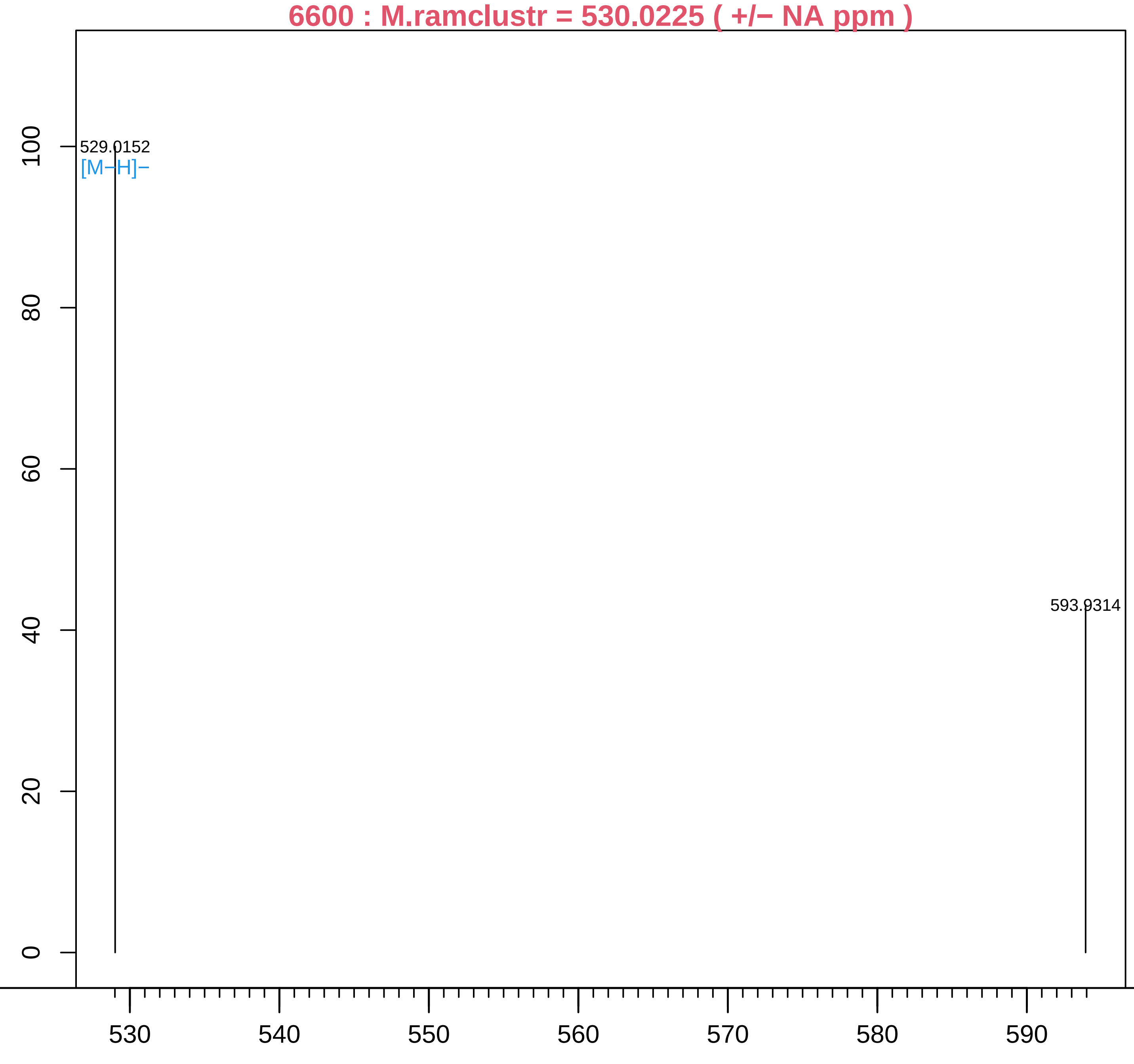

6600 : M.ramclustr = 530.0225 (+/− NA ppm)
529.0152
[M−H]−
593.9314
0
20
40
60
80
100
530
540
550
560
570
580
590

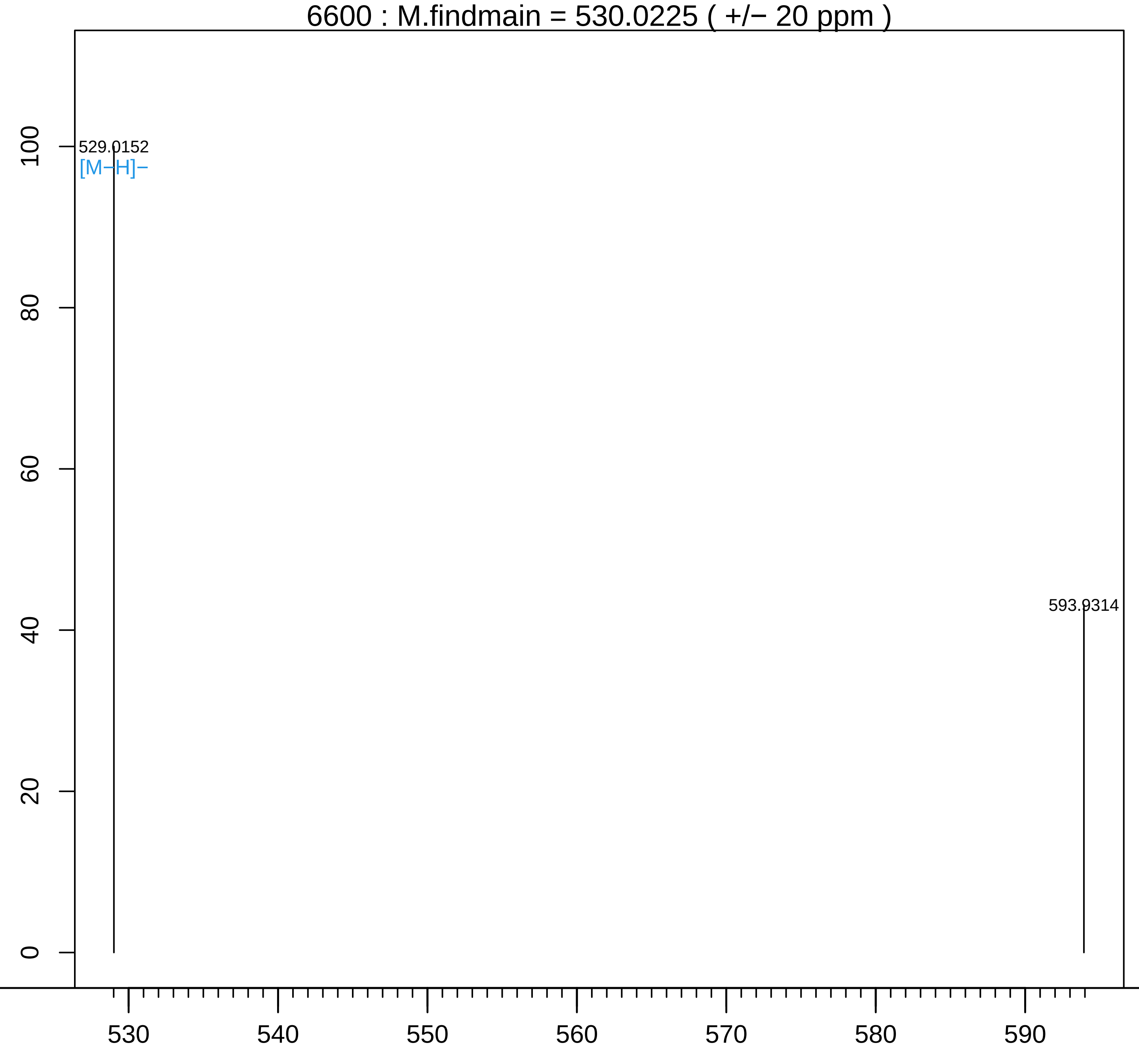

6600 : M.findmain = 530.0225 (+/− 20 ppm)
529.0152
[M−H]−
593.9314
0
20
40
60
80
100
530
540
550
560
570
580
590

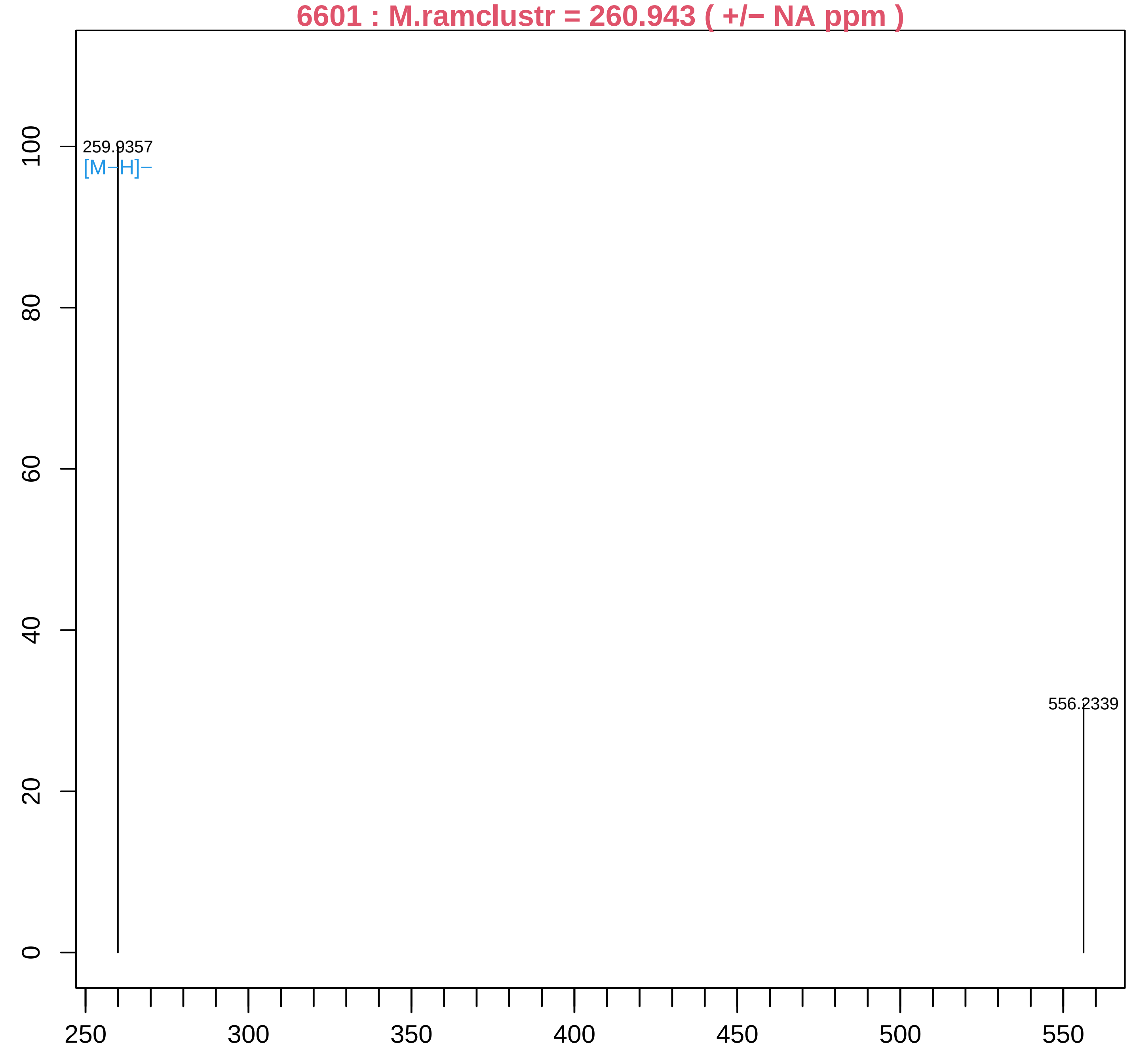

6601 : M.ramclustr = 260.943 (+/− NA ppm)
259.9357
[M−H]−
556.2339

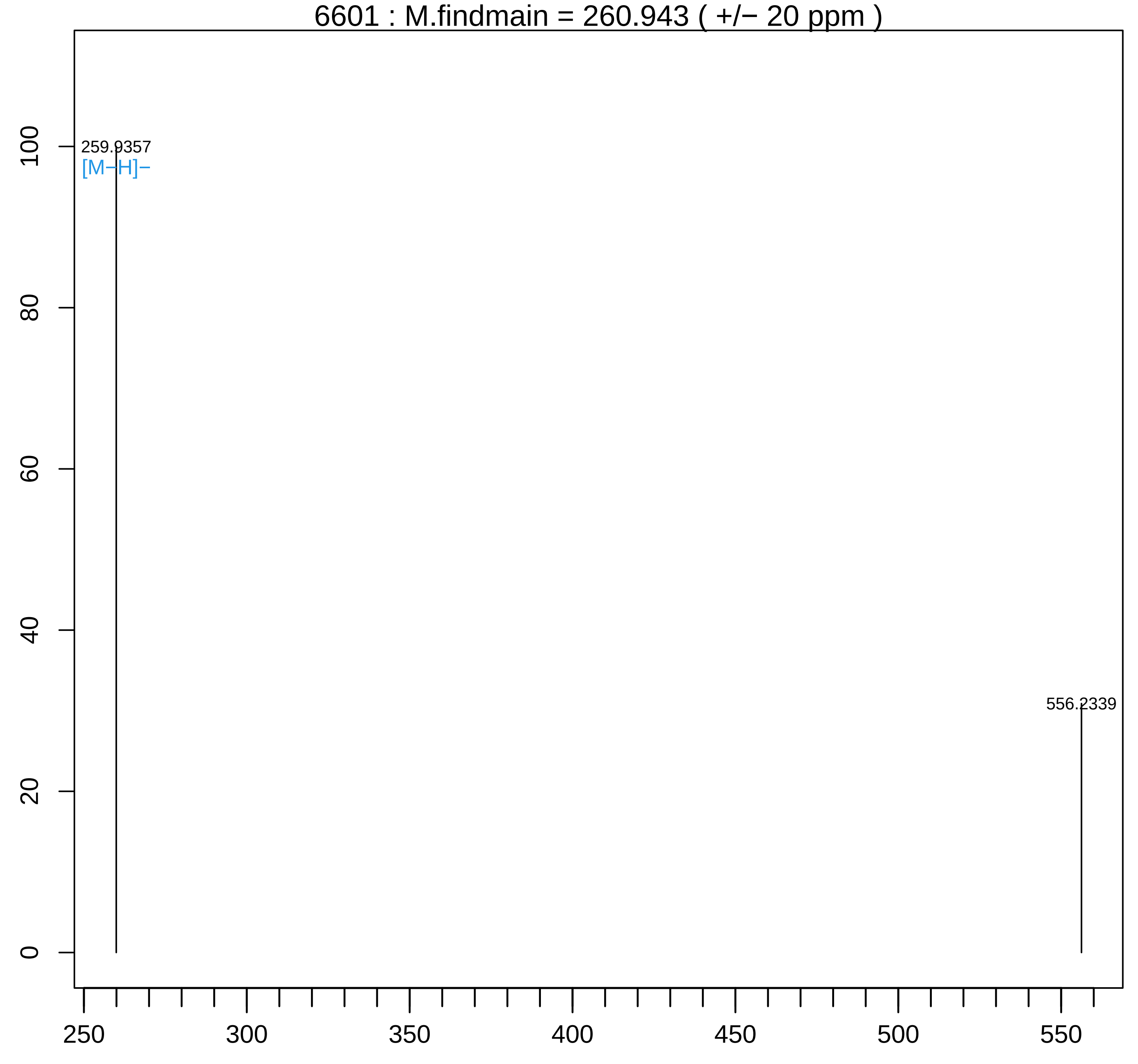

6601 : M.findmain = 260.943 (+/− 20 ppm)
259.9357
[M−H]−
556.2339

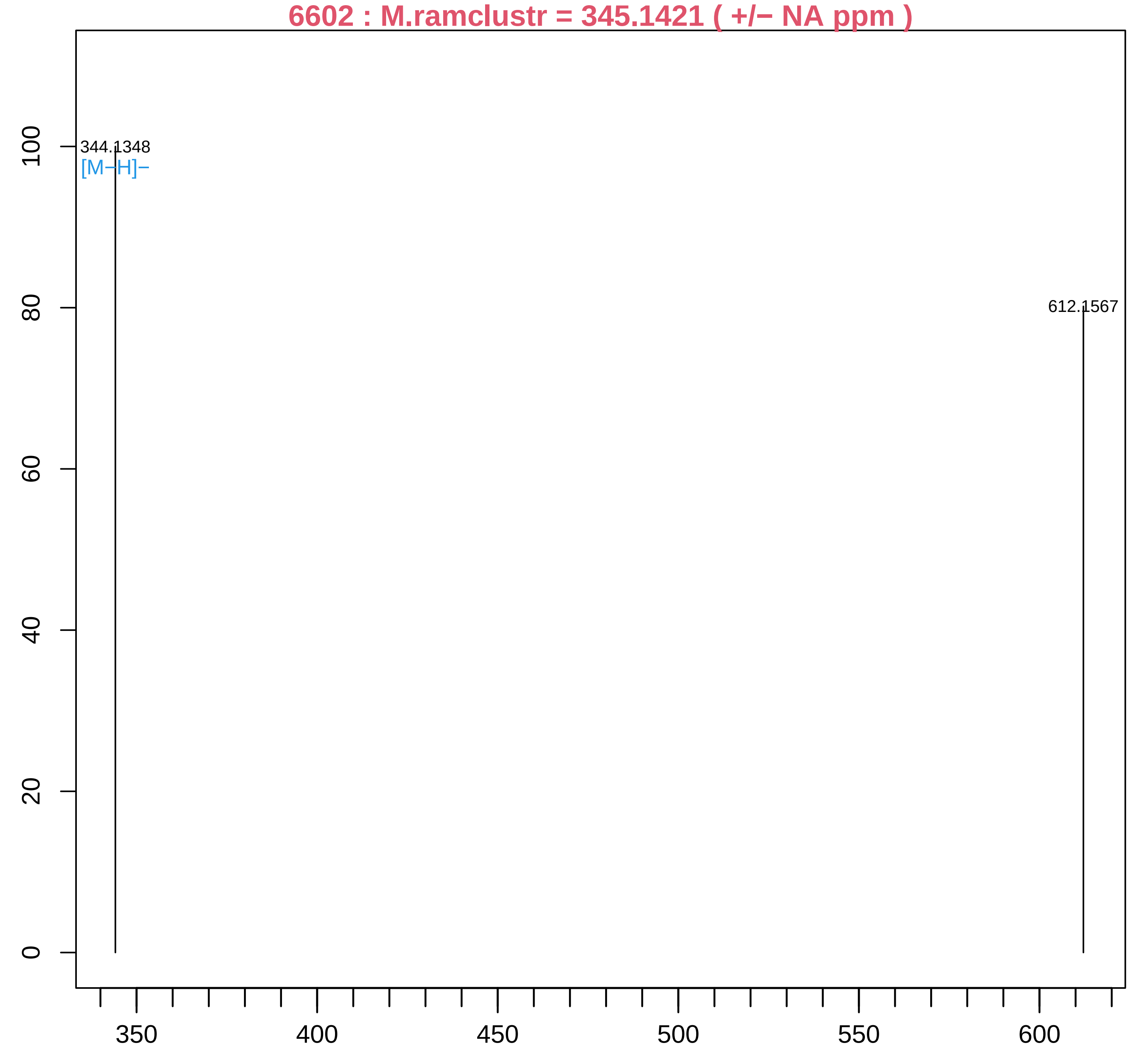
6602 : M.ramclustr = 345.1421 (+/− NA ppm)
344.1348
[M−H]−
612.1567
0
20
40
60
80
100
350
400
450
500
550
600

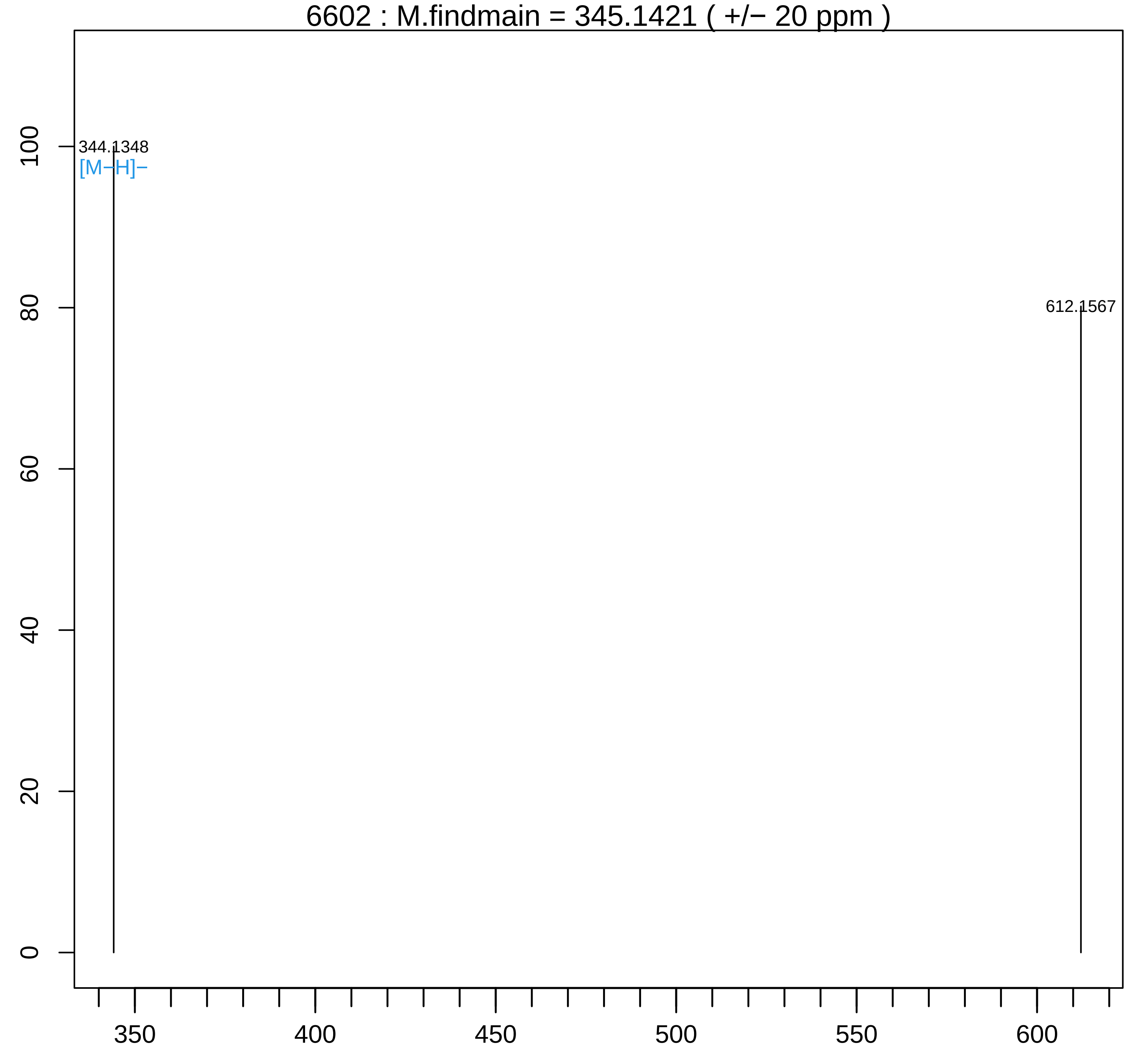
6602 : M.findmain = 345.1421 (+/− 20 ppm)
344.1348
[M−H]−
612.1567
0
20
40
60
80
100
350
400
450
500
550
600

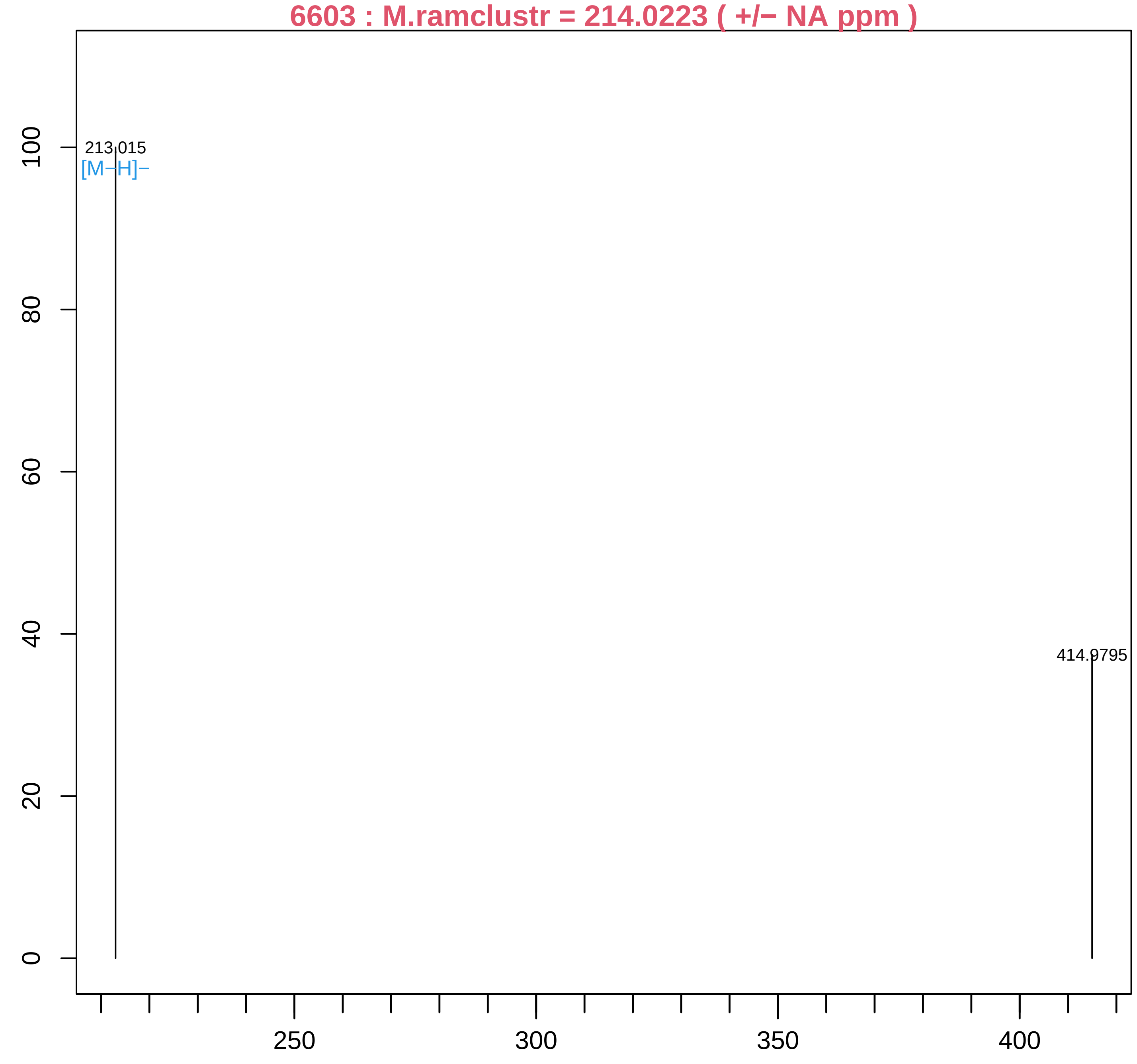
6603 : M.ramclustr = 214.0223 (+/− NA ppm)
213.015
[M−H]−
414.9795
0
20
40
60
80
100
250
300
350
400

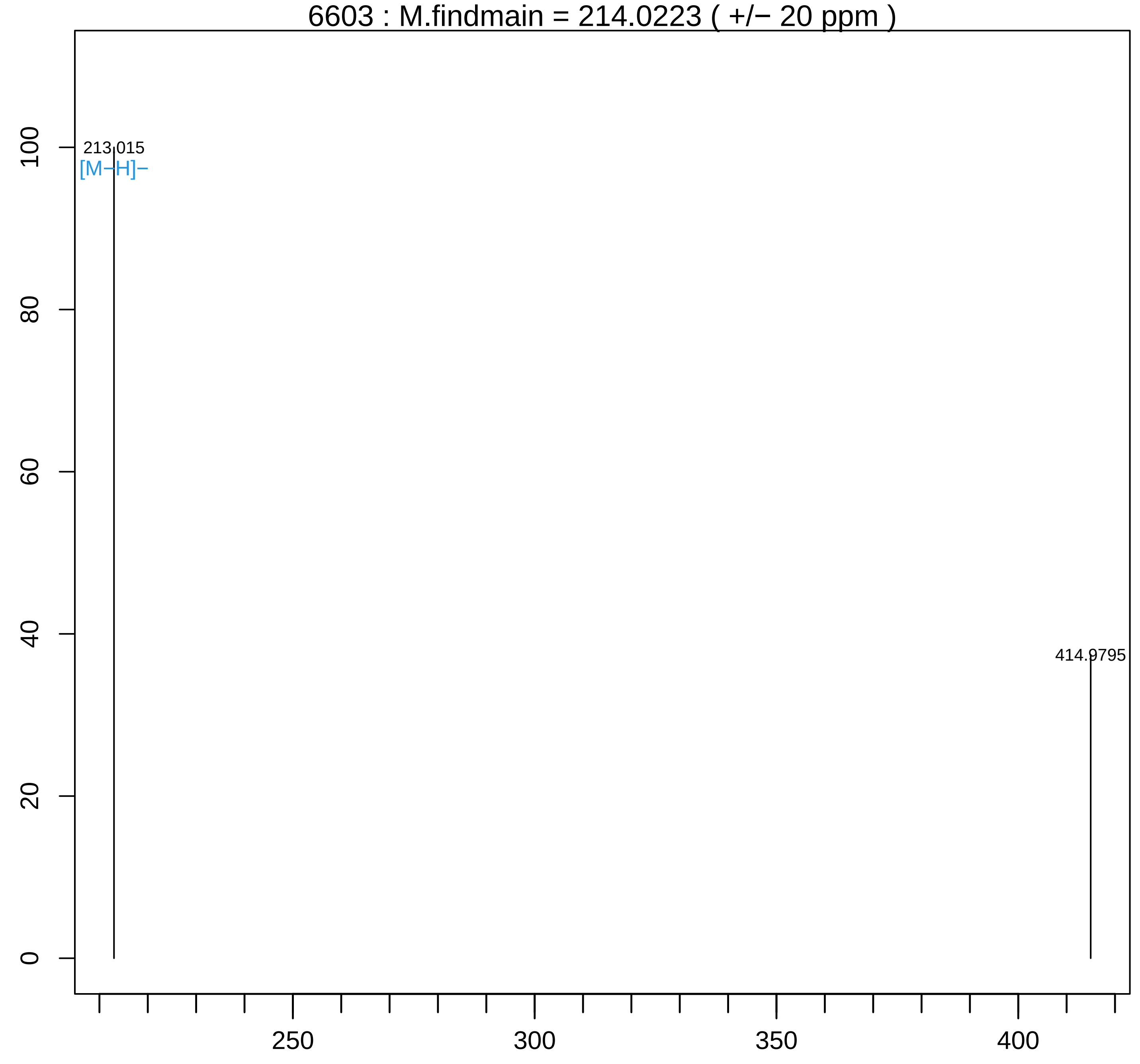
6603 : M.findmain = 214.0223 (+/− 20 ppm)
213.015
[M−H]−
414.9795
0
20
40
60
80
100
250
300
350
400

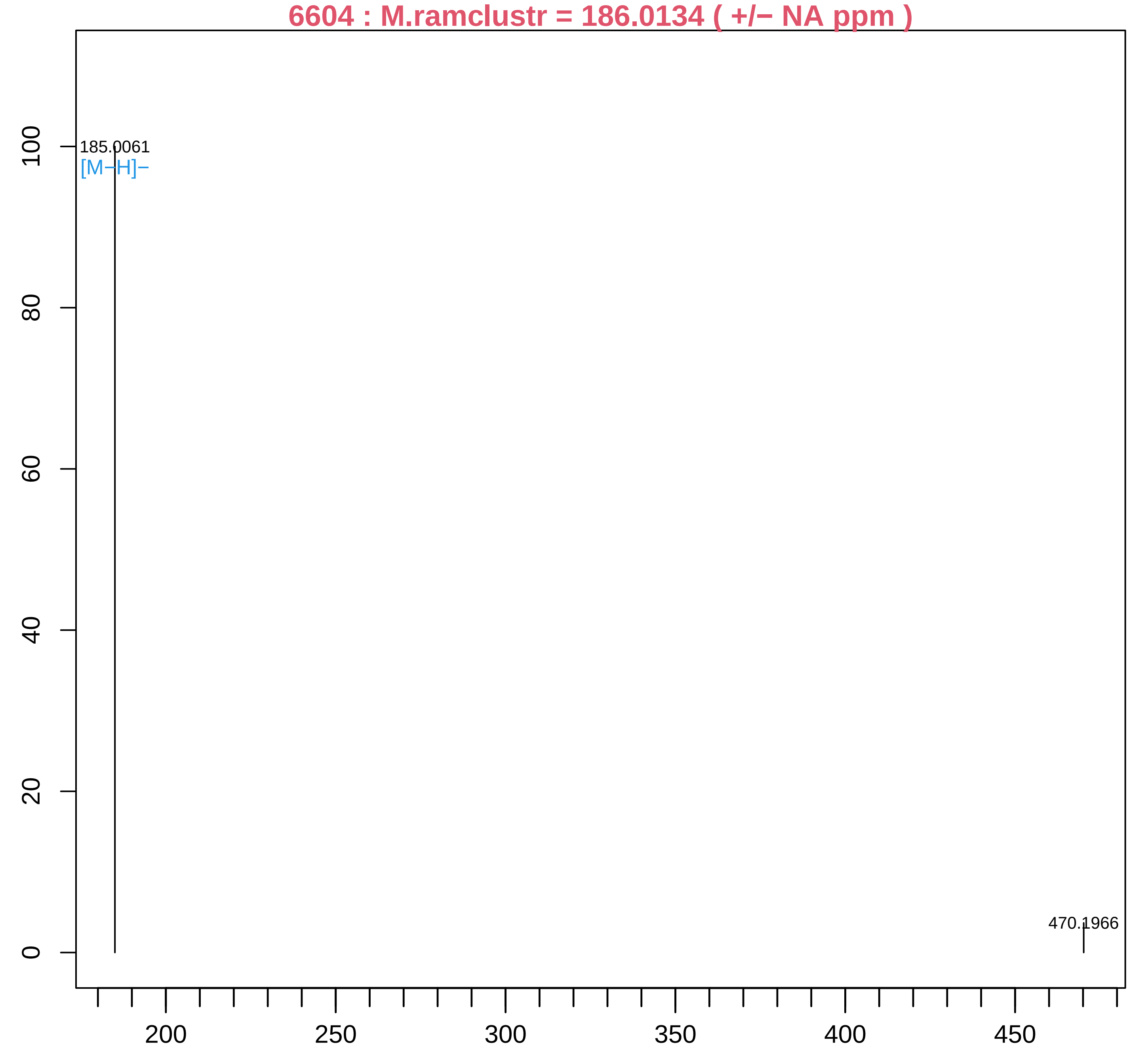
6604 : M.ramclustr = 186.0134 (+/− NA ppm)
185.0061
[M−H]−
470.1966
0
20
40
60
80
100
200
250
300
350
400
450

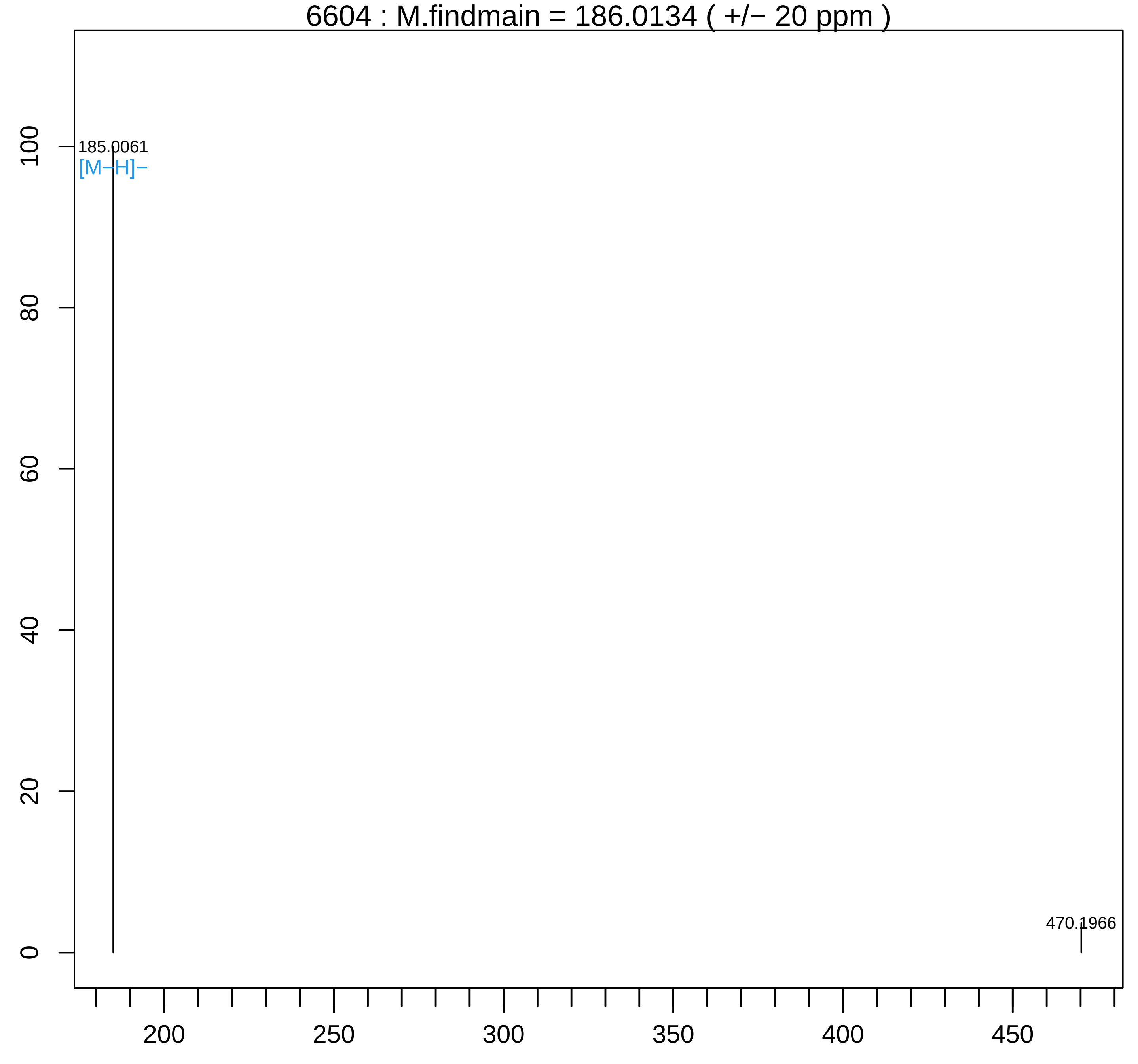
6604 : M.findmain = 186.0134 (+/− 20 ppm)
185.0061
[M−H]−
470.1966
0
20
40
60
80
100
200
250
300
350
400
450

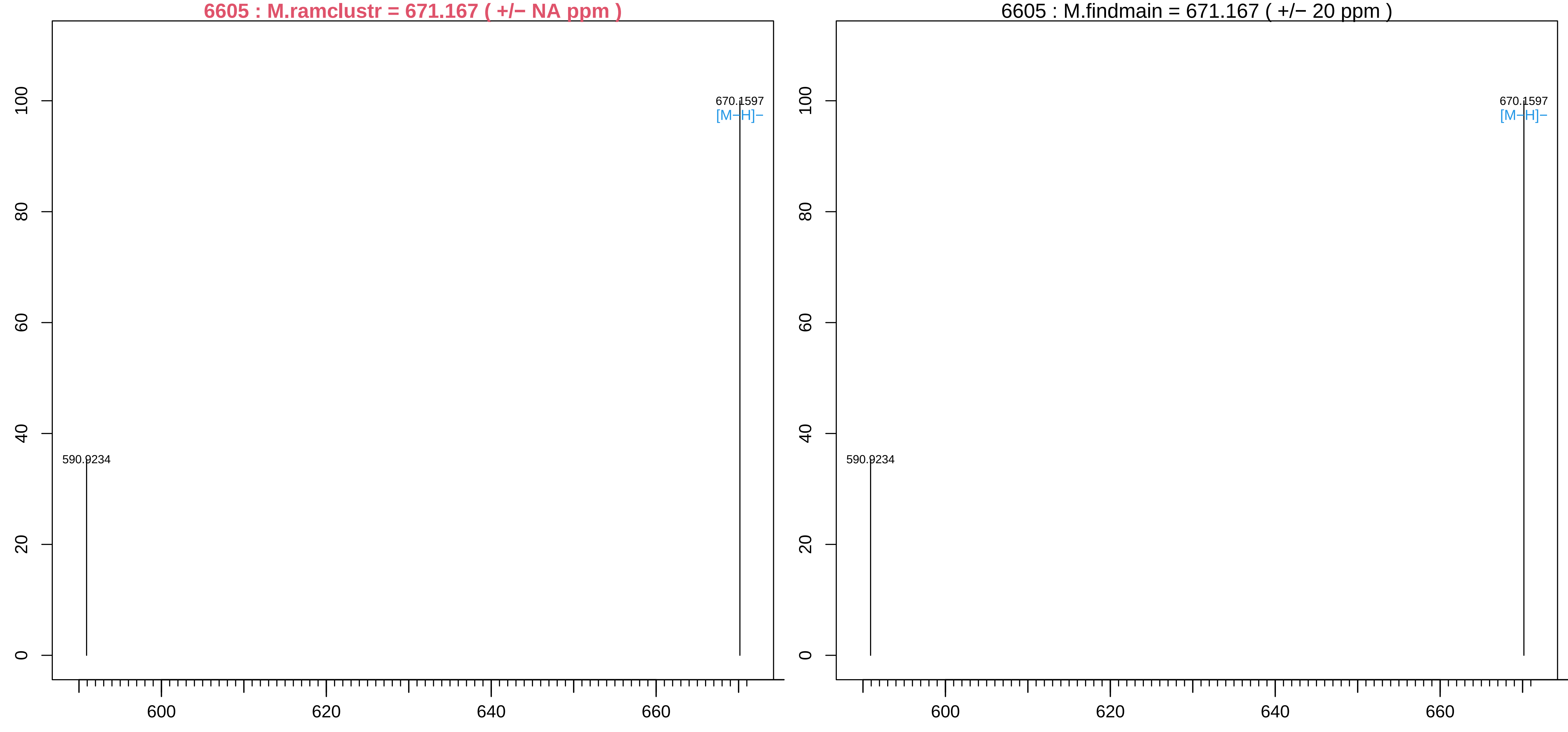
6605 : M.ramclustr = 671.167 (+/− NA ppm)
670.1597
[M−H]−
590.9234
6605 : M.findmain = 671.167 (+/− 20 ppm)
670.1597
[M−H]−
590.9234
0
20
40
60
80
100
600
620
640
660

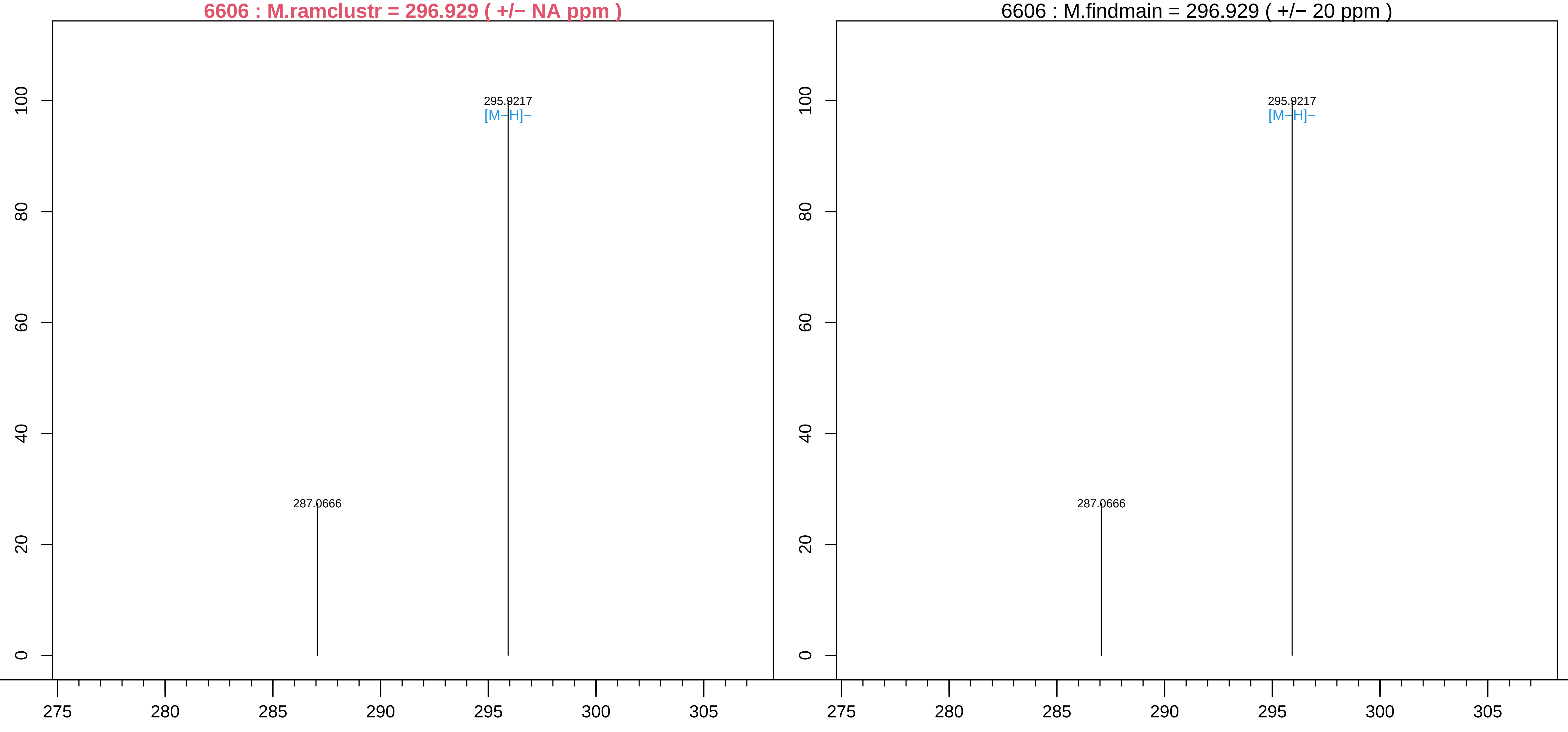

6606 : M.ramclustr = 296.929 (+/− NA ppm)
6606 : M.findmain = 296.929 (+/− 20 ppm)
295.9217
[M−H]−
287.0666
295.9217
[M−H]−
287.0666
0
20
40
60
80
100
275
280
285
290
295
300
305

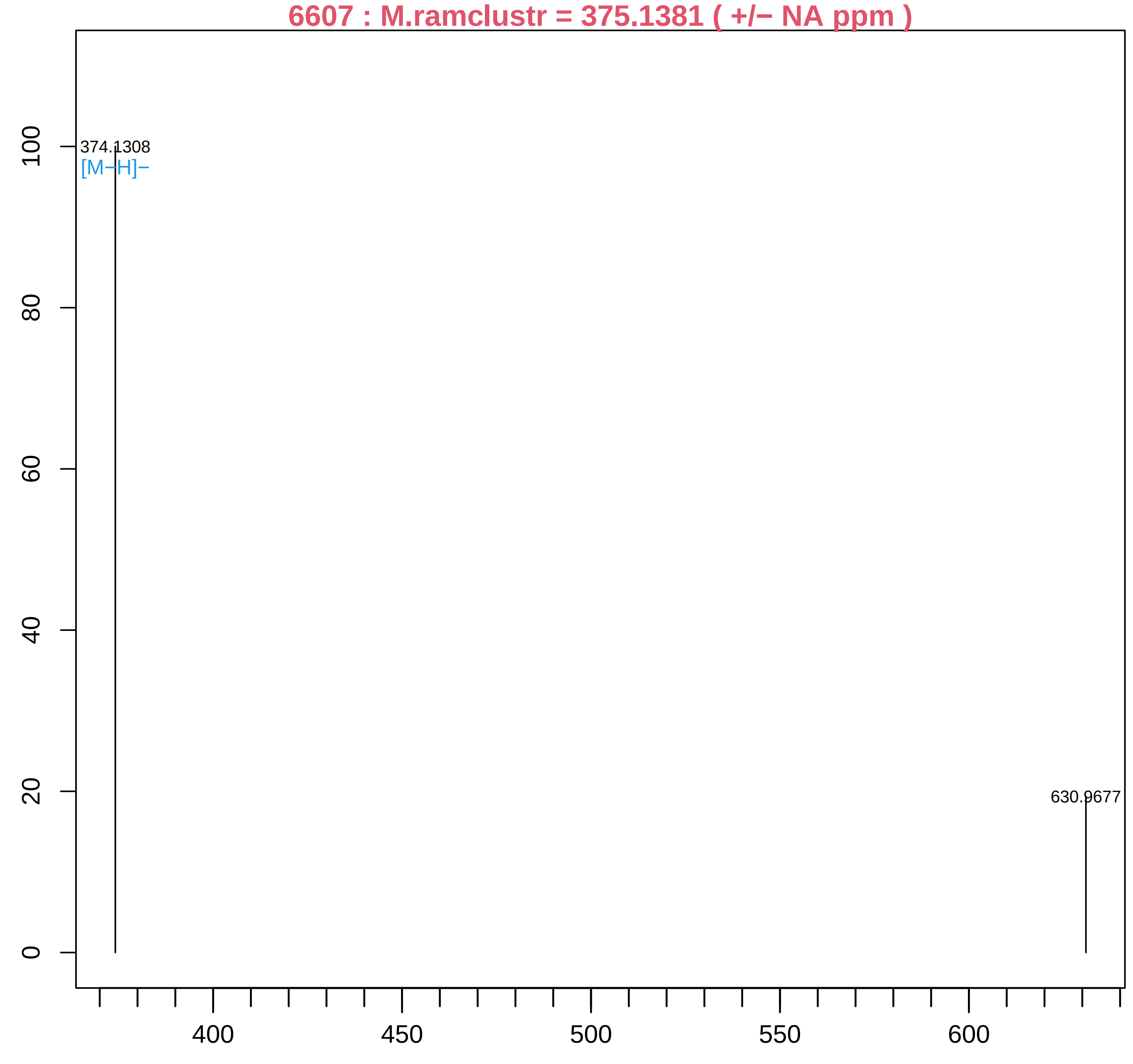
6607 : M.ramclustr = 375.1381 (+/− NA ppm)
374.1308
[M−H]−
630.9677
0
20
40
60
80
100
400
450
500
550
600

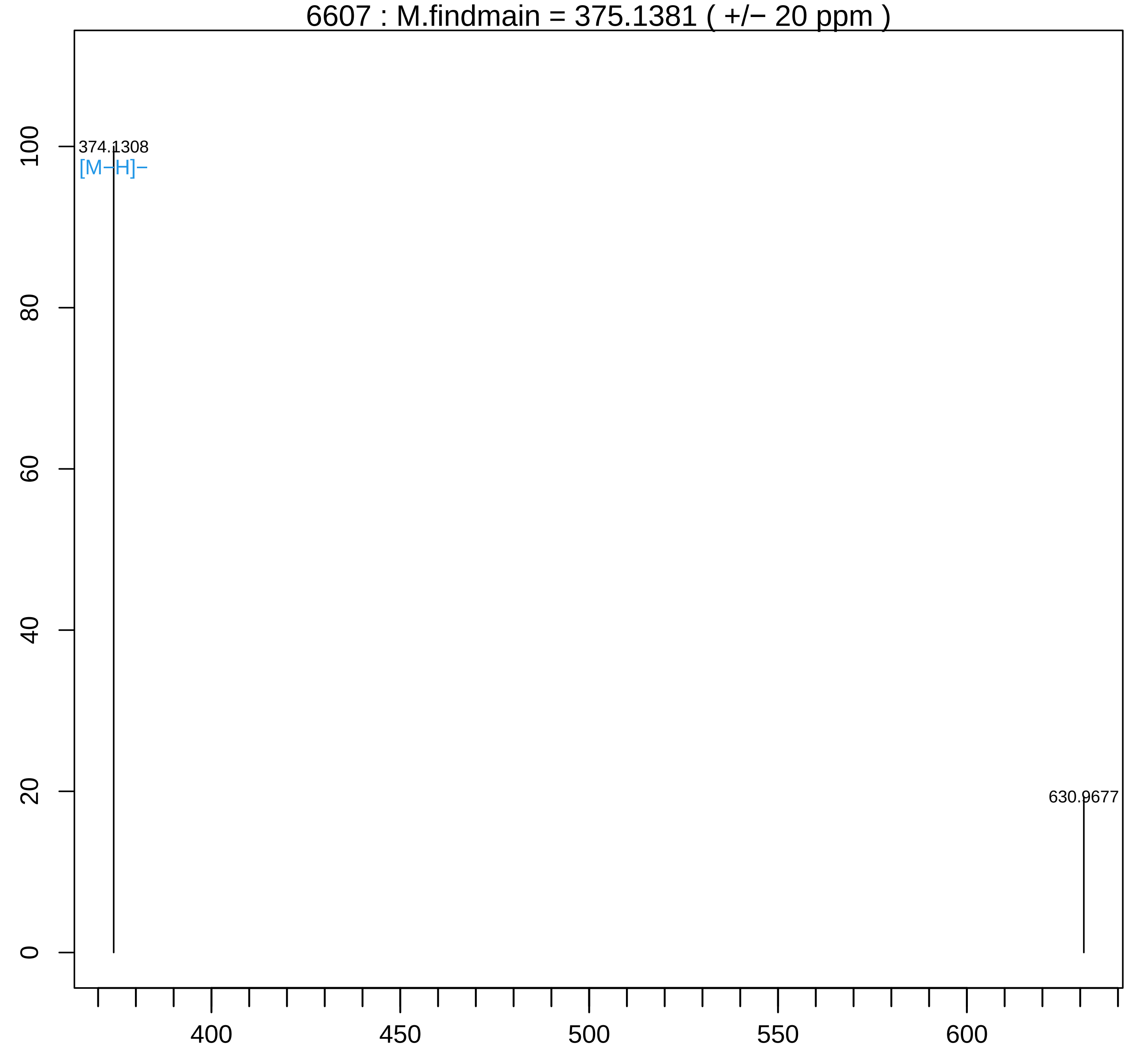
6607 : M.findmain = 375.1381 (+/− 20 ppm)
374.1308
[M−H]−
630.9677
0
20
40
60
80
100
400
450
500
550
600

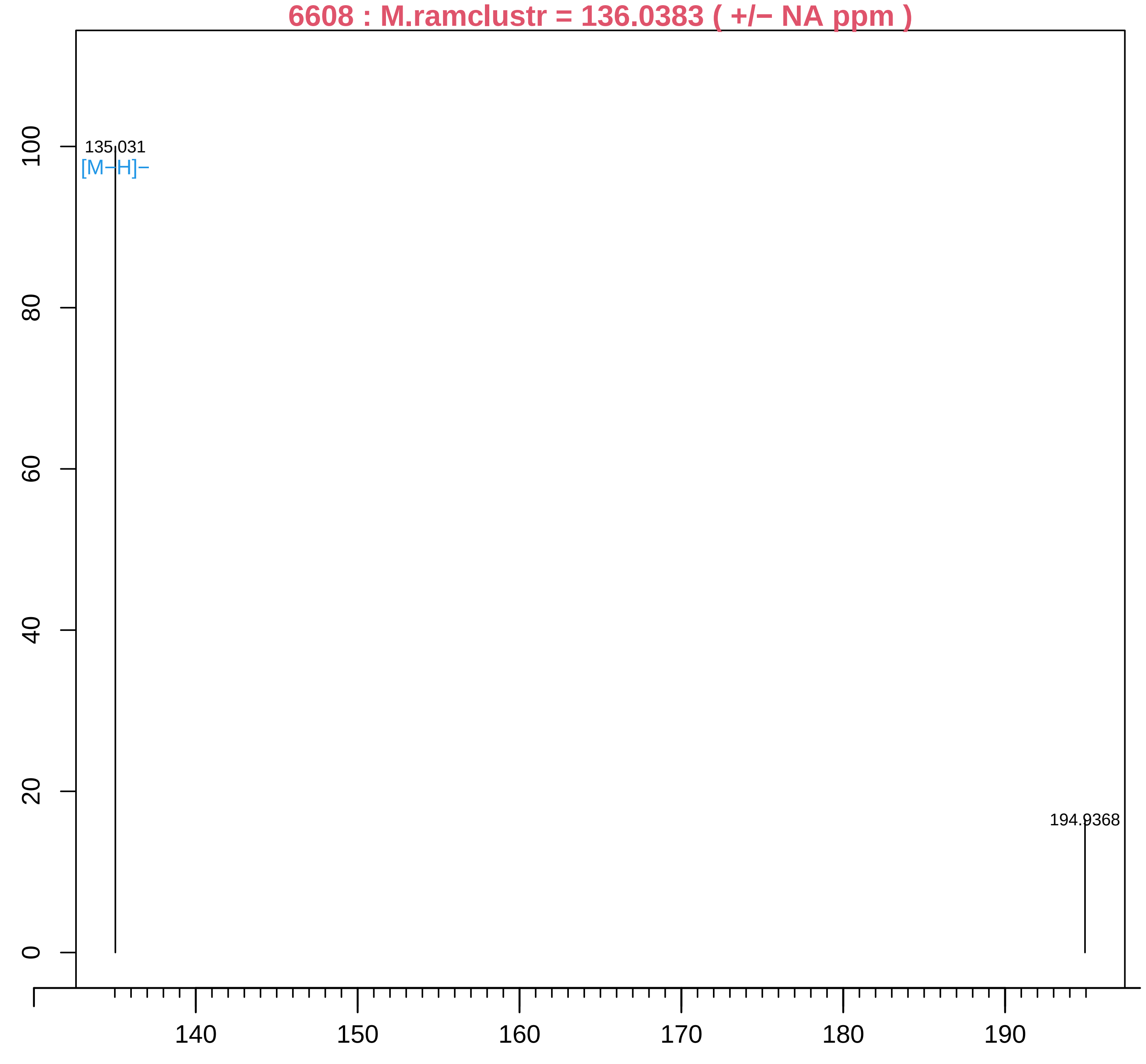
6608 : M.ramclustr = 136.0383 (+/− NA ppm)
135.031
[M−H]−
194.9368
0
20
40
60
80
100
140
150
160
170
180
190

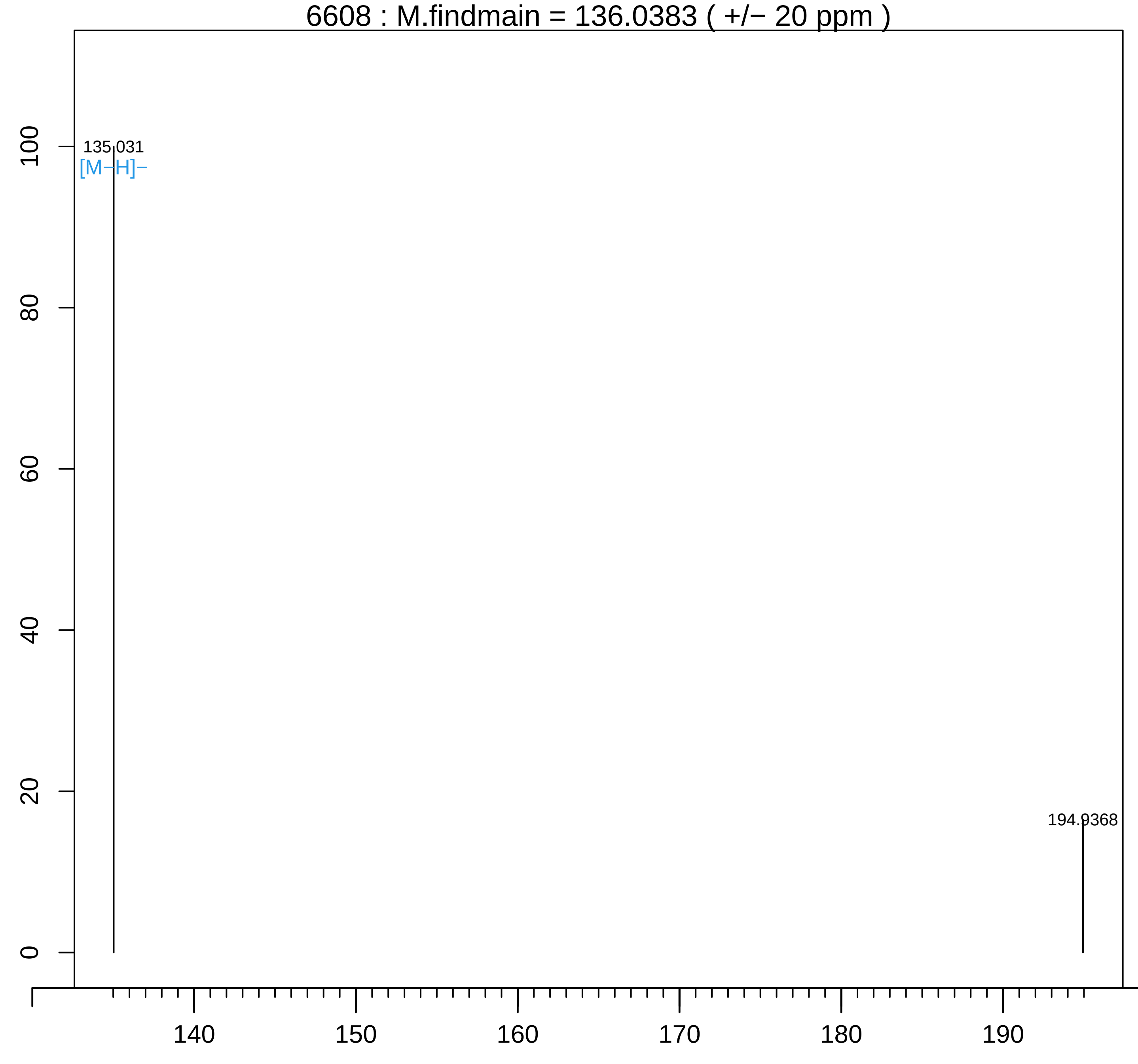
6608 : M.findmain = 136.0383 (+/− 20 ppm)
135.031
[M−H]−
194.9368
0
20
40
60
80
100
140
150
160
170
180
190

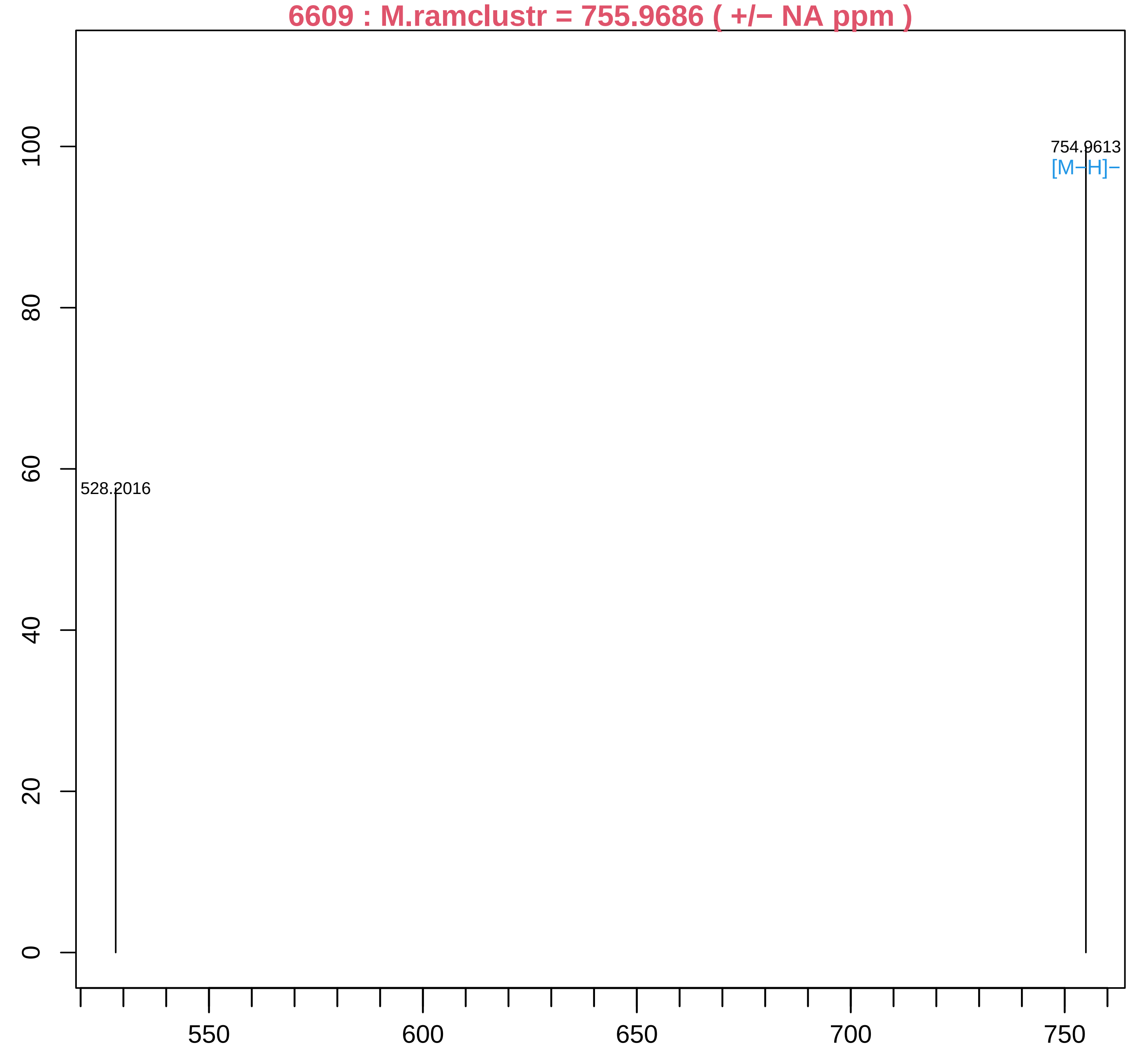
6609 : M.ramclustr = 755.9686 (+/− NA ppm)
754.9613
[M−H]−
528.2016
0
20
40
60
80
100
550
600
650
700
750

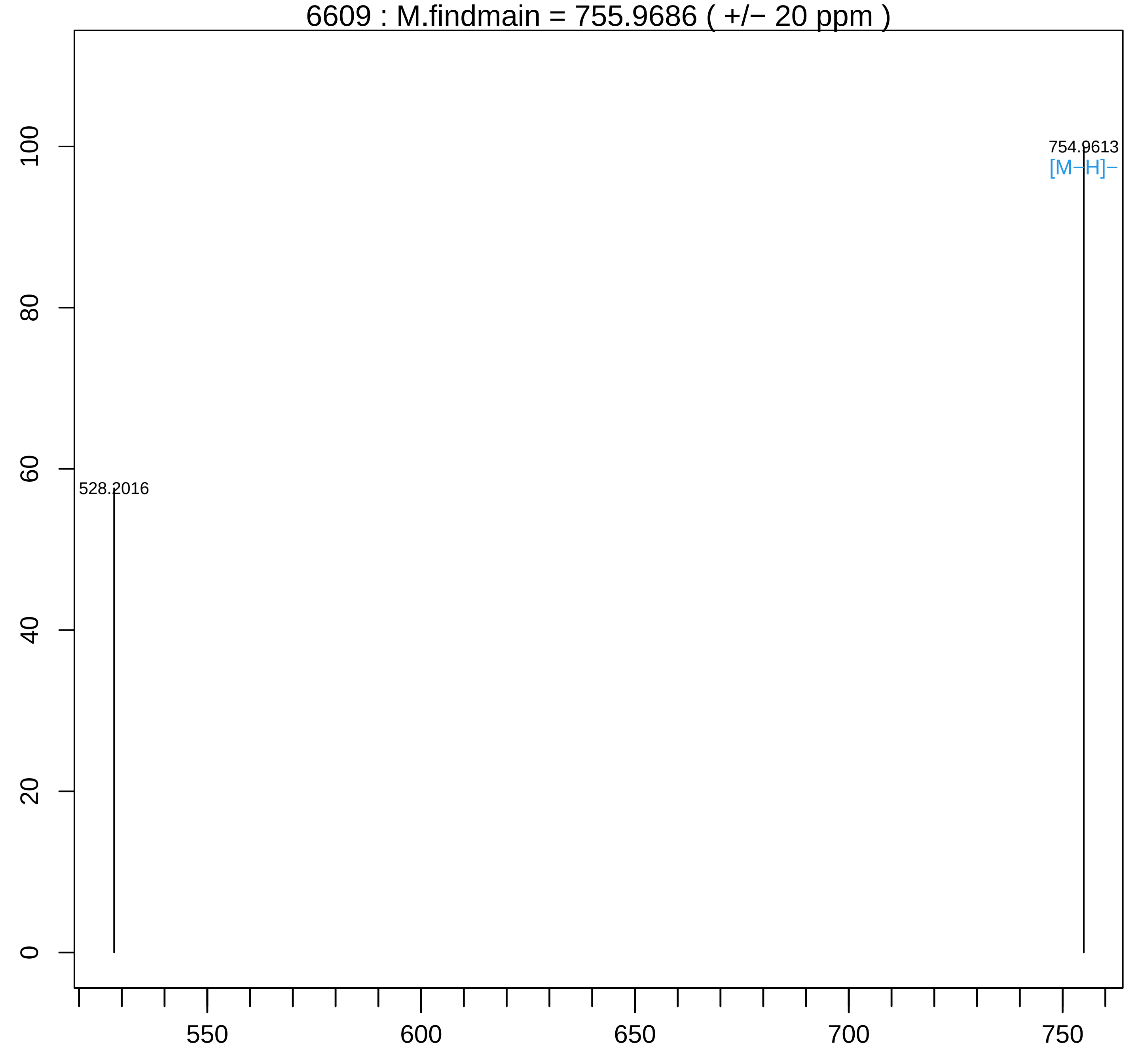
6609 : M.findmain = 755.9686 (+/− 20 ppm)
754.9613
[M−H]−
528.2016
0
20
40
60
80
100
550
600
650
700
750

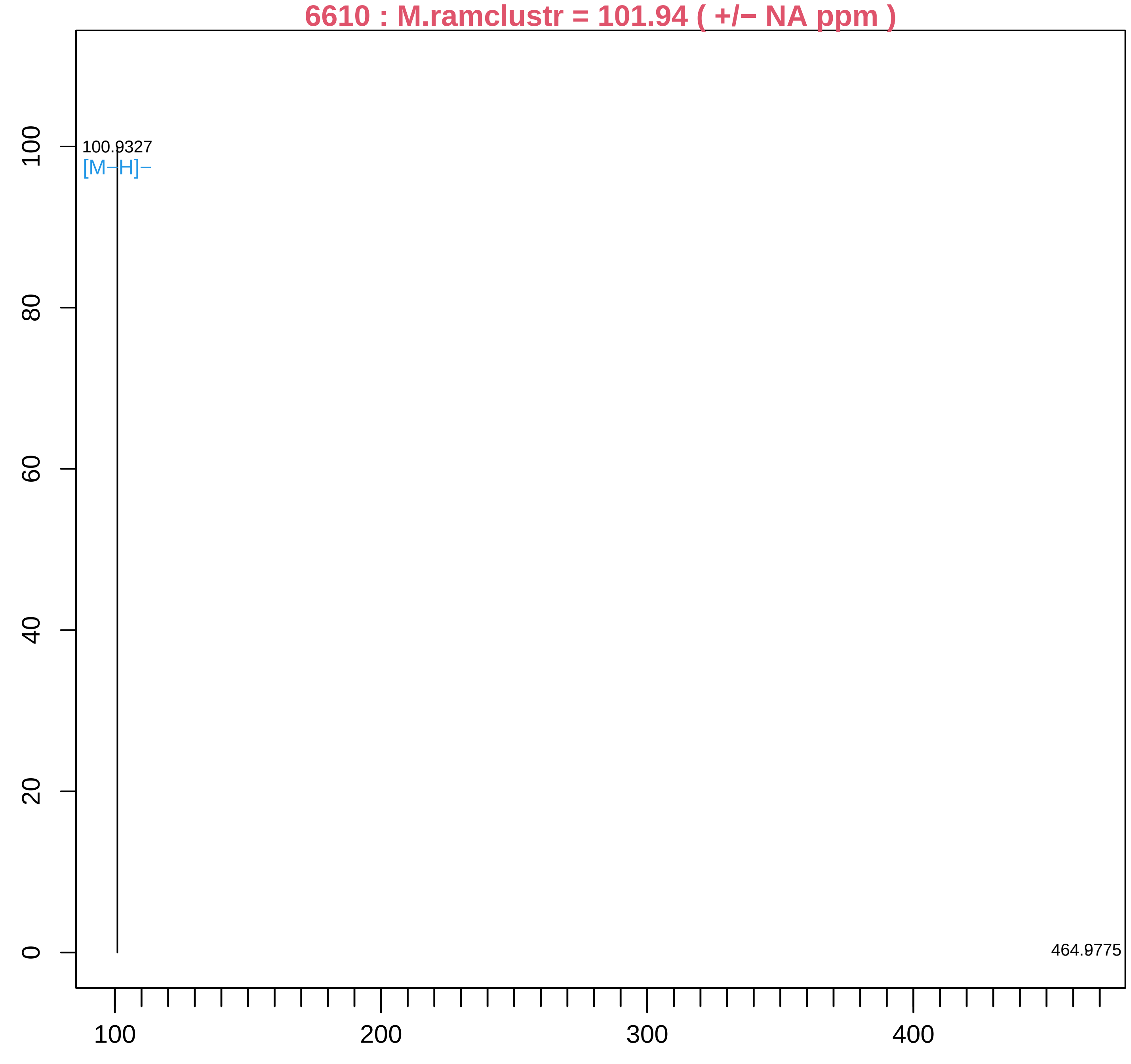
6610 : M.ramclustr = 101.94 (+/− NA ppm)
100.9327
[M−H]−
464.9775
0
20
40
60
80
100
100
200
300
400

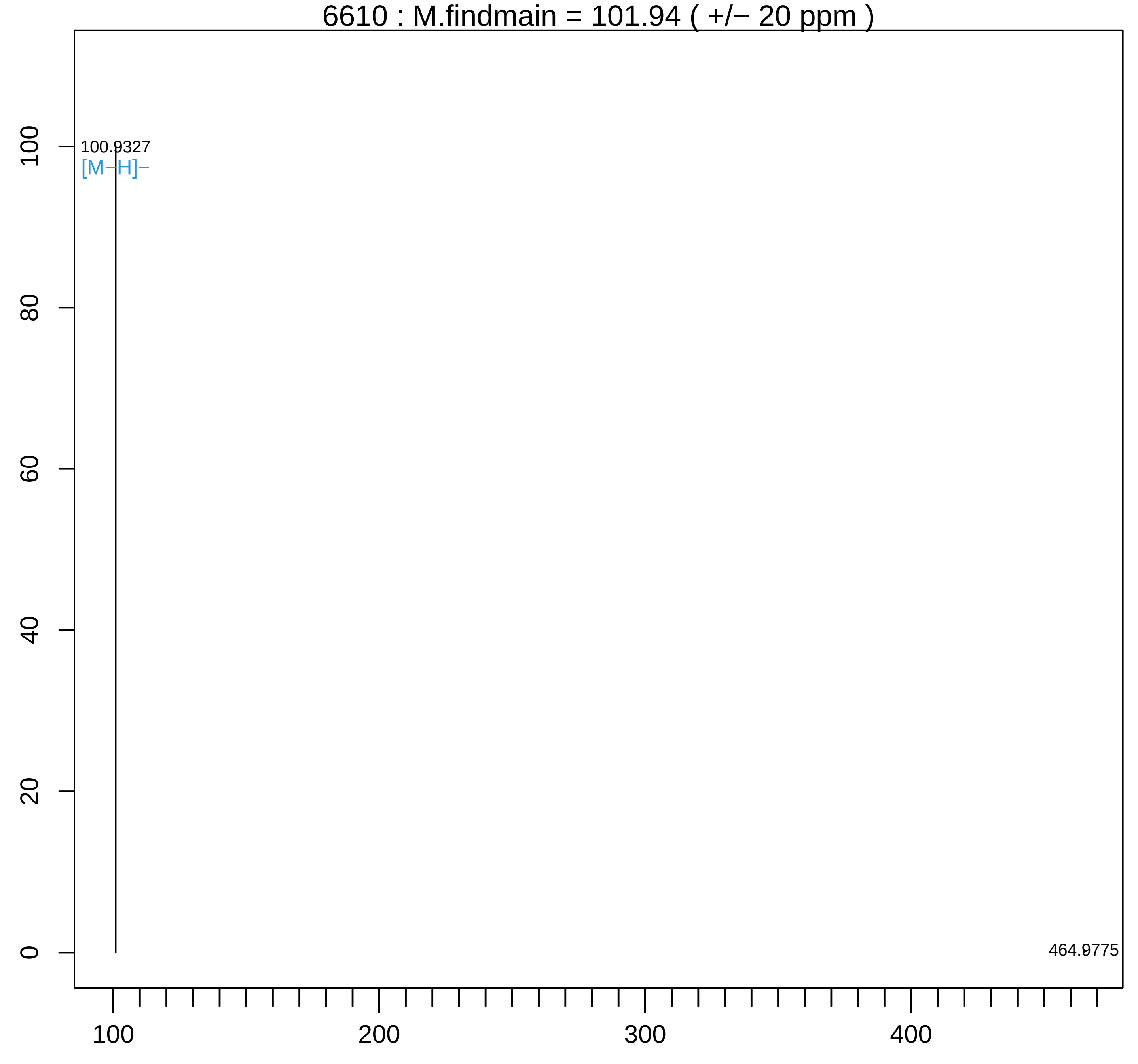
6610 : M.findmain = 101.94 (+/− 20 ppm)
100.9327
[M−H]−
464.9775
0
20
40
60
80
100
100
200
300
400

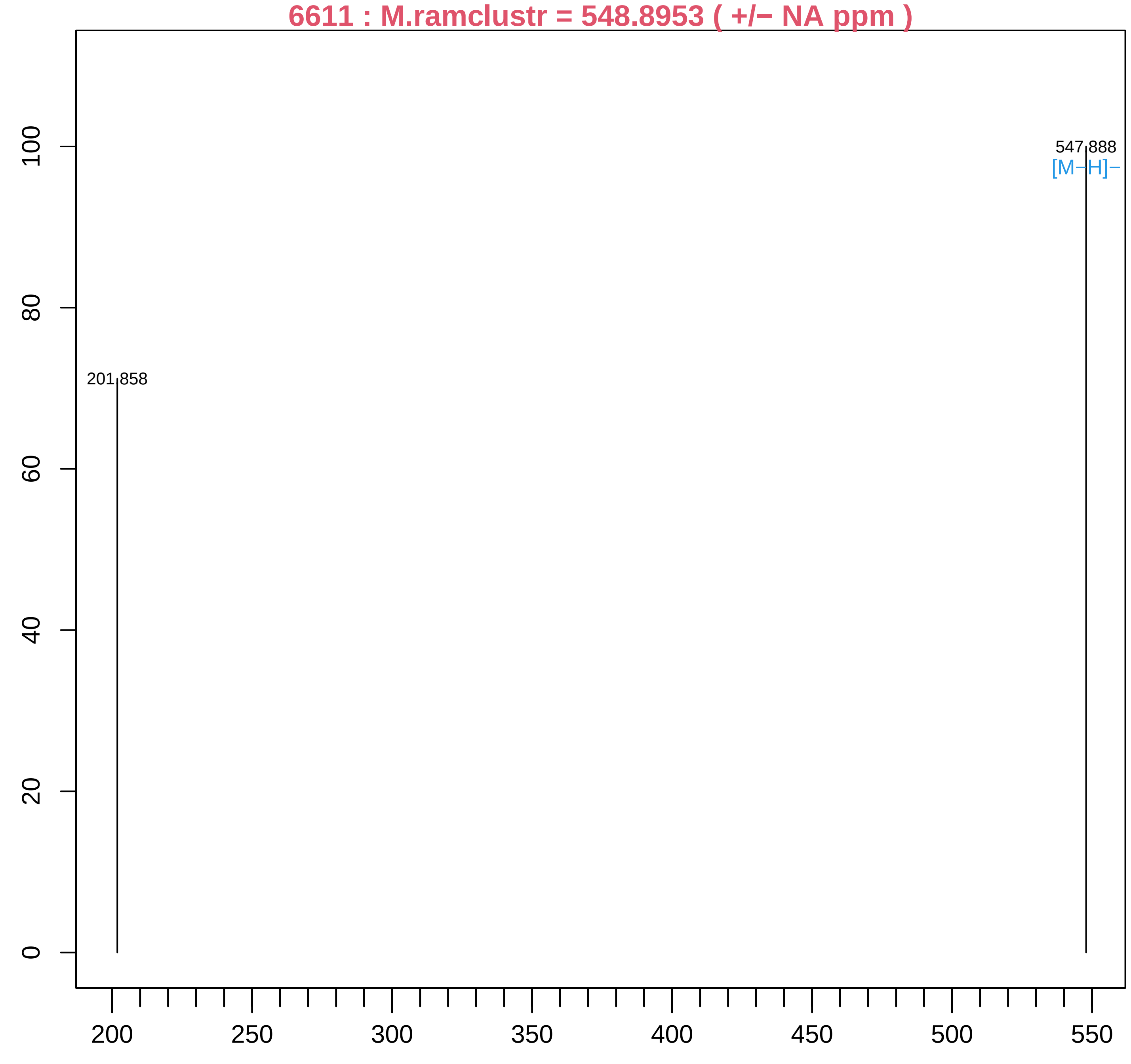
6611 : M.ramclustr = 548.8953 (+/− NA ppm)
547.888
[M−H]−
201.858
0
20
40
60
80
100
200
250
300
350
400
450
500
550

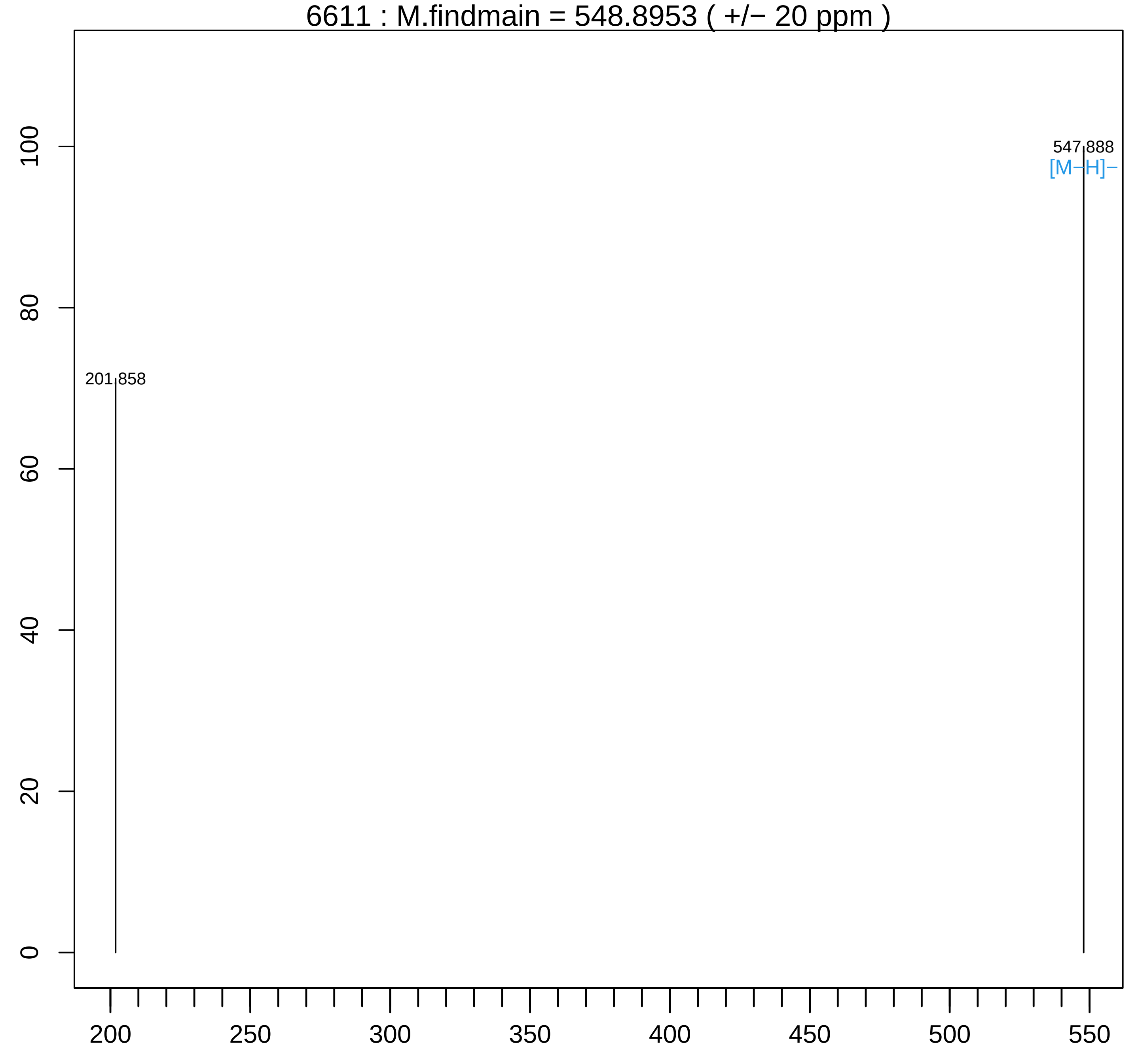
6611 : M.findmain = 548.8953 (+/− 20 ppm)
547.888
[M−H]−
201.858
0
20
40
60
80
100
200
250
300
350
400
450
500
550

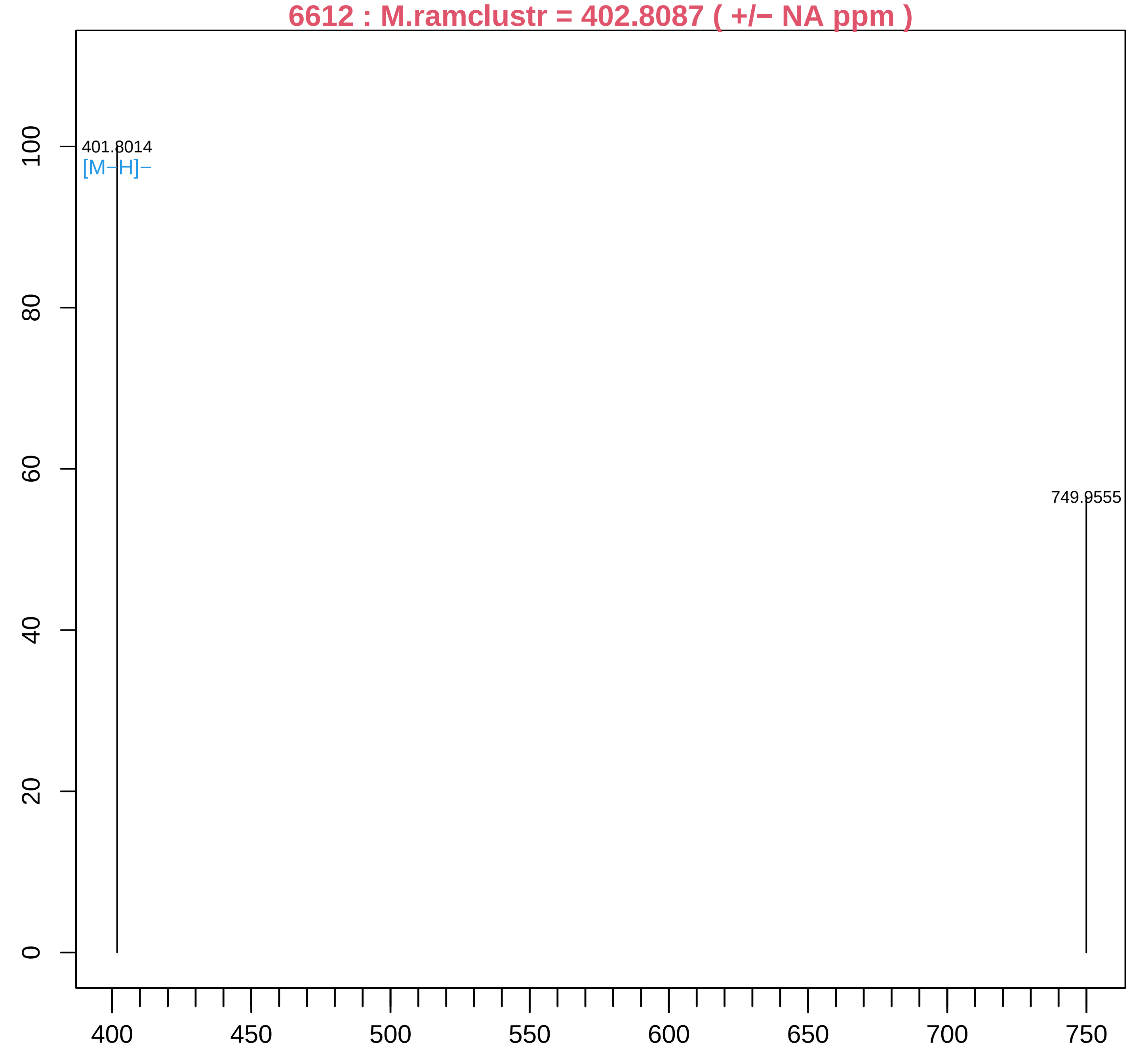
6612 : M.ramclustr = 402.8087 (+/− NA ppm)
401.8014
[M−H]−
749.9555
0
20
40
60
80
100
400
450
500
550
600
650
700
750

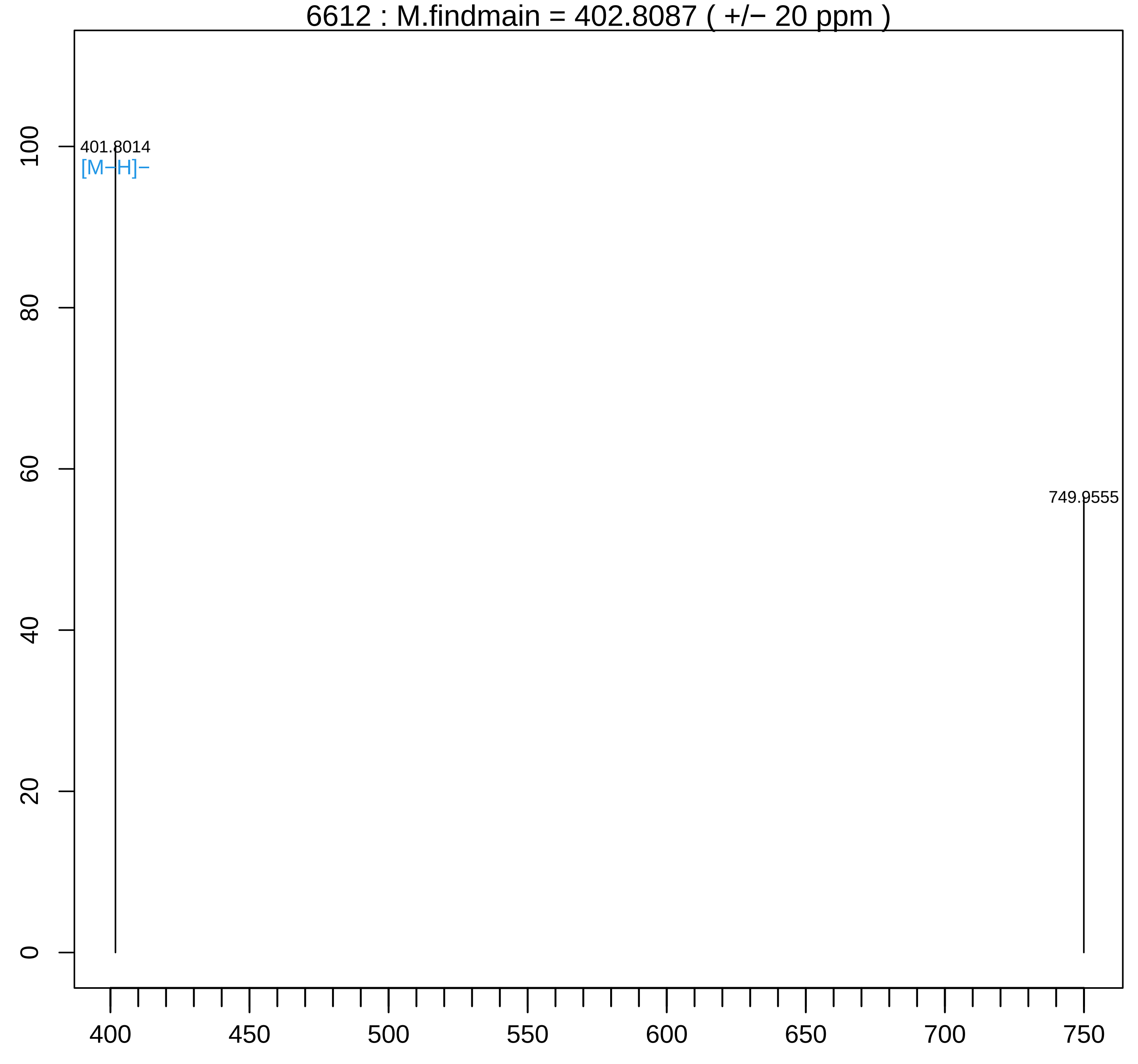
6612 : M.findmain = 402.8087 (+/− 20 ppm)
401.8014
[M−H]−
749.9555
0
20
40
60
80
100
400
450
500
550
600
650
700
750

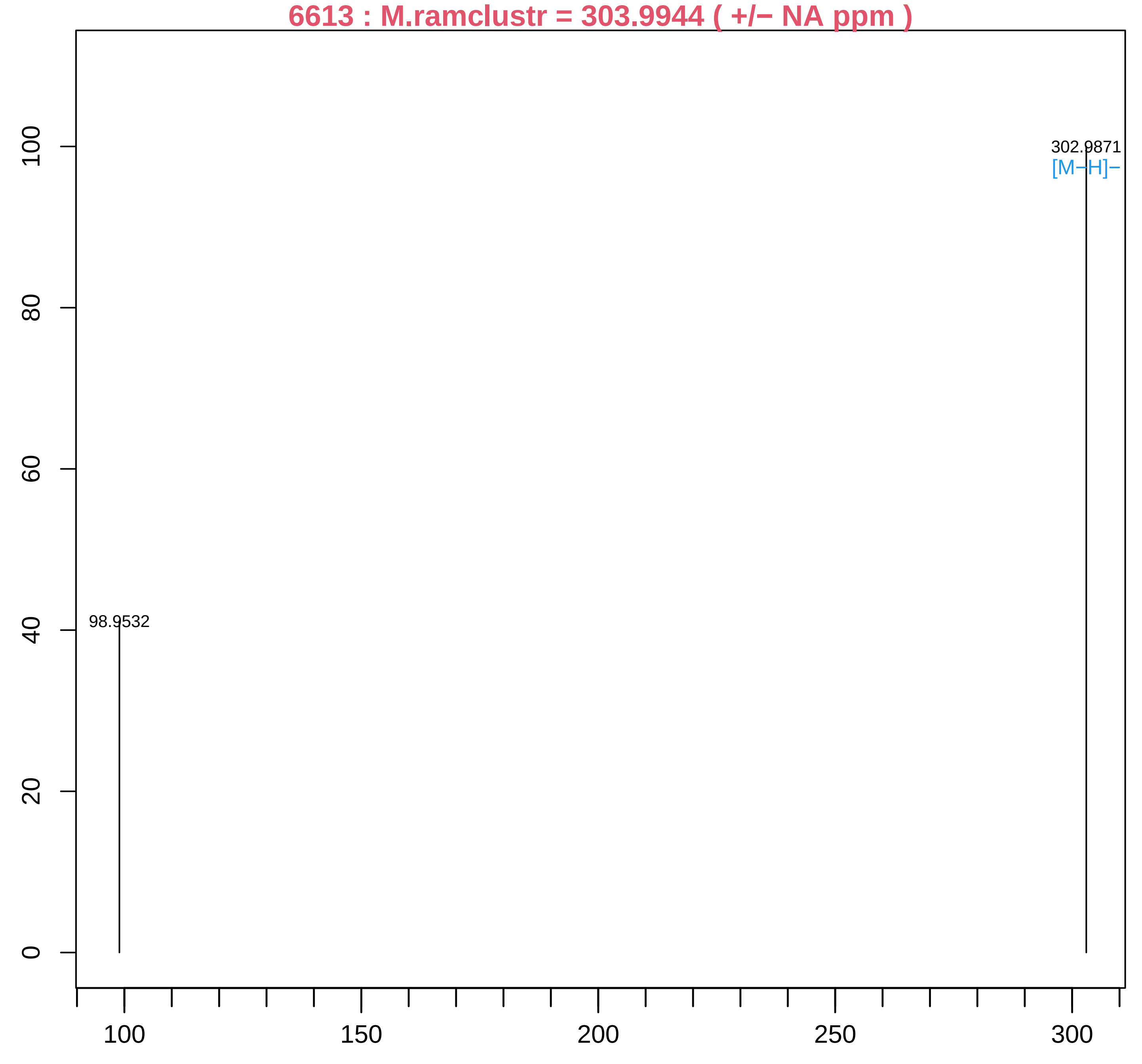
6613 : M.ramclustr = 303.9944 (+/− NA ppm)
302.9871
[M−H]−
98.9532
0
20
40
60
80
100
100
150
200
250
300

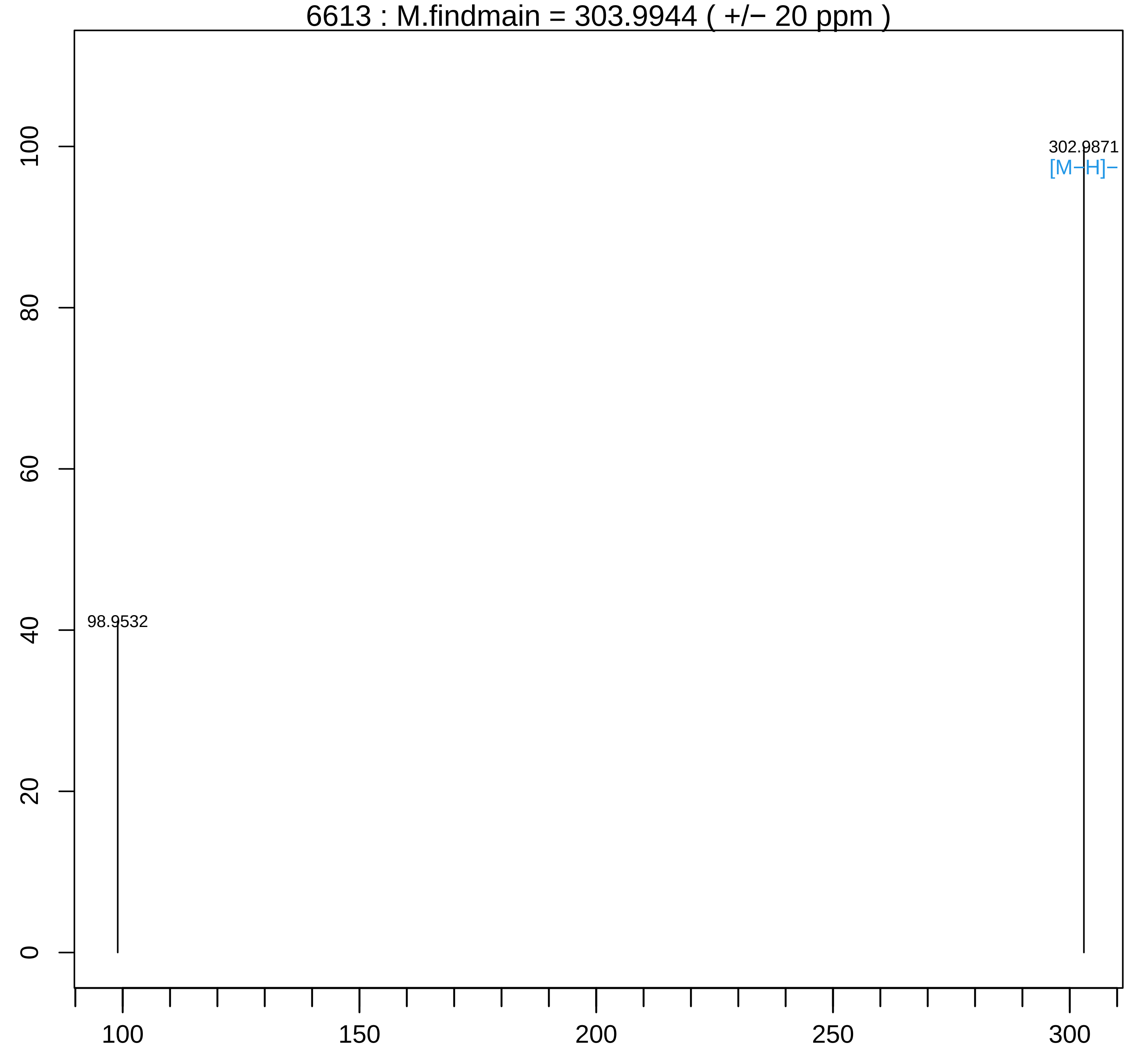
6613 : M.findmain = 303.9944 (+/− 20 ppm)
302.9871
[M−H]−
98.9532
0
20
40
60
80
100
100
150
200
250
300

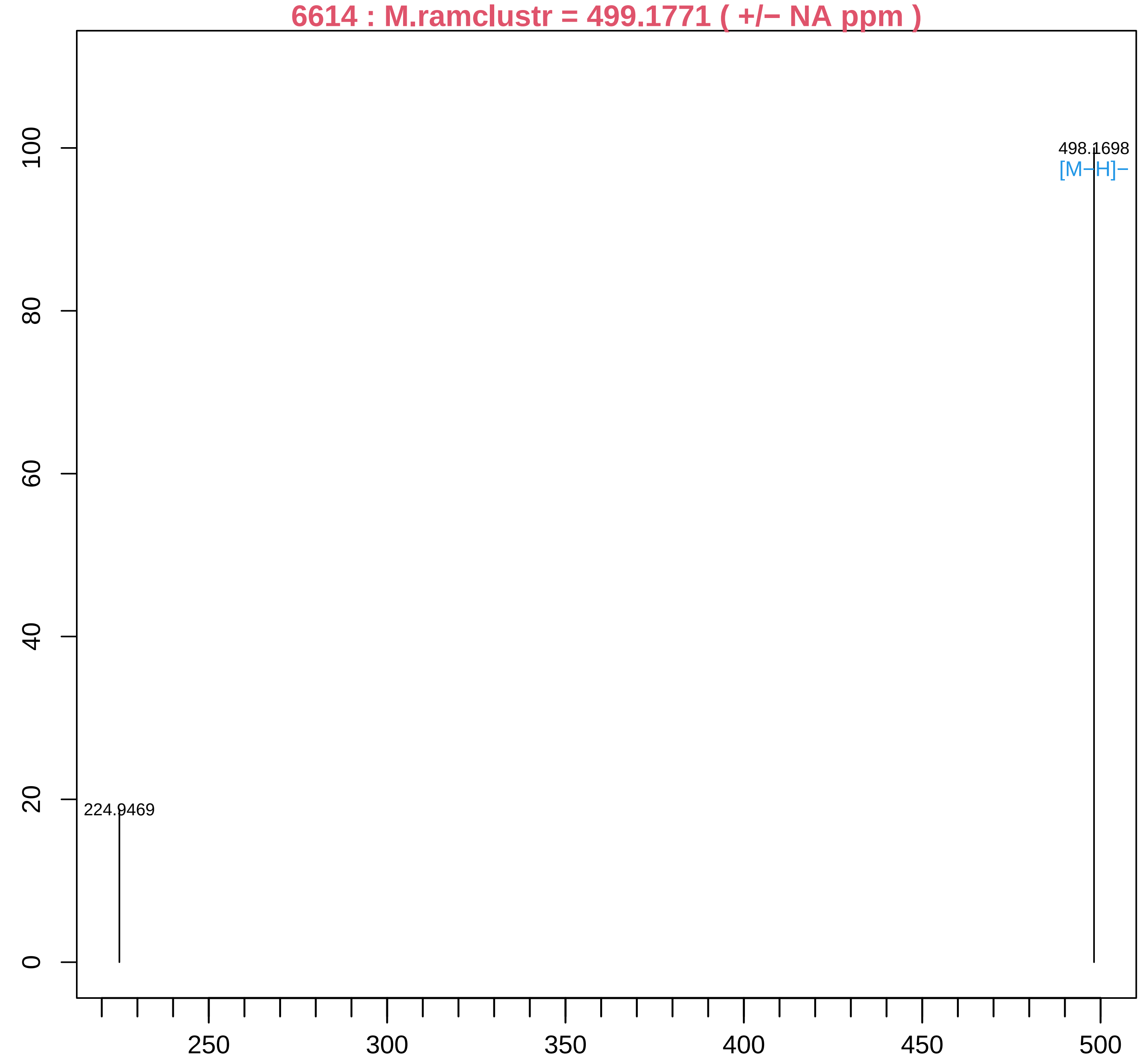

6614 : M.ramclustr = 499.1771 (+/− NA ppm)
498.1698
[M−H]−
224.9469
0
20
40
60
80
100
250
300
350
400
450
500

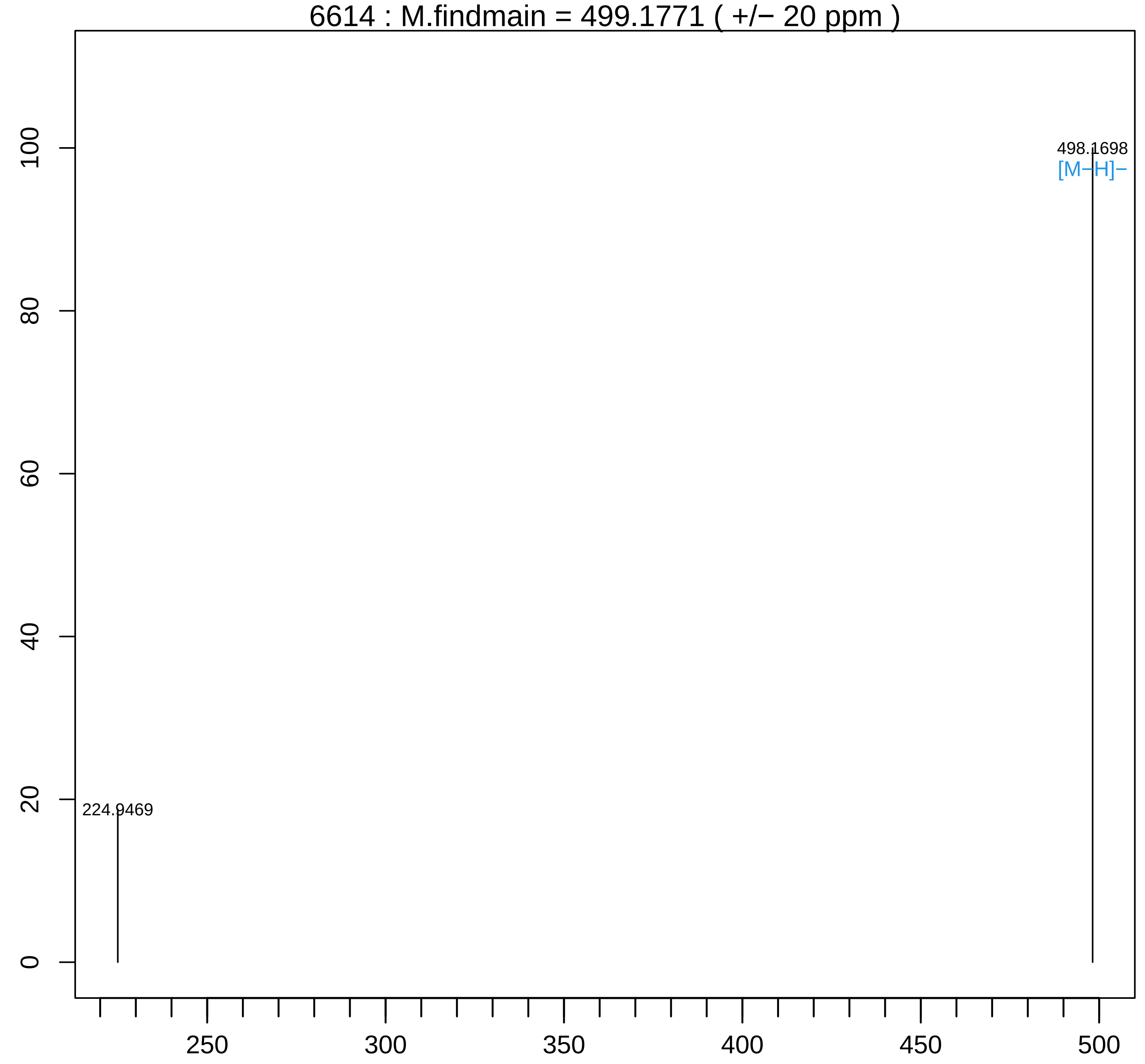

6614 : M.findmain = 499.1771 (+/− 20 ppm)
498.1698
[M−H]−
224.9469
0
20
40
60
80
100
250
300
350
400
450
500

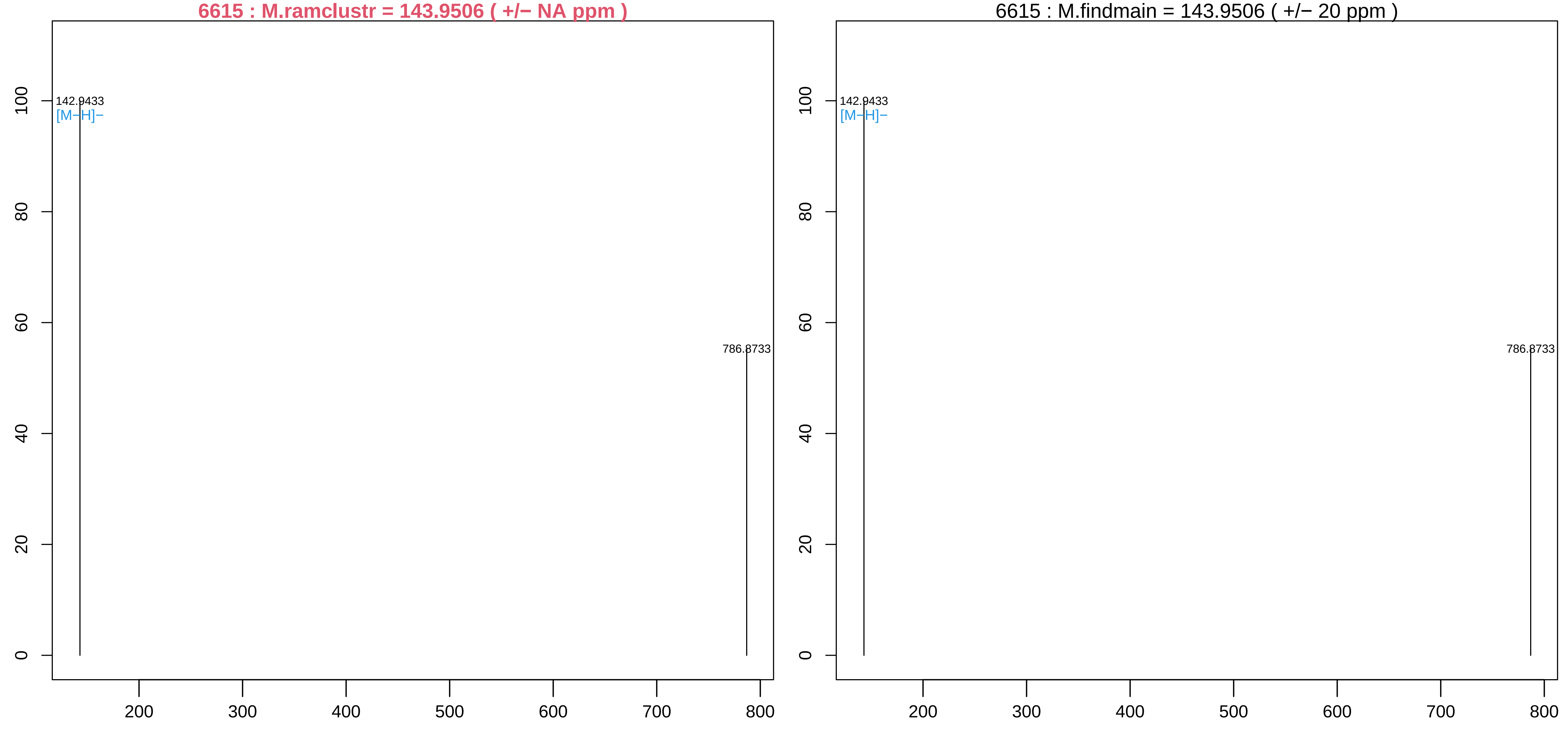
6615 : M.ramclustr = 143.9506 (+/− NA ppm)
142.9433
[M−H]−
786.8733
6615 : M.findmain = 143.9506 (+/− 20 ppm)
142.9433
[M−H]−
786.8733
0
20
40
60
80
100
200
300
400
500
600
700
800

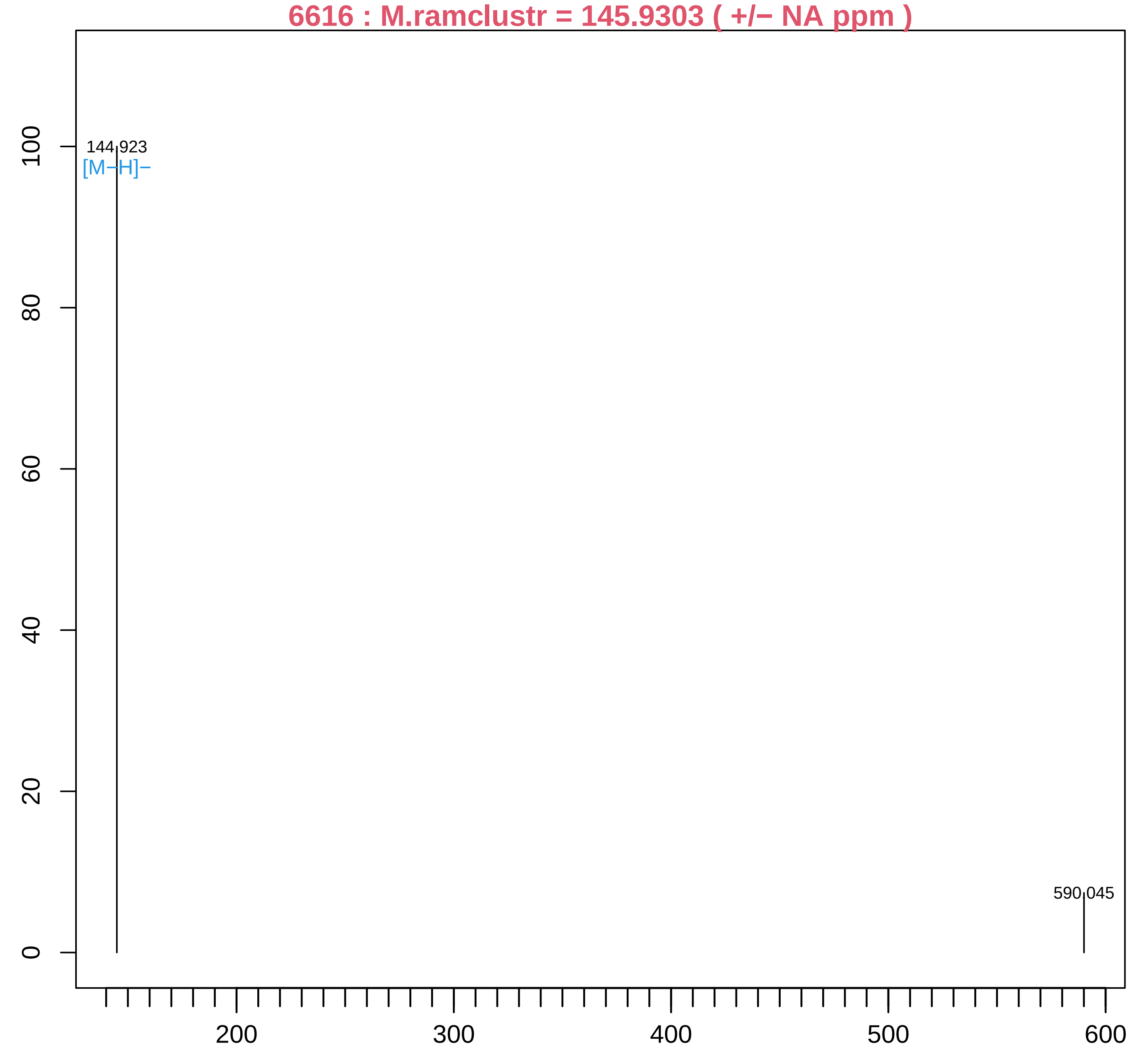
6616 : M.ramclustr = 145.9303 (+/− NA ppm)
144.923
[M−H]−
590.045
0
20
40
60
80
100
200
300
400
500
600

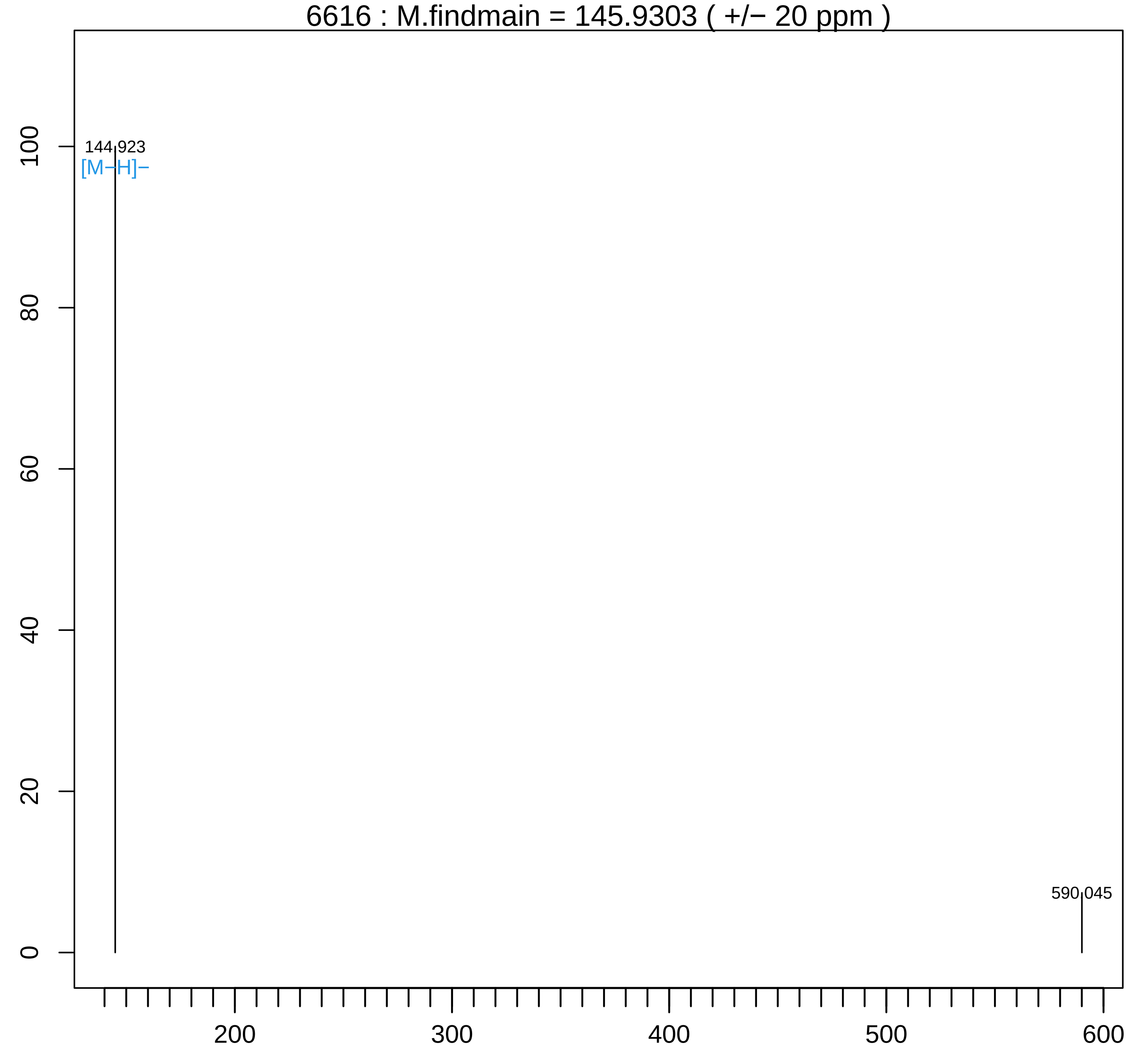
6616 : M.findmain = 145.9303 (+/− 20 ppm)
144.923
[M−H]−
590.045
0
20
40
60
80
100
200
300
400
500
600

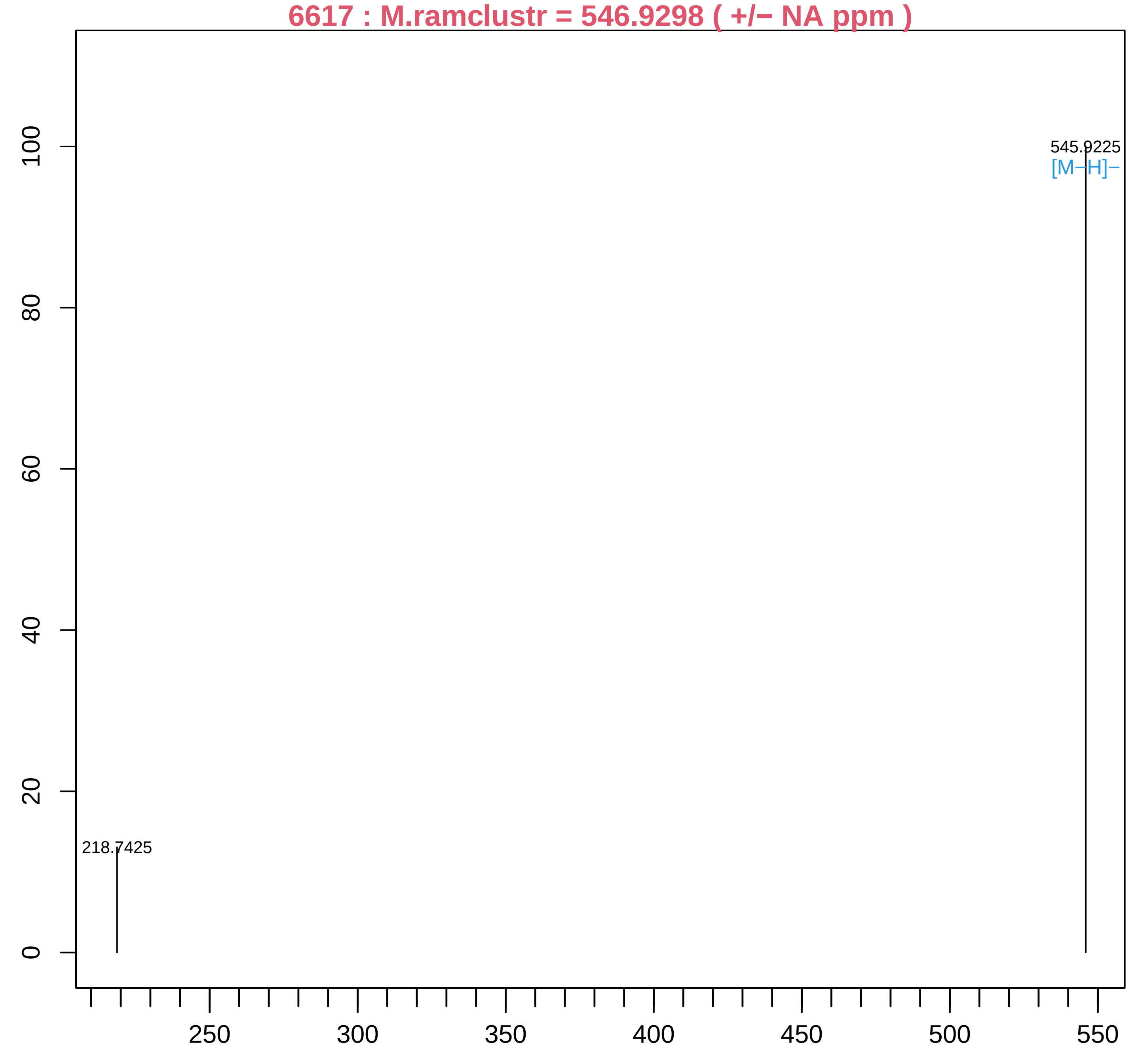
6617 : M.ramclustr = 546.9298 (+/− NA ppm)
545.9225
[M−H]−
218.7425
0
20
40
60
80
100
250
300
350
400
450
500
550

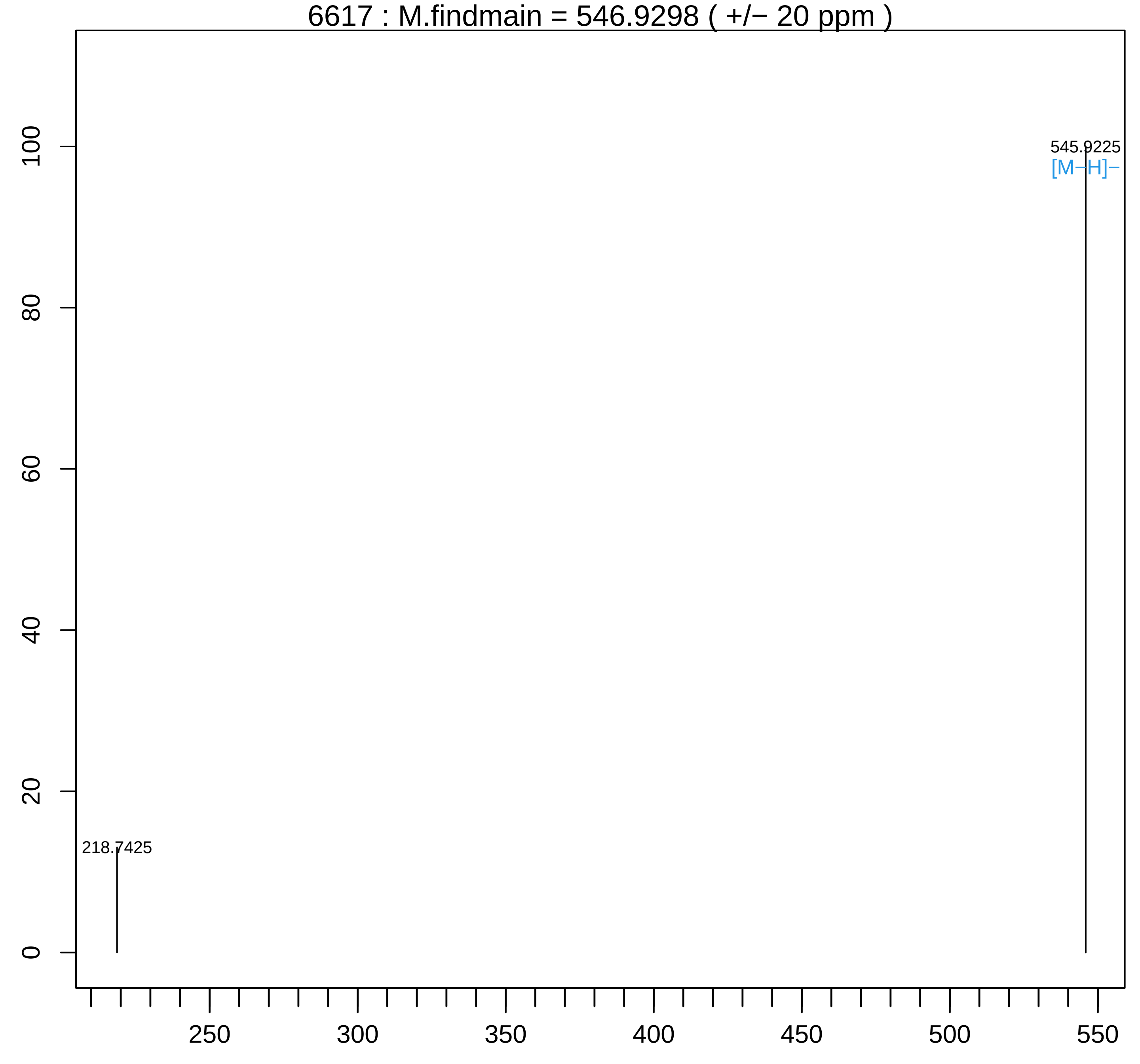
6617 : M.findmain = 546.9298 (+/− 20 ppm)
545.9225
[M−H]−
218.7425
0
20
40
60
80
100
250
300
350
400
450
500
550

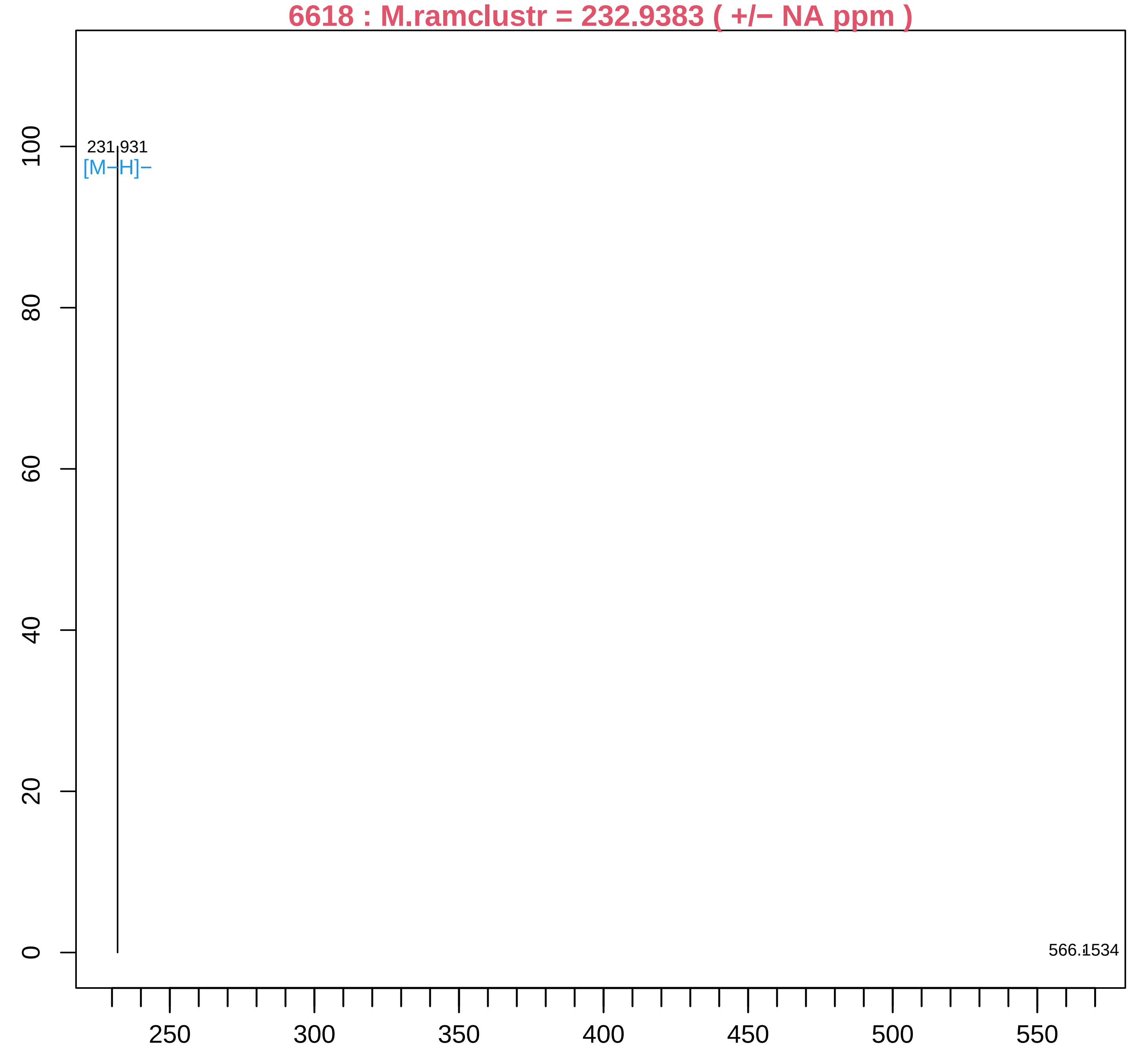
6618 : M.ramclustr = 232.9383 (+/− NA ppm)
231.931
[M−H]−
566.1534
0
20
40
60
80
100
250
300
350
400
450
500
550

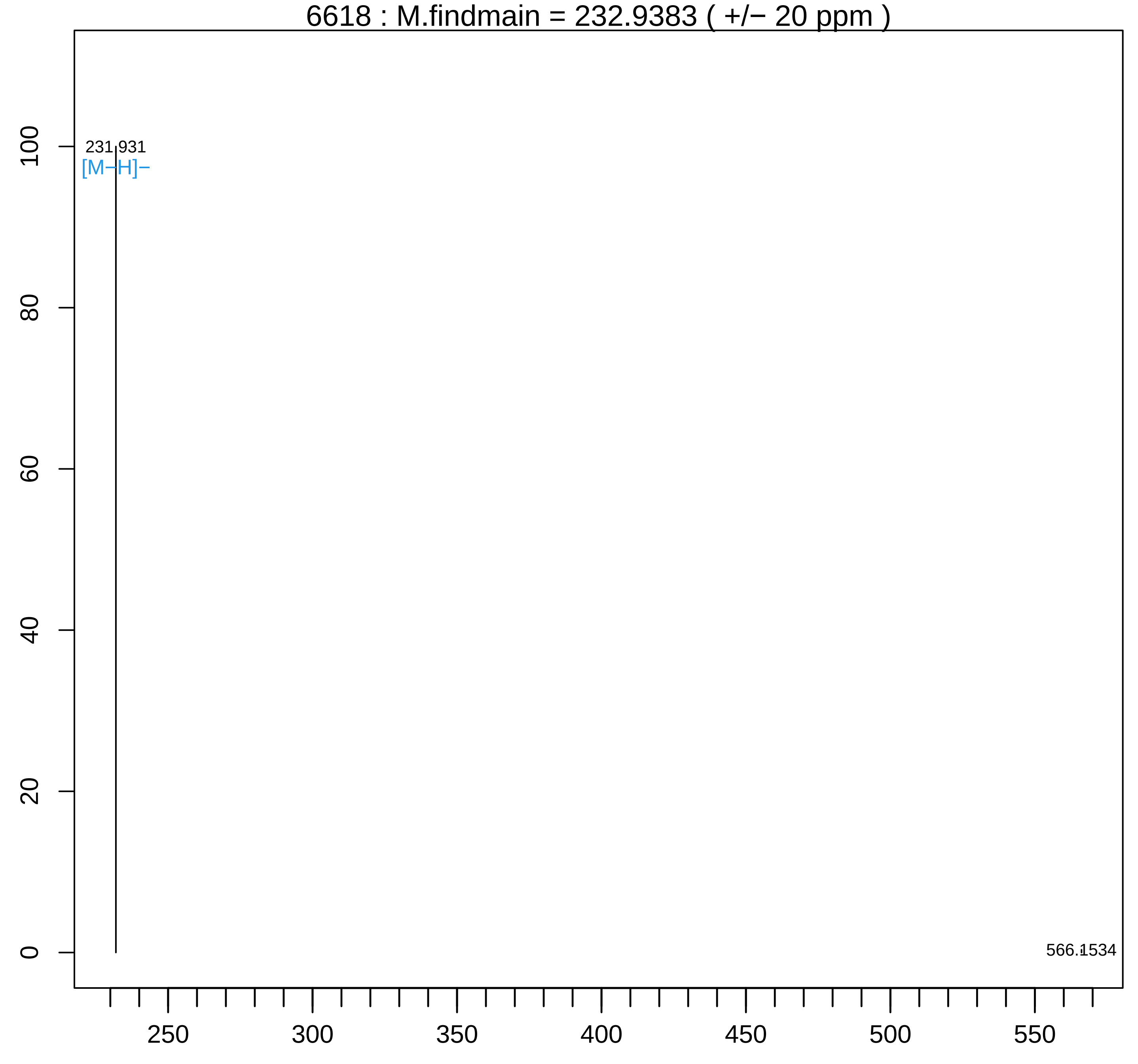
6618 : M.findmain = 232.9383 (+/− 20 ppm)
231.931
[M−H]−
566.1534
0
20
40
60
80
100
250
300
350
400
450
500
550

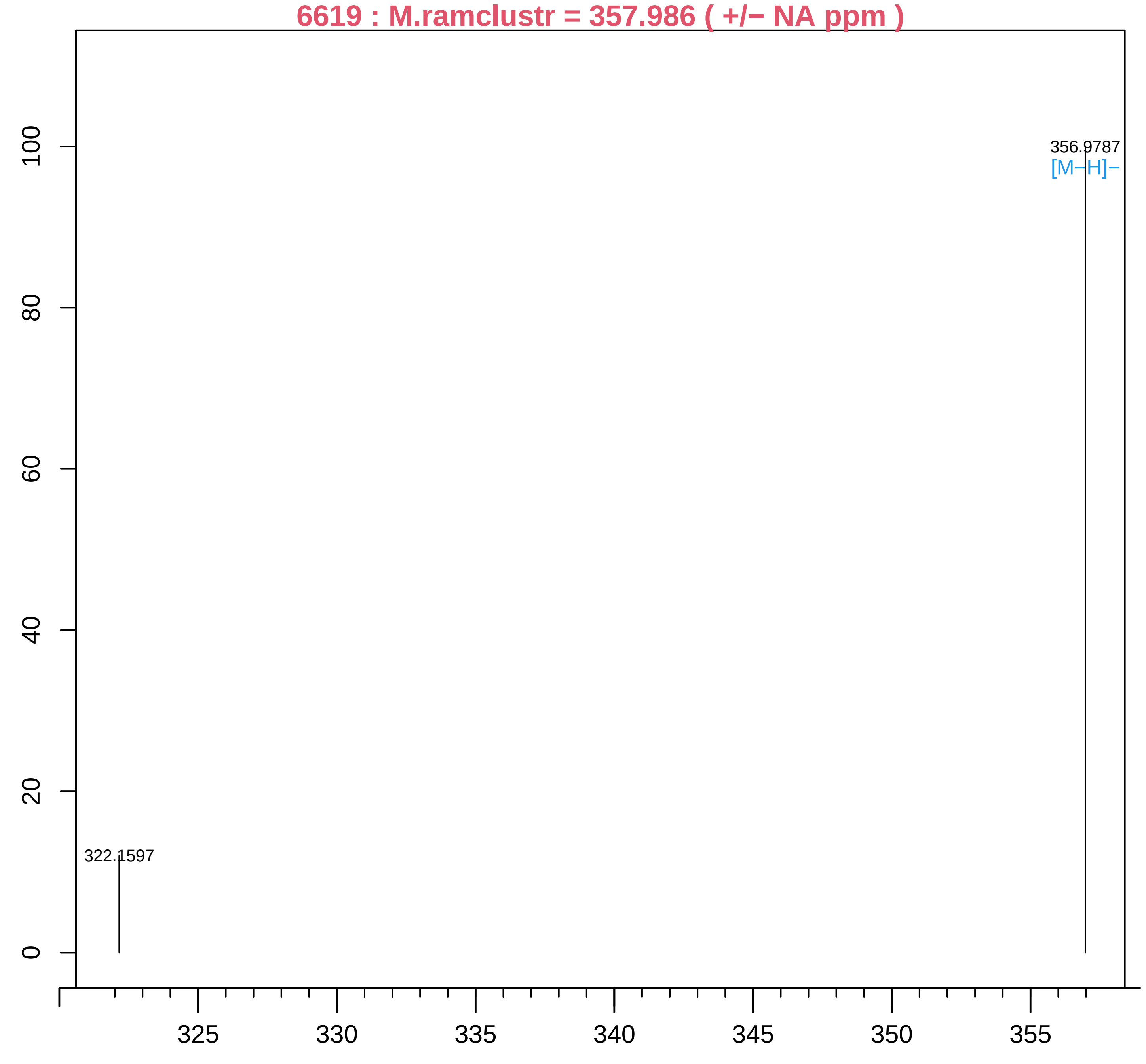
6619 : M.ramclustr = 357.986 (+/− NA ppm)
356.9787
[M−H]−
322.1597

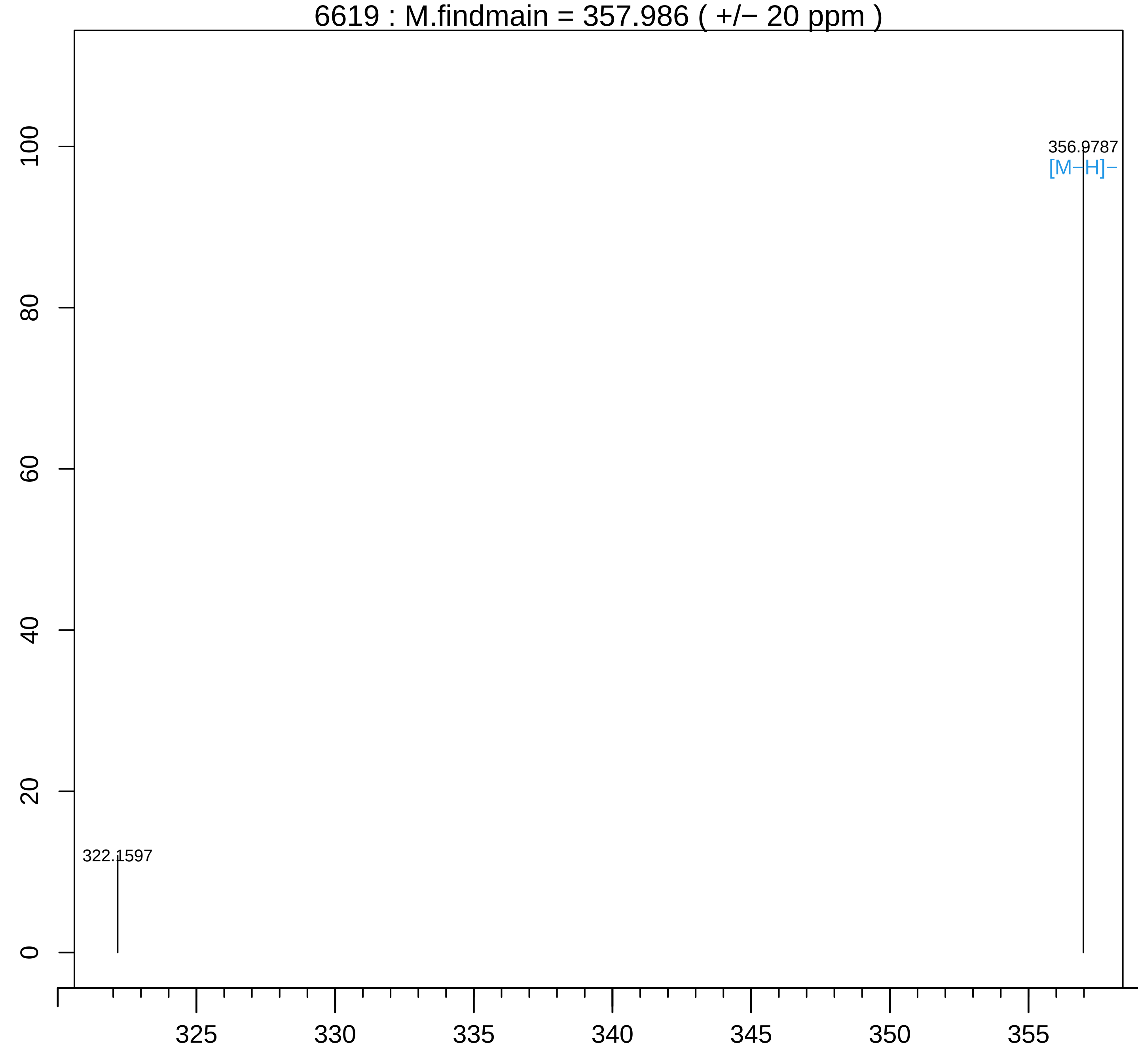
6619 : M.findmain = 357.986 (+/− 20 ppm)
356.9787
[M−H]−
322.1597

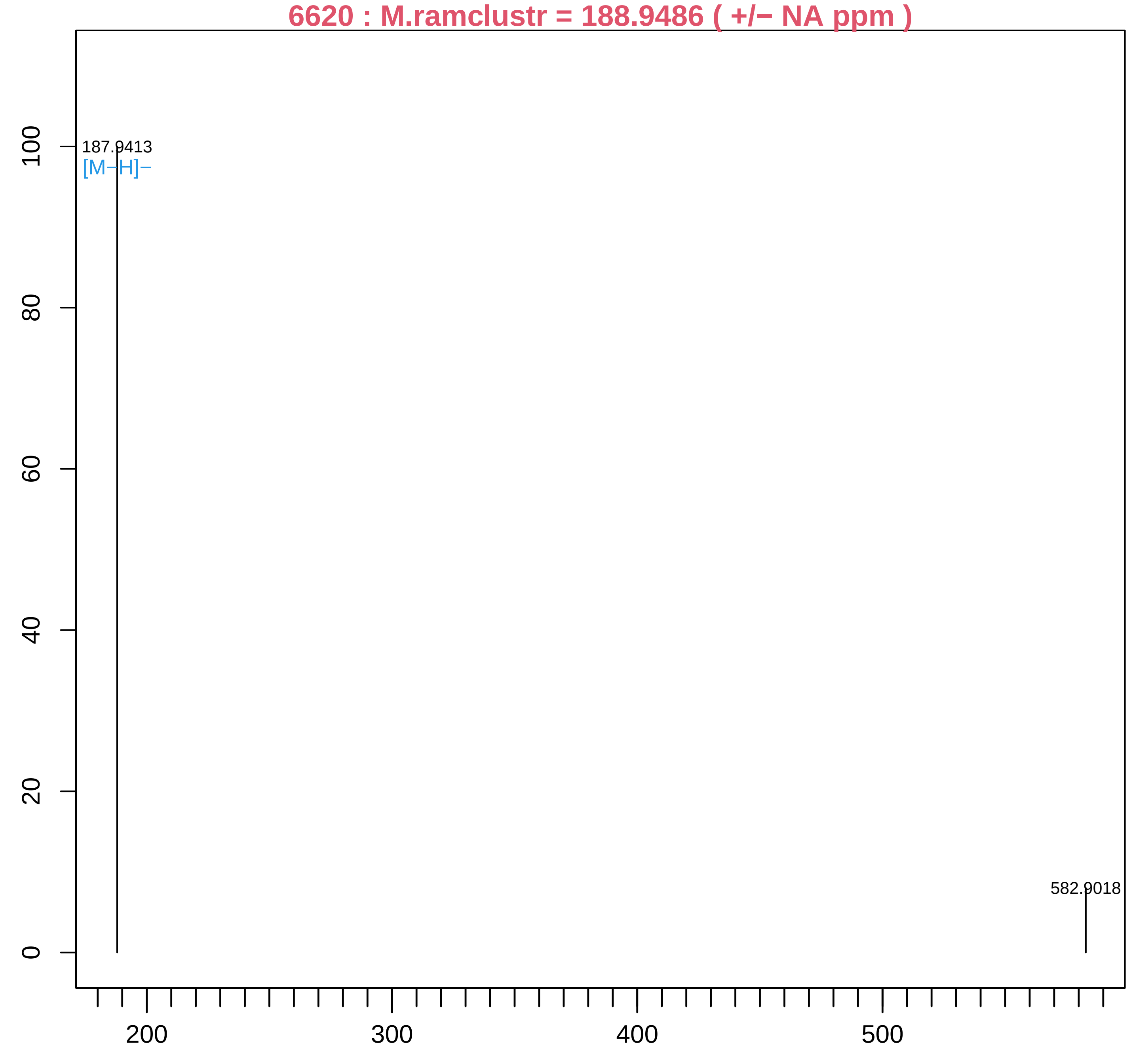
6620 : M.ramclustr = 188.9486 (+/− NA ppm)
187.9413
[M−H]−
582.9018
0
20
40
60
80
100
200
300
400
500

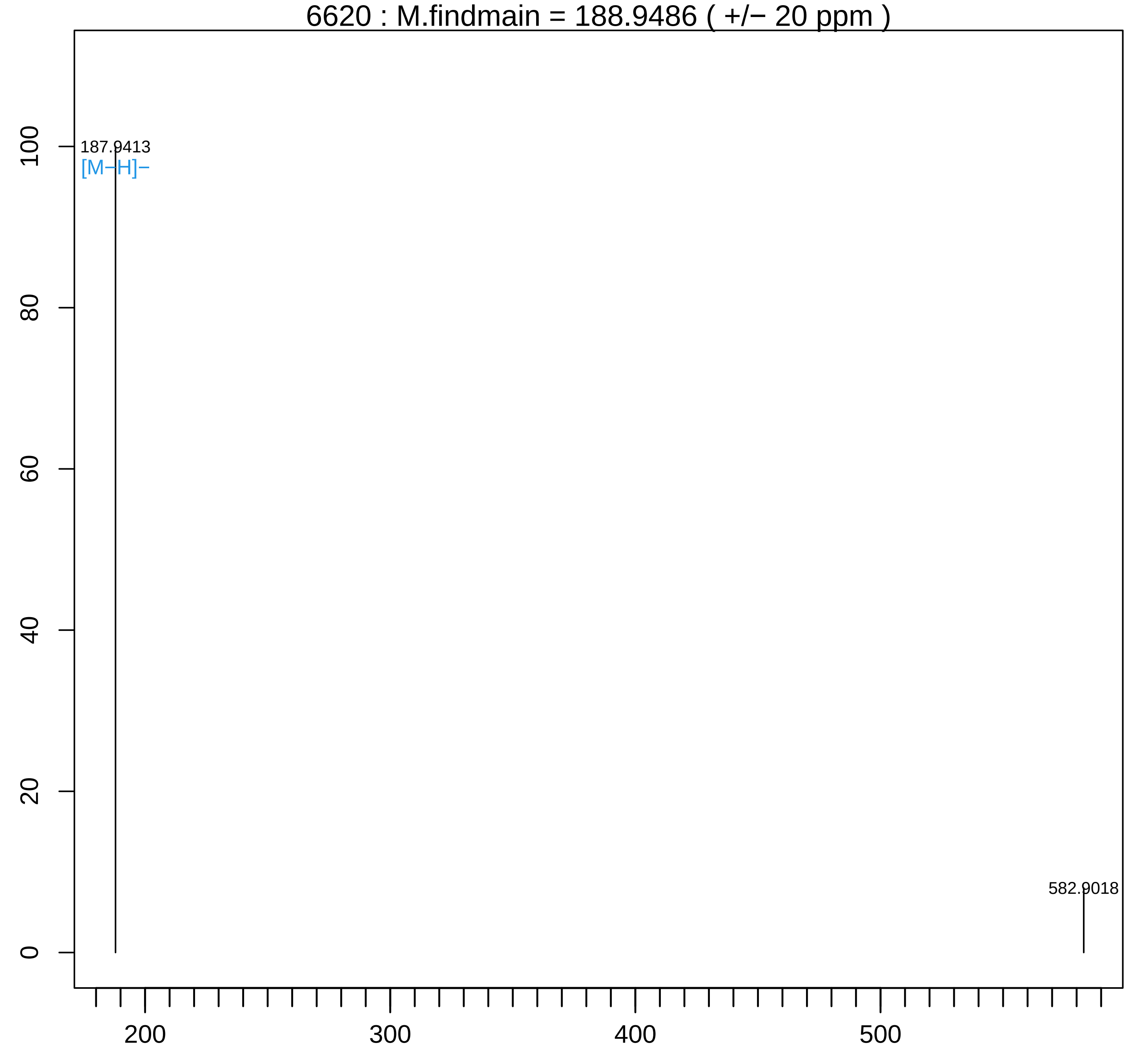
6620 : M.findmain = 188.9486 (+/− 20 ppm)
187.9413
[M−H]−
582.9018
0
20
40
60
80
100
200
300
400
500

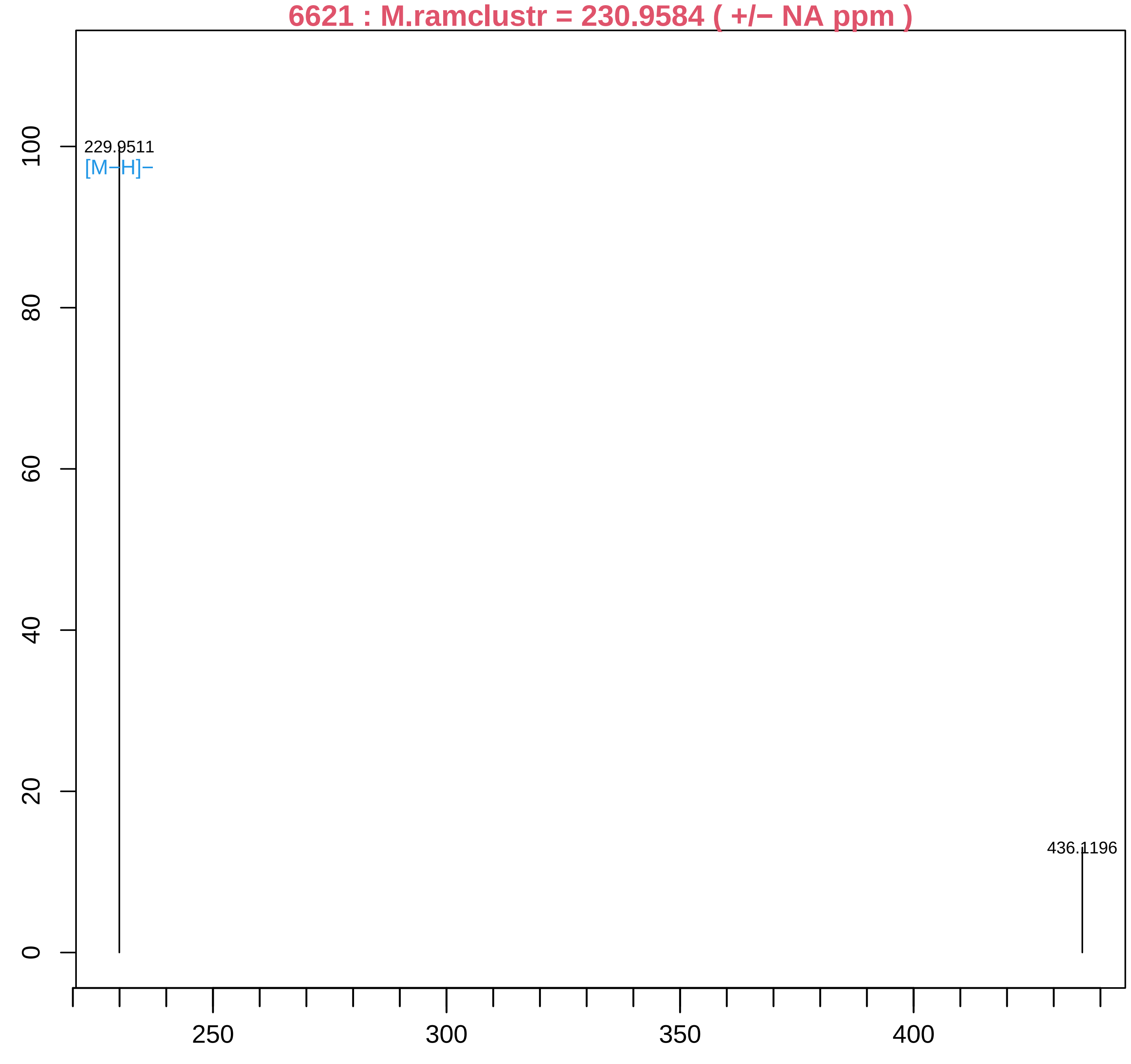
6621 : M.ramclustr = 230.9584 (+/− NA ppm)
229.9511
[M−H]−
436.1196
0
20
40
60
80
100
250
300
350
400

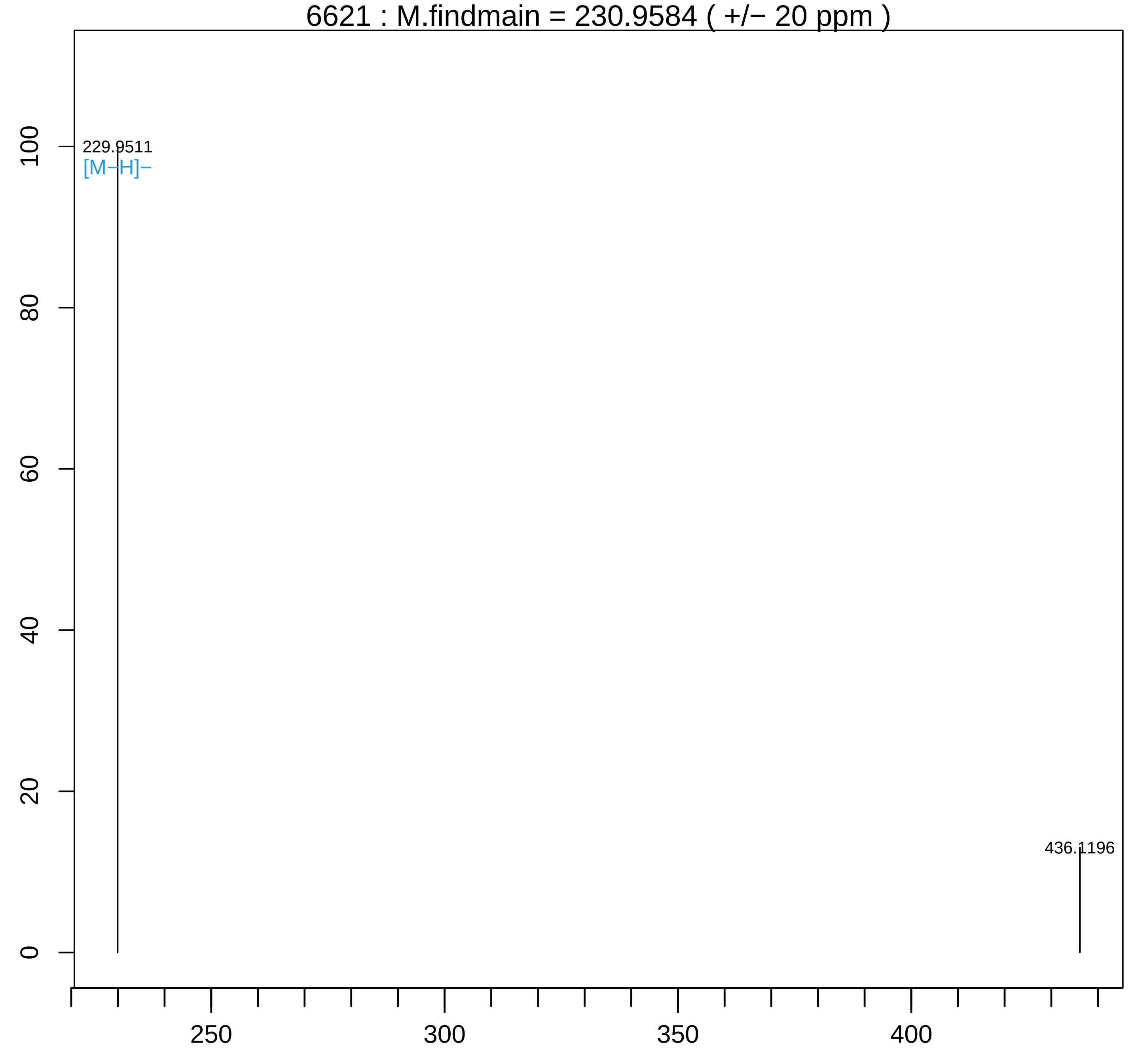
6621 : M.findmain = 230.9584 (+/− 20 ppm)
229.9511
[M−H]−
436.1196
0
20
40
60
80
100
250
300
350
400

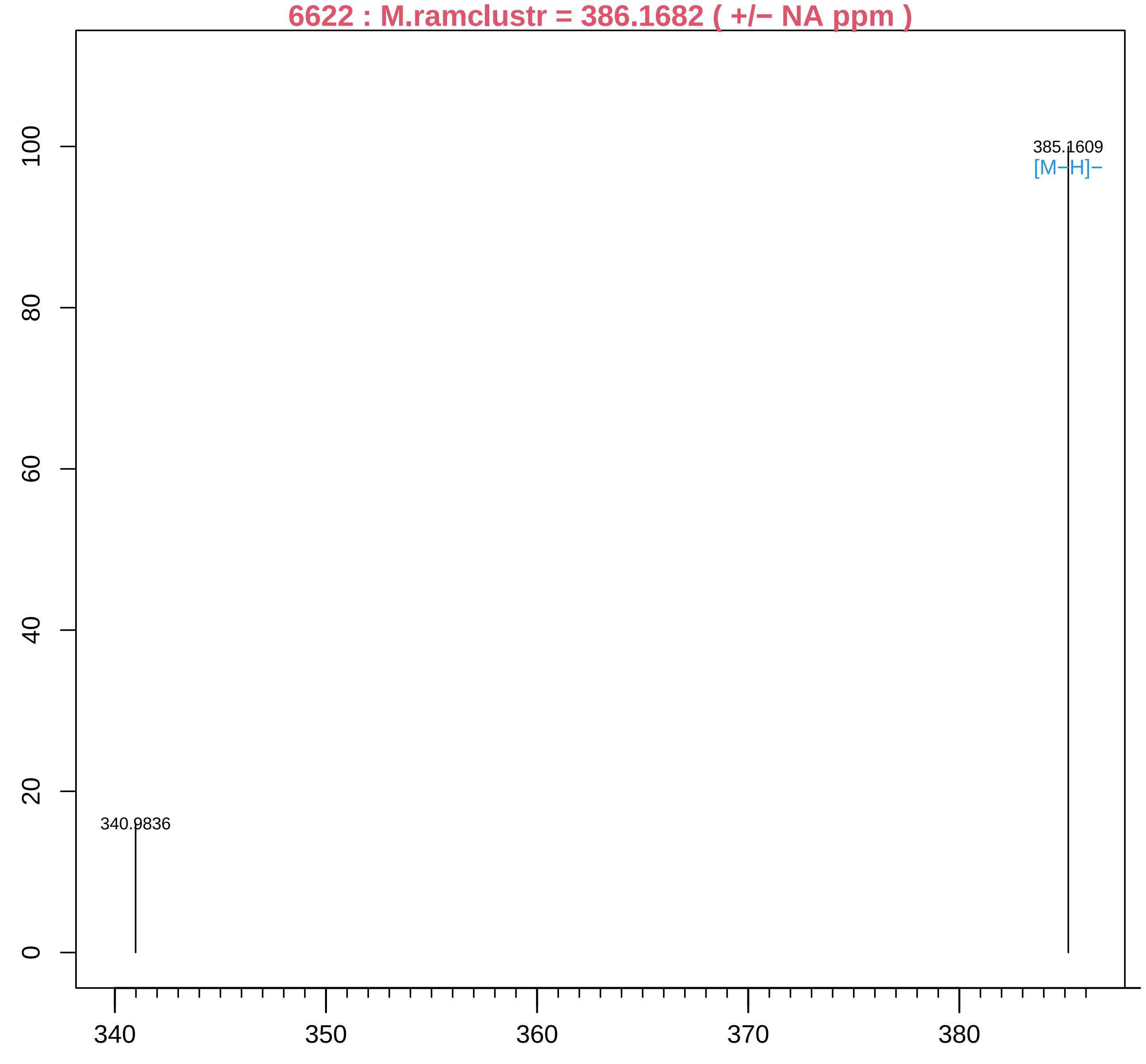
6622 : M.ramclustr = 386.1682 (+/− NA ppm)
385.1609
[M−H]−
340.9836
0
20
40
60
80
100
340
350
360
370
380

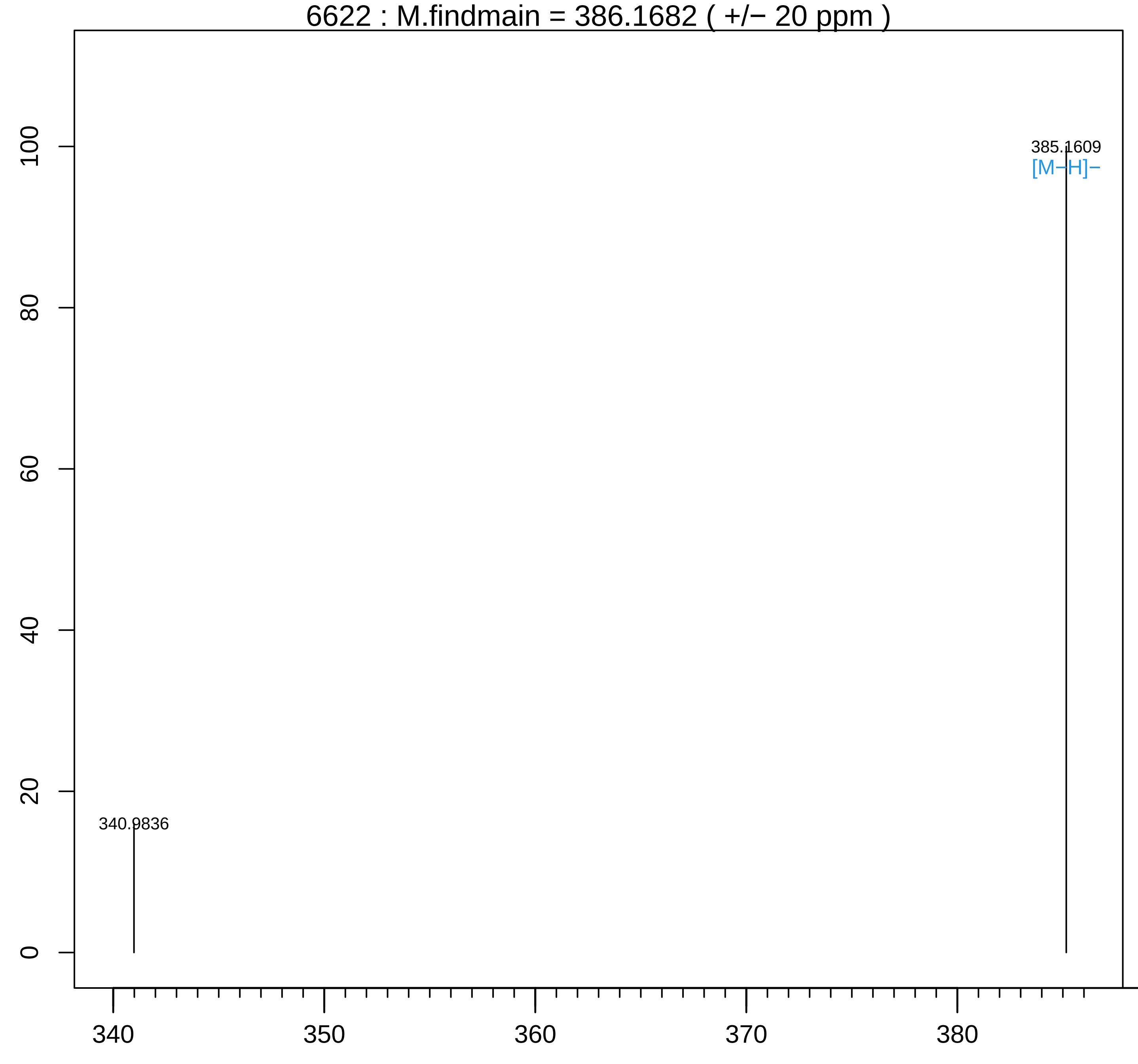
6622 : M.findmain = 386.1682 (+/− 20 ppm)
385.1609
[M−H]−
340.9836
0
20
40
60
80
100
340
350
360
370
380

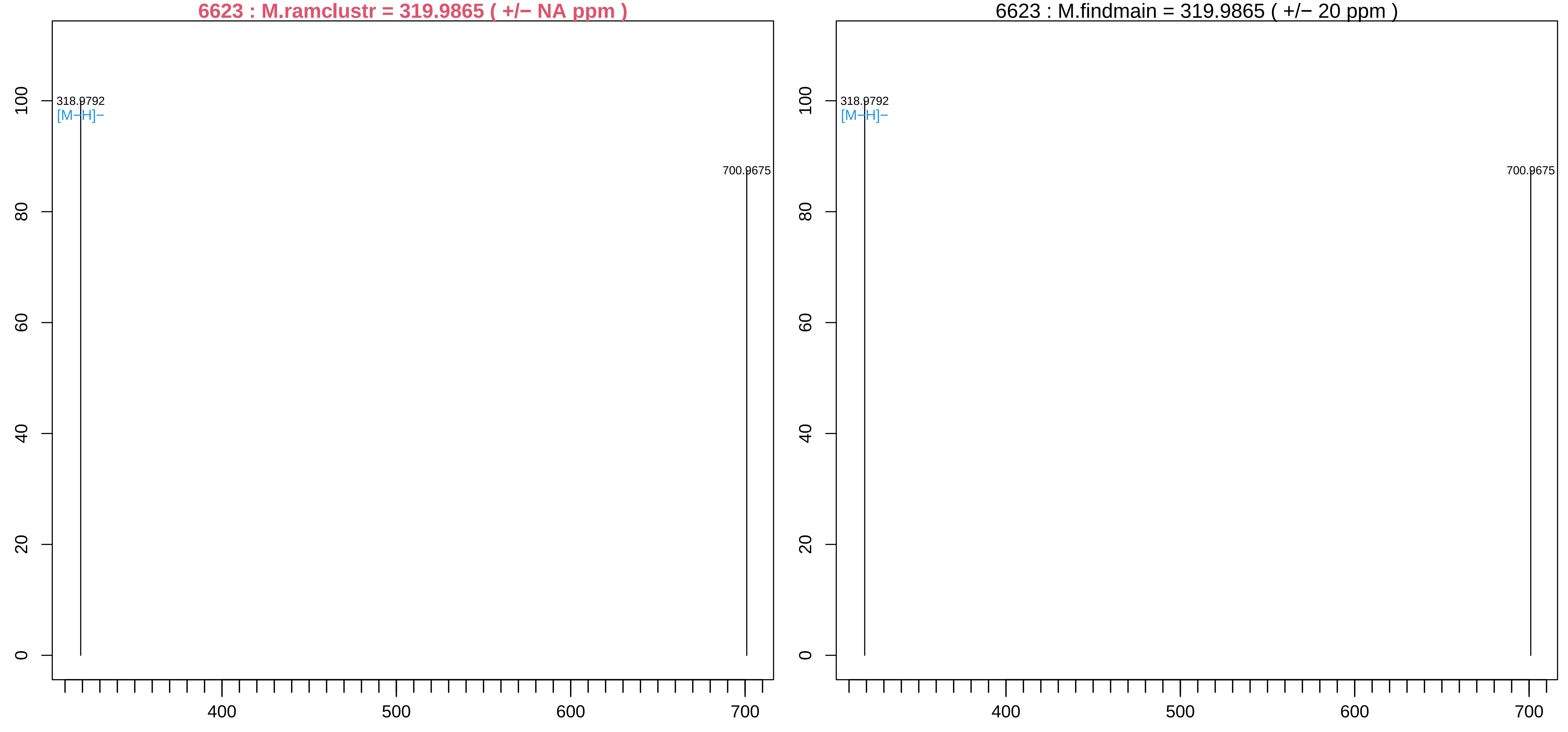
6623 : M.ramclustr = 319.9865 (+/− NA ppm)
318.9792
[M−H]−
700.9675
0
20
40
60
80
100
400
500
600
700
6623 : M.findmain = 319.9865 (+/− 20 ppm)
318.9792
[M−H]−
700.9675
0
20
40
60
80
100
400
500
600
700

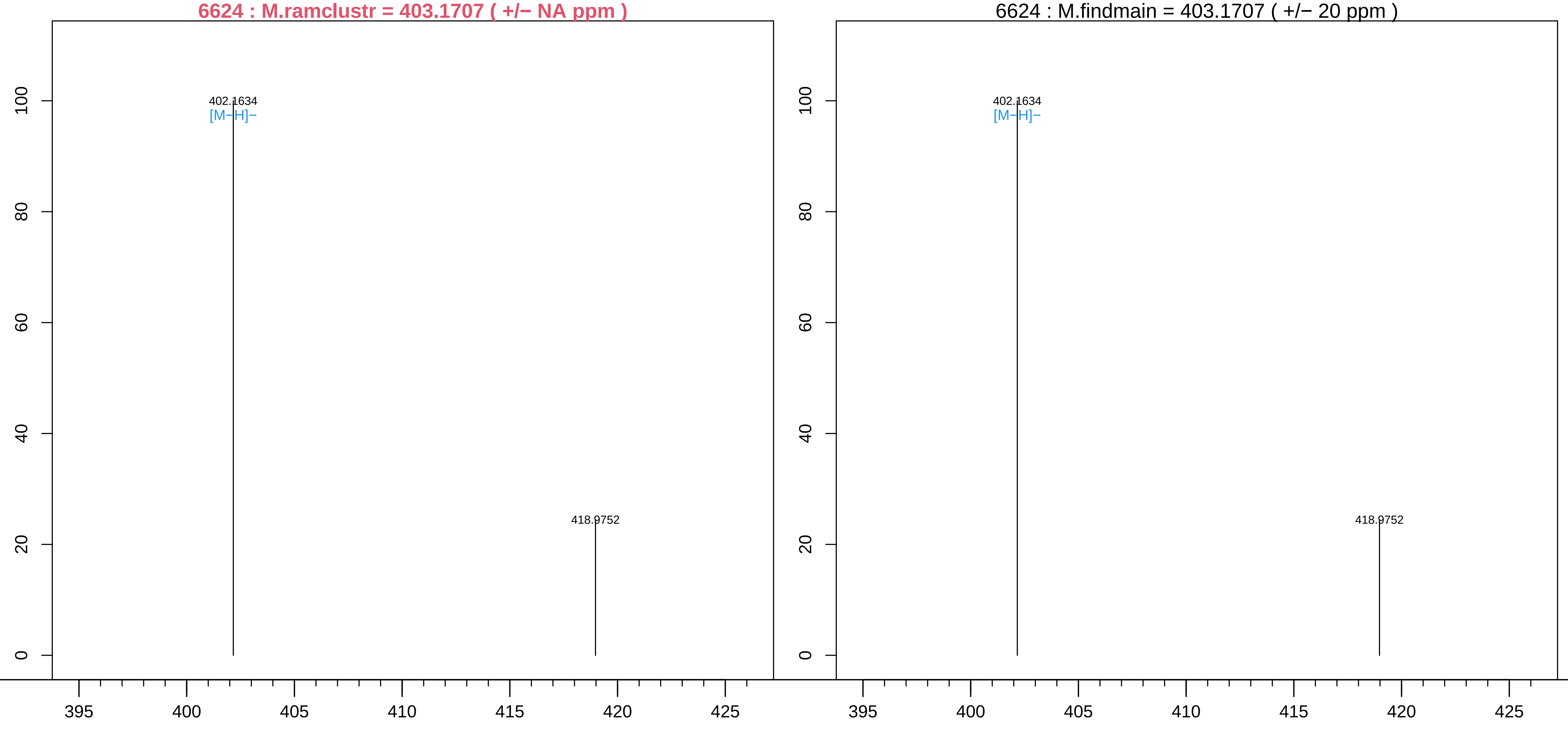
6624 : M.ramclustr = 403.1707 (+/− NA ppm)
402.1634
[M−H]−
418.9752
6624 : M.findmain = 403.1707 (+/− 20 ppm)
402.1634
[M−H]−
418.9752
0
20
40
60
80
100
395
400
405
410
415
420
425

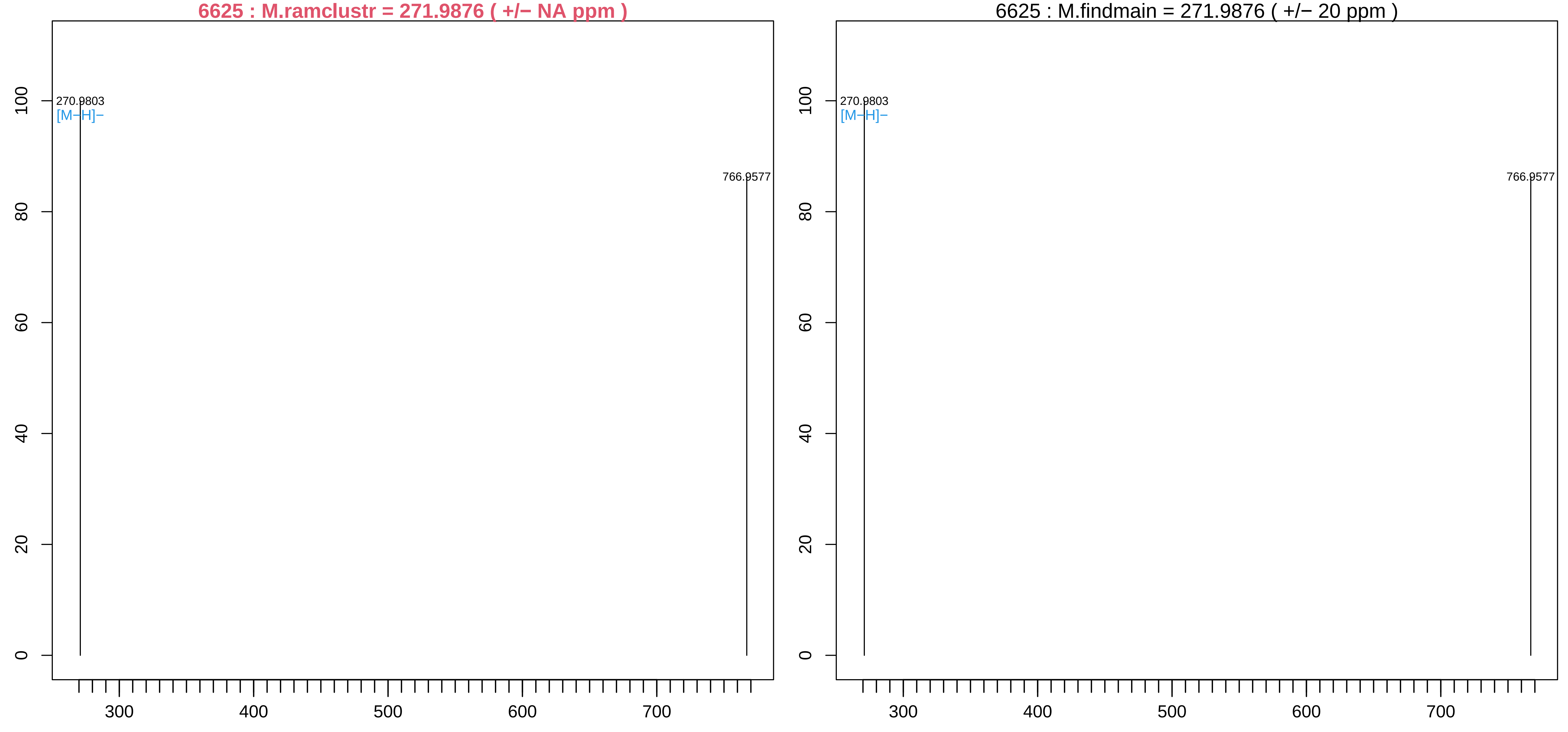

6625 : M.ramclustr = 271.9876 (+/− NA ppm)
270.9803
[M−H]−
766.9577
6625 : M.findmain = 271.9876 (+/− 20 ppm)
270.9803
[M−H]−
766.9577
0
20
40
60
80
100
300
400
500
600
700

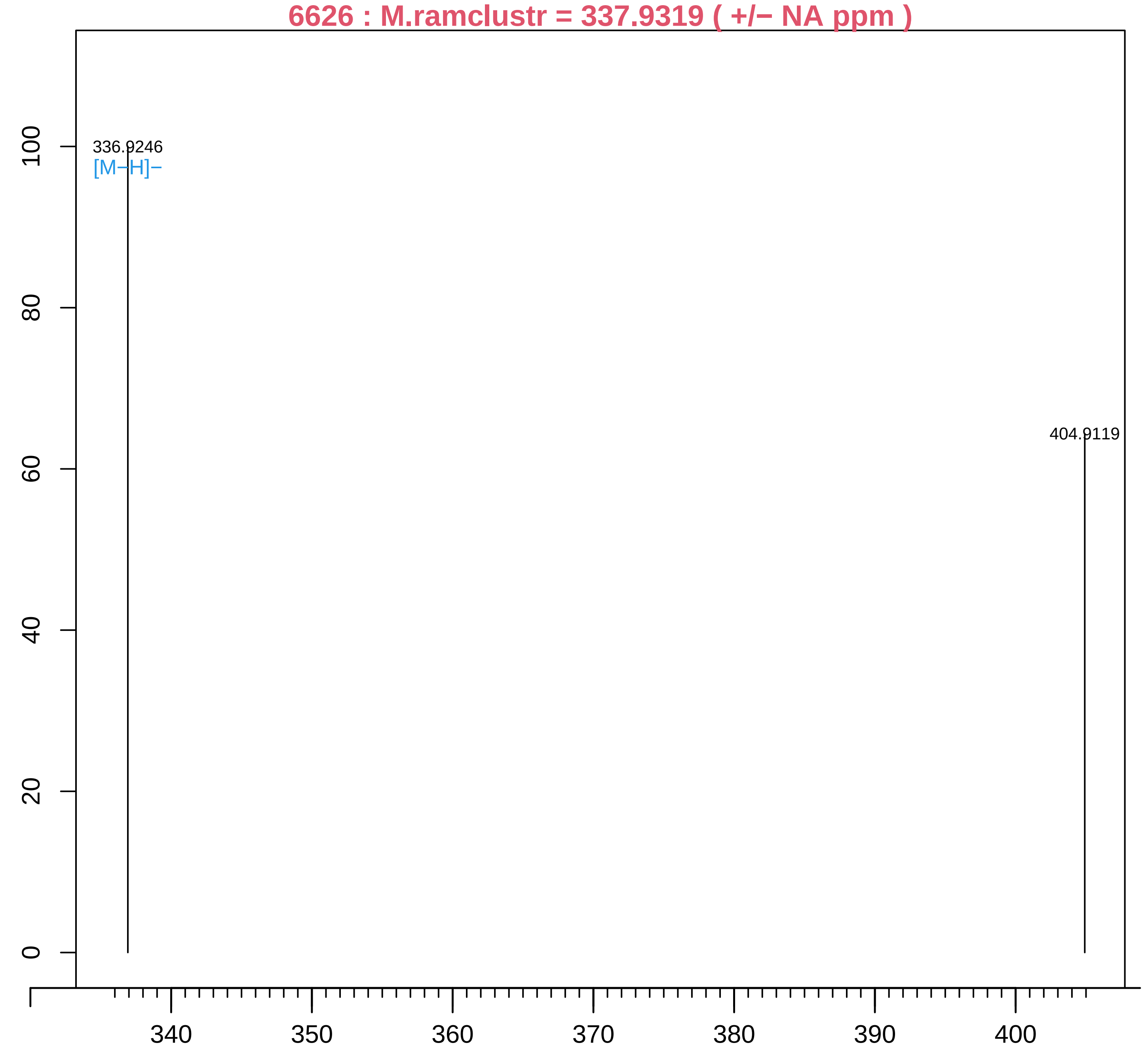
6626 : M.ramclustr = 337.9319 (+/− NA ppm)
336.9246
[M−H]−
404.9119
0
20
40
60
80
100
340
350
360
370
380
390
400

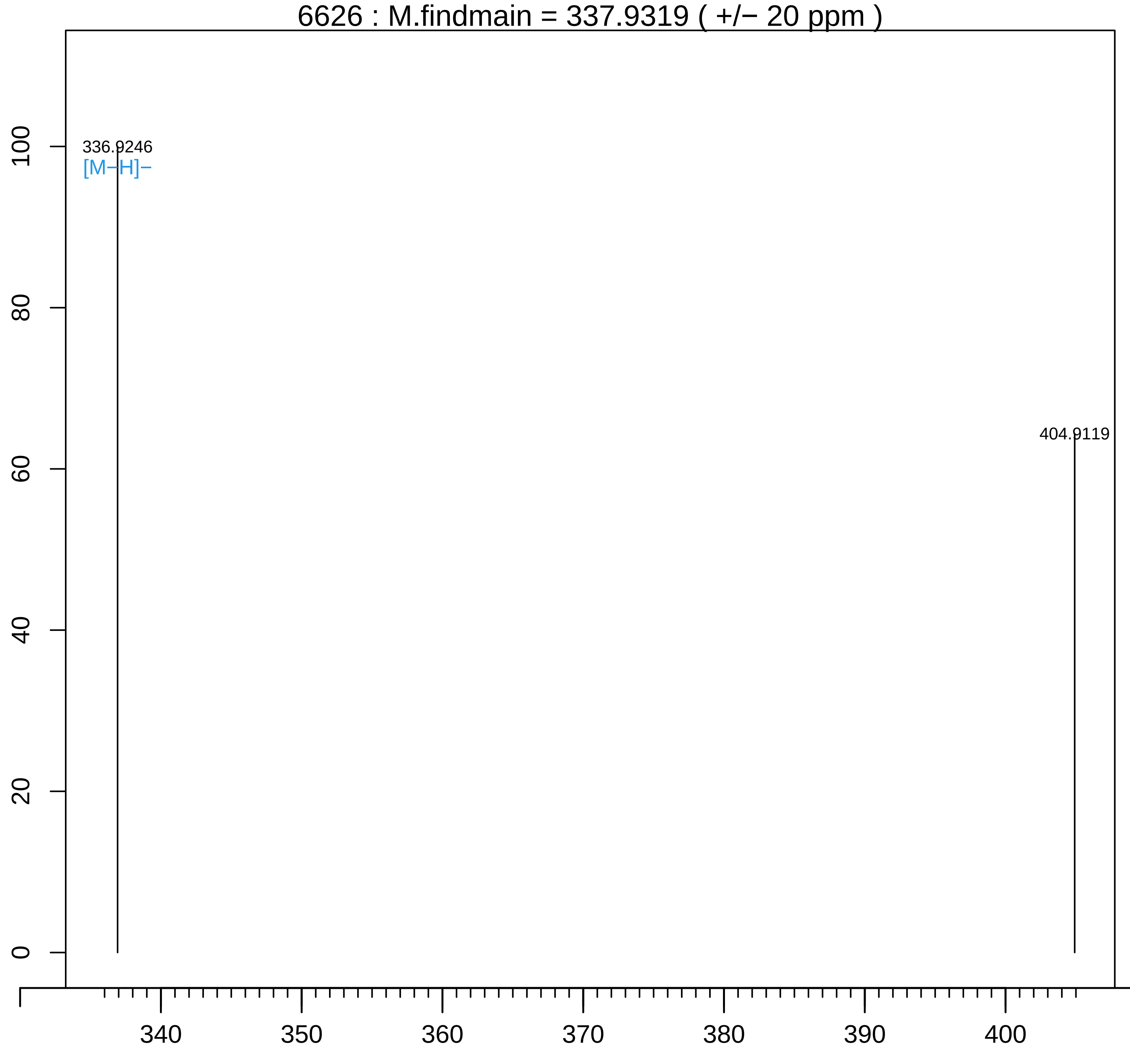
6626 : M.findmain = 337.9319 (+/− 20 ppm)
336.9246
[M−H]−
404.9119
0
20
40
60
80
100
340
350
360
370
380
390
400

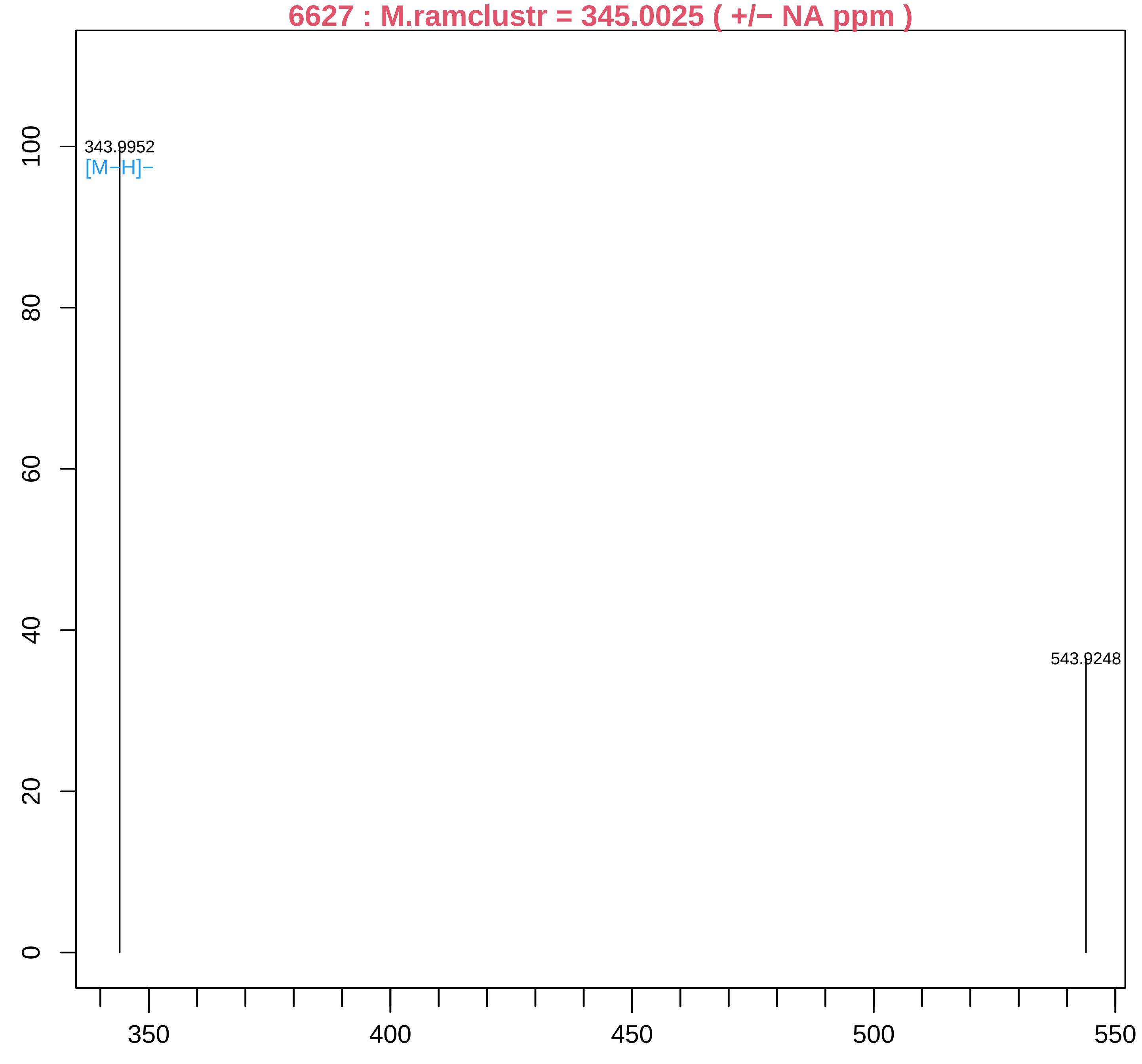
6627 : M.ramclustr = 345.0025 (+/− NA ppm)
343.9952
[M−H]−
543.9248
0
20
40
60
80
100
350
400
450
500
550

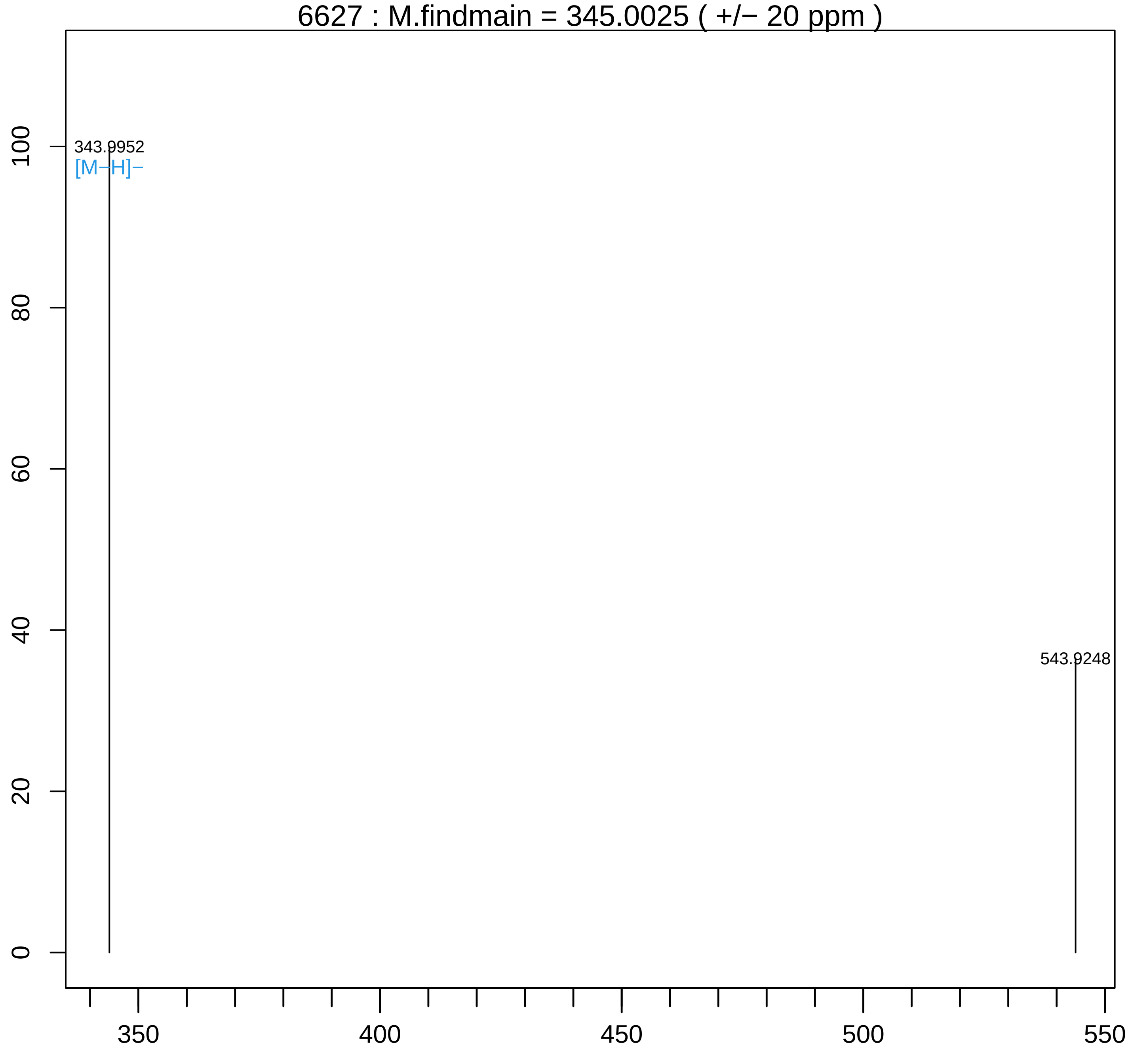
6627 : M.findmain = 345.0025 (+/− 20 ppm)
343.9952
[M−H]−
543.9248
0
20
40
60
80
100
350
400
450
500
550

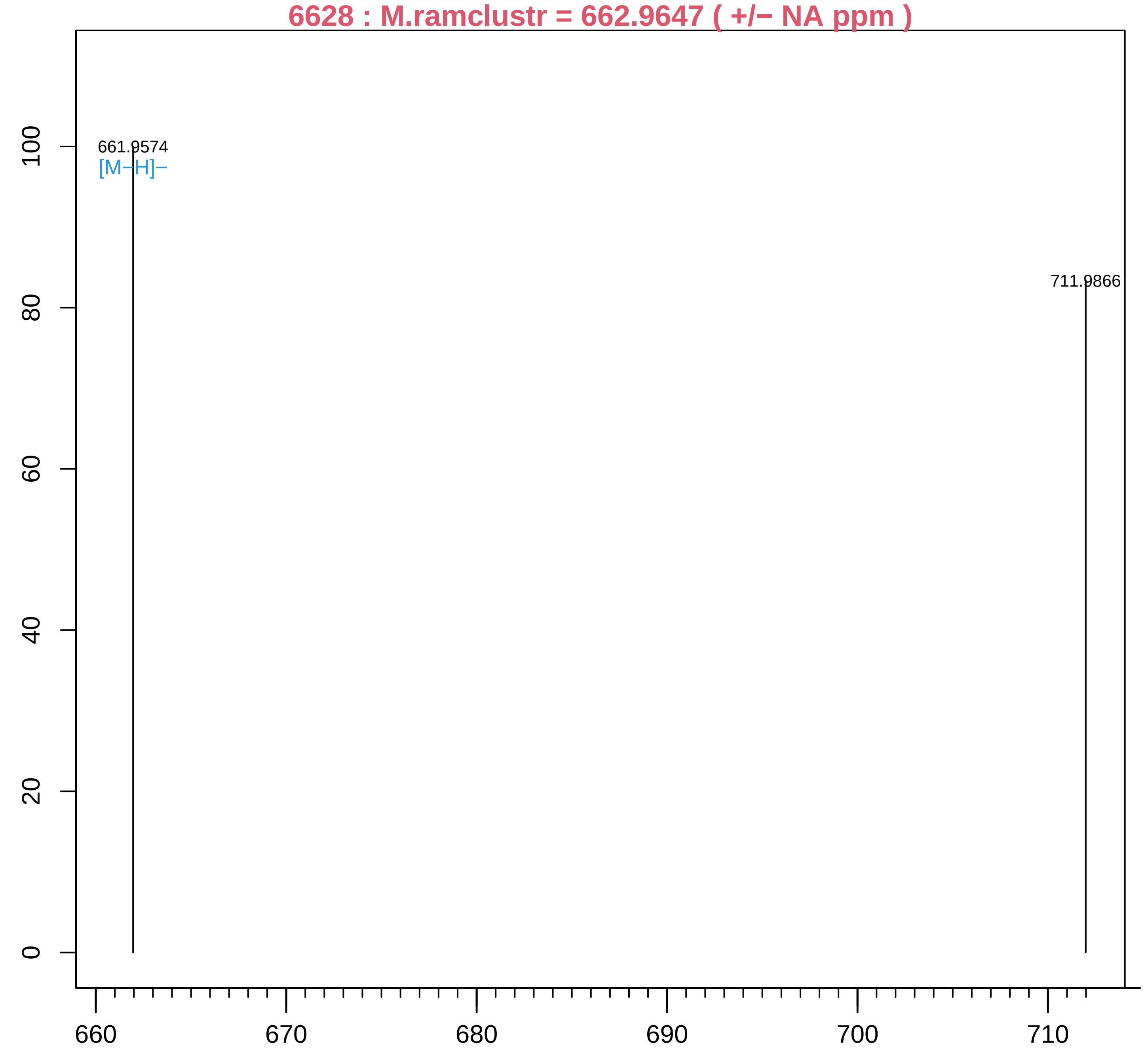
6628 : M.ramclustr = 662.9647 (+/− NA ppm)
661.9574
[M−H]−
711.9866
0
20
40
60
80
100
660
670
680
690
700
710

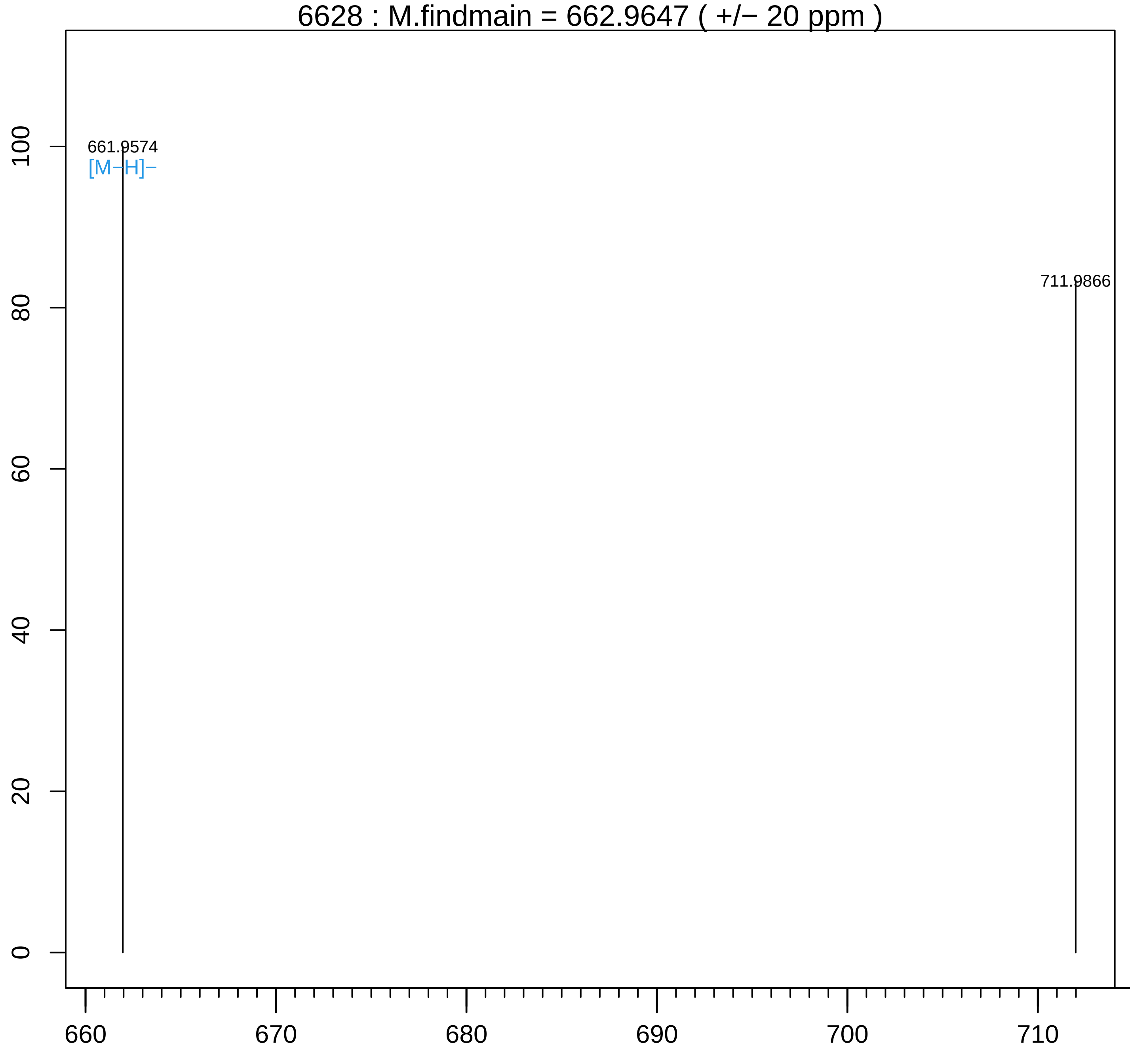
6628 : M.findmain = 662.9647 (+/− 20 ppm)
661.9574
[M−H]−
711.9866
0
20
40
60
80
100
660
670
680
690
700
710

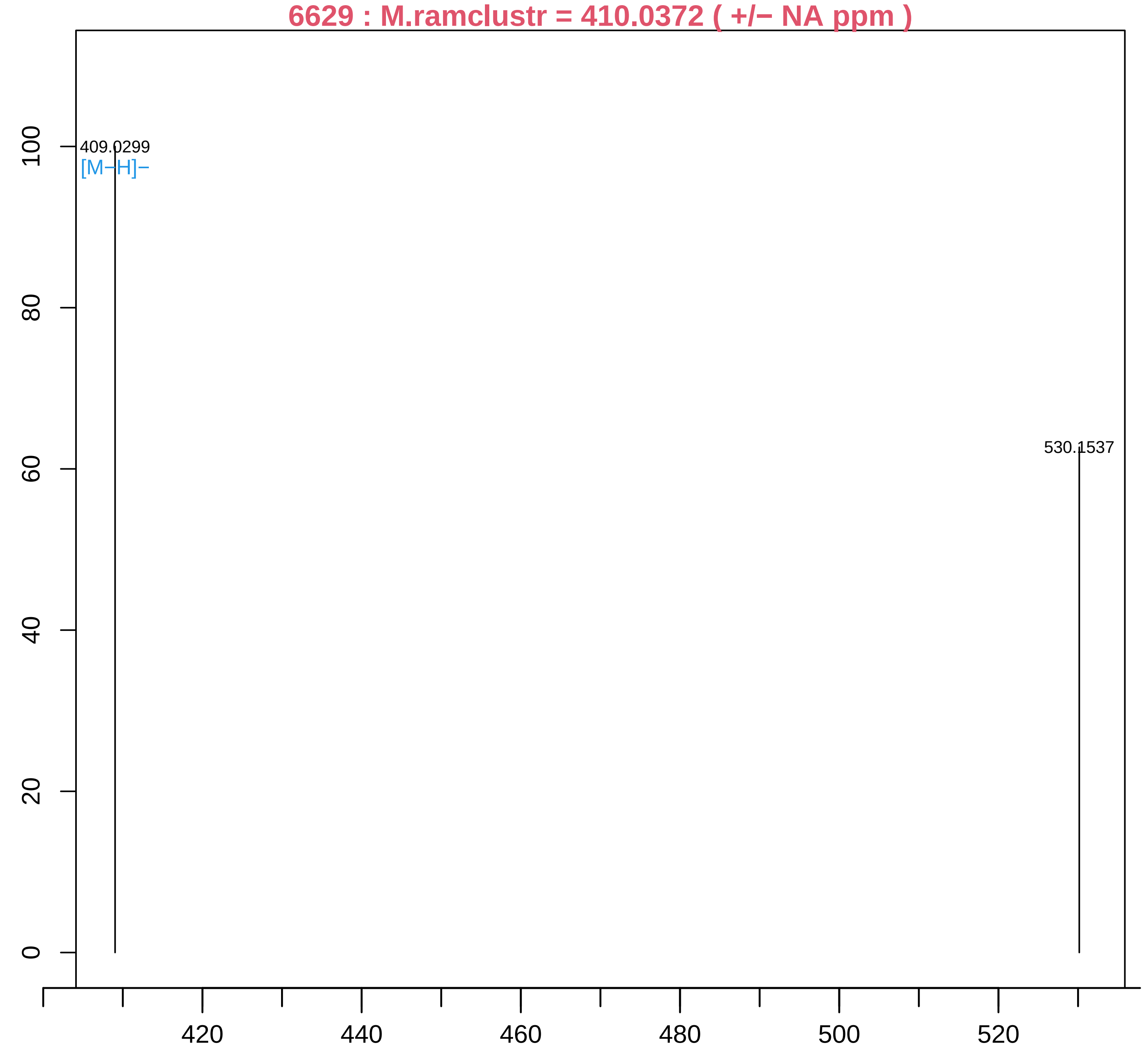
6629 : M.ramclustr = 410.0372 (+/− NA ppm)
409.0299
[M−H]−
530.1537
0
20
40
60
80
100
420
440
460
480
500
520

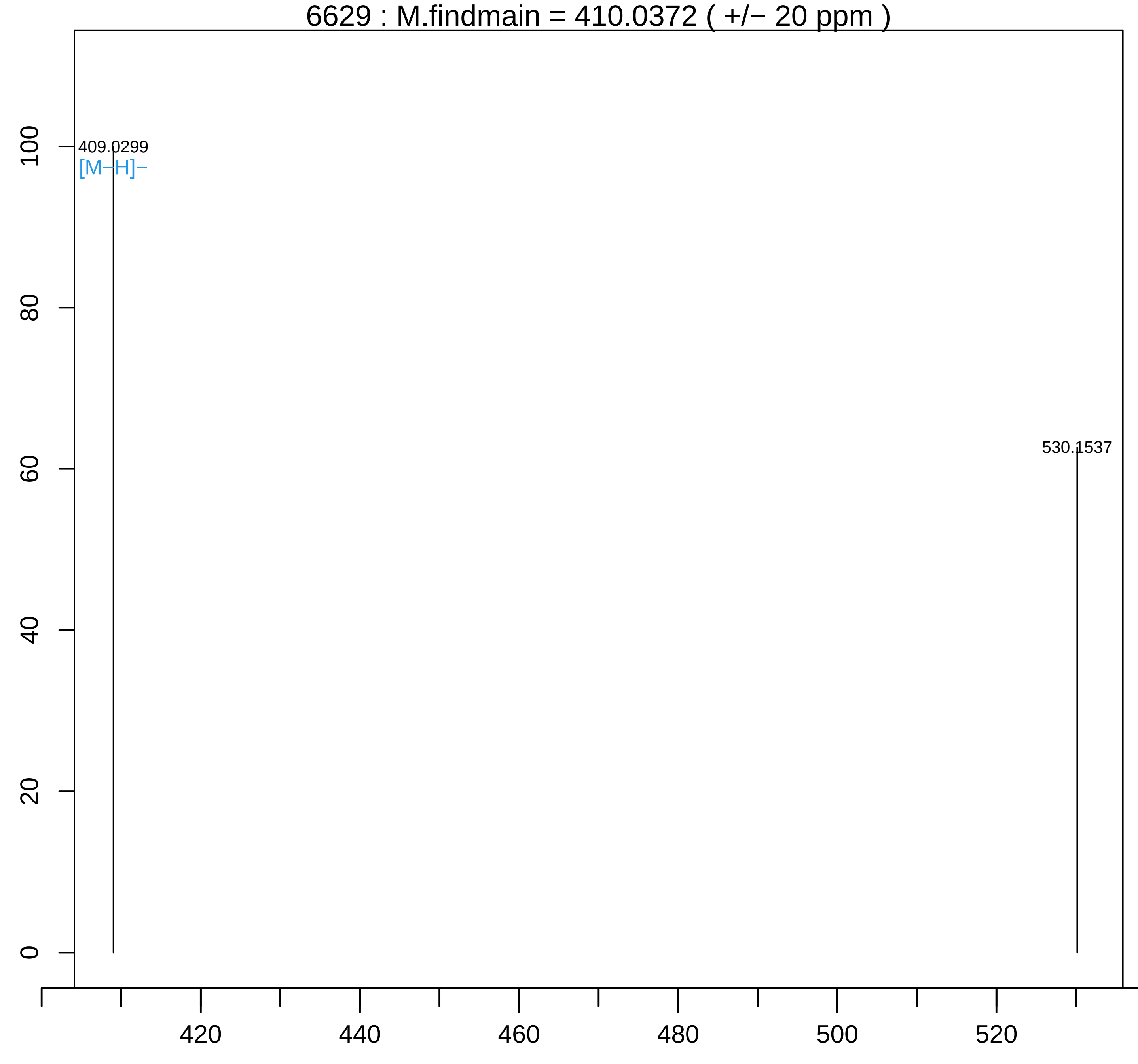
6629 : M.findmain = 410.0372 (+/− 20 ppm)
409.0299
[M−H]−
530.1537
0
20
40
60
80
100
420
440
460
480
500
520

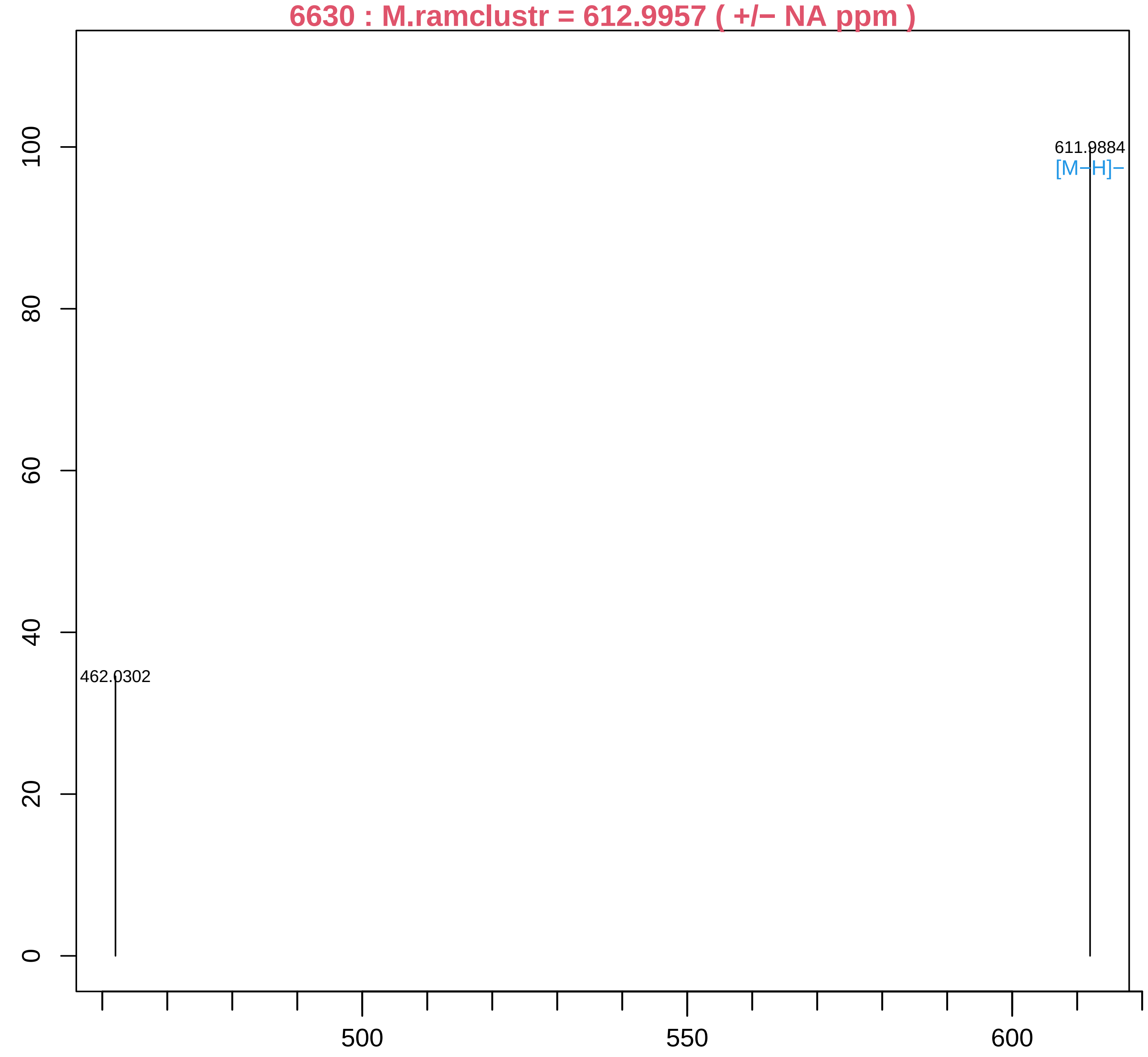
6630 : M.ramclustr = 612.9957 (+/− NA ppm)
611.9884
[M−H]−
462.0302
0
20
40
60
80
100
500
550
600

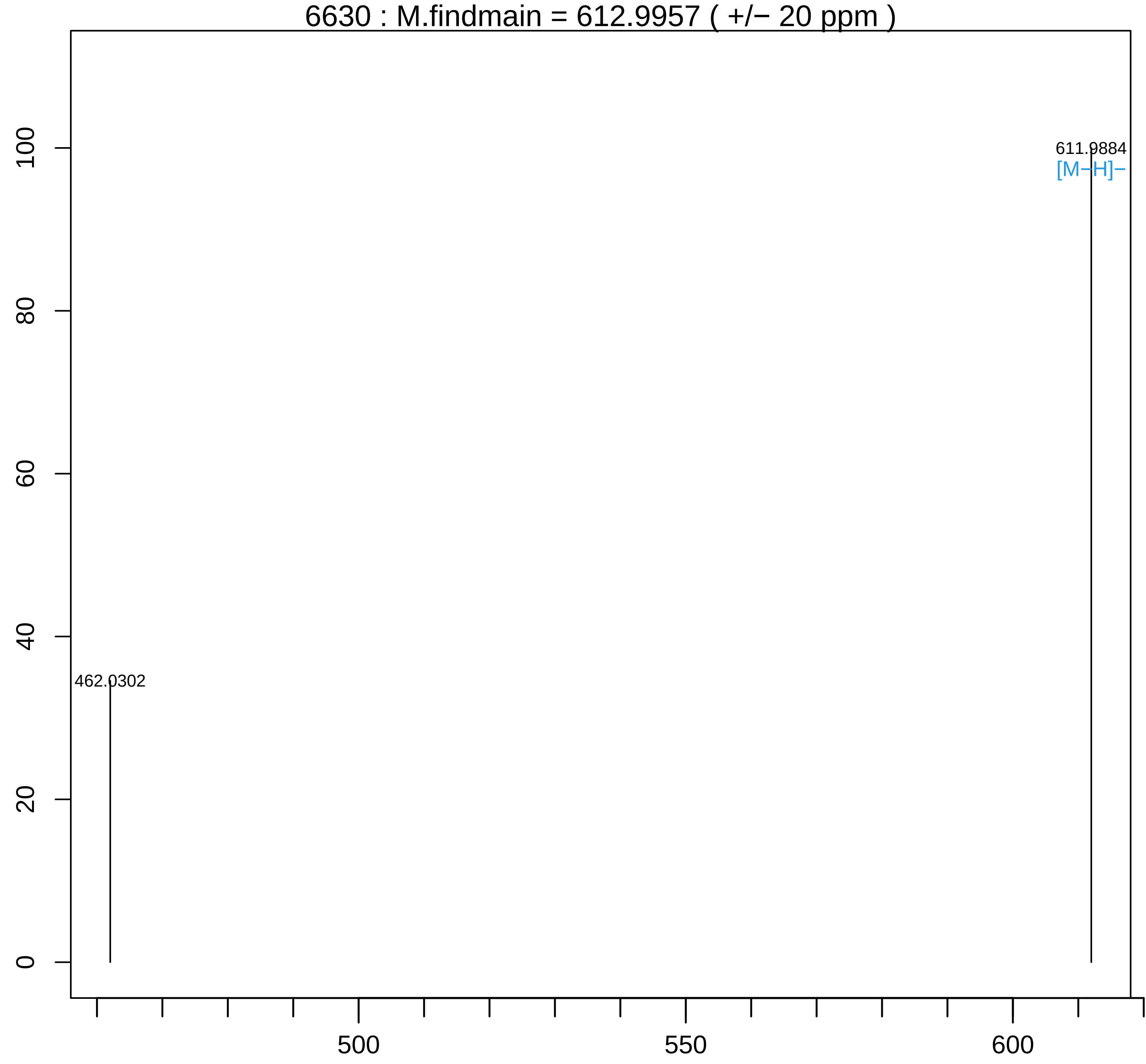
6630 : M.findmain = 612.9957 (+/− 20 ppm)
611.9884
[M−H]−
462.0302
0
20
40
60
80
100
500
550
600

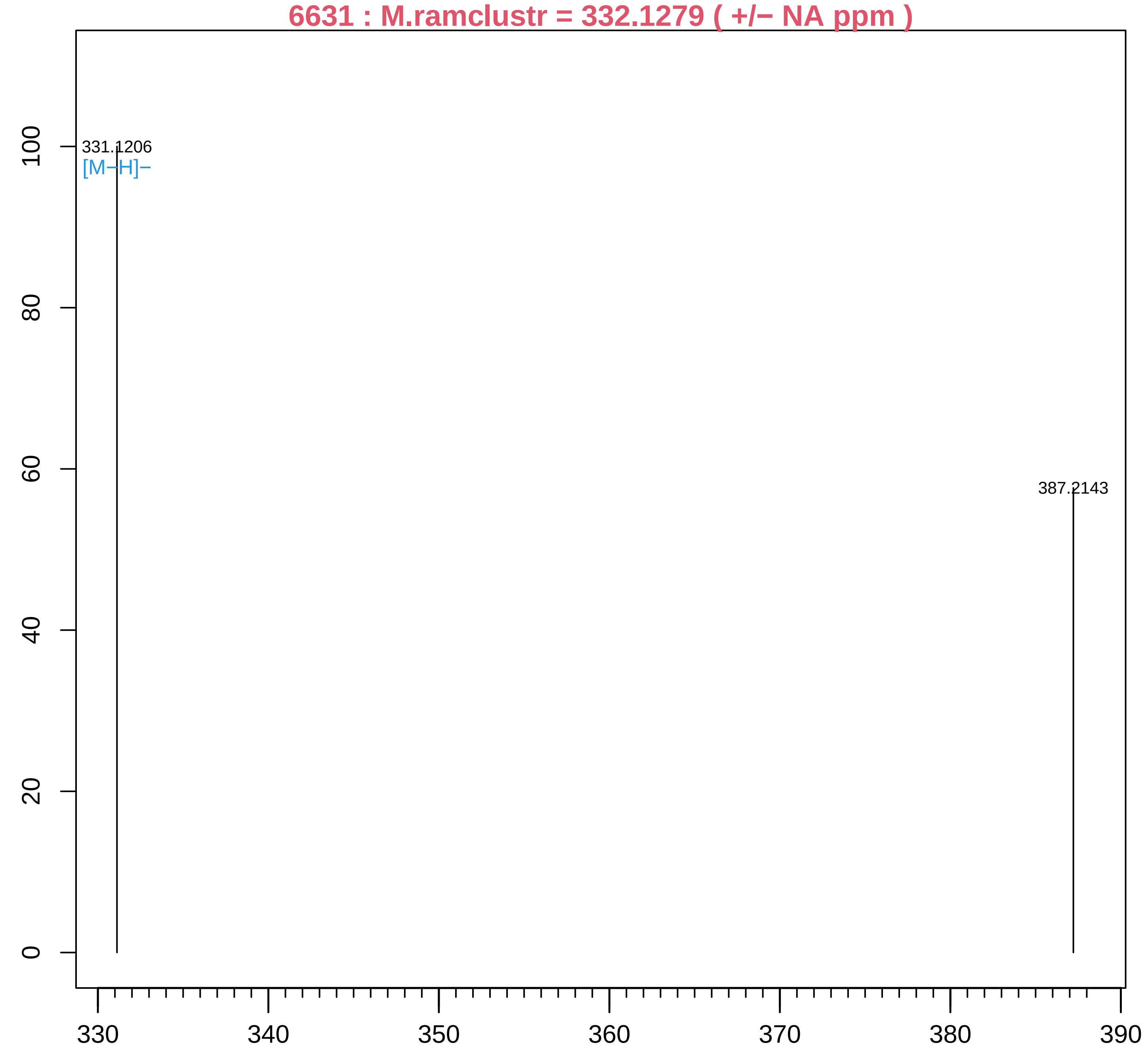
6631 : M.ramclustr = 332.1279 (+/− NA ppm)
331.1206
[M−H]−
387.2143
0
20
40
60
80
100
330
340
350
360
370
380
390

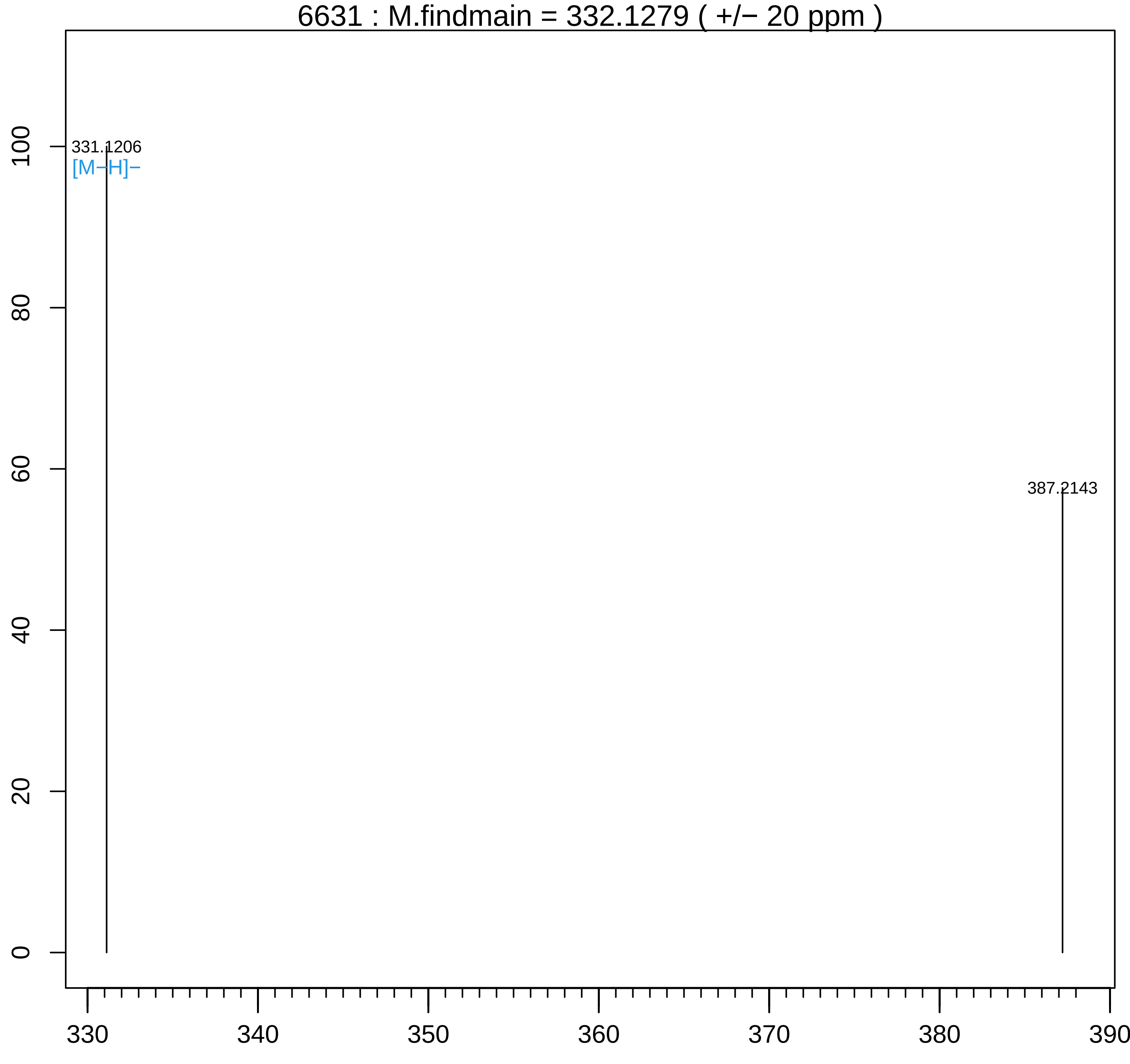
6631 : M.findmain = 332.1279 (+/− 20 ppm)
331.1206
[M−H]−
387.2143
0
20
40
60
80
100
330
340
350
360
370
380
390

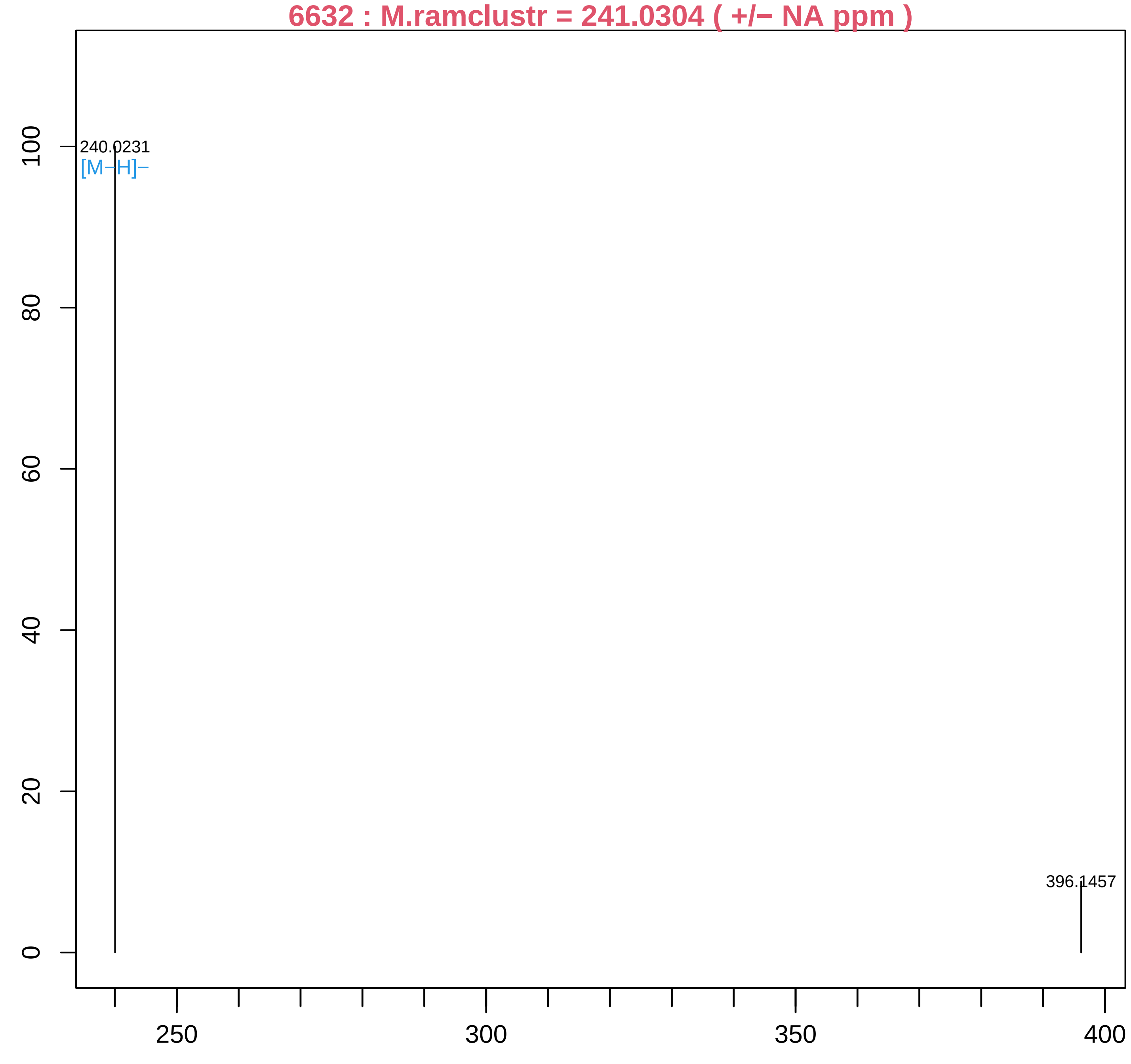

6632 : M.ramclustr = 241.0304 (+/− NA ppm)
240.0231
[M−H]−
396.1457
0
20
40
60
80
100
250
300
350
400

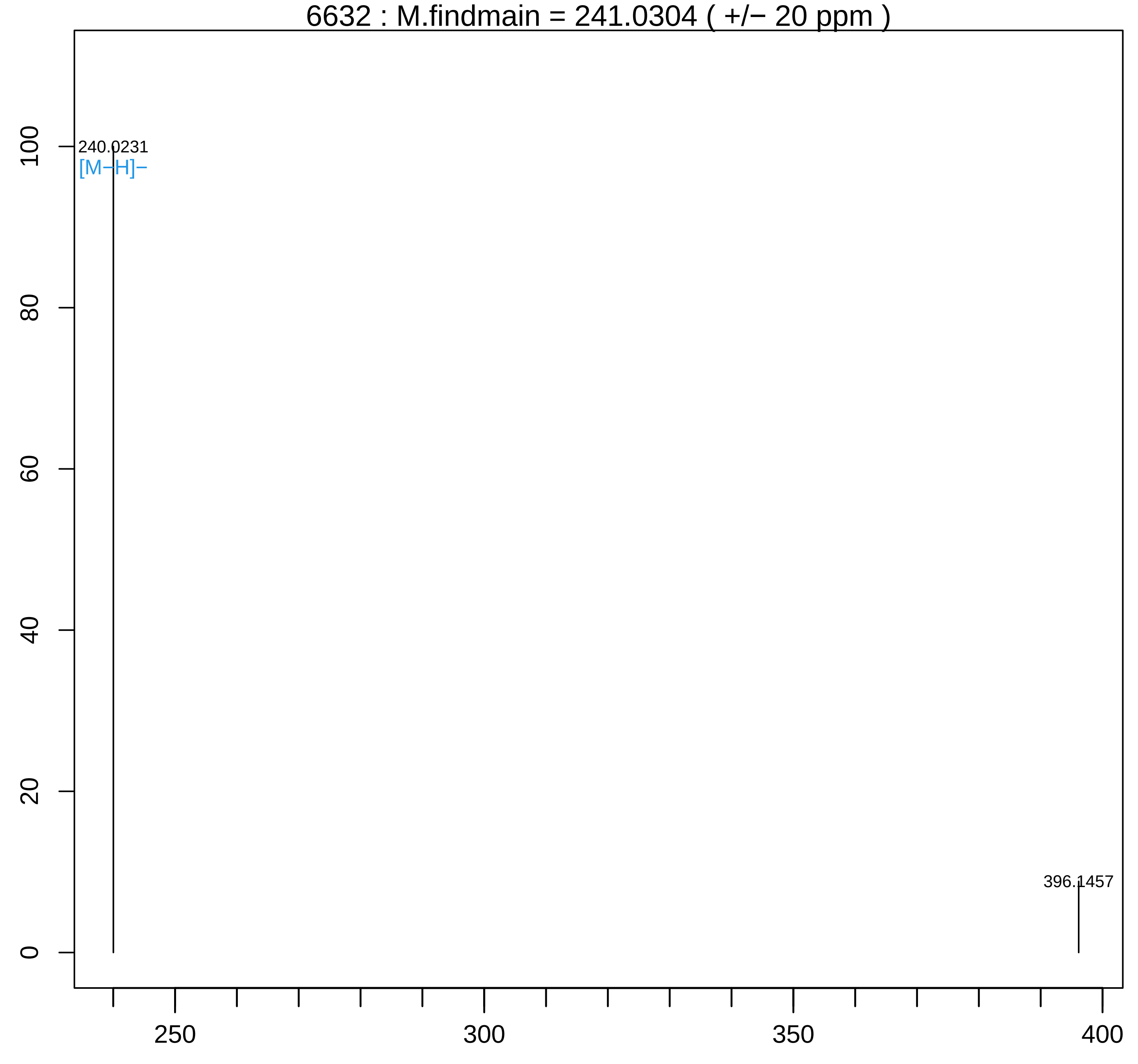

6632 : M.findmain = 241.0304 (+/− 20 ppm)
240.0231
[M−H]−
396.1457
0
20
40
60
80
100
250
300
350
400

6633 : M.ramclustr = 897.0438 (+/− NA ppm)

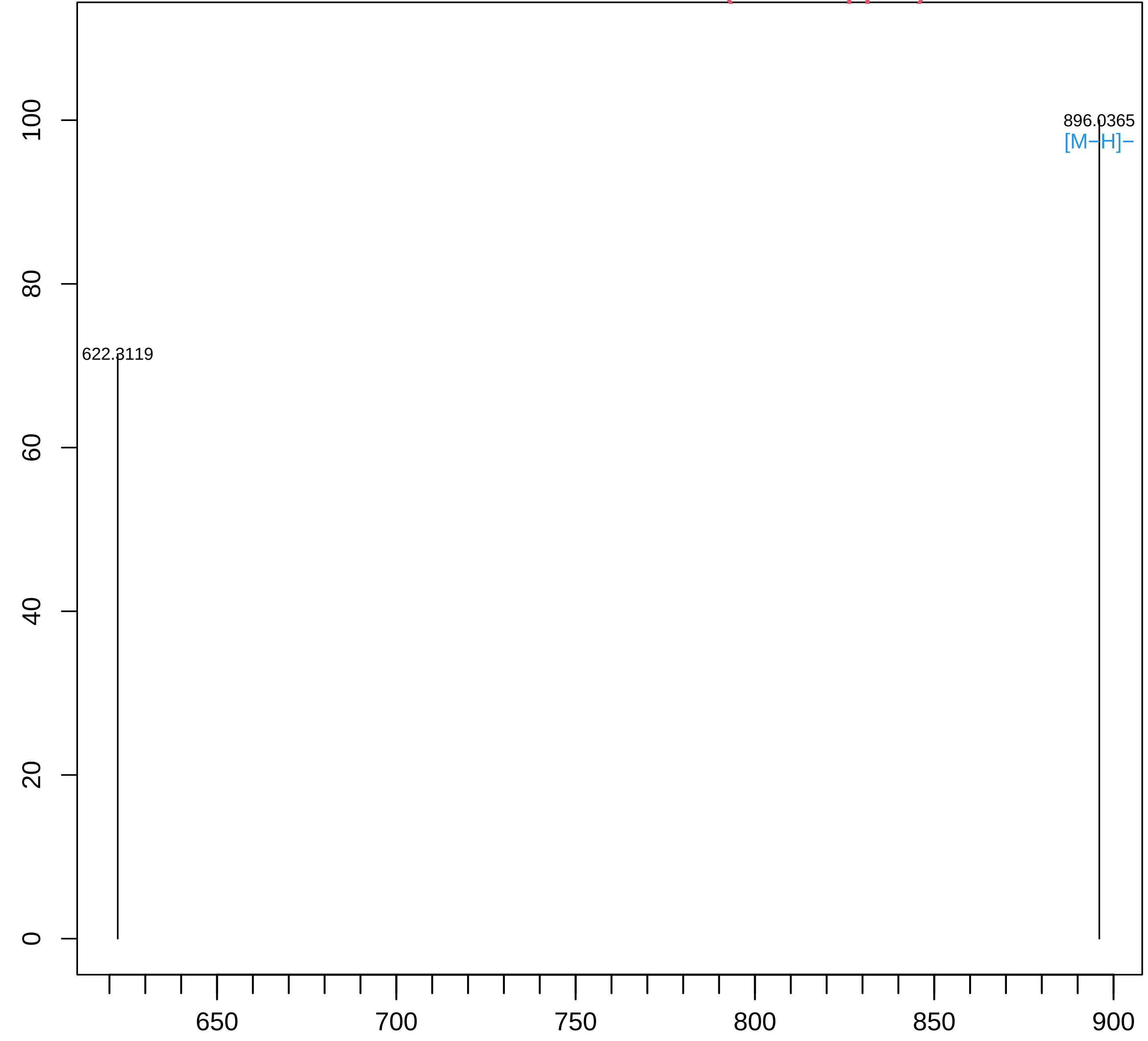

6633 : M.findmain = 897.0438 (+/− 20 ppm)

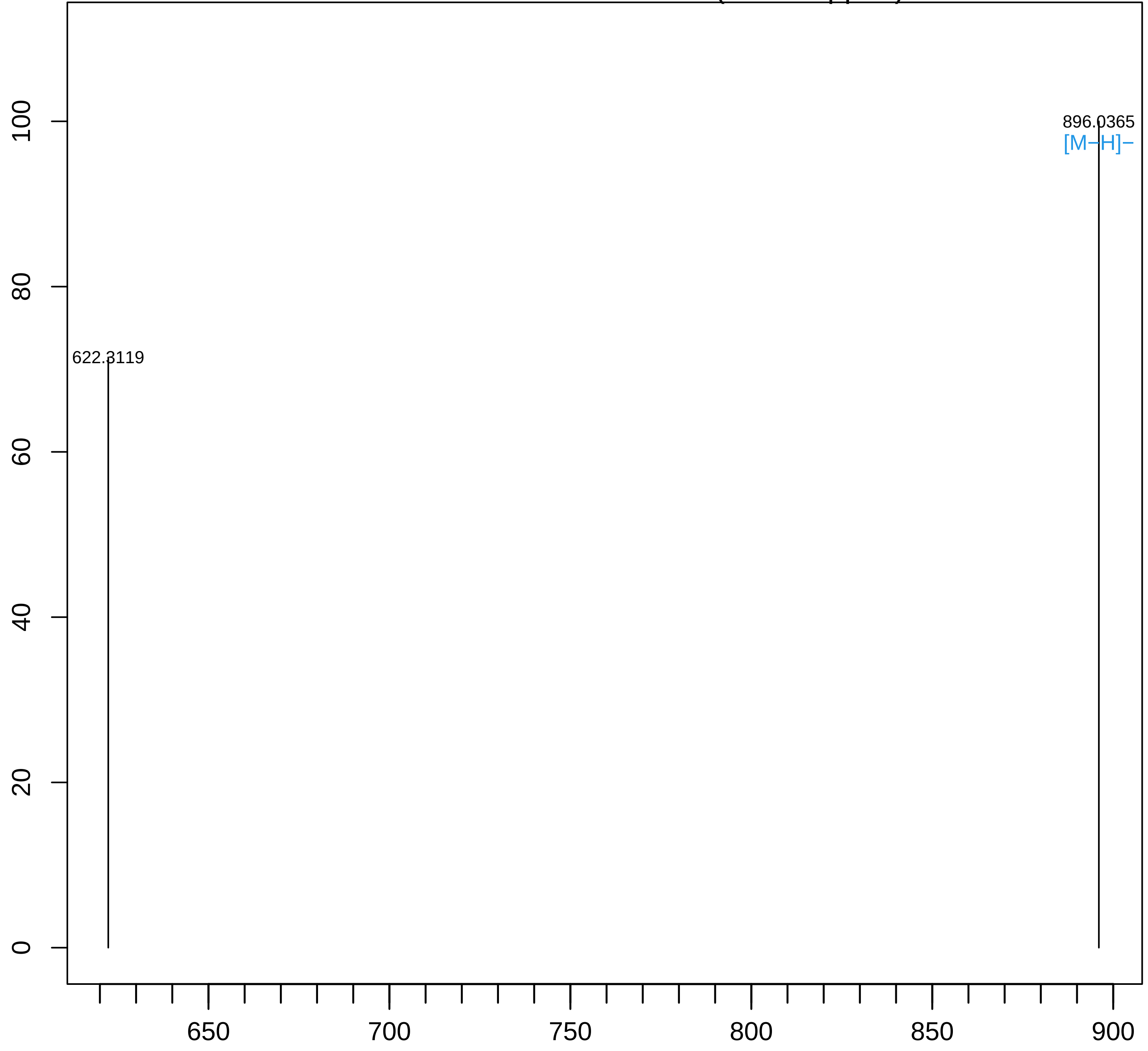

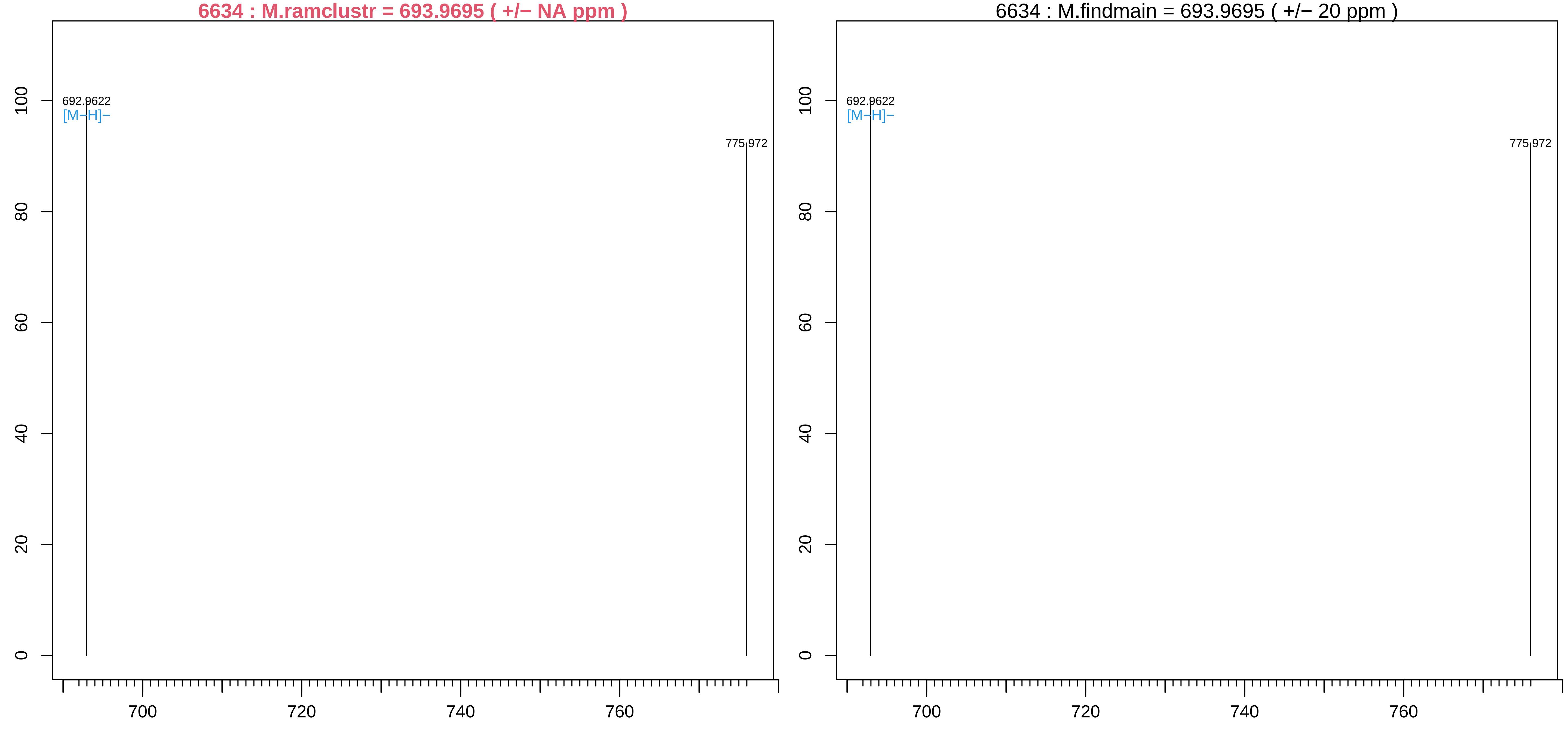
6634 : M.ramclustr = 693.9695 (+/− NA ppm)
692.9622
[M−H]−
775.972
6634 : M.findmain = 693.9695 (+/− 20 ppm)
692.9622
[M−H]−
775.972
0
20
40
60
80
100
700
720
740
760

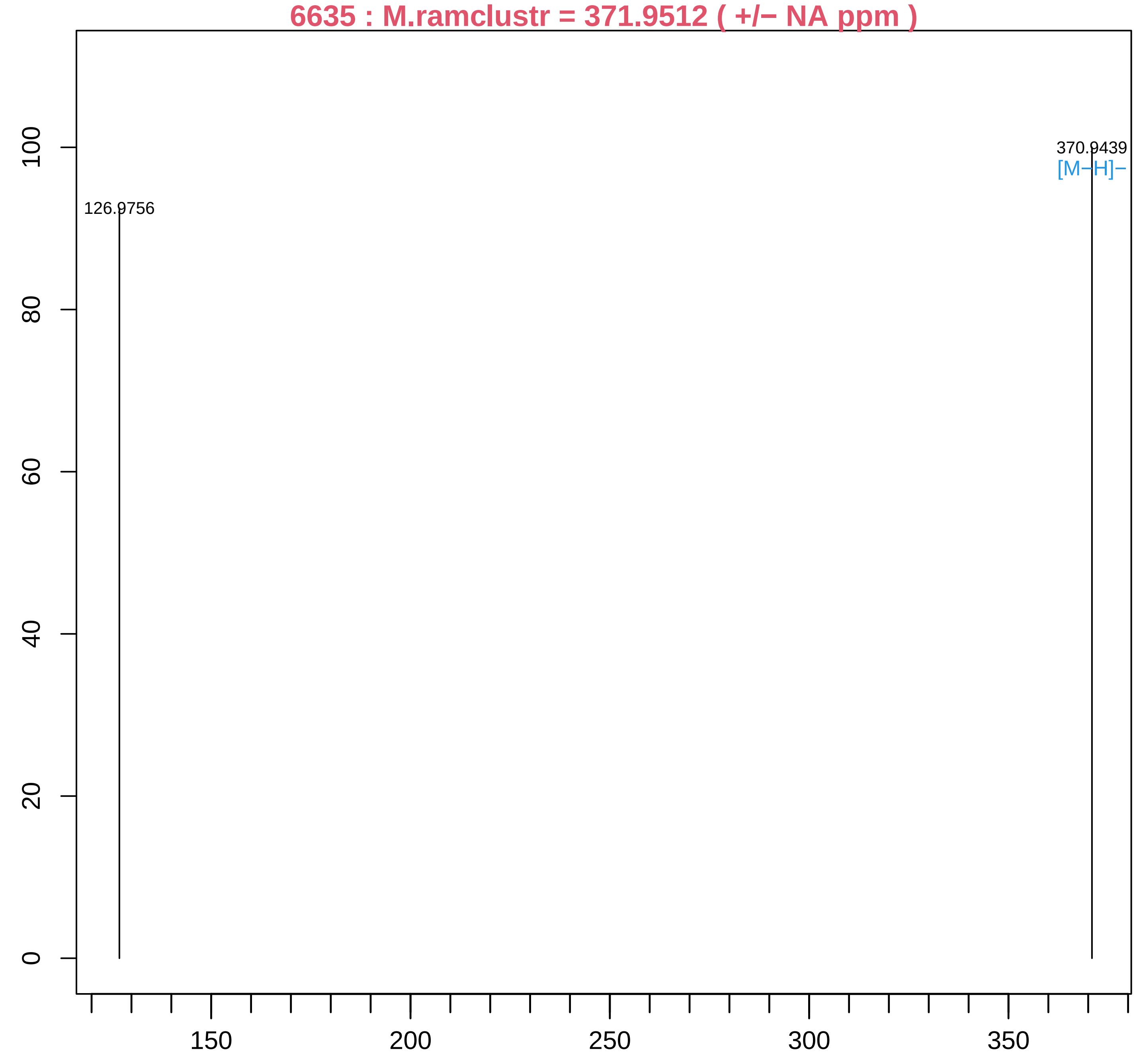

6635 : M.ramclustr = 371.9512 (+/− NA ppm)
370.9439
[M−H]−
126.9756
0
20
40
60
80
100
150
200
250
300
350

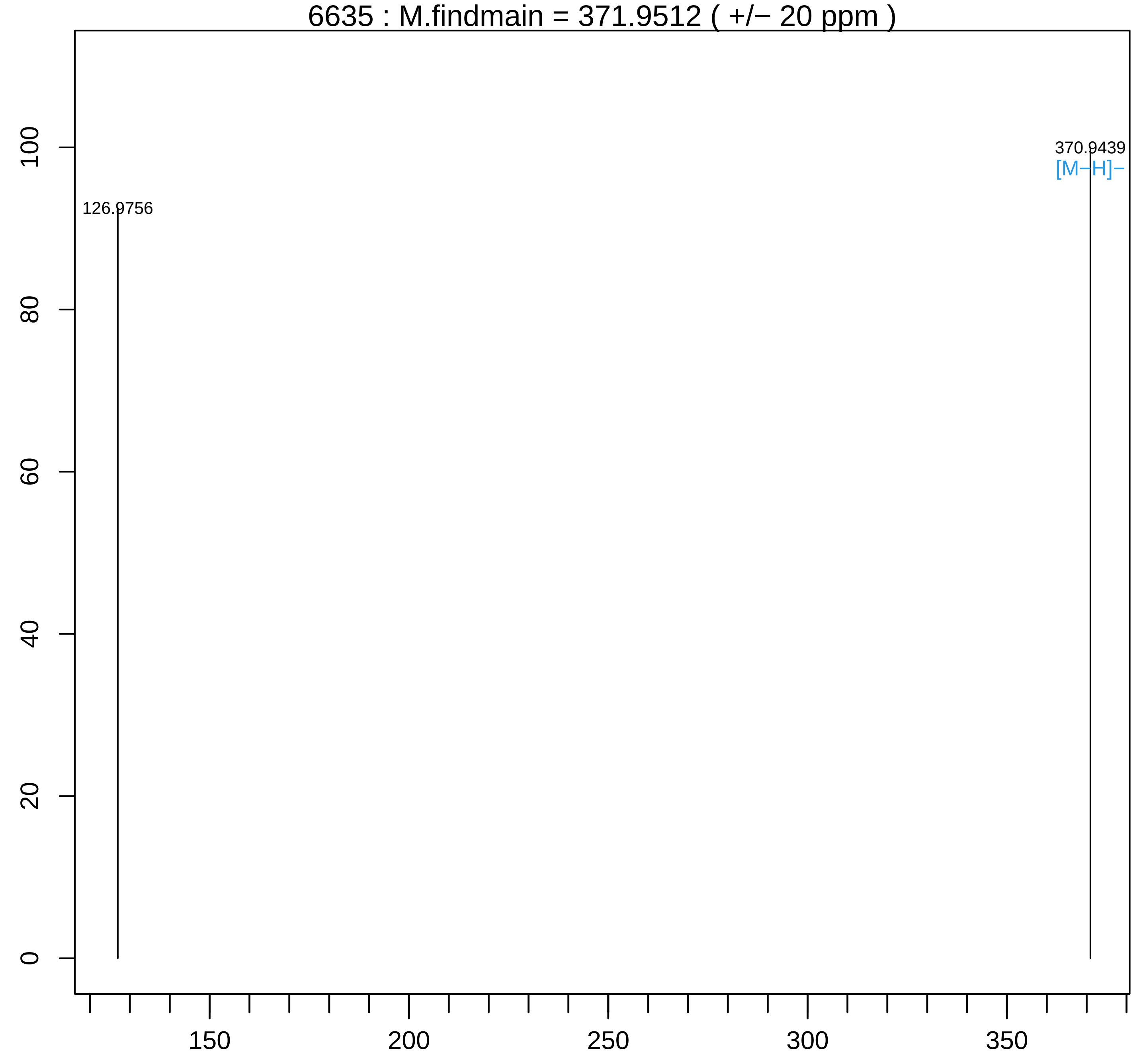

6635 : M.findmain = 371.9512 (+/− 20 ppm)
370.9439
[M−H]−
126.9756
0
20
40
60
80
100
150
200
250
300
350

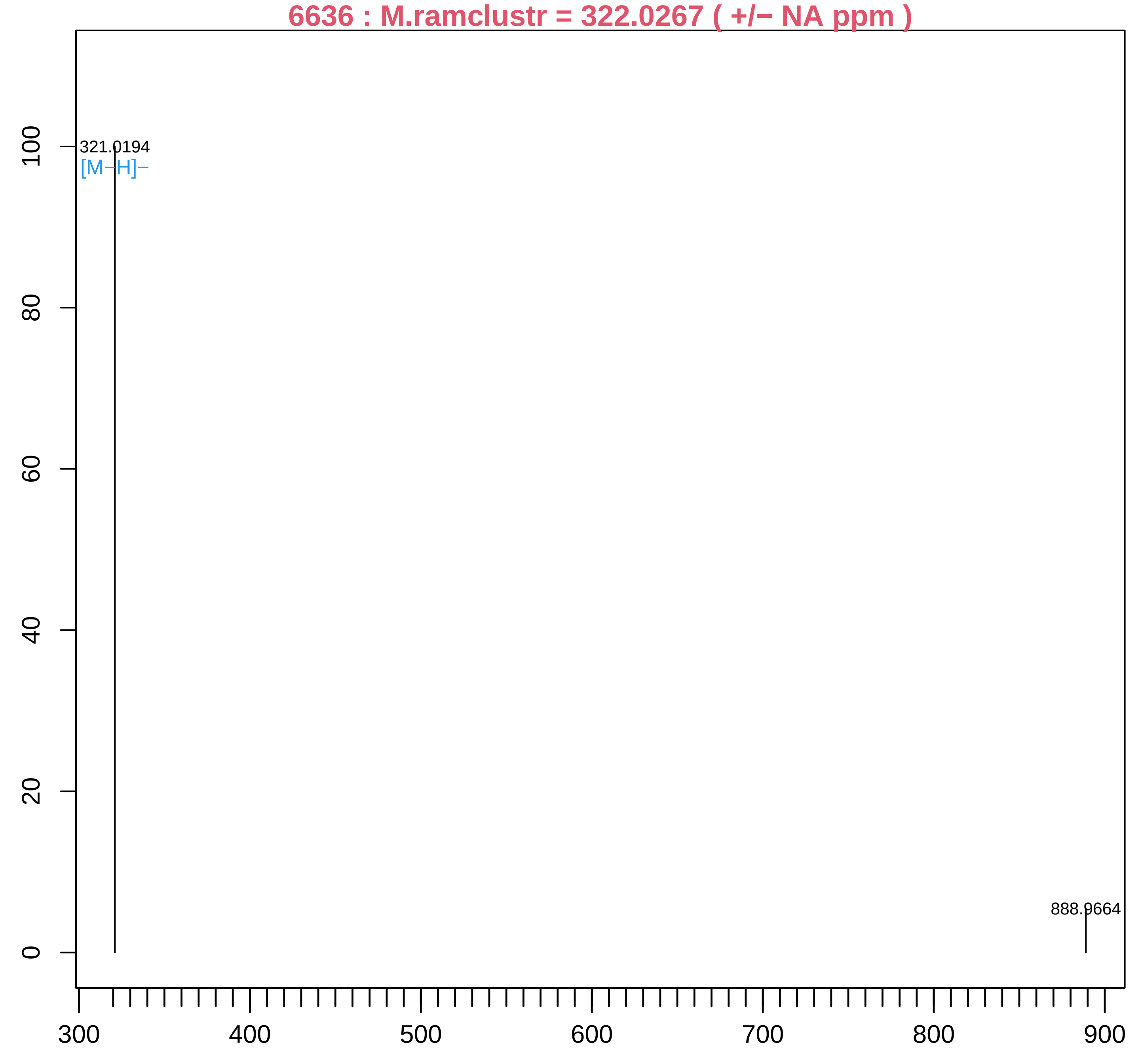
6636 : M.ramclustr = 322.0267 (+/− NA ppm)
321.0194
[M−H]−
888.9664
0
20
40
60
80
100
300
400
500
600
700
800
900

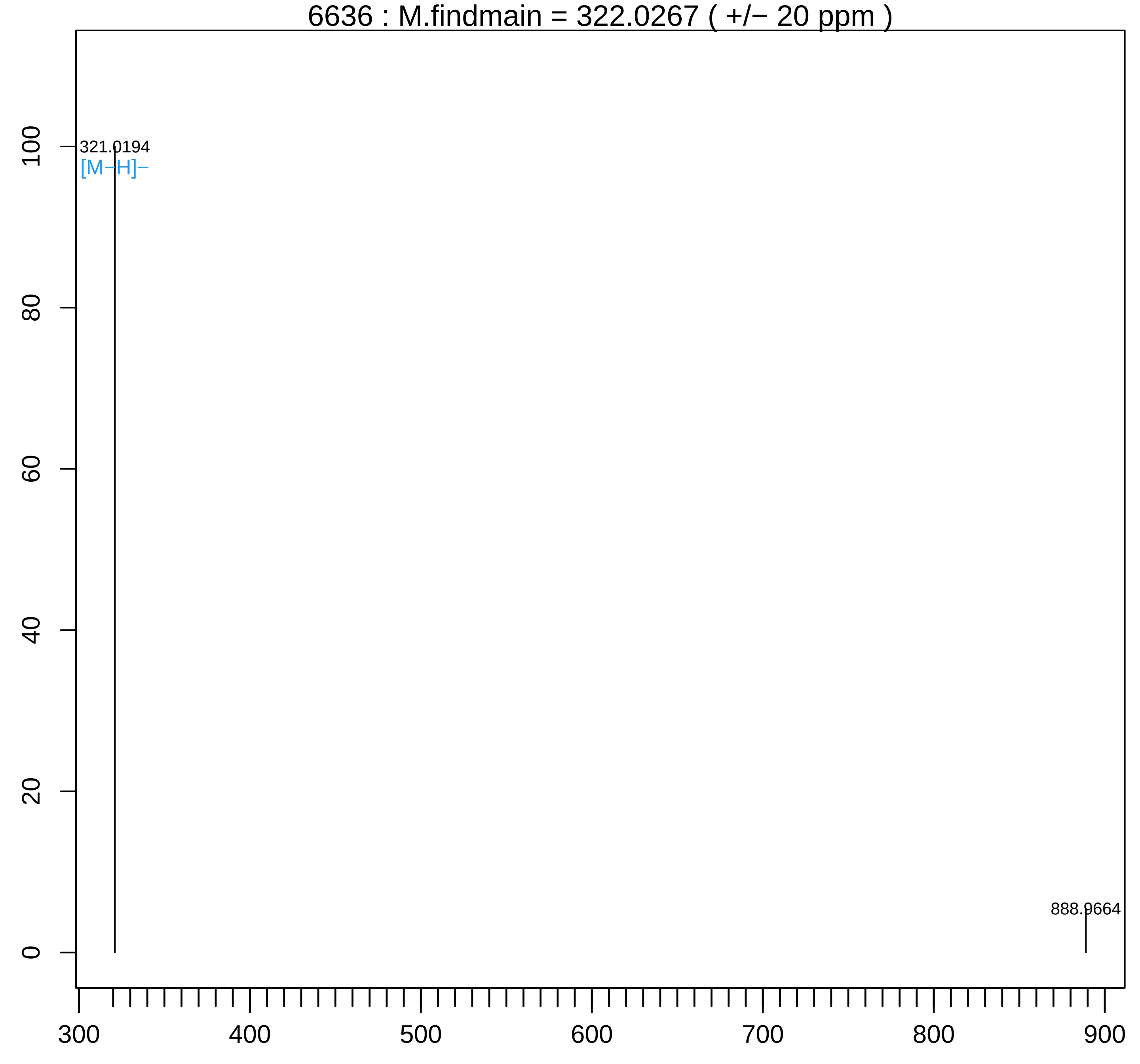
6636 : M.findmain = 322.0267 (+/− 20 ppm)
321.0194
[M−H]−
888.9664
0
20
40
60
80
100
300
400
500
600
700
800
900

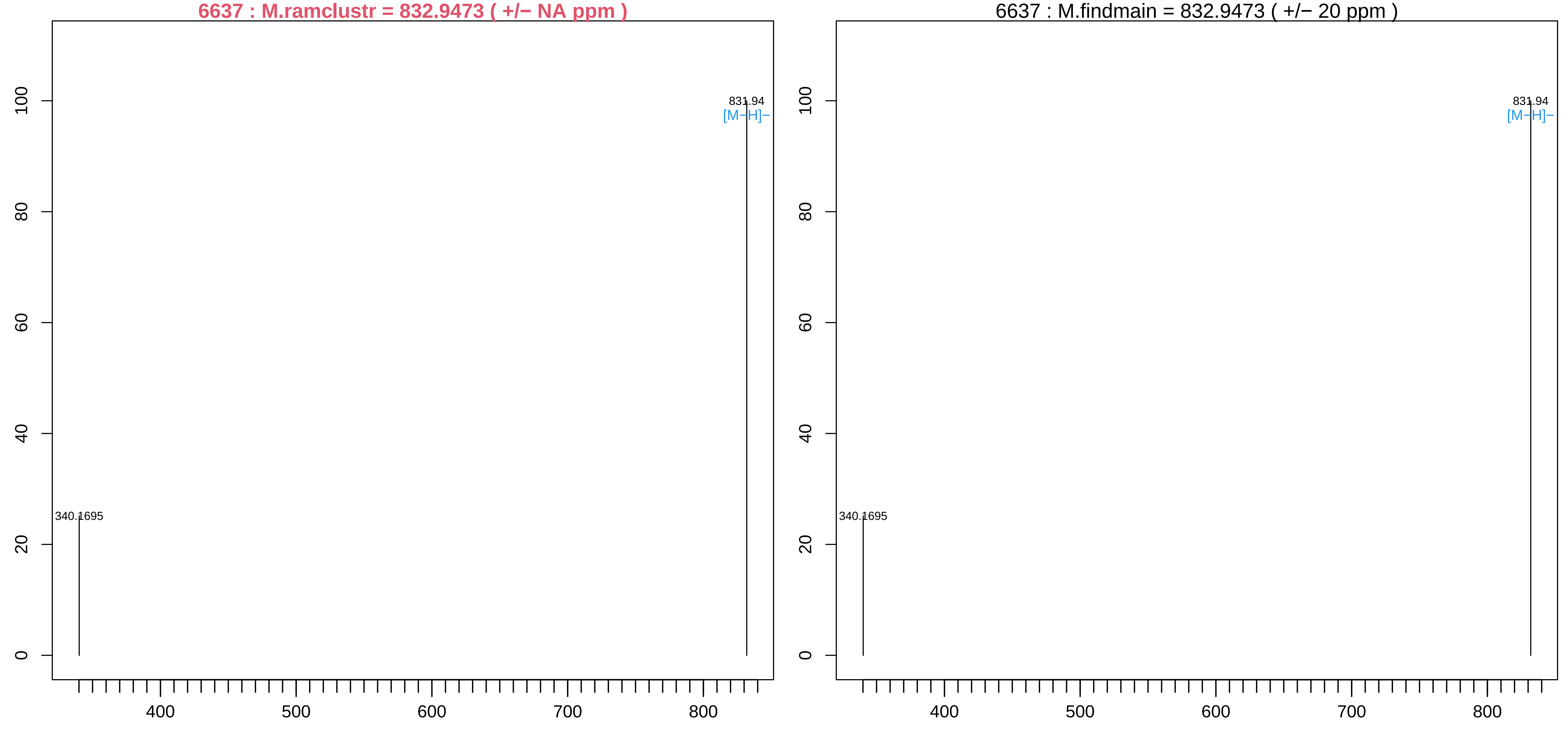

6637 : M.ramclustr = 832.9473 (+/− NA ppm)
6637 : M.findmain = 832.9473 (+/− 20 ppm)
831.94
[M−H]−
340.1695
0
20
40
60
80
100
400
500
600
700
800

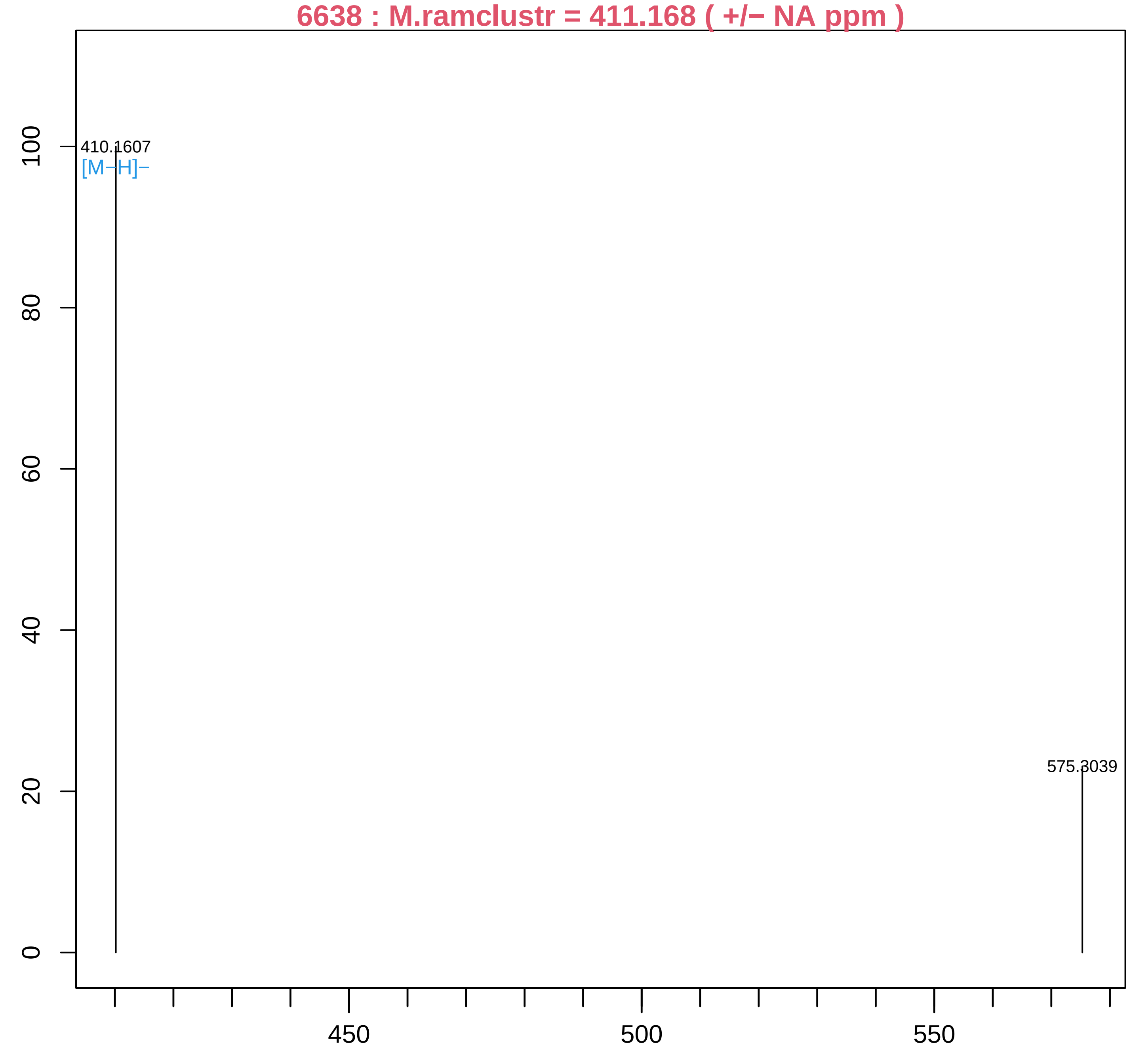

6638 : M.ramclustr = 411.168 (+/− NA ppm)
410.1607
[M−H]−
575.3039
0
20
40
60
80
100
450
500
550

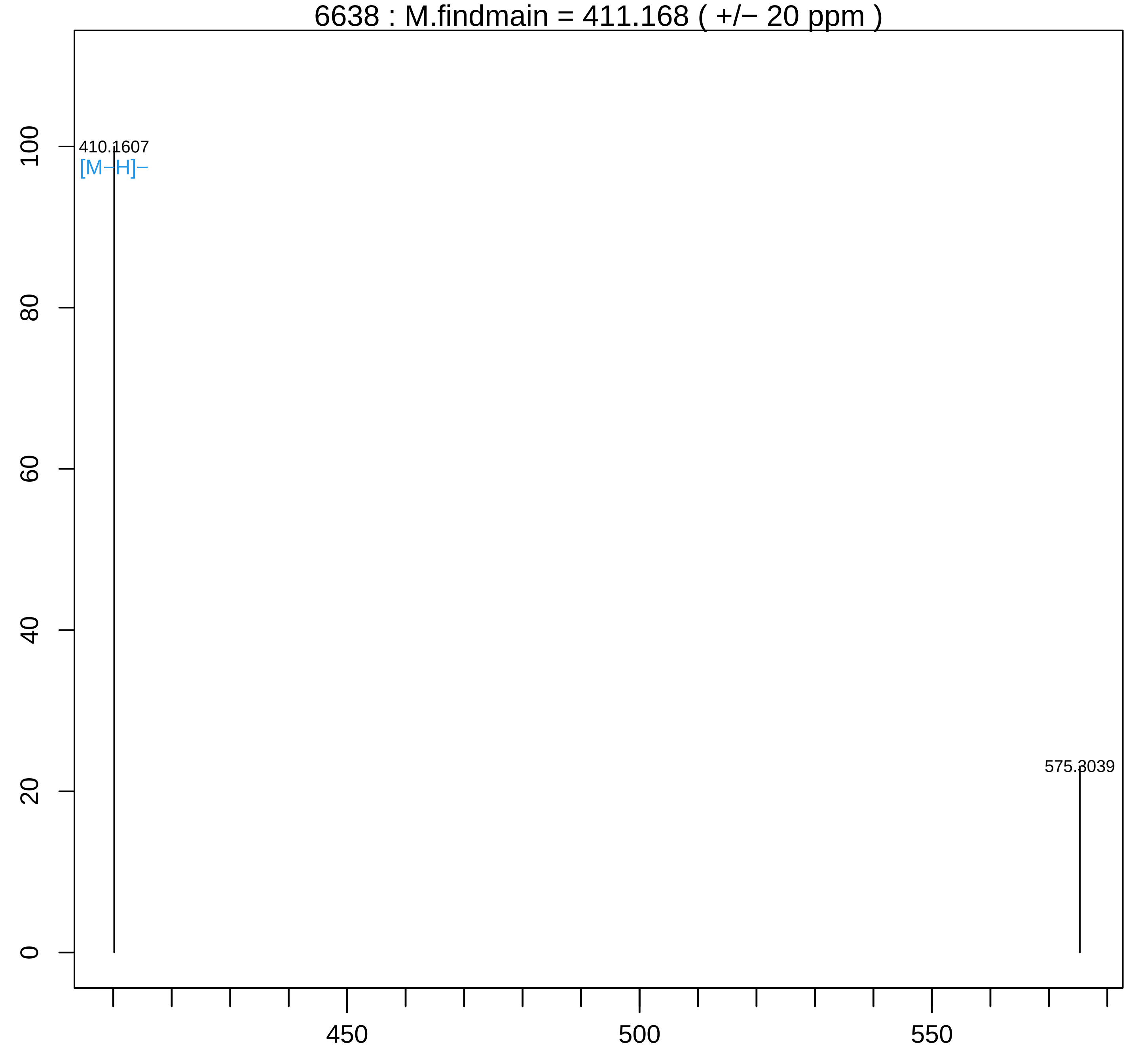

6638 : M.findmain = 411.168 (+/− 20 ppm)
410.1607
[M−H]−
575.3039
0
20
40
60
80
100
450
500
550

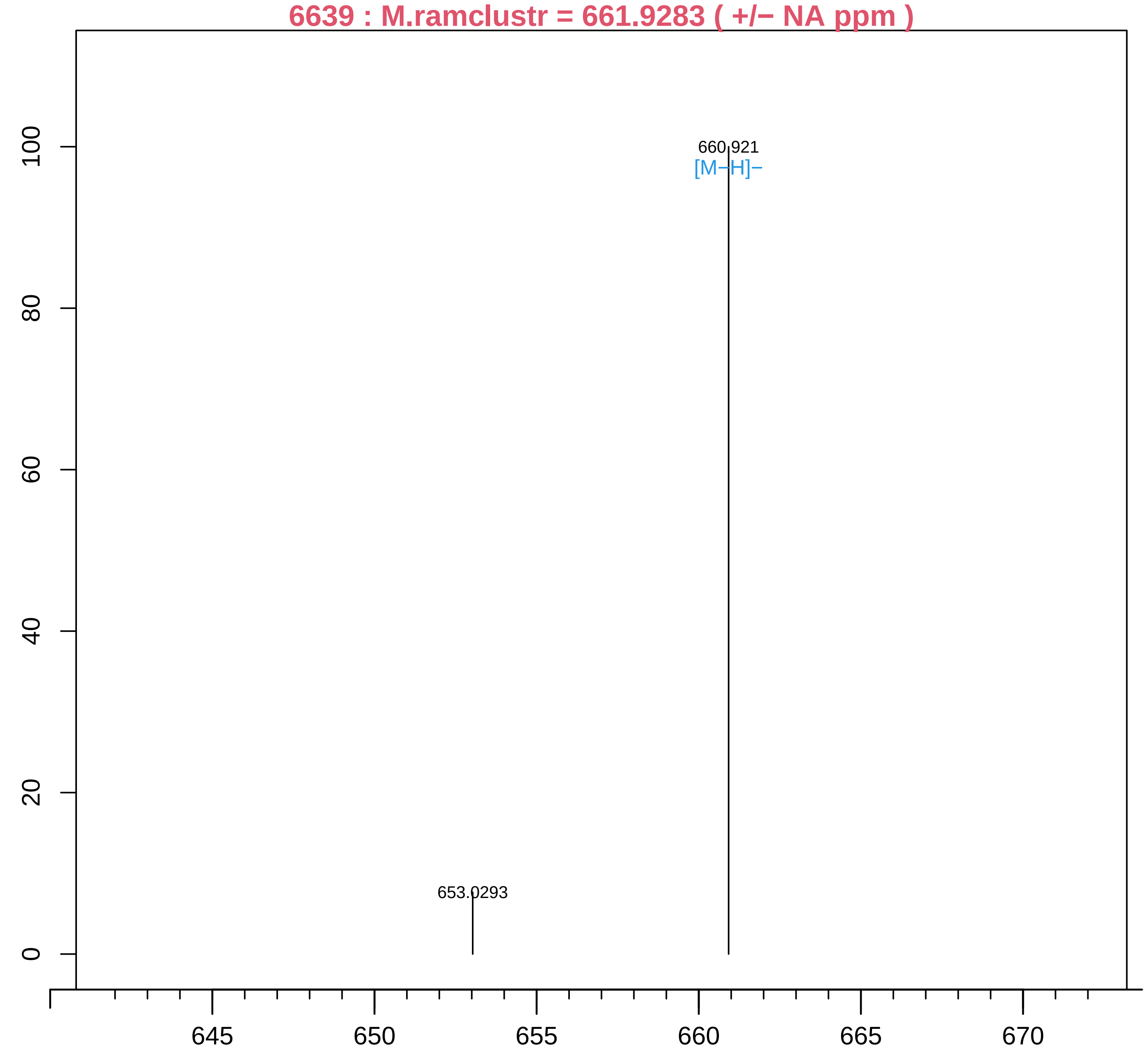
6639 : M.ramclustr = 661.9283 (+/− NA ppm)
660.921
[M−H]−
653.0293
0
20
40
60
80
100
645
650
655
660
665
670

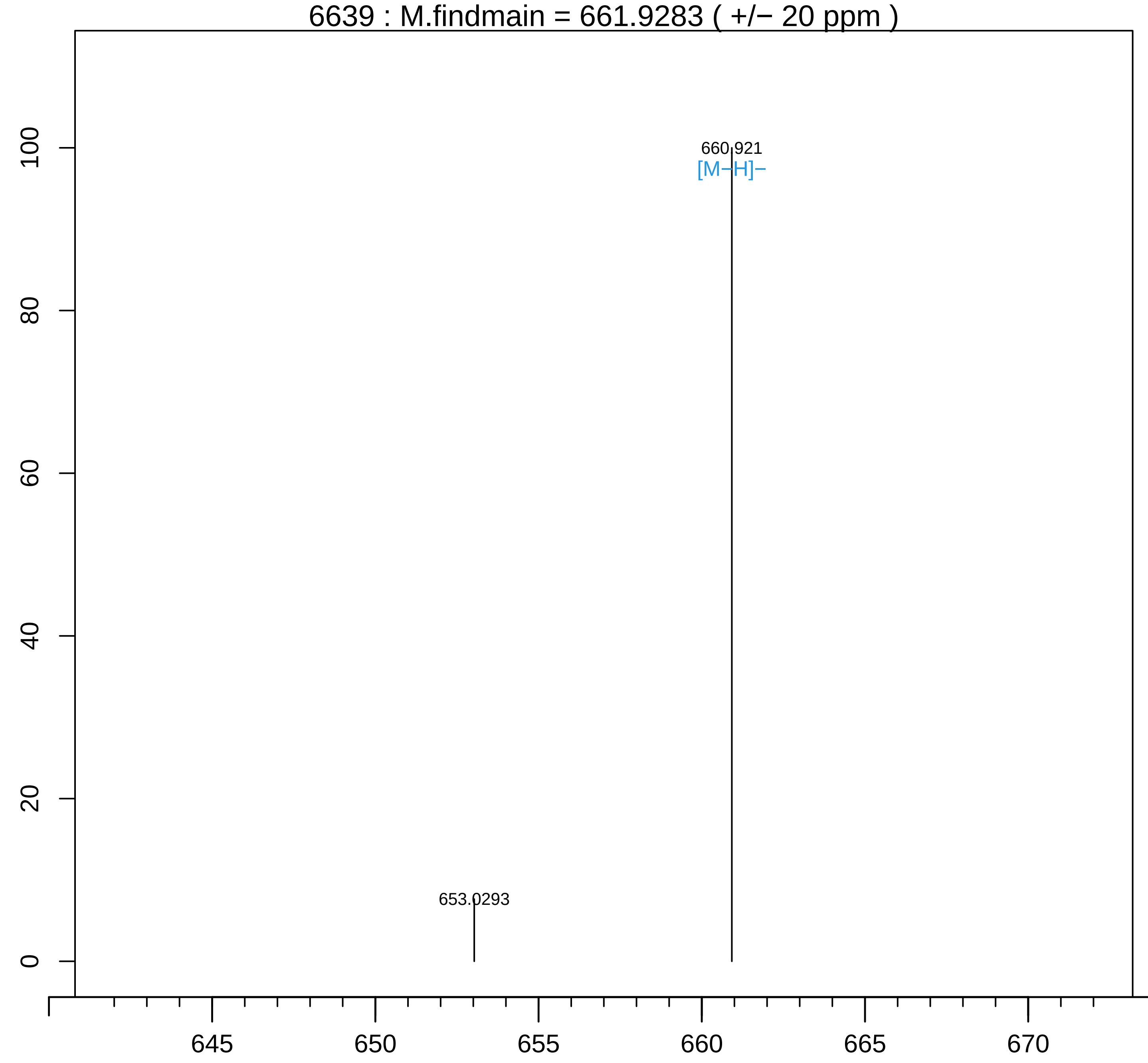
6639 : M.findmain = 661.9283 (+/− 20 ppm)
660.921
[M−H]−
653.0293
0
20
40
60
80
100
645
650
655
660
665
670

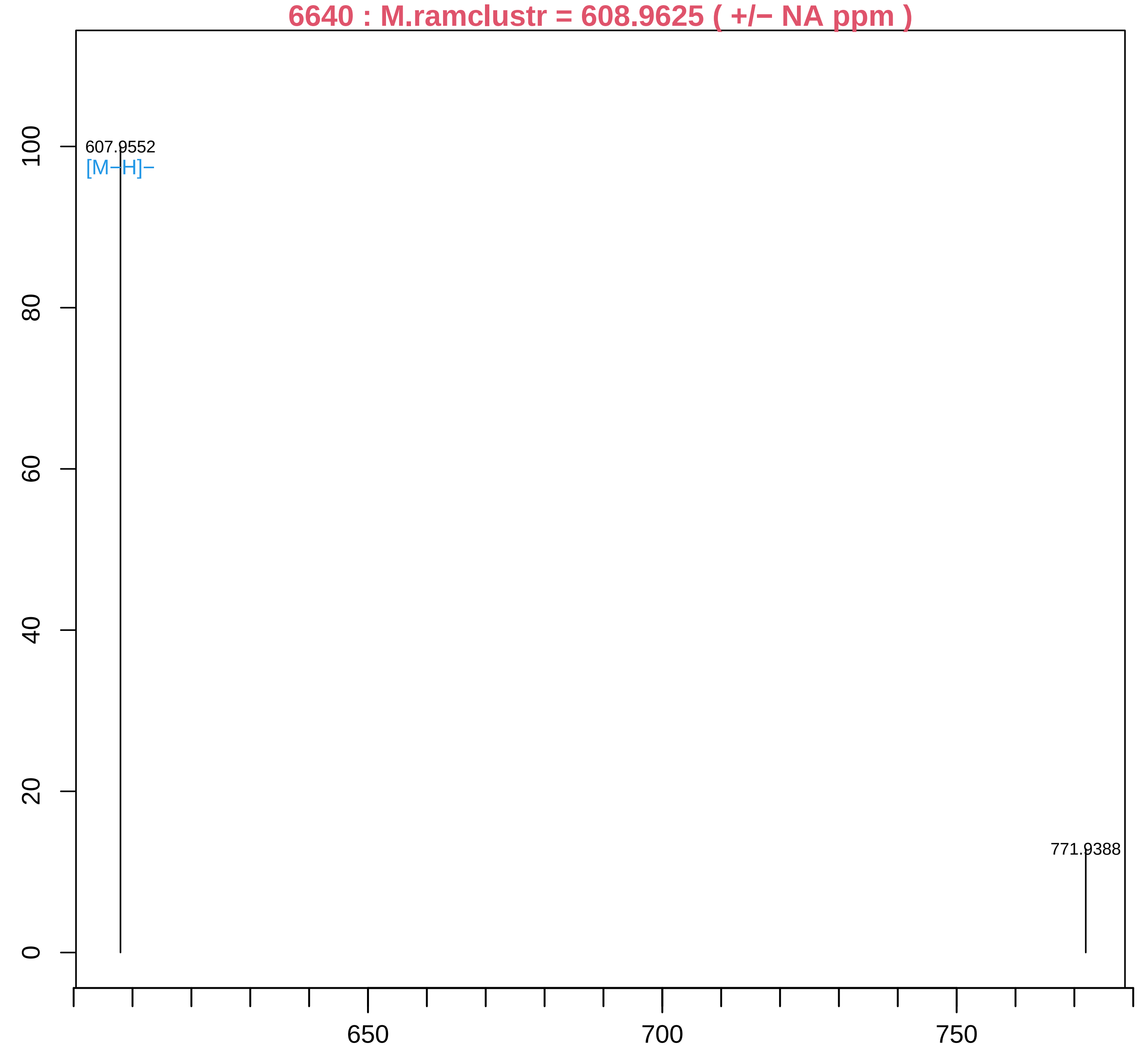

6640 : M.ramclustr = 608.9625 (+/− NA ppm)
607.9552
[M−H]−
771.9388
0
20
40
60
80
100
650
700
750

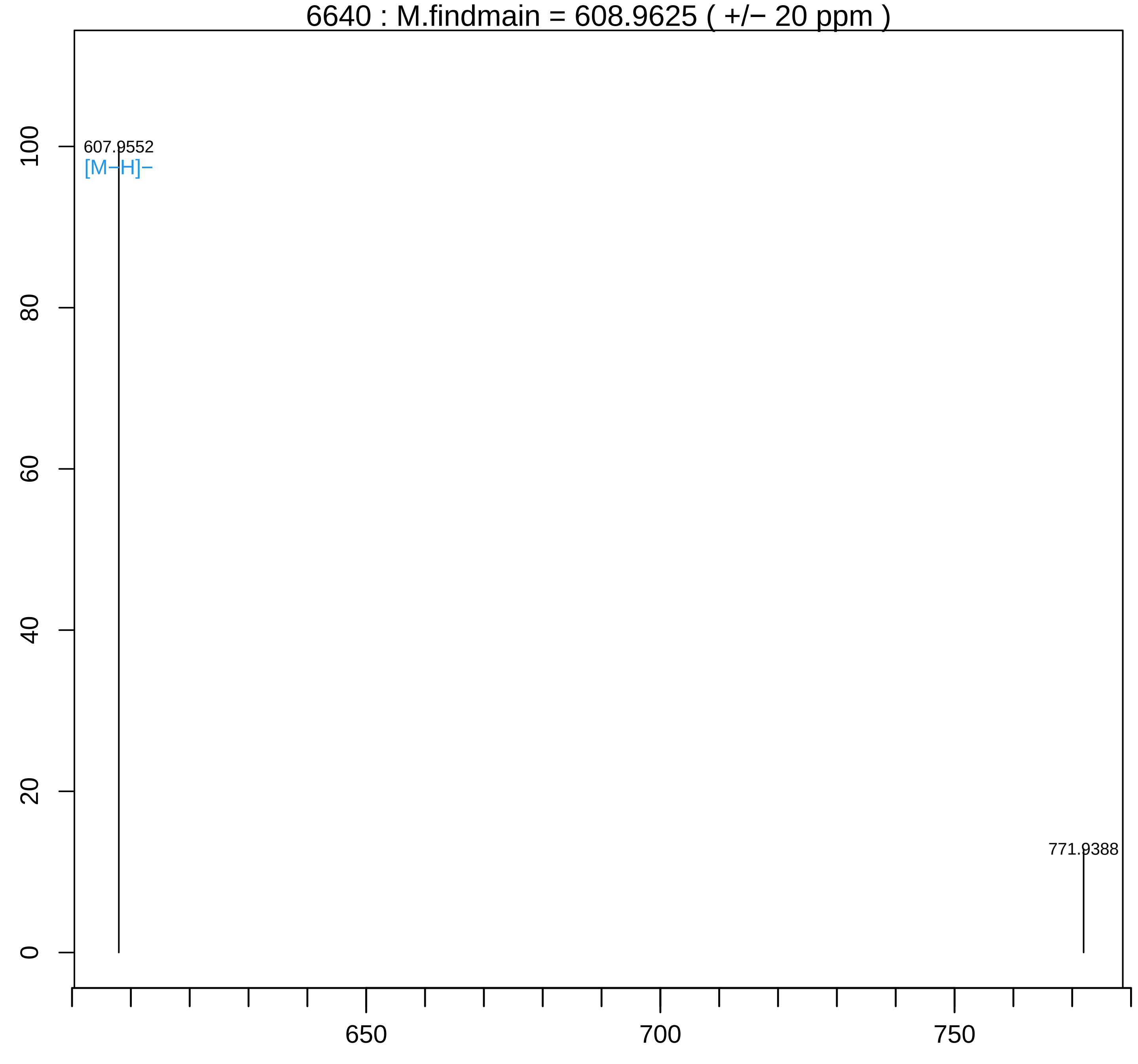

6640 : M.findmain = 608.9625 (+/− 20 ppm)
607.9552
[M−H]−
771.9388
0
20
40
60
80
100
650
700
750

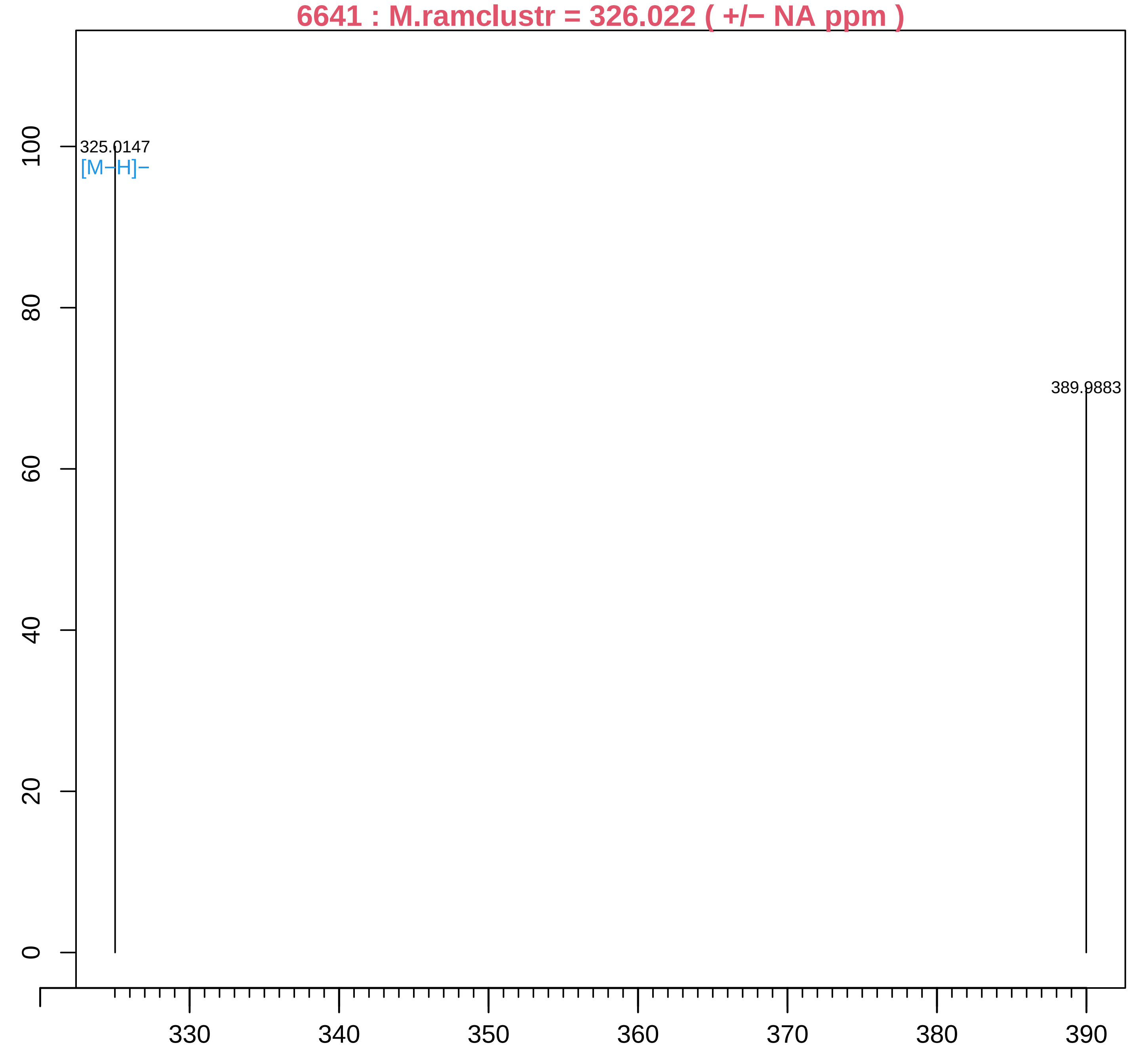

6641 : M.ramclustr = 326.022 (+/− NA ppm)
325.0147
[M−H]−
389.9883
0
20
40
60
80
100
330
340
350
360
370
380
390

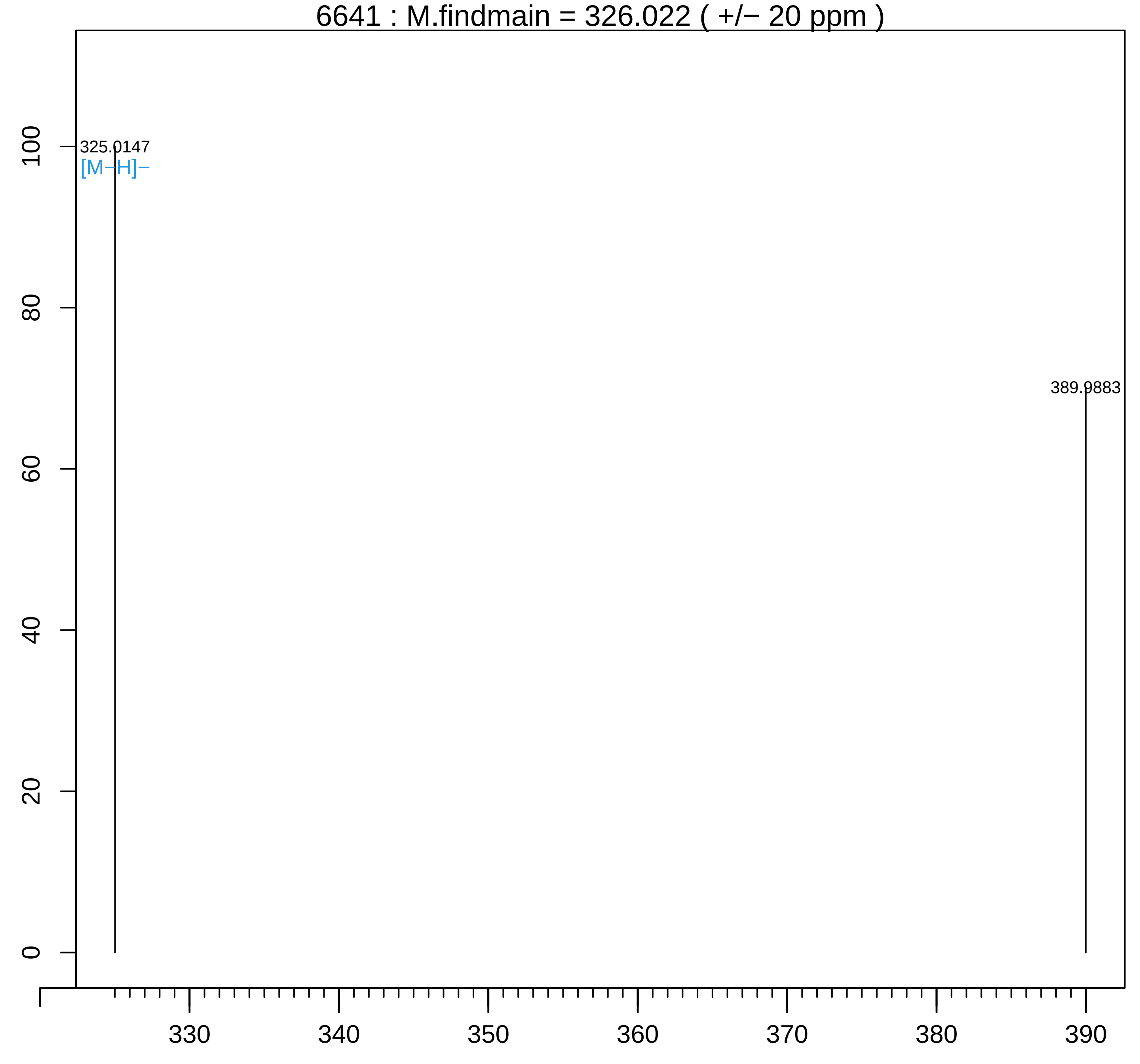

6641 : M.findmain = 326.022 (+/− 20 ppm)
325.0147
[M−H]−
389.9883
0
20
40
60
80
100
330
340
350
360
370
380
390

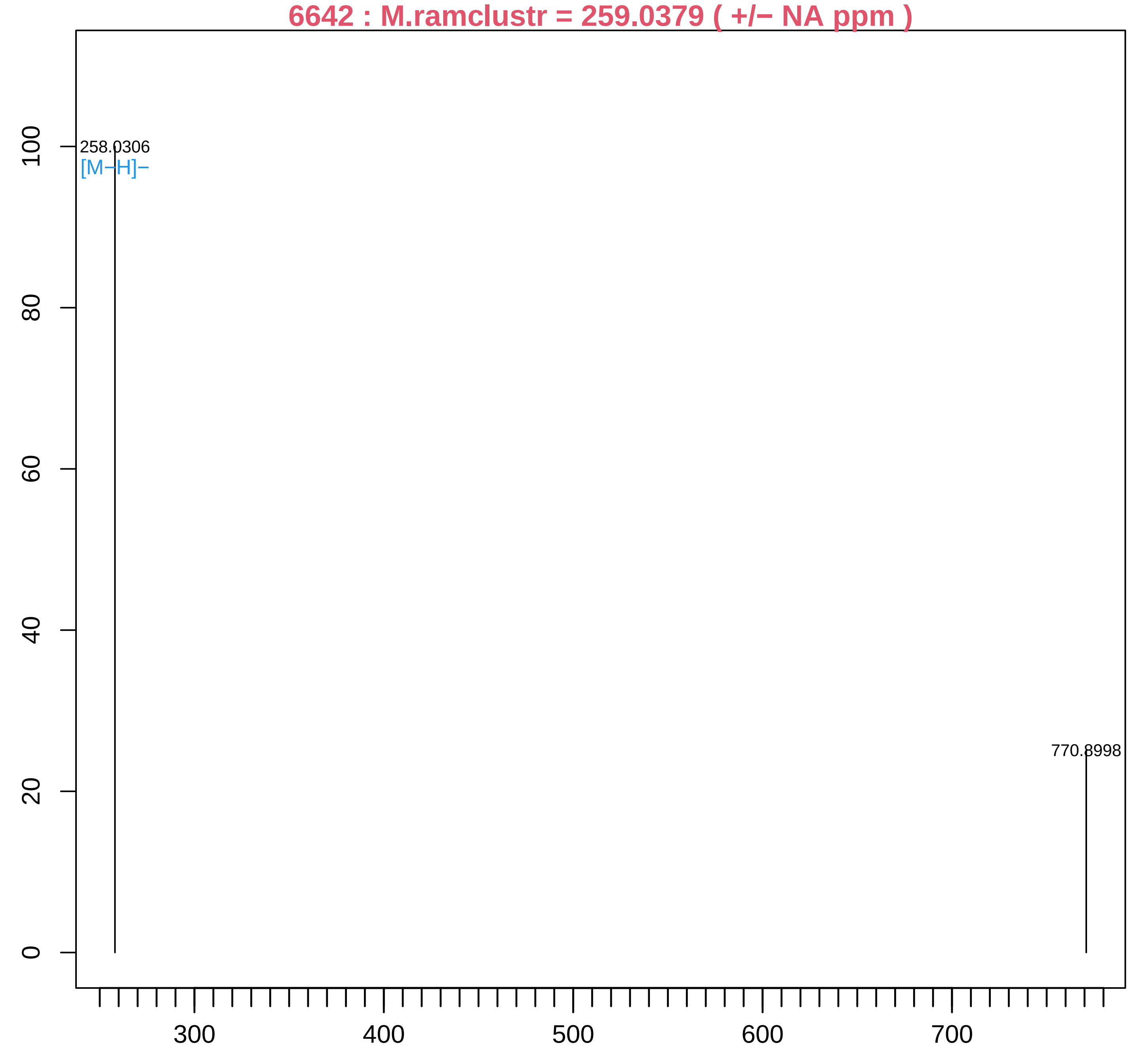
6642 : M.ramclustr = 259.0379 (+/− NA ppm)
258.0306
[M−H]−
770.8998
0
20
40
60
80
100
300
400
500
600
700

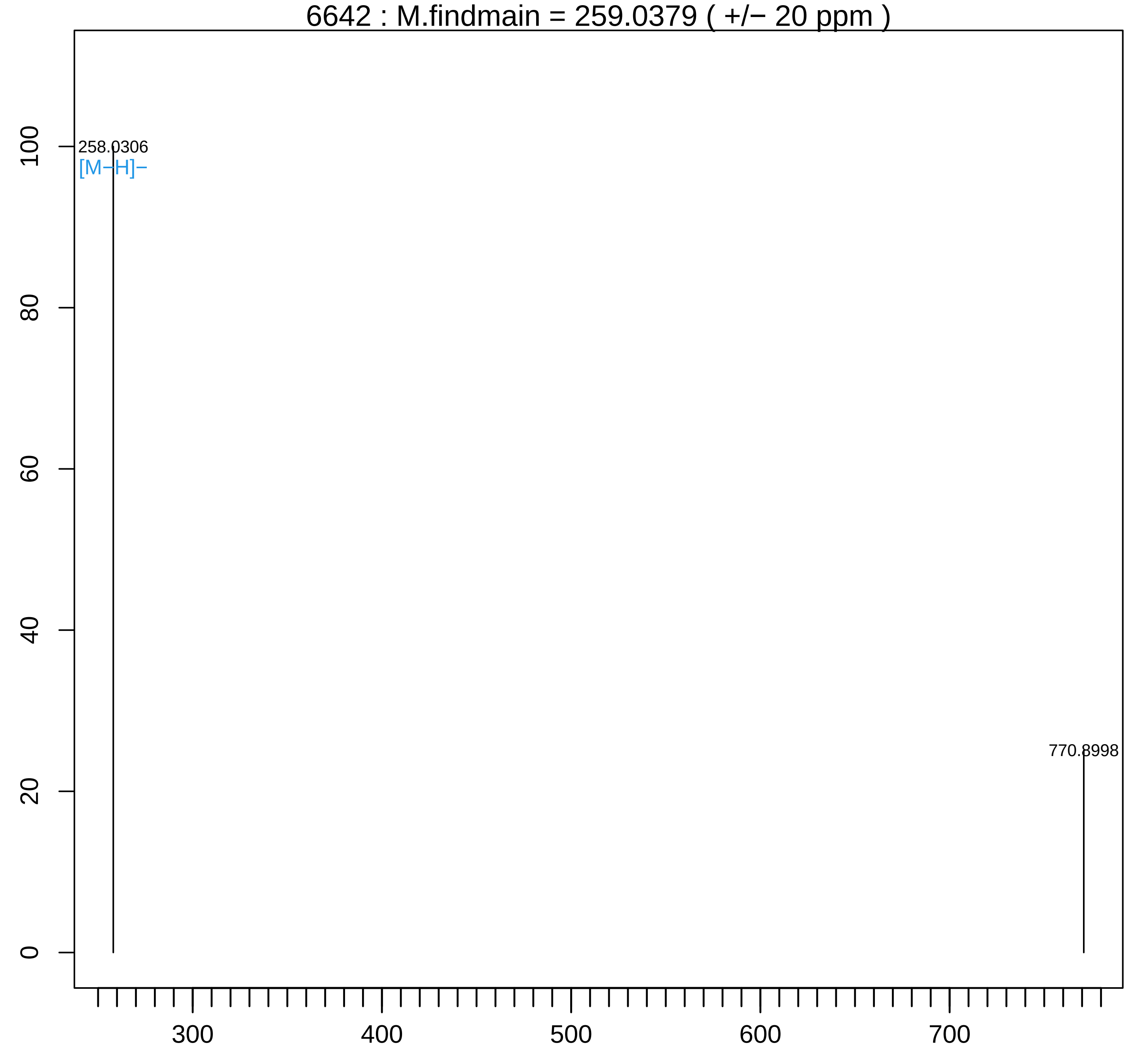
6642 : M.findmain = 259.0379 (+/− 20 ppm)
258.0306
[M−H]−
770.8998
0
20
40
60
80
100
300
400
500
600
700

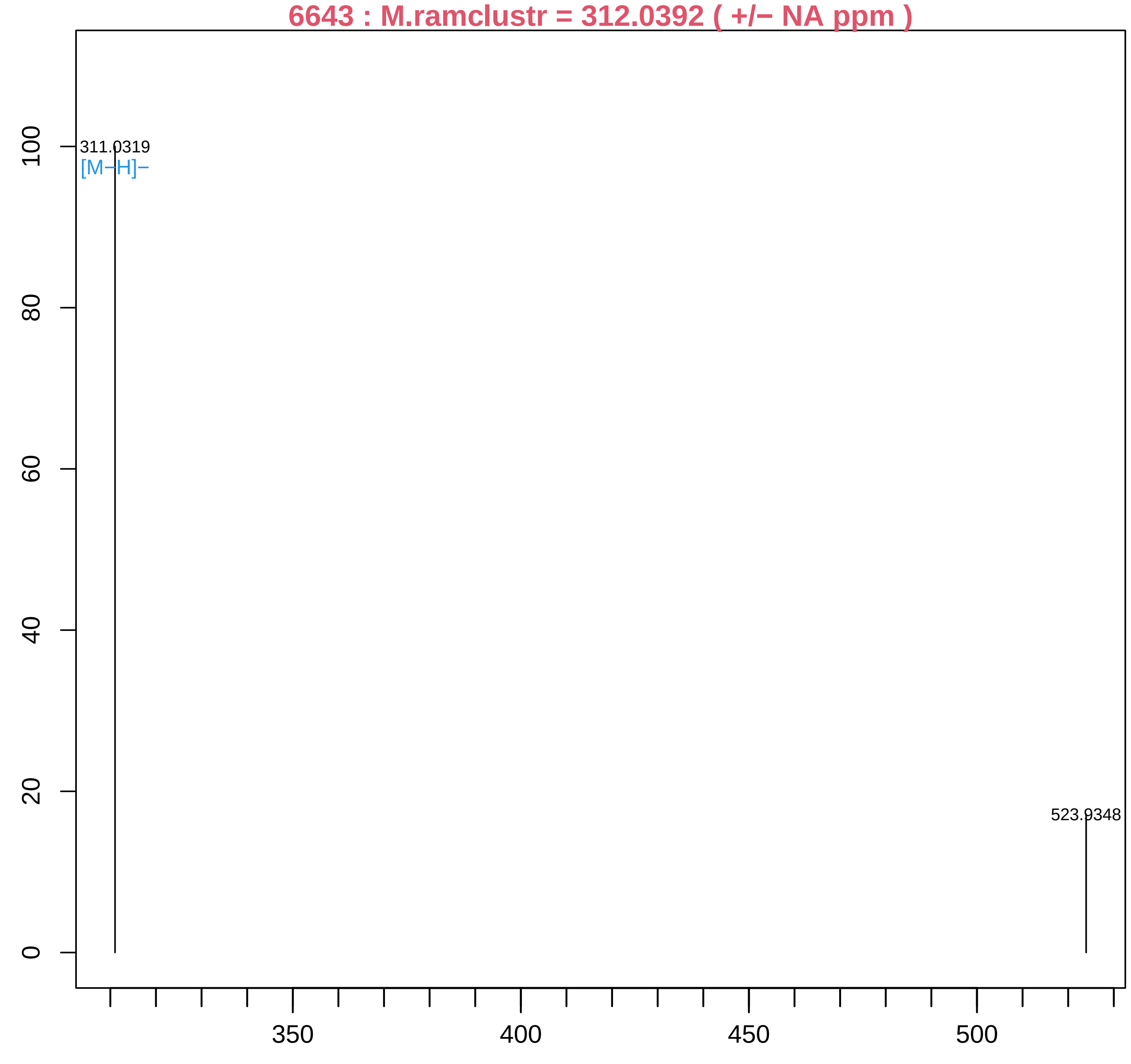

6643 : M.ramclustr = 312.0392 (+/− NA ppm)
311.0319
[M−H]−
523.9348
0
20
40
60
80
100
350
400
450
500

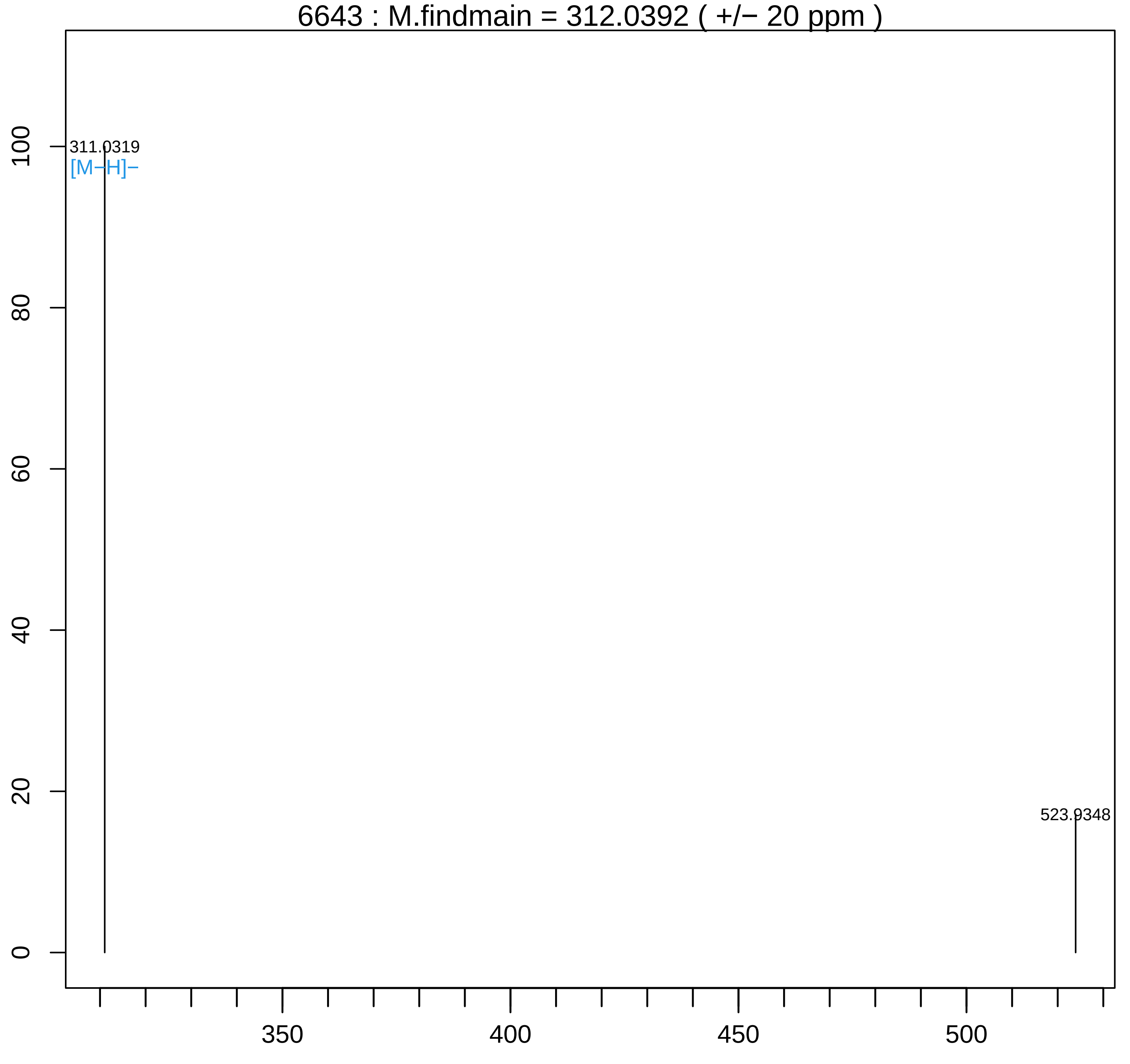

6643 : M.findmain = 312.0392 (+/− 20 ppm)
311.0319
[M−H]−
523.9348
0
20
40
60
80
100
350
400
450
500

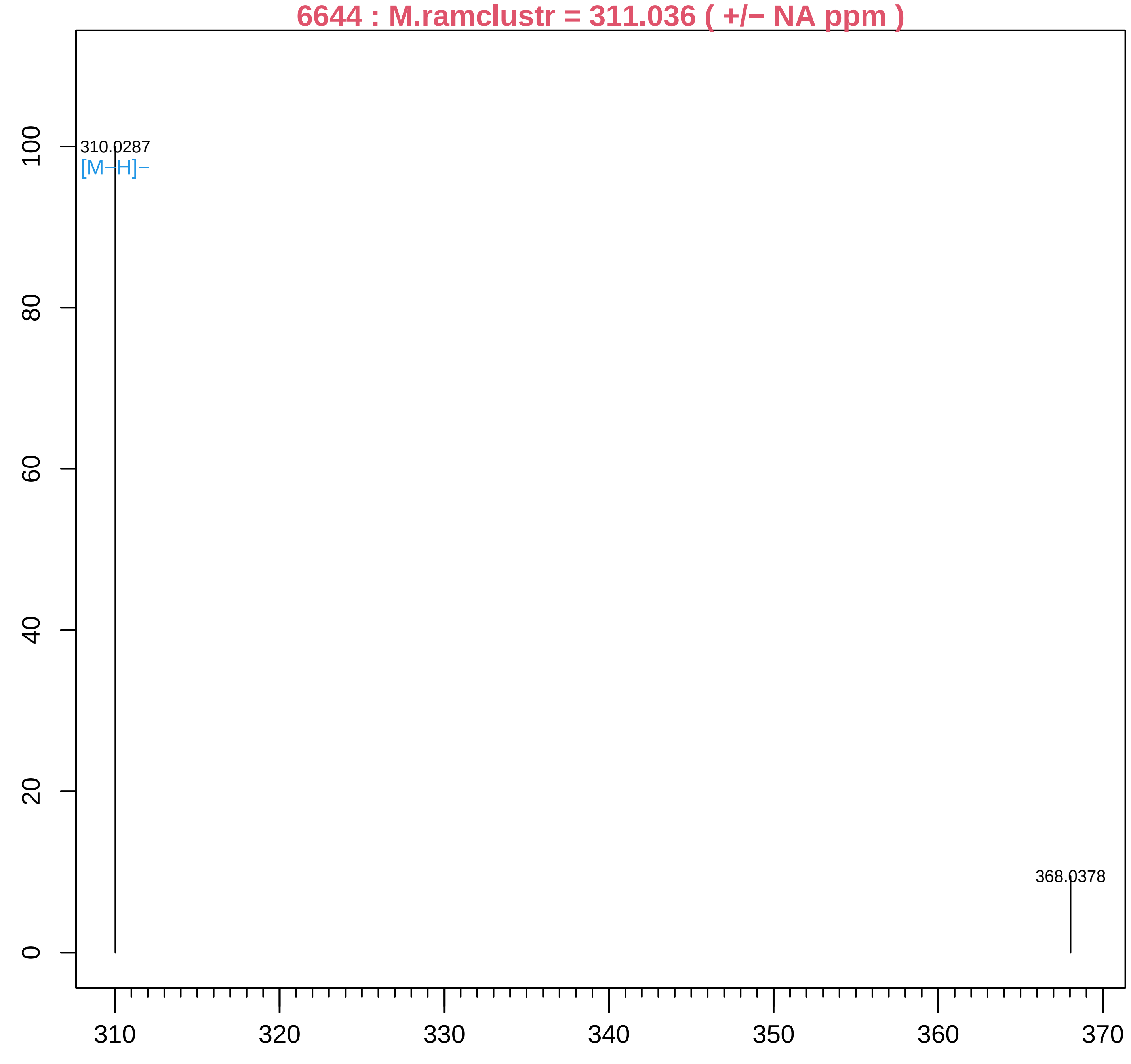
6644 : M.ramclustr = 311.036 (+/− NA ppm)
310.0287
[M−H]−
368.0378
0
20
40
60
80
100
310
320
330
340
350
360
370

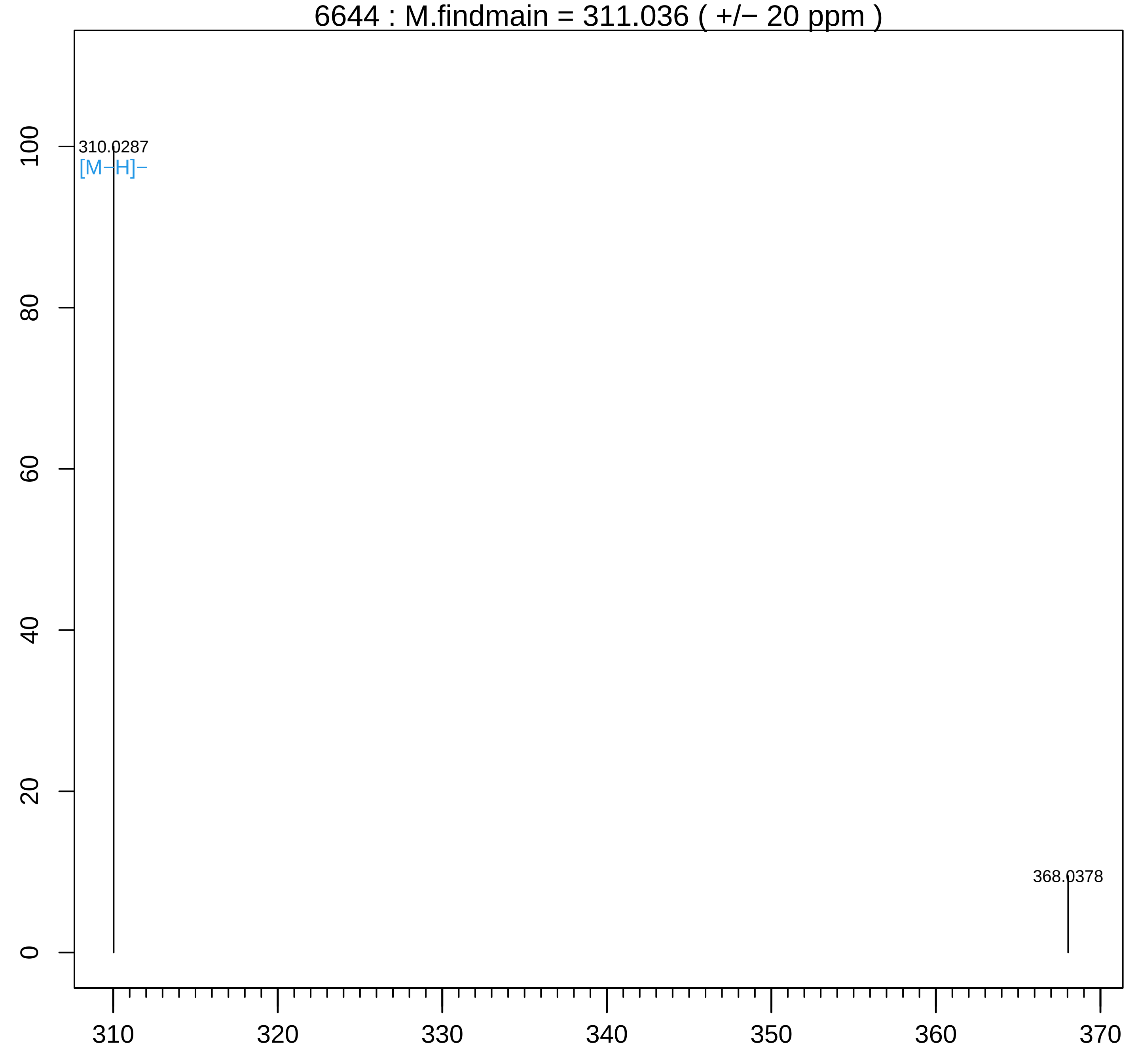
6644 : M.findmain = 311.036 (+/− 20 ppm)
310.0287
[M−H]−
368.0378
0
20
40
60
80
100
310
320
330
340
350
360
370

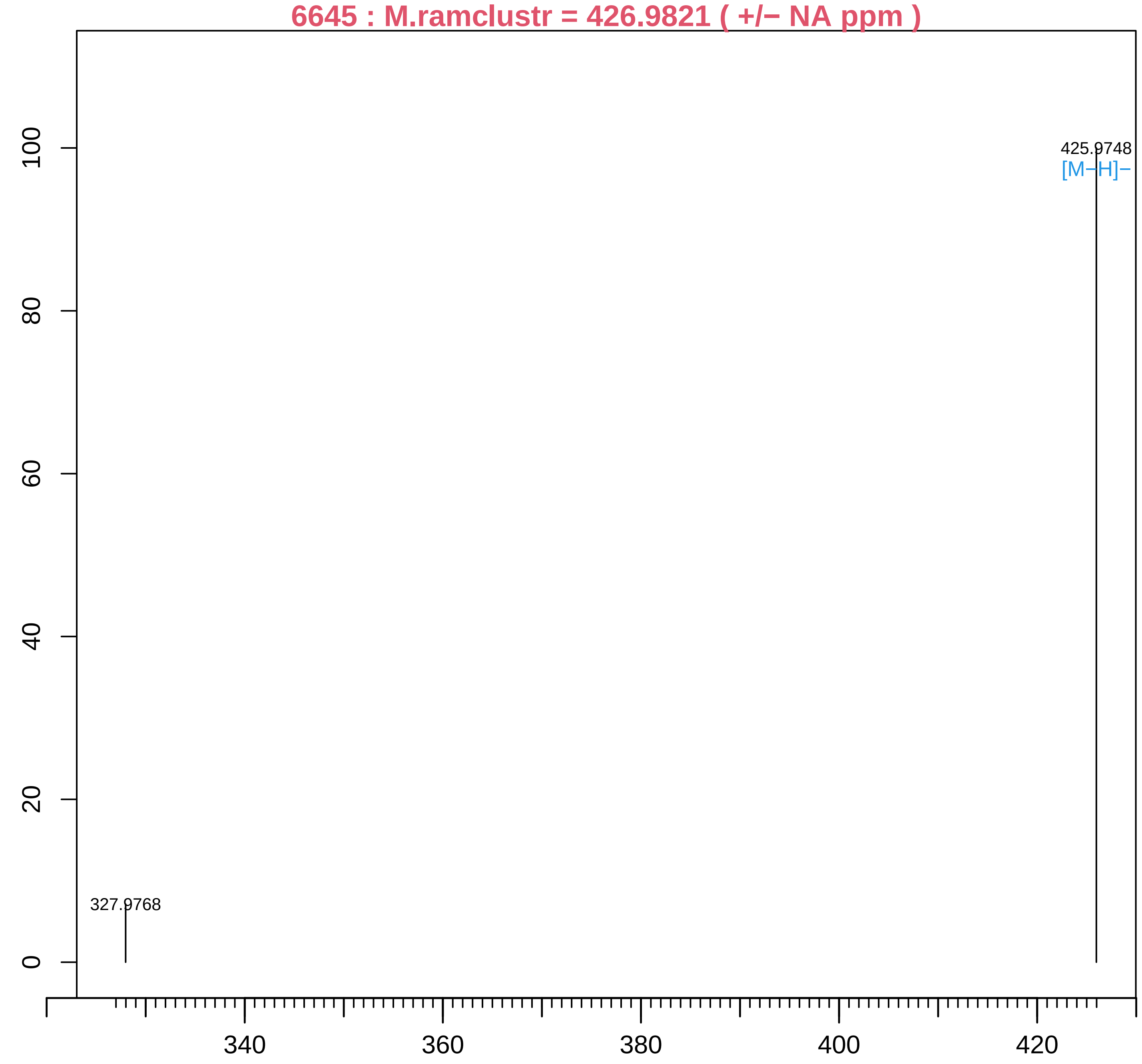
6645 : M.ramclustr = 426.9821 (+/− NA ppm)
425.9748
[M−H]−
327.9768
0
20
40
60
80
100
340
360
380
400
420

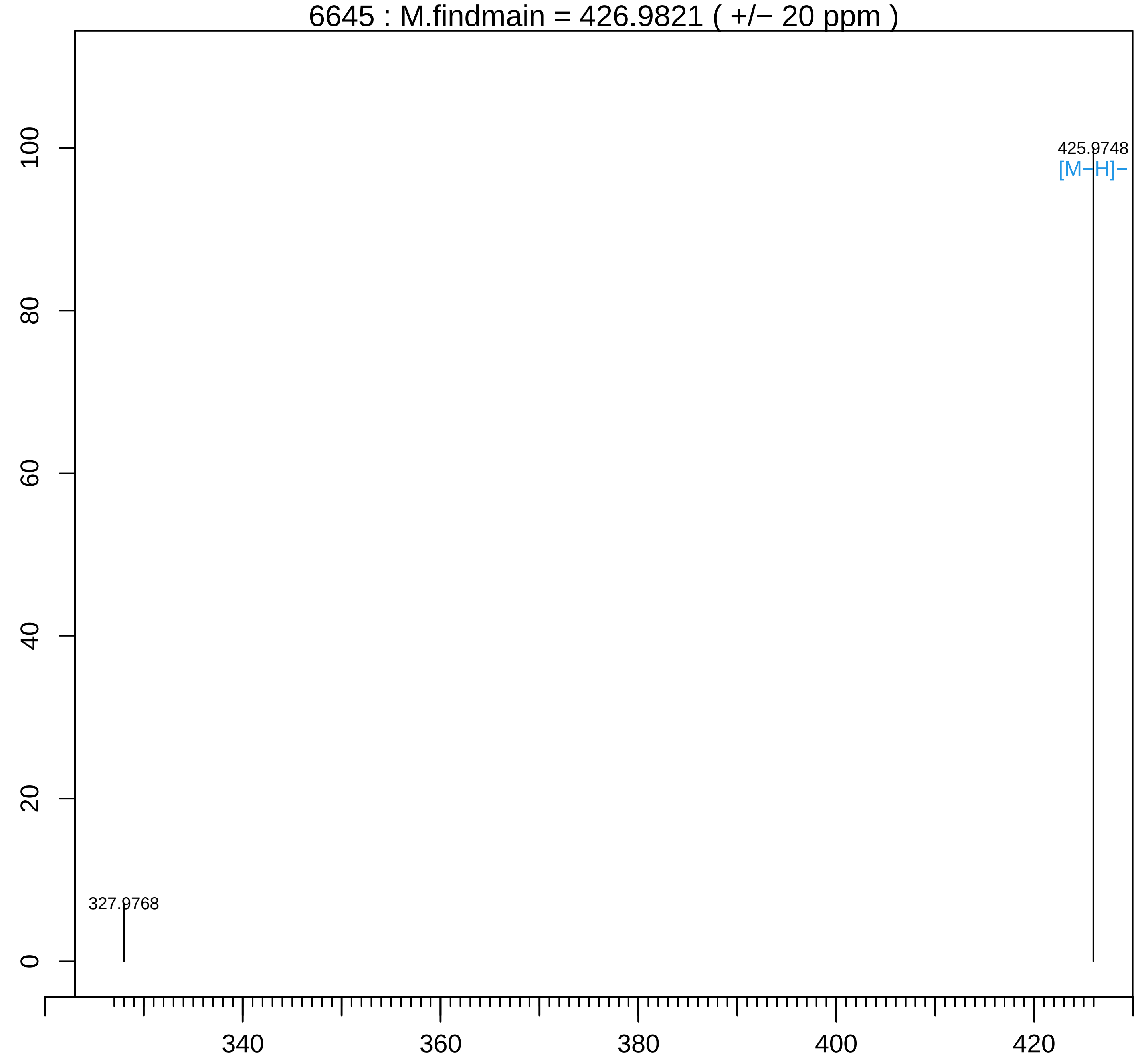
6645 : M.findmain = 426.9821 (+/− 20 ppm)
425.9748
[M−H]−
327.9768
0
20
40
60
80
100
340
360
380
400
420

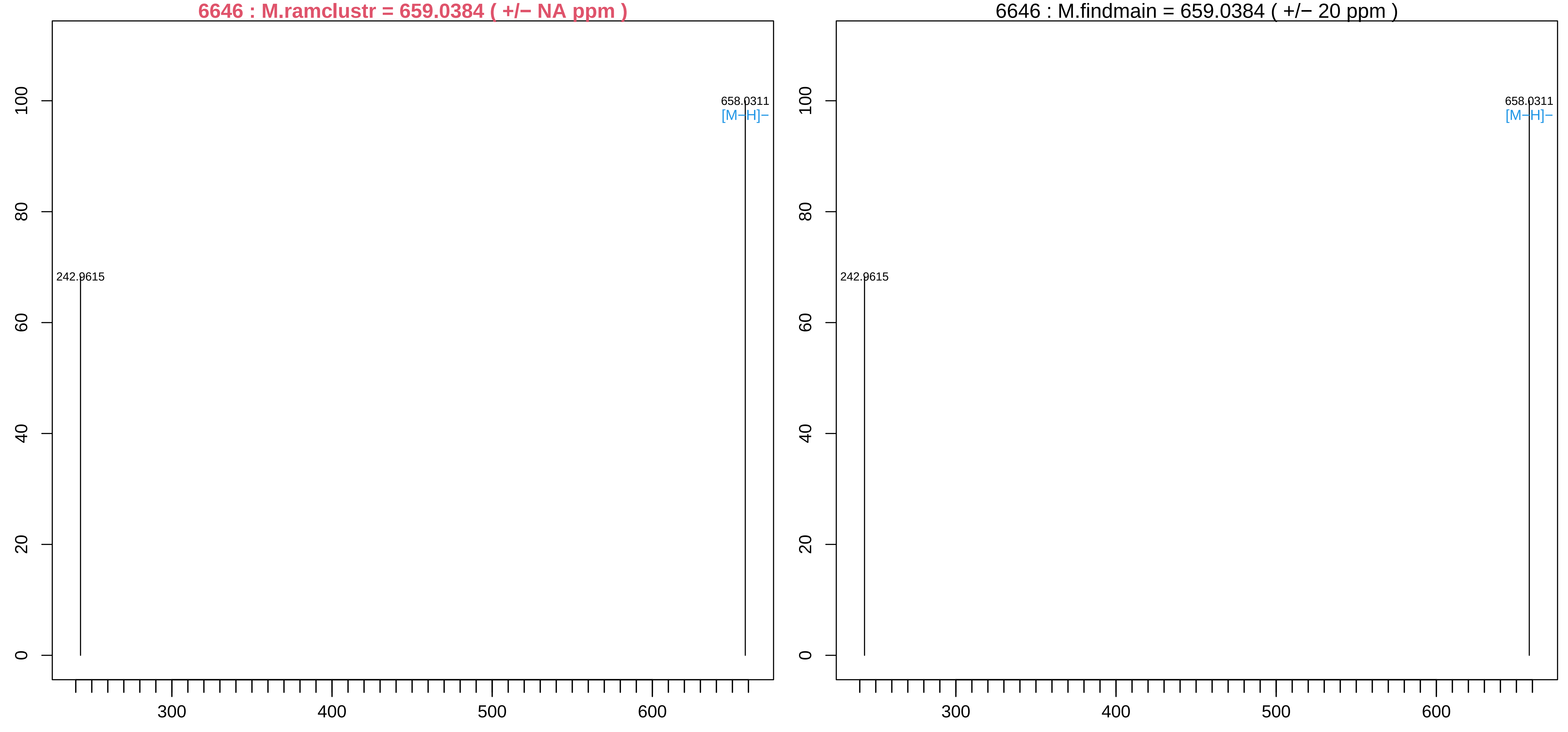
6646 : M.ramclustr = 659.0384 (+/− NA ppm)
6646 : M.findmain = 659.0384 (+/− 20 ppm)
658.0311
[M−H]−
242.9615
658.0311
[M−H]−
242.9615
0
20
40
60
80
100
300
400
500
600

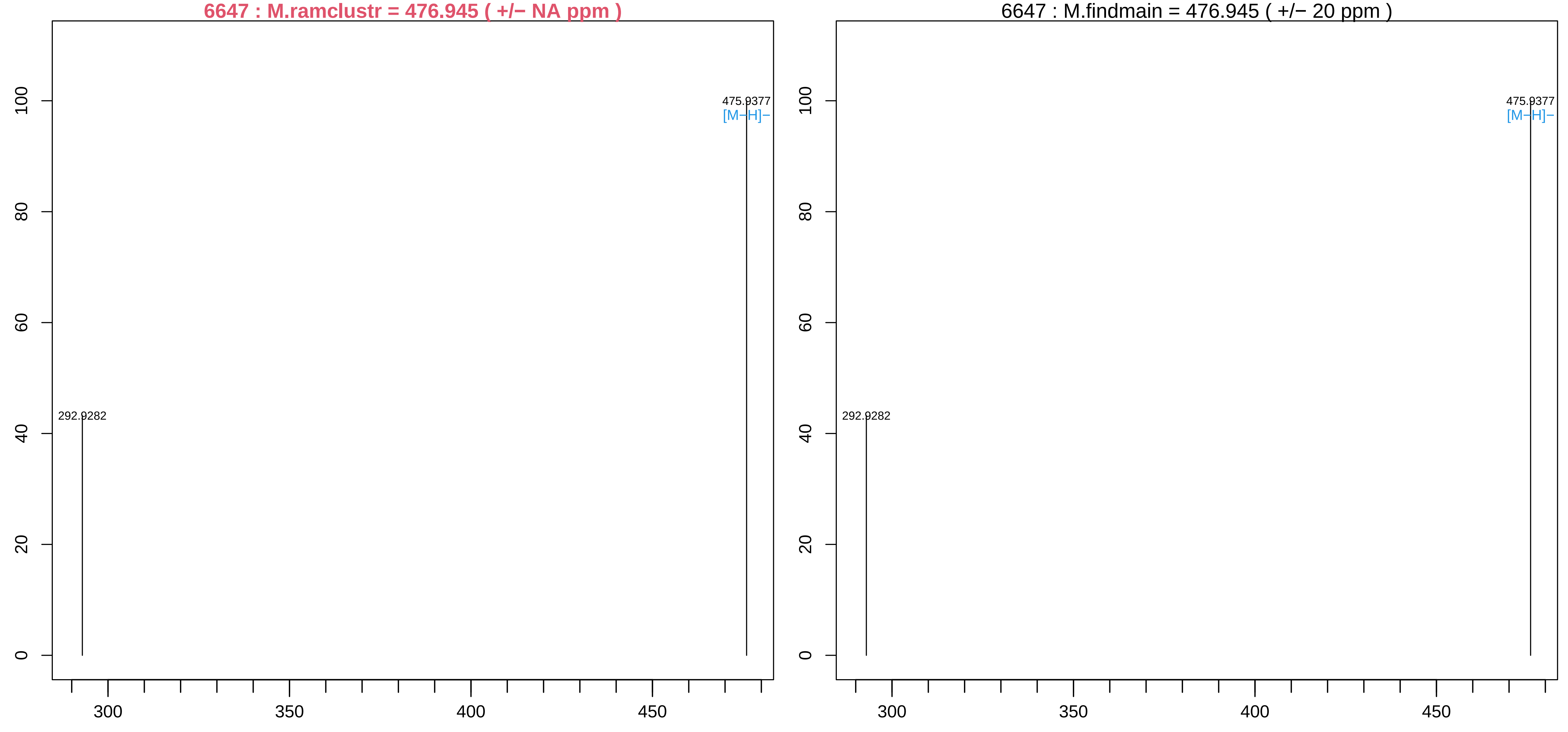
6647 : M.ramclustr = 476.945 (+/− NA ppm)
475.9377
[M−H]−
292.9282
6647 : M.findmain = 476.945 (+/− 20 ppm)
475.9377
[M−H]−
292.9282
0
20
40
60
80
100
300
350
400
450

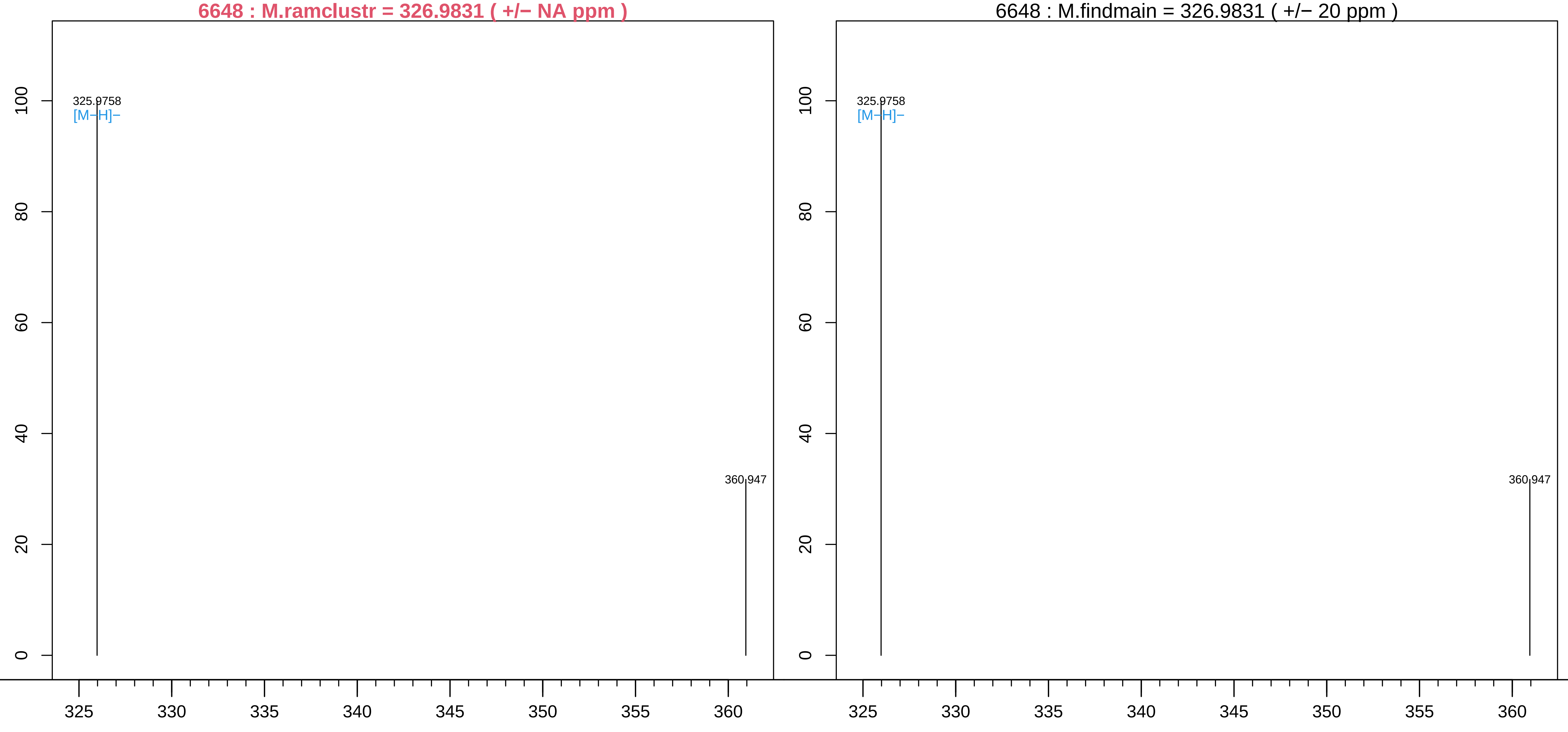

6648 : M.ramclustr = 326.9831 (+/− NA ppm)
325.9758
[M−H]−
360.947
6648 : M.findmain = 326.9831 (+/− 20 ppm)
325.9758
[M−H]−
360.947
0
20
40
60
80
100
325
330
335
340
345
350
355
360

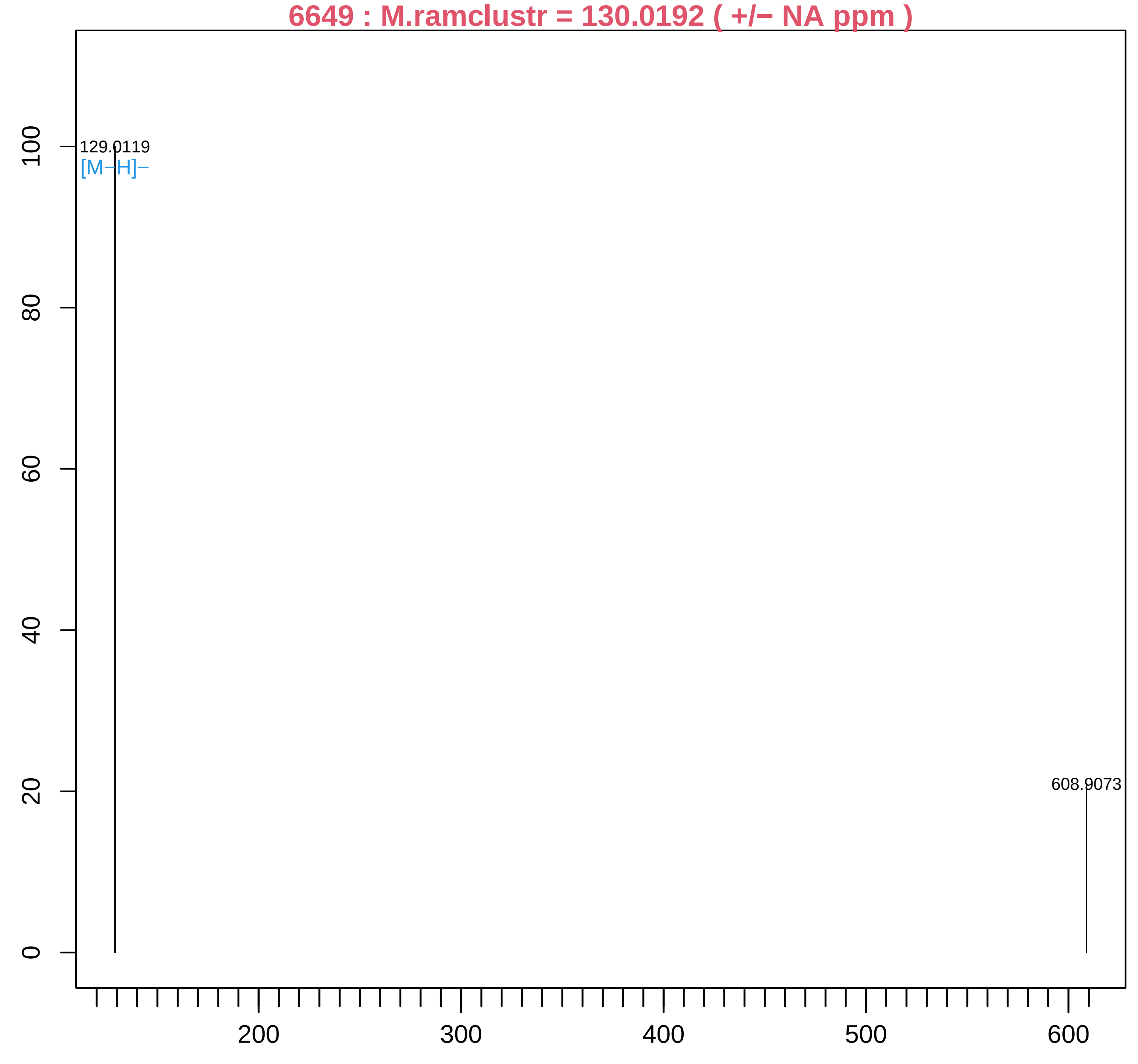
6649 : M.ramclustr = 130.0192 (+/− NA ppm)
129.0119
[M−H]−
608.9073
0
20
40
60
80
100
200
300
400
500
600

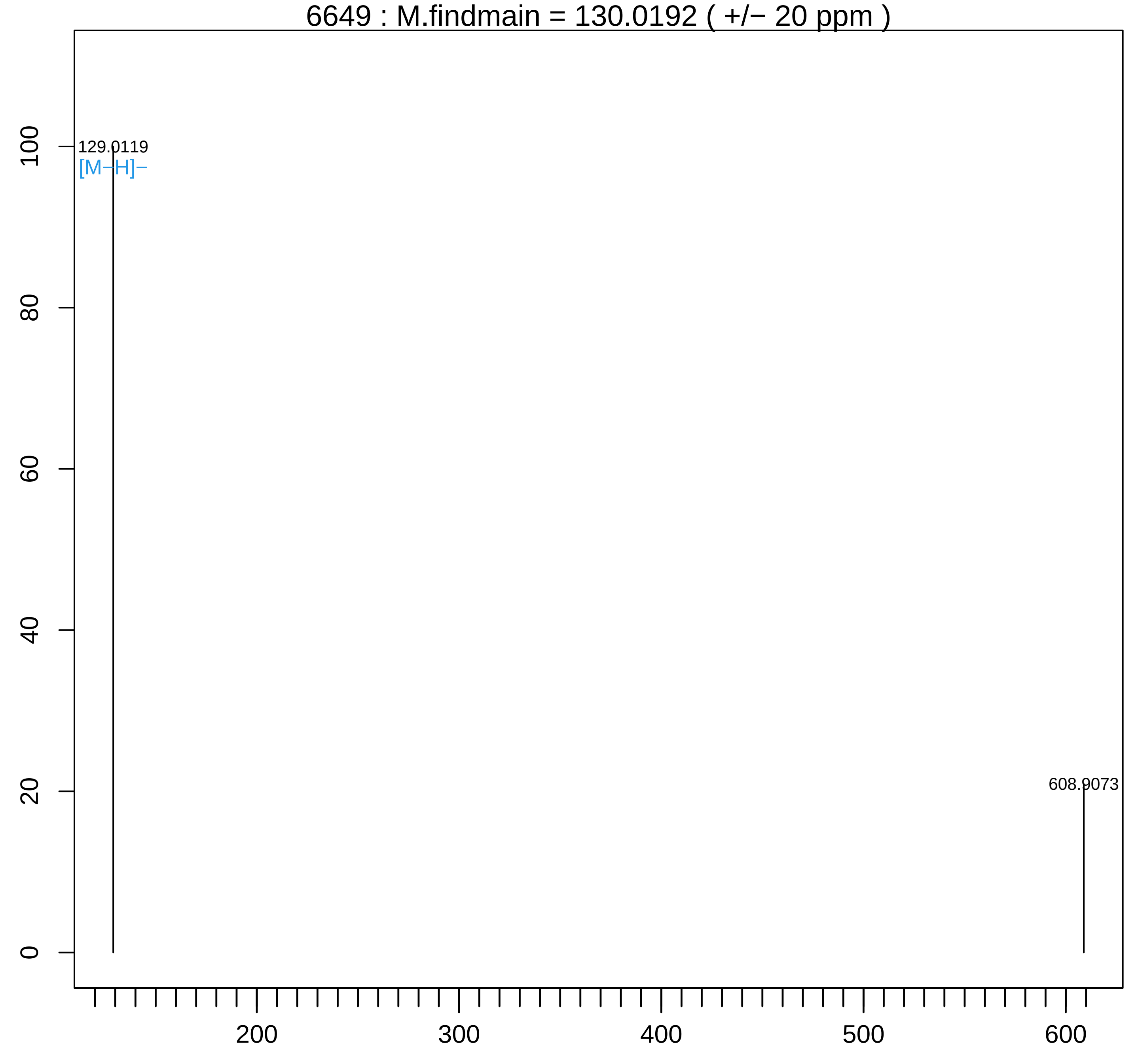
6649 : M.findmain = 130.0192 (+/− 20 ppm)
129.0119
[M−H]−
608.9073
0
20
40
60
80
100
200
300
400
500
600

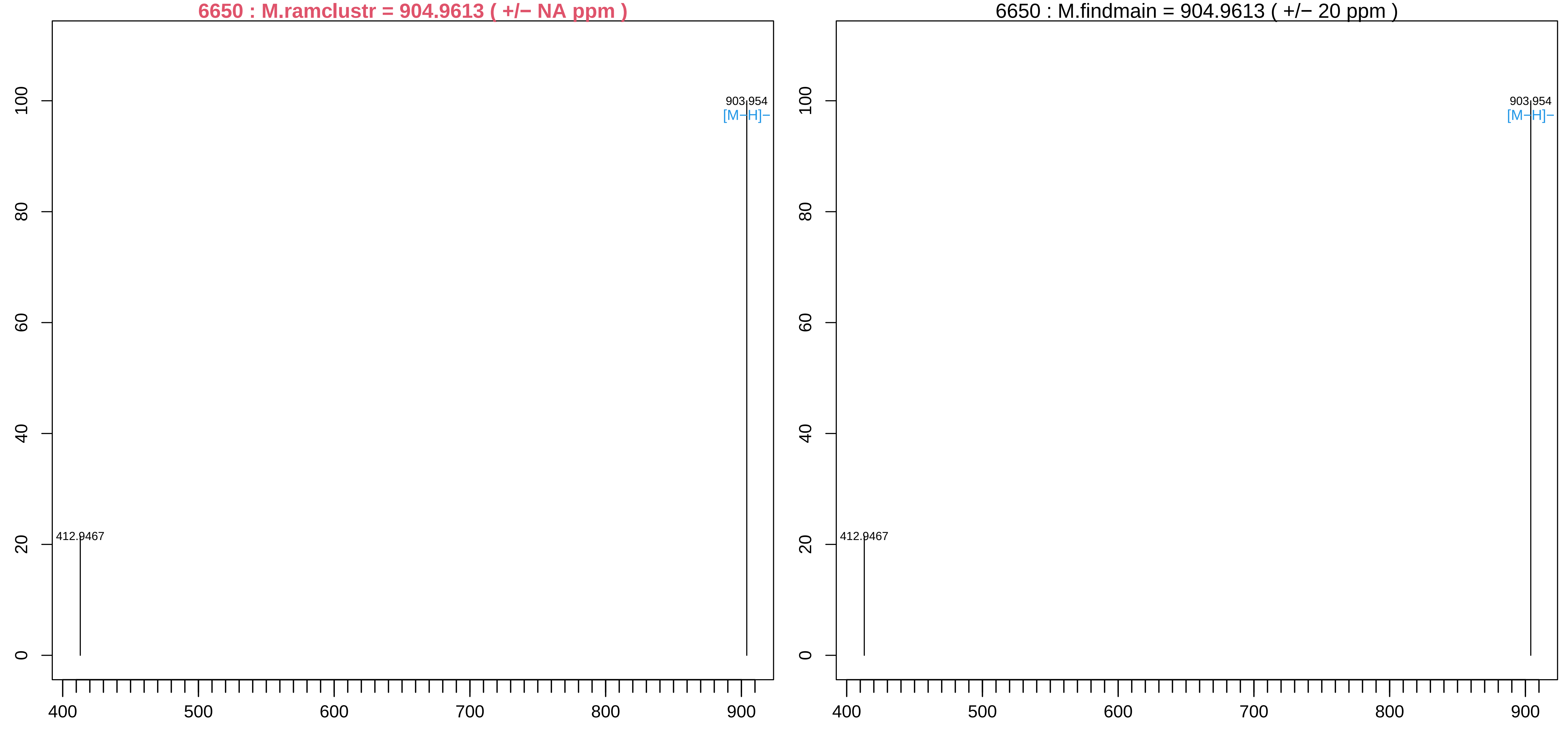
6650 : M.ramclustr = 904.9613 (+/− NA ppm)
6650 : M.findmain = 904.9613 (+/− 20 ppm)
903.954
[M−H]−
412.9467
0
20
40
60
80
100
400
500
600
700
800
900

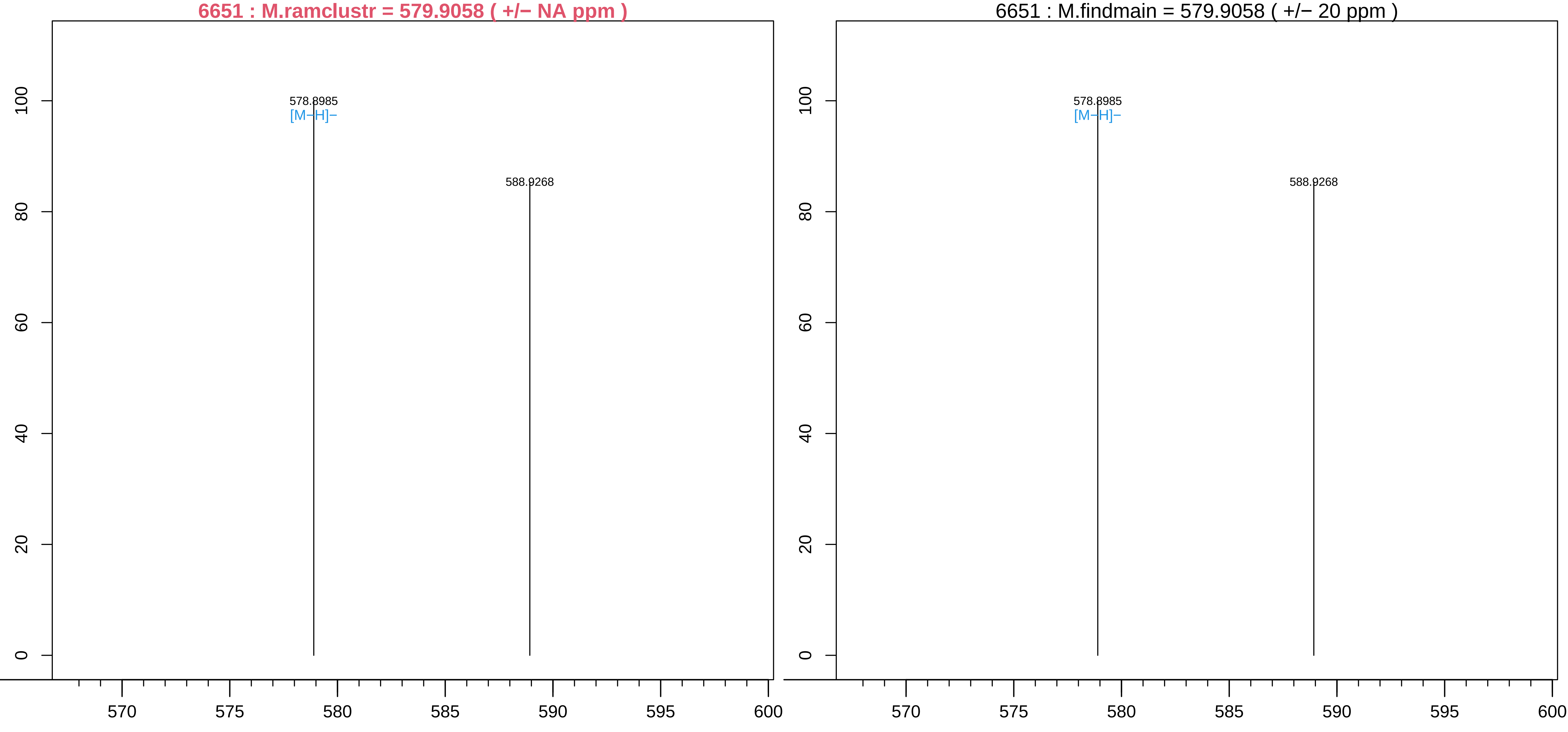

6651 : M.ramclustr = 579.9058 (+/− NA ppm)
578.8985
[M−H]−
588.9268
6651 : M.findmain = 579.9058 (+/− 20 ppm)
578.8985
[M−H]−
588.9268
0
20
40
60
80
100
570
575
580
585
590
595
600

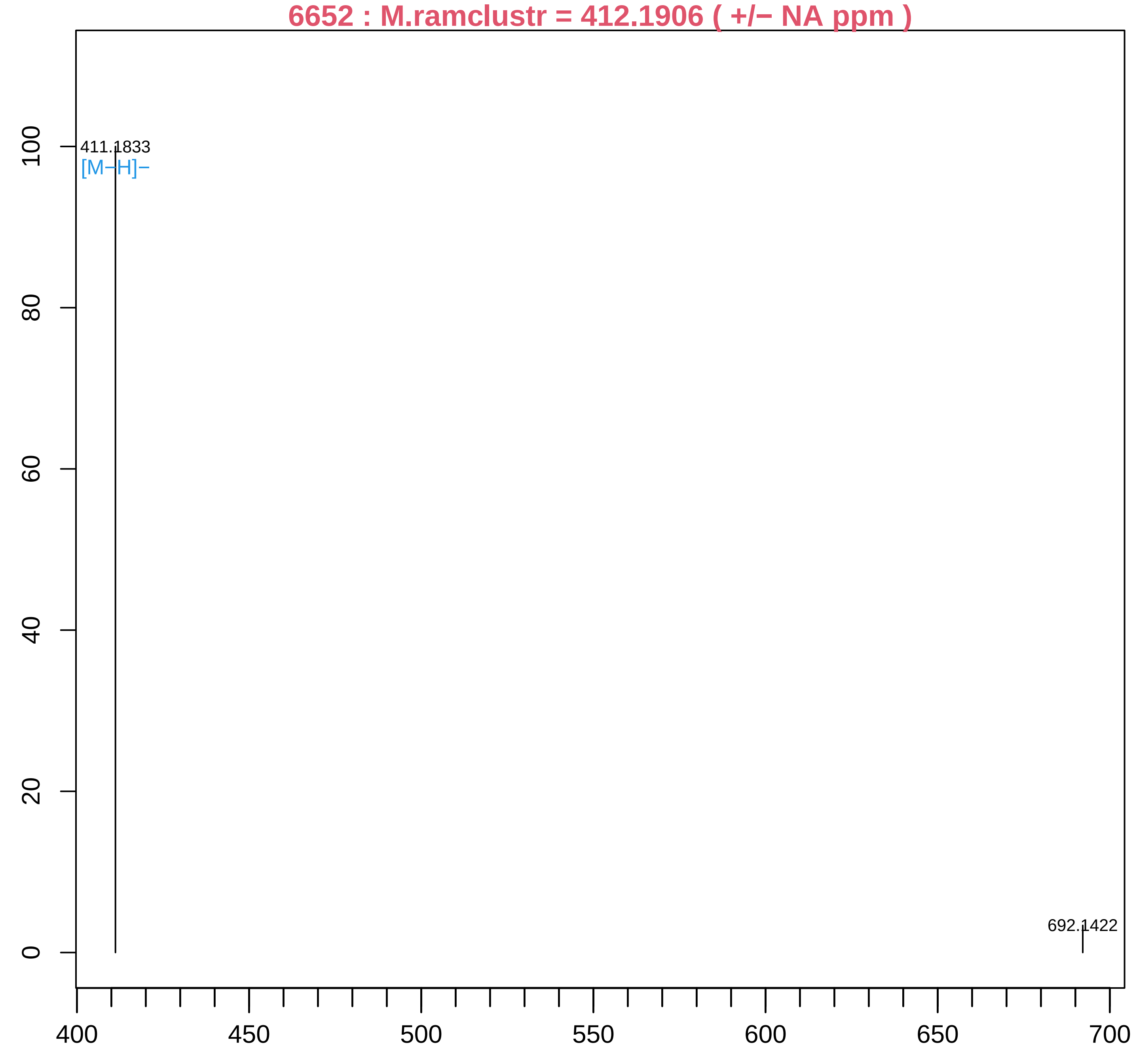

6652 : M.ramclustr = 412.1906 (+/− NA ppm)
411.1833
[M−H]−
692.1422
0
20
40
60
80
100
400
450
500
550
600
650
700

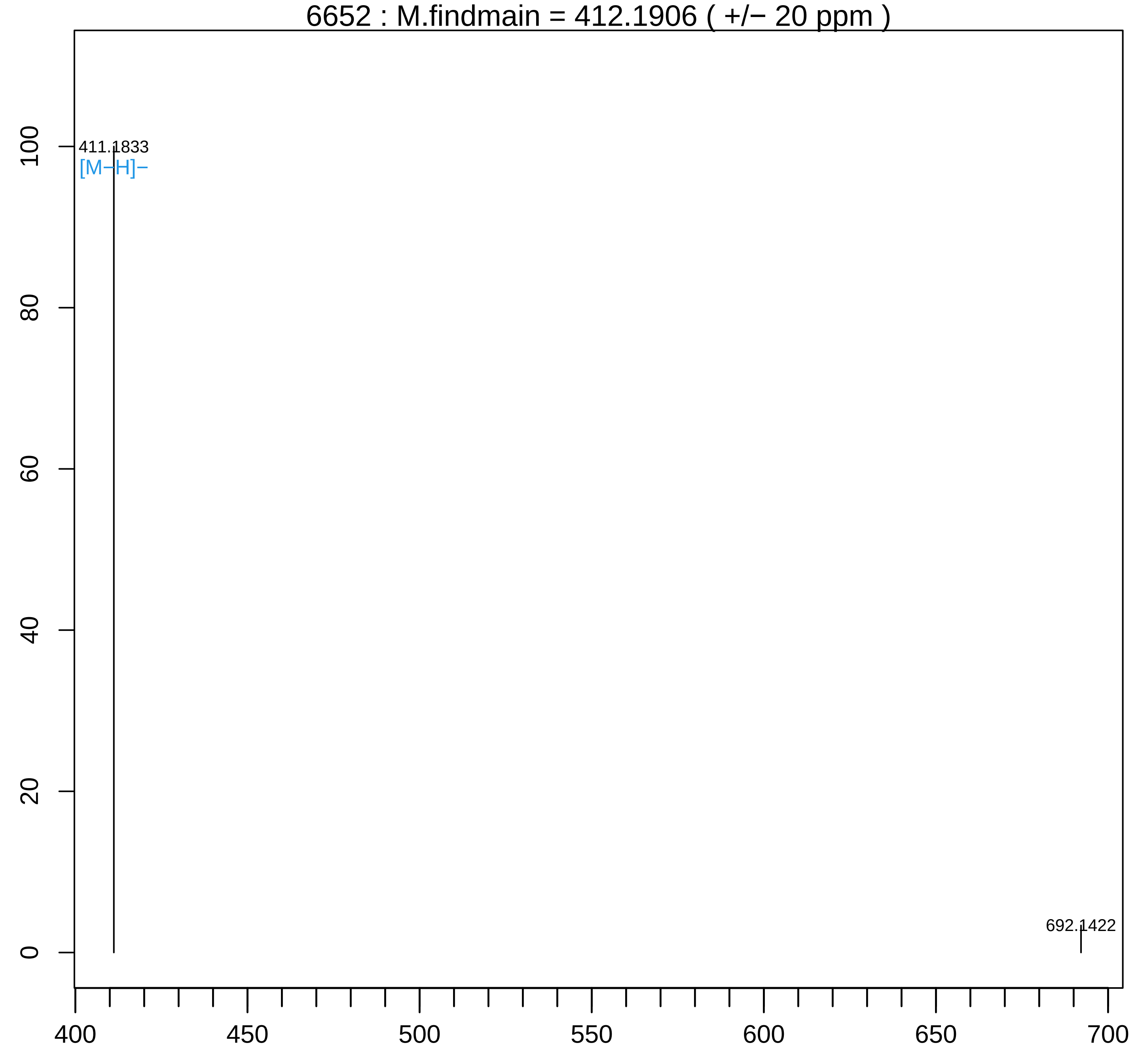

6652 : M.findmain = 412.1906 (+/− 20 ppm)
411.1833
[M−H]−
692.1422
0
20
40
60
80
100
400
450
500
550
600
650
700

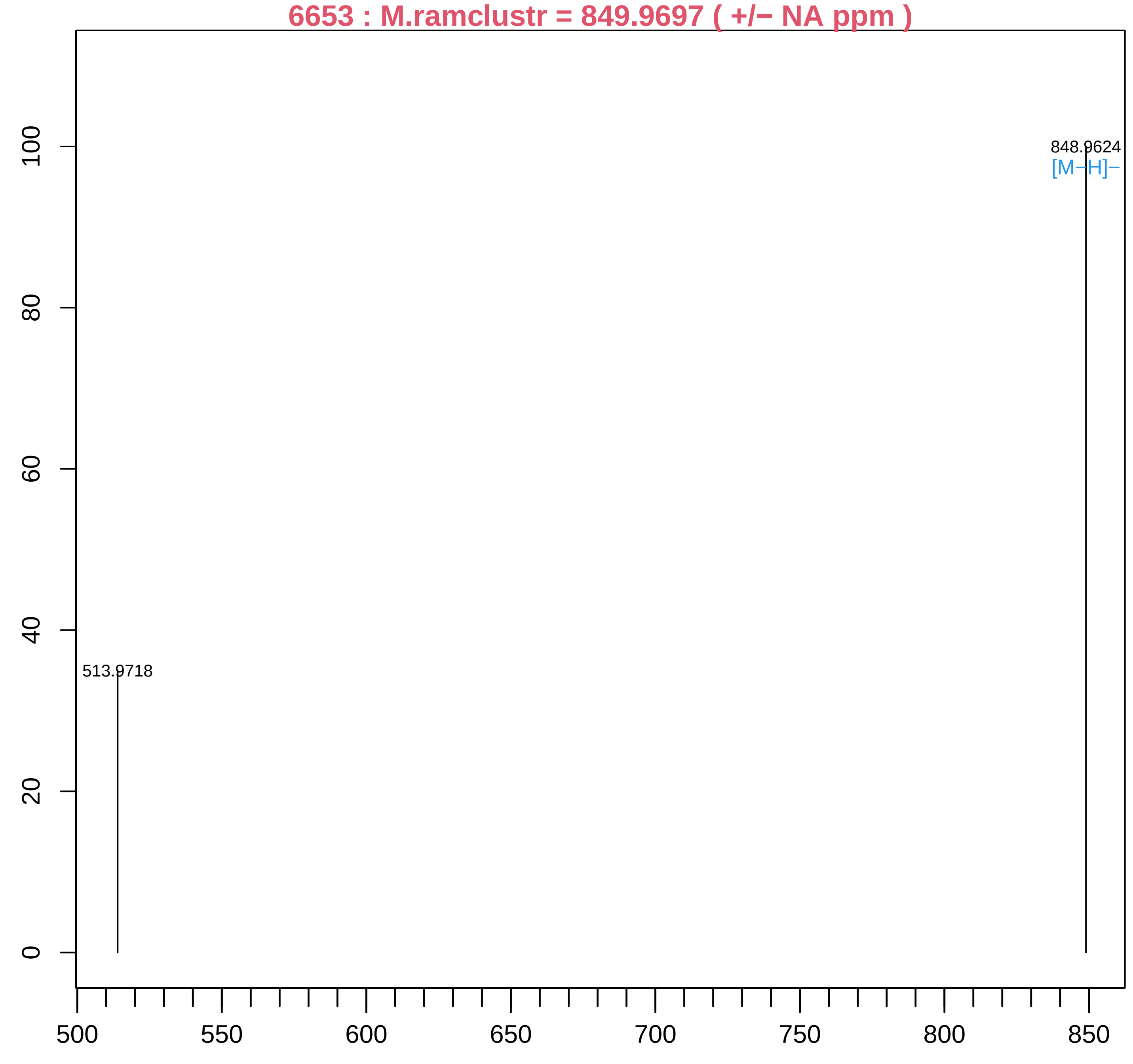
6653 : M.ramclustr = 849.9697 (+/− NA ppm)
848.9624
[M−H]−
513.9718
0
20
40
60
80
100
500
550
600
650
700
750
800
850

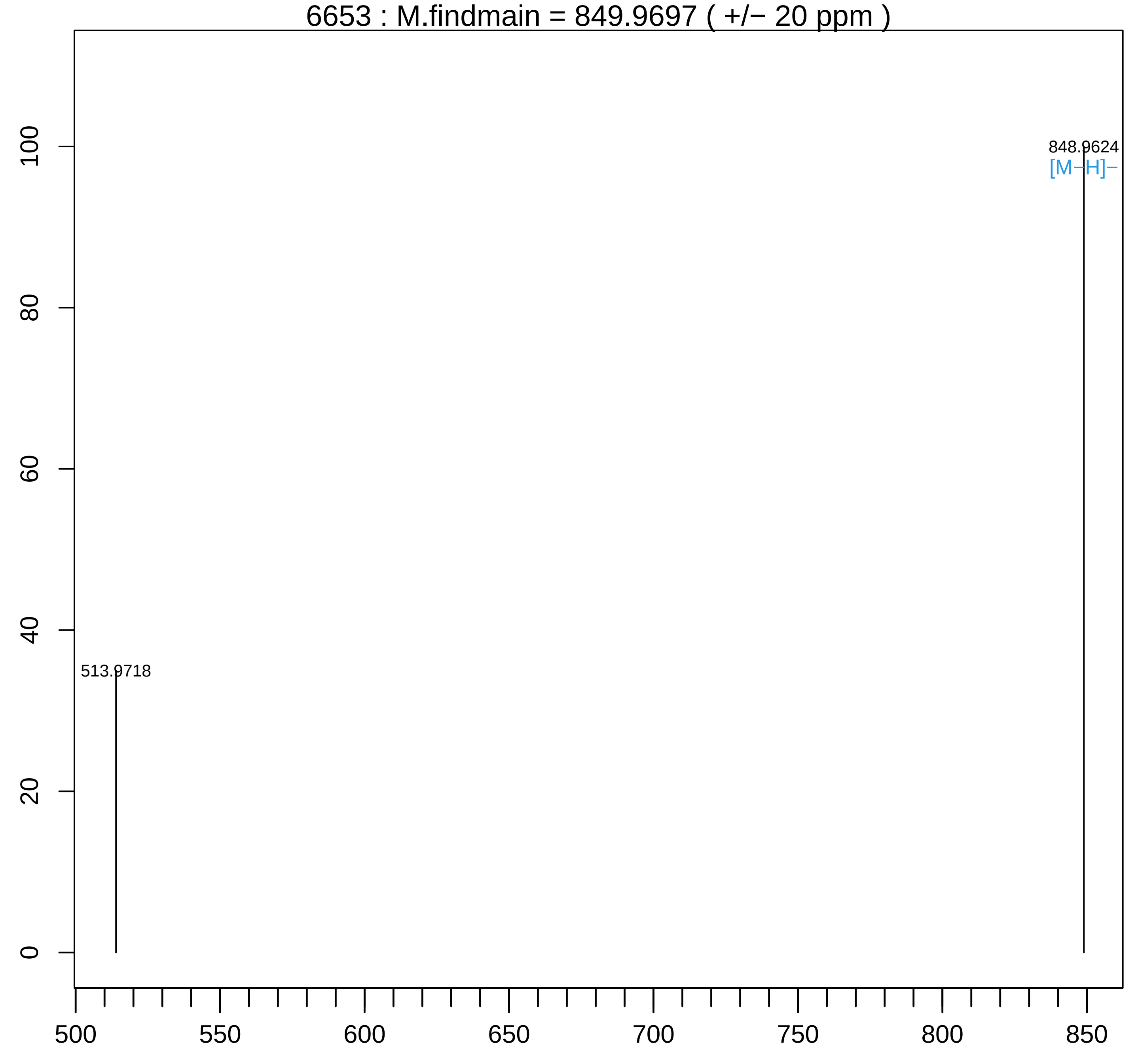
6653 : M.findmain = 849.9697 (+/− 20 ppm)
848.9624
[M−H]−
513.9718
0
20
40
60
80
100
500
550
600
650
700
750
800
850

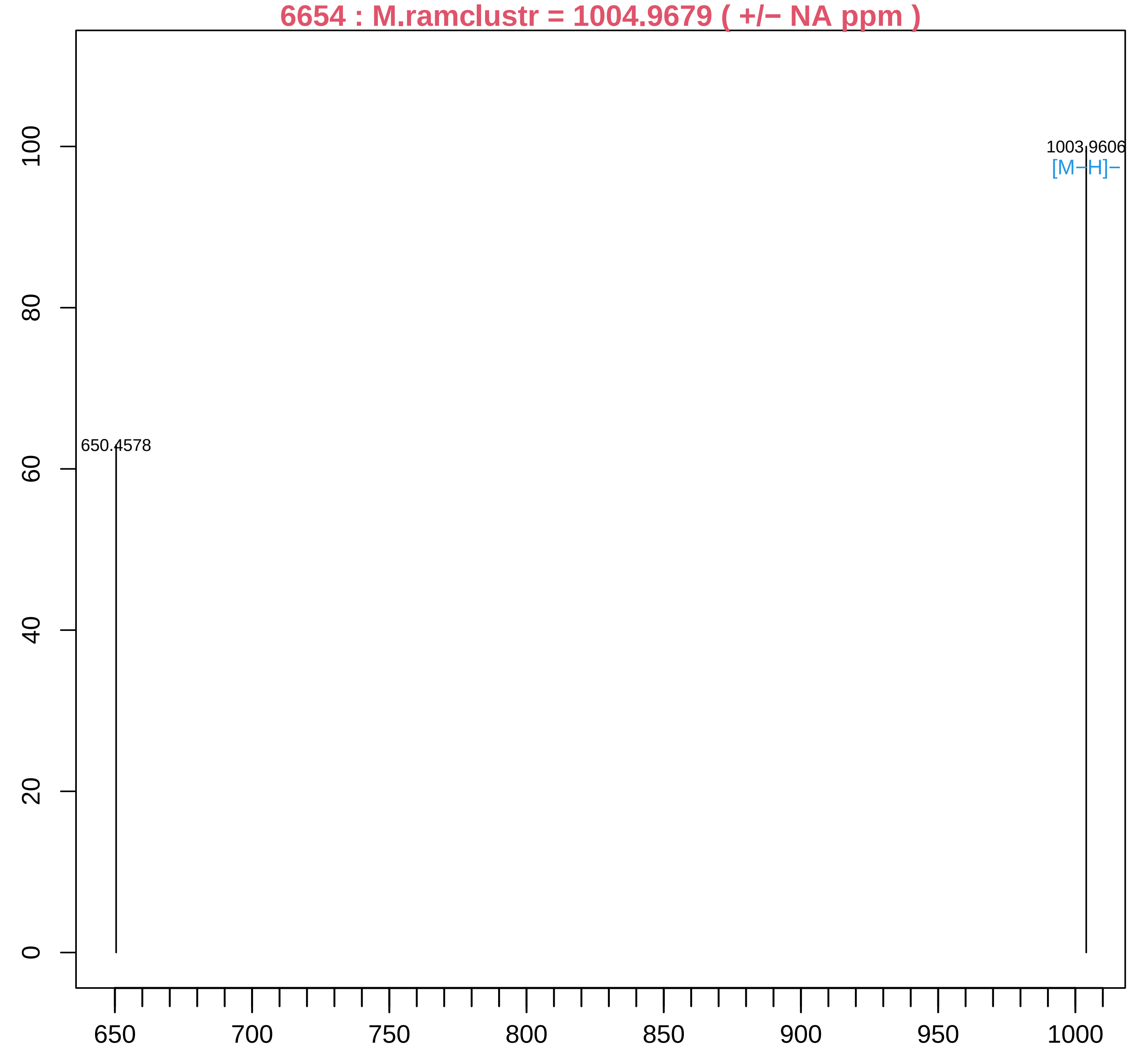

6654 : M.ramclustr = 1004.9679 (+/− NA ppm)
1003.9606
[M−H]−
650.4578
0
20
40
60
80
100
650
700
750
800
850
900
950
1000

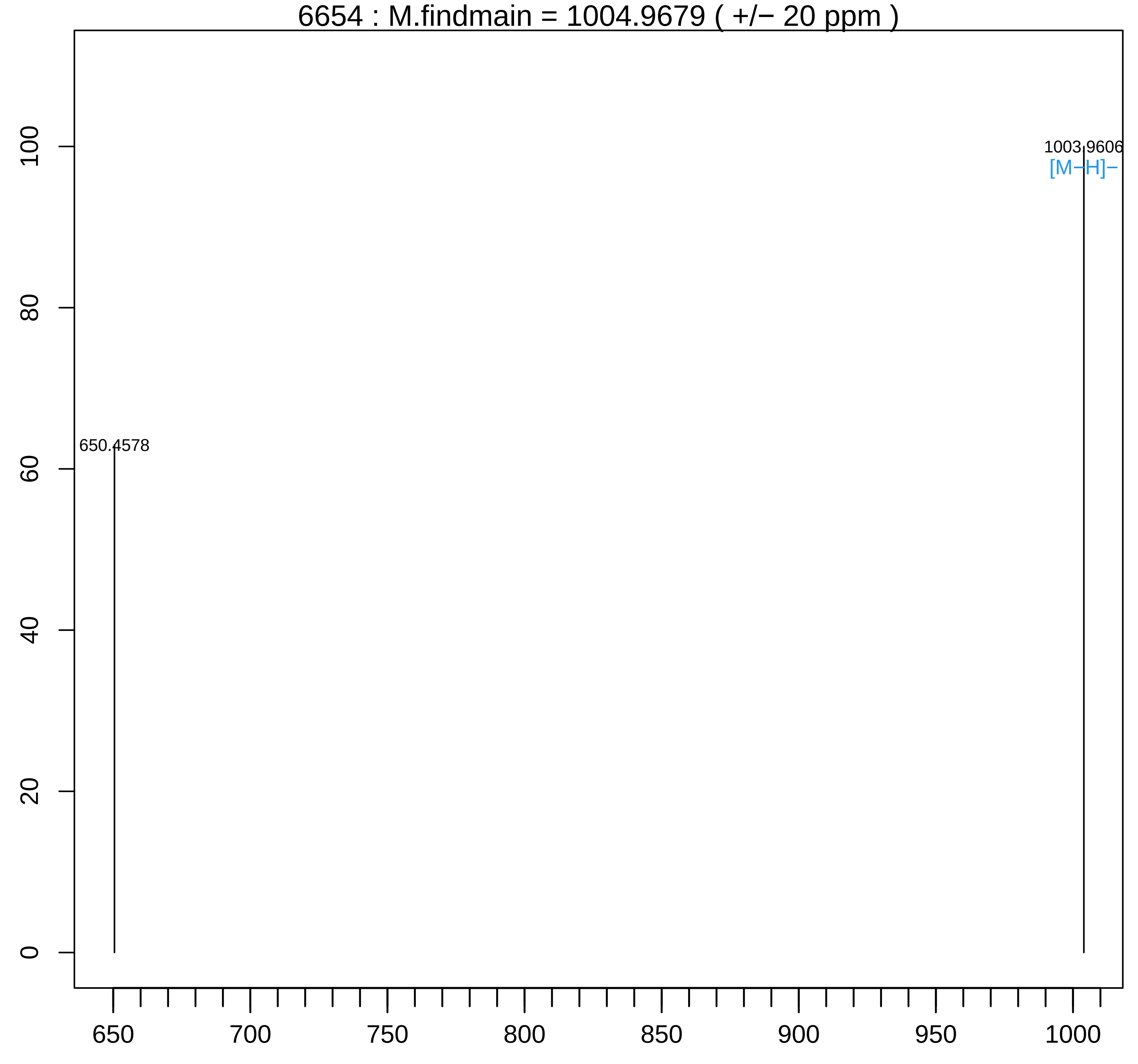

6654 : M.findmain = 1004.9679 (+/− 20 ppm)
1003.9606
[M−H]−
650.4578
0
20
40
60
80
100
650
700
750
800
850
900
950
1000

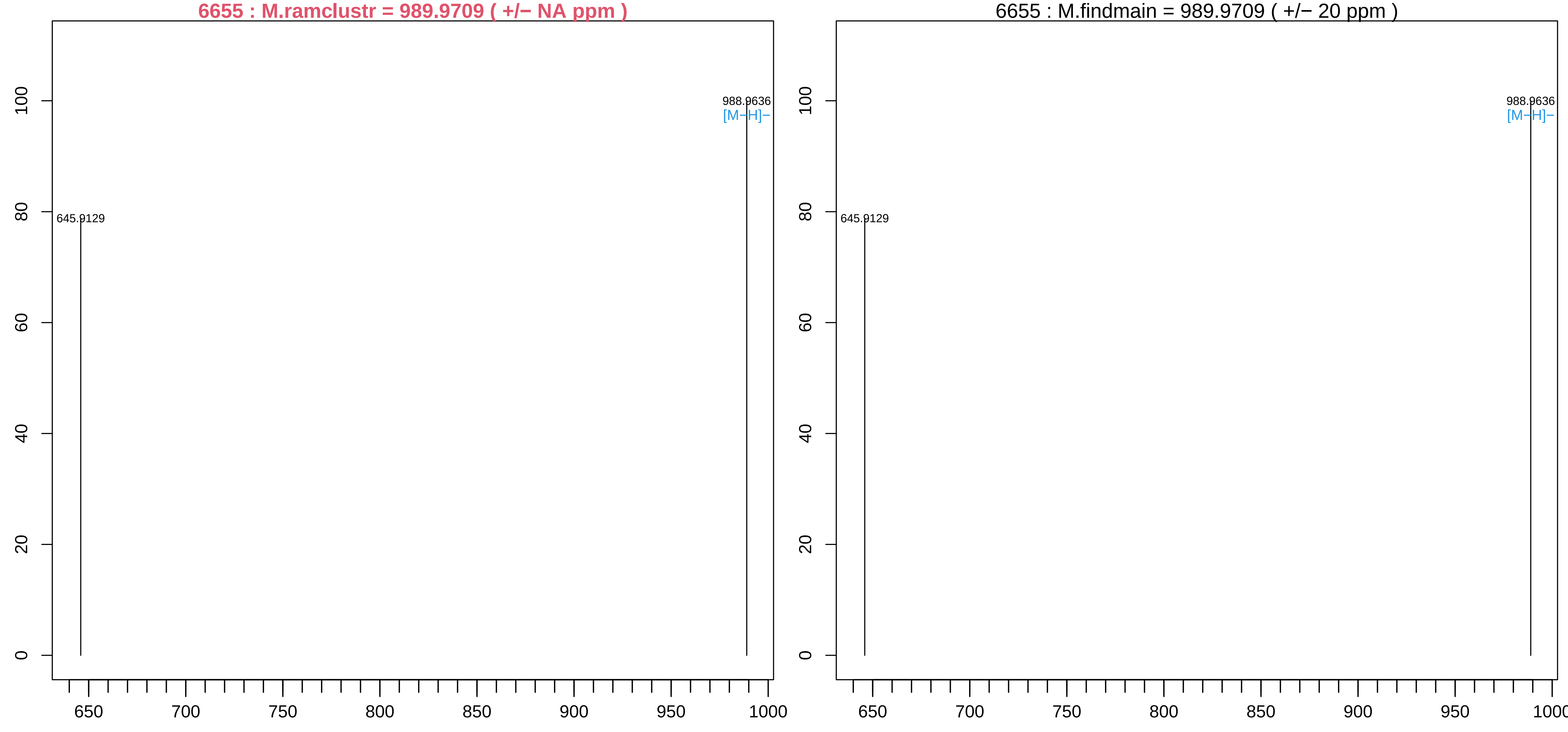

6655 : M.ramclustr = 989.9709 (+/− NA ppm)
645.9129
988.9636
[M−H]−
0
20
40
60
80
100
650
700
750
800
850
900
950
1000
6655 : M.findmain = 989.9709 (+/− 20 ppm)
645.9129
988.9636
[M−H]−
0
20
40
60
80
100
650
700
750
800
850
900
950
1000

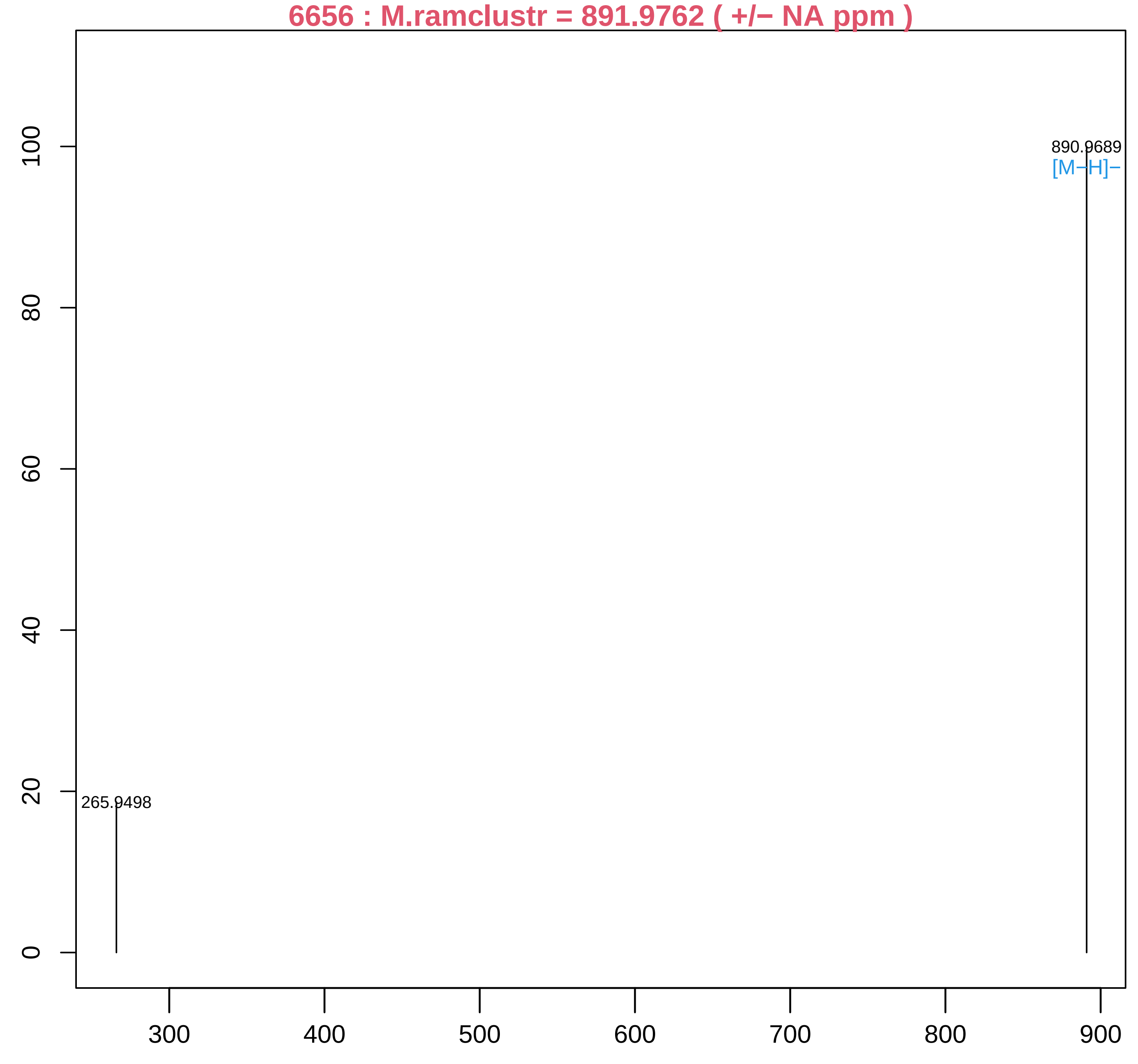
6656 : M.ramclustr = 891.9762 (+/− NA ppm)
890.9689
[M−H]−
265.9498
0
20
40
60
80
100
300
400
500
600
700
800
900

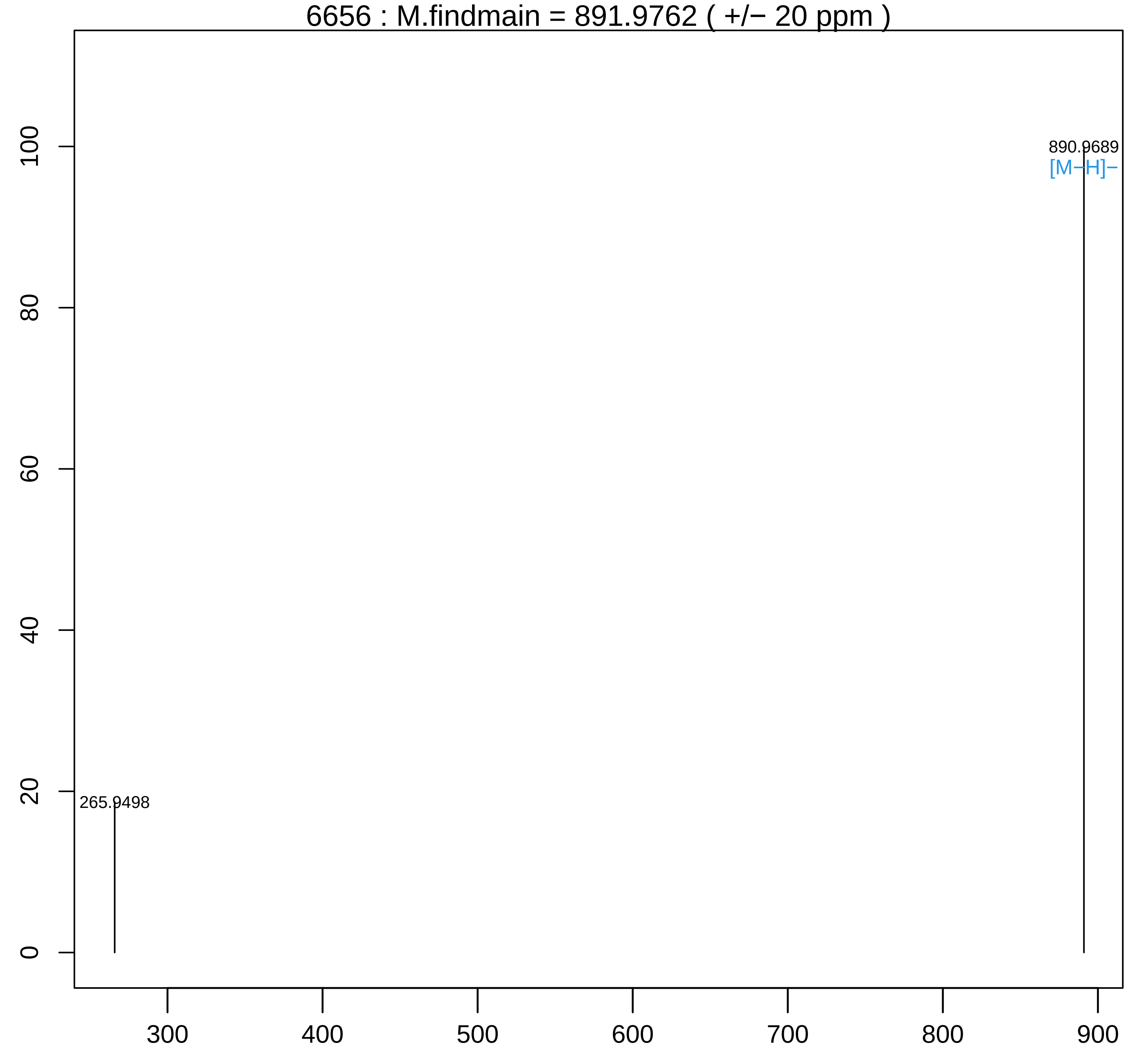
6656 : M.findmain = 891.9762 (+/− 20 ppm)
890.9689
[M−H]−
265.9498
0
20
40
60
80
100
300
400
500
600
700
800
900

6657 : M.ramclustr = 1137.9637 (+/− NA ppm)

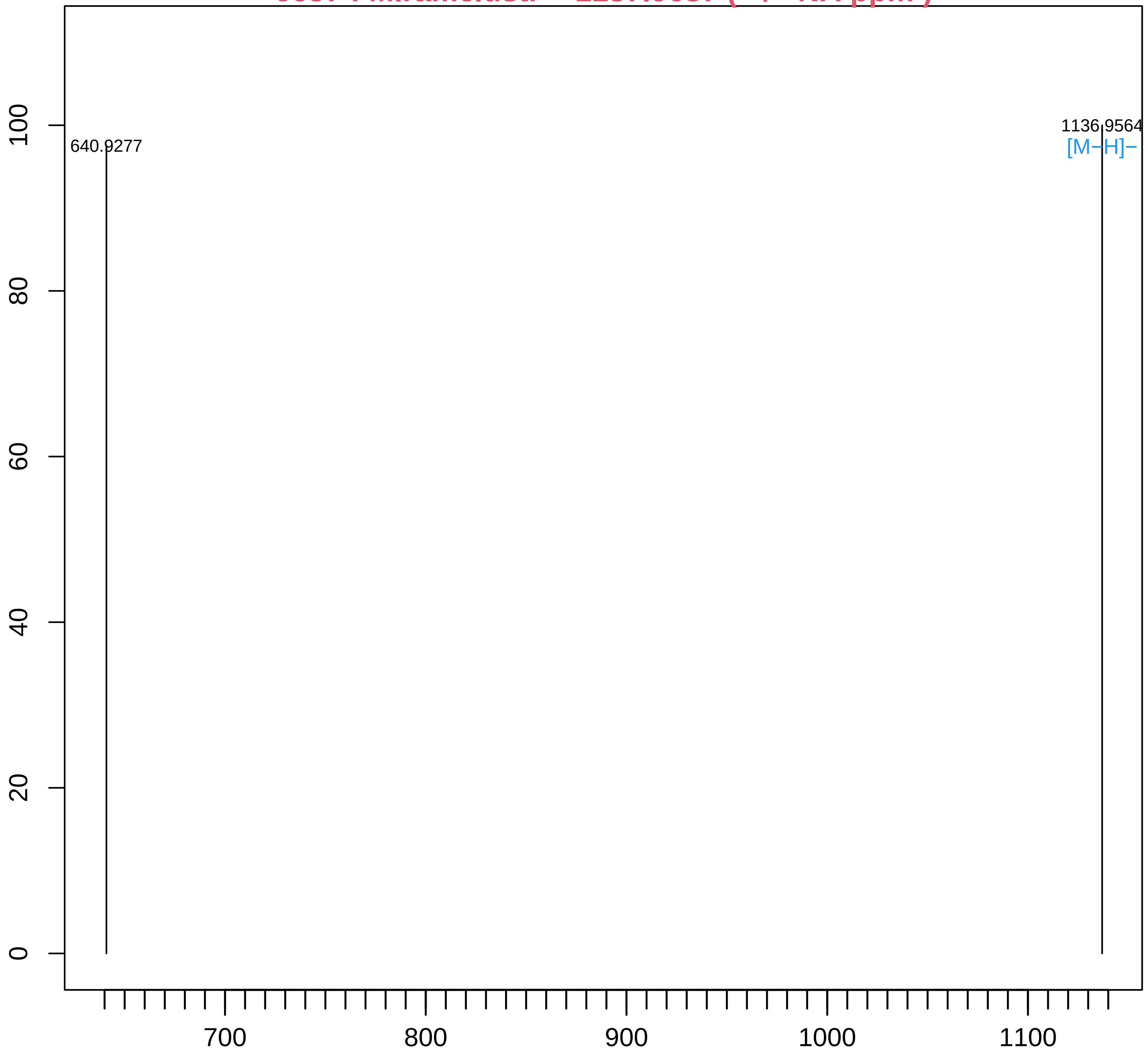

6657 : M.findmain = 1137.9637 (+/− 20 ppm)

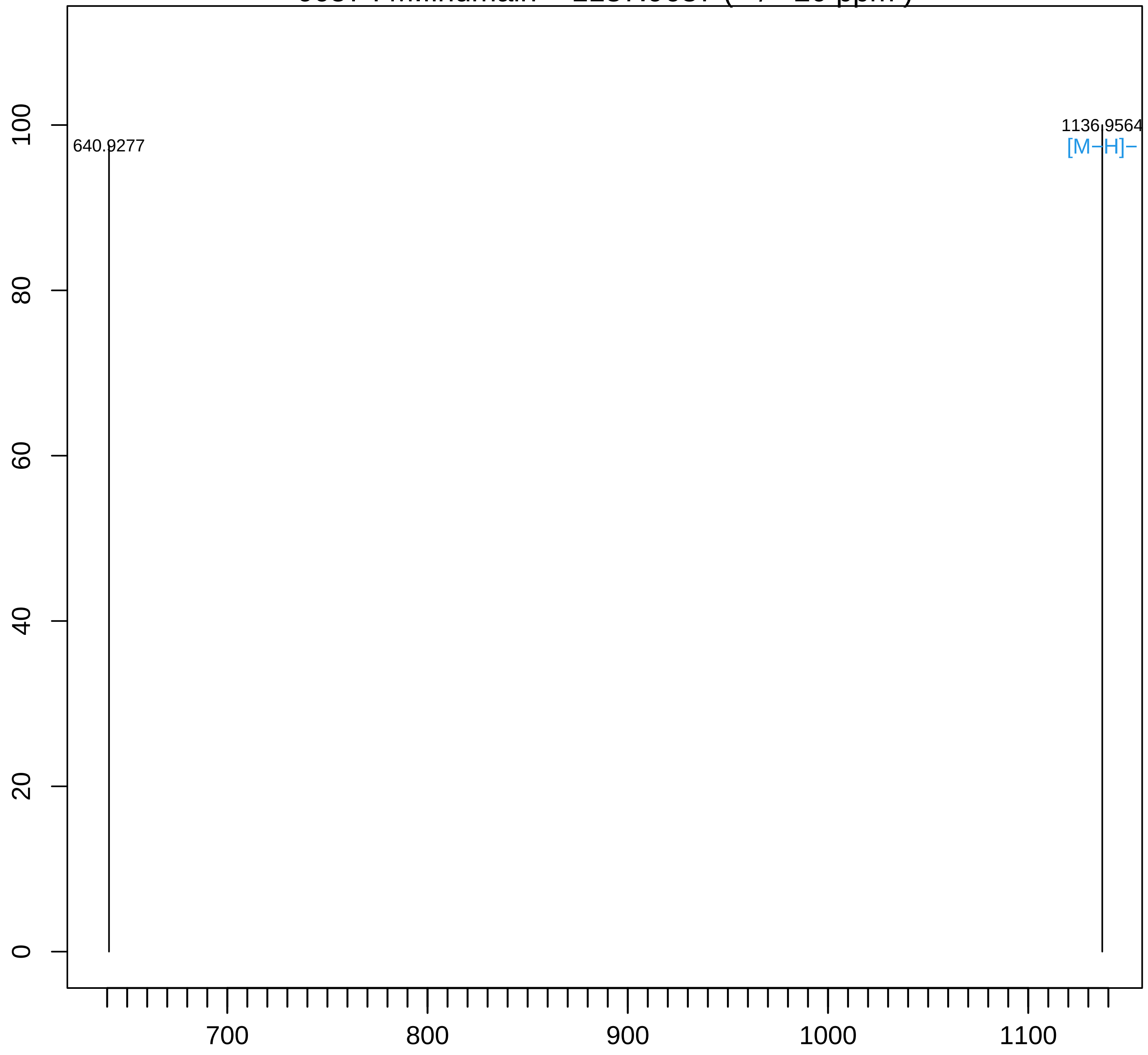

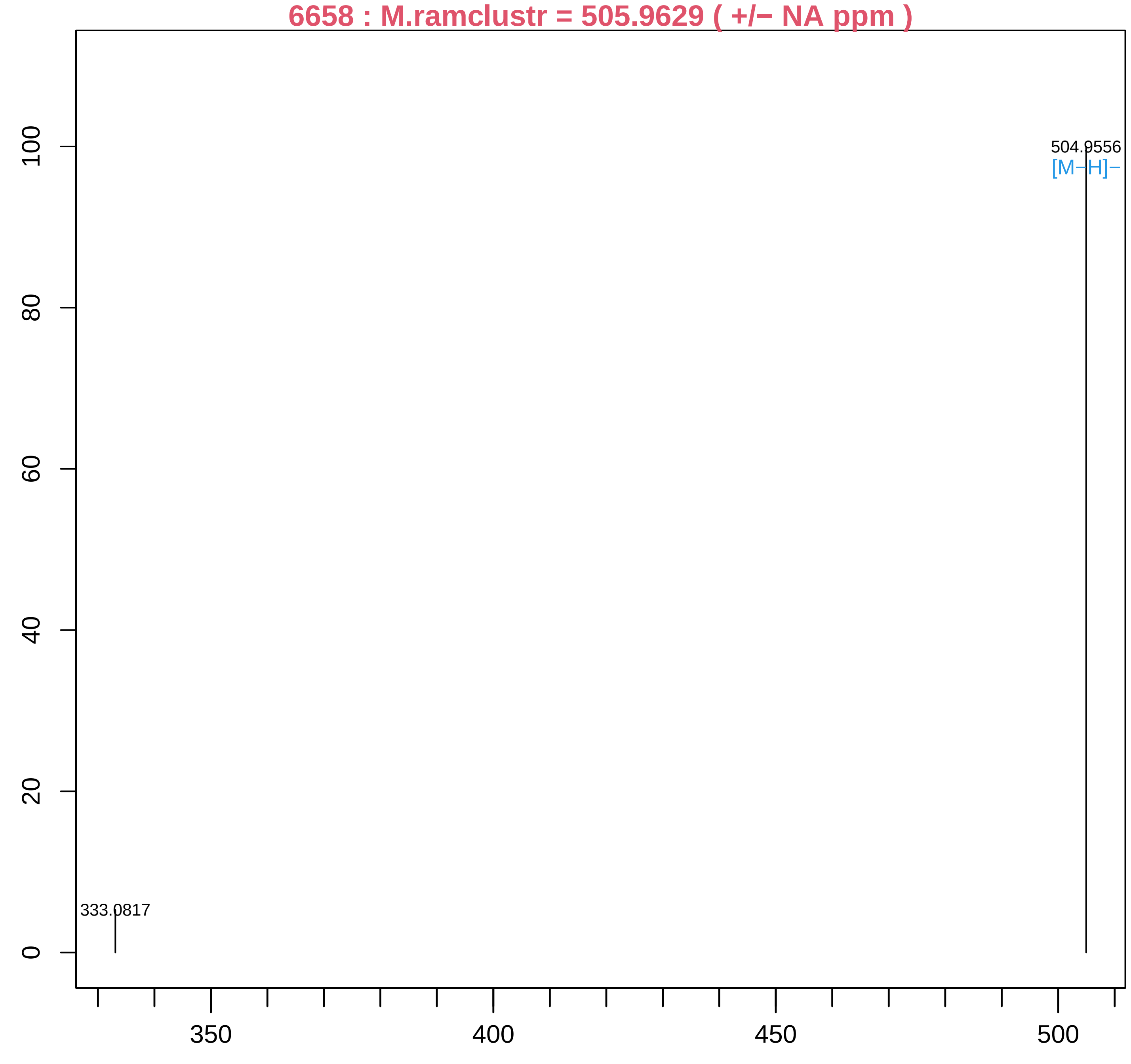
6658 : M.ramclustr = 505.9629 (+/− NA ppm)
504.9556
[M−H]−
333.0817
0
20
40
60
80
100
350
400
450
500

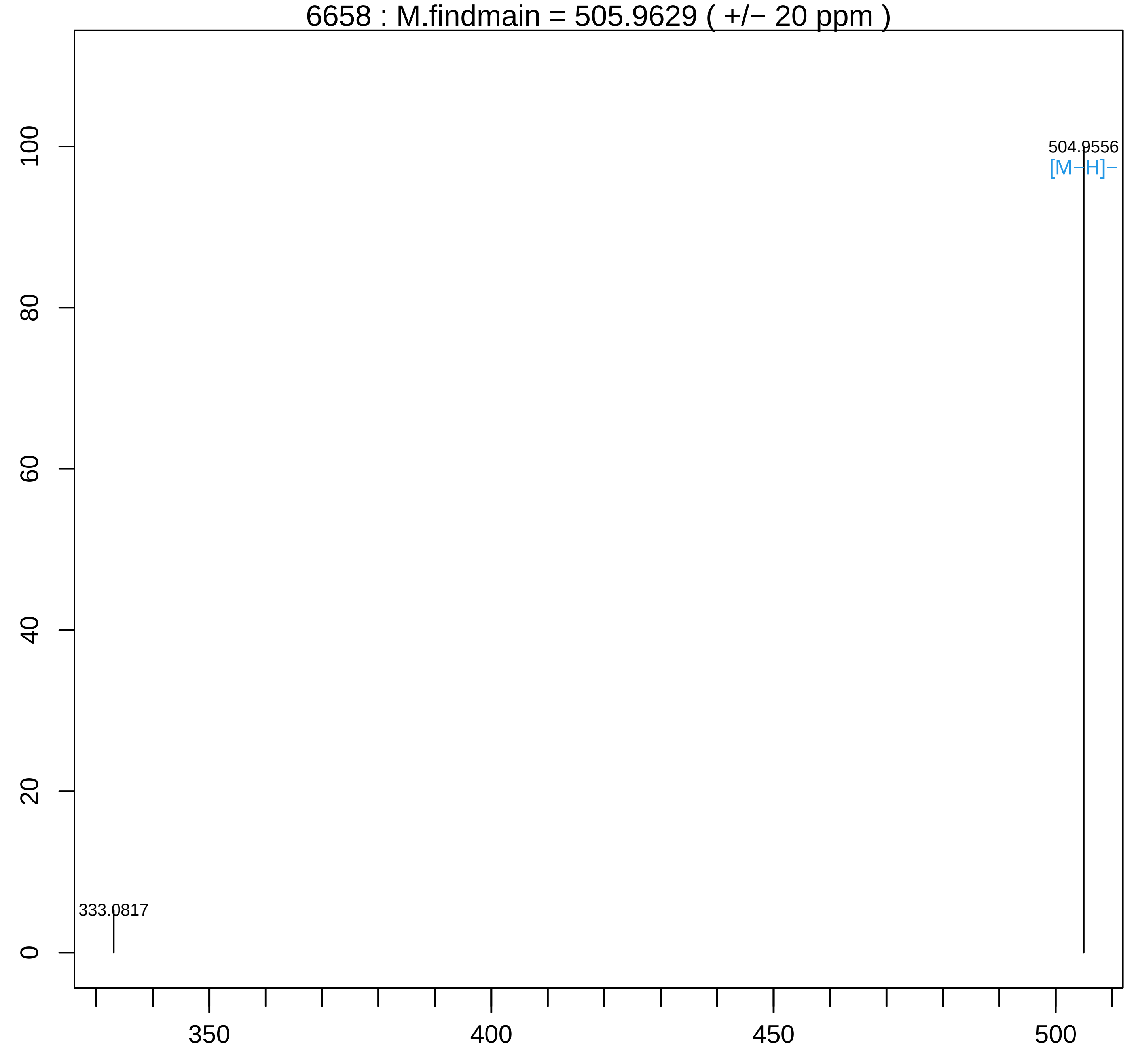
6658 : M.findmain = 505.9629 (+/− 20 ppm)
504.9556
[M−H]−
333.0817
0
20
40
60
80
100
350
400
450
500

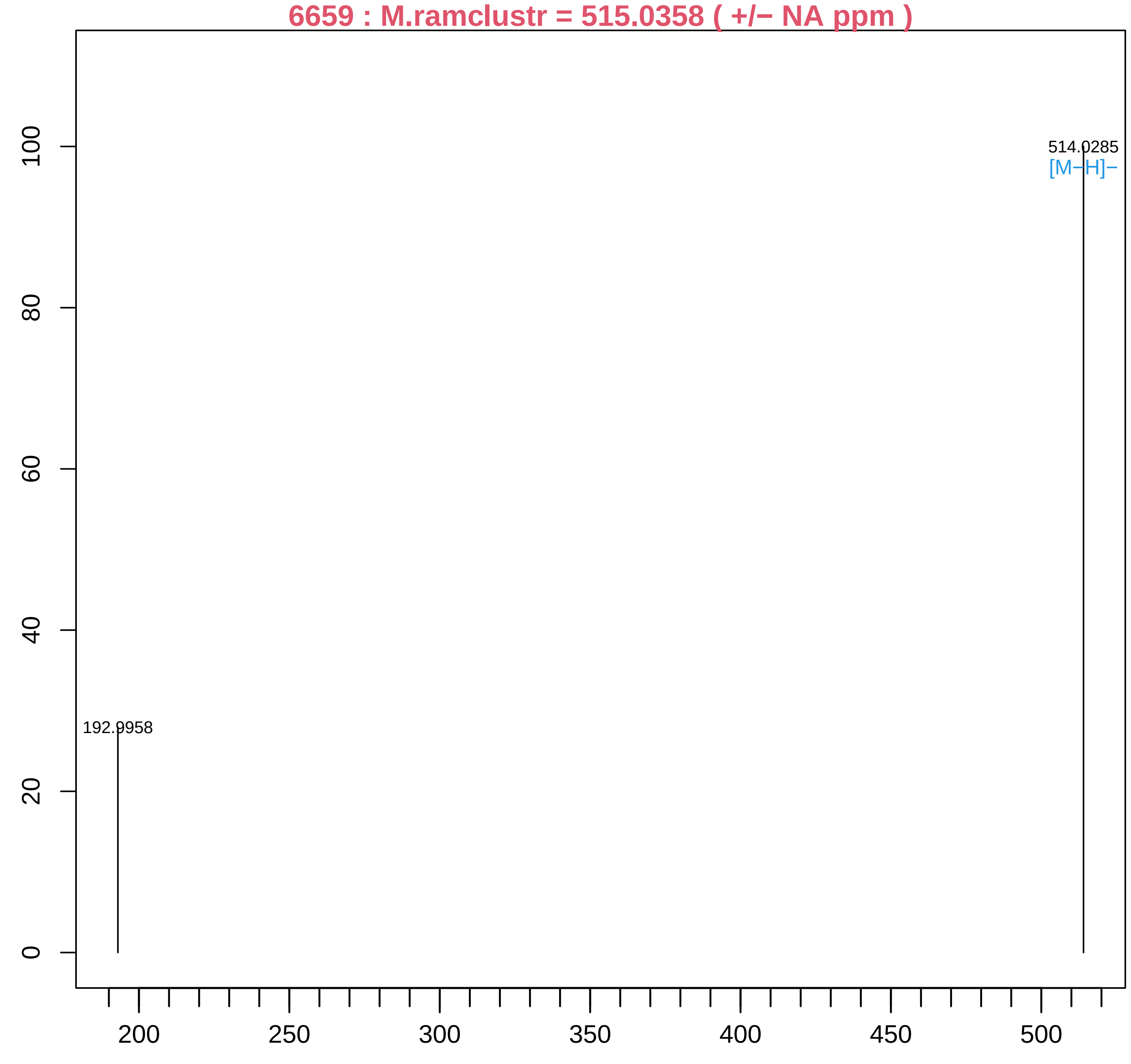
6659 : M.ramclustr = 515.0358 (+/− NA ppm)
514.0285
[M−H]−
192.9958
0
20
40
60
80
100
200
250
300
350
400
450
500

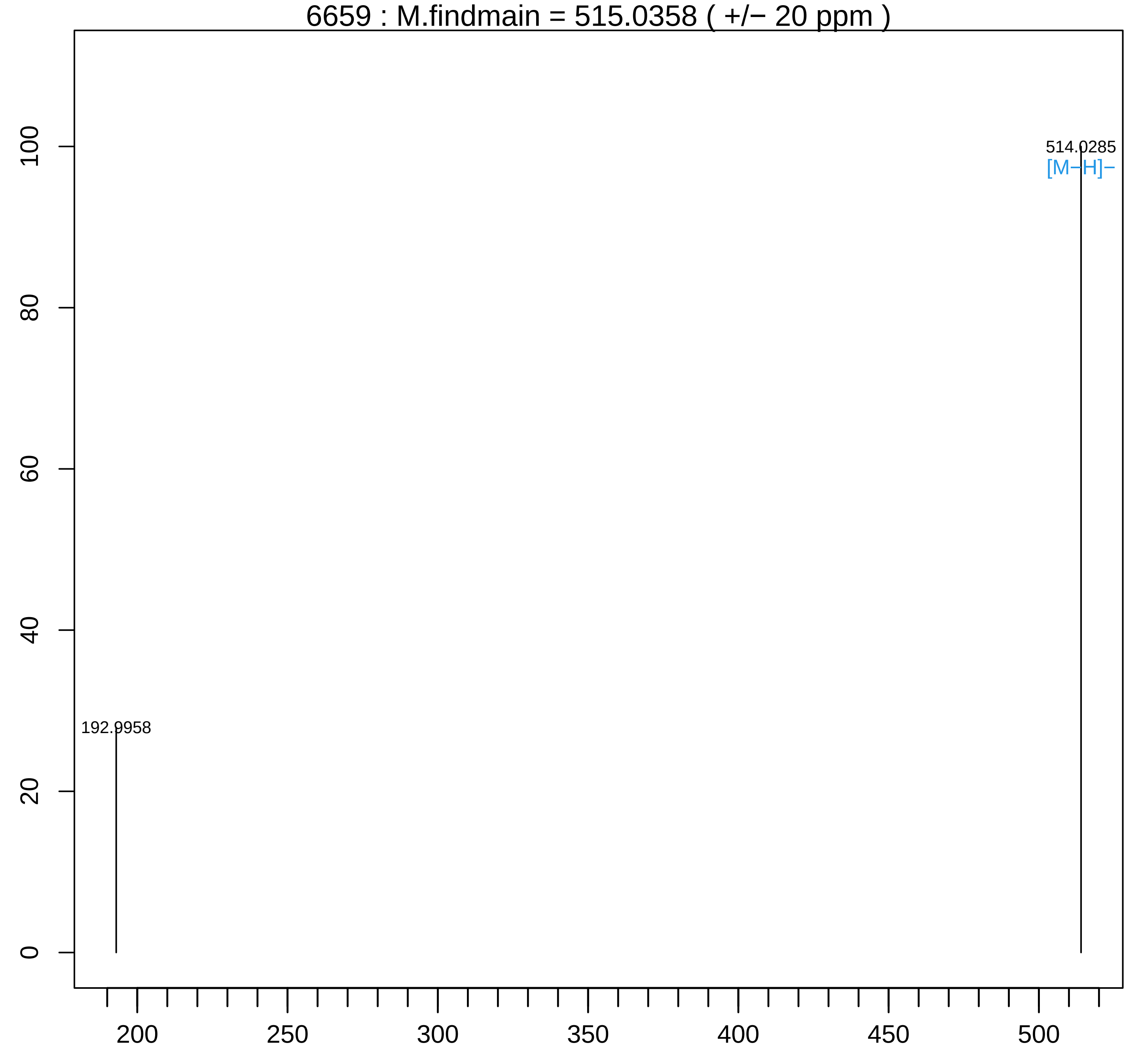
6659 : M.findmain = 515.0358 (+/− 20 ppm)
514.0285
[M−H]−
192.9958
0
20
40
60
80
100
200
250
300
350
400
450
500

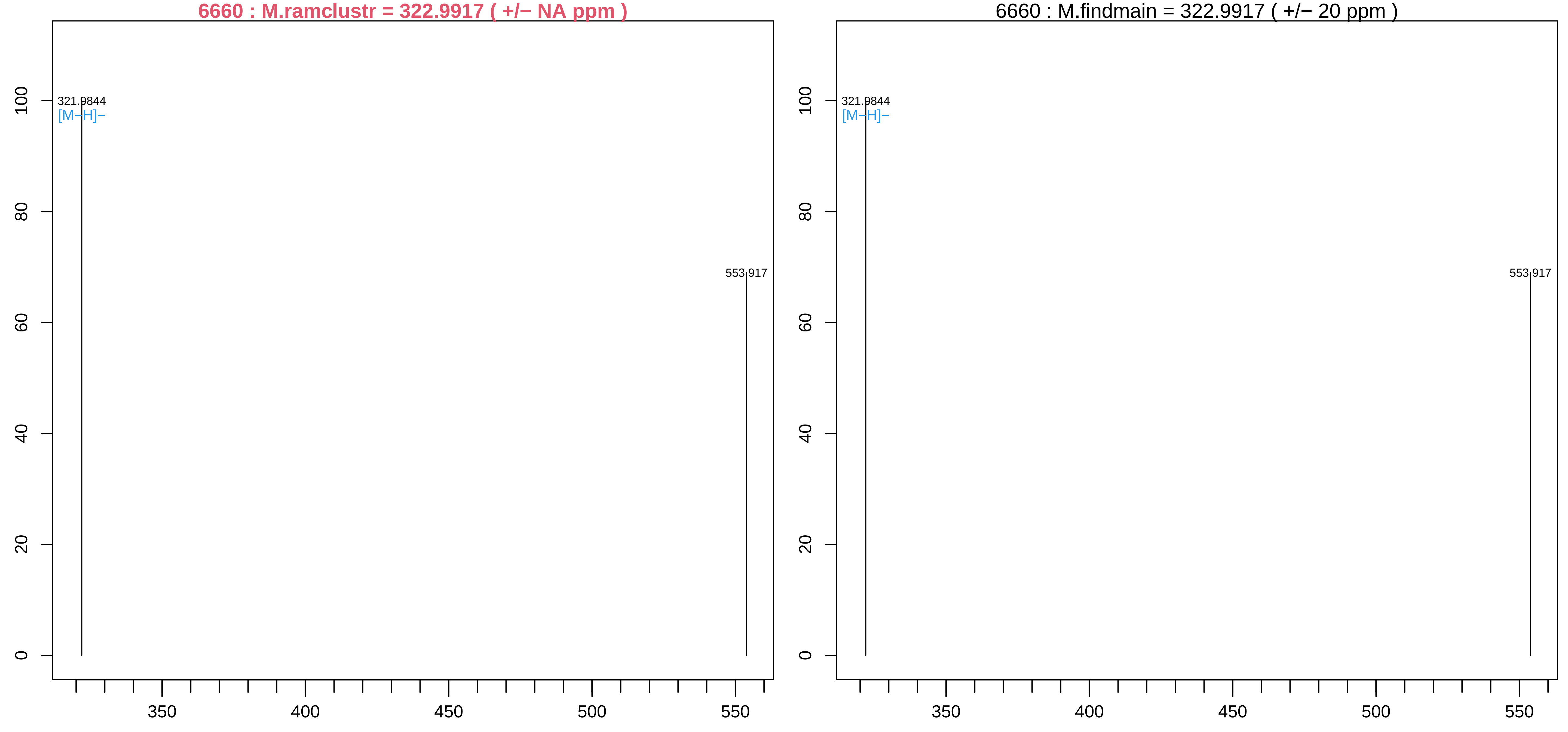

6660 : M.ramclustr = 322.9917 (+/− NA ppm)
321.9844
[M−H]−
553.917
0
20
40
60
80
100
350
400
450
500
550
6660 : M.findmain = 322.9917 (+/− 20 ppm)
321.9844
[M−H]−
553.917
0
20
40
60
80
100
350
400
450
500
550

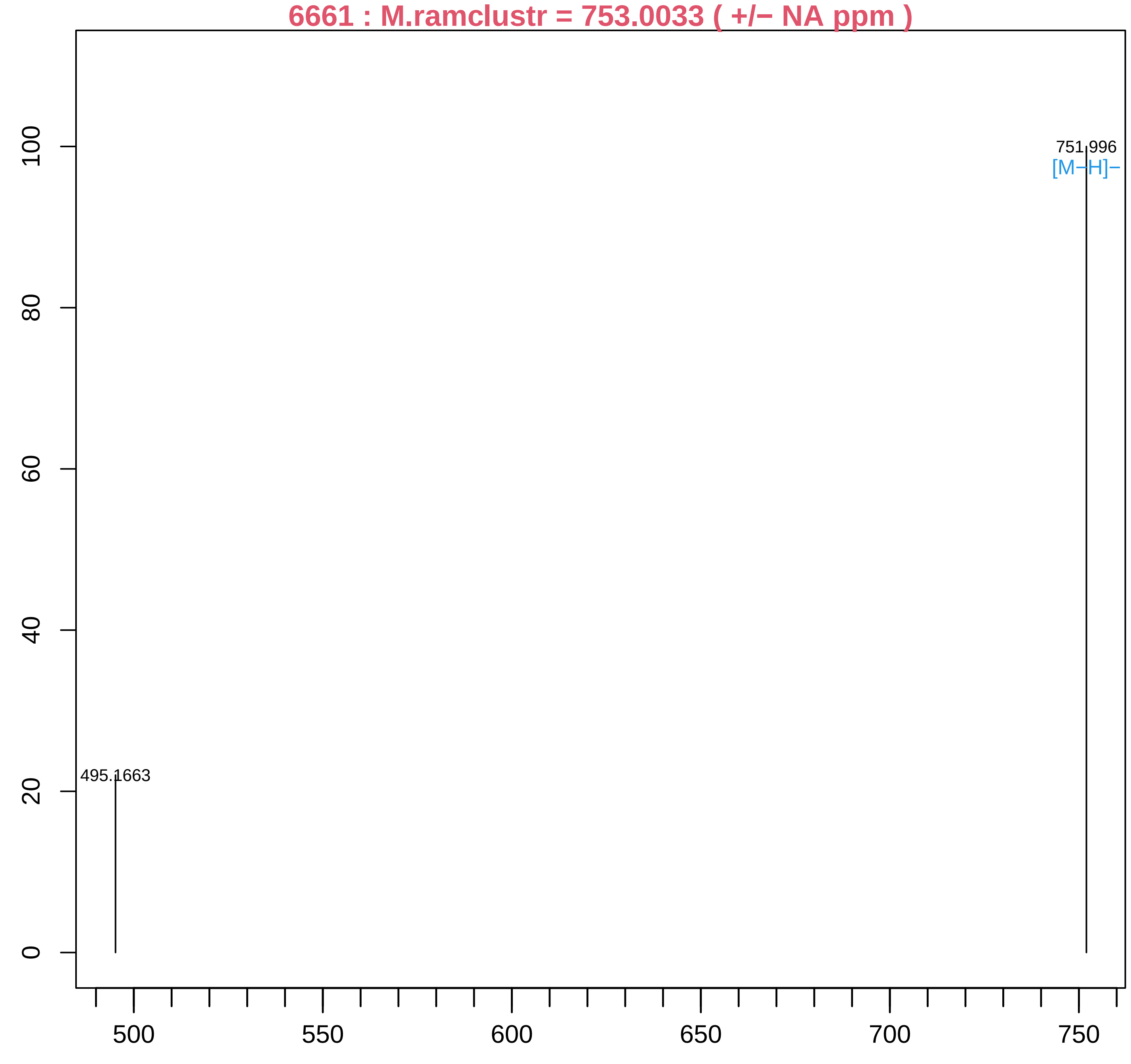

6661 : M.ramclustr = 753.0033 (+/− NA ppm)
751.996
[M−H]−
495.1663
0
20
40
60
80
100
500
550
600
650
700
750

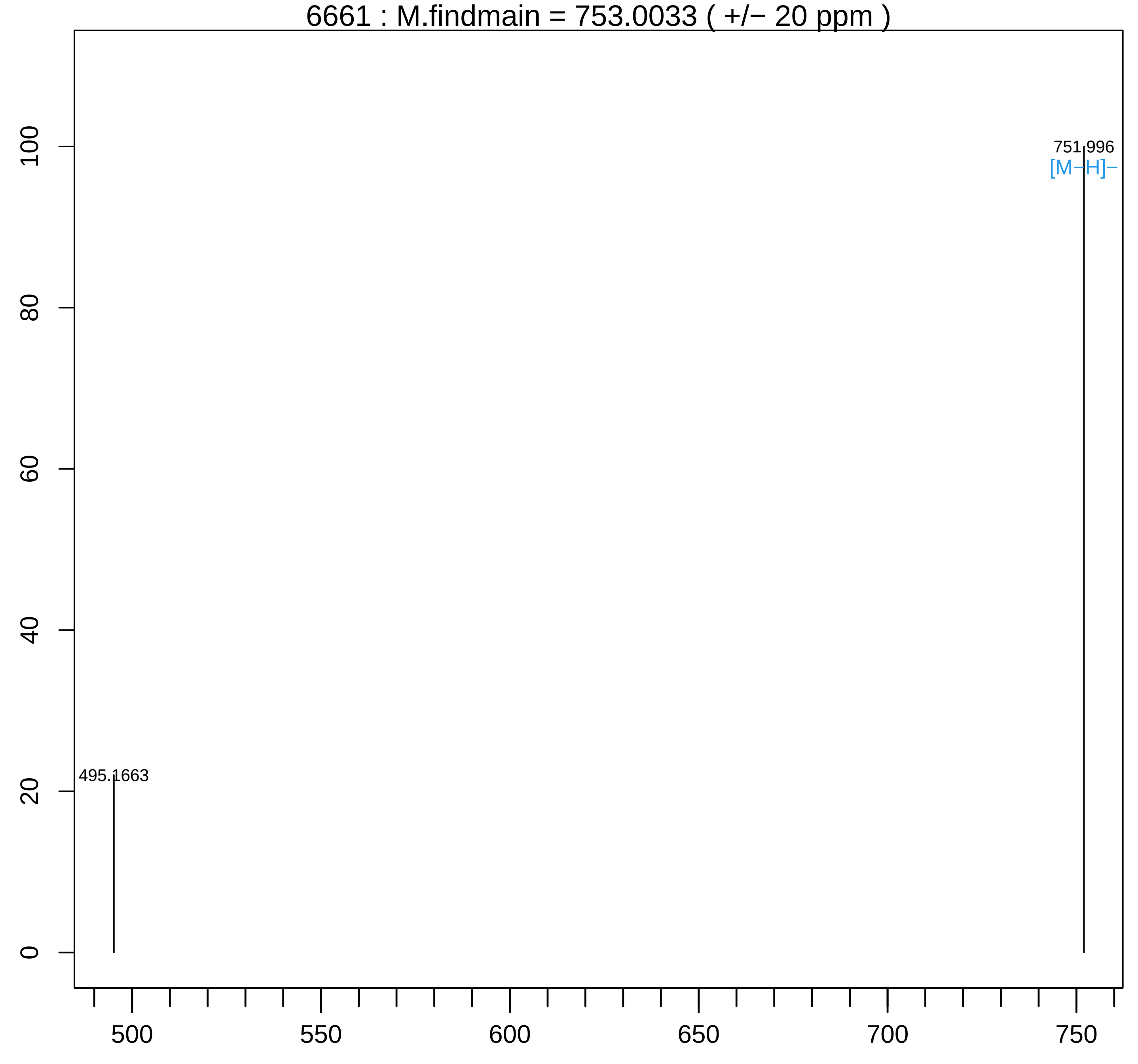

6661 : M.findmain = 753.0033 (+/− 20 ppm)
751.996
[M−H]−
495.1663
0
20
40
60
80
100
500
550
600
650
700
750

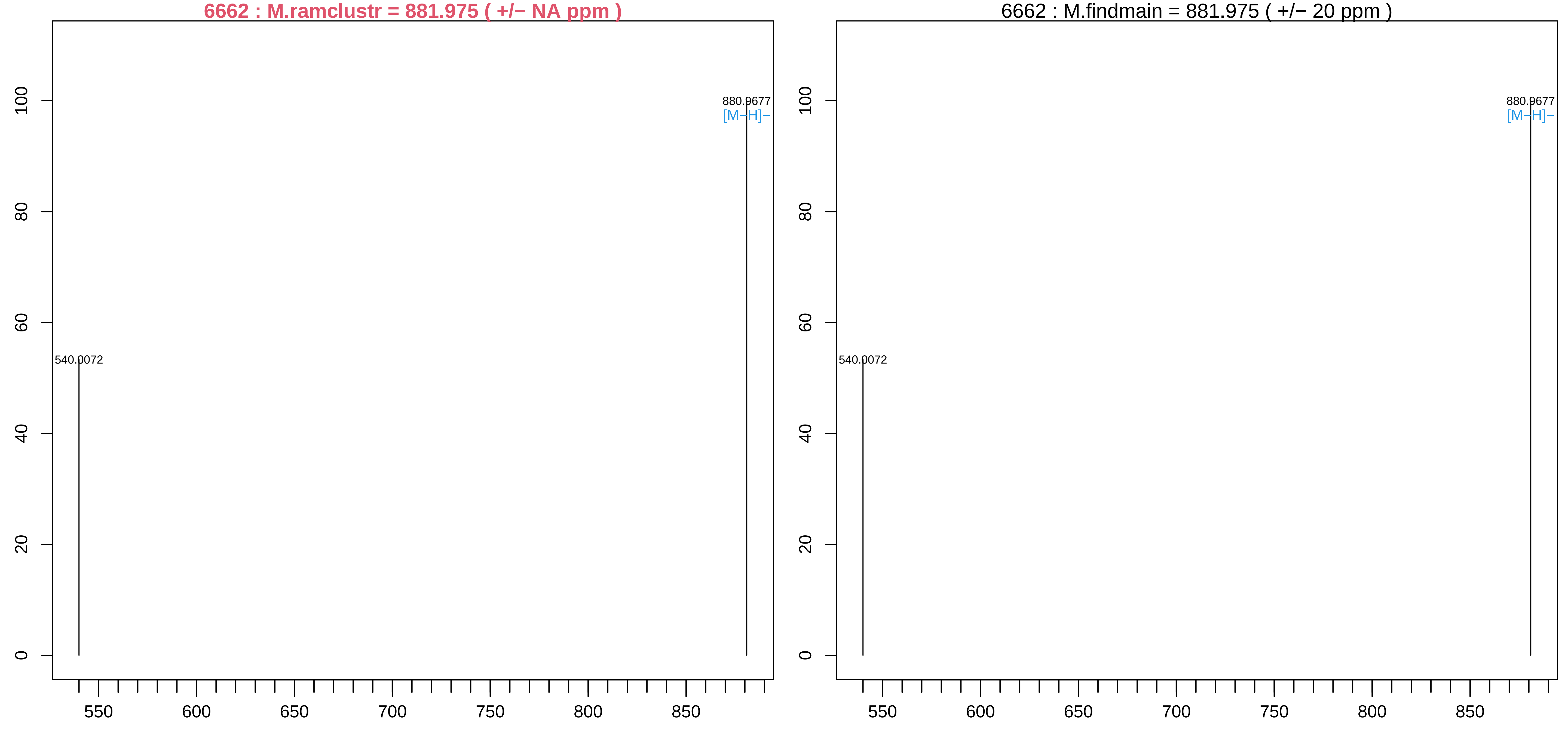
6662 : M.ramclustr = 881.975 (+/− NA ppm)
6662 : M.findmain = 881.975 (+/− 20 ppm)
880.9677
[M−H]−
540.0072
0
20
40
60
80
100
550
600
650
700
750
800
850

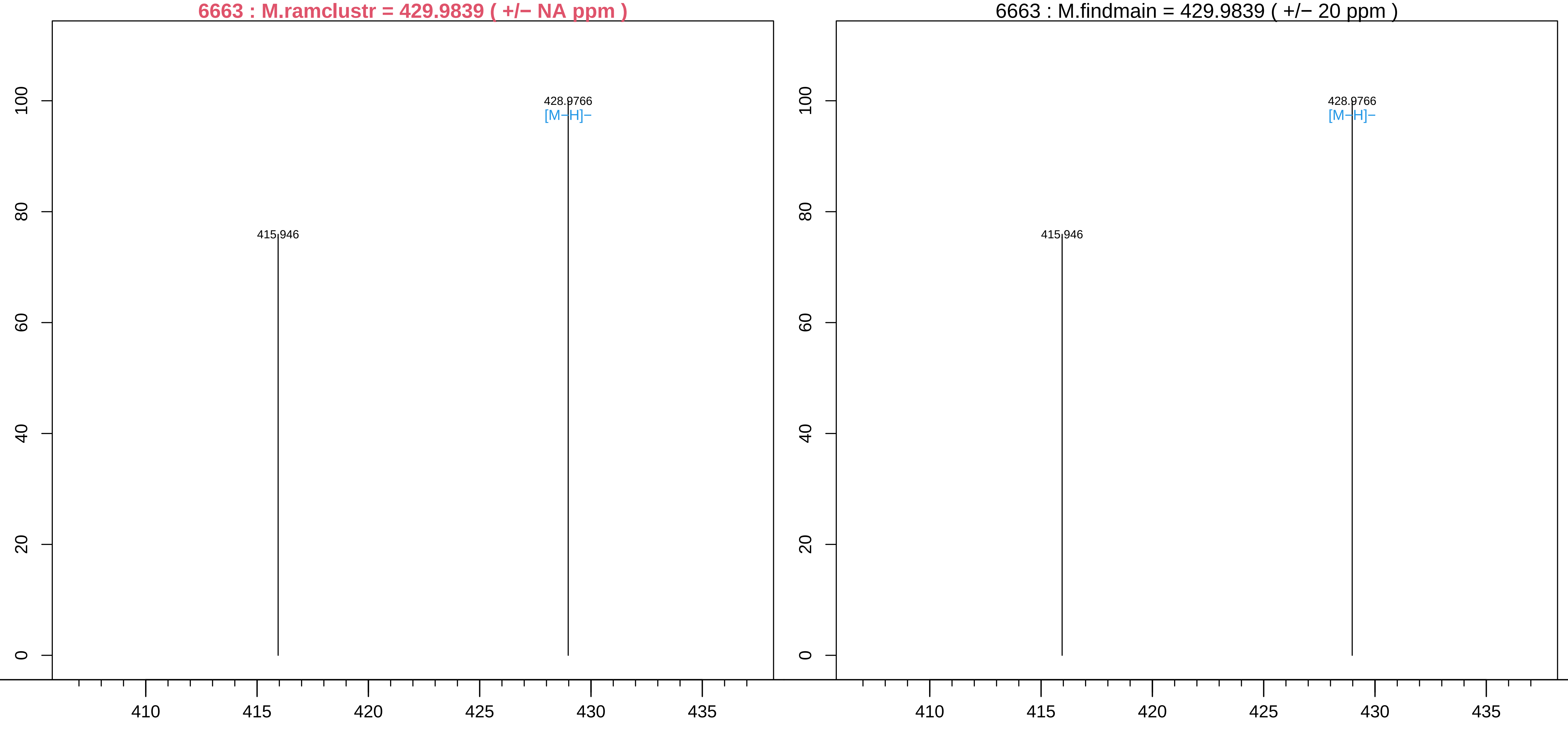

6663 : M.ramclustr = 429.9839 (+/− NA ppm)
428.9766
[M−H]−
415.946
6663 : M.findmain = 429.9839 (+/− 20 ppm)
428.9766
[M−H]−
415.946
0
20
40
60
80
100
410
415
420
425
430
435

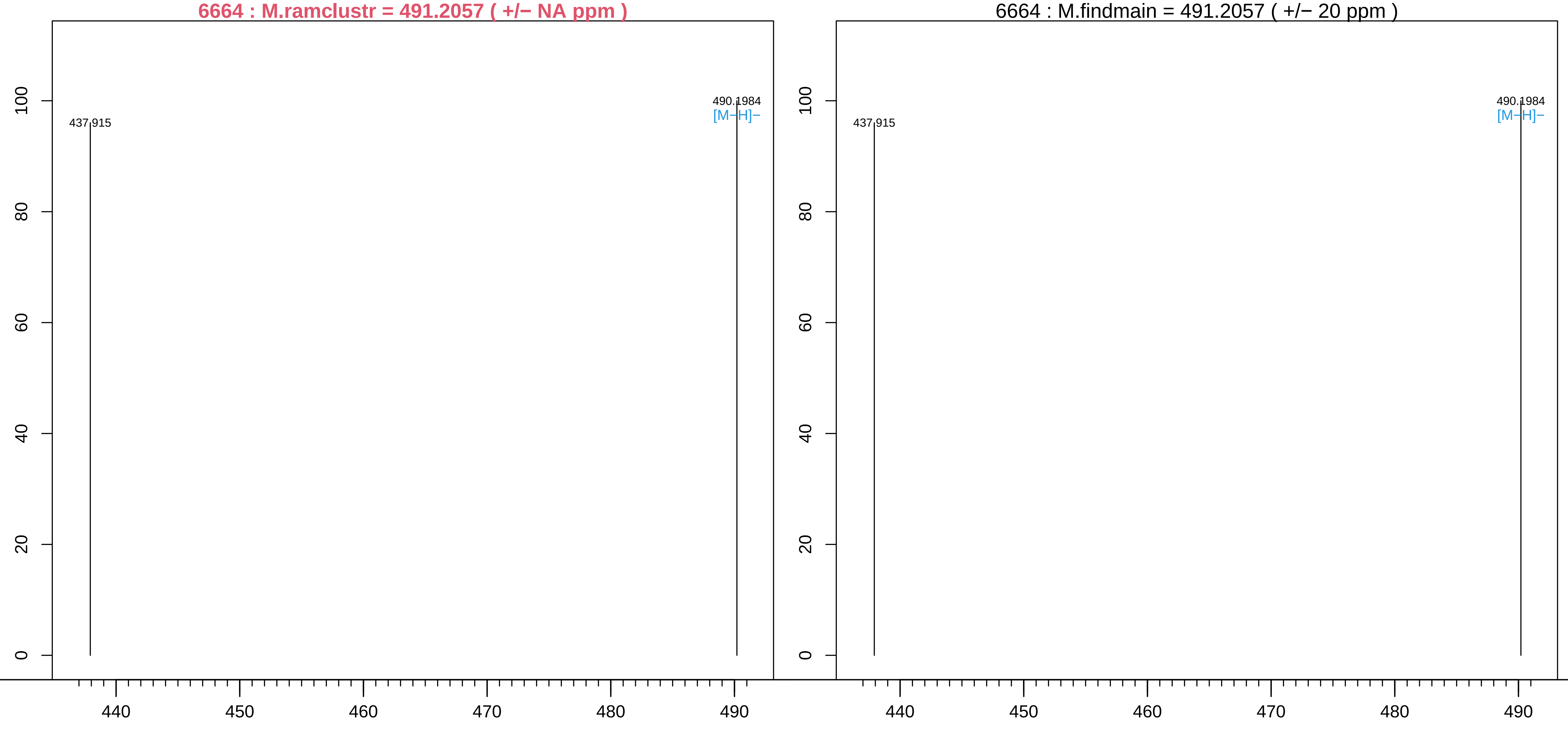

6664 : M.ramclustr = 491.2057 (+/− NA ppm)
437.915
490.1984
[M−H]−
6664 : M.findmain = 491.2057 (+/− 20 ppm)
437.915
490.1984
[M−H]−

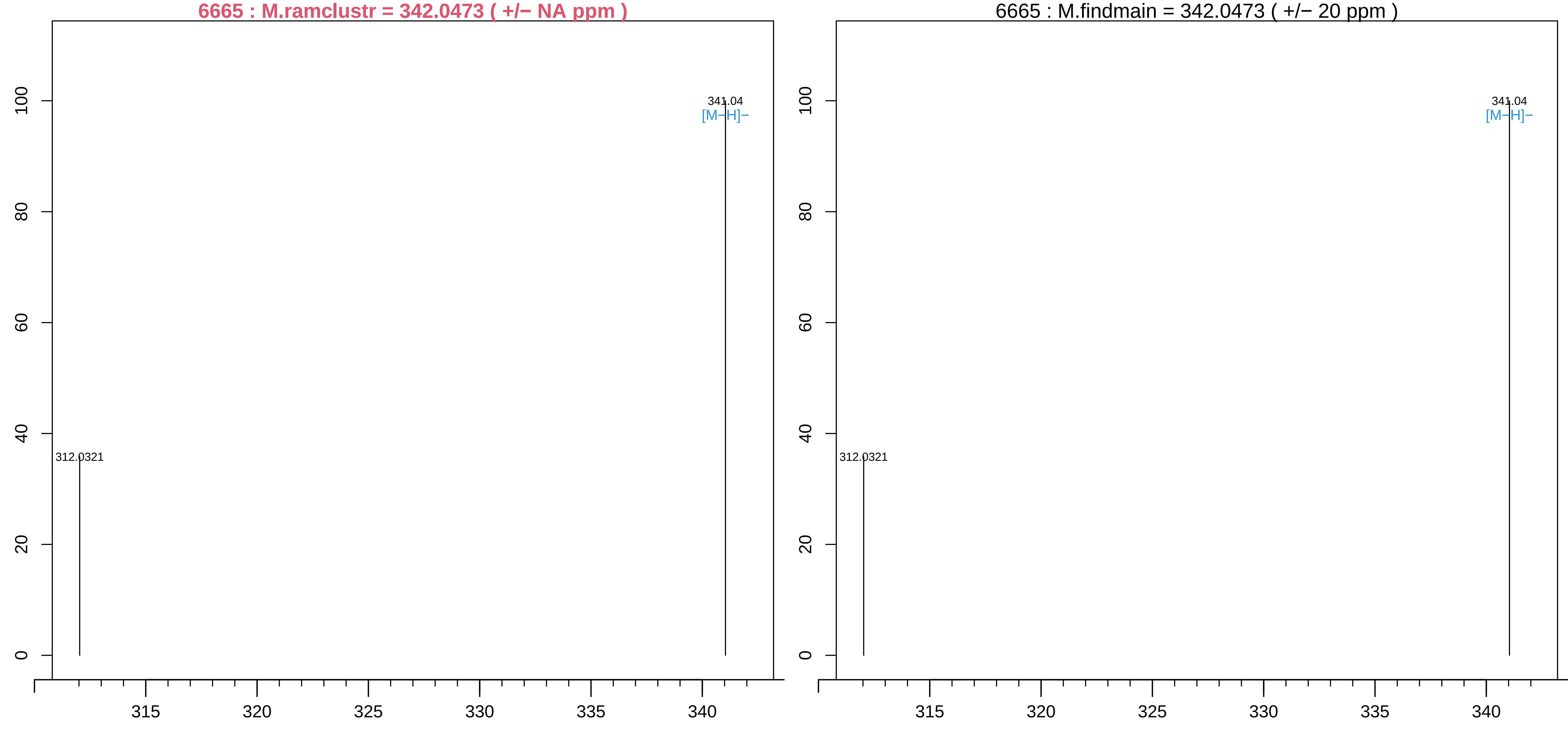

6665 : M.ramclustr = 342.0473 (+/− NA ppm)
341.04
[M−H]−
312.0321
0
20
40
60
80
100
315
320
325
330
335
340
6665 : M.findmain = 342.0473 (+/− 20 ppm)
341.04
[M−H]−
312.0321
0
20
40
60
80
100
315
320
325
330
335
340

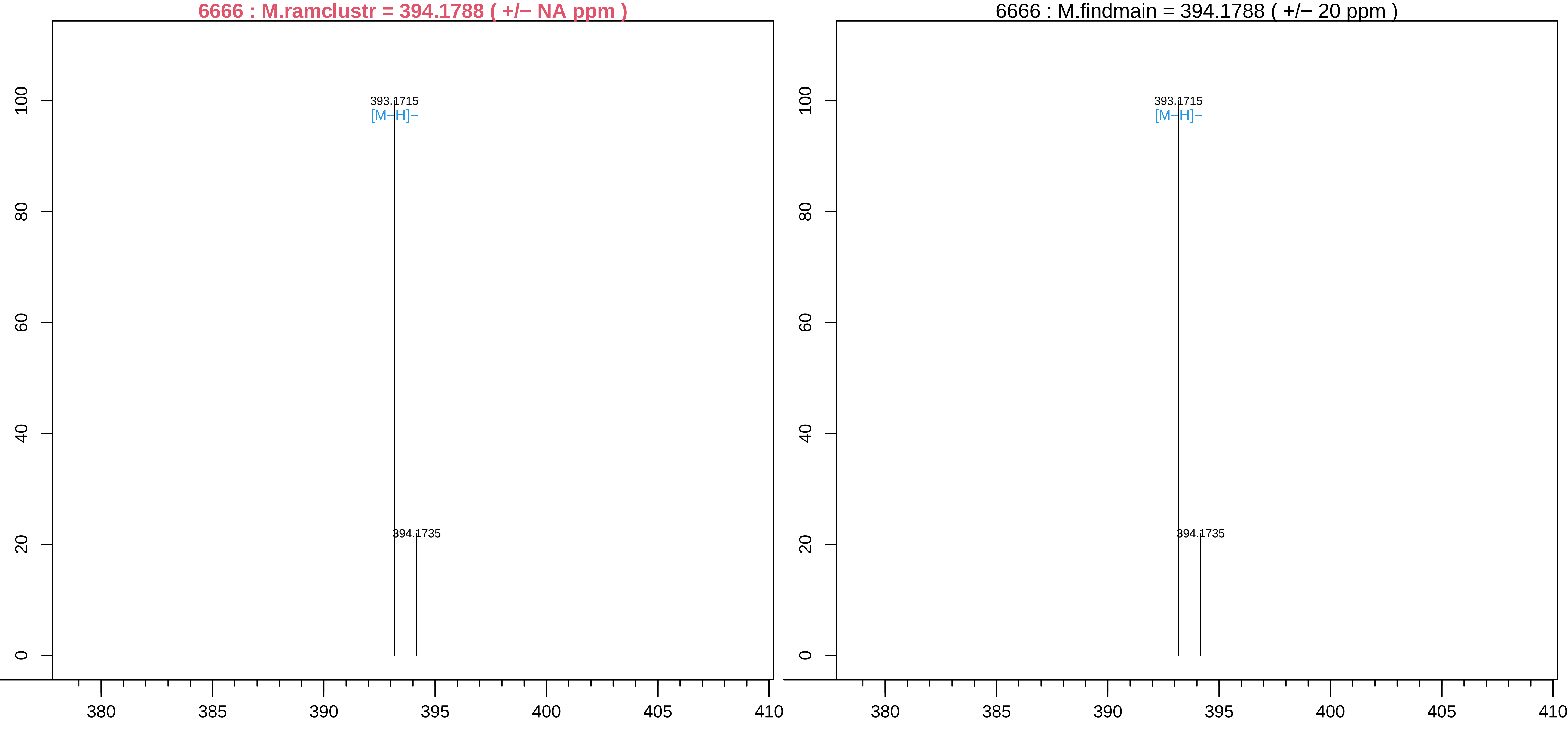
6666 : M.ramclustr = 394.1788 (+/− NA ppm)
393.1715
[M−H]−
394.1735
6666 : M.findmain = 394.1788 (+/− 20 ppm)
393.1715
[M−H]−
394.1735
0
20
40
60
80
100
380
385
390
395
400
405
410

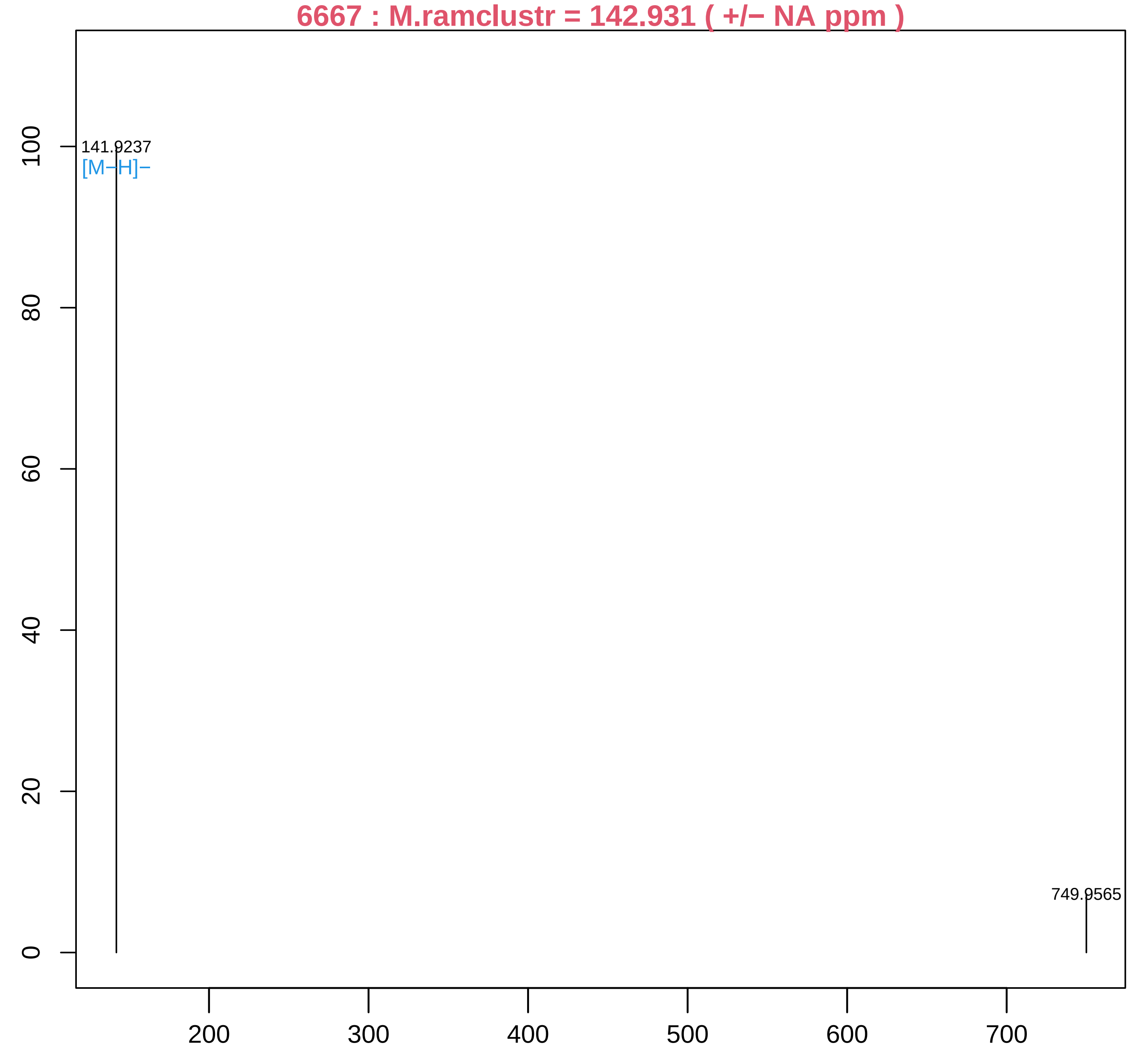
6667 : M.ramclustr = 142.931 (+/− NA ppm)
141.9237
[M−H]−
749.9565
0
20
40
60
80
100
200
300
400
500
600
700

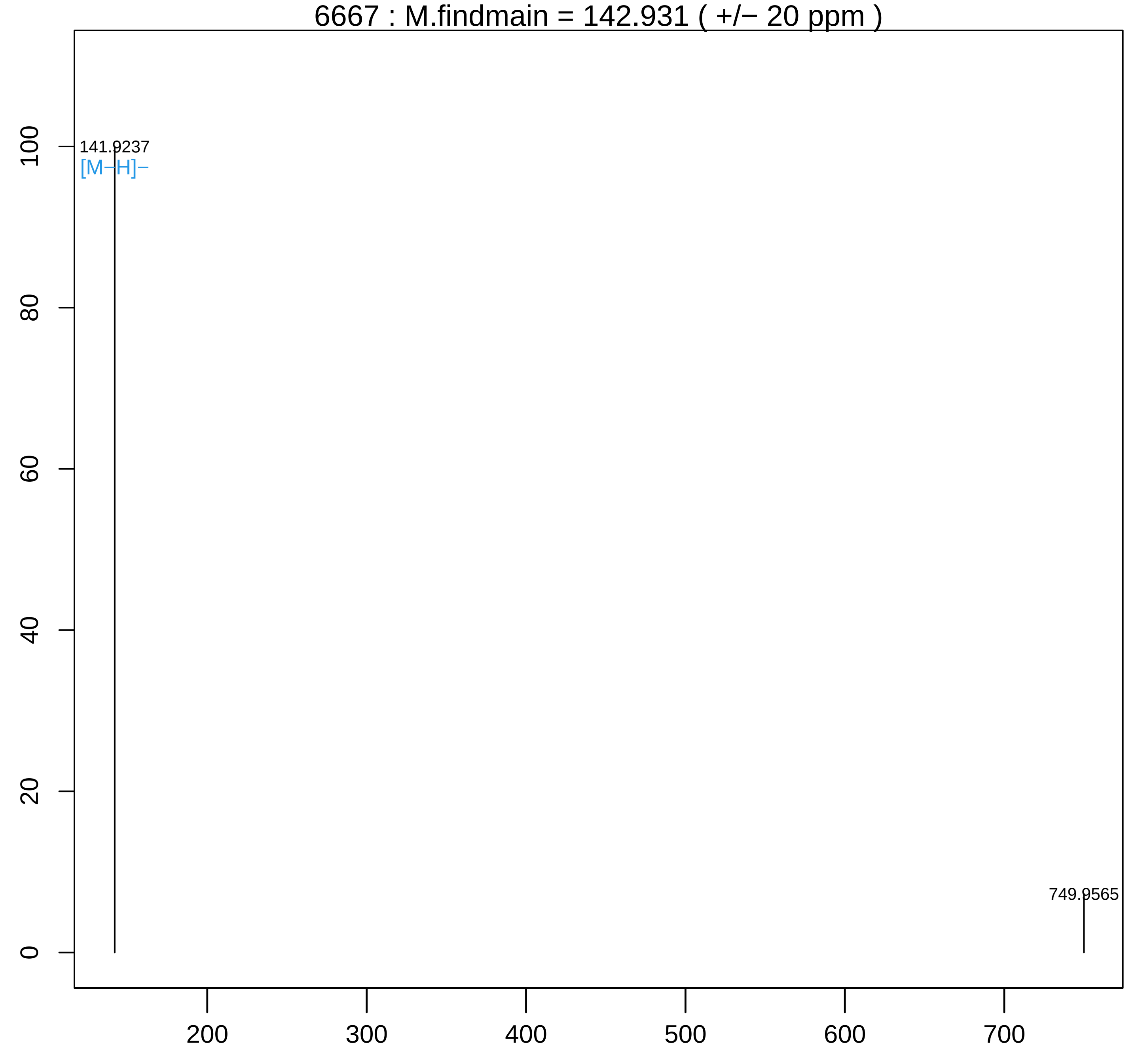
6667 : M.findmain = 142.931 (+/− 20 ppm)
141.9237
[M−H]−
749.9565
0
20
40
60
80
100
200
300
400
500
600
700

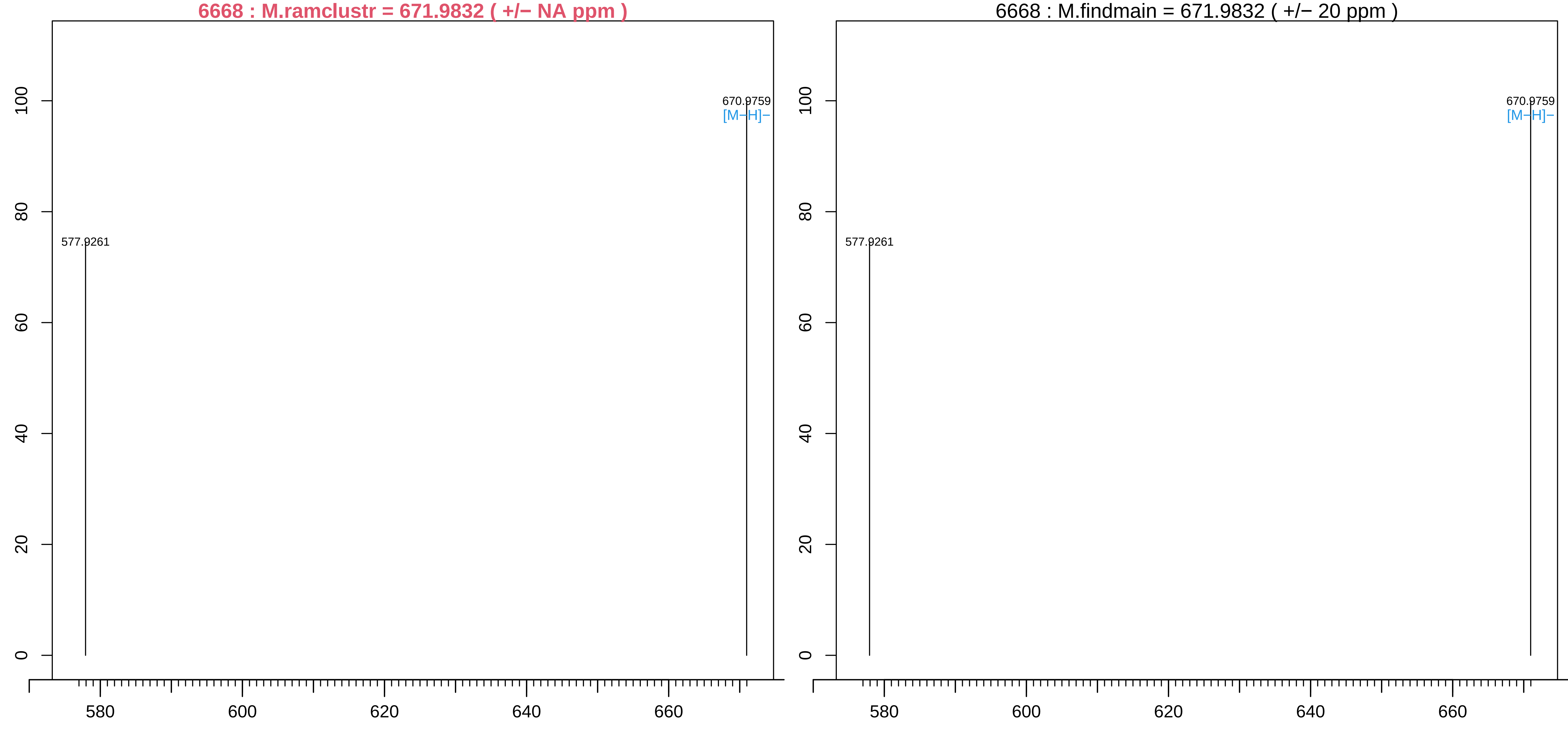
6668 : M.ramclustr = 671.9832 (+/− NA ppm)
670.9759
[M−H]−
577.9261
0
20
40
60
80
100
580
600
620
640
660
6668 : M.findmain = 671.9832 (+/− 20 ppm)
670.9759
[M−H]−
577.9261
0
20
40
60
80
100
580
600
620
640
660

6669 : M.ramclustr = 898.8874 (+/− NA ppm)

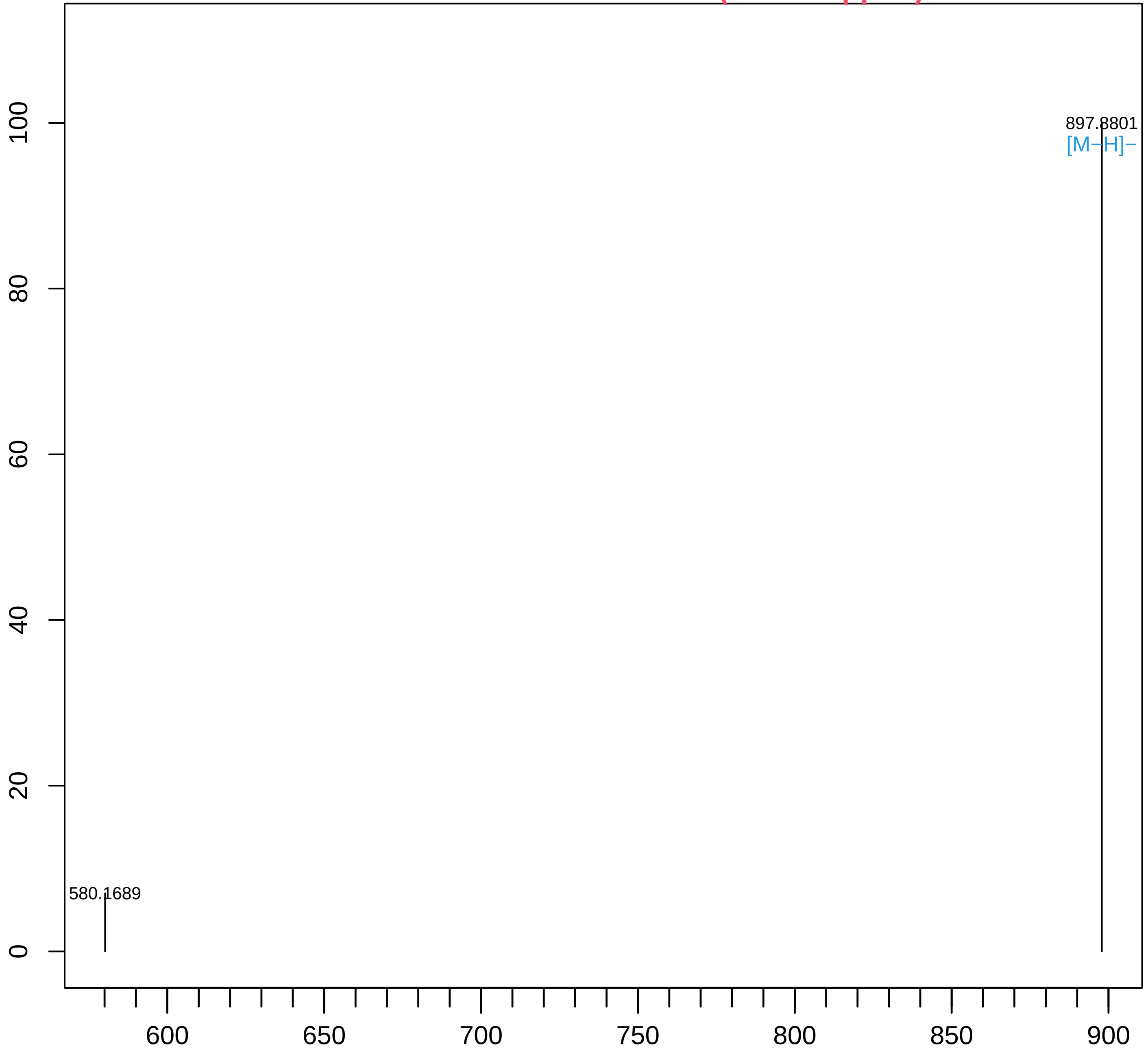

6669 : M.findmain = 898.8874 (+/− 20 ppm)

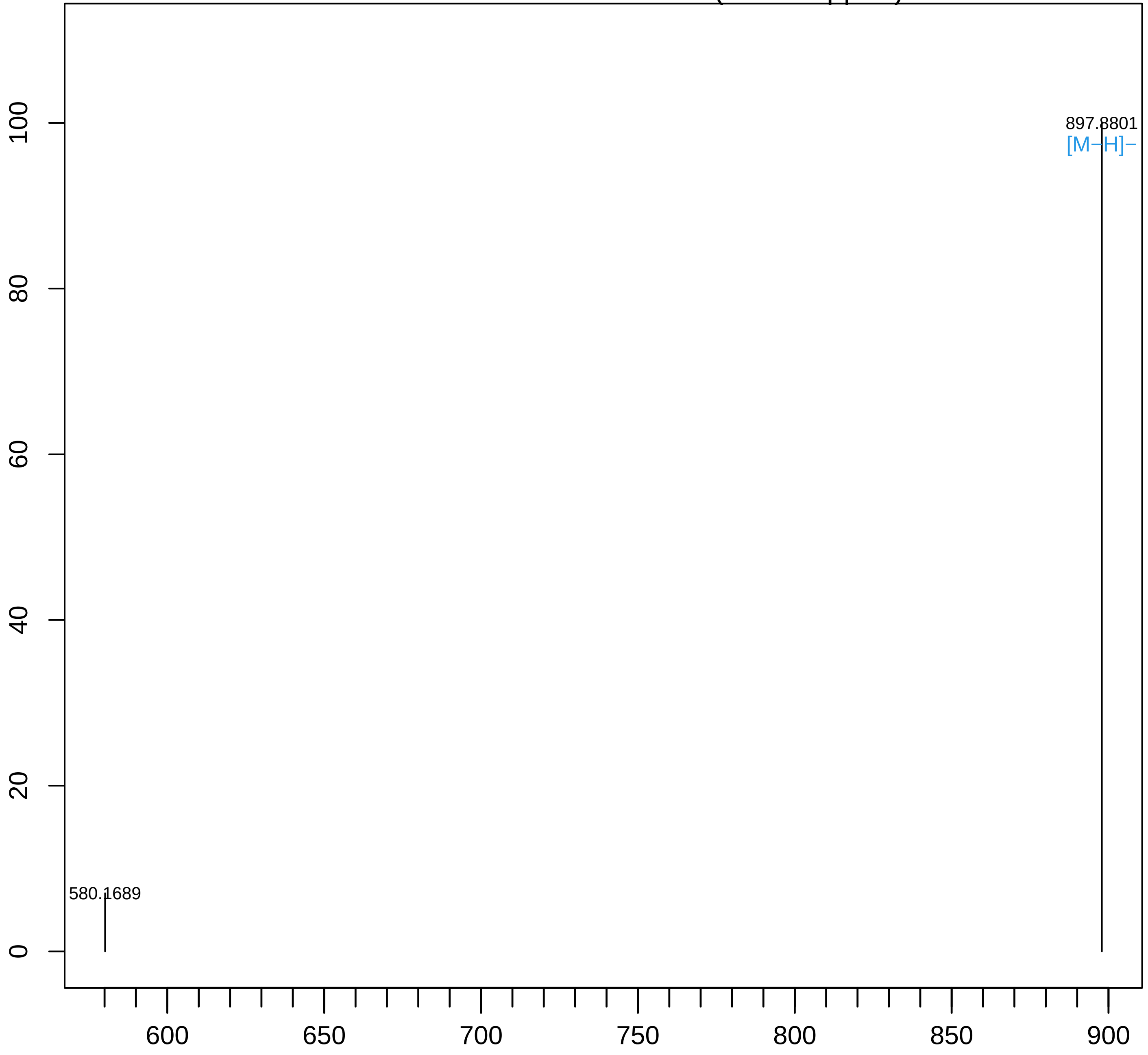

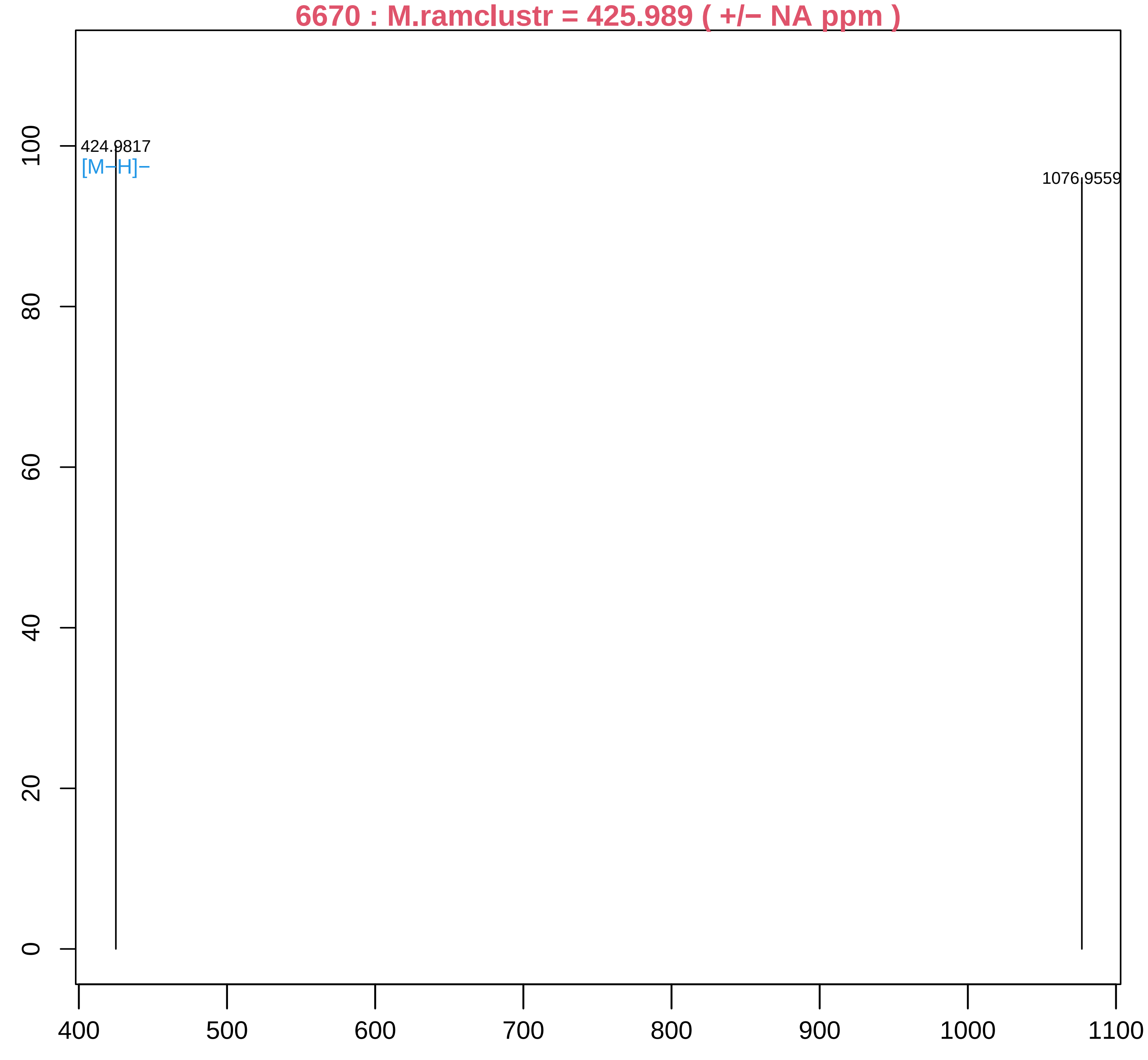
6670 : M.ramclustr = 425.989 (+/− NA ppm)
424.9817
[M−H]−
1076.9559
0
20
40
60
80
100
400
500
600
700
800
900
1000
1100

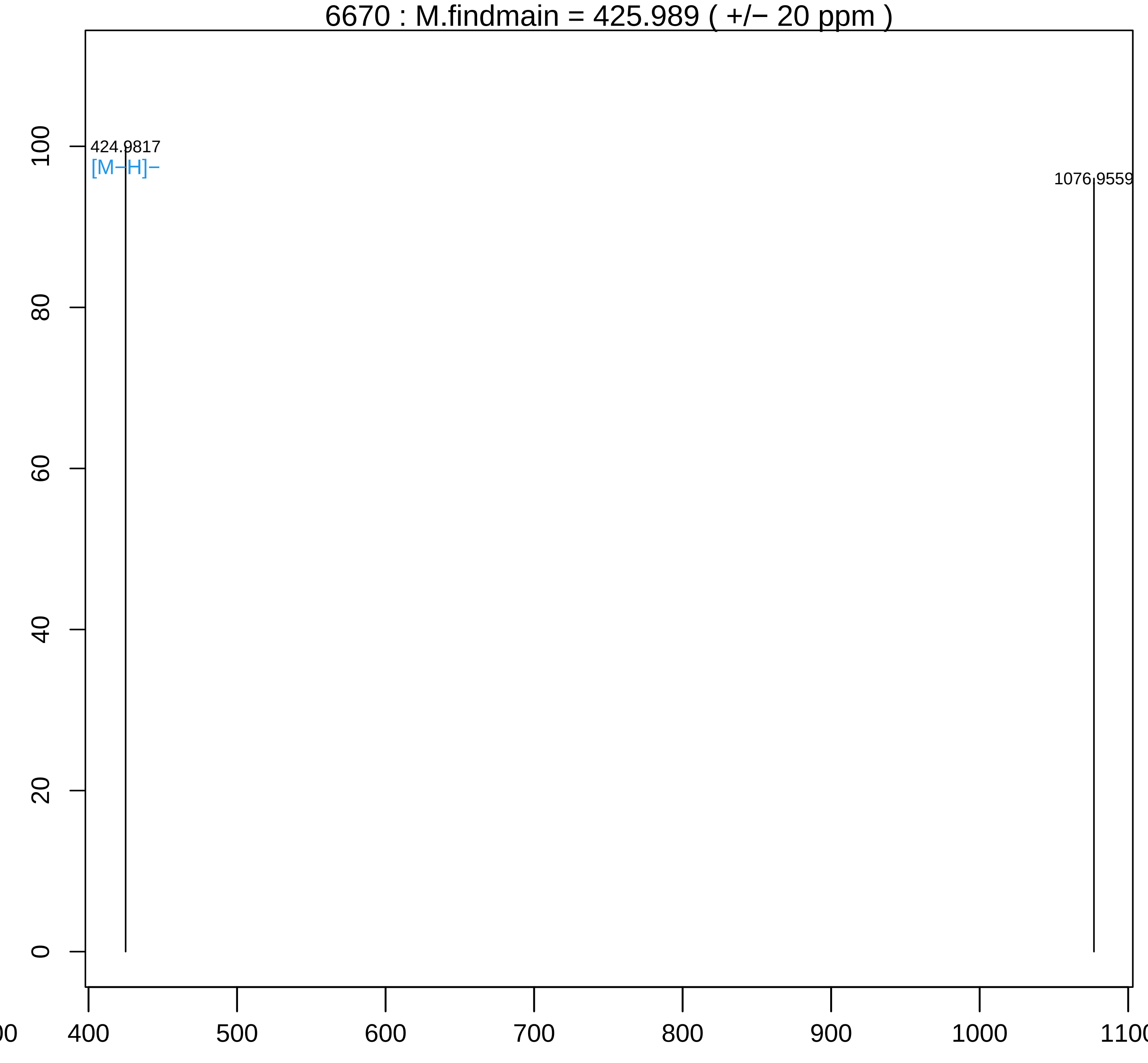
6670 : M.findmain = 425.989 (+/− 20 ppm)
424.9817
[M−H]−
1076.9559
0
20
40
60
80
100
400
500
600
700
800
900
1000
1100

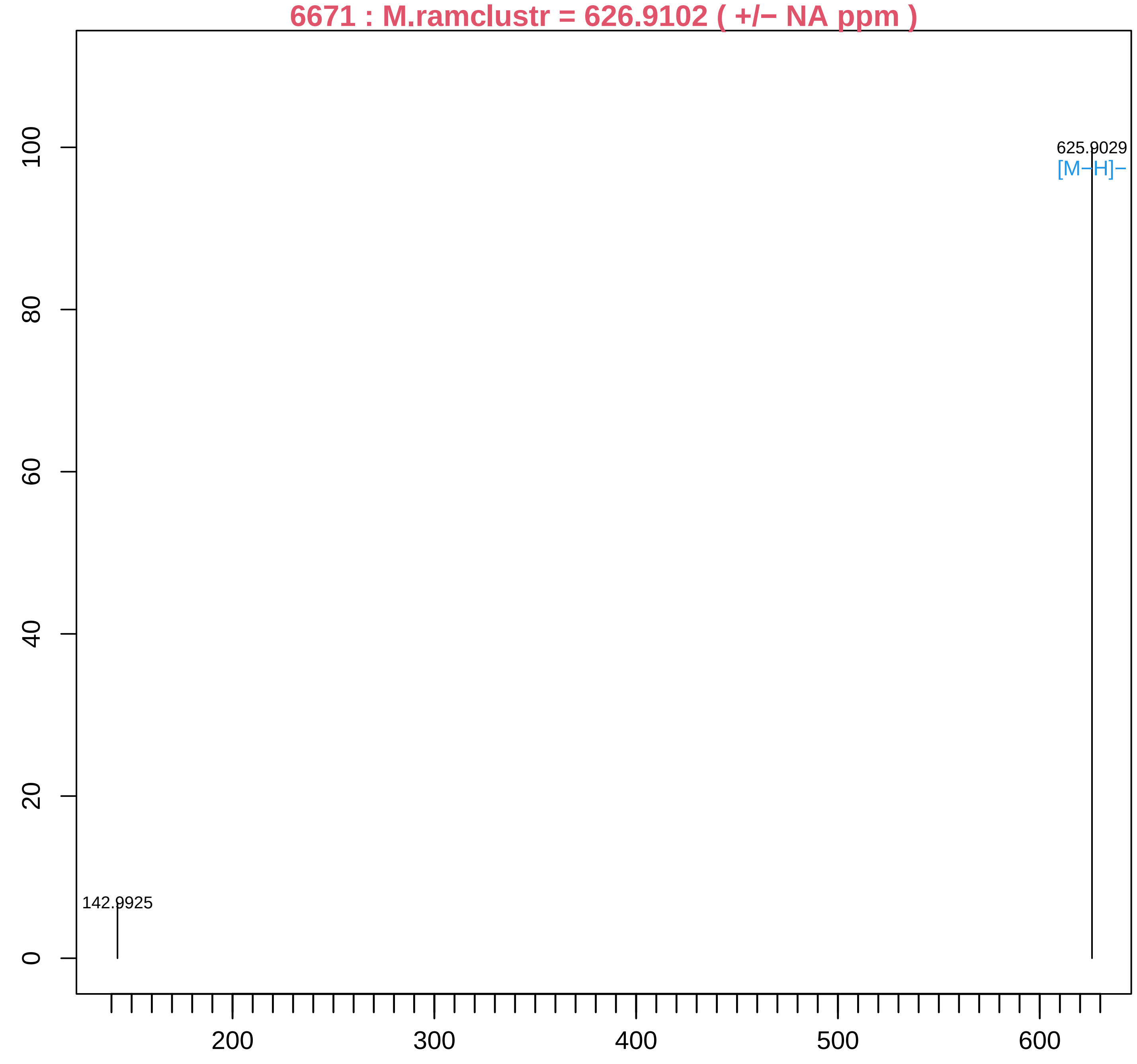
6671 : M.ramclustr = 626.9102 (+/− NA ppm)
625.9029
[M−H]−
142.9925
0
20
40
60
80
100
200
300
400
500
600

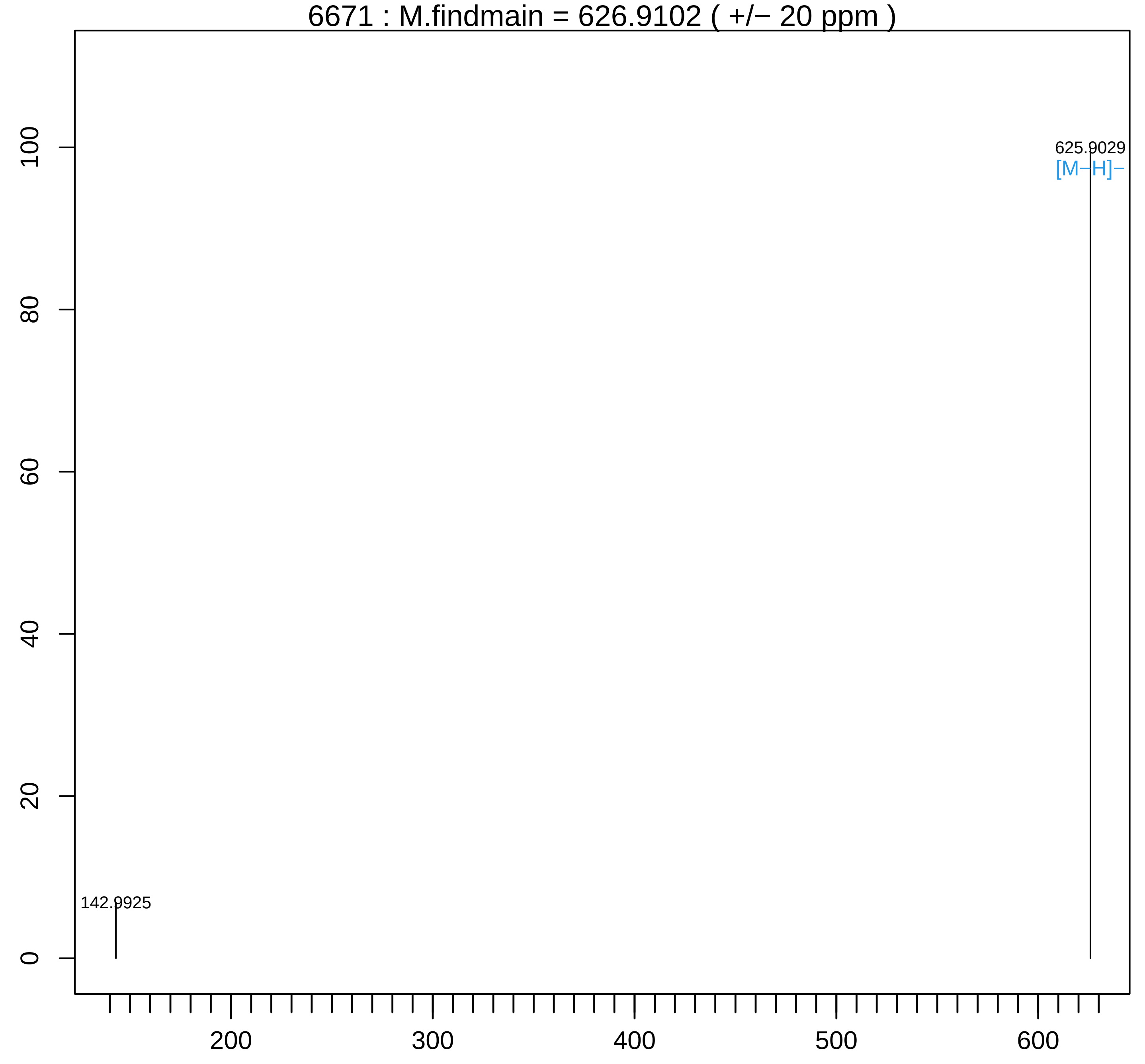
6671 : M.findmain = 626.9102 (+/− 20 ppm)
625.9029
[M−H]−
142.9925
0
20
40
60
80
100
200
300
400
500
600

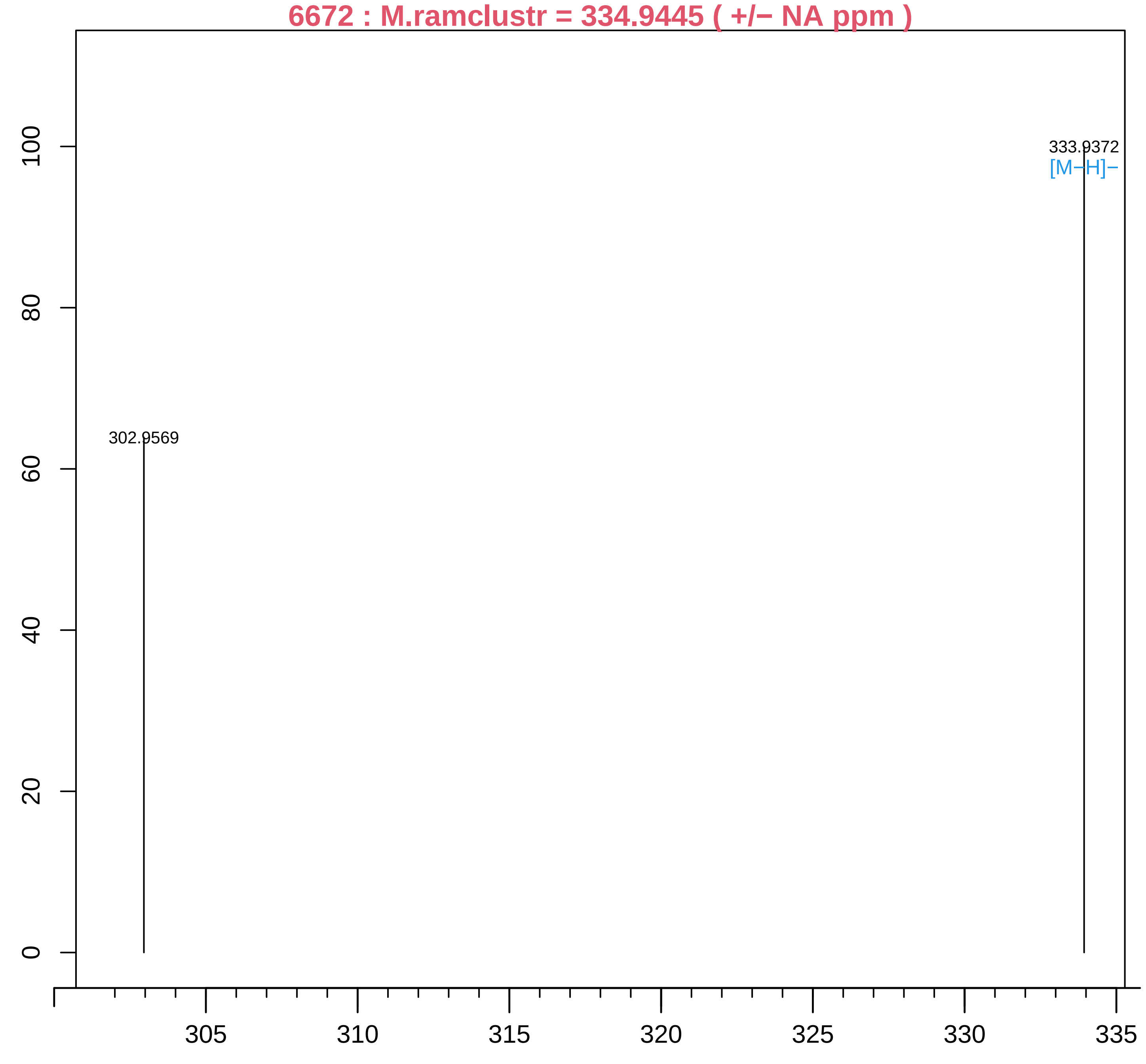
6672 : M.ramclustr = 334.9445 (+/− NA ppm)
333.9372
[M−H]−
302.9569

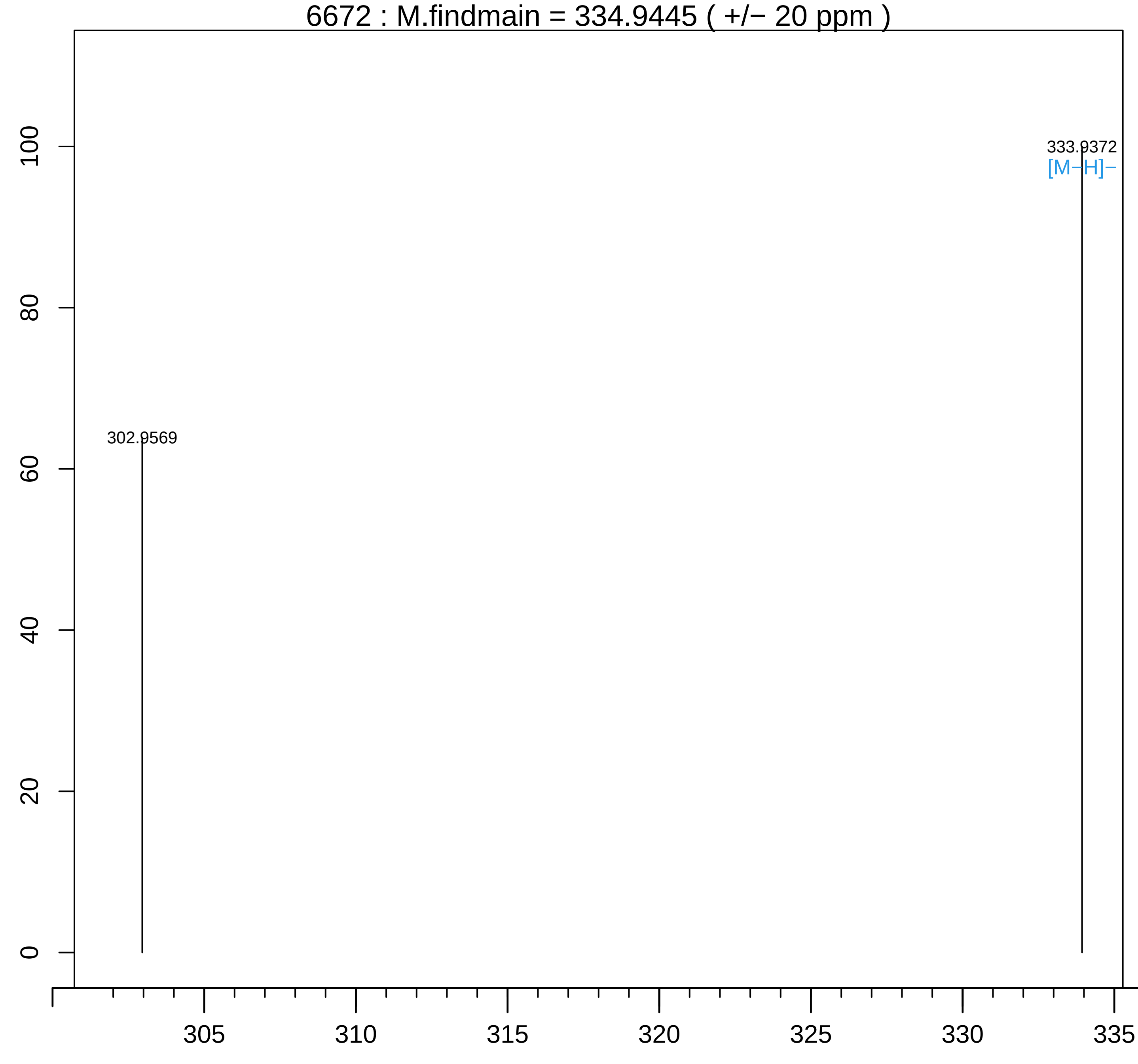
6672 : M.findmain = 334.9445 (+/− 20 ppm)
333.9372
[M−H]−
302.9569

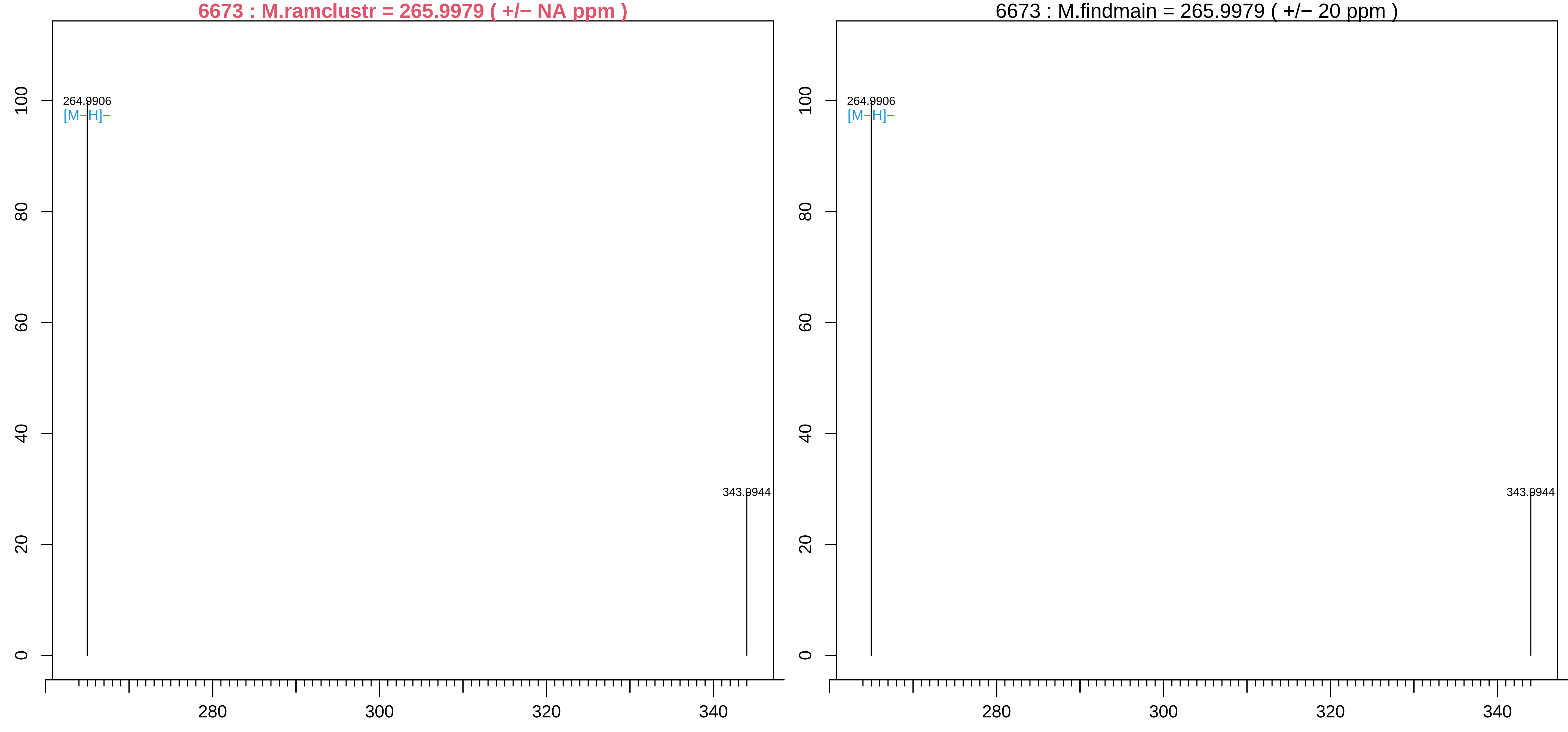

6673 : M.ramclustr = 265.9979 (+/− NA ppm)
264.9906
[M−H]−
343.9944
0
20
40
60
80
100
280
300
320
340
6673 : M.findmain = 265.9979 (+/− 20 ppm)
264.9906
[M−H]−
343.9944
0
20
40
60
80
100
280
300
320
340

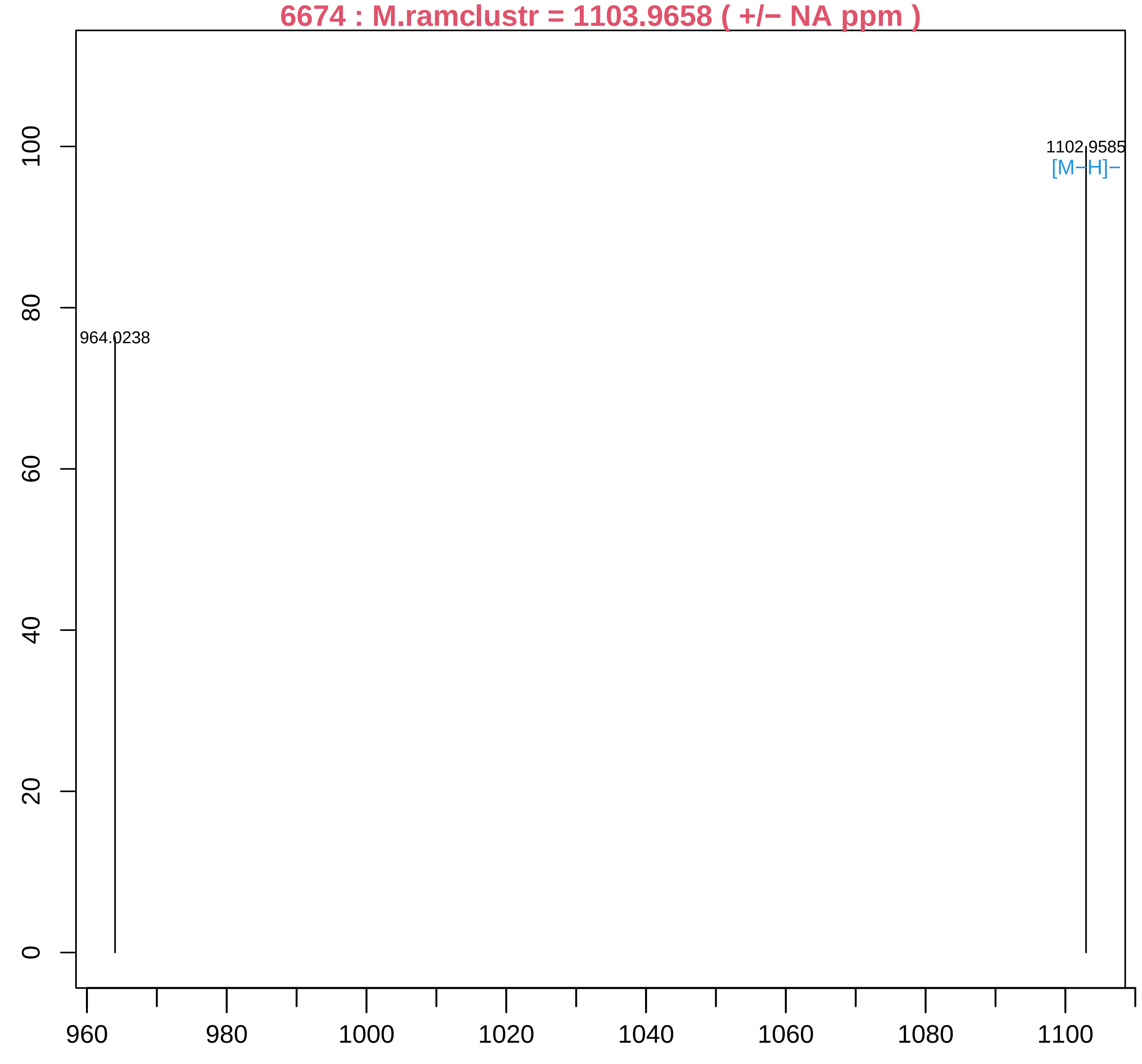

6674 : M.ramclustr = 1103.9658 (+/− NA ppm)
1102.9585
[M−H]−
964.0238
0
20
40
60
80
100
960
980
1000
1020
1040
1060
1080
1100

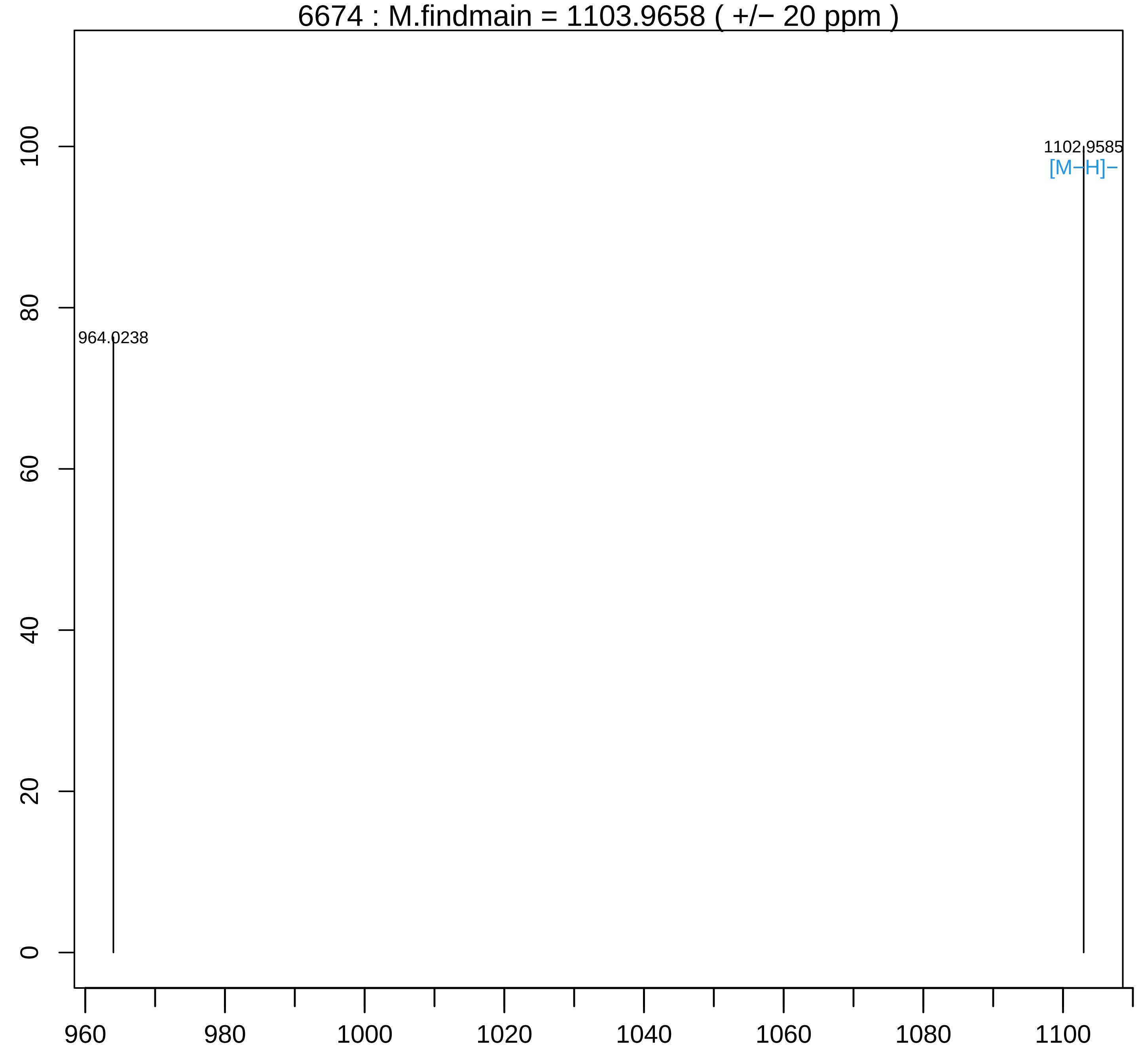

6674 : M.findmain = 1103.9658 (+/− 20 ppm)
1102.9585
[M−H]−
964.0238
0
20
40
60
80
100
960
980
1000
1020
1040
1060
1080
1100

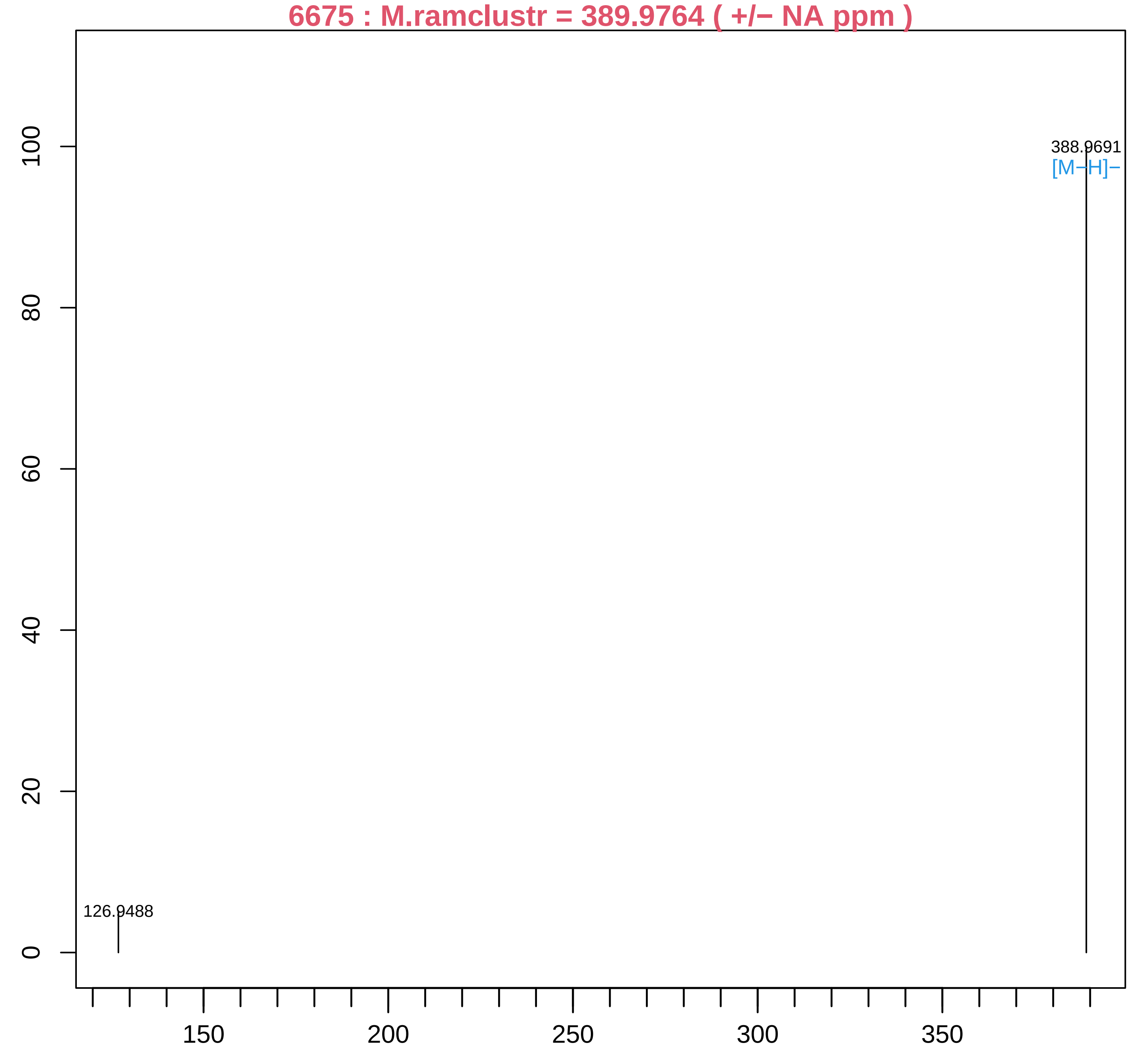
6675 : M.ramclustr = 389.9764 (+/− NA ppm)
388.9691
[M−H]−
126.9488
0
20
40
60
80
100
150
200
250
300
350

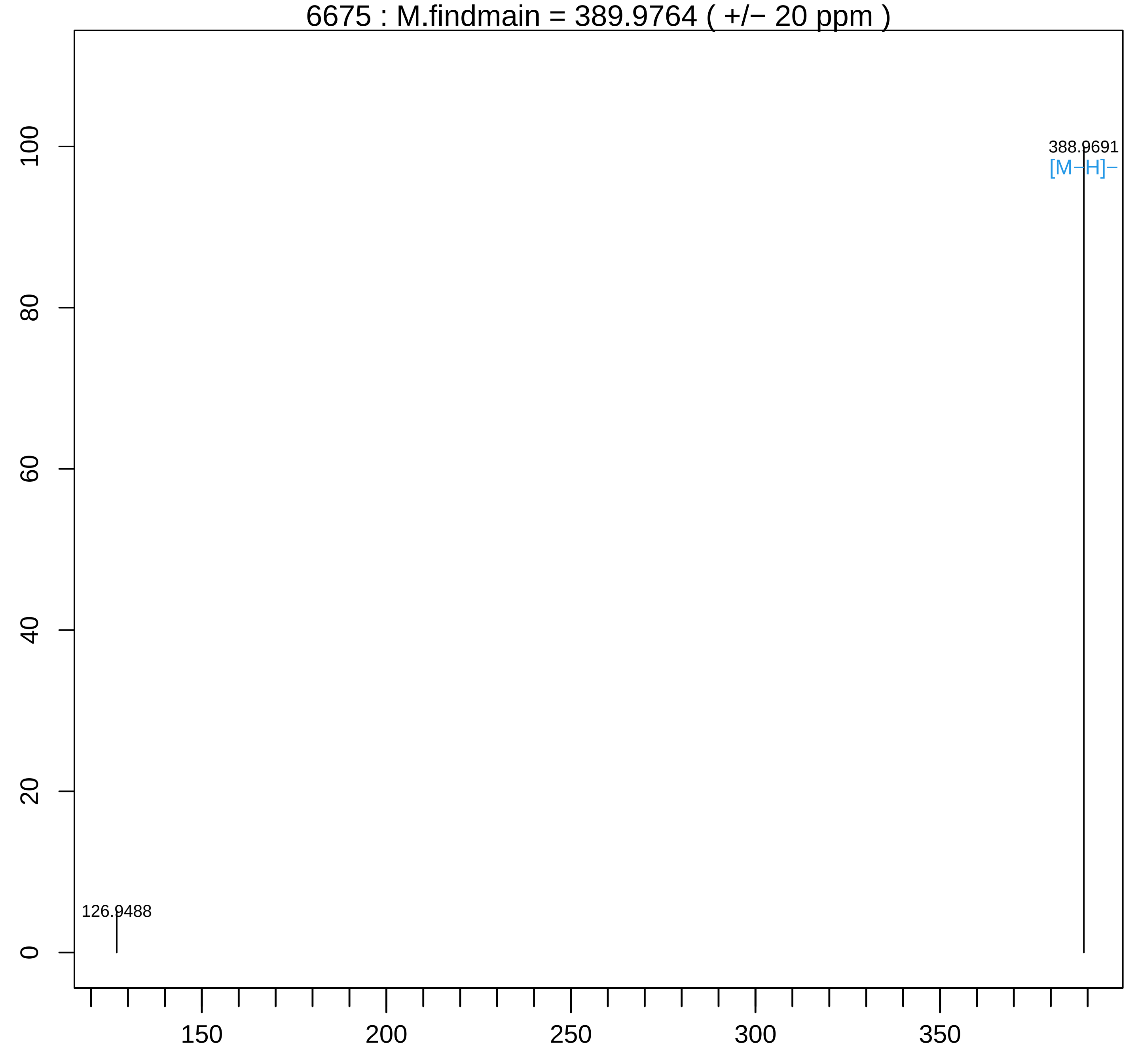
6675 : M.findmain = 389.9764 (+/− 20 ppm)
388.9691
[M−H]−
126.9488
0
20
40
60
80
100
150
200
250
300
350

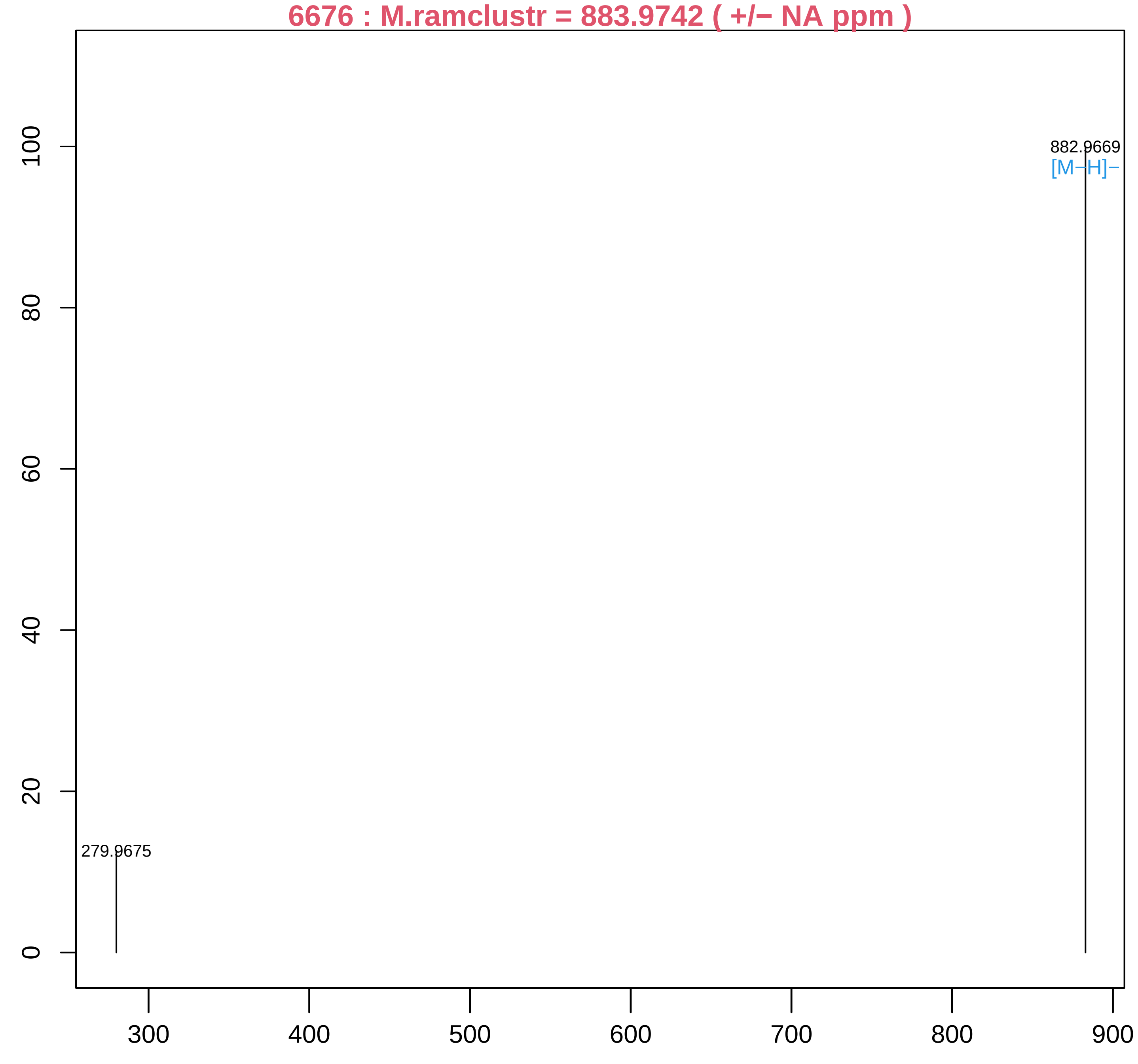
6676 : M.ramclustr = 883.9742 (+/− NA ppm)
882.9669
[M−H]−
279.9675
0
20
40
60
80
100
300
400
500
600
700
800
900

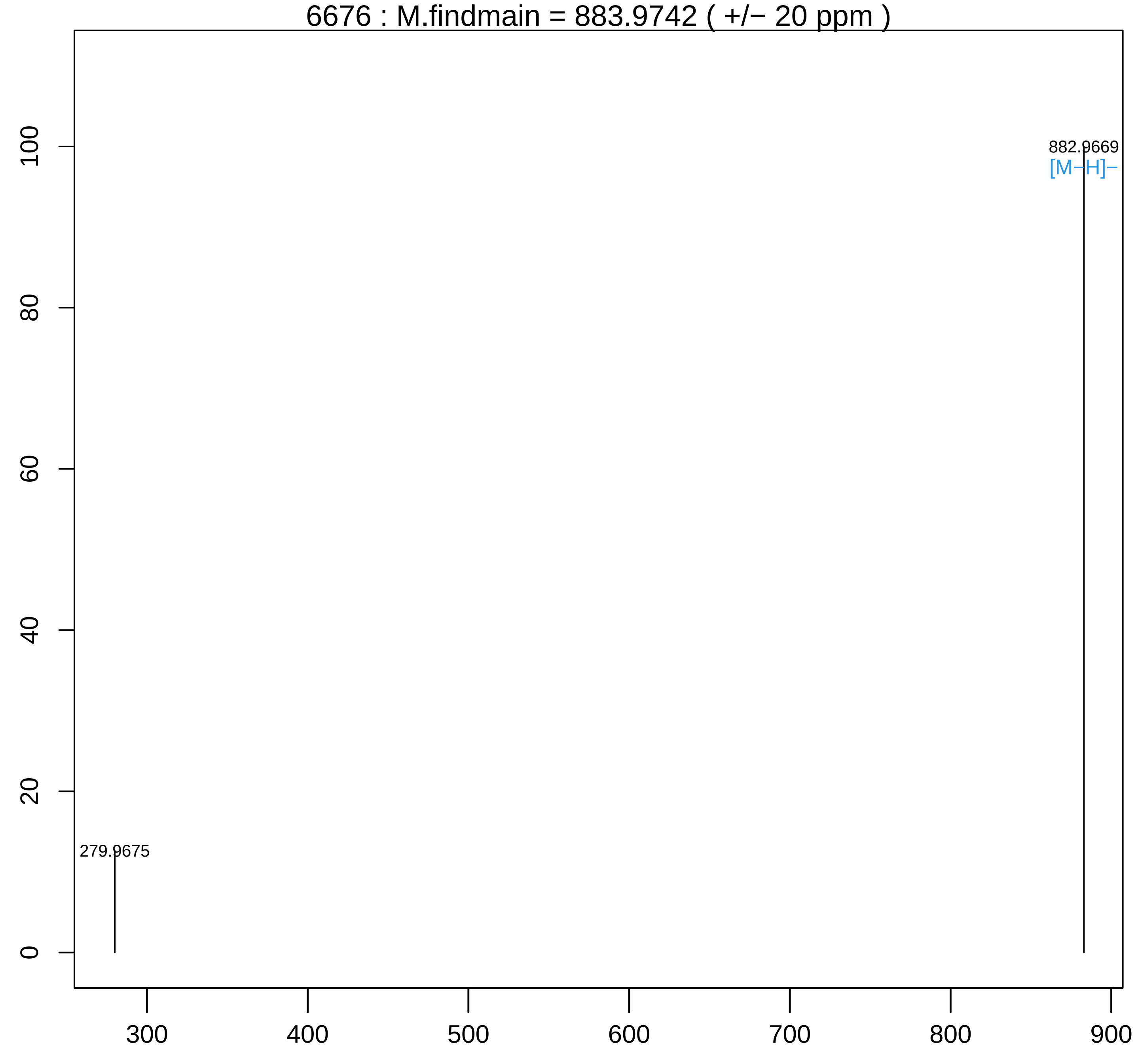
6676 : M.findmain = 883.9742 (+/− 20 ppm)
882.9669
[M−H]−
279.9675
0
20
40
60
80
100
300
400
500
600
700
800
900

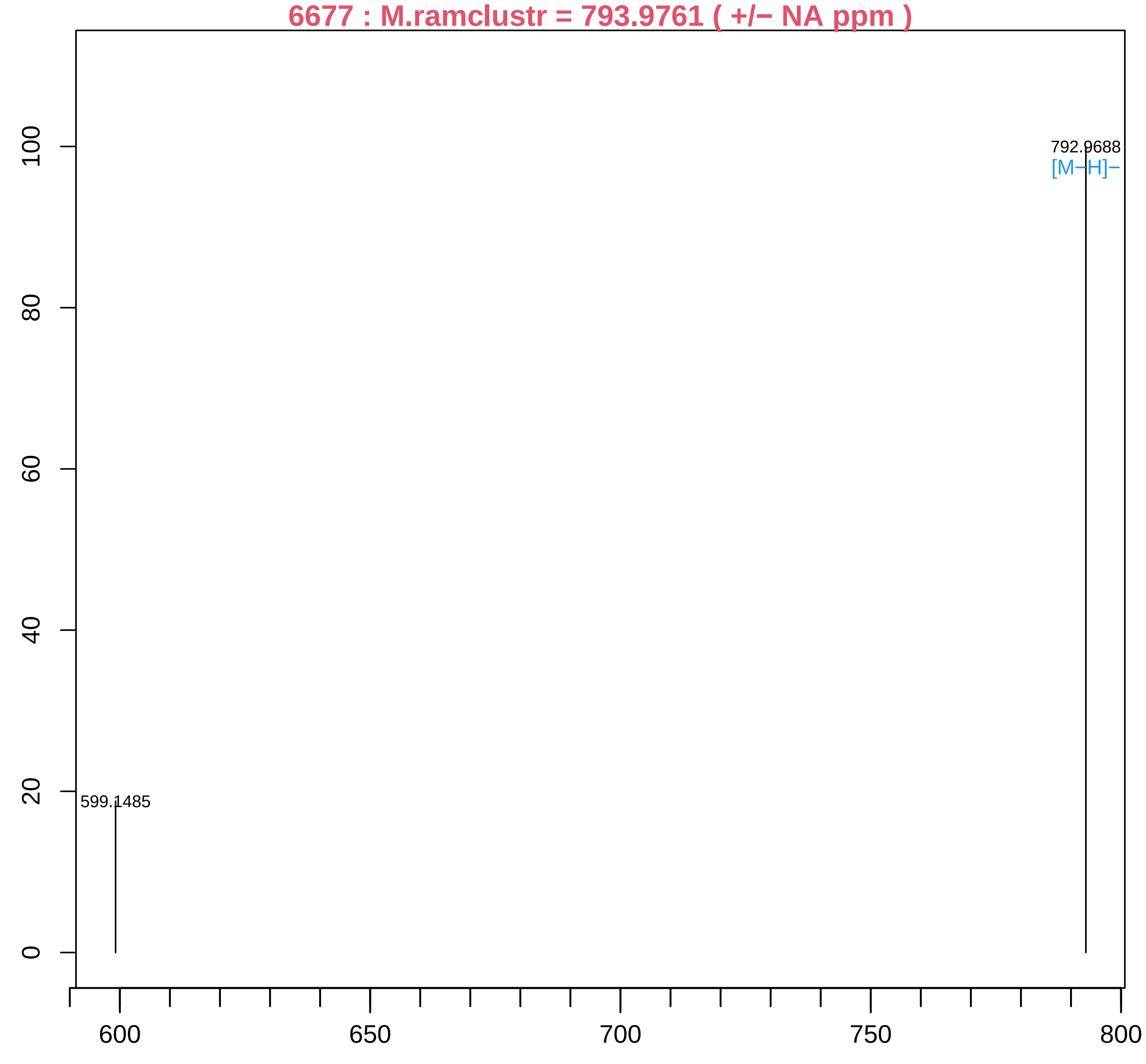
6677 : M.ramclustr = 793.9761 (+/− NA ppm)
792.9688
[M−H]−
599.1485

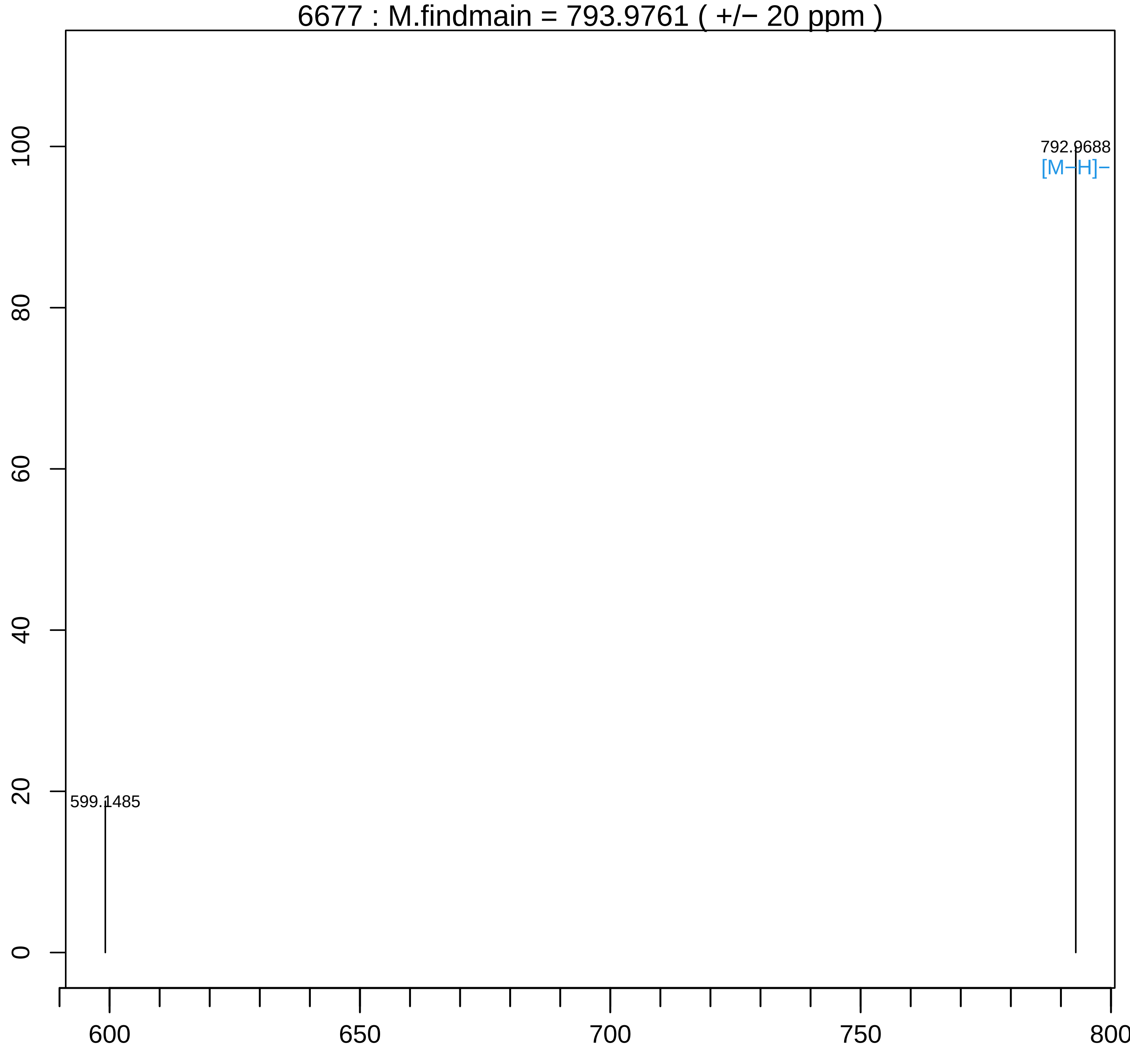
6677 : M.findmain = 793.9761 (+/− 20 ppm)
792.9688
[M−H]−
599.1485

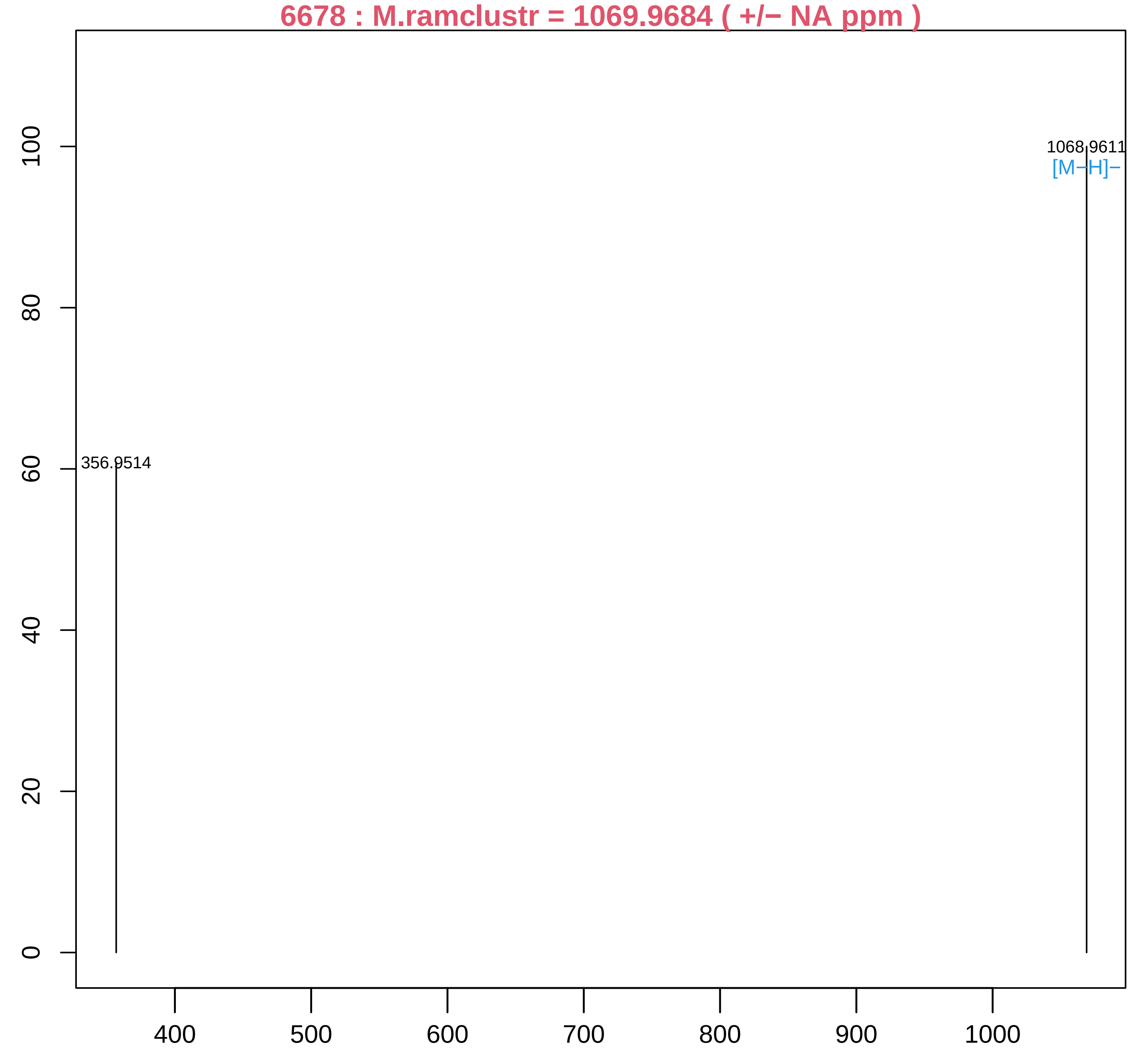
6678 : M.ramclustr = 1069.9684 (+/− NA ppm)
1068.9611
[M−H]−
356.9514
0
20
40
60
80
100
400
500
600
700
800
900
1000

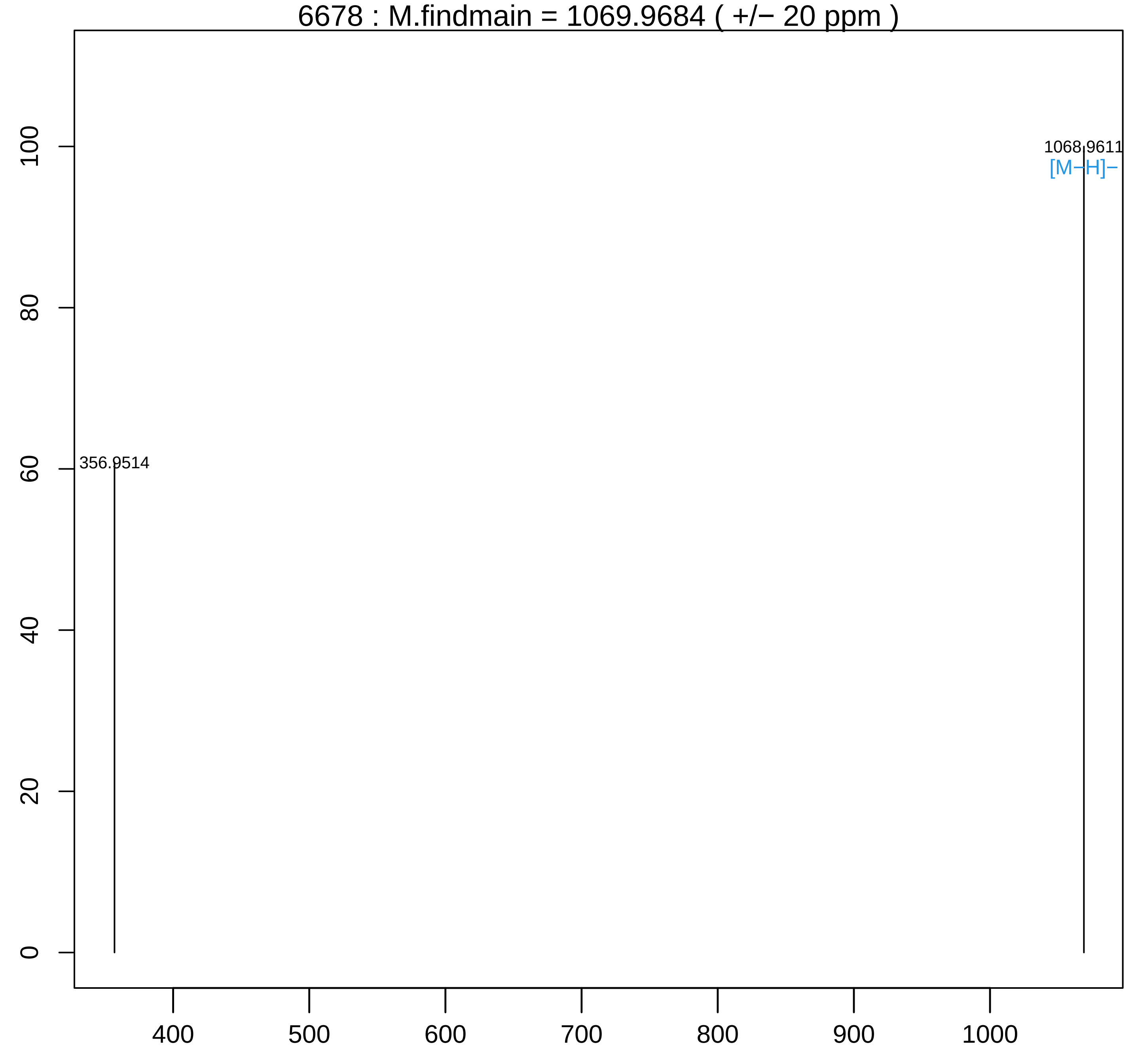
6678 : M.findmain = 1069.9684 (+/− 20 ppm)
1068.9611
[M−H]−
356.9514
0
20
40
60
80
100
400
500
600
700
800
900
1000

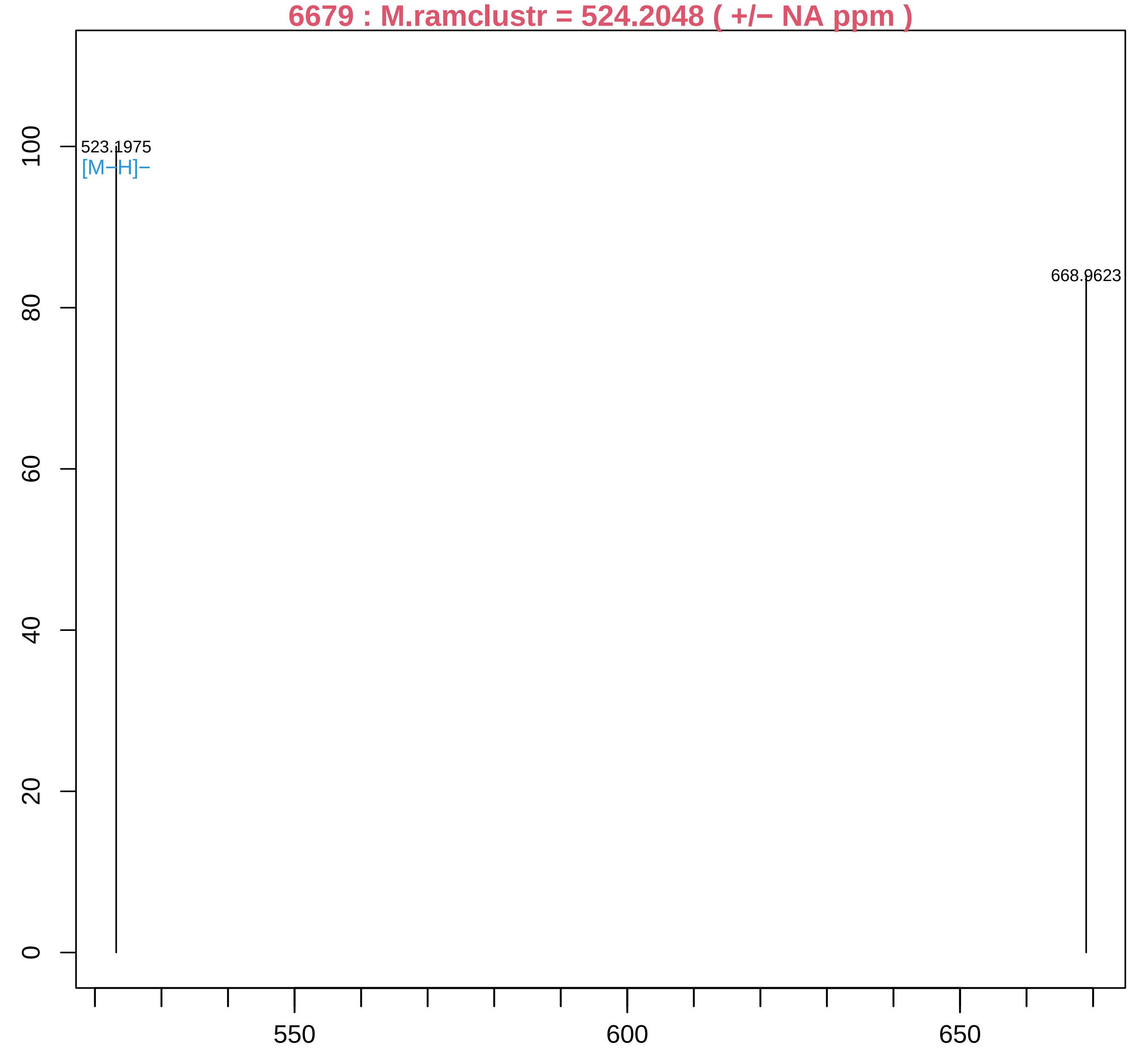

6679 : M.ramclustr = 524.2048 (+/− NA ppm)
523.1975
[M−H]−
668.9623
0
20
40
60
80
100
550
600
650

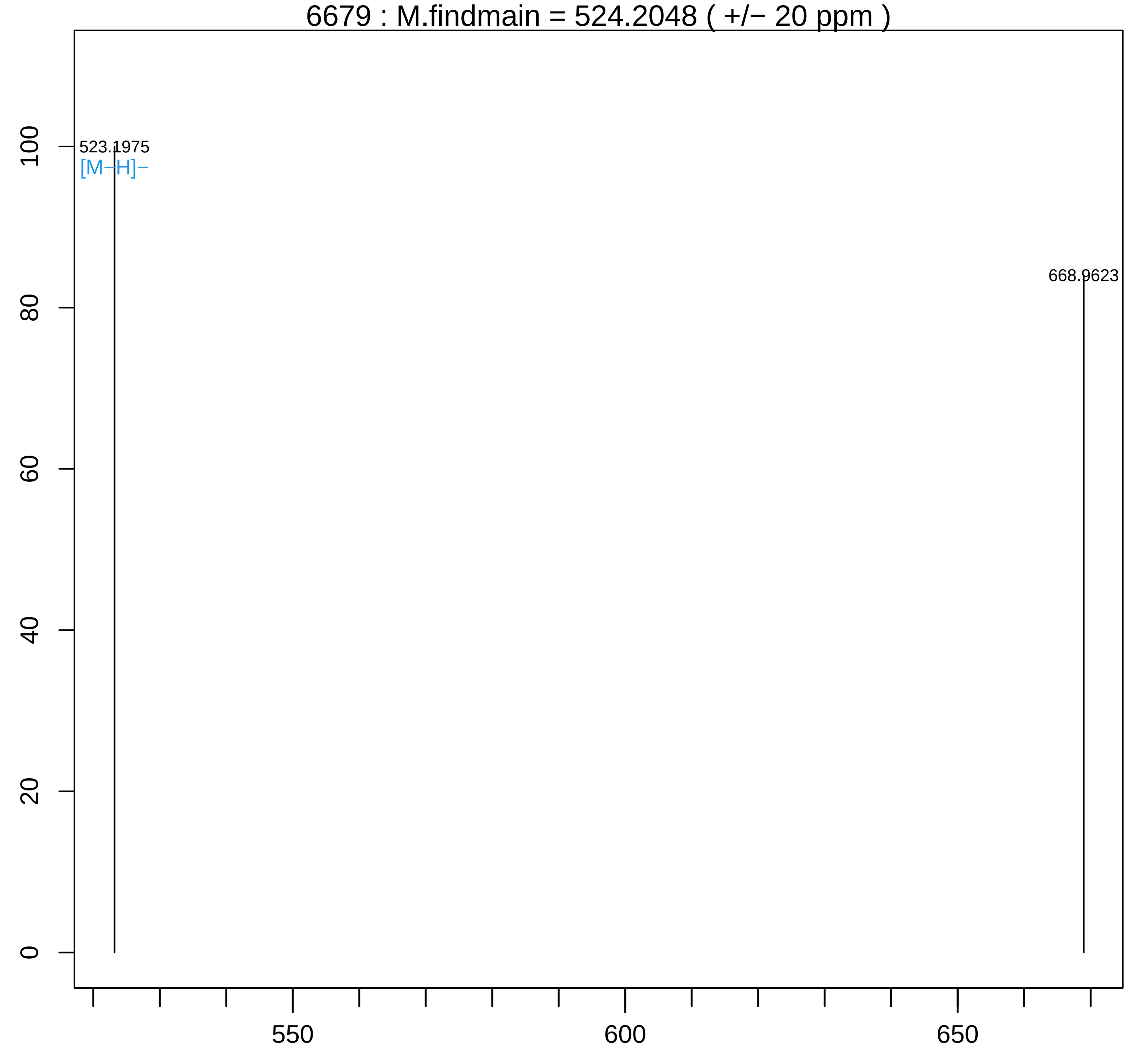

6679 : M.findmain = 524.2048 (+/− 20 ppm)
523.1975
[M−H]−
668.9623
0
20
40
60
80
100
550
600
650

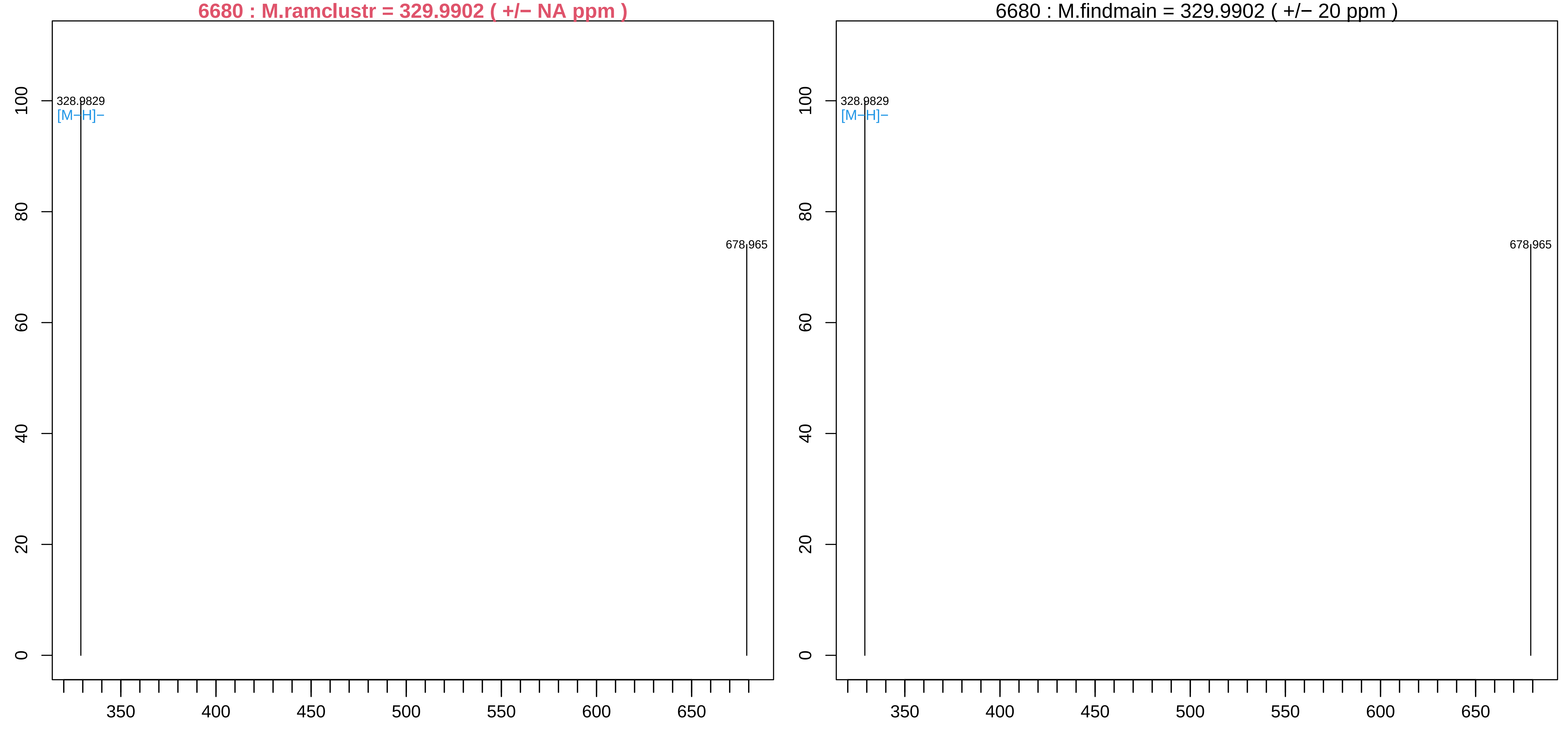
6680 : M.ramclustr = 329.9902 (+/− NA ppm)
328.9829
[M−H]−
678.965
6680 : M.findmain = 329.9902 (+/− 20 ppm)
328.9829
[M−H]−
678.965
0
20
40
60
80
100
350
400
450
500
550
600
650

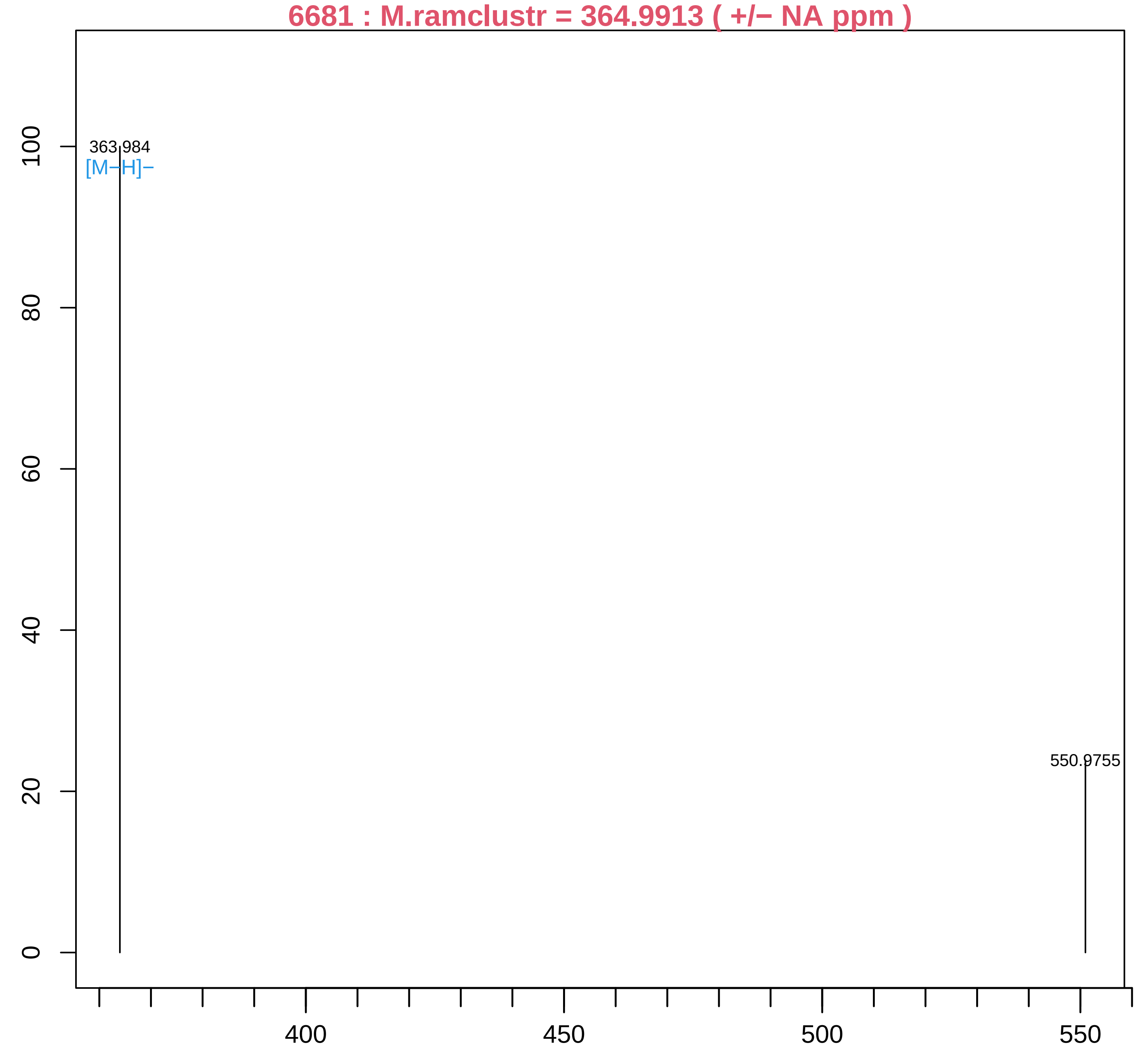
6681 : M.ramclustr = 364.9913 (+/− NA ppm)
363.984
[M−H]−
550.9755
0
20
40
60
80
100
400
450
500
550
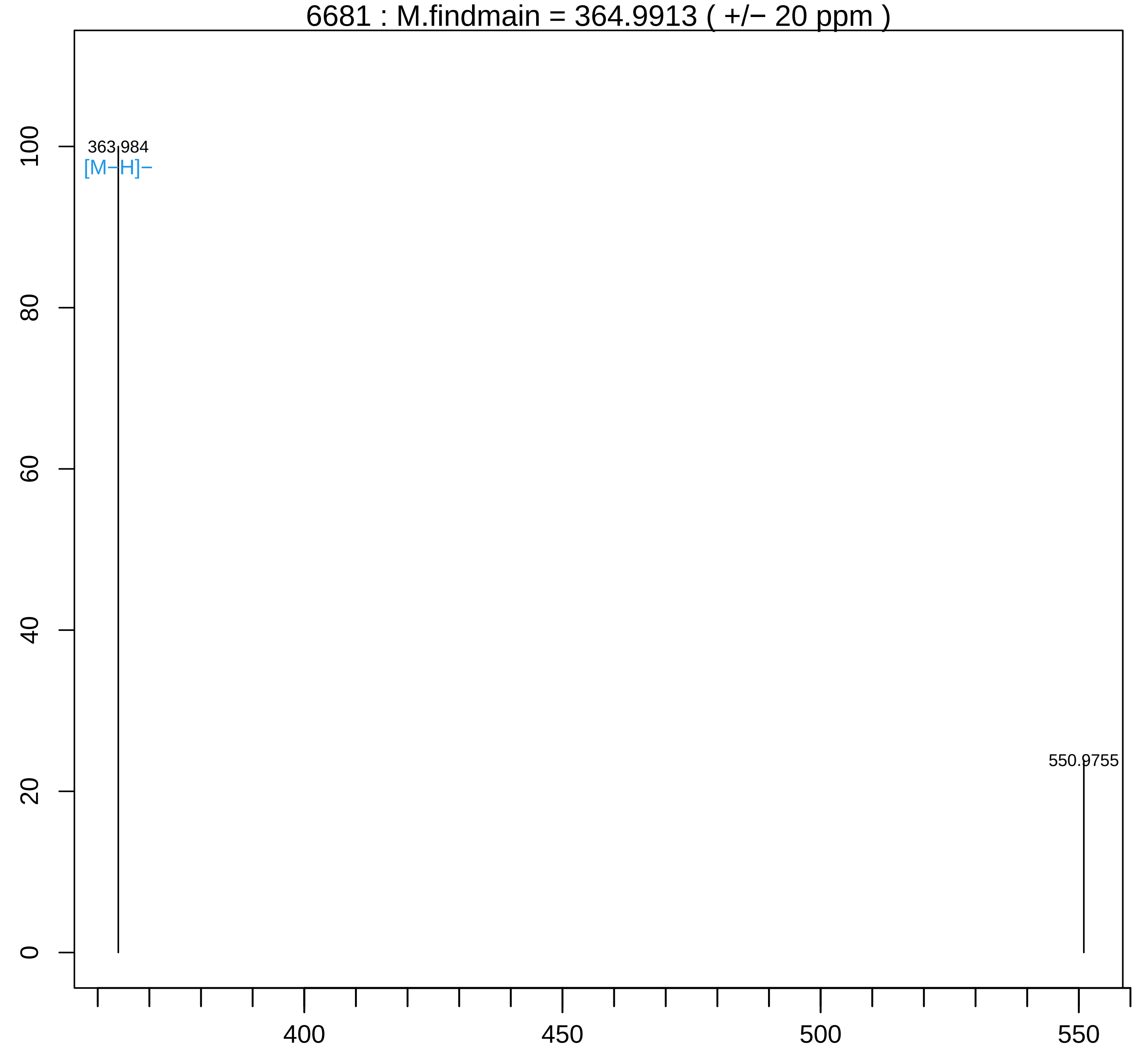
6681 : M.findmain = 364.9913 (+/− 20 ppm)
363.984
[M−H]−
550.9755
0
20
40
60
80
100
400
450
500
550

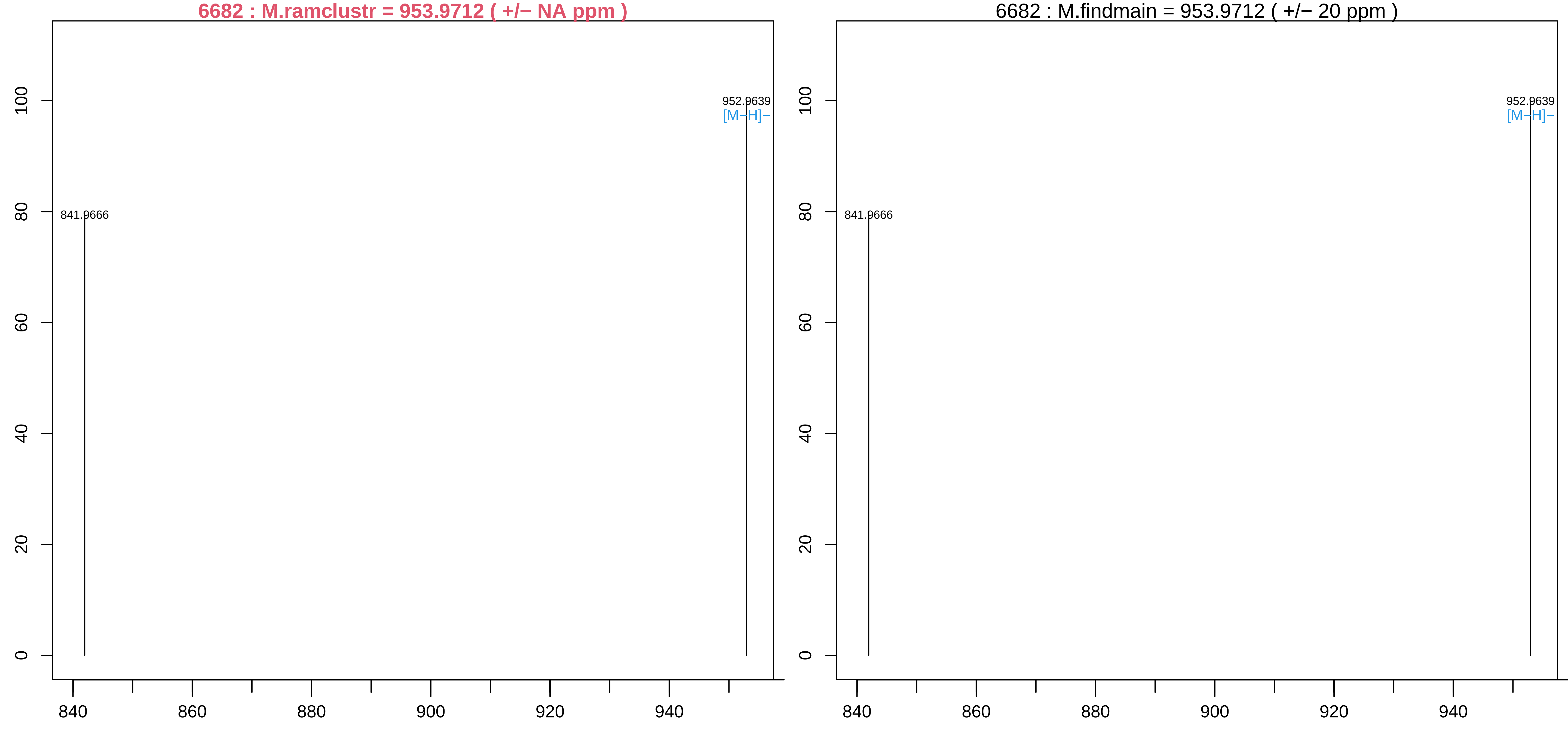
6682 : M.ramclustr = 953.9712 (+/− NA ppm)
952.9639
[M−H]−
841.9666
6682 : M.findmain = 953.9712 (+/− 20 ppm)
952.9639
[M−H]−
841.9666
0
20
40
60
80
100
840
860
880
900
920
940

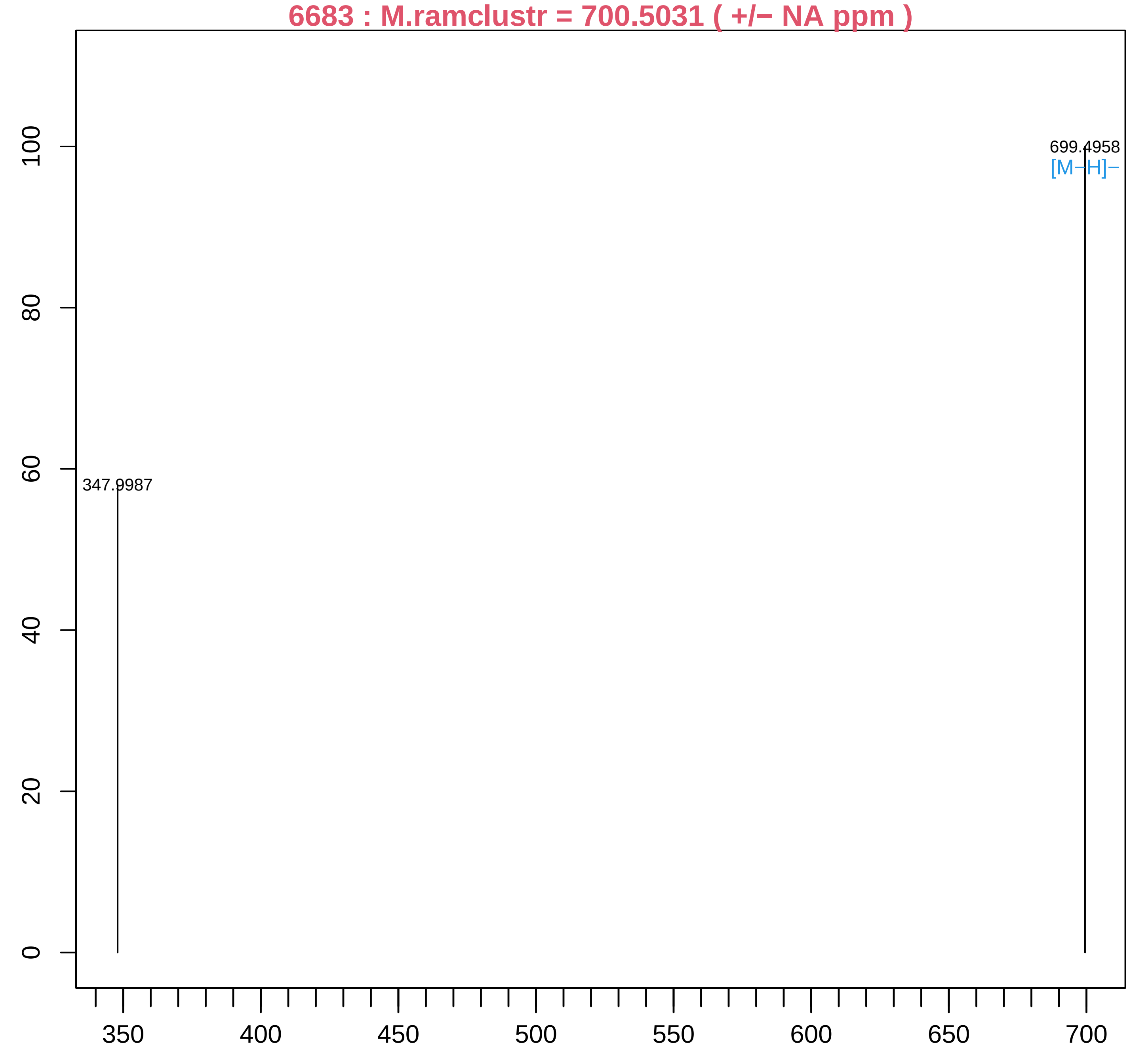
6683 : M.ramclustr = 700.5031 (+/− NA ppm)
699.4958
[M−H]−
347.9987
0
20
40
60
80
100
350
400
450
500
550
600
650
700

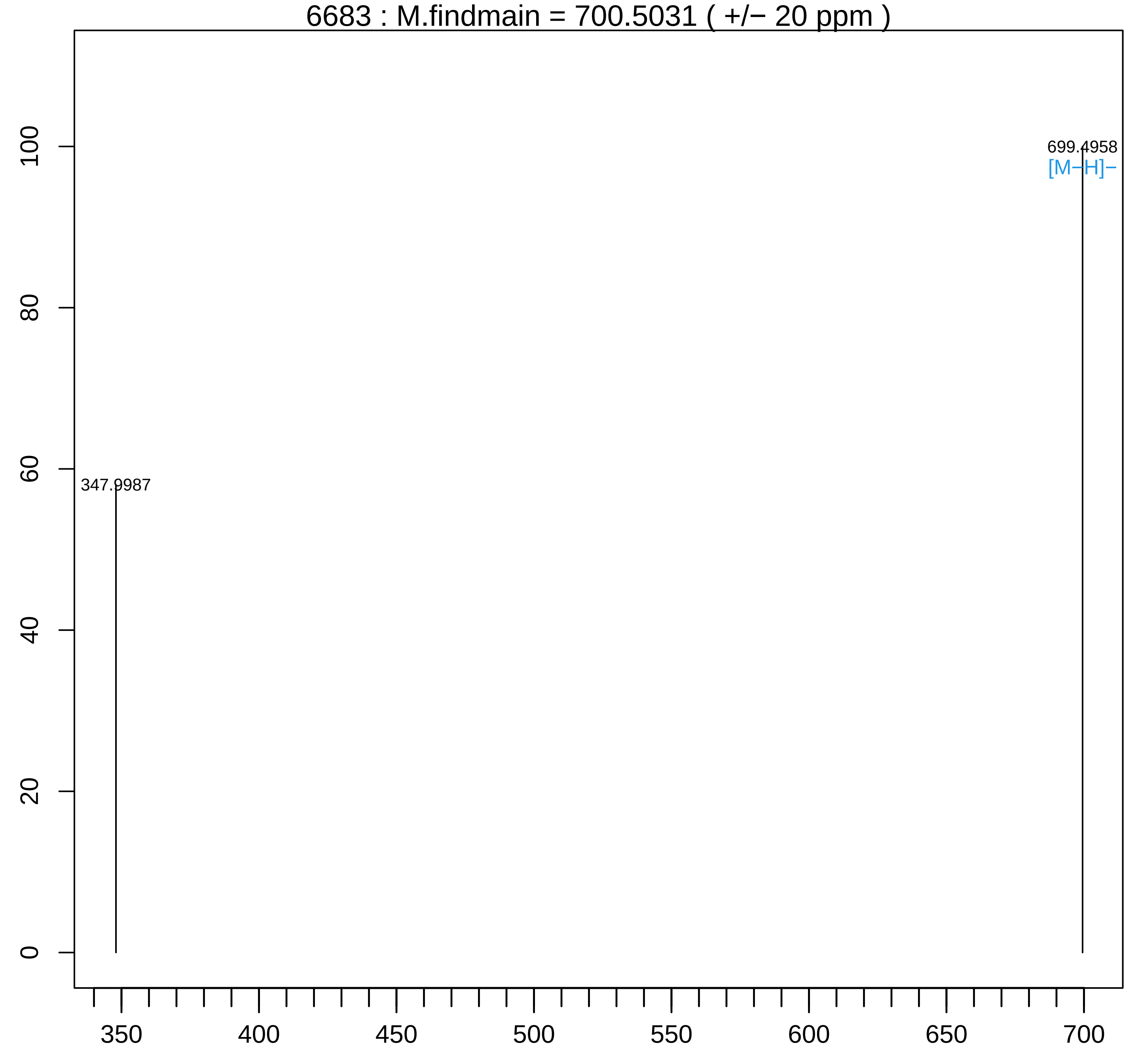
6683 : M.findmain = 700.5031 (+/− 20 ppm)
699.4958
[M−H]−
347.9987
0
20
40
60
80
100
350
400
450
500
550
600
650
700

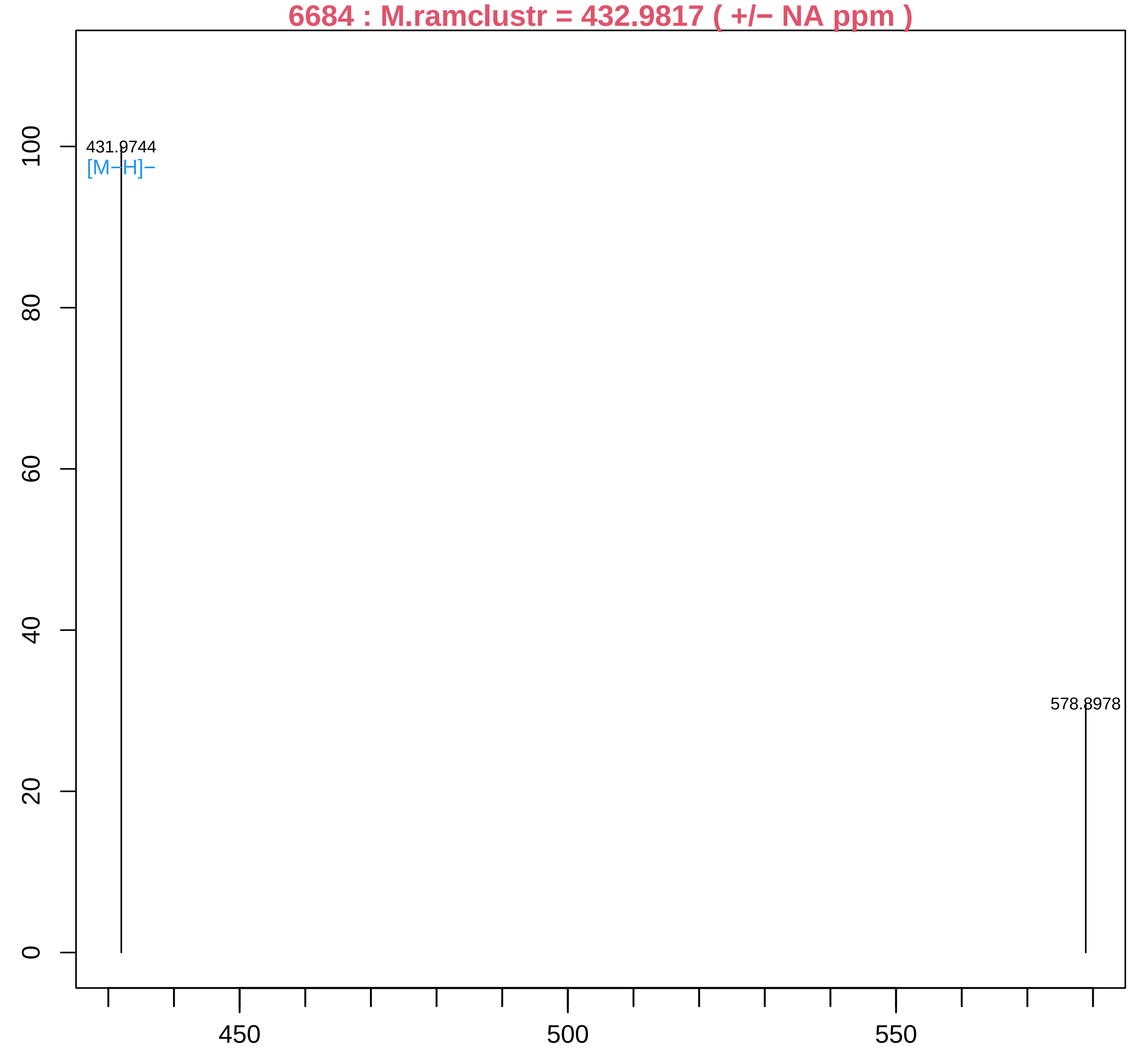
6684 : M.ramclustr = 432.9817 (+/− NA ppm)
431.9744
[M−H]−
578.8978
0
20
40
60
80
100
450
500
550

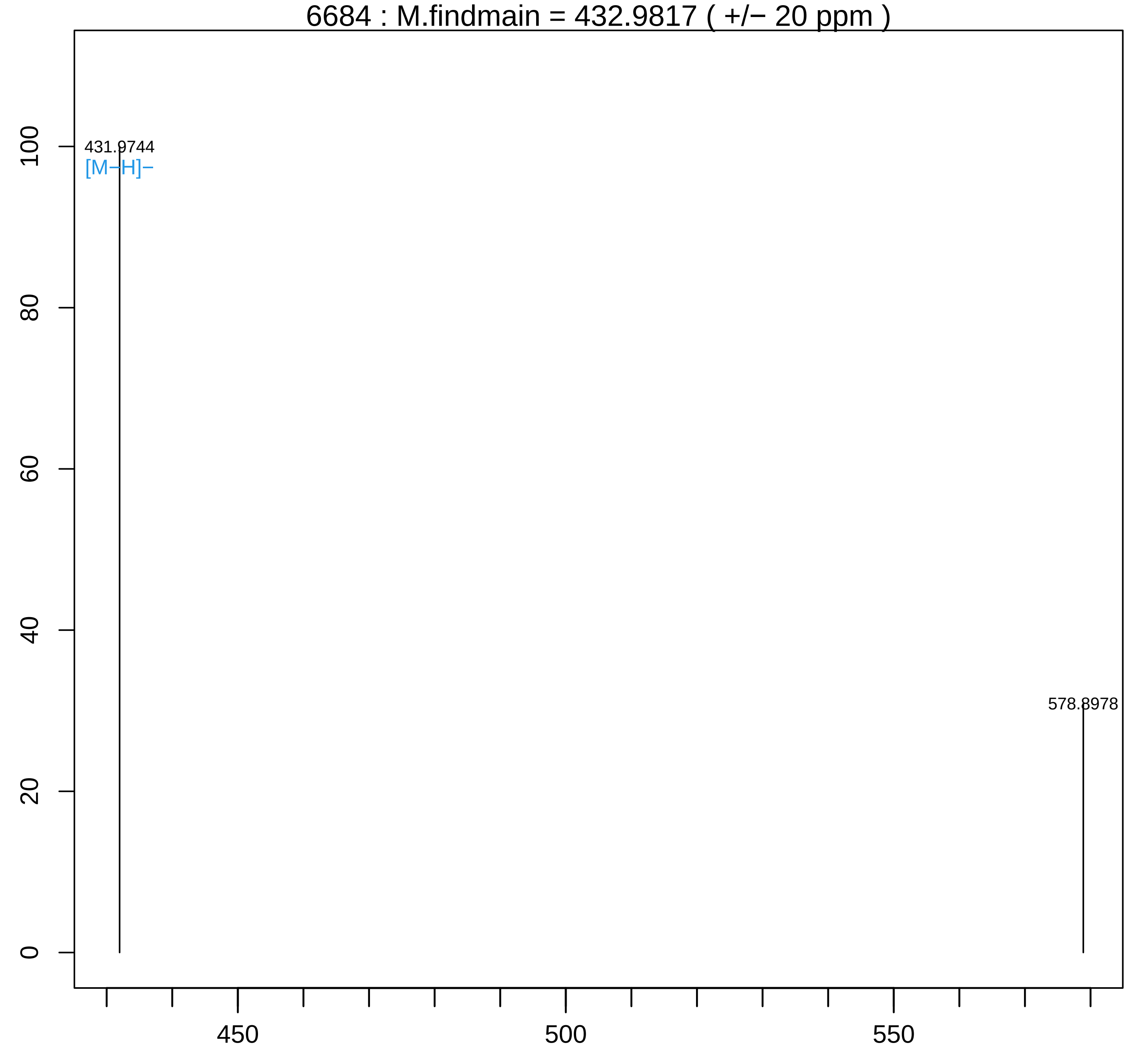
6684 : M.findmain = 432.9817 (+/− 20 ppm)
431.9744
[M−H]−
578.8978
0
20
40
60
80
100
450
500
550

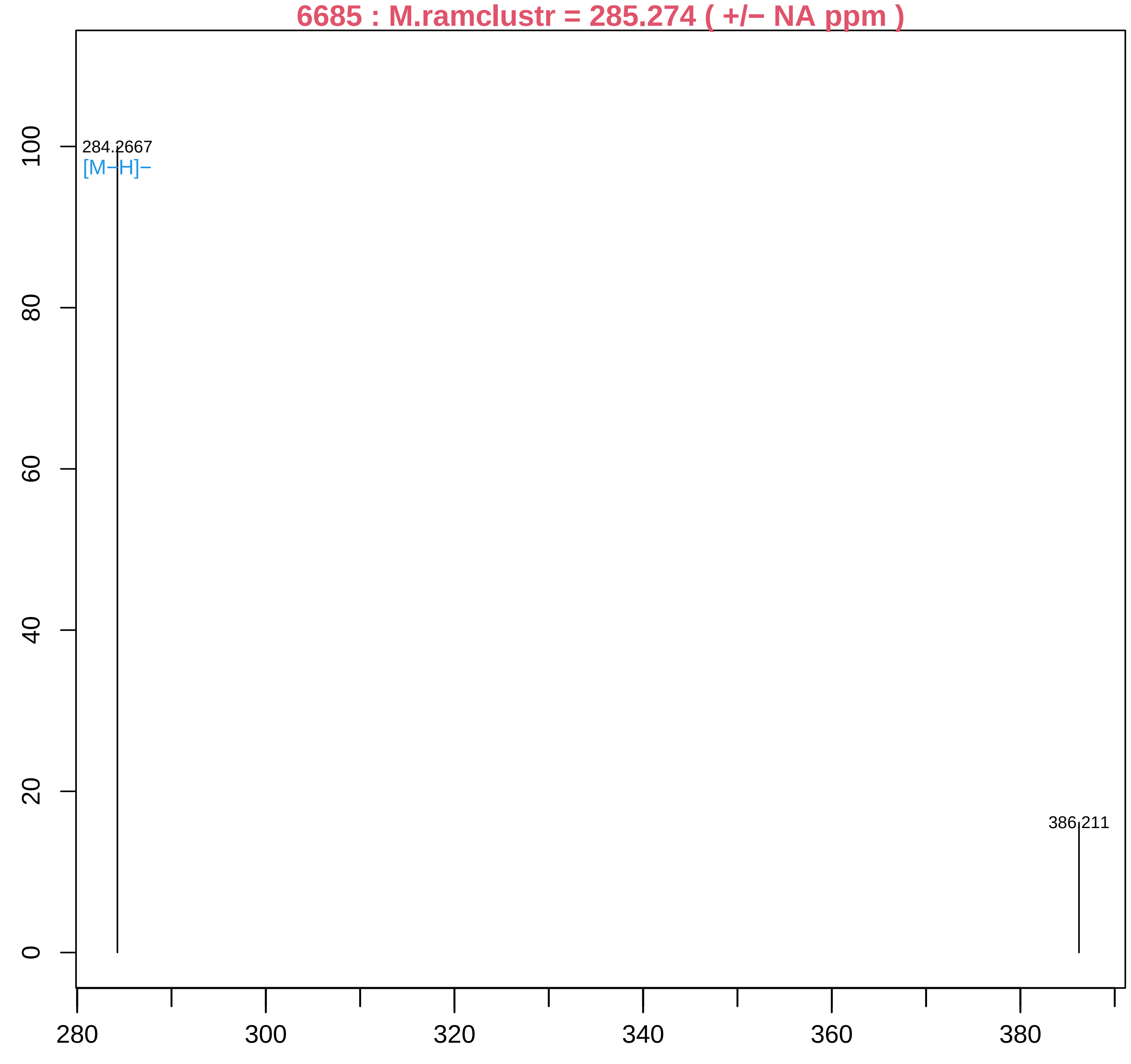

6685 : M.ramclustr = 285.274 (+/− NA ppm)
284.2667
[M−H]−
386.211

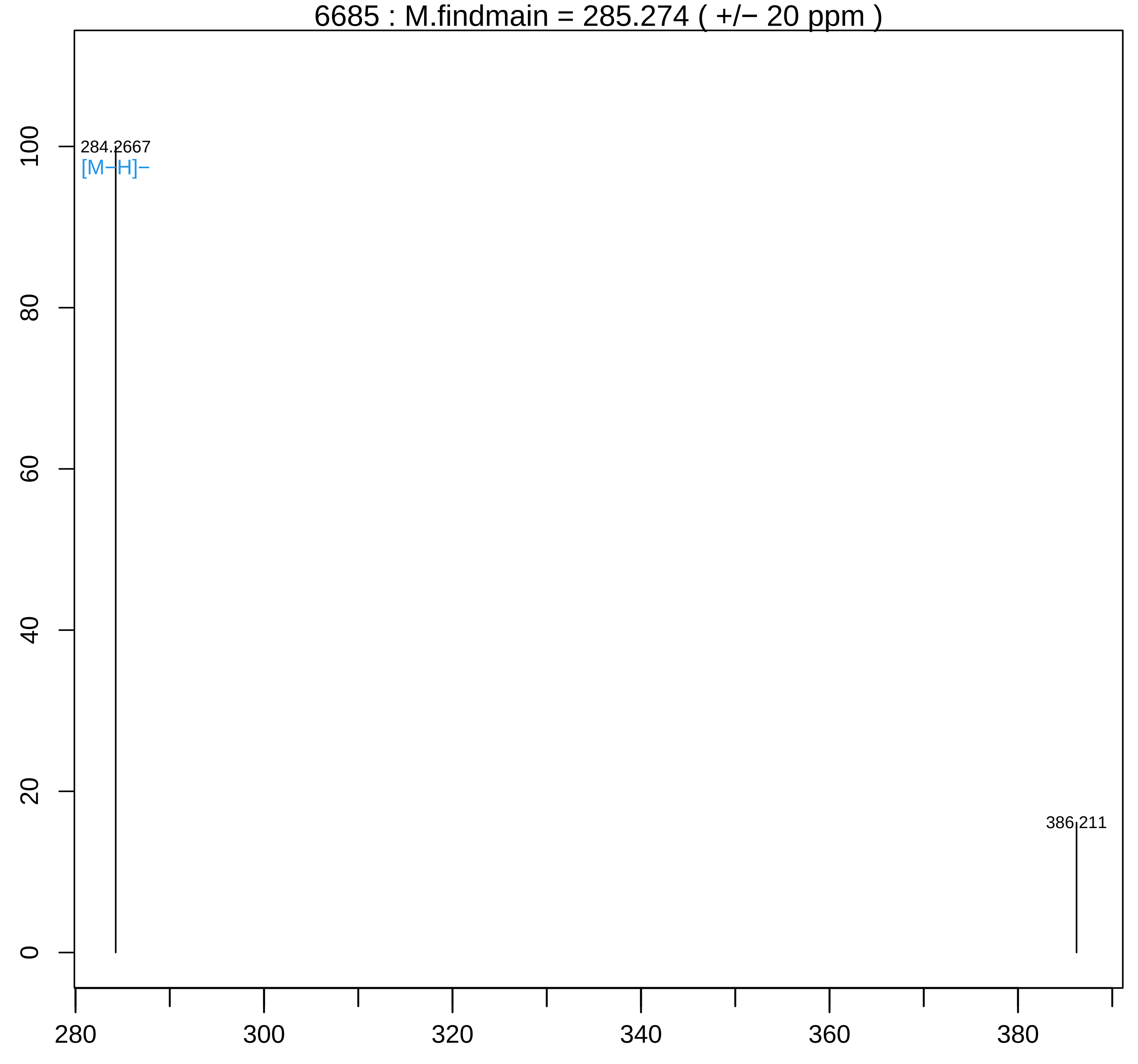

6685 : M.findmain = 285.274 (+/− 20 ppm)
284.2667
[M−H]−
386.211

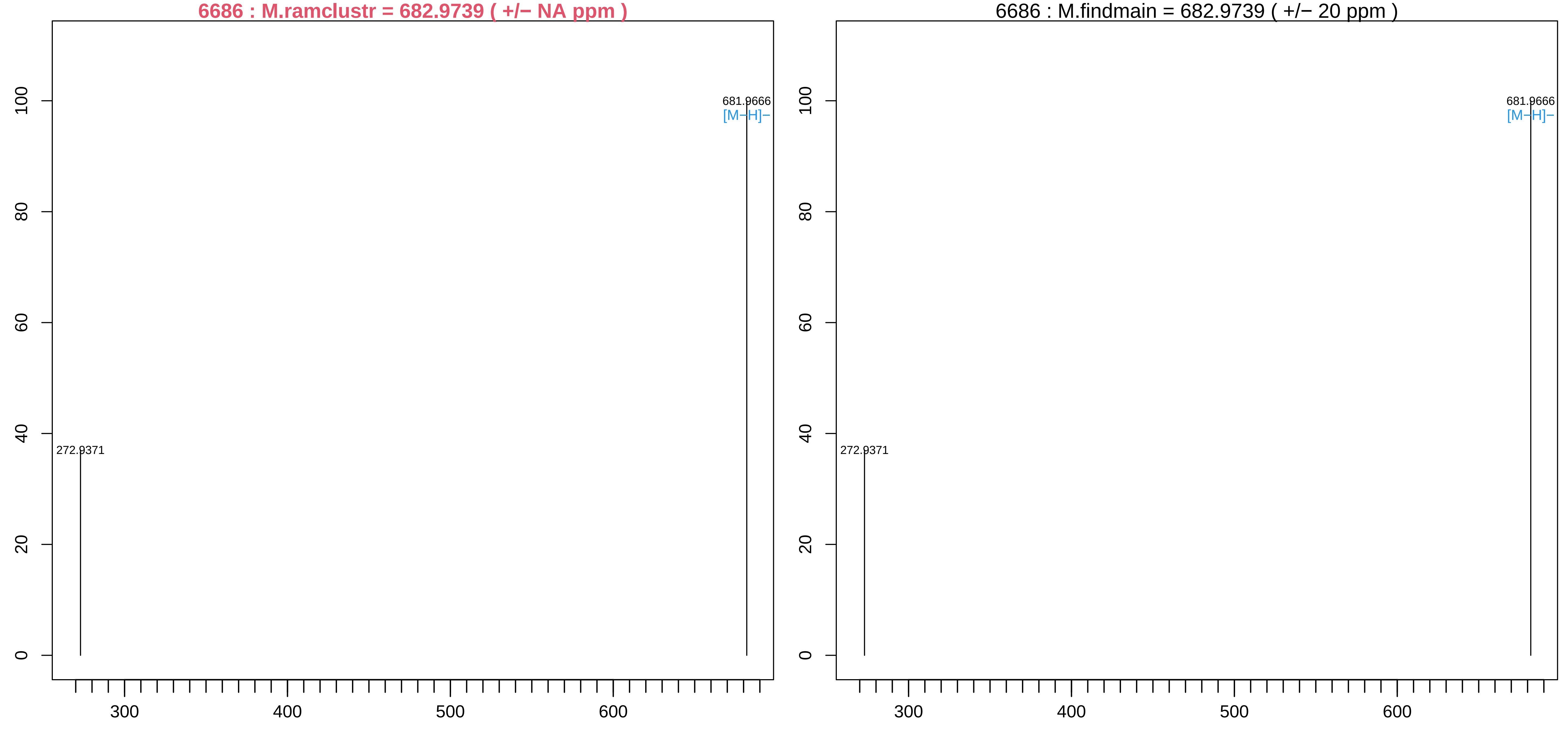

6686 : M.ramclustr = 682.9739 (+/− NA ppm)
681.9666
[M−H]−
272.9371
0
20
40
60
80
100
300
400
500
600
6686 : M.findmain = 682.9739 (+/− 20 ppm)
681.9666
[M−H]−
272.9371
0
20
40
60
80
100
300
400
500
600

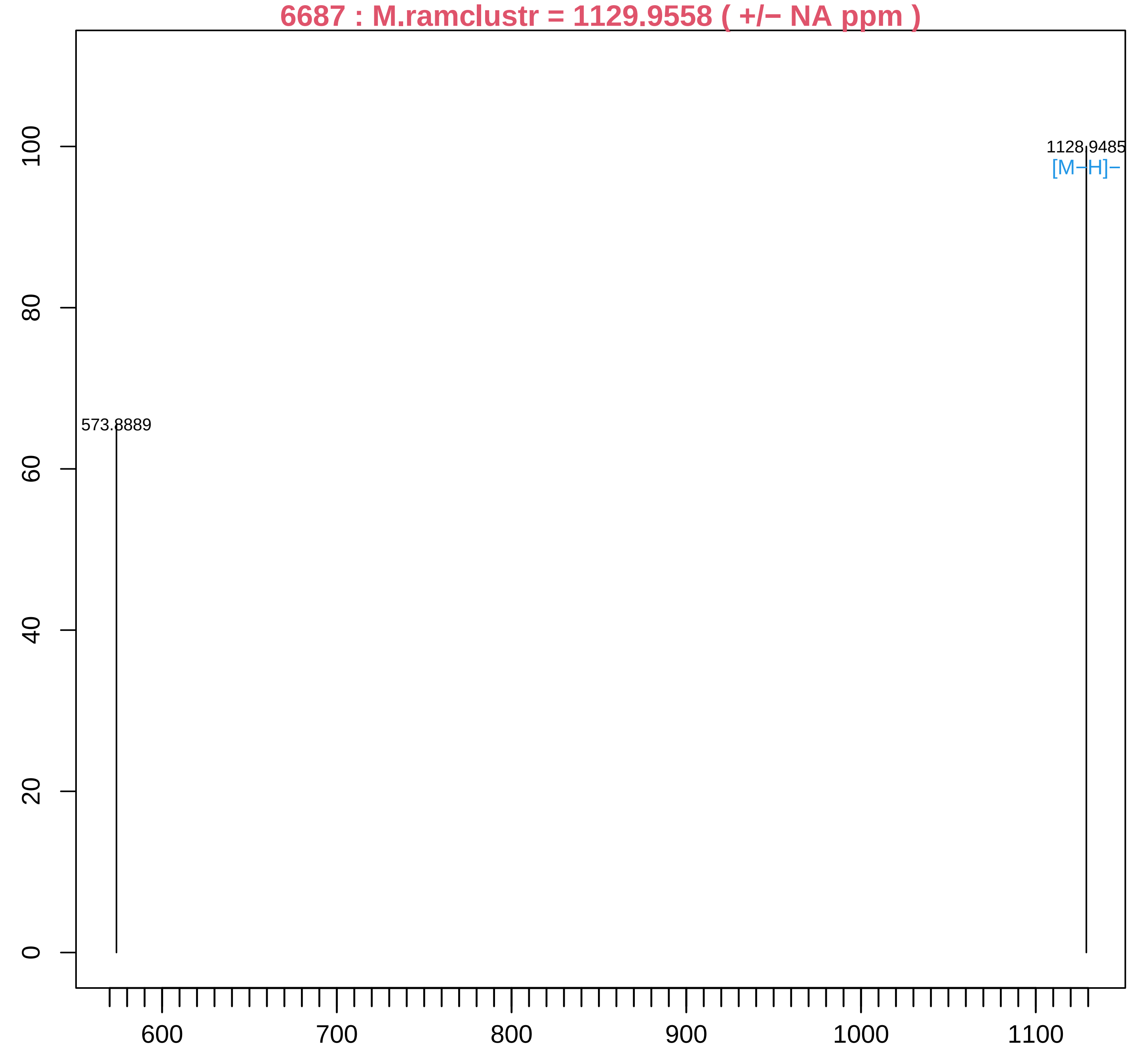
6687 : M.ramclustr = 1129.9558 (+/− NA ppm)
1128.9485
[M−H]−
573.8889
0
20
40
60
80
100
600
700
800
900
1000
1100

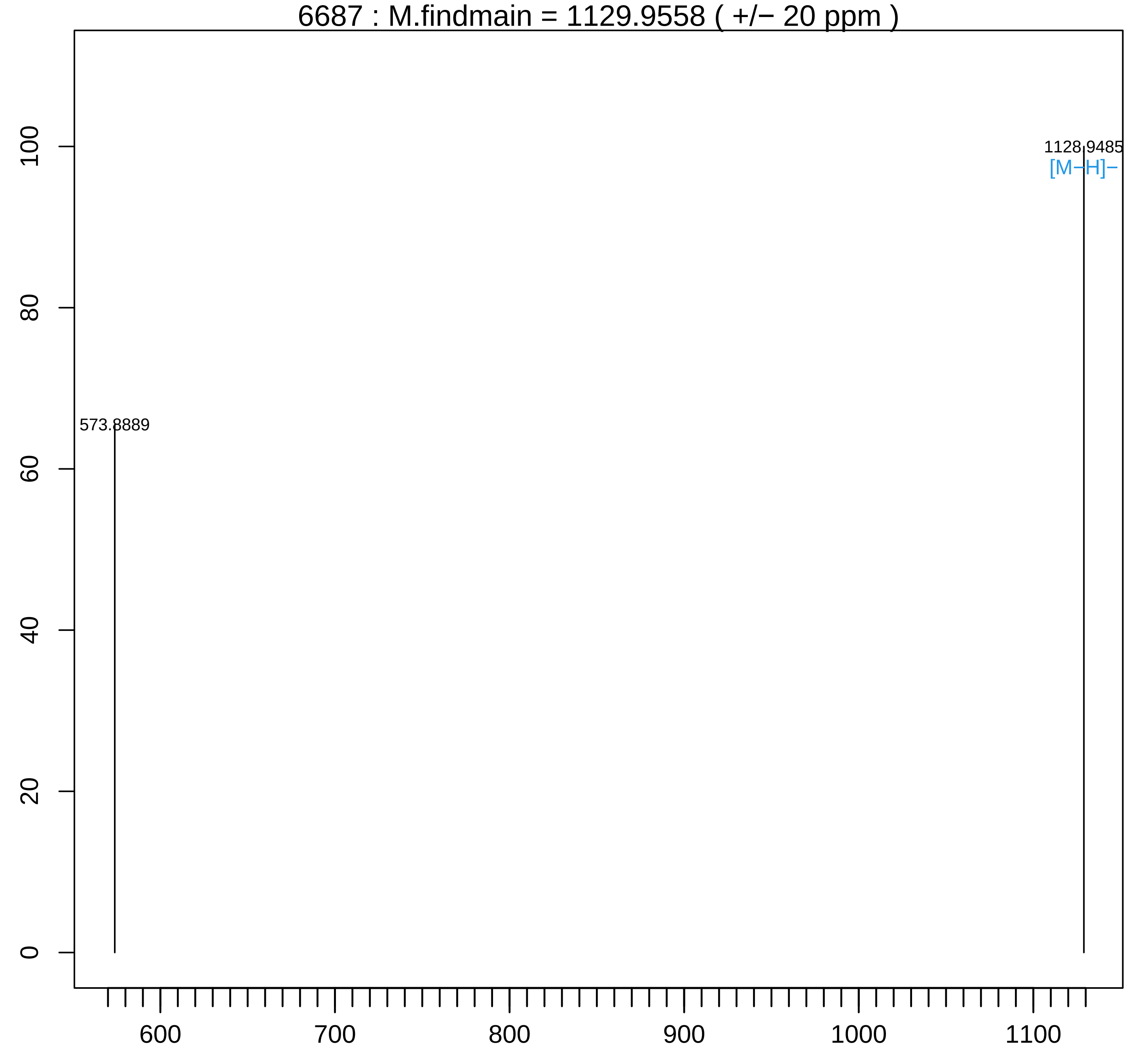
6687 : M.findmain = 1129.9558 (+/− 20 ppm)
1128.9485
[M−H]−
573.8889
0
20
40
60
80
100
600
700
800
900
1000
1100

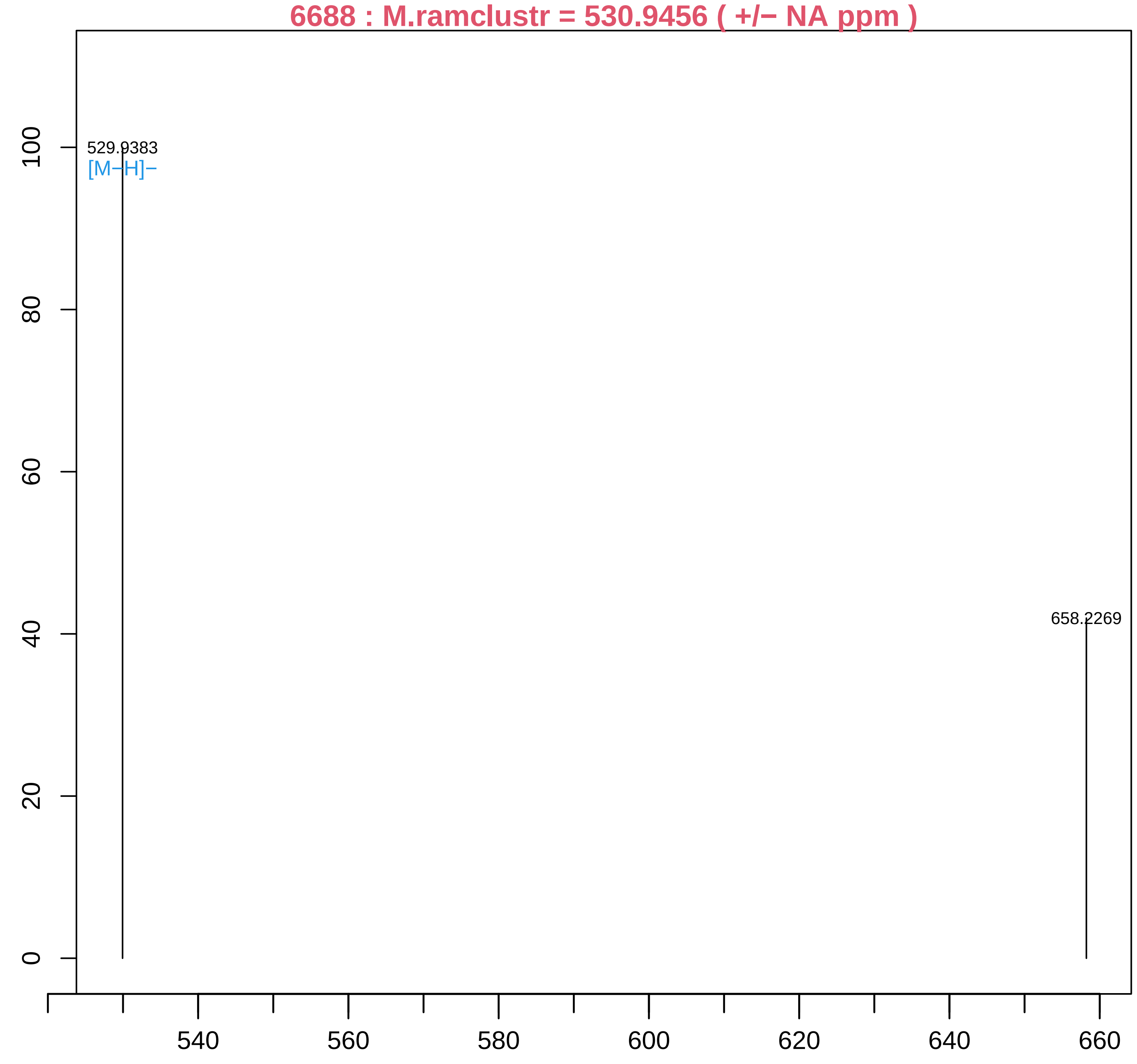
6688 : M.ramclustr = 530.9456 (+/− NA ppm)
529.9383
[M−H]−
658.2269
0
20
40
60
80
100
540
560
580
600
620
640
660

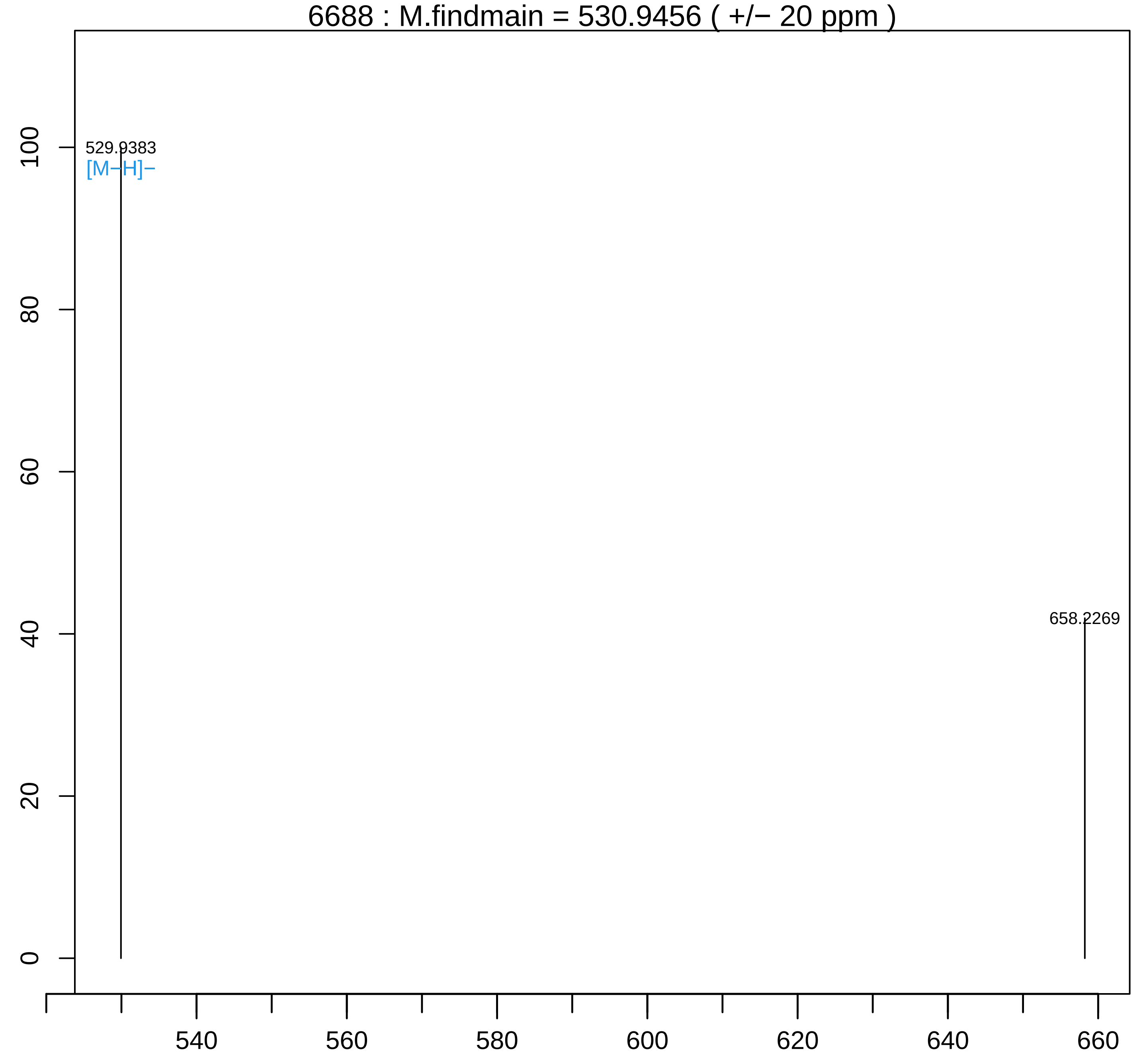
6688 : M.findmain = 530.9456 (+/− 20 ppm)
529.9383
[M−H]−
658.2269
0
20
40
60
80
100
540
560
580
600
620
640
660

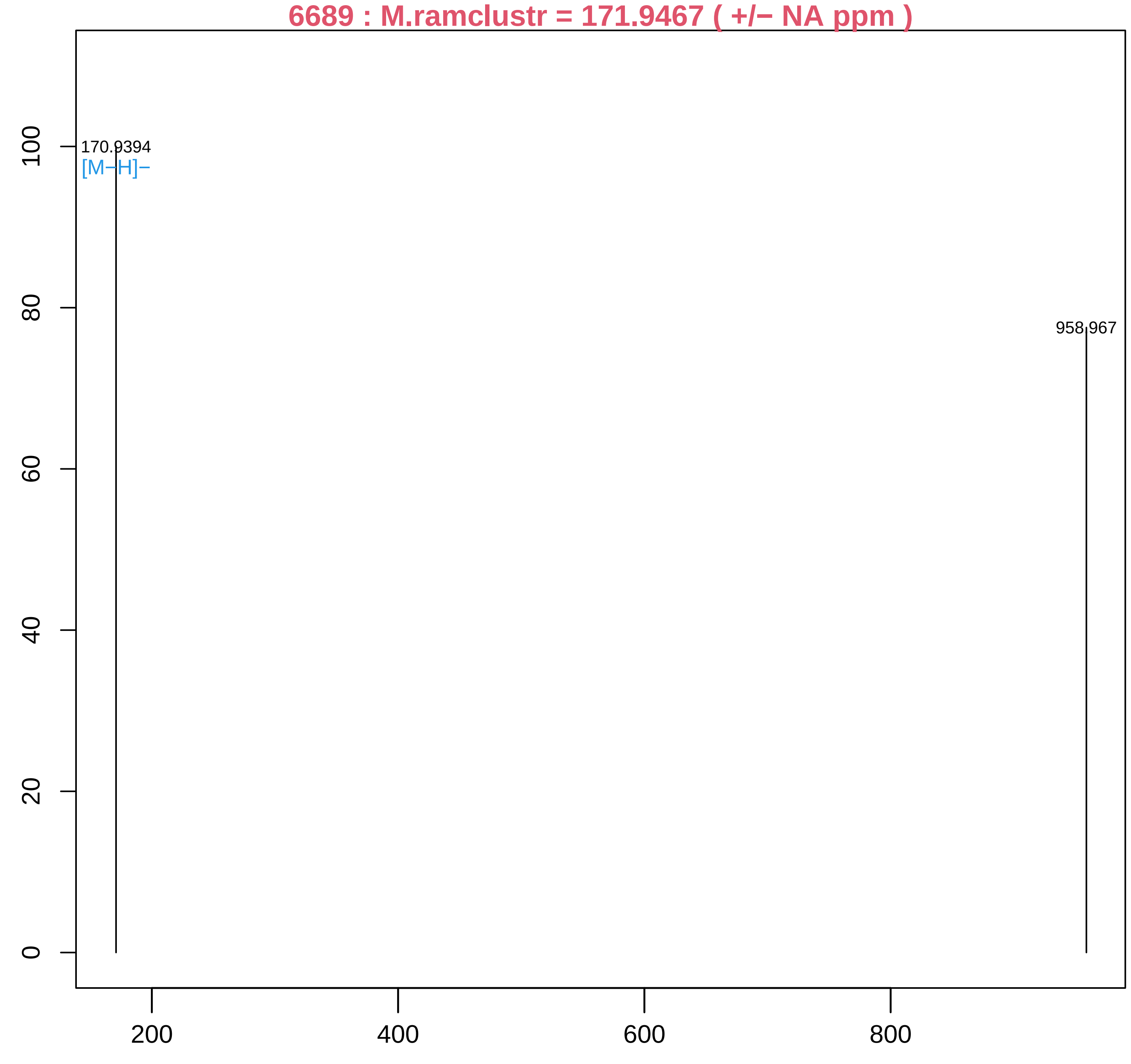

6689 : M.ramclustr = 171.9467 (+/− NA ppm)
170.9394
[M−H]−
958.967
0
20
40
60
80
100
200
400
600
800

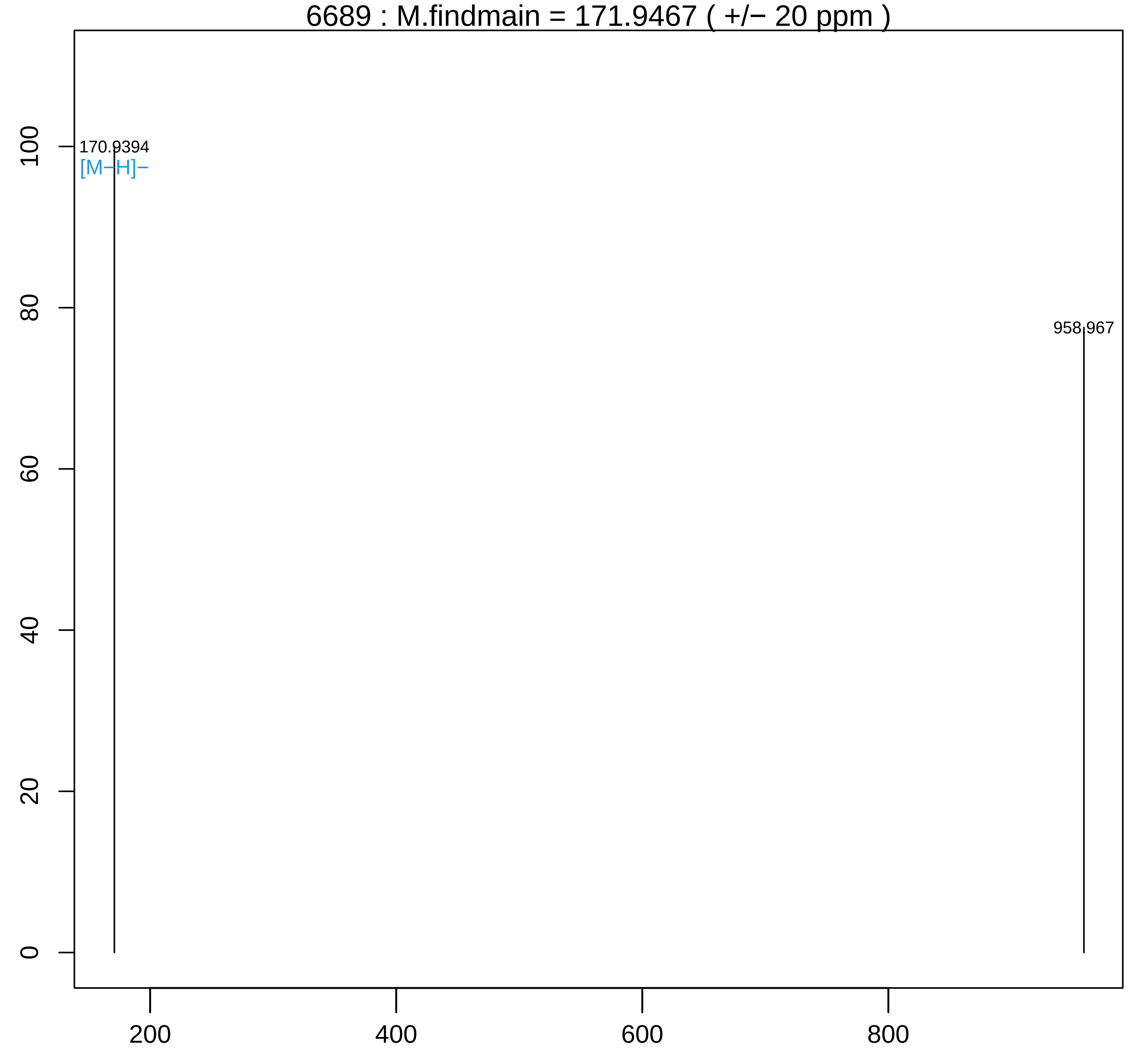

6689 : M.findmain = 171.9467 (+/− 20 ppm)
170.9394
[M−H]−
958.967
0
20
40
60
80
100
200
400
600
800

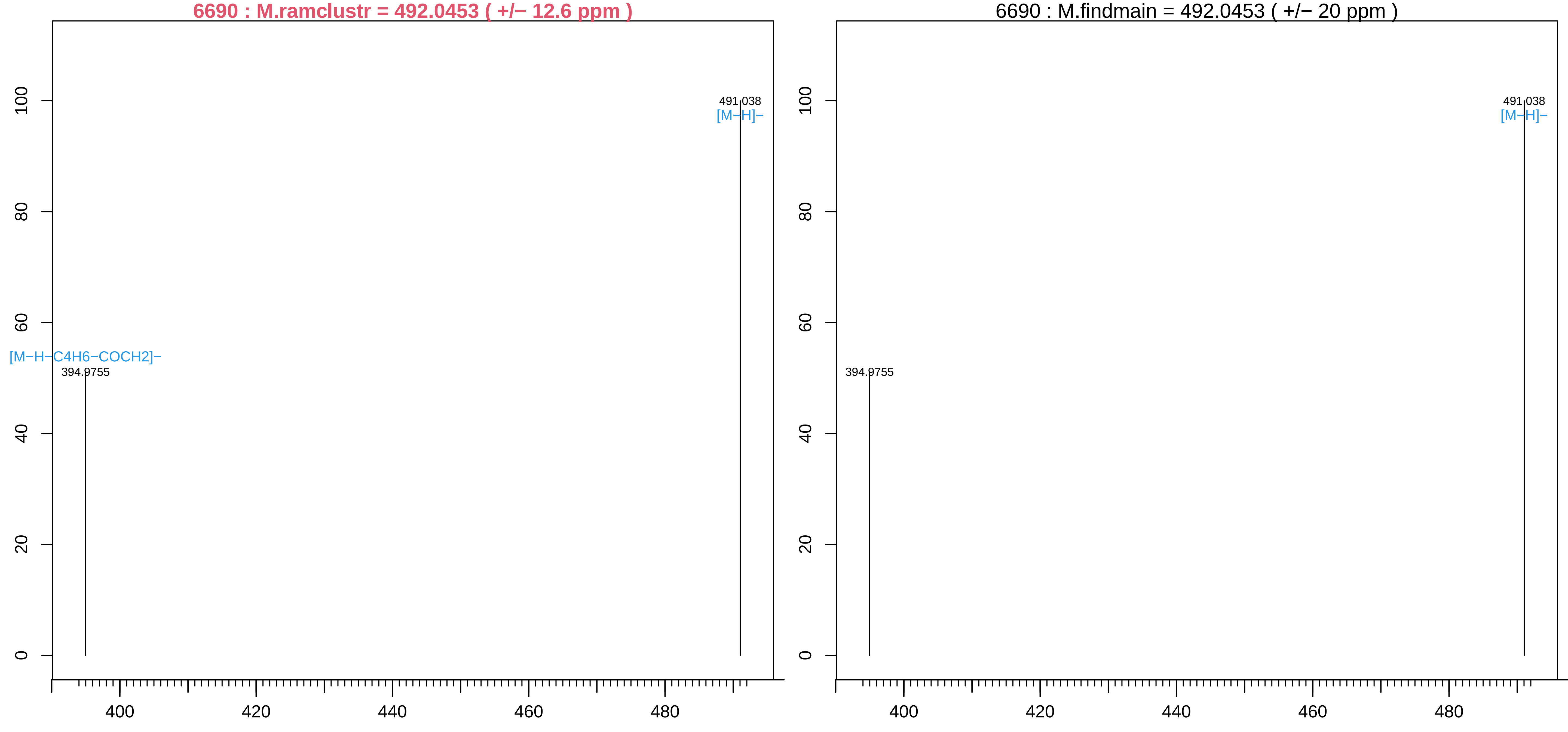
6690 : M.ramclustr = 492.0453 (+/− 12.6 ppm)
491.038
[M−H]−
[M−H−C4H6−COCH2]−
394.9755
6690 : M.findmain = 492.0453 (+/− 20 ppm)
491.038
[M−H]−
394.9755

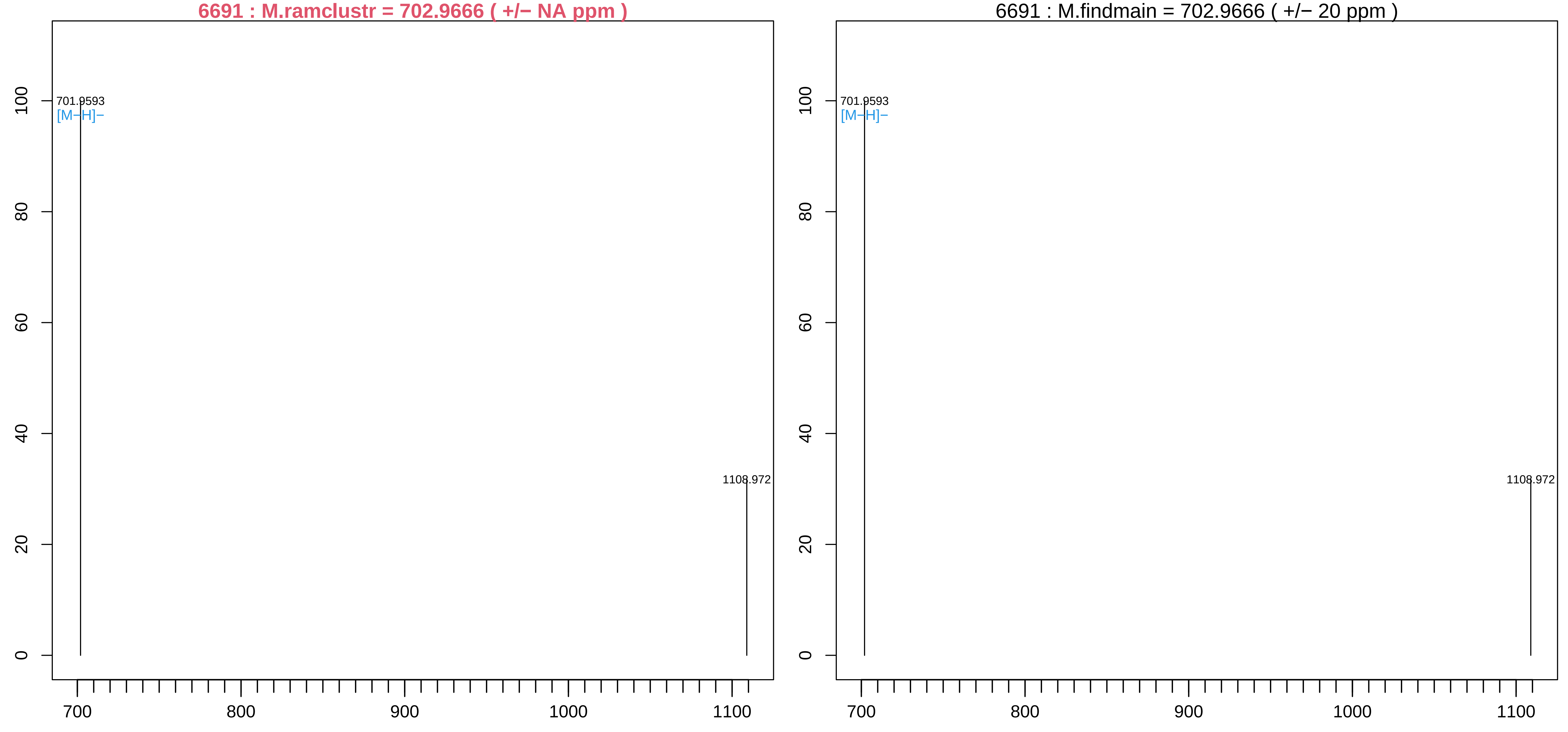
6691 : M.ramclustr = 702.9666 (+/− NA ppm)
701.9593
[M−H]−
1108.972
6691 : M.findmain = 702.9666 (+/− 20 ppm)
701.9593
[M−H]−
1108.972
0
20
40
60
80
100
700
800
900
1000
1100

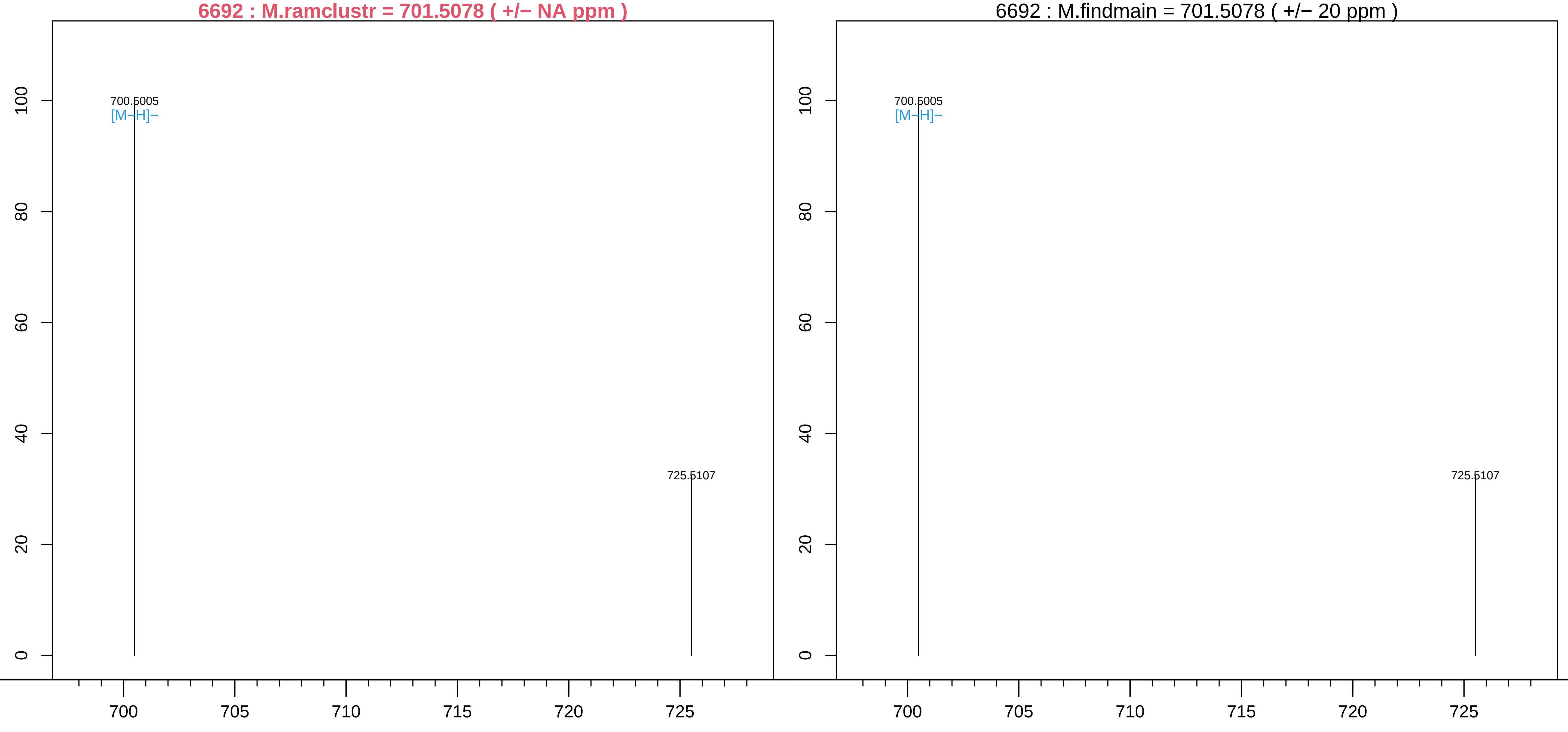
6692 : M.ramclustr = 701.5078 (+/− NA ppm)
700.5005
[M−H]−
725.5107
6692 : M.findmain = 701.5078 (+/− 20 ppm)
700.5005
[M−H]−
725.5107
0
20
40
60
80
100
700
705
710
715
720
725

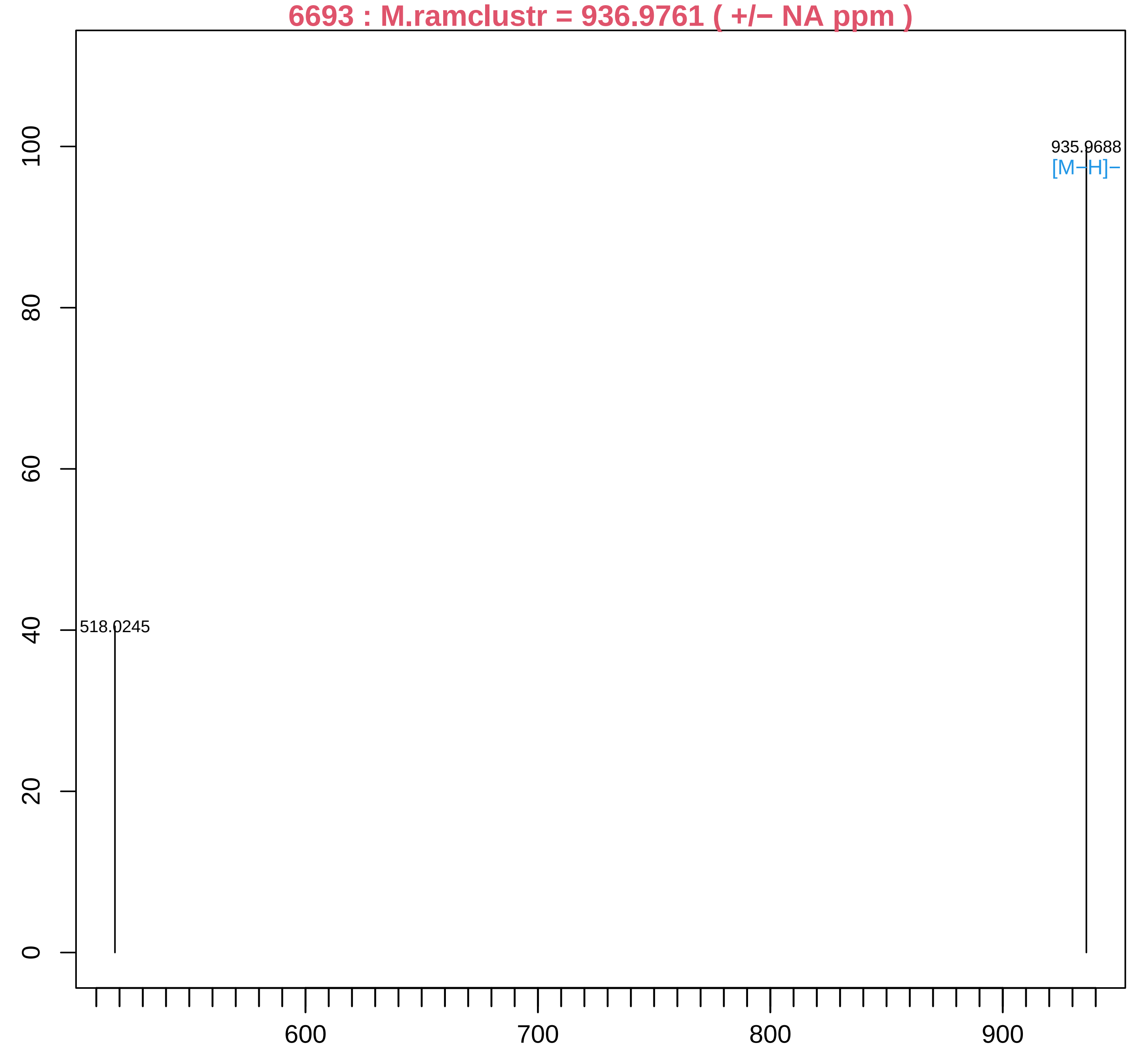
6693 : M.ramclustr = 936.9761 (+/− NA ppm)
935.9688
[M−H]−
518.0245
0
20
40
60
80
100
600
700
800
900

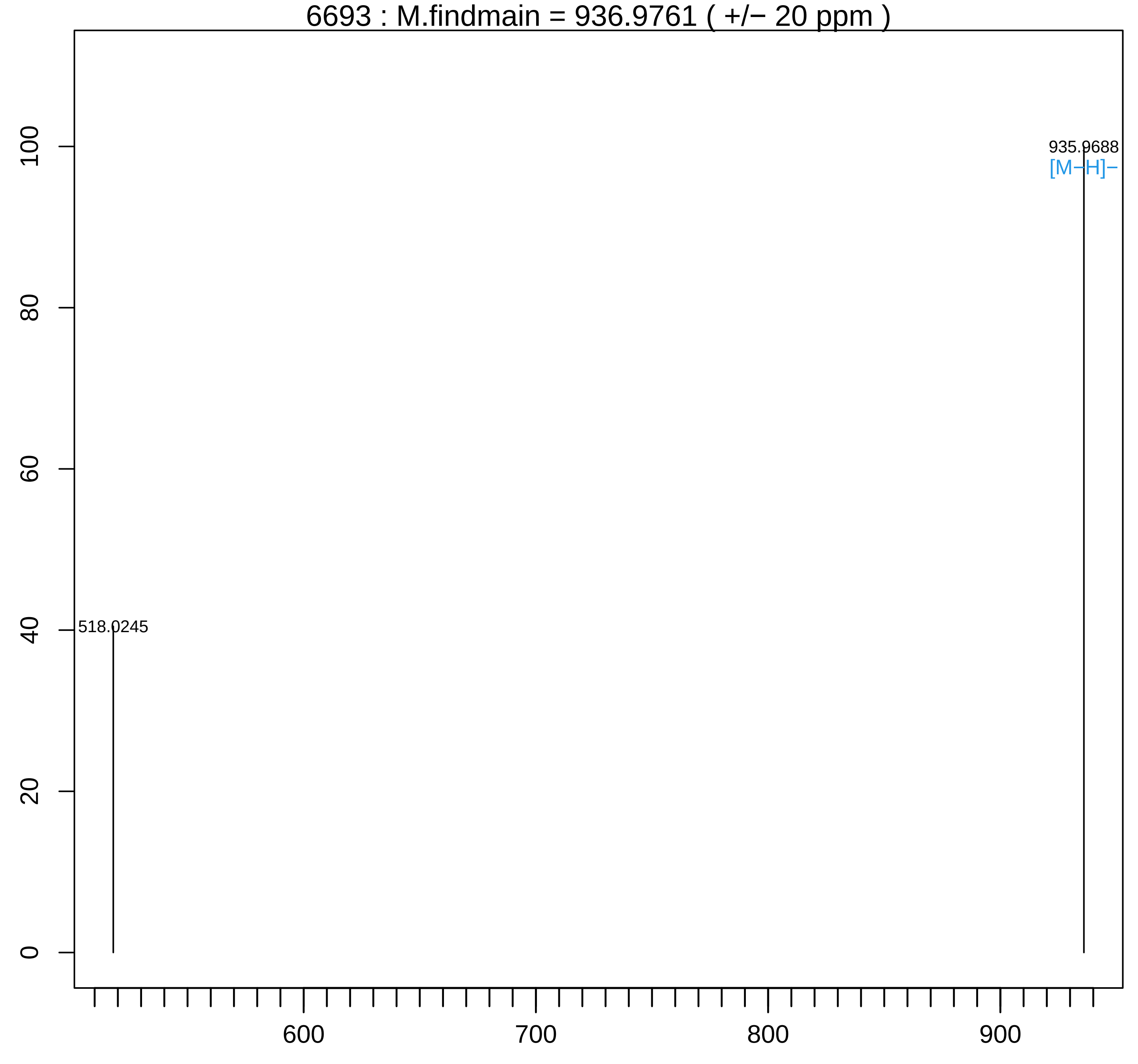
6693 : M.findmain = 936.9761 (+/− 20 ppm)
935.9688
[M−H]−
518.0245
0
20
40
60
80
100
600
700
800
900

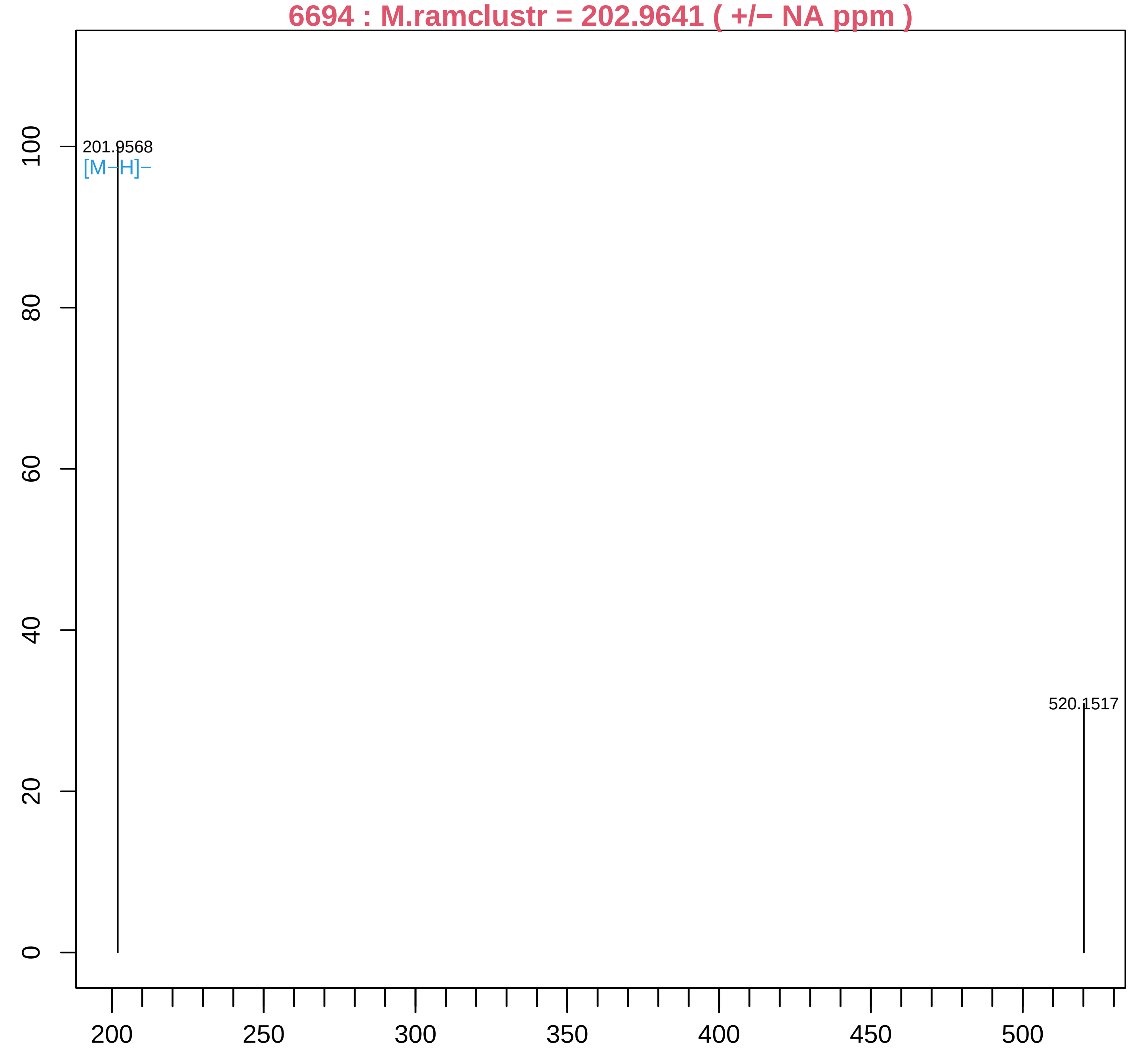
6694 : M.ramclustr = 202.9641 (+/− NA ppm)
201.9568
[M−H]−
520.1517
0
20
40
60
80
100
200
250
300
350
400
450
500

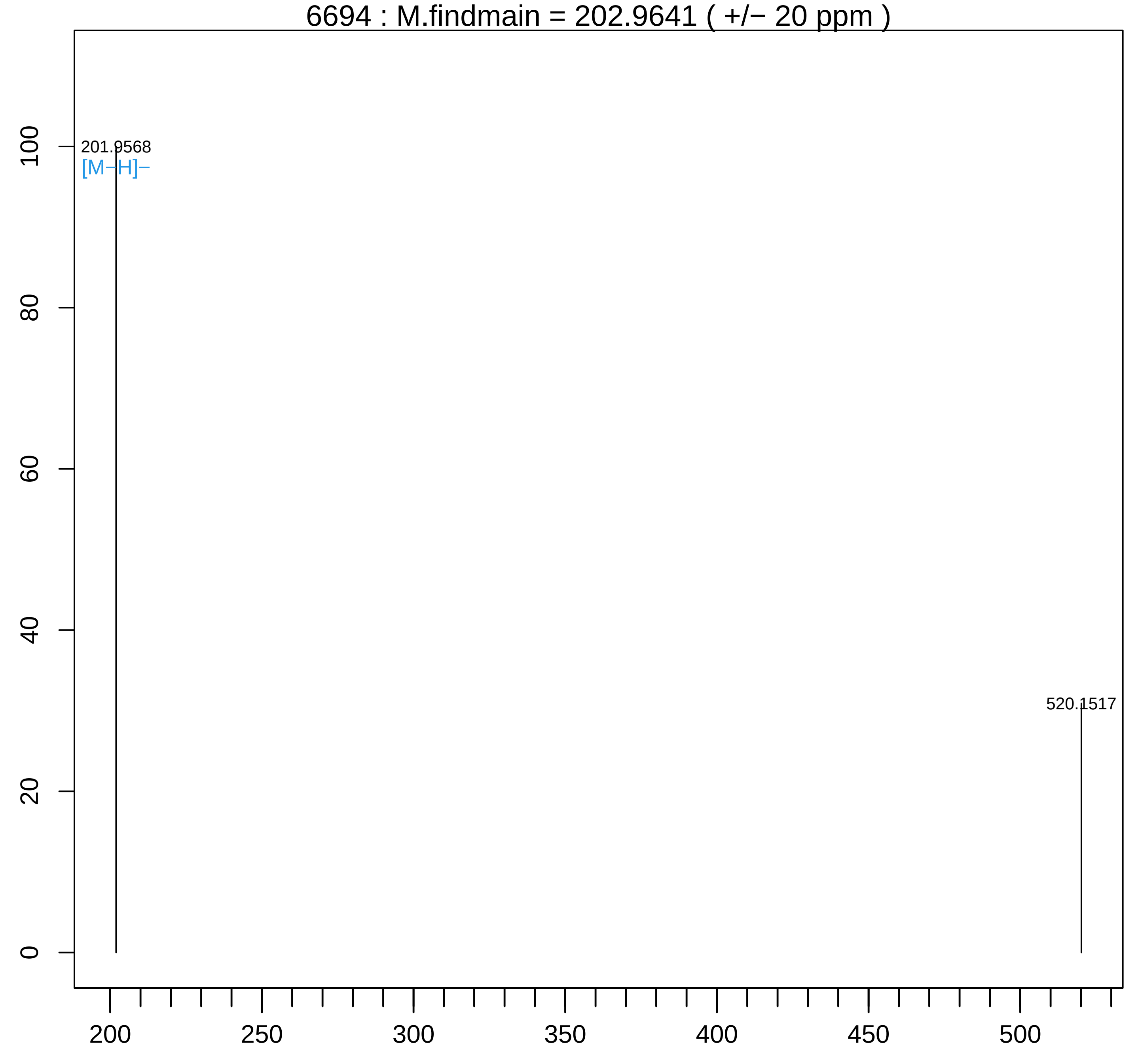
6694 : M.findmain = 202.9641 (+/− 20 ppm)
201.9568
[M−H]−
520.1517
0
20
40
60
80
100
200
250
300
350
400
450
500

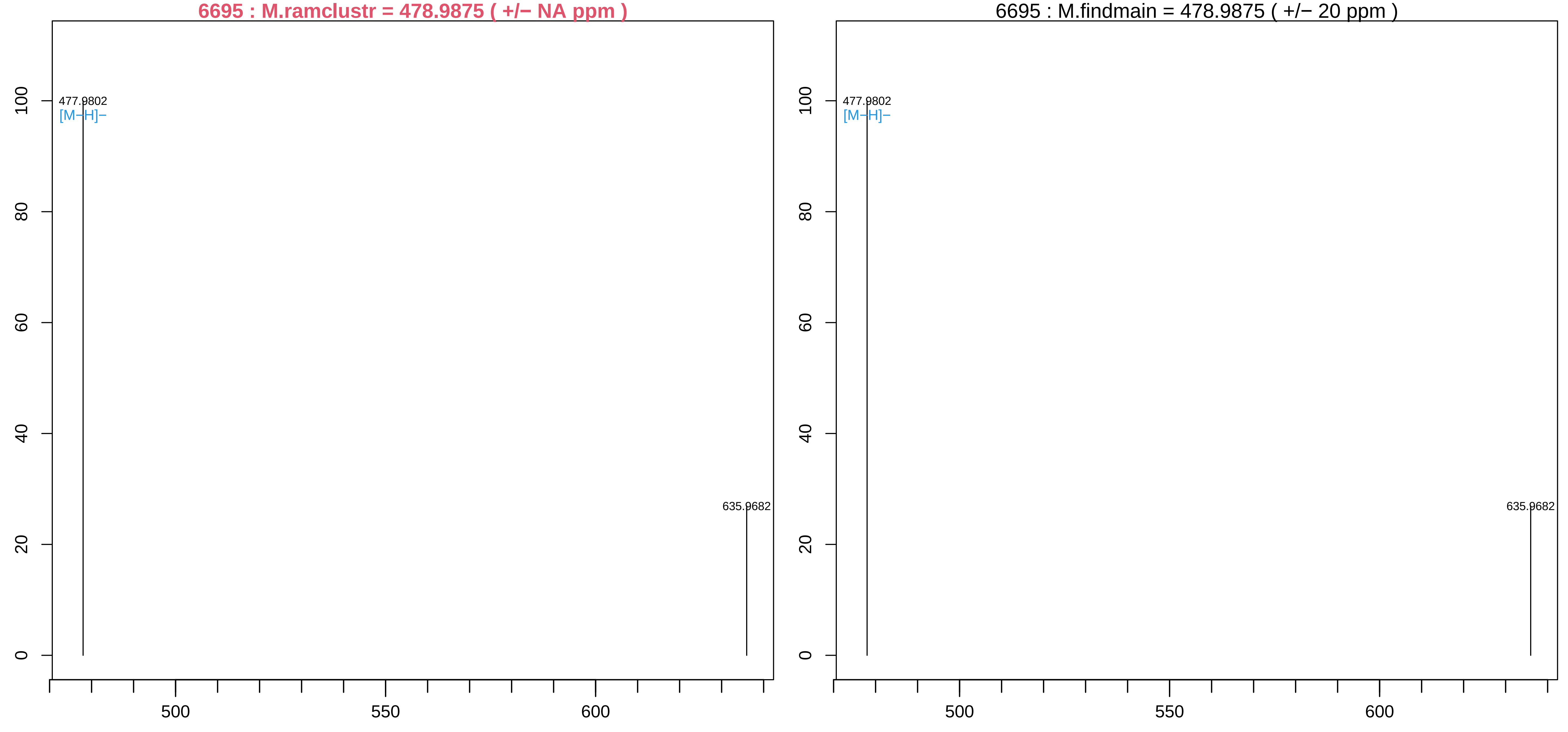
6695 : M.ramclustr = 478.9875 (+/− NA ppm)
477.9802
[M−H]−
635.9682
6695 : M.findmain = 478.9875 (+/− 20 ppm)
477.9802
[M−H]−
635.9682
0
20
40
60
80
100
500
550
600

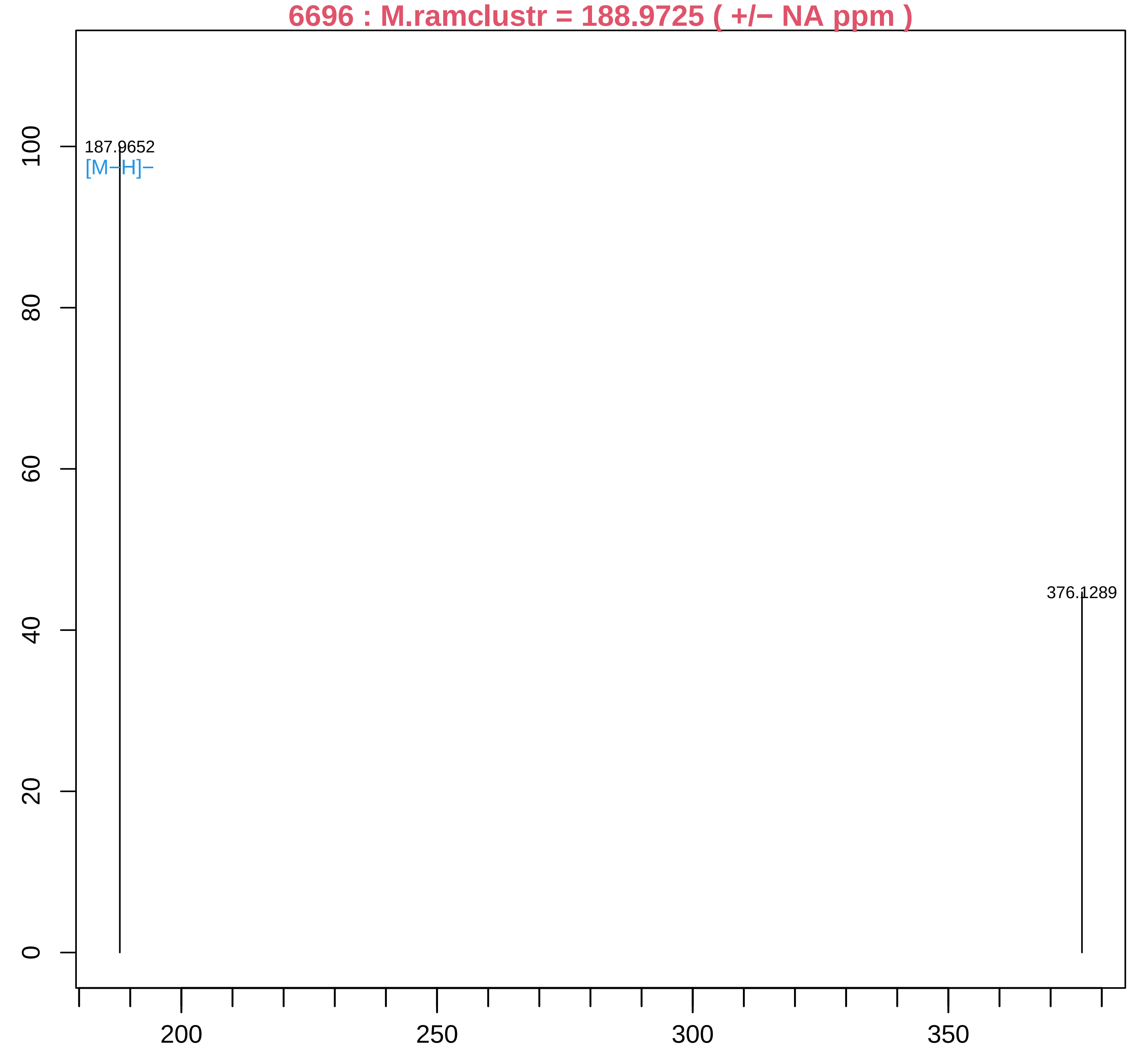
6696 : M.ramclustr = 188.9725 (+/− NA ppm)
187.9652
[M−H]−
376.1289
0
20
40
60
80
100
200
250
300
350
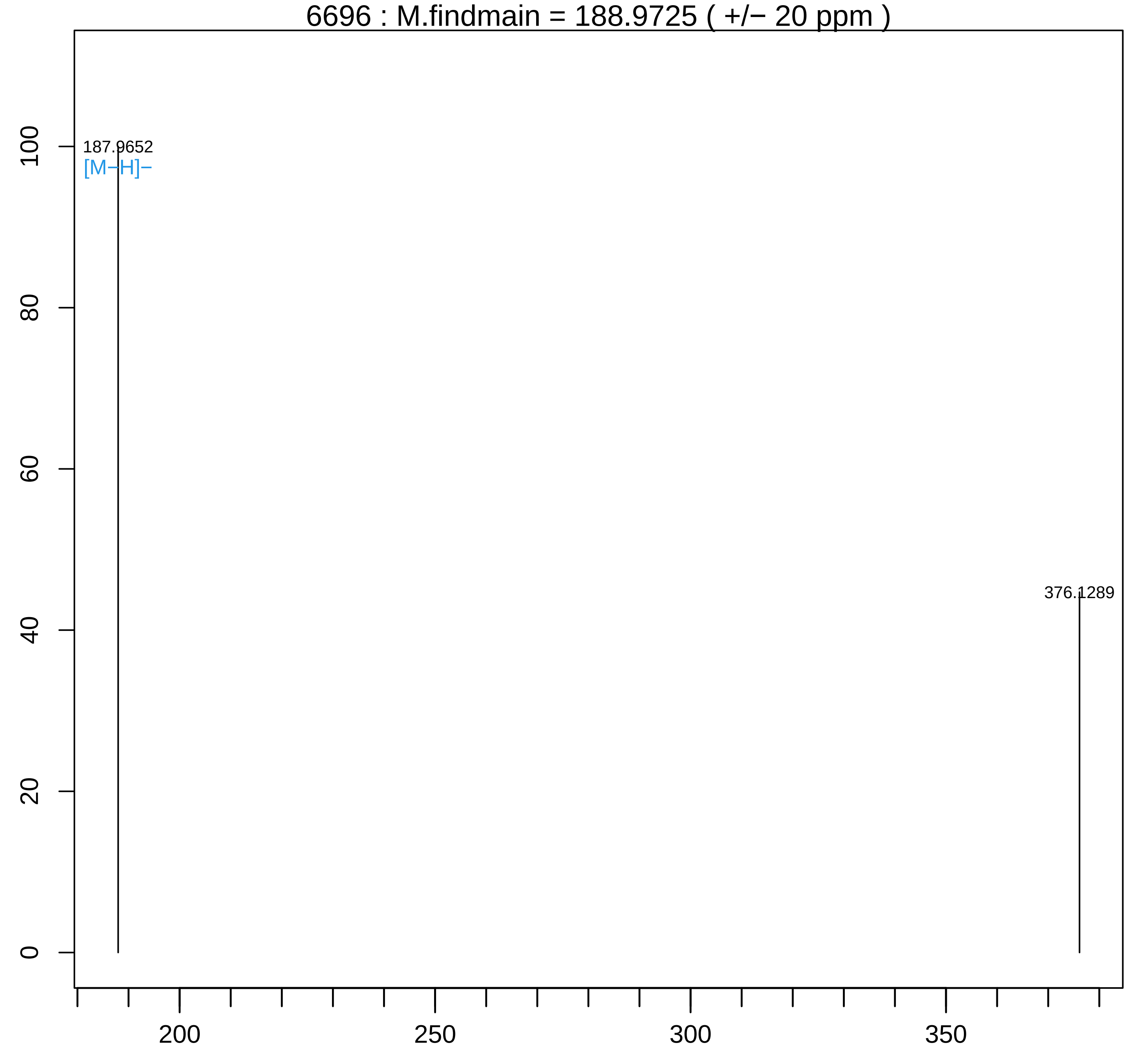
6696 : M.findmain = 188.9725 (+/− 20 ppm)
187.9652
[M−H]−
376.1289
0
20
40
60
80
100
200
250
300
350

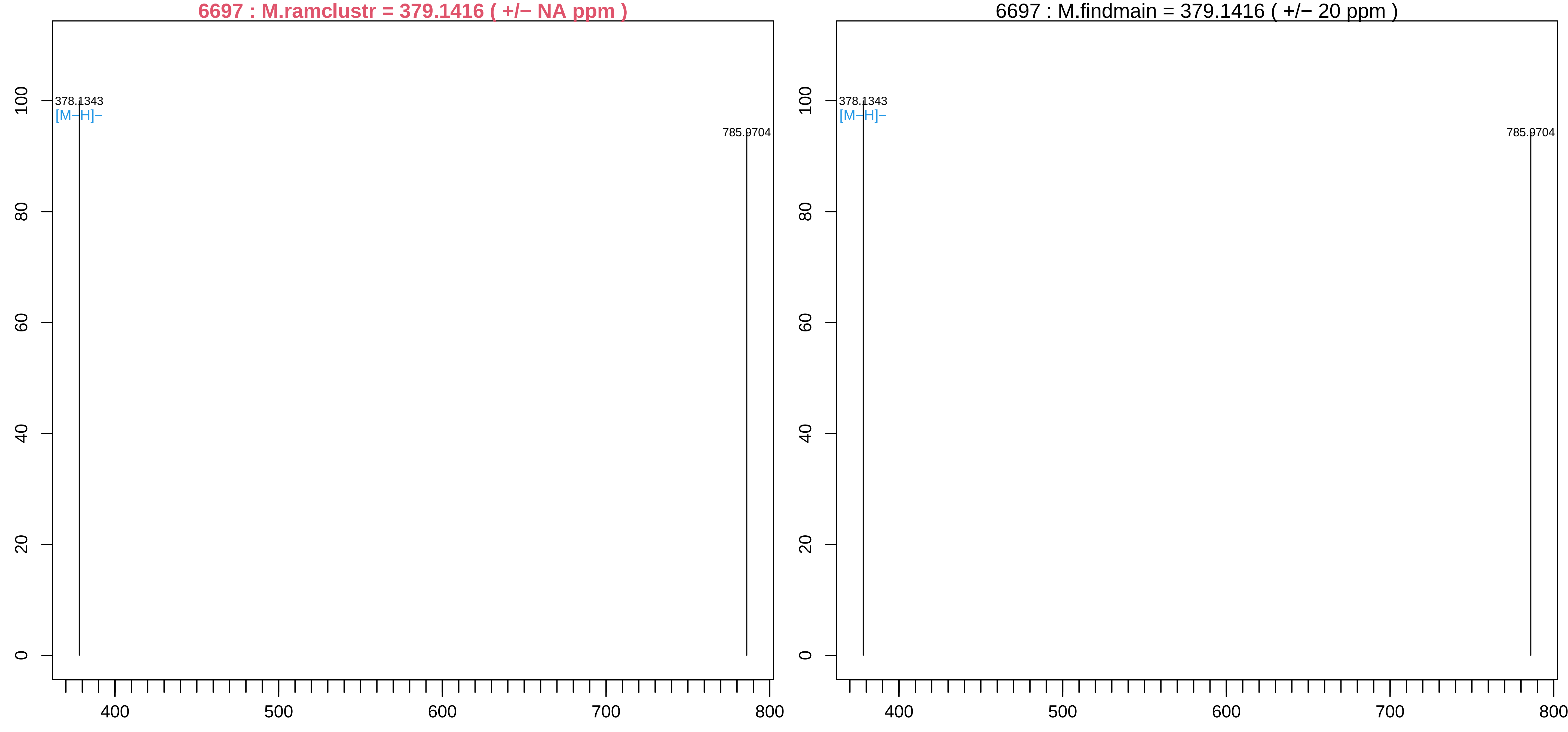
6697 : M.ramclustr = 379.1416 (+/− NA ppm)
378.1343
[M−H]−
785.9704
0
20
40
60
80
100
400
500
600
700
800
6697 : M.findmain = 379.1416 (+/− 20 ppm)
378.1343
[M−H]−
785.9704
0
20
40
60
80
100
400
500
600
700
800

6698 : M.ramclustr = 491.2059 (+/− NA ppm)

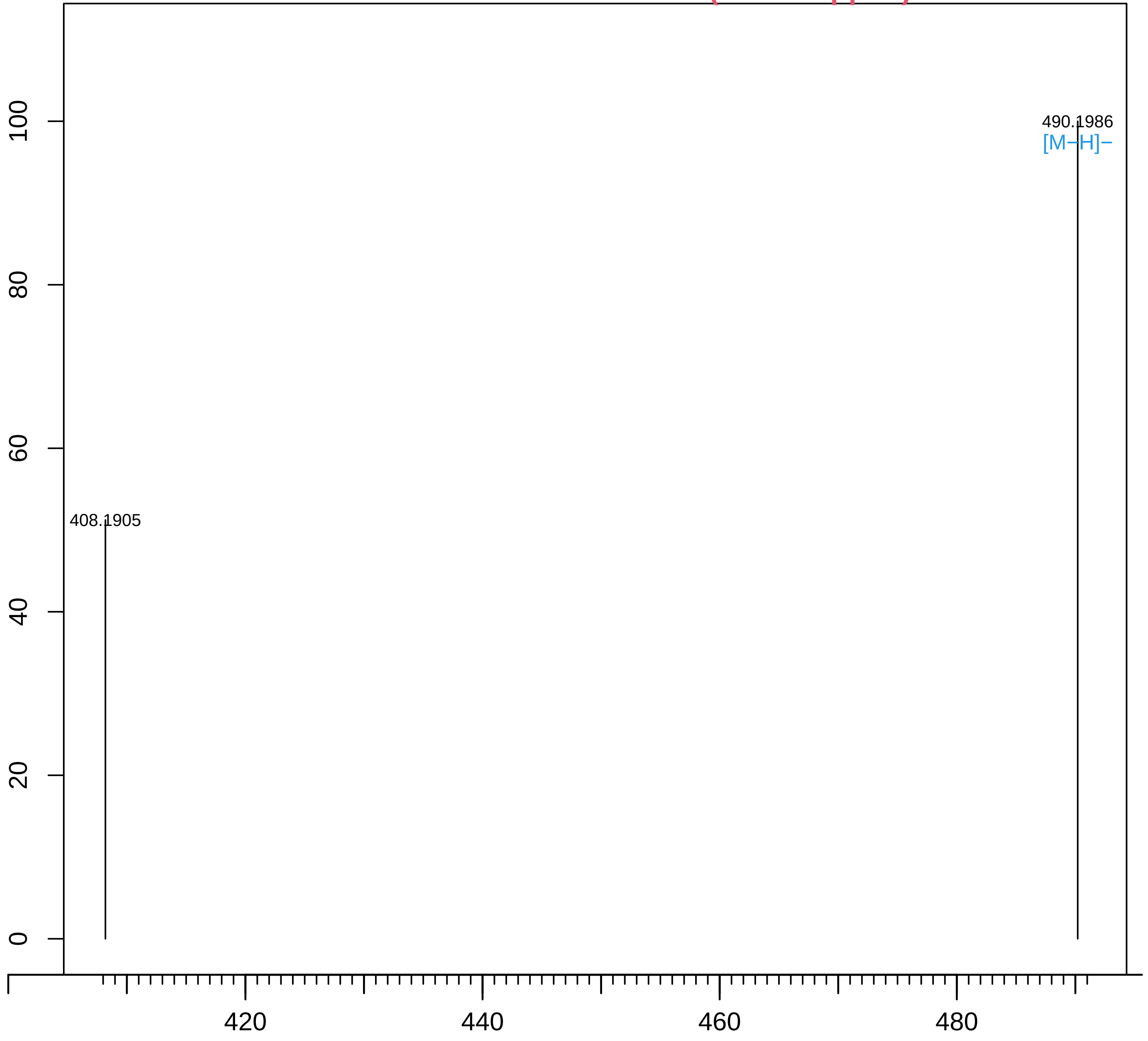

6698 : M.findmain = 491.2059 (+/− 20 ppm)

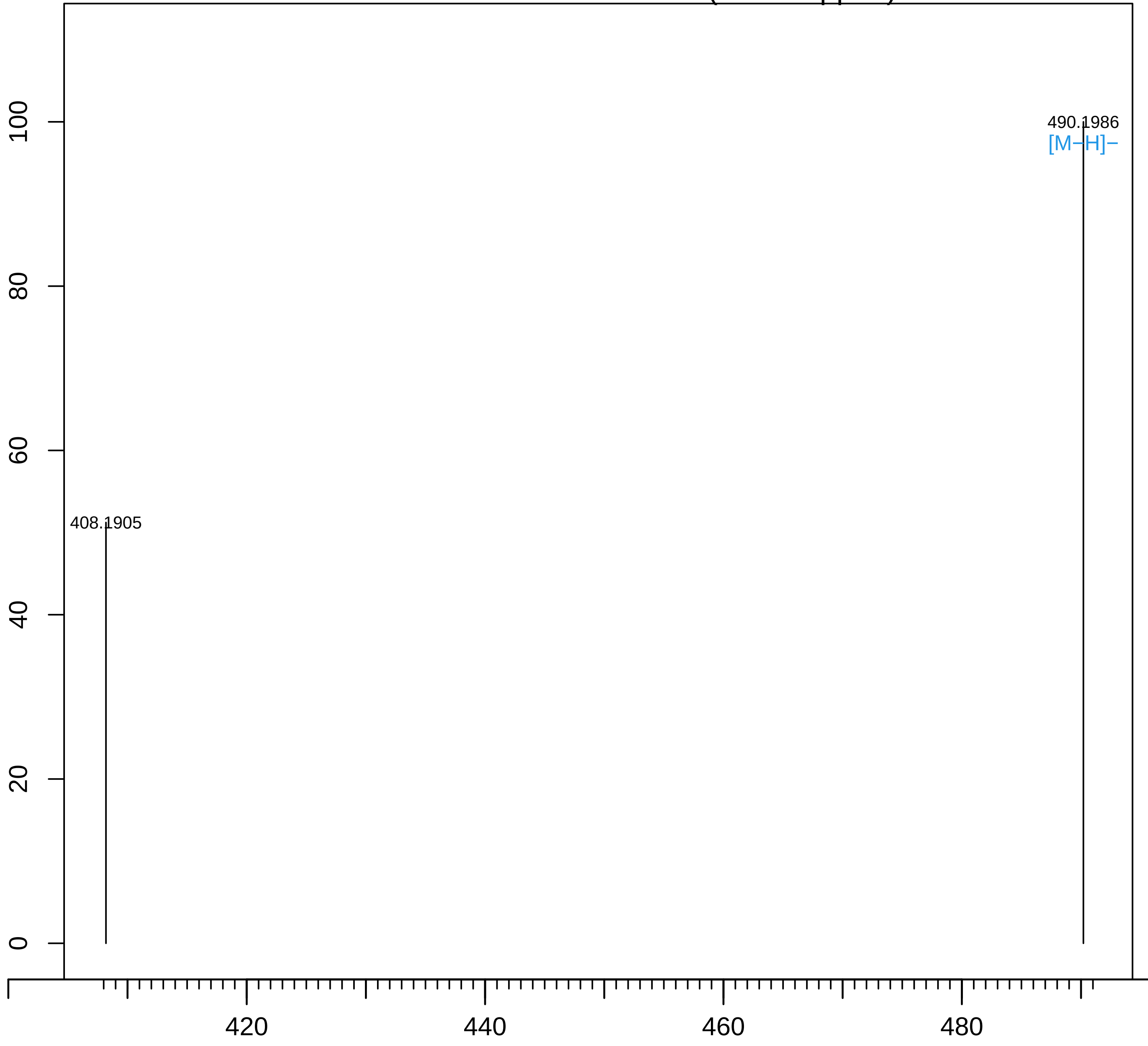

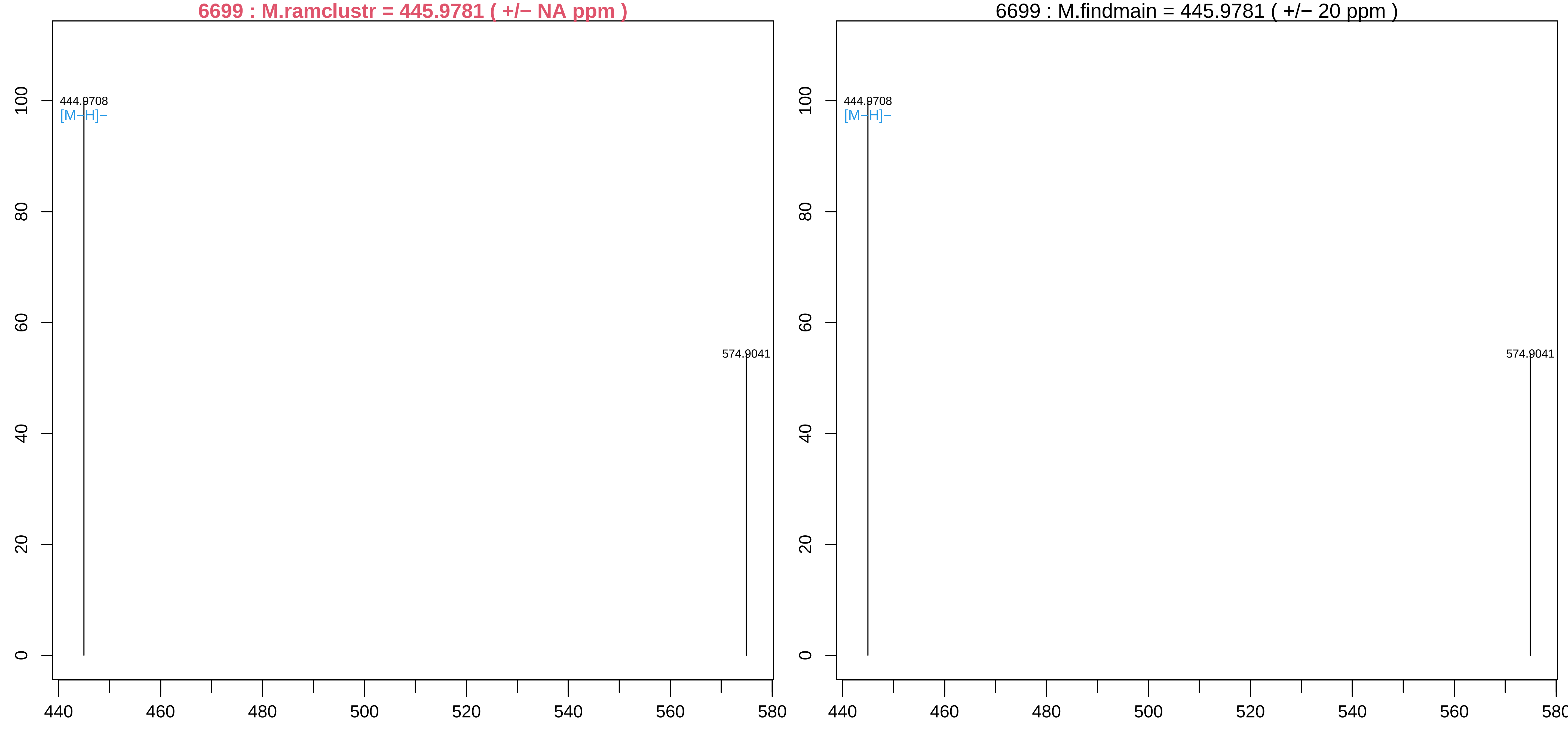
6699 : M.ramclustr = 445.9781 (+/− NA ppm)
444.9708
[M−H]−
574.9041
6699 : M.findmain = 445.9781 (+/− 20 ppm)
444.9708
[M−H]−
574.9041
0
20
40
60
80
100
440
460
480
500
520
540
560
580

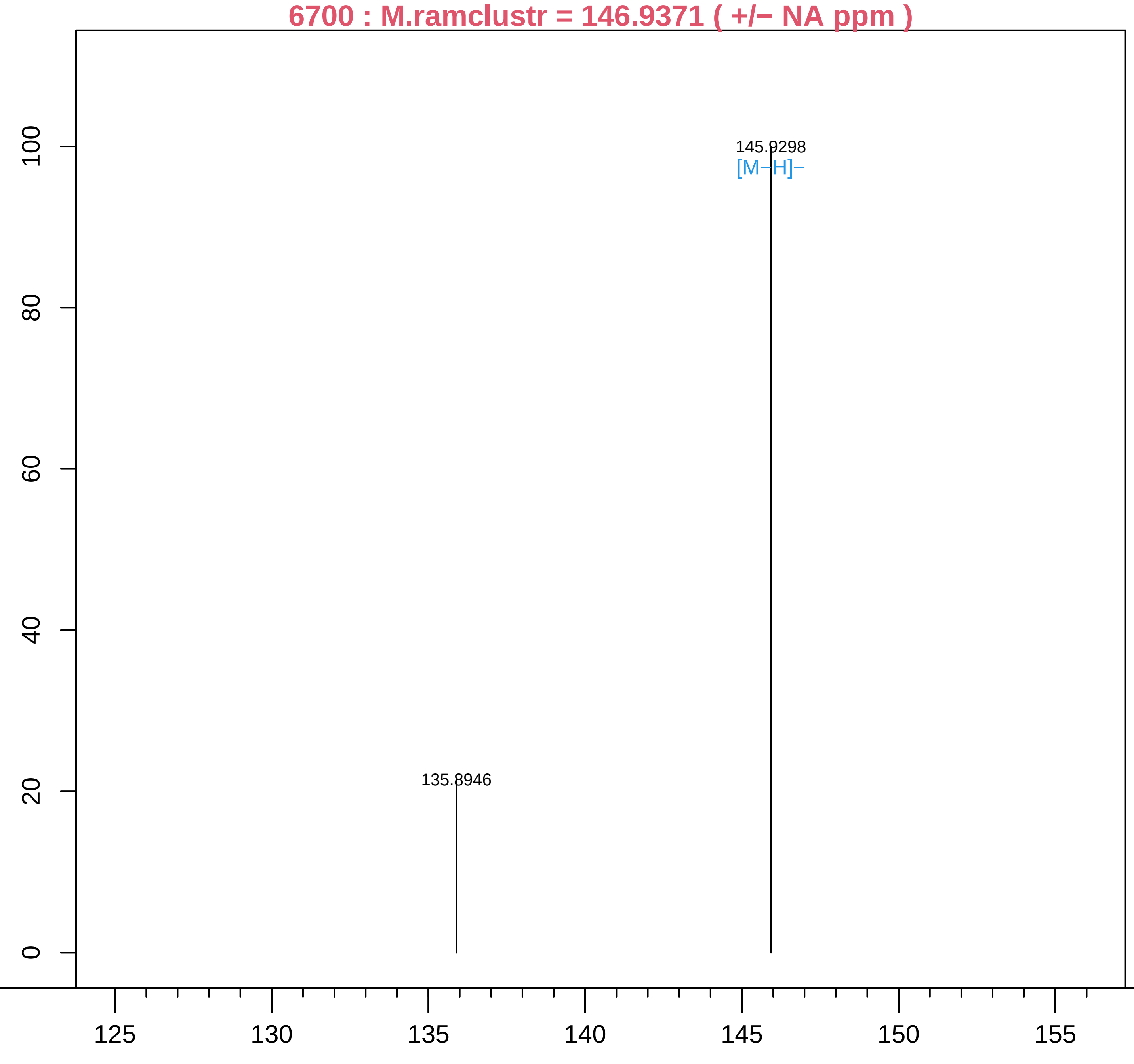

6700 : M.ramclustr = 146.9371 (+/− NA ppm)
145.9298
[M−H]−
135.8946
0
20
40
60
80
100
125
130
135
140
145
150
155

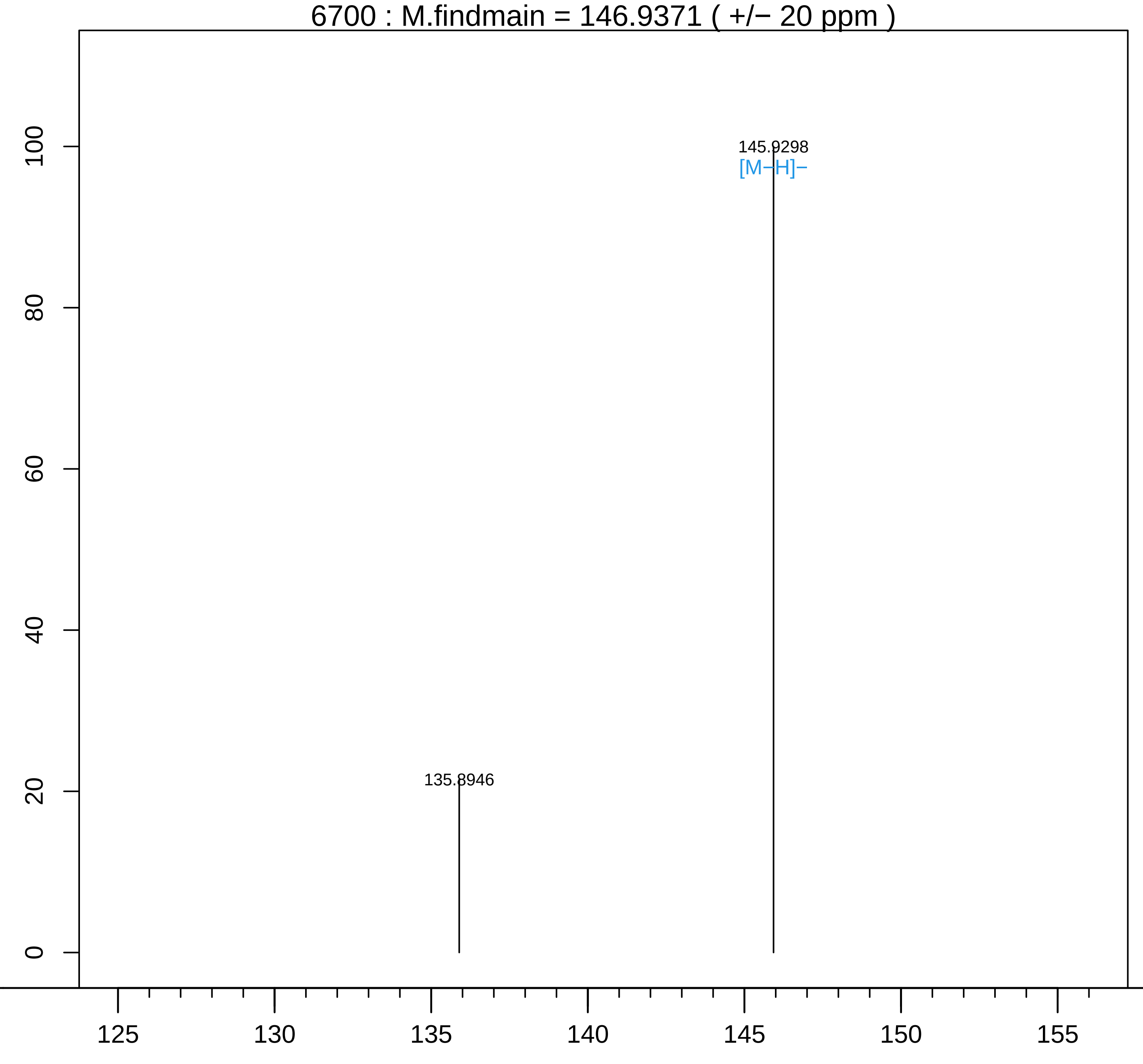

6700 : M.findmain = 146.9371 (+/− 20 ppm)
145.9298
[M−H]−
135.8946
0
20
40
60
80
100
125
130
135
140
145
150
155

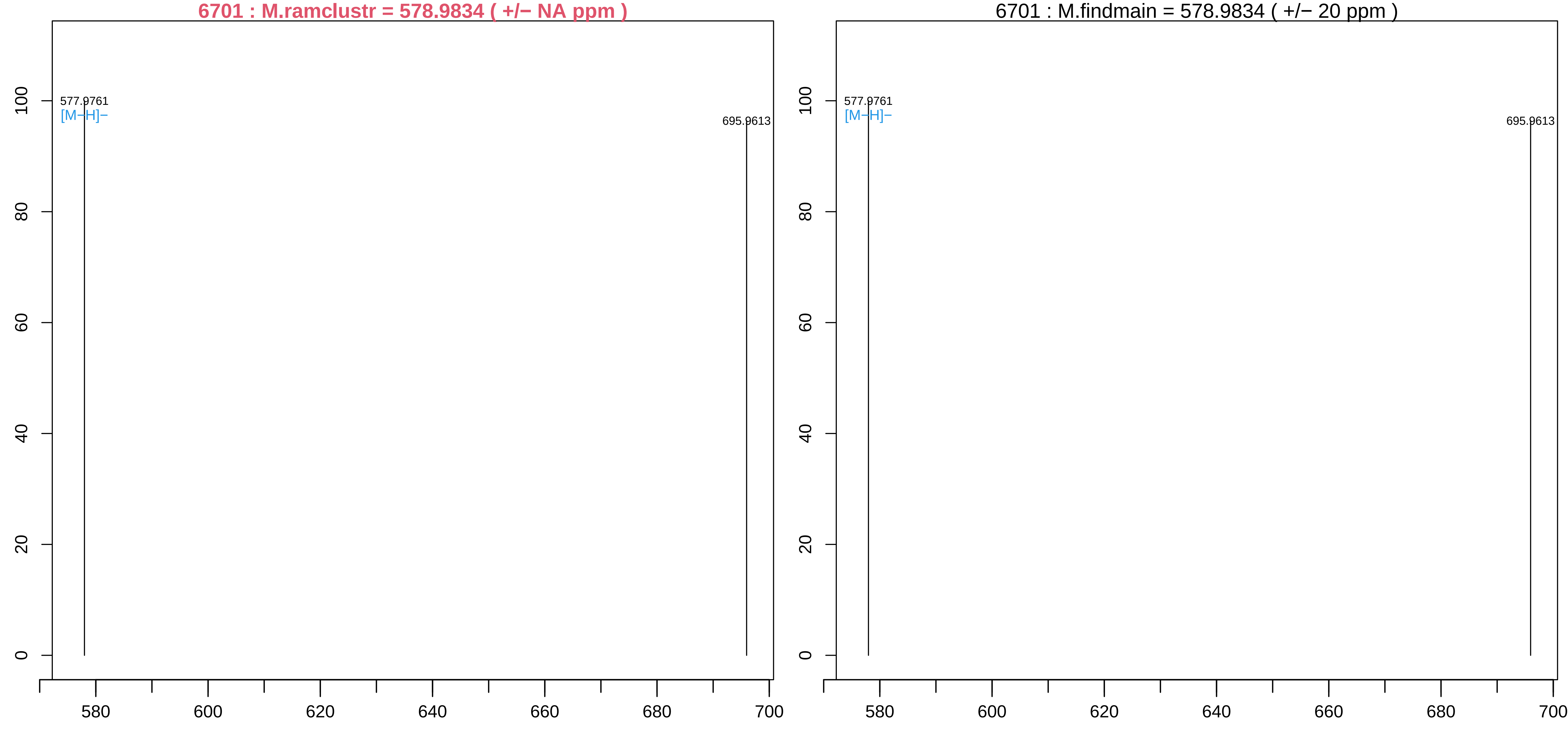

6701 : M.ramclustr = 578.9834 (+/− NA ppm)
577.9761
[M−H]−
695.9613
0
20
40
60
80
100
580
600
620
640
660
680
700
6701 : M.findmain = 578.9834 (+/− 20 ppm)
577.9761
[M−H]−
695.9613
0
20
40
60
80
100
580
600
620
640
660
680
700

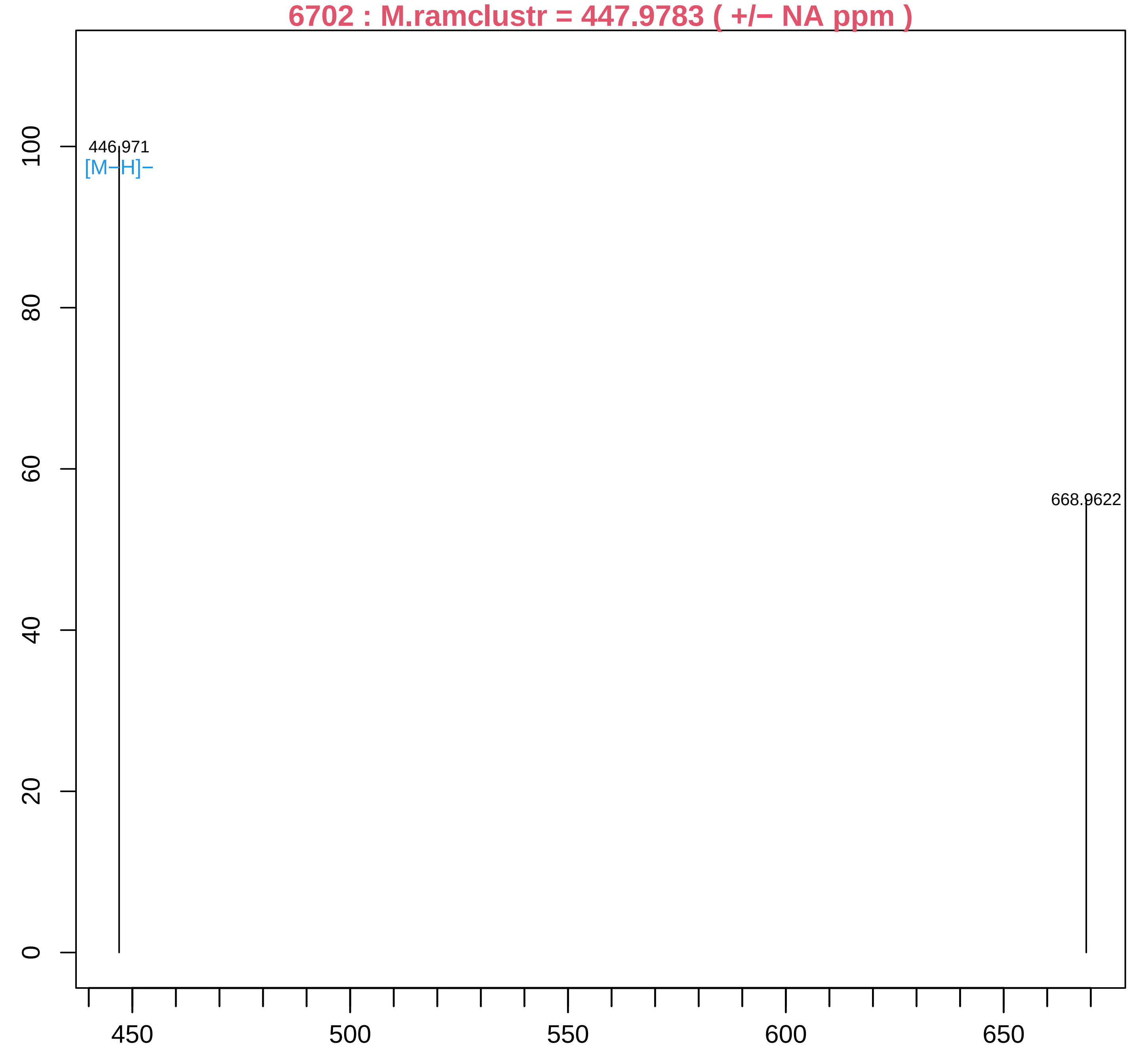
6702 : M.ramclustr = 447.9783 (+/− NA ppm)
446.971
[M−H]−
668.9622
0
20
40
60
80
100
450
500
550
600
650

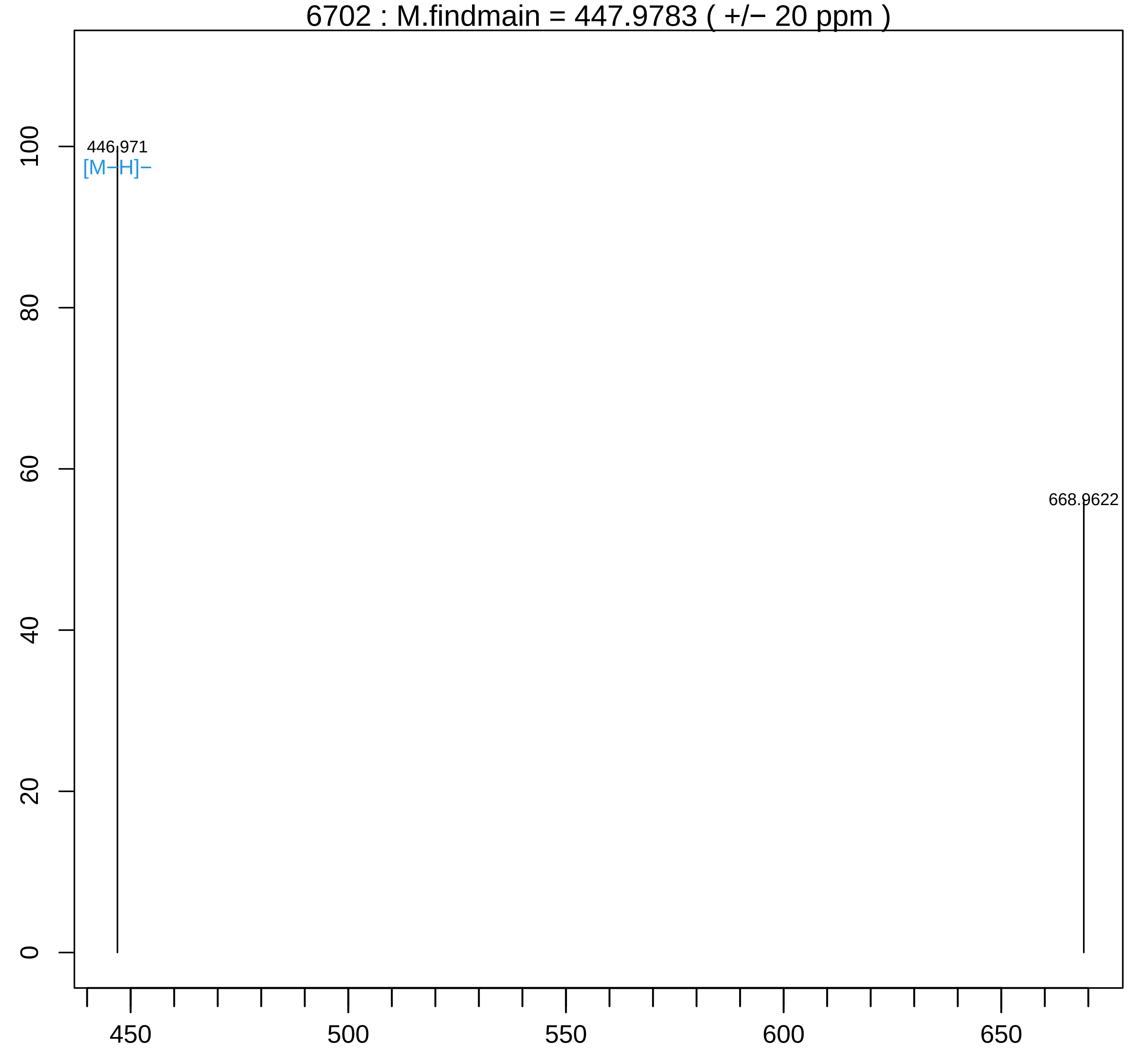
6702 : M.findmain = 447.9783 (+/− 20 ppm)
446.971
[M−H]−
668.9622
0
20
40
60
80
100
450
500
550
600
650

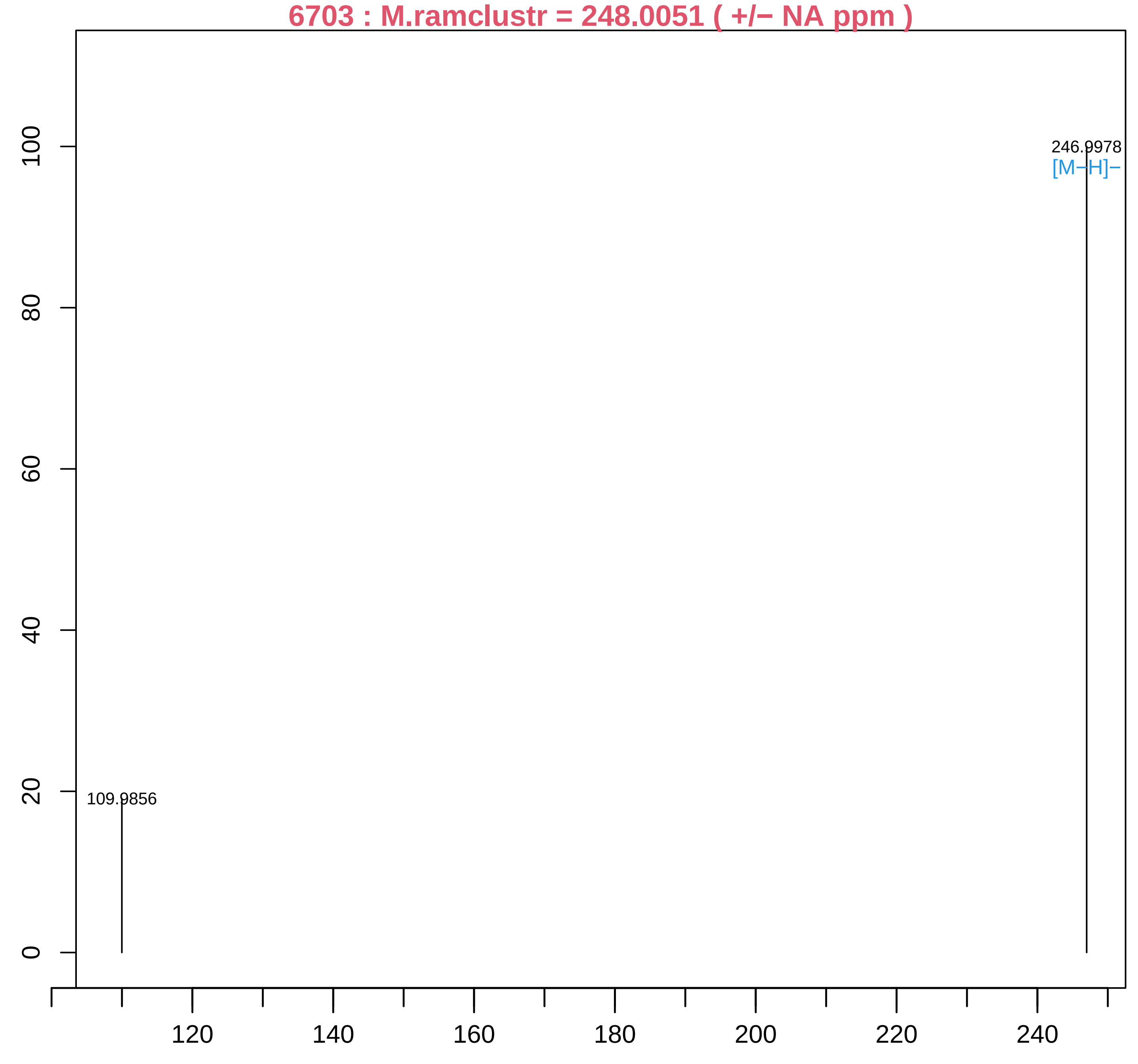

6703 : M.ramclustr = 248.0051 (+/− NA ppm)
246.9978
[M−H]−
109.9856
0
20
40
60
80
100
120
140
160
180
200
220
240

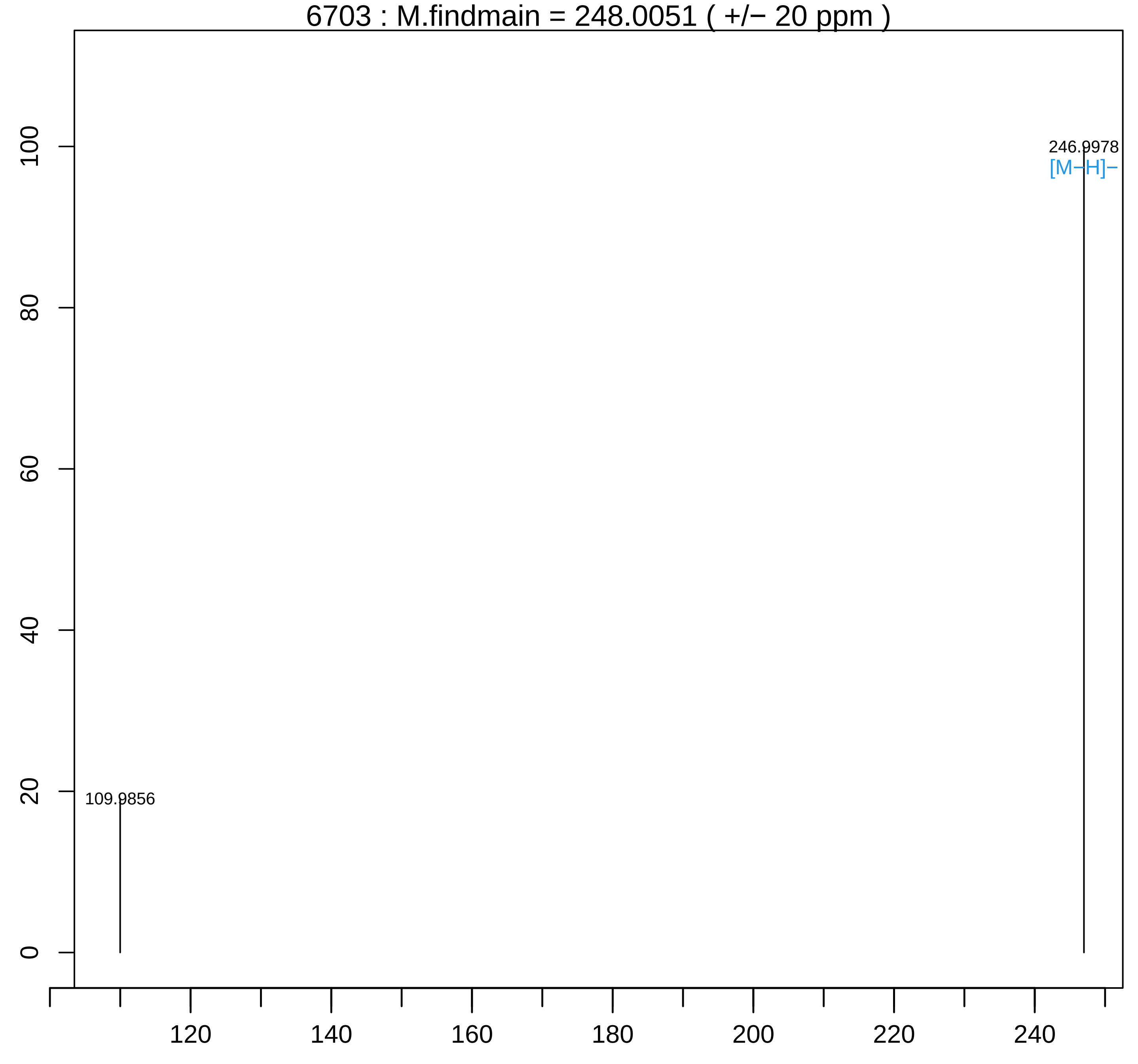

6703 : M.findmain = 248.0051 (+/− 20 ppm)
246.9978
[M−H]−
109.9856
0
20
40
60
80
100
120
140
160
180
200
220
240

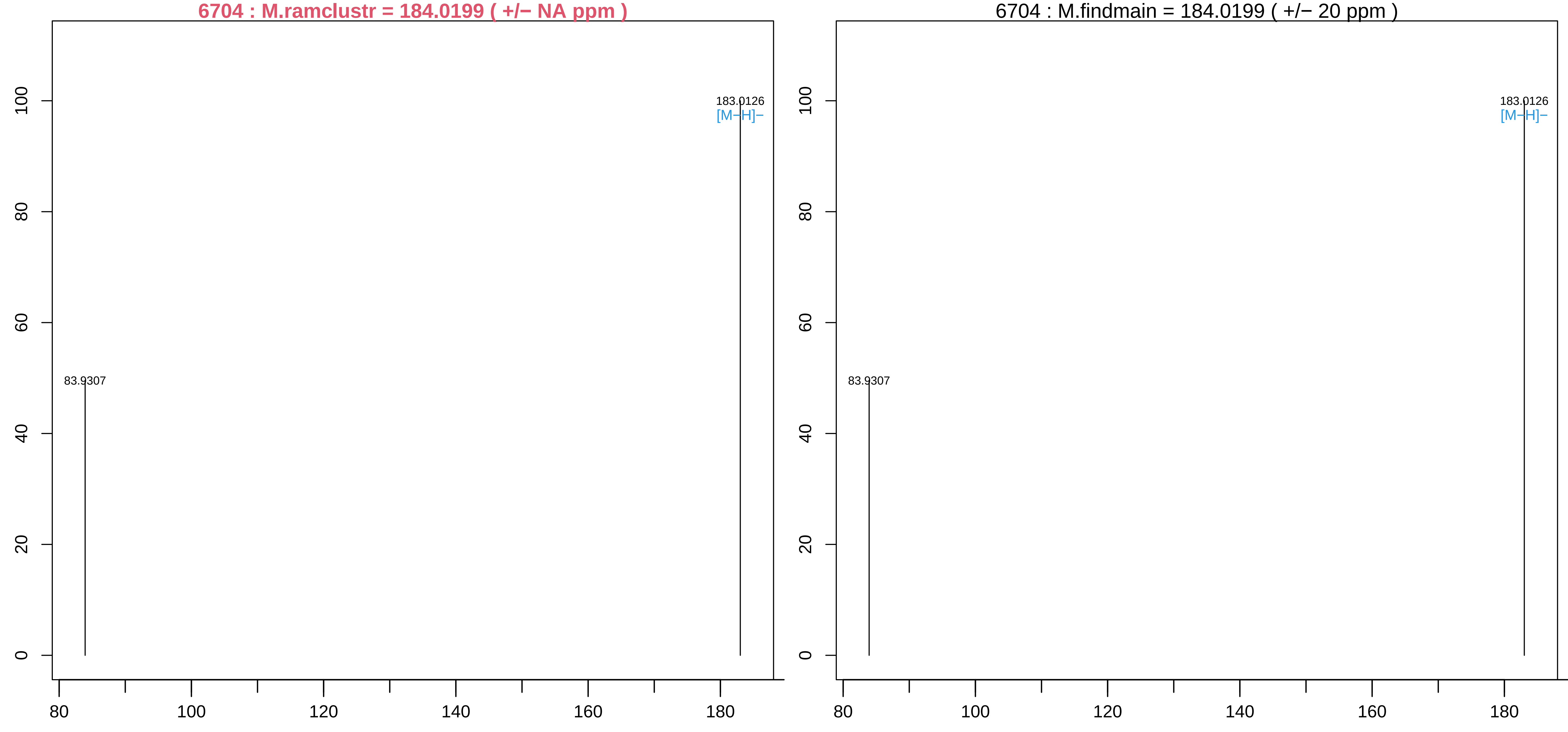

6704 : M.ramclustr = 184.0199 (+/− NA ppm)
6704 : M.findmain = 184.0199 (+/− 20 ppm)
183.0126
[M−H]−
83.9307
0
20
40
60
80
100
80
100
120
140
160
180

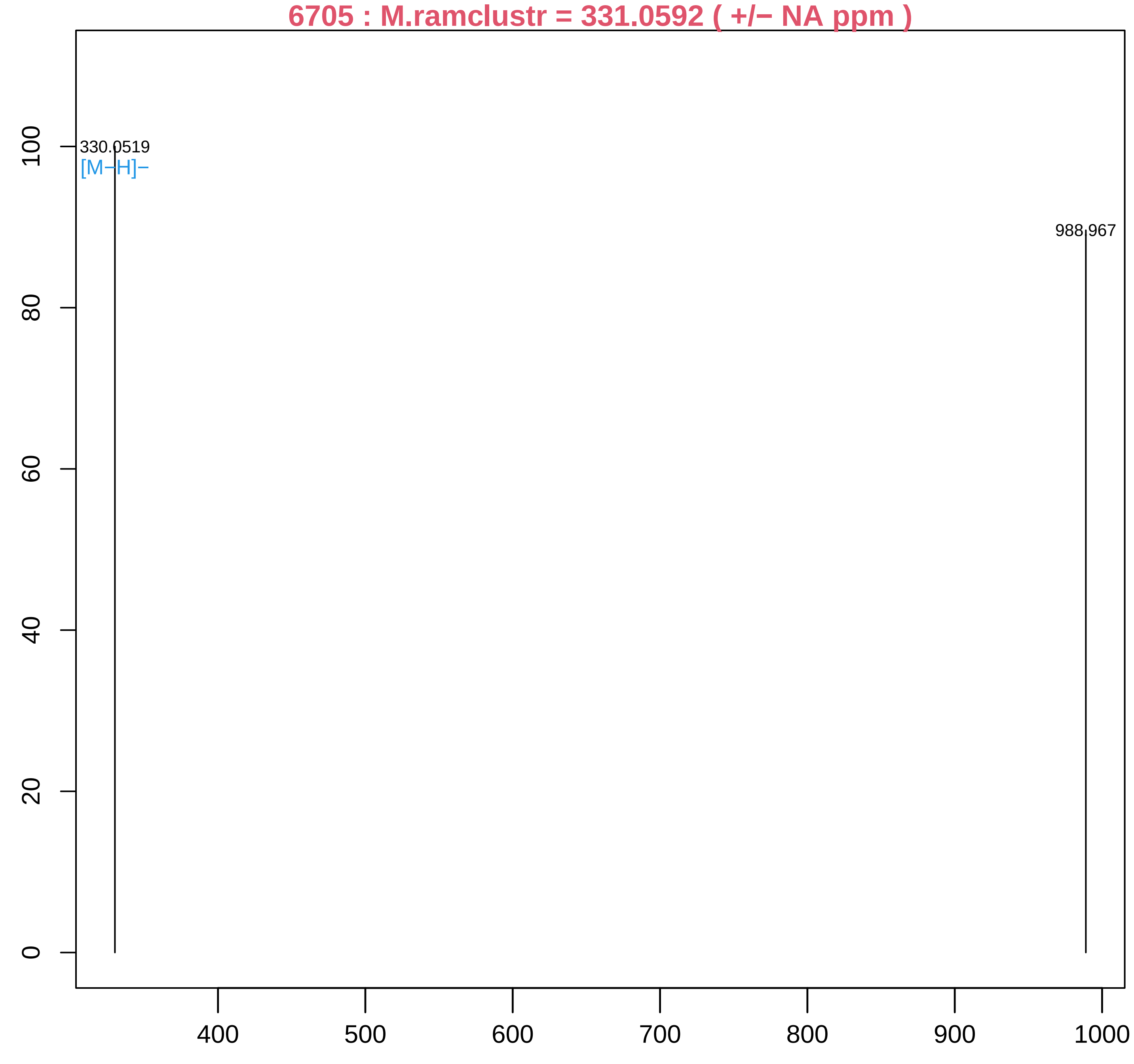
6705 : M.ramclustr = 331.0592 (+/− NA ppm)
330.0519
[M−H]−
988.967
0
20
40
60
80
100
400
500
600
700
800
900
1000

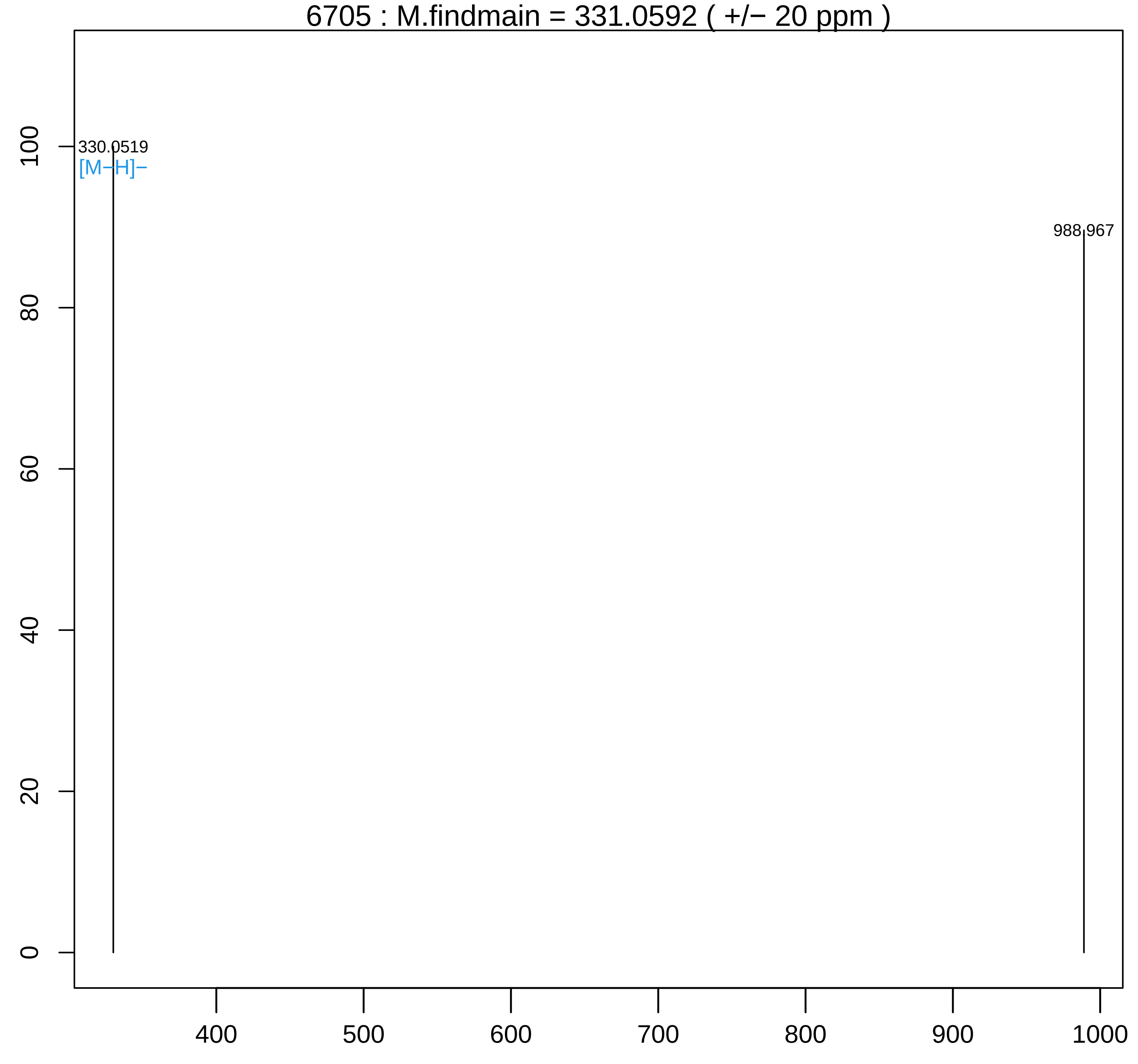
6705 : M.findmain = 331.0592 (+/− 20 ppm)
330.0519
[M−H]−
988.967
0
20
40
60
80
100
400
500
600
700
800
900
1000

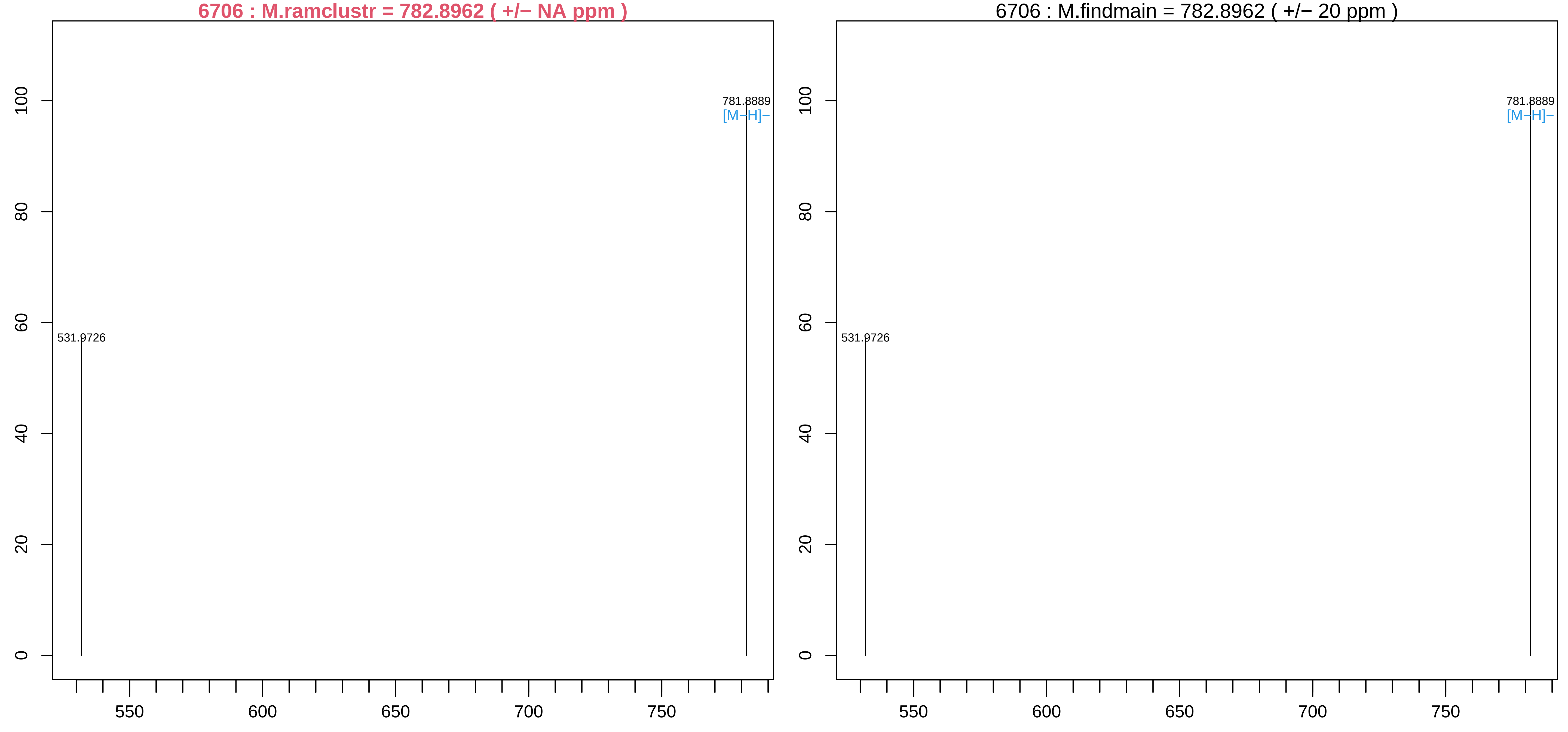
6706 : M.ramclustr = 782.8962 (+/− NA ppm)
531.9726
781.8889
[M−H]−
0
20
40
60
80
100
550
600
650
700
750
6706 : M.findmain = 782.8962 (+/− 20 ppm)
531.9726
781.8889
[M−H]−
0
20
40
60
80
100
550
600
650
700
750

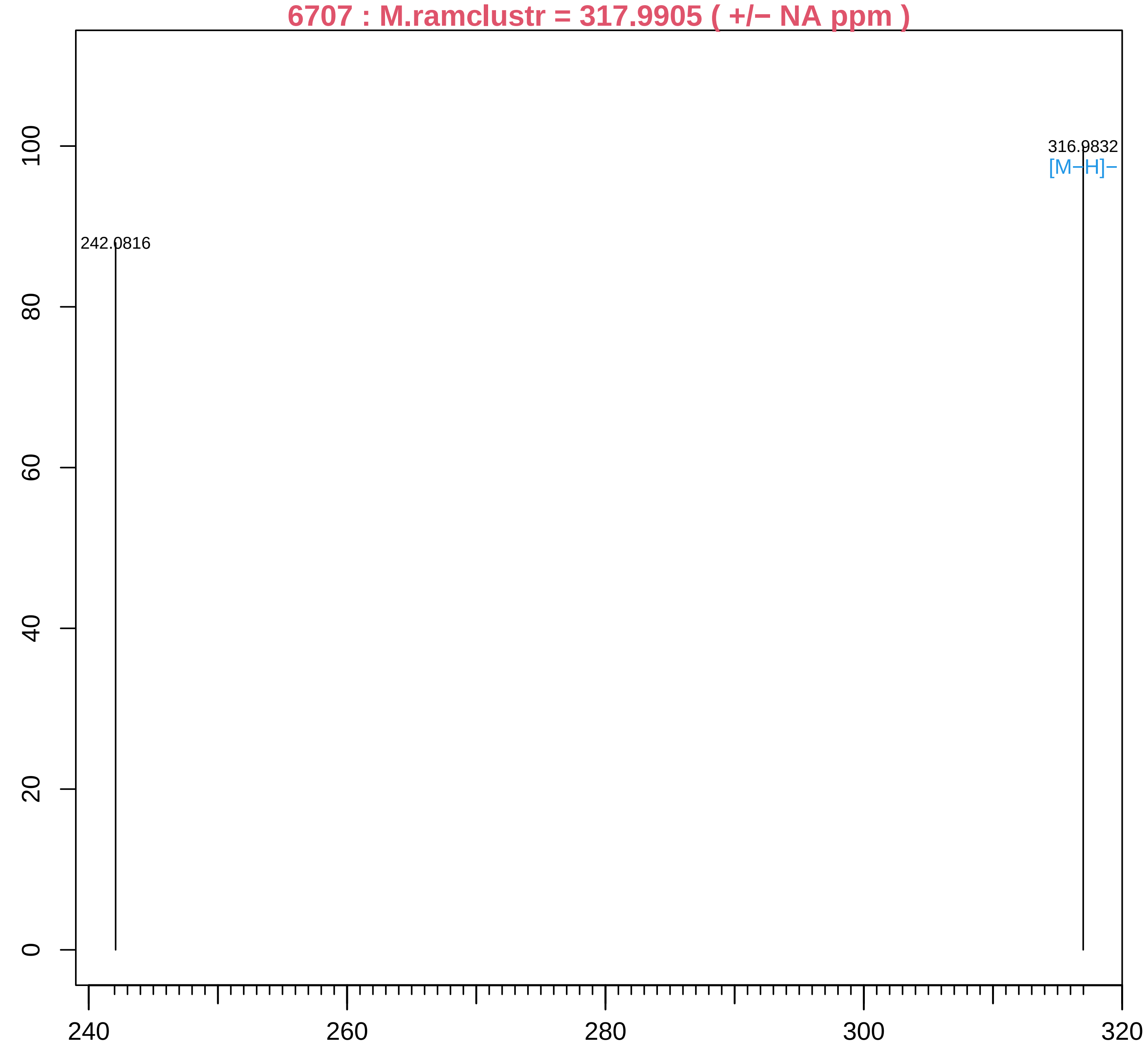
6707 : M.ramclustr = 317.9905 (+/− NA ppm)
316.9832
[M−H]−
242.0816
0
20
40
60
80
100
240
260
280
300
320

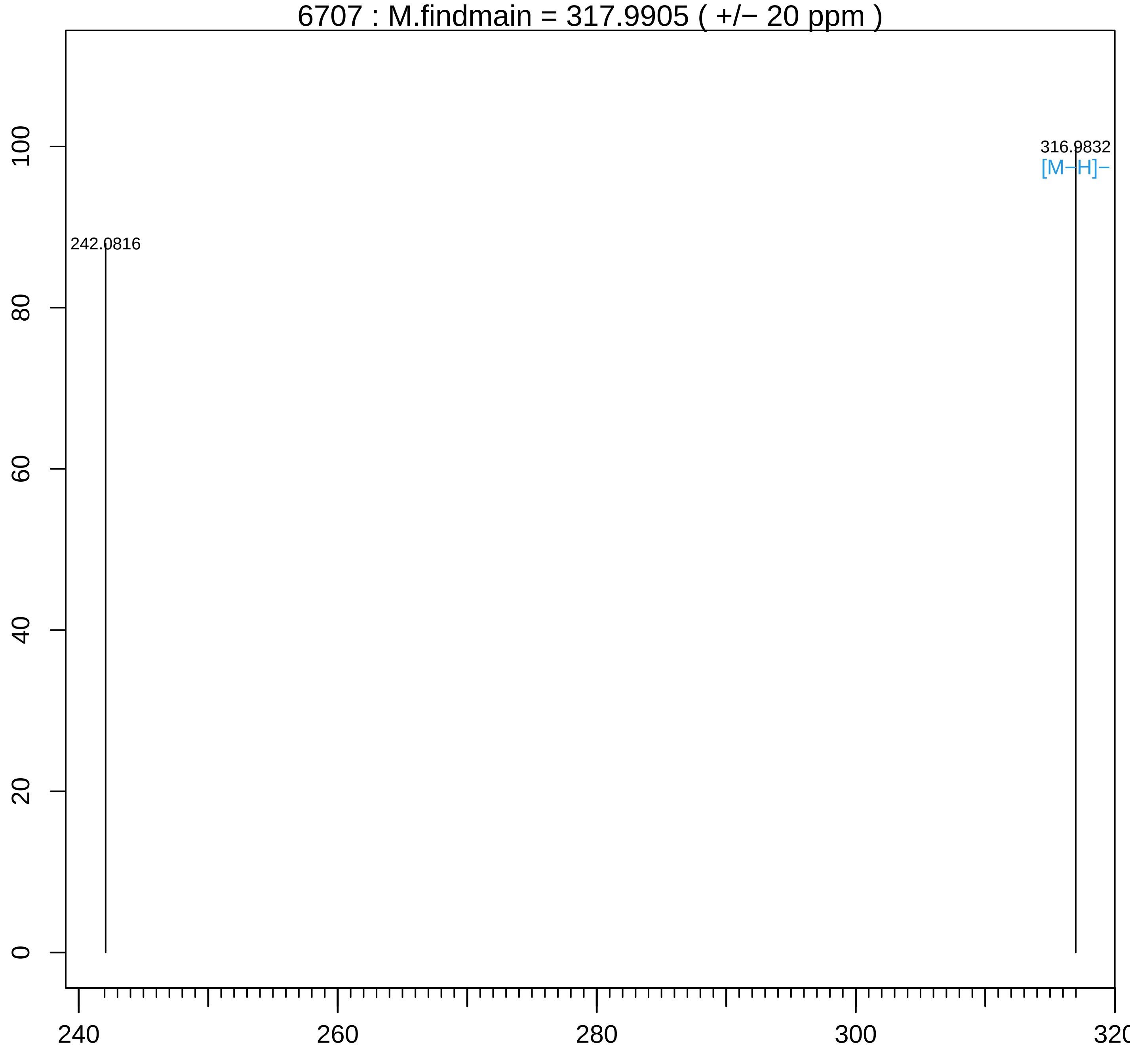
6707 : M.findmain = 317.9905 (+/− 20 ppm)
316.9832
[M−H]−
242.0816
0
20
40
60
80
100
240
260
280
300
320

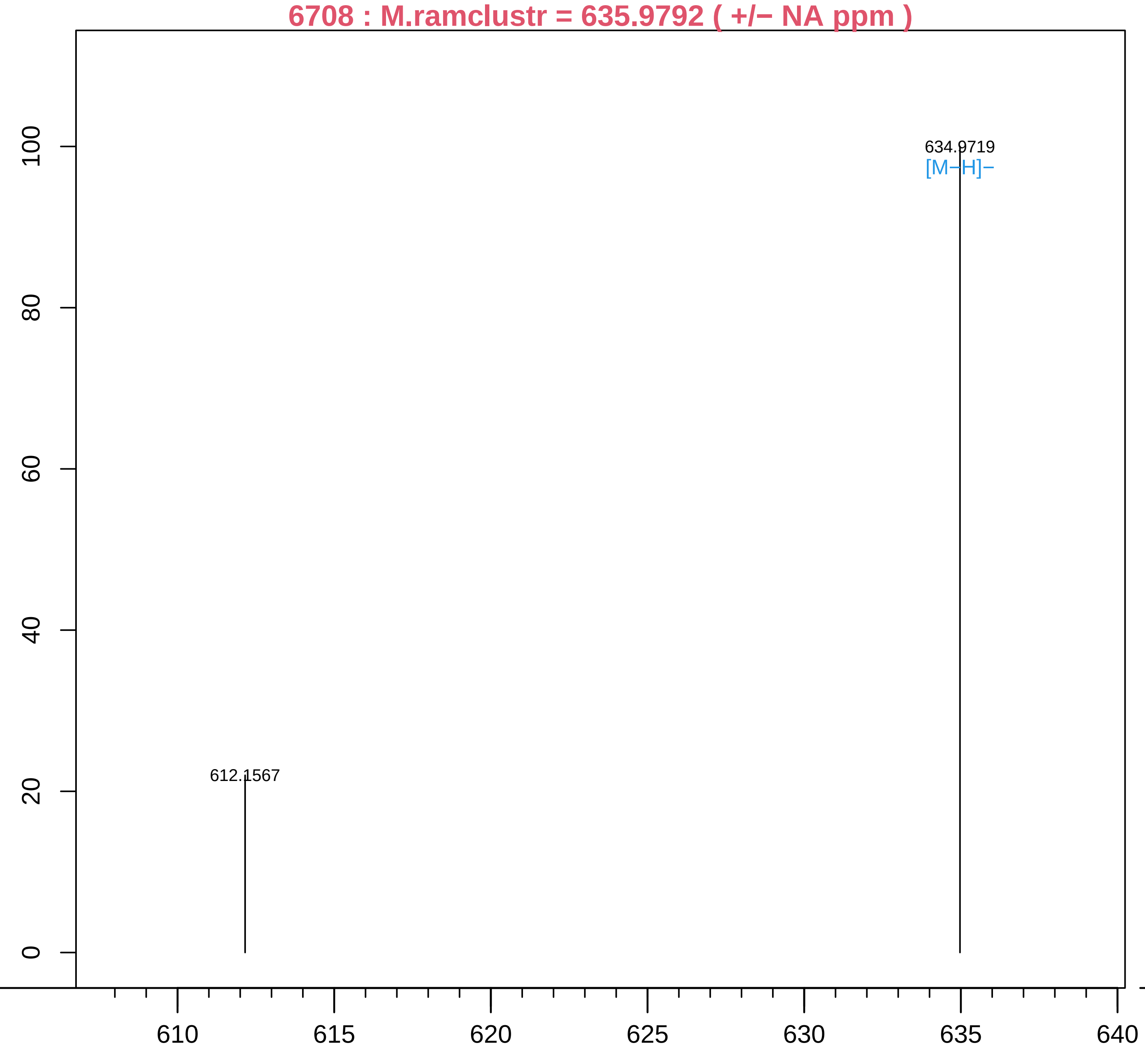

6708 : M.ramclustr = 635.9792 (+/− NA ppm)
634.9719
[M−H]−
612.1567
0
20
40
60
80
100
610
615
620
625
630
635
640

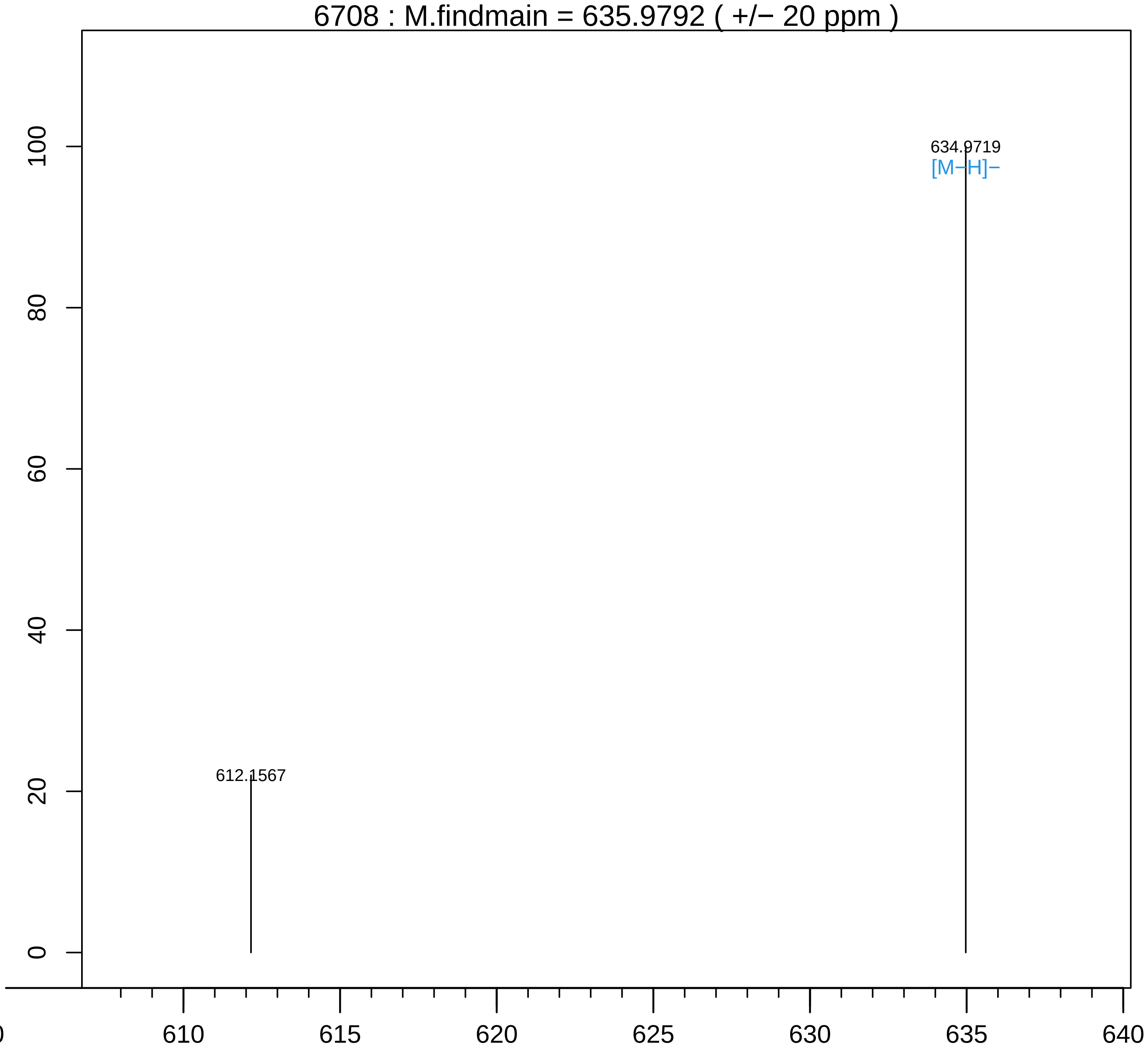

6708 : M.findmain = 635.9792 (+/− 20 ppm)
634.9719
[M−H]−
612.1567
0
20
40
60
80
100
610
615
620
625
630
635
640

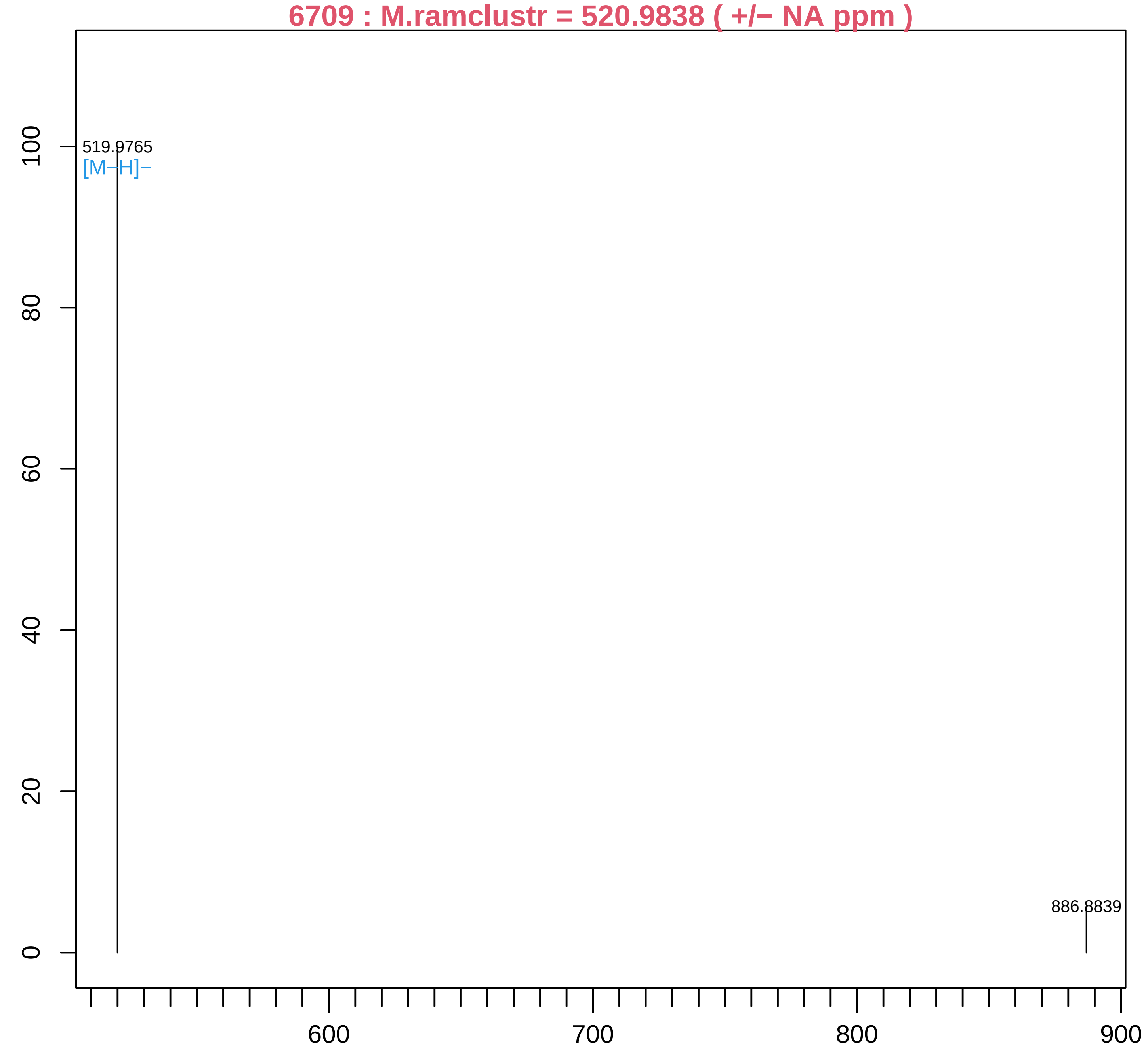
6709 : M.ramclustr = 520.9838 (+/− NA ppm)
519.9765
[M−H]−
886.8839
0
20
40
60
80
100
600
700
800
900

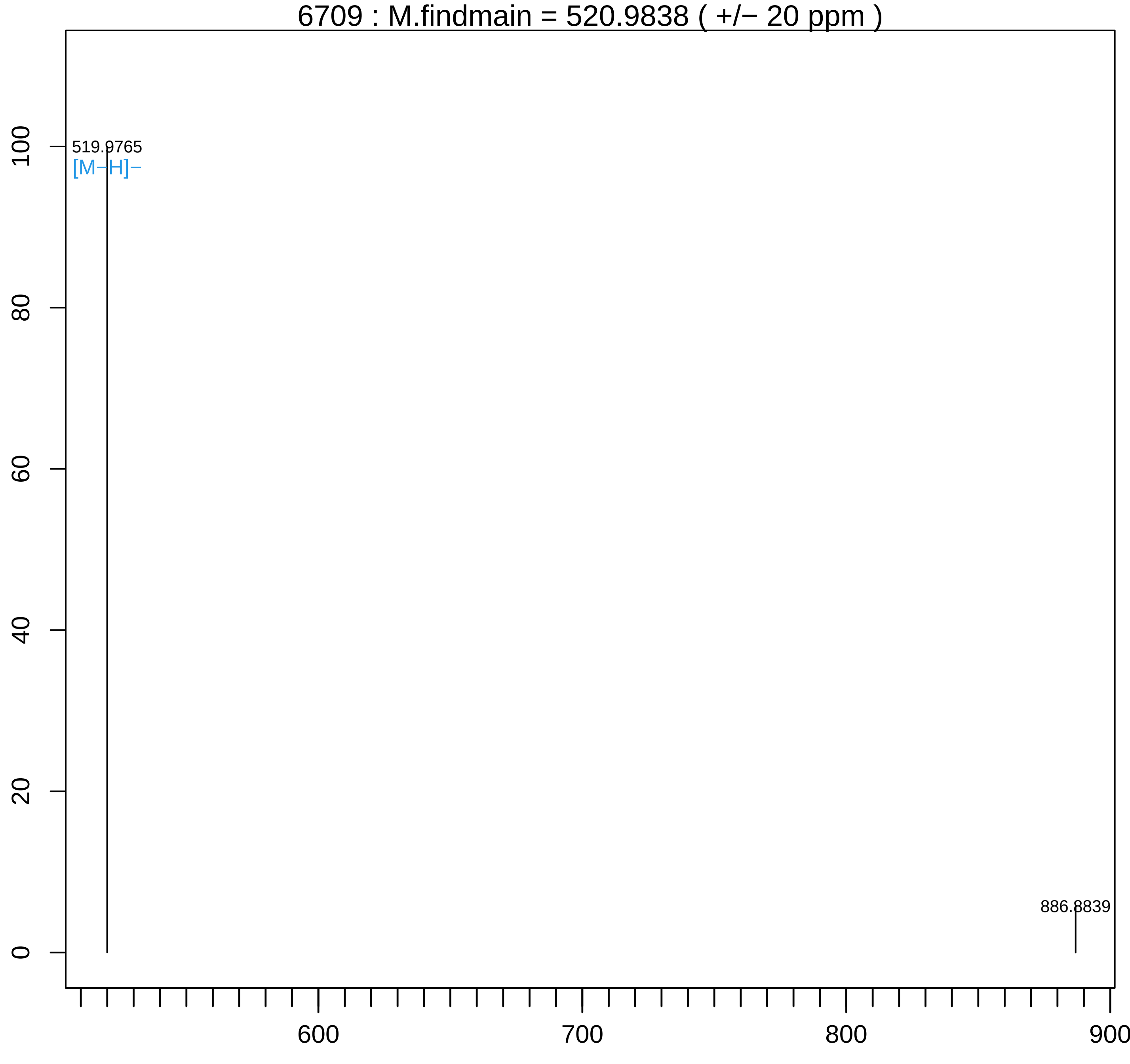
6709 : M.findmain = 520.9838 (+/− 20 ppm)
519.9765
[M−H]−
886.8839
0
20
40
60
80
100
600
700
800
900

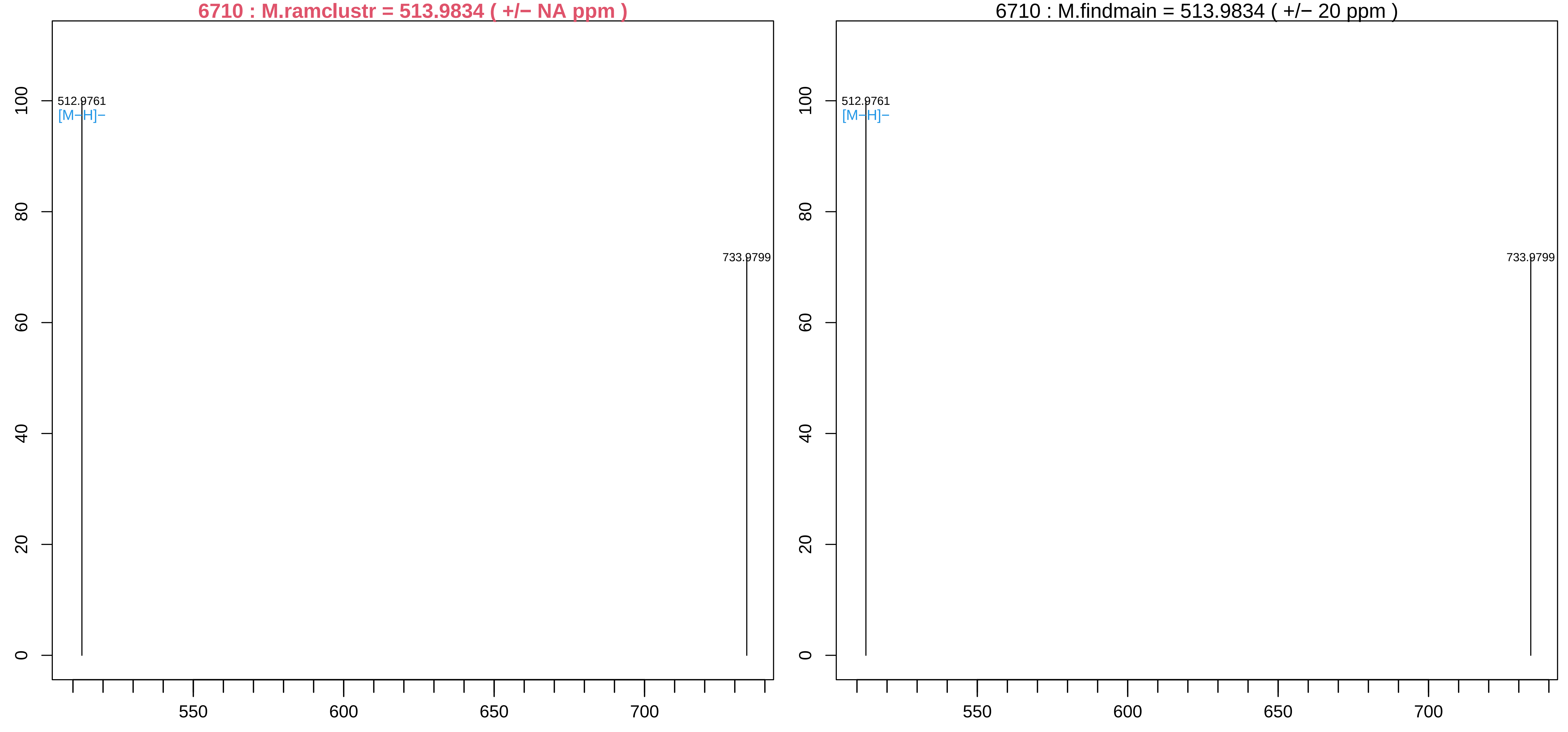
6710 : M.ramclustr = 513.9834 (+/− NA ppm)
512.9761
[M−H]−
733.9799
0
20
40
60
80
100
550
600
650
700
6710 : M.findmain = 513.9834 (+/− 20 ppm)
512.9761
[M−H]−
733.9799
0
20
40
60
80
100
550
600
650
700

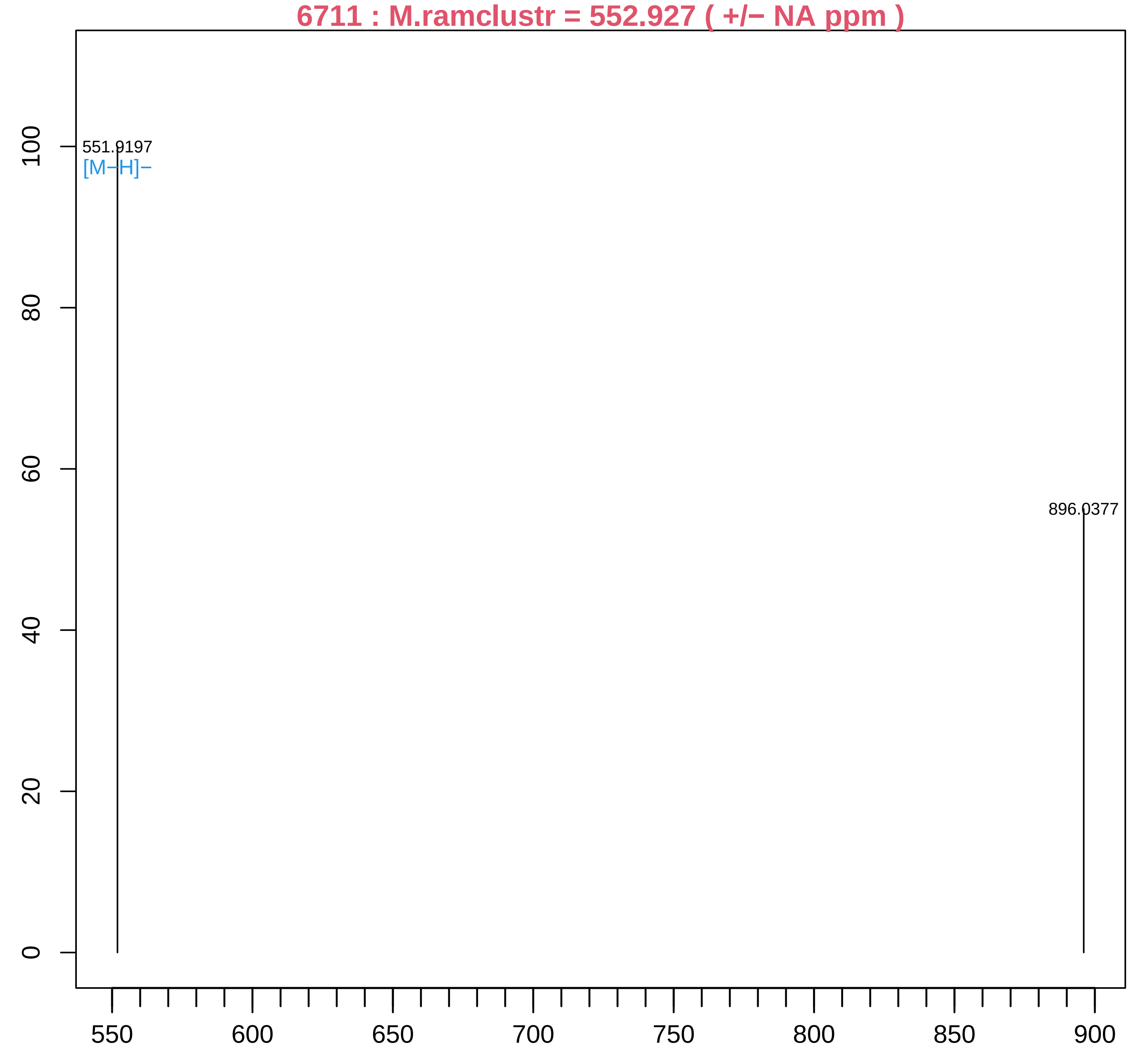
6711 : M.ramclustr = 552.927 (+/− NA ppm)
551.9197
[M−H]−
896.0377
0
20
40
60
80
100
550
600
650
700
750
800
850
900

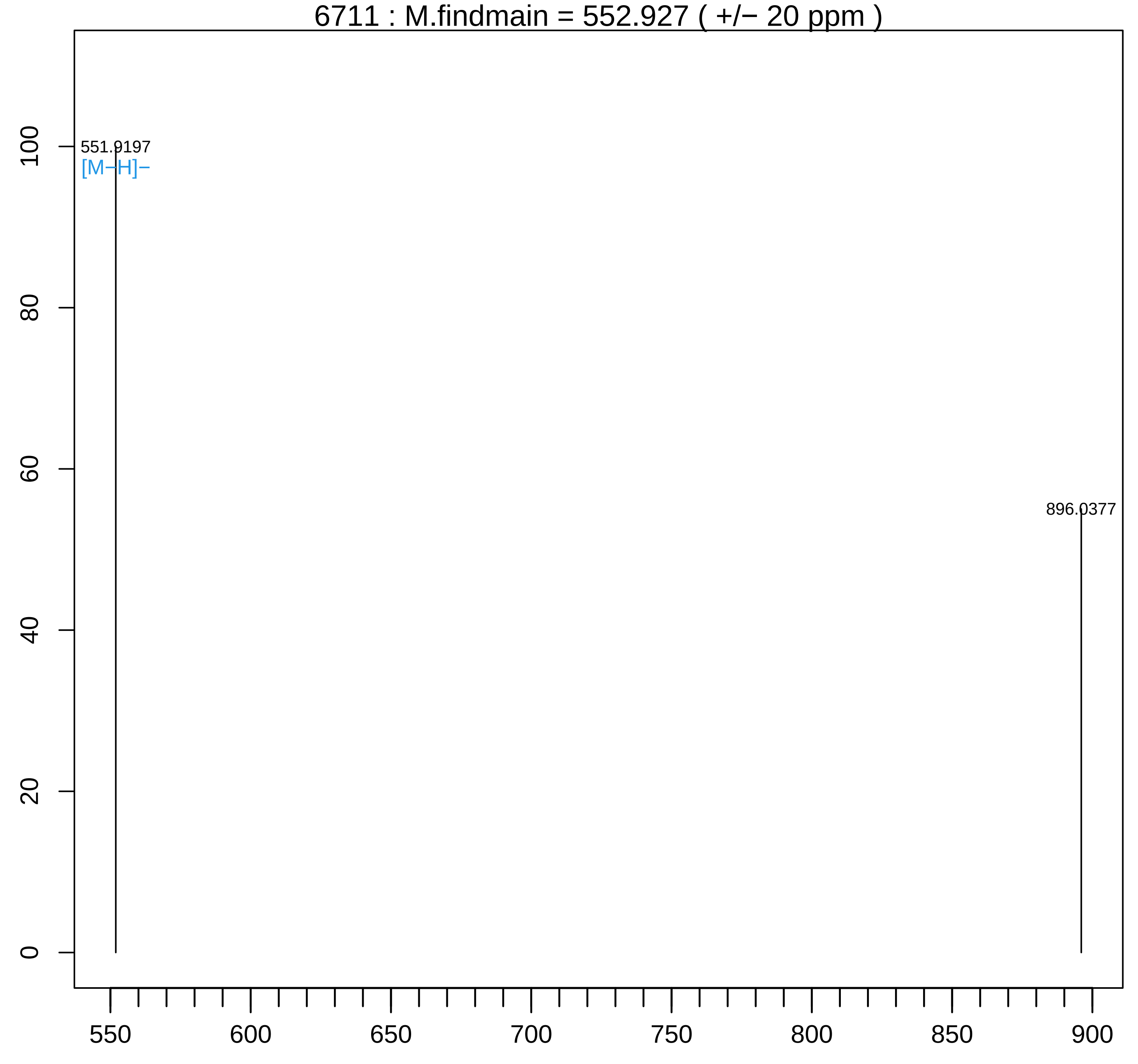
6711 : M.findmain = 552.927 (+/− 20 ppm)
551.9197
[M−H]−
896.0377
0
20
40
60
80
100
550
600
650
700
750
800
850
900

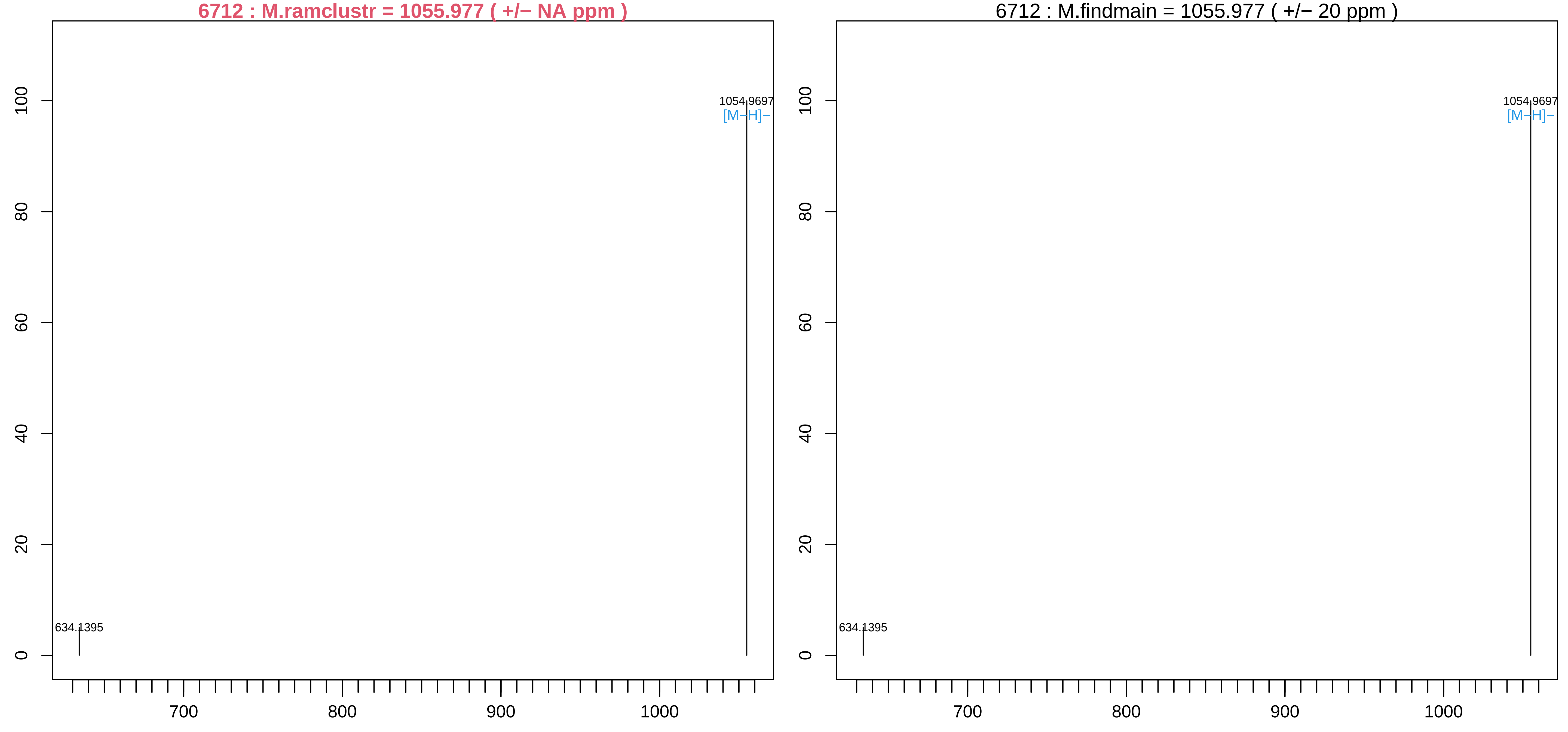
6712 : M.ramclustr = 1055.977 (+/− NA ppm)
1054.9697
[M−H]−
634.1395
0
20
40
60
80
100
700
800
900
1000
6712 : M.findmain = 1055.977 (+/− 20 ppm)
1054.9697
[M−H]−
634.1395
0
20
40
60
80
100
700
800
900
1000

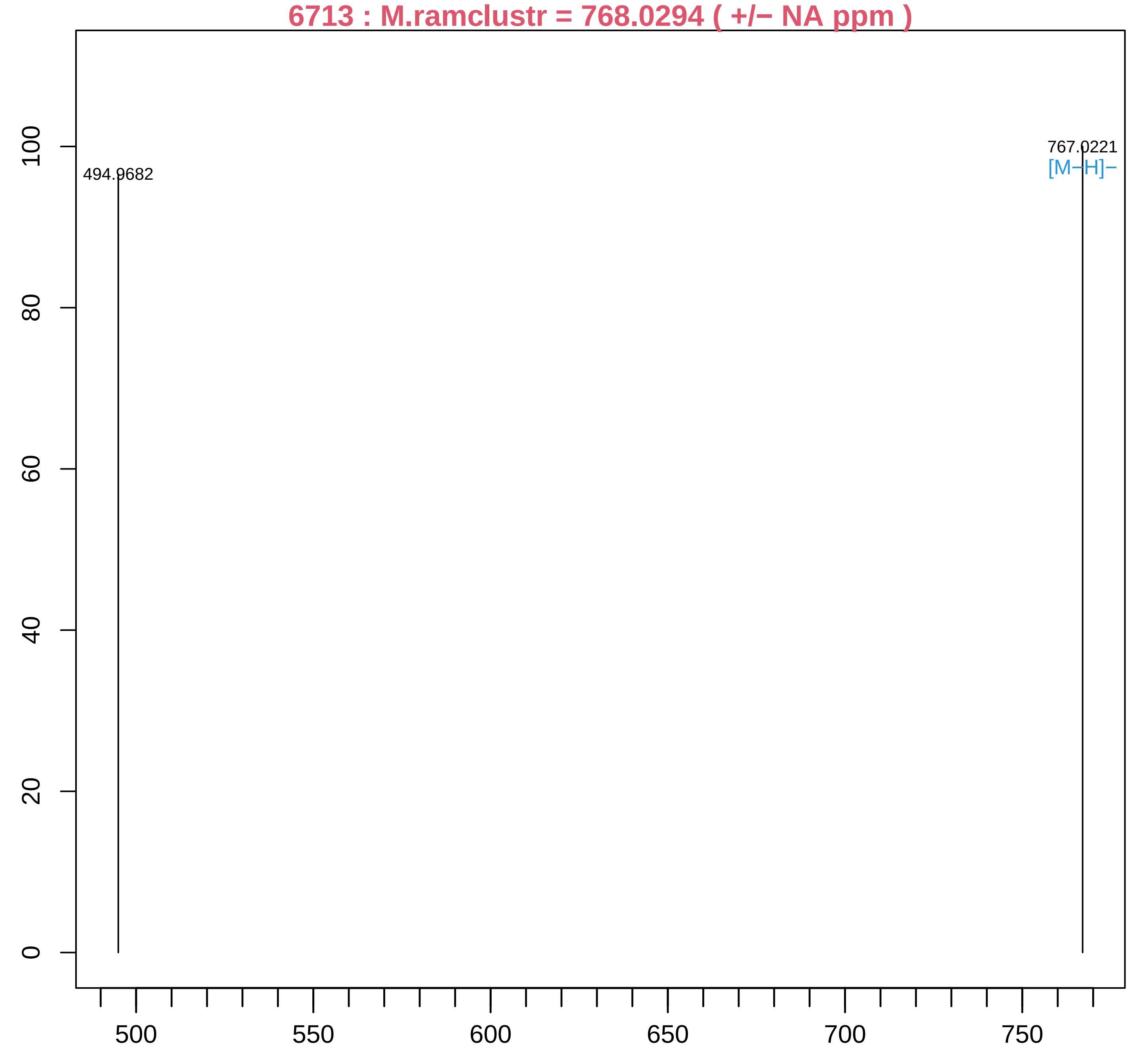
6713 : M.ramclustr = 768.0294 (+/− NA ppm)
494.9682
767.0221
[M−H]−
0
20
40
60
80
100
500
550
600
650
700
750

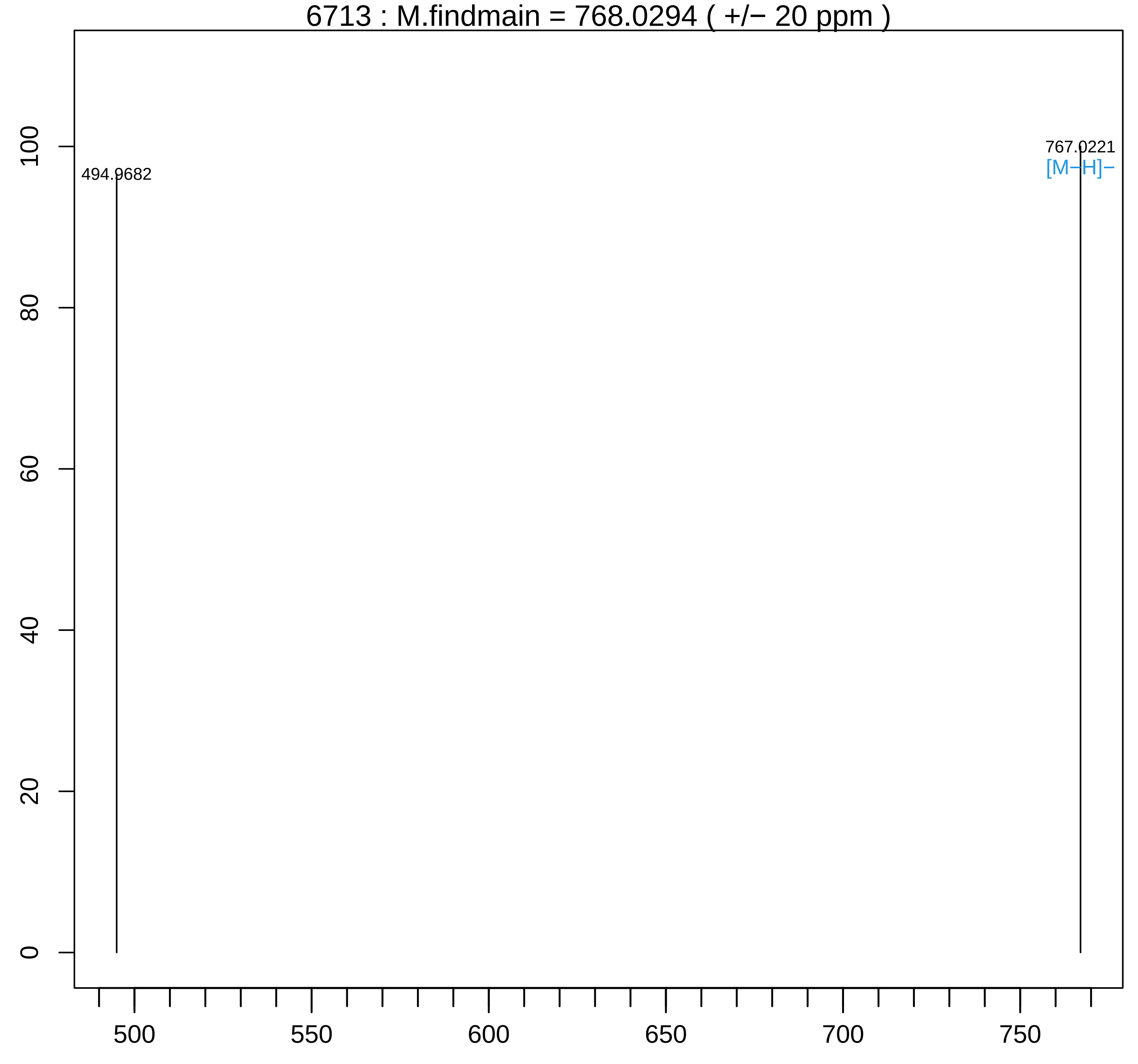
6713 : M.findmain = 768.0294 (+/− 20 ppm)
494.9682
767.0221
[M−H]−
0
20
40
60
80
100
500
550
600
650
700
750

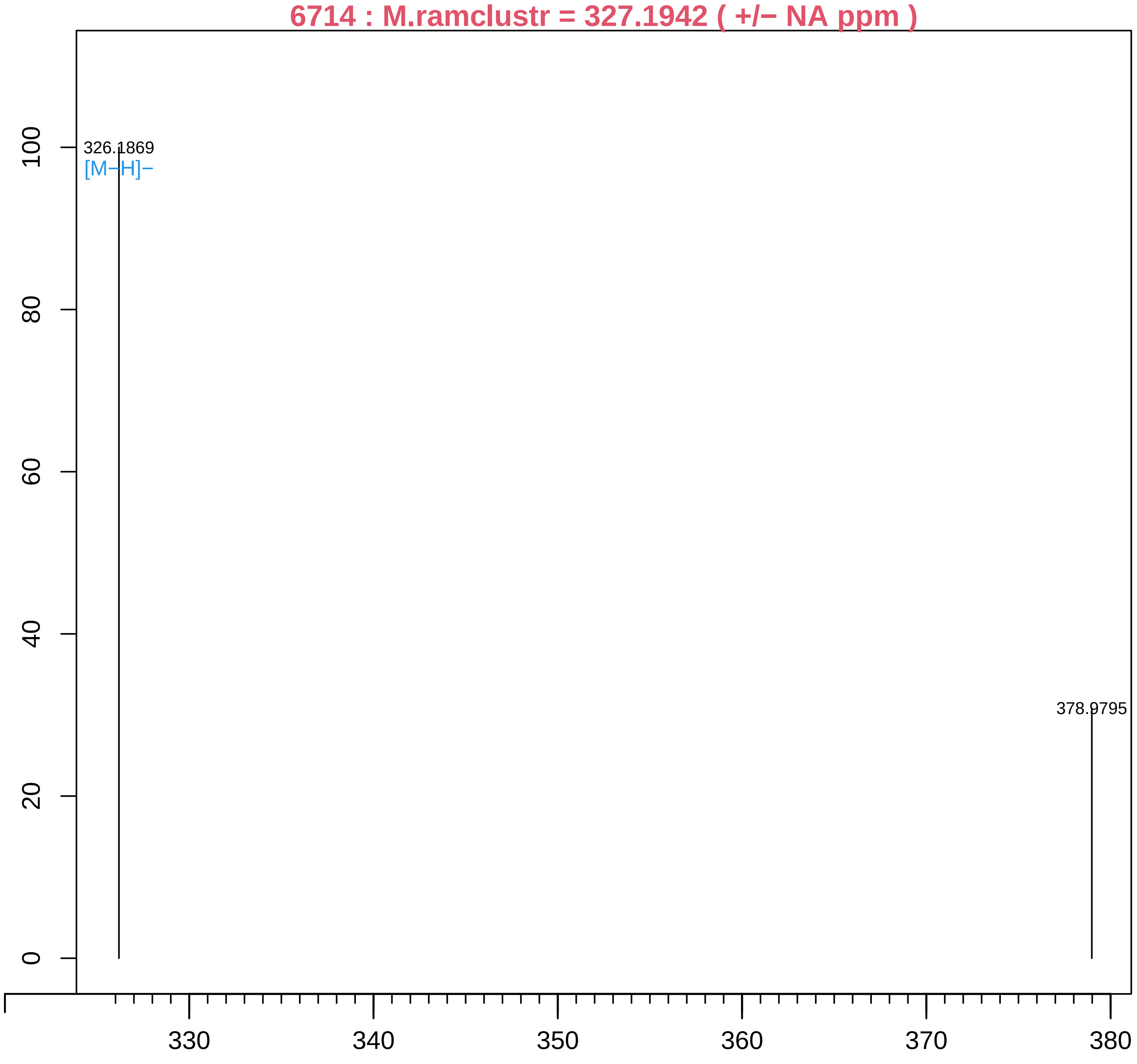

6714 : M.ramclustr = 327.1942 (+/− NA ppm)
326.1869
[M−H]−
378.9795
0
20
40
60
80
100
330
340
350
360
370
380

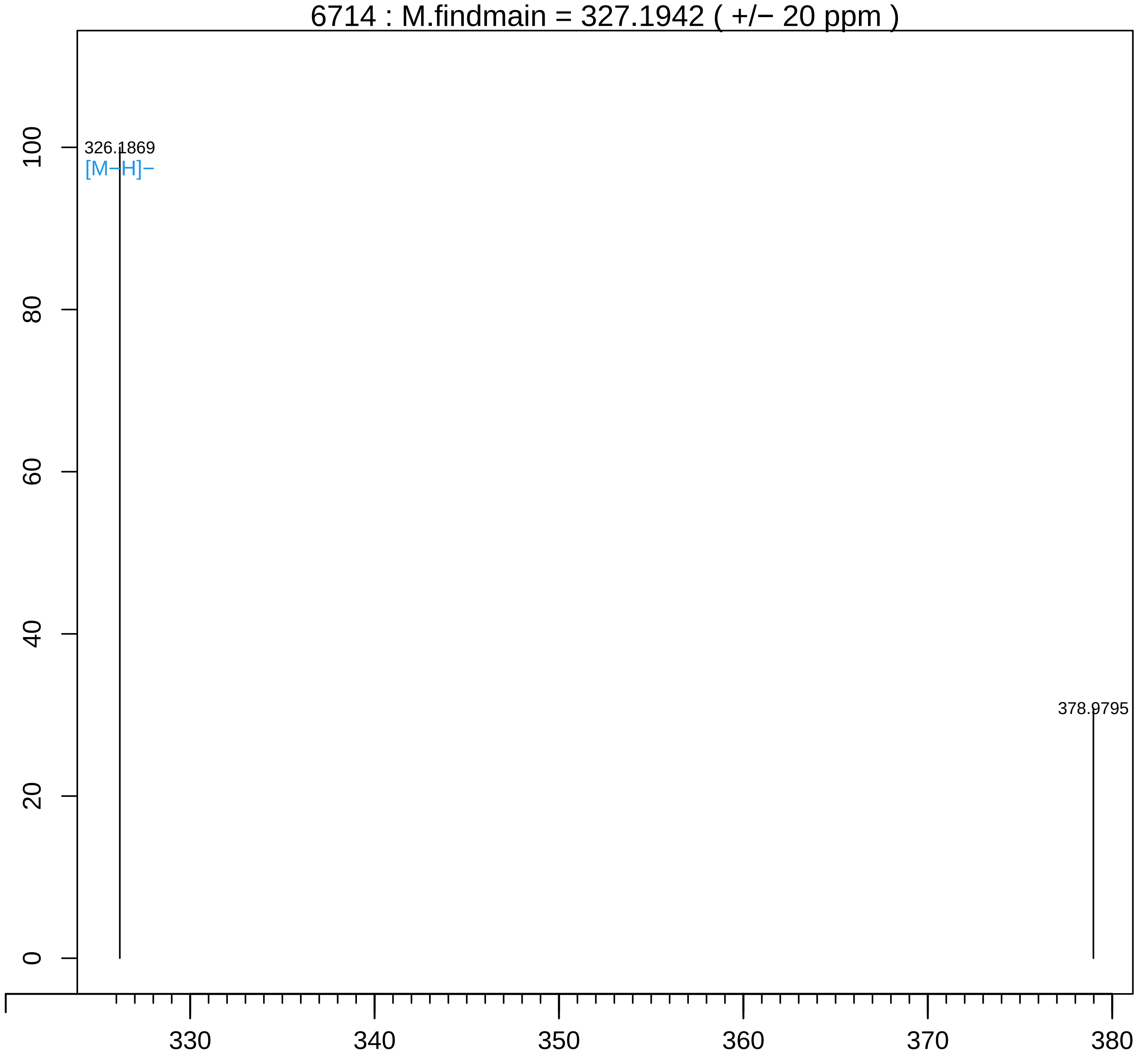

6714 : M.findmain = 327.1942 (+/− 20 ppm)
326.1869
[M−H]−
378.9795
0
20
40
60
80
100
330
340
350
360
370
380

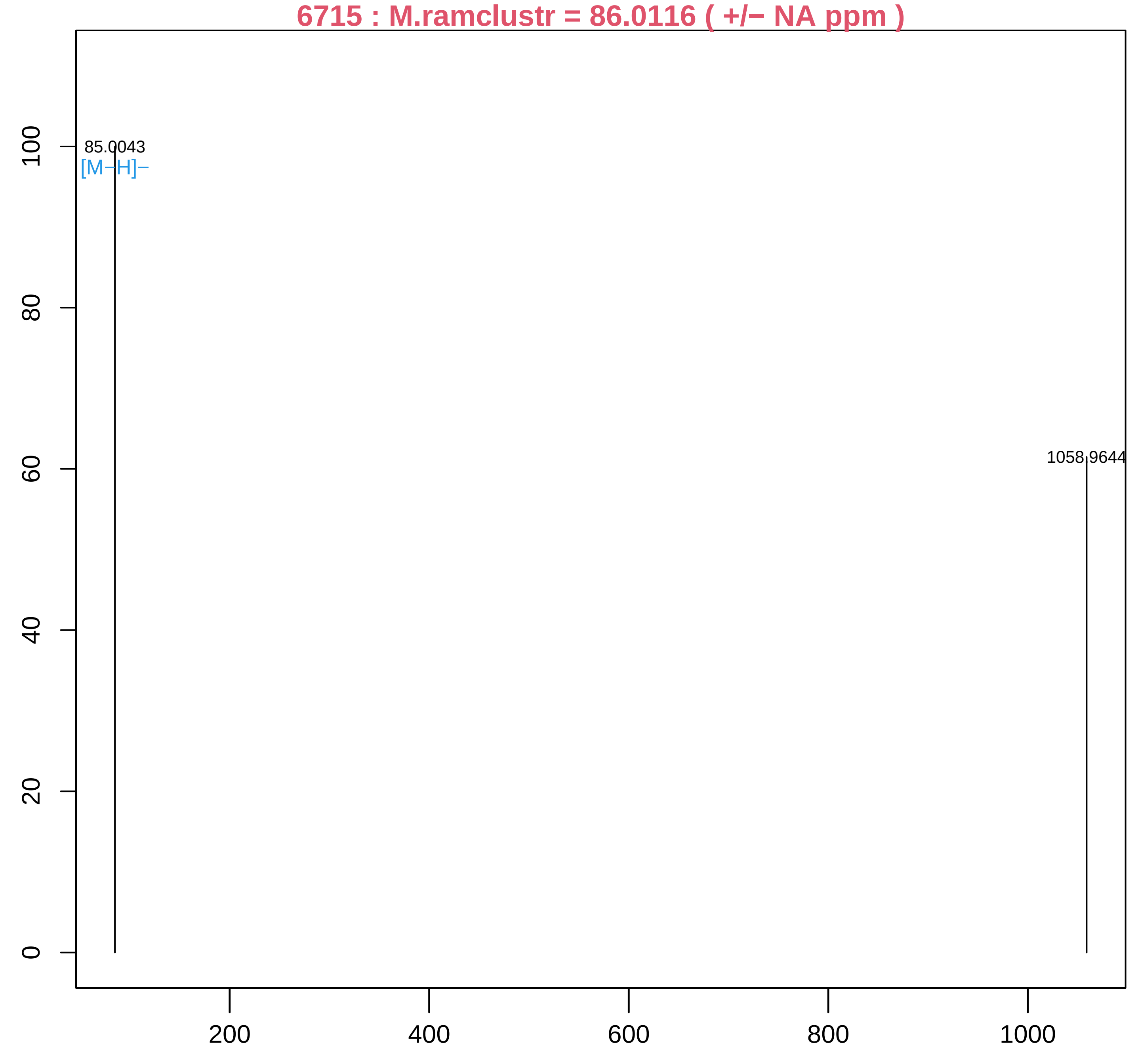
6715 : M.ramclustr = 86.0116 (+/− NA ppm)
85.0043
[M−H]−
1058.9644
0
20
40
60
80
100
200
400
600
800
1000

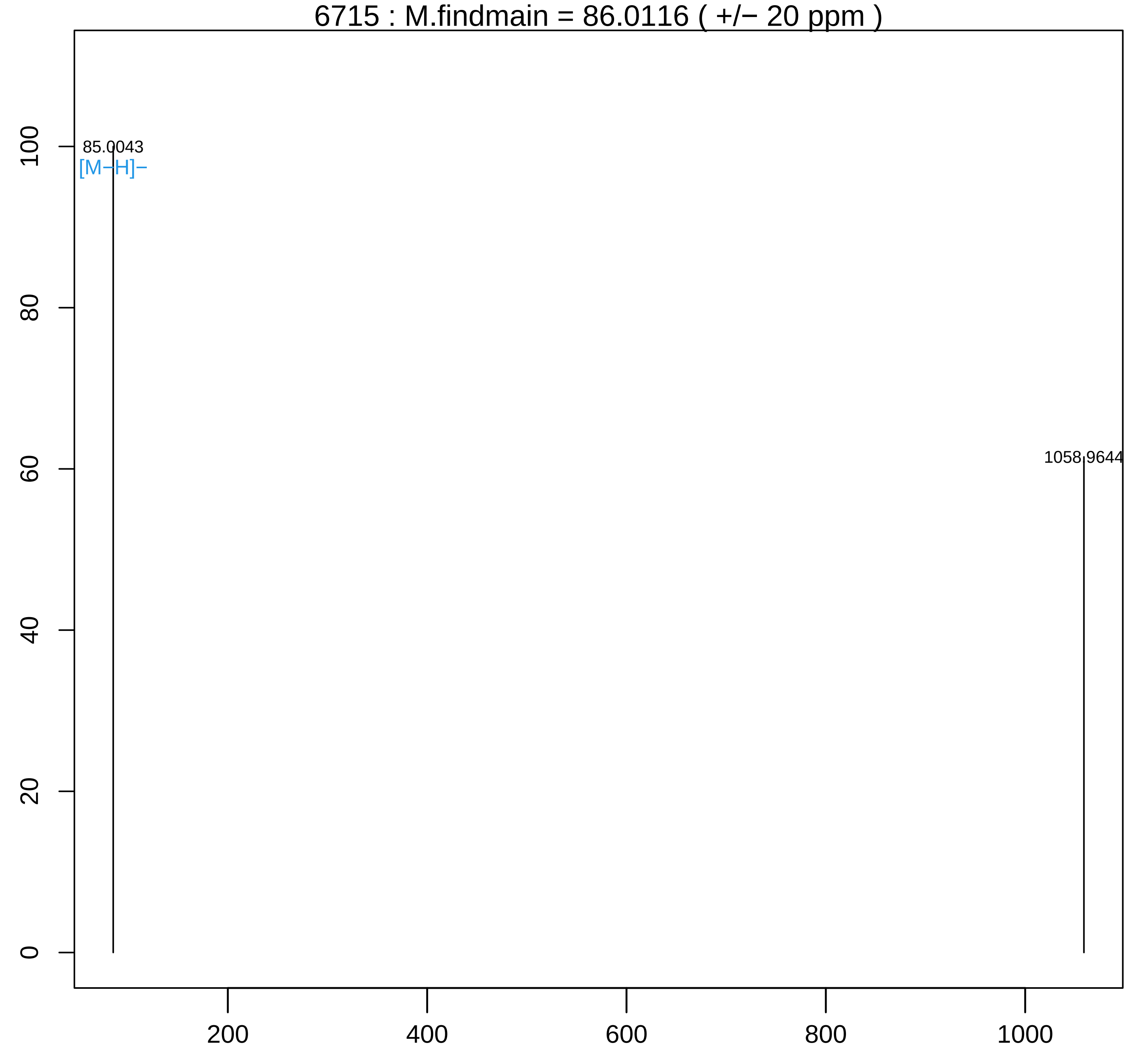
6715 : M.findmain = 86.0116 (+/− 20 ppm)
85.0043
[M−H]−
1058.9644
0
20
40
60
80
100
200
400
600
800
1000

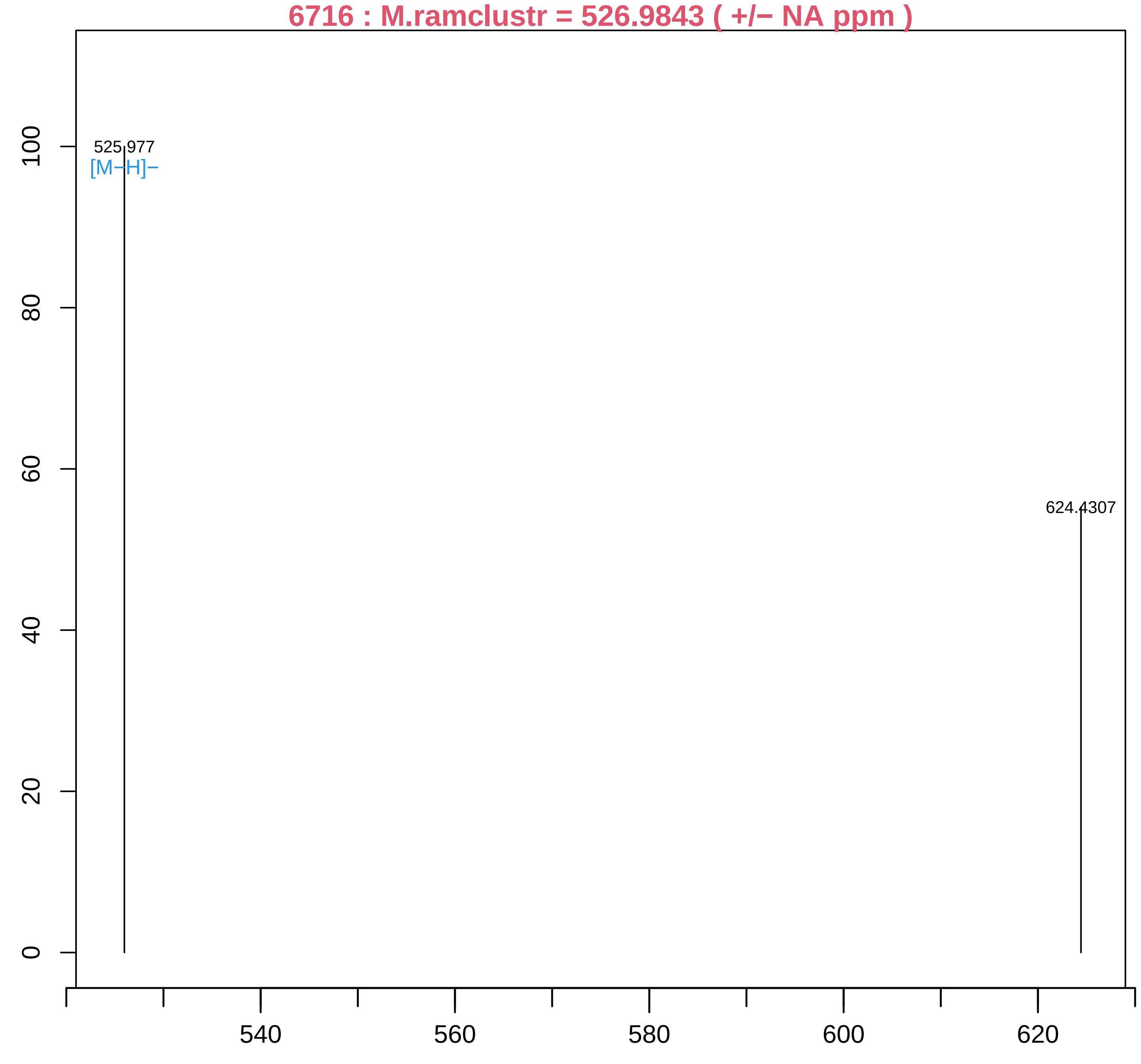
6716 : M.ramclustr = 526.9843 (+/− NA ppm)
525.977
[M−H]−
624.4307
0
20
40
60
80
100
540
560
580
600
620

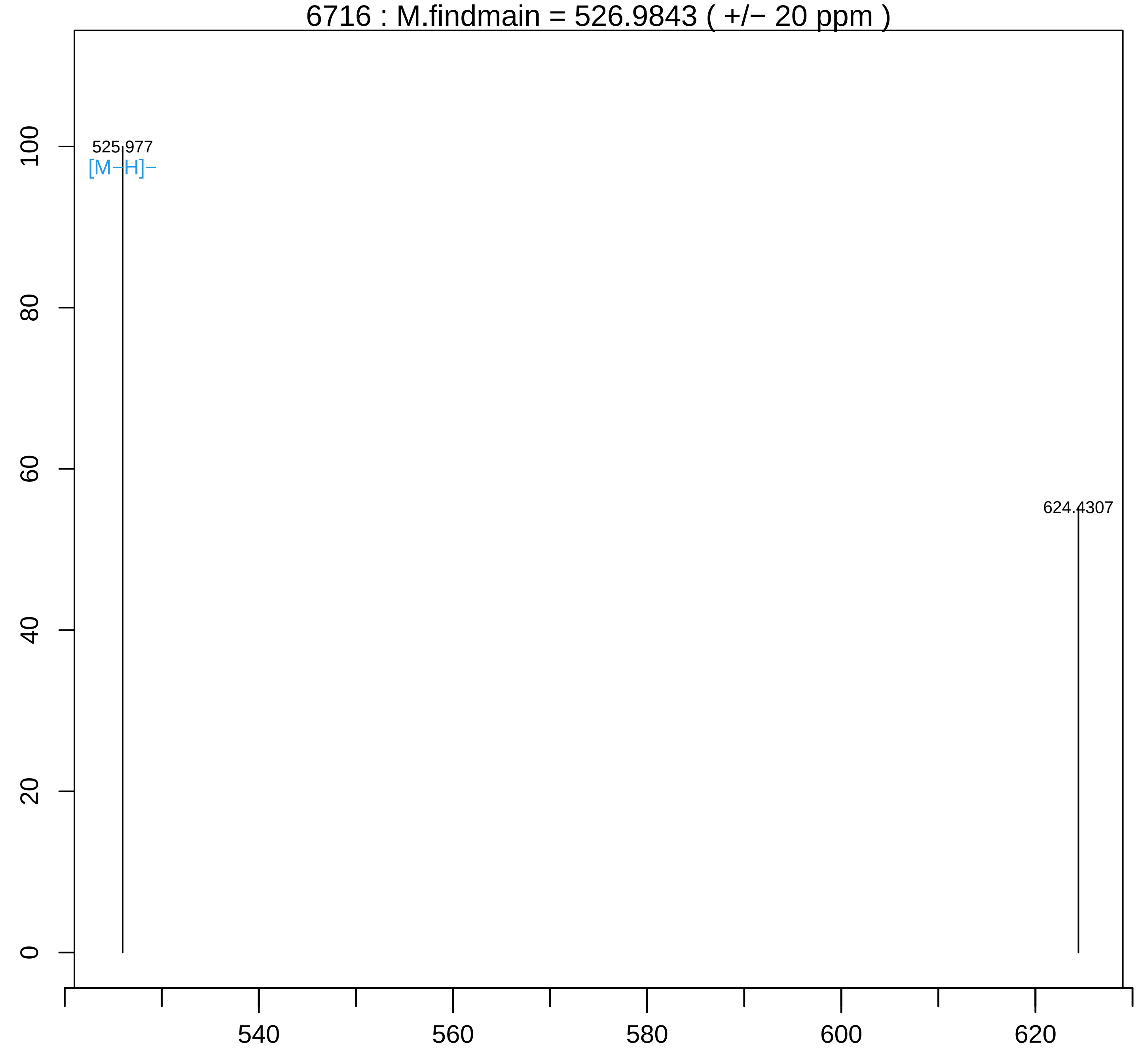
6716 : M.findmain = 526.9843 (+/− 20 ppm)
525.977
[M−H]−
624.4307
0
20
40
60
80
100
540
560
580
600
620

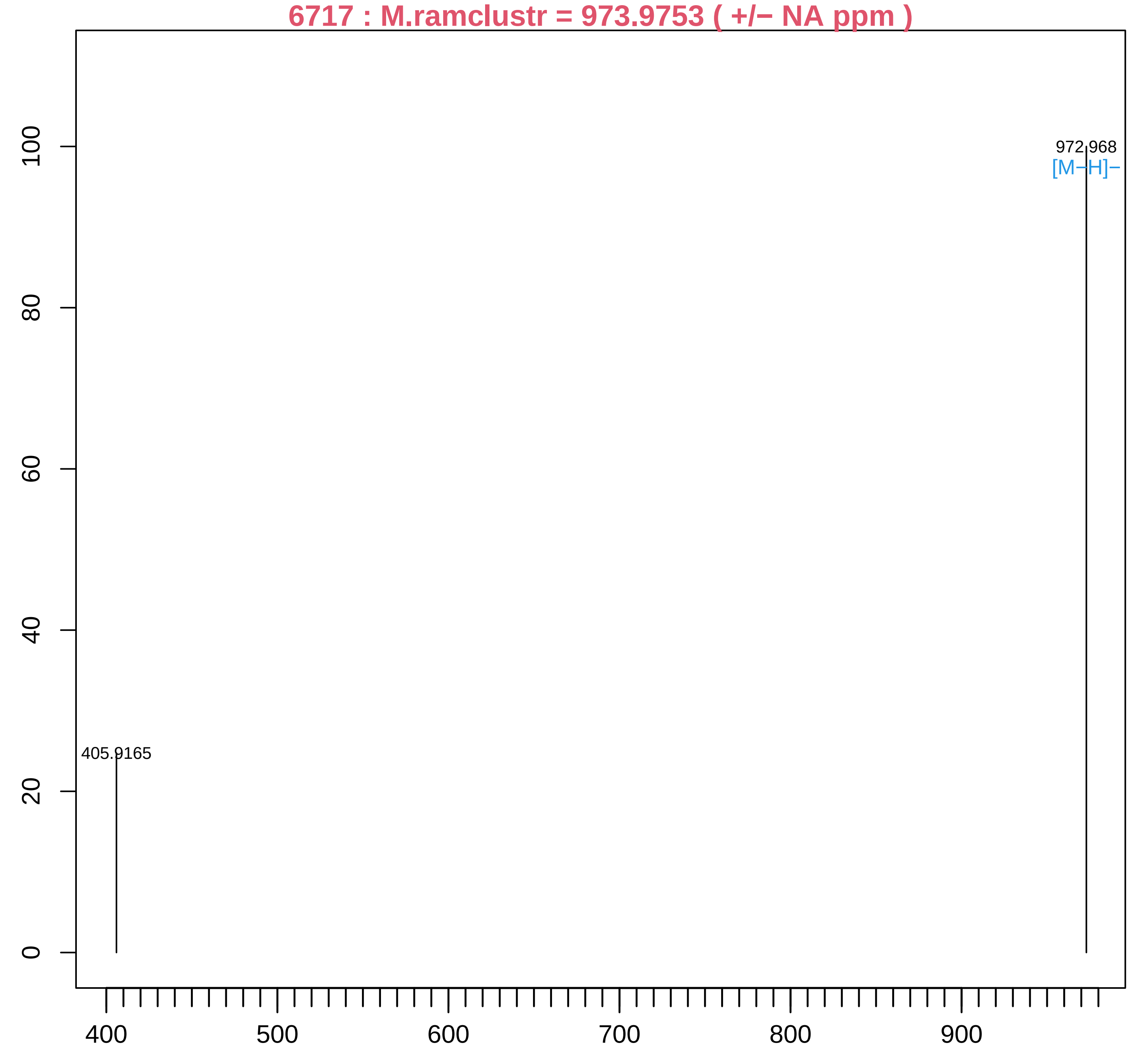
6717 : M.ramclustr = 973.9753 (+/− NA ppm)
972.968
[M−H]−
405.9165
0
20
40
60
80
100
400
500
600
700
800
900

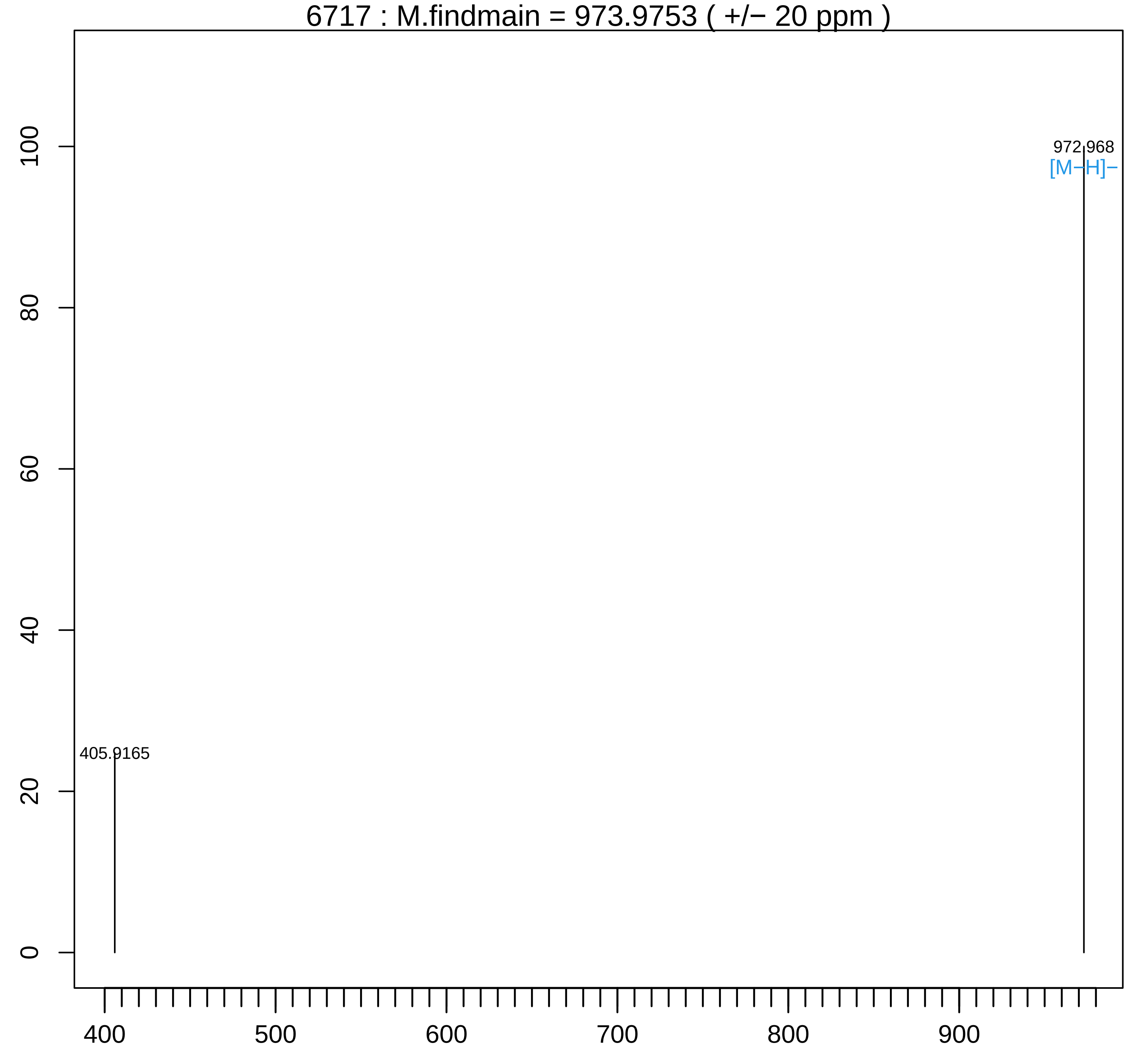
6717 : M.findmain = 973.9753 (+/− 20 ppm)
972.968
[M−H]−
405.9165
0
20
40
60
80
100
400
500
600
700
800
900

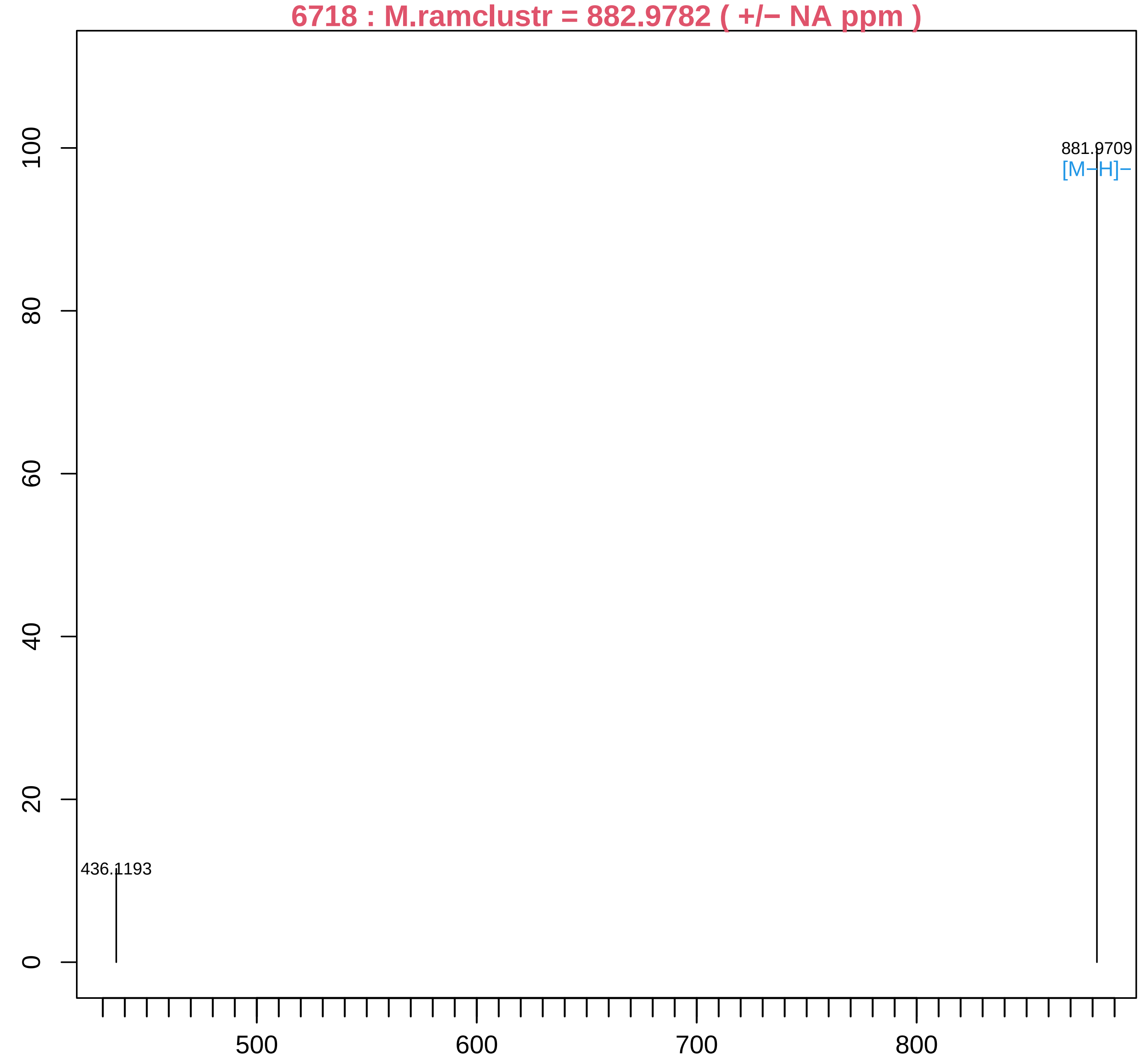
6718 : M.ramclustr = 882.9782 (+/− NA ppm)
0
20
40
60
80
100
500
600
700
800
436.1193
881.9709
[M−H]−

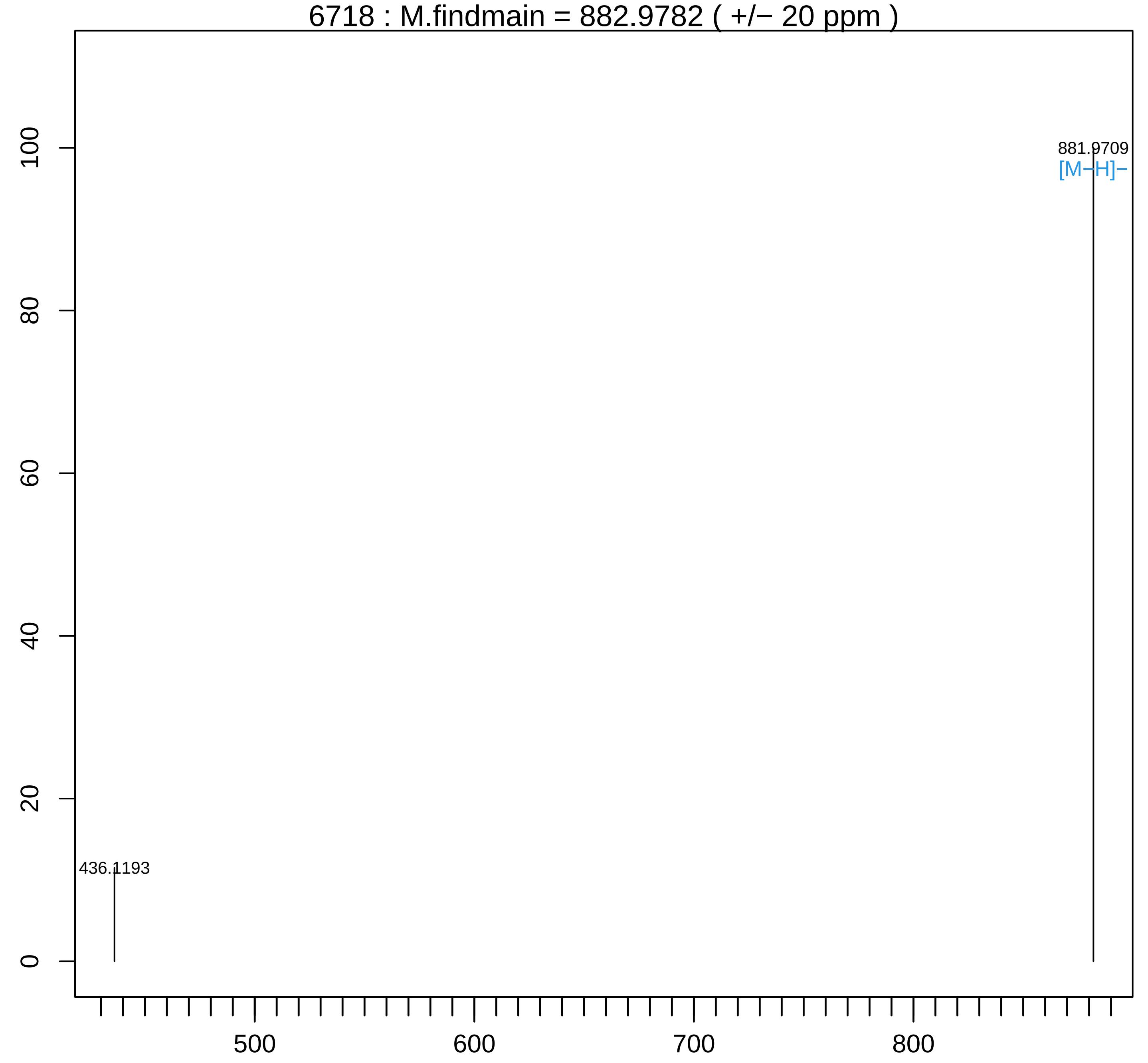
6718 : M.findmain = 882.9782 (+/− 20 ppm)
0
20
40
60
80
100
500
600
700
800
436.1193
881.9709
[M−H]−

6719 : M.ramclustr = 676.9865 (+/− NA ppm)

6719 : M.findmain = 676.9865 (+/− 20 ppm)

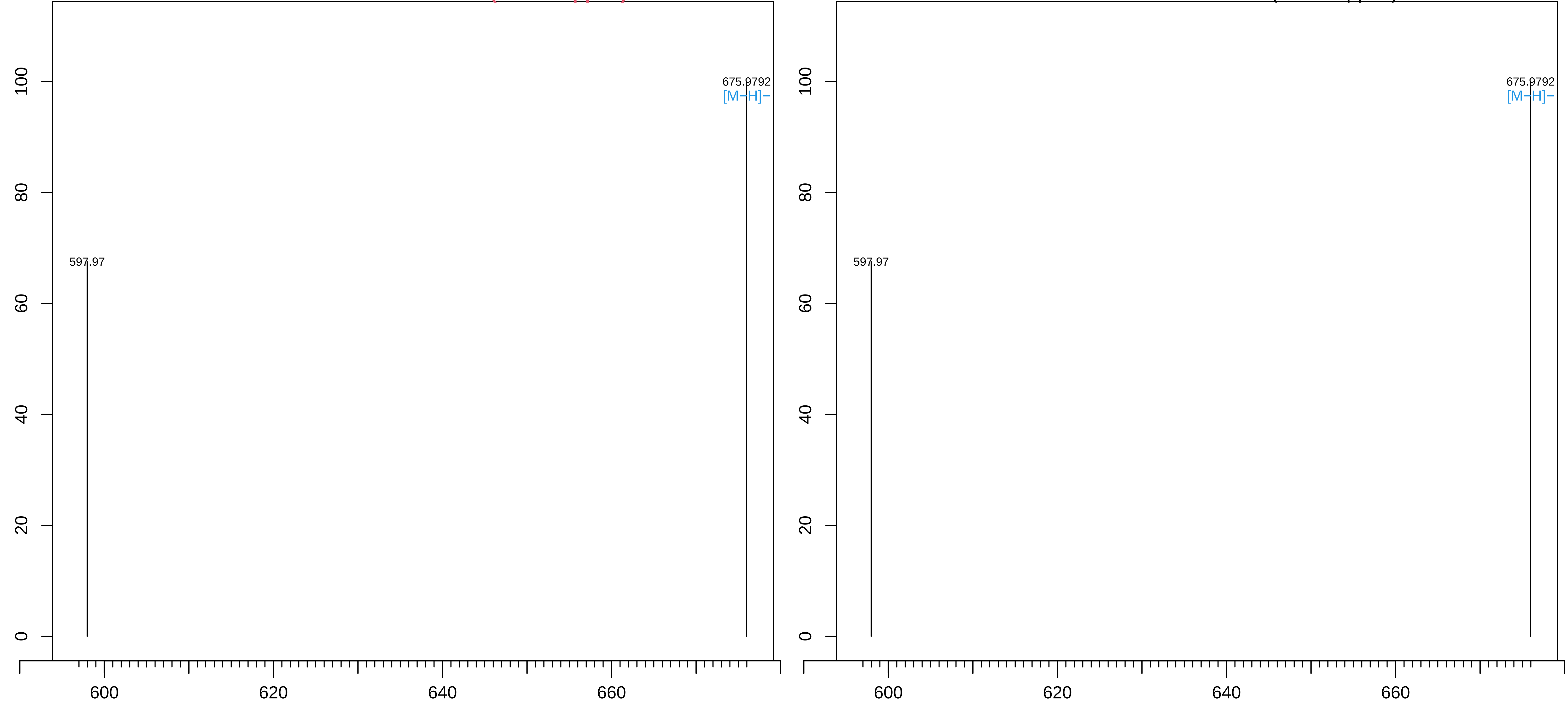

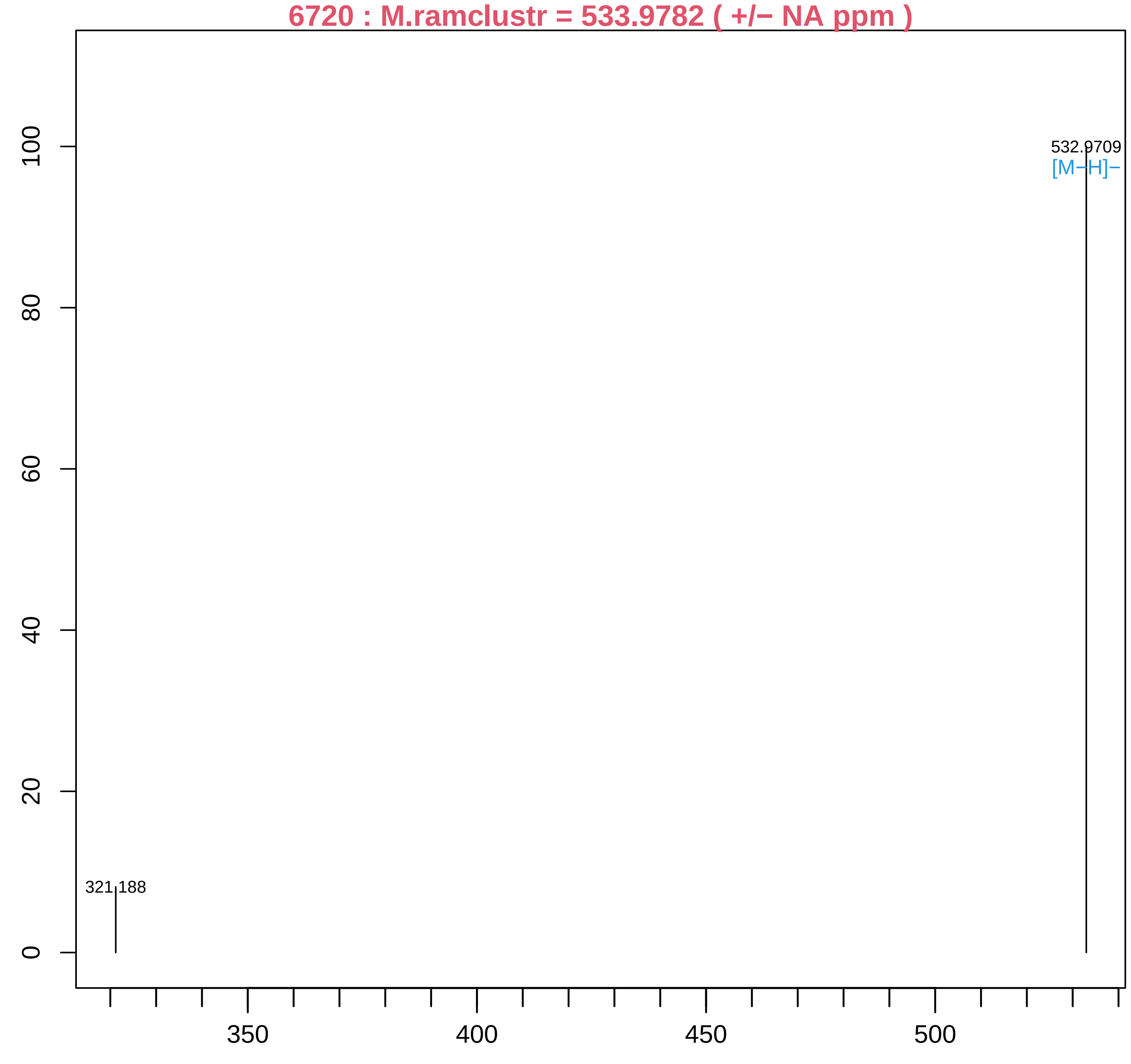
6720 : M.ramclustr = 533.9782 (+/− NA ppm)
532.9709
[M−H]−
321.188
0
20
40
60
80
100
350
400
450
500

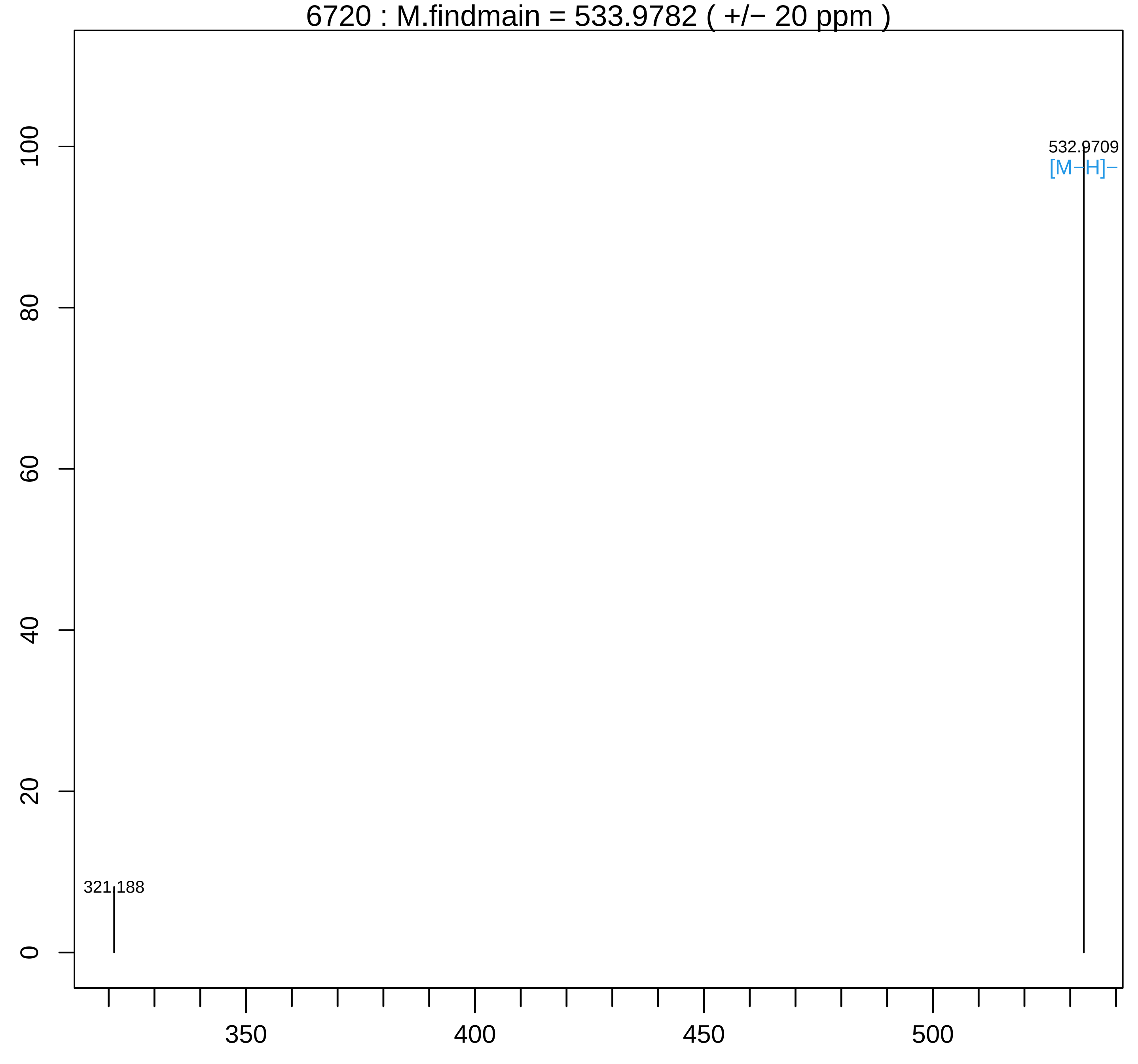
6720 : M.findmain = 533.9782 (+/− 20 ppm)
532.9709
[M−H]−
321.188
0
20
40
60
80
100
350
400
450
500

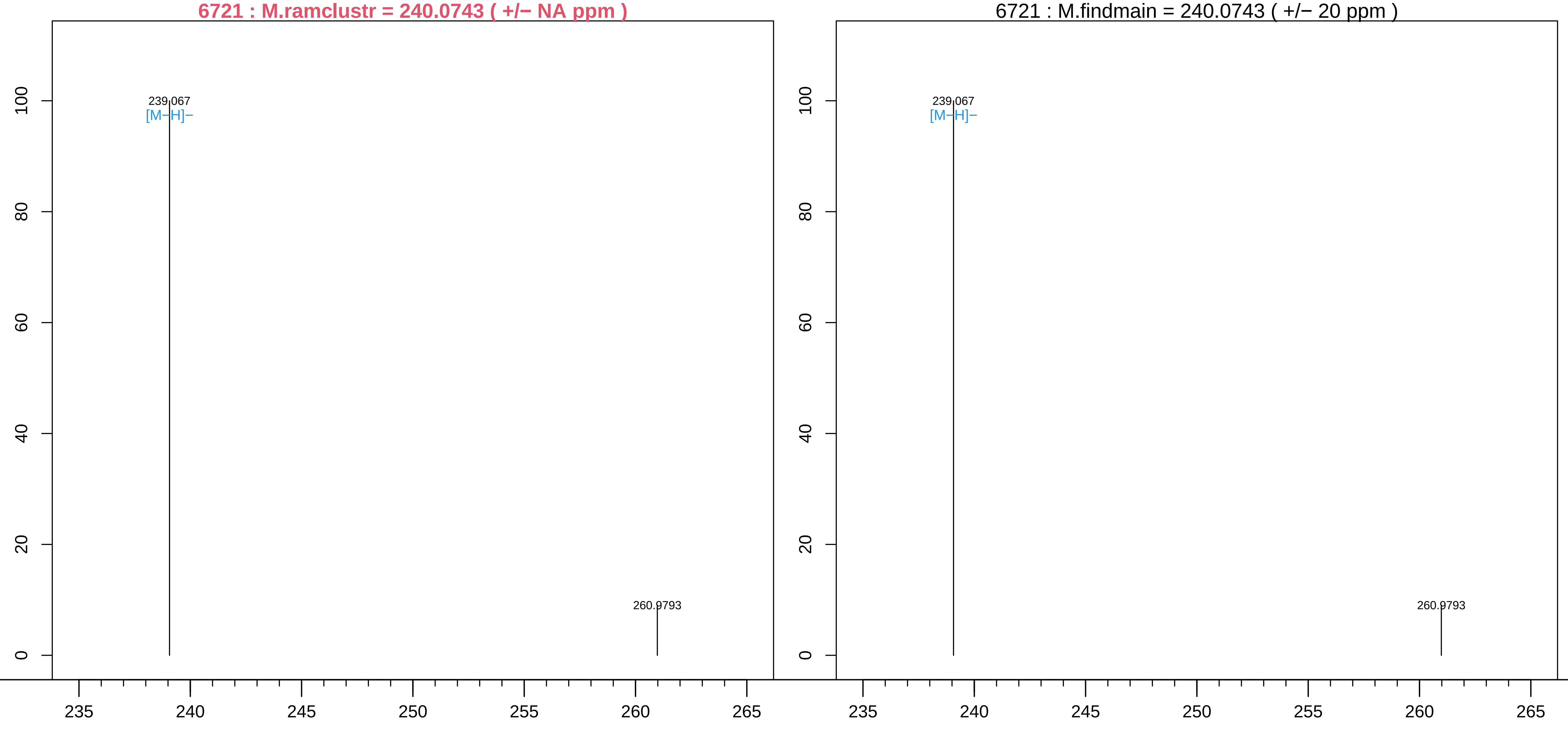

6721 : M.ramclustr = 240.0743 (+/− NA ppm)
239.067
[M−H]−
260.9793
6721 : M.findmain = 240.0743 (+/− 20 ppm)
239.067
[M−H]−
260.9793
0
20
40
60
80
100
235
240
245
250
255
260
265

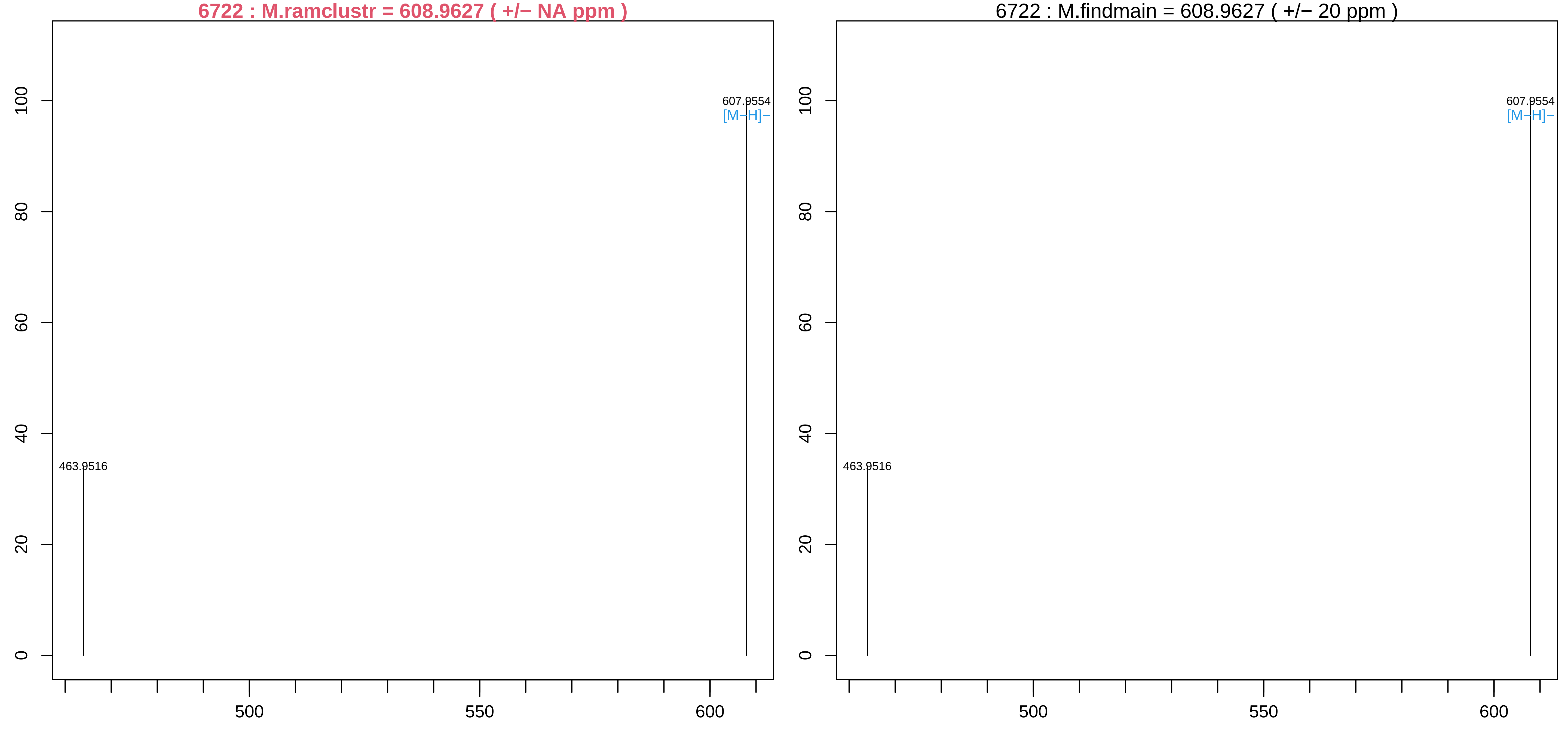

6722 : M.ramclustr = 608.9627 (+/− NA ppm)
6722 : M.findmain = 608.9627 (+/− 20 ppm)
607.9554
[M−H]−
463.9516
607.9554
[M−H]−
463.9516
0
20
40
60
80
100
500
550
600

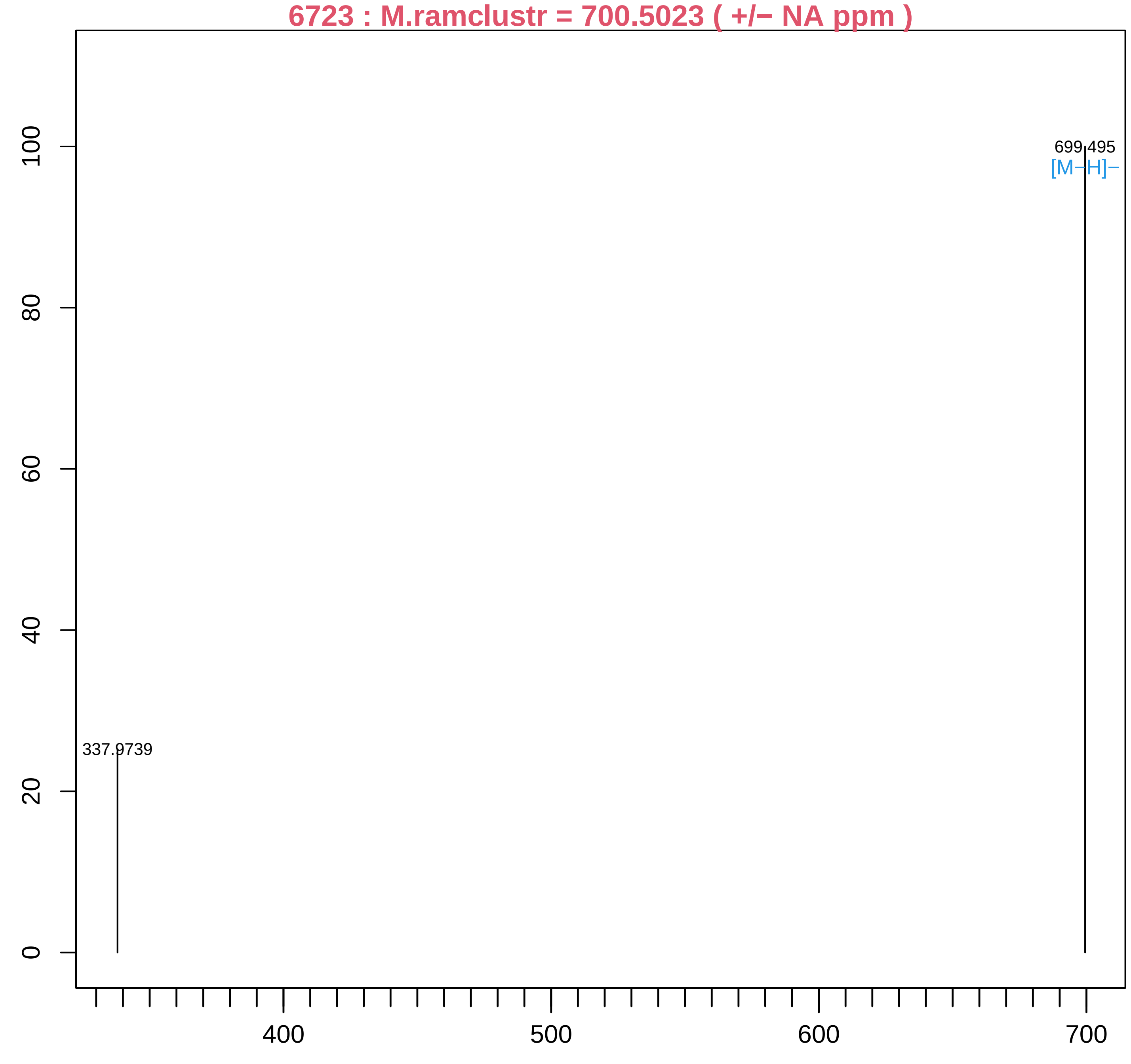
6723 : M.ramclustr = 700.5023 (+/− NA ppm)
699.495
[M−H]−
337.9739
0
20
40
60
80
100
400
500
600
700

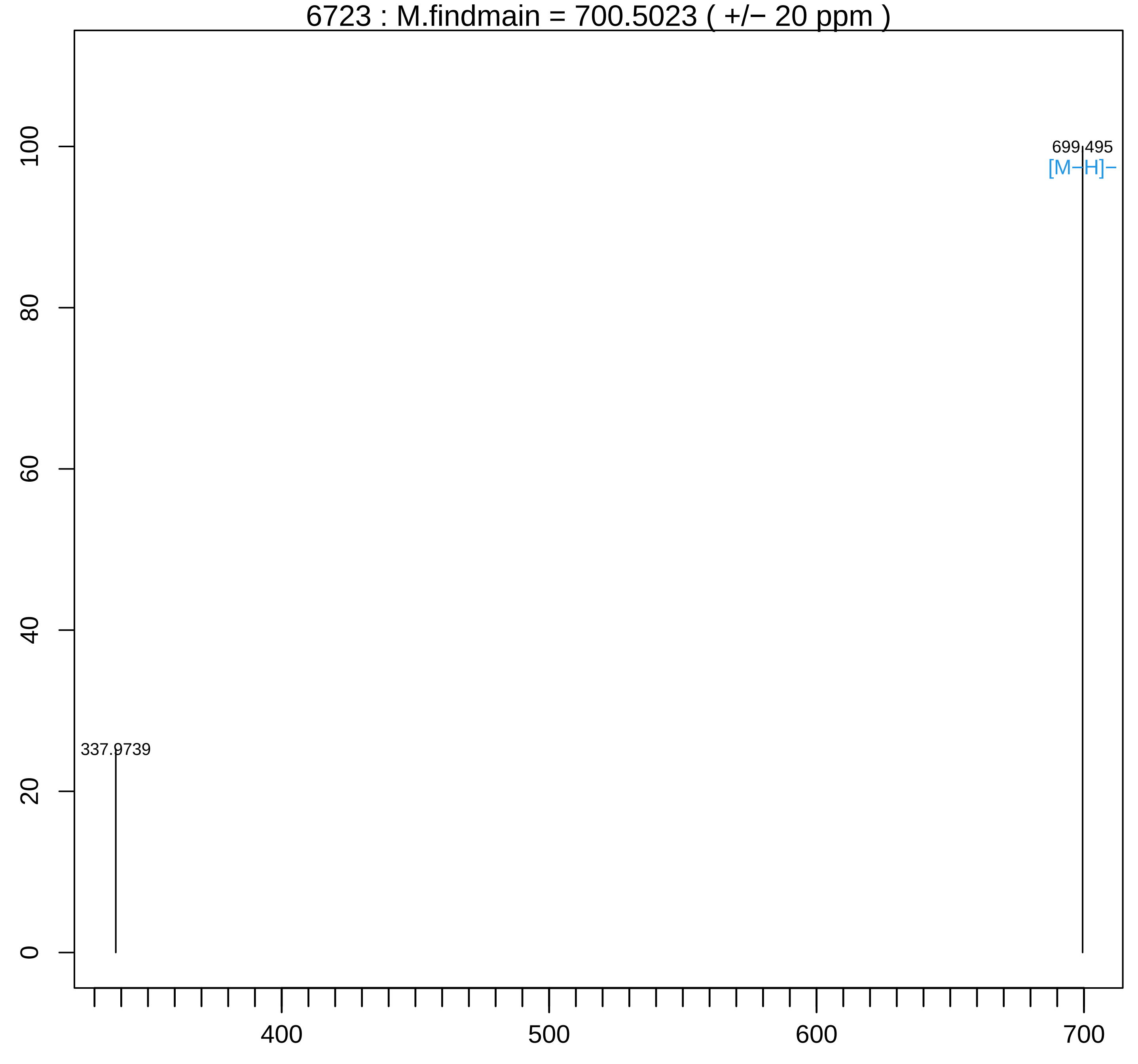
6723 : M.findmain = 700.5023 (+/− 20 ppm)
699.495
[M−H]−
337.9739
0
20
40
60
80
100
400
500
600
700

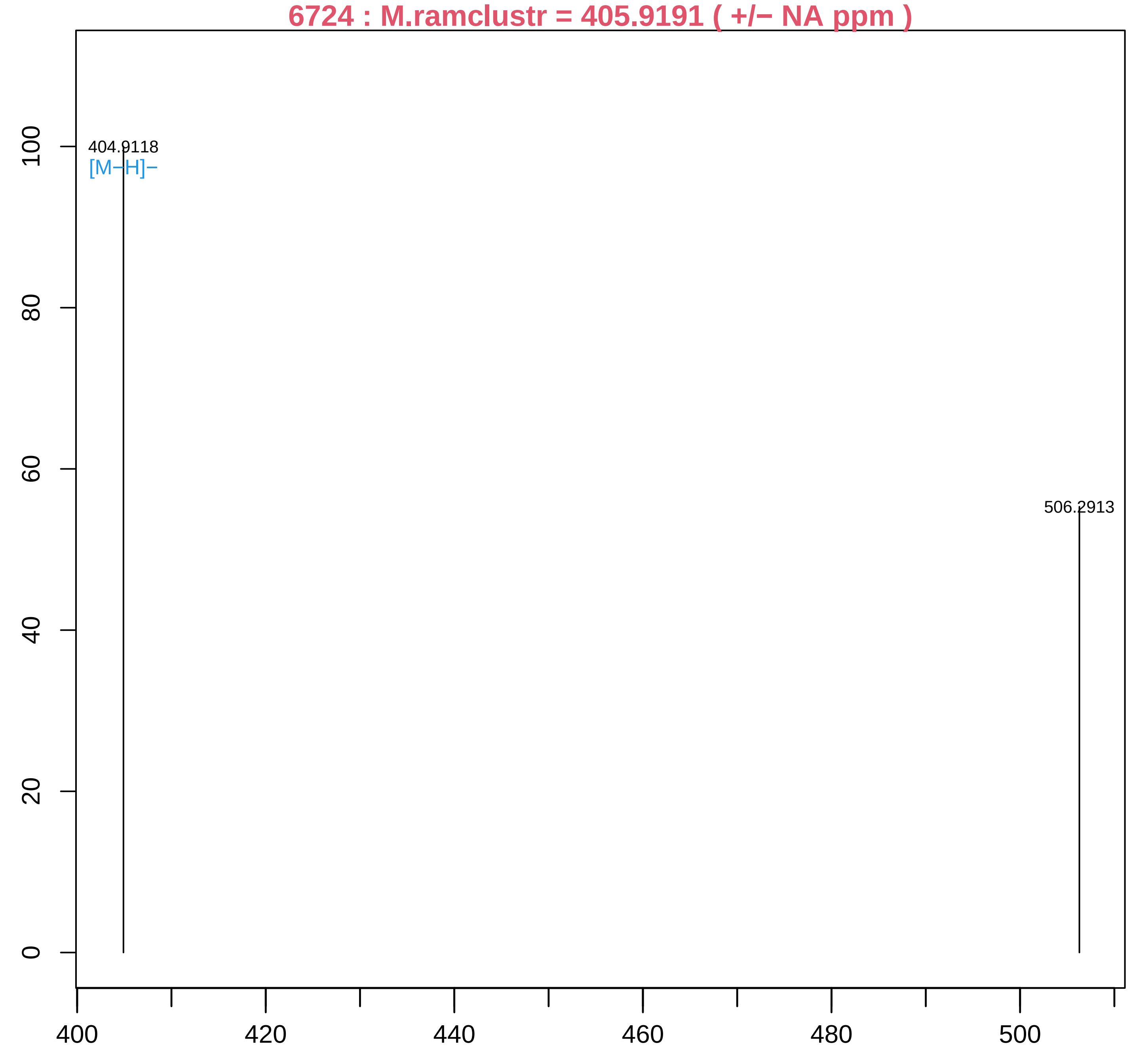
6724 : M.ramclustr = 405.9191 (+/− NA ppm)
404.9118
[M−H]−
506.2913
0
20
40
60
80
100
400
420
440
460
480
500

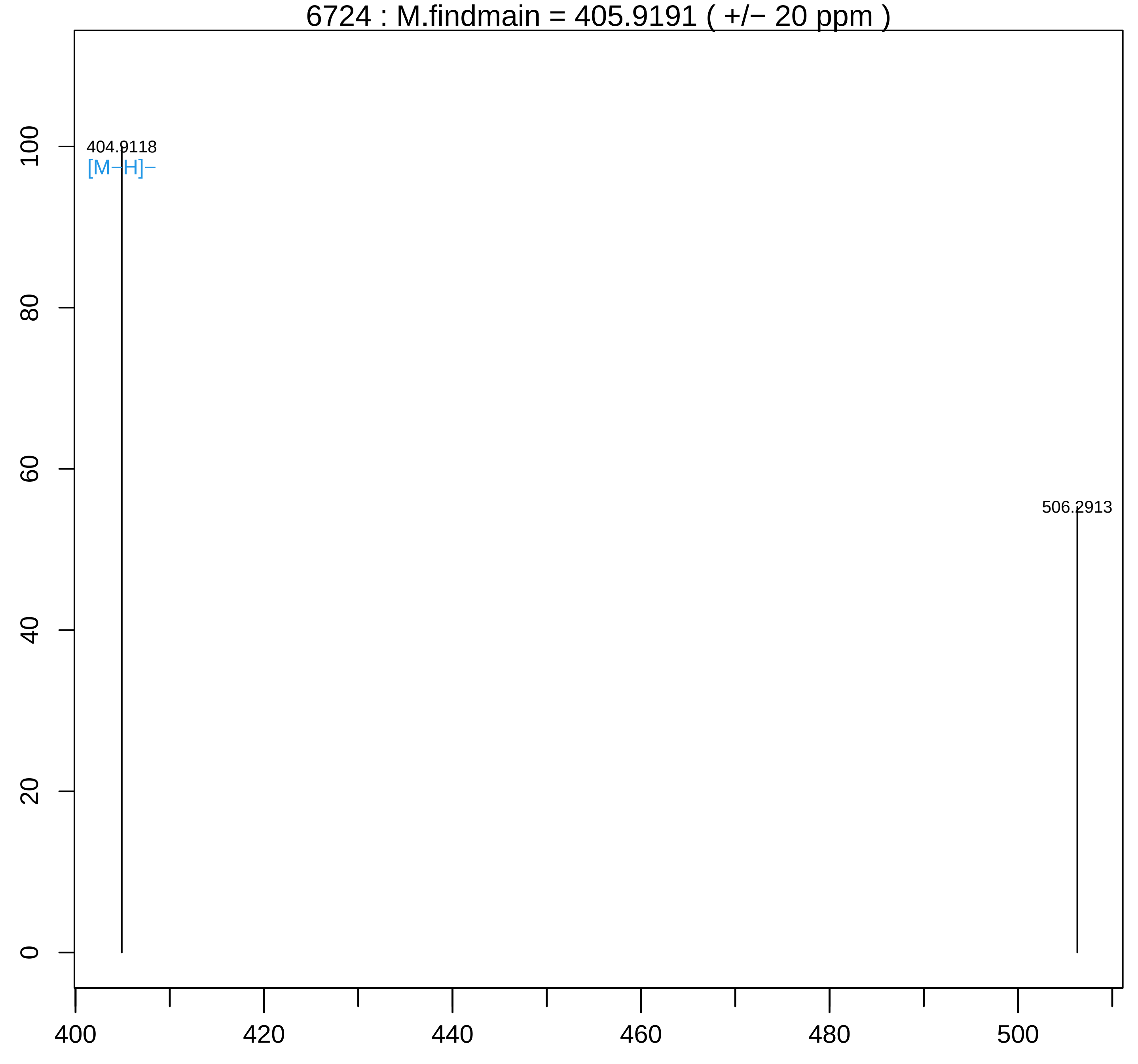
6724 : M.findmain = 405.9191 (+/− 20 ppm)
404.9118
[M−H]−
506.2913
0
20
40
60
80
100
400
420
440
460
480
500

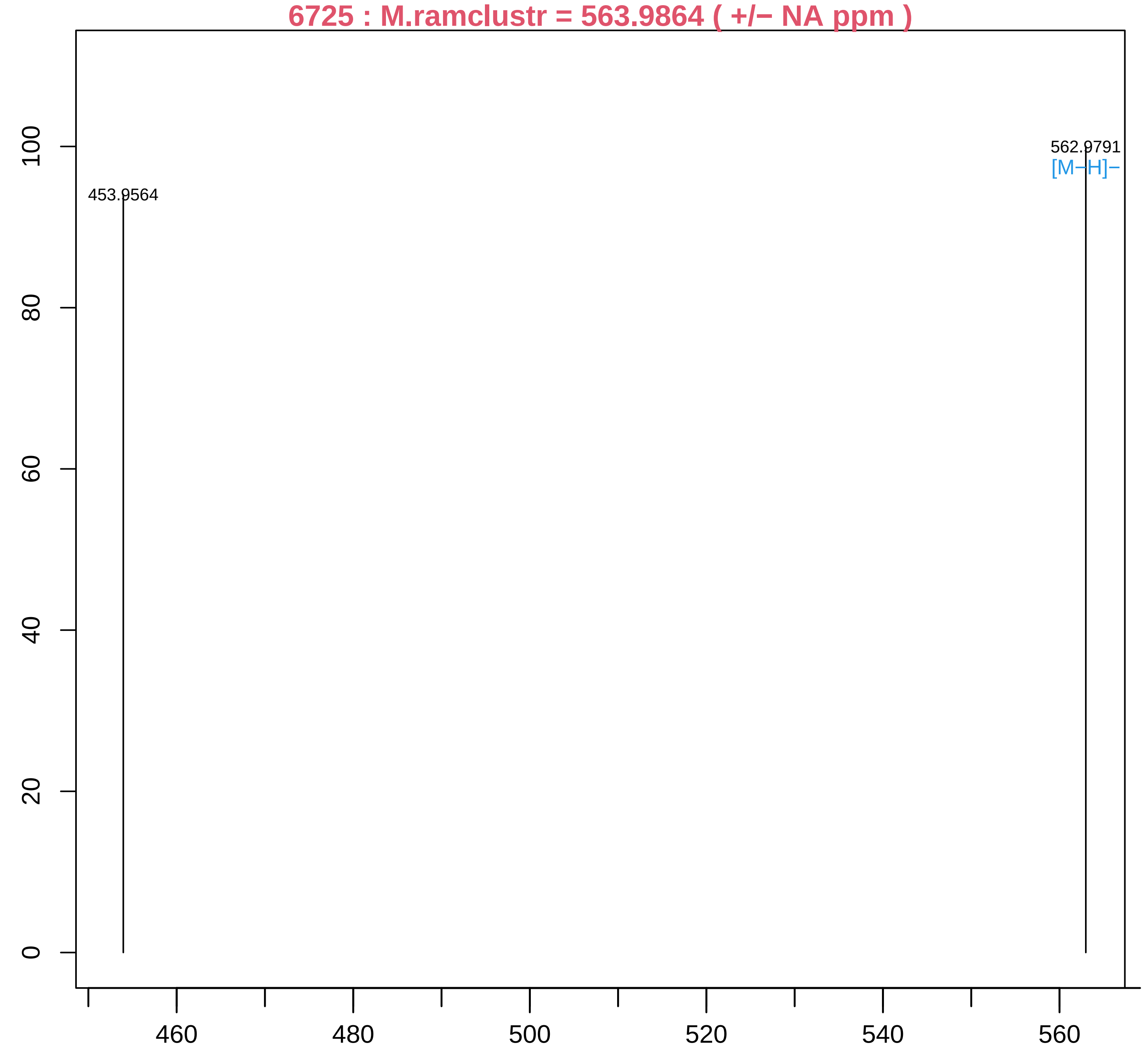

6725 : M.ramclustr = 563.9864 (+/− NA ppm)
453.9564
562.9791
[M−H]−
0
20
40
60
80
100
460
480
500
520
540
560

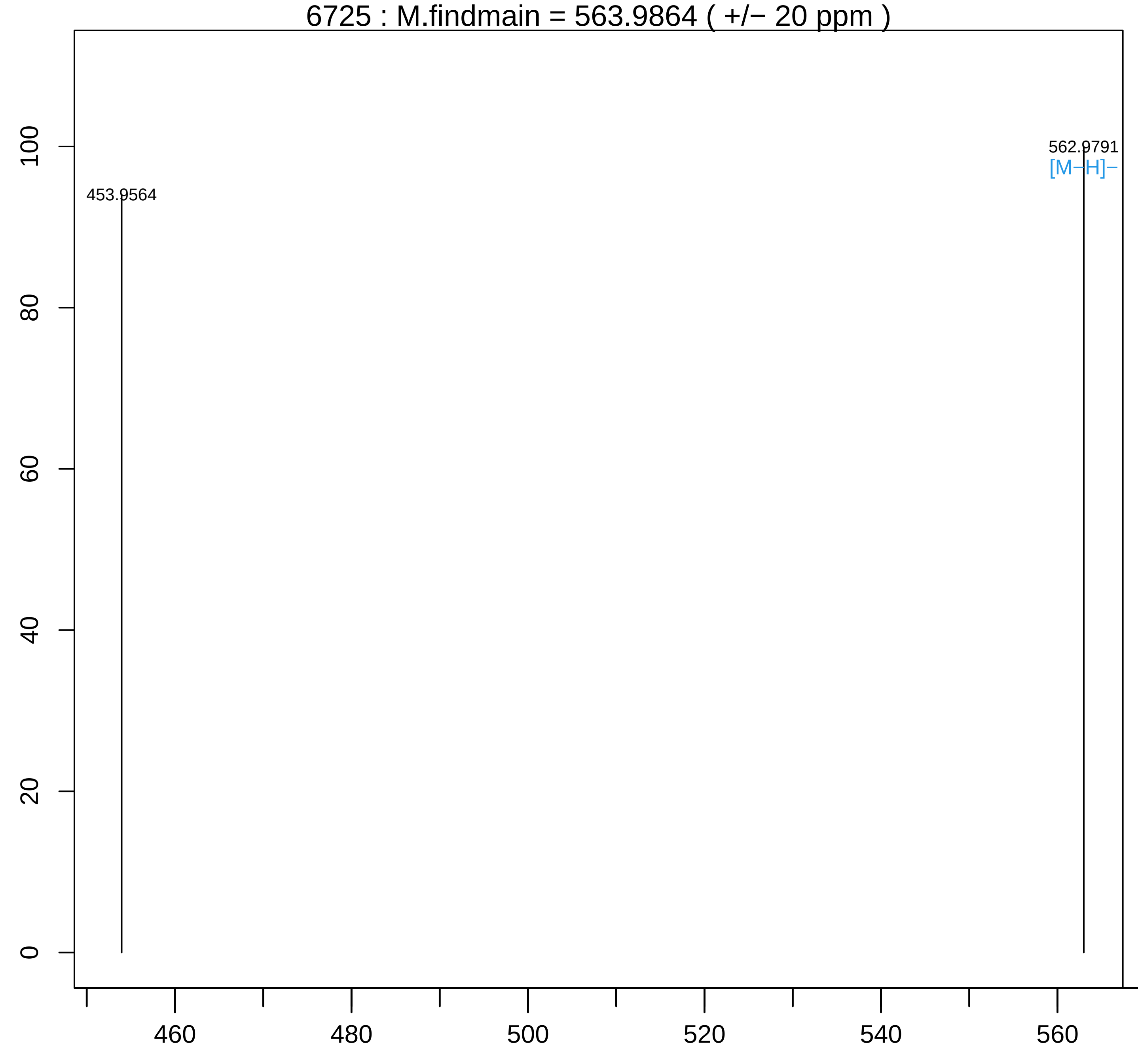

6725 : M.findmain = 563.9864 (+/− 20 ppm)
453.9564
562.9791
[M−H]−
0
20
40
60
80
100
460
480
500
520
540
560

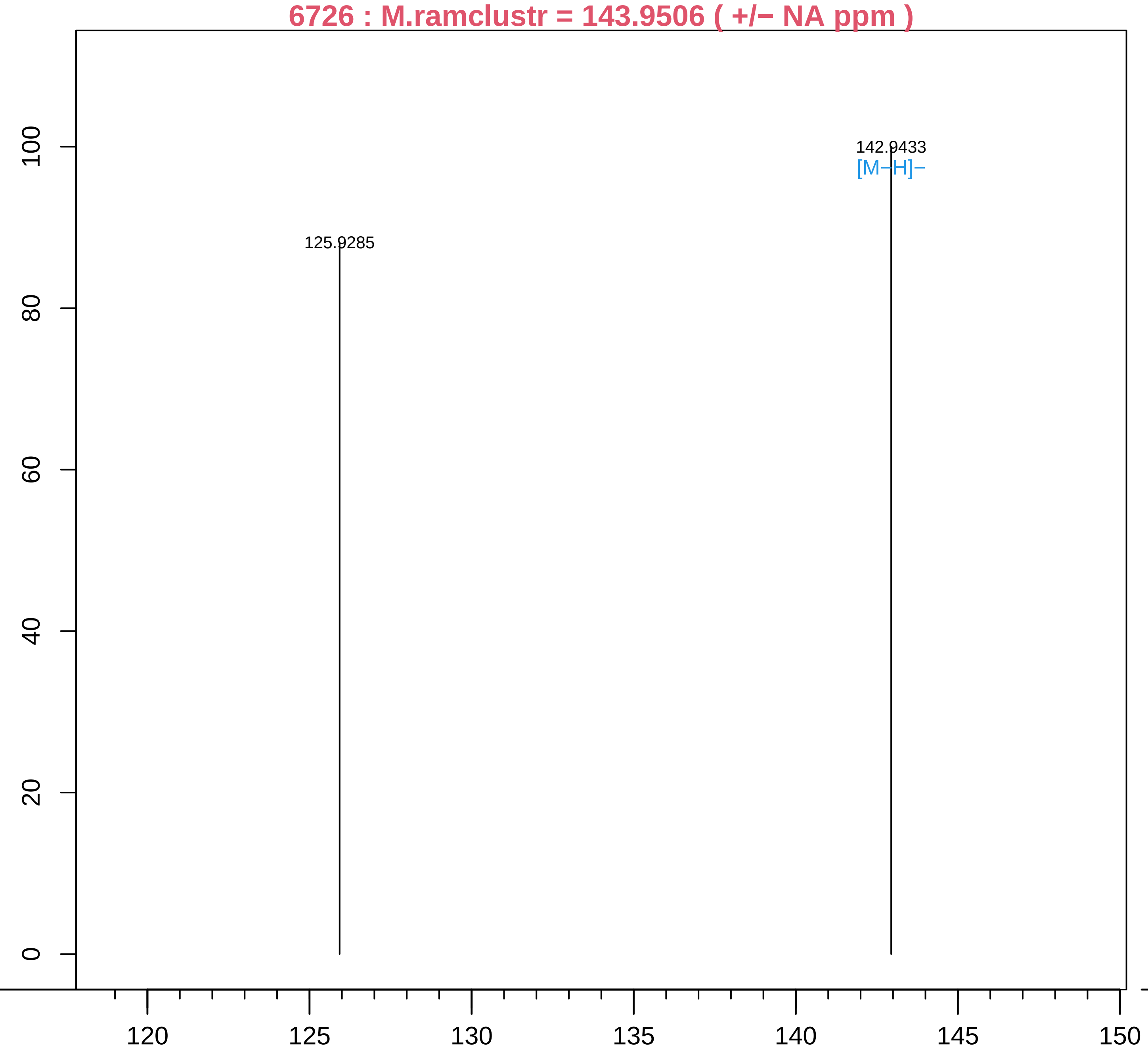
6726 : M.ramclustr = 143.9506 (+/− NA ppm)
142.9433
[M−H]−
125.9285
0
20
40
60
80
100
120
125
130
135
140
145
150

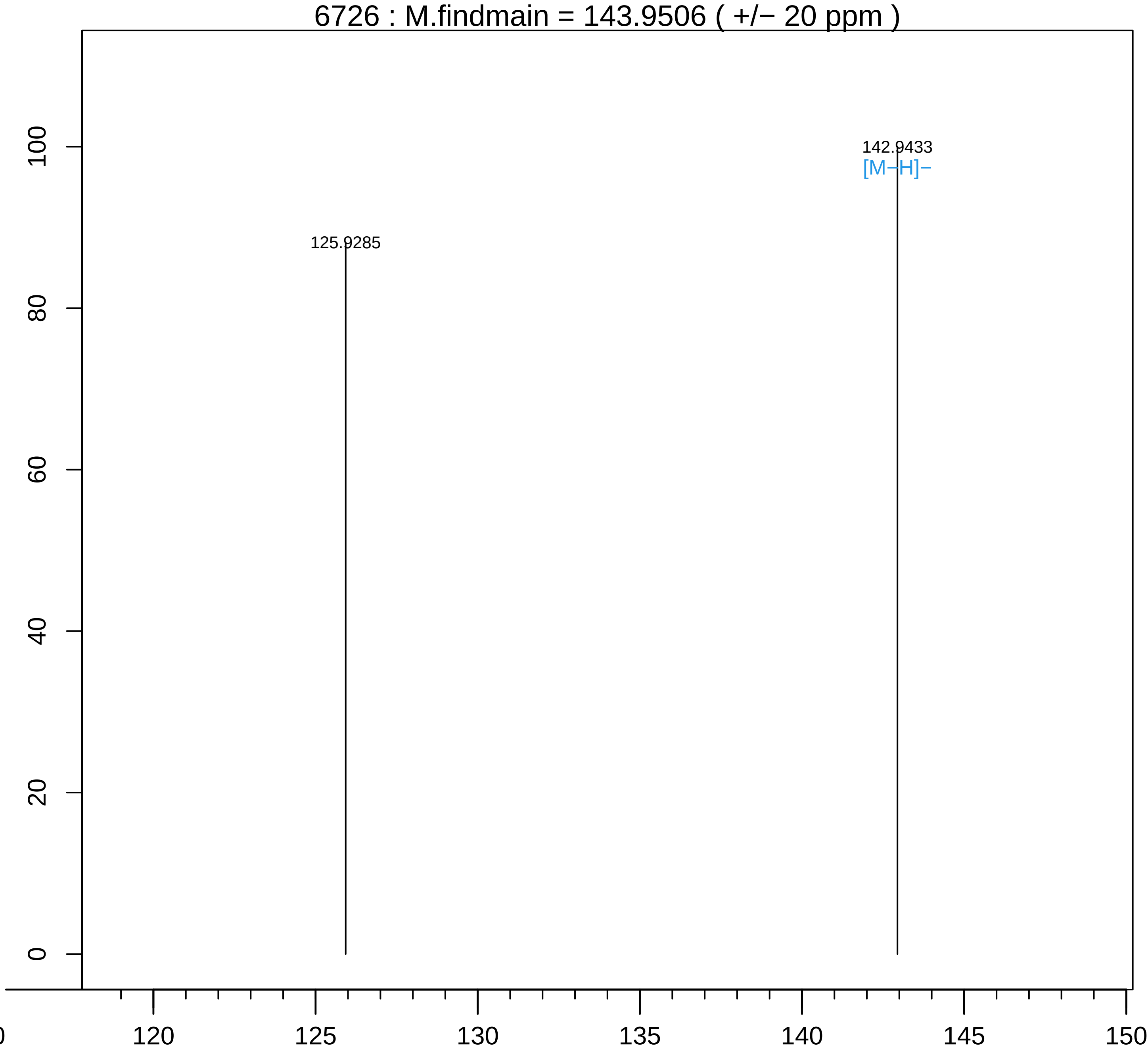
6726 : M.findmain = 143.9506 (+/− 20 ppm)
142.9433
[M−H]−
125.9285
0
20
40
60
80
100
120
125
130
135
140
145
150

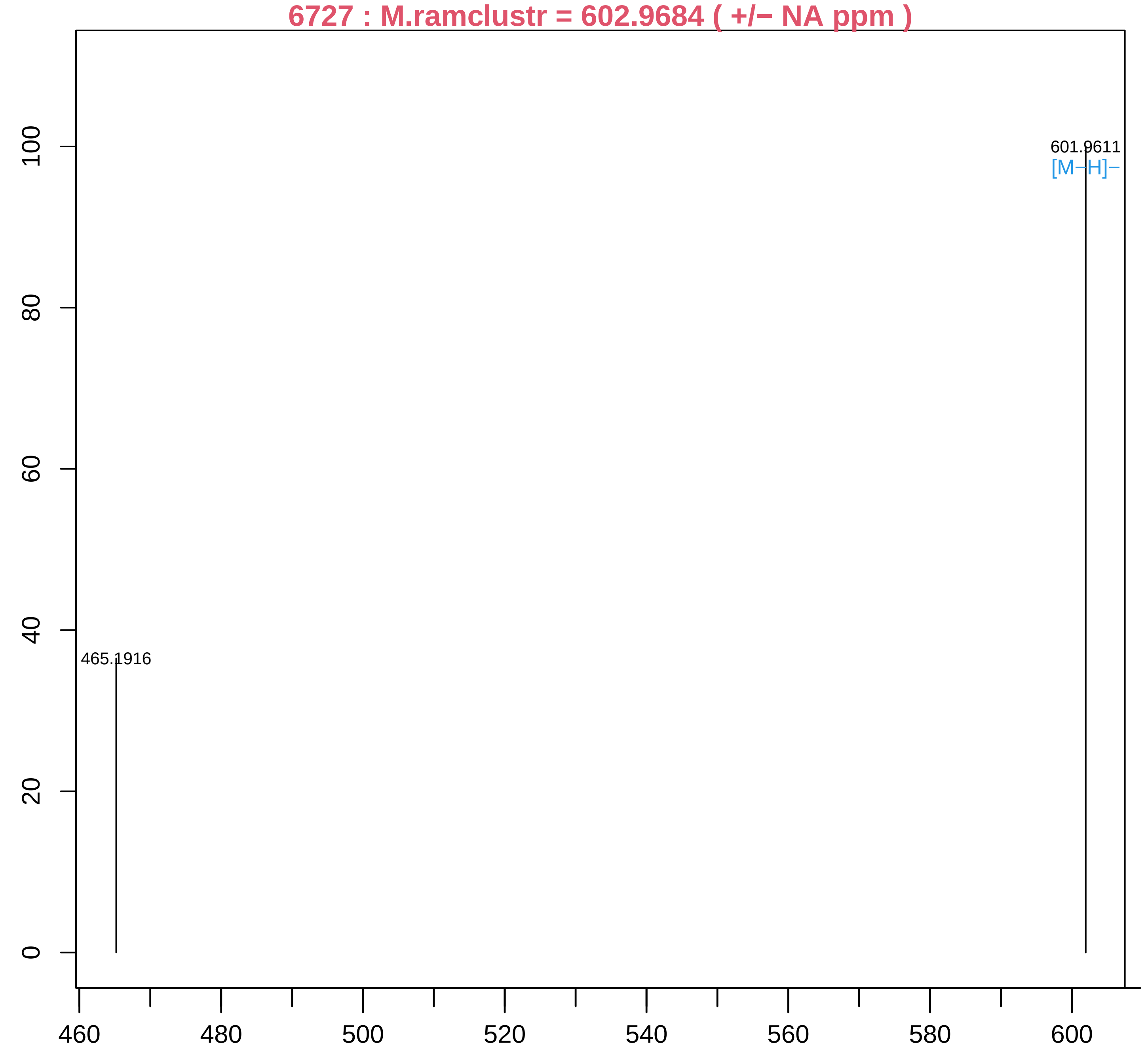
6727 : M.ramclustr = 602.9684 (+/− NA ppm)
601.9611
[M−H]−
465.1916
0
20
40
60
80
100
460
480
500
520
540
560
580
600
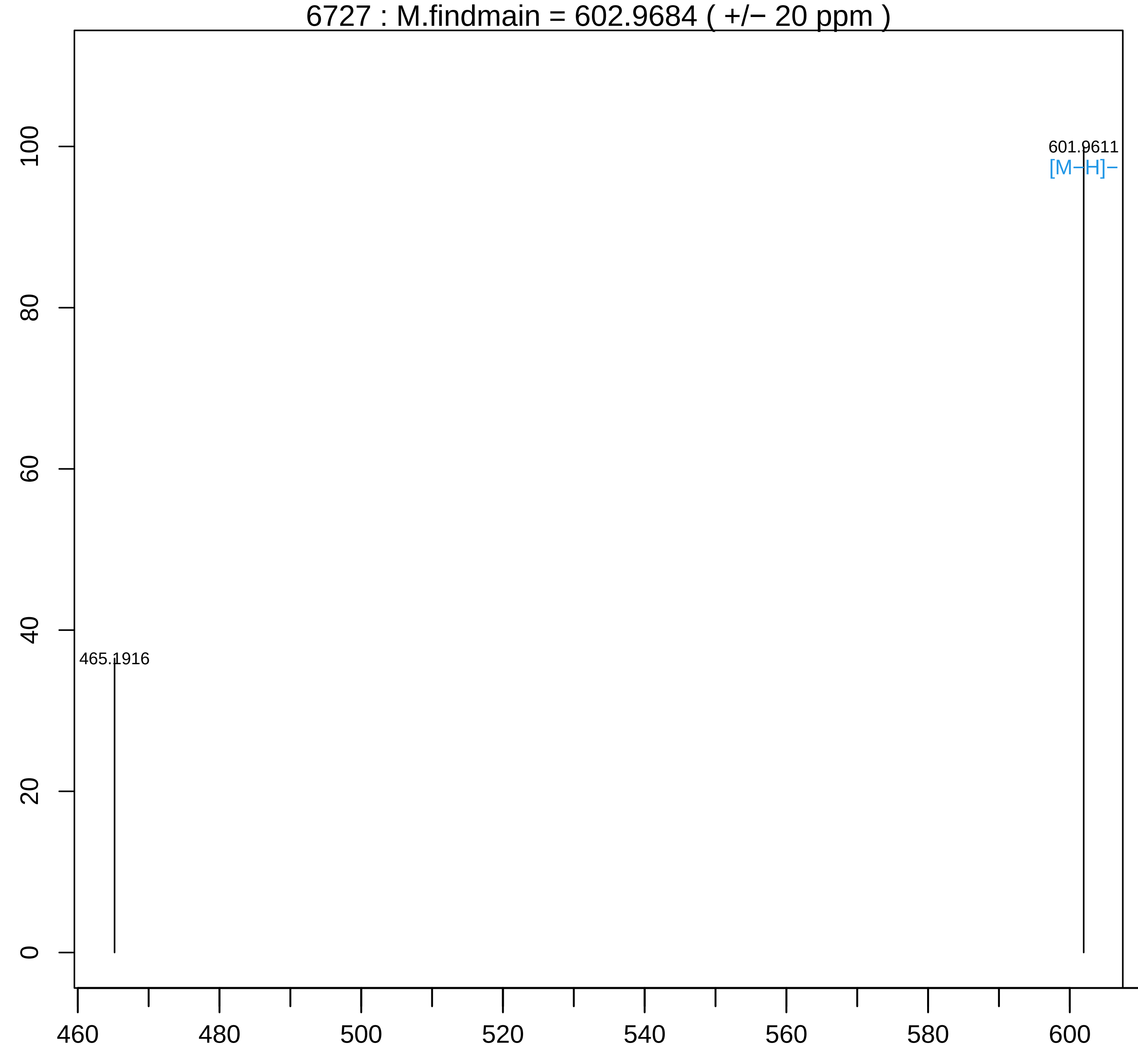
6727 : M.findmain = 602.9684 (+/− 20 ppm)
601.9611
[M−H]−
465.1916
0
20
40
60
80
100
460
480
500
520
540
560
580
600

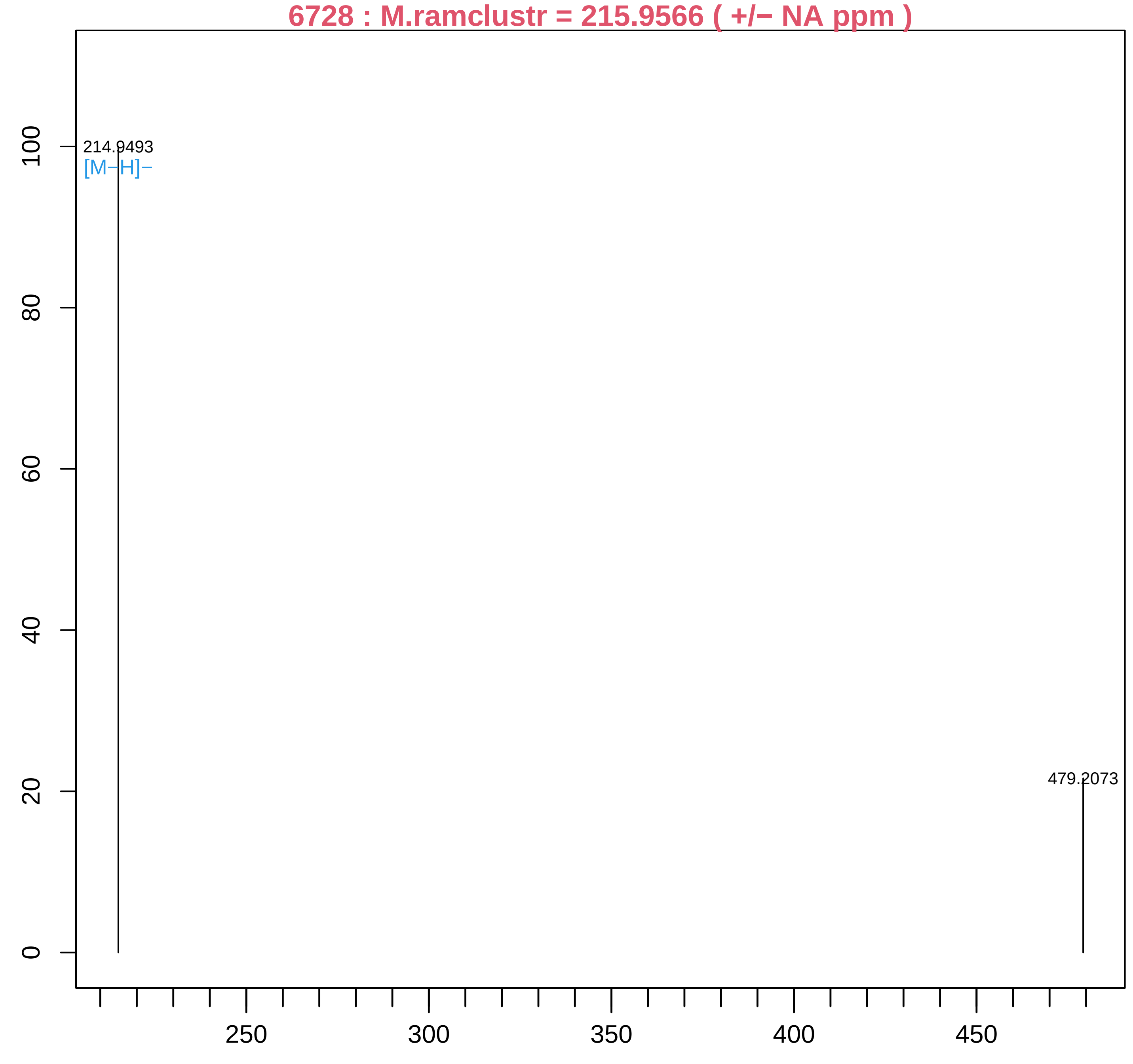
6728 : M.ramclustr = 215.9566 (+/− NA ppm)
214.9493
[M−H]−
479.2073
0
20
40
60
80
100
250
300
350
400
450

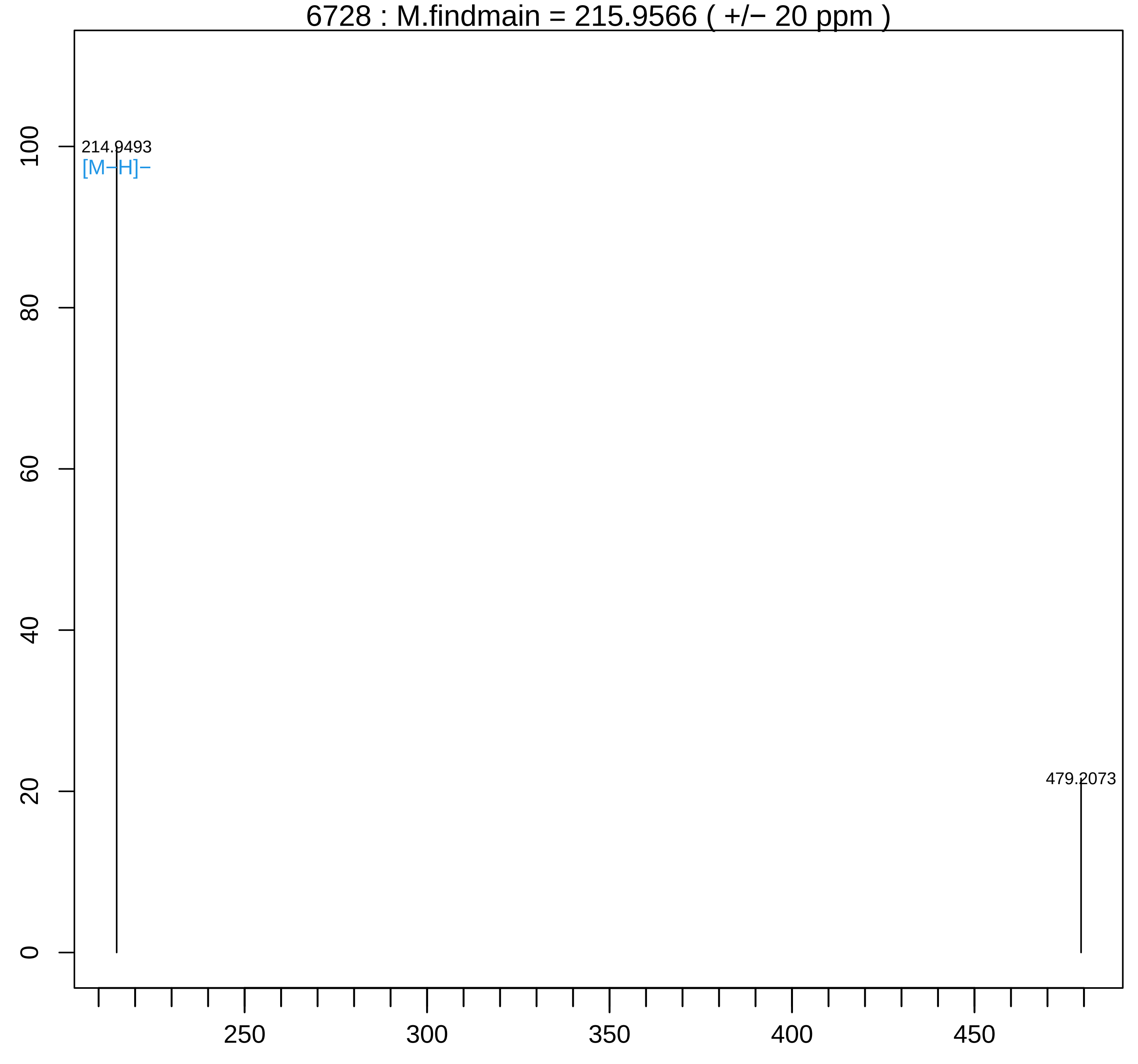
6728 : M.findmain = 215.9566 (+/− 20 ppm)
214.9493
[M−H]−
479.2073
0
20
40
60
80
100
250
300
350
400
450

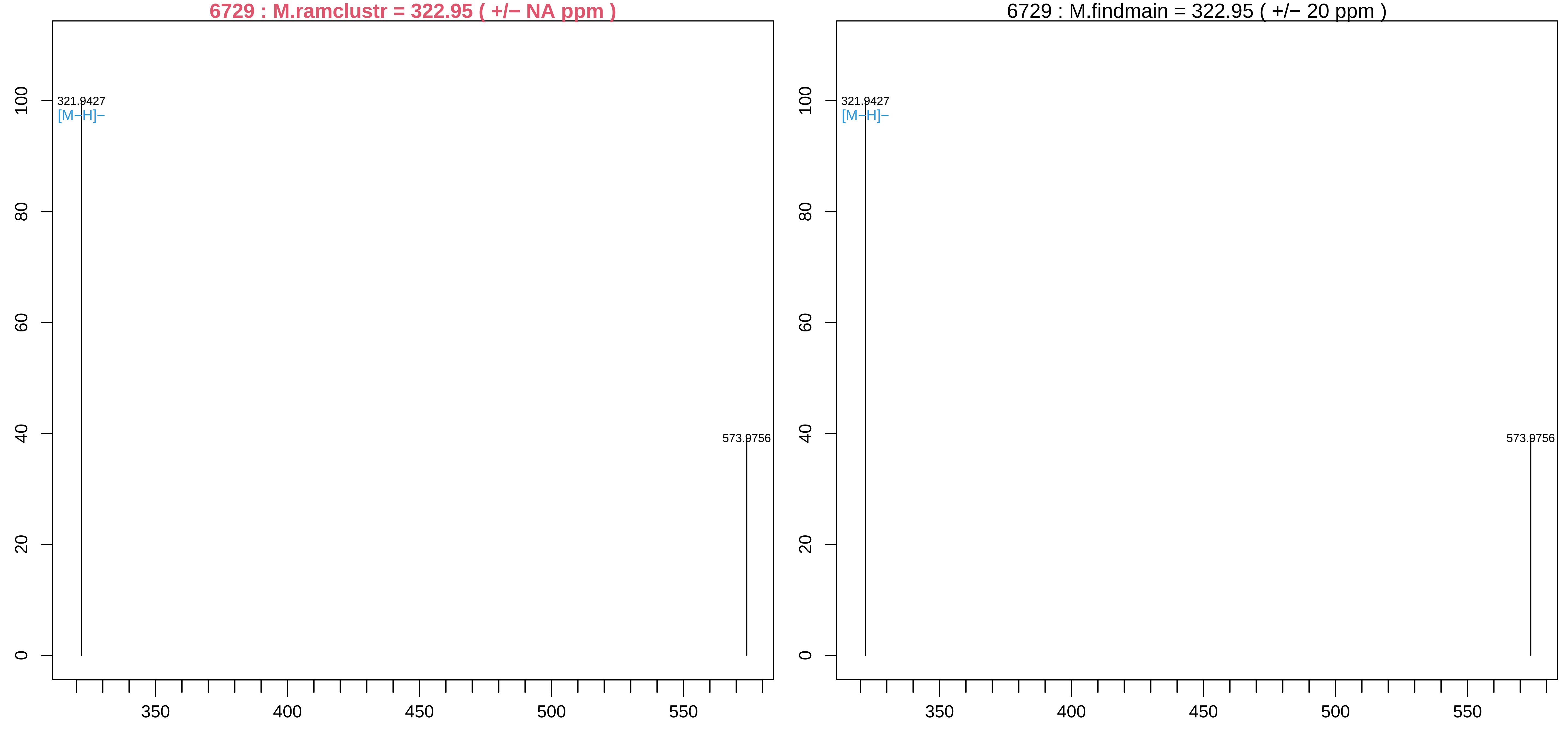
6729 : M.ramclustr = 322.95 (+/− NA ppm)
321.9427
[M−H]−
573.9756
6729 : M.findmain = 322.95 (+/− 20 ppm)
321.9427
[M−H]−
573.9756
0
20
40
60
80
100
350
400
450
500
550

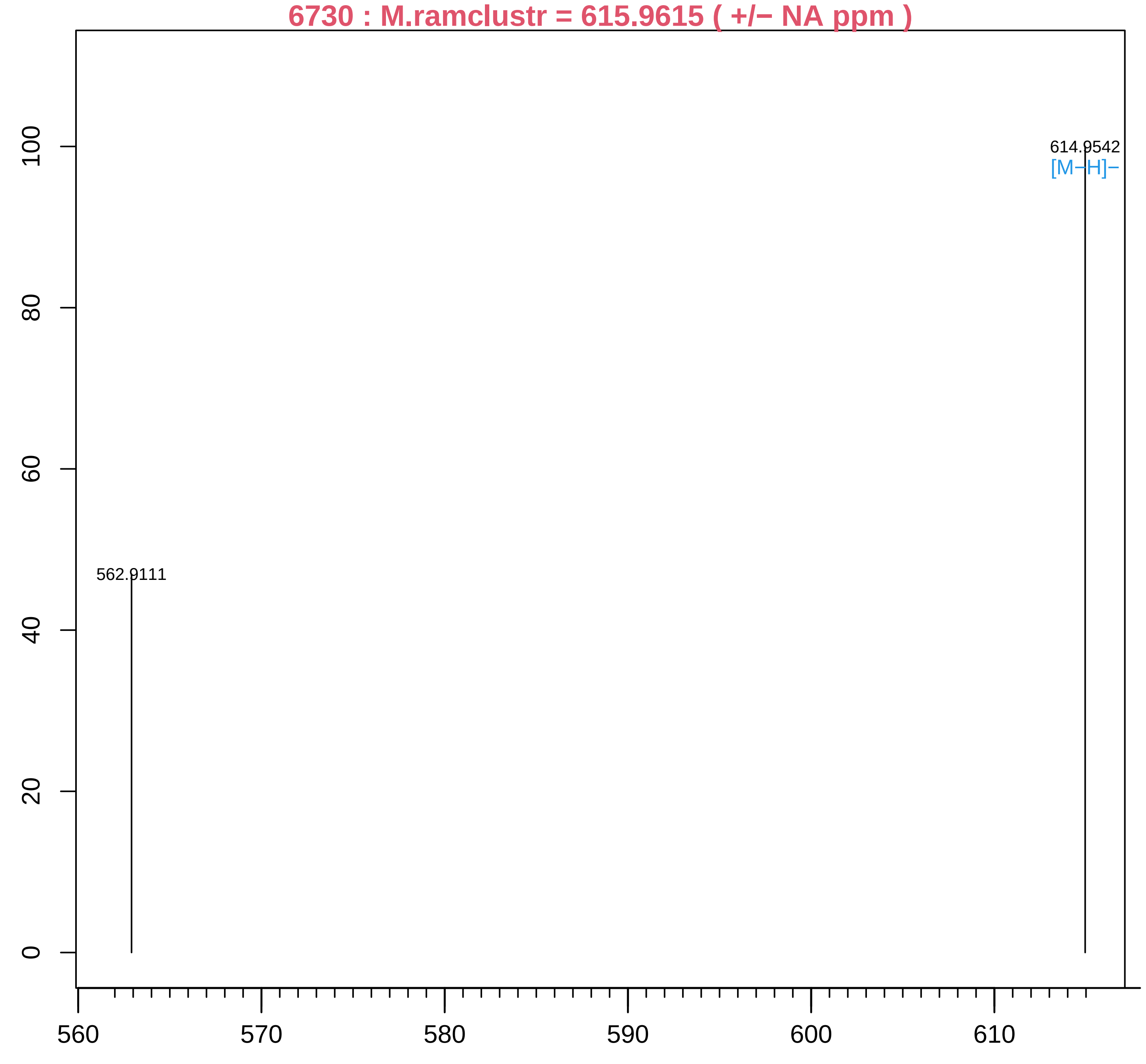
6730 : M.ramclustr = 615.9615 (+/− NA ppm)
614.9542
[M−H]−
562.9111
0
20
40
60
80
100
560
570
580
590
600
610

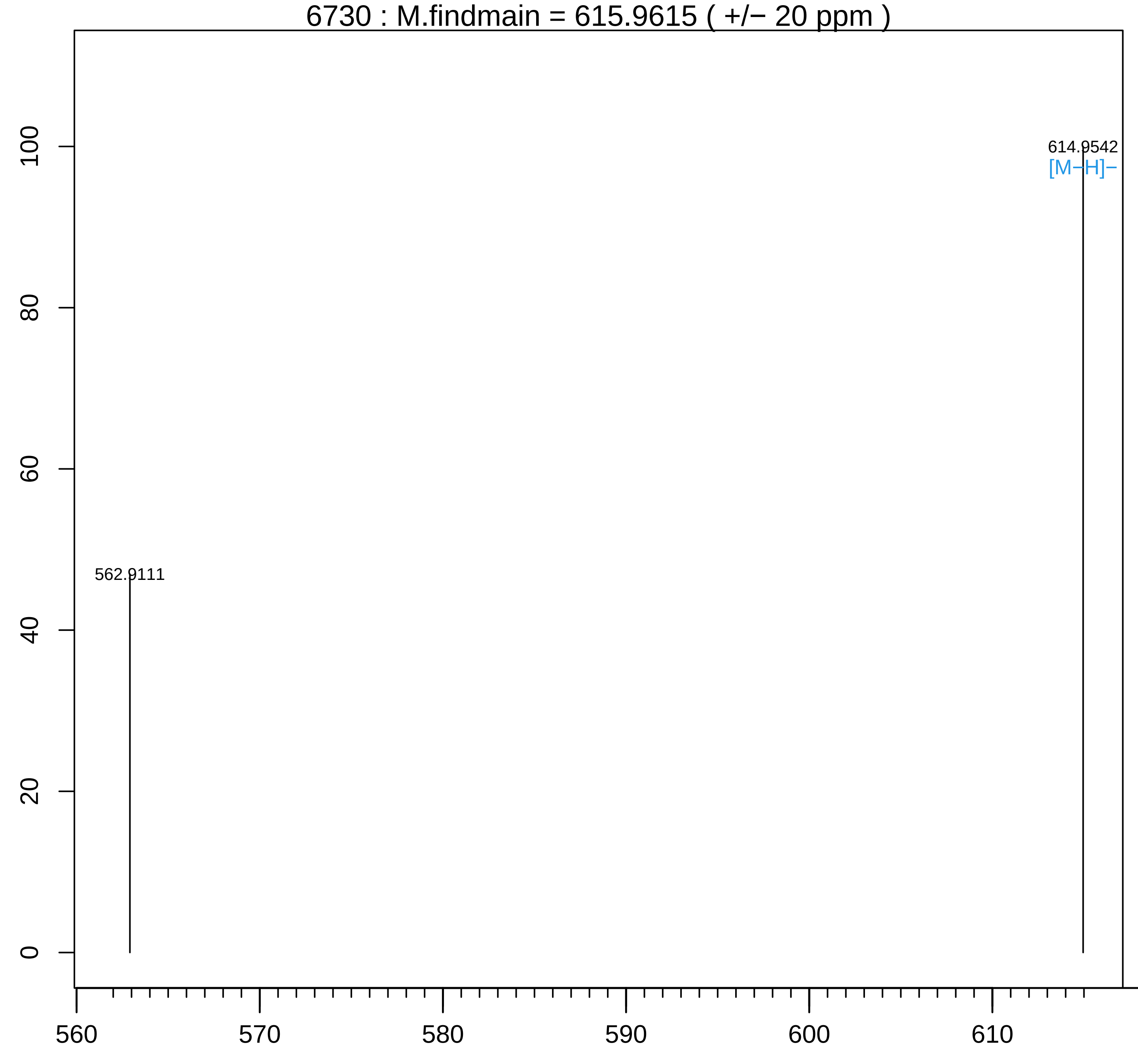
6730 : M.findmain = 615.9615 (+/− 20 ppm)
614.9542
[M−H]−
562.9111
0
20
40
60
80
100
560
570
580
590
600
610

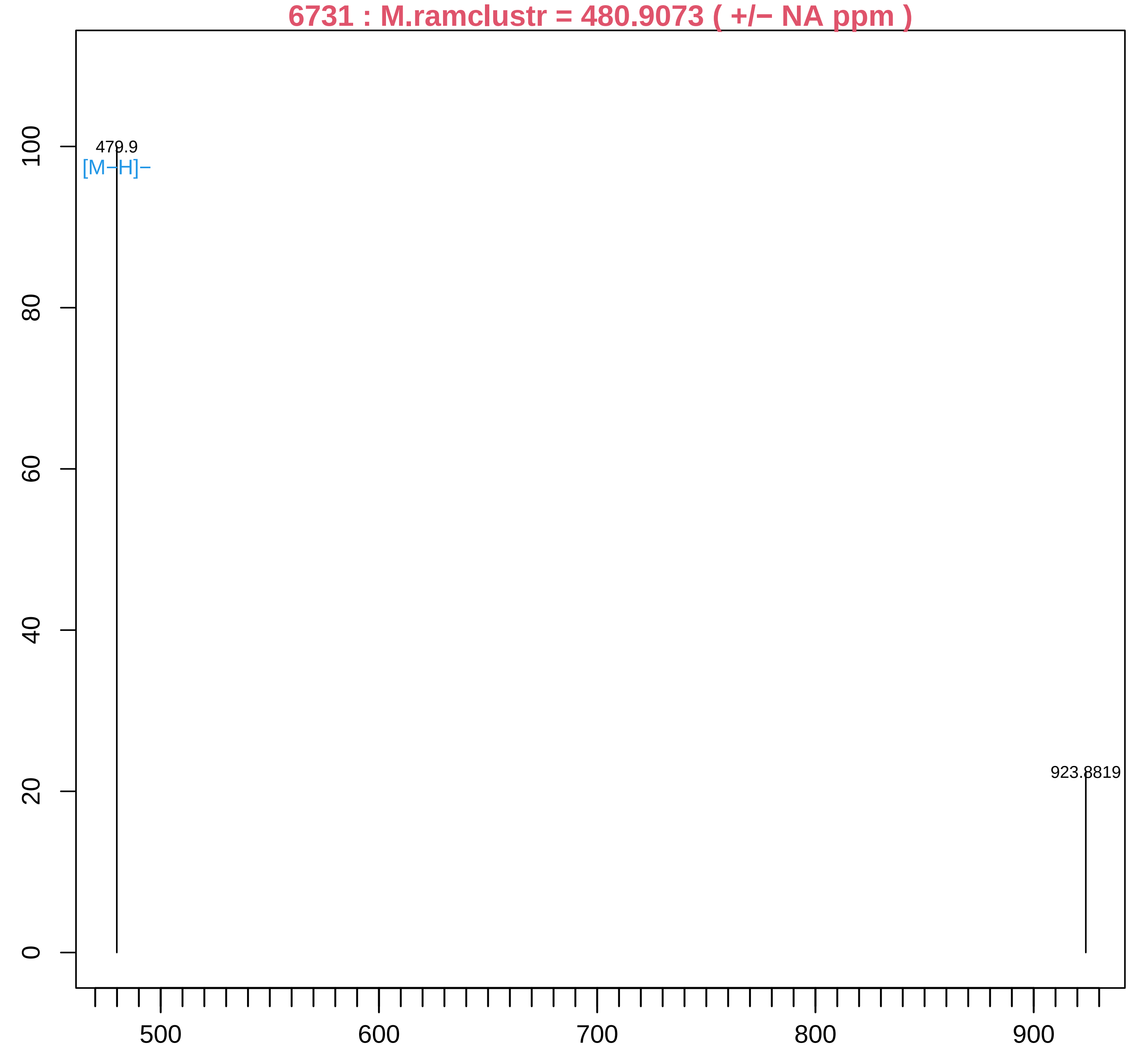

6731 : M.ramclustr = 480.9073 (+/− NA ppm)
479.9
[M−H]−
923.8819
0
20
40
60
80
100
500
600
700
800
900

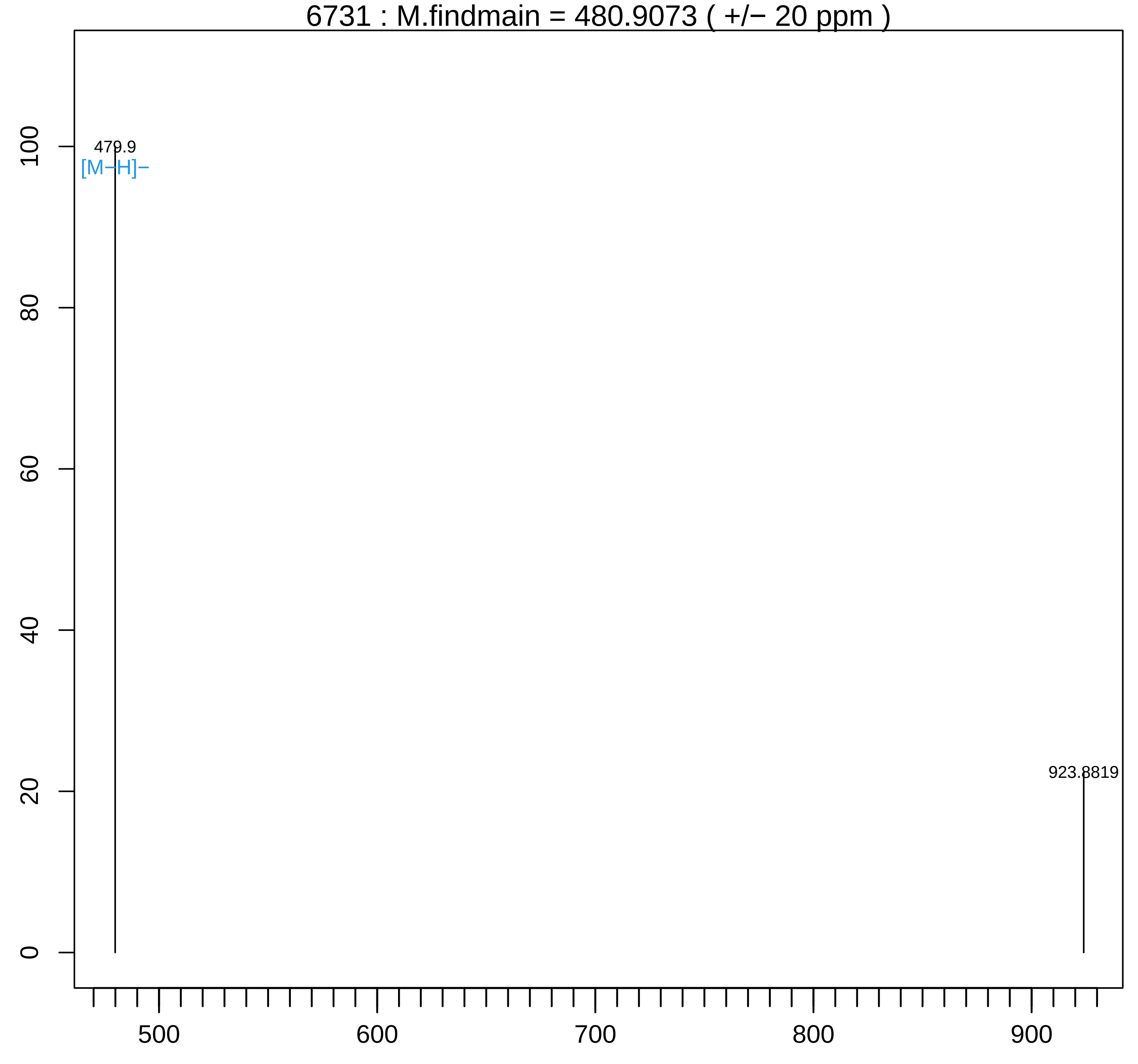

6731 : M.findmain = 480.9073 (+/− 20 ppm)
479.9
[M−H]−
923.8819
0
20
40
60
80
100
500
600
700
800
900

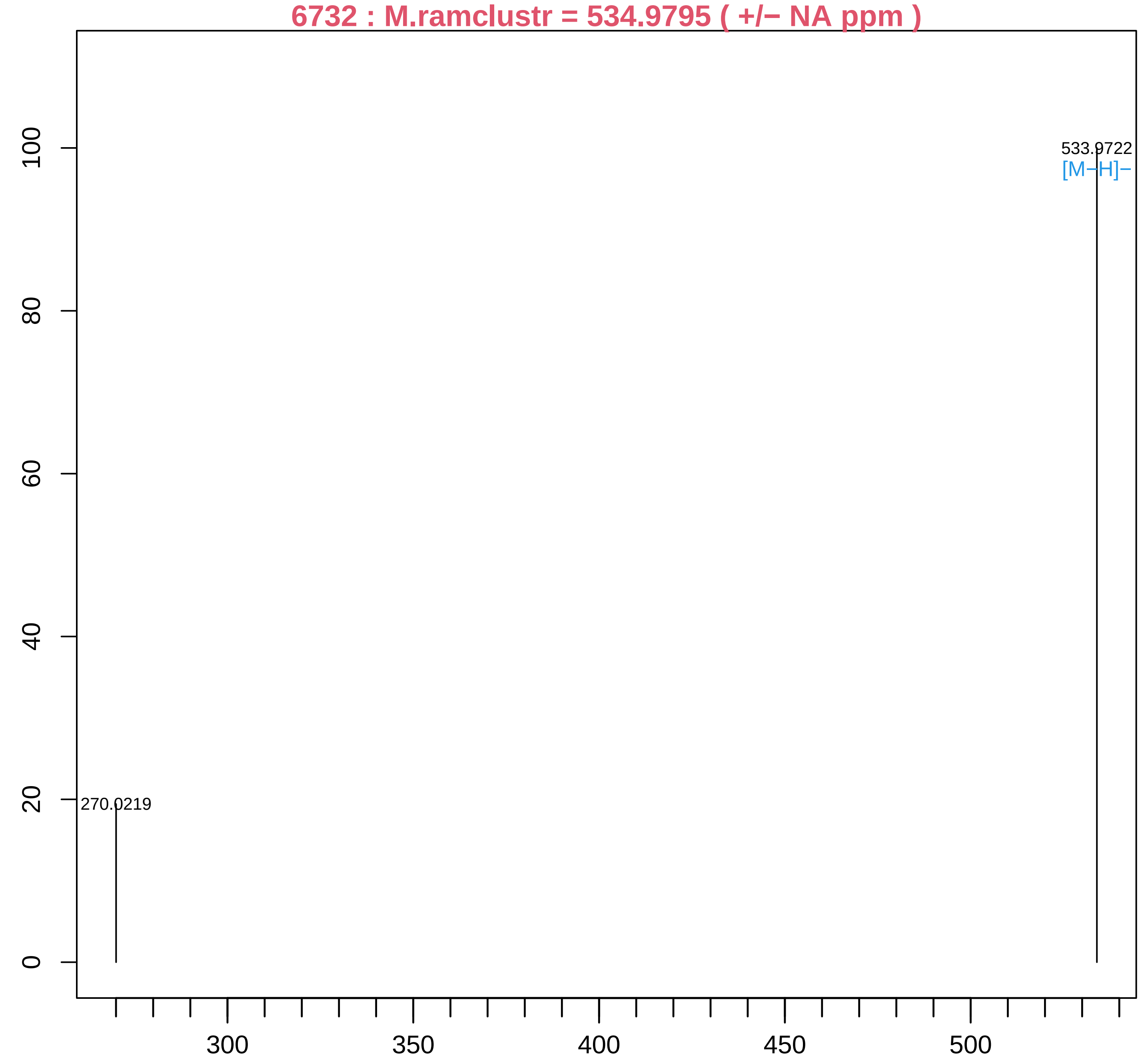
6732 : M.ramclustr = 534.9795 (+/− NA ppm)
533.9722
[M−H]−
270.0219
0
20
40
60
80
100
300
350
400
450
500

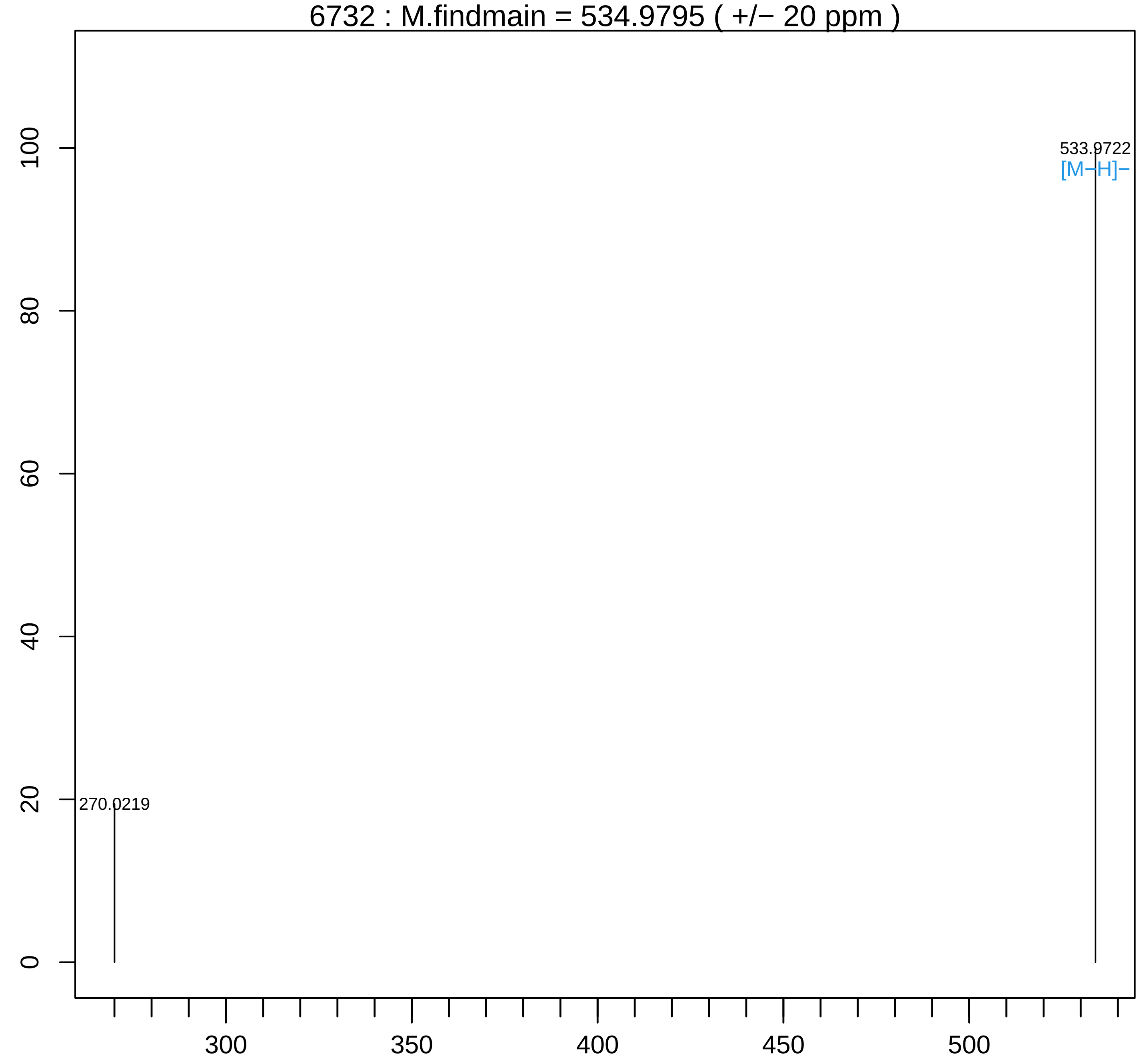
6732 : M.findmain = 534.9795 (+/− 20 ppm)
533.9722
[M−H]−
270.0219
0
20
40
60
80
100
300
350
400
450
500

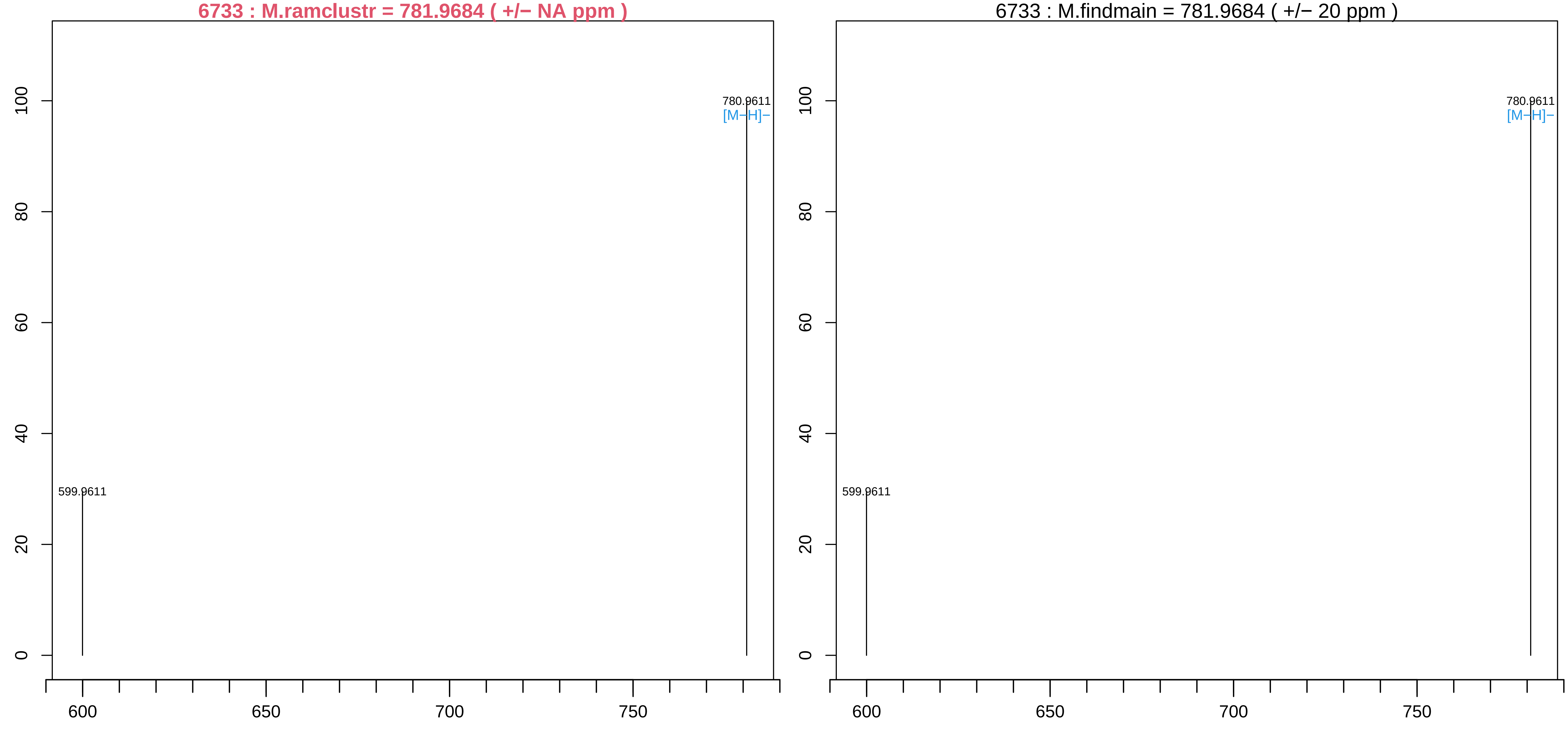
6733 : M.ramclustr = 781.9684 (+/− NA ppm)
6733 : M.findmain = 781.9684 (+/− 20 ppm)
780.9611
[M−H]−
599.9611
780.9611
[M−H]−
599.9611
0
20
40
60
80
100
600
650
700
750

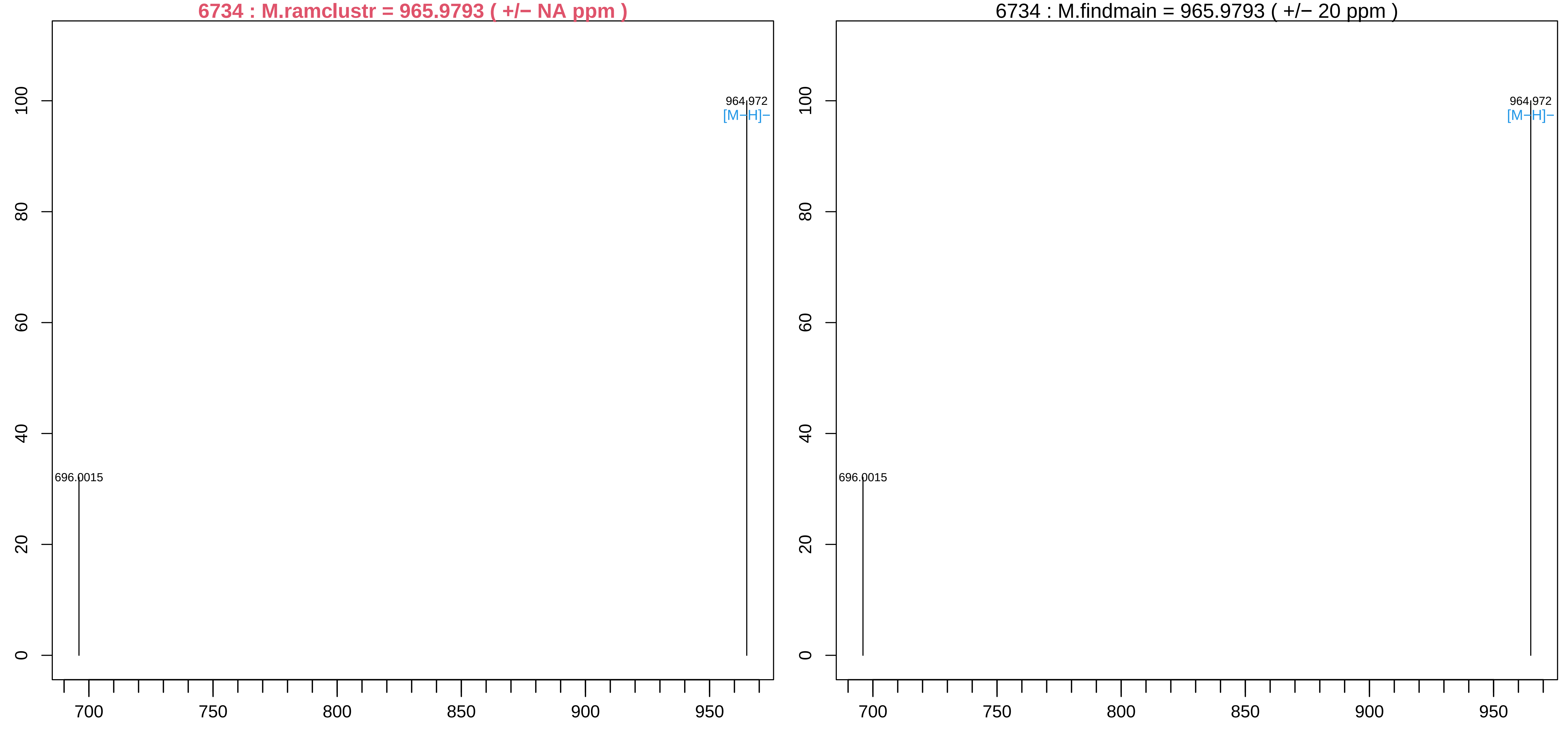
6734 : M.ramclustr = 965.9793 (+/− NA ppm)
6734 : M.findmain = 965.9793 (+/− 20 ppm)
964.972
[M−H]−
696.0015
964.972
[M−H]−
696.0015
0
20
40
60
80
100
700
750
800
850
900
950

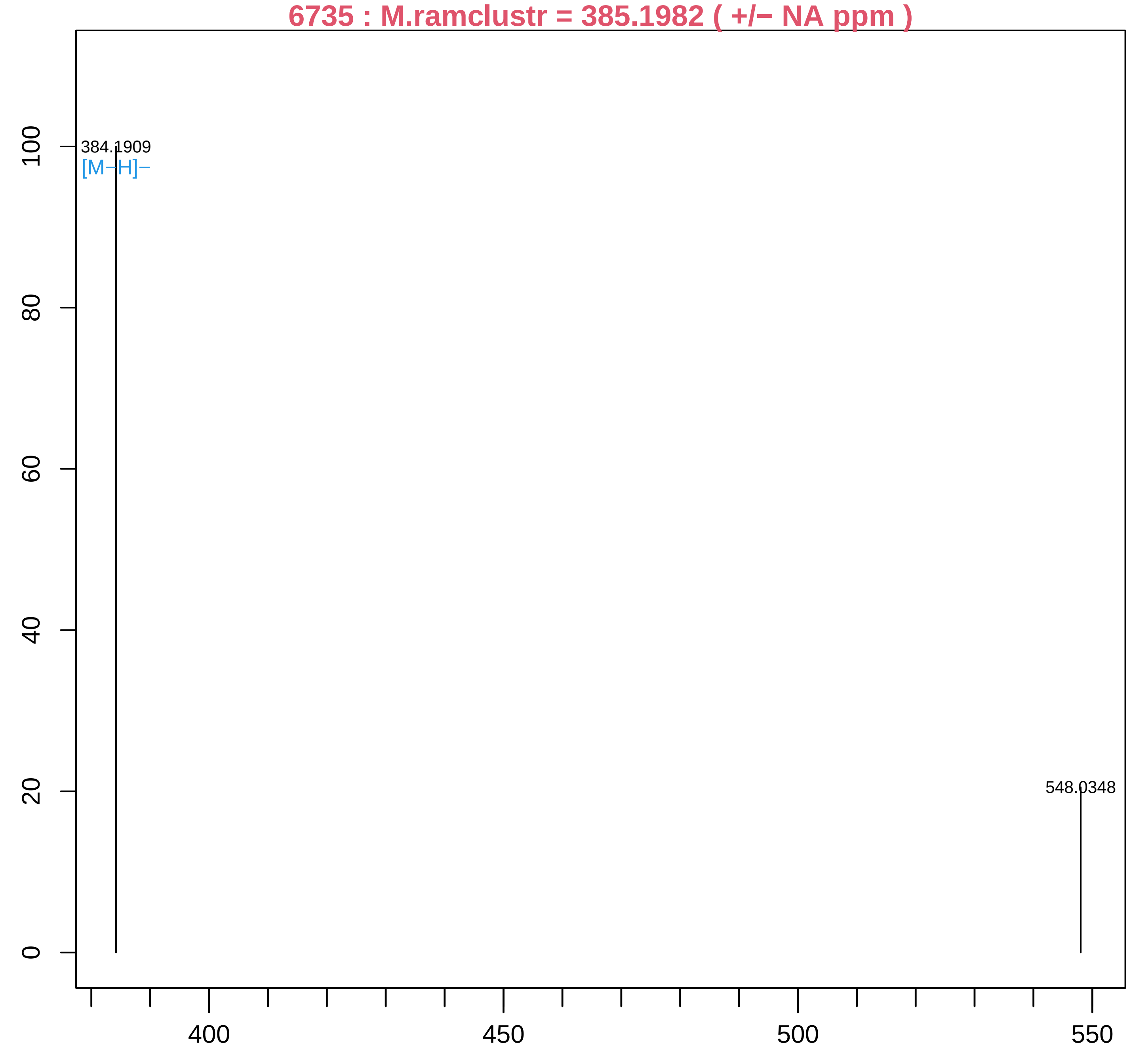
6735 : M.ramclustr = 385.1982 (+/− NA ppm)
384.1909
[M−H]−
548.0348
0
20
40
60
80
100
400
450
500
550
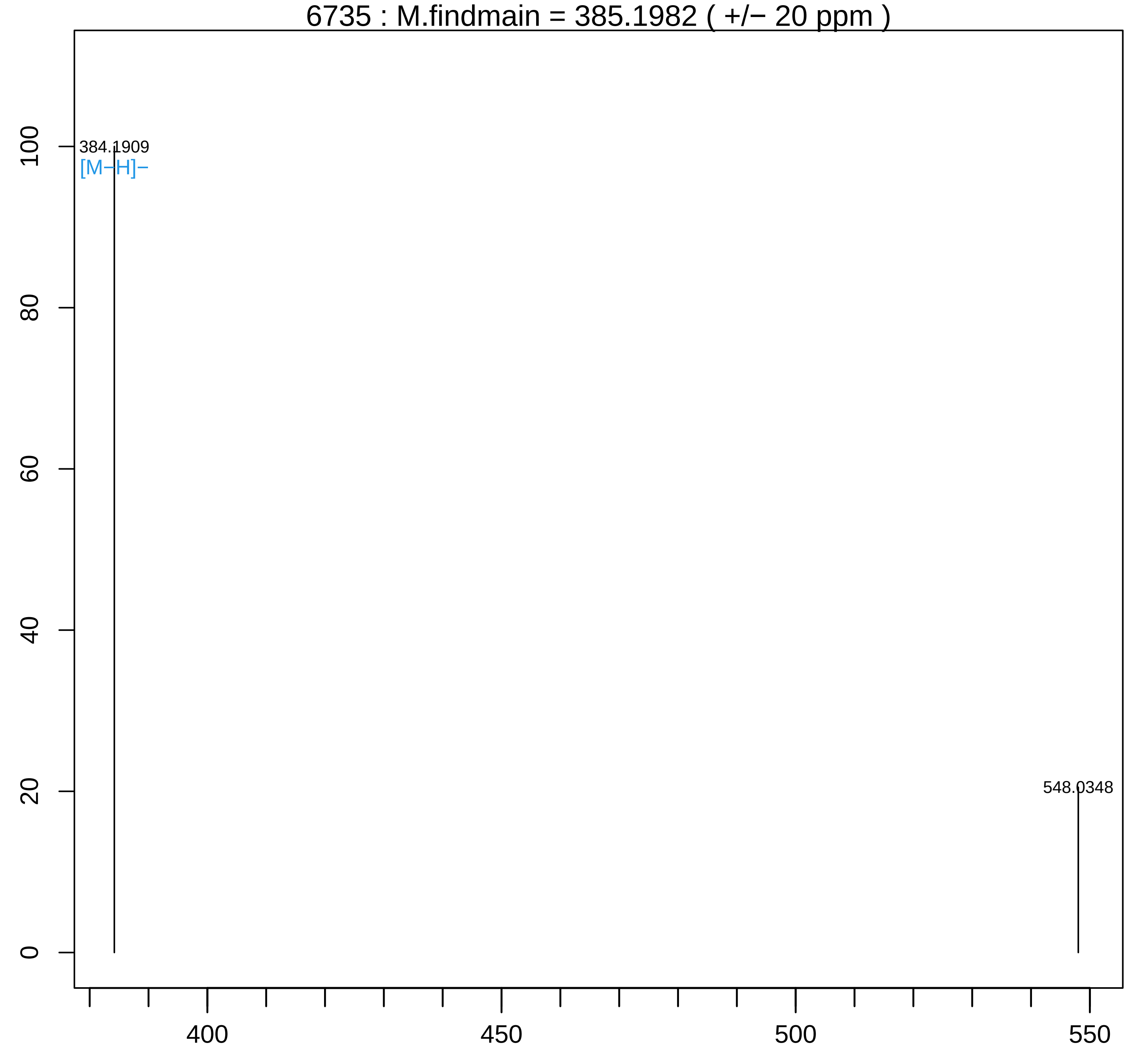
6735 : M.findmain = 385.1982 (+/− 20 ppm)
384.1909
[M−H]−
548.0348
0
20
40
60
80
100
400
450
500
550

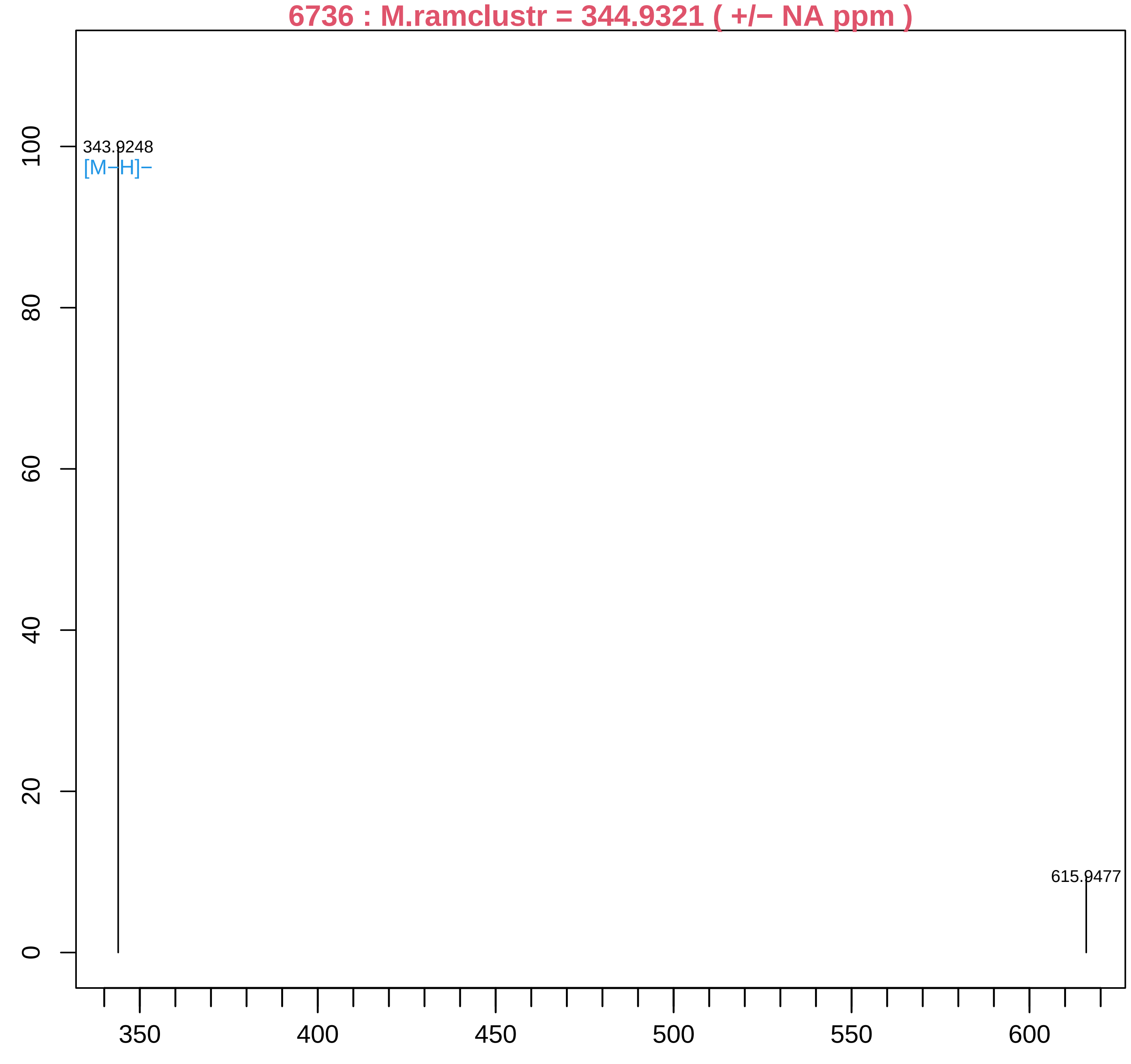
6736 : M.ramclustr = 344.9321 (+/− NA ppm)
343.9248
[M−H]−
615.9477
0
20
40
60
80
100
350
400
450
500
550
600

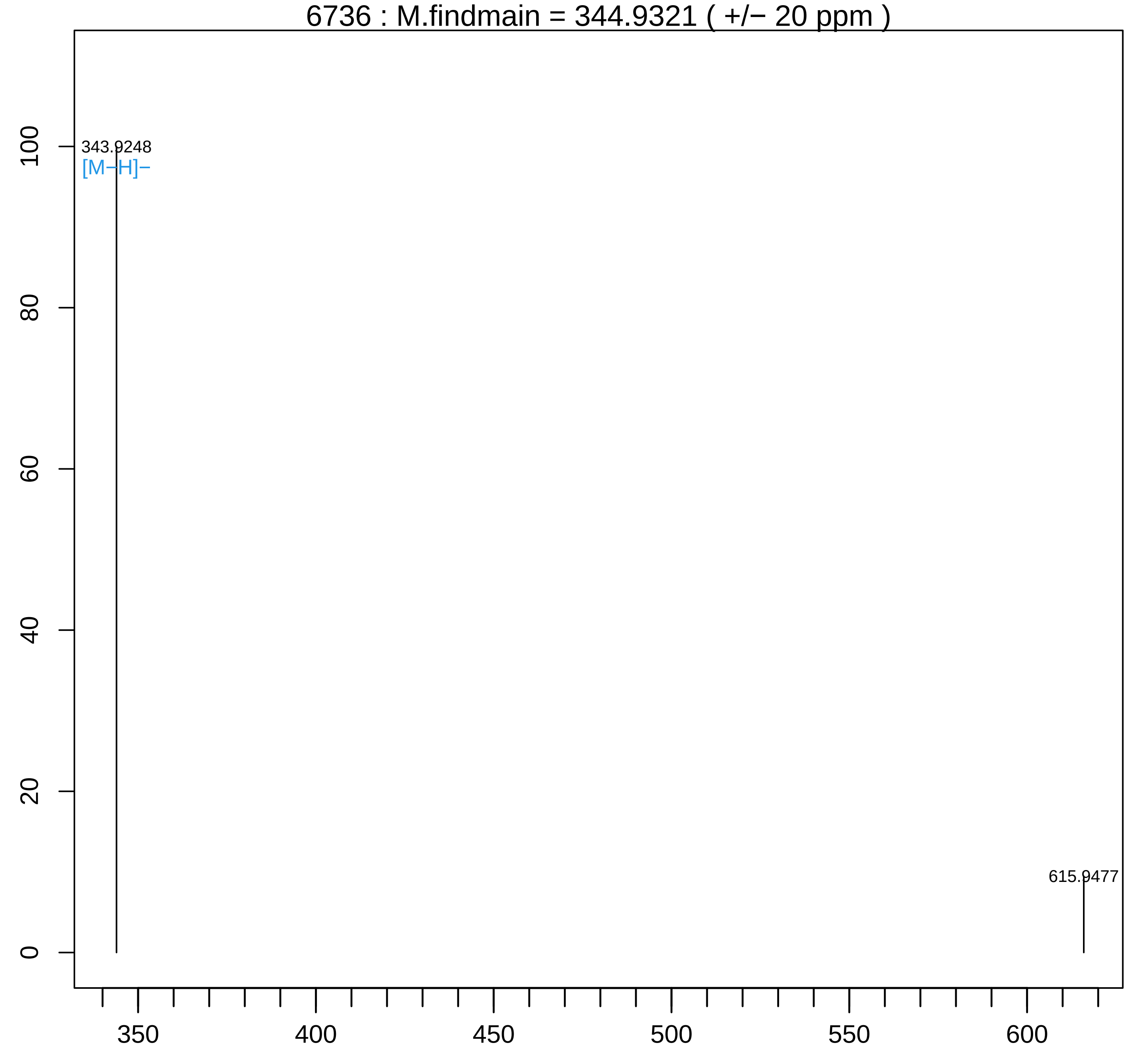
6736 : M.findmain = 344.9321 (+/− 20 ppm)
343.9248
[M−H]−
615.9477
0
20
40
60
80
100
350
400
450
500
550
600

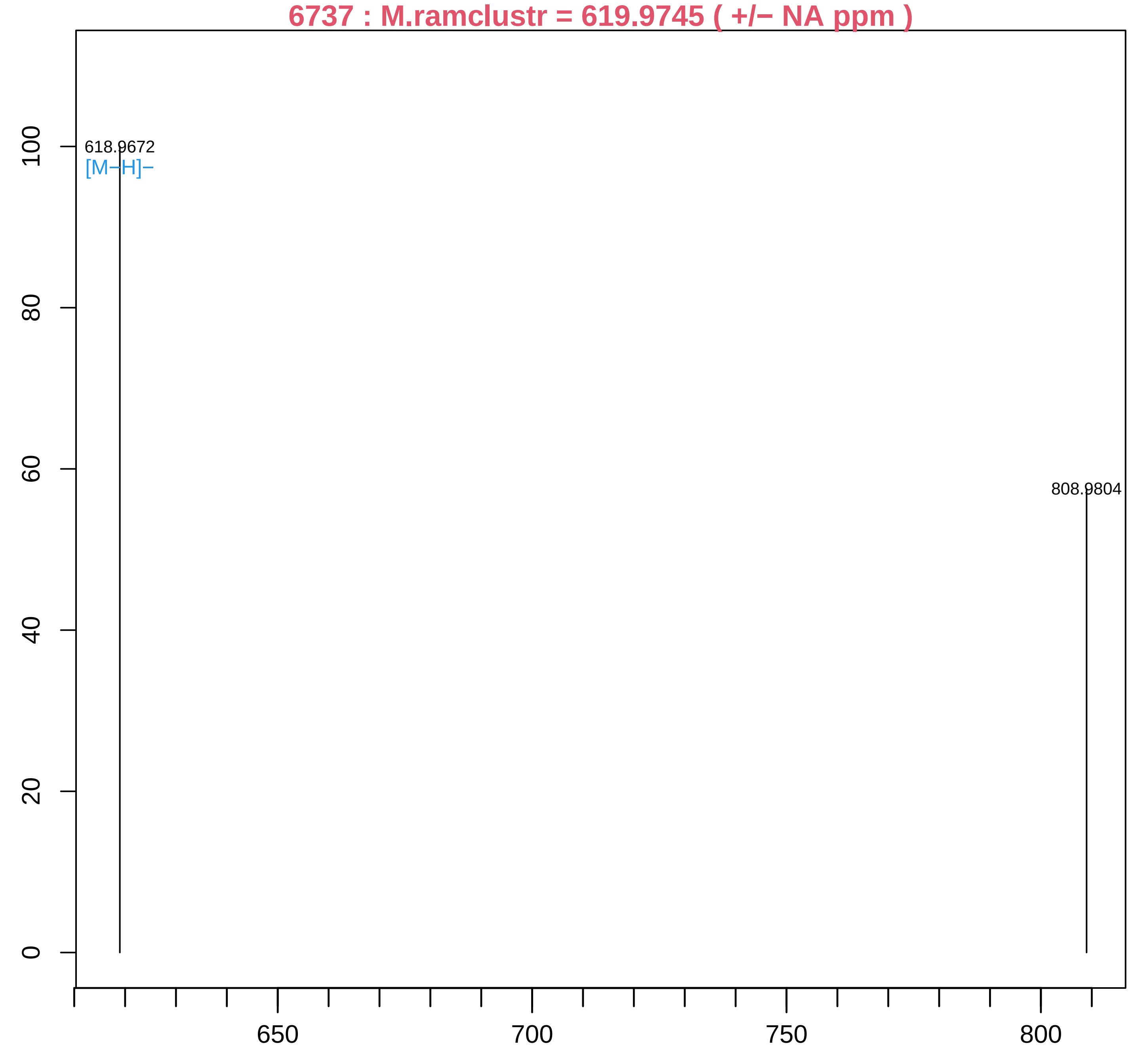
6737 : M.ramclustr = 619.9745 (+/− NA ppm)
618.9672
[M−H]−
808.9804
0
20
40
60
80
100
650
700
750
800

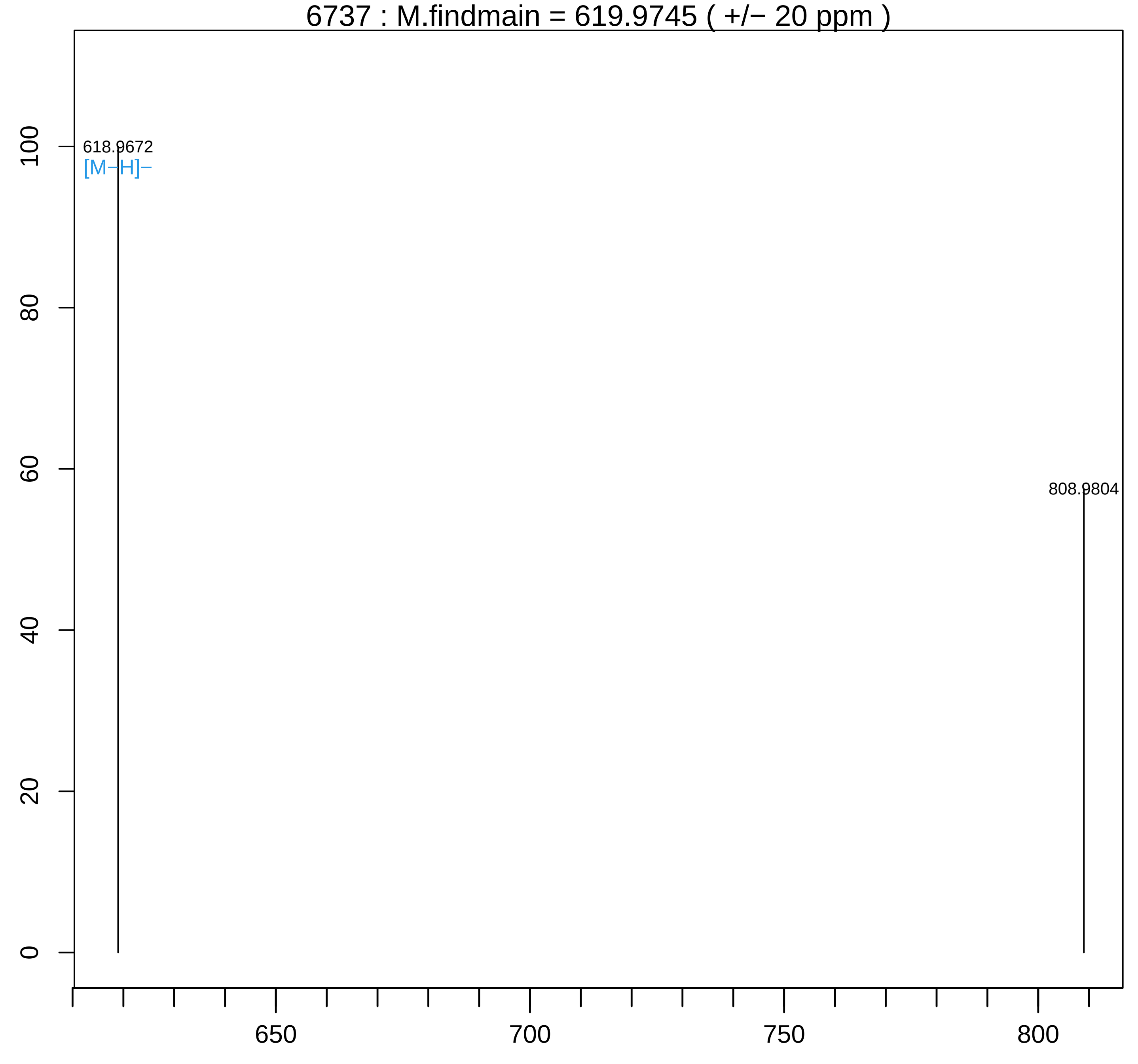
6737 : M.findmain = 619.9745 (+/− 20 ppm)
618.9672
[M−H]−
808.9804
0
20
40
60
80
100
650
700
750
800

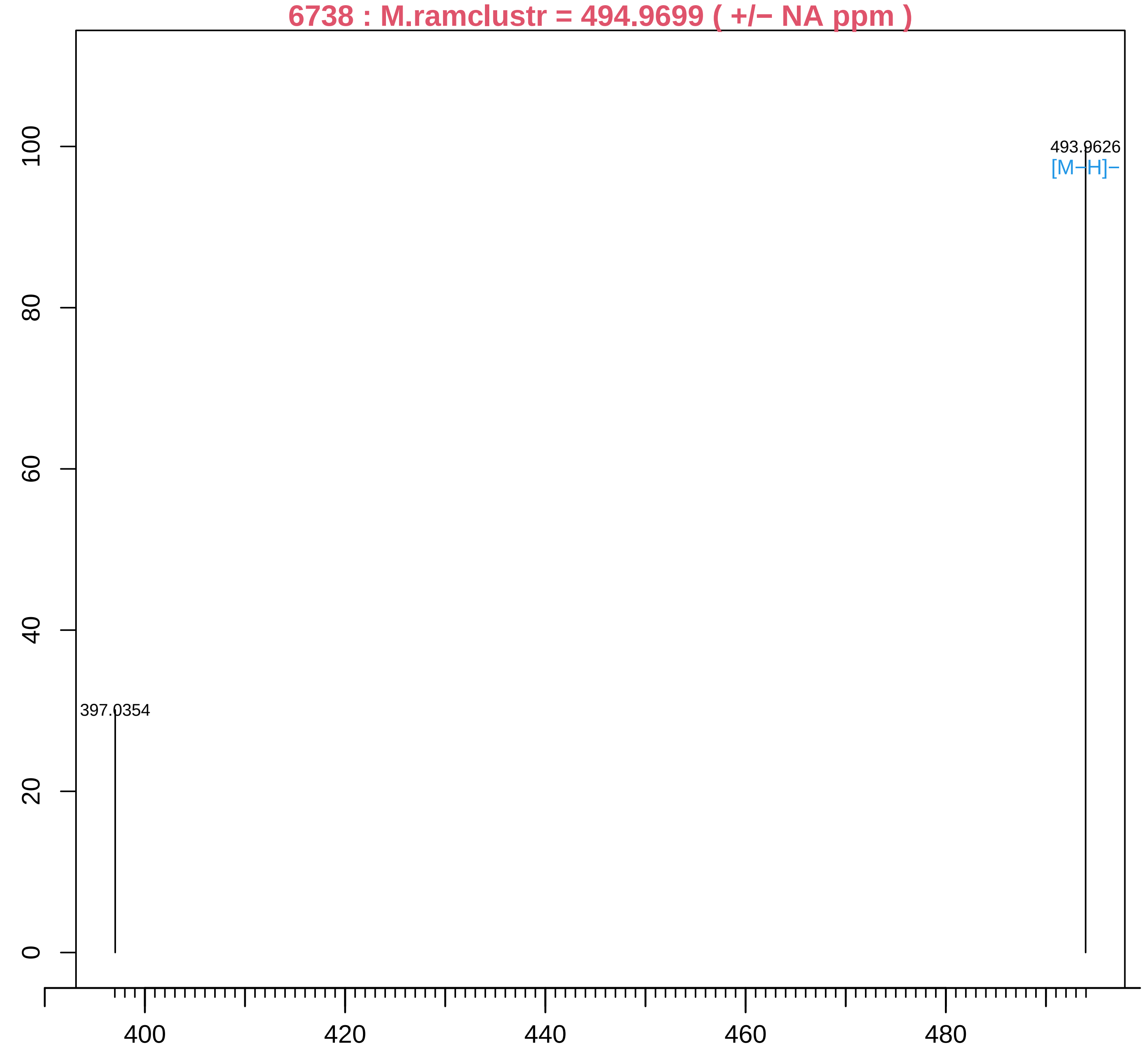
6738 : M.ramclustr = 494.9699 (+/− NA ppm)
493.9626
[M−H]−
397.0354
0
20
40
60
80
100
400
420
440
460
480

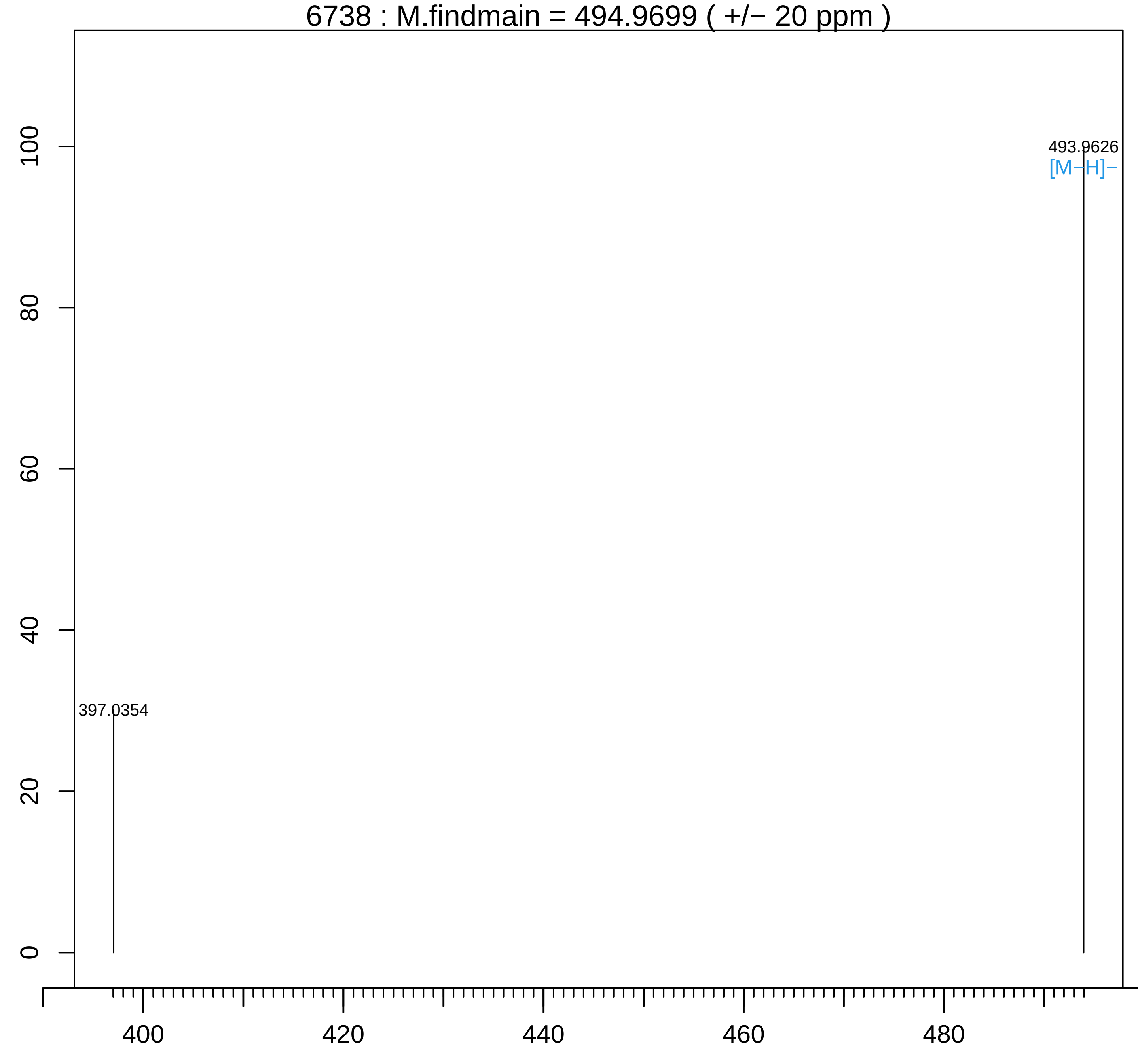
6738 : M.findmain = 494.9699 (+/− 20 ppm)
493.9626
[M−H]−
397.0354
0
20
40
60
80
100
400
420
440
460
480

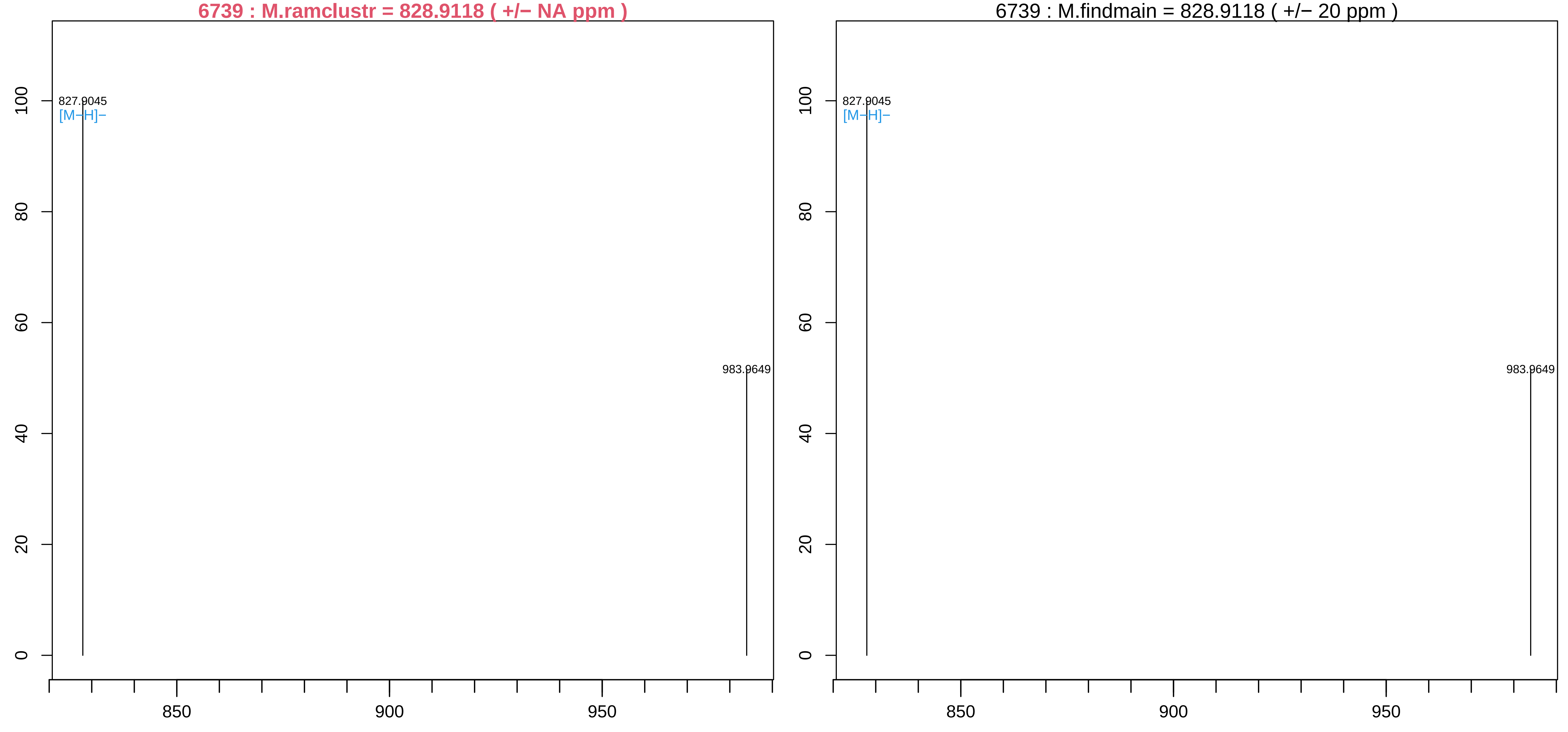
6739 : M.ramclustr = 828.9118 (+/− NA ppm)
827.9045
[M−H]−
983.9649
0
20
40
60
80
100
850
900
950
6739 : M.findmain = 828.9118 (+/− 20 ppm)
827.9045
[M−H]−
983.9649
0
20
40
60
80
100
850
900
950

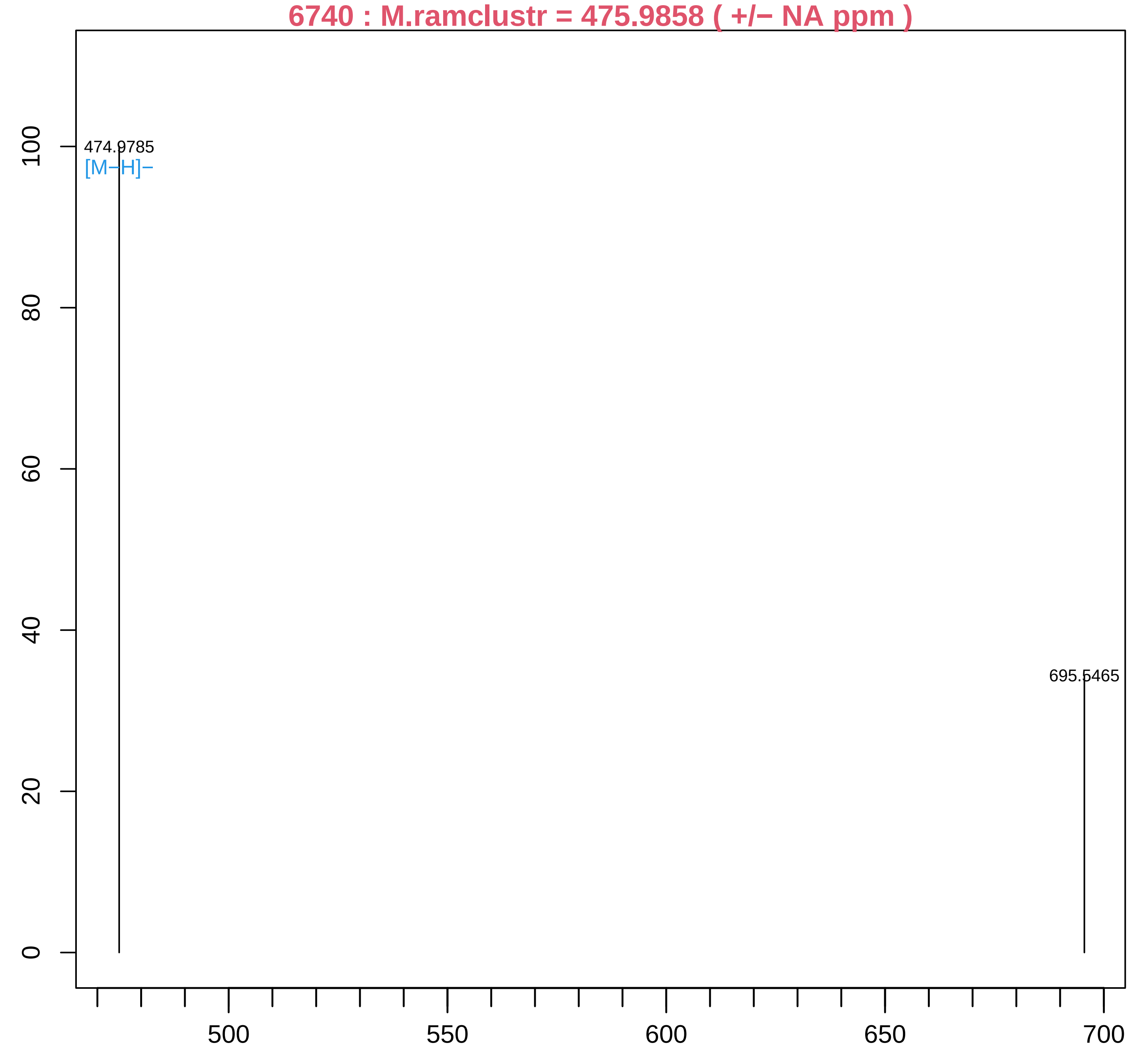
6740 : M.ramclustr = 475.9858 (+/− NA ppm)
474.9785
[M−H]−
695.5465
0
20
40
60
80
100
500
550
600
650
700

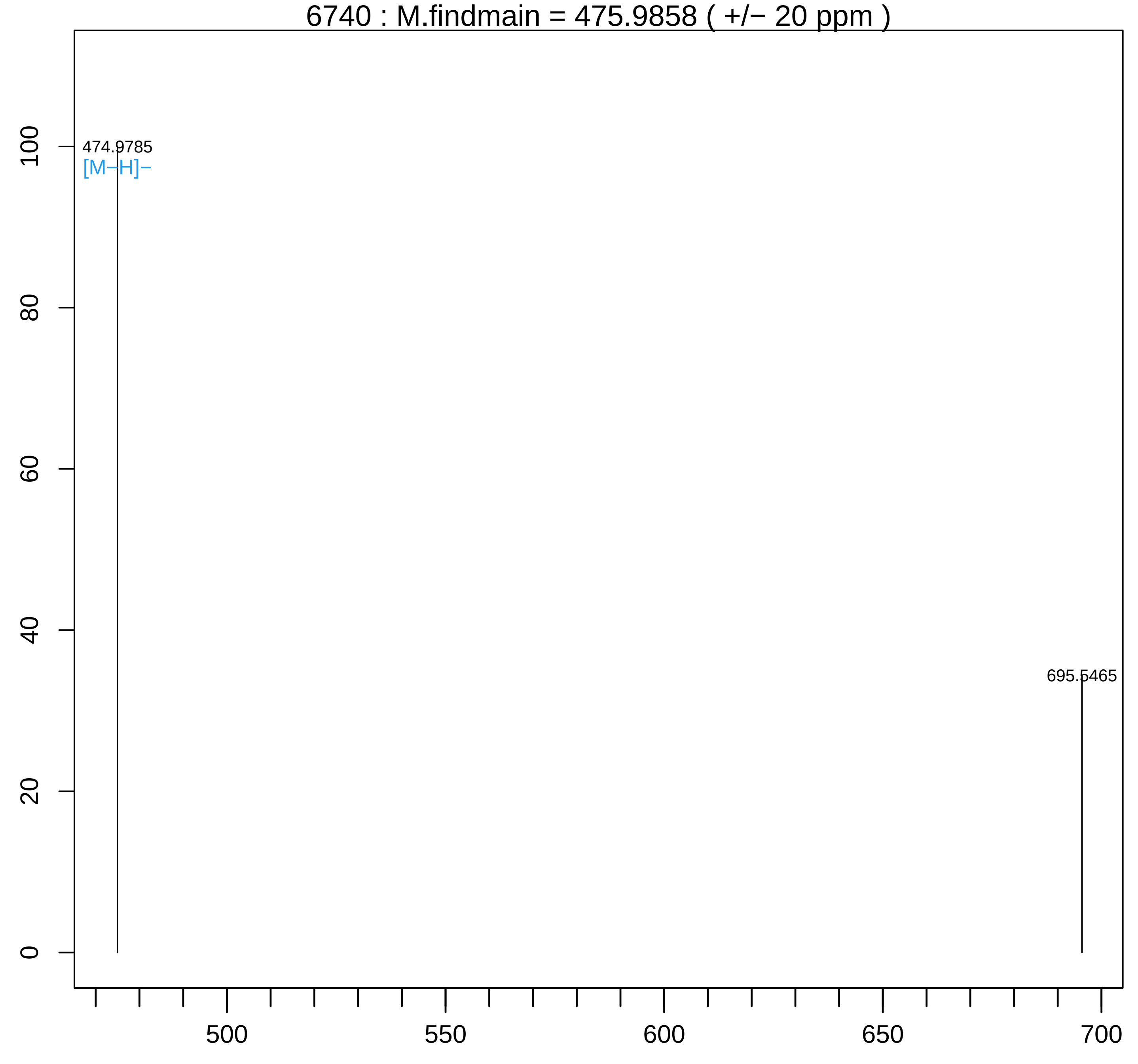
6740 : M.findmain = 475.9858 (+/− 20 ppm)
474.9785
[M−H]−
695.5465
0
20
40
60
80
100
500
550
600
650
700

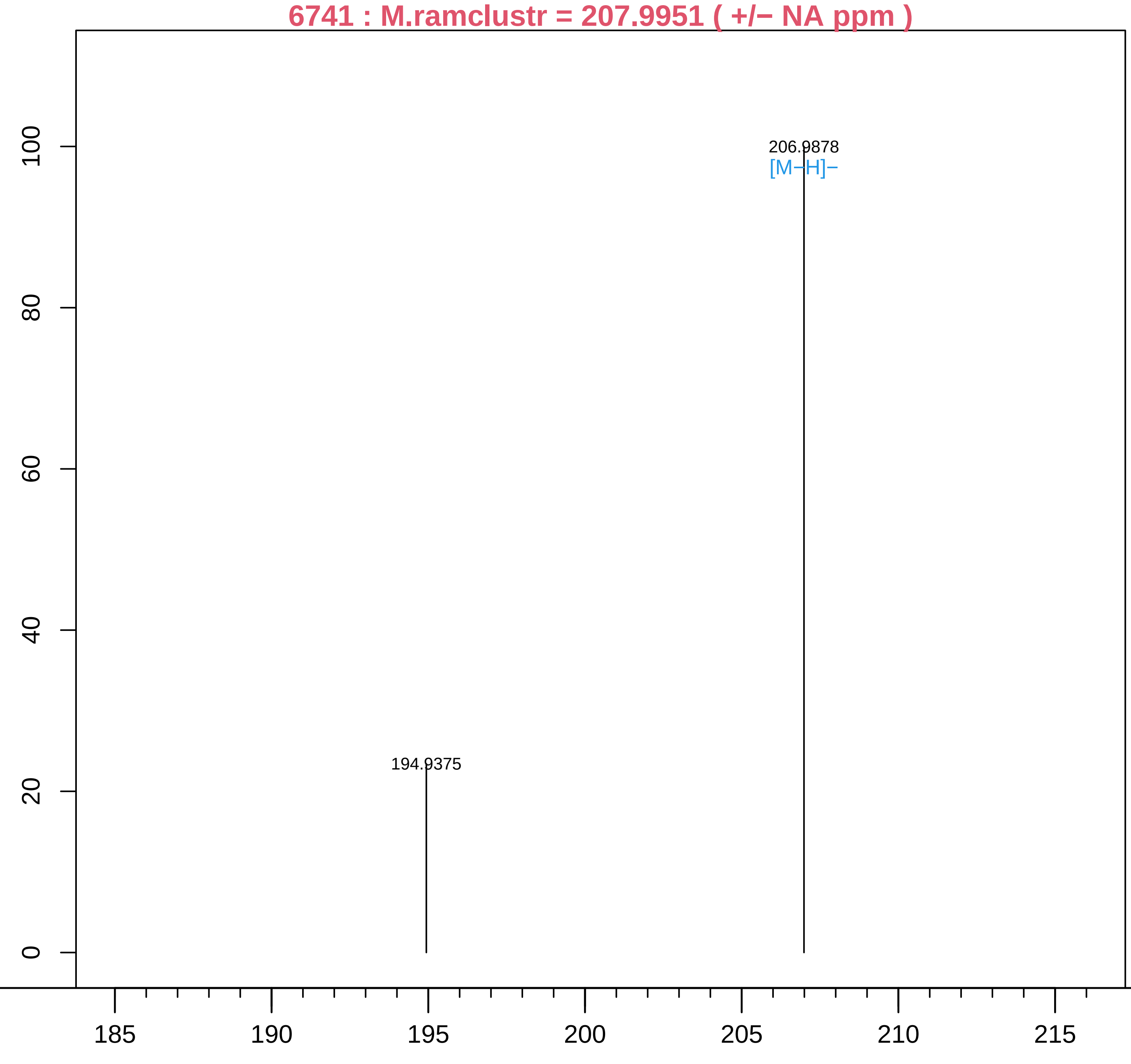
6741 : M.ramclustr = 207.9951 (+/− NA ppm)
206.9878
[M−H]−
194.9375
0
20
40
60
80
100
185
190
195
200
205
210
215

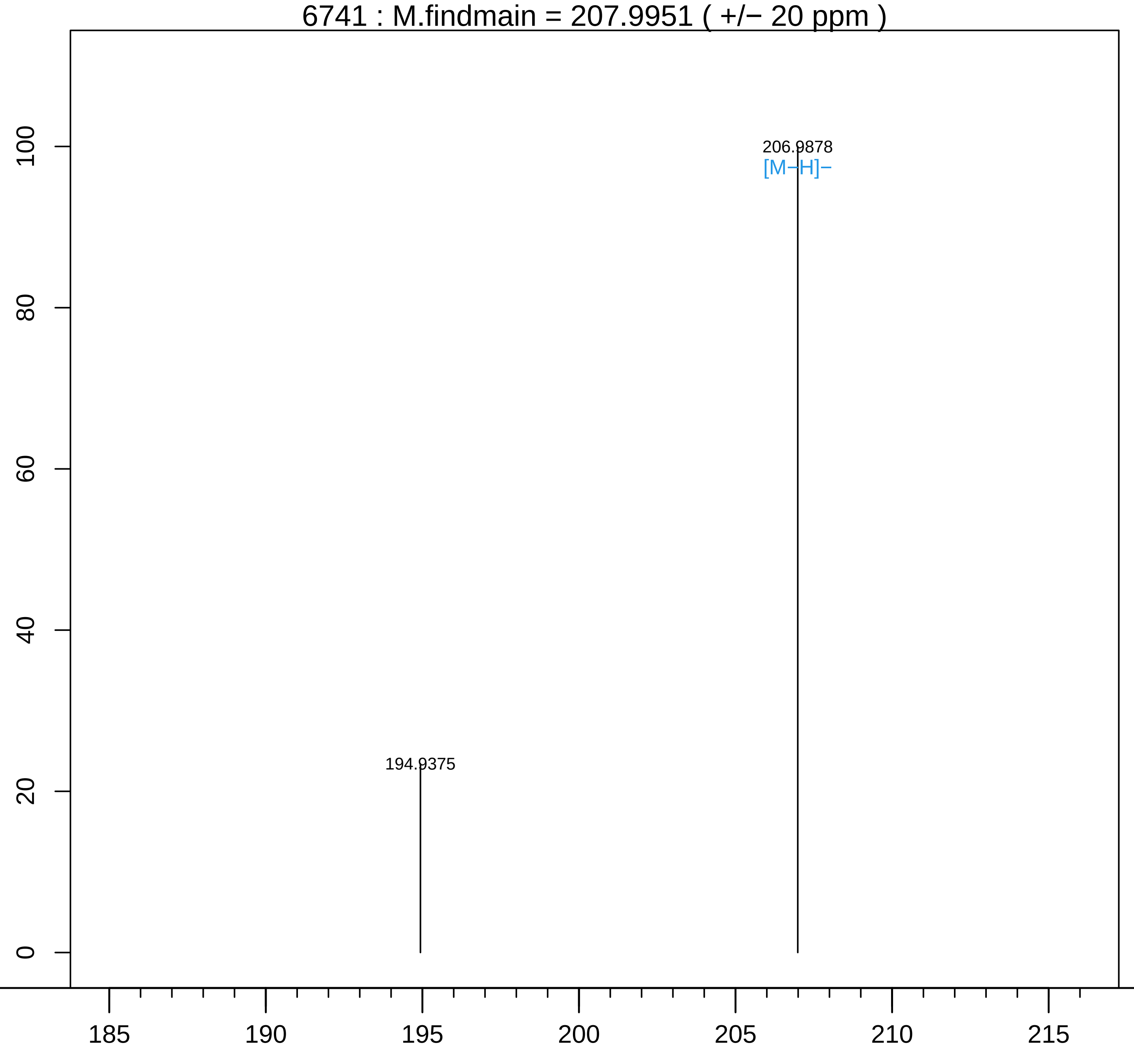
6741 : M.findmain = 207.9951 (+/− 20 ppm)
206.9878
[M−H]−
194.9375
0
20
40
60
80
100
185
190
195
200
205
210
215

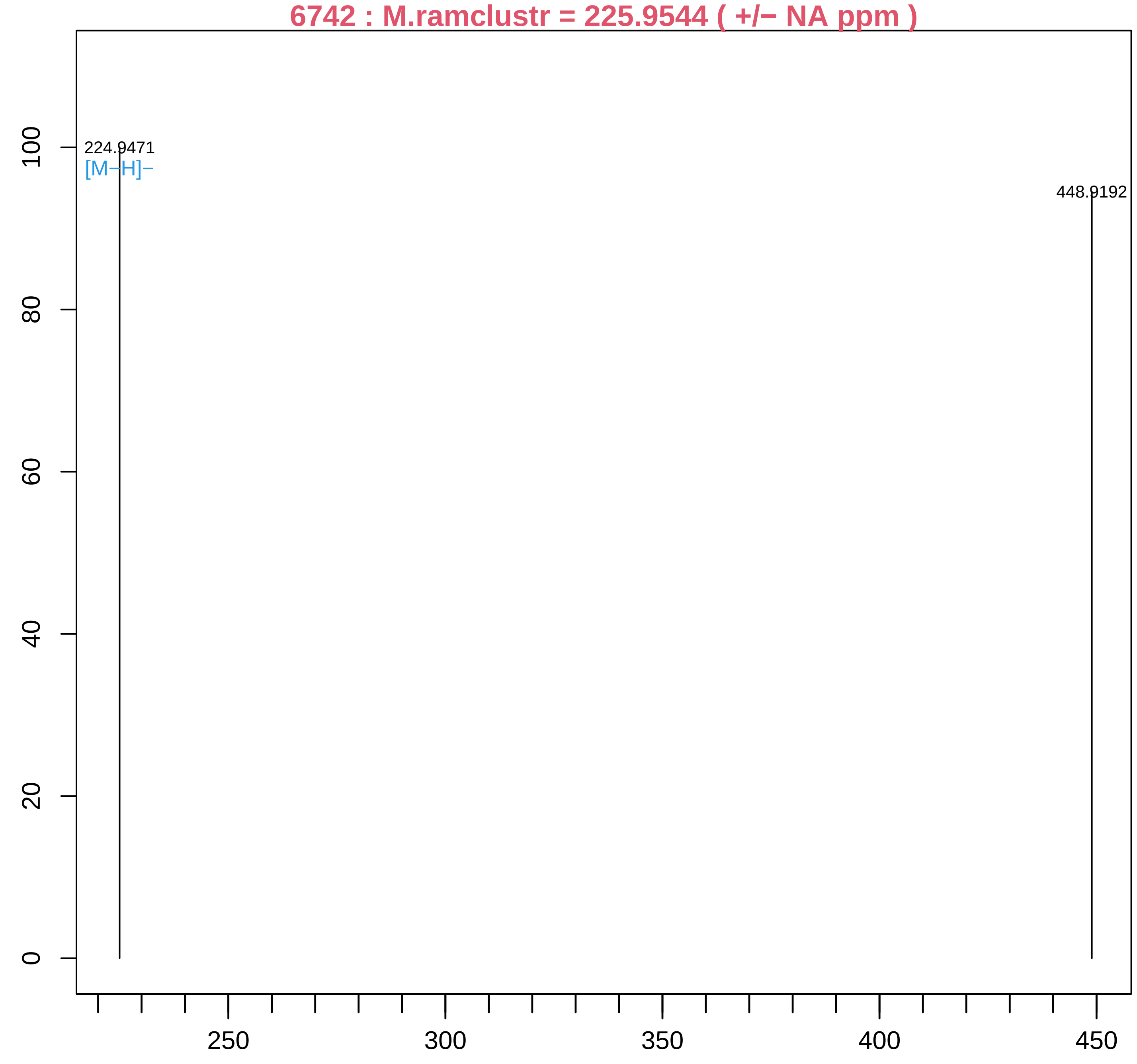
6742 : M.ramclustr = 225.9544 (+/− NA ppm)
224.9471
[M−H]−
448.9192
0
20
40
60
80
100
250
300
350
400
450

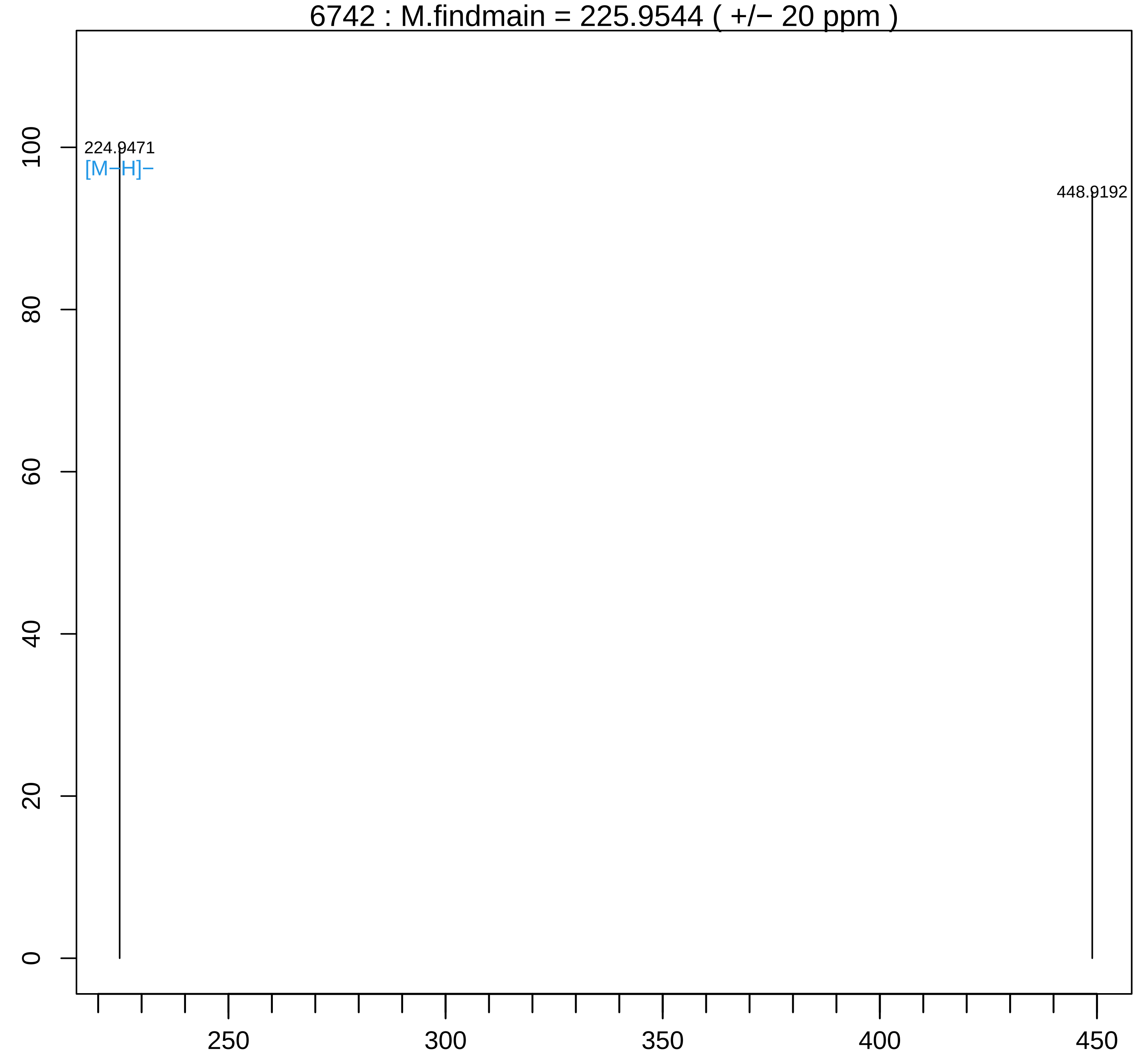
6742 : M.findmain = 225.9544 (+/− 20 ppm)
224.9471
[M−H]−
448.9192
0
20
40
60
80
100
250
300
350
400
450

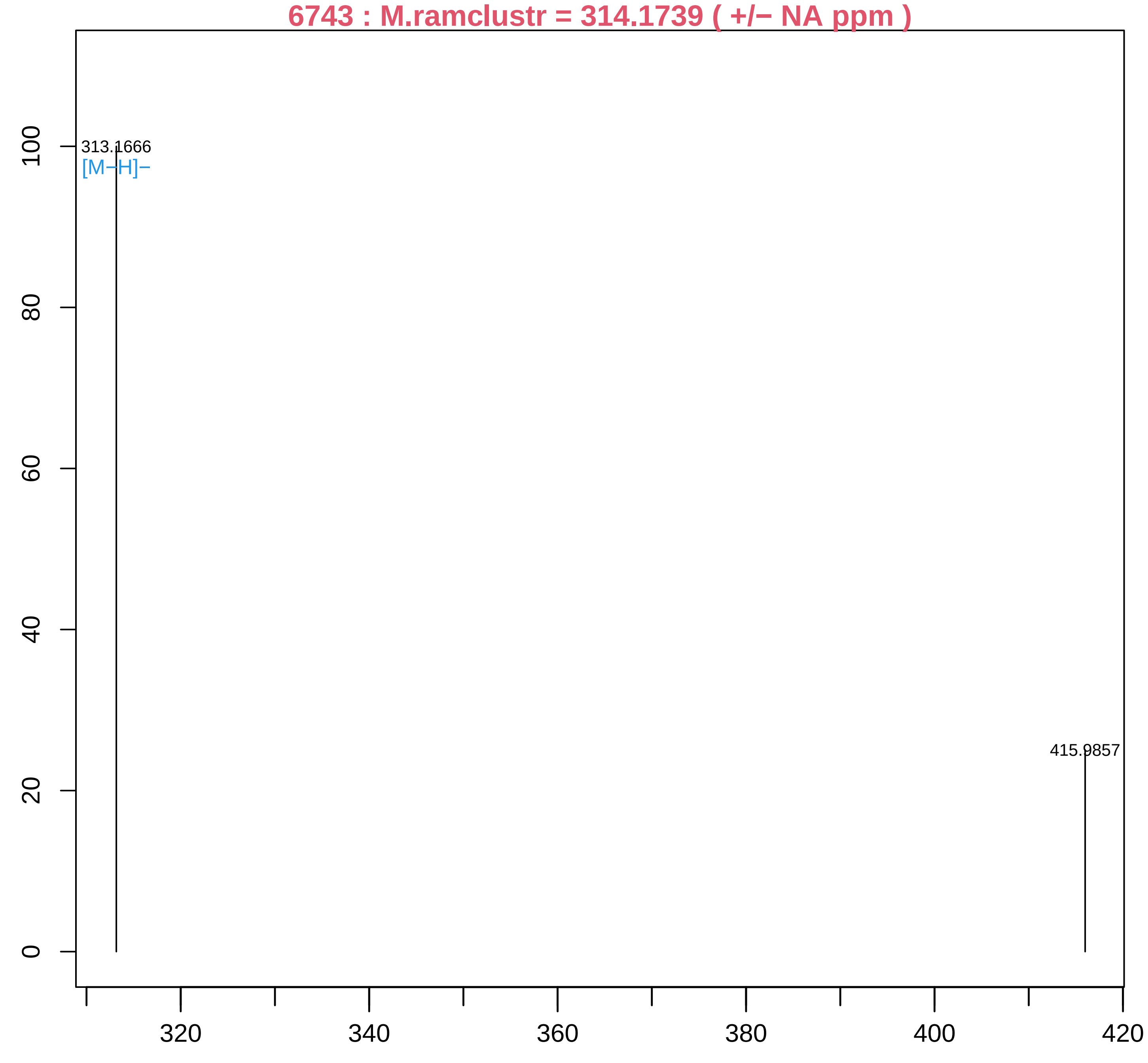
6743 : M.ramclustr = 314.1739 (+/− NA ppm)
313.1666
[M−H]−
415.9857
0
20
40
60
80
100
320
340
360
380
400
420

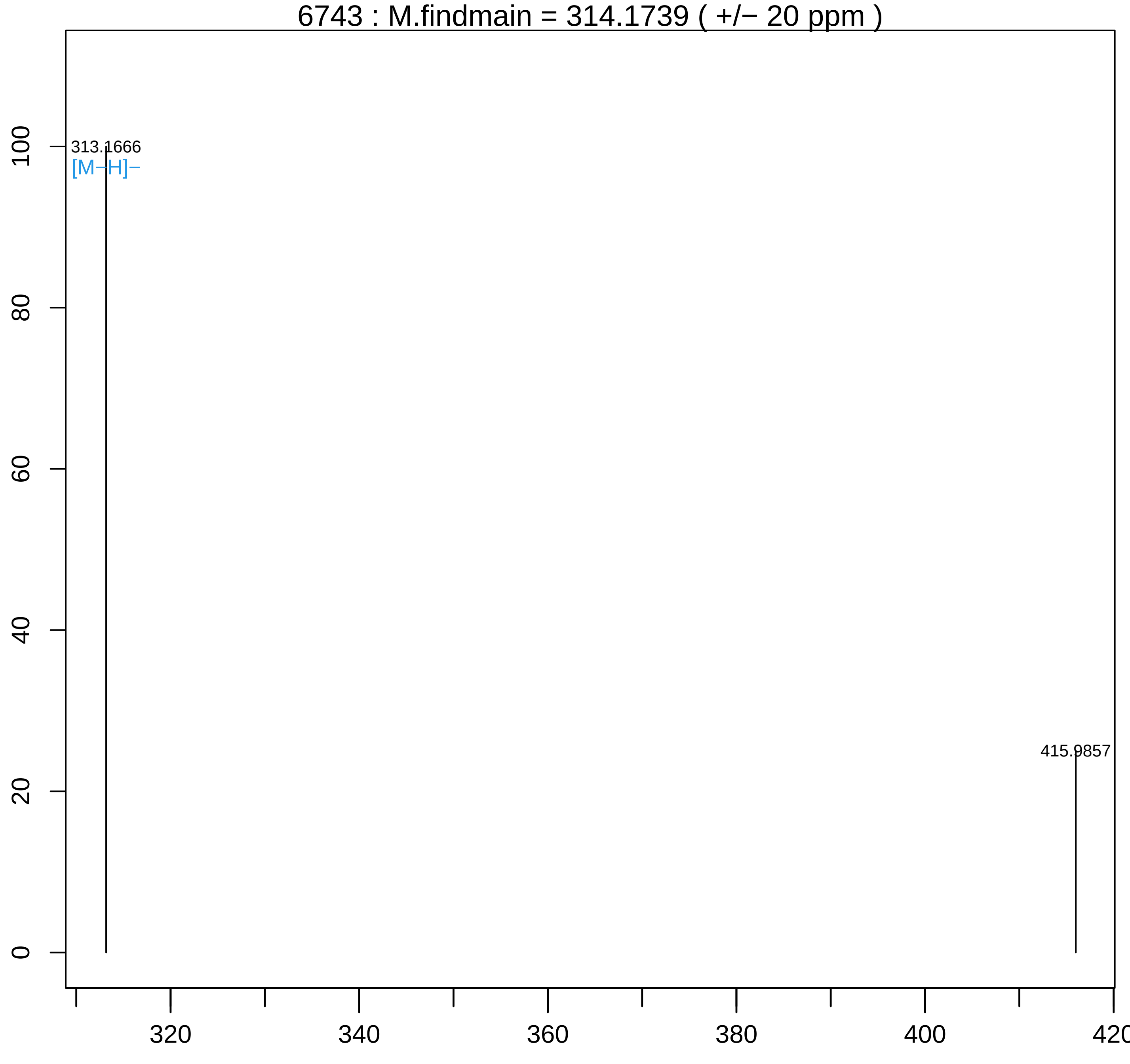
6743 : M.findmain = 314.1739 (+/− 20 ppm)
313.1666
[M−H]−
415.9857
0
20
40
60
80
100
320
340
360
380
400
420

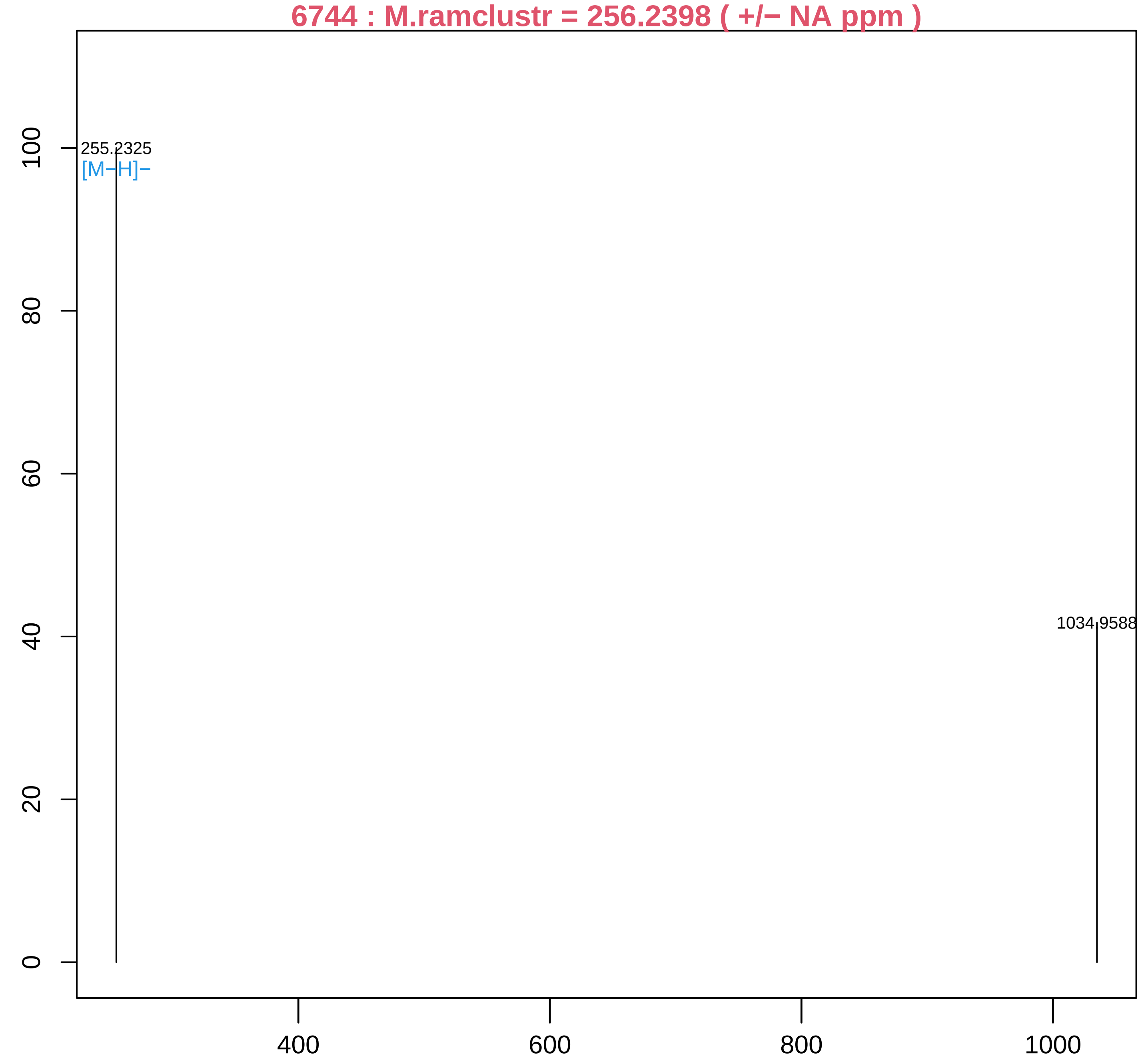
6744 : M.ramclustr = 256.2398 (+/− NA ppm)
255.2325
[M−H]−
1034.9588
0
20
40
60
80
100
400
600
800
1000

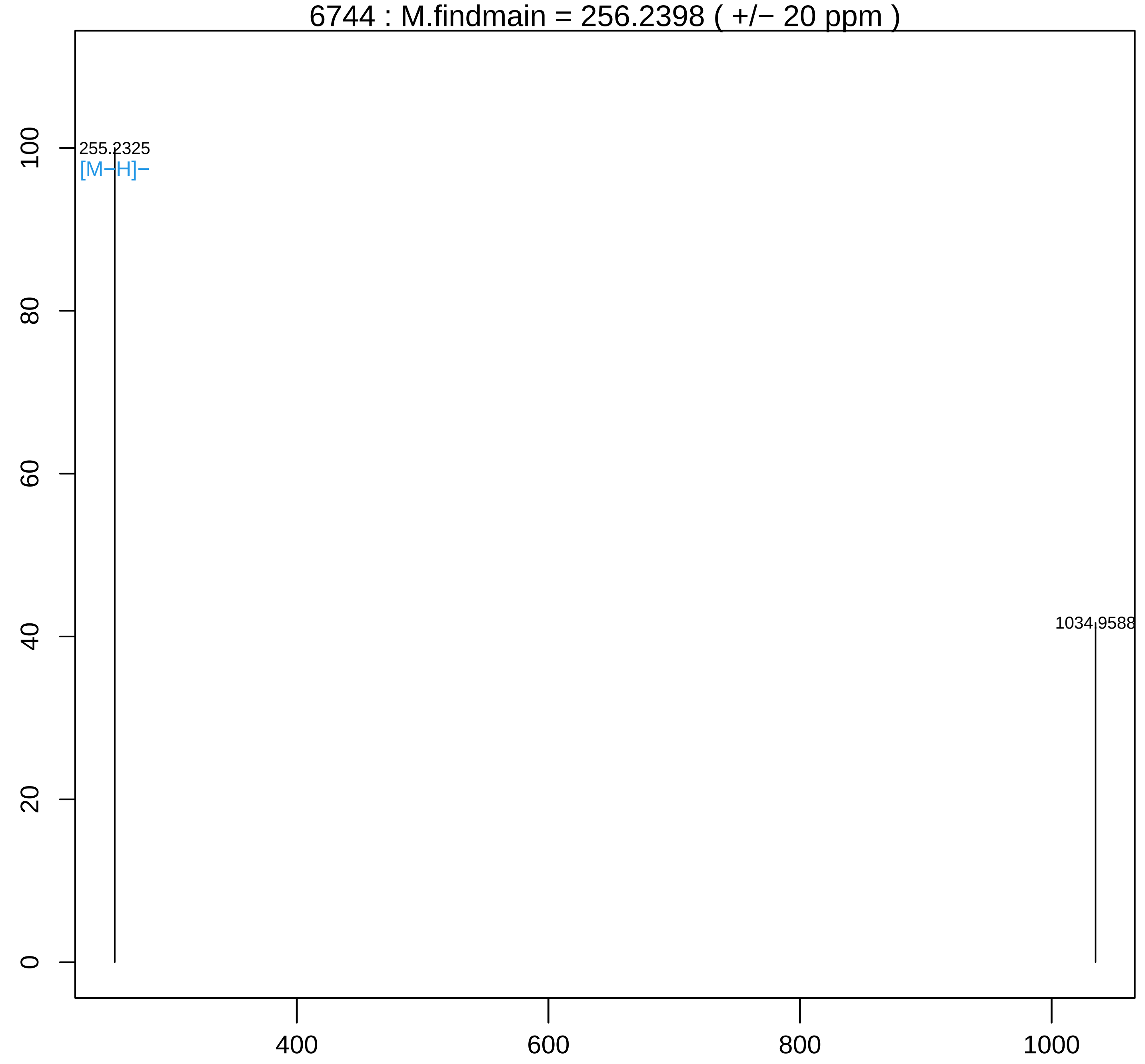
6744 : M.findmain = 256.2398 (+/− 20 ppm)
255.2325
[M−H]−
1034.9588
0
20
40
60
80
100
400
600
800
1000

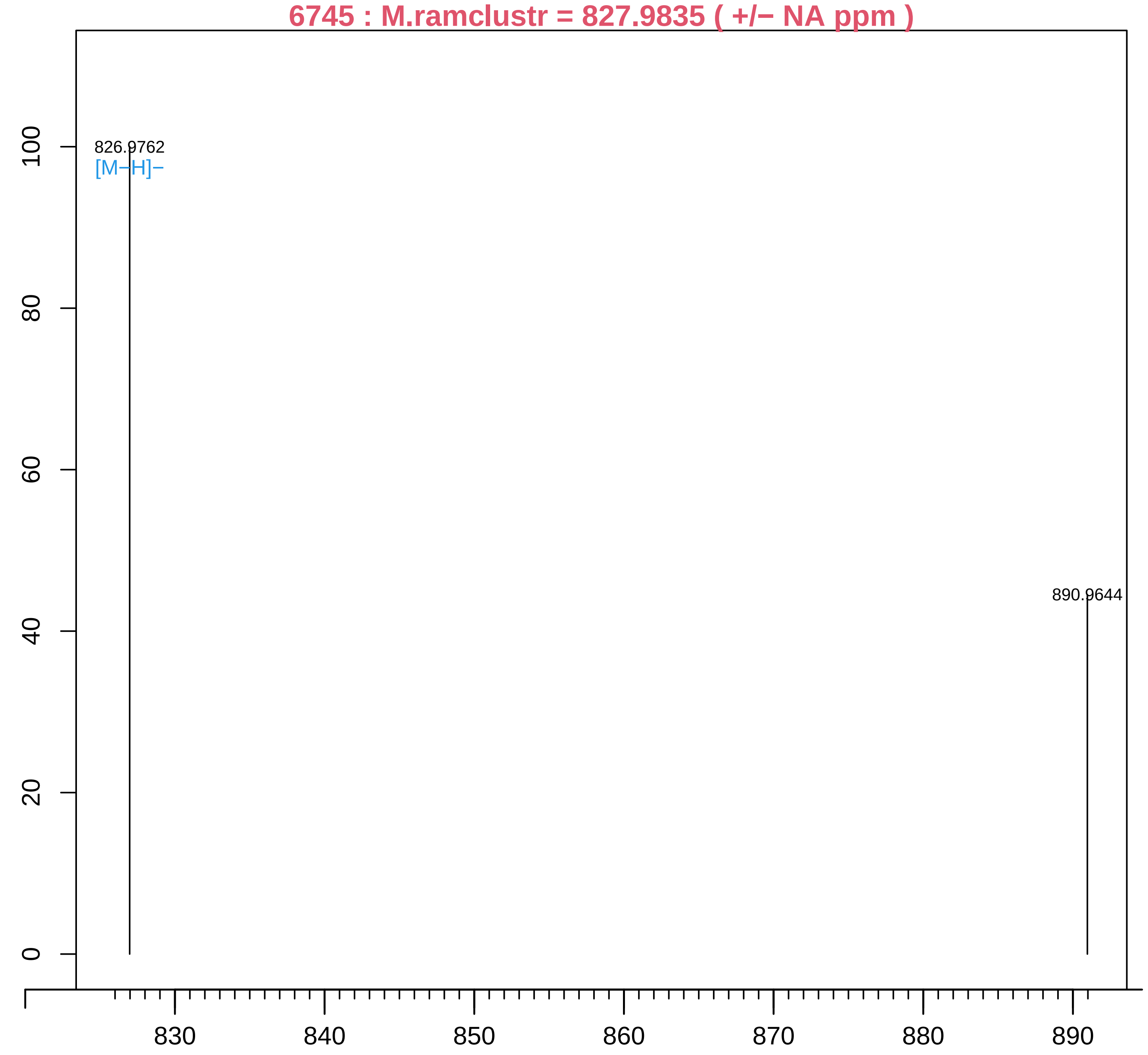
6745 : M.ramclustr = 827.9835 (+/− NA ppm)
826.9762
[M−H]−
890.9644
0
20
40
60
80
100
830
840
850
860
870
880
890

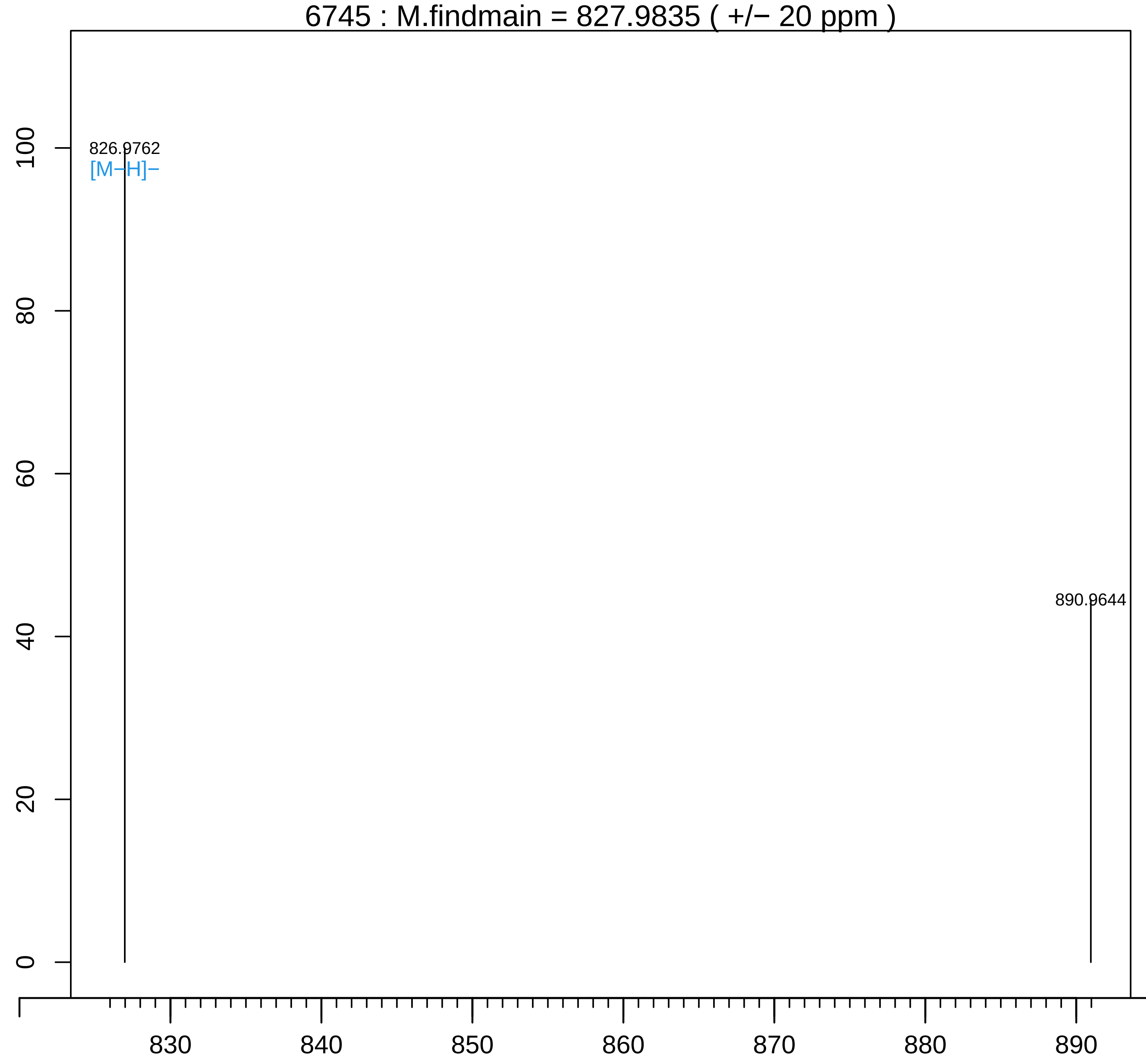
6745 : M.findmain = 827.9835 (+/− 20 ppm)
826.9762
[M−H]−
890.9644
0
20
40
60
80
100
830
840
850
860
870
880
890

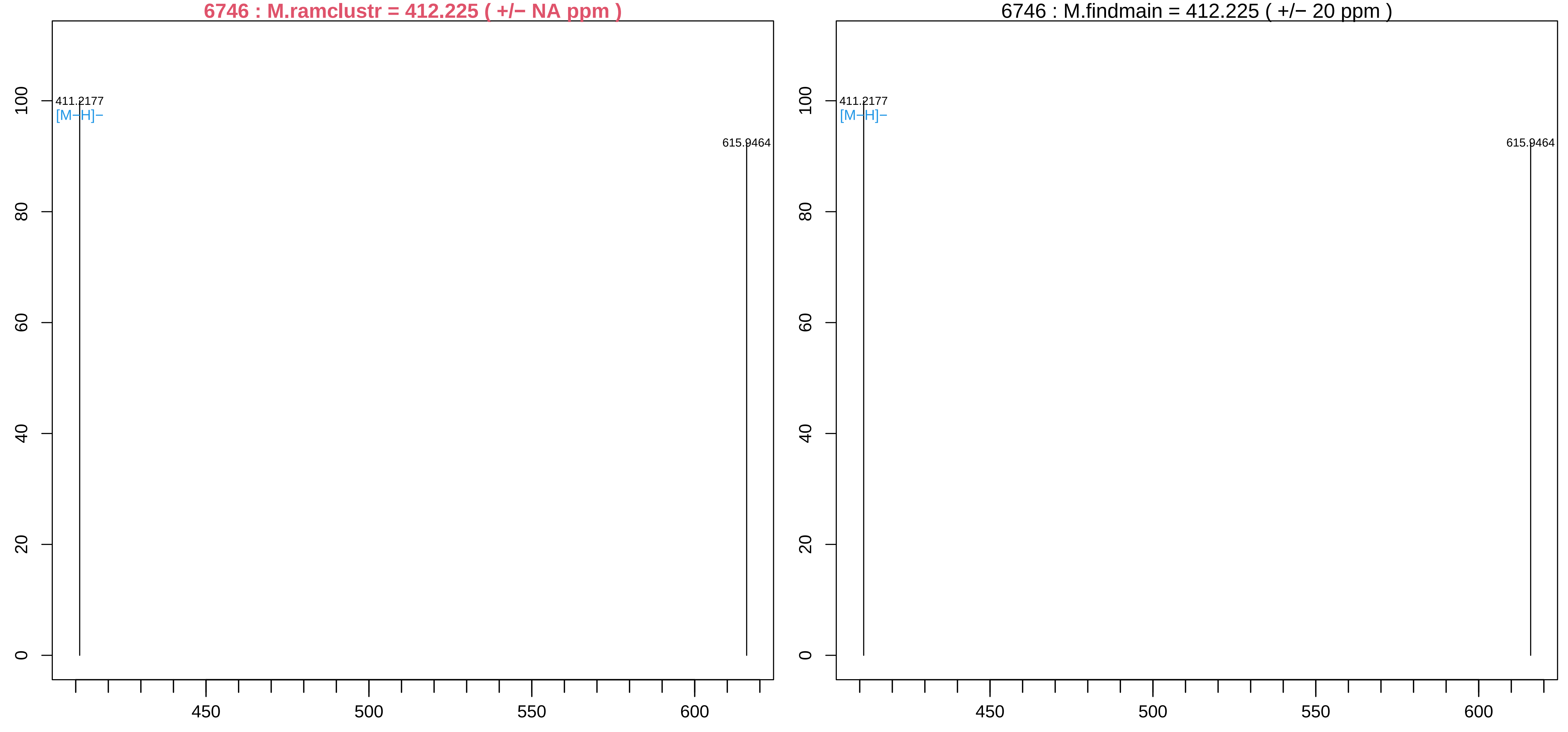
6746 : M.ramclustr = 412.225 (+/− NA ppm)
411.2177
[M−H]−
615.9464
6746 : M.findmain = 412.225 (+/− 20 ppm)
411.2177
[M−H]−
615.9464
0
20
40
60
80
100
450
500
550
600

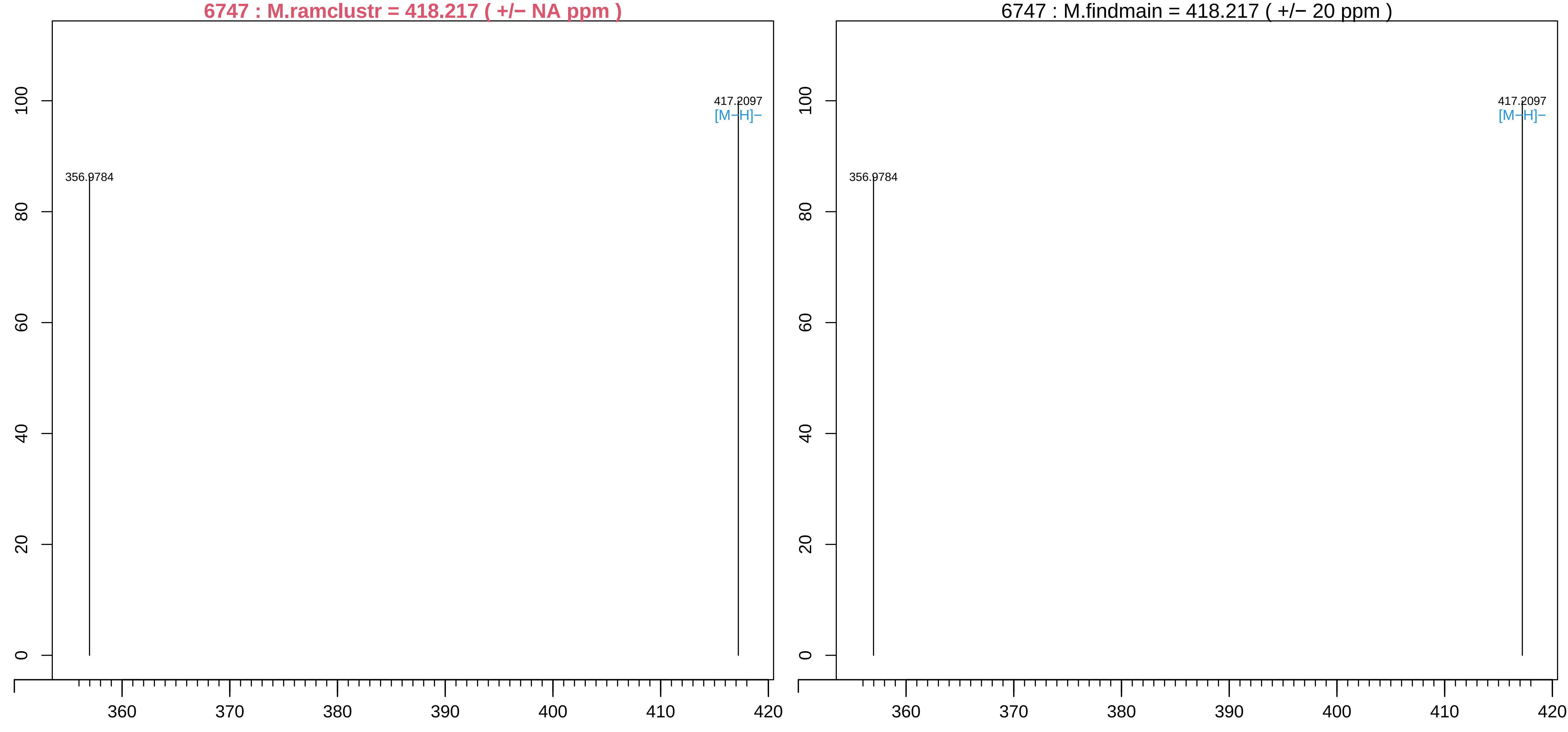
6747 : M.ramclustr = 418.217 (+/− NA ppm)
417.2097
[M−H]−
356.9784
6747 : M.findmain = 418.217 (+/− 20 ppm)
417.2097
[M−H]−
356.9784
0
20
40
60
80
100
360
370
380
390
400
410
420

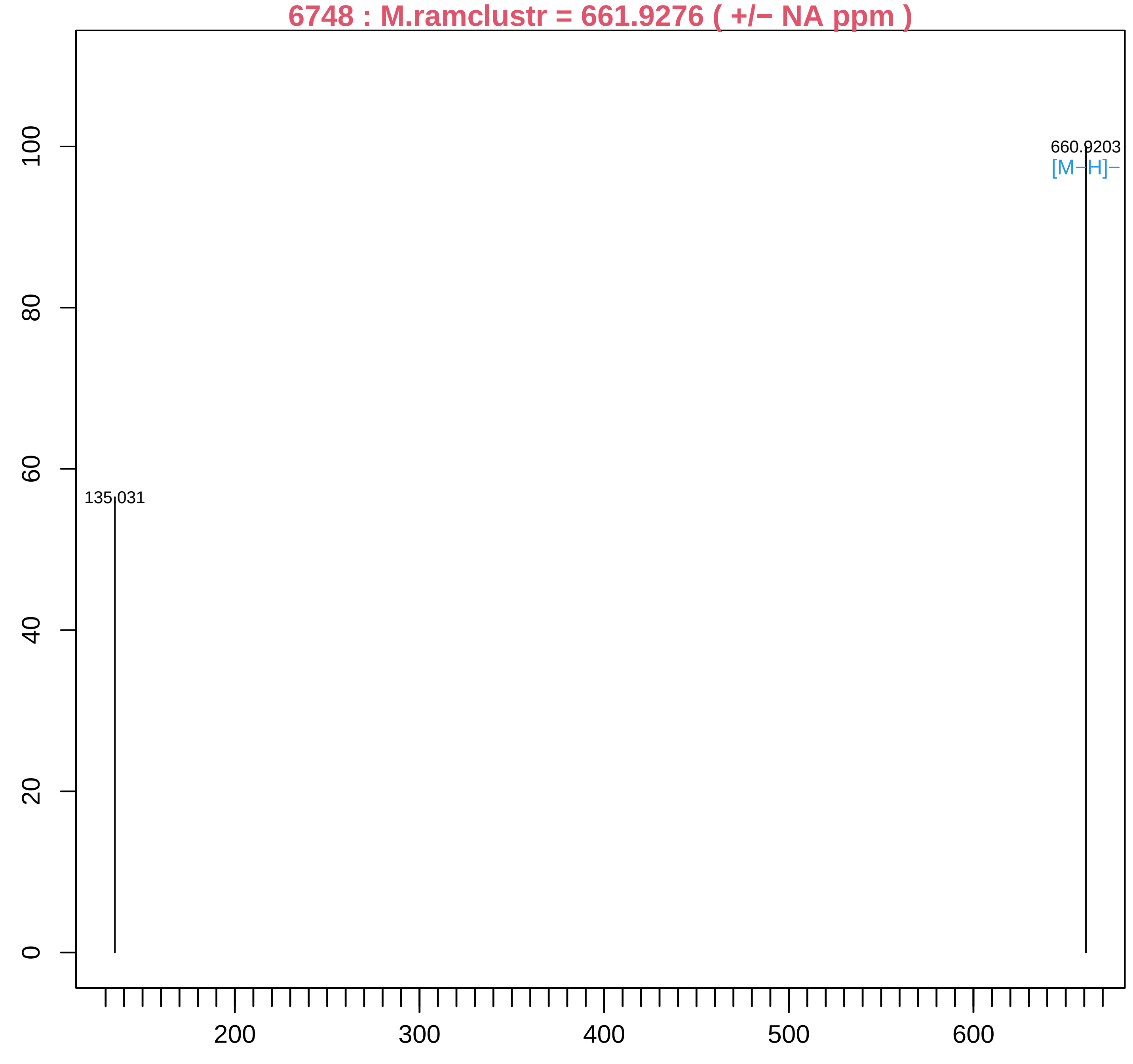
6748 : M.ramclustr = 661.9276 (+/− NA ppm)
660.9203
[M−H]−
135.031
0
20
40
60
80
100
200
300
400
500
600

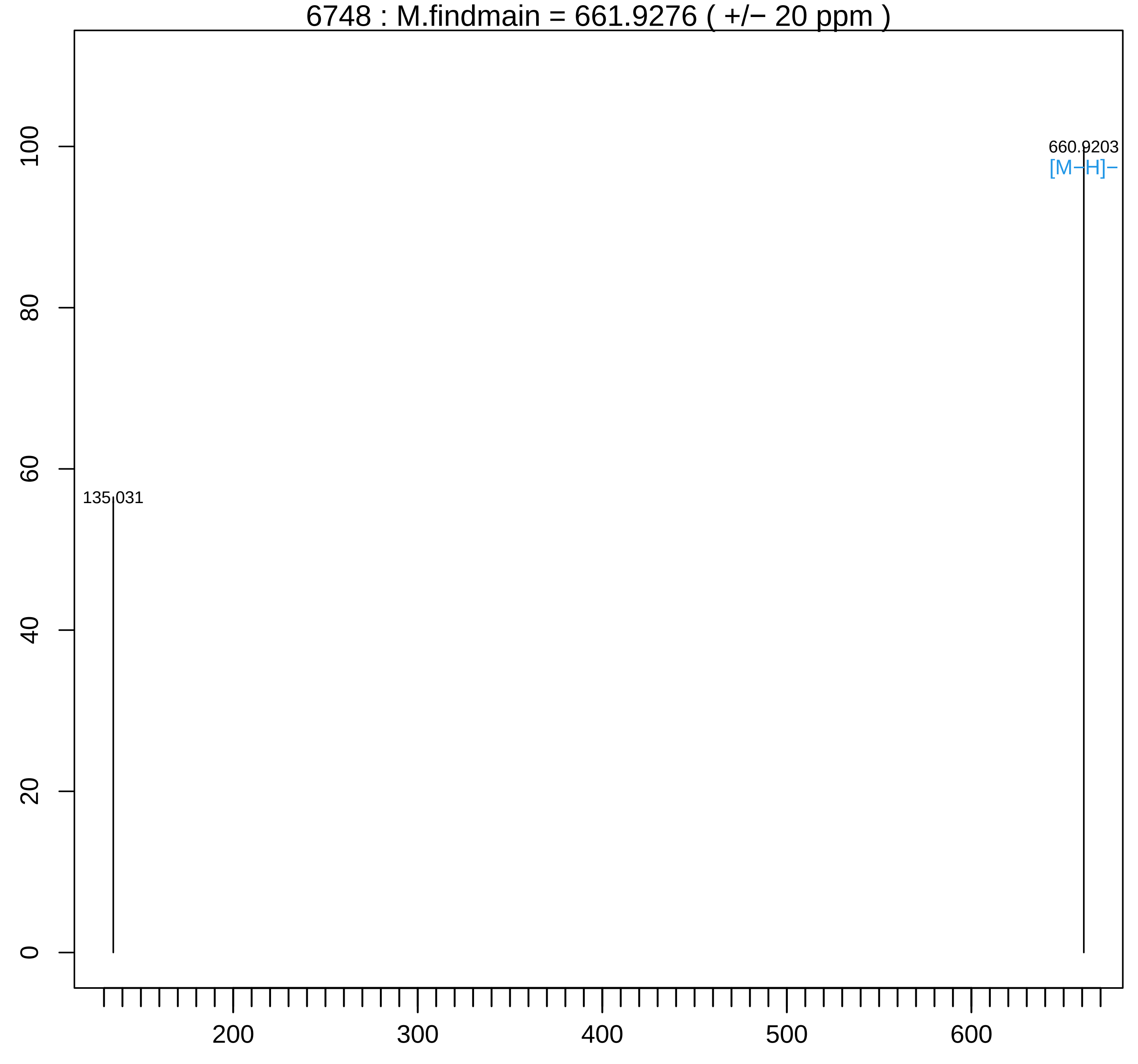
6748 : M.findmain = 661.9276 (+/− 20 ppm)
660.9203
[M−H]−
135.031
0
20
40
60
80
100
200
300
400
500
600

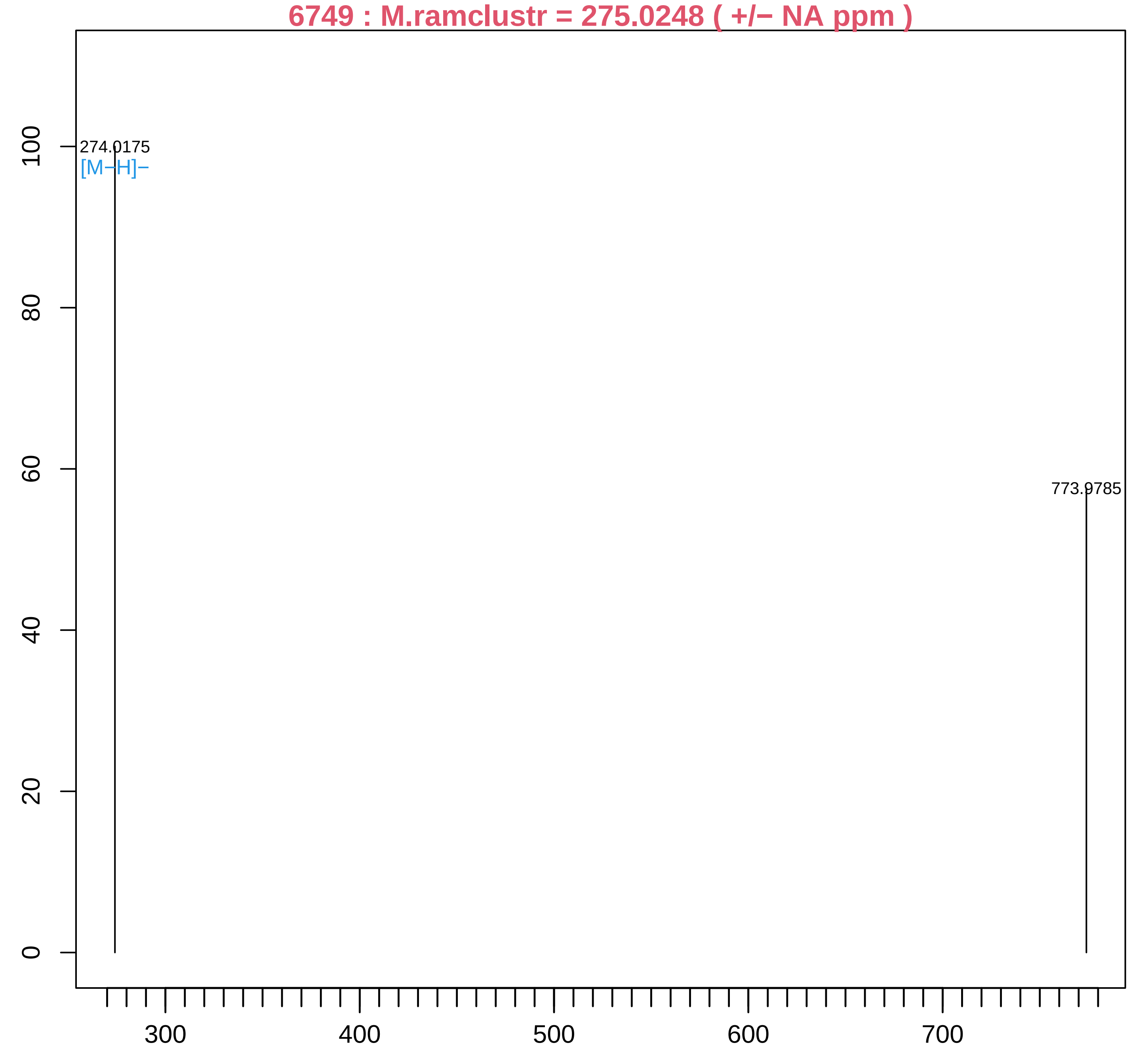
6749 : M.ramclustr = 275.0248 (+/− NA ppm)
274.0175
[M−H]−
773.9785
0
20
40
60
80
100
300
400
500
600
700

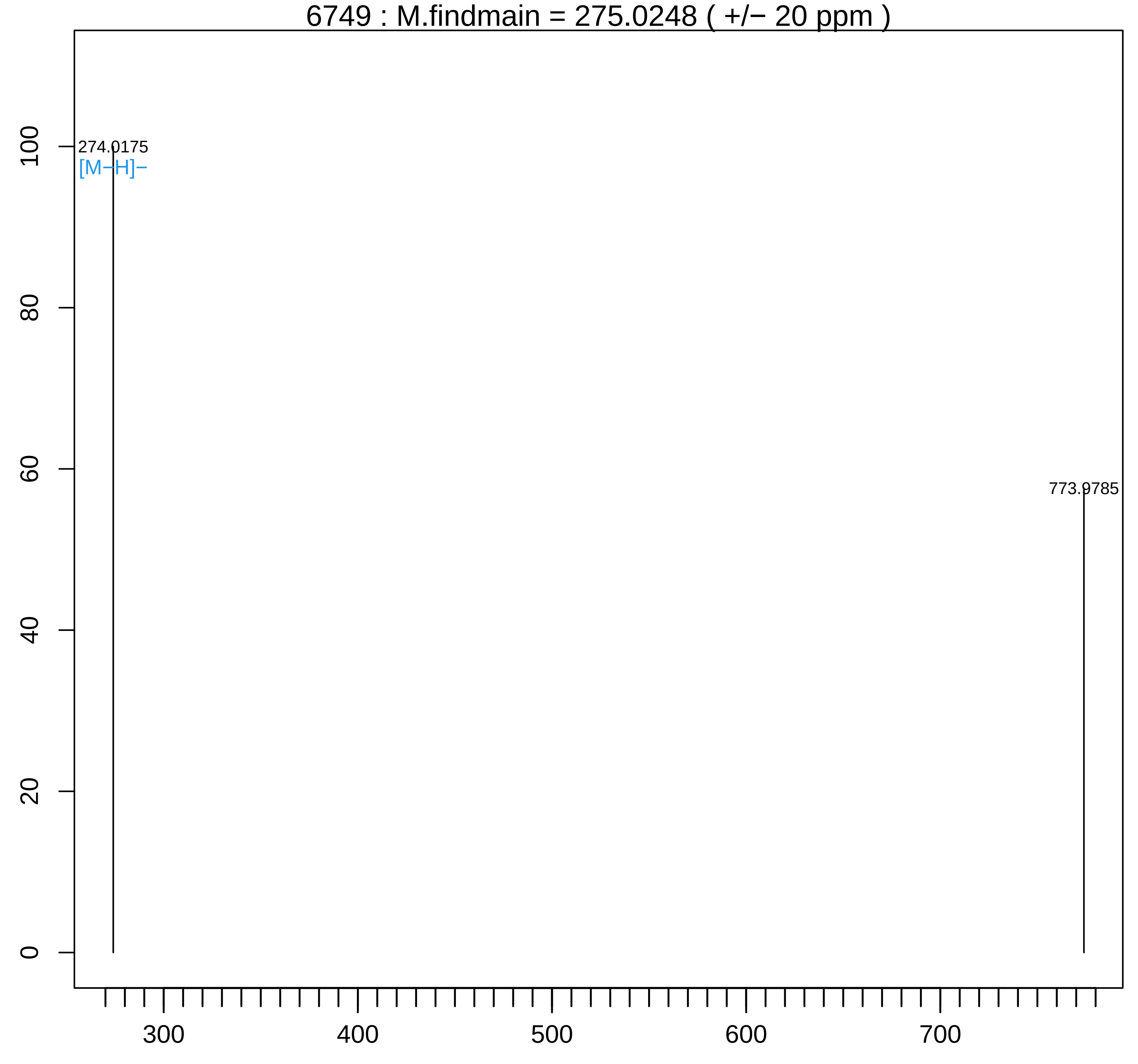
6749 : M.findmain = 275.0248 (+/− 20 ppm)
274.0175
[M−H]−
773.9785
0
20
40
60
80
100
300
400
500
600
700

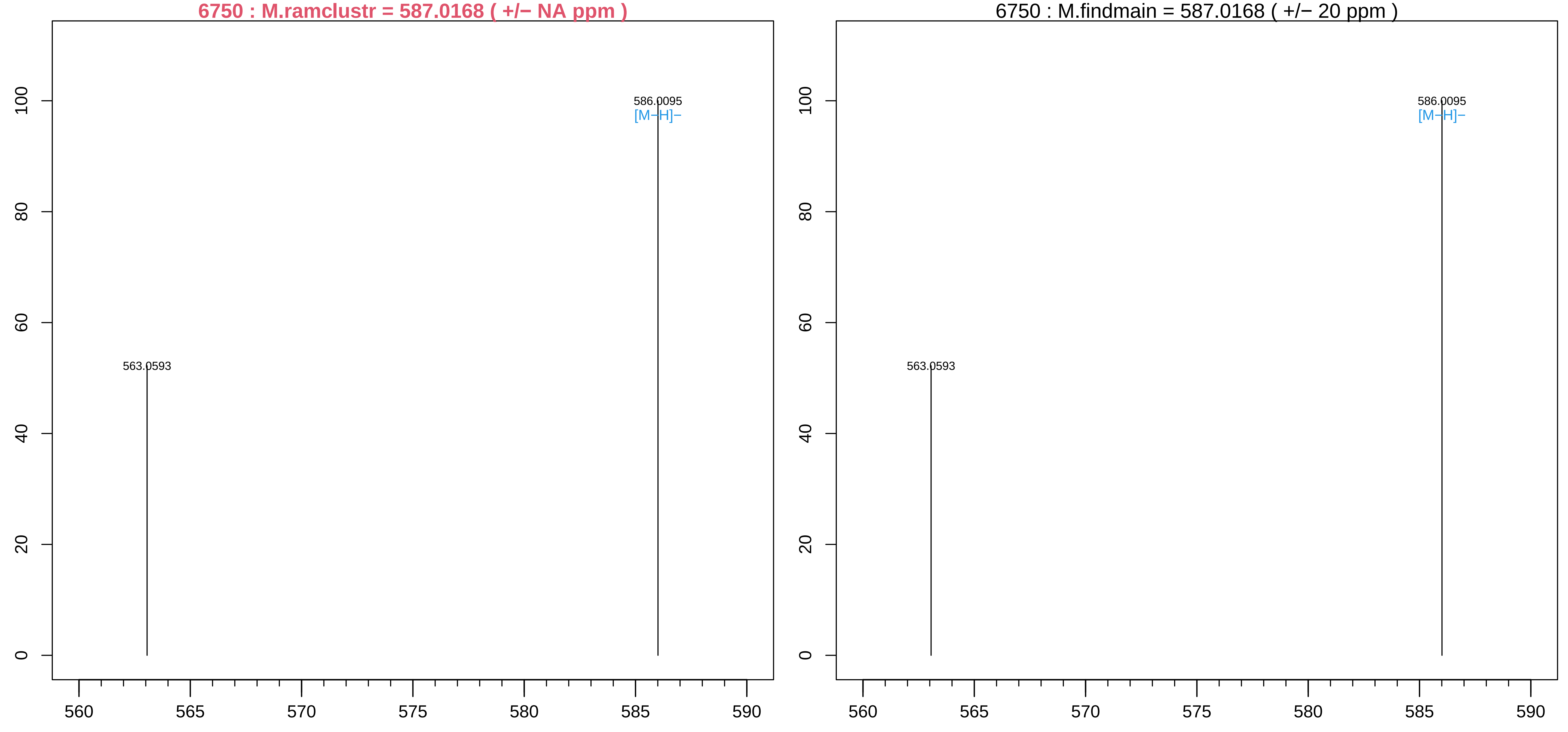
6750 : M.ramclustr = 587.0168 (+/− NA ppm)
586.0095
[M−H]−
563.0593
0
20
40
60
80
100
560
565
570
575
580
585
590
6750 : M.findmain = 587.0168 (+/− 20 ppm)
586.0095
[M−H]−
563.0593
0
20
40
60
80
100
560
565
570
575
580
585
590

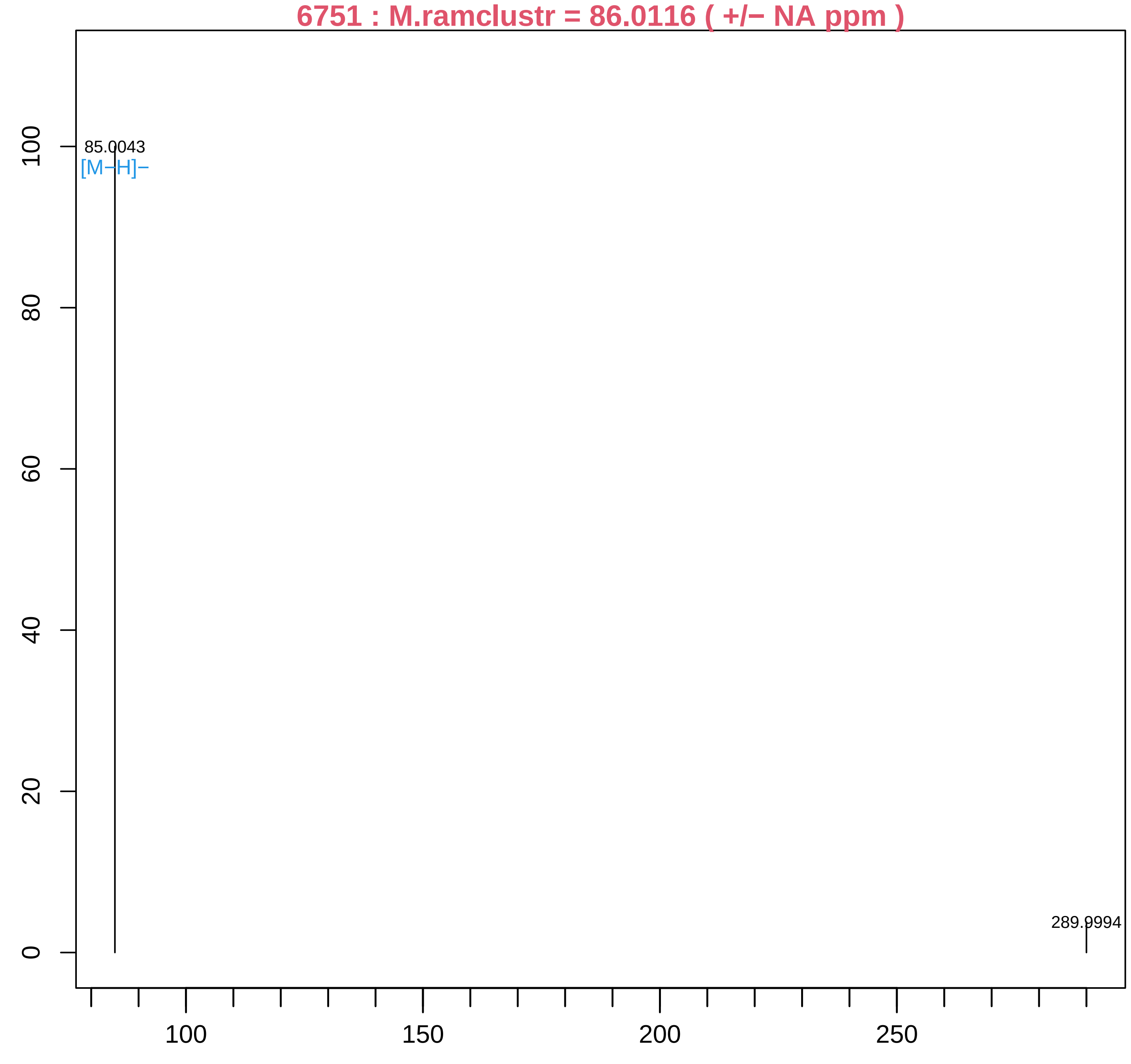
6751 : M.ramclustr = 86.0116 (+/− NA ppm)
85.0043
[M−H]−
289.9994
0
20
40
60
80
100
100
150
200
250

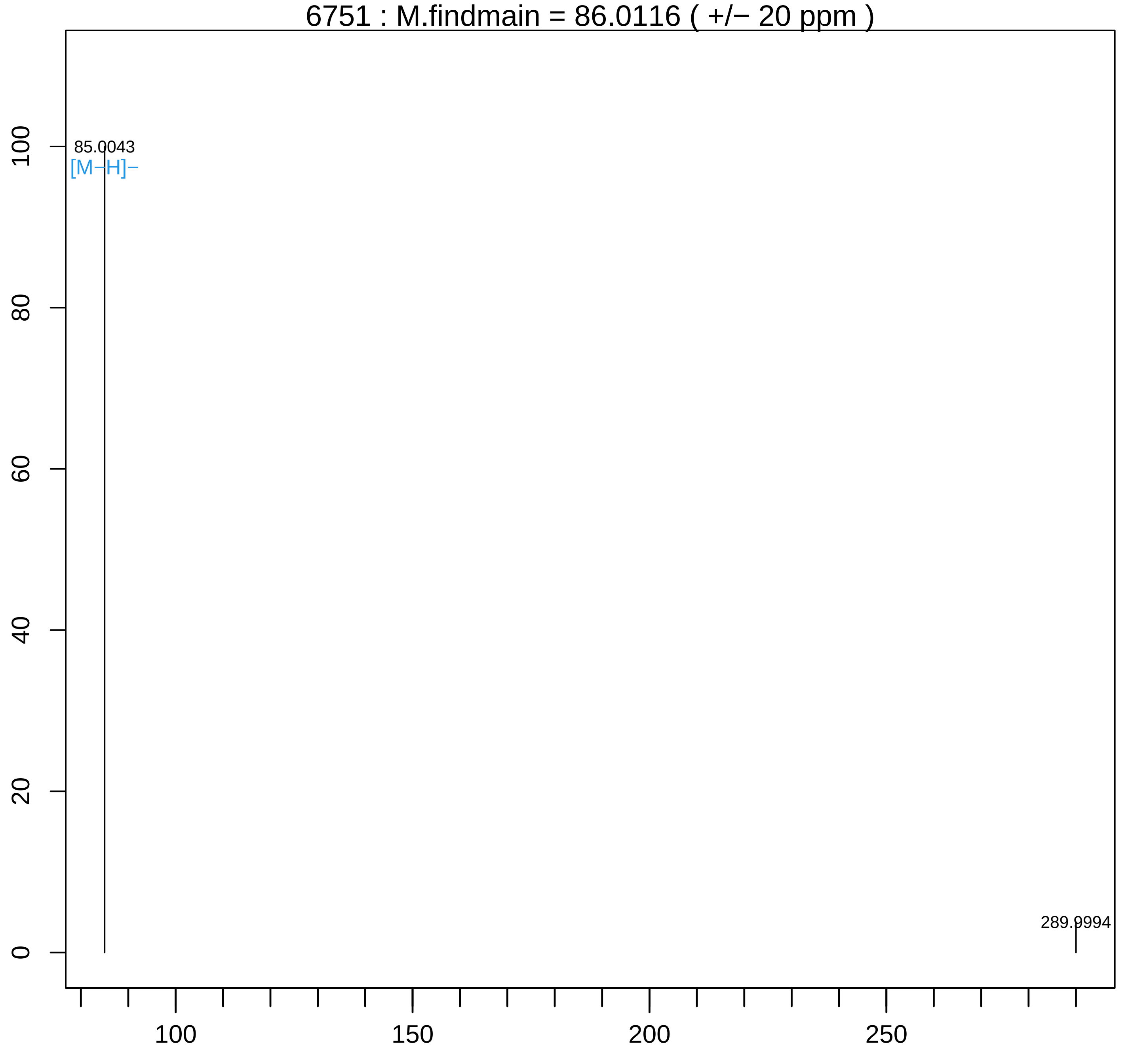
6751 : M.findmain = 86.0116 (+/− 20 ppm)
85.0043
[M−H]−
289.9994
0
20
40
60
80
100
100
150
200
250

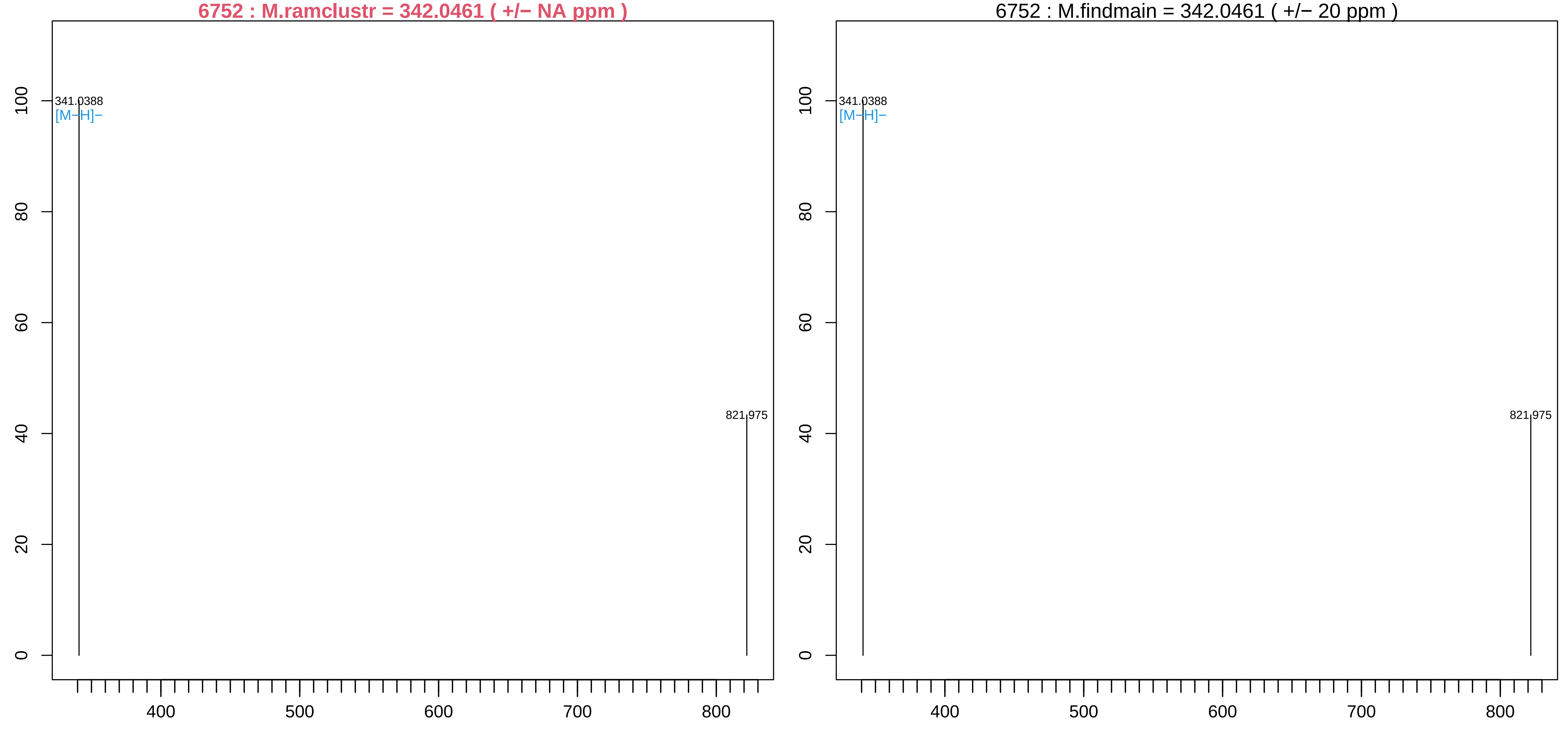
6752 : M.ramclustr = 342.0461 (+/− NA ppm)
341.0388
[M−H]−
821.975
6752 : M.findmain = 342.0461 (+/− 20 ppm)
341.0388
[M−H]−
821.975
0
20
40
60
80
100
400
500
600
700
800

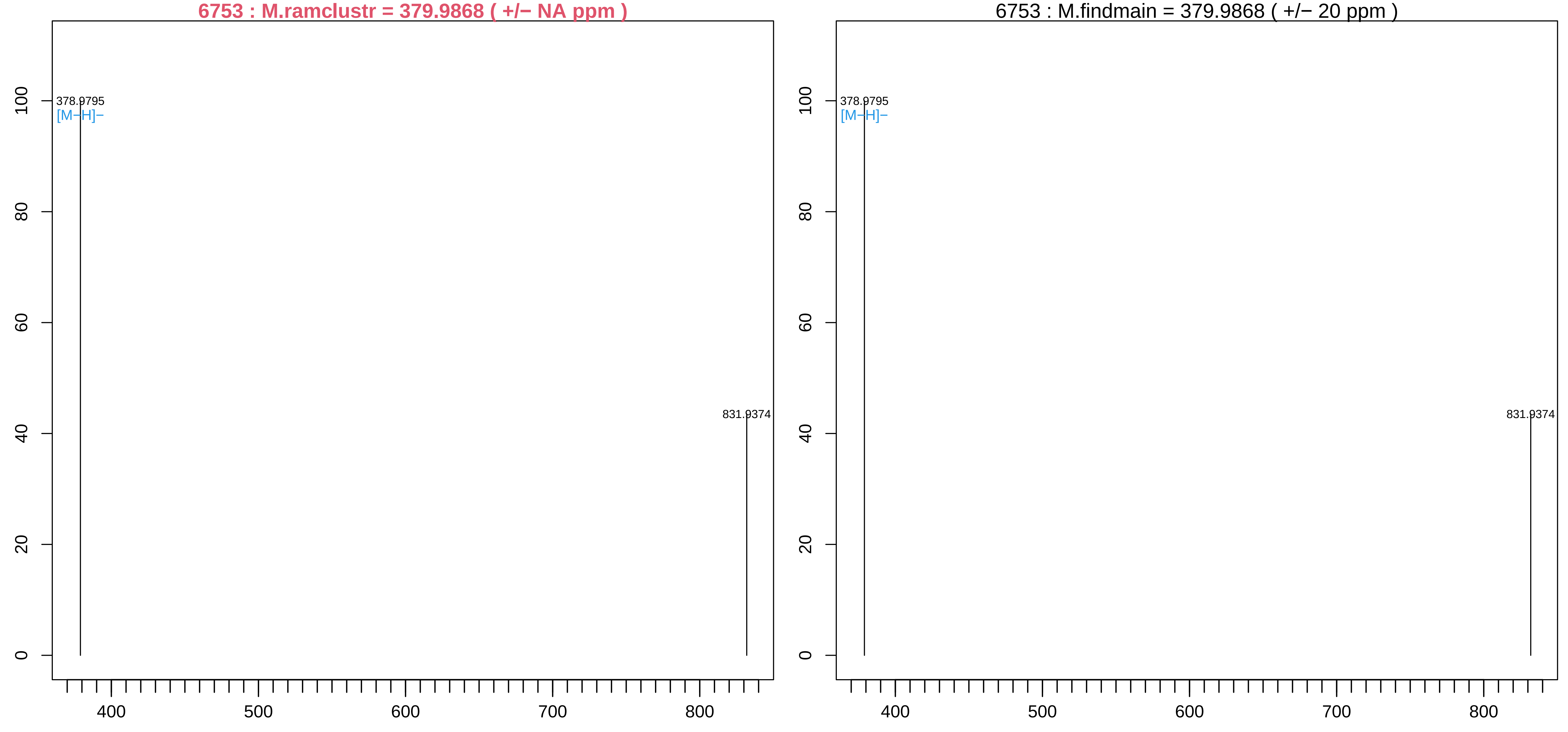

6753 : M.ramclustr = 379.9868 (+/− NA ppm)
378.9795
[M−H]−
831.9374
6753 : M.findmain = 379.9868 (+/− 20 ppm)
378.9795
[M−H]−
831.9374
0
20
40
60
80
100
400
500
600
700
800

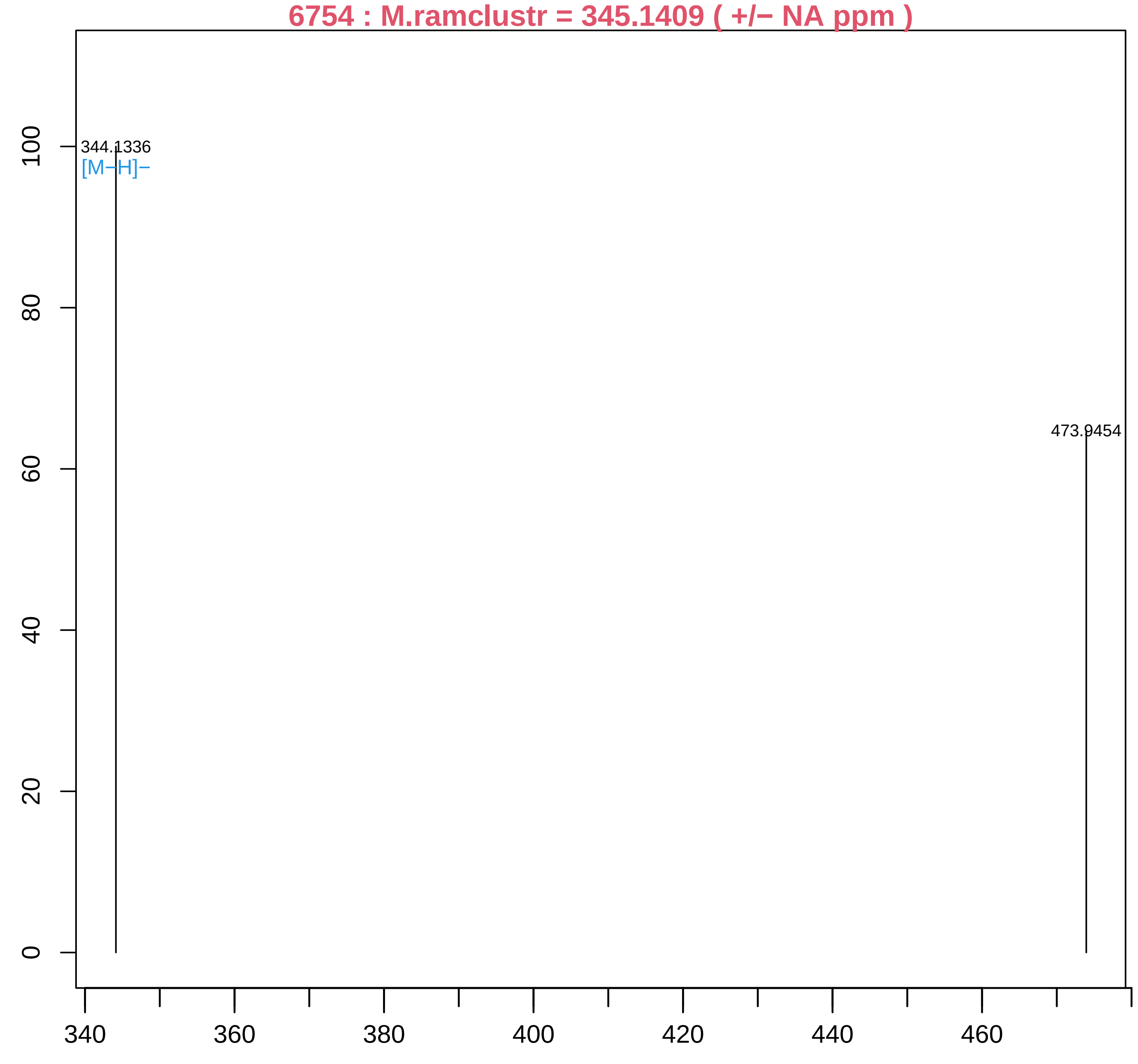
6754 : M.ramclustr = 345.1409 (+/− NA ppm)
344.1336
[M−H]−
473.9454
0
20
40
60
80
100
340
360
380
400
420
440
460

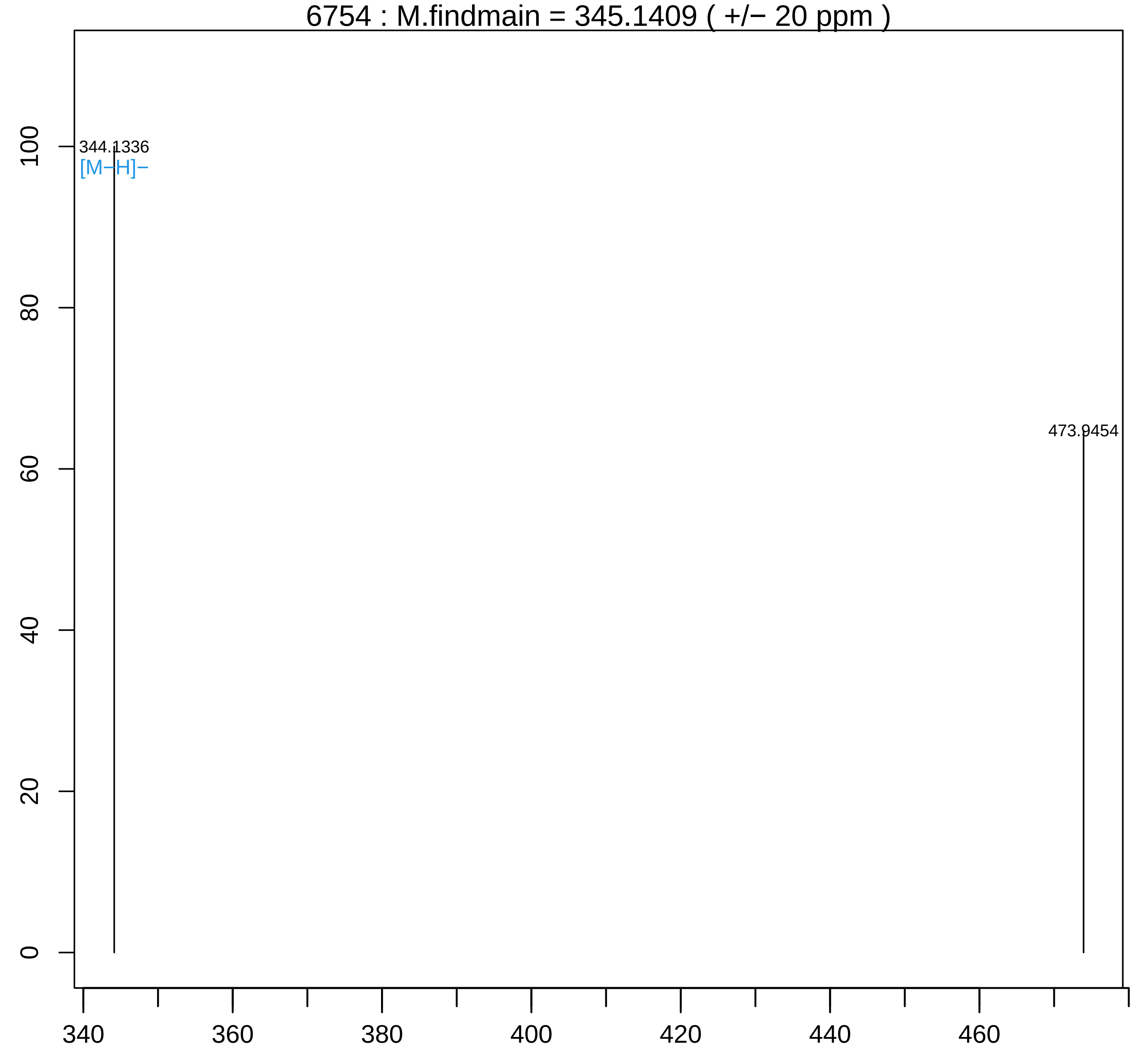
6754 : M.findmain = 345.1409 (+/− 20 ppm)
344.1336
[M−H]−
473.9454
0
20
40
60
80
100
340
360
380
400
420
440
460

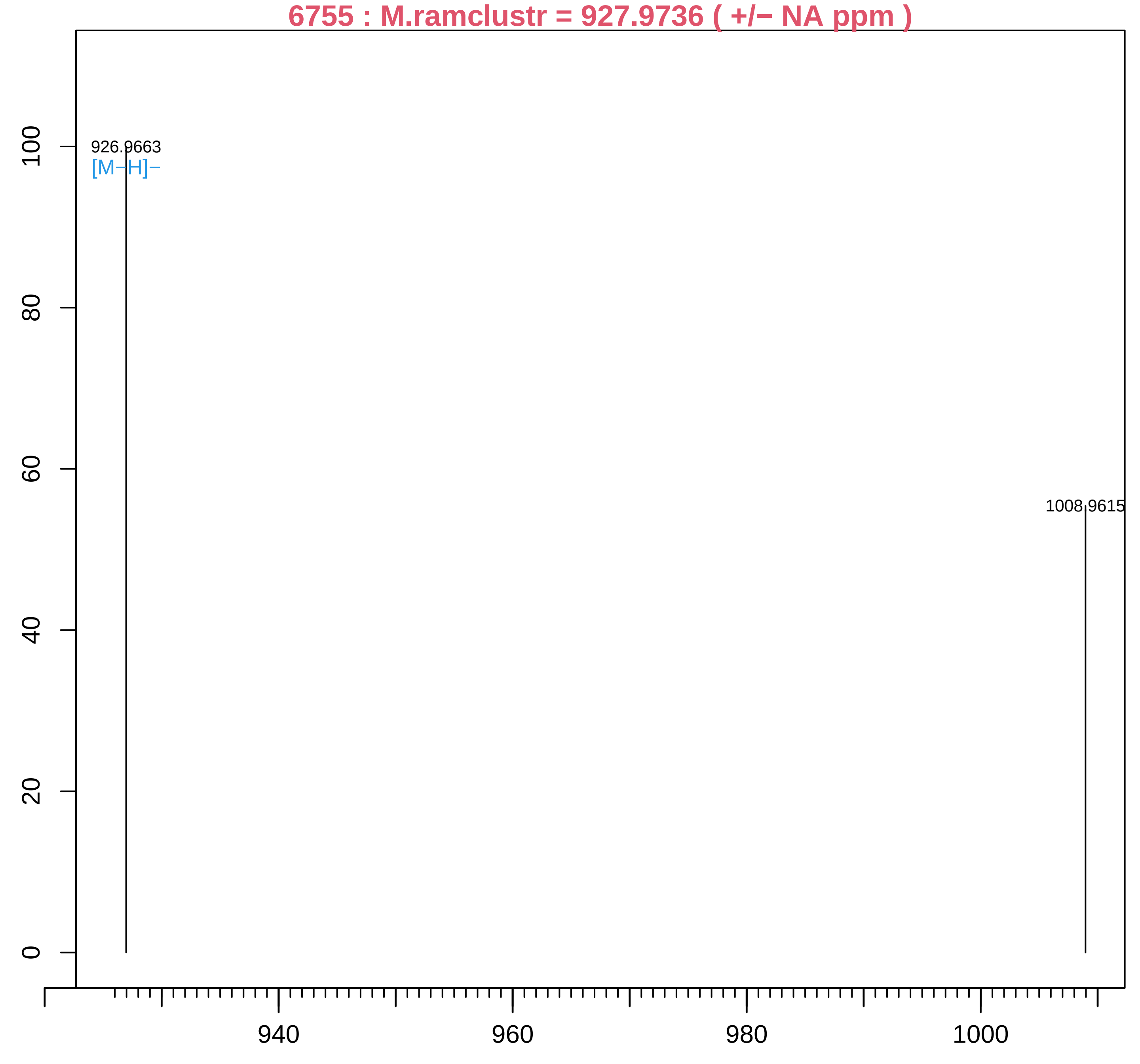
6755 : M.ramclustr = 927.9736 (+/− NA ppm)
926.9663
[M−H]−
1008.9615
0
20
40
60
80
100
940
960
980
1000

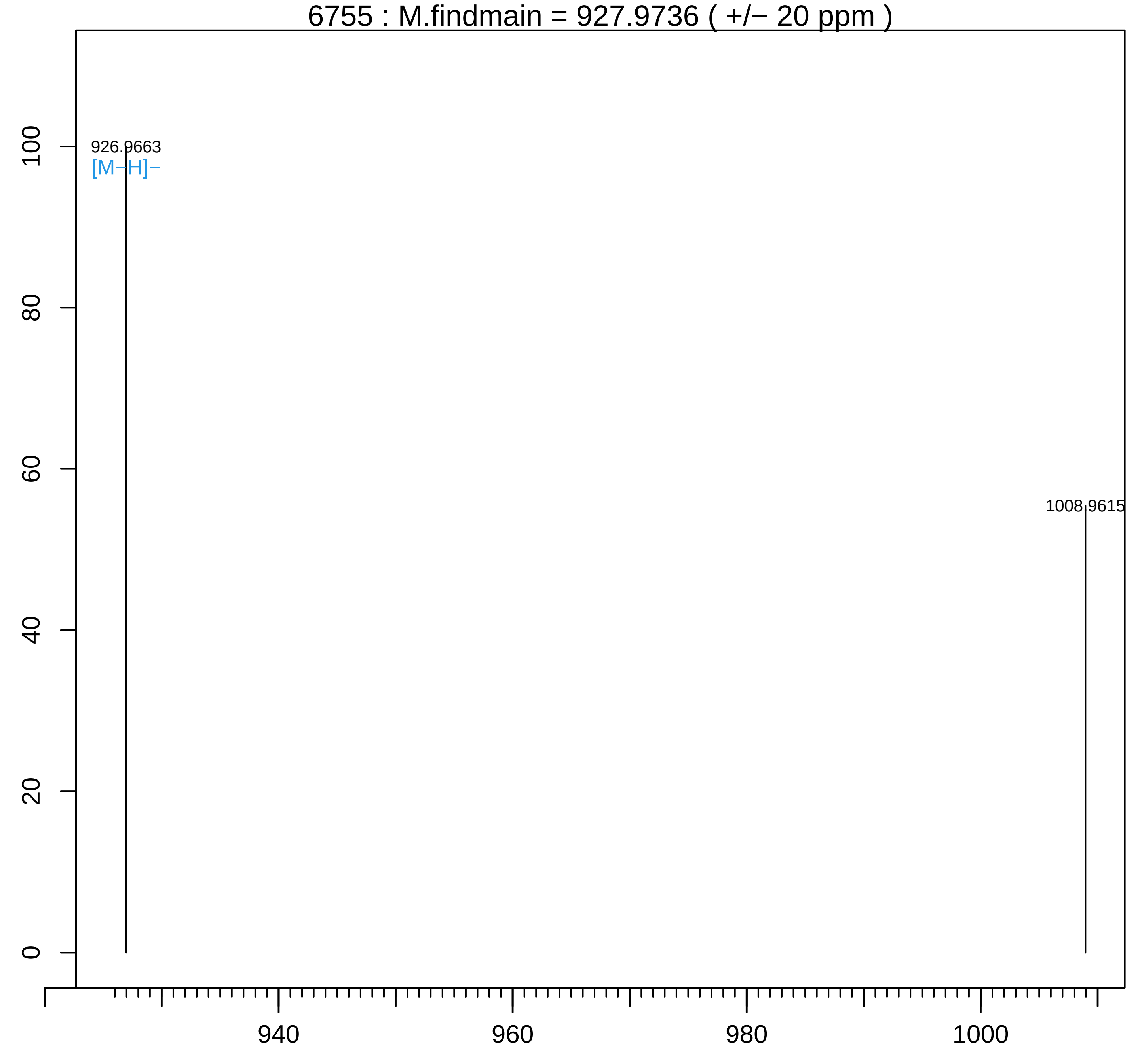
6755 : M.findmain = 927.9736 (+/− 20 ppm)
926.9663
[M−H]−
1008.9615
0
20
40
60
80
100
940
960
980
1000

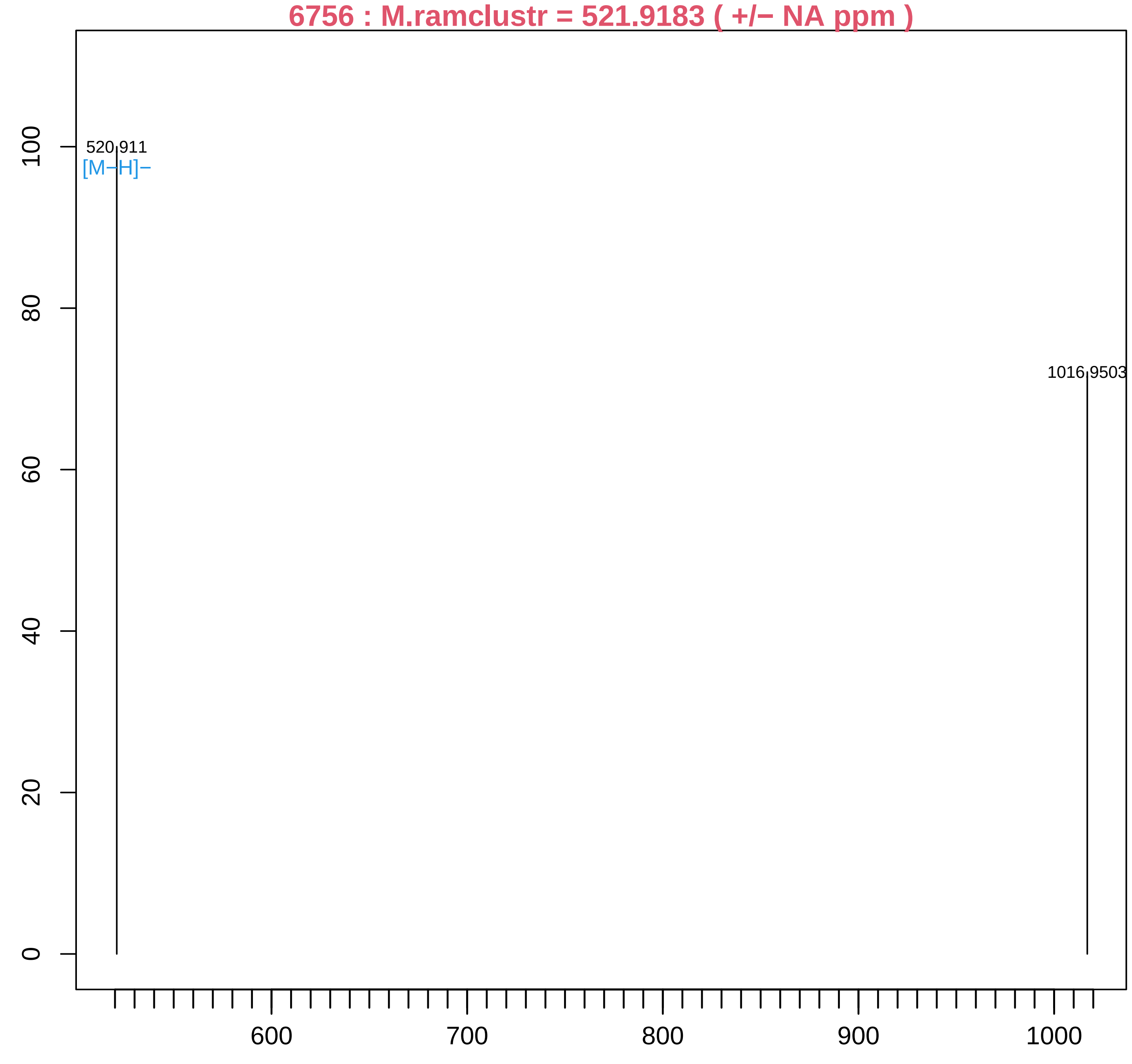
6756 : M.ramclustr = 521.9183 (+/− NA ppm)
520.911
[M−H]−
1016.9503
0
20
40
60
80
100
600
700
800
900
1000

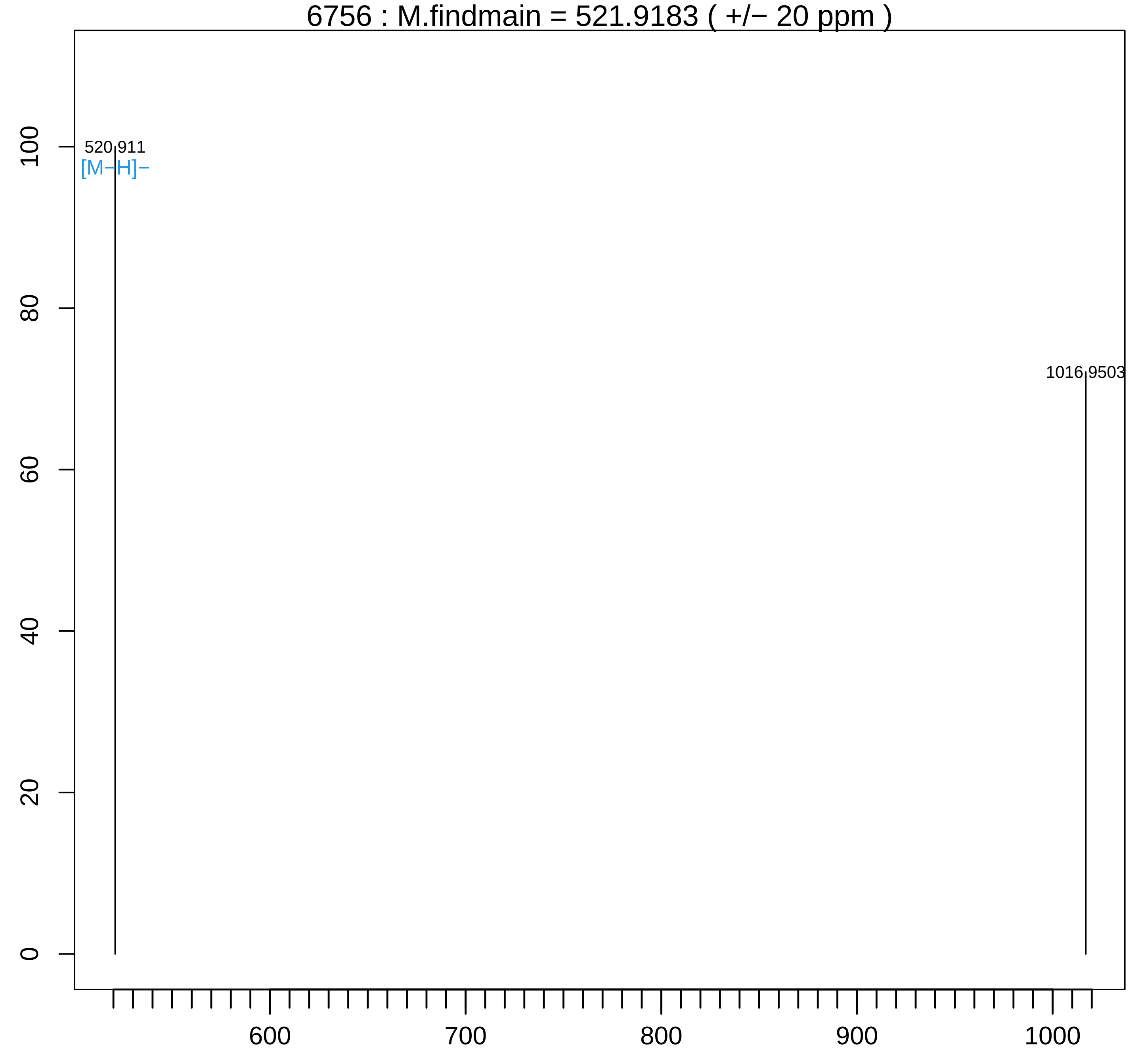
6756 : M.findmain = 521.9183 (+/− 20 ppm)
520.911
[M−H]−
1016.9503
0
20
40
60
80
100
600
700
800
900
1000

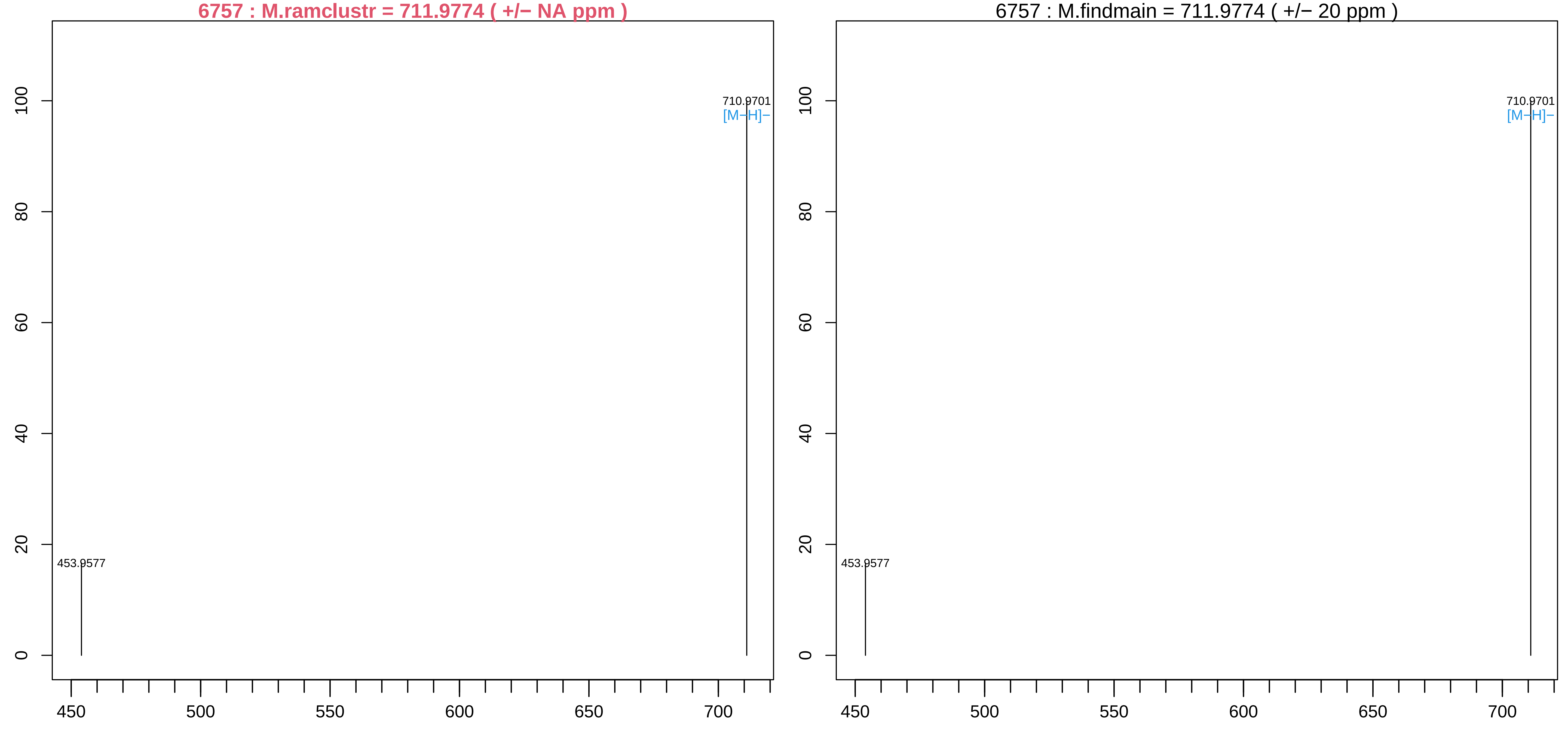

6757 : M.ramclustr = 711.9774 (+/− NA ppm)
710.9701
[M−H]−
453.9577
6757 : M.findmain = 711.9774 (+/− 20 ppm)
710.9701
[M−H]−
453.9577
0
20
40
60
80
100
450
500
550
600
650
700

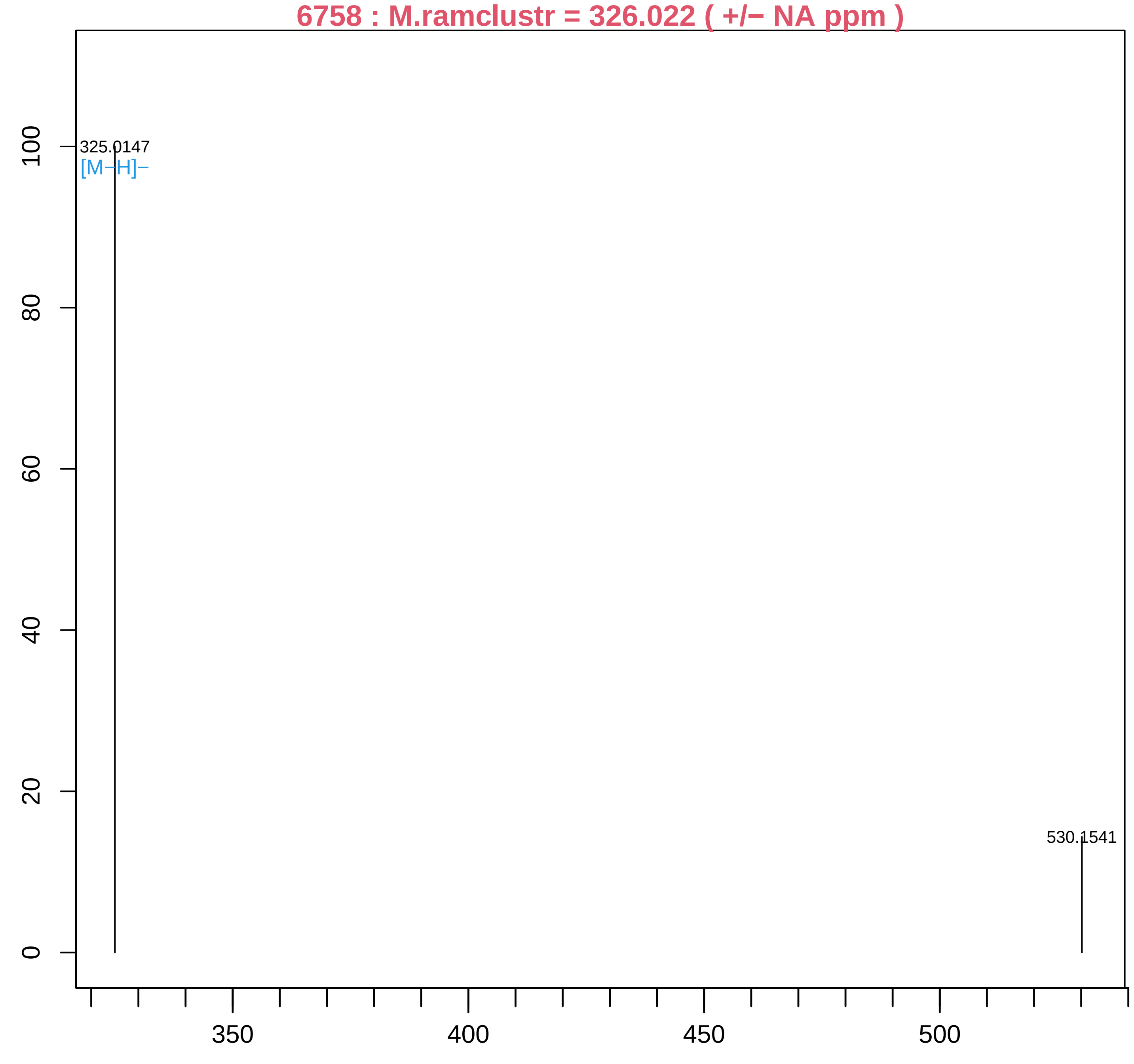

6758 : M.ramclustr = 326.022 (+/− NA ppm)
325.0147
[M−H]−
530.1541
0
20
40
60
80
100
350
400
450
500

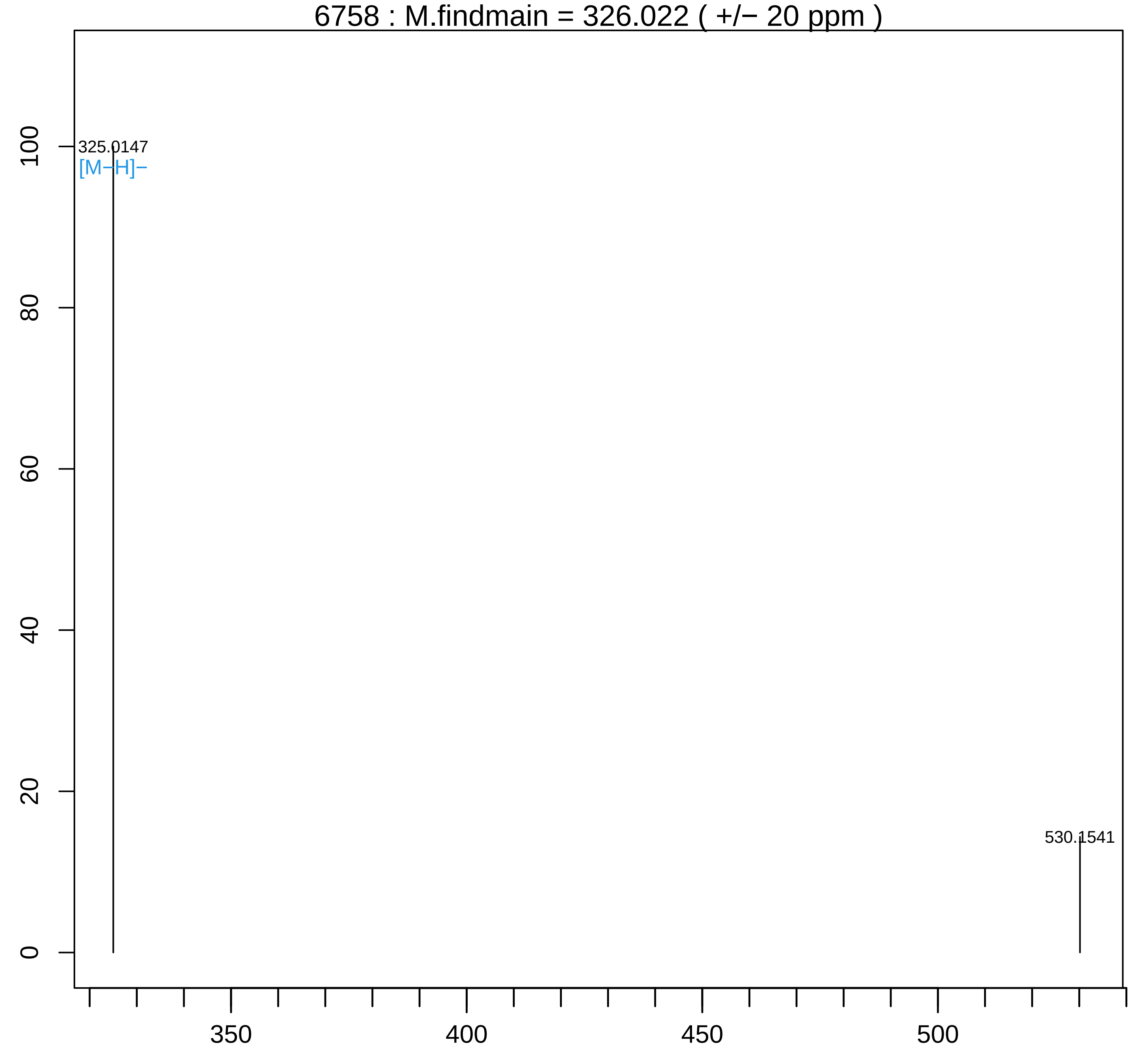

6758 : M.findmain = 326.022 (+/− 20 ppm)
325.0147
[M−H]−
530.1541
0
20
40
60
80
100
350
400
450
500

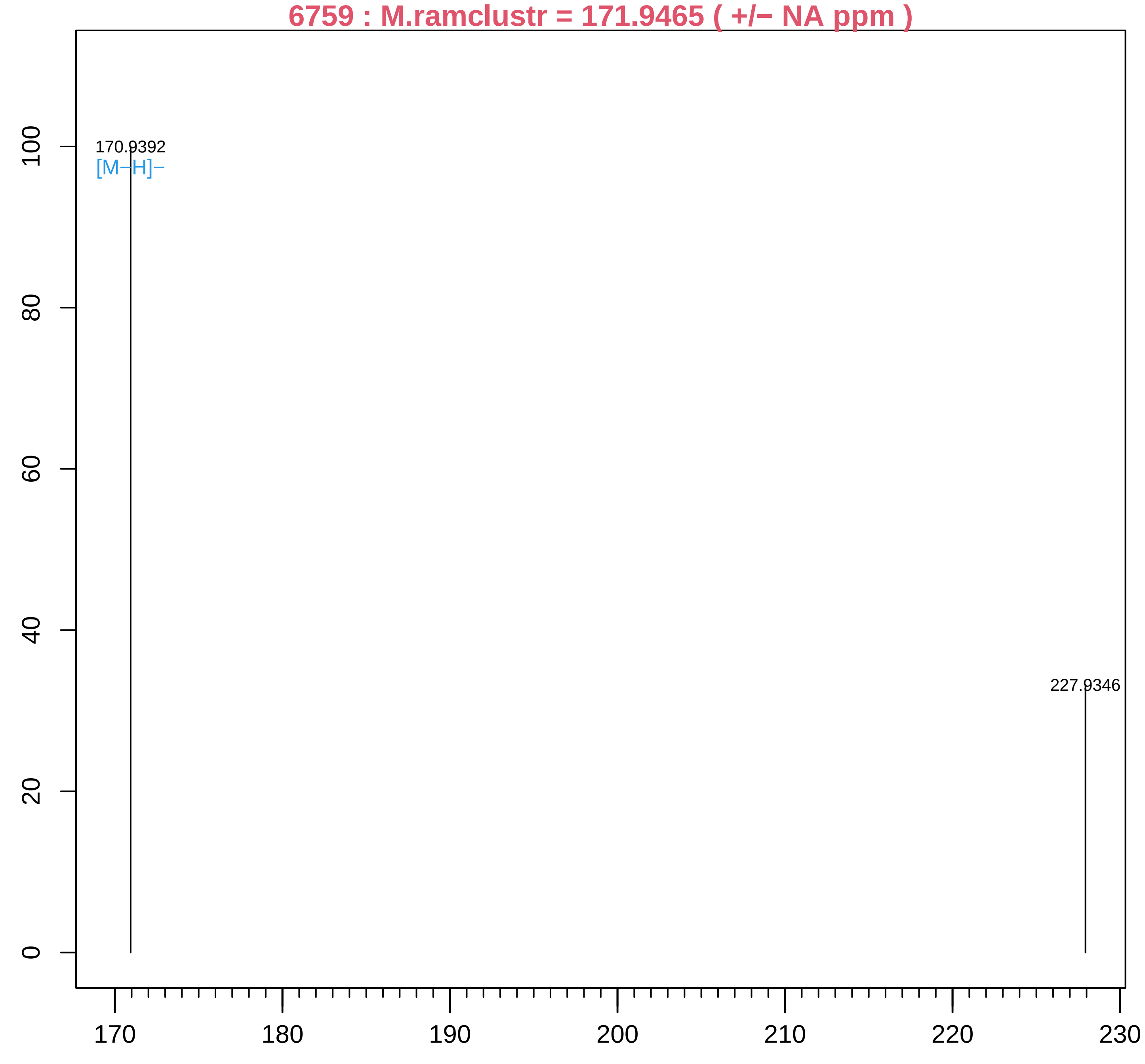
6759 : M.ramclustr = 171.9465 (+/− NA ppm)
170.9392
[M−H]−
227.9346
0
20
40
60
80
100
170
180
190
200
210
220
230

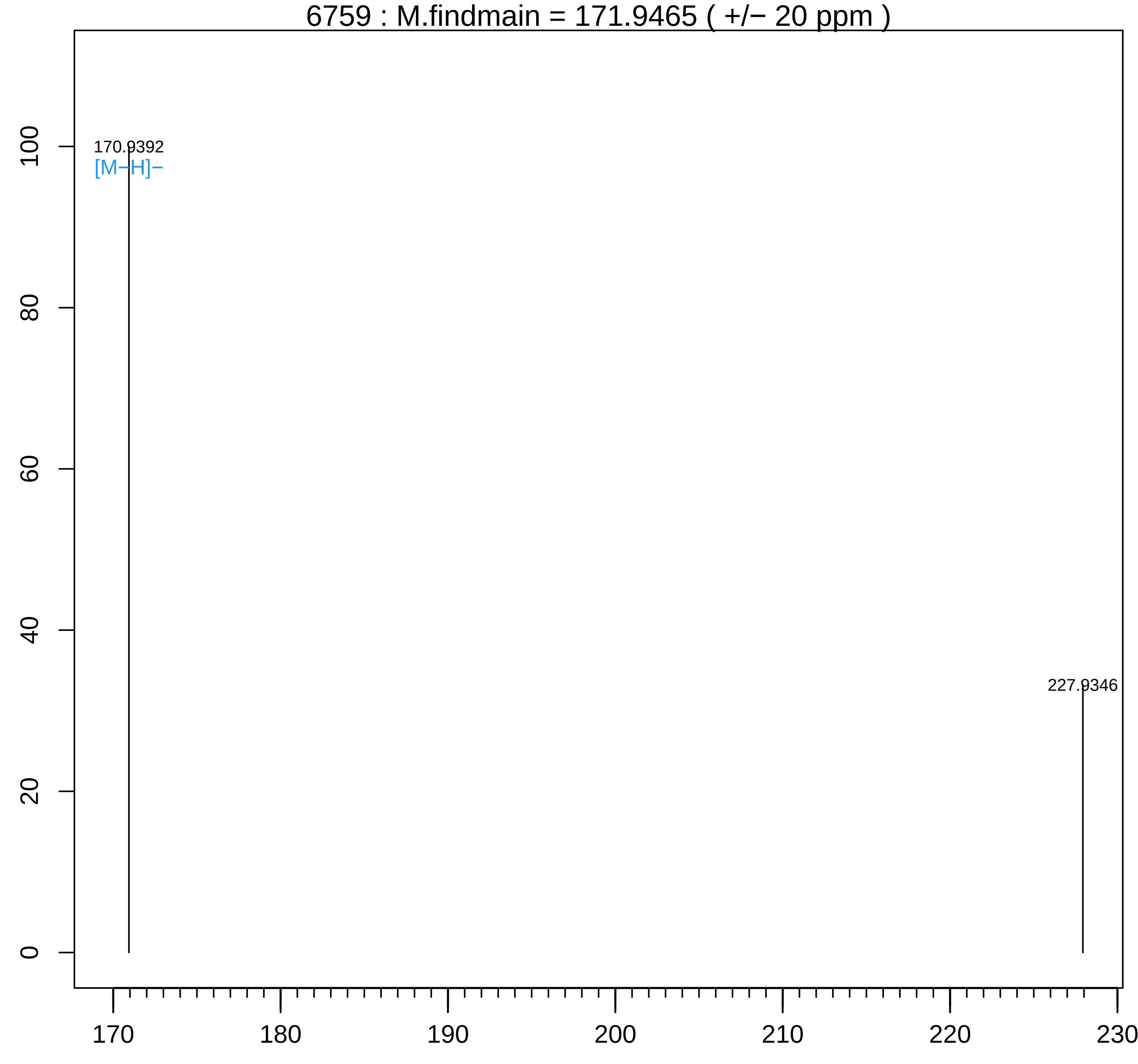
6759 : M.findmain = 171.9465 (+/− 20 ppm)
170.9392
[M−H]−
227.9346
0
20
40
60
80
100
170
180
190
200
210
220
230

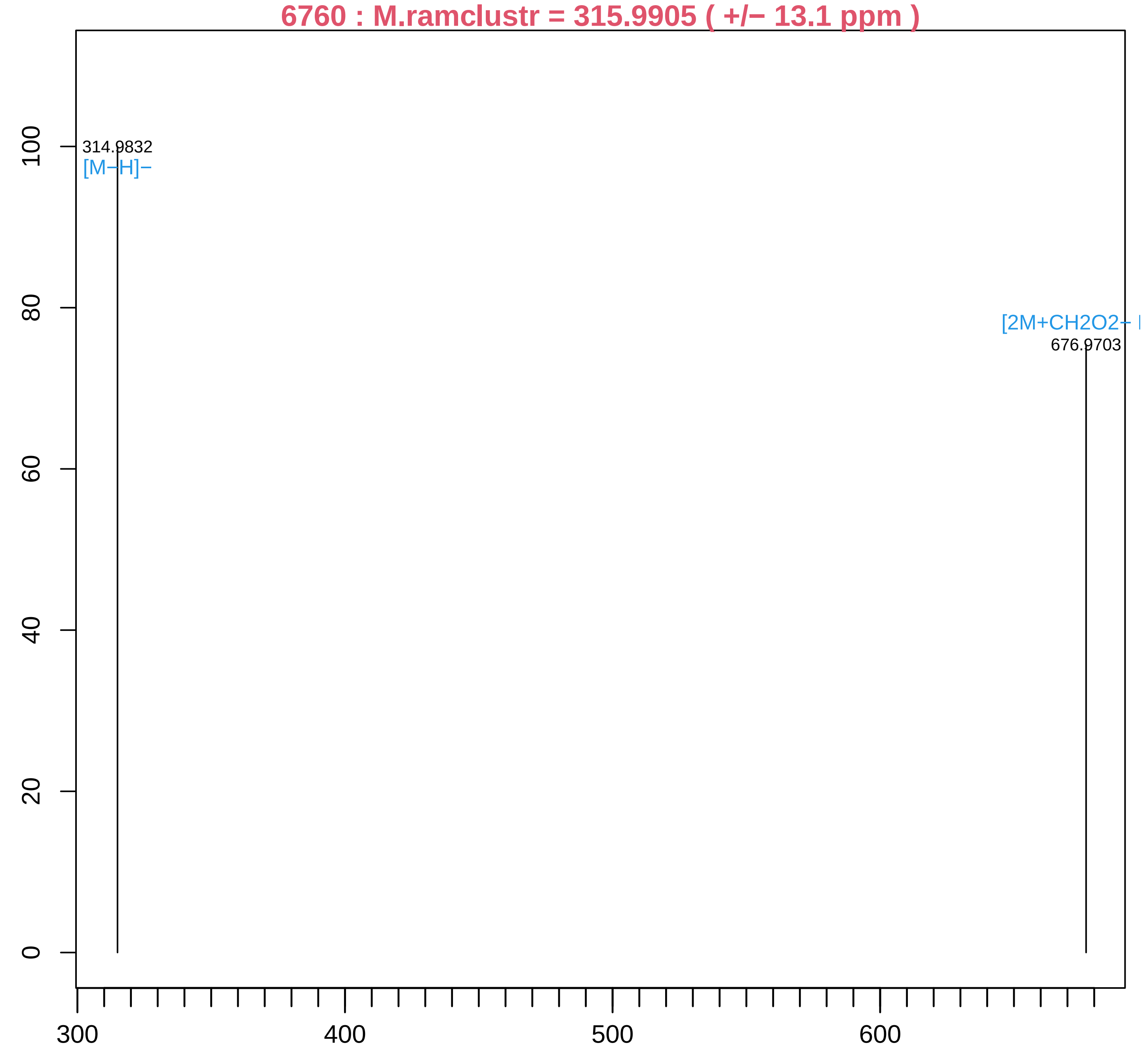
6760 : M.ramclustr = 315.9905 (+/− 13.1 ppm)
314.9832
[M−H]−
[2M+CH2O2−
676.9703
0
20
40
60
80
100
300
400
500
600

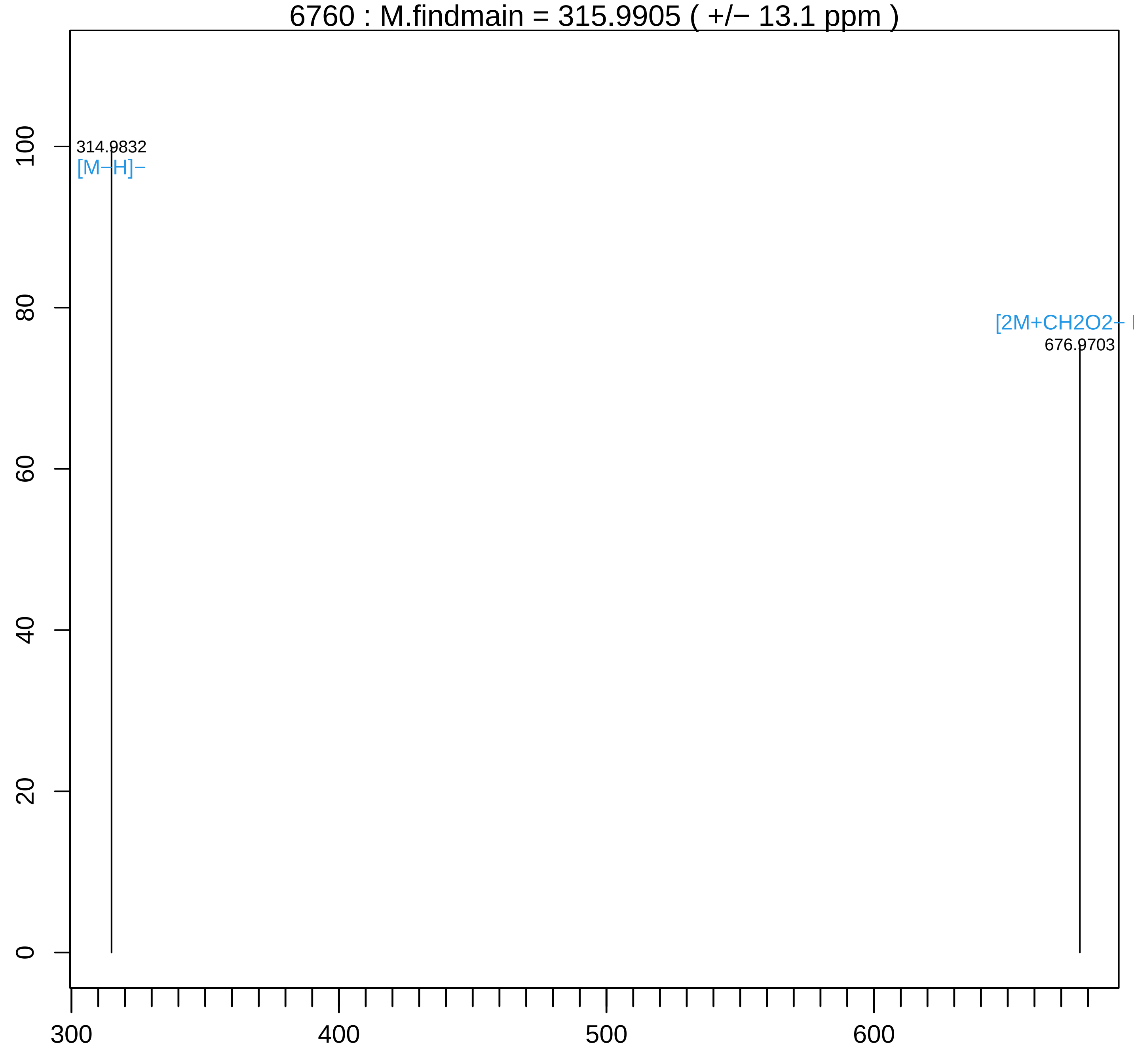
6760 : M.findmain = 315.9905 (+/− 13.1 ppm)
314.9832
[M−H]−
[2M+CH2O2−
676.9703
0
20
40
60
80
100
300
400
500
600

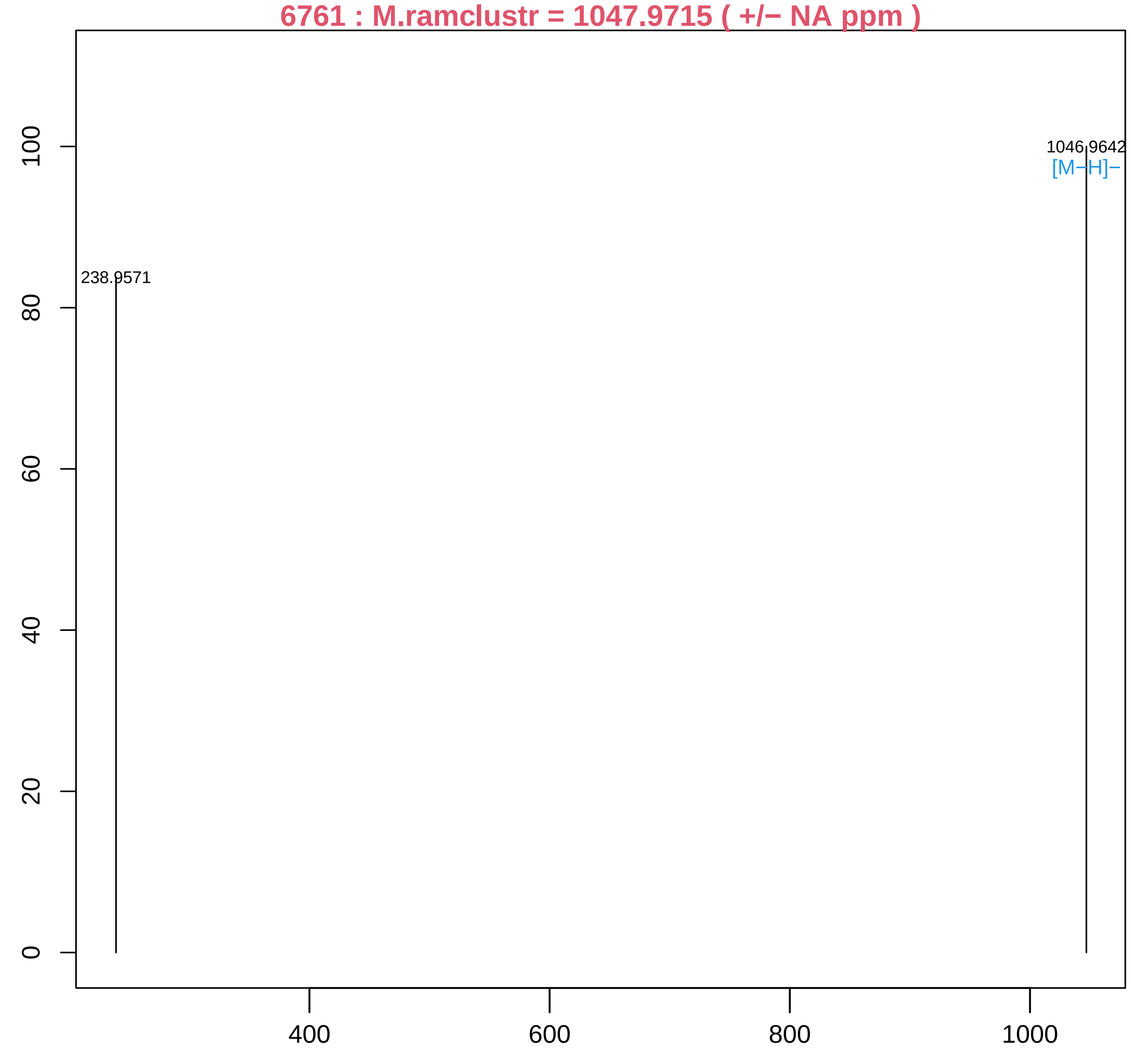
6761 : M.ramclustr = 1047.9715 (+/− NA ppm)
238.9571
1046.9642
[M−H]−
0
20
40
60
80
100
400
600
800
1000

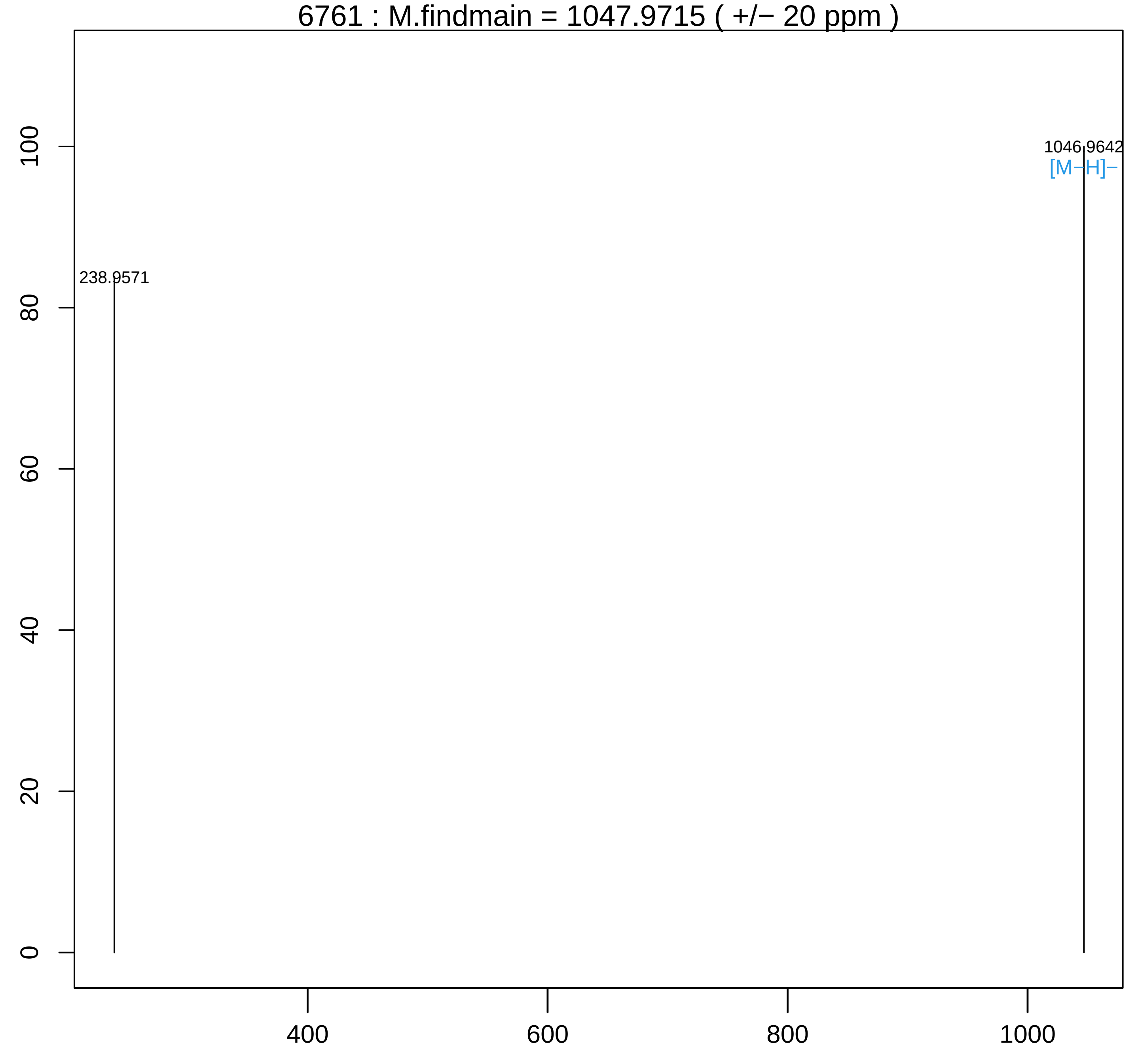
6761 : M.findmain = 1047.9715 (+/− 20 ppm)
238.9571
1046.9642
[M−H]−
0
20
40
60
80
100
400
600
800
1000

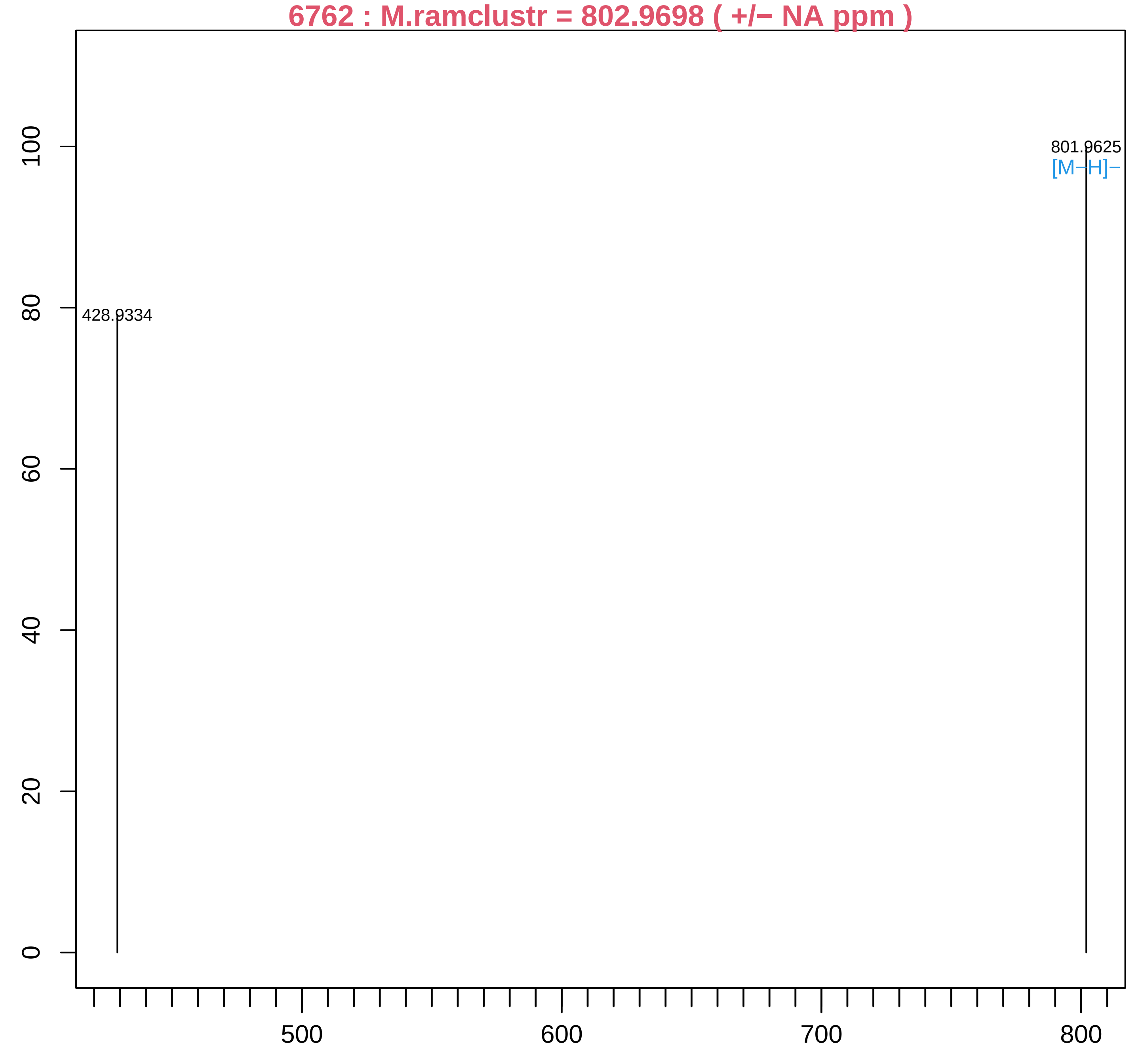
6762 : M.ramclustr = 802.9698 (+/− NA ppm)
428.9334
801.9625
[M−H]−
0
20
40
60
80
100
500
600
700
800

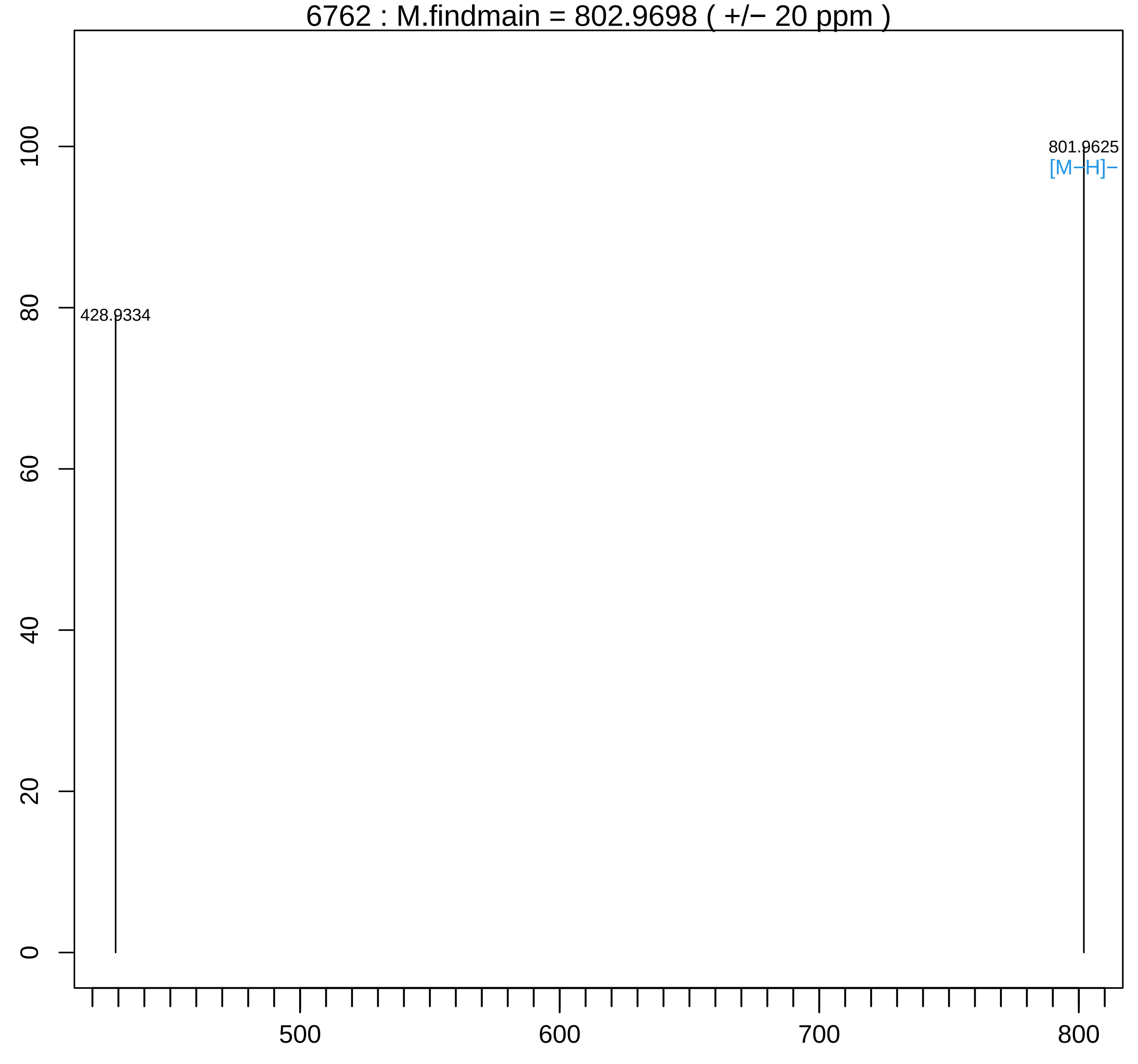
6762 : M.findmain = 802.9698 (+/− 20 ppm)
428.9334
801.9625
[M−H]−
0
20
40
60
80
100
500
600
700
800

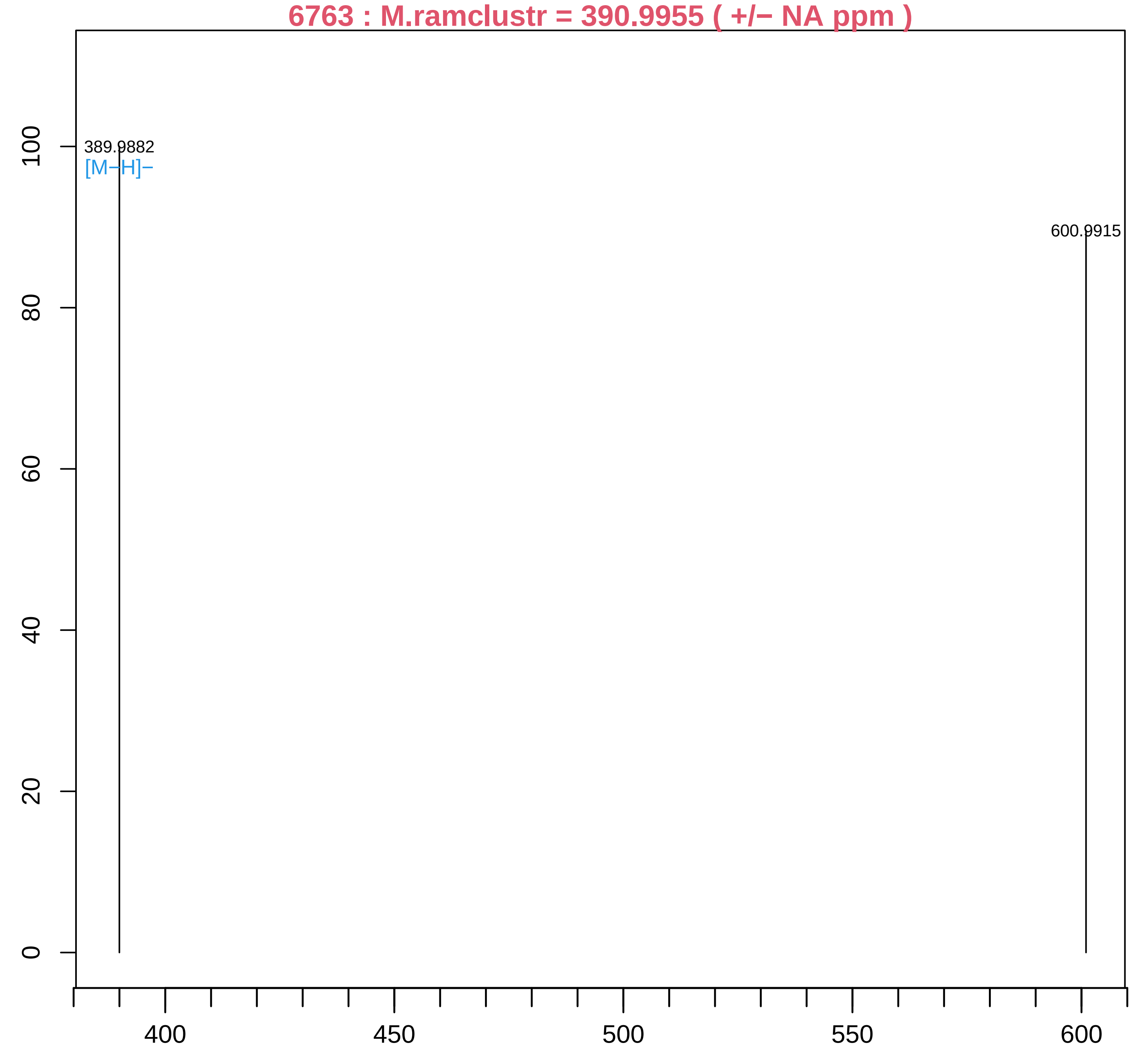

6763 : M.ramclustr = 390.9955 (+/− NA ppm)
389.9882
[M−H]−
600.9915
0
20
40
60
80
100
400
450
500
550
600

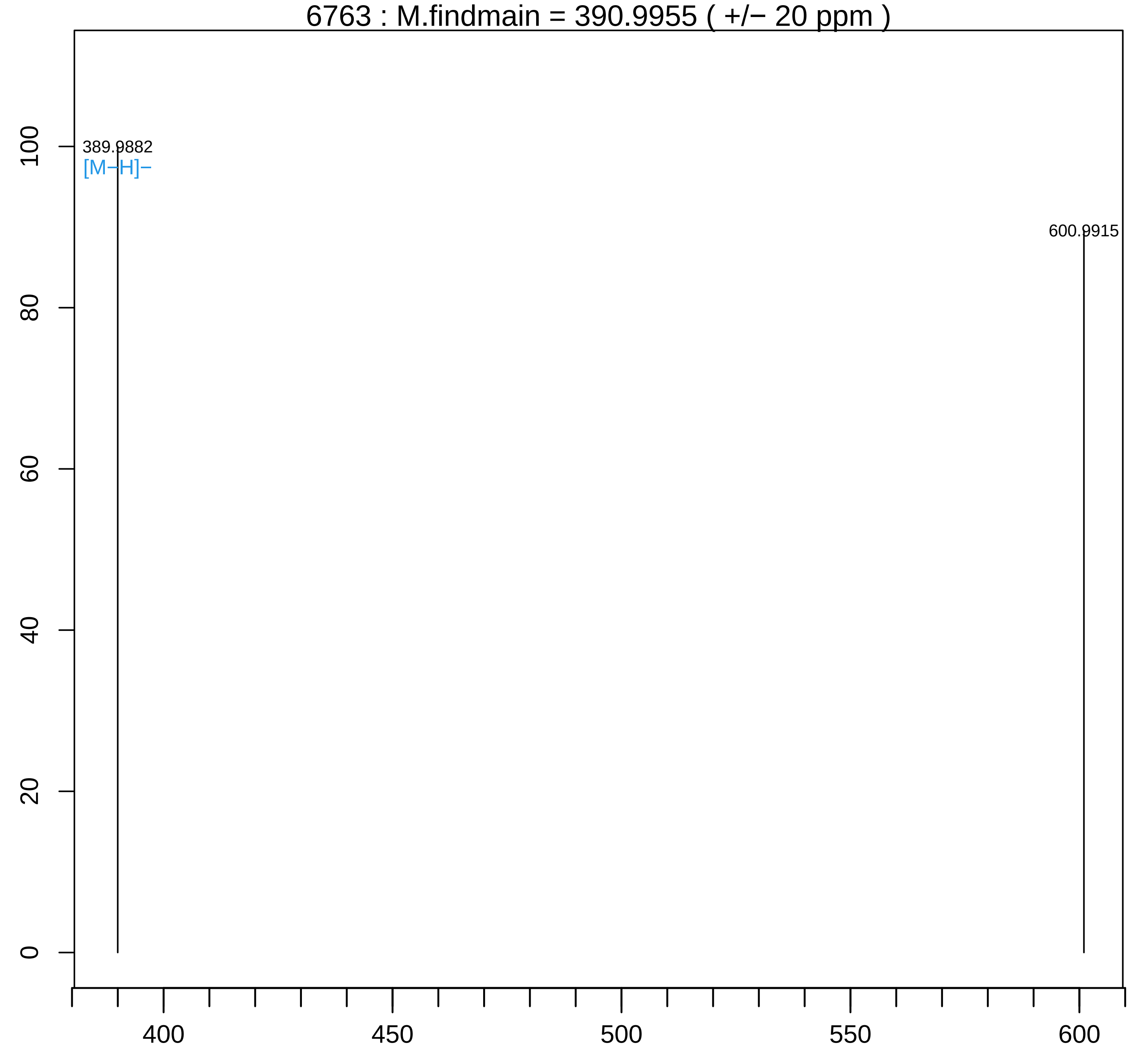

6763 : M.findmain = 390.9955 (+/− 20 ppm)
389.9882
[M−H]−
600.9915
0
20
40
60
80
100
400
450
500
550
600

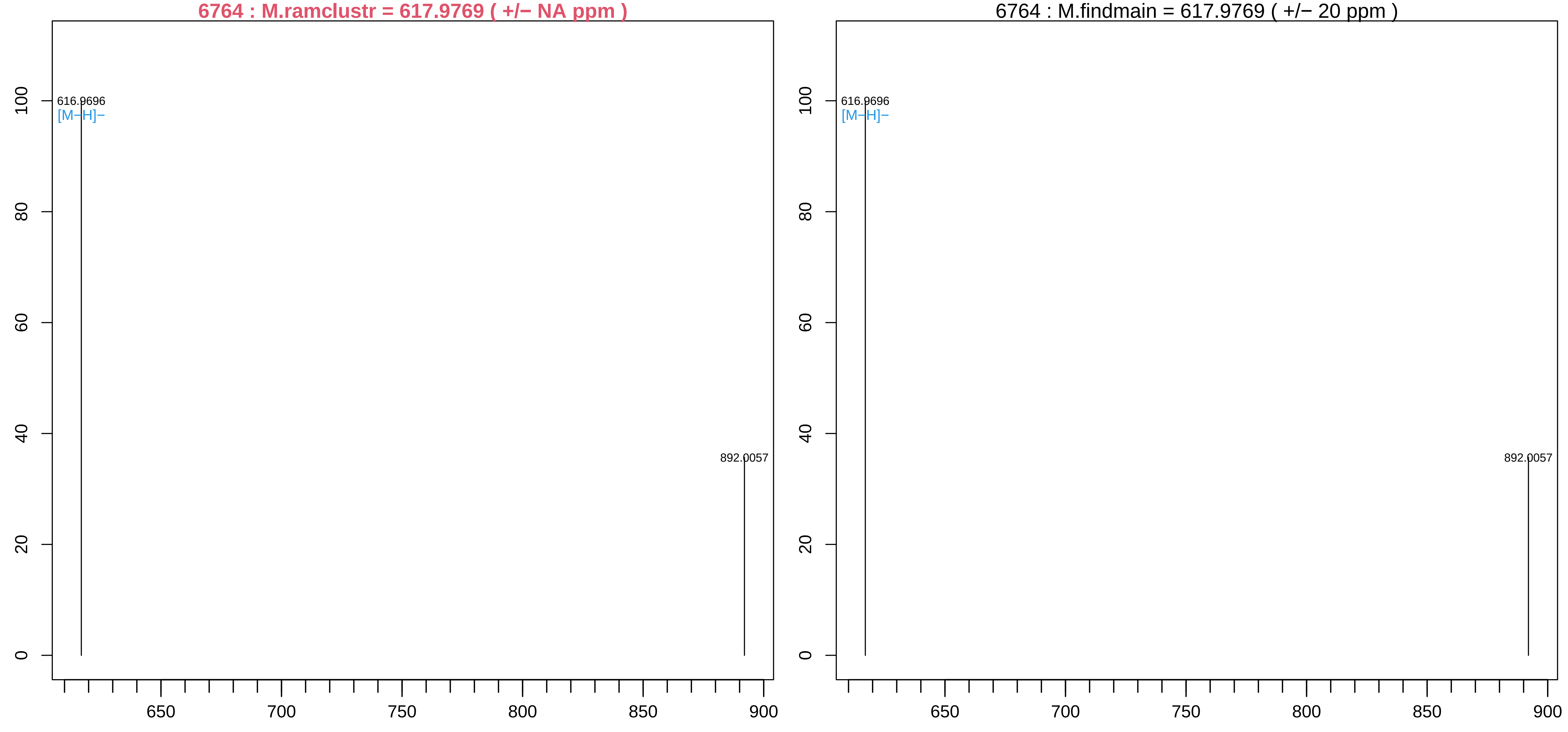
6764 : M.ramclustr = 617.9769 (+/− NA ppm)
6764 : M.findmain = 617.9769 (+/− 20 ppm)
616.9696
[M−H]−
892.0057
0
20
40
60
80
100
650
700
750
800
850
900

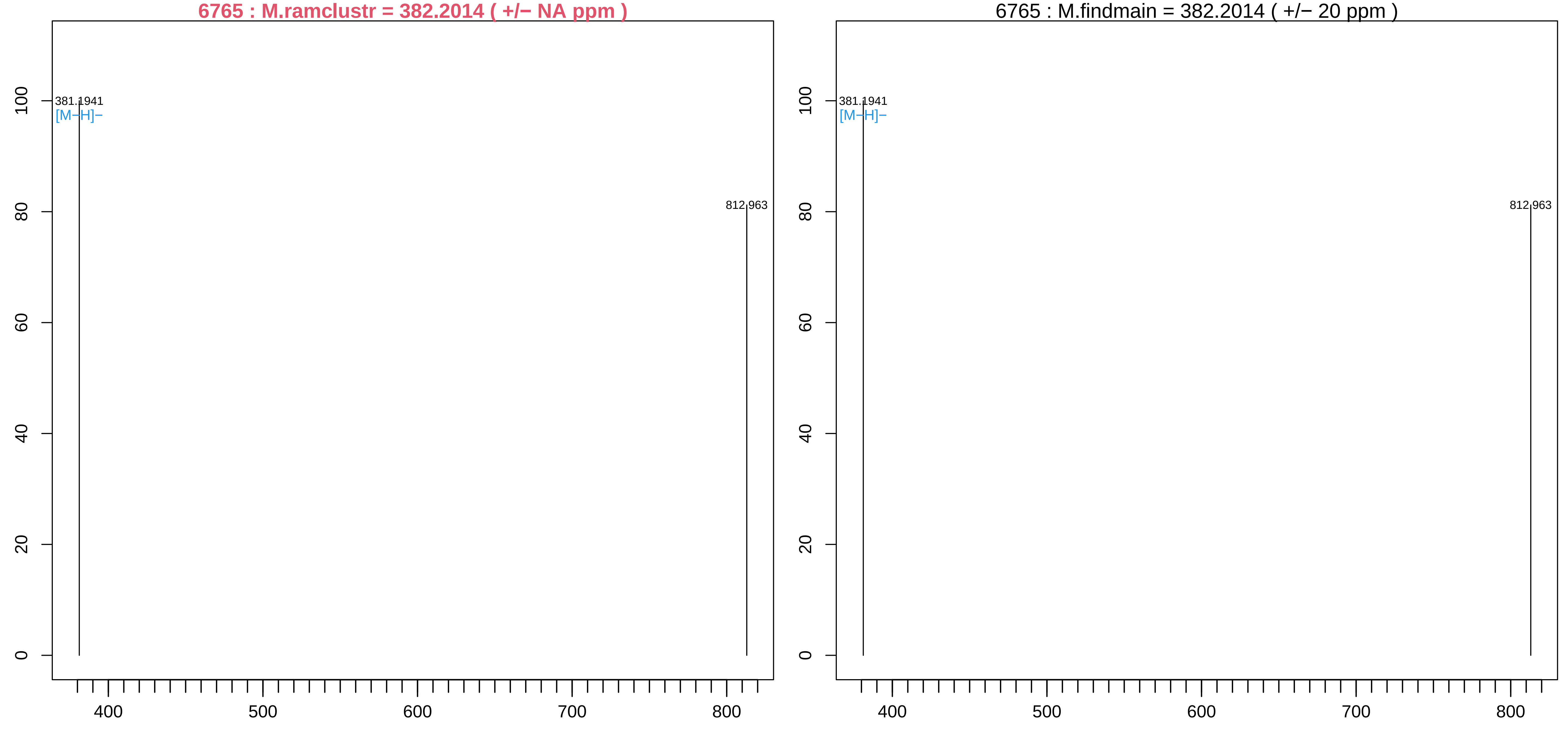
6765 : M.ramclustr = 382.2014 (+/− NA ppm)
381.1941
[M−H]−
812.963
6765 : M.findmain = 382.2014 (+/− 20 ppm)
381.1941
[M−H]−
812.963
0
20
40
60
80
100
400
500
600
700
800

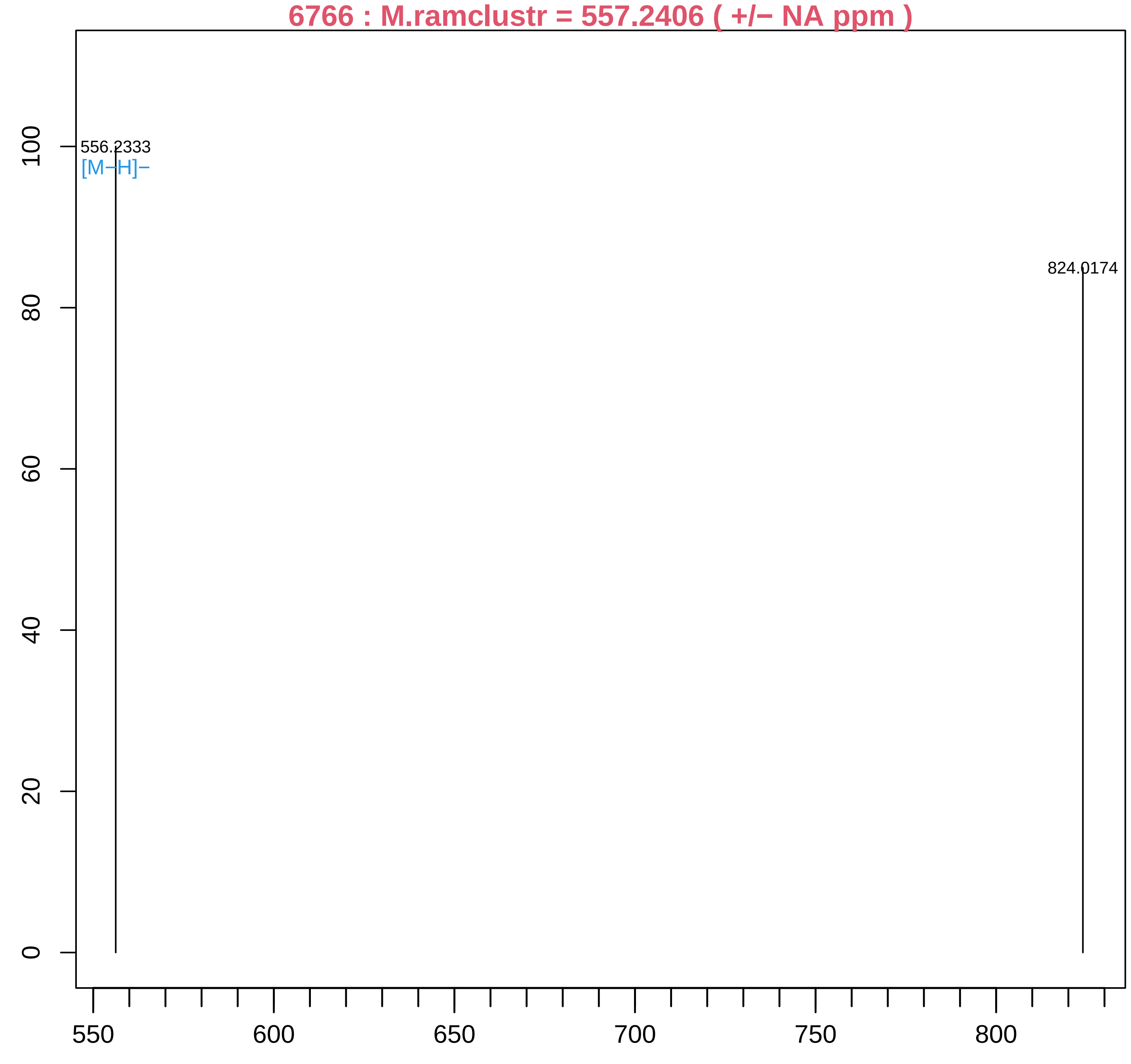
6766 : M.ramclustr = 557.2406 (+/− NA ppm)
556.2333
[M−H]−
824.0174
0
20
40
60
80
100
550
600
650
700
750
800

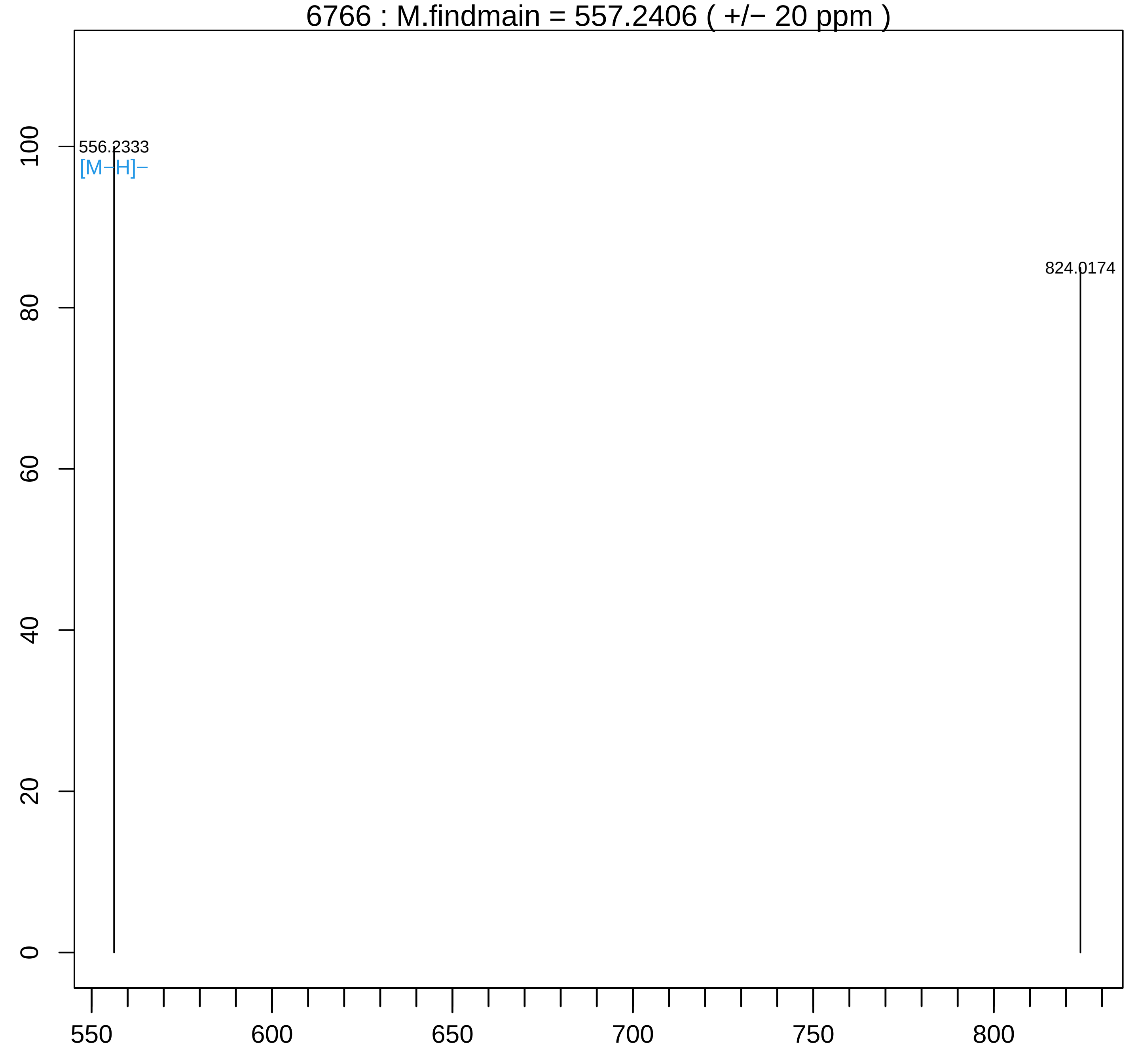
6766 : M.findmain = 557.2406 (+/− 20 ppm)
556.2333
[M−H]−
824.0174
0
20
40
60
80
100
550
600
650
700
750
800

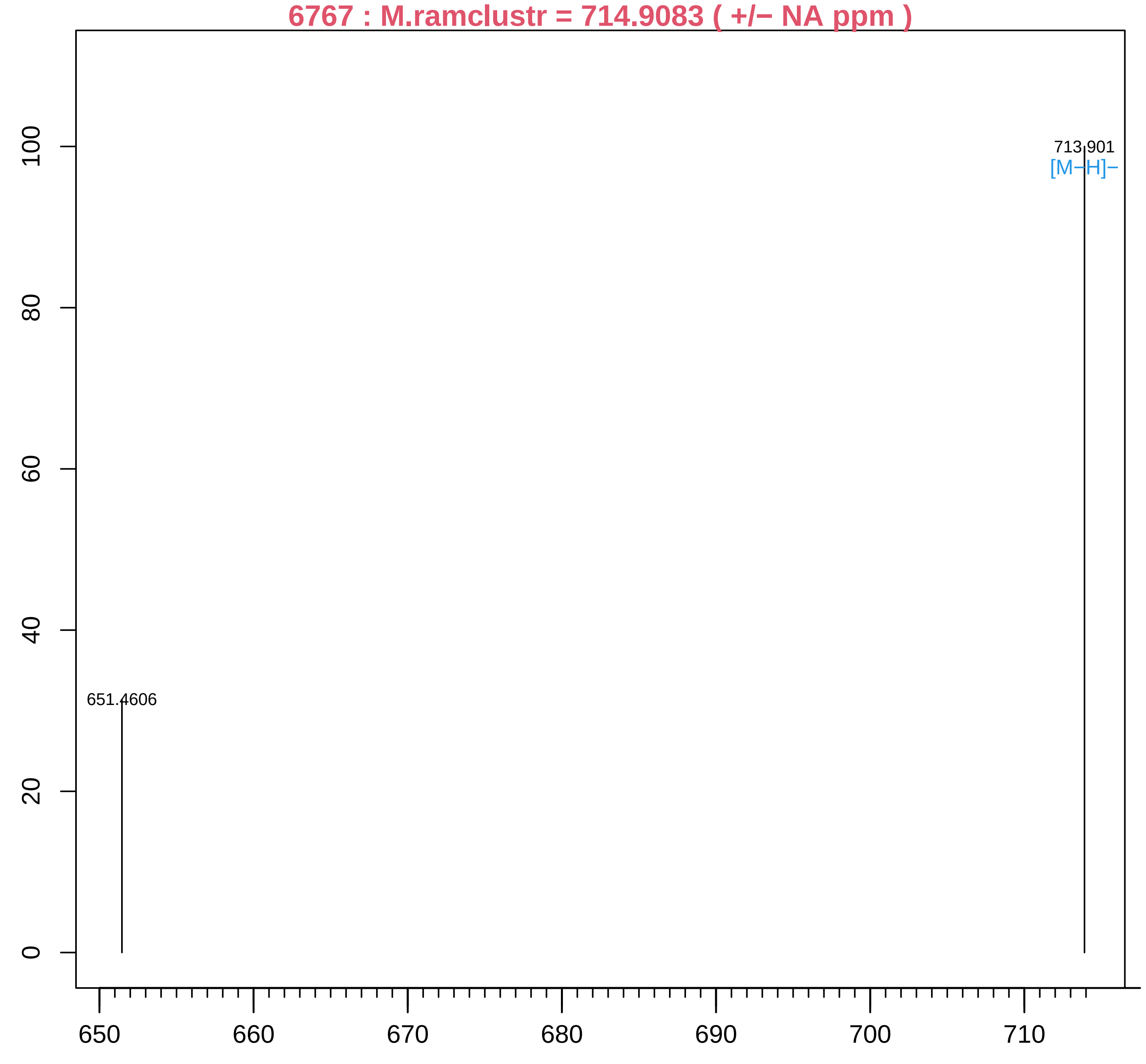
6767 : M.ramclustr = 714.9083 (+/− NA ppm)
713.901
[M−H]−
651.4606
0
20
40
60
80
100
650
660
670
680
690
700
710

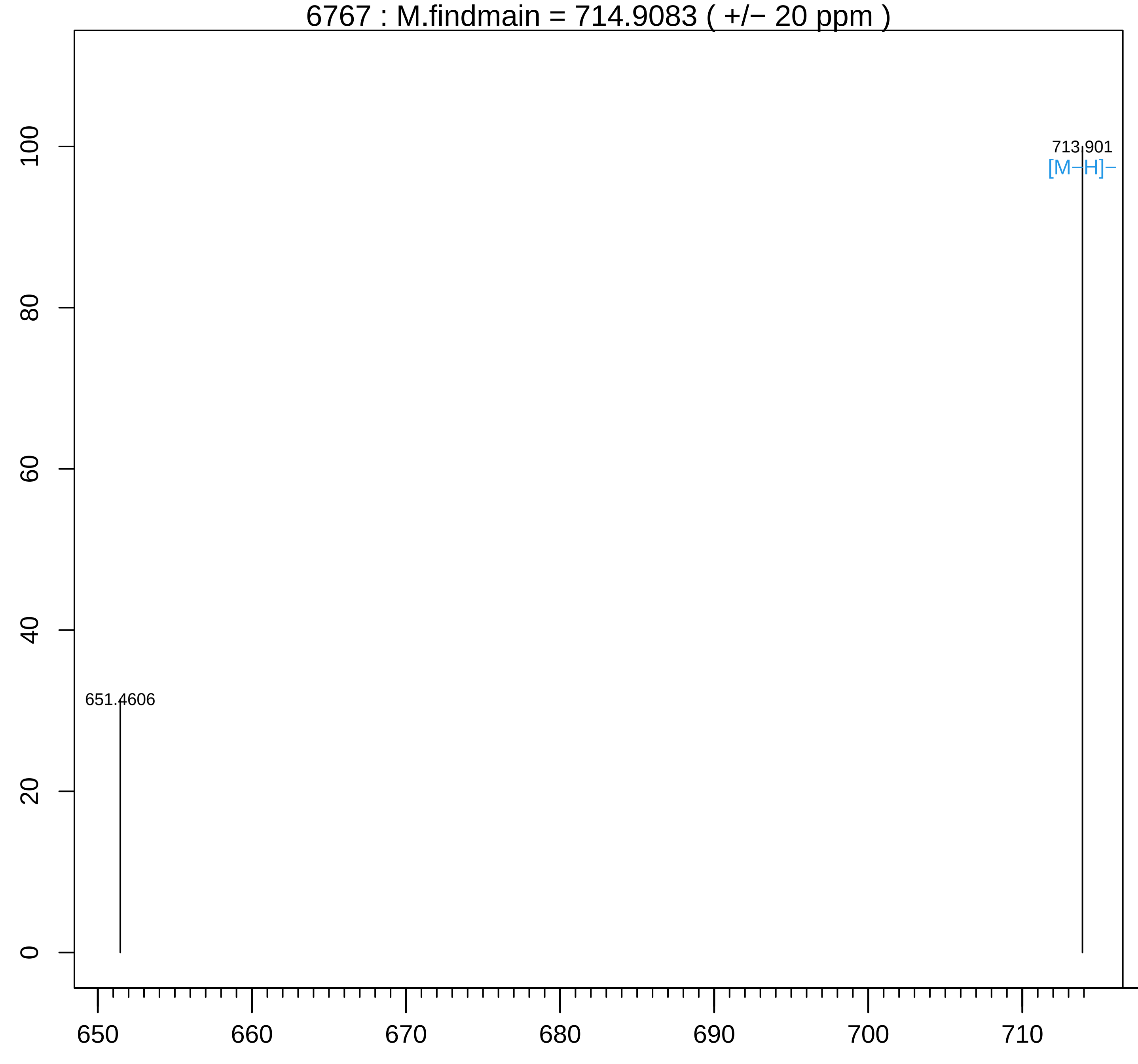
6767 : M.findmain = 714.9083 (+/− 20 ppm)
713.901
[M−H]−
651.4606
0
20
40
60
80
100
650
660
670
680
690
700
710

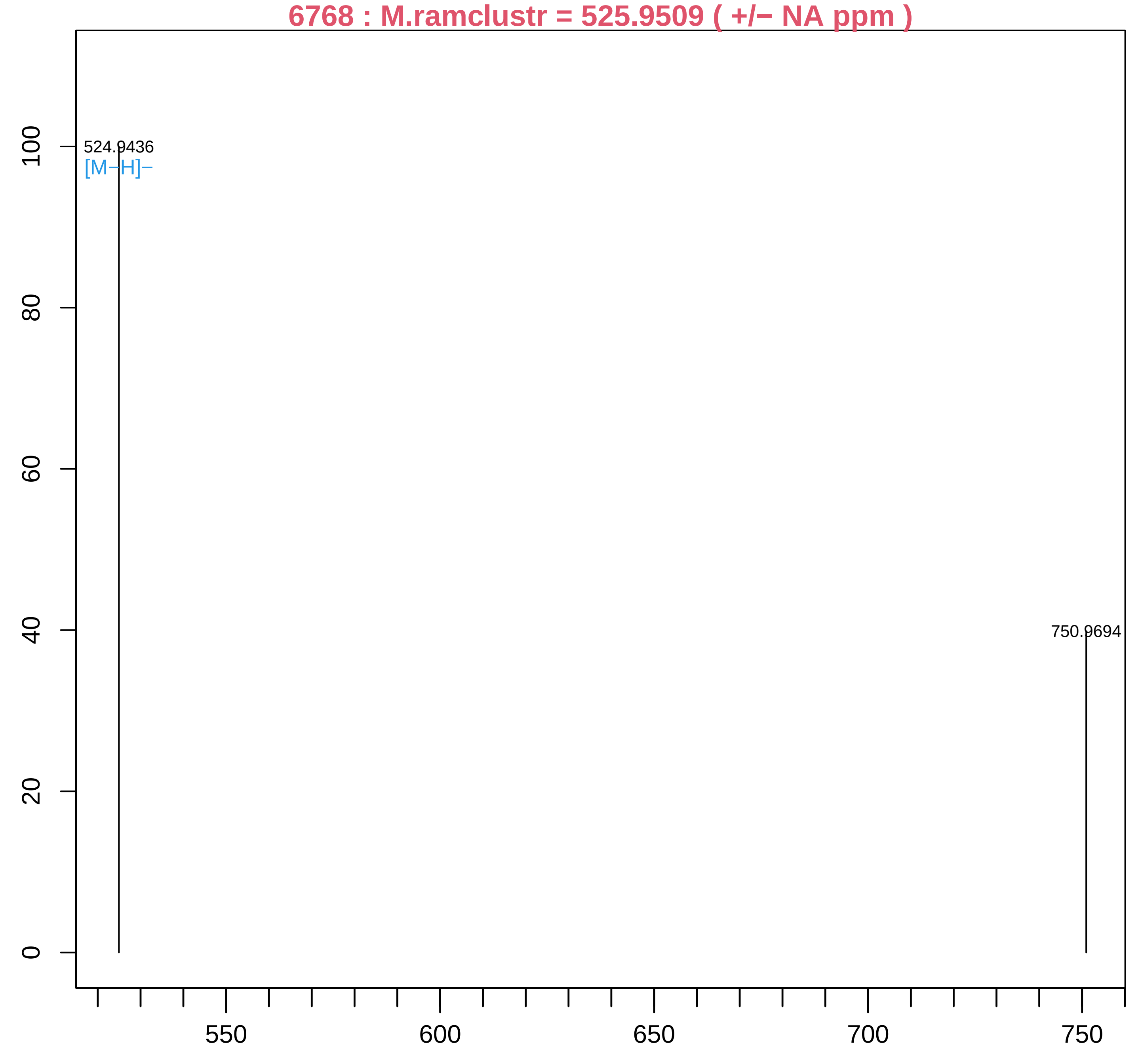
6768 : M.ramclustr = 525.9509 (+/− NA ppm)
524.9436
[M−H]−
750.9694
0
20
40
60
80
100
550
600
650
700
750

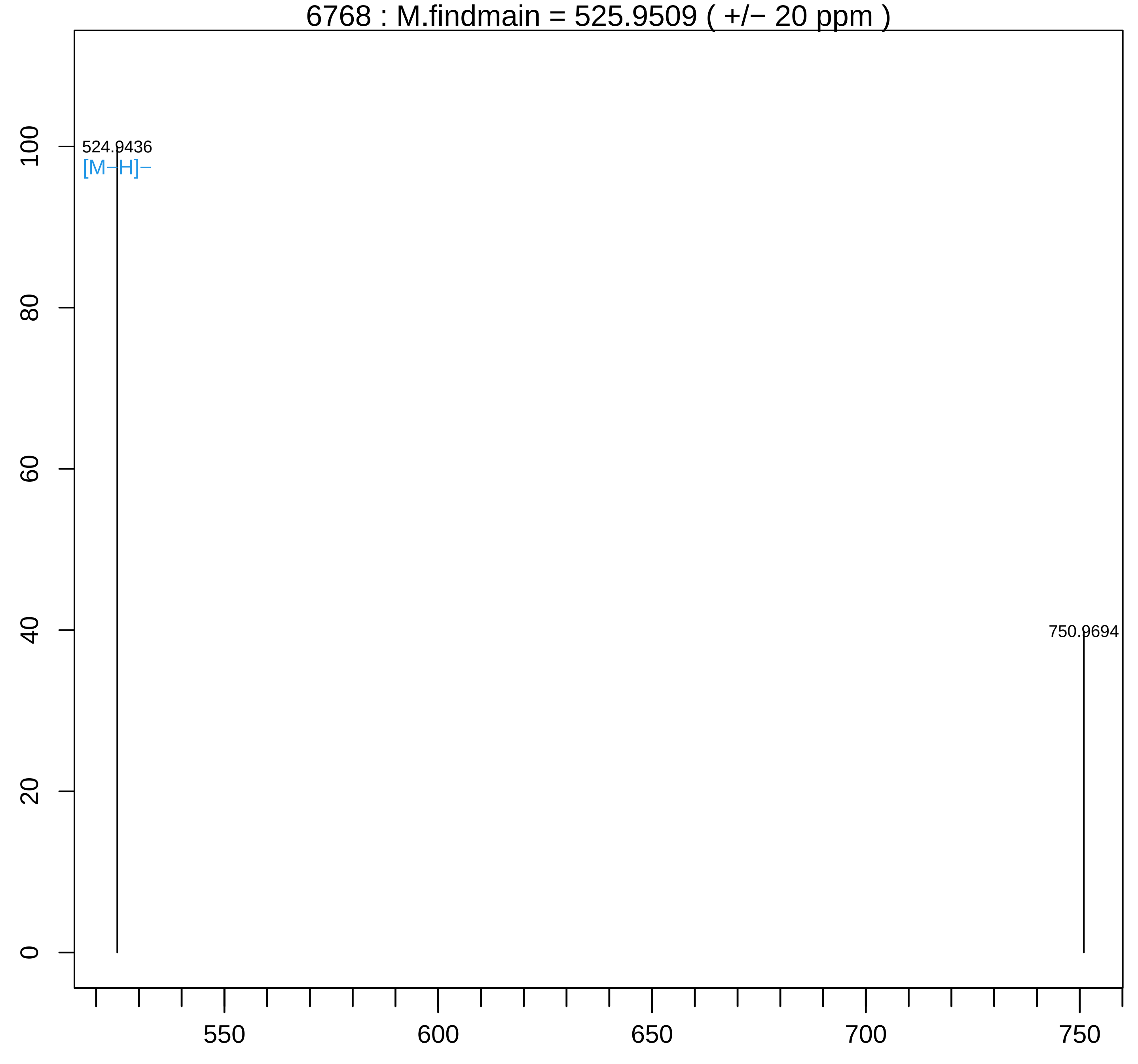
6768 : M.findmain = 525.9509 (+/− 20 ppm)
524.9436
[M−H]−
750.9694
0
20
40
60
80
100
550
600
650
700
750

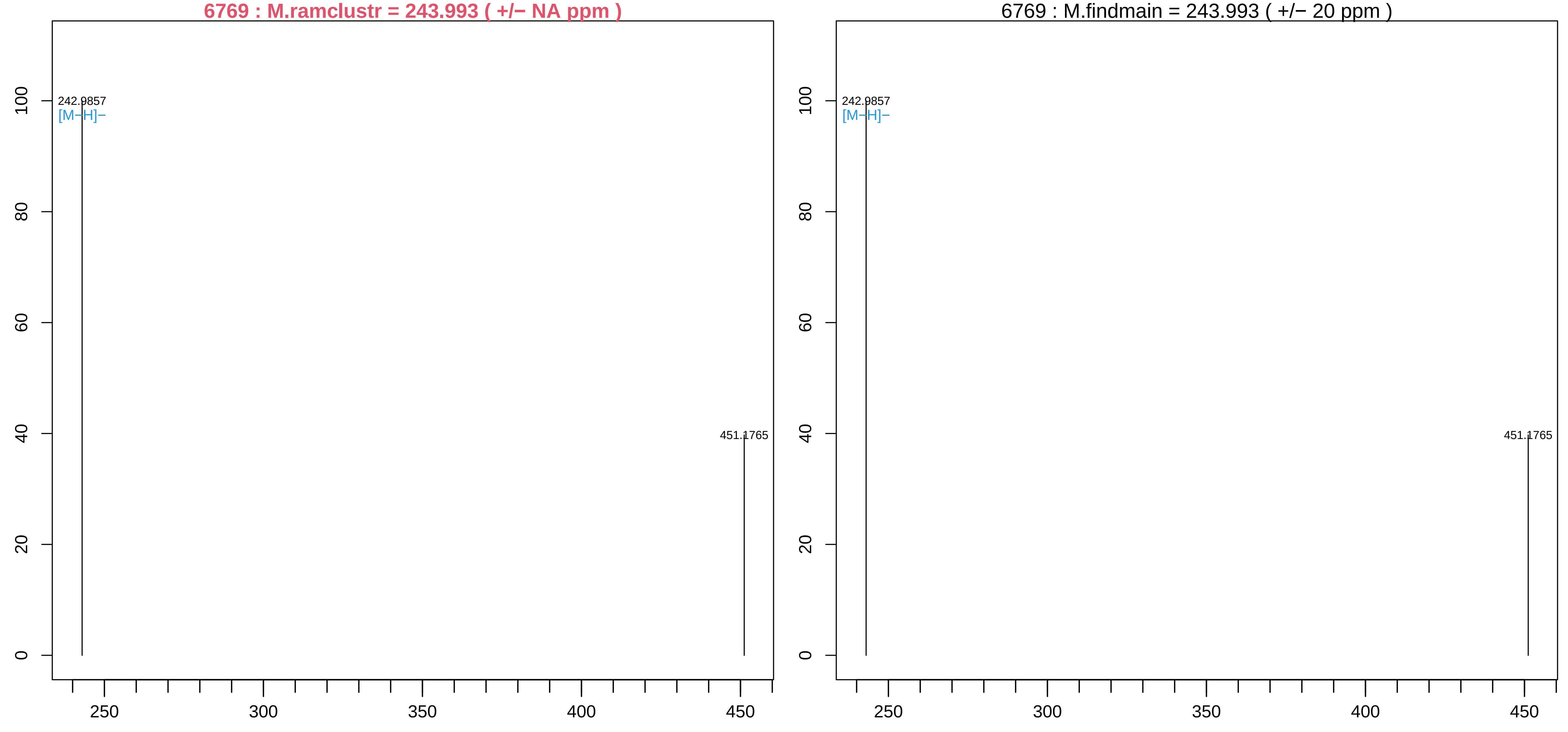

6769 : M.ramclustr = 243.993 (+/− NA ppm)
242.9857
[M−H]−
451.1765
0
20
40
60
80
100
250
300
350
400
450
6769 : M.findmain = 243.993 (+/− 20 ppm)
242.9857
[M−H]−
451.1765
0
20
40
60
80
100
250
300
350
400
450

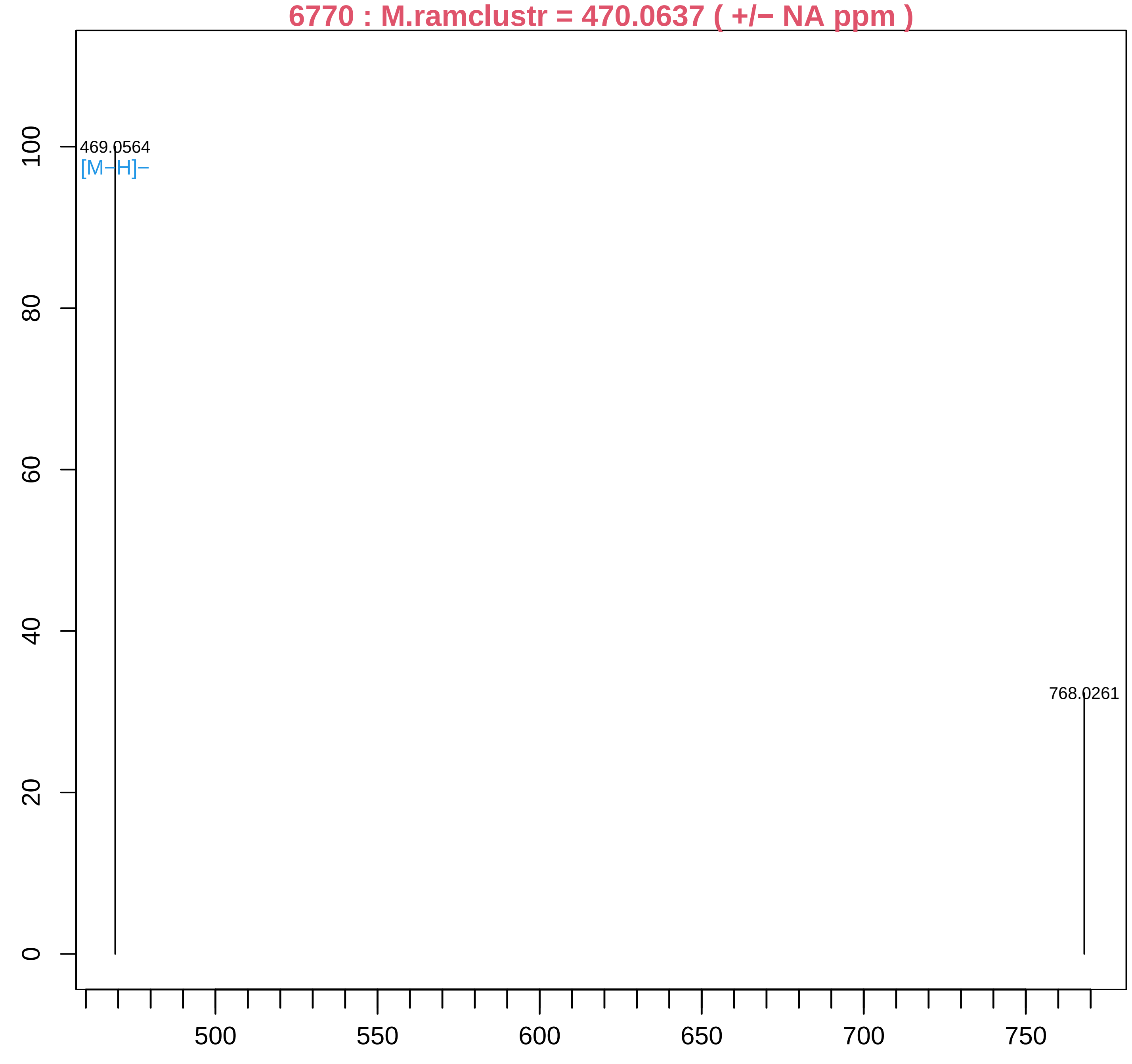
6770 : M.ramclustr = 470.0637 (+/− NA ppm)
469.0564
[M−H]−
768.0261
0
20
40
60
80
100
500
550
600
650
700
750

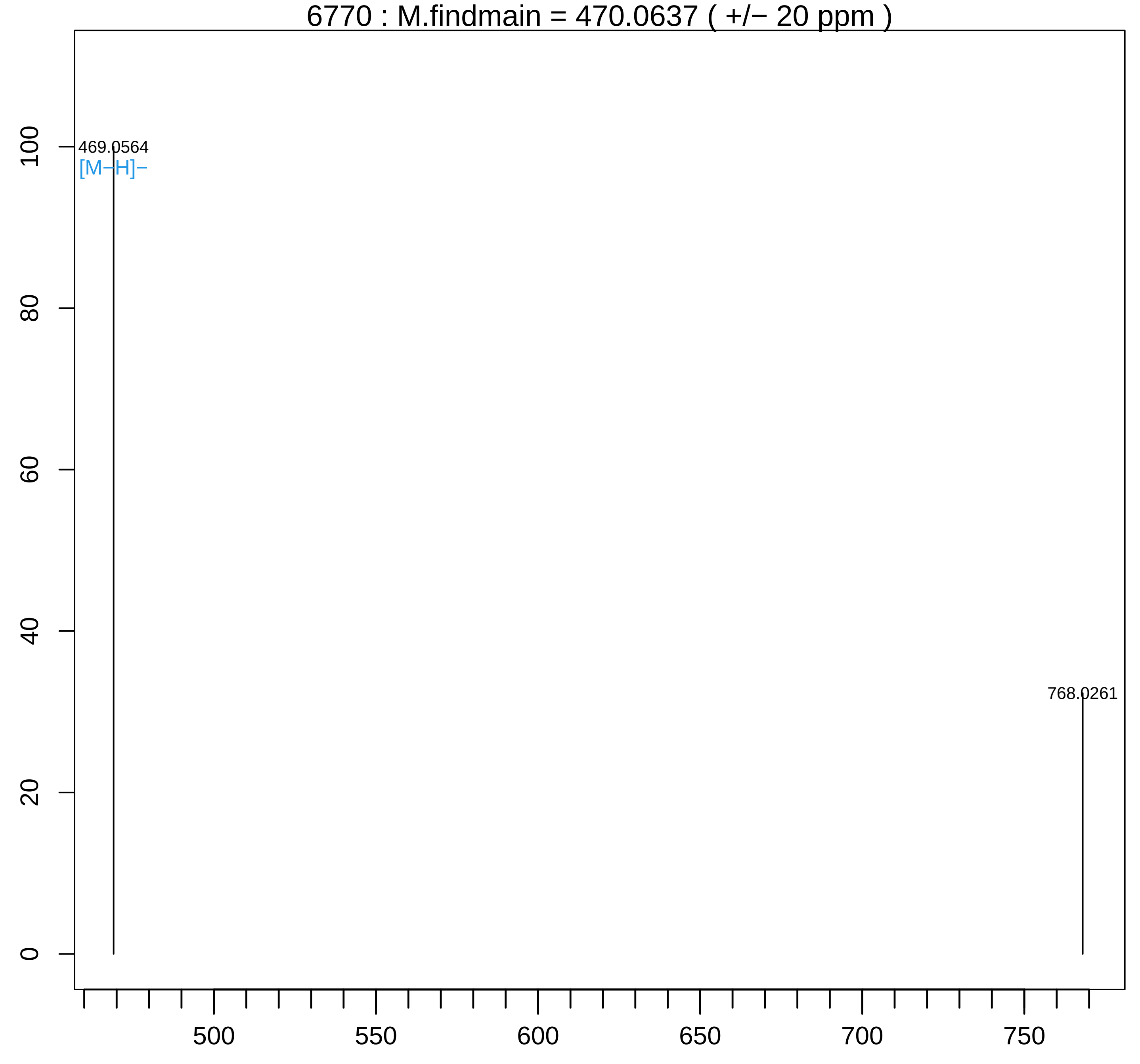
6770 : M.findmain = 470.0637 (+/− 20 ppm)
469.0564
[M−H]−
768.0261
0
20
40
60
80
100
500
550
600
650
700
750

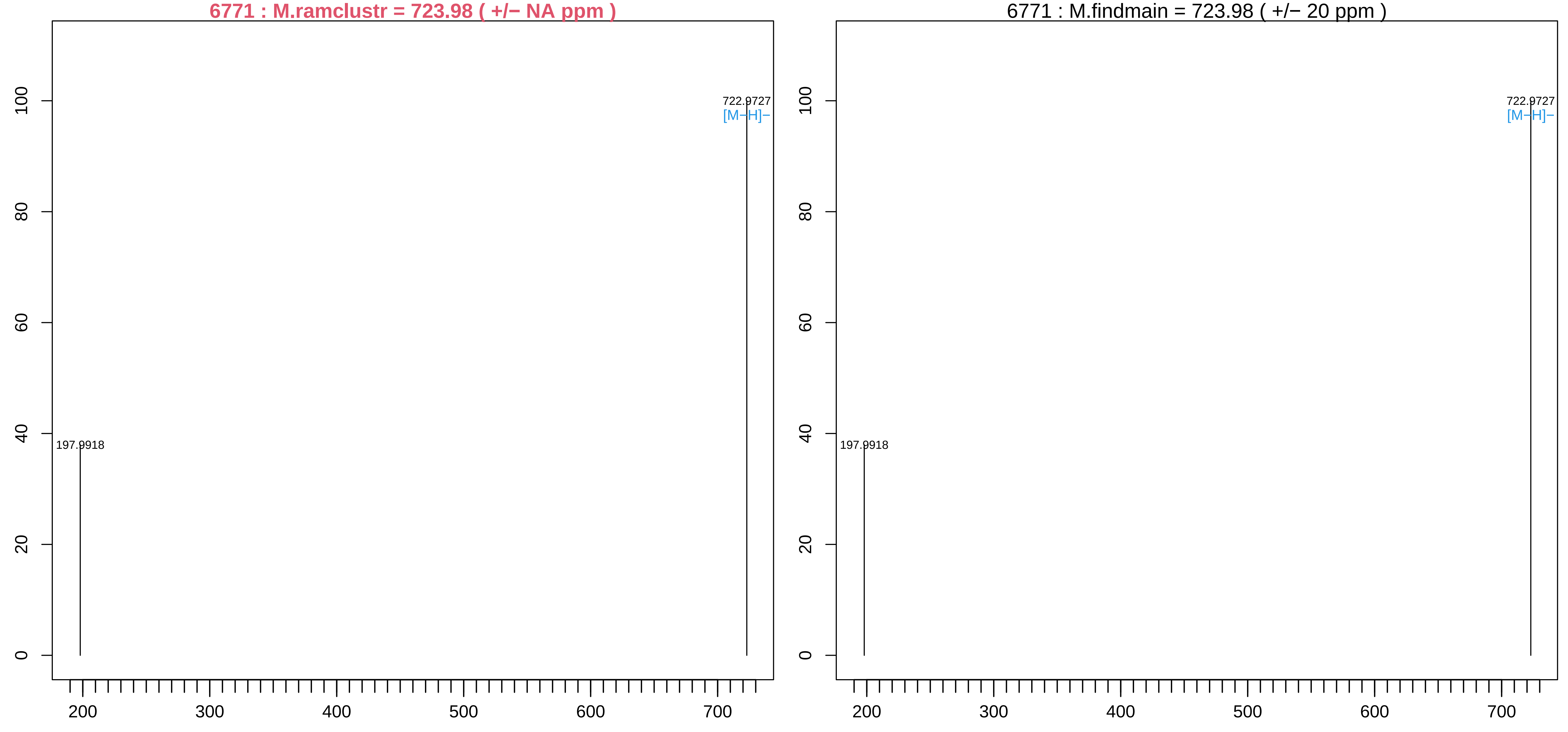
6771 : M.ramclustr = 723.98 (+/− NA ppm)
722.9727
[M−H]−
197.9918
0
20
40
60
80
100
200
300
400
500
600
700
6771 : M.findmain = 723.98 (+/− 20 ppm)
722.9727
[M−H]−
197.9918

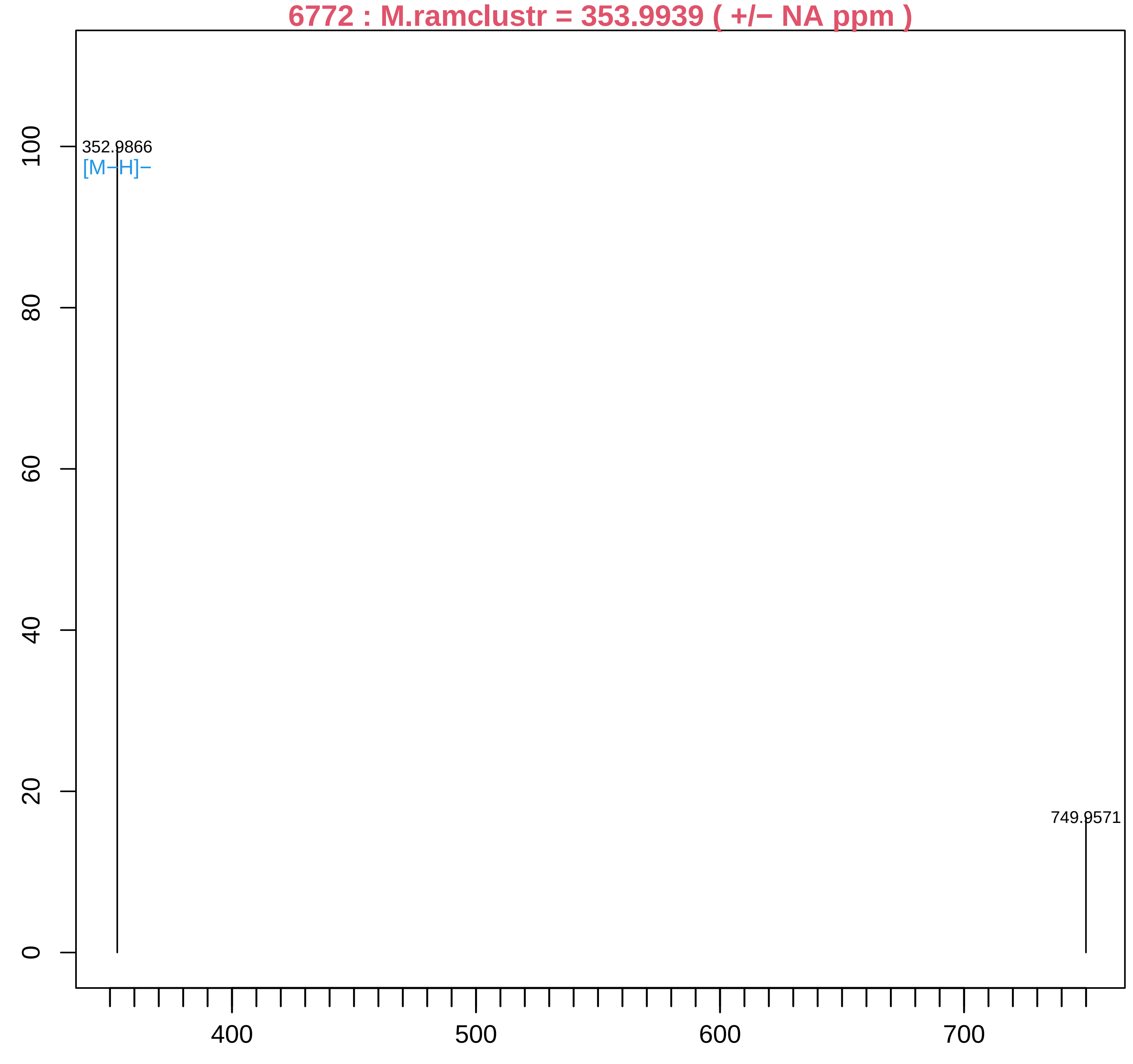
6772 : M.ramclustr = 353.9939 (+/− NA ppm)
352.9866
[M−H]−
749.9571
0
20
40
60
80
100
400
500
600
700

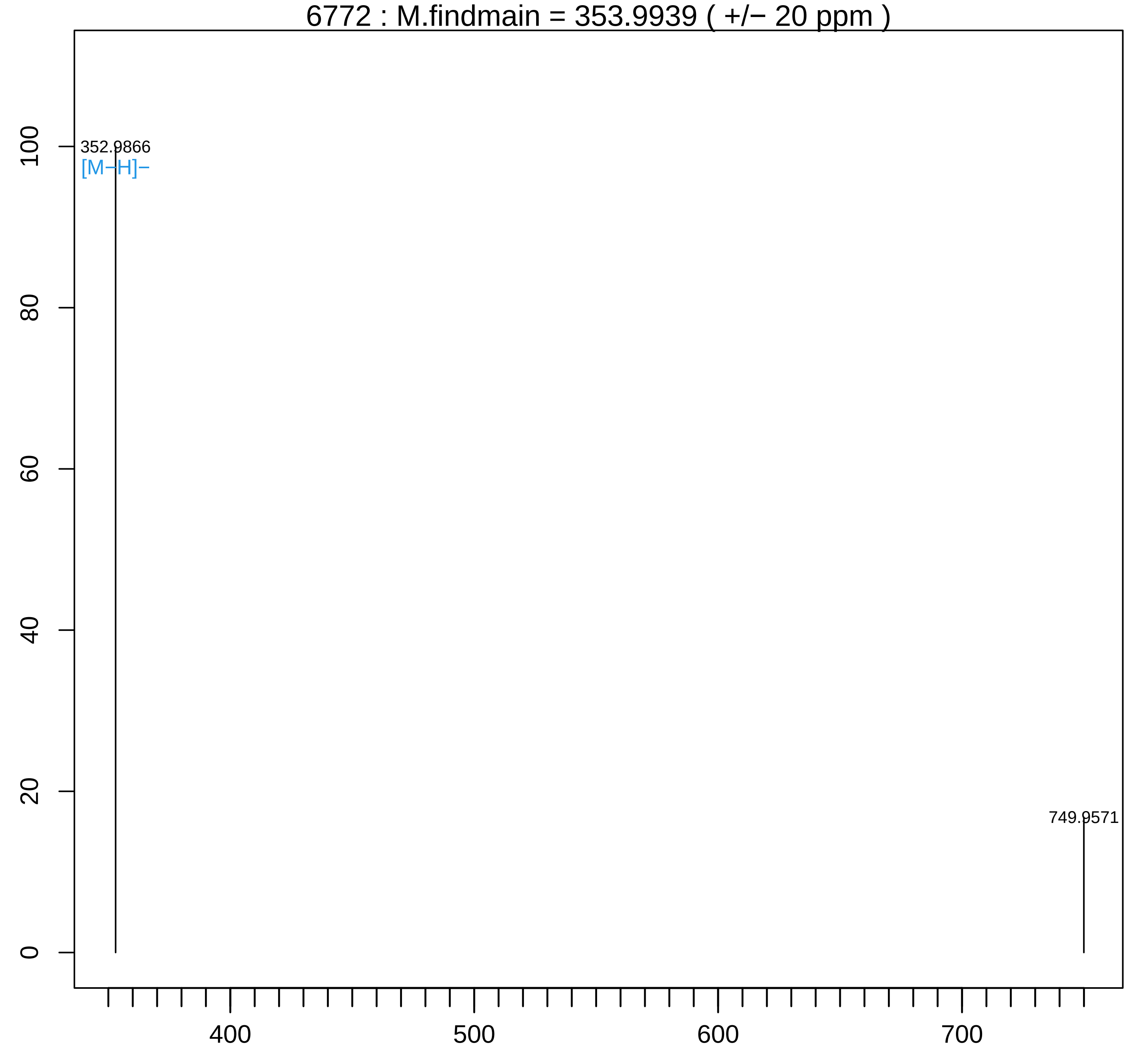
6772 : M.findmain = 353.9939 (+/− 20 ppm)
352.9866
[M−H]−
749.9571
0
20
40
60
80
100
400
500
600
700

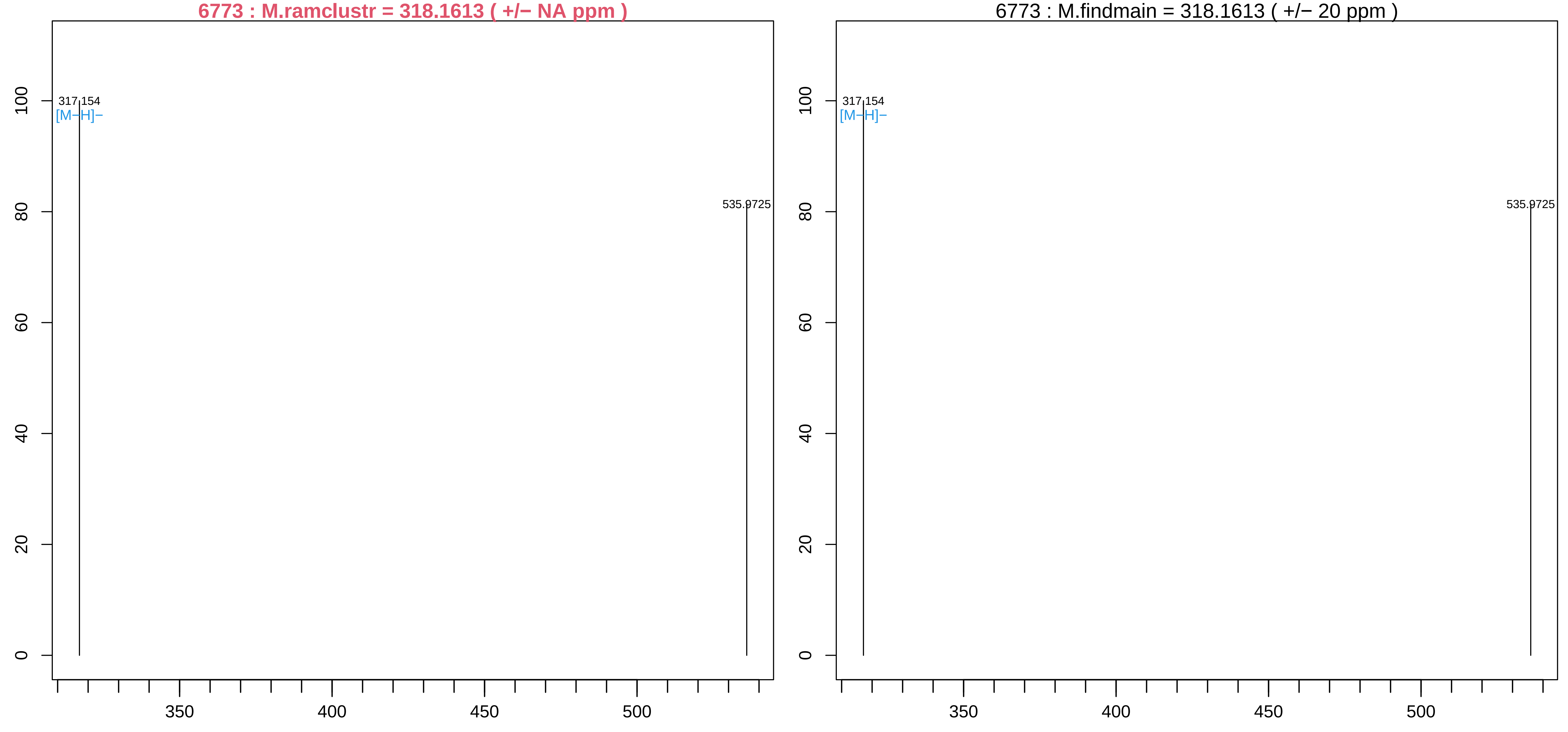

6773 : M.ramclustr = 318.1613 (+/− NA ppm)
317.154
[M−H]−
535.9725
6773 : M.findmain = 318.1613 (+/− 20 ppm)
317.154
[M−H]−
535.9725
0
20
40
60
80
100
350
400
450
500

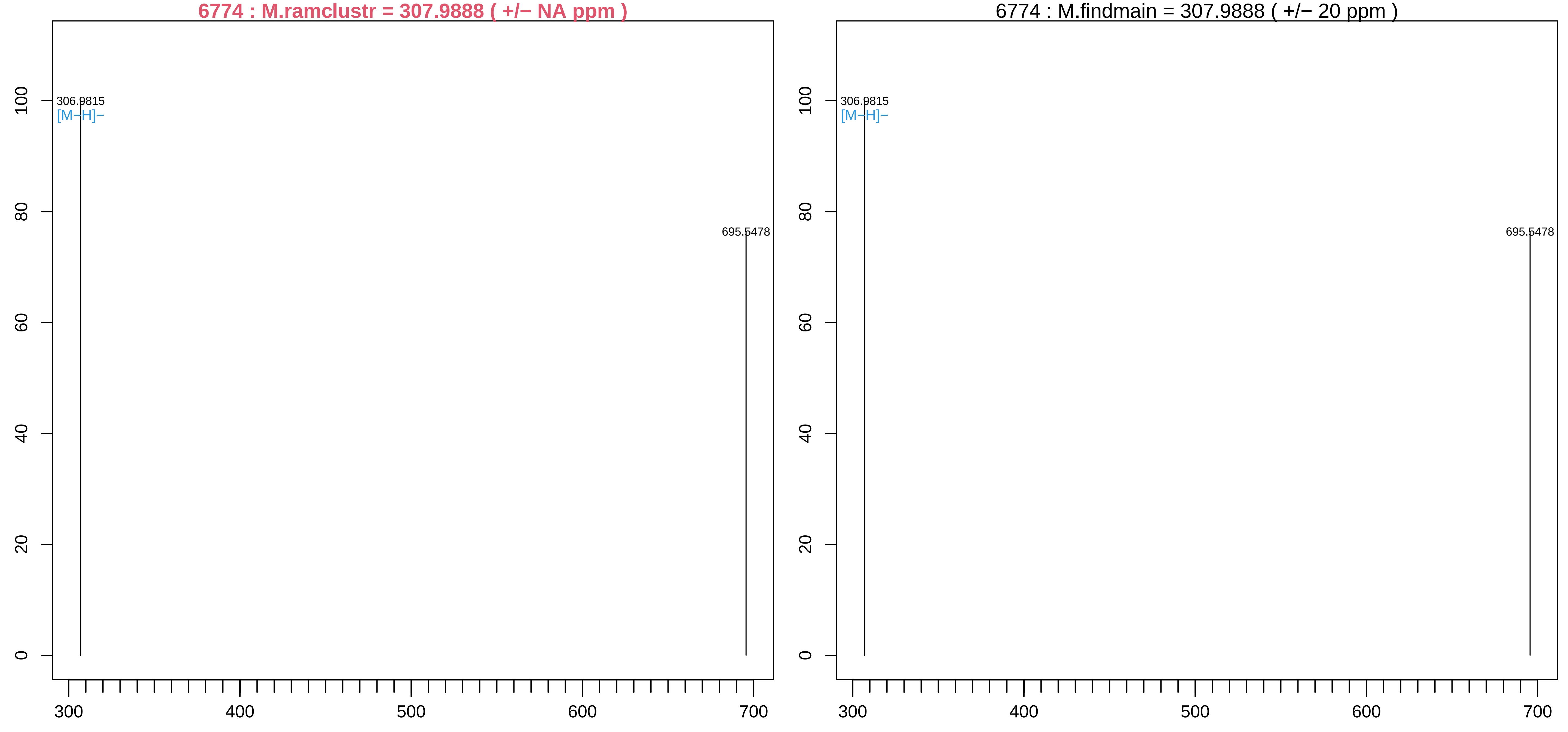
6774 : M.ramclustr = 307.9888 (+/− NA ppm)
306.9815
[M−H]−
695.5478
6774 : M.findmain = 307.9888 (+/− 20 ppm)
306.9815
[M−H]−
695.5478
0
20
40
60
80
100
300
400
500
600
700

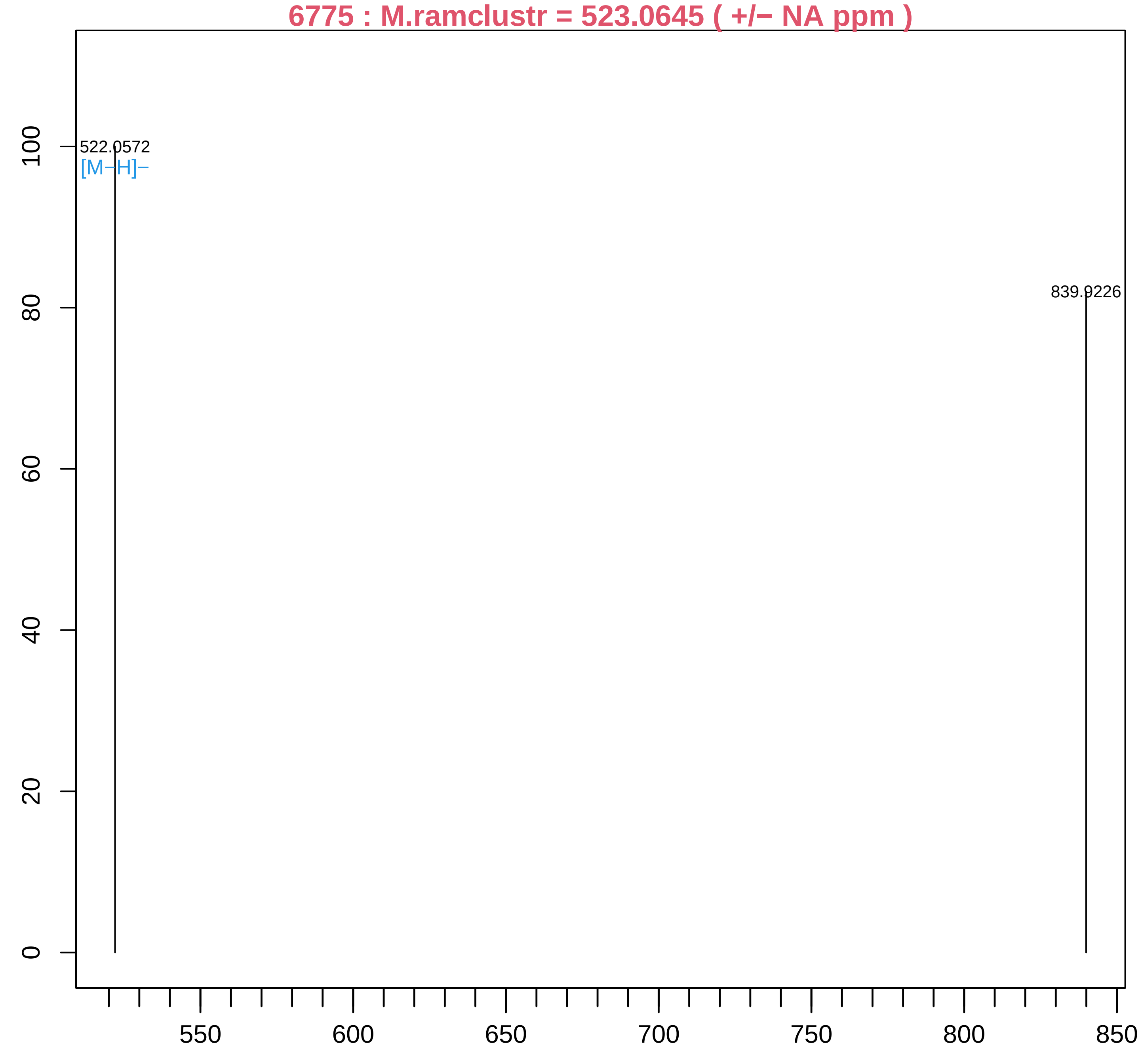
6775 : M.ramclustr = 523.0645 (+/− NA ppm)
522.0572
[M−H]−
839.9226
0
20
40
60
80
100
550
600
650
700
750
800
850

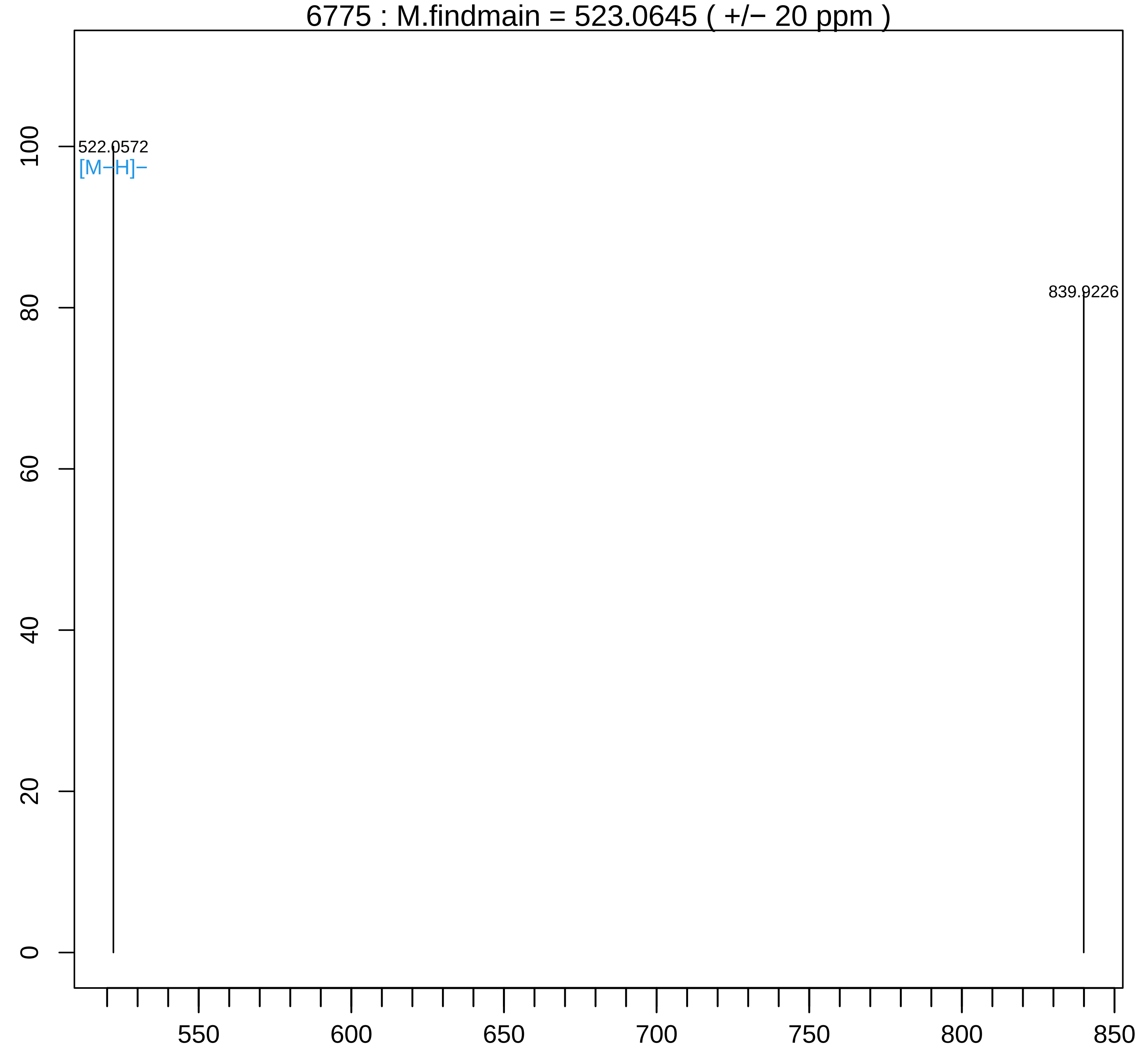
6775 : M.findmain = 523.0645 (+/− 20 ppm)
522.0572
[M−H]−
839.9226
0
20
40
60
80
100
550
600
650
700
750
800
850

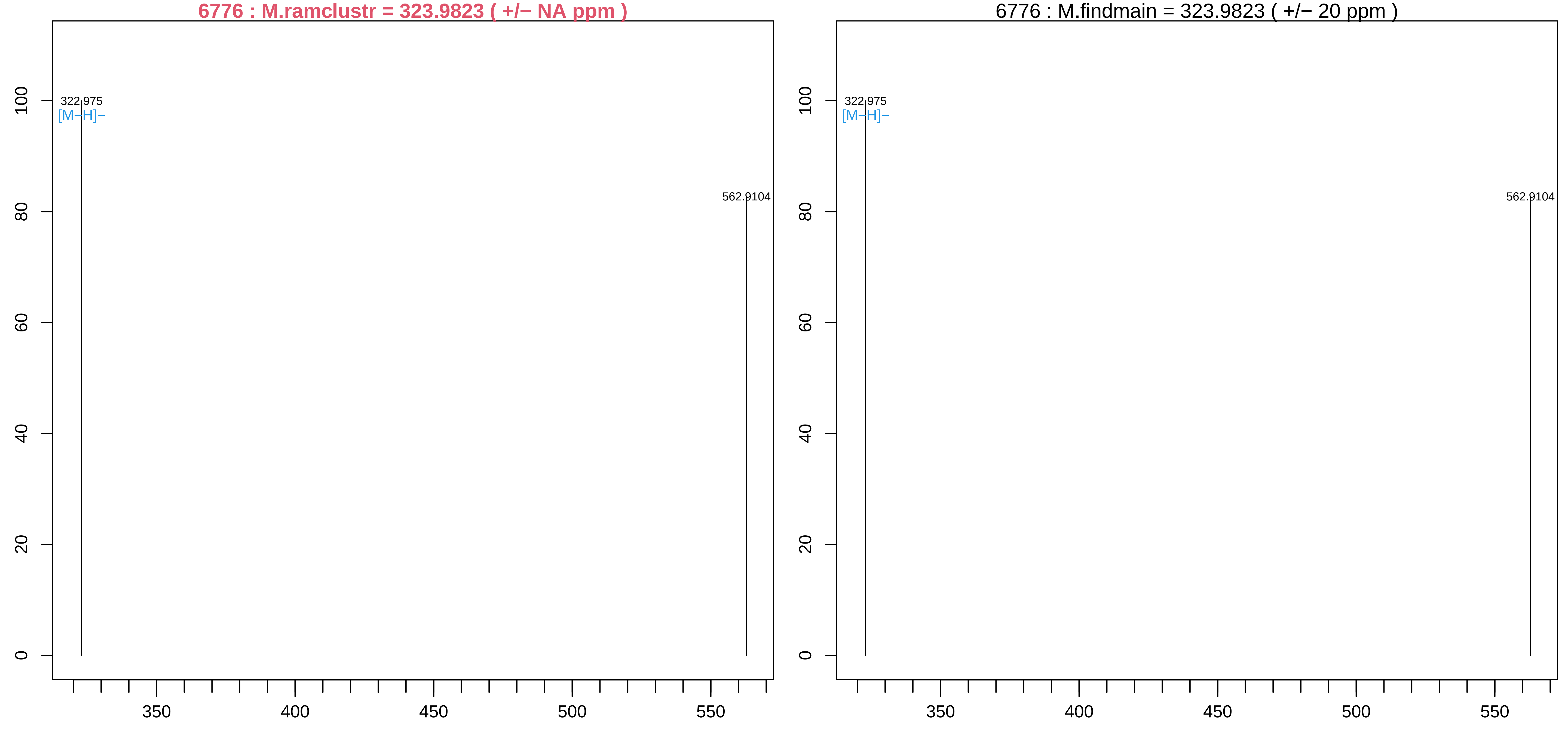
6776 : M.ramclustr = 323.9823 (+/− NA ppm)
322.975
[M−H]−
562.9104
0
20
40
60
80
100
350
400
450
500
550
6776 : M.findmain = 323.9823 (+/− 20 ppm)
322.975
[M−H]−
562.9104
0
20
40
60
80
100
350
400
450
500
550

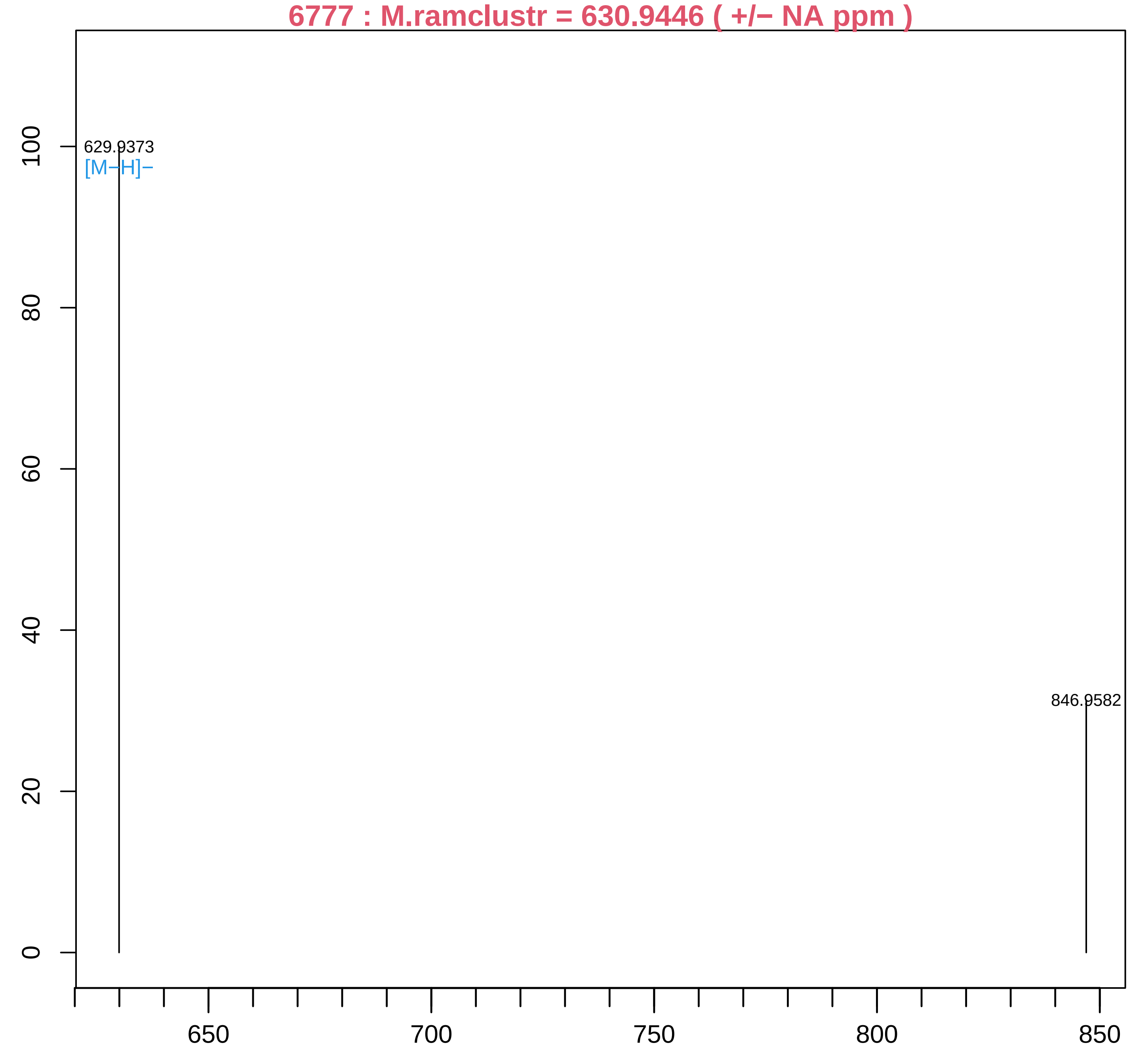
6777 : M.ramclustr = 630.9446 (+/− NA ppm)
629.9373
[M−H]−
846.9582
0
20
40
60
80
100
650
700
750
800
850

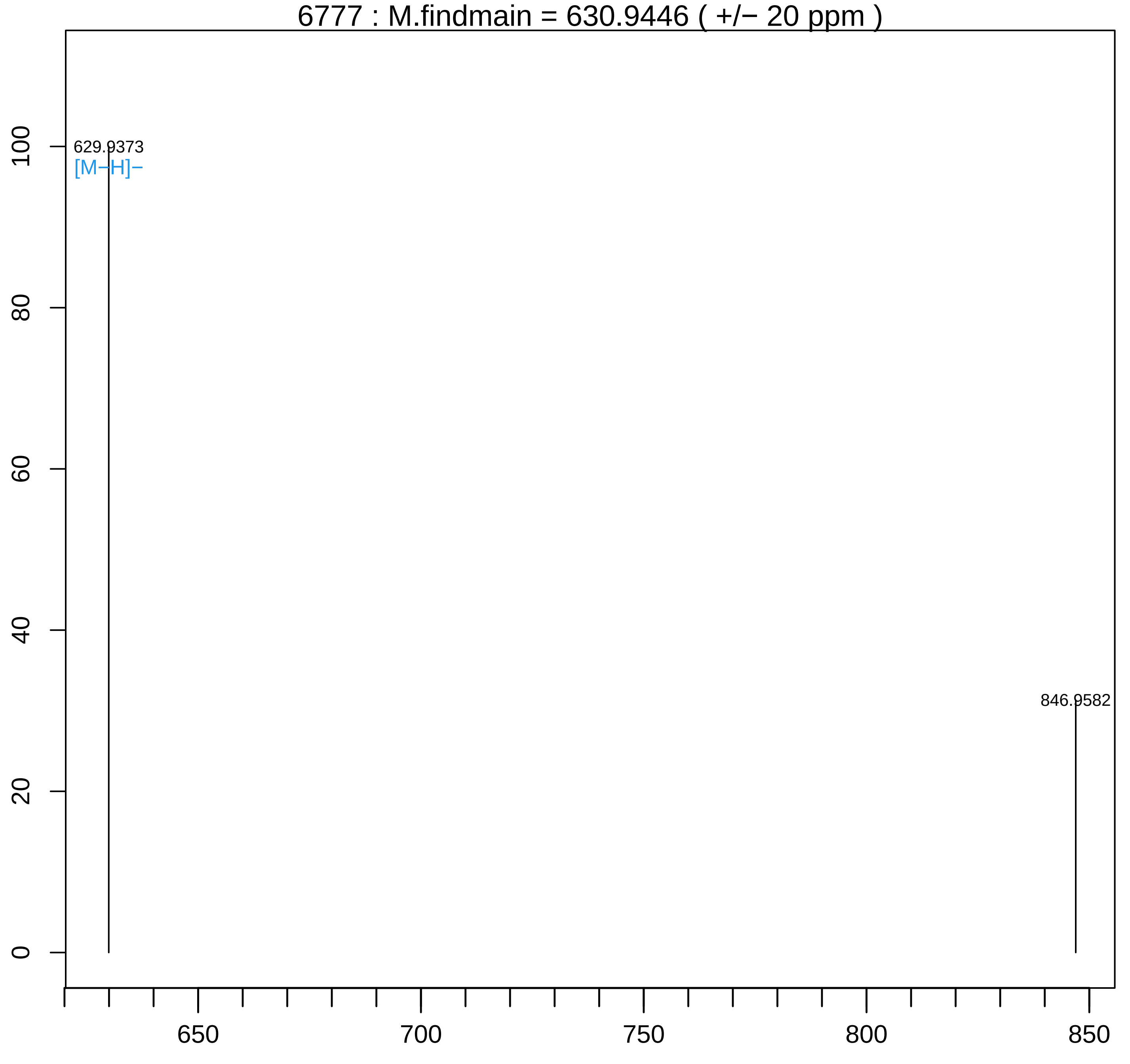
6777 : M.findmain = 630.9446 (+/− 20 ppm)
629.9373
[M−H]−
846.9582
0
20
40
60
80
100
650
700
750
800
850

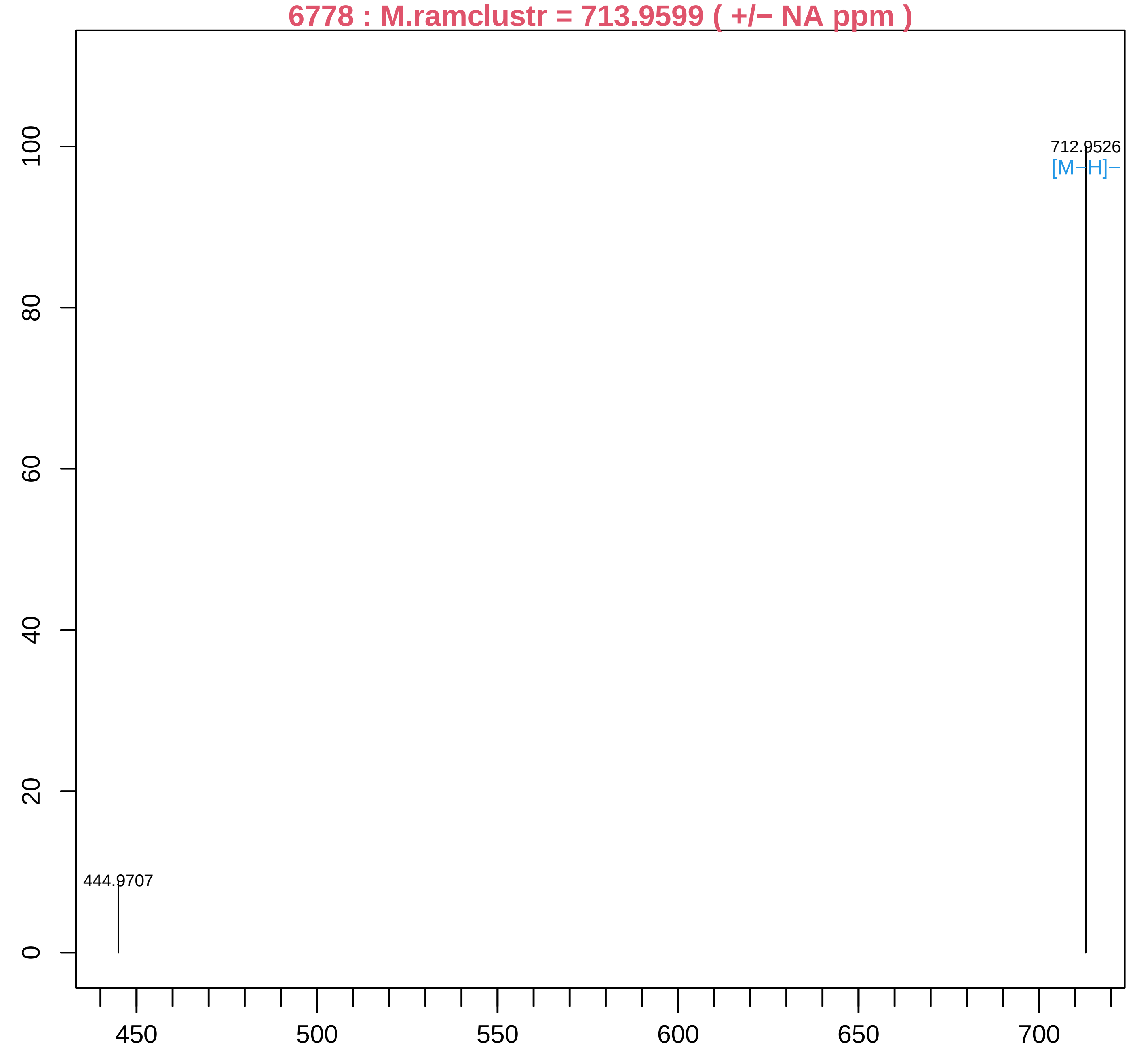

6778 : M.ramclustr = 713.9599 (+/− NA ppm)
712.9526
[M−H]−
444.9707
0
20
40
60
80
100
450
500
550
600
650
700

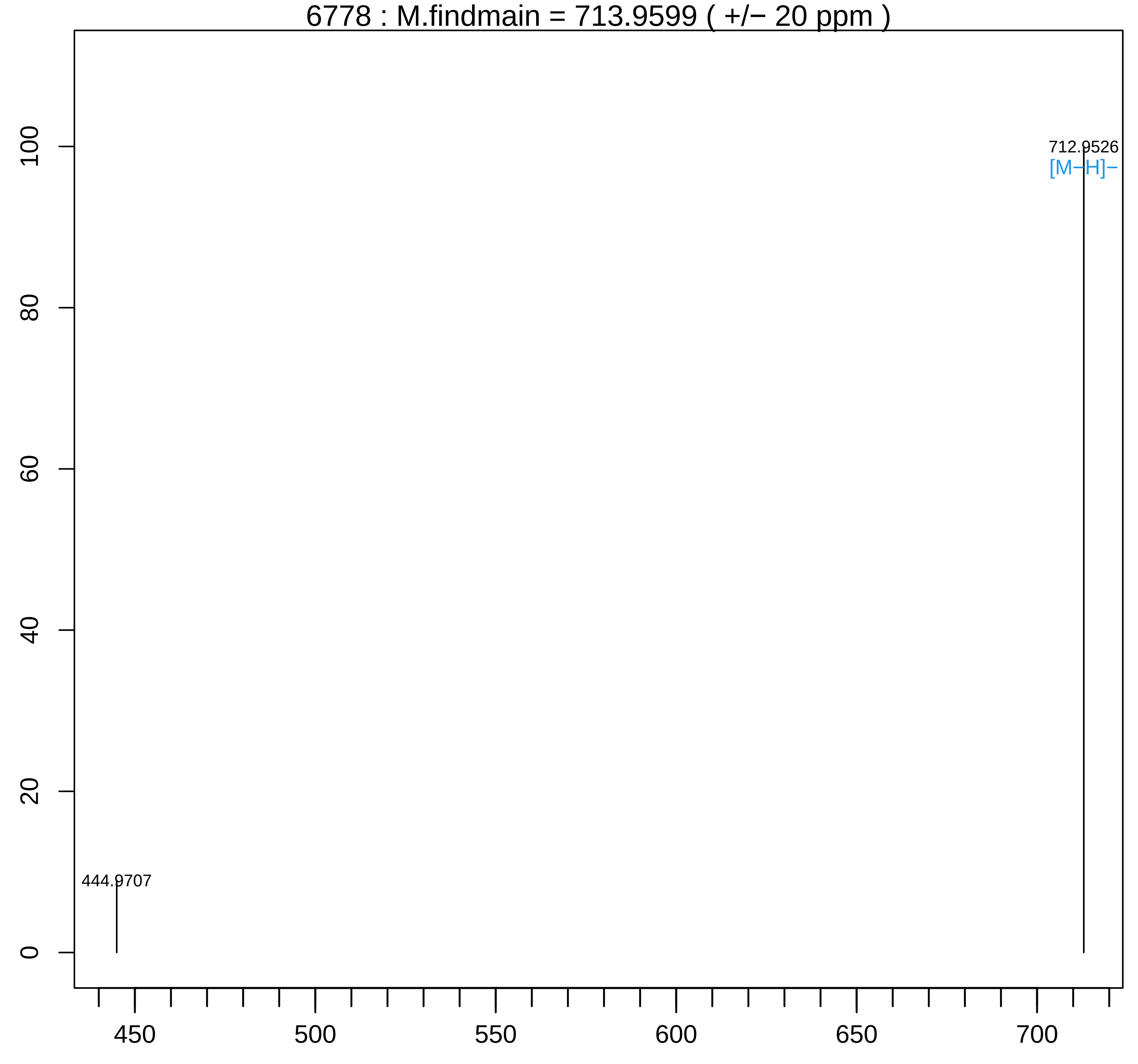

6778 : M.findmain = 713.9599 (+/− 20 ppm)
712.9526
[M−H]−
444.9707
0
20
40
60
80
100
450
500
550
600
650
700

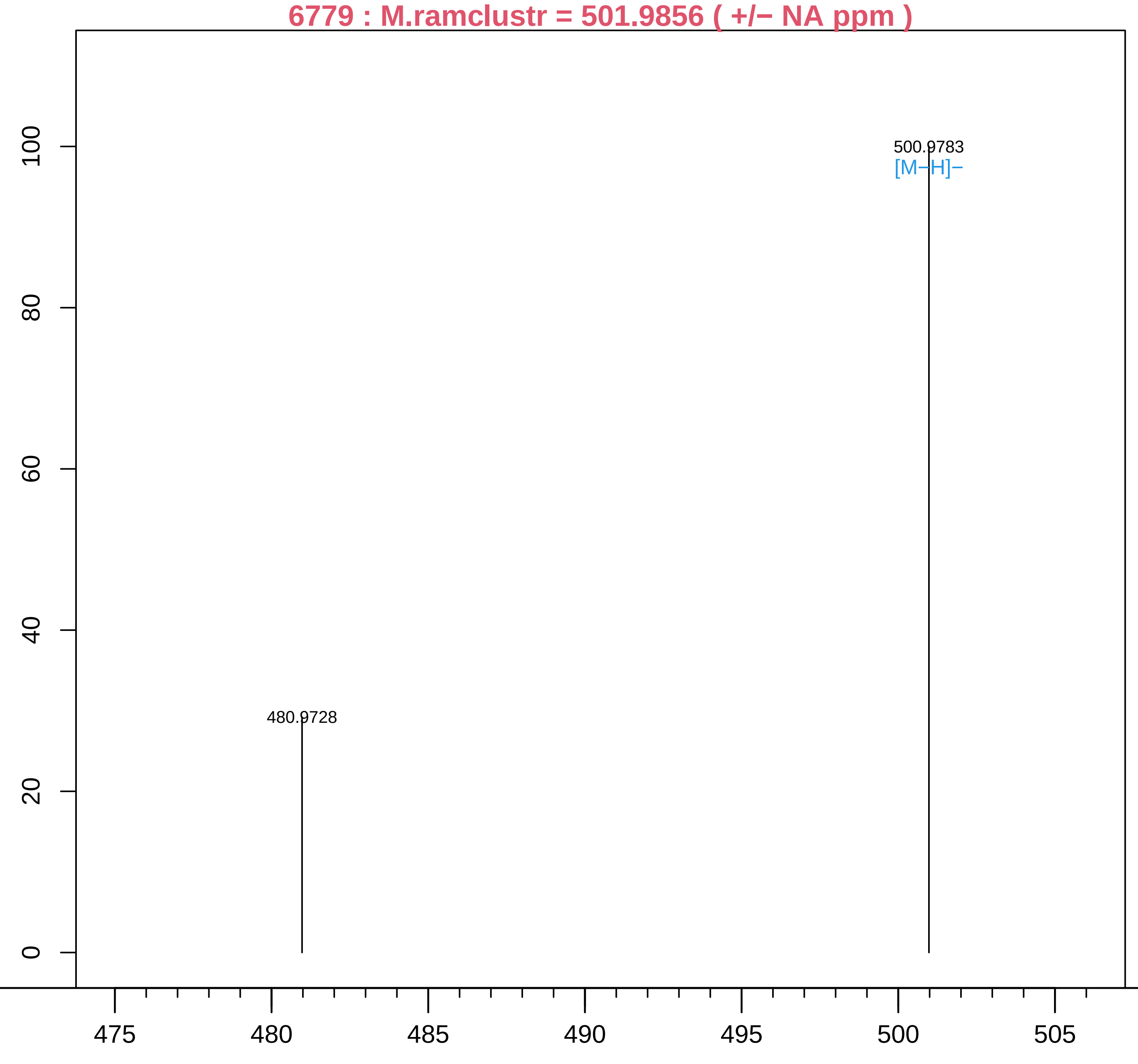
6779 : M.ramclustr = 501.9856 (+/− NA ppm)
500.9783
[M−H]−
480.9728
0
20
40
60
80
100
475
480
485
490
495
500
505

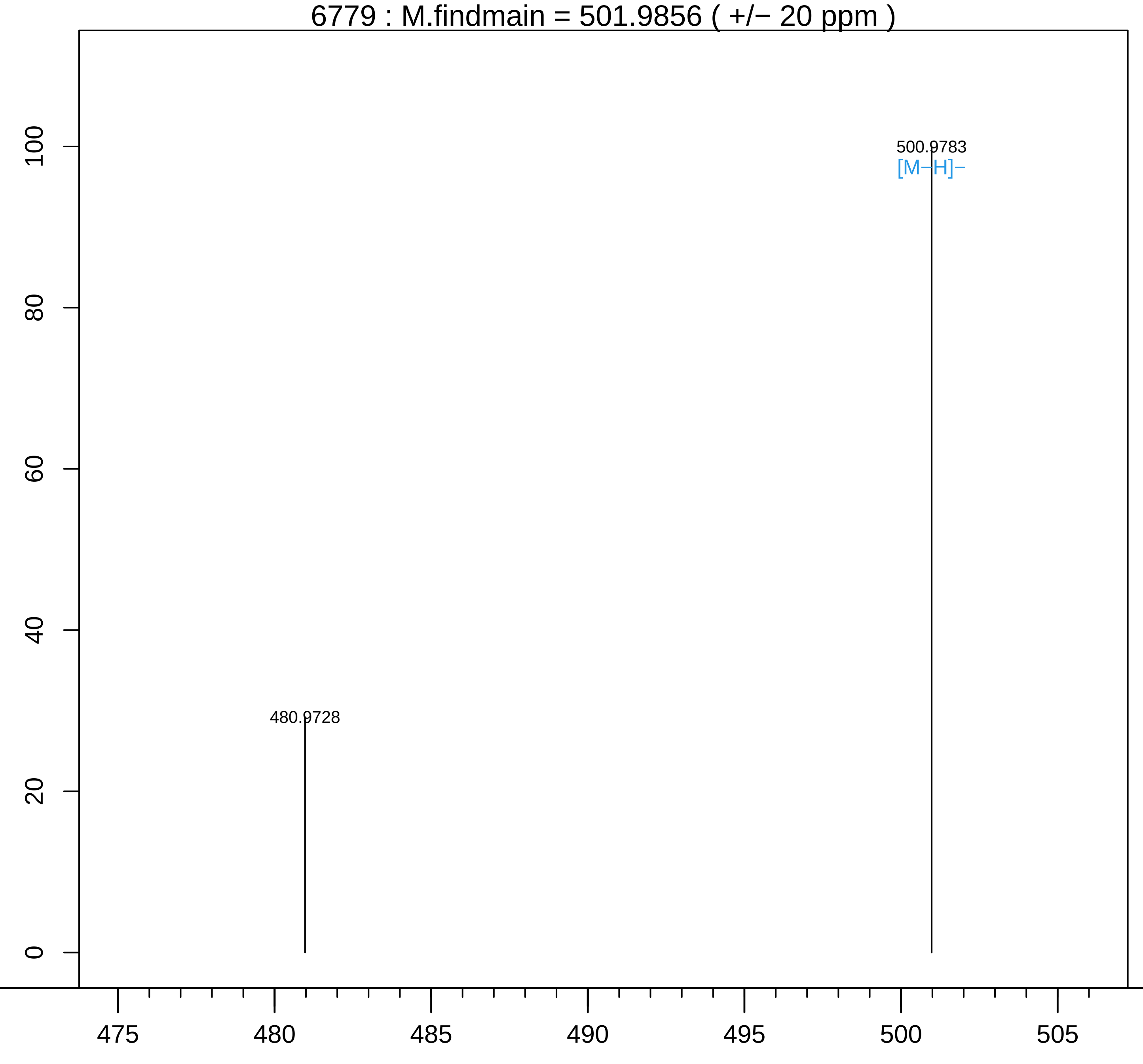
6779 : M.findmain = 501.9856 (+/− 20 ppm)
500.9783
[M−H]−
480.9728
0
20
40
60
80
100
475
480
485
490
495
500
505

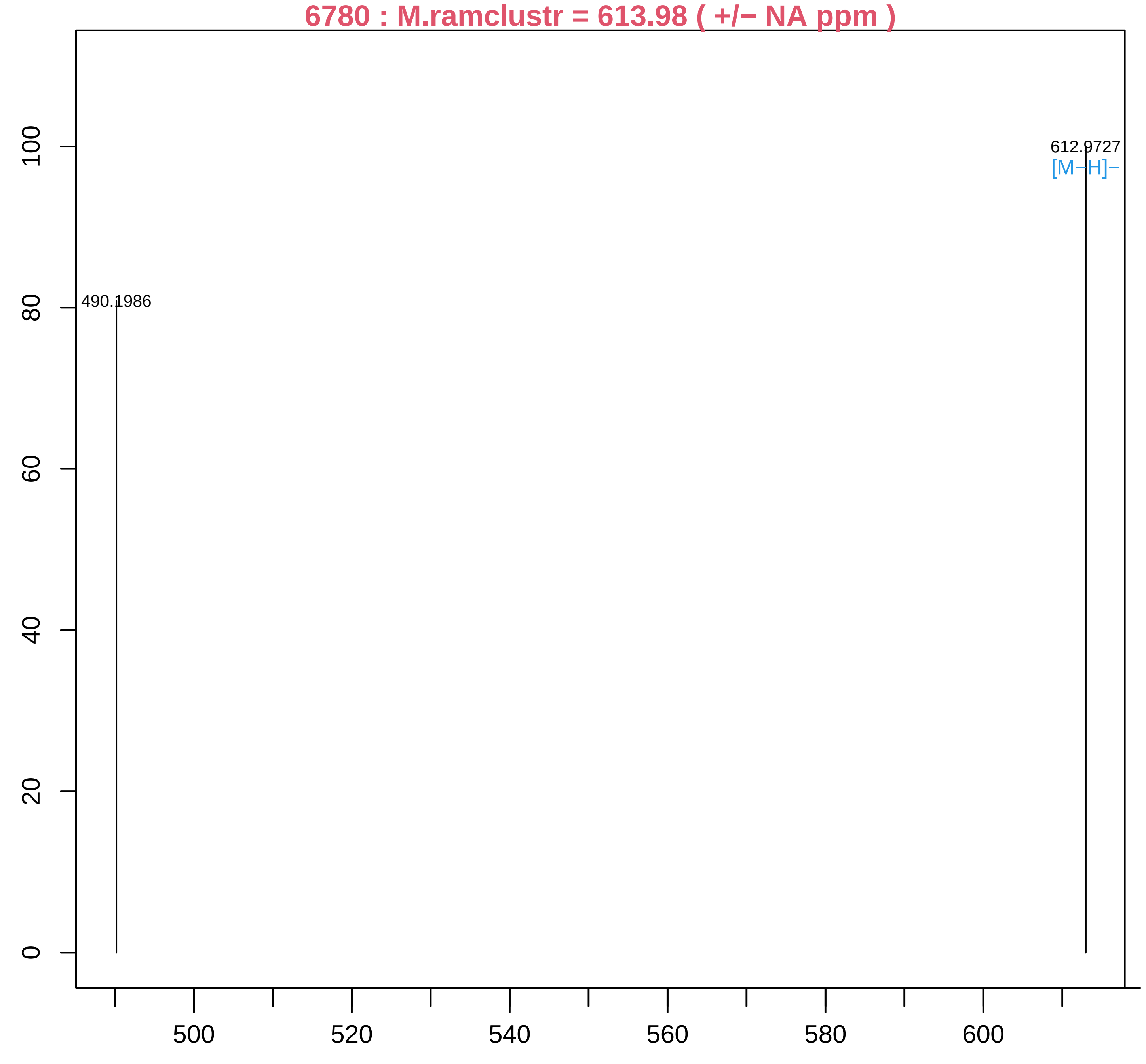
6780 : M.ramclustr = 613.98 (+/− NA ppm)
490.1986
612.9727
[M−H]−
0
20
40
60
80
100
500
520
540
560
580
600

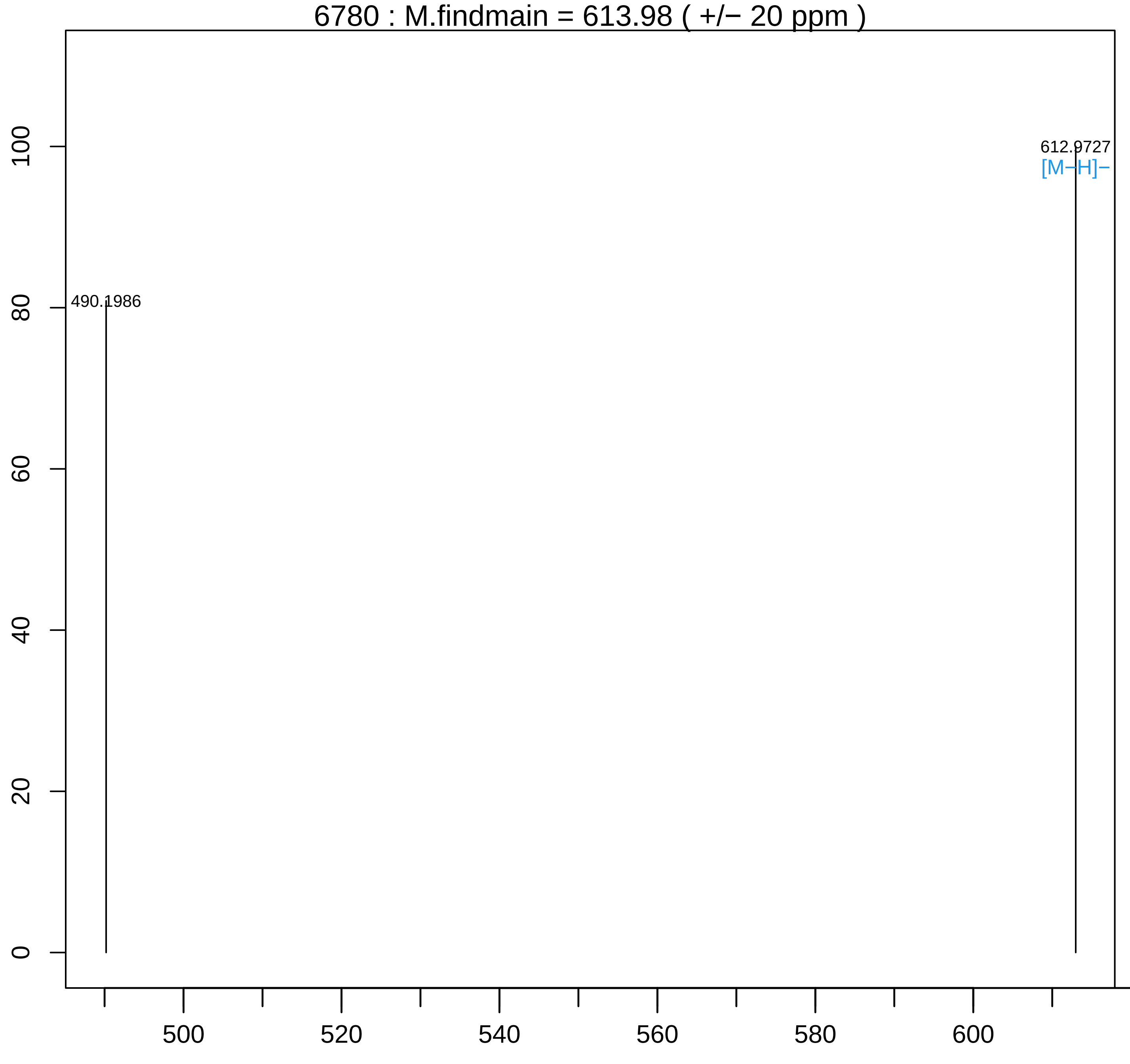
6780 : M.findmain = 613.98 (+/− 20 ppm)
490.1986
612.9727
[M−H]−
0
20
40
60
80
100
500
520
540
560
580
600

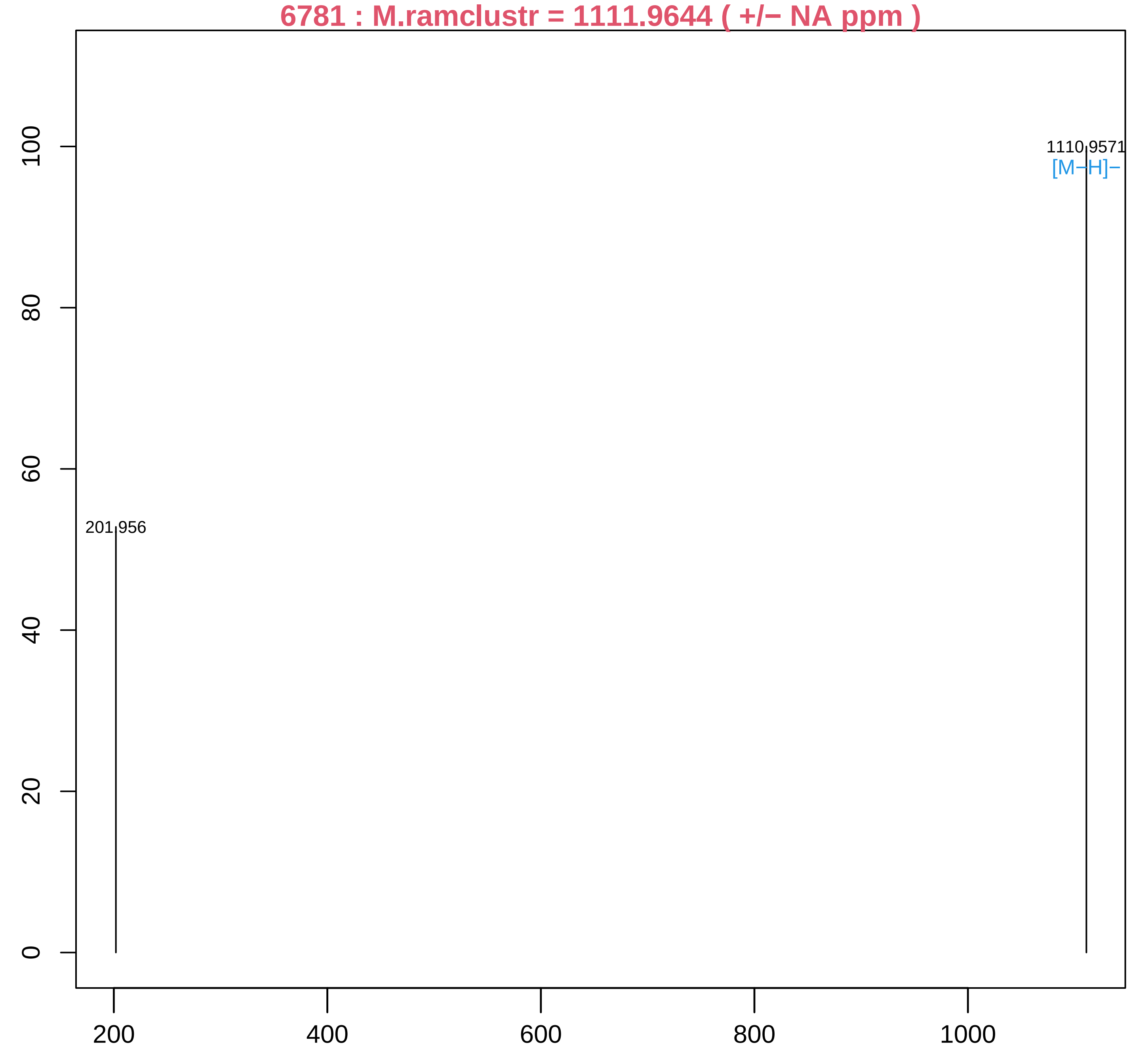

6781 : M.ramclustr = 1111.9644 (+/− NA ppm)
1110.9571
[M−H]−
201.956
0
20
40
60
80
100
200
400
600
800
1000

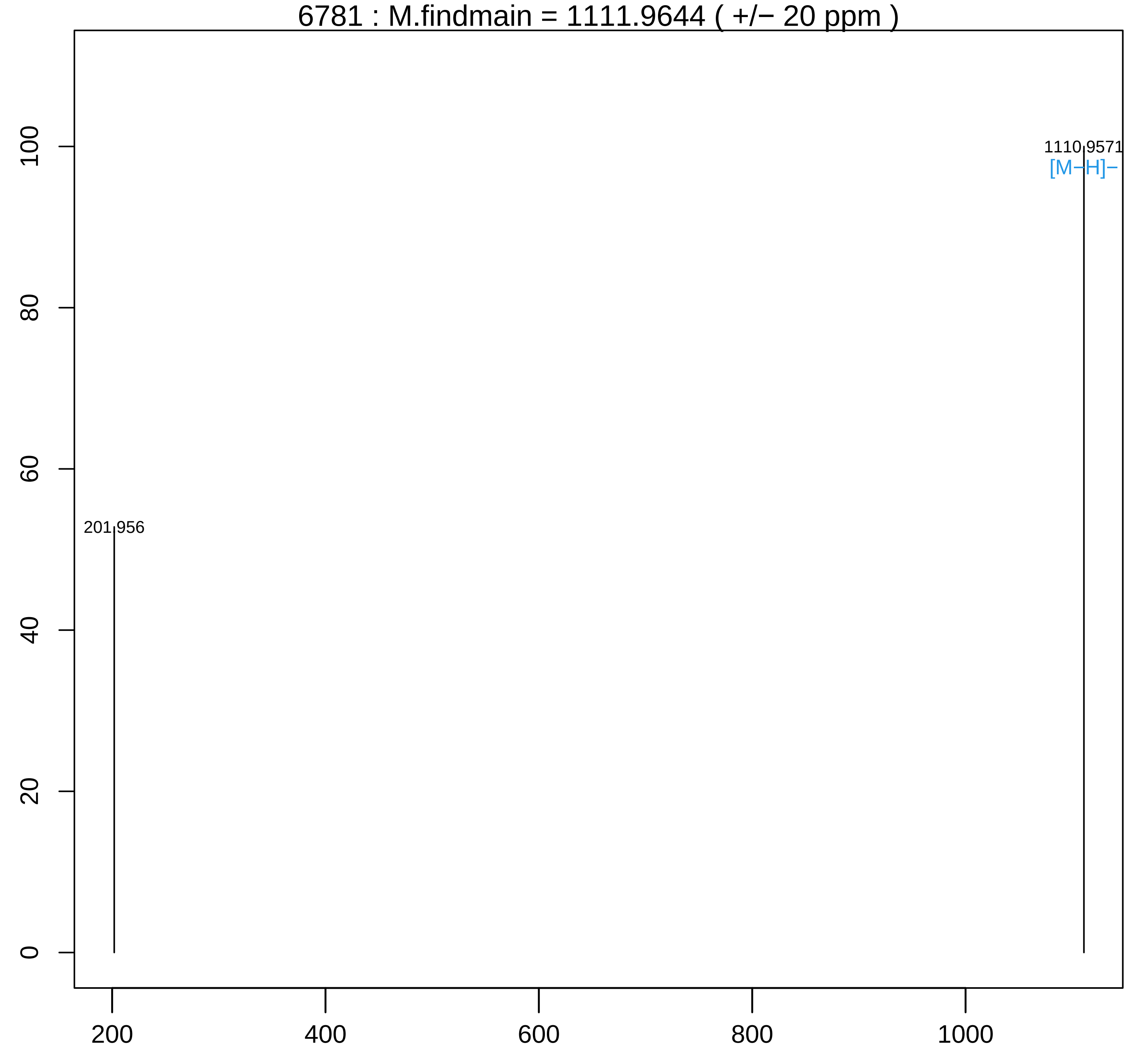

6781 : M.findmain = 1111.9644 (+/− 20 ppm)
1110.9571
[M−H]−
201.956
0
20
40
60
80
100
200
400
600
800
1000

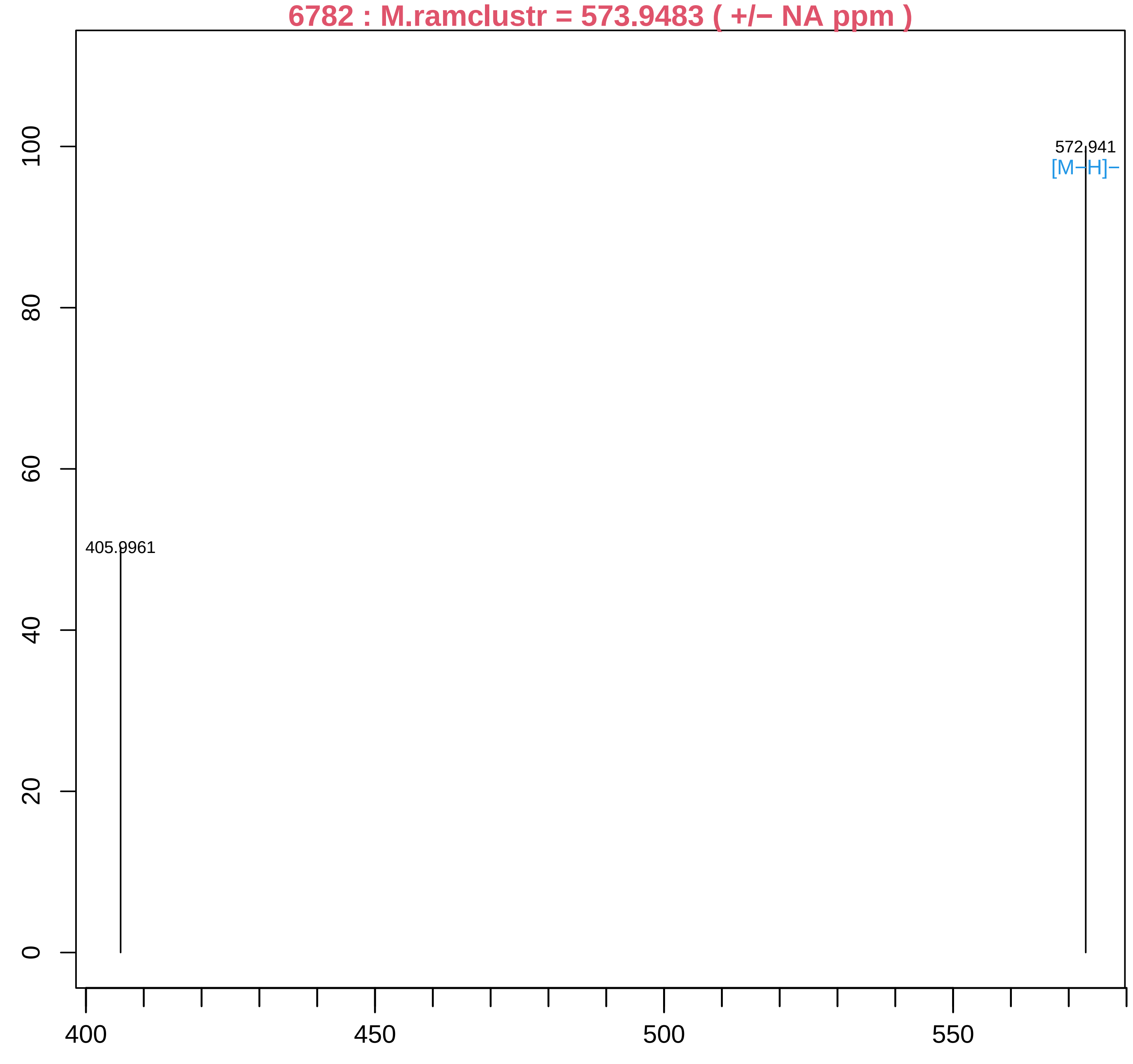
6782 : M.ramclustr = 573.9483 (+/− NA ppm)
572.941
[M−H]−
405.9961
0
20
40
60
80
100
400
450
500
550

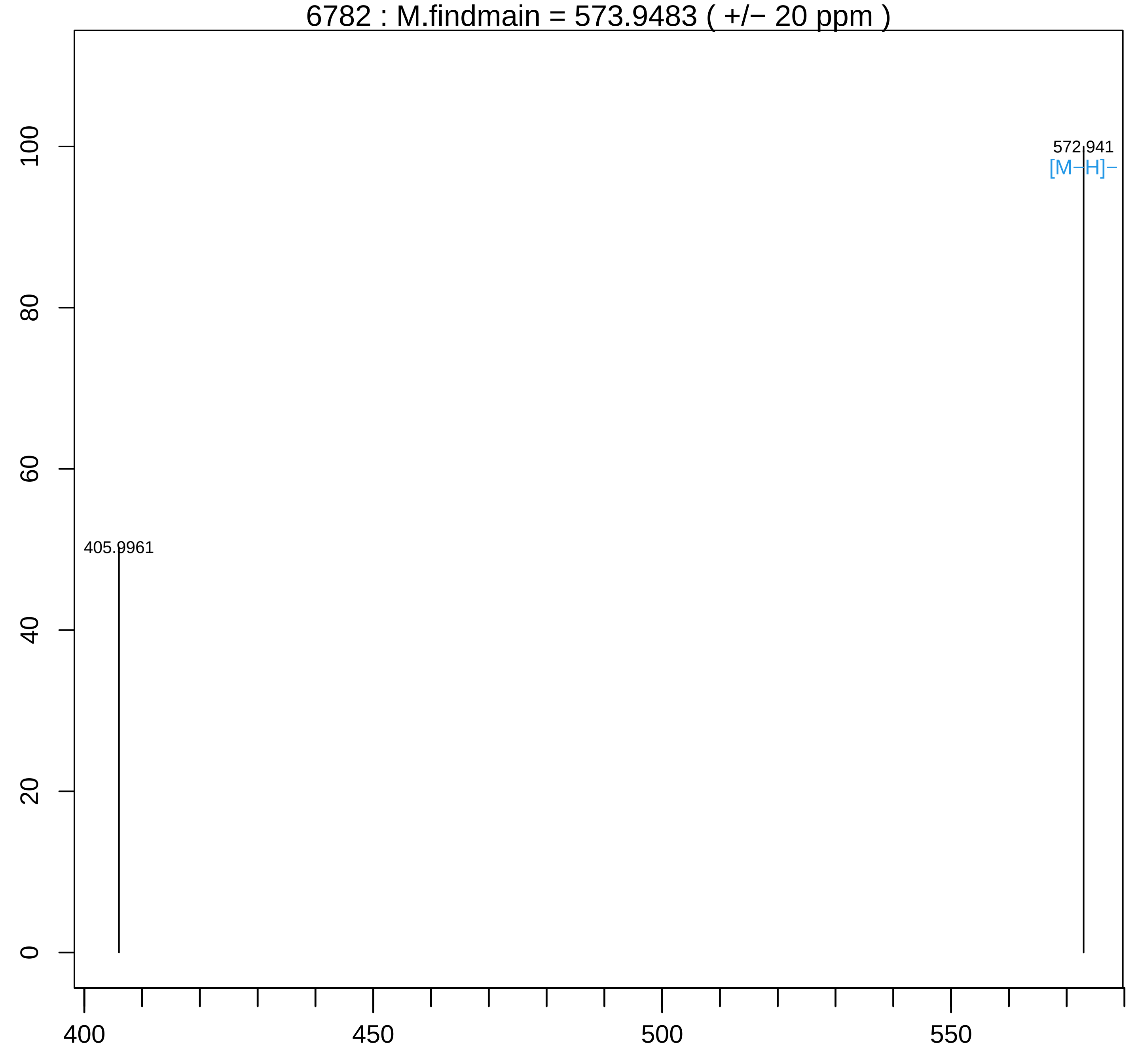
6782 : M.findmain = 573.9483 (+/− 20 ppm)
572.941
[M−H]−
405.9961
0
20
40
60
80
100
400
450
500
550

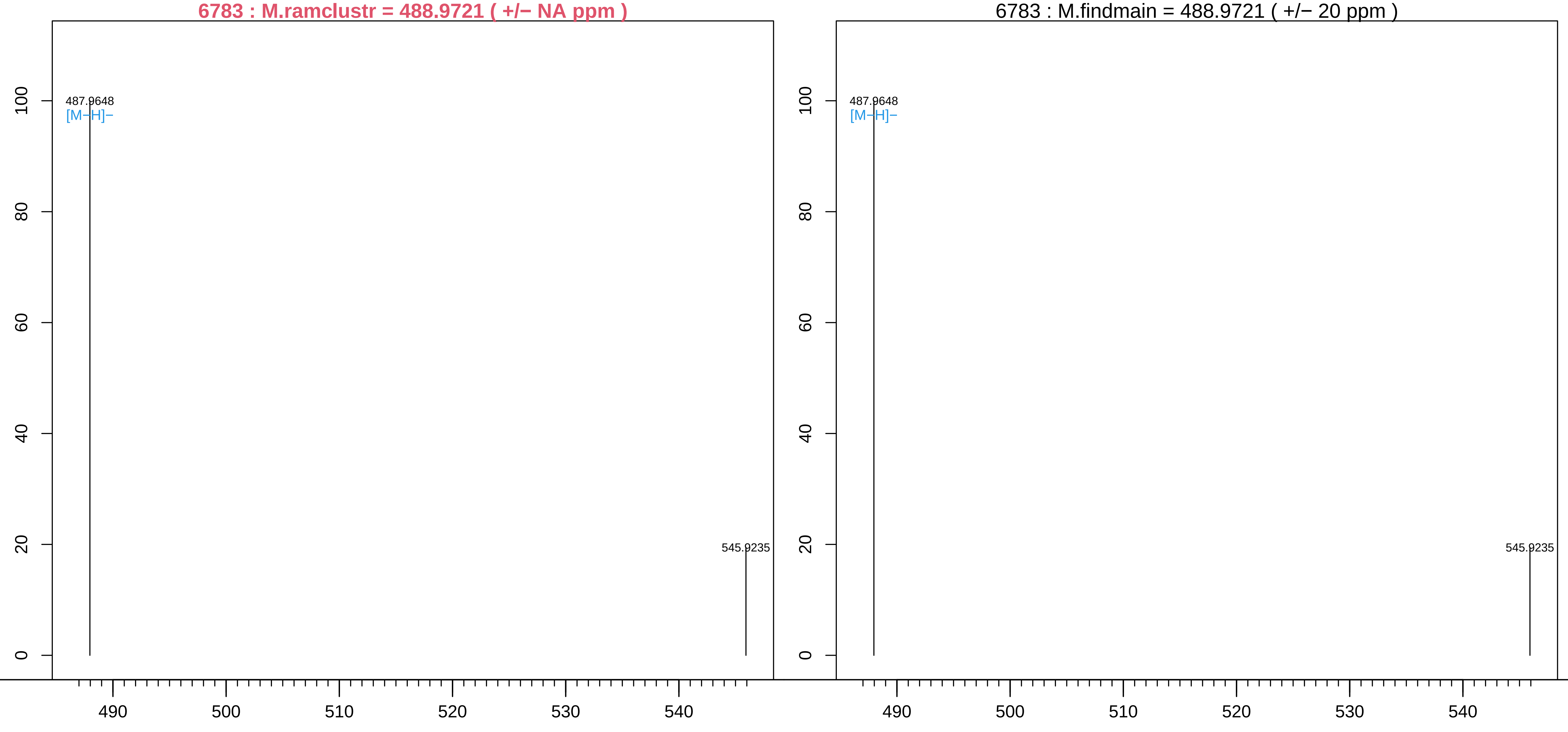
6783 : M.ramclustr = 488.9721 (+/− NA ppm)
487.9648
[M−H]−
545.9235
6783 : M.findmain = 488.9721 (+/− 20 ppm)
487.9648
[M−H]−
545.9235
0
20
40
60
80
100
490
500
510
520
530
540

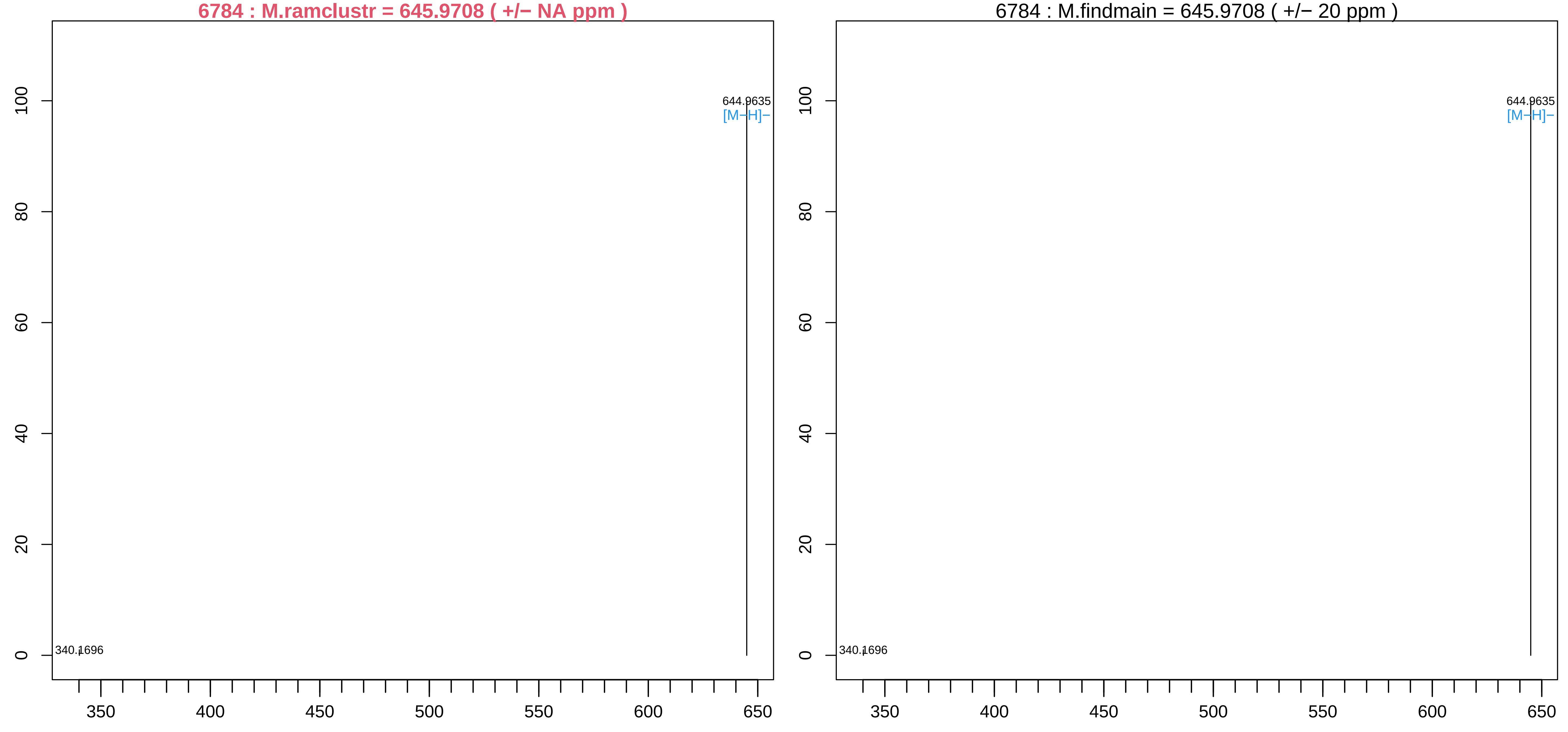
6784 : M.ramclustr = 645.9708 (+/− NA ppm)
644.9635
[M−H]−
340.1696
0
20
40
60
80
100
350
400
450
500
550
600
650
6784 : M.findmain = 645.9708 (+/− 20 ppm)
644.9635
[M−H]−
340.1696
0
20
40
60
80
100
350
400
450
500
550
600
650

6785 : M.ramclustr = 499.9667 (+/− NA ppm)

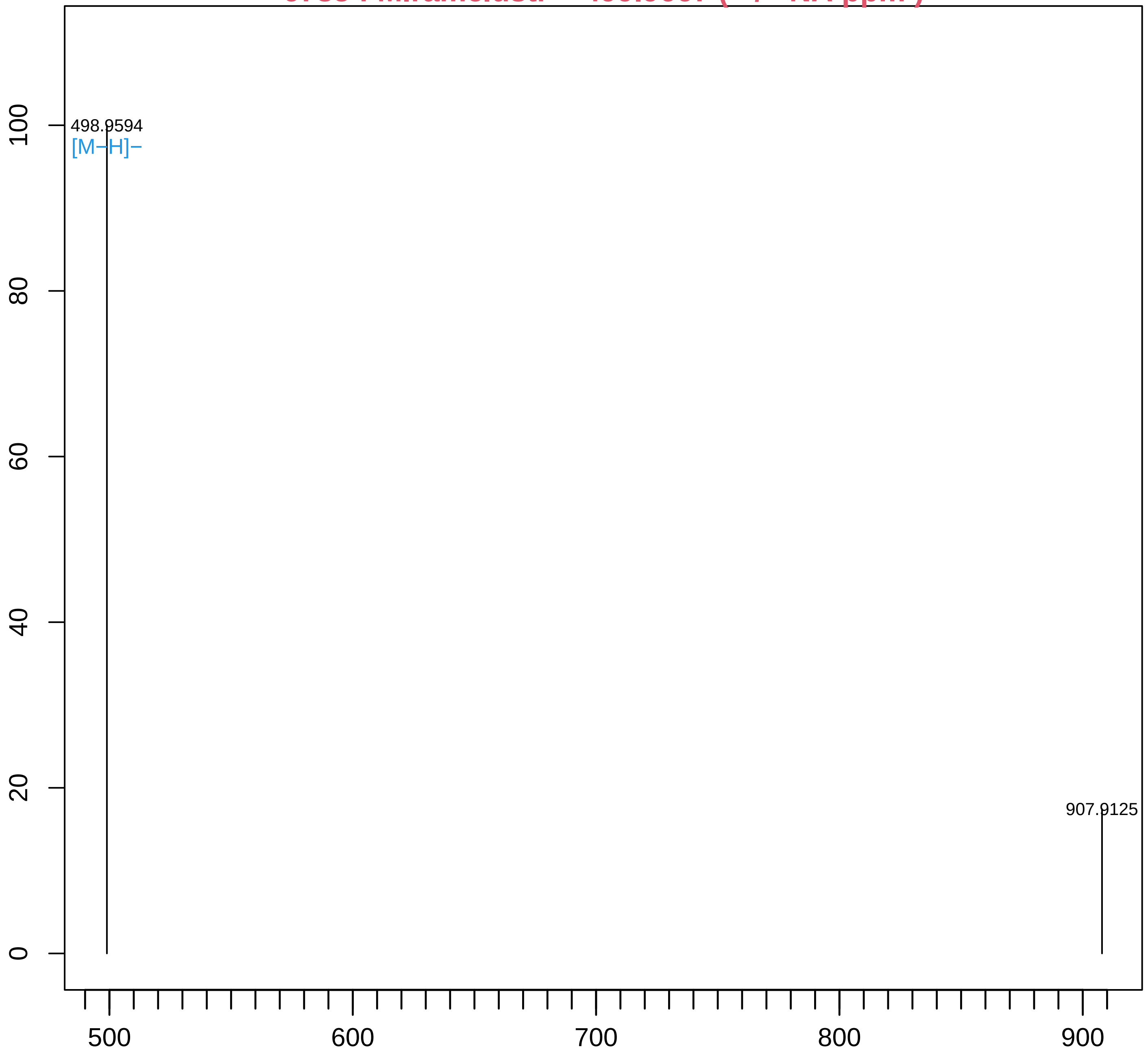

6785 : M.findmain = 499.9667 (+/− 20 ppm)

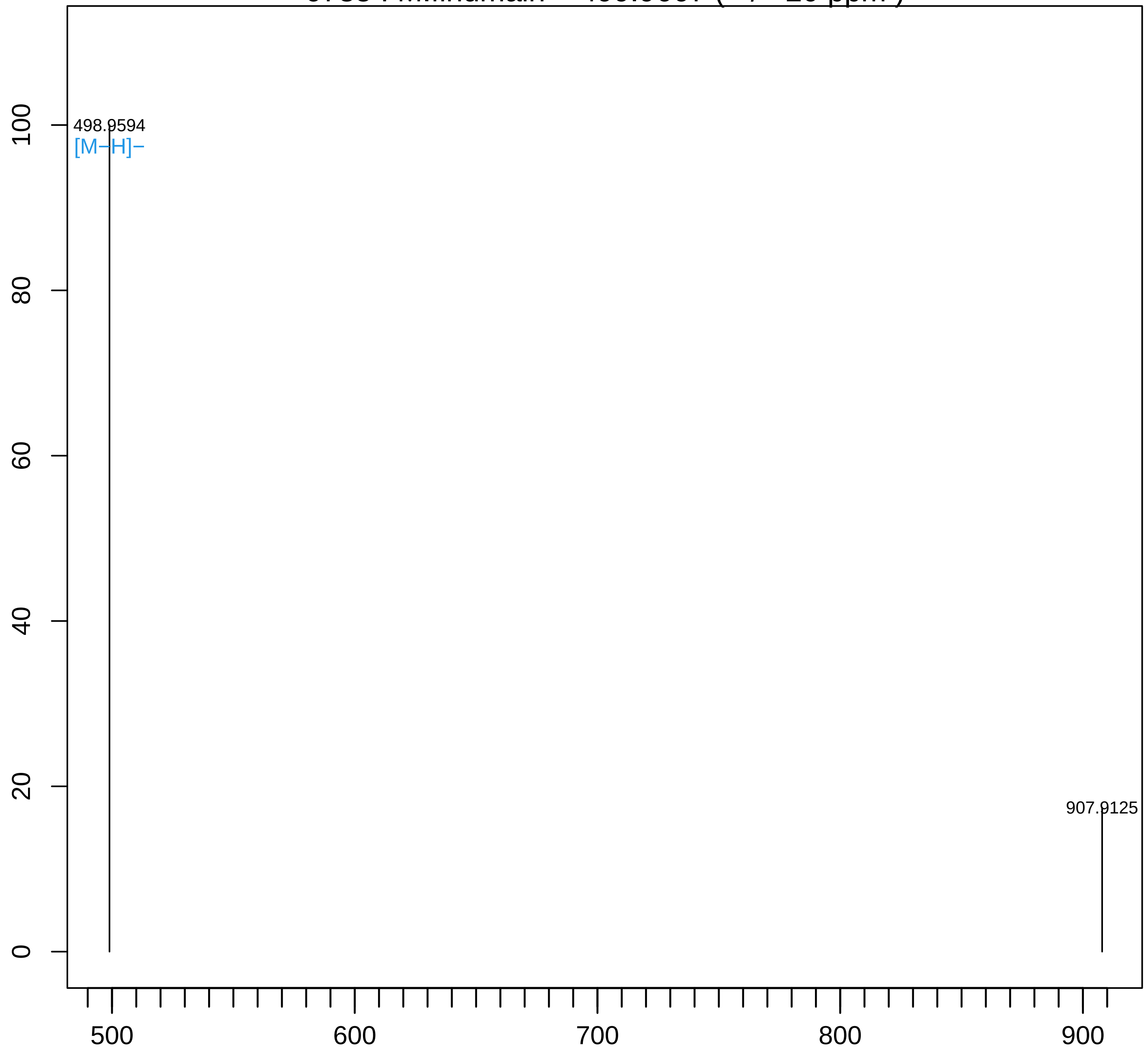

6786 : M.ramclustr = 869.9598 (+/− NA ppm)

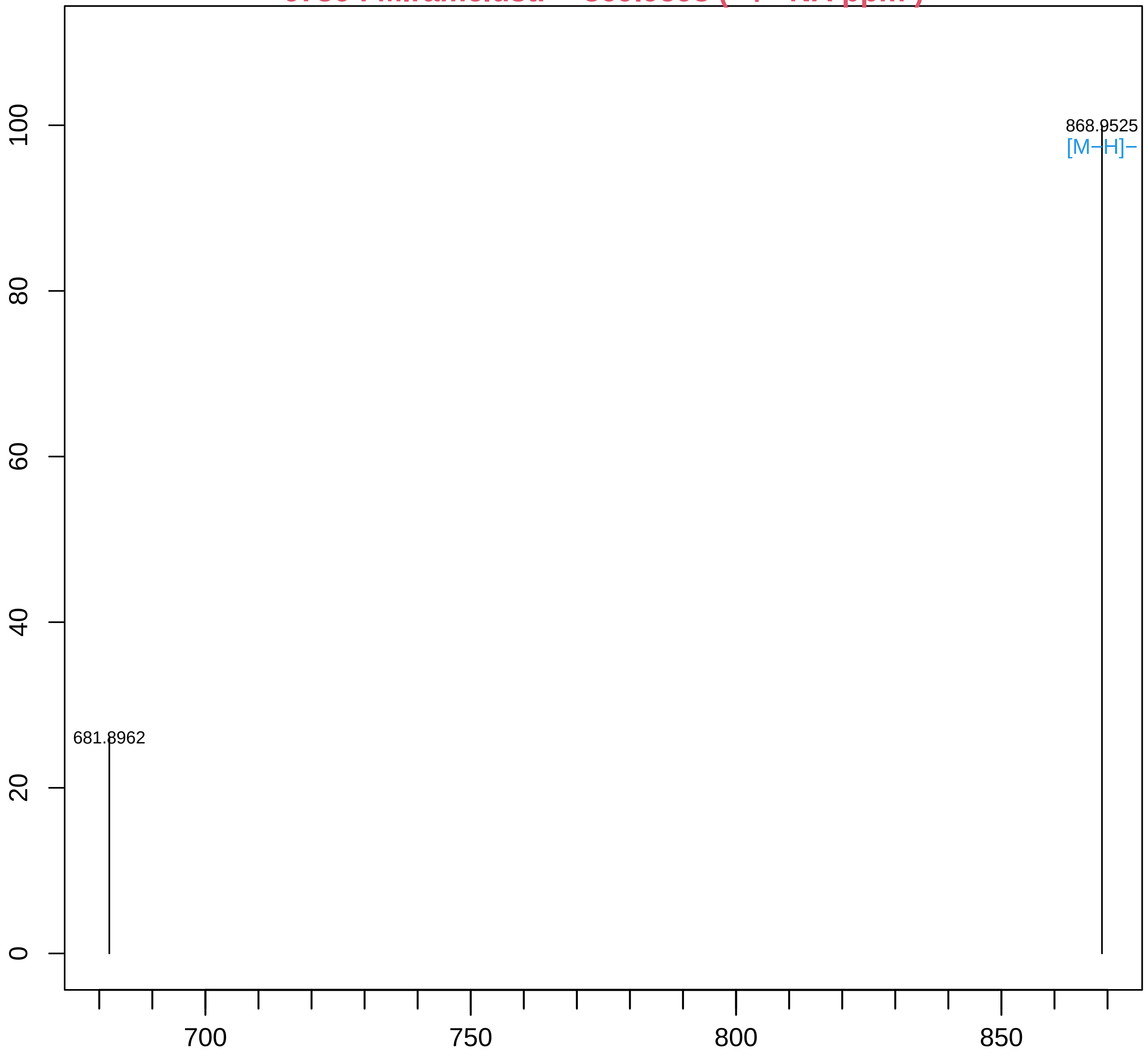

6786 : M.findmain = 869.9598 (+/− 20 ppm)

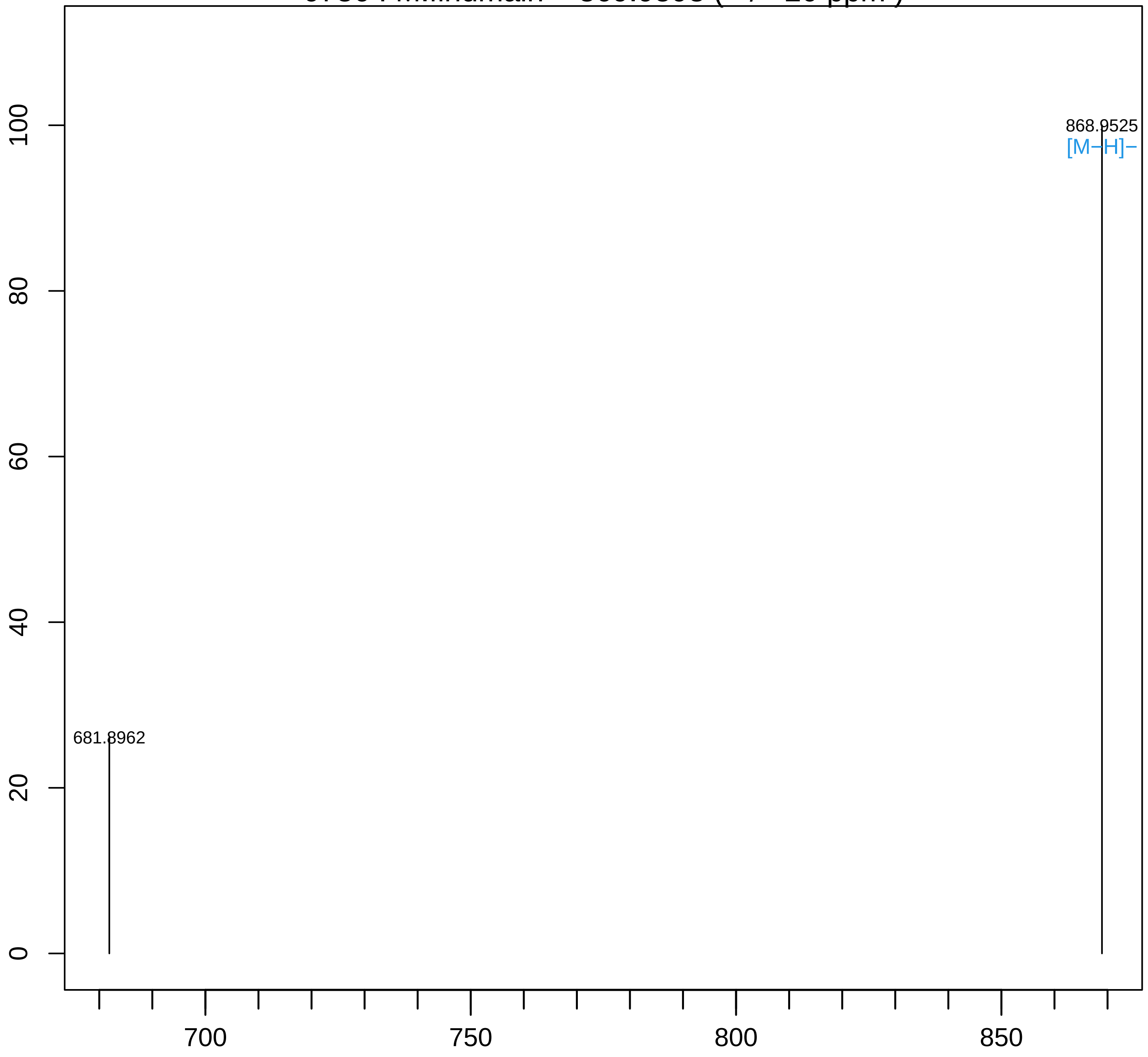

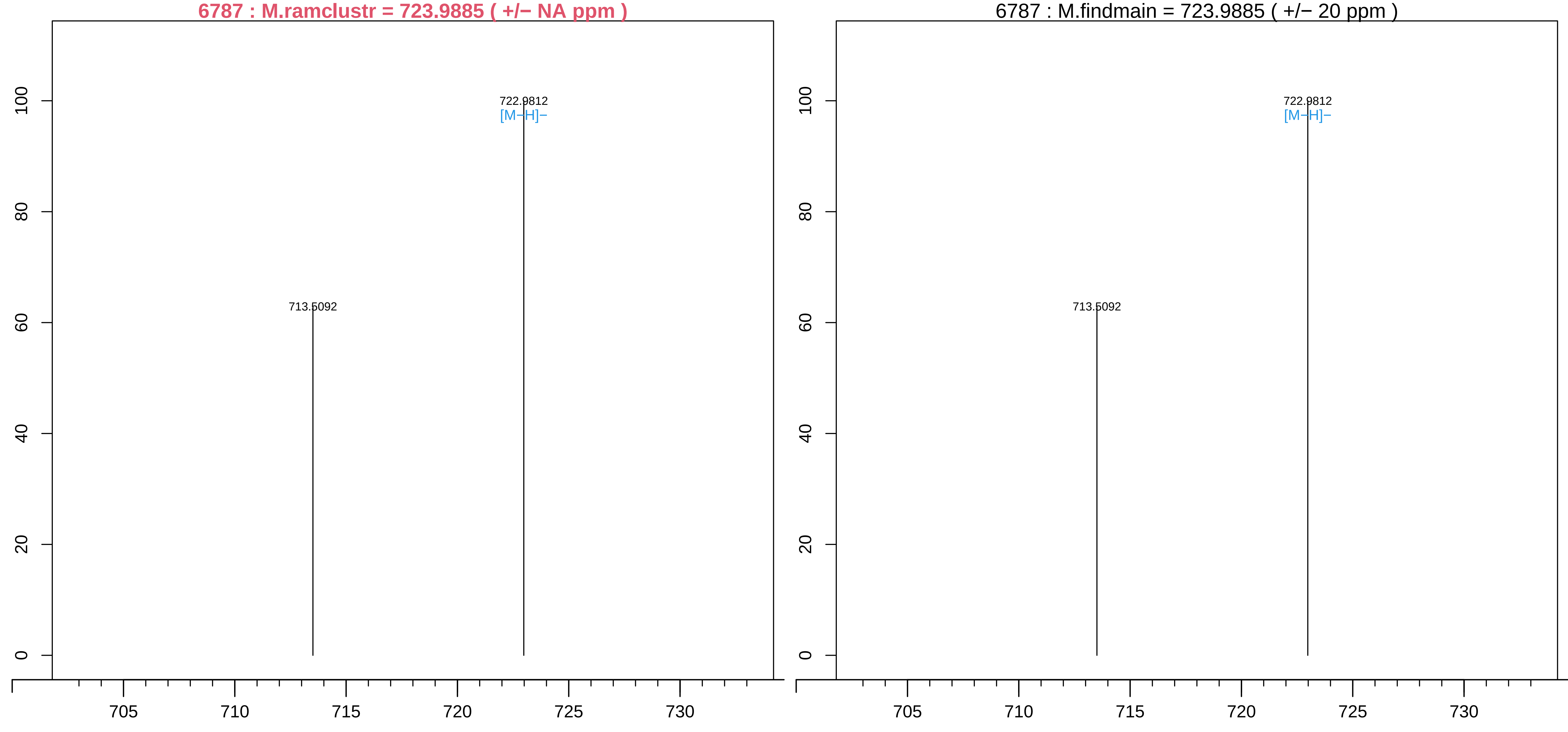
6787 : M.ramclustr = 723.9885 (+/− NA ppm)
722.9812
[M−H]−
713.5092
0
20
40
60
80
100
705
710
715
720
725
730
6787 : M.findmain = 723.9885 (+/− 20 ppm)
722.9812
[M−H]−
713.5092
0
20
40
60
80
100
705
710
715
720
725
730

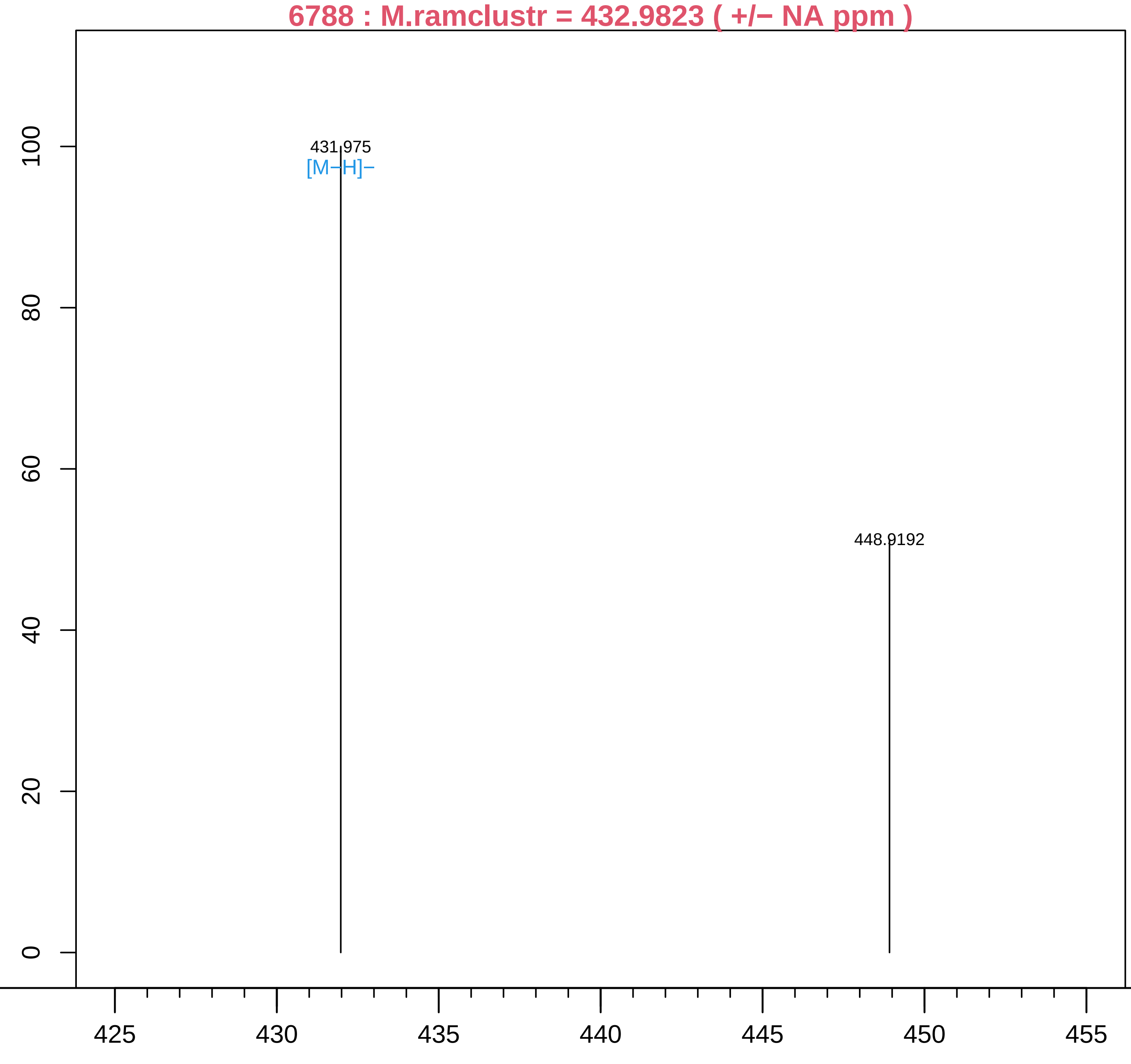

6788 : M.ramclustr = 432.9823 (+/− NA ppm)
431.975
[M−H]−
448.9192
0
20
40
60
80
100
425
430
435
440
445
450
455

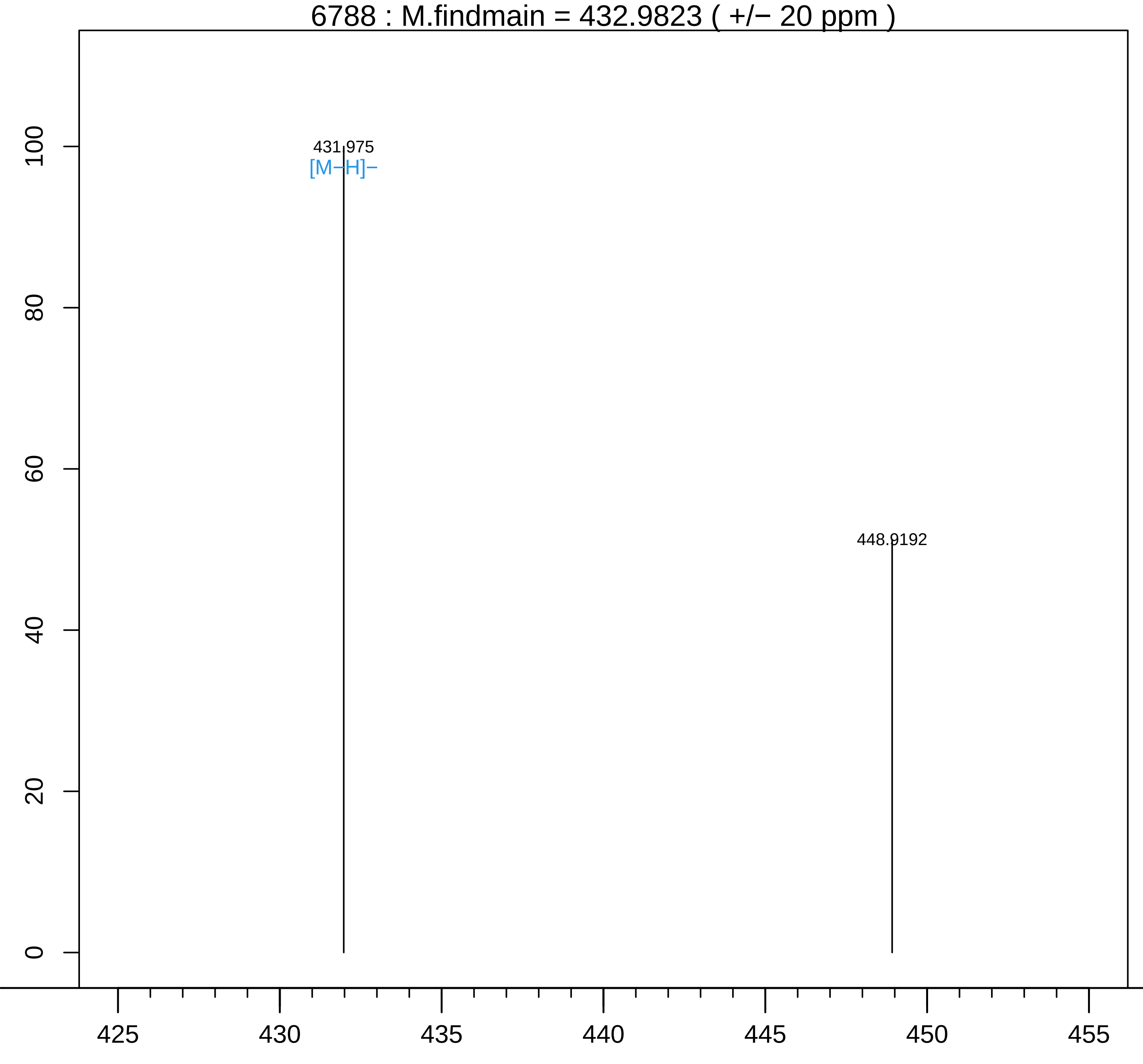

6788 : M.findmain = 432.9823 (+/− 20 ppm)
431.975
[M−H]−
448.9192
0
20
40
60
80
100
425
430
435
440
445
450
455

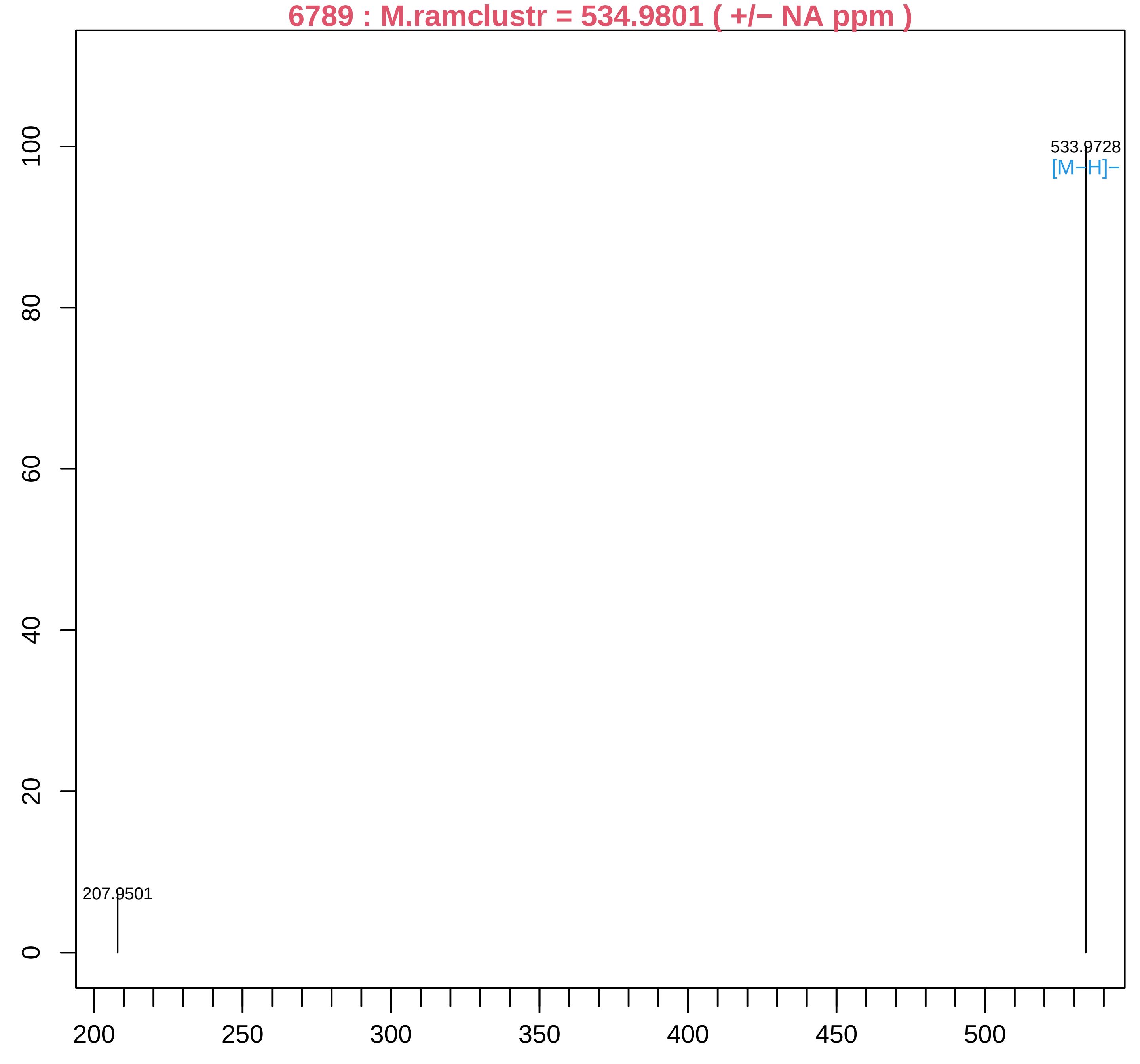
6789 : M.ramclustr = 534.9801 (+/− NA ppm)
533.9728
[M−H]−
207.9501
0
20
40
60
80
100
200
250
300
350
400
450
500

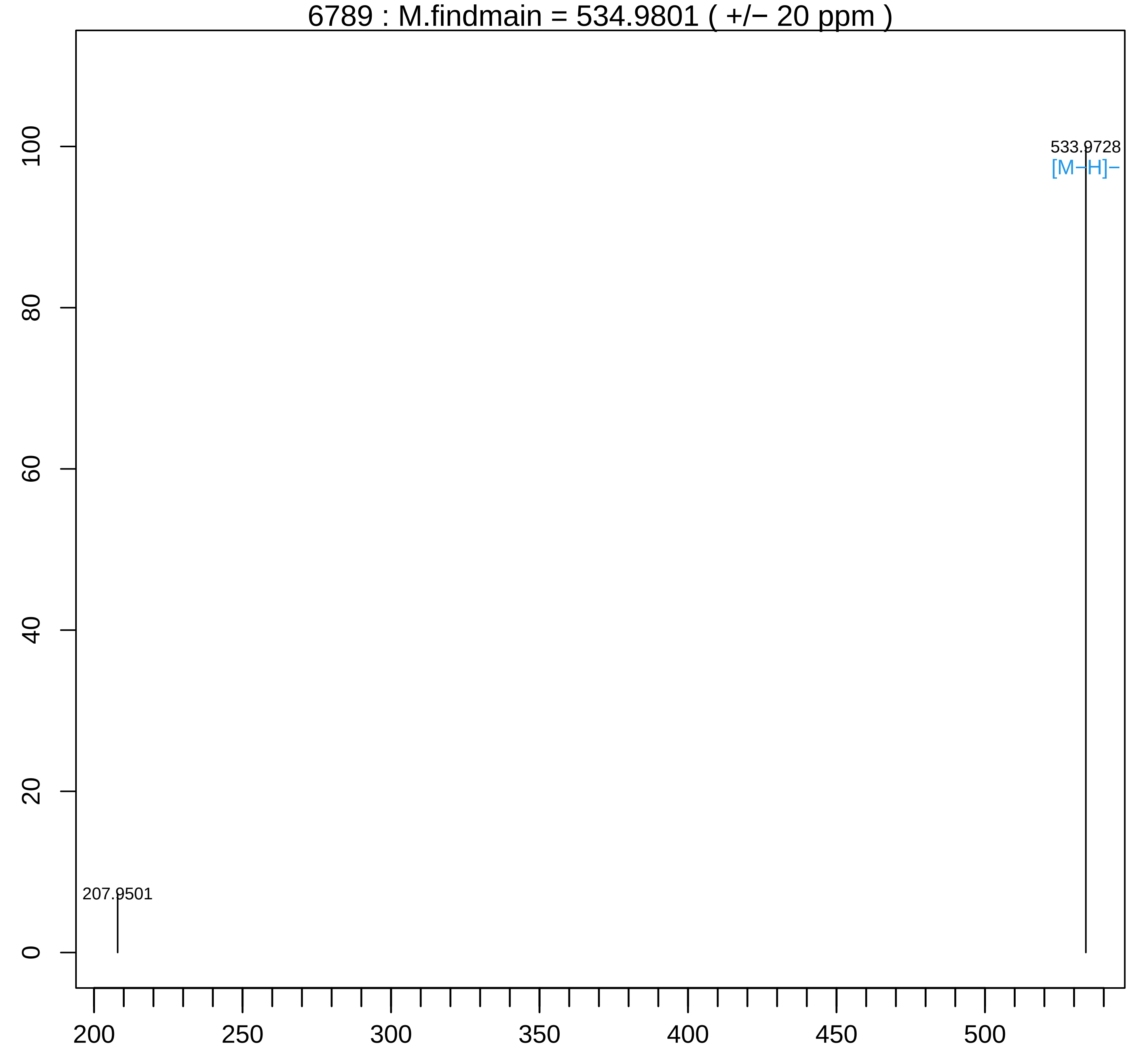
6789 : M.findmain = 534.9801 (+/− 20 ppm)
533.9728
[M−H]−
207.9501
0
20
40
60
80
100
200
250
300
350
400
450
500

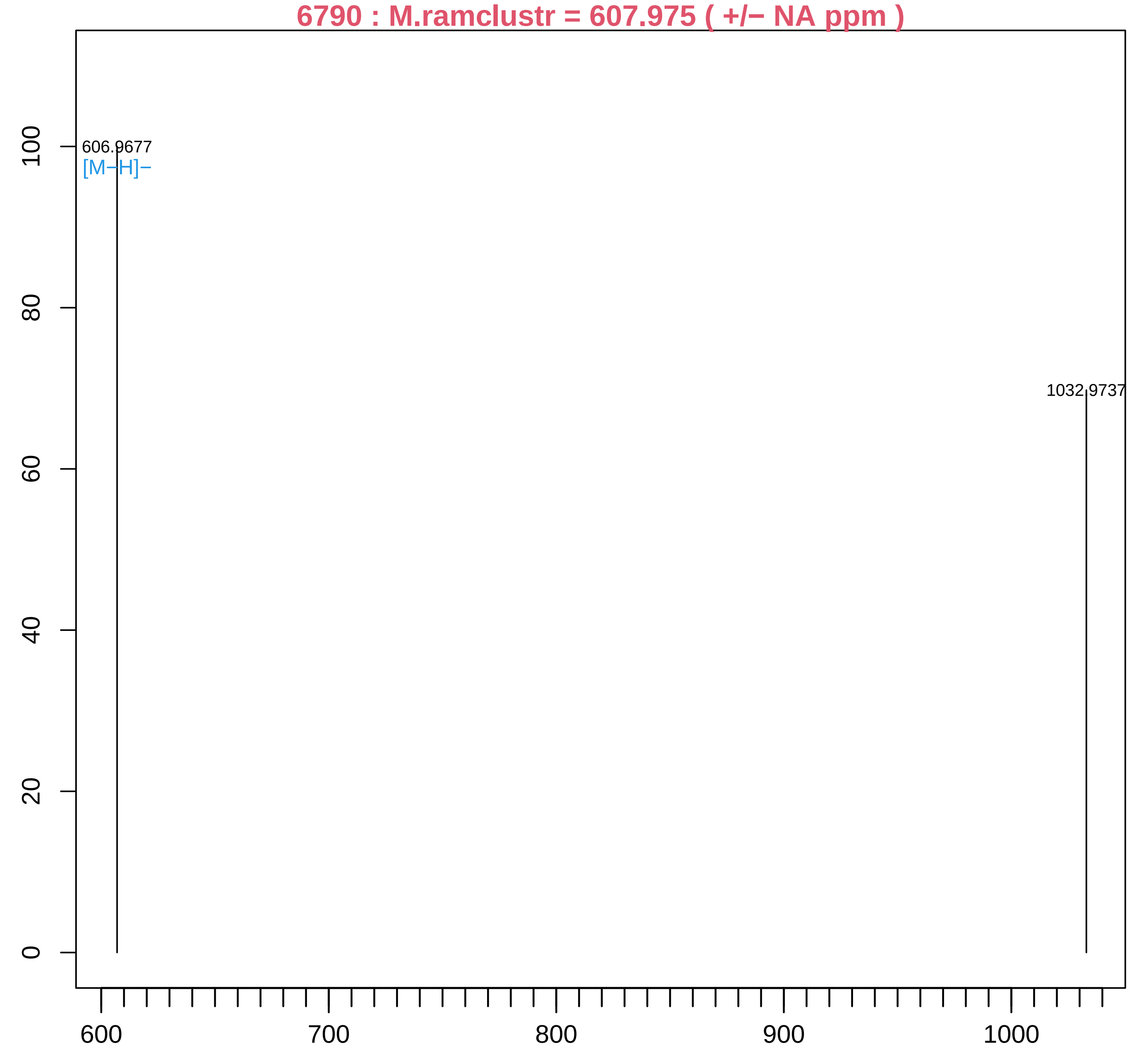
6790 : M.ramclustr = 607.975 (+/− NA ppm)
606.9677
[M−H]−
1032.9737
0
20
40
60
80
100
600
700
800
900
1000

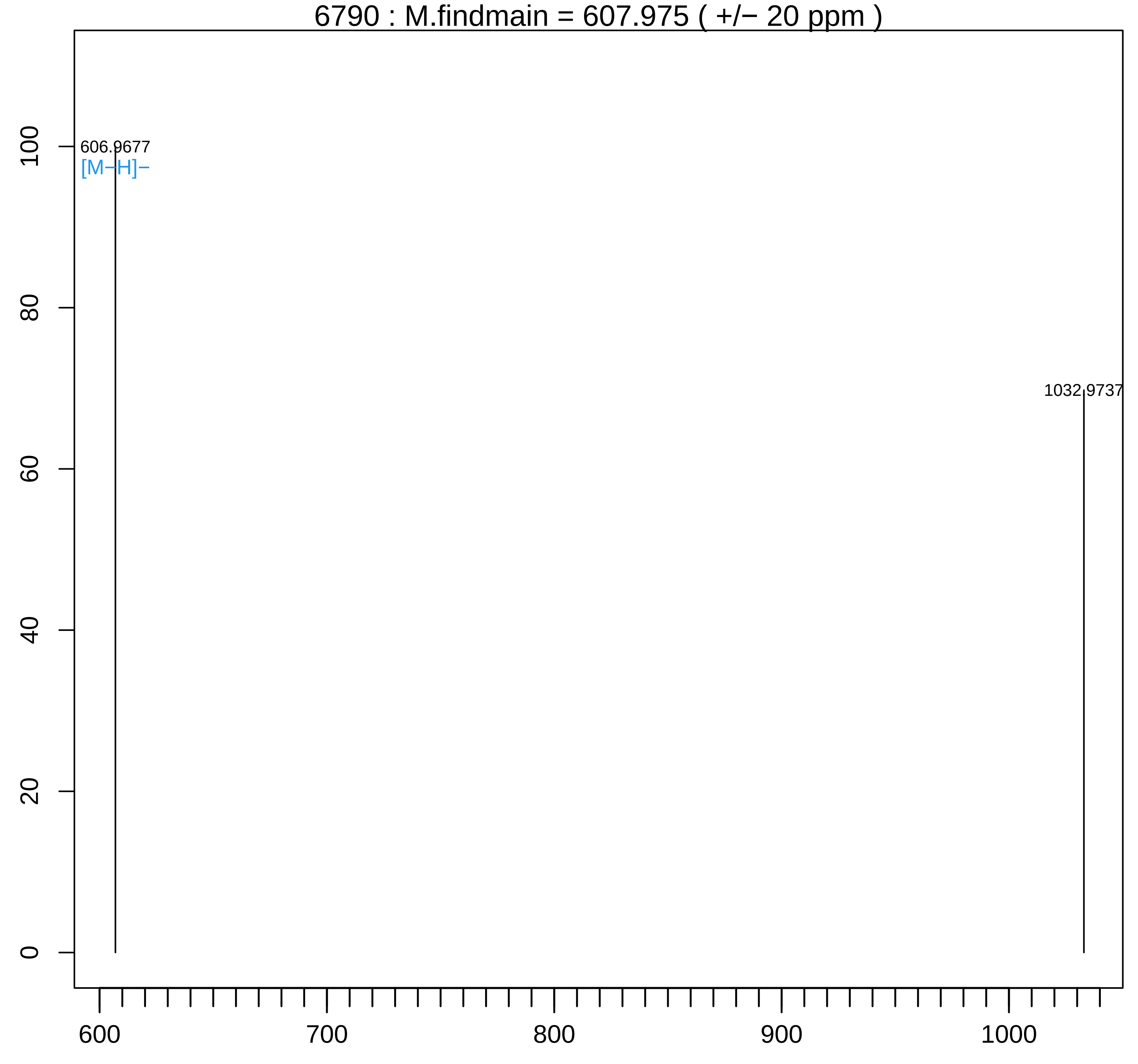
6790 : M.findmain = 607.975 (+/− 20 ppm)
606.9677
[M−H]−
1032.9737
0
20
40
60
80
100
600
700
800
900
1000

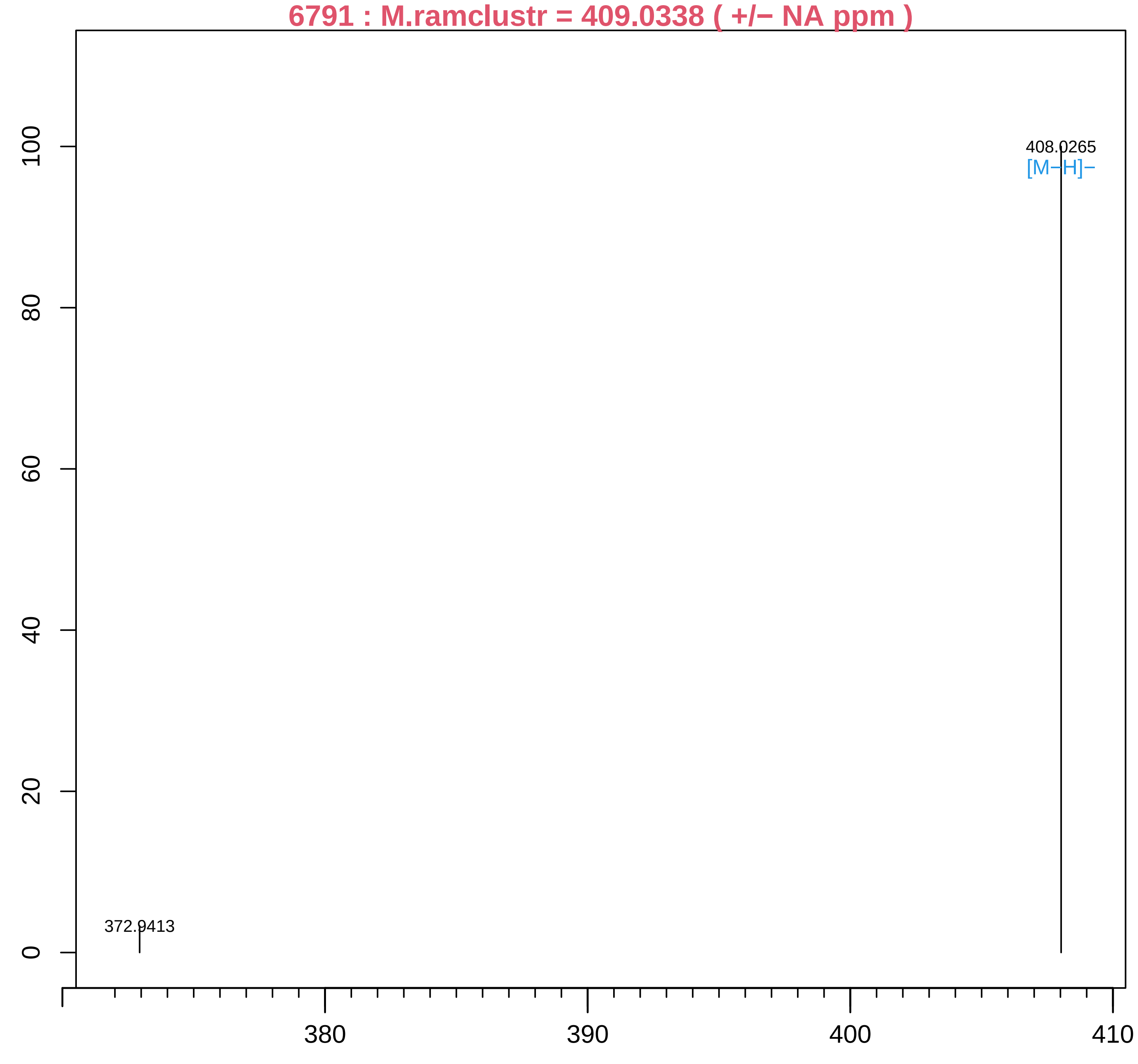
6791 : M.ramclustr = 409.0338 (+/− NA ppm)
408.0265
[M−H]−
372.9413
0
20
40
60
80
100
380
390
400
410

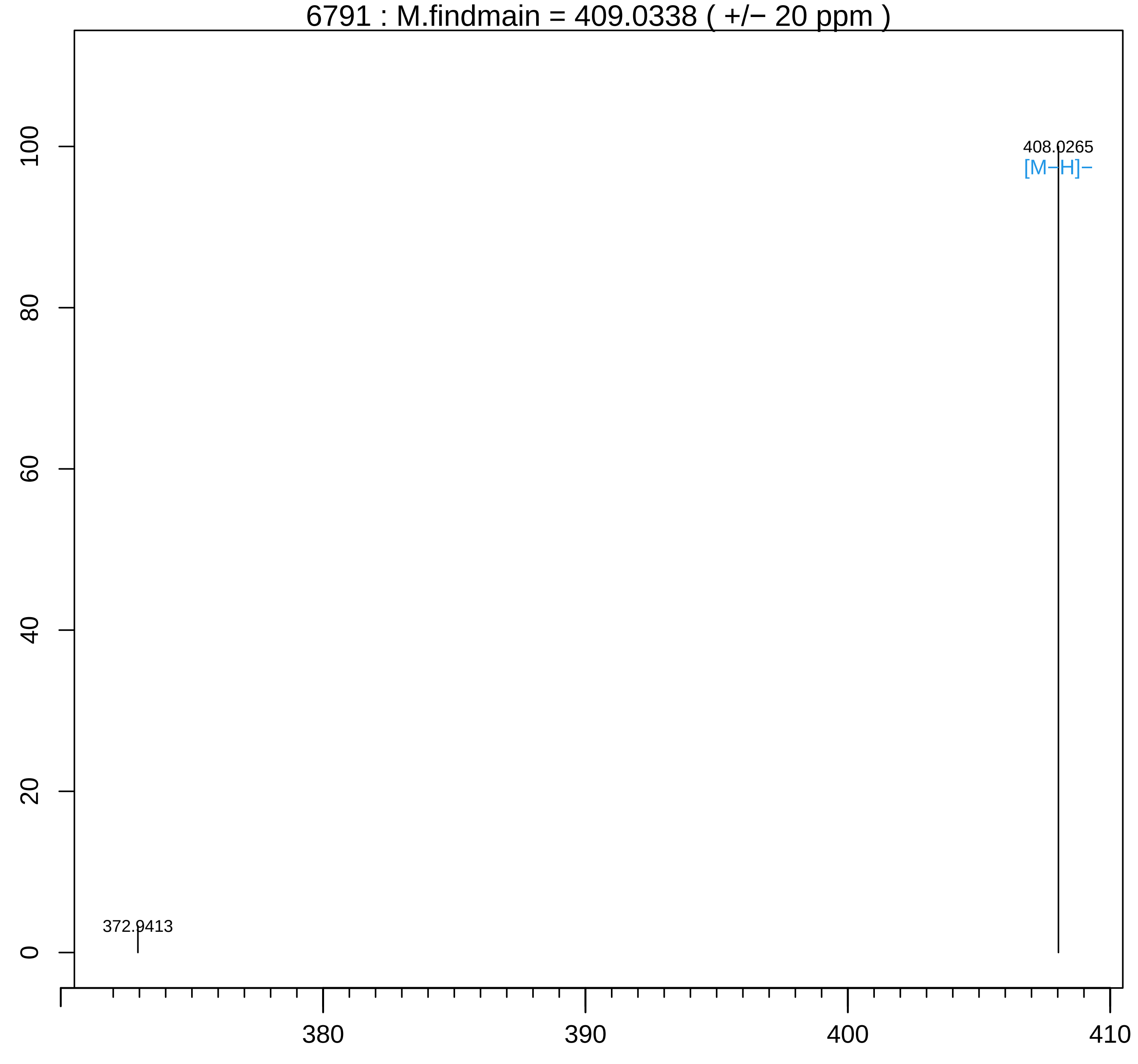
6791 : M.findmain = 409.0338 (+/− 20 ppm)
408.0265
[M−H]−
372.9413
0
20
40
60
80
100
380
390
400
410

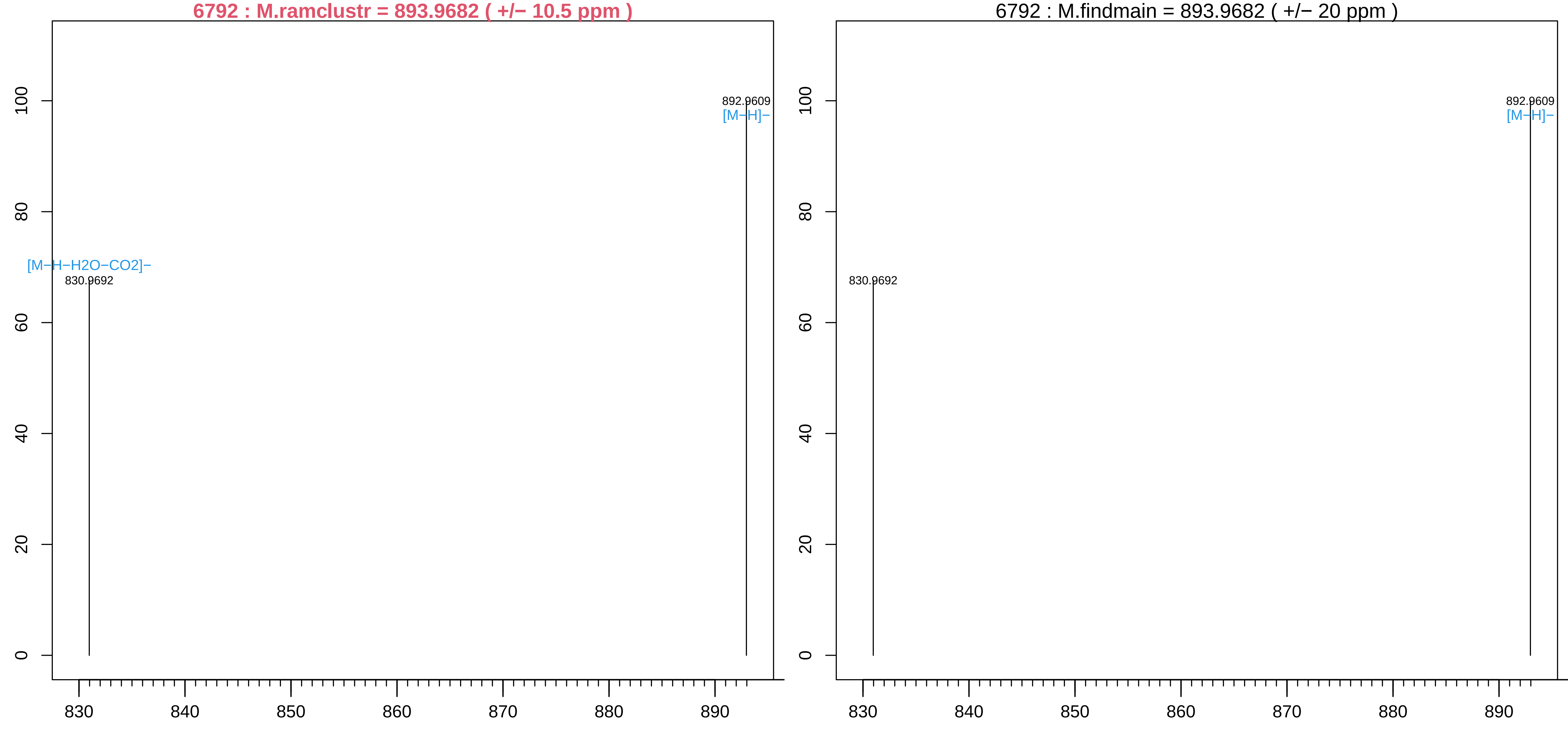
6792 : M.ramclustr = 893.9682 (+/− 10.5 ppm)
6792 : M.findmain = 893.9682 (+/− 20 ppm)
892.9609
[M−H]−
[M−H−H2O−CO2]−
830.9692
892.9609
[M−H]−
830.9692
0
20
40
60
80
100
830
840
850
860
870
880
890

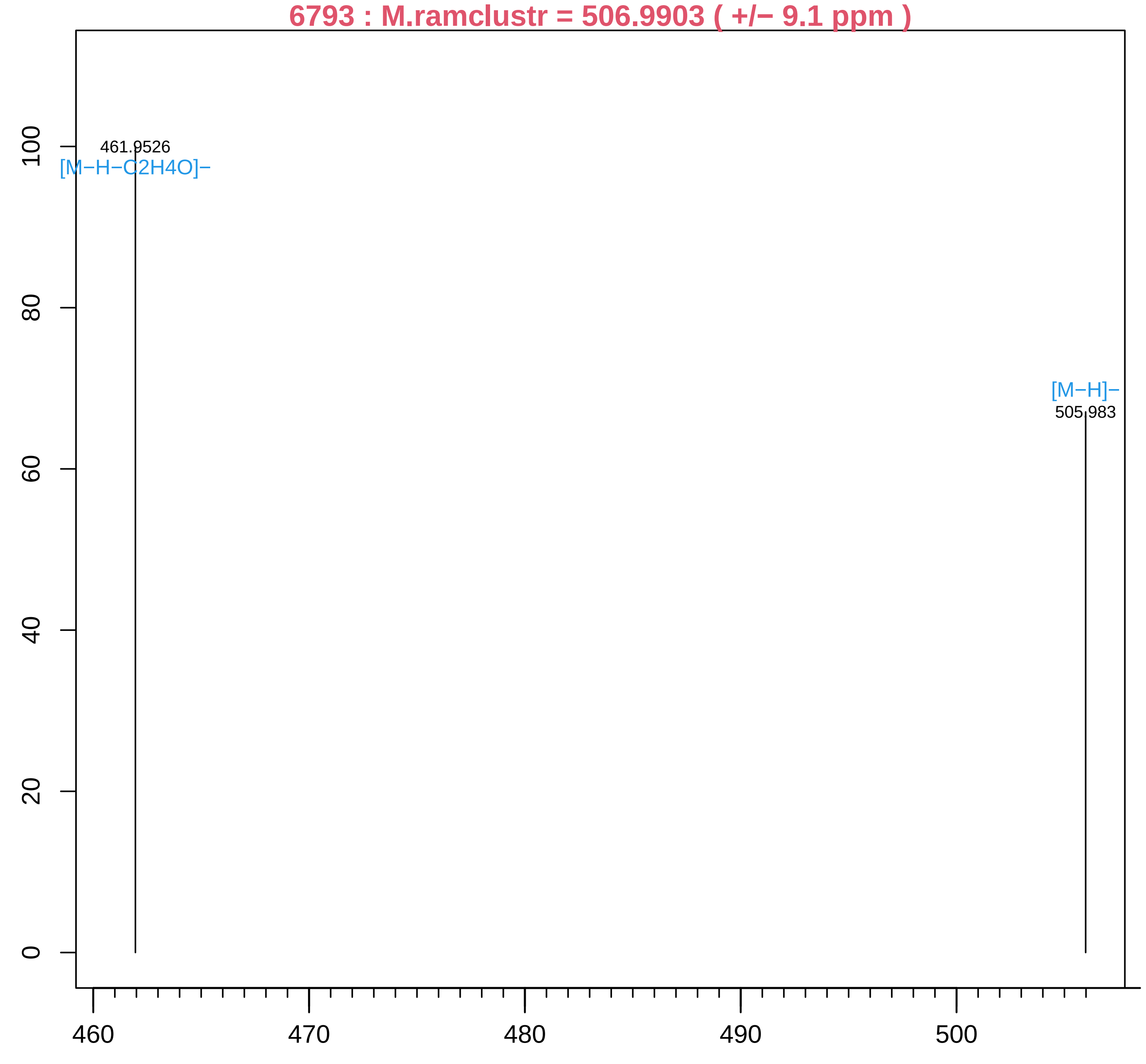
6793 : M.ramclustr = 506.9903 (+/− 9.1 ppm)
461.9526
[M−H−C2H4O]−
[M−H]−
505.983
0
20
40
60
80
100
460
470
480
490
500

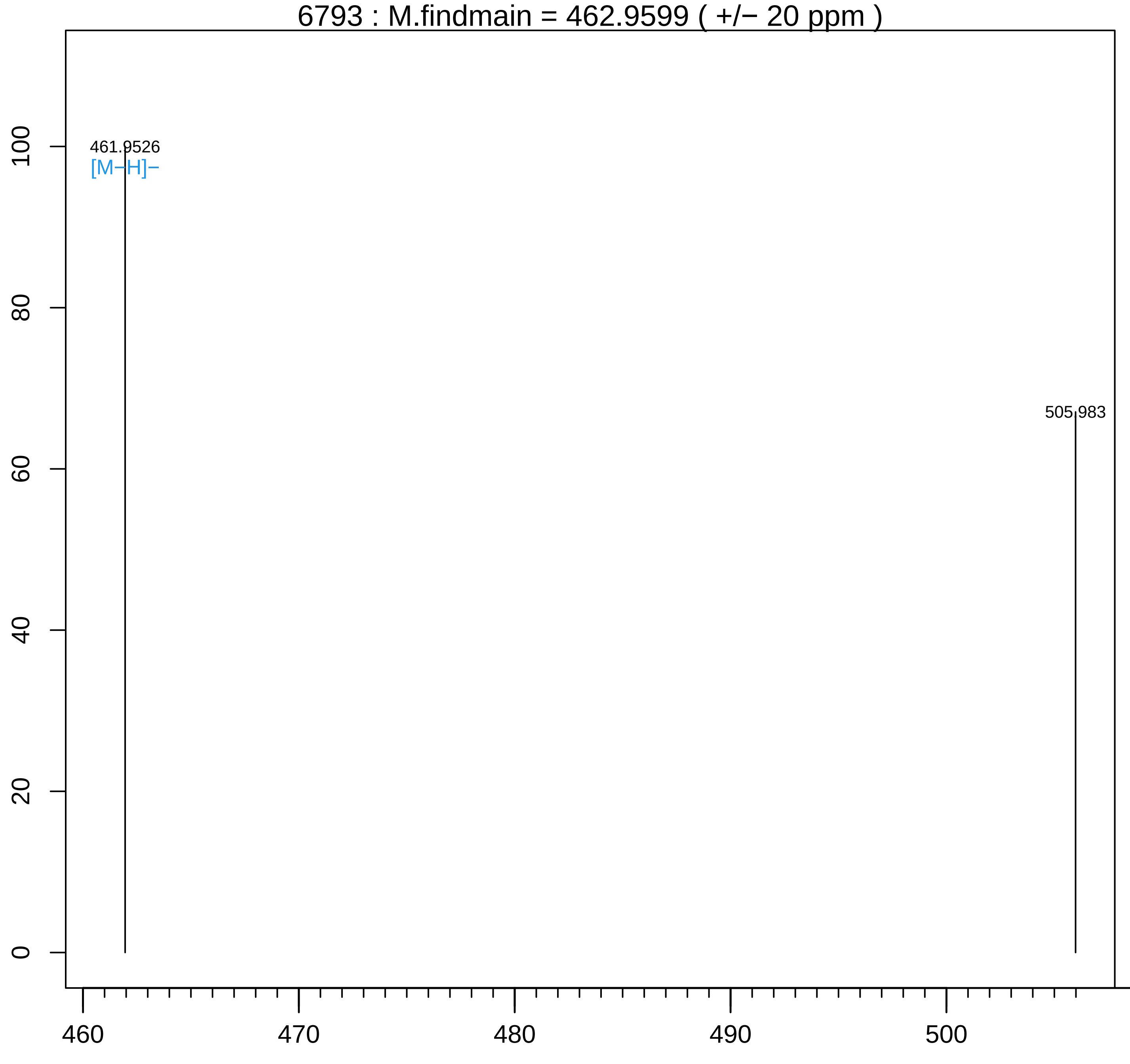
6793 : M.findmain = 462.9599 (+/− 20 ppm)
461.9526
[M−H]−
505.983
0
20
40
60
80
100
460
470
480
490
500

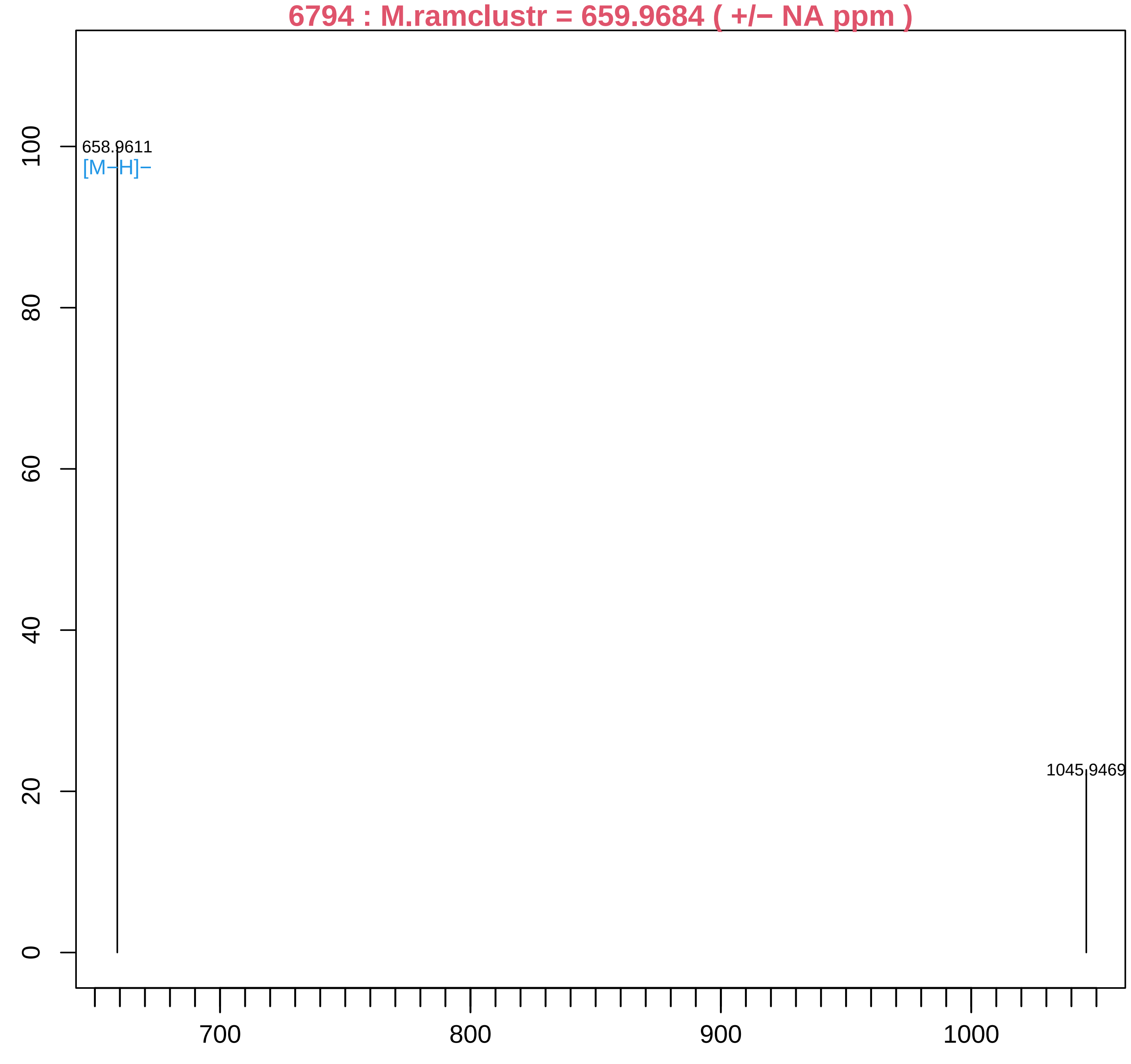

6794 : M.ramclustr = 659.9684 (+/− NA ppm)
658.9611
[M−H]−
1045.9469
0
20
40
60
80
100
700
800
900
1000

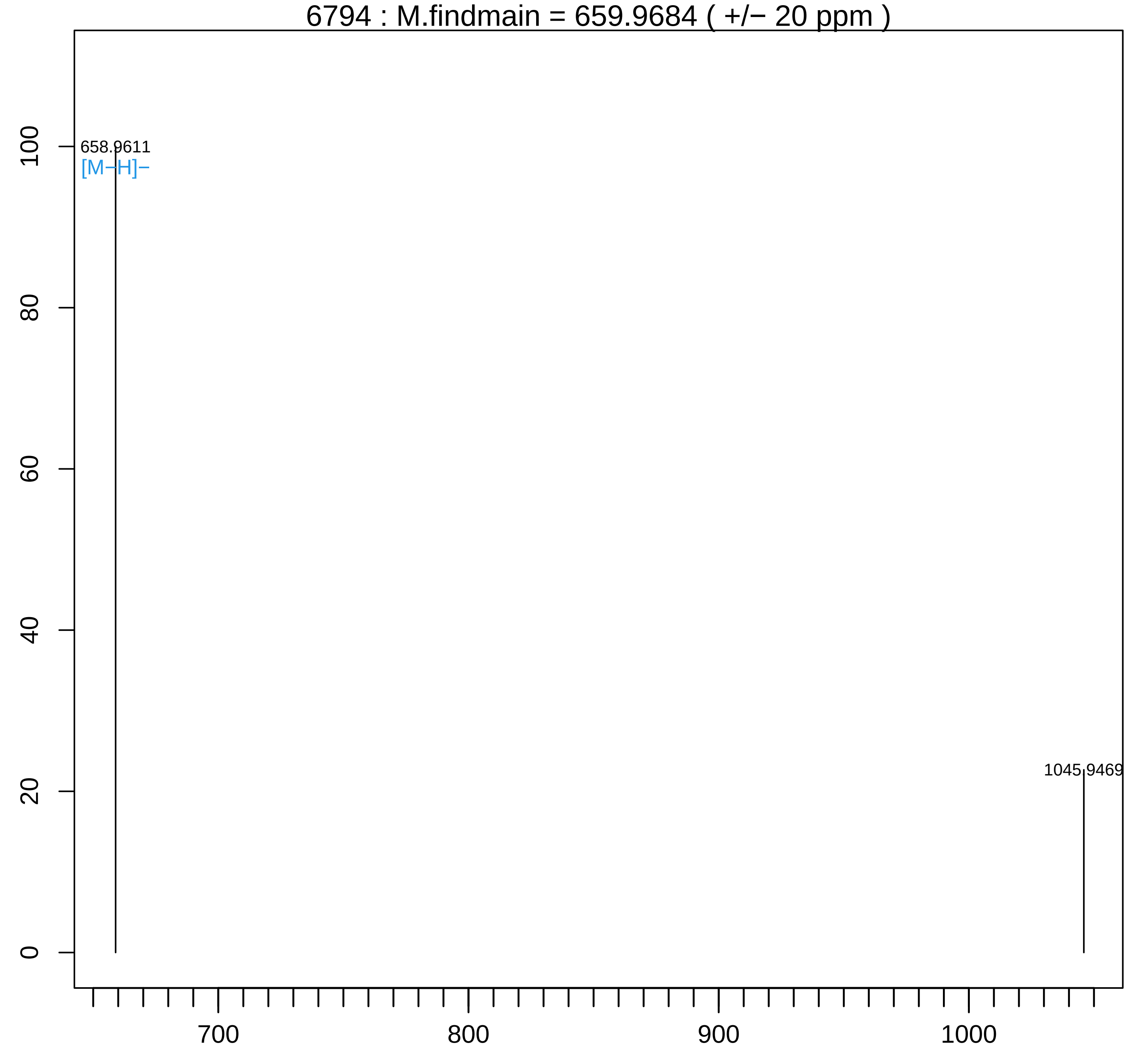

6794 : M.findmain = 659.9684 (+/− 20 ppm)
658.9611
[M−H]−
1045.9469
0
20
40
60
80
100
700
800
900
1000

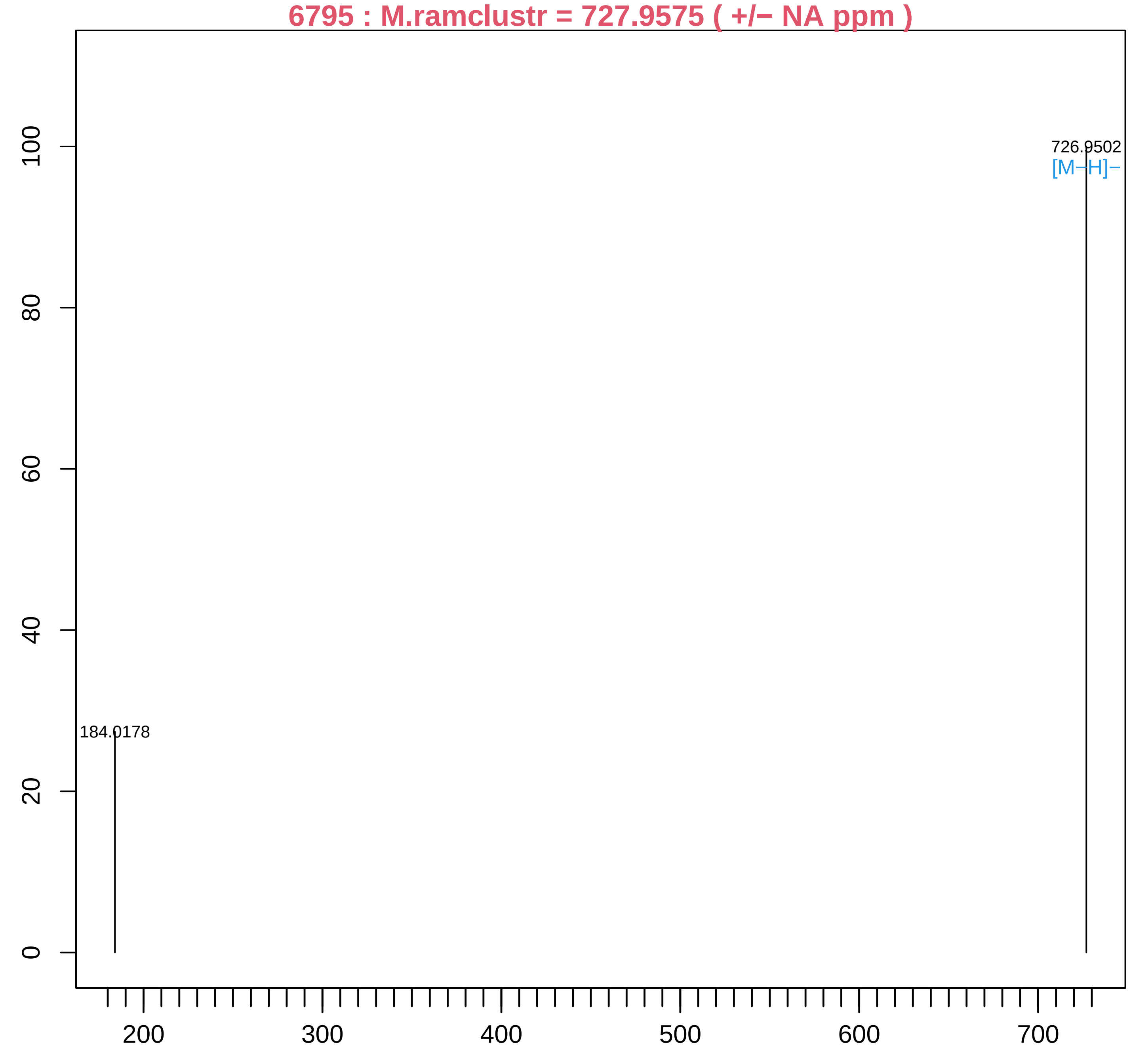
6795 : M.ramclustr = 727.9575 (+/− NA ppm)
726.9502
[M−H]−
184.0178
0
20
40
60
80
100
200
300
400
500
600
700

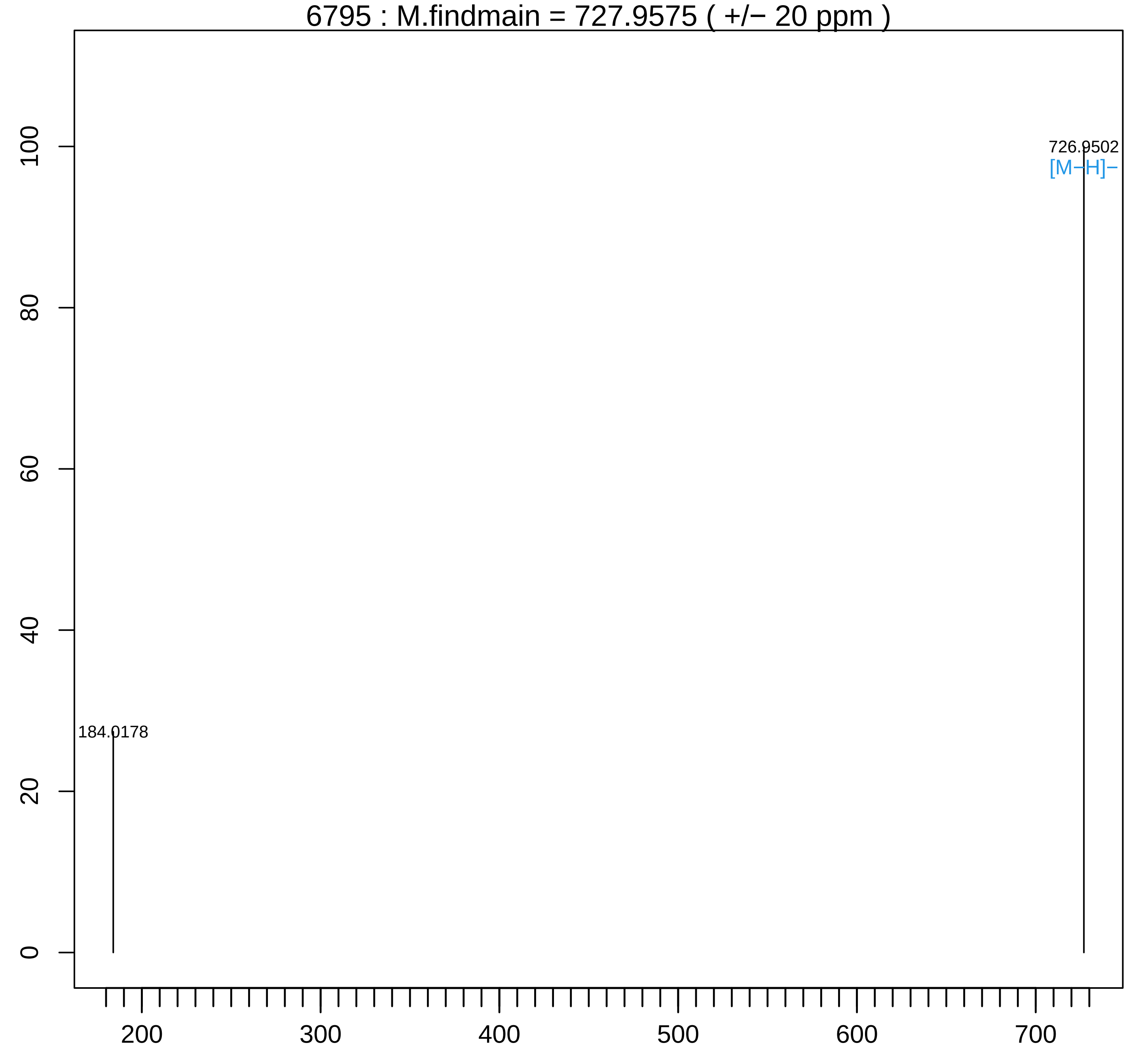
6795 : M.findmain = 727.9575 (+/− 20 ppm)
726.9502
[M−H]−
184.0178
0
20
40
60
80
100
200
300
400
500
600
700

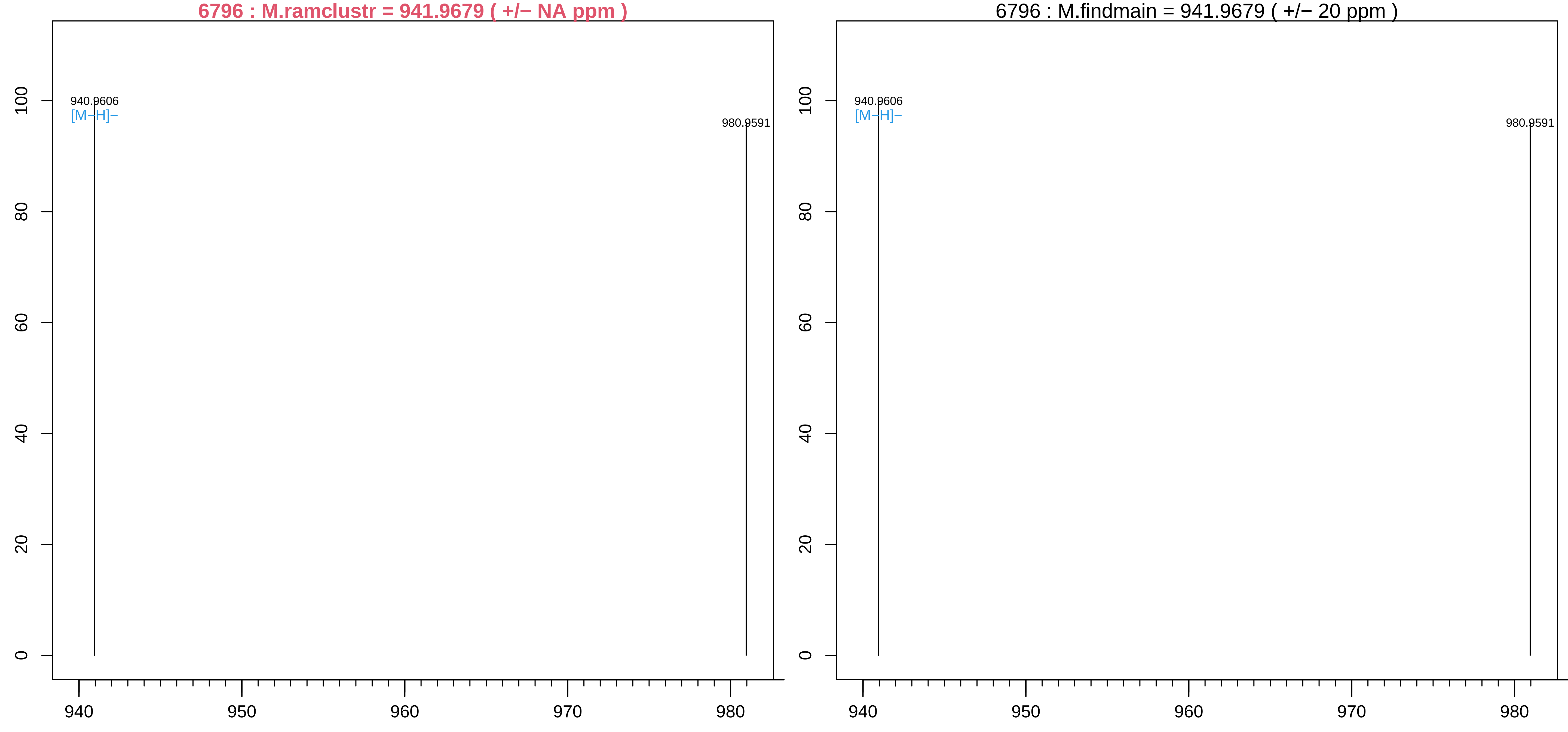

6796 : M.ramclustr = 941.9679 (+/− NA ppm)
940.9606
[M−H]−
980.9591
6796 : M.findmain = 941.9679 (+/− 20 ppm)
940.9606
[M−H]−
980.9591
0
20
40
60
80
100
940
950
960
970
980

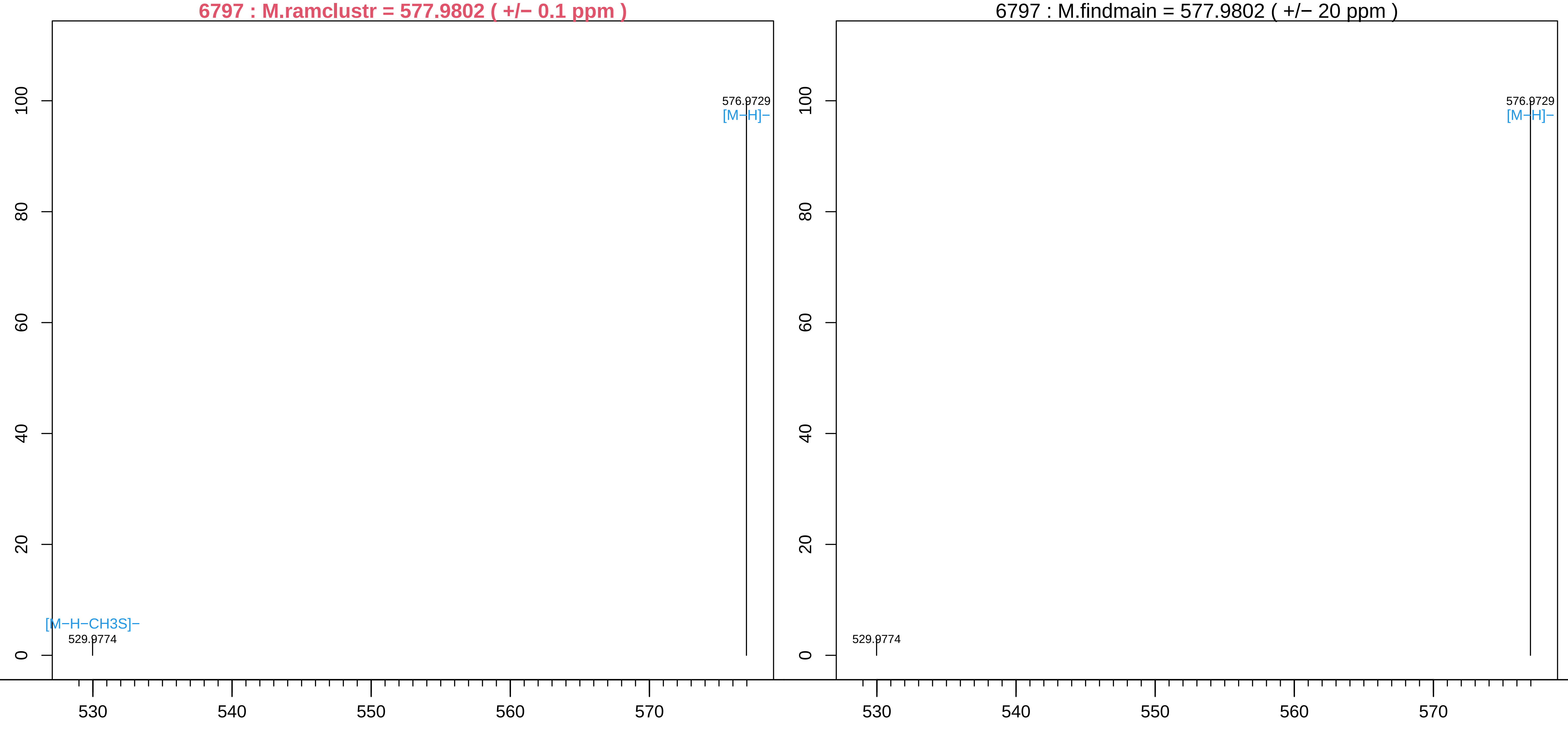
6797 : M.ramclustr = 577.9802 (+/− 0.1 ppm)
576.9729
[M−H]−
[M−H−CH3S]−
529.9774
6797 : M.findmain = 577.9802 (+/− 20 ppm)
576.9729
[M−H]−
529.9774
0
20
40
60
80
100
530
540
550
560
570

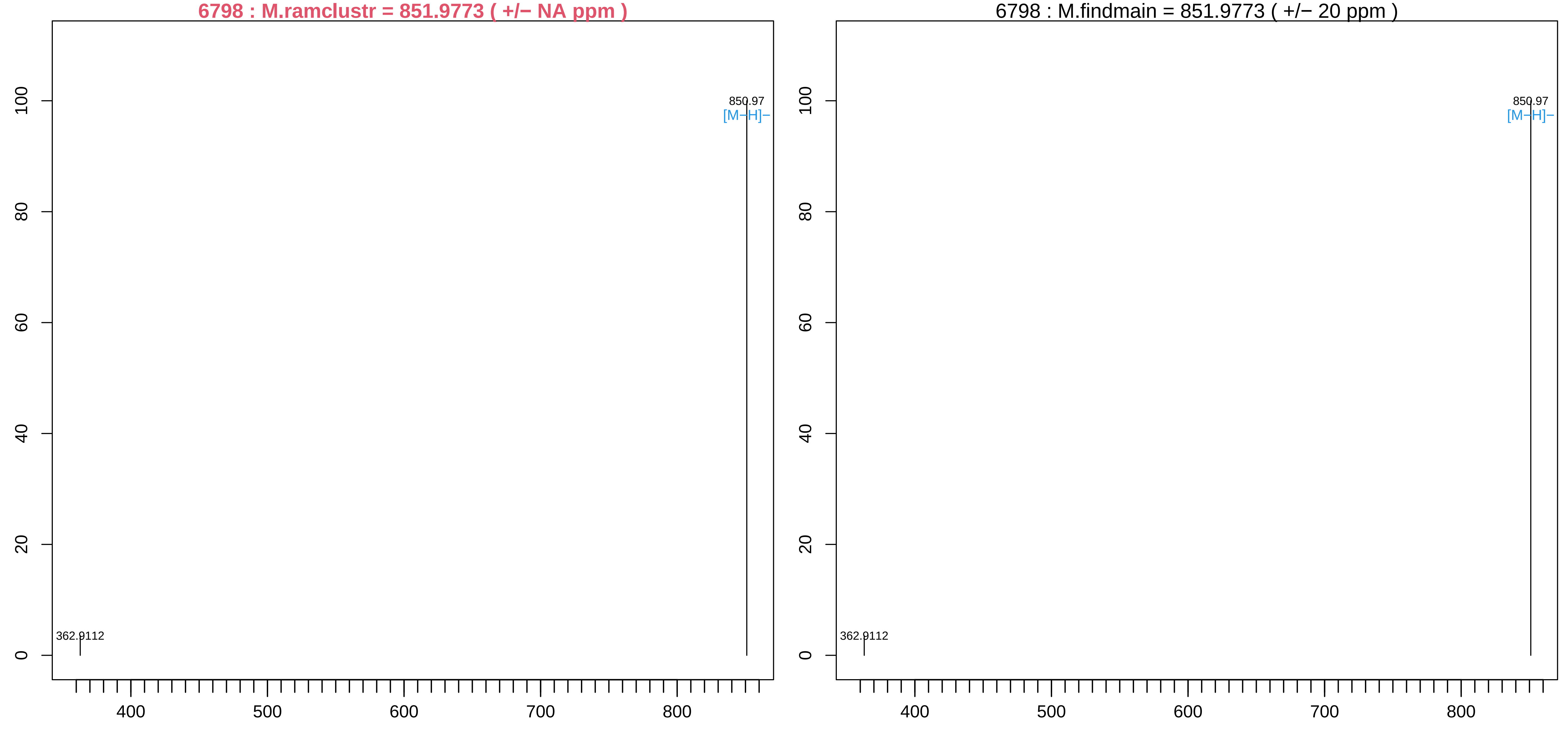
6798 : M.ramclustr = 851.9773 (+/− NA ppm)
850.97
[M−H]−
362.9112
6798 : M.findmain = 851.9773 (+/− 20 ppm)
850.97
[M−H]−
362.9112
0
20
40
60
80
100
400
500
600
700
800

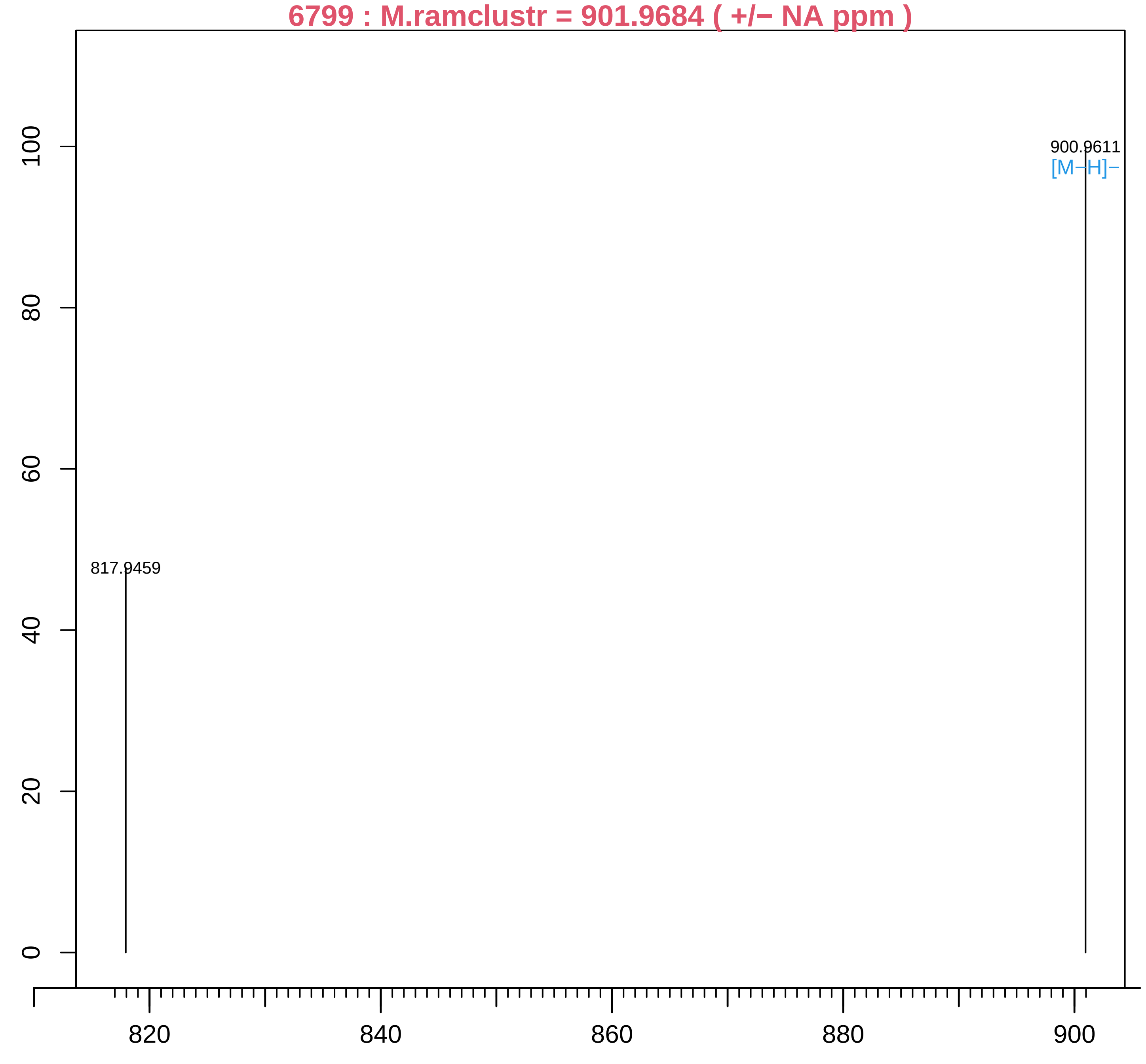
6799 : M.ramclustr = 901.9684 (+/− NA ppm)
900.9611
[M−H]−
817.9459
0
20
40
60
80
100
820
840
860
880
900

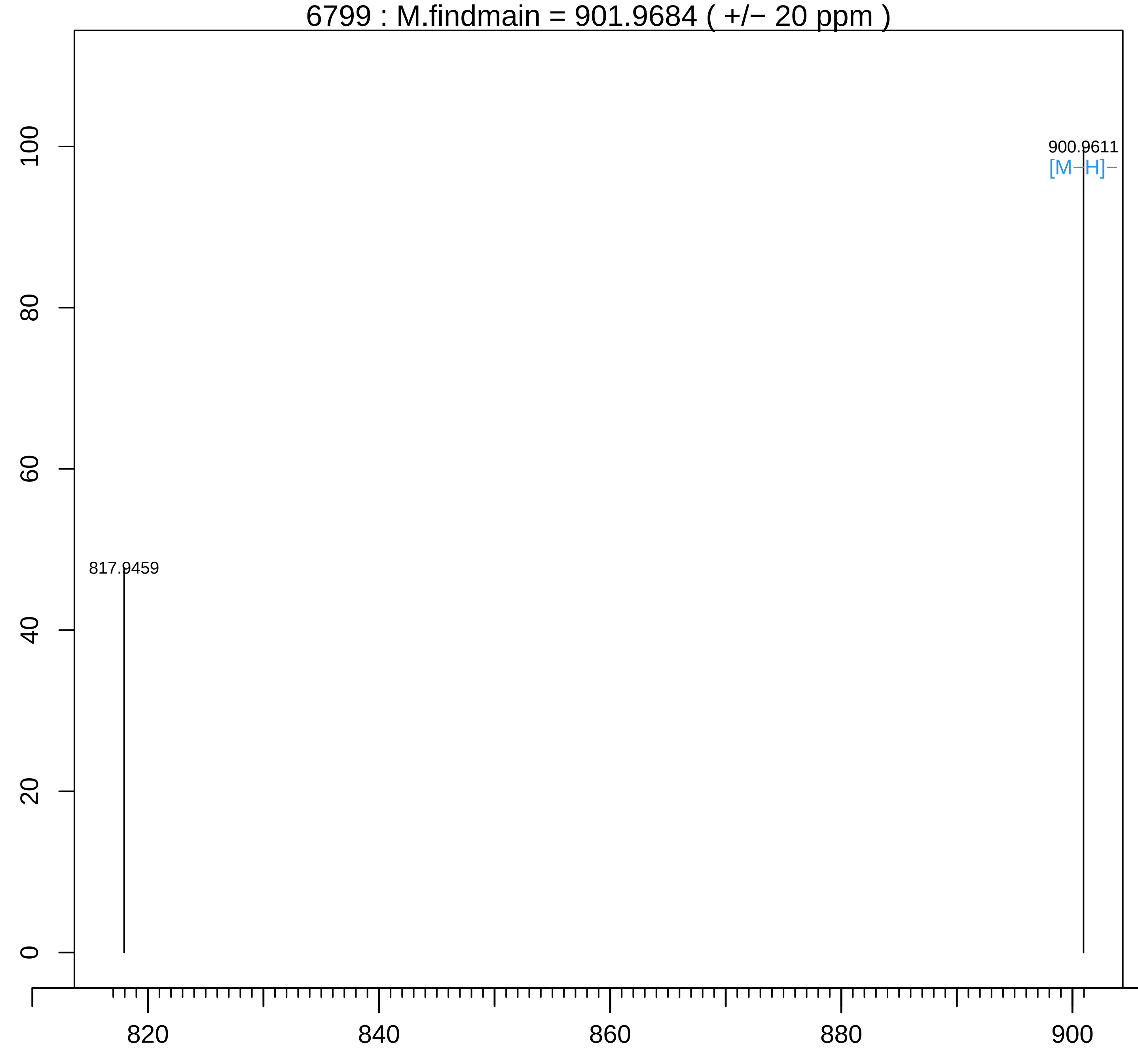
6799 : M.findmain = 901.9684 (+/− 20 ppm)
900.9611
[M−H]−
817.9459
0
20
40
60
80
100
820
840
860
880
900

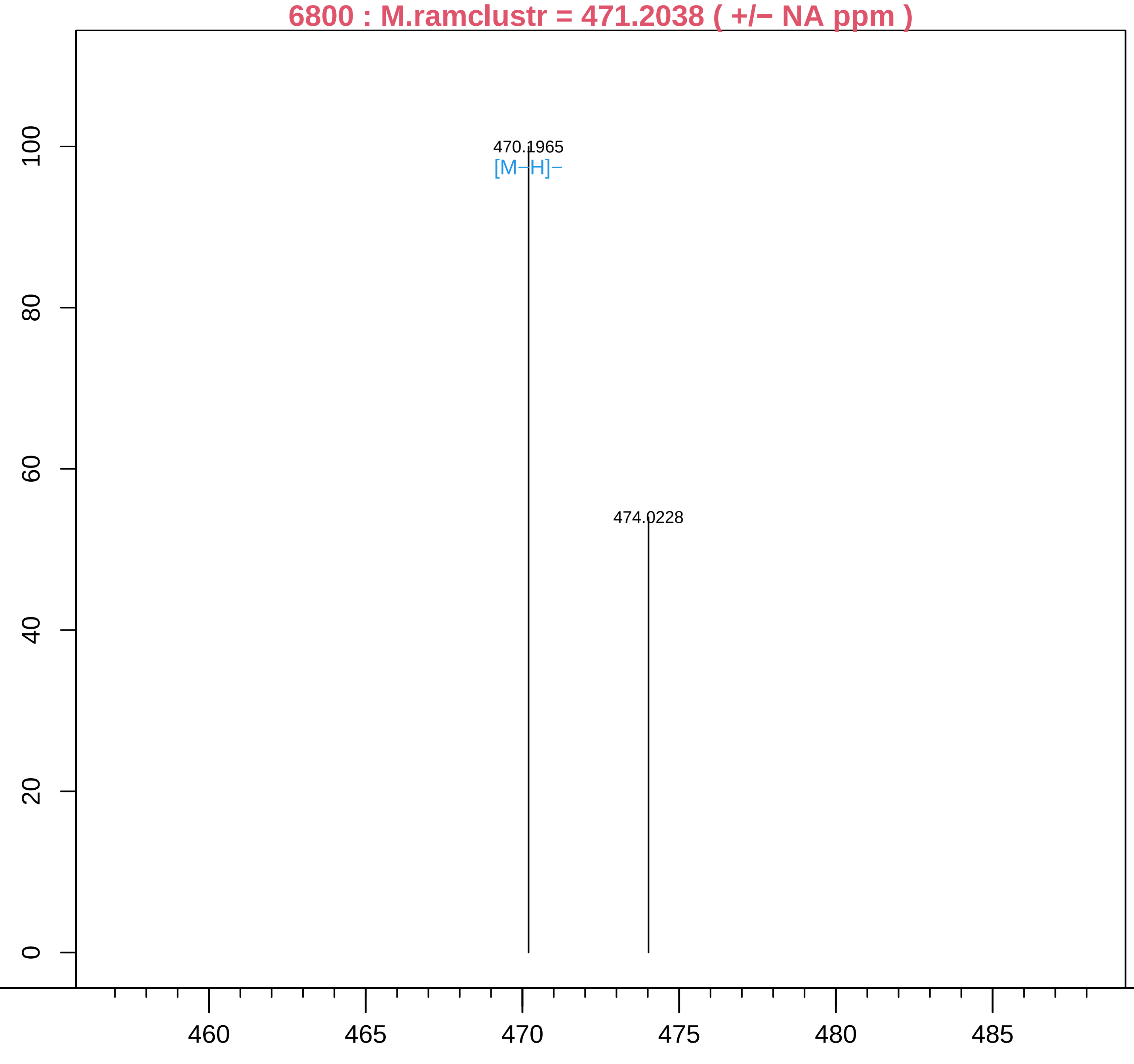
6800 : M.ramclustr = 471.2038 (+/− NA ppm)
470.1965
[M−H]−
474.0228
0
20
40
60
80
100
460
465
470
475
480
485

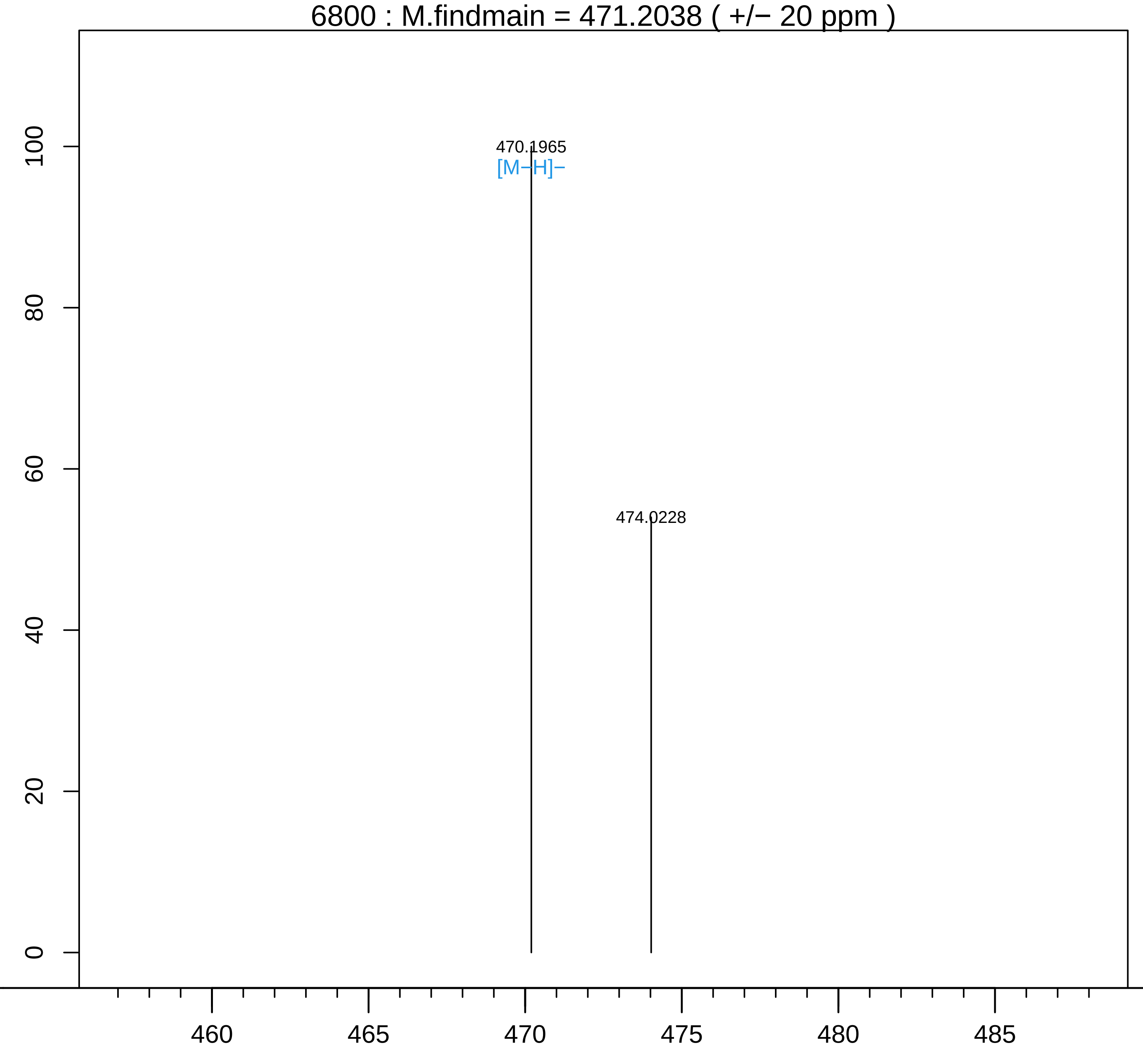
6800 : M.findmain = 471.2038 (+/− 20 ppm)
470.1965
[M−H]−
474.0228
0
20
40
60
80
100
460
465
470
475
480
485

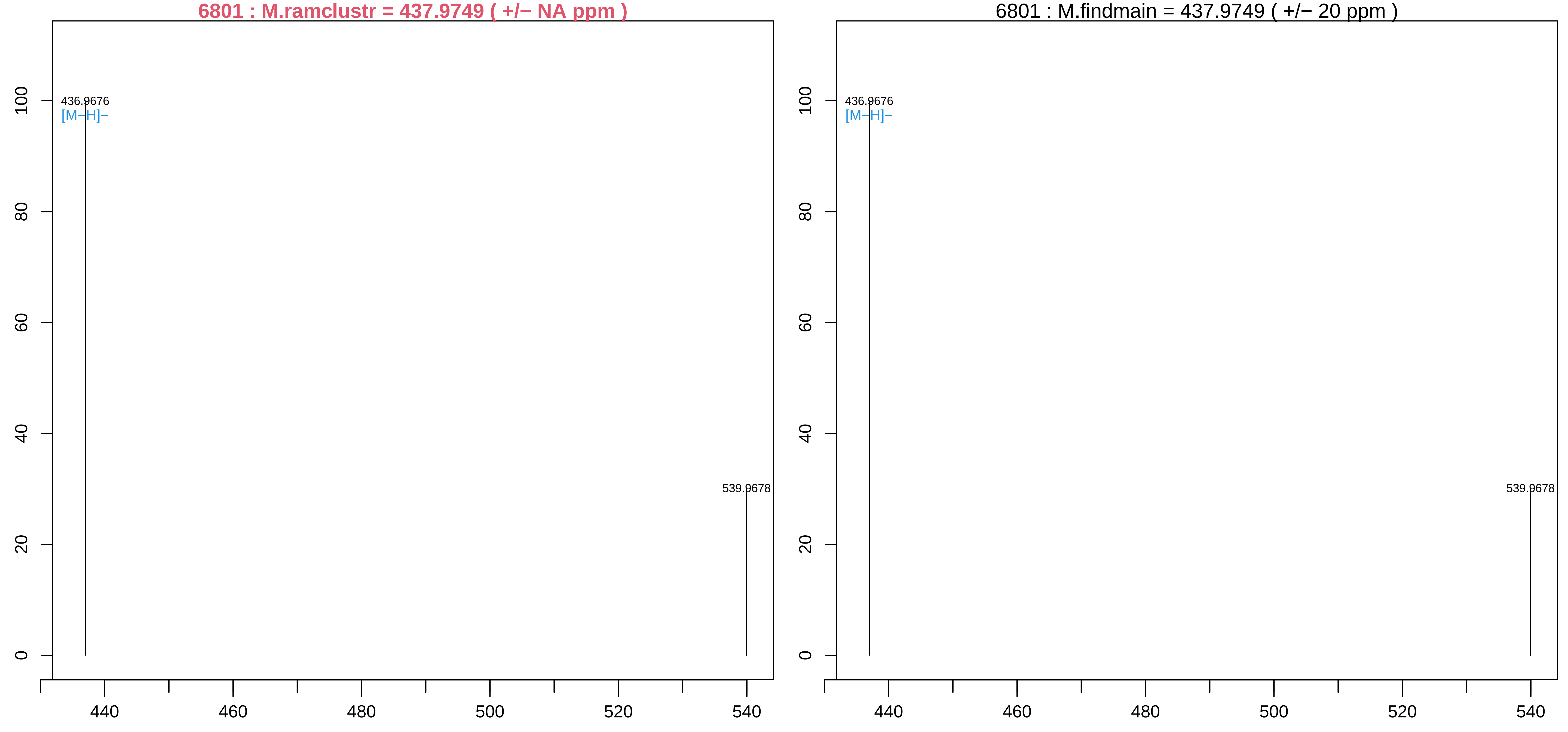
6801 : M.ramclustr = 437.9749 (+/− NA ppm)
436.9676
[M−H]−
539.9678
6801 : M.findmain = 437.9749 (+/− 20 ppm)
436.9676
[M−H]−
539.9678
0
20
40
60
80
100
440
460
480
500
520
540

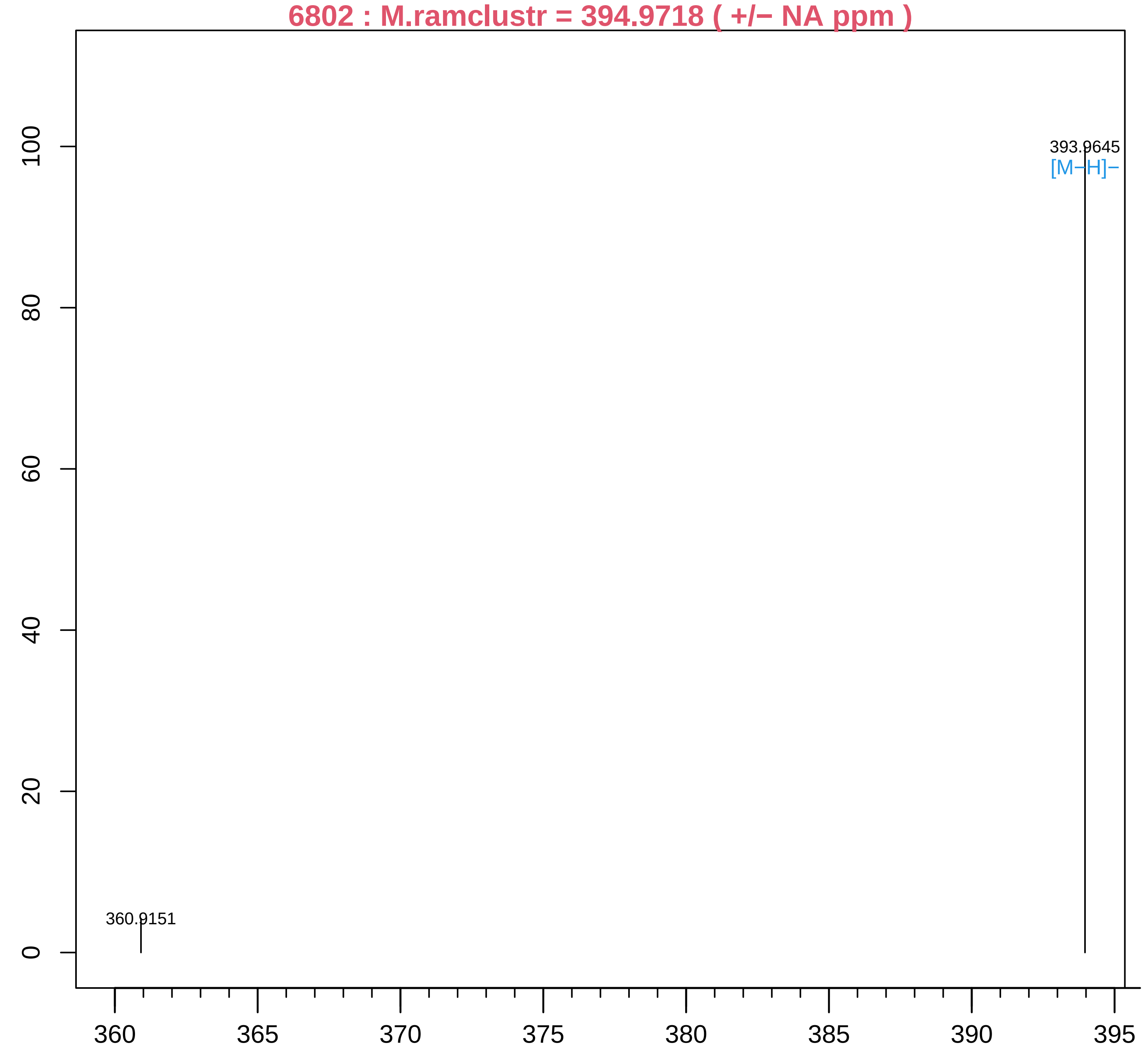
6802 : M.ramclustr = 394.9718 (+/− NA ppm)
393.9645
[M−H]−
360.9151

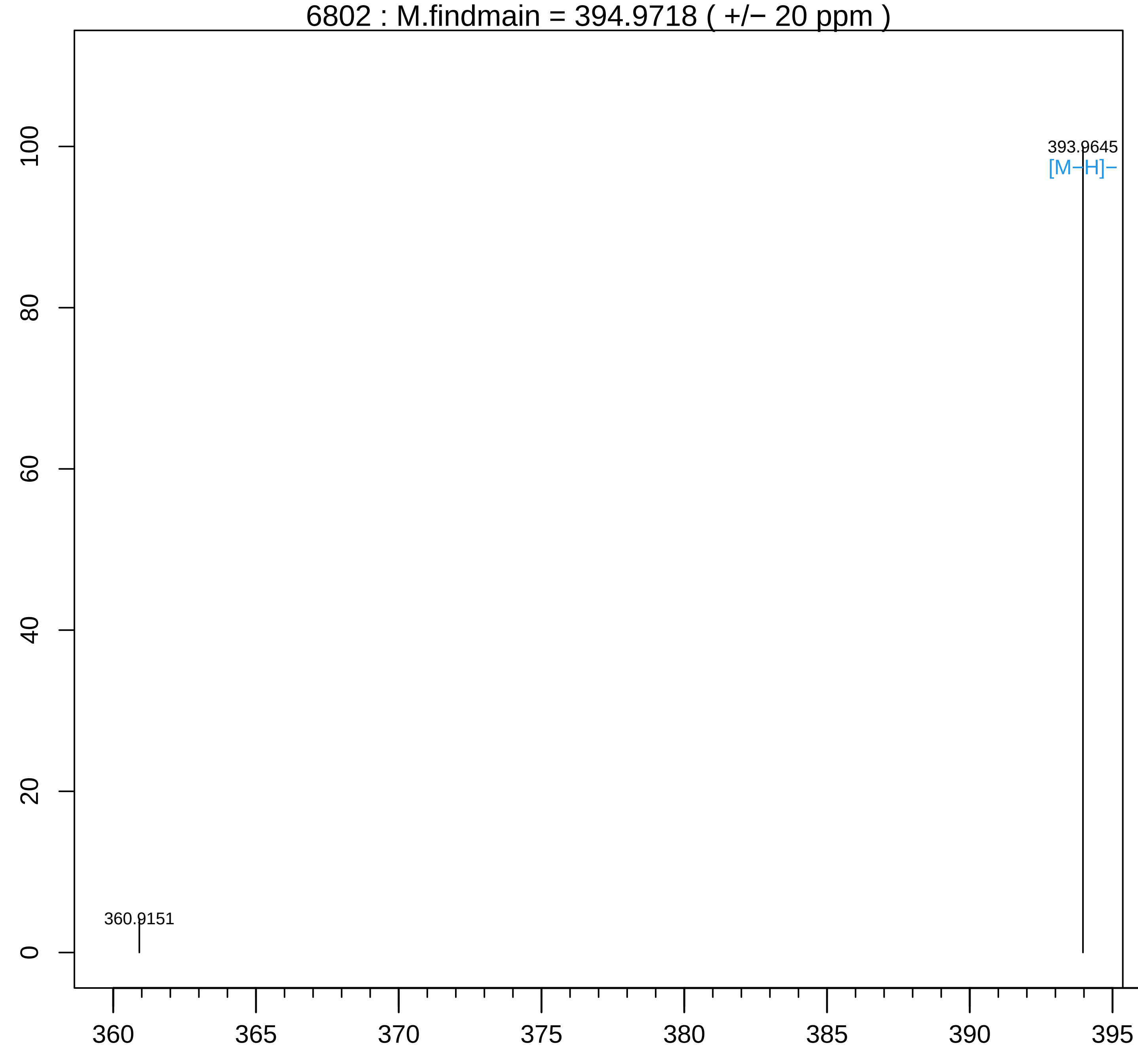
6802 : M.findmain = 394.9718 (+/− 20 ppm)
393.9645
[M−H]−
360.9151

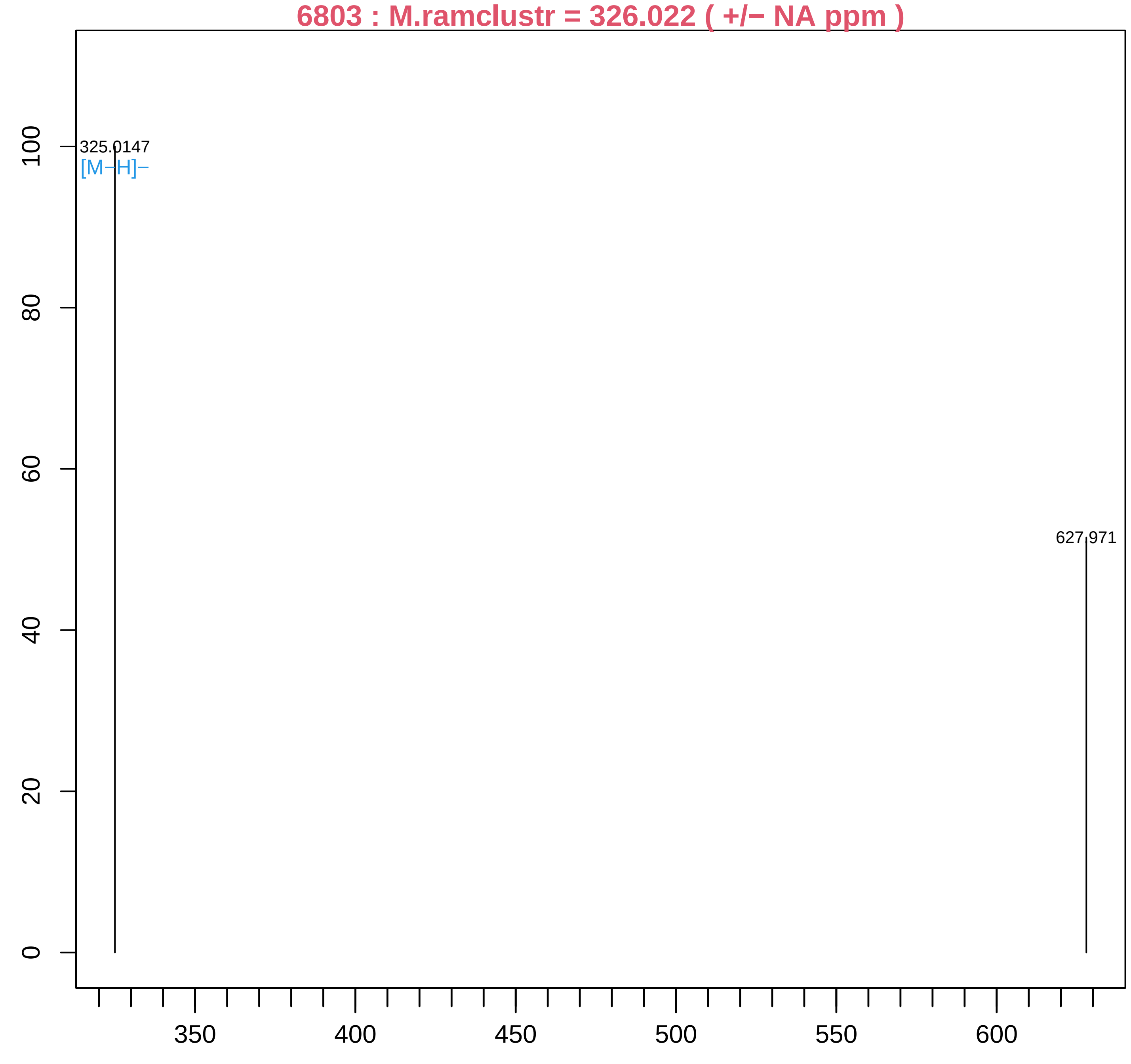
6803 : M.ramclustr = 326.022 (+/− NA ppm)
325.0147
[M−H]−
627.971
0
20
40
60
80
100
350
400
450
500
550
600

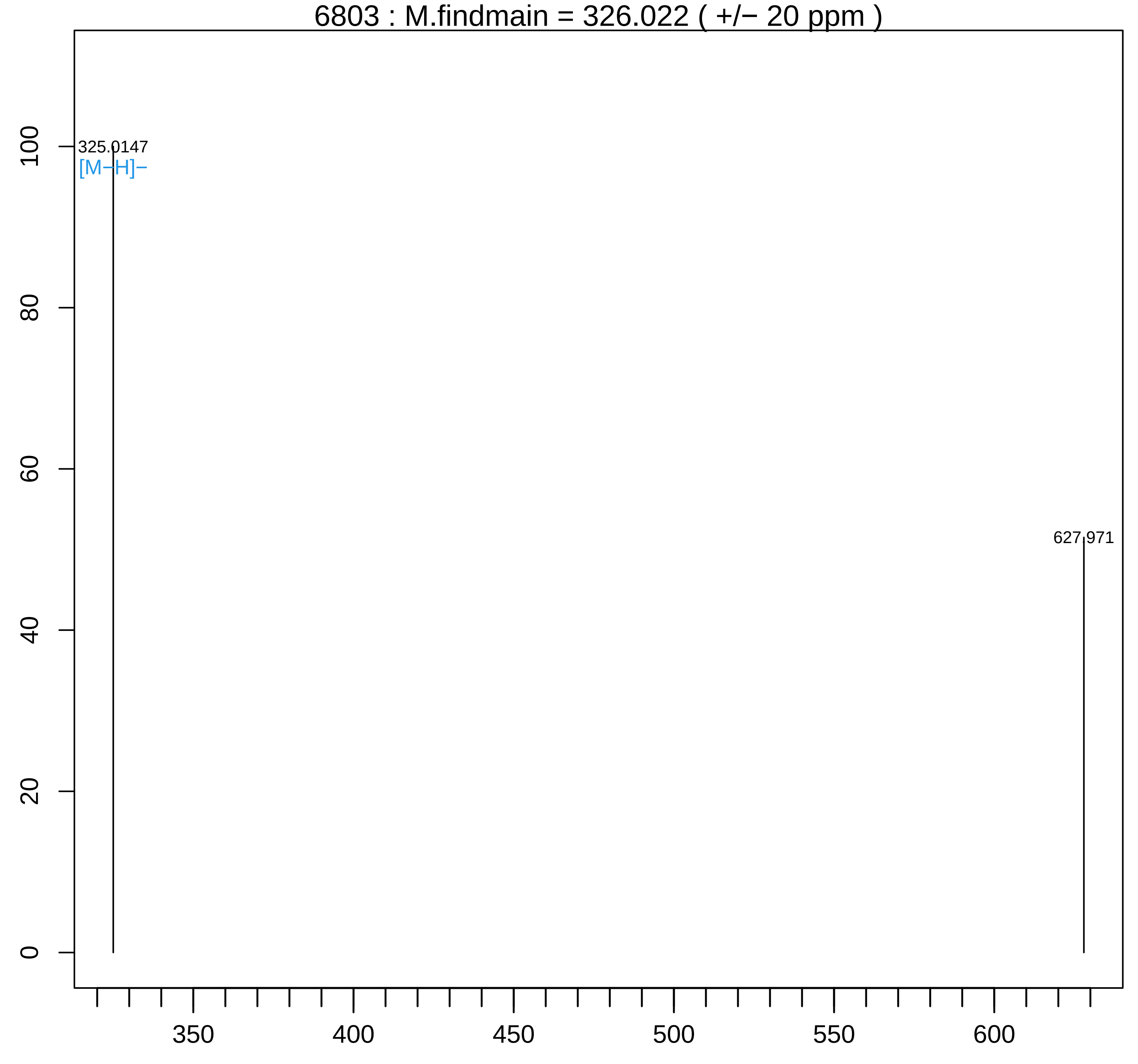
6803 : M.findmain = 326.022 (+/− 20 ppm)
325.0147
[M−H]−
627.971
0
20
40
60
80
100
350
400
450
500
550
600

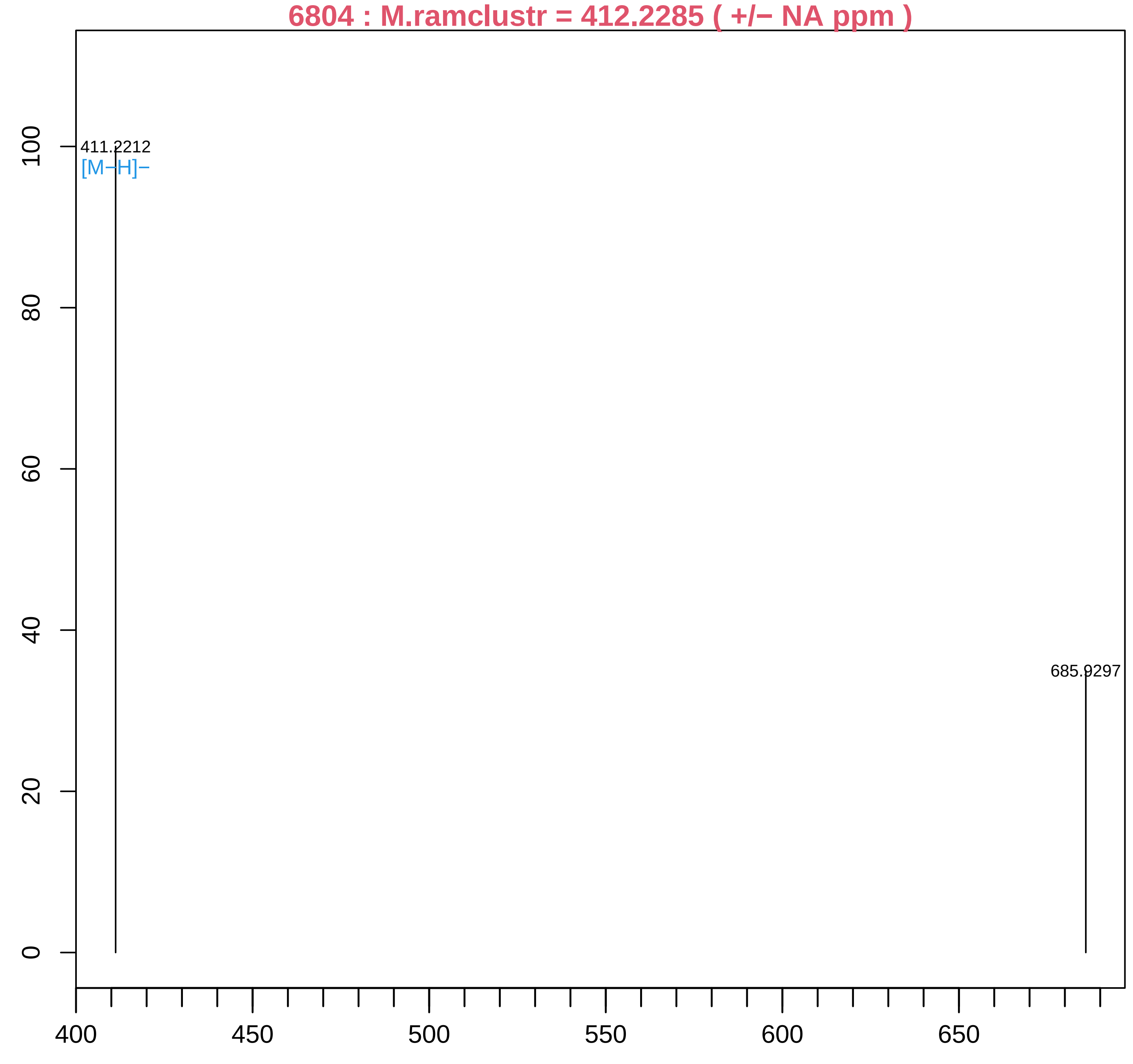
6804 : M.ramclustr = 412.2285 (+/− NA ppm)
411.2212
[M−H]−
685.9297
0
20
40
60
80
100
400
450
500
550
600
650

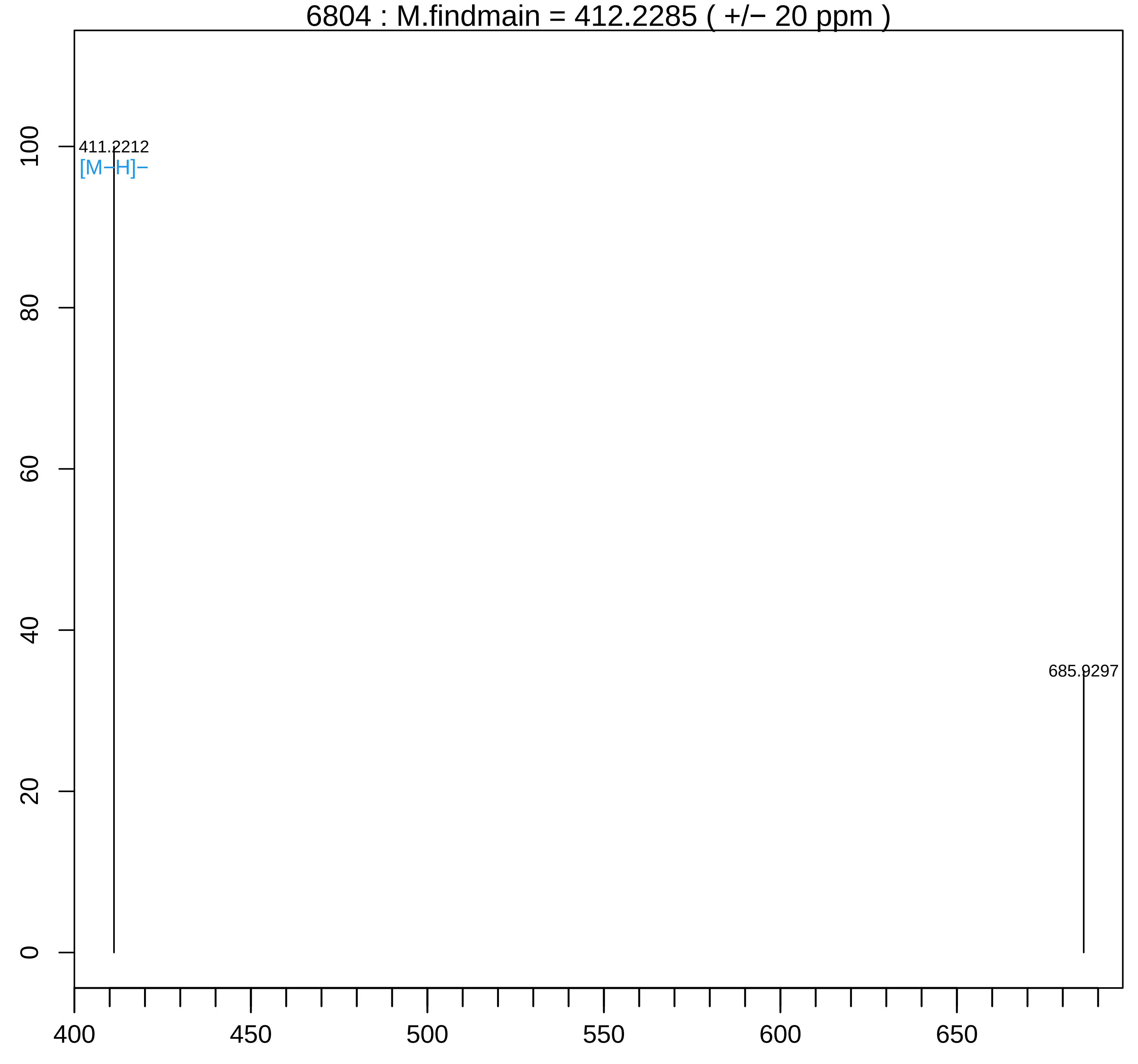
6804 : M.findmain = 412.2285 (+/− 20 ppm)
411.2212
[M−H]−
685.9297
0
20
40
60
80
100
400
450
500
550
600
650

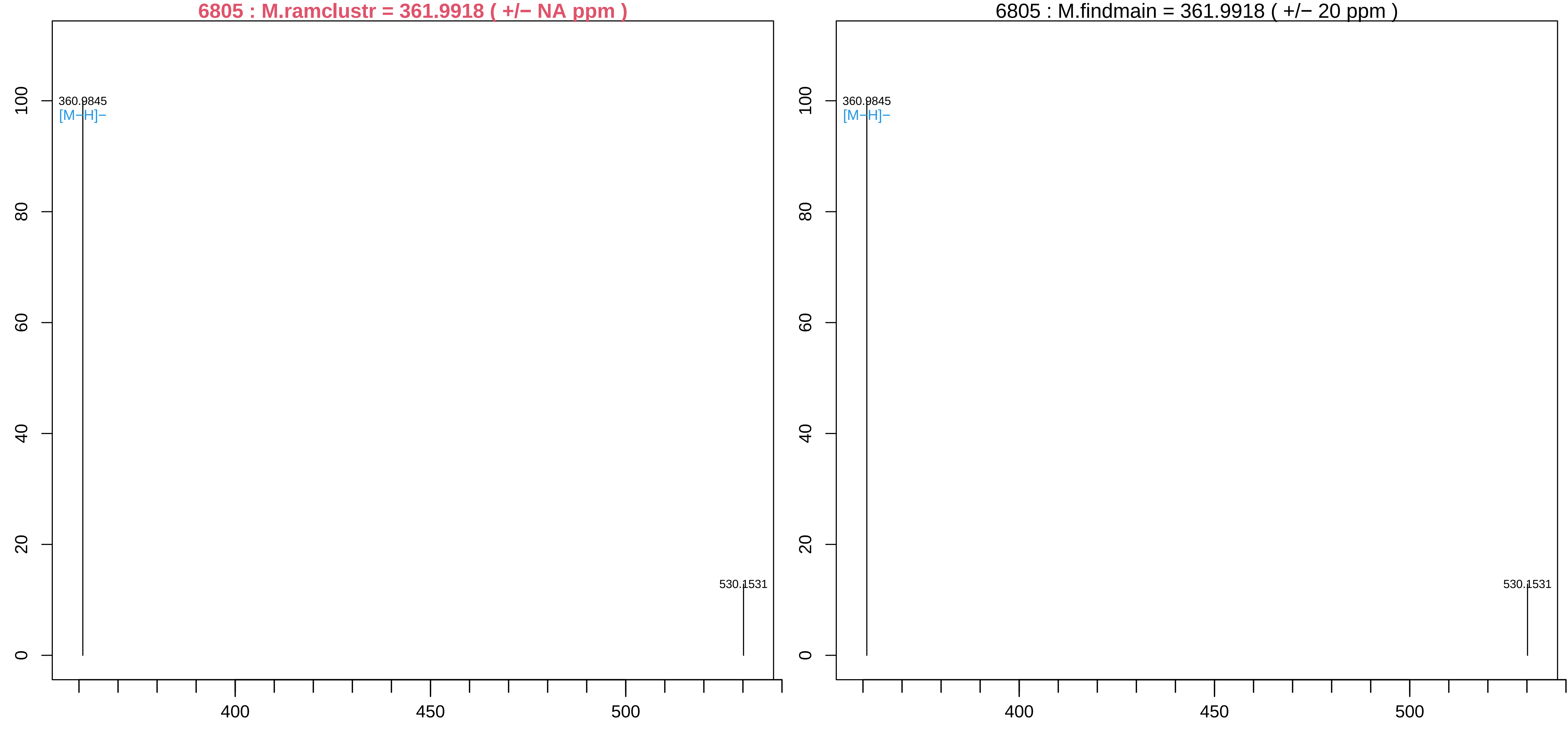
6805 : M.ramclustr = 361.9918 (+/− NA ppm)
360.9845
[M−H]−
530.1531
6805 : M.findmain = 361.9918 (+/− 20 ppm)
360.9845
[M−H]−
530.1531
0
20
40
60
80
100
400
450
500

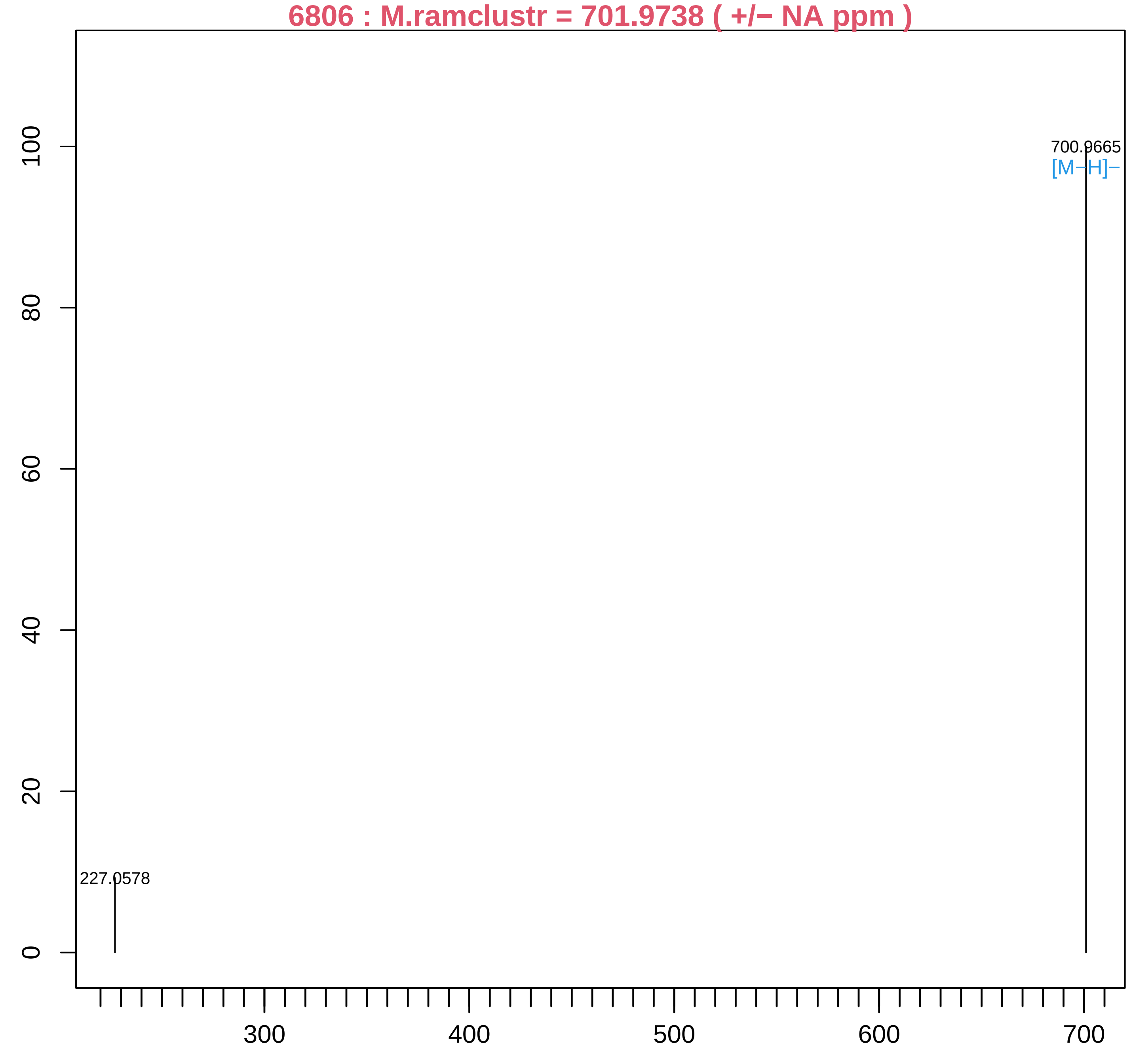
6806 : M.ramclustr = 701.9738 (+/− NA ppm)
700.9665
[M−H]−
227.0578
0
20
40
60
80
100
300
400
500
600
700

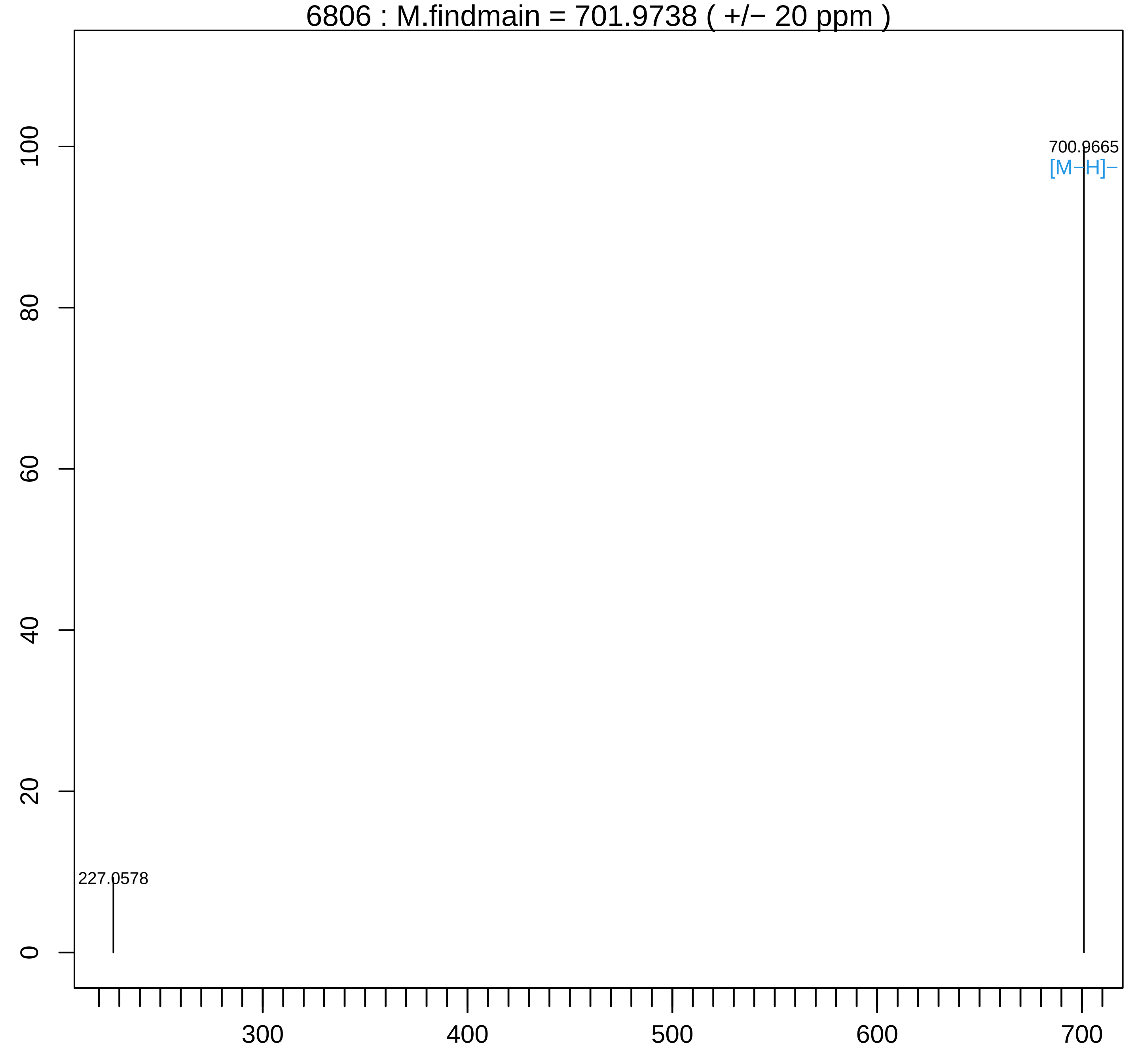
6806 : M.findmain = 701.9738 (+/− 20 ppm)
700.9665
[M−H]−
227.0578
0
20
40
60
80
100
300
400
500
600
700

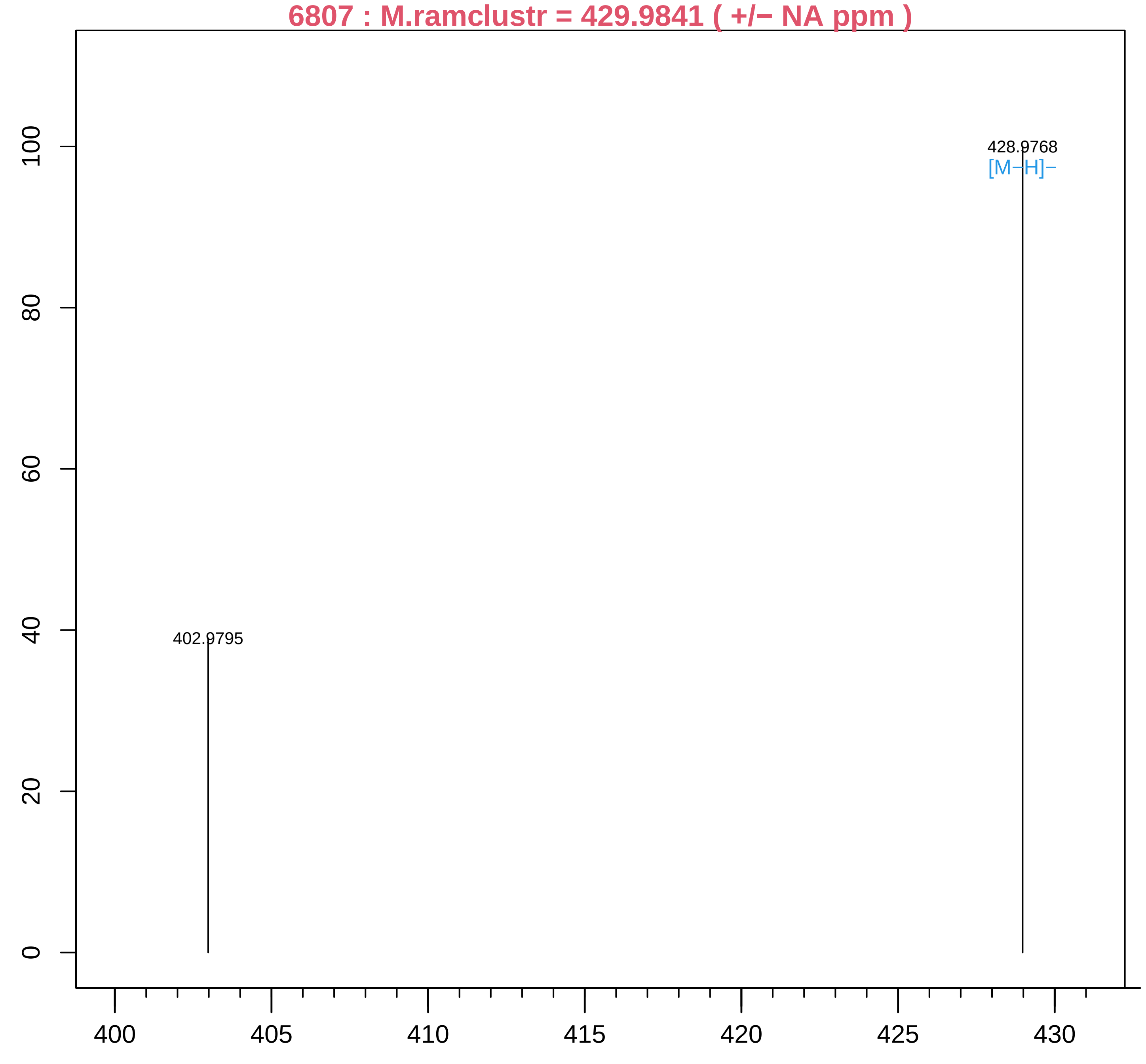
6807 : M.ramclustr = 429.9841 (+/− NA ppm)
428.9768
[M−H]−
402.9795
0
20
40
60
80
100
400
405
410
415
420
425
430

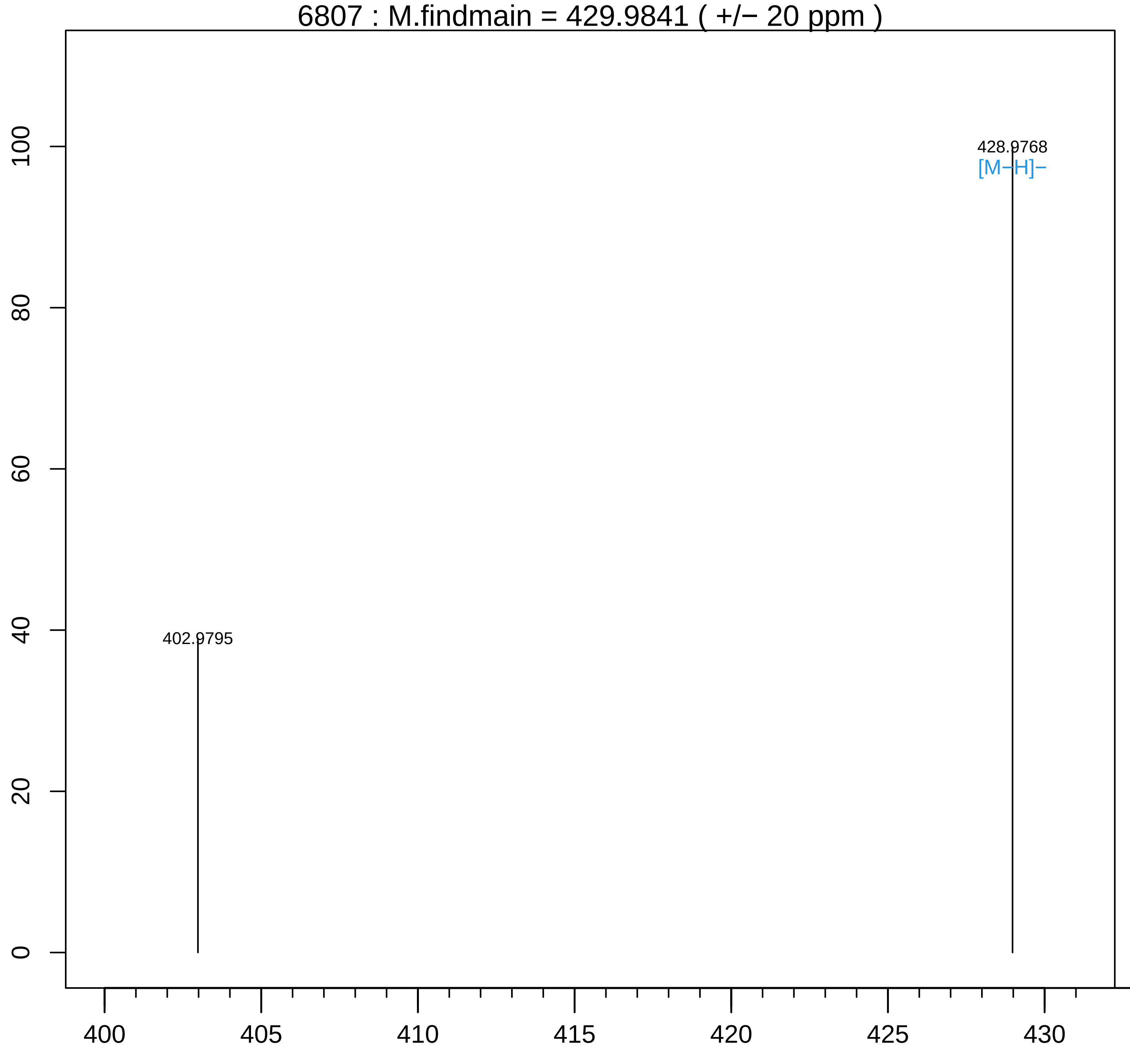
6807 : M.findmain = 429.9841 (+/− 20 ppm)
428.9768
[M−H]−
402.9795
0
20
40
60
80
100
400
405
410
415
420
425
430

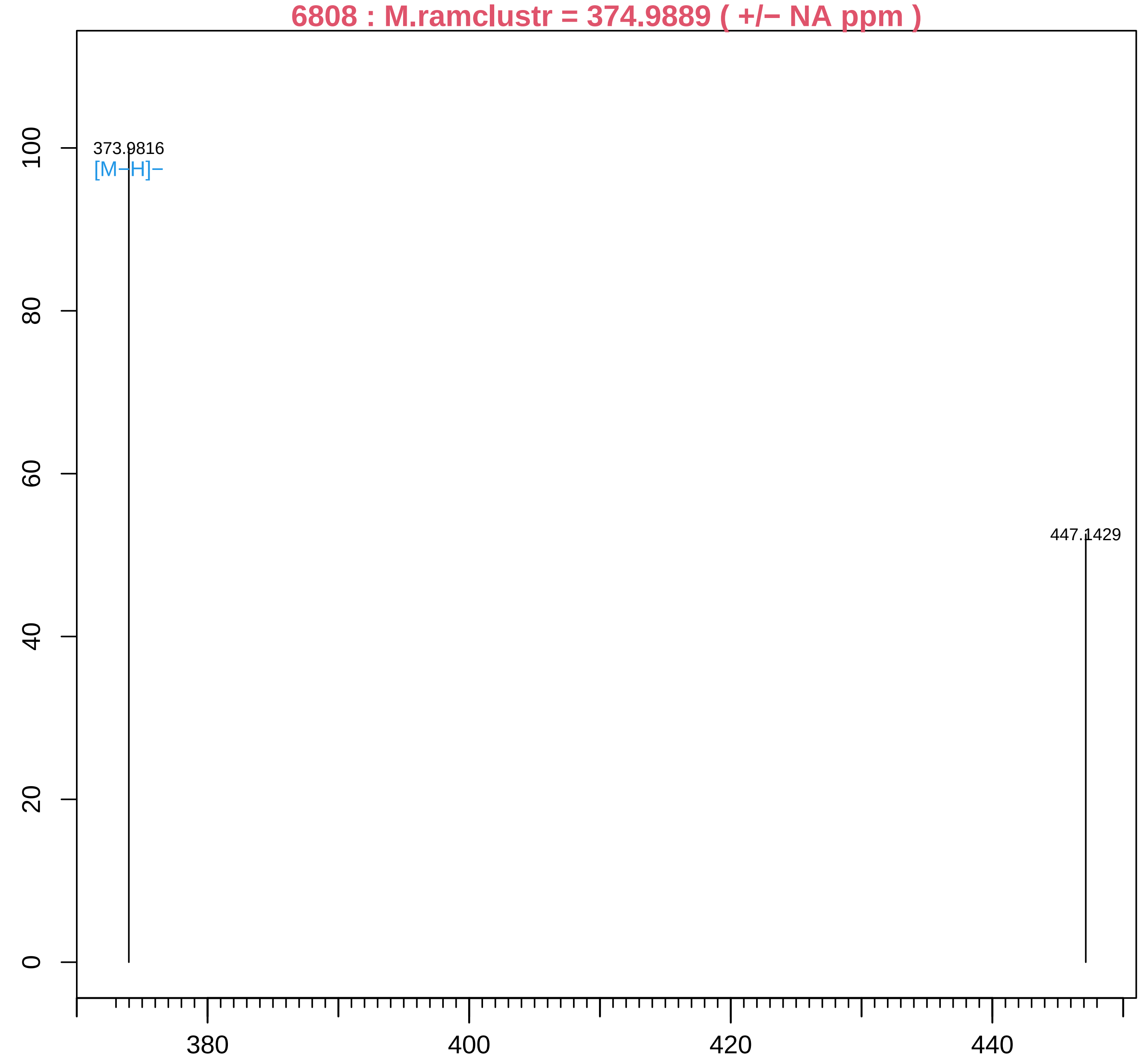

6808 : M.ramclustr = 374.9889 (+/− NA ppm)
373.9816
[M−H]−
447.1429
0
20
40
60
80
100
380
400
420
440

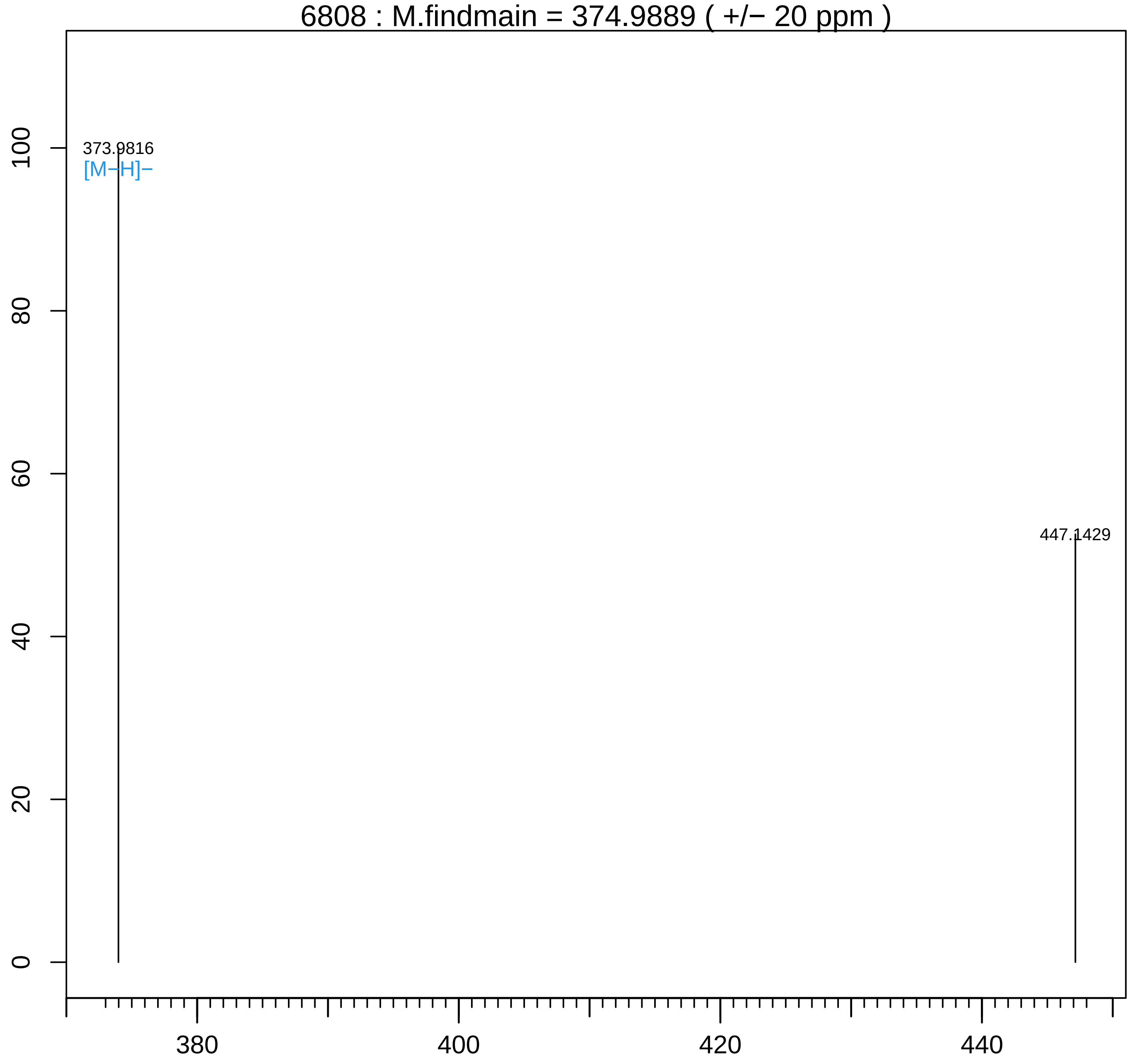

6808 : M.findmain = 374.9889 (+/− 20 ppm)
373.9816
[M−H]−
447.1429
0
20
40
60
80
100
380
400
420
440

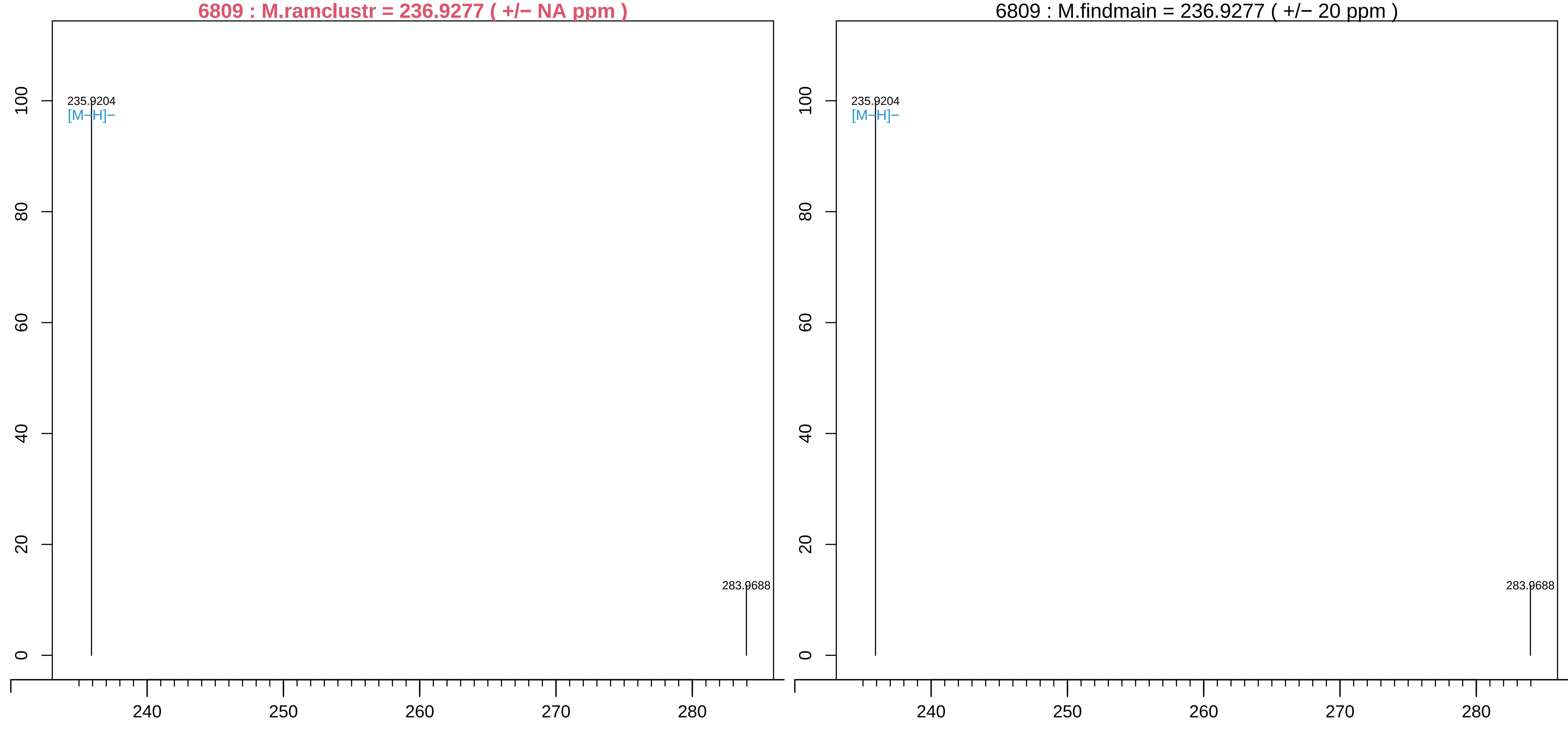
6809 : M.ramclustr = 236.9277 (+/− NA ppm)
235.9204
[M−H]−
283.9688
0
20
40
60
80
100
240
250
260
270
280
6809 : M.findmain = 236.9277 (+/− 20 ppm)
235.9204
[M−H]−
283.9688
0
20
40
60
80
100
240
250
260
270
280

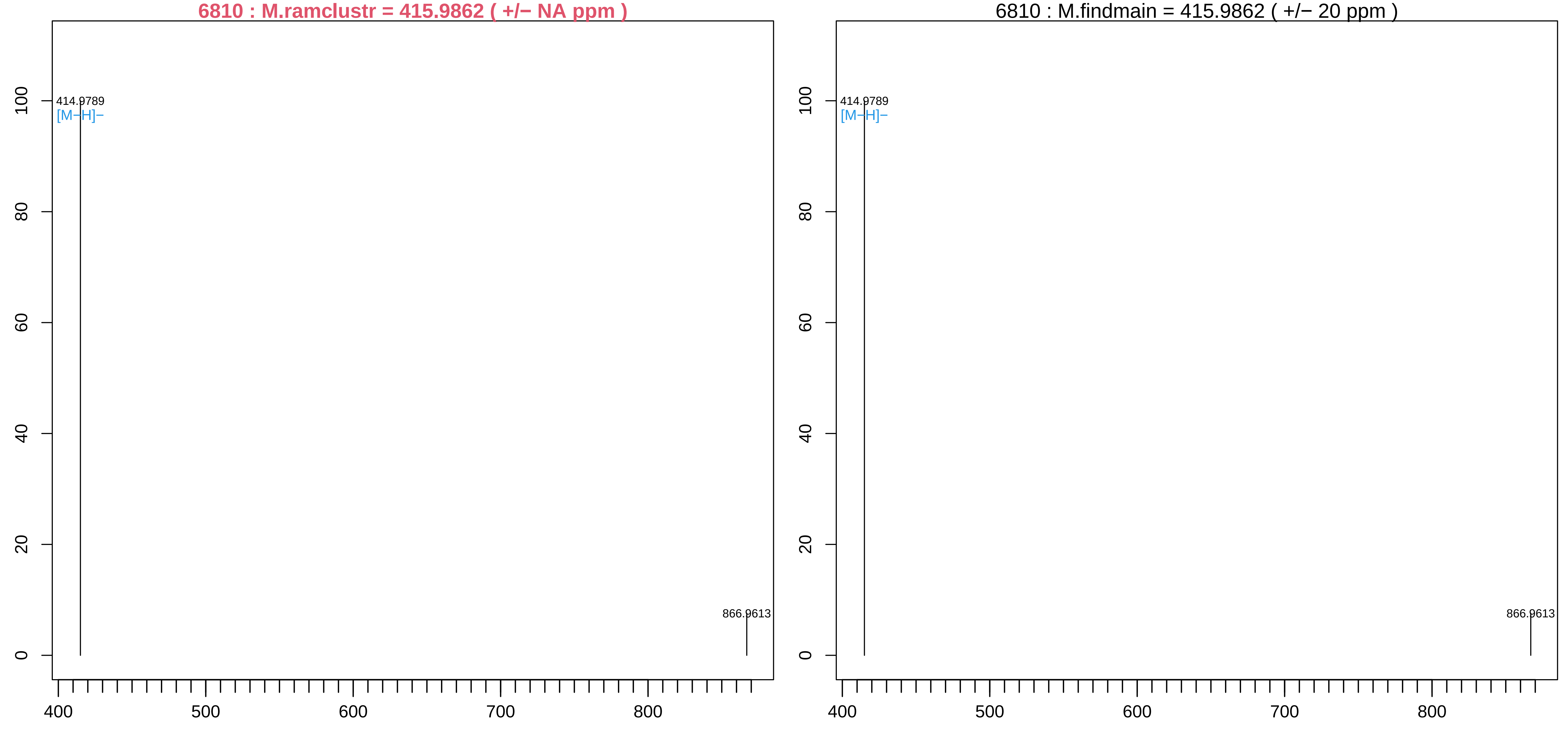
6810 : M.ramclustr = 415.9862 (+/− NA ppm)
414.9789
[M−H]−
866.9613
0
20
40
60
80
100
400
500
600
700
800
6810 : M.findmain = 415.9862 (+/− 20 ppm)
414.9789
[M−H]−
866.9613
0
20
40
60
80
100
400
500
600
700
800

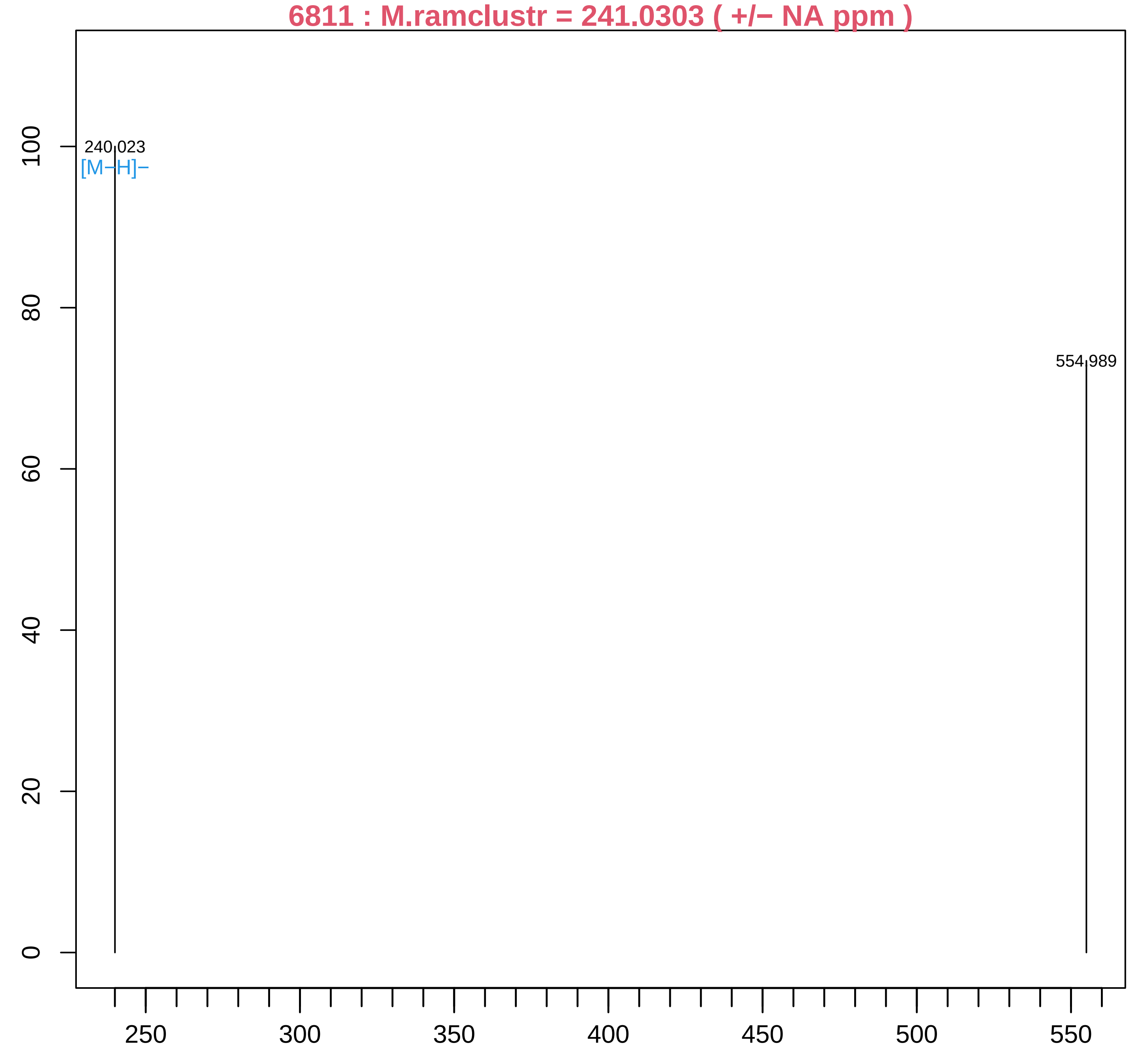
6811 : M.ramclustr = 241.0303 (+/− NA ppm)
240.023
[M−H]−
554.989
0
20
40
60
80
100
250
300
350
400
450
500
550

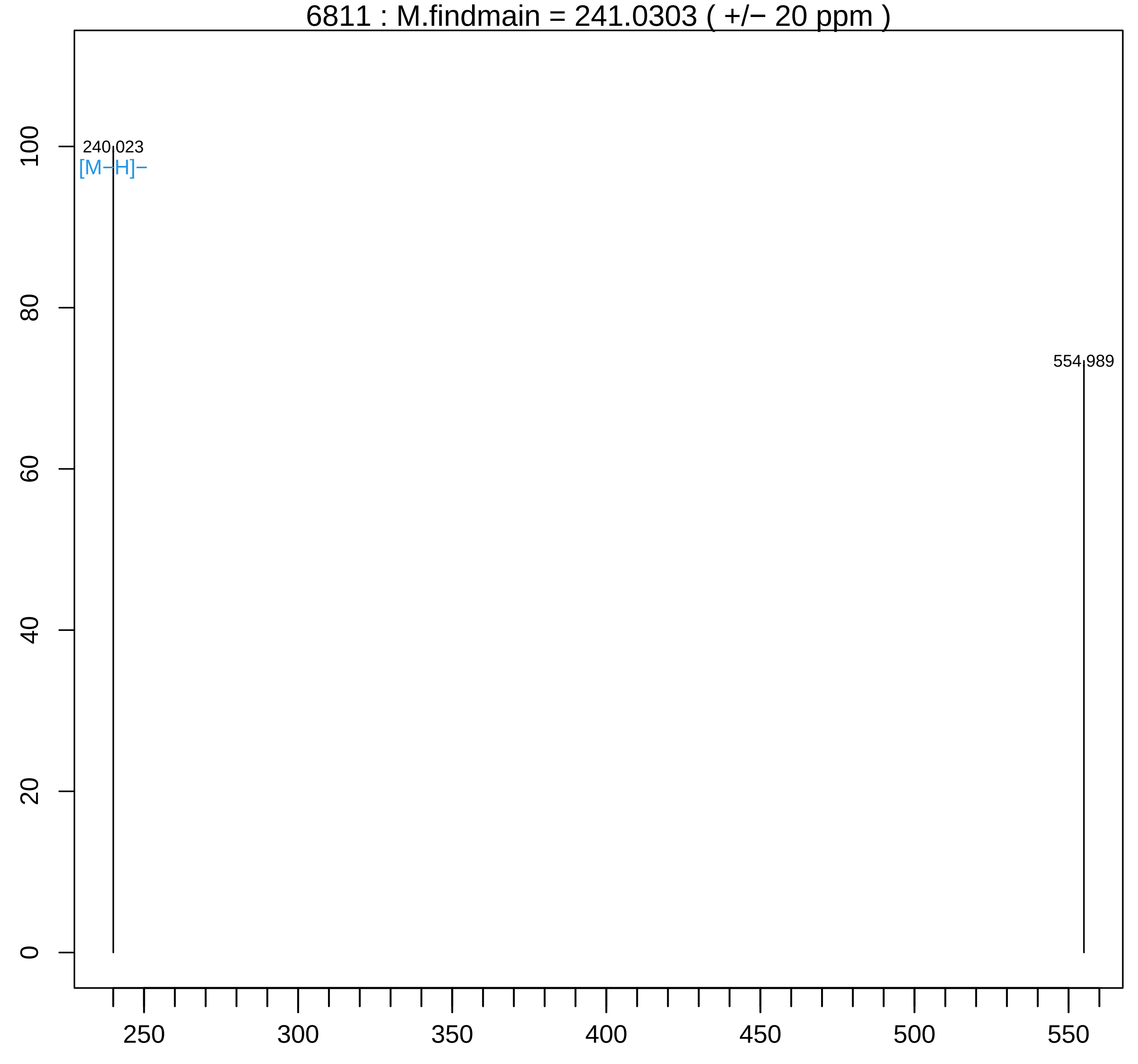
6811 : M.findmain = 241.0303 (+/− 20 ppm)
240.023
[M−H]−
554.989
0
20
40
60
80
100
250
300
350
400
450
500
550

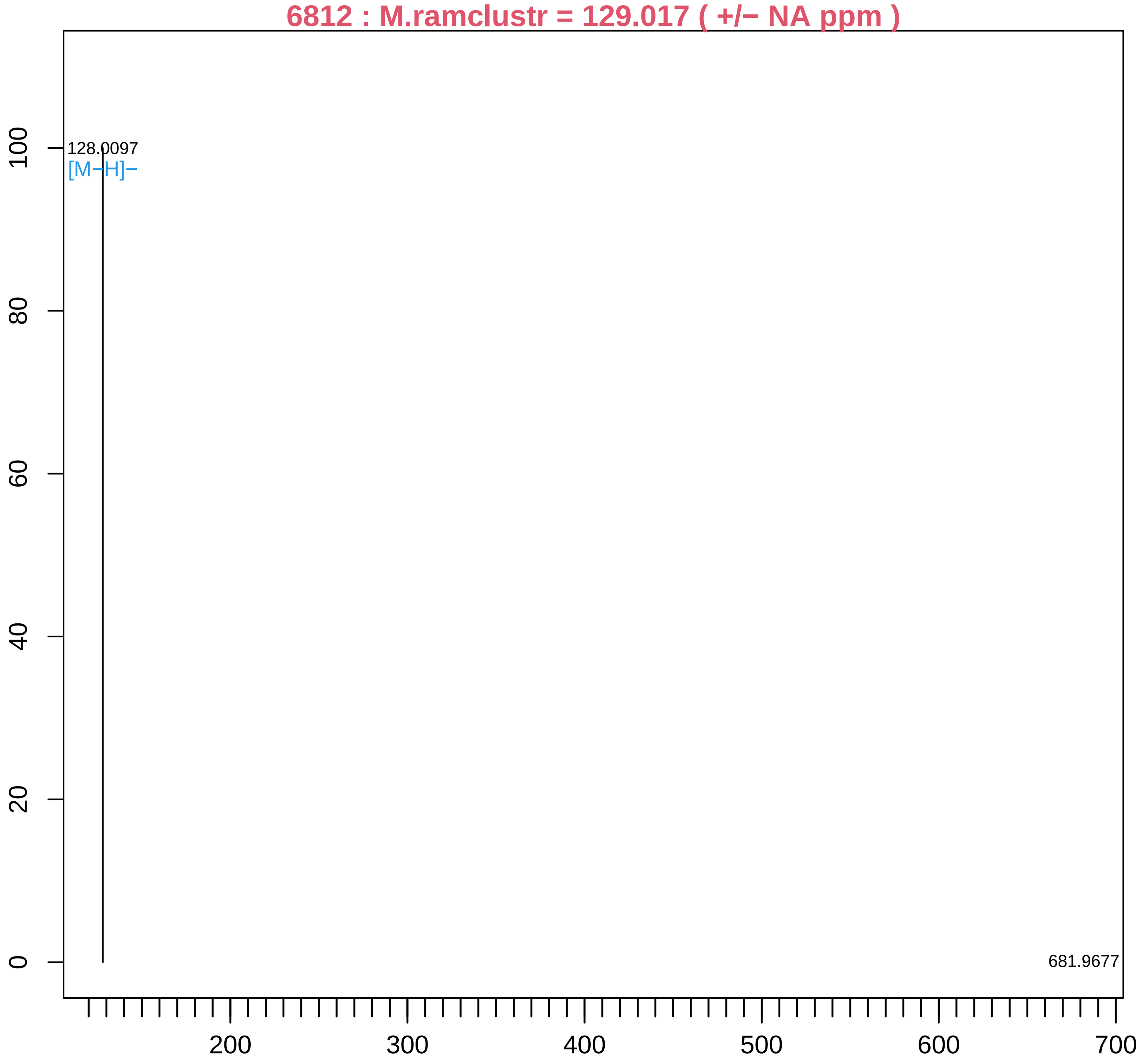
6812 : M.ramclustr = 129.017 (+/− NA ppm)
128.0097
[M−H]−
681.9677
0
20
40
60
80
100
200
300
400
500
600
700

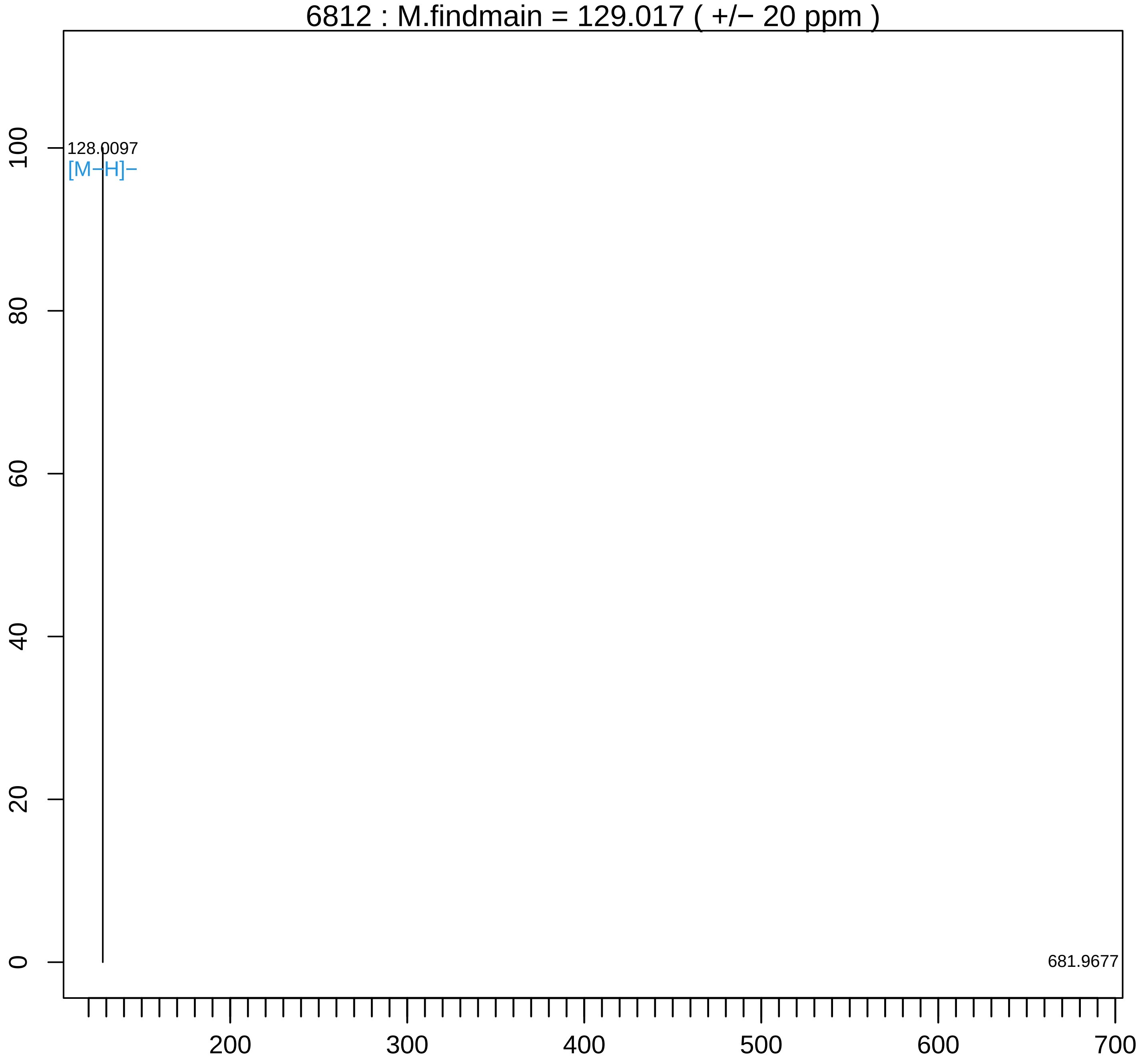
6812 : M.findmain = 129.017 (+/− 20 ppm)
128.0097
[M−H]−
681.9677
0
20
40
60
80
100
200
300
400
500
600
700

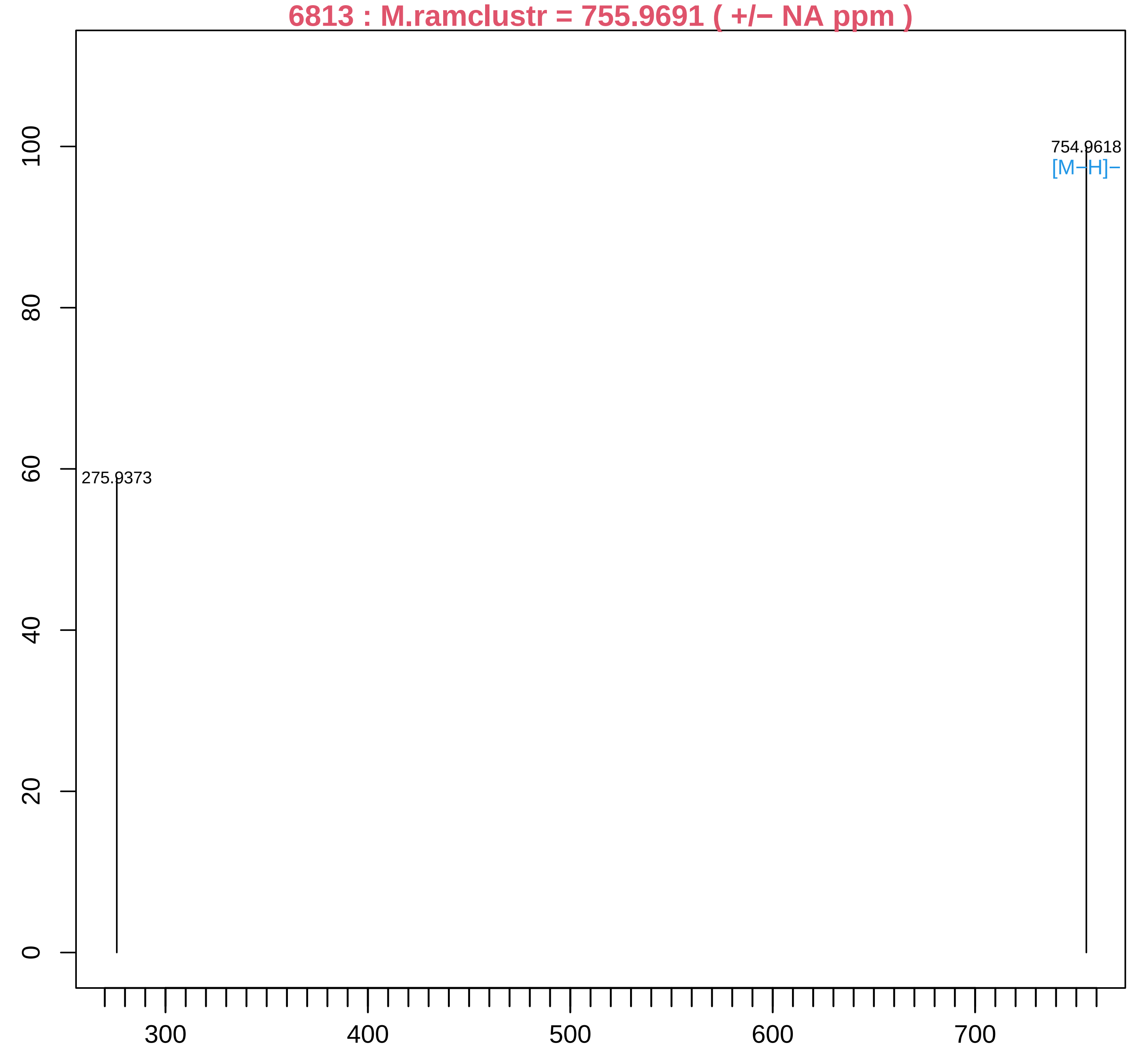

6813 : M.ramclustr = 755.9691 (+/− NA ppm)
754.9618
[M−H]−
275.9373
0
20
40
60
80
100
300
400
500
600
700

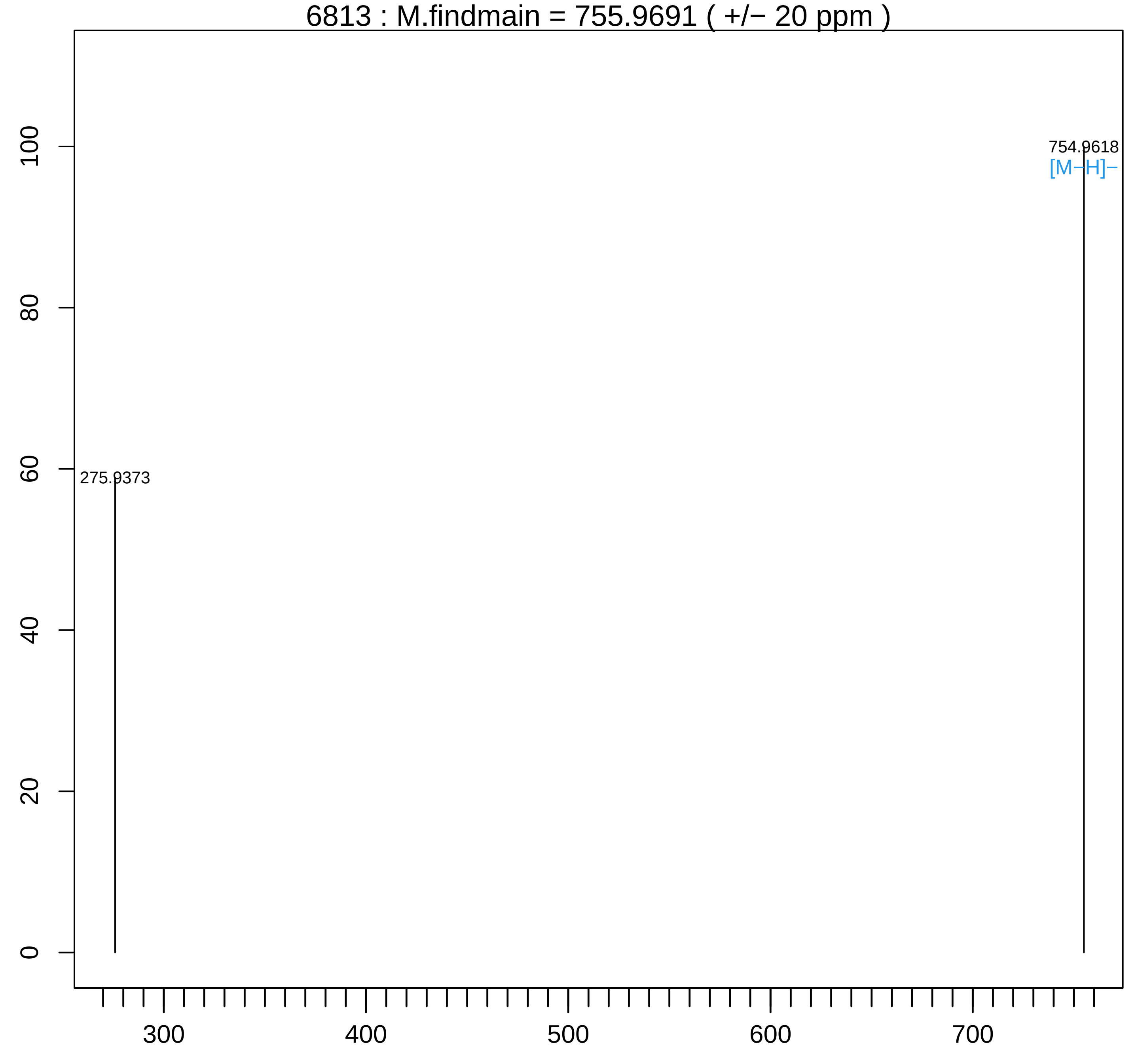

6813 : M.findmain = 755.9691 (+/− 20 ppm)
754.9618
[M−H]−
275.9373
0
20
40
60
80
100
300
400
500
600
700

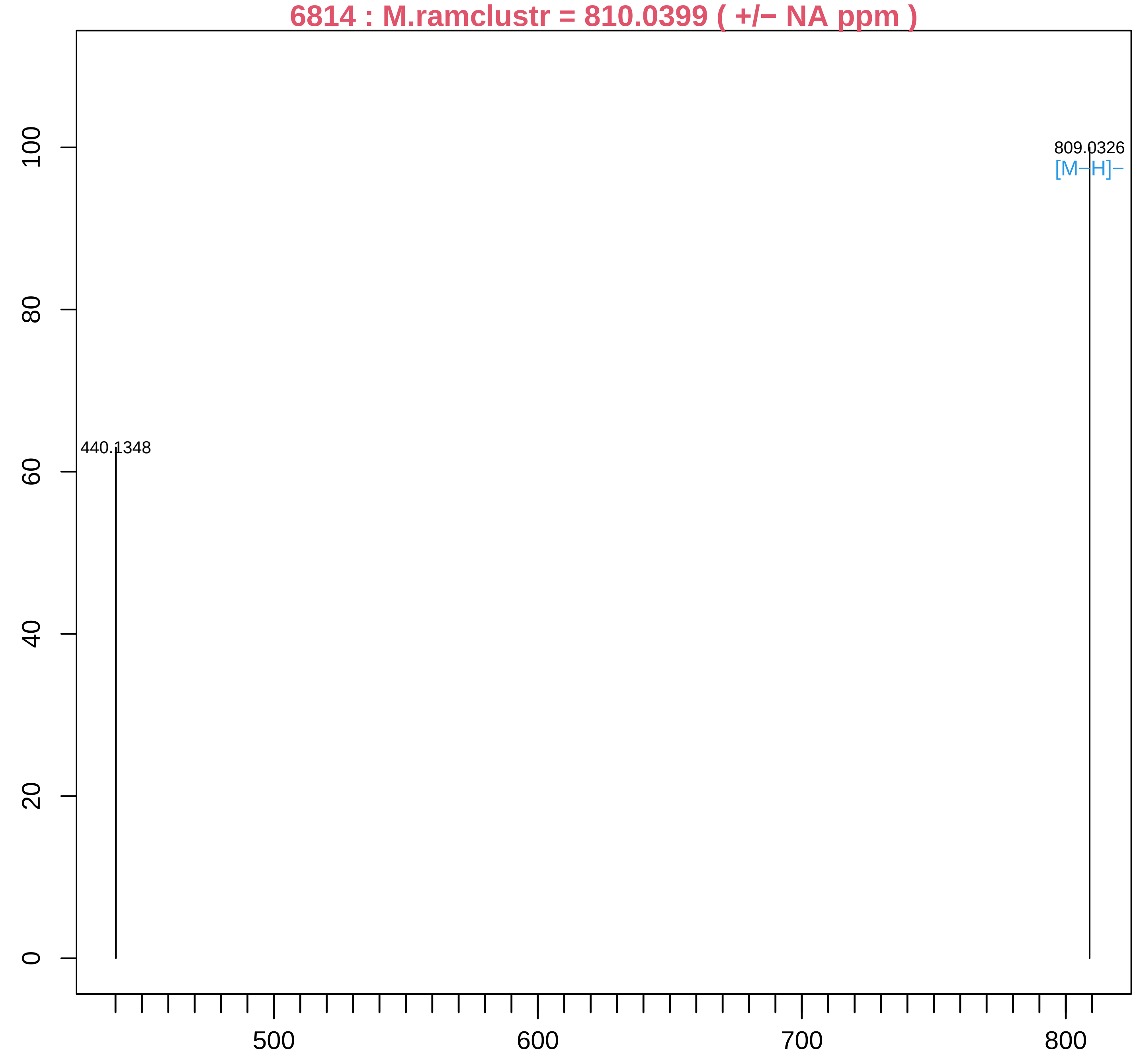
6814 : M.ramclustr = 810.0399 (+/− NA ppm)
440.1348
809.0326
[M−H]−
0
20
40
60
80
100
500
600
700
800

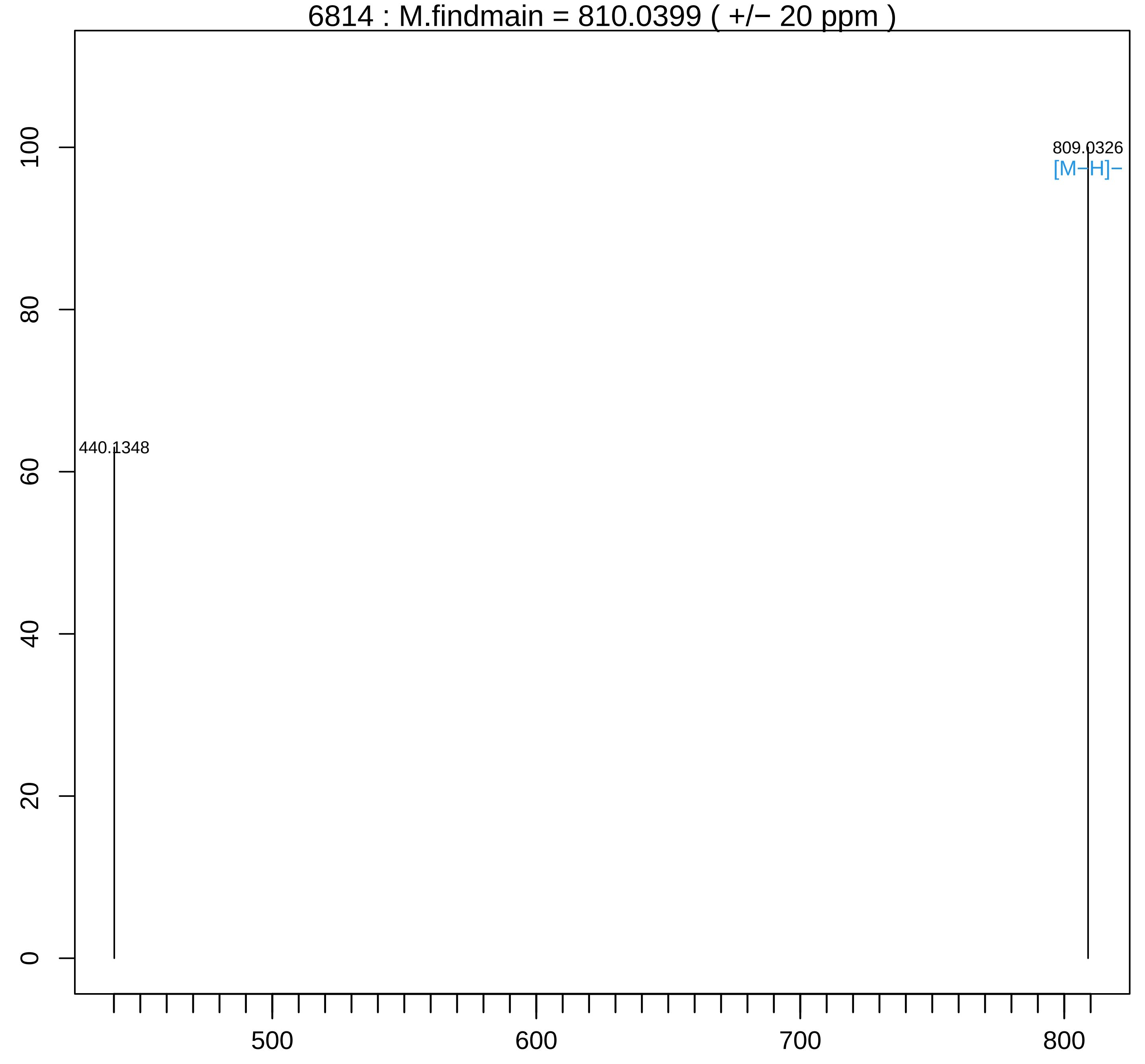
6814 : M.findmain = 810.0399 (+/− 20 ppm)
440.1348
809.0326
[M−H]−
0
20
40
60
80
100
500
600
700
800

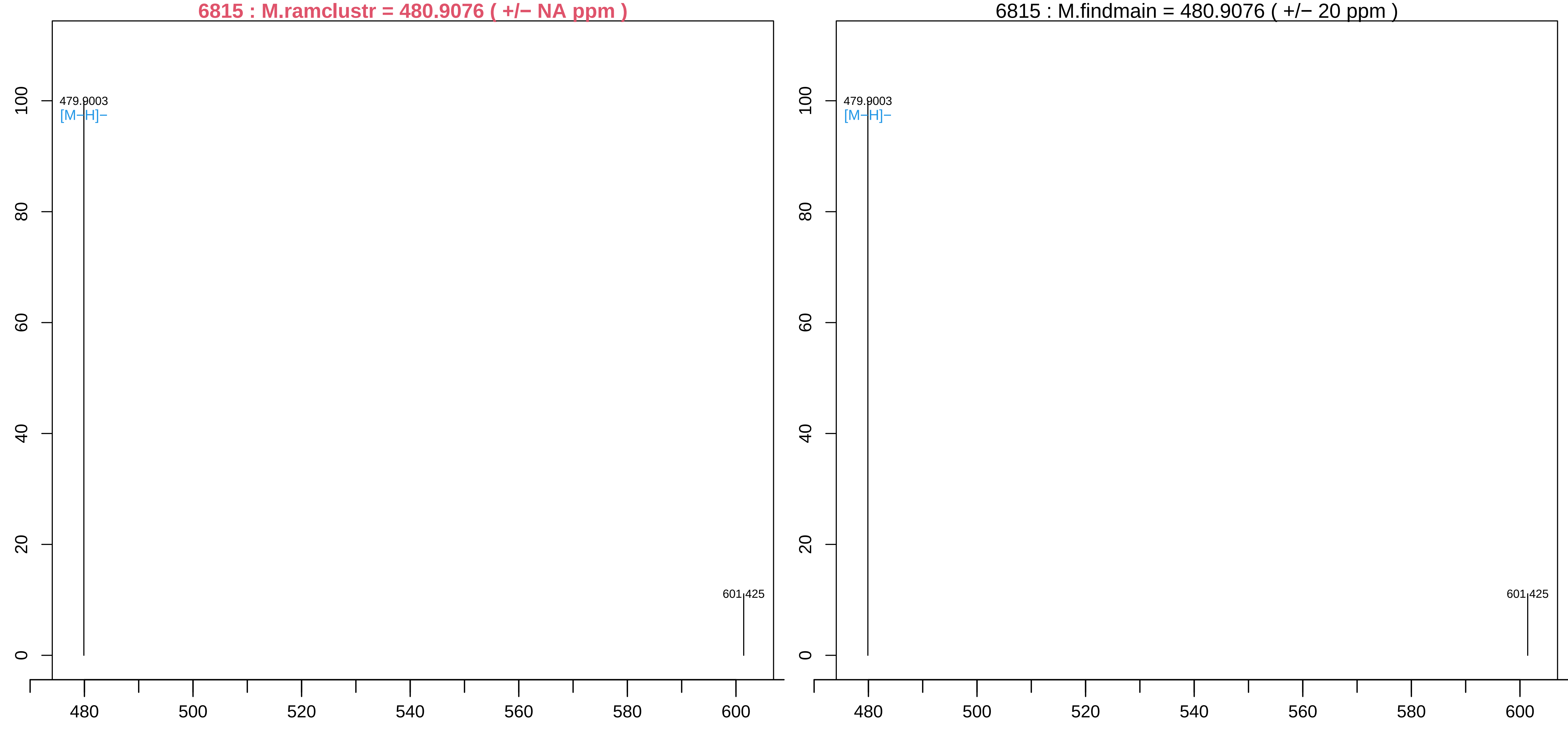

6815 : M.ramclustr = 480.9076 (+/− NA ppm)
479.9003
[M−H]−
601.425
0
20
40
60
80
100
480
500
520
540
560
580
600
6815 : M.findmain = 480.9076 (+/− 20 ppm)
479.9003
[M−H]−
601.425
0
20
40
60
80
100
480
500
520
540
560
580
600

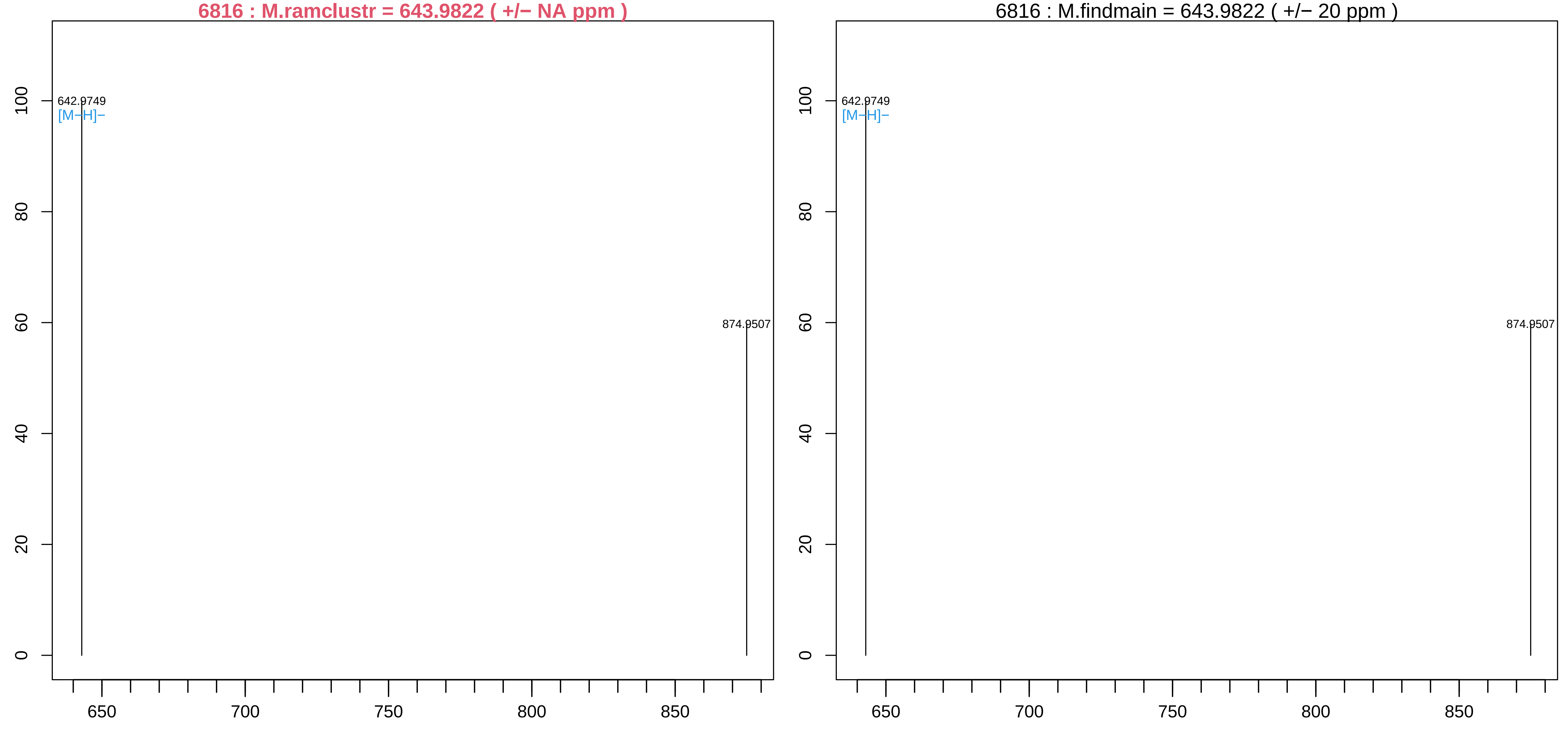
6816 : M.ramclustr = 643.9822 (+/− NA ppm)
642.9749
[M−H]−
874.9507
0
20
40
60
80
100
650
700
750
800
850
6816 : M.findmain = 643.9822 (+/− 20 ppm)
642.9749
[M−H]−
874.9507
0
20
40
60
80
100
650
700
750
800
850

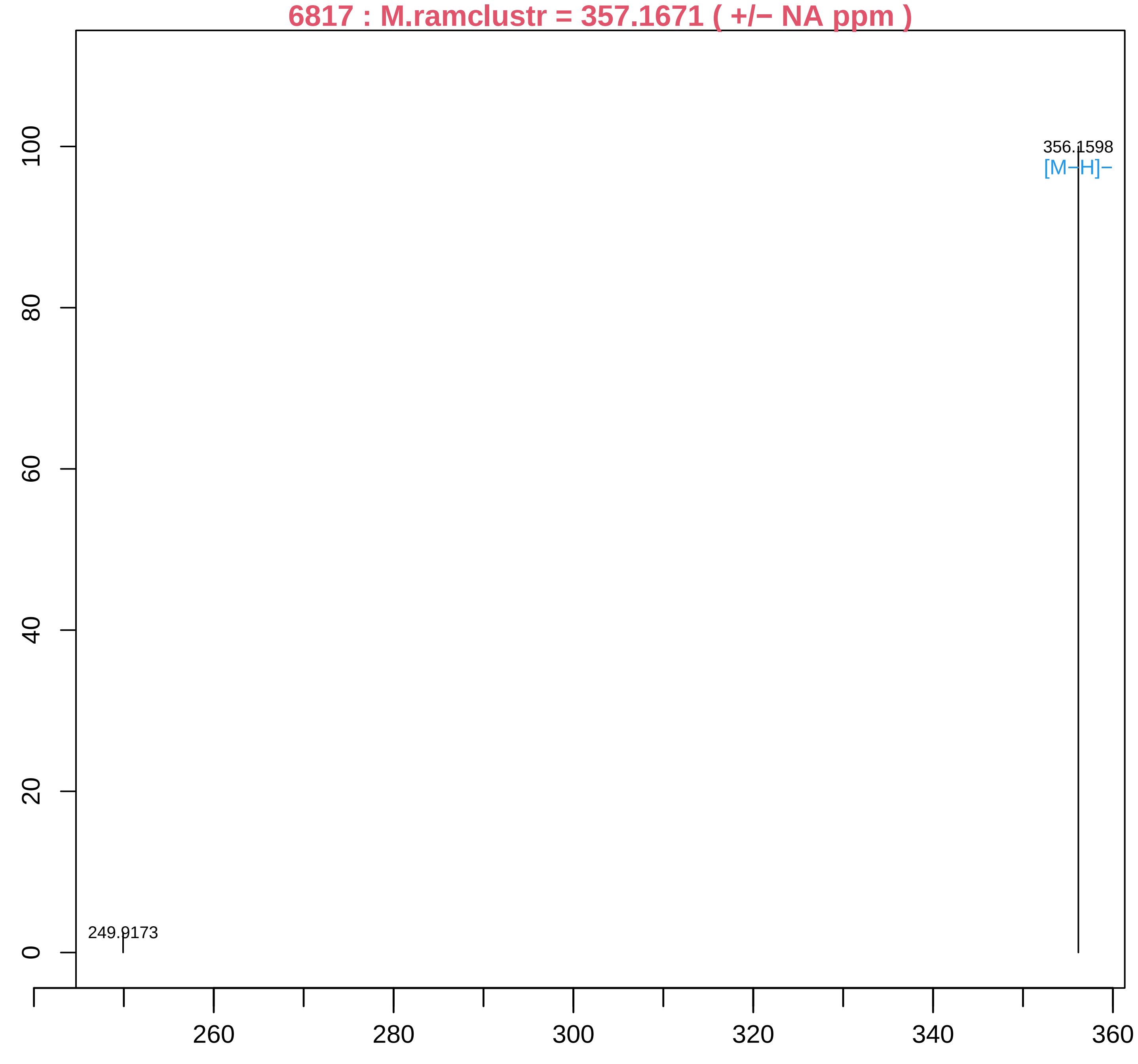
6817 : M.ramclustr = 357.1671 (+/− NA ppm)
356.1598
[M−H]−
249.9173
0
20
40
60
80
100
260
280
300
320
340
360

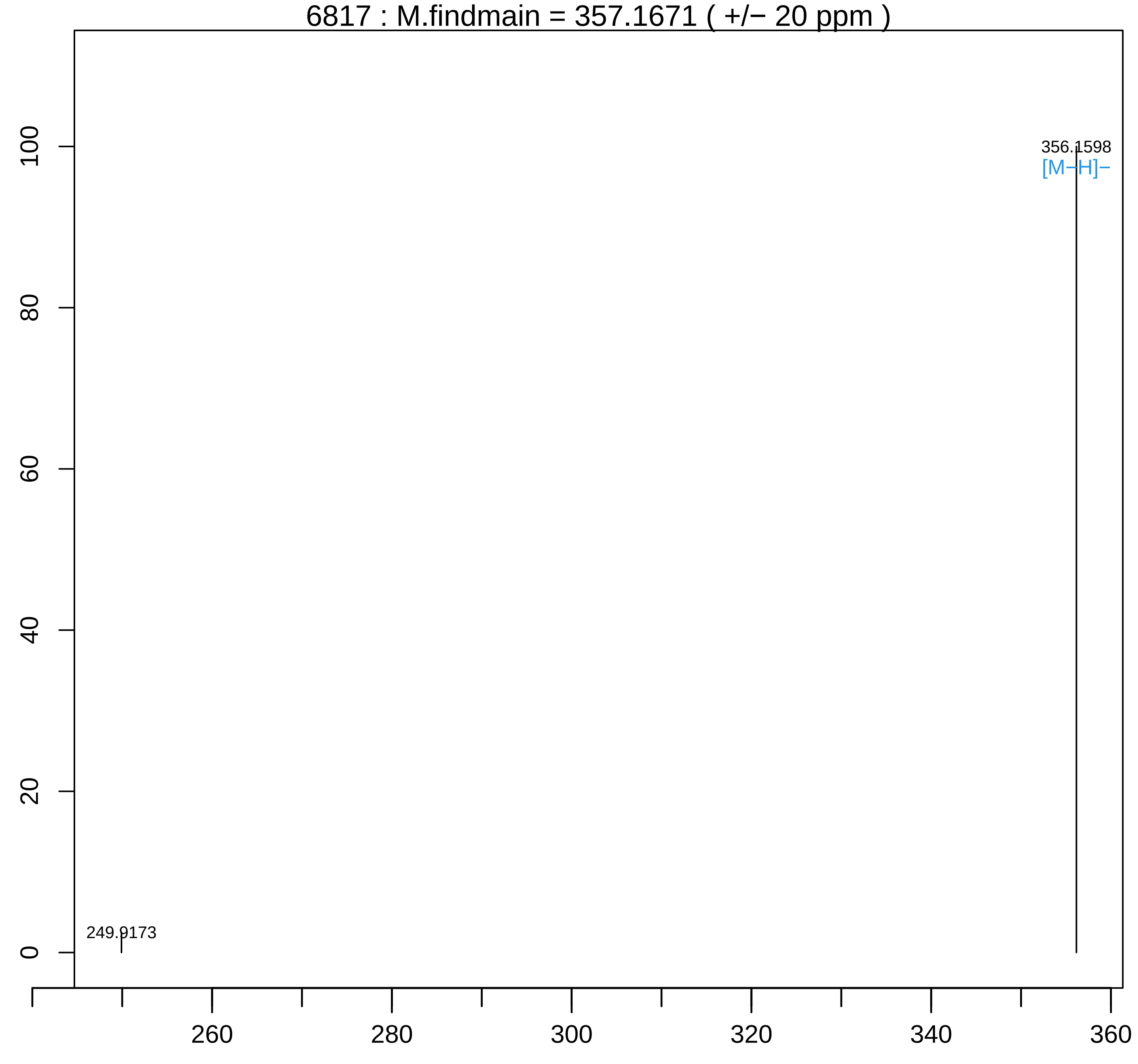
6817 : M.findmain = 357.1671 (+/− 20 ppm)
356.1598
[M−H]−
249.9173
0
20
40
60
80
100
260
280
300
320
340
360

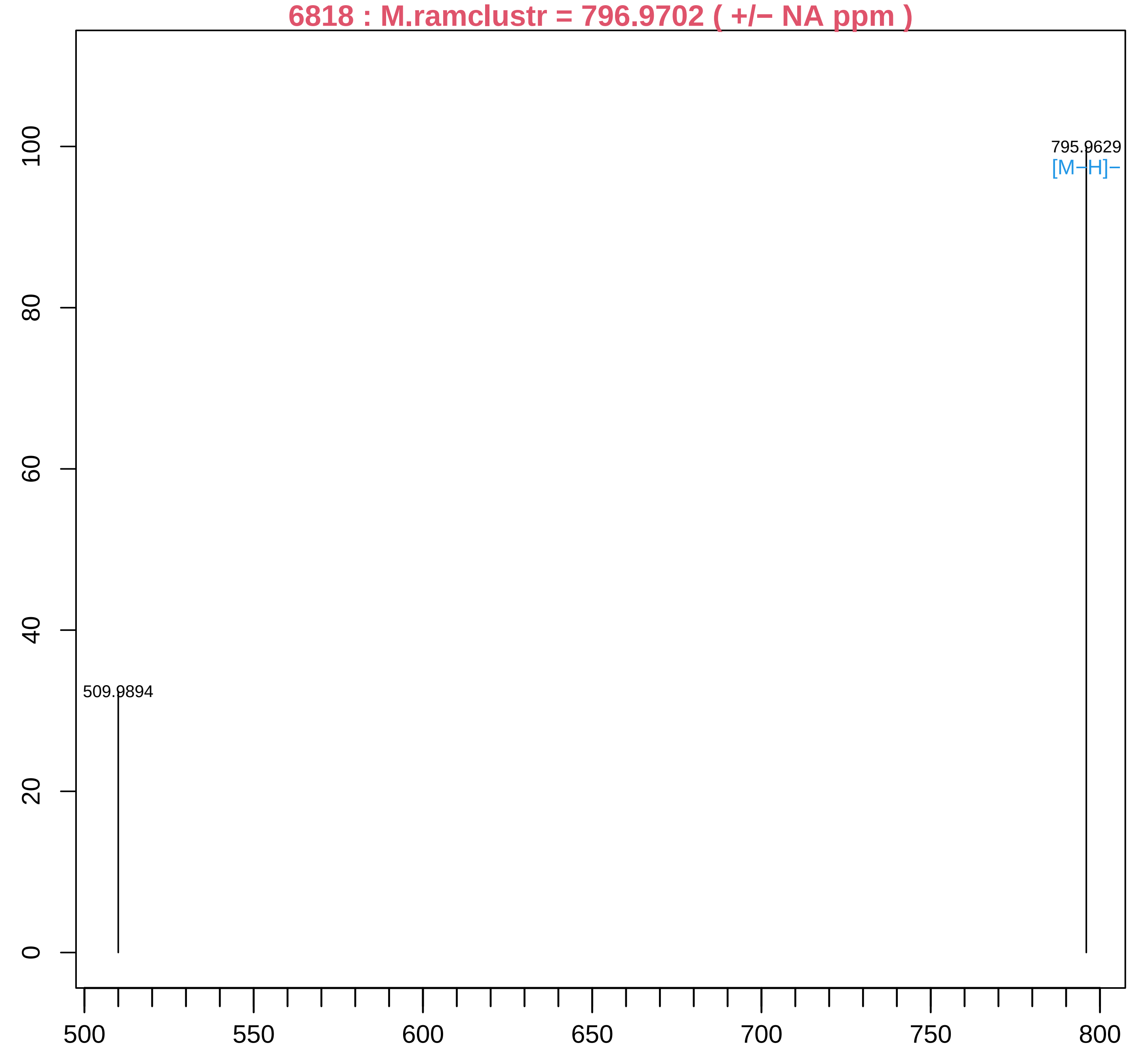
6818 : M.ramclustr = 796.9702 (+/− NA ppm)
795.9629
[M−H]−
509.9894
0
20
40
60
80
100
500
550
600
650
700
750
800

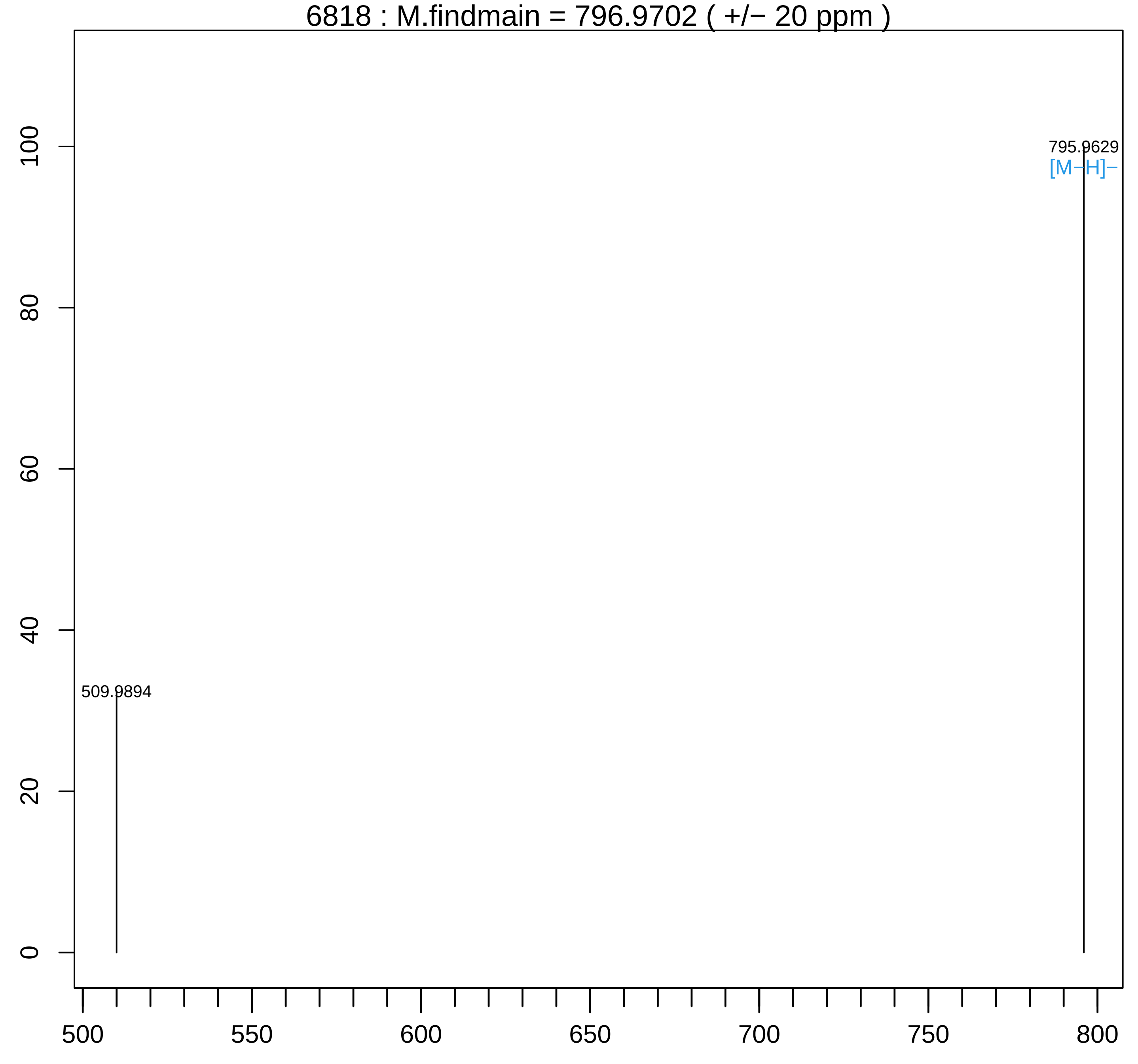
6818 : M.findmain = 796.9702 (+/− 20 ppm)
795.9629
[M−H]−
509.9894
0
20
40
60
80
100
500
550
600
650
700
750
800

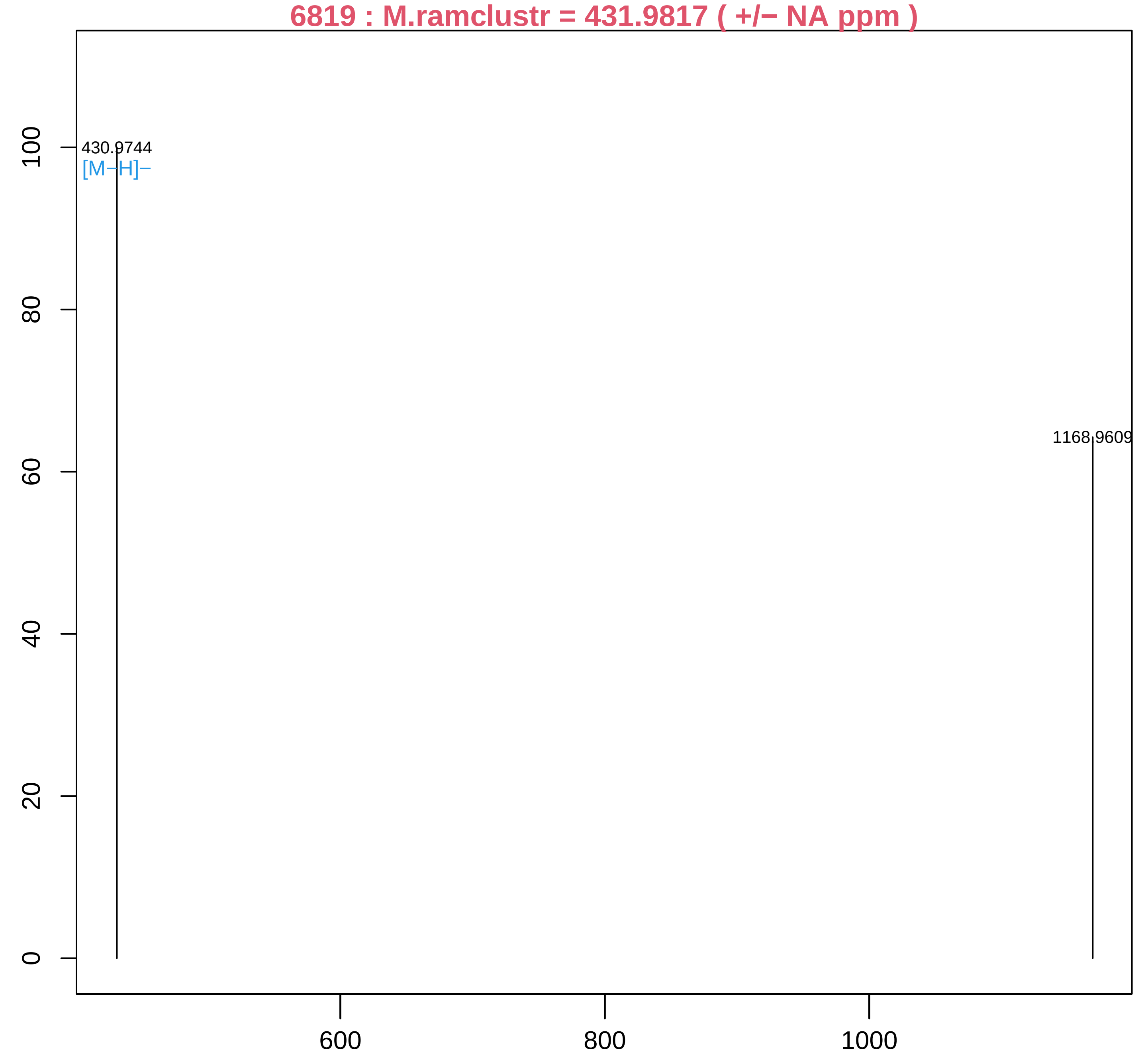
6819 : M.ramclustr = 431.9817 (+/− NA ppm)
430.9744
[M−H]−
1168.9609
0
20
40
60
80
100
600
800
1000

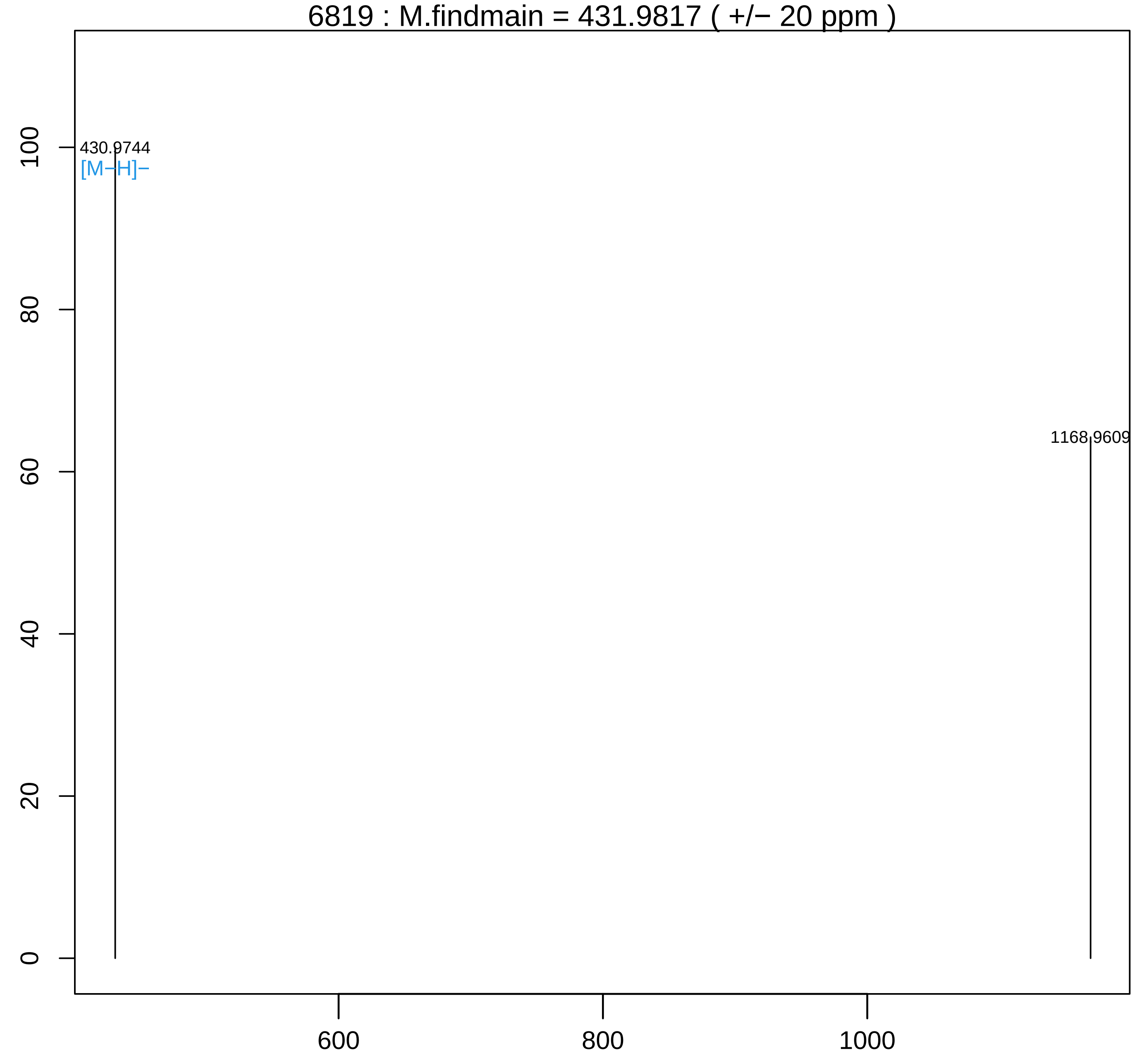
6819 : M.findmain = 431.9817 (+/− 20 ppm)
430.9744
[M−H]−
1168.9609
0
20
40
60
80
100
600
800
1000

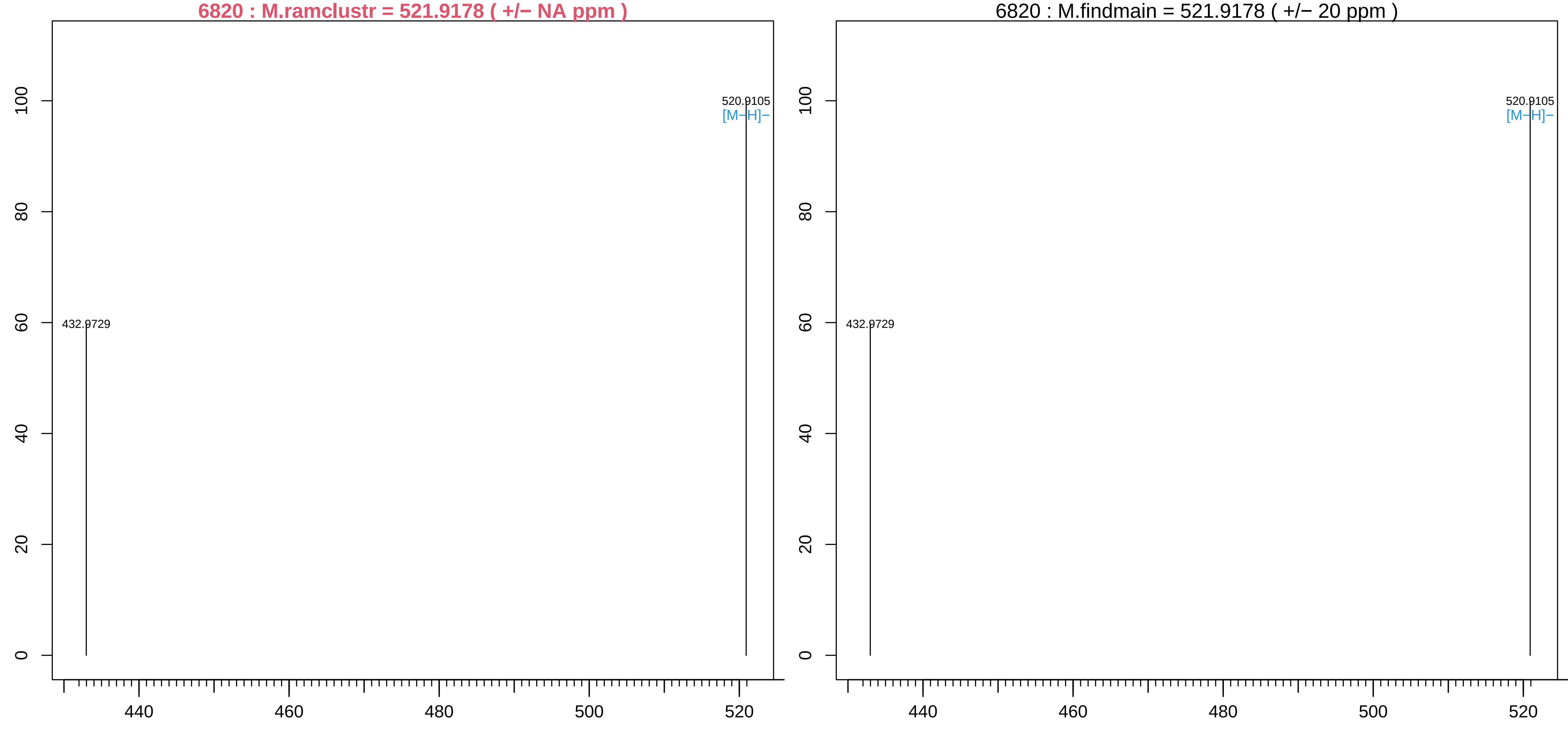
6820 : M.ramclustr = 521.9178 (+/− NA ppm)
432.9729
520.9105
[M−H]−
0
20
40
60
80
100
440
460
480
500
520
6820 : M.findmain = 521.9178 (+/− 20 ppm)
432.9729
520.9105
[M−H]−
0
20
40
60
80
100
440
460
480
500
520

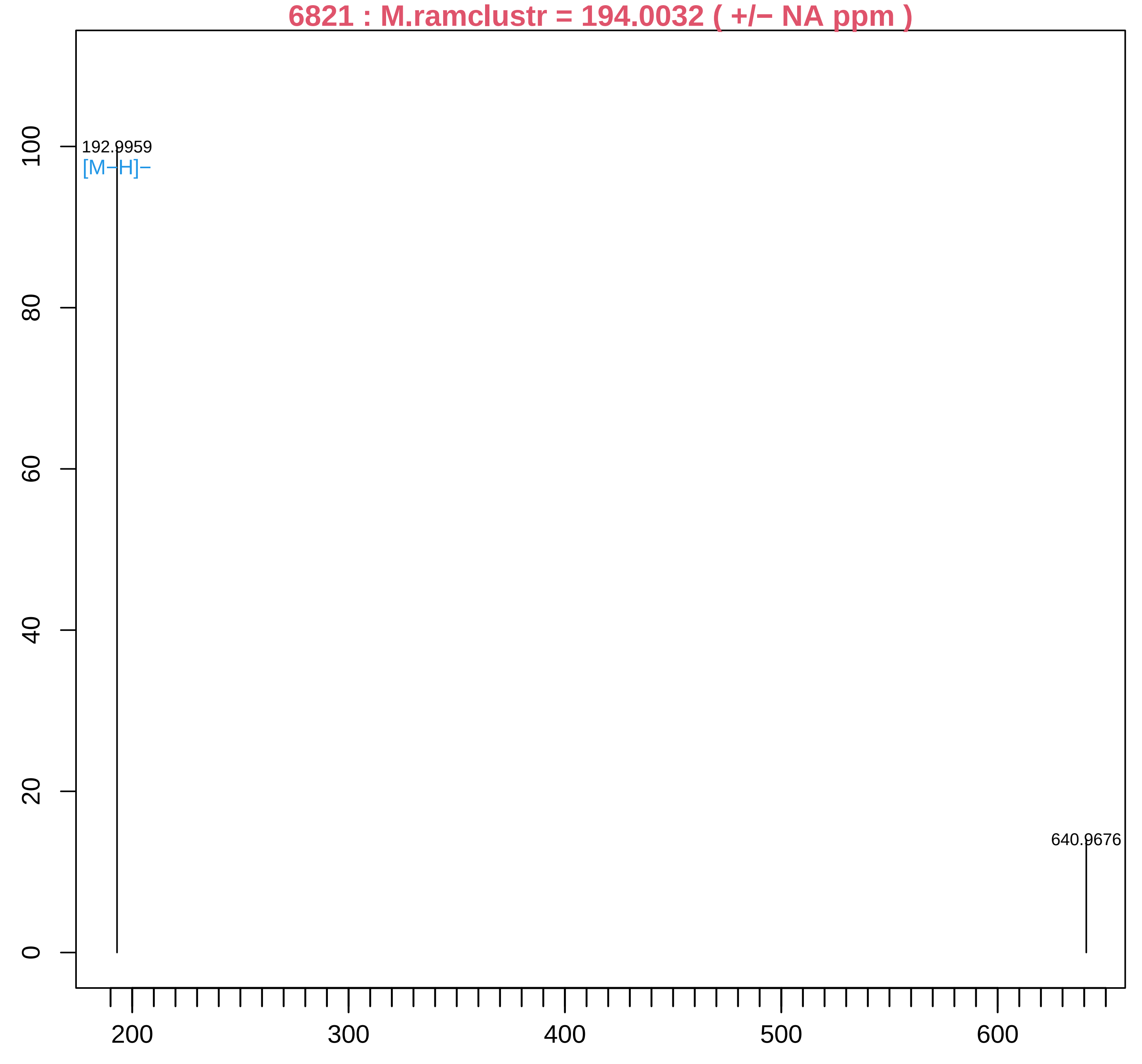
6821 : M.ramclustr = 194.0032 (+/− NA ppm)
192.9959
[M−H]−
640.9676
0
20
40
60
80
100
200
300
400
500
600

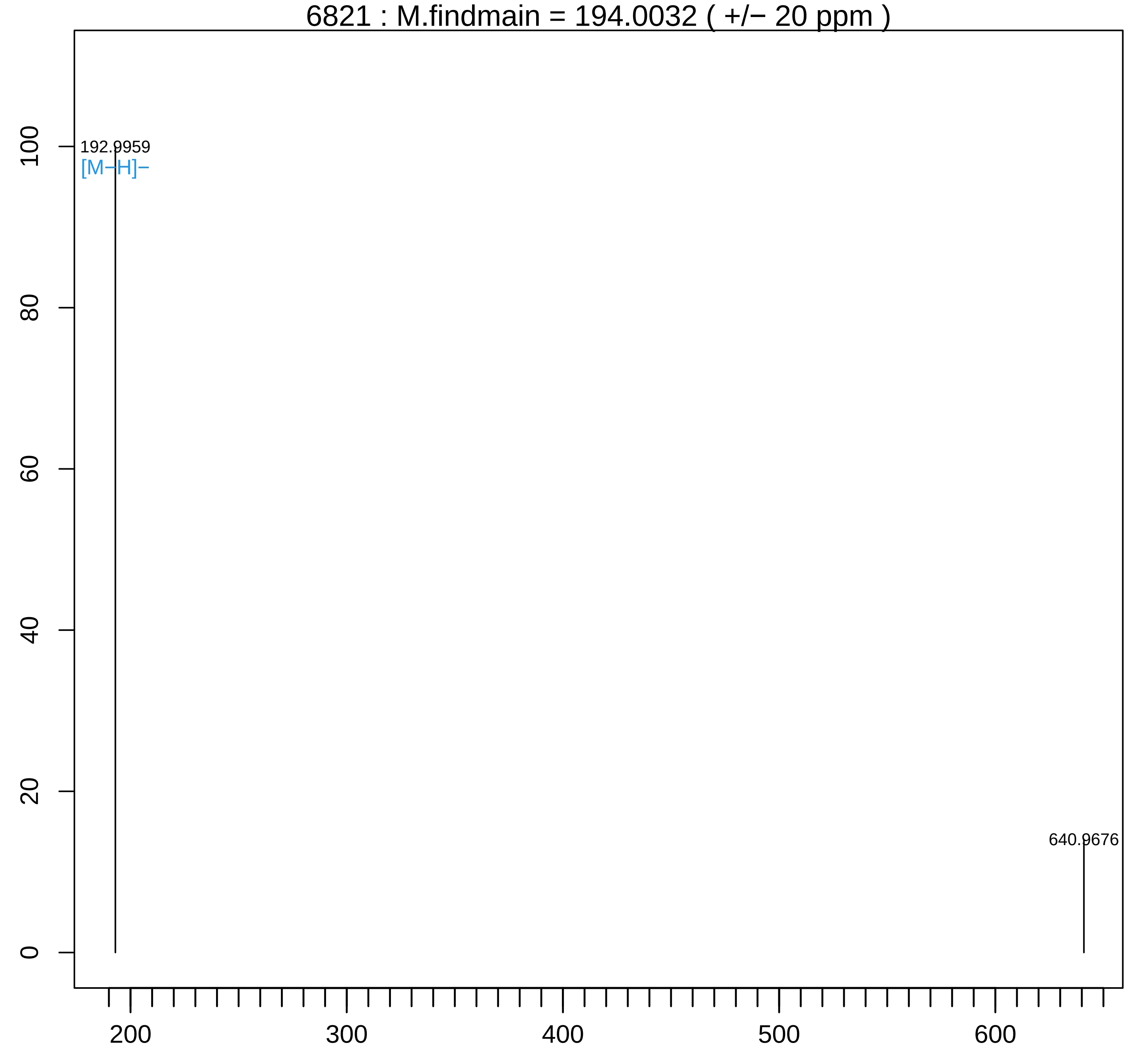
6821 : M.findmain = 194.0032 (+/− 20 ppm)
192.9959
[M−H]−
640.9676
0
20
40
60
80
100
200
300
400
500
600

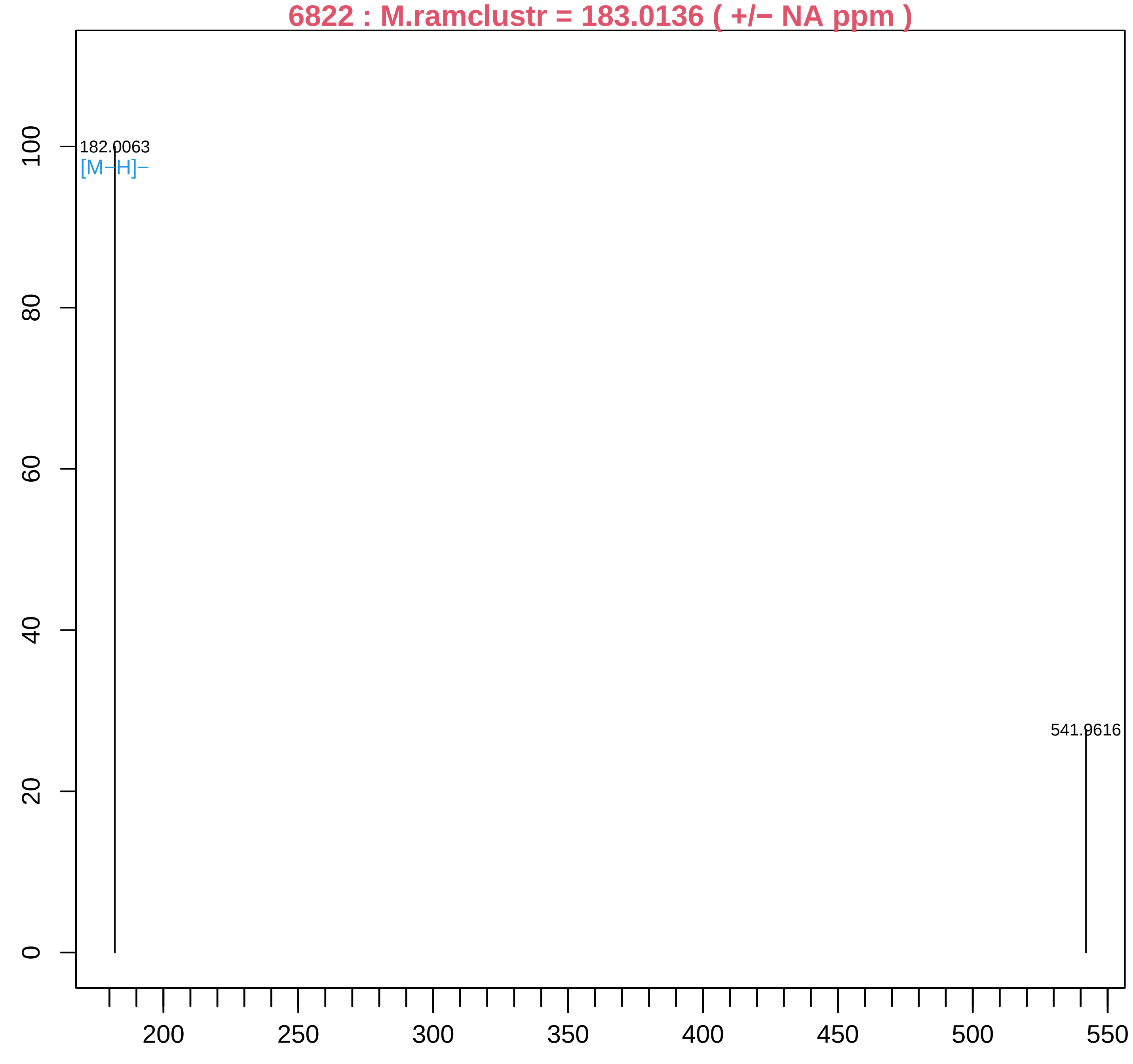
6822 : M.ramclustr = 183.0136 (+/− NA ppm)
182.0063
[M−H]−
541.9616
0
20
40
60
80
100
200
250
300
350
400
450
500
550

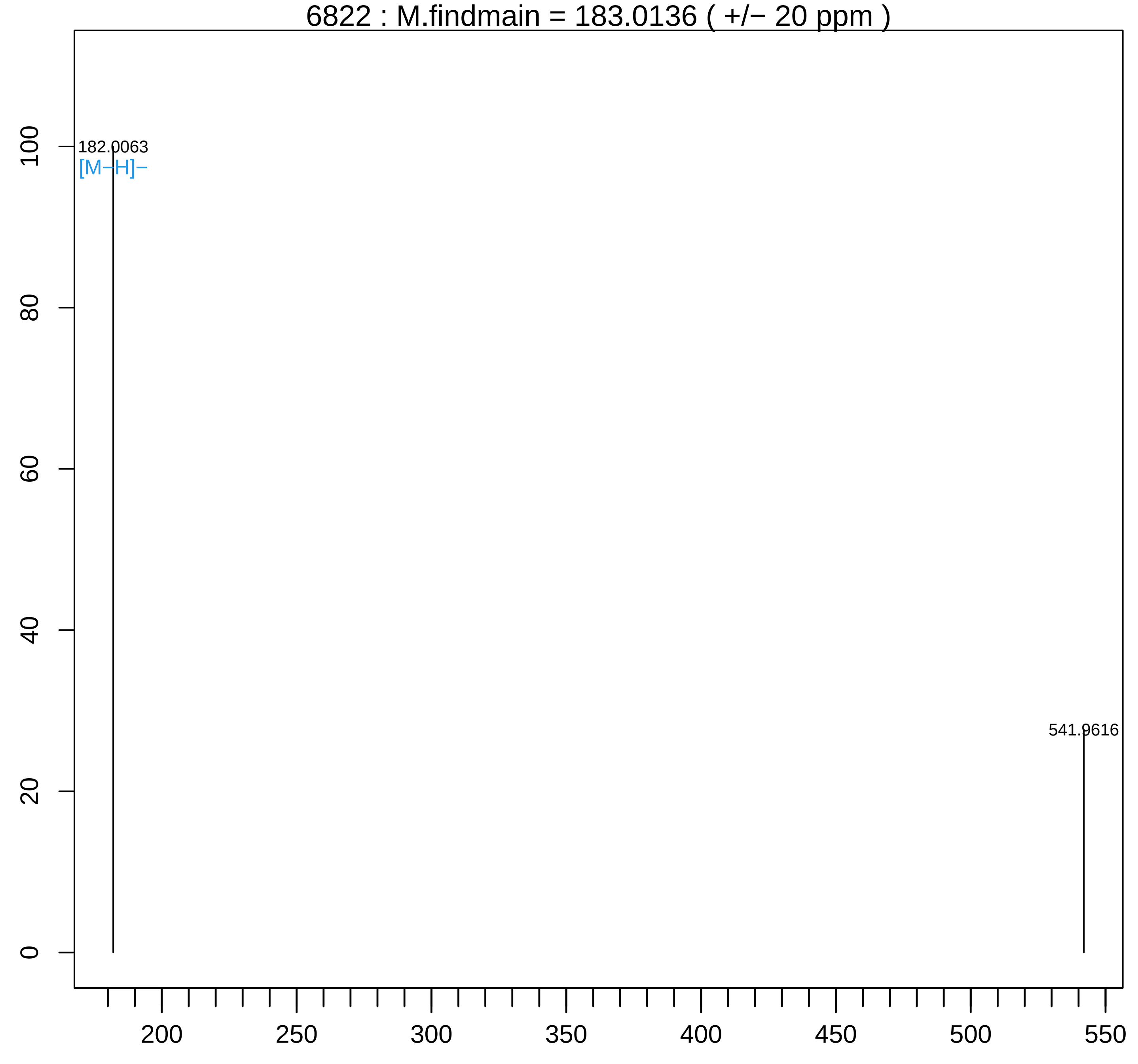
6822 : M.findmain = 183.0136 (+/− 20 ppm)
182.0063
[M−H]−
541.9616
0
20
40
60
80
100
200
250
300
350
400
450
500
550

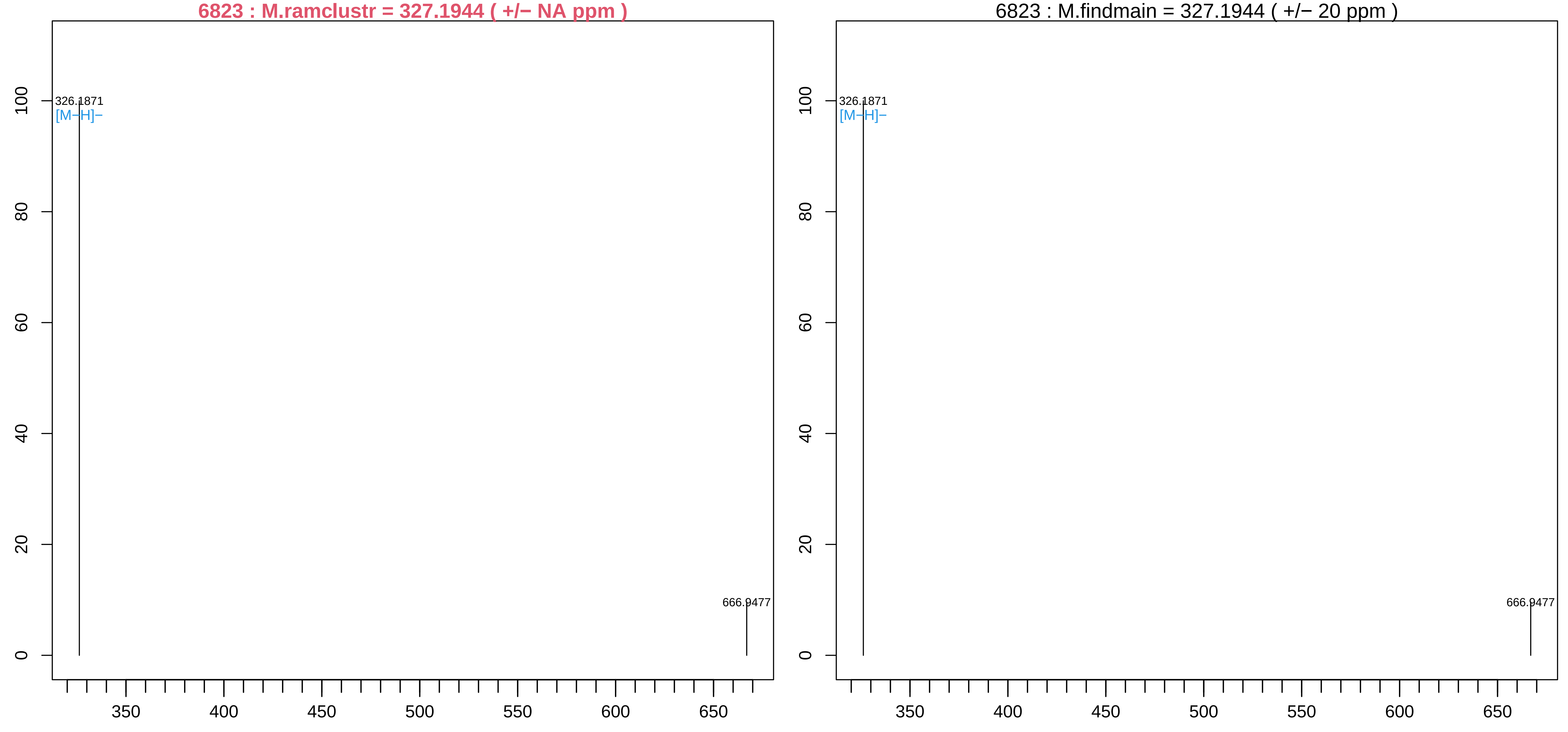
6823 : M.ramclustr = 327.1944 (+/− NA ppm)
326.1871
[M−H]−
666.9477
0
20
40
60
80
100
350
400
450
500
550
600
650
6823 : M.findmain = 327.1944 (+/− 20 ppm)
326.1871
[M−H]−
666.9477
0
20
40
60
80
100
350
400
450
500
550
600
650

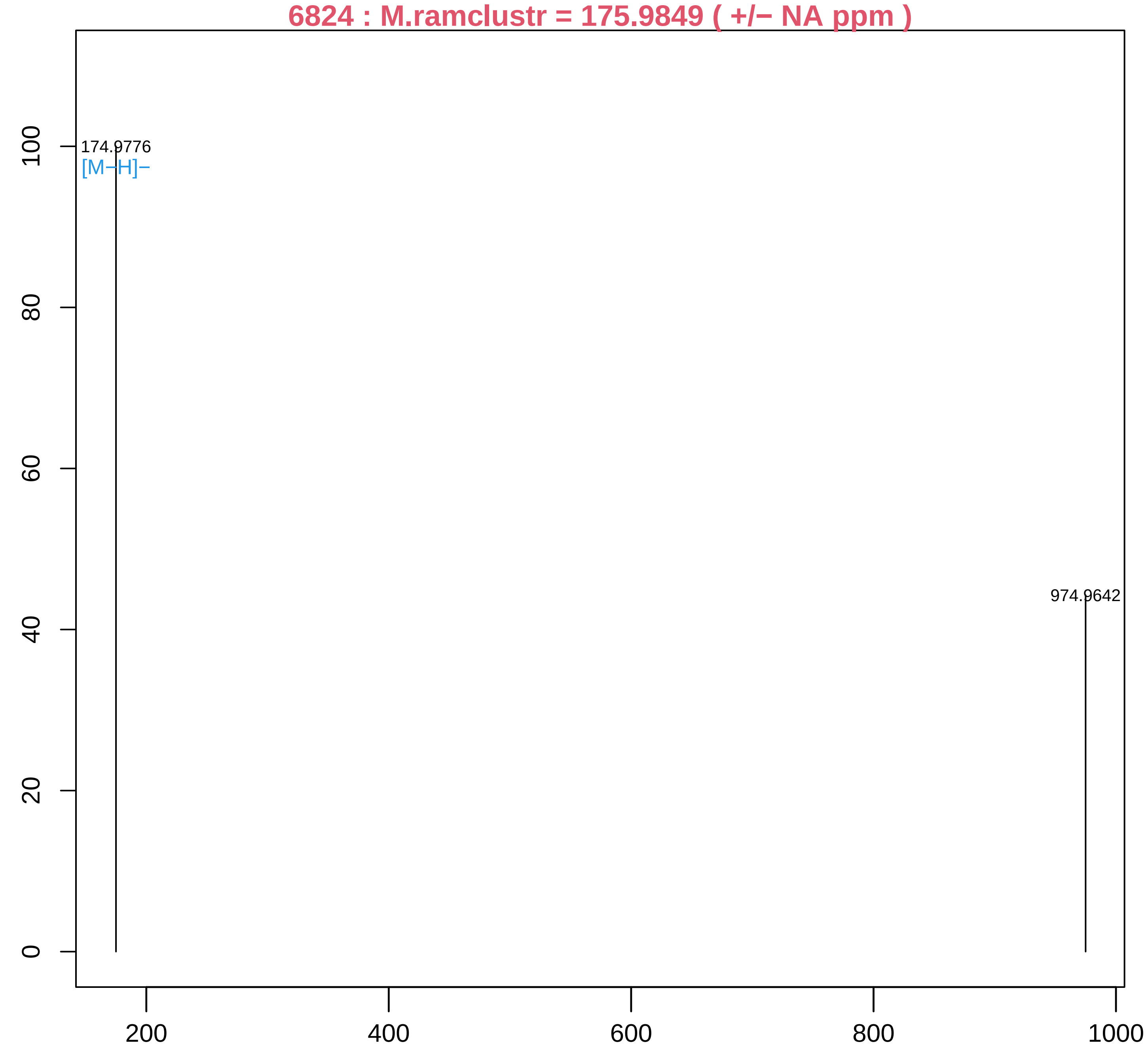
6824 : M.ramclustr = 175.9849 (+/− NA ppm)
174.9776
[M−H]−
974.9642
0
20
40
60
80
100
200
400
600
800
1000

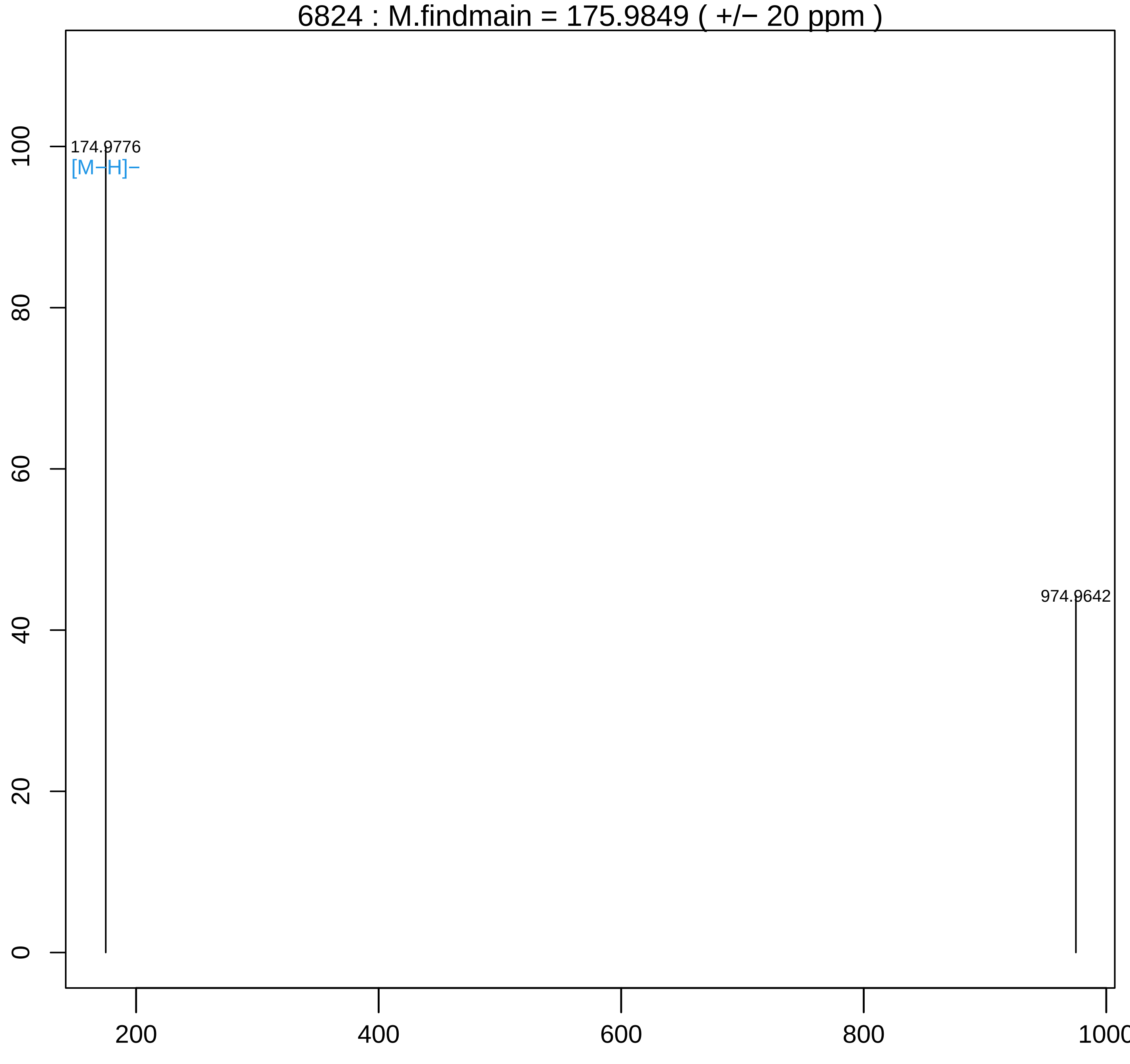
6824 : M.findmain = 175.9849 (+/− 20 ppm)
174.9776
[M−H]−
974.9642
0
20
40
60
80
100
200
400
600
800
1000

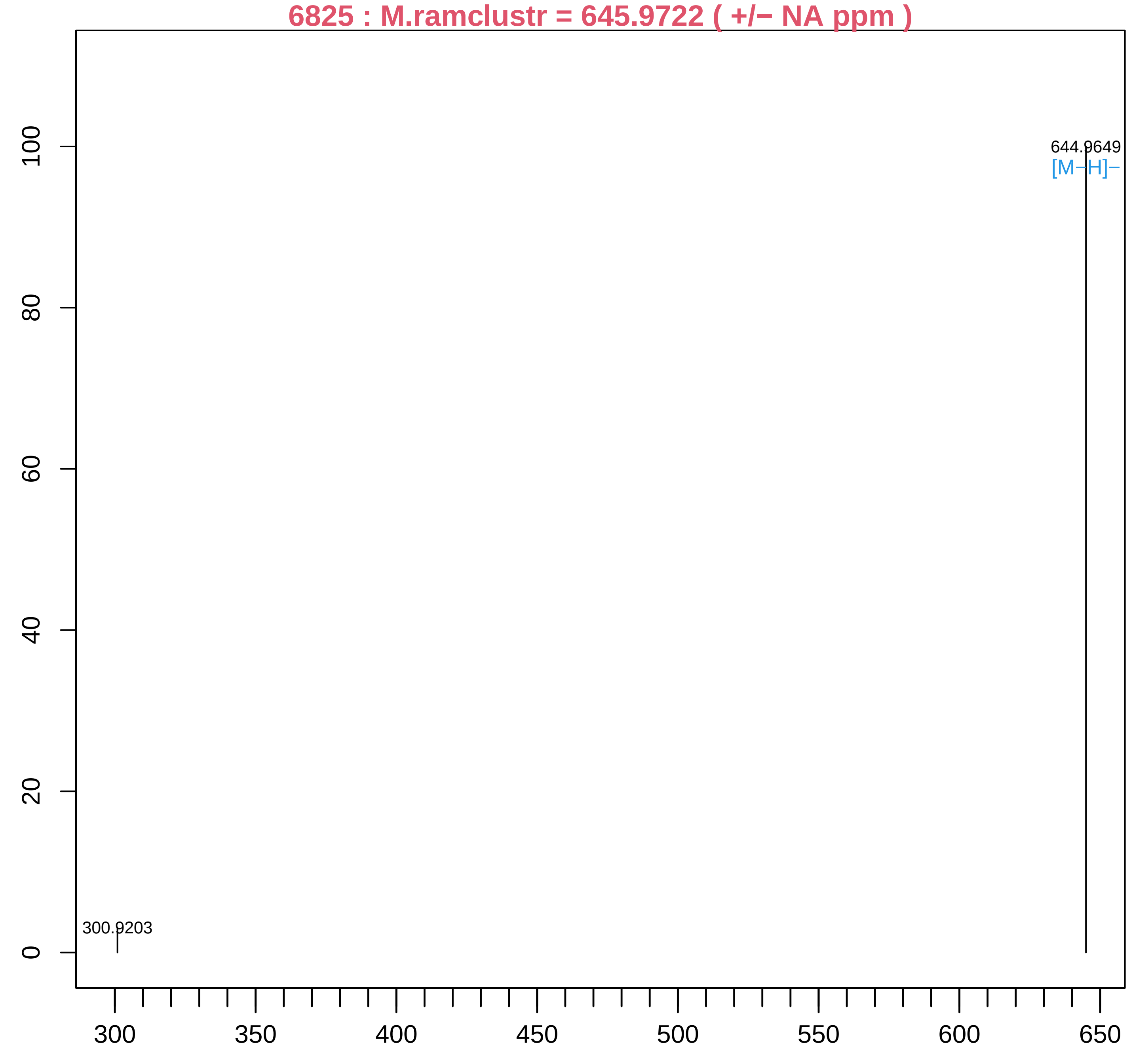
6825 : M.ramclustr = 645.9722 (+/− NA ppm)
644.9649
[M−H]−
300.9203
0
20
40
60
80
100
300
350
400
450
500
550
600
650

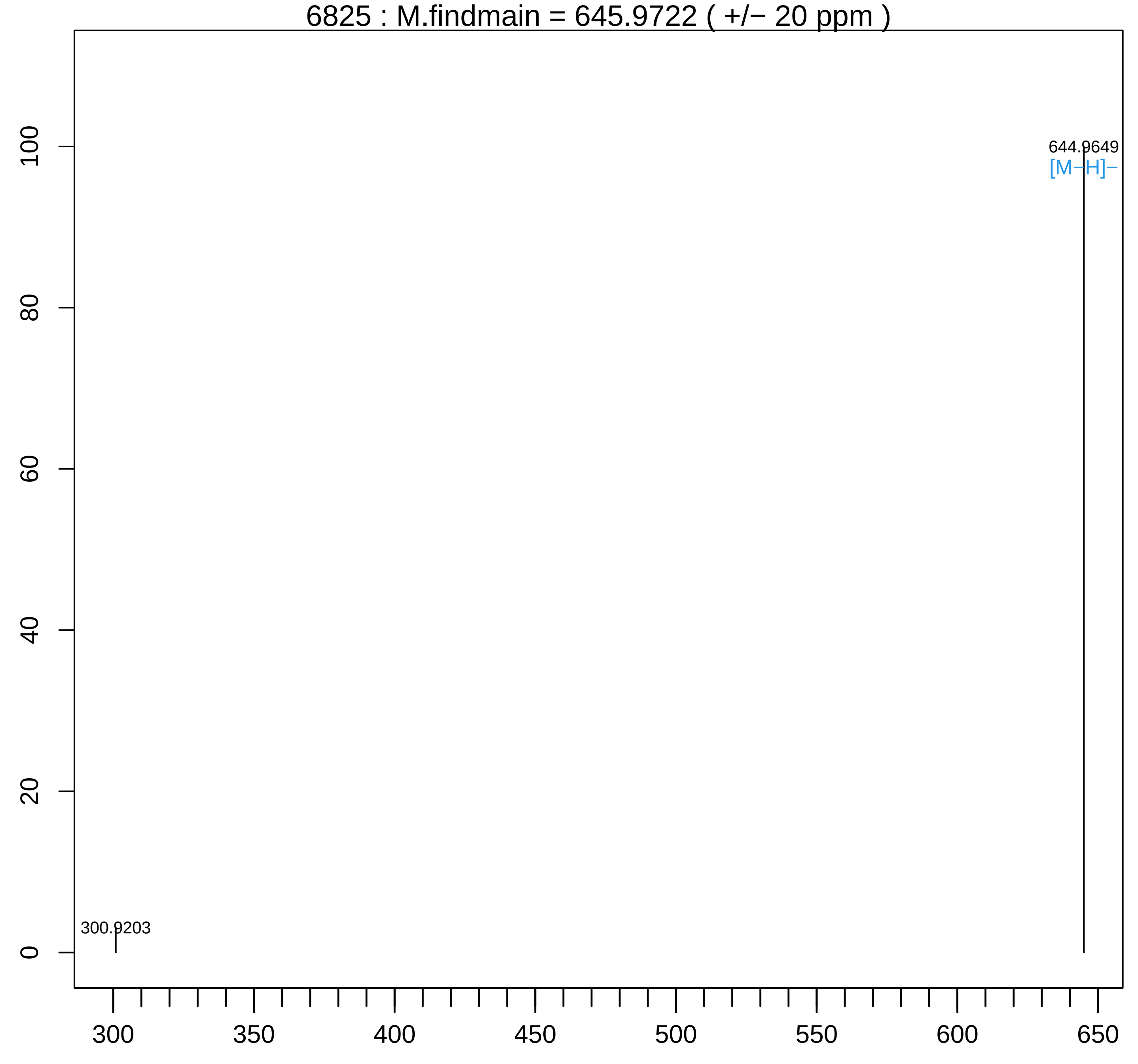
6825 : M.findmain = 645.9722 (+/− 20 ppm)
644.9649
[M−H]−
300.9203
0
20
40
60
80
100
300
350
400
450
500
550
600
650

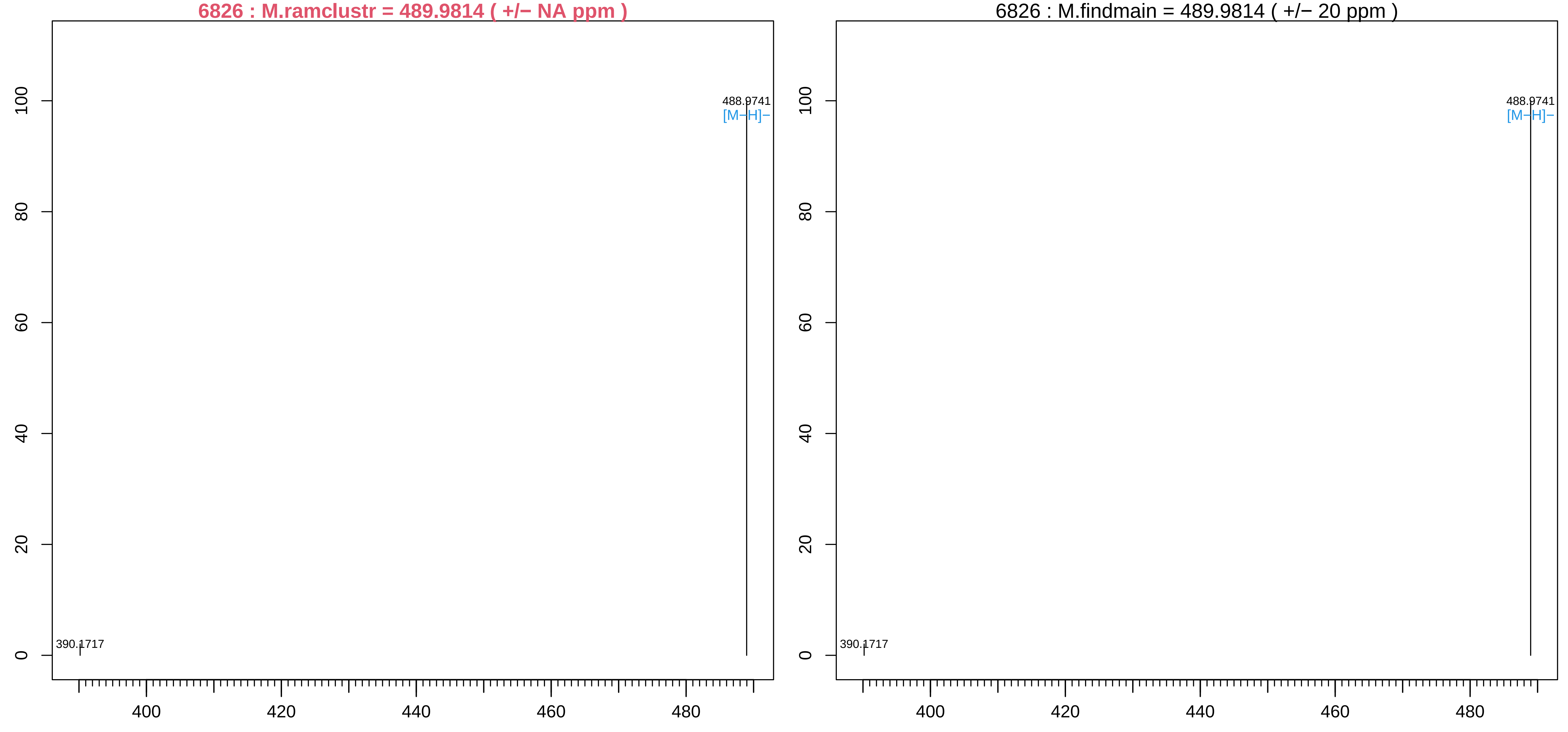
6826 : M.ramclustr = 489.9814 (+/− NA ppm)
488.9741
[M−H]−
390.1717
6826 : M.findmain = 489.9814 (+/− 20 ppm)
488.9741
[M−H]−
390.1717

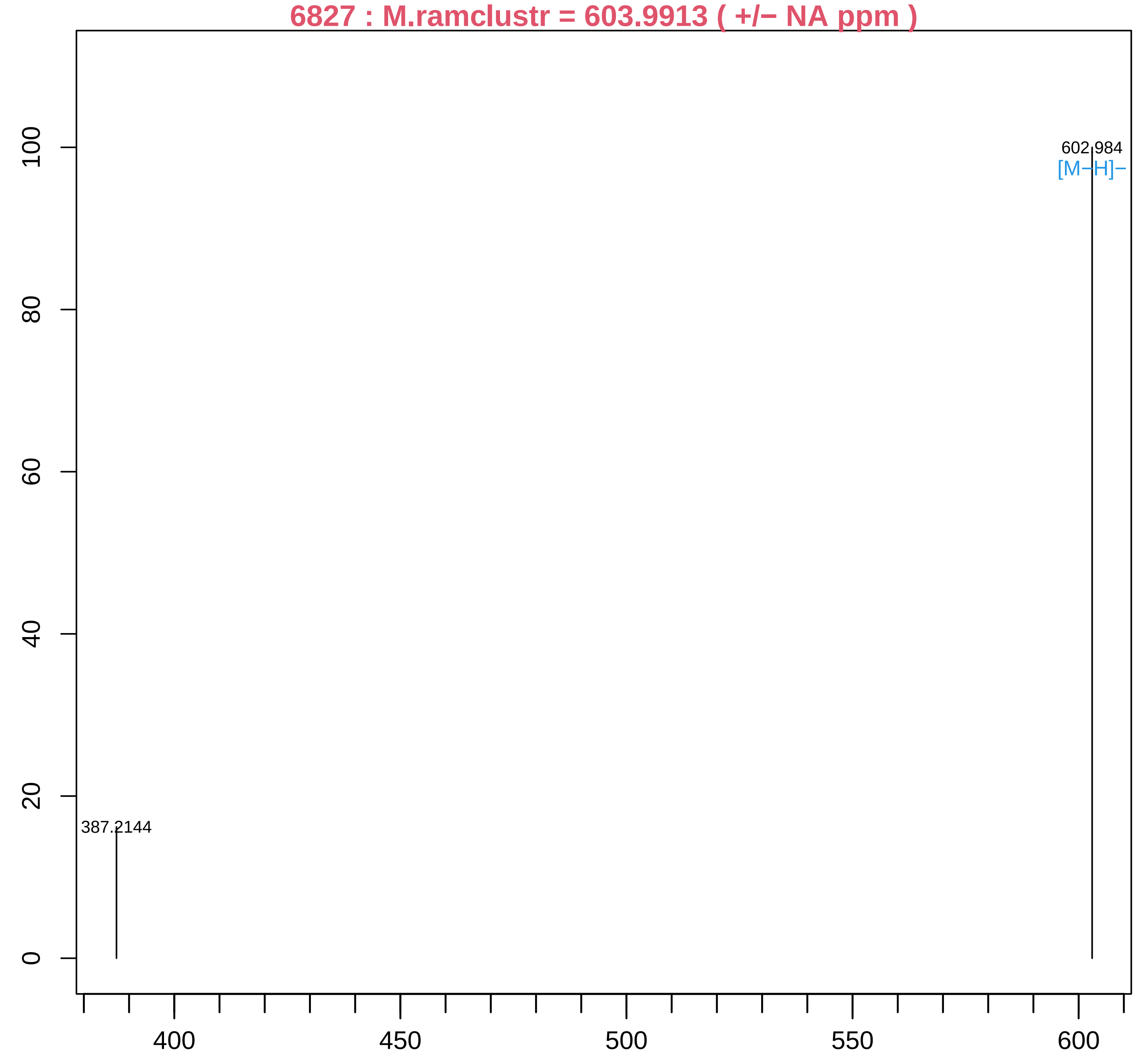

6827 : M.ramclustr = 603.9913 (+/− NA ppm)
602.984
[M−H]−
387.2144
0
20
40
60
80
100
400
450
500
550
600

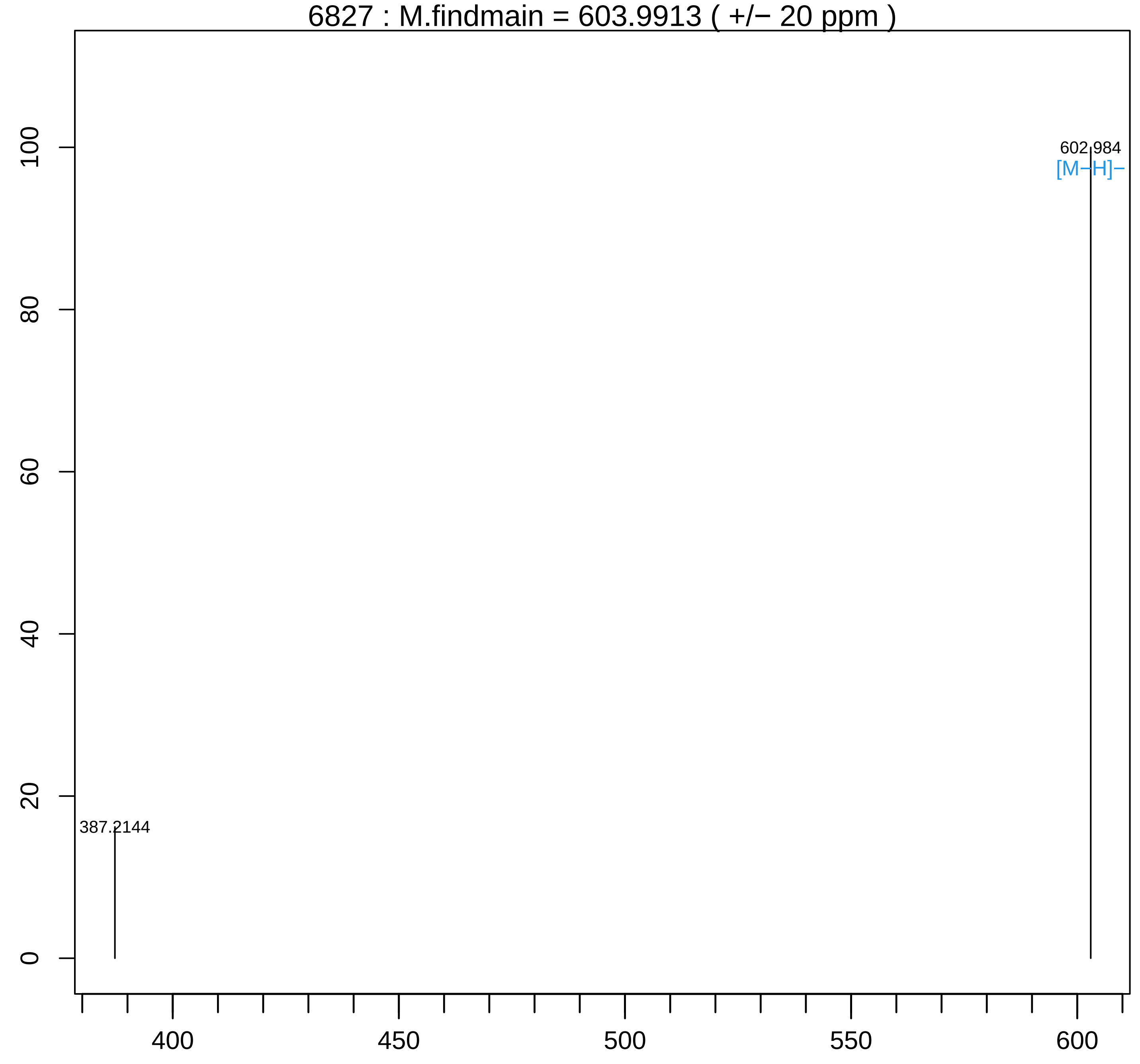

6827 : M.findmain = 603.9913 (+/− 20 ppm)
602.984
[M−H]−
387.2144
0
20
40
60
80
100
400
450
500
550
600

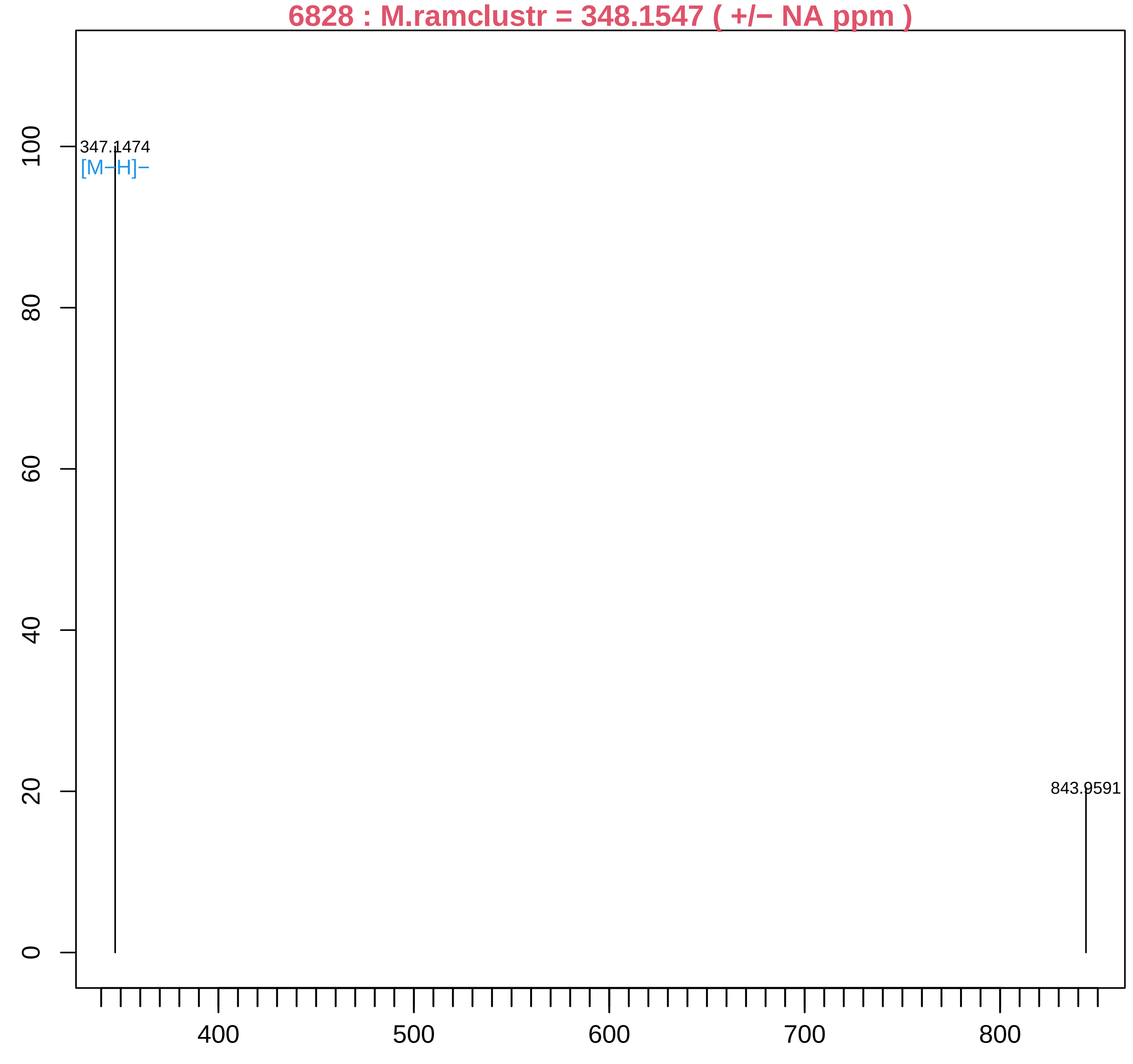
6828 : M.ramclustr = 348.1547 (+/− NA ppm)
347.1474
[M−H]−
843.9591
0
20
40
60
80
100
400
500
600
700
800

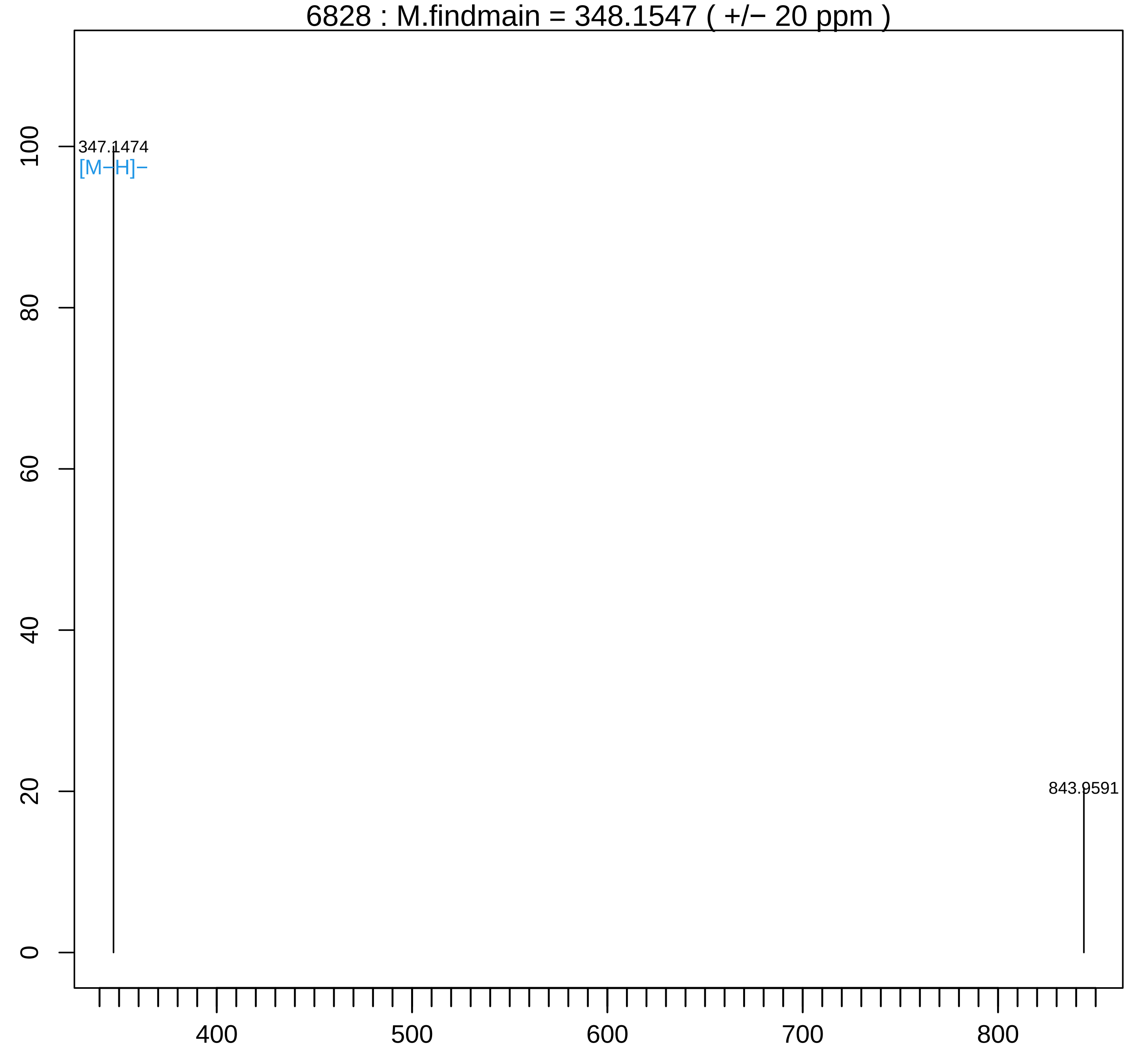
6828 : M.findmain = 348.1547 (+/− 20 ppm)
347.1474
[M−H]−
843.9591
0
20
40
60
80
100
400
500
600
700
800

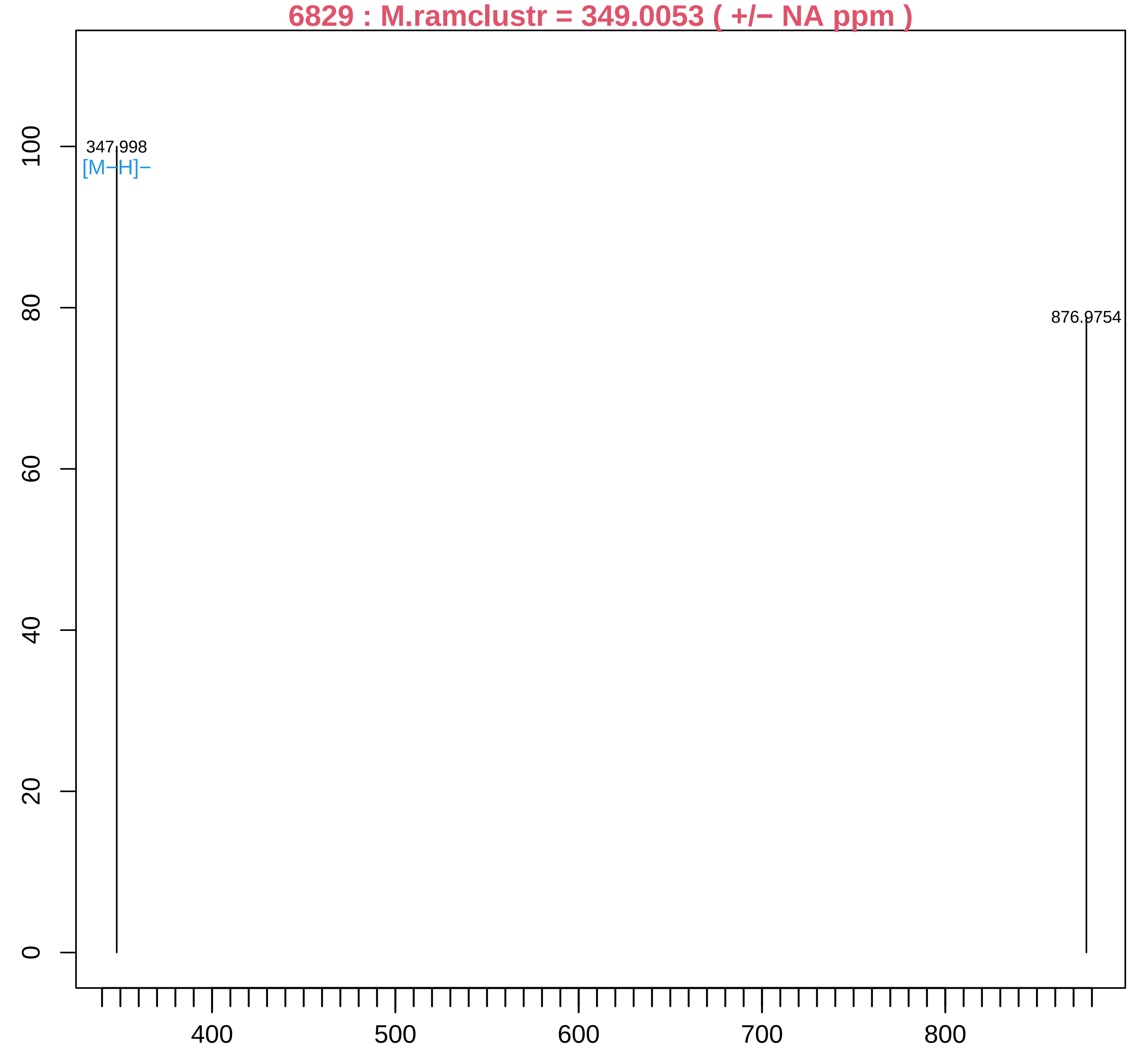
6829 : M.ramclustr = 349.0053 (+/− NA ppm)
347.998
[M−H]−
876.9754
0
20
40
60
80
100
400
500
600
700
800

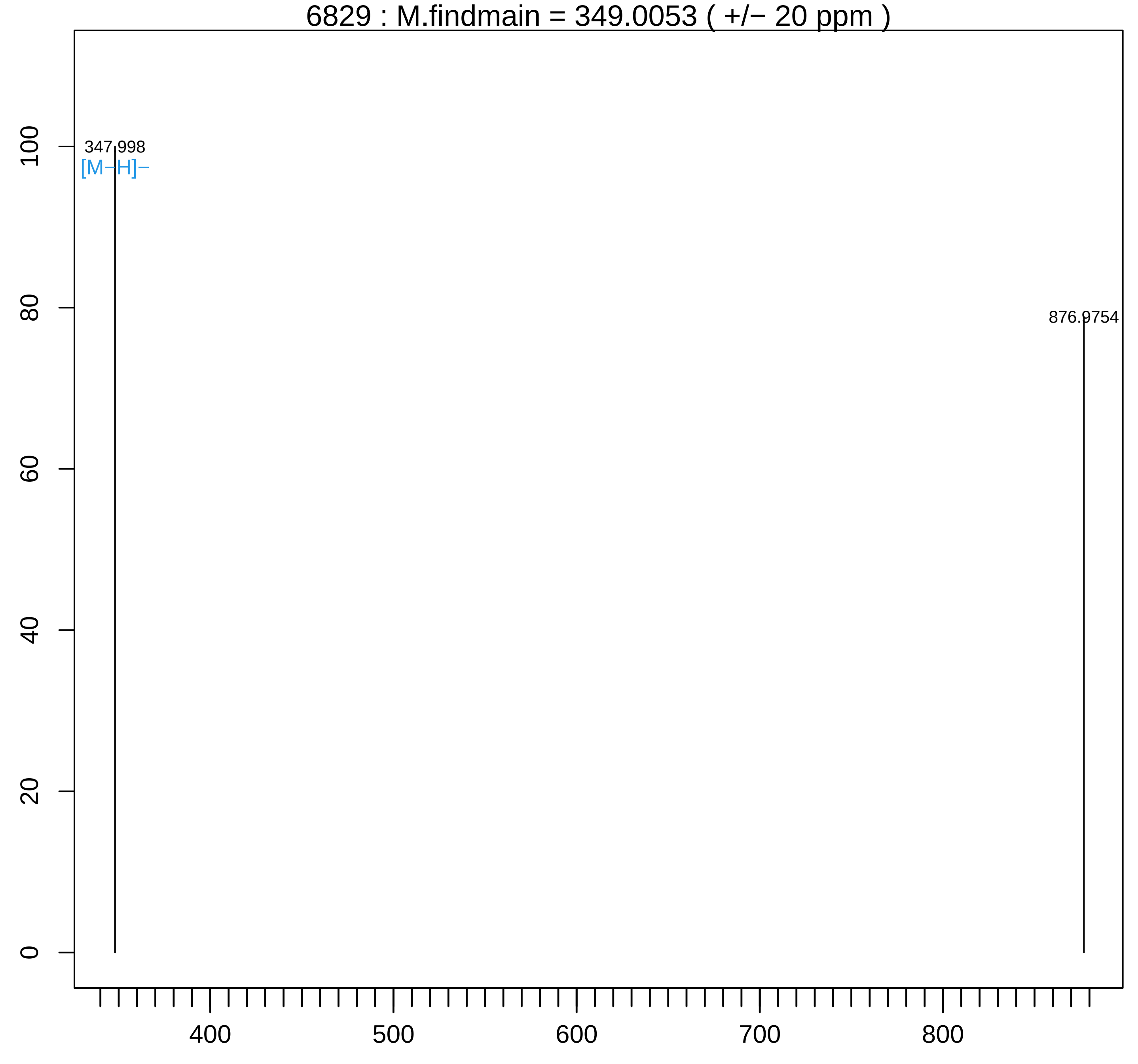
6829 : M.findmain = 349.0053 (+/− 20 ppm)
347.998
[M−H]−
876.9754
0
20
40
60
80
100
400
500
600
700
800

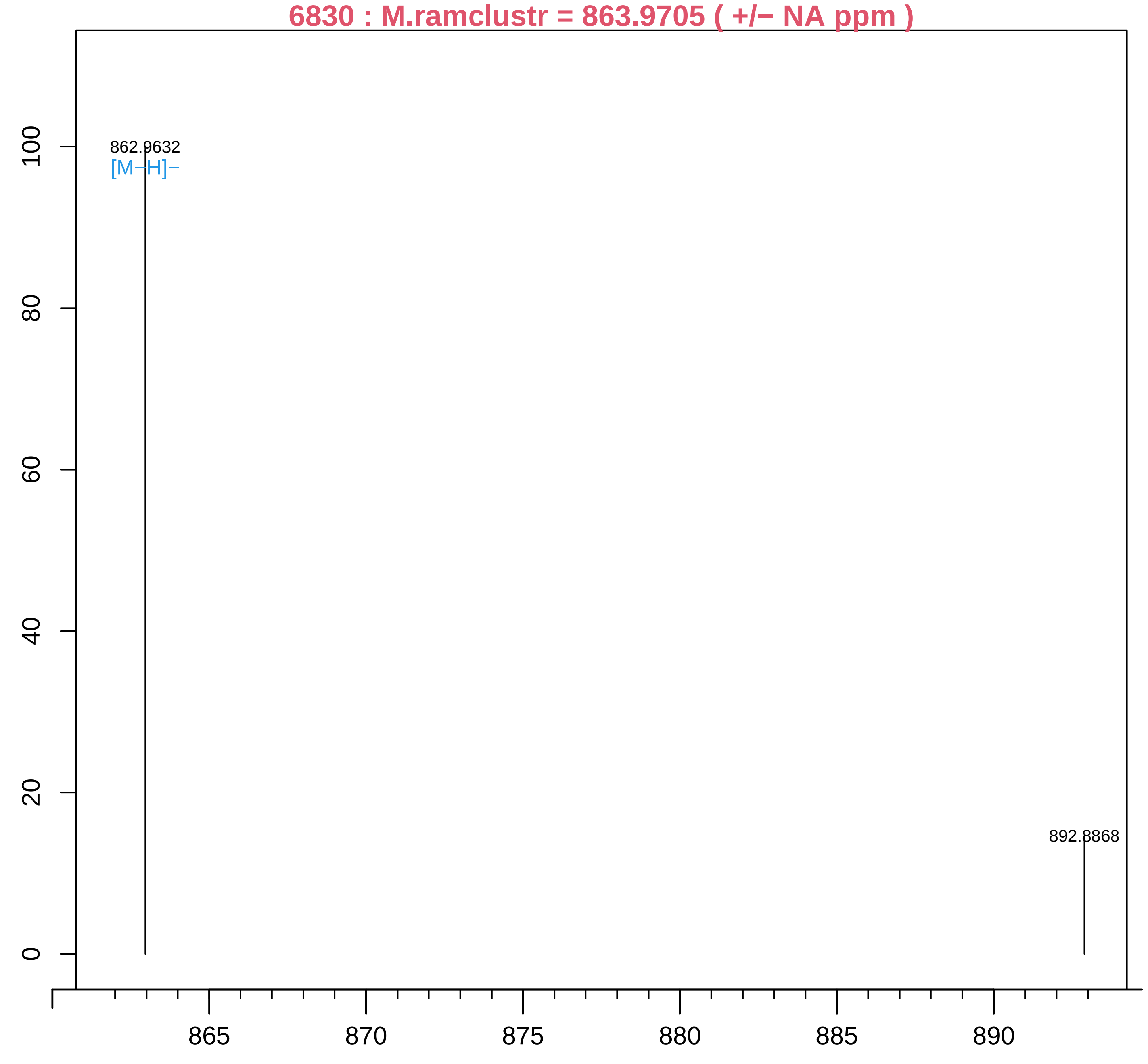
6830 : M.ramclustr = 863.9705 (+/− NA ppm)
862.9632
[M−H]−
892.8868
0
20
40
60
80
100
865
870
875
880
885
890

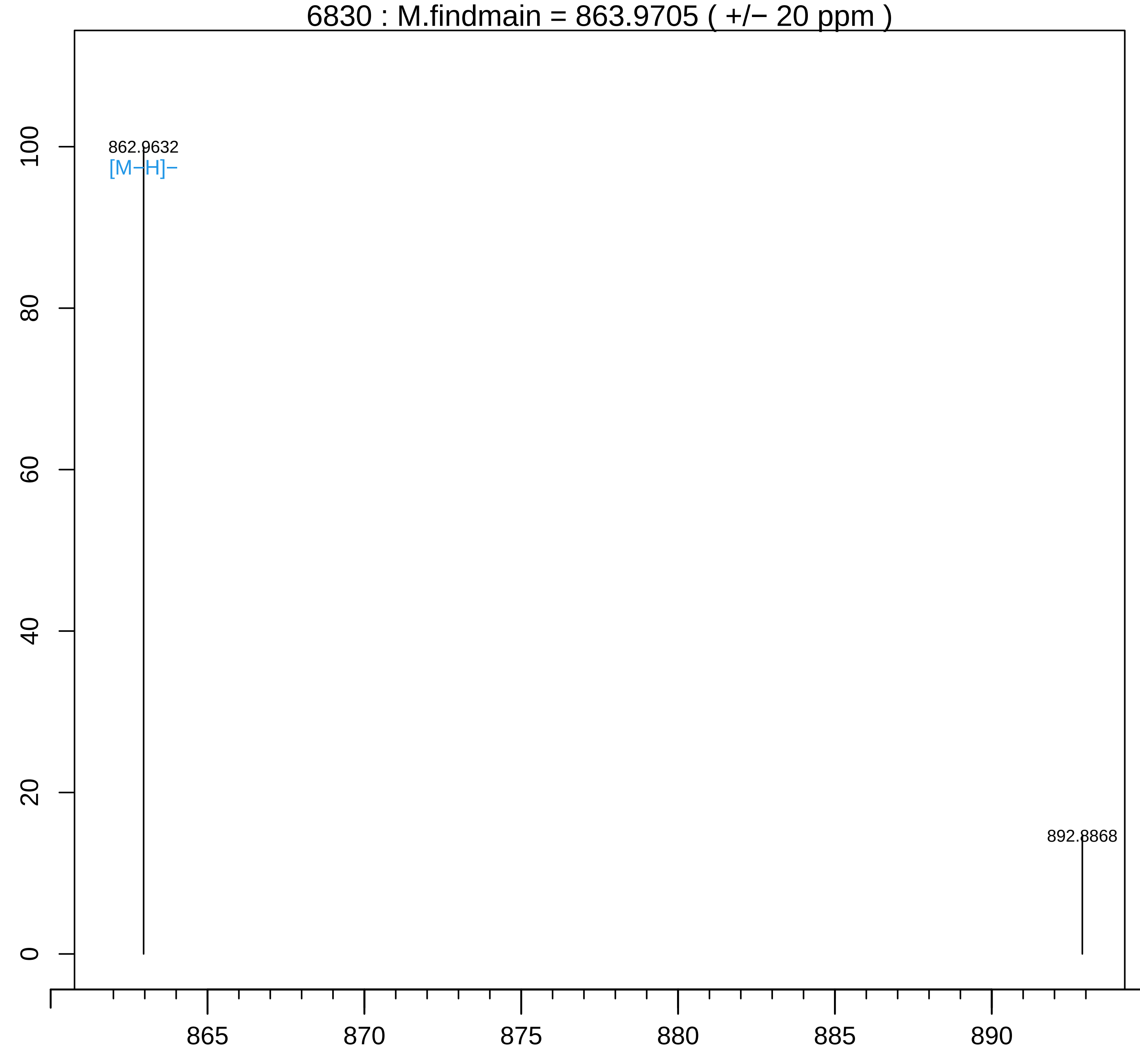
6830 : M.findmain = 863.9705 (+/− 20 ppm)
862.9632
[M−H]−
892.8868
0
20
40
60
80
100
865
870
875
880
885
890

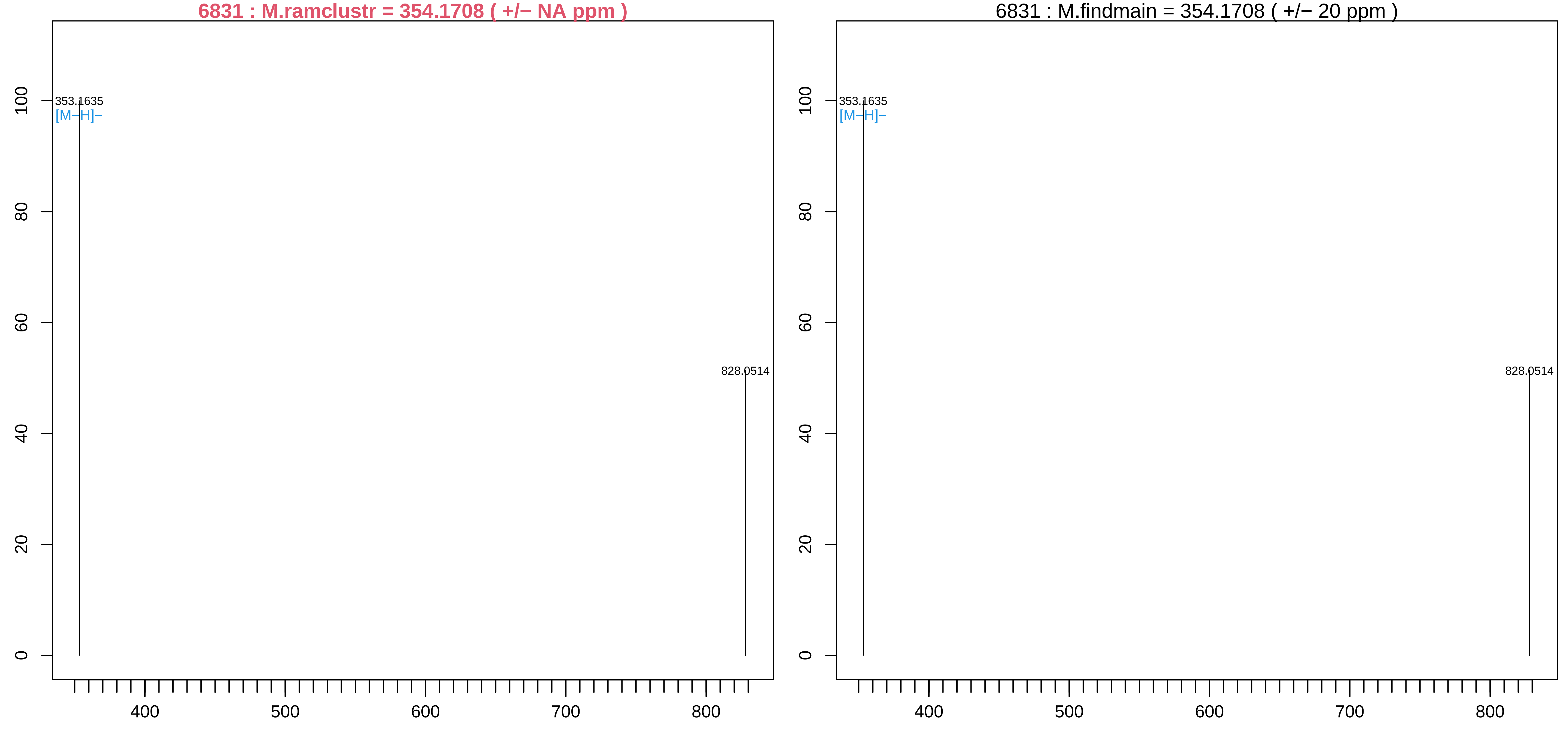

6831 : M.ramclustr = 354.1708 (+/− NA ppm)
6831 : M.findmain = 354.1708 (+/− 20 ppm)
353.1635
[M−H]−
828.0514
0
20
40
60
80
100
400
500
600
700
800

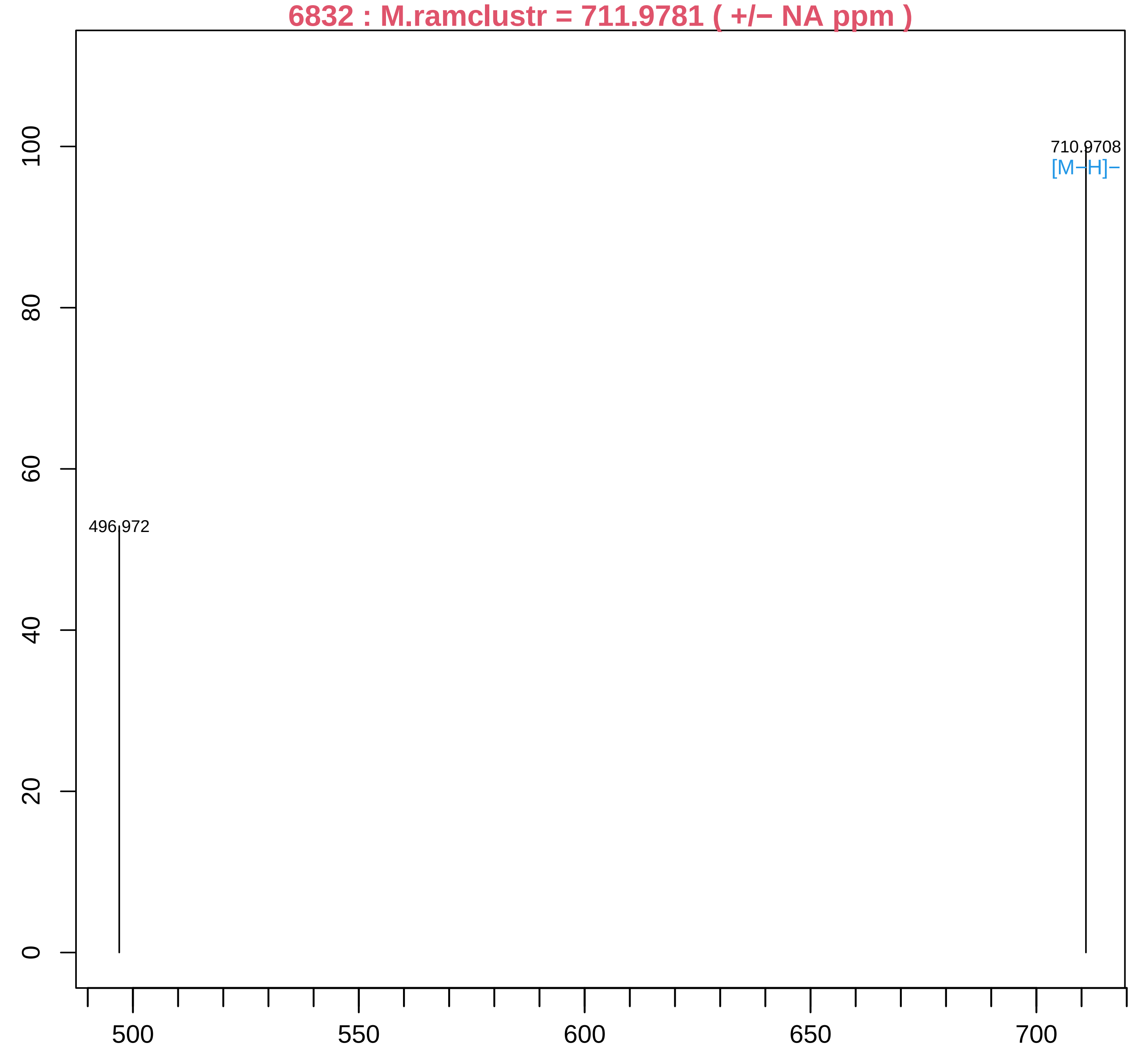
6832 : M.ramclustr = 711.9781 (+/− NA ppm)
710.9708
[M−H]−
496.972
0
20
40
60
80
100
500
550
600
650
700

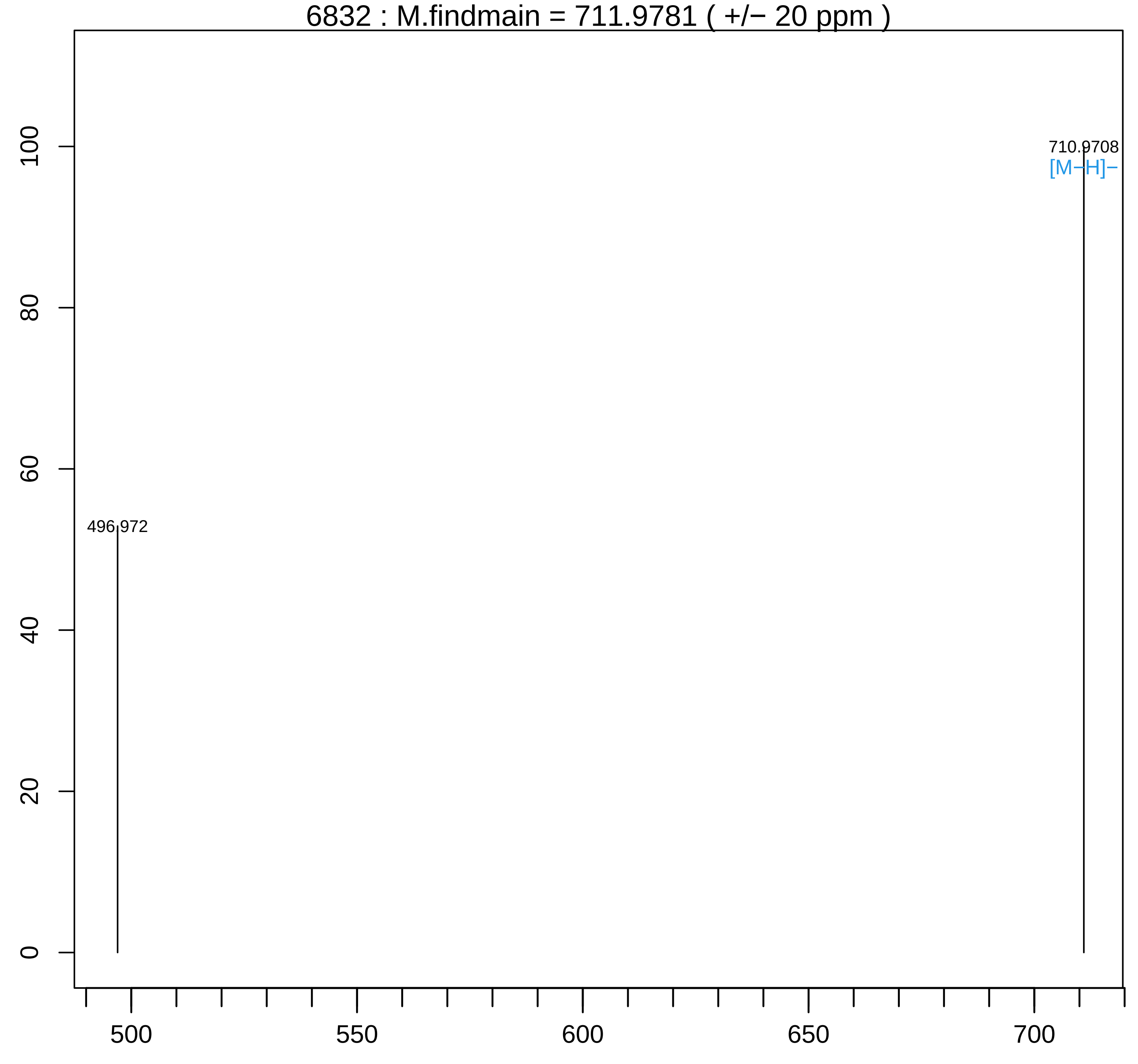
6832 : M.findmain = 711.9781 (+/− 20 ppm)
710.9708
[M−H]−
496.972
0
20
40
60
80
100
500
550
600
650
700

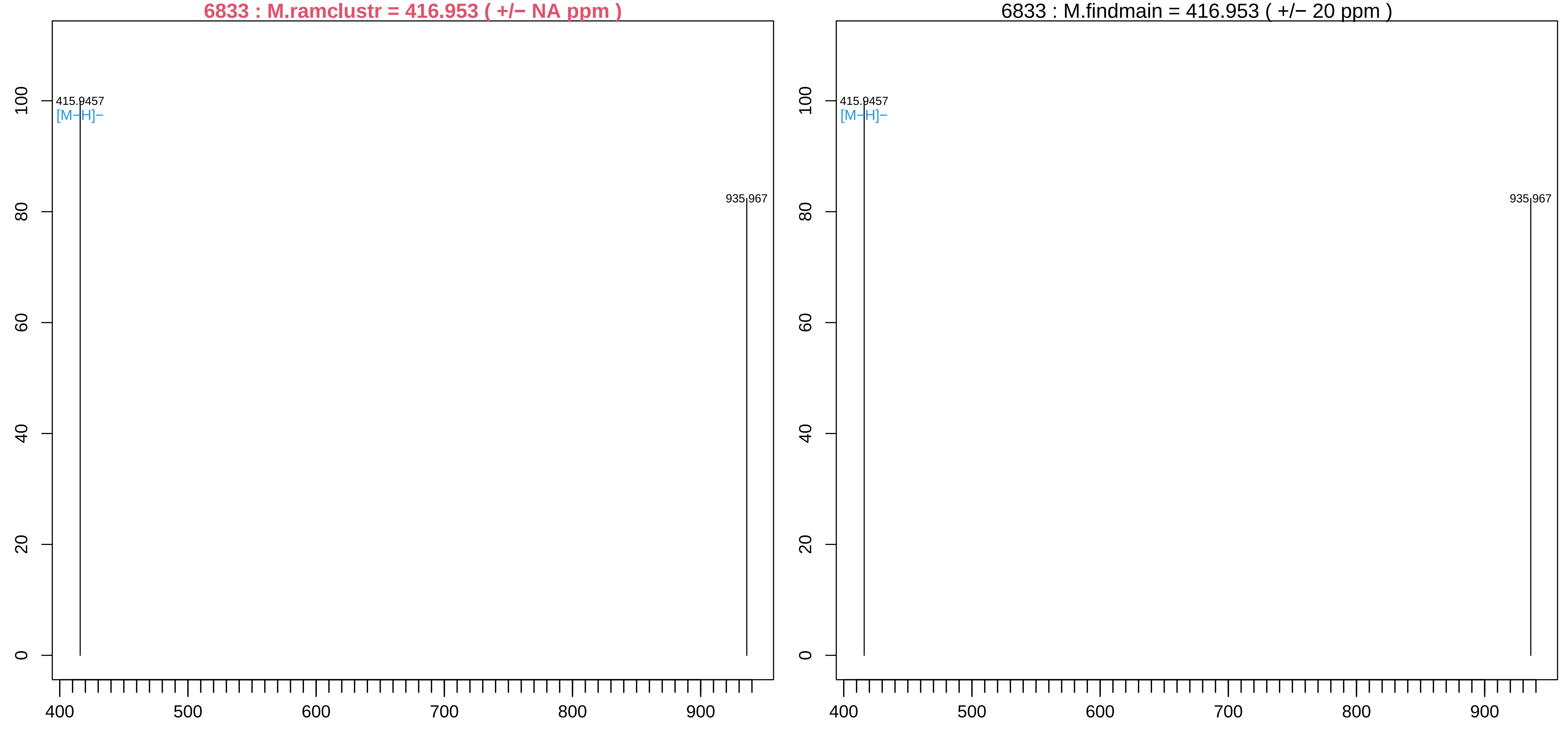
6833 : M.ramclustr = 416.953 (+/− NA ppm)
6833 : M.findmain = 416.953 (+/− 20 ppm)
415.9457
[M−H]−
935.967
0
20
40
60
80
100
400
500
600
700
800
900

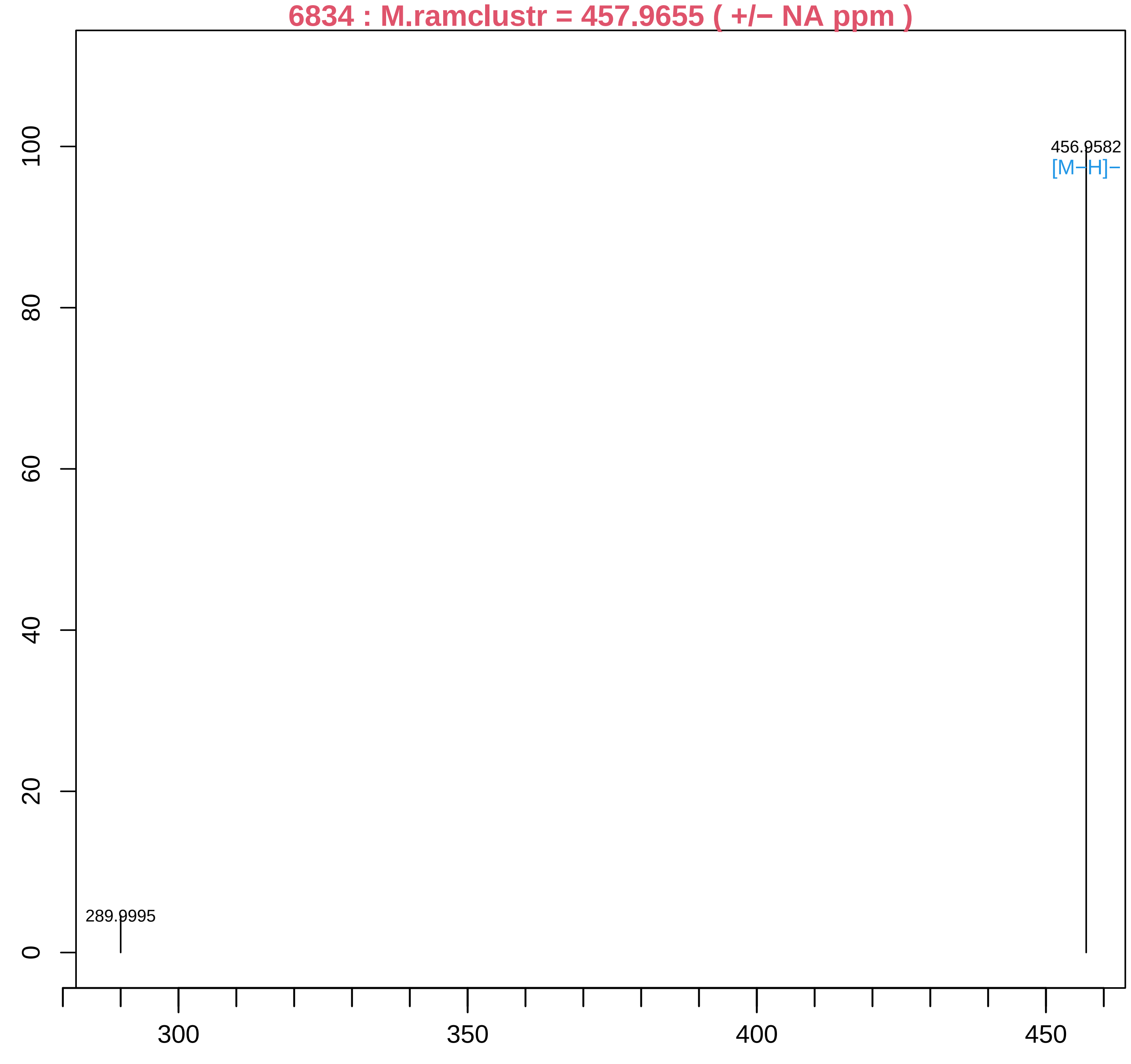
6834 : M.ramclustr = 457.9655 (+/− NA ppm)
456.9582
[M−H]−
289.9995
0
20
40
60
80
100
300
350
400
450

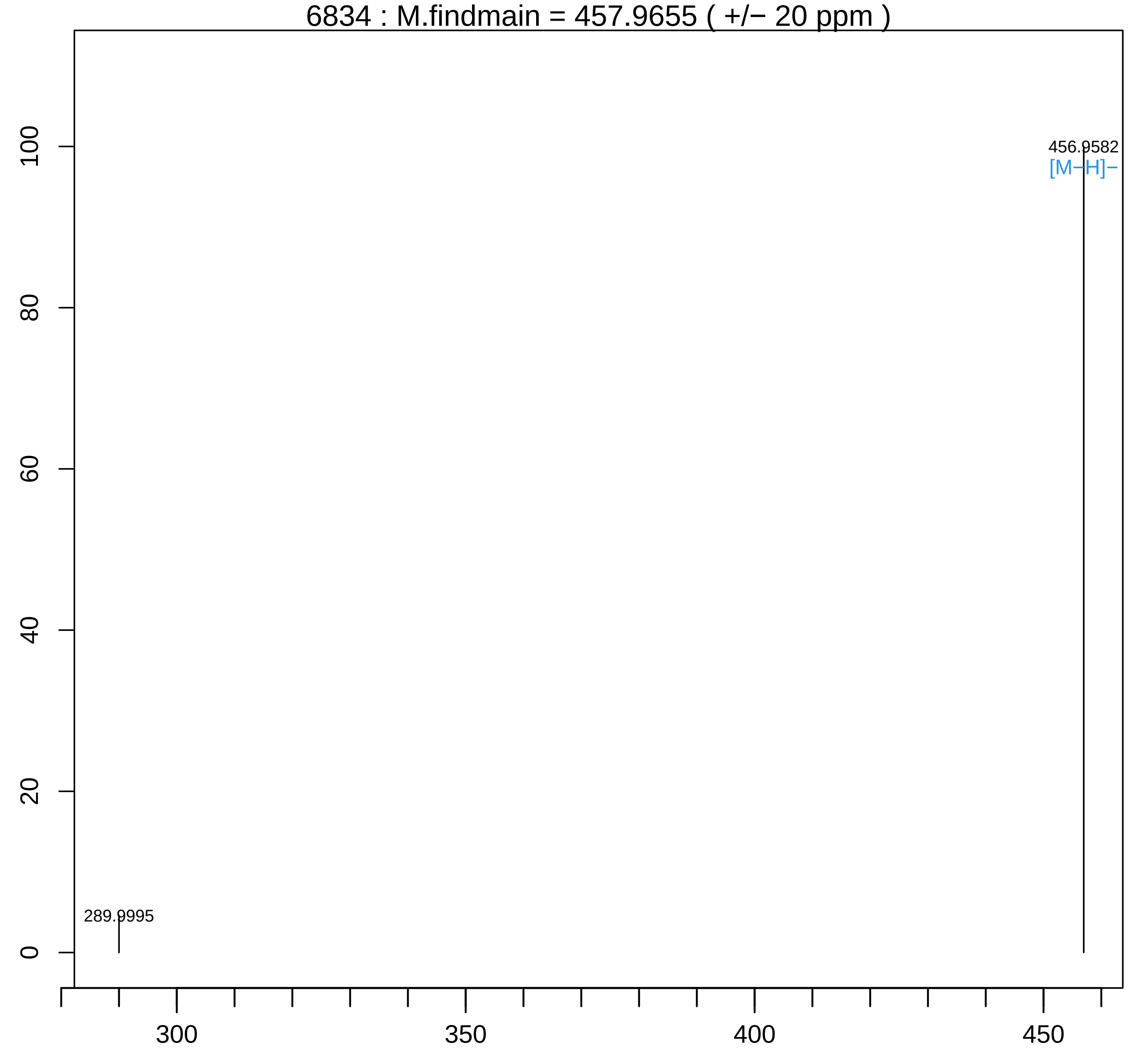
6834 : M.findmain = 457.9655 (+/− 20 ppm)
456.9582
[M−H]−
289.9995
0
20
40
60
80
100
300
350
400
450

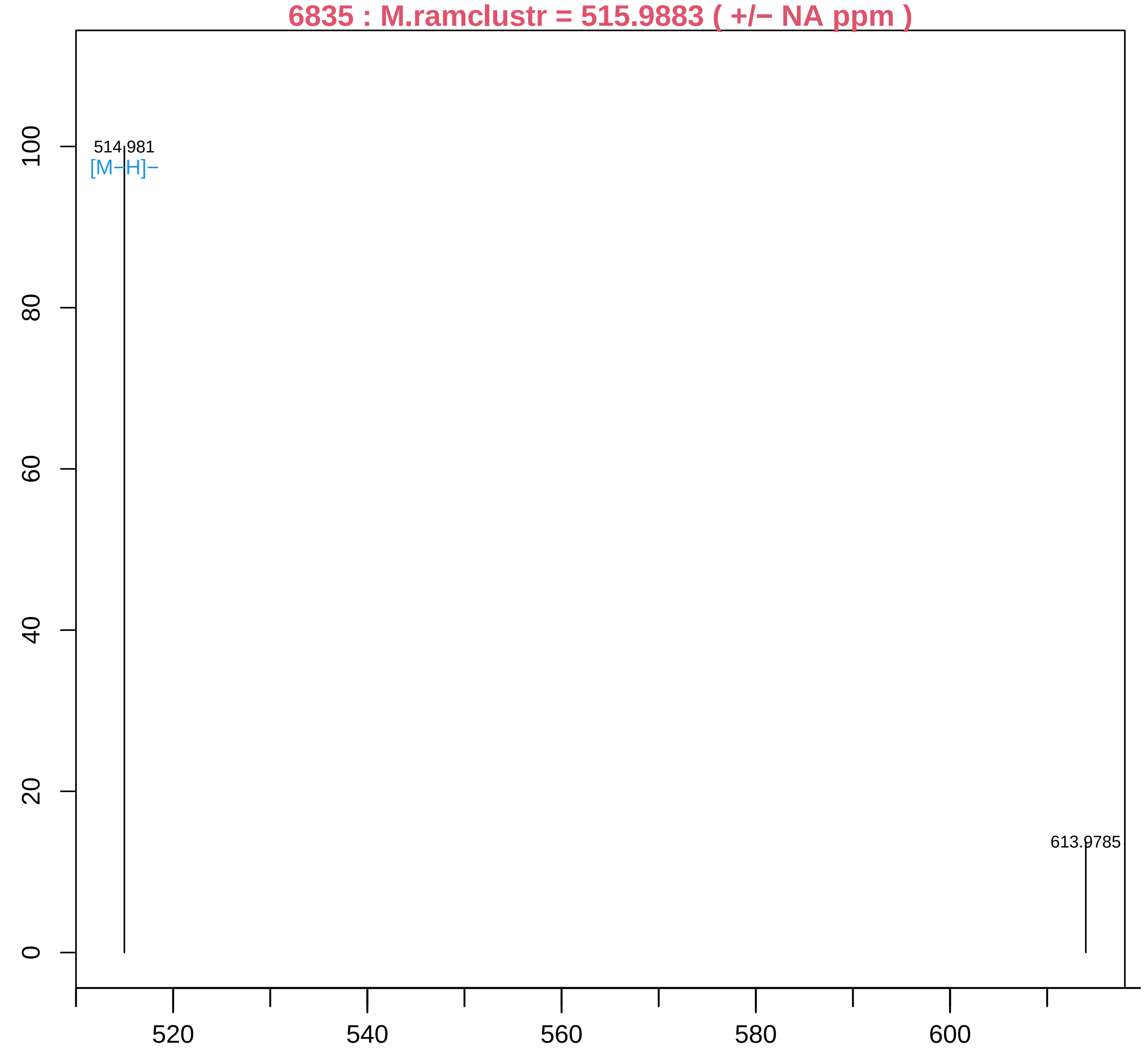
6835 : M.ramclustr = 515.9883 (+/− NA ppm)
514.981
[M−H]−
613.9785
0
20
40
60
80
100
520
540
560
580
600

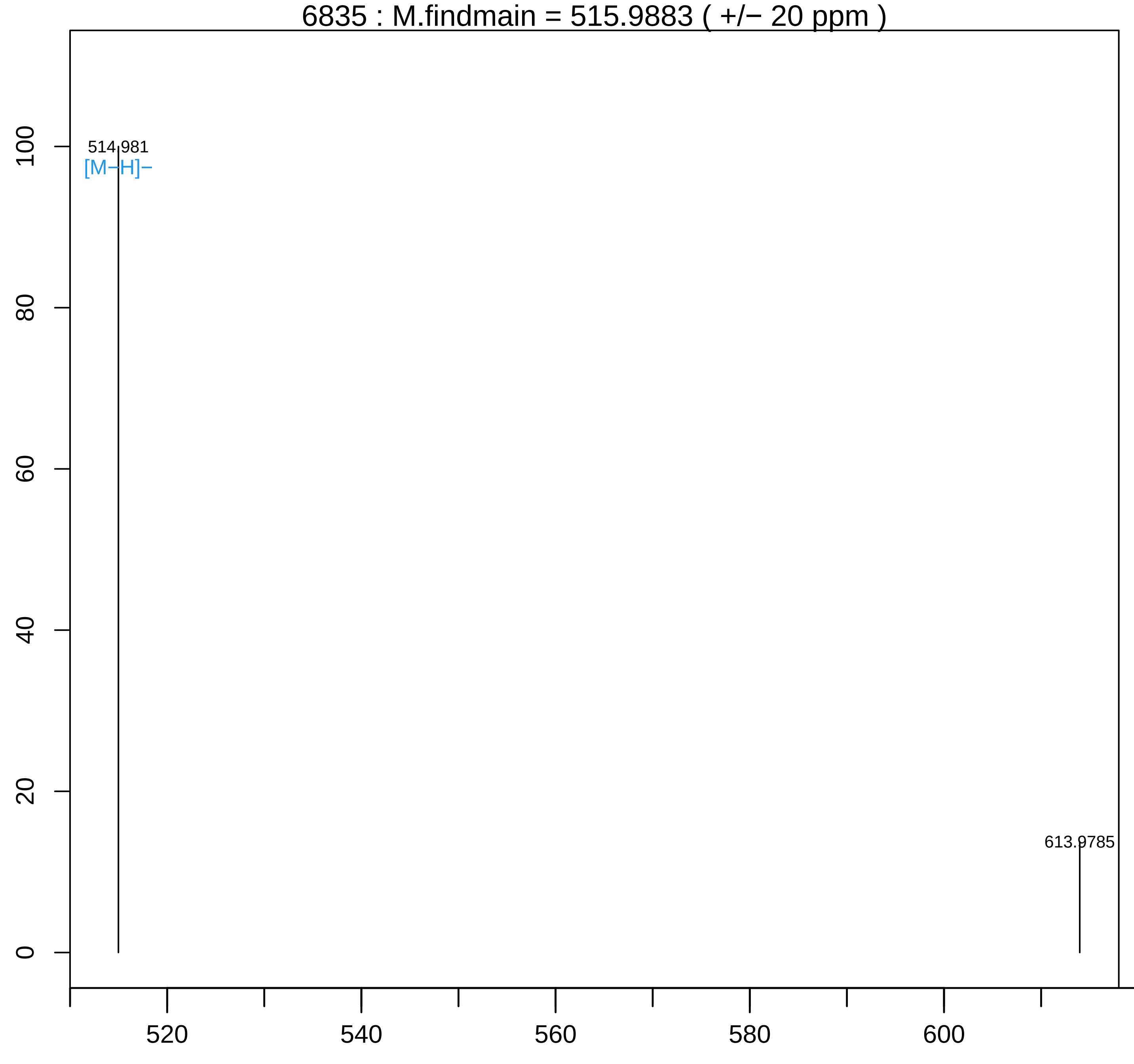
6835 : M.findmain = 515.9883 (+/− 20 ppm)
514.981
[M−H]−
613.9785
0
20
40
60
80
100
520
540
560
580
600

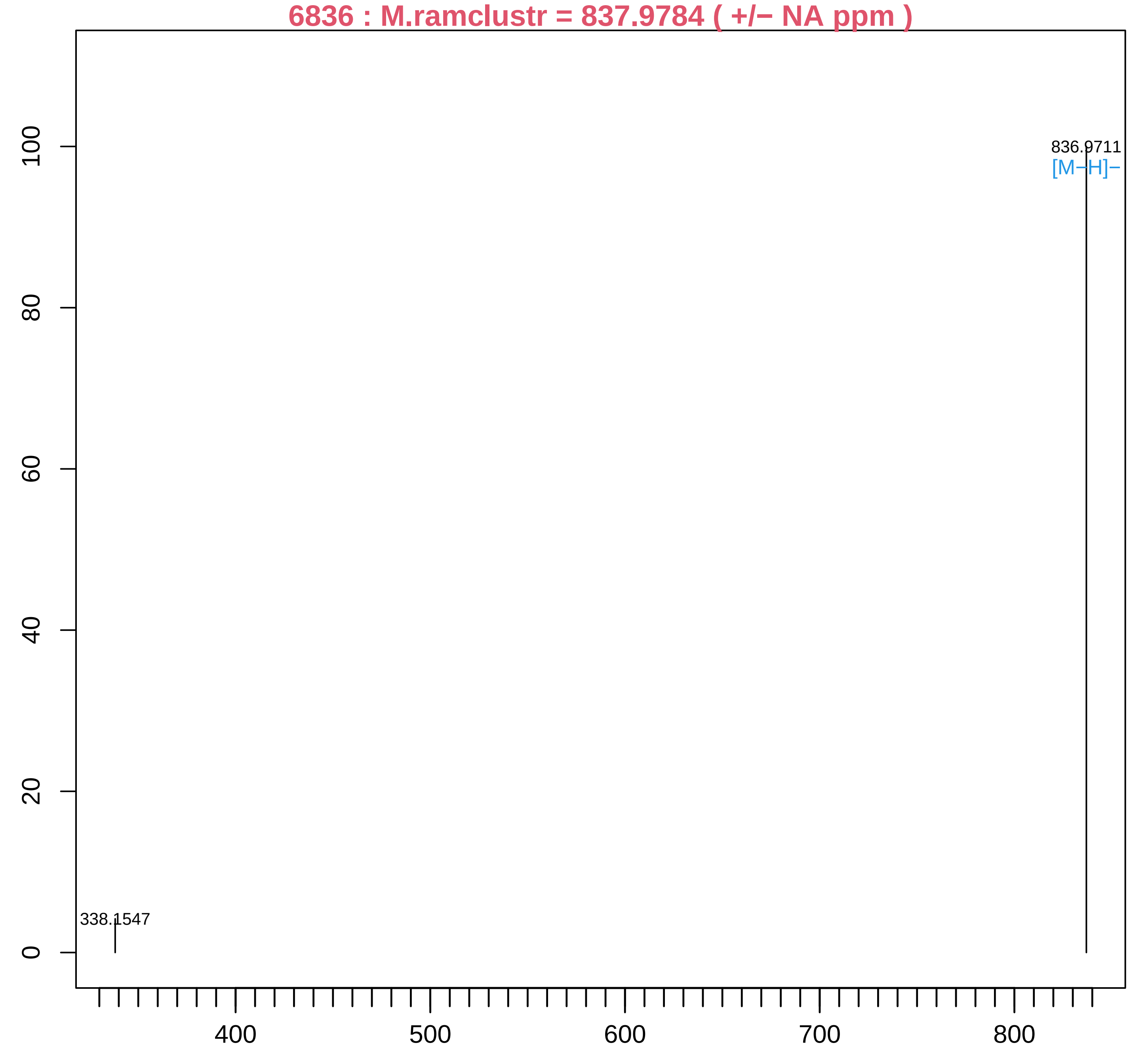
6836 : M.ramclustr = 837.9784 (+/− NA ppm)
836.9711
[M−H]−
338.1547
0
20
40
60
80
100
400
500
600
700
800

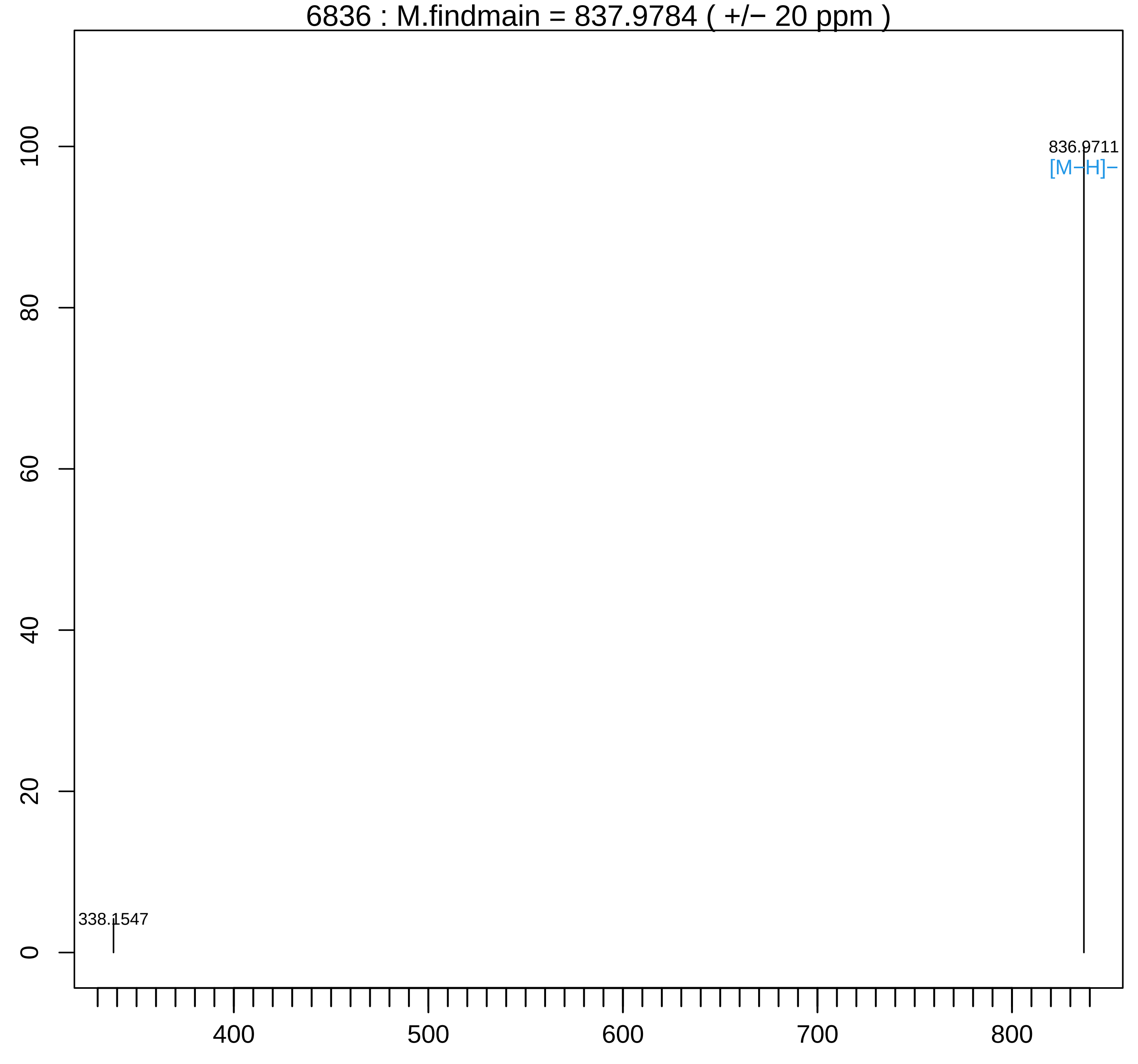
6836 : M.findmain = 837.9784 (+/− 20 ppm)
836.9711
[M−H]−
338.1547
0
20
40
60
80
100
400
500
600
700
800

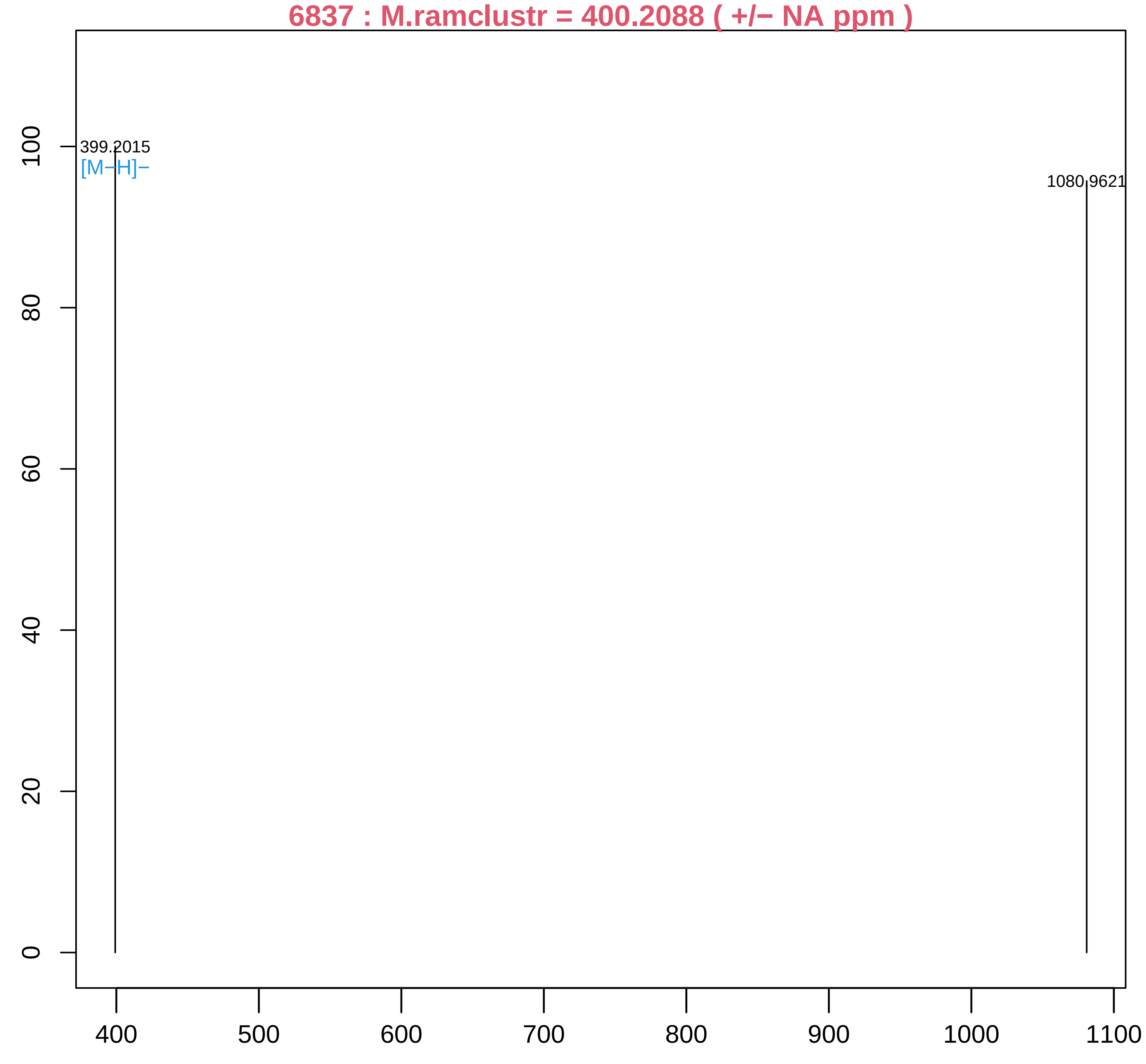
6837 : M.ramclustr = 400.2088 (+/− NA ppm)
399.2015
[M−H]−
1080.9621
0
20
40
60
80
100
400
500
600
700
800
900
1000
1100
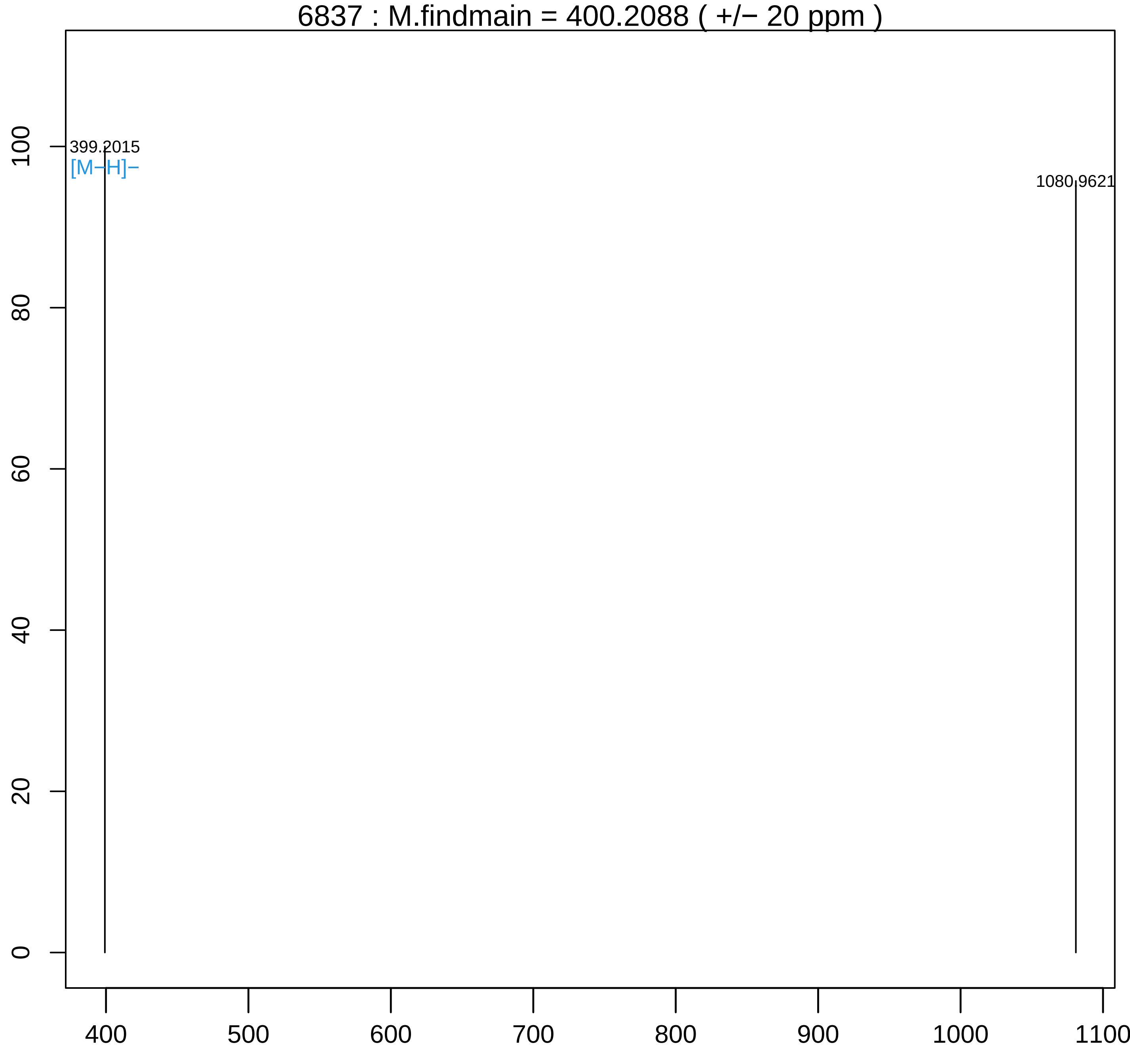
6837 : M.findmain = 400.2088 (+/− 20 ppm)
399.2015
[M−H]−
1080.9621
0
20
40
60
80
100
400
500
600
700
800
900
1000
1100

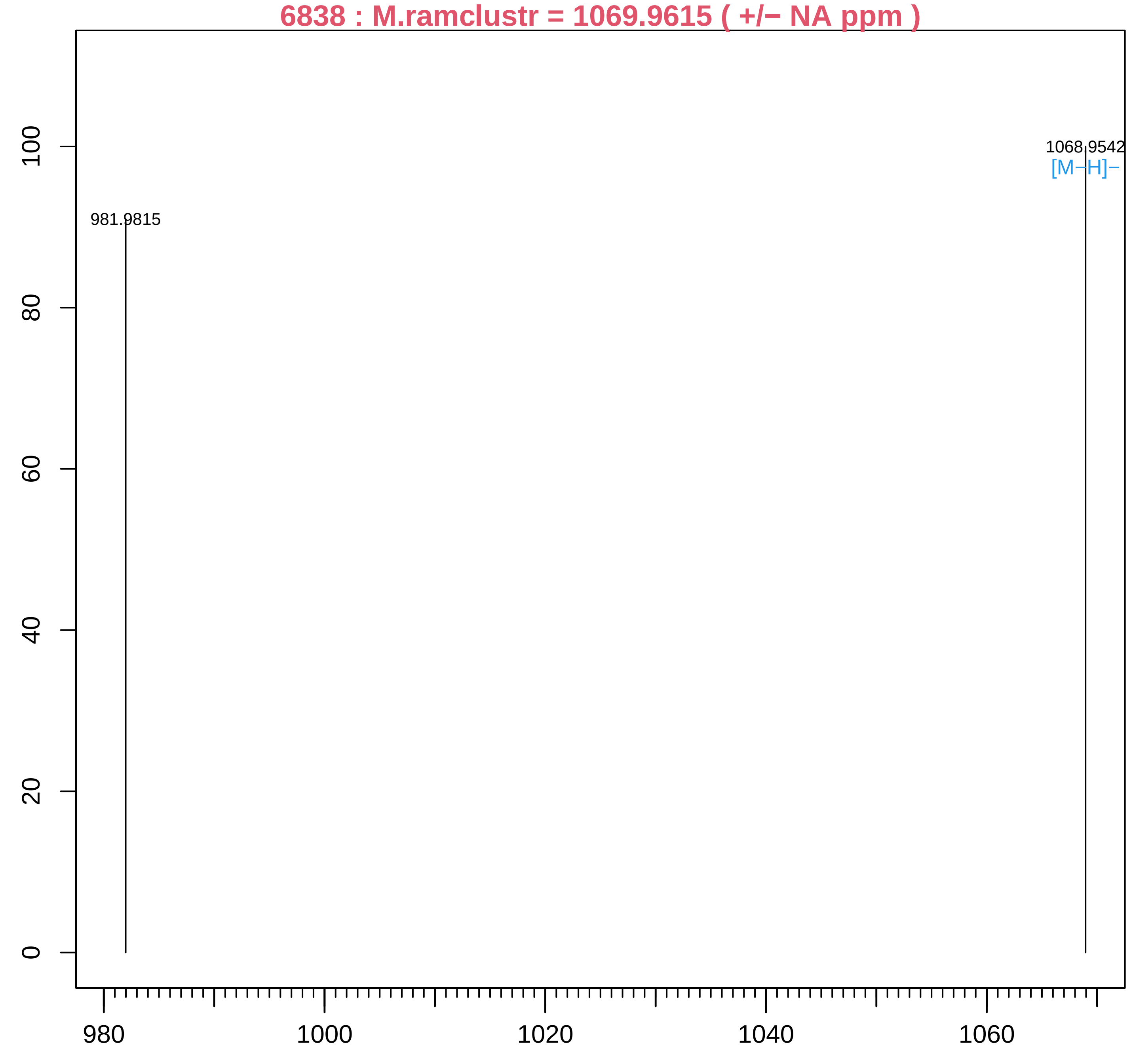
6838 : M.ramclustr = 1069.9615 (+/− NA ppm)
1068.9542
[M−H]−
981.9815
0
20
40
60
80
100
980
1000
1020
1040
1060

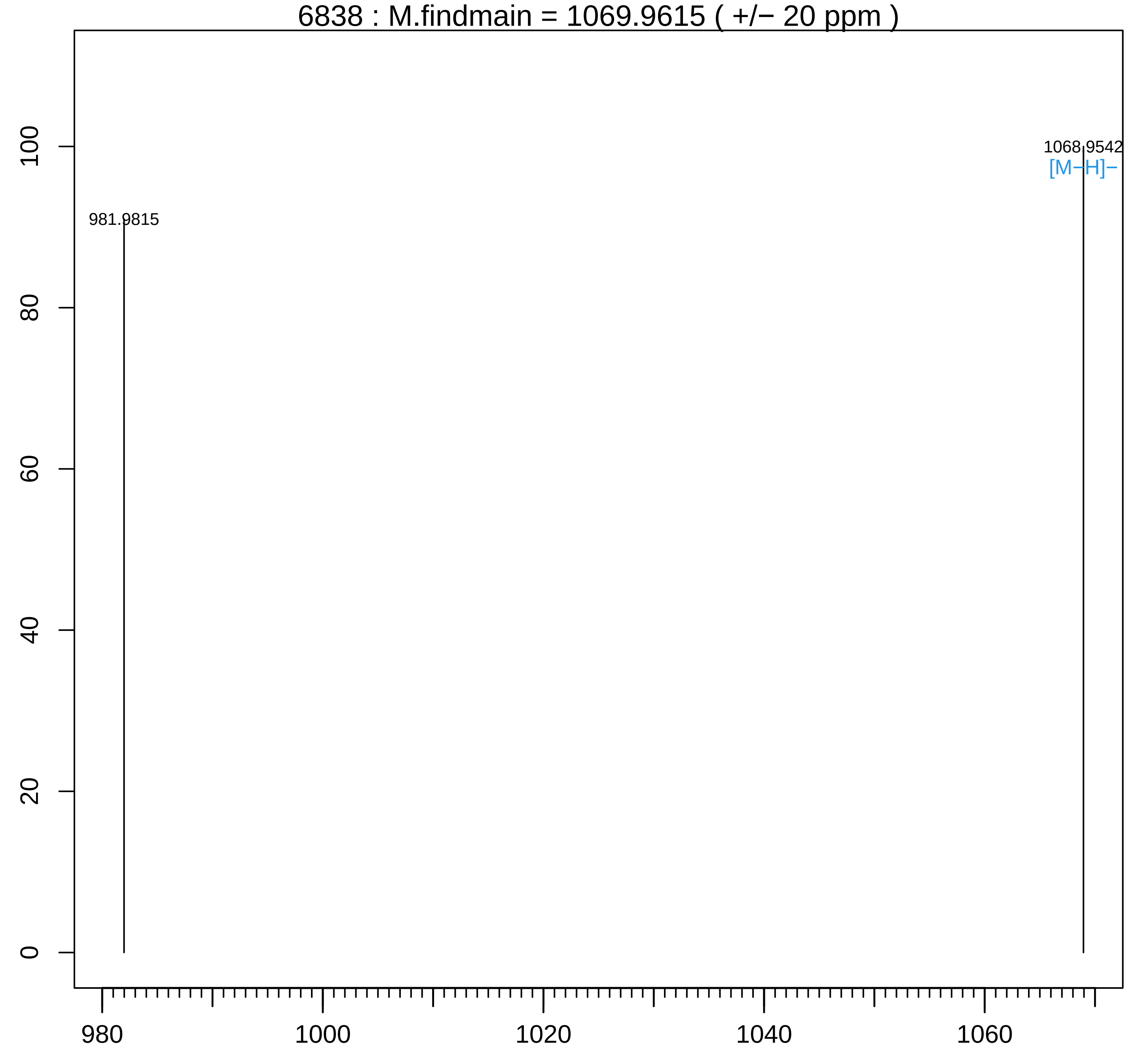
6838 : M.findmain = 1069.9615 (+/− 20 ppm)
1068.9542
[M−H]−
981.9815
0
20
40
60
80
100
980
1000
1020
1040
1060

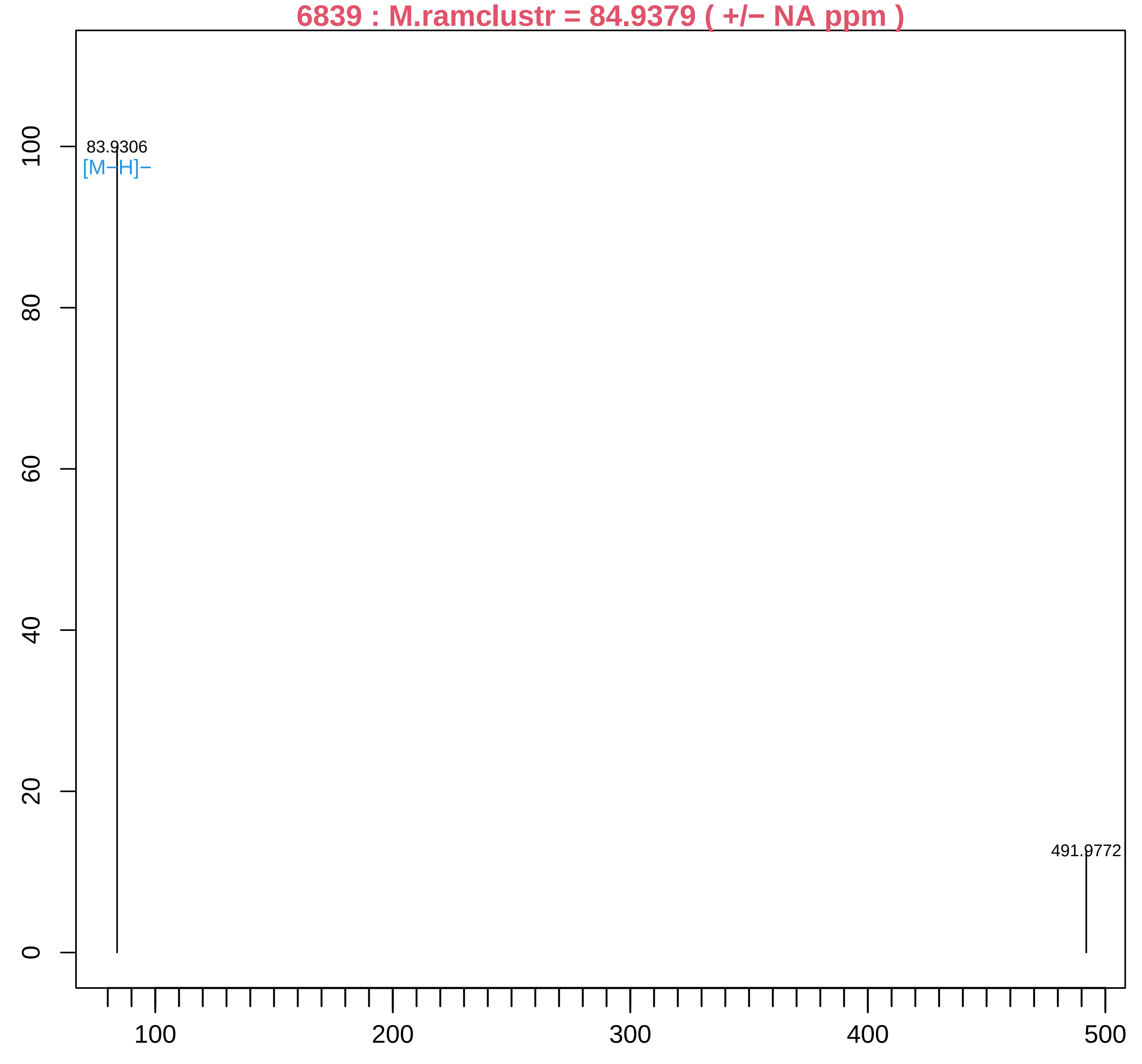

6839 : M.ramclustr = 84.9379 (+/− NA ppm)
83.9306
[M−H]−
491.9772
0
20
40
60
80
100
100
200
300
400
500

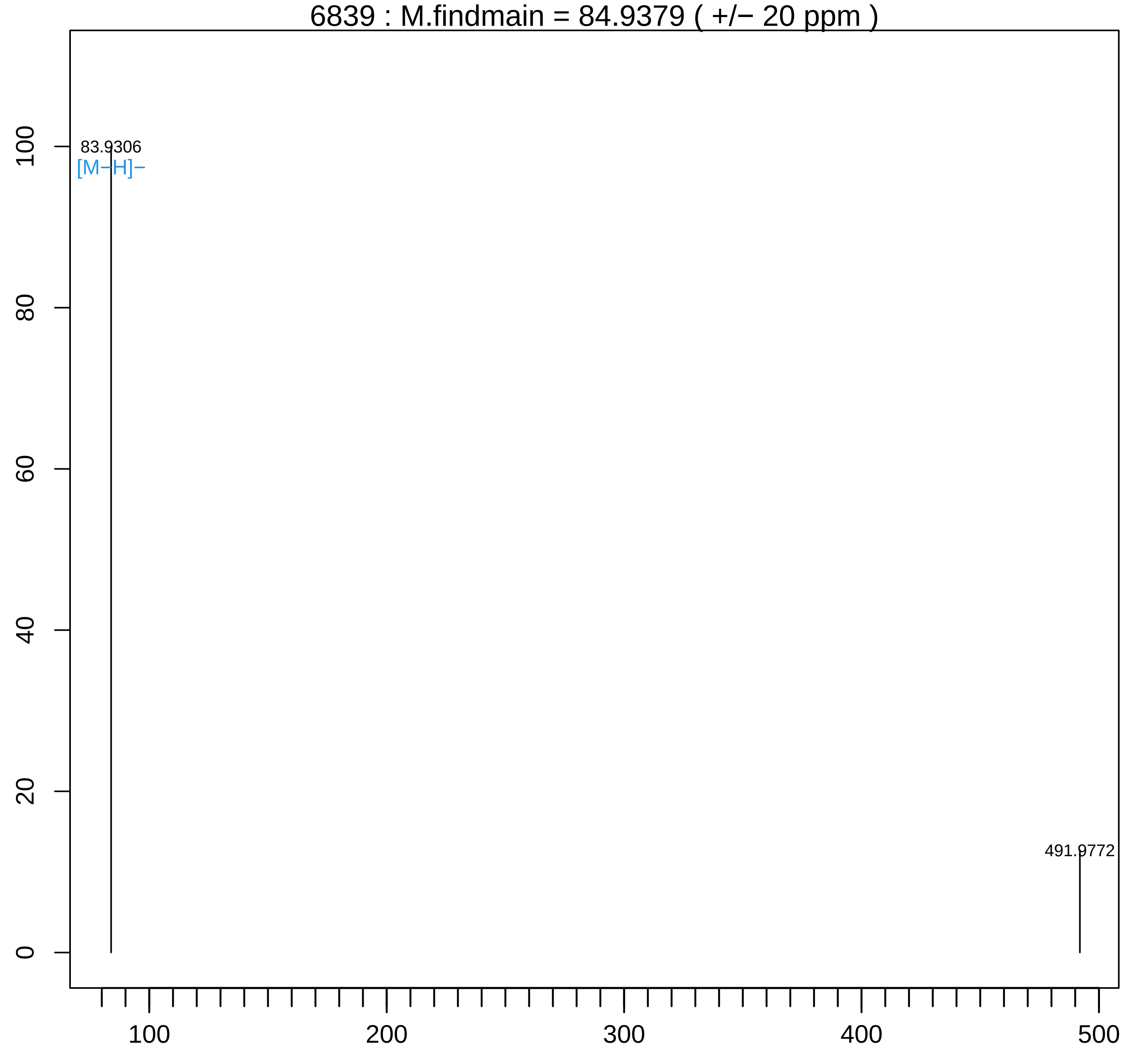

6839 : M.findmain = 84.9379 (+/− 20 ppm)
83.9306
[M−H]−
491.9772
0
20
40
60
80
100
100
200
300
400
500

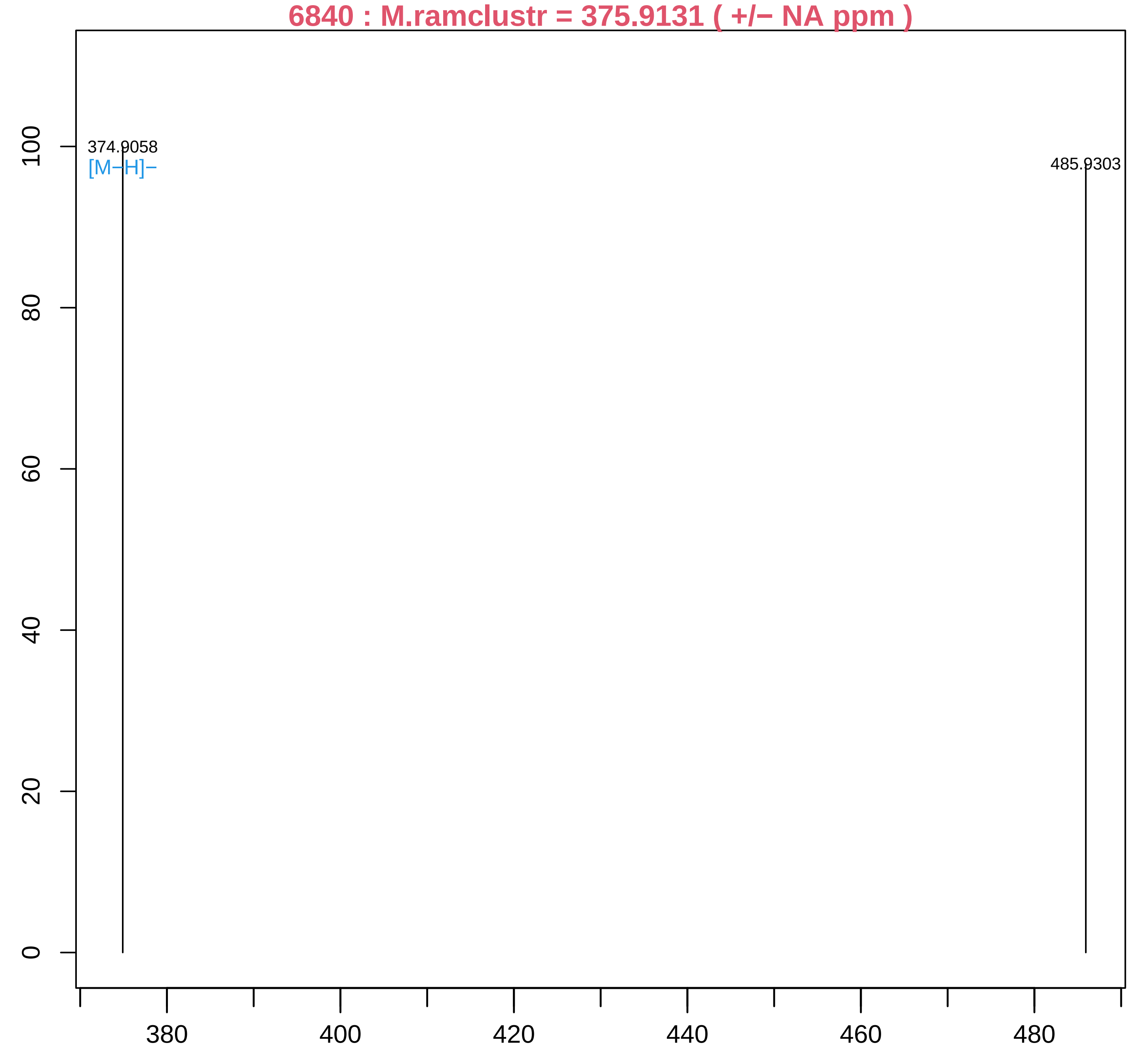

6840 : M.ramclustr = 375.9131 (+/− NA ppm)
374.9058
[M−H]−
485.9303
0
20
40
60
80
100
380
400
420
440
460
480

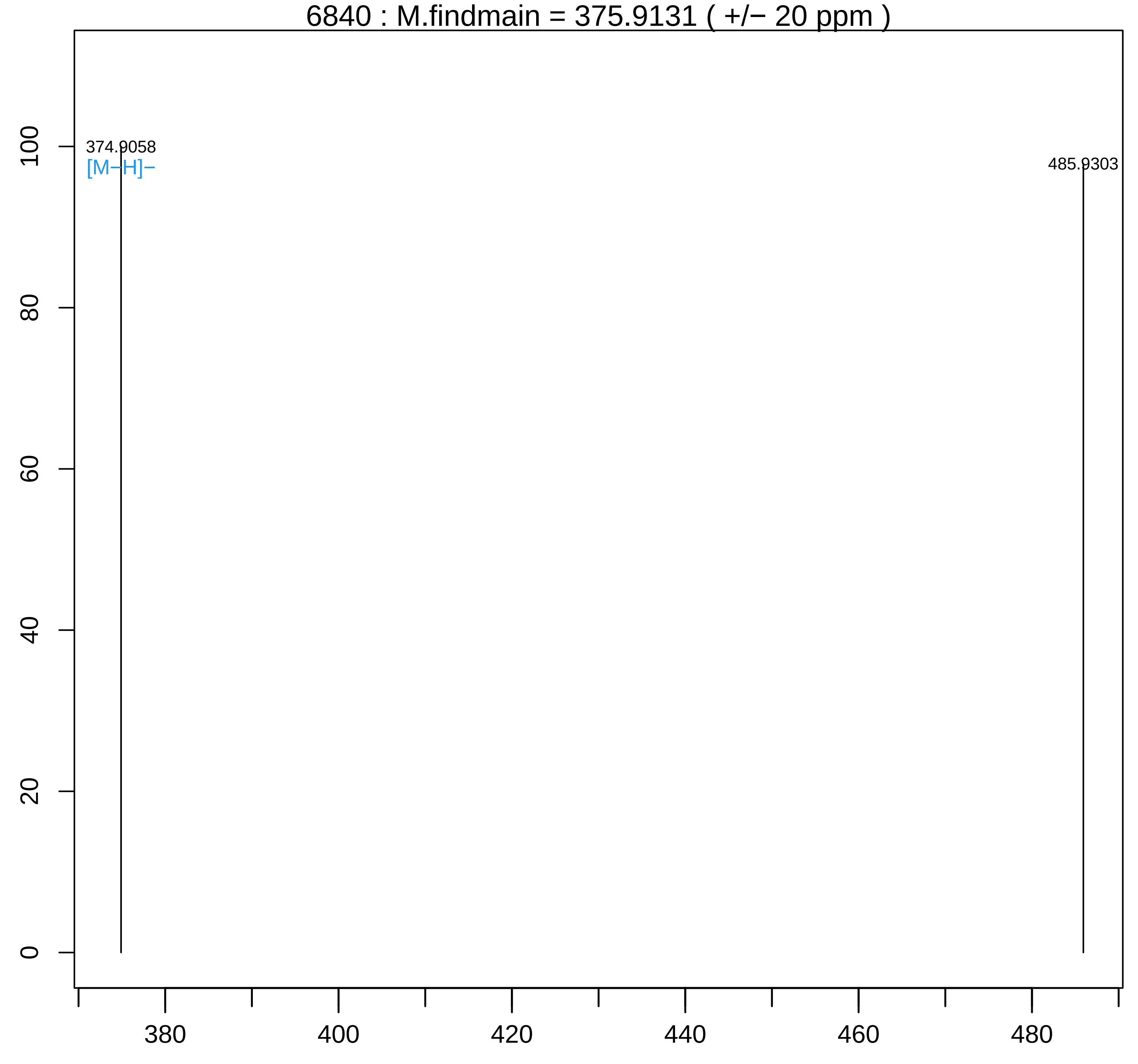

6840 : M.findmain = 375.9131 (+/− 20 ppm)
374.9058
[M−H]−
485.9303
0
20
40
60
80
100
380
400
420
440
460
480

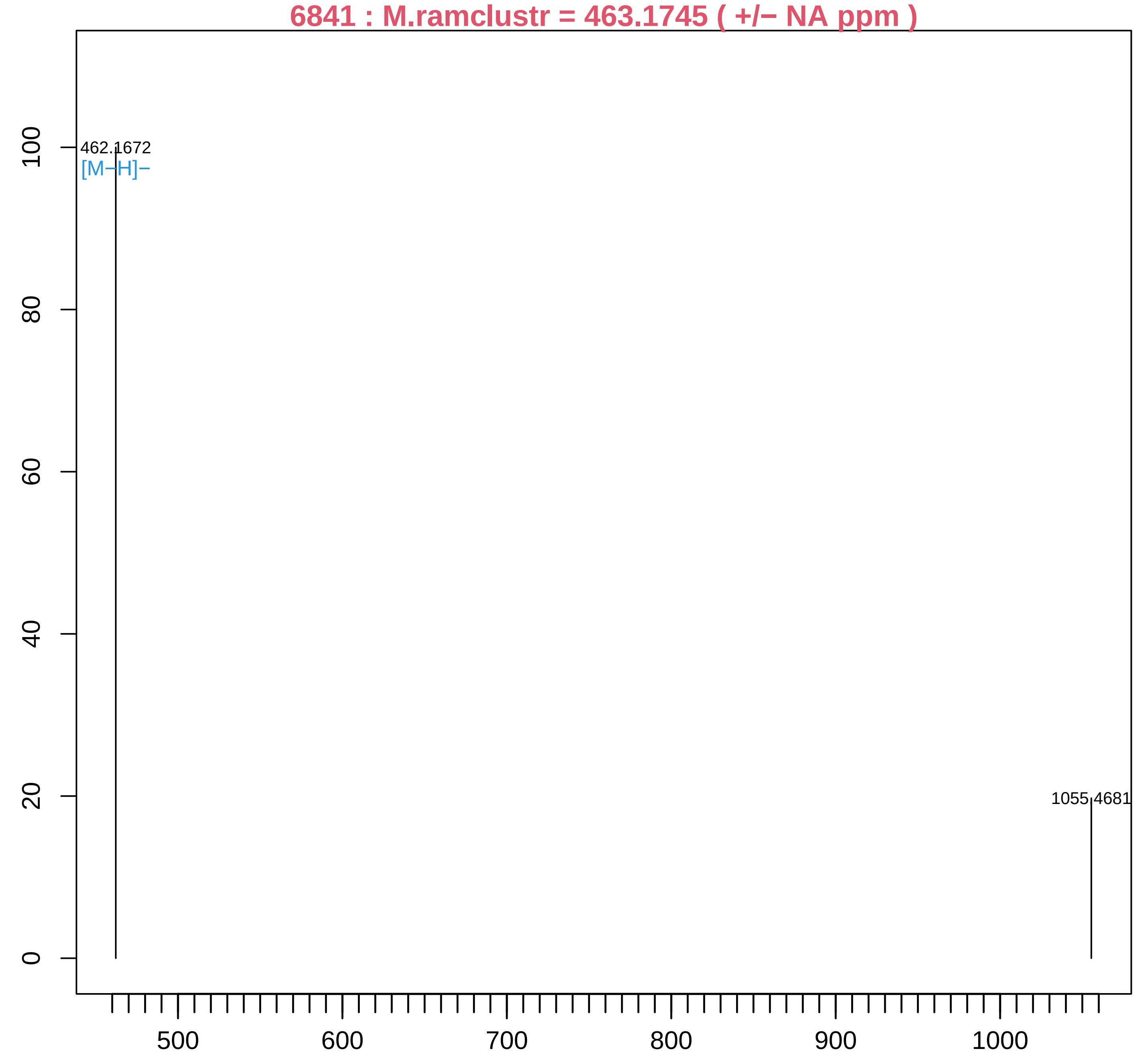
6841 : M.ramclustr = 463.1745 (+/− NA ppm)
462.1672
[M−H]−
1055.4681
0
20
40
60
80
100
500
600
700
800
900
1000

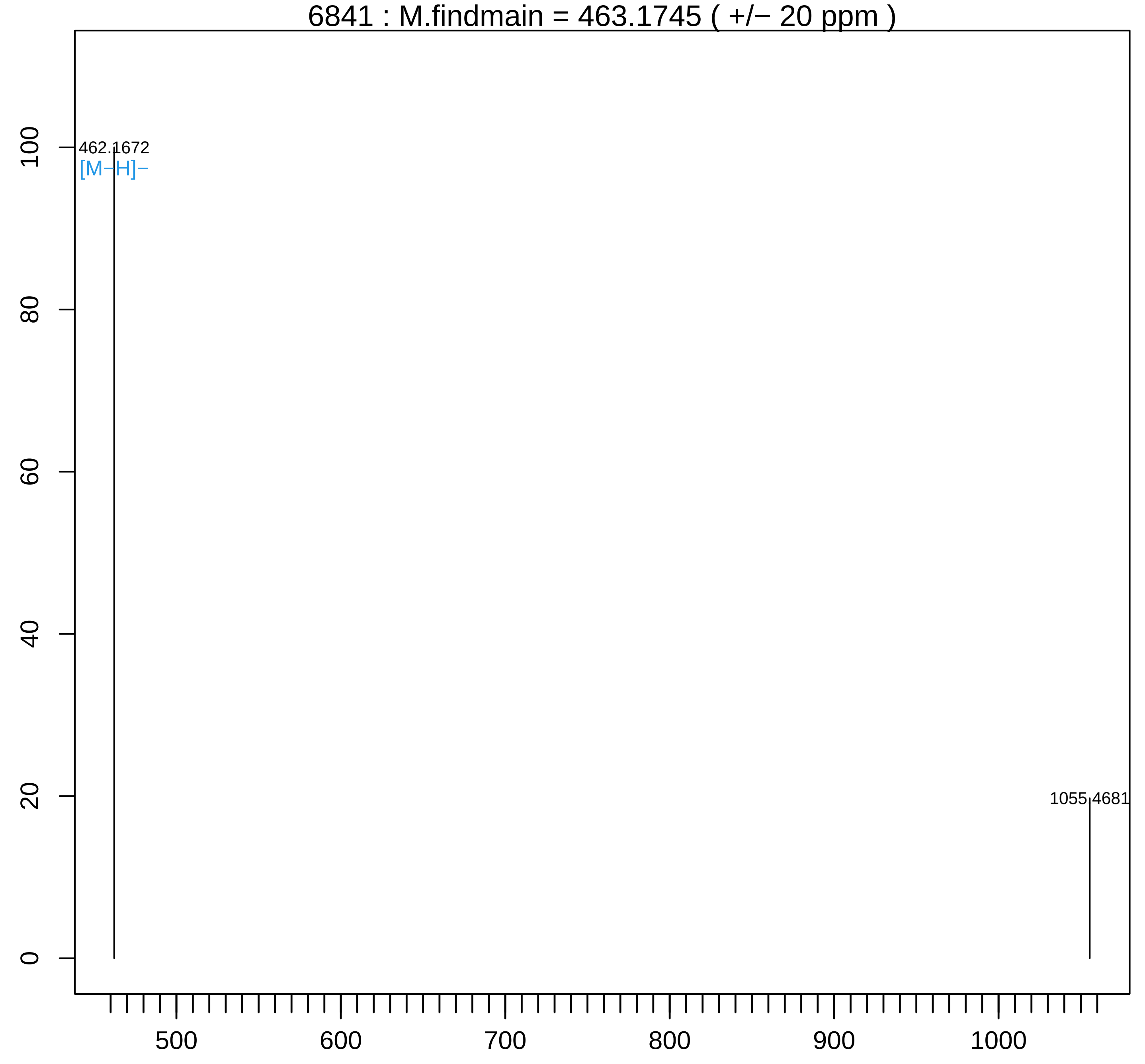
6841 : M.findmain = 463.1745 (+/− 20 ppm)
462.1672
[M−H]−
1055.4681
0
20
40
60
80
100
500
600
700
800
900
1000

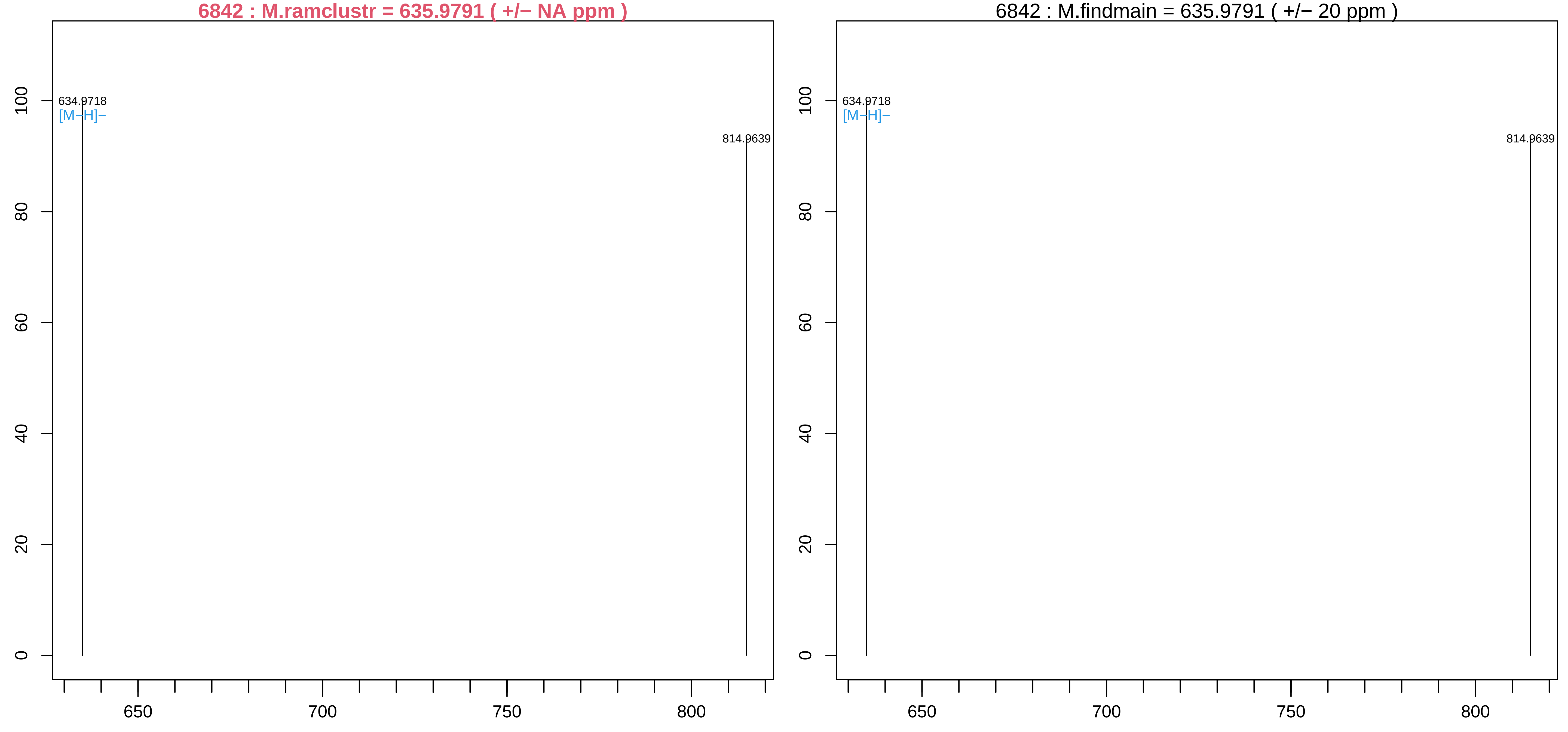

6842 : M.ramclustr = 635.9791 (+/− NA ppm)
634.9718
[M−H]−
814.9639
6842 : M.findmain = 635.9791 (+/− 20 ppm)
634.9718
[M−H]−
814.9639
0
20
40
60
80
100
650
700
750
800

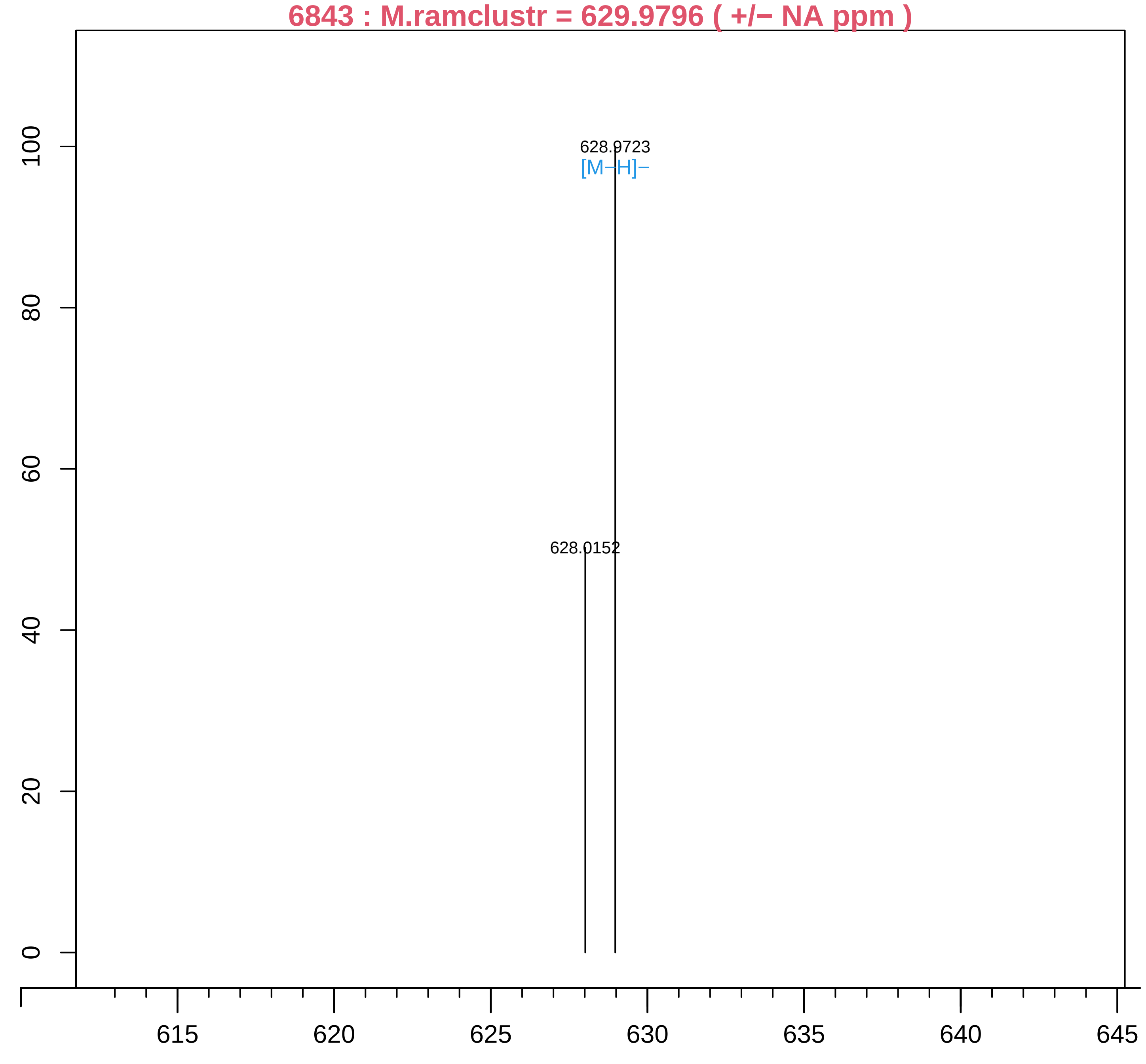
6843 : M.ramclustr = 629.9796 (+/− NA ppm)
628.9723
[M−H]−
628.0152
0
20
40
60
80
100
615
620
625
630
635
640
645

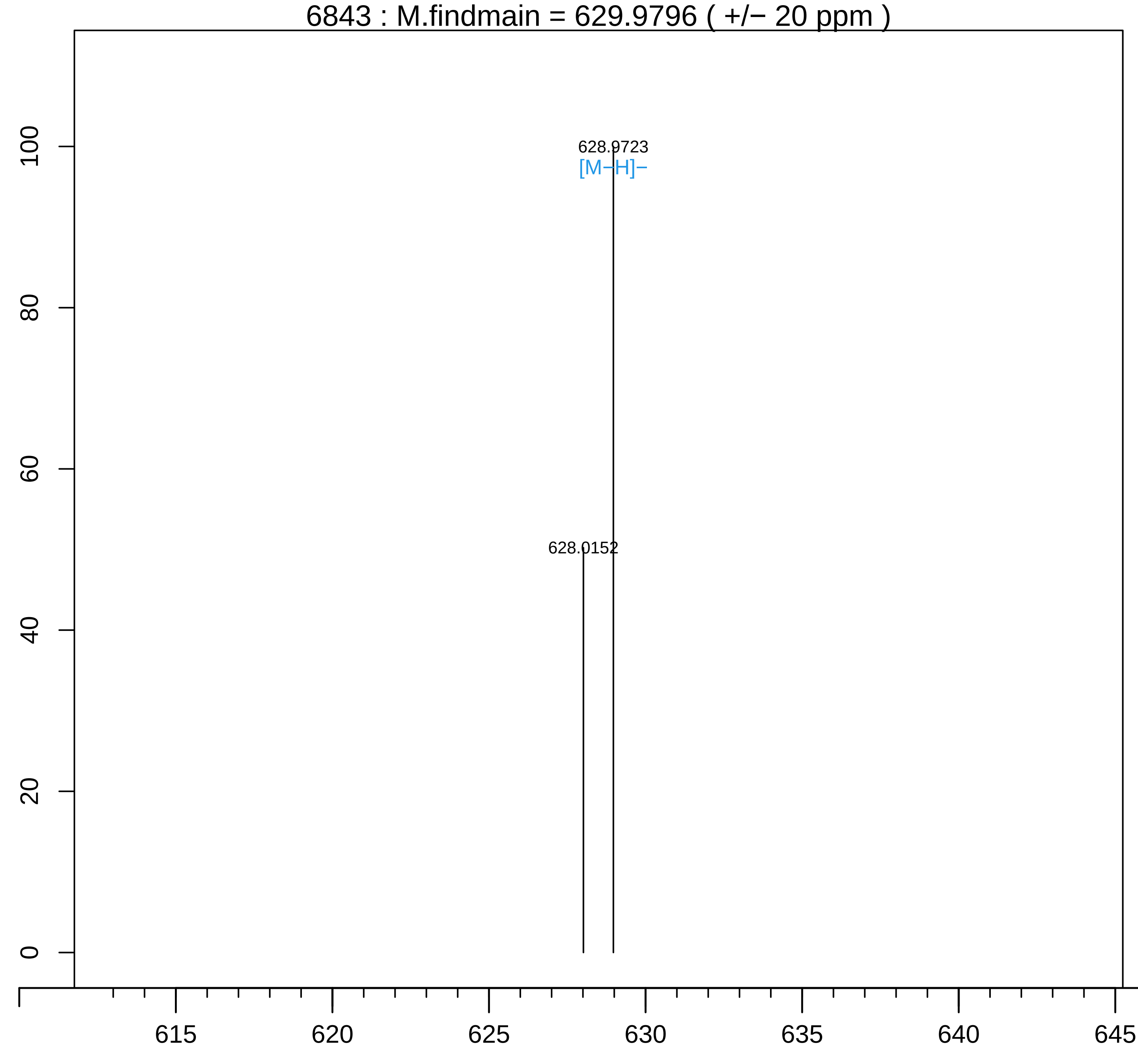
6843 : M.findmain = 629.9796 (+/− 20 ppm)
628.9723
[M−H]−
628.0152
0
20
40
60
80
100
615
620
625
630
635
640
645

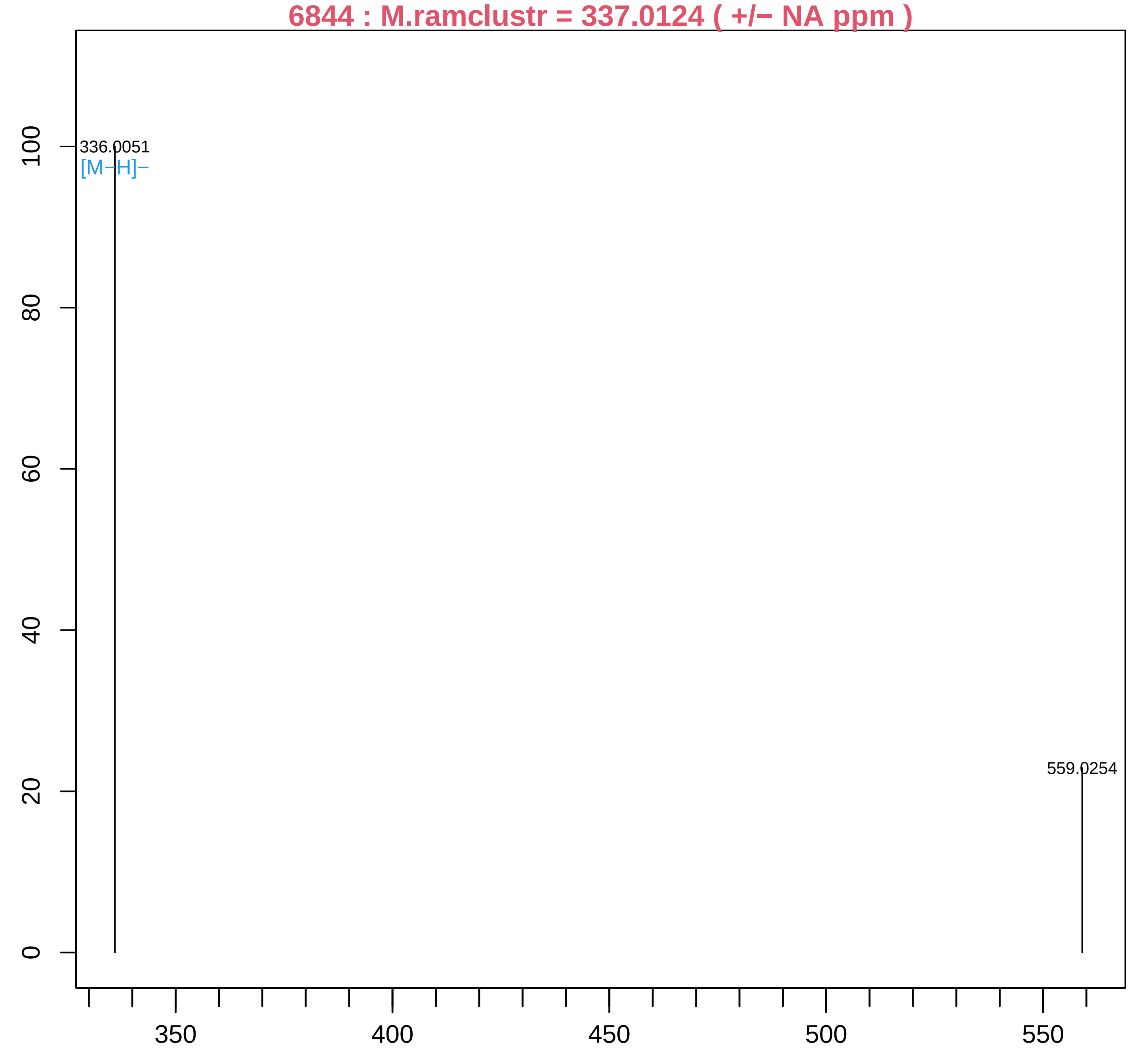
6844 : M.ramclustr = 337.0124 (+/− NA ppm)
336.0051
[M−H]−
559.0254
0
20
40
60
80
100
350
400
450
500
550

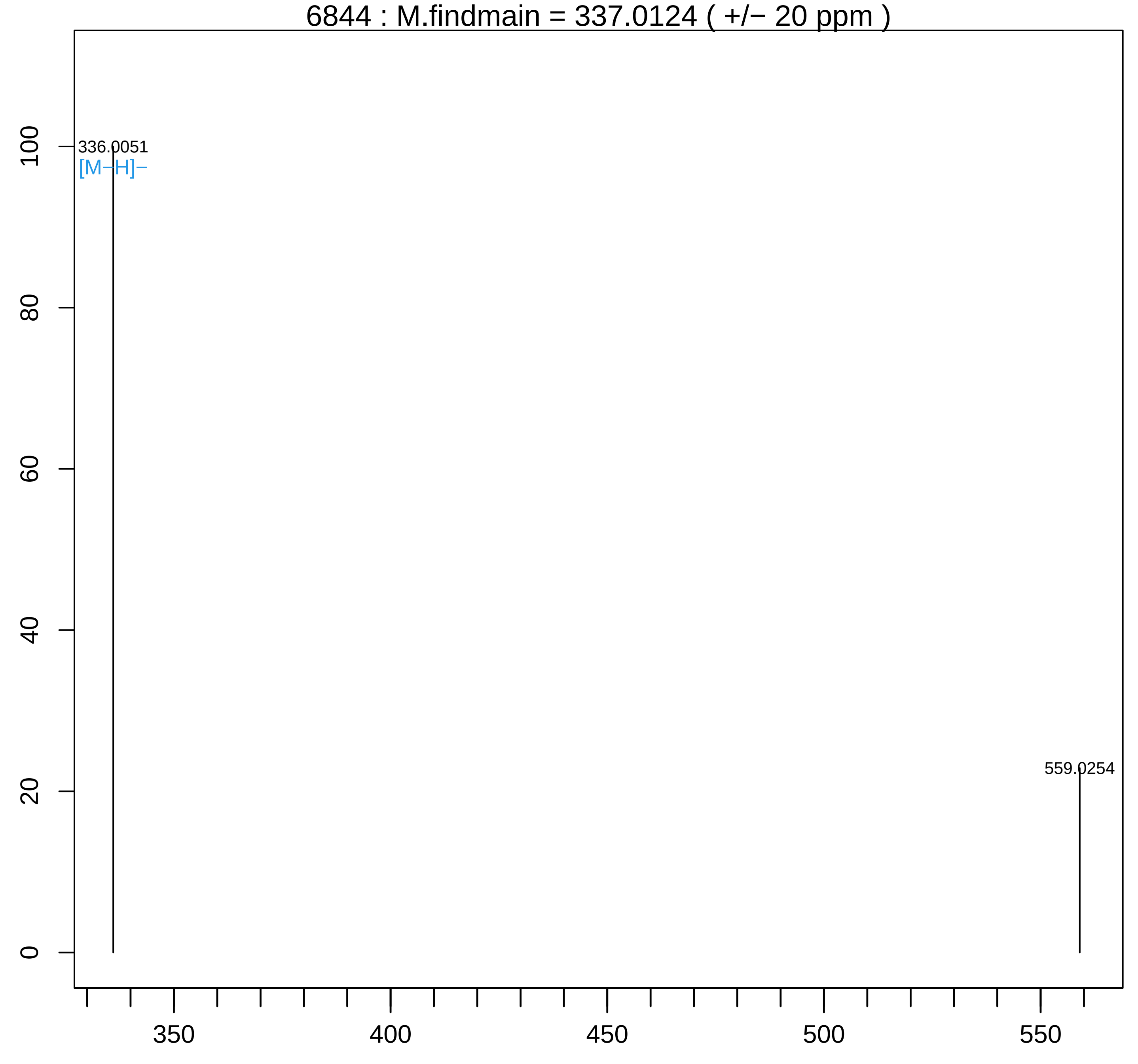
6844 : M.findmain = 337.0124 (+/− 20 ppm)
336.0051
[M−H]−
559.0254
0
20
40
60
80
100
350
400
450
500
550

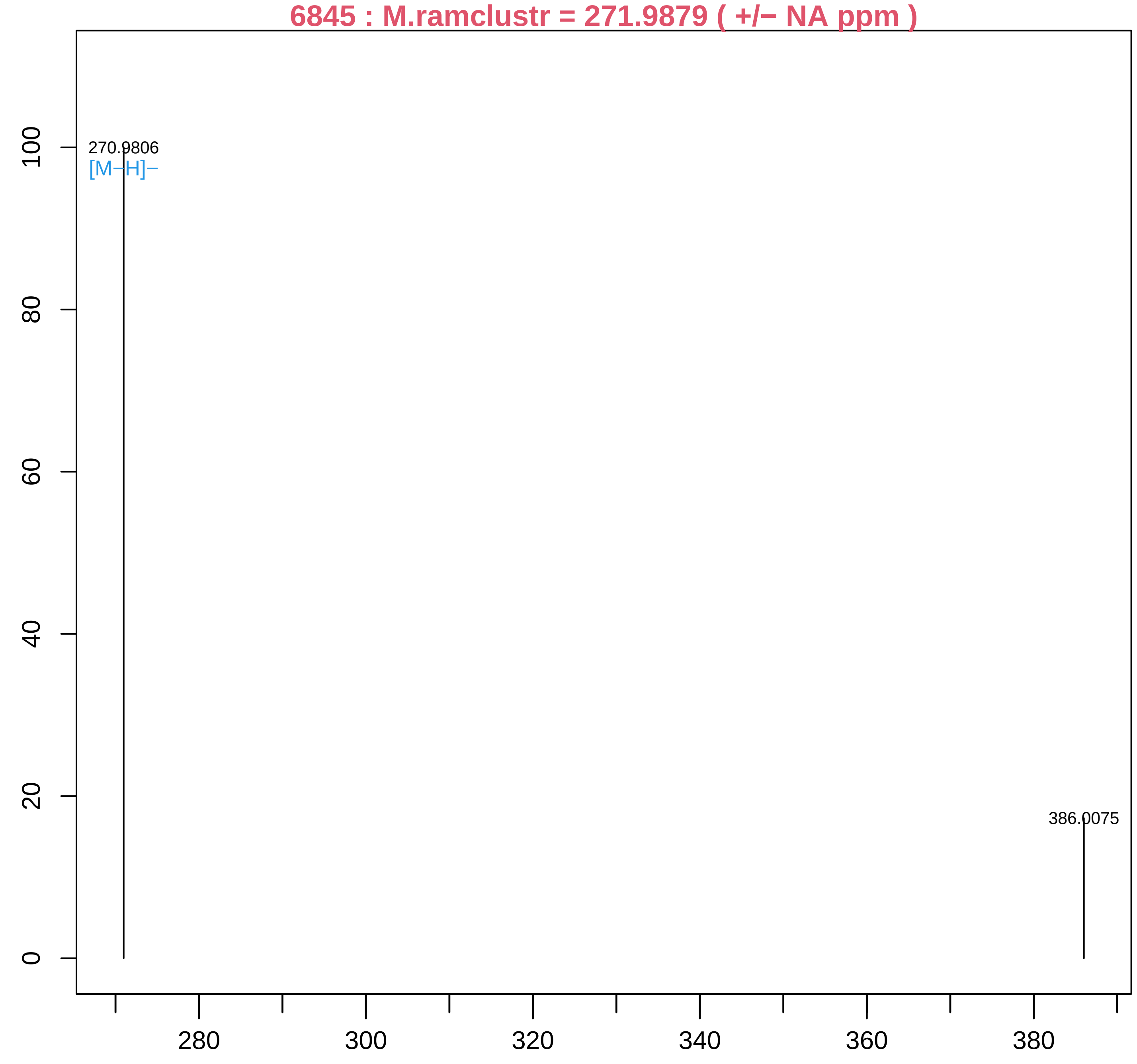
6845 : M.ramclustr = 271.9879 (+/− NA ppm)
270.9806
[M−H]−
386.0075
0
20
40
60
80
100
280
300
320
340
360
380

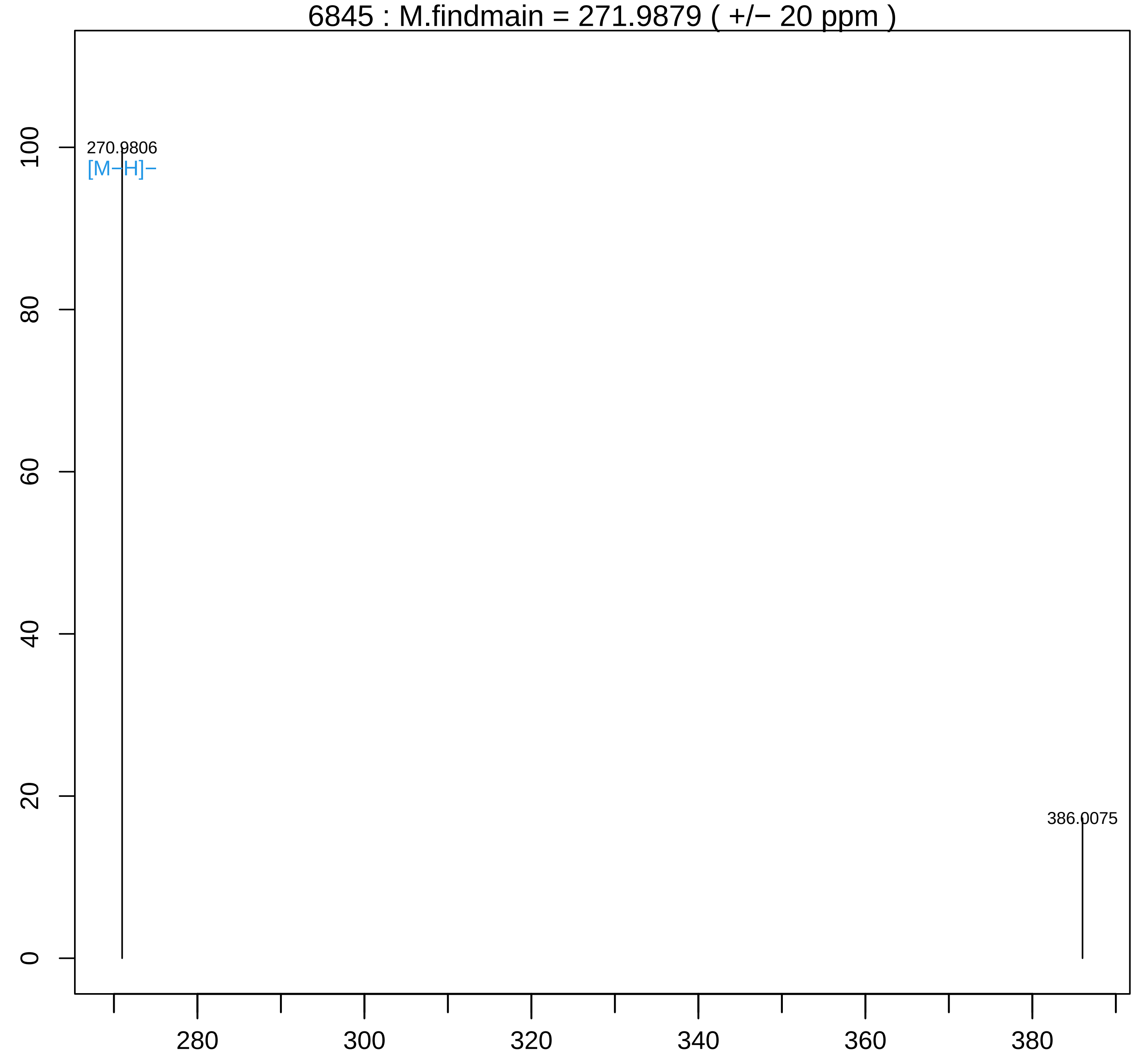
6845 : M.findmain = 271.9879 (+/− 20 ppm)
270.9806
[M−H]−
386.0075
0
20
40
60
80
100
280
300
320
340
360
380

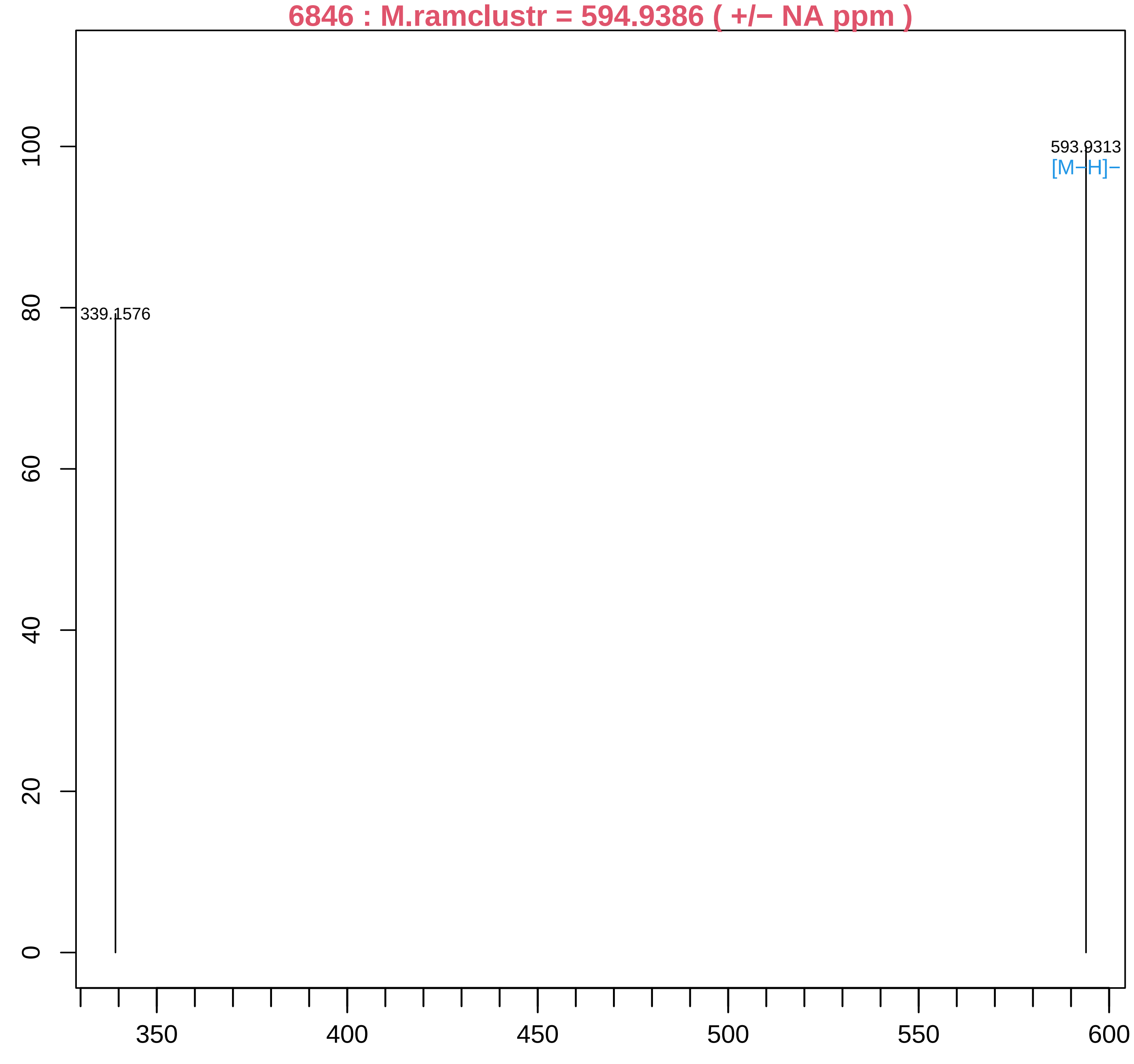
6846 : M.ramclustr = 594.9386 (+/− NA ppm)
593.9313
[M−H]−
339.1576
0
20
40
60
80
100
350
400
450
500
550
600

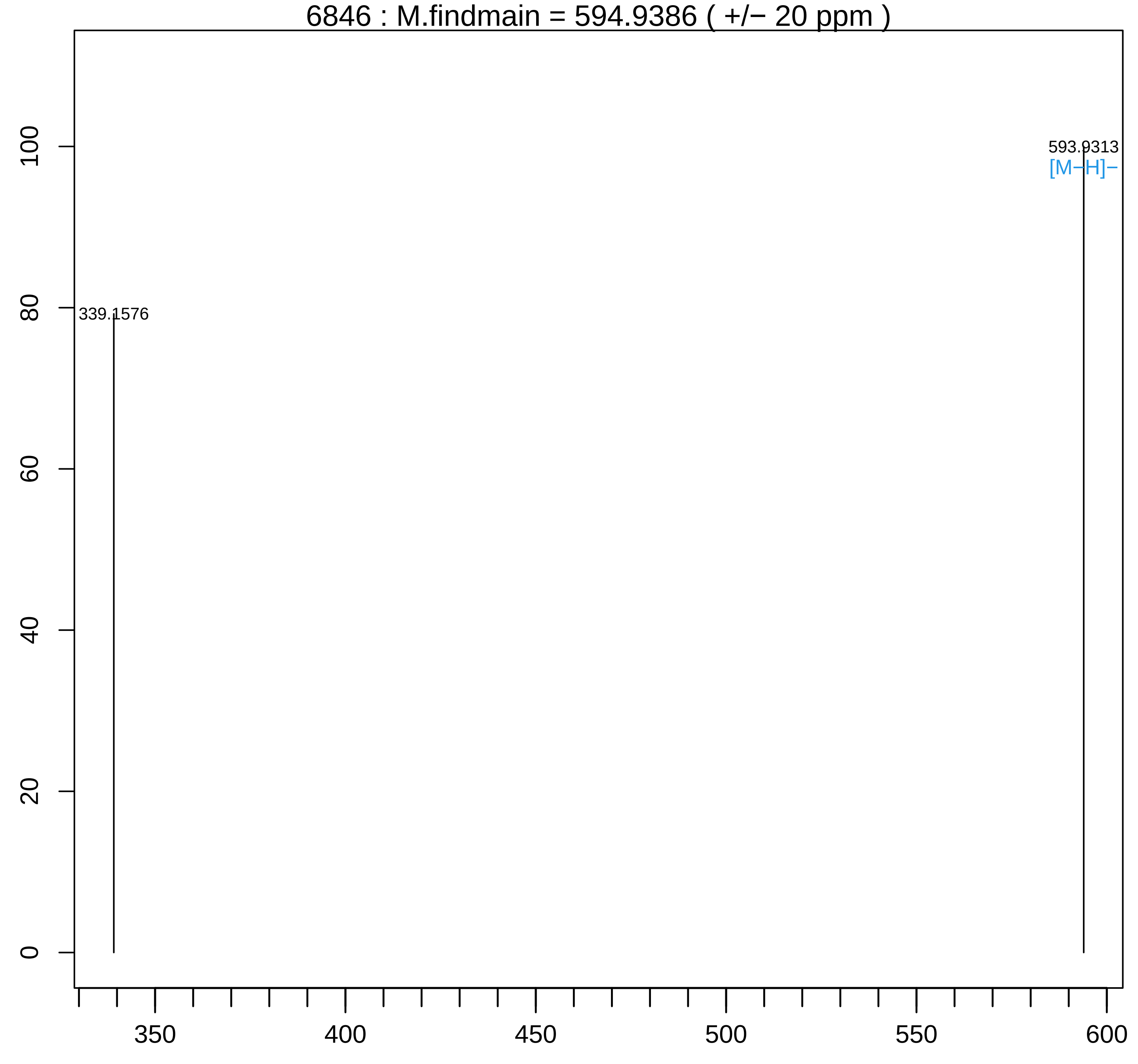
6846 : M.findmain = 594.9386 (+/− 20 ppm)
593.9313
[M−H]−
339.1576
0
20
40
60
80
100
350
400
450
500
550
600

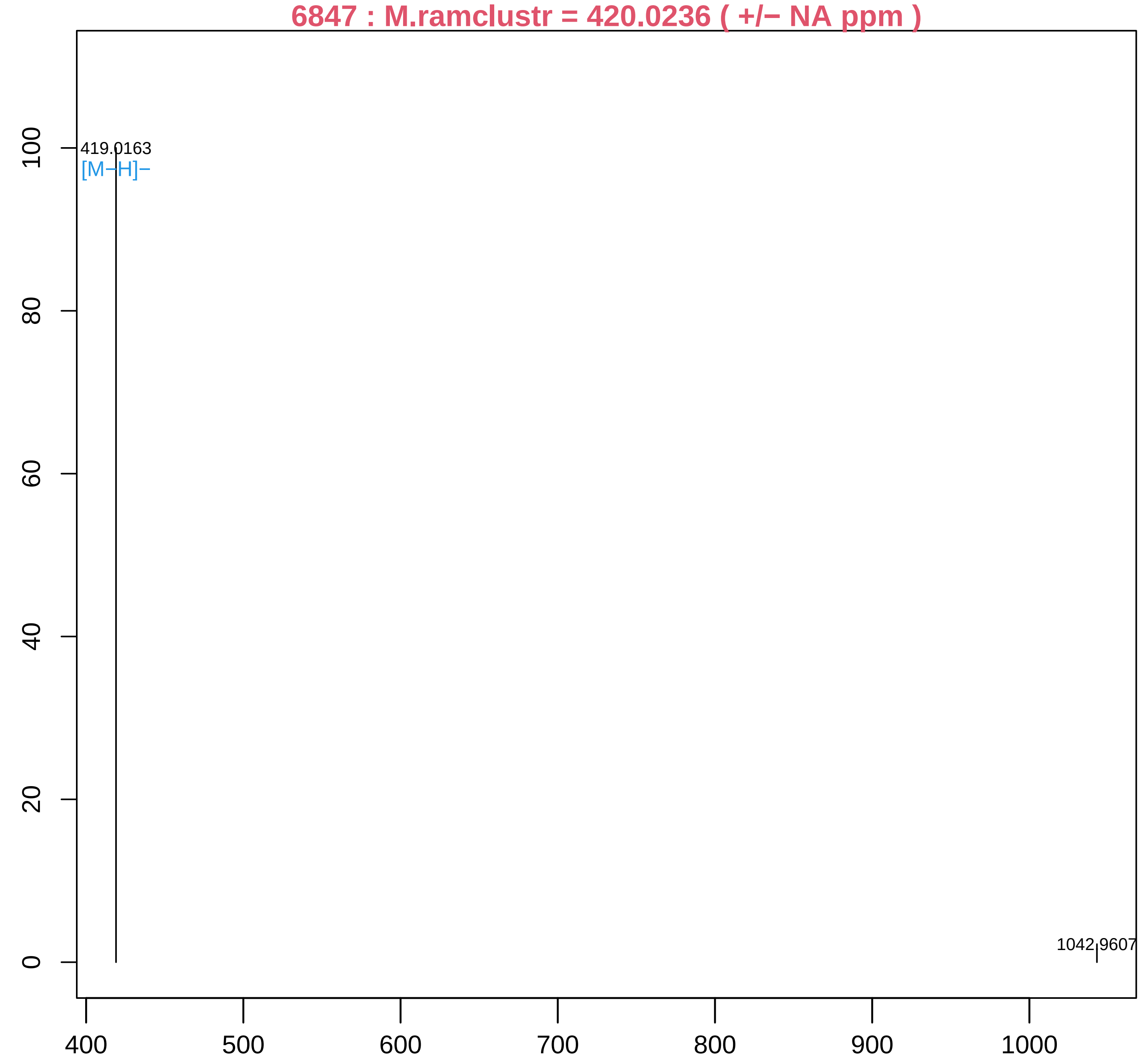
6847 : M.ramclustr = 420.0236 (+/− NA ppm)
419.0163
[M−H]−
1042.9607
0
20
40
60
80
100
400
500
600
700
800
900
1000

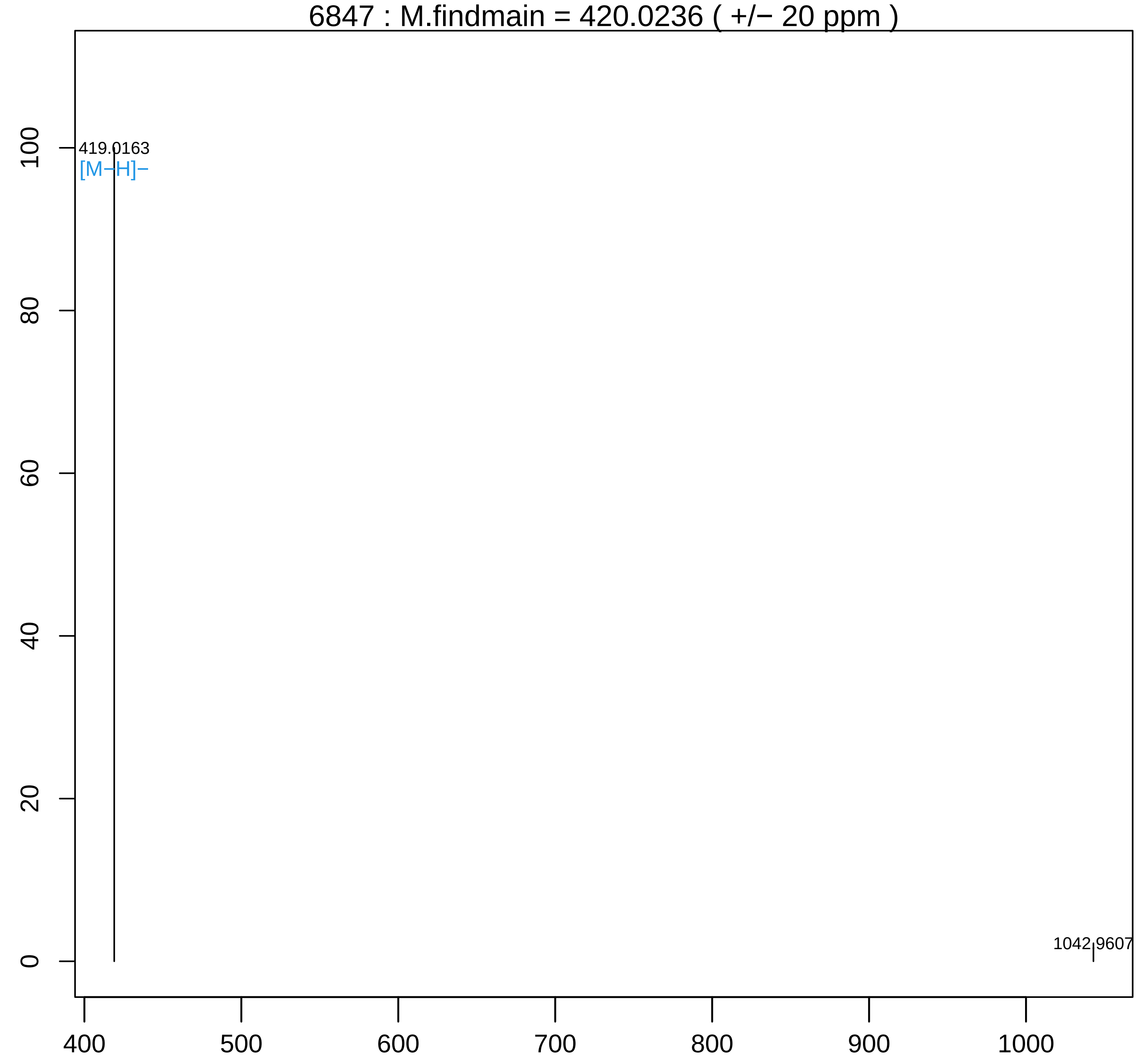
6847 : M.findmain = 420.0236 (+/− 20 ppm)
419.0163
[M−H]−
1042.9607
0
20
40
60
80
100
400
500
600
700
800
900
1000

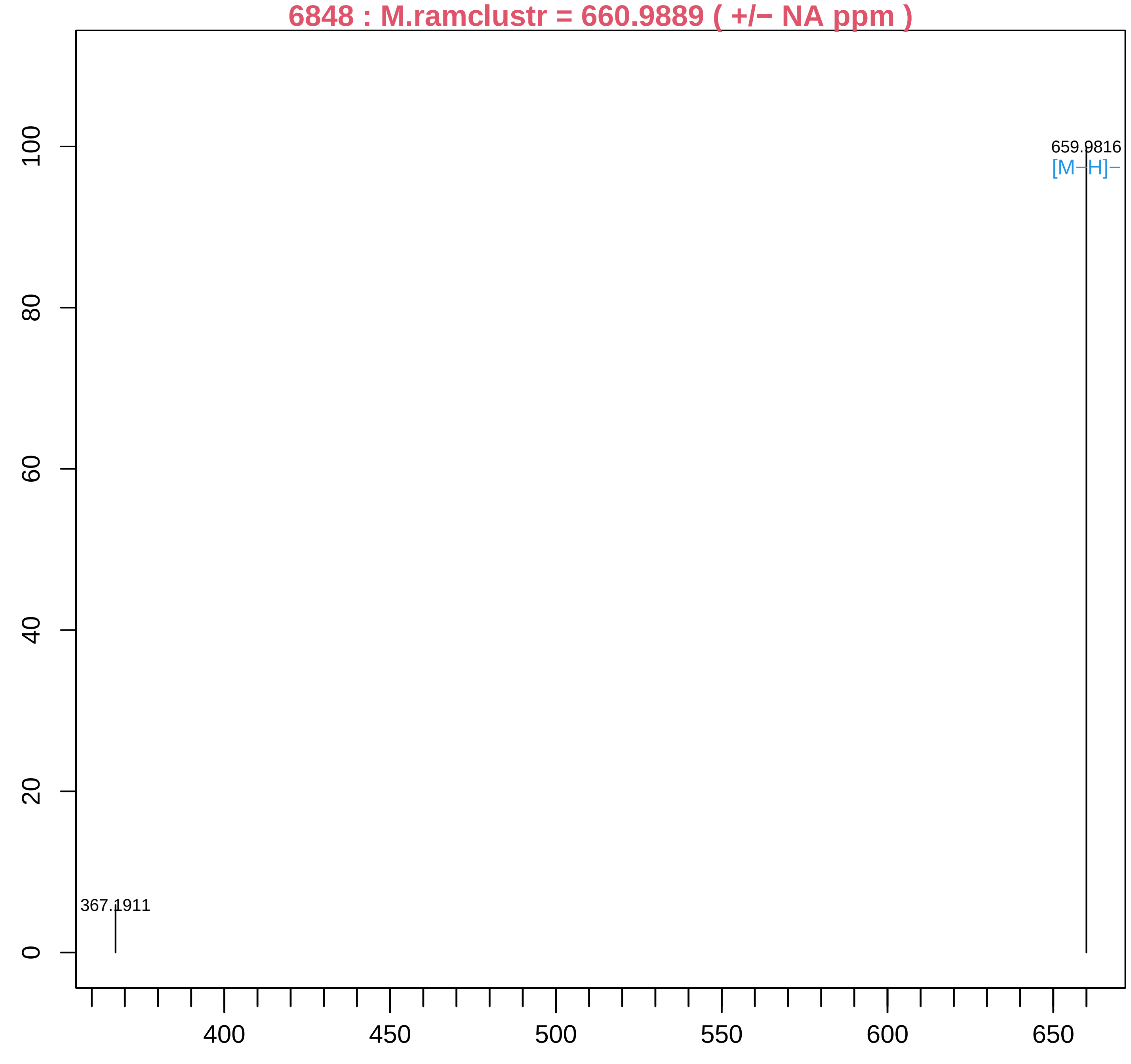
6848 : M.ramclustr = 660.9889 (+/− NA ppm)
659.9816
[M−H]−
367.1911
0
20
40
60
80
100
400
450
500
550
600
650

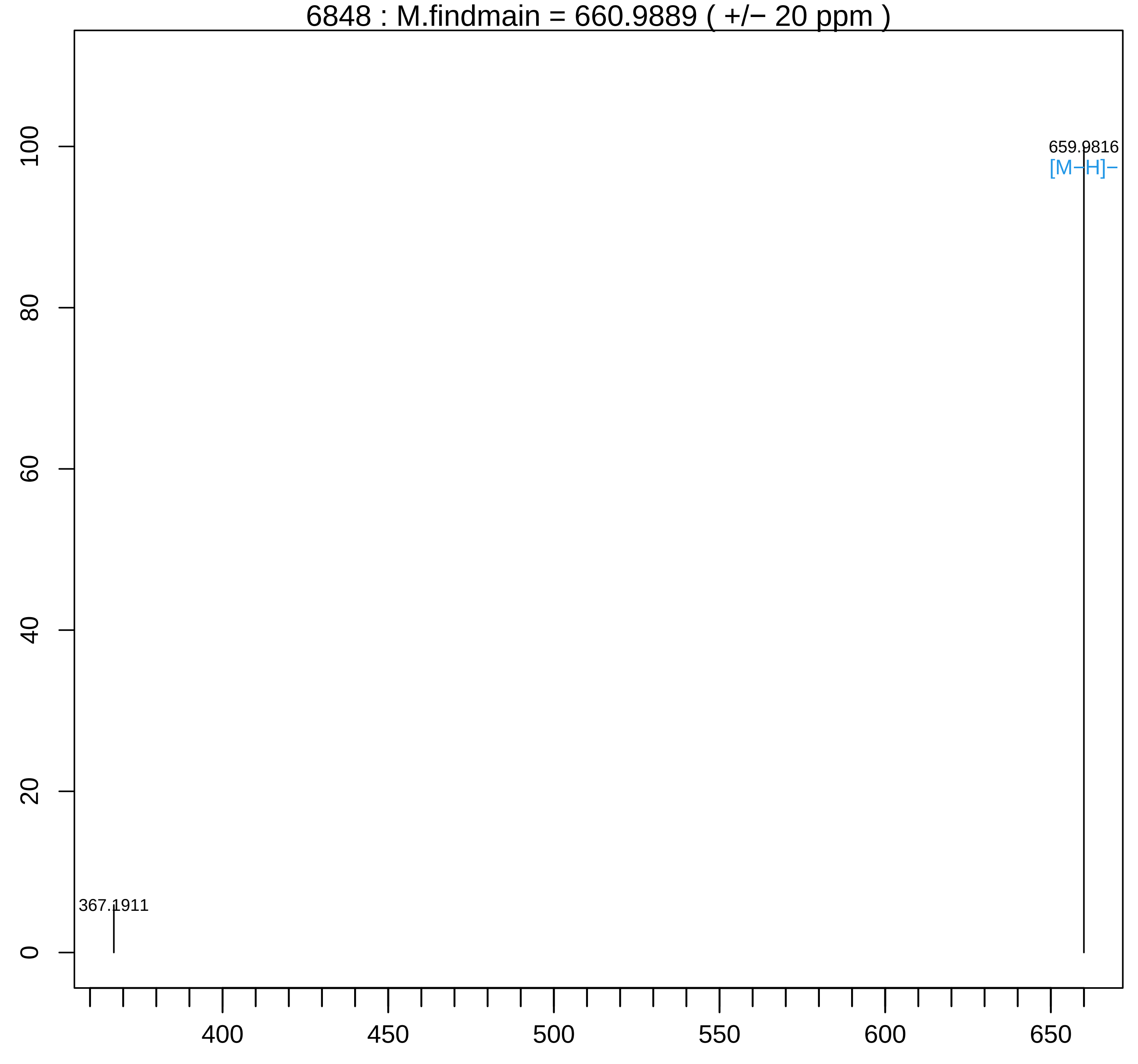
6848 : M.findmain = 660.9889 (+/− 20 ppm)
659.9816
[M−H]−
367.1911
0
20
40
60
80
100
400
450
500
550
600
650

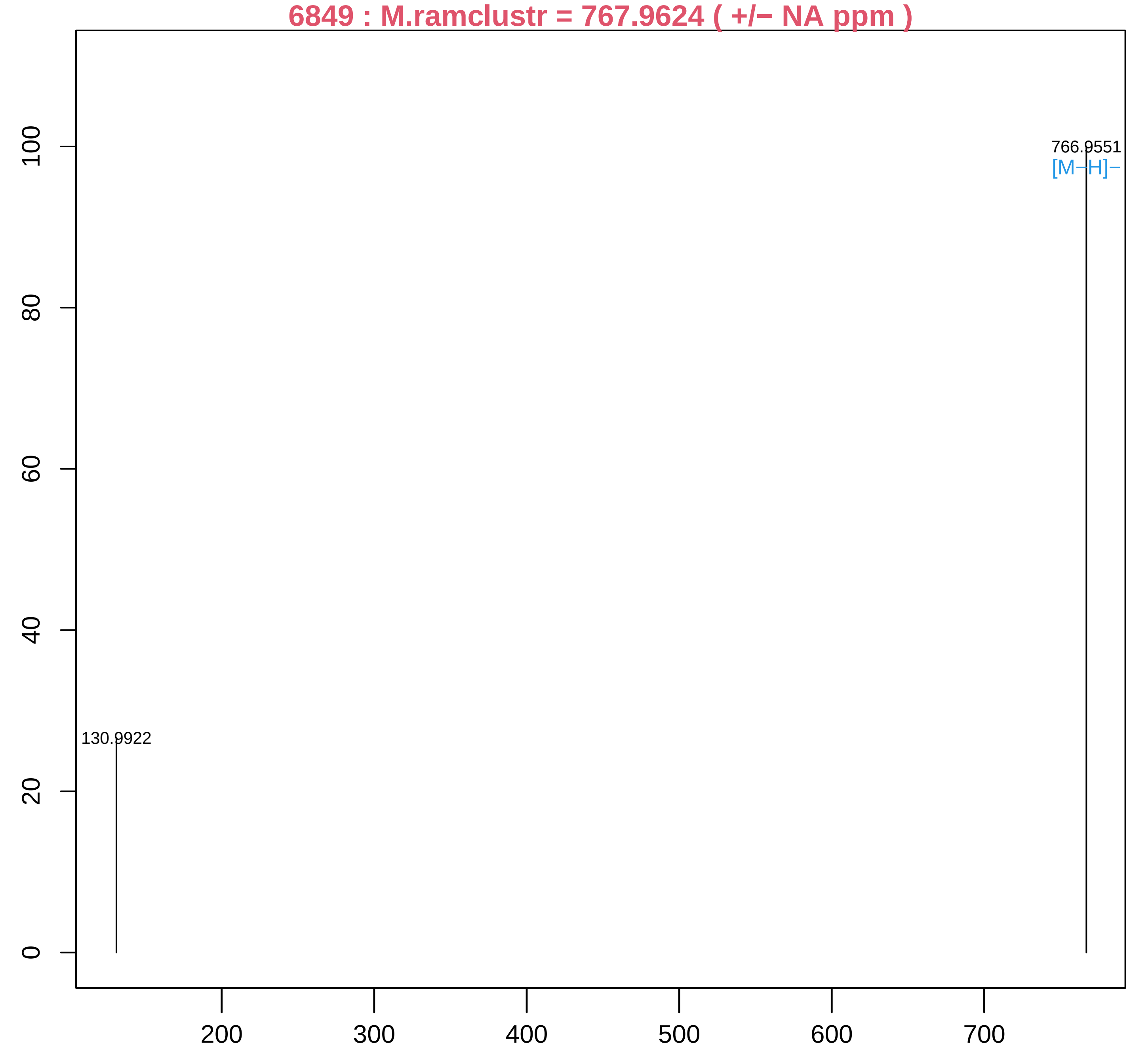

6849 : M.ramclustr = 767.9624 (+/− NA ppm)
766.9551
[M−H]−
130.9922
0
20
40
60
80
100
200
300
400
500
600
700

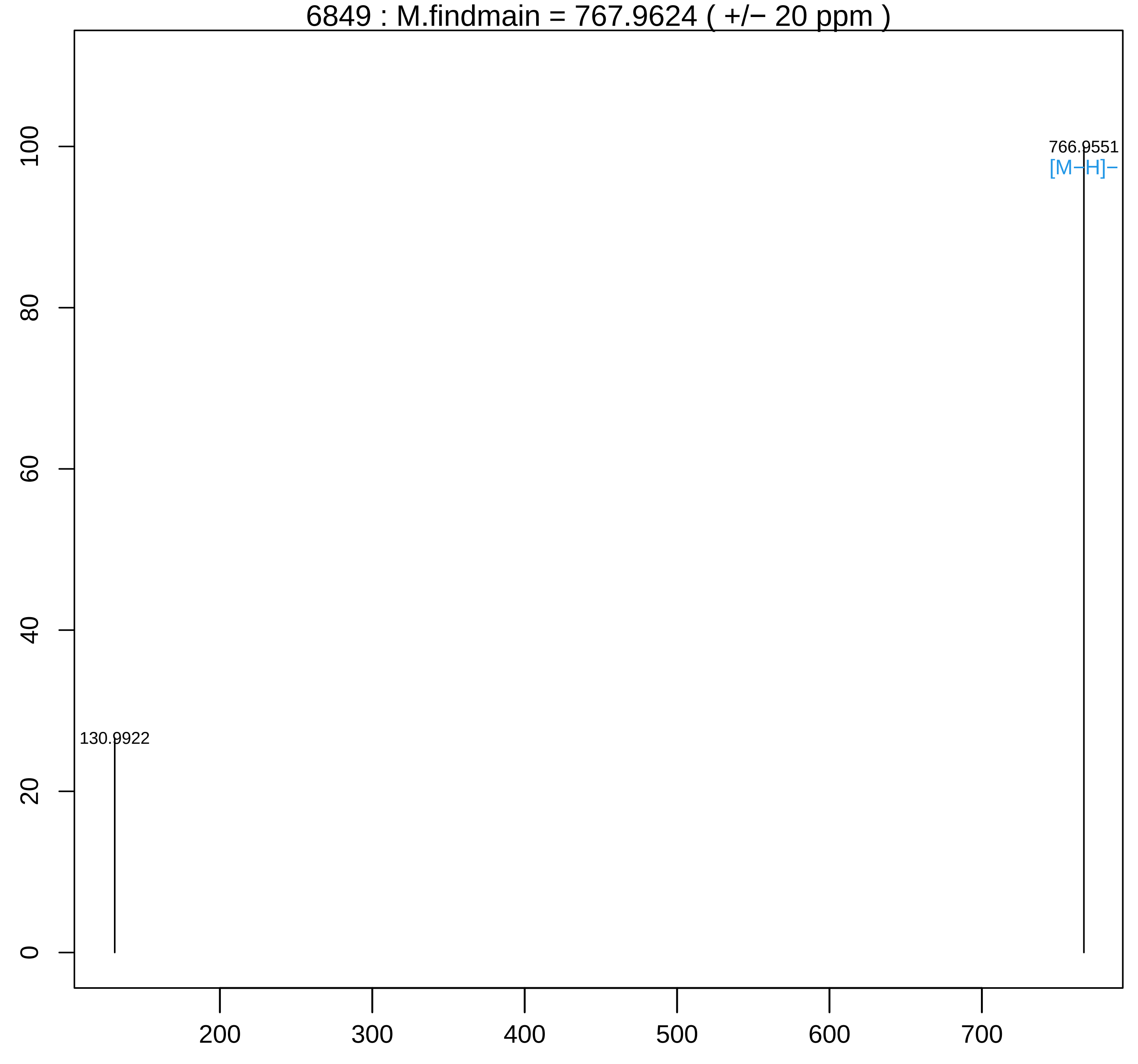

6849 : M.findmain = 767.9624 (+/− 20 ppm)
766.9551
[M−H]−
130.9922
0
20
40
60
80
100
200
300
400
500
600
700

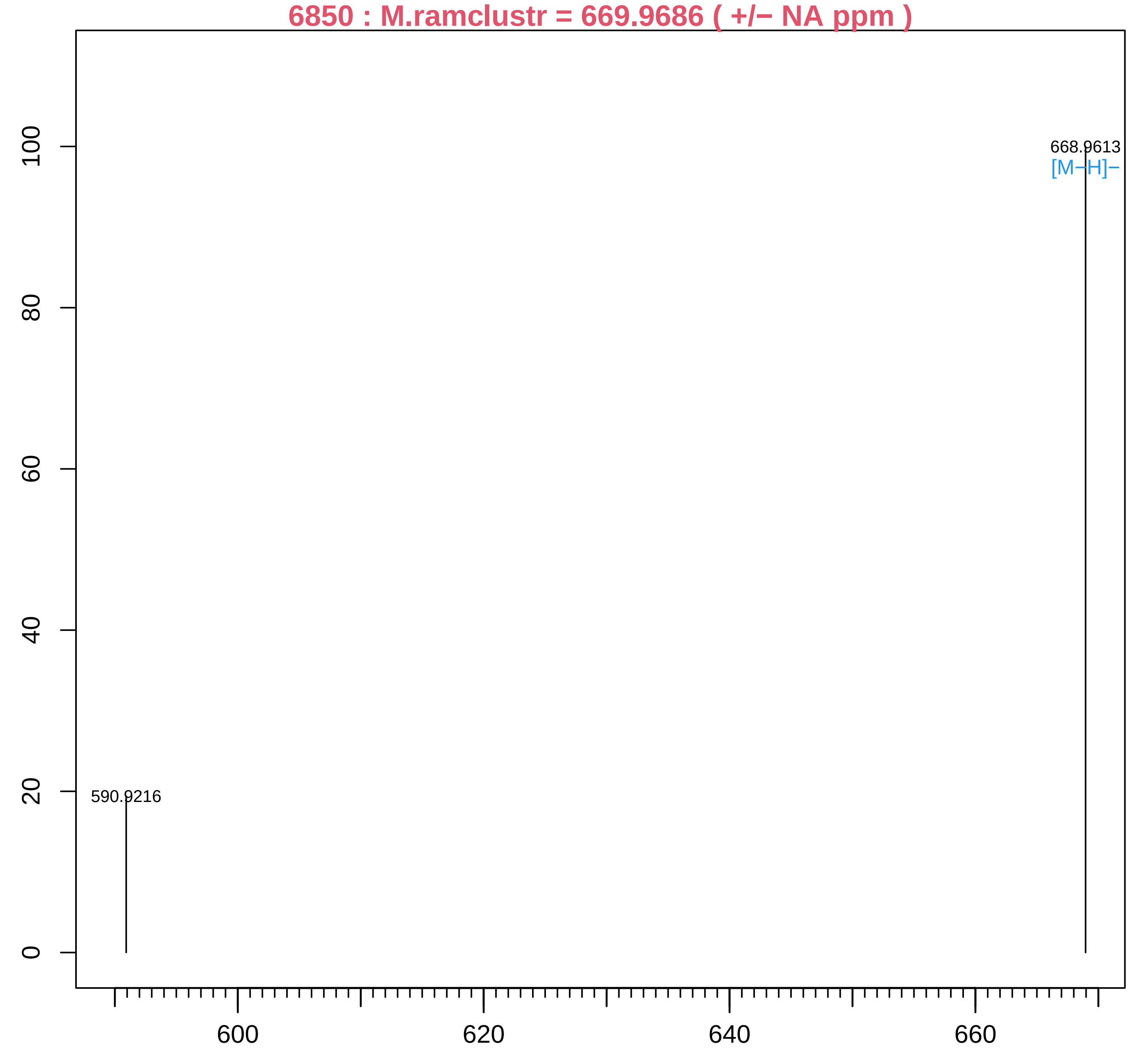
6850 : M.ramclustr = 669.9686 (+/− NA ppm)
668.9613
[M−H]−
590.9216
0
20
40
60
80
100
600
620
640
660

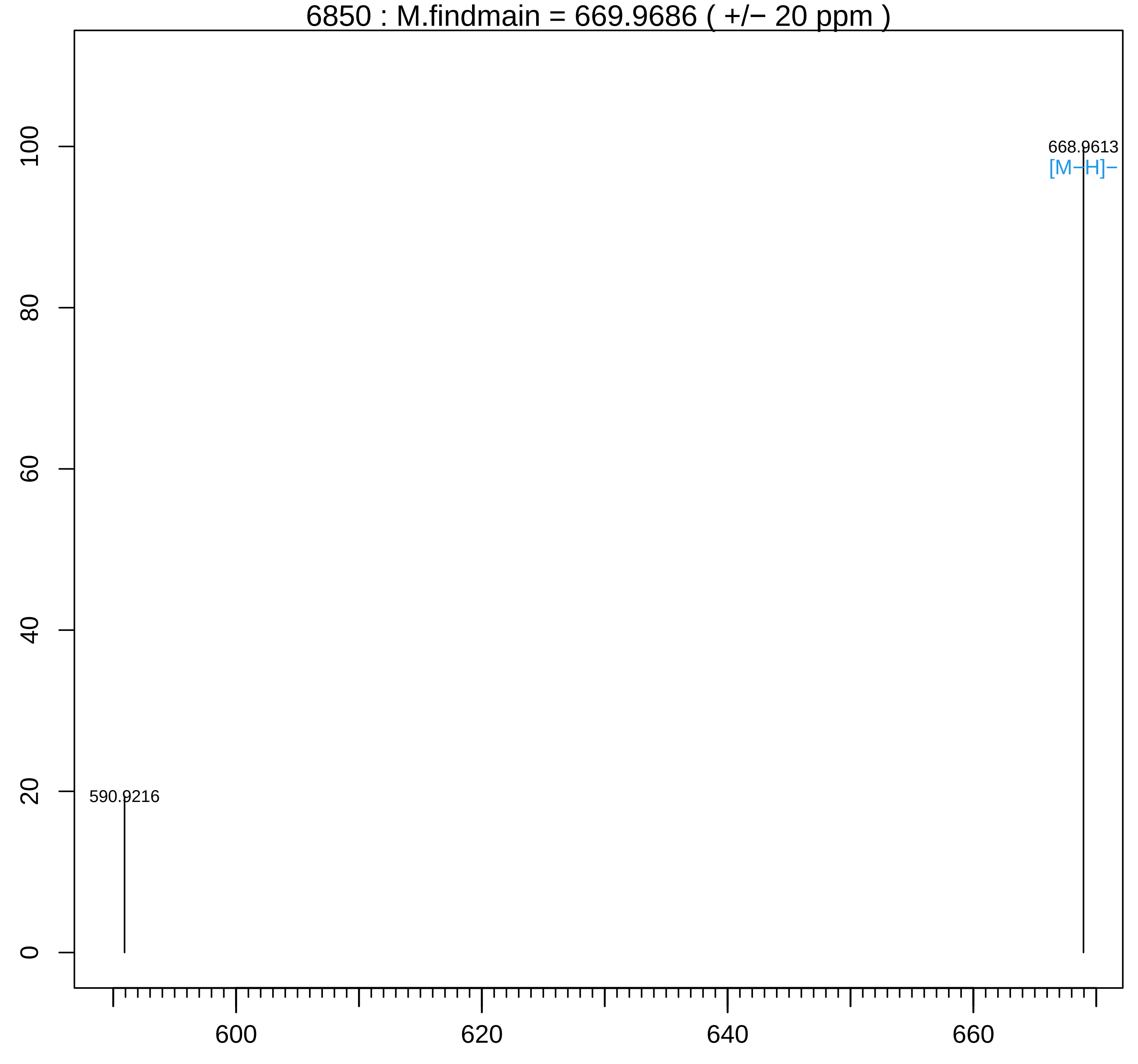
6850 : M.findmain = 669.9686 (+/− 20 ppm)
668.9613
[M−H]−
590.9216
0
20
40
60
80
100
600
620
640
660

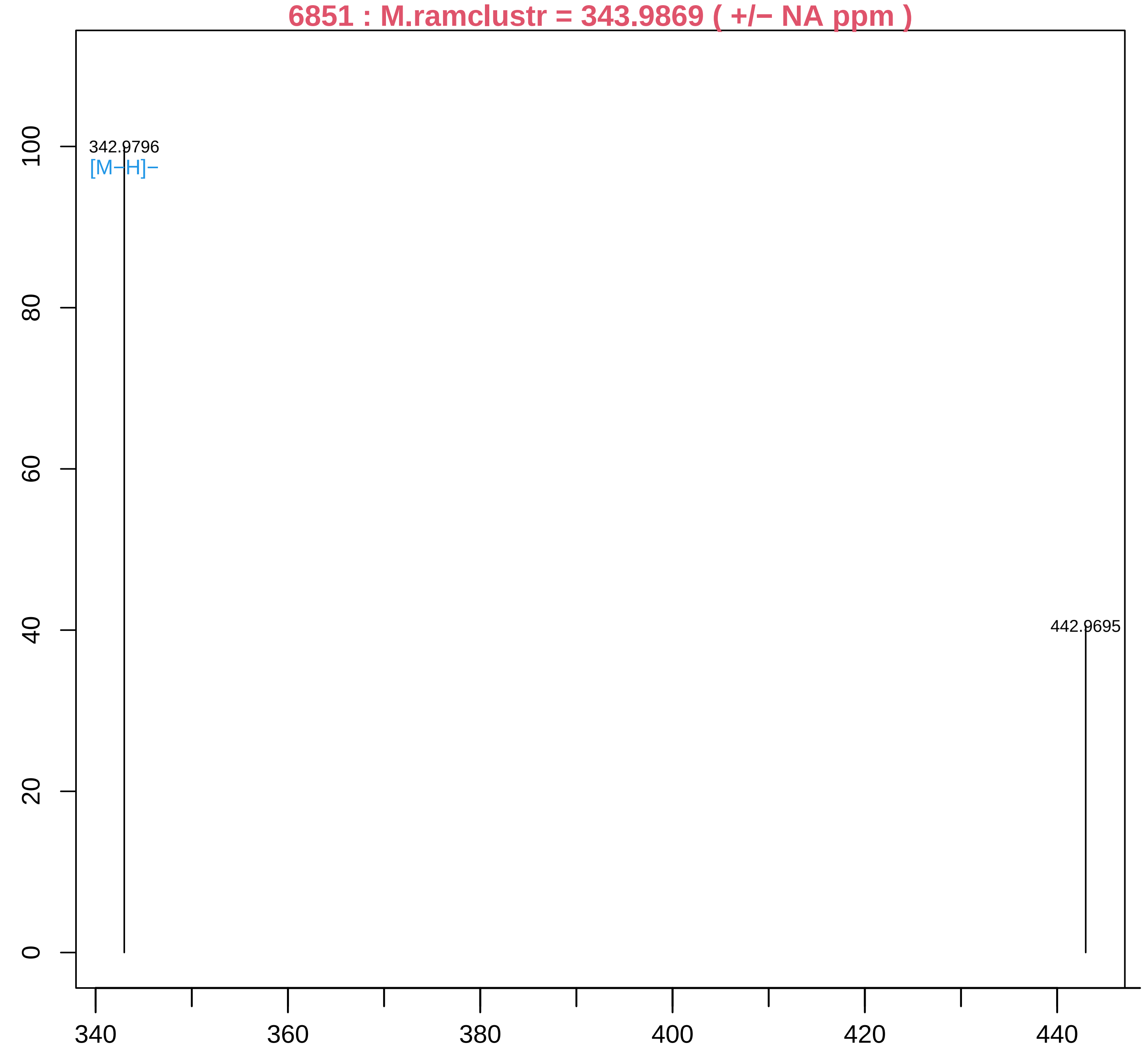
6851 : M.ramclustr = 343.9869 (+/− NA ppm)
342.9796
[M−H]−
442.9695
0
20
40
60
80
100
340
360
380
400
420
440

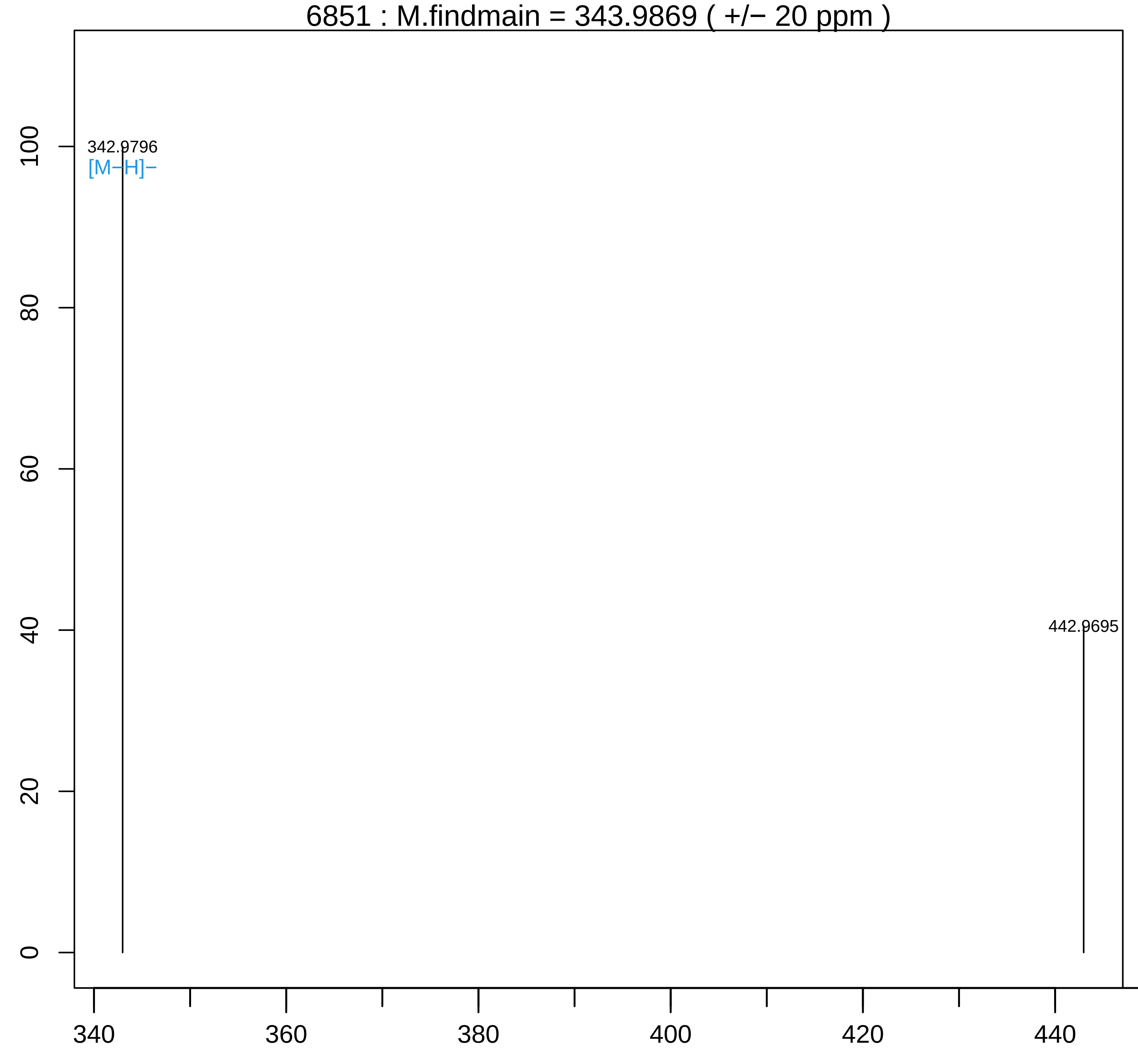
6851 : M.findmain = 343.9869 (+/− 20 ppm)
342.9796
[M−H]−
442.9695
0
20
40
60
80
100
340
360
380
400
420
440

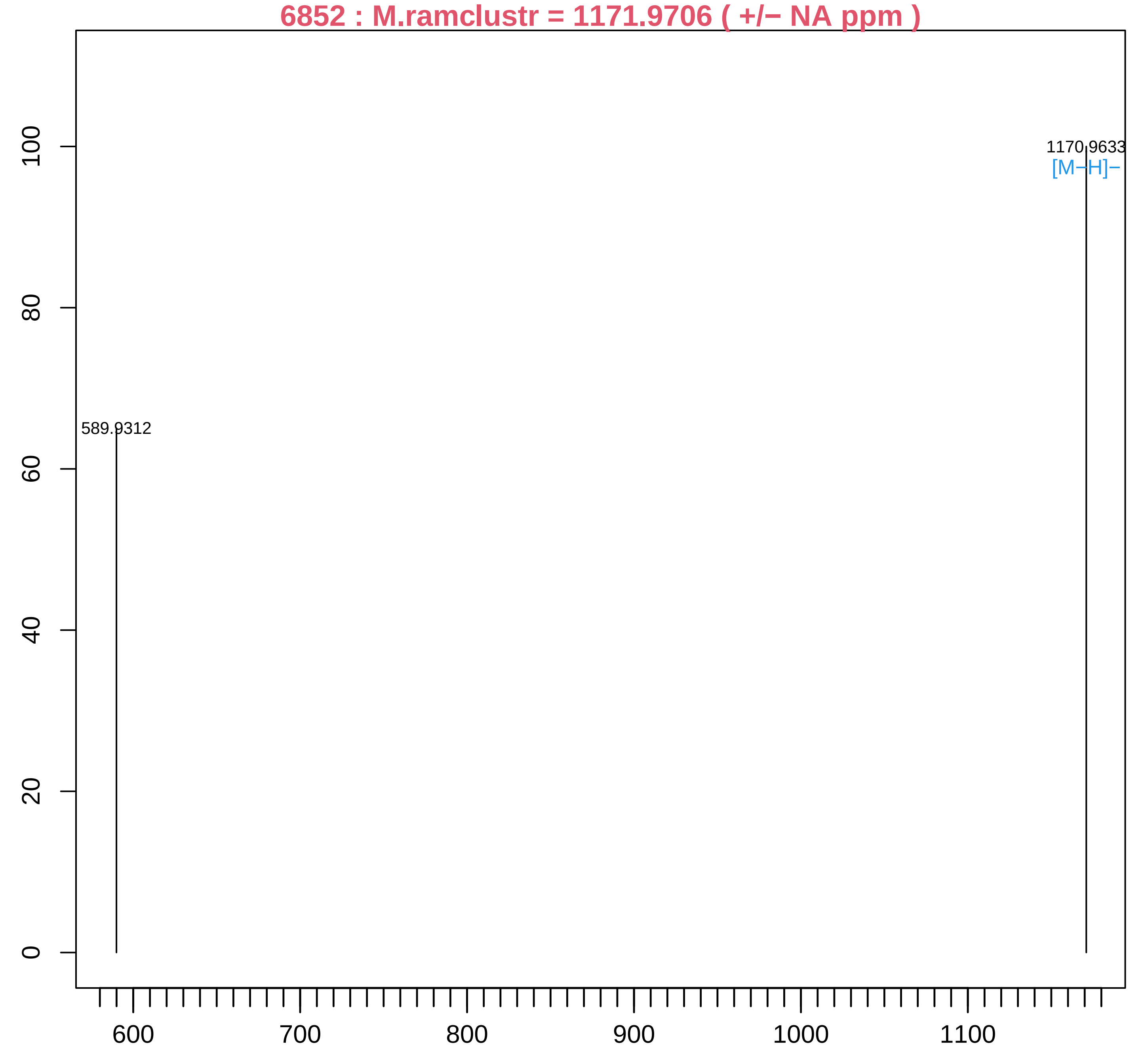
6852 : M.ramclustr = 1171.9706 (+/− NA ppm)
1170.9633
[M−H]−
589.9312
0
20
40
60
80
100
600
700
800
900
1000
1100

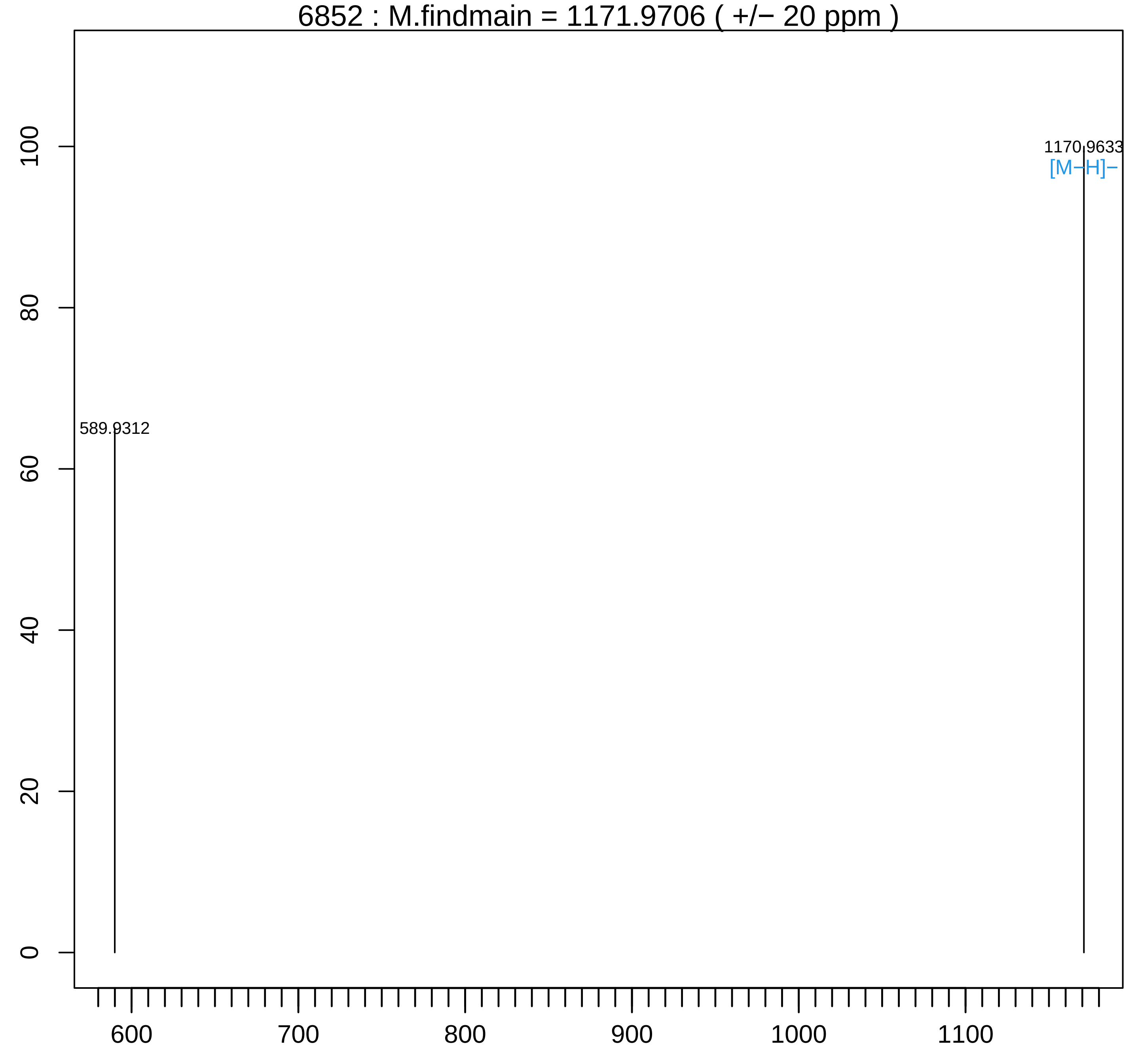
6852 : M.findmain = 1171.9706 (+/− 20 ppm)
1170.9633
[M−H]−
589.9312
0
20
40
60
80
100
600
700
800
900
1000
1100

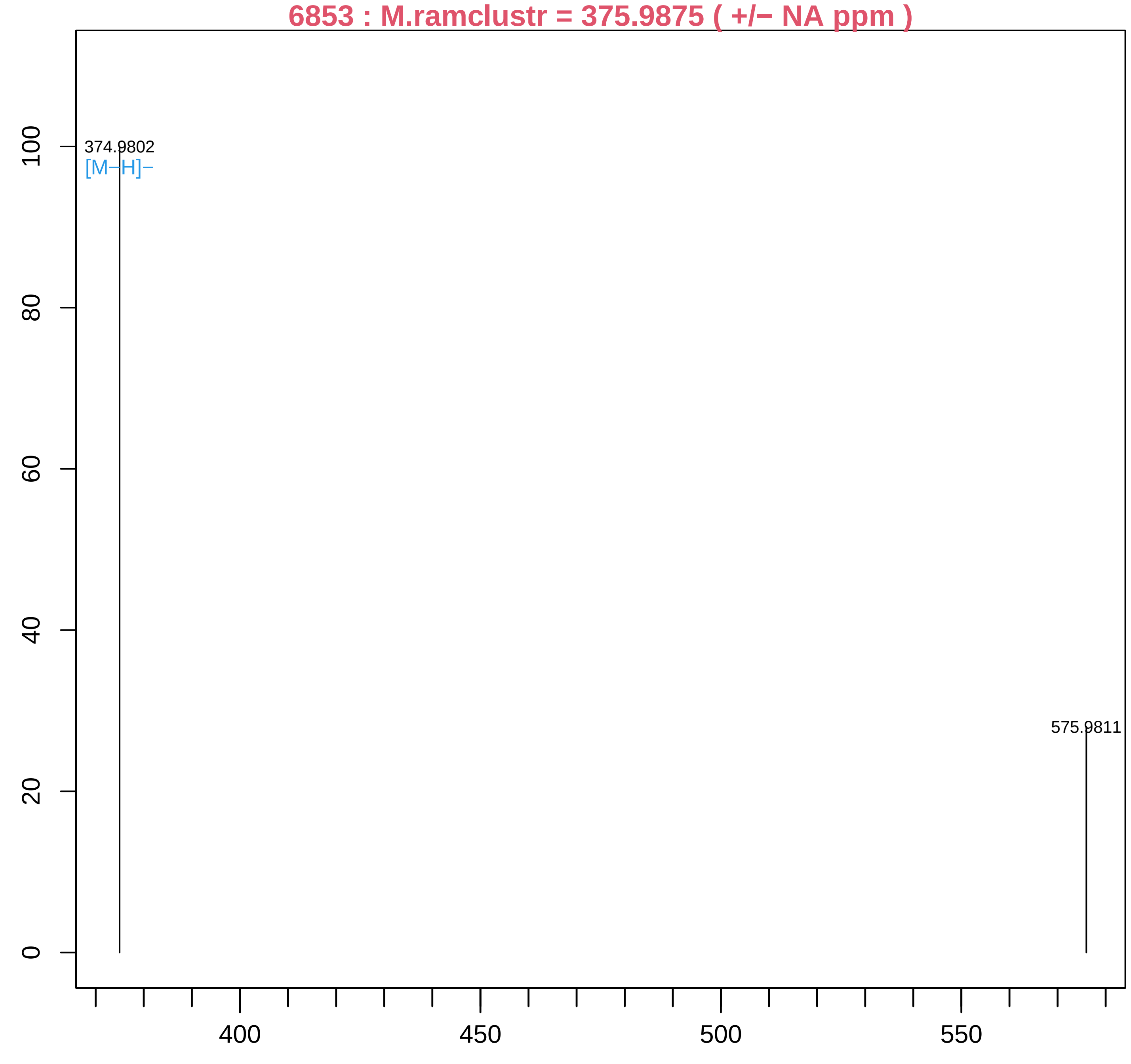
6853 : M.ramclustr = 375.9875 (+/− NA ppm)
374.9802
[M−H]−
575.9811
0
20
40
60
80
100
400
450
500
550

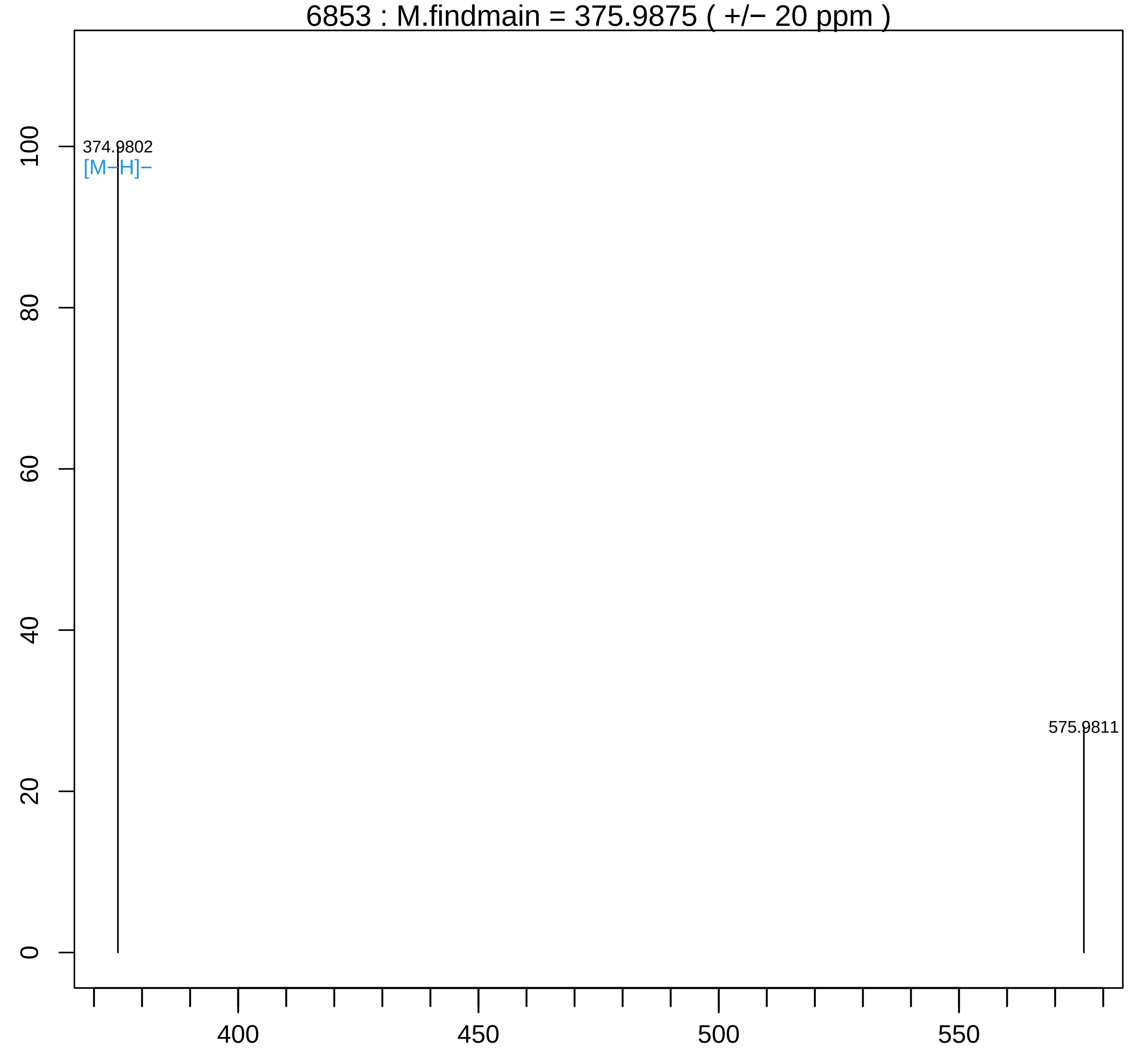
6853 : M.findmain = 375.9875 (+/− 20 ppm)
374.9802
[M−H]−
575.9811
0
20
40
60
80
100
400
450
500
550

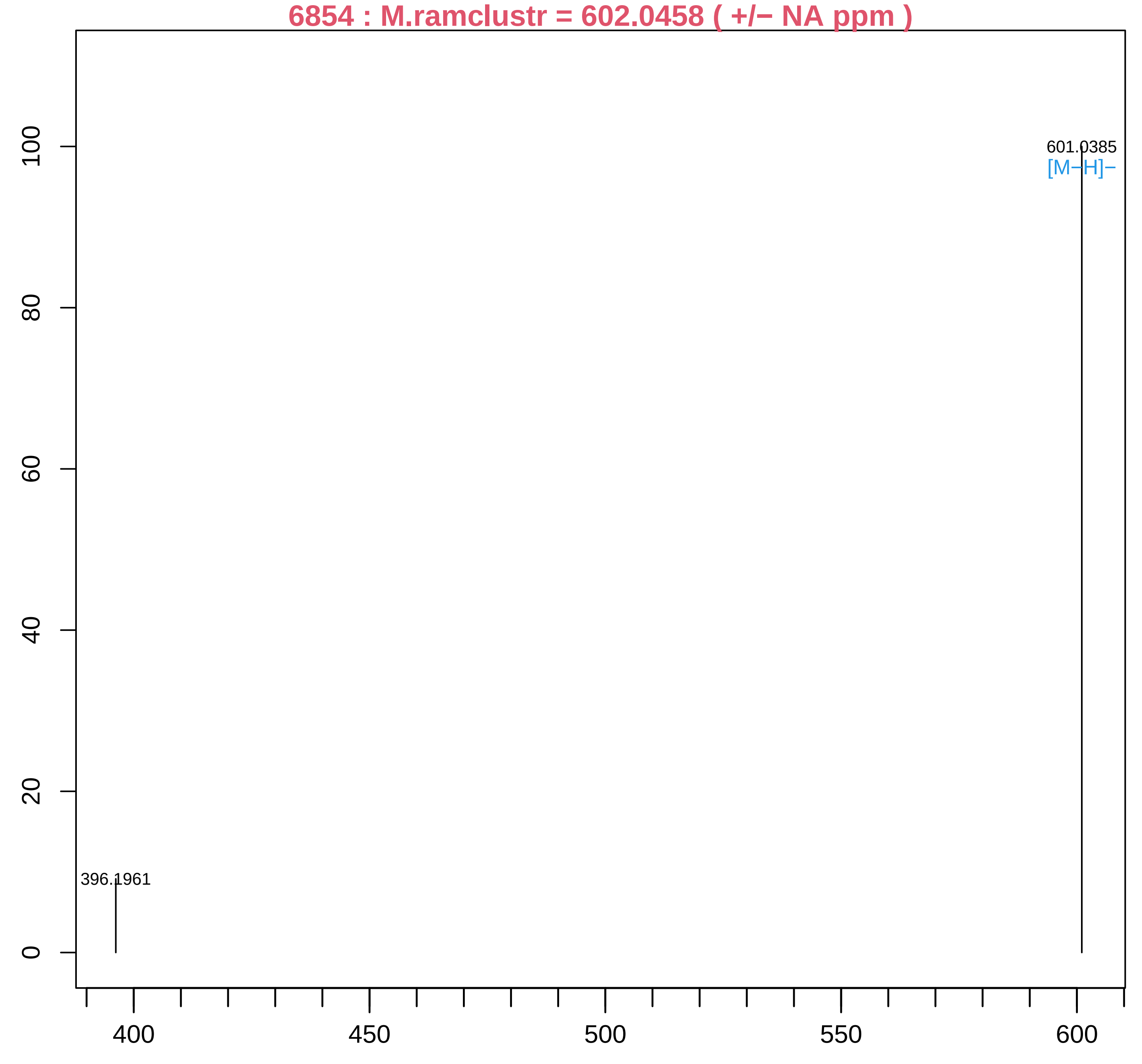

6854 : M.ramclustr = 602.0458 (+/− NA ppm)
601.0385
[M−H]−
396.1961
0
20
40
60
80
100
400
450
500
550
600

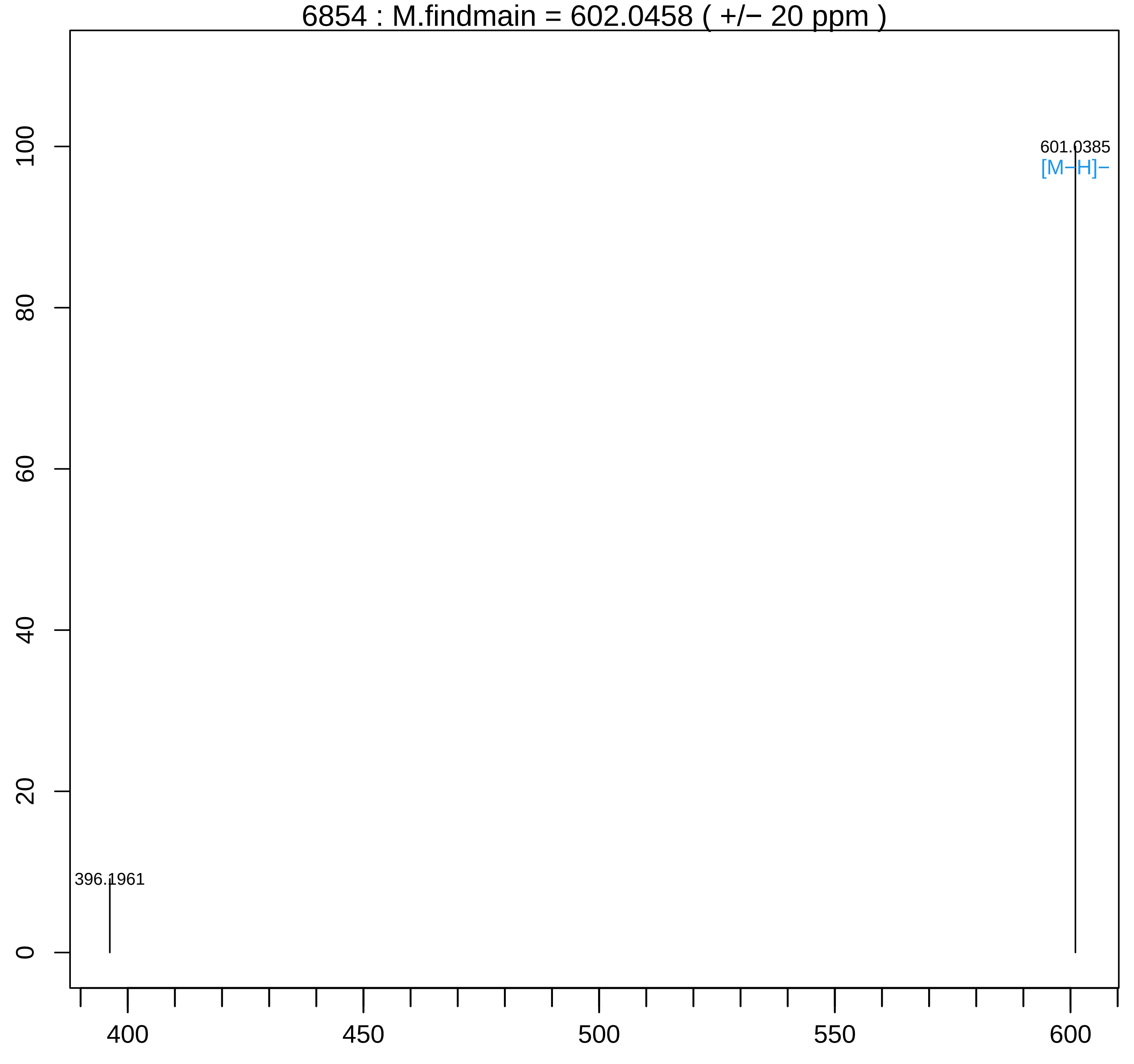

6854 : M.findmain = 602.0458 (+/− 20 ppm)
601.0385
[M−H]−
396.1961
0
20
40
60
80
100
400
450
500
550
600

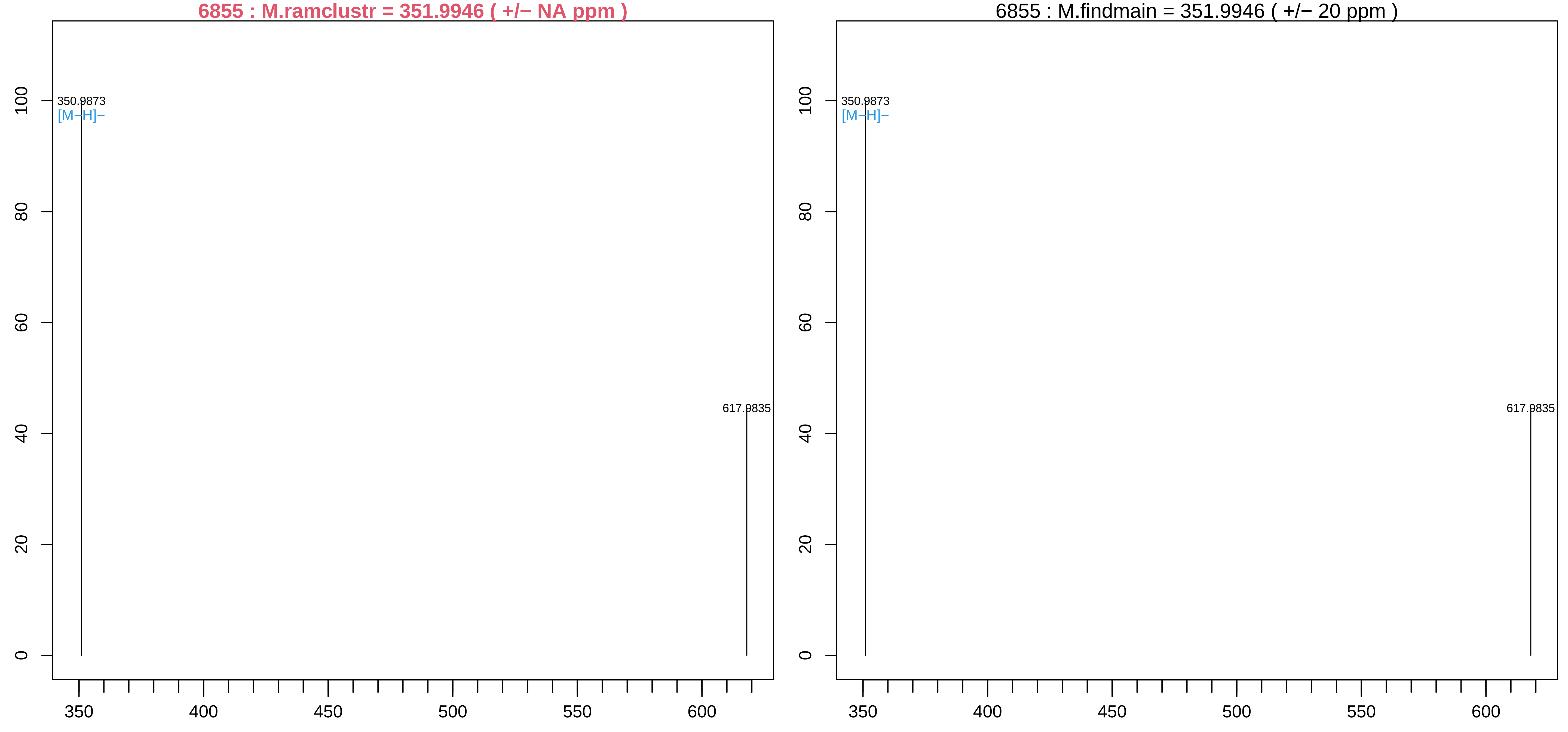

6855 : M.ramclustr = 351.9946 (+/− NA ppm)
350.9873
[M−H]−
617.9835
6855 : M.findmain = 351.9946 (+/− 20 ppm)
350.9873
[M−H]−
617.9835
0
20
40
60
80
100
350
400
450
500
550
600

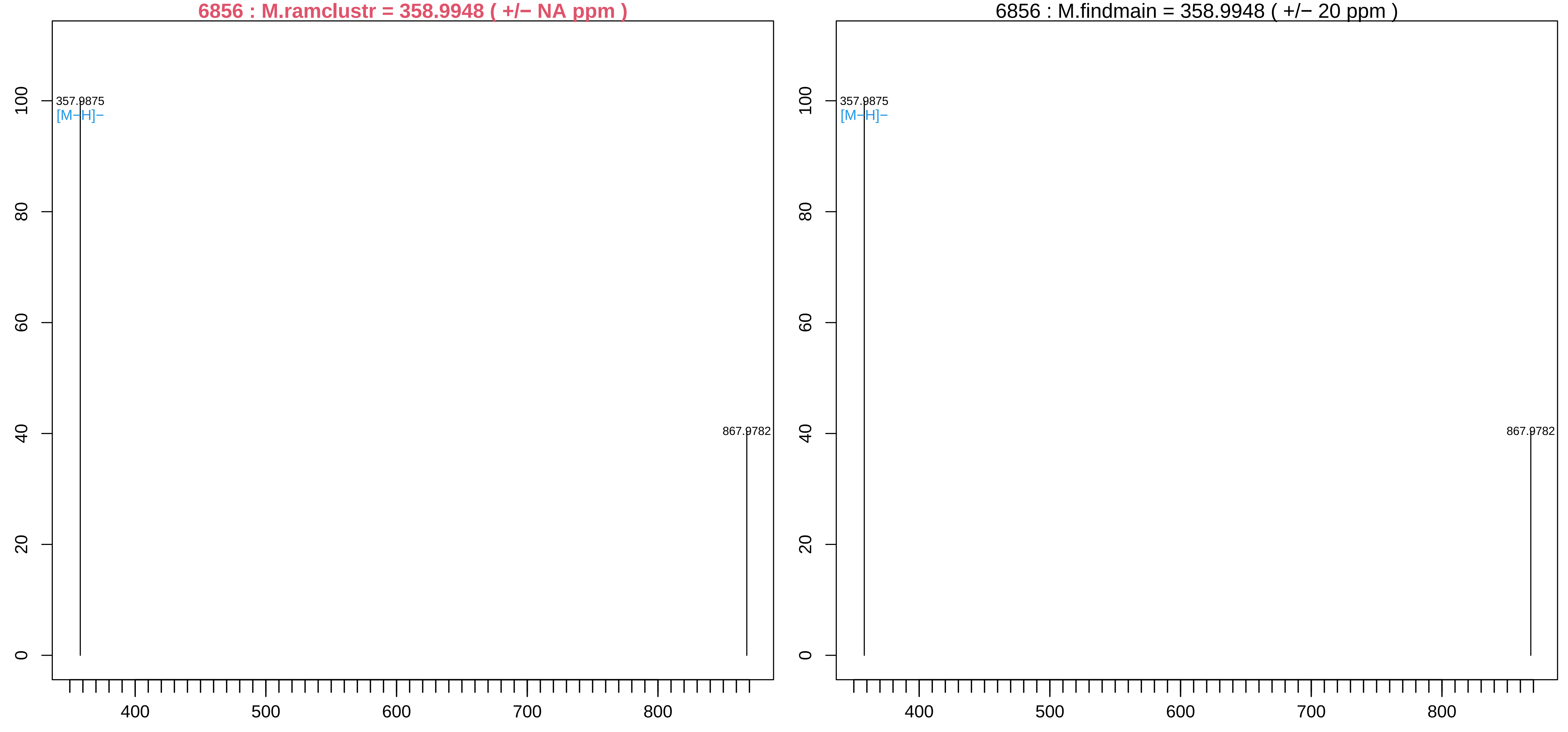
6856 : M.ramclustr = 358.9948 (+/− NA ppm)
357.9875
[M−H]−
867.9782
0
20
40
60
80
100
400
500
600
700
800
6856 : M.findmain = 358.9948 (+/− 20 ppm)
357.9875
[M−H]−
867.9782
0
20
40
60
80
100
400
500
600
700
800

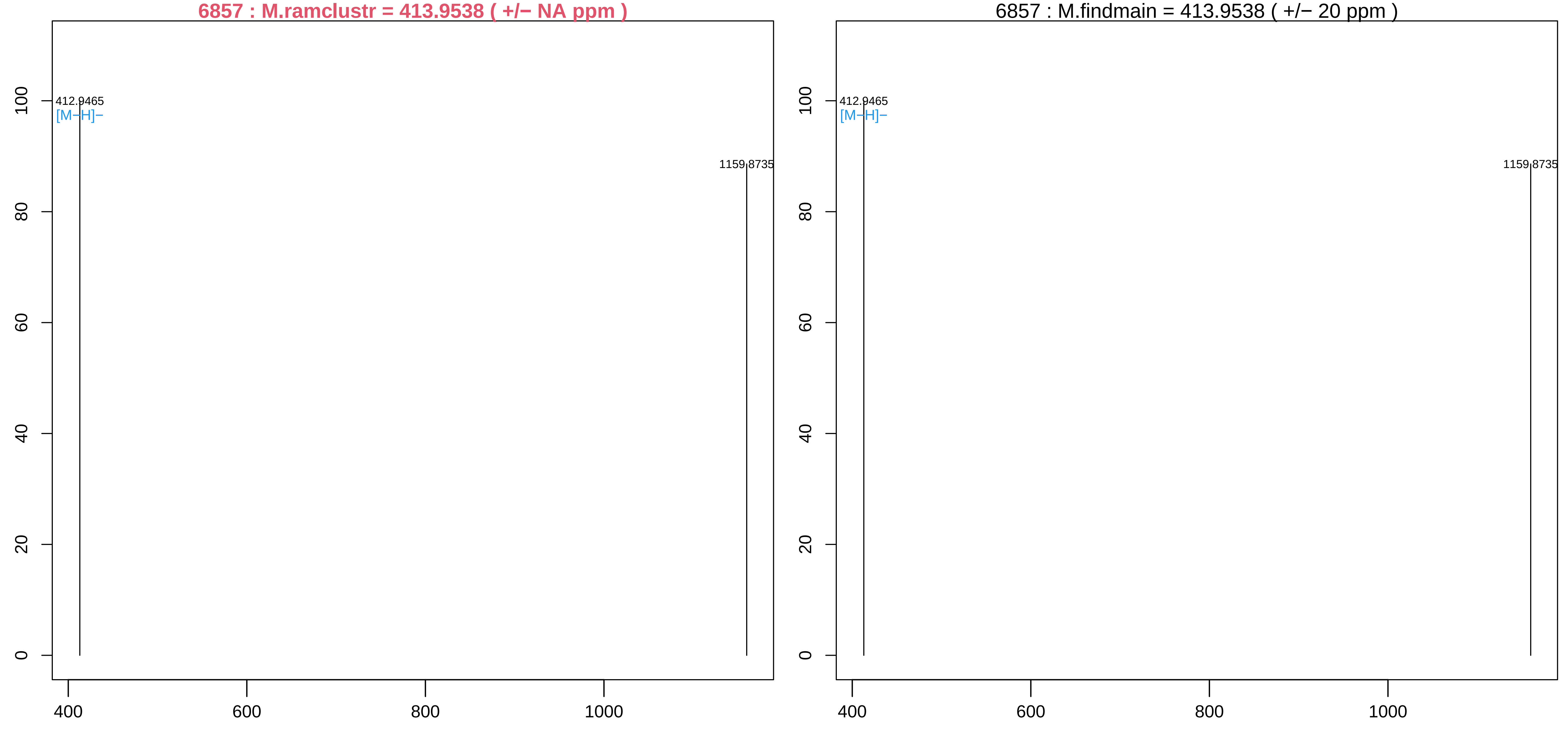
6857 : M.ramclustr = 413.9538 (+/− NA ppm)
412.9465
[M−H]−
1159.8735
0
20
40
60
80
100
400
600
800
1000
6857 : M.findmain = 413.9538 (+/− 20 ppm)
412.9465
[M−H]−
1159.8735
0
20
40
60
80
100
400
600
800
1000

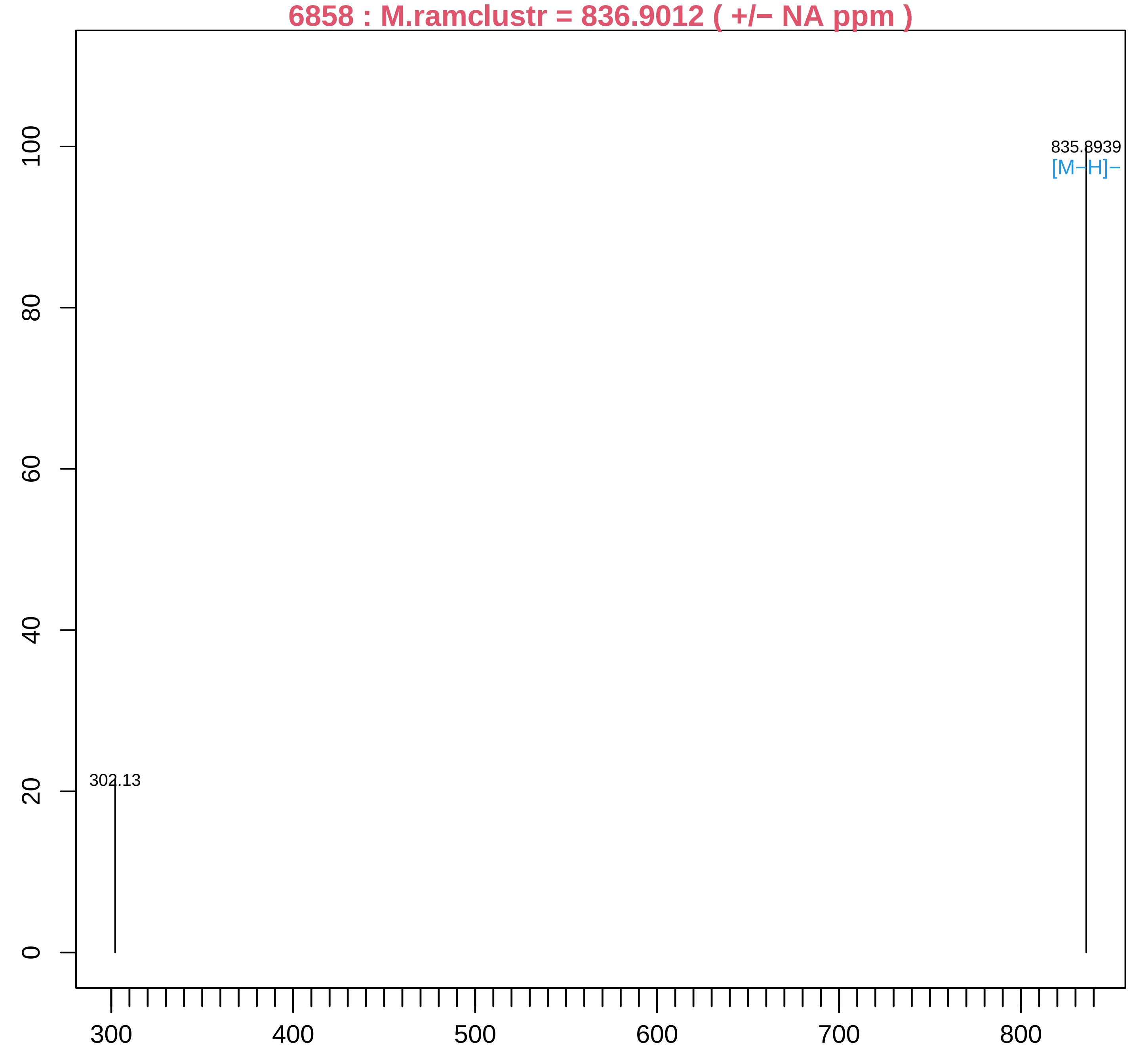
6858 : M.ramclustr = 836.9012 (+/− NA ppm)
835.8939
[M−H]−
302.13
0
20
40
60
80
100
300
400
500
600
700
800

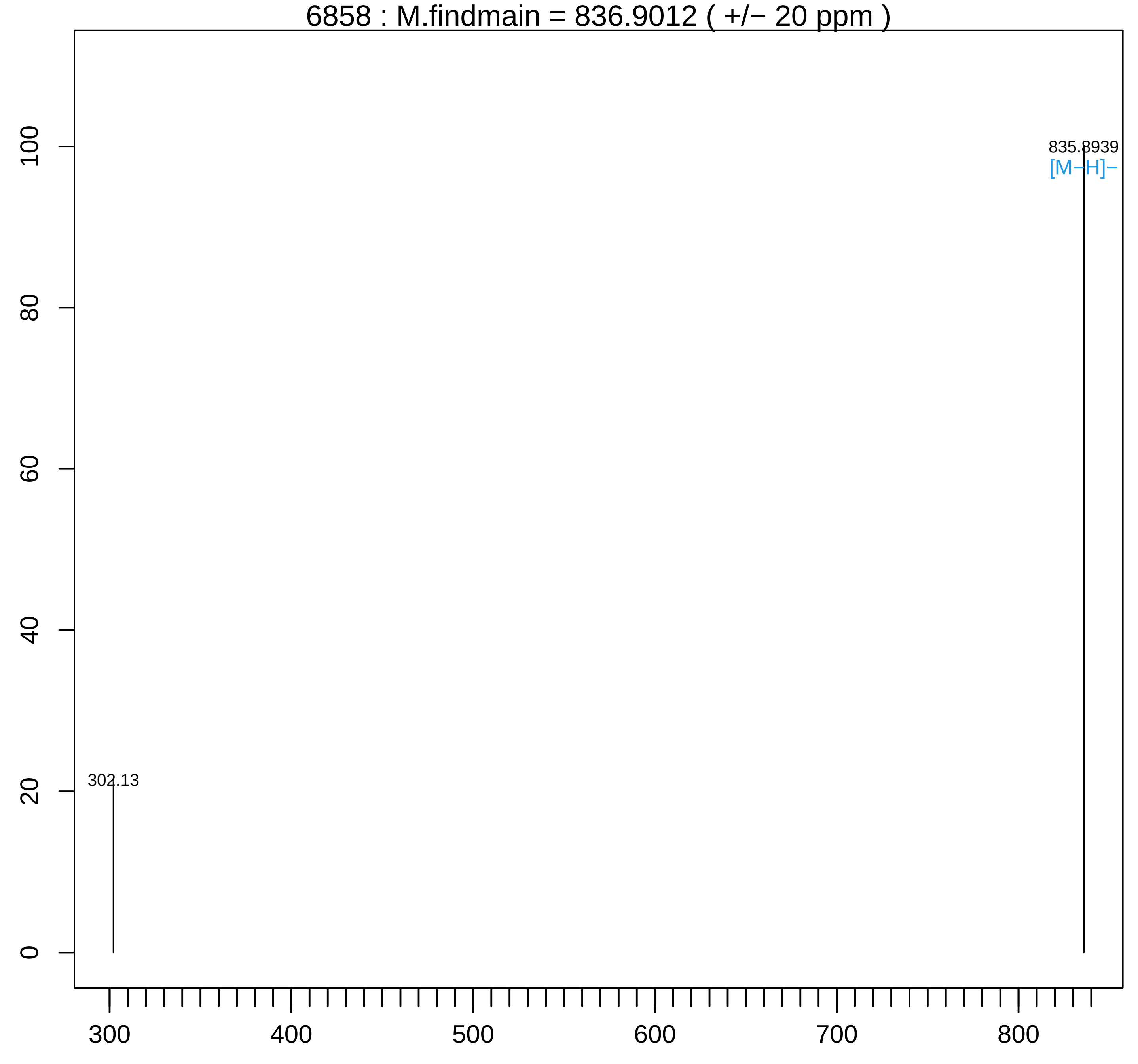
6858 : M.findmain = 836.9012 (+/− 20 ppm)
835.8939
[M−H]−
302.13
0
20
40
60
80
100
300
400
500
600
700
800

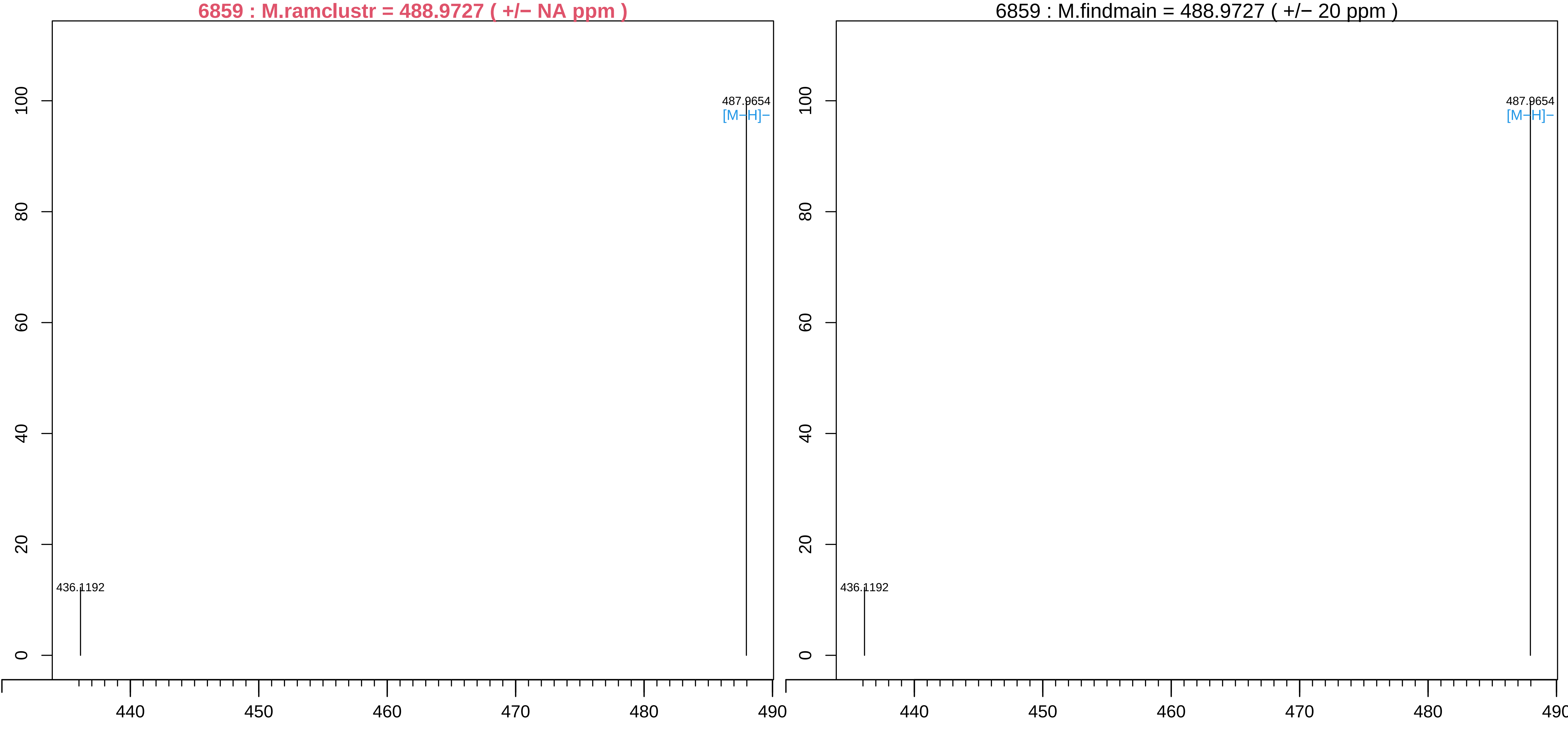
6859 : M.ramclustr = 488.9727 (+/− NA ppm)
487.9654
[M−H]−
436.1192
6859 : M.findmain = 488.9727 (+/− 20 ppm)
487.9654
[M−H]−
436.1192
0
20
40
60
80
100
440
450
460
470
480
490

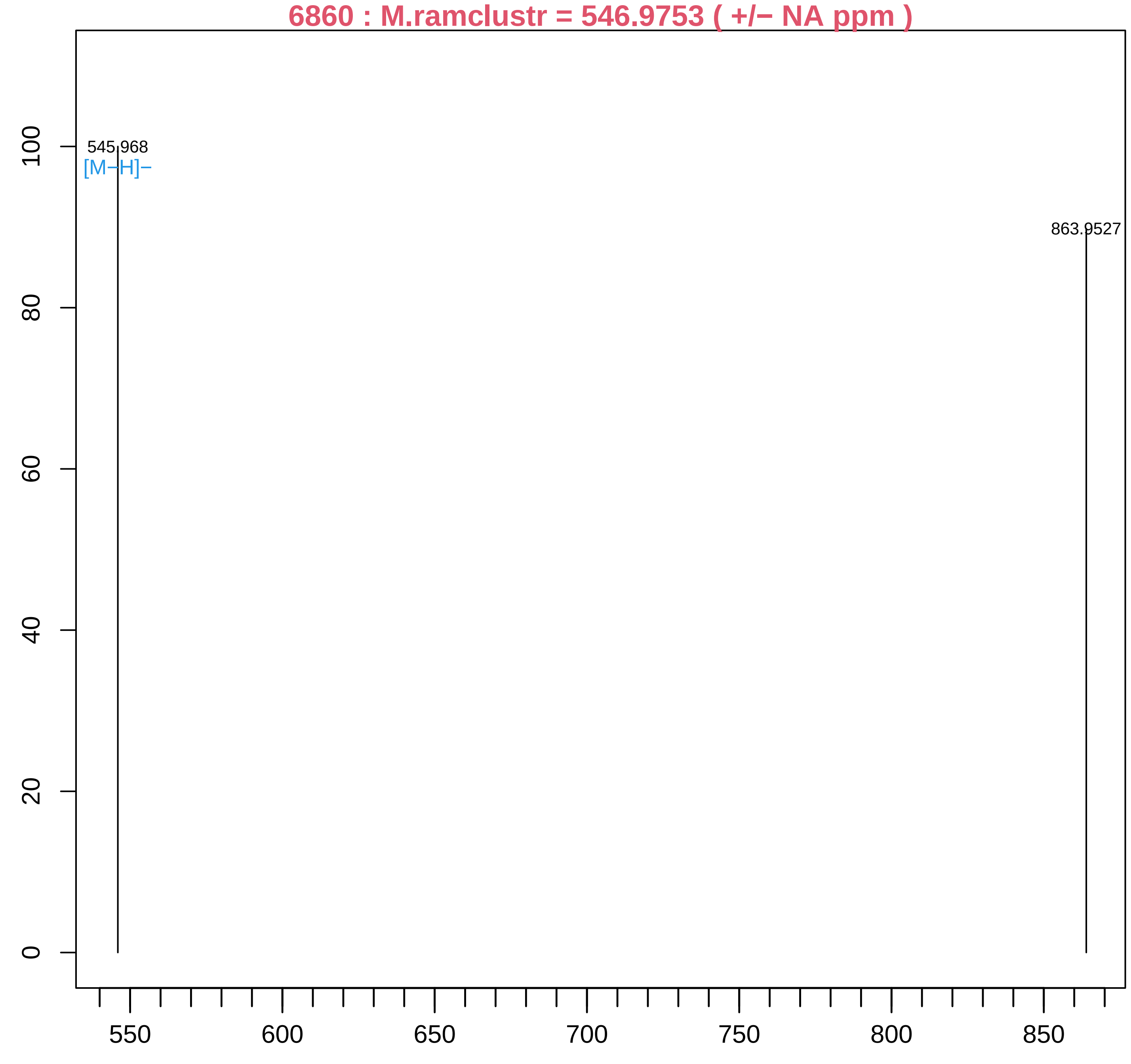
6860 : M.ramclustr = 546.9753 (+/− NA ppm)
545.968
[M−H]−
863.9527
0
20
40
60
80
100
550
600
650
700
750
800
850

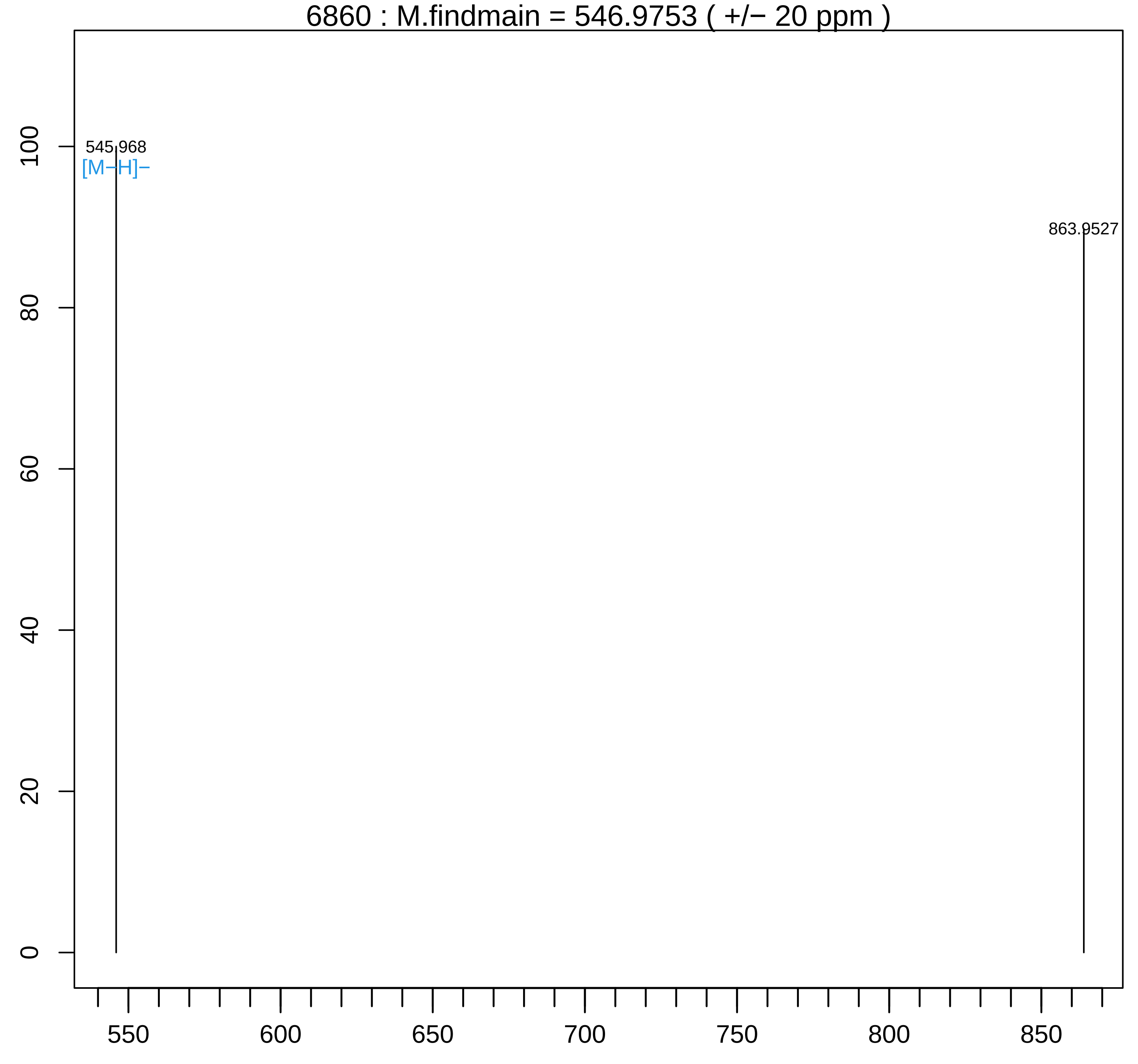
6860 : M.findmain = 546.9753 (+/− 20 ppm)
545.968
[M−H]−
863.9527
0
20
40
60
80
100
550
600
650
700
750
800
850

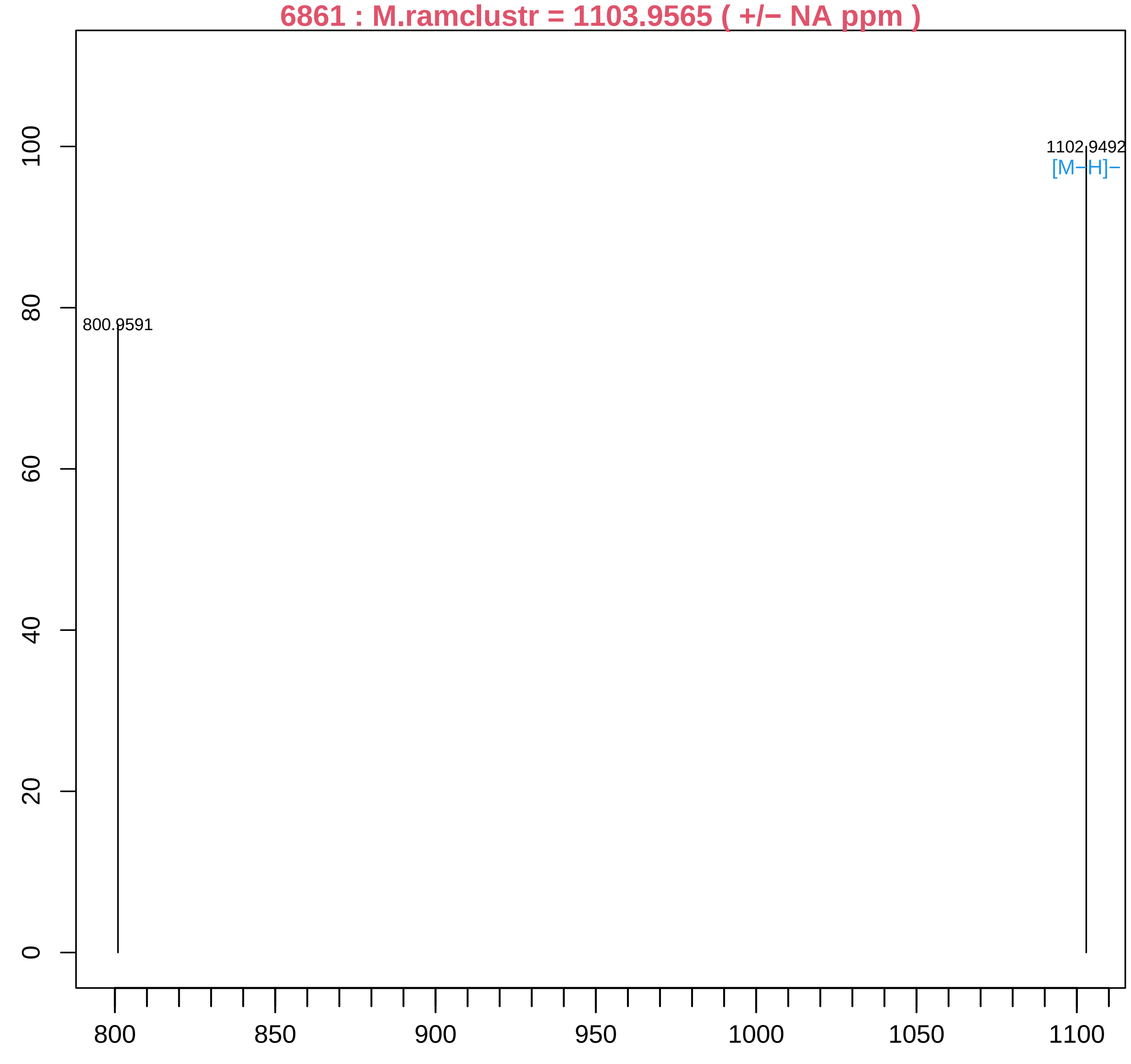
6861 : M.ramclustr = 1103.9565 (+/− NA ppm)
1102.9492
[M−H]−
800.9591
0
20
40
60
80
100
800
850
900
950
1000
1050
1100

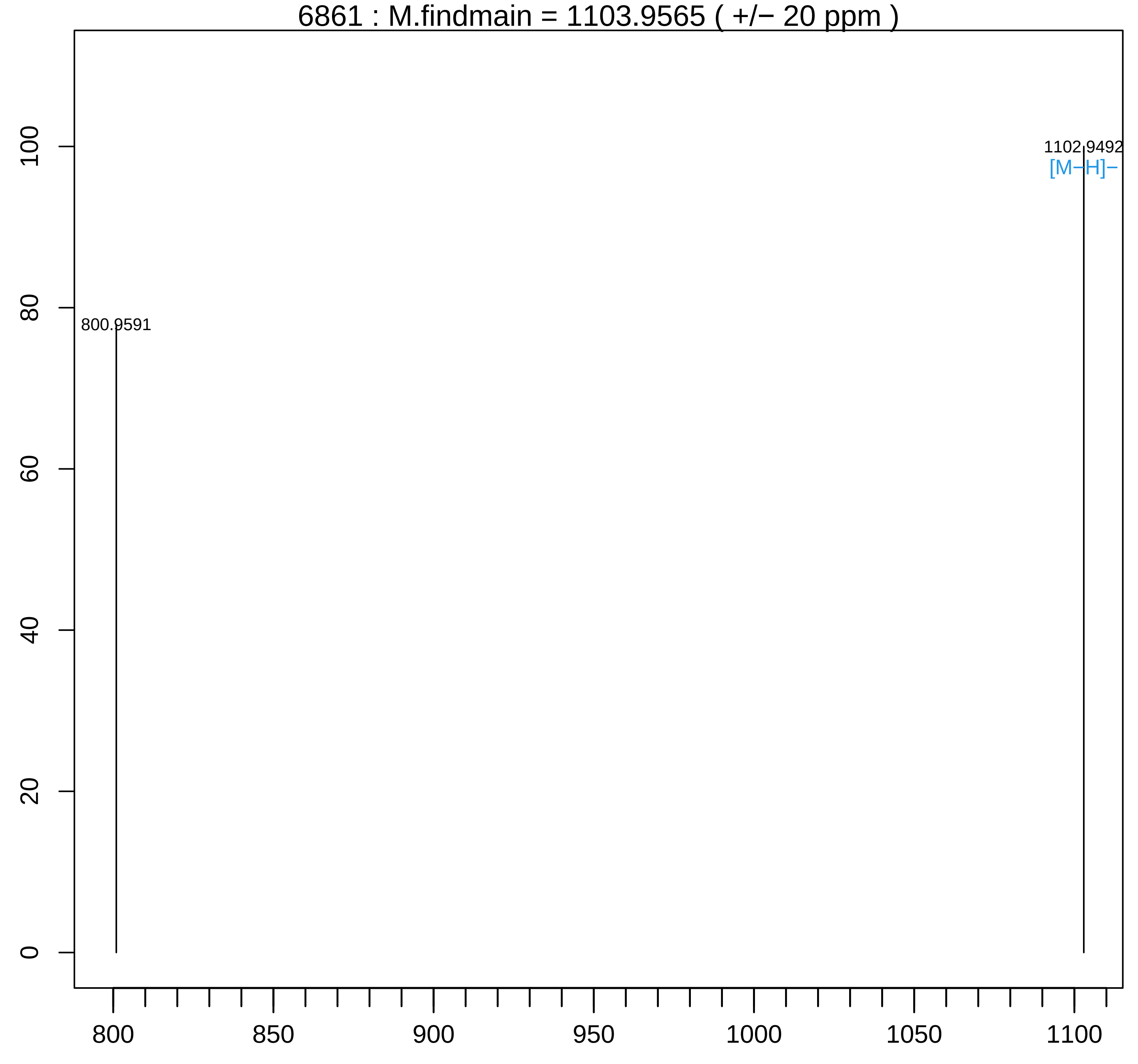
6861 : M.findmain = 1103.9565 (+/− 20 ppm)
1102.9492
[M−H]−
800.9591
0
20
40
60
80
100
800
850
900
950
1000
1050
1100

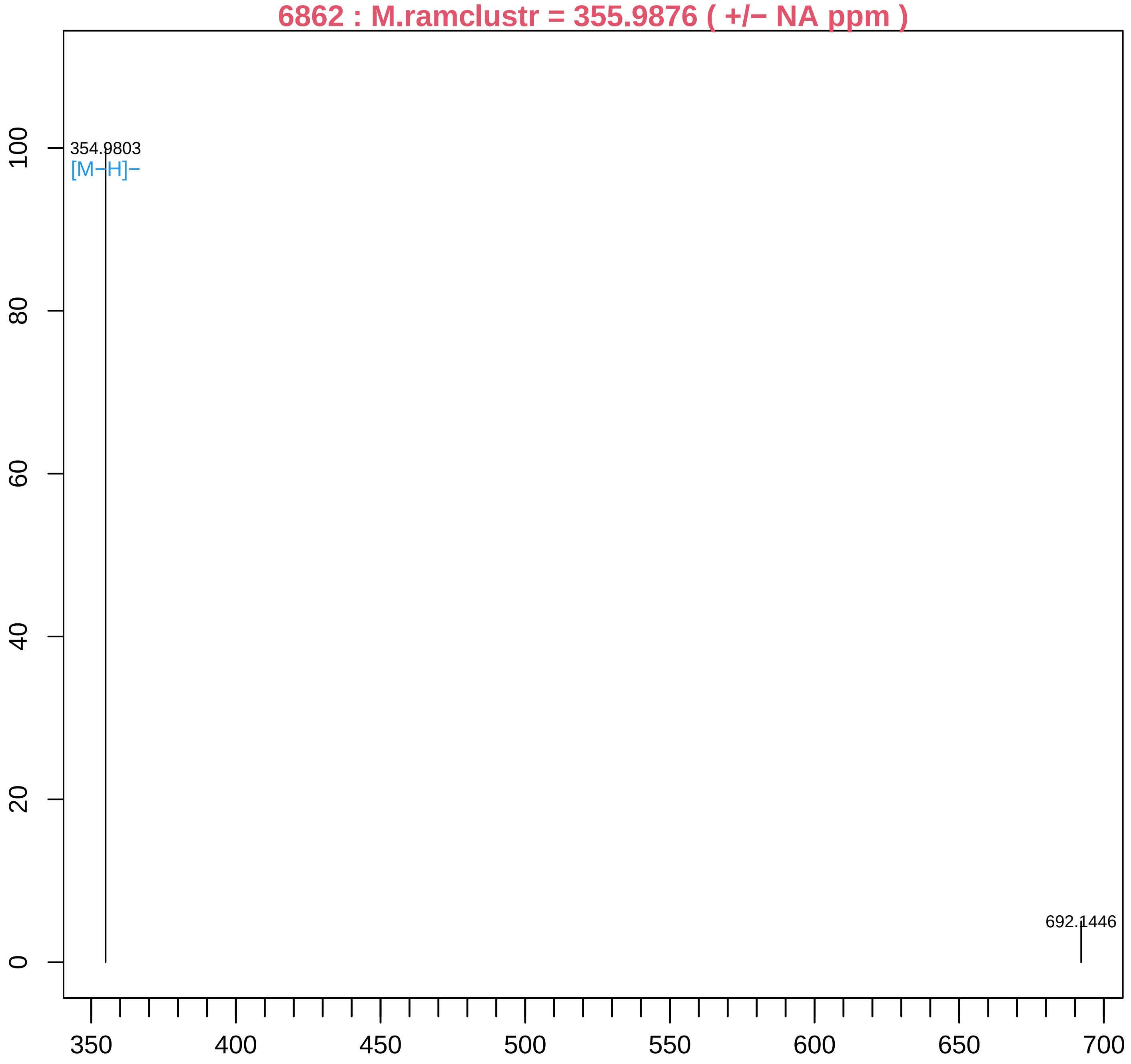
6862 : M.ramclustr = 355.9876 (+/− NA ppm)
354.9803
[M−H]−
692.1446
0
20
40
60
80
100
350
400
450
500
550
600
650
700

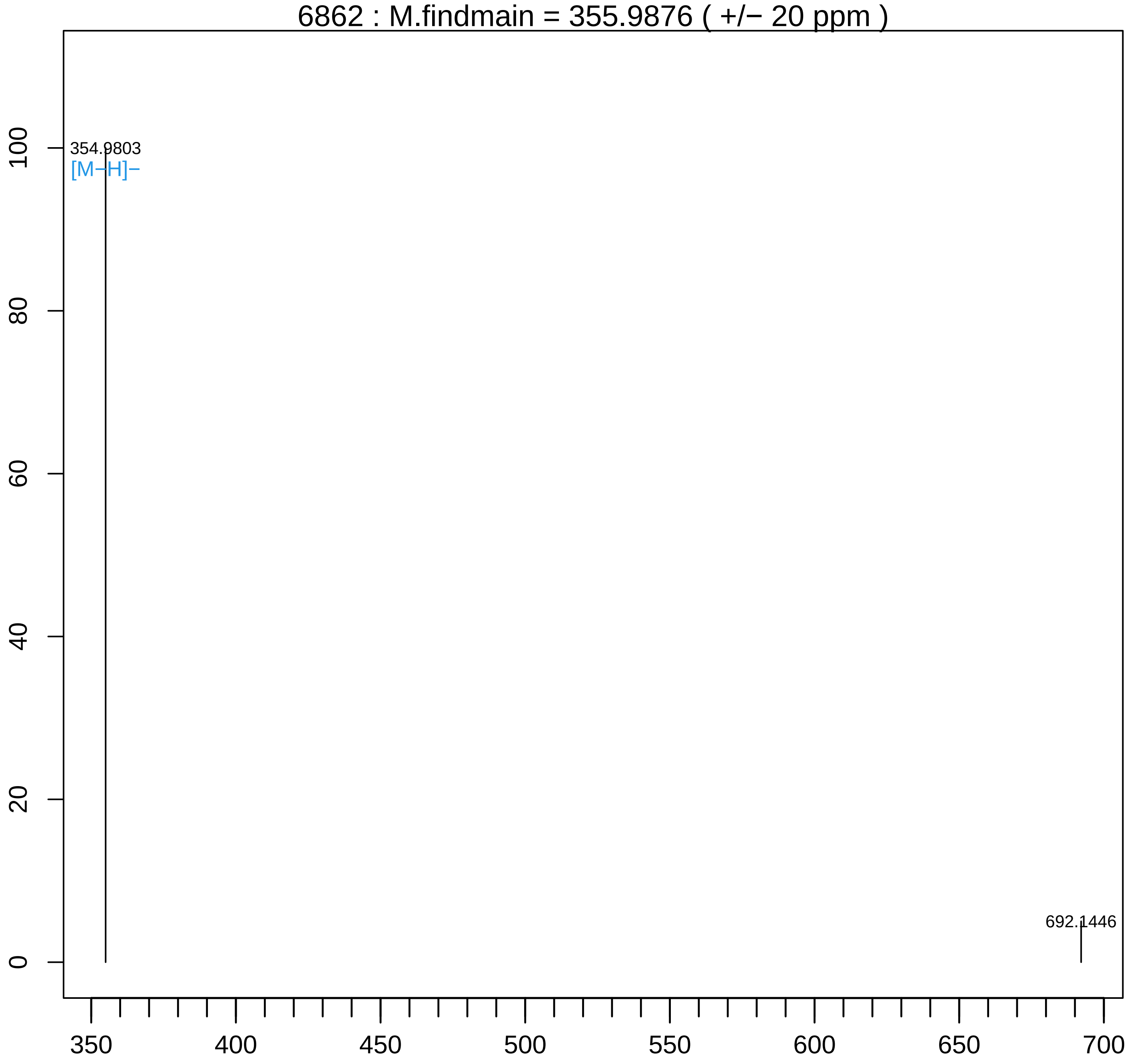
6862 : M.findmain = 355.9876 (+/− 20 ppm)
354.9803
[M−H]−
692.1446
0
20
40
60
80
100
350
400
450
500
550
600
650
700

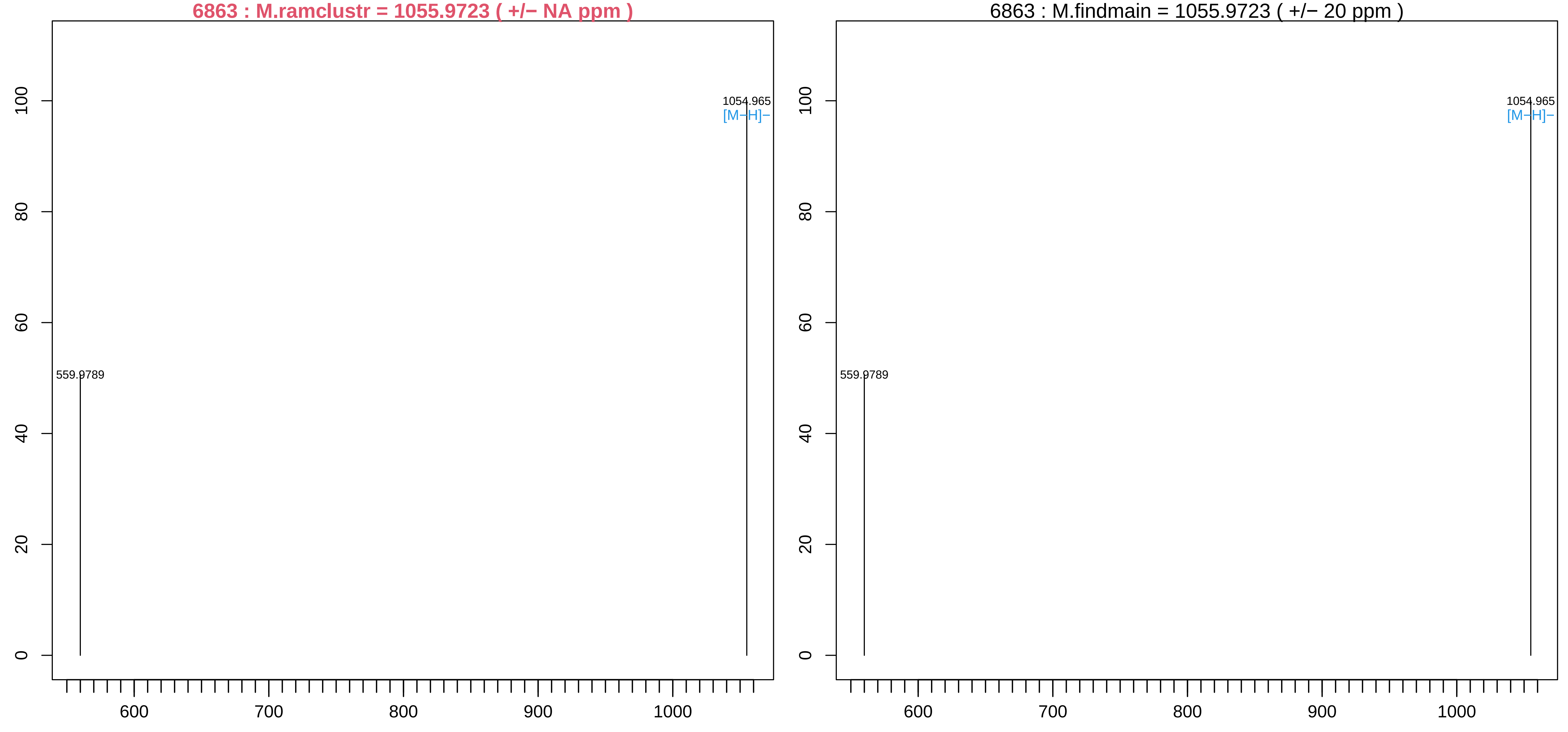

6863 : M.ramclustr = 1055.9723 (+/− NA ppm)
6863 : M.findmain = 1055.9723 (+/− 20 ppm)
1054.965
[M−H]−
559.9789
1054.965
[M−H]−
559.9789

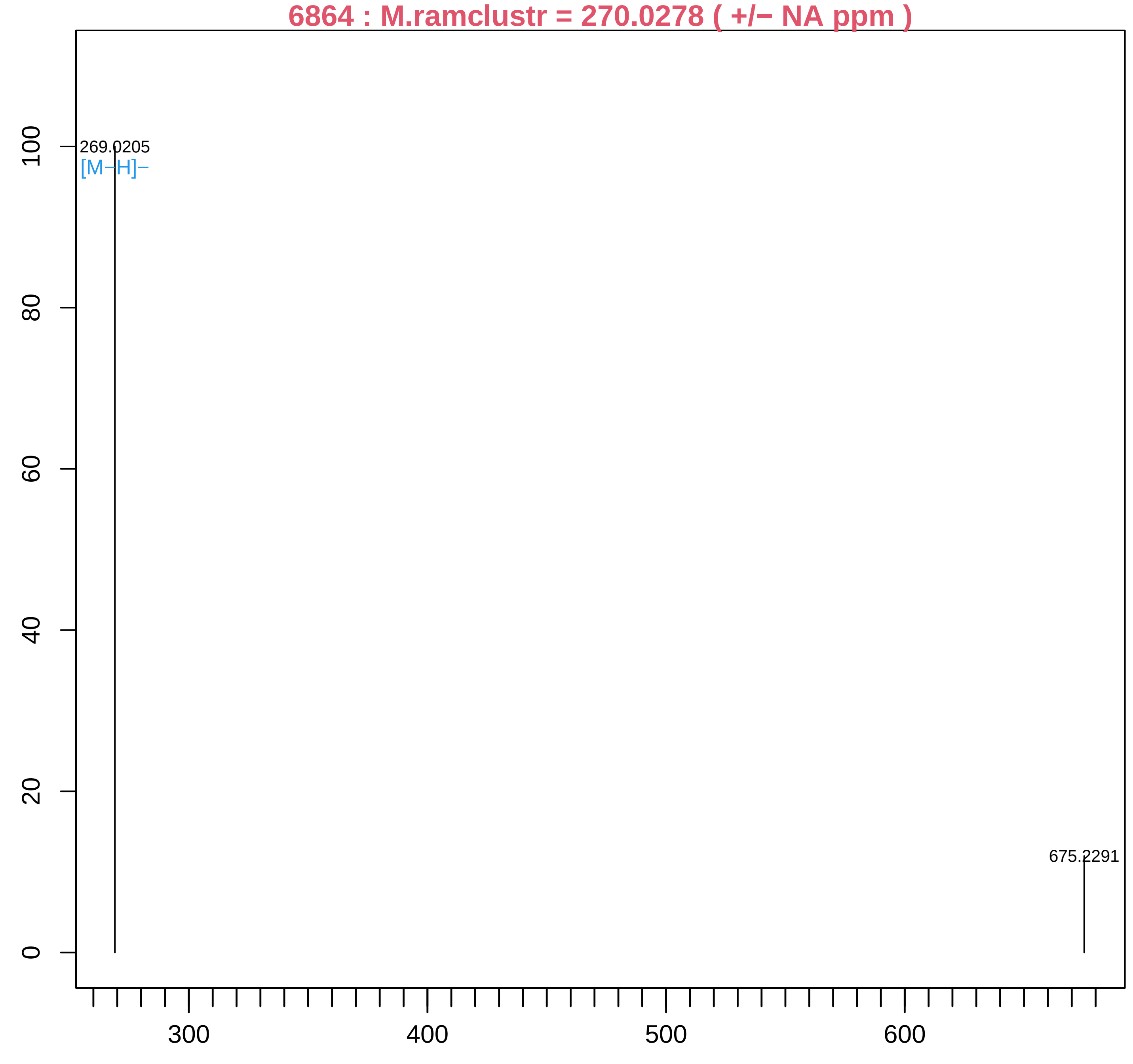
6864 : M.ramclustr = 270.0278 (+/− NA ppm)
269.0205
[M−H]−
675.2291
0
20
40
60
80
100
300
400
500
600

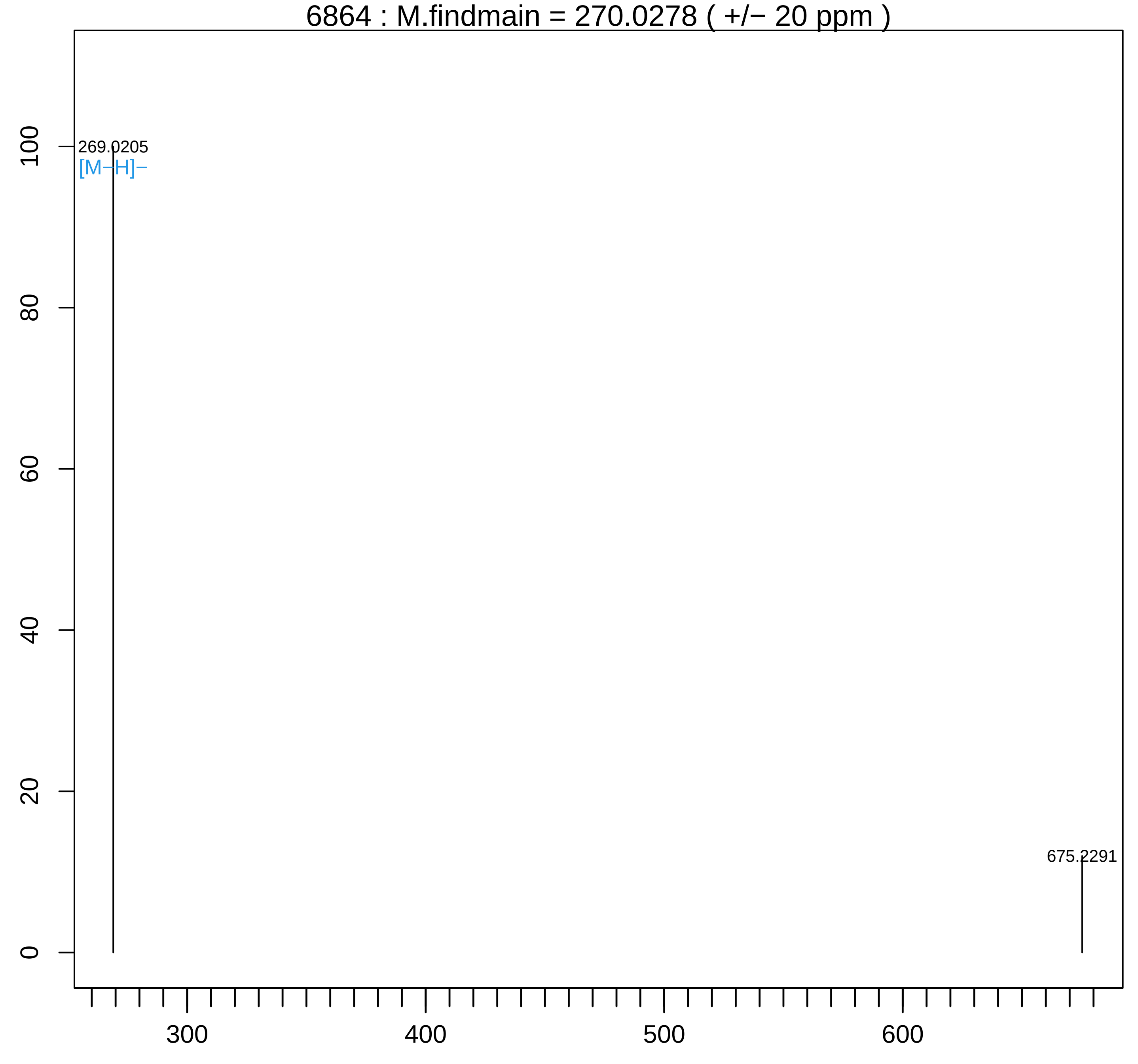
6864 : M.findmain = 270.0278 (+/− 20 ppm)
269.0205
[M−H]−
675.2291
0
20
40
60
80
100
300
400
500
600

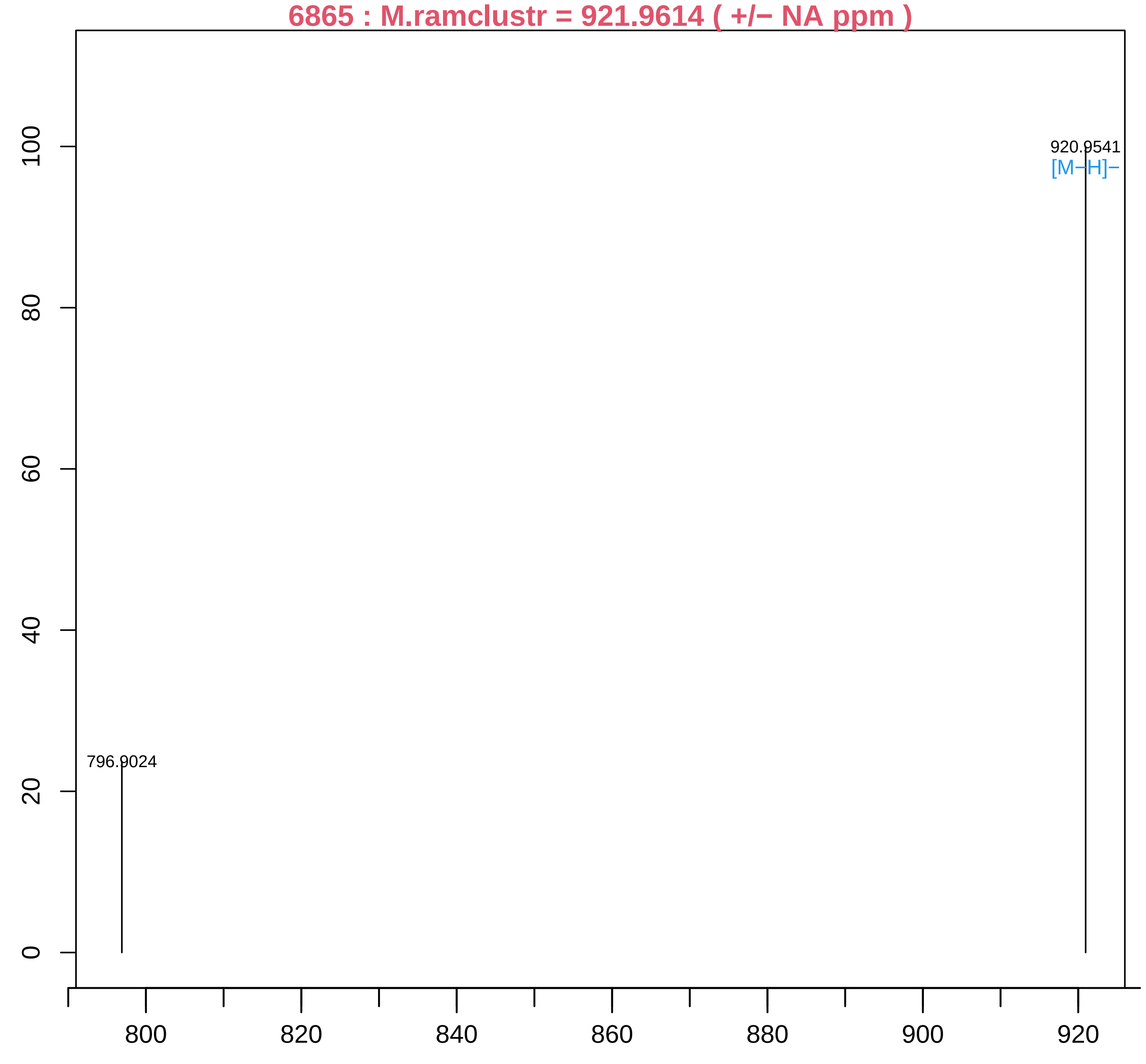
6865 : M.ramclustr = 921.9614 (+/− NA ppm)
920.9541
[M−H]−
796.9024
0
20
40
60
80
100
800
820
840
860
880
900
920

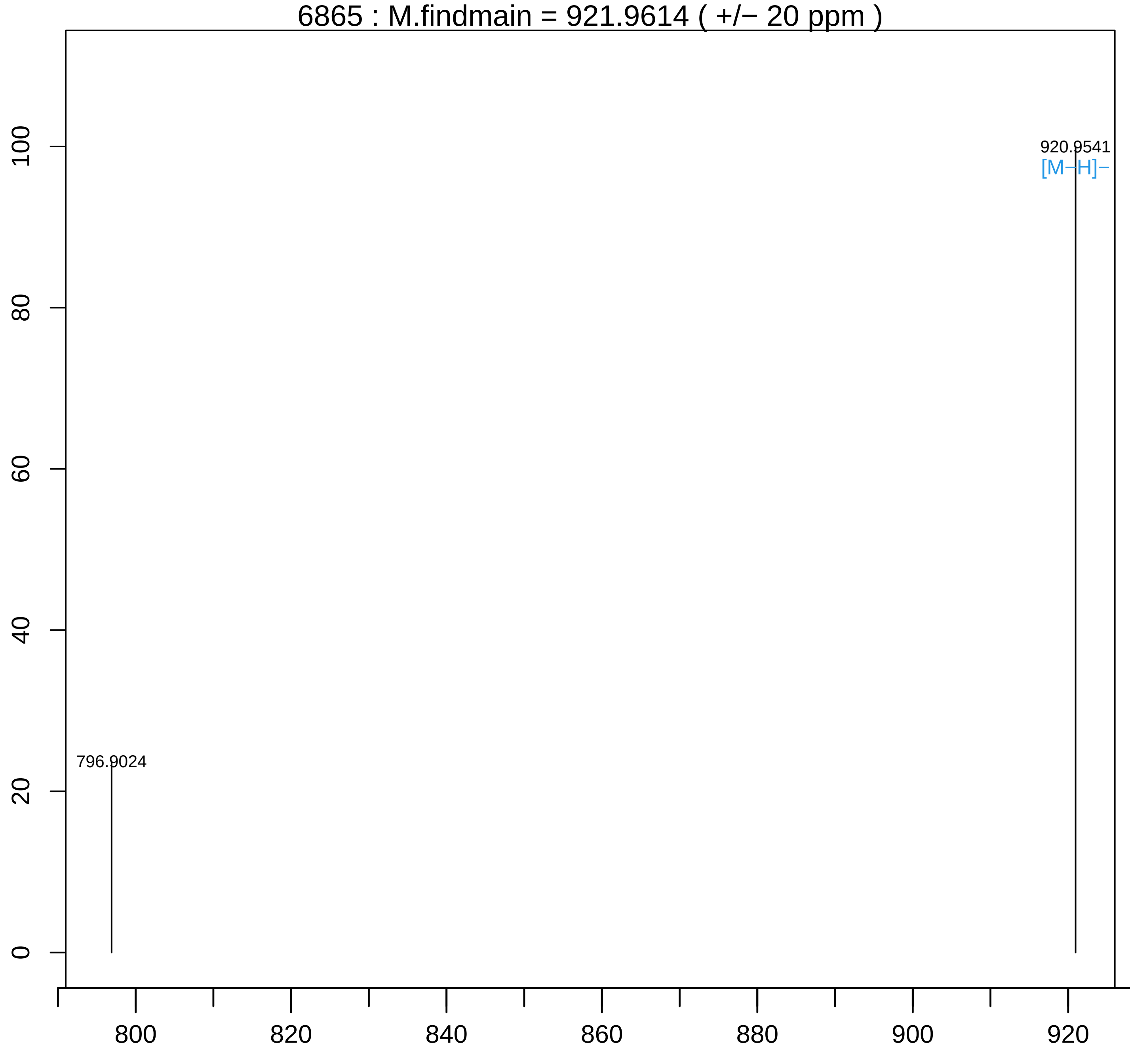
6865 : M.findmain = 921.9614 (+/− 20 ppm)
920.9541
[M−H]−
796.9024
0
20
40
60
80
100
800
820
840
860
880
900
920

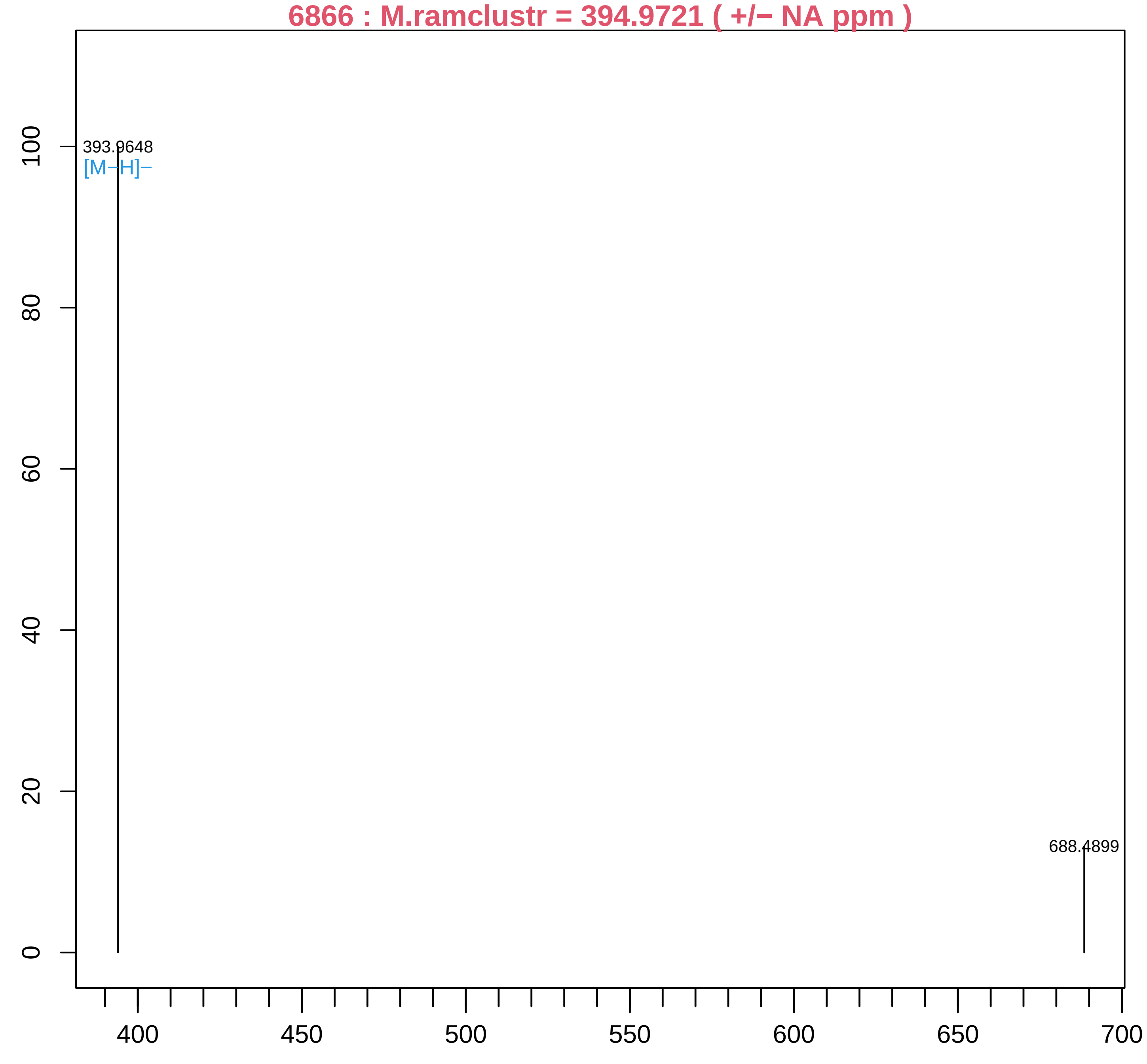
6866 : M.ramclustr = 394.9721 (+/− NA ppm)
393.9648
[M−H]−
688.4899
0
20
40
60
80
100
400
450
500
550
600
650
700

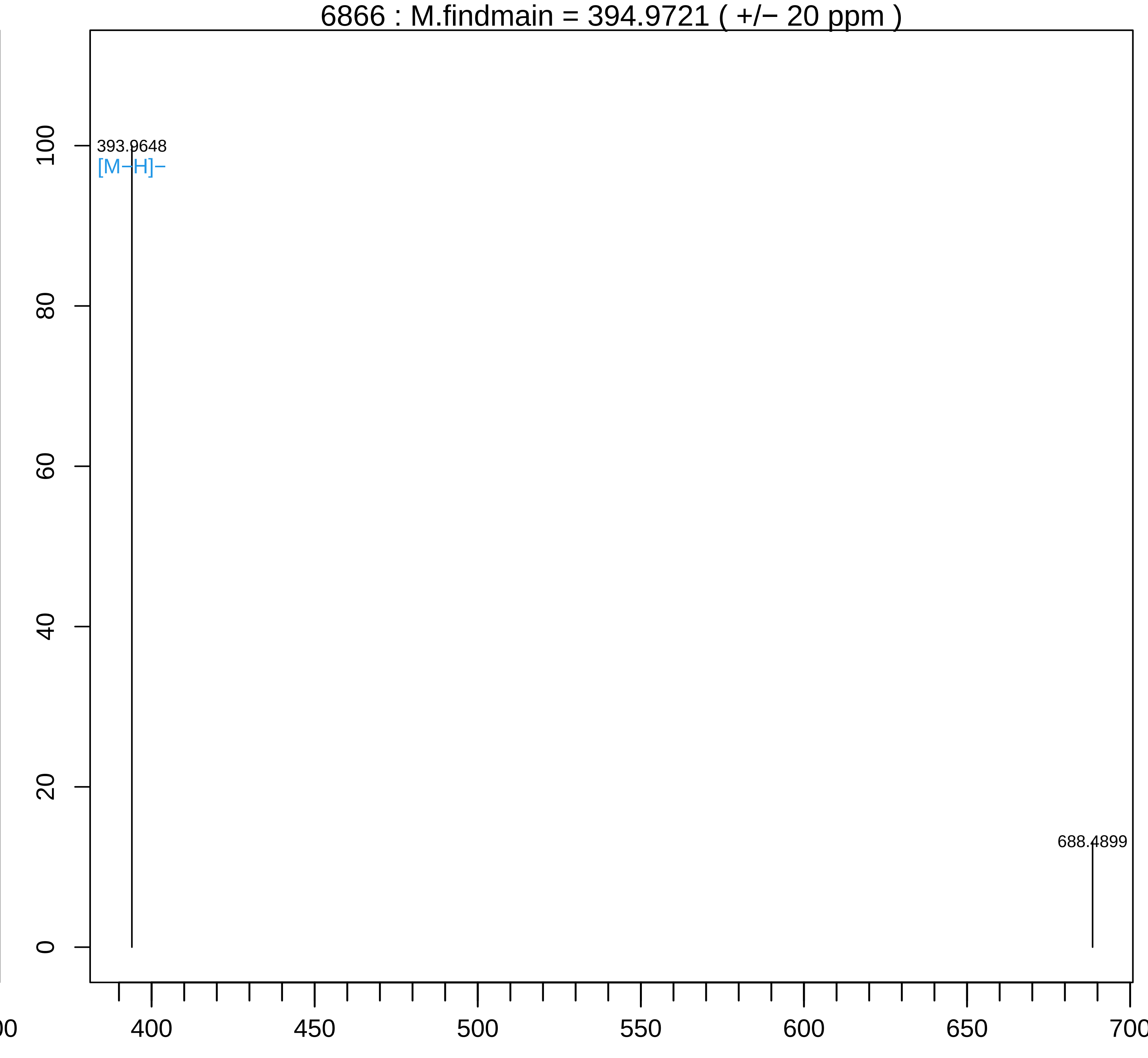
6866 : M.findmain = 394.9721 (+/− 20 ppm)
393.9648
[M−H]−
688.4899
0
20
40
60
80
100
400
450
500
550
600
650
700

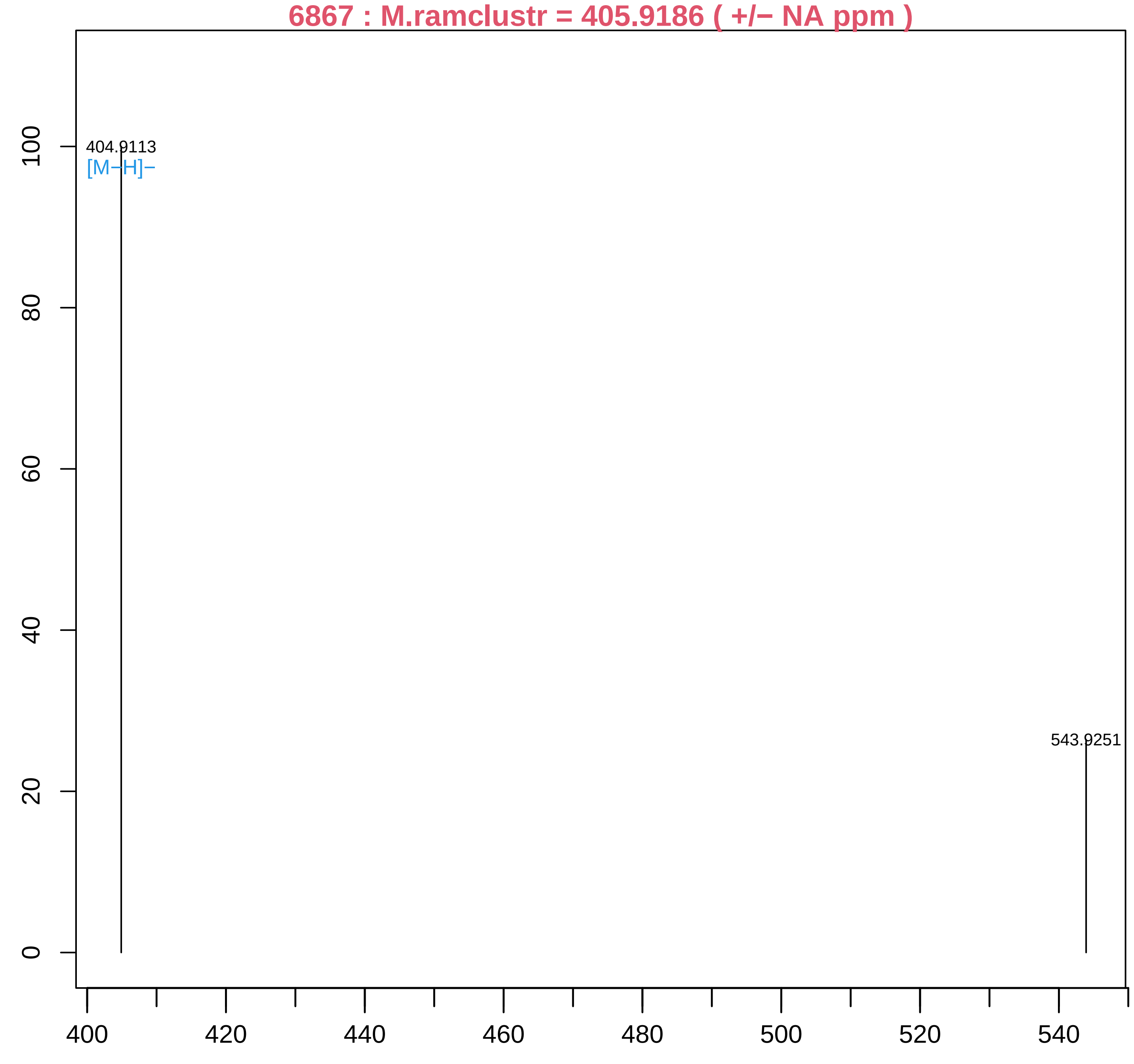
6867 : M.ramclustr = 405.9186 (+/− NA ppm)
404.9113
[M−H]−
543.9251
0
20
40
60
80
100
400
420
440
460
480
500
520
540

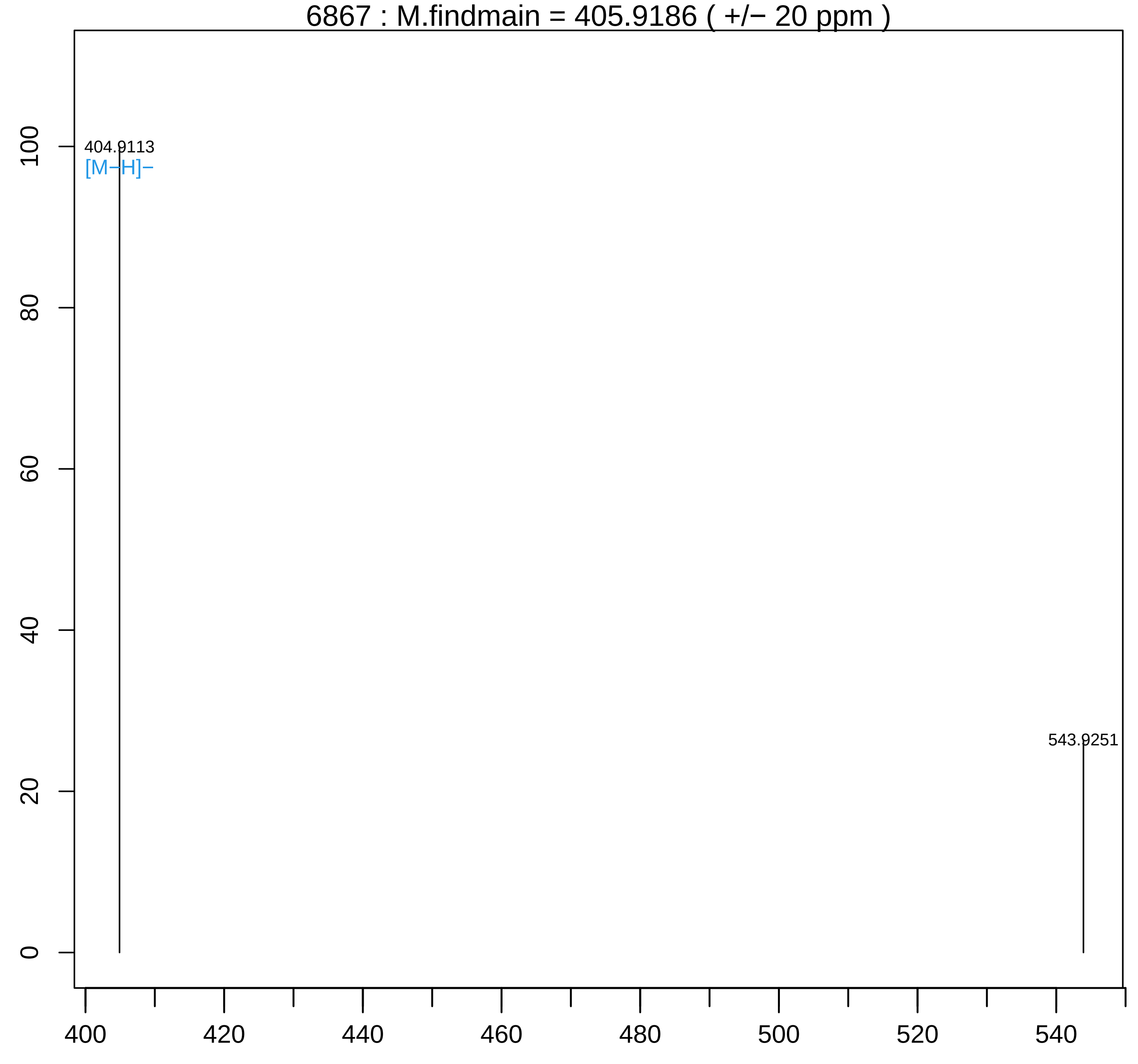
6867 : M.findmain = 405.9186 (+/− 20 ppm)
404.9113
[M−H]−
543.9251
0
20
40
60
80
100
400
420
440
460
480
500
520
540

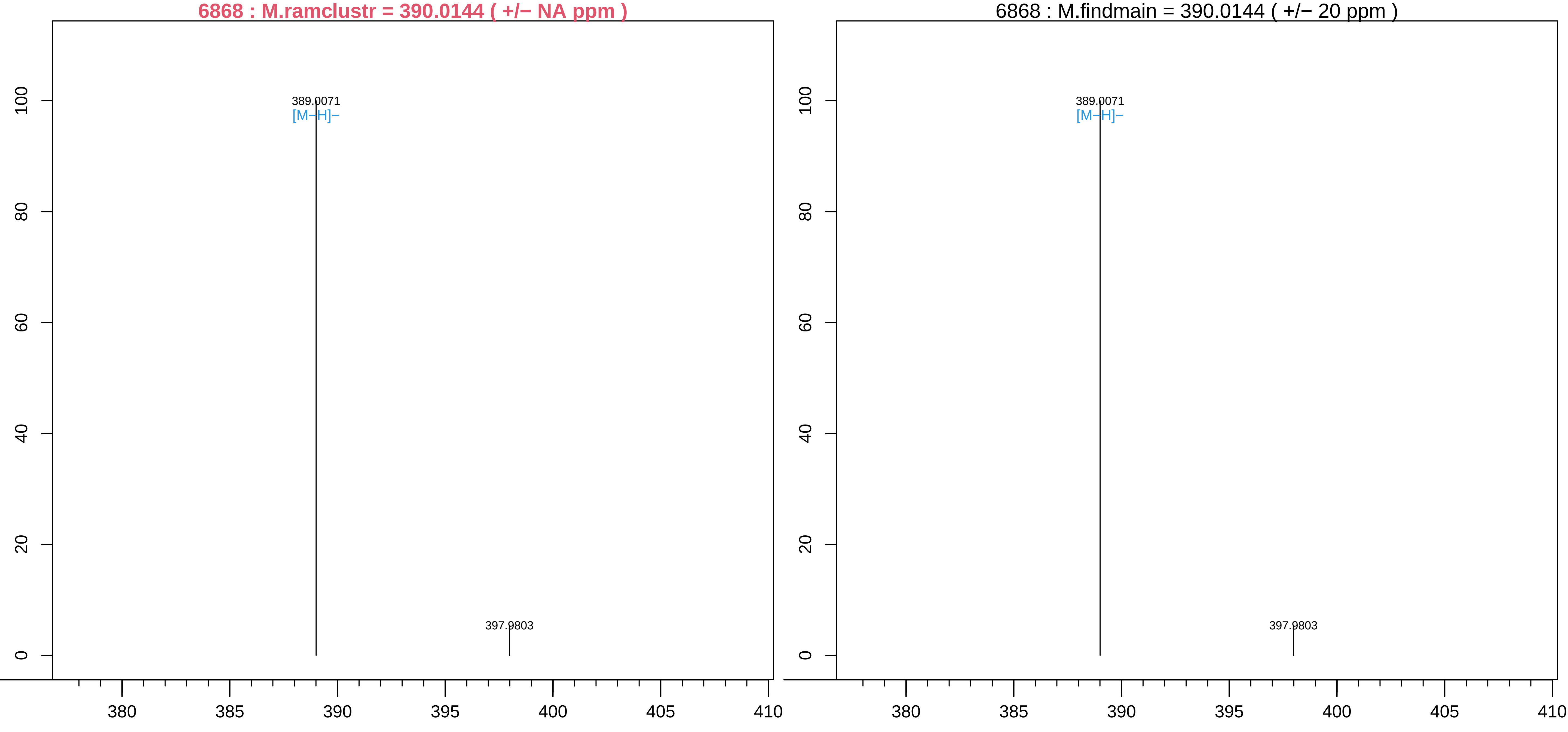

6868 : M.ramclustr = 390.0144 (+/− NA ppm)
389.0071
[M−H]−
397.9803
6868 : M.findmain = 390.0144 (+/− 20 ppm)
389.0071
[M−H]−
397.9803
0
20
40
60
80
100
380
385
390
395
400
405
410

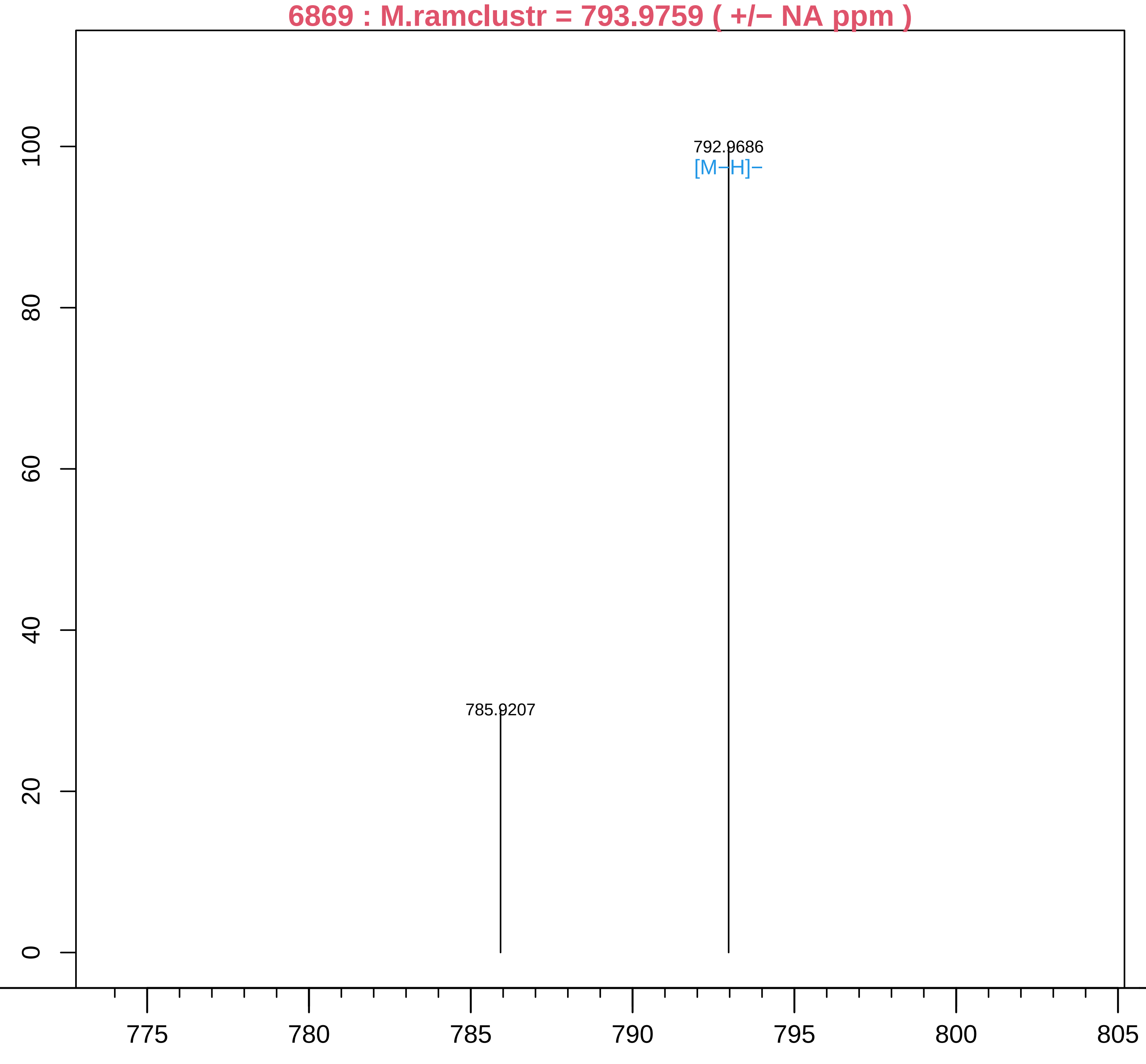

6869 : M.ramclustr = 793.9759 (+/− NA ppm)
792.9686
[M−H]−
785.9207
0
20
40
60
80
100
775
780
785
790
795
800
805

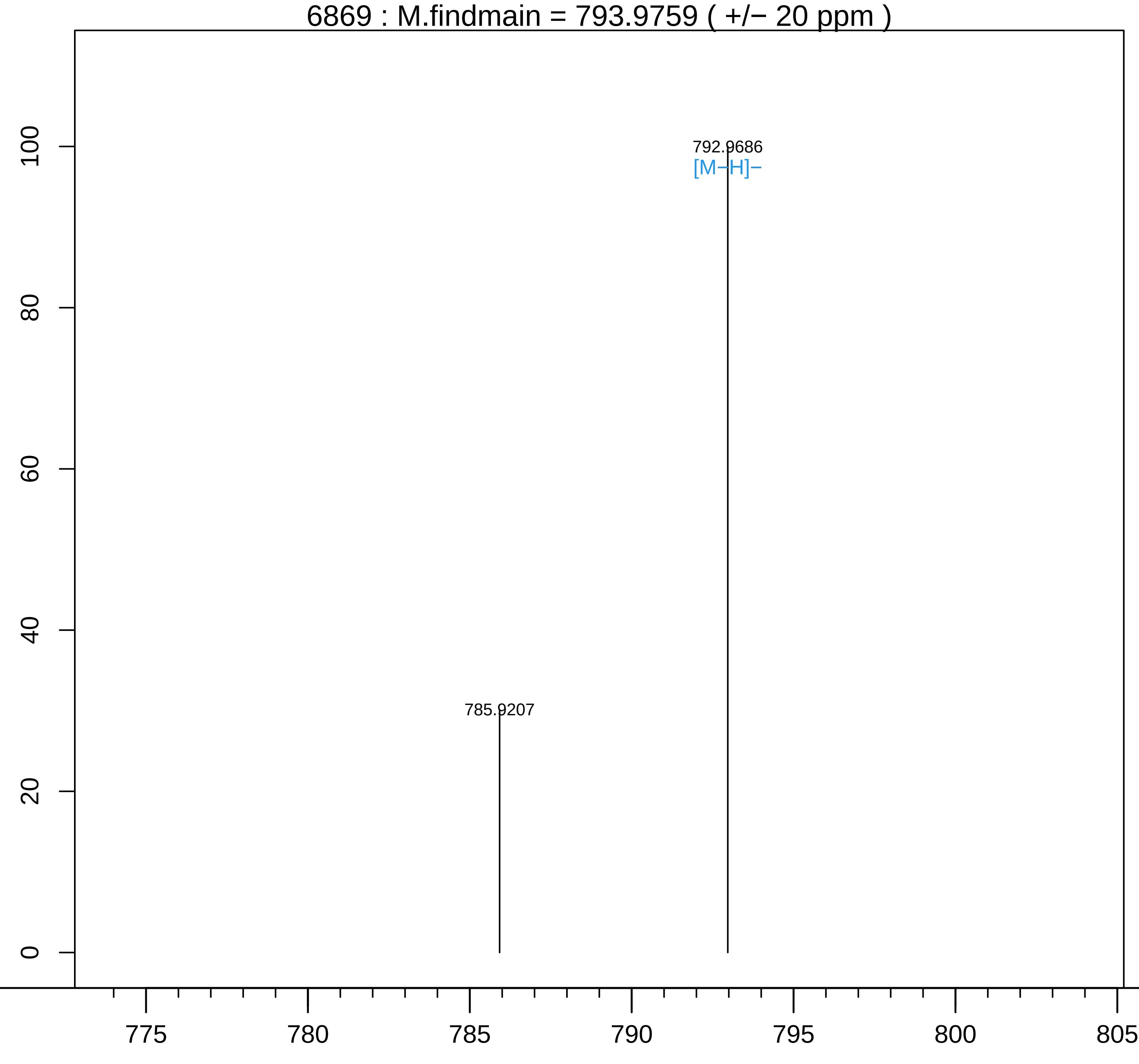

6869 : M.findmain = 793.9759 (+/− 20 ppm)
792.9686
[M−H]−
785.9207
0
20
40
60
80
100
775
780
785
790
795
800
805

6870 : M.ramclustr = 256.0034 (+/− NA ppm)

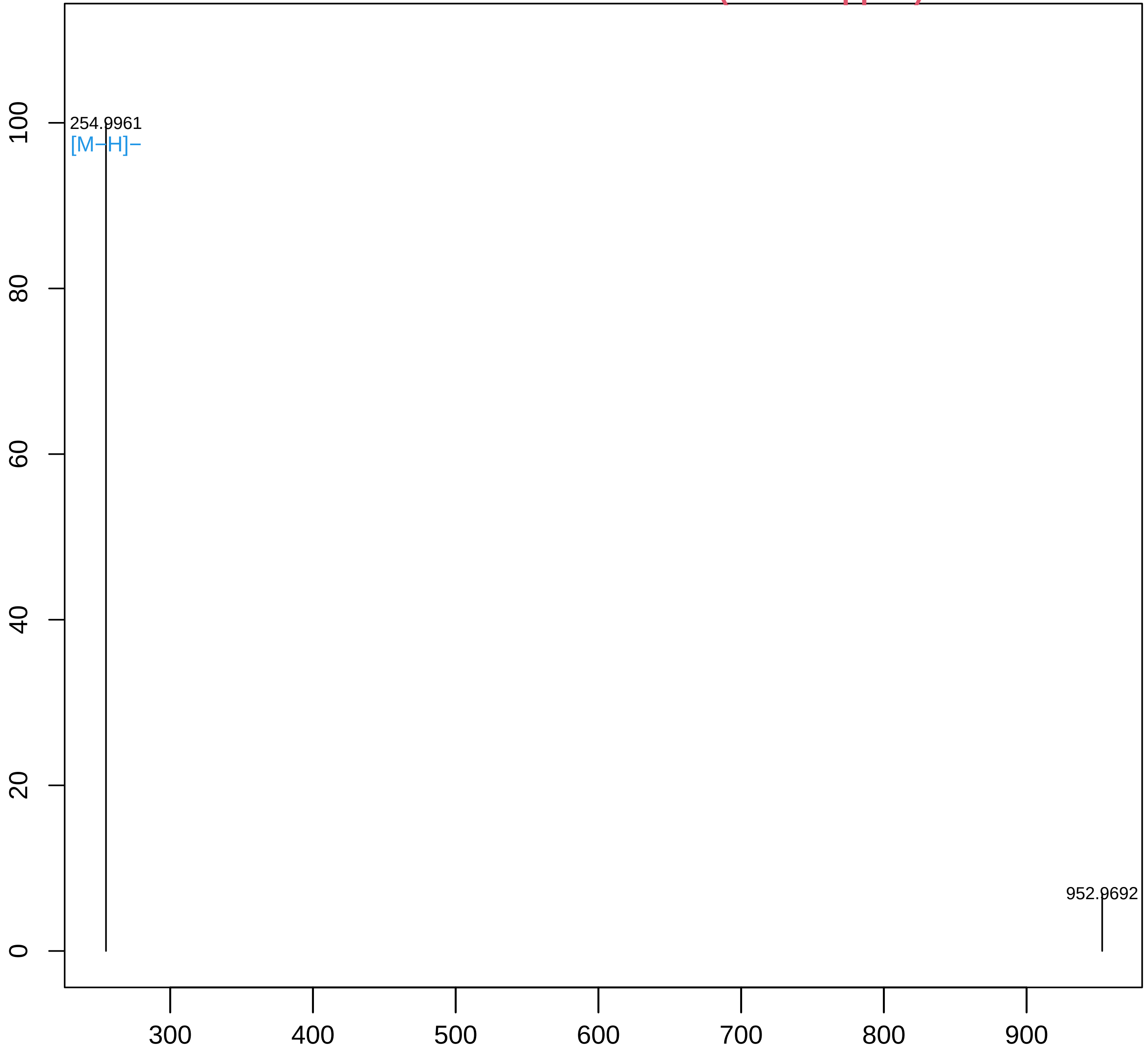

6870 : M.findmain = 256.0034 (+/− 20 ppm)

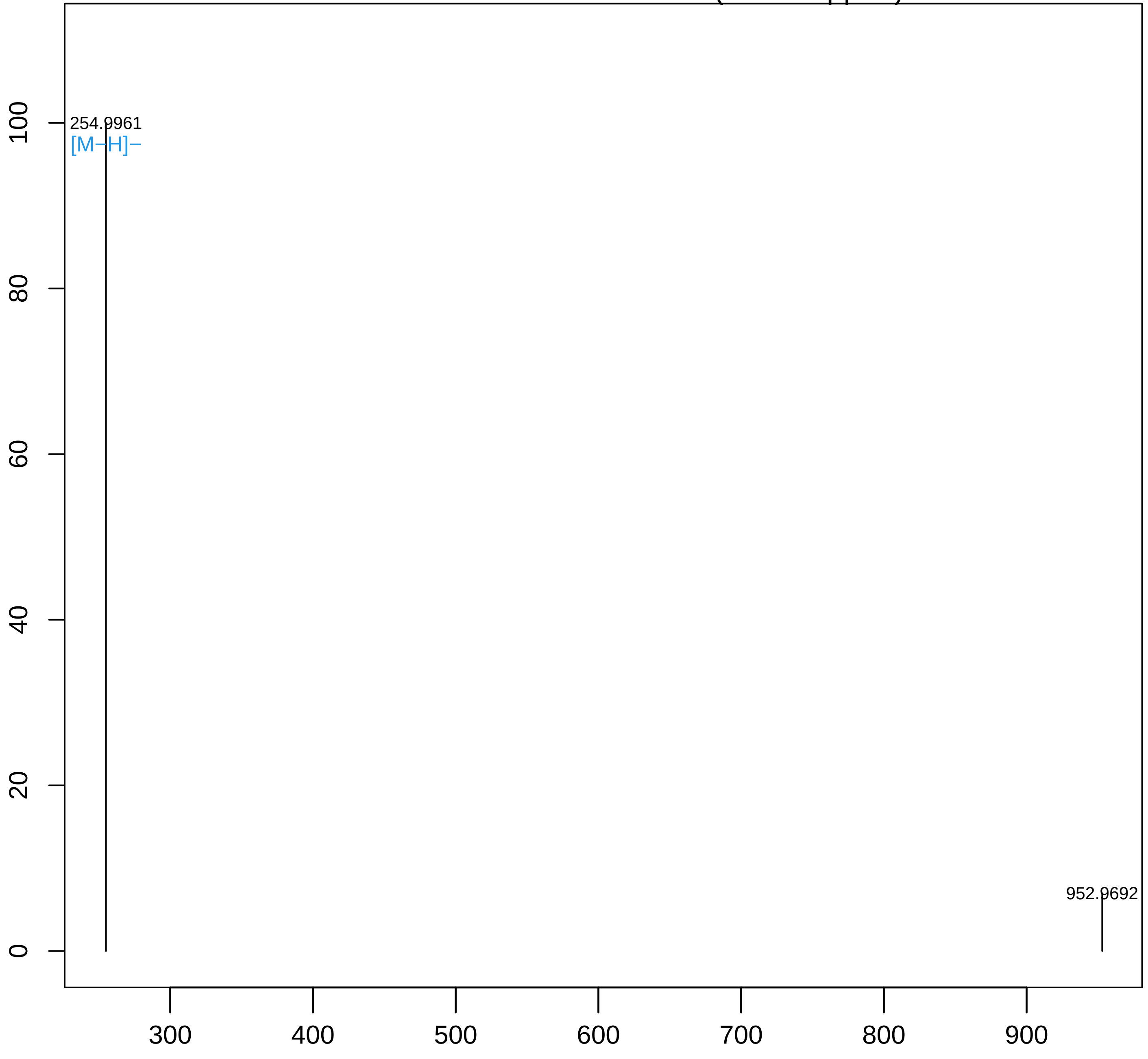

6871 : M.ramclustr = 175.9848 (+/− NA ppm)

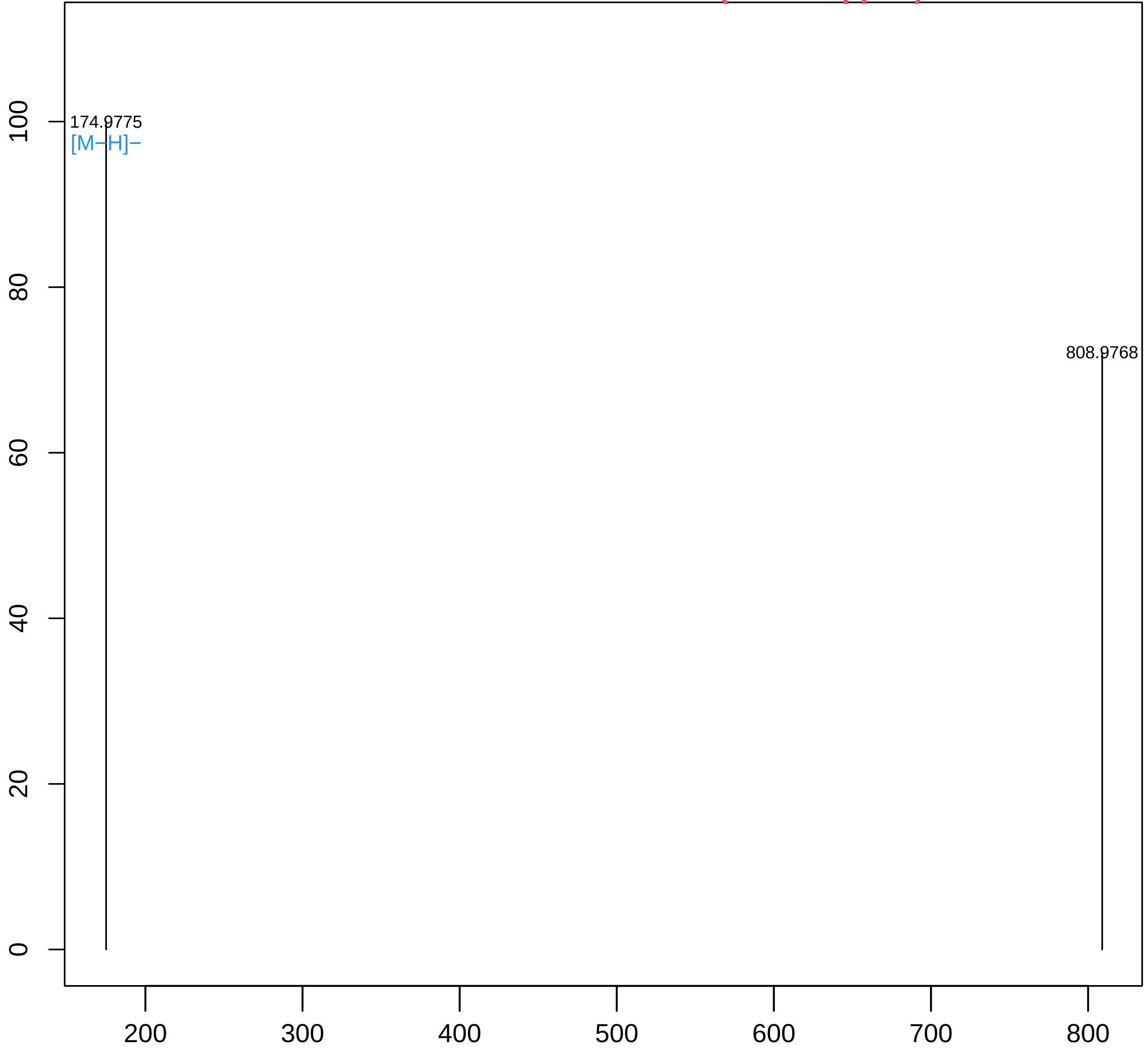

6871 : M.findmain = 175.9848 (+/− 20 ppm)

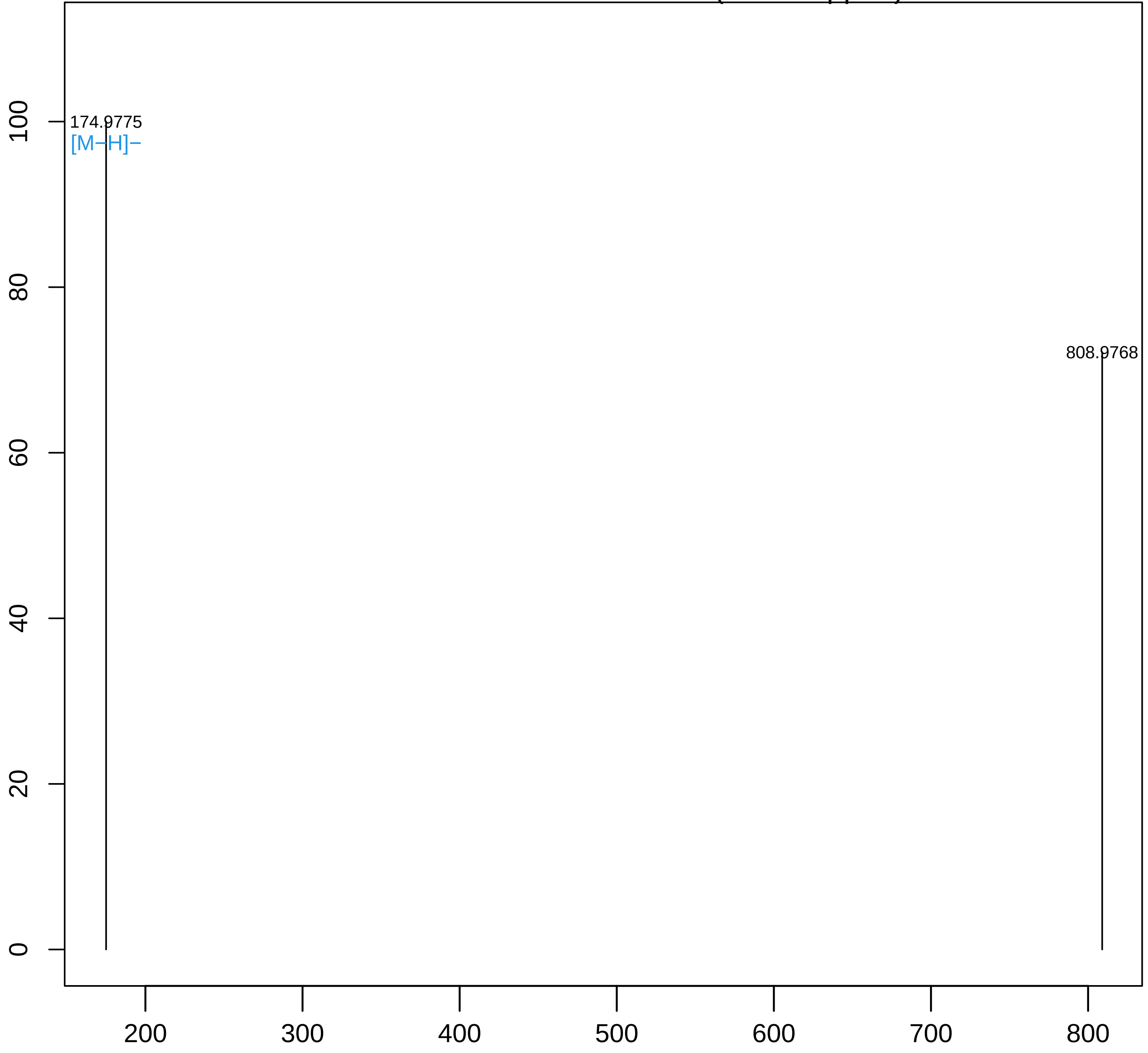

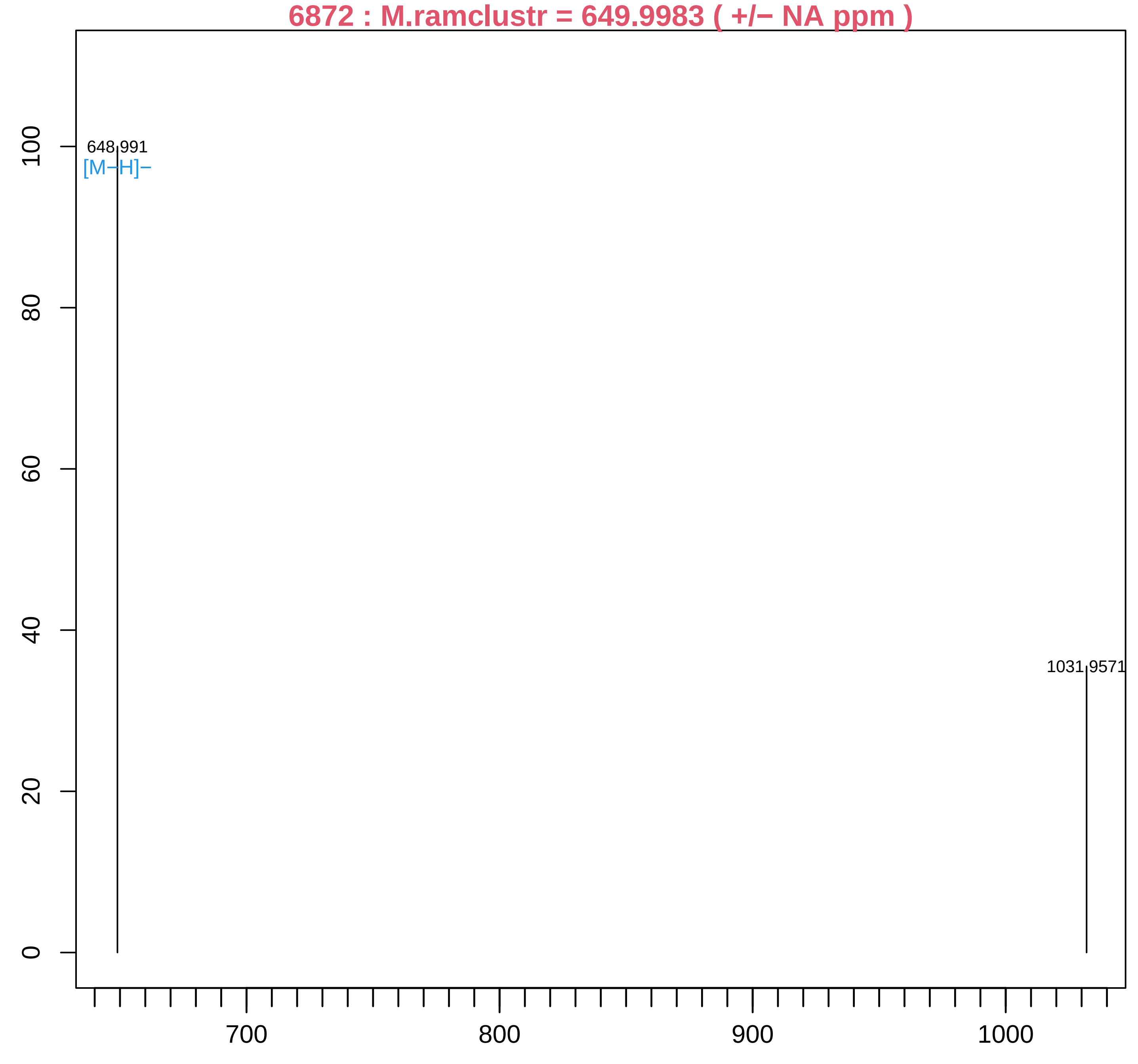
6872 : M.ramclustr = 649.9983 (+/− NA ppm)
648.991
[M−H]−
1031.9571
0
20
40
60
80
100
700
800
900
1000

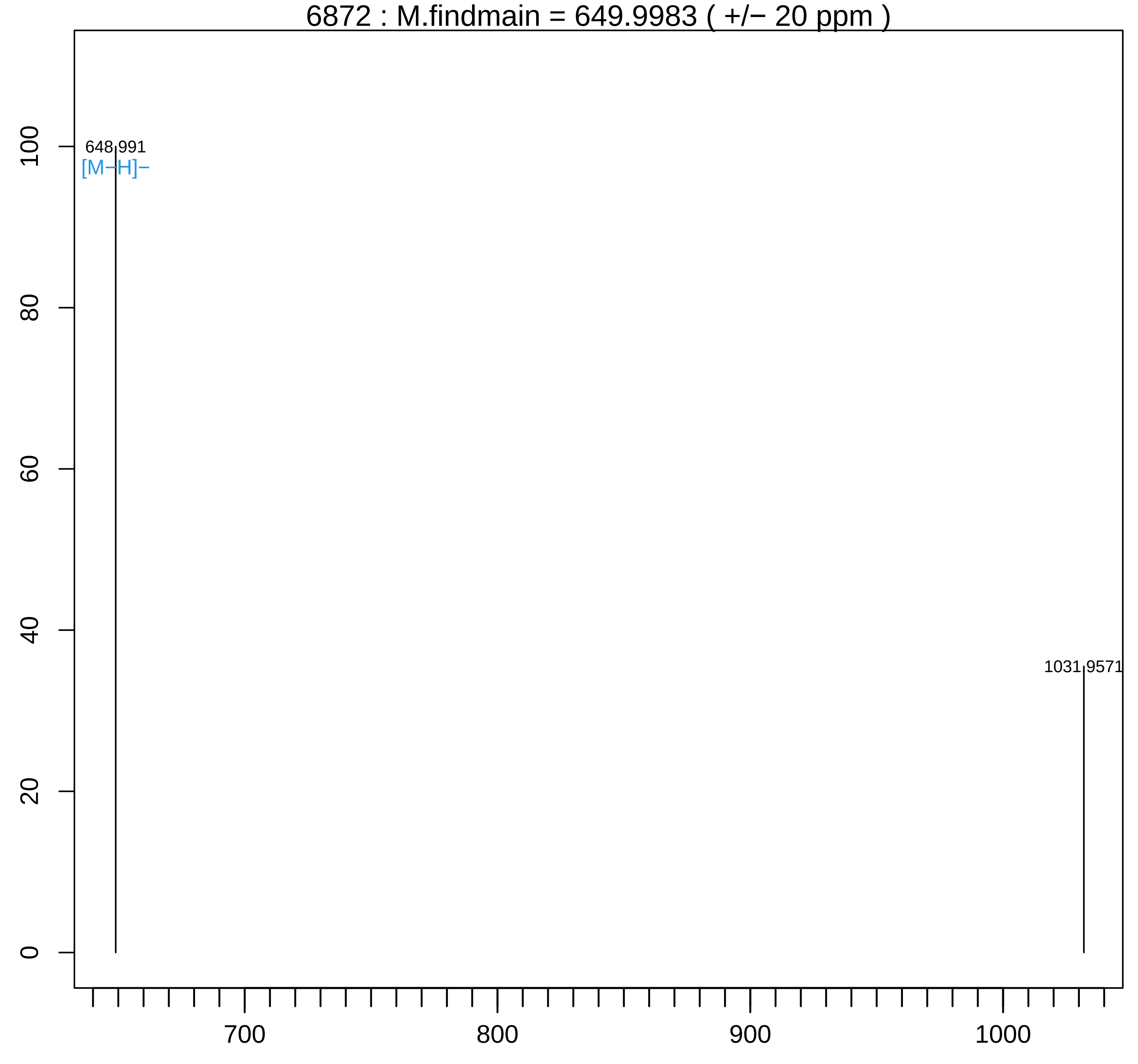
6872 : M.findmain = 649.9983 (+/− 20 ppm)
648.991
[M−H]−
1031.9571
0
20
40
60
80
100
700
800
900
1000

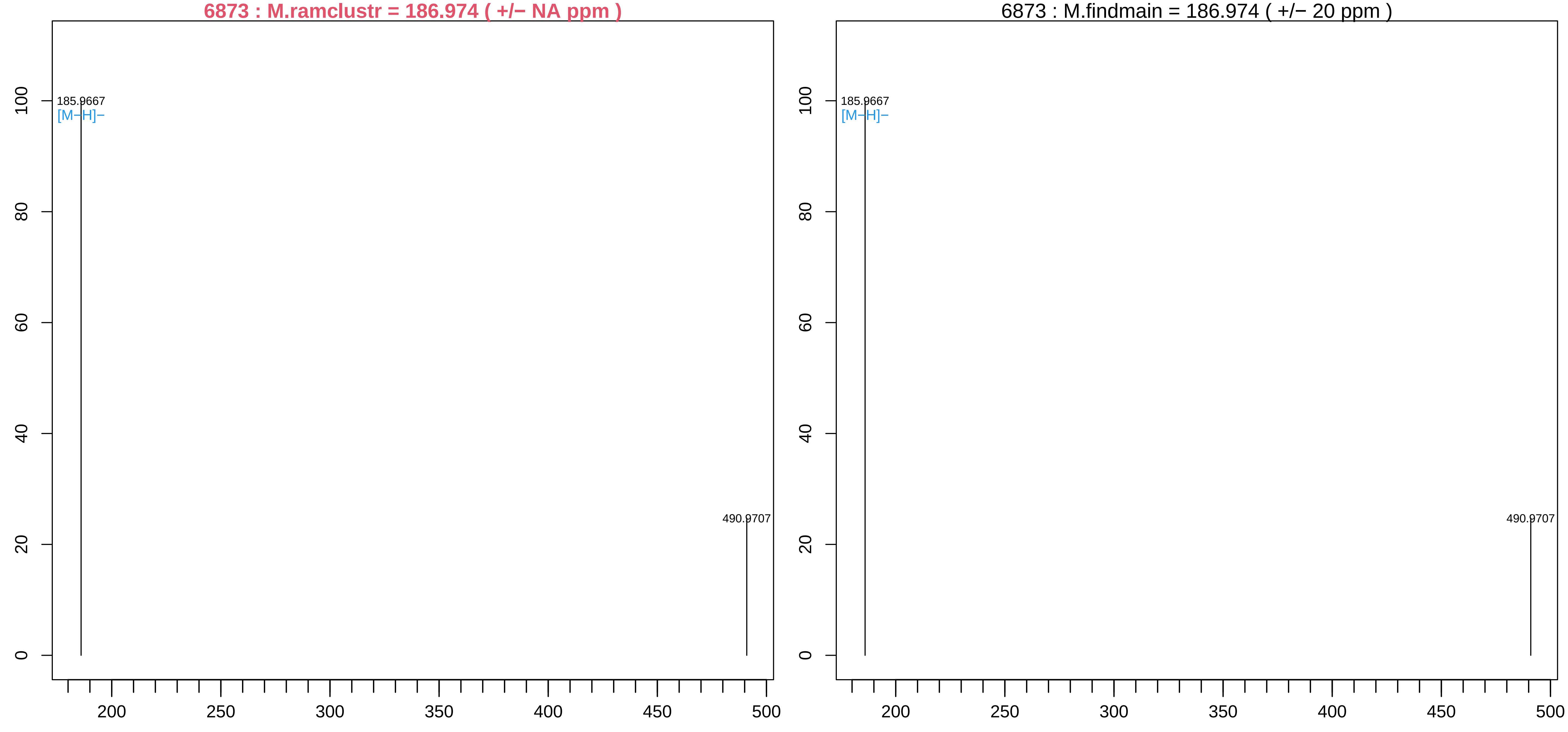
6873 : M.ramclustr = 186.974 (+/− NA ppm)
185.9667
[M−H]−
490.9707
6873 : M.findmain = 186.974 (+/− 20 ppm)
185.9667
[M−H]−
490.9707

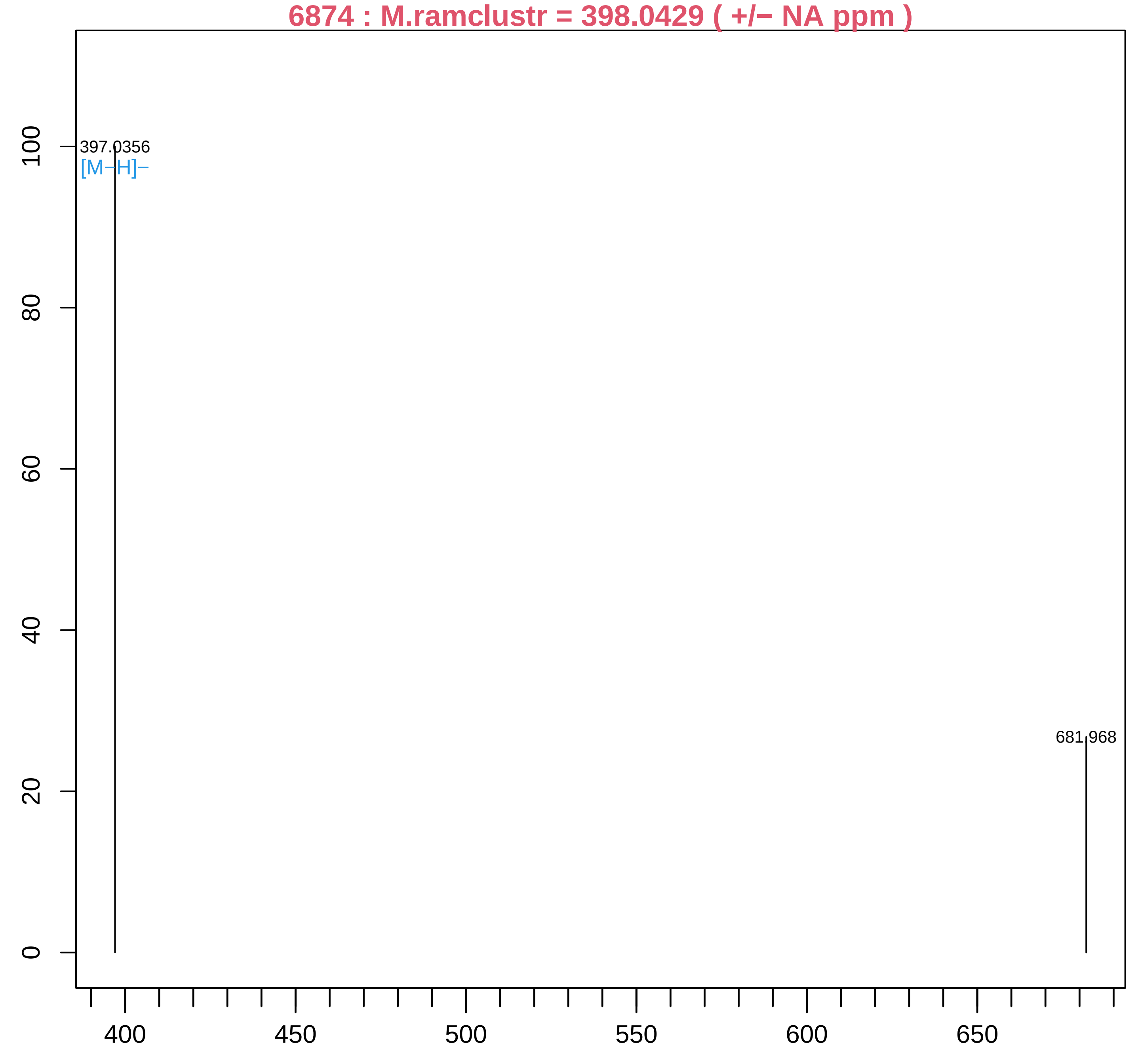
6874 : M.ramclustr = 398.0429 (+/− NA ppm)
397.0356
[M−H]−
681.968
0
20
40
60
80
100
400
450
500
550
600
650

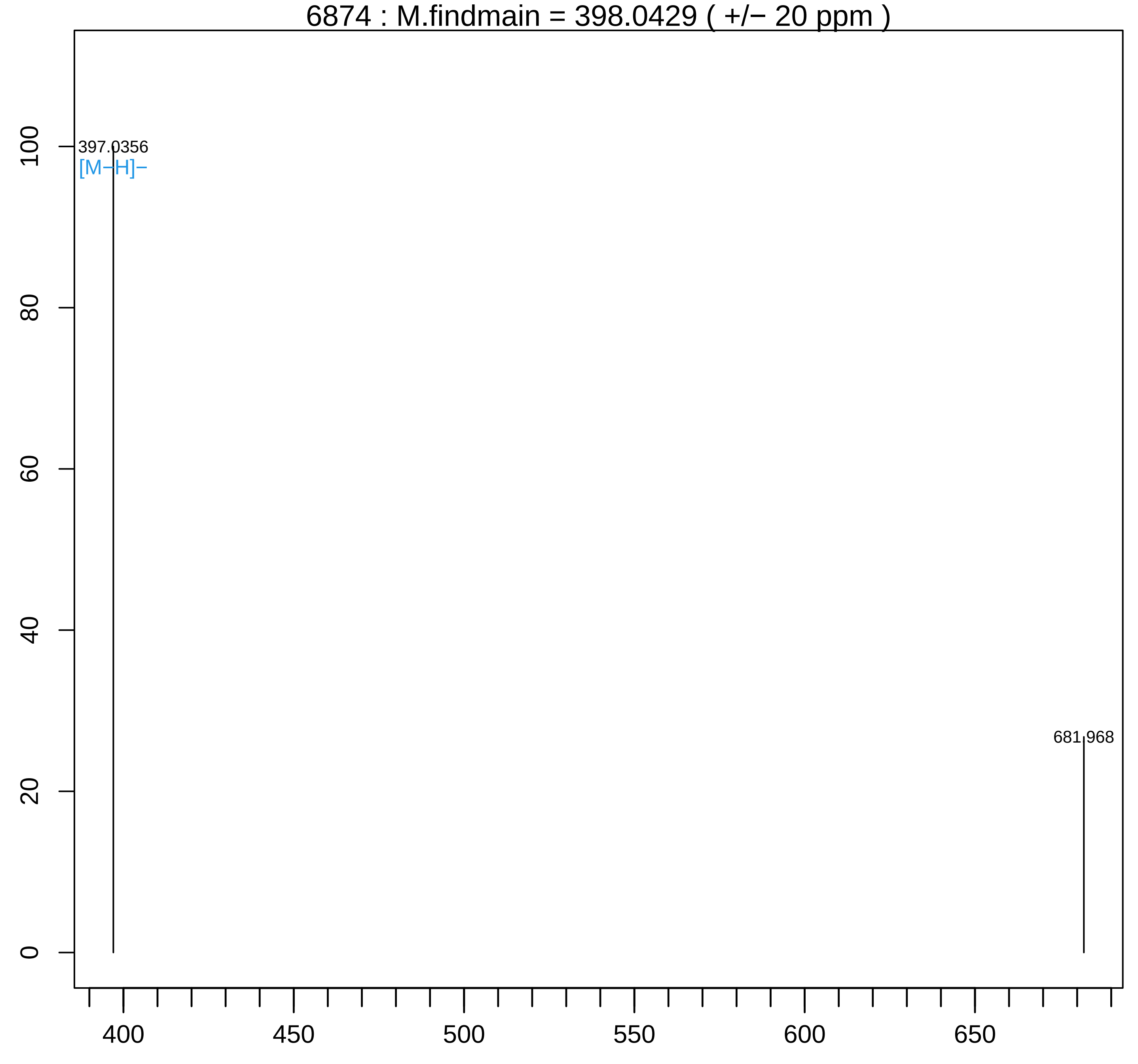
6874 : M.findmain = 398.0429 (+/− 20 ppm)
397.0356
[M−H]−
681.968
0
20
40
60
80
100
400
450
500
550
600
650

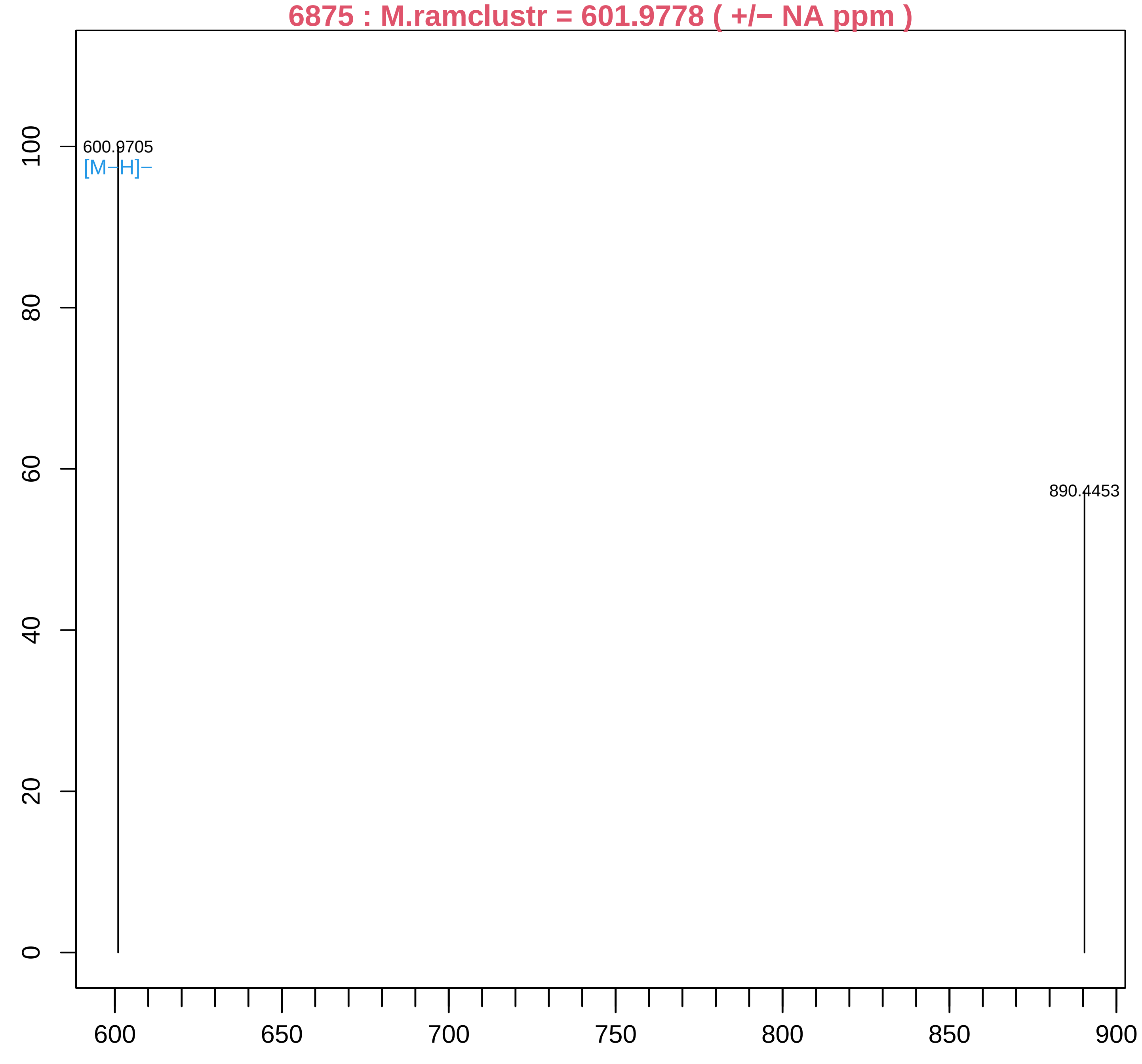
6875 : M.ramclustr = 601.9778 (+/− NA ppm)
600.9705
[M−H]−
890.4453
0
20
40
60
80
100
600
650
700
750
800
850
900

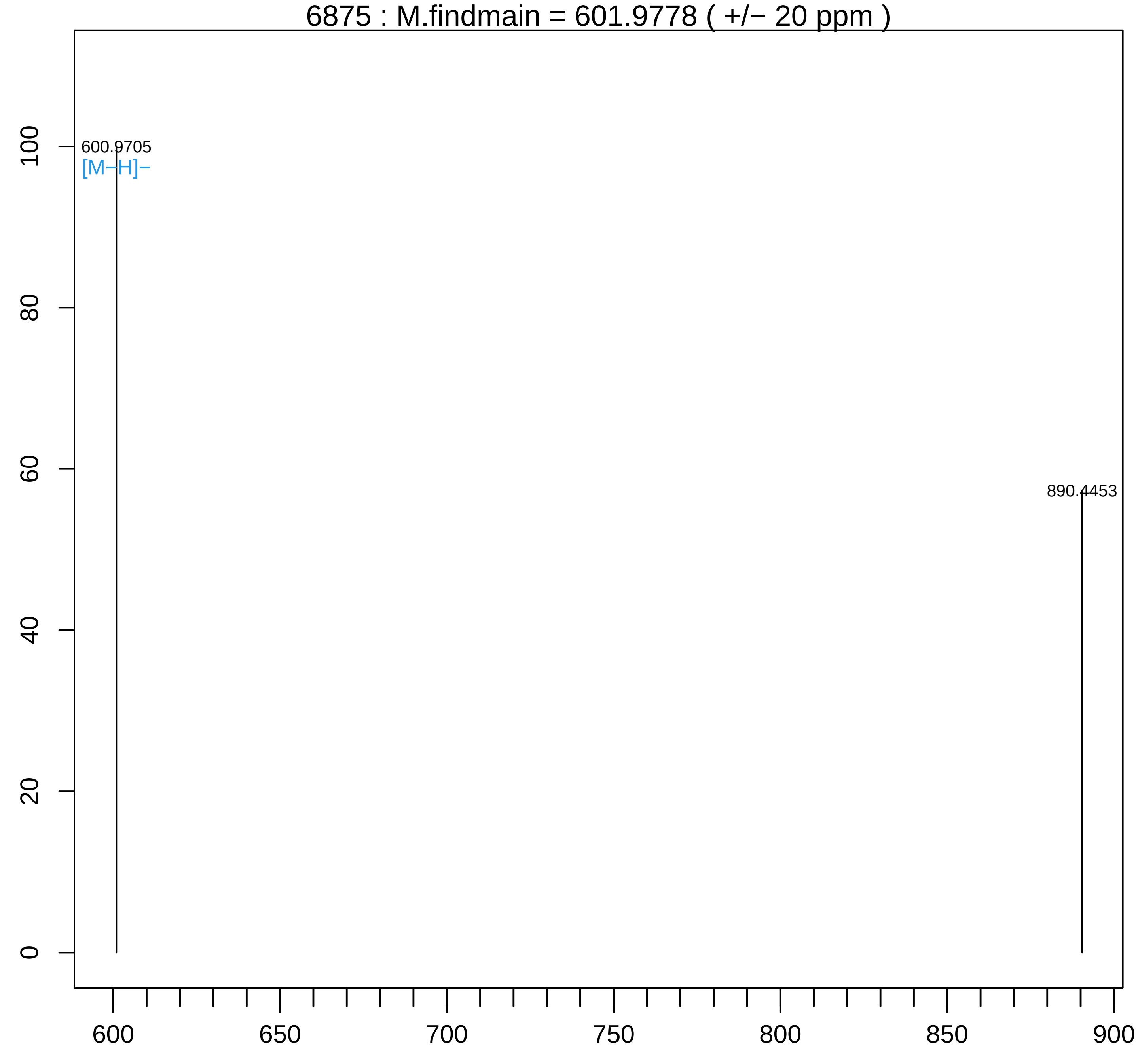
6875 : M.findmain = 601.9778 (+/− 20 ppm)
600.9705
[M−H]−
890.4453
0
20
40
60
80
100
600
650
700
750
800
850
900

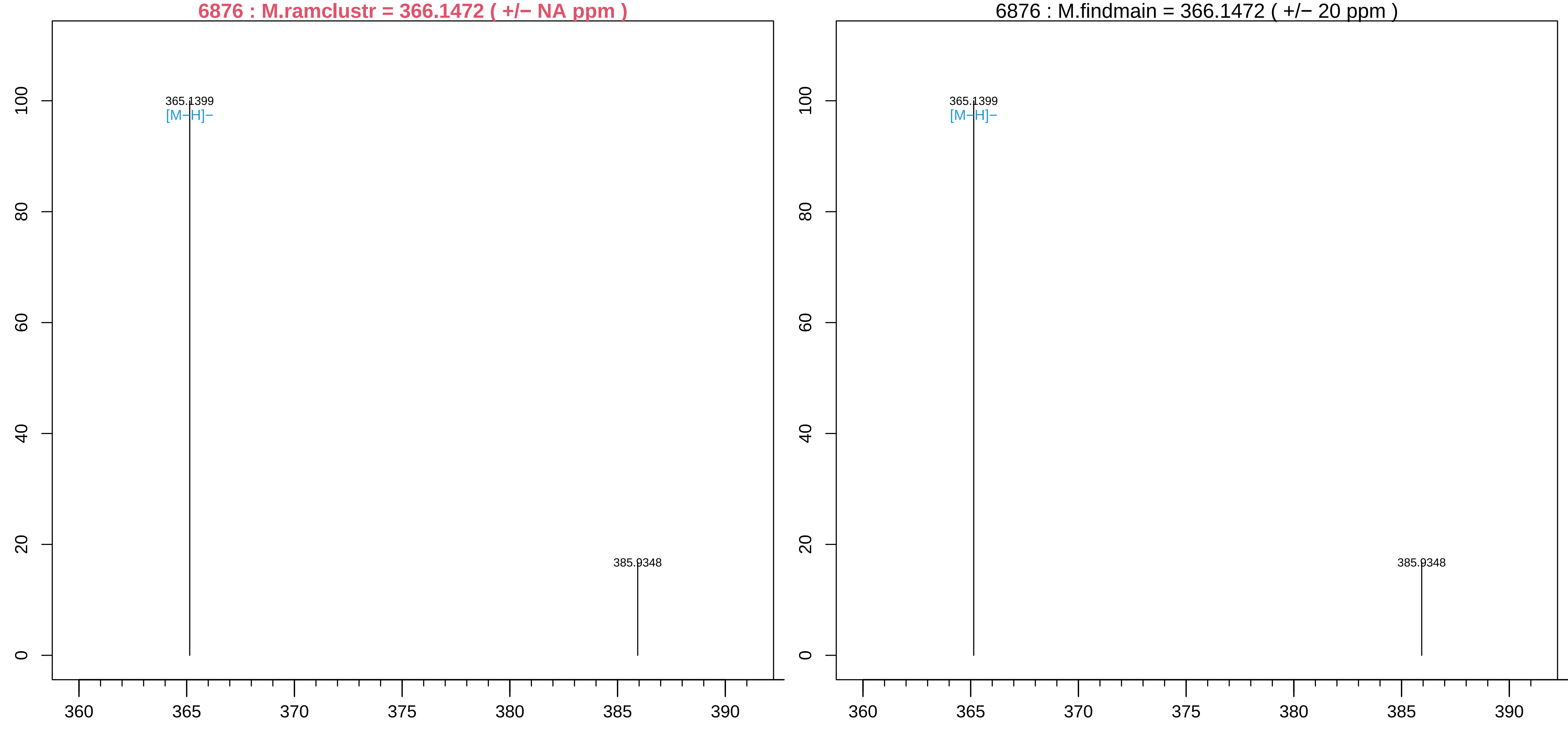
6876 : M.ramclustr = 366.1472 (+/− NA ppm)
365.1399
[M−H]−
385.9348
6876 : M.findmain = 366.1472 (+/− 20 ppm)
365.1399
[M−H]−
385.9348
0
20
40
60
80
100
360
365
370
375
380
385
390

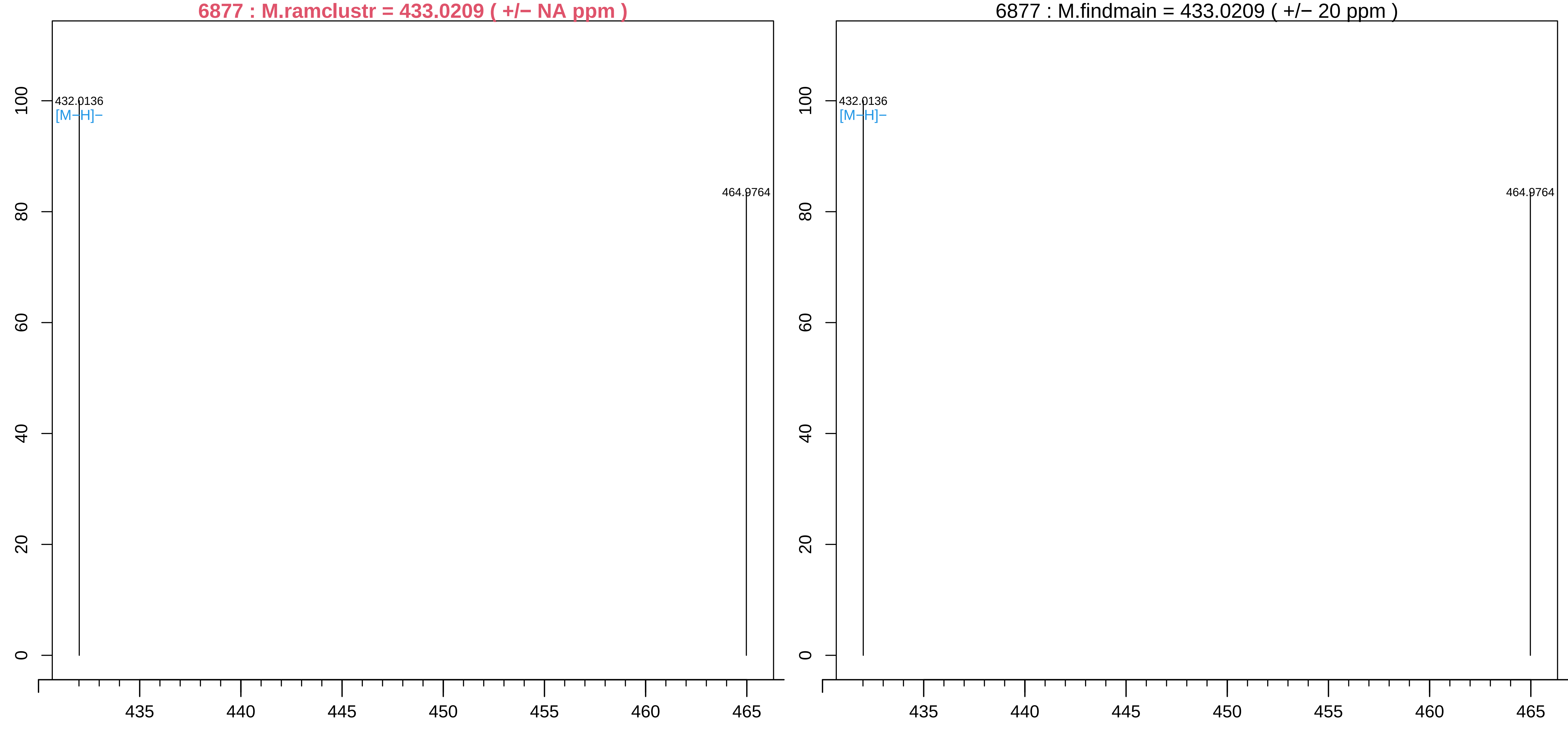
6877 : M.ramclustr = 433.0209 (+/− NA ppm)
432.0136
[M−H]−
464.9764
6877 : M.findmain = 433.0209 (+/− 20 ppm)
432.0136
[M−H]−
464.9764
0
20
40
60
80
100
435
440
445
450
455
460
465

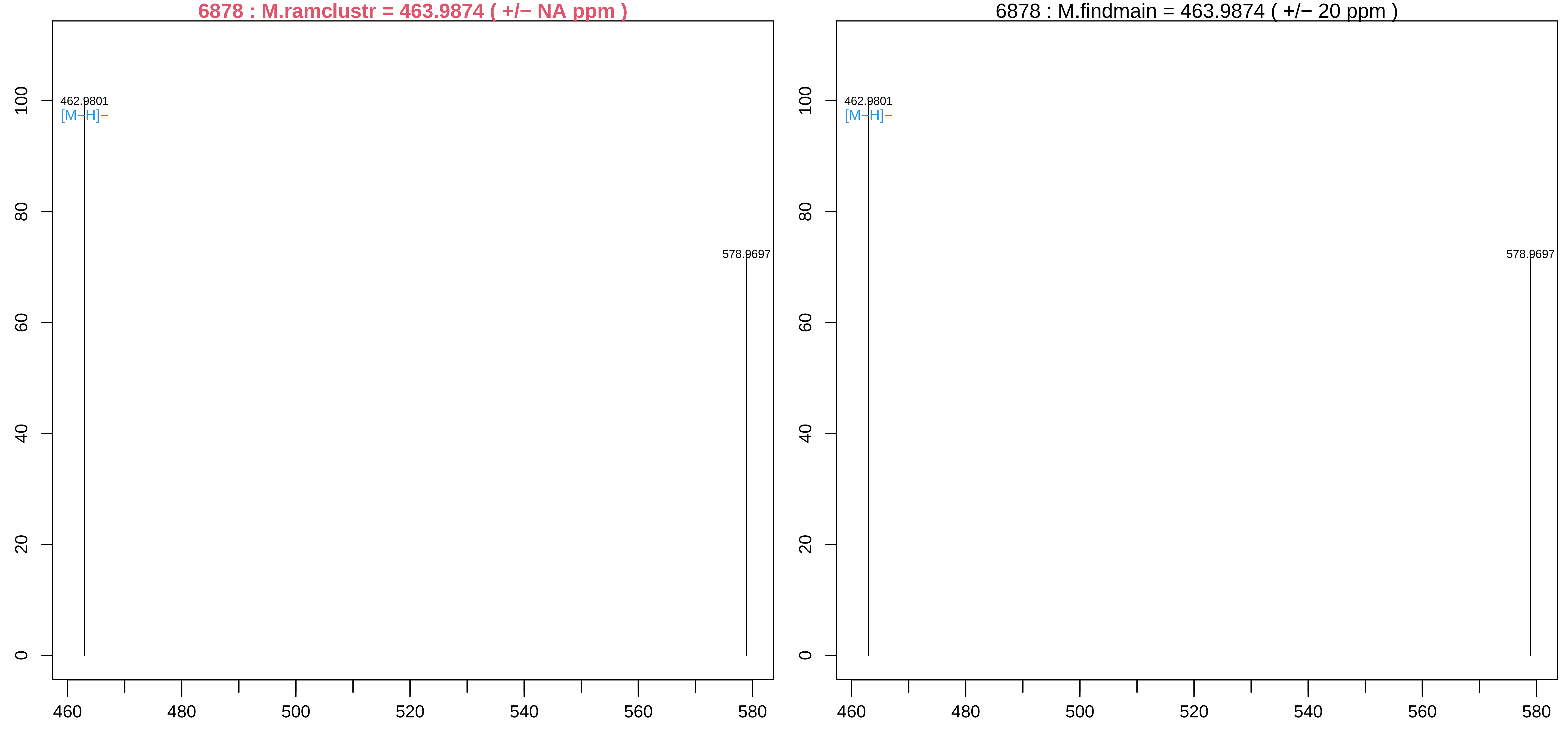

6878 : M.ramclustr = 463.9874 (+/− NA ppm)
462.9801
[M−H]−
578.9697
6878 : M.findmain = 463.9874 (+/− 20 ppm)
462.9801
[M−H]−
578.9697
0
20
40
60
80
100
460
480
500
520
540
560
580

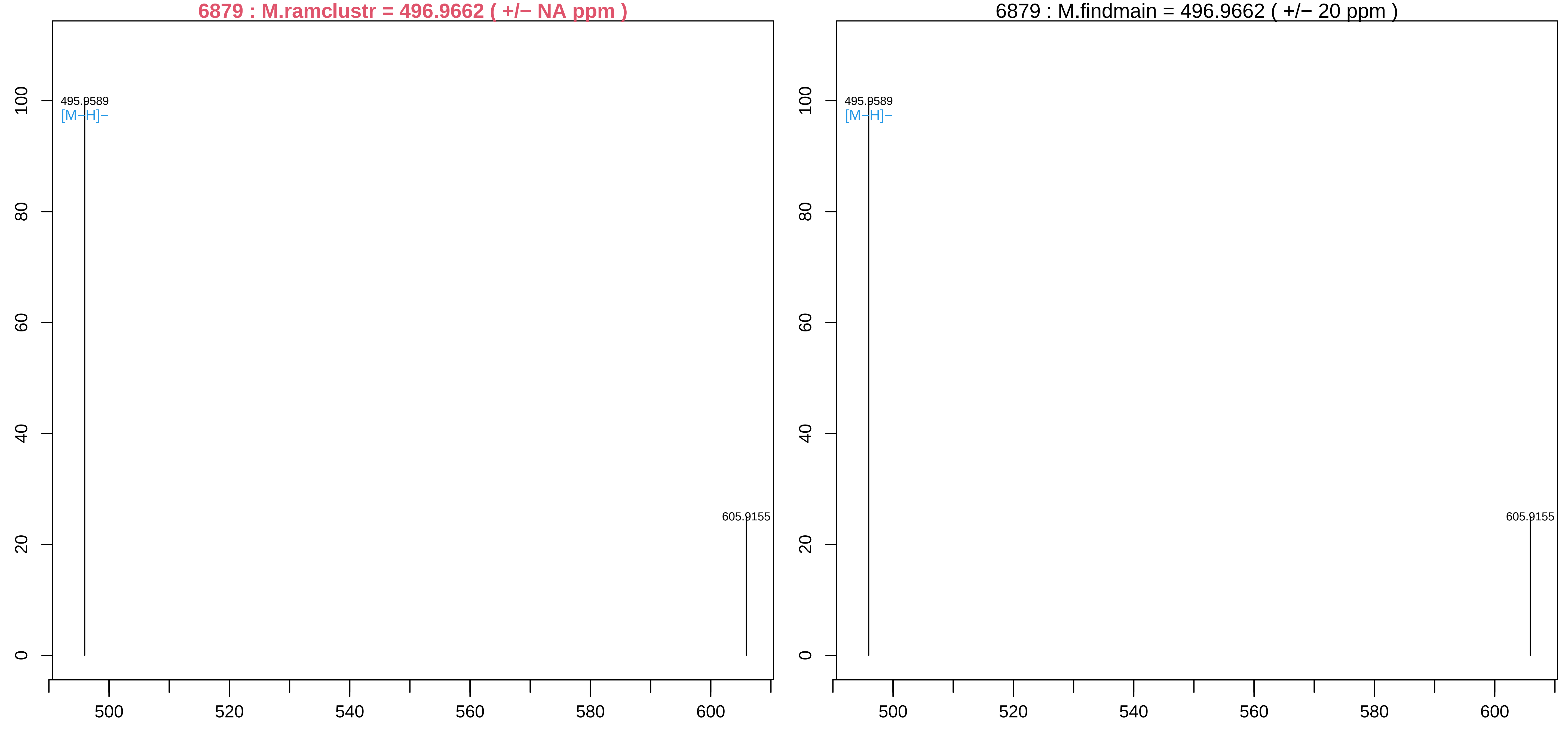
6879 : M.ramclustr = 496.9662 (+/− NA ppm)
495.9589
[M−H]−
605.9155
6879 : M.findmain = 496.9662 (+/− 20 ppm)
495.9589
[M−H]−
605.9155
0
20
40
60
80
100
500
520
540
560
580
600

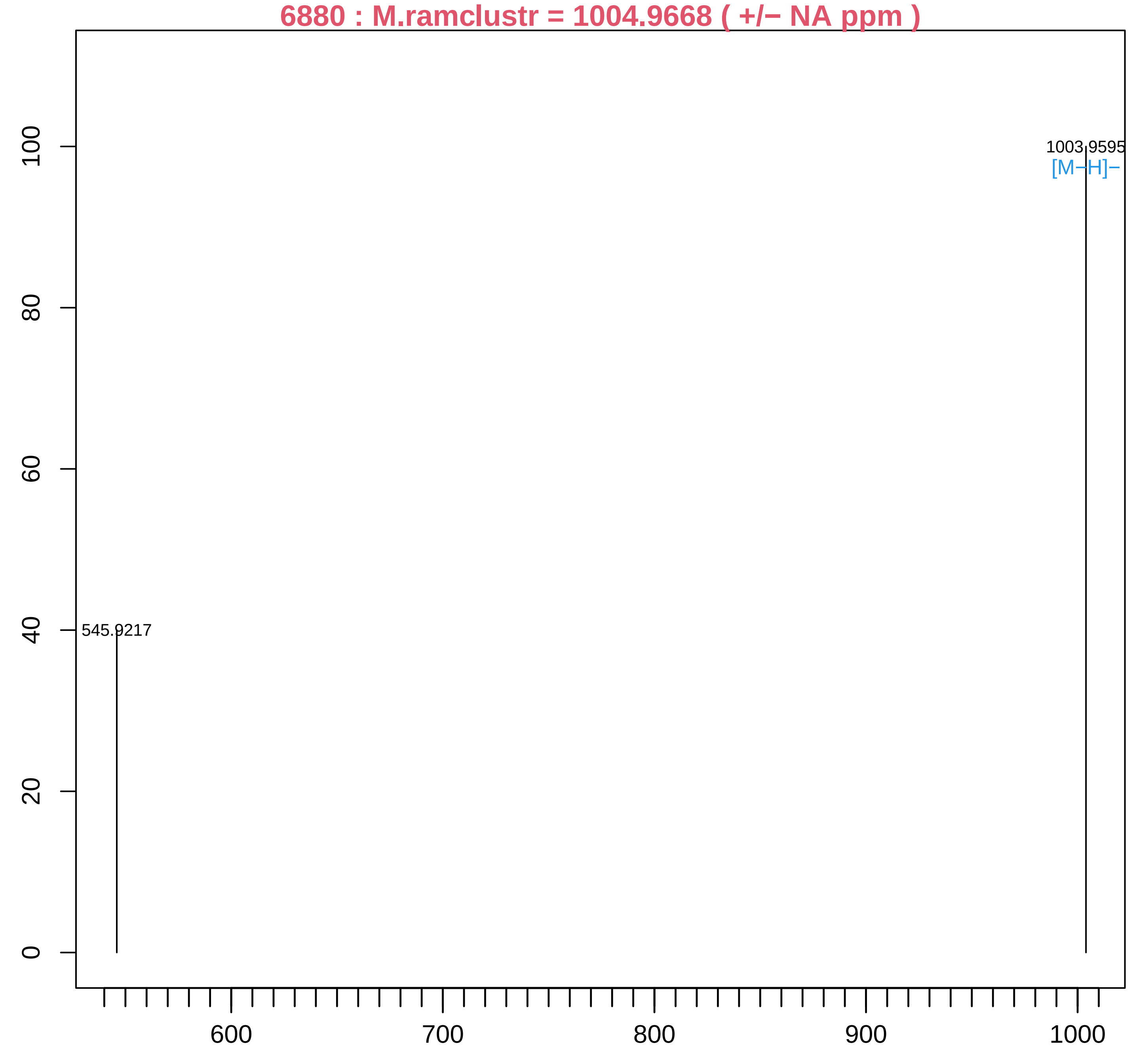

6880 : M.ramclustr = 1004.9668 (+/− NA ppm)
1003.9595
[M−H]−
545.9217
0
20
40
60
80
100
600
700
800
900
1000

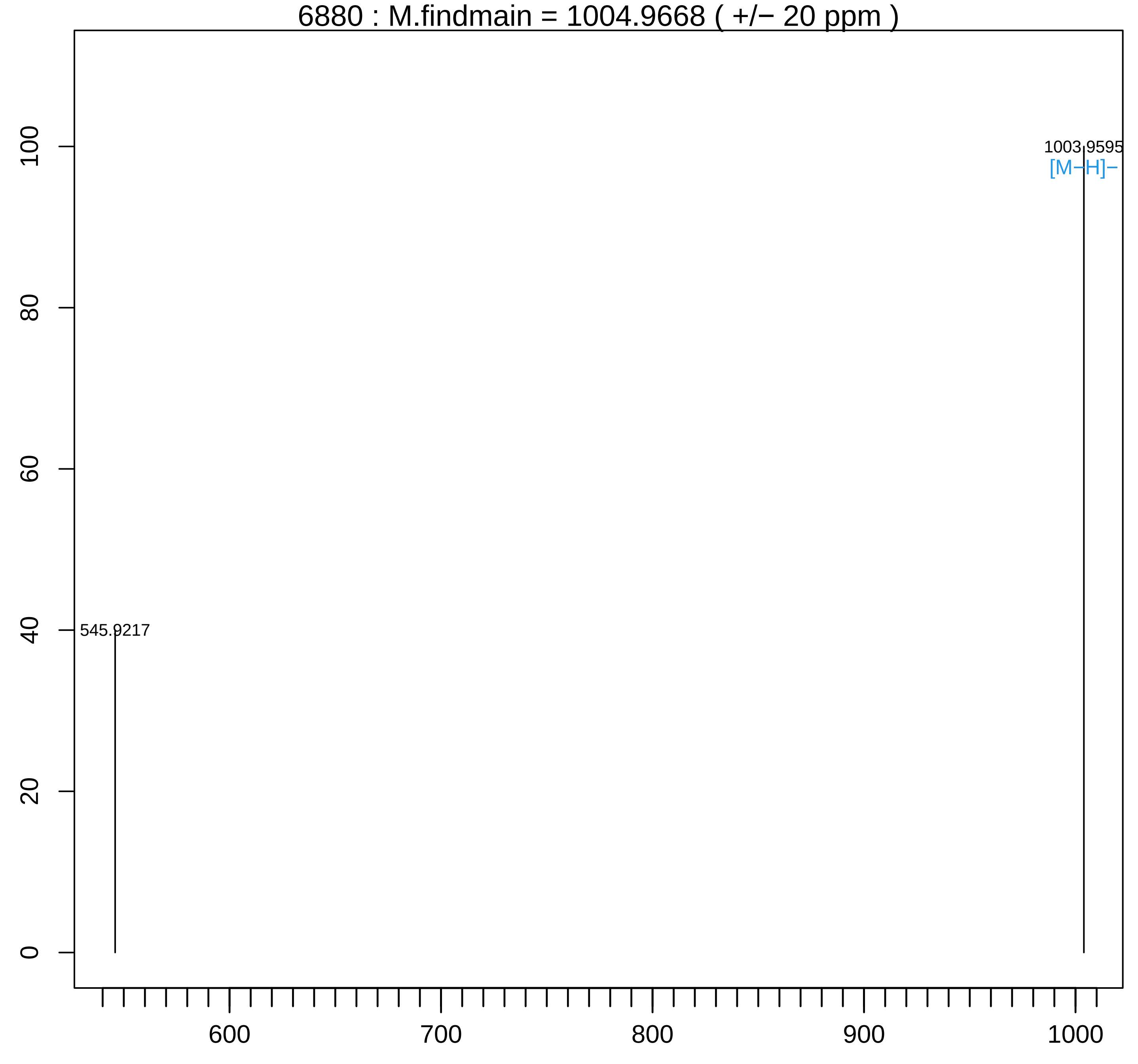

6880 : M.findmain = 1004.9668 (+/− 20 ppm)
1003.9595
[M−H]−
545.9217
0
20
40
60
80
100
600
700
800
900
1000

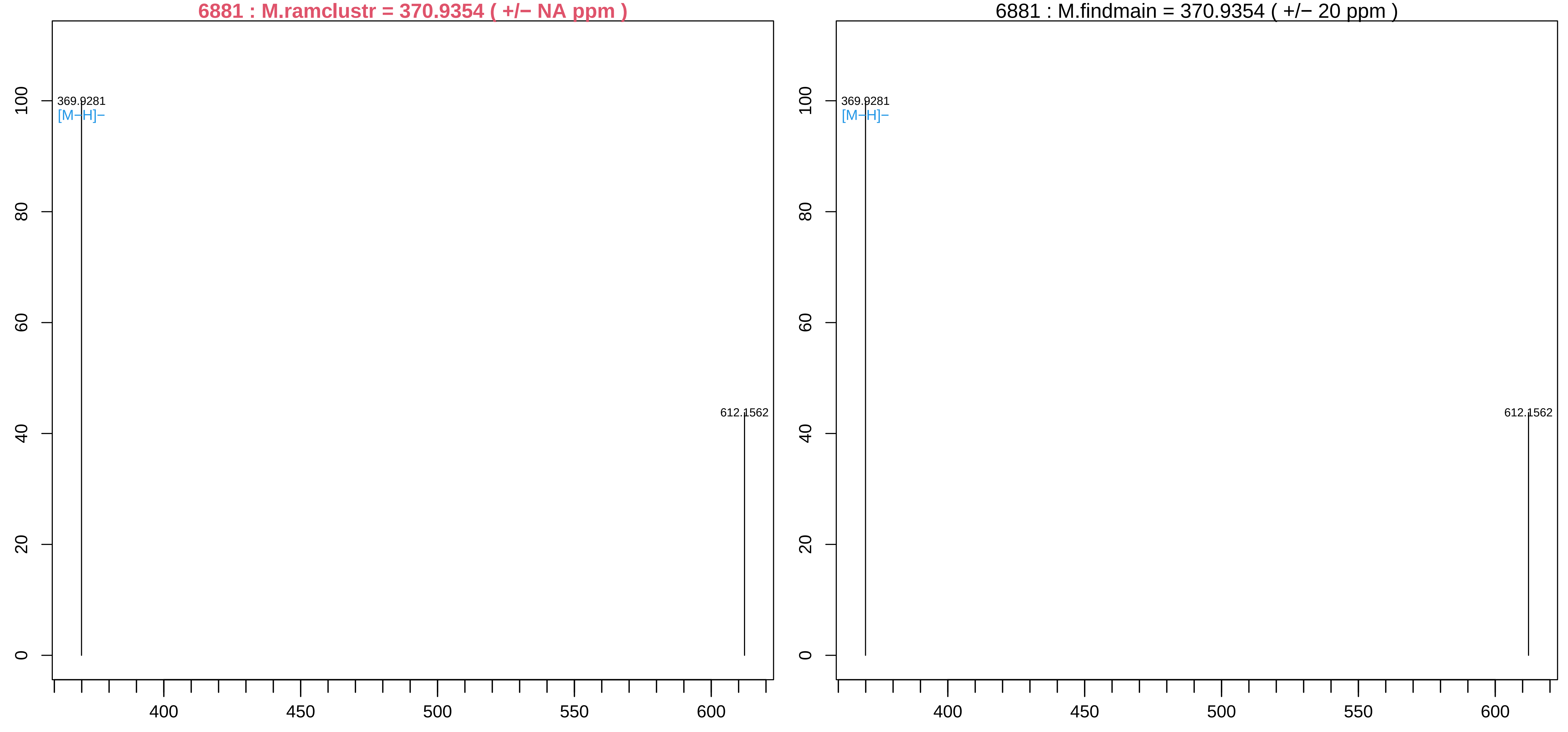

6881 : M.ramclustr = 370.9354 (+/− NA ppm)
369.9281
[M−H]−
612.1562
6881 : M.findmain = 370.9354 (+/− 20 ppm)
369.9281
[M−H]−
612.1562
0
20
40
60
80
100
400
450
500
550
600

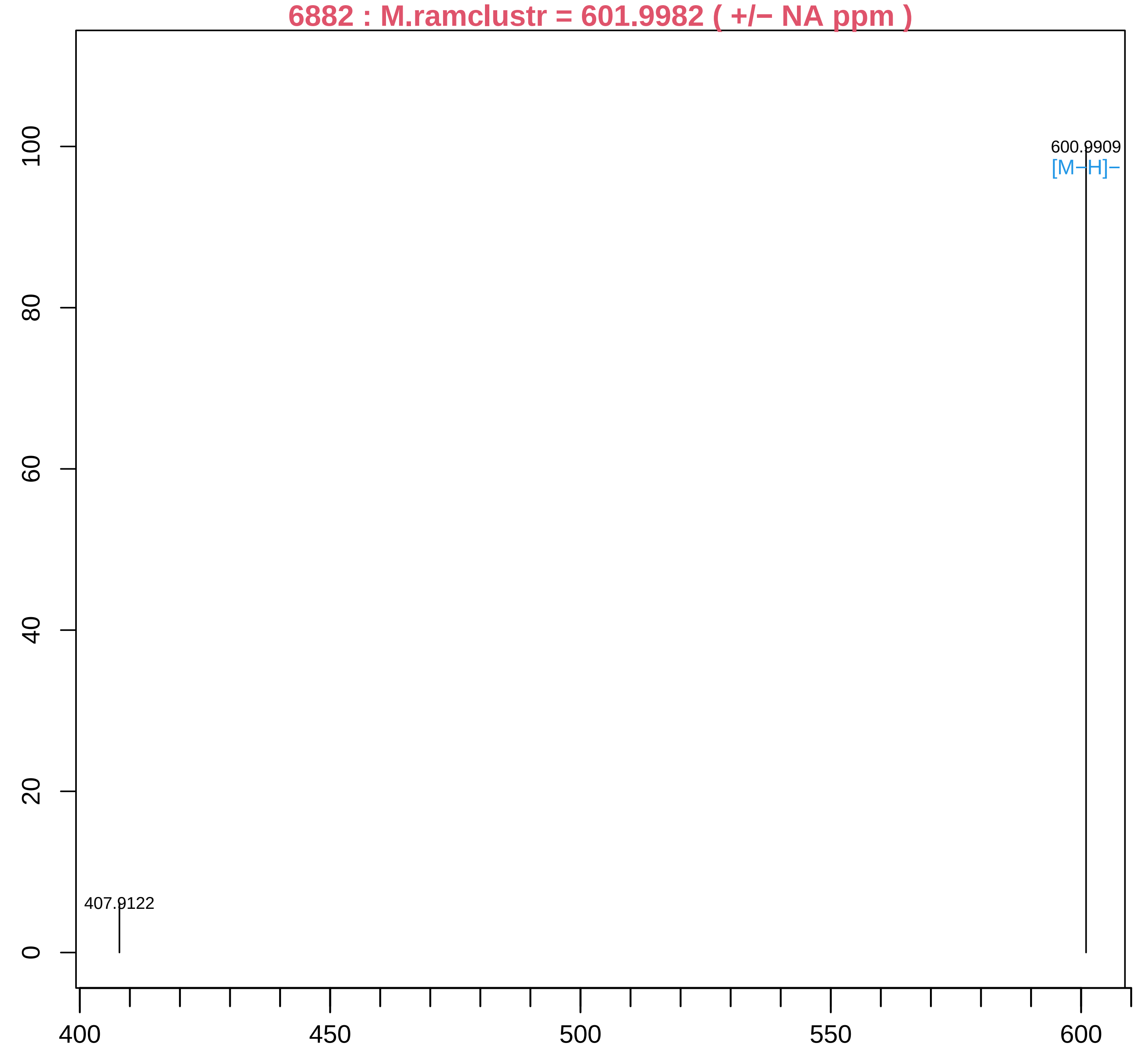

6882 : M.ramclustr = 601.9982 (+/− NA ppm)
600.9909
[M−H]−
407.9122
0
20
40
60
80
100
400
450
500
550
600

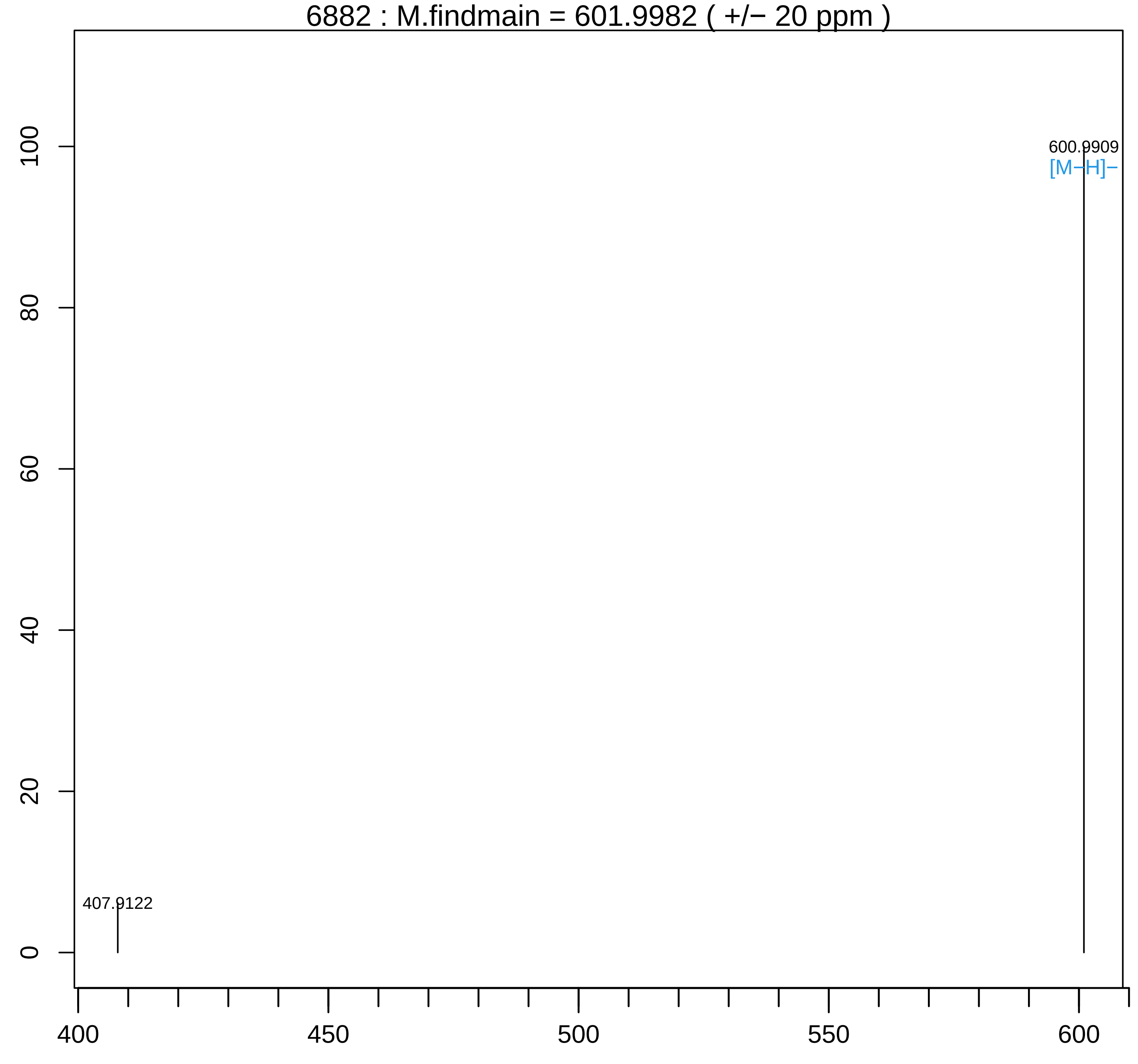

6882 : M.findmain = 601.9982 (+/− 20 ppm)
600.9909
[M−H]−
407.9122
0
20
40
60
80
100
400
450
500
550
600

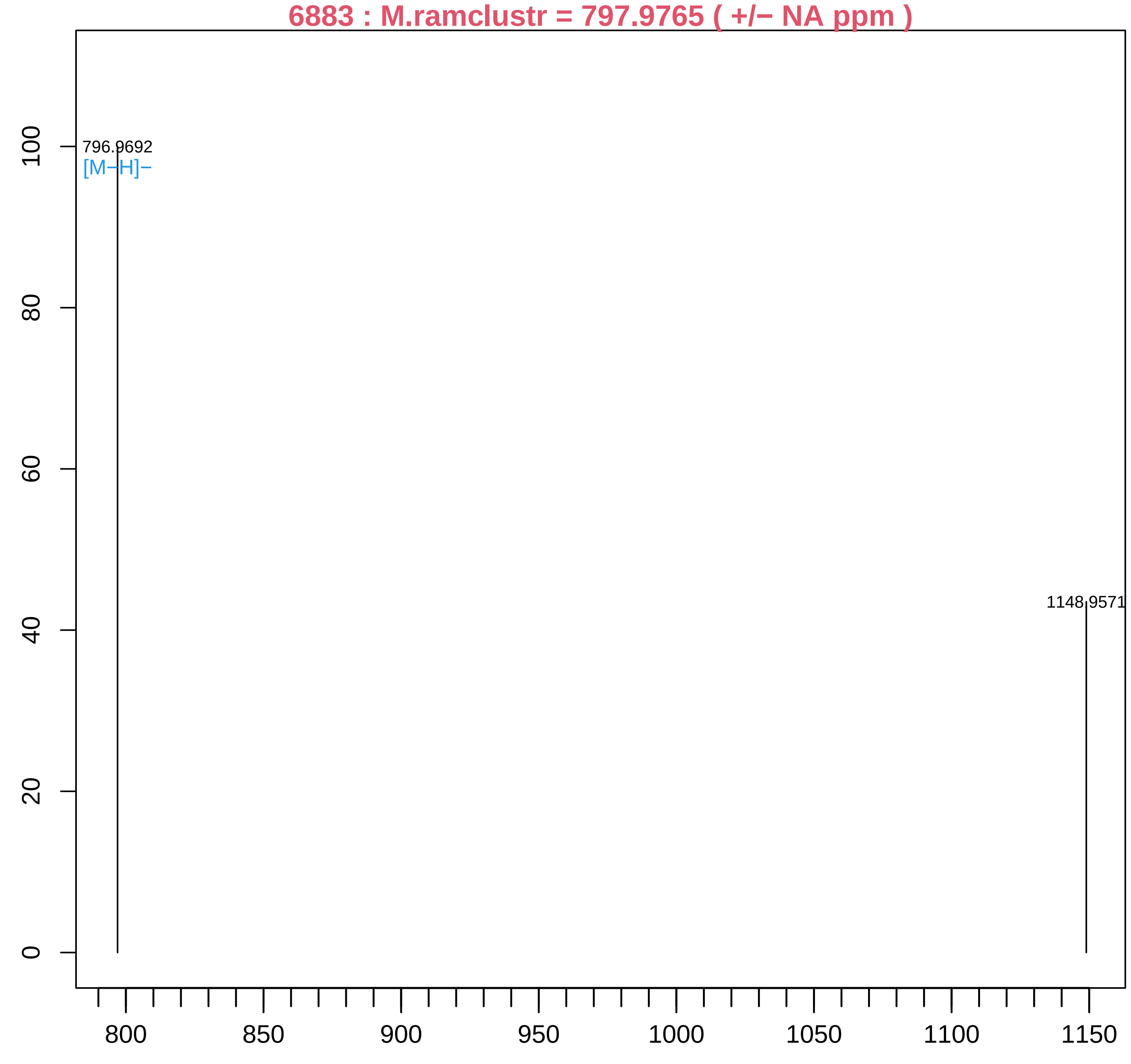

6883 : M.ramclustr = 797.9765 (+/− NA ppm)
796.9692
[M−H]−
1148.9571

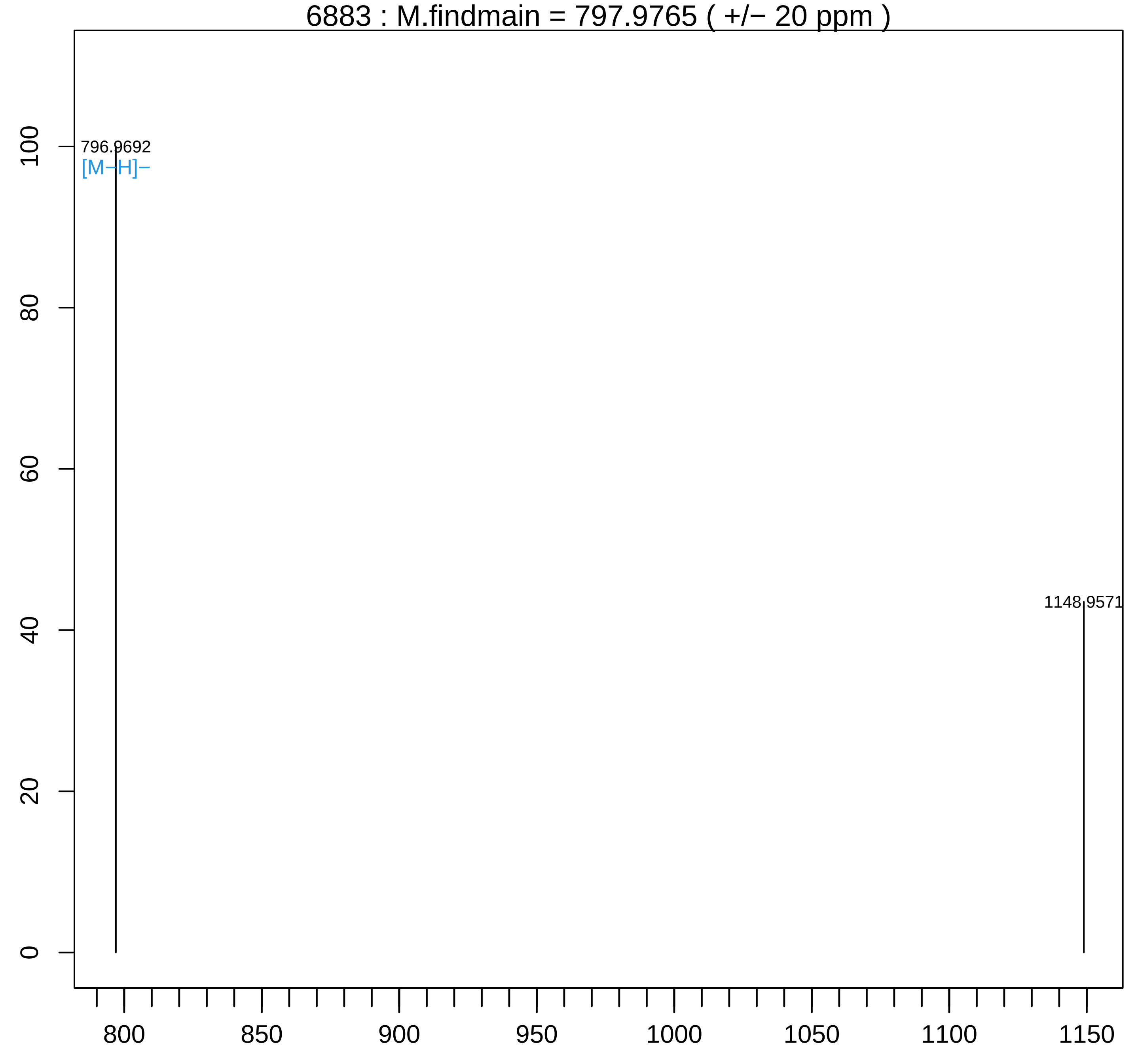

6883 : M.findmain = 797.9765 (+/− 20 ppm)
796.9692
[M−H]−
1148.9571

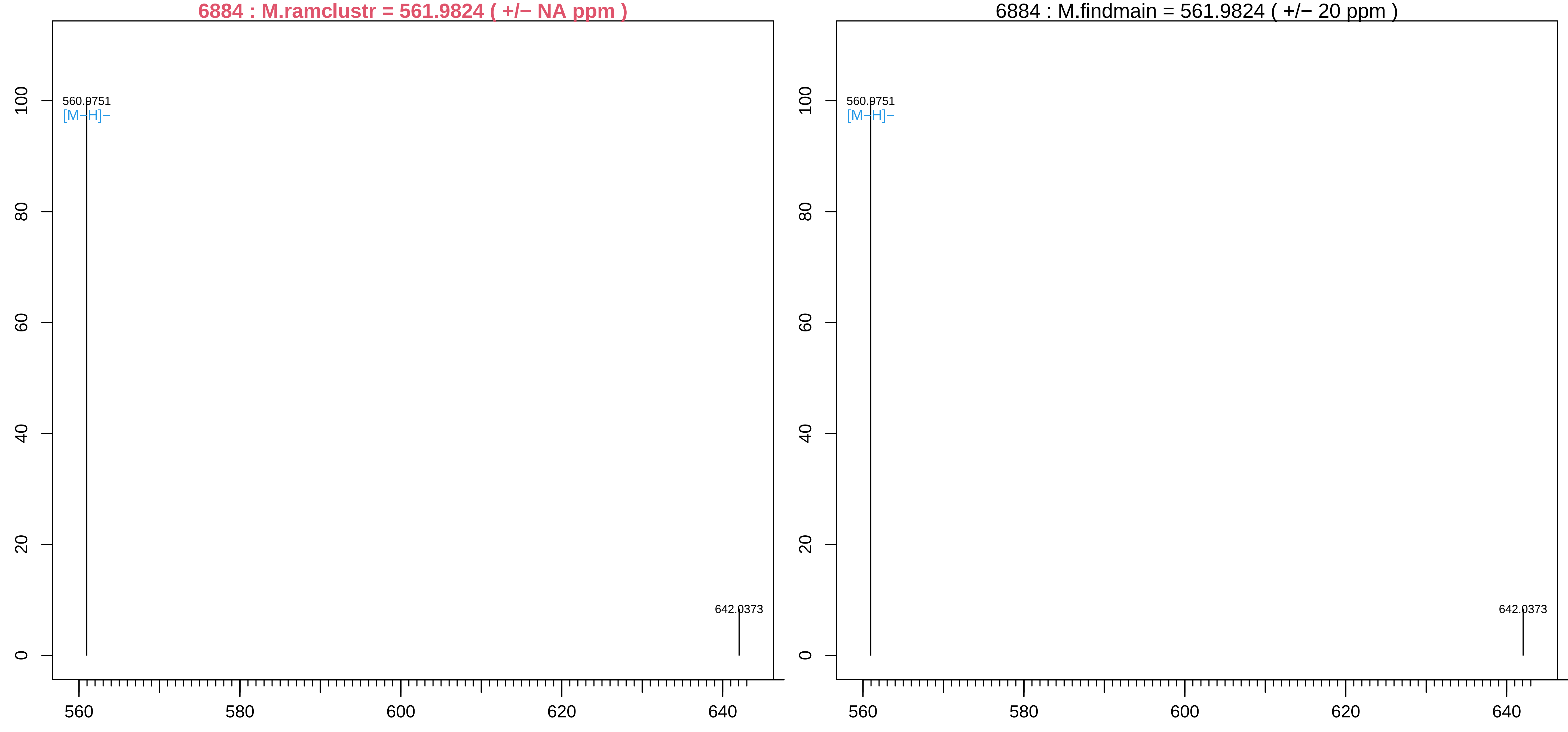
6884 : M.ramclustr = 561.9824 (+/− NA ppm)
560.9751
[M−H]−
642.0373
0
20
40
60
80
100
560
580
600
620
640
6884 : M.findmain = 561.9824 (+/− 20 ppm)
560.9751
[M−H]−
642.0373
0
20
40
60
80
100
560
580
600
620
640

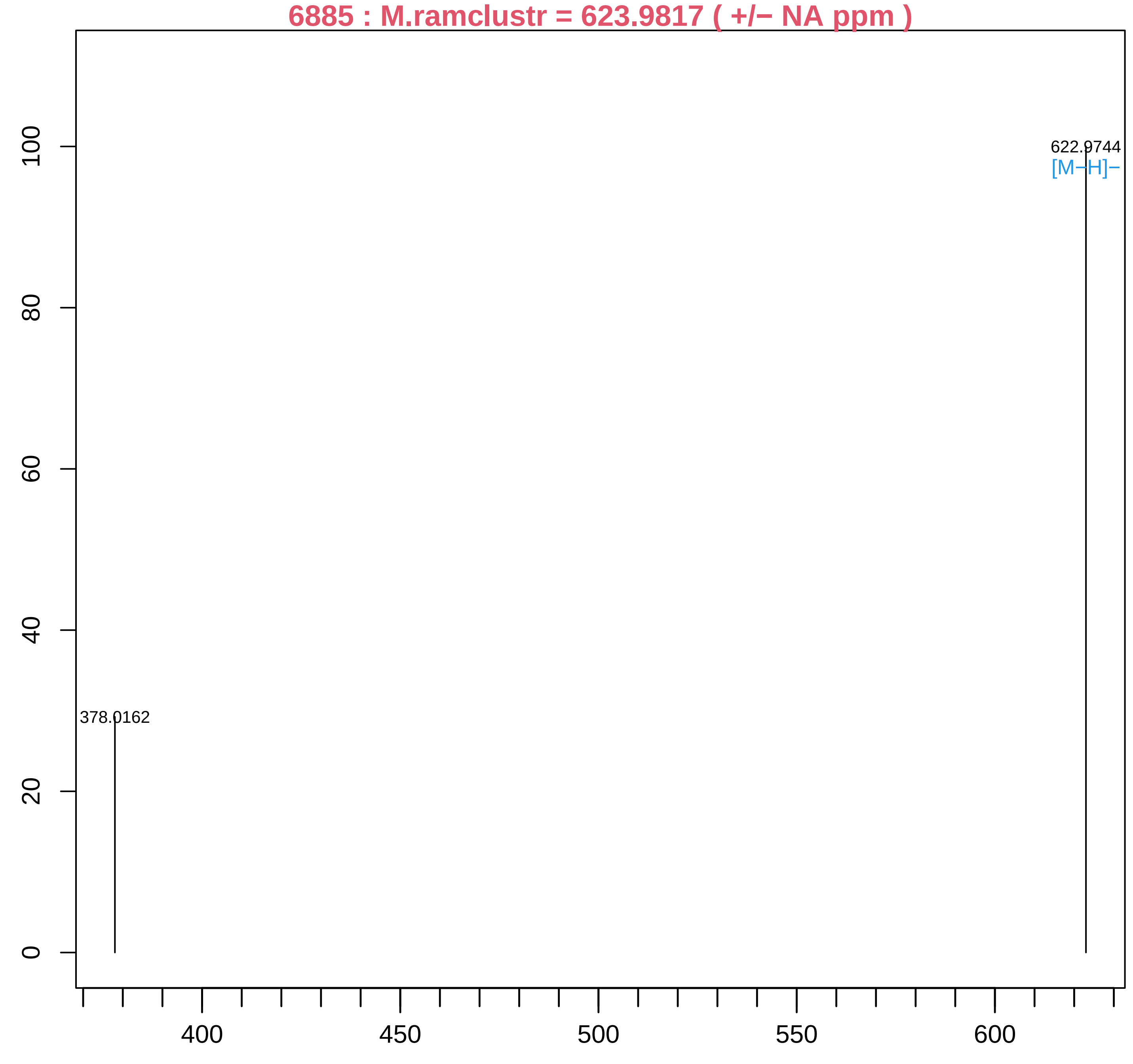

6885 : M.ramclustr = 623.9817 (+/− NA ppm)
622.9744
[M−H]−
378.0162
0
20
40
60
80
100
400
450
500
550
600

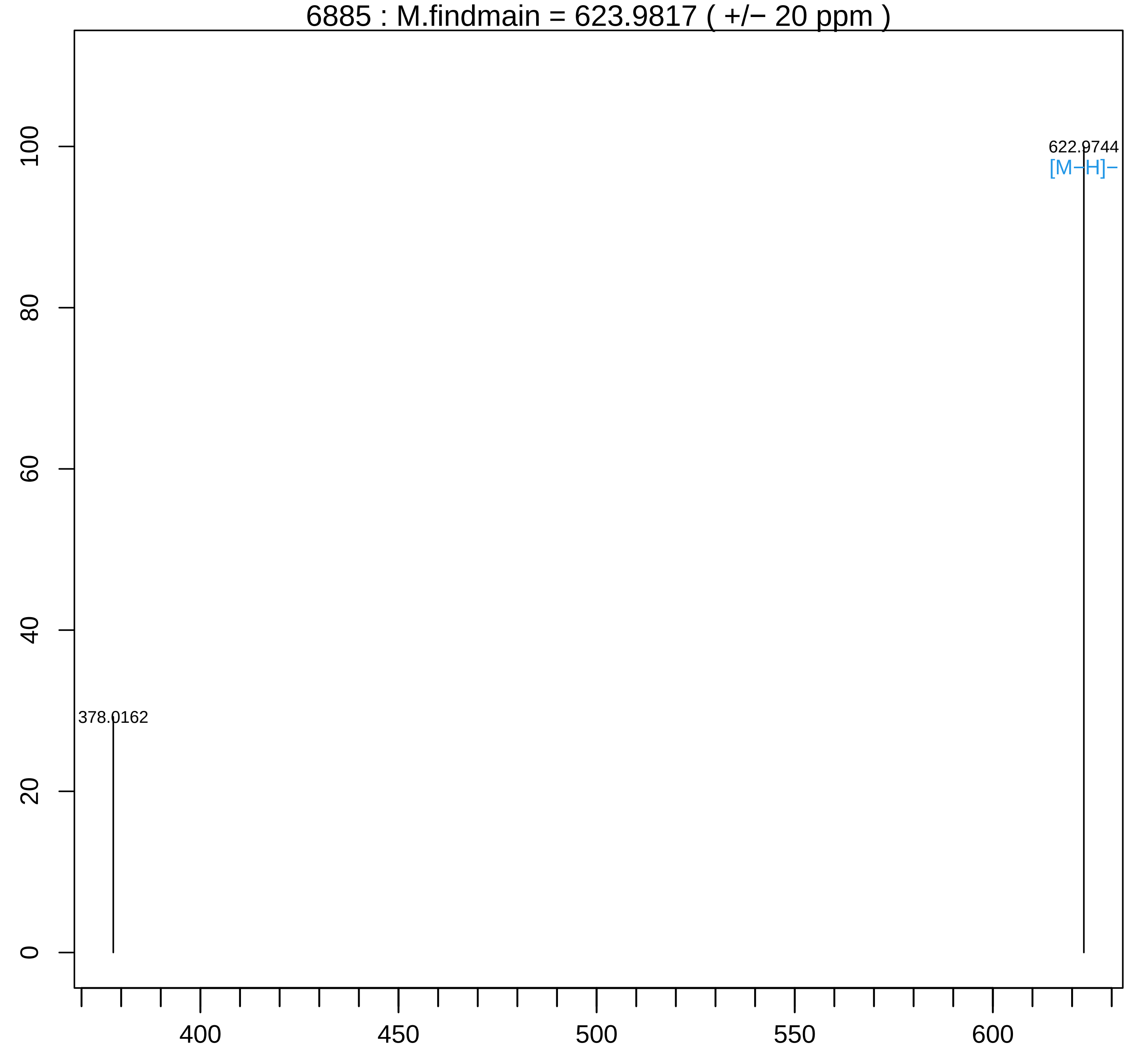

6885 : M.findmain = 623.9817 (+/− 20 ppm)
622.9744
[M−H]−
378.0162
0
20
40
60
80
100
400
450
500
550
600

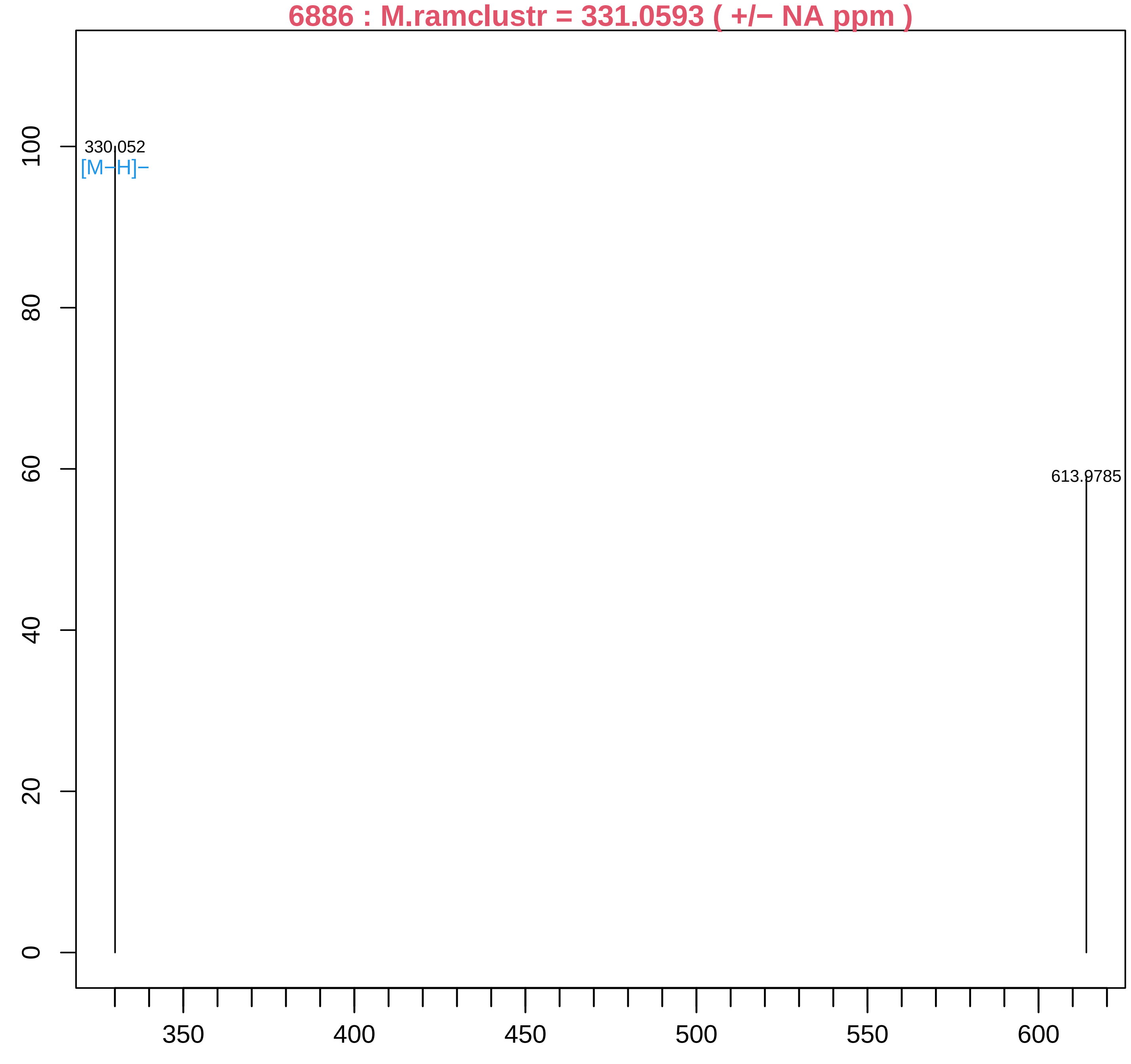
6886 : M.ramclustr = 331.0593 (+/− NA ppm)
330.052
[M−H]−
613.9785
0
20
40
60
80
100
350
400
450
500
550
600

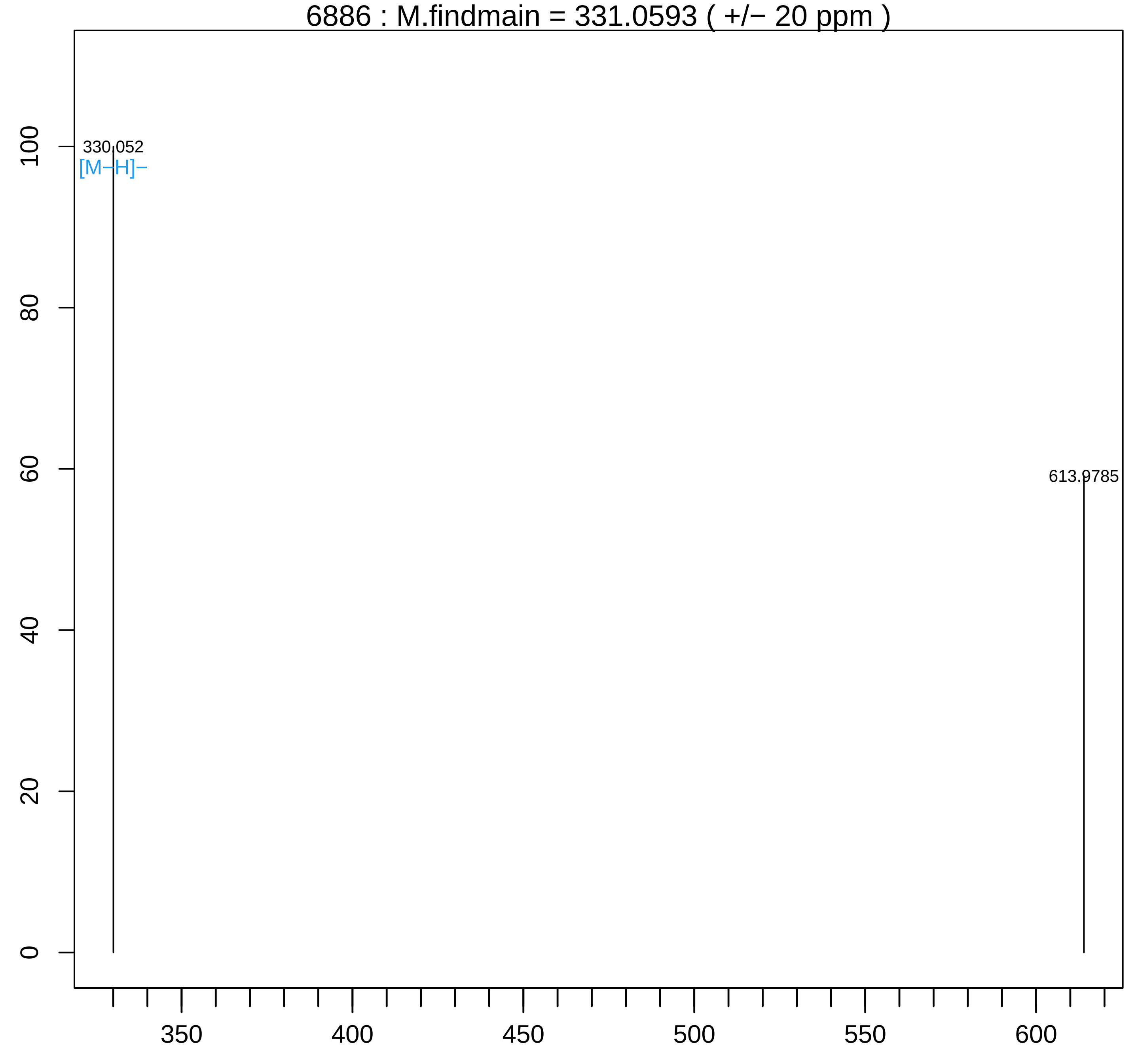
6886 : M.findmain = 331.0593 (+/− 20 ppm)
330.052
[M−H]−
613.9785
0
20
40
60
80
100
350
400
450
500
550
600

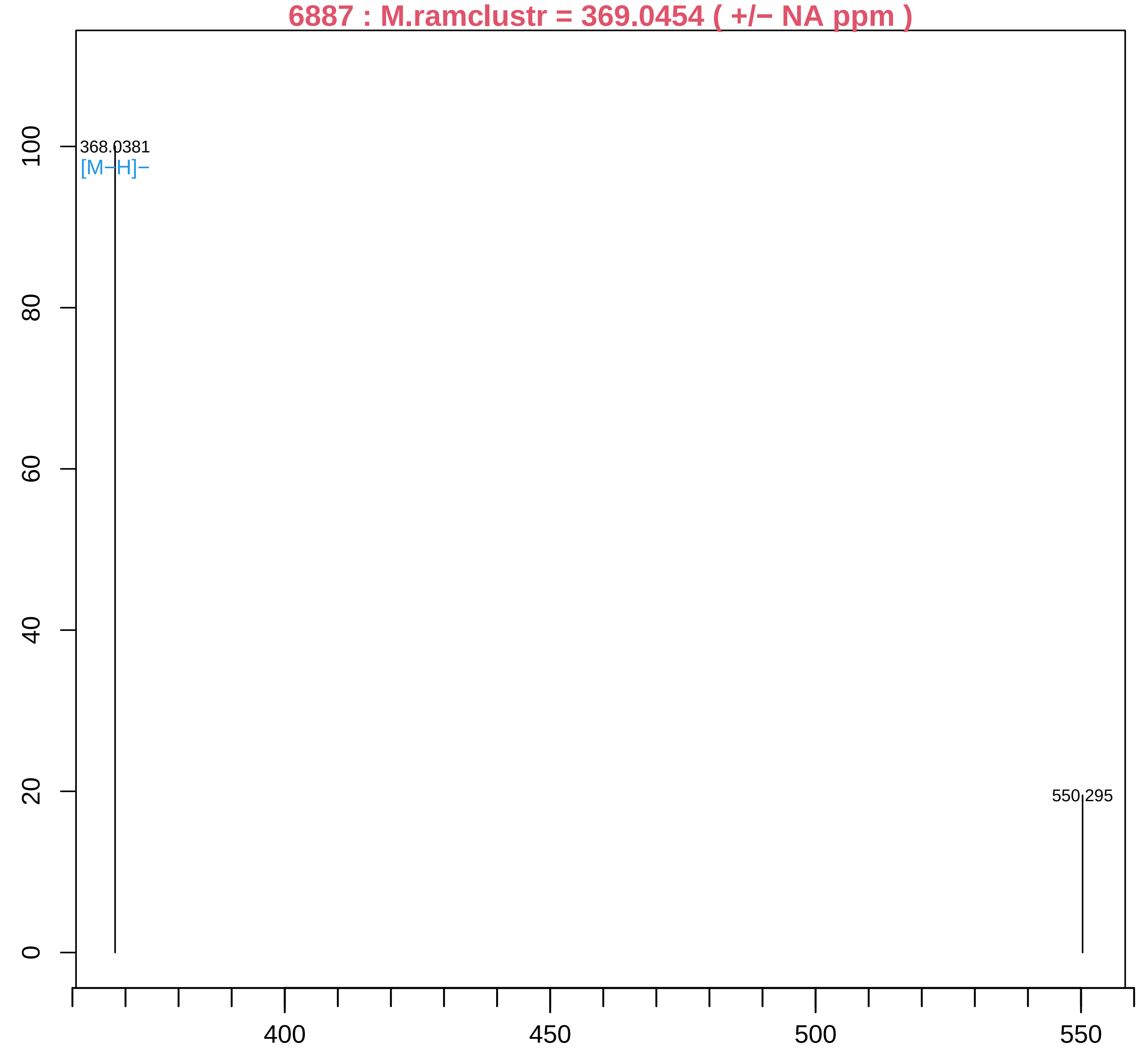
6887 : M.ramclustr = 369.0454 (+/− NA ppm)
368.0381
[M−H]−
550.295
0
20
40
60
80
100
400
450
500
550

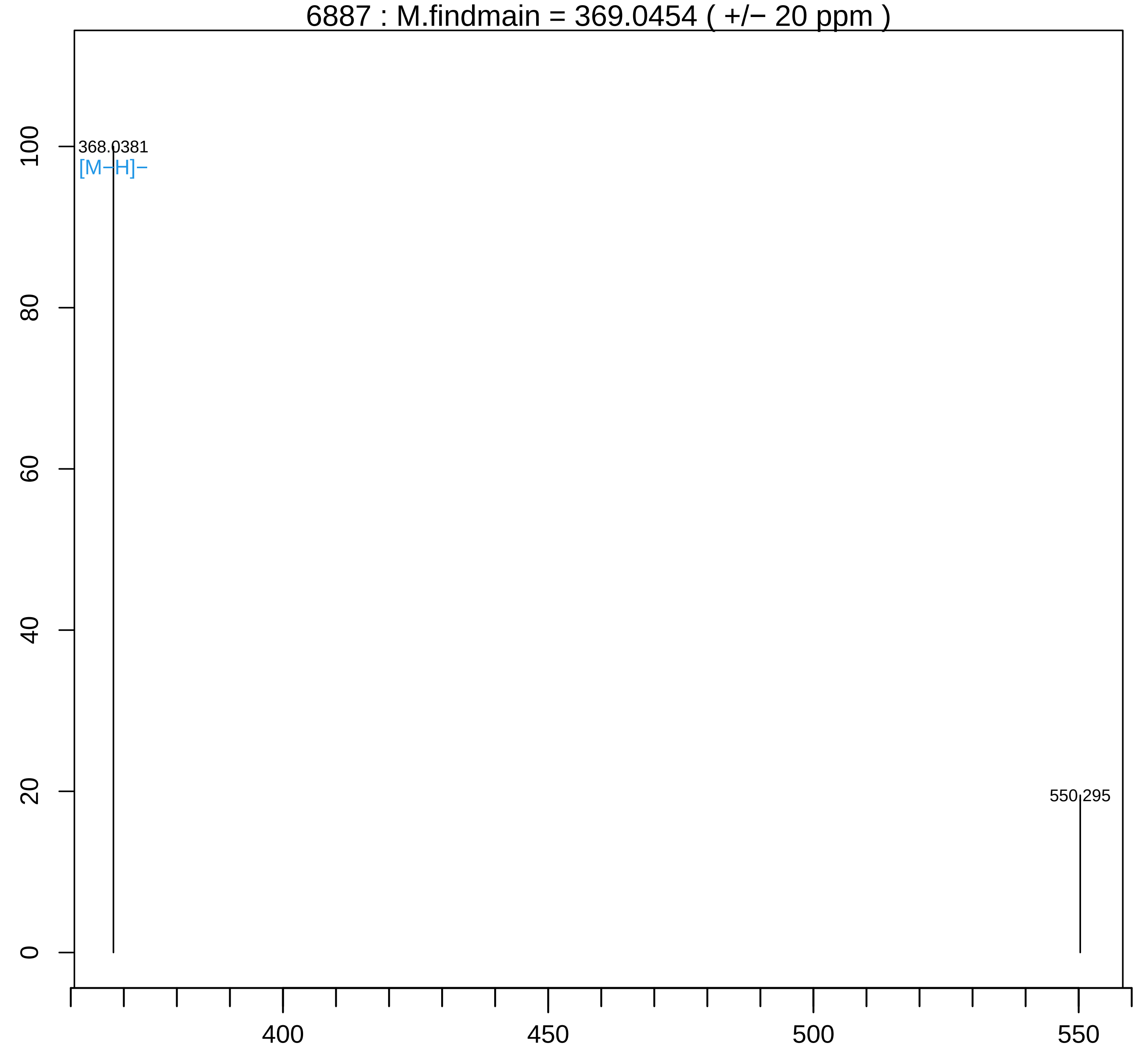
6887 : M.findmain = 369.0454 (+/− 20 ppm)
368.0381
[M−H]−
550.295
0
20
40
60
80
100
400
450
500
550

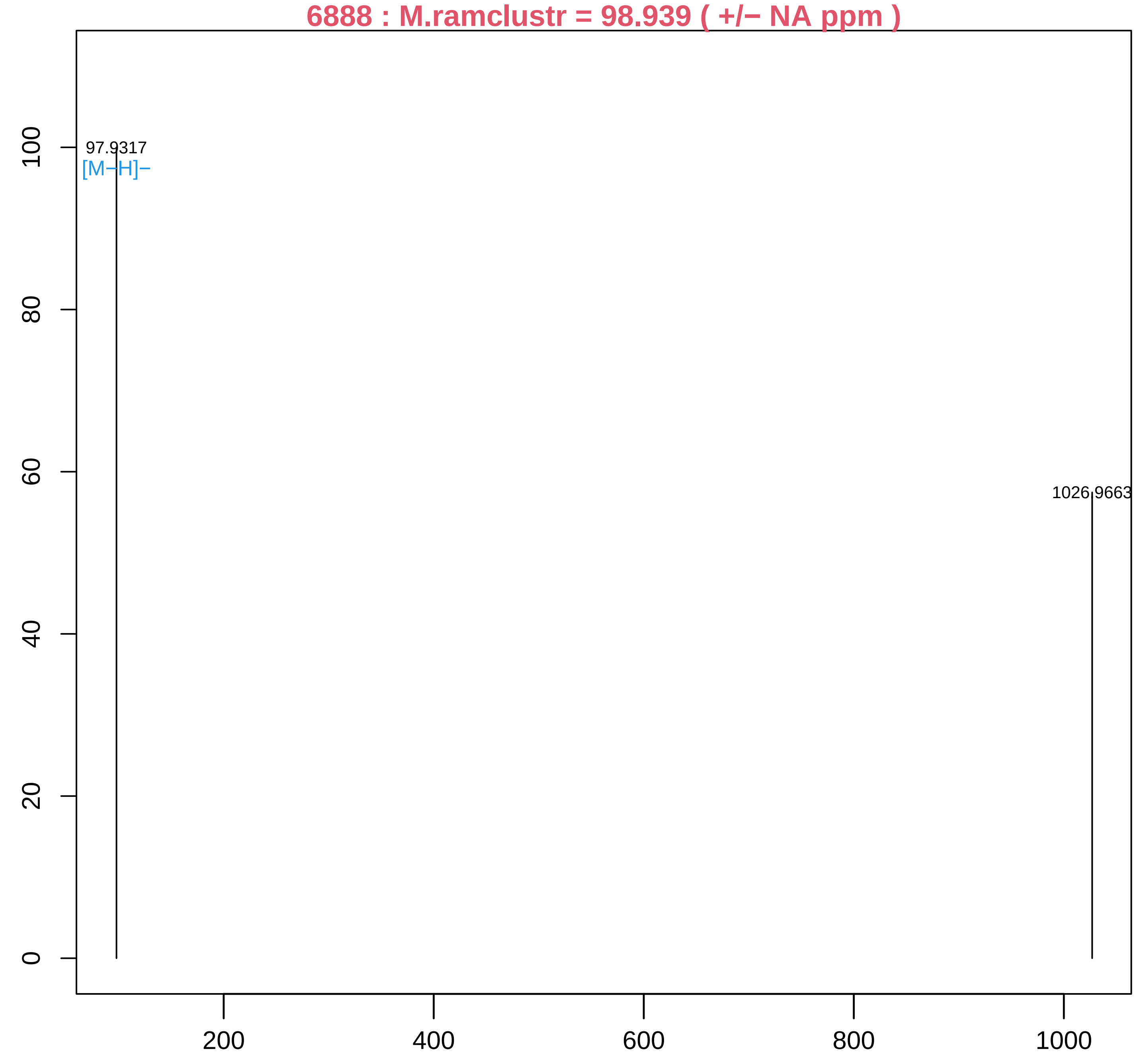
6888 : M.ramclustr = 98.939 (+/− NA ppm)
97.9317
[M−H]−
1026.9663
0
20
40
60
80
100
200
400
600
800
1000

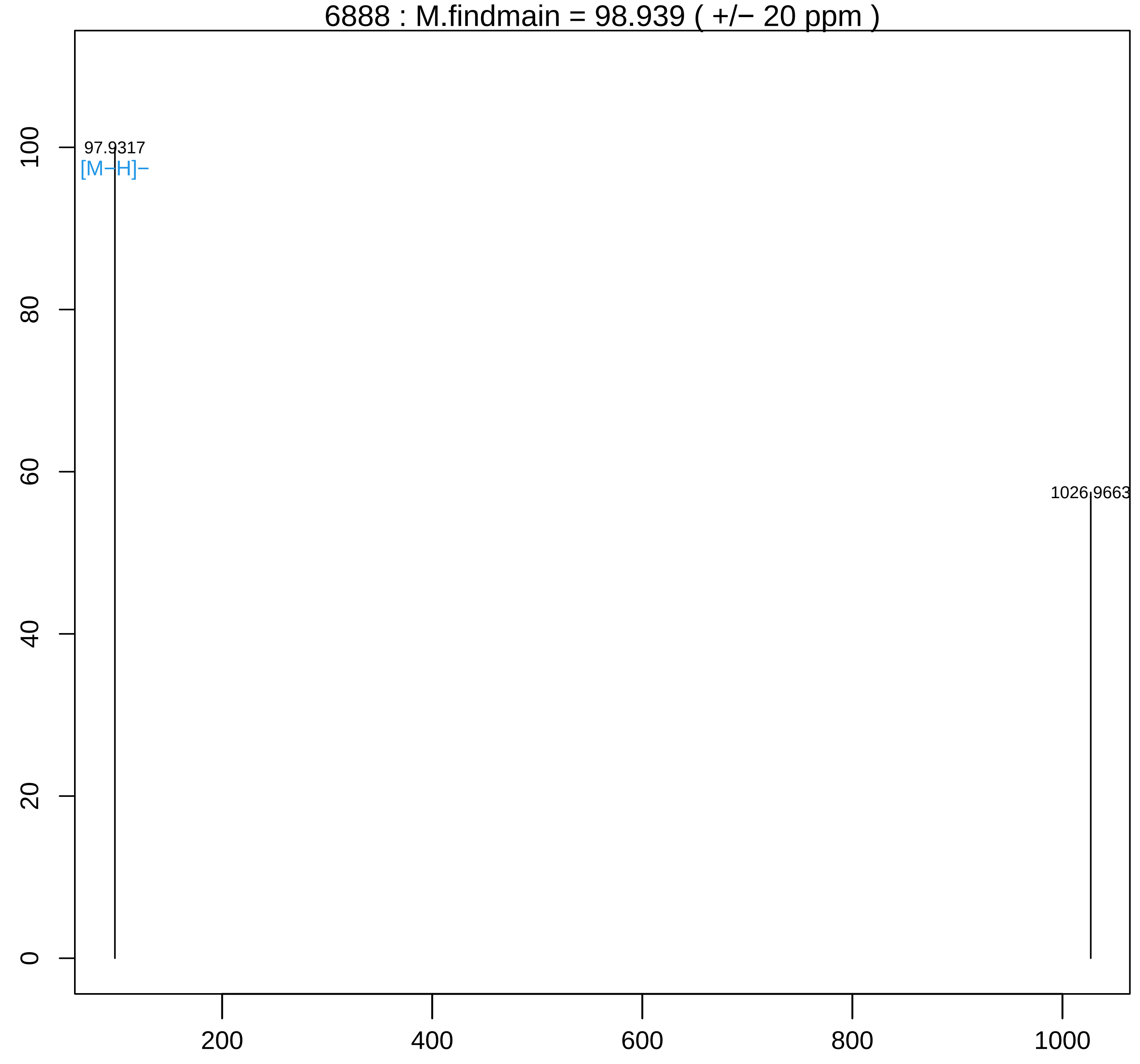
6888 : M.findmain = 98.939 (+/− 20 ppm)
97.9317
[M−H]−
1026.9663
0
20
40
60
80
100
200
400
600
800
1000

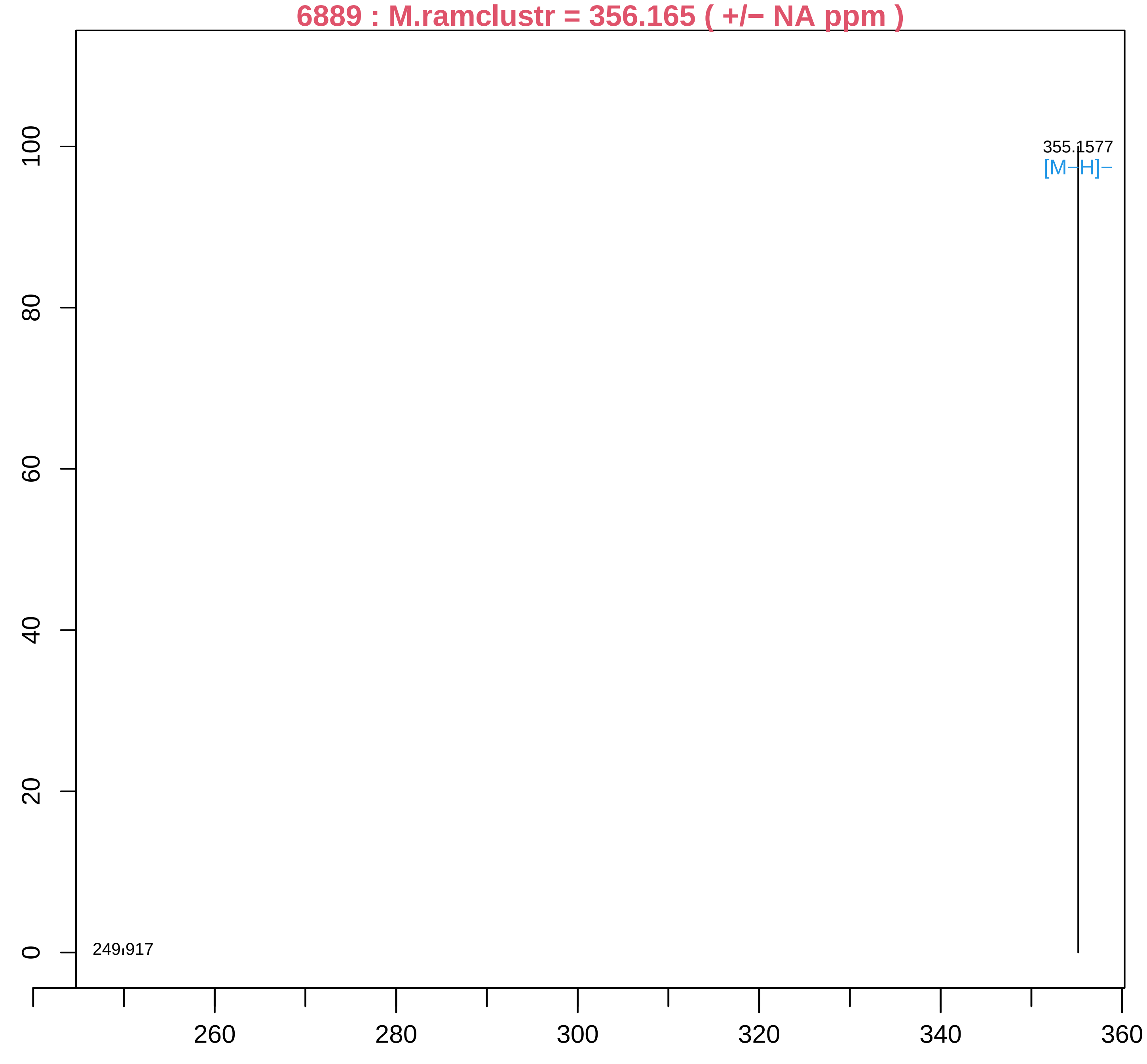
6889 : M.ramclustr = 356.165 (+/− NA ppm)
355.1577
[M−H]−
249.917
0
20
40
60
80
100
260
280
300
320
340
360

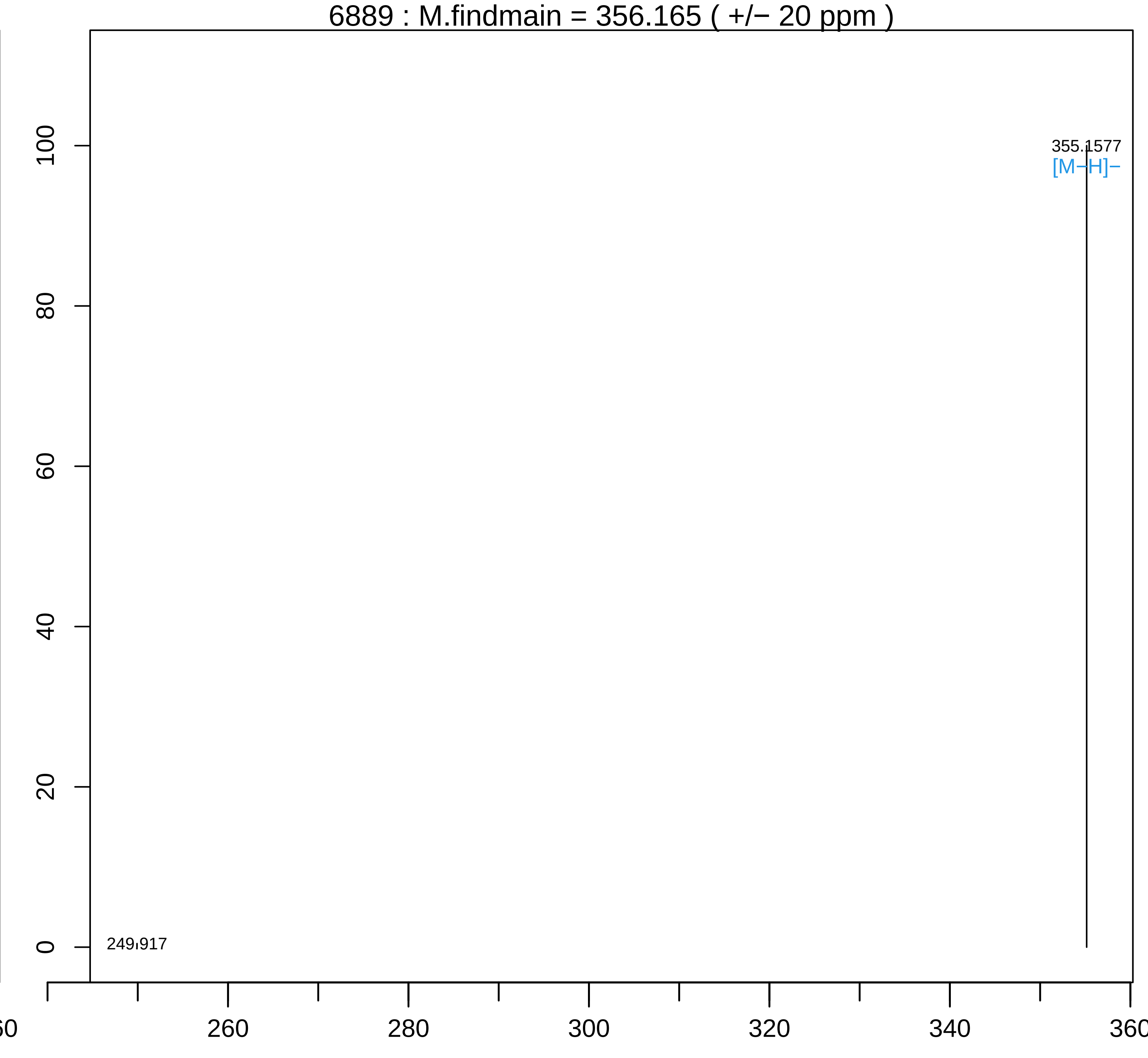
6889 : M.findmain = 356.165 (+/− 20 ppm)
355.1577
[M−H]−
249.917
0
20
40
60
80
100
260
280
300
320
340
360

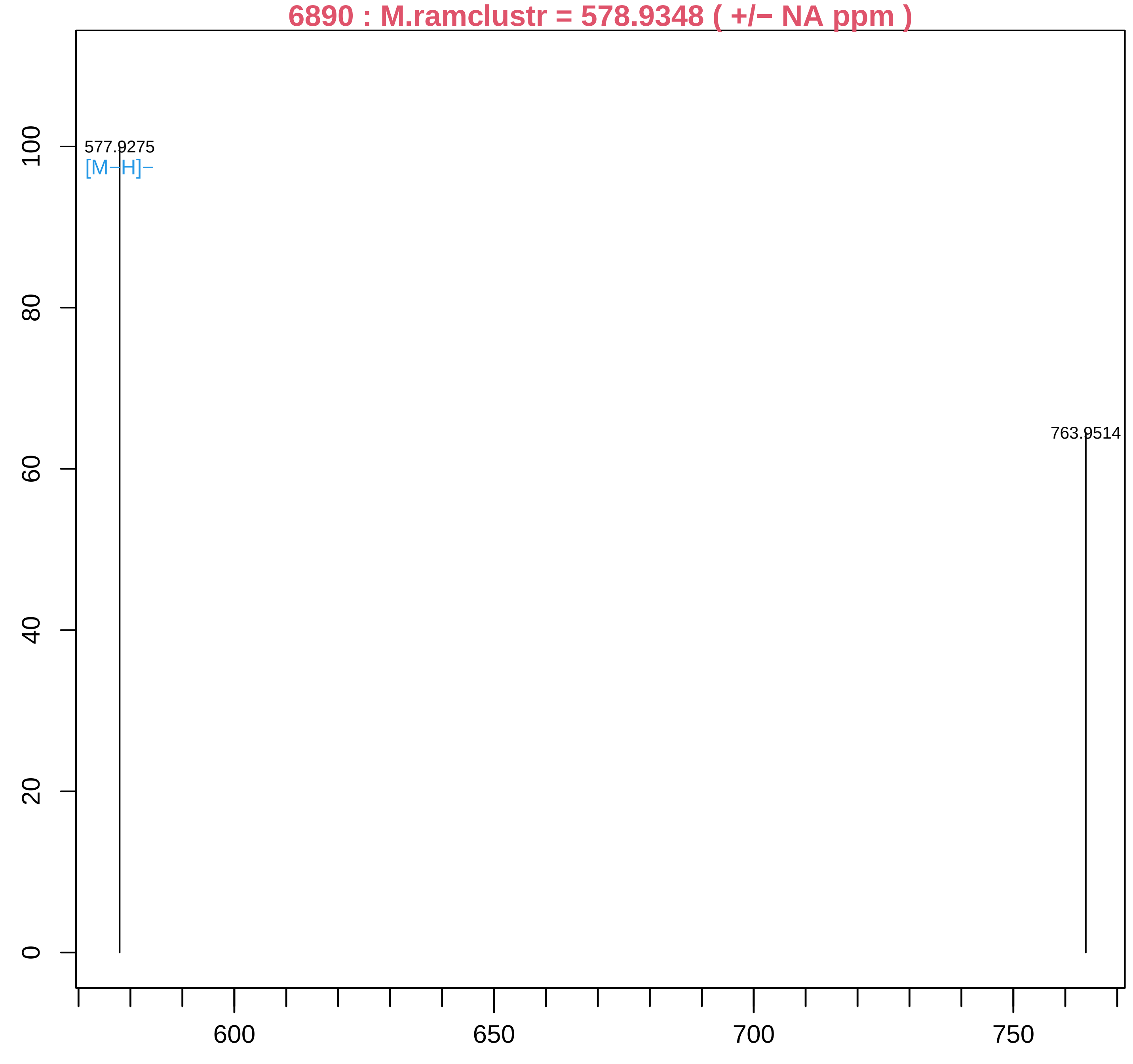
6890 : M.ramclustr = 578.9348 (+/− NA ppm)
577.9275
[M−H]−
763.9514
0
20
40
60
80
100
600
650
700
750

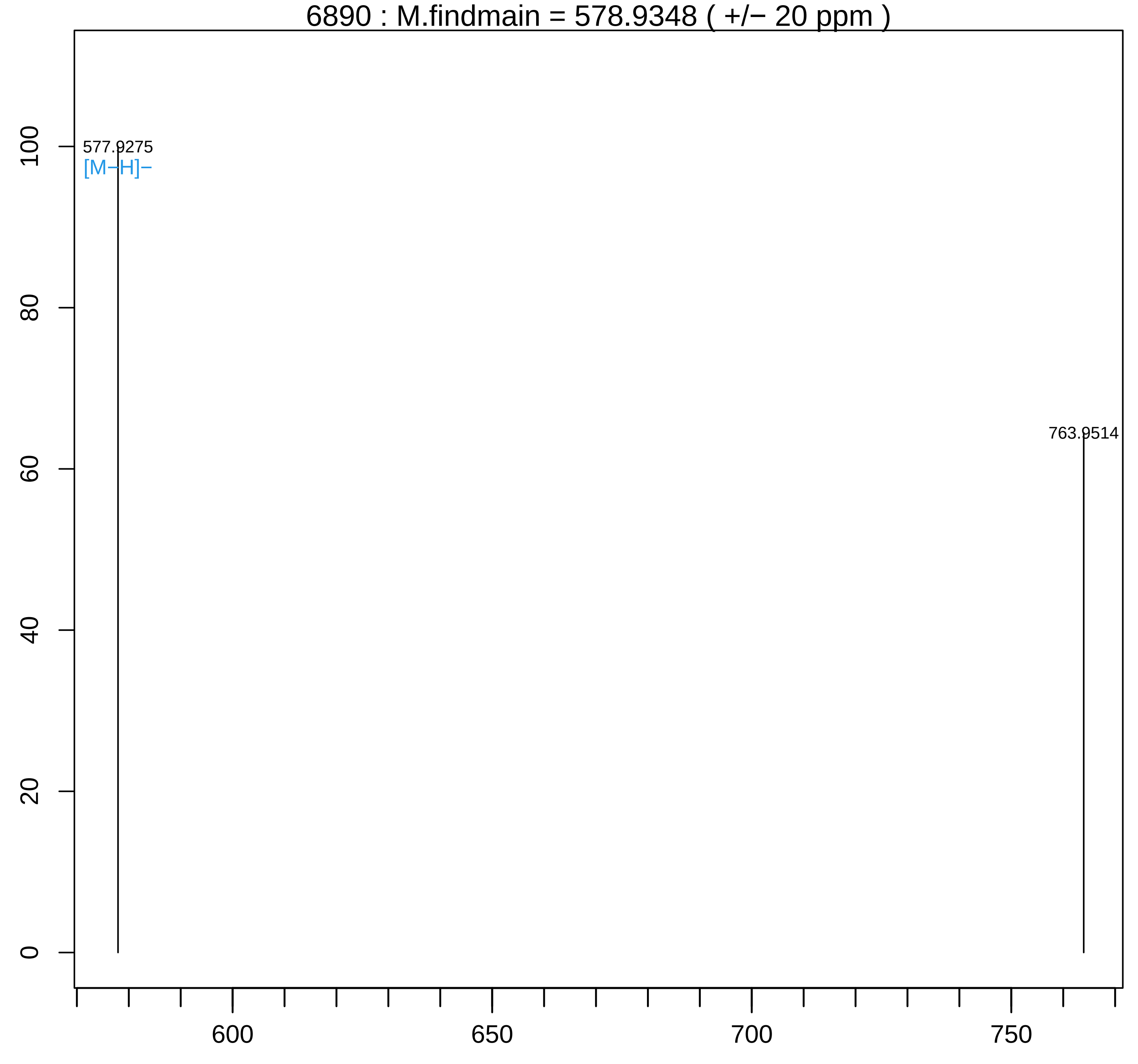
6890 : M.findmain = 578.9348 (+/− 20 ppm)
577.9275
[M−H]−
763.9514
0
20
40
60
80
100
600
650
700
750

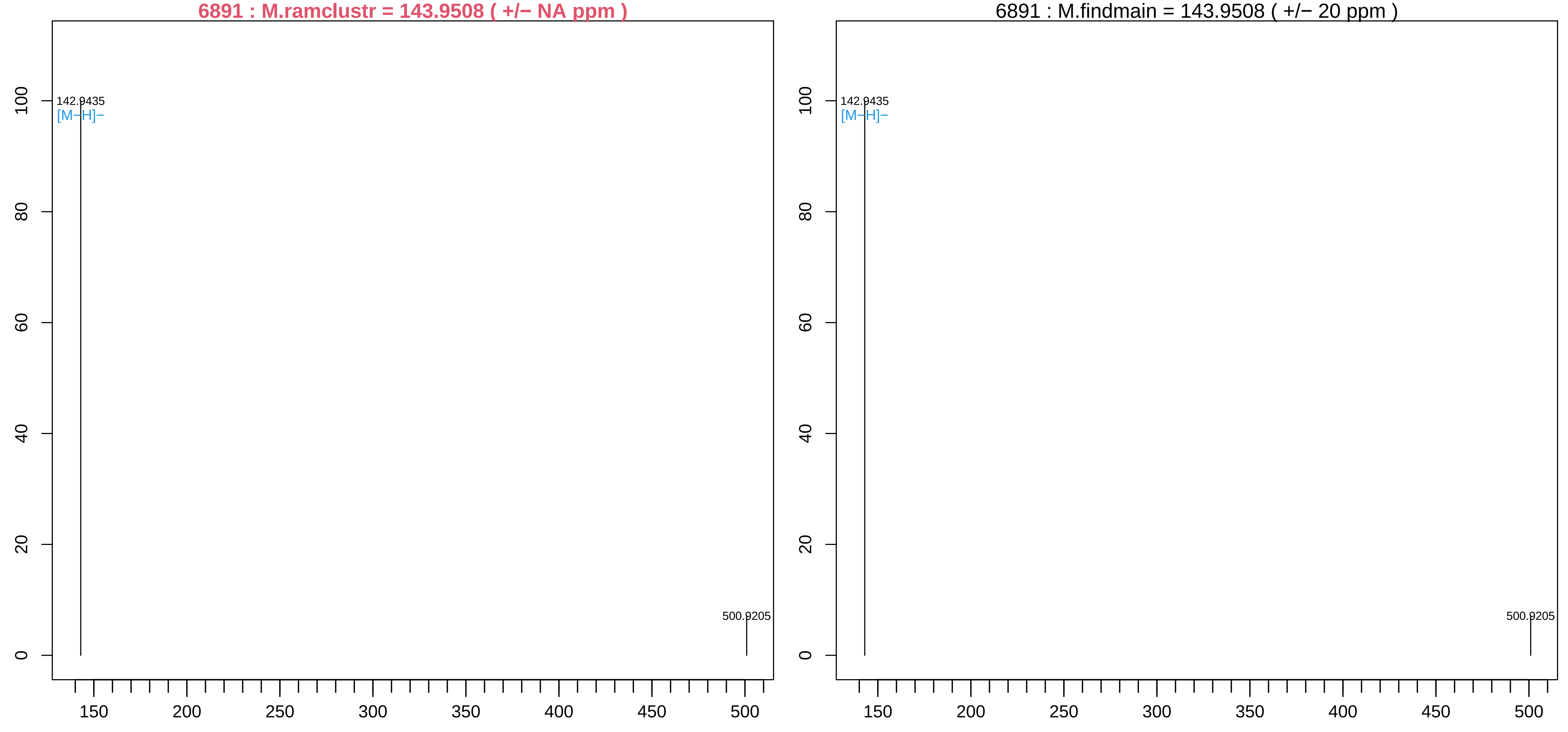

6891 : M.ramclustr = 143.9508 (+/− NA ppm)
142.9435
[M−H]−
500.9205
0
20
40
60
80
100
150
200
250
300
350
400
450
500
6891 : M.findmain = 143.9508 (+/− 20 ppm)
142.9435
[M−H]−
500.9205
0
20
40
60
80
100
150
200
250
300
350
400
450
500

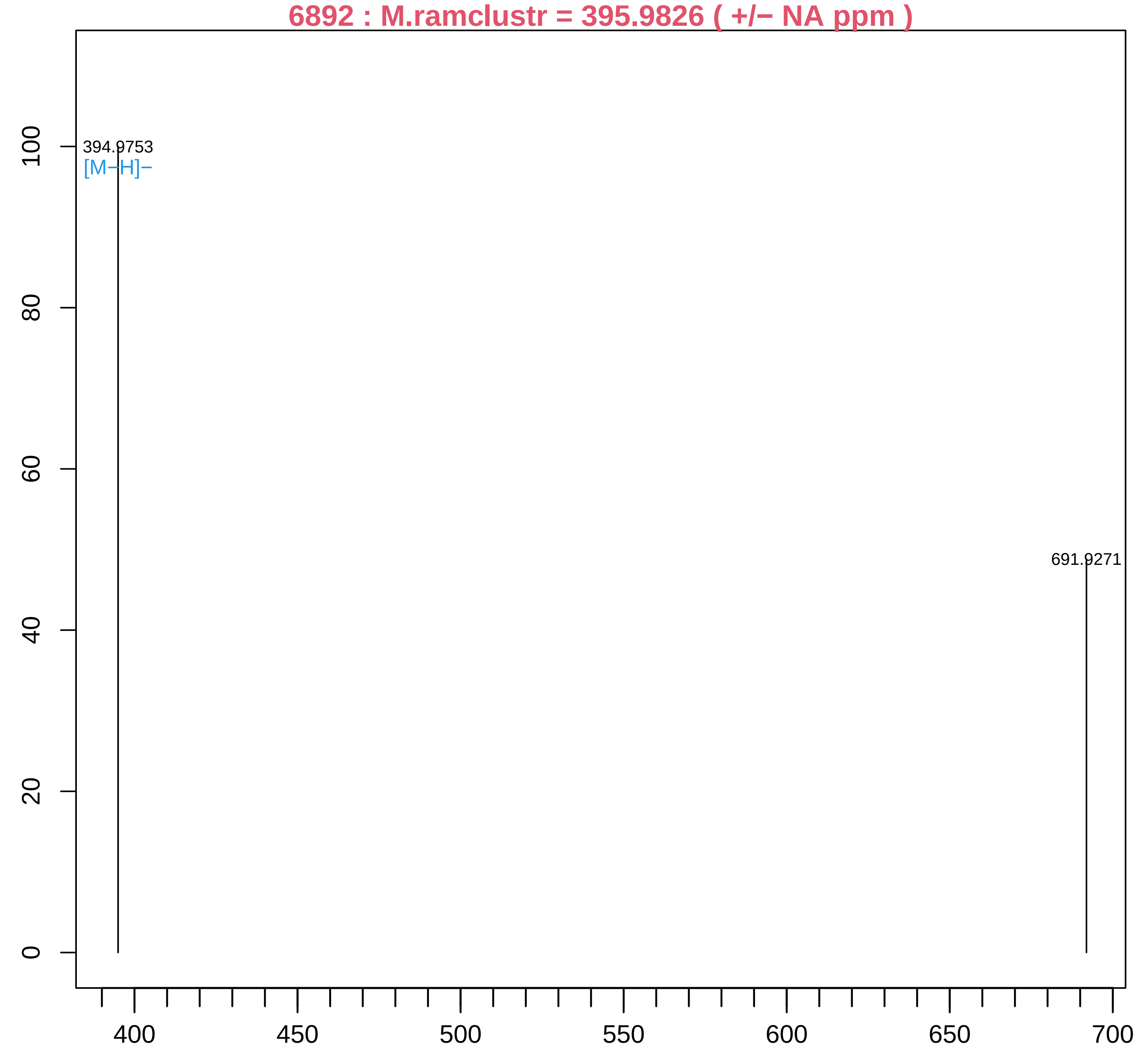

6892 : M.ramclustr = 395.9826 (+/− NA ppm)
394.9753
[M−H]−
691.9271
0
20
40
60
80
100
400
450
500
550
600
650
700

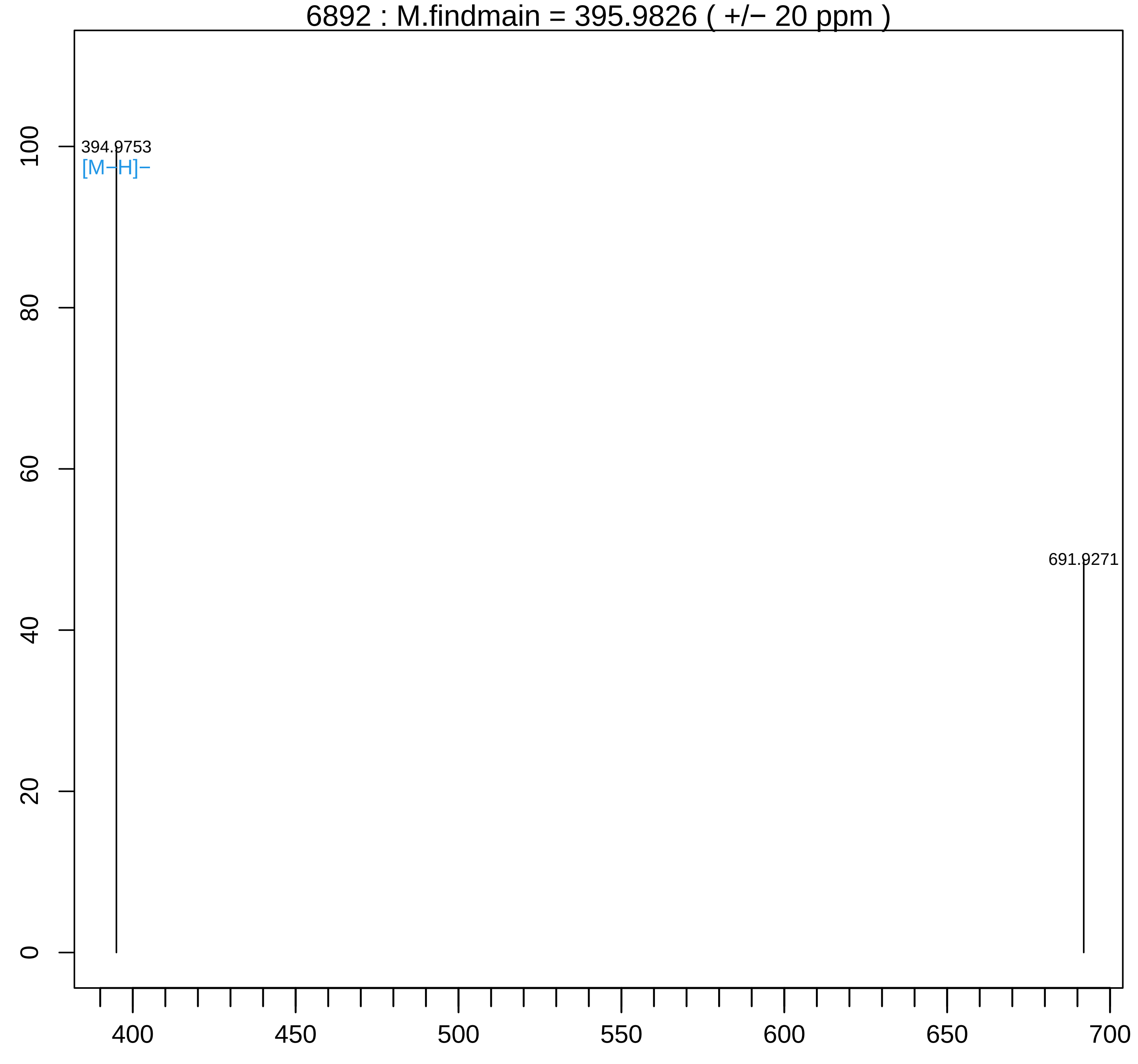

6892 : M.findmain = 395.9826 (+/− 20 ppm)
394.9753
[M−H]−
691.9271
0
20
40
60
80
100
400
450
500
550
600
650
700

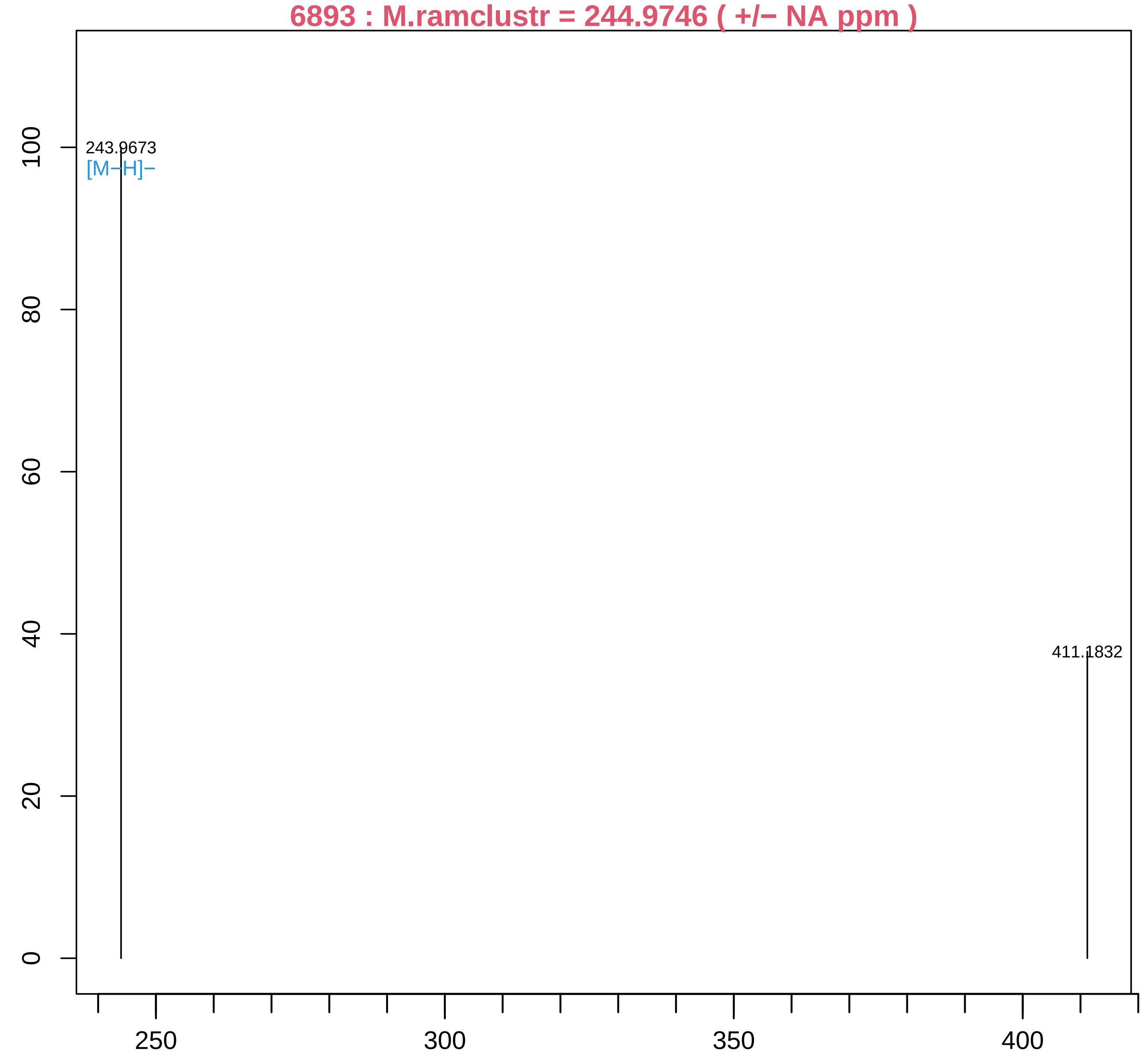
6893 : M.ramclustr = 244.9746 (+/− NA ppm)
243.9673
[M−H]−
411.1832
0
20
40
60
80
100
250
300
350
400

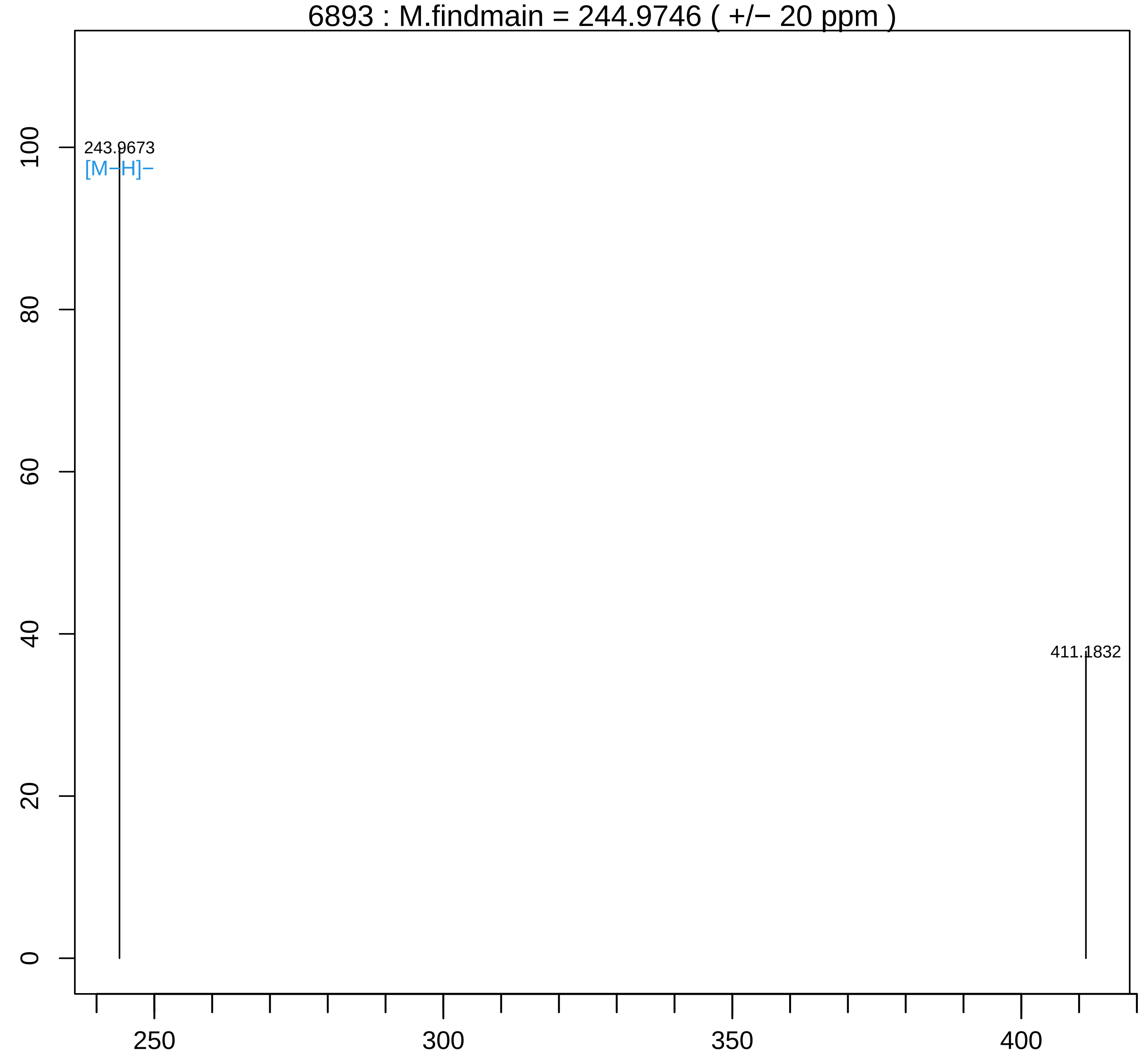
6893 : M.findmain = 244.9746 (+/− 20 ppm)
243.9673
[M−H]−
411.1832
0
20
40
60
80
100
250
300
350
400

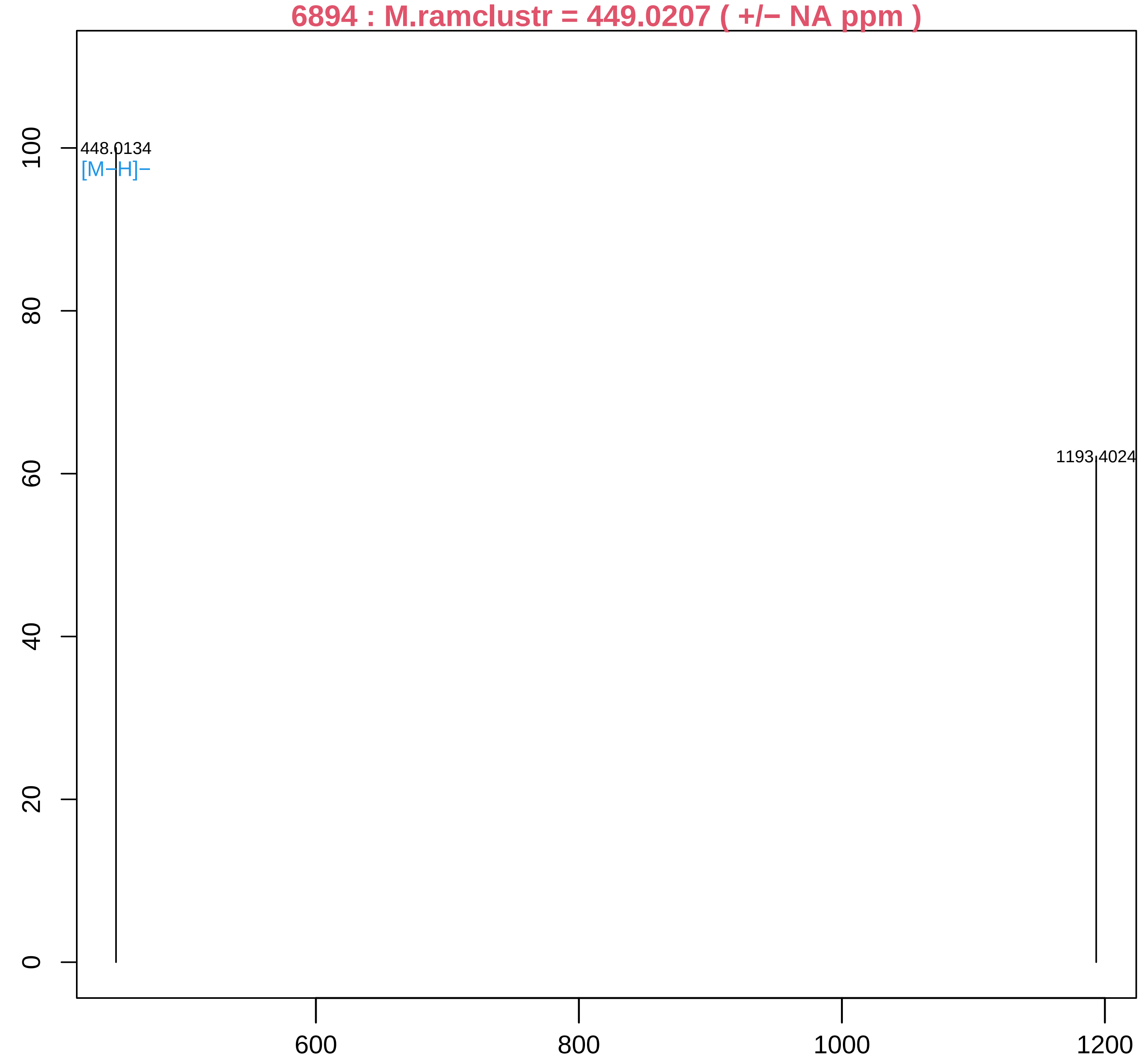
6894 : M.ramclustr = 449.0207 (+/− NA ppm)
448.0134
[M−H]−
1193.4024
0
20
40
60
80
100
600
800
1000
1200

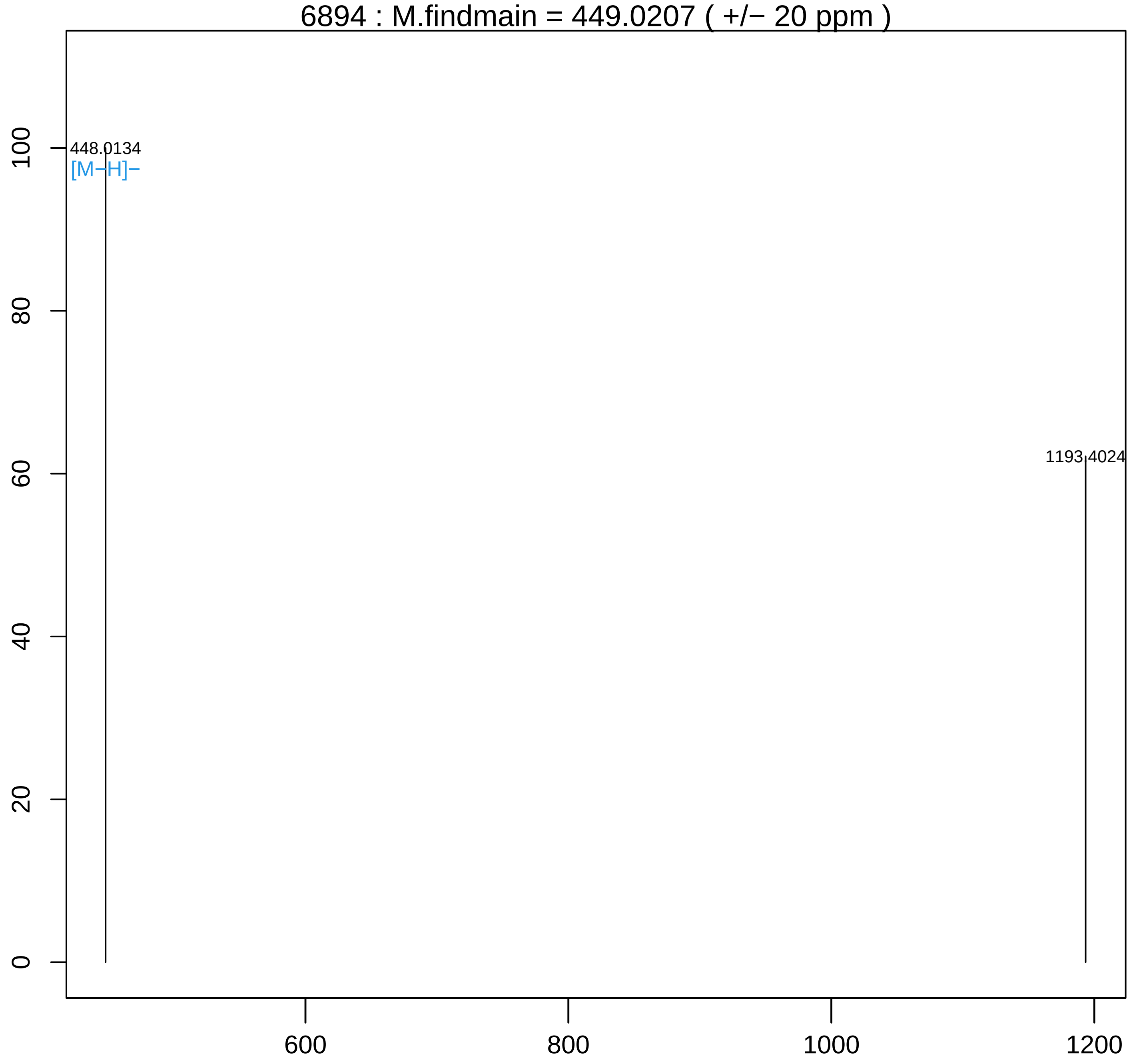
6894 : M.findmain = 449.0207 (+/− 20 ppm)
448.0134
[M−H]−
1193.4024
0
20
40
60
80
100
600
800
1000
1200

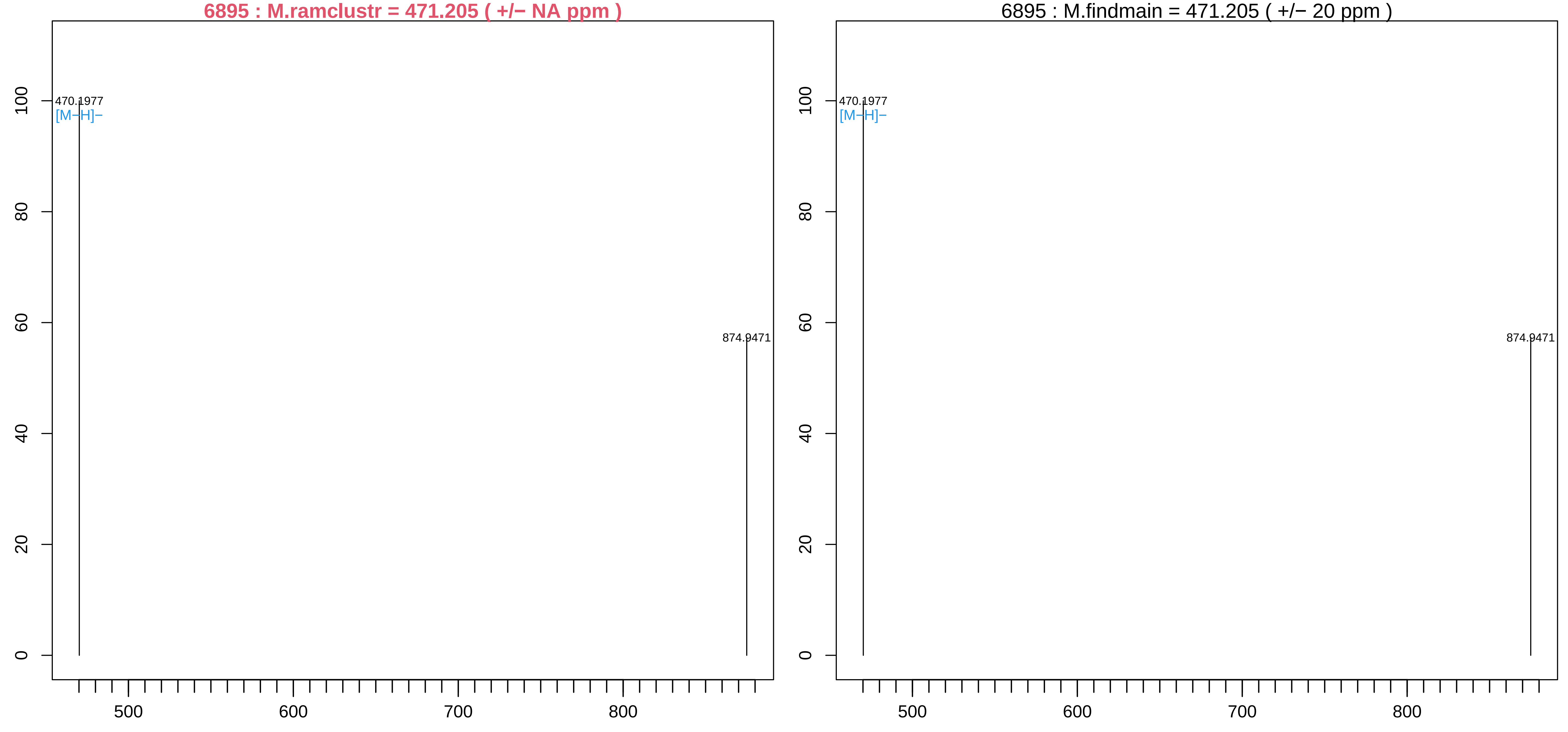
6895 : M.ramclustr = 471.205 (+/− NA ppm)
470.1977
[M−H]−
874.9471
6895 : M.findmain = 471.205 (+/− 20 ppm)
470.1977
[M−H]−
874.9471
0
20
40
60
80
100
500
600
700
800

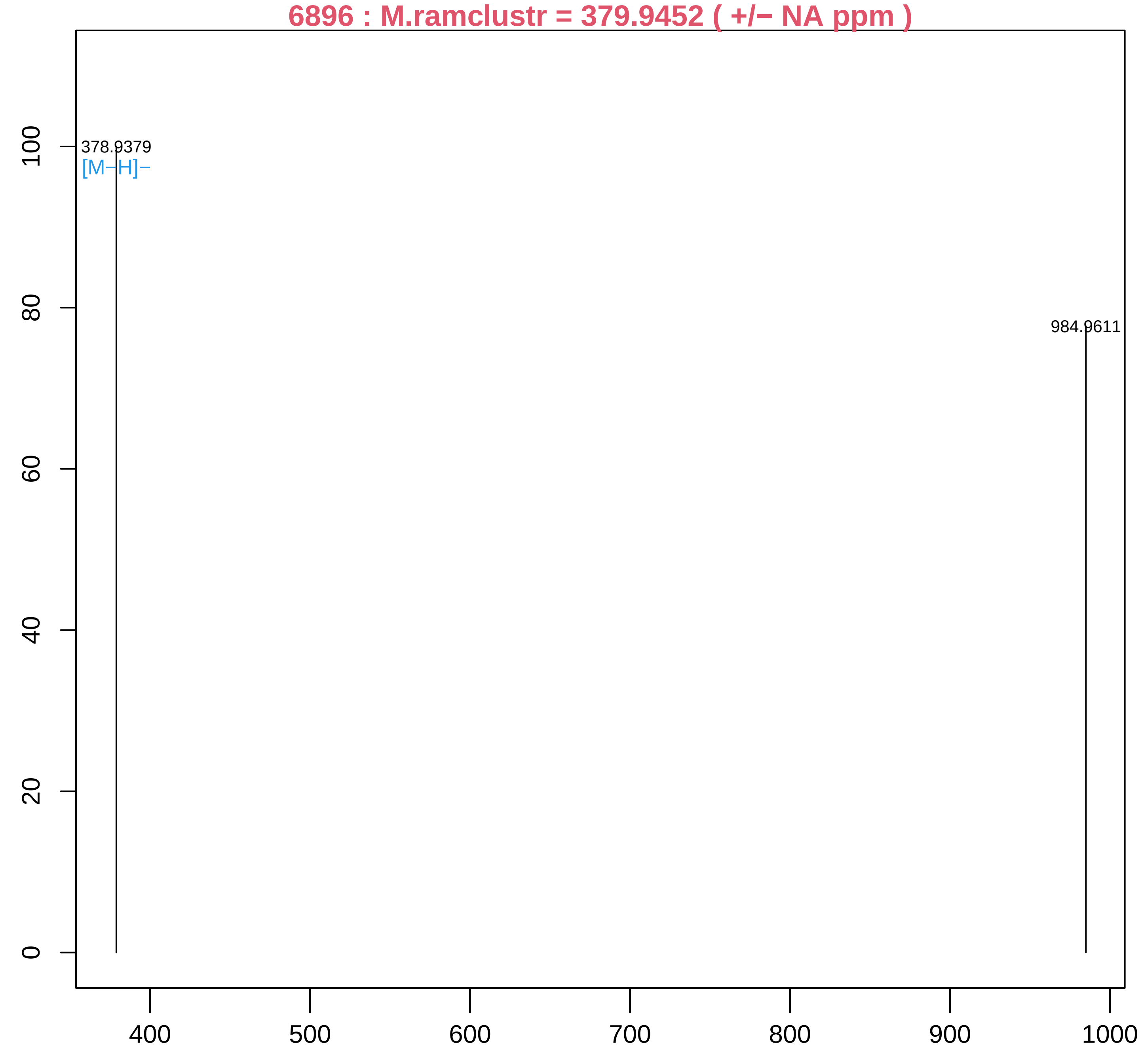
6896 : M.ramclustr = 379.9452 (+/− NA ppm)
378.9379
[M−H]−
984.9611
0
20
40
60
80
100
400
500
600
700
800
900
1000

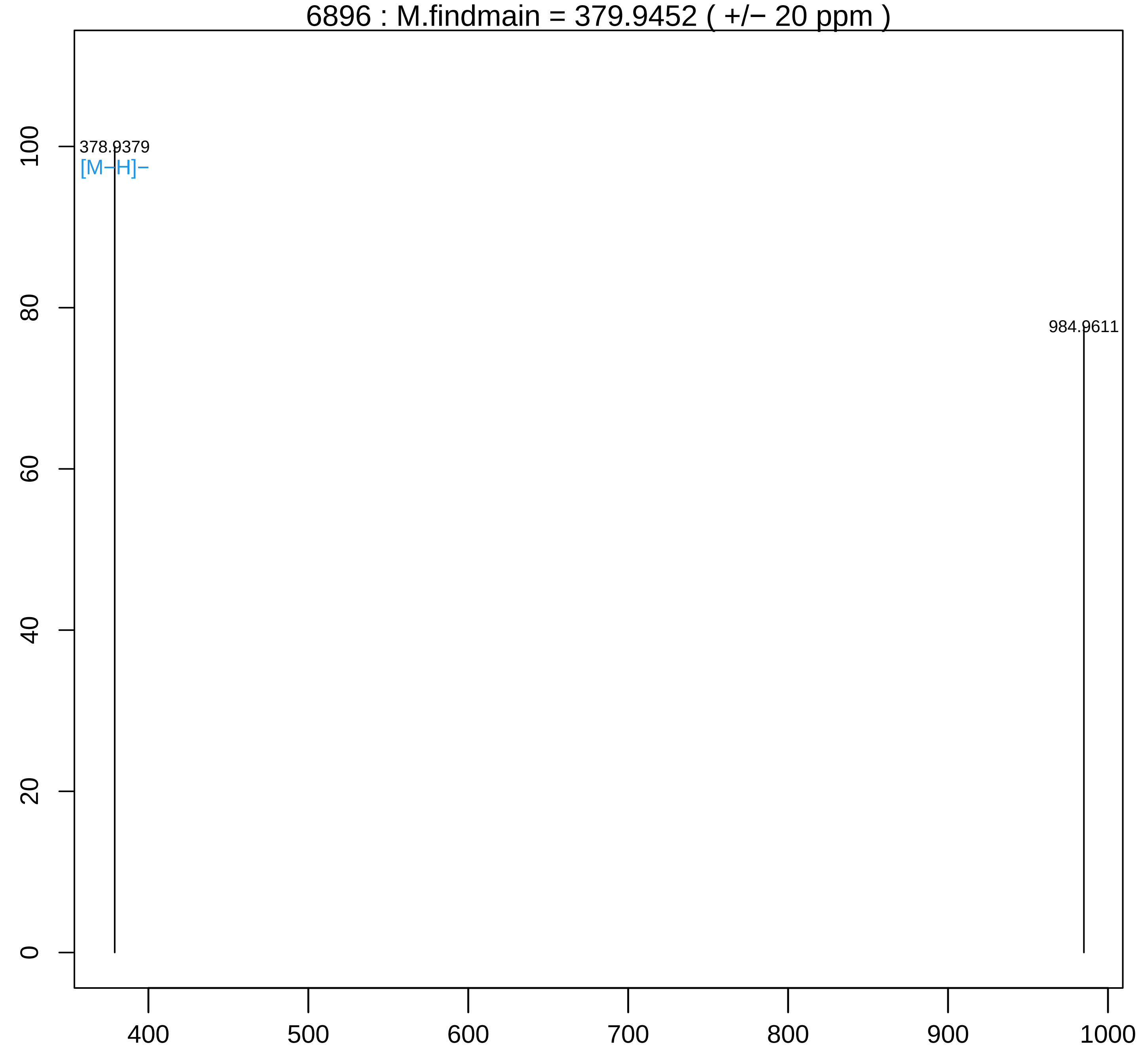
6896 : M.findmain = 379.9452 (+/− 20 ppm)
378.9379
[M−H]−
984.9611
0
20
40
60
80
100
400
500
600
700
800
900
1000

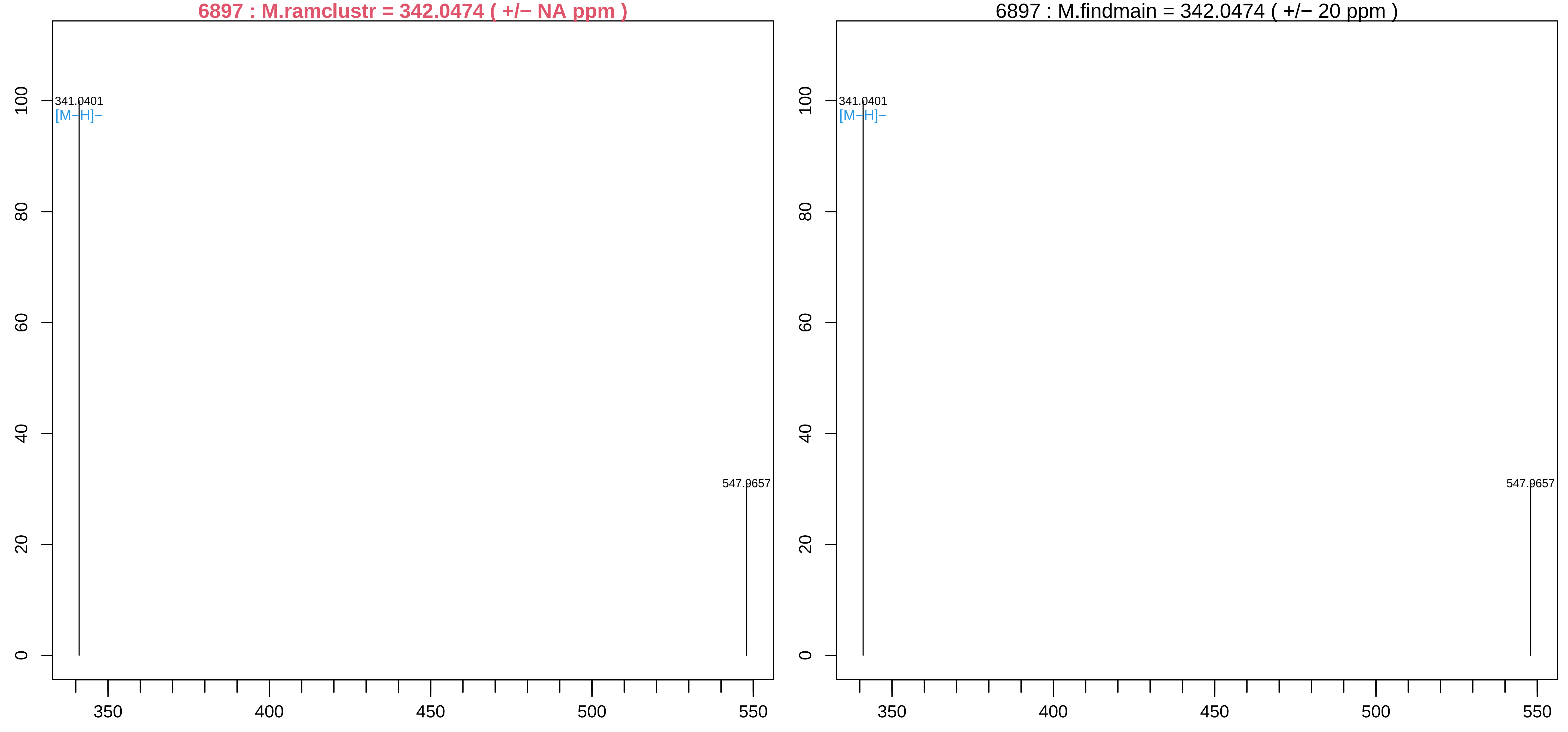

6897 : M.ramclustr = 342.0474 (+/− NA ppm)
341.0401
[M−H]−
547.9657
0
20
40
60
80
100
350
400
450
500
550
6897 : M.findmain = 342.0474 (+/− 20 ppm)
341.0401
[M−H]−
547.9657
0
20
40
60
80
100
350
400
450
500
550

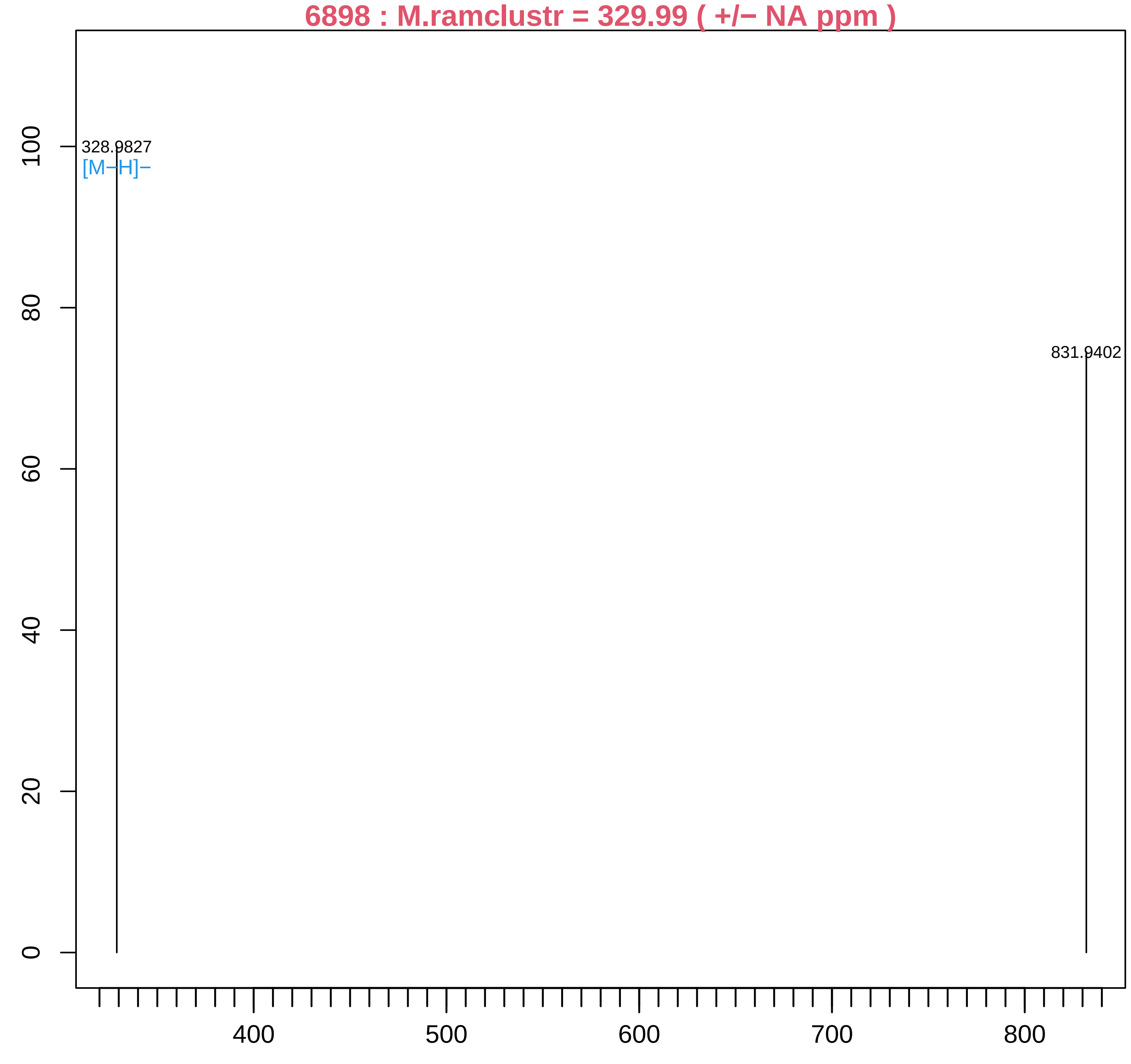
6898 : M.ramclustr = 329.99 (+/− NA ppm)
328.9827
[M−H]−
831.9402

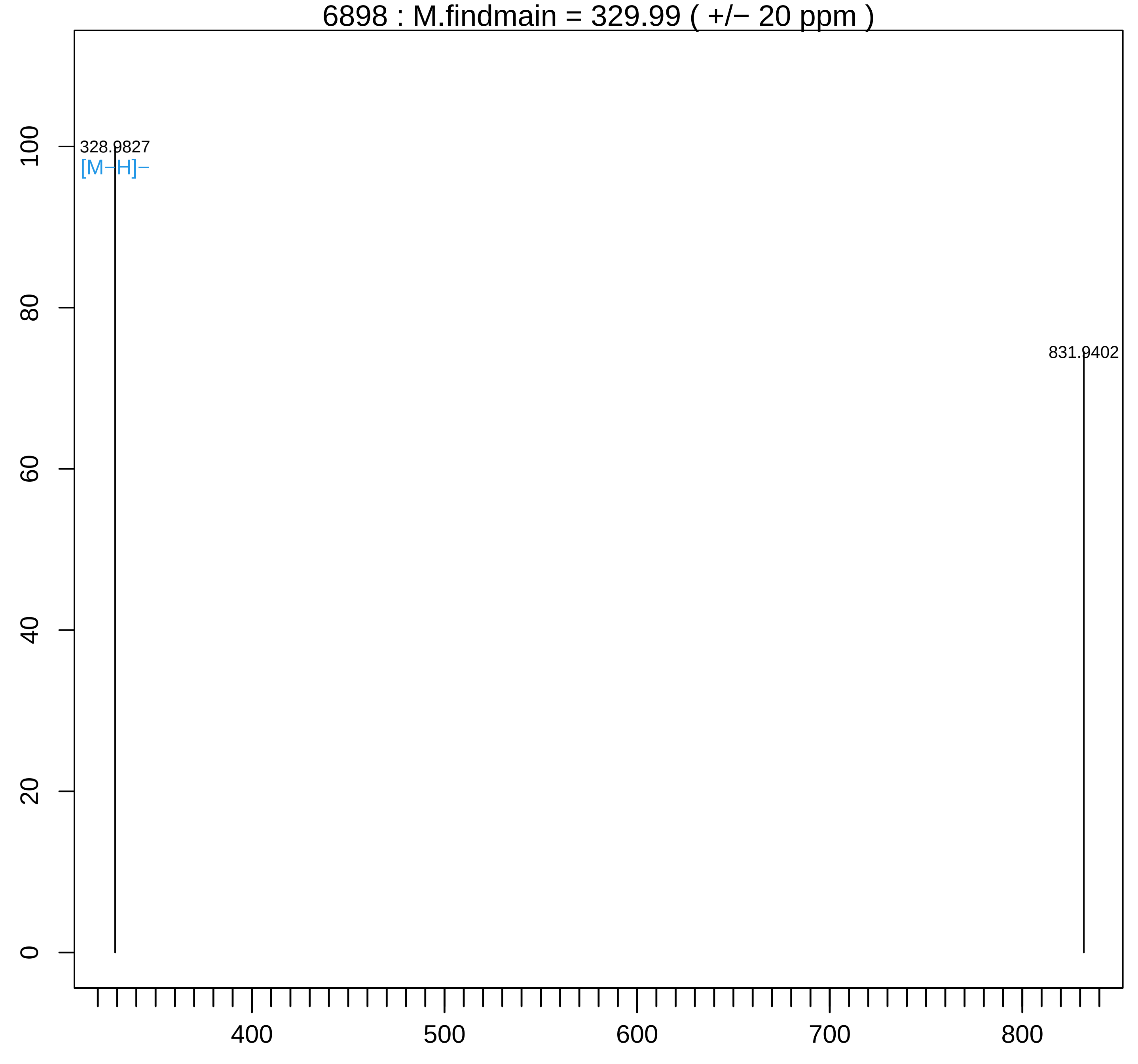
6898 : M.findmain = 329.99 (+/− 20 ppm)
328.9827
[M−H]−
831.9402

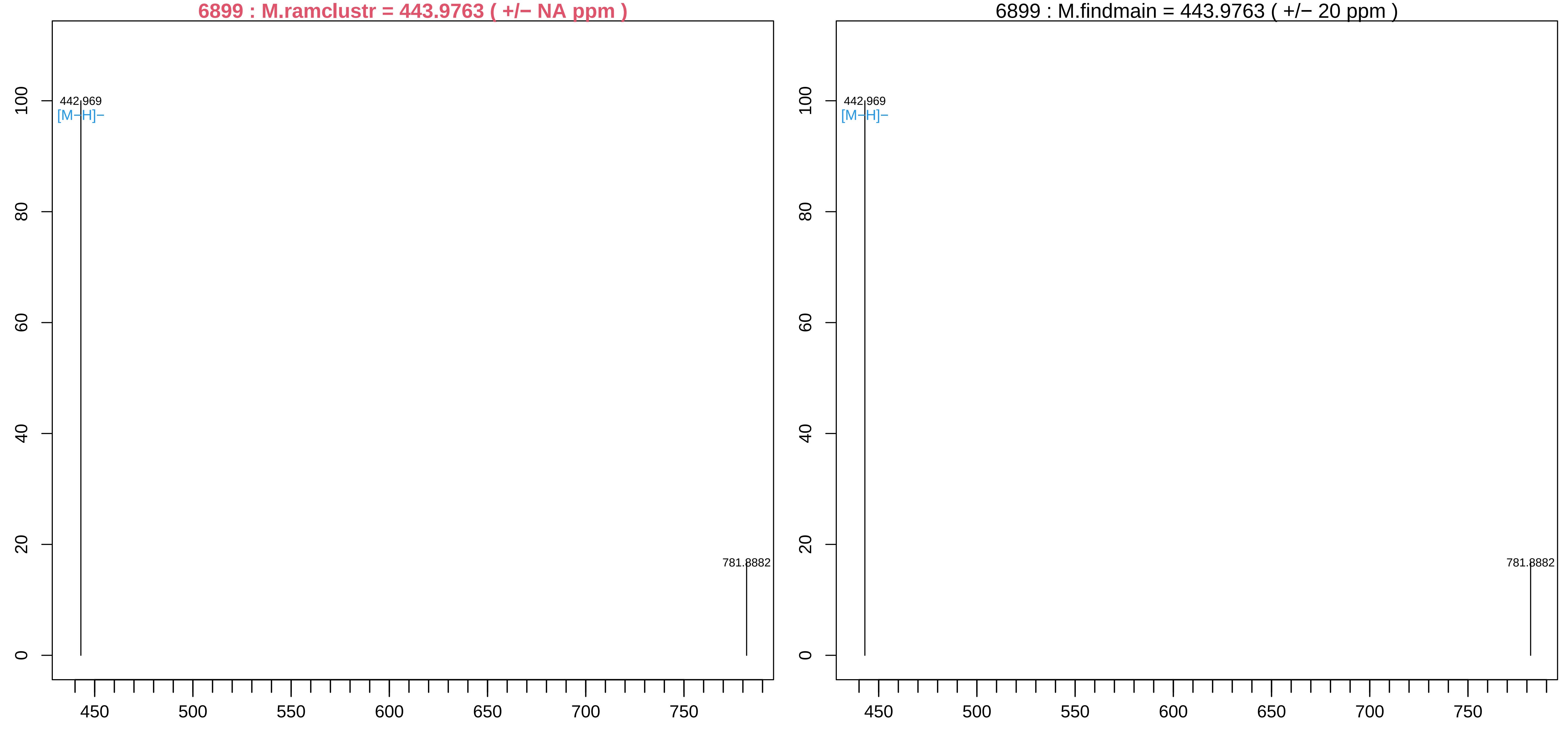

6899 : M.ramclustr = 443.9763 (+/− NA ppm)
442.969
[M−H]−
781.8882
0
20
40
60
80
100
450
500
550
600
650
700
750
6899 : M.findmain = 443.9763 (+/− 20 ppm)
442.969
[M−H]−
781.8882
0
20
40
60
80
100
450
500
550
600
650
700
750

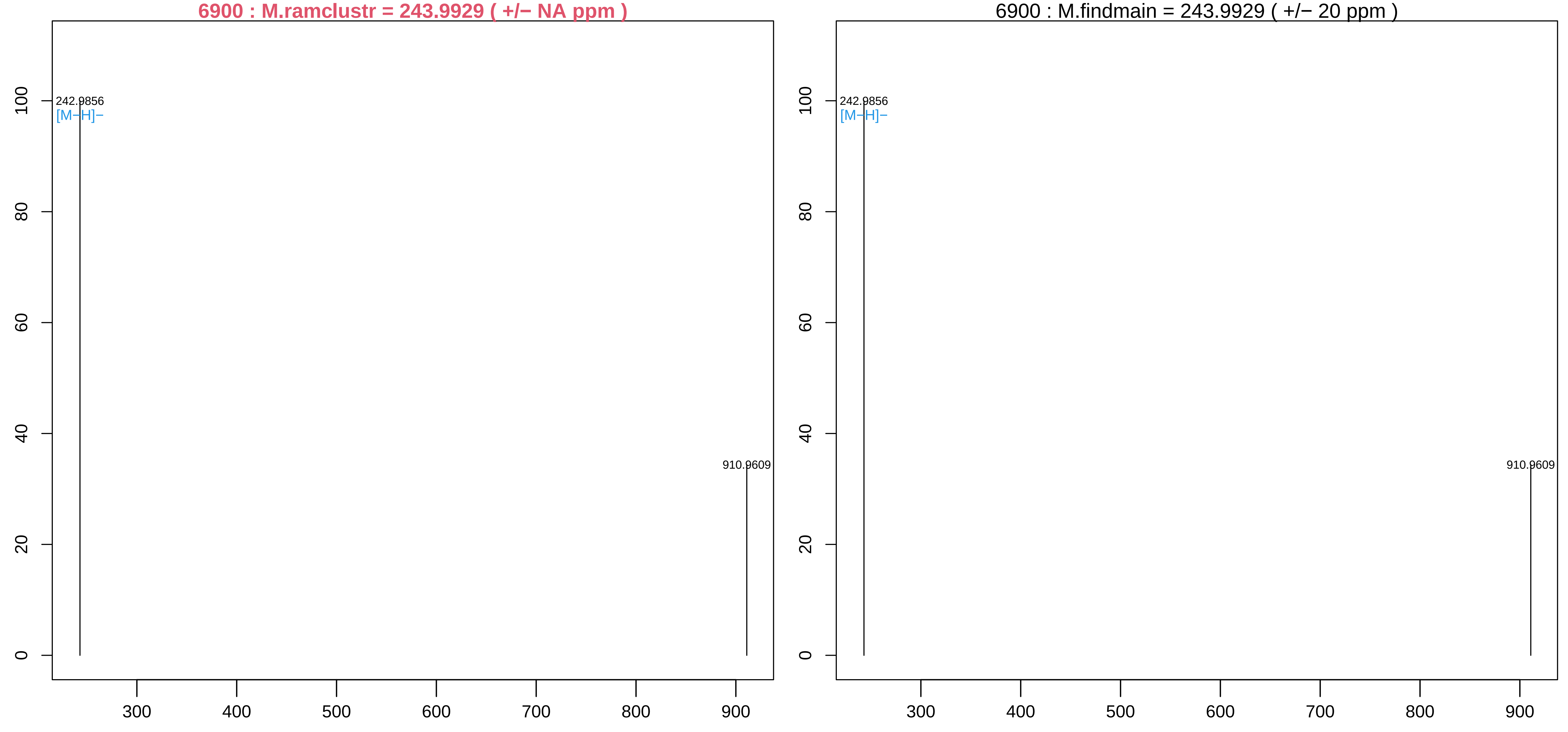
6900 : M.ramclustr = 243.9929 (+/− NA ppm)
6900 : M.findmain = 243.9929 (+/− 20 ppm)
242.9856
[M−H]−
910.9609
0
20
40
60
80
100
300
400
500
600
700
800
900

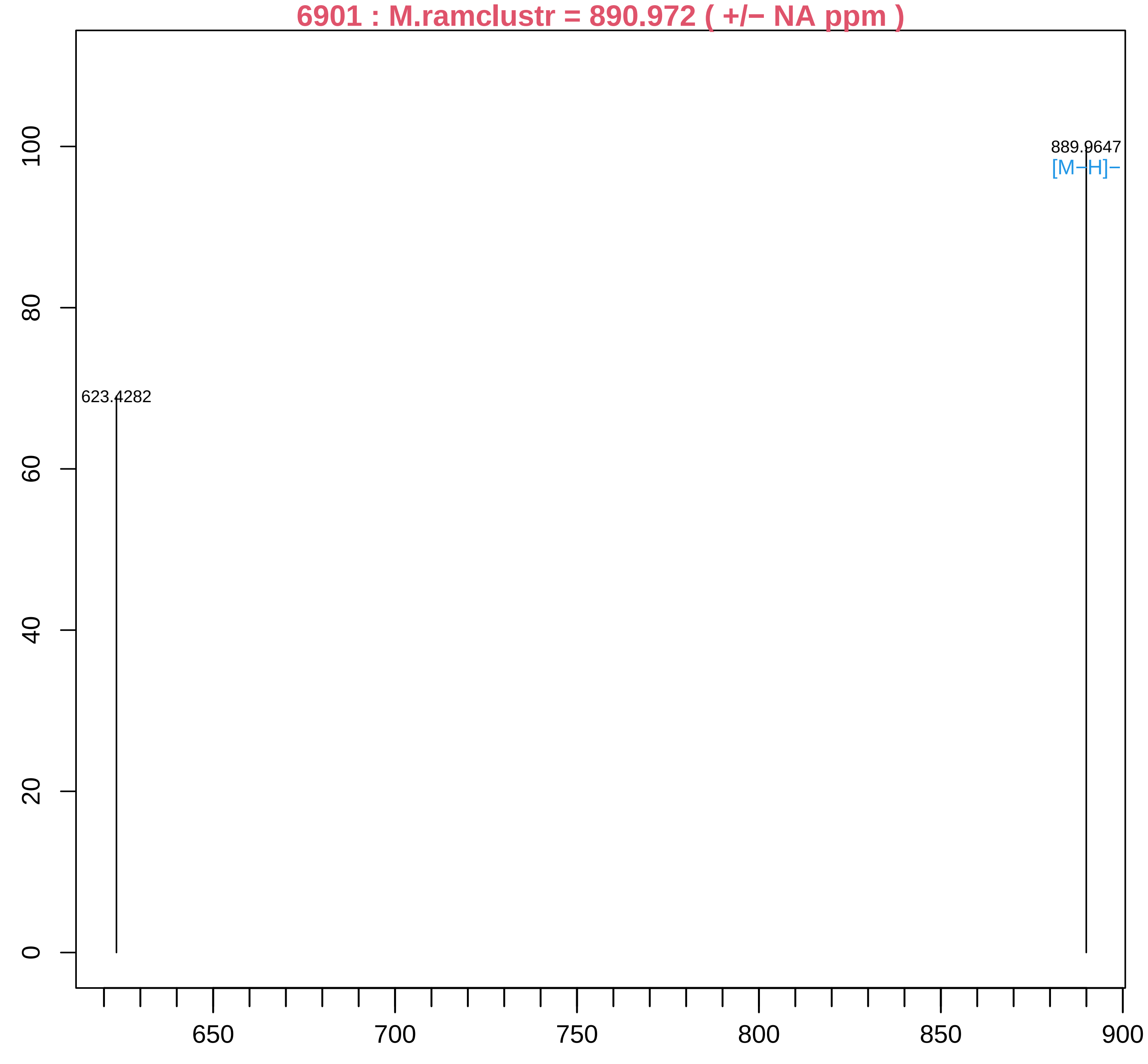

6901 : M.ramclustr = 890.972 (+/− NA ppm)
889.9647
[M−H]−
623.4282
0
20
40
60
80
100
650
700
750
800
850
900

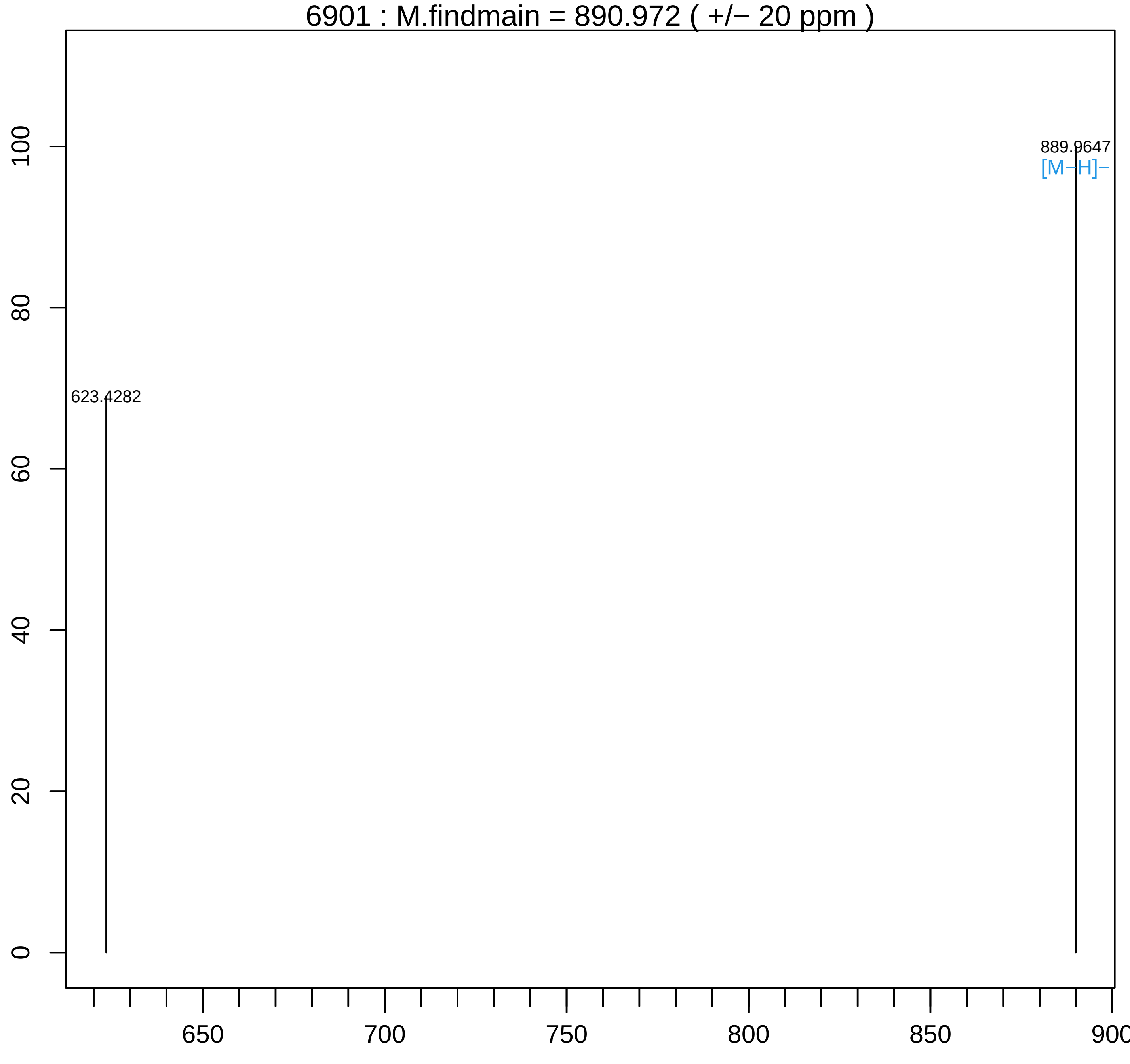

6901 : M.findmain = 890.972 (+/− 20 ppm)
889.9647
[M−H]−
623.4282
0
20
40
60
80
100
650
700
750
800
850
900

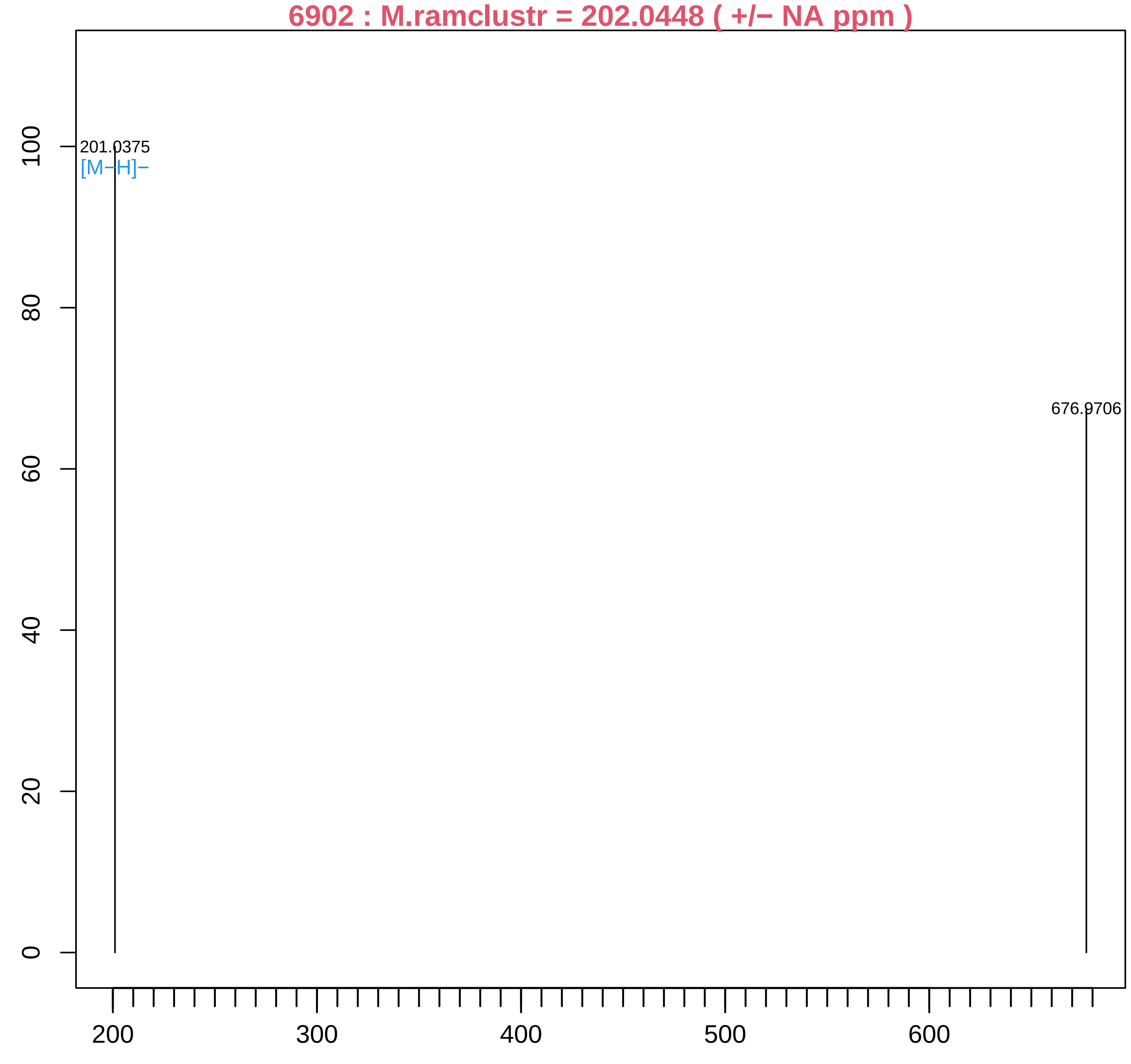
6902 : M.ramclustr = 202.0448 (+/− NA ppm)
201.0375
[M−H]−
676.9706
0
20
40
60
80
100
200
300
400
500
600
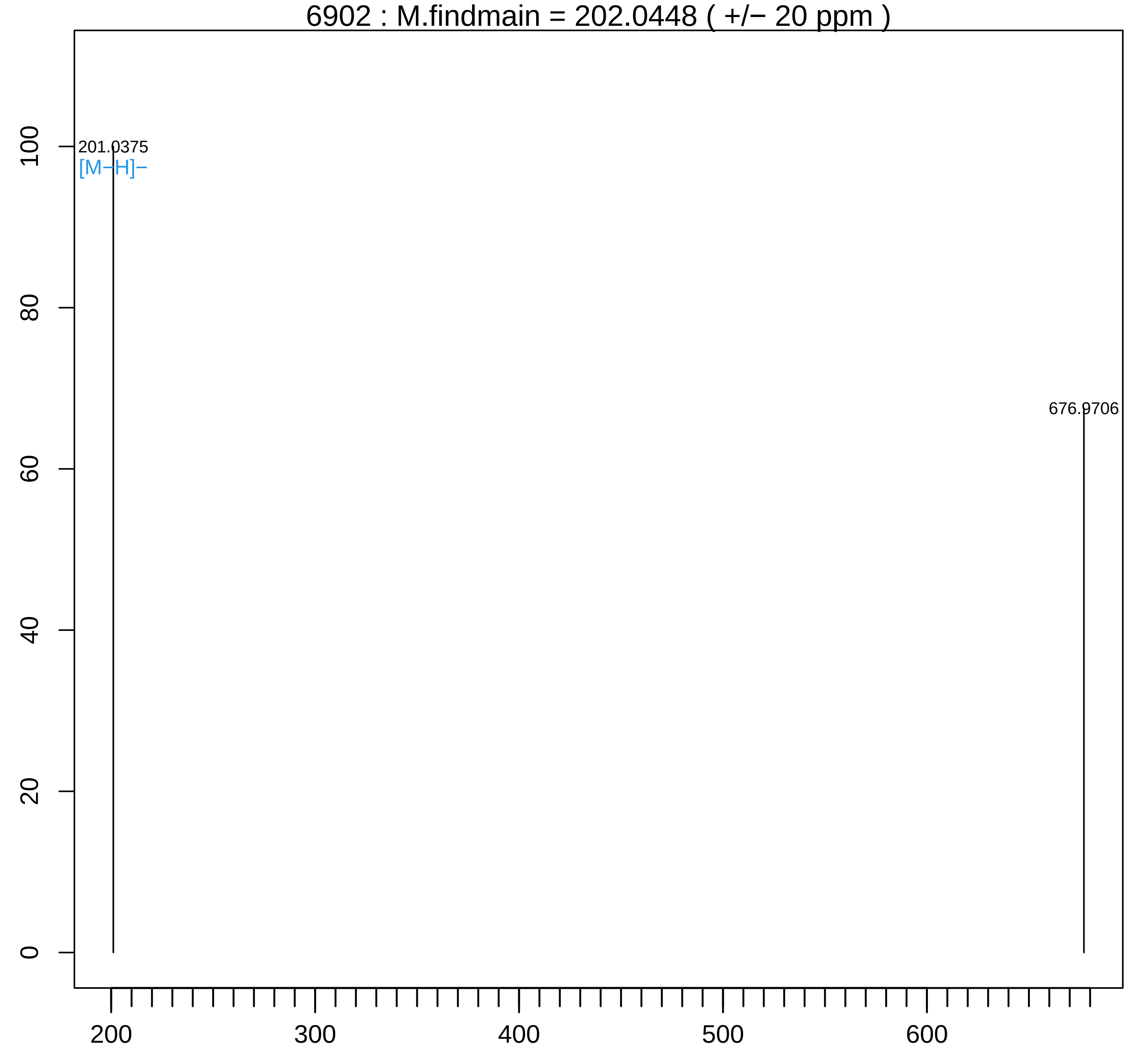
6902 : M.findmain = 202.0448 (+/− 20 ppm)
201.0375
[M−H]−
676.9706
0
20
40
60
80
100
200
300
400
500
600

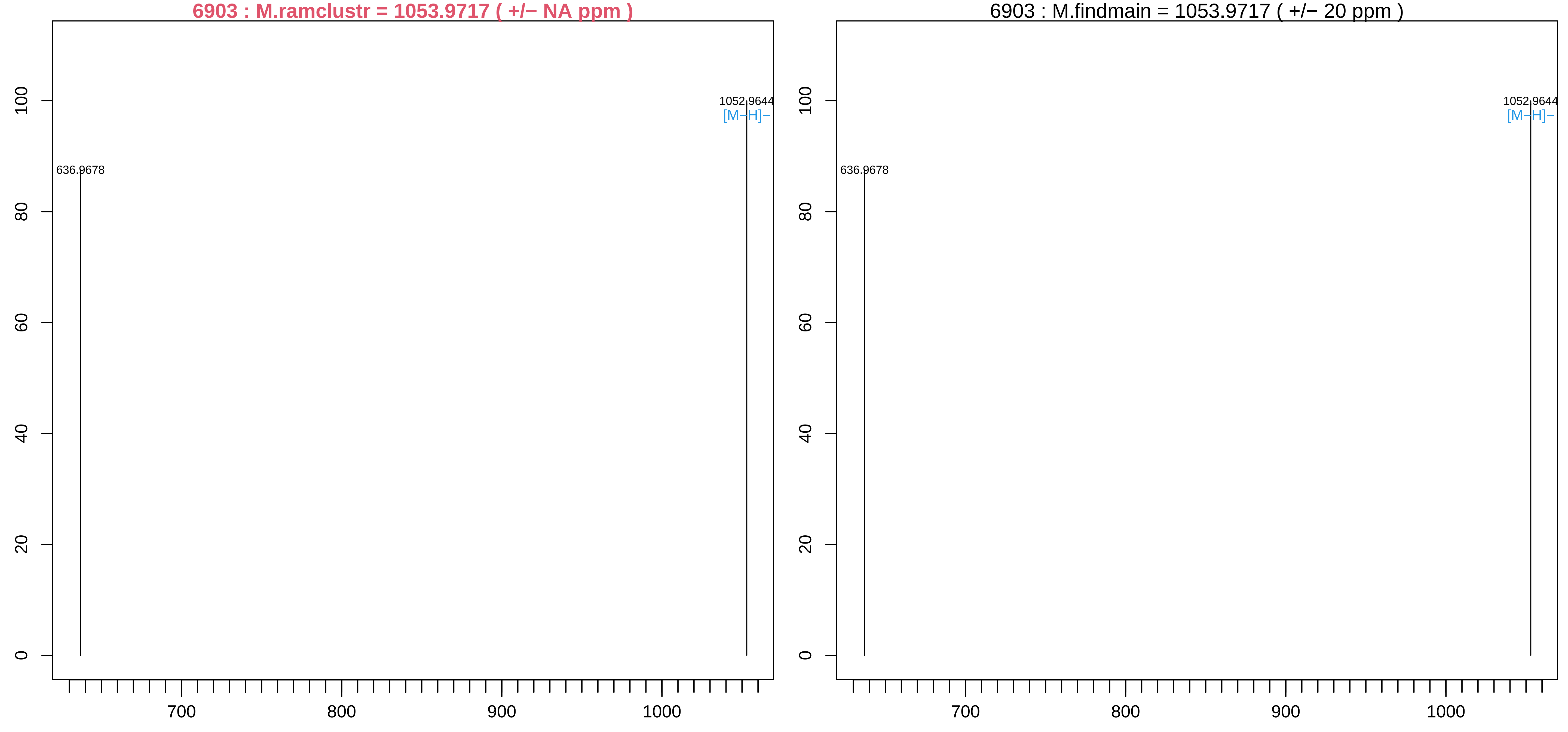
6903 : M.ramclustr = 1053.9717 (+/− NA ppm)
636.9678
1052.9644
[M−H]−
0
20
40
60
80
100
700
800
900
1000
6903 : M.findmain = 1053.9717 (+/− 20 ppm)
636.9678
1052.9644
[M−H]−
0
20
40
60
80
100
700
800
900
1000

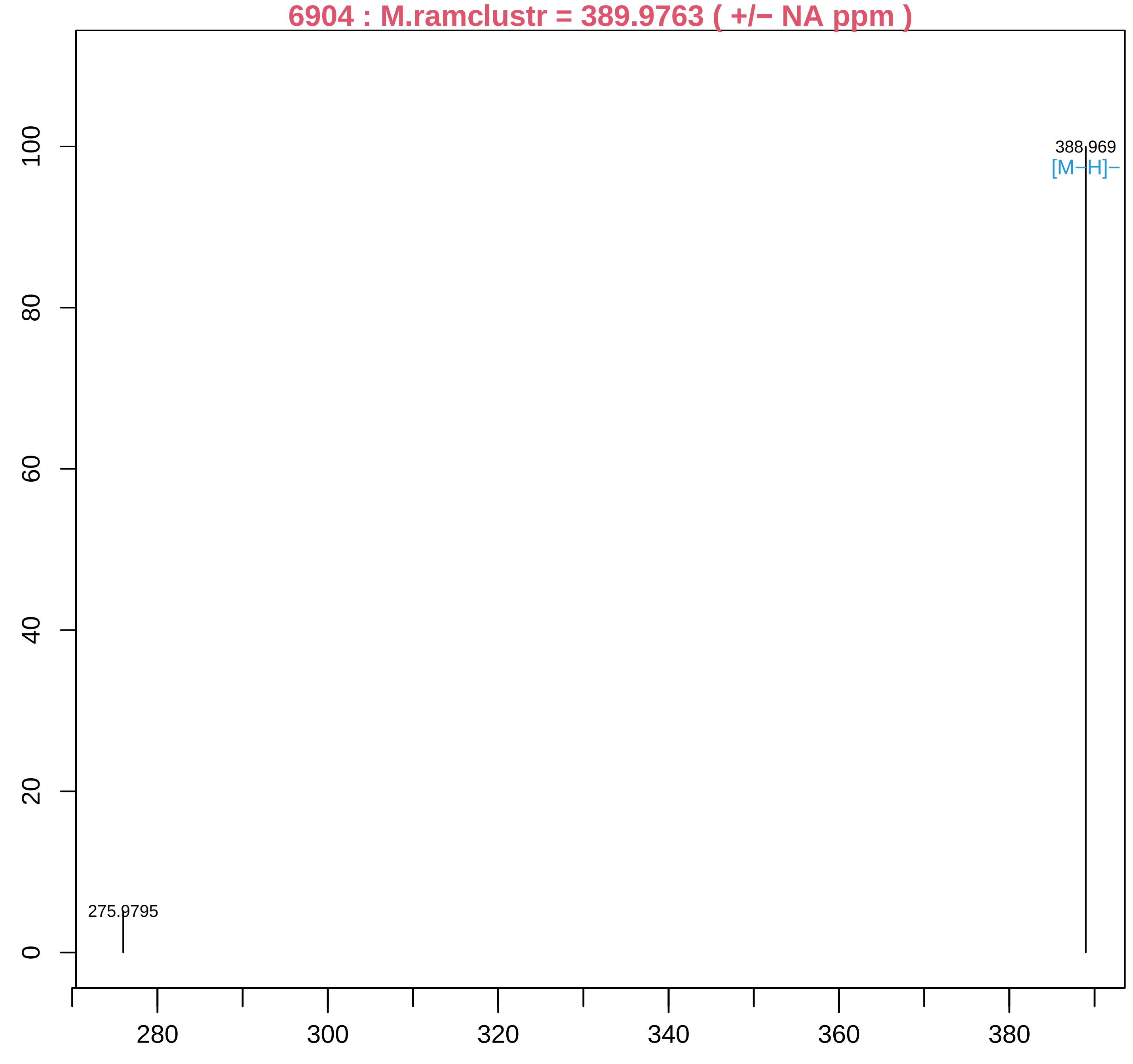
6904 : M.ramclustr = 389.9763 (+/− NA ppm)
388.969
[M−H]−
275.9795
0
20
40
60
80
100
280
300
320
340
360
380

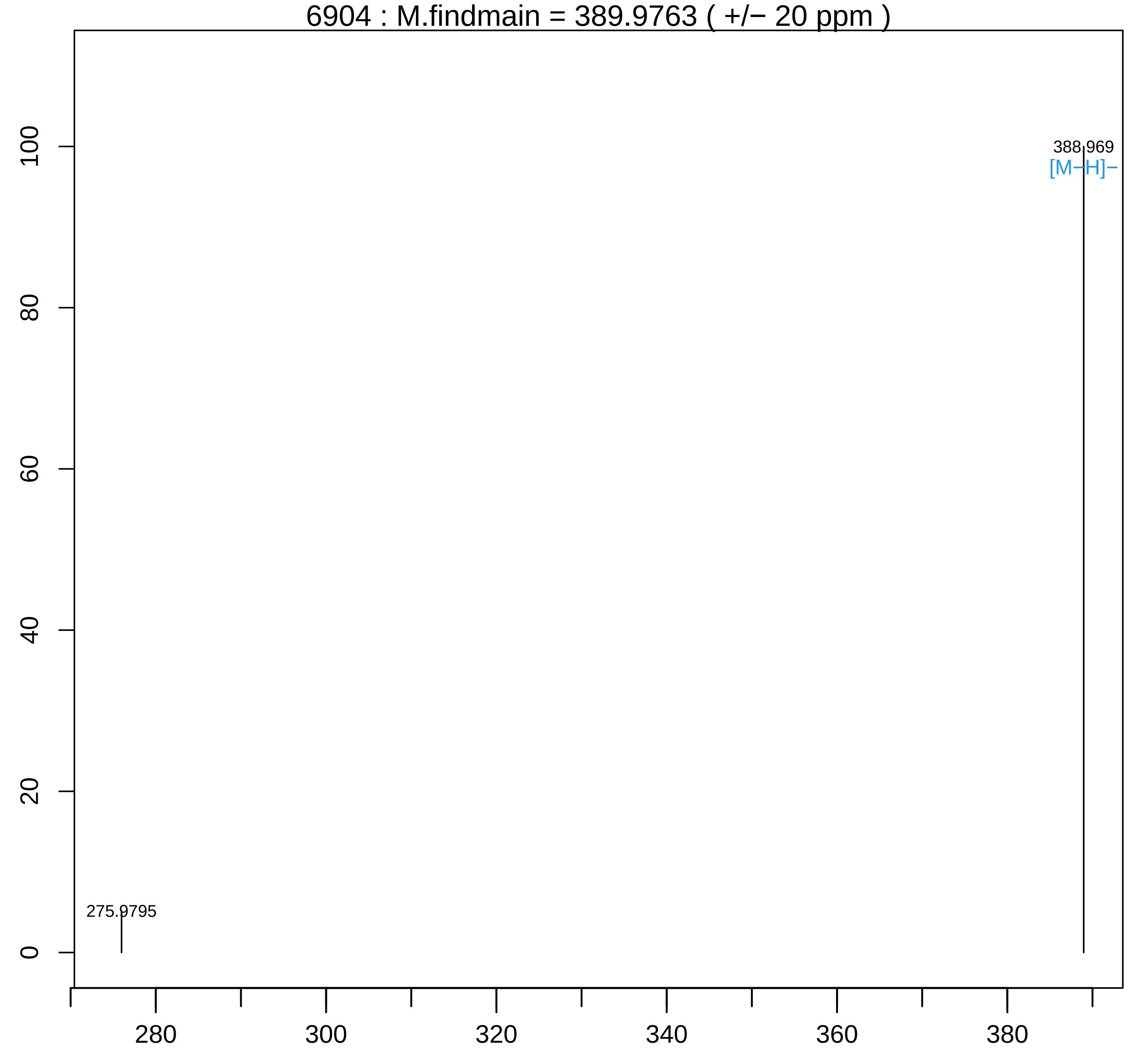
6904 : M.findmain = 389.9763 (+/− 20 ppm)
388.969
[M−H]−
275.9795
0
20
40
60
80
100
280
300
320
340
360
380

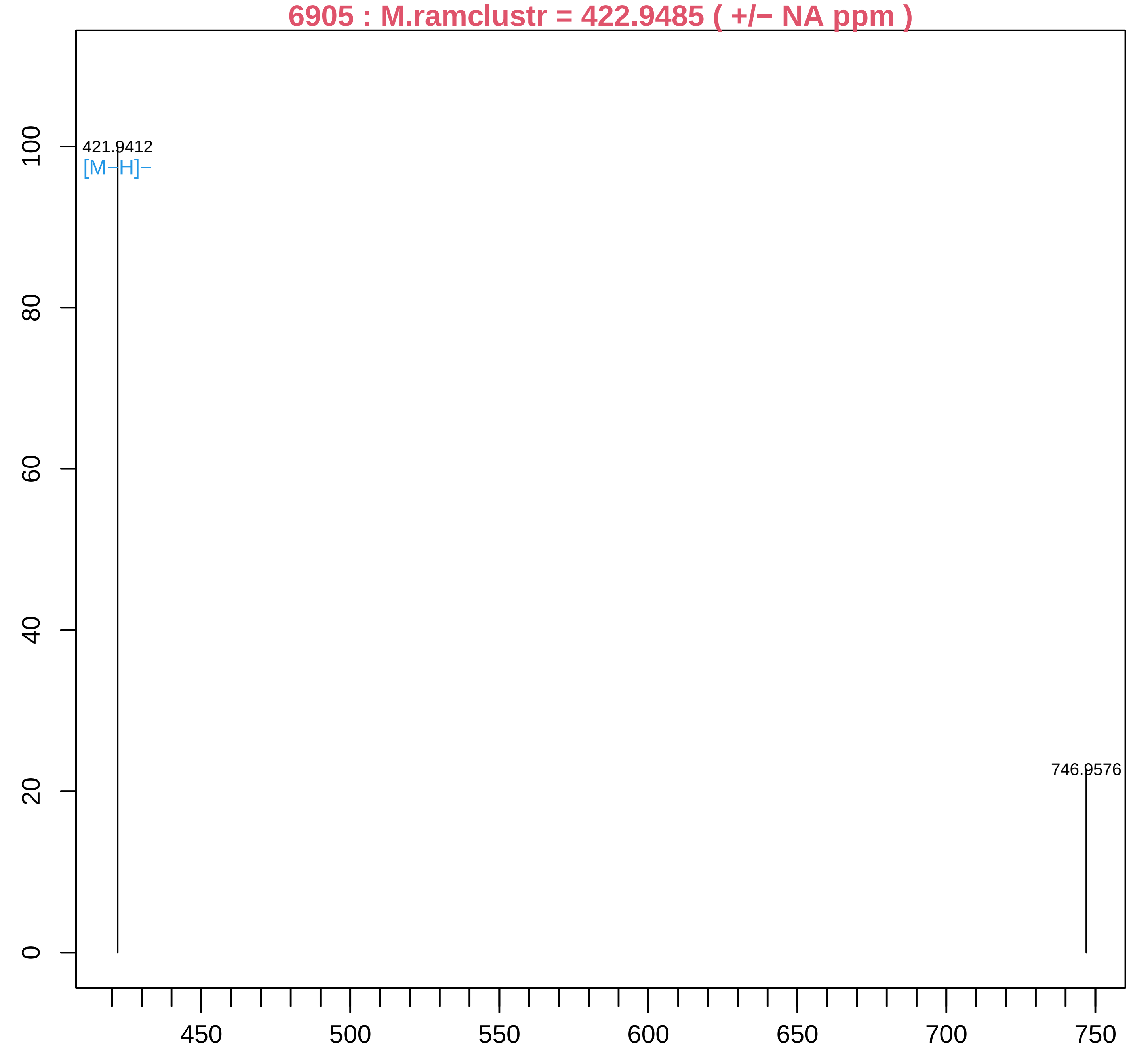
6905 : M.ramclustr = 422.9485 (+/− NA ppm)
421.9412
[M−H]−
746.9576
0
20
40
60
80
100
450
500
550
600
650
700
750

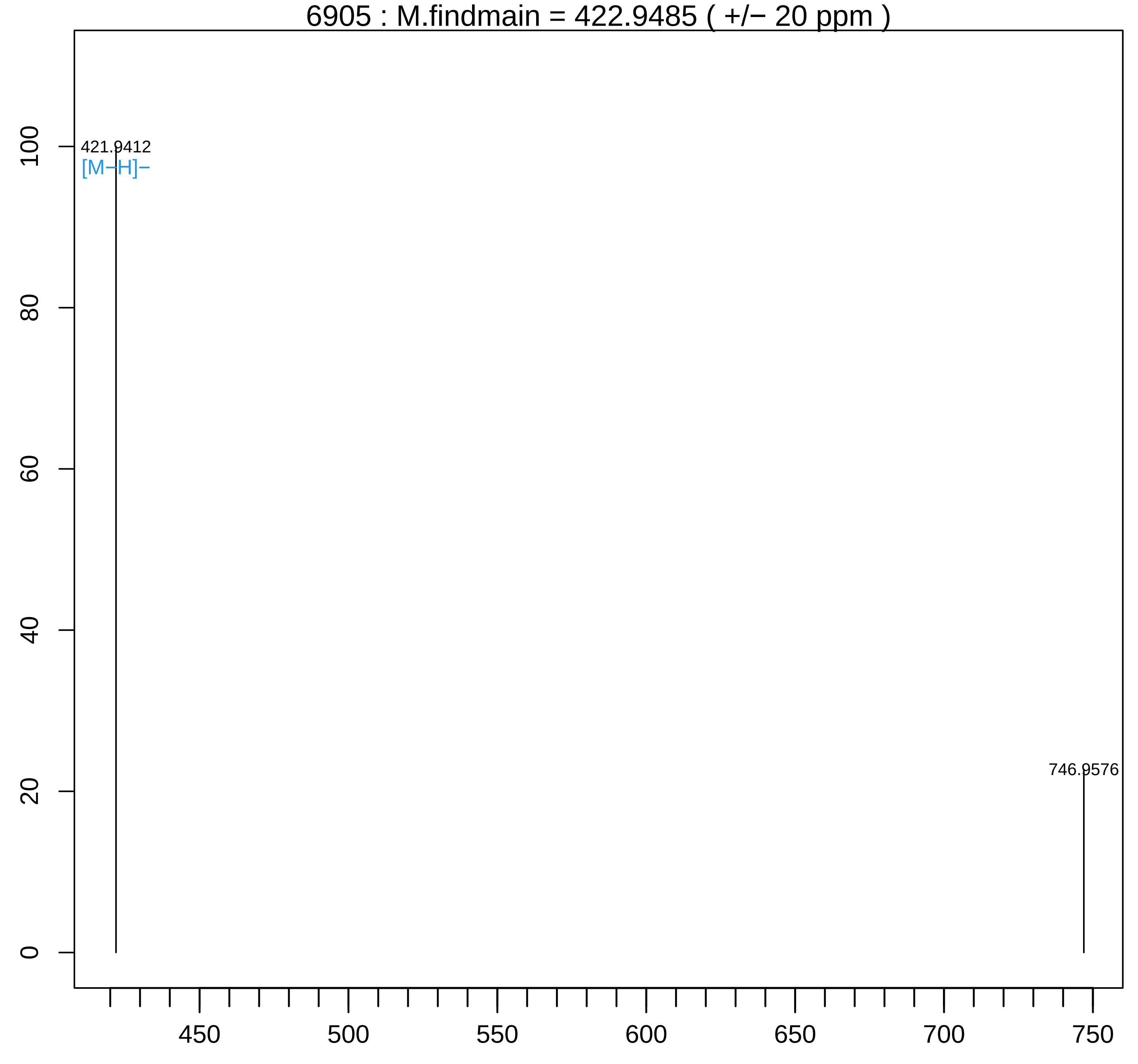
6905 : M.findmain = 422.9485 (+/− 20 ppm)
421.9412
[M−H]−
746.9576
0
20
40
60
80
100
450
500
550
600
650
700
750

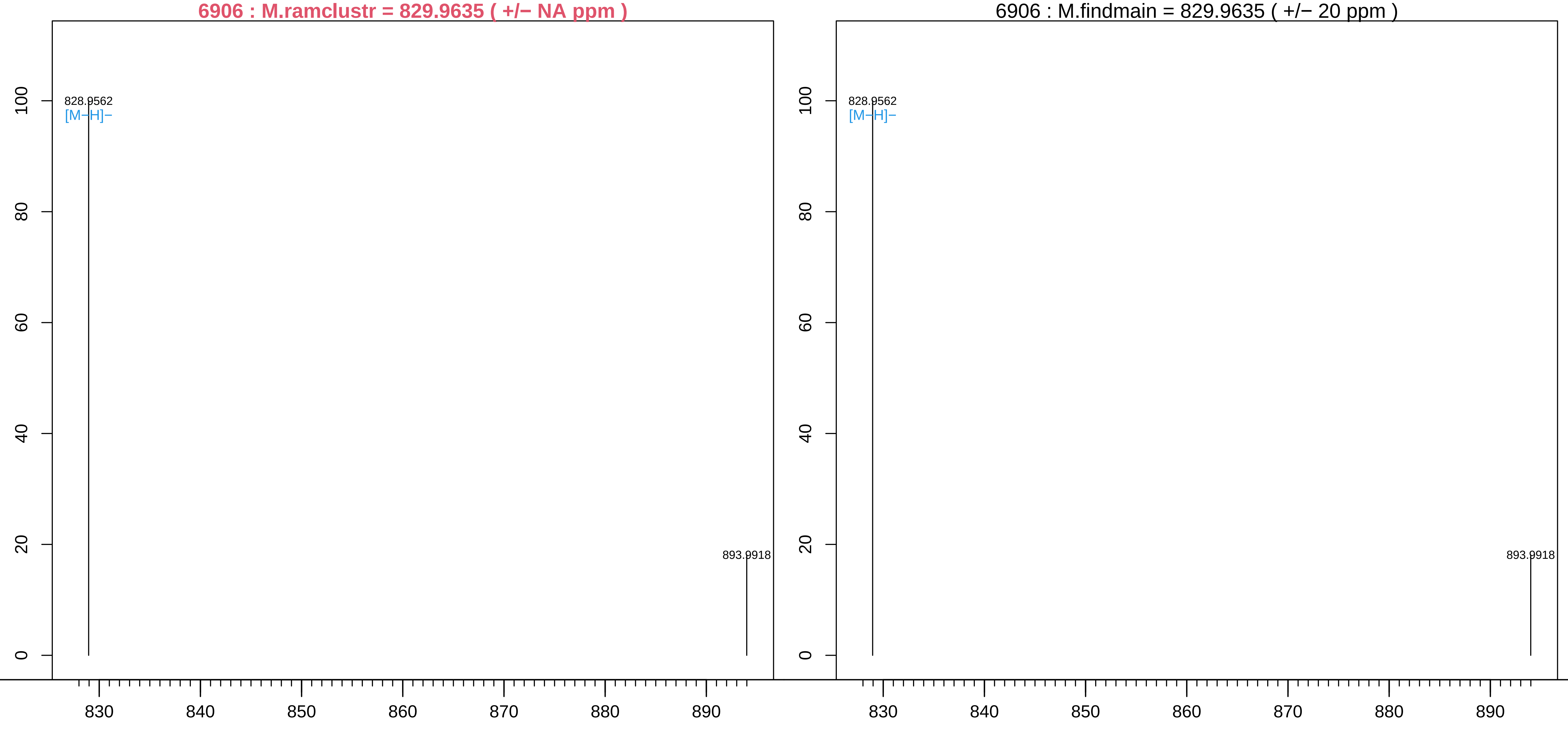
6906 : M.ramclustr = 829.9635 (+/− NA ppm)
6906 : M.findmain = 829.9635 (+/− 20 ppm)
828.9562
[M−H]−
893.9918
0
20
40
60
80
100
830
840
850
860
870
880
890

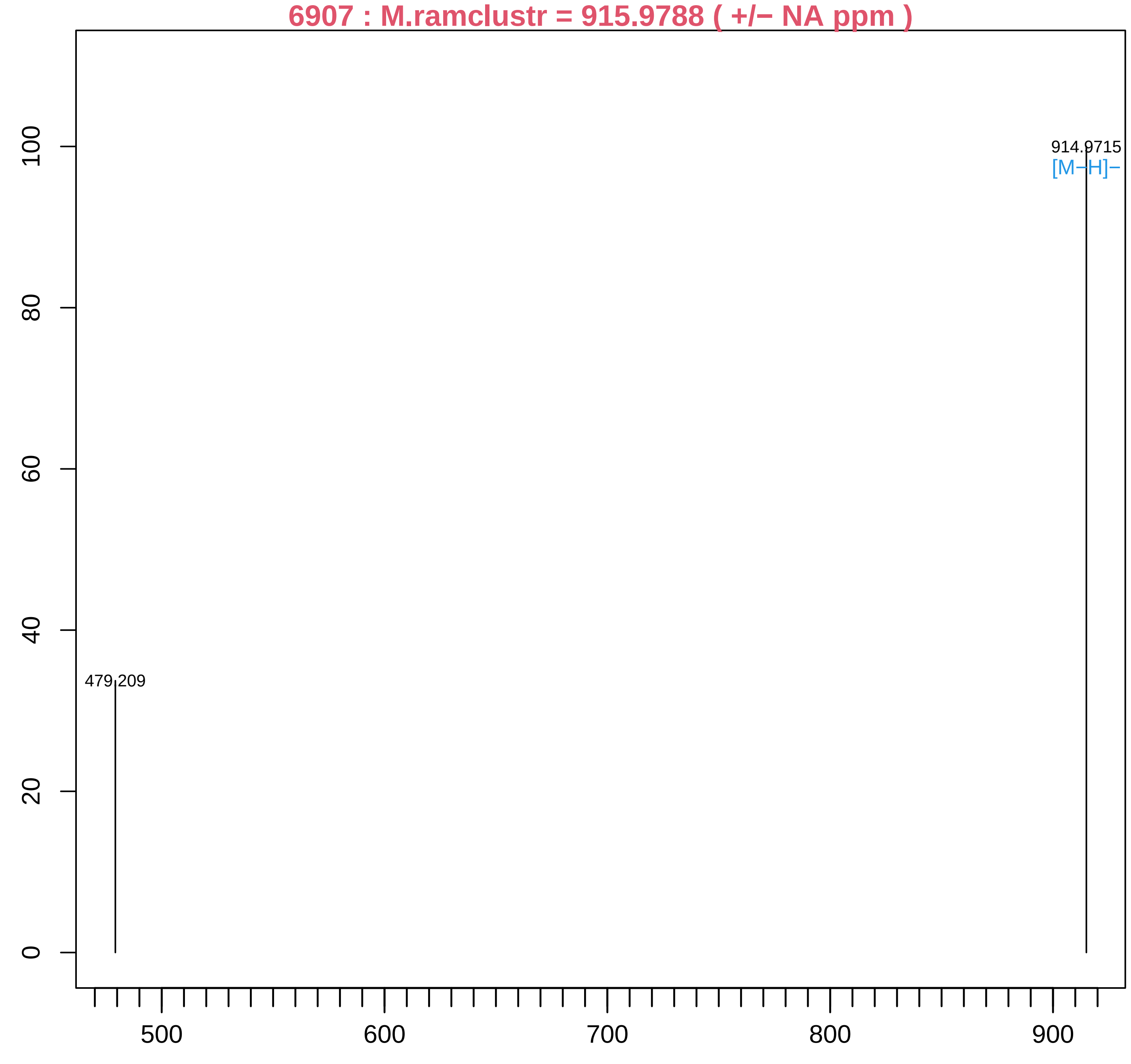
6907 : M.ramclustr = 915.9788 (+/− NA ppm)
914.9715
[M−H]−
479.209
0
20
40
60
80
100
500
600
700
800
900

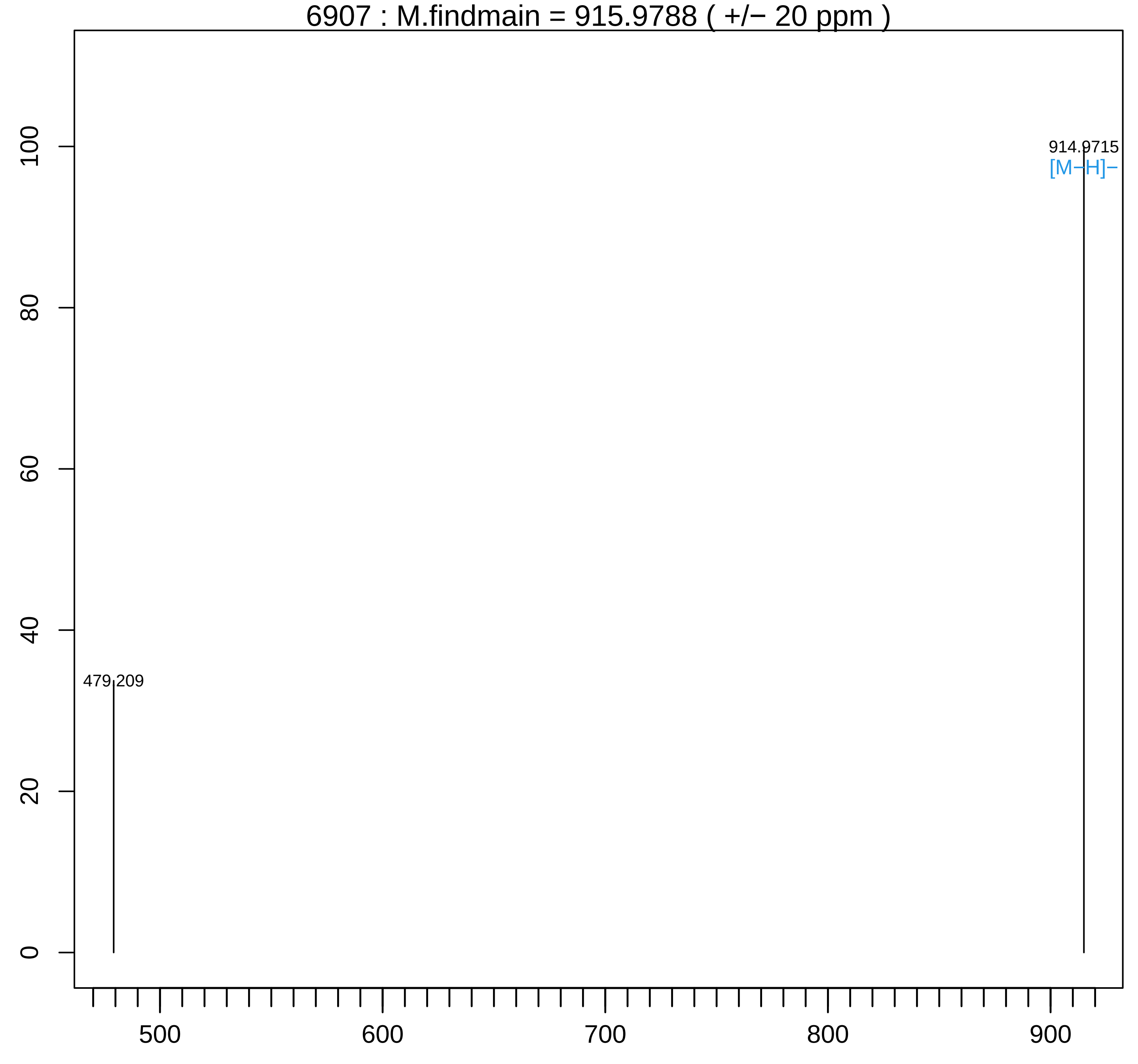
6907 : M.findmain = 915.9788 (+/− 20 ppm)
914.9715
[M−H]−
479.209
0
20
40
60
80
100
500
600
700
800
900

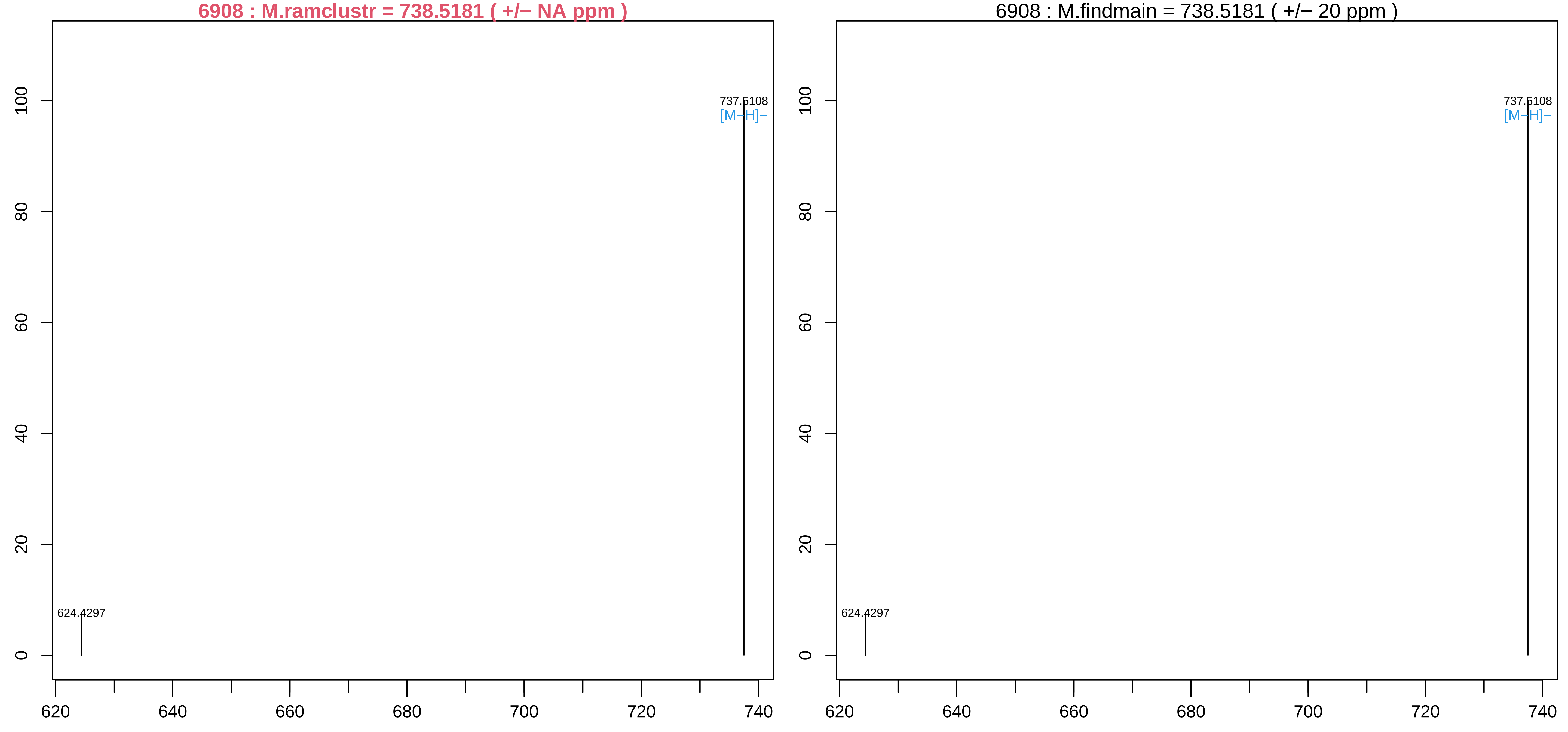
6908 : M.ramclustr = 738.5181 (+/− NA ppm)
737.5108
[M−H]−
624.4297
6908 : M.findmain = 738.5181 (+/− 20 ppm)
737.5108
[M−H]−
624.4297
0
20
40
60
80
100
620
640
660
680
700
720
740

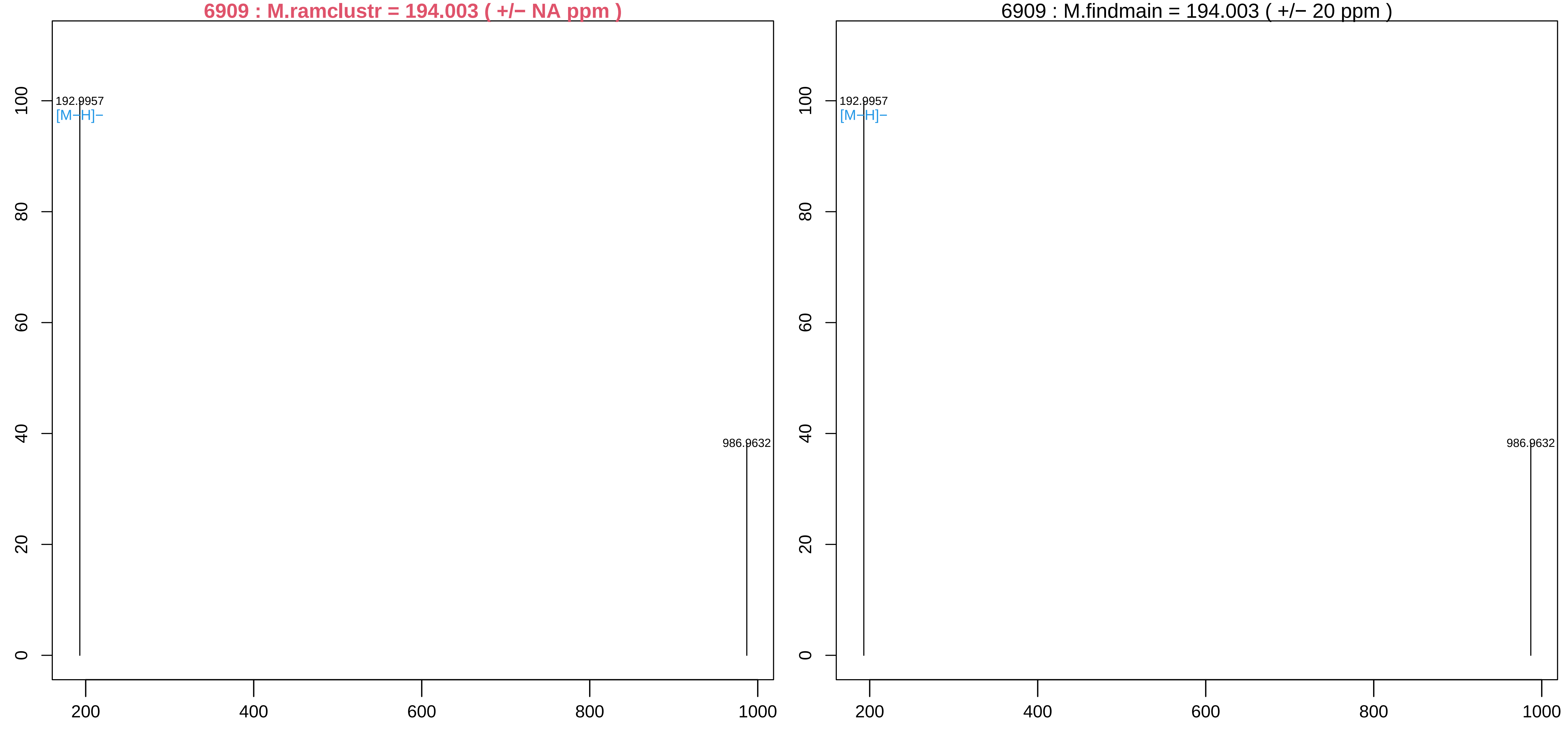
6909 : M.ramclustr = 194.003 (+/− NA ppm)
6909 : M.findmain = 194.003 (+/− 20 ppm)
192.9957
[M−H]−
986.9632
0
20
40
60
80
100
200
400
600
800
1000

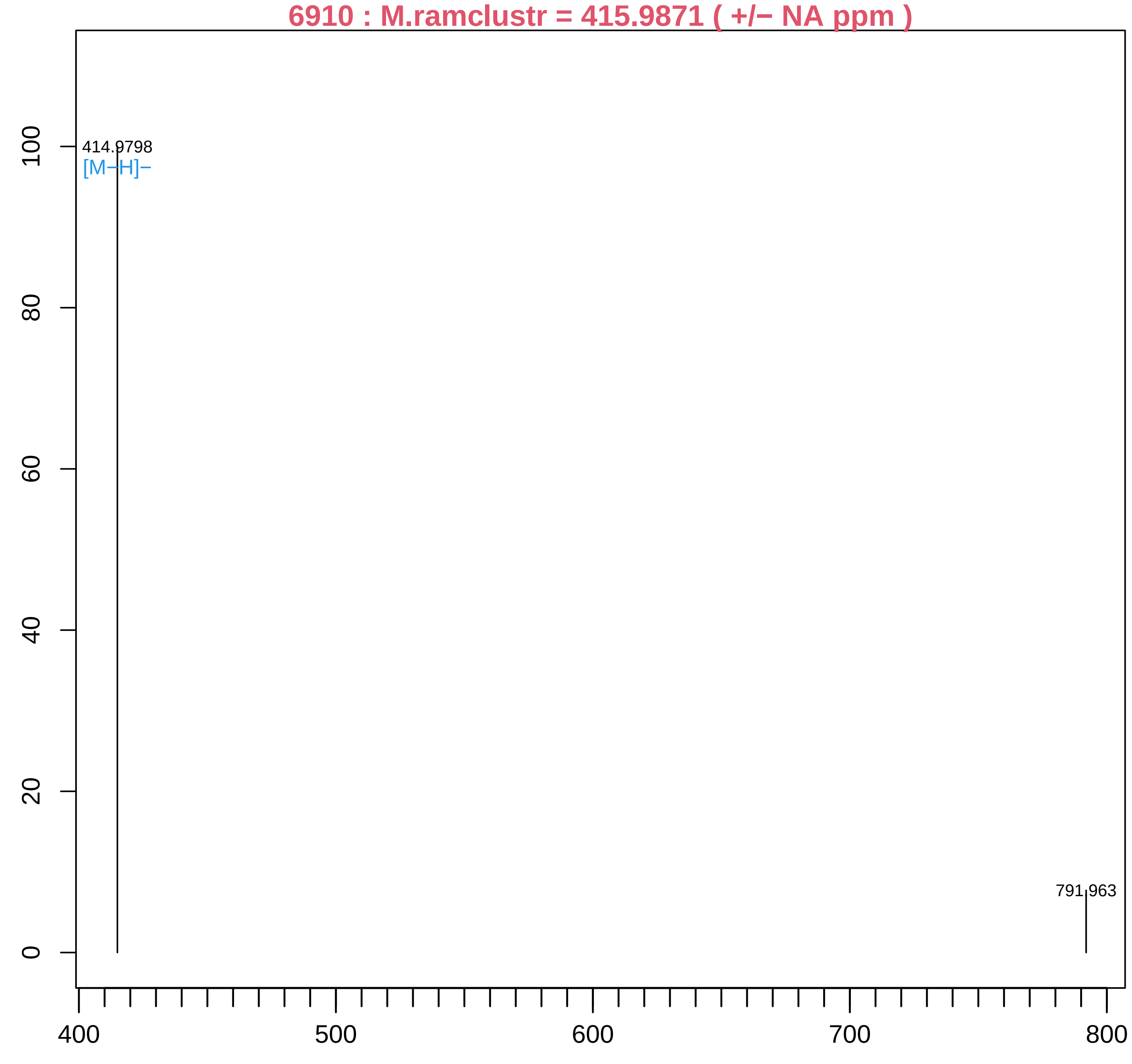
6910 : M.ramclustr = 415.9871 (+/− NA ppm)
414.9798
[M−H]−
791.963
0
20
40
60
80
100
400
500
600
700
800

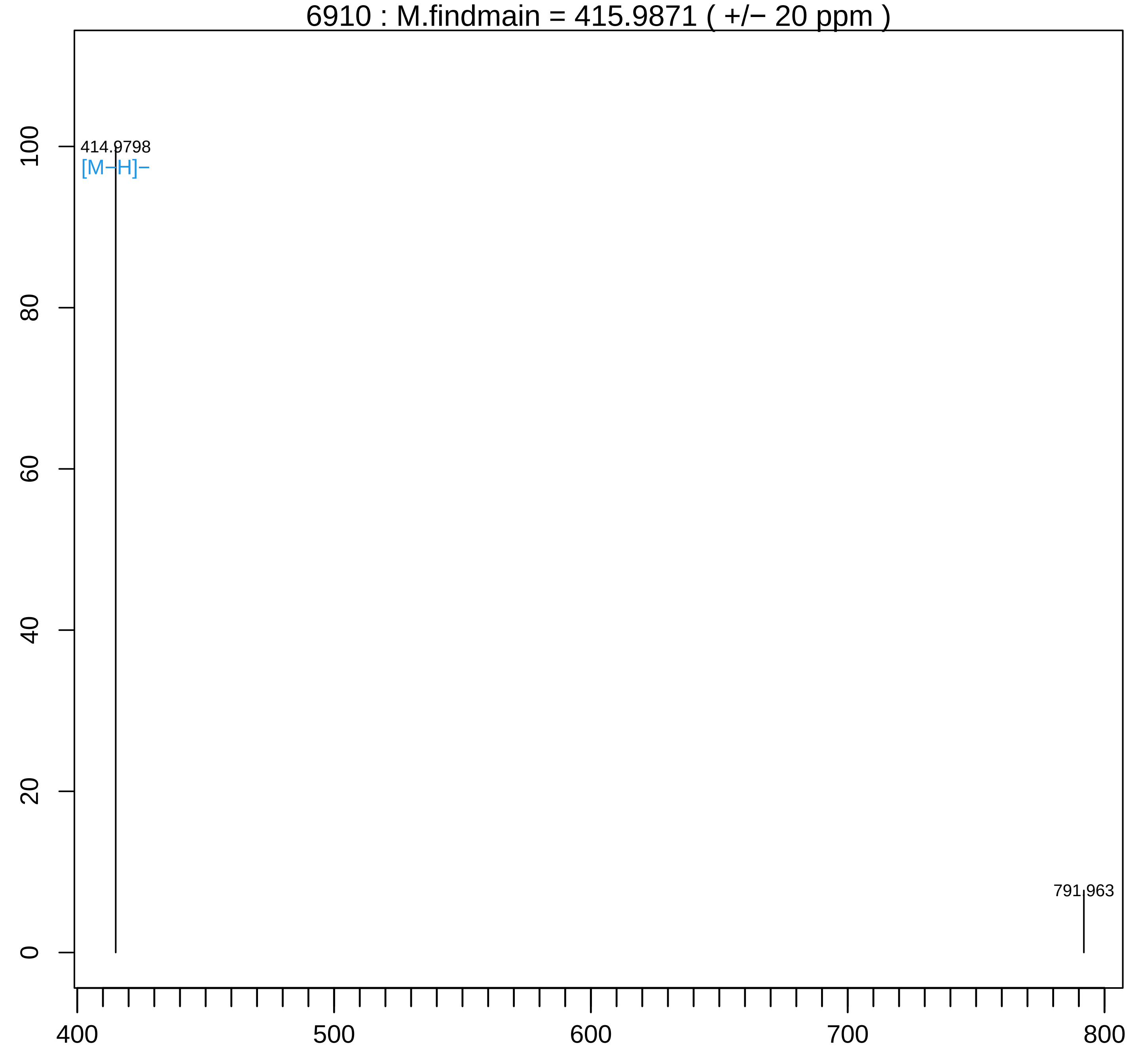
6910 : M.findmain = 415.9871 (+/− 20 ppm)
414.9798
[M−H]−
791.963
0
20
40
60
80
100
400
500
600
700
800

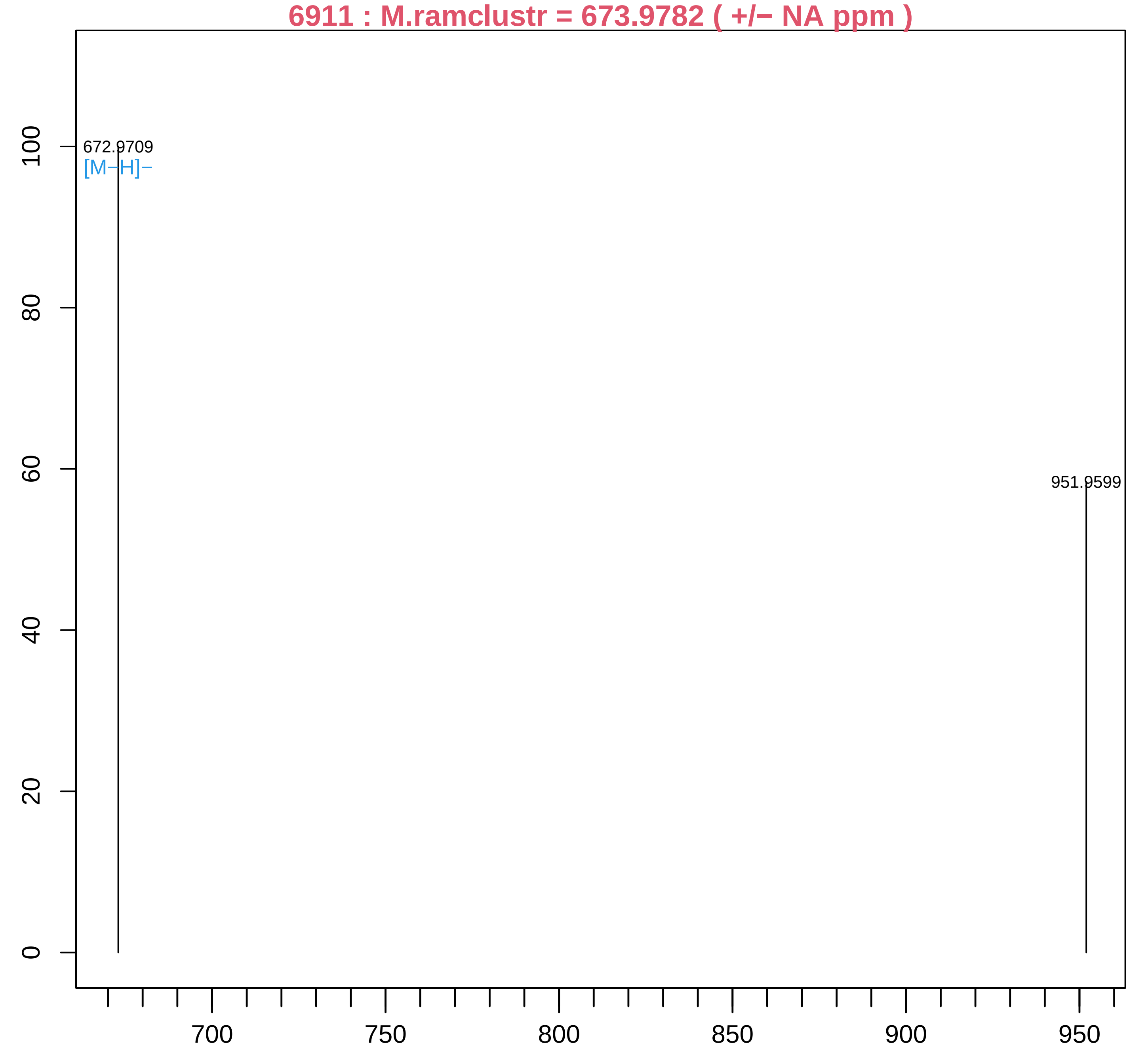
6911 : M.ramclustr = 673.9782 (+/− NA ppm)
672.9709
[M−H]−
951.9599
0
20
40
60
80
100
700
750
800
850
900
950

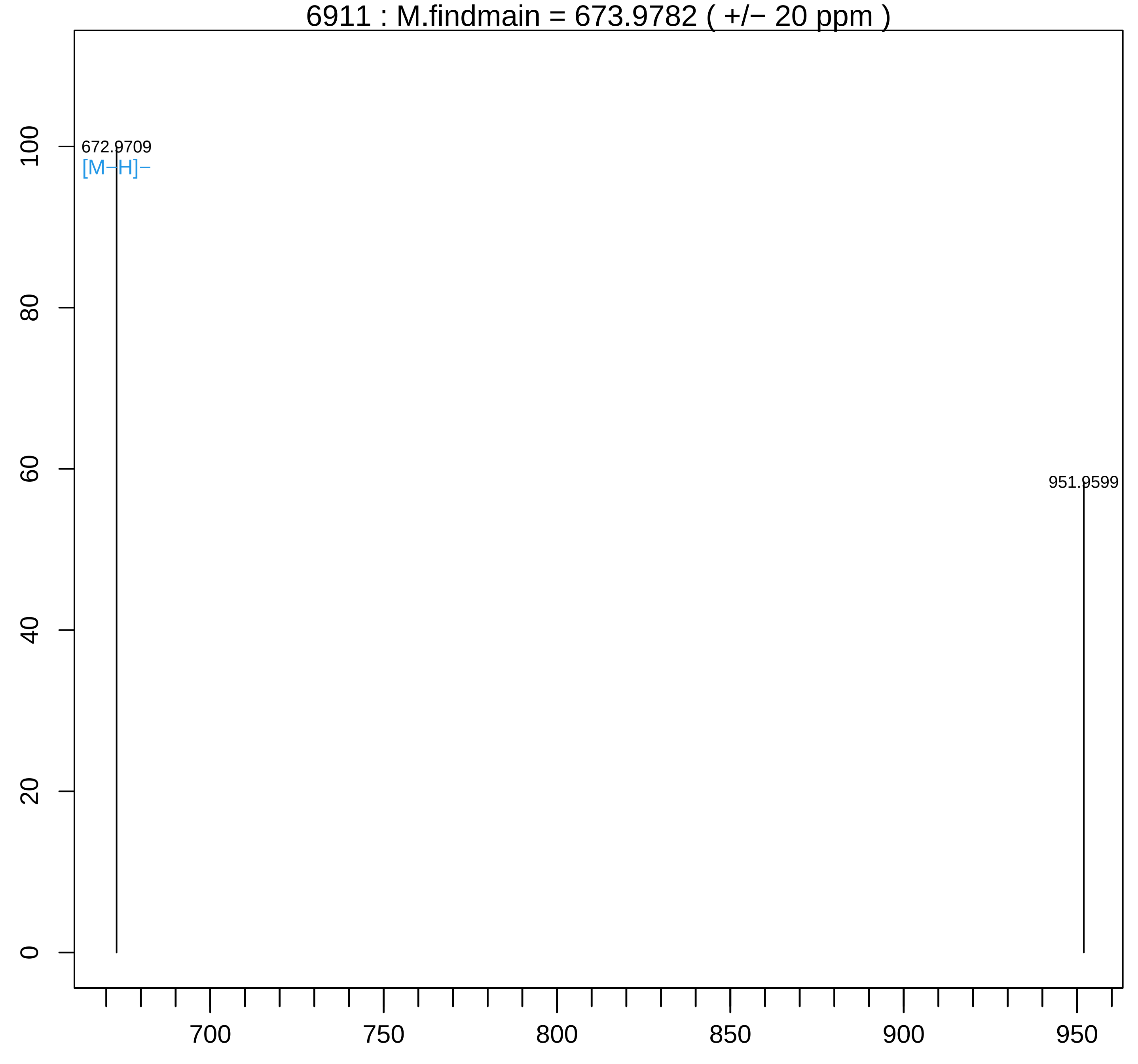
6911 : M.findmain = 673.9782 (+/− 20 ppm)
672.9709
[M−H]−
951.9599
0
20
40
60
80
100
700
750
800
850
900
950

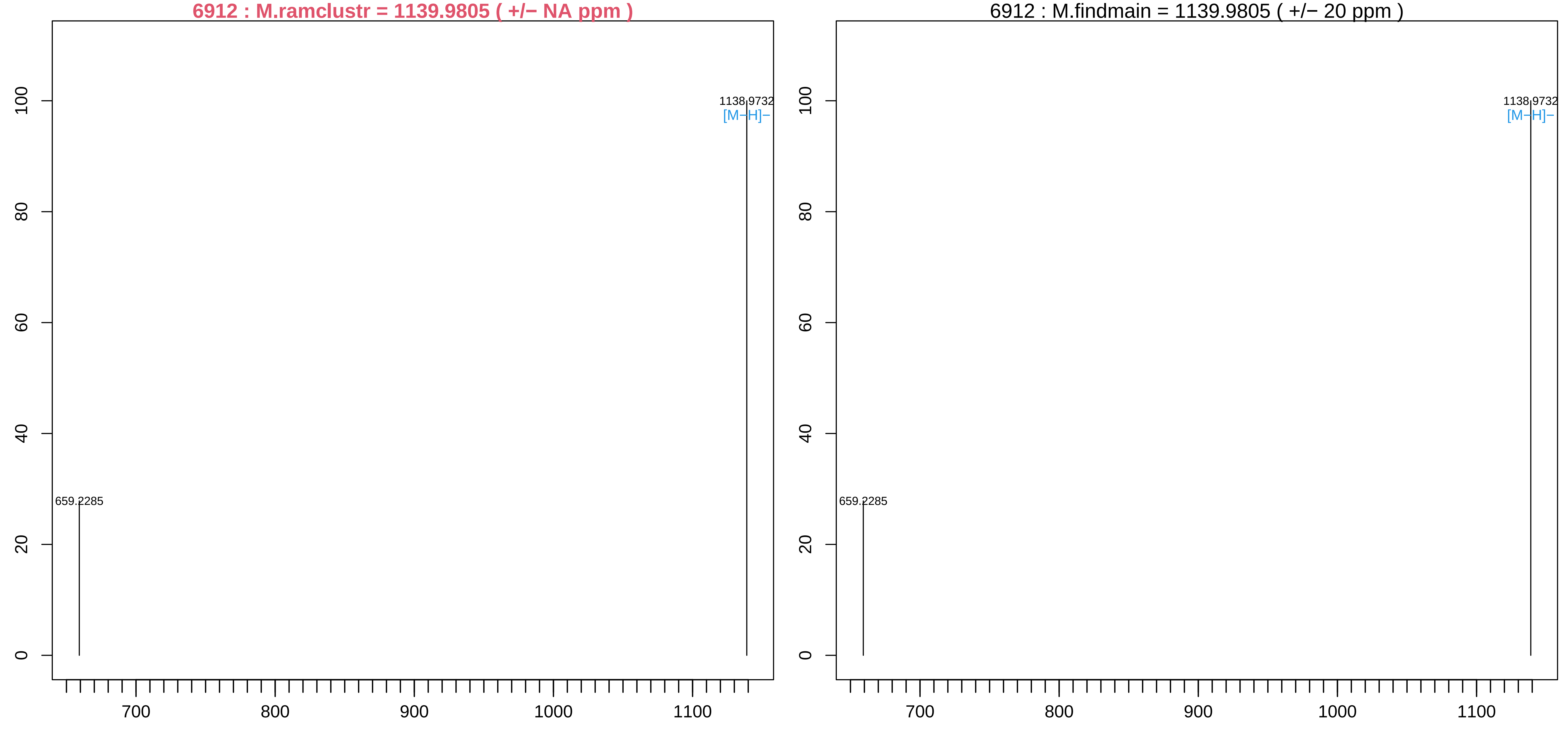
6912 : M.ramclustr = 1139.9805 (+/− NA ppm)
6912 : M.findmain = 1139.9805 (+/− 20 ppm)
1138.9732
[M−H]−
659.2285
0
20
40
60
80
100
700
800
900
1000
1100

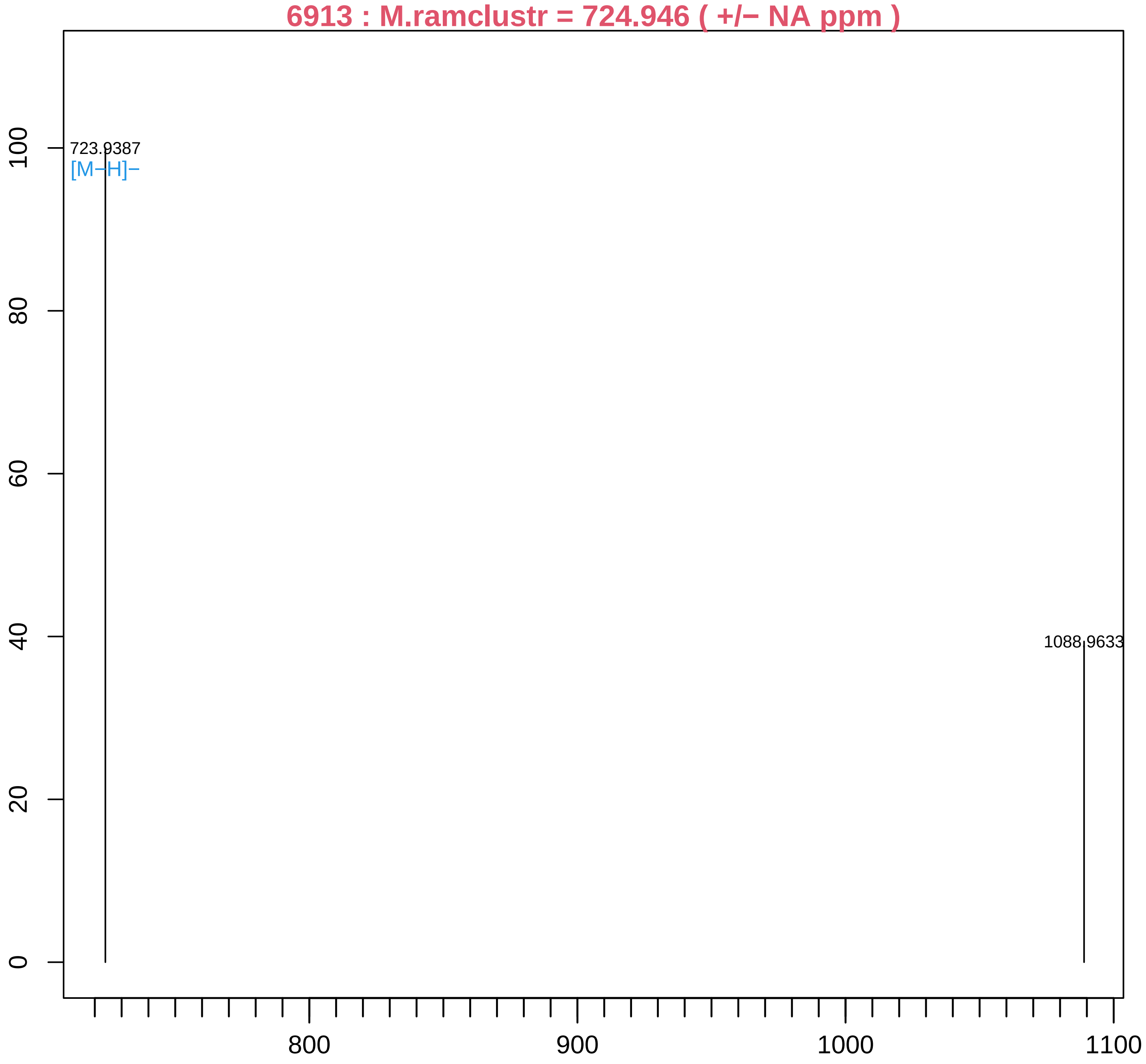
6913 : M.ramclustr = 724.946 (+/− NA ppm)
723.9387
[M−H]−
1088.9633
0
20
40
60
80
100
800
900
1000
1100

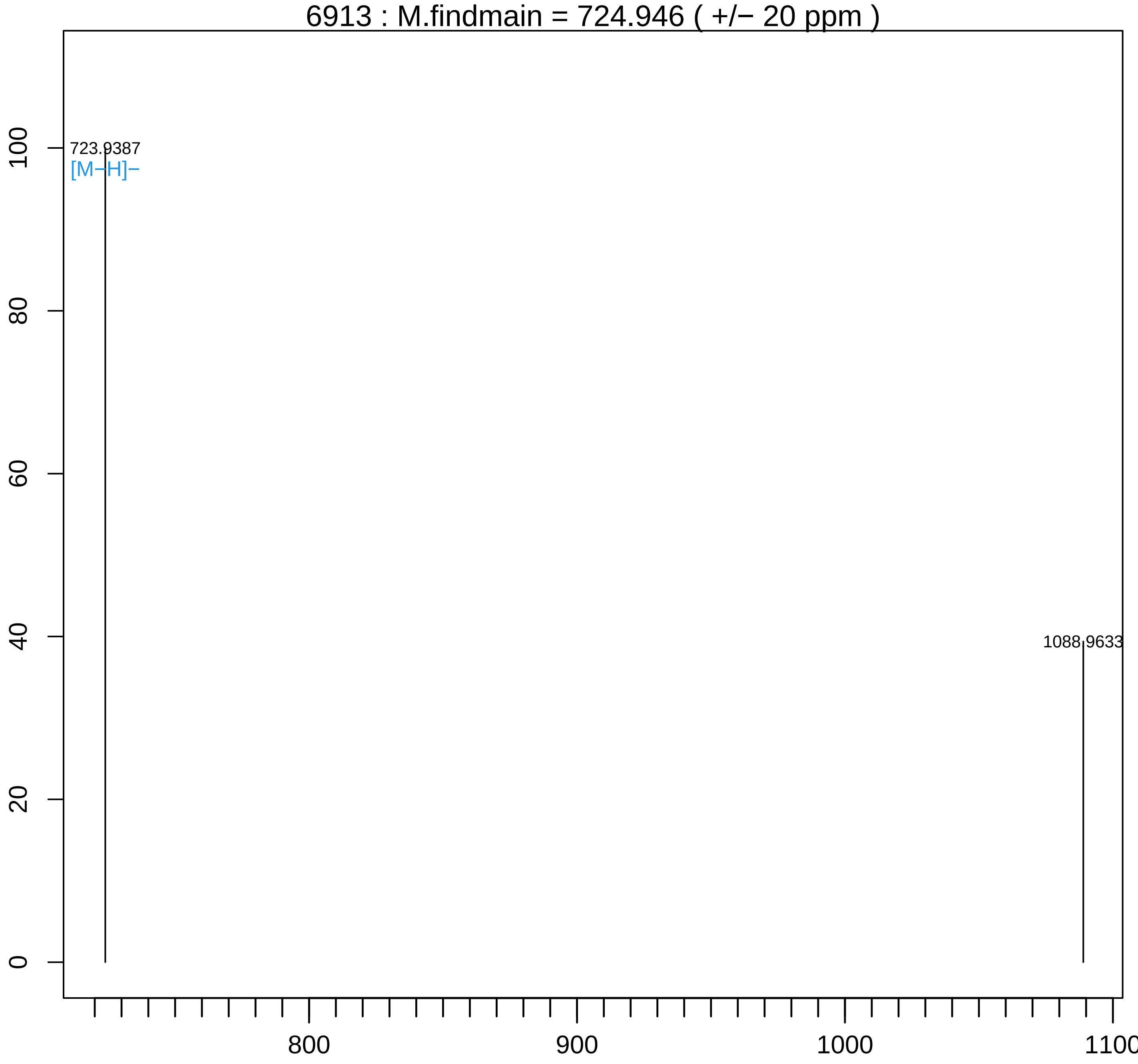
6913 : M.findmain = 724.946 (+/− 20 ppm)
723.9387
[M−H]−
1088.9633
0
20
40
60
80
100
800
900
1000
1100

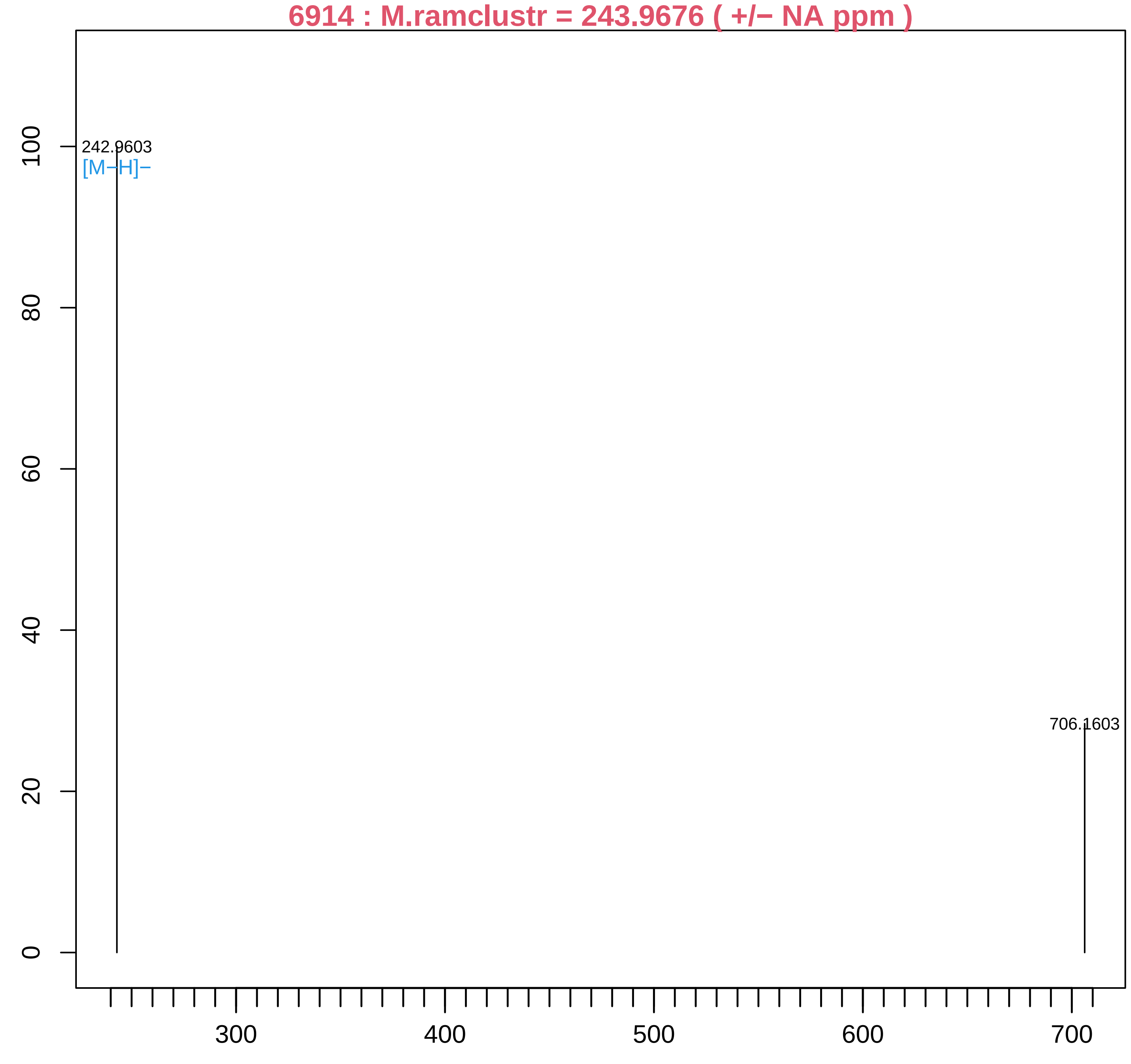
6914 : M.ramclustr = 243.9676 (+/− NA ppm)
242.9603
[M−H]−
706.1603
0
20
40
60
80
100
300
400
500
600
700

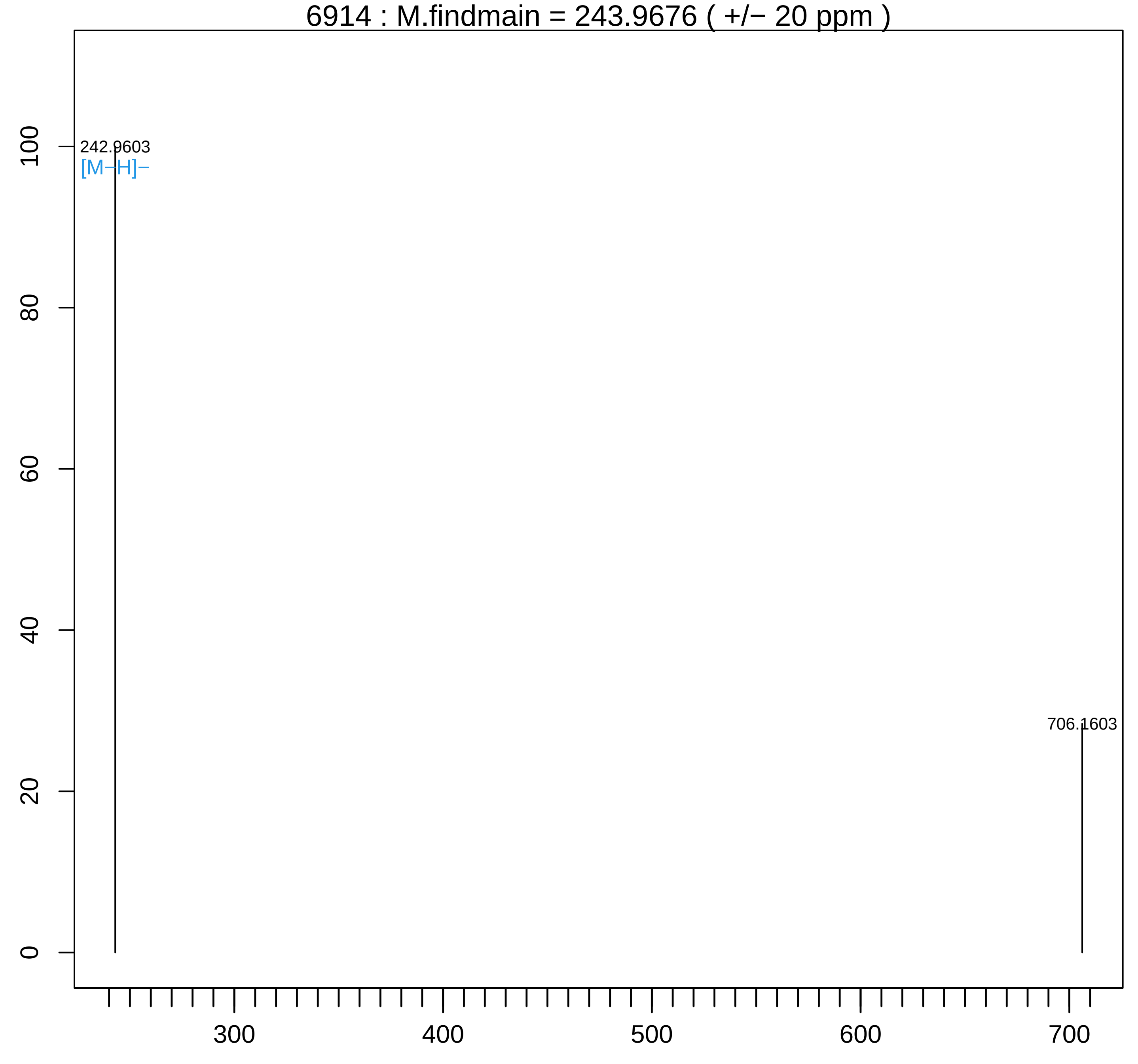
6914 : M.findmain = 243.9676 (+/− 20 ppm)
242.9603
[M−H]−
706.1603
0
20
40
60
80
100
300
400
500
600
700

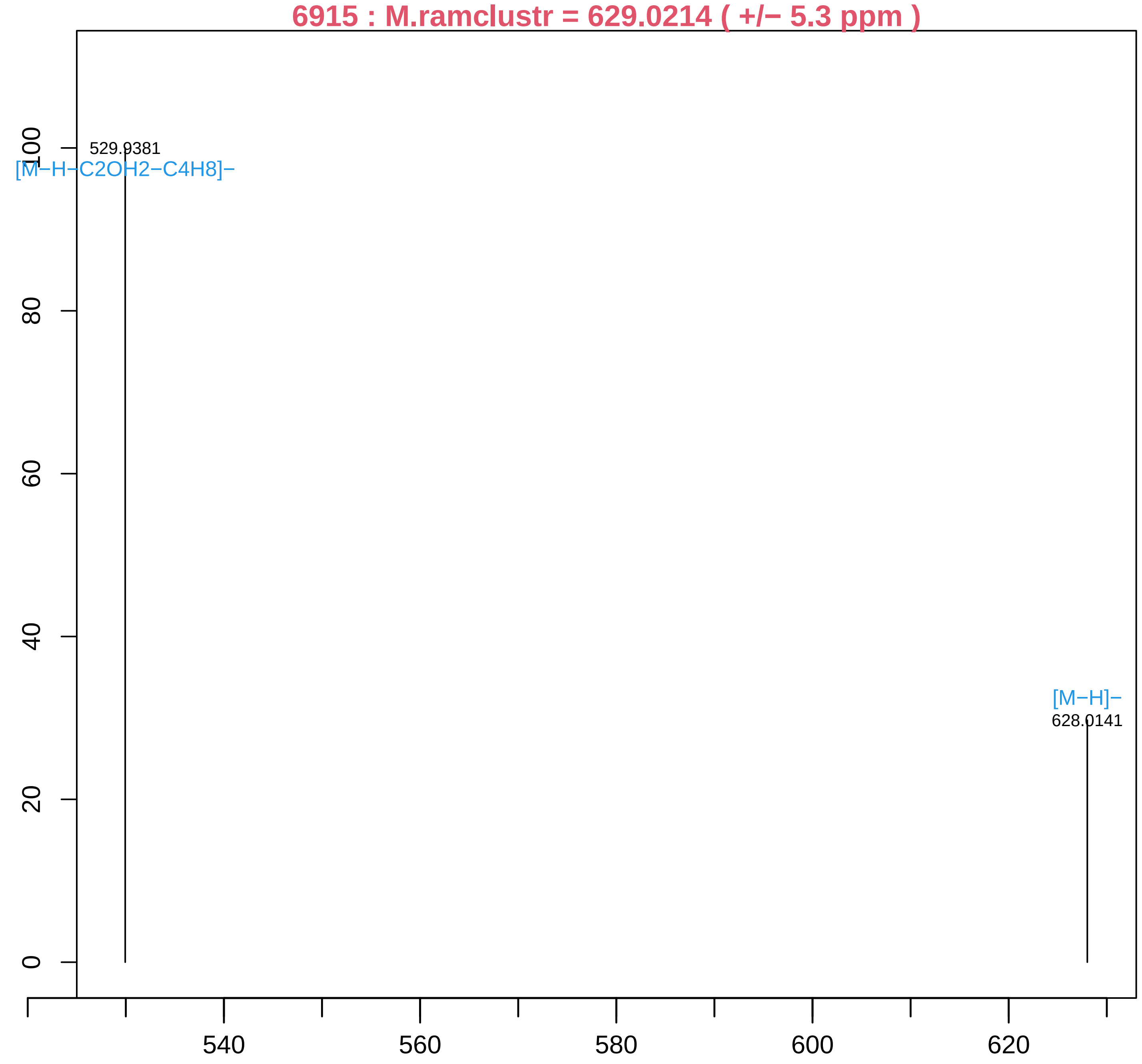
6915 : M.ramclustr = 629.0214 (+/− 5.3 ppm)
529.9381
[M−H−C2OH2−C4H8]−
[M−H]−
628.0141
0
20
40
60
80
100
540
560
580
600
620

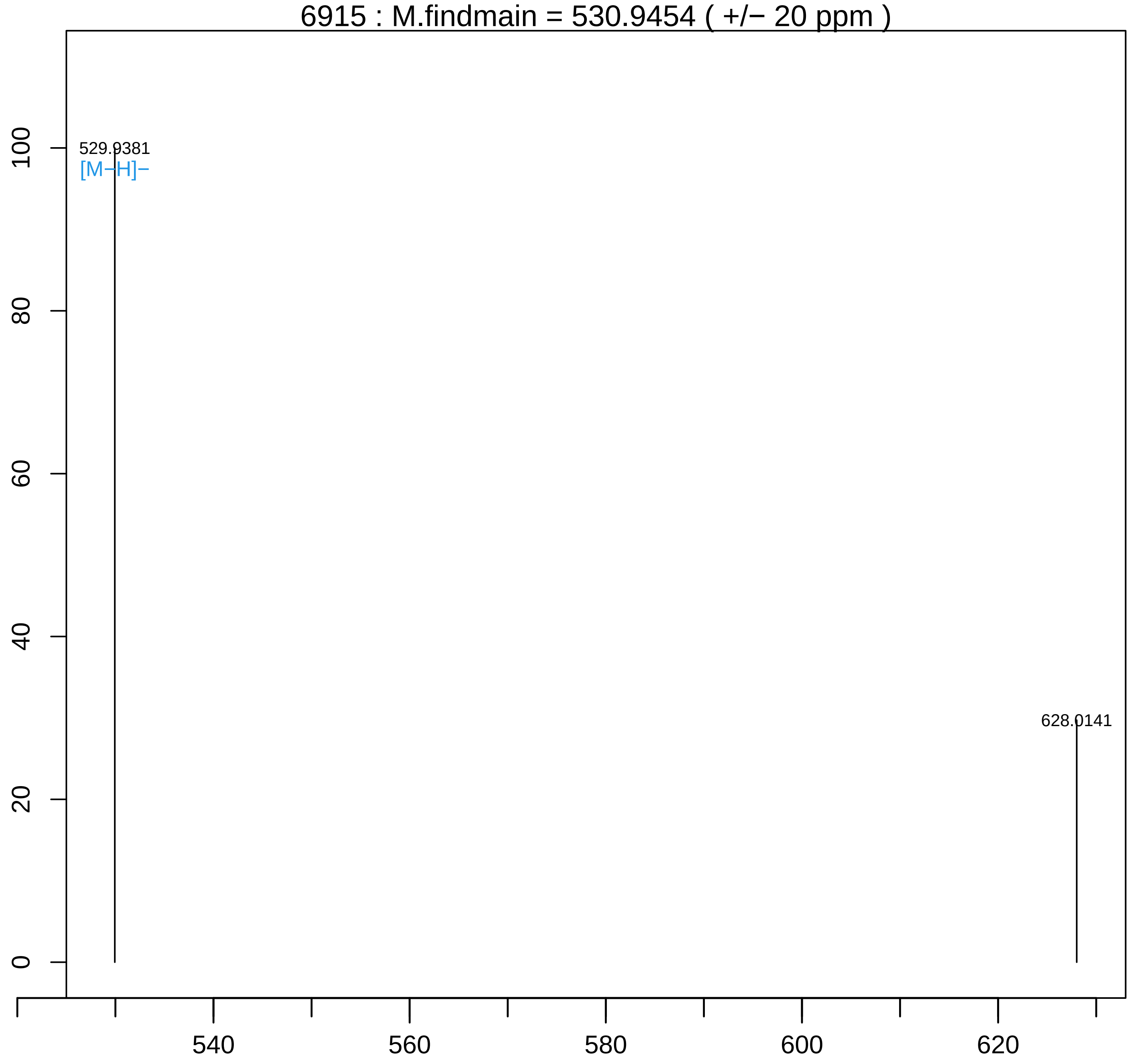
6915 : M.findmain = 530.9454 (+/− 20 ppm)
529.9381
[M−H]−
628.0141
0
20
40
60
80
100
540
560
580
600
620

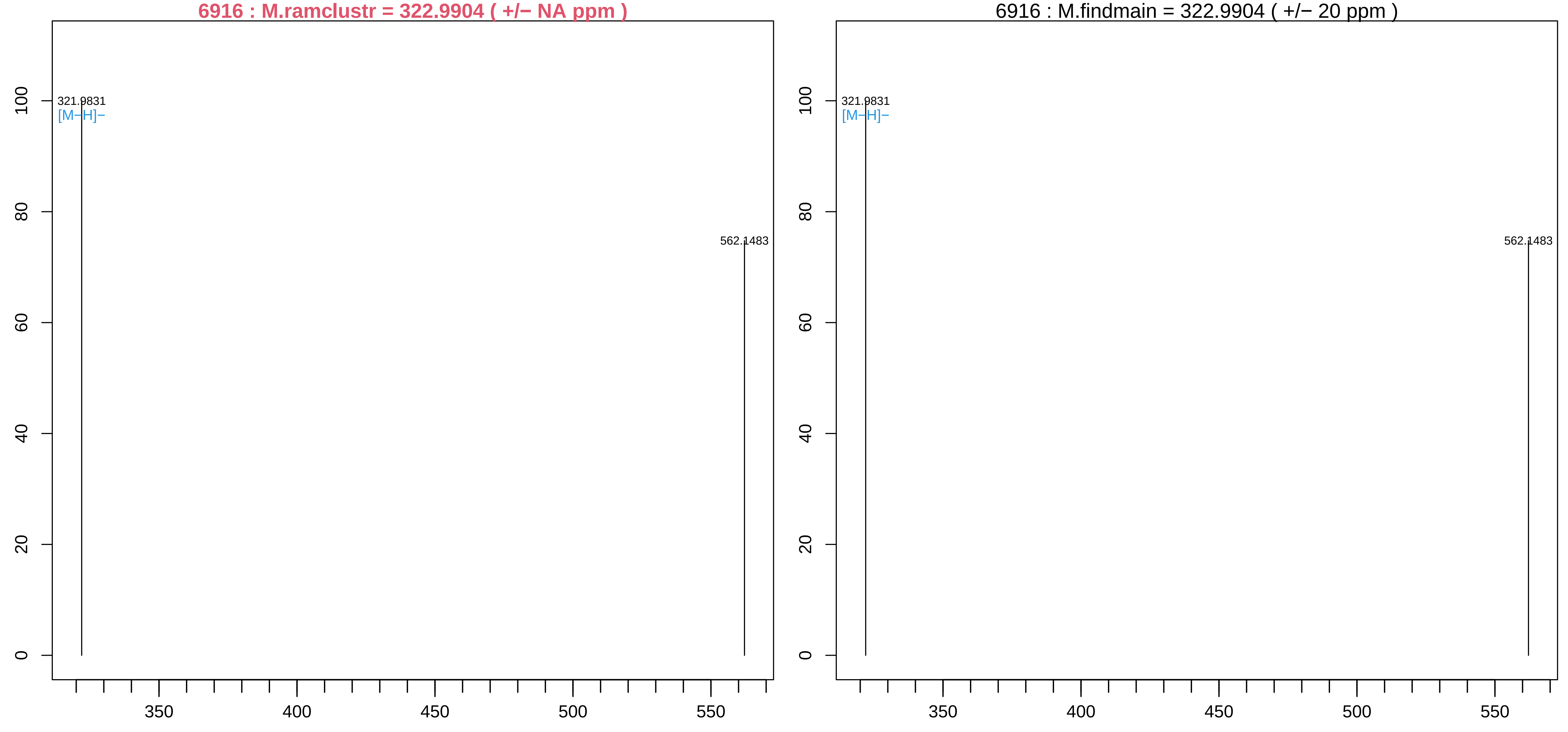

6916 : M.ramclustr = 322.9904 (+/− NA ppm)
321.9831
[M−H]−
562.1483
6916 : M.findmain = 322.9904 (+/− 20 ppm)
321.9831
[M−H]−
562.1483
0
20
40
60
80
100
350
400
450
500
550

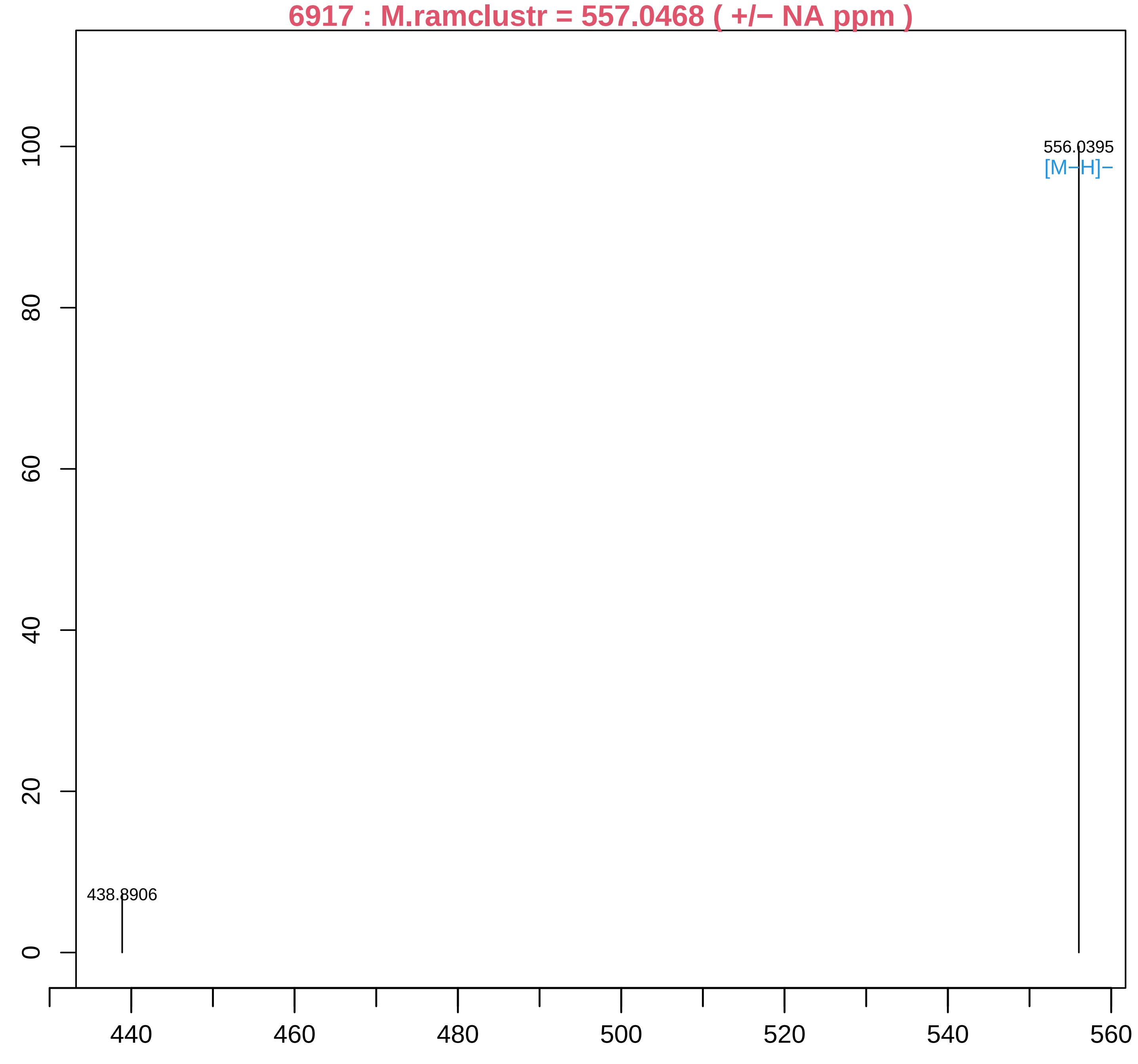
6917 : M.ramclustr = 557.0468 (+/− NA ppm)
556.0395
[M−H]−
438.8906
0
20
40
60
80
100
440
460
480
500
520
540
560

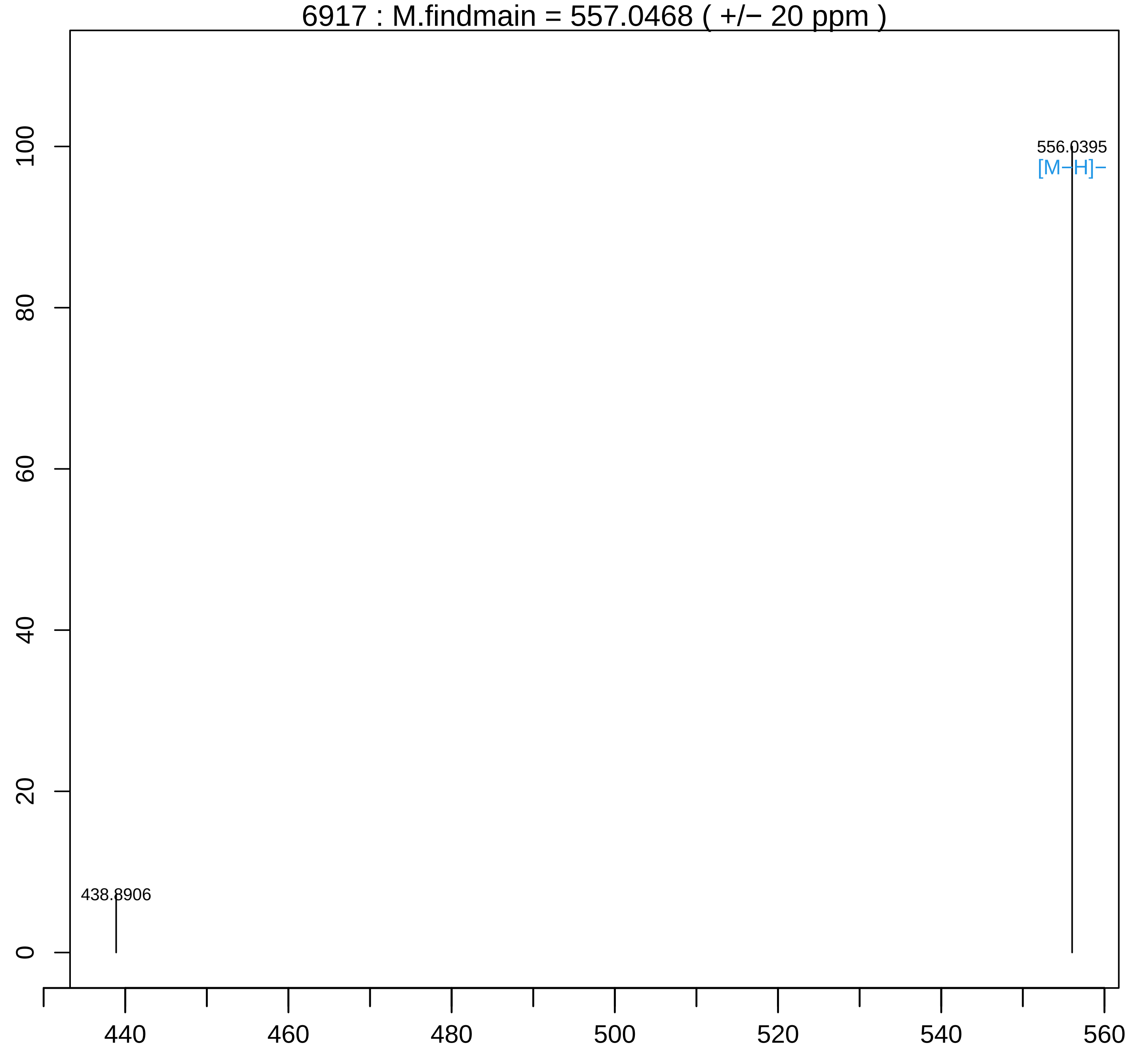
6917 : M.findmain = 557.0468 (+/− 20 ppm)
556.0395
[M−H]−
438.8906
0
20
40
60
80
100
440
460
480
500
520
540
560

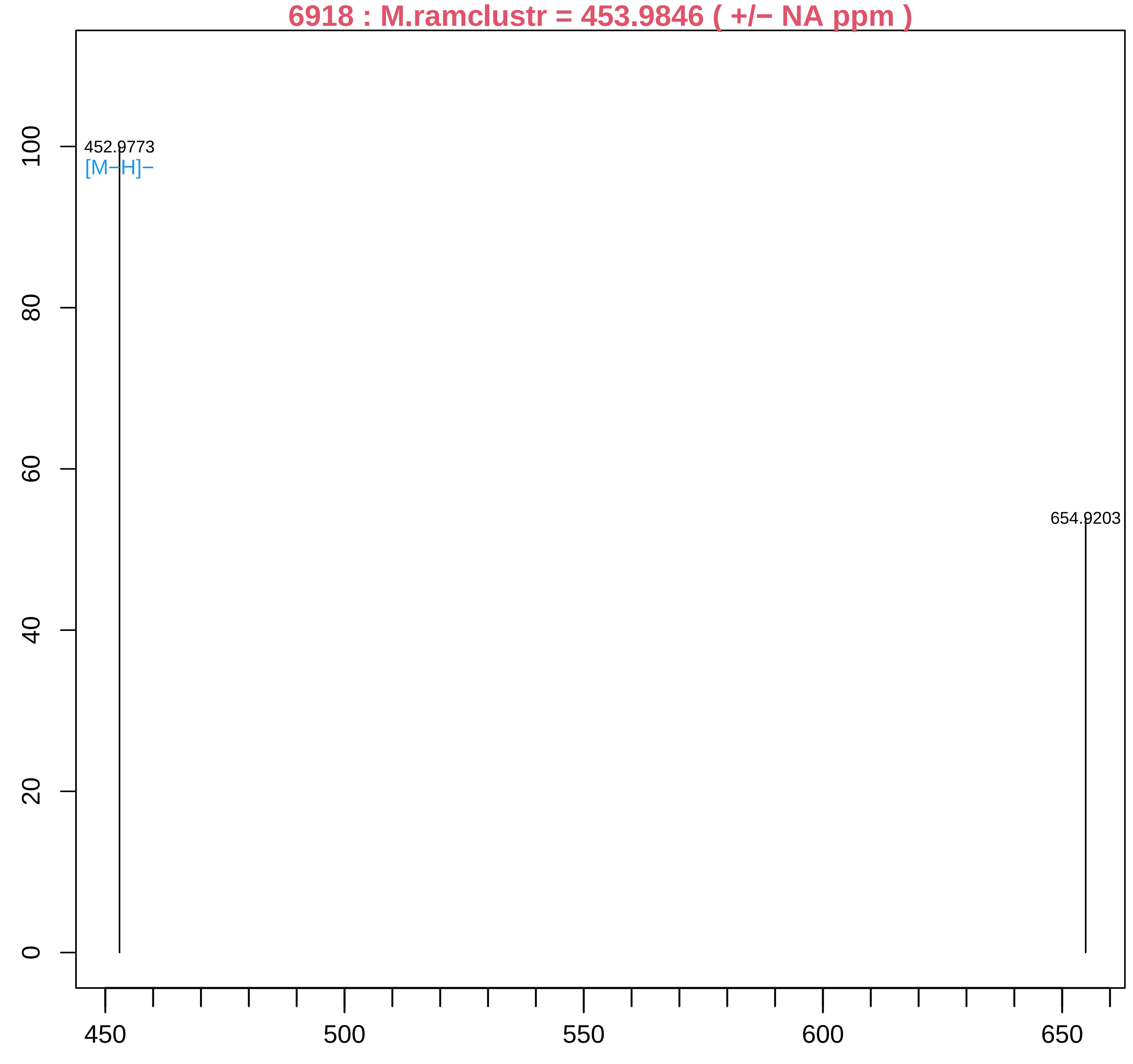

6918 : M.ramclustr = 453.9846 (+/− NA ppm)
452.9773
[M−H]−
654.9203
0
20
40
60
80
100
450
500
550
600
650

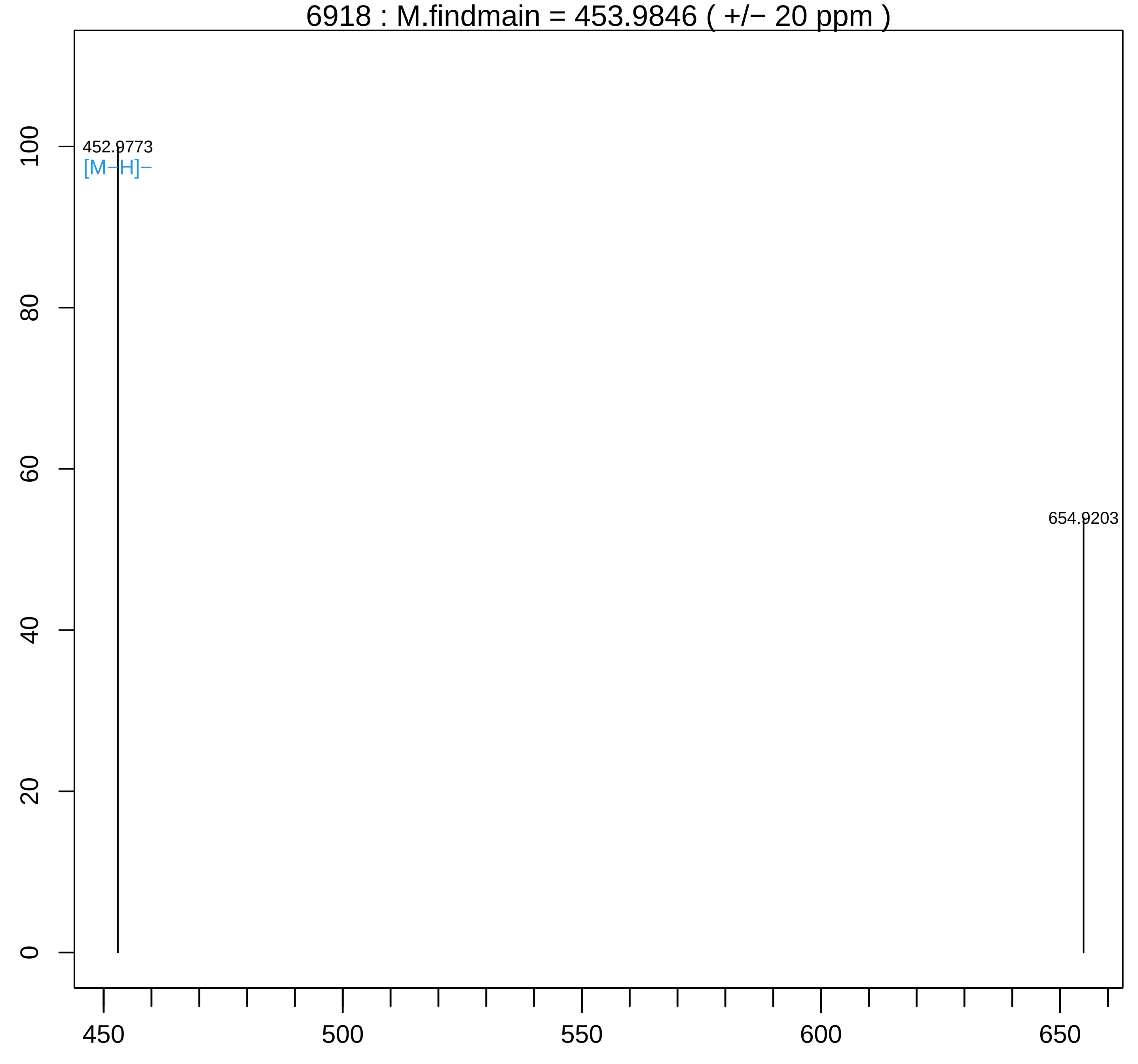

6918 : M.findmain = 453.9846 (+/− 20 ppm)
452.9773
[M−H]−
654.9203
0
20
40
60
80
100
450
500
550
600
650

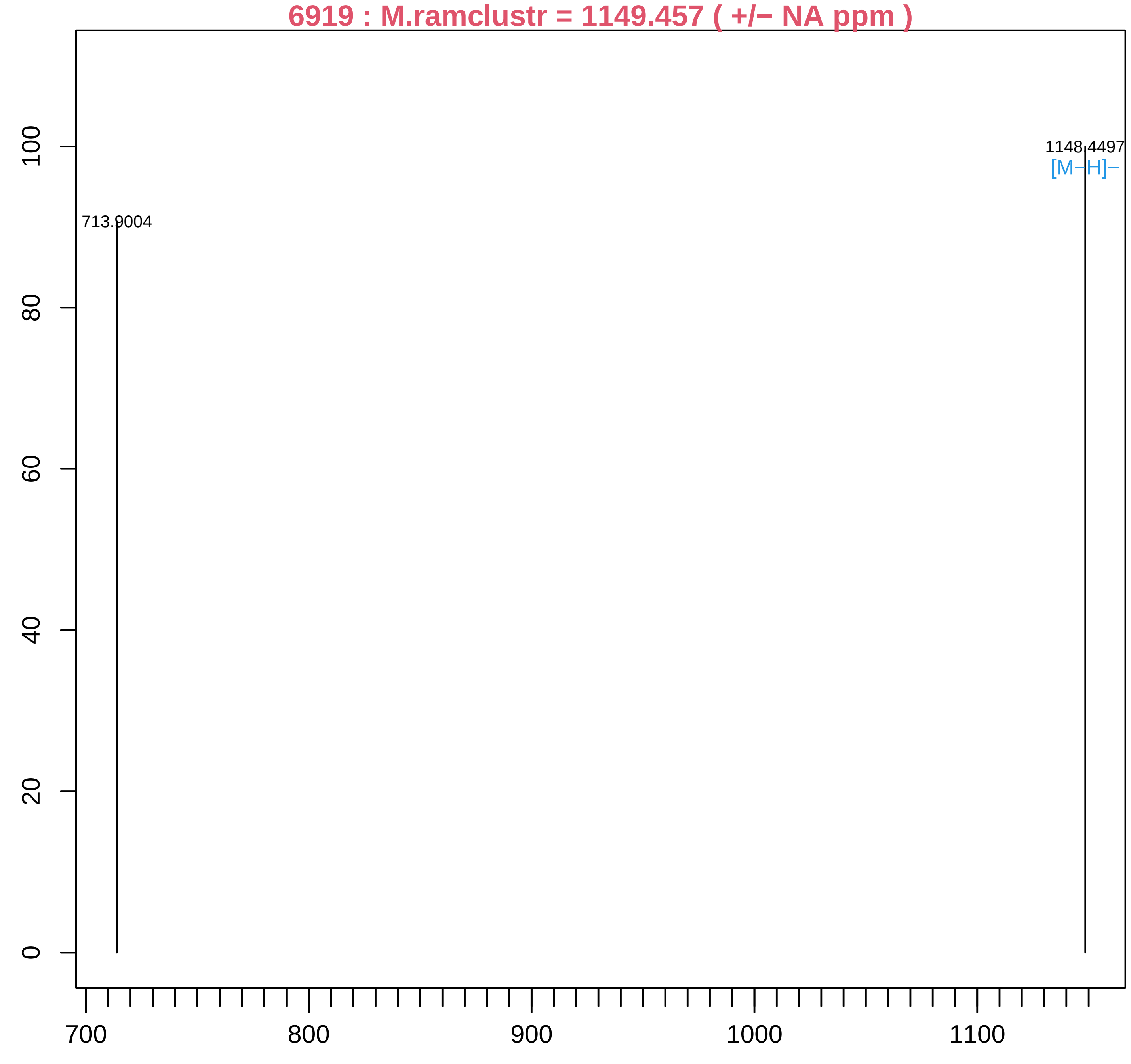
6919 : M.ramclustr = 1149.457 (+/− NA ppm)
713.9004
1148.4497
[M−H]−
0
20
40
60
80
100
700
800
900
1000
1100

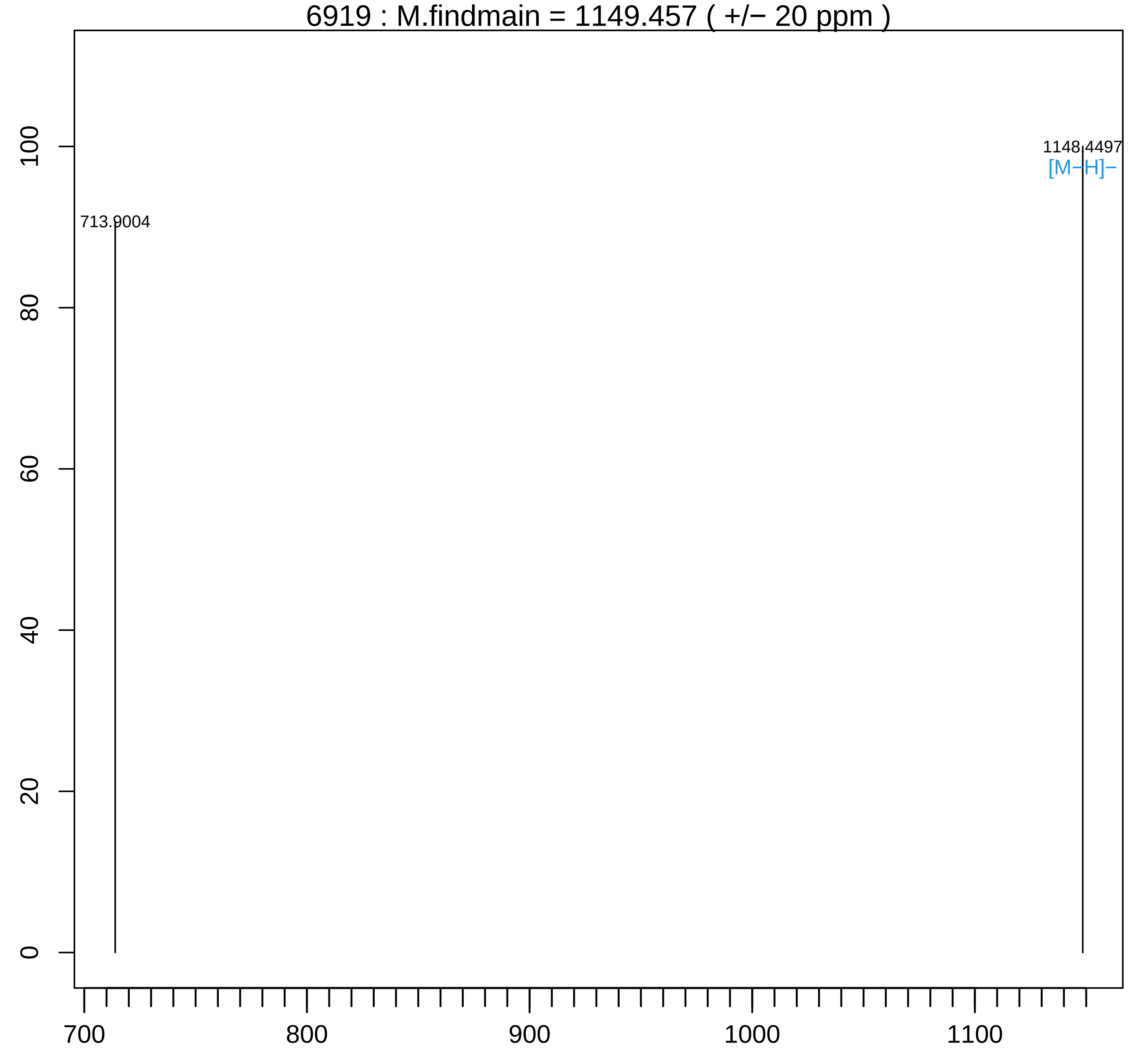
6919 : M.findmain = 1149.457 (+/− 20 ppm)
713.9004
1148.4497
[M−H]−
0
20
40
60
80
100
700
800
900
1000
1100

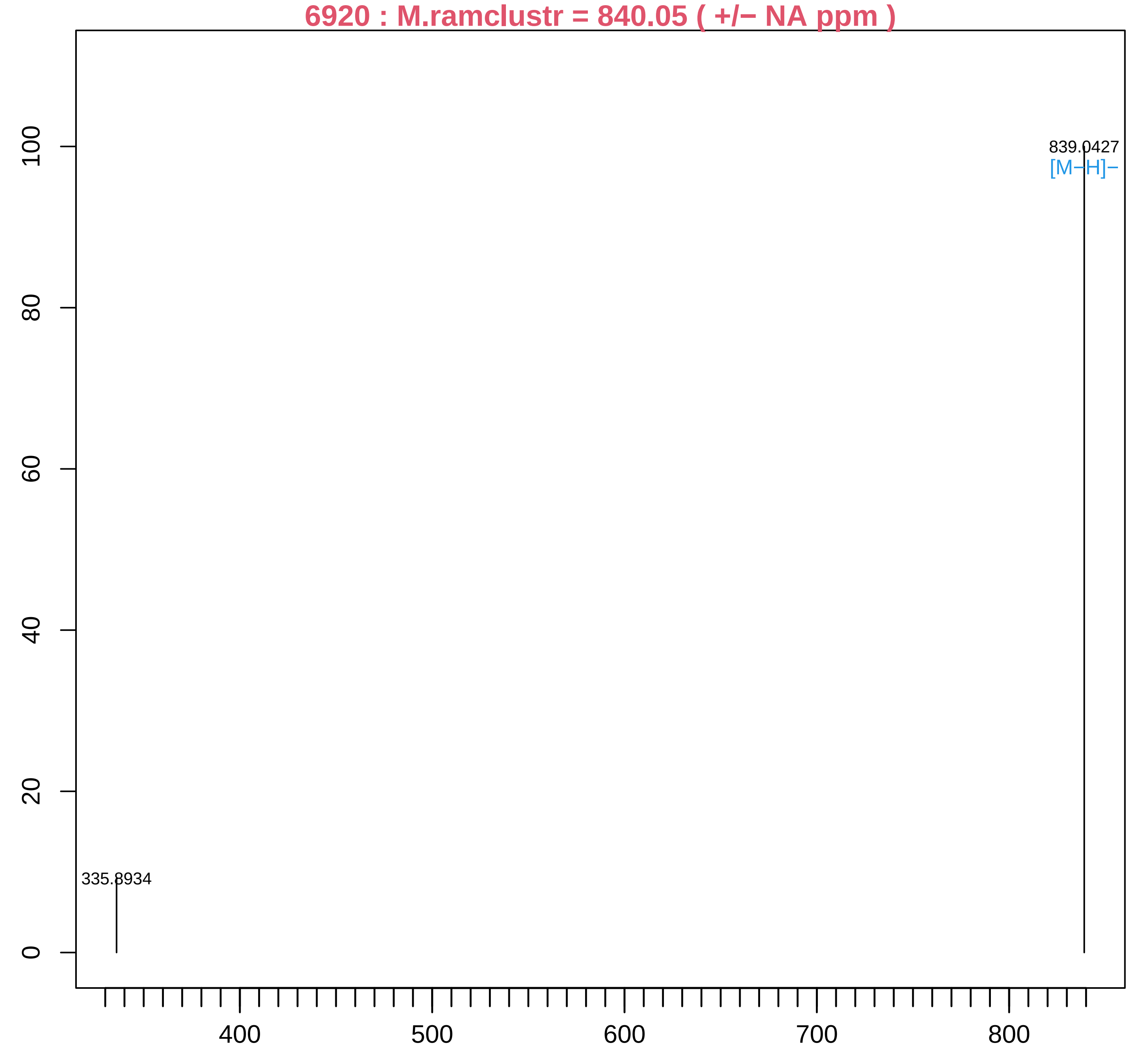
6920 : M.ramclustr = 840.05 (+/− NA ppm)
839.0427
[M−H]−
335.8934
0
20
40
60
80
100
400
500
600
700
800

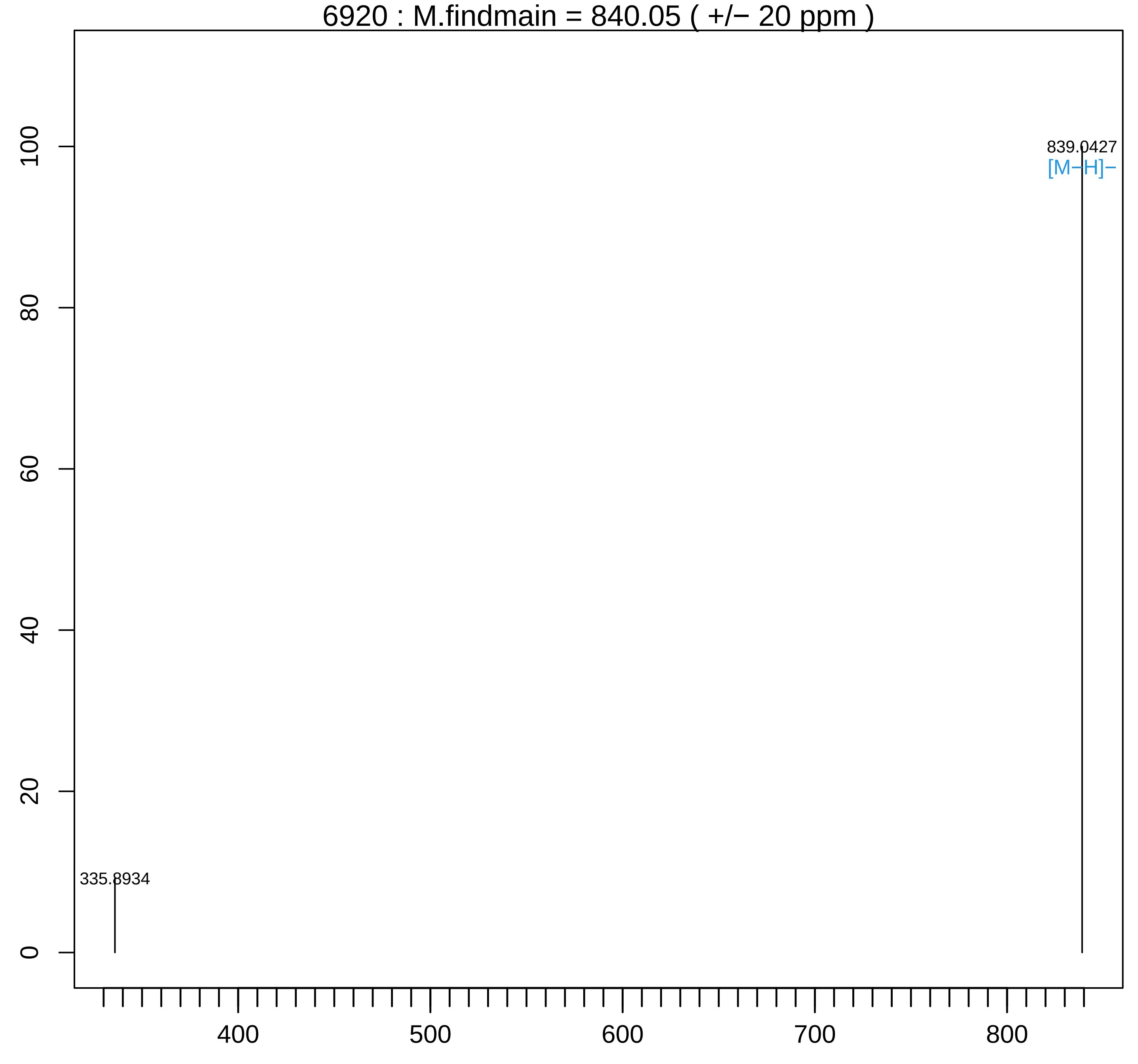
6920 : M.findmain = 840.05 (+/− 20 ppm)
839.0427
[M−H]−
335.8934
0
20
40
60
80
100
400
500
600
700
800

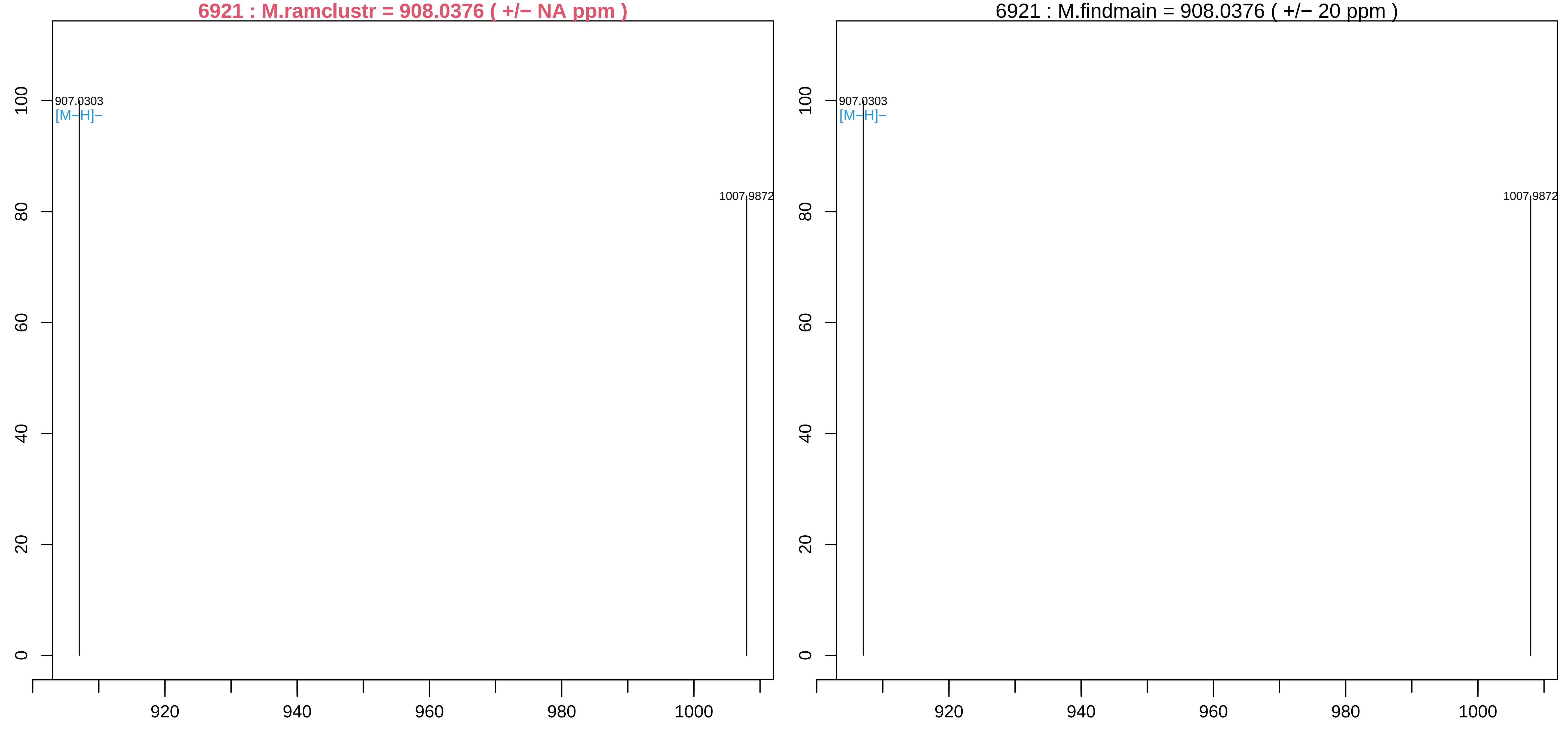
6921 : M.ramclustr = 908.0376 (+/− NA ppm)
907.0303
[M−H]−
1007.9872
6921 : M.findmain = 908.0376 (+/− 20 ppm)
907.0303
[M−H]−
1007.9872
0
20
40
60
80
100
920
940
960
980
1000

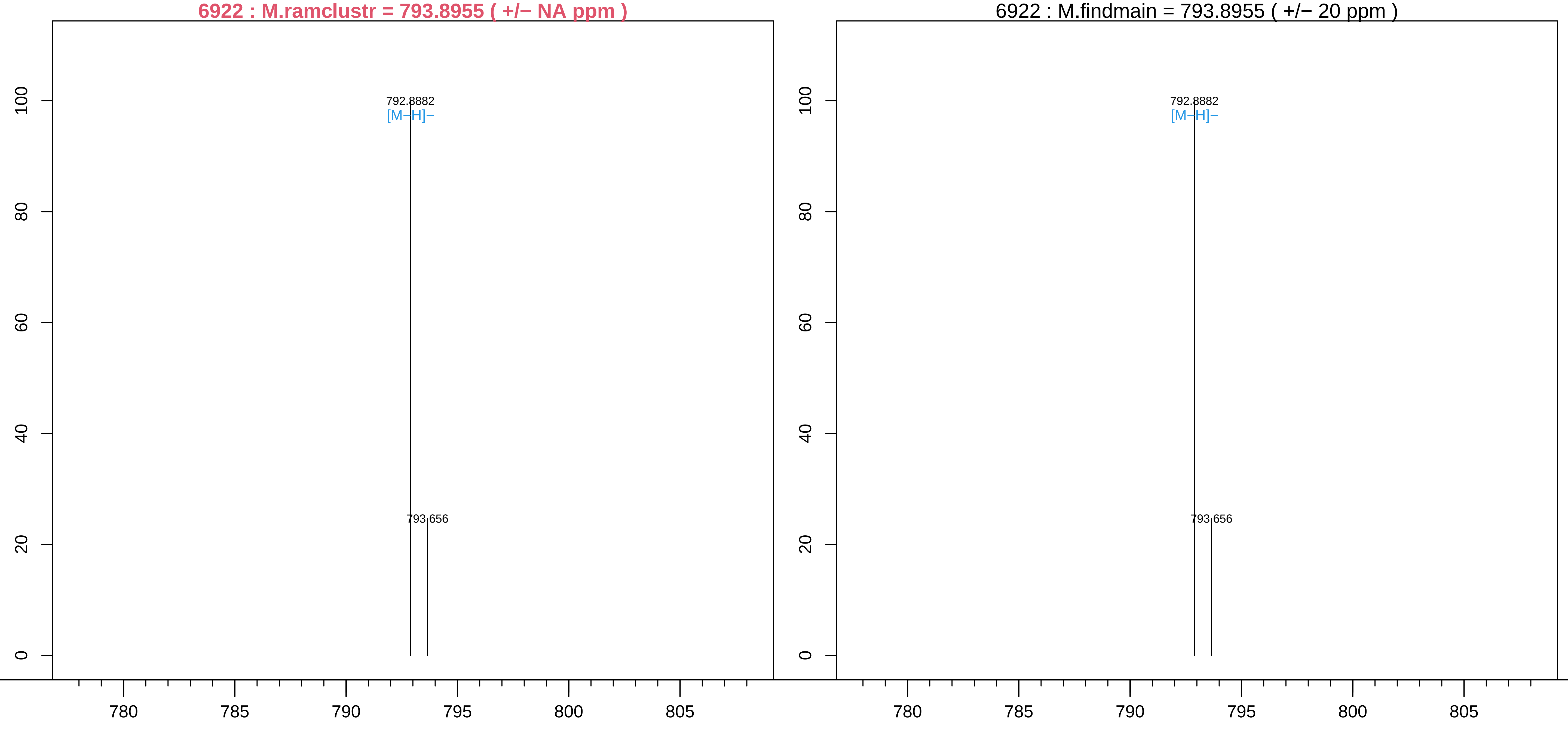

6922 : M.ramclustr = 793.8955 (+/− NA ppm)
792.8882
[M−H]−
793.656
6922 : M.findmain = 793.8955 (+/− 20 ppm)
792.8882
[M−H]−
793.656
0
20
40
60
80
100
780
785
790
795
800
805

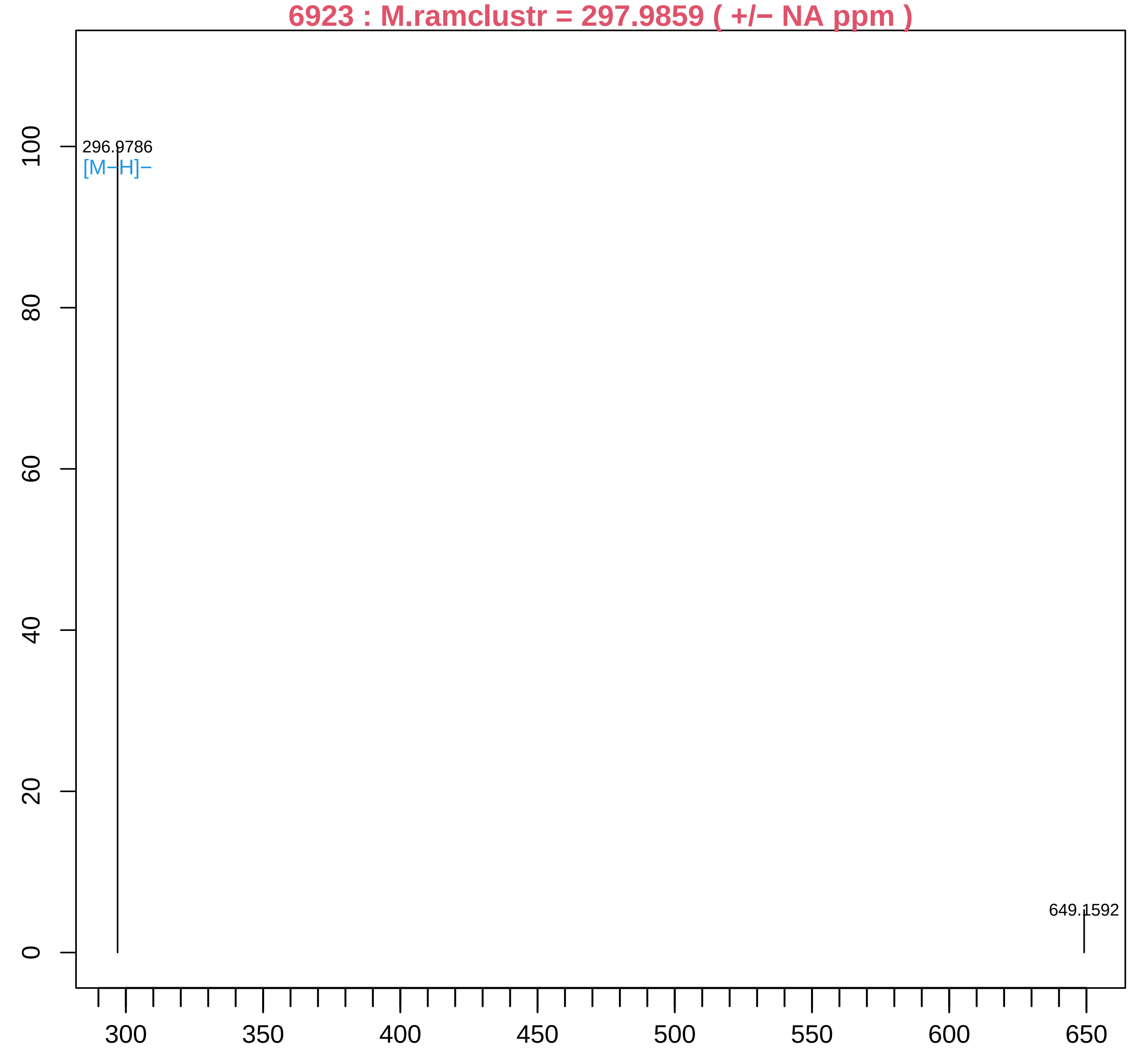
6923 : M.ramclustr = 297.9859 (+/− NA ppm)
296.9786
[M−H]−
649.1592
0
20
40
60
80
100
300
350
400
450
500
550
600
650

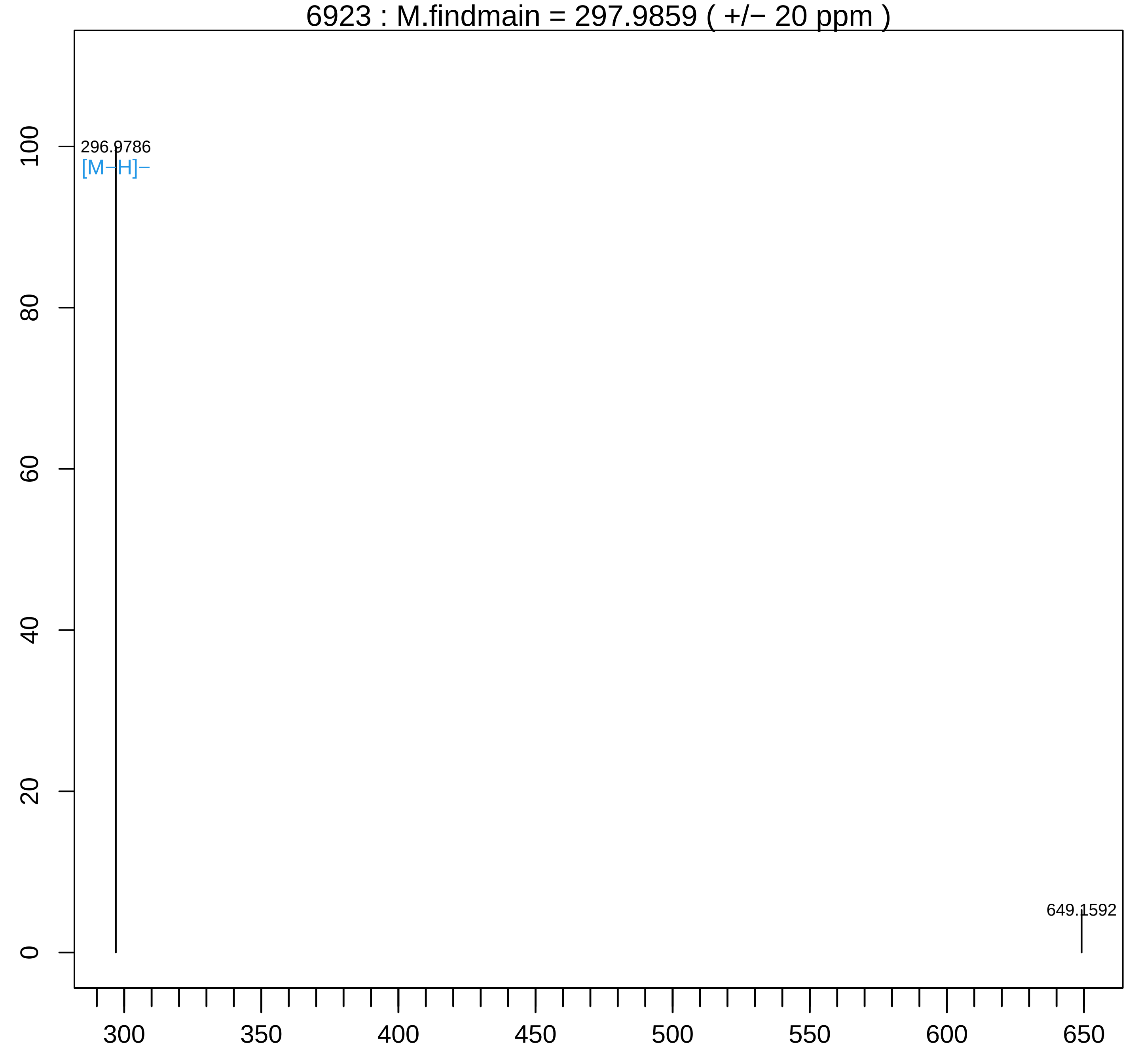
6923 : M.findmain = 297.9859 (+/− 20 ppm)
296.9786
[M−H]−
649.1592
0
20
40
60
80
100
300
350
400
450
500
550
600
650

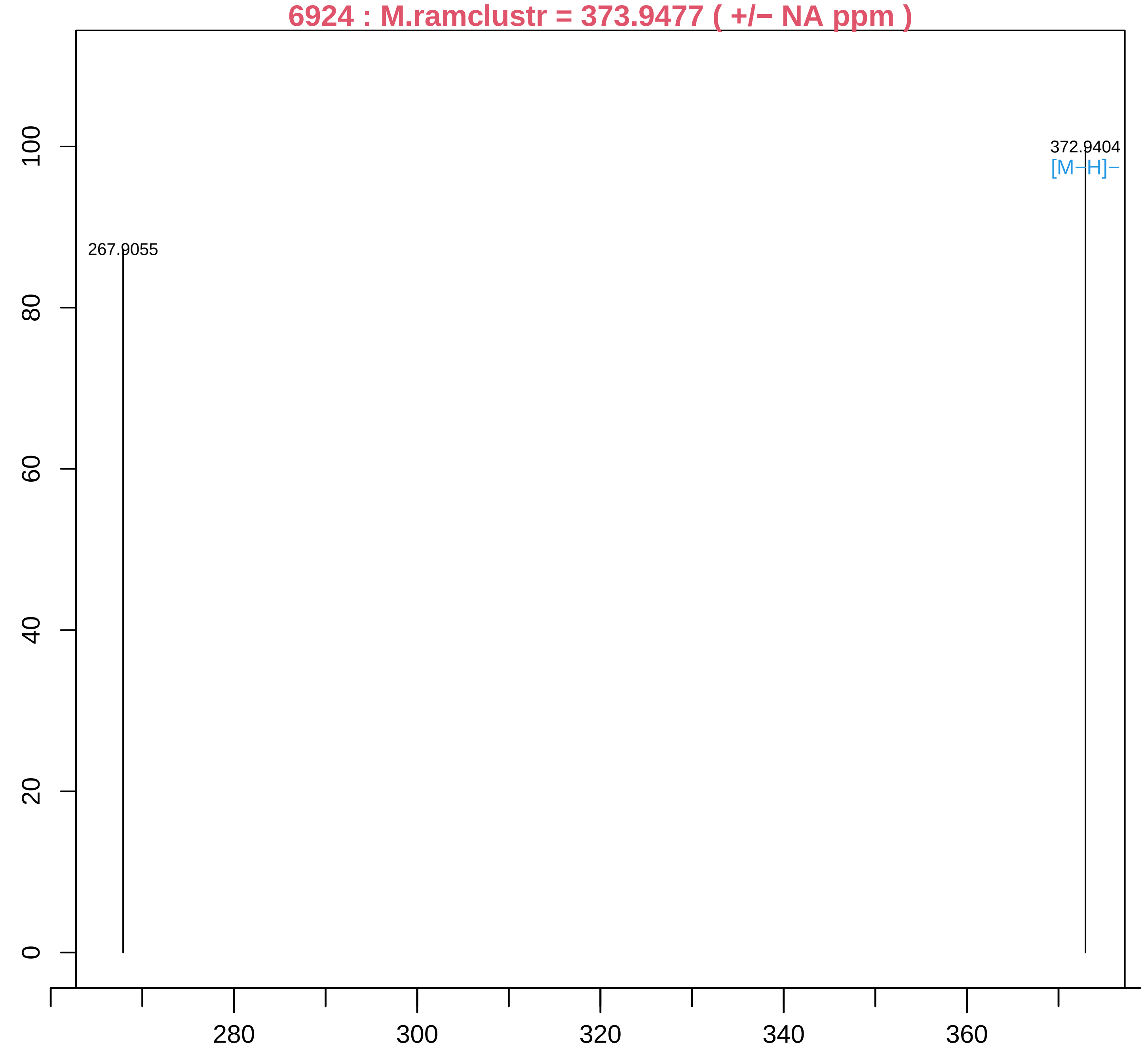
6924 : M.ramclustr = 373.9477 (+/− NA ppm)
100
80
60
40
20
0
280
300
320
340
360
267.9055
372.9404
[M−H]−

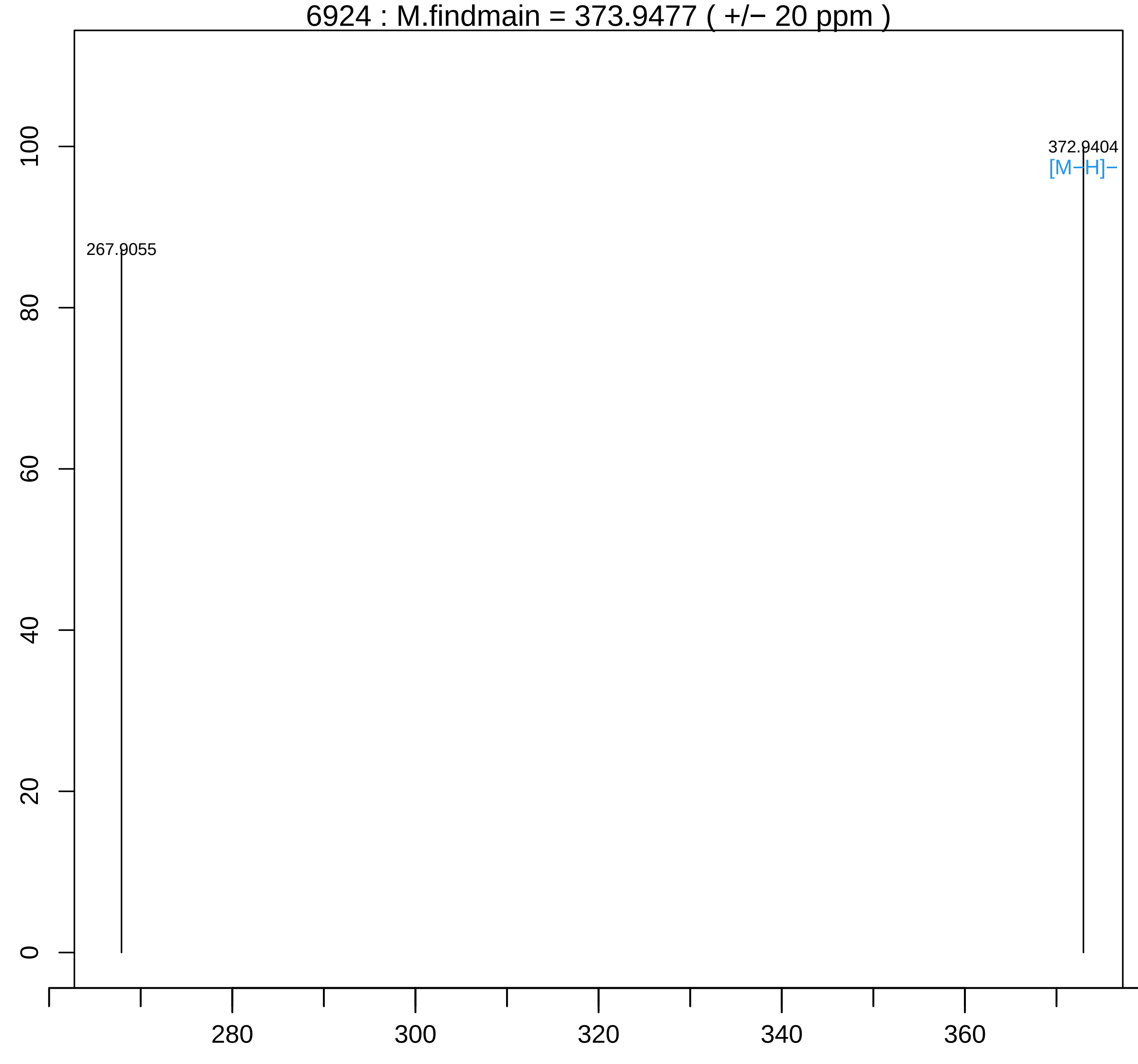
6924 : M.findmain = 373.9477 (+/− 20 ppm)
100
80
60
40
20
0
280
300
320
340
360
267.9055
372.9404
[M−H]−

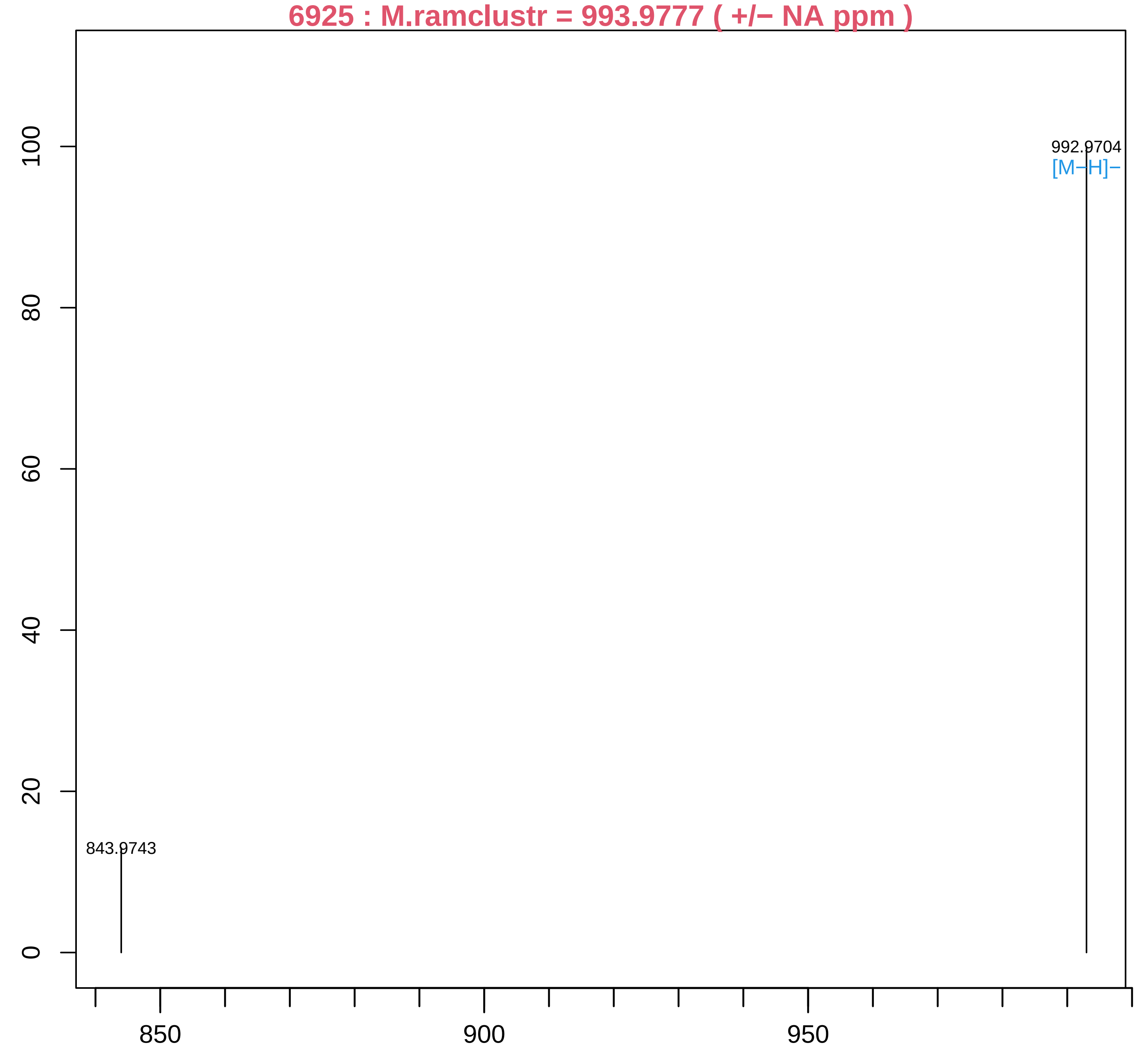
6925 : M.ramclustr = 993.9777 (+/− NA ppm)
992.9704
[M−H]−
843.9743
0
20
40
60
80
100
850
900
950

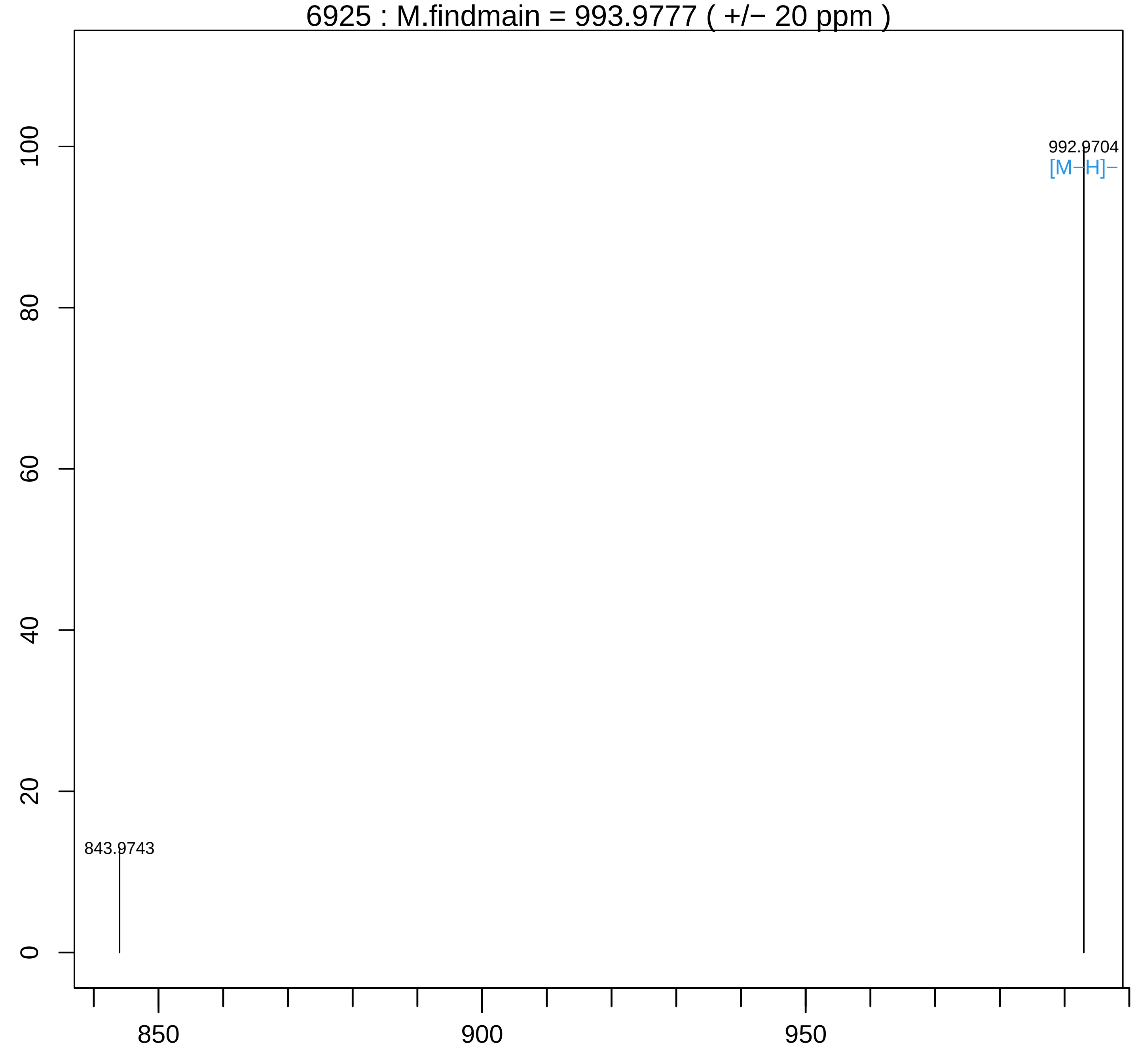
6925 : M.findmain = 993.9777 (+/− 20 ppm)
992.9704
[M−H]−
843.9743
0
20
40
60
80
100
850
900
950

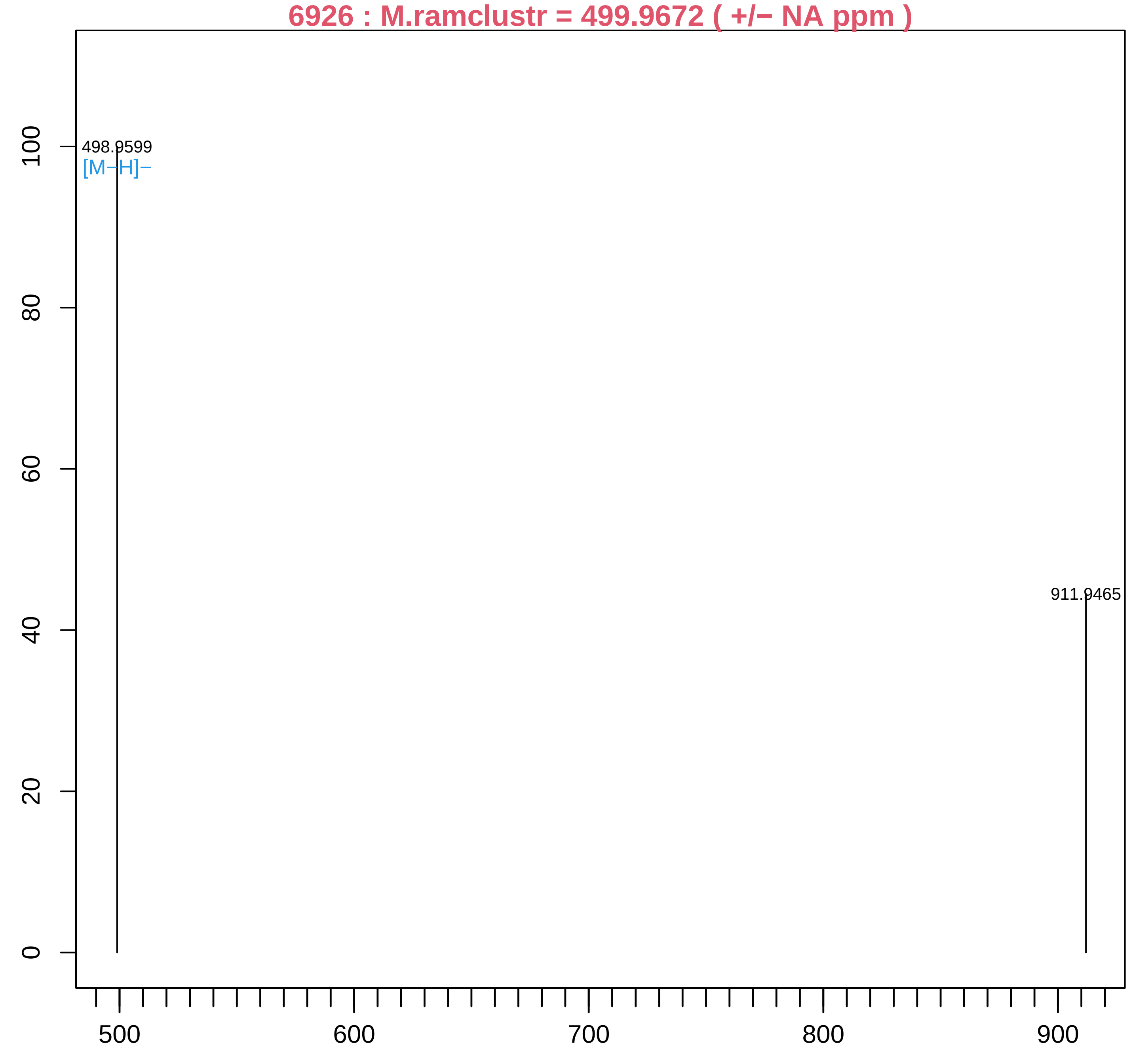
6926 : M.ramclustr = 499.9672 (+/− NA ppm)
498.9599
[M−H]−
911.9465
0
20
40
60
80
100
500
600
700
800
900

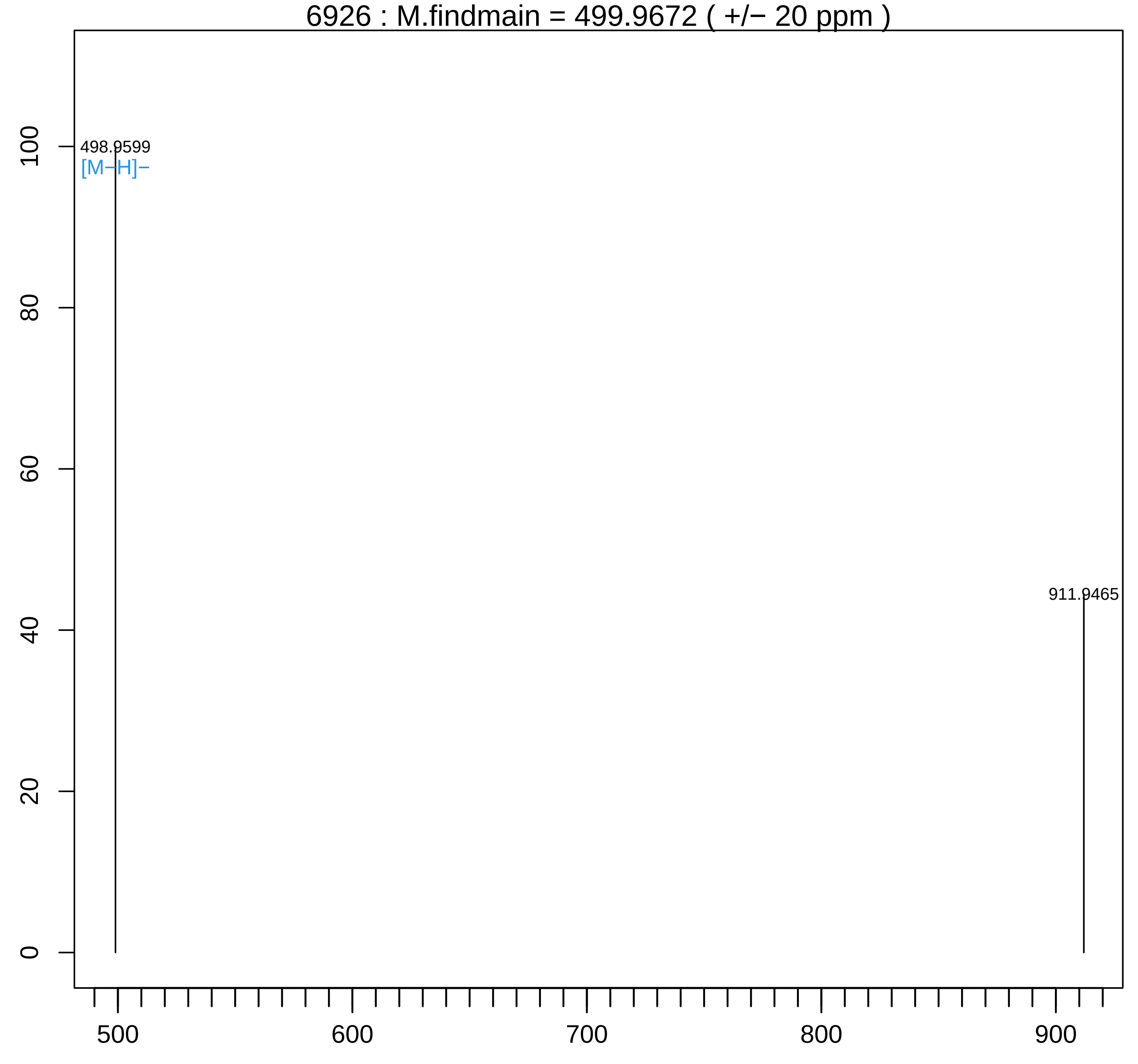
6926 : M.findmain = 499.9672 (+/− 20 ppm)
498.9599
[M−H]−
911.9465
0
20
40
60
80
100
500
600
700
800
900

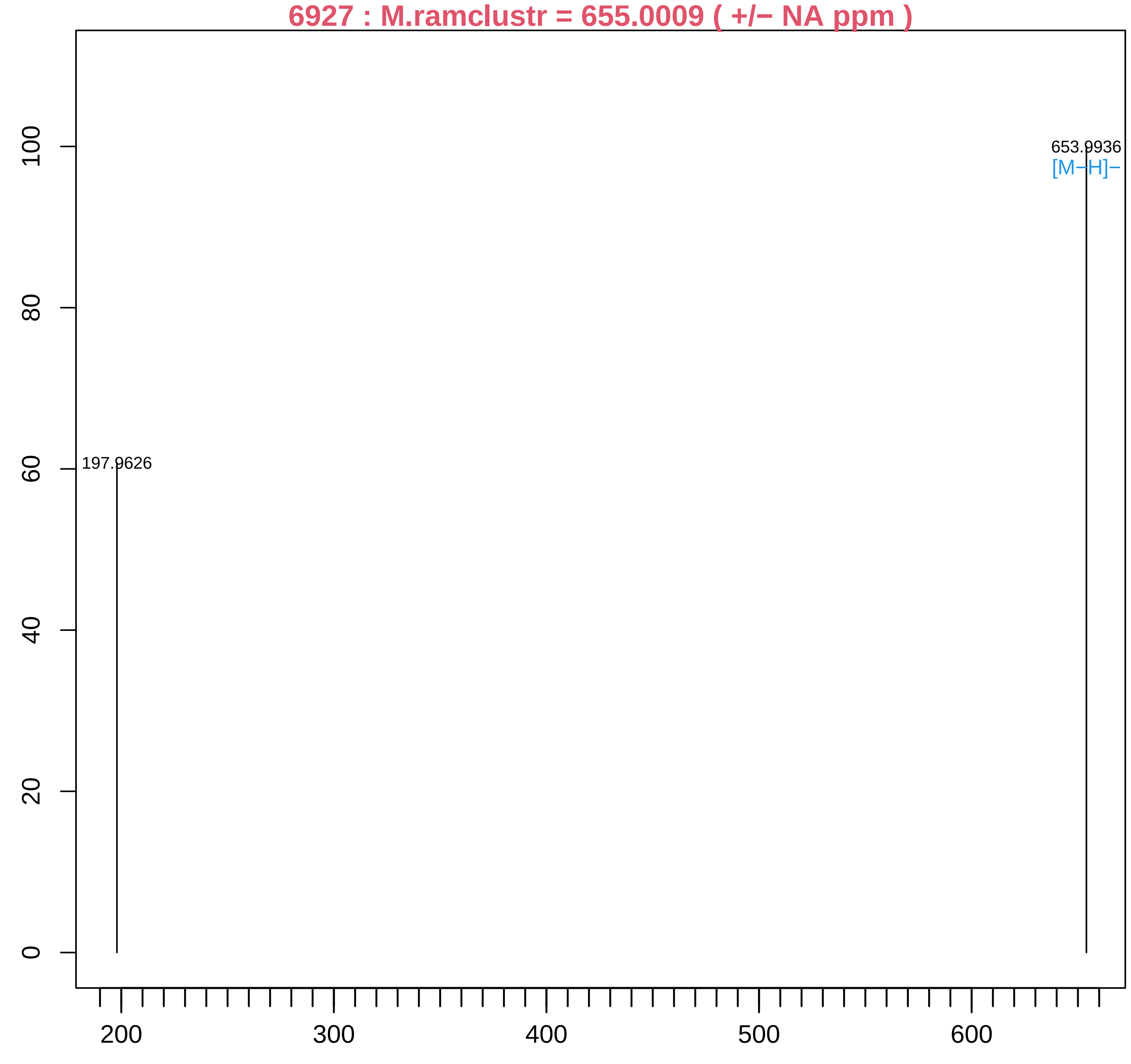
6927 : M.ramclustr = 655.0009 (+/− NA ppm)
653.9936
[M−H]−
197.9626
0
20
40
60
80
100
200
300
400
500
600

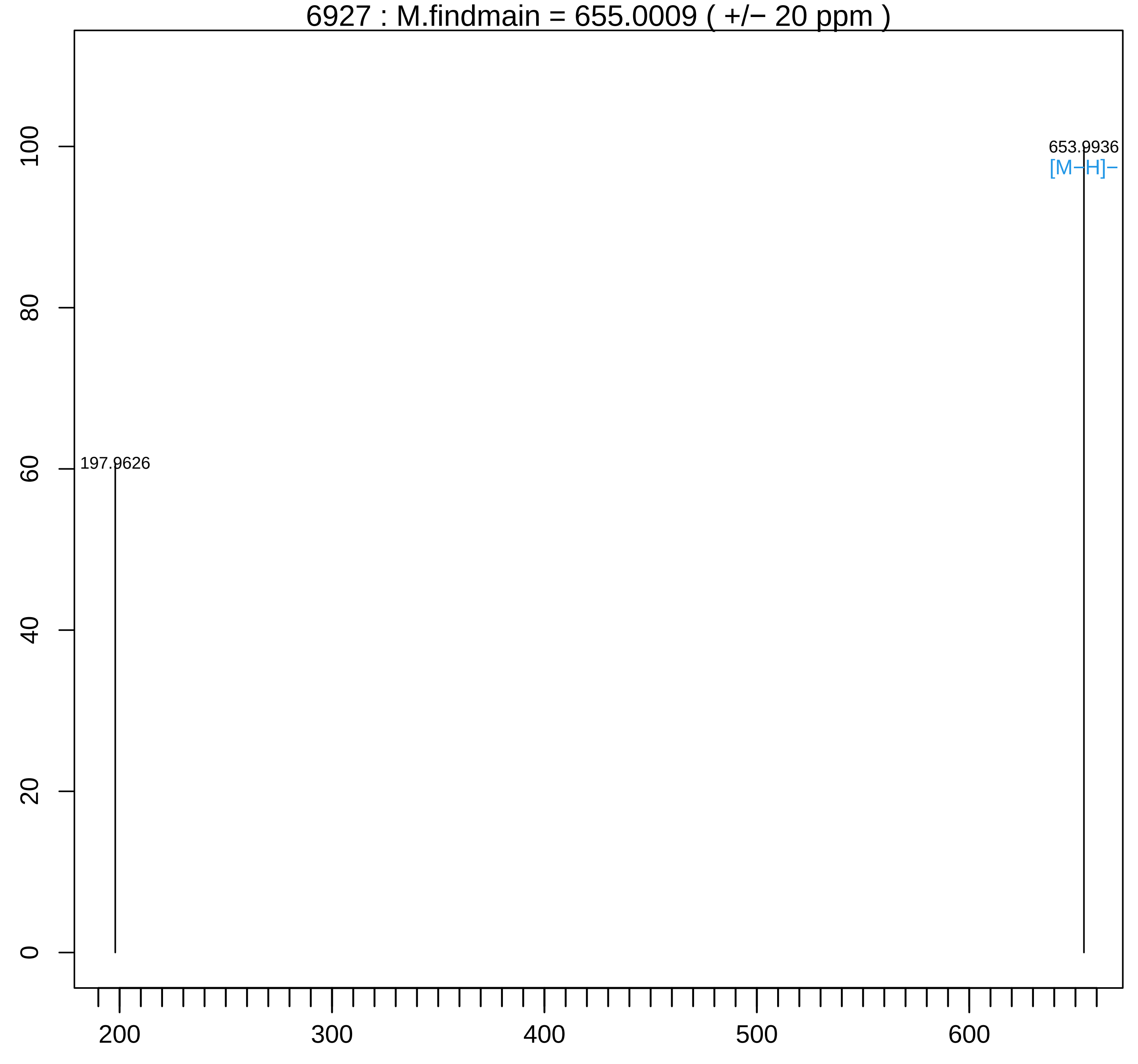
6927 : M.findmain = 655.0009 (+/− 20 ppm)
653.9936
[M−H]−
197.9626
0
20
40
60
80
100
200
300
400
500
600

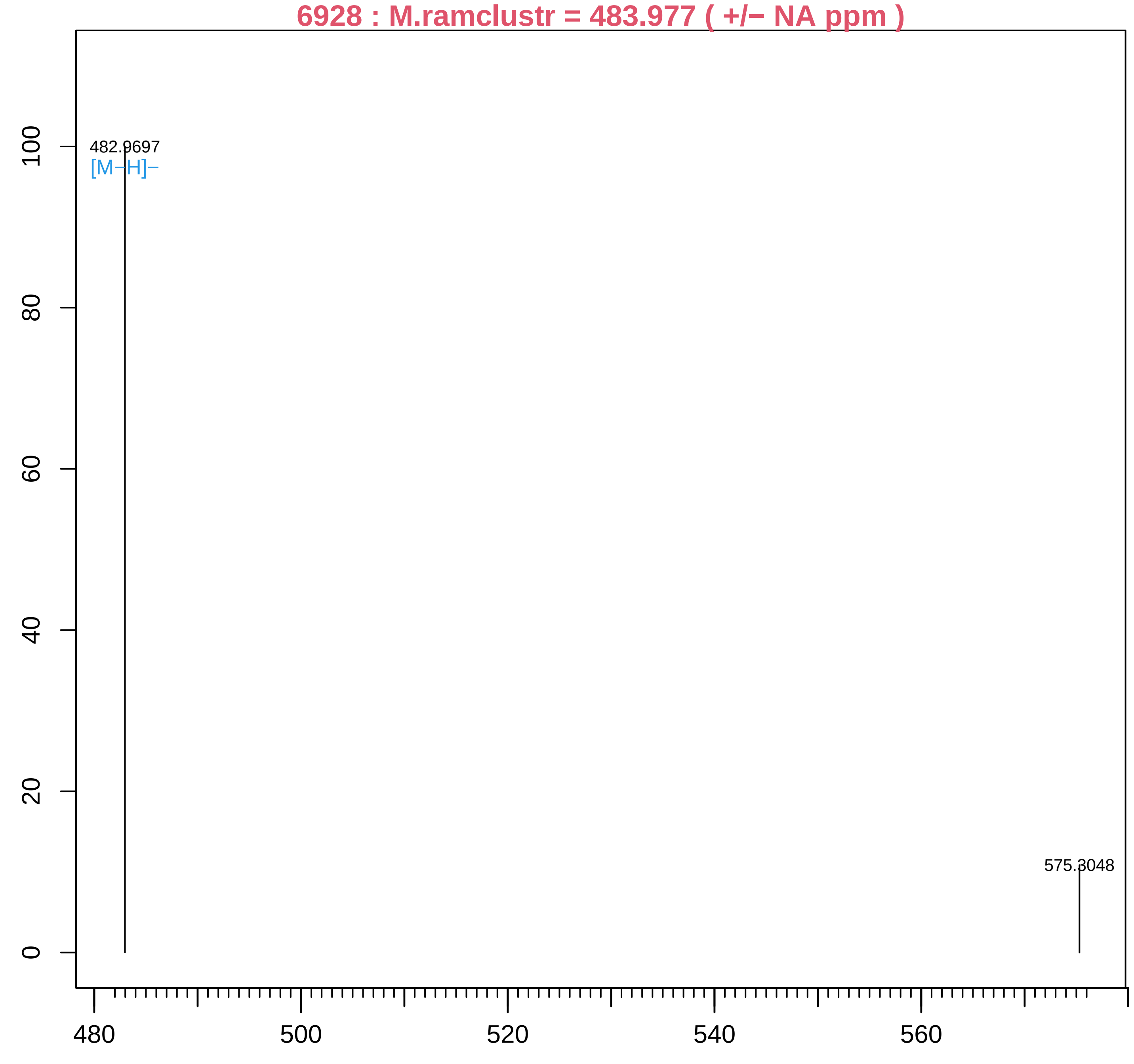
6928 : M.ramclustr = 483.977 (+/− NA ppm)
482.9697
[M−H]−
575.3048
0
20
40
60
80
100
480
500
520
540
560

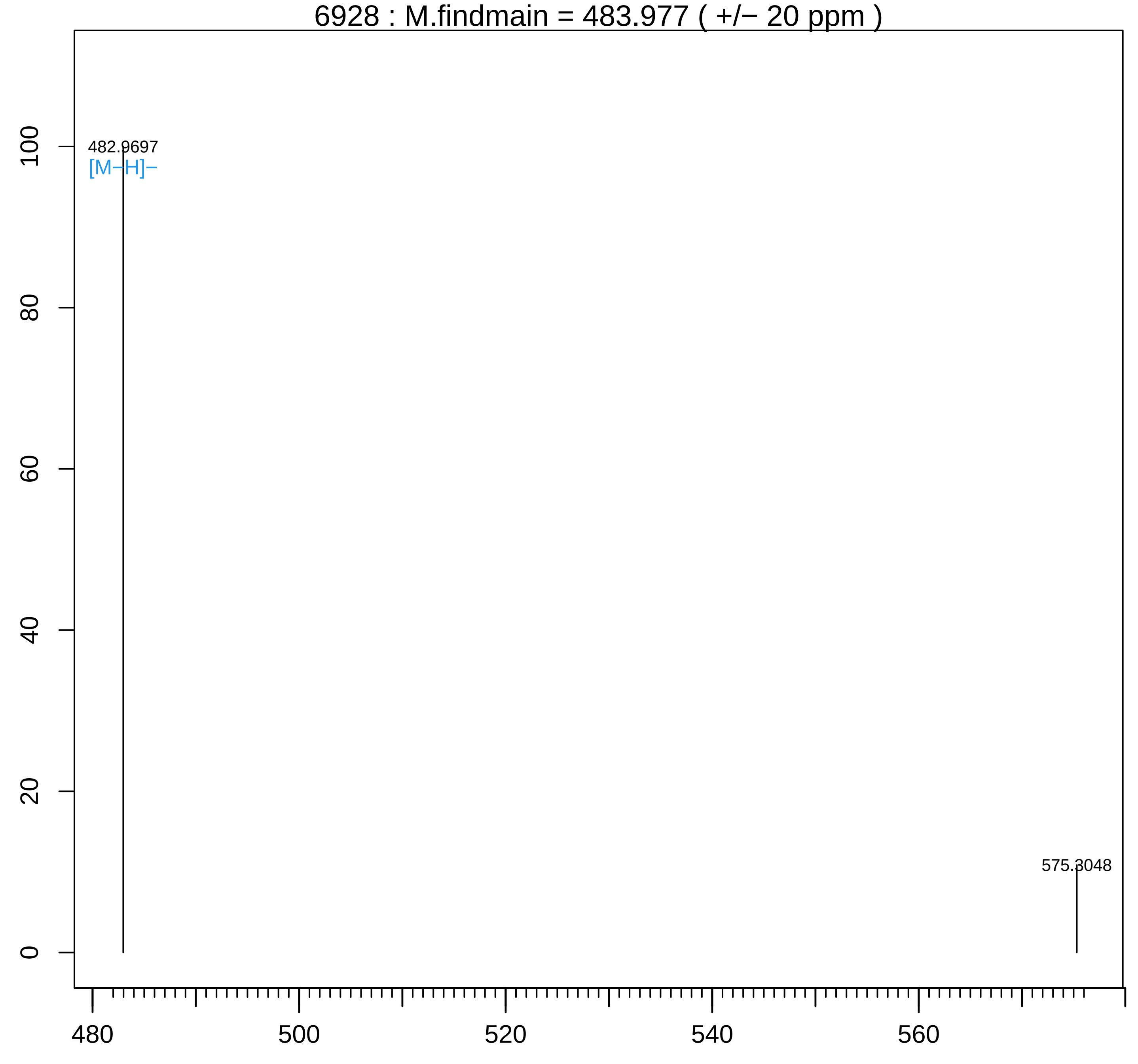
6928 : M.findmain = 483.977 (+/− 20 ppm)
482.9697
[M−H]−
575.3048
0
20
40
60
80
100
480
500
520
540
560

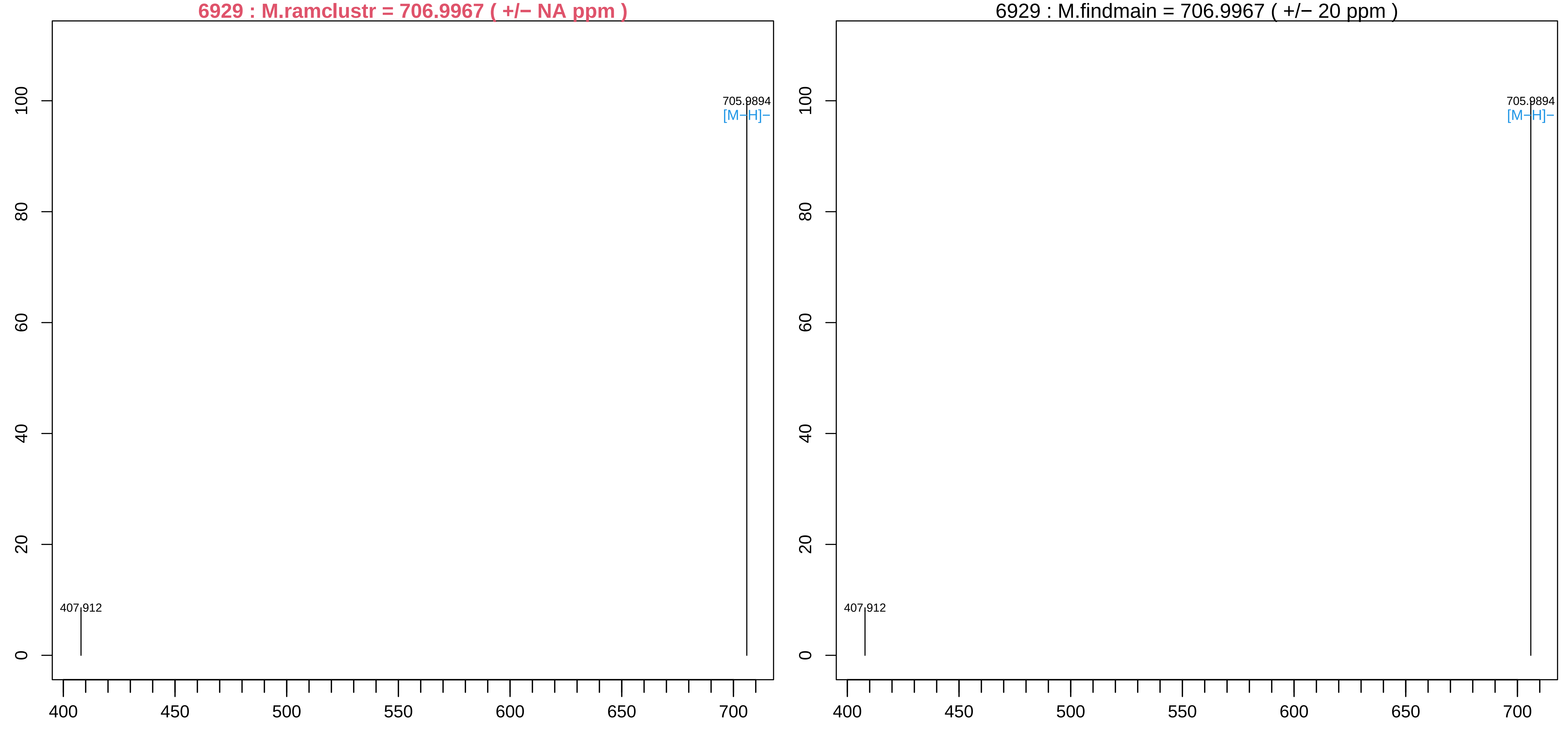
6929 : M.ramclustr = 706.9967 (+/− NA ppm)
705.9894
[M−H]−
407.912
6929 : M.findmain = 706.9967 (+/− 20 ppm)
705.9894
[M−H]−
407.912
0
20
40
60
80
100
400
450
500
550
600
650
700

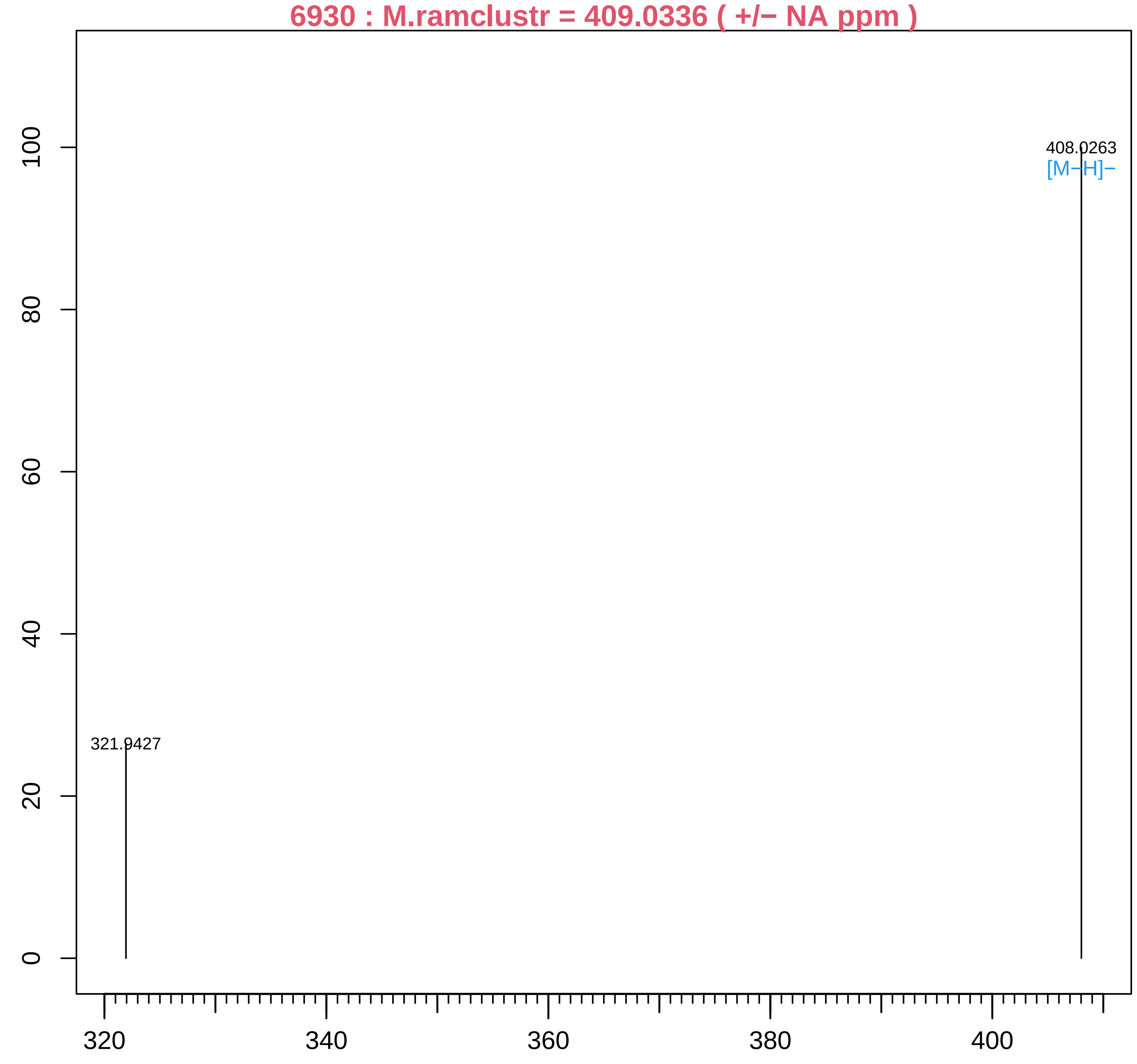
6930 : M.ramclustr = 409.0336 (+/− NA ppm)
408.0263
[M−H]−
321.9427
0
20
40
60
80
100
320
340
360
380
400

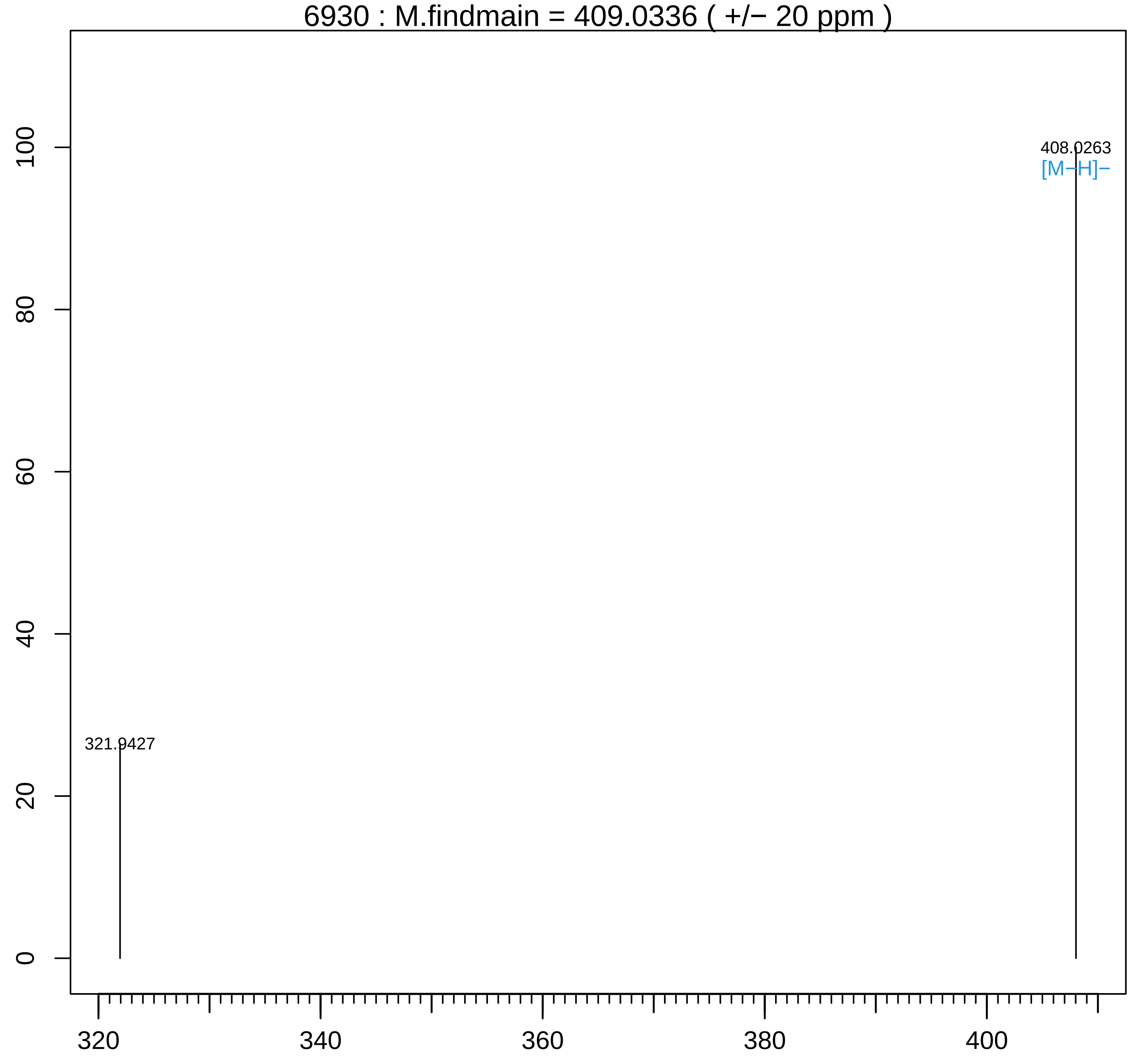
6930 : M.findmain = 409.0336 (+/− 20 ppm)
408.0263
[M−H]−
321.9427
0
20
40
60
80
100
320
340
360
380
400

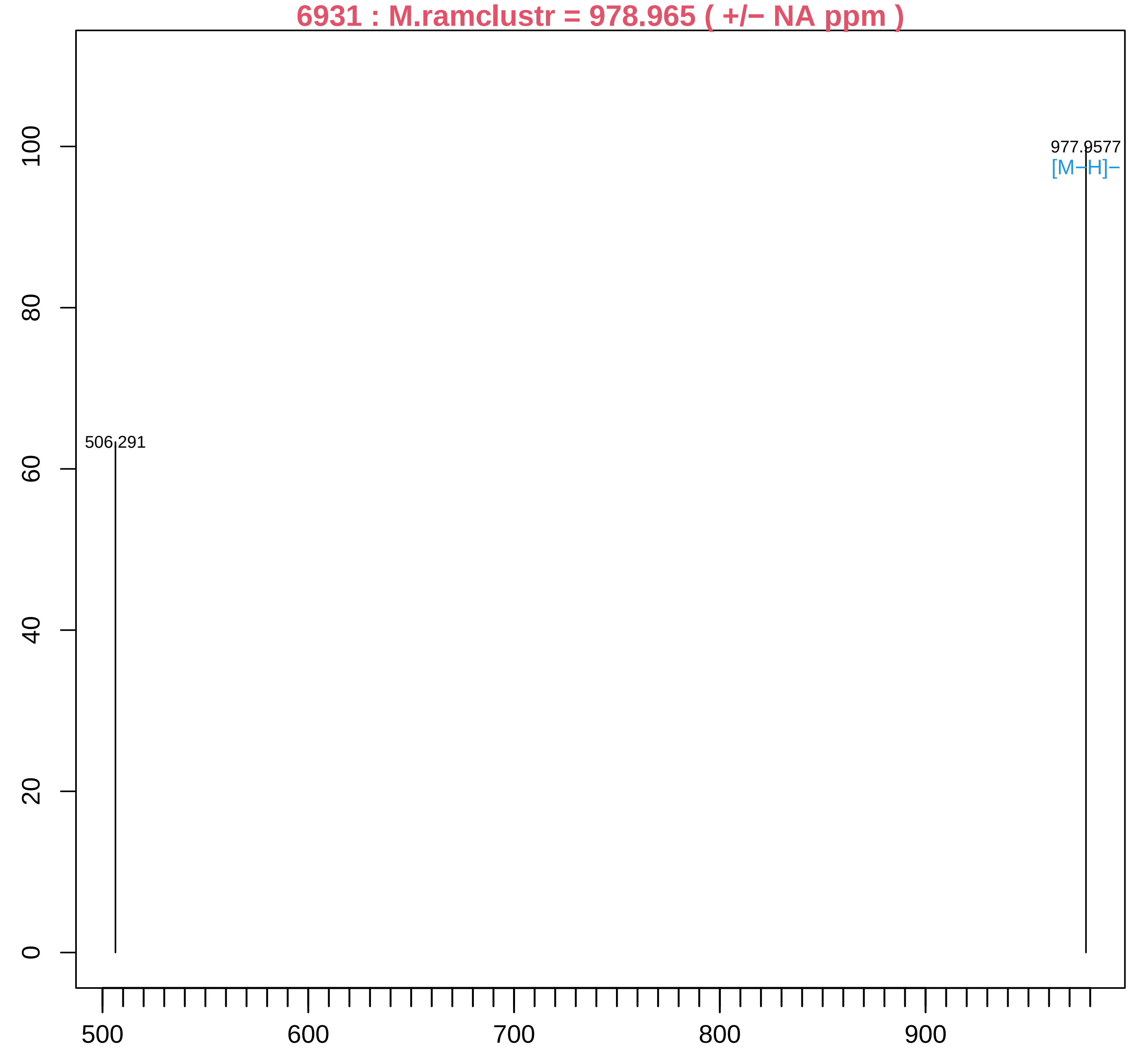

6931 : M.ramclustr = 978.965 (+/− NA ppm)
977.9577
[M−H]−
506.291
0
20
40
60
80
100
500
600
700
800
900

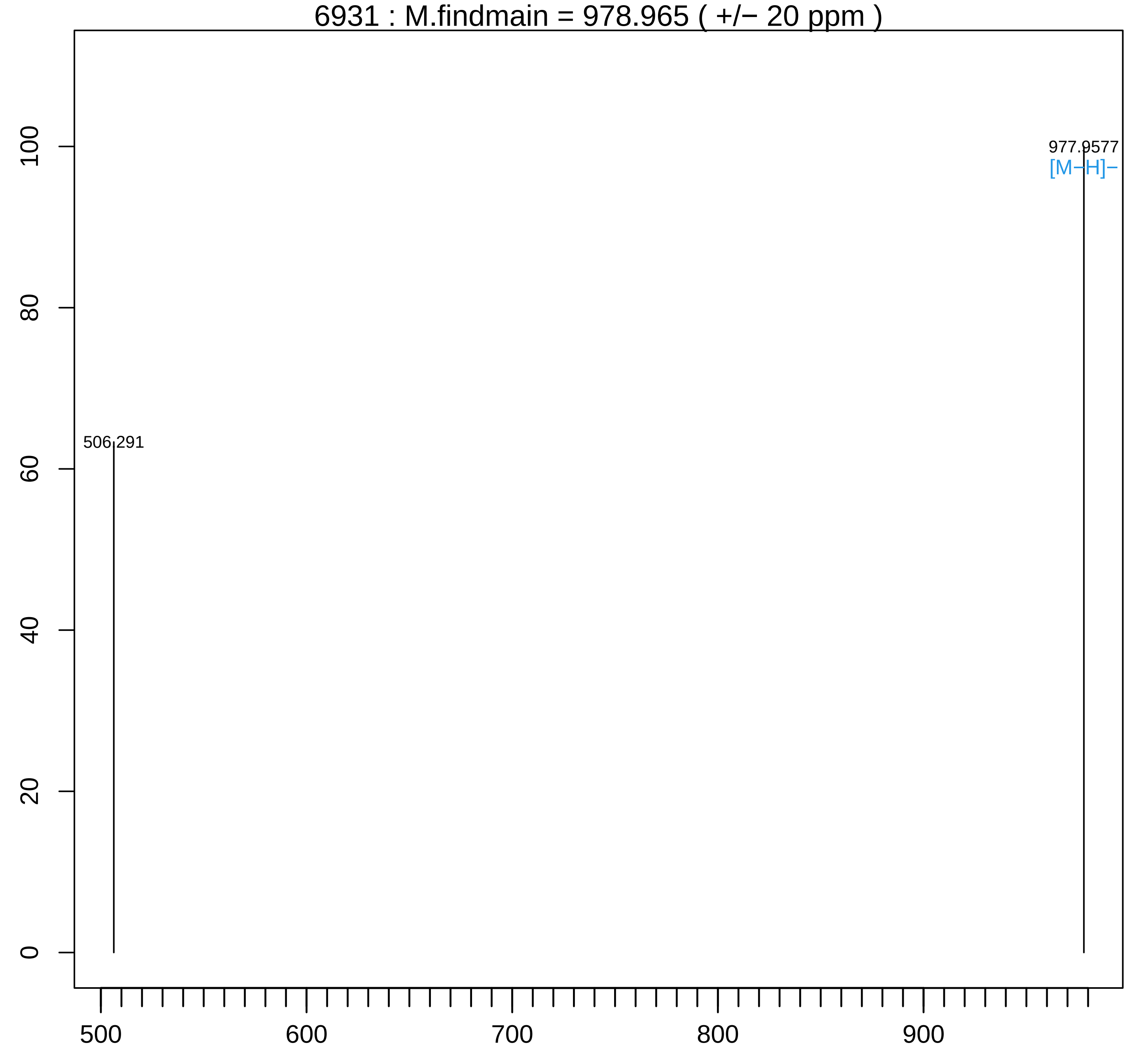

6931 : M.findmain = 978.965 (+/− 20 ppm)
977.9577
[M−H]−
506.291
0
20
40
60
80
100
500
600
700
800
900

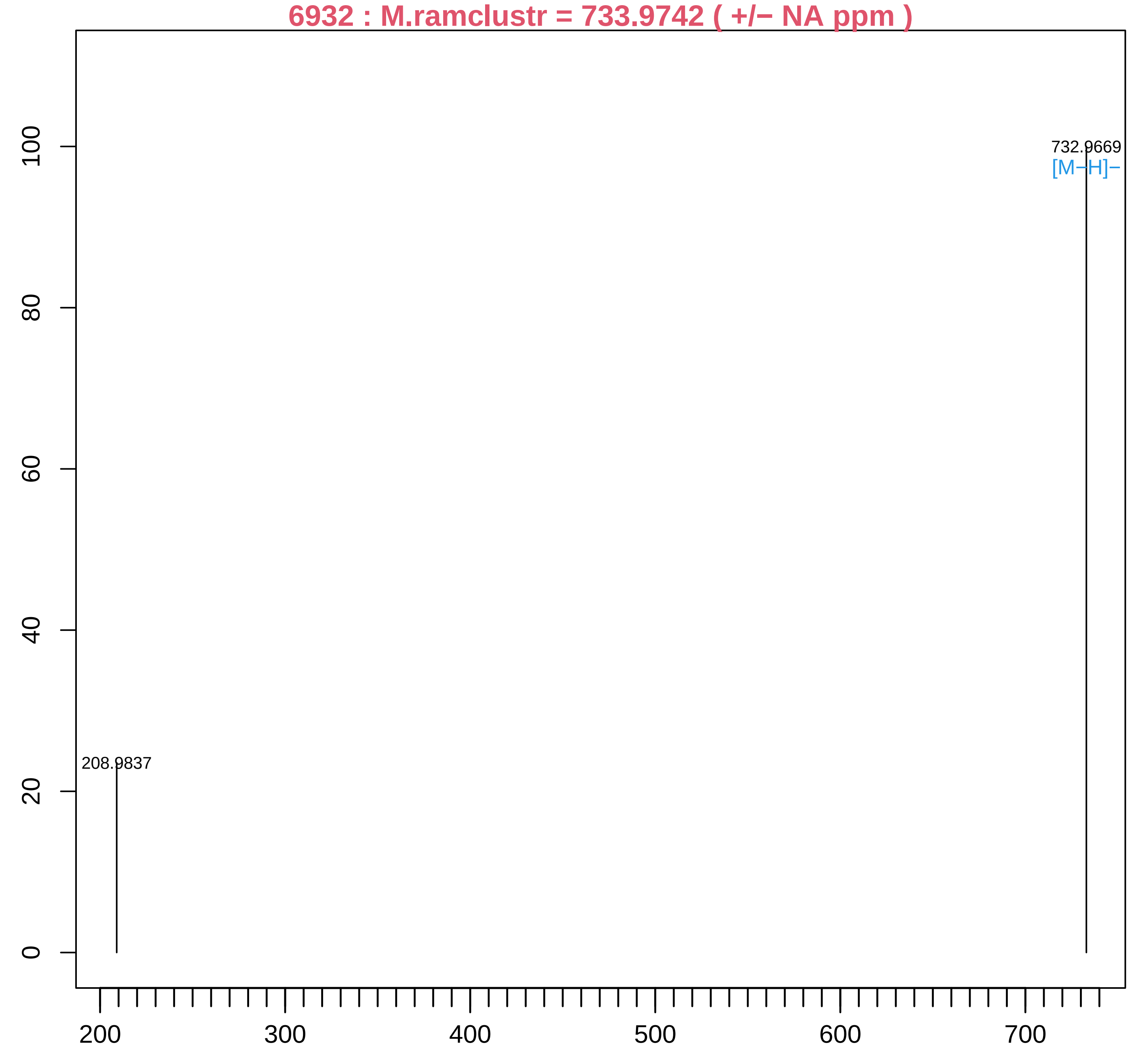
6932 : M.ramclustr = 733.9742 (+/− NA ppm)
732.9669
[M−H]−
208.9837
0
20
40
60
80
100
200
300
400
500
600
700

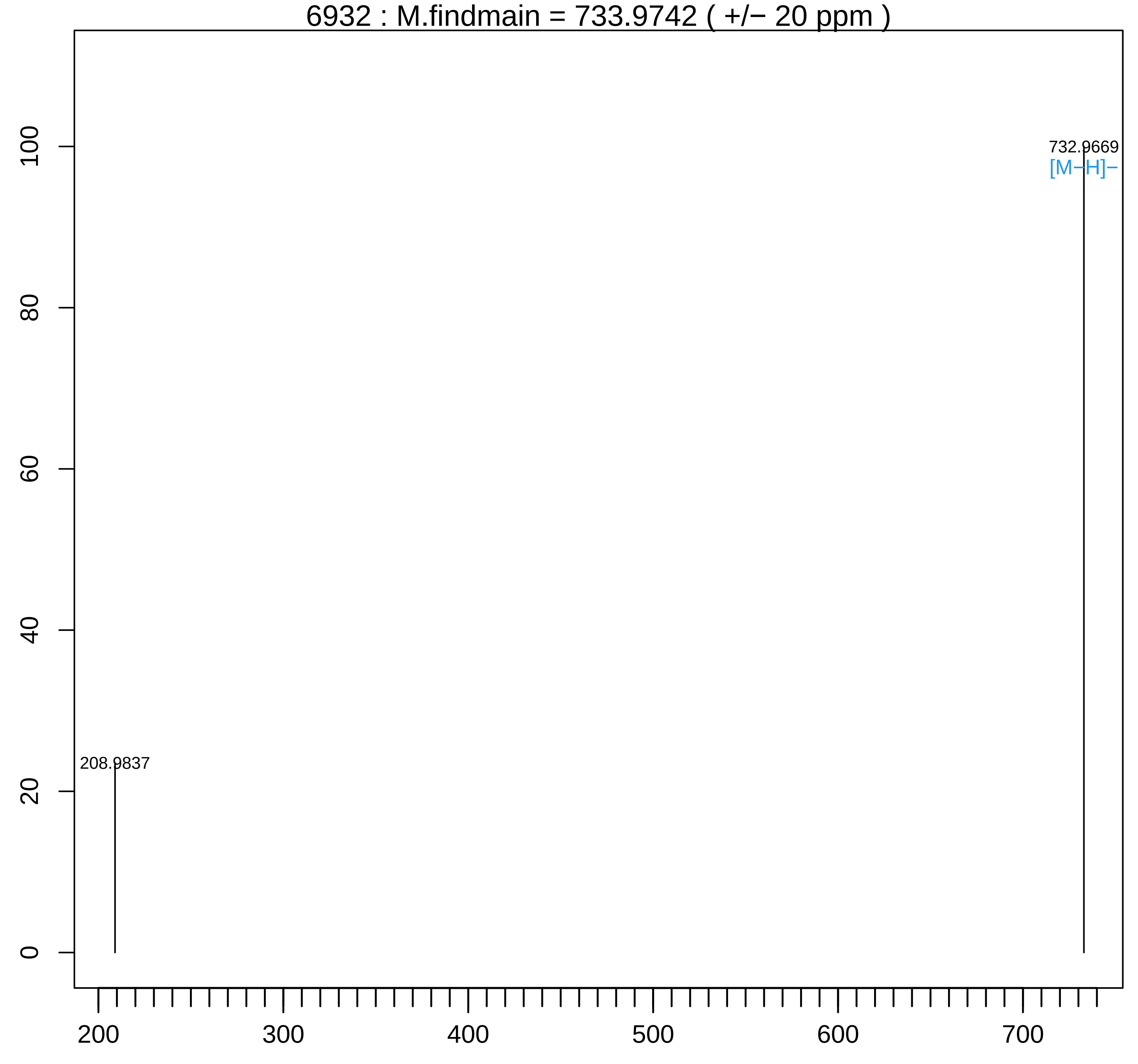
6932 : M.findmain = 733.9742 (+/− 20 ppm)
732.9669
[M−H]−
208.9837
0
20
40
60
80
100
200
300
400
500
600
700

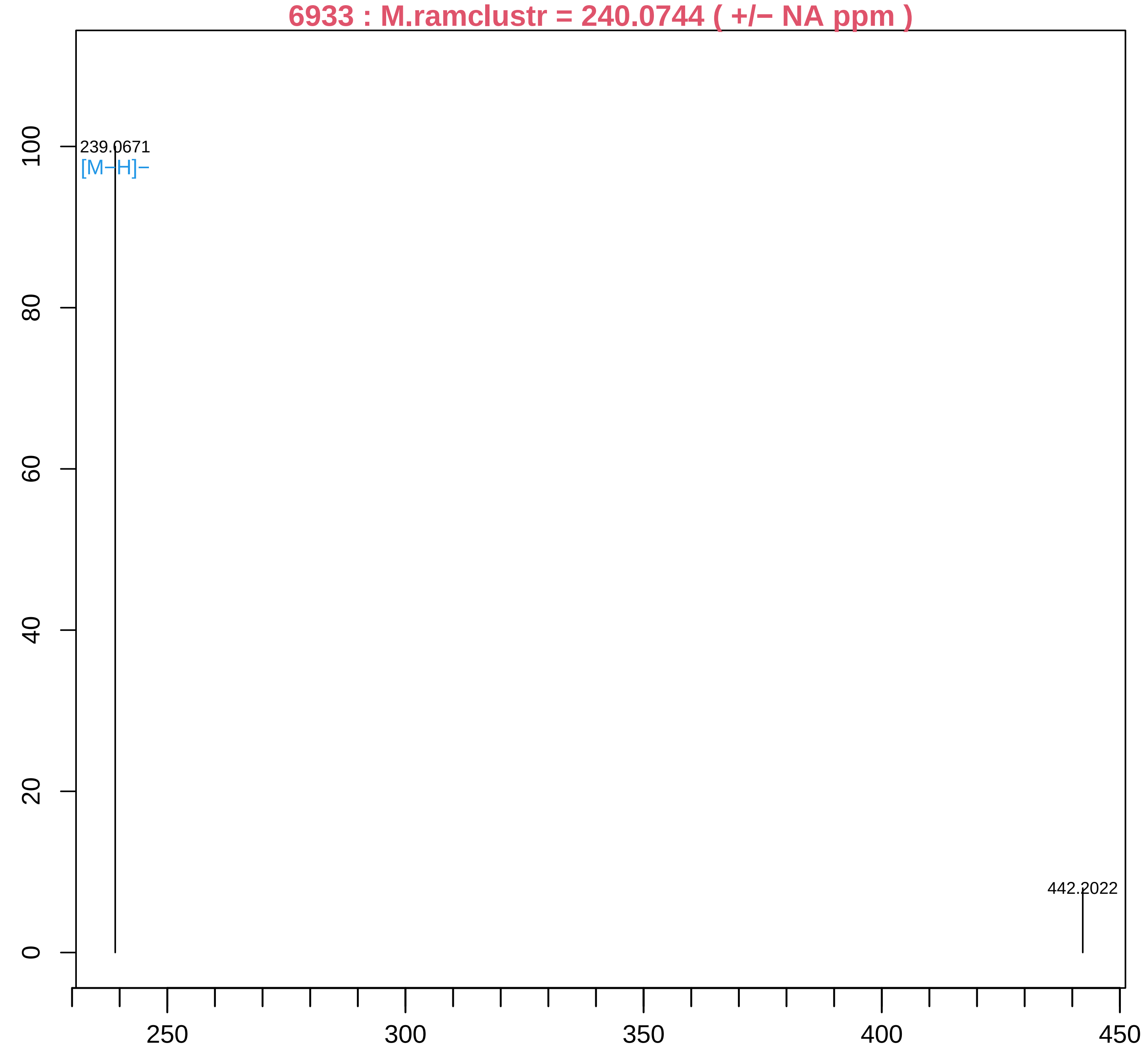
6933 : M.ramclustr = 240.0744 (+/− NA ppm)
239.0671
[M−H]−
442.2022
0
20
40
60
80
100
250
300
350
400
450

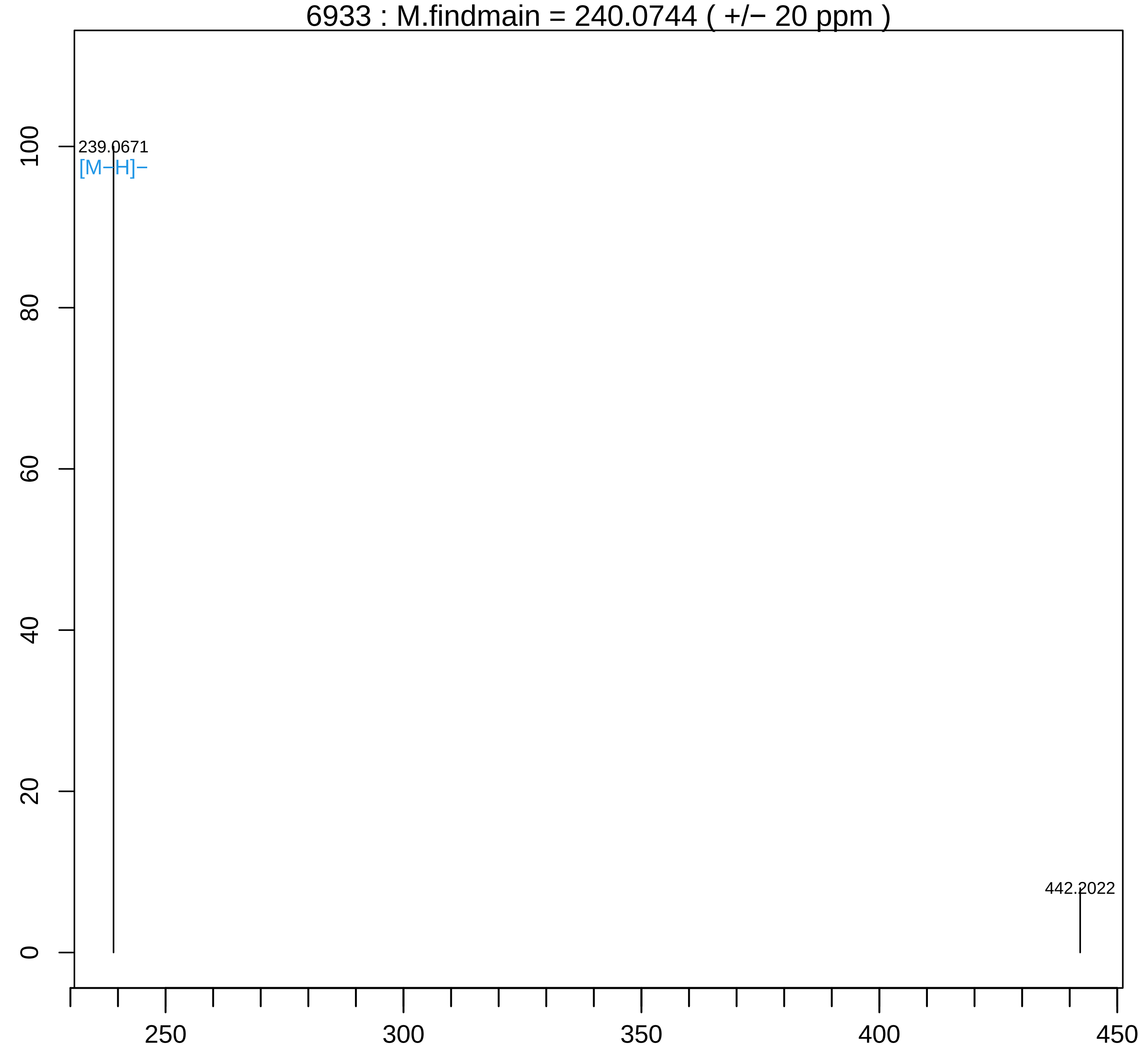
6933 : M.findmain = 240.0744 (+/− 20 ppm)
239.0671
[M−H]−
442.2022
0
20
40
60
80
100
250
300
350
400
450

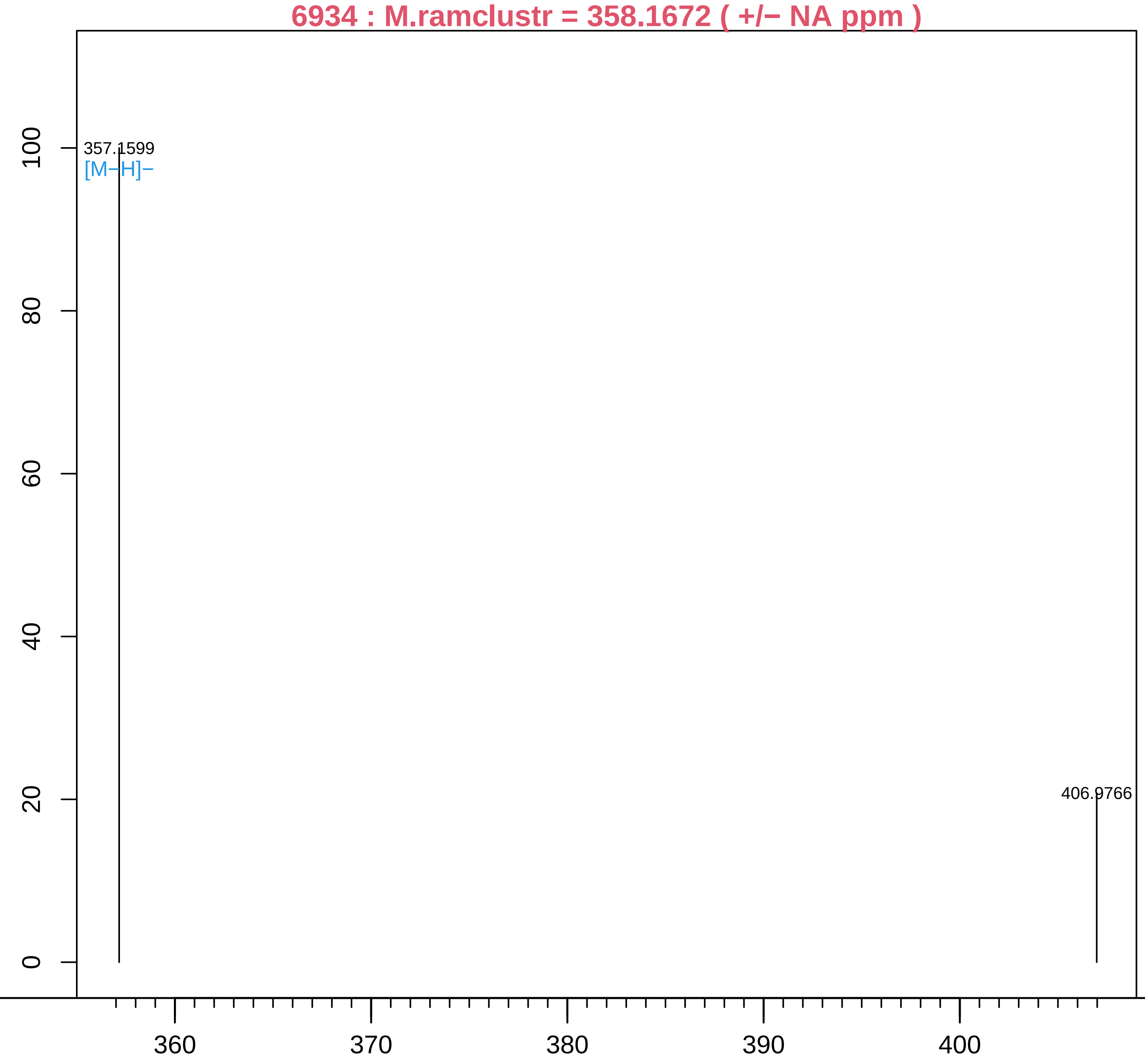
6934 : M.ramclustr = 358.1672 (+/− NA ppm)
357.1599
[M−H]−
406.9766
0
20
40
60
80
100
360
370
380
390
400

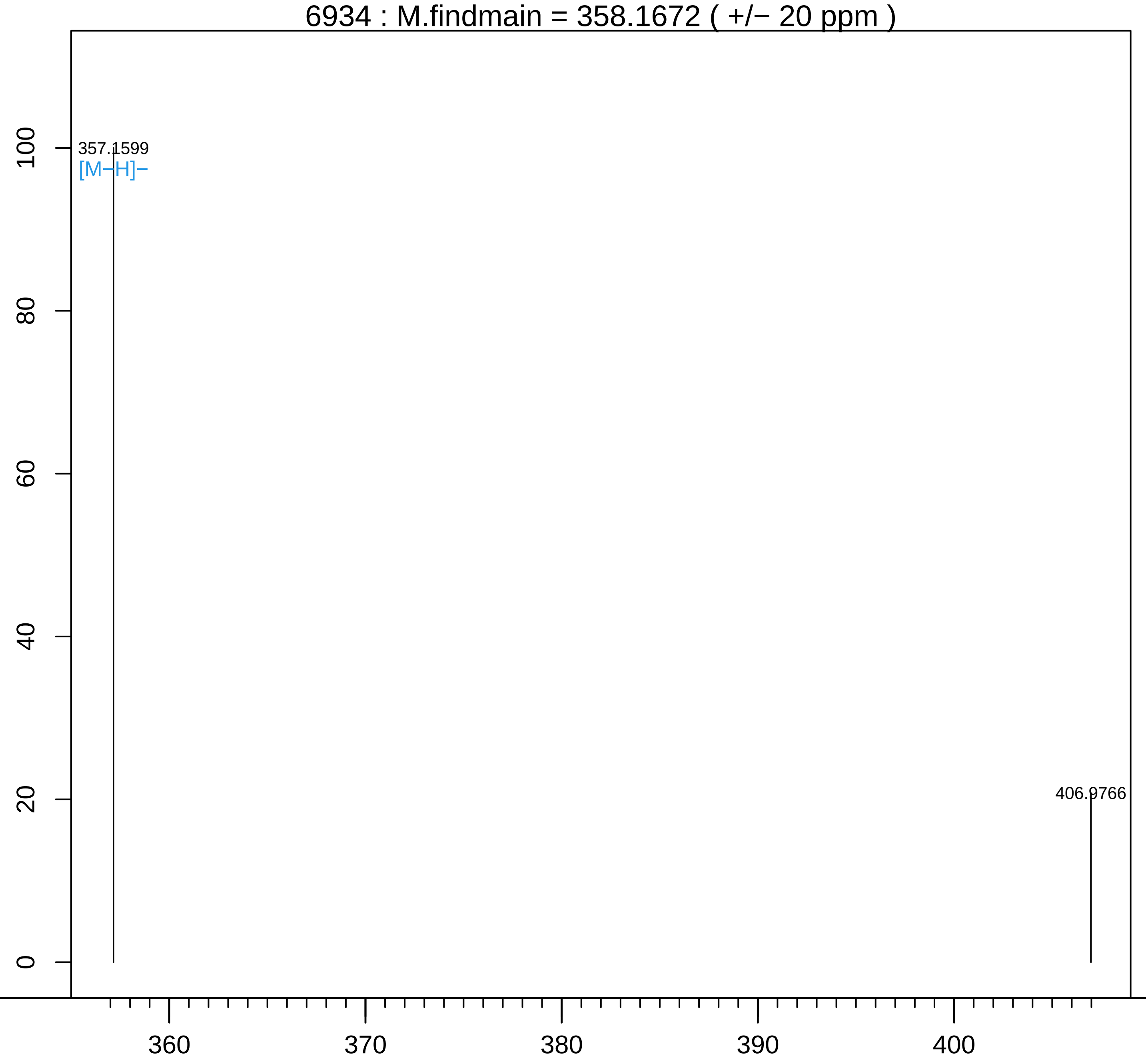
6934 : M.findmain = 358.1672 (+/− 20 ppm)
357.1599
[M−H]−
406.9766
0
20
40
60
80
100
360
370
380
390
400

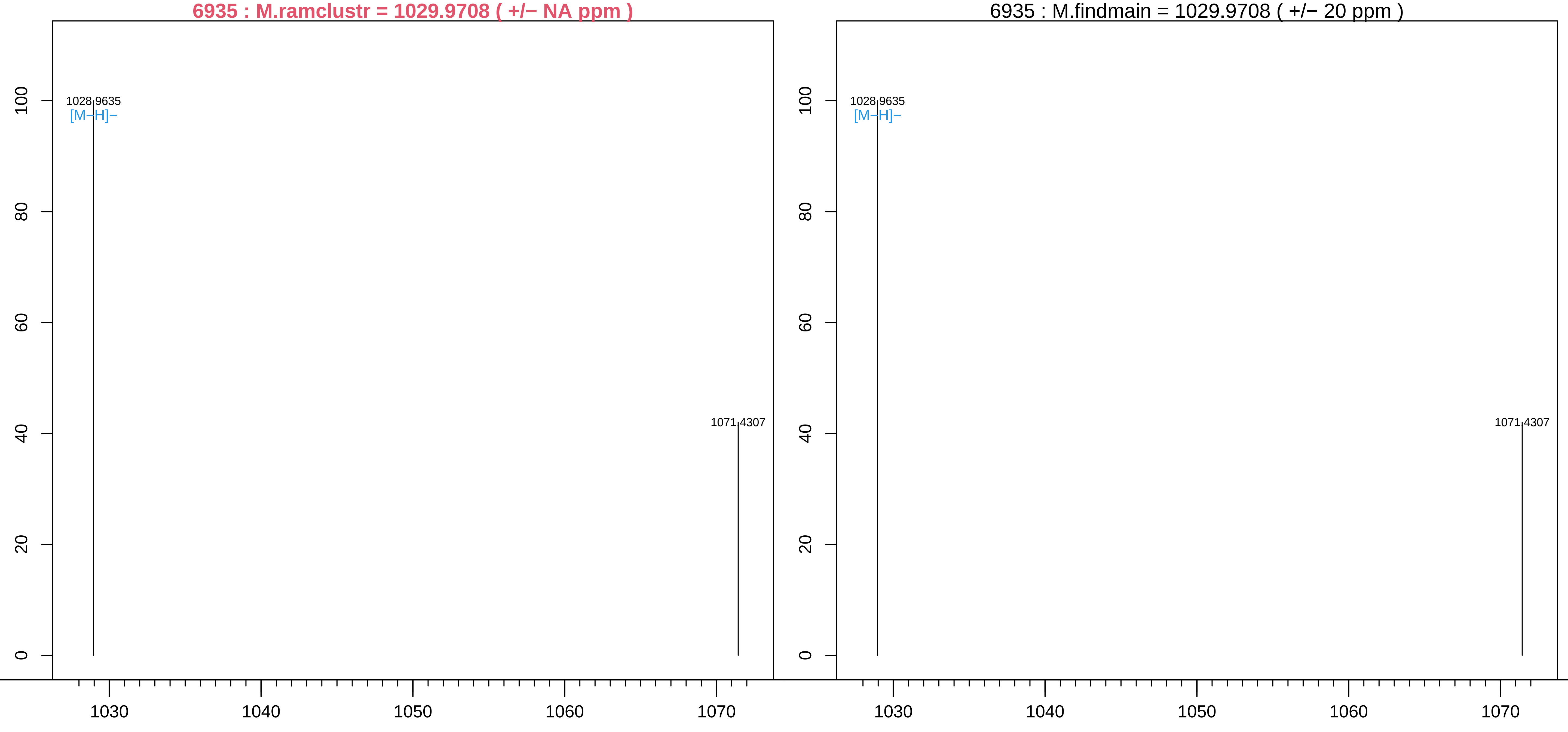

6935 : M.ramclustr = 1029.9708 (+/− NA ppm)
1028.9635
[M−H]−
1071.4307
6935 : M.findmain = 1029.9708 (+/− 20 ppm)
1028.9635
[M−H]−
1071.4307

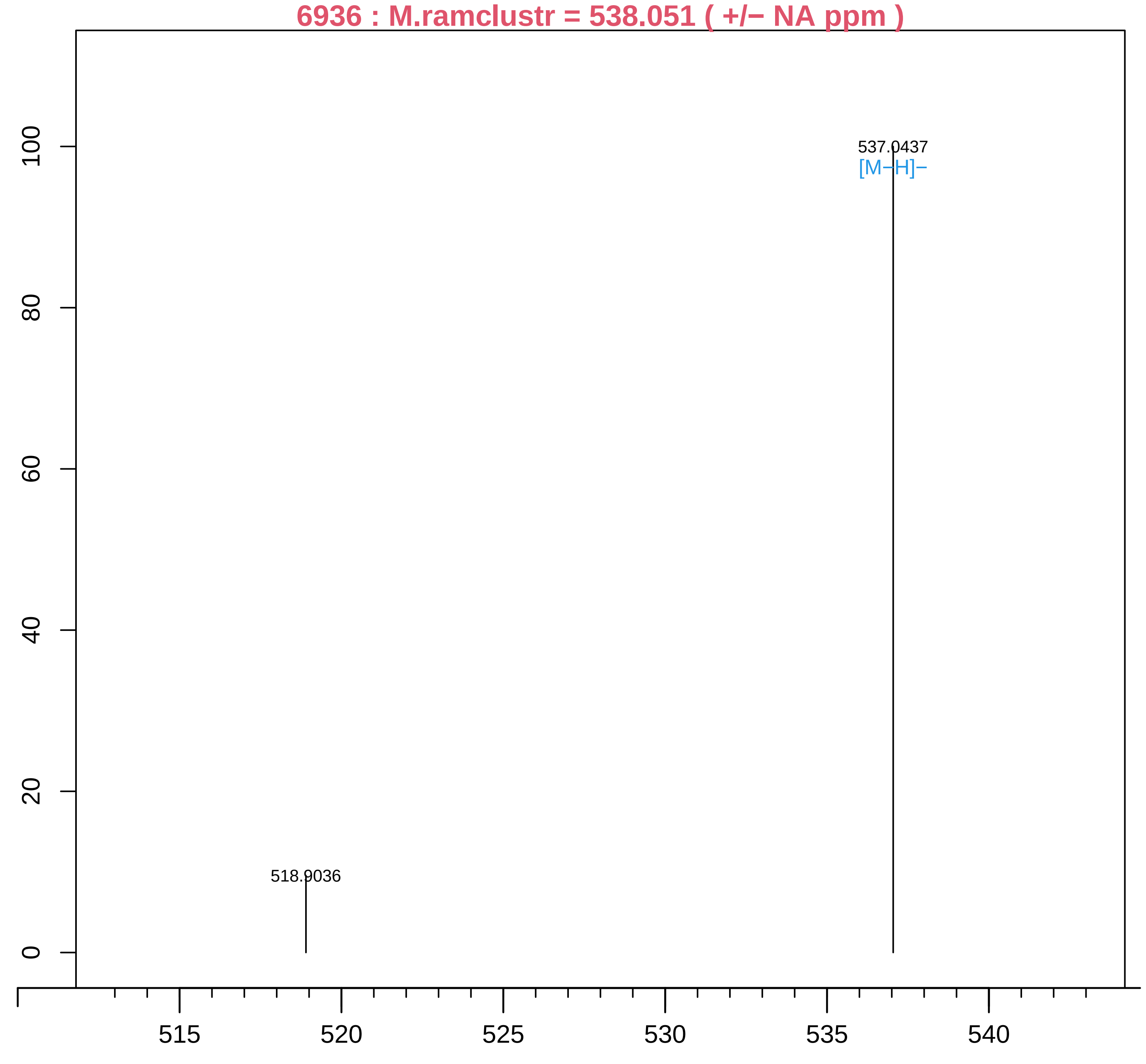
6936 : M.ramclustr = 538.051 (+/− NA ppm)
537.0437
[M−H]−
518.9036
0
20
40
60
80
100
515
520
525
530
535
540

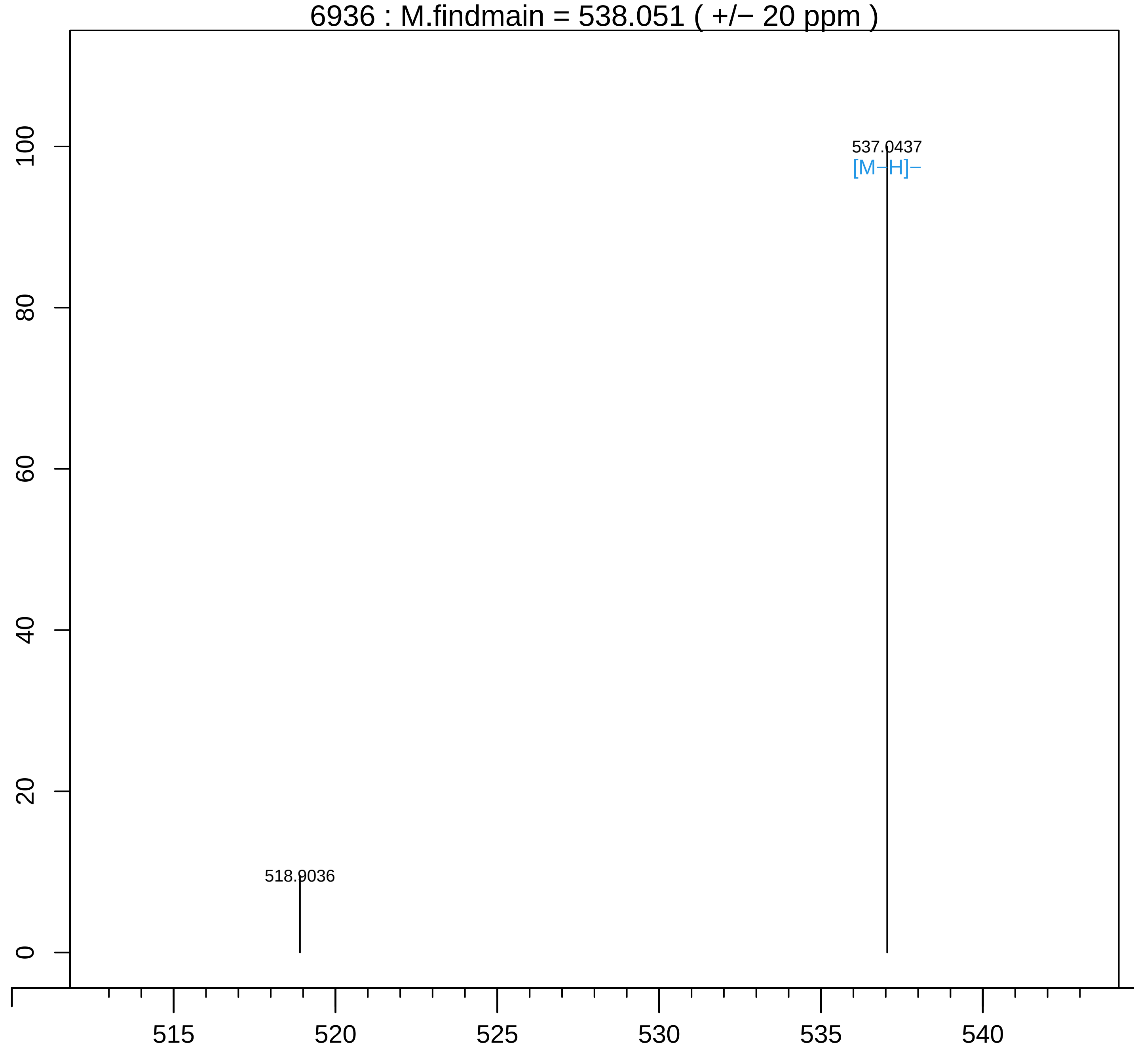
6936 : M.findmain = 538.051 (+/− 20 ppm)
537.0437
[M−H]−
518.9036
0
20
40
60
80
100
515
520
525
530
535
540

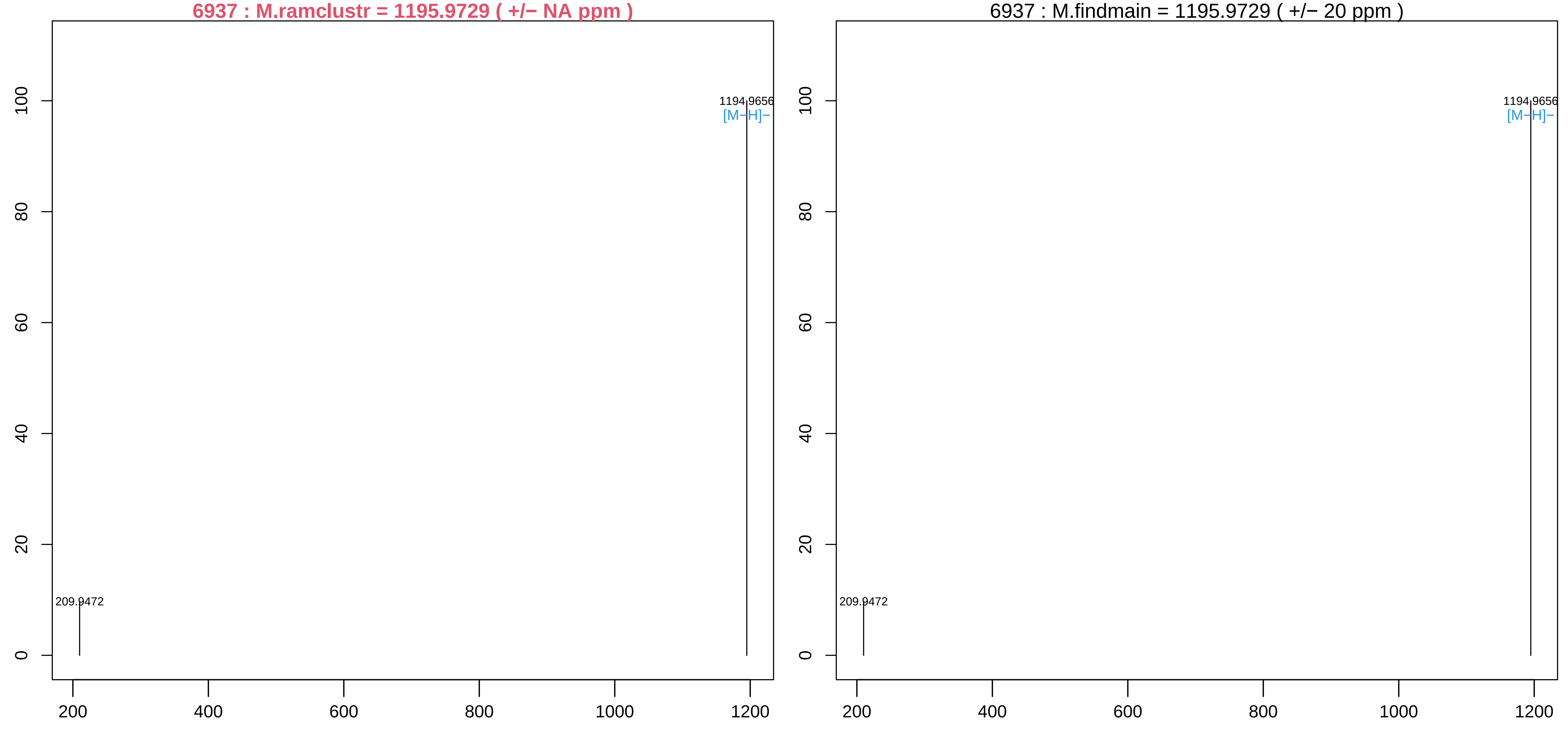
6937 : M.ramclustr = 1195.9729 (+/− NA ppm)
1194.9656
[M−H]−
209.9472
0
20
40
60
80
100
200
400
600
800
1000
1200
6937 : M.findmain = 1195.9729 (+/− 20 ppm)
1194.9656
[M−H]−
209.9472
0
20
40
60
80
100
200
400
600
800
1000
1200

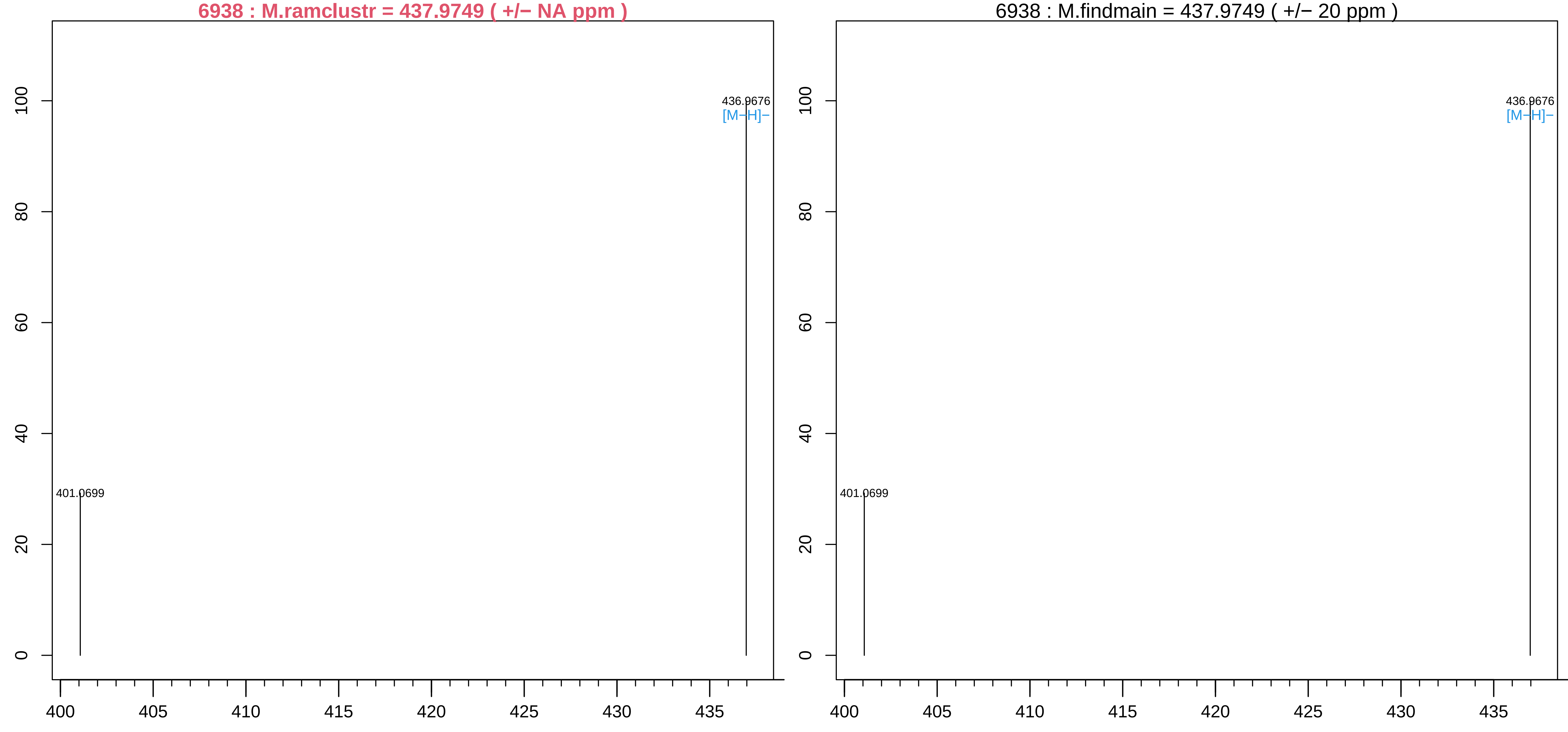
6938 : M.ramclustr = 437.9749 (+/− NA ppm)
436.9676
[M−H]−
401.0699
0
20
40
60
80
100
400
405
410
415
420
425
430
435
6938 : M.findmain = 437.9749 (+/− 20 ppm)
436.9676
[M−H]−
401.0699
0
20
40
60
80
100
400
405
410
415
420
425
430
435

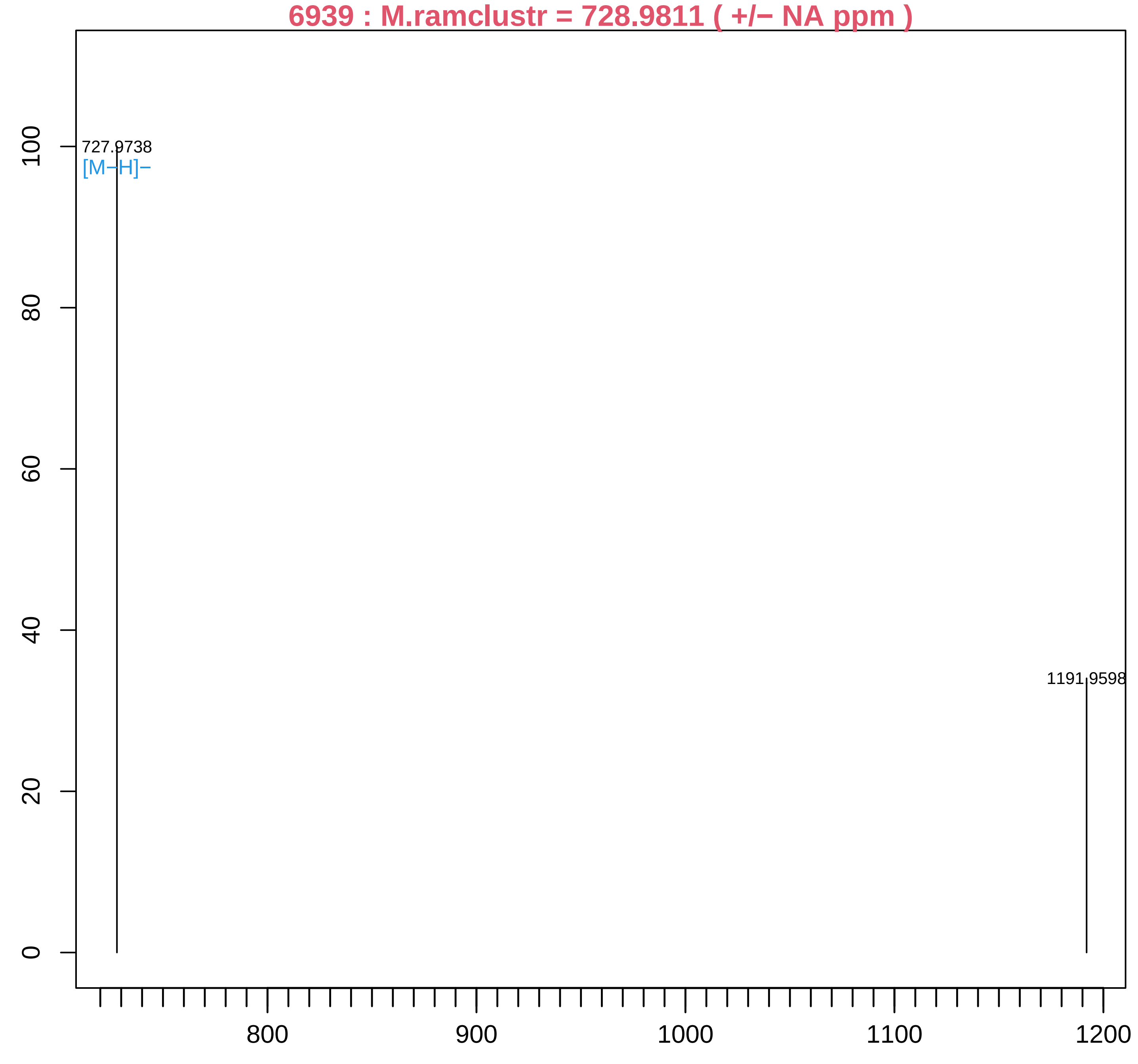
6939 : M.ramclustr = 728.9811 (+/− NA ppm)
727.9738
[M−H]−
1191.9598
0
20
40
60
80
100
800
900
1000
1100
1200

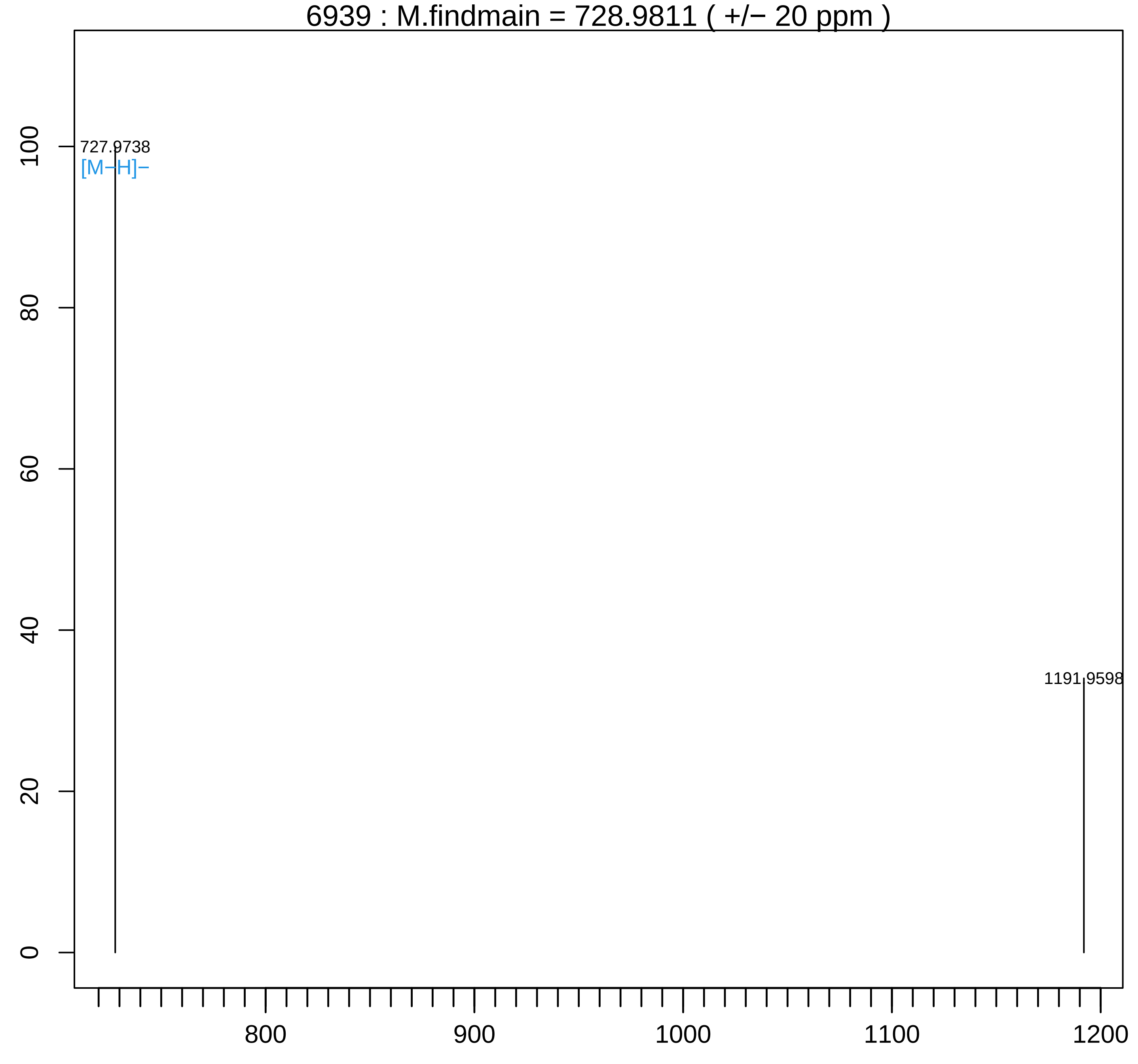
6939 : M.findmain = 728.9811 (+/− 20 ppm)
727.9738
[M−H]−
1191.9598
0
20
40
60
80
100
800
900
1000
1100
1200

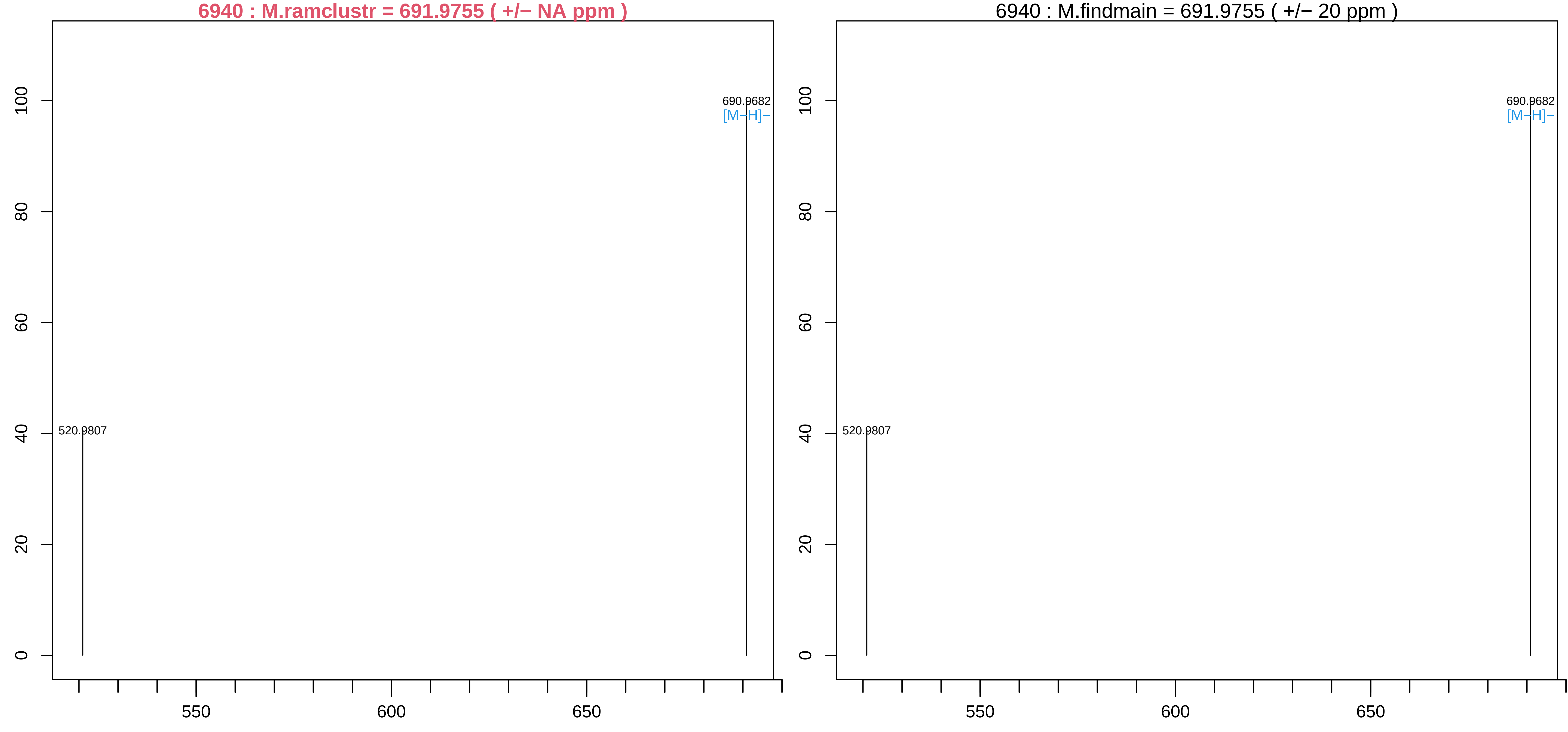

6940 : M.ramclustr = 691.9755 (+/− NA ppm)
690.9682
[M−H]−
520.9807
0
20
40
60
80
100
550
600
650
6940 : M.findmain = 691.9755 (+/− 20 ppm)
690.9682
[M−H]−
520.9807
0
20
40
60
80
100
550
600
650

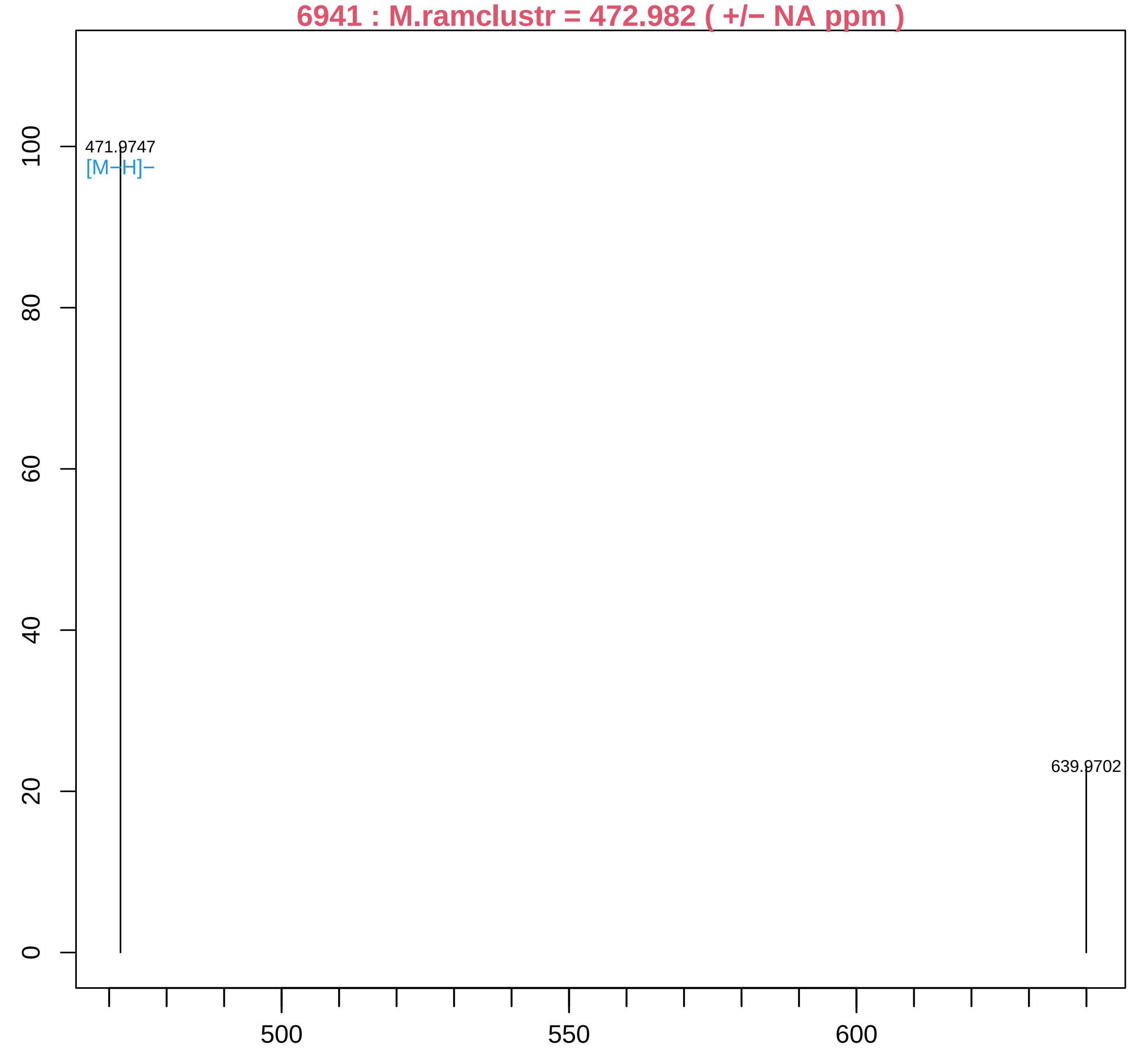
6941 : M.ramclustr = 472.982 (+/− NA ppm)
471.9747
[M−H]−
639.9702
0
20
40
60
80
100
500
550
600

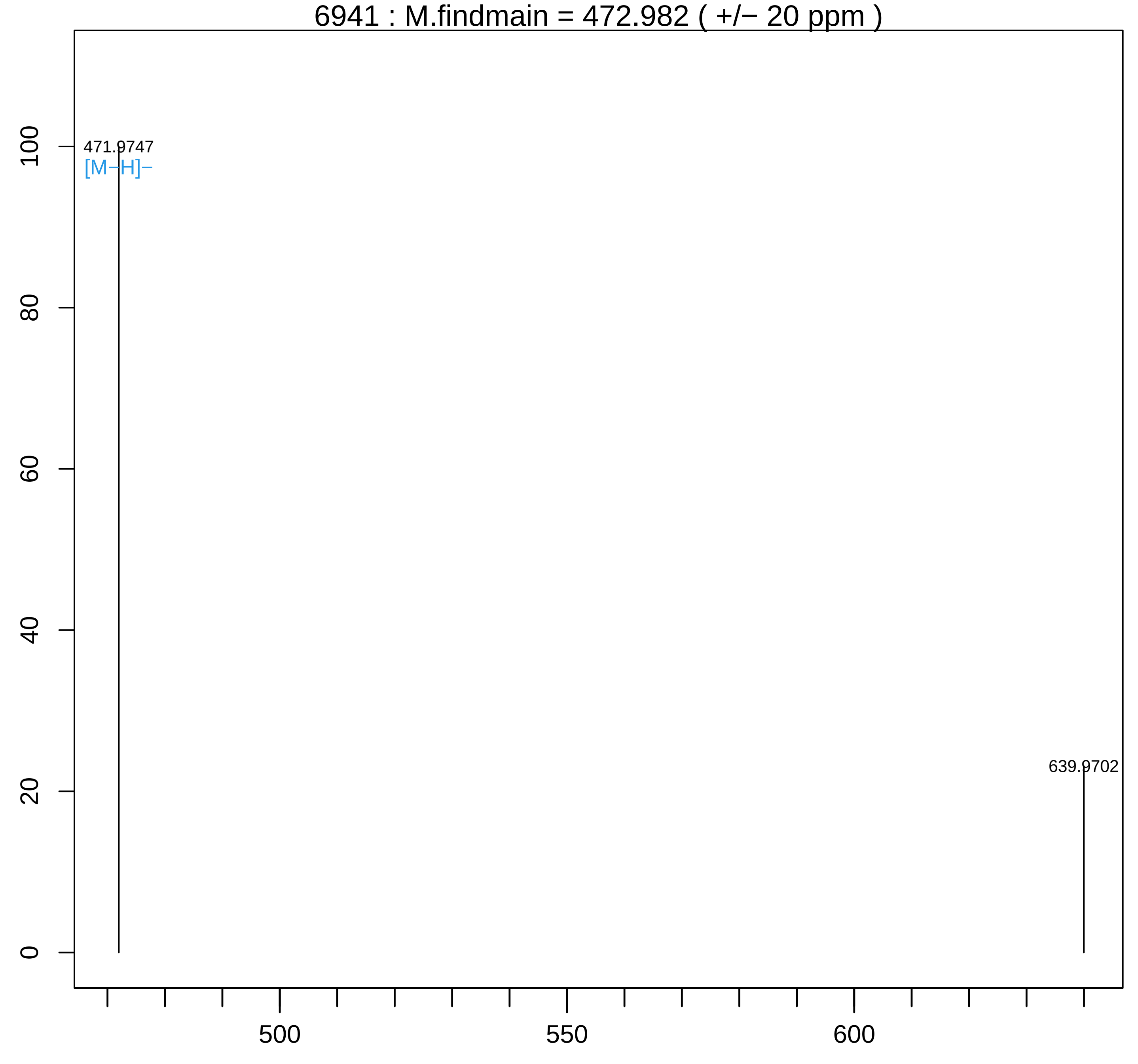
6941 : M.findmain = 472.982 (+/− 20 ppm)
471.9747
[M−H]−
639.9702
0
20
40
60
80
100
500
550
600

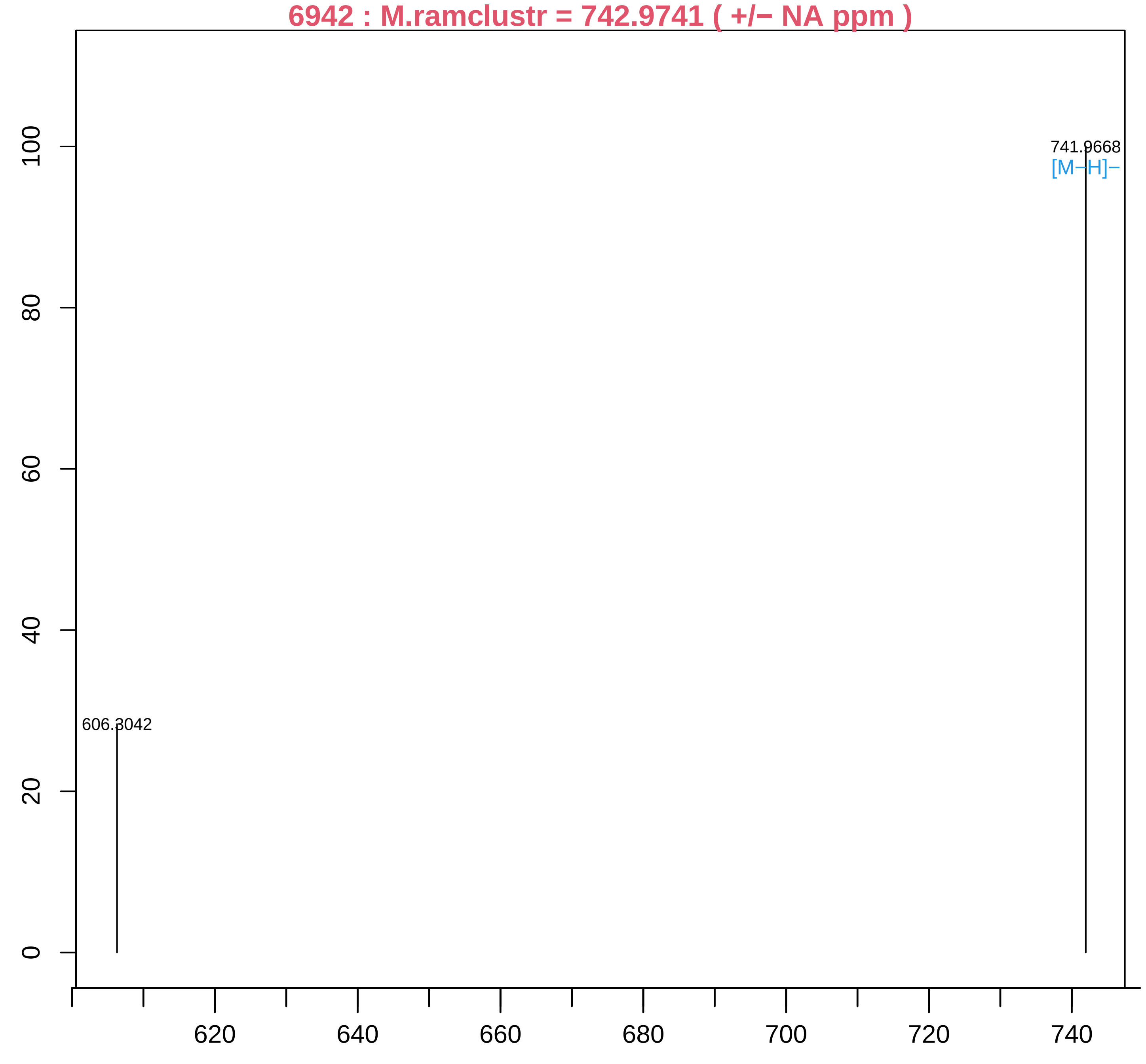
6942 : M.ramclustr = 742.9741 (+/− NA ppm)
741.9668
[M−H]−
606.3042
0
20
40
60
80
100
620
640
660
680
700
720
740

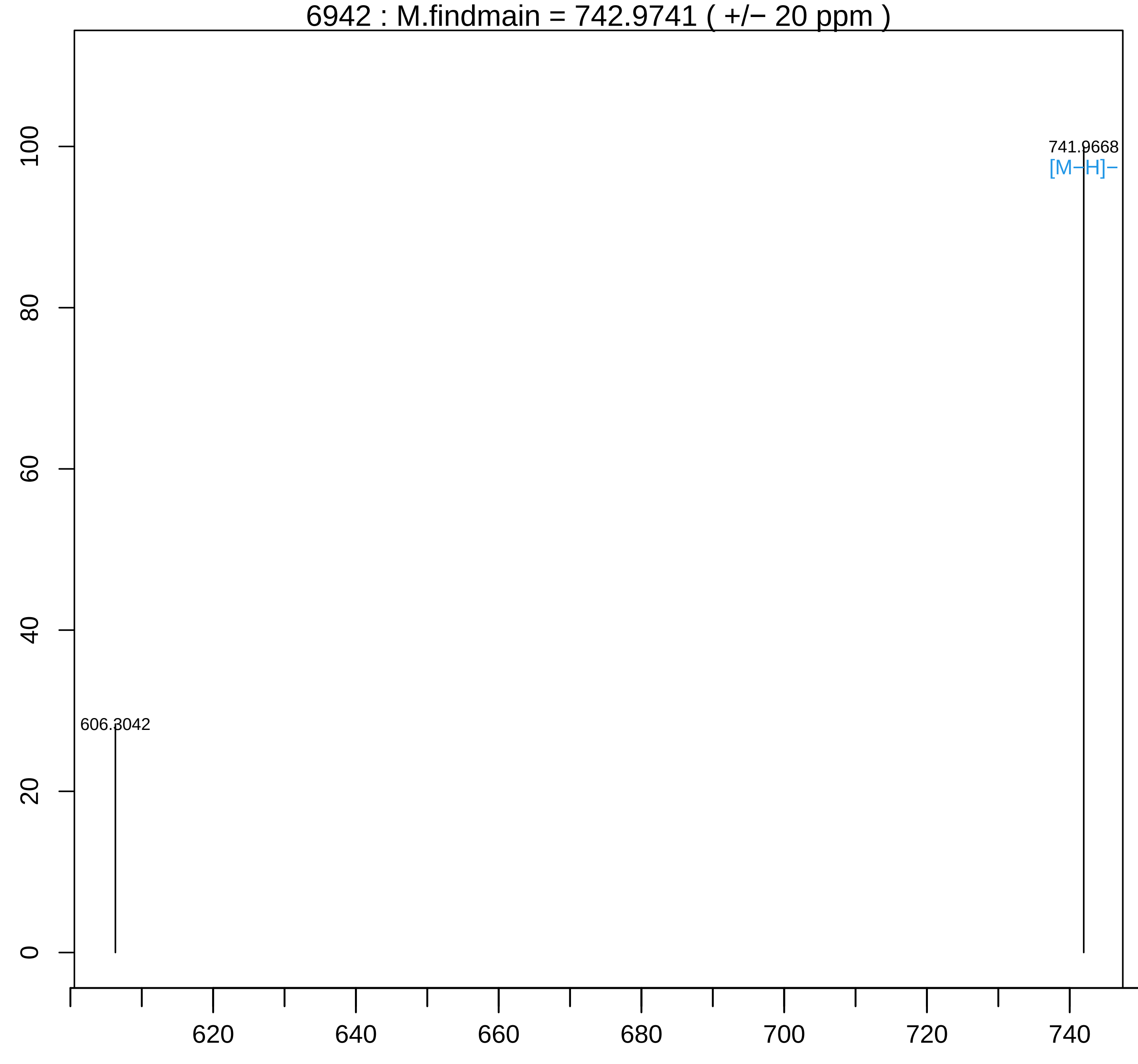
6942 : M.findmain = 742.9741 (+/− 20 ppm)
741.9668
[M−H]−
606.3042
0
20
40
60
80
100
620
640
660
680
700
720
740

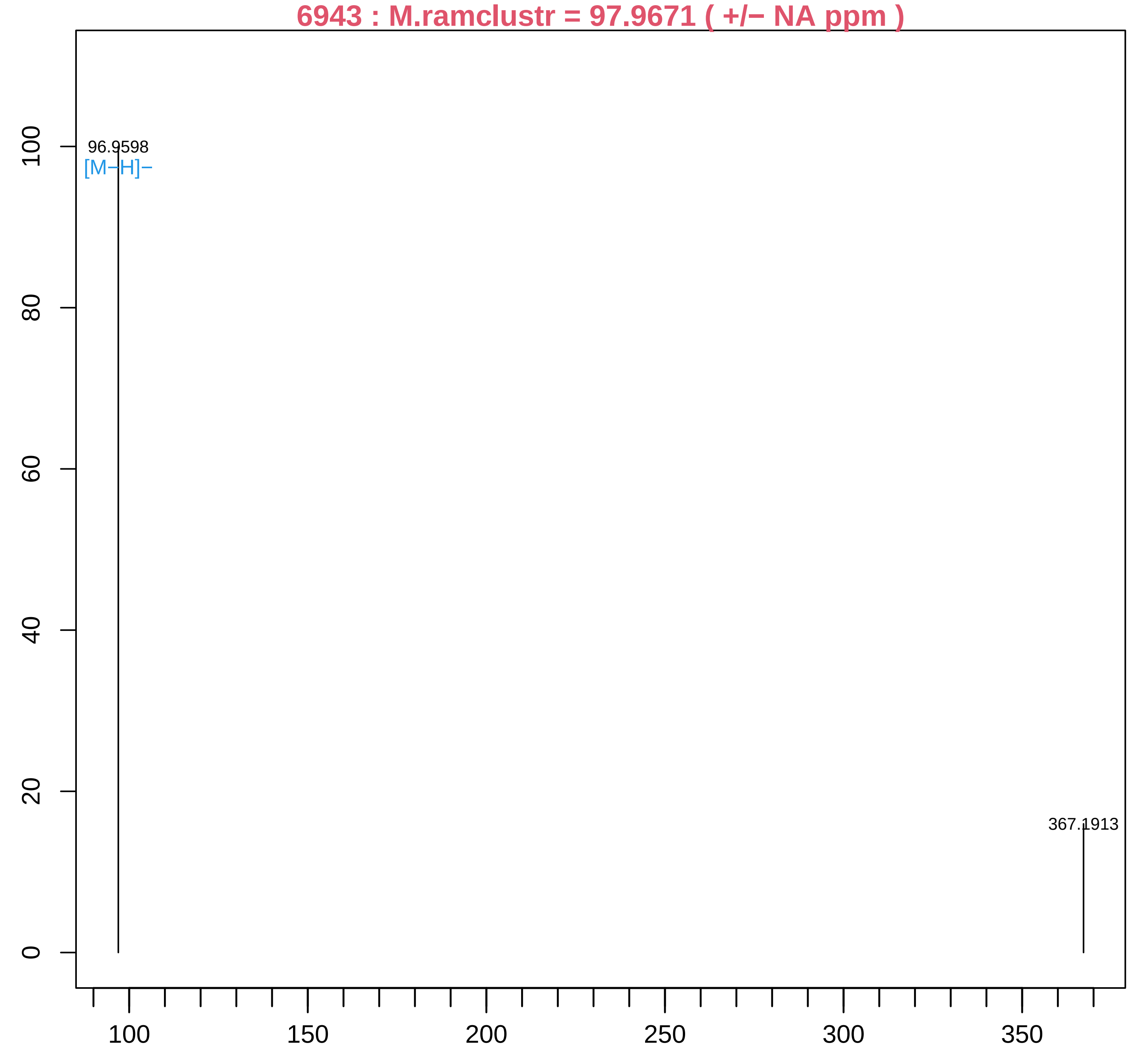
6943 : M.ramclustr = 97.9671 (+/− NA ppm)
96.9598
[M−H]−
367.1913
0
20
40
60
80
100
100
150
200
250
300
350

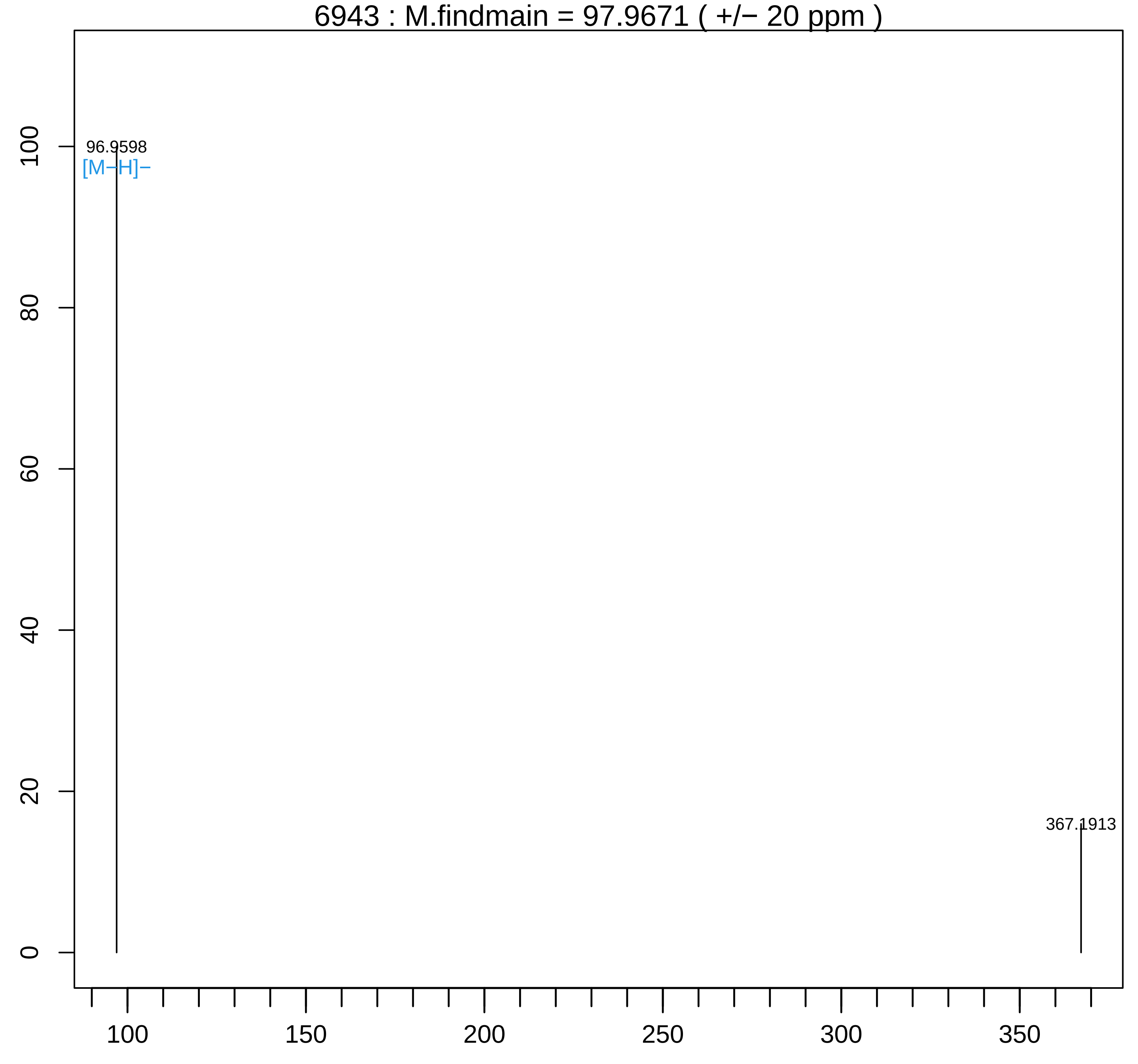
6943 : M.findmain = 97.9671 (+/− 20 ppm)
96.9598
[M−H]−
367.1913
0
20
40
60
80
100
100
150
200
250
300
350

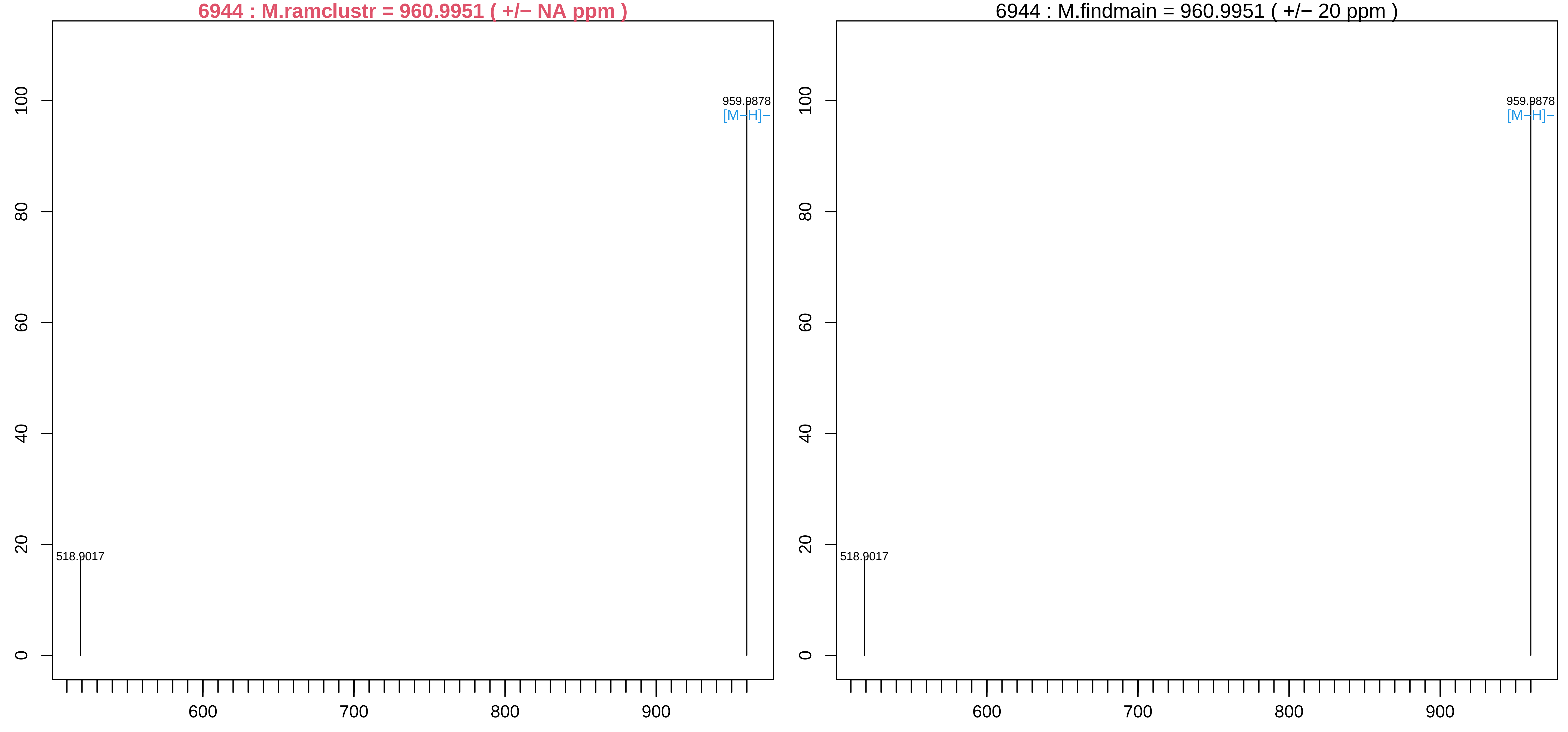
6944 : M.ramclustr = 960.9951 (+/− NA ppm)
6944 : M.findmain = 960.9951 (+/− 20 ppm)
959.9878
[M−H]−
518.9017
0
20
40
60
80
100
600
700
800
900

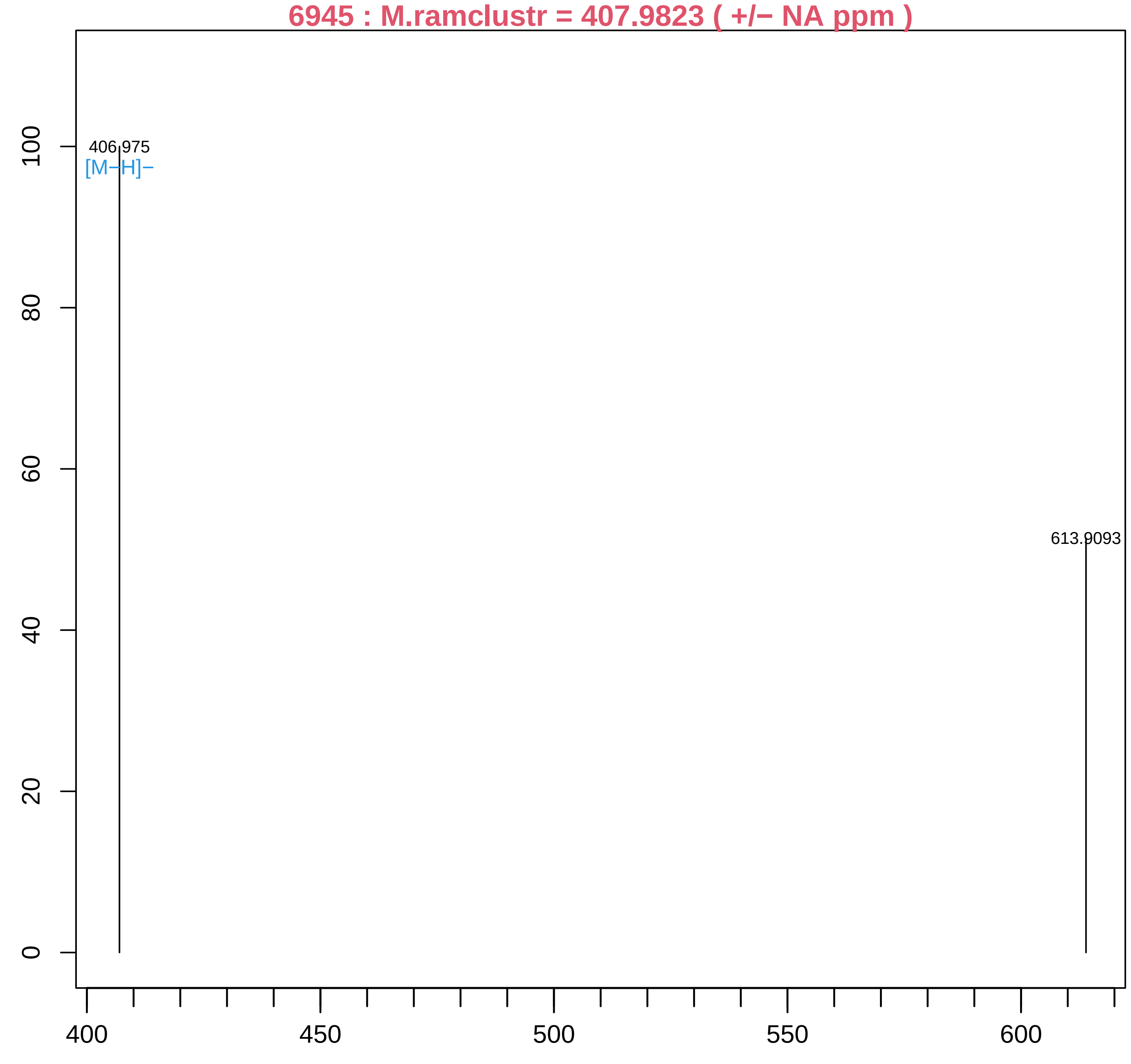
6945 : M.ramclustr = 407.9823 (+/− NA ppm)
406.975
[M−H]−
613.9093
0
20
40
60
80
100
400
450
500
550
600

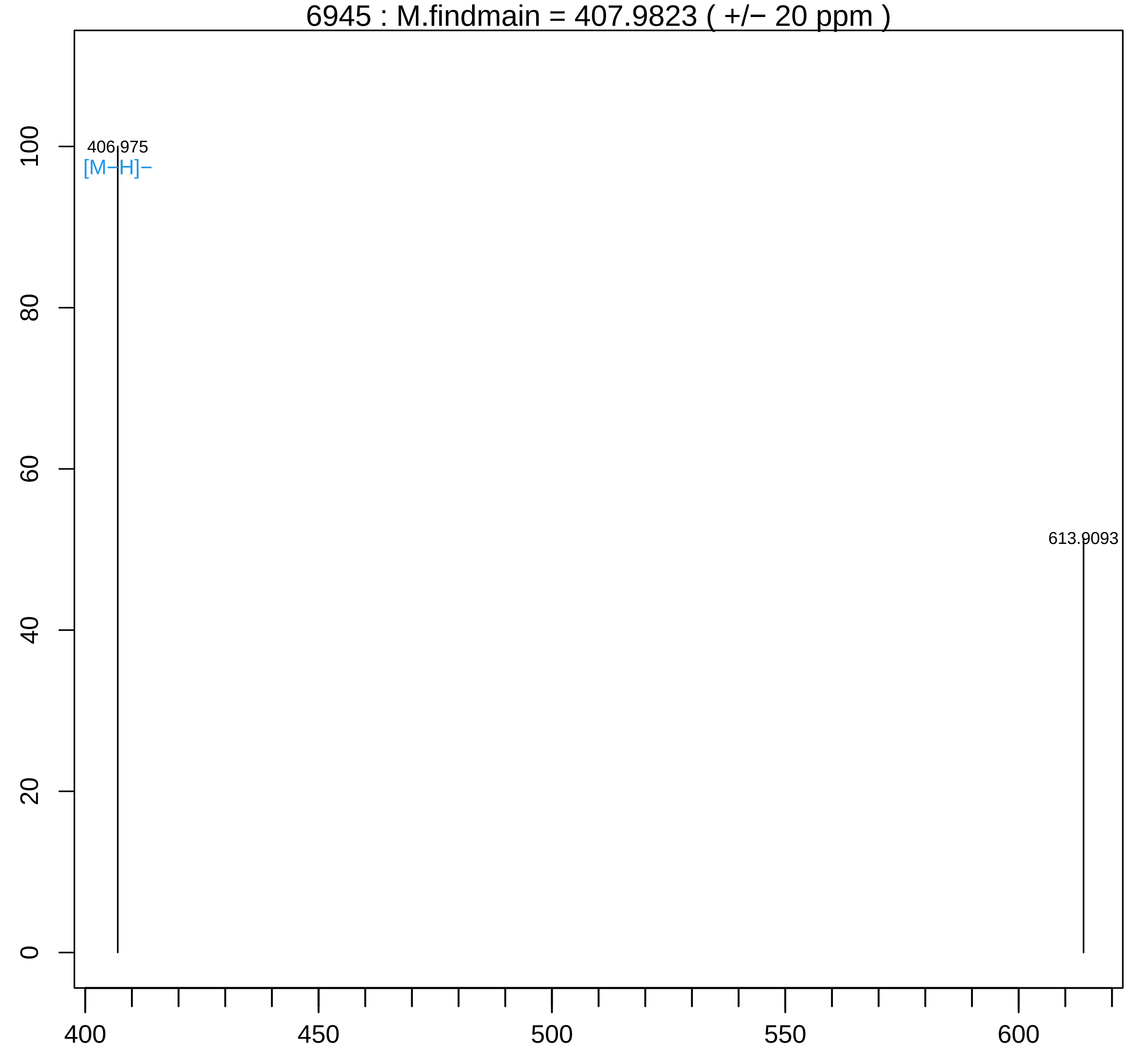
6945 : M.findmain = 407.9823 (+/− 20 ppm)
406.975
[M−H]−
613.9093
0
20
40
60
80
100
400
450
500
550
600

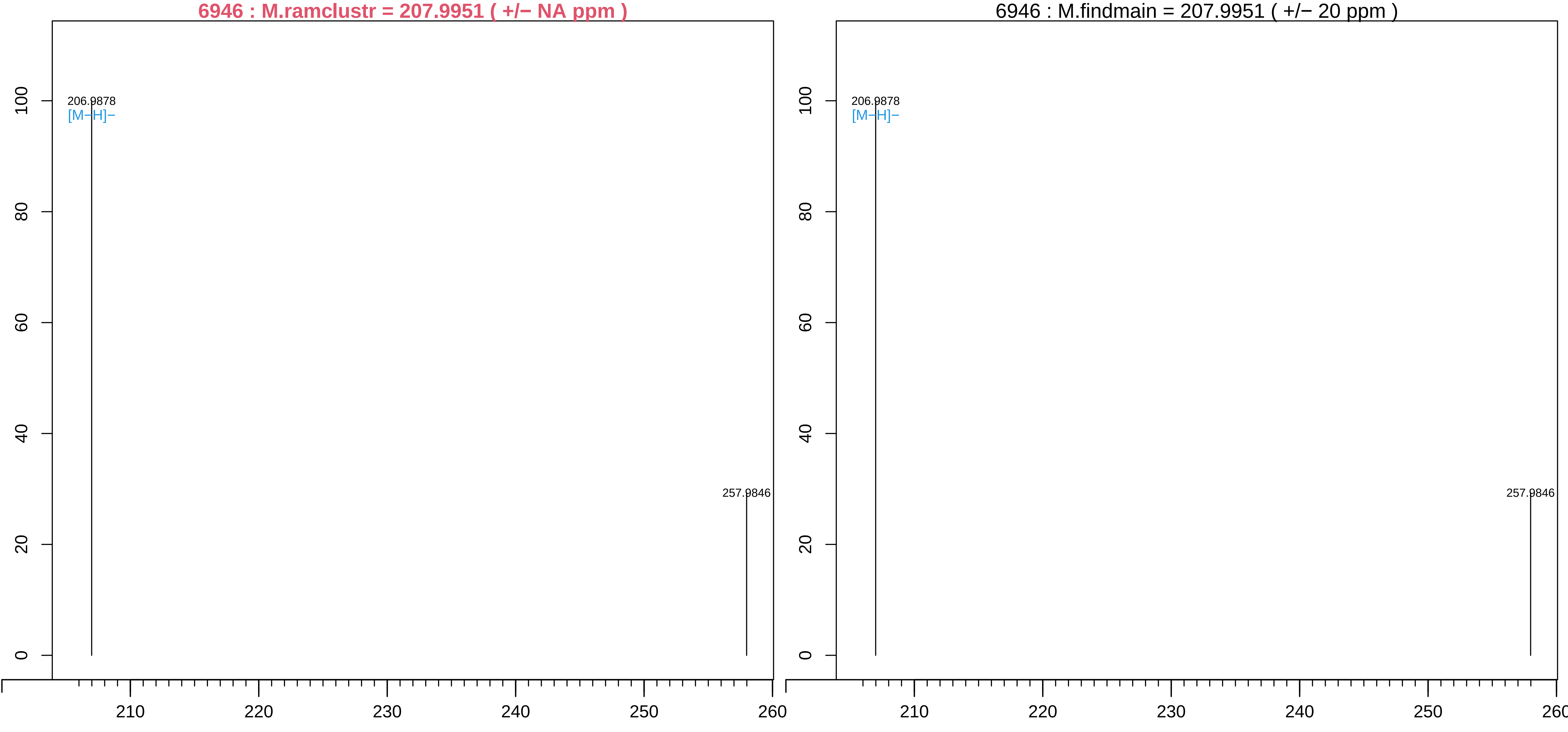
6946 : M.ramclustr = 207.9951 (+/− NA ppm)
206.9878
[M−H]−
257.9846
6946 : M.findmain = 207.9951 (+/− 20 ppm)
206.9878
[M−H]−
257.9846
0
20
40
60
80
100
210
220
230
240
250
260

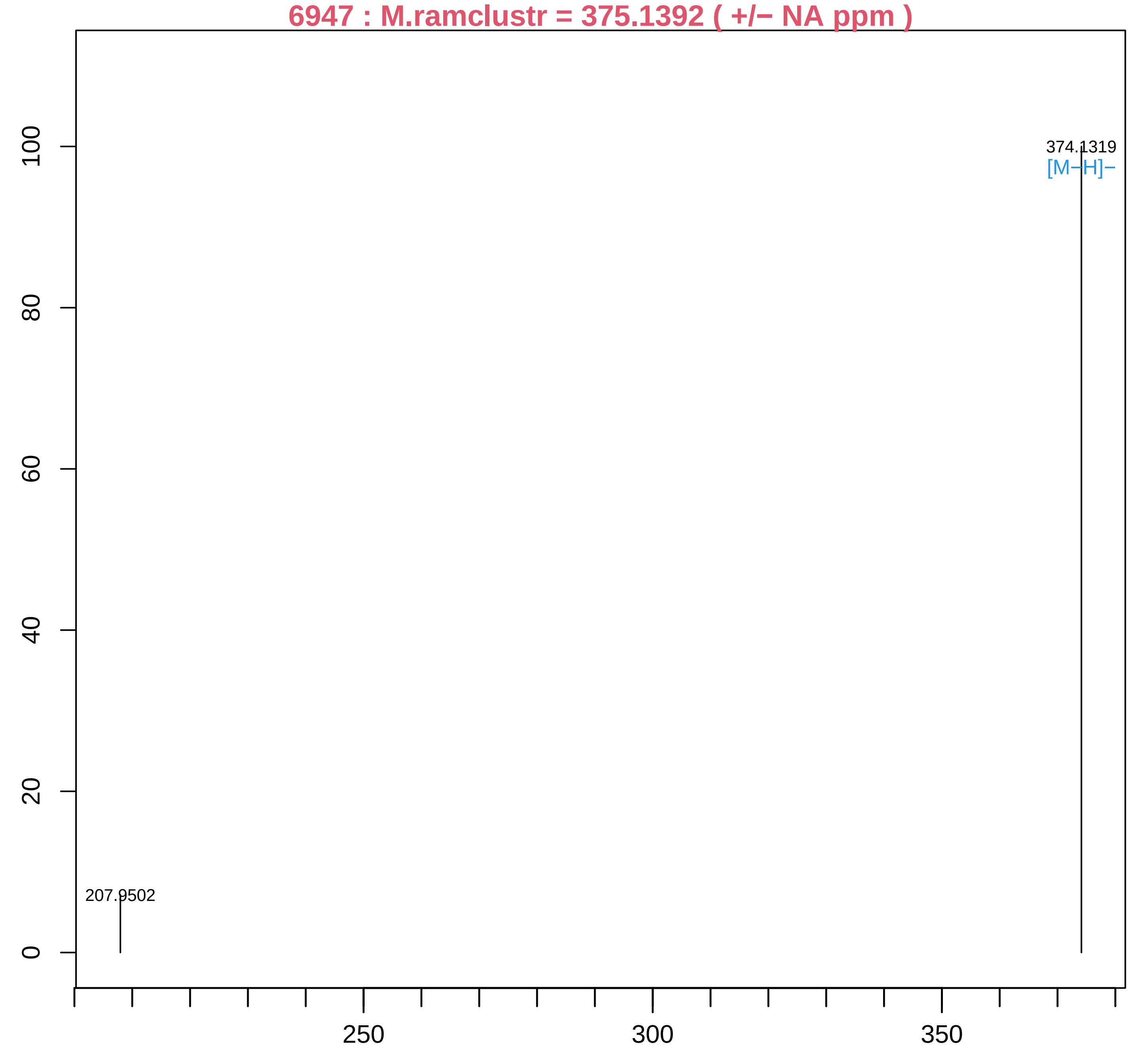
6947 : M.ramclustr = 375.1392 (+/− NA ppm)
374.1319
[M−H]−
207.9502
0
20
40
60
80
100
250
300
350

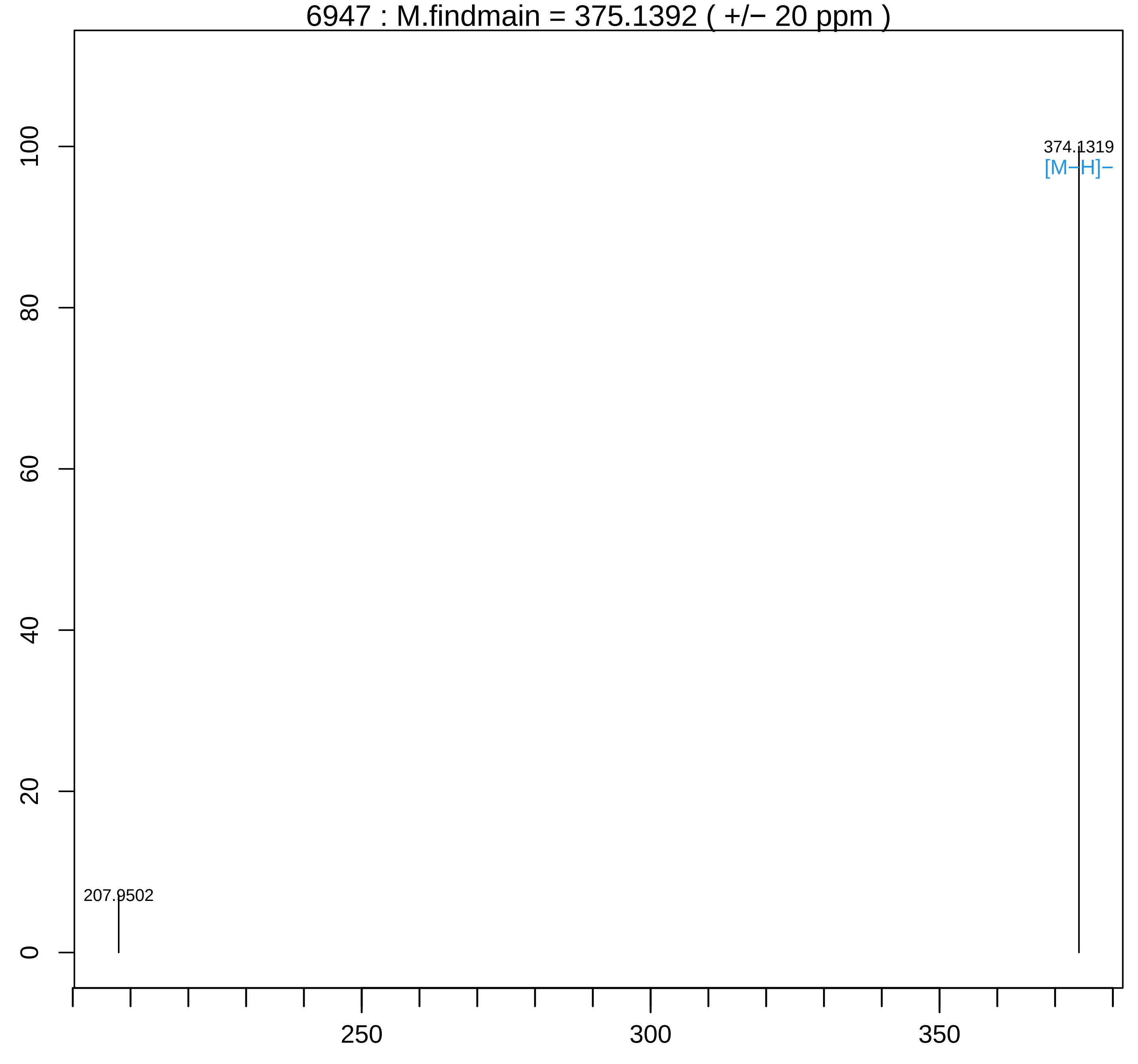
6947 : M.findmain = 375.1392 (+/− 20 ppm)
374.1319
[M−H]−
207.9502
0
20
40
60
80
100
250
300
350

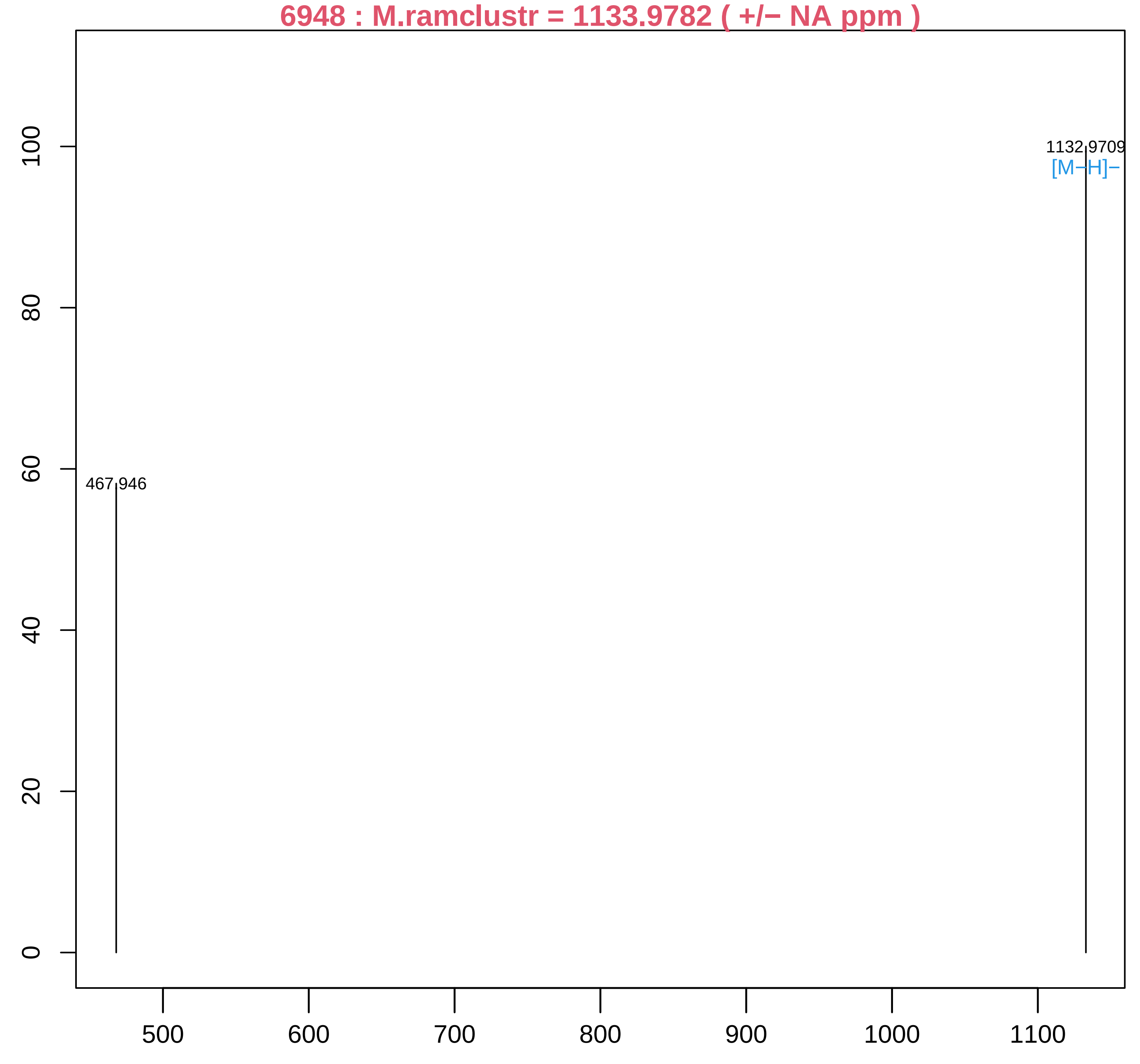
6948 : M.ramclustr = 1133.9782 (+/− NA ppm)
1132.9709
[M−H]−
467.946
0
20
40
60
80
100
500
600
700
800
900
1000
1100

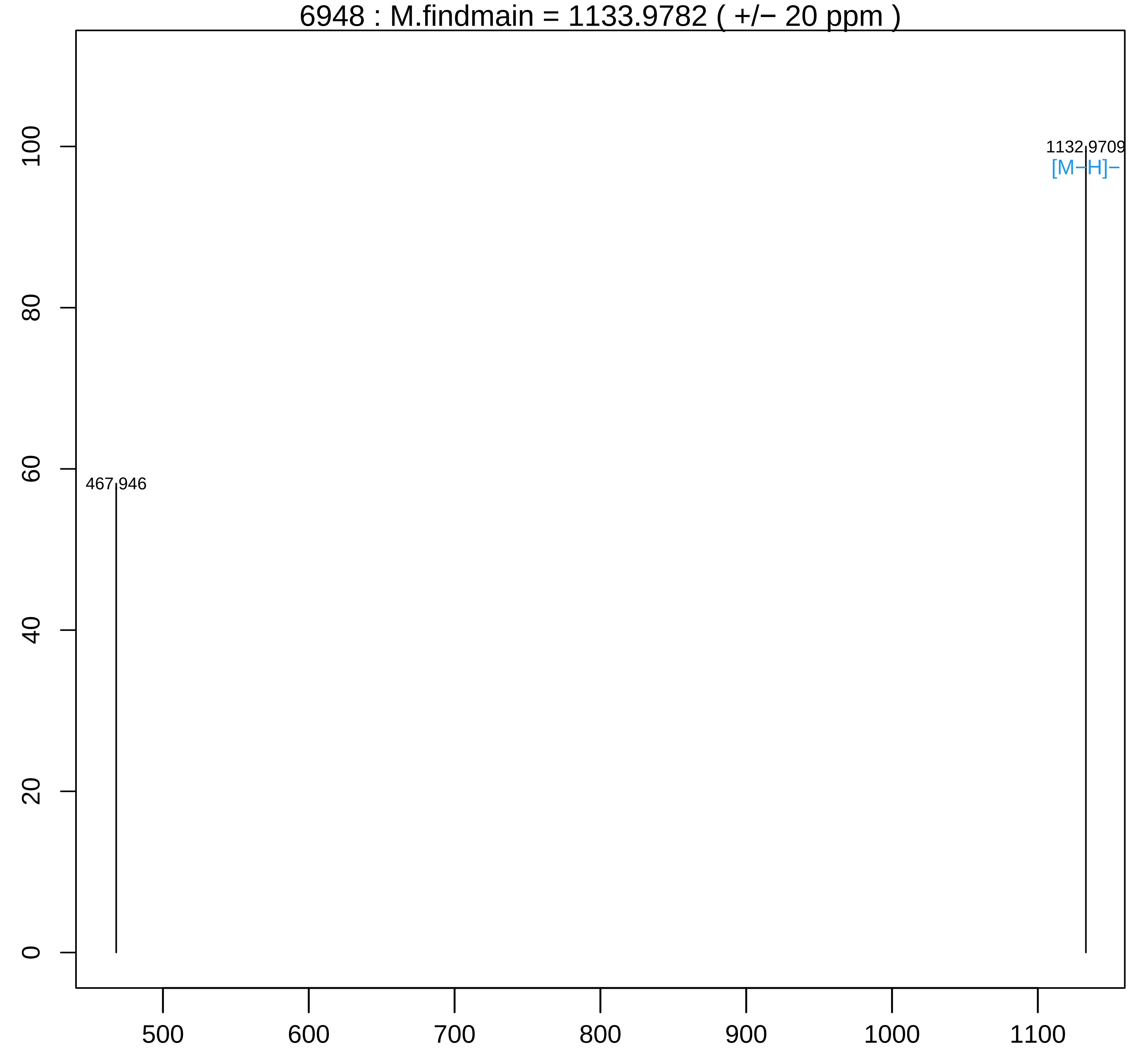
6948 : M.findmain = 1133.9782 (+/− 20 ppm)
1132.9709
[M−H]−
467.946
0
20
40
60
80
100
500
600
700
800
900
1000
1100

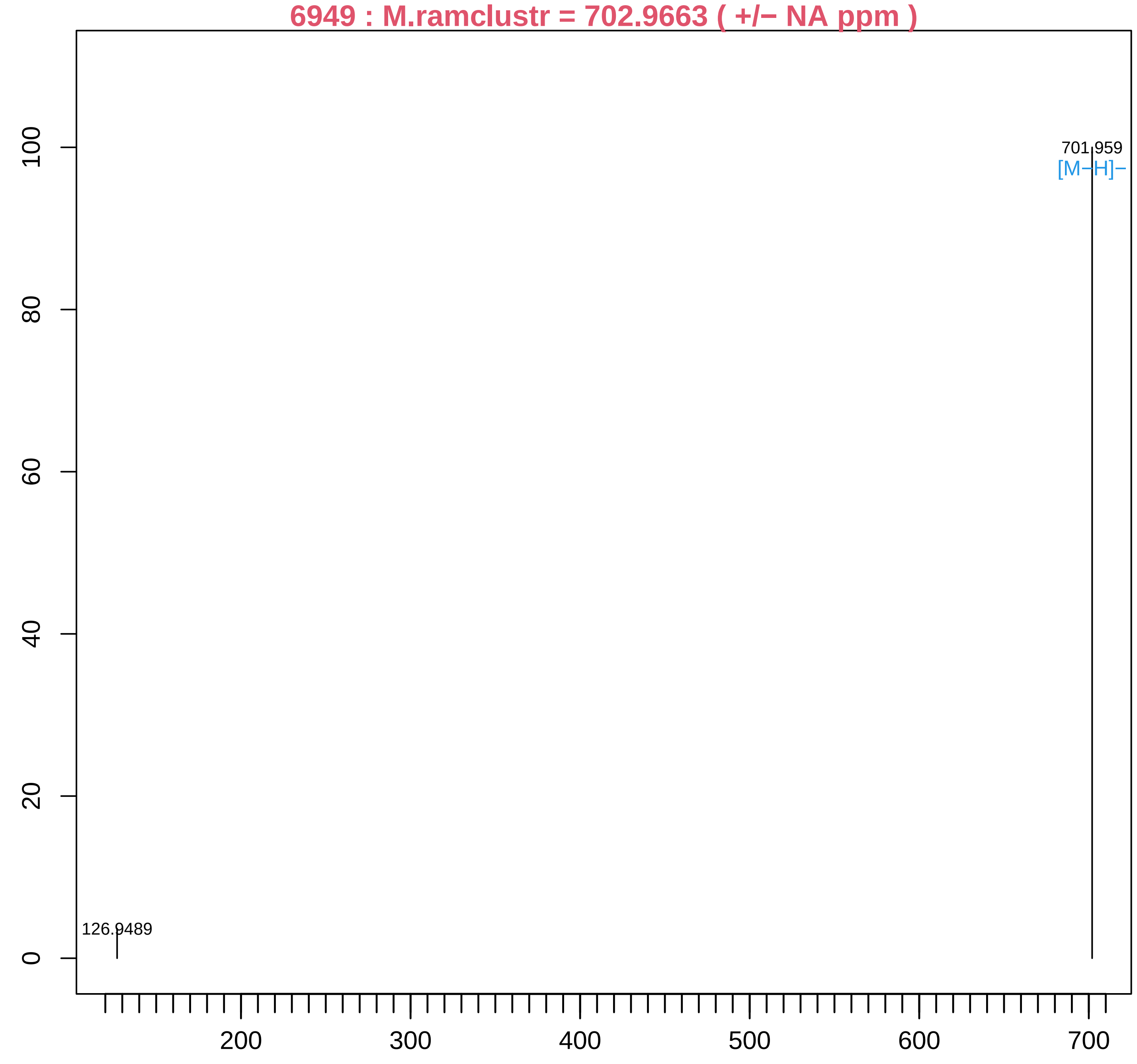
6949 : M.ramclustr = 702.9663 (+/− NA ppm)
701.959
[M−H]−
126.9489
0
20
40
60
80
100
200
300
400
500
600
700

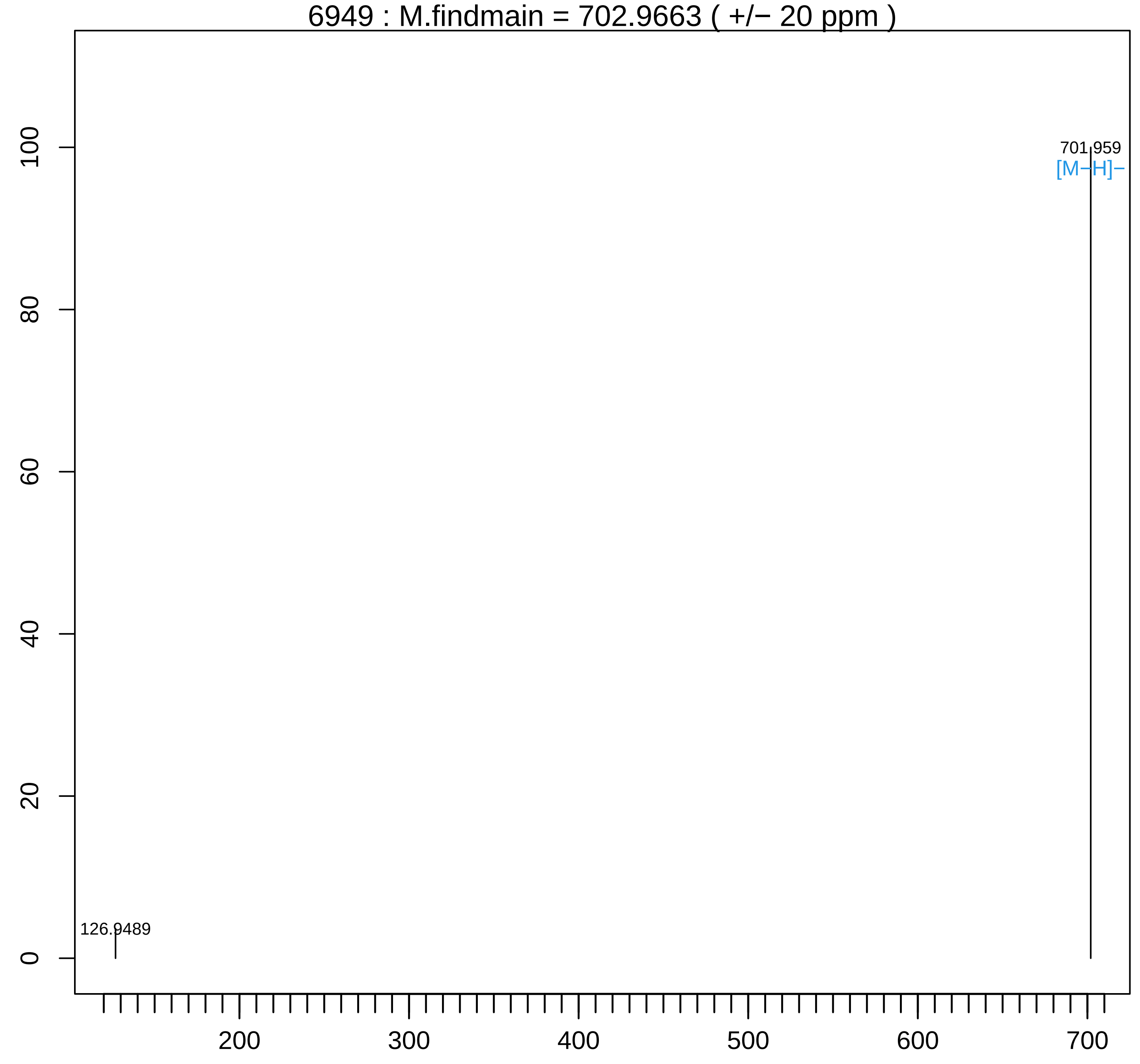
6949 : M.findmain = 702.9663 (+/− 20 ppm)
701.959
[M−H]−
126.9489
0
20
40
60
80
100
200
300
400
500
600
700

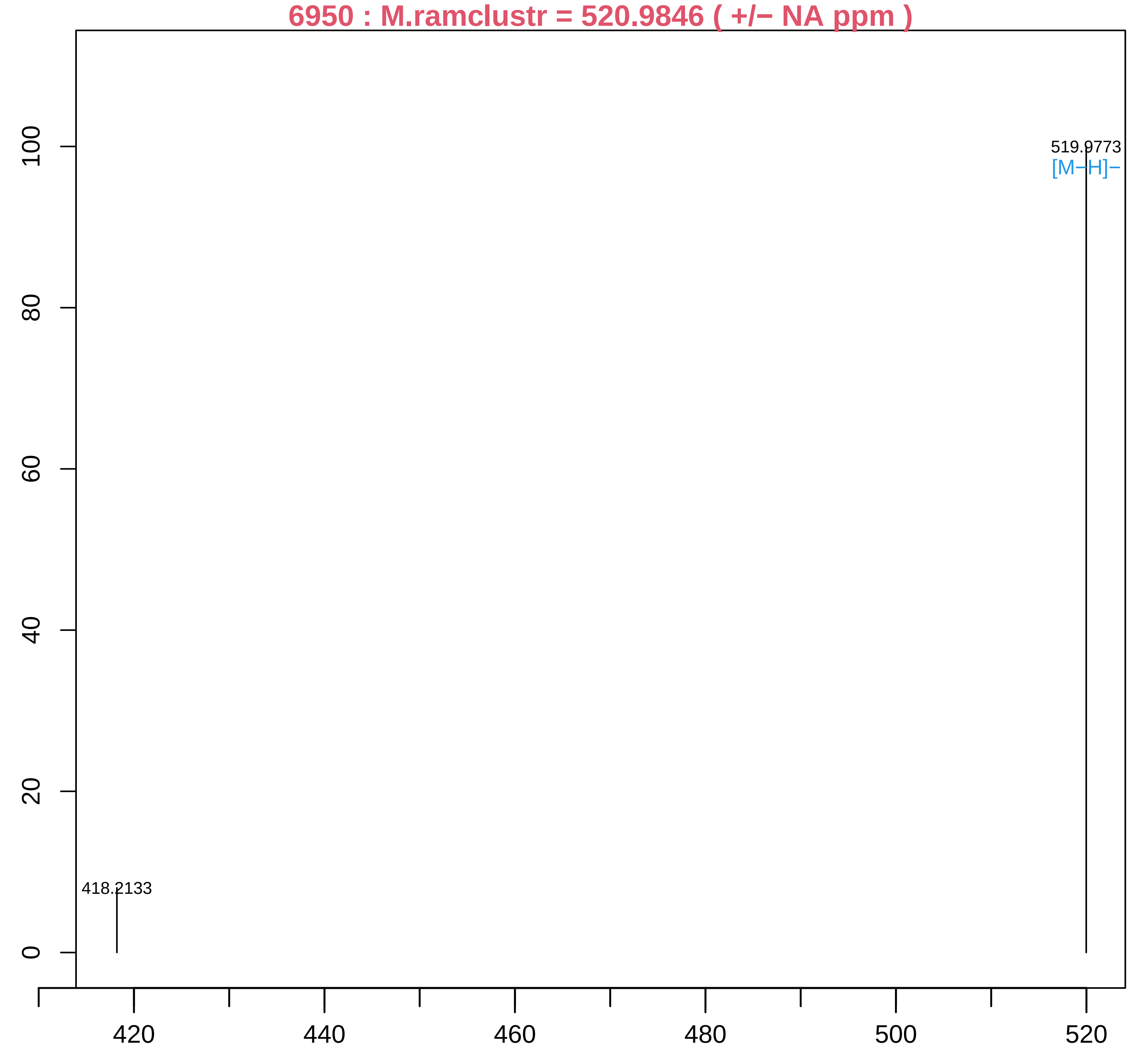

6950 : M.ramclustr = 520.9846 (+/− NA ppm)
519.9773
[M−H]−
418.2133
0
20
40
60
80
100
420
440
460
480
500
520

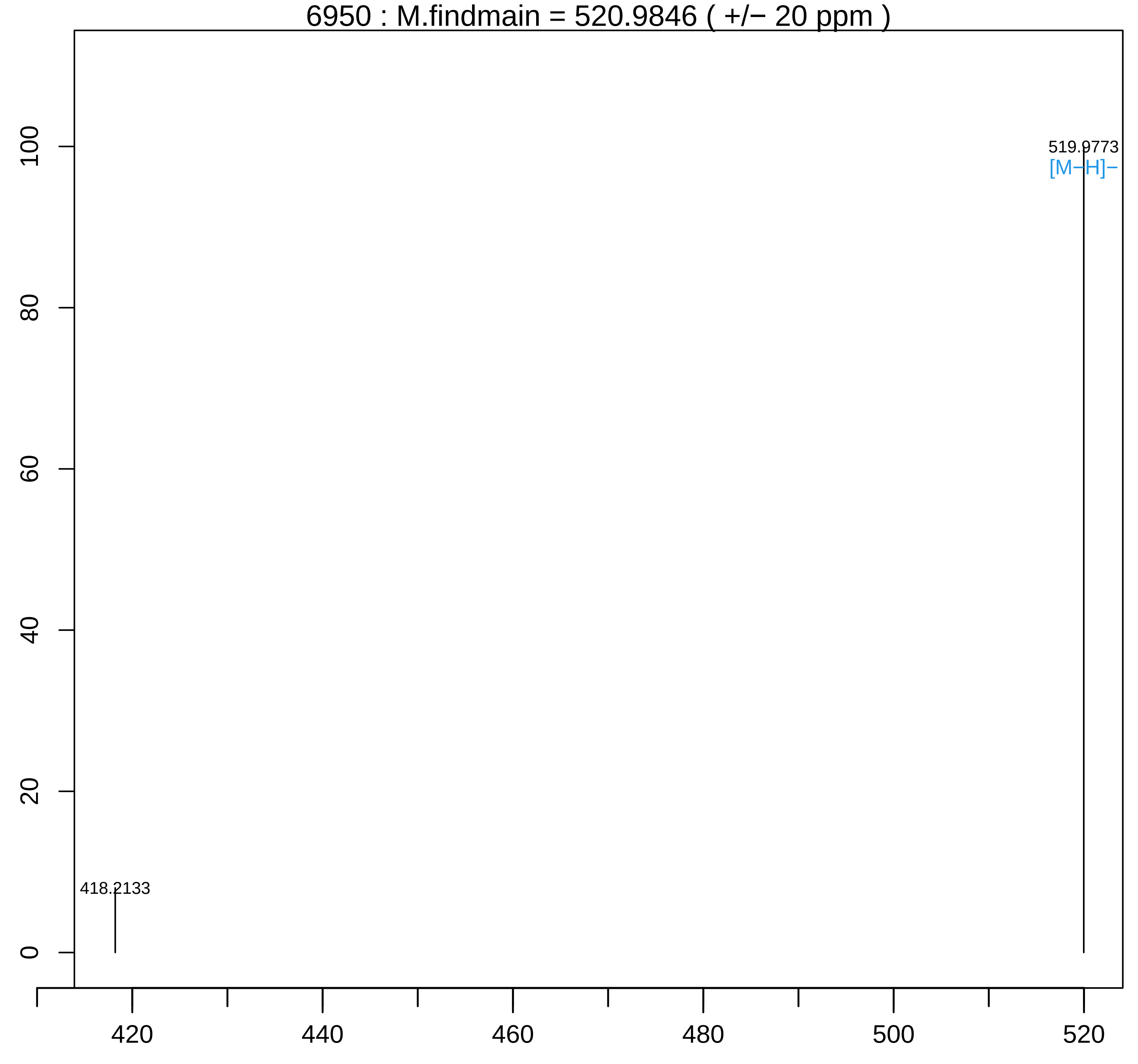

6950 : M.findmain = 520.9846 (+/− 20 ppm)
519.9773
[M−H]−
418.2133
0
20
40
60
80
100
420
440
460
480
500
520

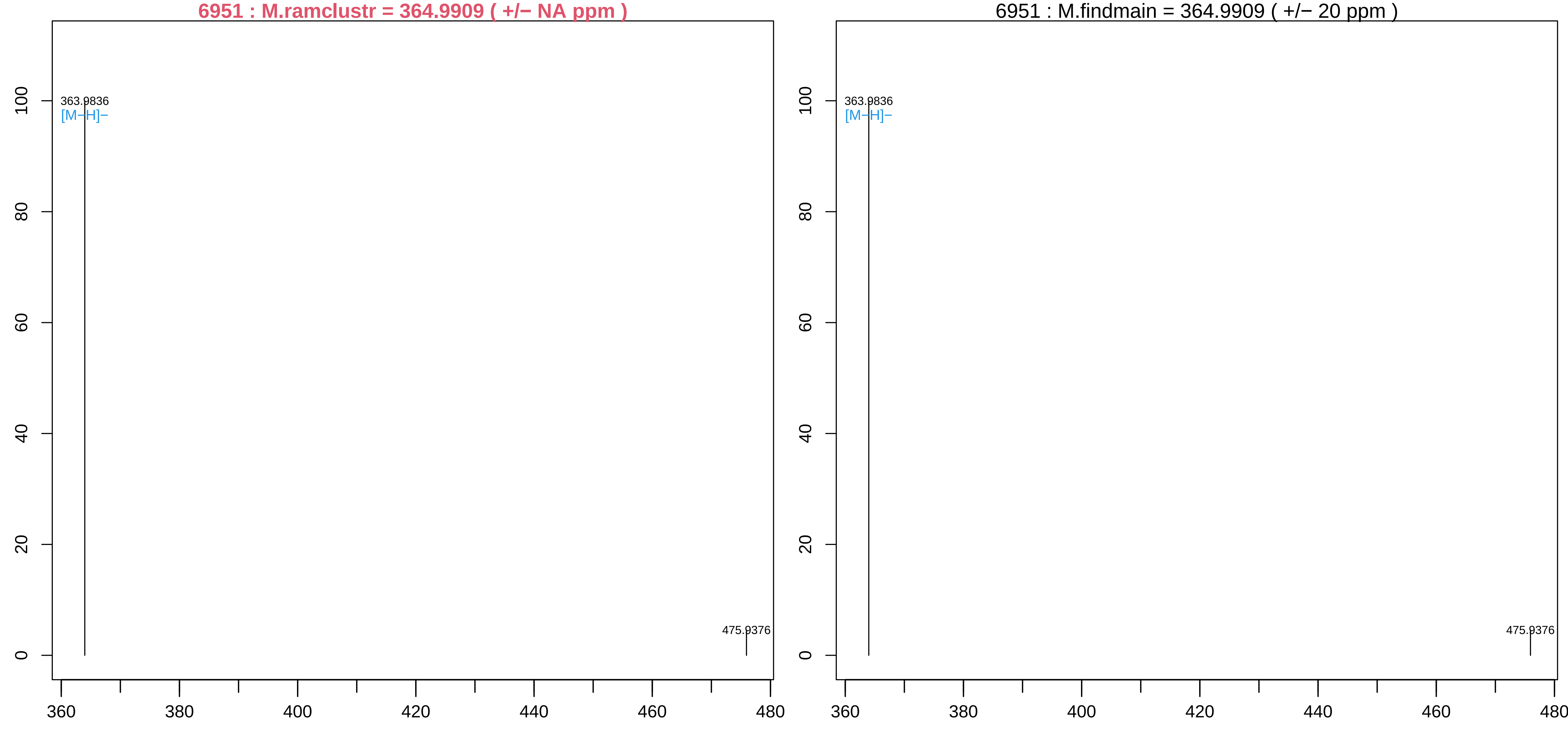
6951 : M.ramclustr = 364.9909 (+/− NA ppm)
363.9836
[M−H]−
475.9376
6951 : M.findmain = 364.9909 (+/− 20 ppm)
363.9836
[M−H]−
475.9376
0
20
40
60
80
100
360
380
400
420
440
460
480

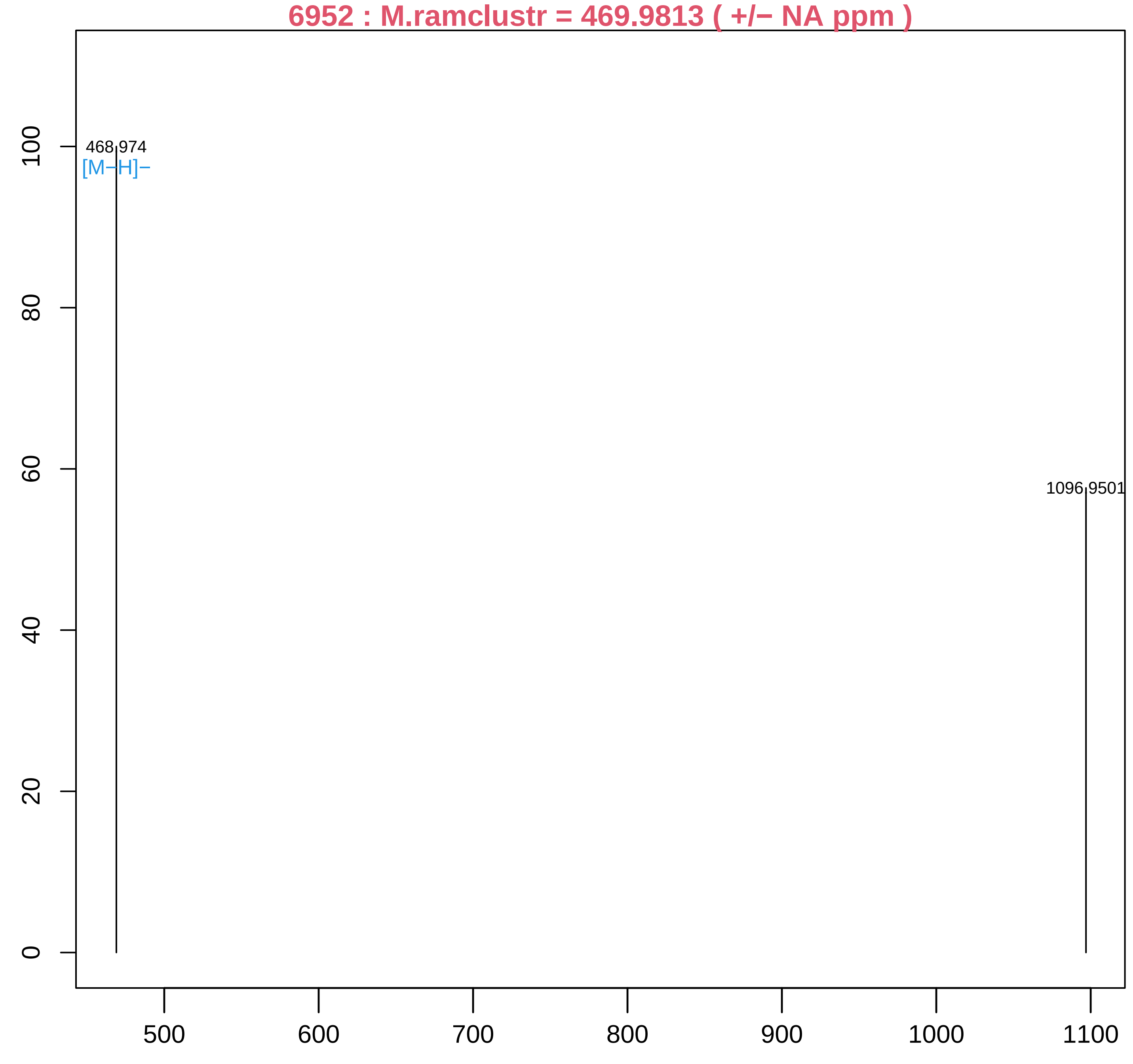
6952 : M.ramclustr = 469.9813 (+/− NA ppm)
468.974
[M−H]−
1096.9501
0
20
40
60
80
100
500
600
700
800
900
1000
1100
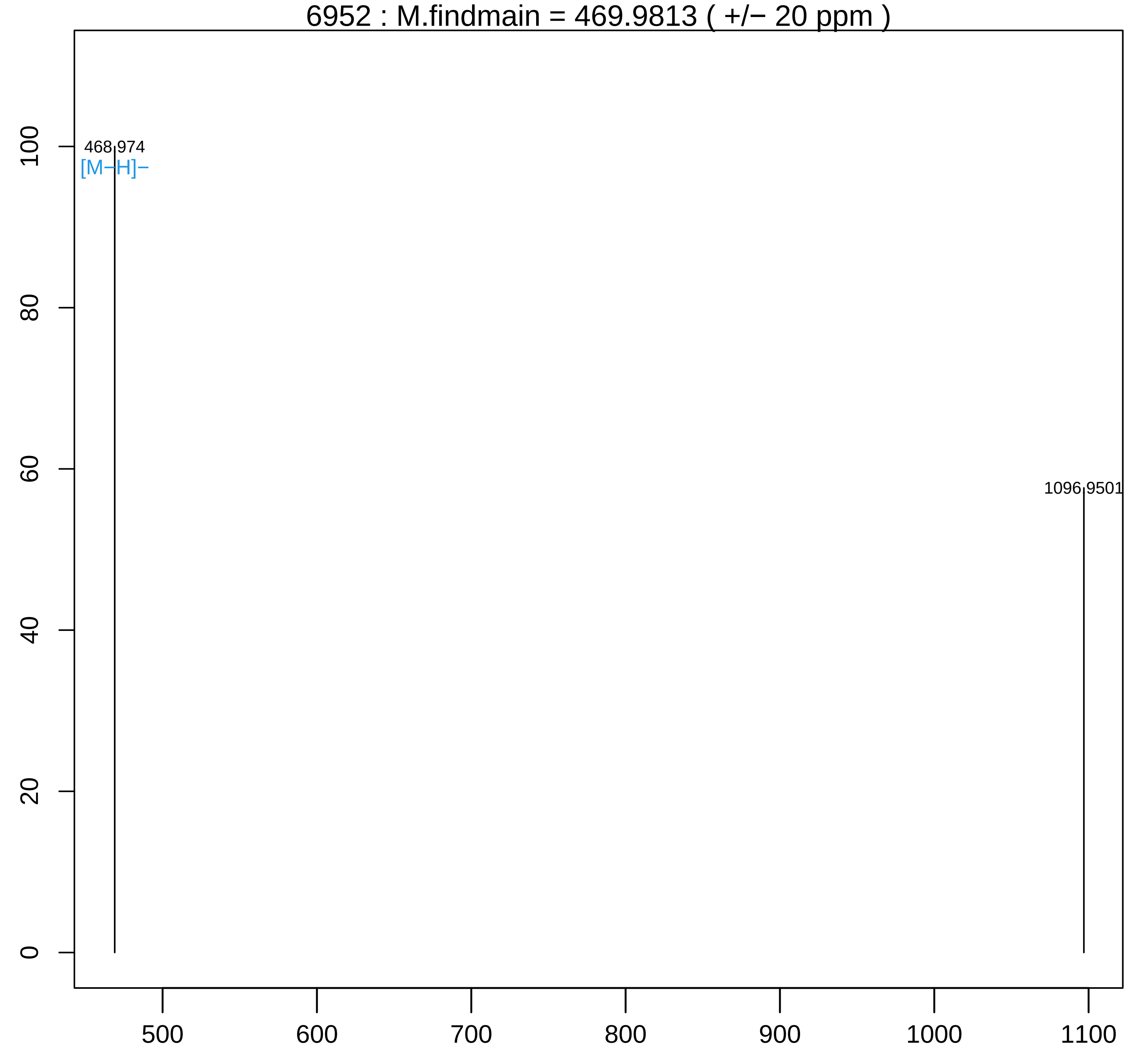
6952 : M.findmain = 469.9813 (+/− 20 ppm)
468.974
[M−H]−
1096.9501
0
20
40
60
80
100
500
600
700
800
900
1000
1100

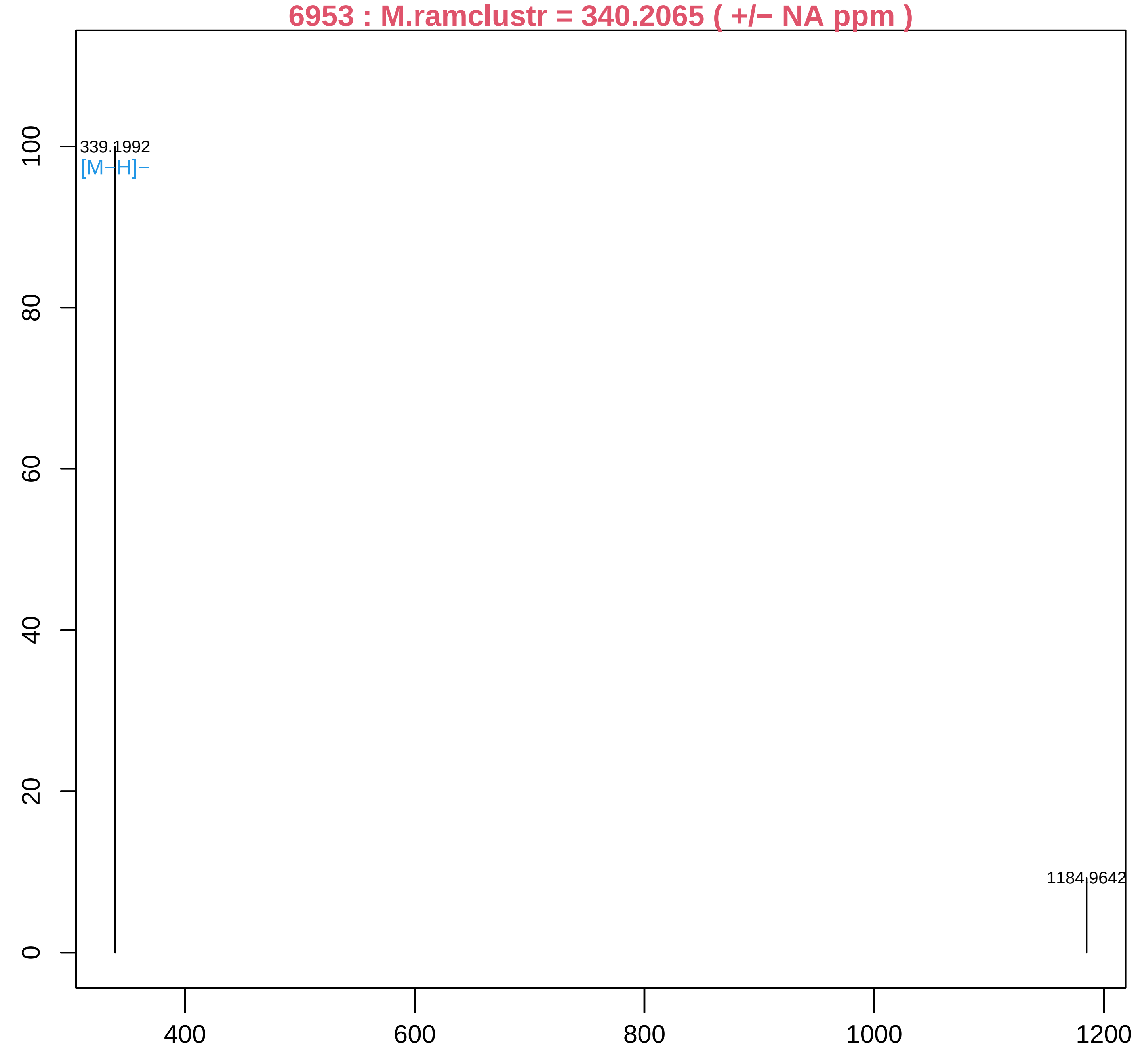
6953 : M.ramclustr = 340.2065 (+/− NA ppm)
339.1992
[M−H]−
1184.9642
0
20
40
60
80
100
400
600
800
1000
1200

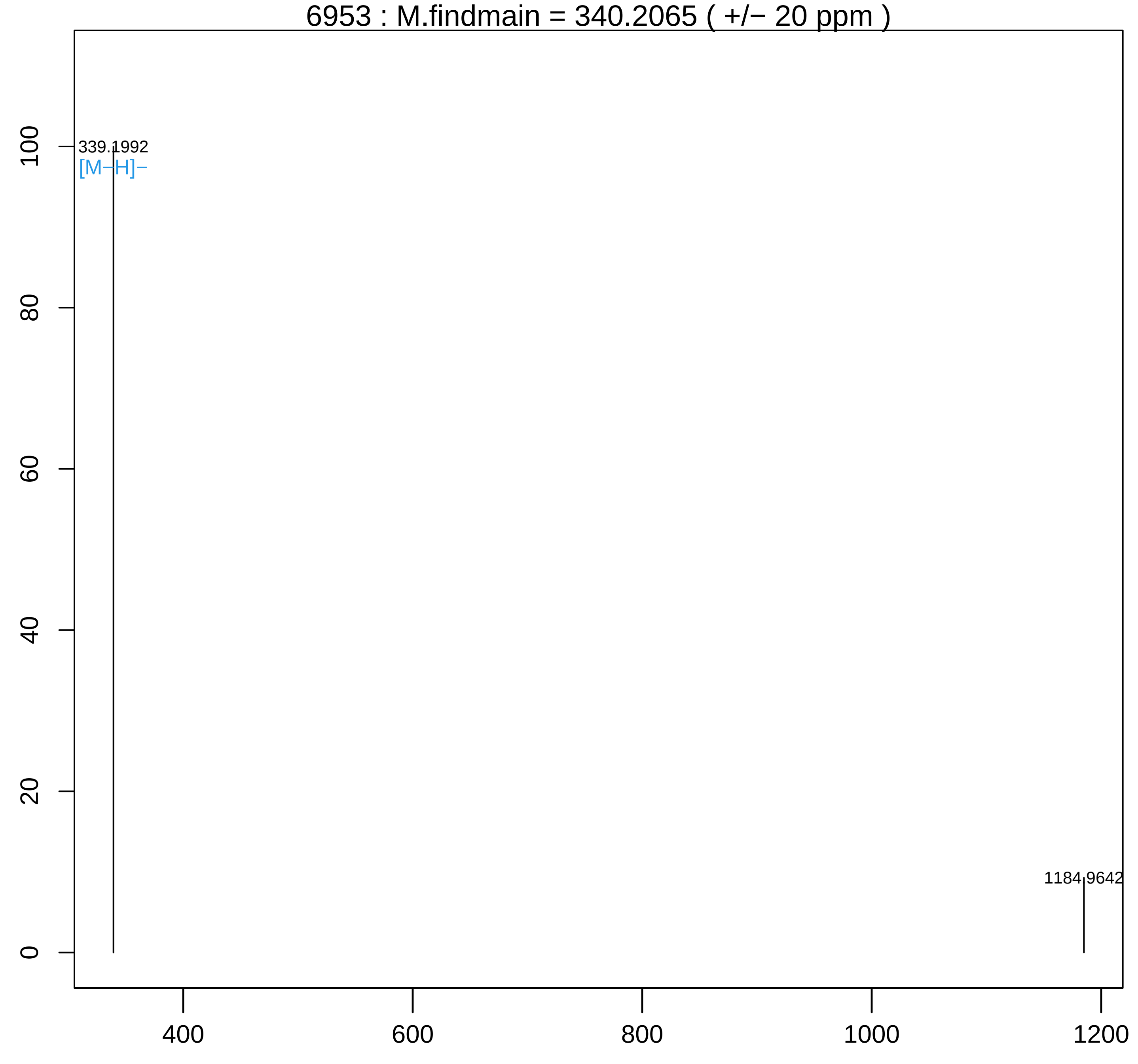
6953 : M.findmain = 340.2065 (+/− 20 ppm)
339.1992
[M−H]−
1184.9642
0
20
40
60
80
100
400
600
800
1000
1200

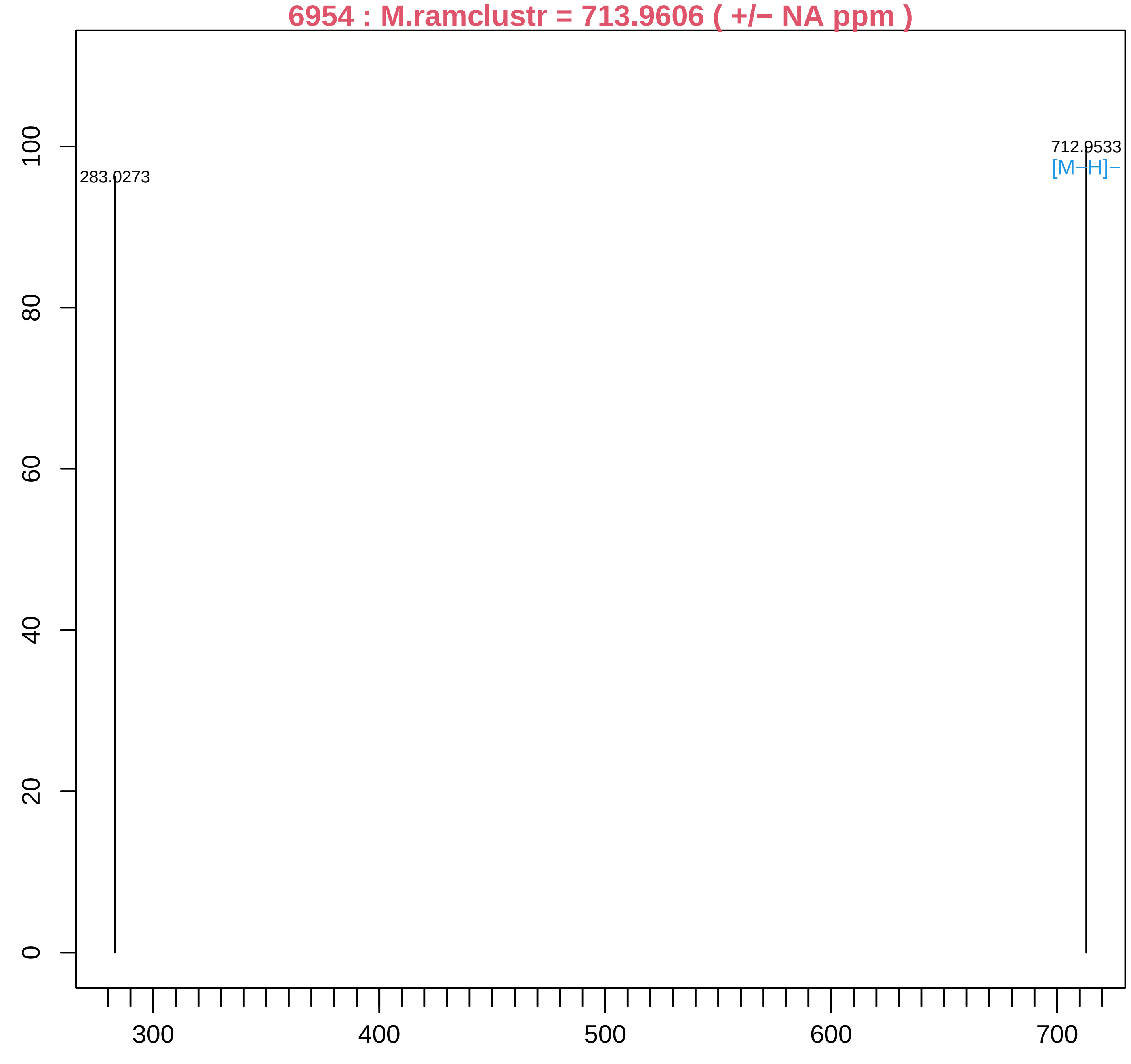
6954 : M.ramclustr = 713.9606 (+/− NA ppm)
100
80
60
40
20
0
283.0273
712.9533
[M−H]−
300
400
500
600
700

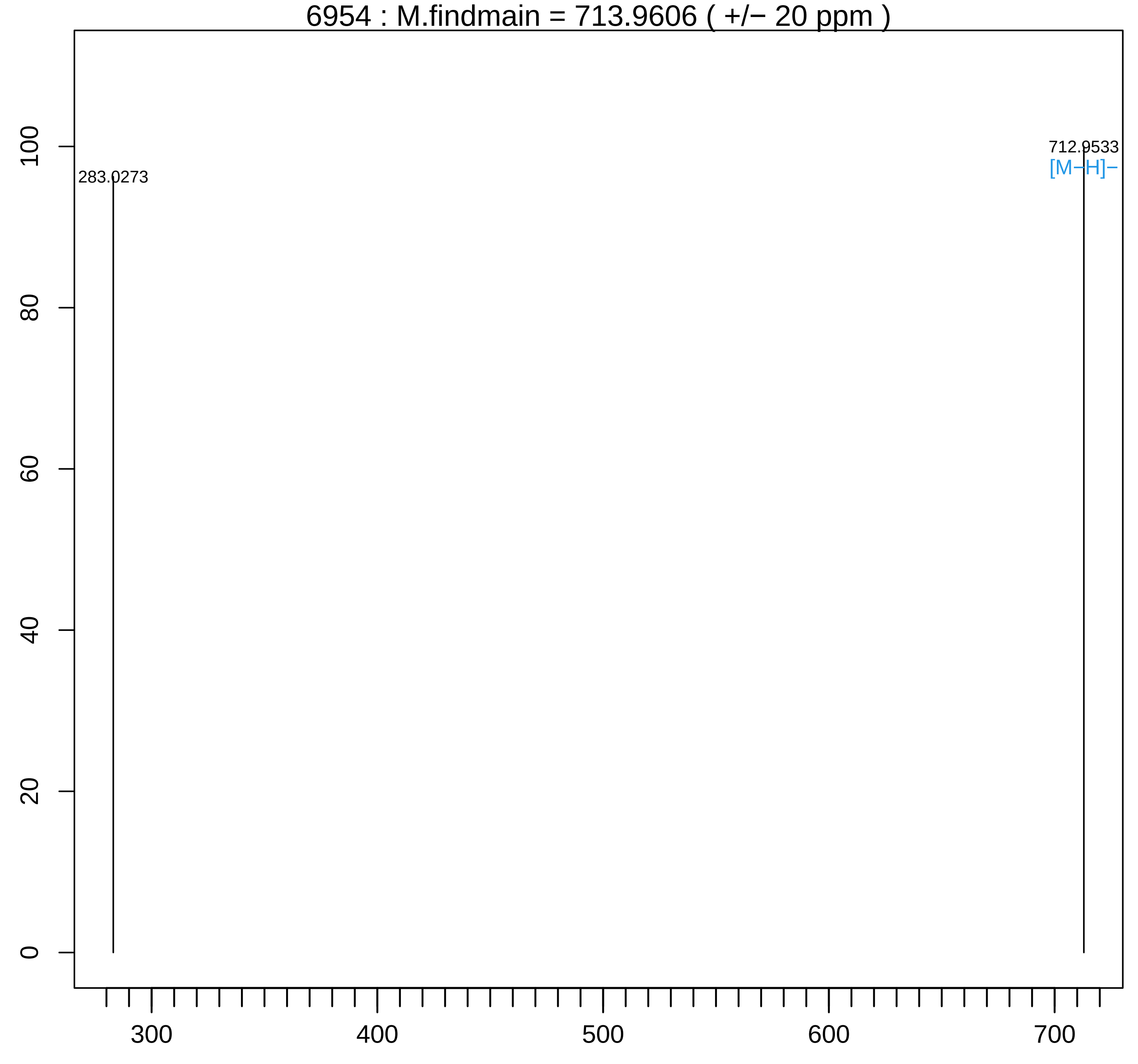
6954 : M.findmain = 713.9606 (+/− 20 ppm)
100
80
60
40
20
0
283.0273
712.9533
[M−H]−
300
400
500
600
700

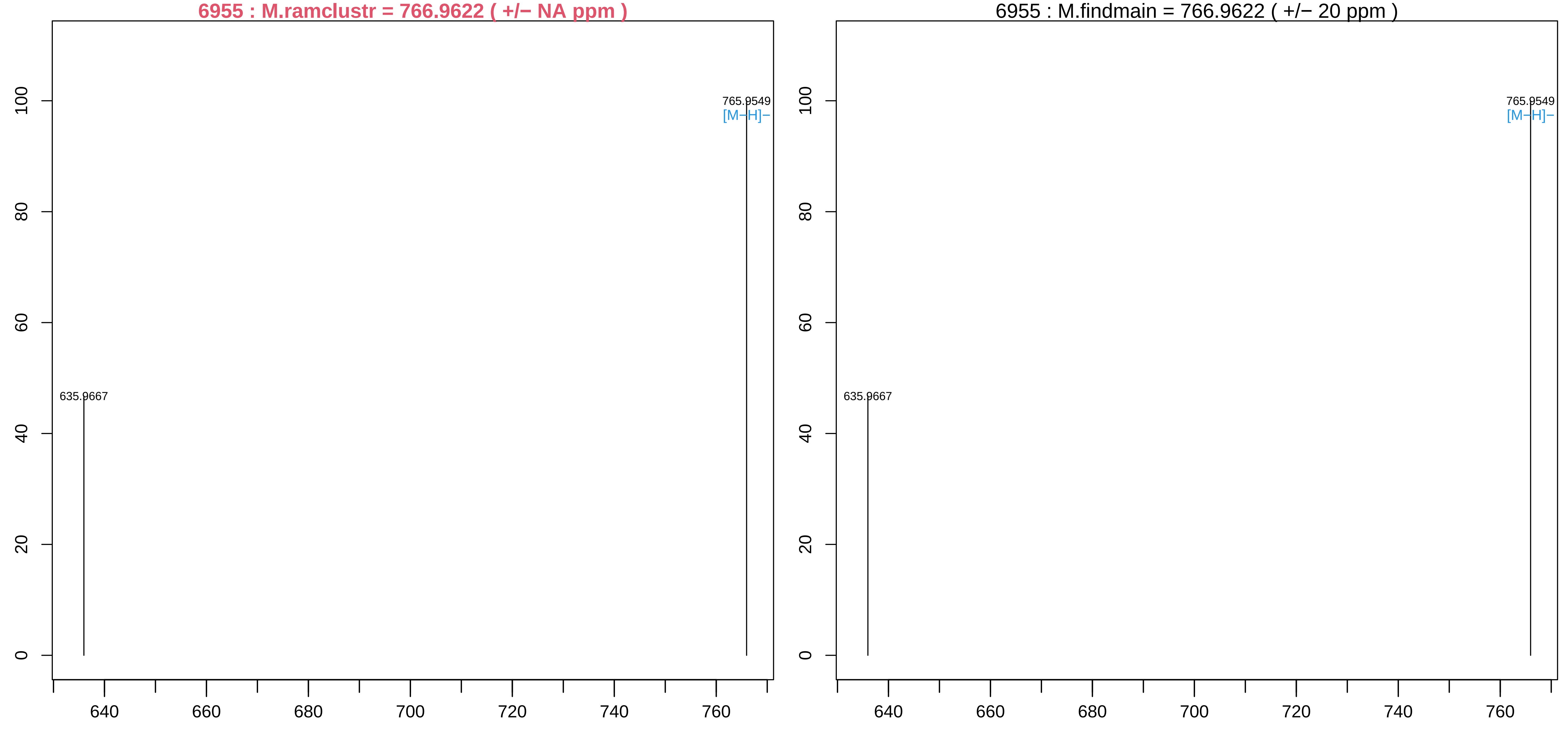
6955 : M.ramclustr = 766.9622 (+/− NA ppm)
765.9549
[M−H]−
635.9667
0
20
40
60
80
100
640
660
680
700
720
740
760
6955 : M.findmain = 766.9622 (+/− 20 ppm)
765.9549
[M−H]−
635.9667
0
20
40
60
80
100
640
660
680
700
720
740
760

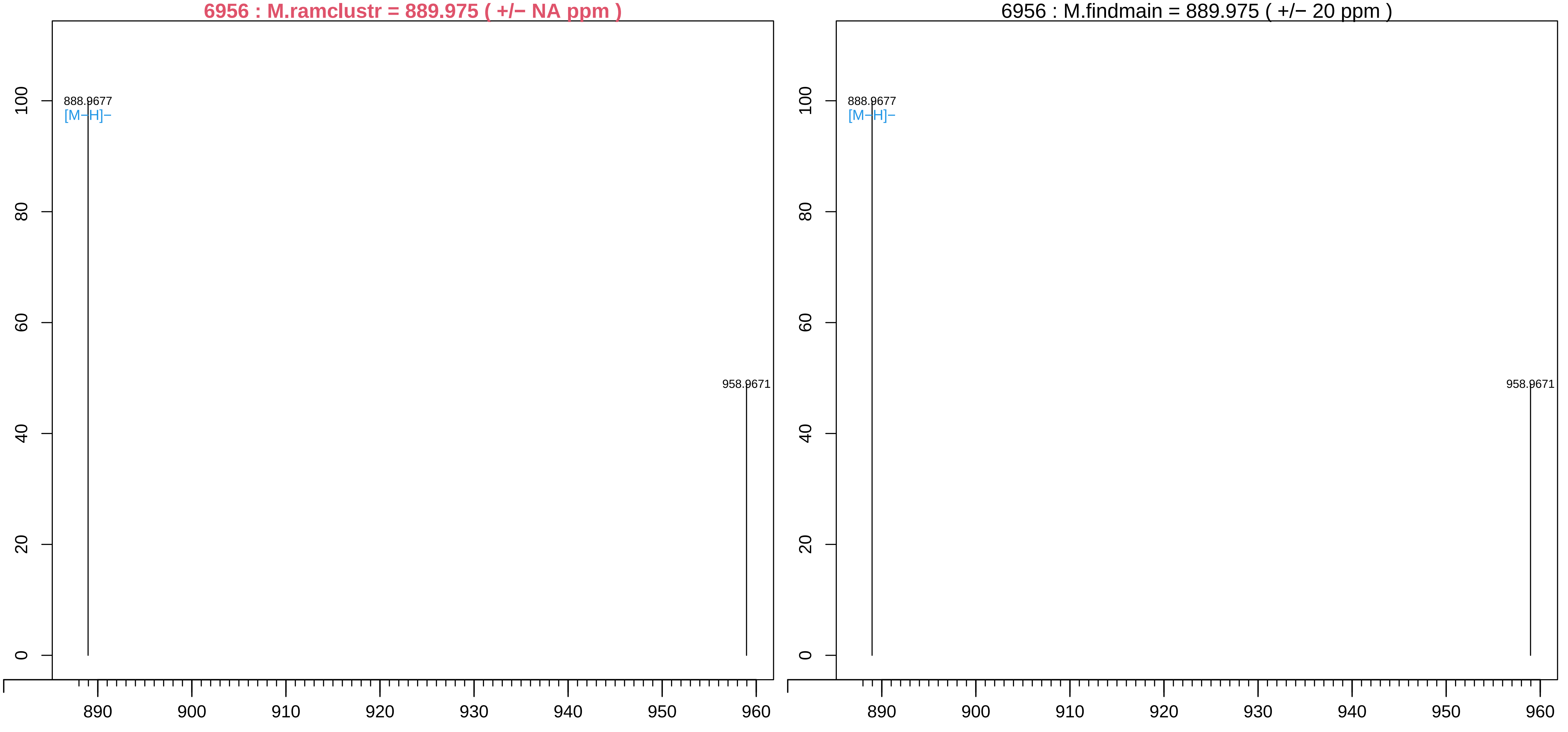
6956 : M.ramclustr = 889.975 (+/− NA ppm)
888.9677
[M−H]−
958.9671
6956 : M.findmain = 889.975 (+/− 20 ppm)
888.9677
[M−H]−
958.9671
0
20
40
60
80
100
890
900
910
920
930
940
950
960

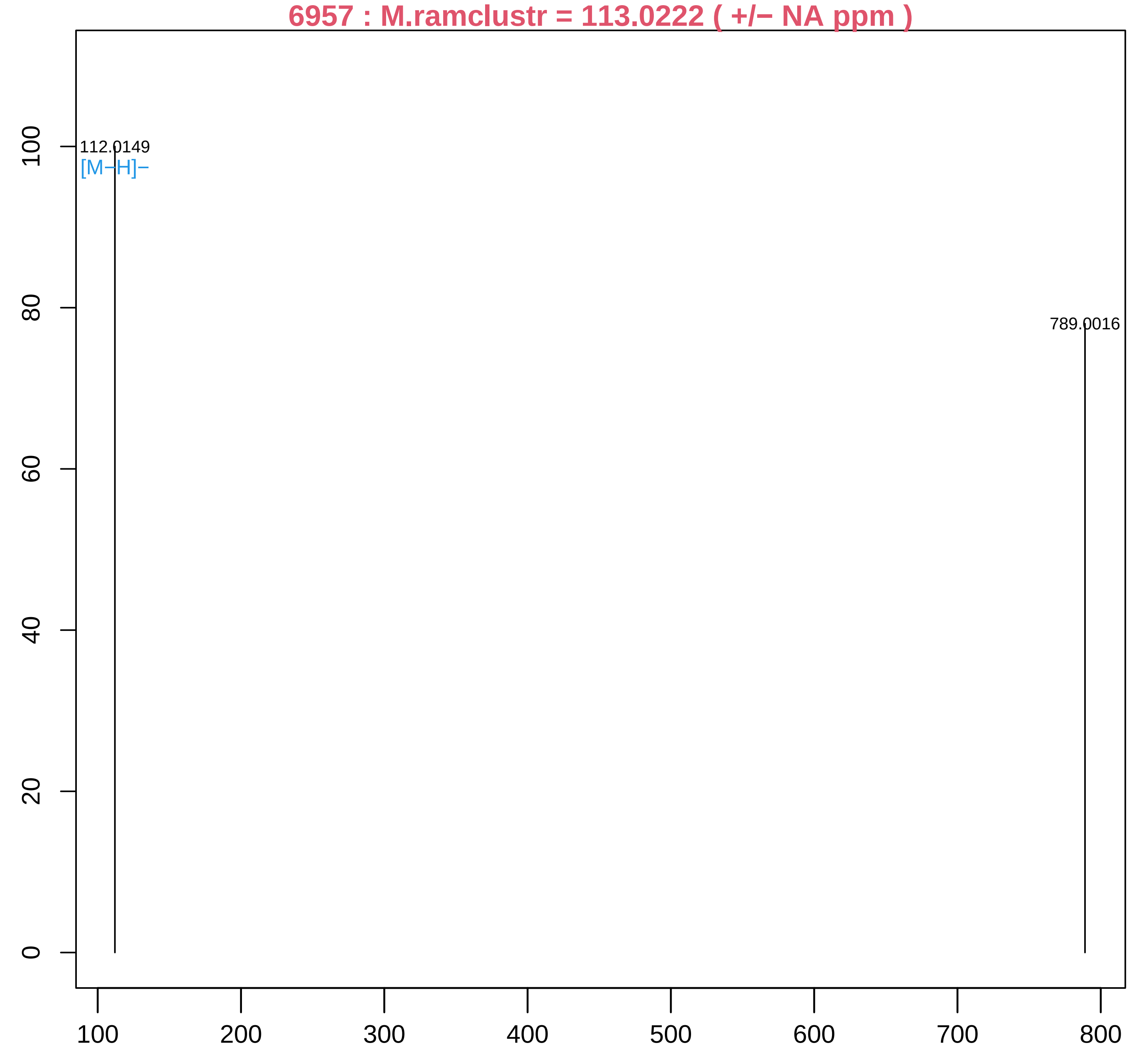
6957 : M.ramclustr = 113.0222 (+/− NA ppm)
112.0149
[M−H]−
789.0016
0
20
40
60
80
100
100
200
300
400
500
600
700
800

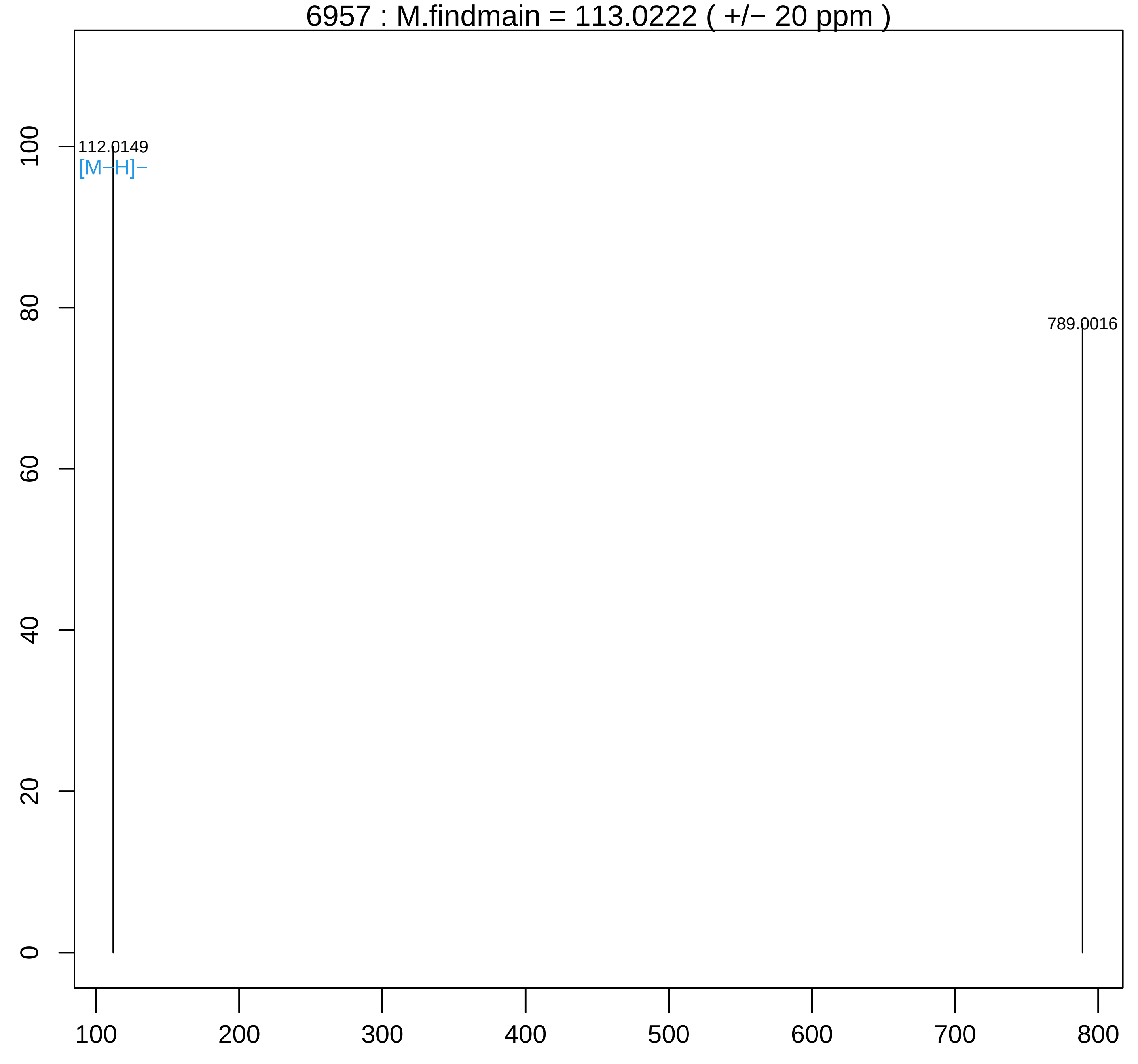
6957 : M.findmain = 113.0222 (+/− 20 ppm)
112.0149
[M−H]−
789.0016
0
20
40
60
80
100
100
200
300
400
500
600
700
800

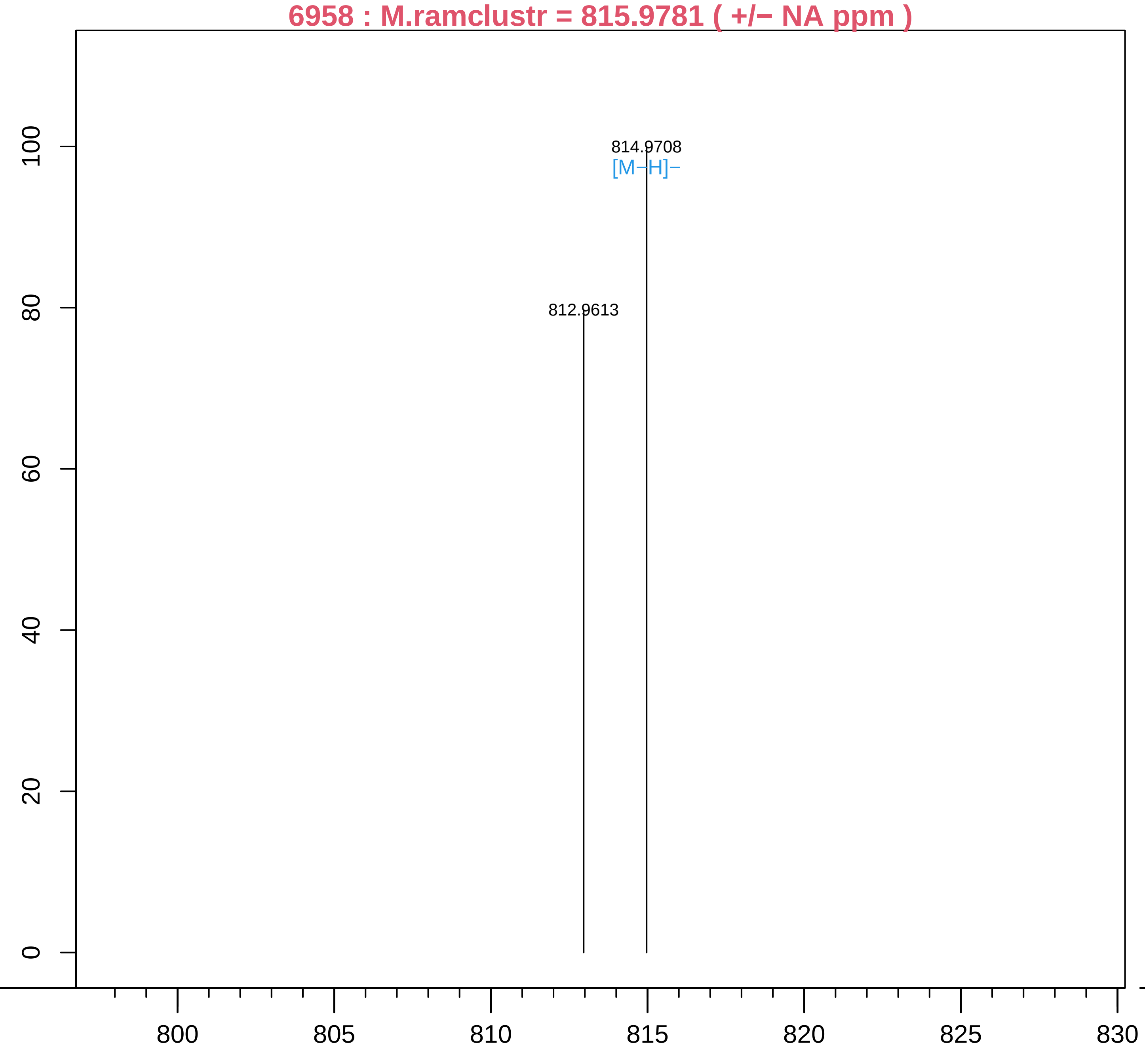

6958 : M.ramclustr = 815.9781 (+/− NA ppm)
814.9708
[M−H]−
812.9613
0
20
40
60
80
100
800
805
810
815
820
825
830

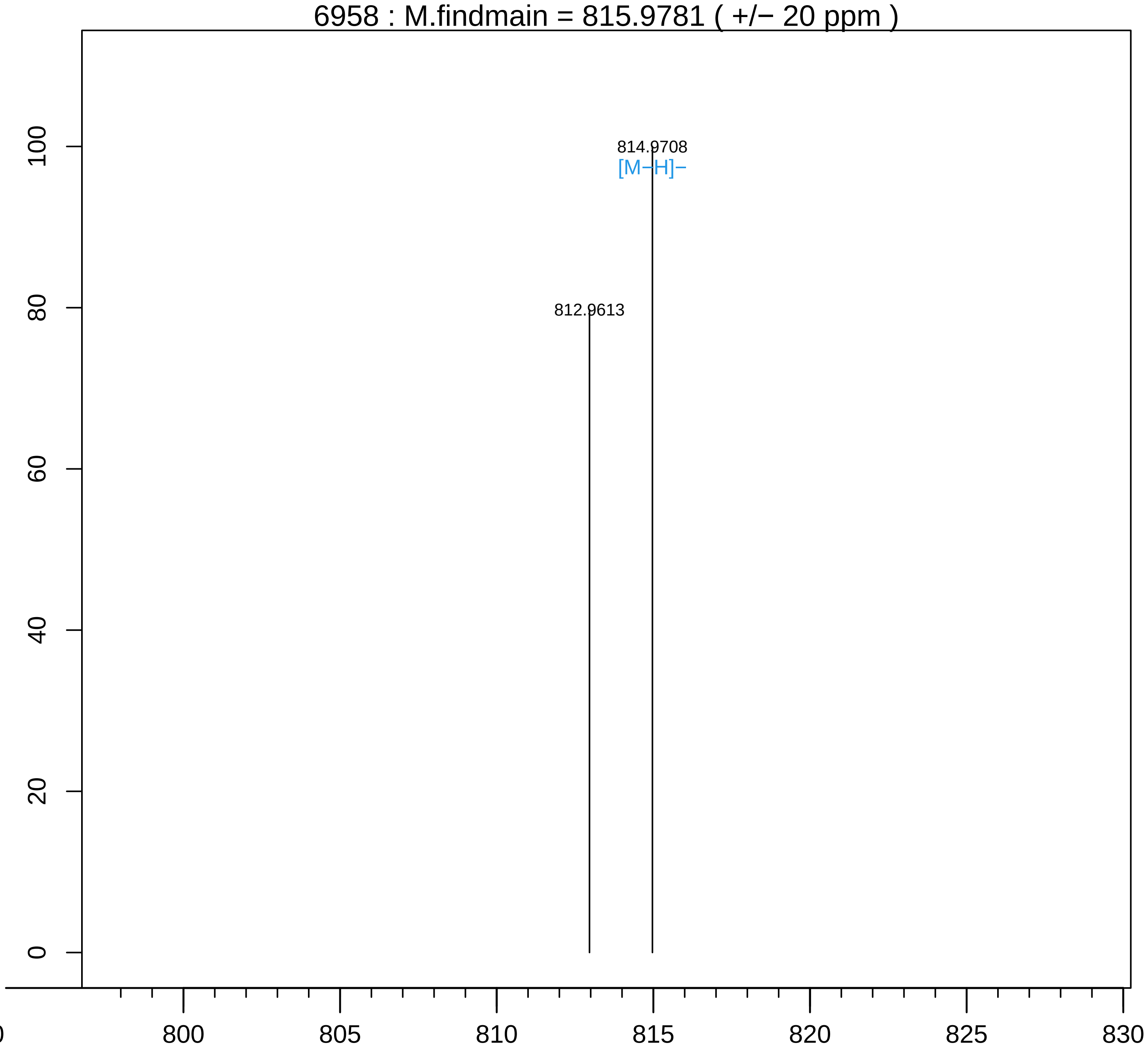

6958 : M.findmain = 815.9781 (+/− 20 ppm)
814.9708
[M−H]−
812.9613
0
20
40
60
80
100
800
805
810
815
820
825
830

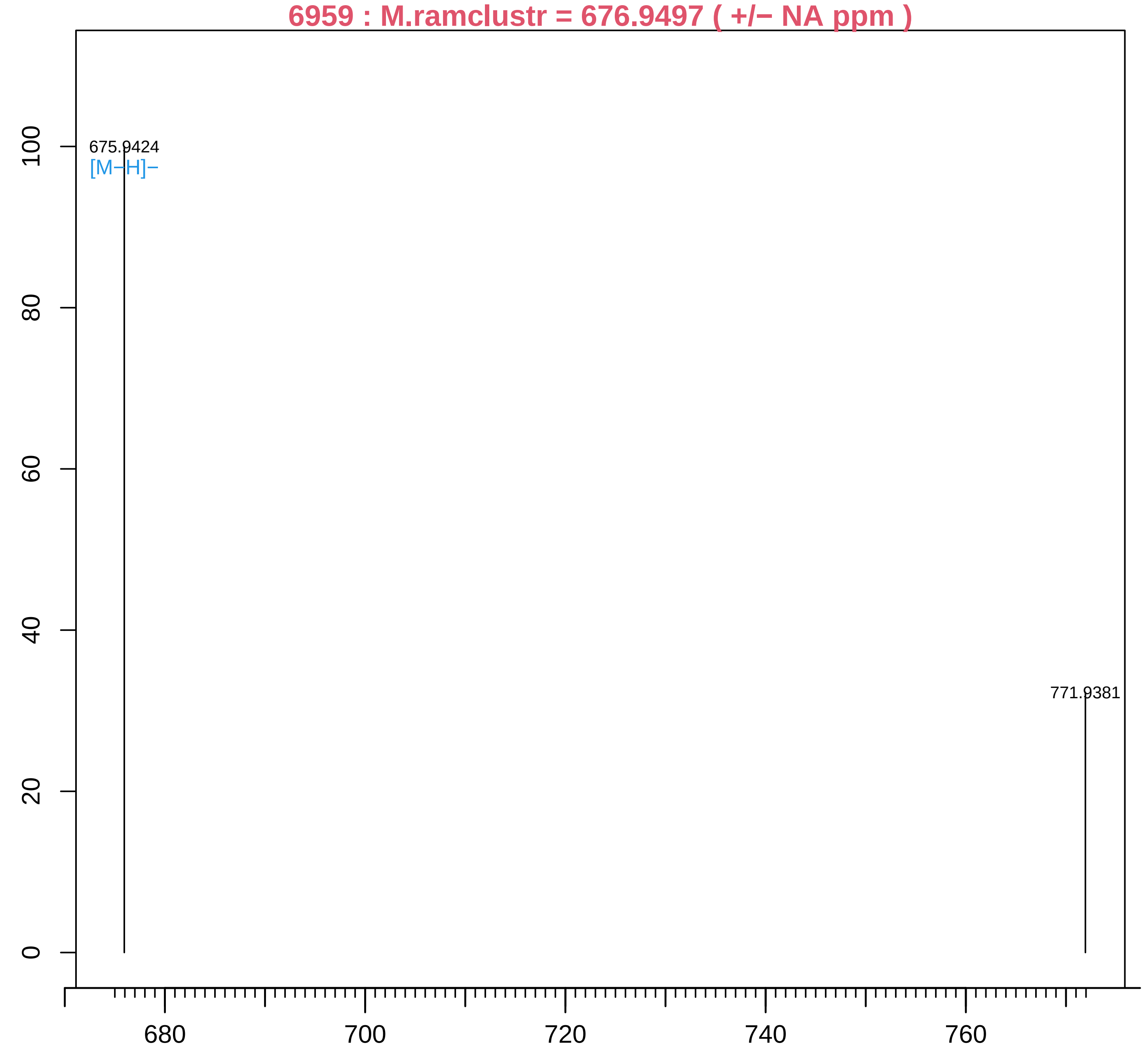
6959 : M.ramclustr = 676.9497 (+/− NA ppm)
675.9424
[M−H]−
771.9381
0
20
40
60
80
100
680
700
720
740
760

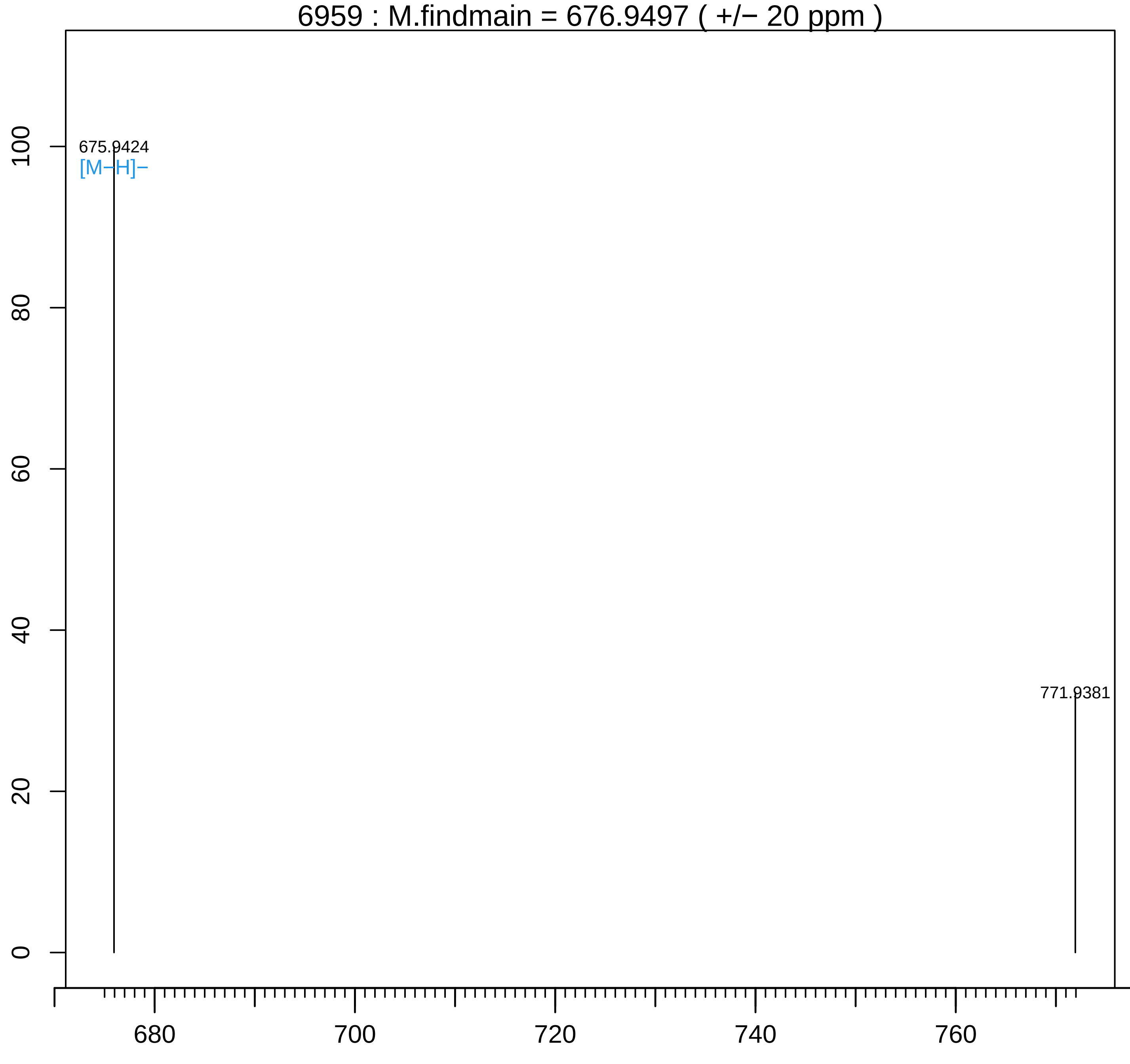
6959 : M.findmain = 676.9497 (+/− 20 ppm)
675.9424
[M−H]−
771.9381
0
20
40
60
80
100
680
700
720
740
760

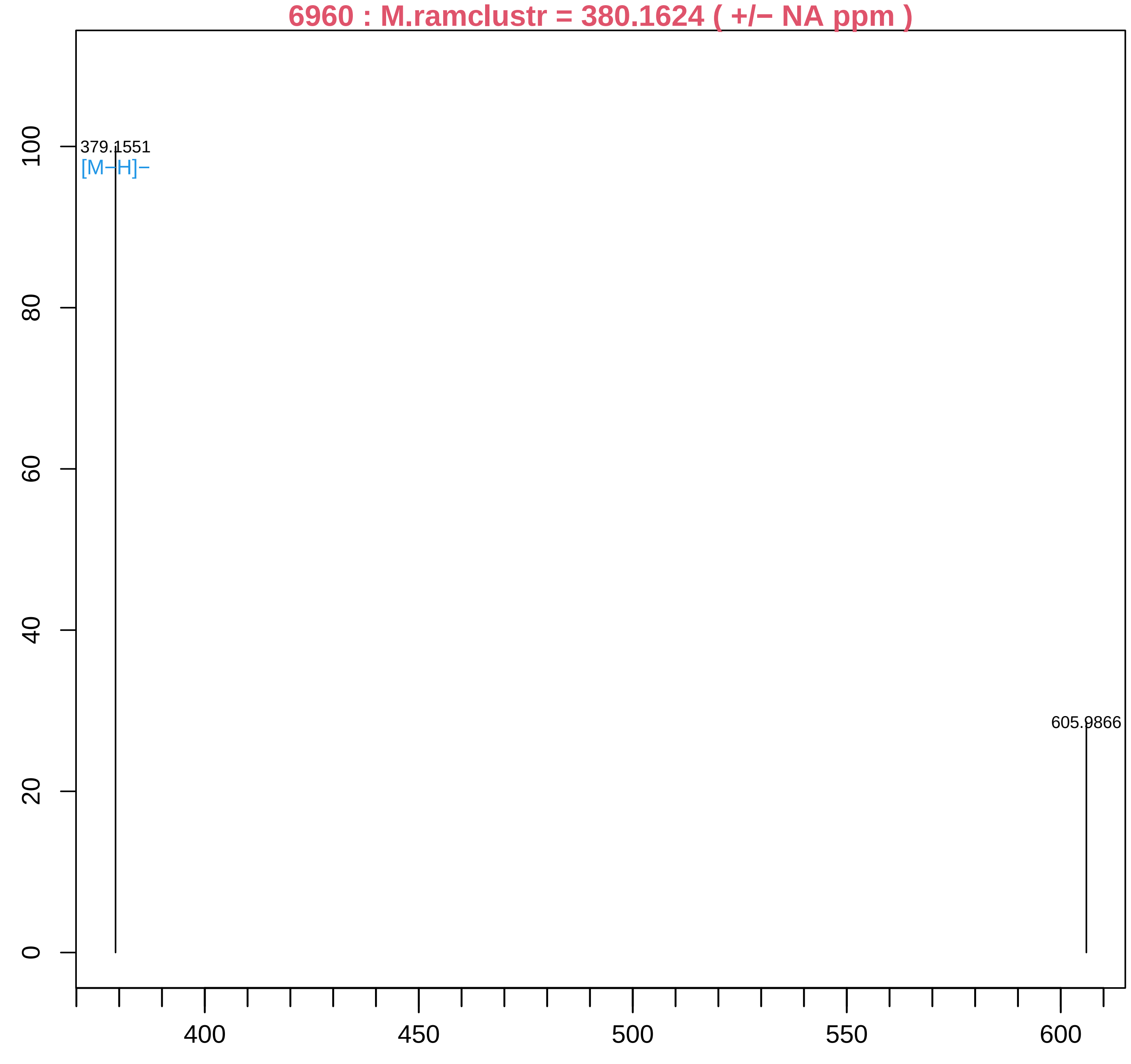
6960 : M.ramclustr = 380.1624 (+/− NA ppm)
379.1551
[M−H]−
605.9866
0
20
40
60
80
100
400
450
500
550
600

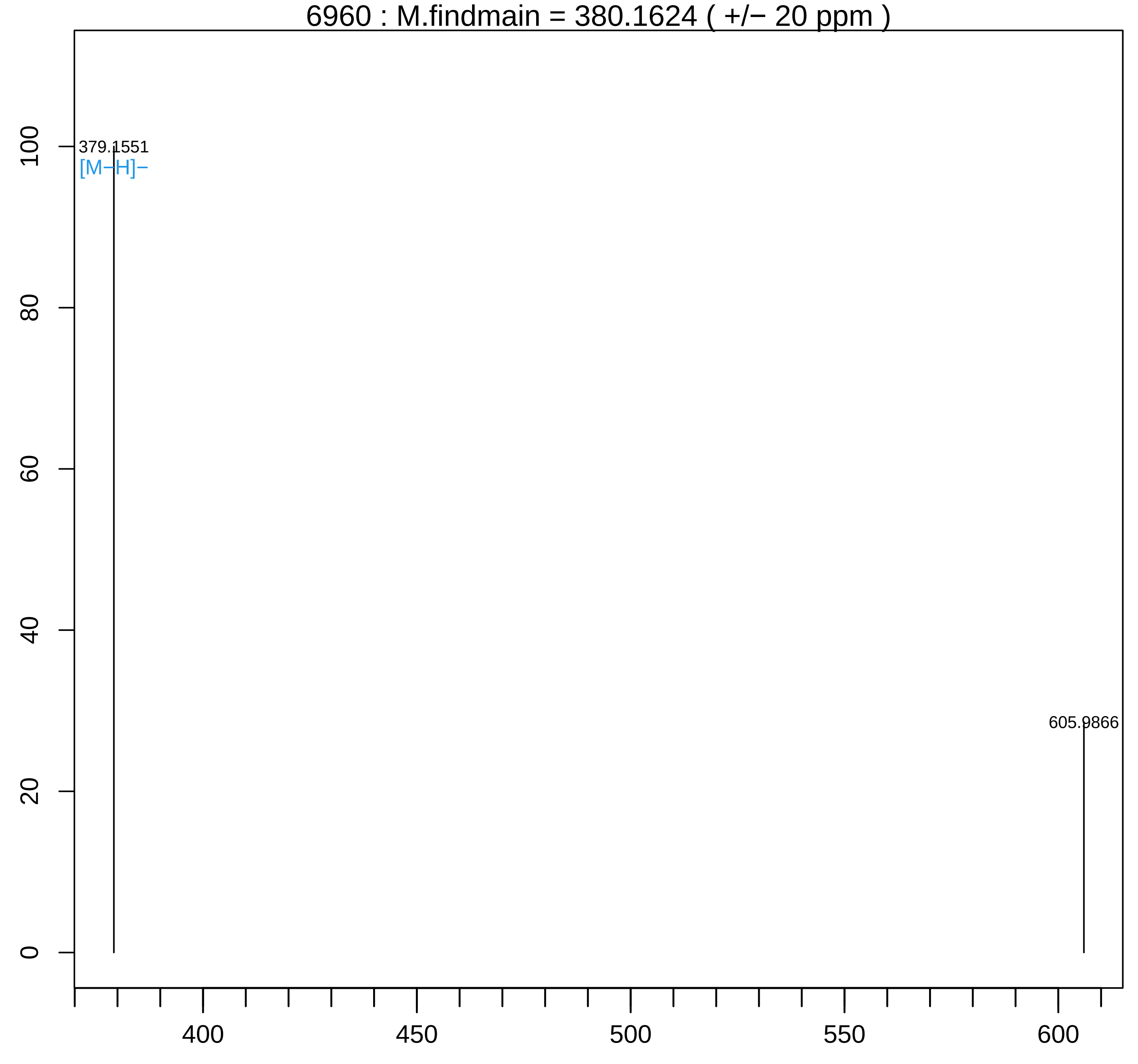
6960 : M.findmain = 380.1624 (+/− 20 ppm)
379.1551
[M−H]−
605.9866
0
20
40
60
80
100
400
450
500
550
600

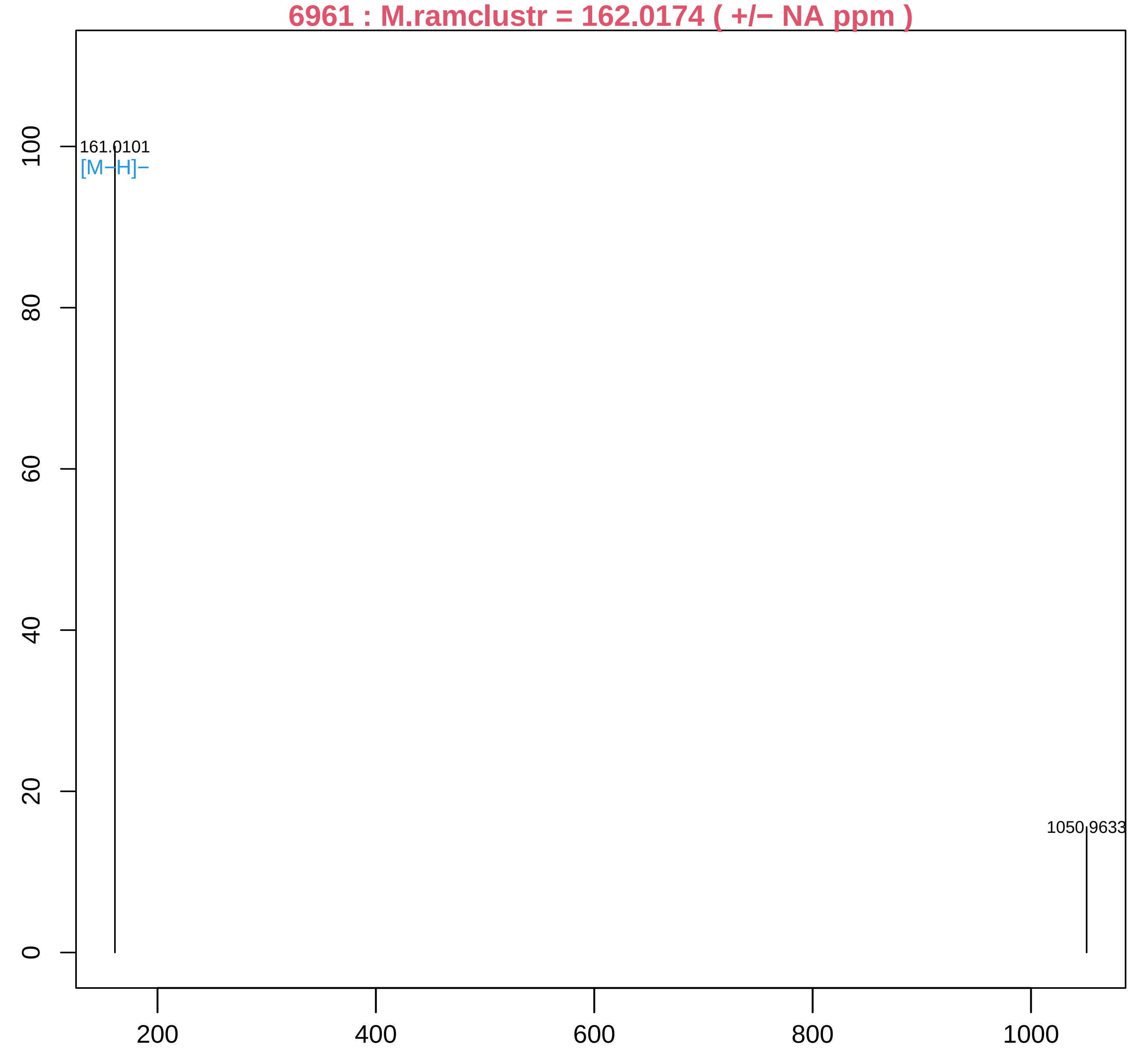
6961 : M.ramclustr = 162.0174 (+/− NA ppm)
161.0101
[M−H]−
1050.9633
0
20
40
60
80
100
200
400
600
800
1000
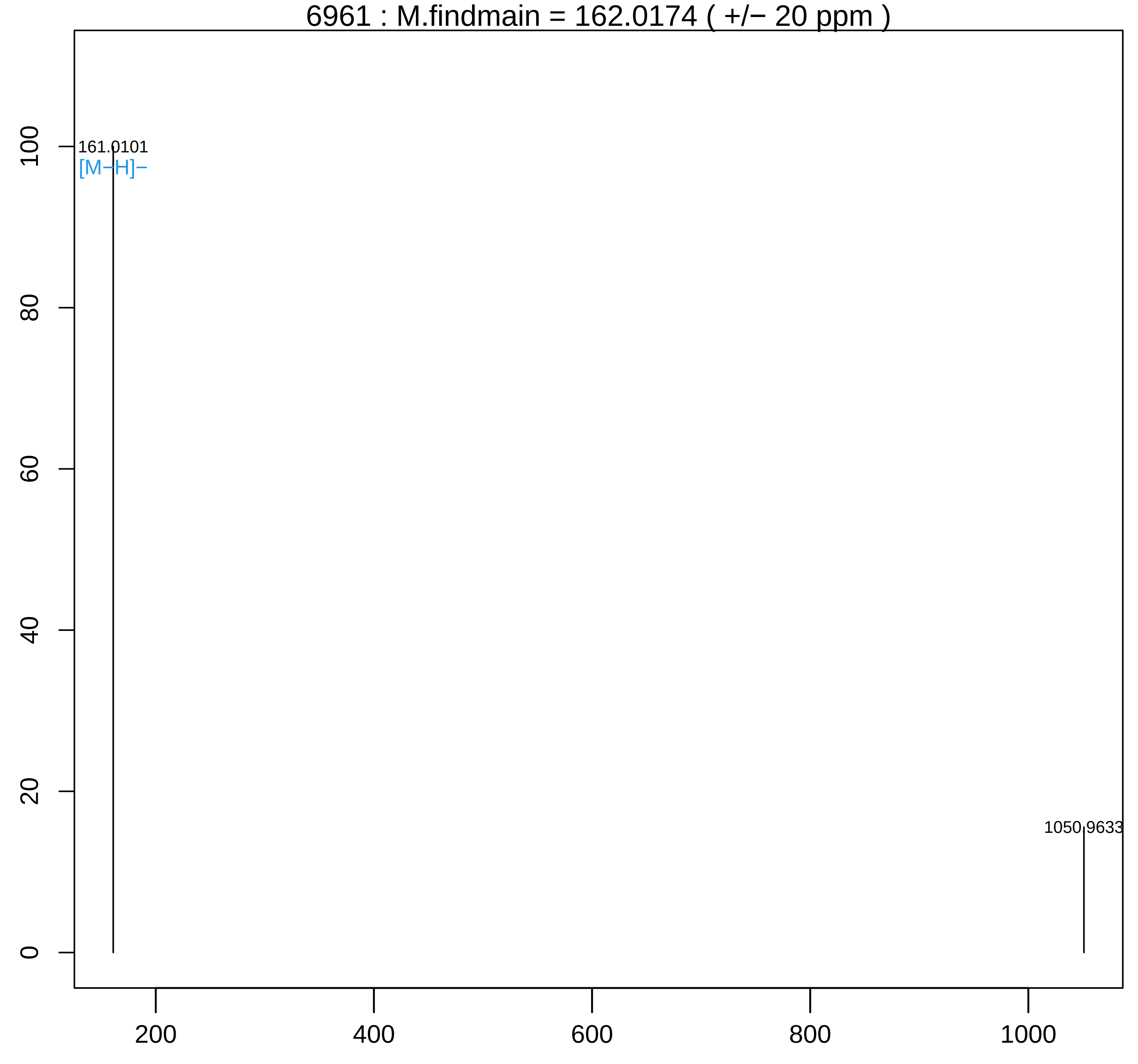
6961 : M.findmain = 162.0174 (+/− 20 ppm)
161.0101
[M−H]−
1050.9633
0
20
40
60
80
100
200
400
600
800
1000

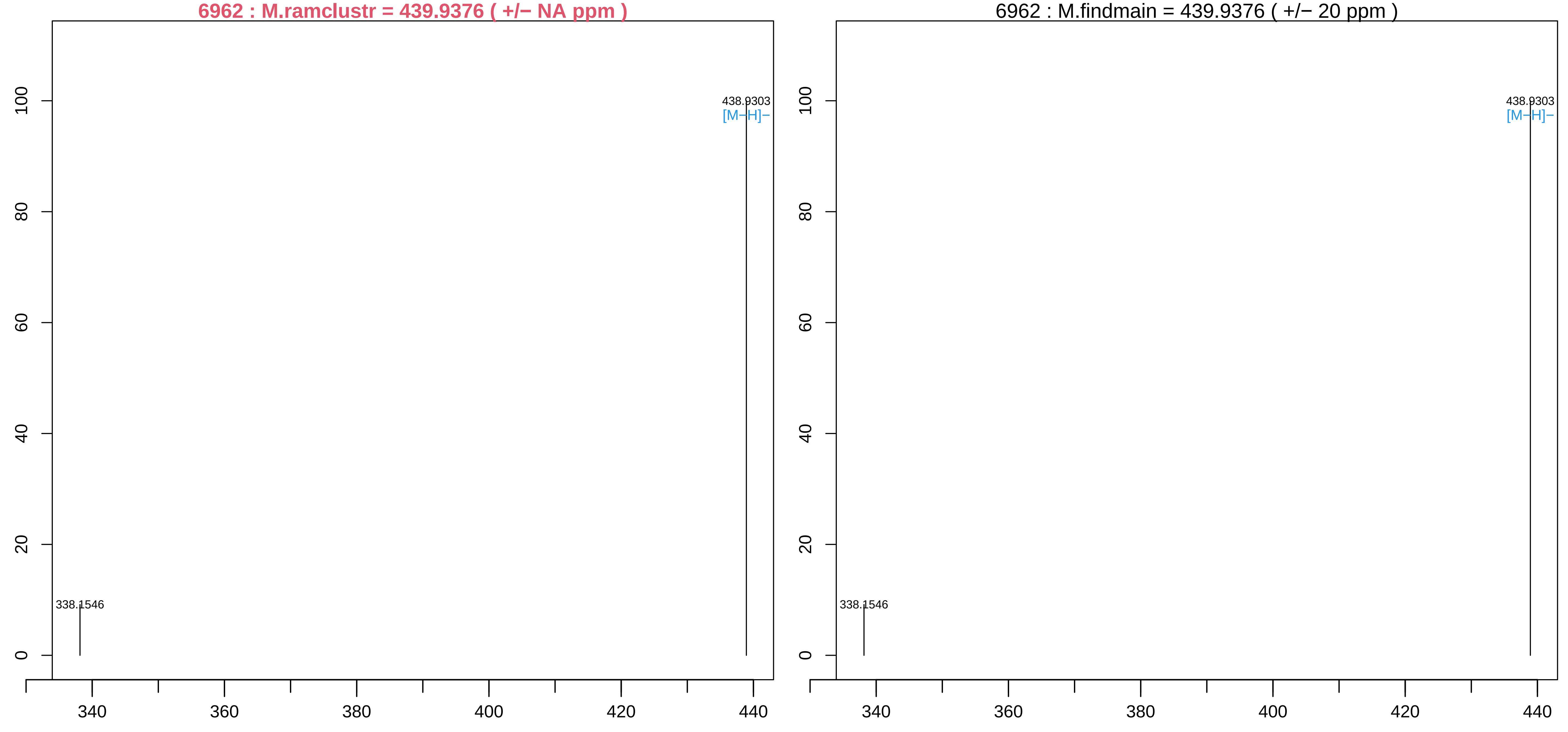
6962 : M.ramclustr = 439.9376 (+/− NA ppm)
6962 : M.findmain = 439.9376 (+/− 20 ppm)
438.9303
[M−H]−
338.1546
0
20
40
60
80
100
340
360
380
400
420
440

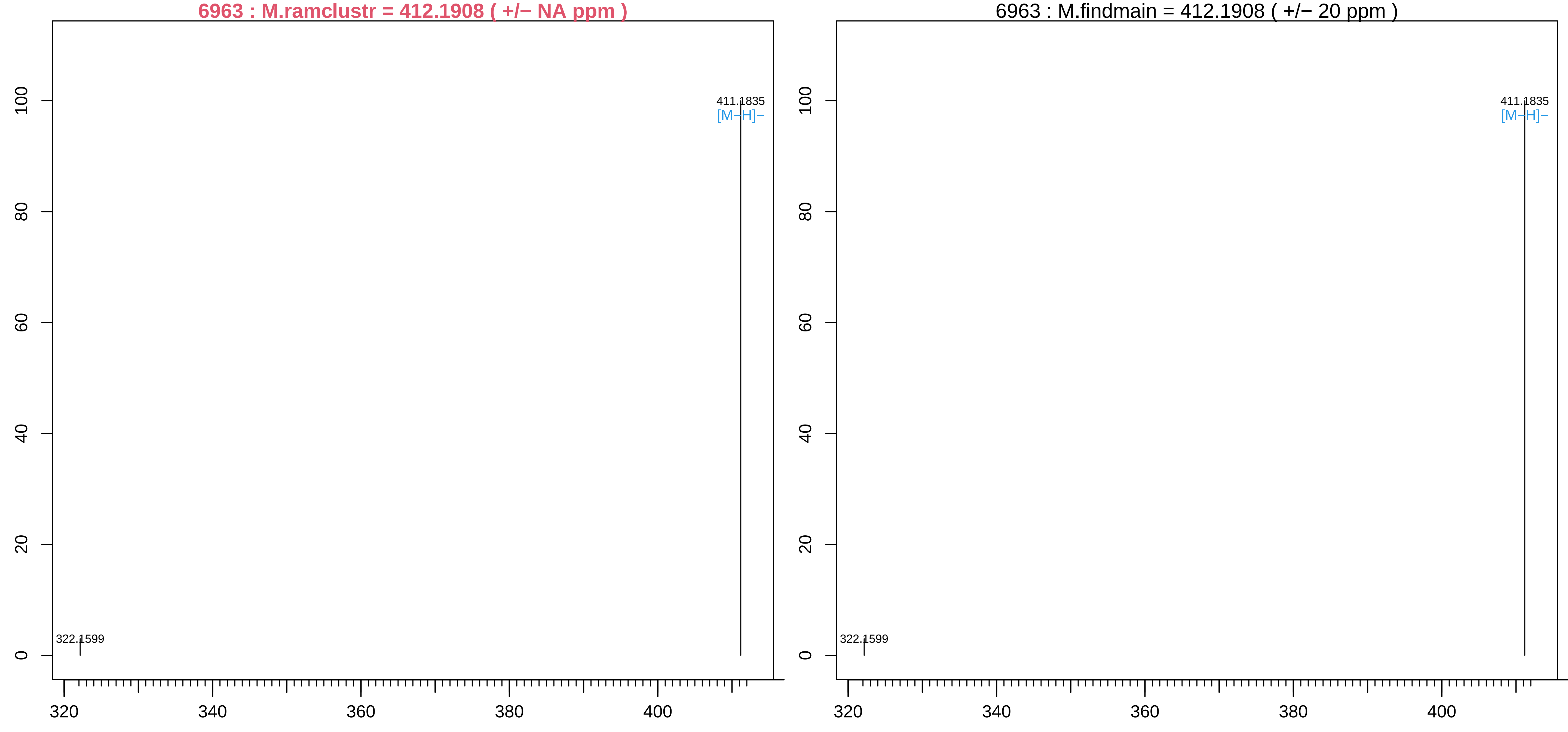

6963 : M.ramclustr = 412.1908 (+/− NA ppm)
411.1835
[M−H]−
322.1599
6963 : M.findmain = 412.1908 (+/− 20 ppm)
411.1835
[M−H]−
322.1599

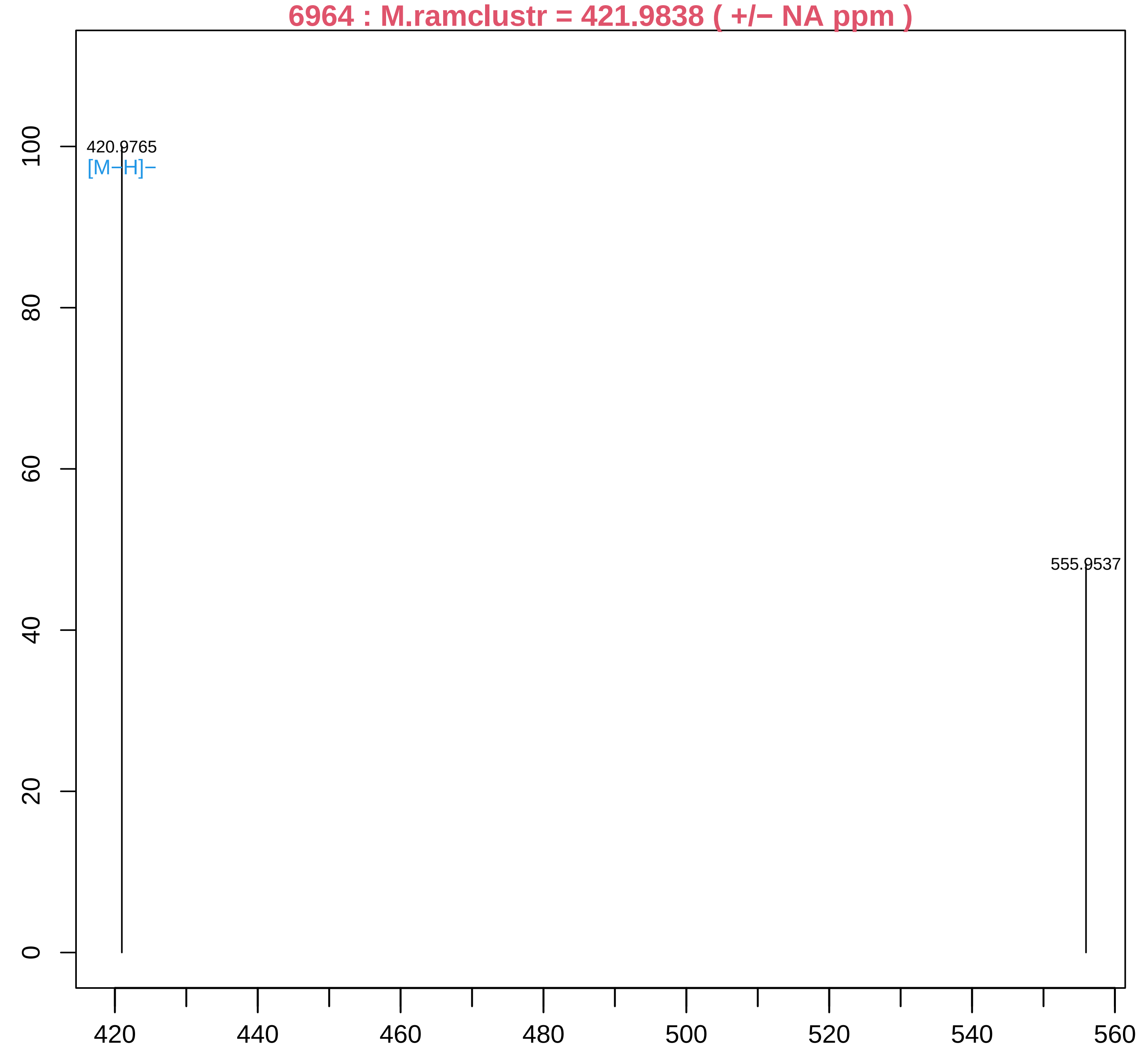
6964 : M.ramclustr = 421.9838 (+/− NA ppm)
420.9765
[M−H]−
555.9537
0
20
40
60
80
100
420
440
460
480
500
520
540
560
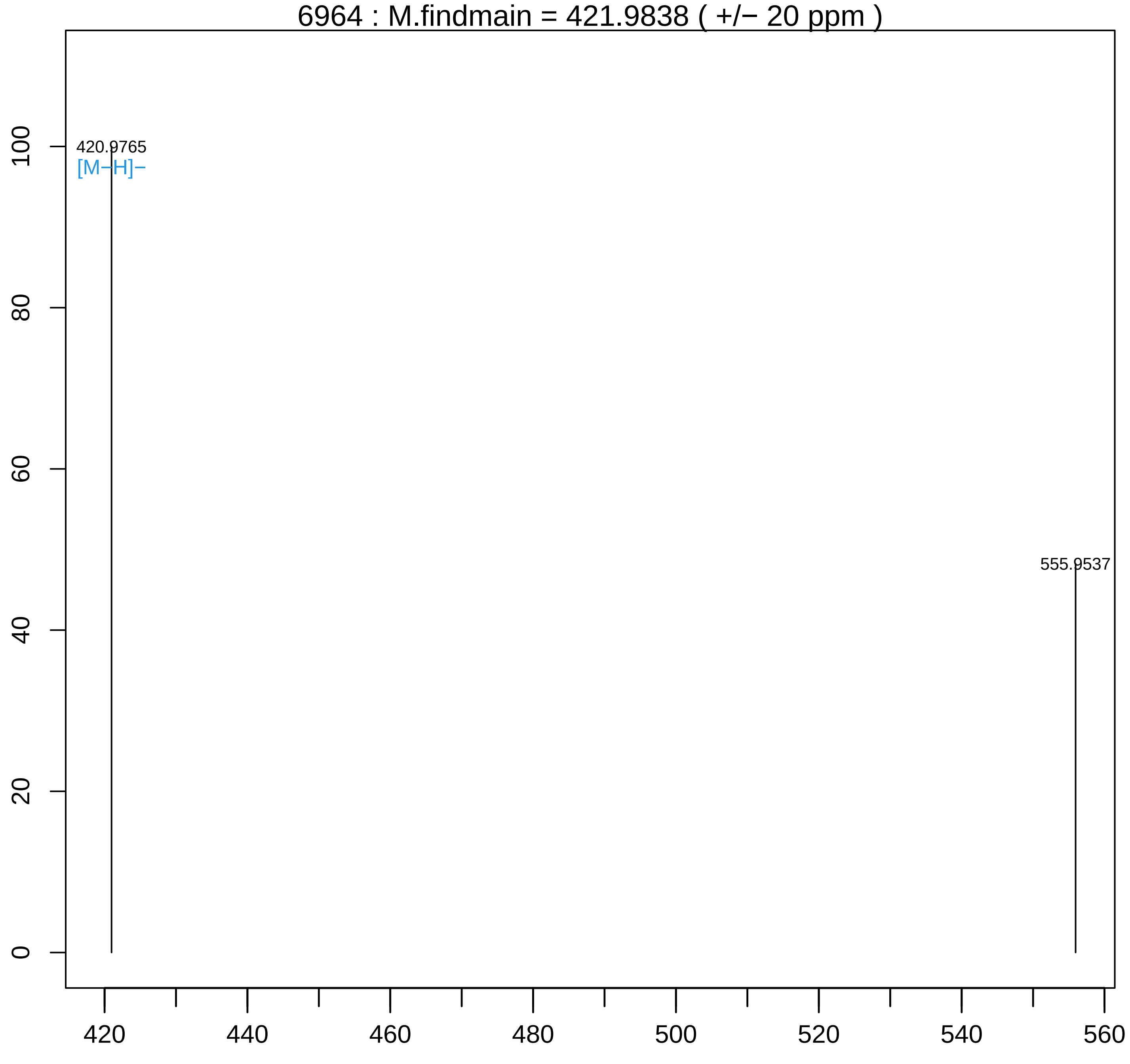
6964 : M.findmain = 421.9838 (+/− 20 ppm)
420.9765
[M−H]−
555.9537
0
20
40
60
80
100
420
440
460
480
500
520
540
560

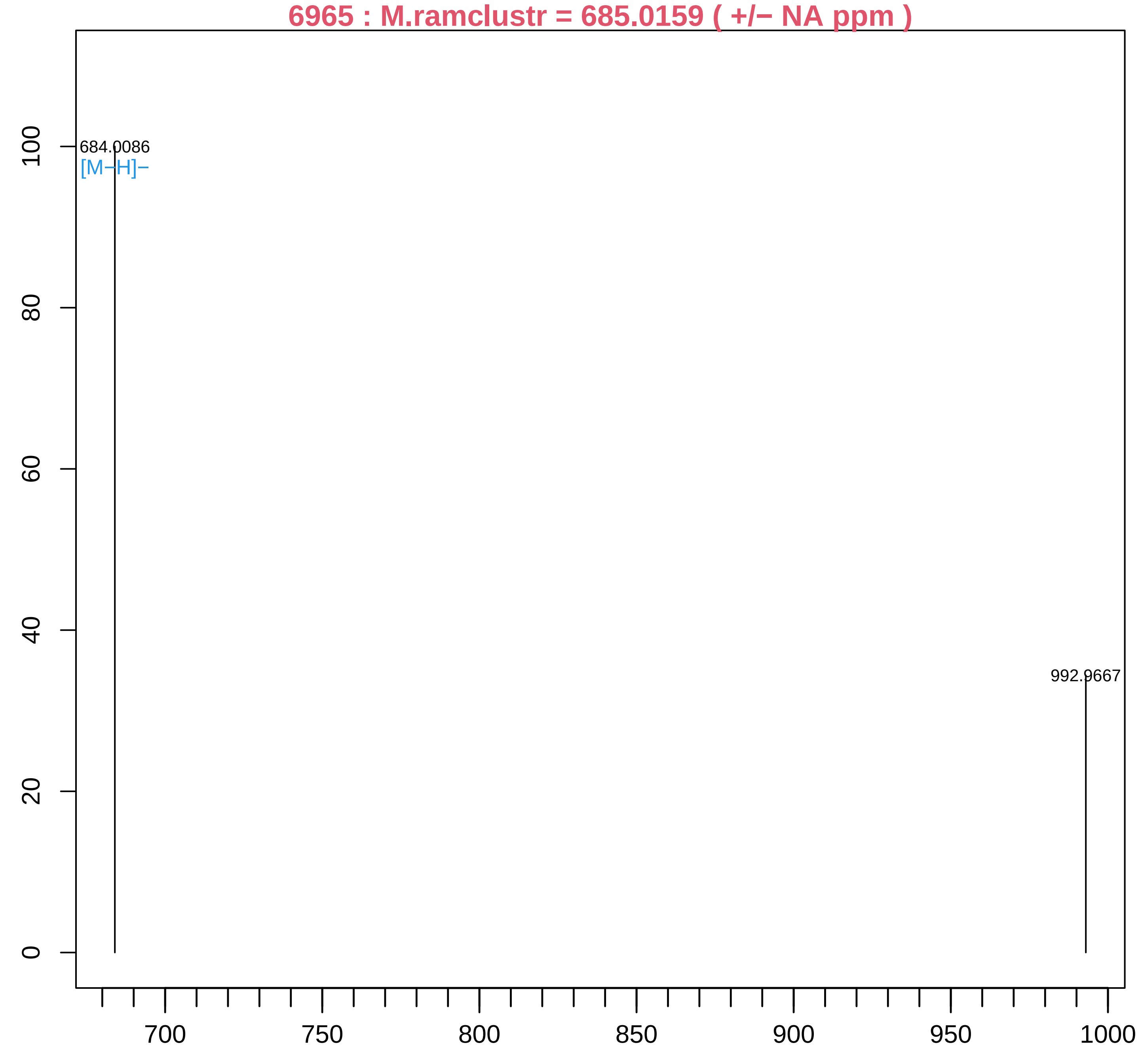
6965 : M.ramclustr = 685.0159 (+/− NA ppm)
684.0086
[M−H]−
992.9667
0
20
40
60
80
100
700
750
800
850
900
950
1000

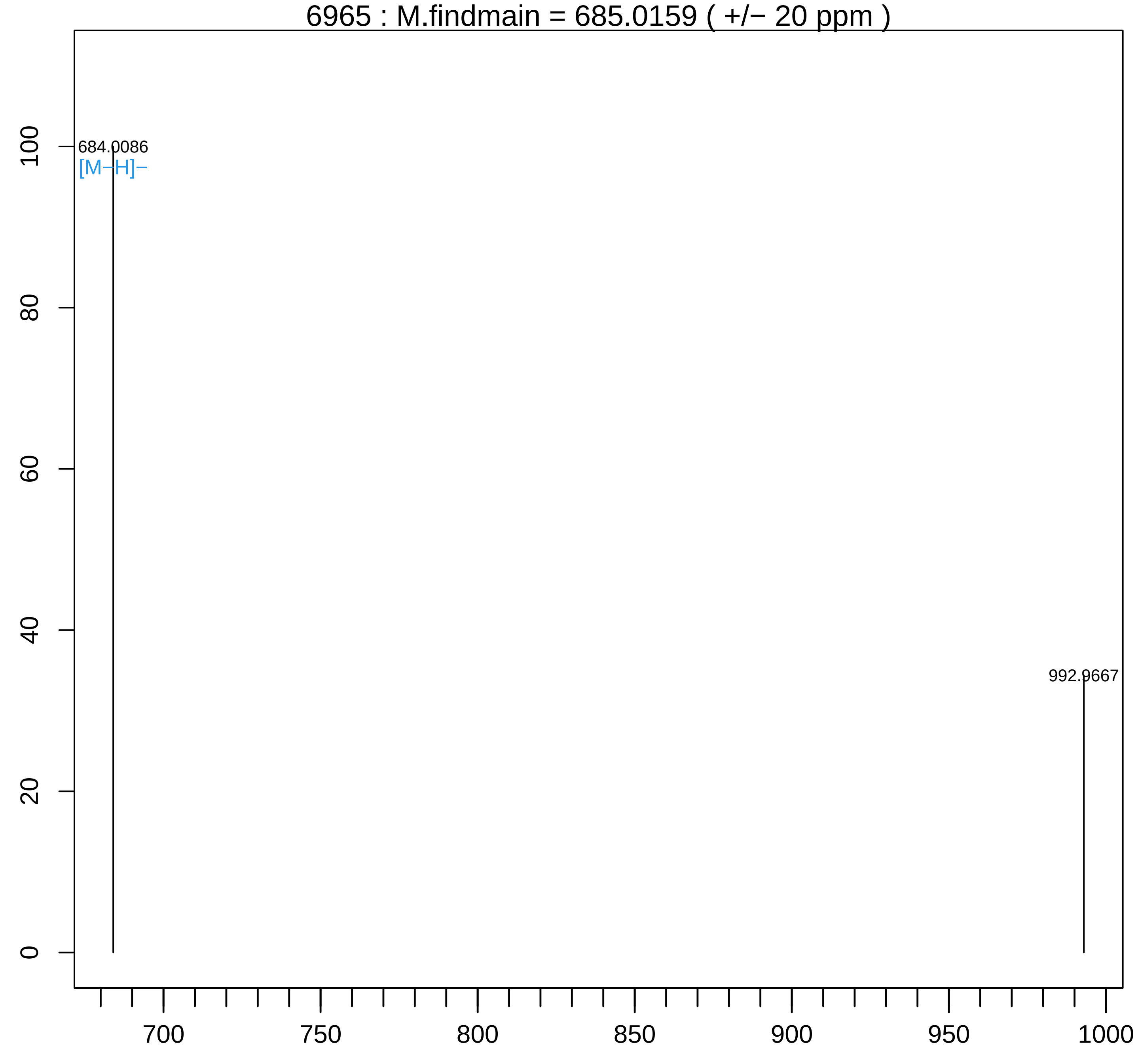
6965 : M.findmain = 685.0159 (+/− 20 ppm)
684.0086
[M−H]−
992.9667
0
20
40
60
80
100
700
750
800
850
900
950
1000

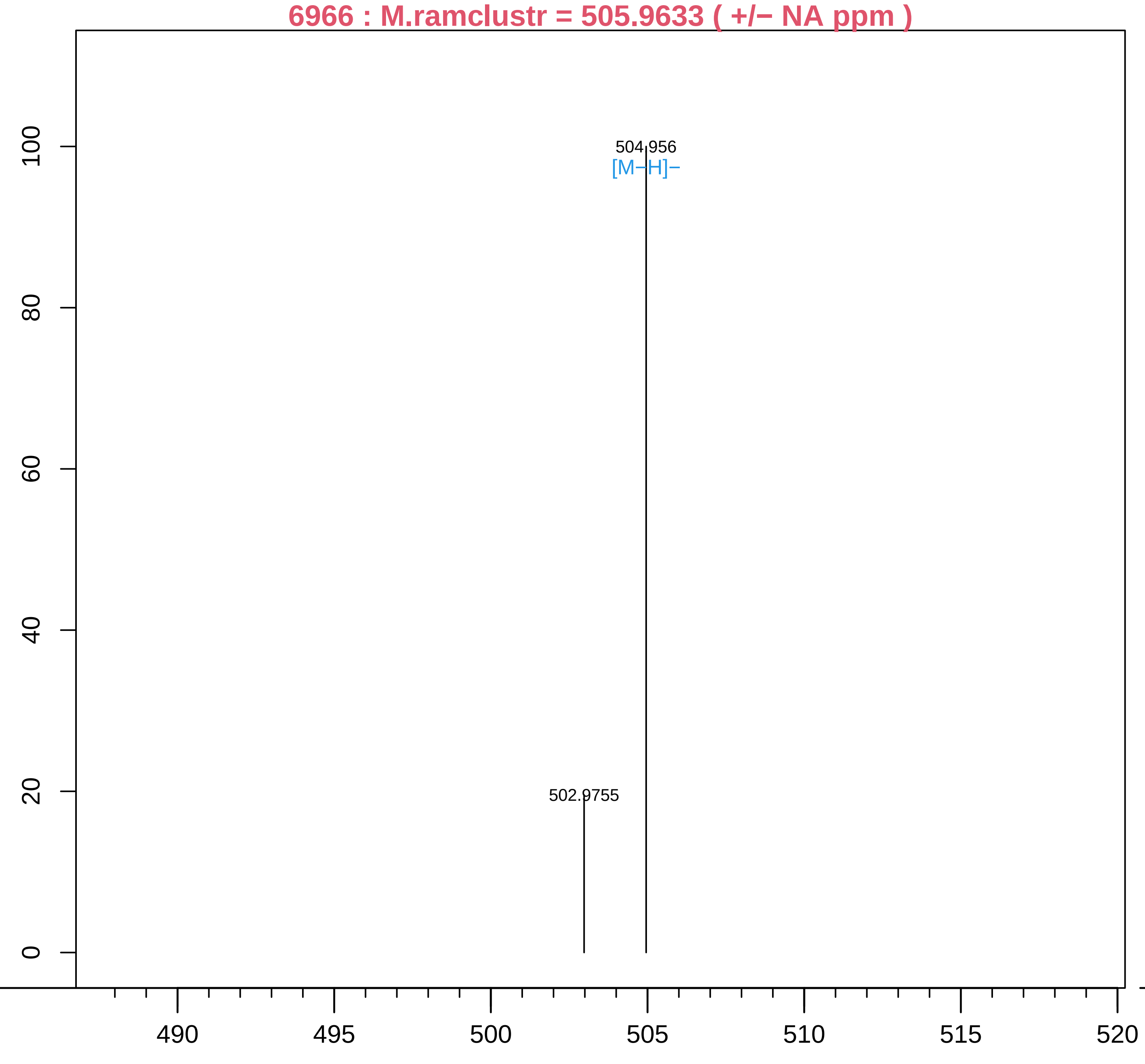
6966 : M.ramclustr = 505.9633 (+/− NA ppm)
504.956
[M−H]−
502.9755
0
20
40
60
80
100
490
495
500
505
510
515
520

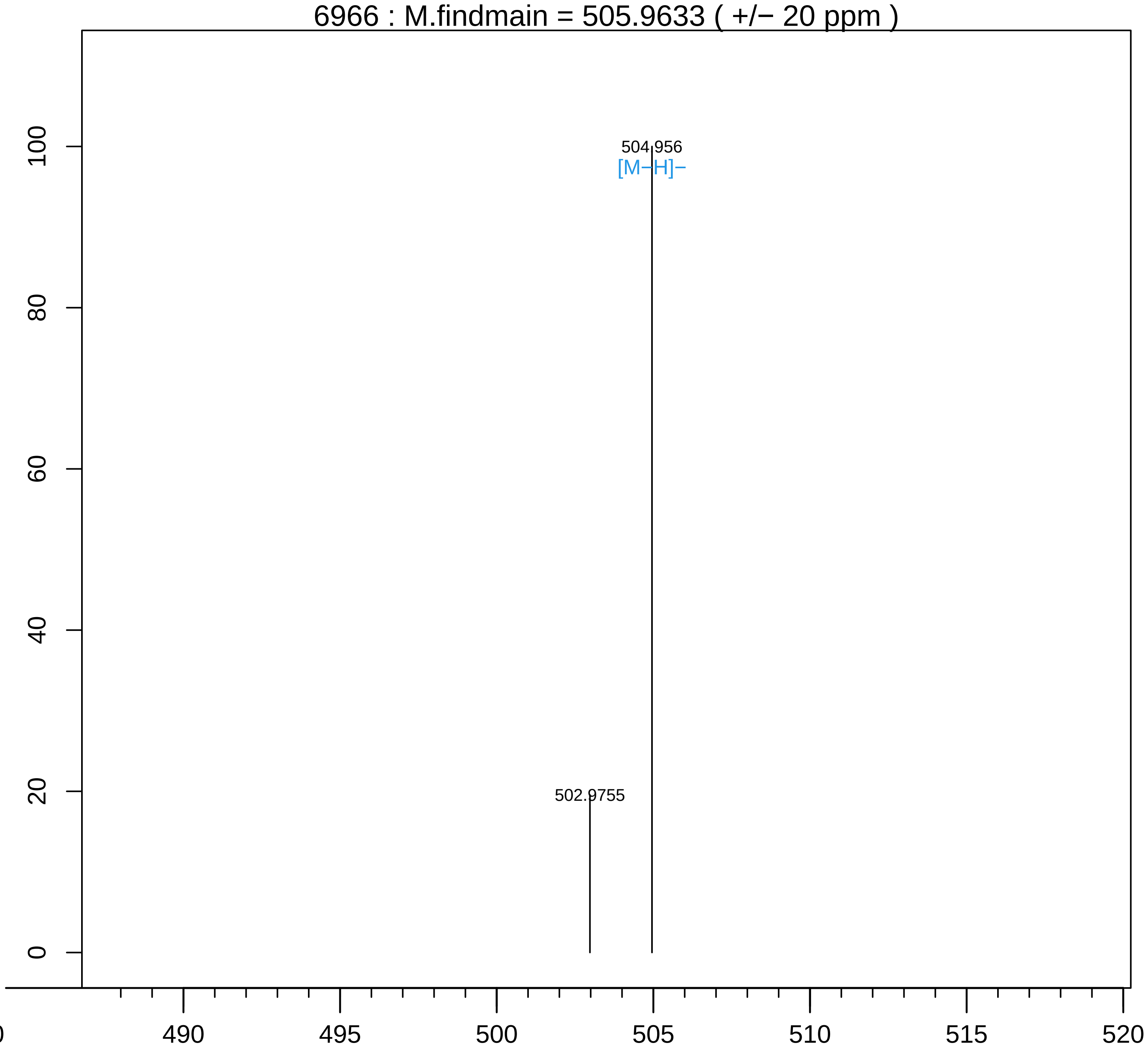
6966 : M.findmain = 505.9633 (+/− 20 ppm)
504.956
[M−H]−
502.9755
0
20
40
60
80
100
490
495
500
505
510
515
520

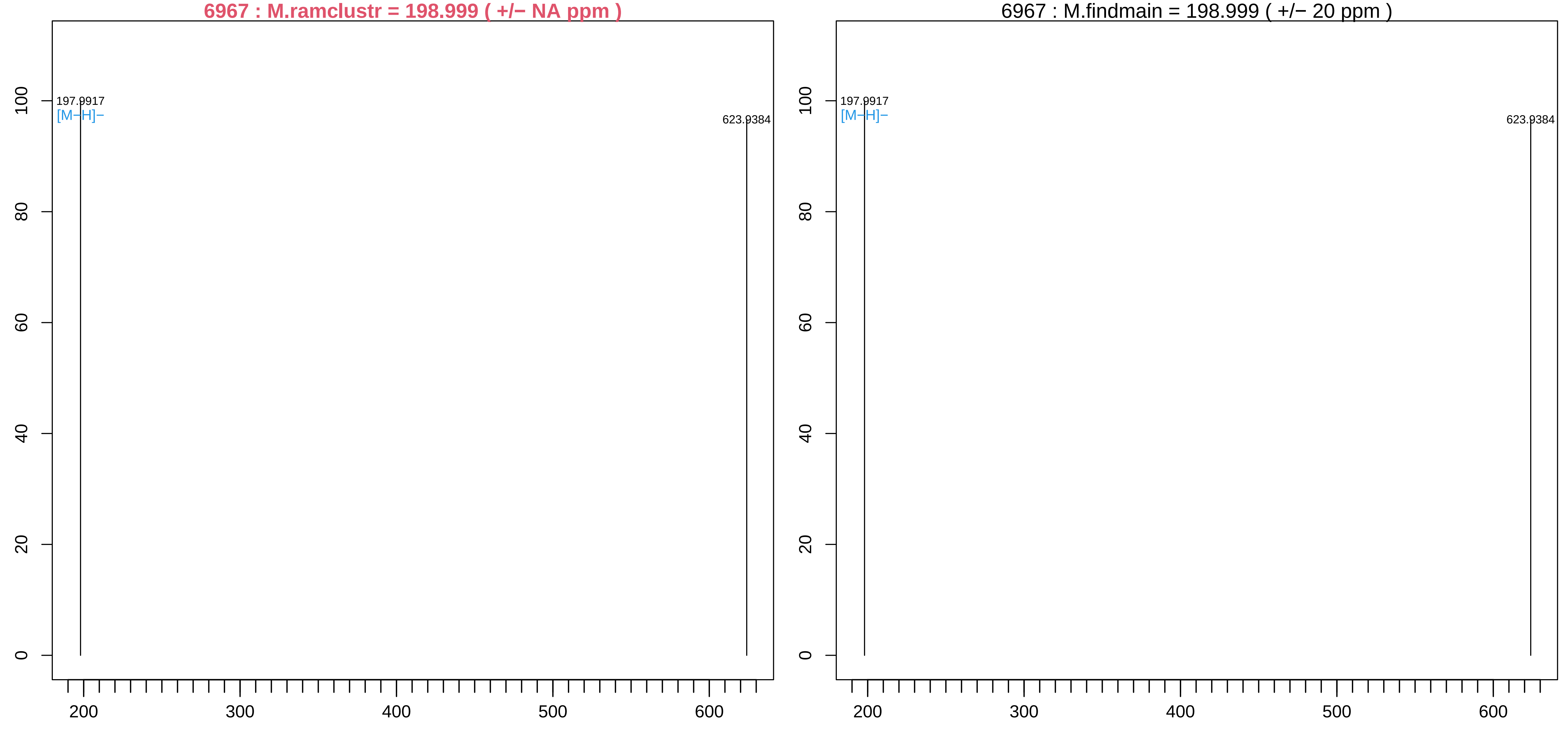
6967 : M.ramclustr = 198.999 (+/− NA ppm)
197.9917
[M−H]−
623.9384
6967 : M.findmain = 198.999 (+/− 20 ppm)
197.9917
[M−H]−
623.9384
0
20
40
60
80
100
200
300
400
500
600

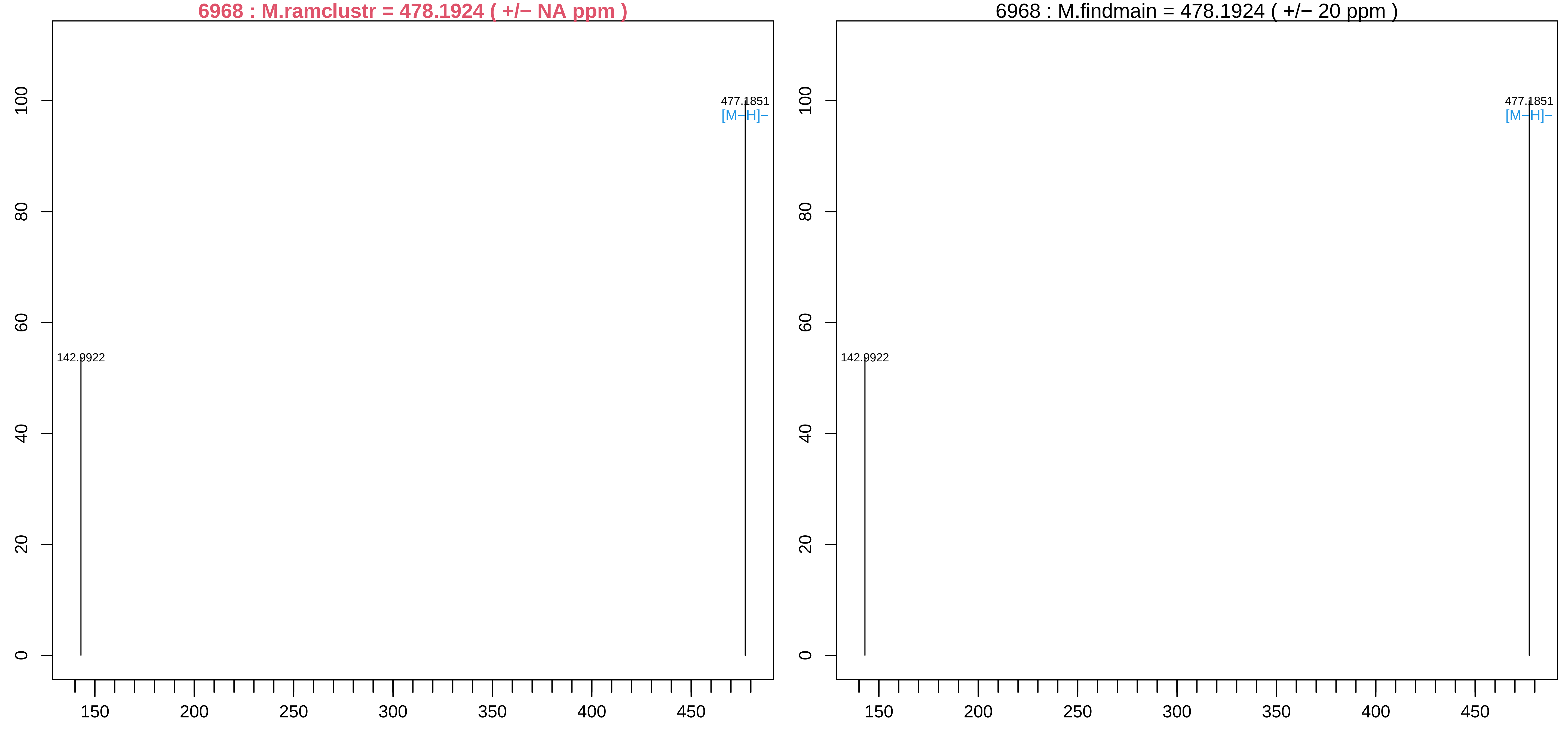
6968 : M.ramclustr = 478.1924 (+/− NA ppm)
6968 : M.findmain = 478.1924 (+/− 20 ppm)
477.1851
[M−H]−
142.9922
477.1851
[M−H]−
142.9922
0
20
40
60
80
100
150
200
250
300
350
400
450

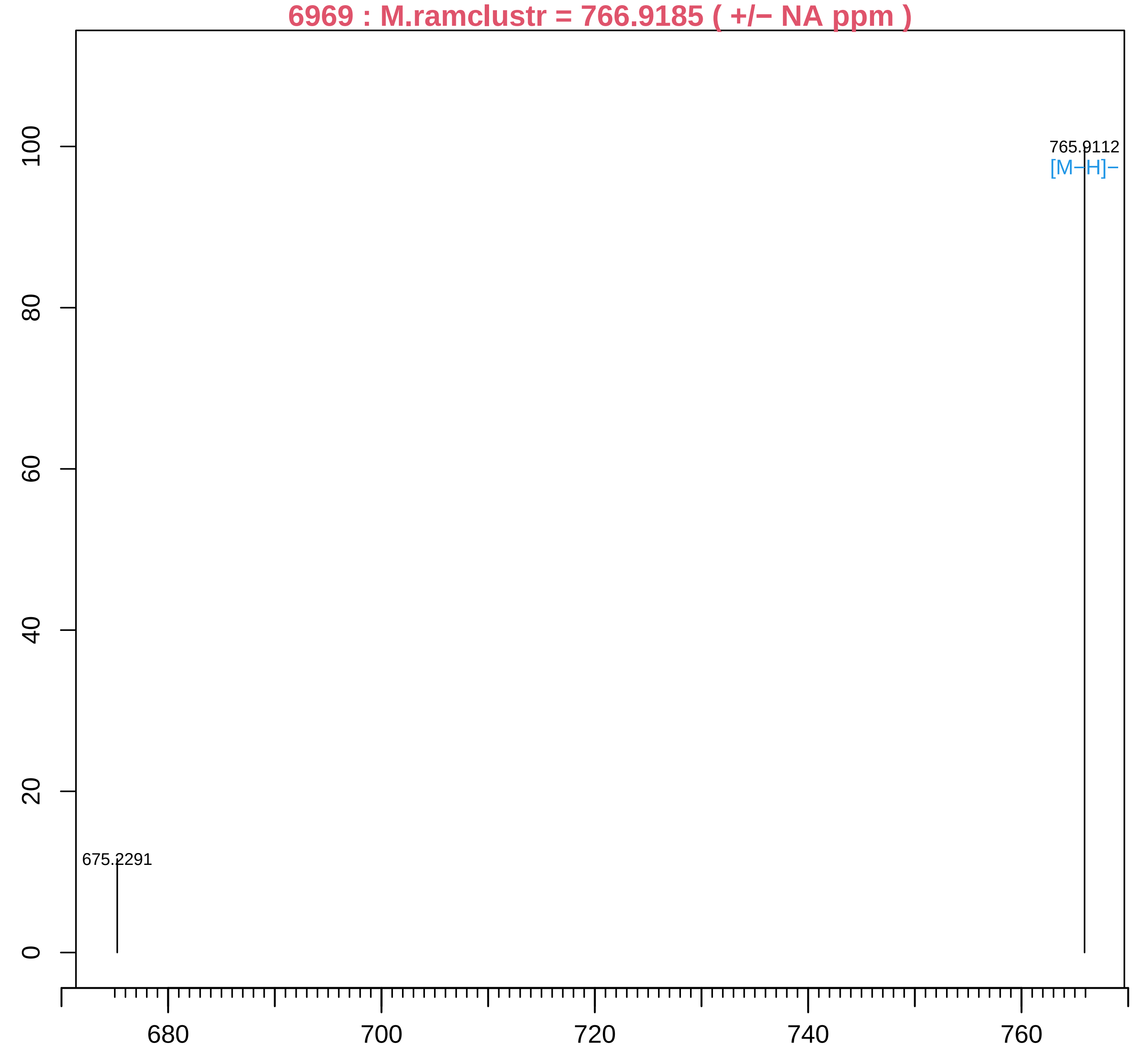
6969 : M.ramclustr = 766.9185 (+/− NA ppm)
765.9112
[M−H]−
675.2291
0
20
40
60
80
100
680
700
720
740
760

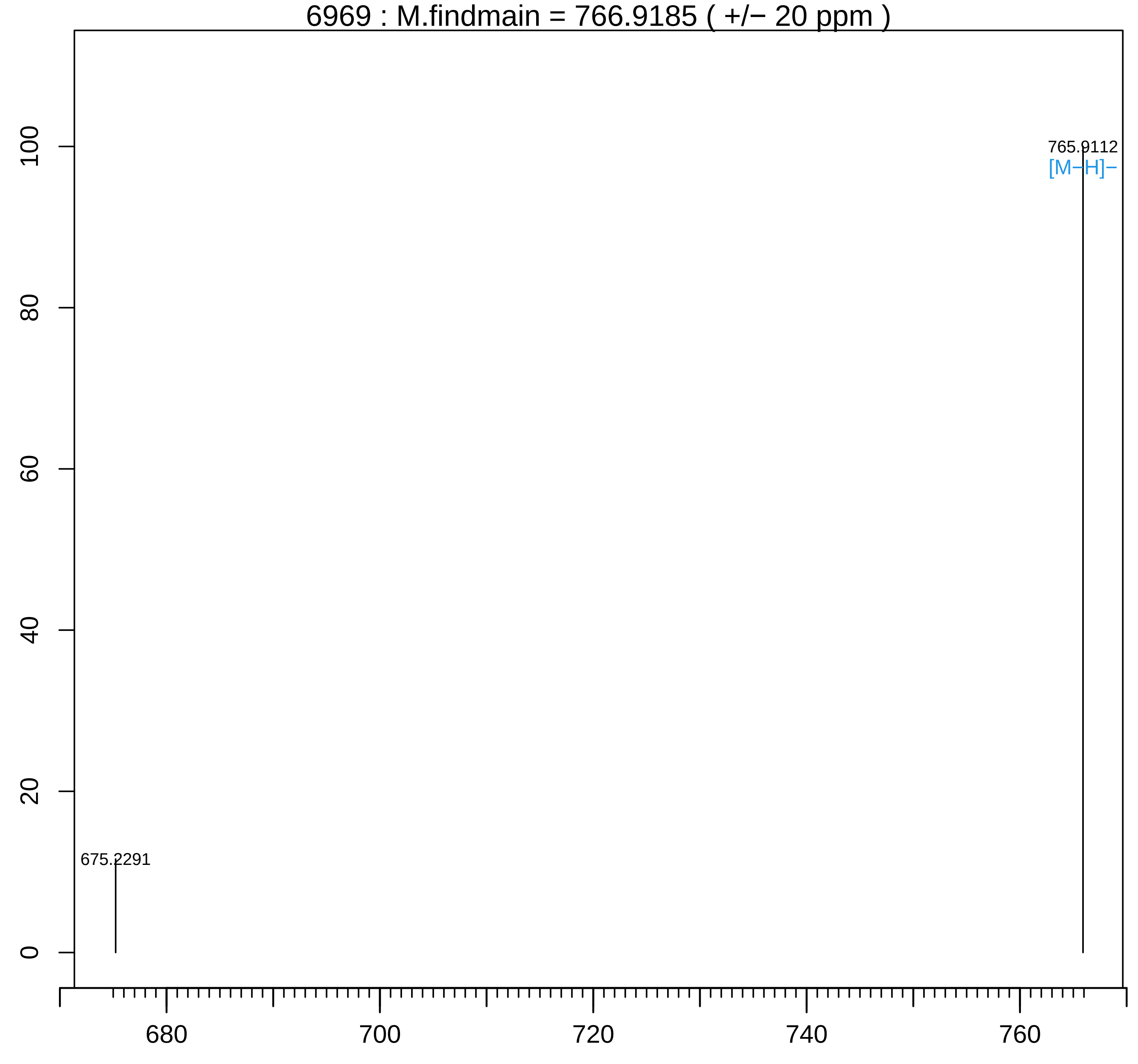
6969 : M.findmain = 766.9185 (+/− 20 ppm)
765.9112
[M−H]−
675.2291
0
20
40
60
80
100
680
700
720
740
760

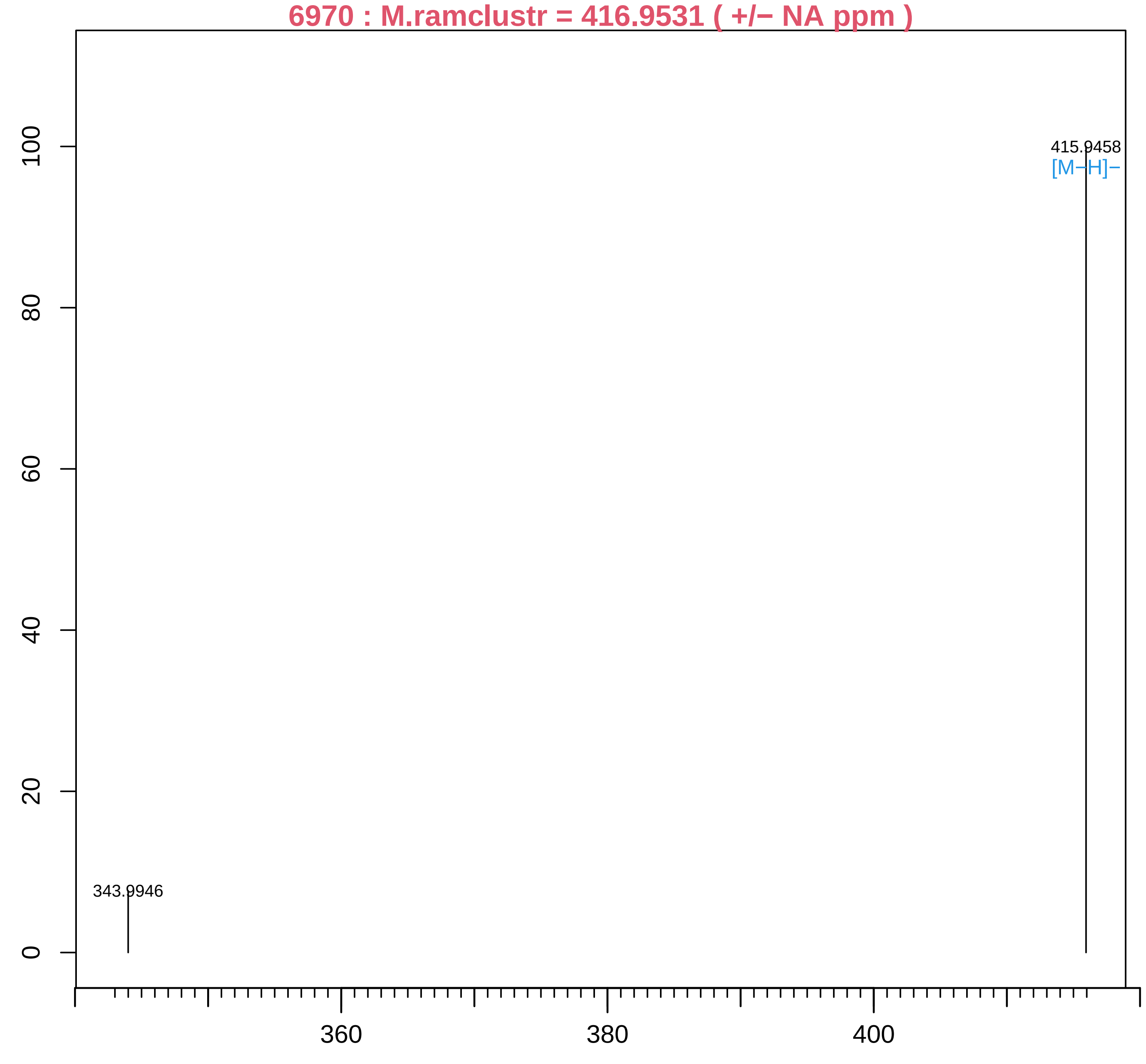

6970 : M.ramclustr = 416.9531 (+/− NA ppm)
415.9458
[M−H]−
343.9946
0
20
40
60
80
100
360
380
400

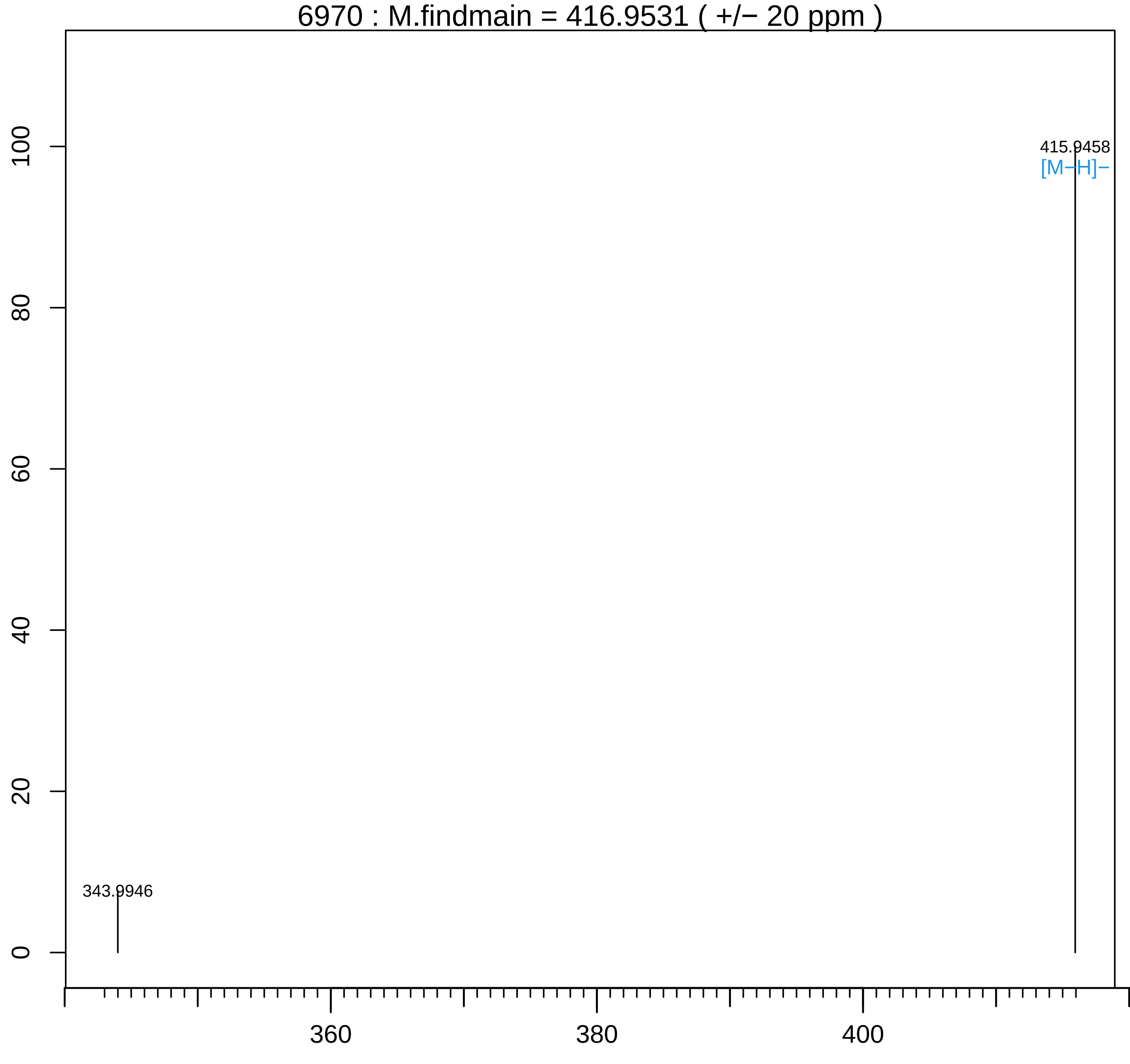

6970 : M.findmain = 416.9531 (+/− 20 ppm)
415.9458
[M−H]−
343.9946
0
20
40
60
80
100
360
380
400

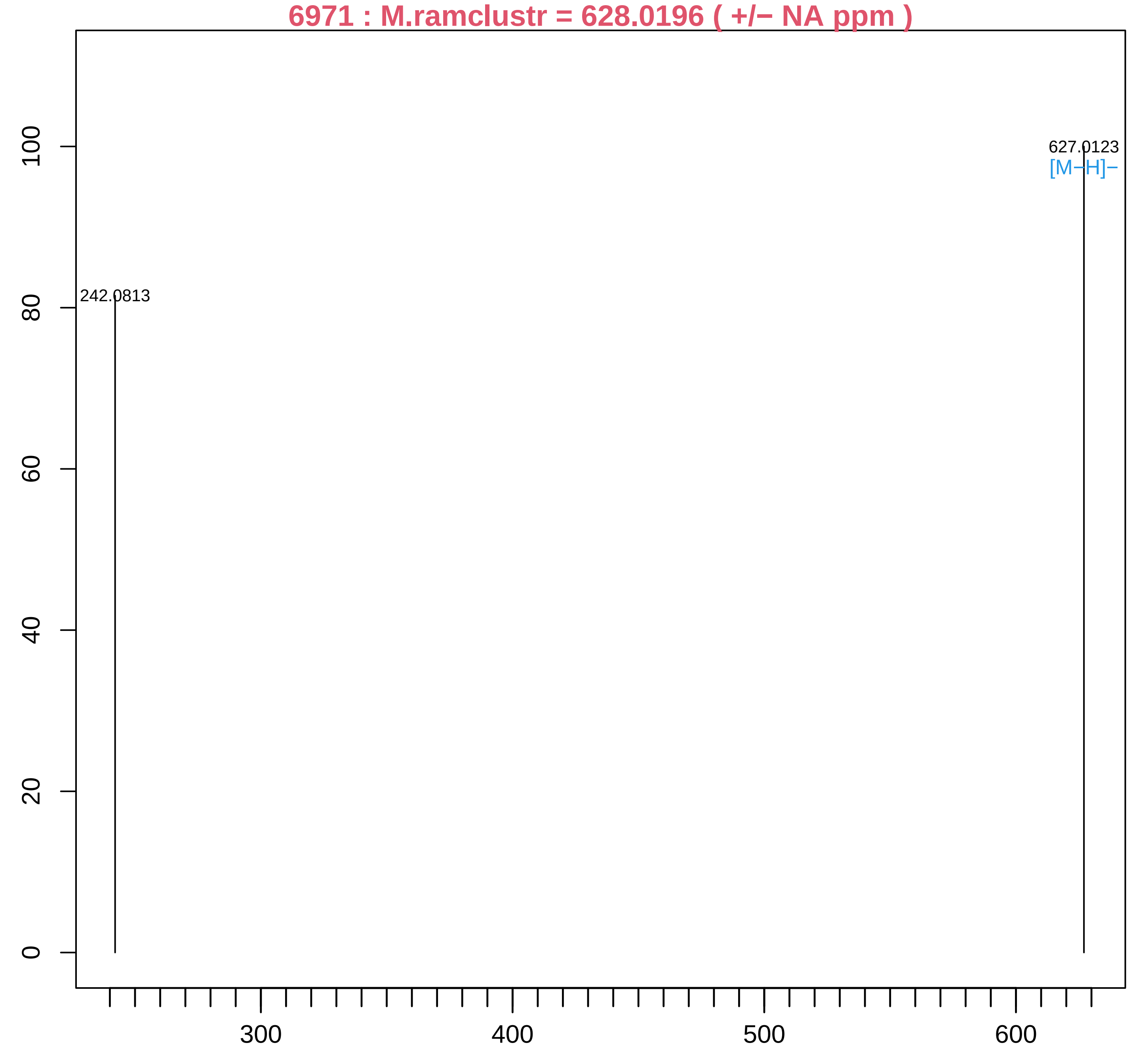
6971 : M.ramclustr = 628.0196 (+/− NA ppm)
627.0123
[M−H]−
242.0813
0
20
40
60
80
100
300
400
500
600

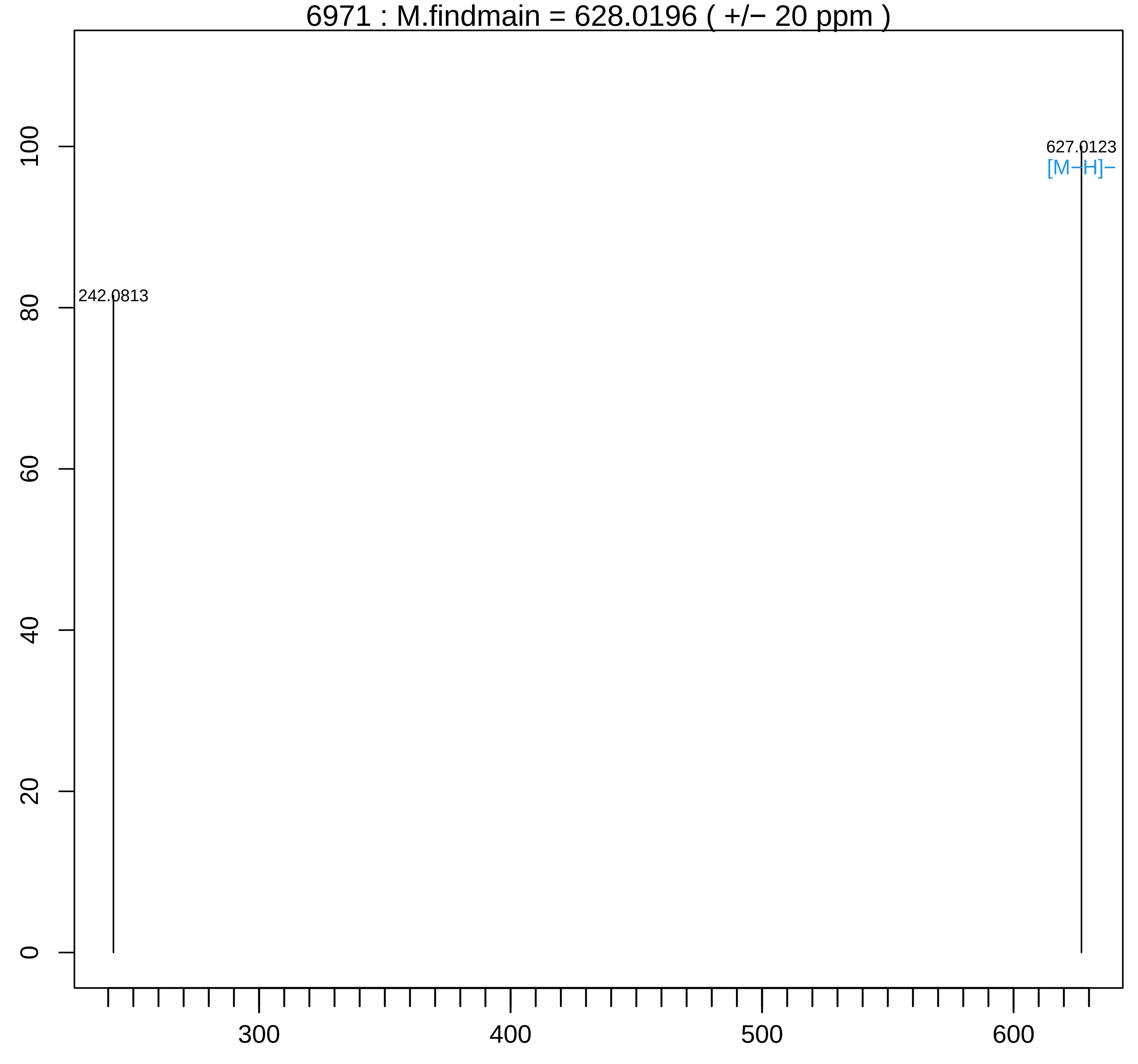
6971 : M.findmain = 628.0196 (+/− 20 ppm)
627.0123
[M−H]−
242.0813
0
20
40
60
80
100
300
400
500
600

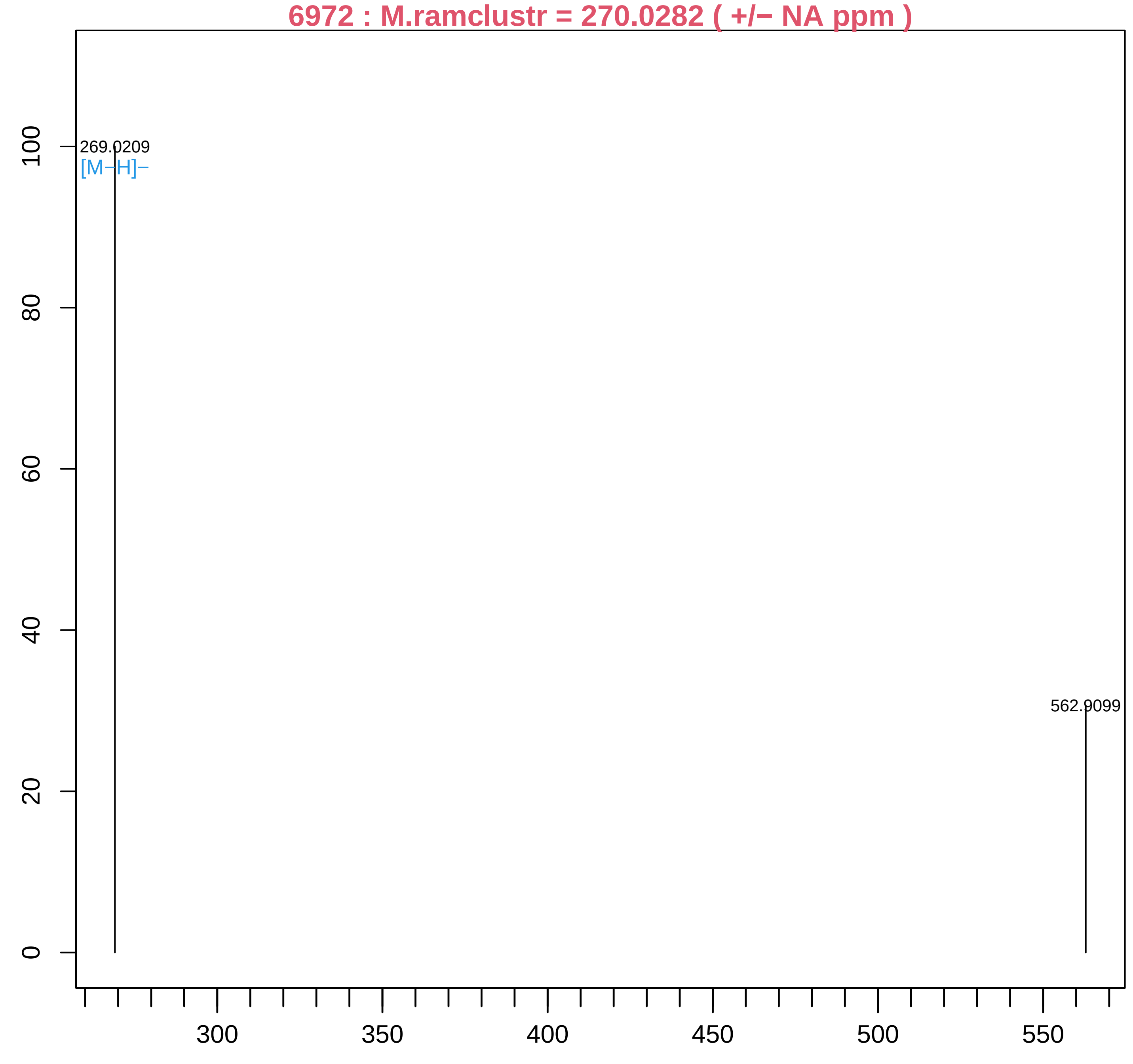
6972 : M.ramclustr = 270.0282 (+/− NA ppm)
269.0209
[M−H]−
562.9099
0
20
40
60
80
100
300
350
400
450
500
550

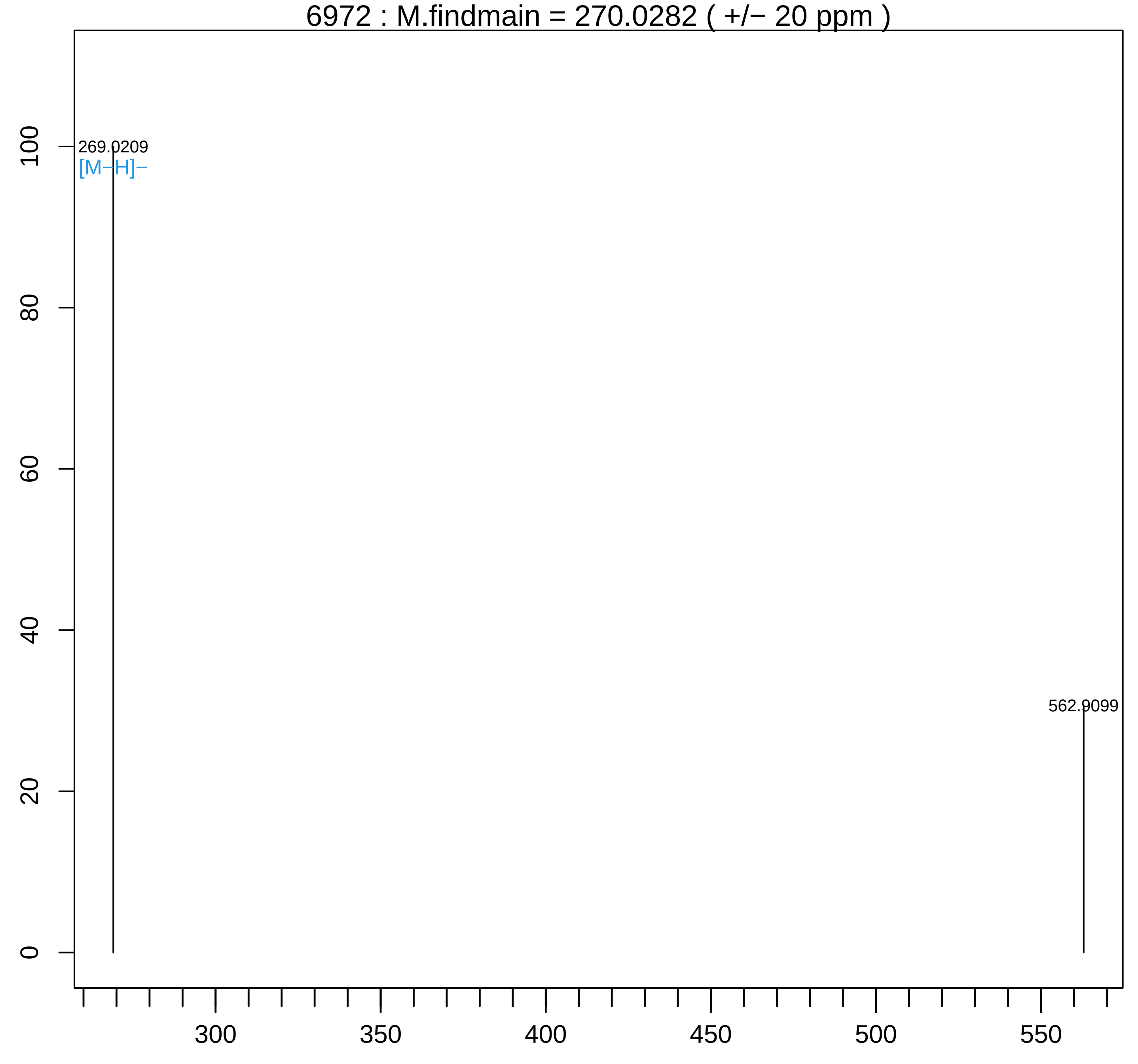
6972 : M.findmain = 270.0282 (+/− 20 ppm)
269.0209
[M−H]−
562.9099
0
20
40
60
80
100
300
350
400
450
500
550

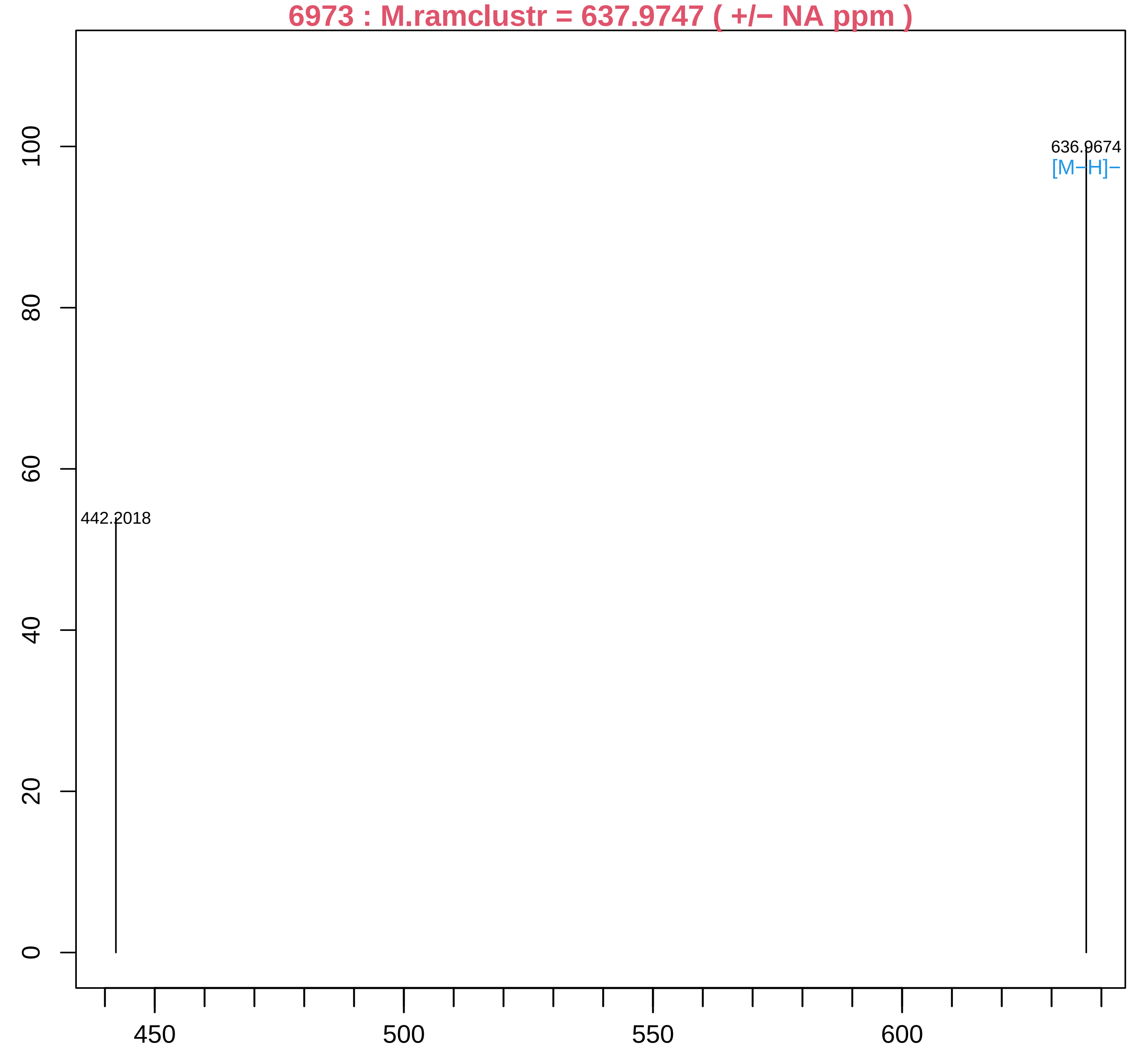
6973 : M.ramclustr = 637.9747 (+/− NA ppm)
636.9674
[M−H]−
442.2018
0
20
40
60
80
100
450
500
550
600

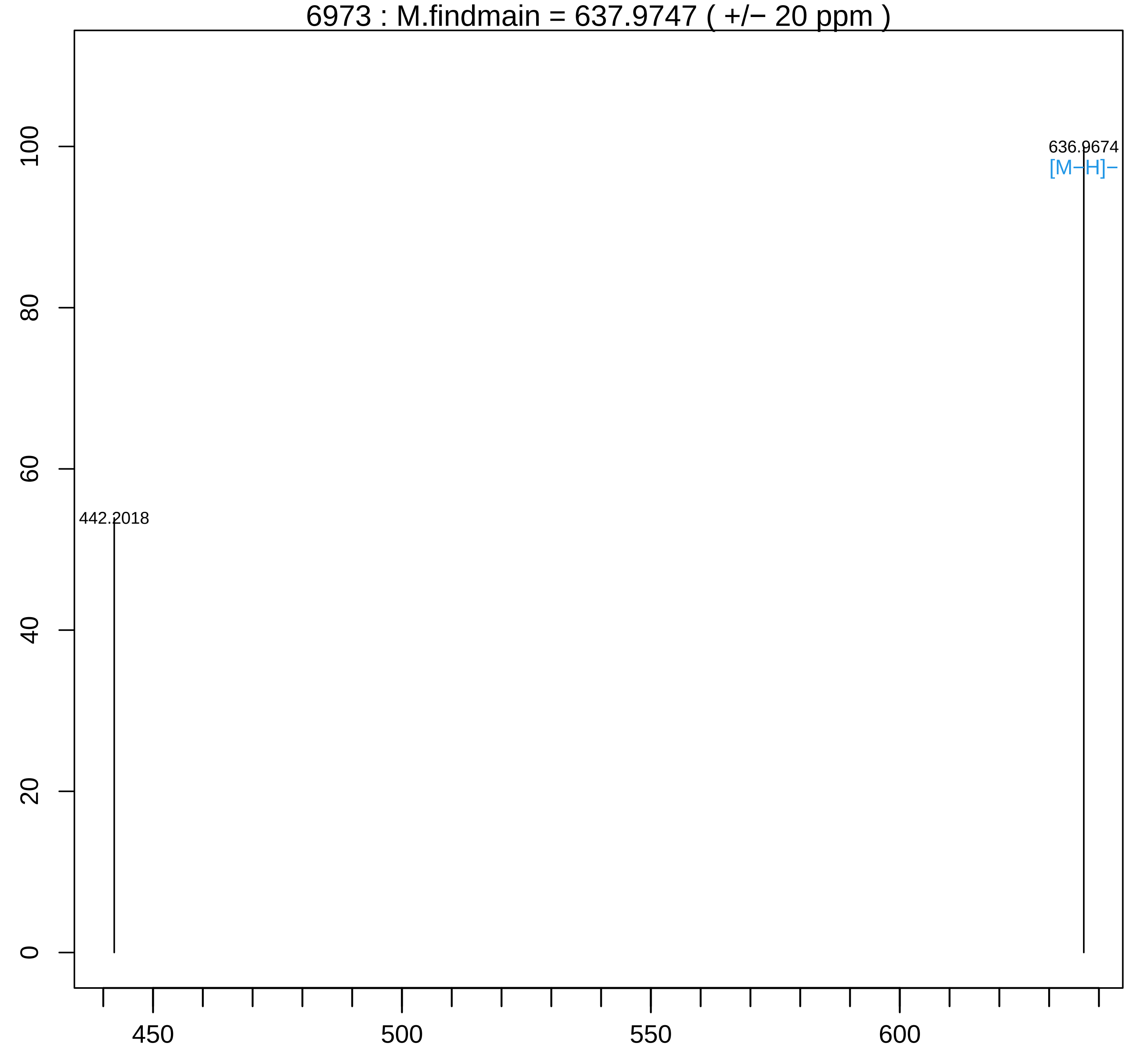
6973 : M.findmain = 637.9747 (+/− 20 ppm)
636.9674
[M−H]−
442.2018
0
20
40
60
80
100
450
500
550
600

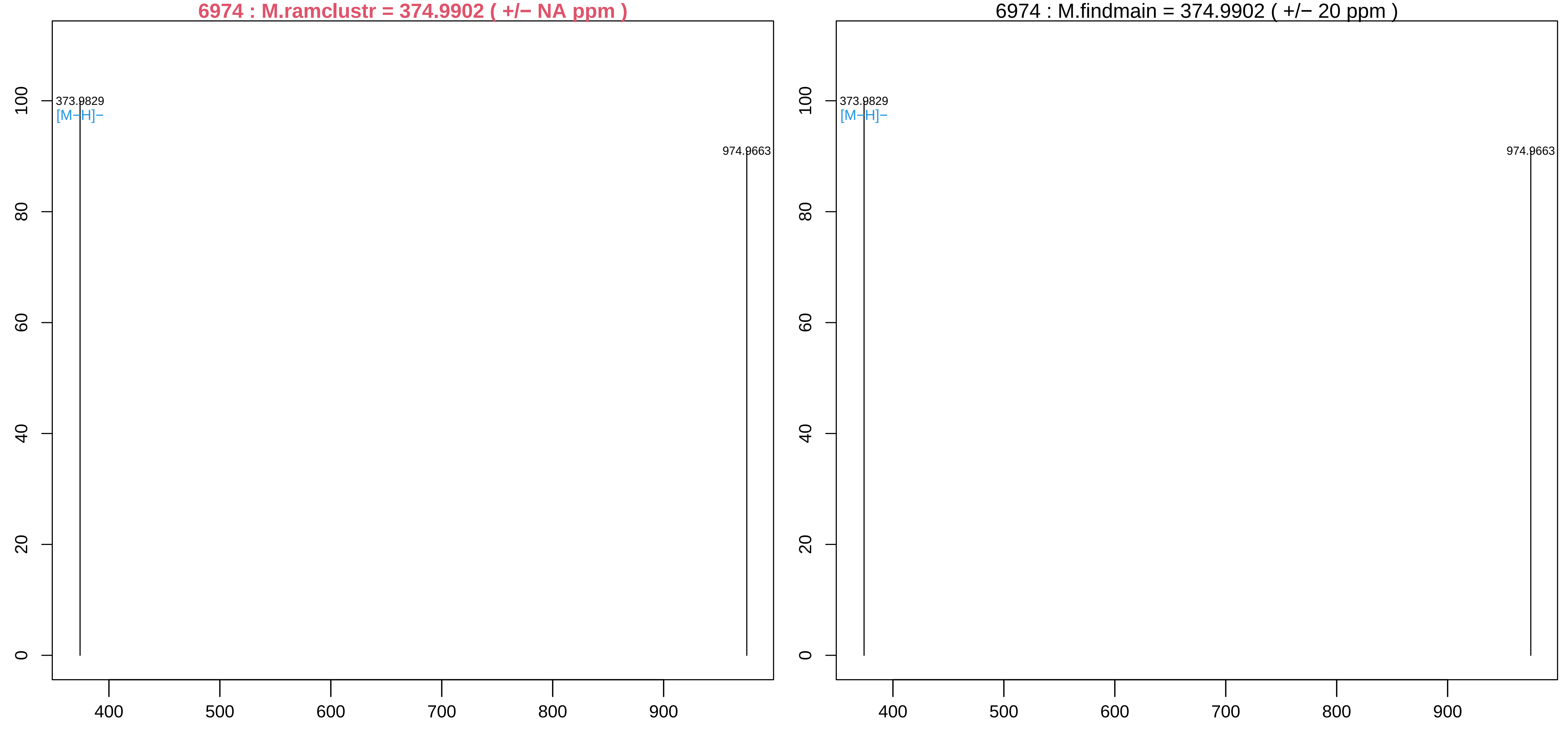
6974 : M.ramclustr = 374.9902 (+/− NA ppm)
373.9829
[M−H]−
974.9663
0
20
40
60
80
100
400
500
600
700
800
900
6974 : M.findmain = 374.9902 (+/− 20 ppm)
373.9829
[M−H]−
974.9663
0
20
40
60
80
100
400
500
600
700
800
900

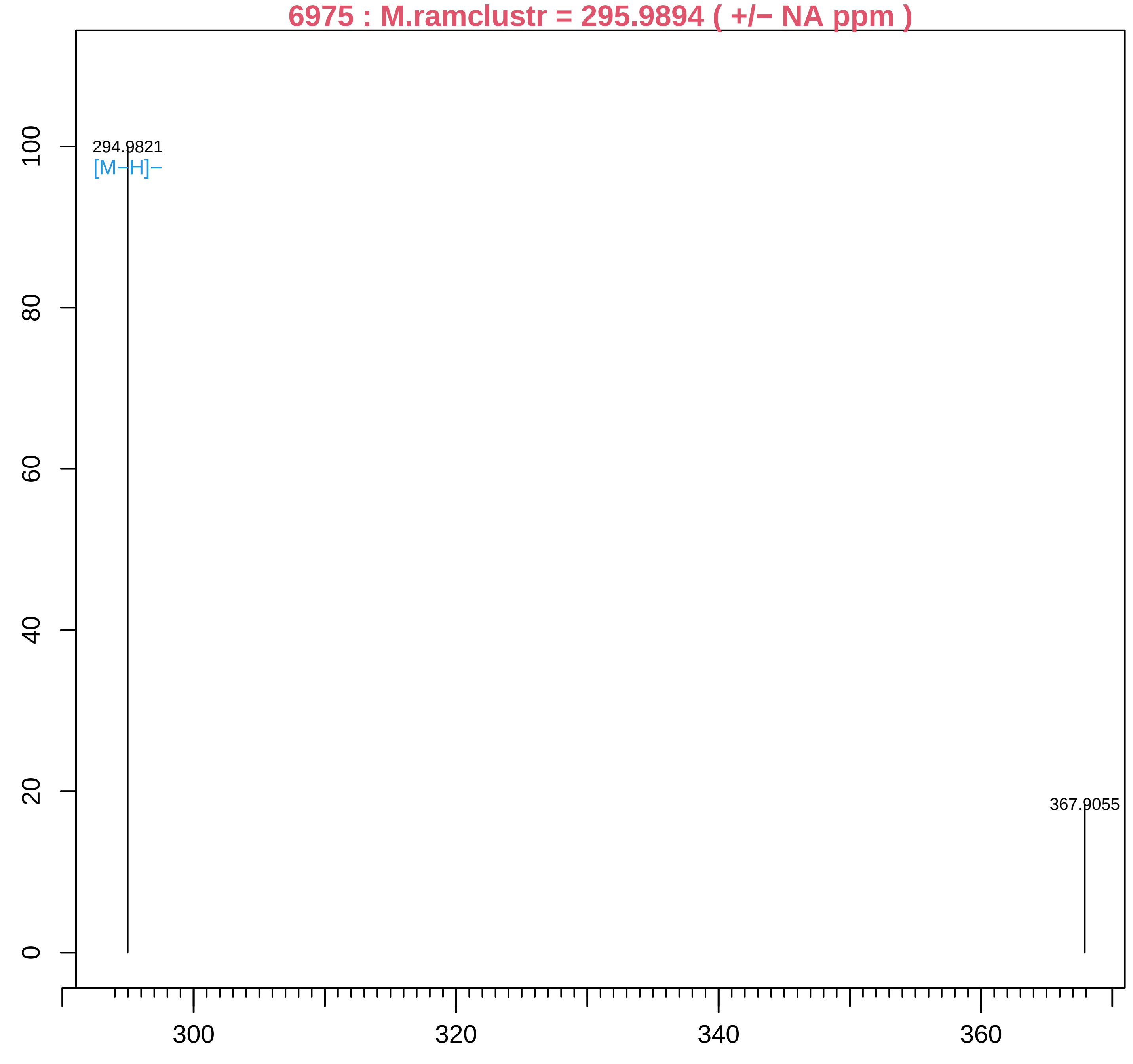

6975 : M.ramclustr = 295.9894 (+/− NA ppm)
294.9821
[M−H]−
367.9055
0
20
40
60
80
100
300
320
340
360

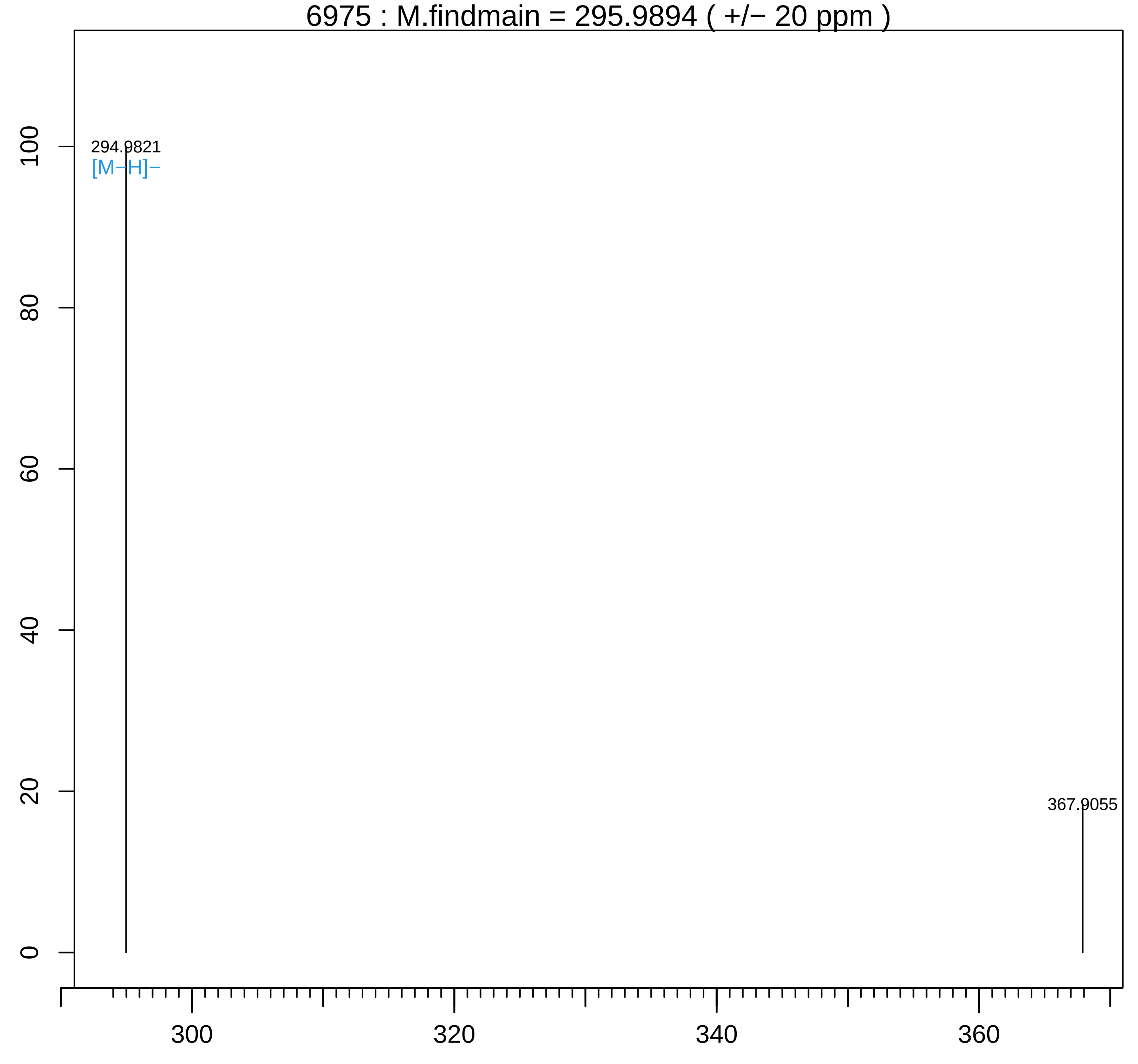

6975 : M.findmain = 295.9894 (+/− 20 ppm)
294.9821
[M−H]−
367.9055
0
20
40
60
80
100
300
320
340
360

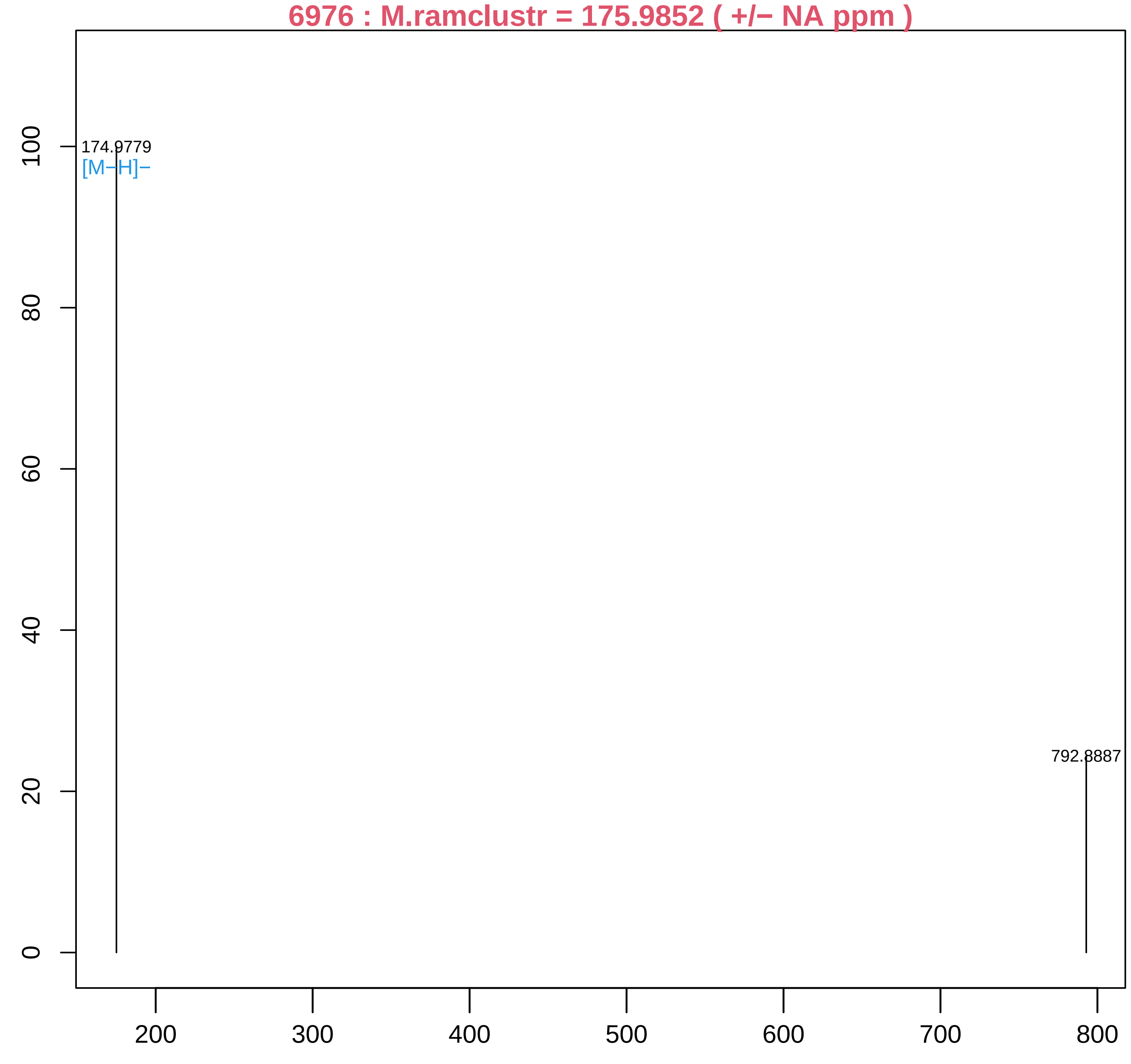
6976 : M.ramclustr = 175.9852 (+/− NA ppm)
174.9779
[M−H]−
792.8887
0
20
40
60
80
100
200
300
400
500
600
700
800

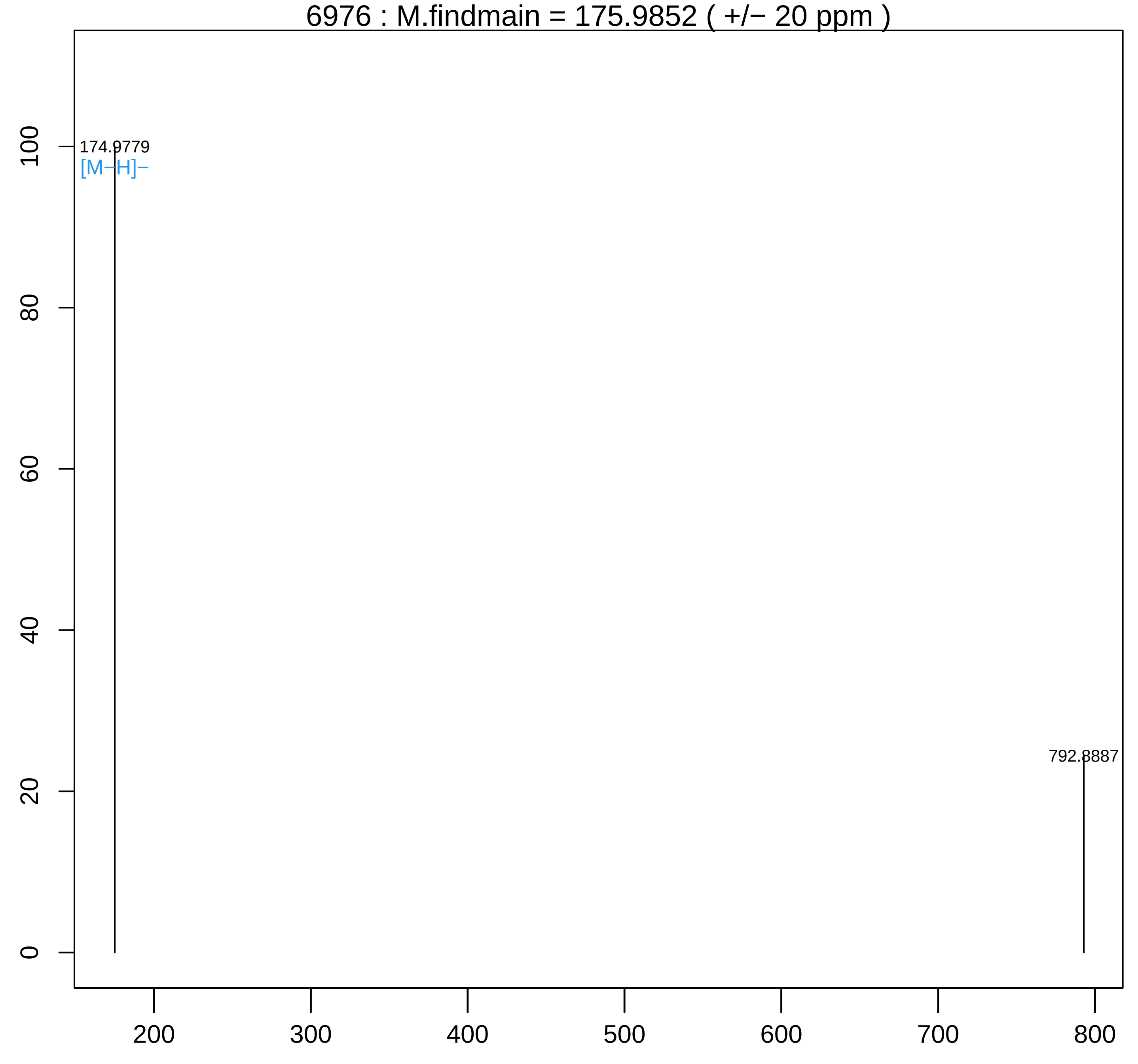
6976 : M.findmain = 175.9852 (+/− 20 ppm)
174.9779
[M−H]−
792.8887
0
20
40
60
80
100
200
300
400
500
600
700
800

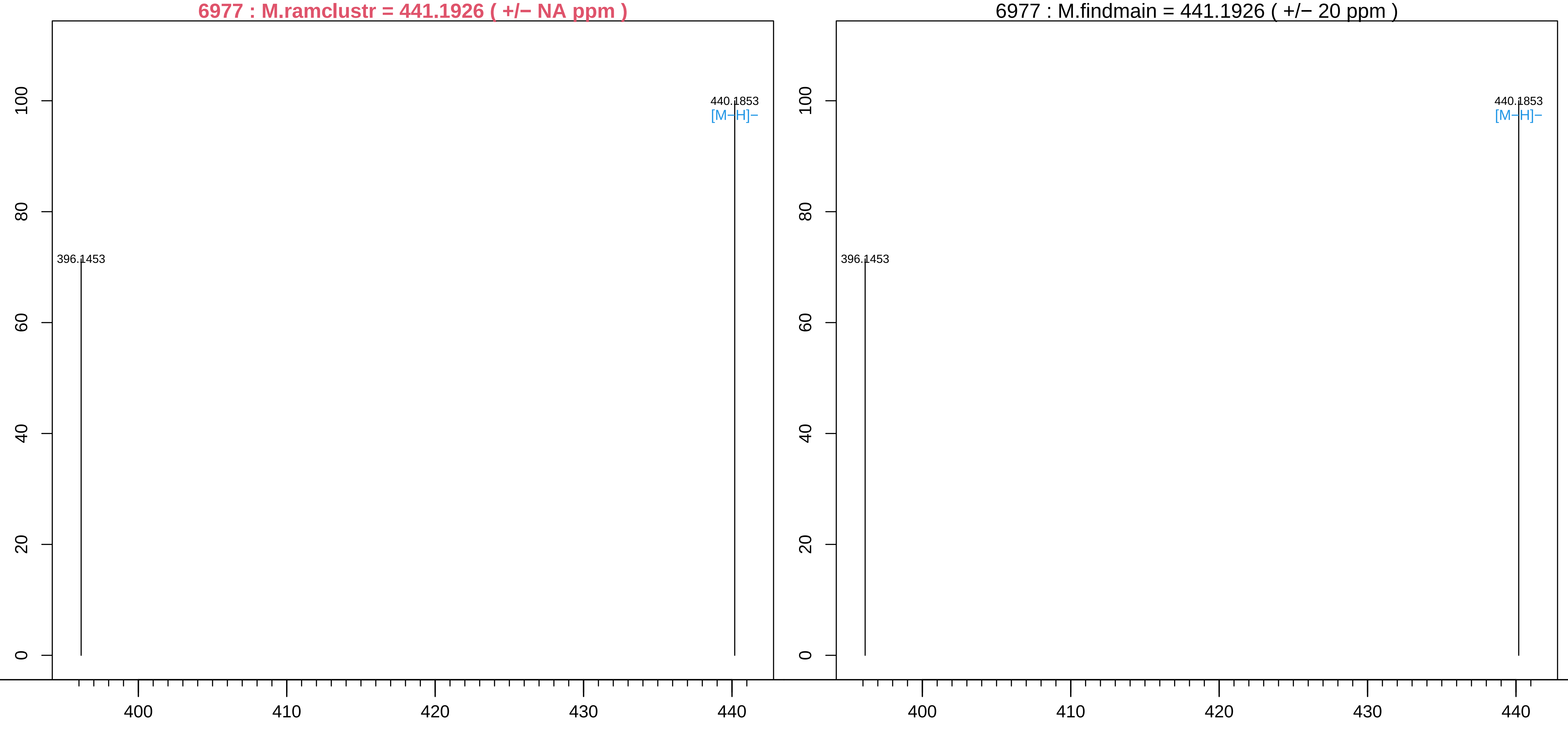
6977 : M.ramclustr = 441.1926 (+/− NA ppm)
440.1853
[M−H]−
396.1453
6977 : M.findmain = 441.1926 (+/− 20 ppm)
440.1853
[M−H]−
396.1453
0
20
40
60
80
100
400
410
420
430
440

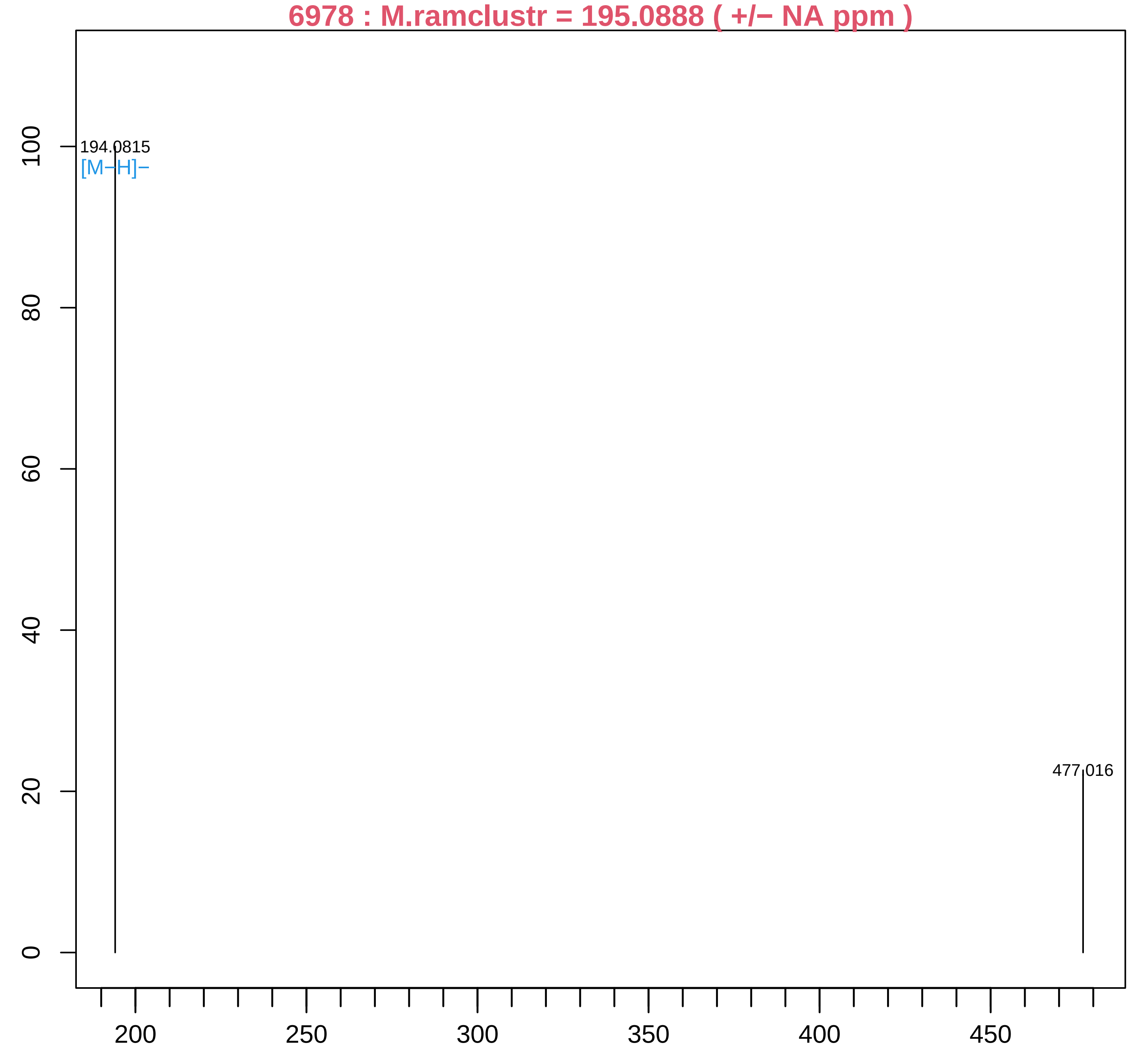
6978 : M.ramclustr = 195.0888 (+/− NA ppm)
194.0815
[M−H]−
477.016
0
20
40
60
80
100
200
250
300
350
400
450

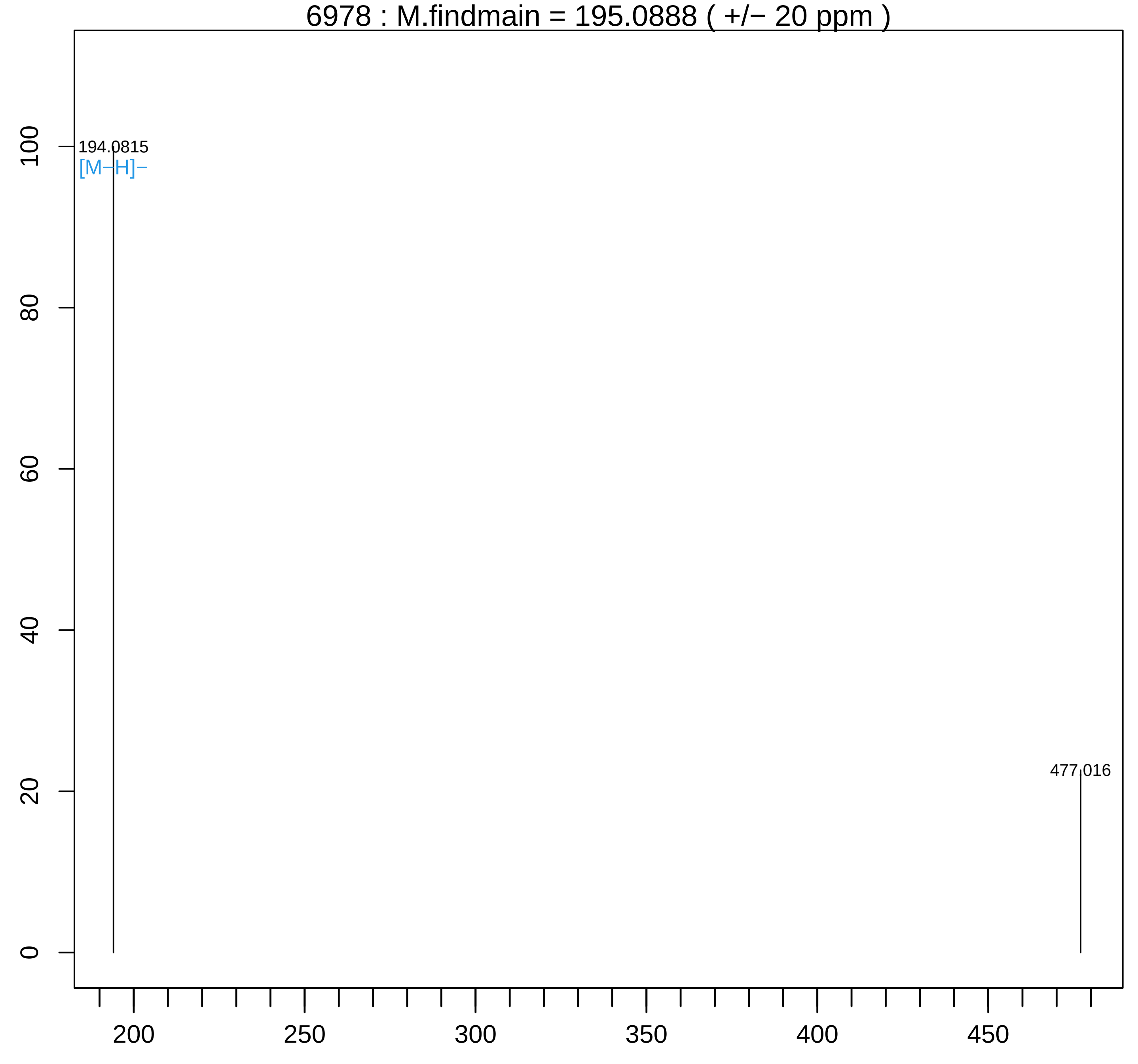
6978 : M.findmain = 195.0888 (+/− 20 ppm)
194.0815
[M−H]−
477.016
0
20
40
60
80
100
200
250
300
350
400
450

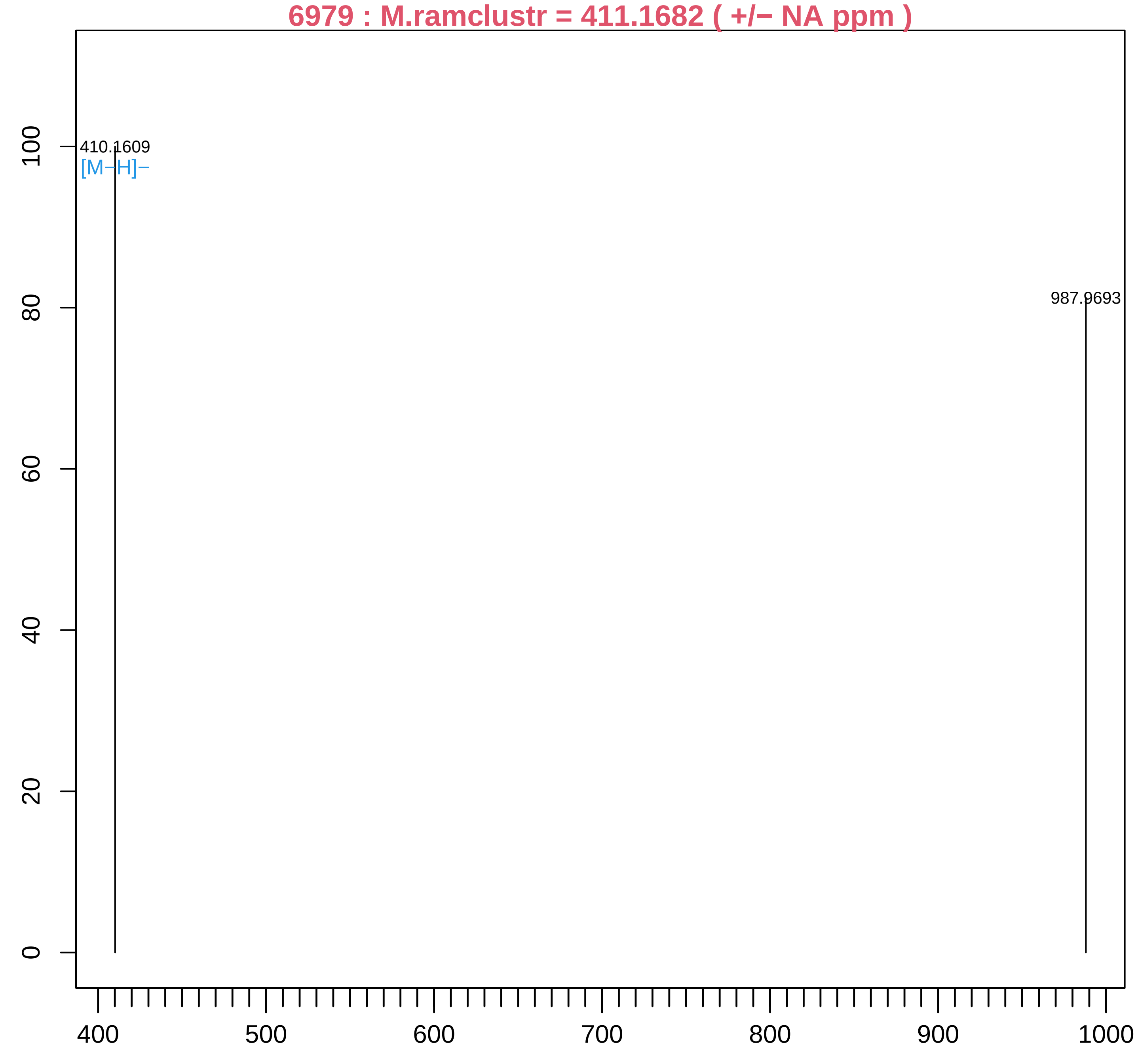
6979 : M.ramclustr = 411.1682 (+/− NA ppm)
410.1609
[M−H]−
987.9693
0
20
40
60
80
100
400
500
600
700
800
900
1000

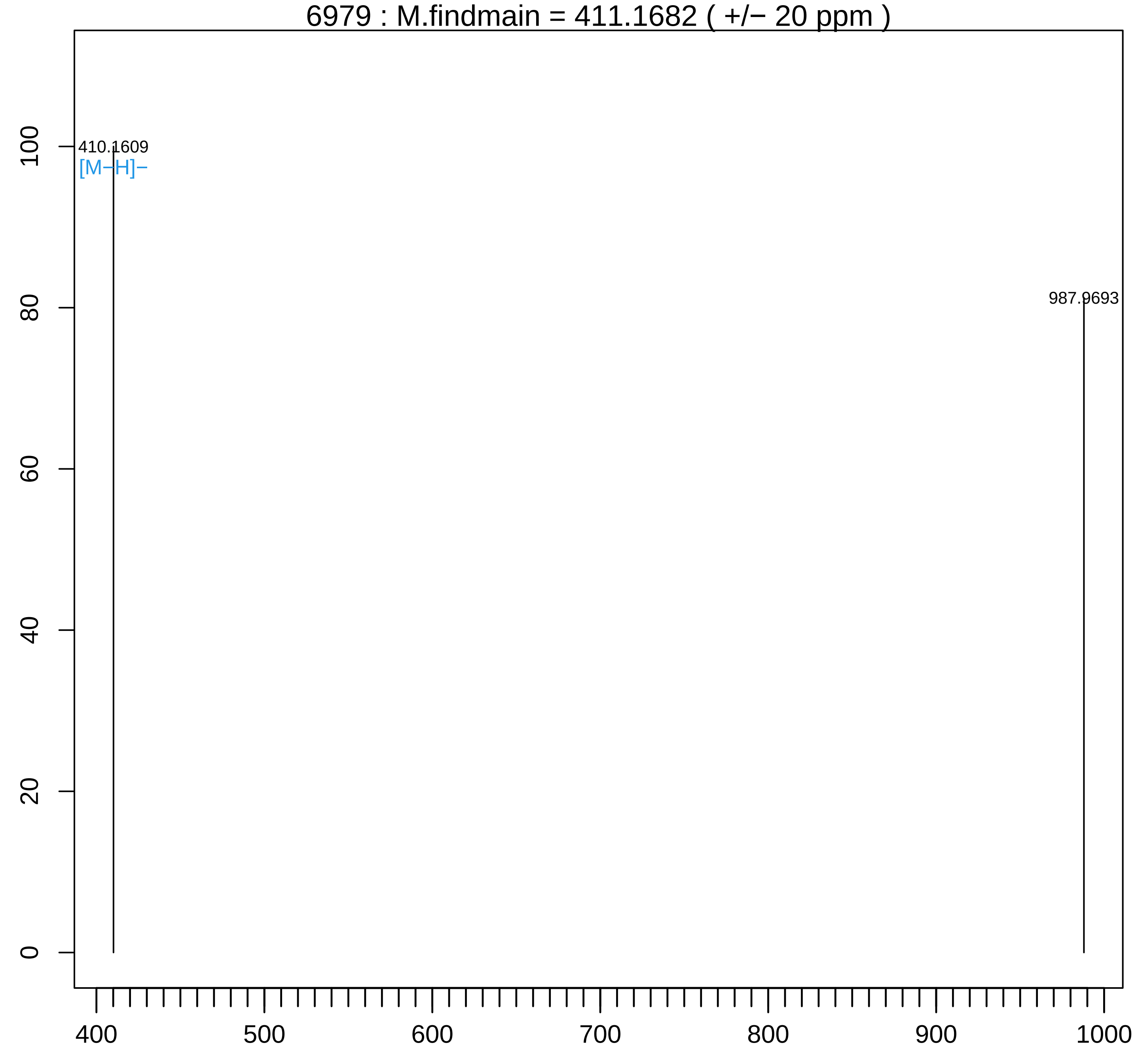
6979 : M.findmain = 411.1682 (+/− 20 ppm)
410.1609
[M−H]−
987.9693
0
20
40
60
80
100
400
500
600
700
800
900
1000

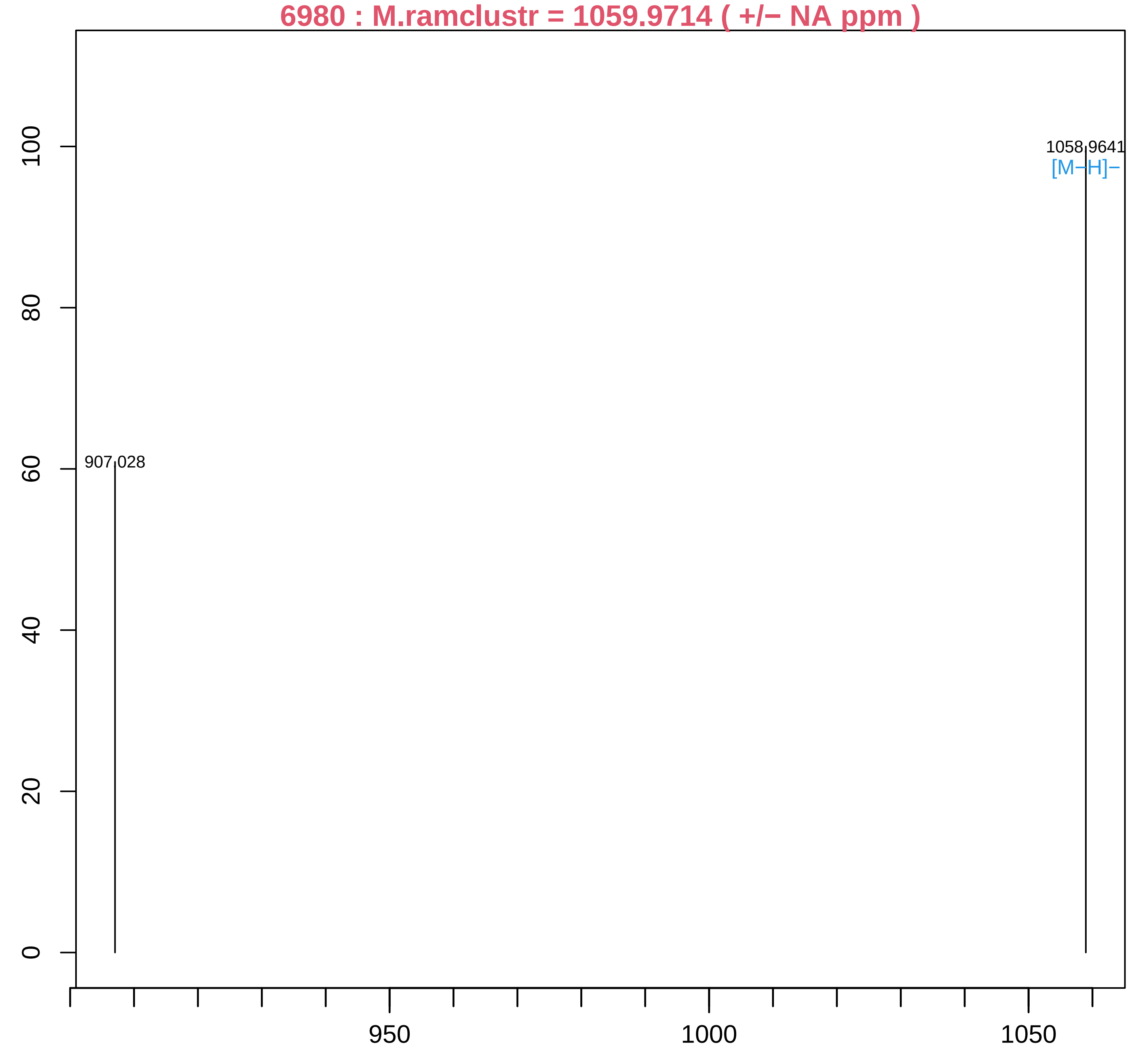
6980 : M.ramclustr = 1059.9714 (+/− NA ppm)
1058.9641
[M−H]−
907.028
0
20
40
60
80
100
950
1000
1050

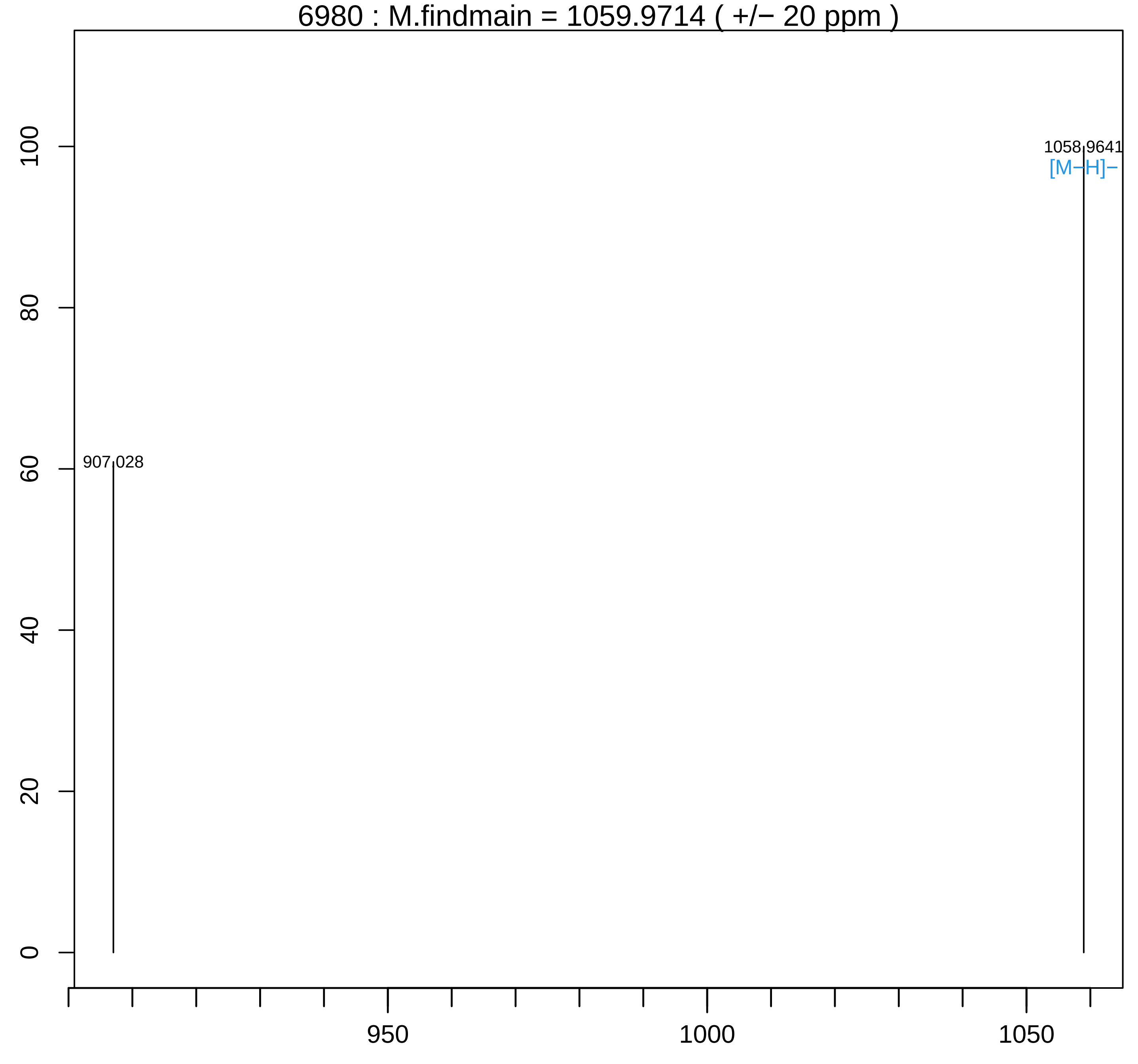
6980 : M.findmain = 1059.9714 (+/− 20 ppm)
1058.9641
[M−H]−
907.028
0
20
40
60
80
100
950
1000
1050

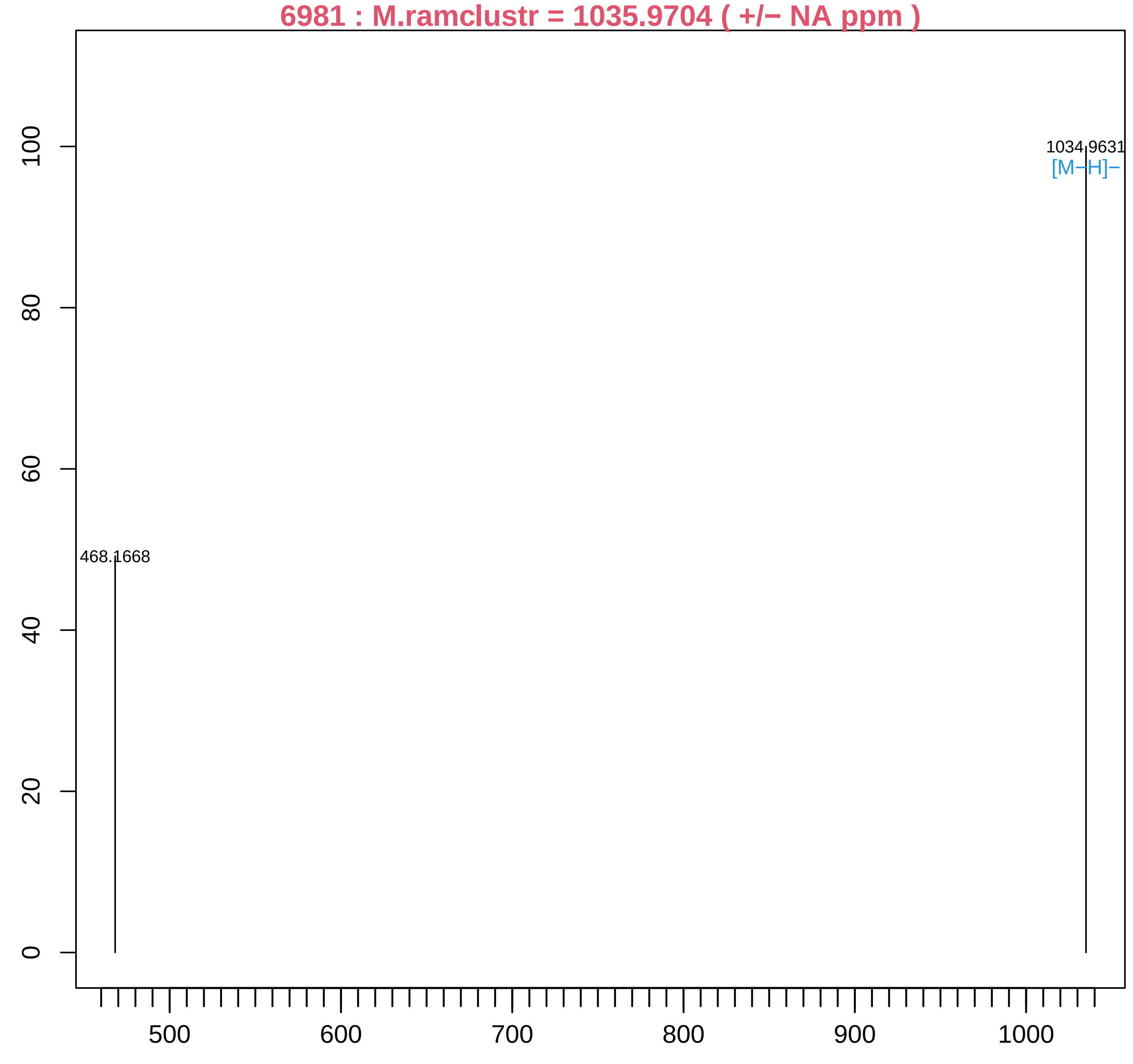
6981 : M.ramclustr = 1035.9704 (+/− NA ppm)
1034.9631
[M−H]−
468.1668
0
20
40
60
80
100
500
600
700
800
900
1000

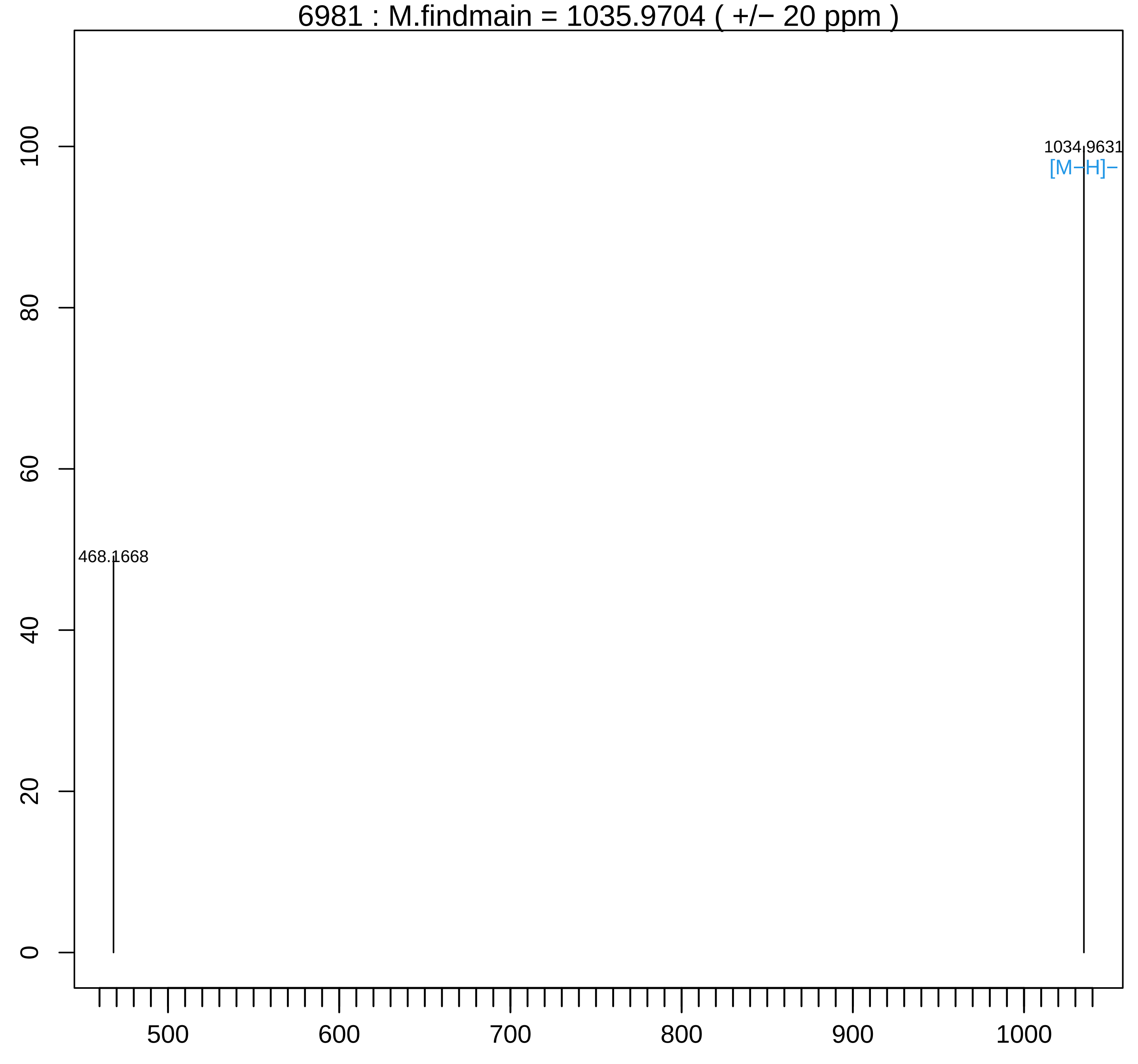
6981 : M.findmain = 1035.9704 (+/− 20 ppm)
1034.9631
[M−H]−
468.1668
0
20
40
60
80
100
500
600
700
800
900
1000

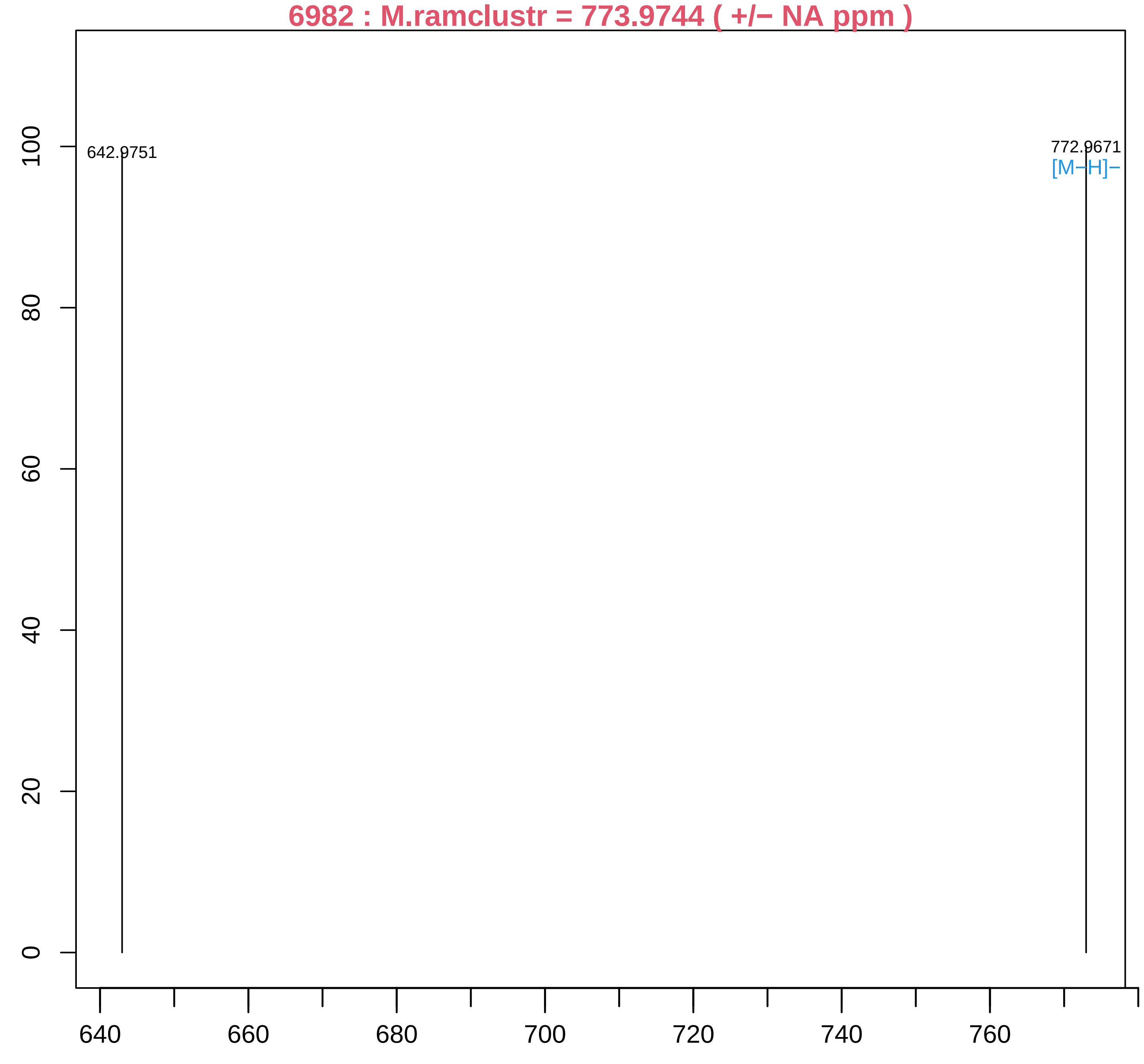
6982 : M.ramclustr = 773.9744 (+/− NA ppm)
642.9751
772.9671
[M−H]−
0
20
40
60
80
100
640
660
680
700
720
740
760

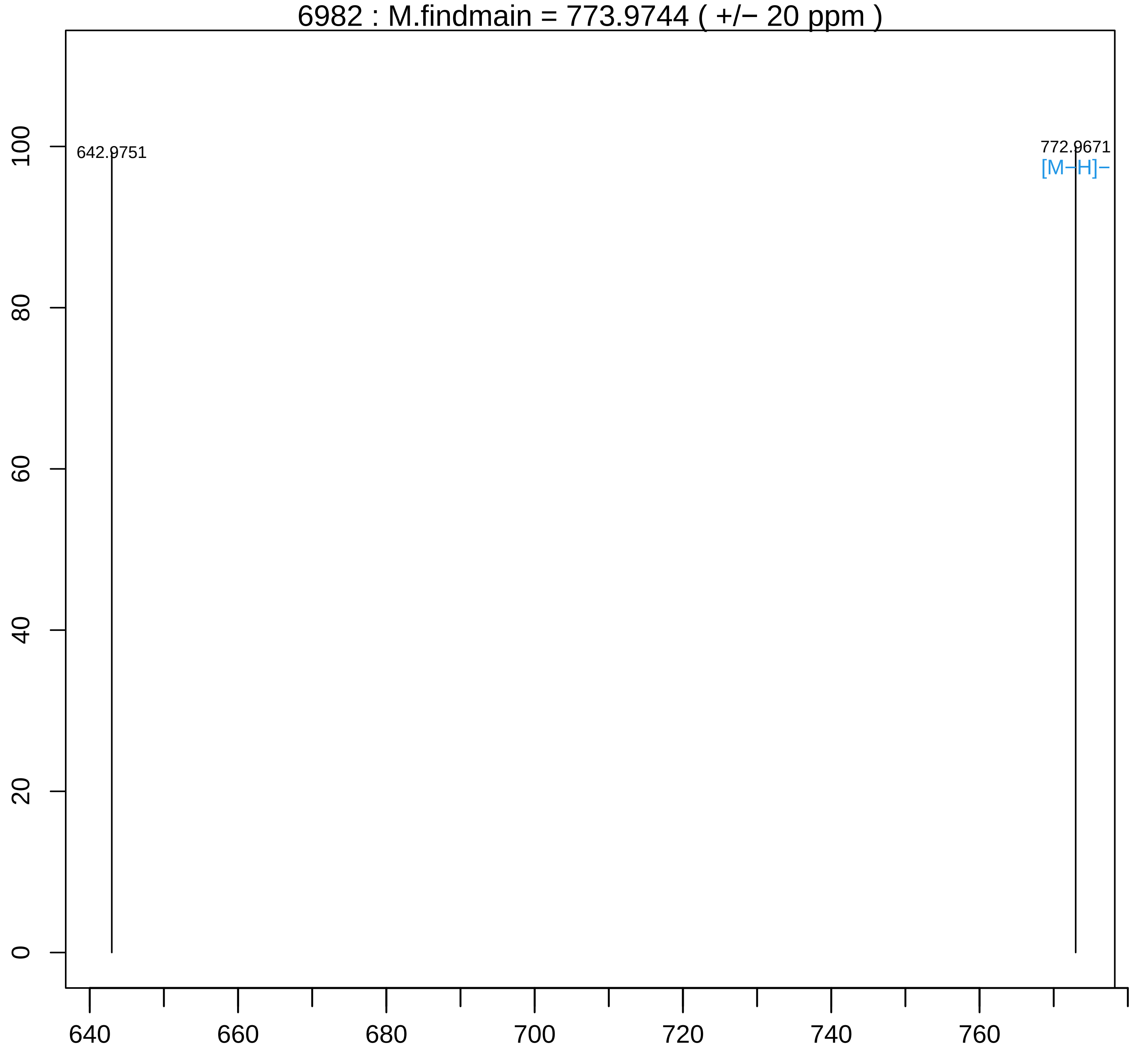
6982 : M.findmain = 773.9744 (+/− 20 ppm)
642.9751
772.9671
[M−H]−
0
20
40
60
80
100
640
660
680
700
720
740
760

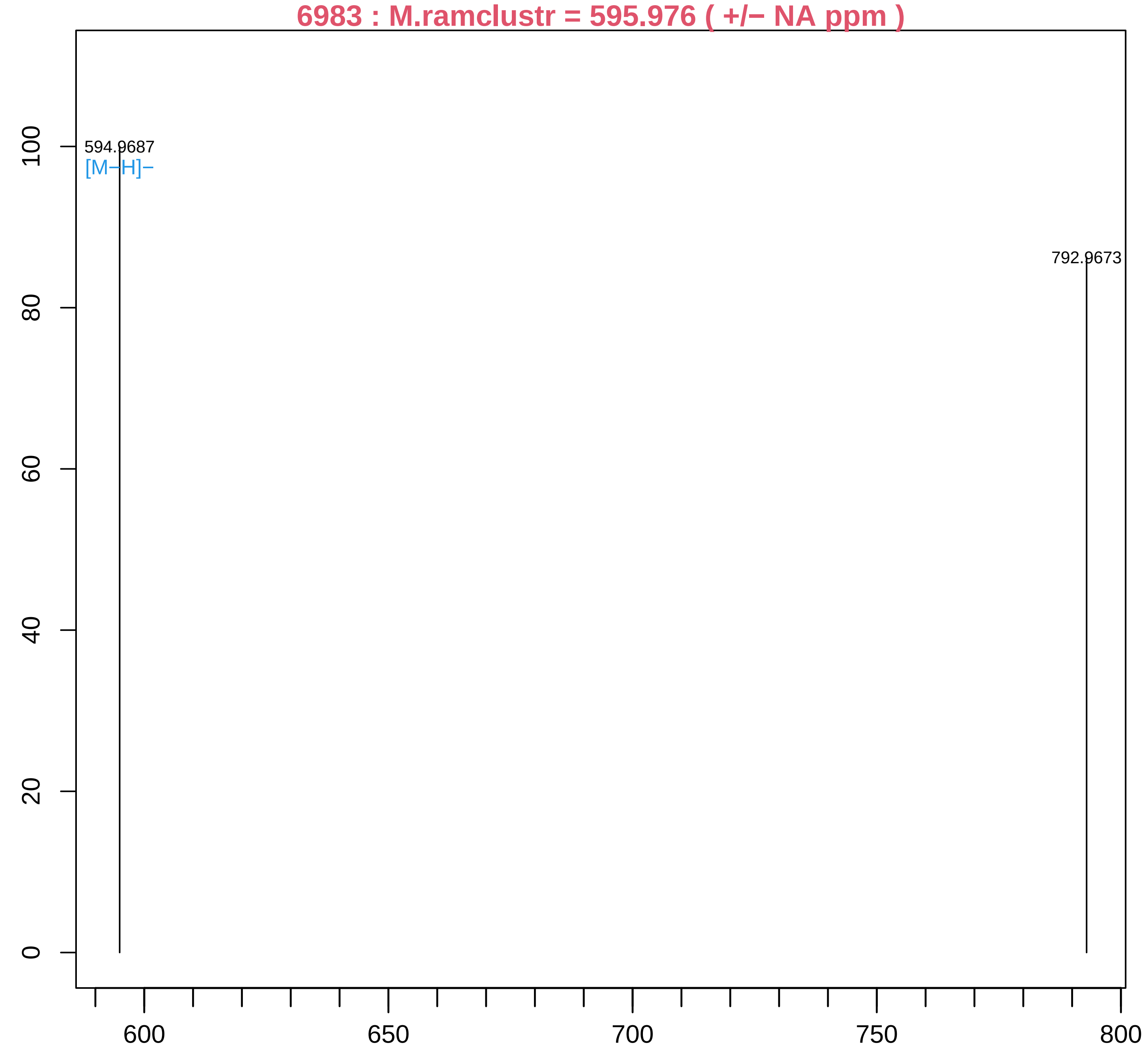
6983 : M.ramclustr = 595.976 (+/− NA ppm)
594.9687
[M−H]−
792.9673
0
20
40
60
80
100
600
650
700
750
800

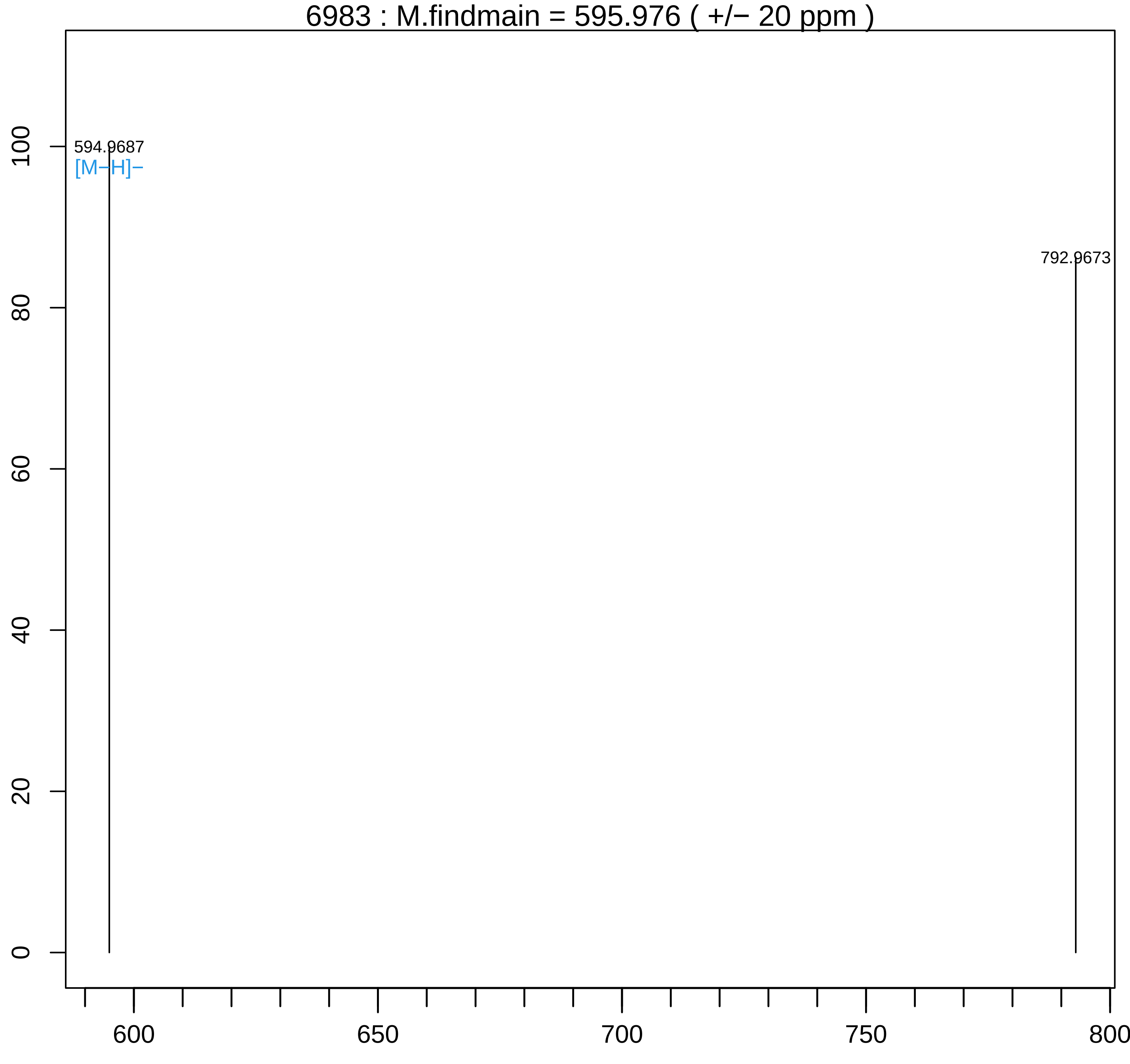
6983 : M.findmain = 595.976 (+/− 20 ppm)
594.9687
[M−H]−
792.9673
0
20
40
60
80
100
600
650
700
750
800

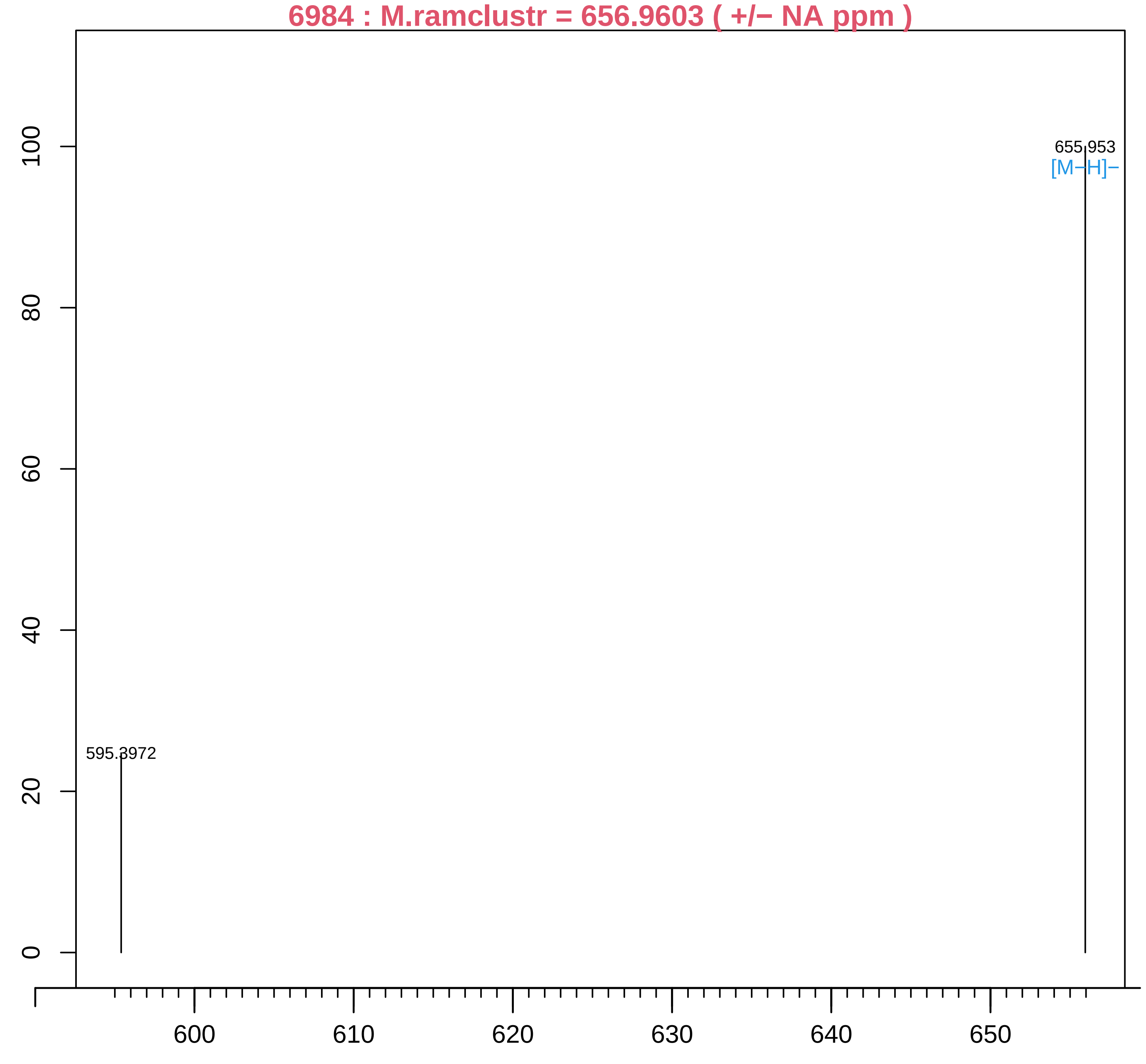
6984 : M.ramclustr = 656.9603 (+/− NA ppm)
655.953
[M−H]−
595.3972
0
20
40
60
80
100
600
610
620
630
640
650

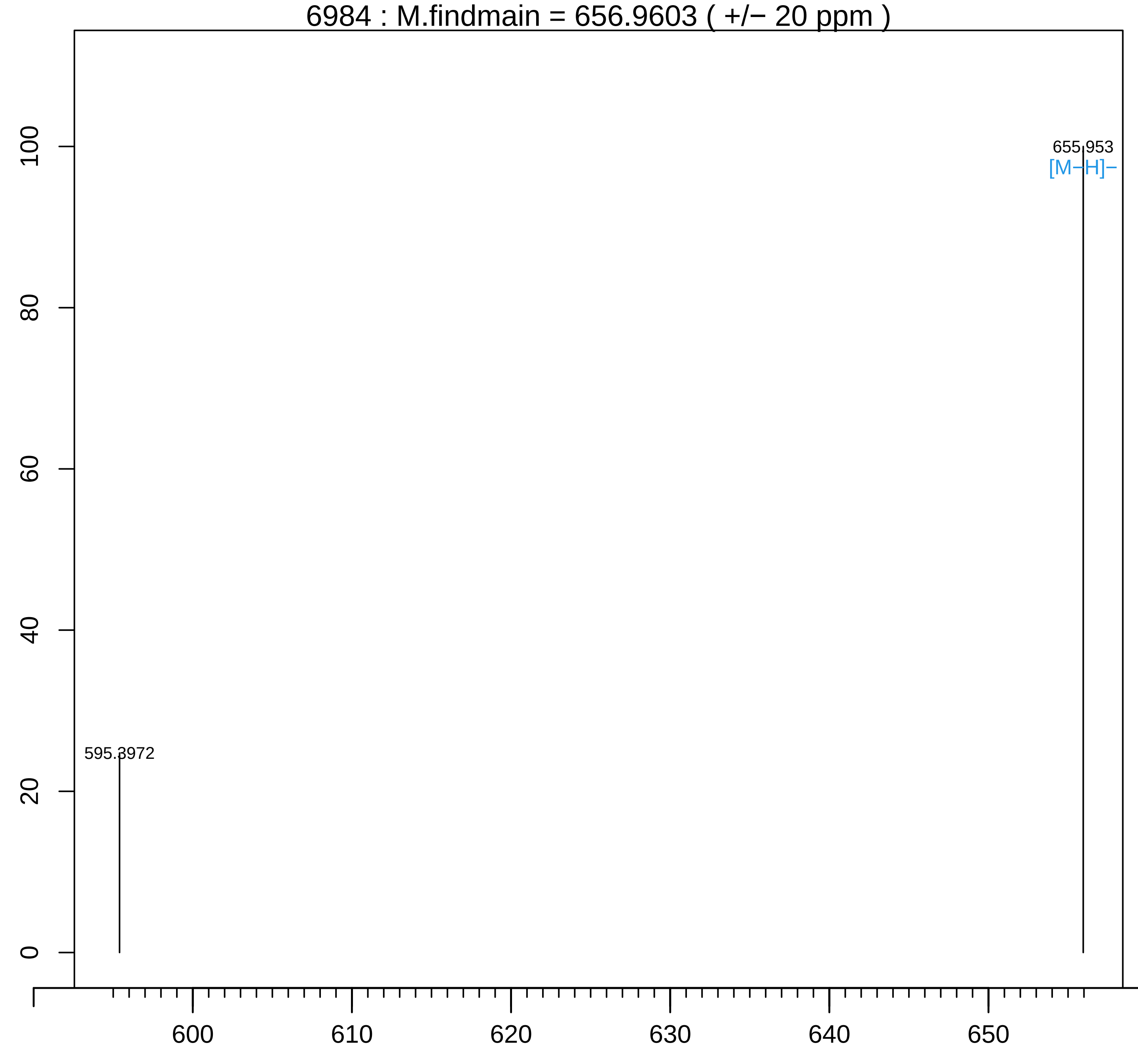
6984 : M.findmain = 656.9603 (+/− 20 ppm)
655.953
[M−H]−
595.3972
0
20
40
60
80
100
600
610
620
630
640
650

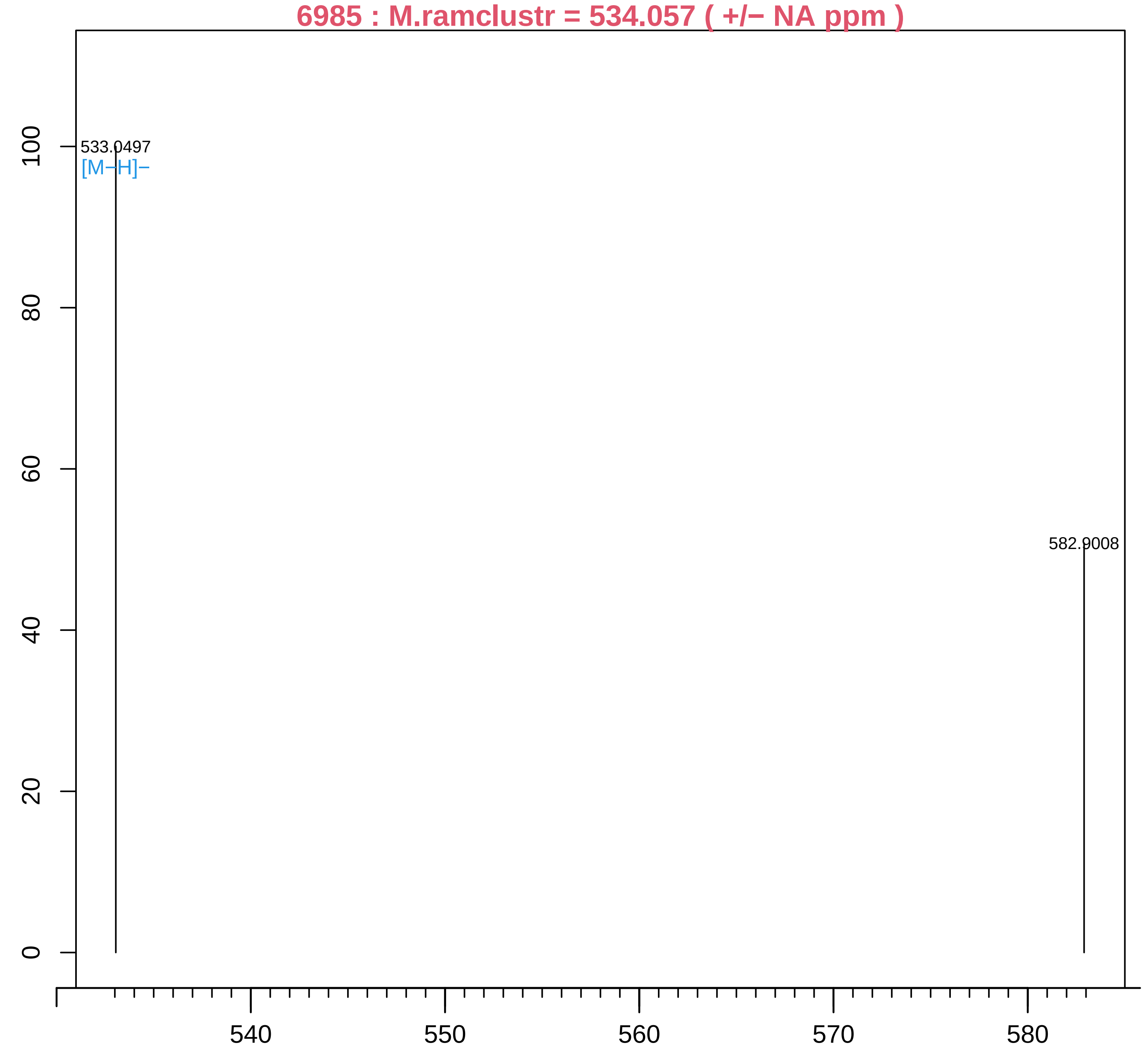
6985 : M.ramclustr = 534.057 (+/− NA ppm)
533.0497
[M−H]−
582.9008
0
20
40
60
80
100
540
550
560
570
580

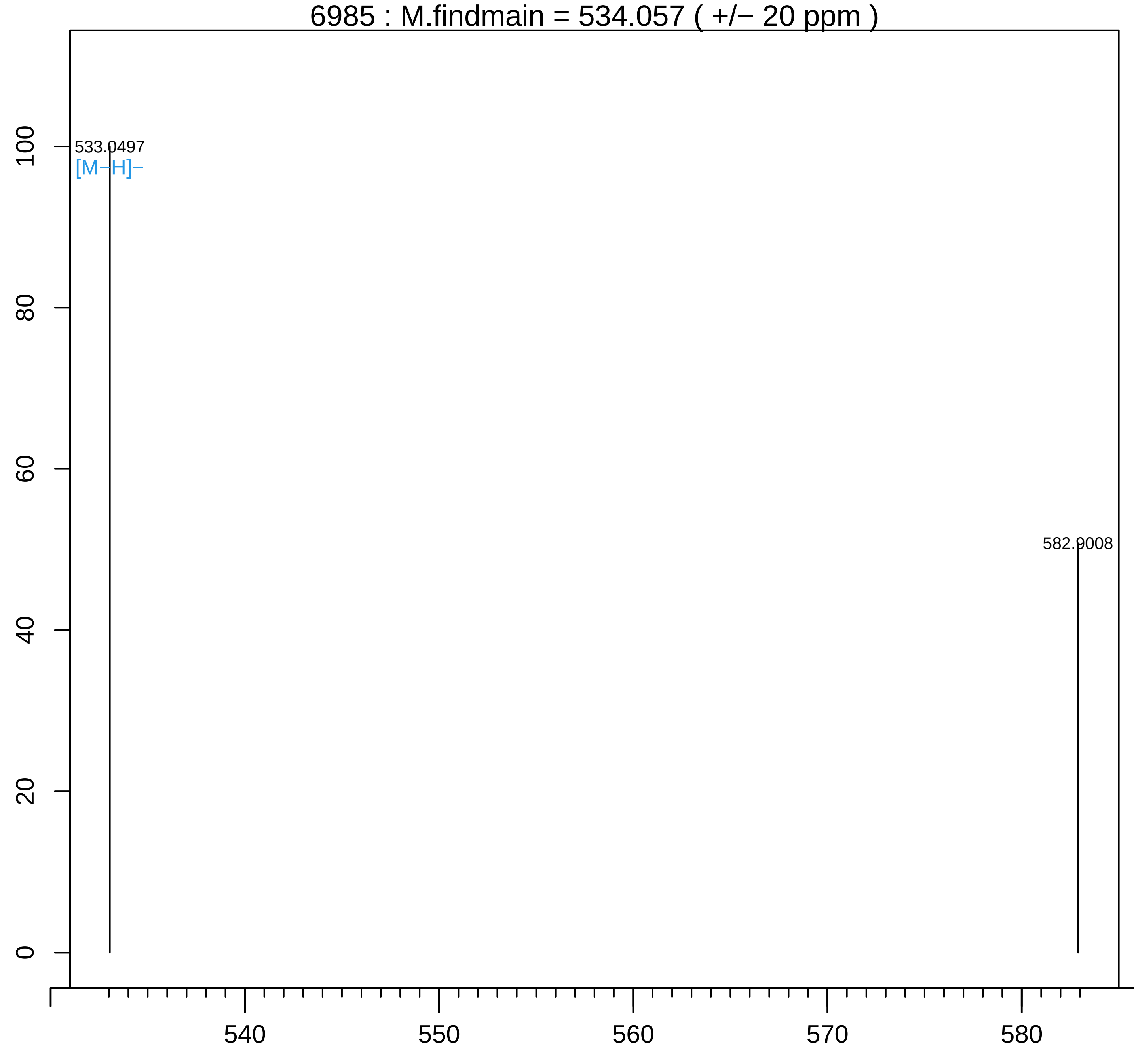
6985 : M.findmain = 534.057 (+/− 20 ppm)
533.0497
[M−H]−
582.9008
0
20
40
60
80
100
540
550
560
570
580

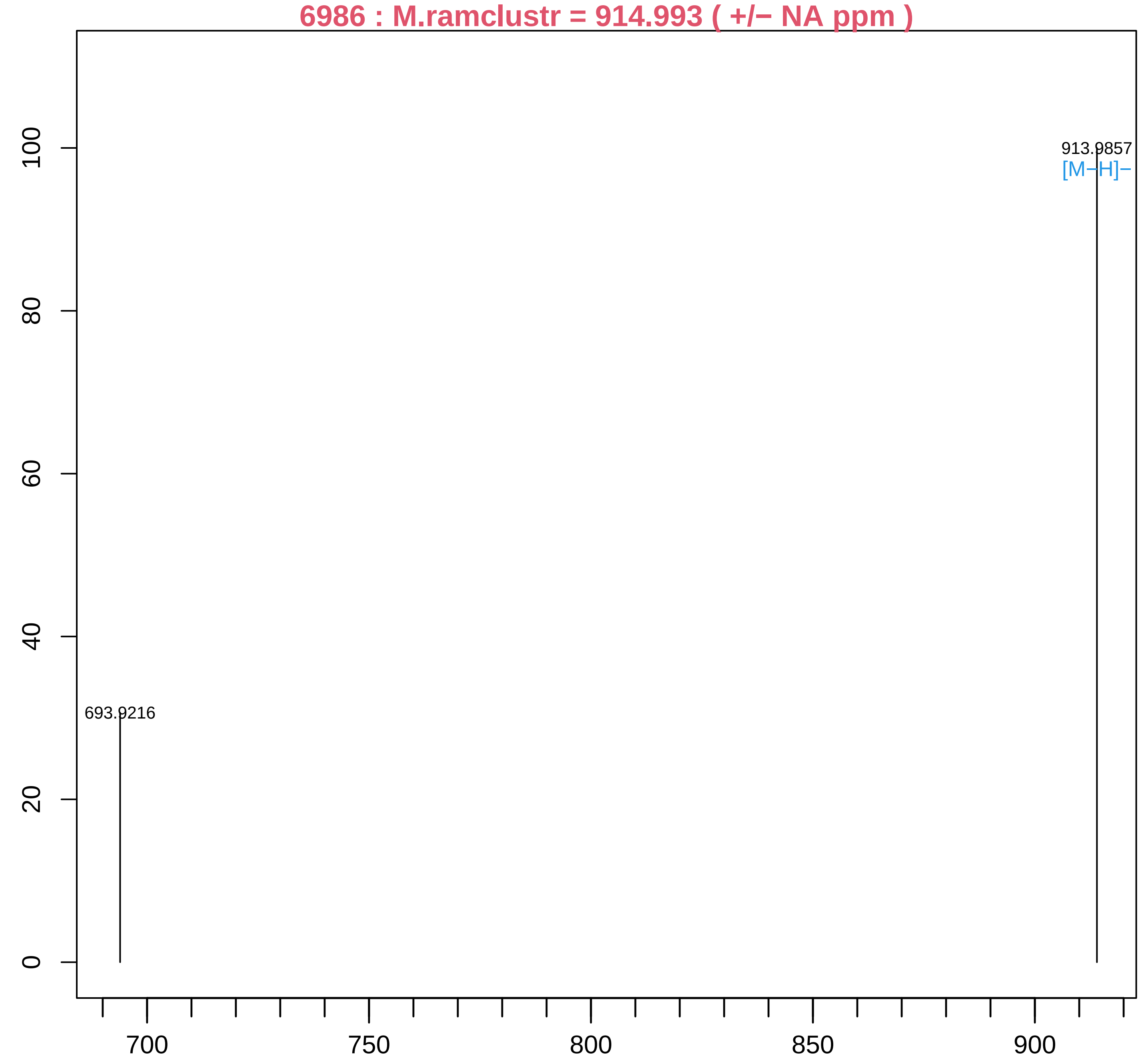
6986 : M.ramclustr = 914.993 (+/− NA ppm)
913.9857
[M−H]−
693.9216
0
20
40
60
80
100
700
750
800
850
900

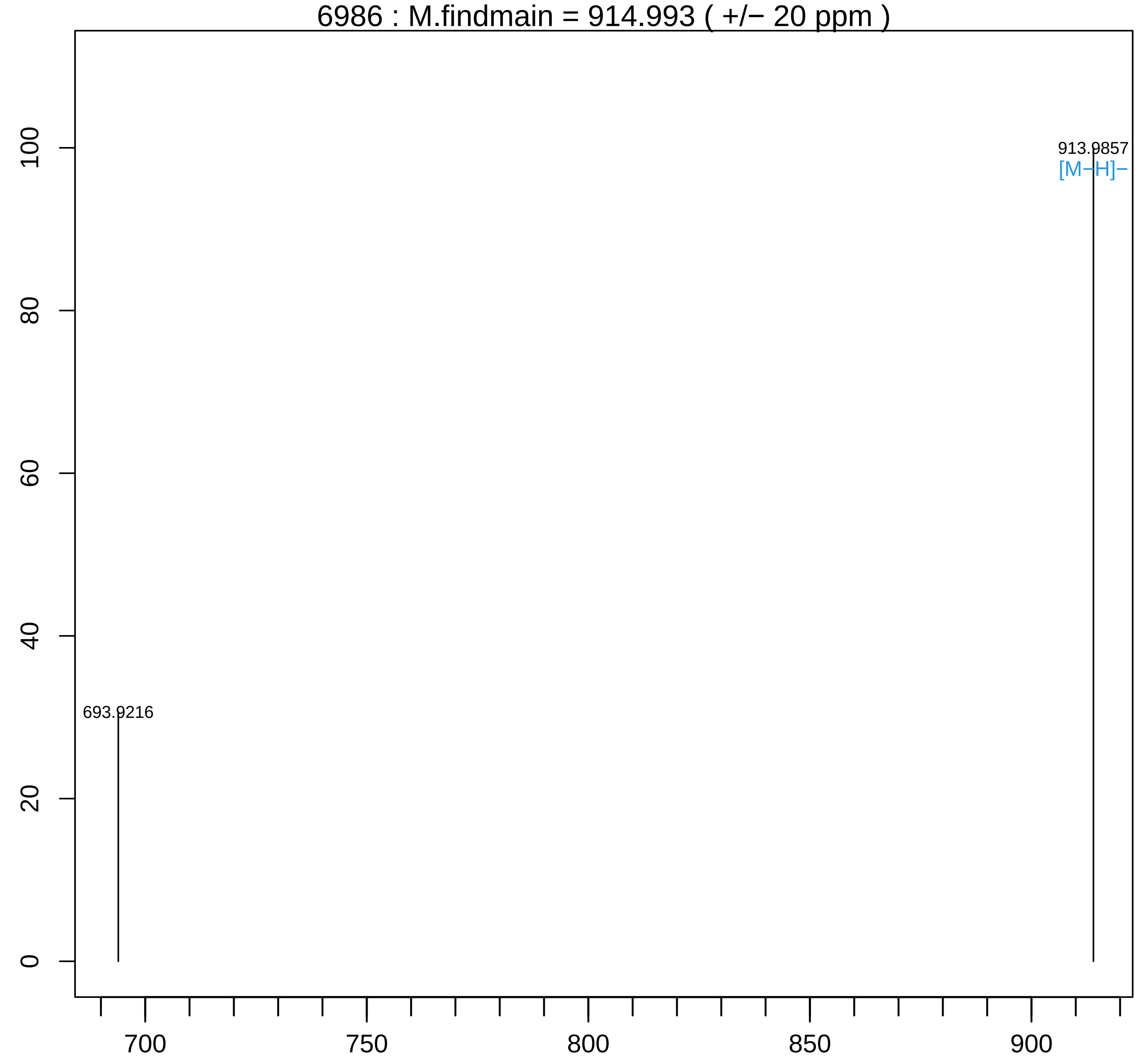
6986 : M.findmain = 914.993 (+/− 20 ppm)
913.9857
[M−H]−
693.9216
0
20
40
60
80
100
700
750
800
850
900

6987 : M.ramclustr = 631.9735 (+/− NA ppm)

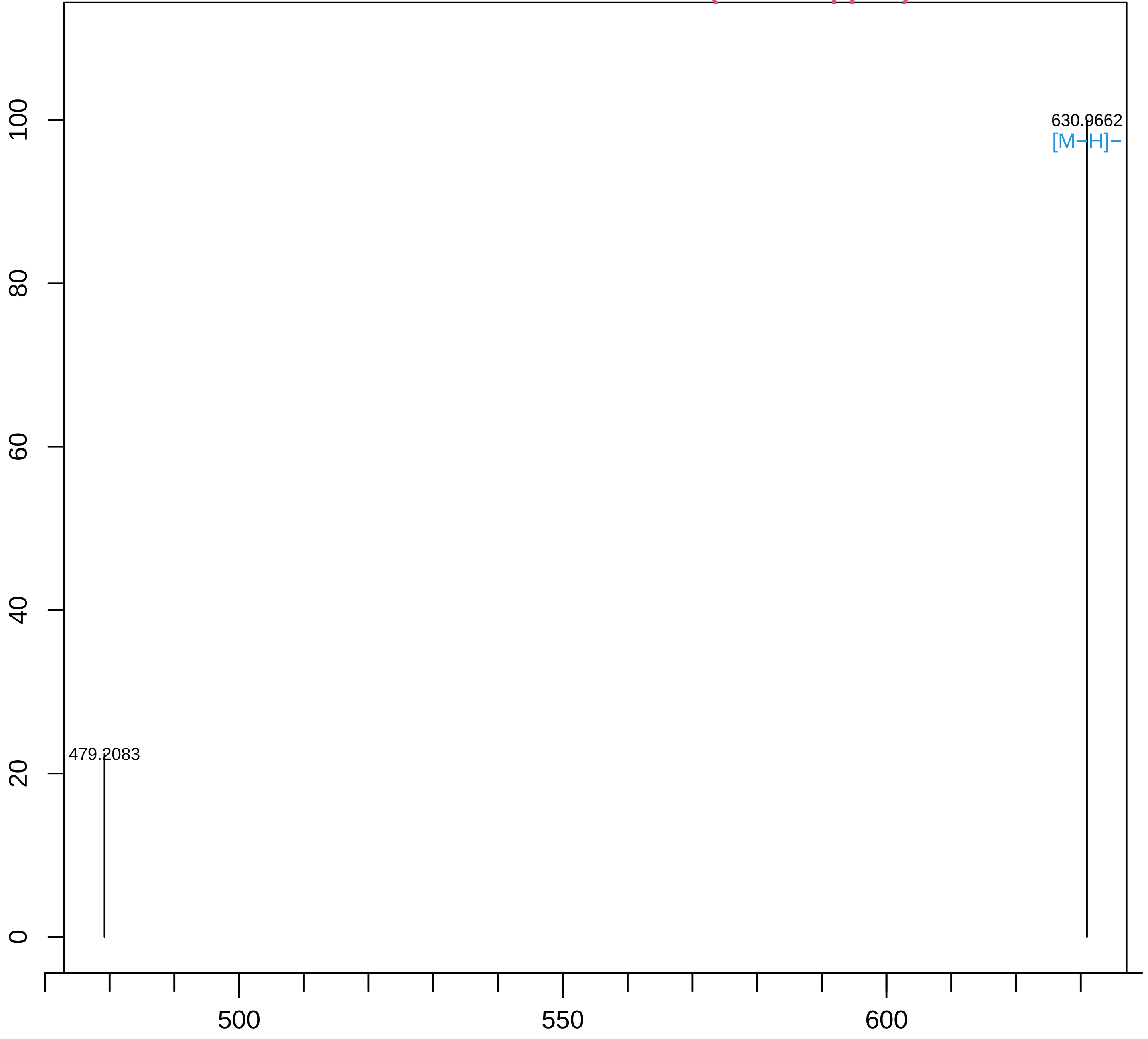

6987 : M.findmain = 631.9735 (+/− 20 ppm)

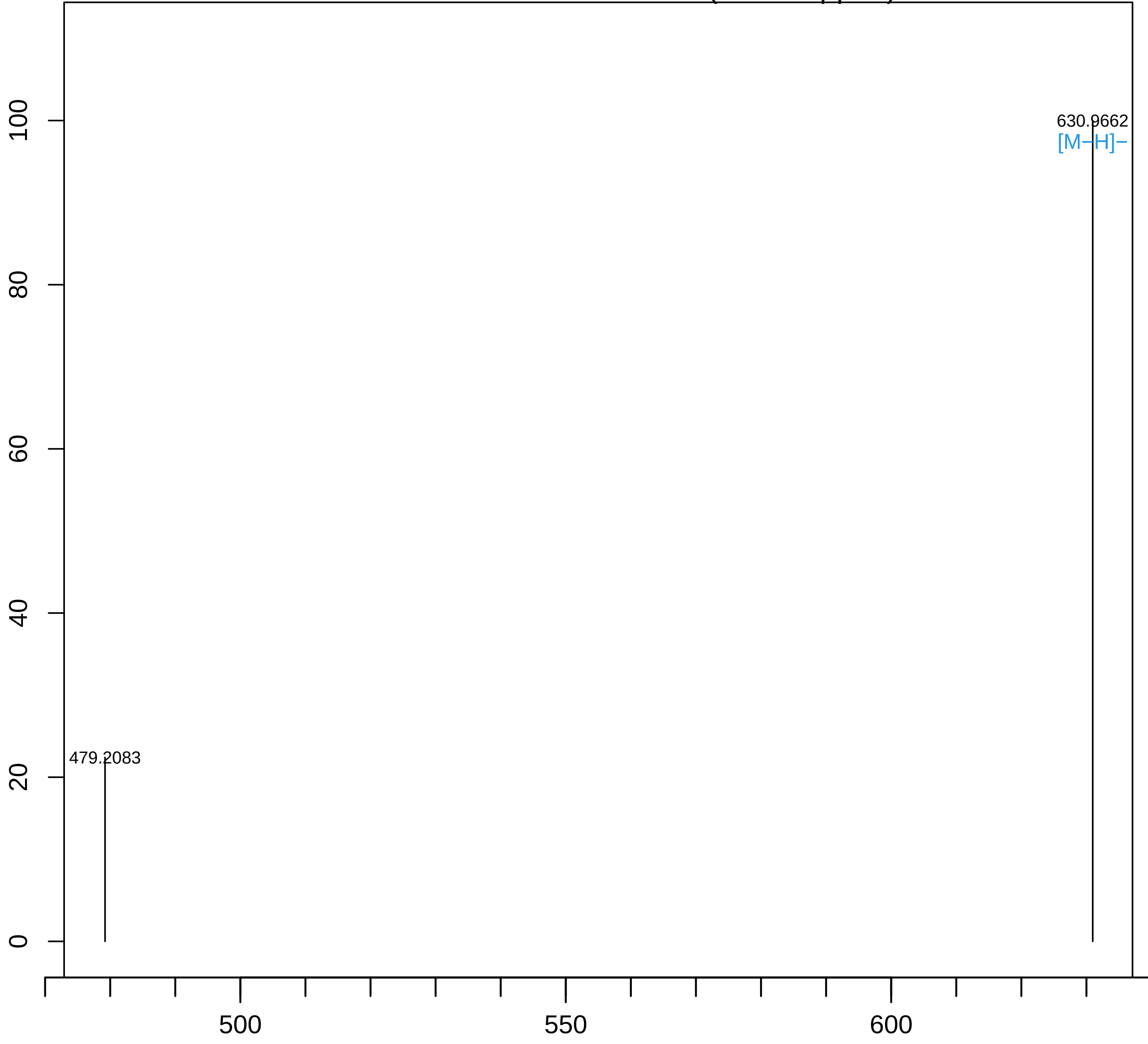

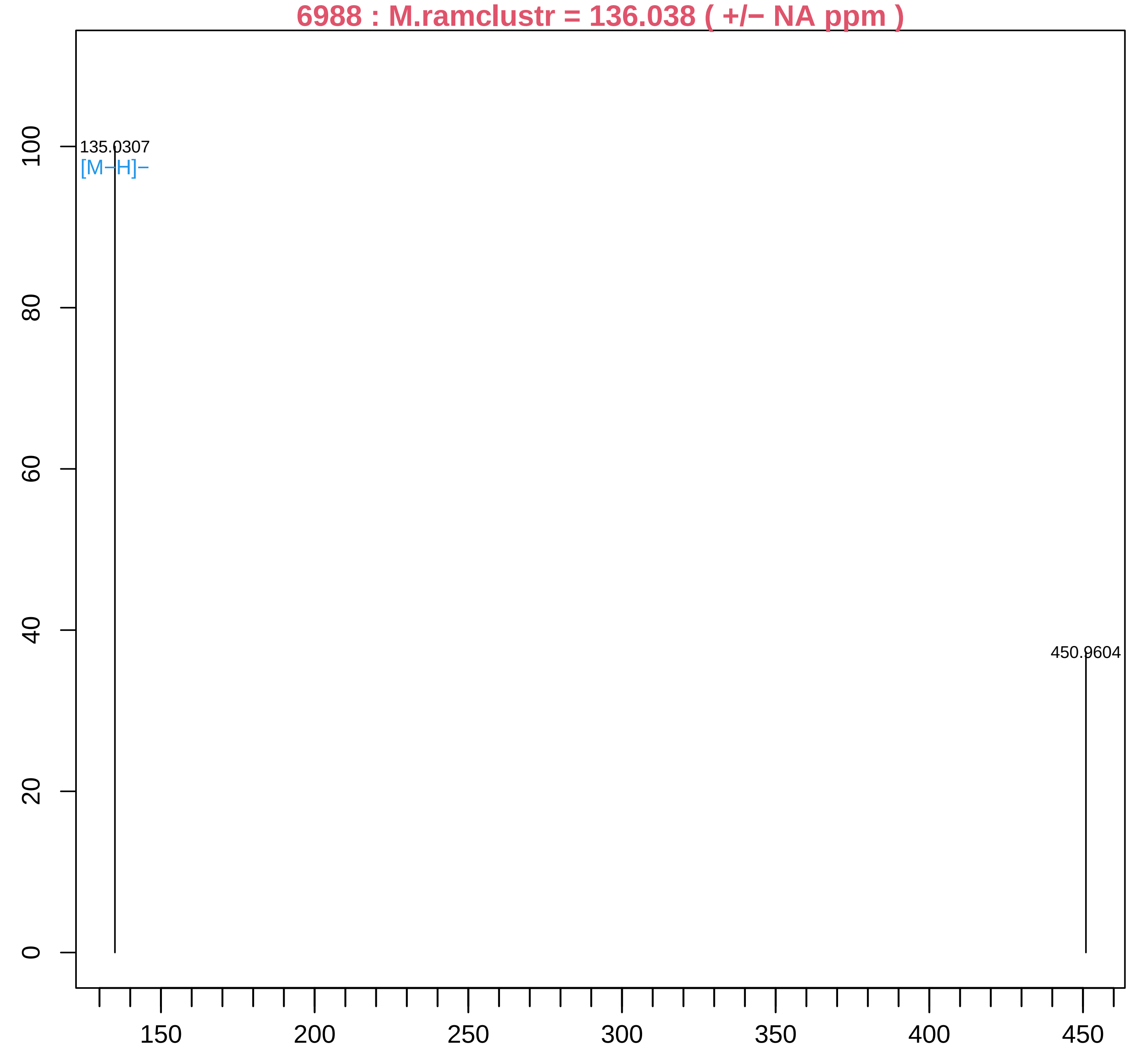
6988 : M.ramclustr = 136.038 (+/− NA ppm)
135.0307
[M−H]−
450.9604
0
20
40
60
80
100
150
200
250
300
350
400
450

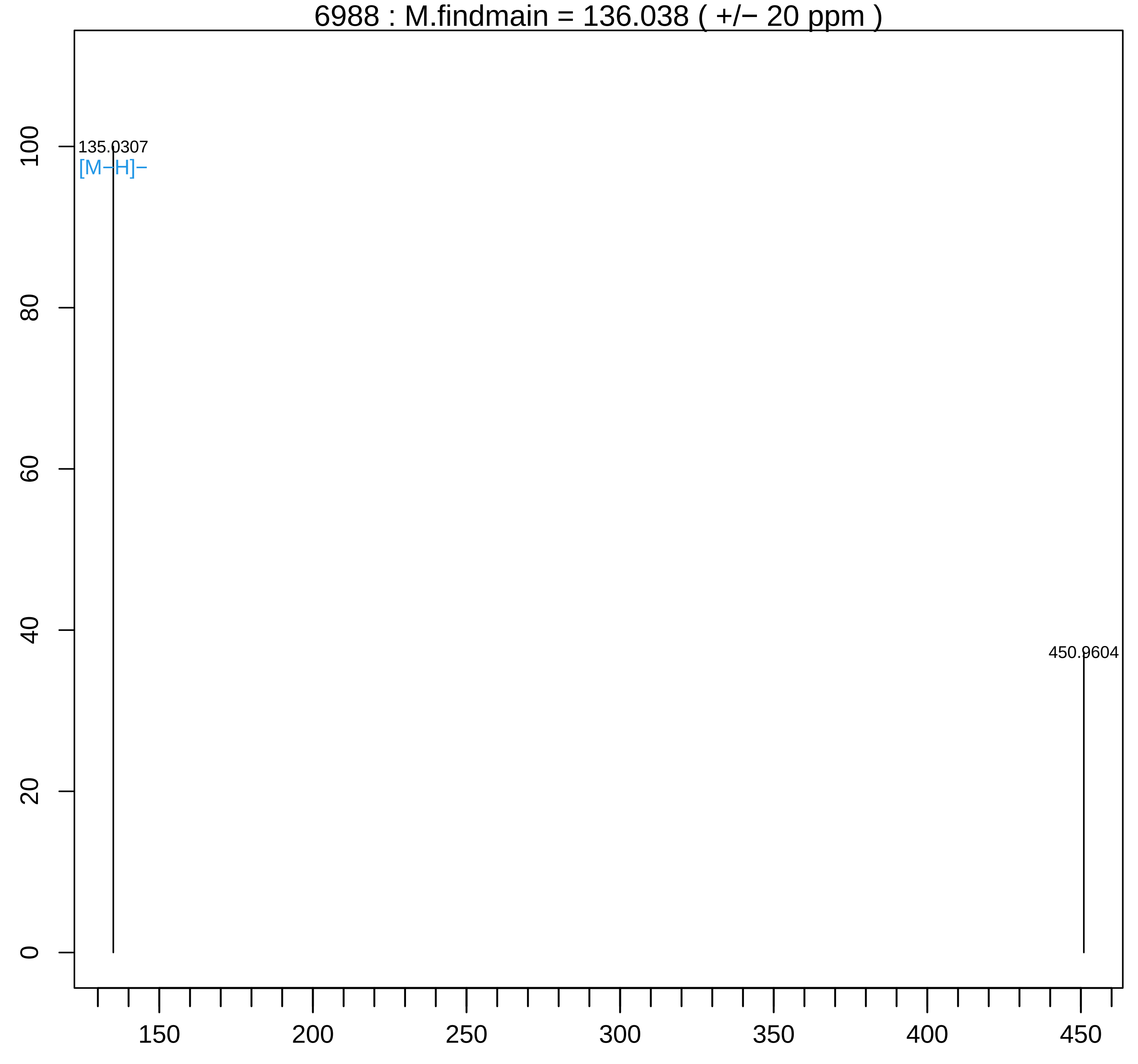
6988 : M.findmain = 136.038 (+/− 20 ppm)
135.0307
[M−H]−
450.9604
0
20
40
60
80
100
150
200
250
300
350
400
450

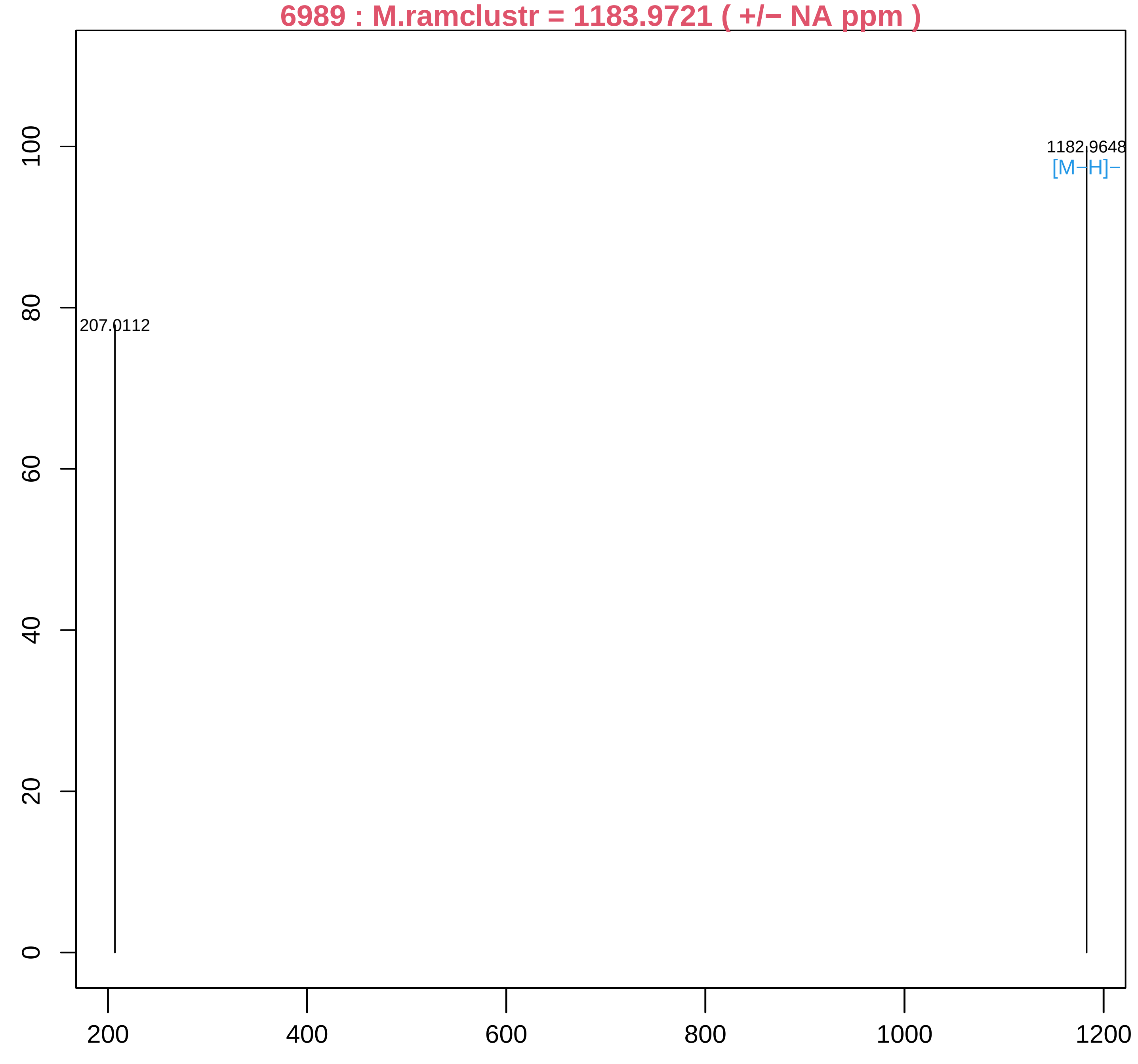

6989 : M.ramclustr = 1183.9721 (+/− NA ppm)
1182.9648
[M−H]−
207.0112
0
20
40
60
80
100
200
400
600
800
1000
1200

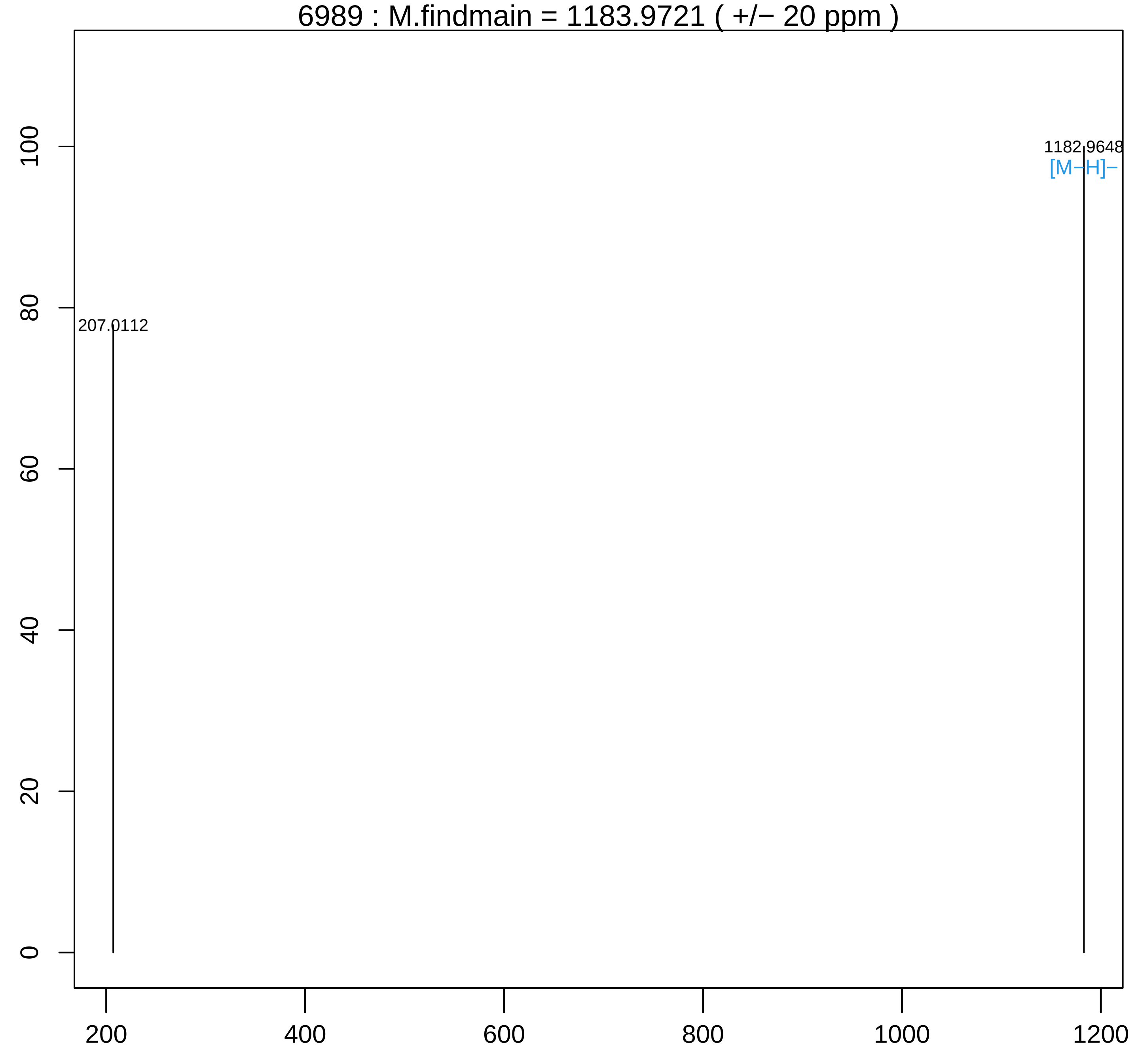

6989 : M.findmain = 1183.9721 (+/− 20 ppm)
1182.9648
[M−H]−
207.0112
0
20
40
60
80
100
200
400
600
800
1000
1200

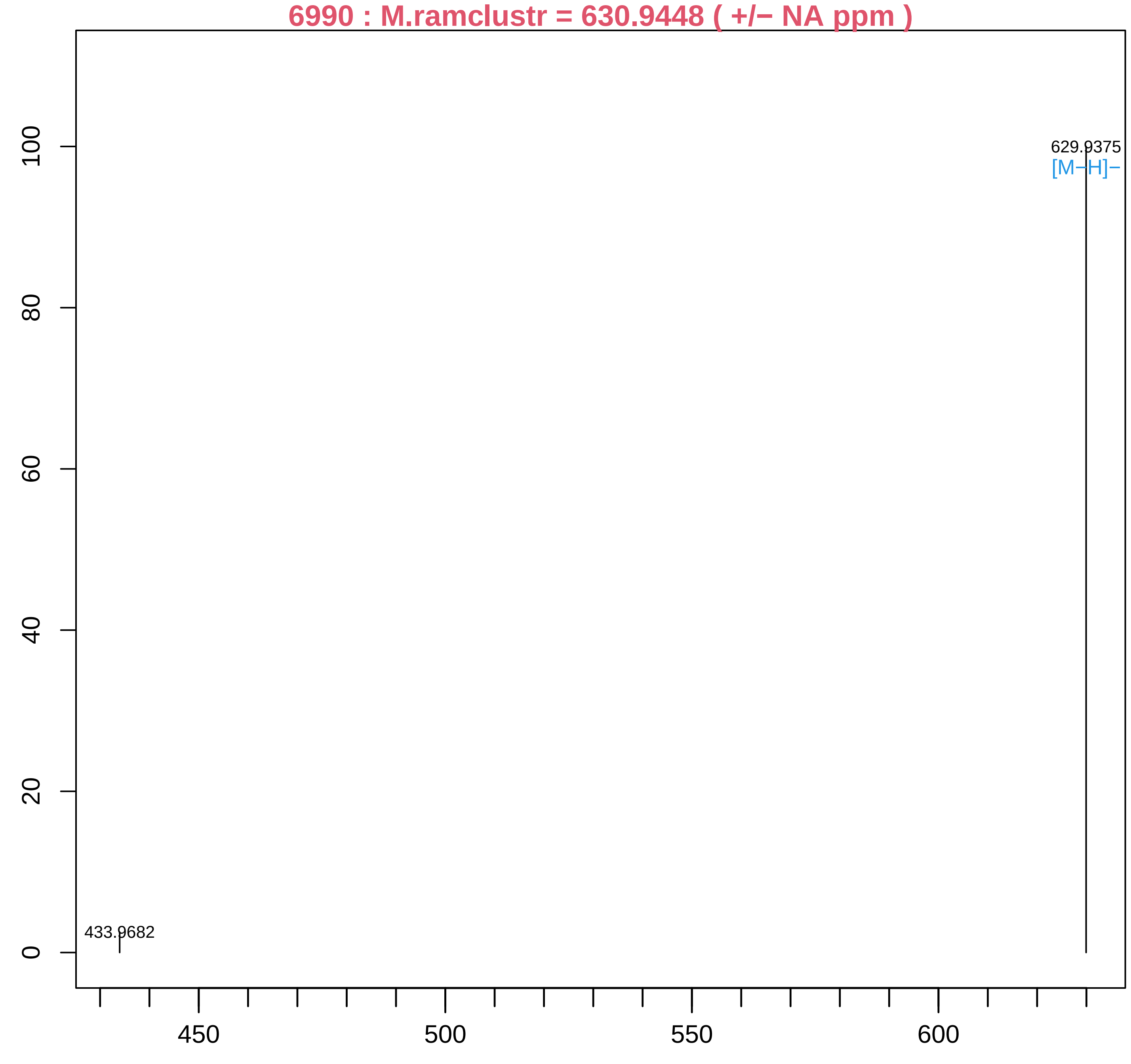
6990 : M.ramclustr = 630.9448 (+/− NA ppm)
629.9375
[M−H]−
433.9682
0
20
40
60
80
100
450
500
550
600

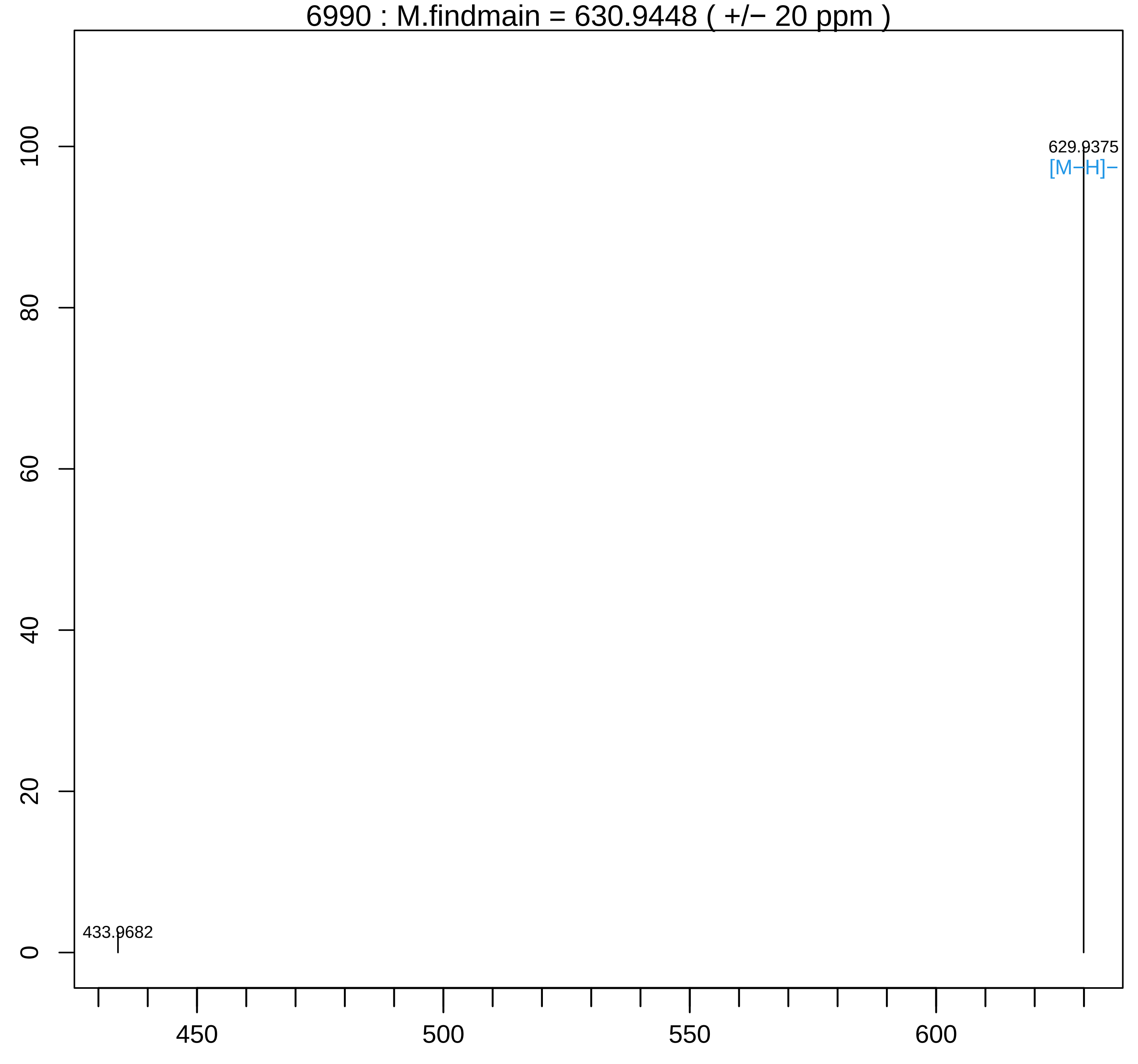
6990 : M.findmain = 630.9448 (+/− 20 ppm)
629.9375
[M−H]−
433.9682
0
20
40
60
80
100
450
500
550
600

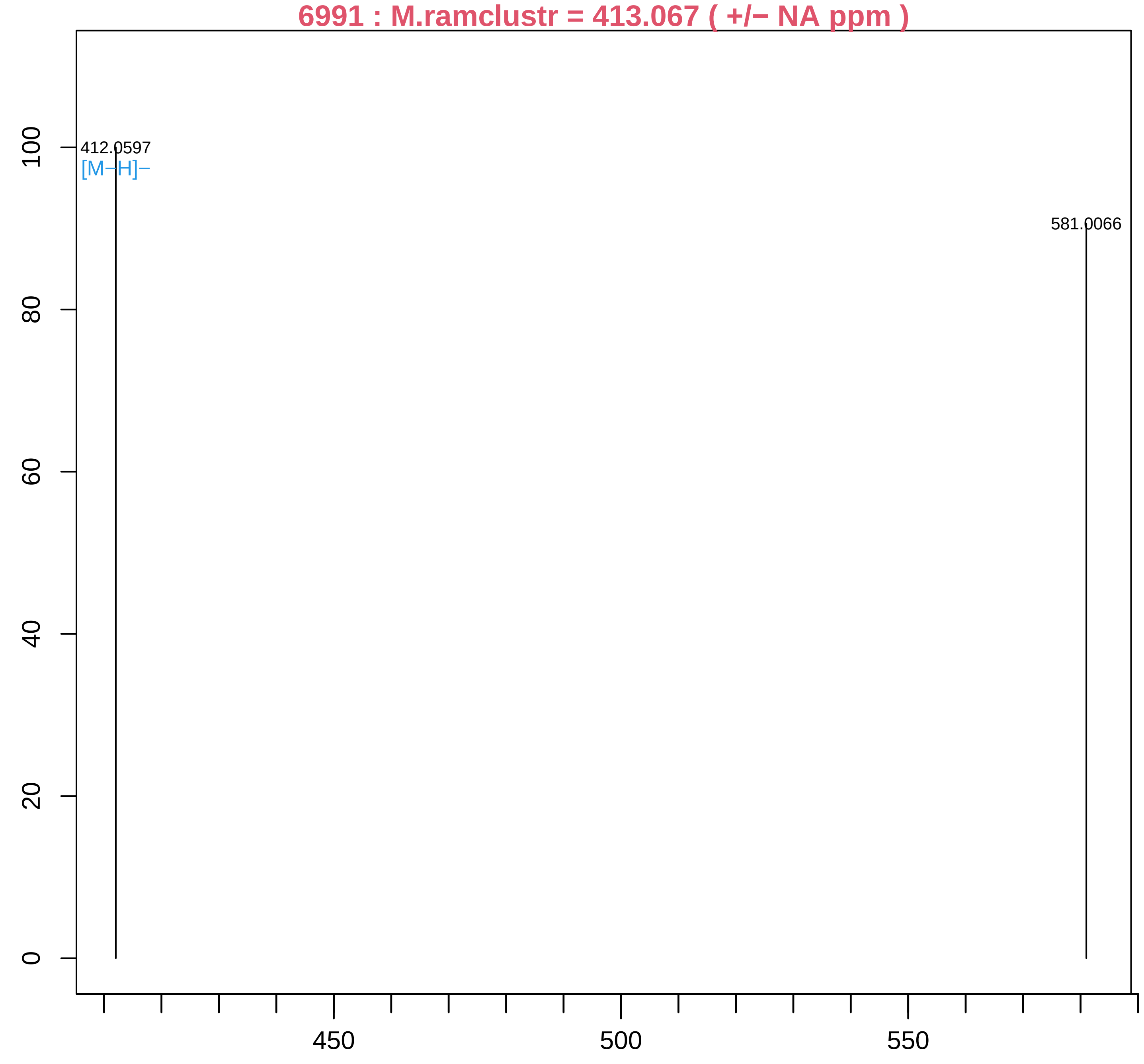
6991 : M.ramclustr = 413.067 (+/− NA ppm)
412.0597
[M−H]−
581.0066
0
20
40
60
80
100
450
500
550

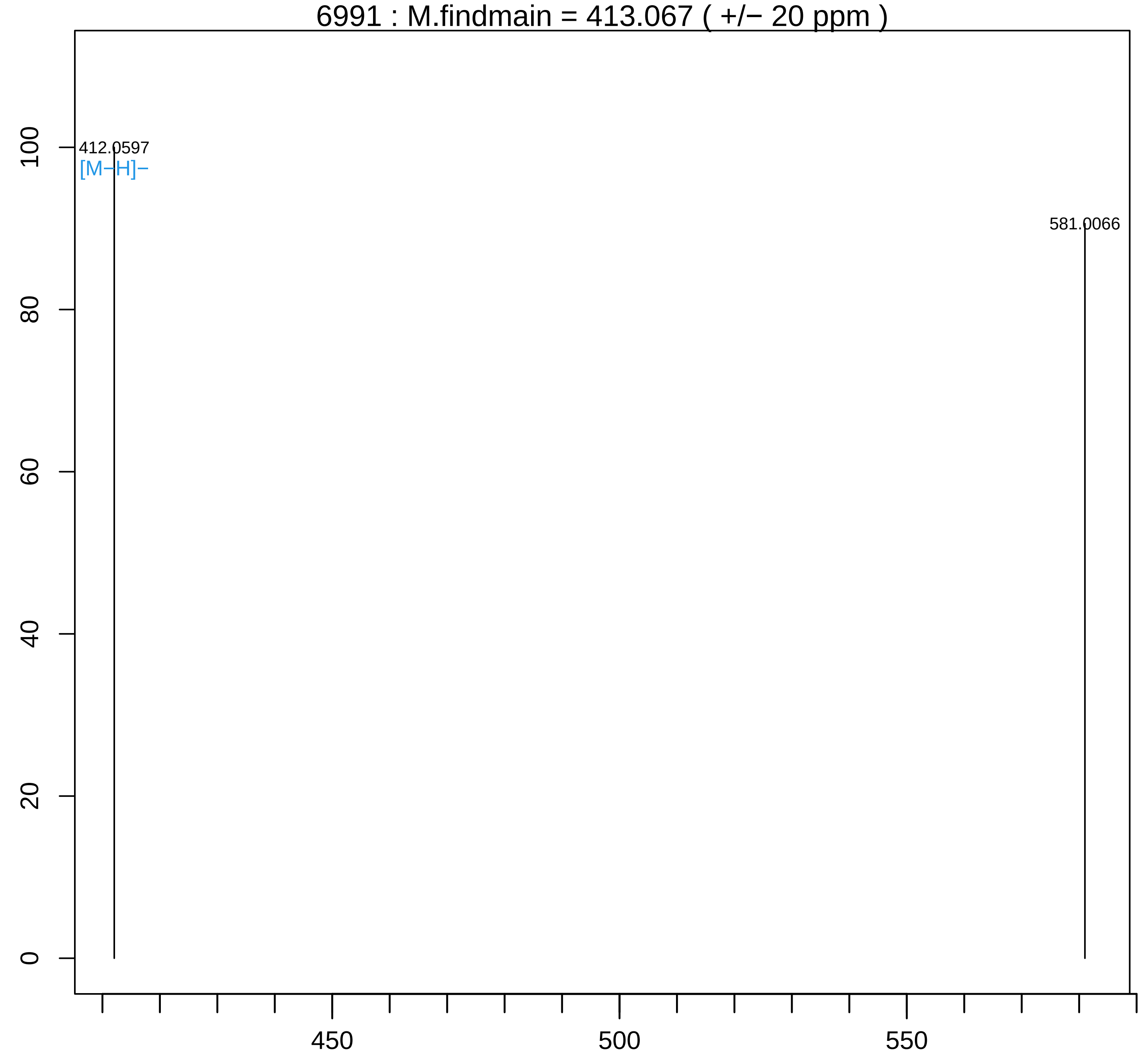
6991 : M.findmain = 413.067 (+/− 20 ppm)
412.0597
[M−H]−
581.0066
0
20
40
60
80
100
450
500
550

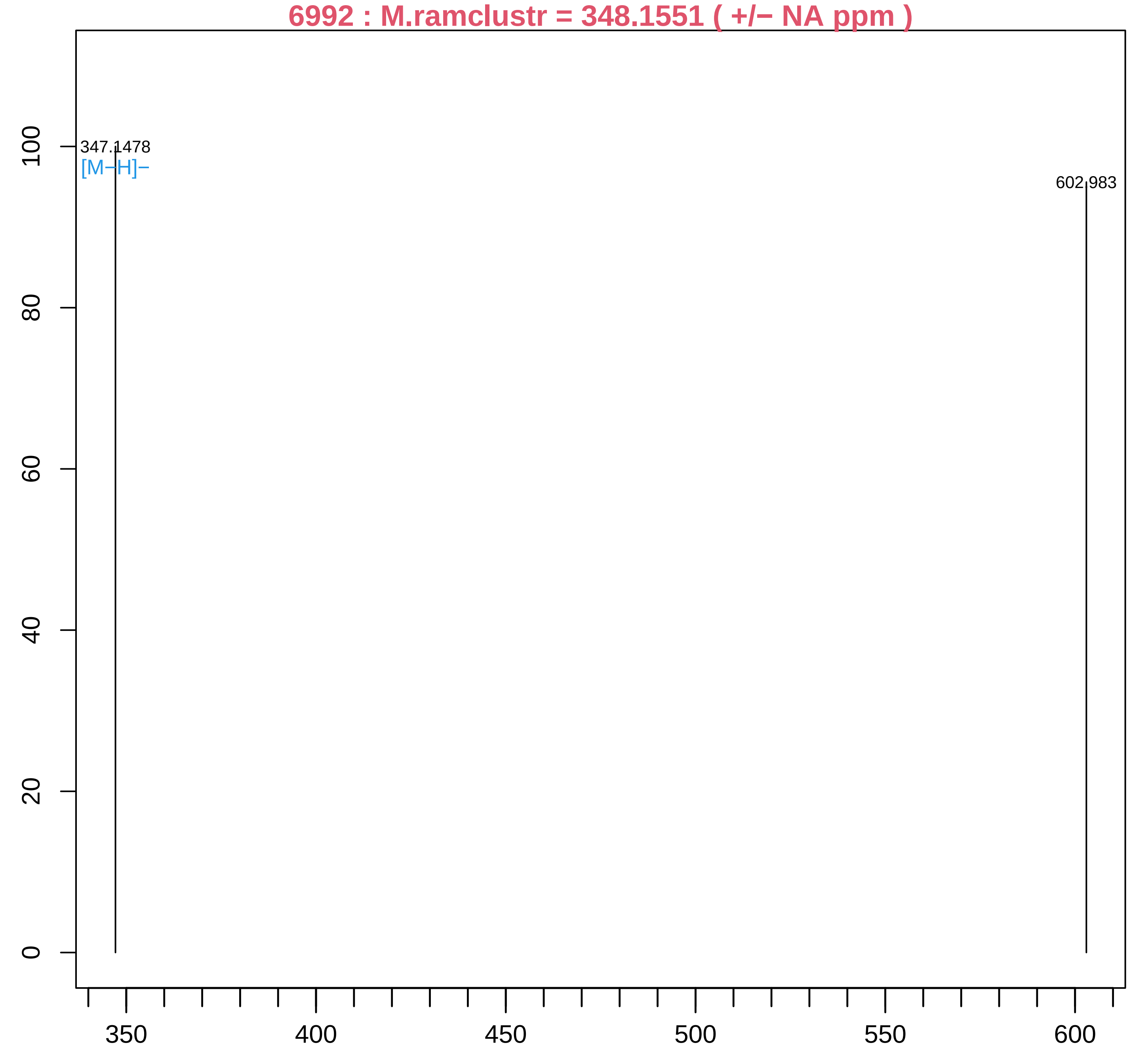
6992 : M.ramclustr = 348.1551 (+/− NA ppm)
347.1478
[M−H]−
602.983
0
20
40
60
80
100
350
400
450
500
550
600

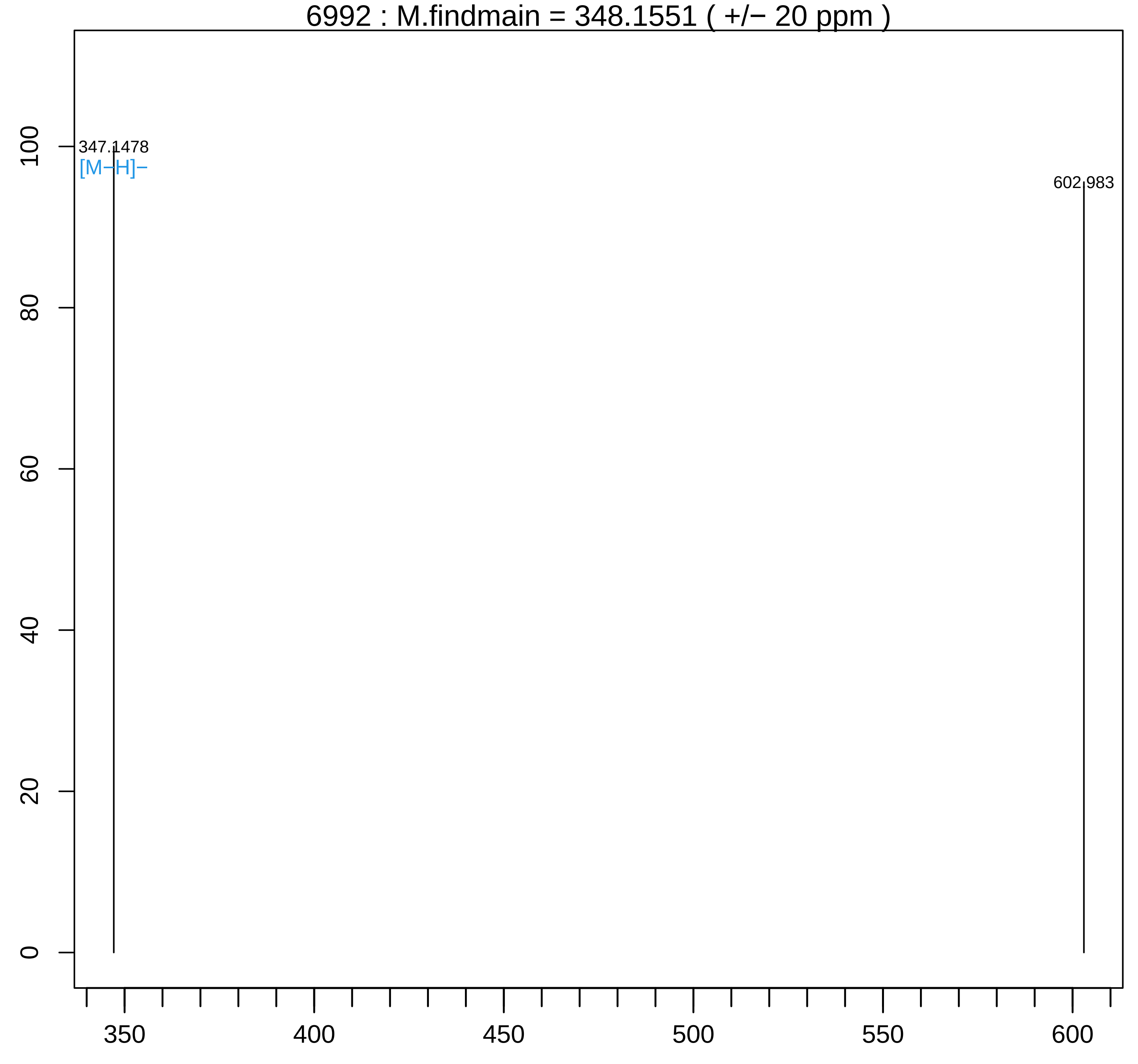
6992 : M.findmain = 348.1551 (+/− 20 ppm)
347.1478
[M−H]−
602.983
0
20
40
60
80
100
350
400
450
500
550
600

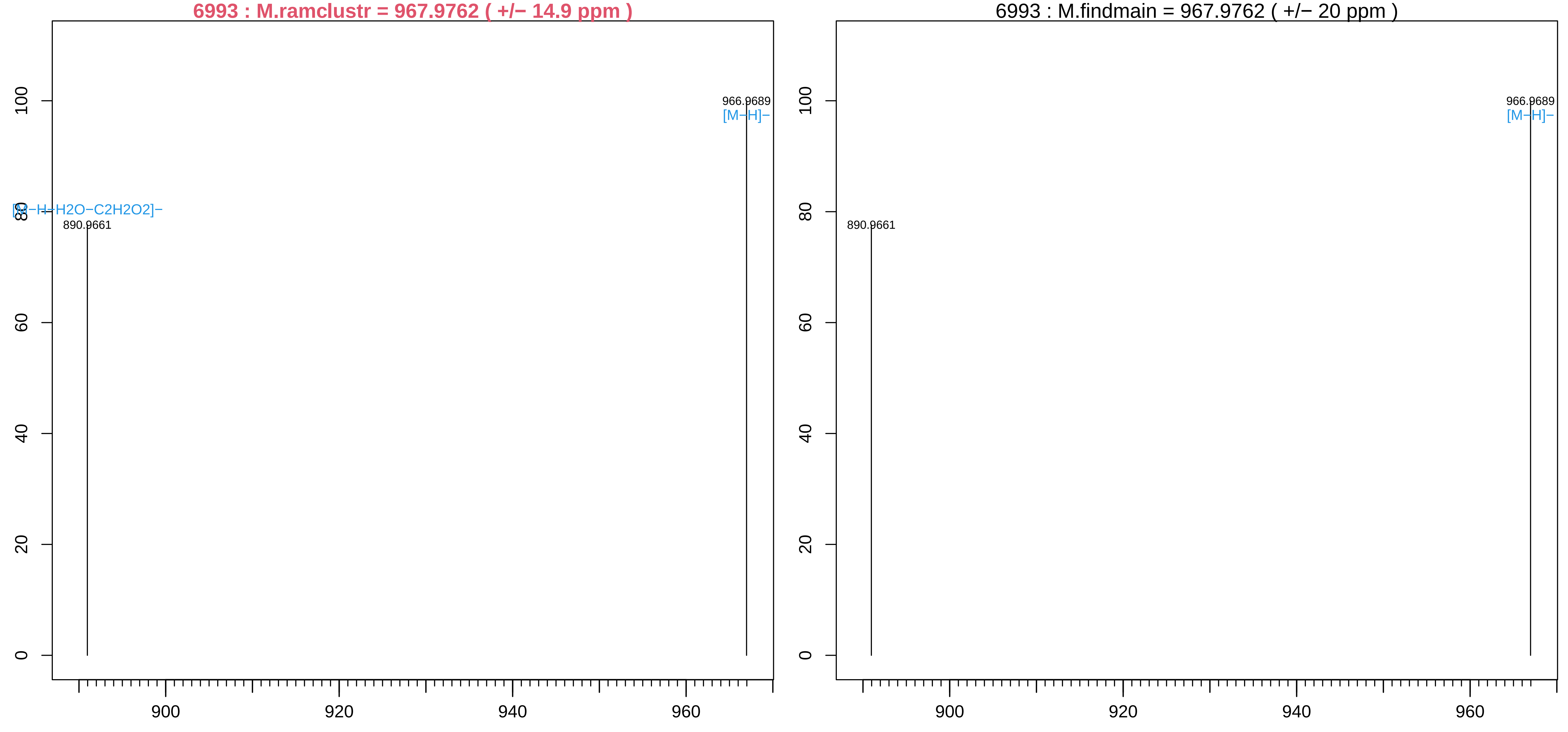
6993 : M.ramclustr = 967.9762 (+/− 14.9 ppm)
966.9689
[M−H]−
[M−H−H2O−C2H2O2]−
890.9661
6993 : M.findmain = 967.9762 (+/− 20 ppm)
966.9689
[M−H]−
890.9661
0
20
40
60
80
100
900
920
940
960

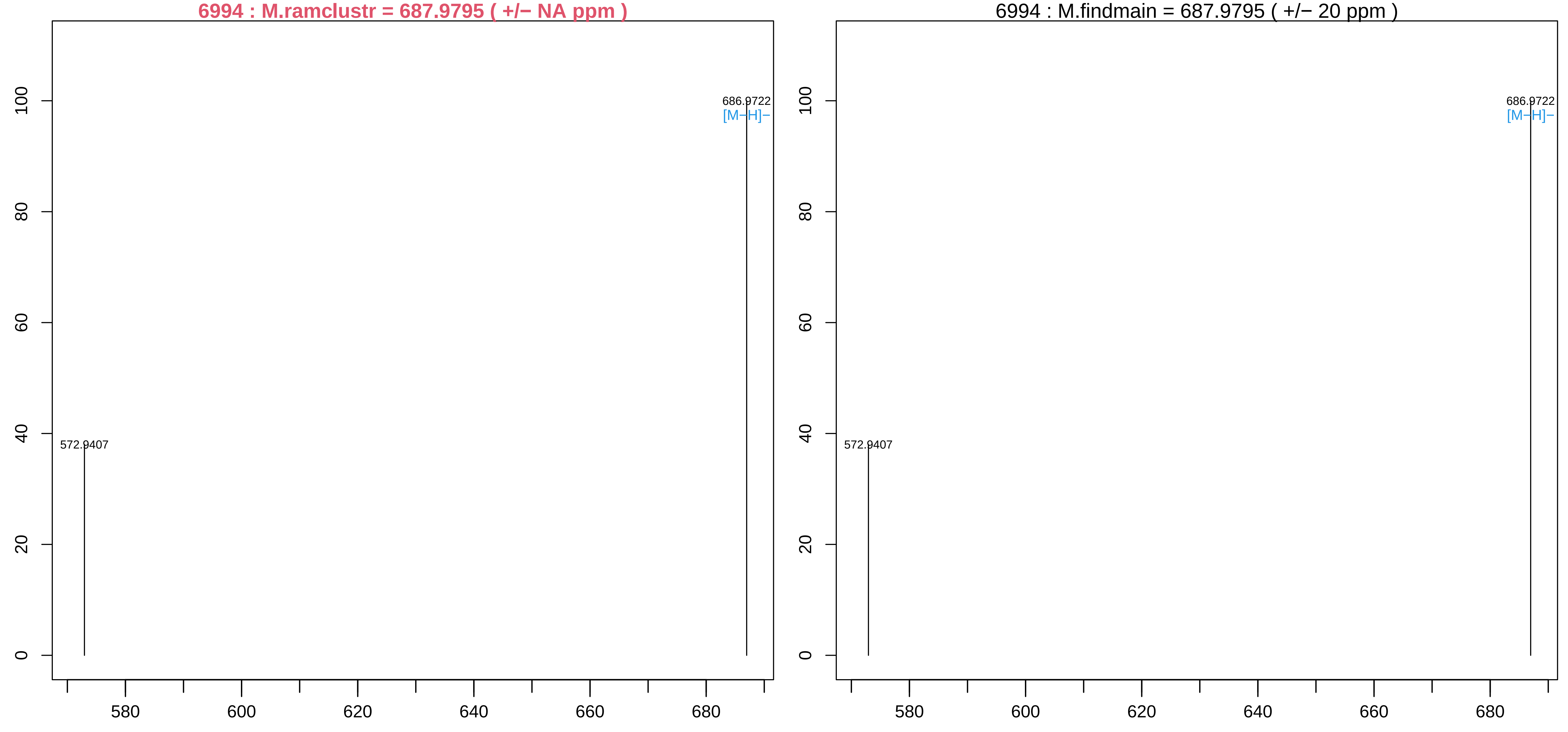
6994 : M.ramclustr = 687.9795 (+/− NA ppm)
686.9722
[M−H]−
572.9407
0
20
40
60
80
100
580
600
620
640
660
680
6994 : M.findmain = 687.9795 (+/− 20 ppm)
686.9722
[M−H]−
572.9407
0
20
40
60
80
100
580
600
620
640
660
680

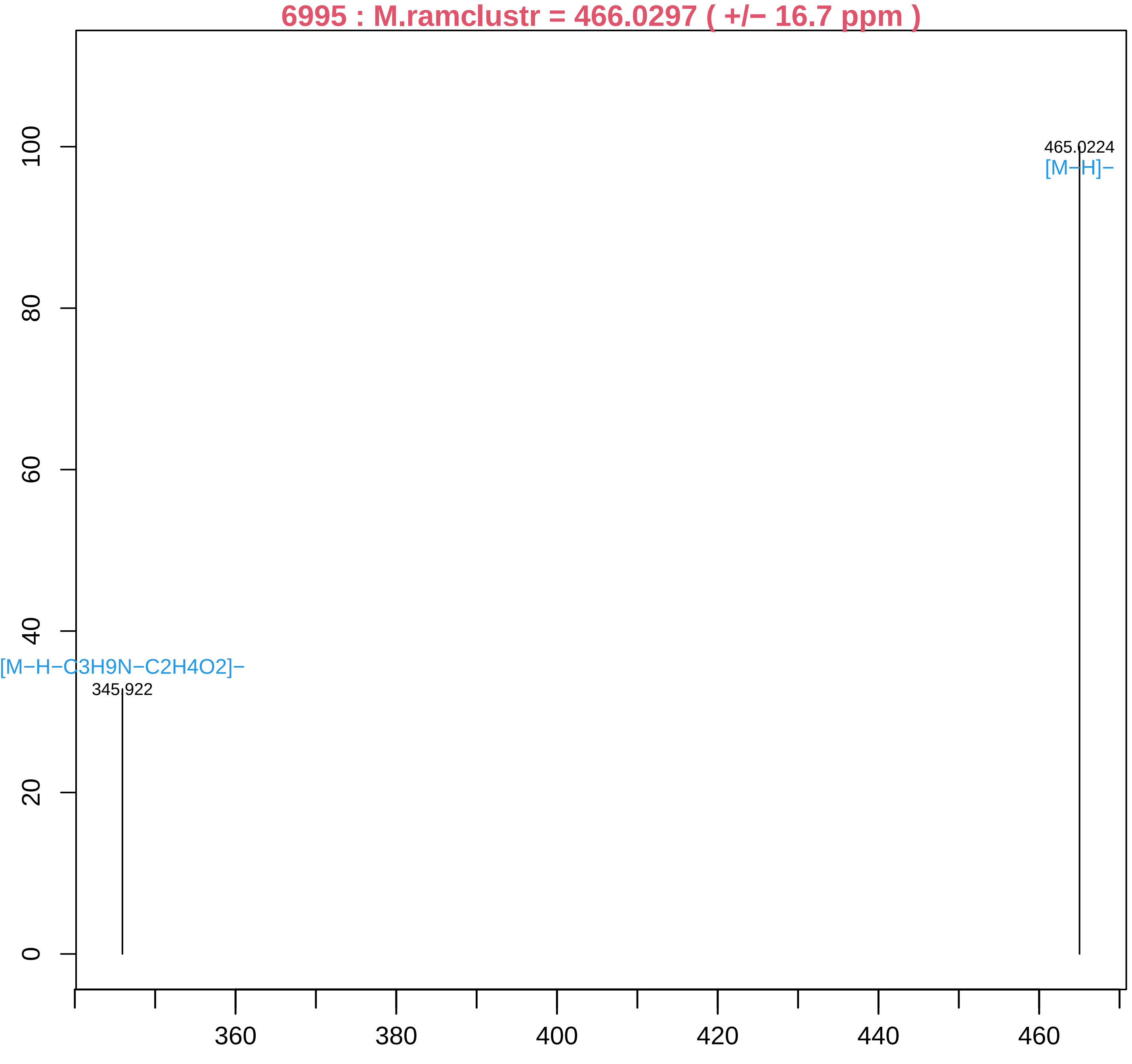
6995 : M.ramclustr = 466.0297 (+/− 16.7 ppm)
465.0224
[M−H]−
[M−H−C3H9N−C2H4O2]−
345.922
0
20
40
60
80
100
360
380
400
420
440
460

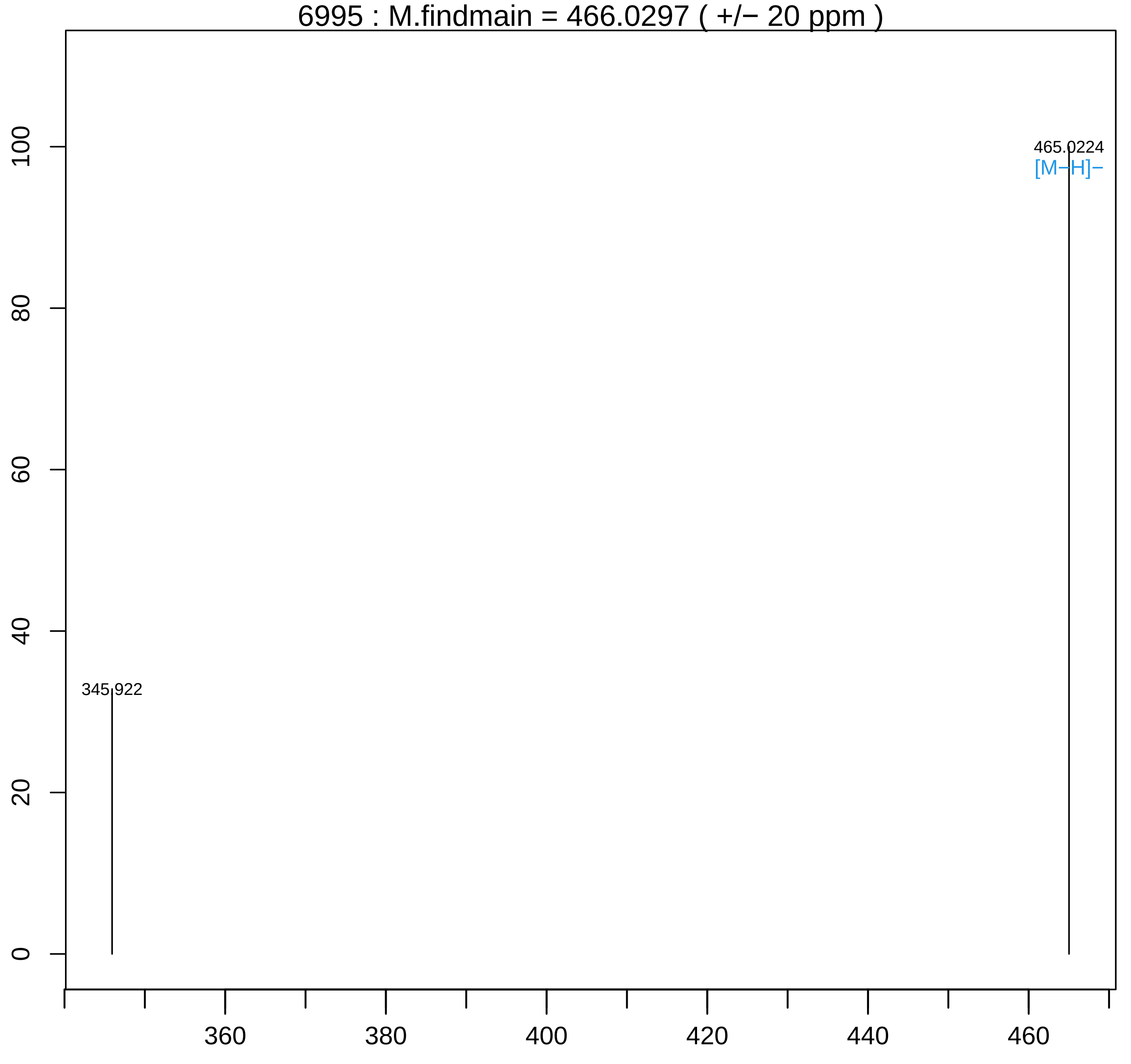
6995 : M.findmain = 466.0297 (+/− 20 ppm)
465.0224
[M−H]−
345.922
0
20
40
60
80
100
360
380
400
420
440
460

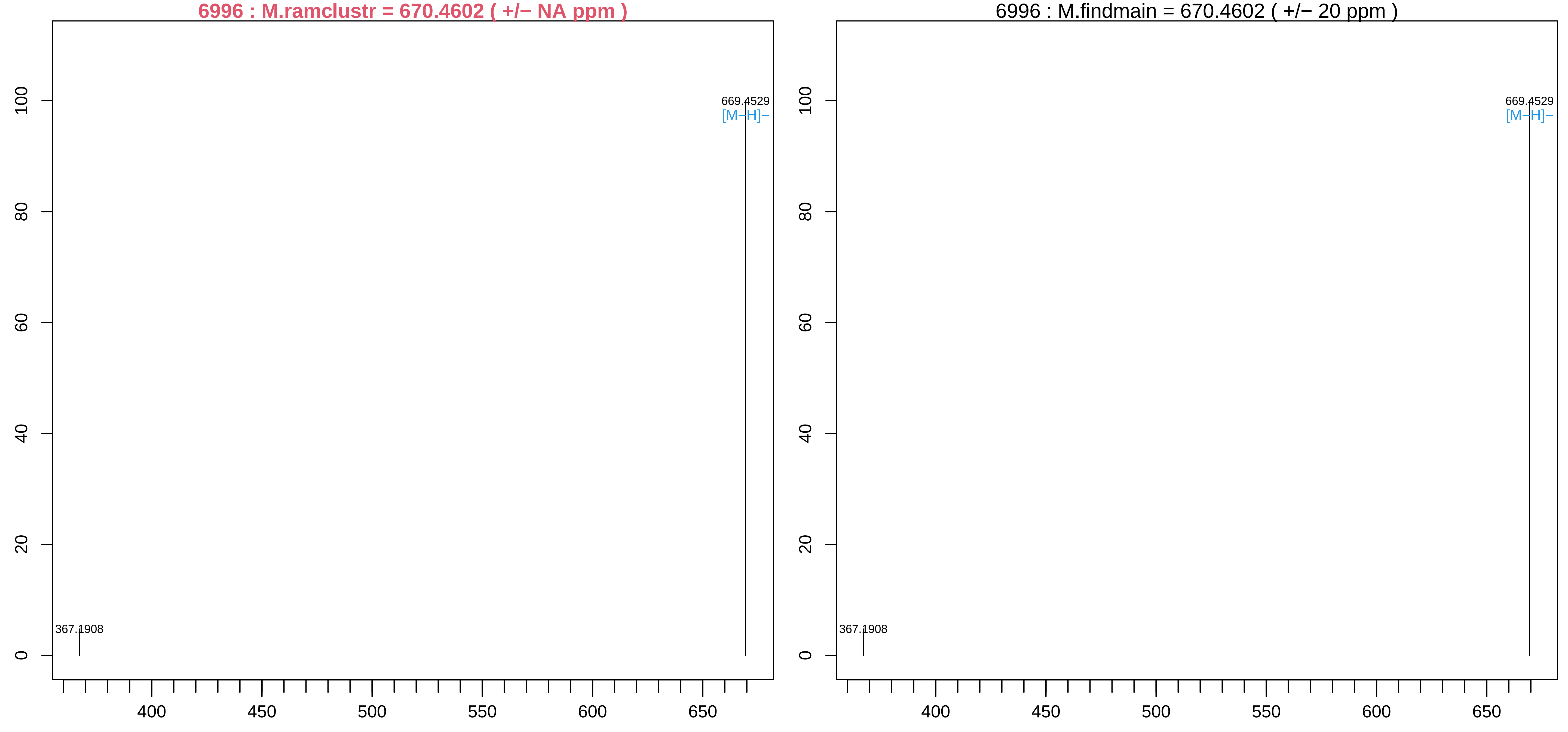
6996 : M.ramclustr = 670.4602 (+/− NA ppm)
669.4529
[M−H]−
367.1908
6996 : M.findmain = 670.4602 (+/− 20 ppm)
669.4529
[M−H]−
367.1908

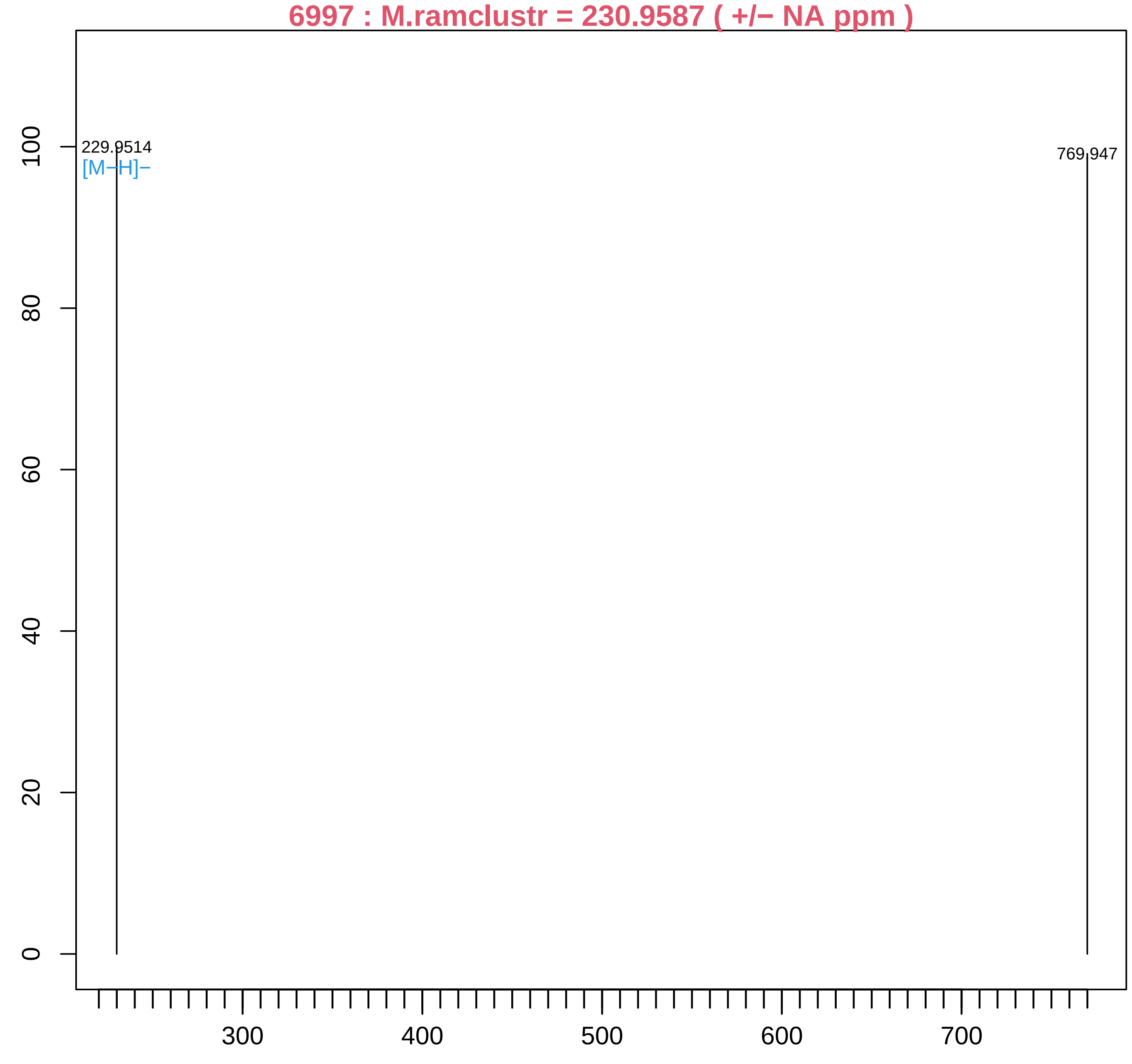
6997 : M.ramclustr = 230.9587 (+/− NA ppm)
229.9514
[M−H]−
769.947
0
20
40
60
80
100
300
400
500
600
700

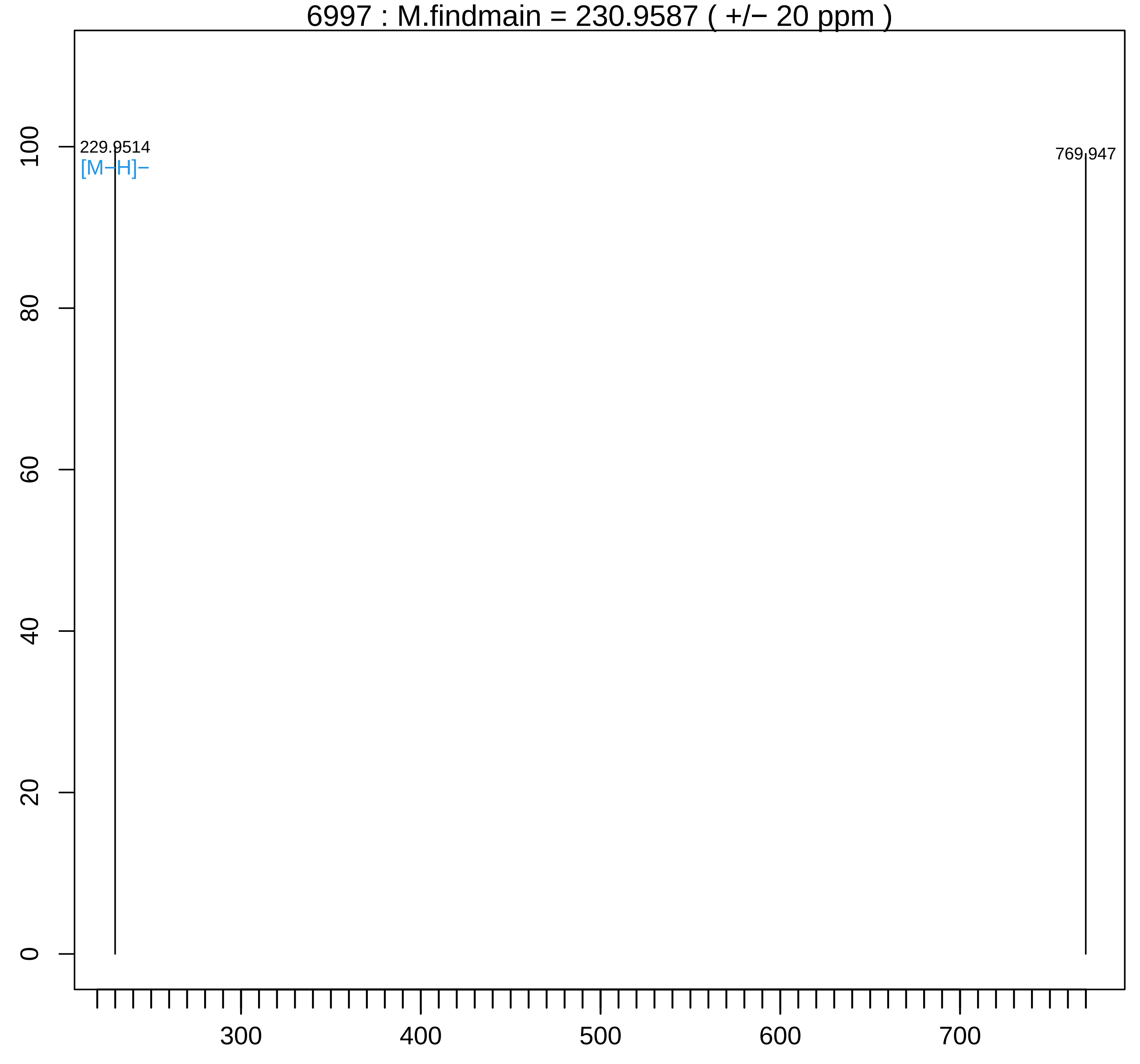
6997 : M.findmain = 230.9587 (+/− 20 ppm)
229.9514
[M−H]−
769.947
0
20
40
60
80
100
300
400
500
600
700

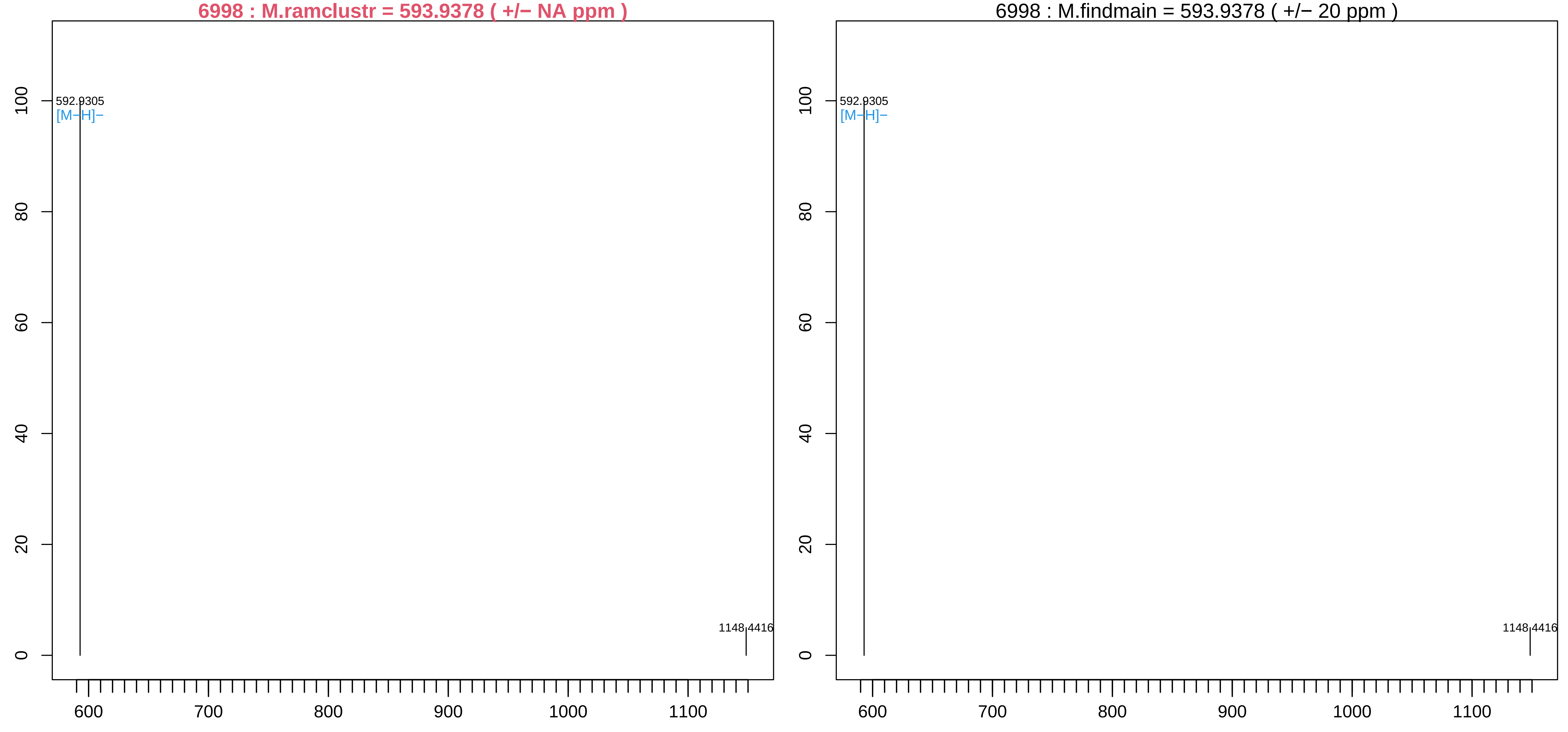
6998 : M.ramclustr = 593.9378 (+/− NA ppm)
592.9305
[M−H]−
1148.4416
6998 : M.findmain = 593.9378 (+/− 20 ppm)
592.9305
[M−H]−
1148.4416
0
20
40
60
80
100
600
700
800
900
1000
1100

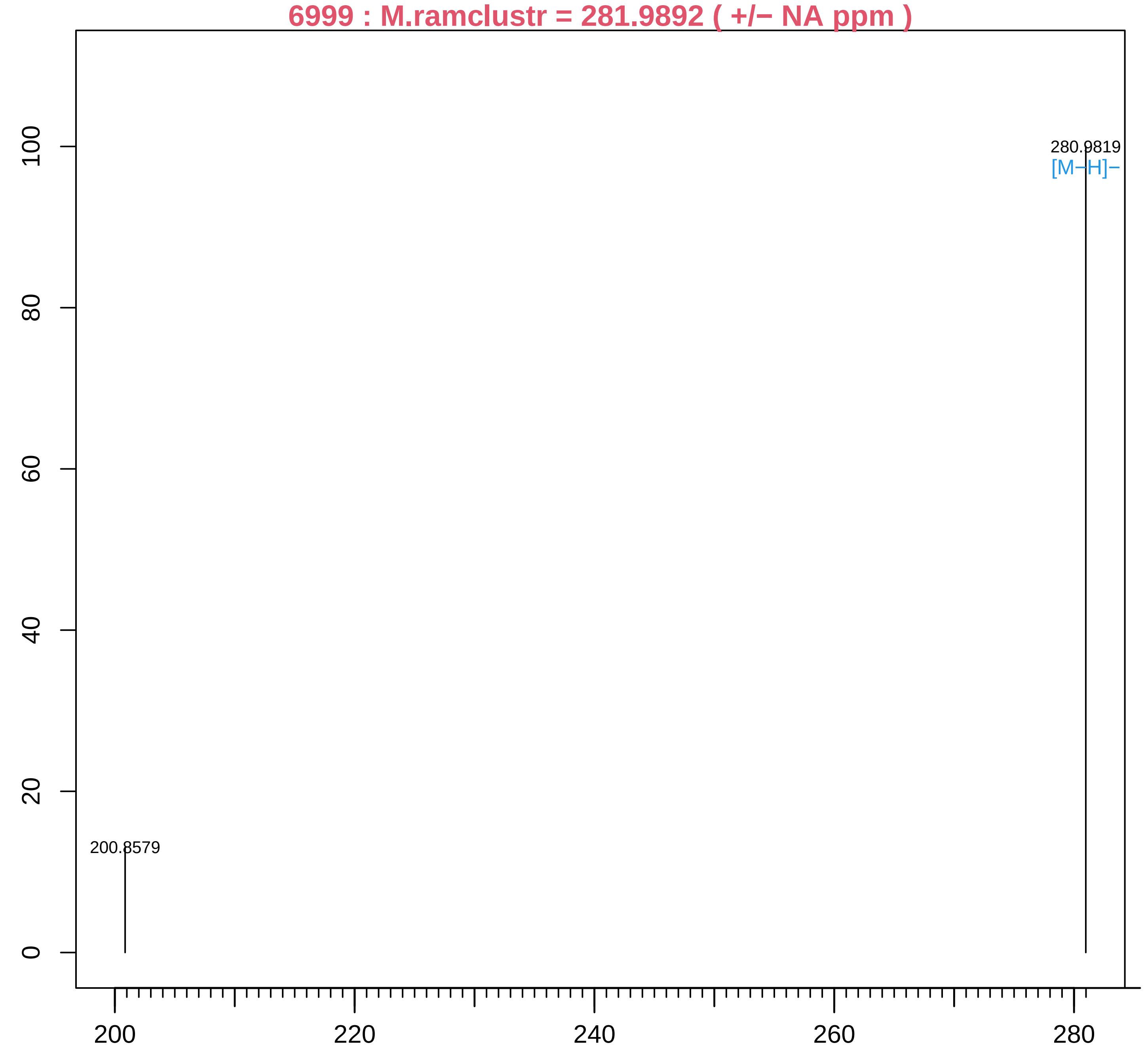
6999 : M.ramclustr = 281.9892 (+/− NA ppm)
280.9819
[M−H]−
200.8579
100
80
60
40
20
0
200
220
240
260
280

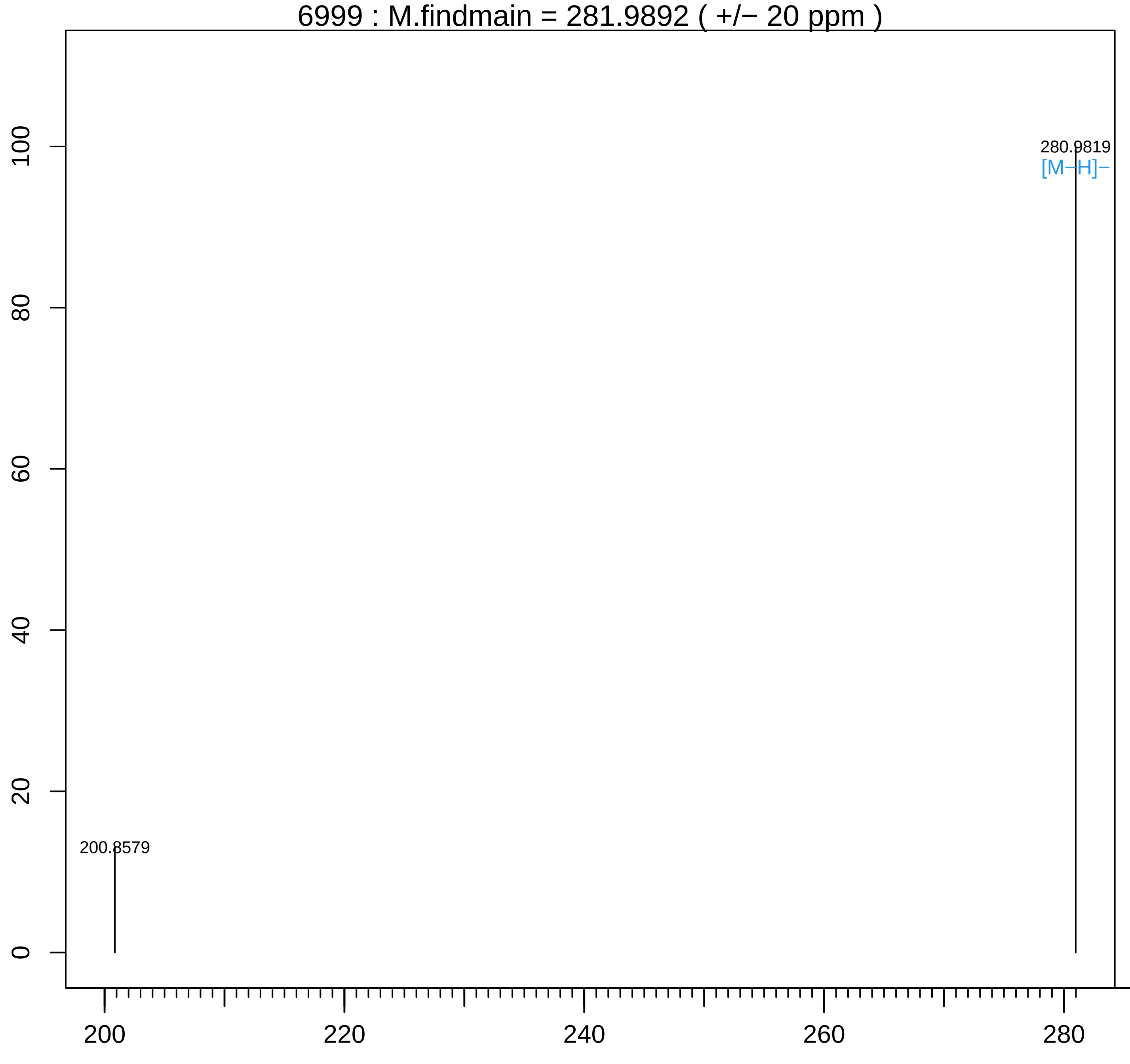
6999 : M.findmain = 281.9892 (+/− 20 ppm)
280.9819
[M−H]−
200.8579
100
80
60
40
20
0
200
220
240
260
280

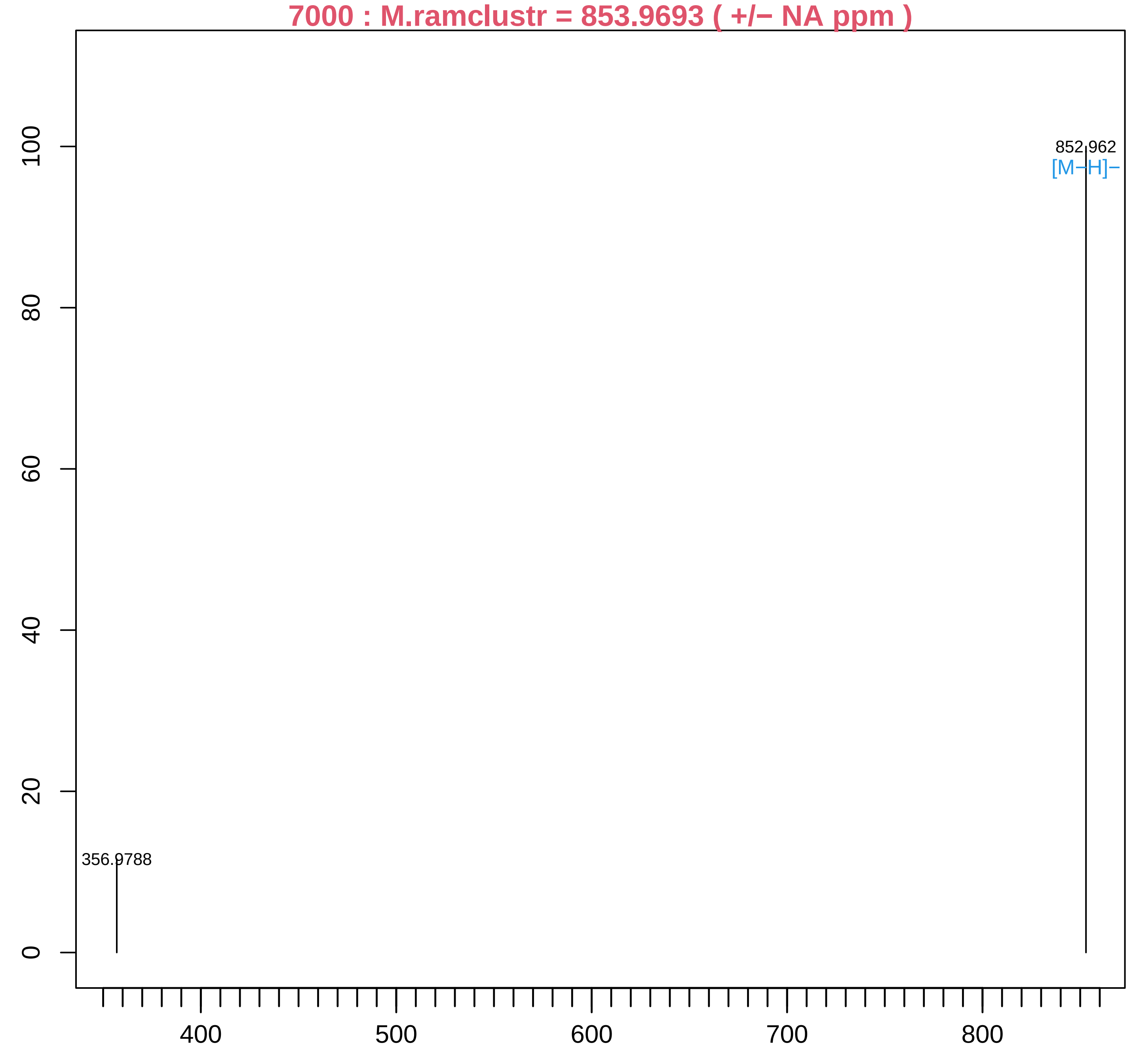
7000 : M.ramclustr = 853.9693 (+/− NA ppm)
852.962
[M−H]−
356.9788
0
20
40
60
80
100
400
500
600
700
800

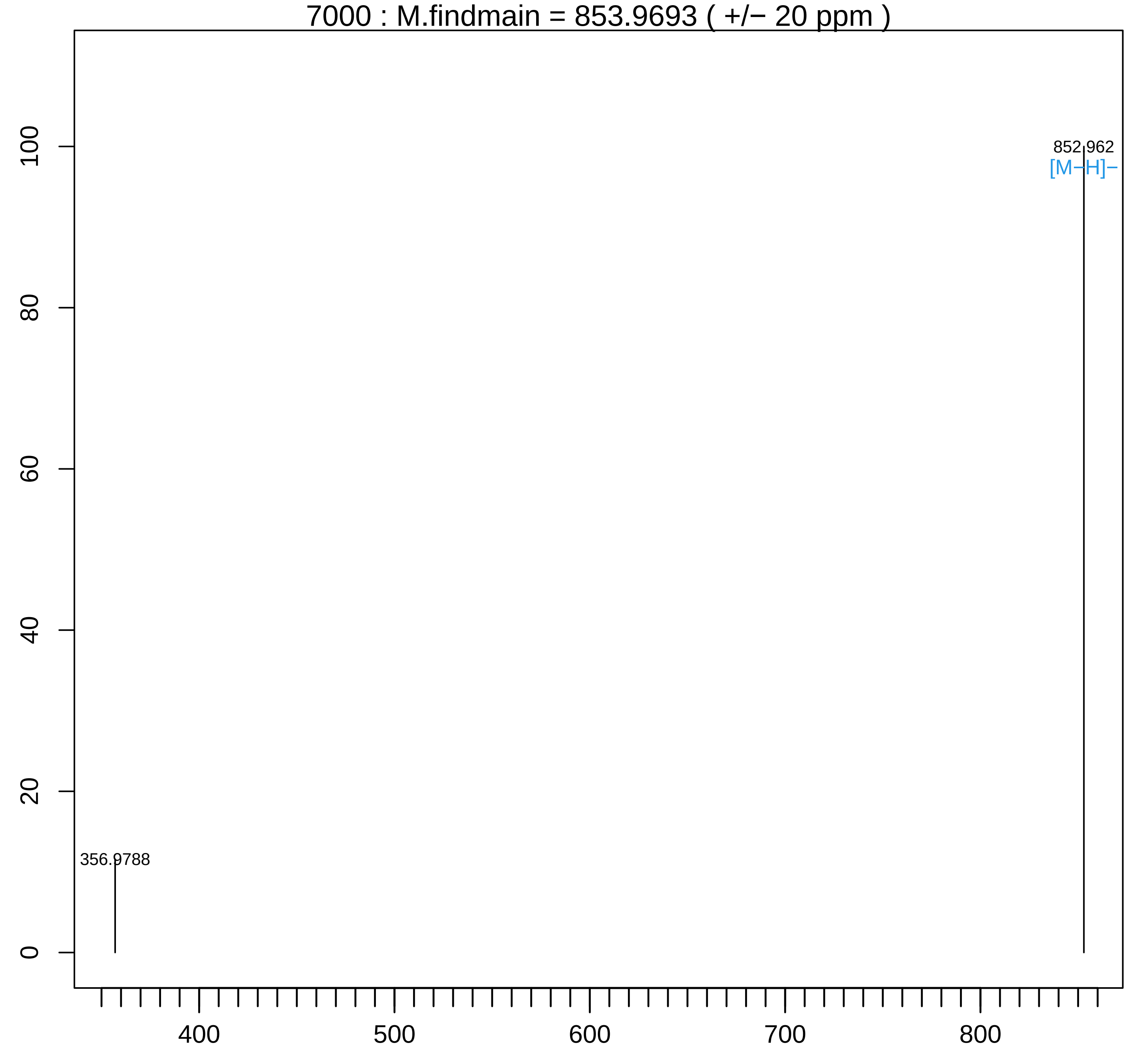
7000 : M.findmain = 853.9693 (+/− 20 ppm)
852.962
[M−H]−
356.9788
0
20
40
60
80
100
400
500
600
700
800

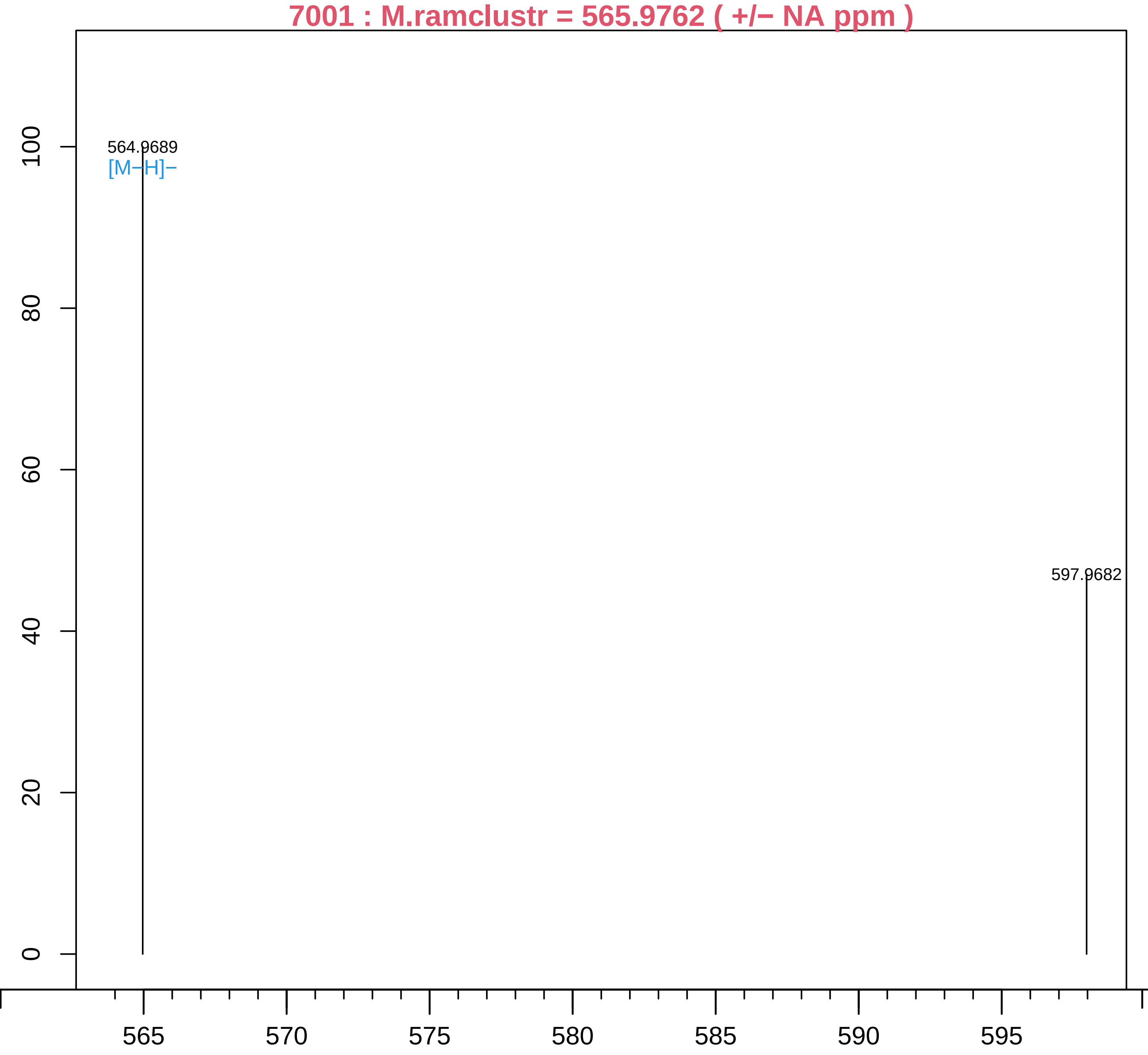
7001 : M.ramclustr = 565.9762 (+/− NA ppm)
564.9689
[M−H]−
597.9682
0
20
40
60
80
100
565
570
575
580
585
590
595

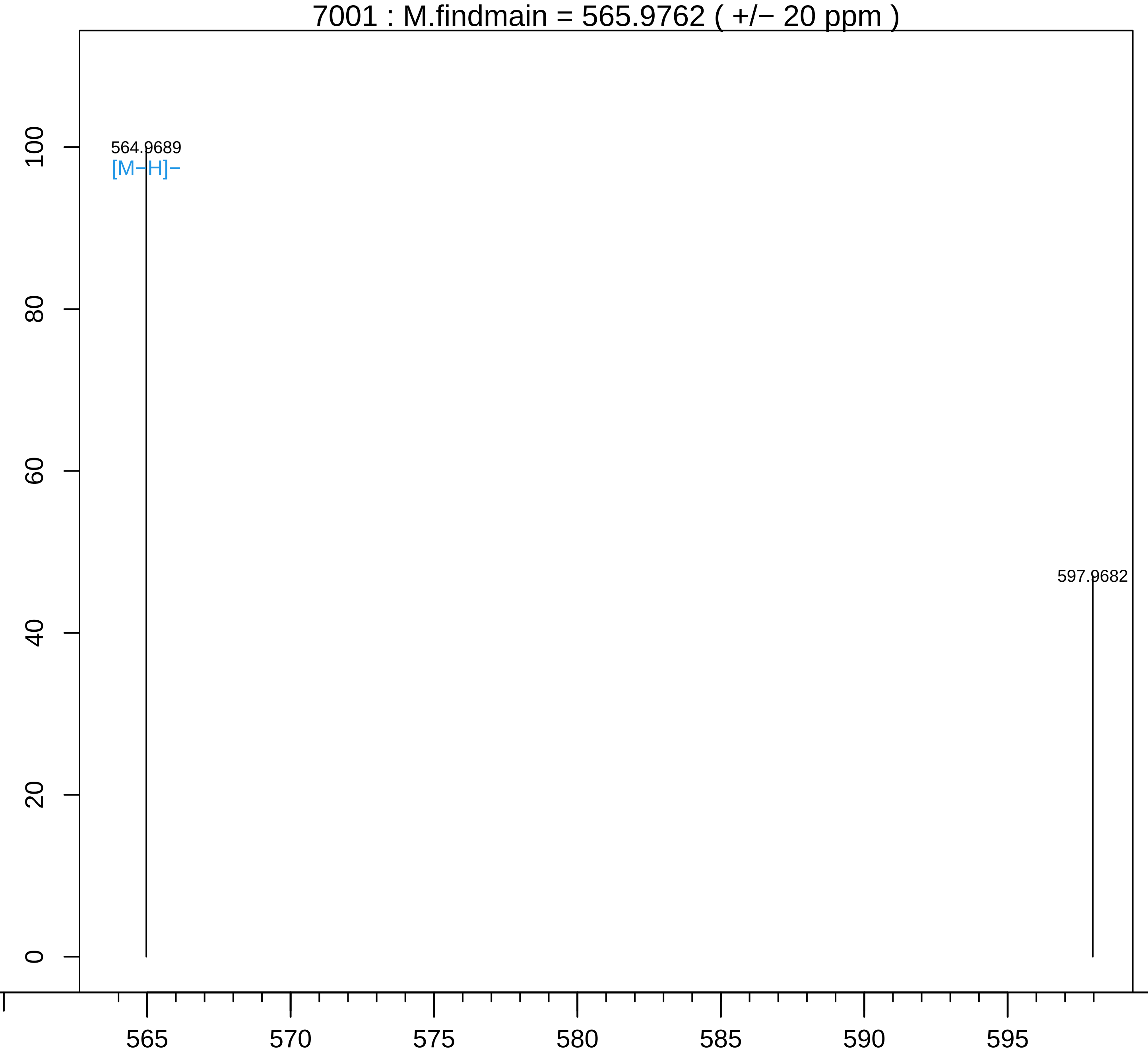
7001 : M.findmain = 565.9762 (+/− 20 ppm)
564.9689
[M−H]−
597.9682
0
20
40
60
80
100
565
570
575
580
585
590
595

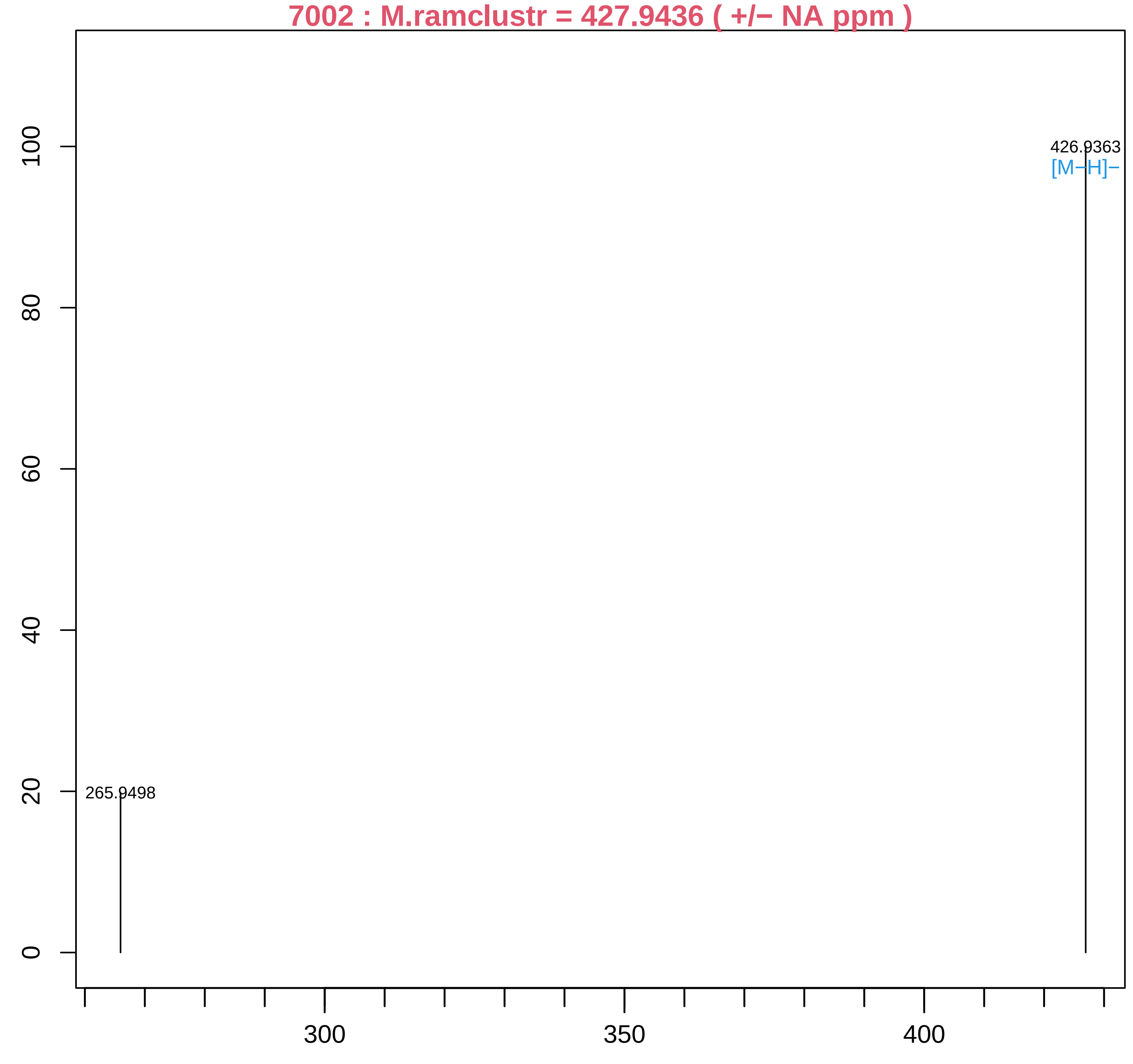

7002 : M.ramclustr = 427.9436 (+/− NA ppm)
426.9363
[M−H]−
265.9498
0
20
40
60
80
100
300
350
400

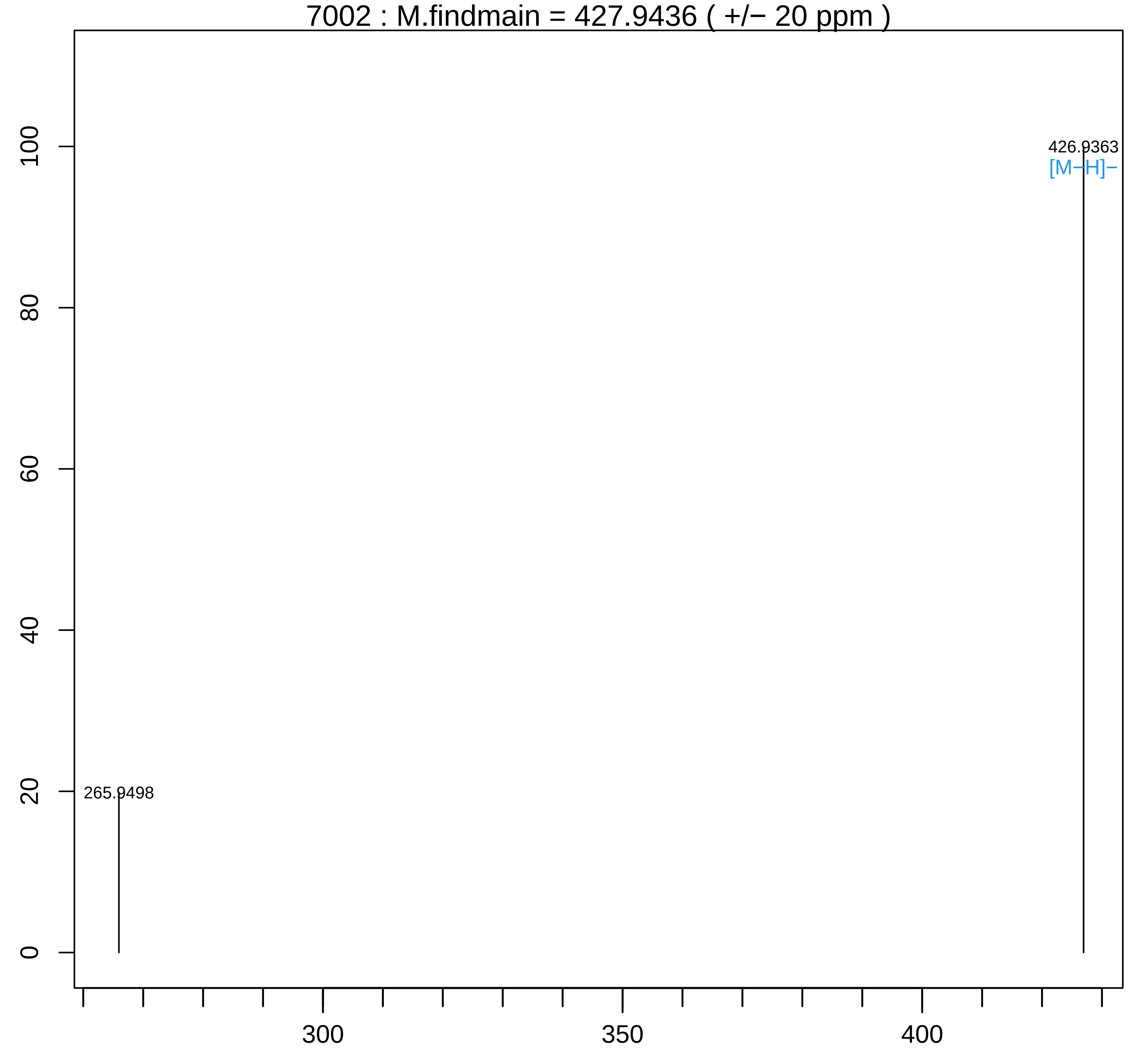

7002 : M.findmain = 427.9436 (+/− 20 ppm)
426.9363
[M−H]−
265.9498
0
20
40
60
80
100
300
350
400

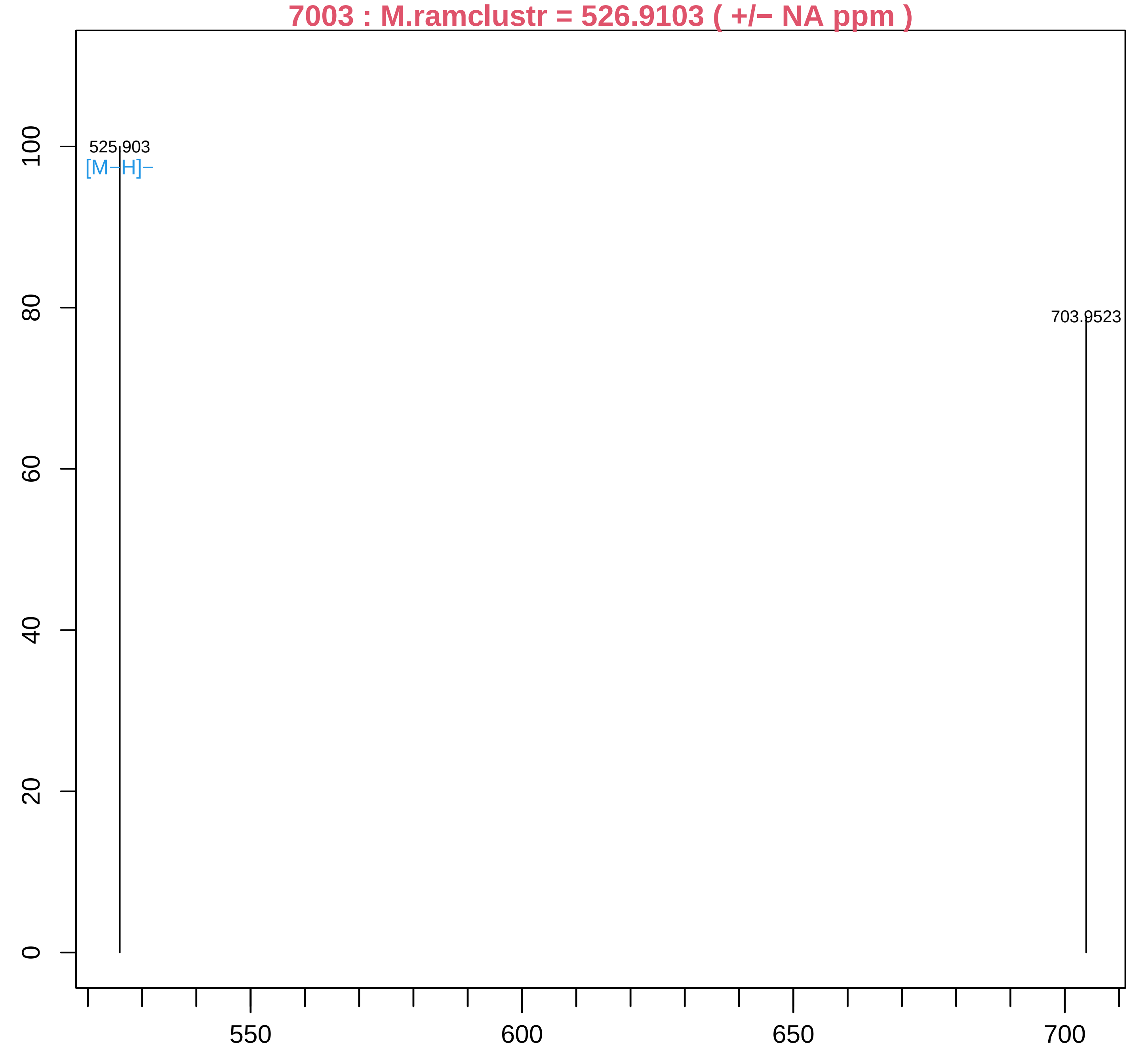
7003 : M.ramclustr = 526.9103 (+/− NA ppm)
525.903
[M−H]−
703.9523
0
20
40
60
80
100
550
600
650
700

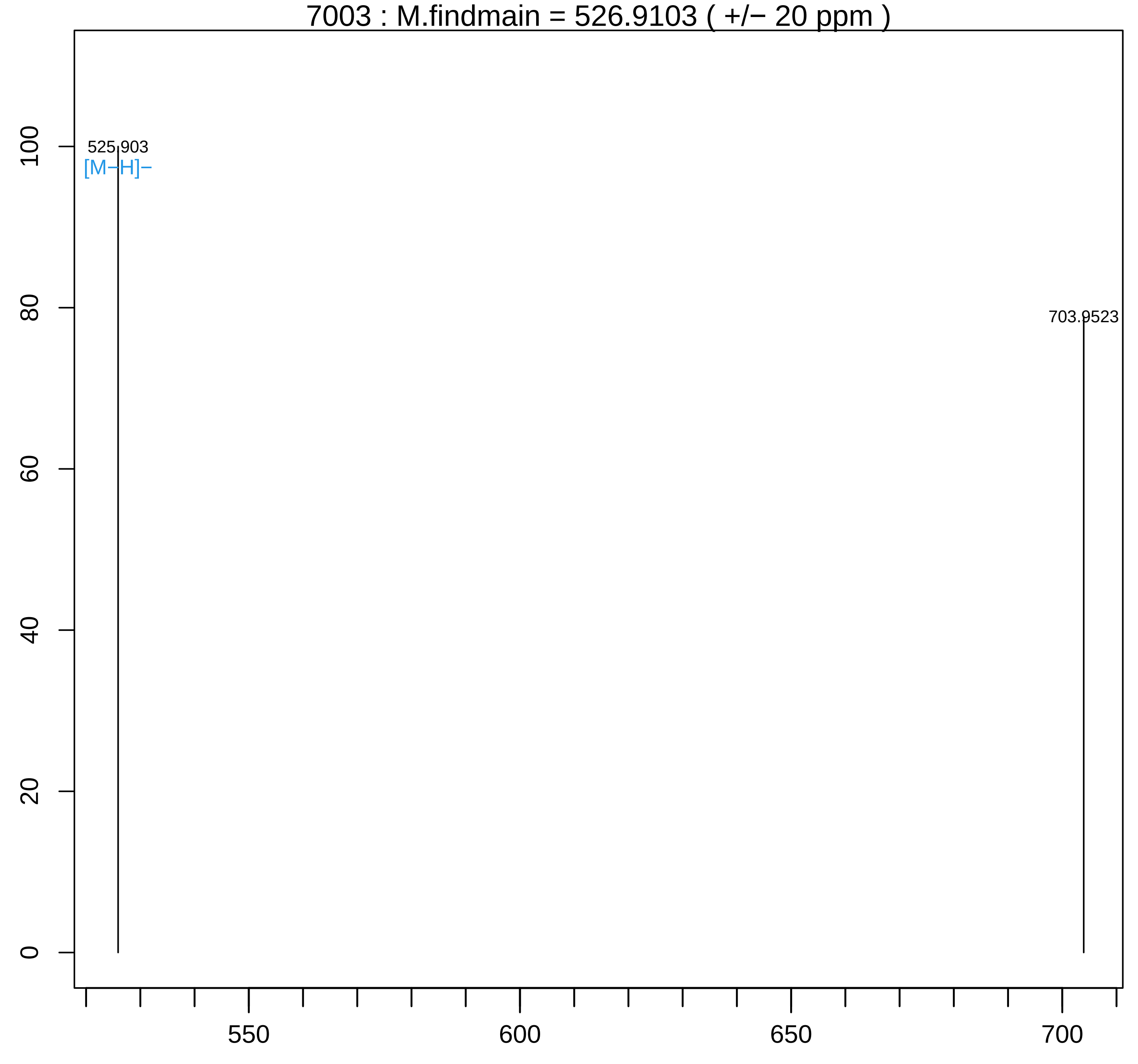
7003 : M.findmain = 526.9103 (+/− 20 ppm)
525.903
[M−H]−
703.9523
0
20
40
60
80
100
550
600
650
700

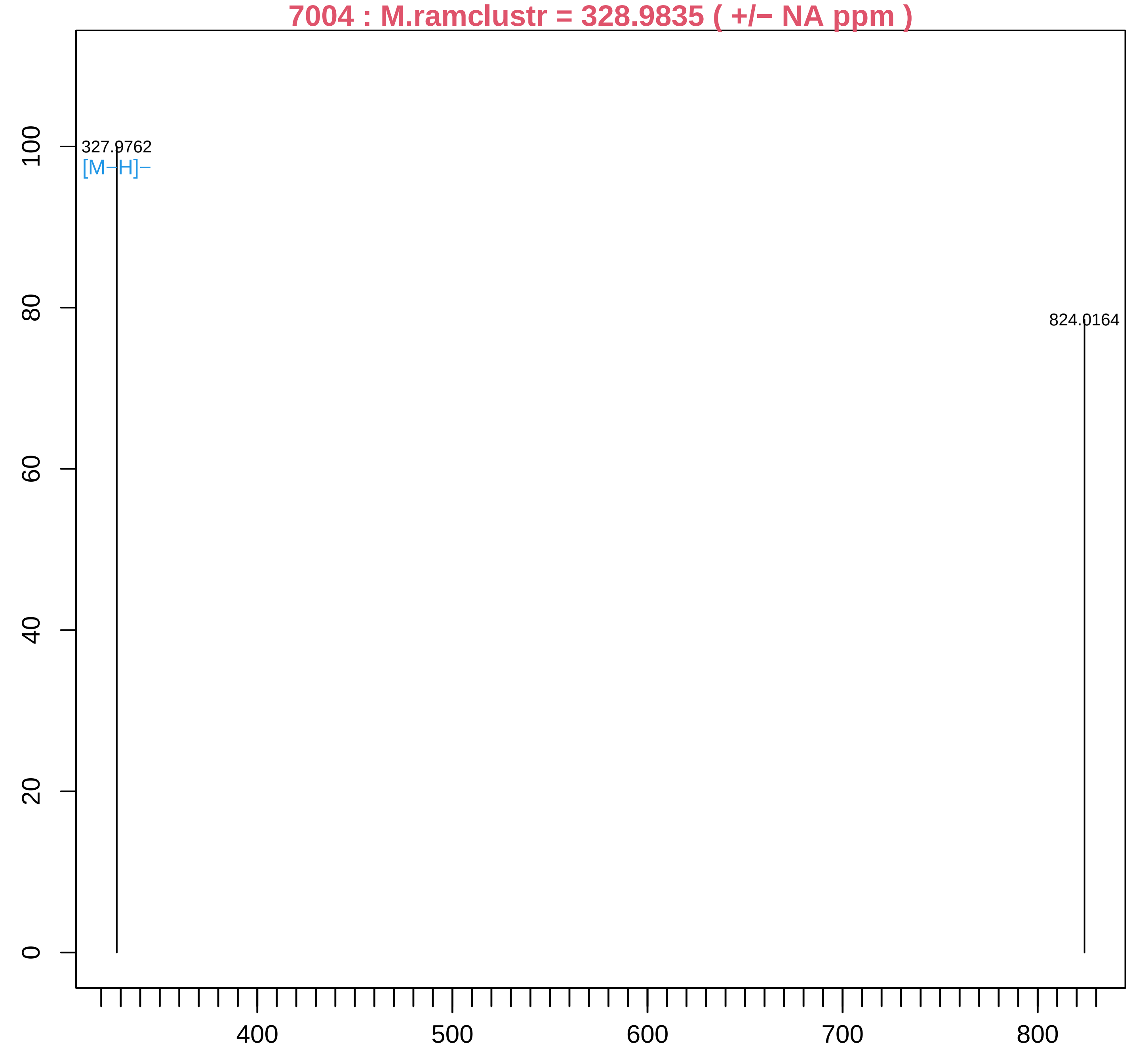
7004 : M.ramclustr = 328.9835 (+/− NA ppm)
327.9762
[M−H]−
824.0164
0
20
40
60
80
100
400
500
600
700
800

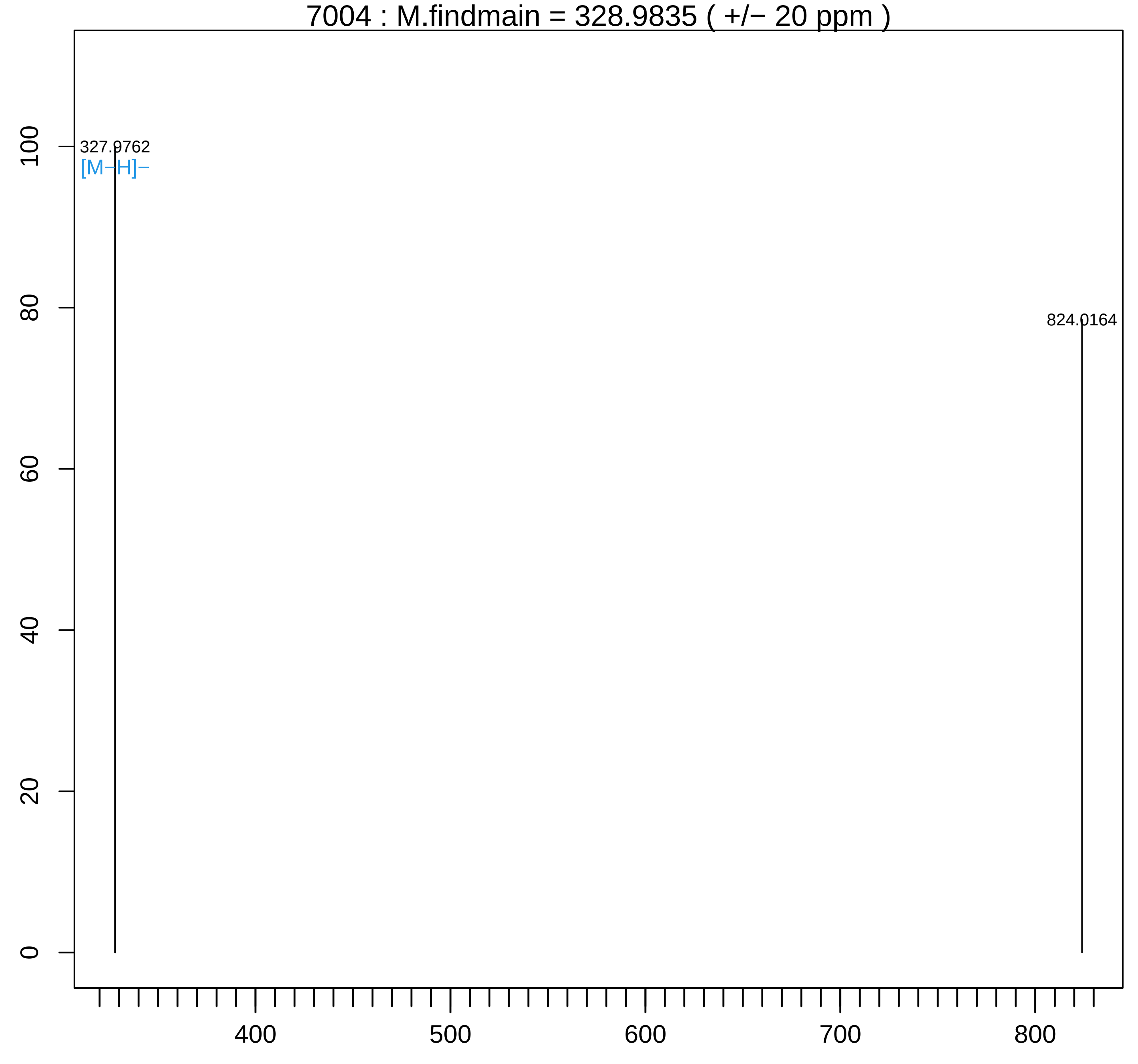
7004 : M.findmain = 328.9835 (+/− 20 ppm)
327.9762
[M−H]−
824.0164
0
20
40
60
80
100
400
500
600
700
800

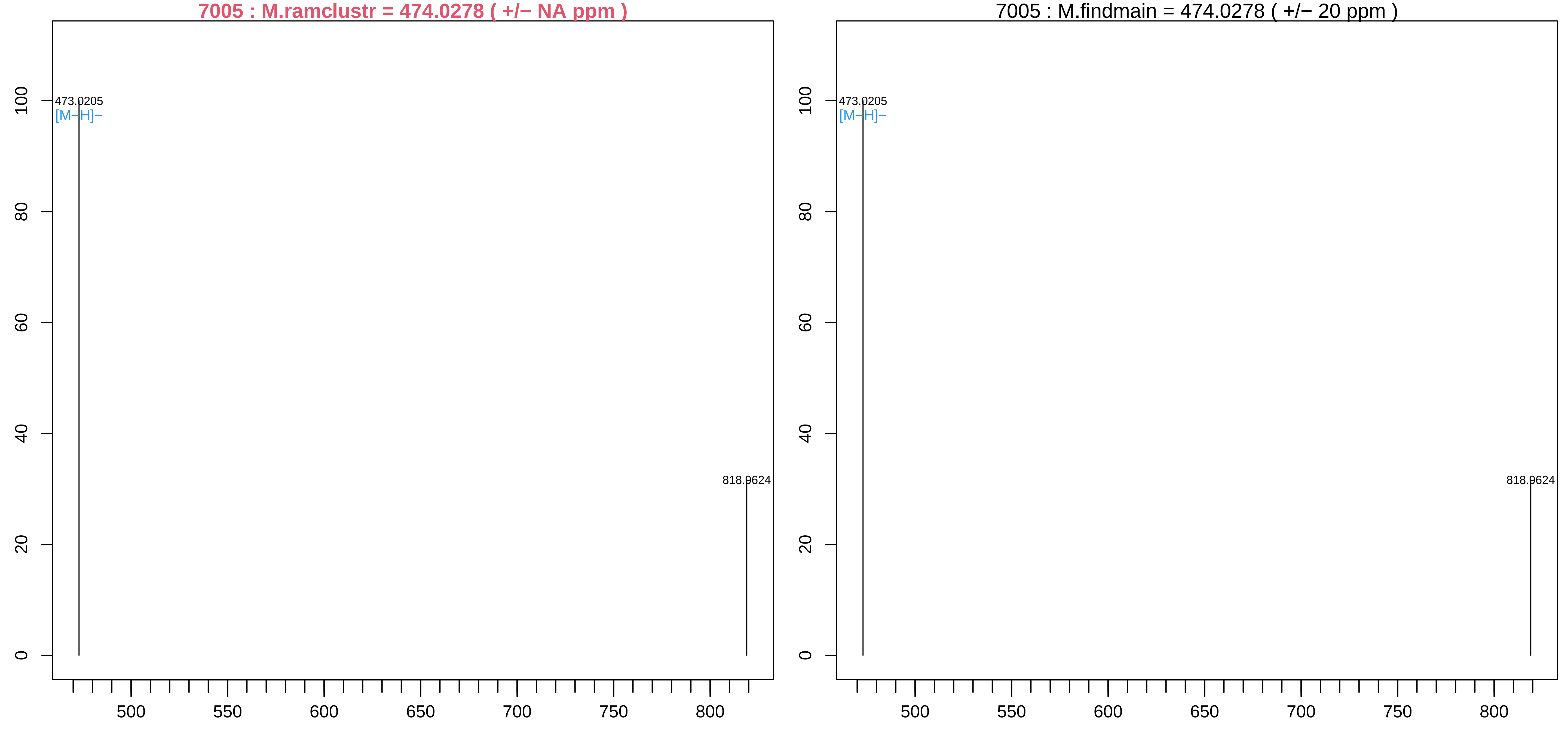

7005 : M.ramclustr = 474.0278 (+/− NA ppm)
473.0205
[M−H]−
818.9624
0
20
40
60
80
100
500
550
600
650
700
750
800
7005 : M.findmain = 474.0278 (+/− 20 ppm)
473.0205
[M−H]−
818.9624
0
20
40
60
80
100
500
550
600
650
700
750
800

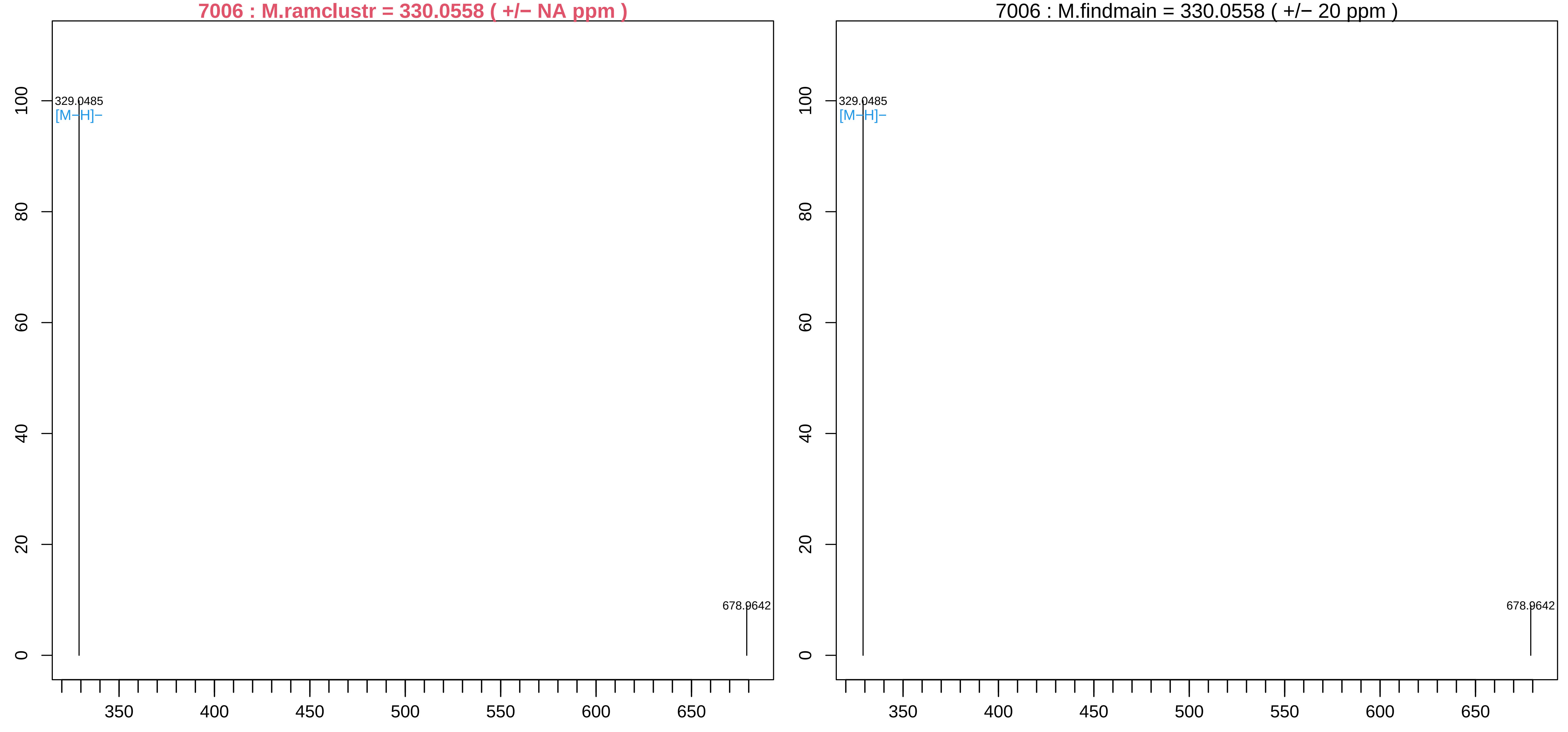
7006 : M.ramclustr = 330.0558 (+/− NA ppm)
7006 : M.findmain = 330.0558 (+/− 20 ppm)
329.0485
[M−H]−
678.9642
0
20
40
60
80
100
350
400
450
500
550
600
650

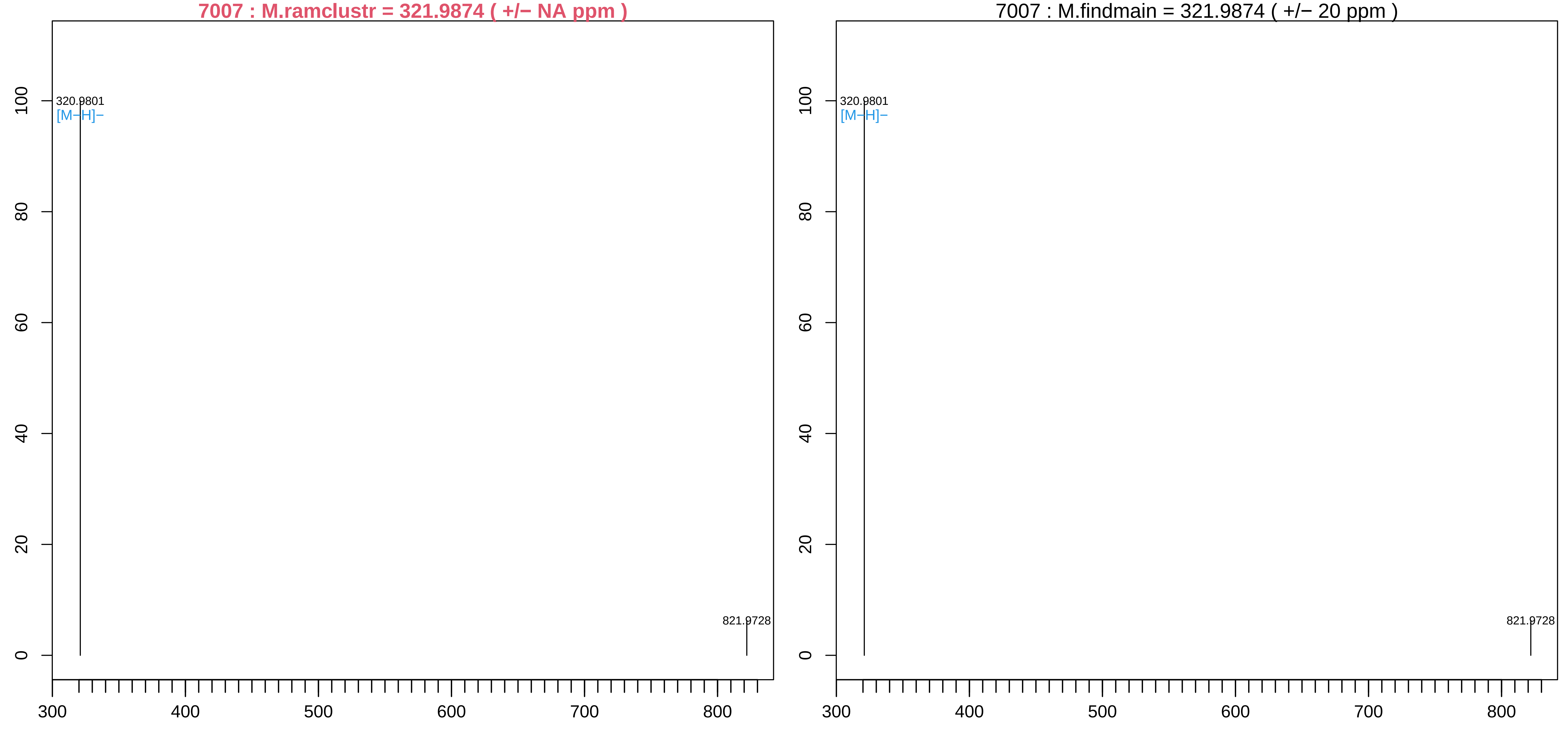
7007 : M.ramclustr = 321.9874 (+/− NA ppm)
320.9801
[M−H]−
821.9728
7007 : M.findmain = 321.9874 (+/− 20 ppm)
320.9801
[M−H]−
821.9728
0
20
40
60
80
100
300
400
500
600
700
800

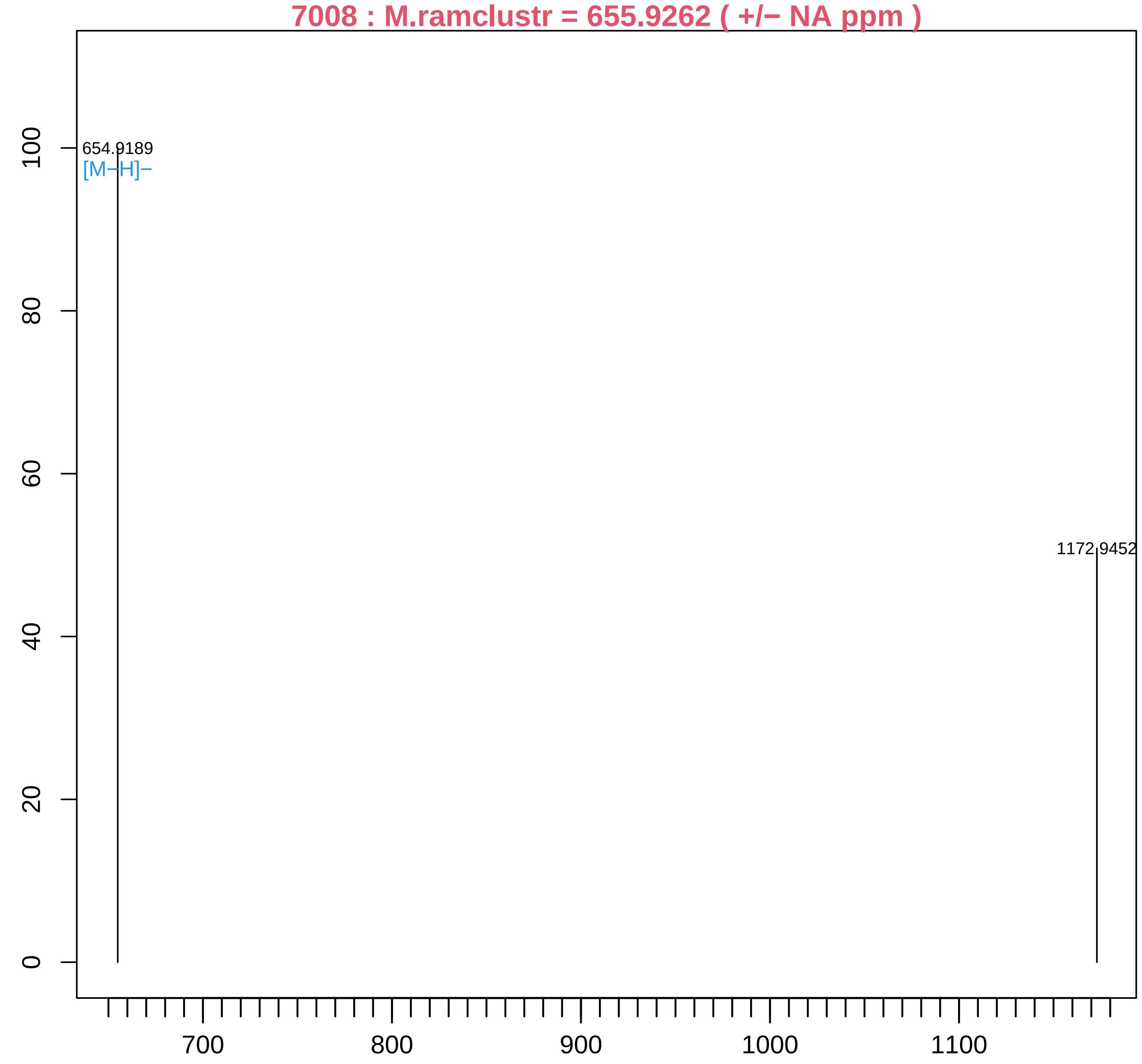
7008 : M.ramclustr = 655.9262 (+/− NA ppm)
654.9189
[M−H]−
1172.9452
0
20
40
60
80
100
700
800
900
1000
1100

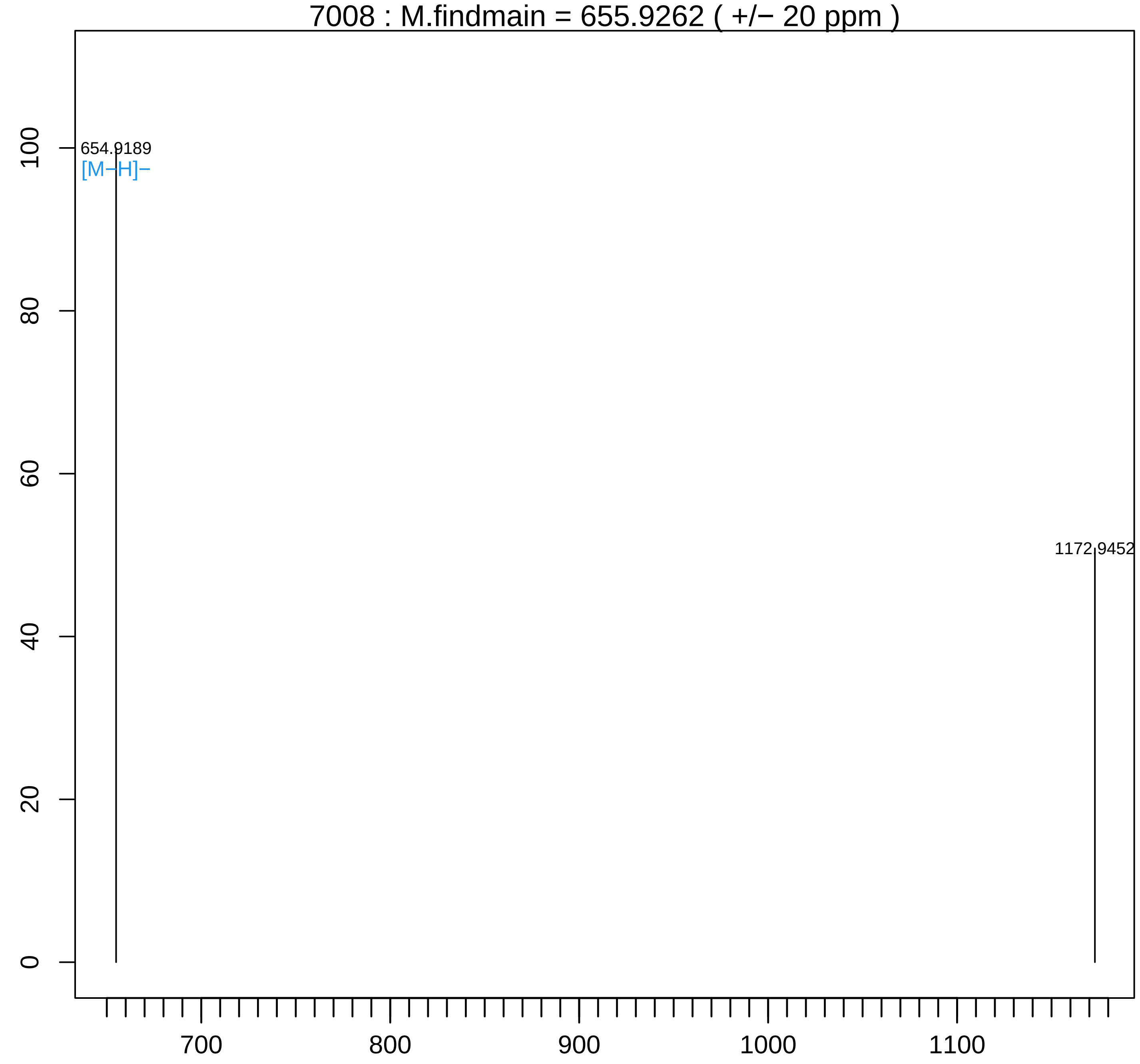
7008 : M.findmain = 655.9262 (+/− 20 ppm)
654.9189
[M−H]−
1172.9452
0
20
40
60
80
100
700
800
900
1000
1100

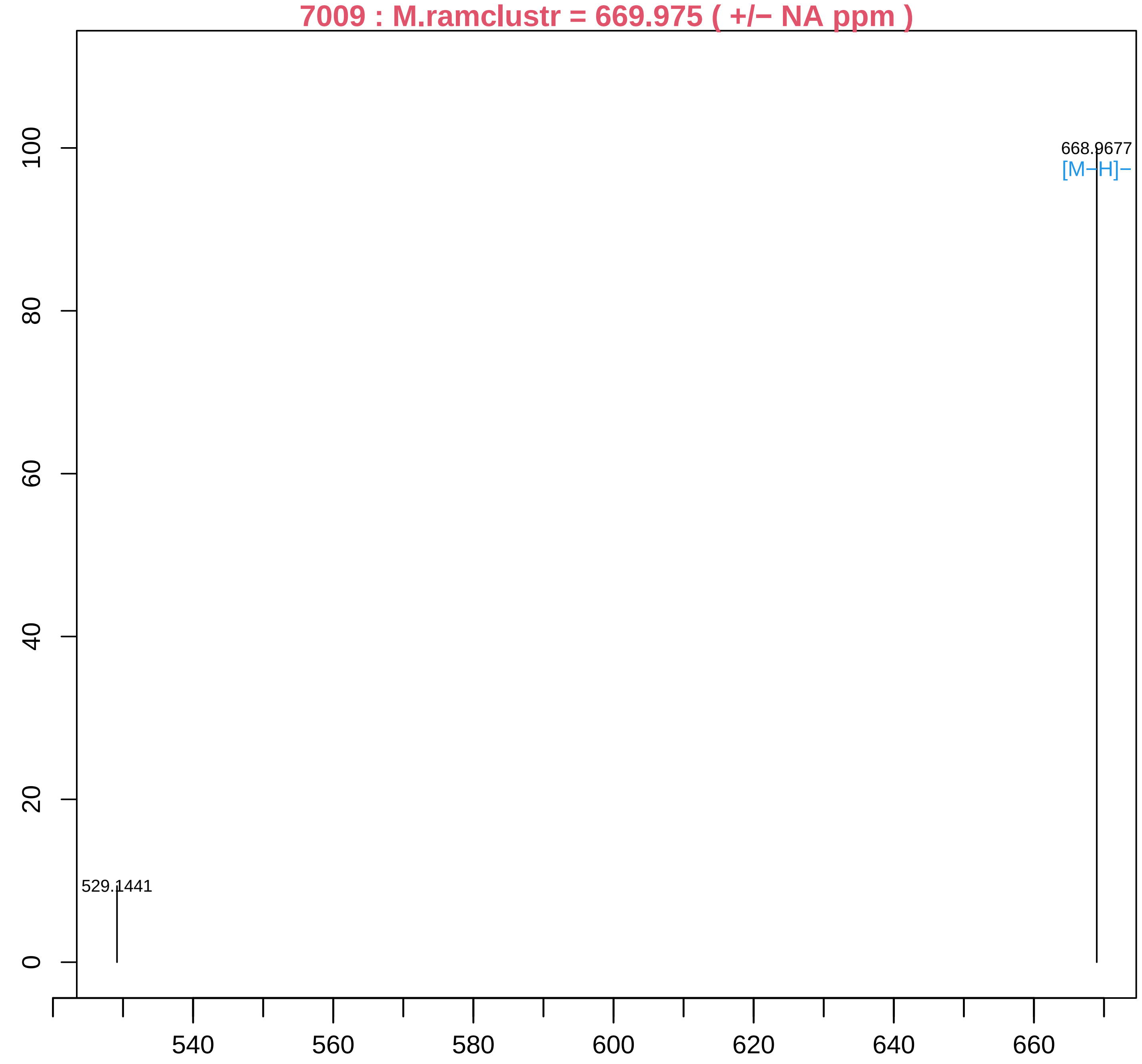
7009 : M.ramclustr = 669.975 (+/− NA ppm)
668.9677
[M−H]−
529.1441
0
20
40
60
80
100
540
560
580
600
620
640
660

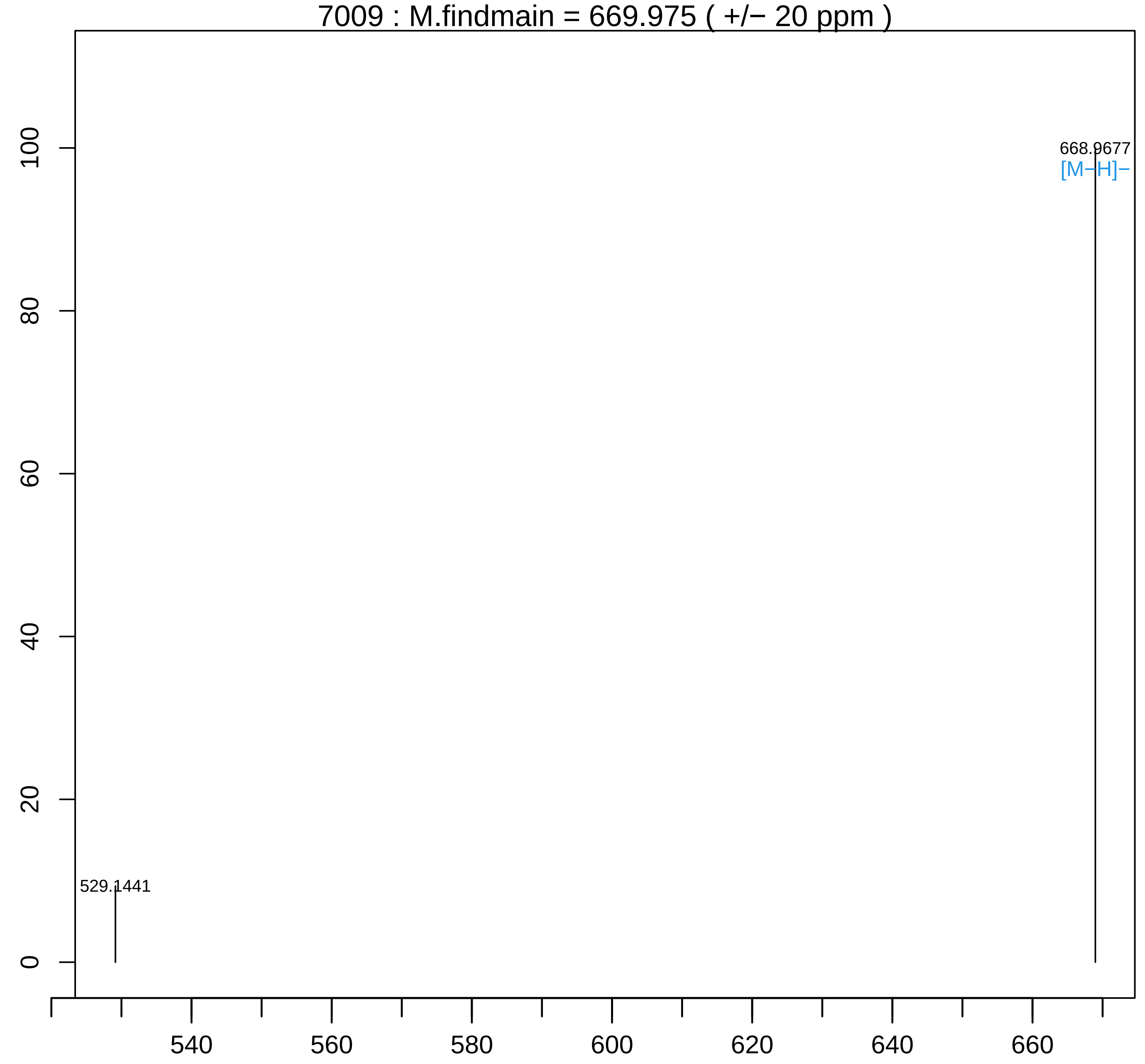
7009 : M.findmain = 669.975 (+/− 20 ppm)
668.9677
[M−H]−
529.1441
0
20
40
60
80
100
540
560
580
600
620
640
660

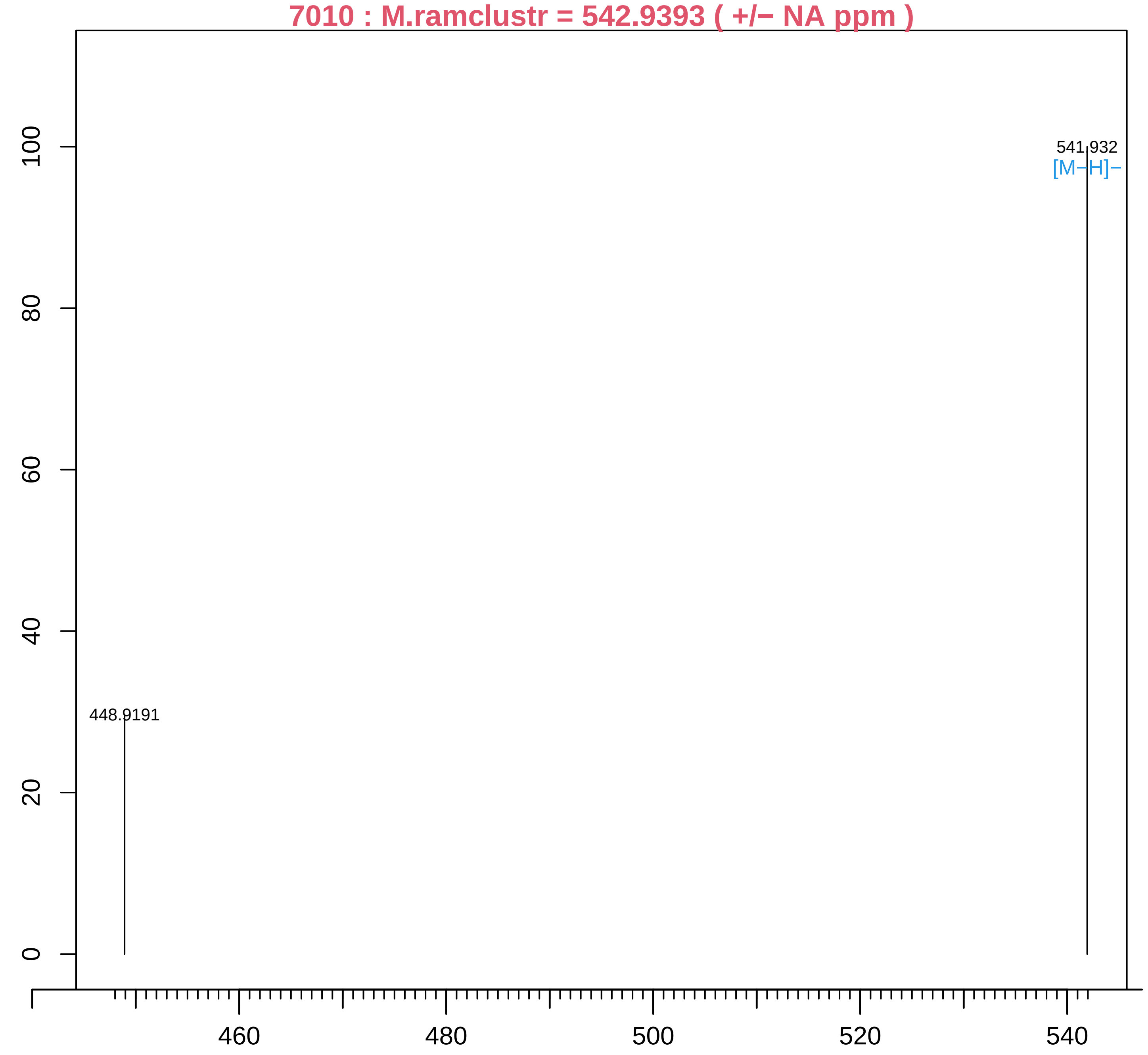
7010 : M.ramclustr = 542.9393 (+/− NA ppm)
541.932
[M−H]−
448.9191
0
20
40
60
80
100
460
480
500
520
540

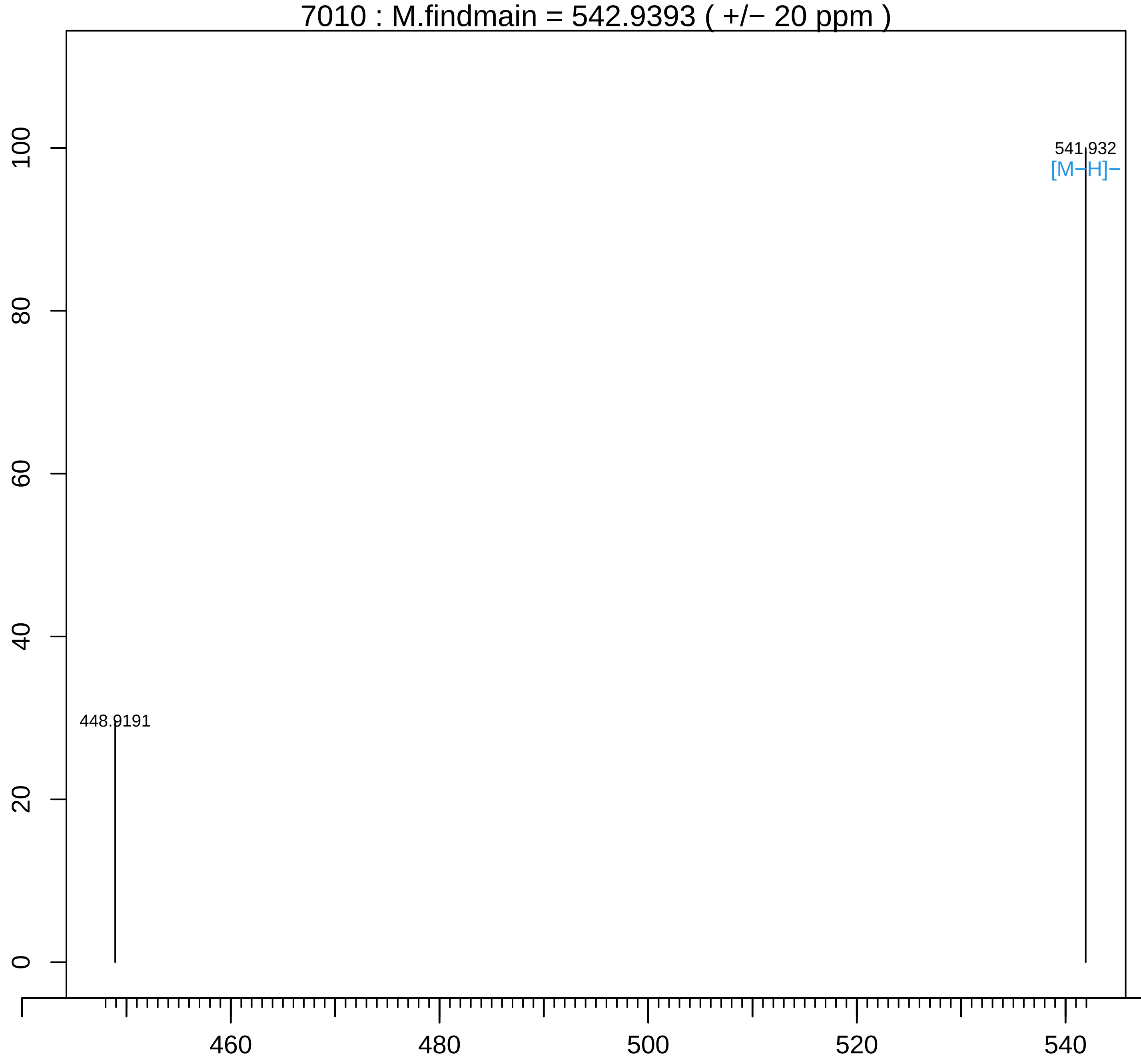
7010 : M.findmain = 542.9393 (+/− 20 ppm)
541.932
[M−H]−
448.9191
0
20
40
60
80
100
460
480
500
520
540

7011 : M.ramclustr = 219.9924 (+/− NA ppm)

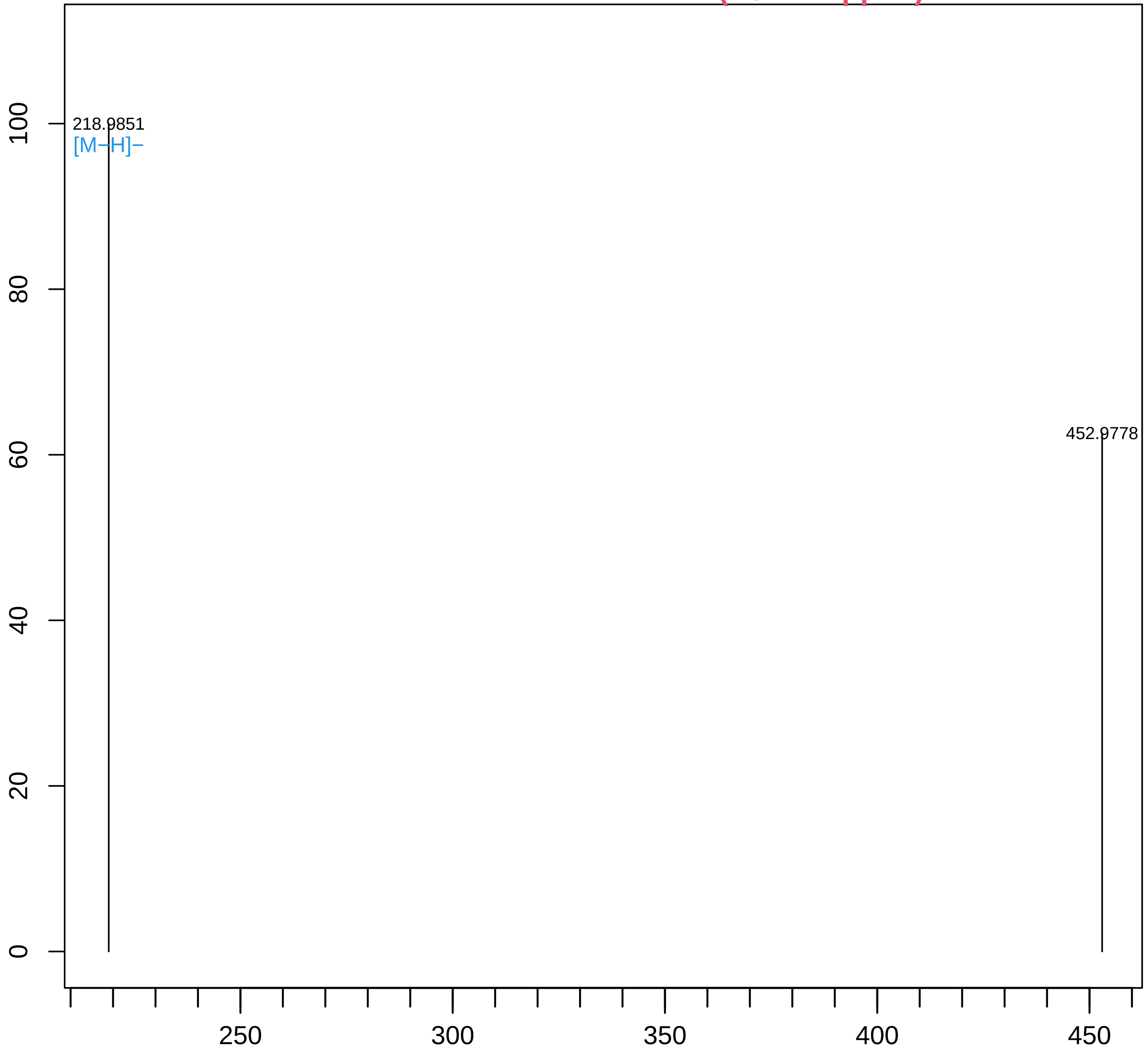

7011 : M.findmain = 219.9924 (+/− 20 ppm)

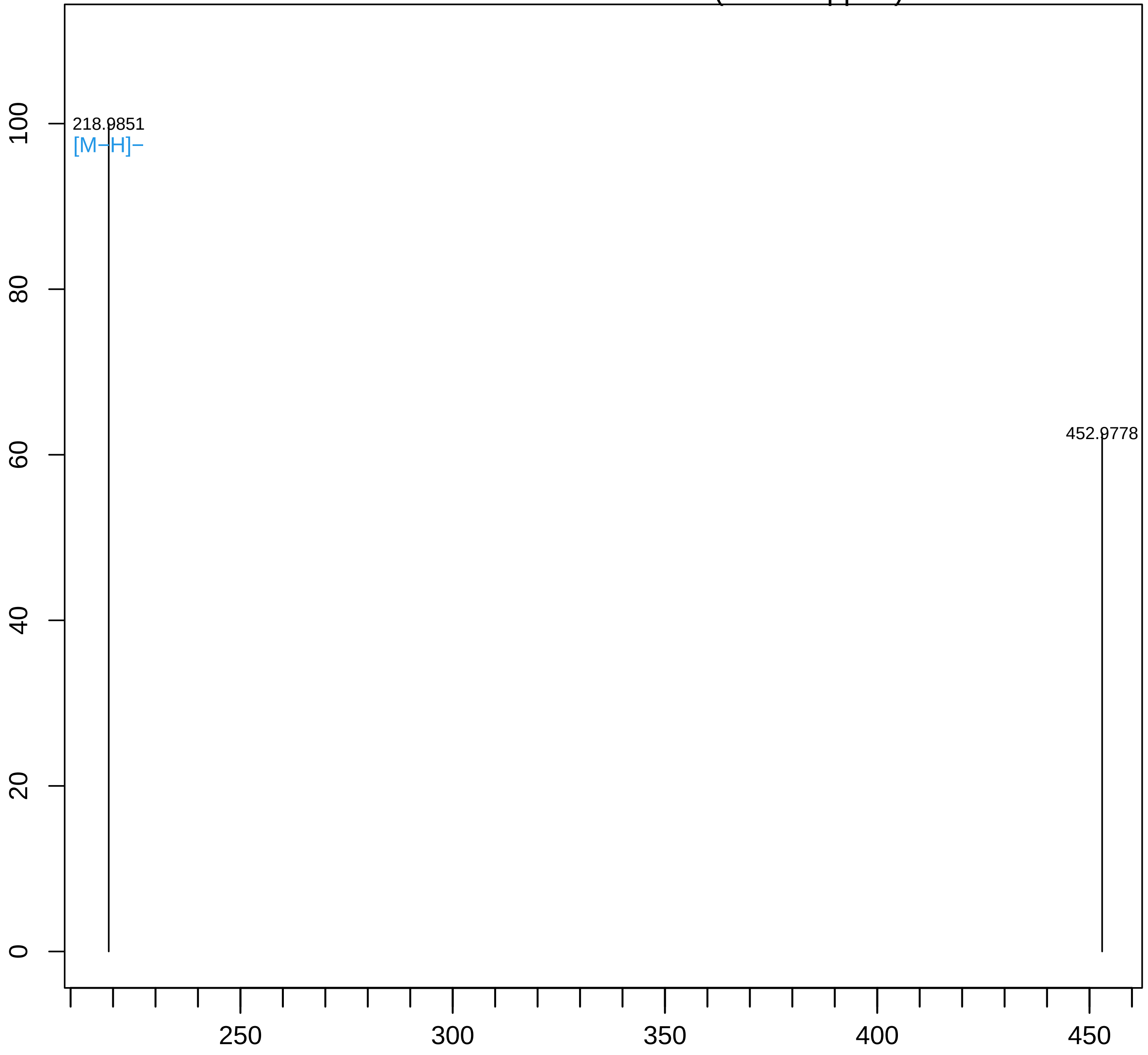

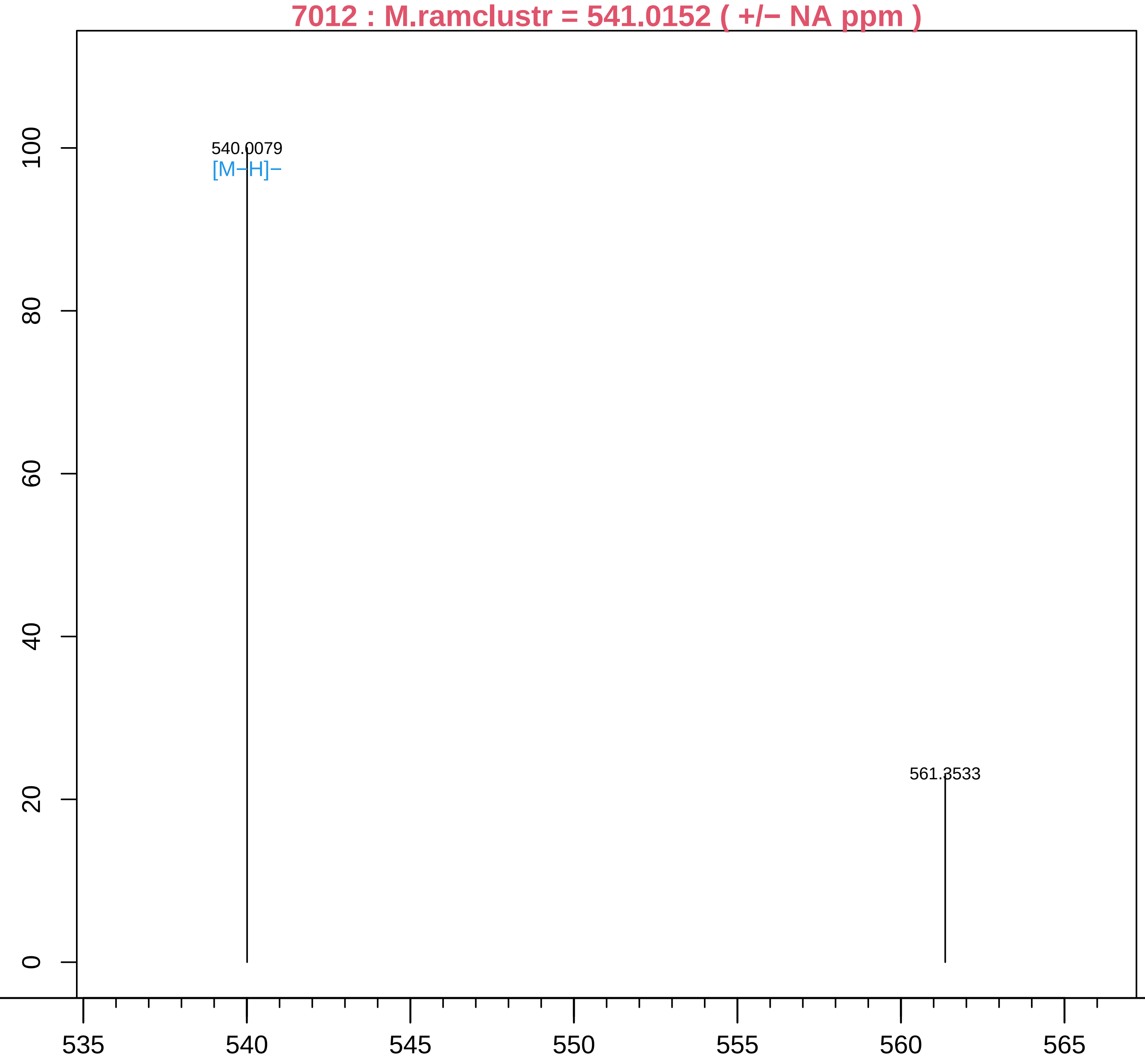
7012 : M.ramclustr = 541.0152 (+/− NA ppm)
540.0079
[M−H]−
561.3533
0
20
40
60
80
100
535
540
545
550
555
560
565

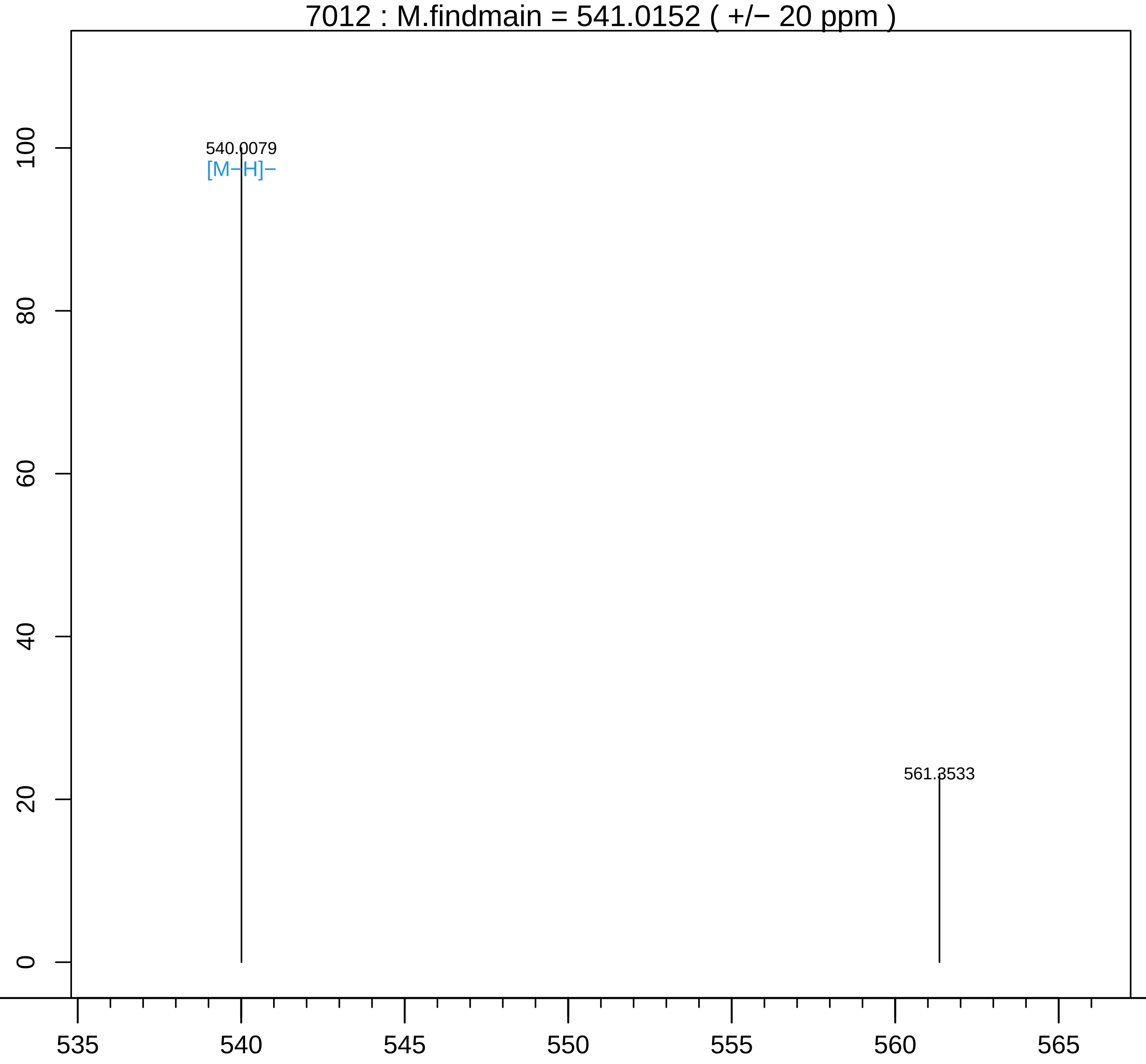
7012 : M.findmain = 541.0152 (+/− 20 ppm)
540.0079
[M−H]−
561.3533
0
20
40
60
80
100
535
540
545
550
555
560
565

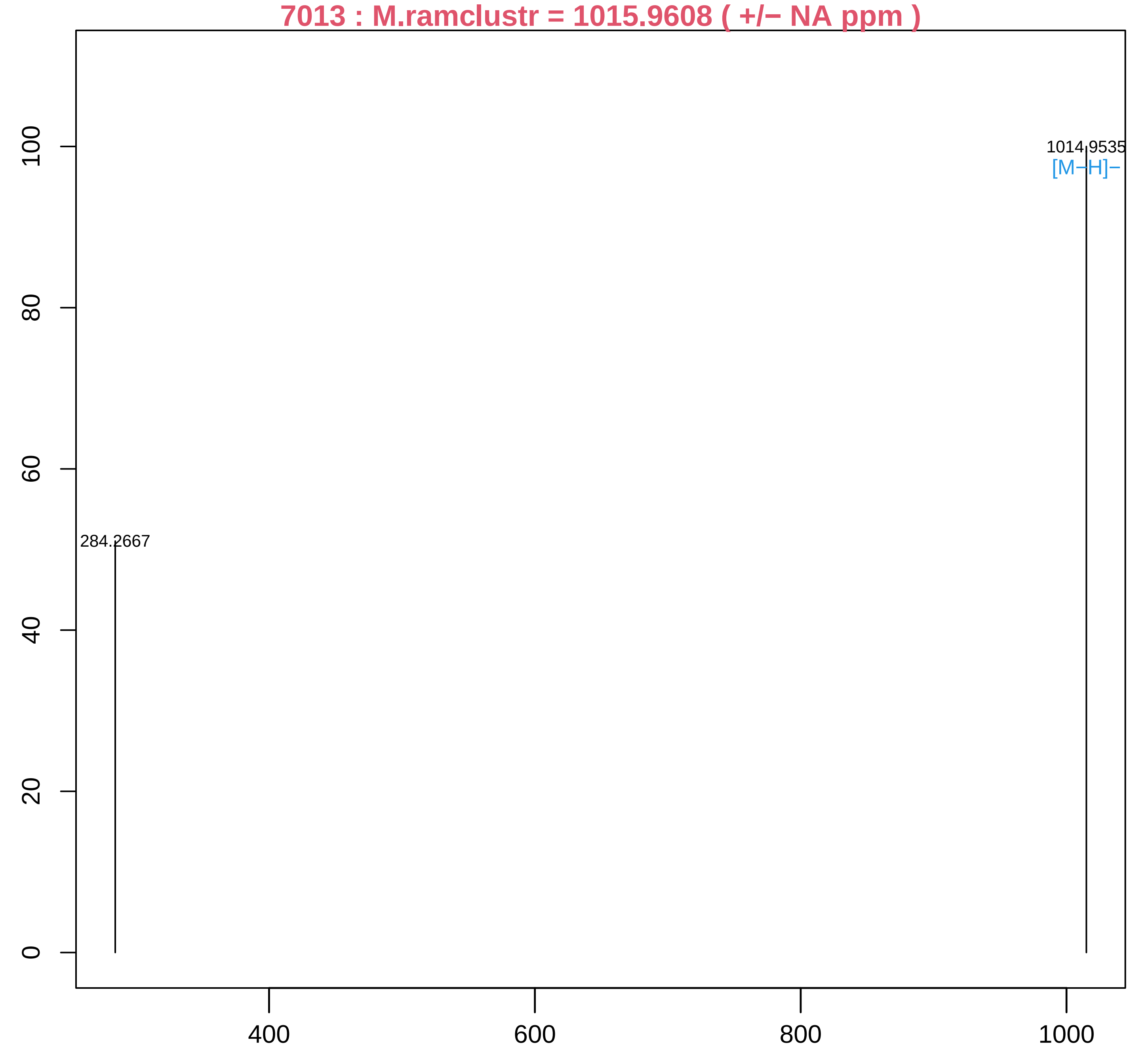
7013 : M.ramclustr = 1015.9608 (+/− NA ppm)
1014.9535
[M−H]−
284.2667
0
20
40
60
80
100
400
600
800
1000

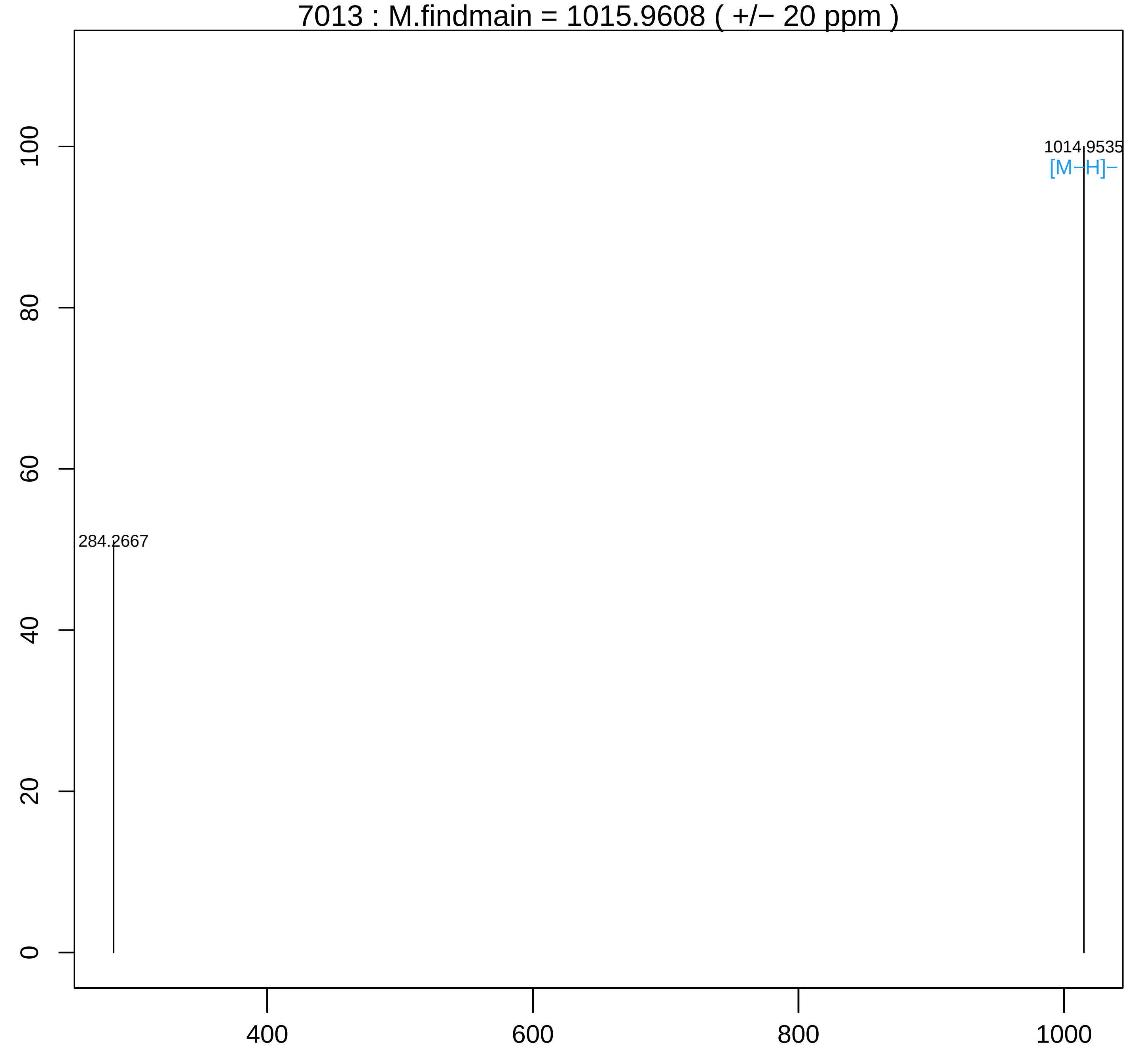
7013 : M.findmain = 1015.9608 (+/− 20 ppm)
1014.9535
[M−H]−
284.2667
0
20
40
60
80
100
400
600
800
1000

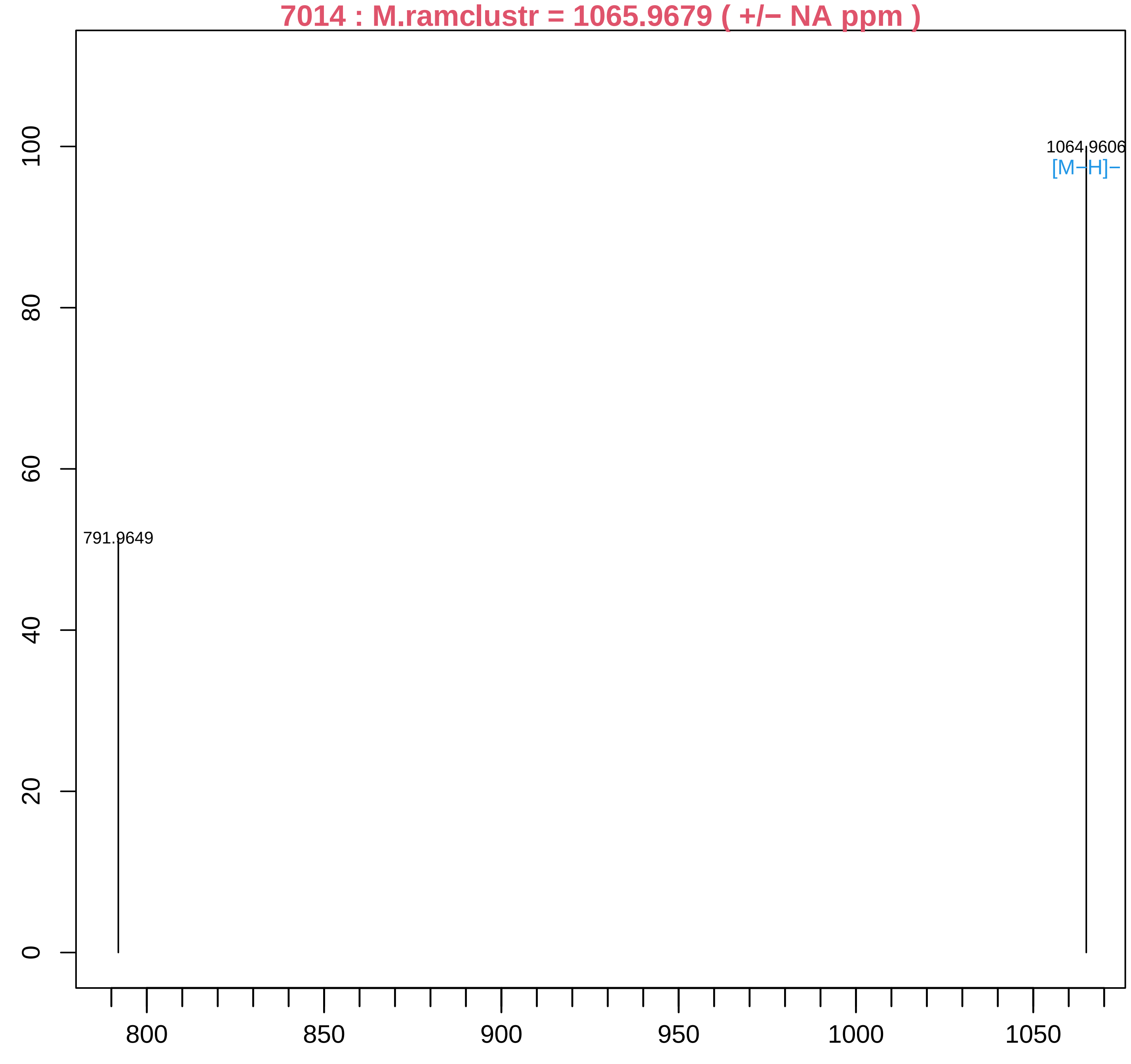

7014 : M.ramclustr = 1065.9679 (+/− NA ppm)
1064.9606
[M−H]−
791.9649
0
20
40
60
80
100
800
850
900
950
1000
1050

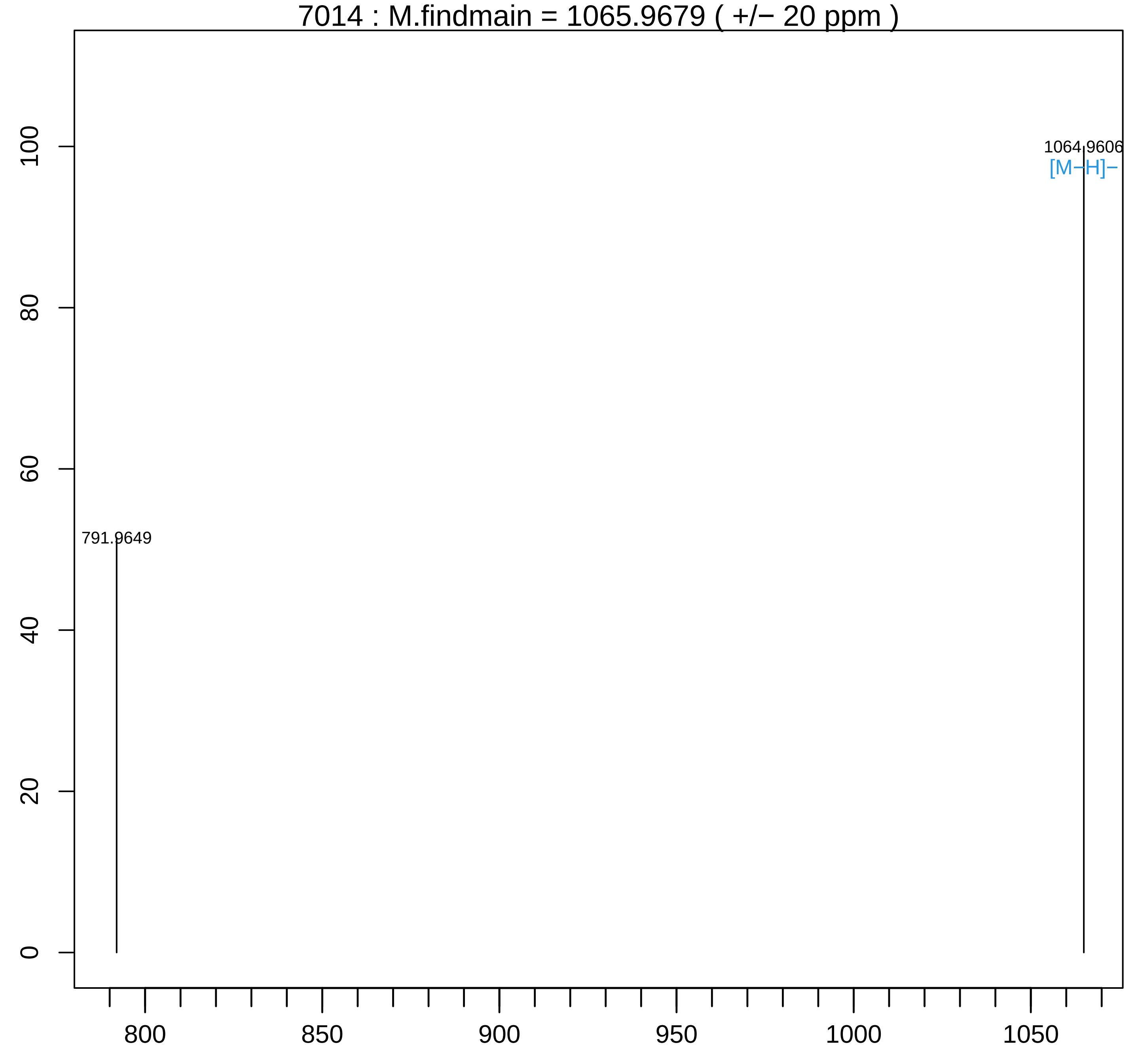

7014 : M.findmain = 1065.9679 (+/− 20 ppm)
1064.9606
[M−H]−
791.9649
0
20
40
60
80
100
800
850
900
950
1000
1050

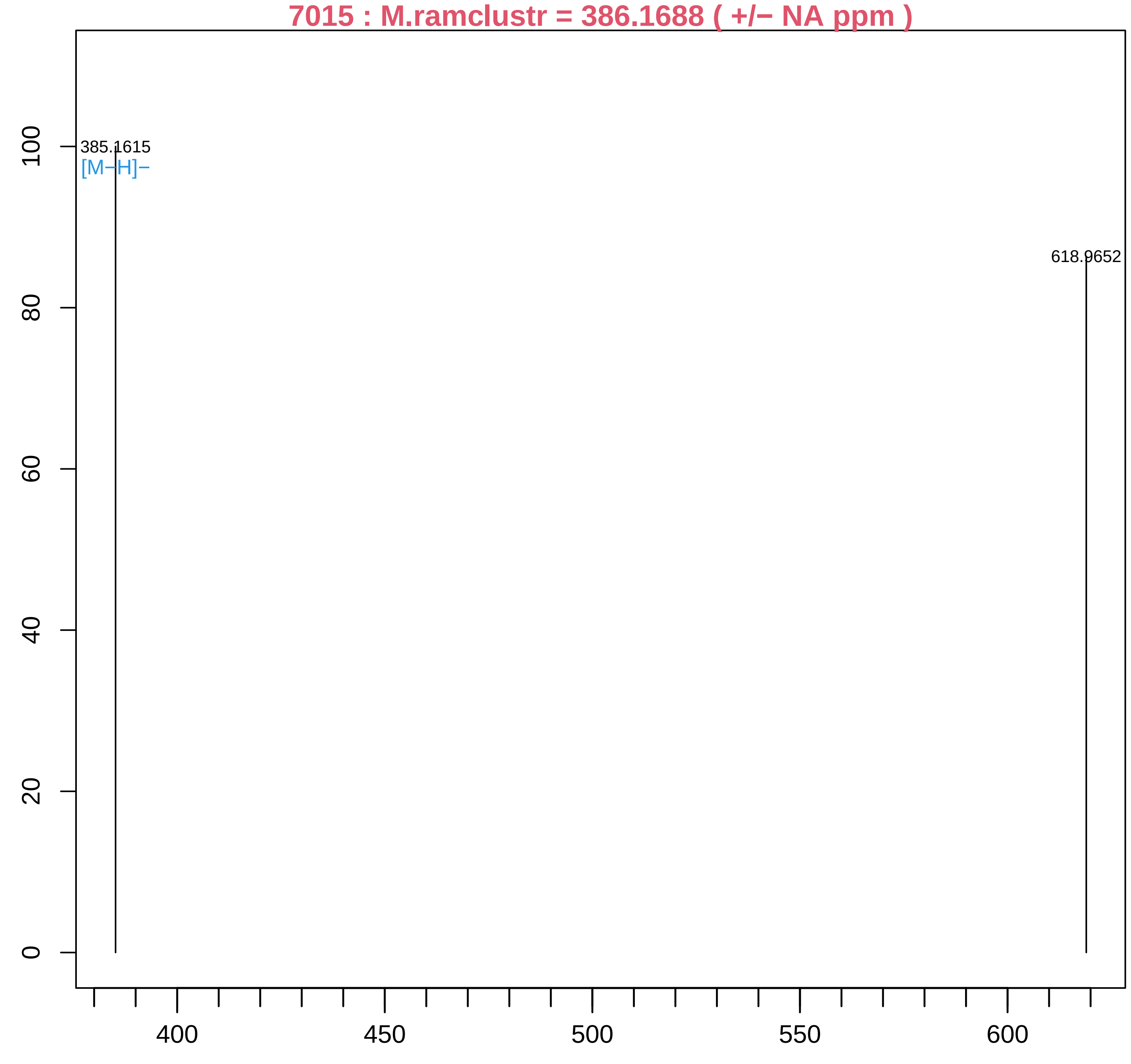
7015 : M.ramclustr = 386.1688 (+/− NA ppm)
385.1615
[M−H]−
618.9652
0
20
40
60
80
100
400
450
500
550
600

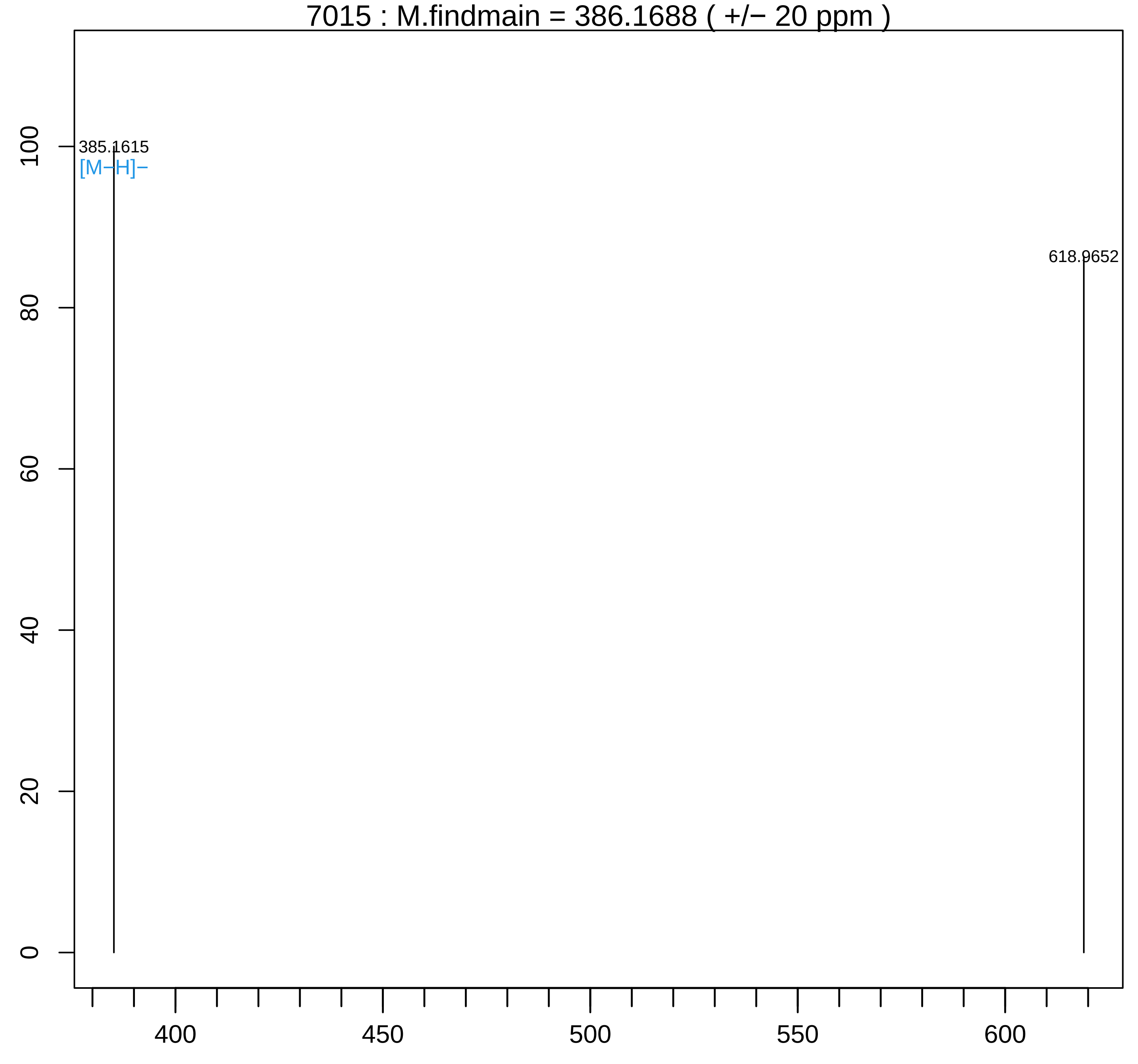
7015 : M.findmain = 386.1688 (+/− 20 ppm)
385.1615
[M−H]−
618.9652
0
20
40
60
80
100
400
450
500
550
600

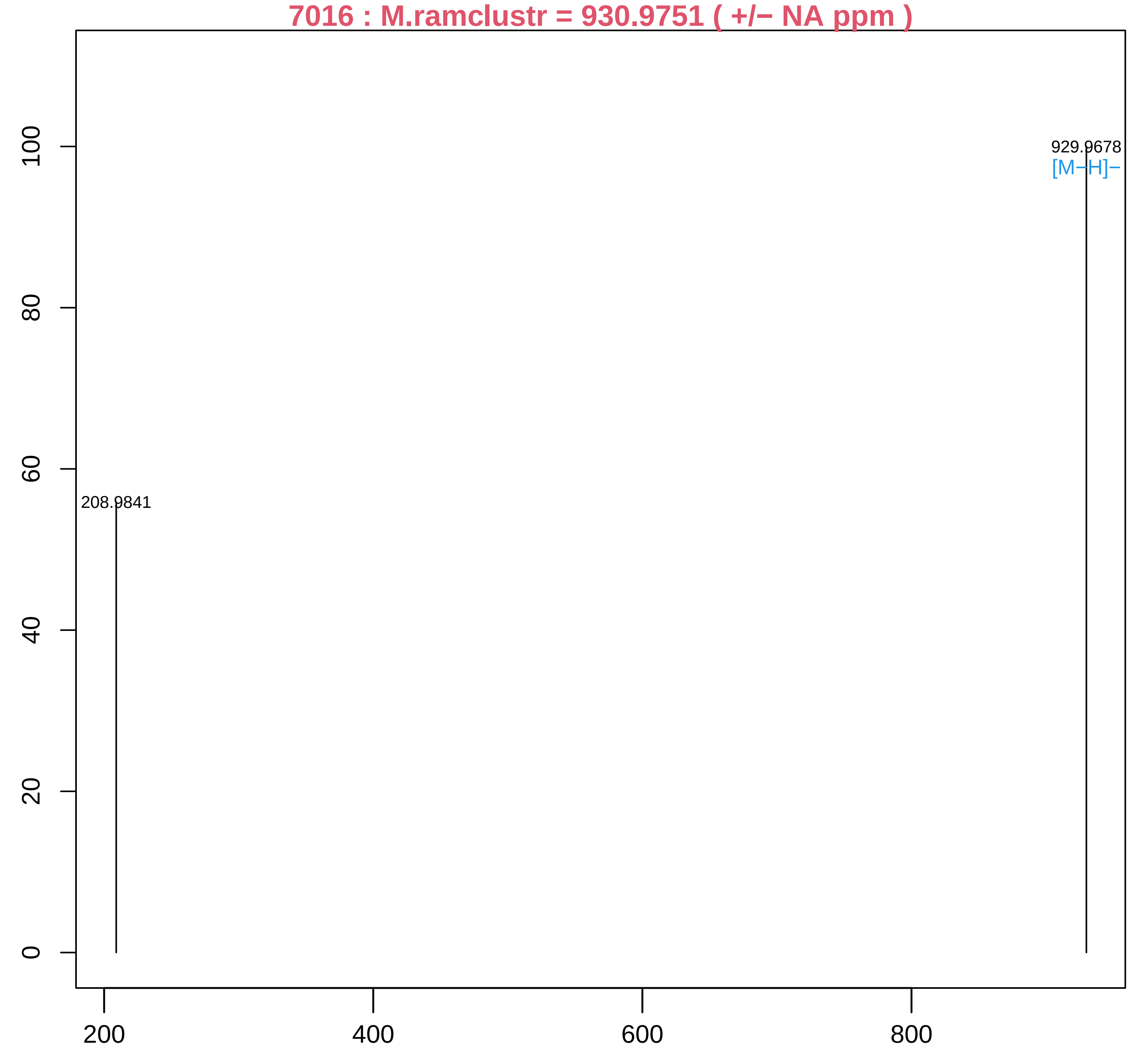

7016 : M.ramclustr = 930.9751 (+/− NA ppm)
929.9678
[M−H]−
208.9841
0
20
40
60
80
100
200
400
600
800

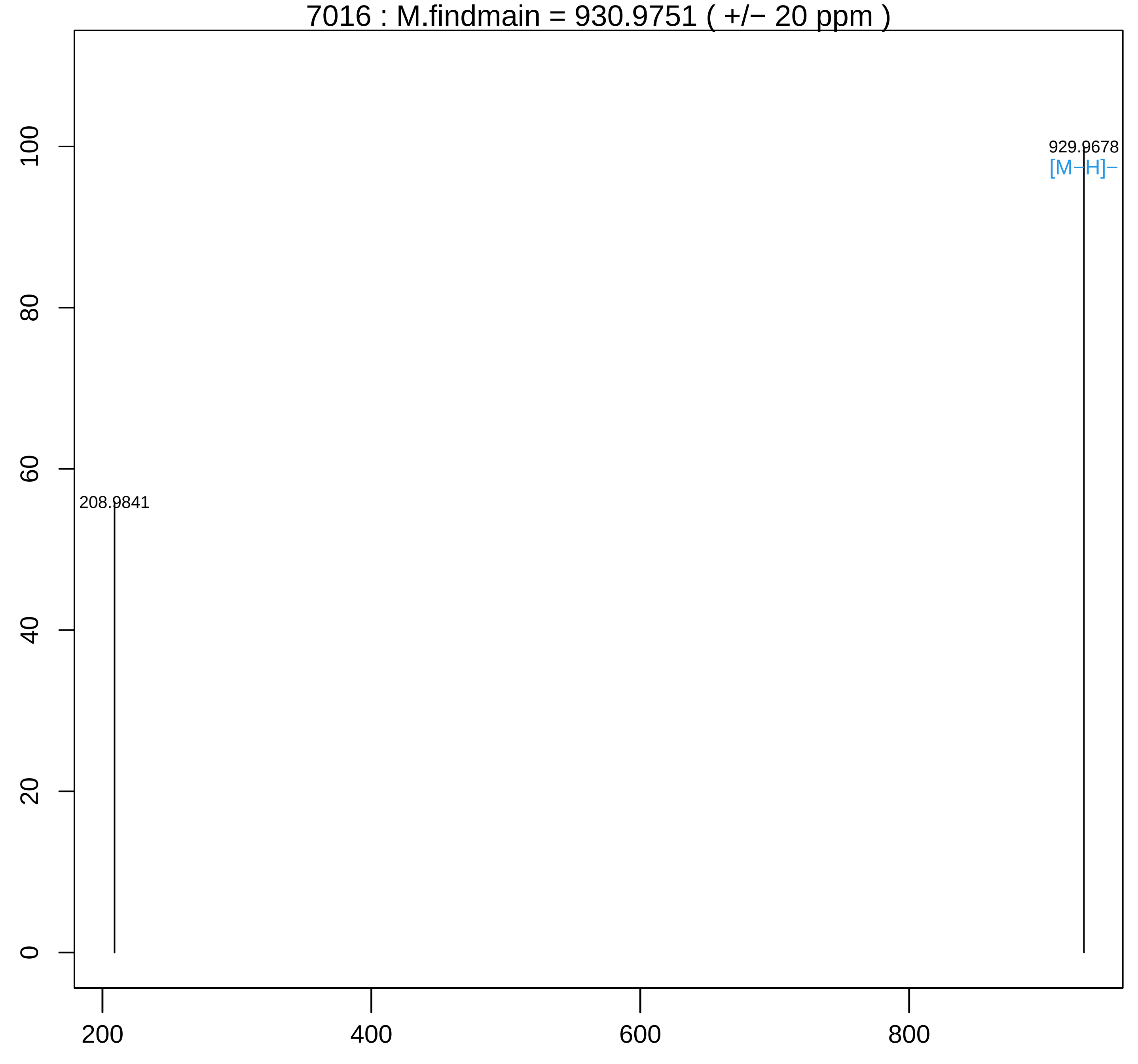

7016 : M.findmain = 930.9751 (+/− 20 ppm)
929.9678
[M−H]−
208.9841
0
20
40
60
80
100
200
400
600
800

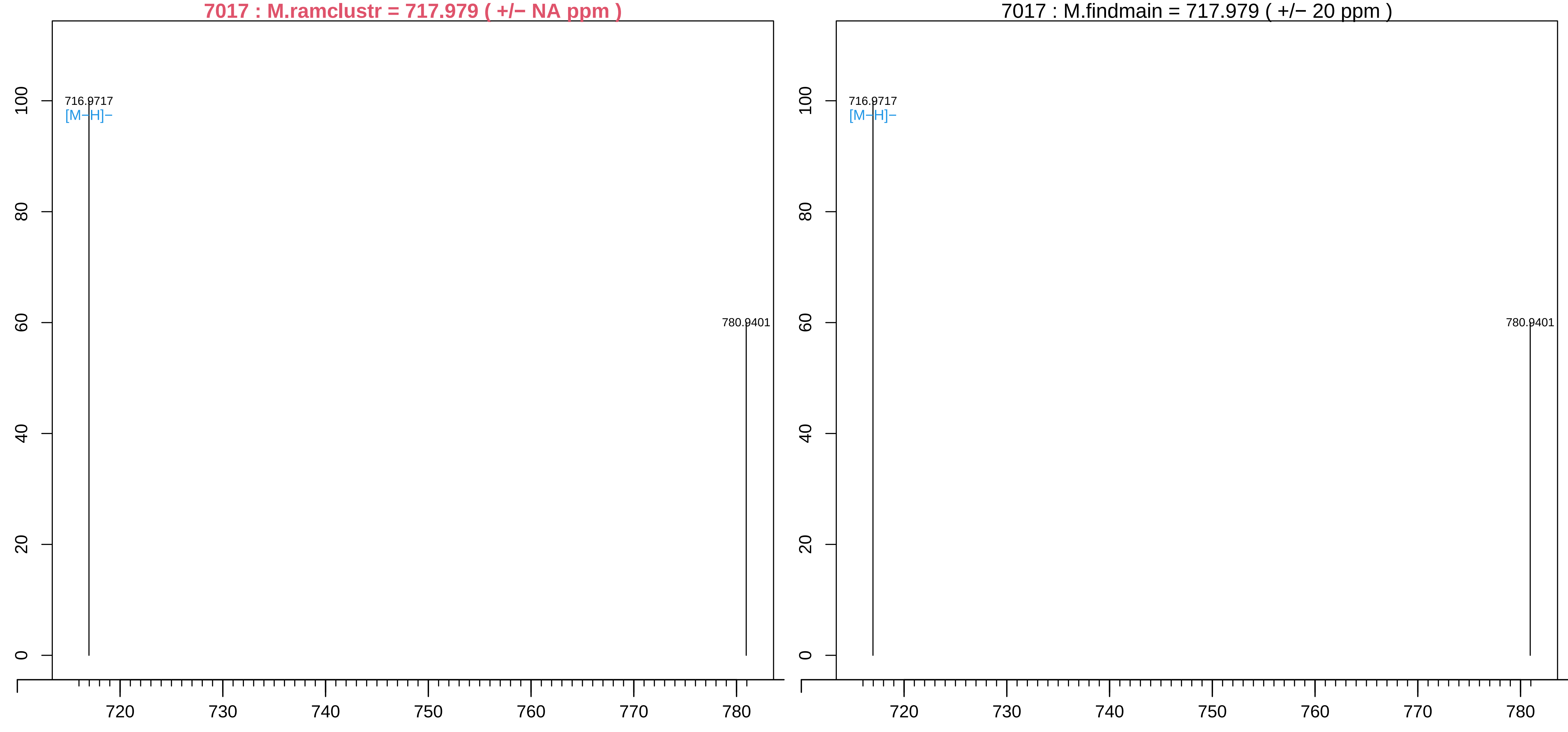
7017 : M.ramclustr = 717.979 (+/− NA ppm)
716.9717
[M−H]−
780.9401
0
20
40
60
80
100
720
730
740
750
760
770
780
7017 : M.findmain = 717.979 (+/− 20 ppm)
716.9717
[M−H]−
780.9401
0
20
40
60
80
100
720
730
740
750
760
770
780

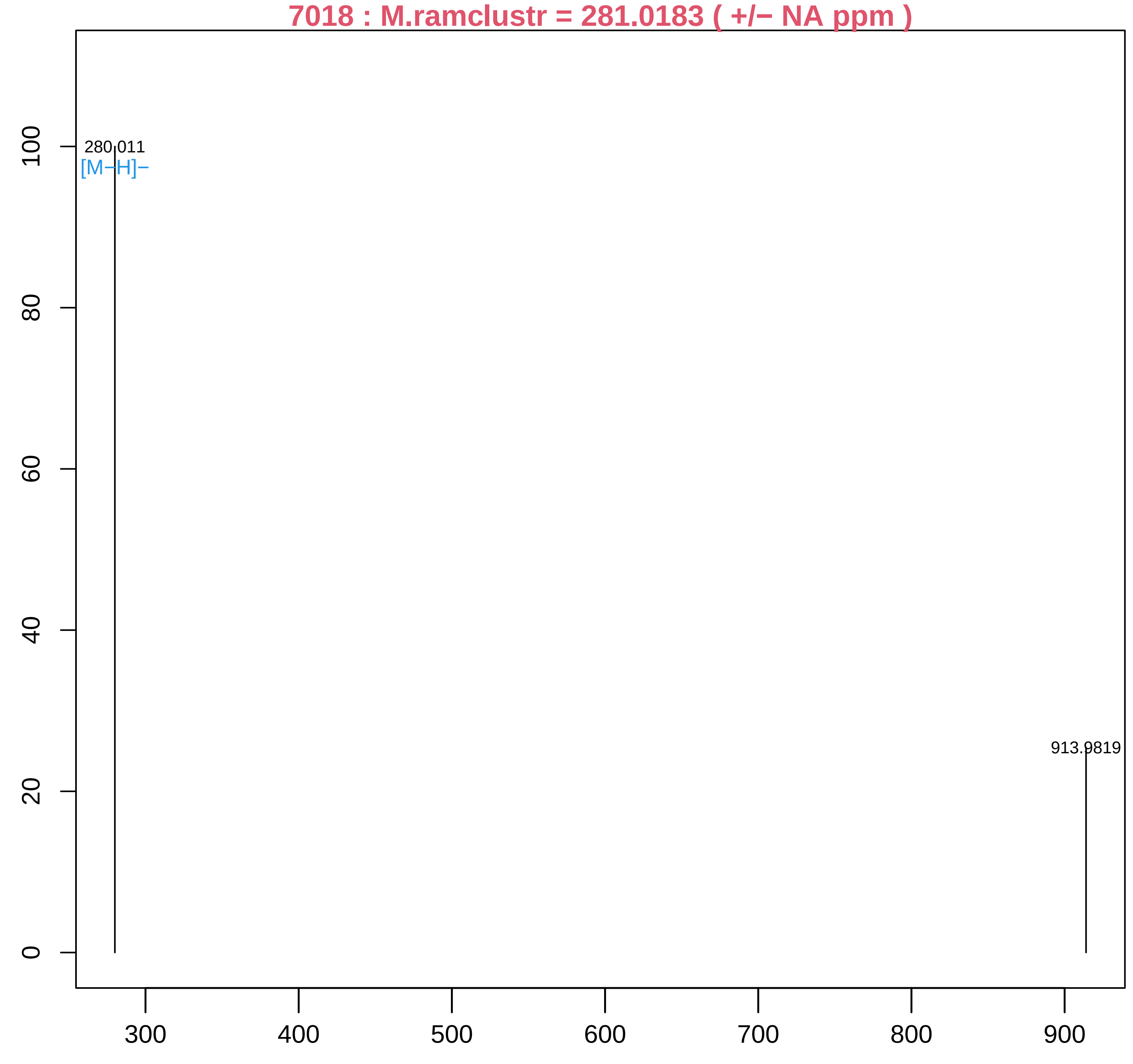

7018 : M.ramclustr = 281.0183 (+/− NA ppm)
280.011
[M−H]−
913.9819
0
20
40
60
80
100
300
400
500
600
700
800
900

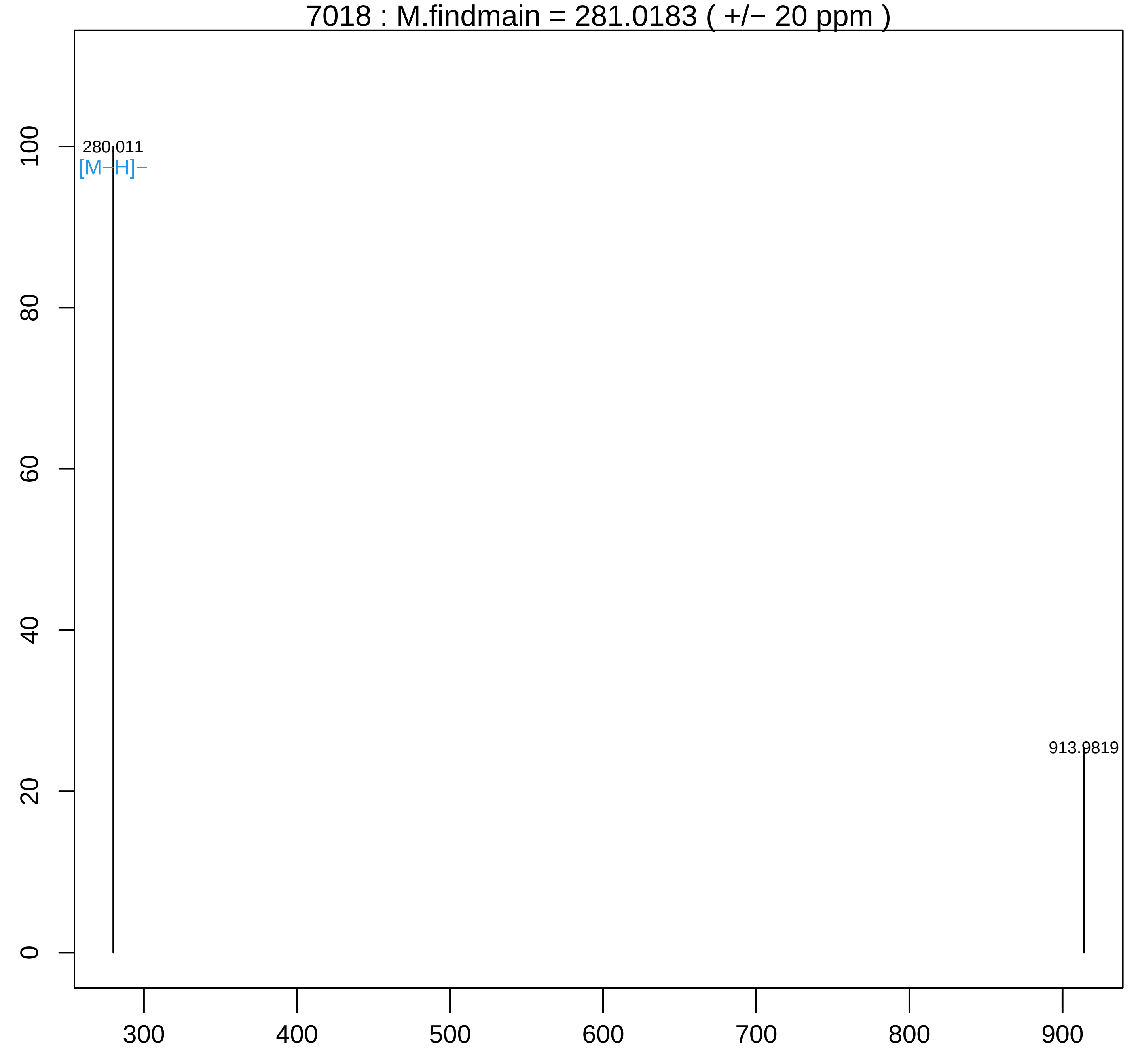

7018 : M.findmain = 281.0183 (+/− 20 ppm)
280.011
[M−H]−
913.9819
0
20
40
60
80
100
300
400
500
600
700
800
900

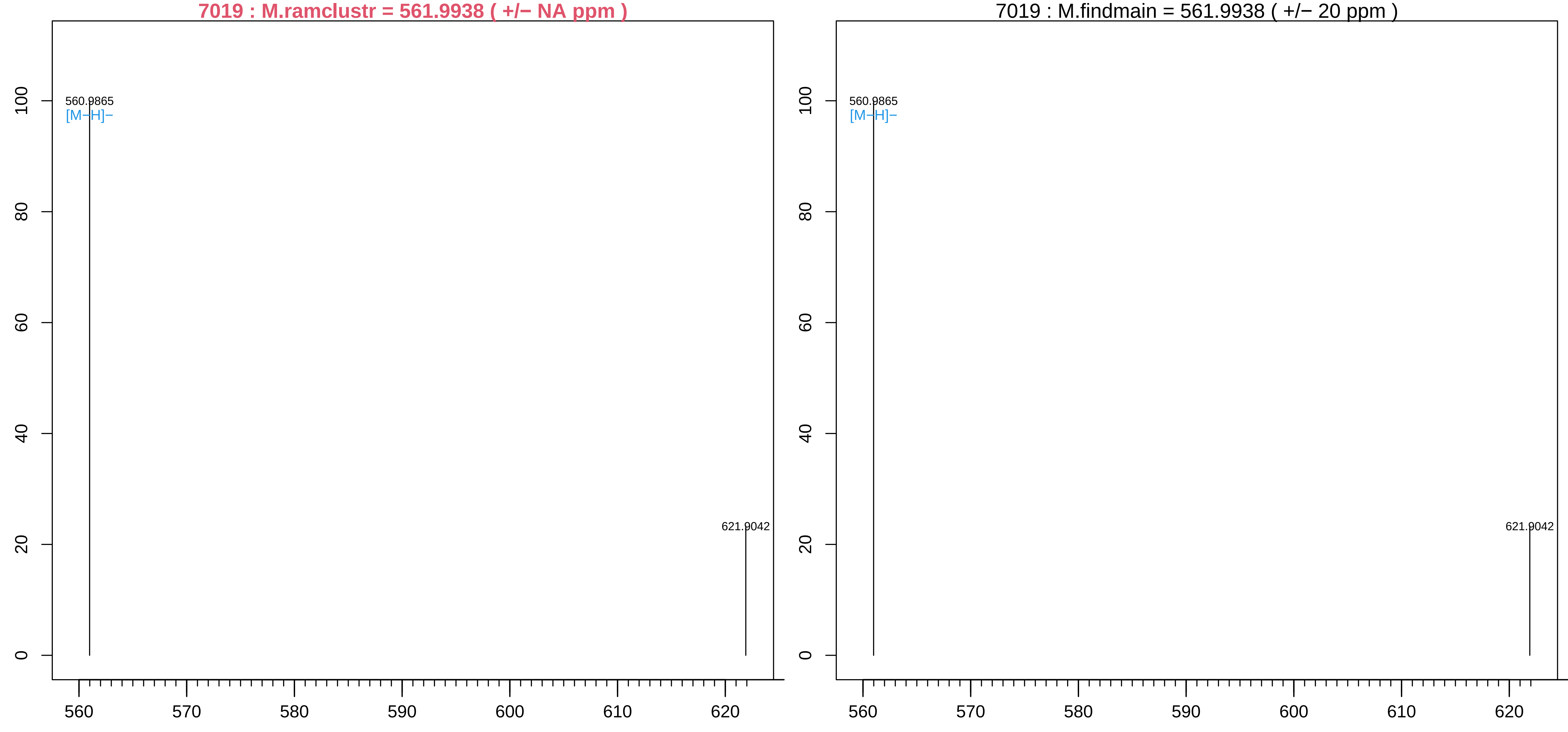

7019 : M.ramclustr = 561.9938 (+/− NA ppm)
560.9865
[M−H]−
621.9042
0
20
40
60
80
100
560
570
580
590
600
610
620
7019 : M.findmain = 561.9938 (+/− 20 ppm)
560.9865
[M−H]−
621.9042
0
20
40
60
80
100
560
570
580
590
600
610
620

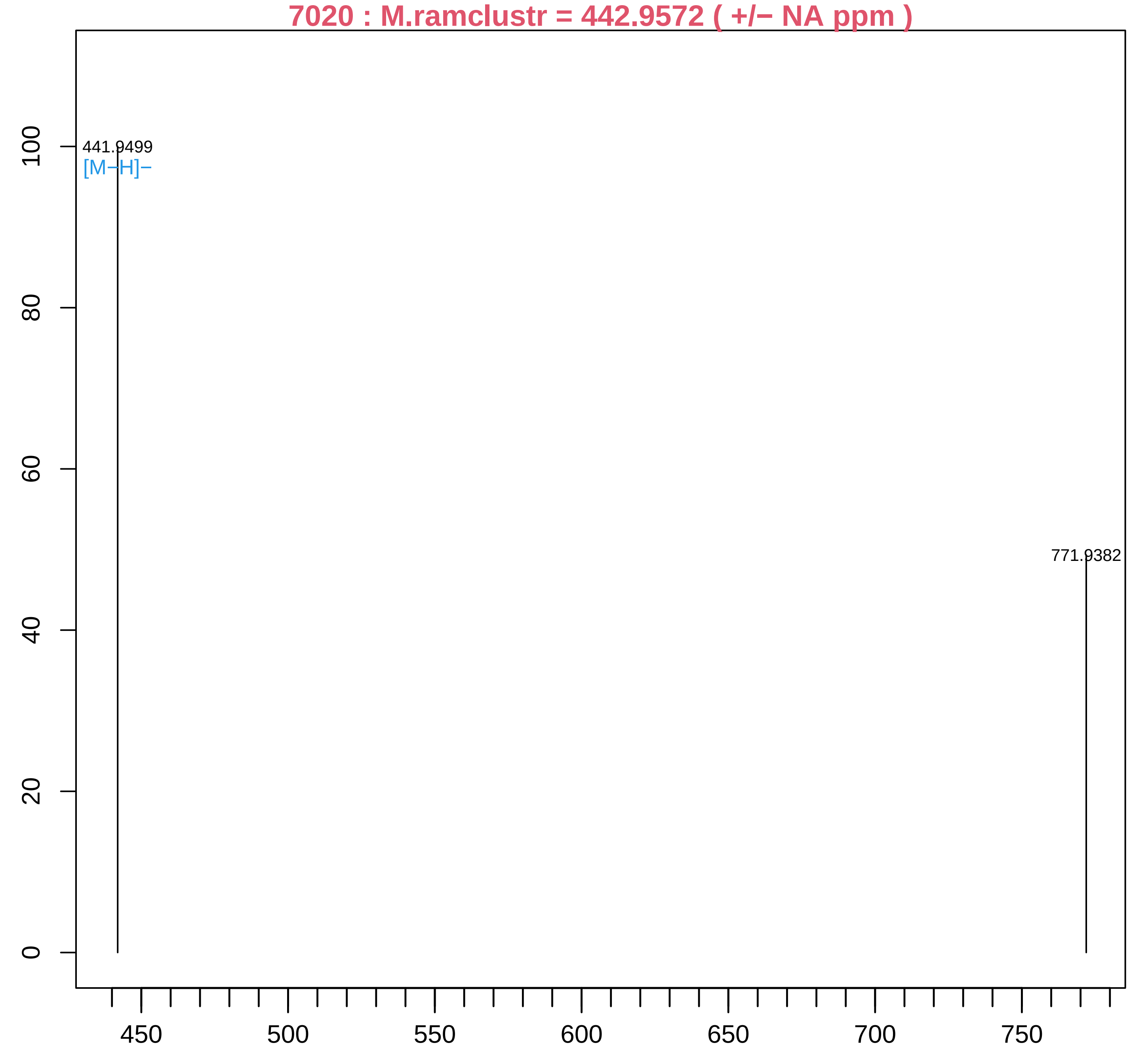
7020 : M.ramclustr = 442.9572 (+/− NA ppm)
441.9499
[M−H]−
771.9382
0
20
40
60
80
100
450
500
550
600
650
700
750

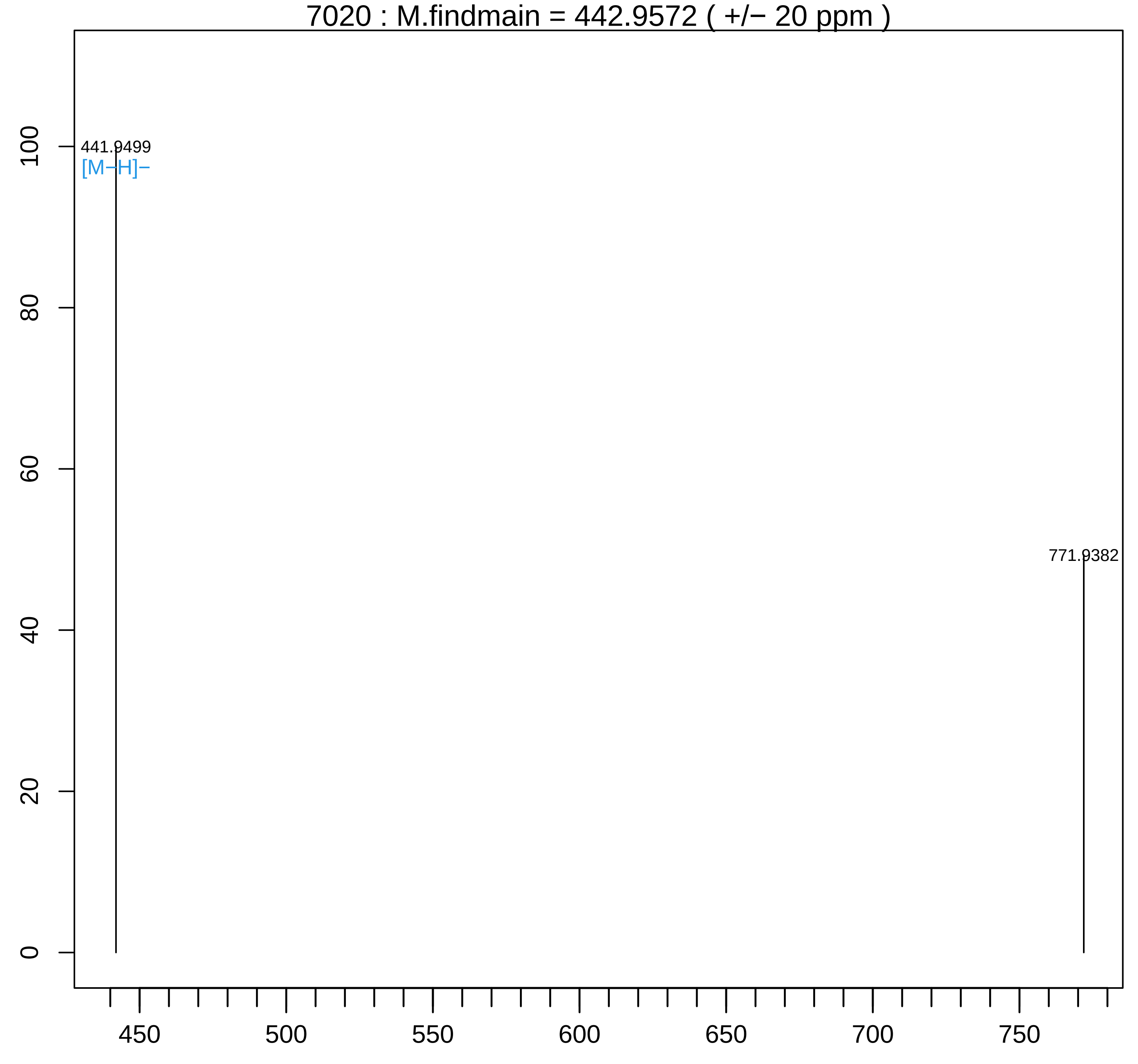
7020 : M.findmain = 442.9572 (+/− 20 ppm)
441.9499
[M−H]−
771.9382
0
20
40
60
80
100
450
500
550
600
650
700
750

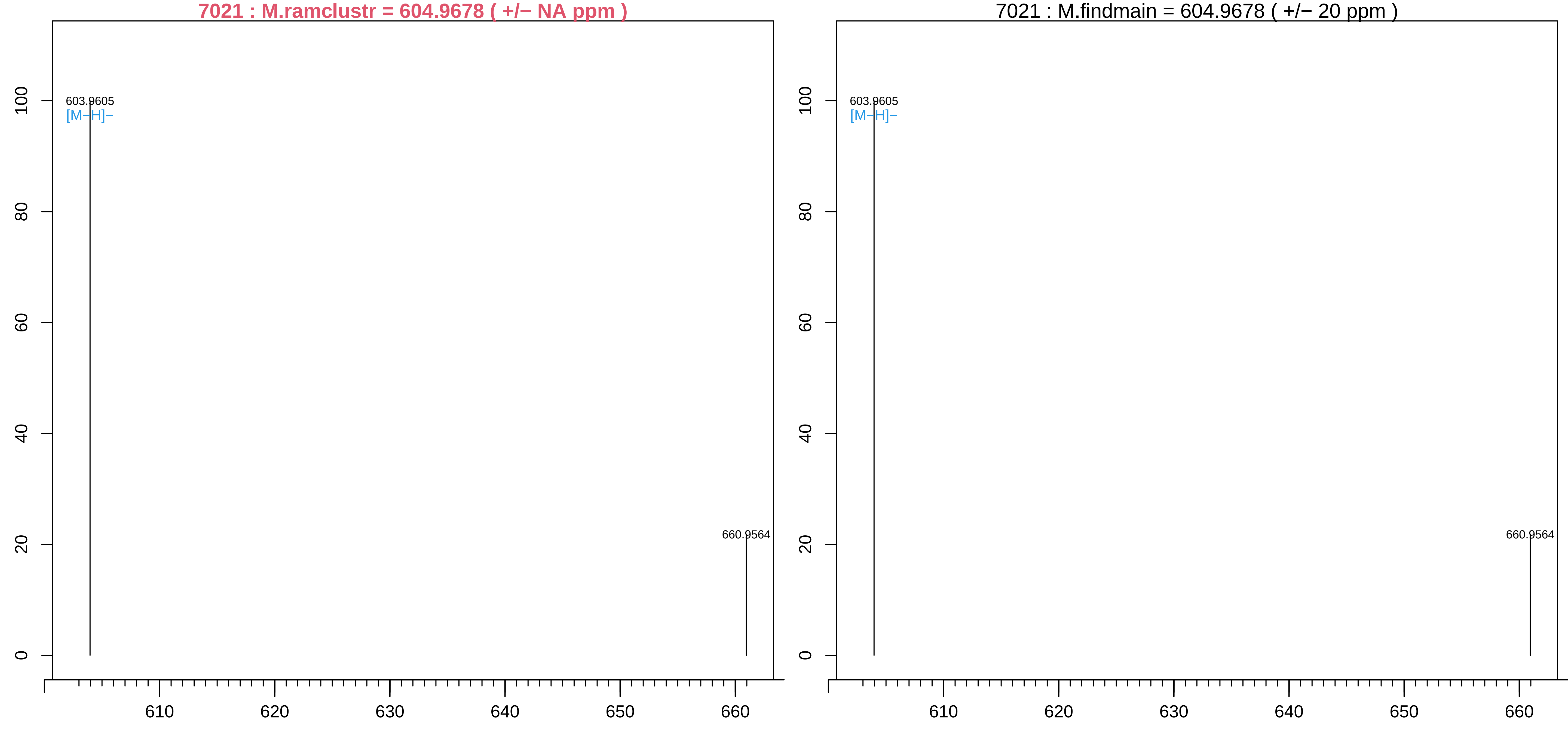
7021 : M.ramclustr = 604.9678 (+/− NA ppm)
603.9605
[M−H]−
660.9564
7021 : M.findmain = 604.9678 (+/− 20 ppm)
603.9605
[M−H]−
660.9564
0
20
40
60
80
100
610
620
630
640
650
660

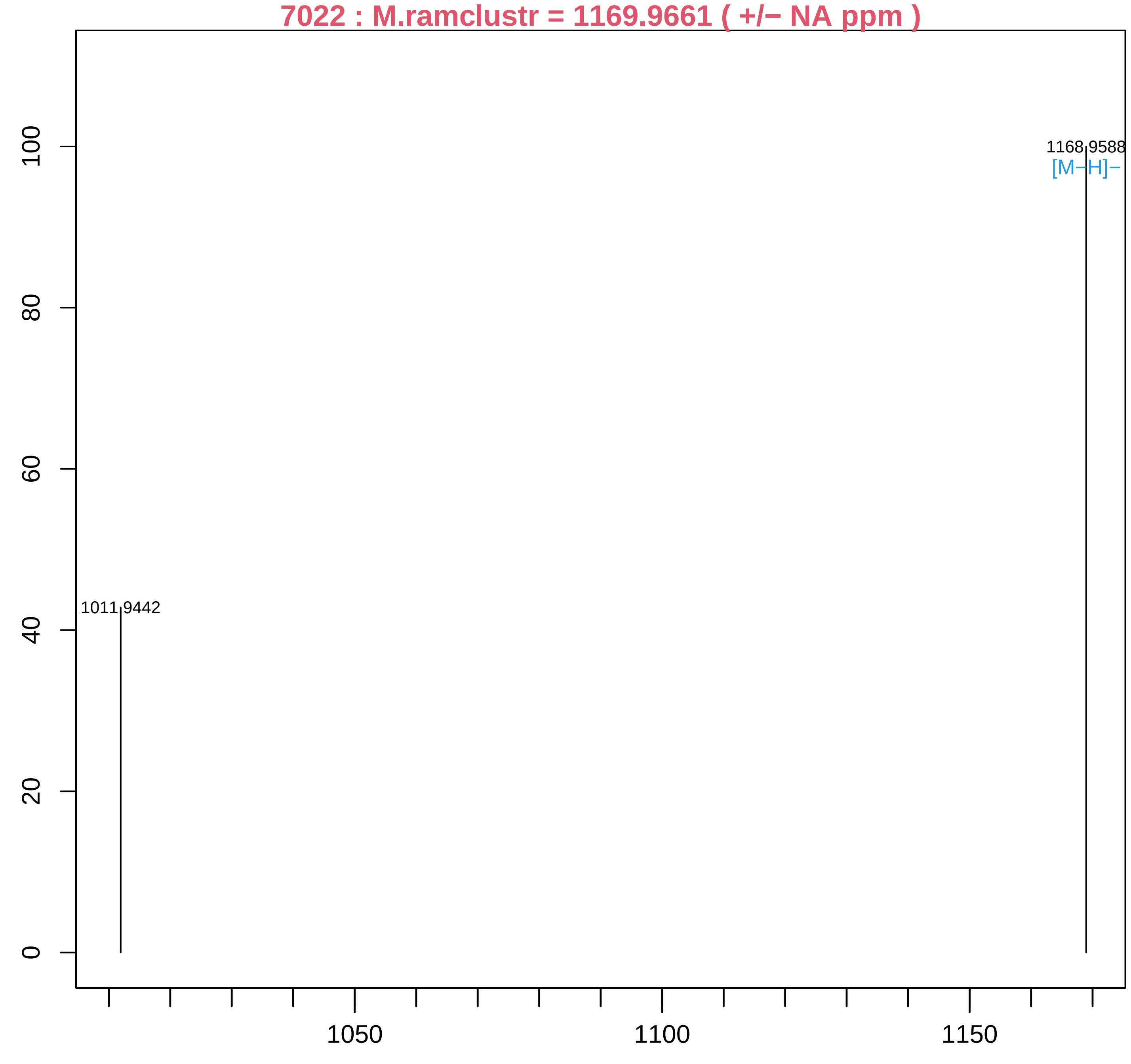
7022 : M.ramclustr = 1169.9661 (+/− NA ppm)
1168.9588
[M−H]−
1011.9442
0
20
40
60
80
100
1050
1100
1150

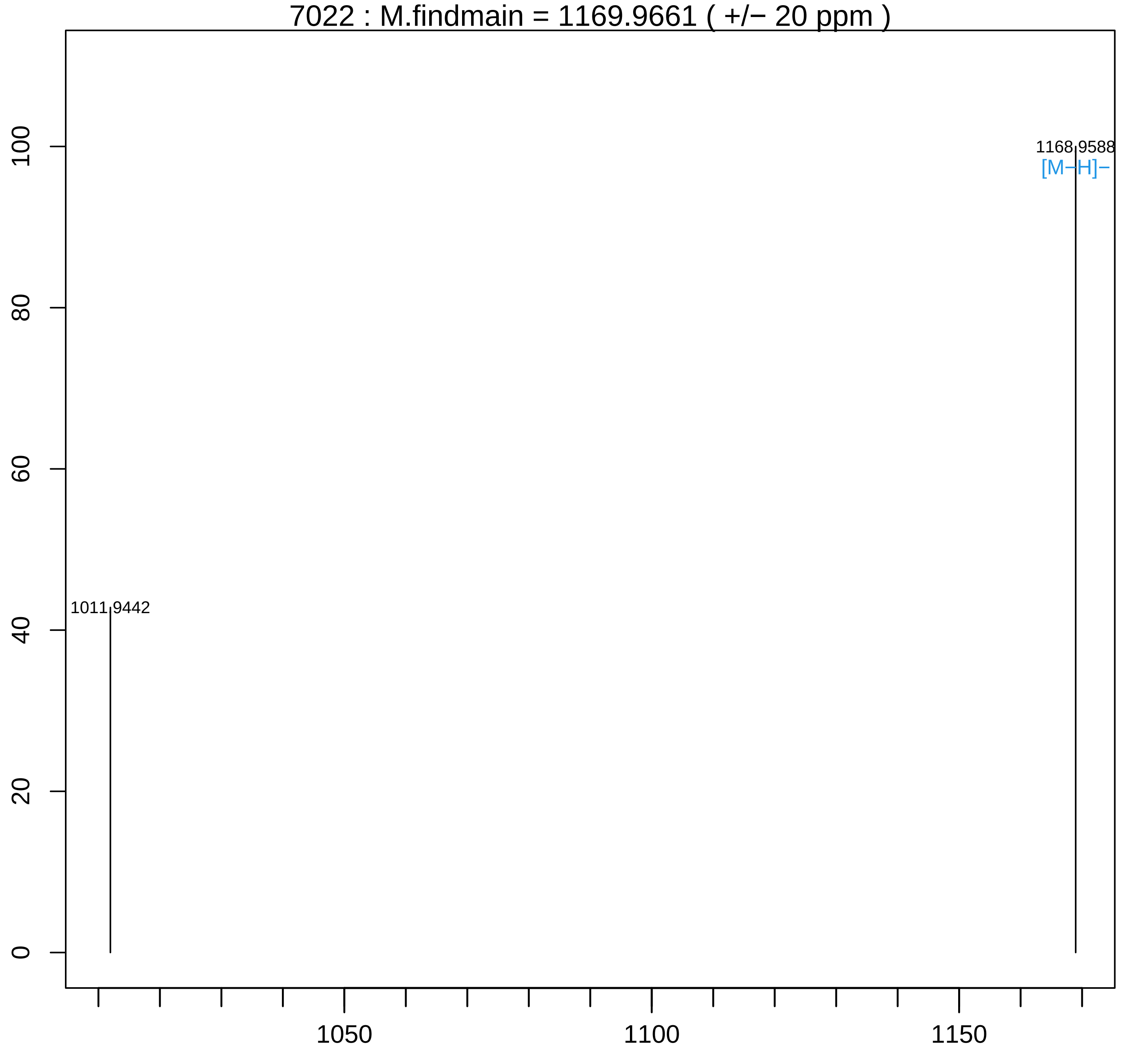
7022 : M.findmain = 1169.9661 (+/− 20 ppm)
1168.9588
[M−H]−
1011.9442
0
20
40
60
80
100
1050
1100
1150

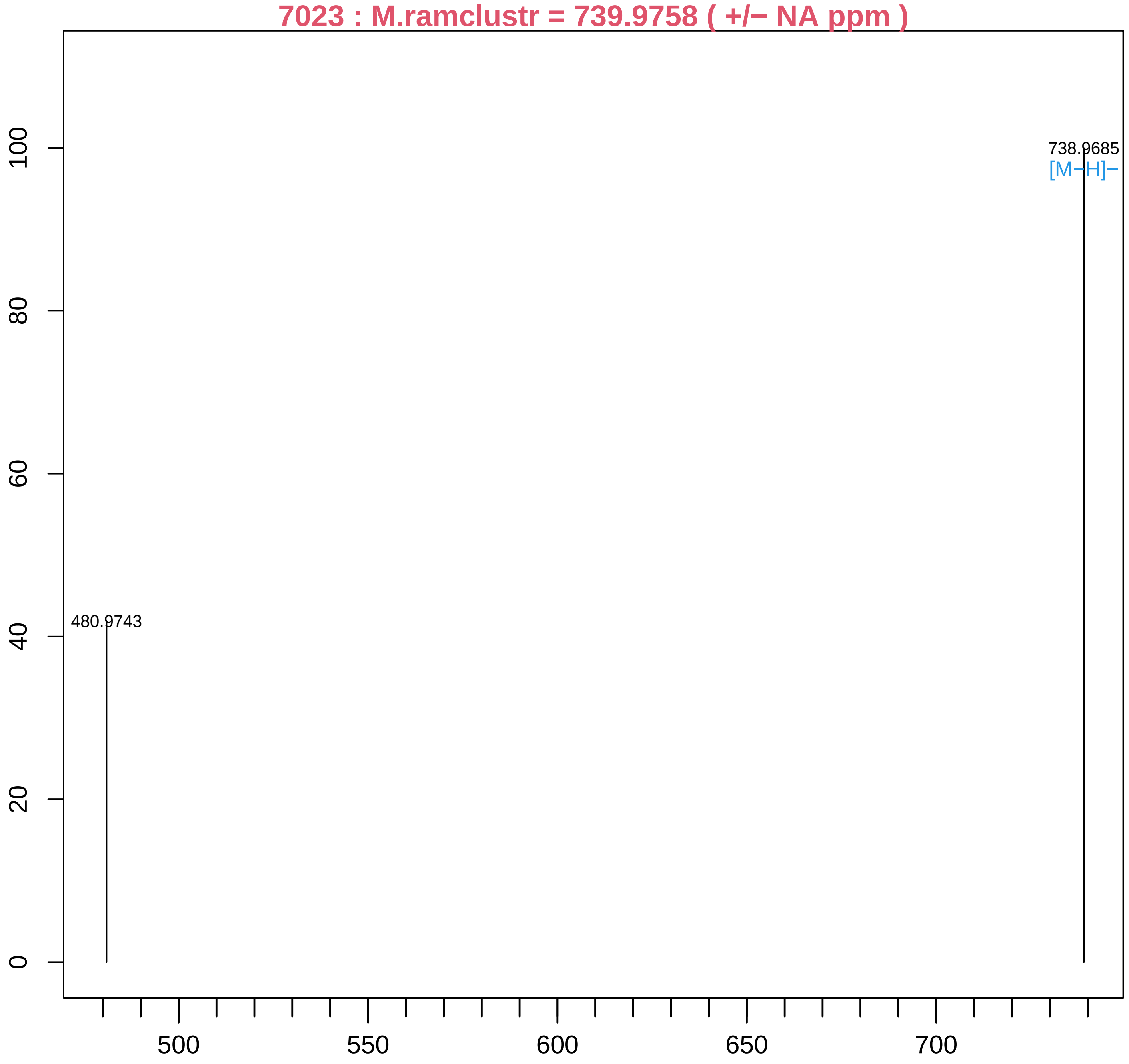
7023 : M.ramclustr = 739.9758 (+/− NA ppm)
738.9685
[M−H]−
480.9743
0
20
40
60
80
100
500
550
600
650
700

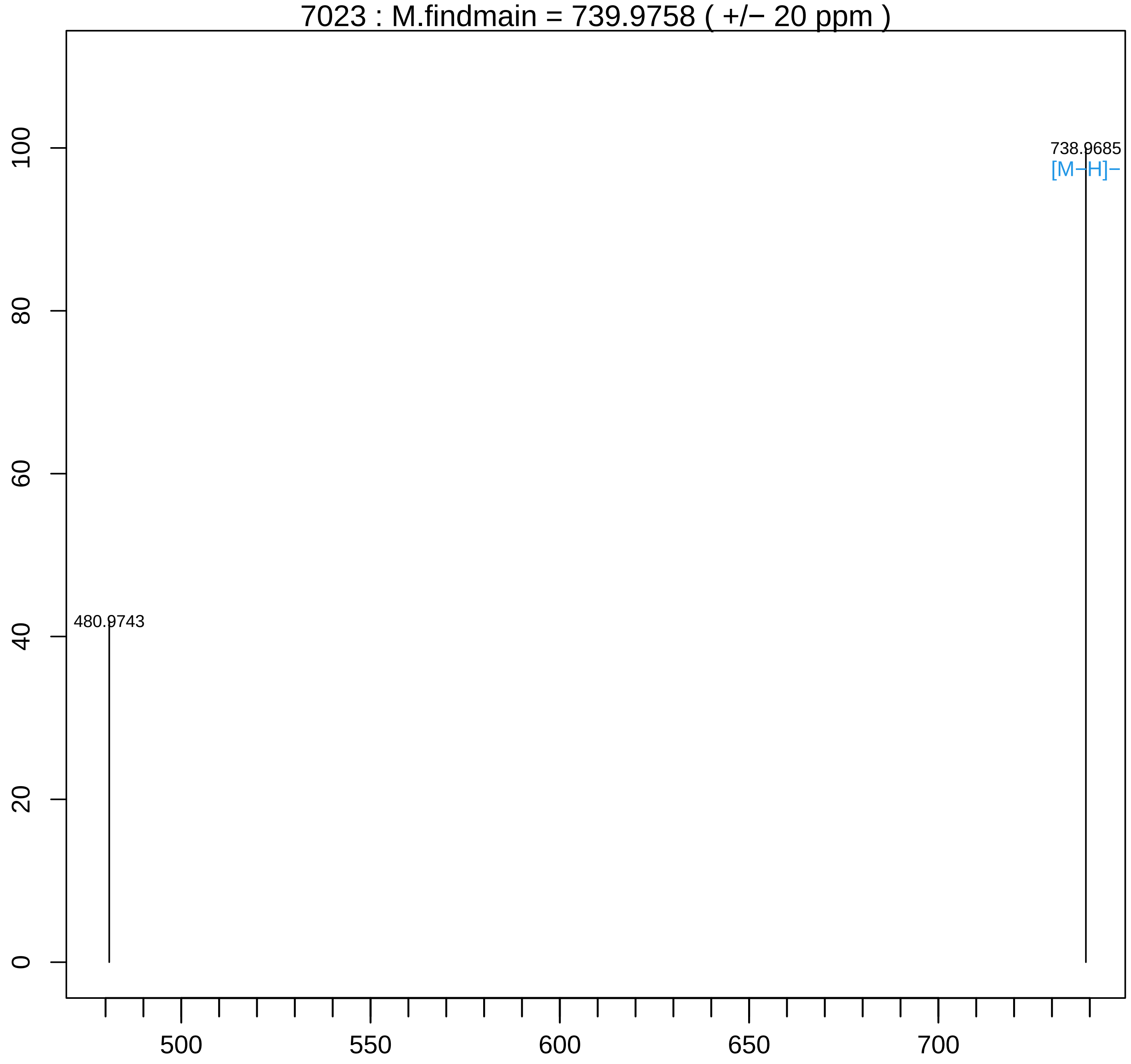
7023 : M.findmain = 739.9758 (+/− 20 ppm)
738.9685
[M−H]−
480.9743
0
20
40
60
80
100
500
550
600
650
700

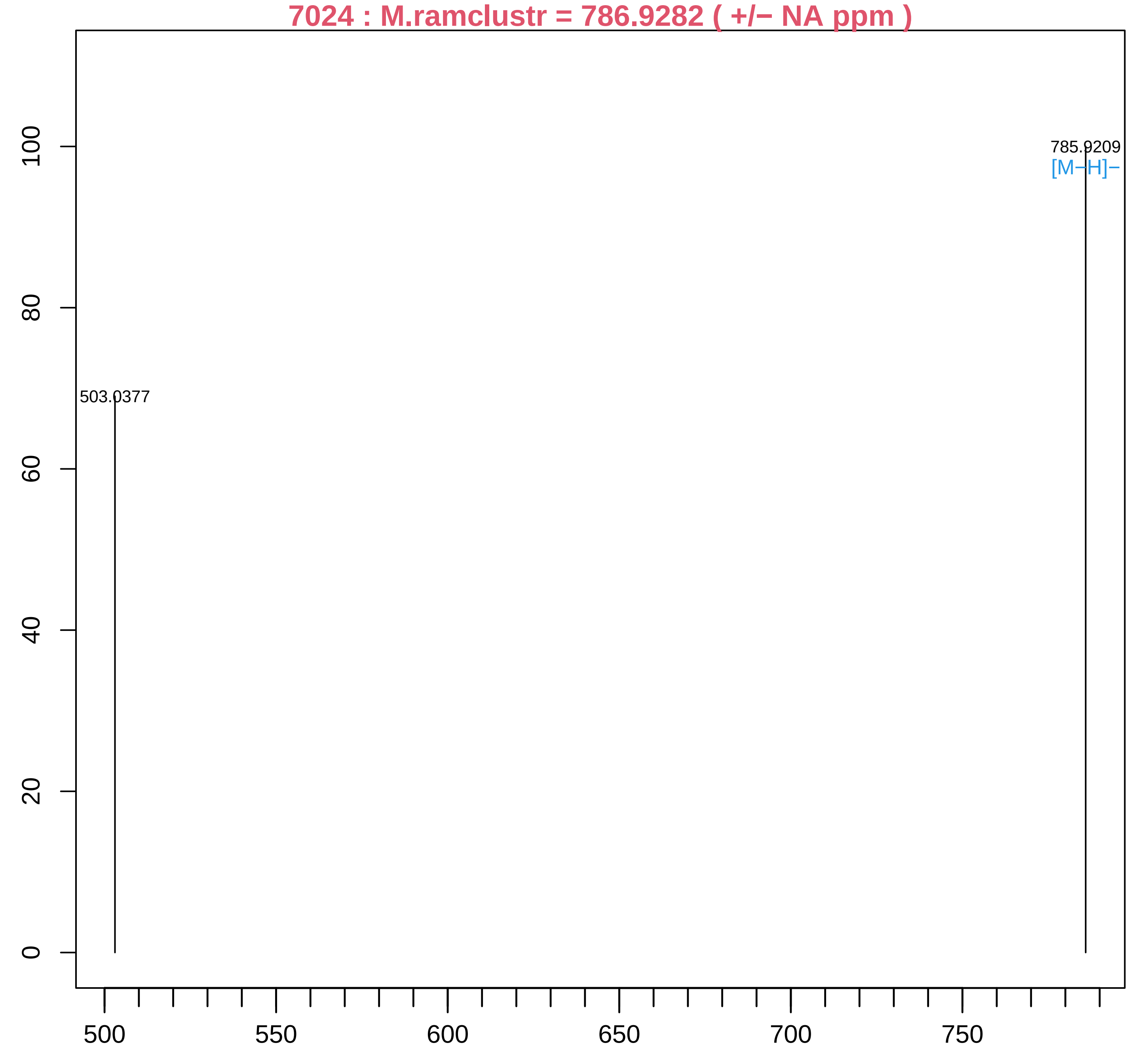
7024 : M.ramclustr = 786.9282 (+/− NA ppm)
785.9209
[M−H]−
503.0377
0
20
40
60
80
100
500
550
600
650
700
750

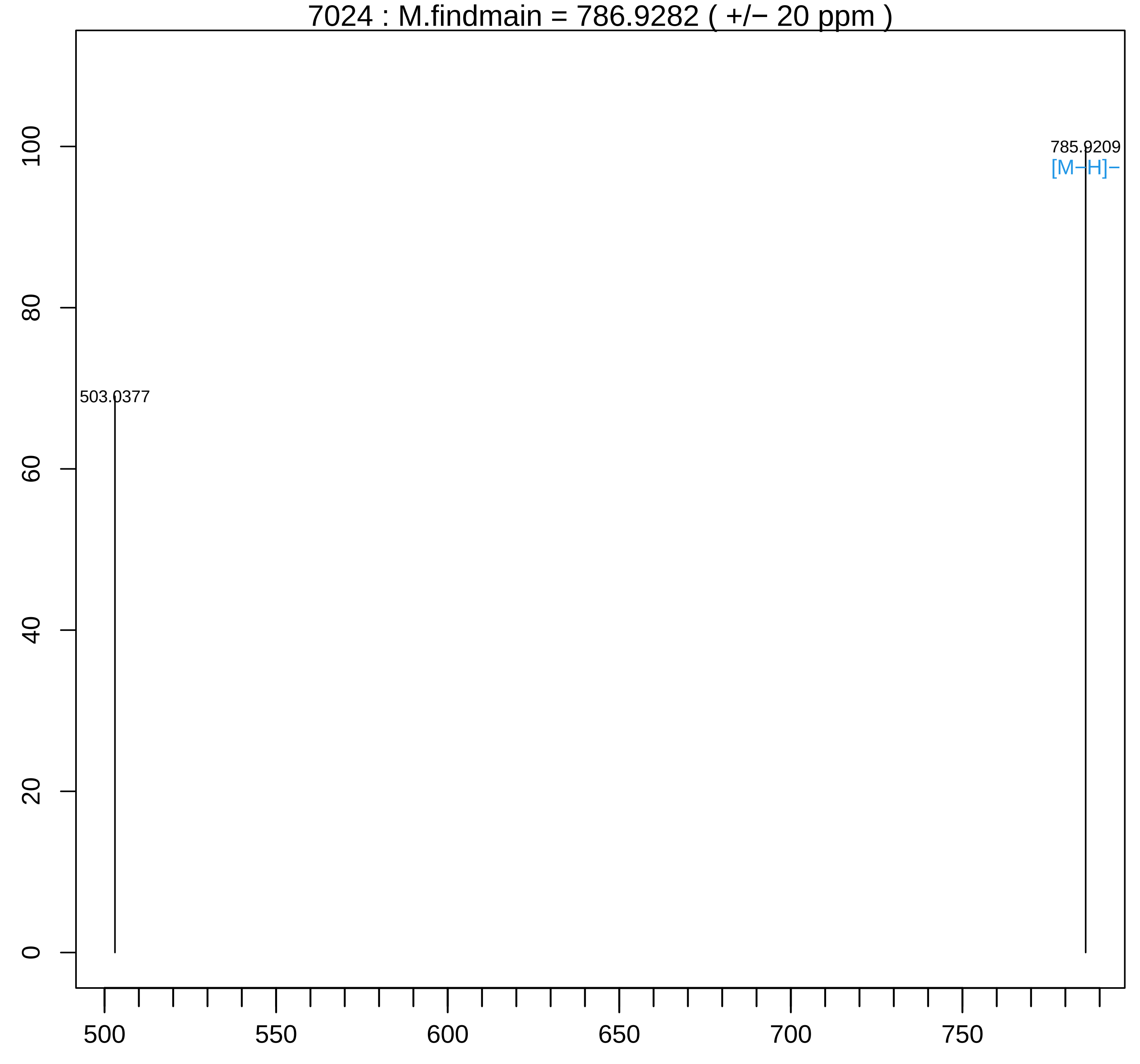
7024 : M.findmain = 786.9282 (+/− 20 ppm)
785.9209
[M−H]−
503.0377
0
20
40
60
80
100
500
550
600
650
700
750

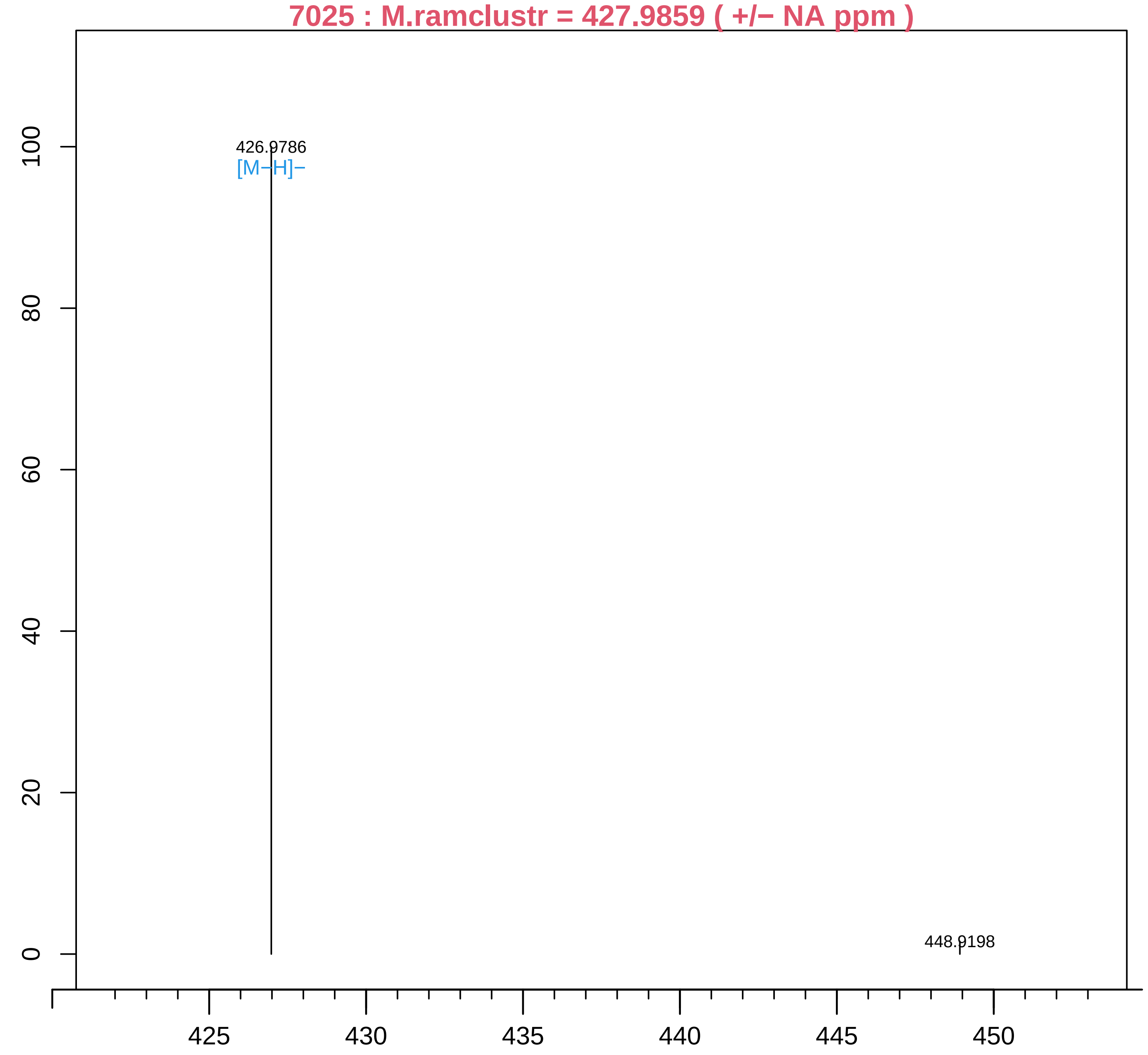
7025 : M.ramclustr = 427.9859 (+/− NA ppm)
426.9786
[M−H]−
448.9198
0
20
40
60
80
100
425
430
435
440
445
450

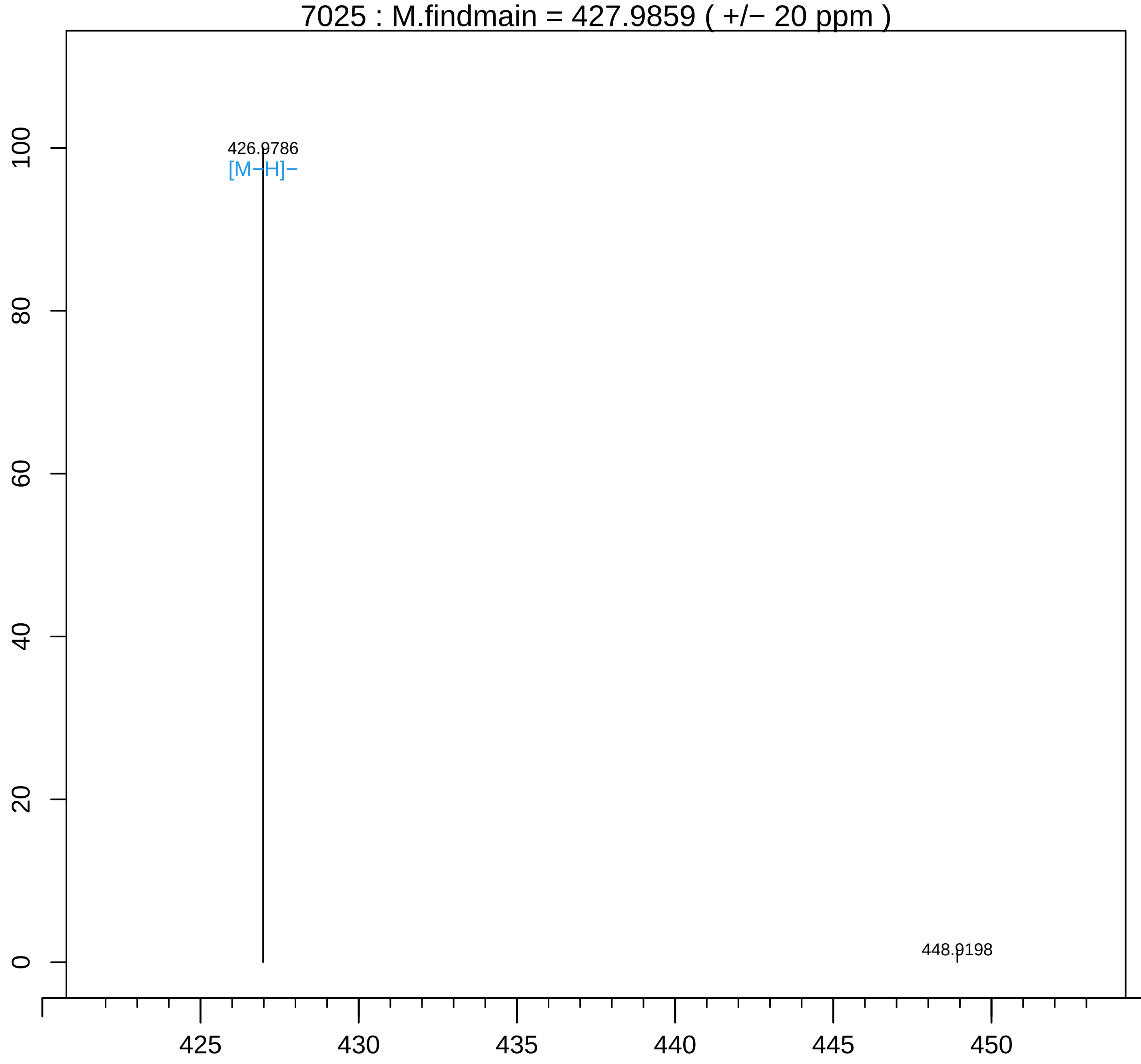
7025 : M.findmain = 427.9859 (+/− 20 ppm)
426.9786
[M−H]−
448.9198
0
20
40
60
80
100
425
430
435
440
445
450

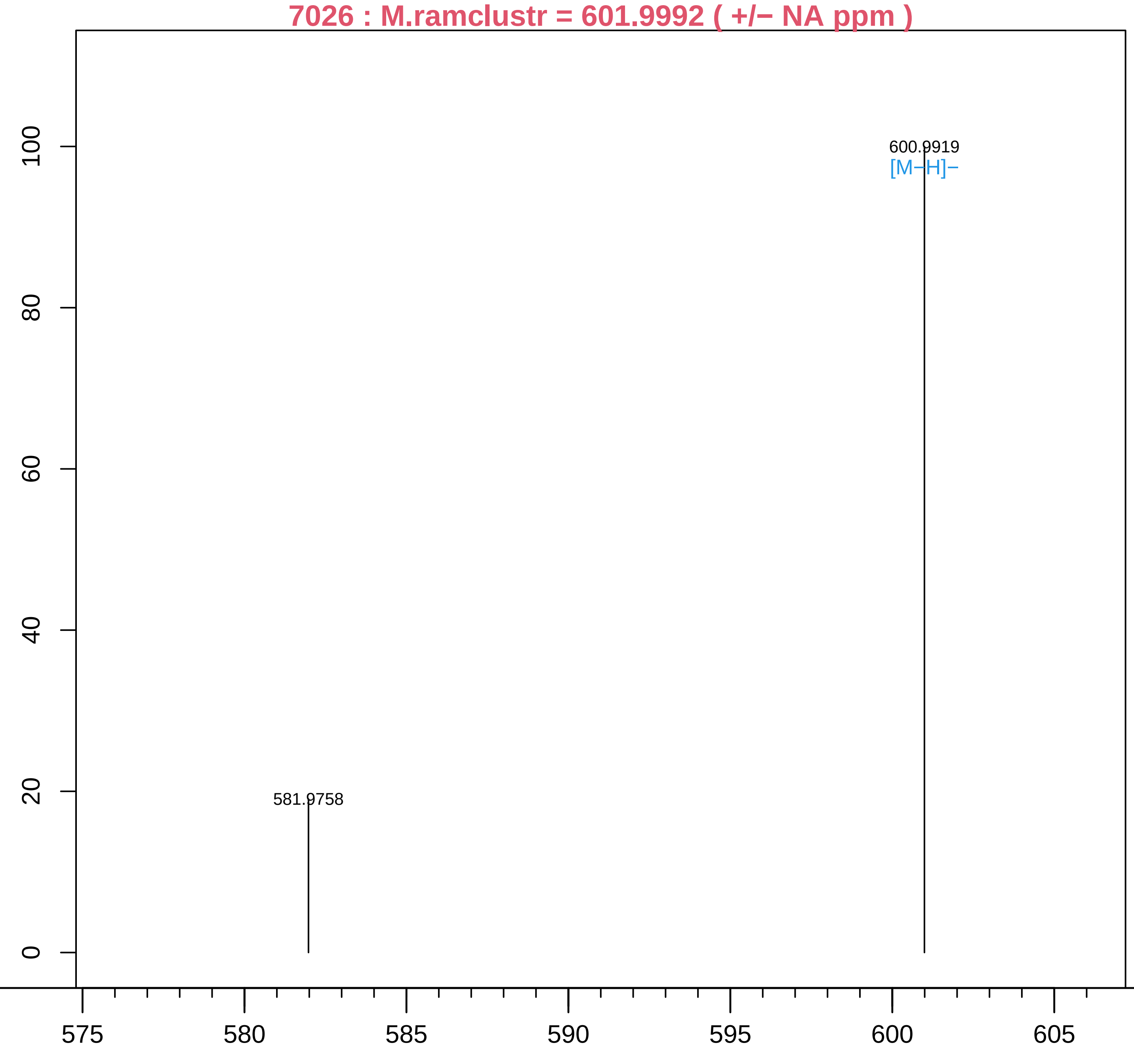
7026 : M.ramclustr = 601.9992 (+/− NA ppm)
600.9919
[M−H]−
581.9758
0
20
40
60
80
100
575
580
585
590
595
600
605

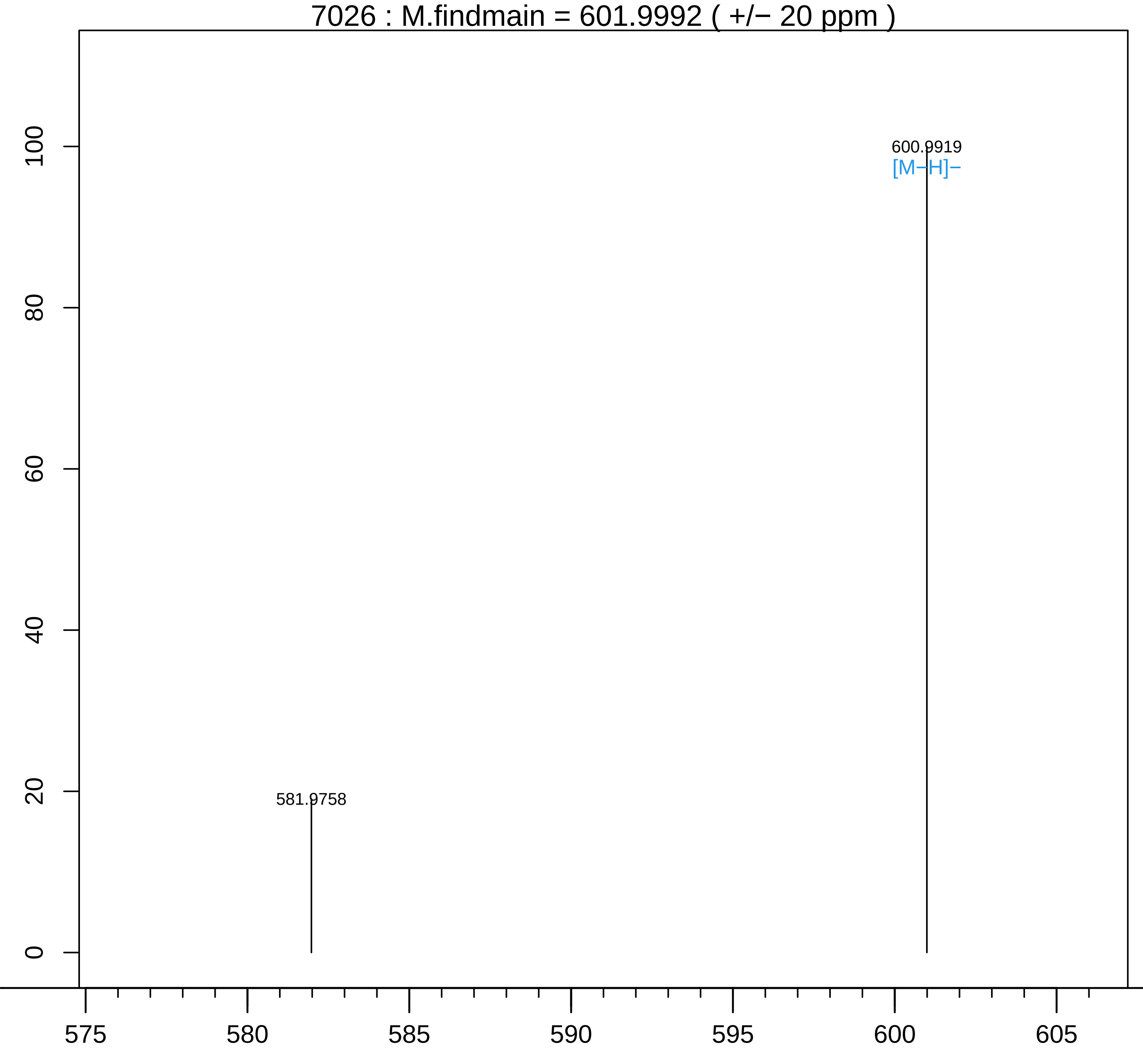
7026 : M.findmain = 601.9992 (+/− 20 ppm)
600.9919
[M−H]−
581.9758
0
20
40
60
80
100
575
580
585
590
595
600
605

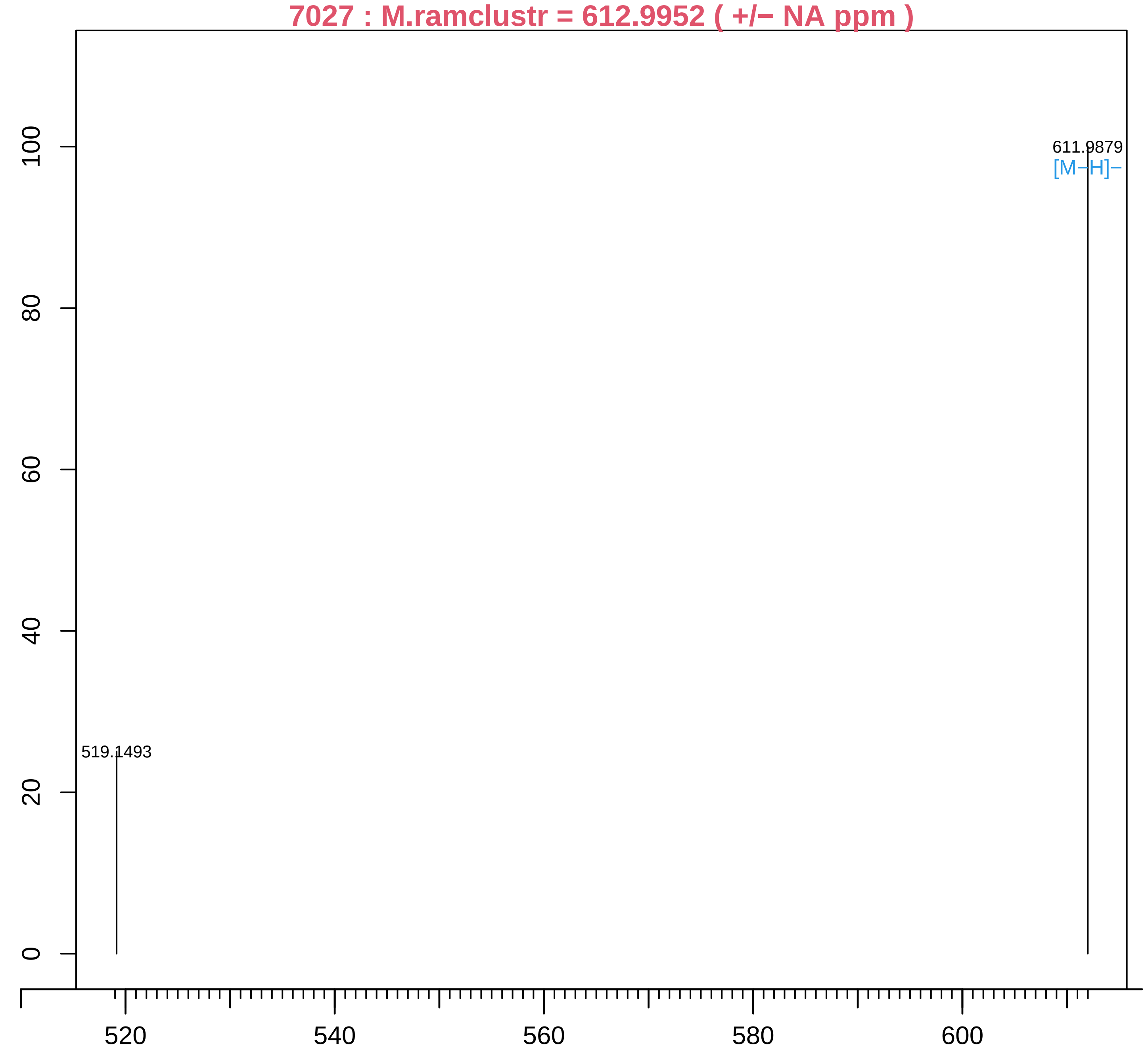
7027 : M.ramclustr = 612.9952 (+/− NA ppm)
611.9879
[M−H]−
519.1493
0
20
40
60
80
100
520
540
560
580
600

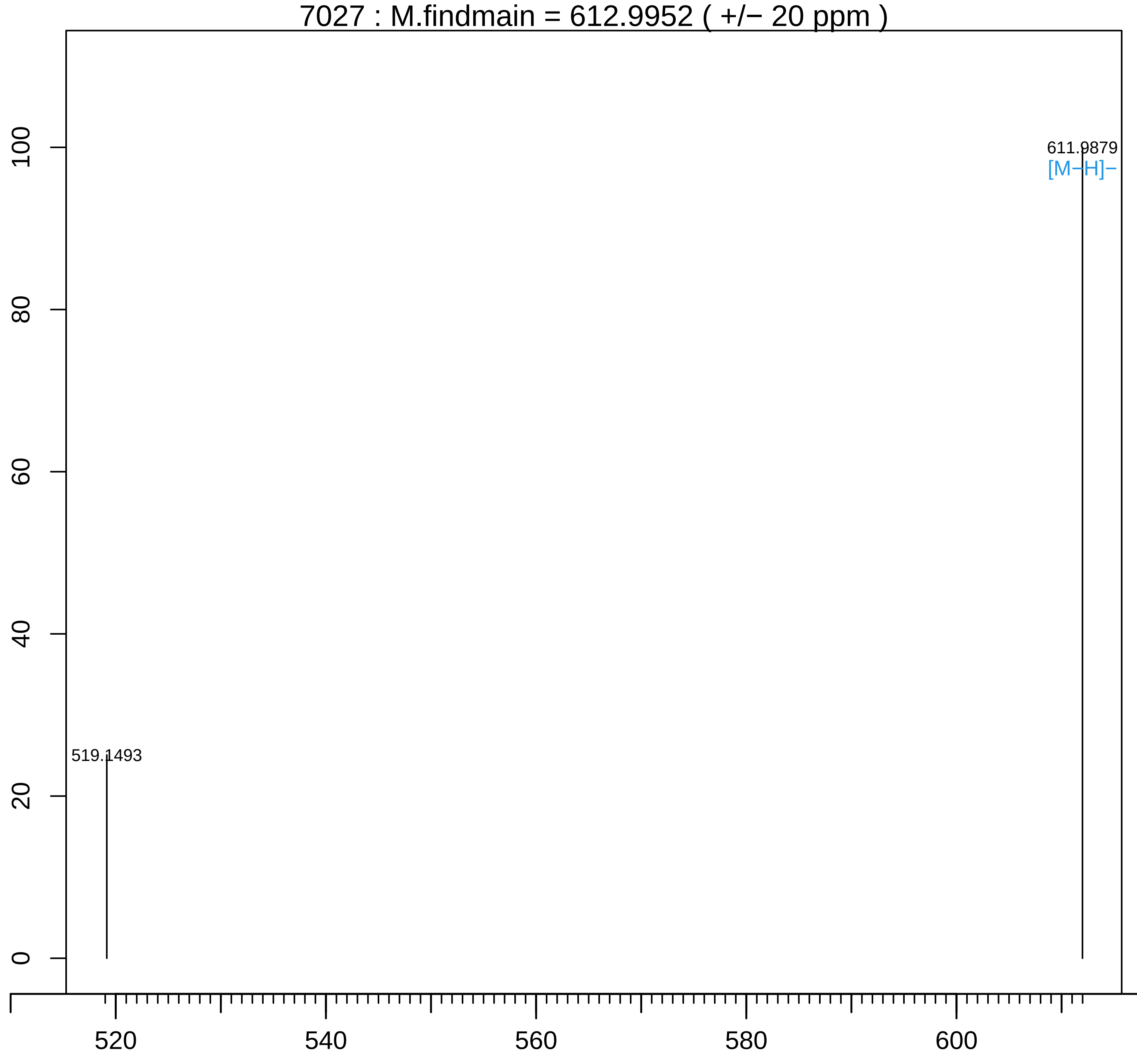
7027 : M.findmain = 612.9952 (+/− 20 ppm)
611.9879
[M−H]−
519.1493
0
20
40
60
80
100
520
540
560
580
600

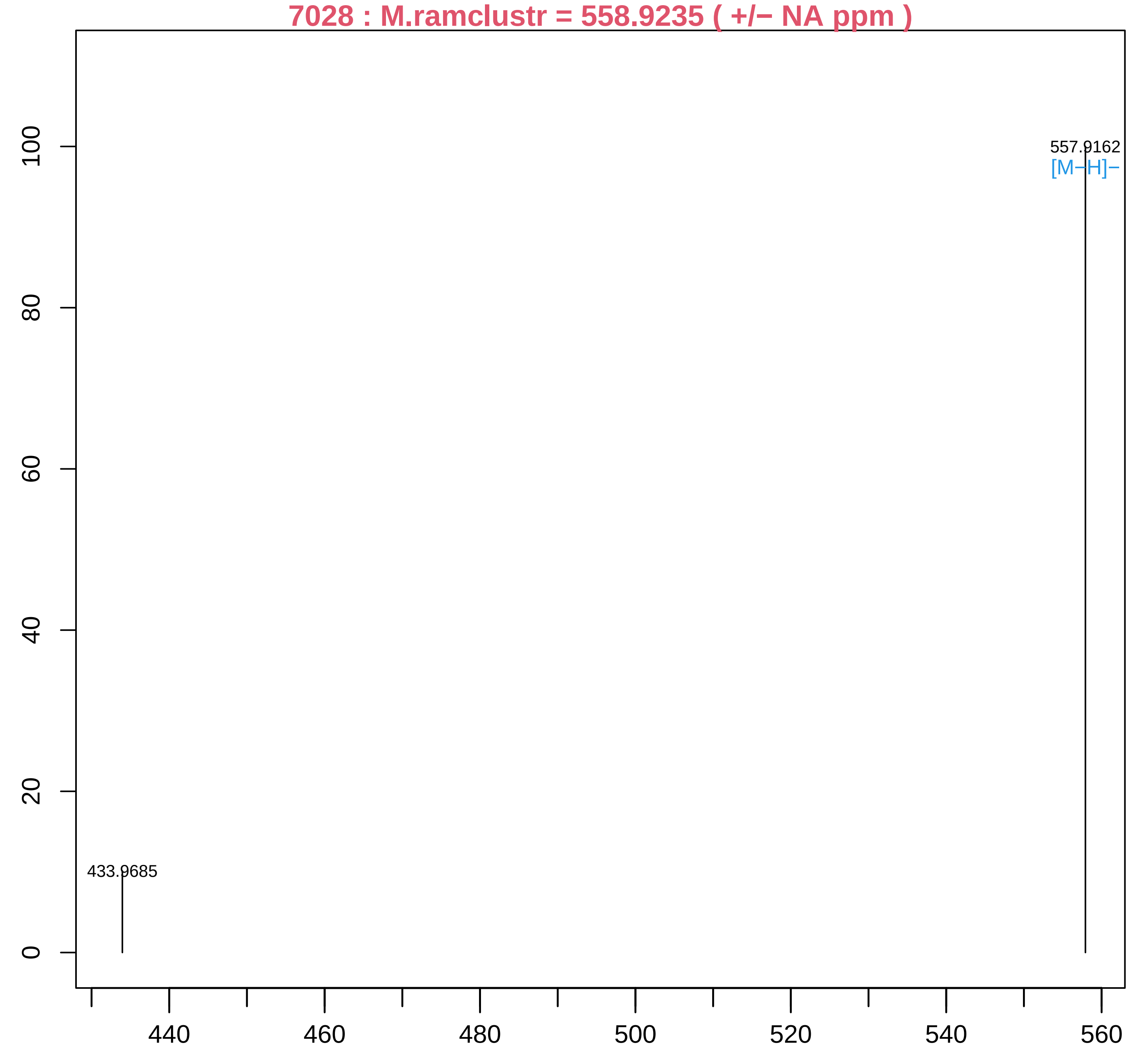
7028 : M.ramclustr = 558.9235 (+/− NA ppm)
557.9162
[M−H]−
433.9685
0
20
40
60
80
100
440
460
480
500
520
540
560

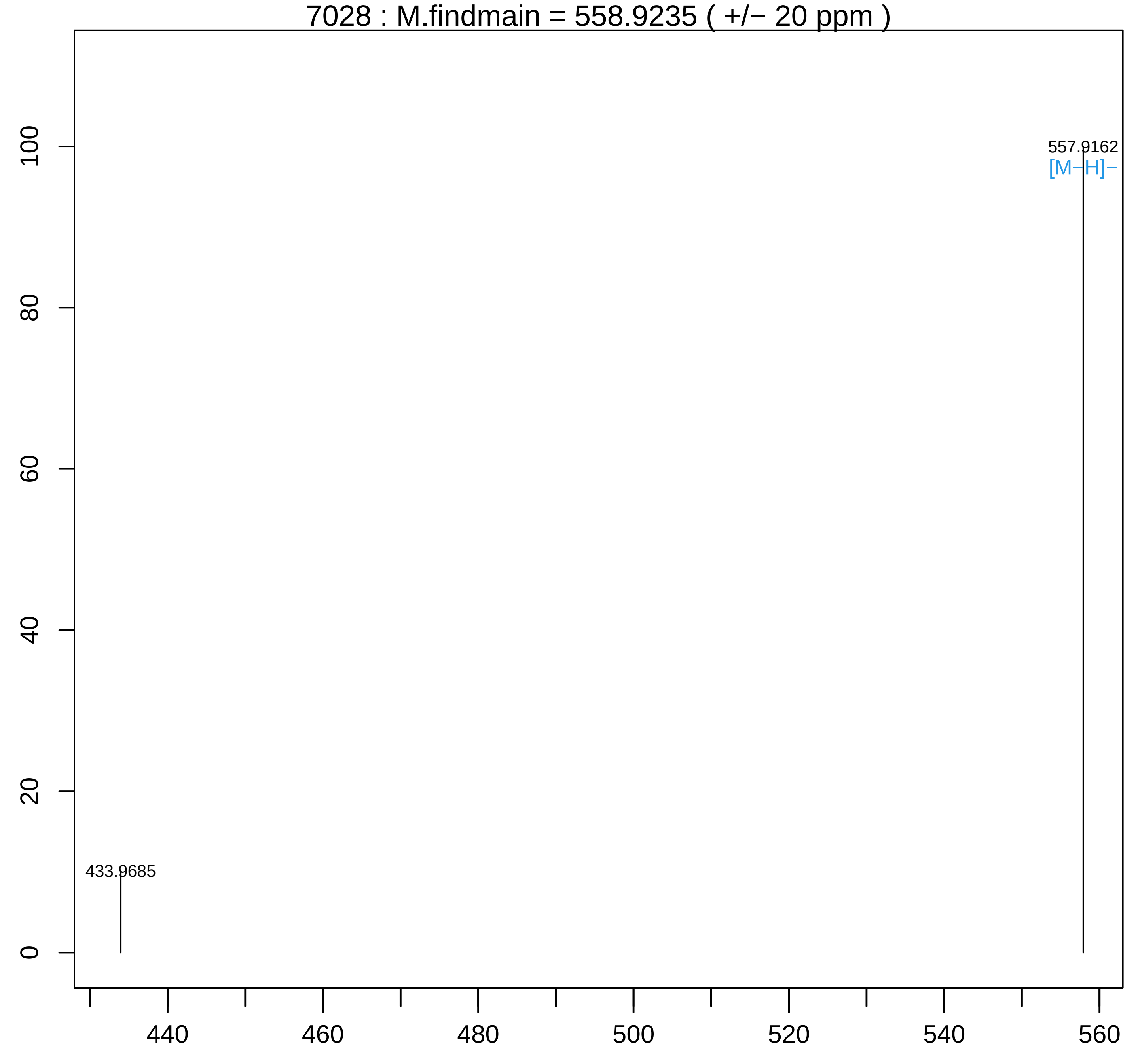
7028 : M.findmain = 558.9235 (+/− 20 ppm)
557.9162
[M−H]−
433.9685
0
20
40
60
80
100
440
460
480
500
520
540
560

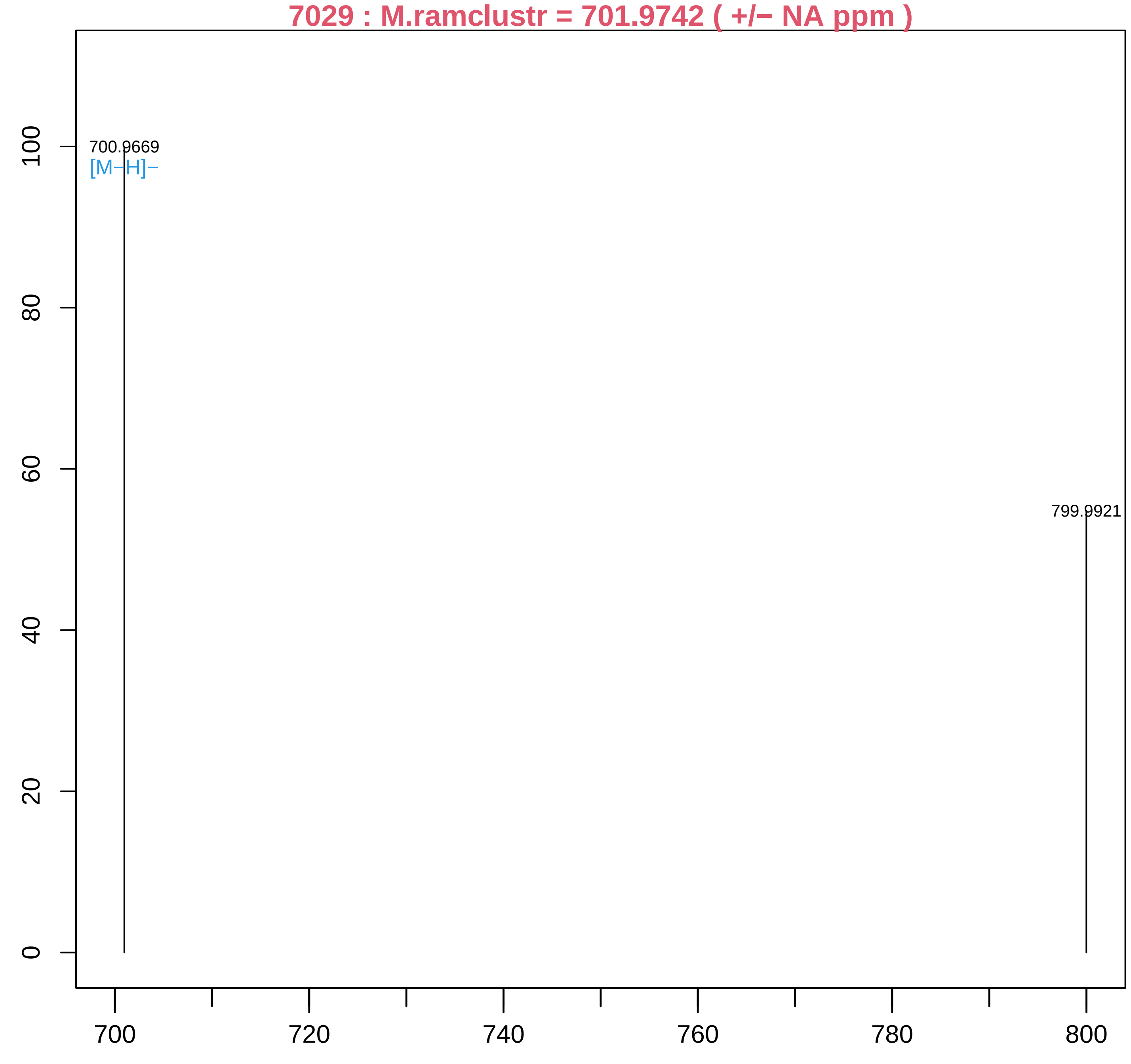

7029 : M.ramclustr = 701.9742 (+/− NA ppm)
700.9669
[M−H]−
799.9921
0
20
40
60
80
100
700
720
740
760
780
800

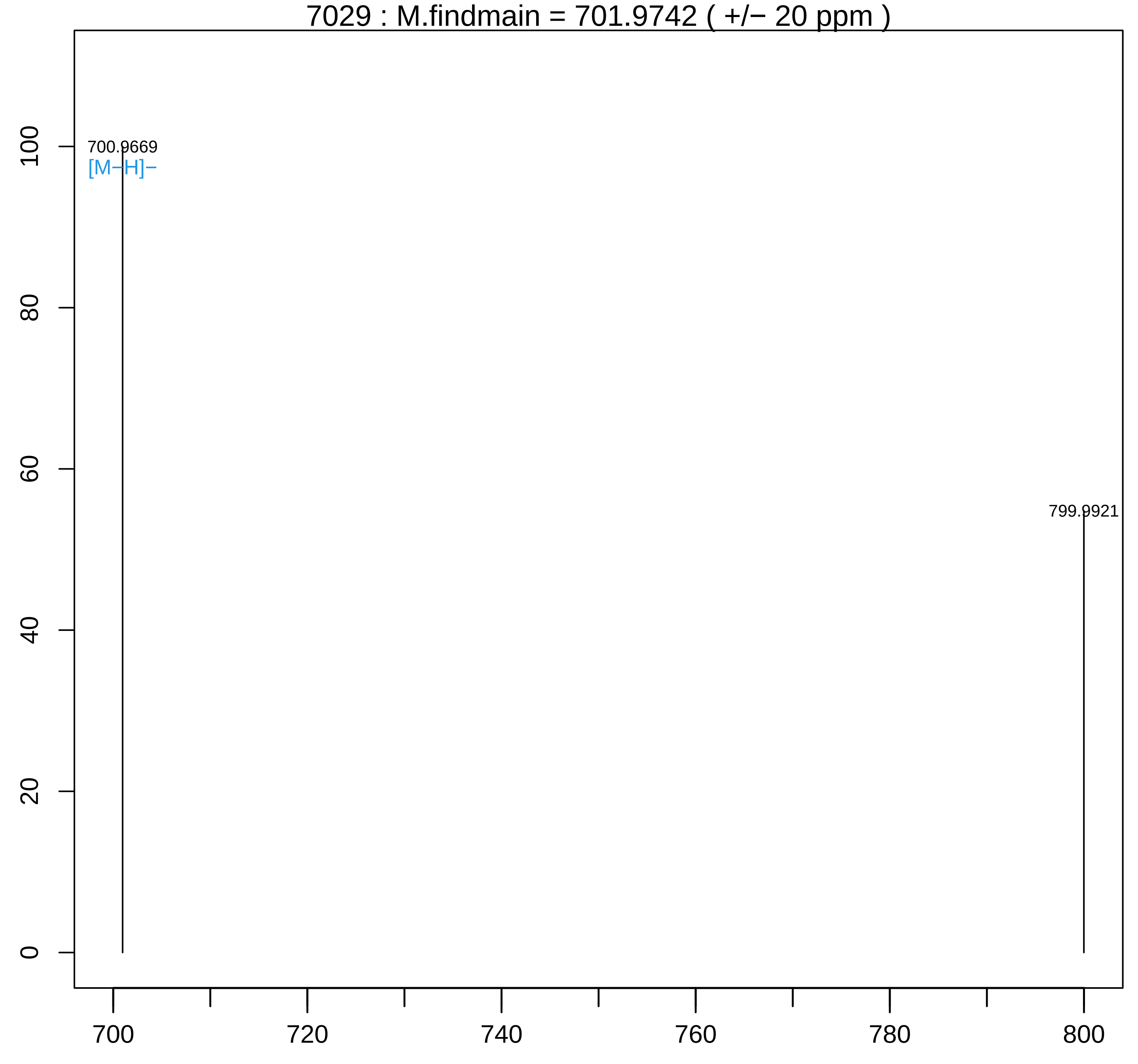

7029 : M.findmain = 701.9742 (+/− 20 ppm)
700.9669
[M−H]−
799.9921
0
20
40
60
80
100
700
720
740
760
780
800

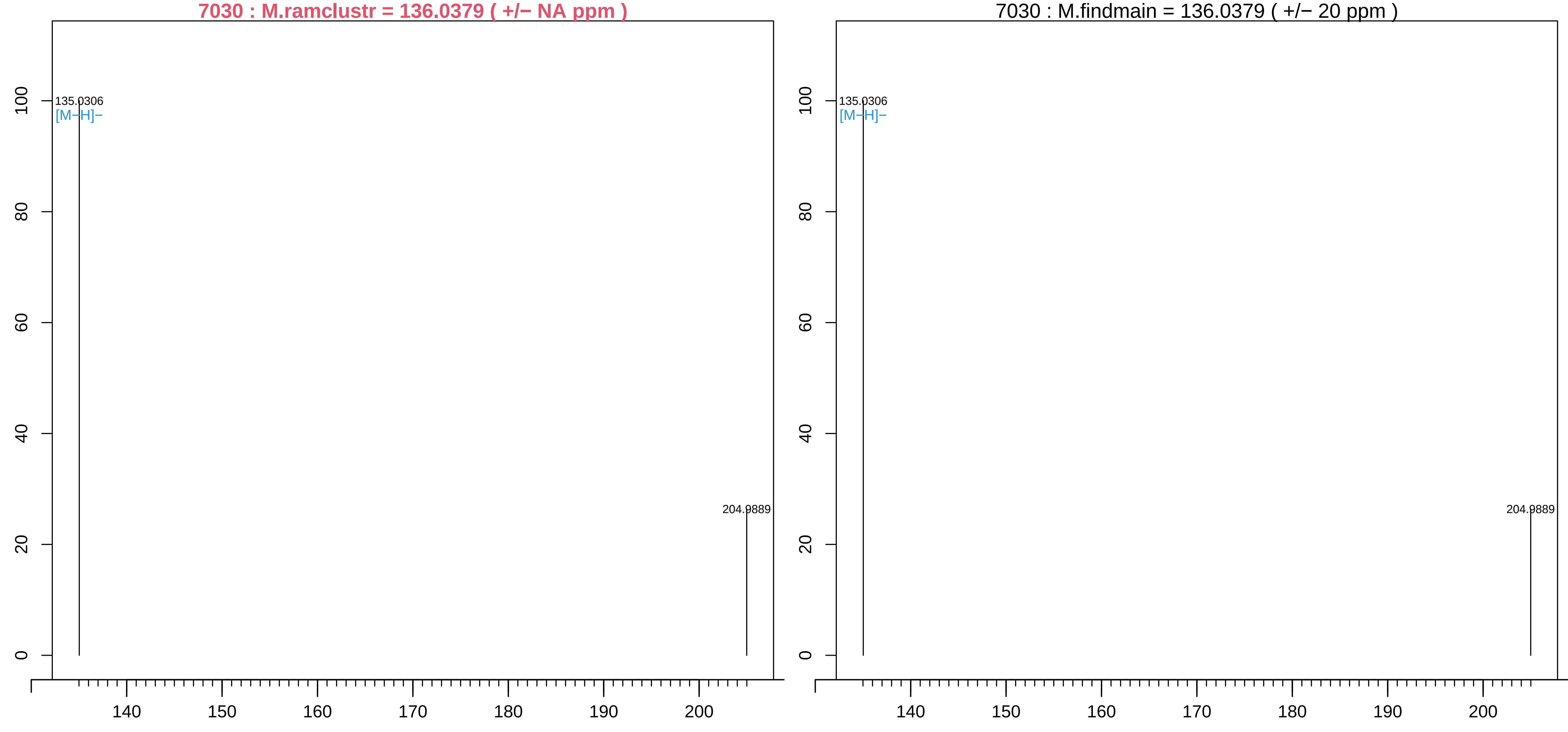

7030 : M.ramclustr = 136.0379 (+/− NA ppm)
135.0306
[M−H]−
204.9889
7030 : M.findmain = 136.0379 (+/− 20 ppm)
135.0306
[M−H]−
204.9889
0
20
40
60
80
100
140
150
160
170
180
190
200

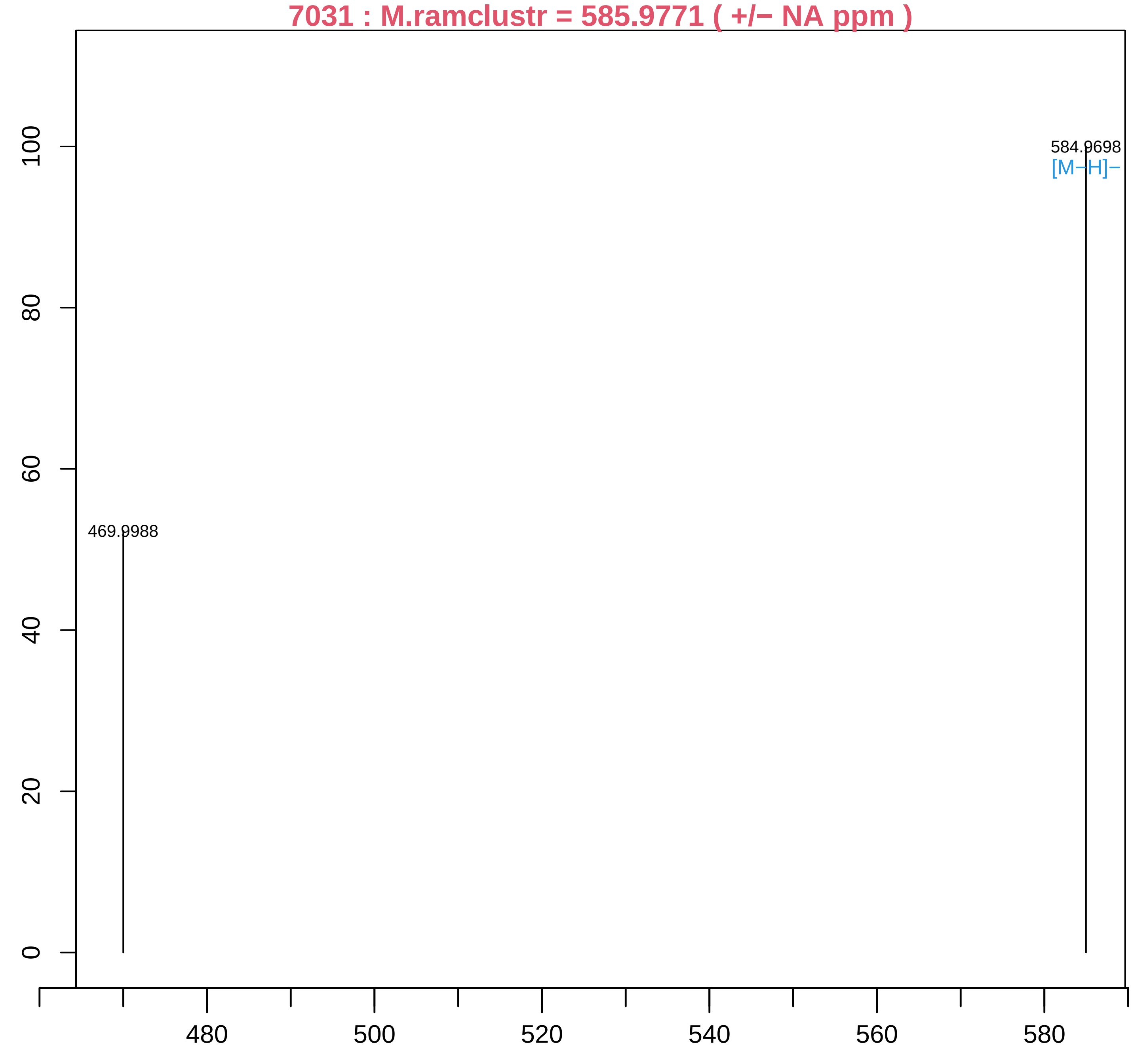
7031 : M.ramclustr = 585.9771 (+/− NA ppm)
584.9698
[M−H]−
469.9988
0
20
40
60
80
100
480
500
520
540
560
580

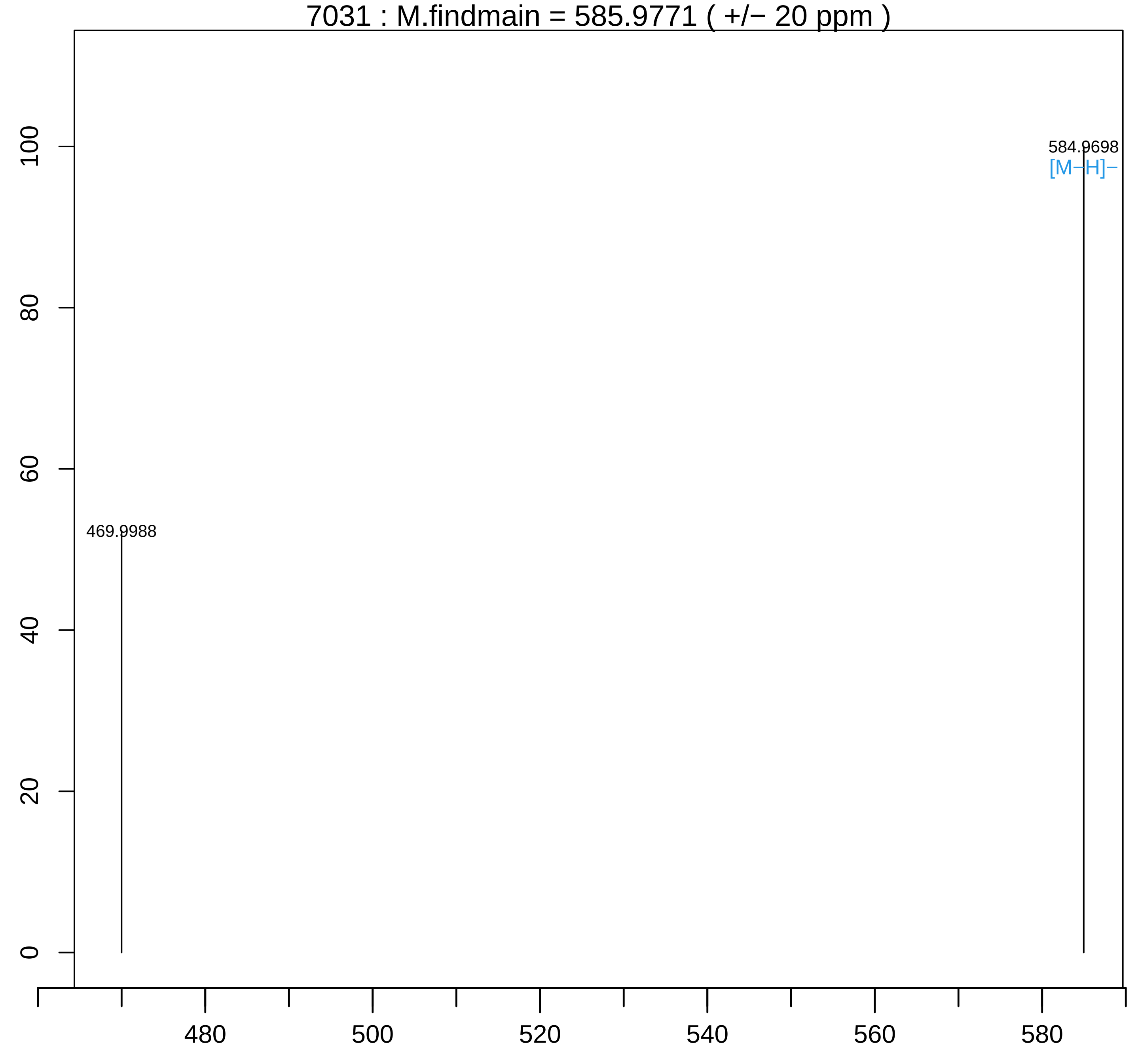
7031 : M.findmain = 585.9771 (+/− 20 ppm)
584.9698
[M−H]−
469.9988
0
20
40
60
80
100
480
500
520
540
560
580

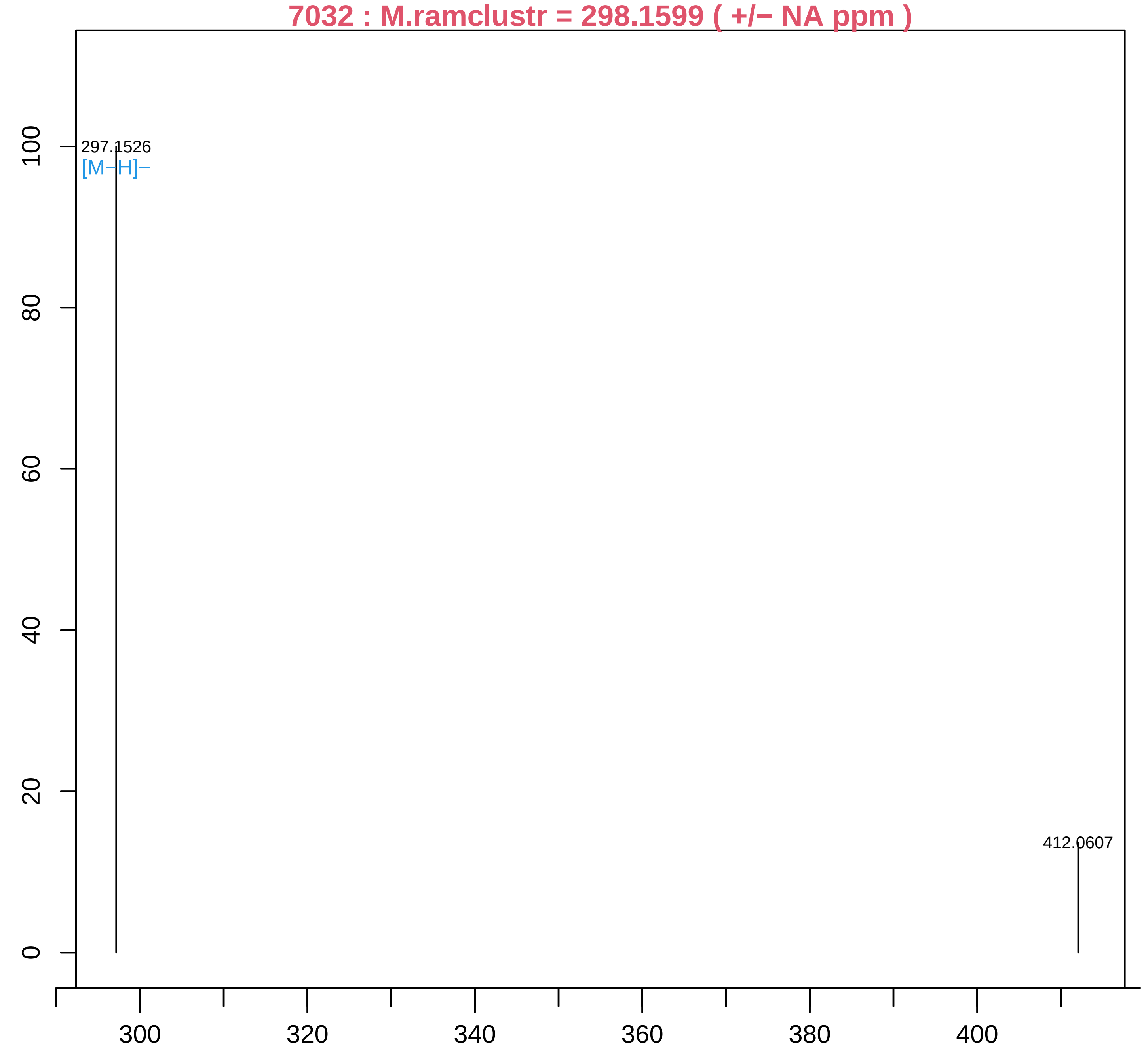
7032 : M.ramclustr = 298.1599 (+/− NA ppm)
297.1526
[M−H]−
412.0607
0
20
40
60
80
100
300
320
340
360
380
400

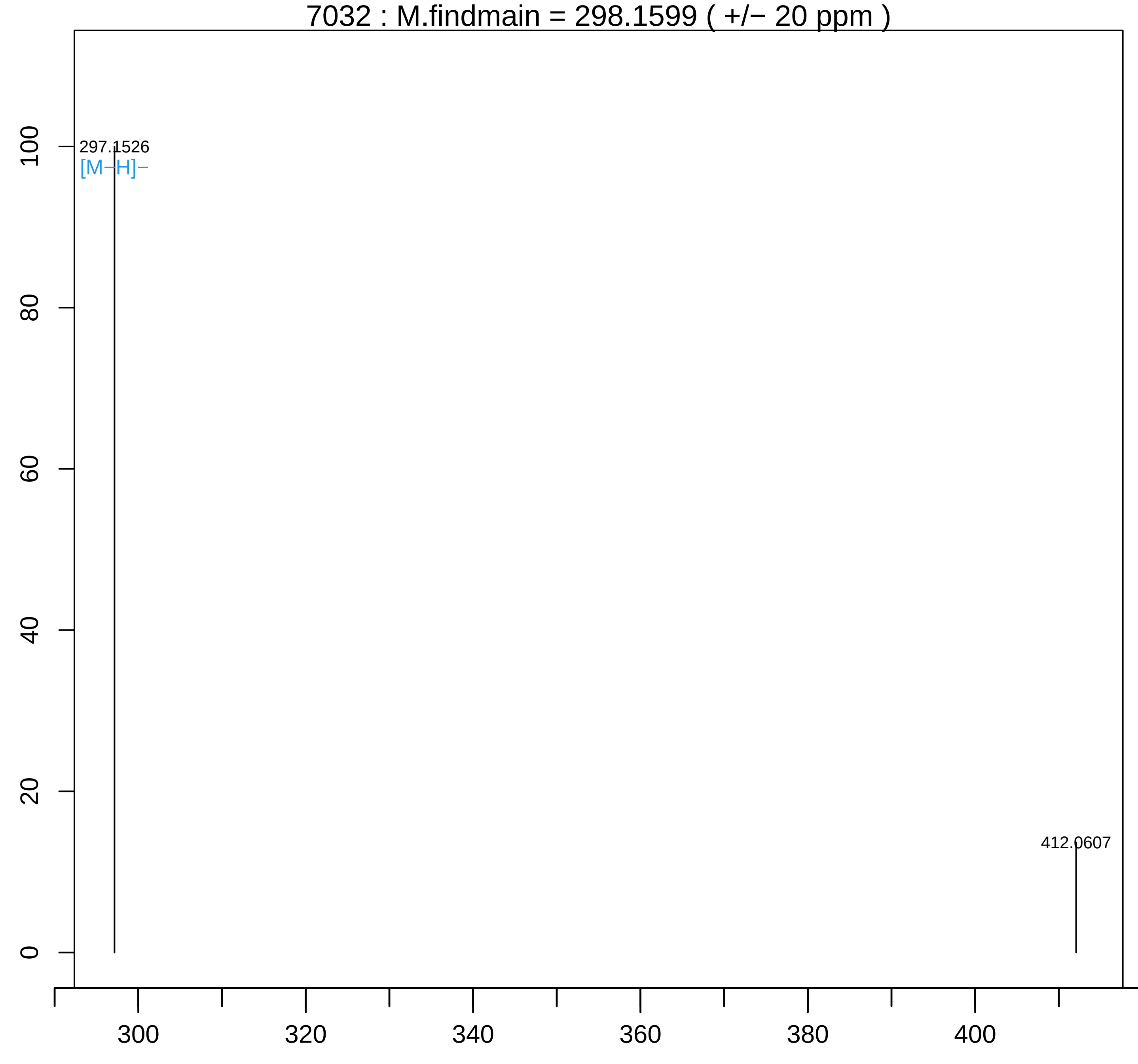
7032 : M.findmain = 298.1599 (+/− 20 ppm)
297.1526
[M−H]−
412.0607
0
20
40
60
80
100
300
320
340
360
380
400

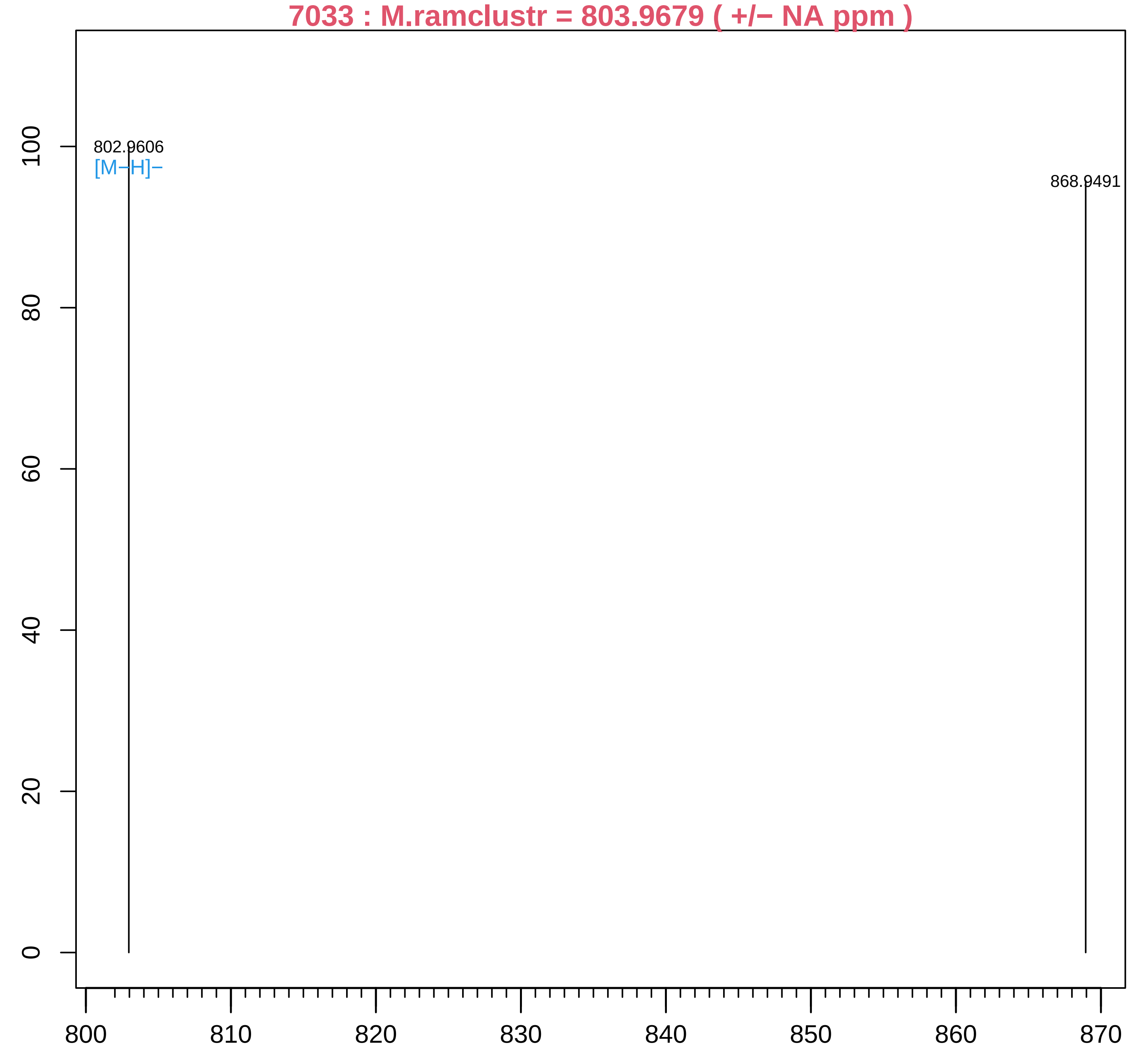
7033 : M.ramclustr = 803.9679 (+/− NA ppm)
802.9606
[M−H]−
868.9491
0
20
40
60
80
100
800
810
820
830
840
850
860
870

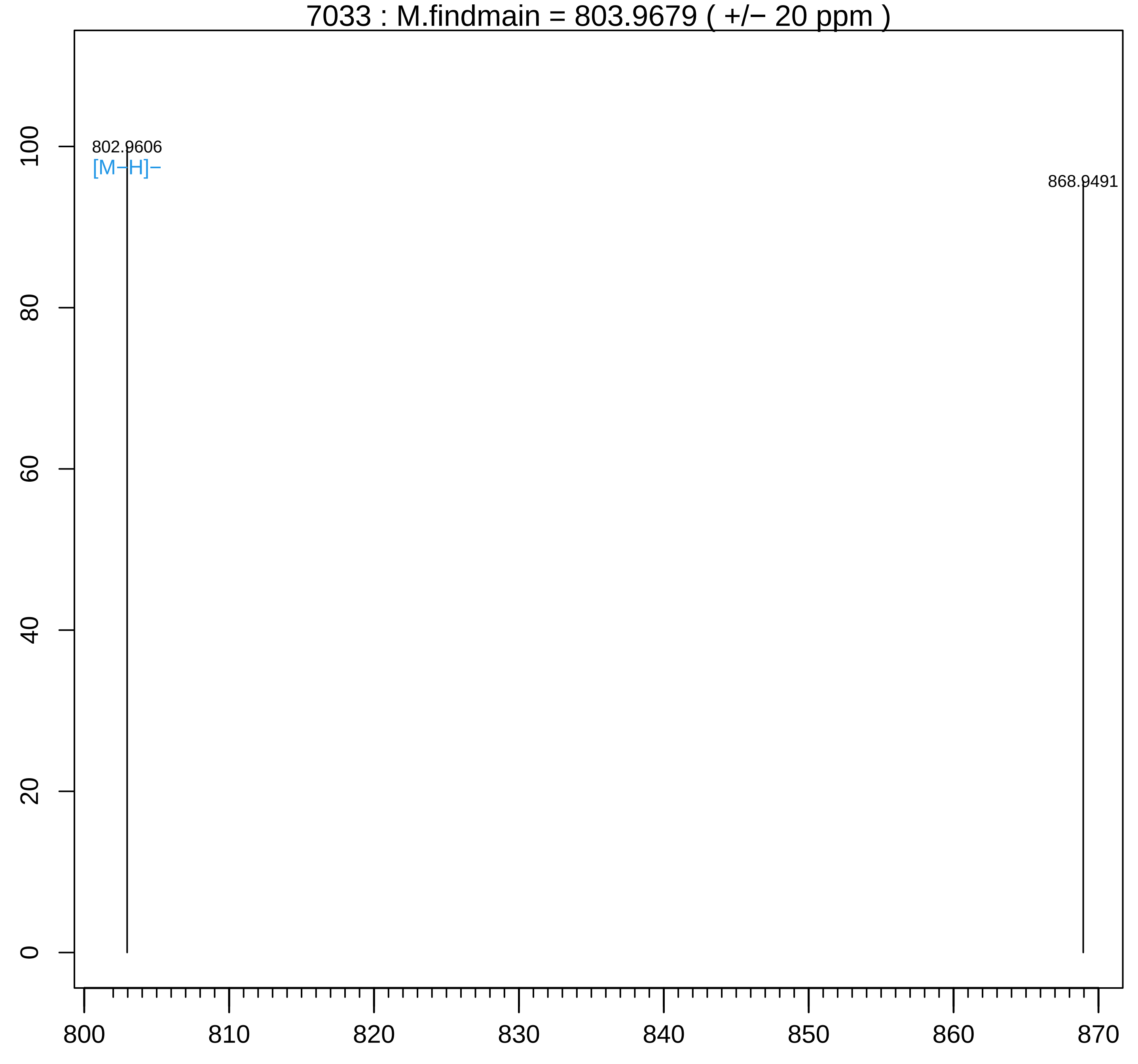
7033 : M.findmain = 803.9679 (+/− 20 ppm)
802.9606
[M−H]−
868.9491
0
20
40
60
80
100
800
810
820
830
840
850
860
870

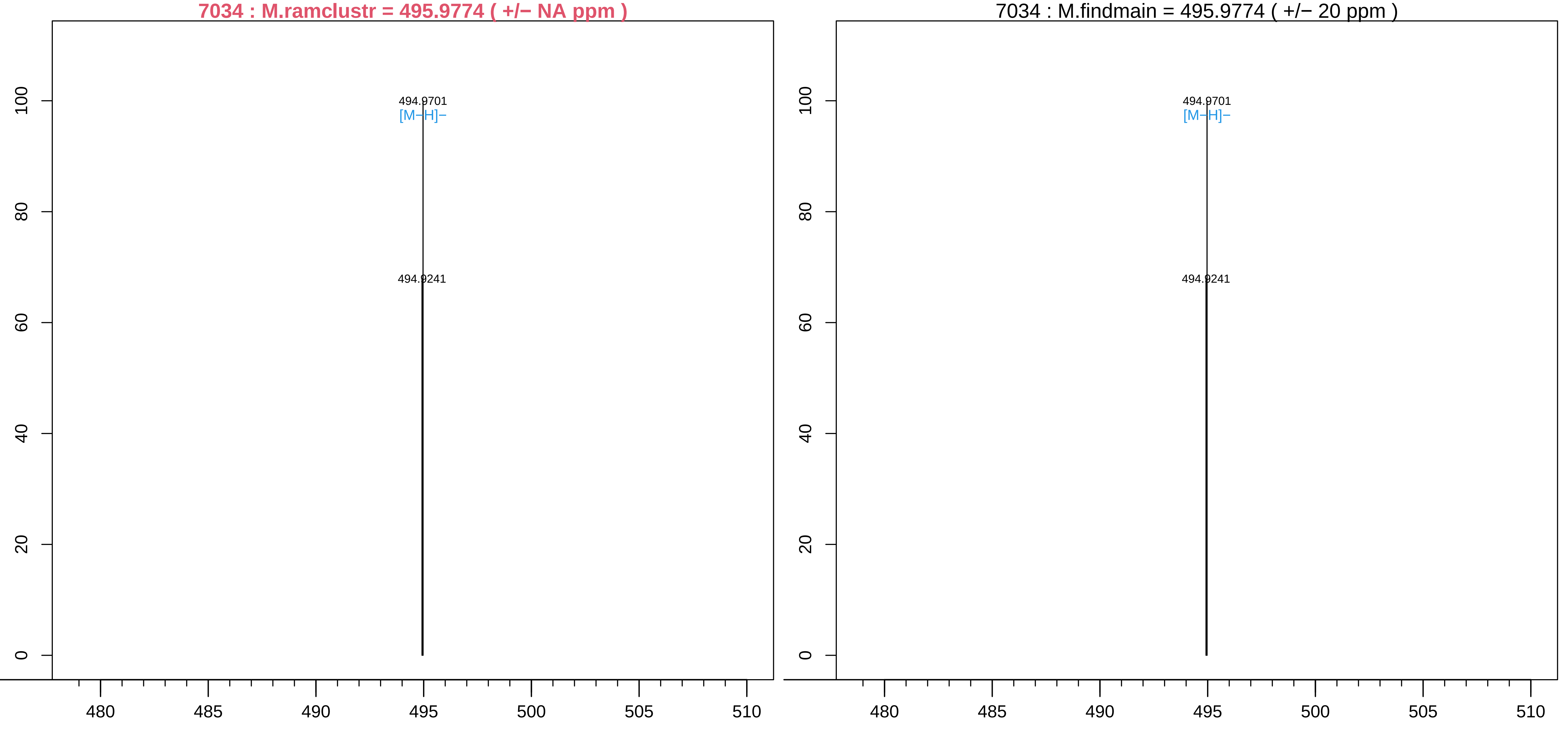

7034 : M.ramclustr = 495.9774 (+/− NA ppm)
494.9701
[M−H]−
494.9241
7034 : M.findmain = 495.9774 (+/− 20 ppm)
494.9701
[M−H]−
494.9241
0
20
40
60
80
100
480
485
490
495
500
505
510

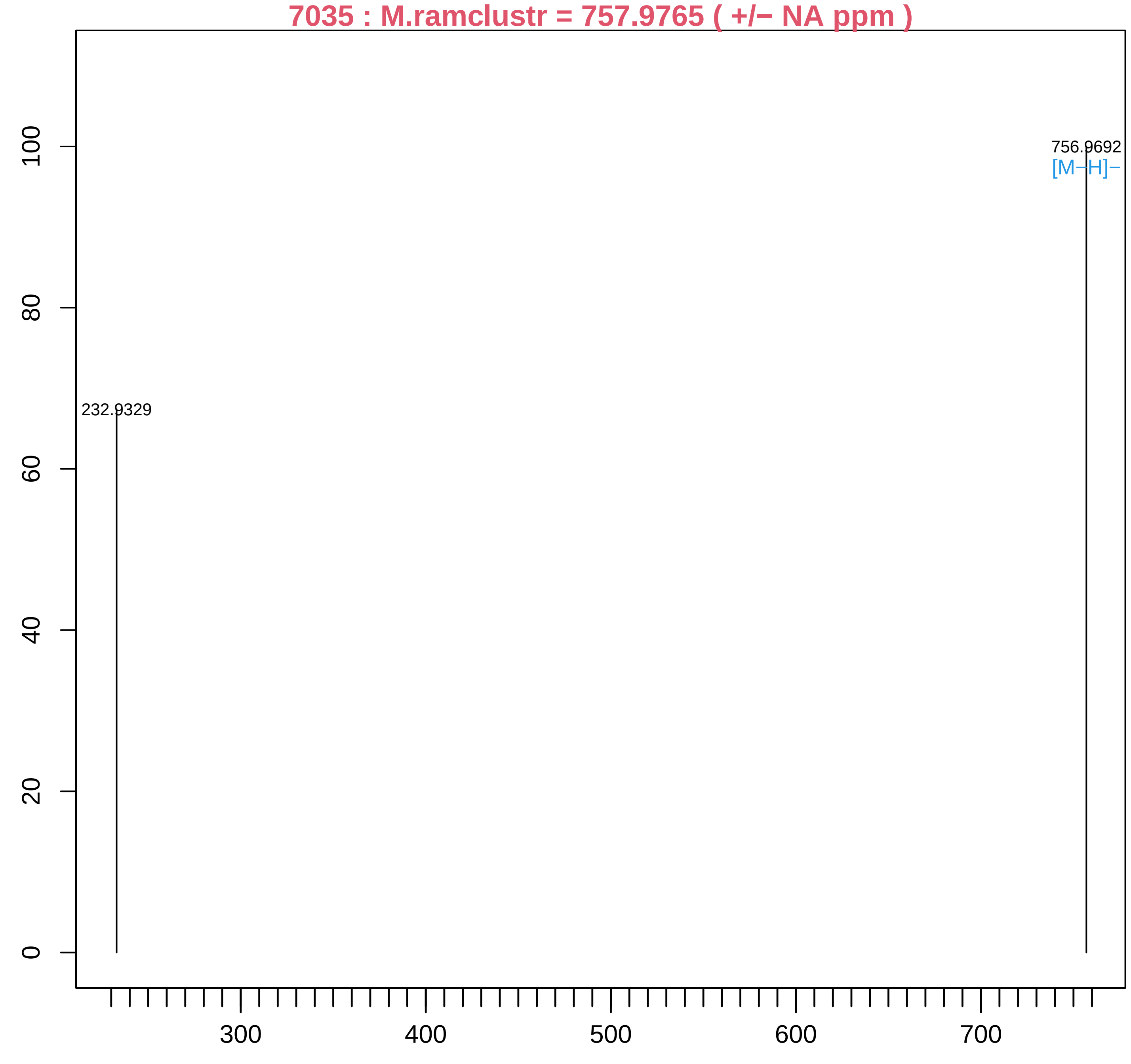

7035 : M.ramclustr = 757.9765 (+/− NA ppm)
756.9692
[M−H]−
232.9329
0
20
40
60
80
100
300
400
500
600
700

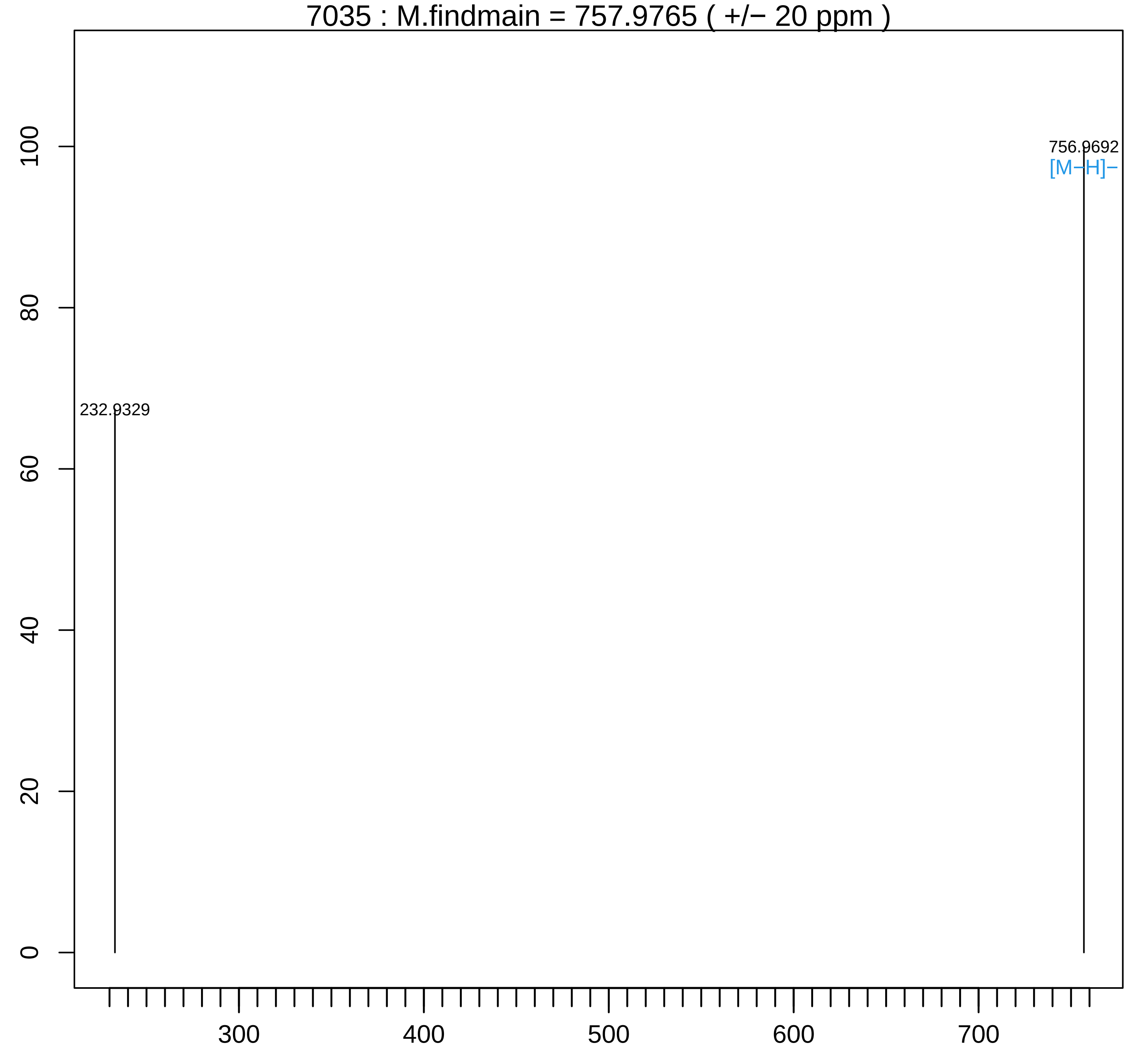

7035 : M.findmain = 757.9765 (+/− 20 ppm)
756.9692
[M−H]−
232.9329
0
20
40
60
80
100
300
400
500
600
700

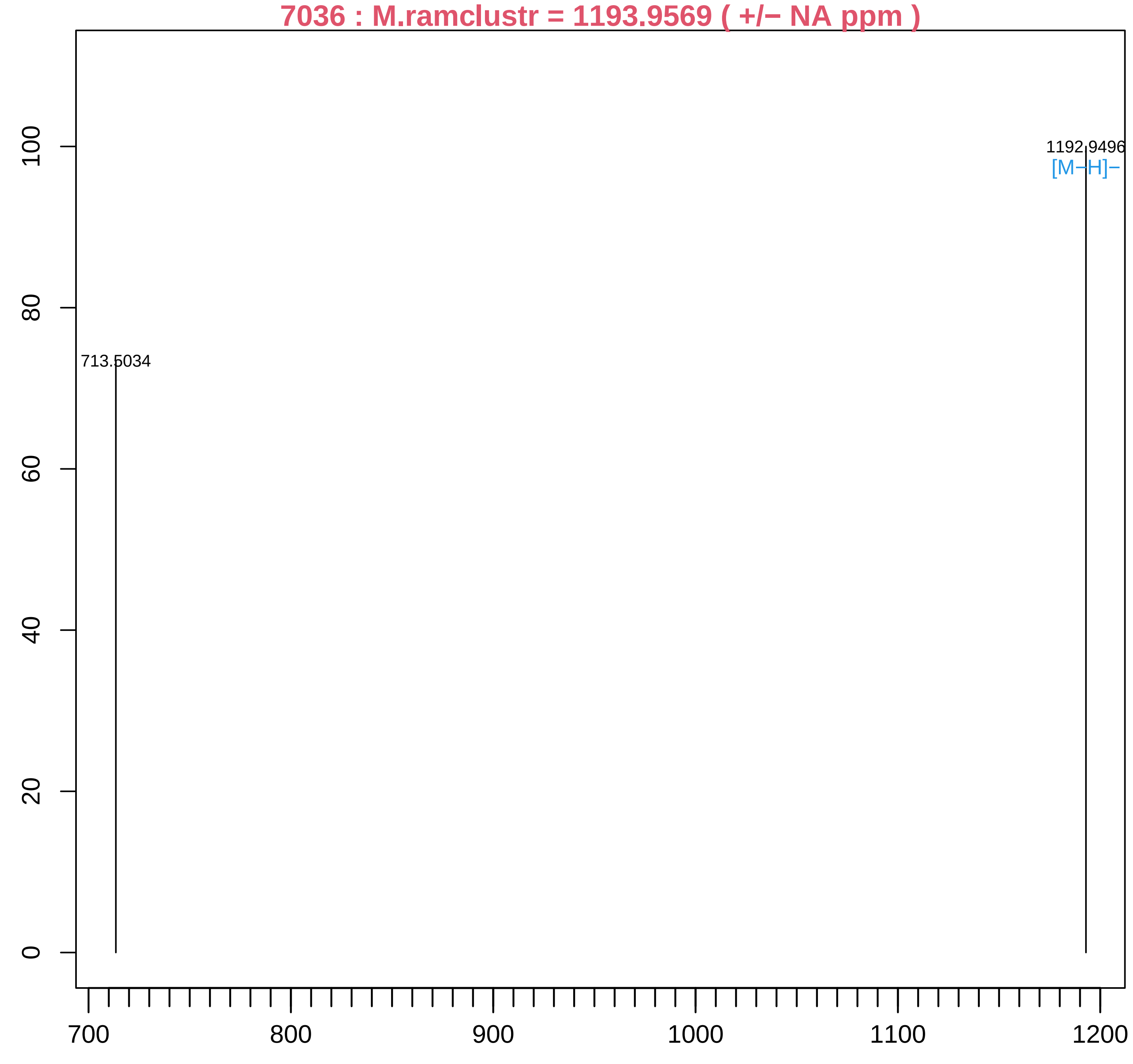
7036 : M.ramclustr = 1193.9569 (+/− NA ppm)
1192.9496
[M−H]−
713.5034
0
20
40
60
80
100
700
800
900
1000
1100
1200

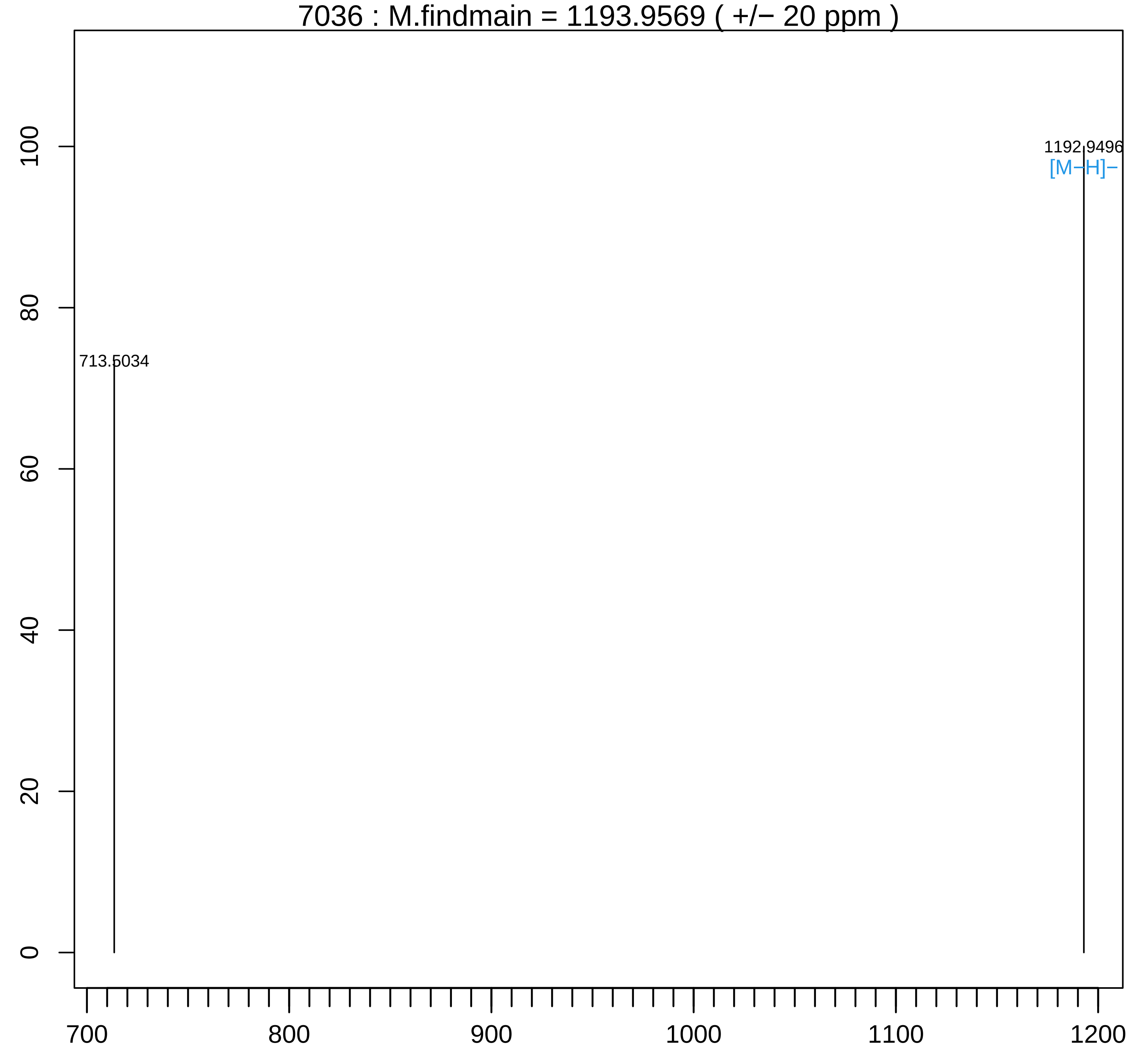
7036 : M.findmain = 1193.9569 (+/− 20 ppm)
1192.9496
[M−H]−
713.5034
0
20
40
60
80
100
700
800
900
1000
1100
1200

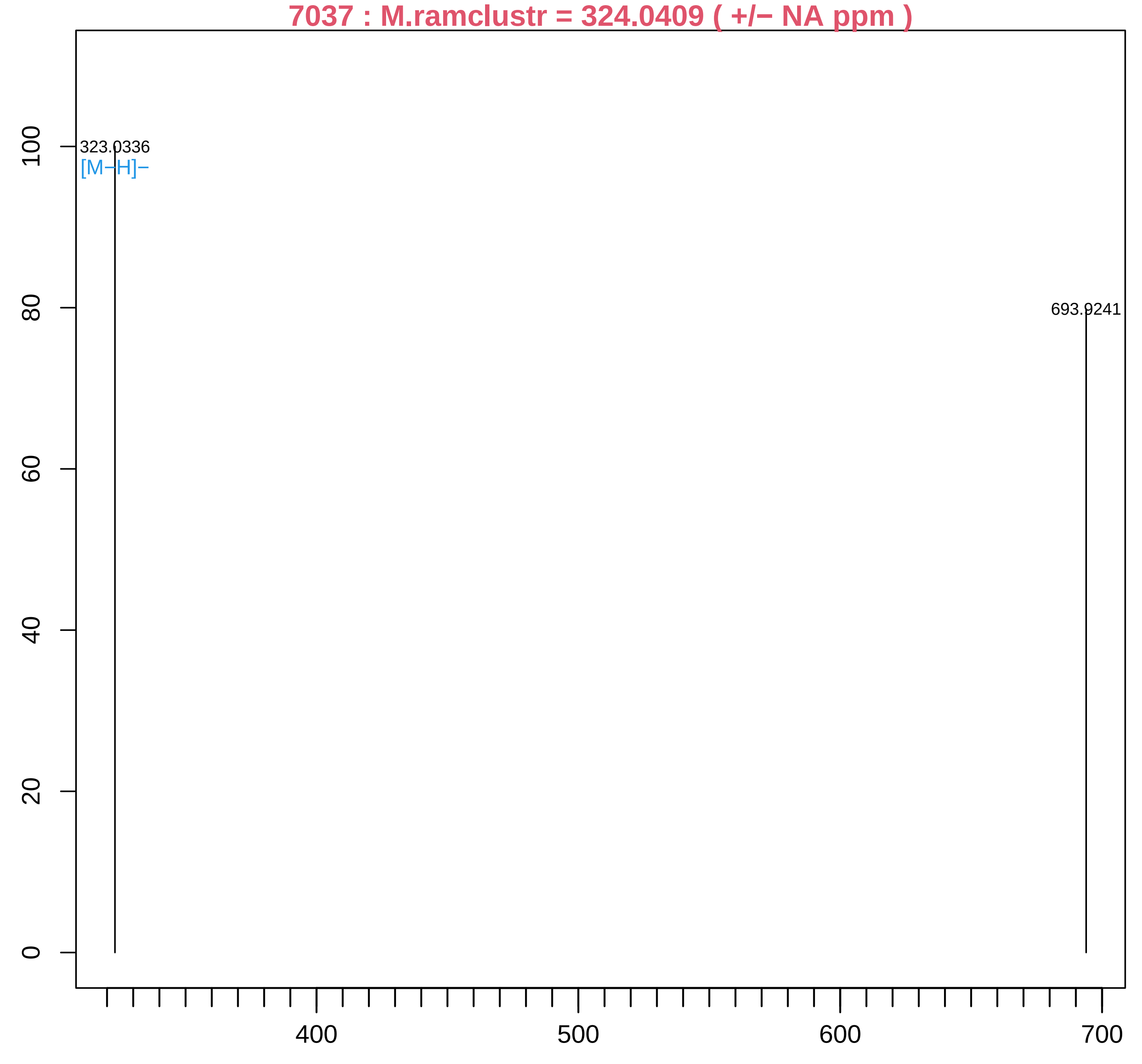
7037 : M.ramclustr = 324.0409 (+/− NA ppm)
323.0336
[M−H]−
693.9241
0
20
40
60
80
100
400
500
600
700

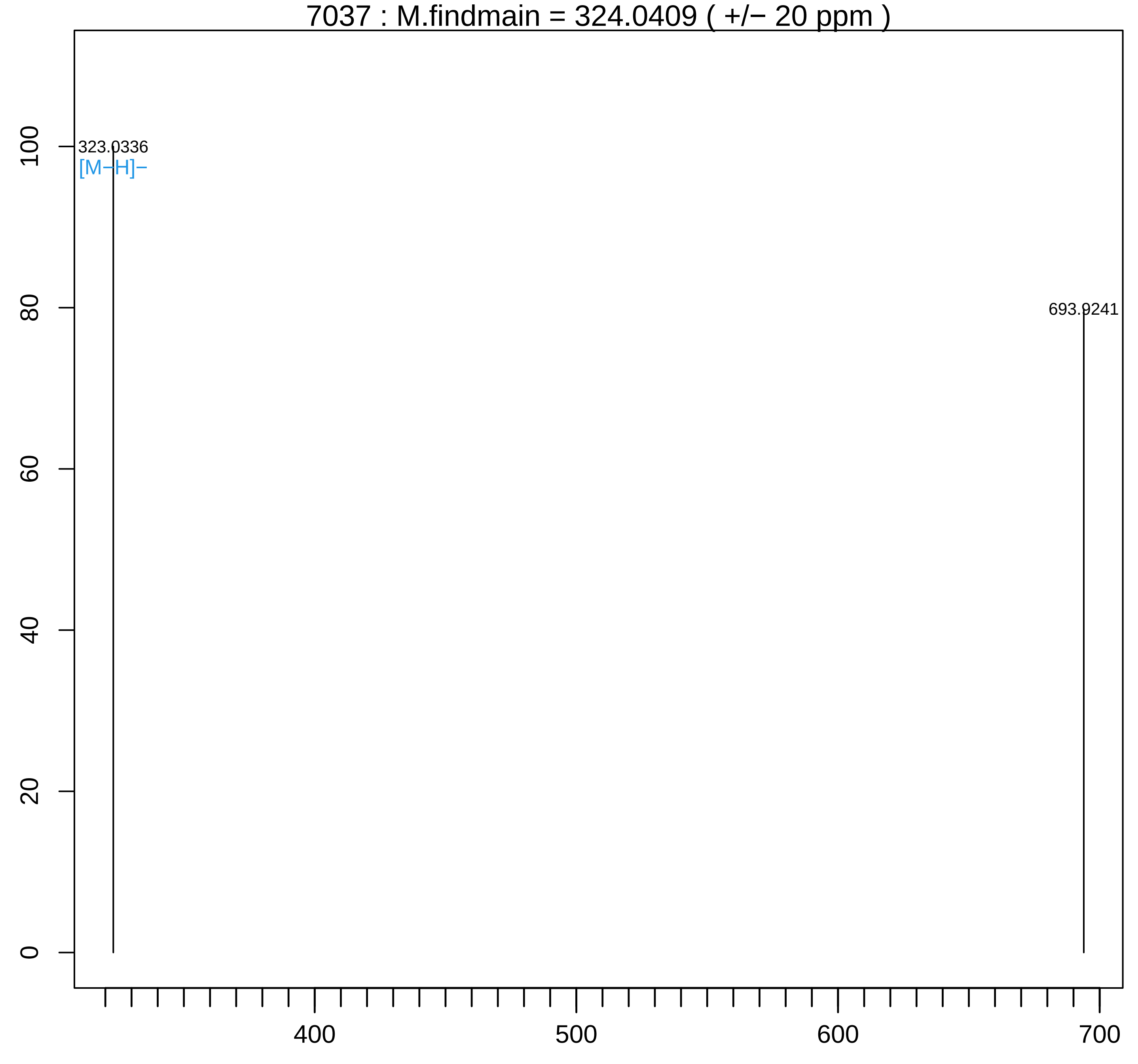
7037 : M.findmain = 324.0409 (+/− 20 ppm)
323.0336
[M−H]−
693.9241
0
20
40
60
80
100
400
500
600
700

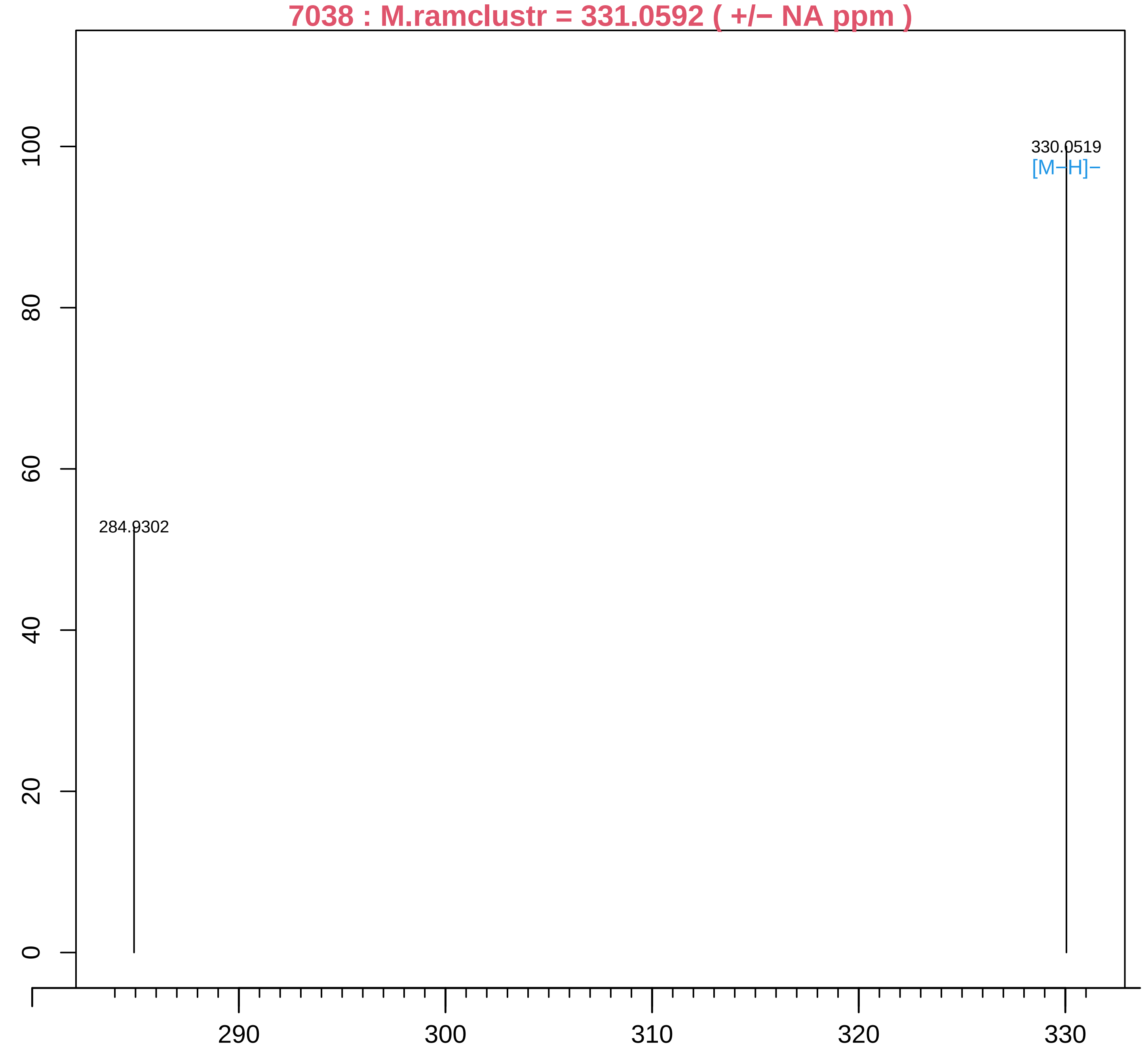
7038 : M.ramclustr = 331.0592 (+/− NA ppm)
330.0519
[M−H]−
284.9302
0
20
40
60
80
100
290
300
310
320
330

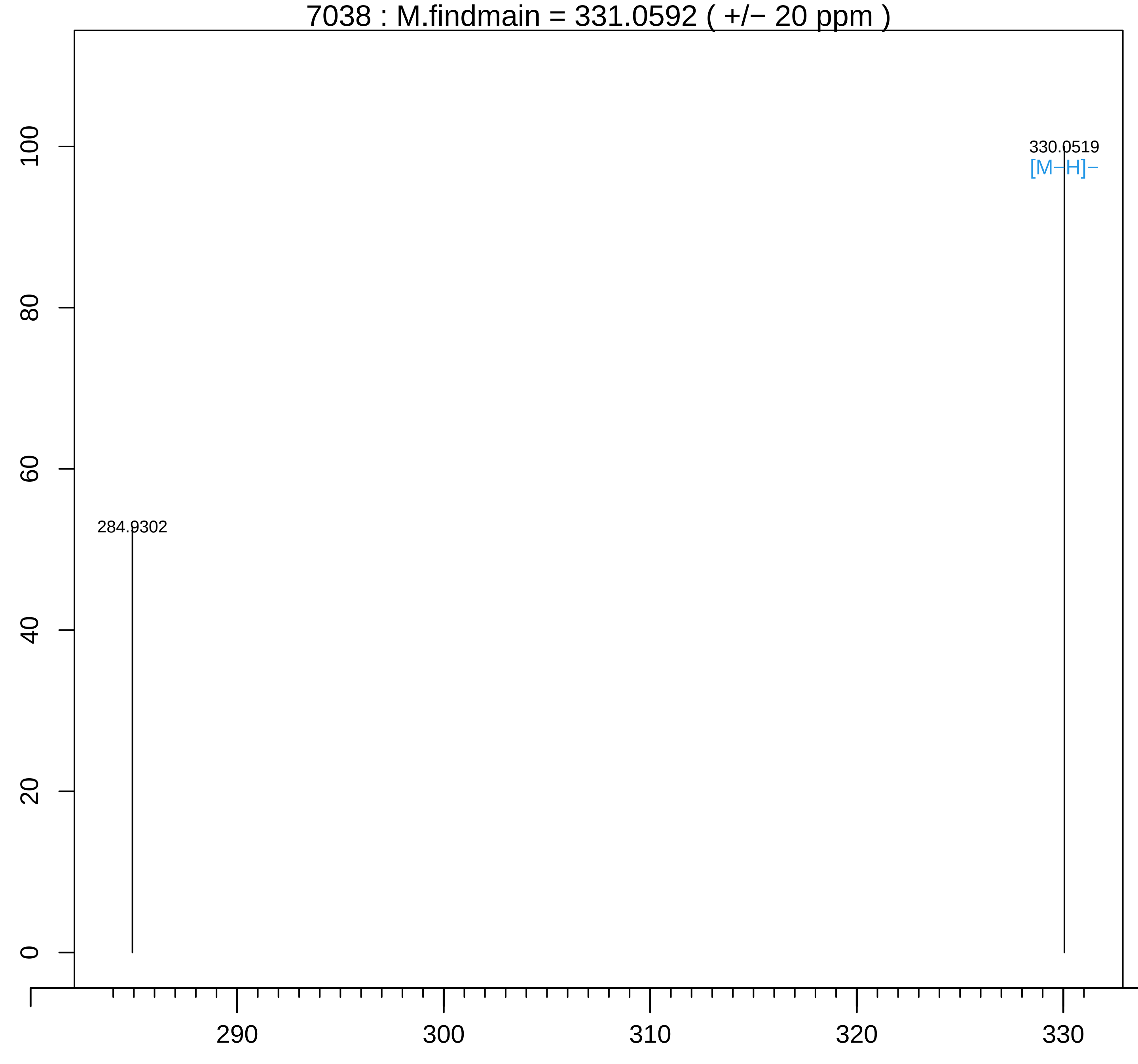
7038 : M.findmain = 331.0592 (+/− 20 ppm)
330.0519
[M−H]−
284.9302
0
20
40
60
80
100
290
300
310
320
330

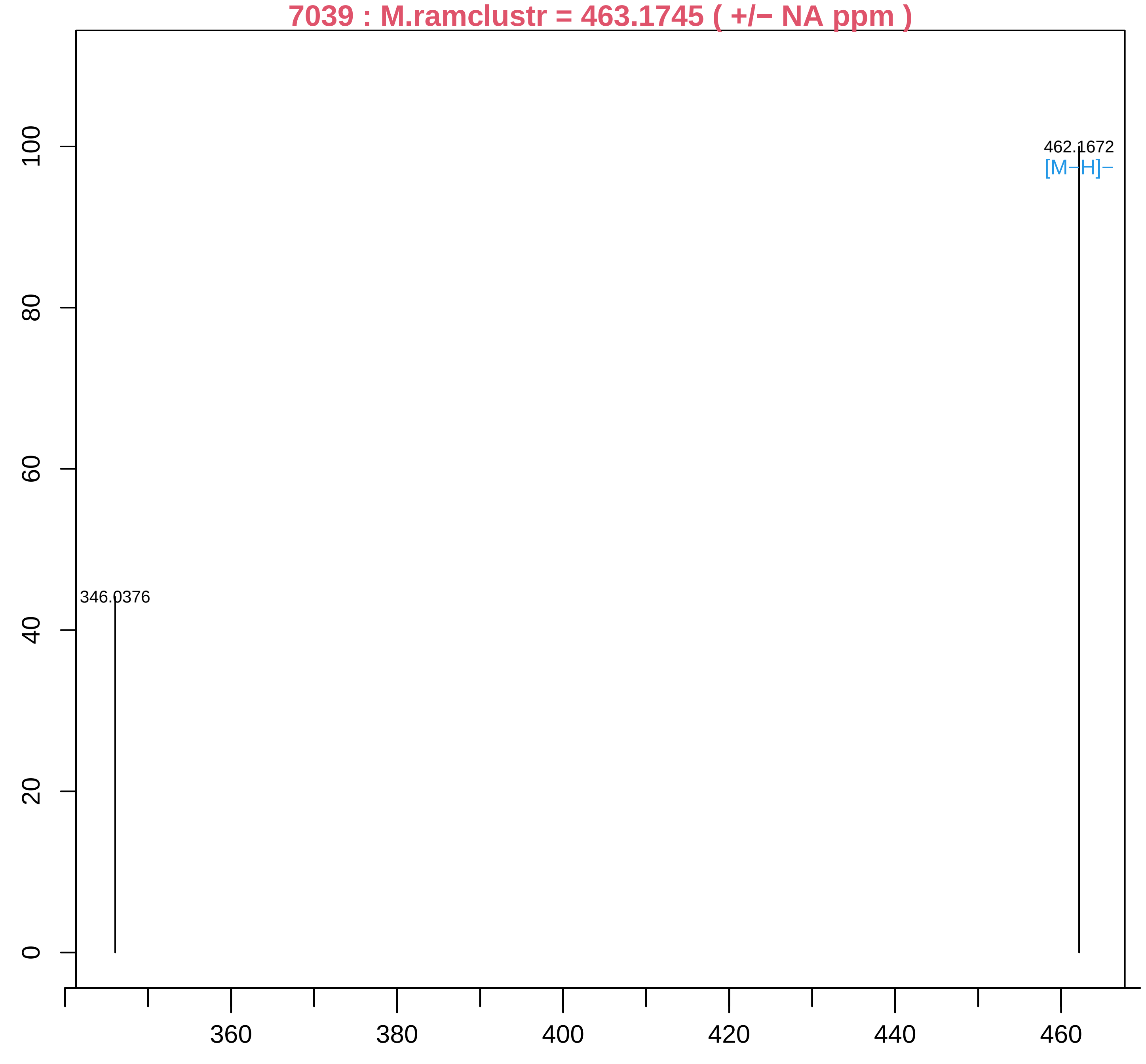
7039 : M.ramclustr = 463.1745 (+/− NA ppm)
462.1672
[M−H]−
346.0376
0
20
40
60
80
100
360
380
400
420
440
460

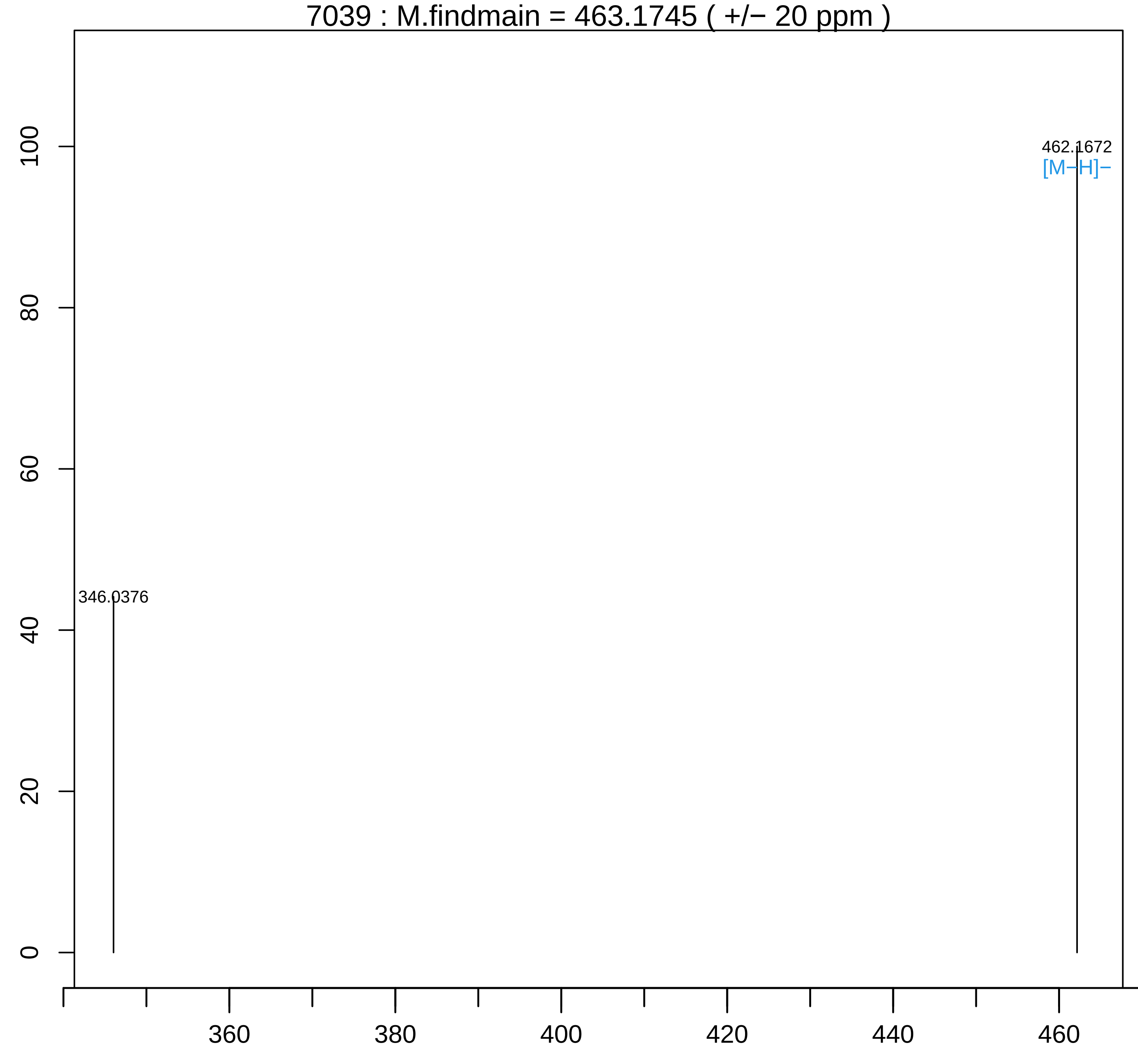
7039 : M.findmain = 463.1745 (+/− 20 ppm)
462.1672
[M−H]−
346.0376
0
20
40
60
80
100
360
380
400
420
440
460

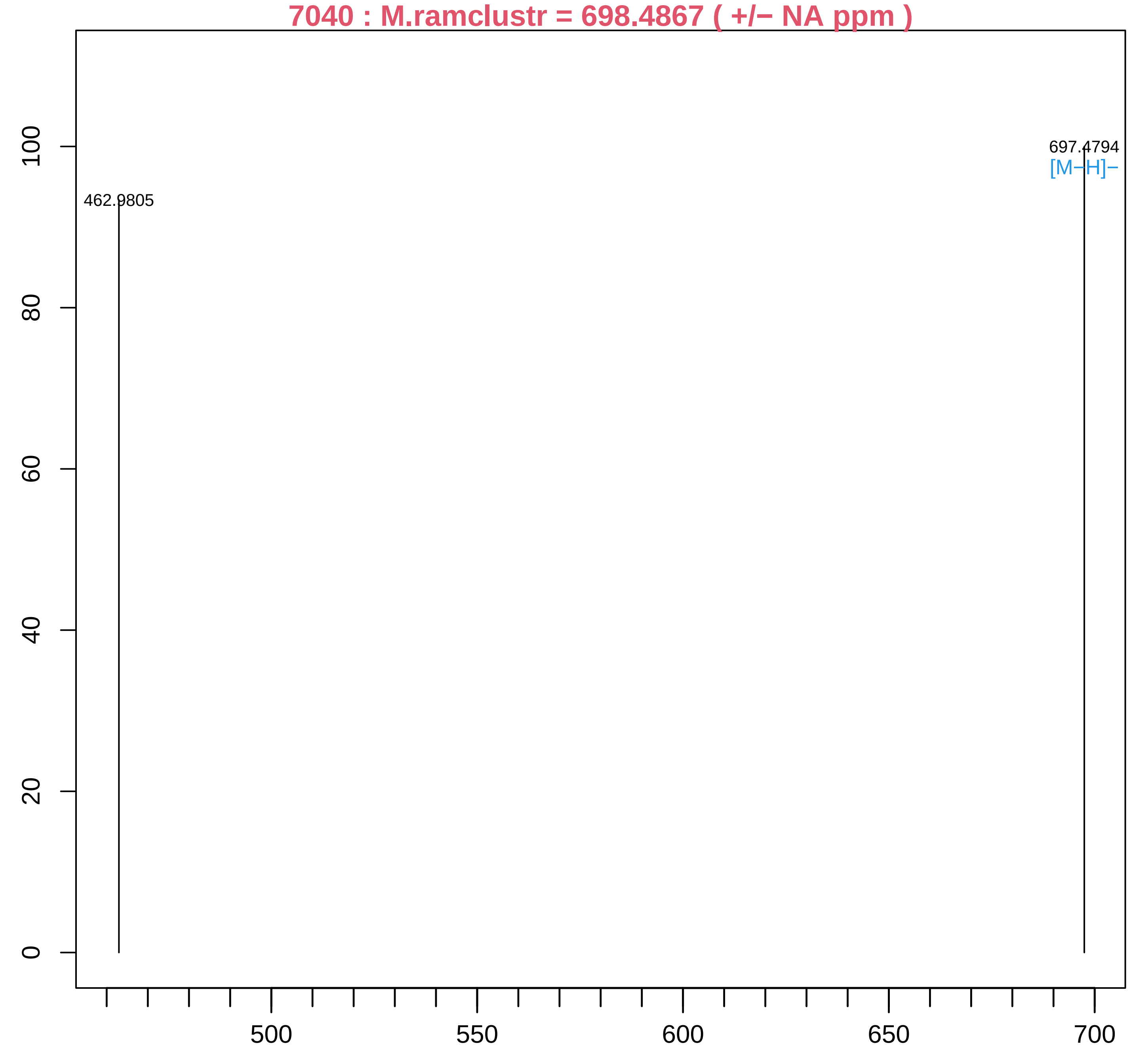
7040 : M.ramclustr = 698.4867 (+/− NA ppm)
697.4794
[M−H]−
462.9805
0
20
40
60
80
100
500
550
600
650
700

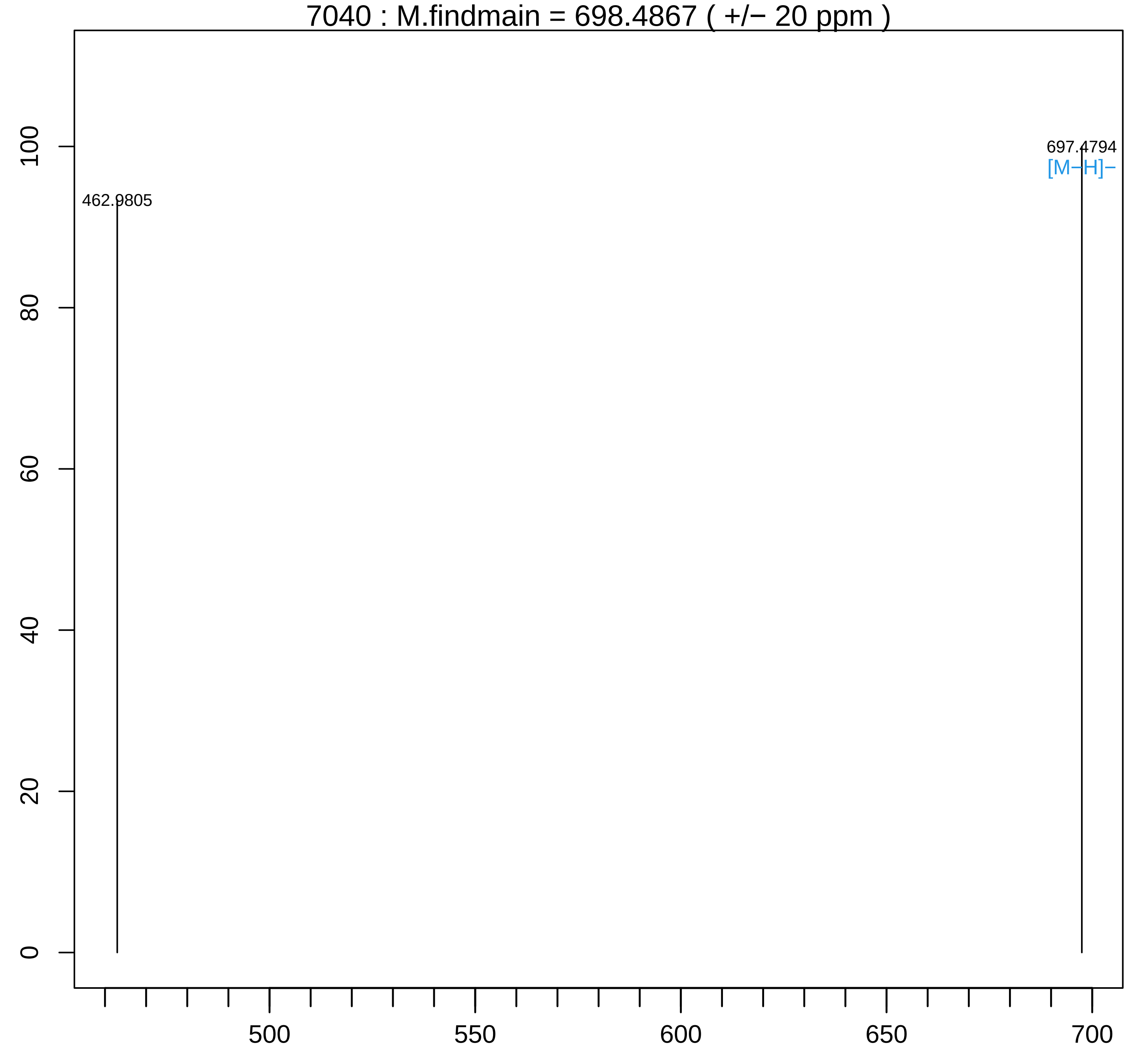
7040 : M.findmain = 698.4867 (+/− 20 ppm)
697.4794
[M−H]−
462.9805
0
20
40
60
80
100
500
550
600
650
700

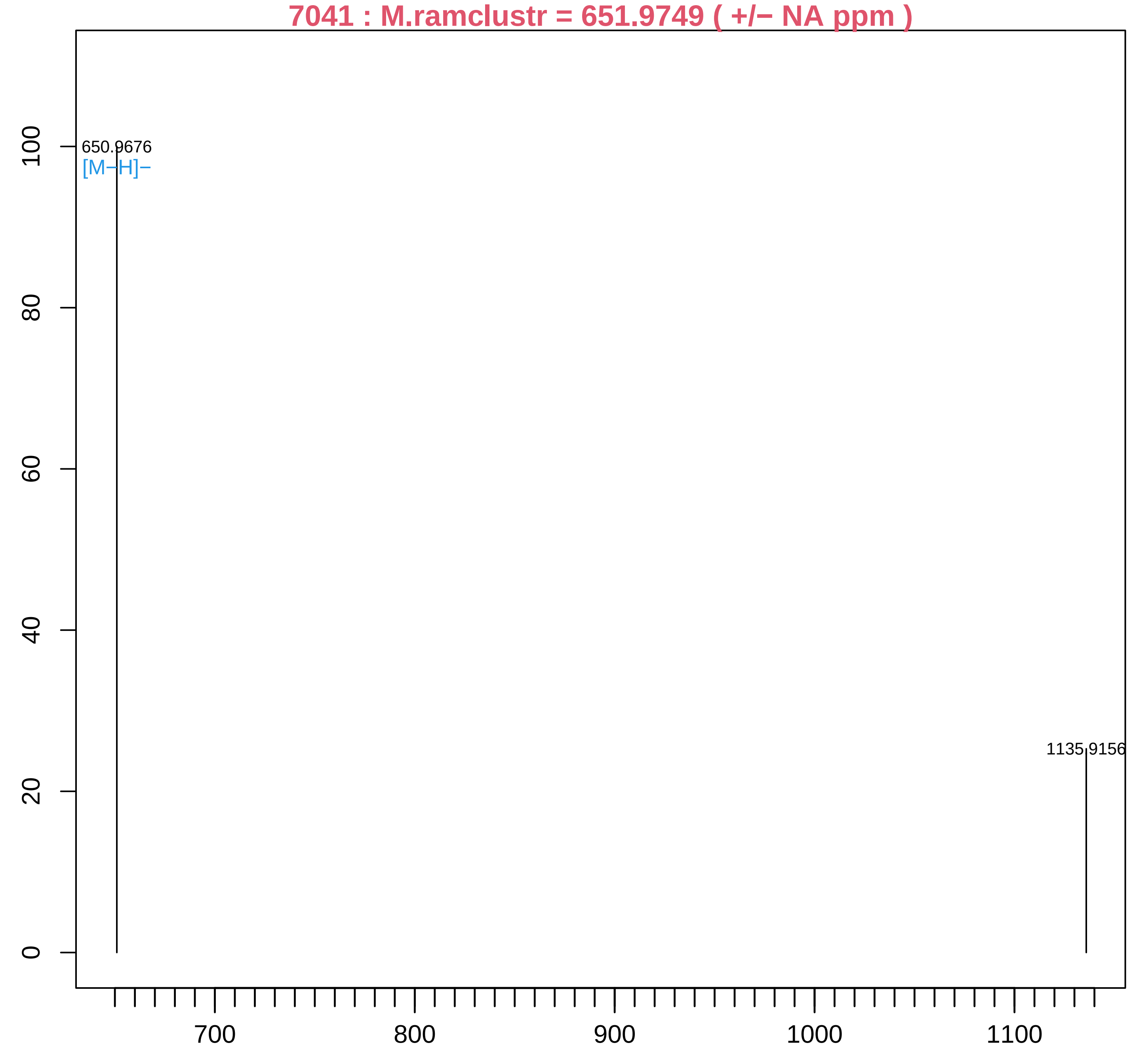

7041 : M.ramclustr = 651.9749 (+/− NA ppm)
650.9676
[M−H]−
1135.9156
0
20
40
60
80
100
700
800
900
1000
1100

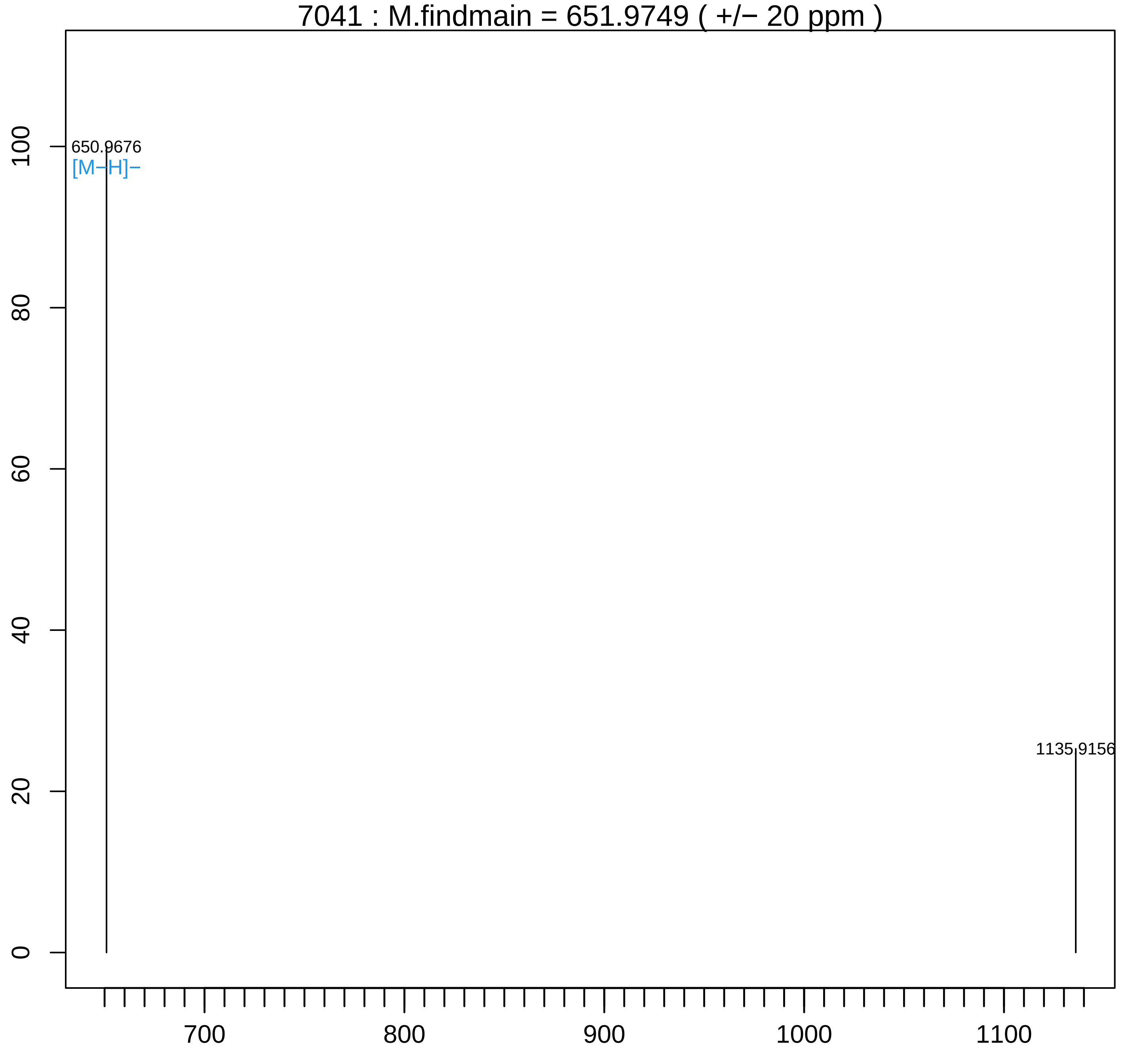

7041 : M.findmain = 651.9749 (+/− 20 ppm)
650.9676
[M−H]−
1135.9156
0
20
40
60
80
100
700
800
900
1000
1100

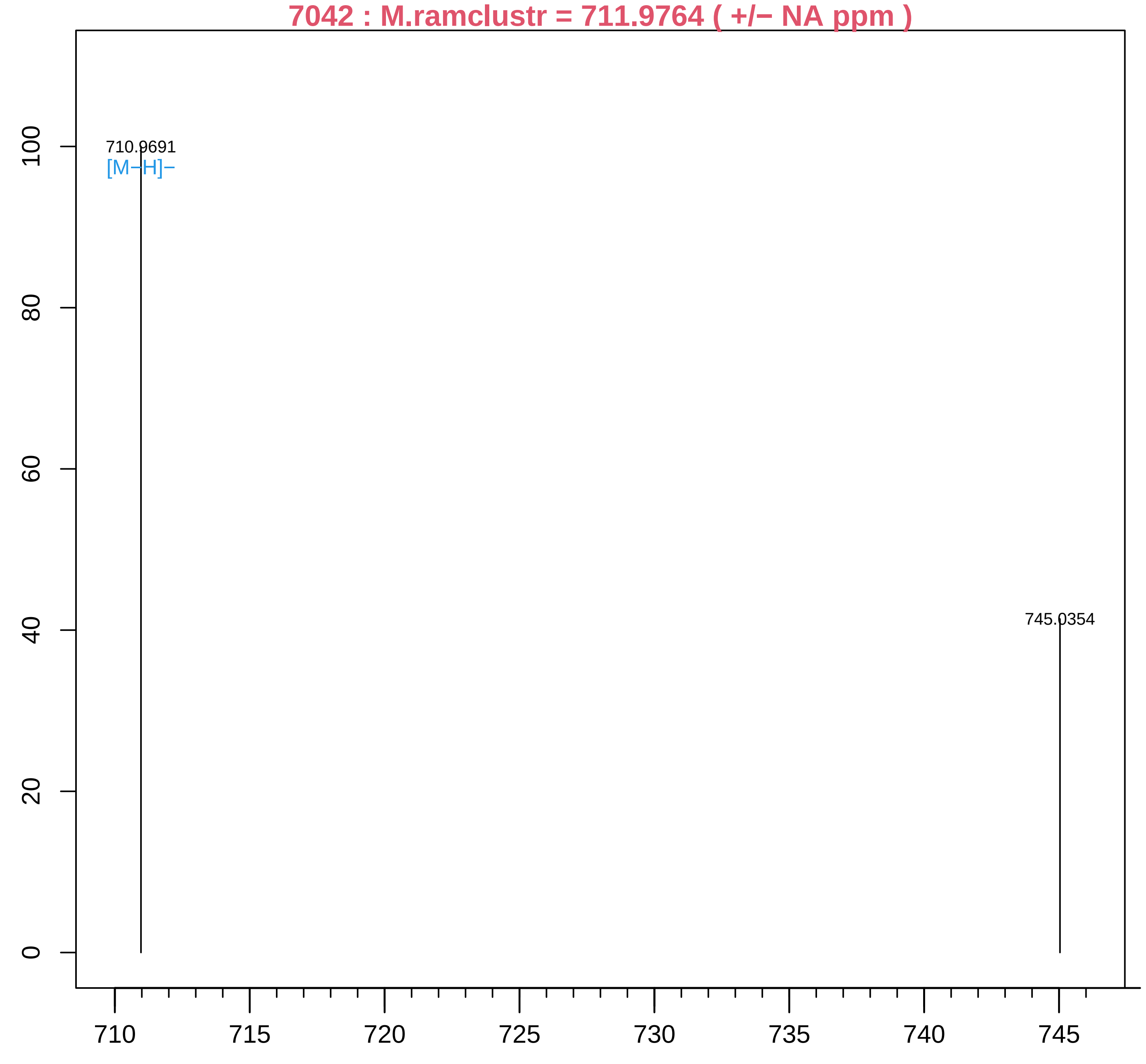
7042 : M.ramclustr = 711.9764 (+/− NA ppm)
710.9691
[M−H]−
745.0354
0
20
40
60
80
100
710
715
720
725
730
735
740
745

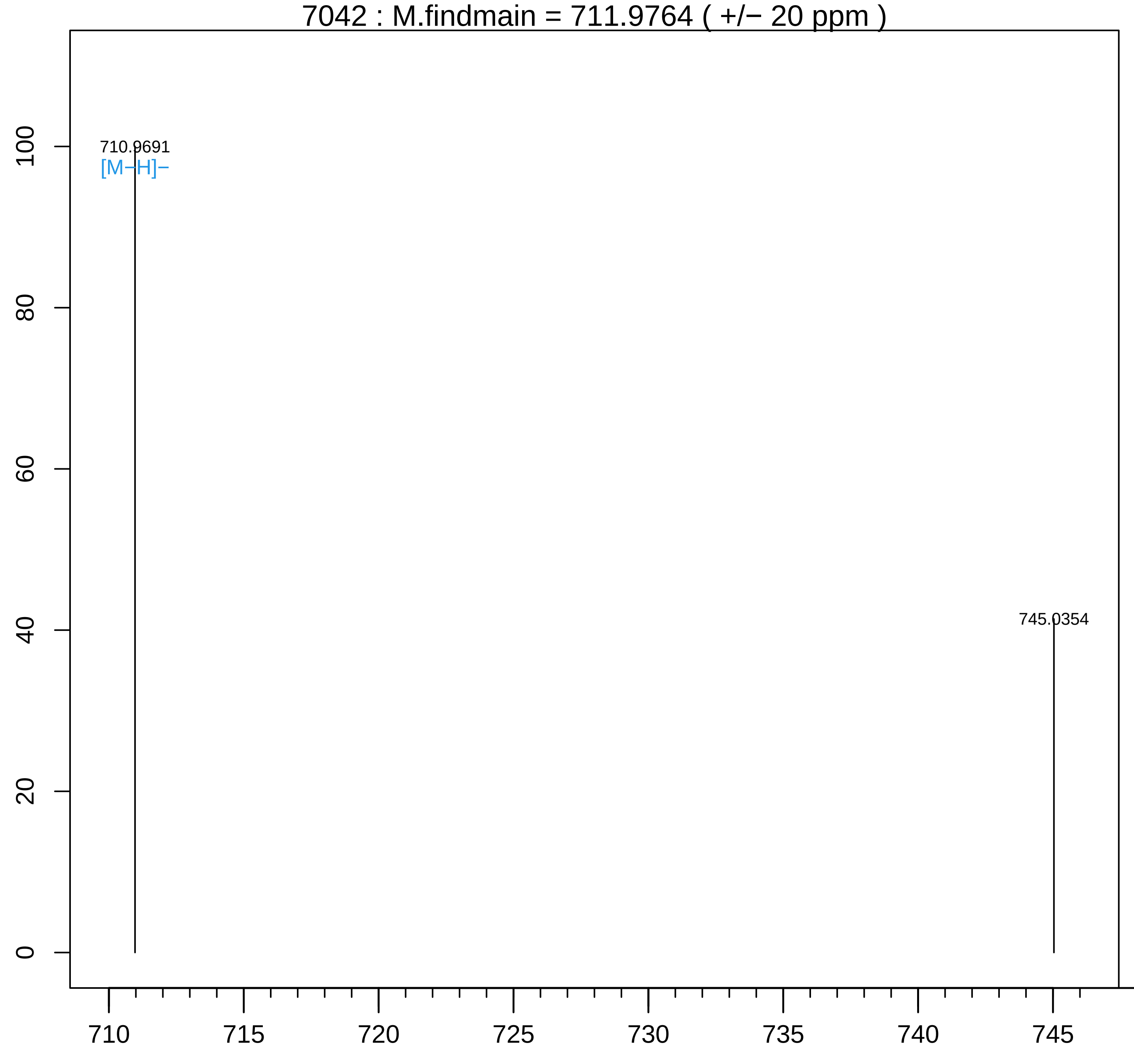
7042 : M.findmain = 711.9764 (+/− 20 ppm)
710.9691
[M−H]−
745.0354
0
20
40
60
80
100
710
715
720
725
730
735
740
745

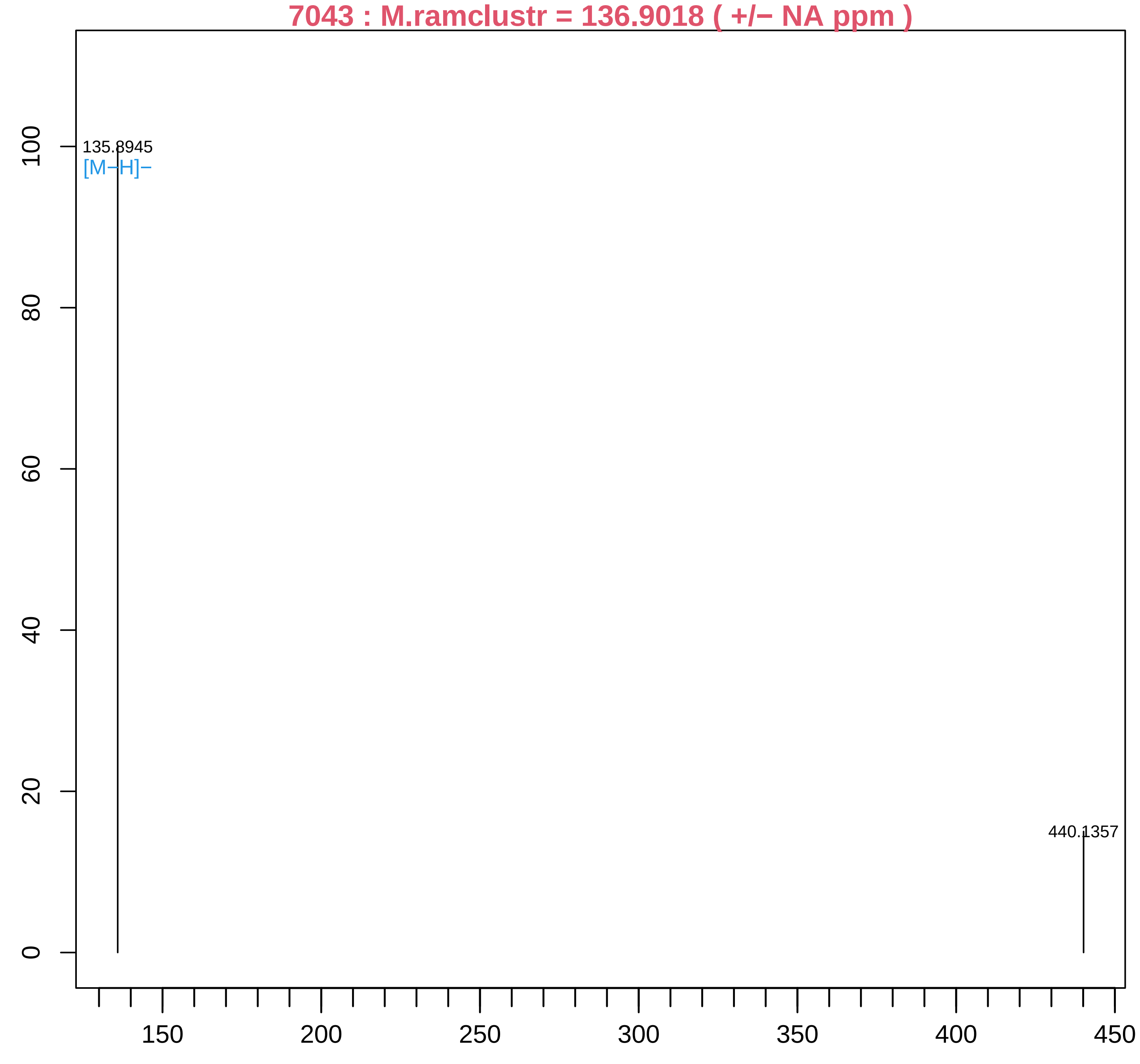

7043 : M.ramclustr = 136.9018 (+/− NA ppm)
135.8945
[M−H]−
440.1357
0
20
40
60
80
100
150
200
250
300
350
400
450

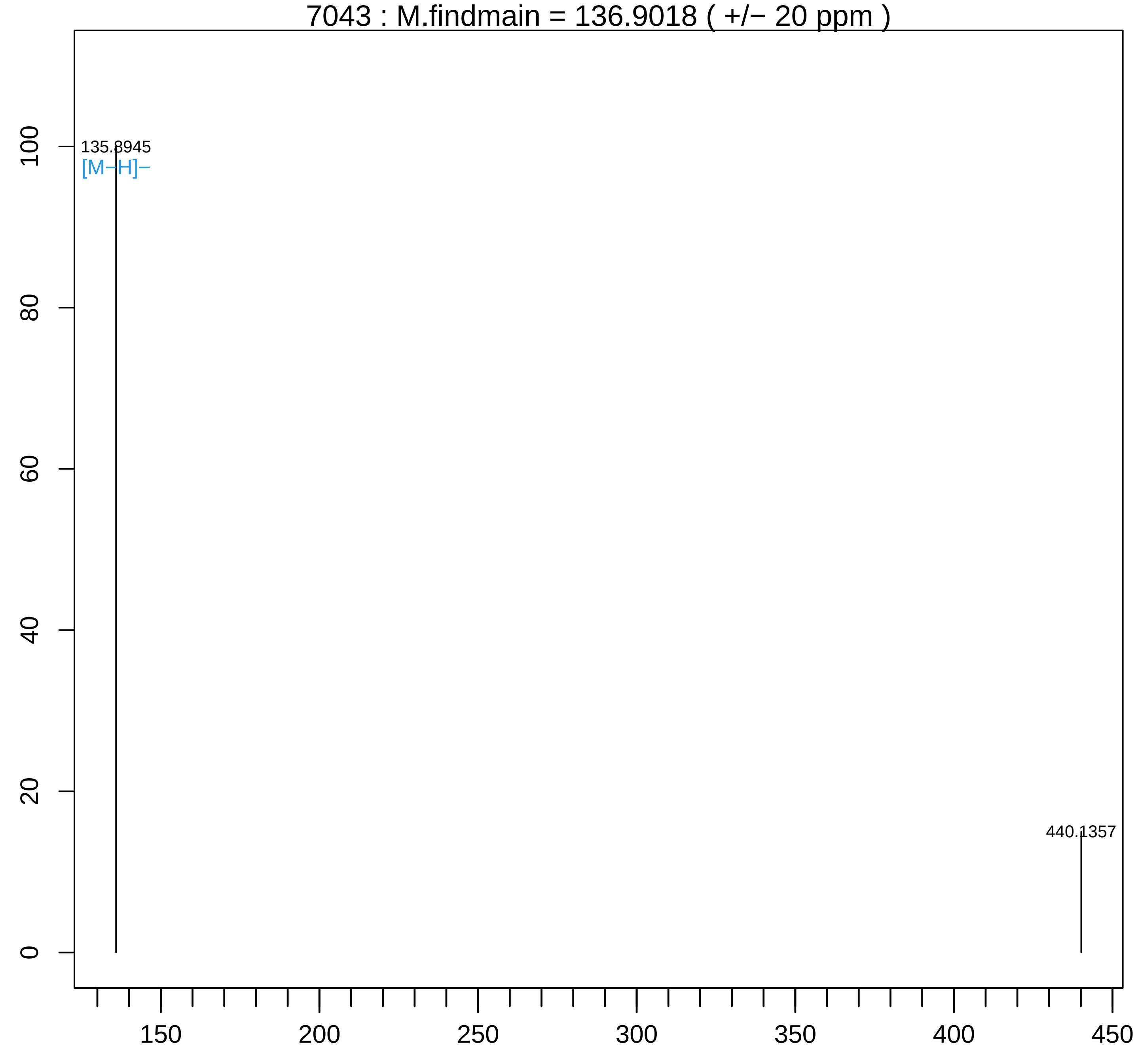

7043 : M.findmain = 136.9018 (+/− 20 ppm)
135.8945
[M−H]−
440.1357
0
20
40
60
80
100
150
200
250
300
350
400
450

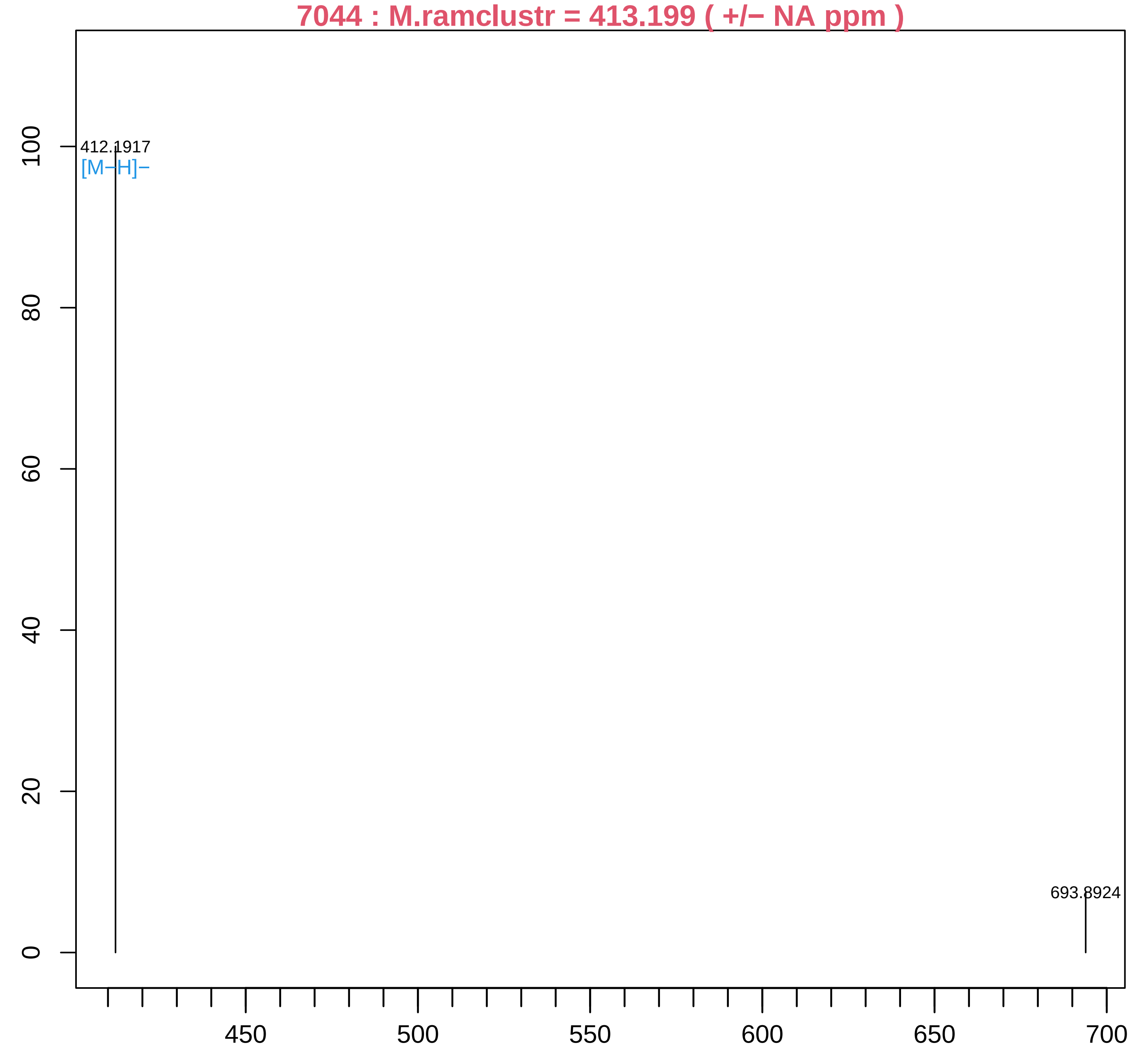

7044 : M.ramclustr = 413.199 (+/− NA ppm)
412.1917
[M−H]−
693.8924
0
20
40
60
80
100
450
500
550
600
650
700

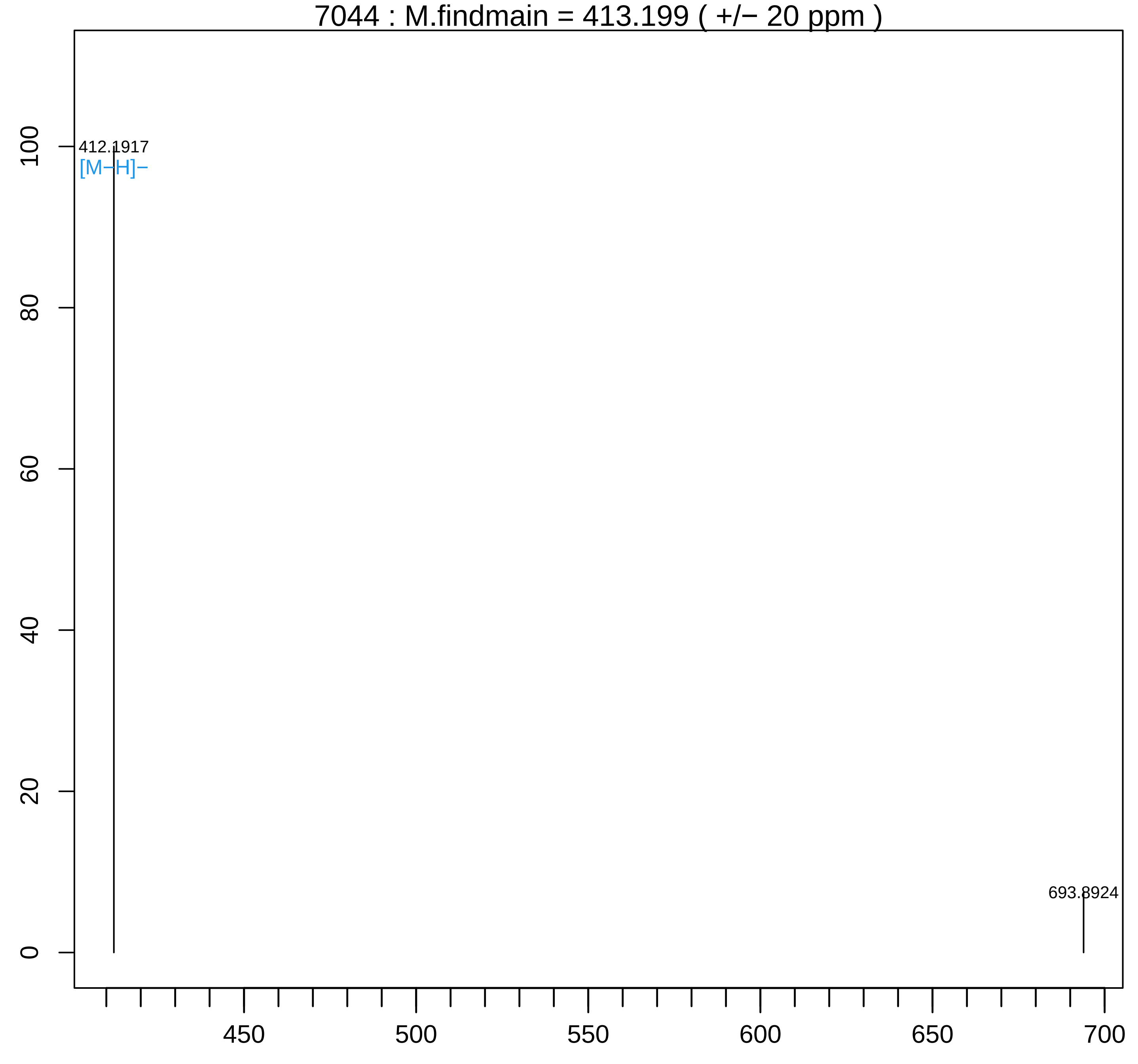

7044 : M.findmain = 413.199 (+/− 20 ppm)
412.1917
[M−H]−
693.8924
0
20
40
60
80
100
450
500
550
600
650
700

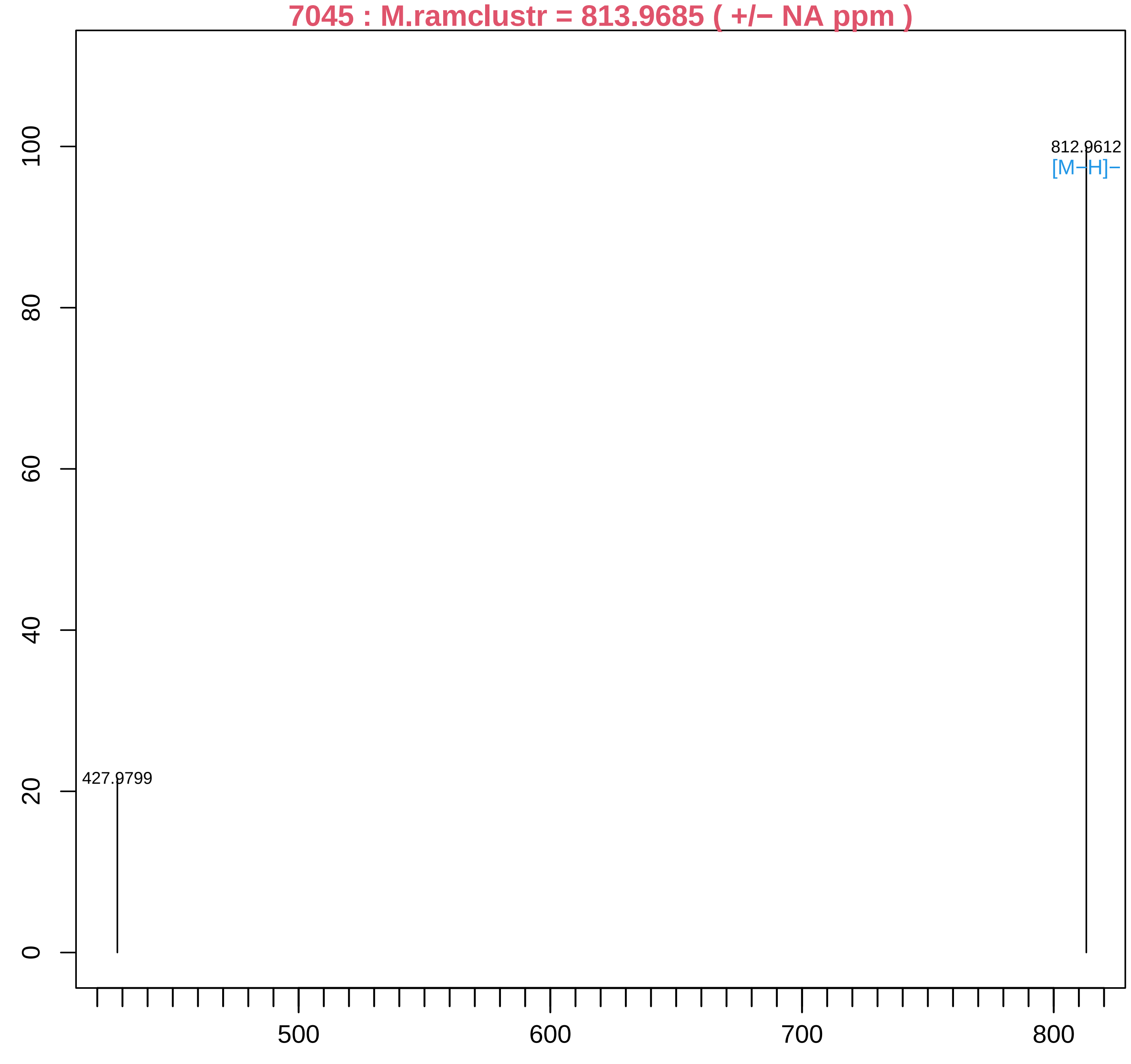
7045 : M.ramclustr = 813.9685 (+/− NA ppm)
812.9612
[M−H]−
427.9799
0
20
40
60
80
100
500
600
700
800

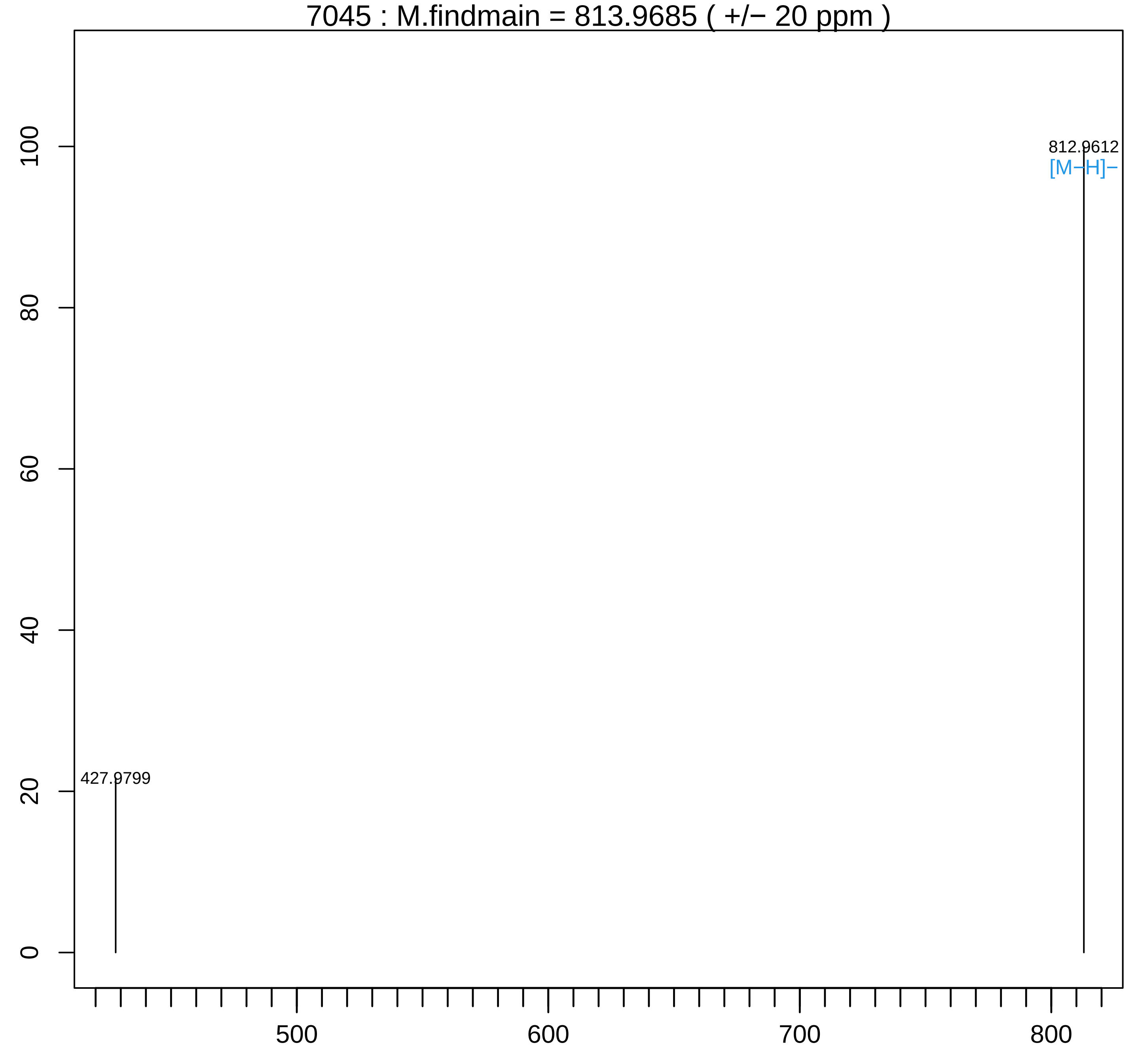
7045 : M.findmain = 813.9685 (+/− 20 ppm)
812.9612
[M−H]−
427.9799
0
20
40
60
80
100
500
600
700
800

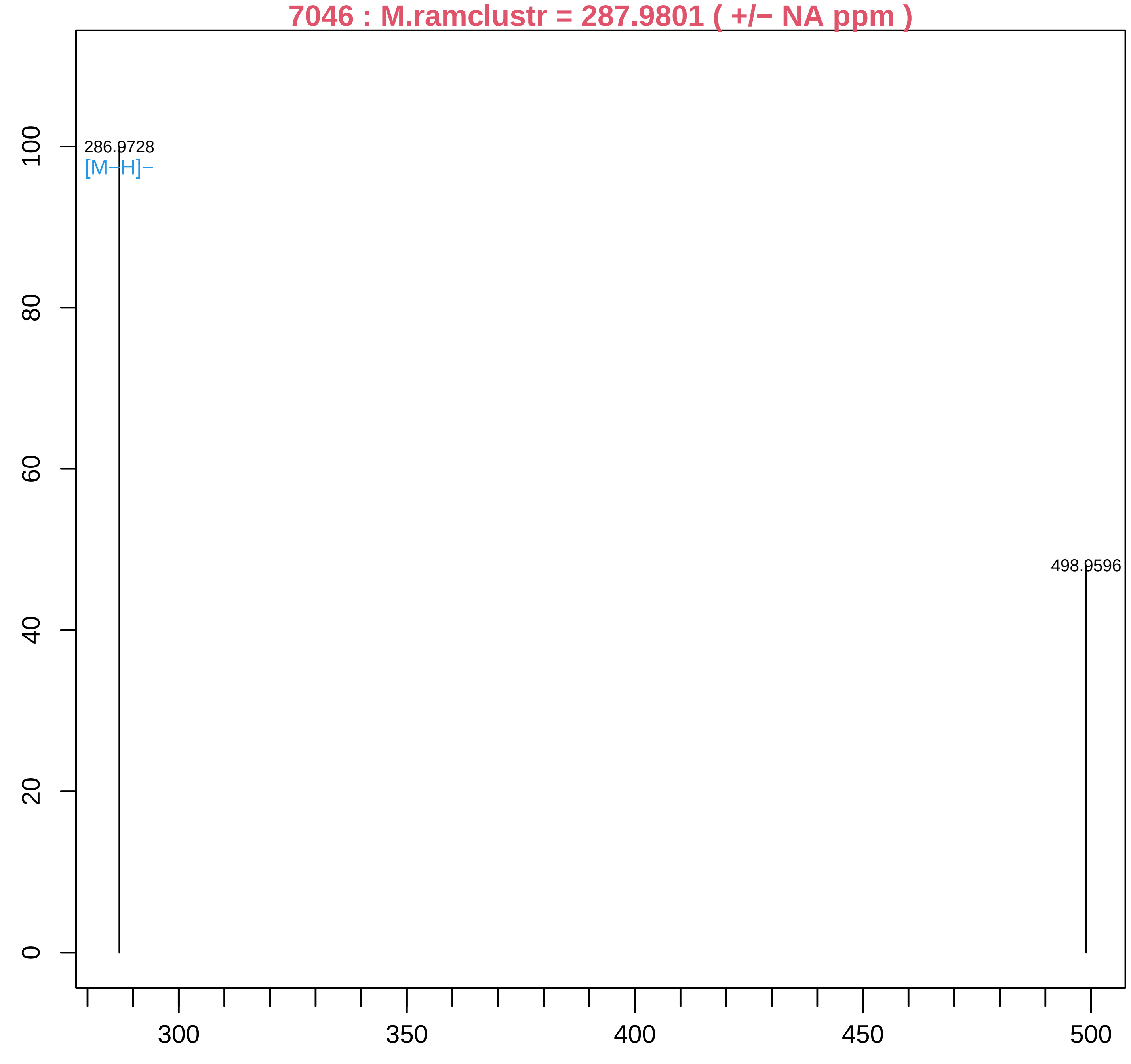
7046 : M.ramclustr = 287.9801 (+/− NA ppm)
286.9728
[M−H]−
498.9596
0
20
40
60
80
100
300
350
400
450
500

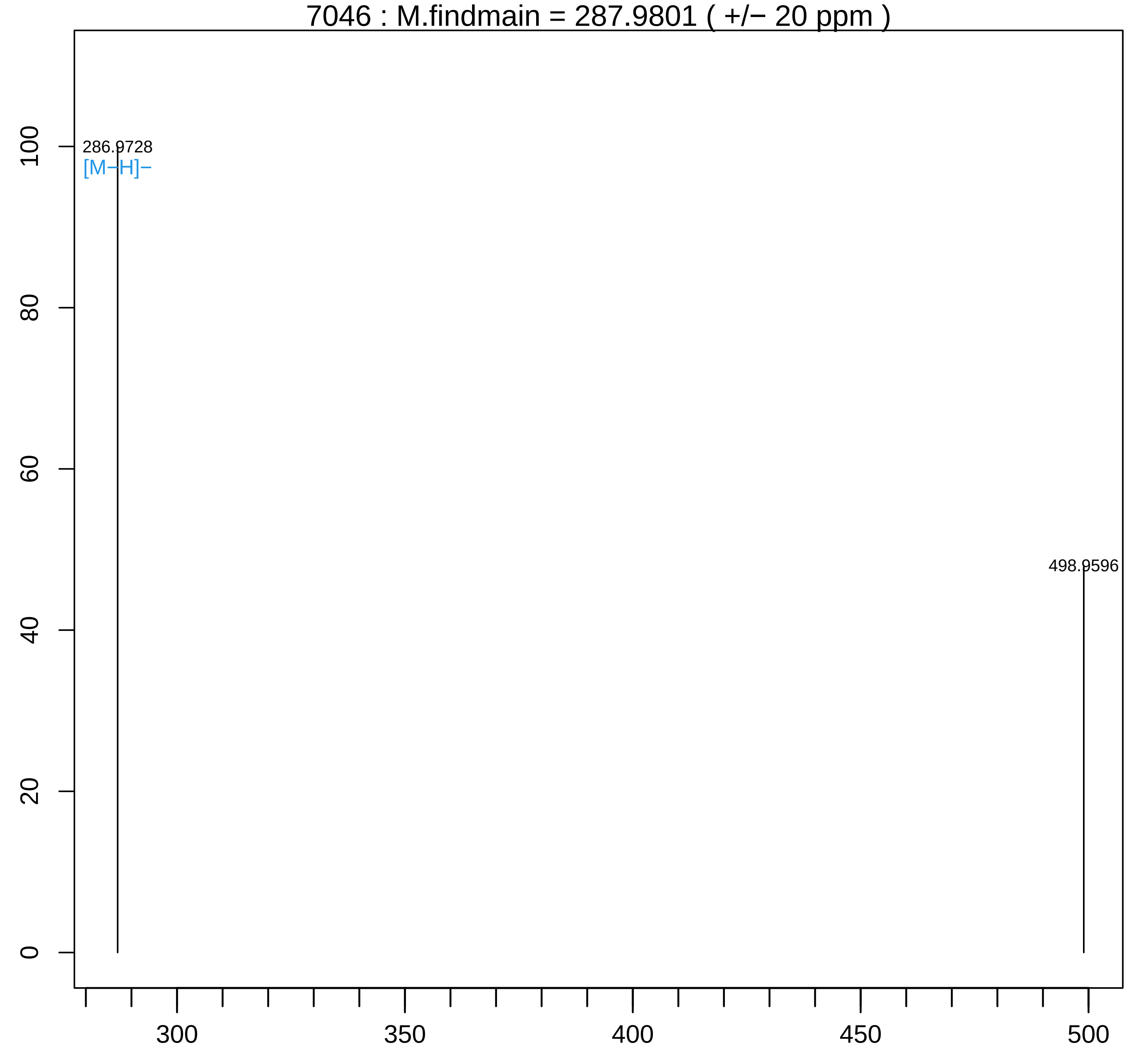
7046 : M.findmain = 287.9801 (+/− 20 ppm)
286.9728
[M−H]−
498.9596
0
20
40
60
80
100
300
350
400
450
500

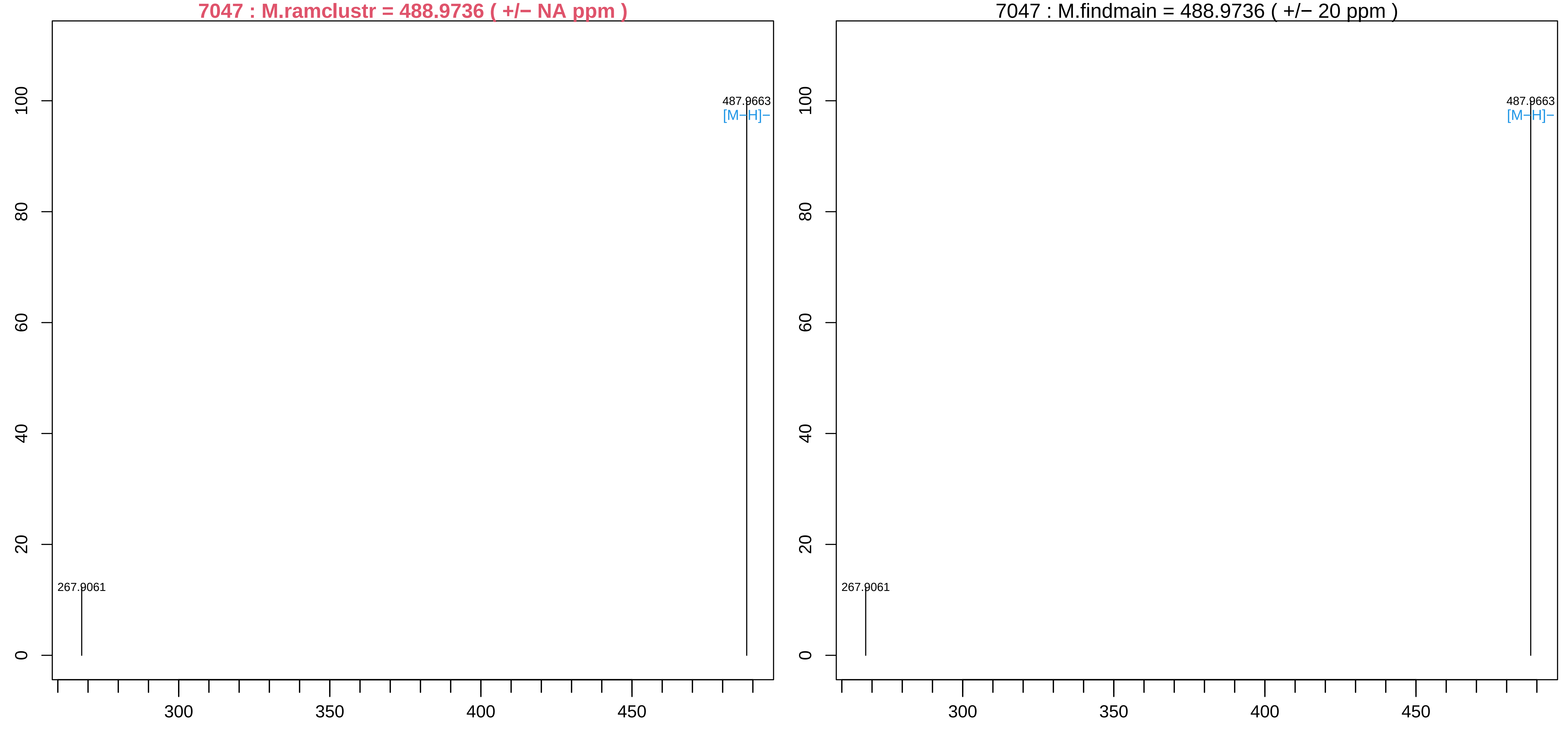
7047 : M.ramclustr = 488.9736 (+/− NA ppm)
487.9663
[M−H]−
267.9061
7047 : M.findmain = 488.9736 (+/− 20 ppm)
487.9663
[M−H]−
267.9061
0
20
40
60
80
100
300
350
400
450

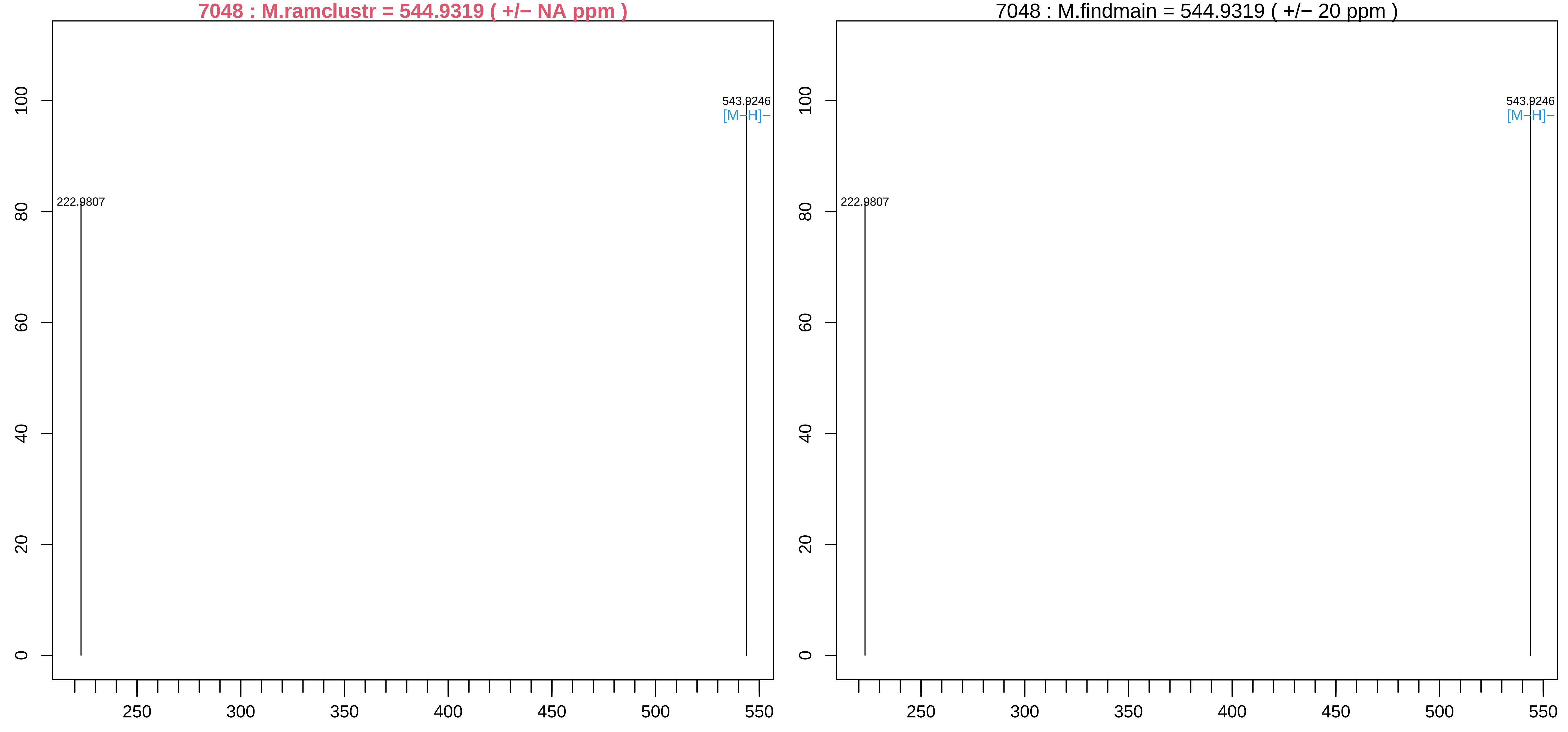

7048 : M.ramclustr = 544.9319 (+/− NA ppm)
543.9246
[M−H]−
222.9807
0
20
40
60
80
100
250
300
350
400
450
500
550
7048 : M.findmain = 544.9319 (+/− 20 ppm)
543.9246
[M−H]−
222.9807
0
20
40
60
80
100
250
300
350
400
450
500
550

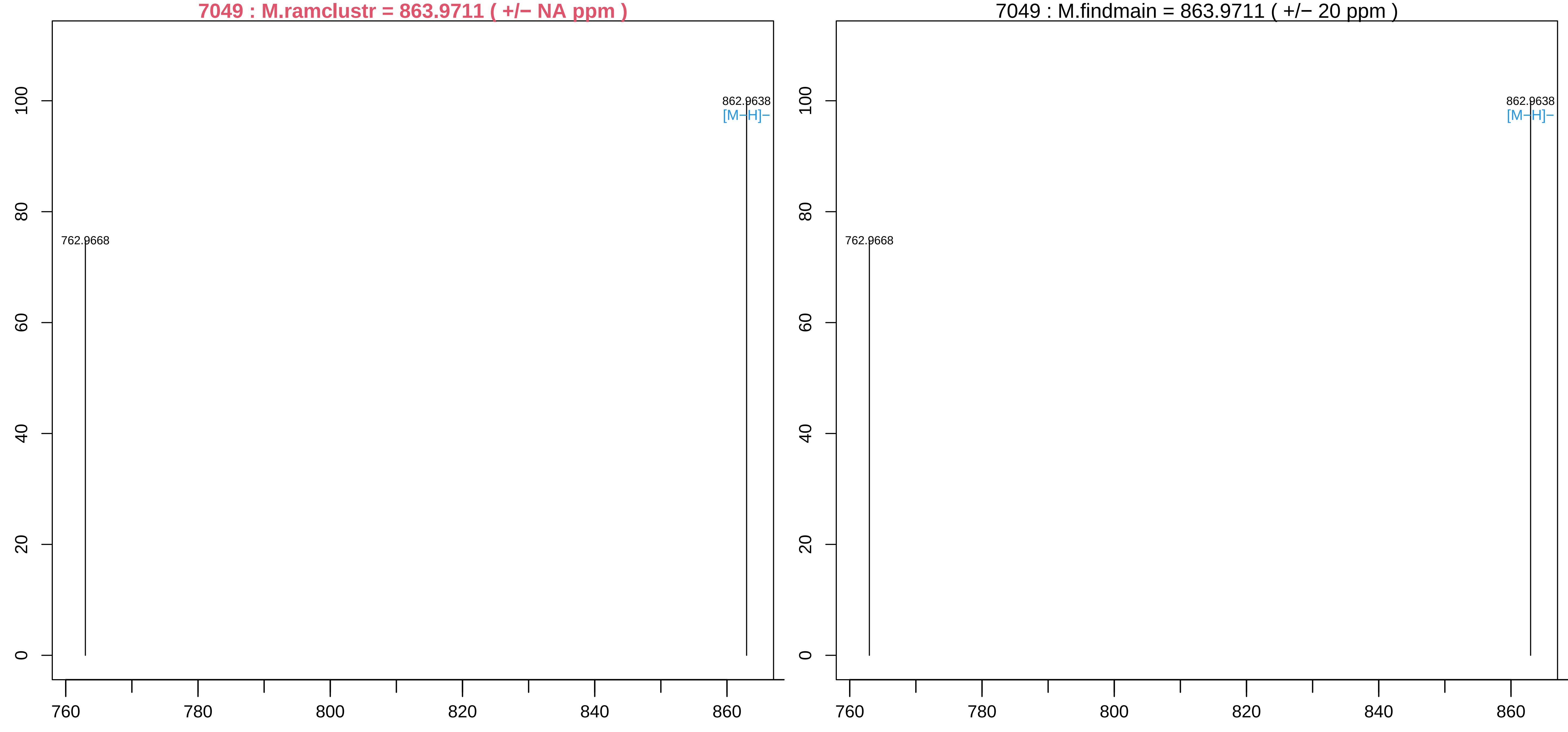
7049 : M.ramclustr = 863.9711 (+/− NA ppm)
762.9668
862.9638
[M−H]−
0
20
40
60
80
100
760
780
800
820
840
860
7049 : M.findmain = 863.9711 (+/− 20 ppm)
762.9668
862.9638
[M−H]−
0
20
40
60
80
100
760
780
800
820
840
860

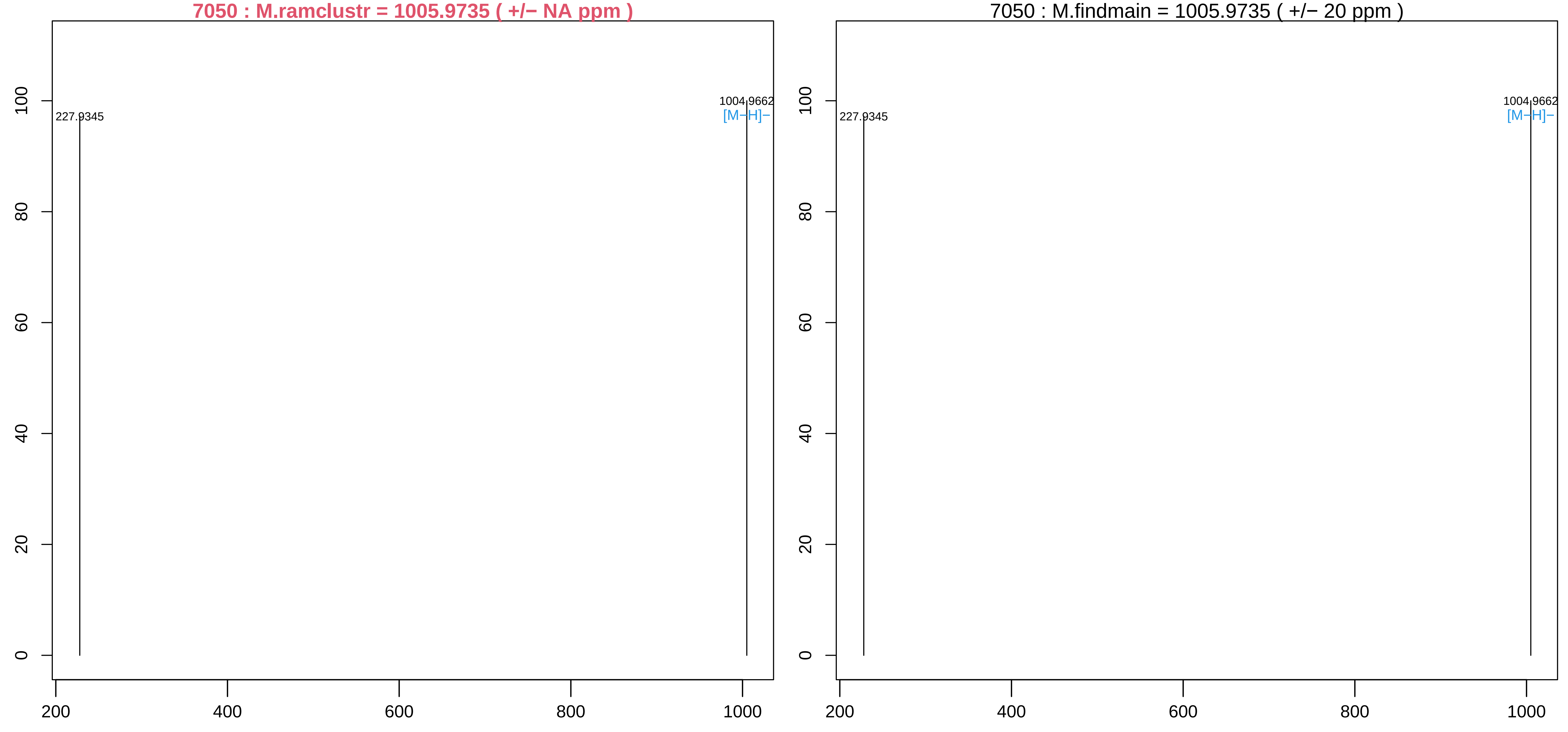
7050 : M.ramclustr = 1005.9735 (+/− NA ppm)
227.9345
1004.9662
[M−H]−
7050 : M.findmain = 1005.9735 (+/− 20 ppm)
227.9345
1004.9662
[M−H]−
0
20
40
60
80
100
200
400
600
800
1000

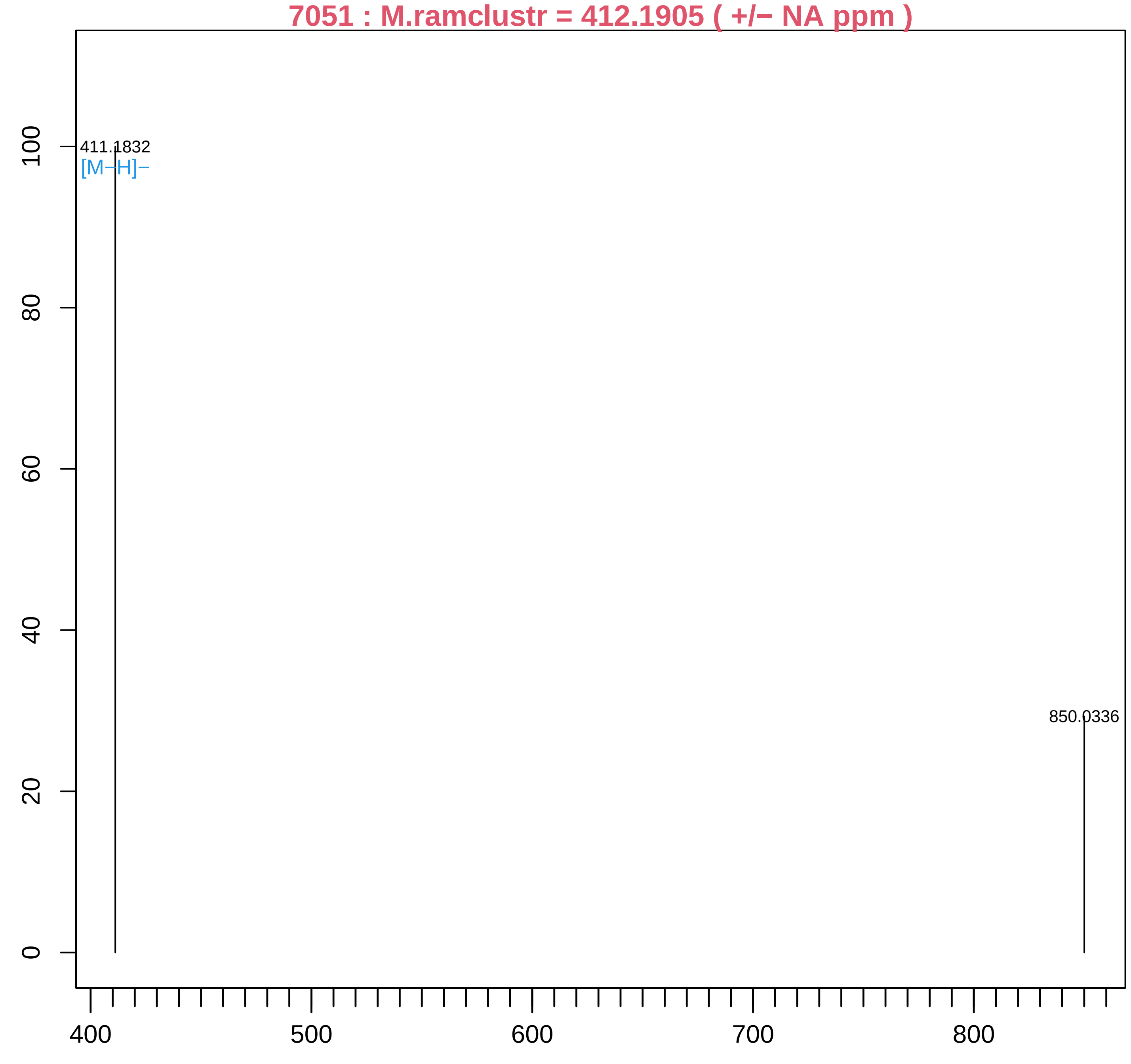
7051 : M.ramclustr = 412.1905 (+/− NA ppm)
411.1832
[M−H]−
850.0336
0
20
40
60
80
100
400
500
600
700
800

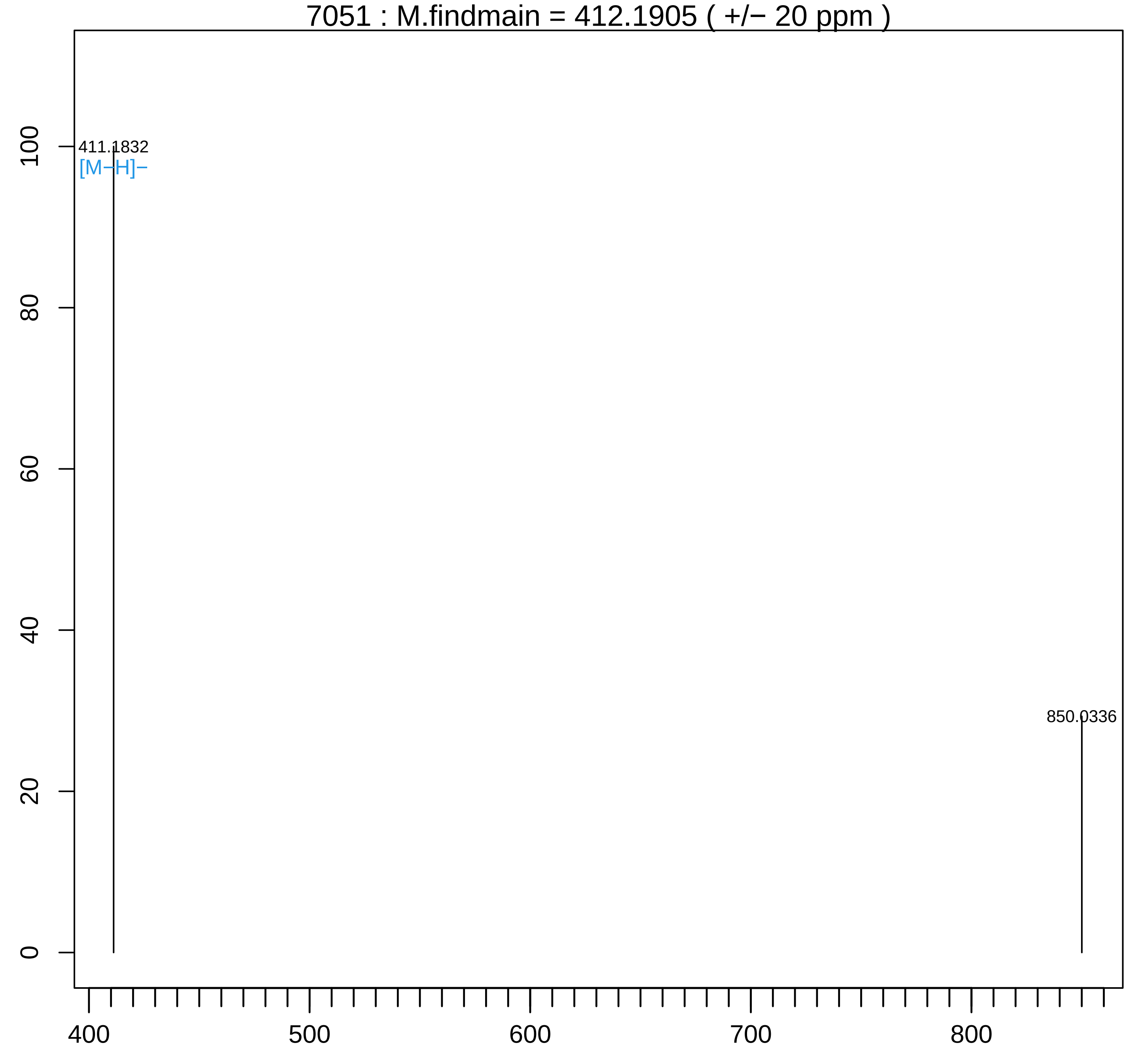
7051 : M.findmain = 412.1905 (+/− 20 ppm)
411.1832
[M−H]−
850.0336
0
20
40
60
80
100
400
500
600
700
800

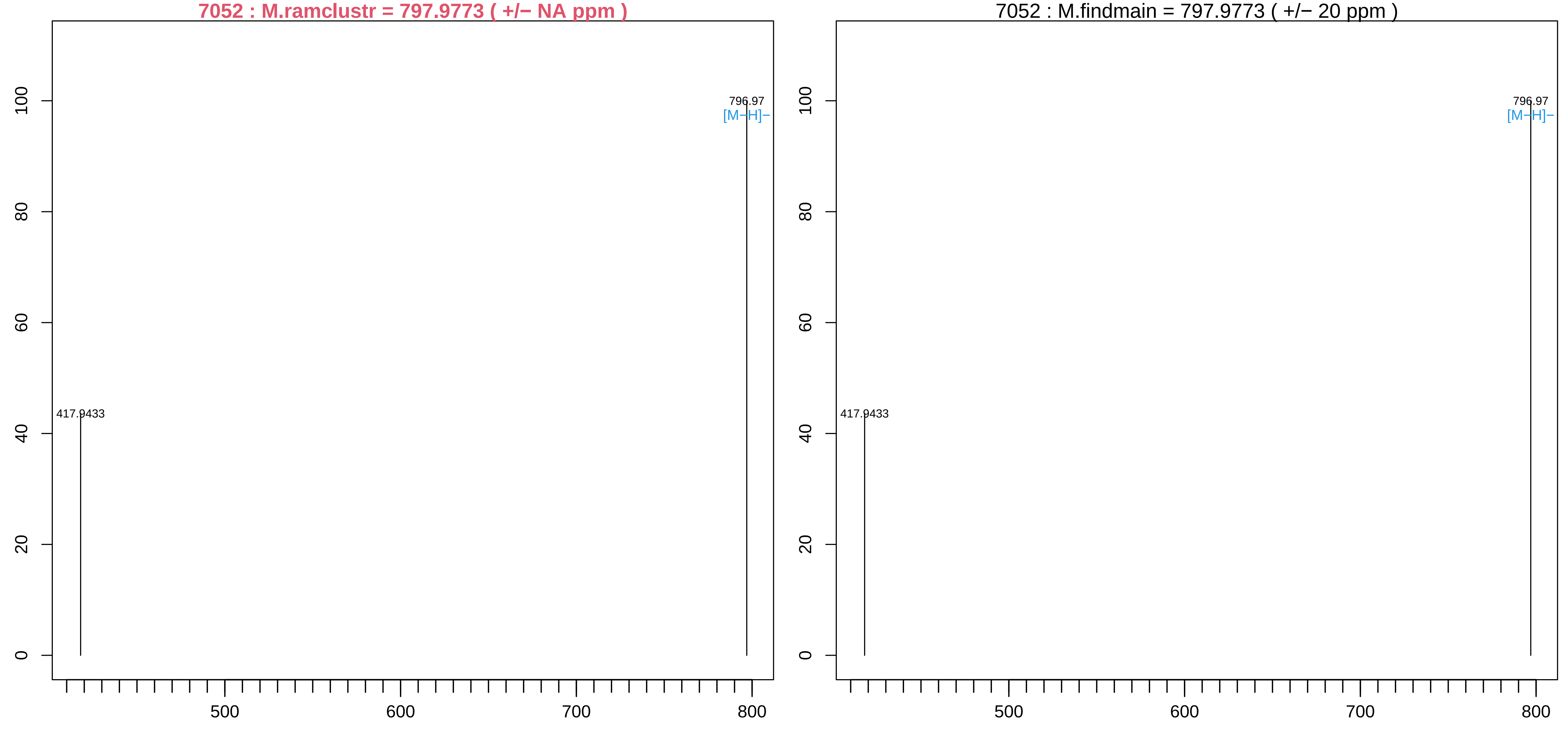

7052 : M.ramclustr = 797.9773 (+/− NA ppm)
100
80
60
40
20
0
500
600
700
800
796.97
[M−H]−
417.9433
7052 : M.findmain = 797.9773 (+/− 20 ppm)
100
80
60
40
20
0
500
600
700
800
796.97
[M−H]−
417.9433

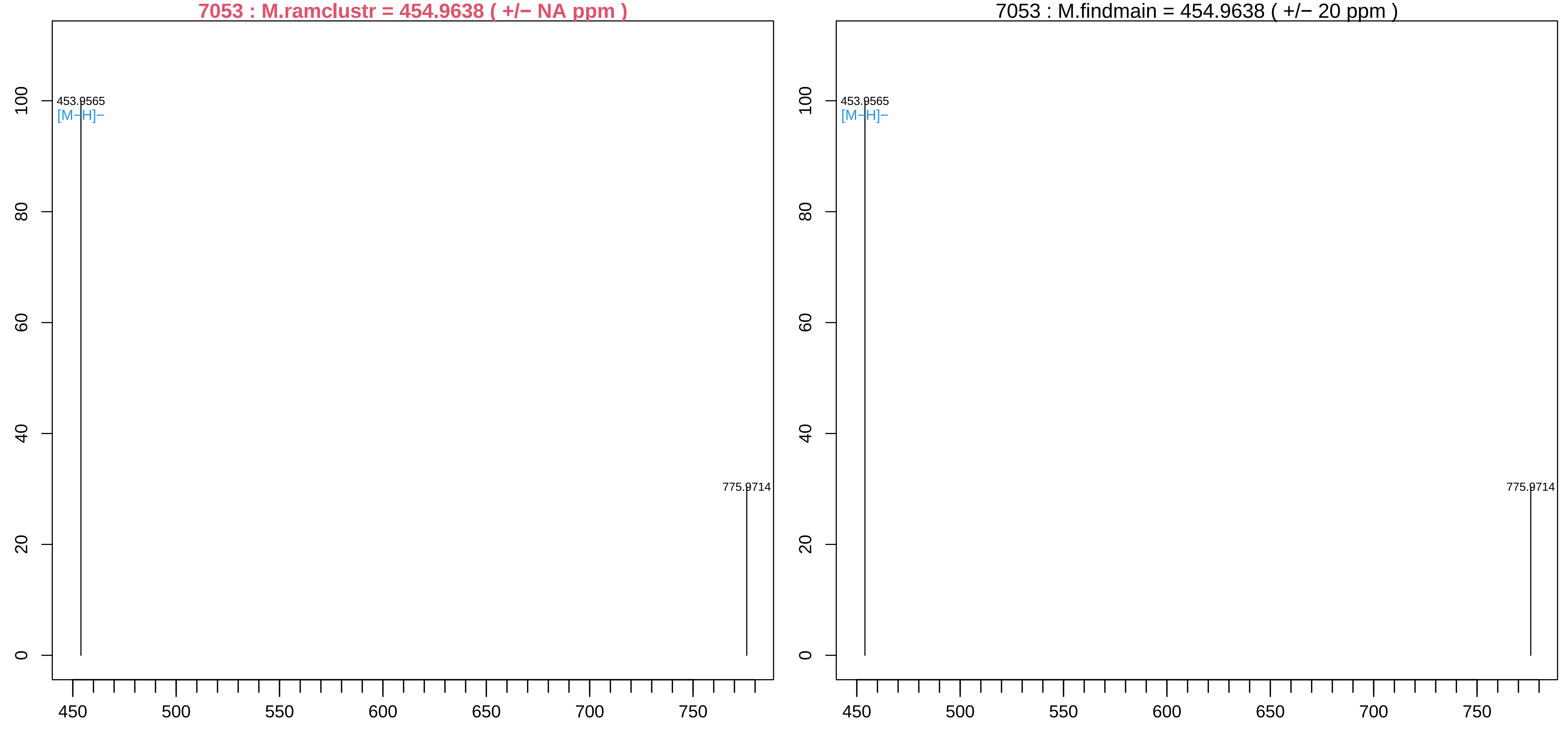

7053 : M.ramclustr = 454.9638 (+/− NA ppm)
453.9565
[M−H]−
775.9714
7053 : M.findmain = 454.9638 (+/− 20 ppm)
453.9565
[M−H]−
775.9714
0
20
40
60
80
100
450
500
550
600
650
700
750

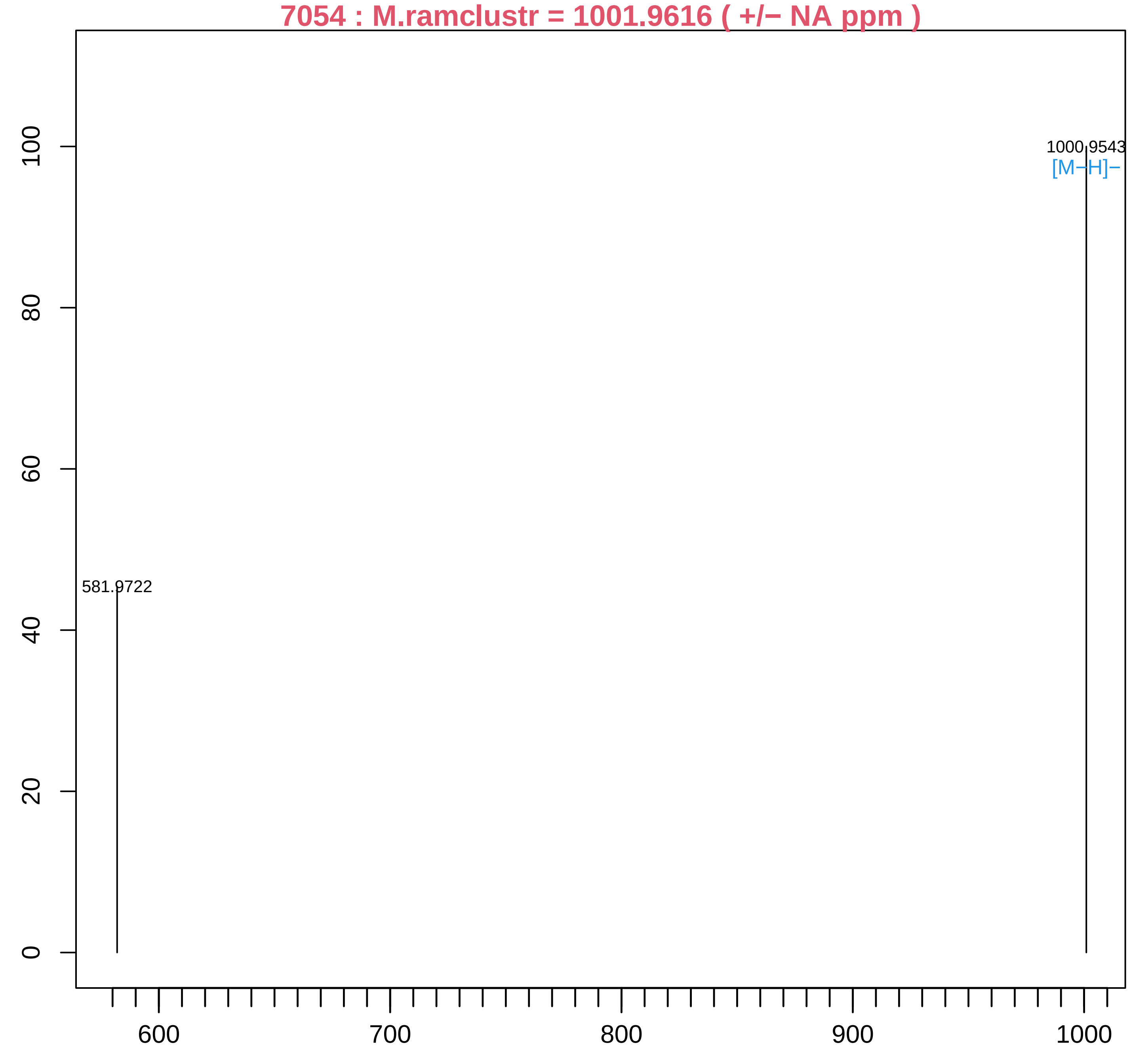
7054 : M.ramclustr = 1001.9616 (+/− NA ppm)
1000.9543
[M−H]−
581.9722
0
20
40
60
80
100
600
700
800
900
1000

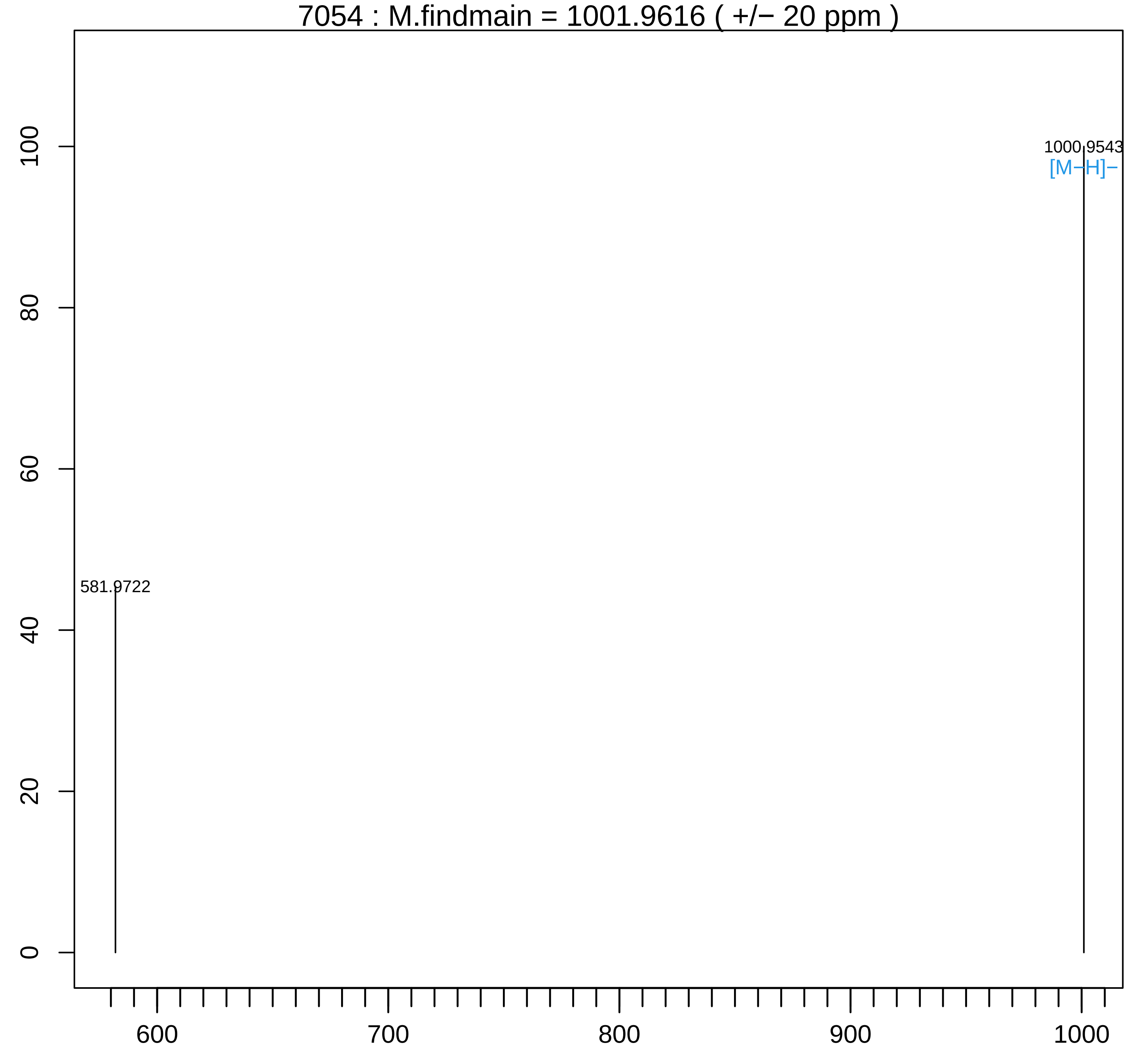
7054 : M.findmain = 1001.9616 (+/− 20 ppm)
1000.9543
[M−H]−
581.9722
0
20
40
60
80
100
600
700
800
900
1000

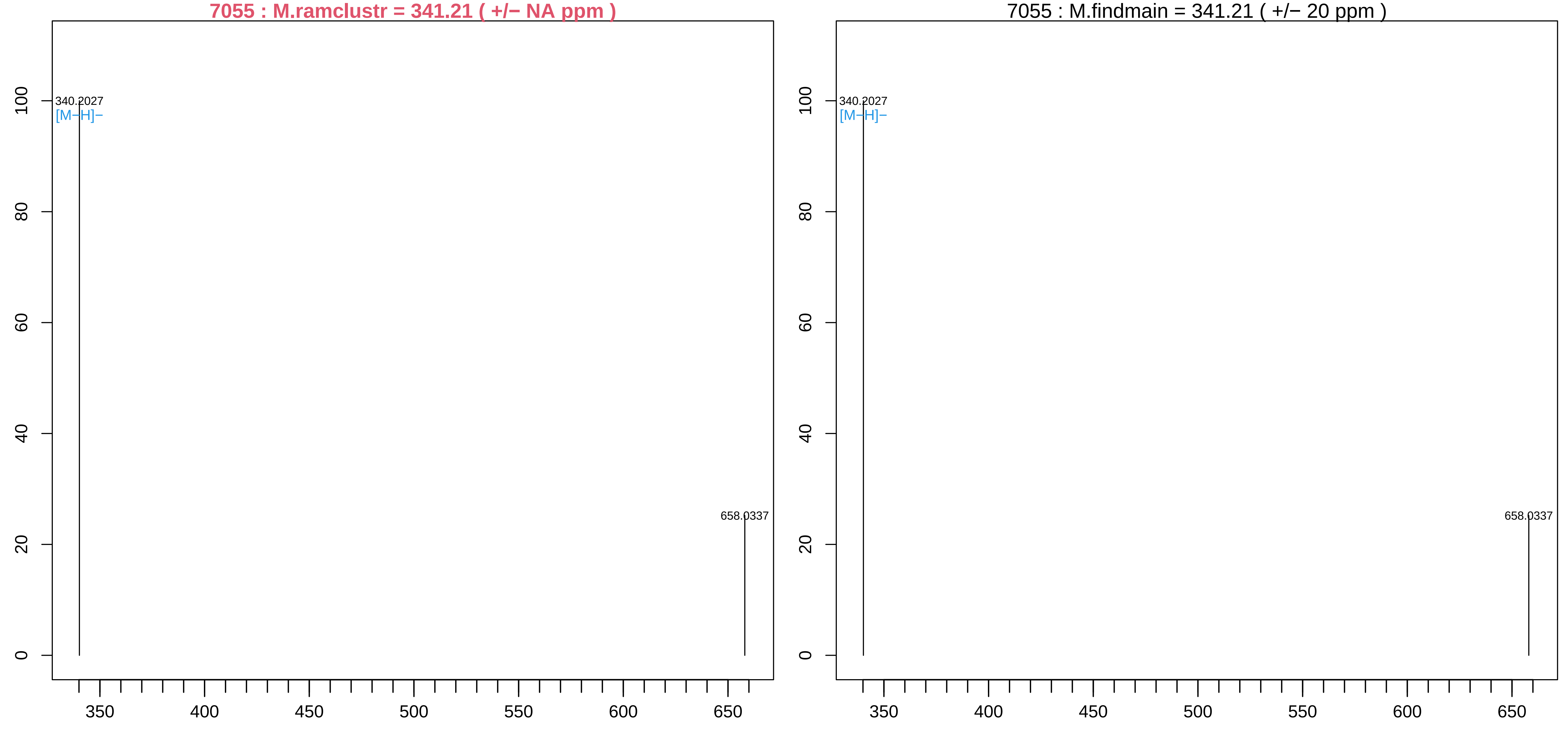
7055 : M.ramclustr = 341.21 (+/− NA ppm)
340.2027
[M−H]−
658.0337
0
20
40
60
80
100
350
400
450
500
550
600
650
7055 : M.findmain = 341.21 (+/− 20 ppm)
340.2027
[M−H]−
658.0337
0
20
40
60
80
100
350
400
450
500
550
600
650

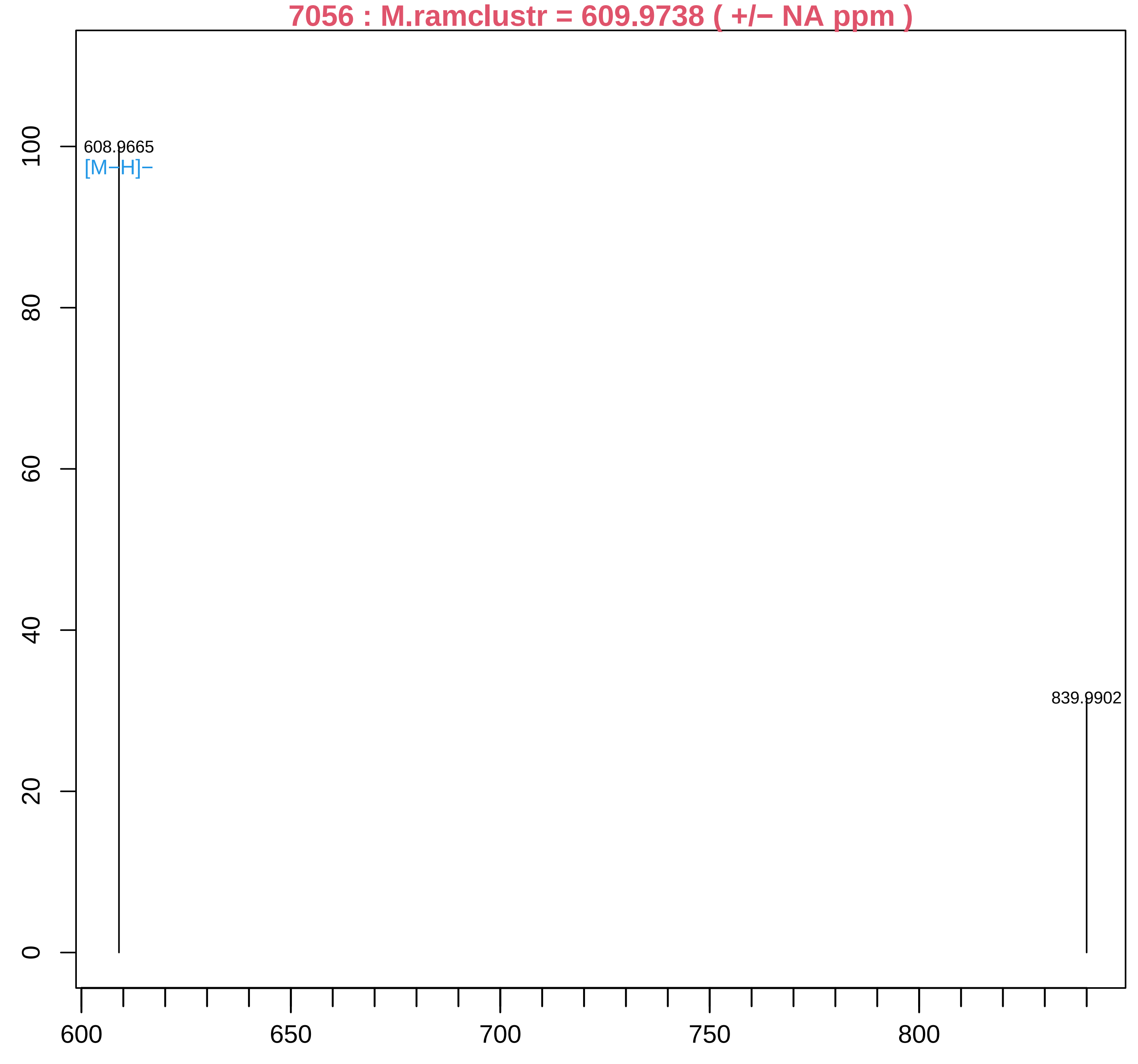
7056 : M.ramclustr = 609.9738 (+/− NA ppm)
608.9665
[M−H]−
839.9902
0
20
40
60
80
100
600
650
700
750
800

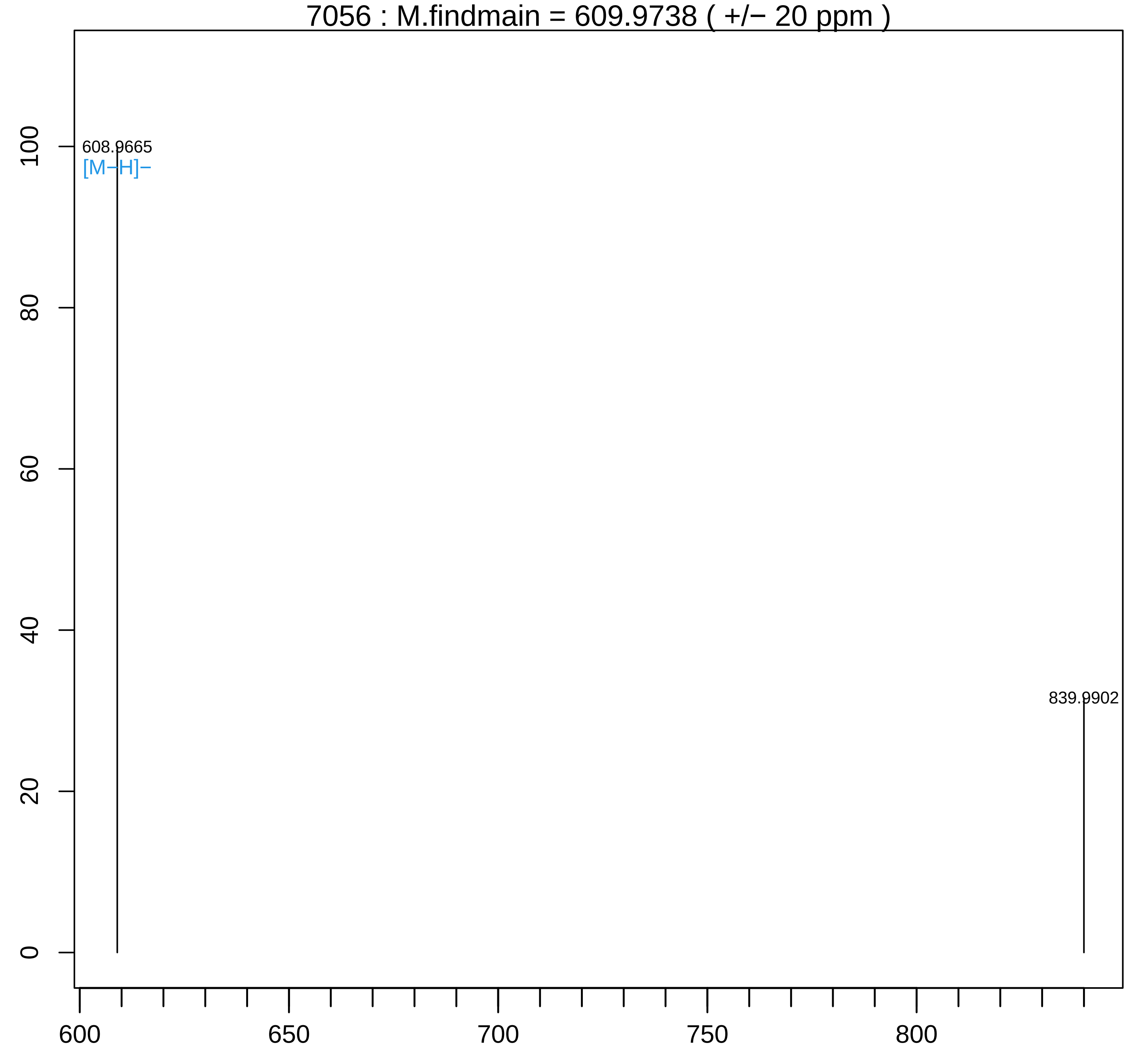
7056 : M.findmain = 609.9738 (+/− 20 ppm)
608.9665
[M−H]−
839.9902
0
20
40
60
80
100
600
650
700
750
800

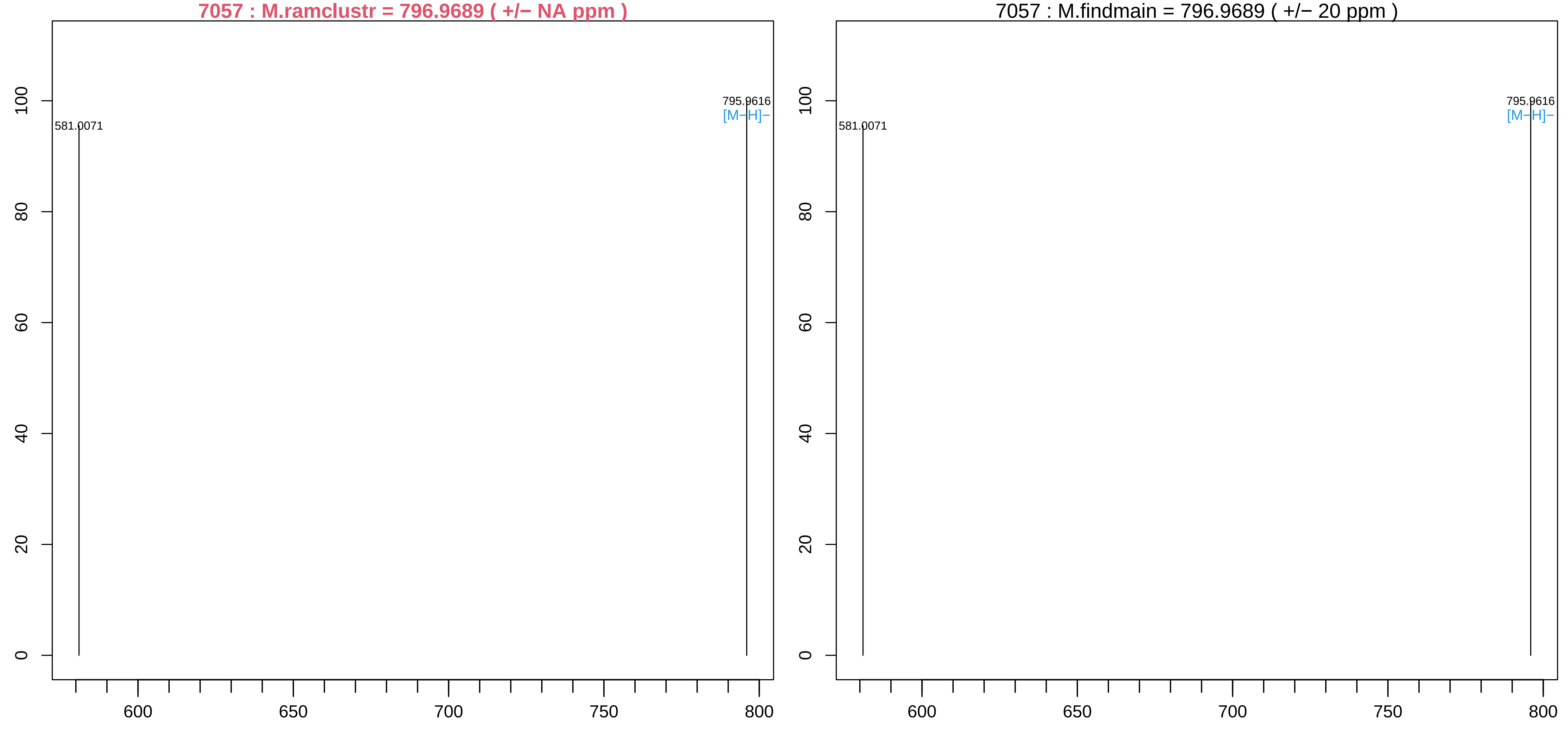
7057 : M.ramclustr = 796.9689 (+/− NA ppm)
581.0071
795.9616
[M−H]−
0
20
40
60
80
100
600
650
700
750
800
7057 : M.findmain = 796.9689 (+/− 20 ppm)
581.0071
795.9616
[M−H]−
0
20
40
60
80
100
600
650
700
750
800

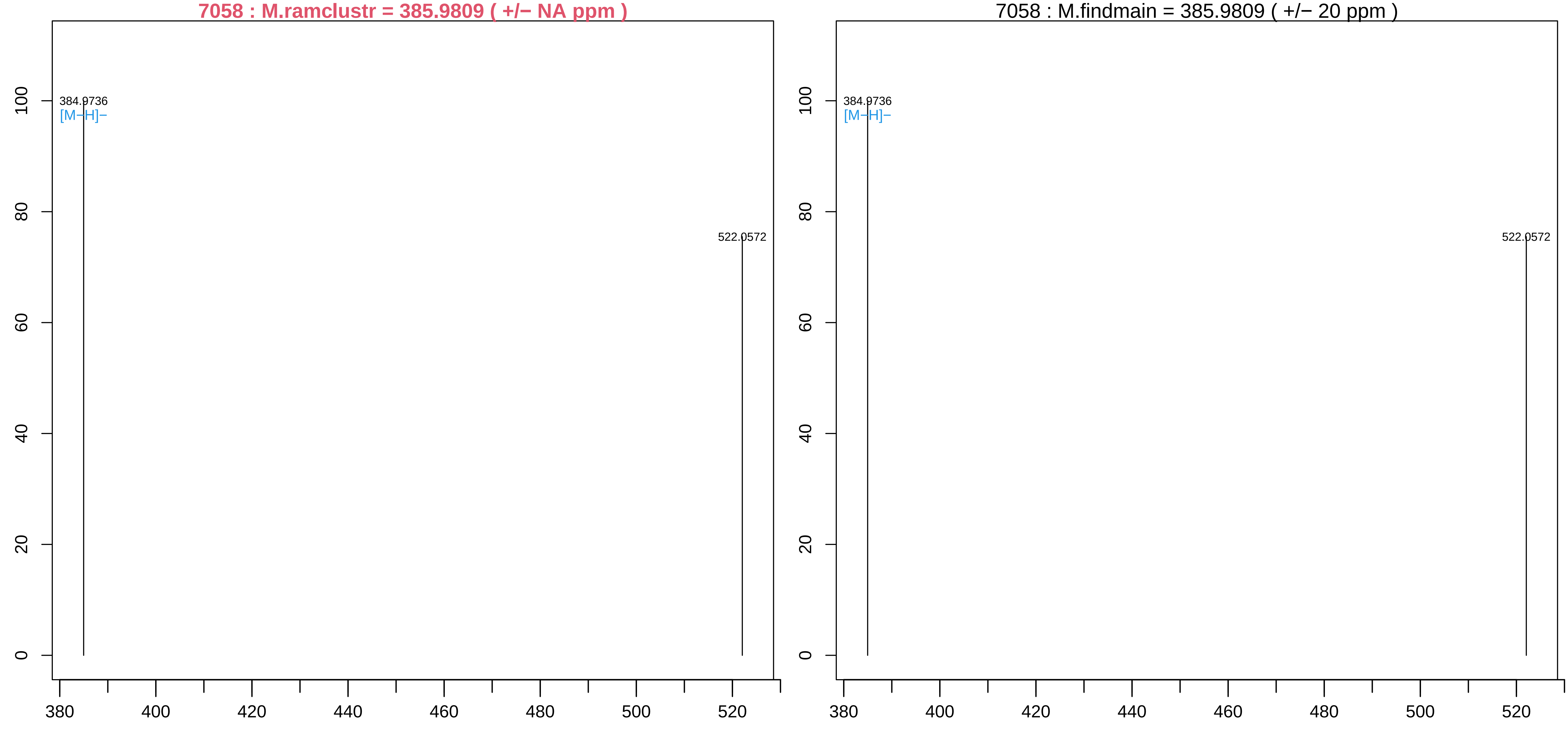
7058 : M.ramclustr = 385.9809 (+/− NA ppm)
7058 : M.findmain = 385.9809 (+/− 20 ppm)
384.9736
[M−H]−
522.0572
0
20
40
60
80
100
380
400
420
440
460
480
500
520

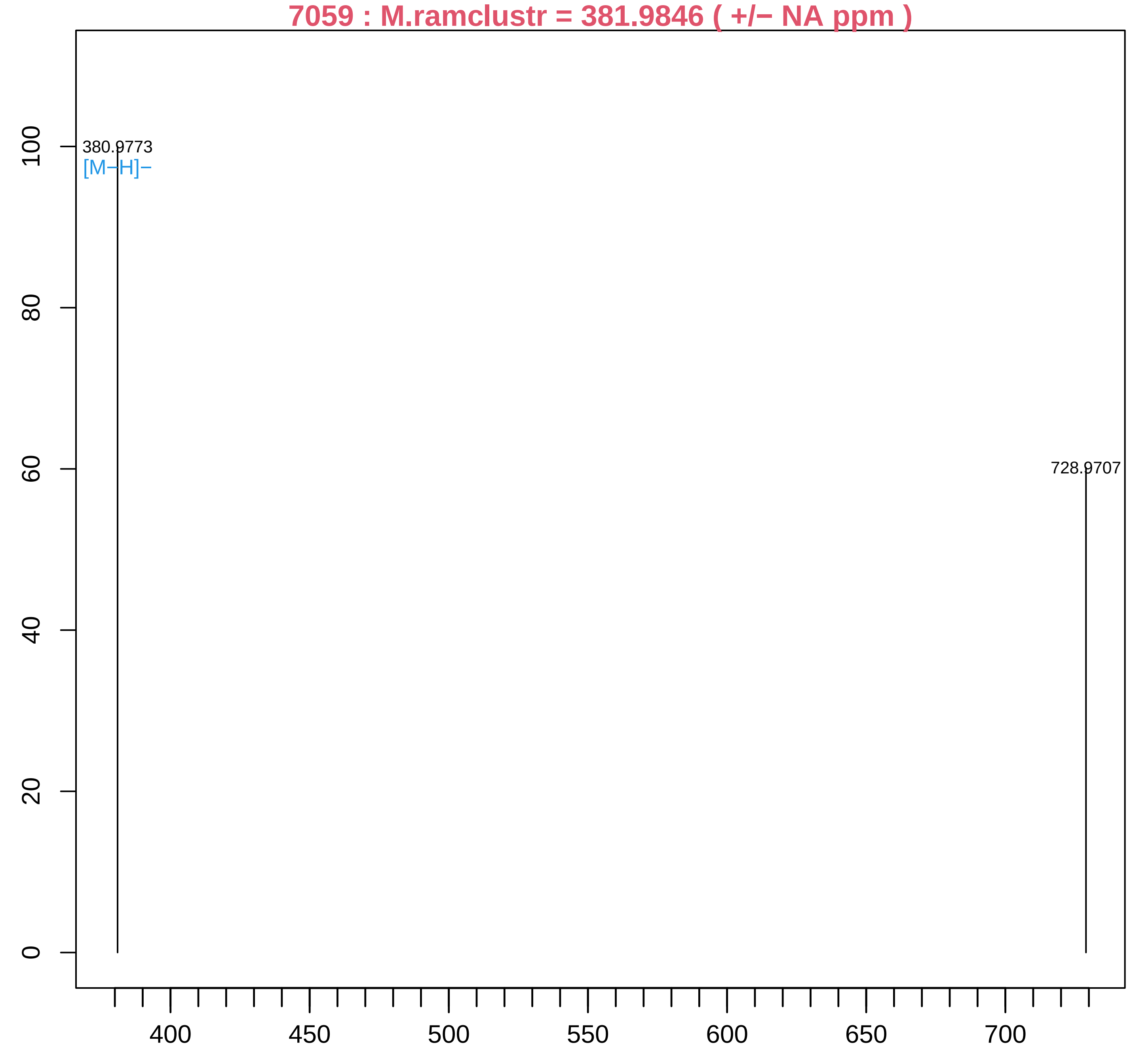
7059 : M.ramclustr = 381.9846 (+/− NA ppm)
380.9773
[M−H]−
728.9707
0
20
40
60
80
100
400
450
500
550
600
650
700

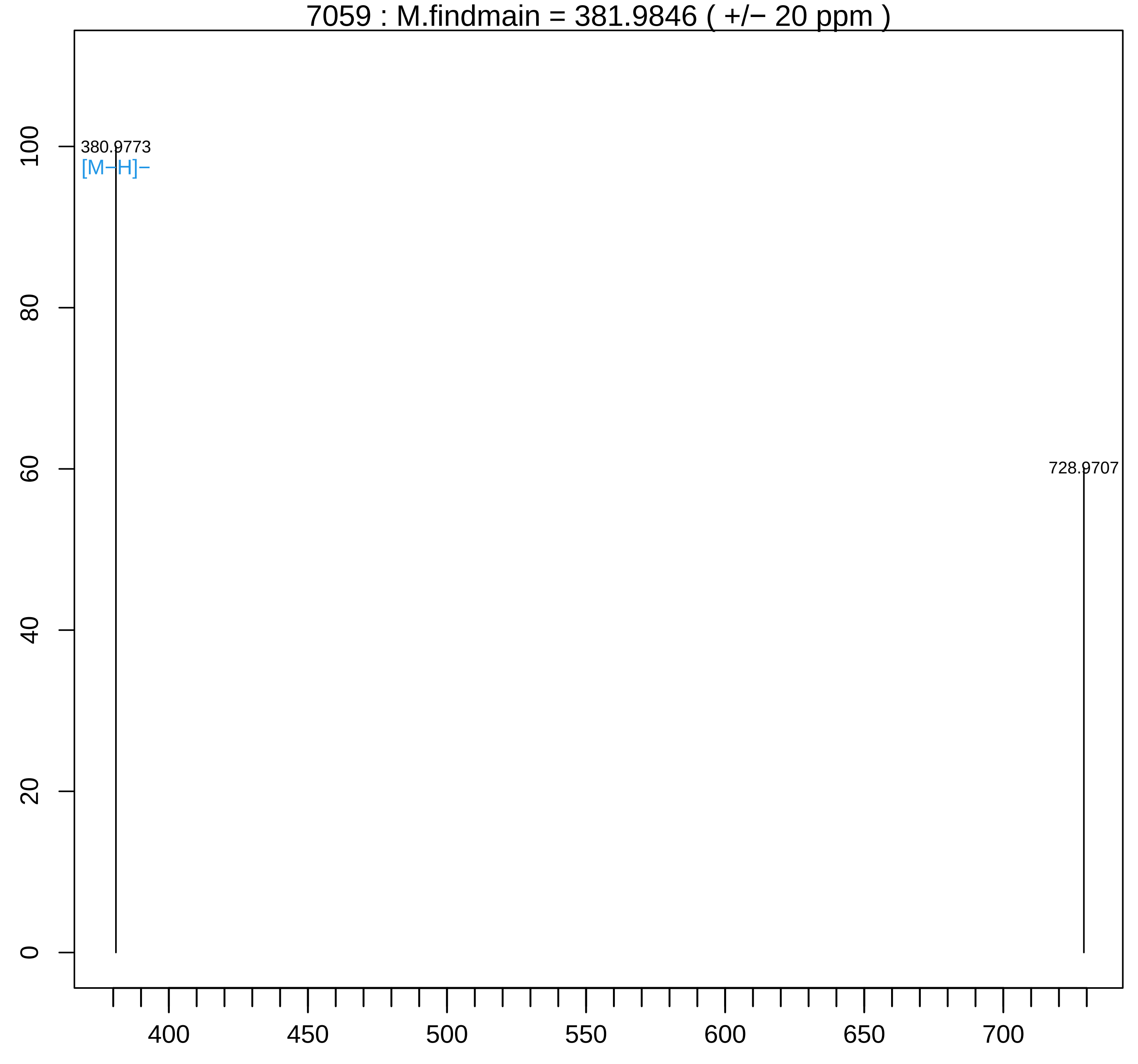
7059 : M.findmain = 381.9846 (+/− 20 ppm)
380.9773
[M−H]−
728.9707
0
20
40
60
80
100
400
450
500
550
600
650
700

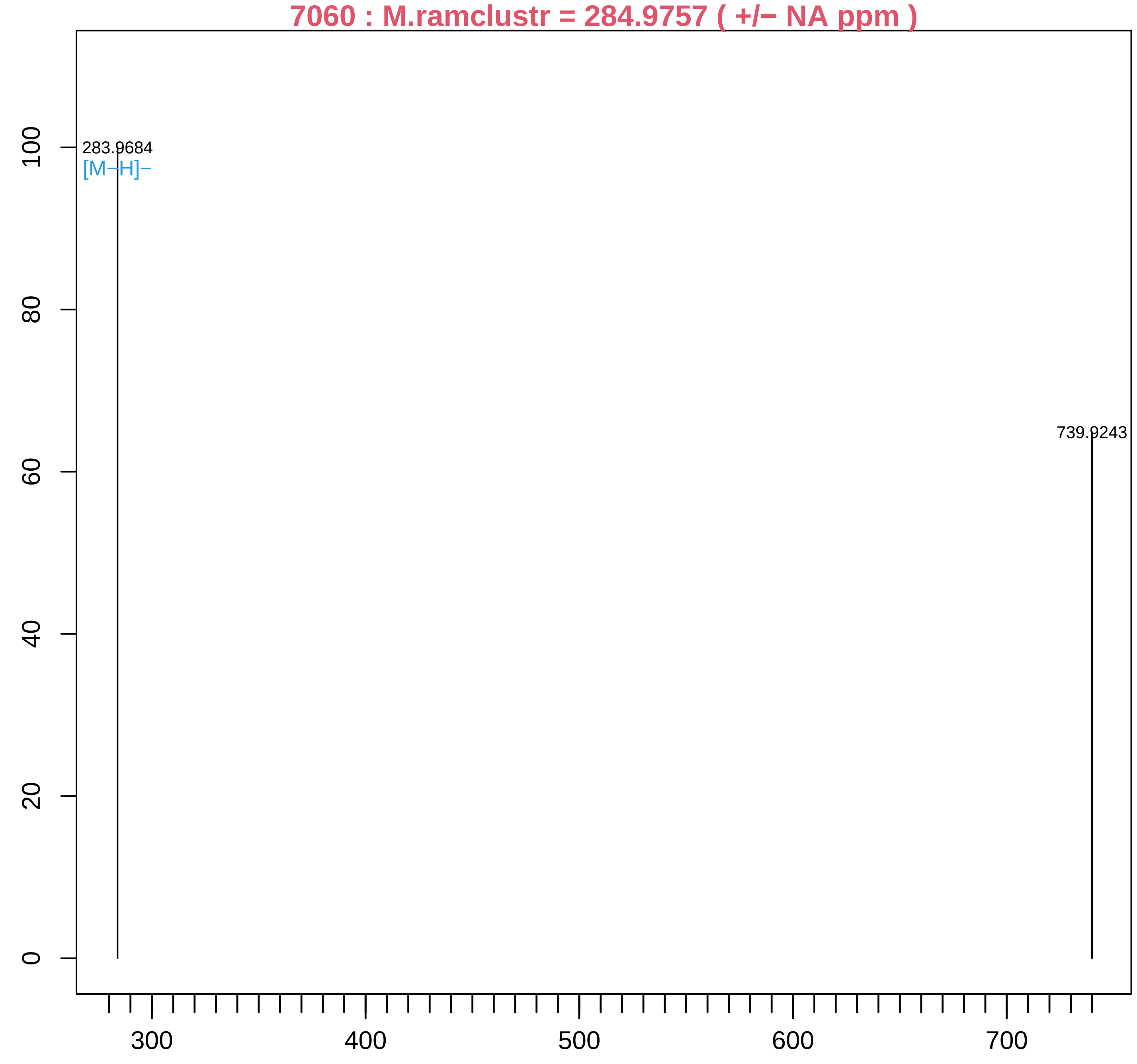
7060 : M.ramclustr = 284.9757 (+/− NA ppm)
283.9684
[M−H]−
739.9243
0
20
40
60
80
100
300
400
500
600
700

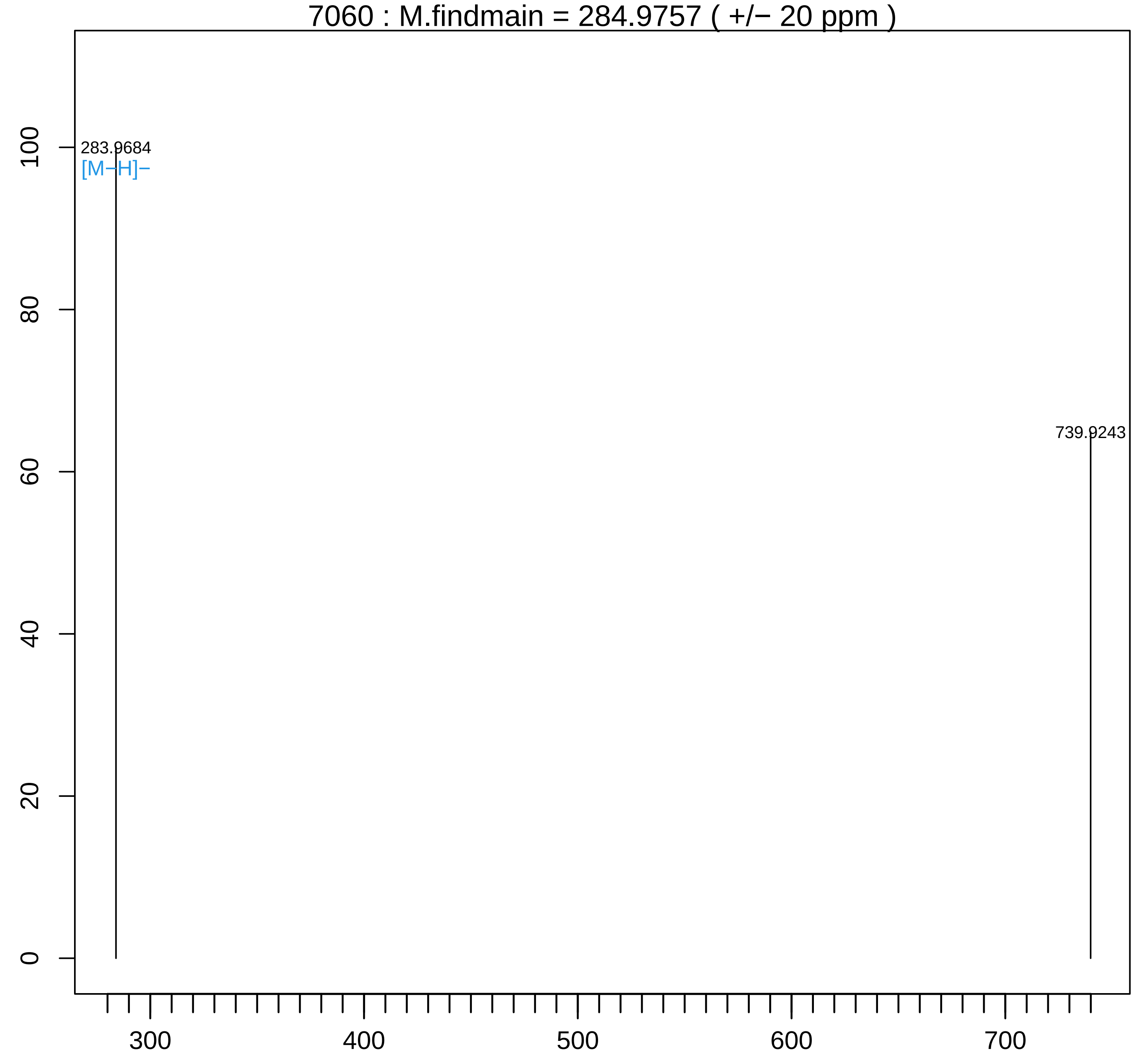
7060 : M.findmain = 284.9757 (+/− 20 ppm)
283.9684
[M−H]−
739.9243
0
20
40
60
80
100
300
400
500
600
700

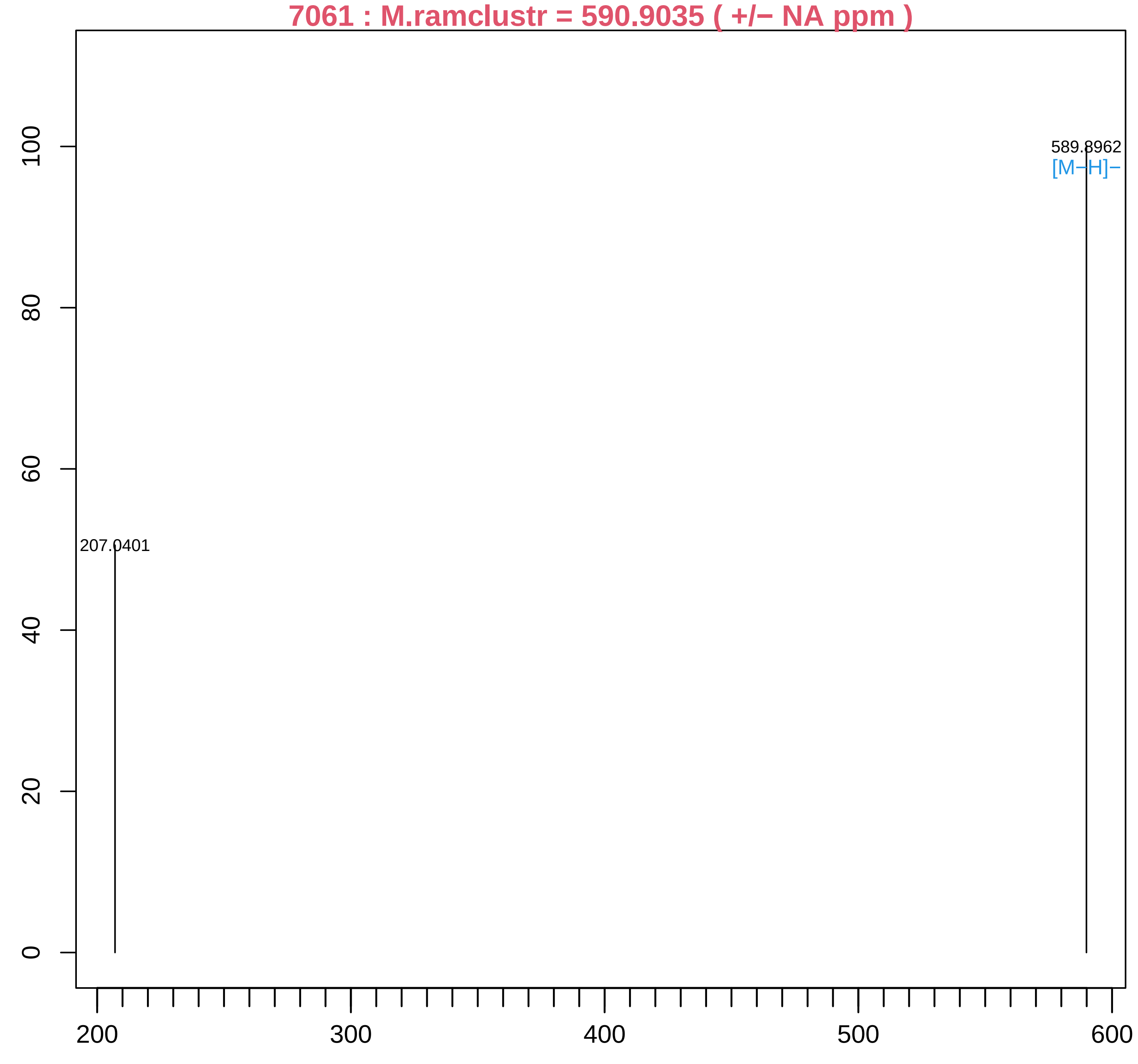
7061 : M.ramclustr = 590.9035 (+/− NA ppm)
589.8962
[M−H]−
207.0401
0
20
40
60
80
100
200
300
400
500
600
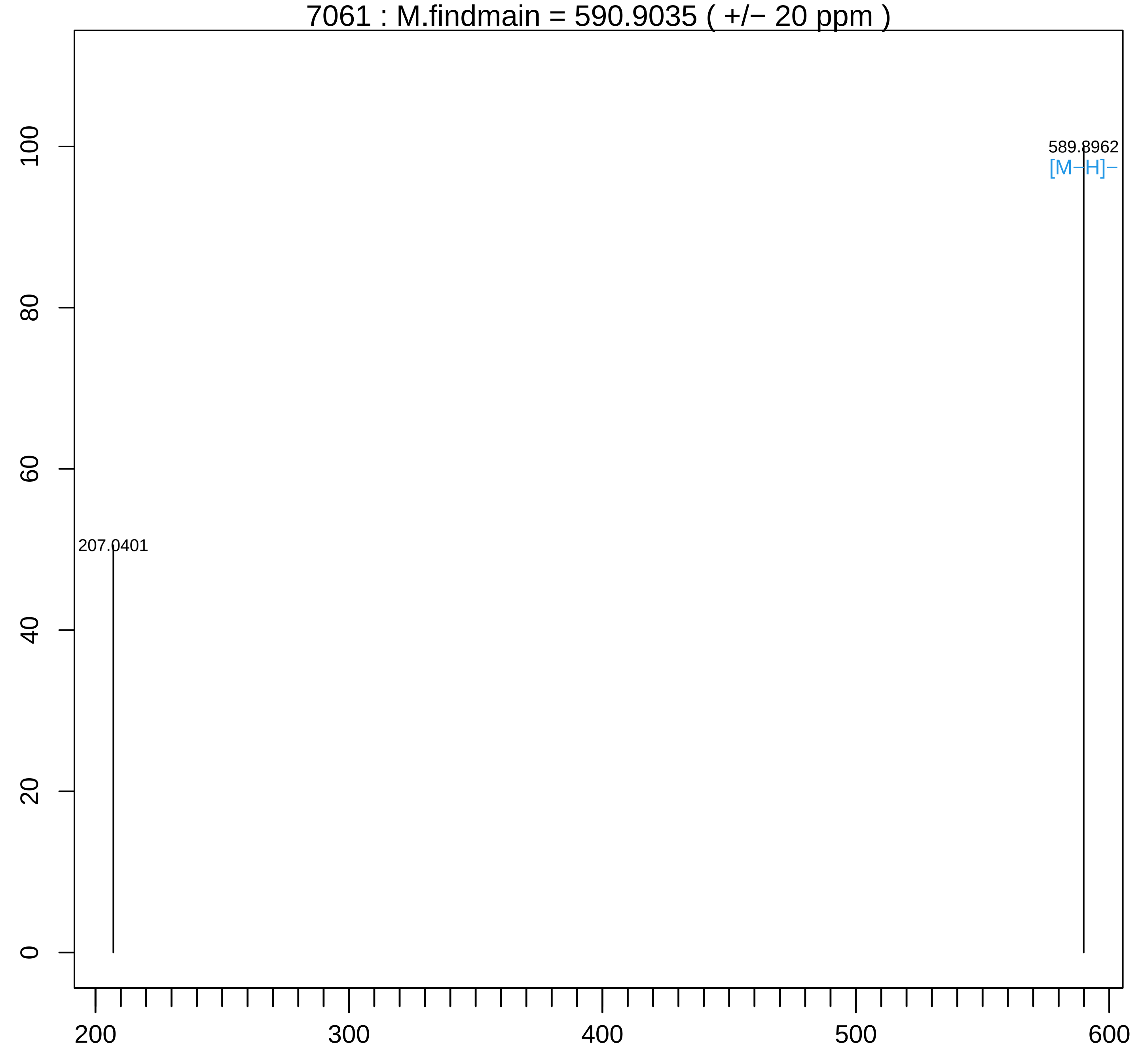
7061 : M.findmain = 590.9035 (+/− 20 ppm)
589.8962
[M−H]−
207.0401
0
20
40
60
80
100
200
300
400
500
600

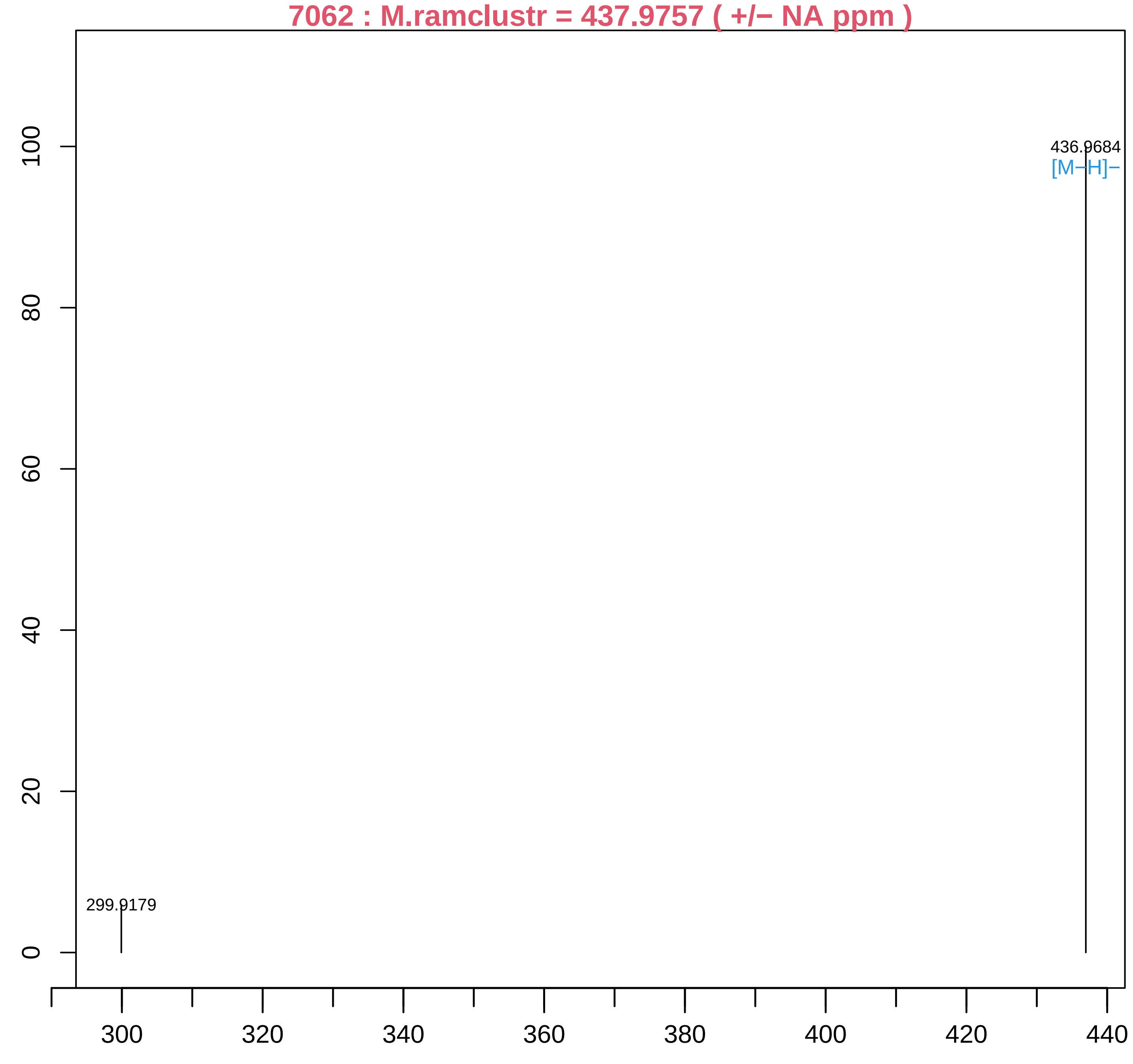
7062 : M.ramclustr = 437.9757 (+/− NA ppm)
436.9684
[M−H]−
299.9179

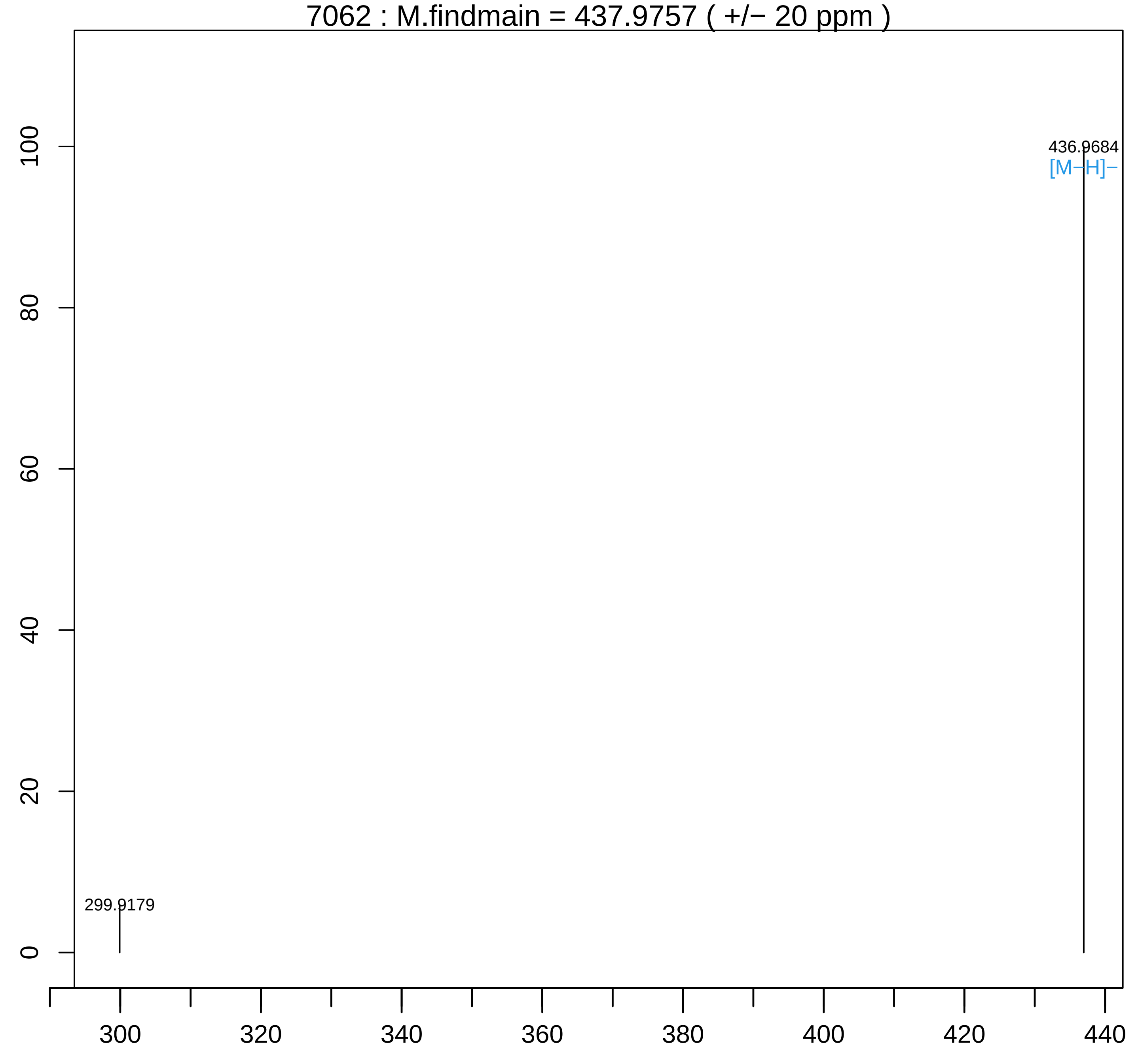
7062 : M.findmain = 437.9757 (+/− 20 ppm)
436.9684
[M−H]−
299.9179

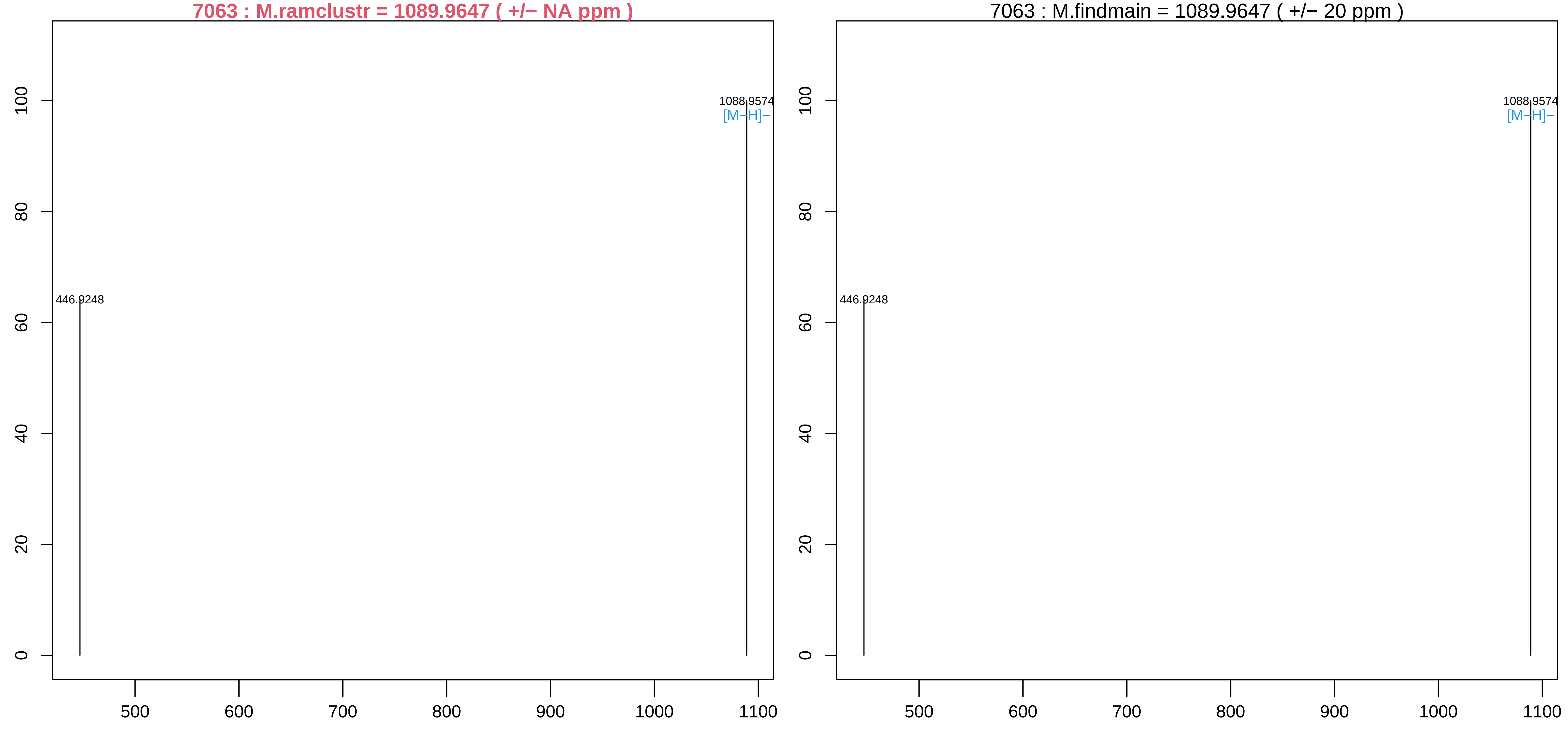
7063 : M.ramclustr = 1089.9647 (+/− NA ppm)
7063 : M.findmain = 1089.9647 (+/− 20 ppm)
1088.9574
[M−H]−
446.9248
1088.9574
[M−H]−
446.9248
0
20
40
60
80
100
500
600
700
800
900
1000
1100

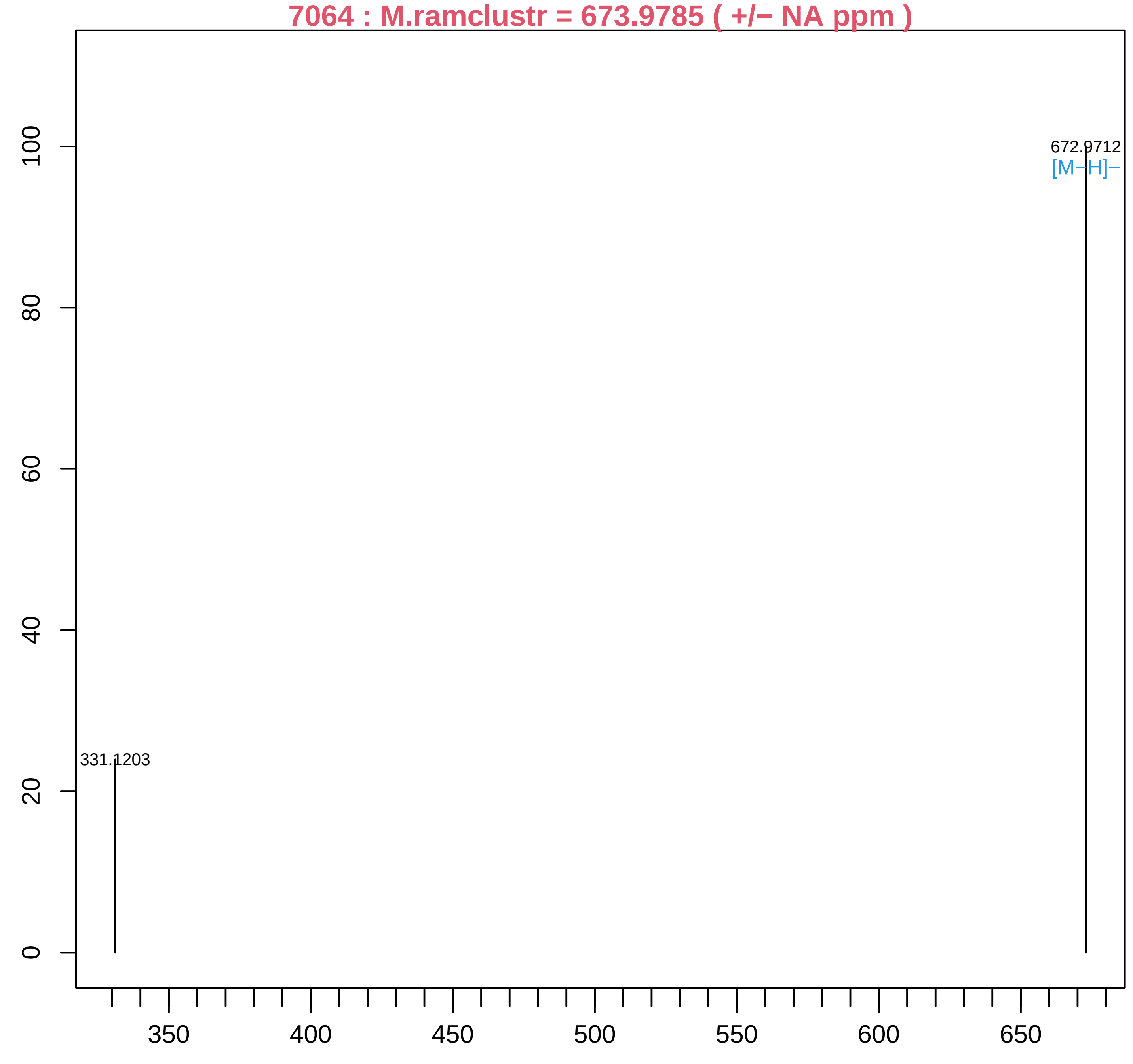
7064 : M.ramclustr = 673.9785 (+/− NA ppm)
672.9712
[M−H]−
331.1203
0
20
40
60
80
100
350
400
450
500
550
600
650

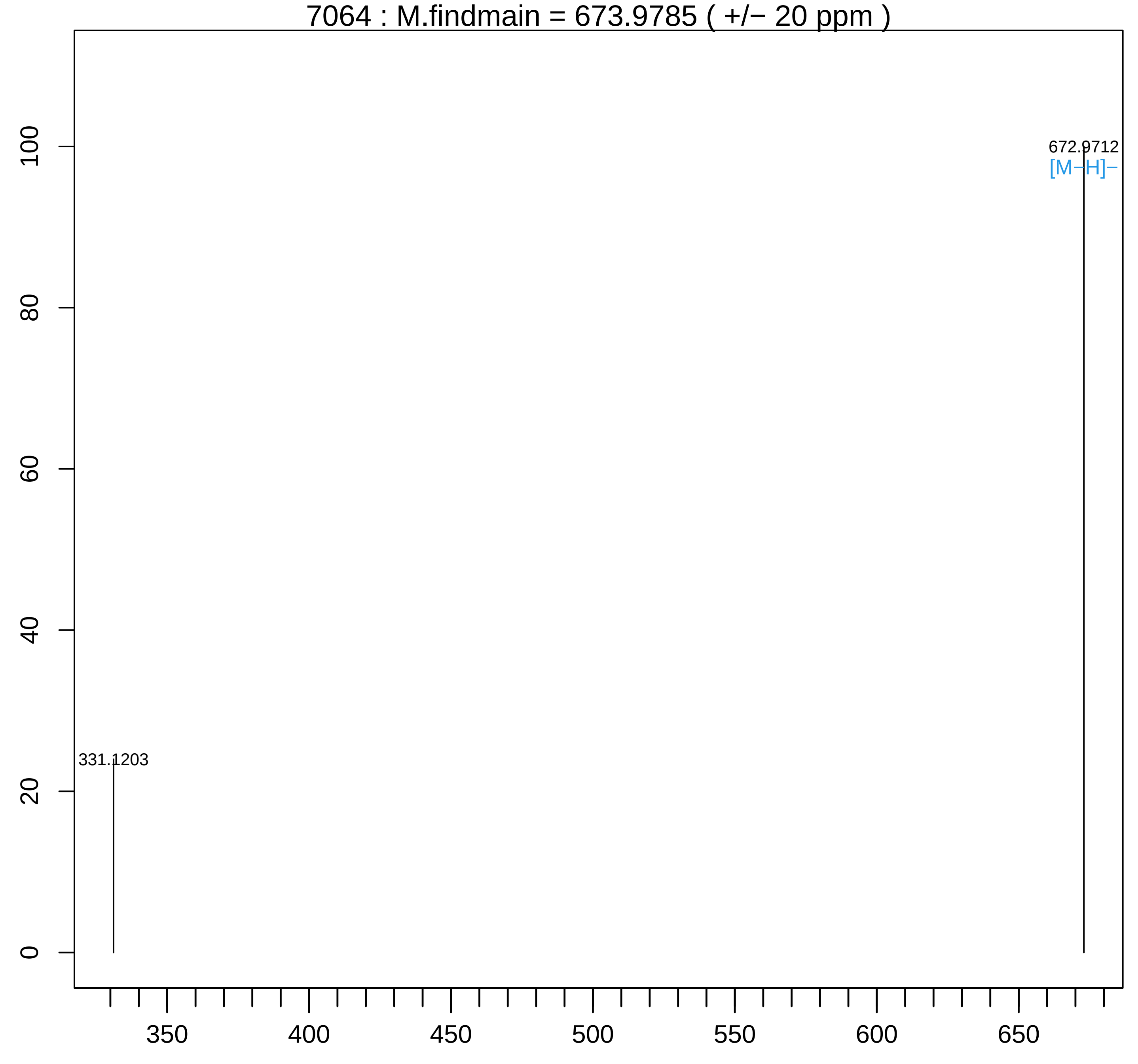
7064 : M.findmain = 673.9785 (+/− 20 ppm)
672.9712
[M−H]−
331.1203
0
20
40
60
80
100
350
400
450
500
550
600
650

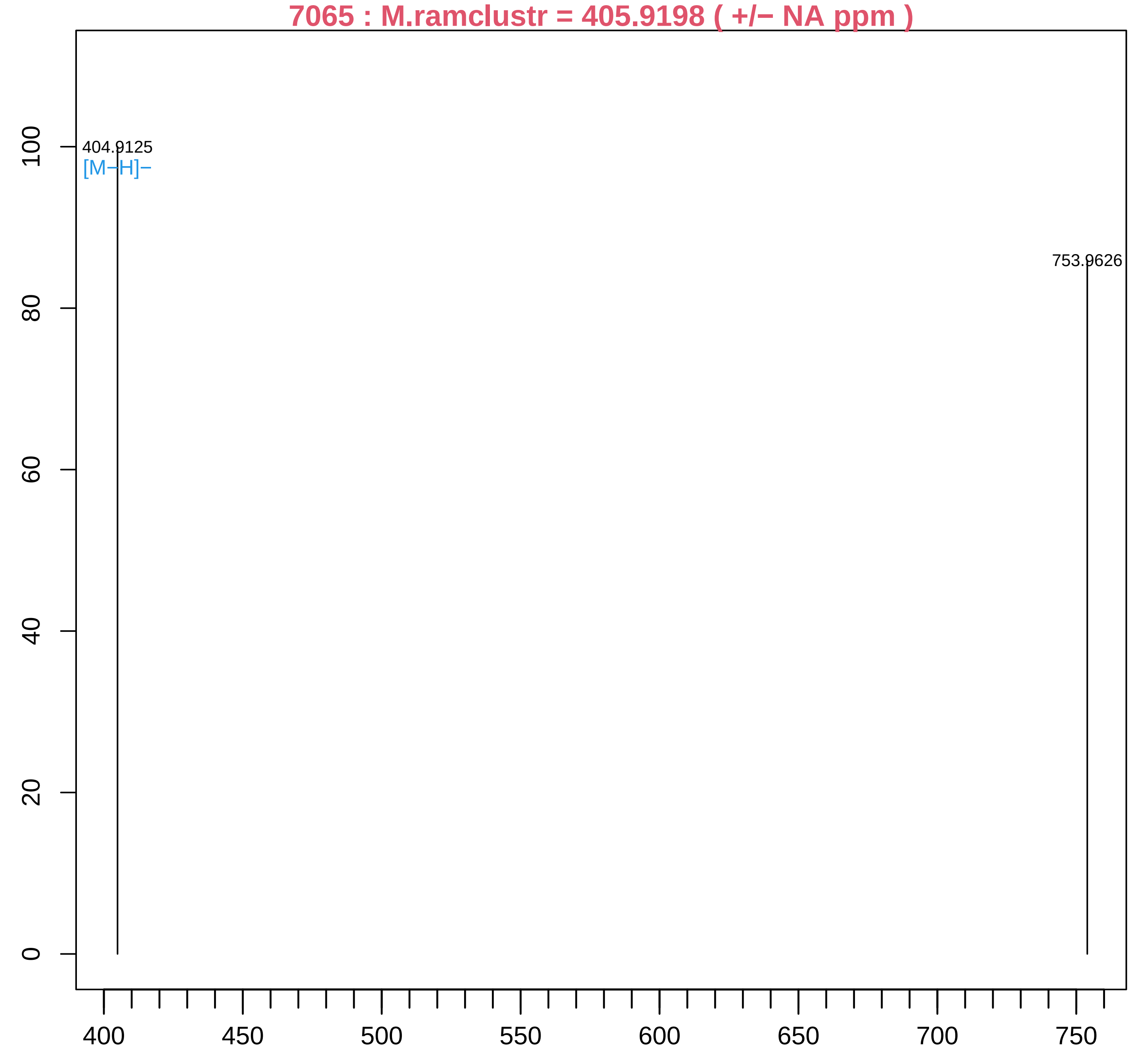
7065 : M.ramclustr = 405.9198 (+/− NA ppm)
404.9125
[M−H]−
753.9626
0
20
40
60
80
100
400
450
500
550
600
650
700
750

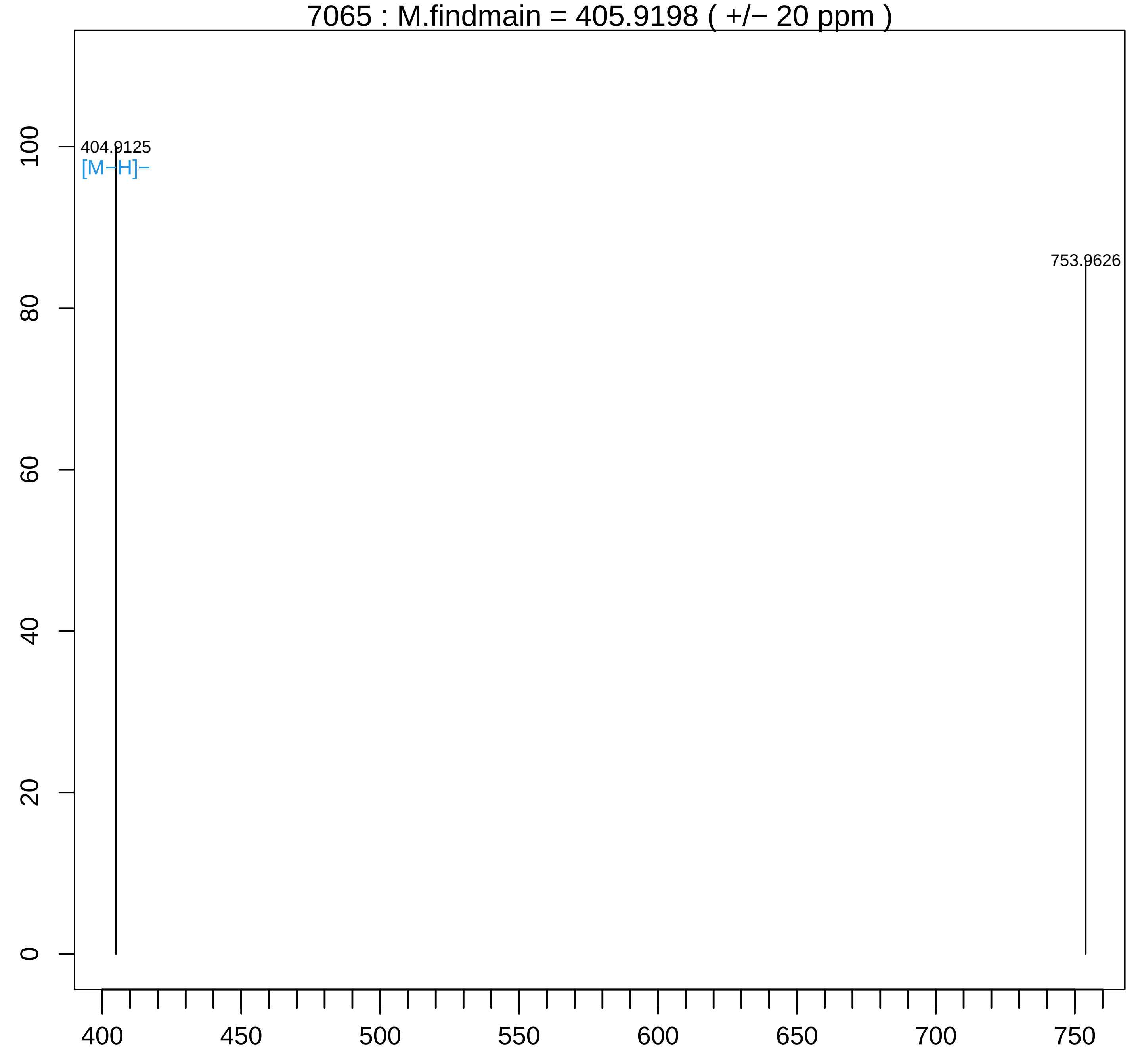
7065 : M.findmain = 405.9198 (+/− 20 ppm)
404.9125
[M−H]−
753.9626
0
20
40
60
80
100
400
450
500
550
600
650
700
750

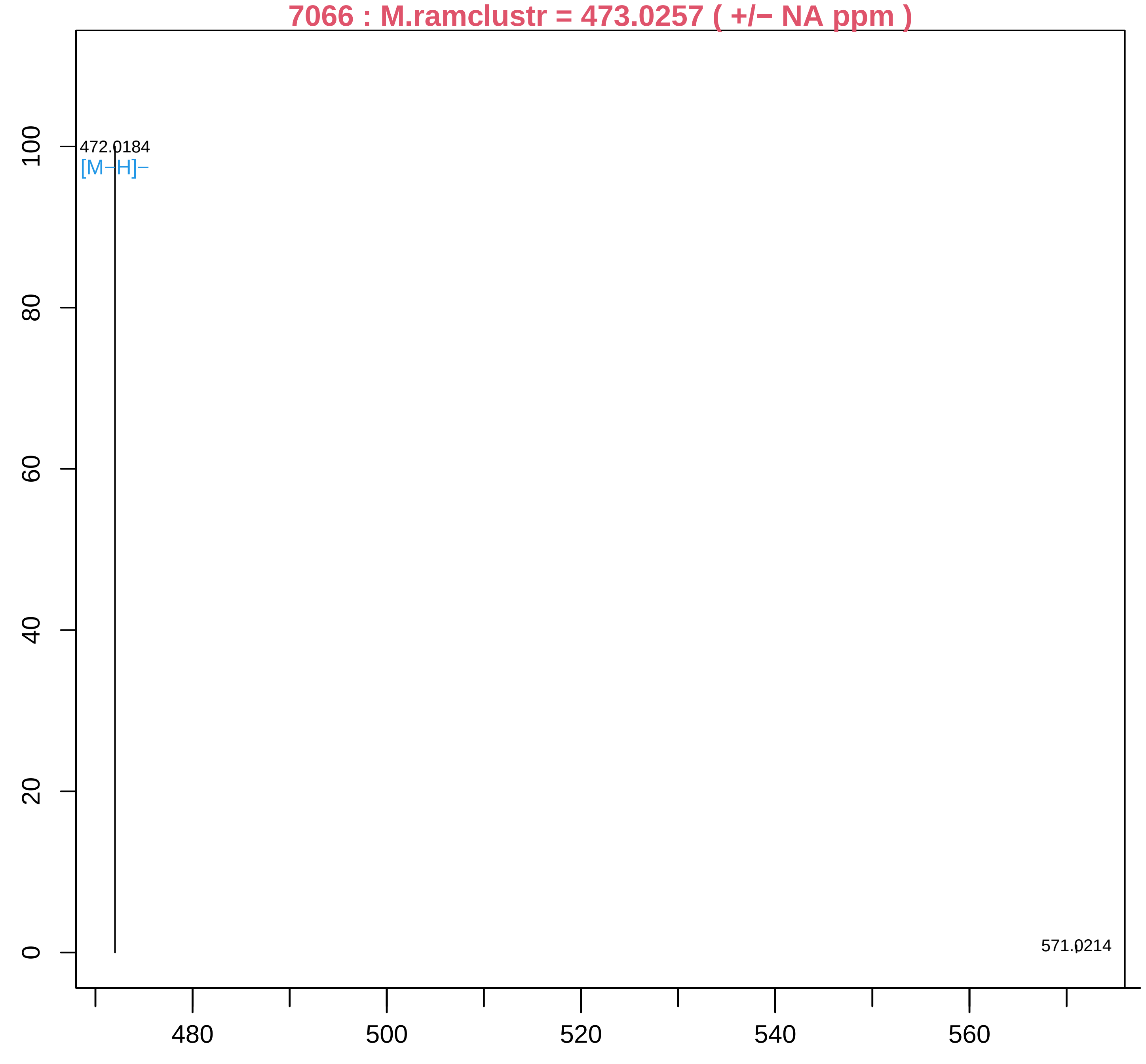
7066 : M.ramclustr = 473.0257 (+/− NA ppm)
472.0184
[M−H]−
571.0214
0
20
40
60
80
100
480
500
520
540
560

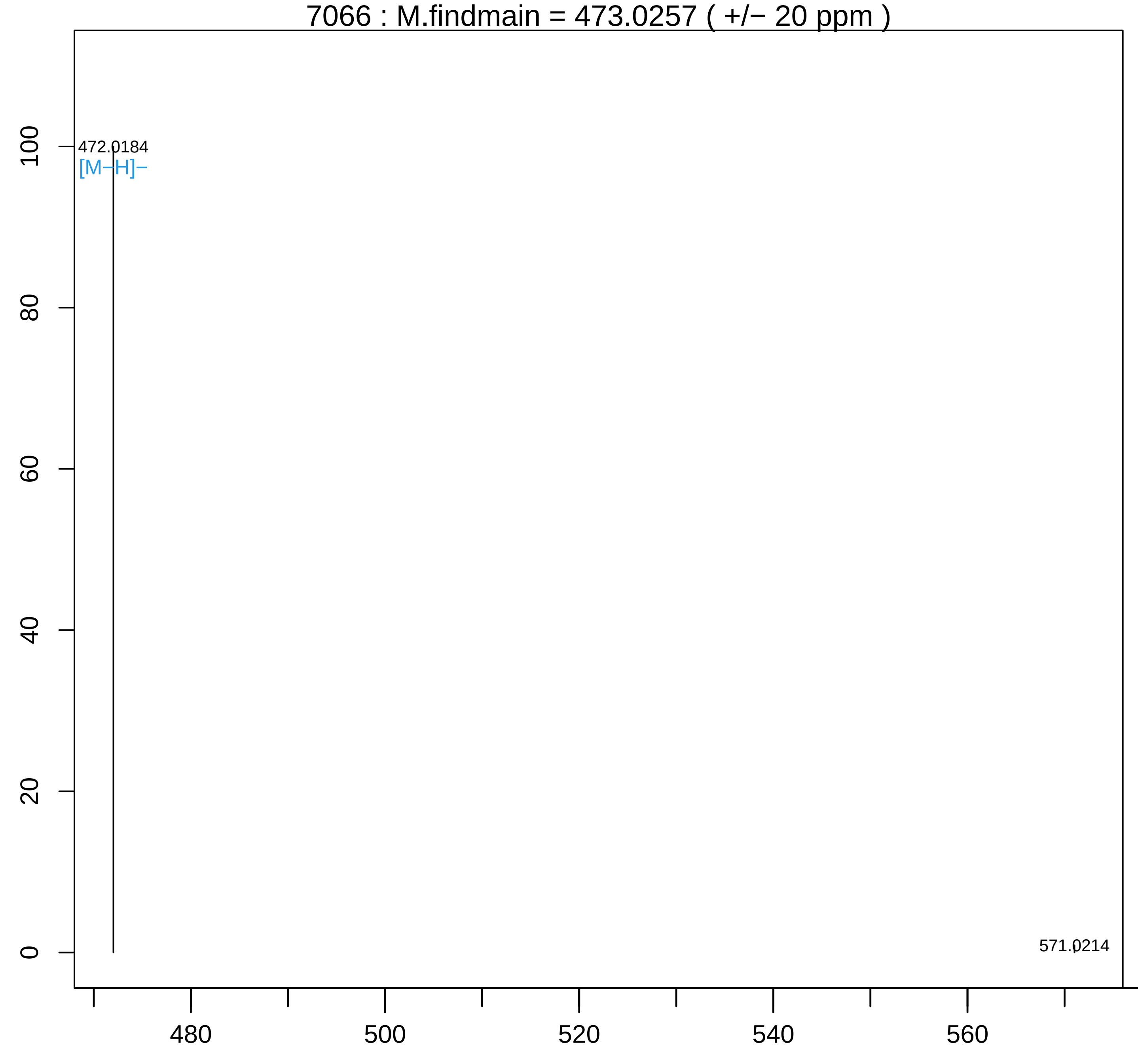
7066 : M.findmain = 473.0257 (+/− 20 ppm)
472.0184
[M−H]−
571.0214
0
20
40
60
80
100
480
500
520
540
560

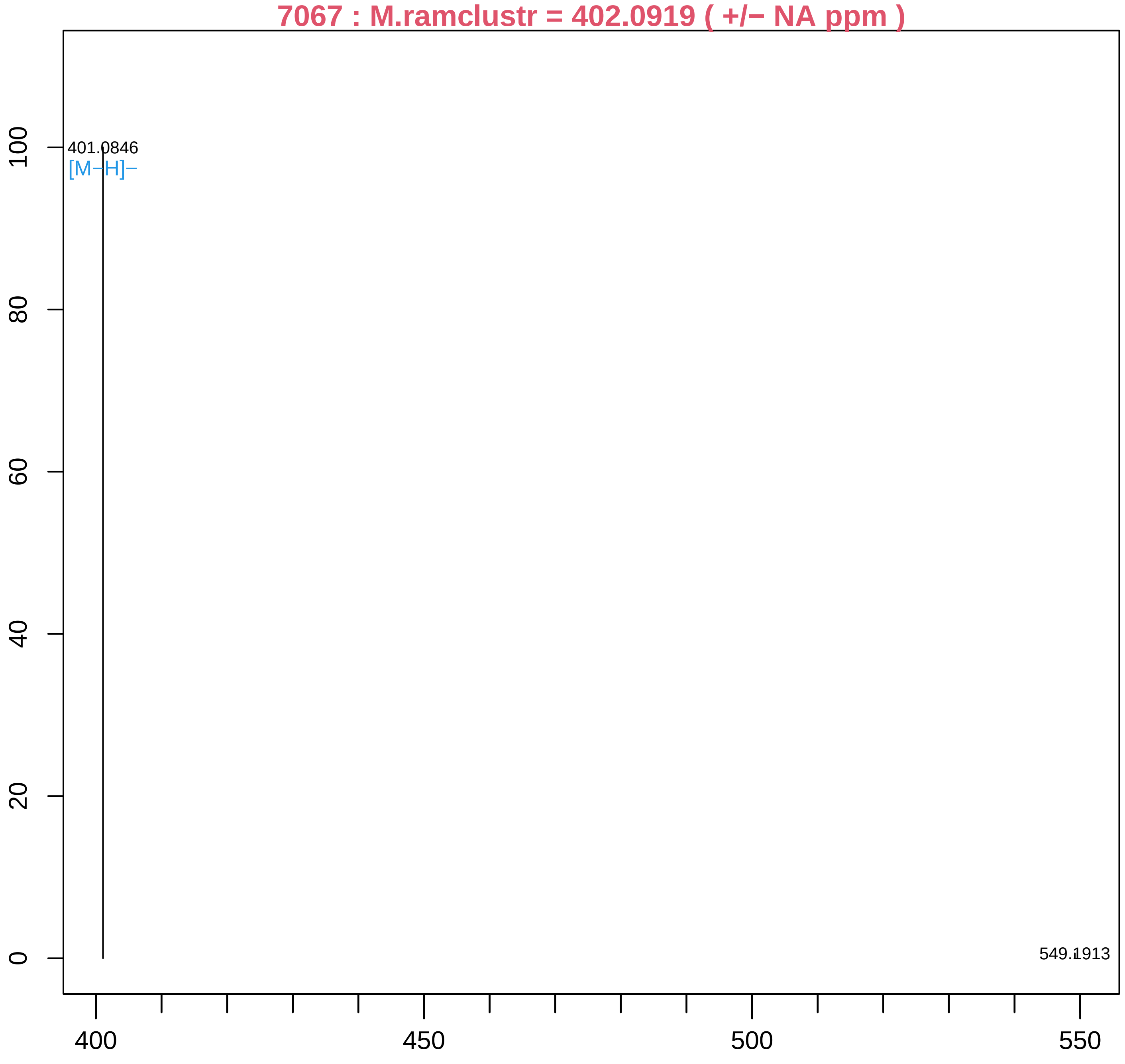
7067 : M.ramclustr = 402.0919 (+/− NA ppm)
401.0846
[M−H]−
549.1913
0
20
40
60
80
100
400
450
500
550
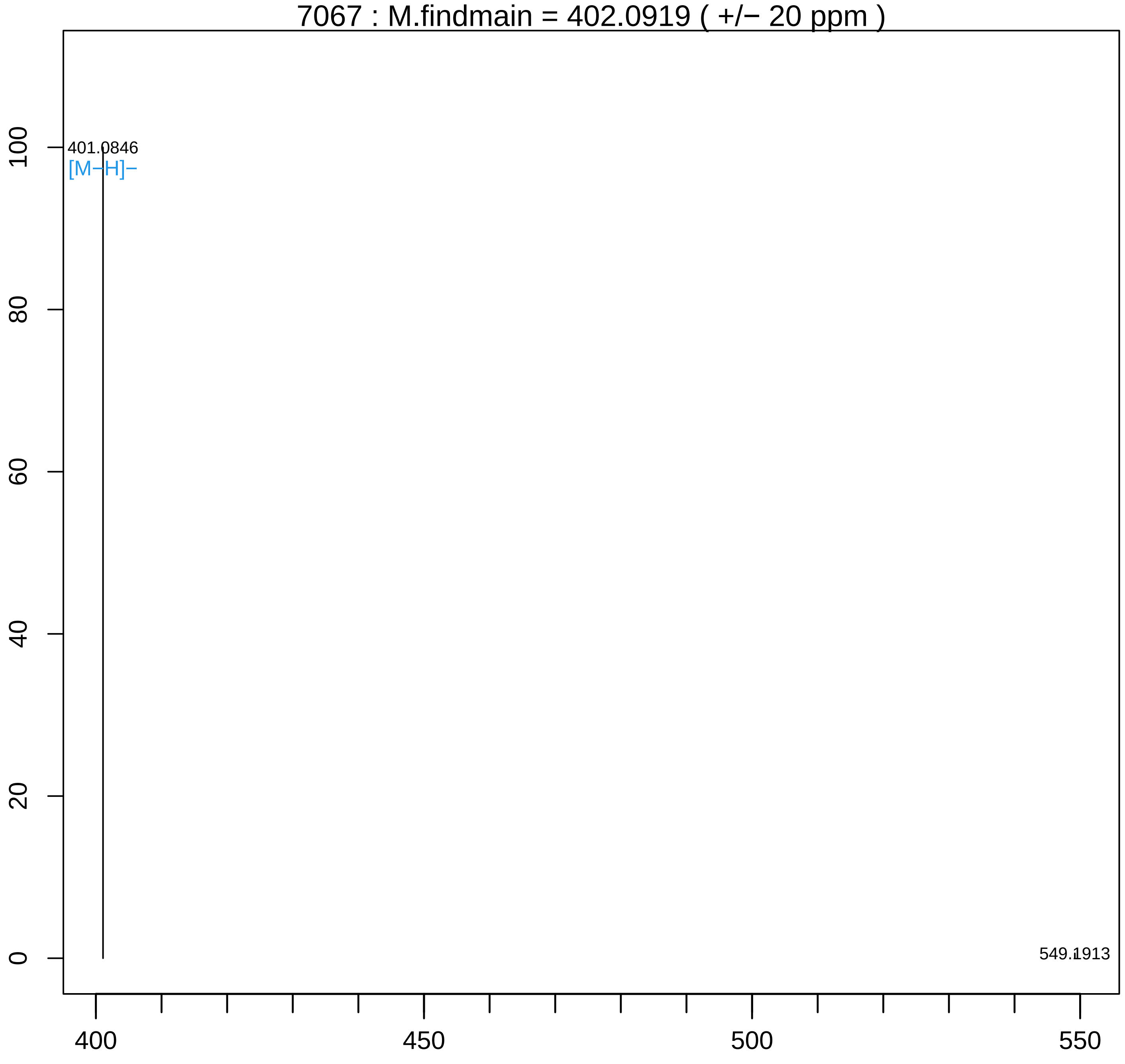
7067 : M.findmain = 402.0919 (+/− 20 ppm)
401.0846
[M−H]−
549.1913
0
20
40
60
80
100
400
450
500
550

7068 : M.ramclustr = 532.1848 (+/− NA ppm)

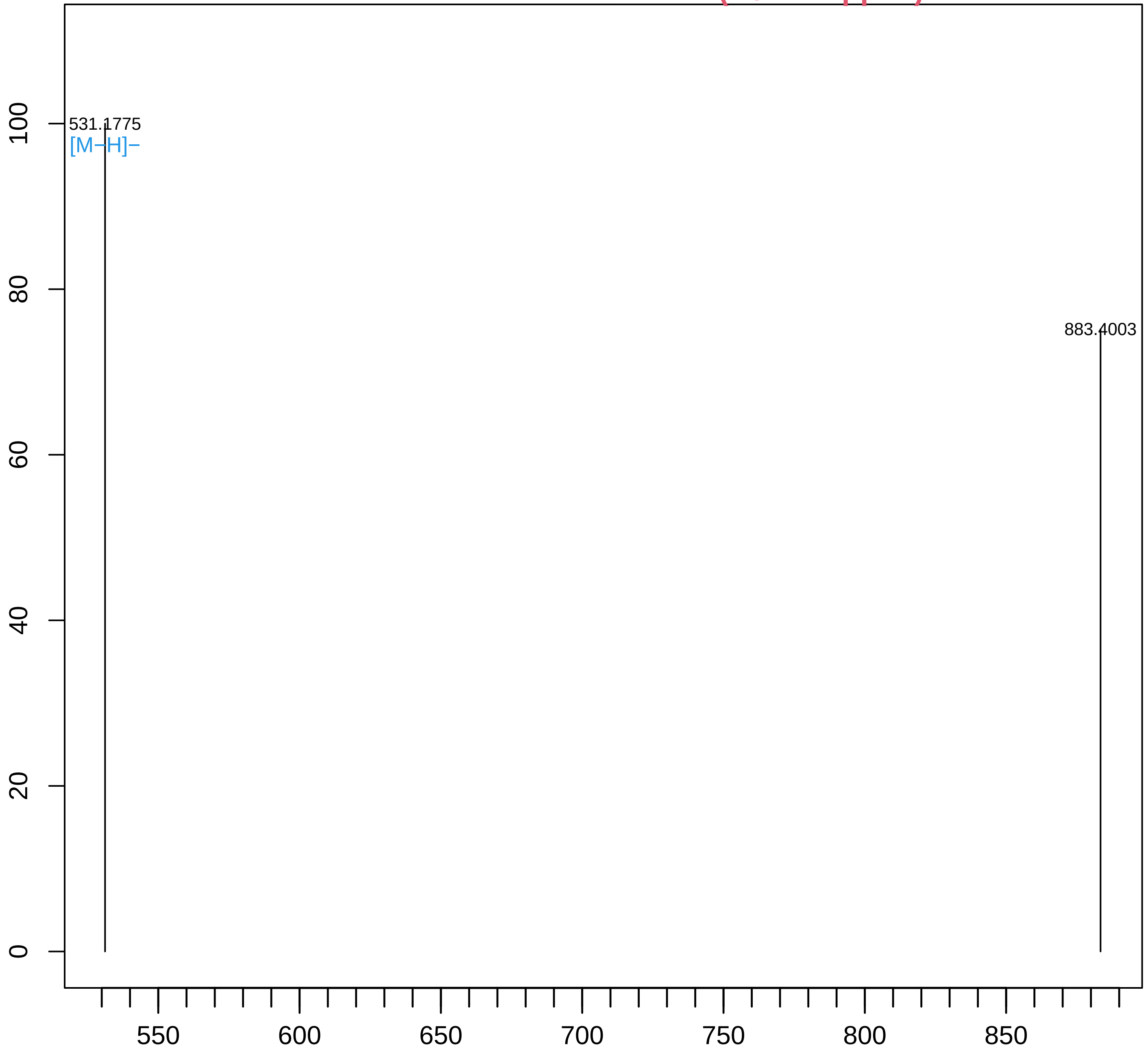

7068 : M.findmain = 532.1848 (+/− 20 ppm)

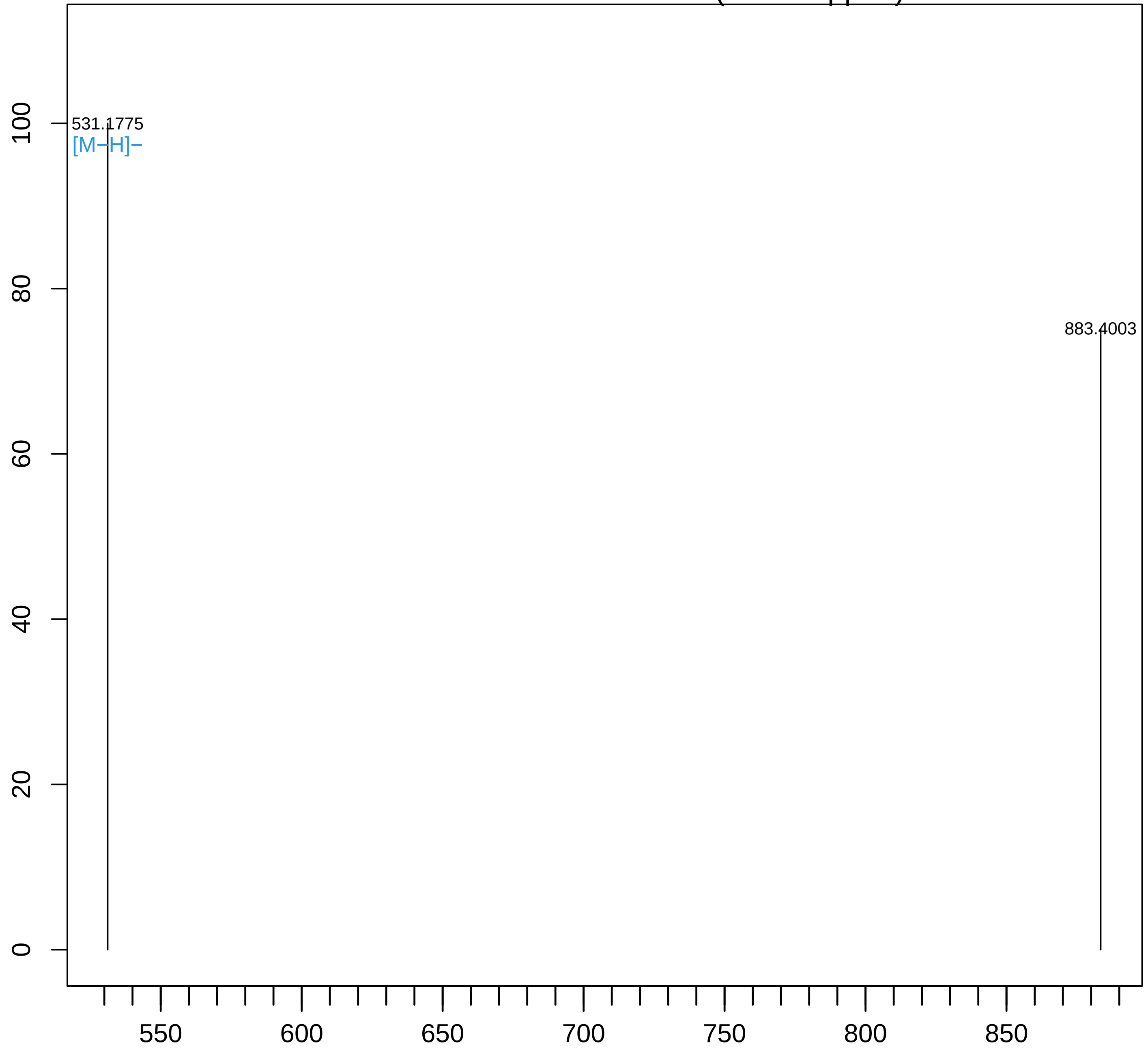

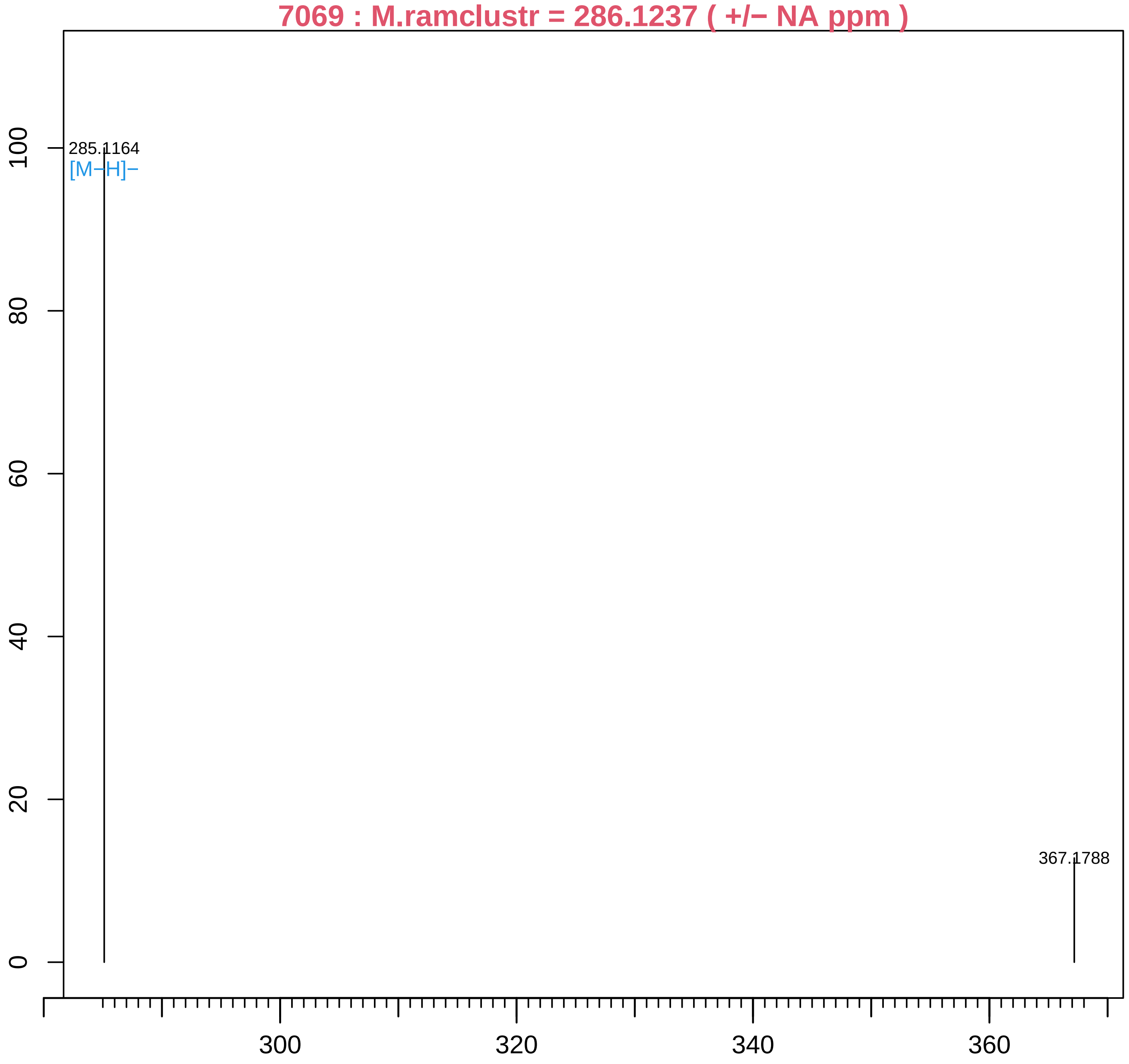
7069 : M.ramclustr = 286.1237 (+/− NA ppm)
285.1164
[M−H]−
367.1788
0
20
40
60
80
100
300
320
340
360

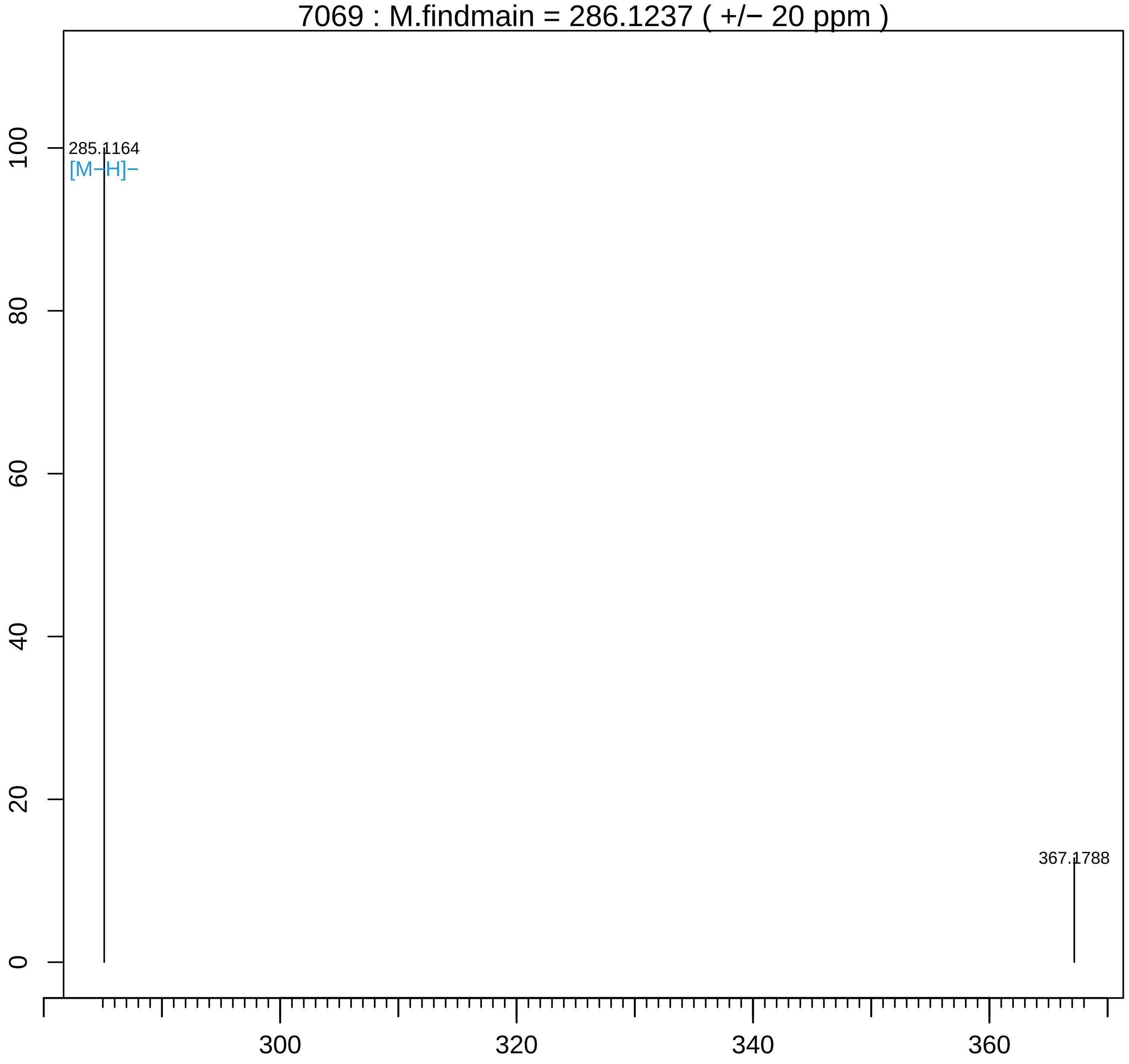
7069 : M.findmain = 286.1237 (+/− 20 ppm)
285.1164
[M−H]−
367.1788
0
20
40
60
80
100
300
320
340
360

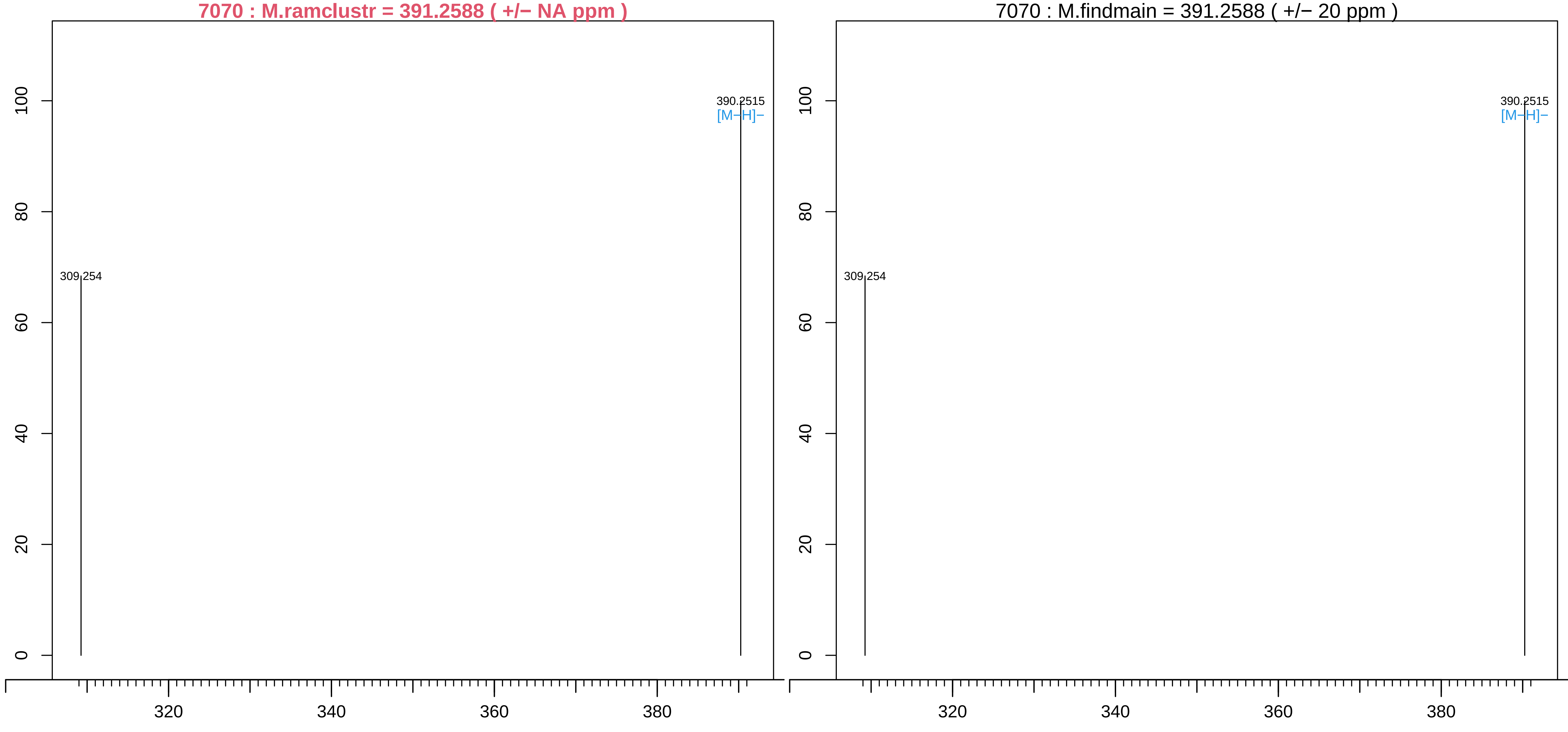
7070 : M.ramclustr = 391.2588 (+/− NA ppm)
7070 : M.findmain = 391.2588 (+/− 20 ppm)
390.2515
[M−H]−
309.254
390.2515
[M−H]−
309.254
0
20
40
60
80
100
320
340
360
380

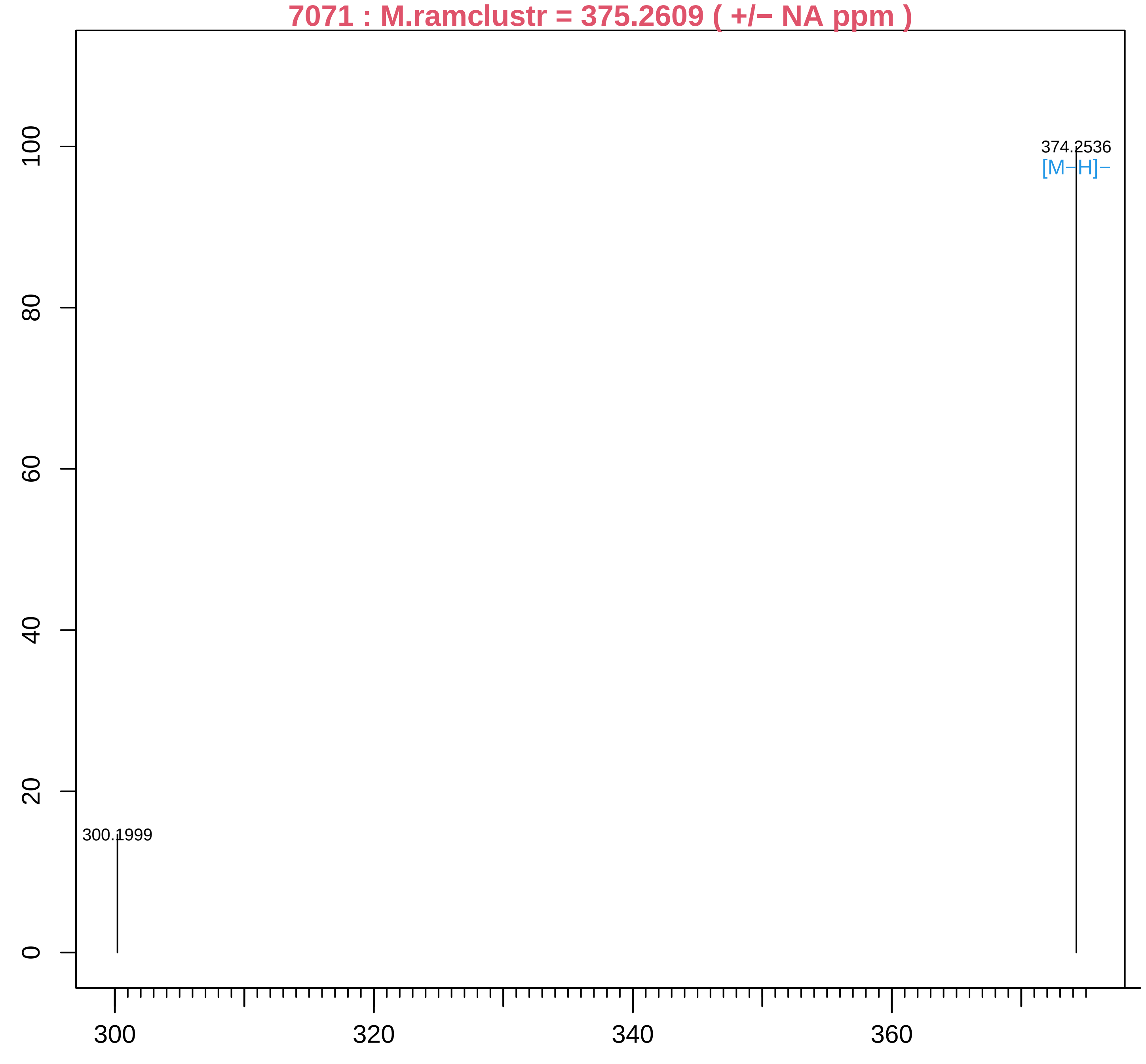
7071 : M.ramclustr = 375.2609 (+/− NA ppm)
374.2536
[M−H]−
300.1999
0
20
40
60
80
100
300
320
340
360
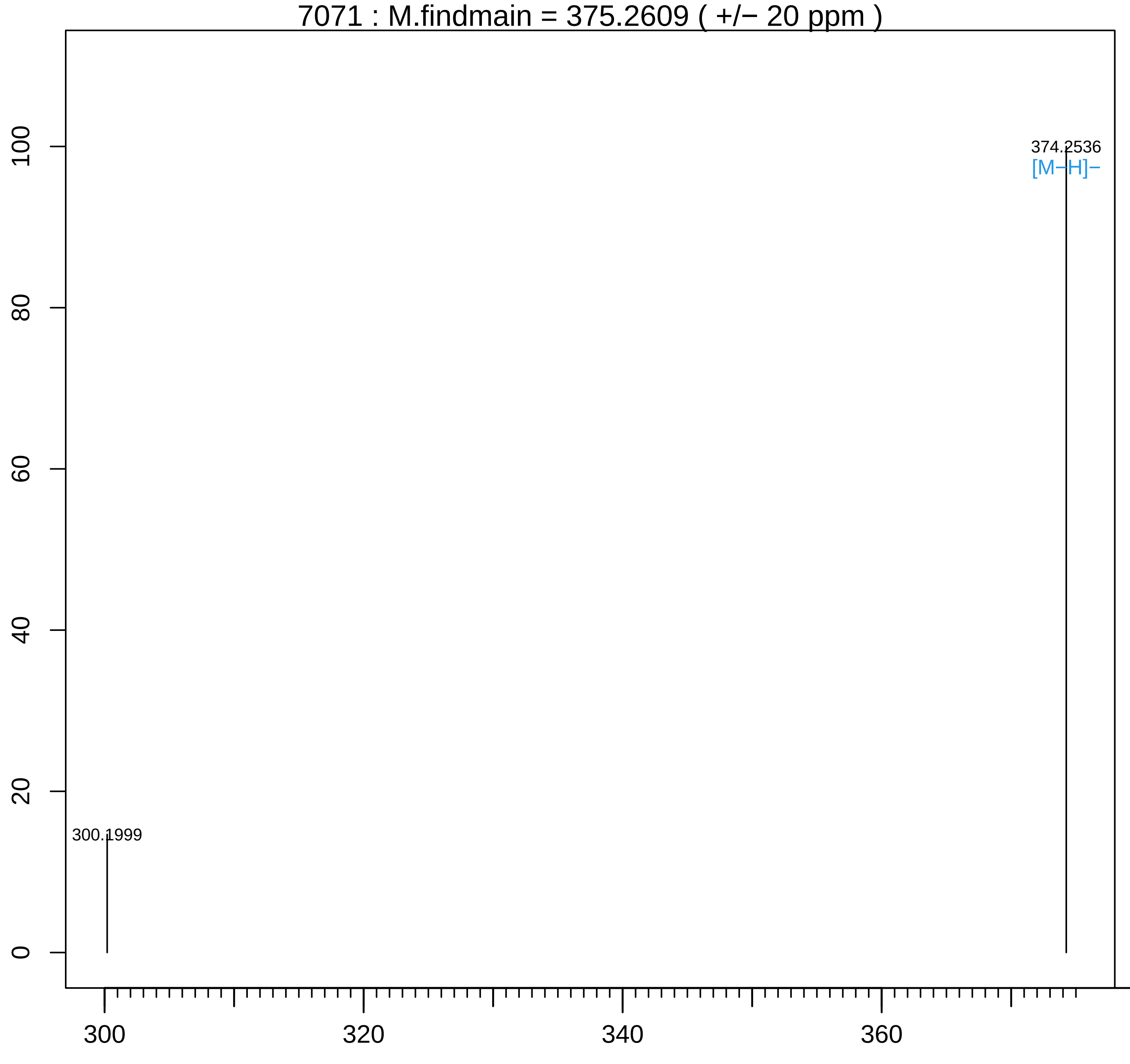
7071 : M.findmain = 375.2609 (+/− 20 ppm)
374.2536
[M−H]−
300.1999
0
20
40
60
80
100
300
320
340
360

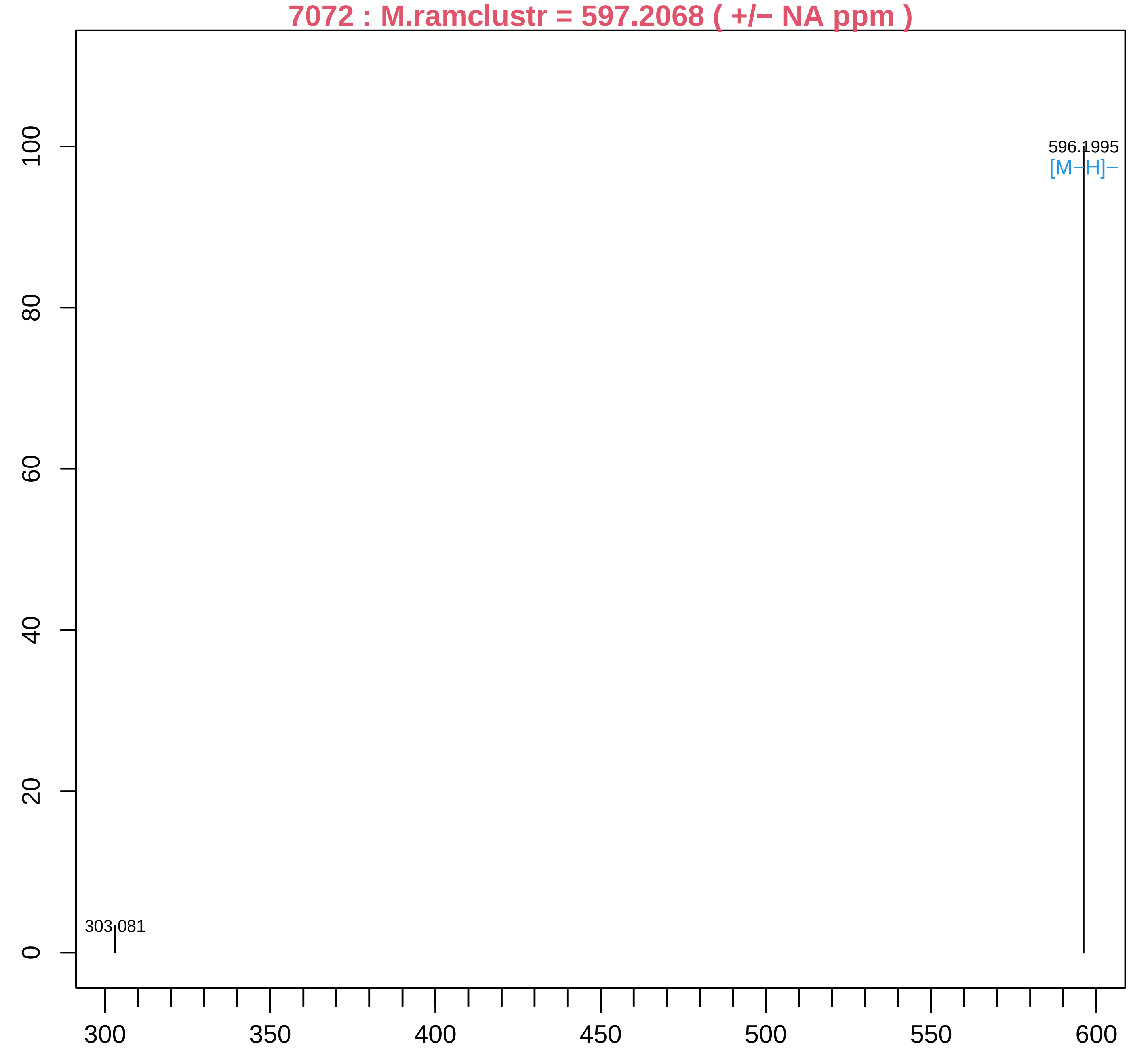
7072 : M.ramclustr = 597.2068 (+/− NA ppm)
596.1995
[M−H]−
303.081
0
20
40
60
80
100
300
350
400
450
500
550
600

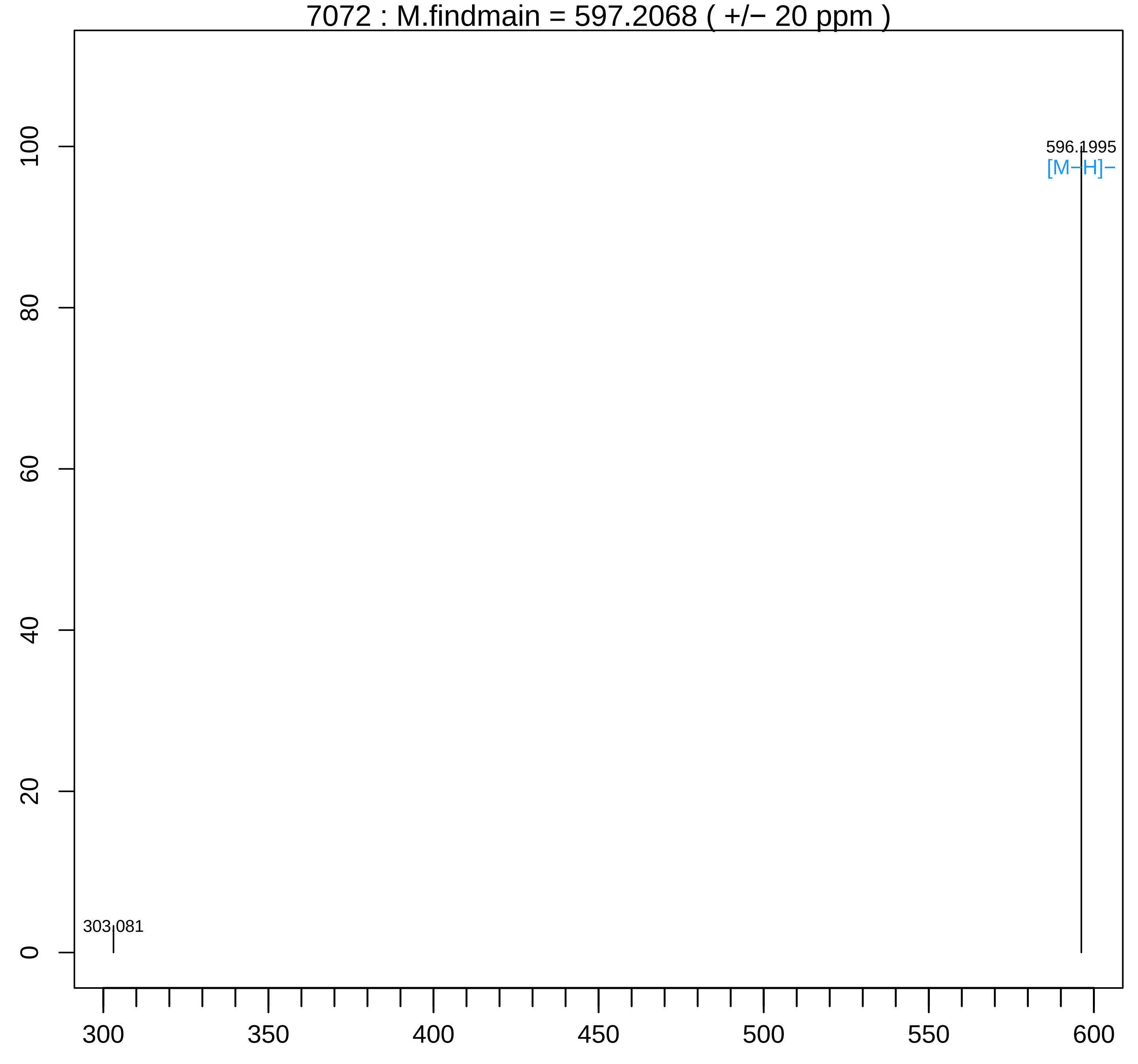
7072 : M.findmain = 597.2068 (+/− 20 ppm)
596.1995
[M−H]−
303.081
0
20
40
60
80
100
300
350
400
450
500
550
600

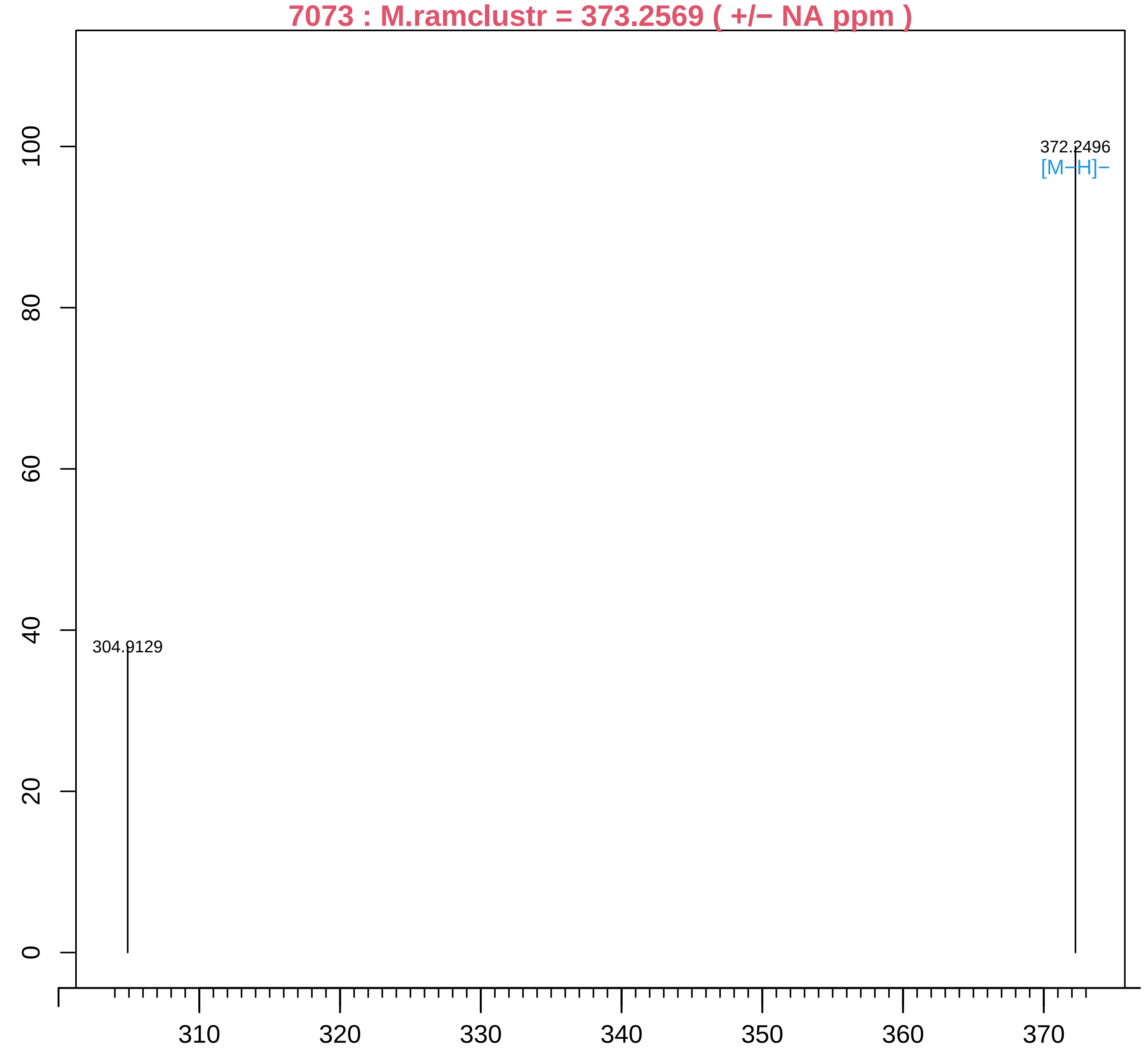
7073 : M.ramclustr = 373.2569 (+/− NA ppm)
372.2496
[M−H]−
304.9129
0
20
40
60
80
100
310
320
330
340
350
360
370

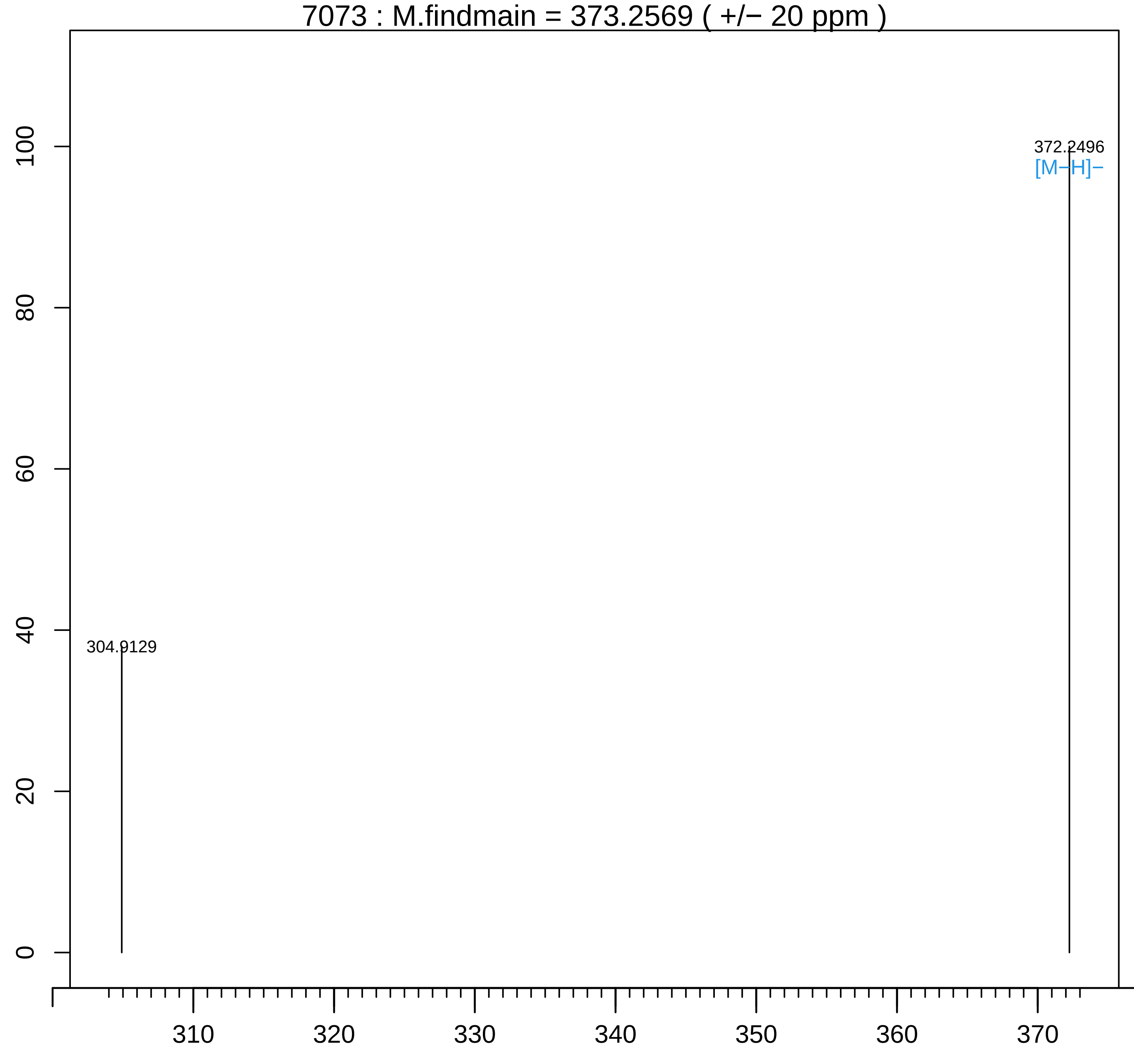
7073 : M.findmain = 373.2569 (+/− 20 ppm)
372.2496
[M−H]−
304.9129
0
20
40
60
80
100
310
320
330
340
350
360
370

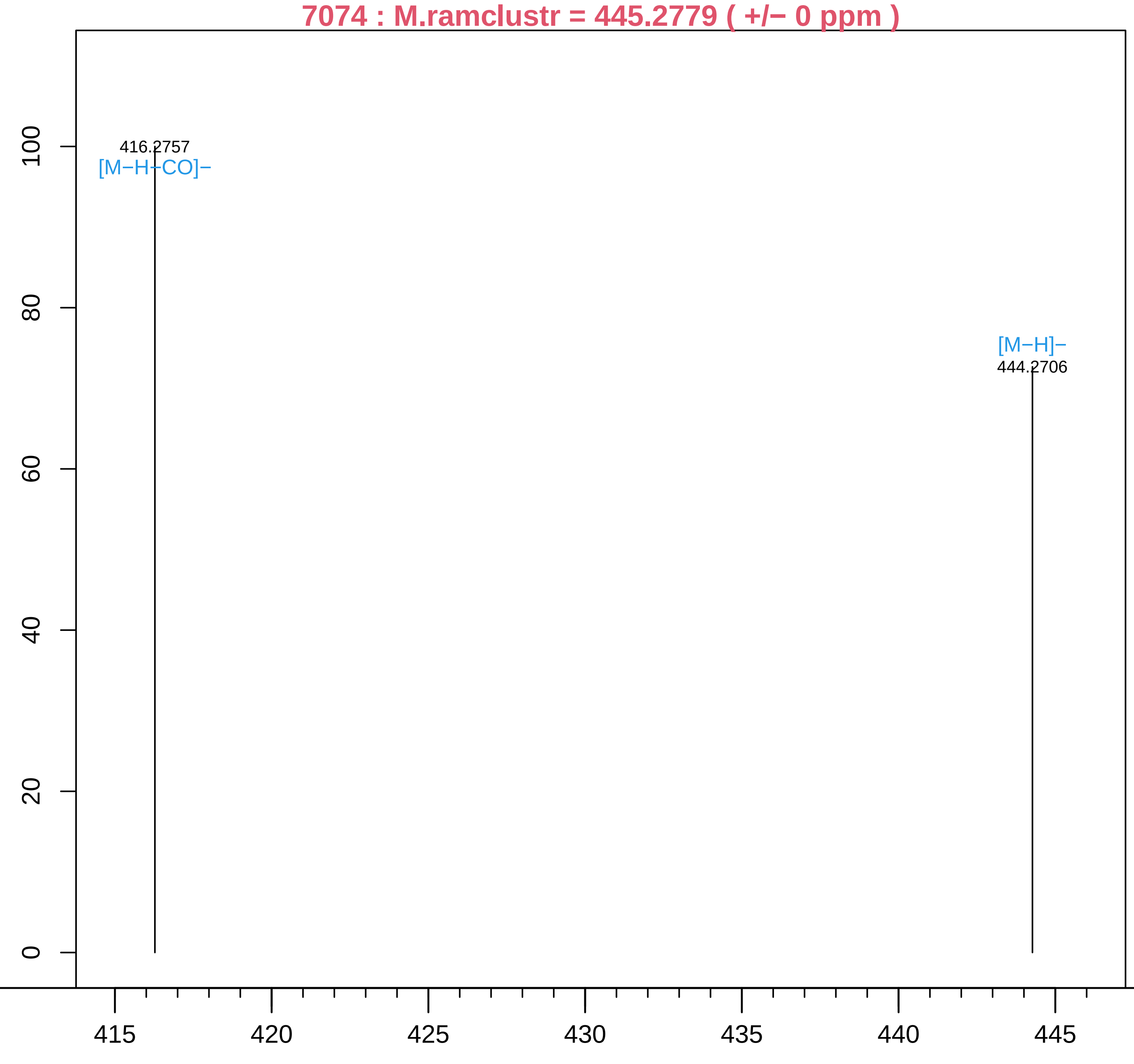
7074 : M.ramclustr = 445.2779 (+/− 0 ppm)
416.2757
[M−H−CO]−
[M−H]−
444.2706
0
20
40
60
80
100
415
420
425
430
435
440
445

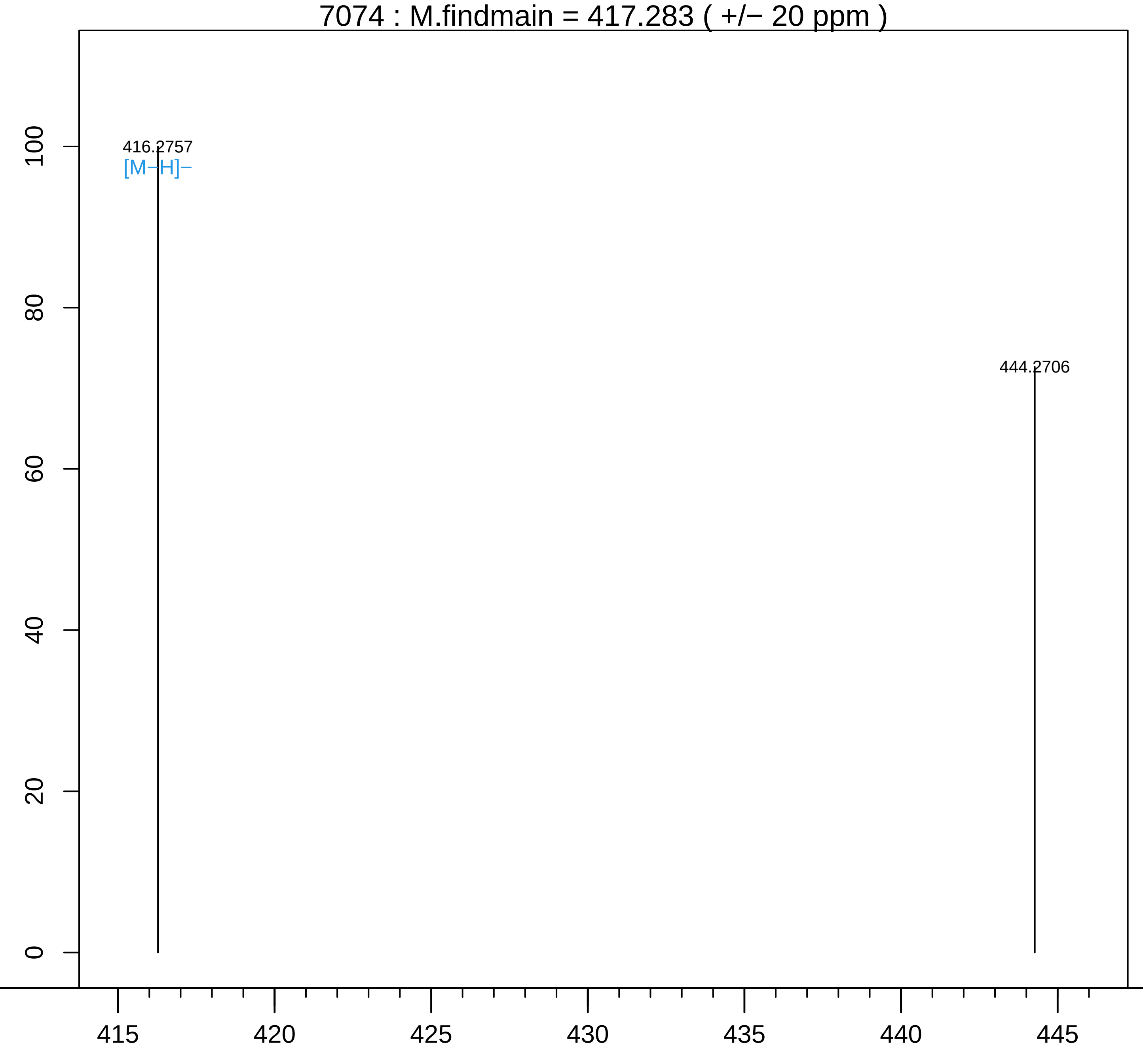
7074 : M.findmain = 417.283 (+/− 20 ppm)
416.2757
[M−H]−
444.2706
0
20
40
60
80
100
415
420
425
430
435
440
445

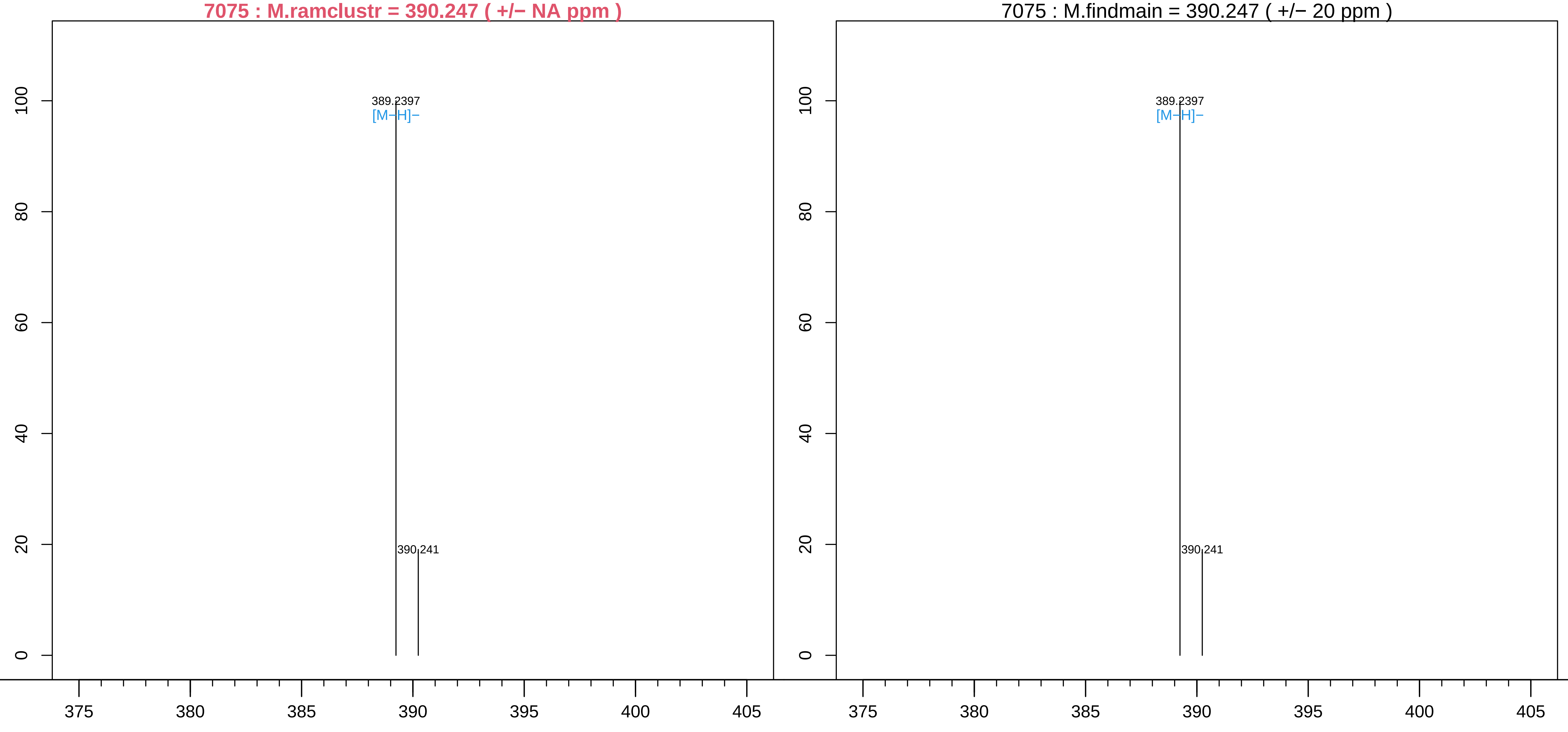

7075 : M.ramclustr = 390.247 (+/− NA ppm)
389.2397
[M−H]−
390.241
7075 : M.findmain = 390.247 (+/− 20 ppm)
389.2397
[M−H]−
390.241

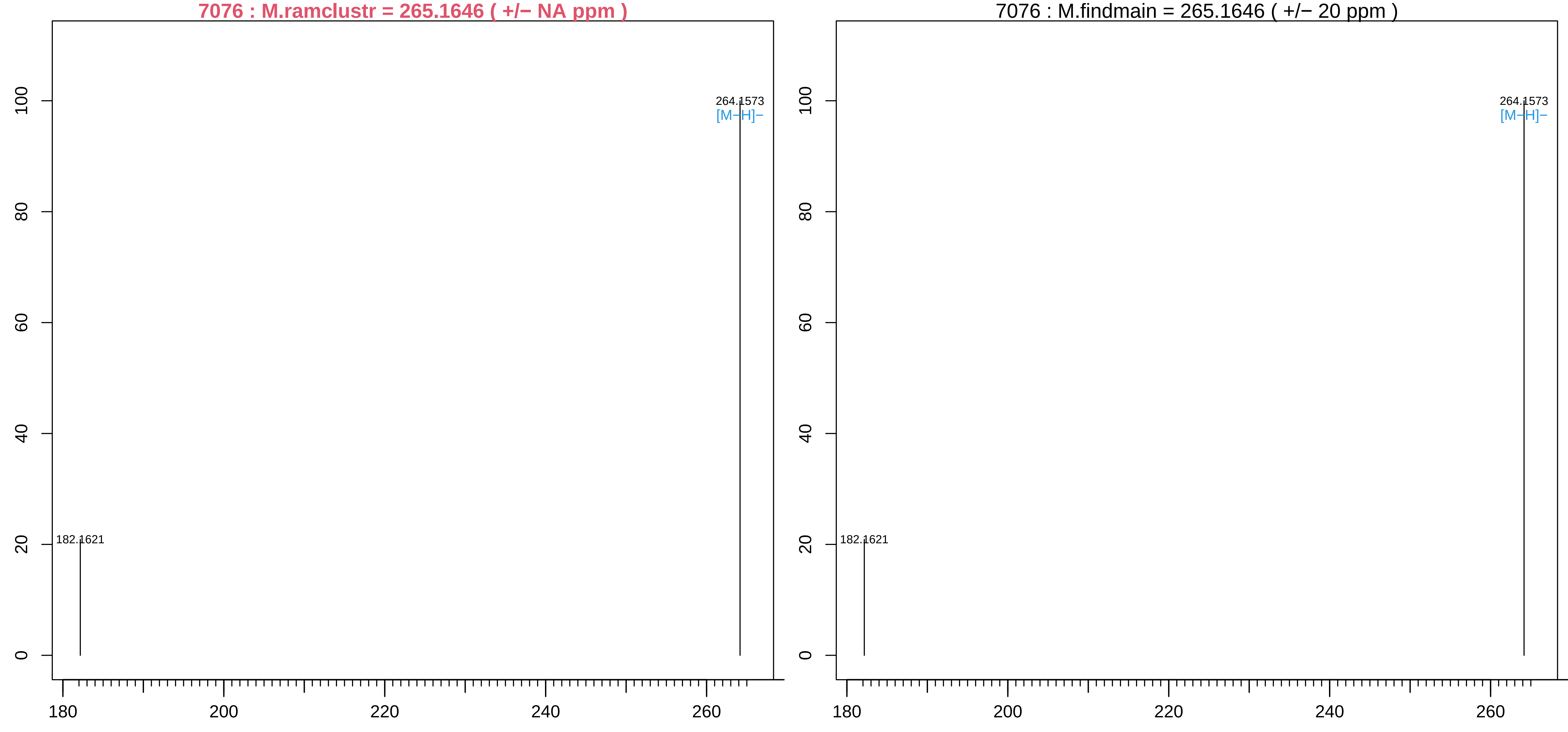

7076 : M.ramclustr = 265.1646 (+/− NA ppm)
264.1573
[M−H]−
182.1621
7076 : M.findmain = 265.1646 (+/− 20 ppm)
264.1573
[M−H]−
182.1621
0
20
40
60
80
100
180
200
220
240
260

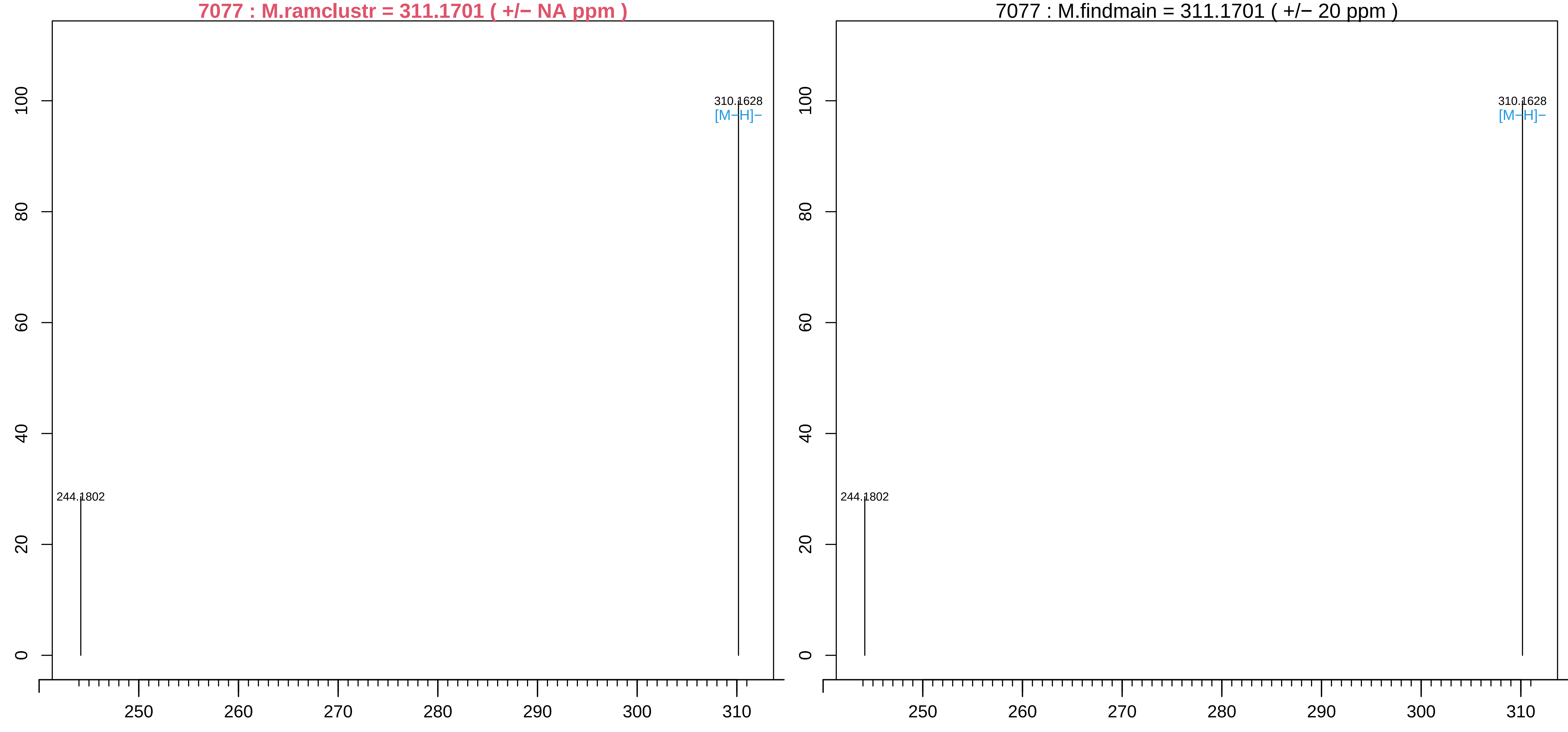

7077 : M.ramclustr = 311.1701 (+/− NA ppm)
7077 : M.findmain = 311.1701 (+/− 20 ppm)
310.1628
[M−H]−
244.1802
0
20
40
60
80
100
250
260
270
280
290
300
310

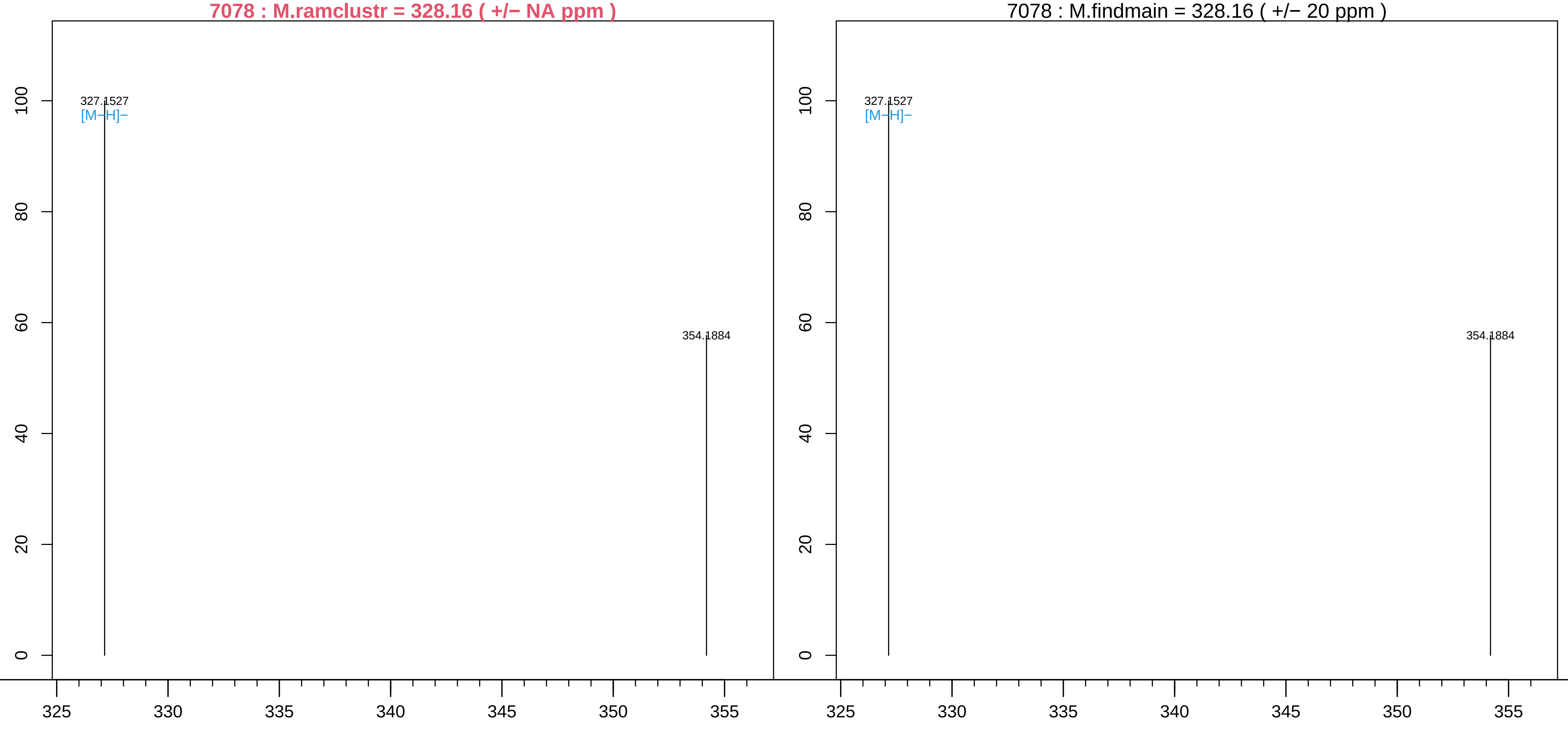
7078 : M.ramclustr = 328.16 (+/− NA ppm)
327.1527
[M−H]−
354.1884
7078 : M.findmain = 328.16 (+/− 20 ppm)
327.1527
[M−H]−
354.1884
0
20
40
60
80
100
325
330
335
340
345
350
355

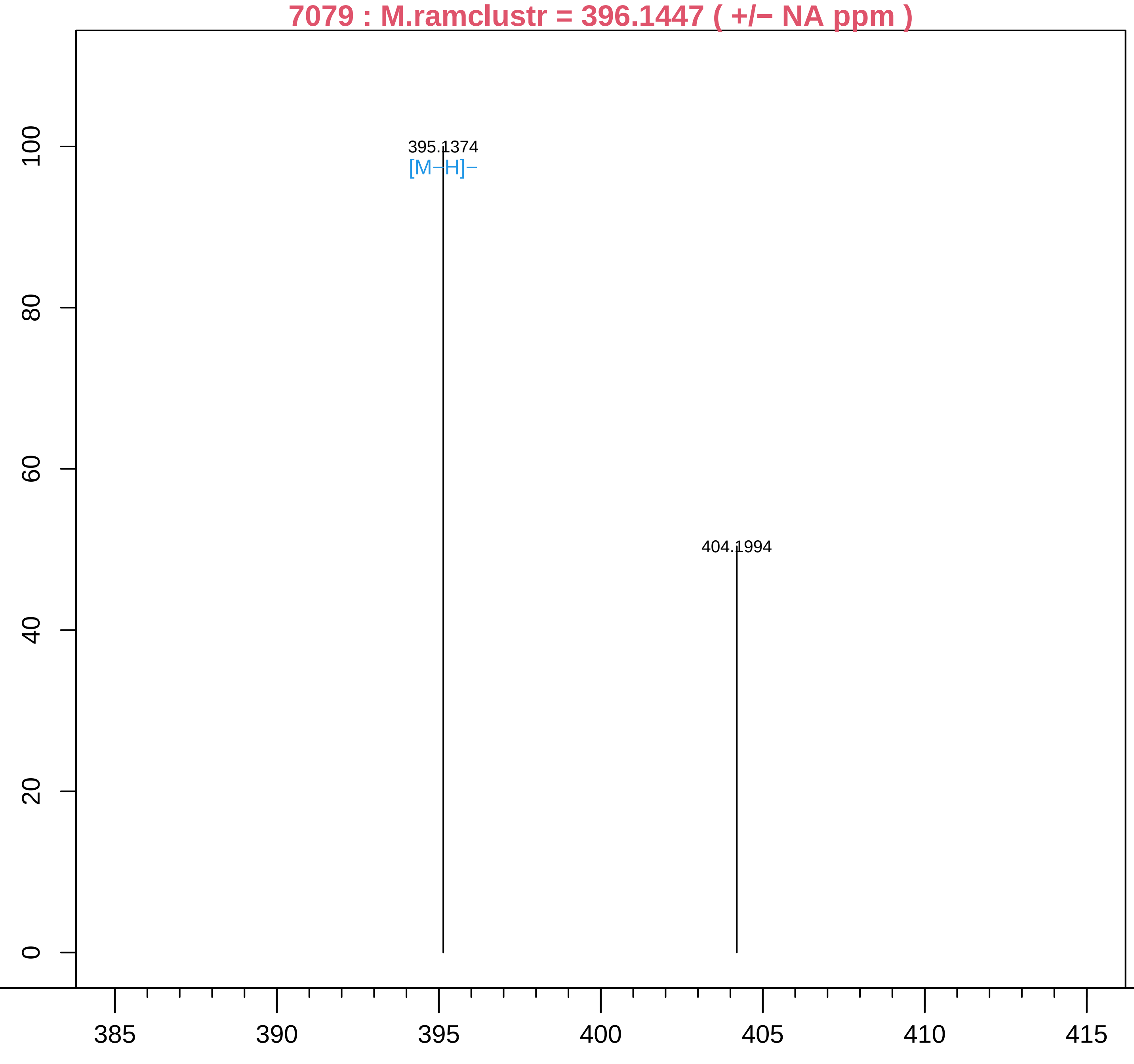

7079 : M.ramclustr = 396.1447 (+/− NA ppm)
395.1374
[M−H]−
404.1994
0
20
40
60
80
100
385
390
395
400
405
410
415

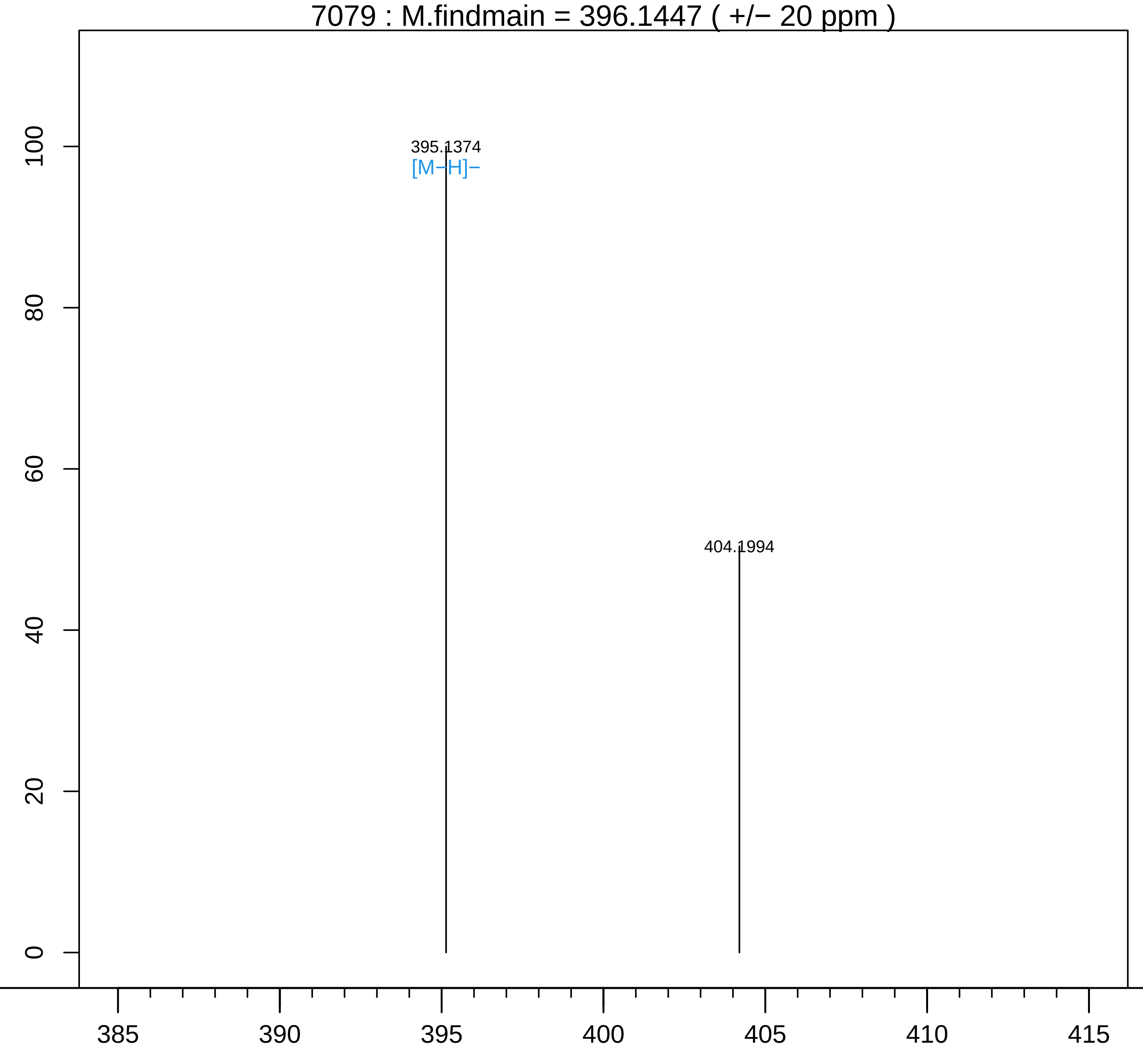

7079 : M.findmain = 396.1447 (+/− 20 ppm)
395.1374
[M−H]−
404.1994
0
20
40
60
80
100
385
390
395
400
405
410
415

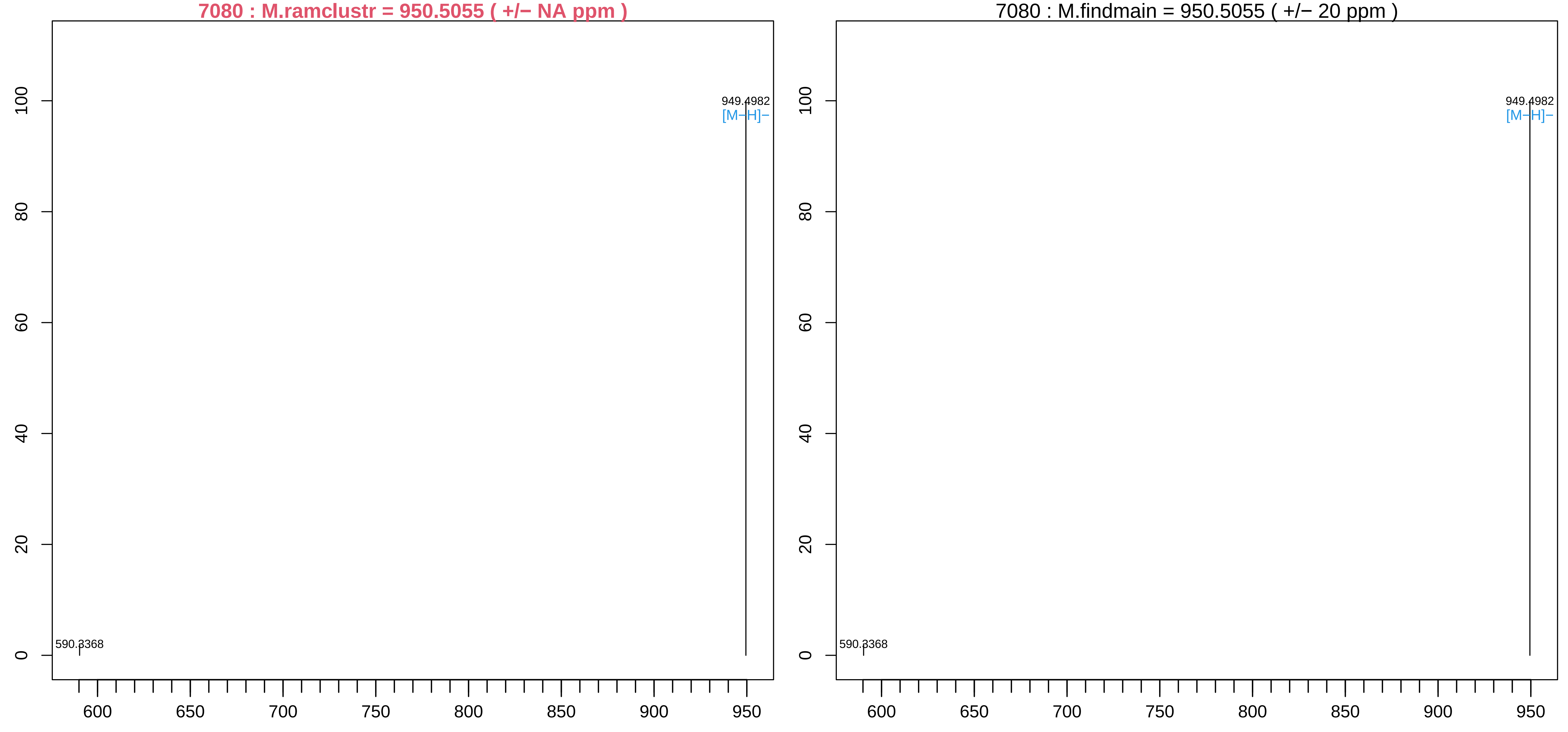

7080 : M.ramclustr = 950.5055 (+/− NA ppm)
7080 : M.findmain = 950.5055 (+/− 20 ppm)
949.4982
[M−H]−
590.3368
949.4982
[M−H]−
590.3368
0
20
40
60
80
100
600
650
700
750
800
850
900
950

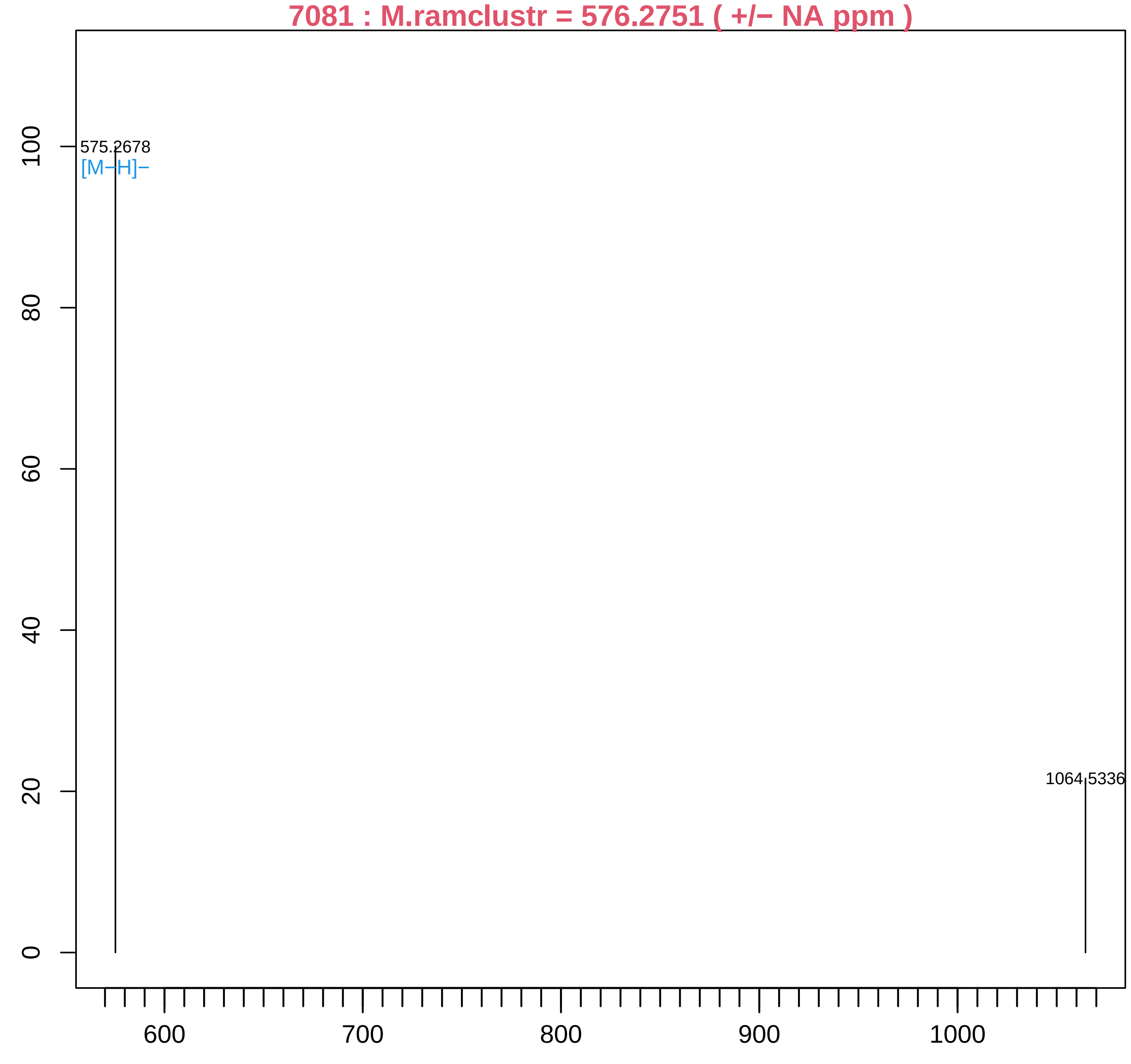

7081 : M.ramclustr = 576.2751 (+/− NA ppm)
575.2678
[M−H]−
1064.5336
0
20
40
60
80
100
600
700
800
900
1000

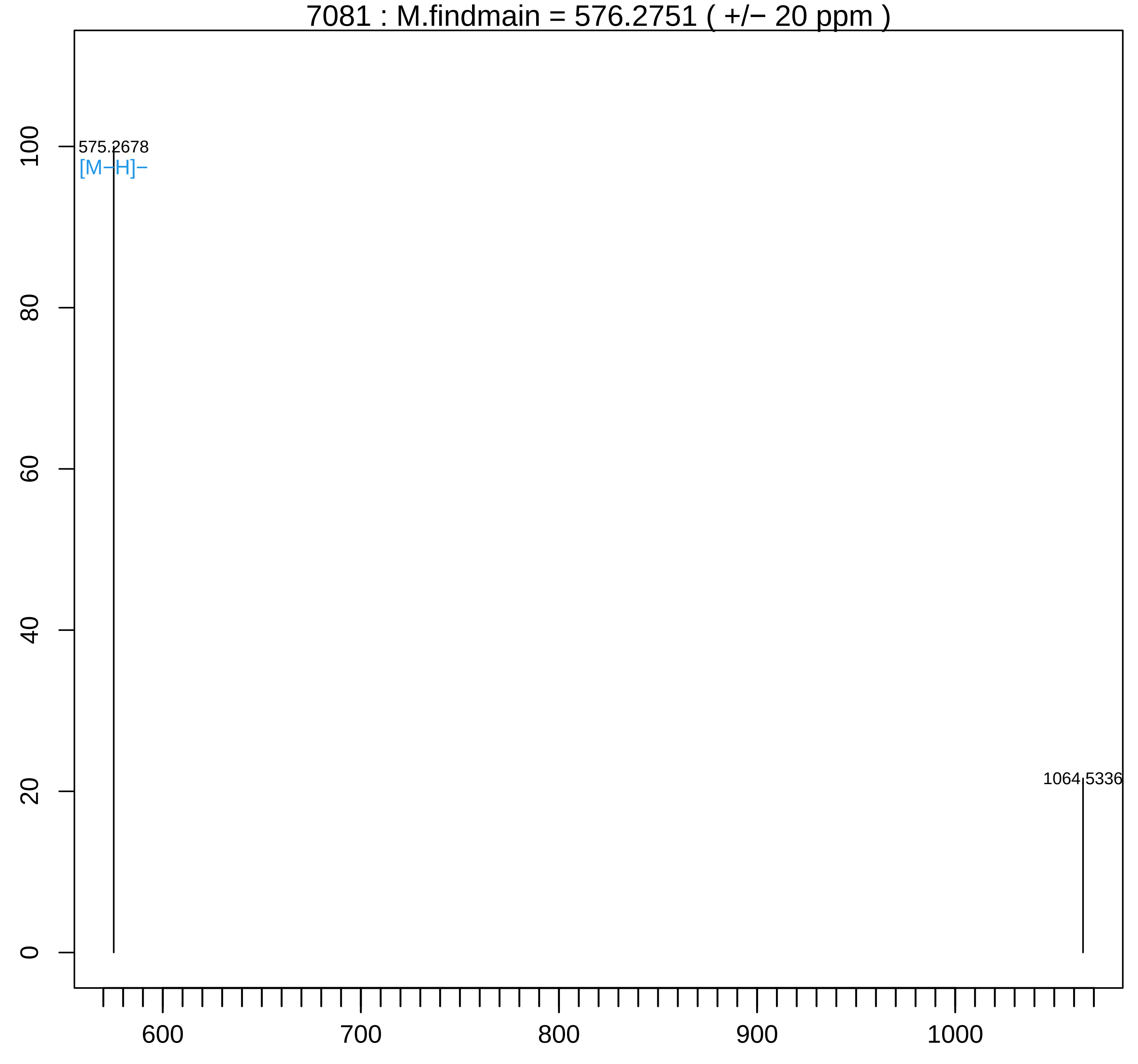

7081 : M.findmain = 576.2751 (+/− 20 ppm)
575.2678
[M−H]−
1064.5336
0
20
40
60
80
100
600
700
800
900
1000

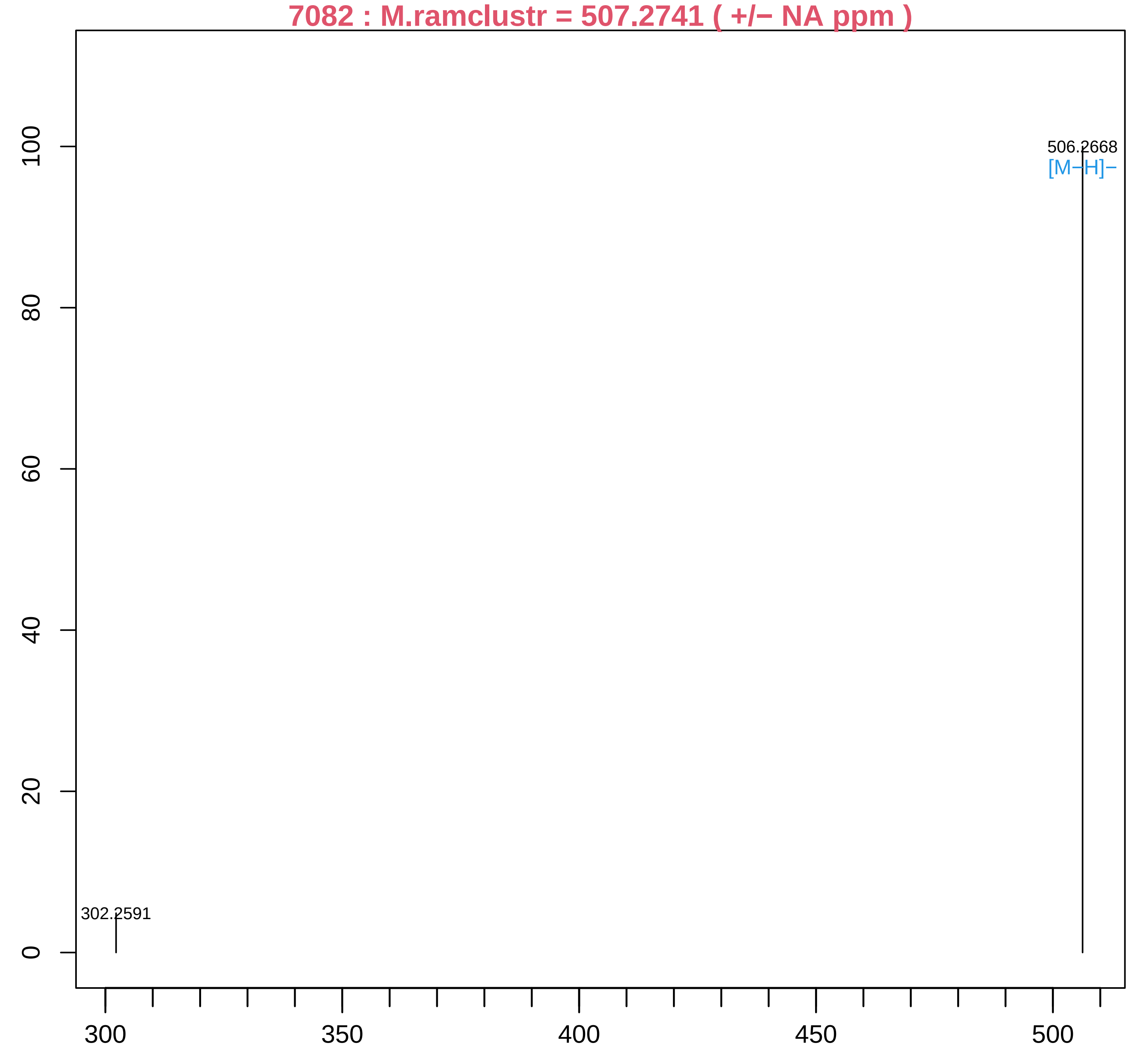
7082 : M.ramclustr = 507.2741 (+/− NA ppm)
506.2668
[M−H]−
302.2591
0
20
40
60
80
100
300
350
400
450
500

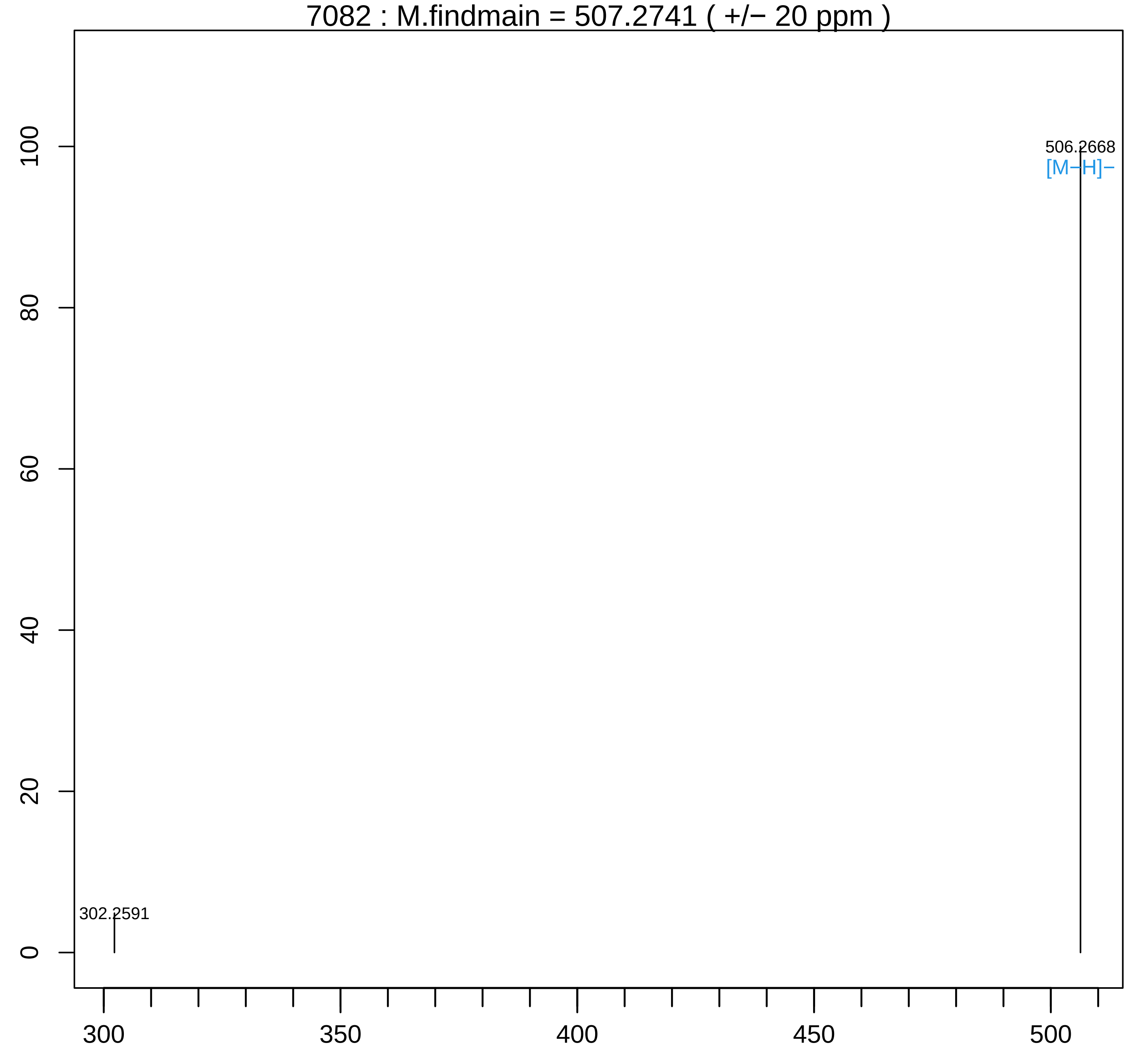
7082 : M.findmain = 507.2741 (+/− 20 ppm)
506.2668
[M−H]−
302.2591
0
20
40
60
80
100
300
350
400
450
500

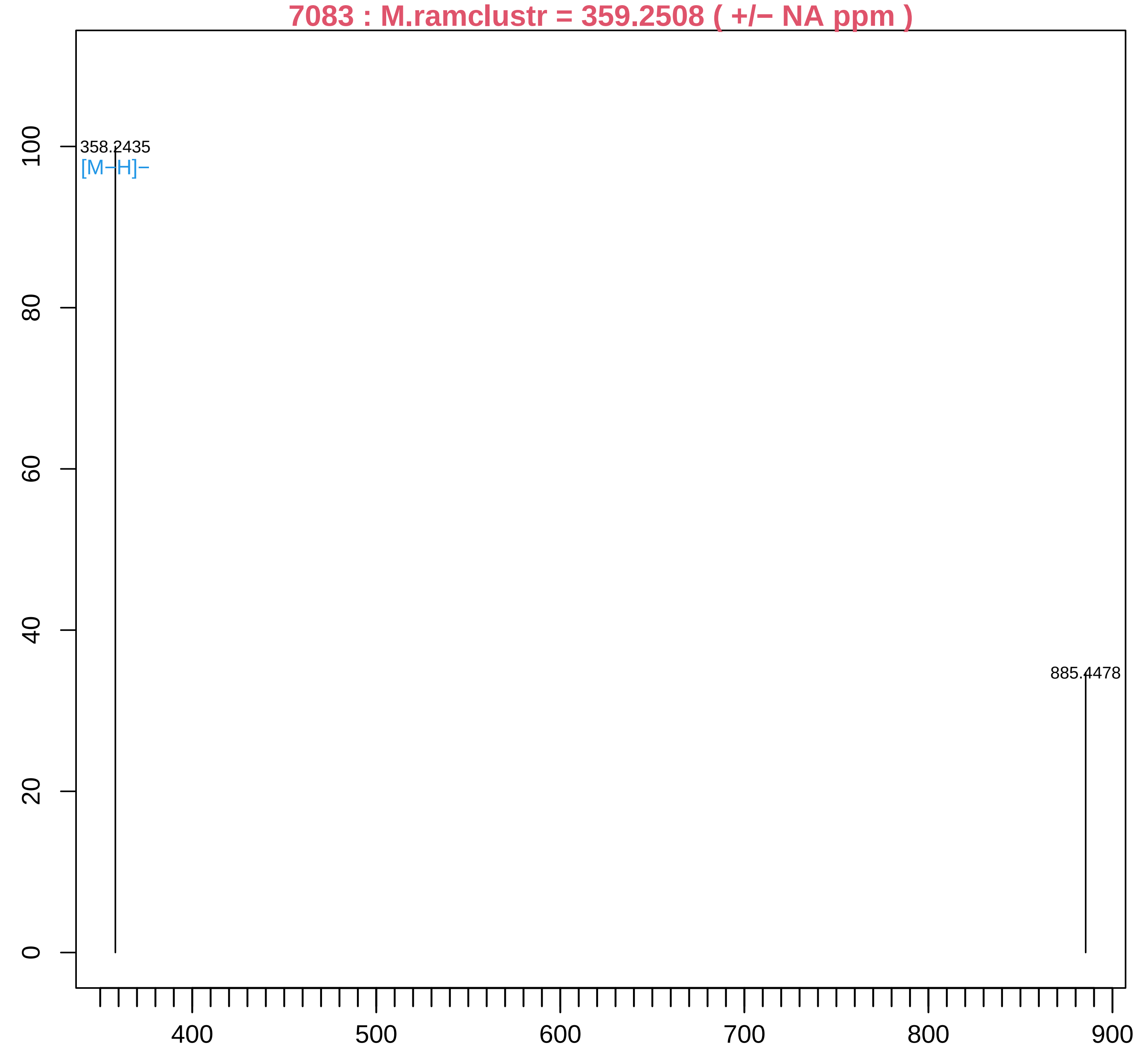
7083 : M.ramclustr = 359.2508 (+/− NA ppm)
358.2435
[M−H]−
885.4478
0
20
40
60
80
100
400
500
600
700
800
900

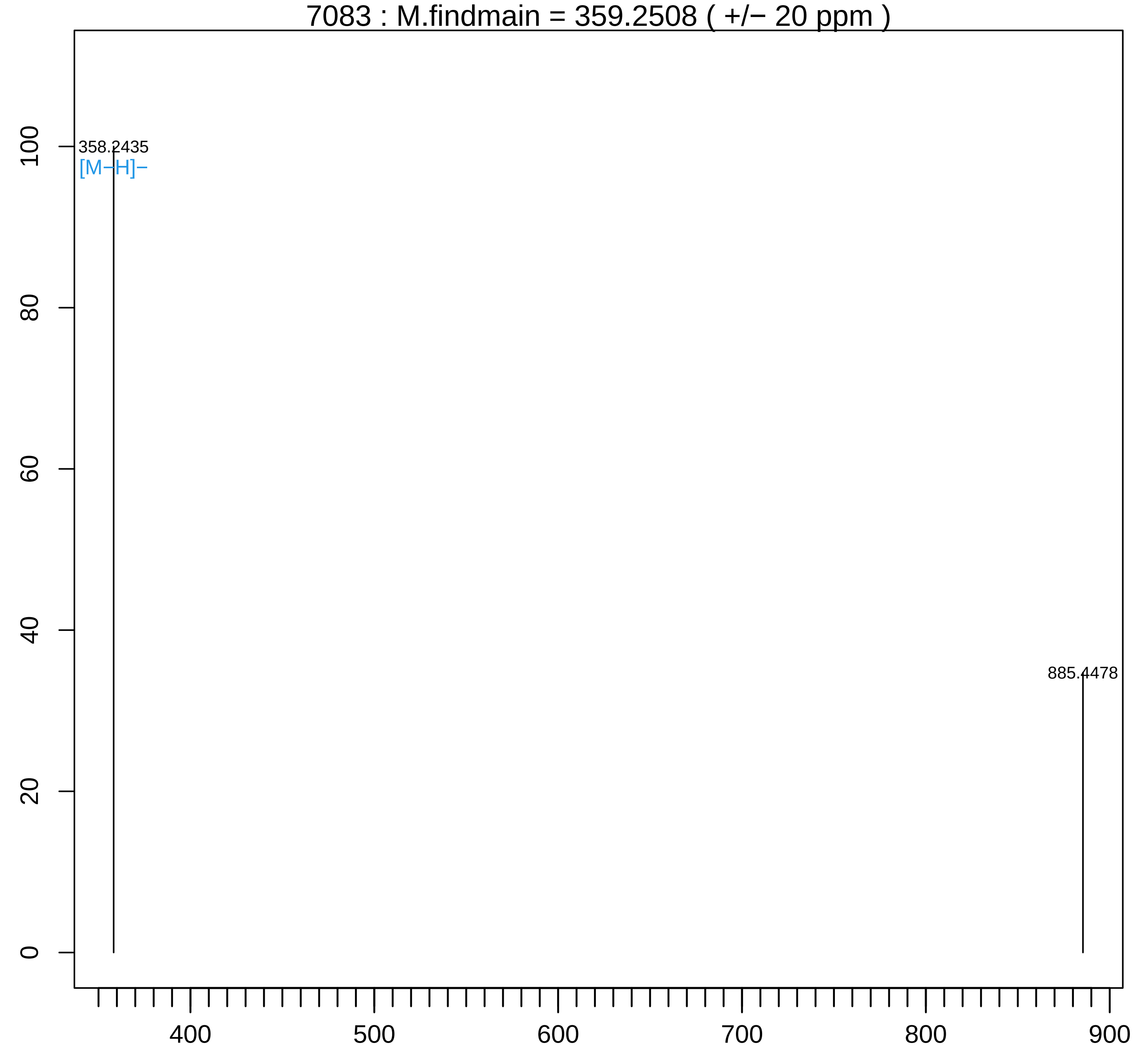
7083 : M.findmain = 359.2508 (+/− 20 ppm)
358.2435
[M−H]−
885.4478
0
20
40
60
80
100
400
500
600
700
800
900

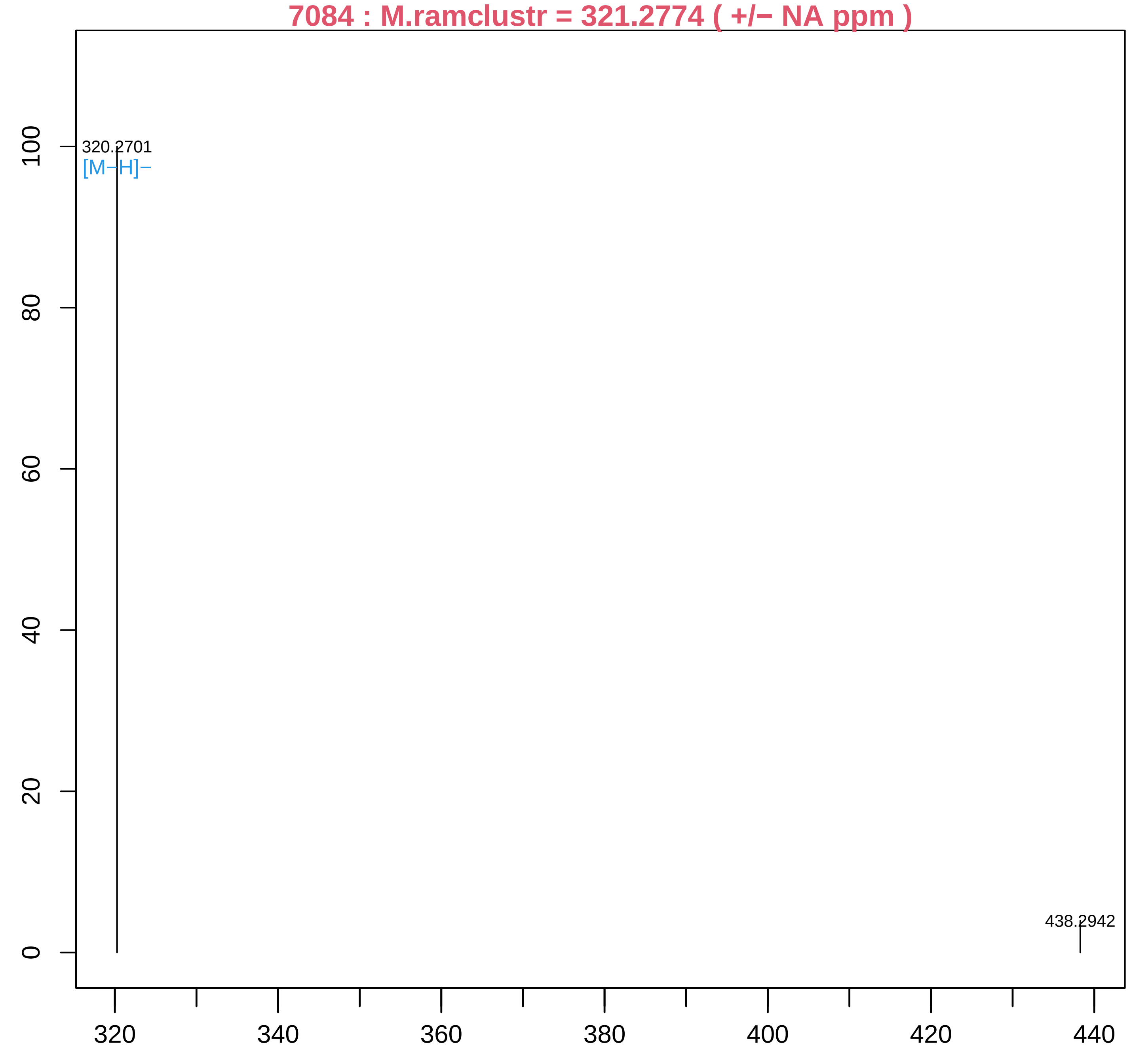
7084 : M.ramclustr = 321.2774 (+/− NA ppm)
320.2701
[M−H]−
438.2942
0
20
40
60
80
100
320
340
360
380
400
420
440

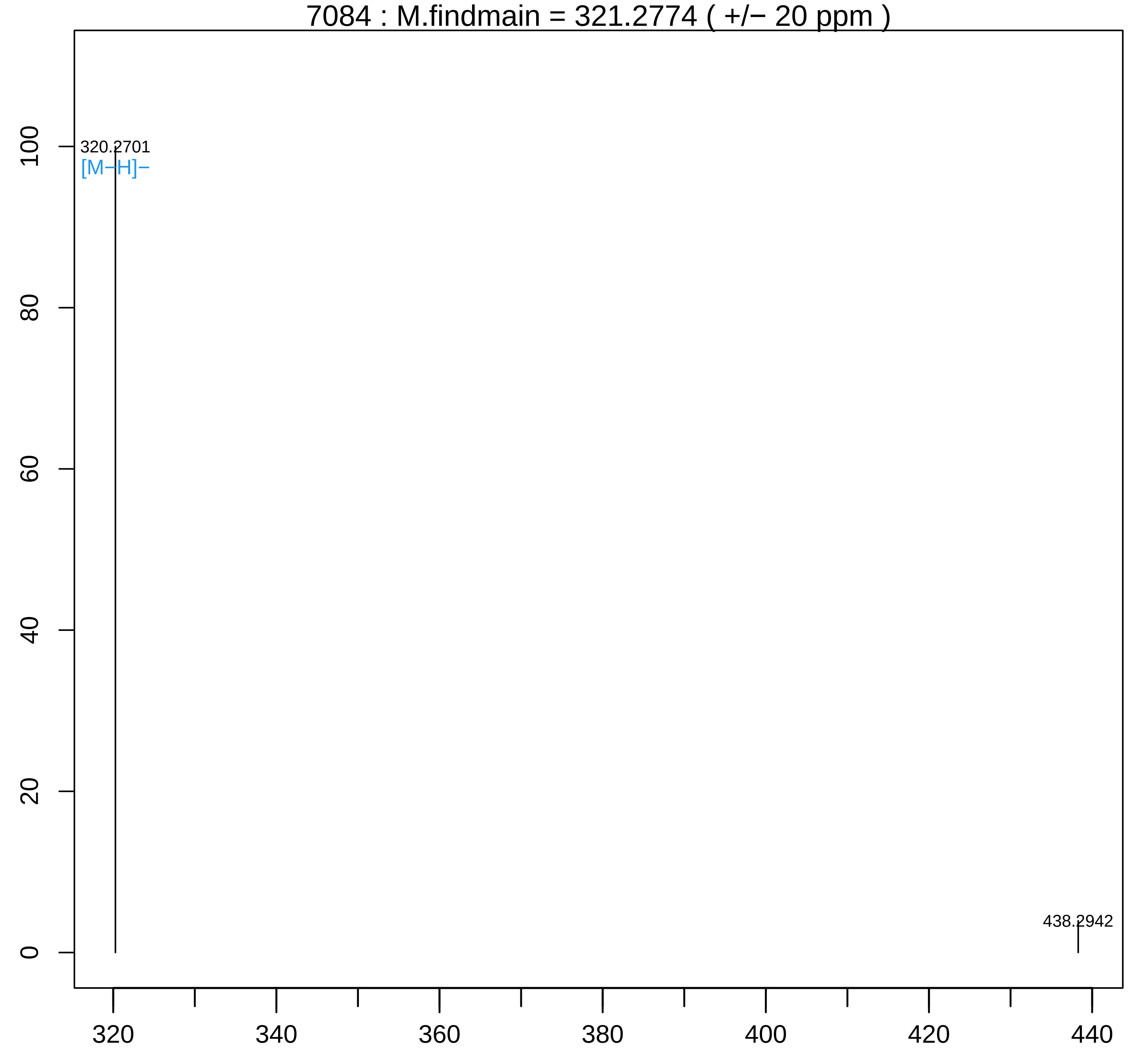
7084 : M.findmain = 321.2774 (+/− 20 ppm)
320.2701
[M−H]−
438.2942
0
20
40
60
80
100
320
340
360
380
400
420
440

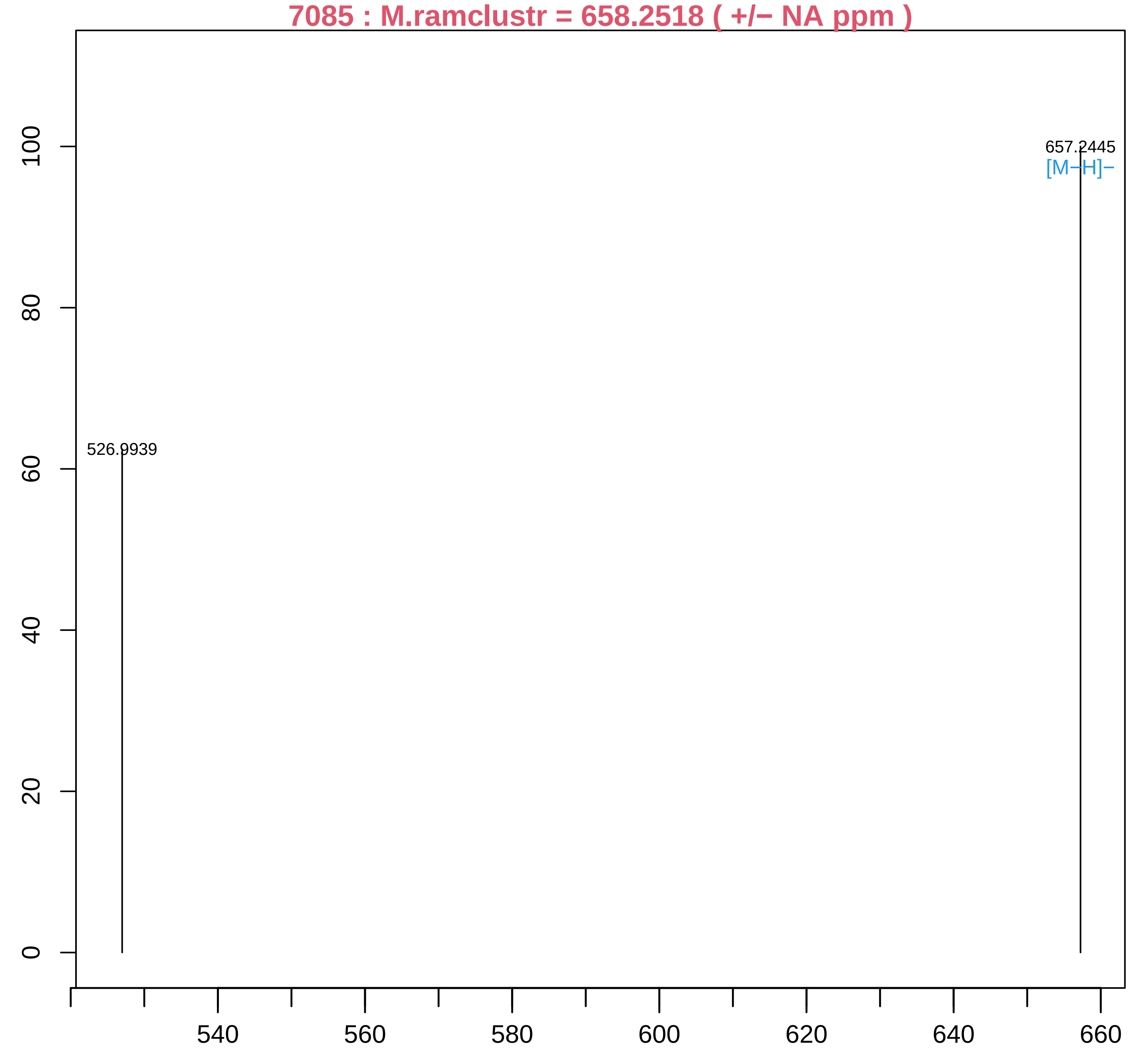
7085 : M.ramclustr = 658.2518 (+/− NA ppm)
657.2445
[M−H]−
526.9939
0
20
40
60
80
100
540
560
580
600
620
640
660
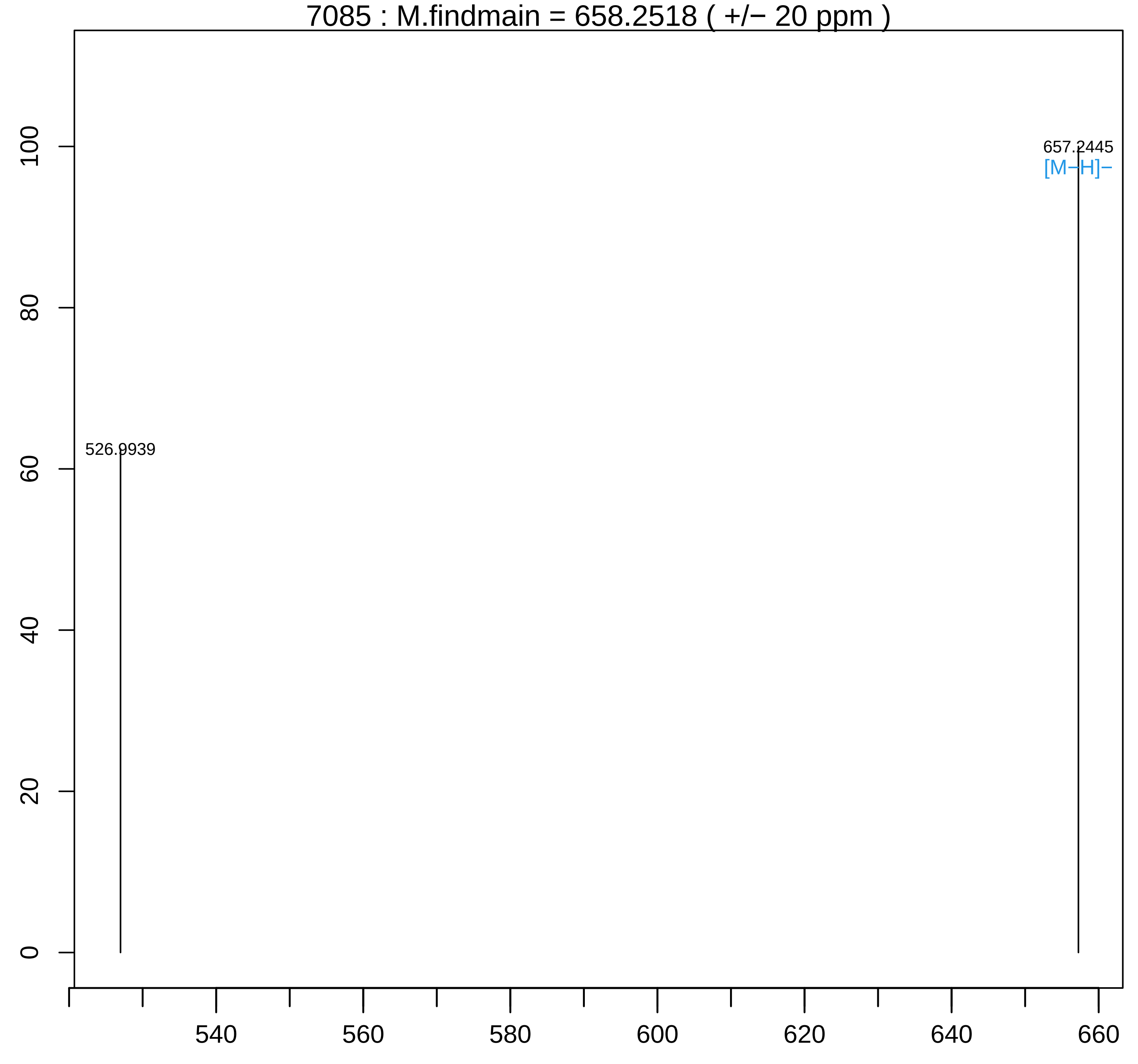
7085 : M.findmain = 658.2518 (+/− 20 ppm)
657.2445
[M−H]−
526.9939
0
20
40
60
80
100
540
560
580
600
620
640
660

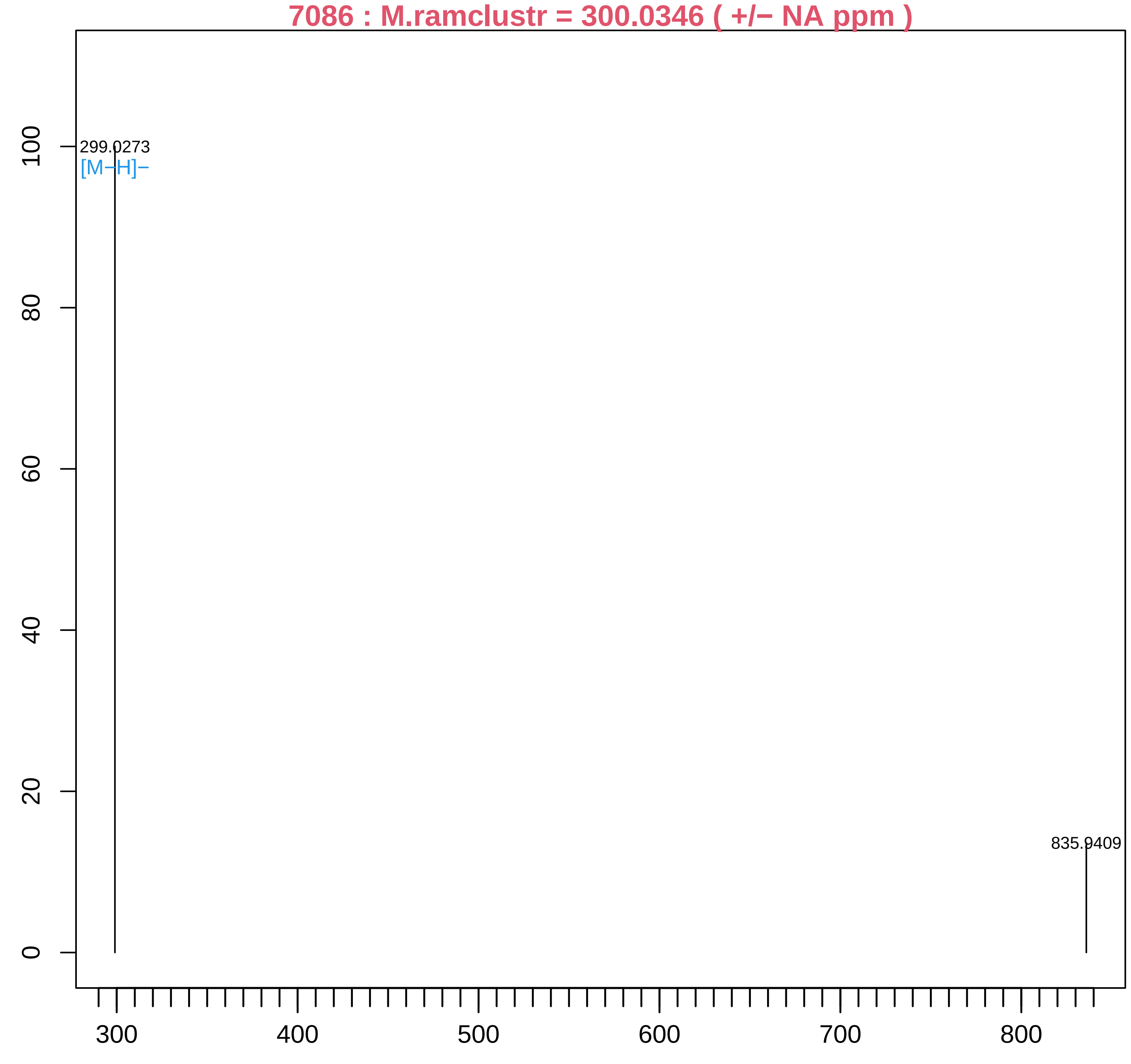
7086 : M.ramclustr = 300.0346 (+/− NA ppm)
299.0273
[M−H]−
835.9409
0
20
40
60
80
100
300
400
500
600
700
800

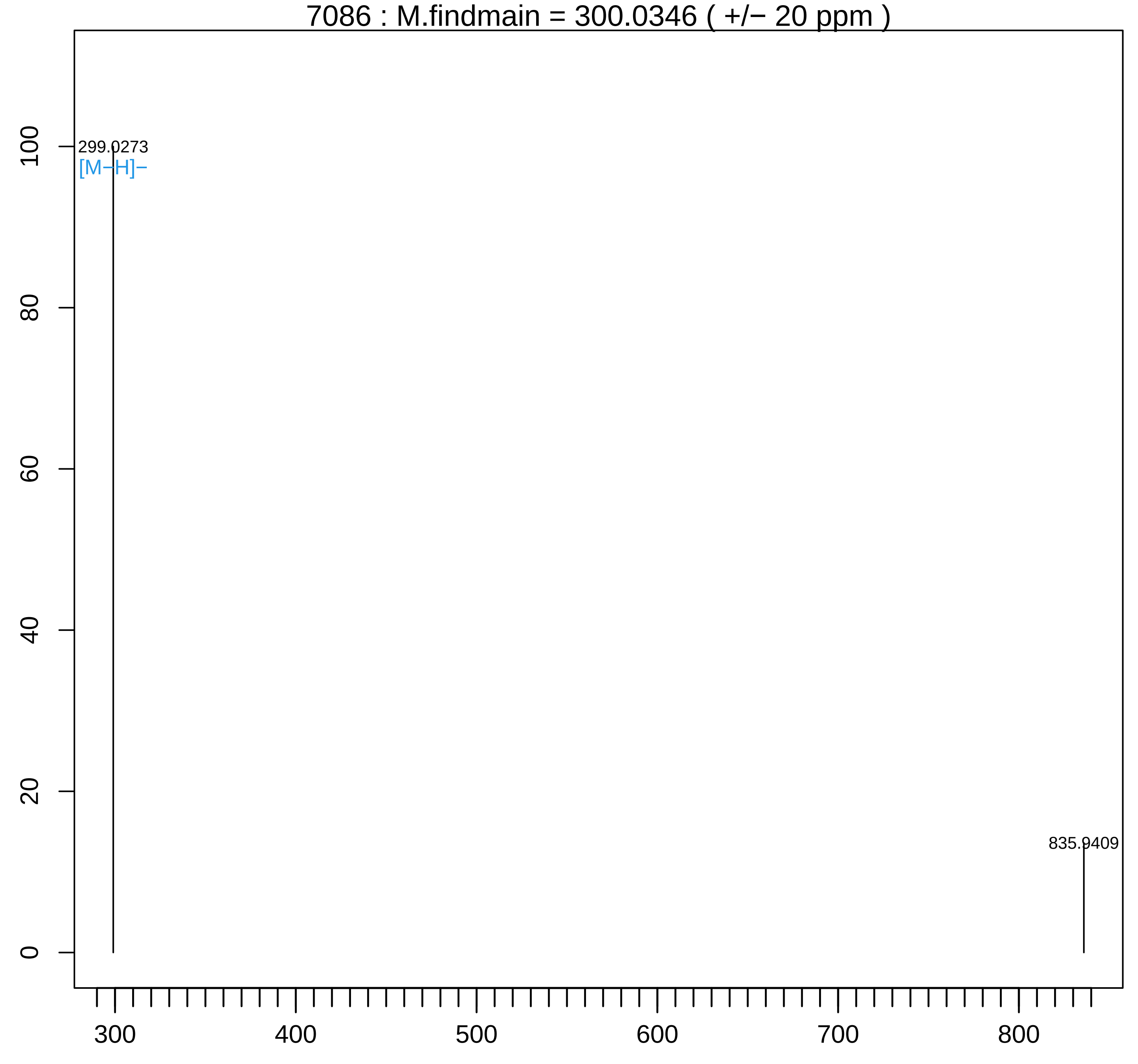
7086 : M.findmain = 300.0346 (+/− 20 ppm)
299.0273
[M−H]−
835.9409
0
20
40
60
80
100
300
400
500
600
700
800

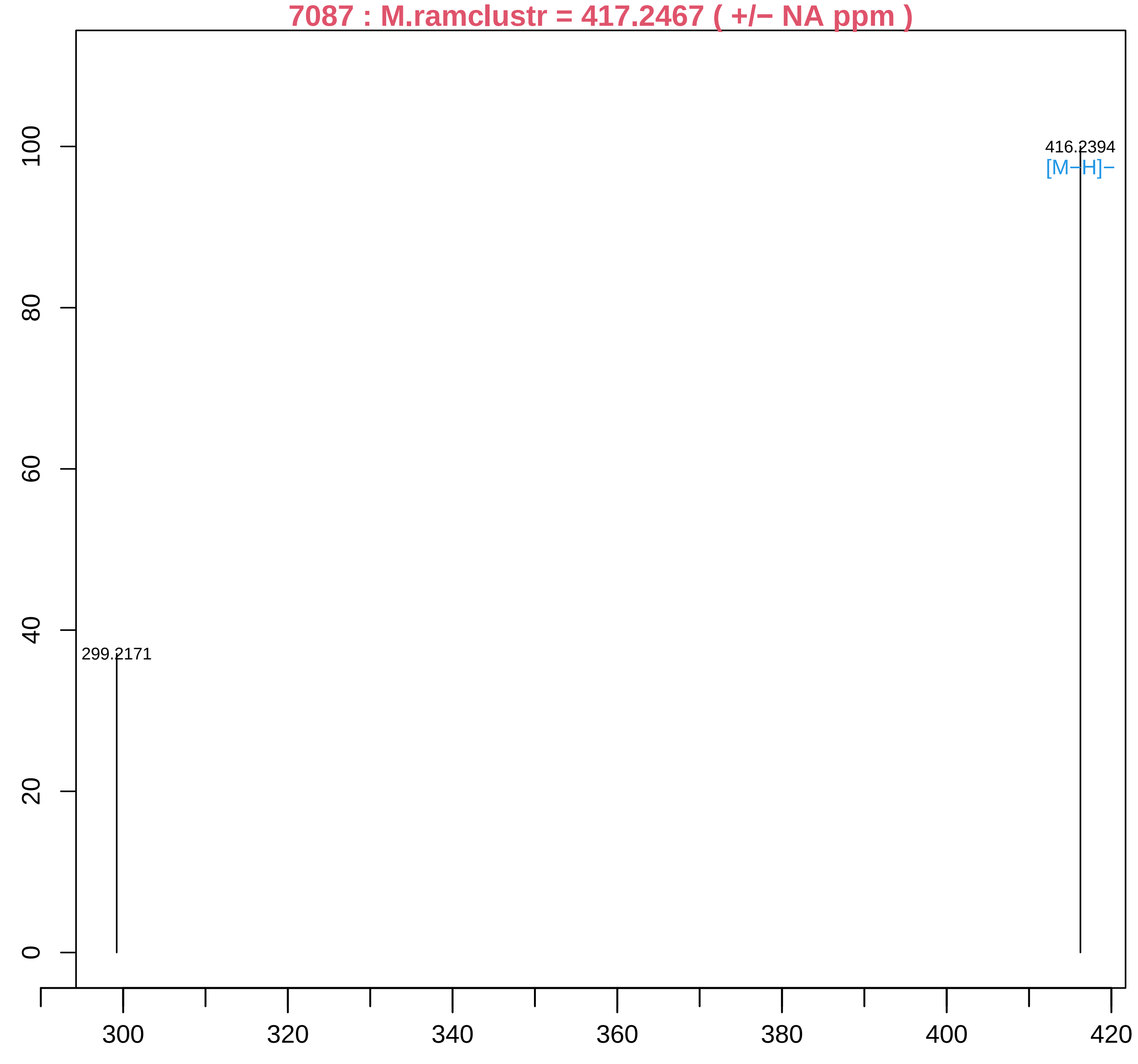
7087 : M.ramclustr = 417.2467 (+/− NA ppm)
416.2394
[M−H]−
299.2171
0
20
40
60
80
100
300
320
340
360
380
400
420

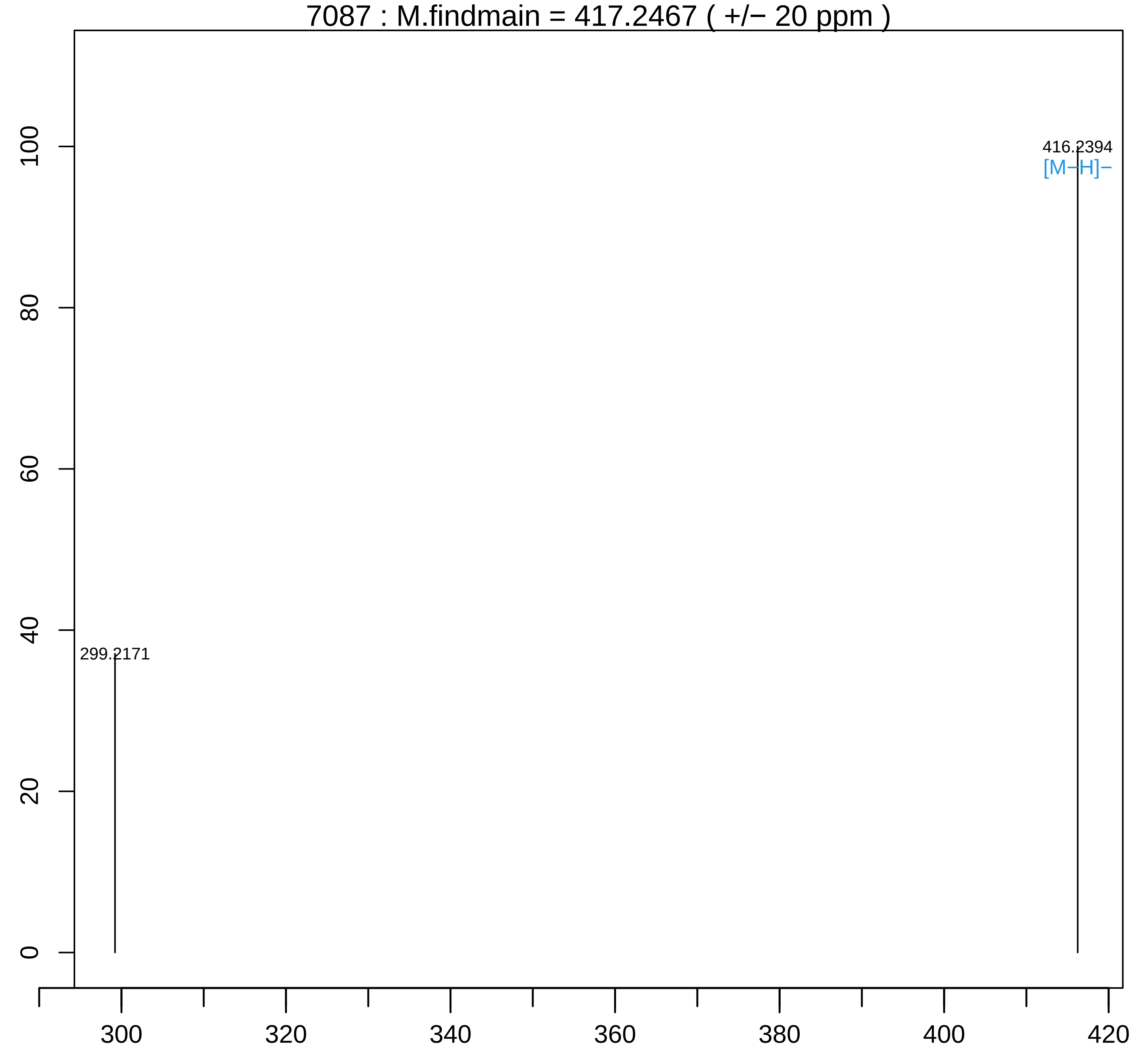
7087 : M.findmain = 417.2467 (+/− 20 ppm)
416.2394
[M−H]−
299.2171
0
20
40
60
80
100
300
320
340
360
380
400
420

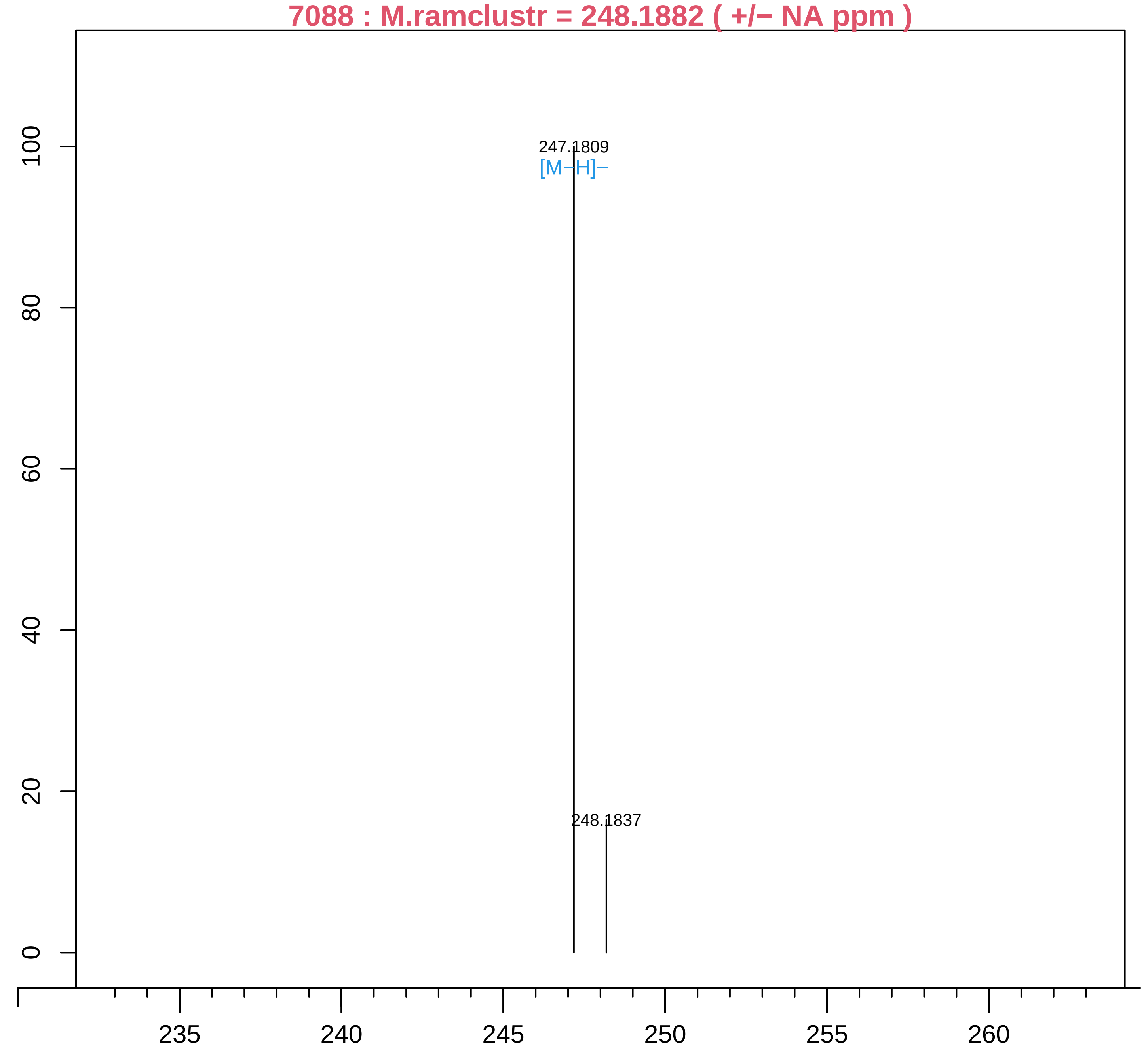
7088 : M.ramclustr = 248.1882 (+/− NA ppm)
247.1809
[M−H]−
248.1837
0
20
40
60
80
100
235
240
245
250
255
260
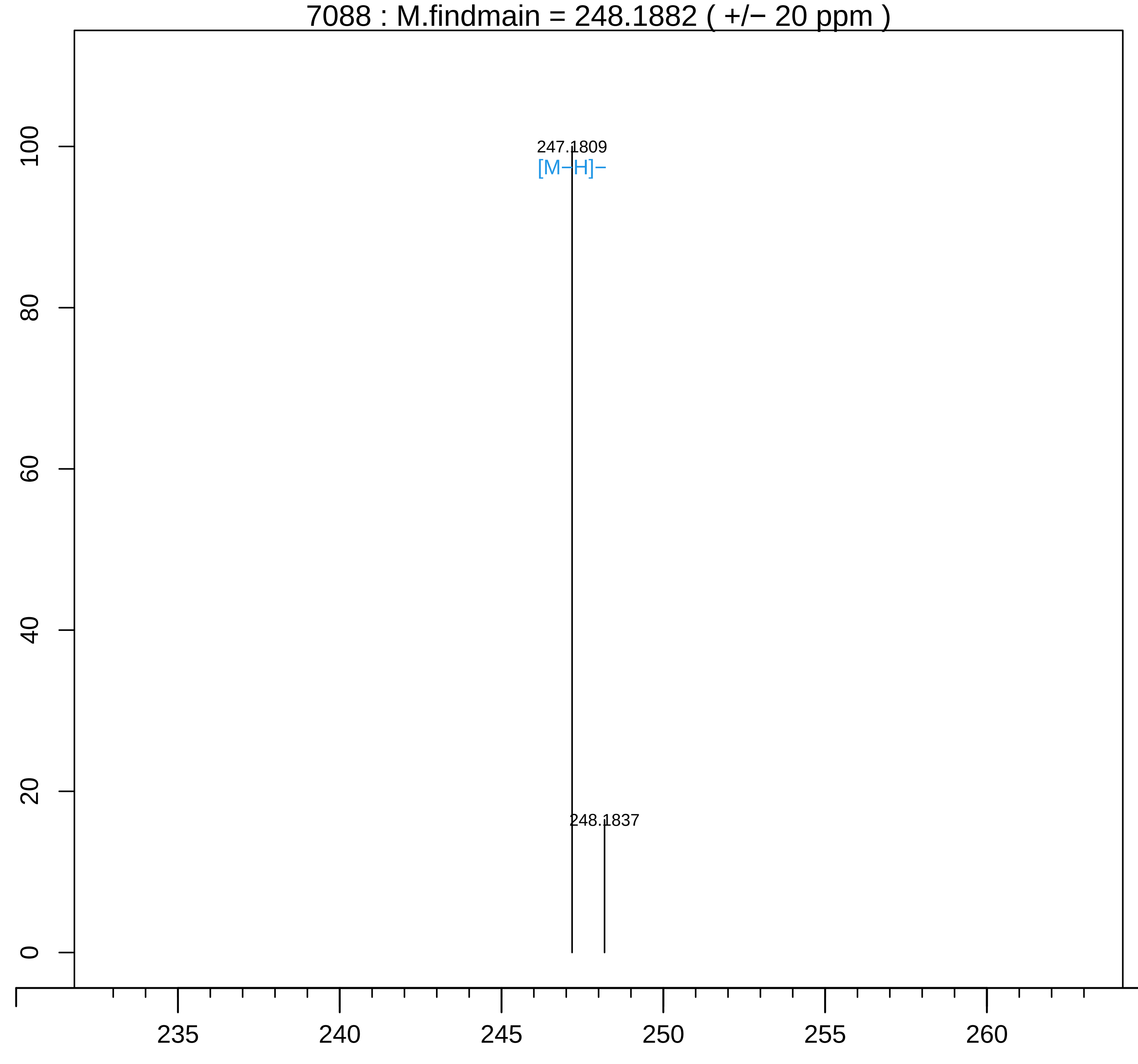
7088 : M.findmain = 248.1882 (+/− 20 ppm)
247.1809
[M−H]−
248.1837
0
20
40
60
80
100
235
240
245
250
255
260

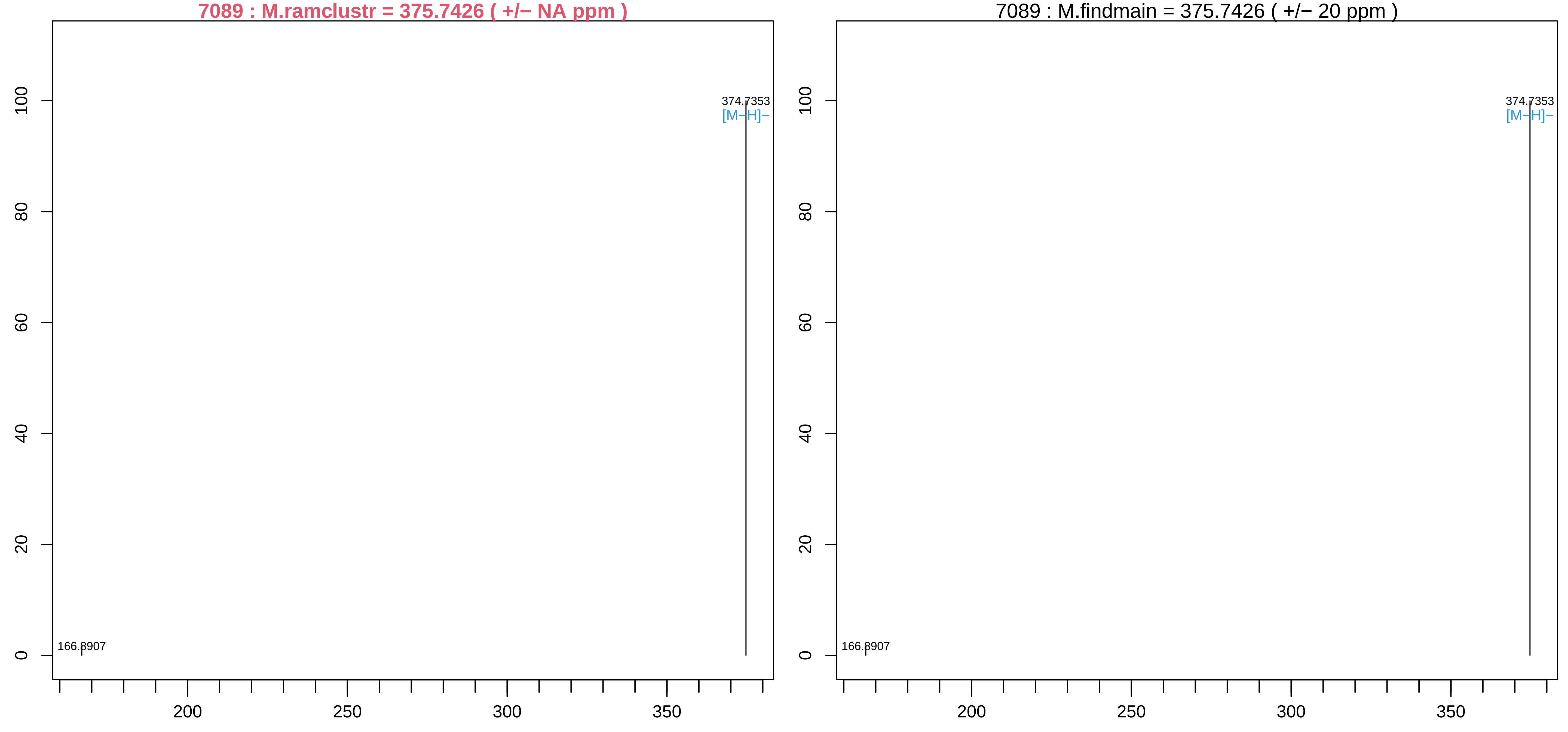
7089 : M.ramclustr = 375.7426 (+/− NA ppm)
374.7353
[M−H]−
166.8907
7089 : M.findmain = 375.7426 (+/− 20 ppm)
374.7353
[M−H]−
166.8907
0
20
40
60
80
100
200
250
300
350

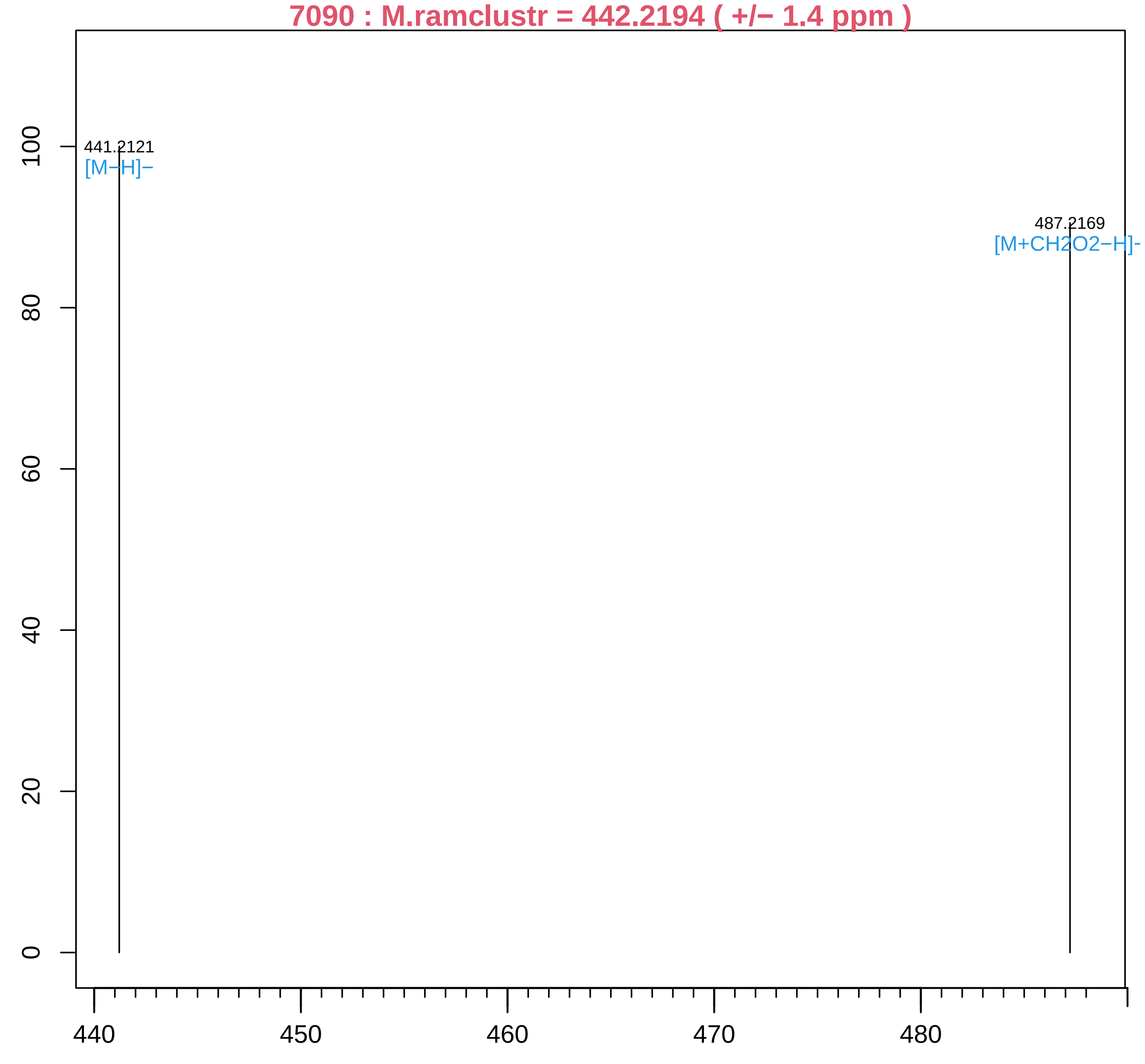
7090 : M.ramclustr = 442.2194 (+/− 1.4 ppm)
441.2121
[M−H]−
487.2169
[M+CH2O2−H]-
0
20
40
60
80
100
440
450
460
470
480

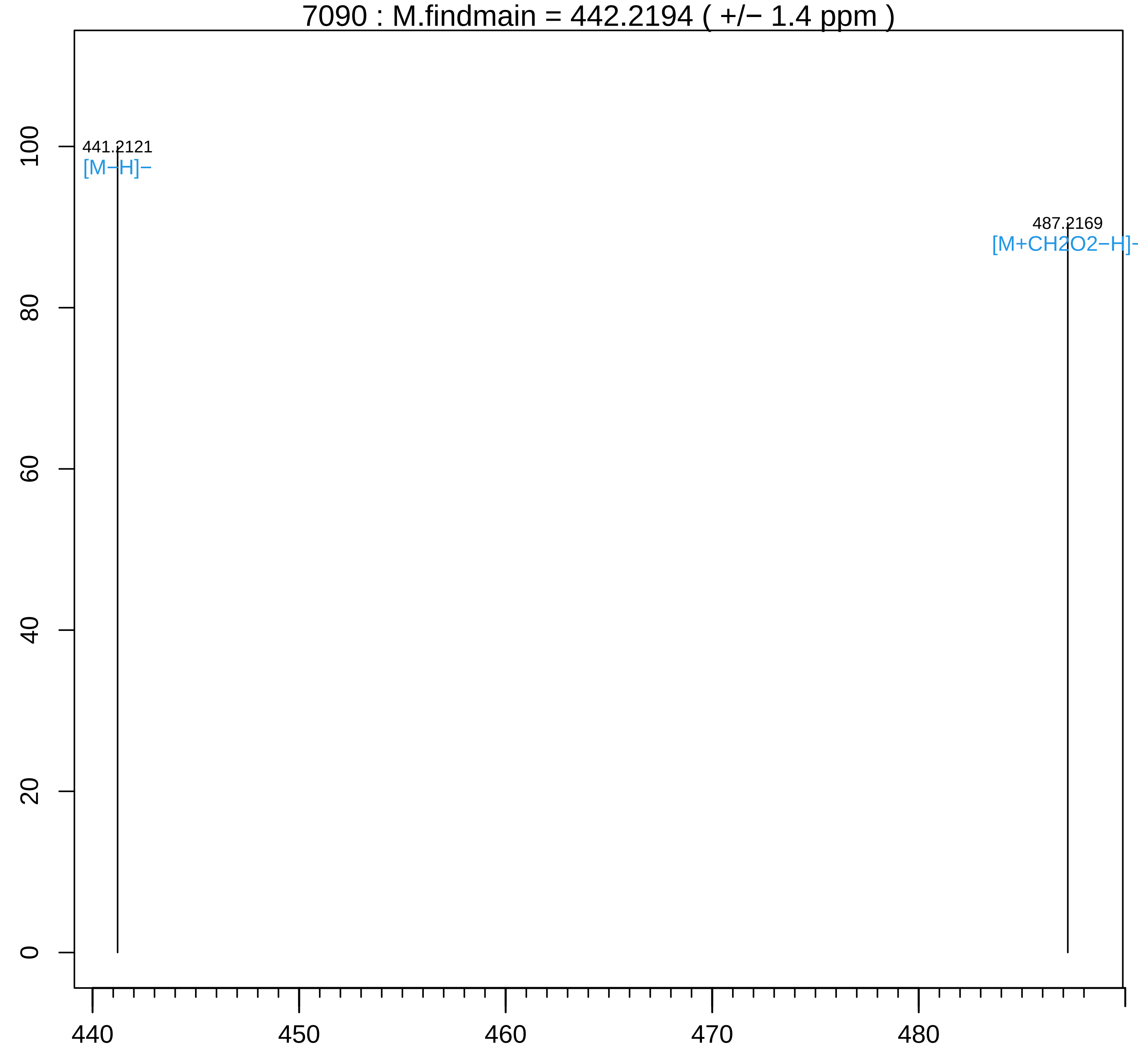
7090 : M.findmain = 442.2194 (+/− 1.4 ppm)
441.2121
[M−H]−
487.2169
[M+CH2O2−H]-
0
20
40
60
80
100
440
450
460
470
480

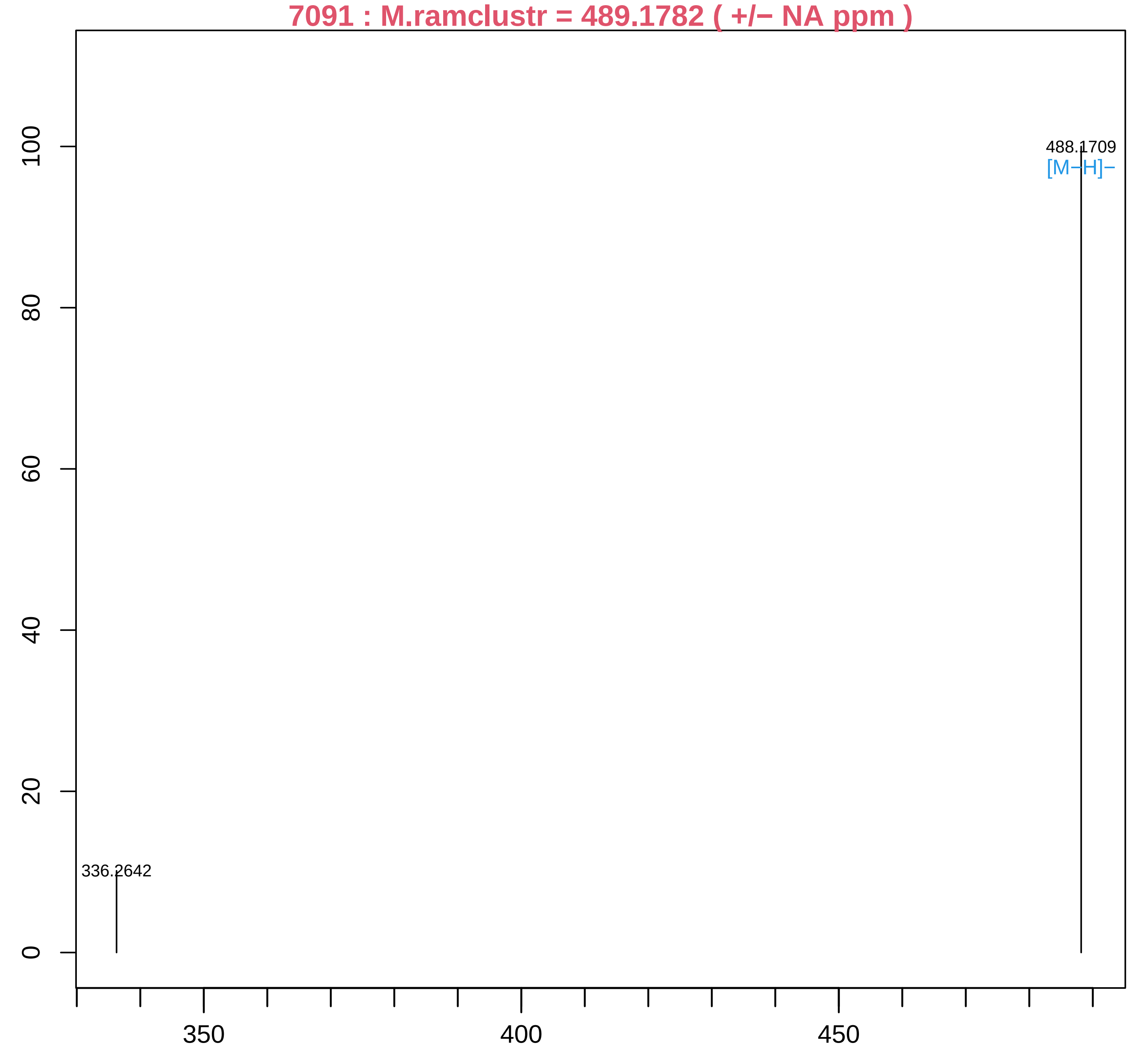

7091 : M.ramclustr = 489.1782 (+/− NA ppm)
488.1709
[M−H]−
336.2642
0
20
40
60
80
100
350
400
450

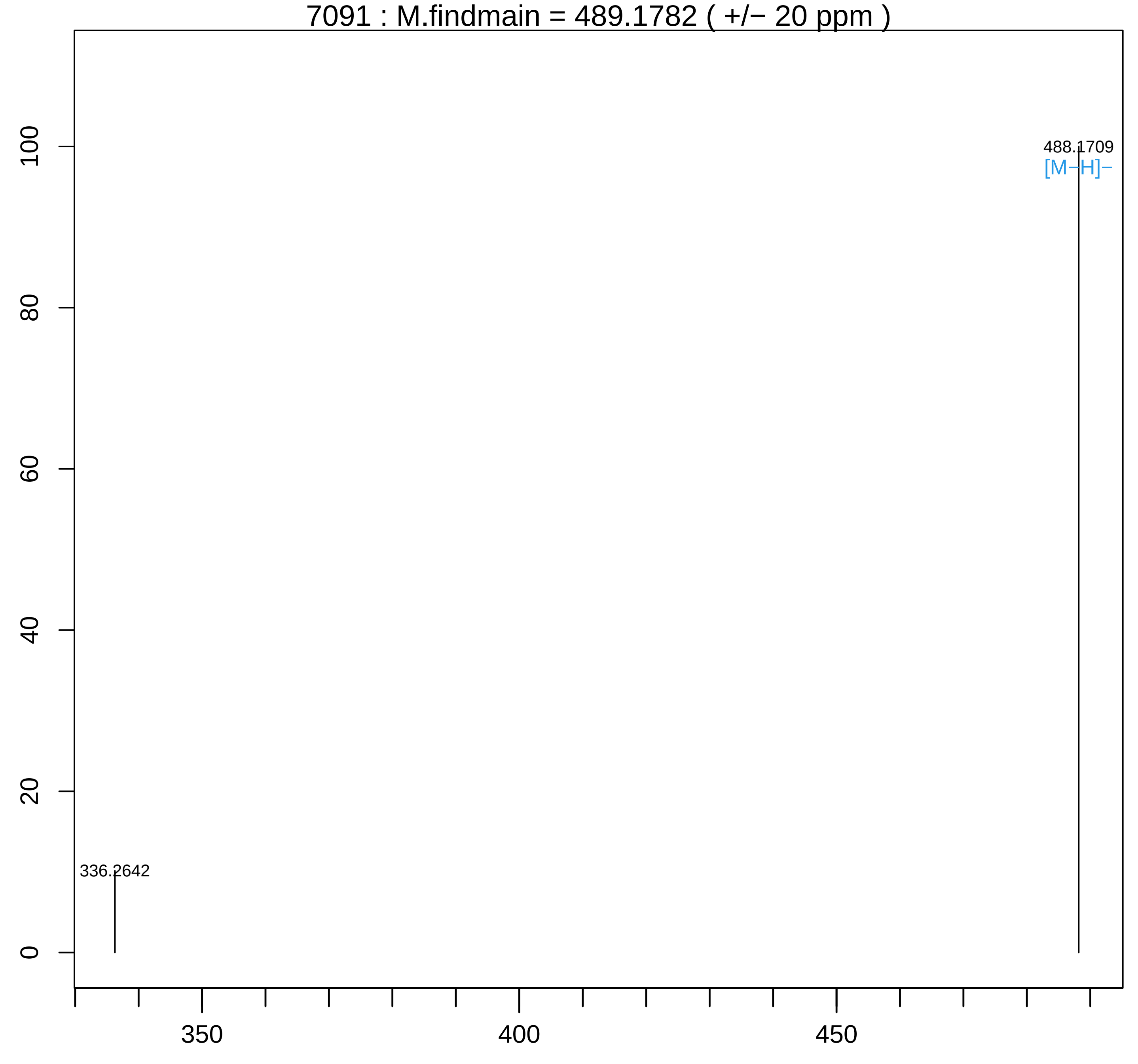

7091 : M.findmain = 489.1782 (+/− 20 ppm)
488.1709
[M−H]−
336.2642
0
20
40
60
80
100
350
400
450

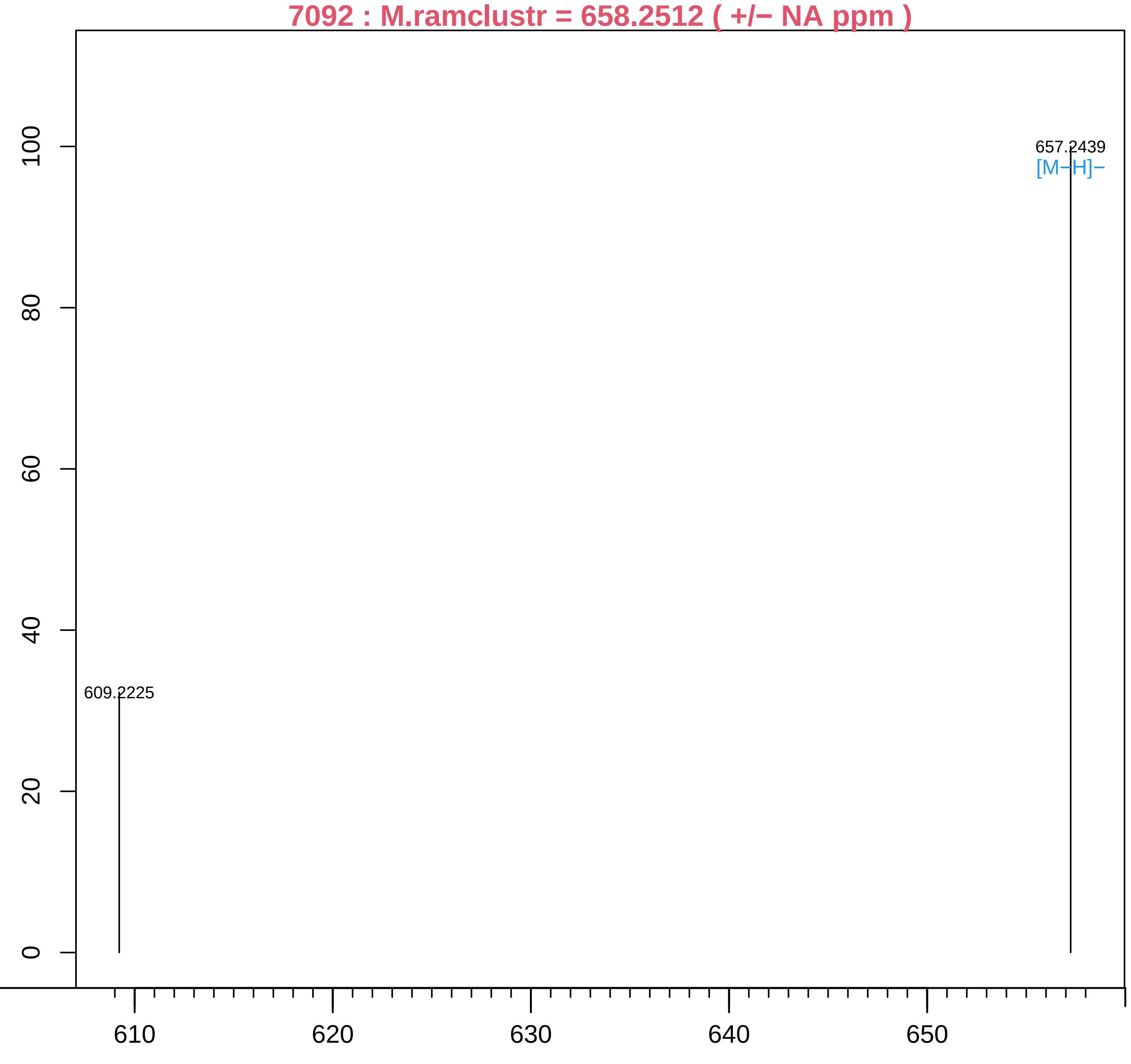
7092 : M.ramclustr = 658.2512 (+/− NA ppm)
657.2439
[M−H]−
609.2225
0
20
40
60
80
100
610
620
630
640
650

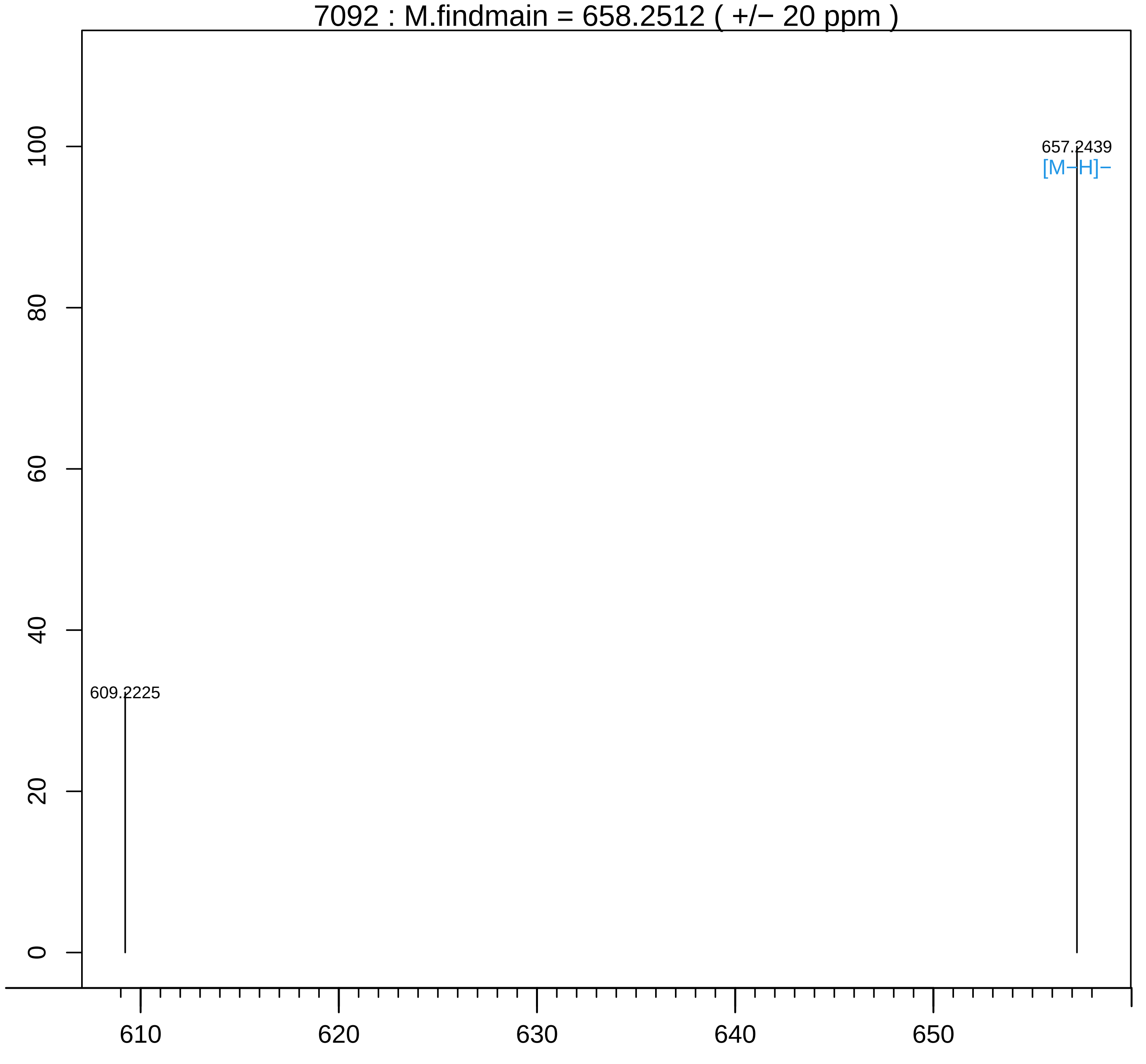
7092 : M.findmain = 658.2512 (+/− 20 ppm)
657.2439
[M−H]−
609.2225
0
20
40
60
80
100
610
620
630
640
650

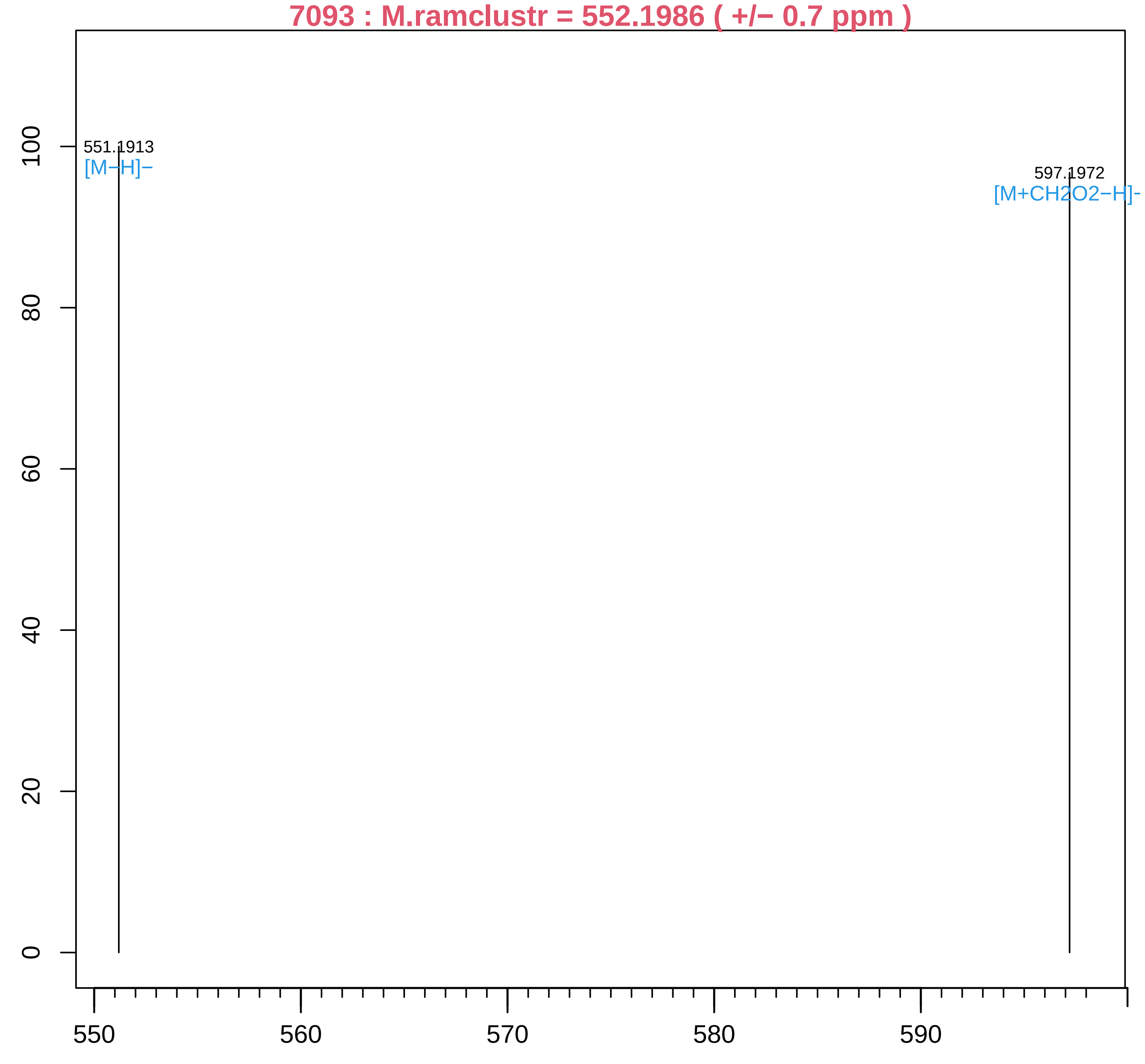
7093 : M.ramclustr = 552.1986 (+/− 0.7 ppm)
551.1913
[M−H]−
597.1972
[M+CH2O2−H]-
0
20
40
60
80
100
550
560
570
580
590

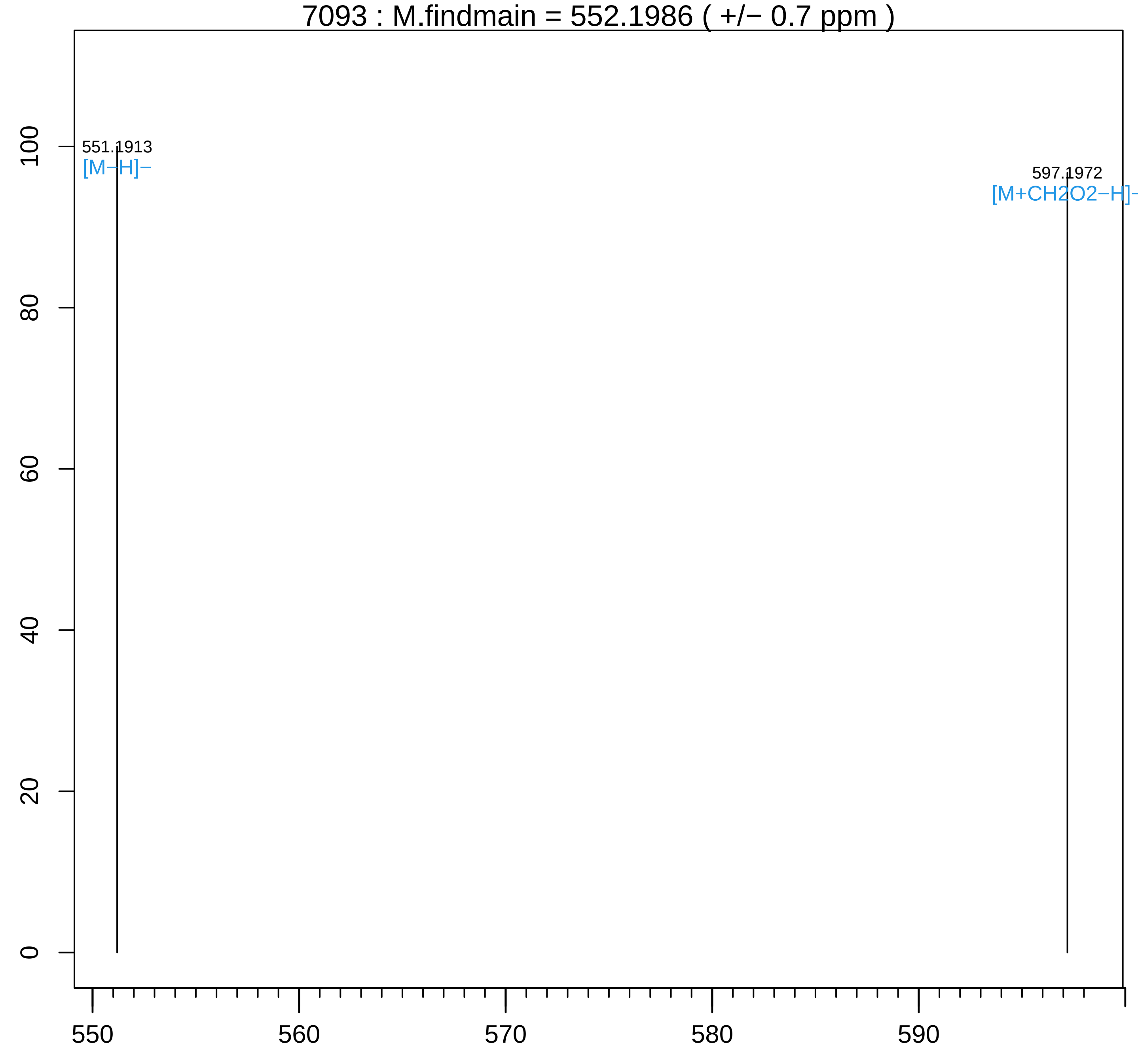
7093 : M.findmain = 552.1986 (+/− 0.7 ppm)
551.1913
[M−H]−
597.1972
[M+CH2O2−H]-
0
20
40
60
80
100
550
560
570
580
590

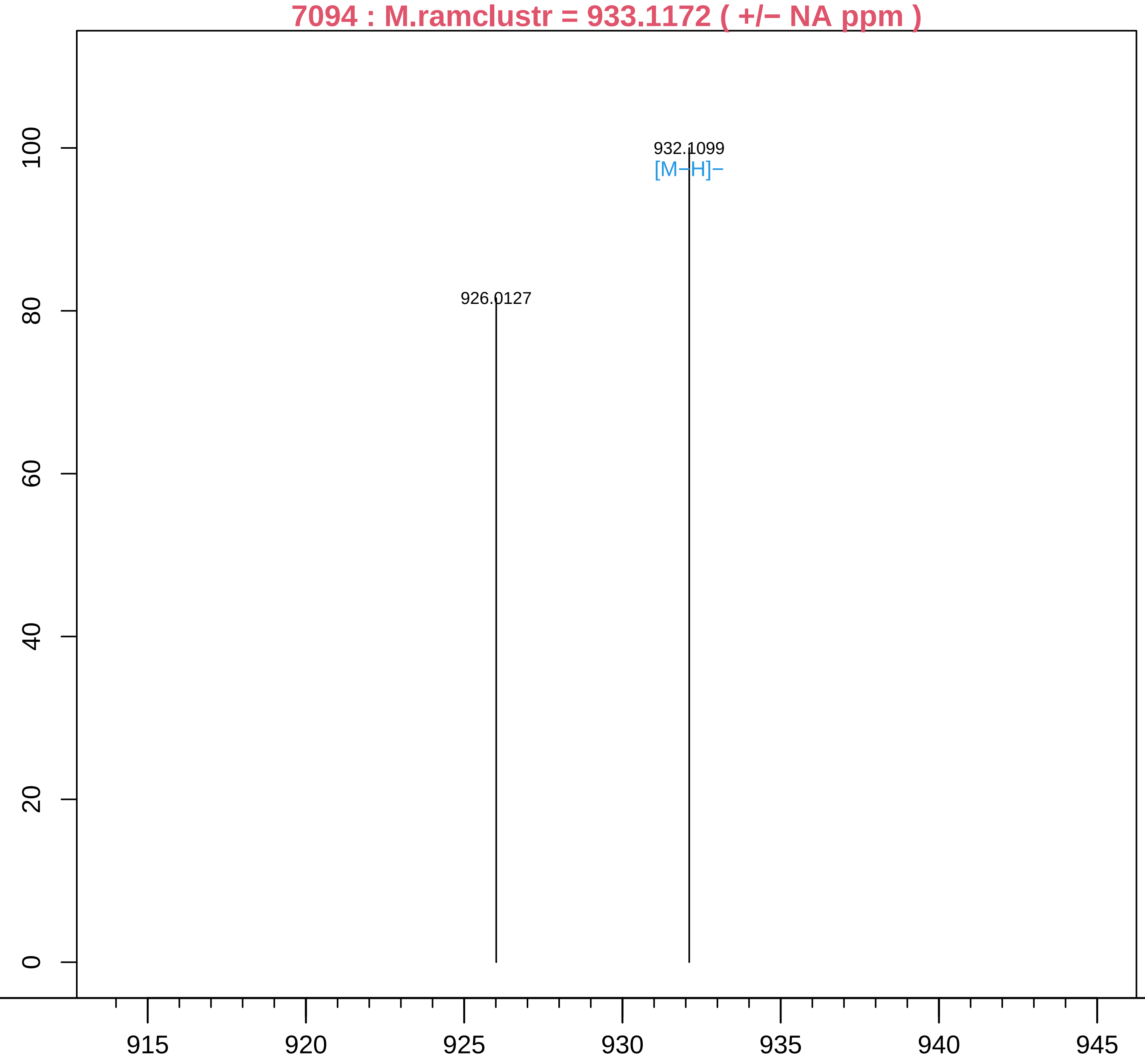
7094 : M.ramclustr = 933.1172 (+/− NA ppm)
932.1099
[M−H]−
926.0127
0
20
40
60
80
100
915
920
925
930
935
940
945

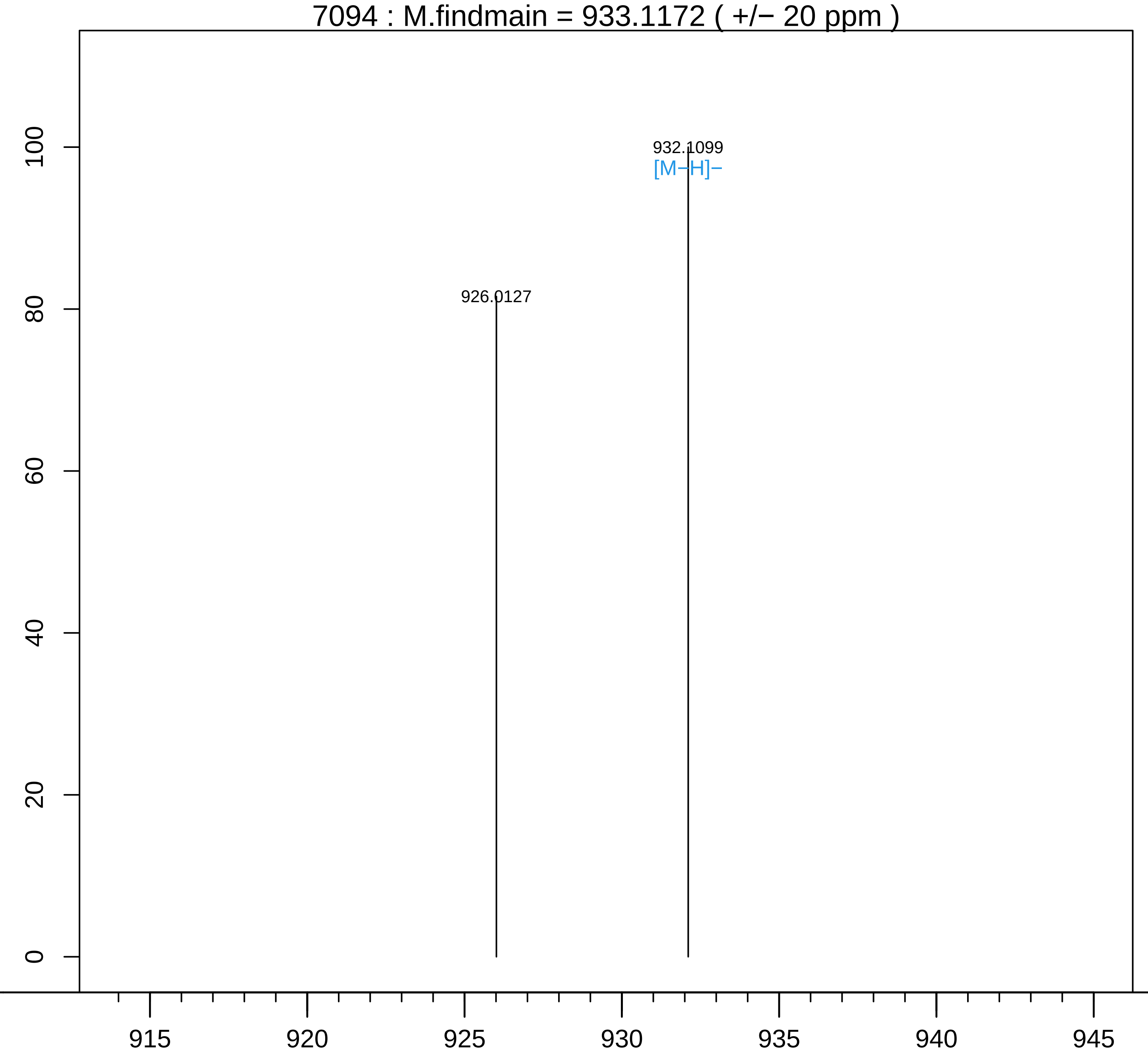
7094 : M.findmain = 933.1172 (+/− 20 ppm)
932.1099
[M−H]−
926.0127
0
20
40
60
80
100
915
920
925
930
935
940
945

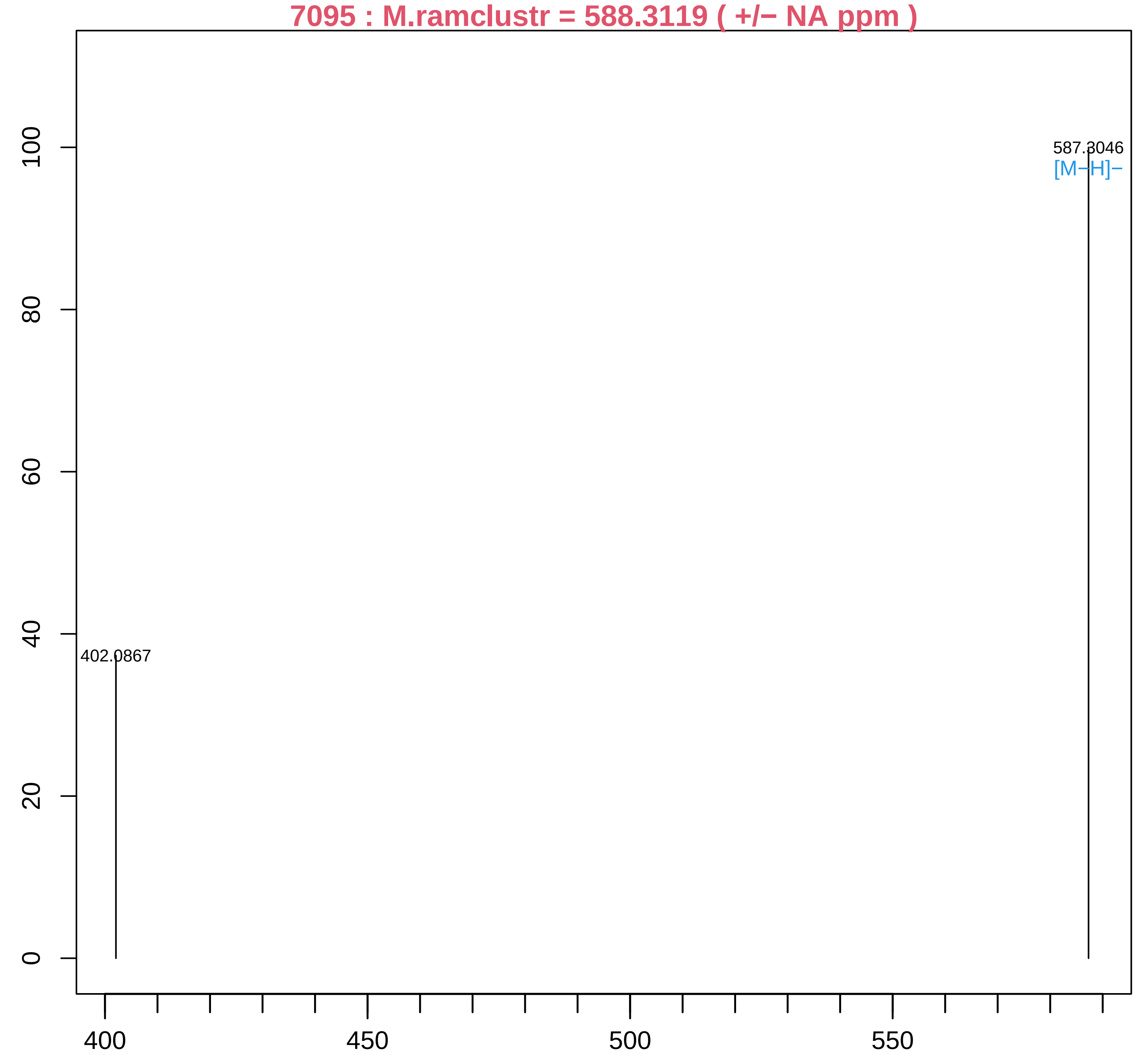

7095 : M.ramclustr = 588.3119 (+/− NA ppm)
587.3046
[M−H]−
402.0867
0
20
40
60
80
100
400
450
500
550

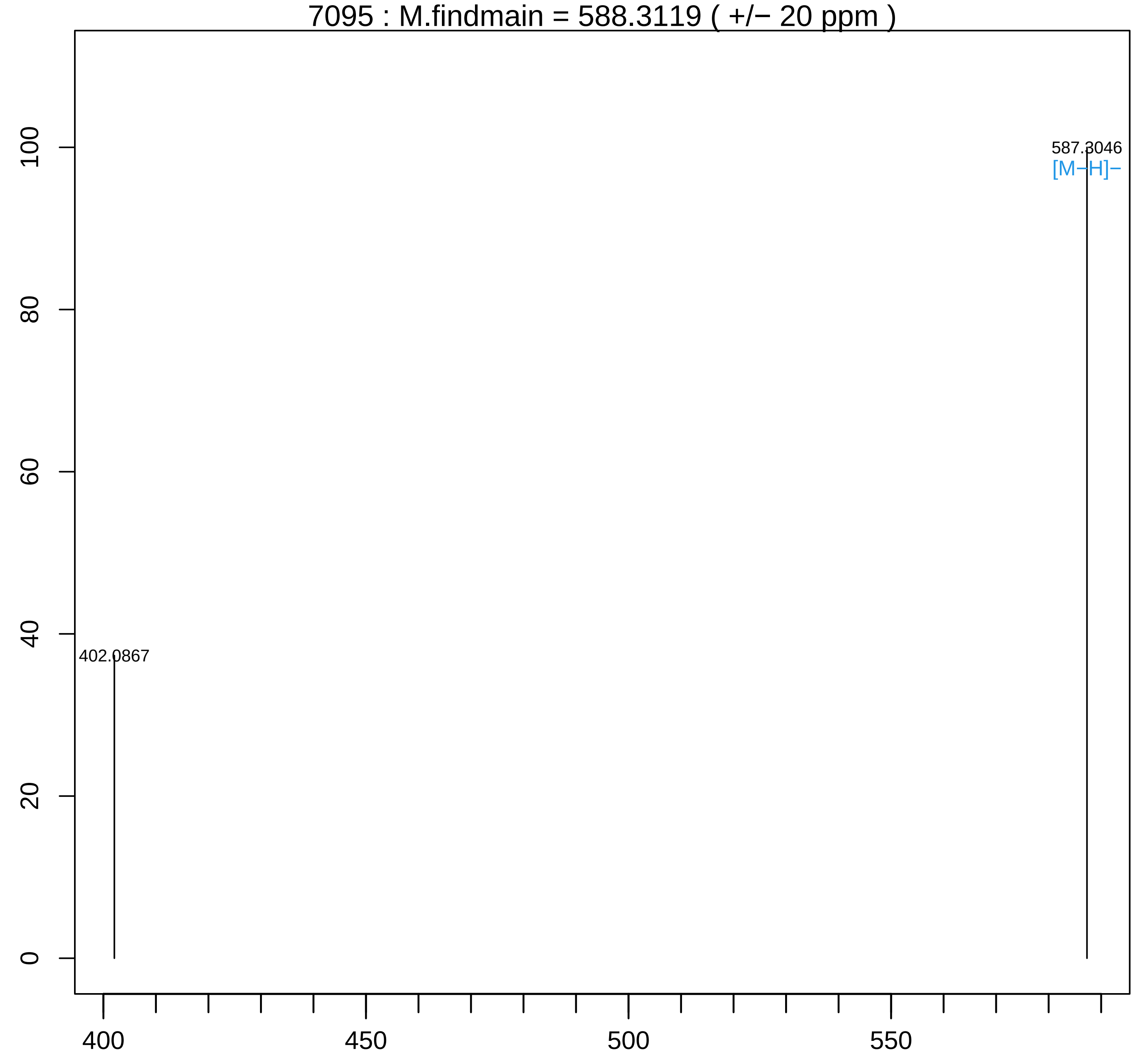

7095 : M.findmain = 588.3119 (+/− 20 ppm)
587.3046
[M−H]−
402.0867
0
20
40
60
80
100
400
450
500
550

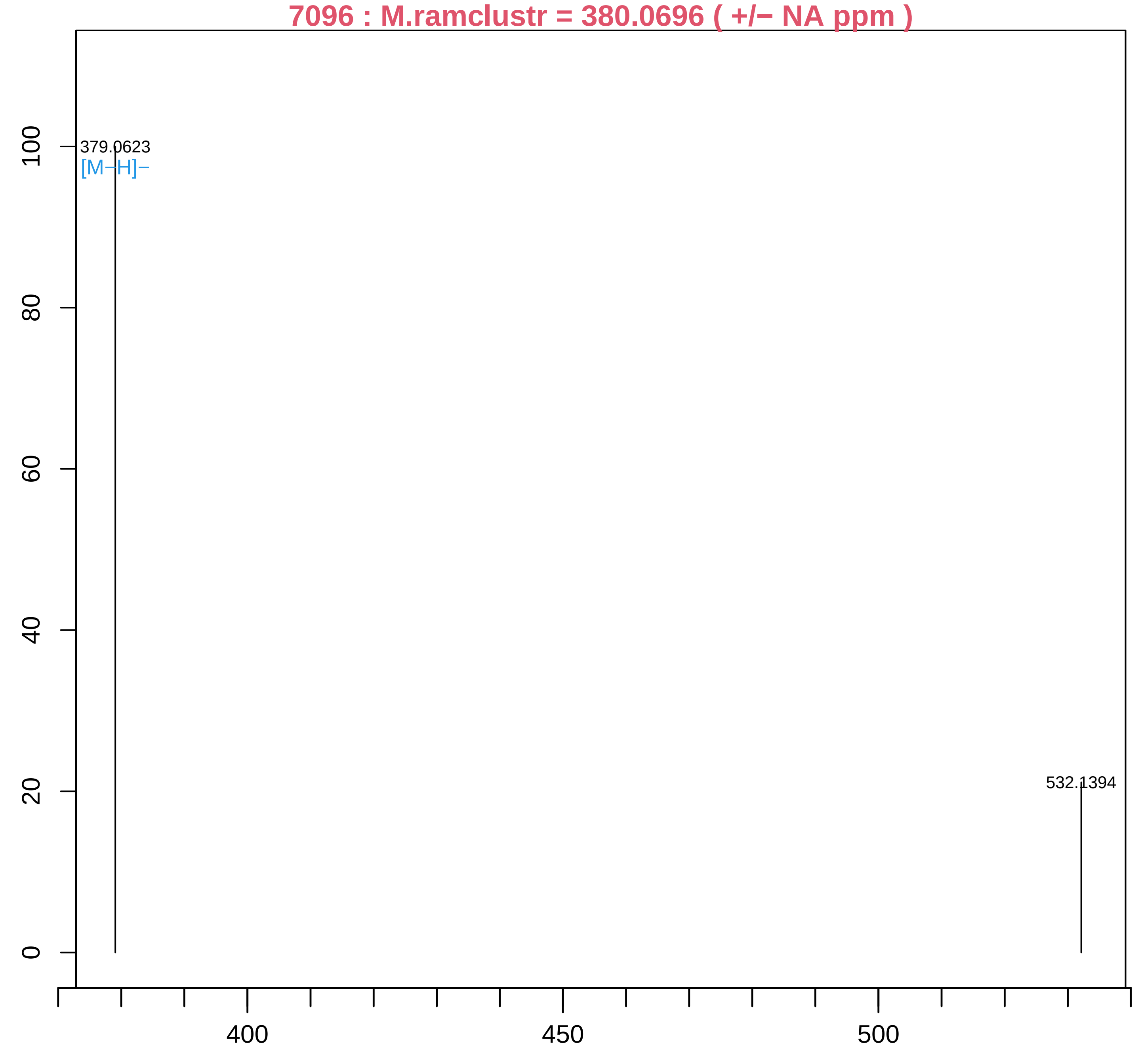

7096 : M.ramclustr = 380.0696 (+/− NA ppm)
379.0623
[M−H]−
532.1394
0
20
40
60
80
100
400
450
500

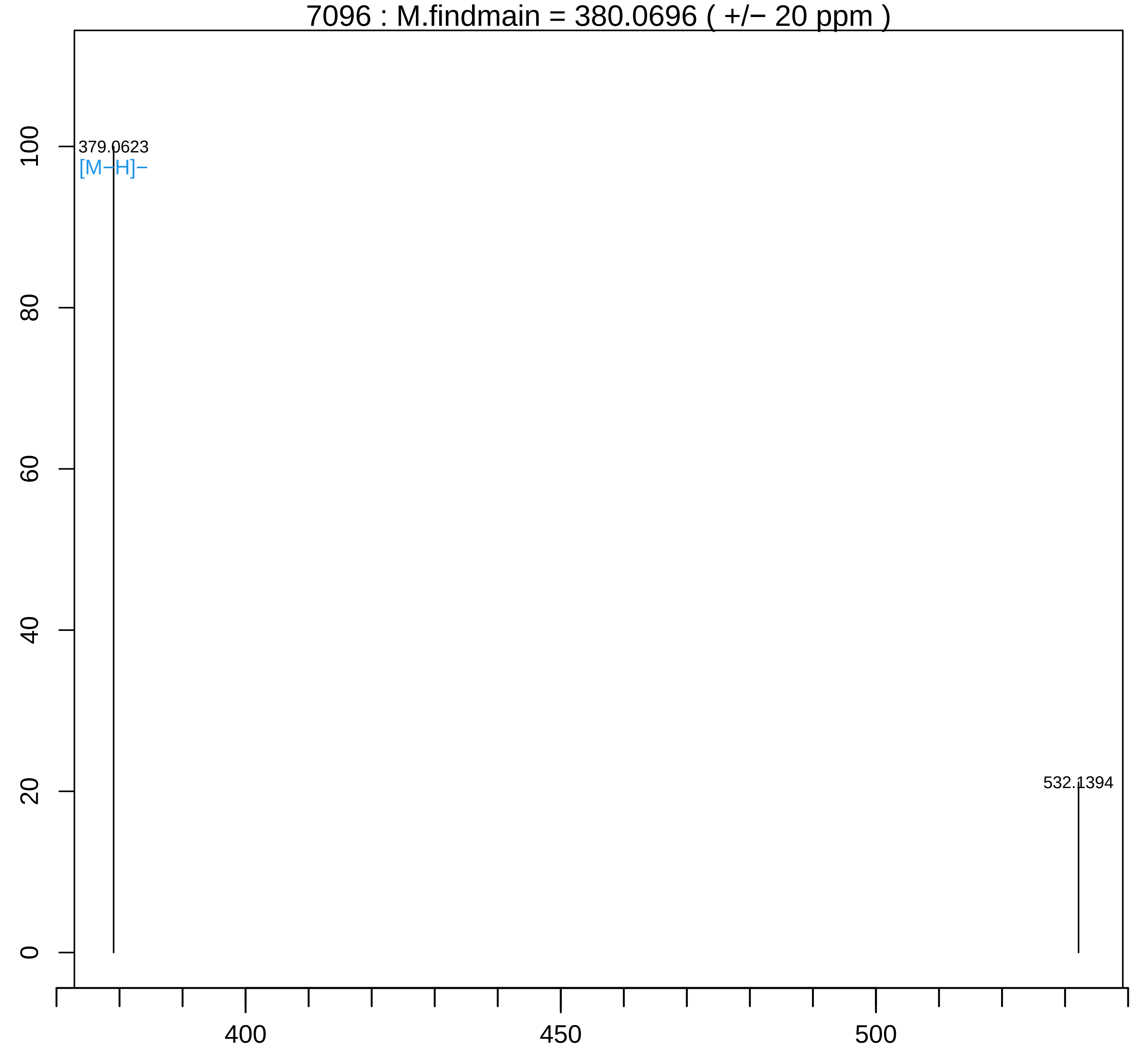

7096 : M.findmain = 380.0696 (+/− 20 ppm)
379.0623
[M−H]−
532.1394
0
20
40
60
80
100
400
450
500

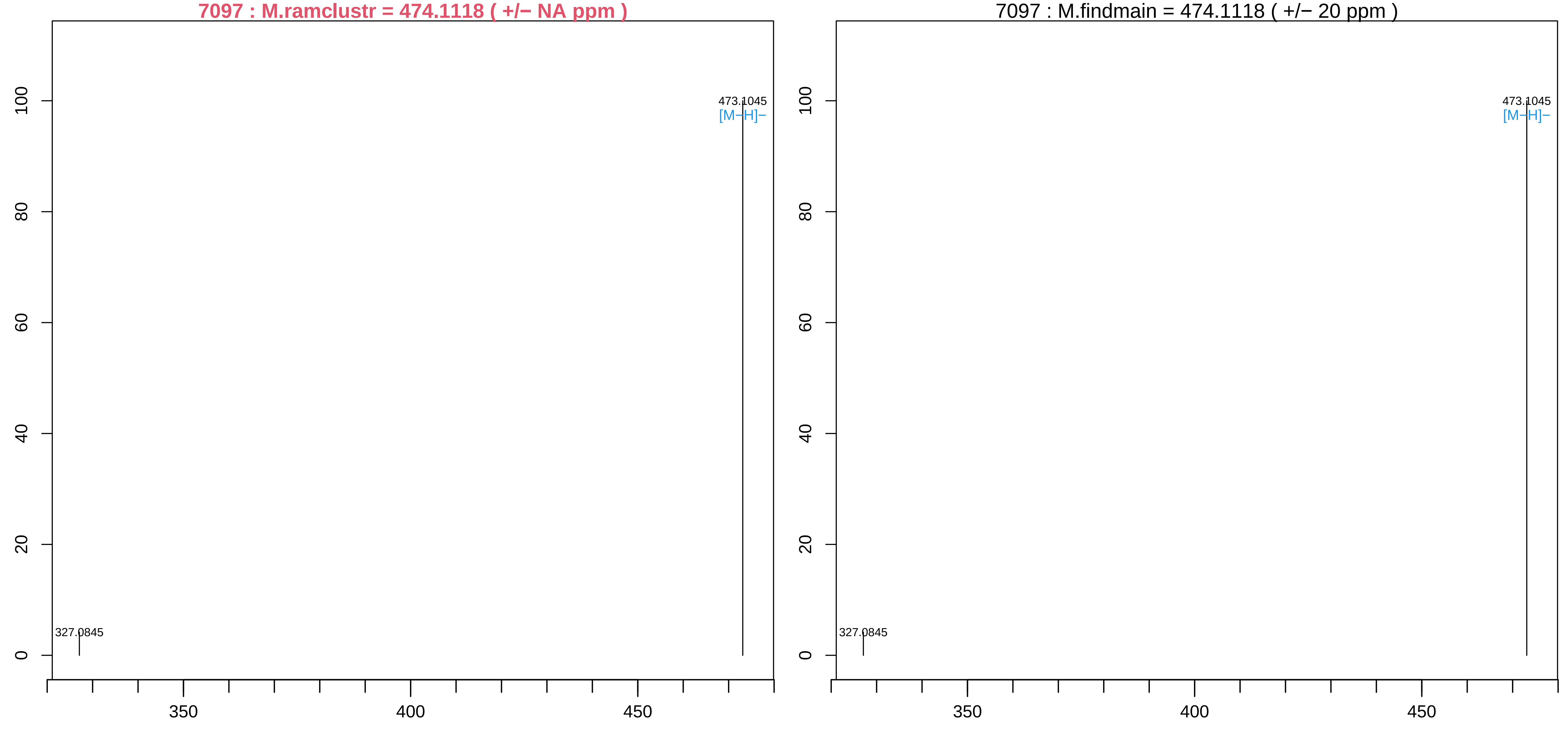
7097 : M.ramclustr = 474.1118 (+/− NA ppm)
473.1045
[M−H]−
327.0845
0
20
40
60
80
100
350
400
450
7097 : M.findmain = 474.1118 (+/− 20 ppm)
473.1045
[M−H]−
327.0845
0
20
40
60
80
100
350
400
450

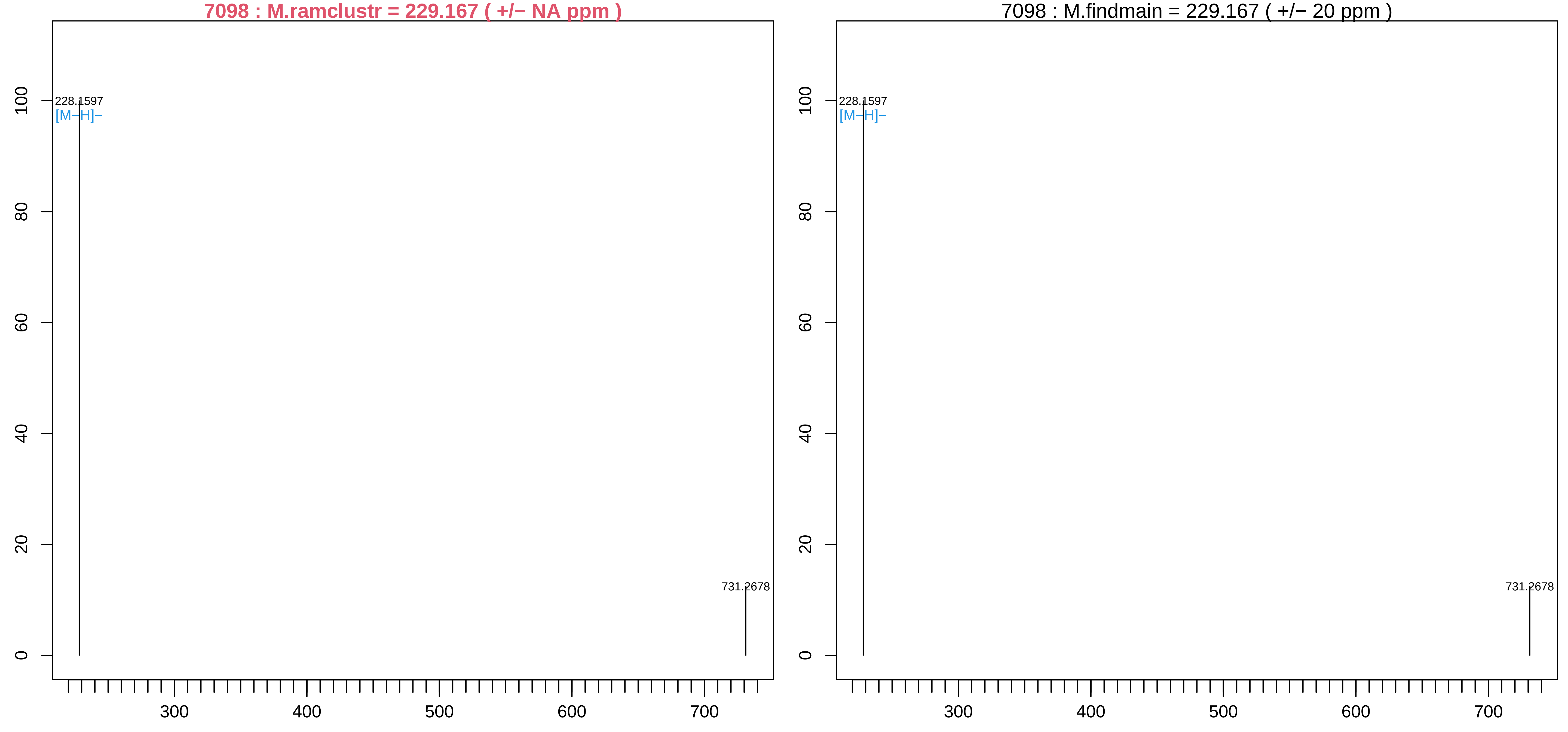
7098 : M.ramclustr = 229.167 (+/− NA ppm)
228.1597
[M−H]−
731.2678
0
20
40
60
80
100
300
400
500
600
700
7098 : M.findmain = 229.167 (+/− 20 ppm)
228.1597
[M−H]−
731.2678
0
20
40
60
80
100
300
400
500
600
700

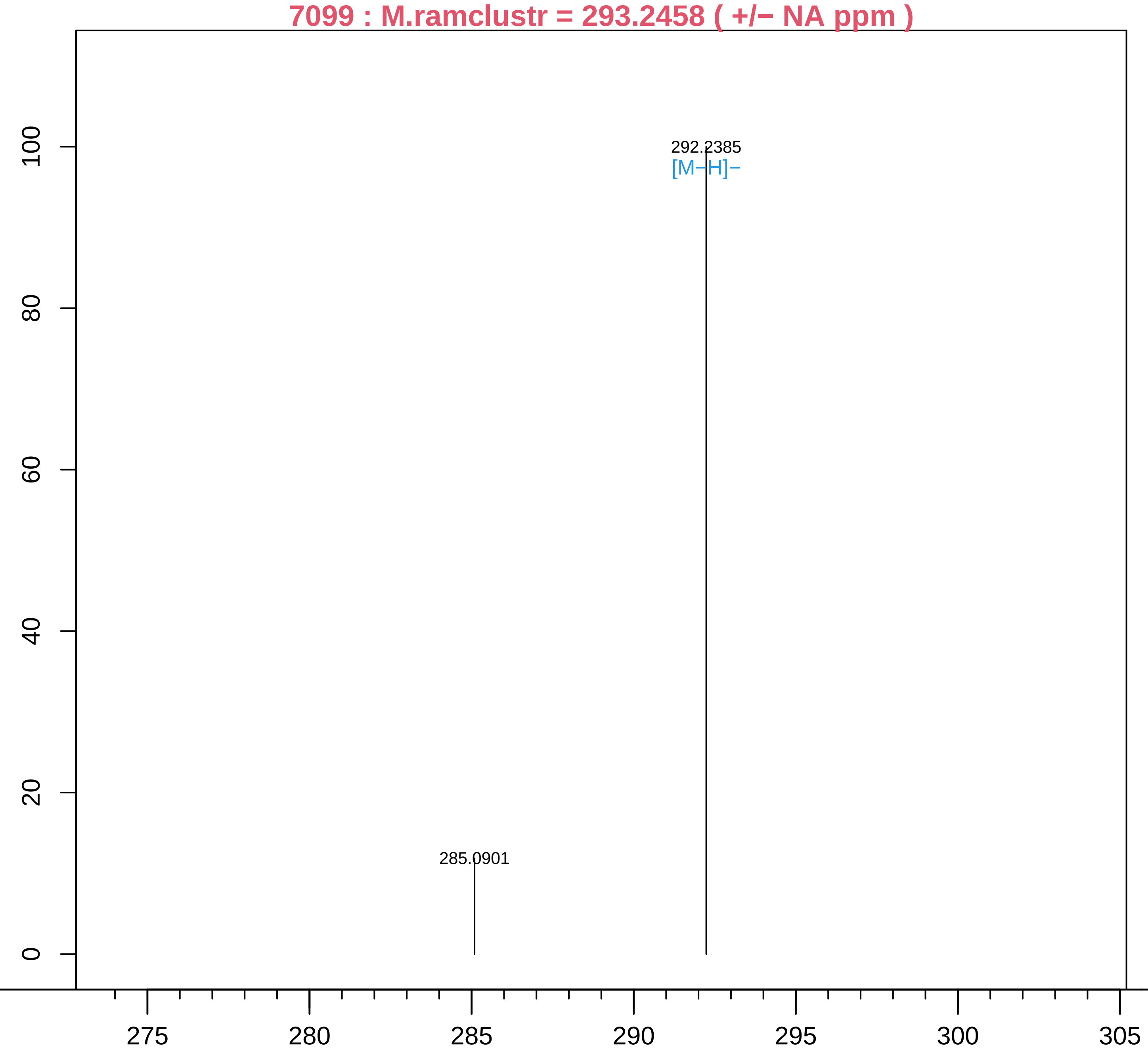

7099 : M.ramclustr = 293.2458 (+/− NA ppm)
292.2385
[M−H]−
285.0901
0
20
40
60
80
100
275
280
285
290
295
300
305

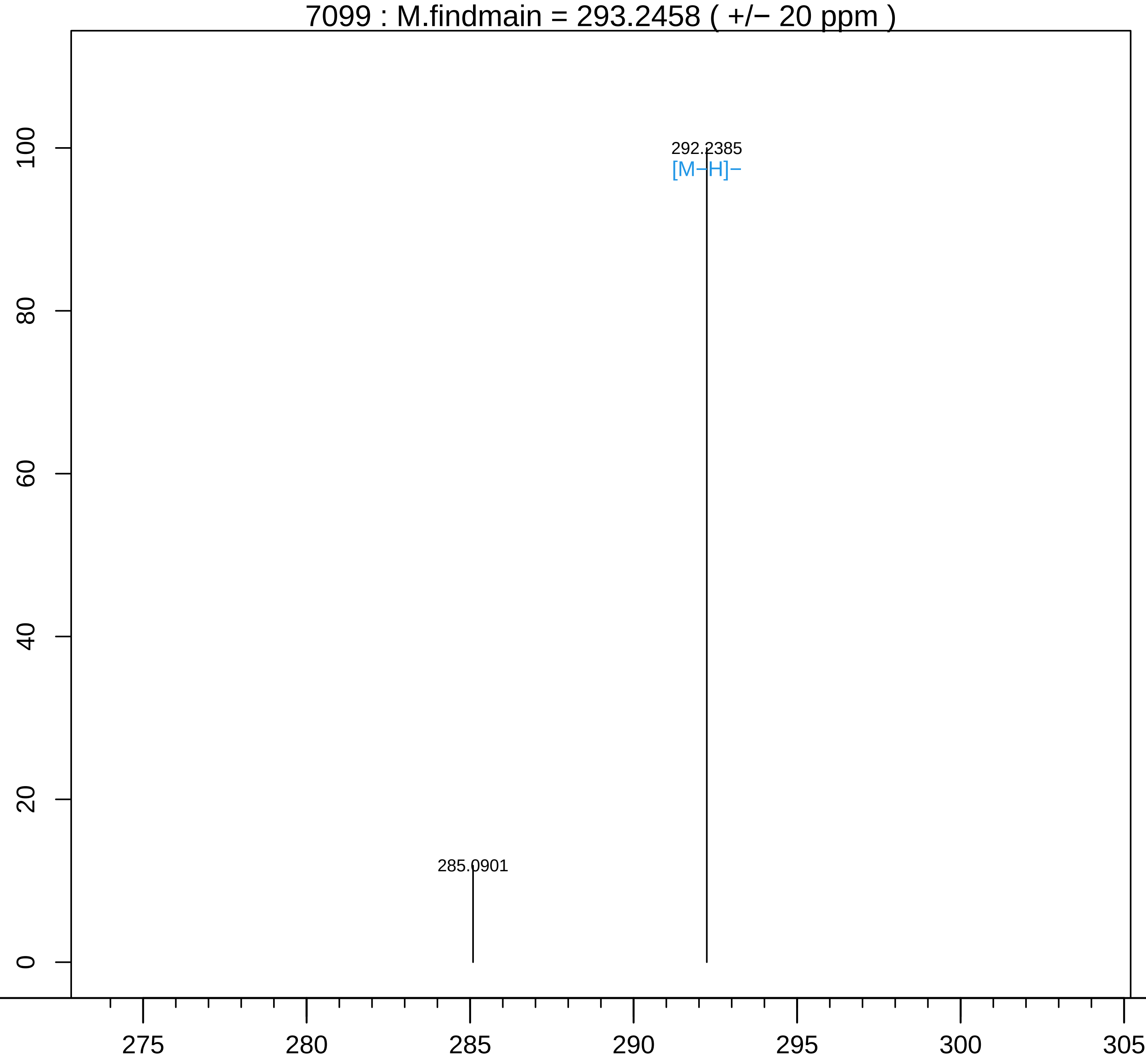

7099 : M.findmain = 293.2458 (+/− 20 ppm)
292.2385
[M−H]−
285.0901
0
20
40
60
80
100
275
280
285
290
295
300
305

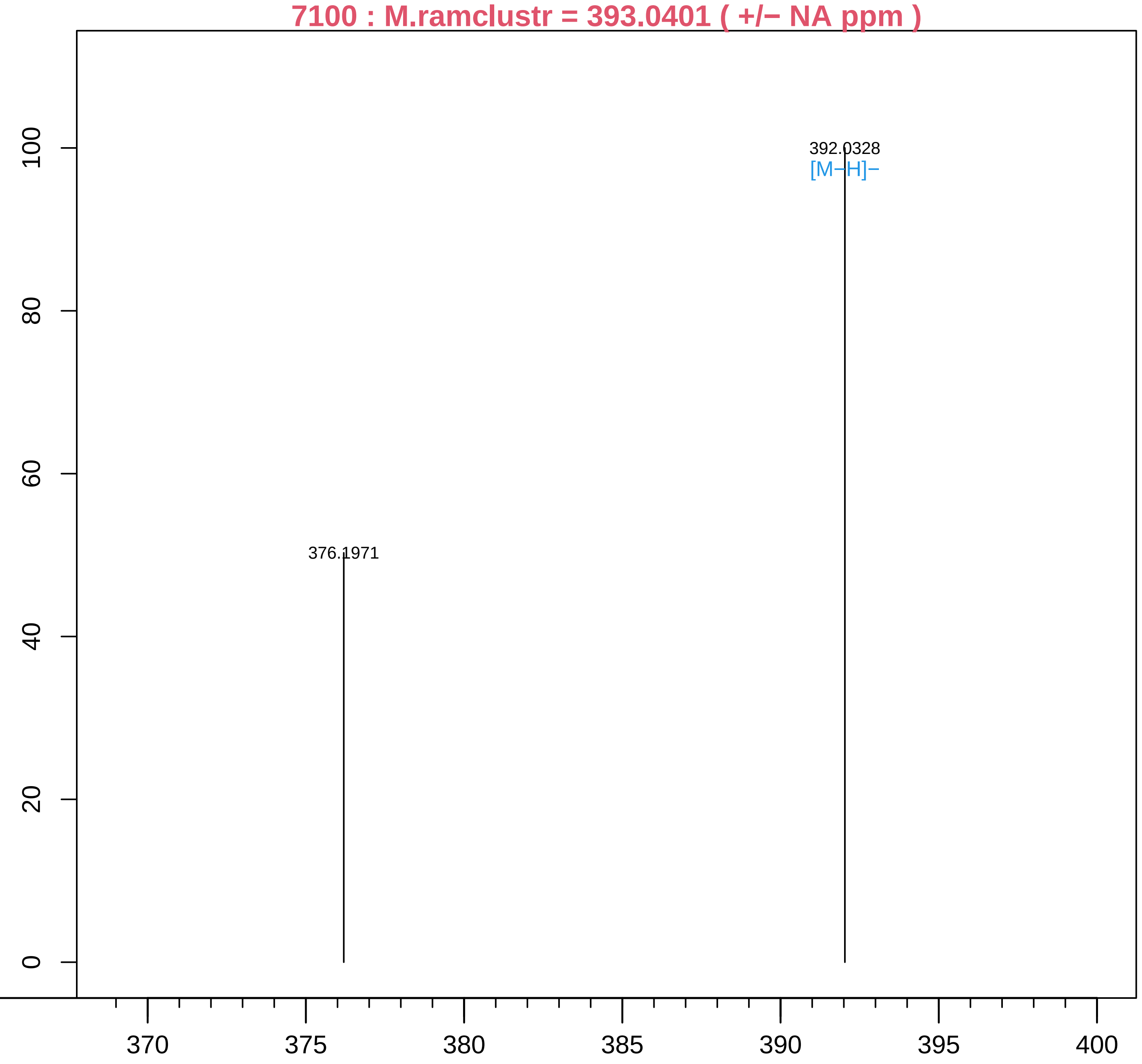
7100 : M.ramclustr = 393.0401 (+/− NA ppm)
392.0328
[M−H]−
376.1971
0
20
40
60
80
100
370
375
380
385
390
395
400

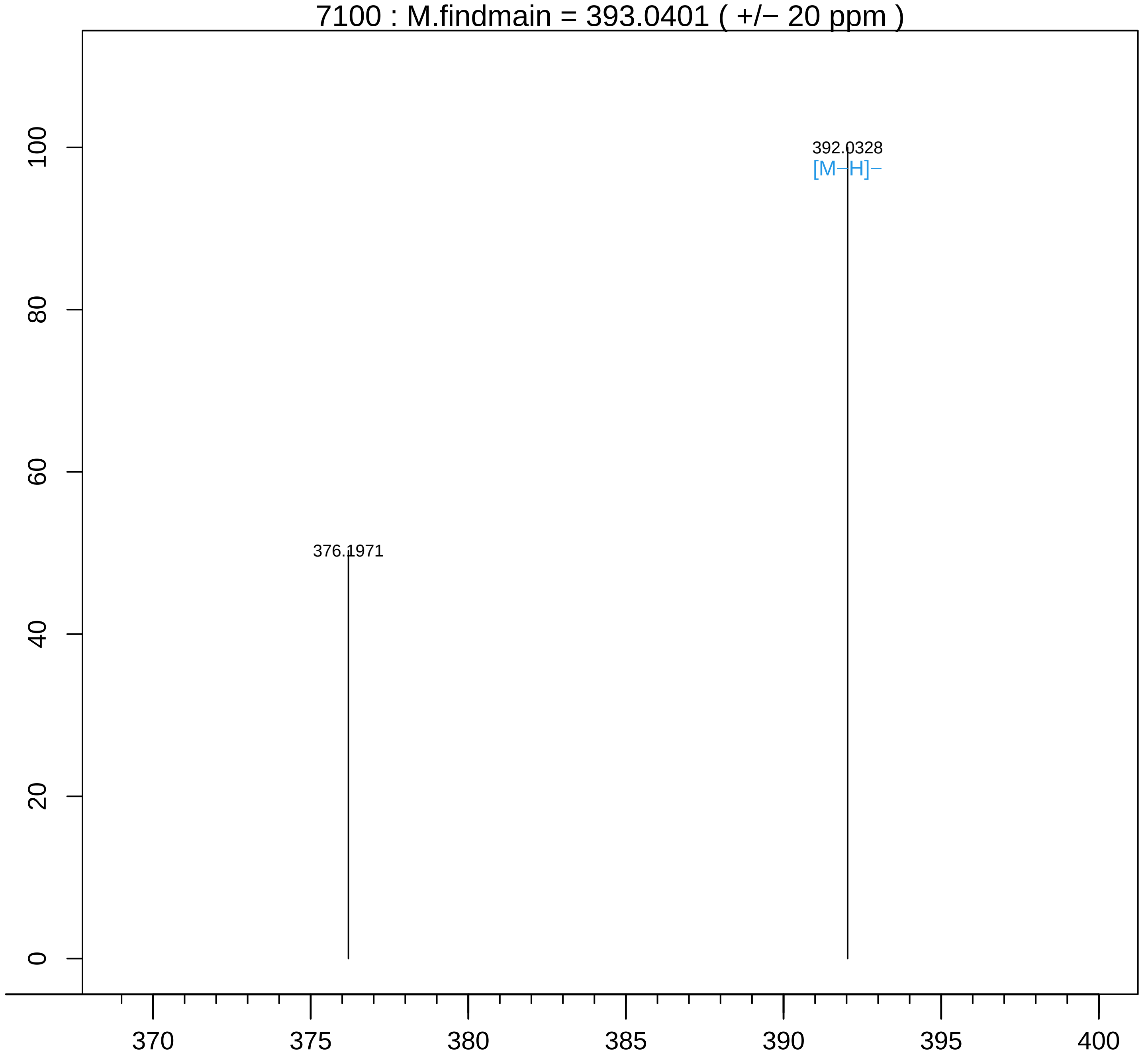
7100 : M.findmain = 393.0401 (+/− 20 ppm)
392.0328
[M−H]−
376.1971
0
20
40
60
80
100
370
375
380
385
390
395
400

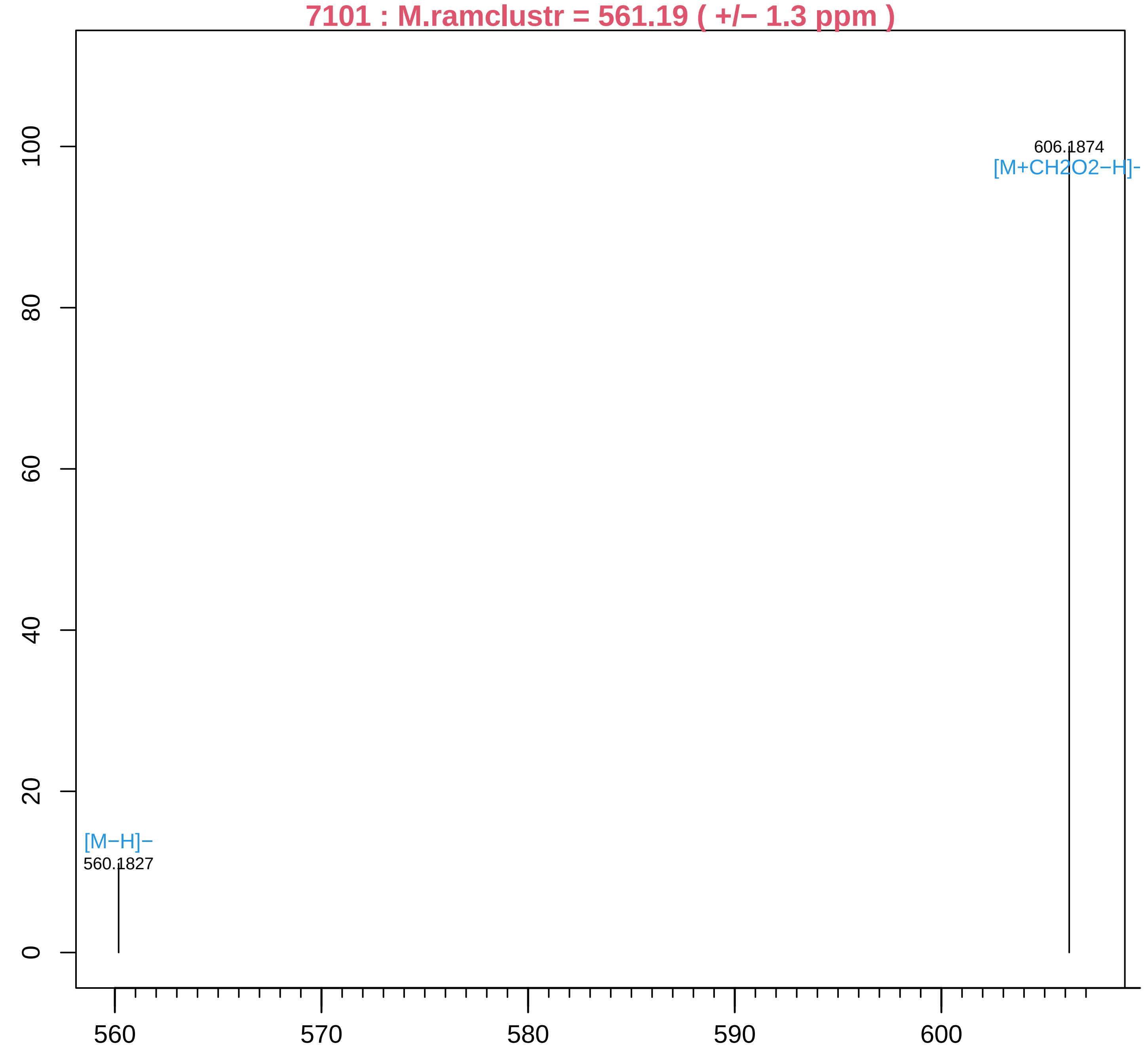
7101 : M.ramclustr = 561.19 (+/− 1.3 ppm)
606.1874
[M+CH2O2−H]-
[M−H]−
560.1827
0
20
40
60
80
100
560
570
580
590
600

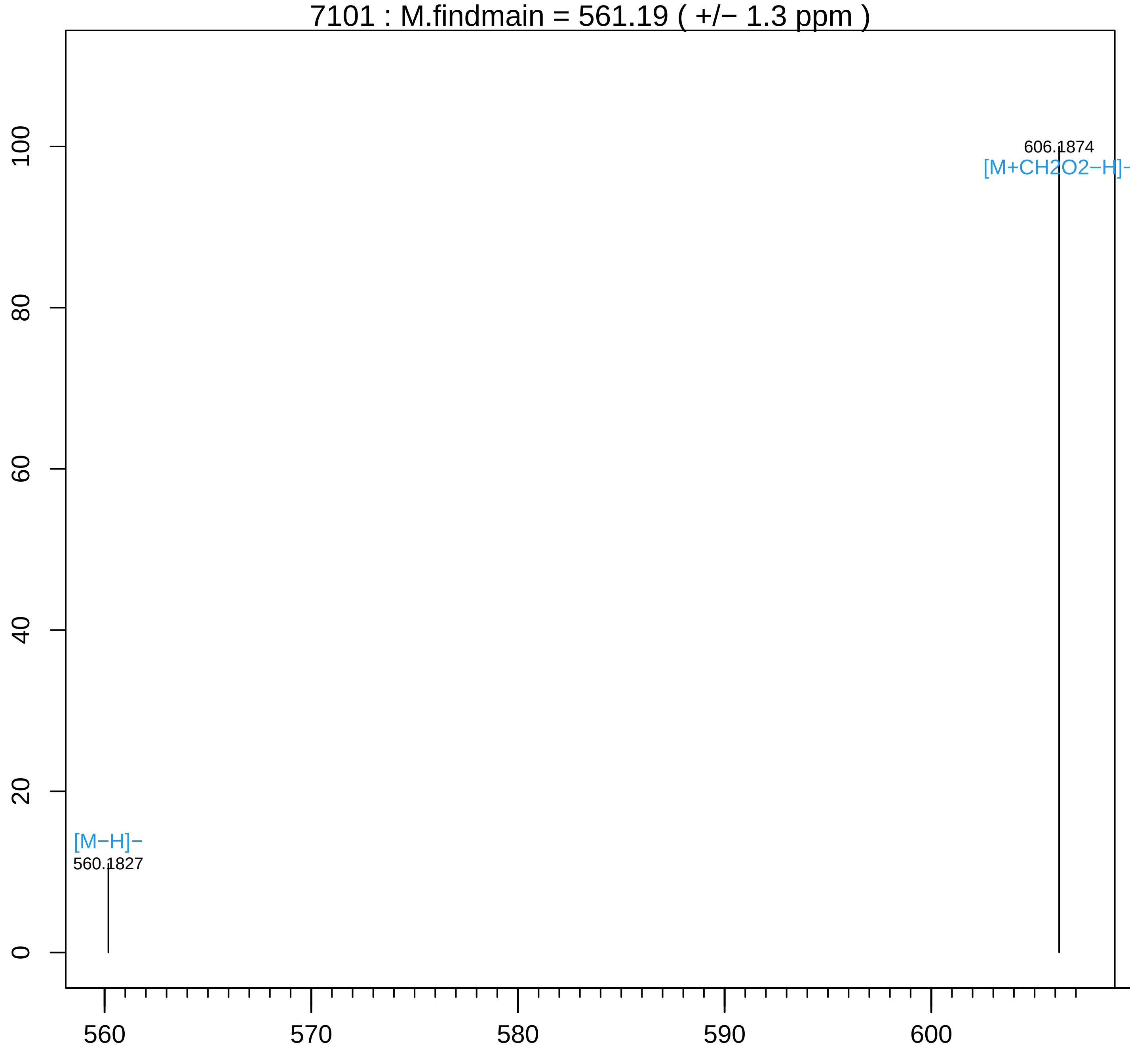
7101 : M.findmain = 561.19 (+/− 1.3 ppm)
606.1874
[M+CH2O2−H]-
[M−H]−
560.1827
0
20
40
60
80
100
560
570
580
590
600

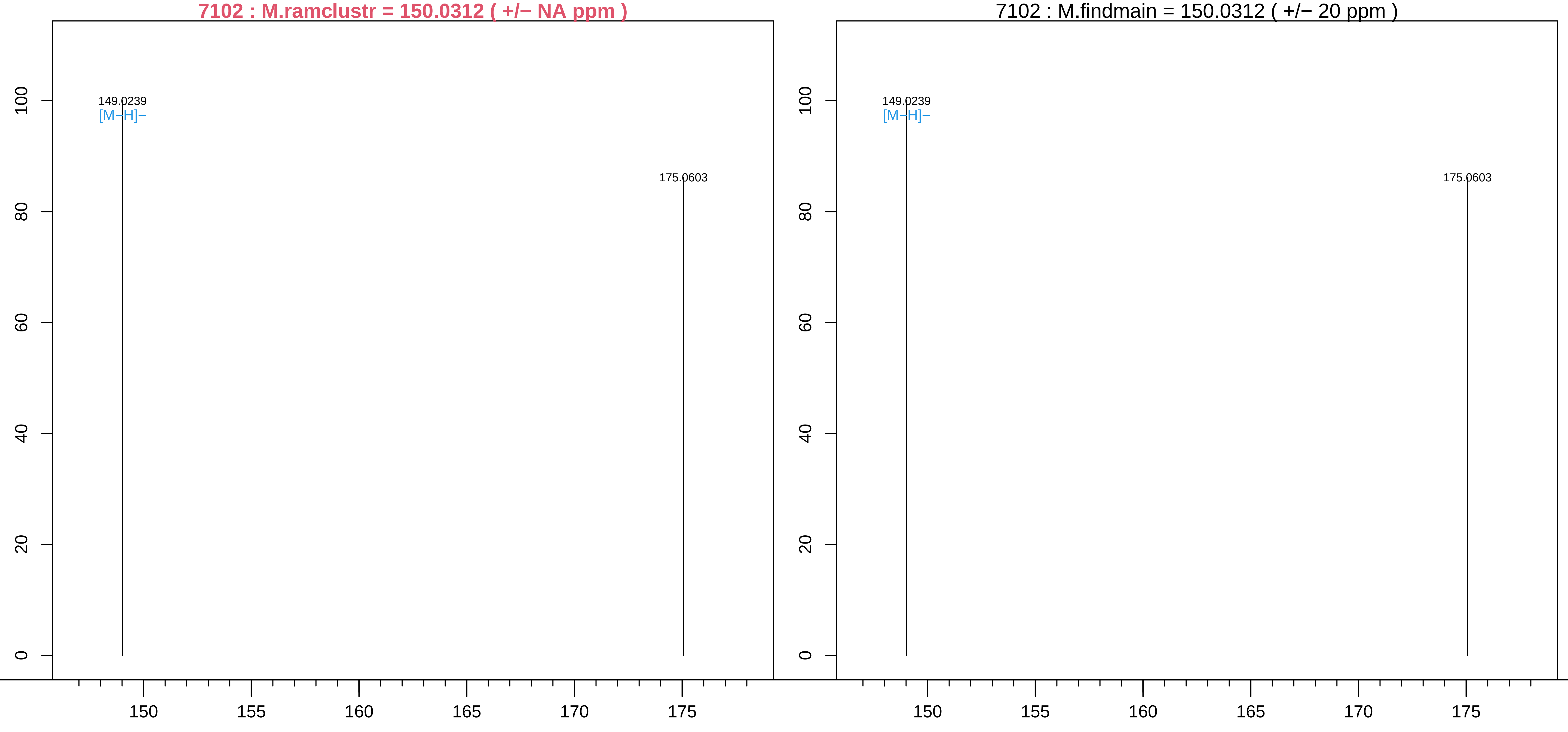
7102 : M.ramclustr = 150.0312 (+/− NA ppm)
149.0239
[M−H]−
175.0603
7102 : M.findmain = 150.0312 (+/− 20 ppm)
149.0239
[M−H]−
175.0603
0
20
40
60
80
100
150
155
160
165
170
175

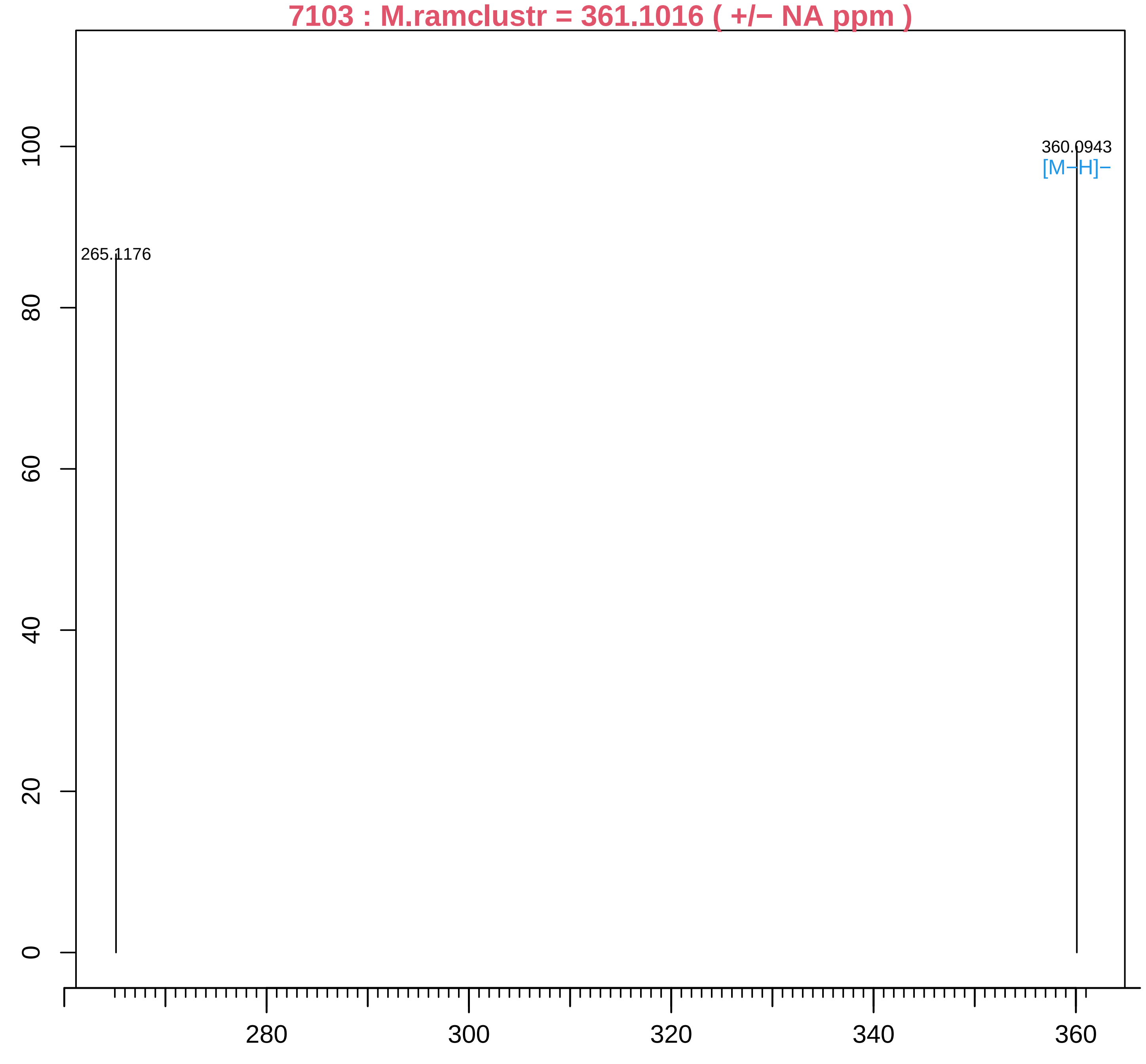
7103 : M.ramclustr = 361.1016 (+/− NA ppm)
360.0943
[M−H]−
265.1176
0
20
40
60
80
100
280
300
320
340
360

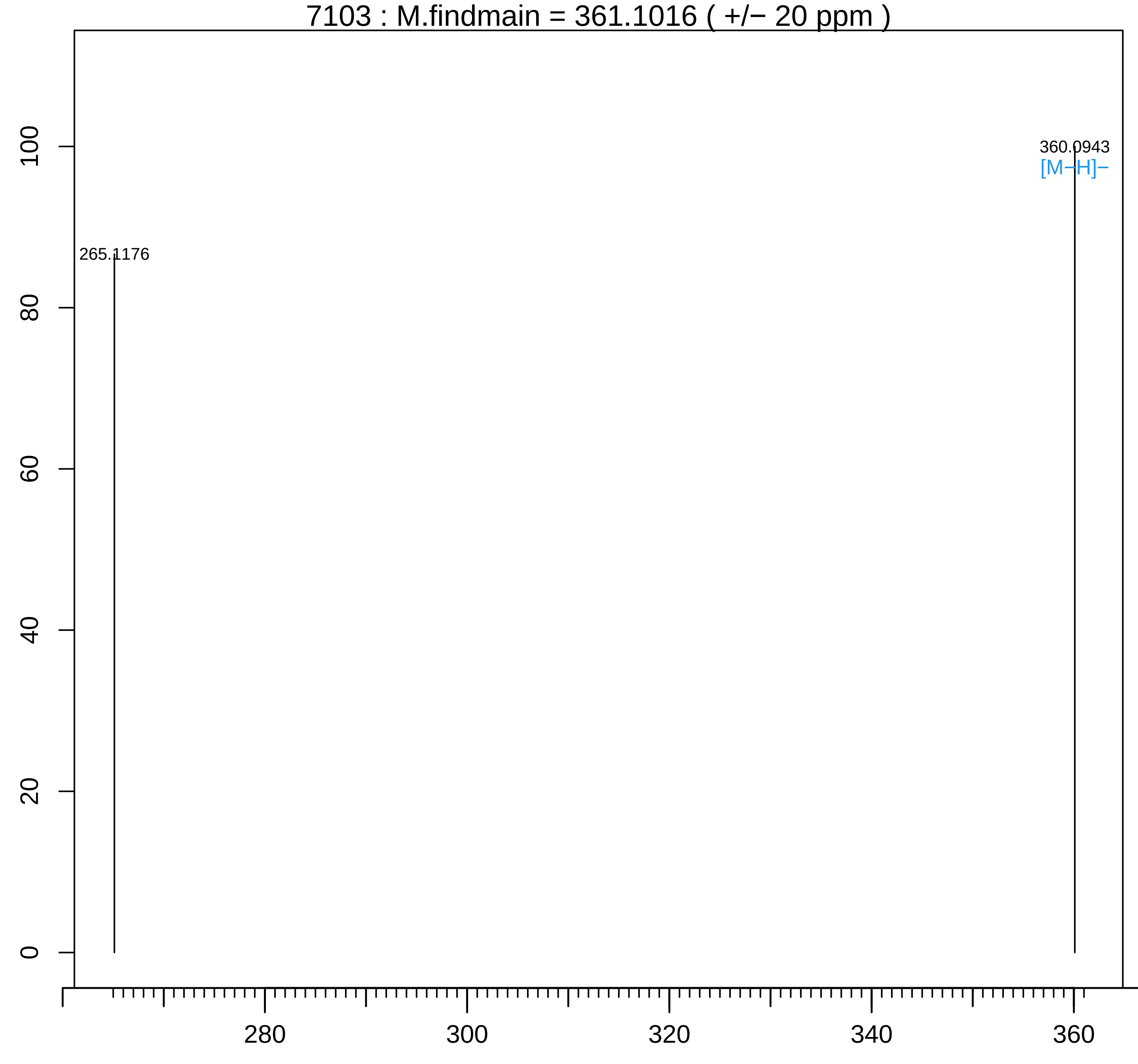
7103 : M.findmain = 361.1016 (+/− 20 ppm)
360.0943
[M−H]−
265.1176
0
20
40
60
80
100
280
300
320
340
360

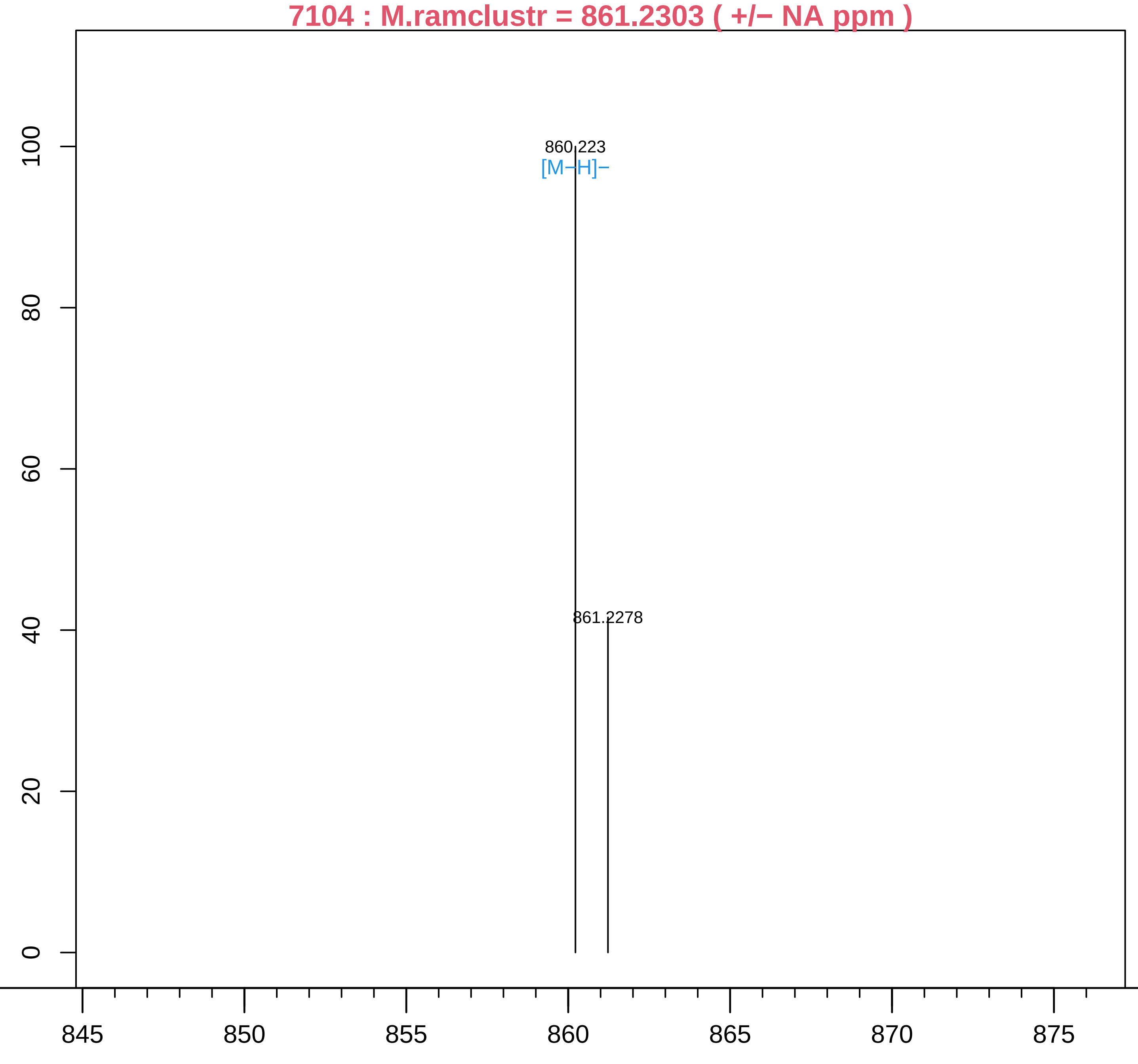
7104 : M.ramclustr = 861.2303 (+/− NA ppm)
860.223
[M−H]−
861.2278
0
20
40
60
80
100
845
850
855
860
865
870
875

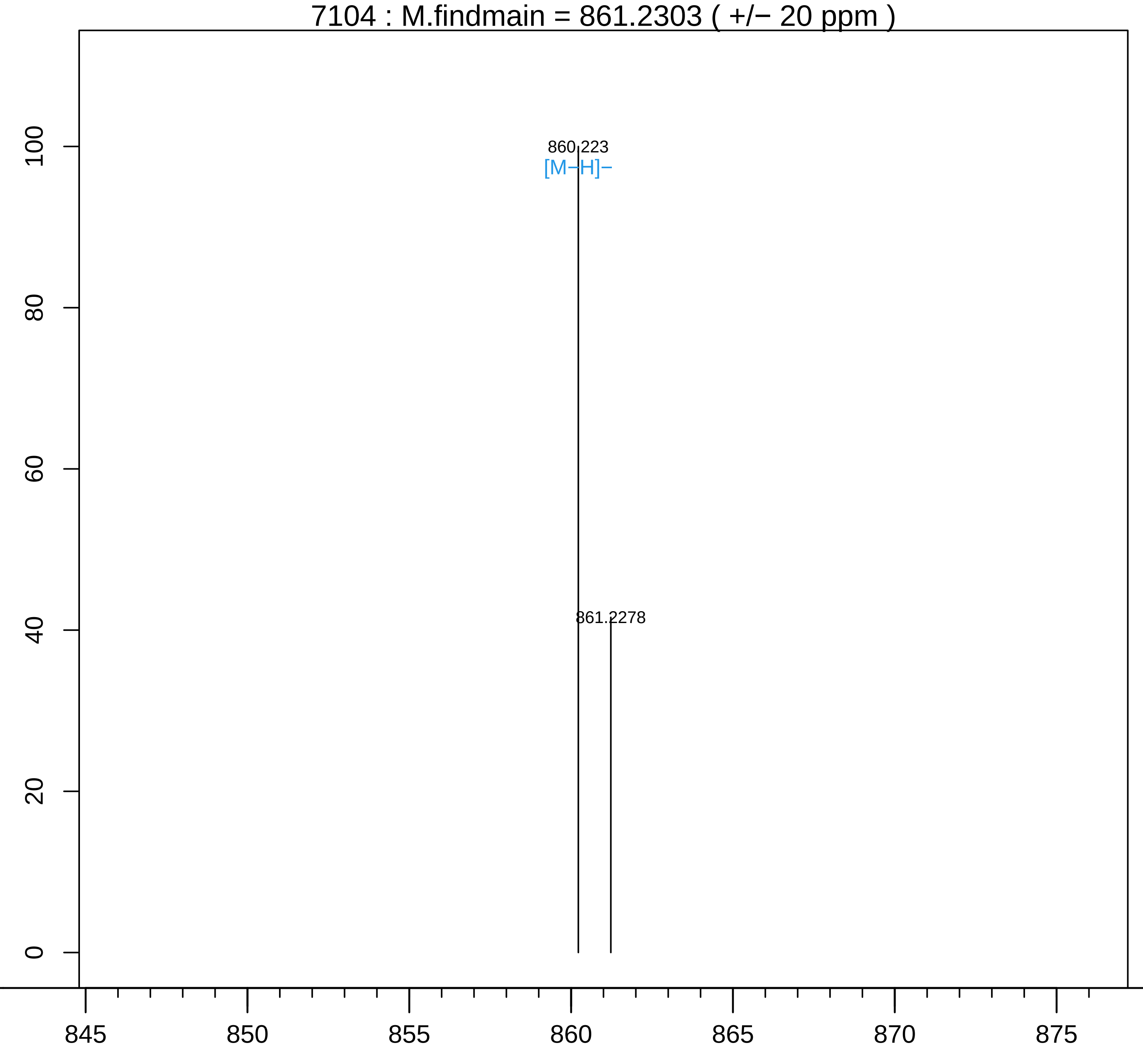
7104 : M.findmain = 861.2303 (+/− 20 ppm)
860.223
[M−H]−
861.2278
0
20
40
60
80
100
845
850
855
860
865
870
875

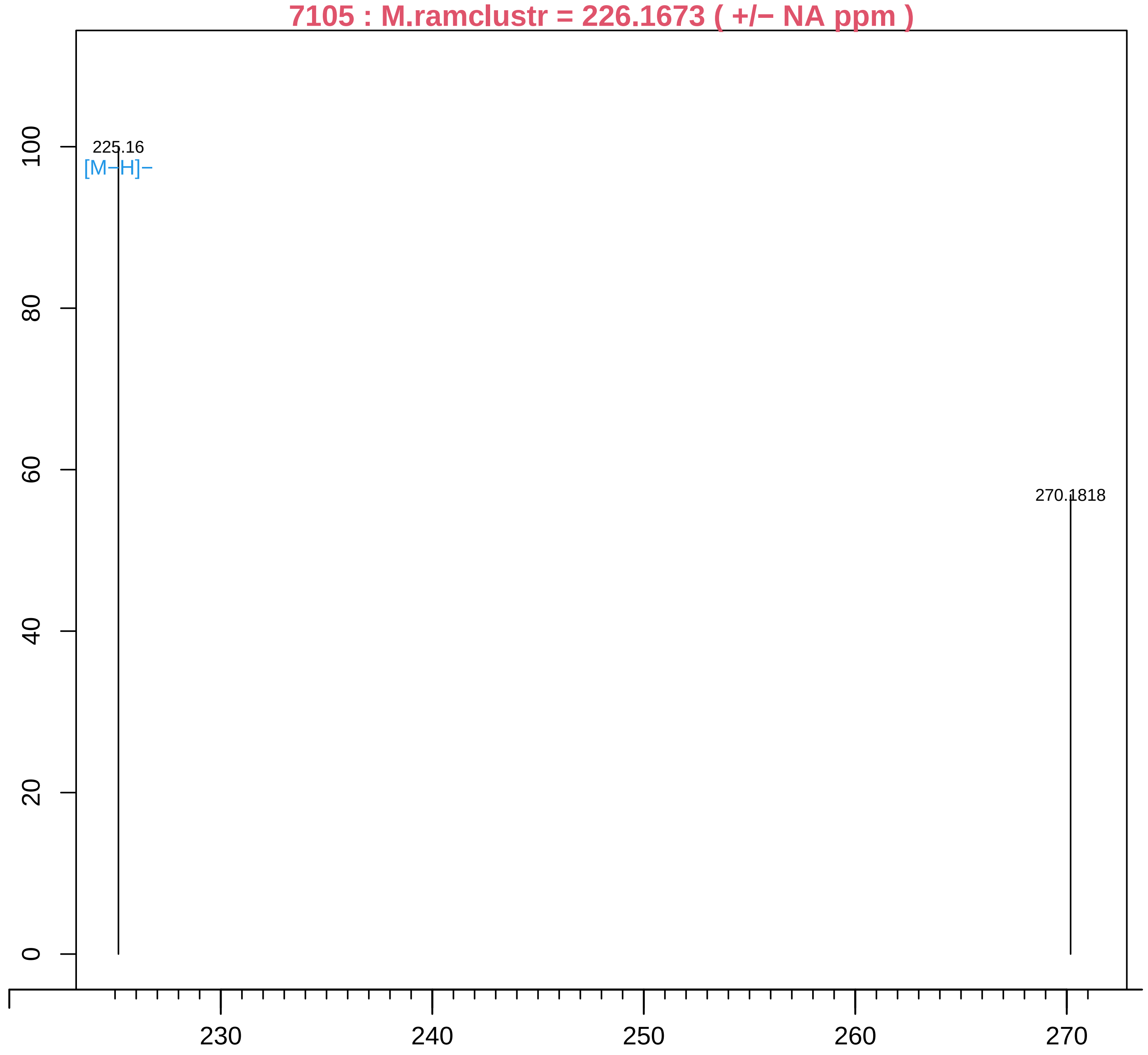
7105 : M.ramclustr = 226.1673 (+/− NA ppm)
225.16
[M−H]−
270.1818
0
20
40
60
80
100
230
240
250
260
270

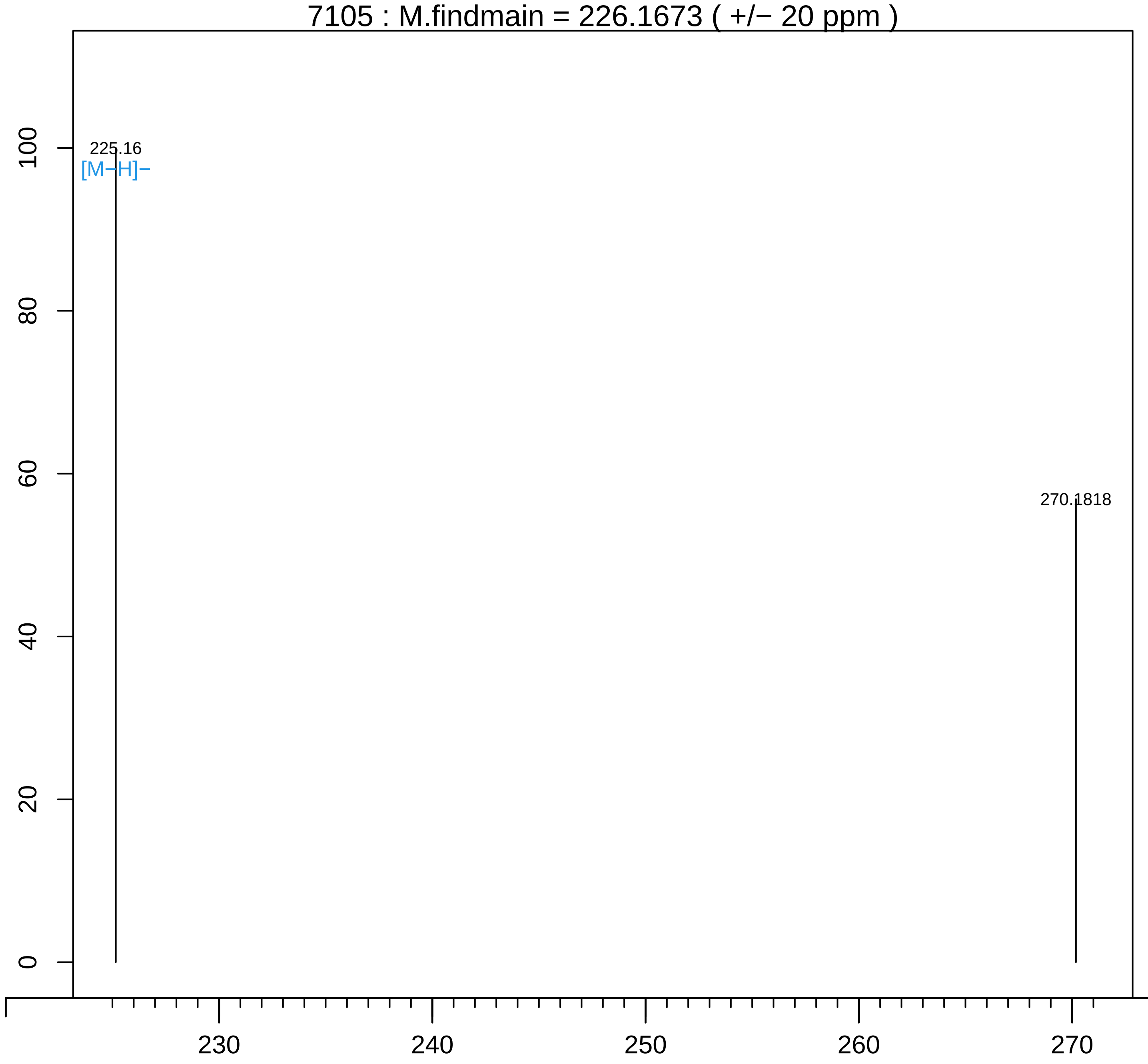
7105 : M.findmain = 226.1673 (+/− 20 ppm)
225.16
[M−H]−
270.1818
0
20
40
60
80
100
230
240
250
260
270

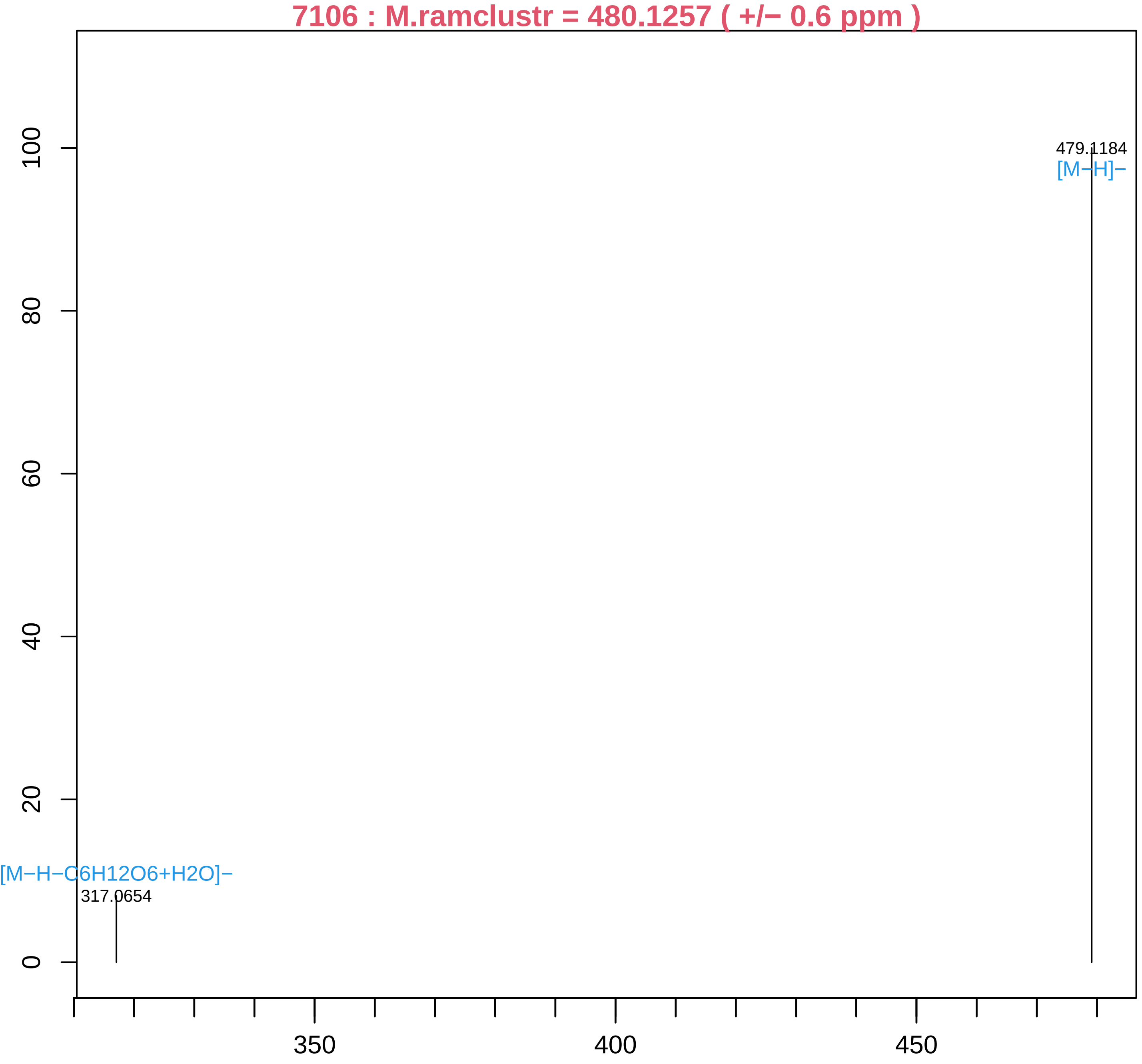
7106 : M.ramclustr = 480.1257 (+/− 0.6 ppm)
479.1184
[M−H]−
[M−H−C6H12O6+H2O]−
317.0654
0
20
40
60
80
100
350
400
450

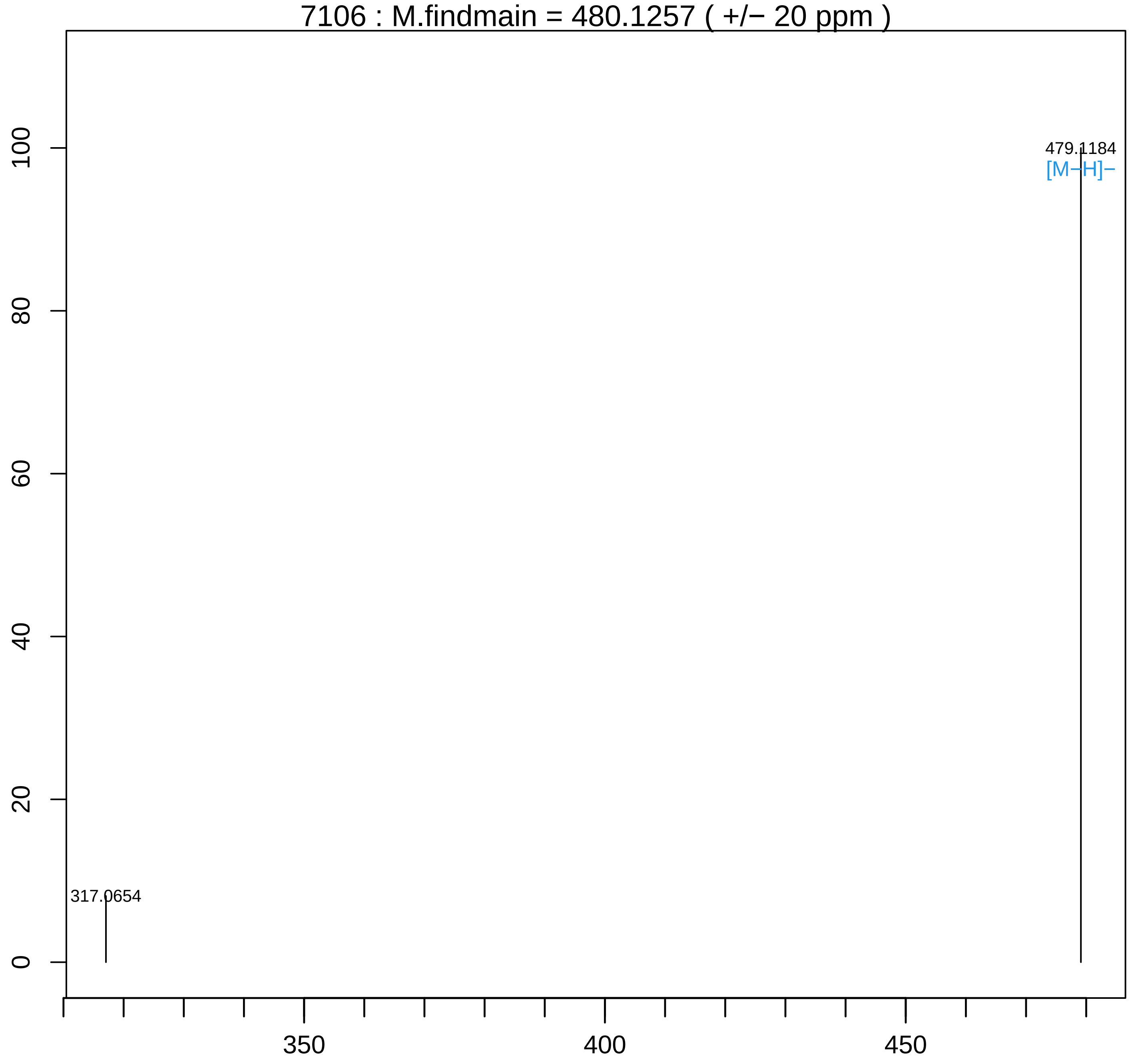
7106 : M.findmain = 480.1257 (+/− 20 ppm)
479.1184
[M−H]−
317.0654
0
20
40
60
80
100
350
400
450

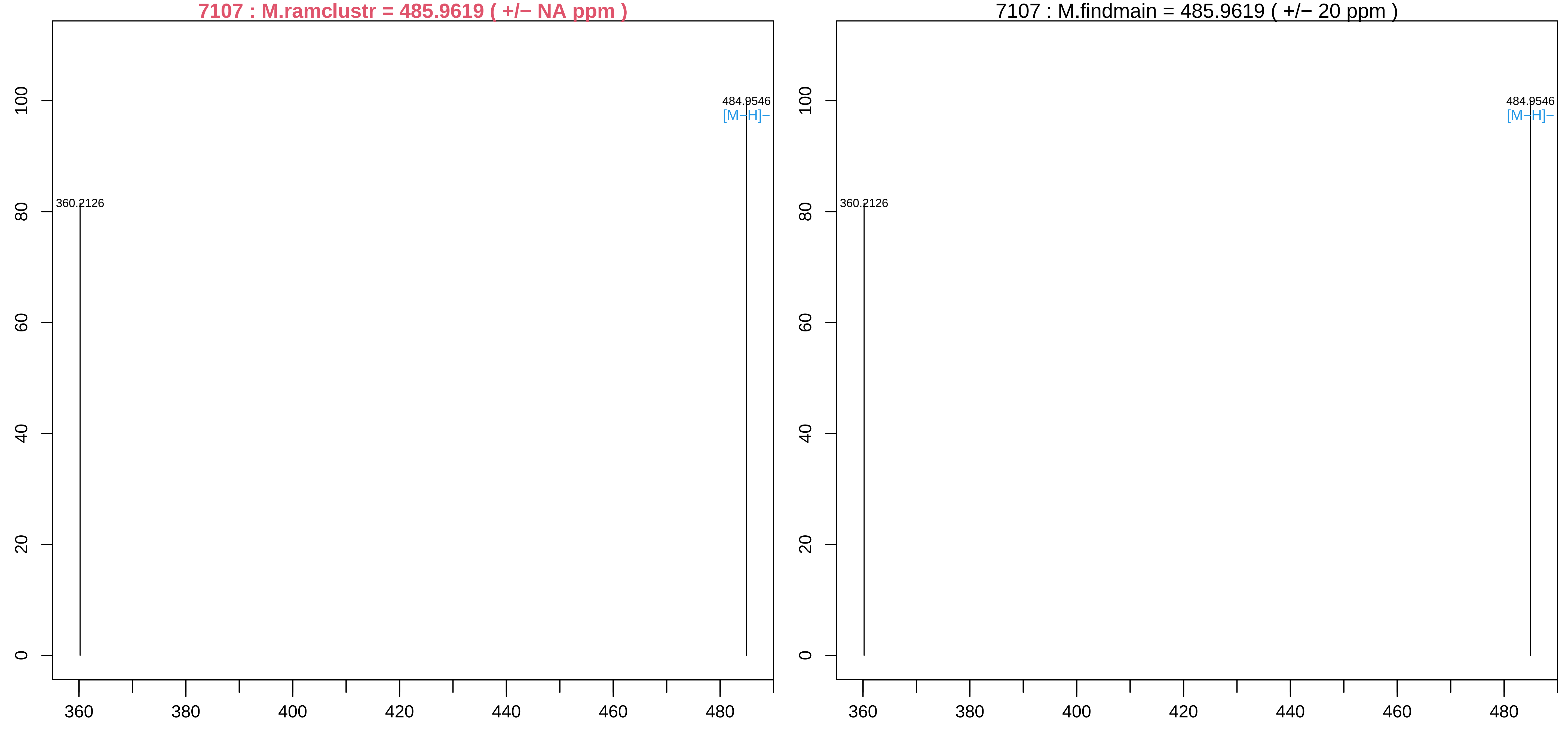
7107 : M.ramclustr = 485.9619 (+/− NA ppm)
484.9546
[M−H]−
360.2126
0
20
40
60
80
100
360
380
400
420
440
460
480
7107 : M.findmain = 485.9619 (+/− 20 ppm)
484.9546
[M−H]−
360.2126
0
20
40
60
80
100
360
380
400
420
440
460
480

7108 : M.ramclustr = 462.2091 (+/− 0.4 ppm)

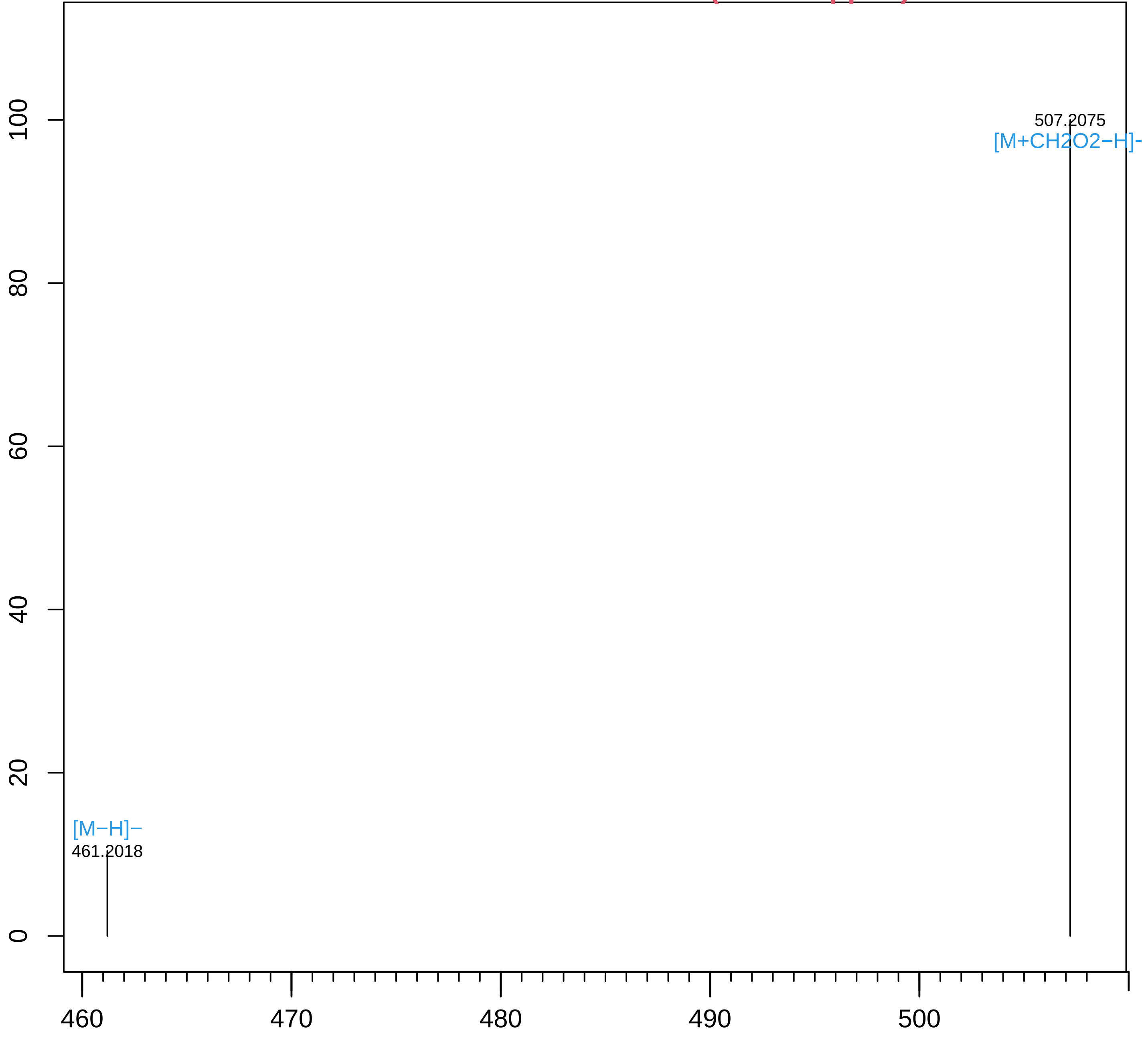

7108 : M.findmain = 462.2091 (+/− 0.4 ppm)

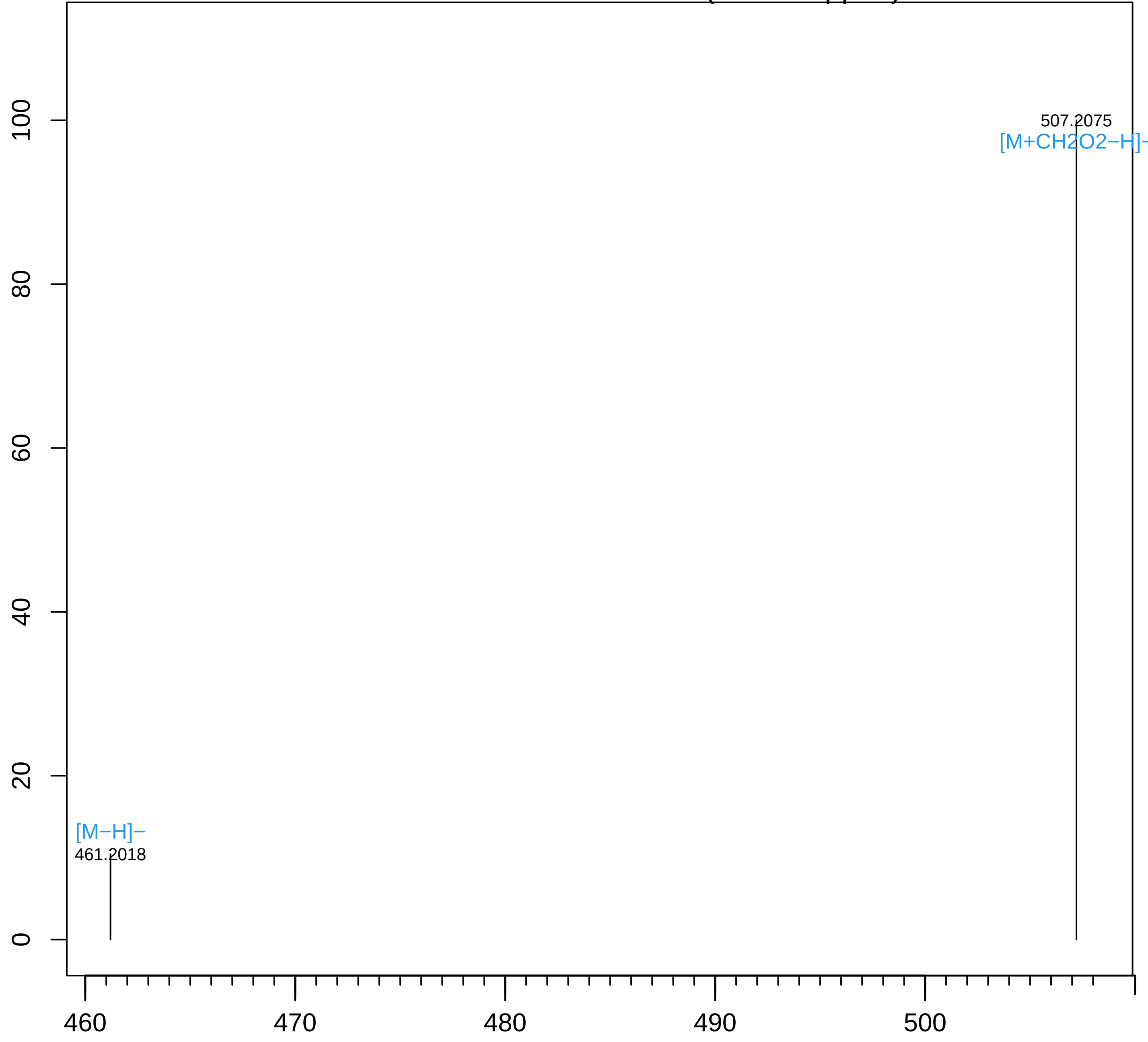

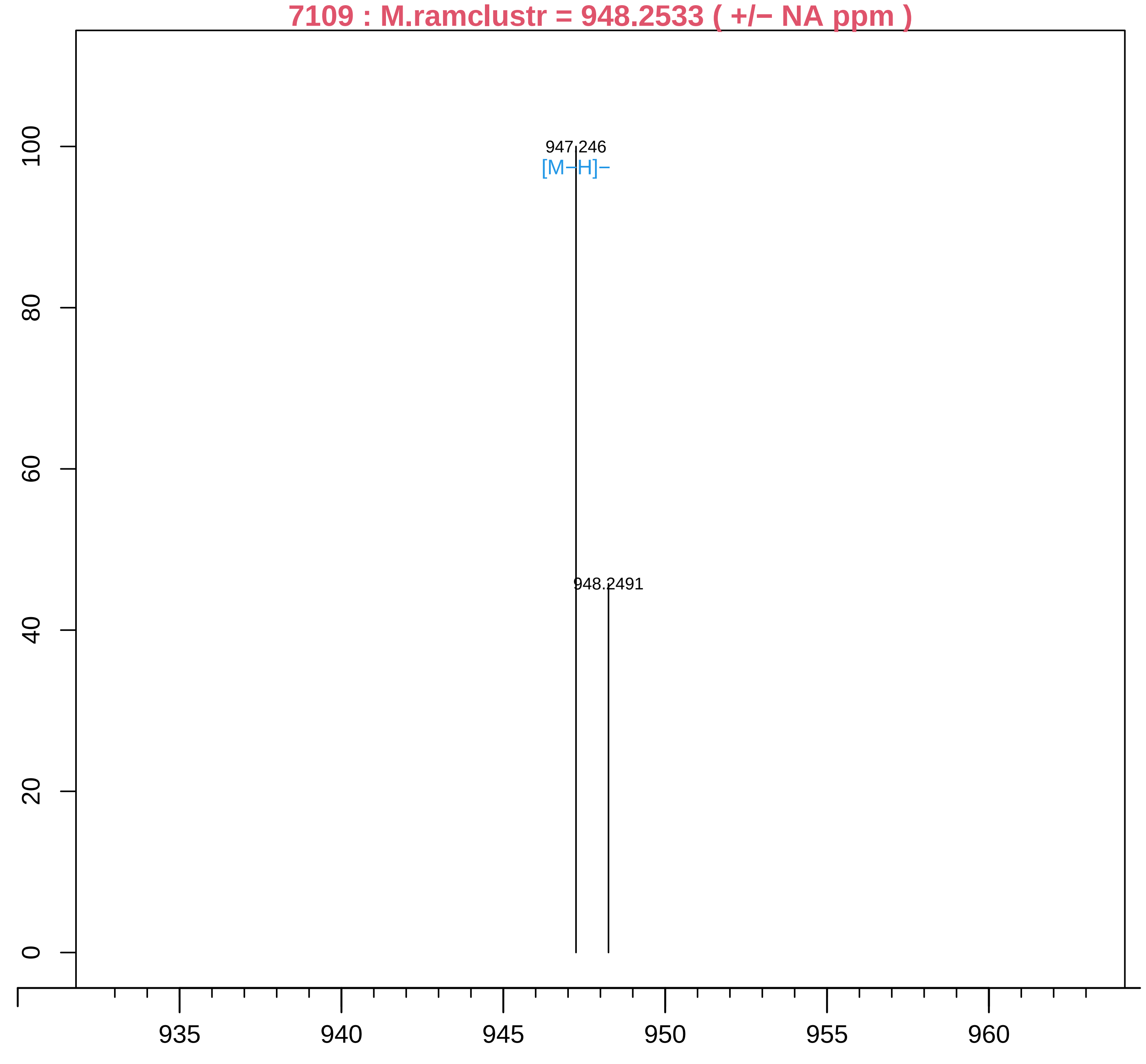
7109 : M.ramclustr = 948.2533 (+/− NA ppm)
947.246
[M−H]−
948.2491
0
20
40
60
80
100
935
940
945
950
955
960

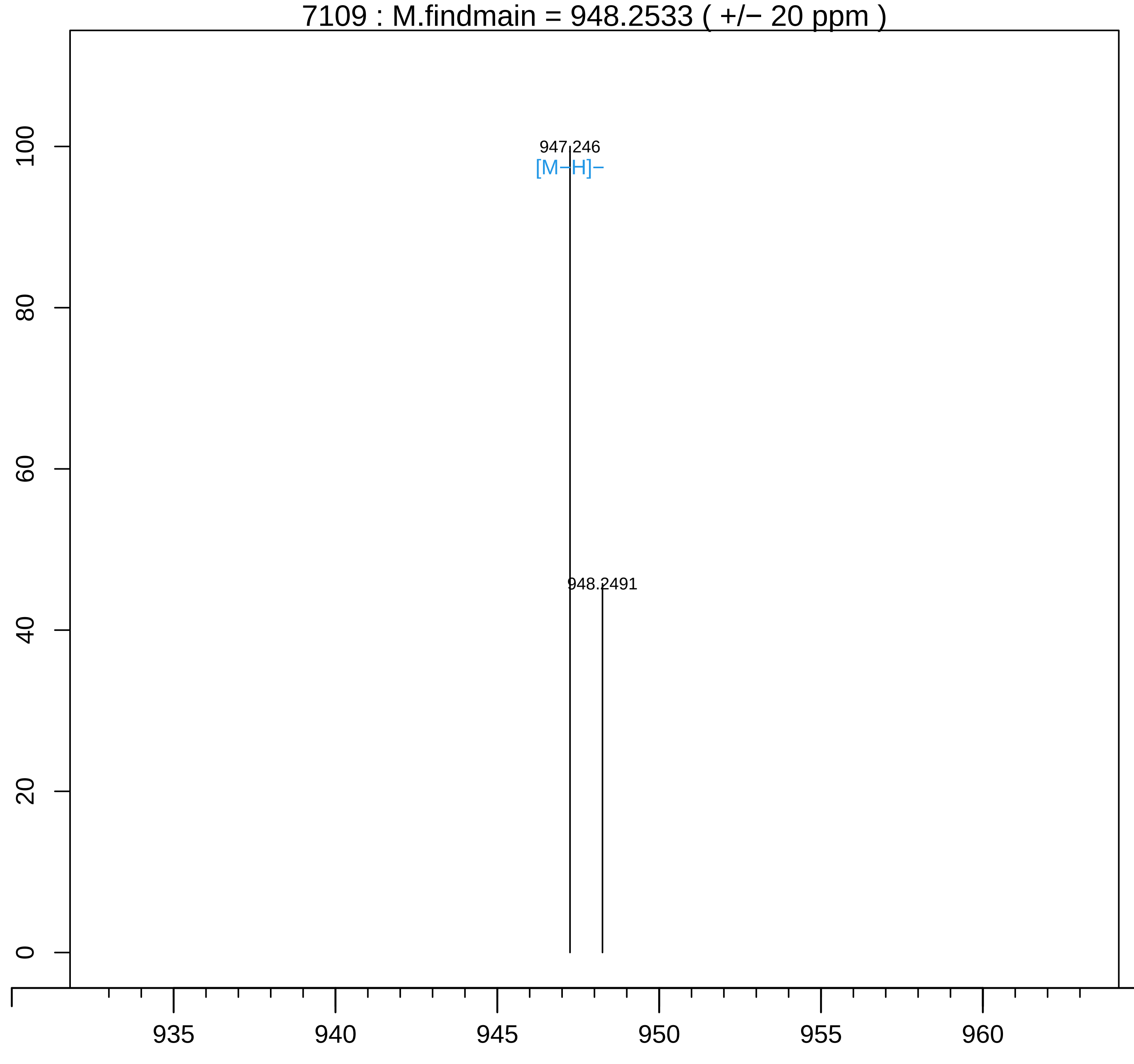
7109 : M.findmain = 948.2533 (+/− 20 ppm)
947.246
[M−H]−
948.2491
0
20
40
60
80
100
935
940
945
950
955
960

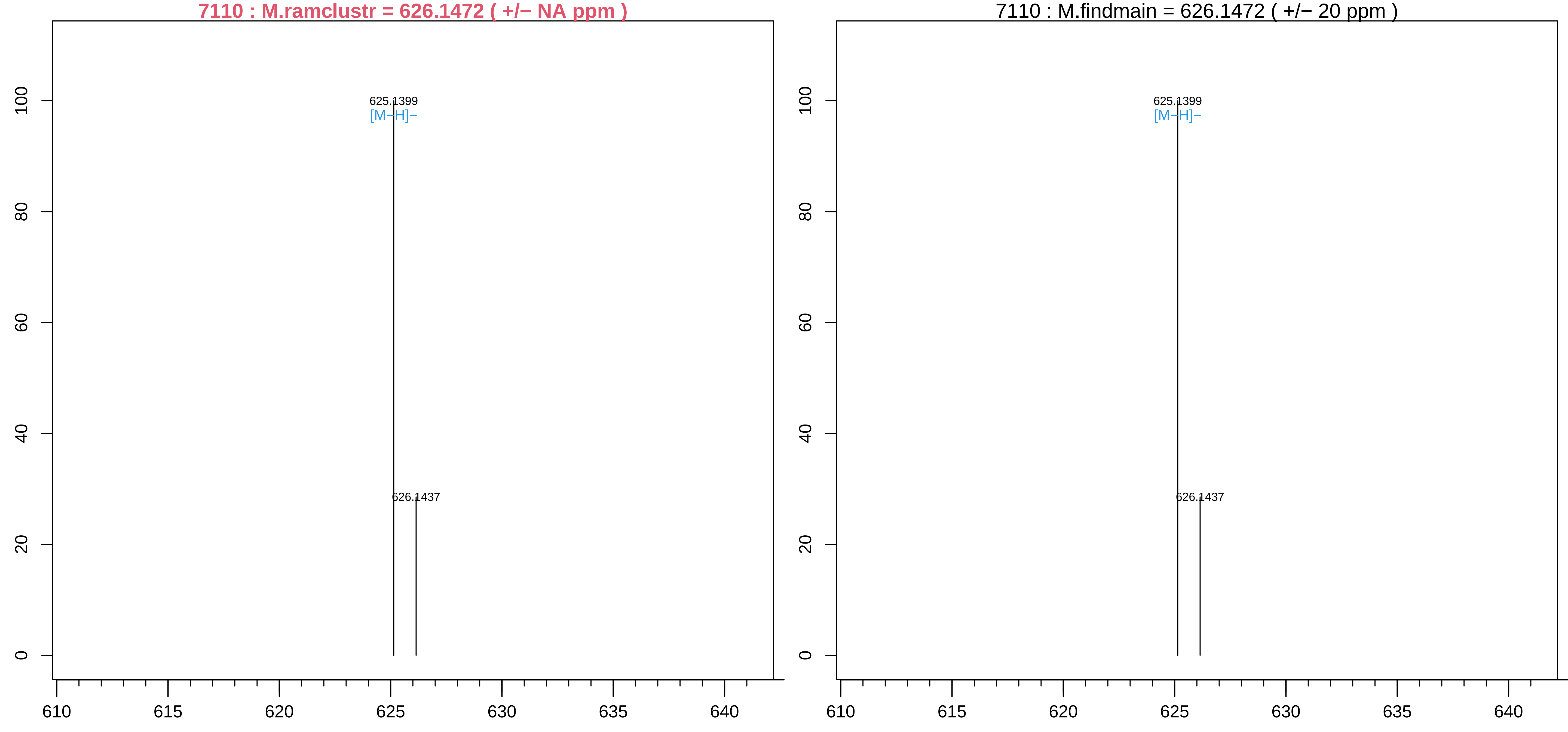
7110 : M.ramclustr = 626.1472 (+/− NA ppm)
625.1399
[M−H]−
626.1437
7110 : M.findmain = 626.1472 (+/− 20 ppm)
625.1399
[M−H]−
626.1437
0
20
40
60
80
100
610
615
620
625
630
635
640

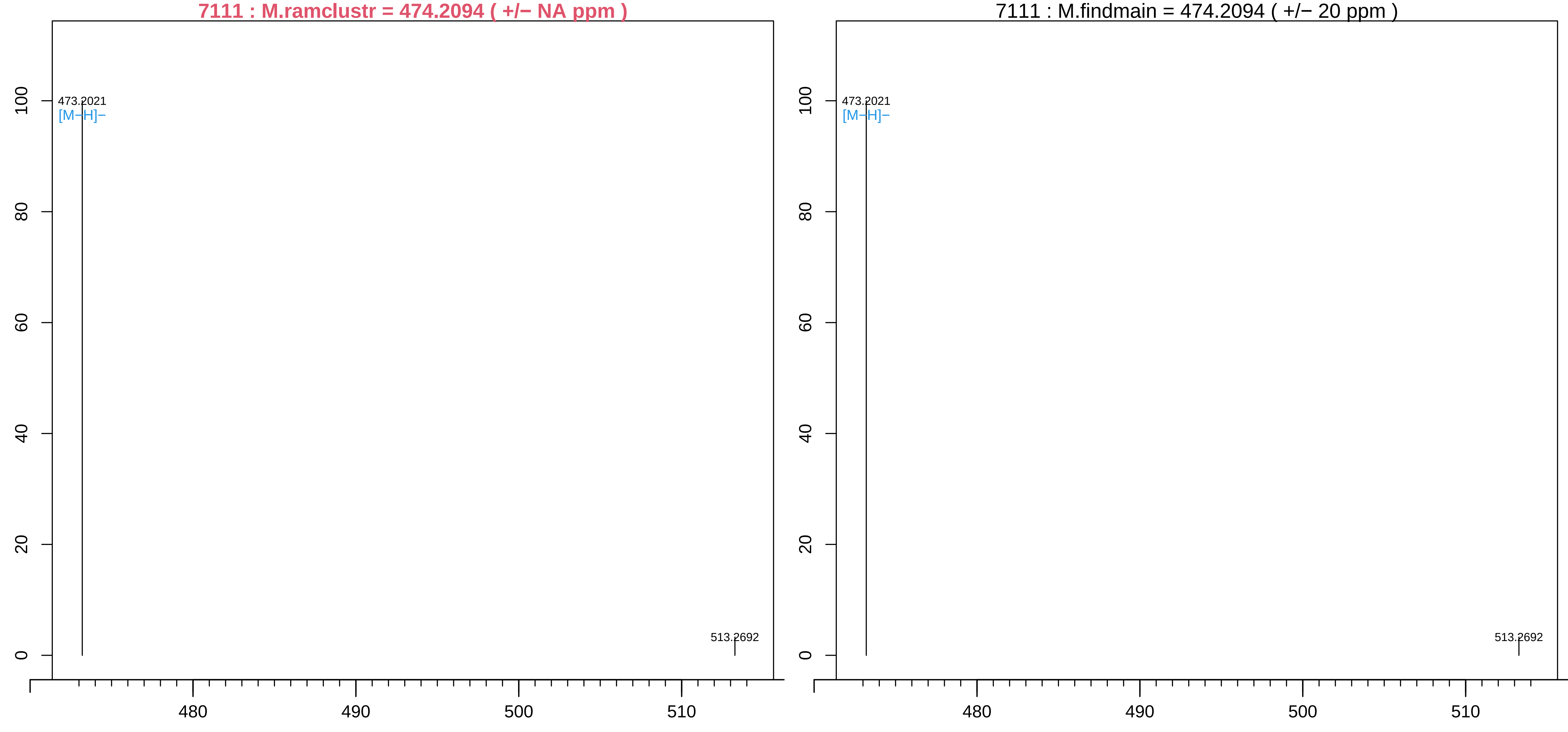
7111 : M.ramclustr = 474.2094 (+/− NA ppm)
7111 : M.findmain = 474.2094 (+/− 20 ppm)
473.2021
[M−H]−
513.2692
0
20
40
60
80
100
480
490
500
510

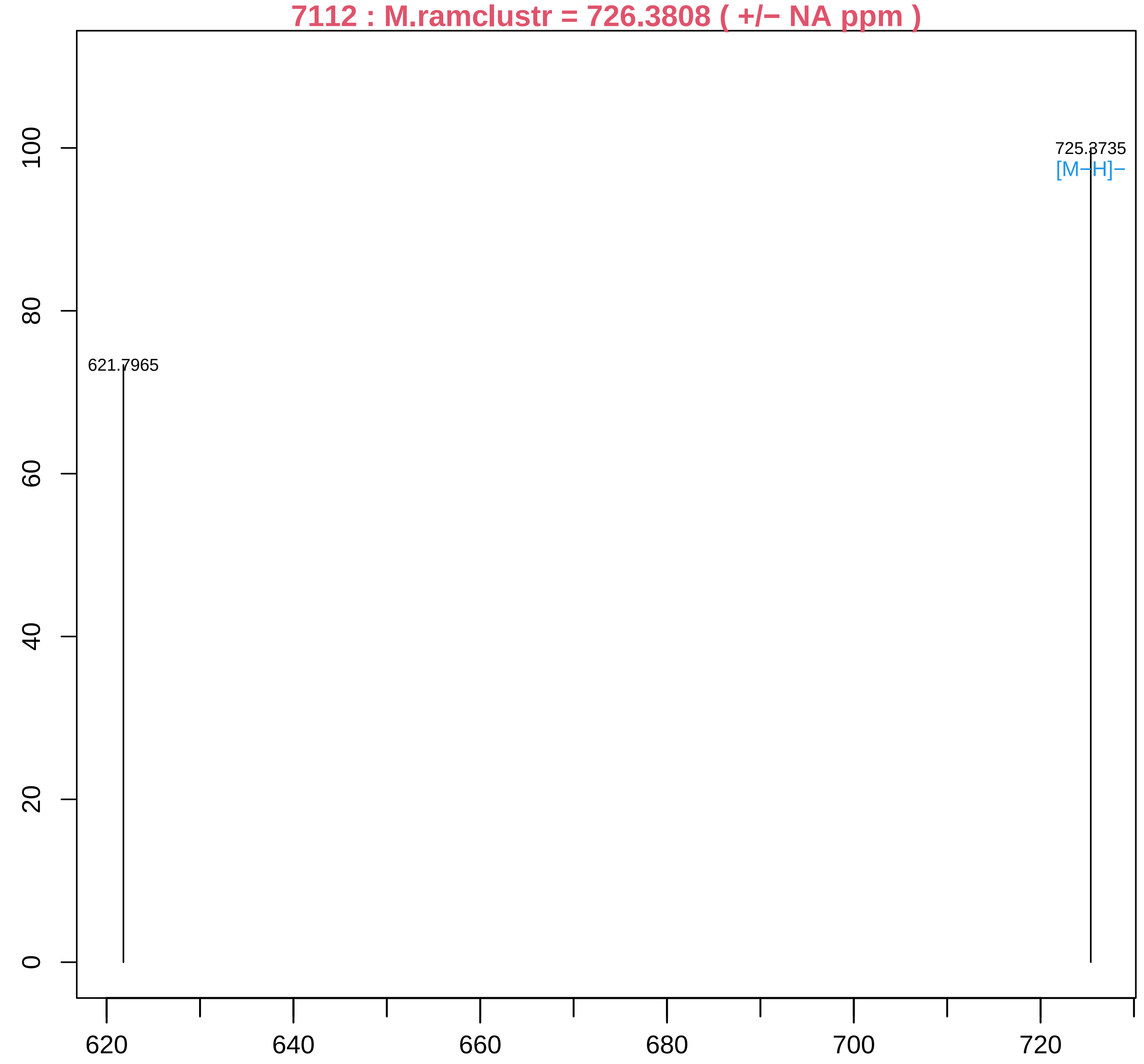

7112 : M.ramclustr = 726.3808 (+/− NA ppm)
725.3735
[M−H]−
621.7965
0
20
40
60
80
100
620
640
660
680
700
720

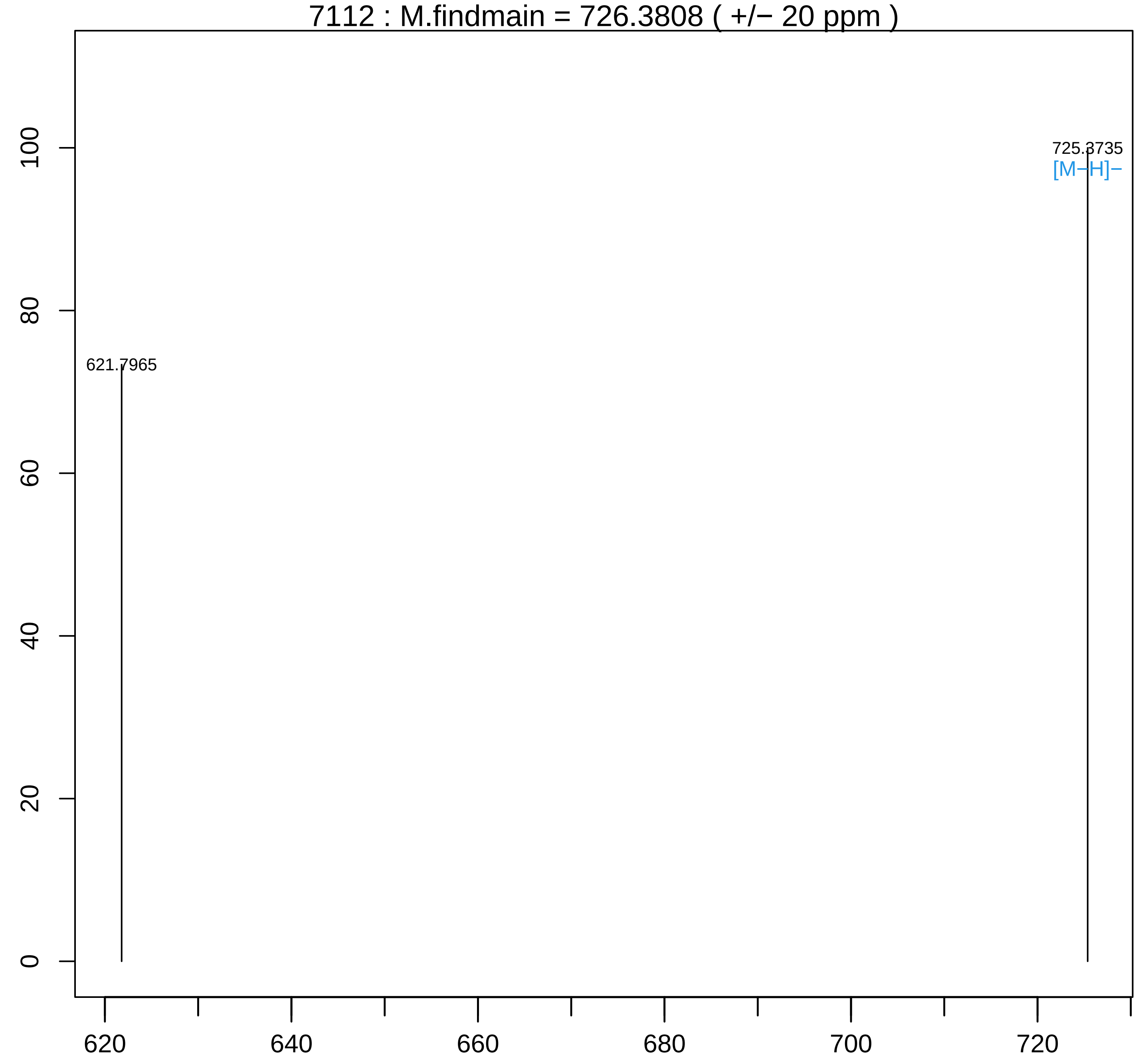

7112 : M.findmain = 726.3808 (+/− 20 ppm)
725.3735
[M−H]−
621.7965
0
20
40
60
80
100
620
640
660
680
700
720

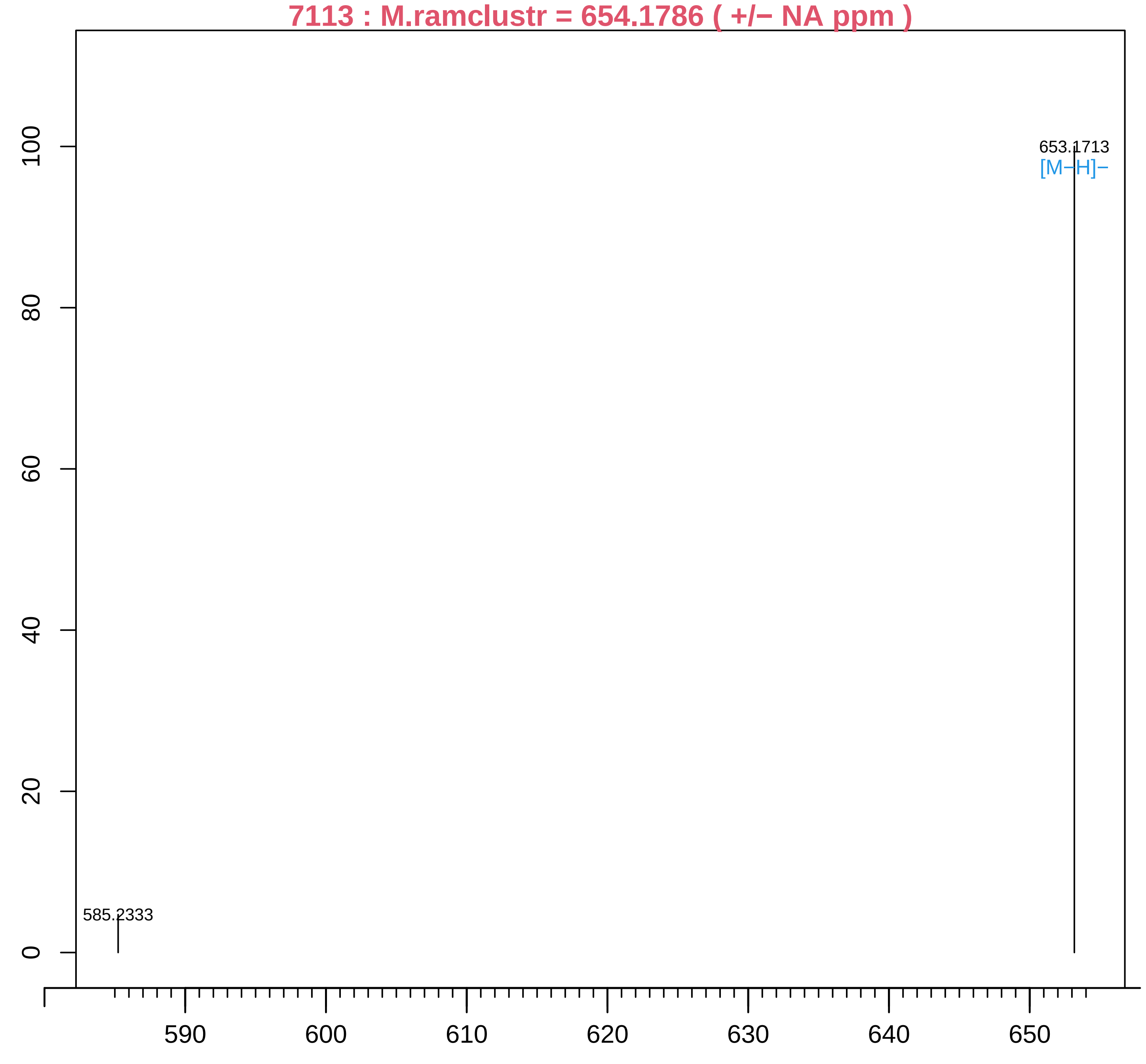
7113 : M.ramclustr = 654.1786 (+/− NA ppm)
653.1713
[M−H]−
585.2333
0
20
40
60
80
100
590
600
610
620
630
640
650

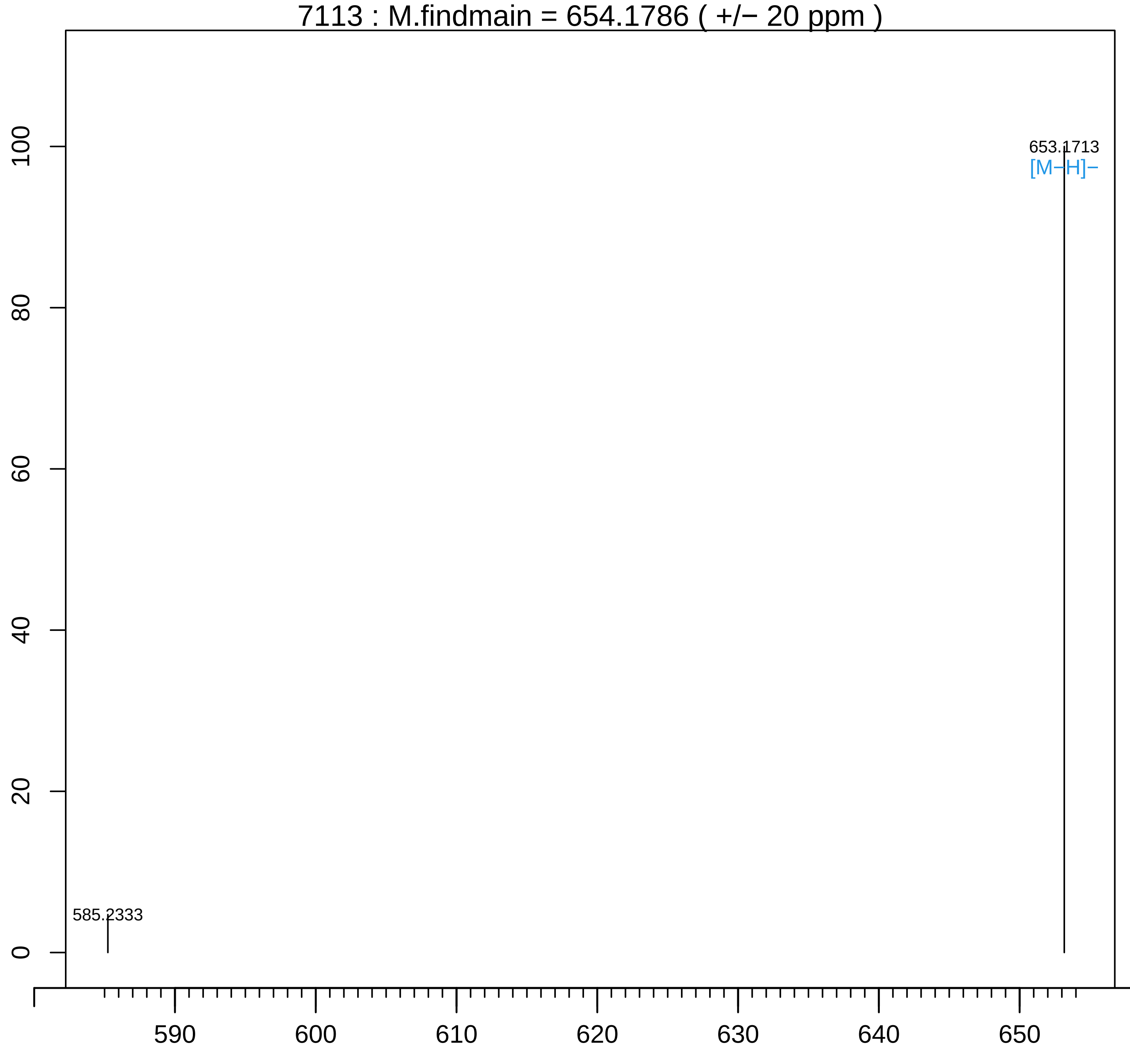
7113 : M.findmain = 654.1786 (+/− 20 ppm)
653.1713
[M−H]−
585.2333
0
20
40
60
80
100
590
600
610
620
630
640
650

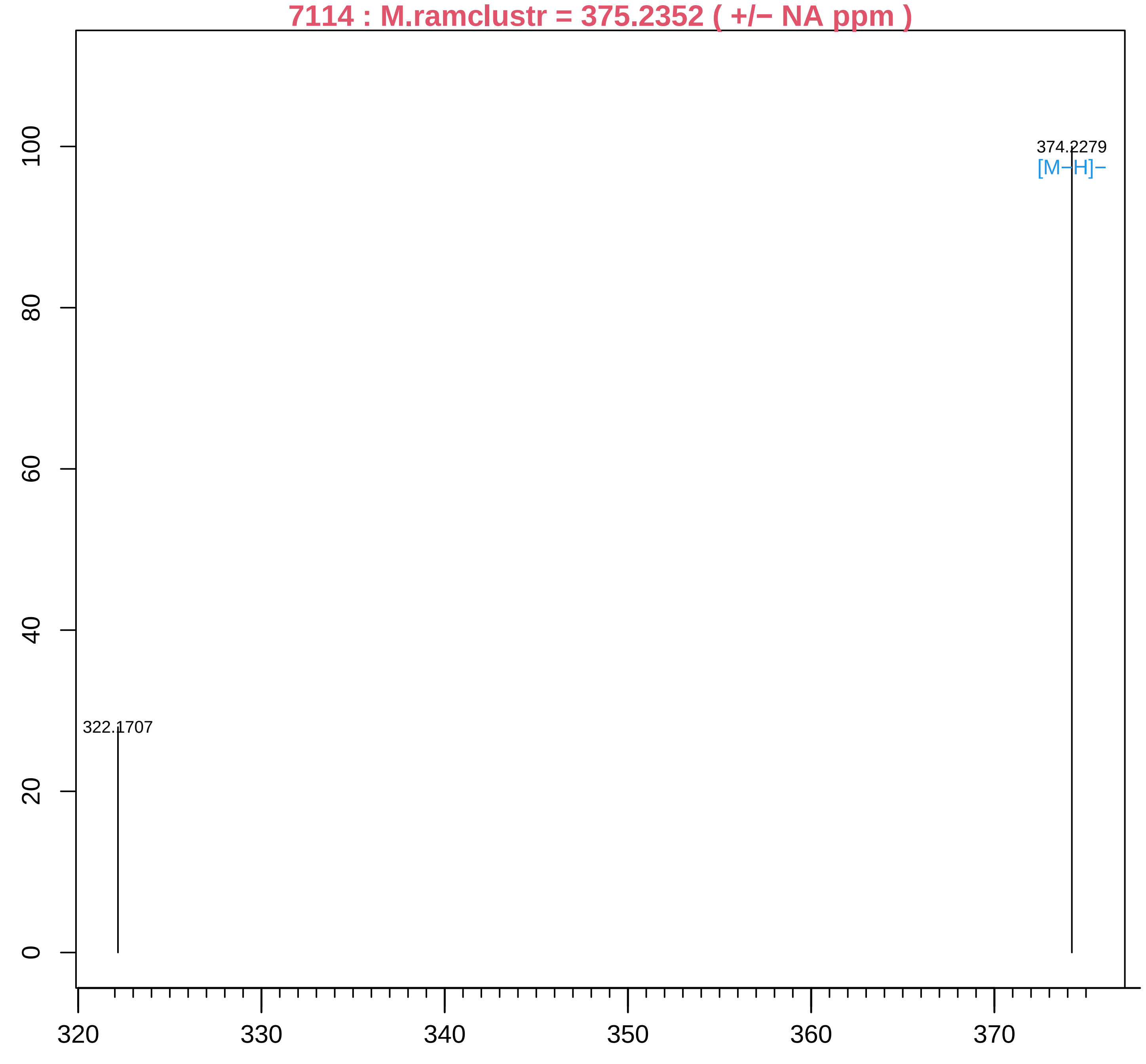
7114 : M.ramclustr = 375.2352 (+/− NA ppm)
374.2279
[M−H]−
322.1707

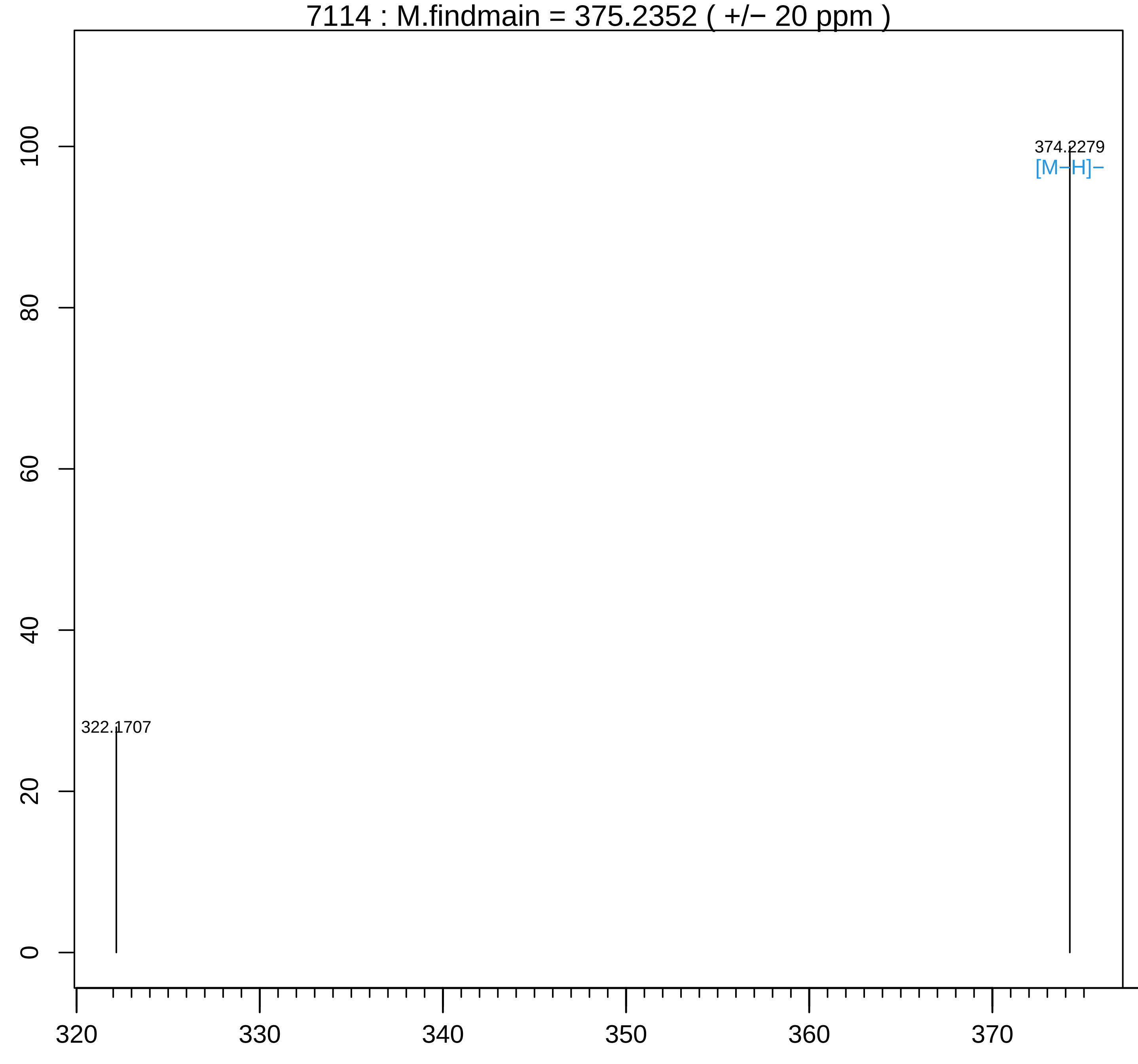
7114 : M.findmain = 375.2352 (+/− 20 ppm)
374.2279
[M−H]−
322.1707

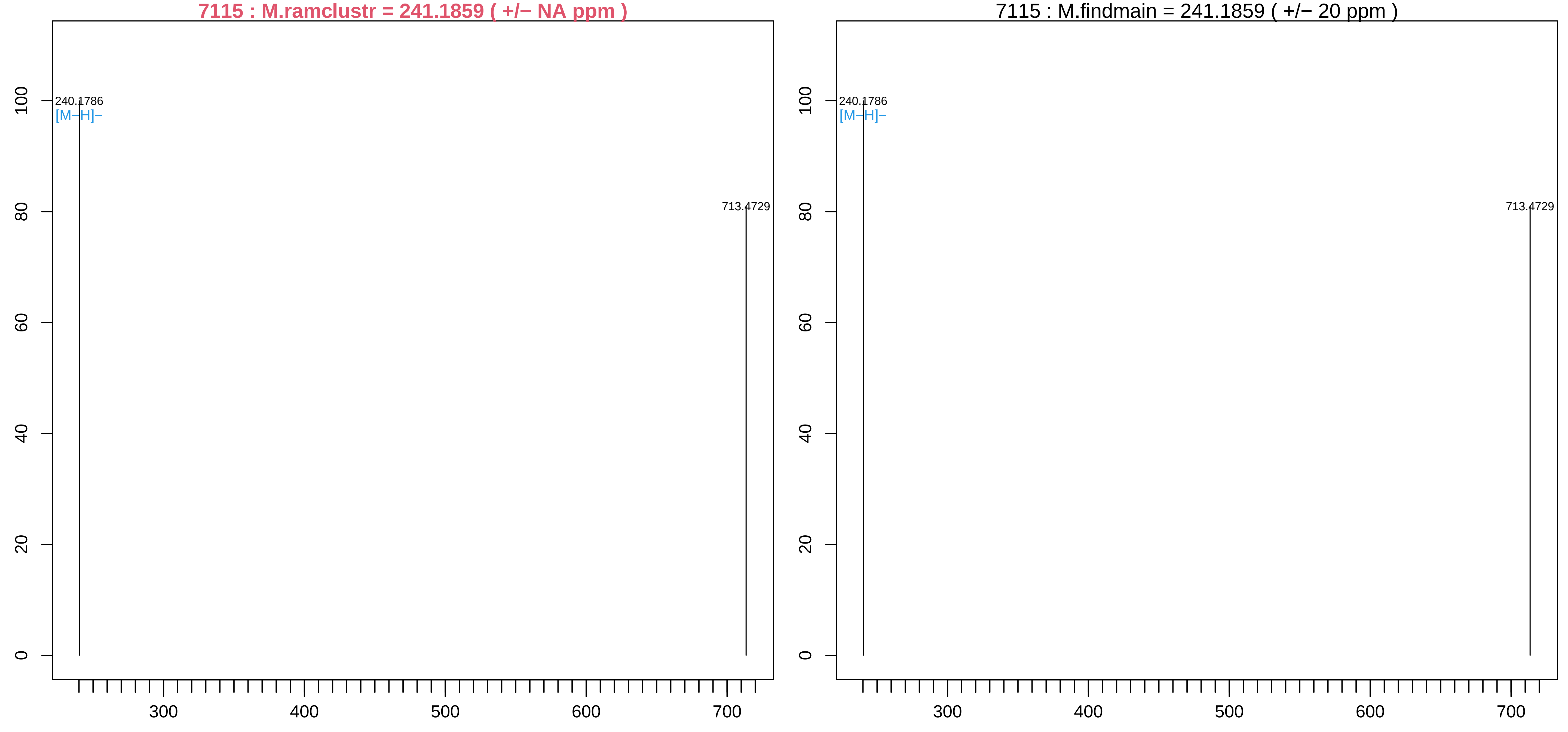
7115 : M.ramclustr = 241.1859 (+/− NA ppm)
240.1786
[M−H]−
713.4729
0
20
40
60
80
100
300
400
500
600
700
7115 : M.findmain = 241.1859 (+/− 20 ppm)
240.1786
[M−H]−
713.4729
0
20
40
60
80
100
300
400
500
600
700

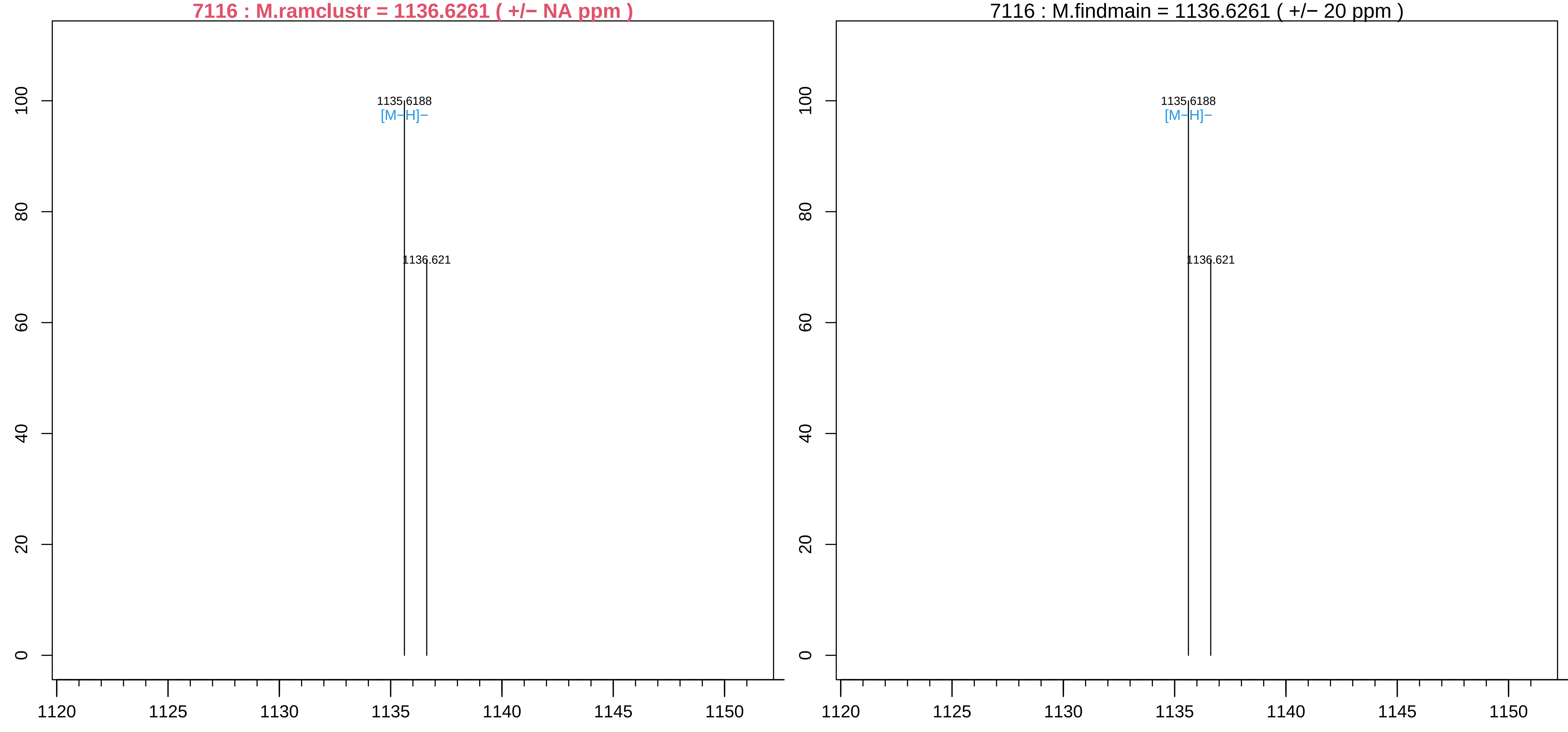
7116 : M.ramclustr = 1136.6261 (+/− NA ppm)
1135.6188
[M−H]−
1136.621
7116 : M.findmain = 1136.6261 (+/− 20 ppm)
1135.6188
[M−H]−
1136.621
0
20
40
60
80
100
1120
1125
1130
1135
1140
1145
1150

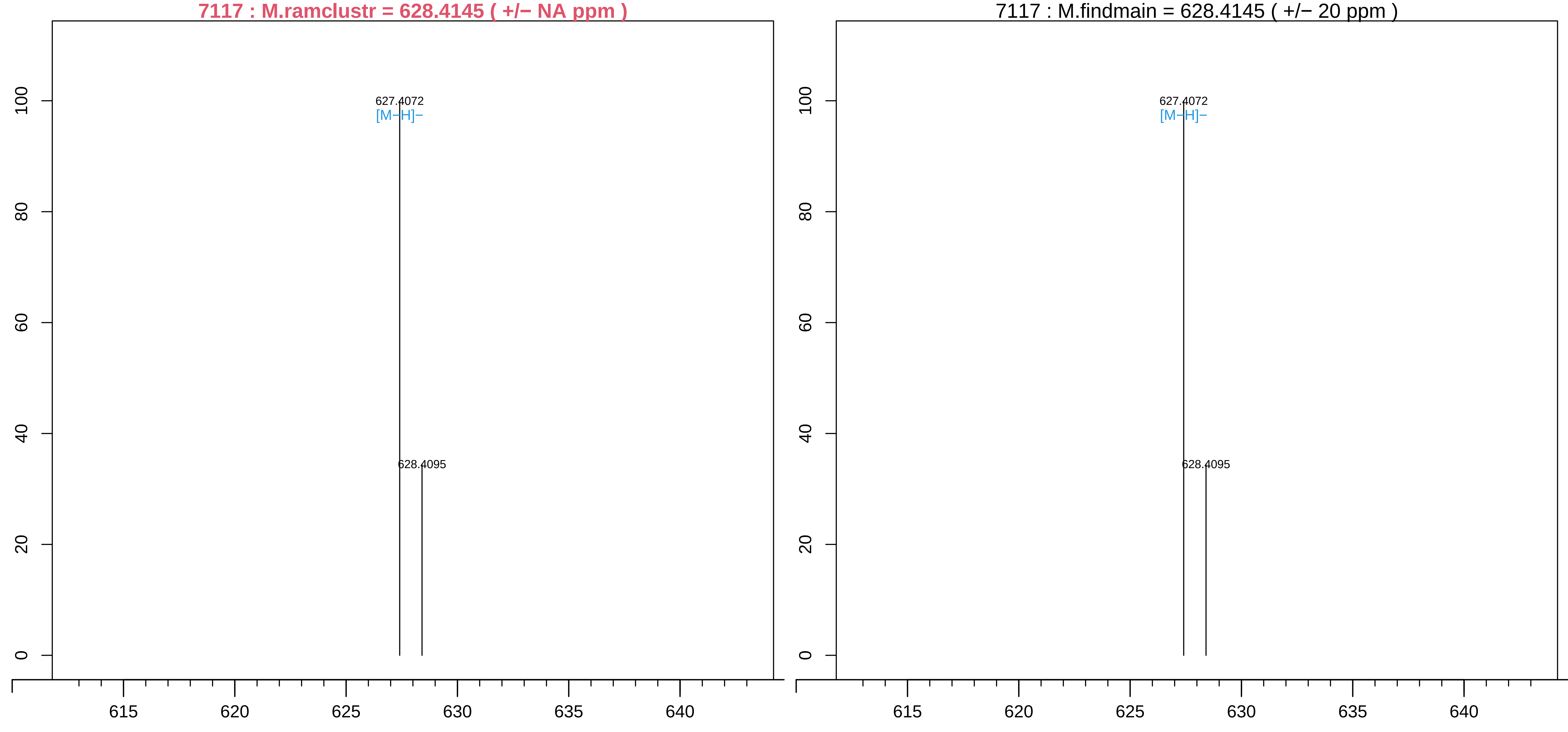

7117 : M.ramclustr = 628.4145 (+/− NA ppm)
627.4072
[M−H]−
628.4095
0
20
40
60
80
100
615
620
625
630
635
640
7117 : M.findmain = 628.4145 (+/− 20 ppm)
627.4072
[M−H]−
628.4095
0
20
40
60
80
100
615
620
625
630
635
640

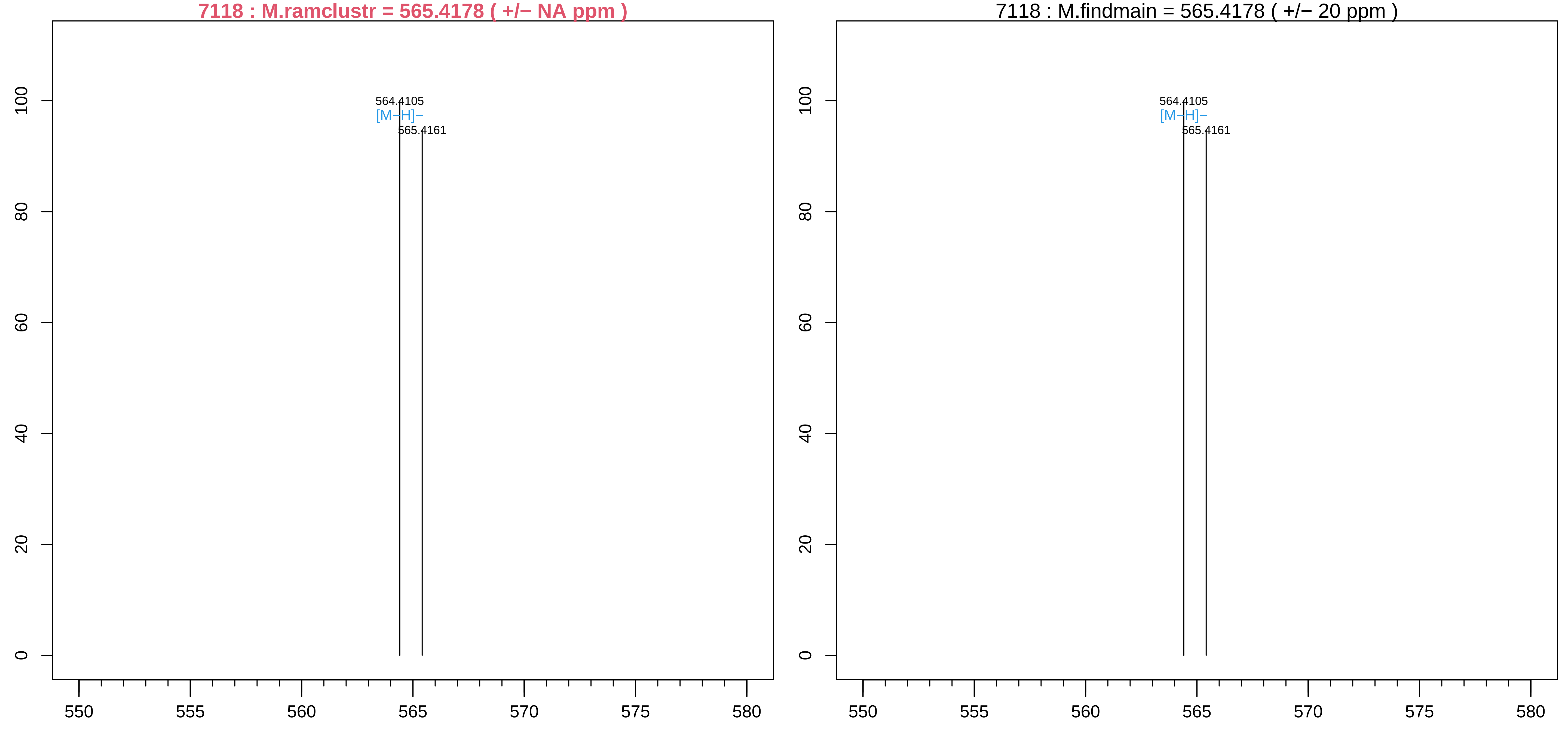
7118 : M.ramclustr = 565.4178 (+/− NA ppm)
564.4105
[M−H]−
565.4161
0
20
40
60
80
100
550
555
560
565
570
575
580
7118 : M.findmain = 565.4178 (+/− 20 ppm)
564.4105
[M−H]−
565.4161
0
20
40
60
80
100
550
555
560
565
570
575
580

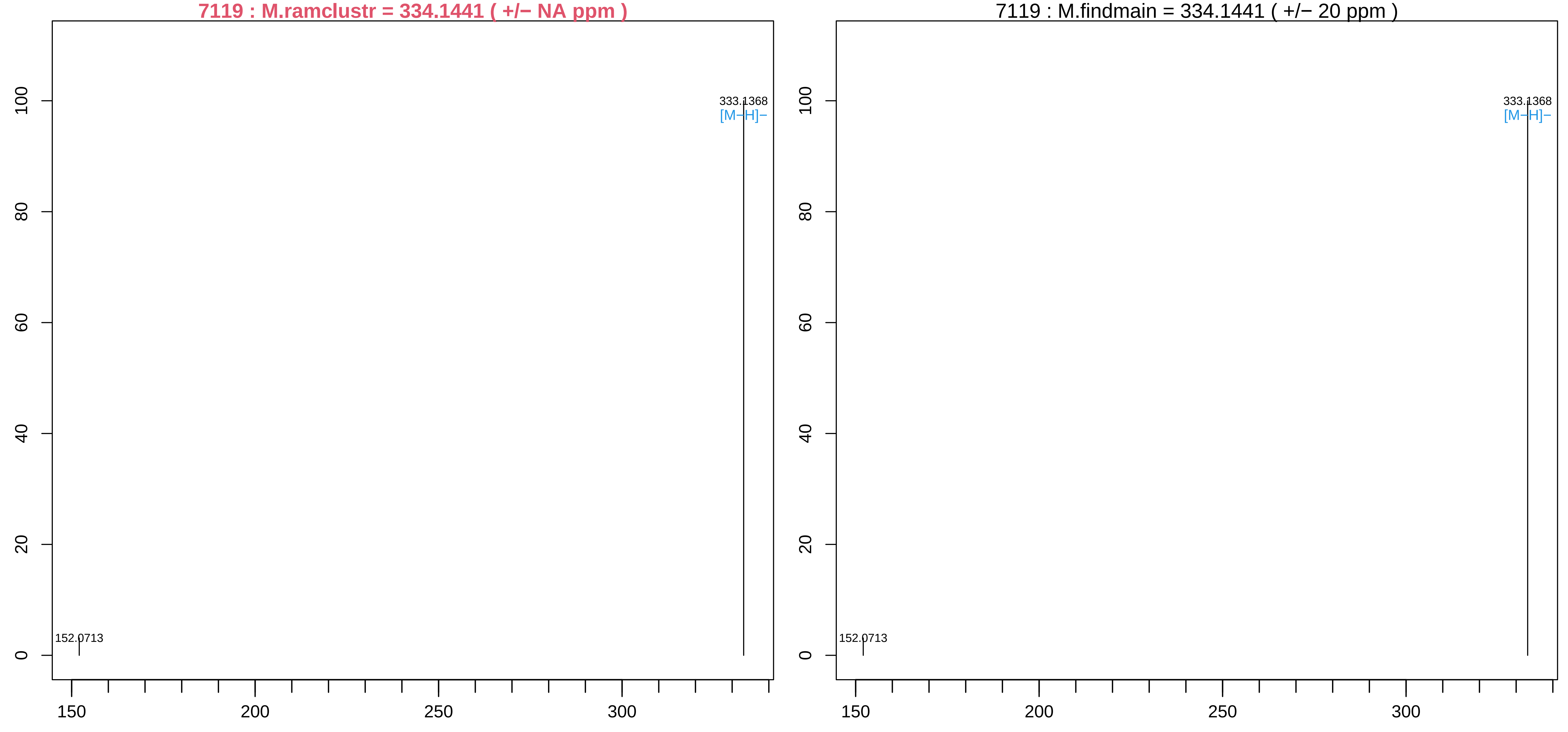
7119 : M.ramclustr = 334.1441 (+/− NA ppm)
333.1368
[M−H]−
152.0713
0
20
40
60
80
100
150
200
250
300
7119 : M.findmain = 334.1441 (+/− 20 ppm)
333.1368
[M−H]−
152.0713
0
20
40
60
80
100
150
200
250
300

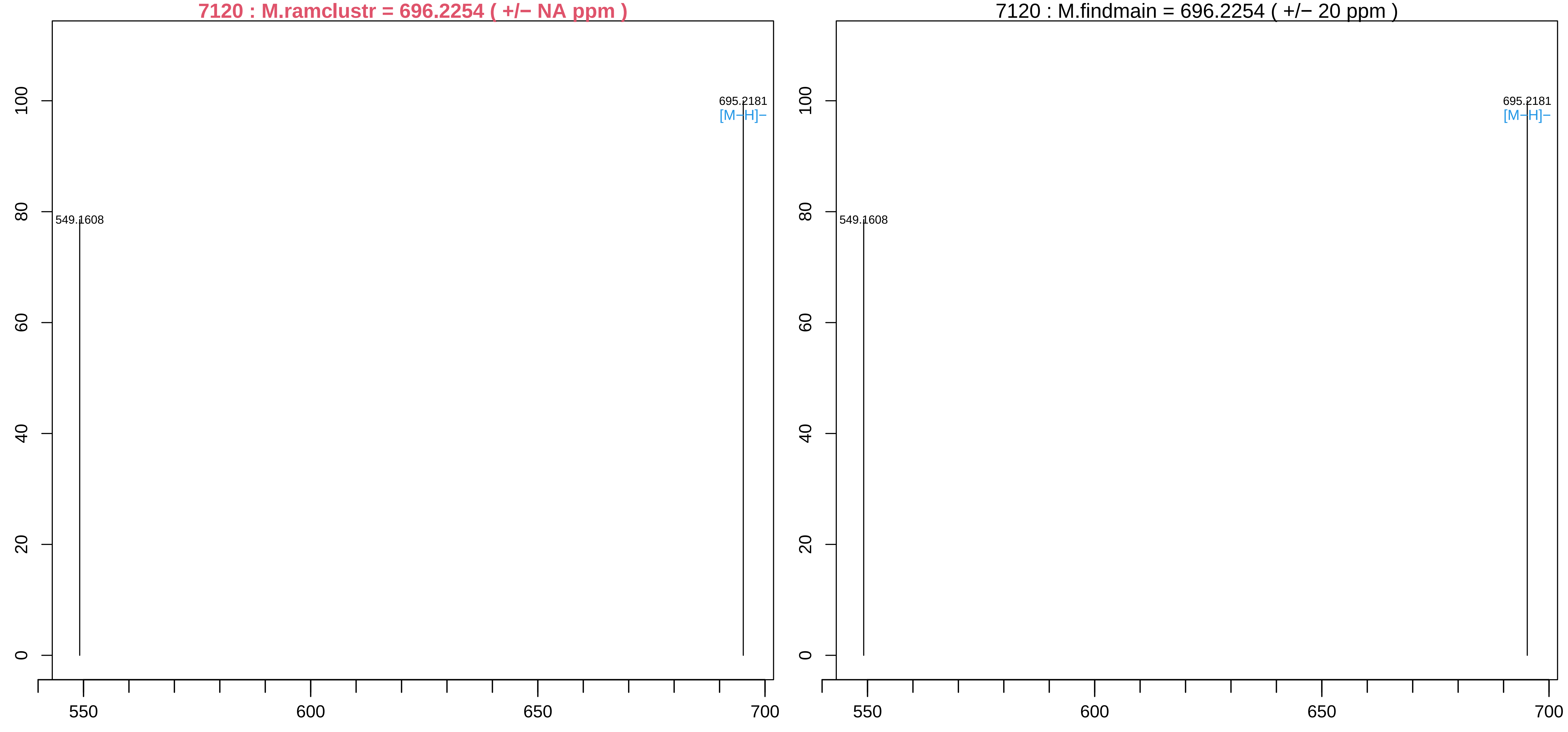

7120 : M.ramclustr = 696.2254 (+/− NA ppm)
695.2181
[M−H]−
549.1608
7120 : M.findmain = 696.2254 (+/− 20 ppm)
695.2181
[M−H]−
549.1608
0
20
40
60
80
100
550
600
650
700

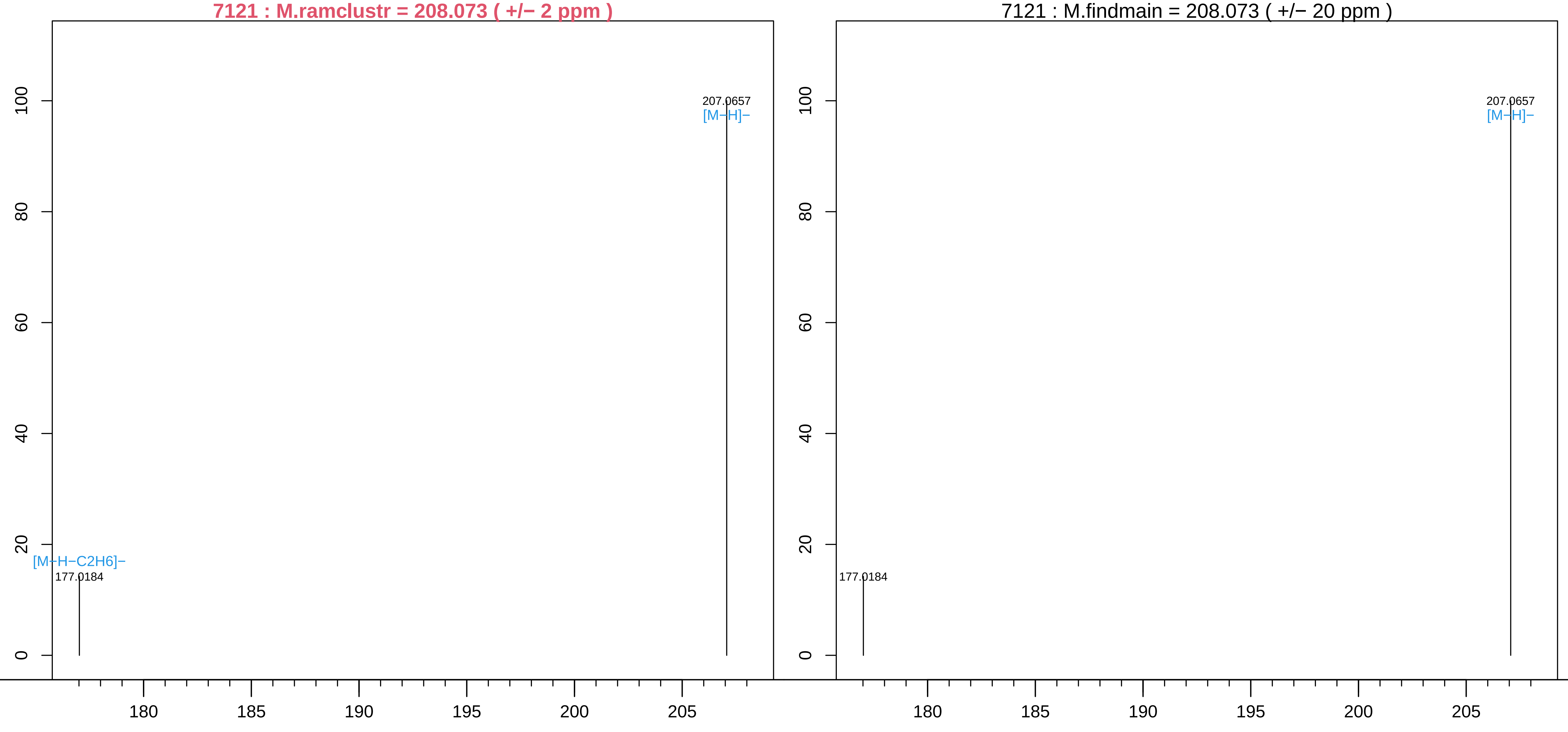
7121 : M.ramclustr = 208.073 (+/− 2 ppm)
207.0657
[M−H]−
[M−H−C2H6]−
177.0184
7121 : M.findmain = 208.073 (+/− 20 ppm)
207.0657
[M−H]−
177.0184
0
20
40
60
80
100
180
185
190
195
200
205

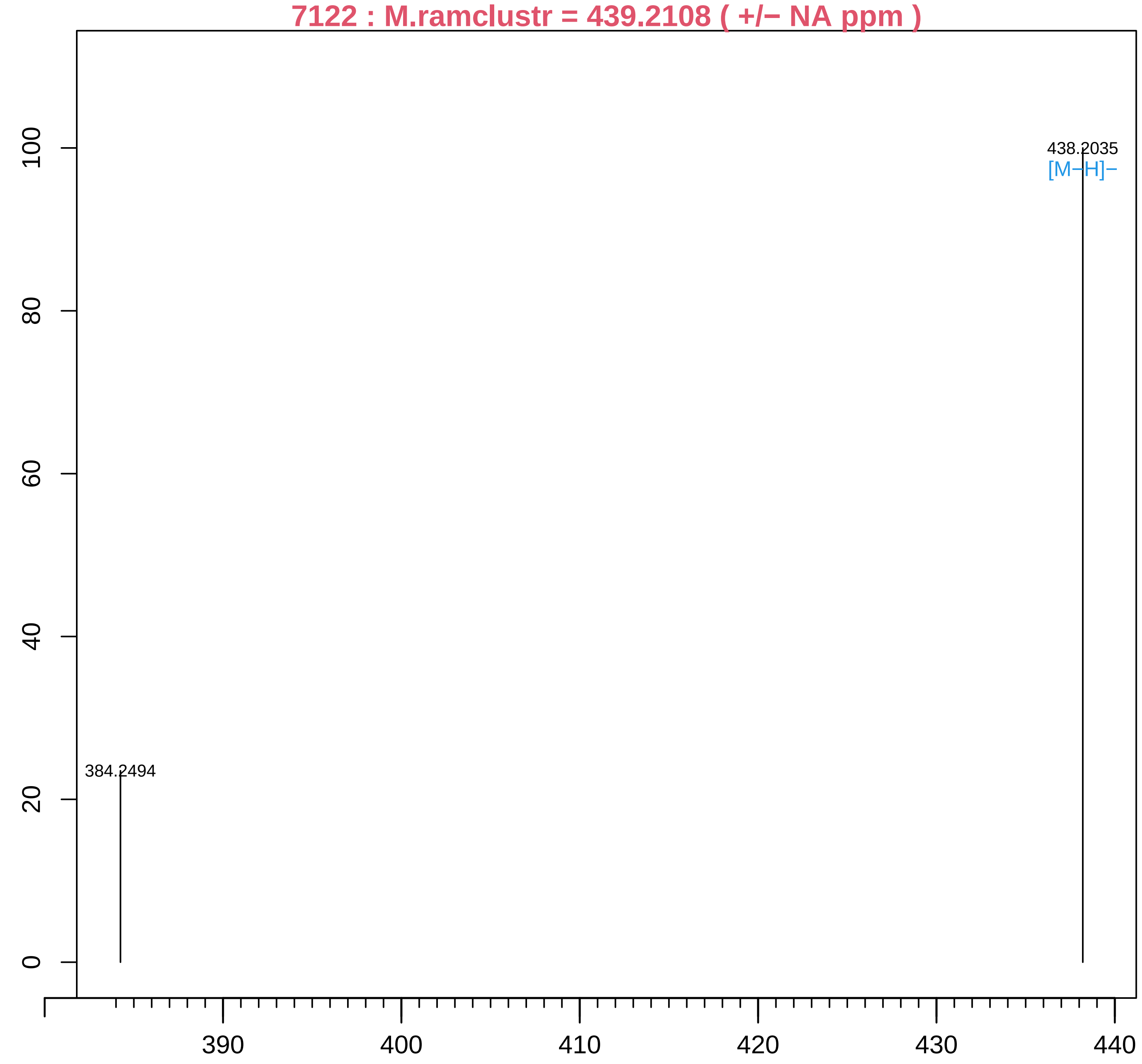
7122 : M.ramclustr = 439.2108 (+/− NA ppm)
438.2035
[M−H]−
384.2494
0
20
40
60
80
100
390
400
410
420
430
440

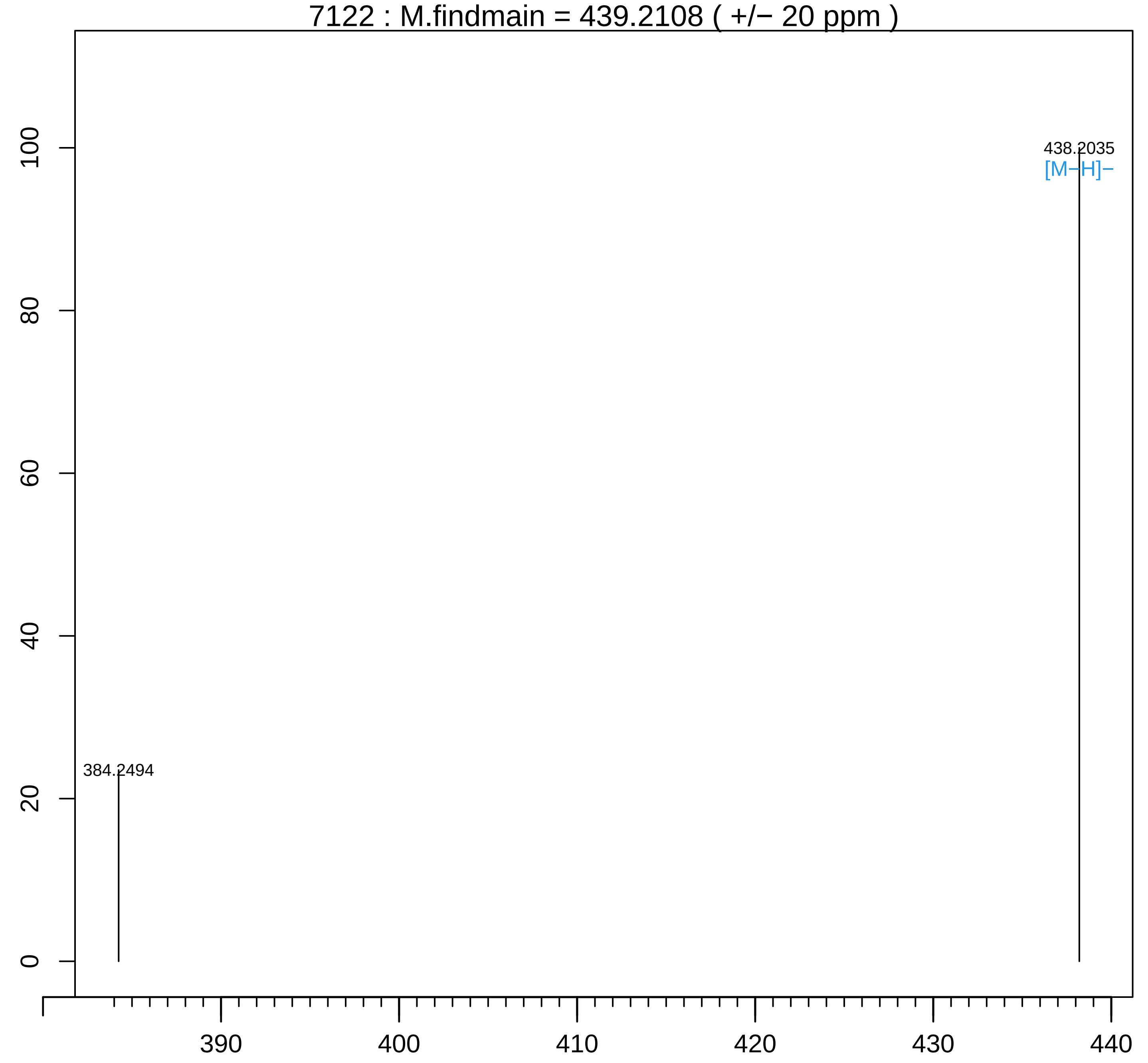
7122 : M.findmain = 439.2108 (+/− 20 ppm)
438.2035
[M−H]−
384.2494
0
20
40
60
80
100
390
400
410
420
430
440

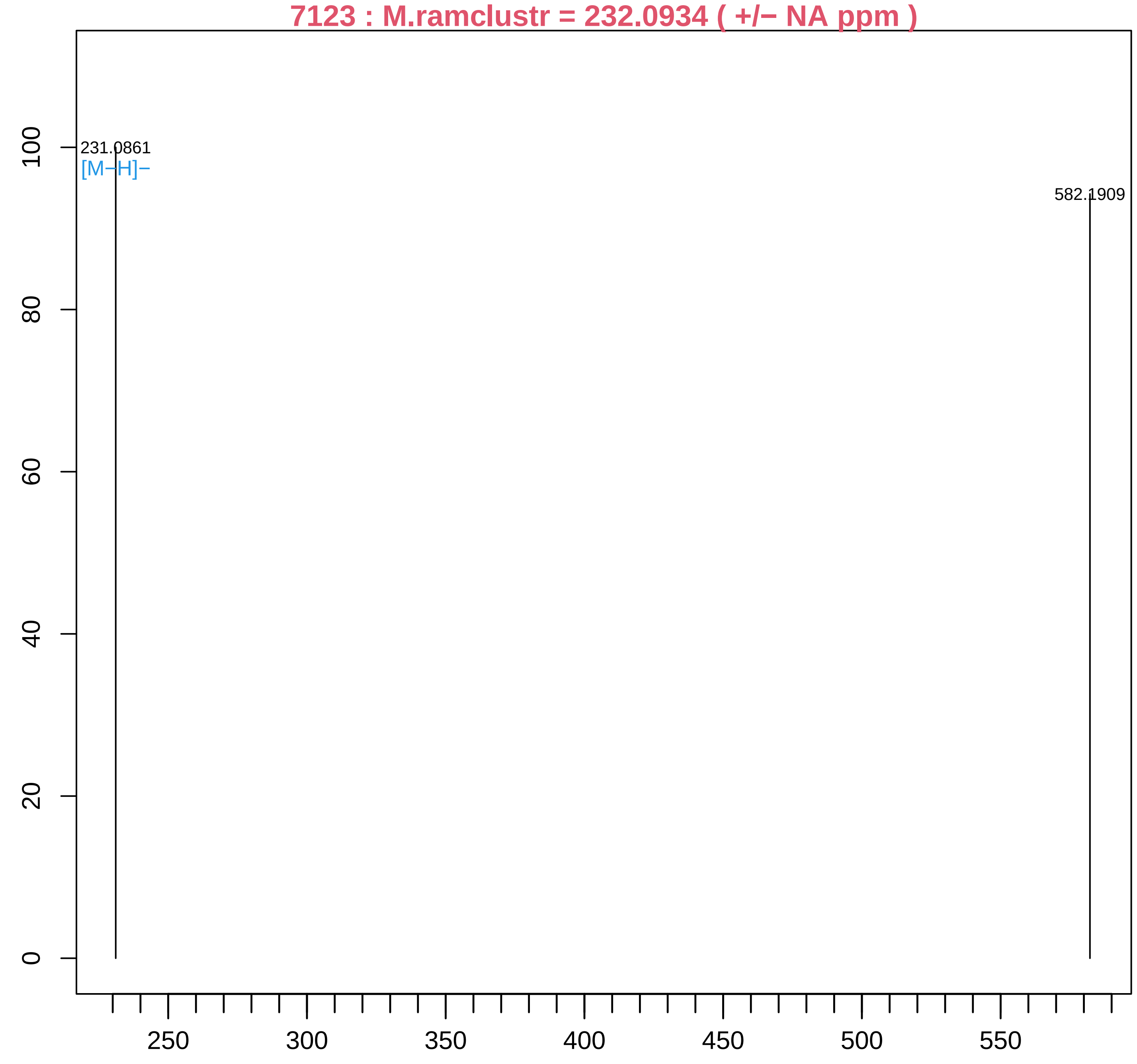

7123 : M.ramclustr = 232.0934 (+/− NA ppm)
231.0861
[M−H]−
582.1909
0
20
40
60
80
100
250
300
350
400
450
500
550

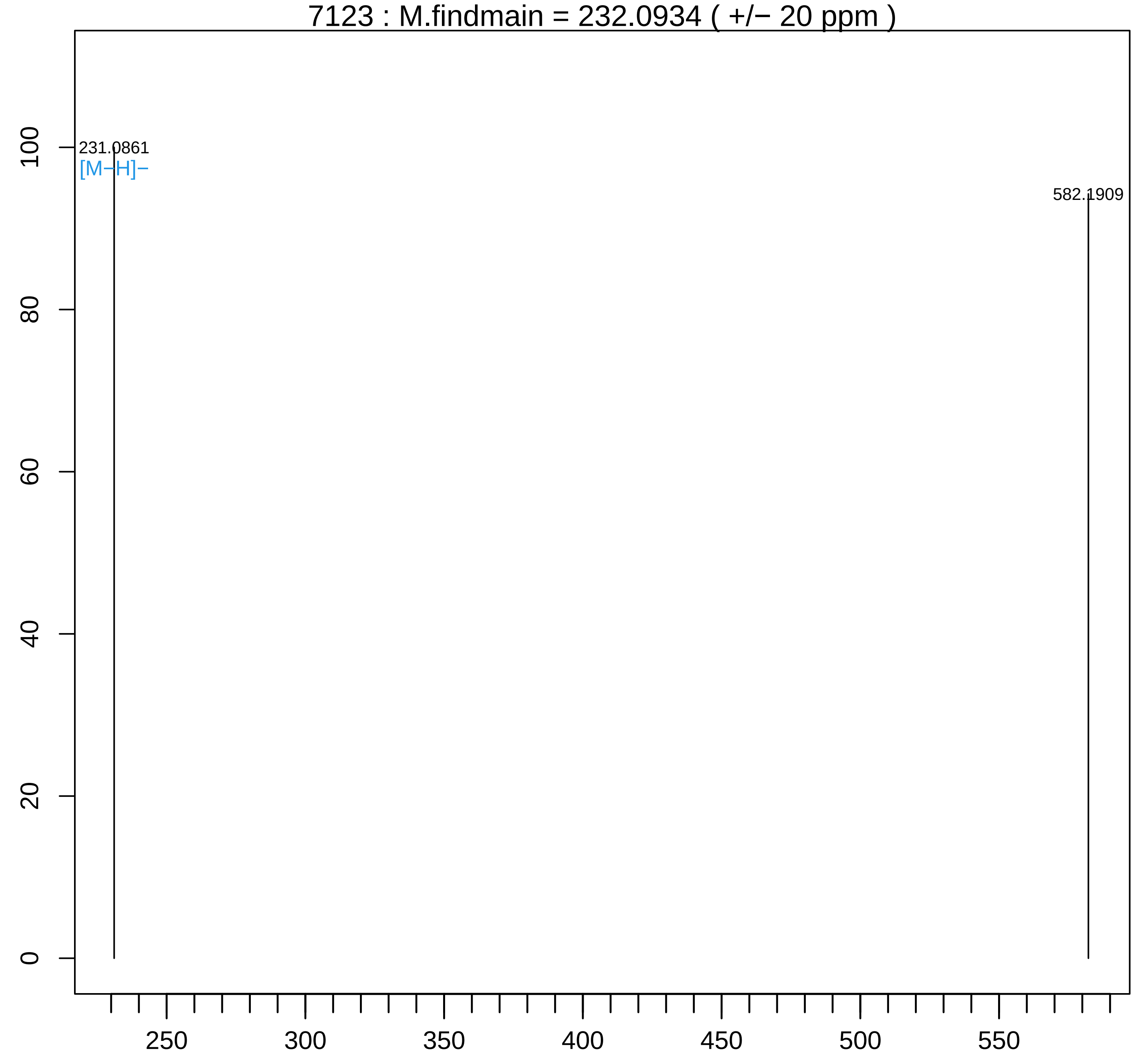

7123 : M.findmain = 232.0934 (+/− 20 ppm)
231.0861
[M−H]−
582.1909
0
20
40
60
80
100
250
300
350
400
450
500
550

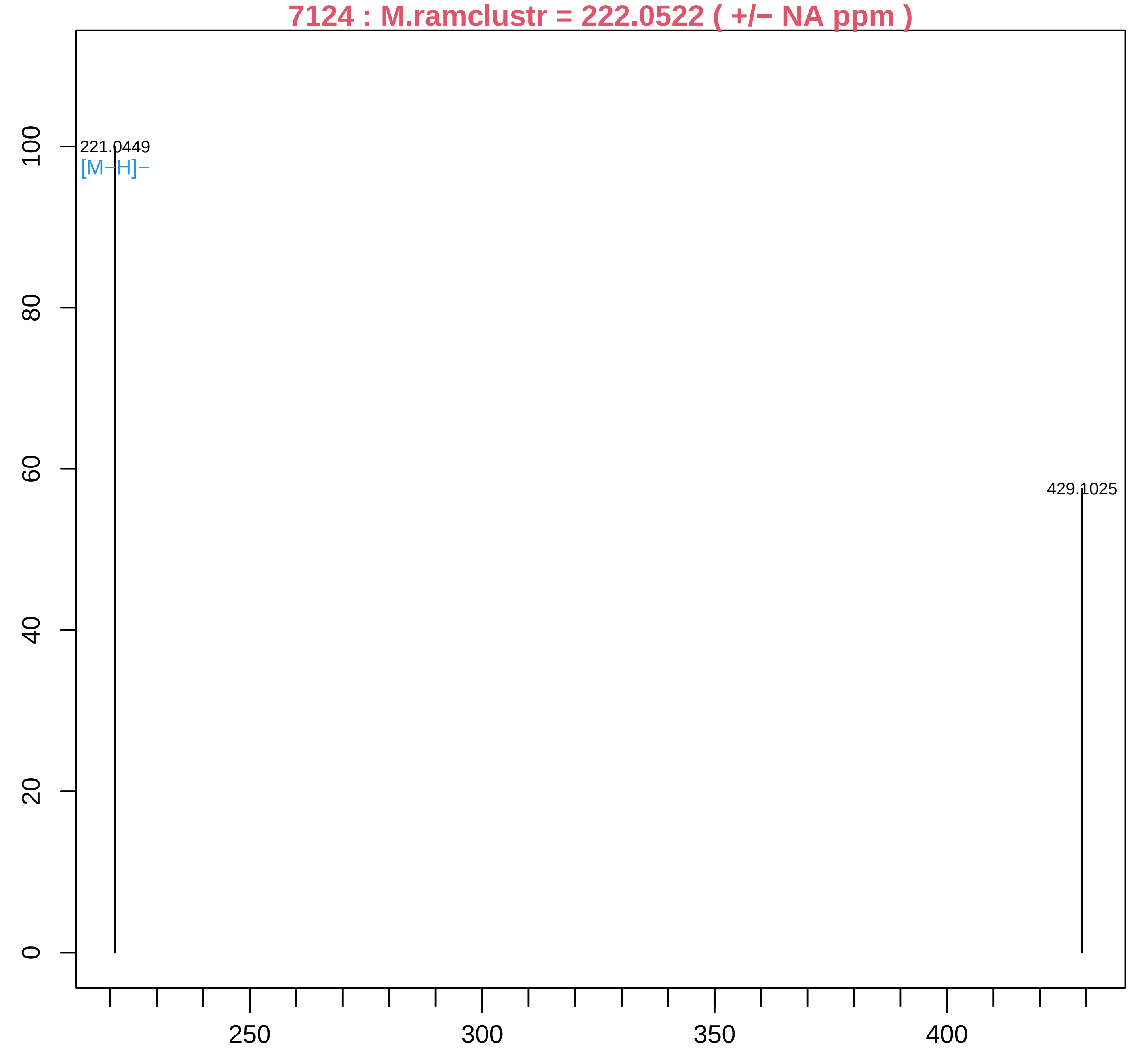
7124 : M.ramclustr = 222.0522 (+/− NA ppm)
221.0449
[M−H]−
429.1025
0
20
40
60
80
100
250
300
350
400

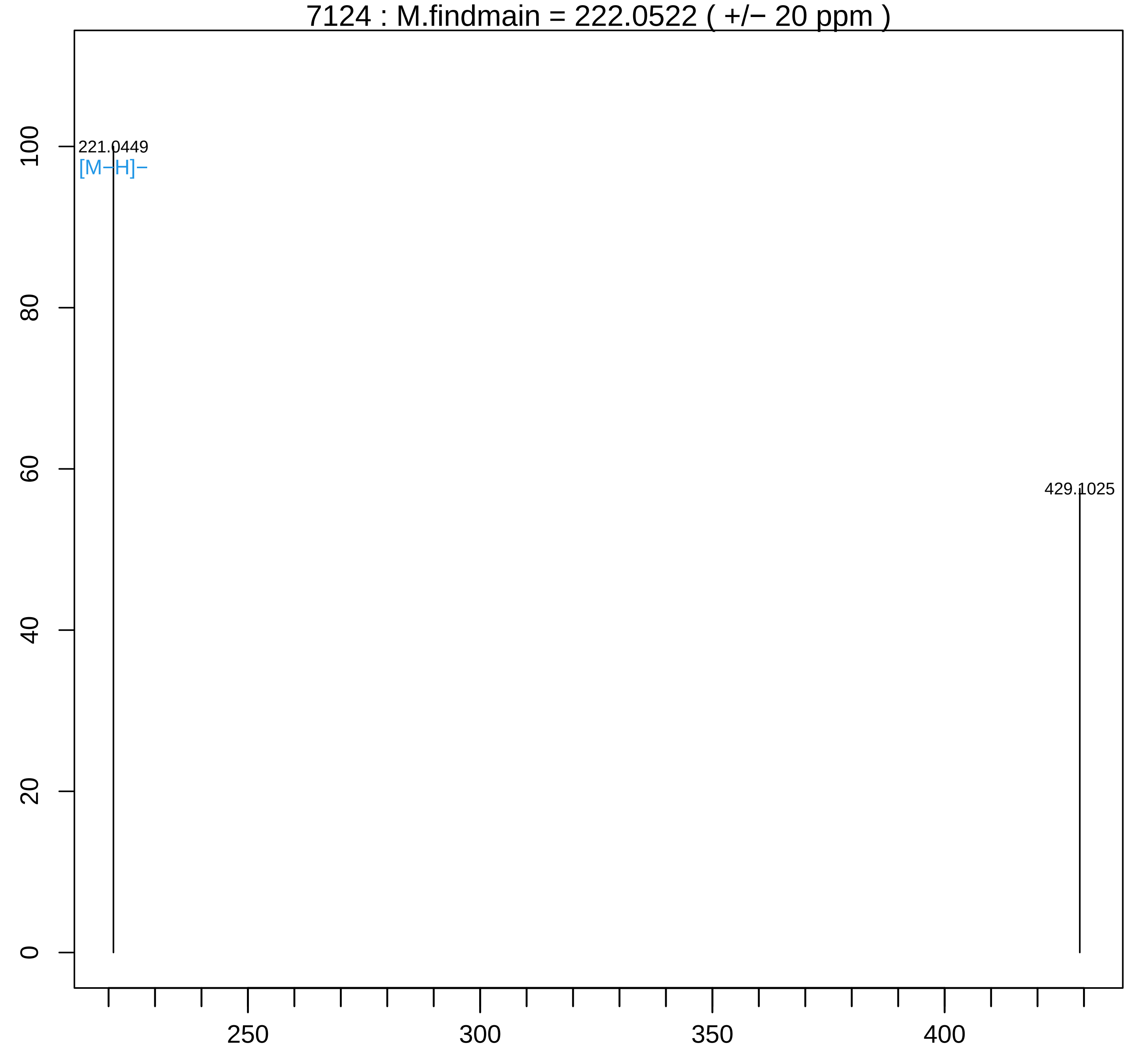
7124 : M.findmain = 222.0522 (+/− 20 ppm)
221.0449
[M−H]−
429.1025
0
20
40
60
80
100
250
300
350
400

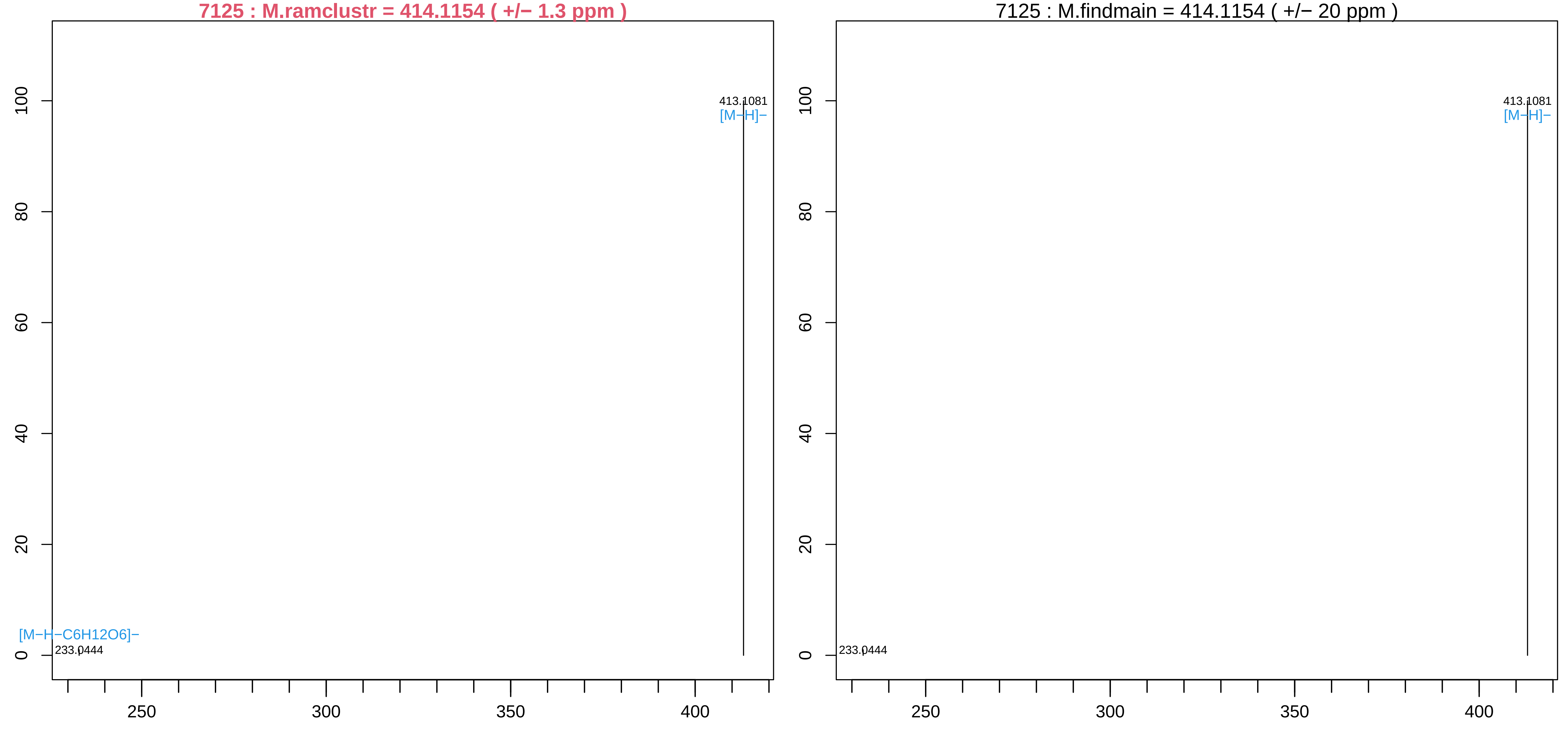
7125 : M.ramclustr = 414.1154 (+/− 1.3 ppm)
413.1081
[M−H]−
[M−H−C6H12O6]−
233.0444
7125 : M.findmain = 414.1154 (+/− 20 ppm)
413.1081
[M−H]−
233.0444
0
20
40
60
80
100
250
300
350
400

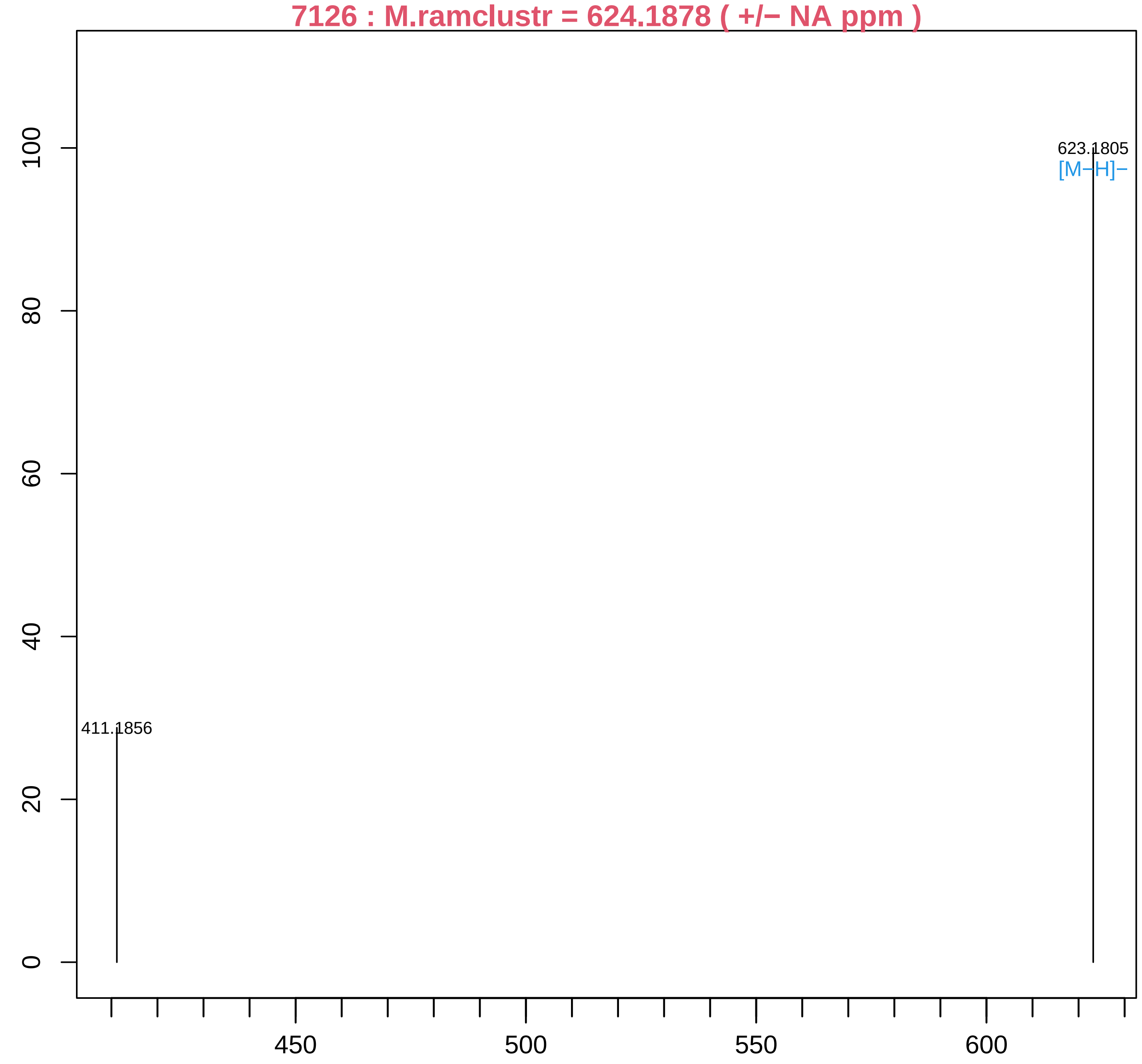

7126 : M.ramclustr = 624.1878 (+/− NA ppm)
623.1805
[M−H]−
411.1856
0
20
40
60
80
100
450
500
550
600

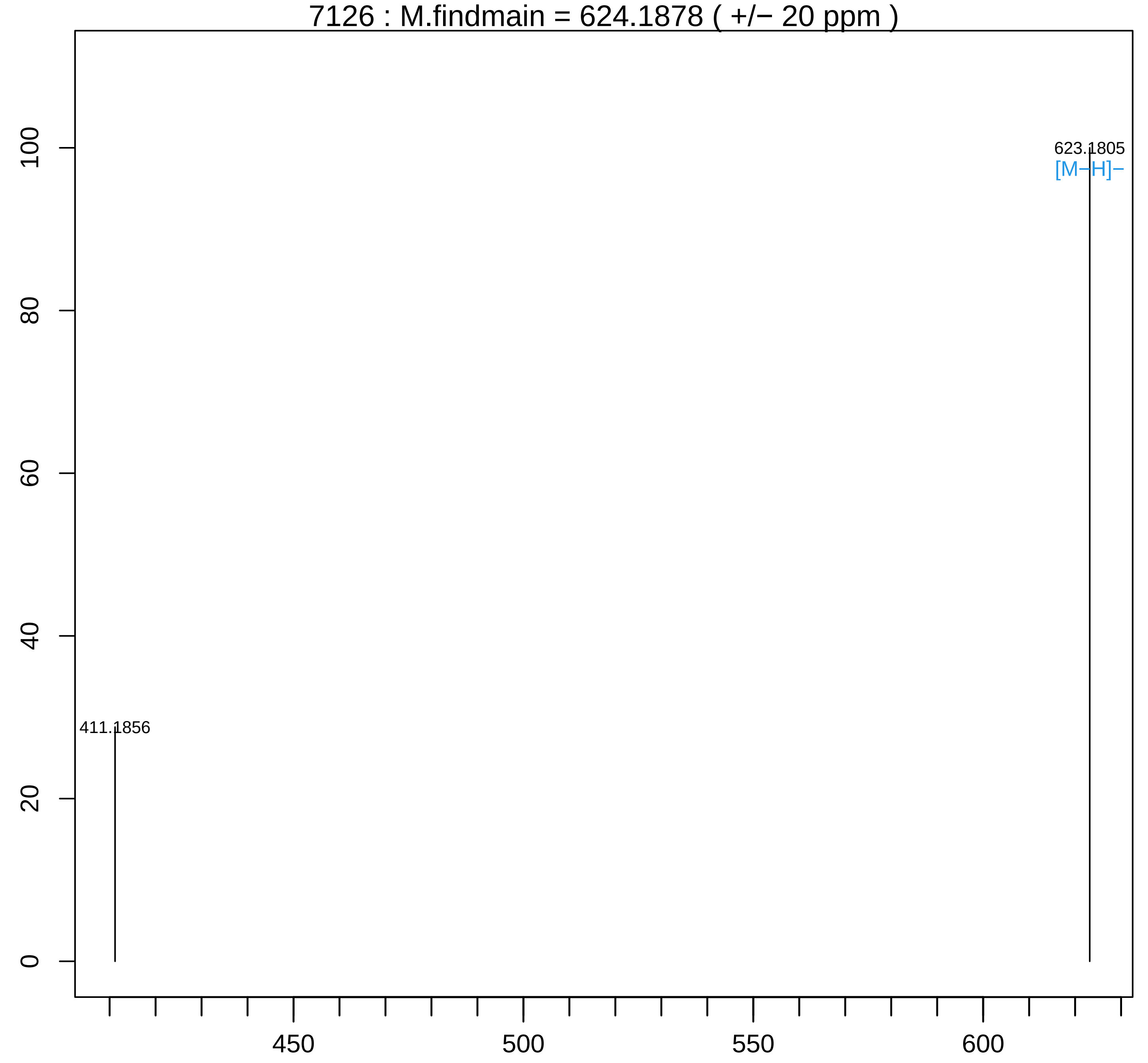

7126 : M.findmain = 624.1878 (+/− 20 ppm)
623.1805
[M−H]−
411.1856
0
20
40
60
80
100
450
500
550
600

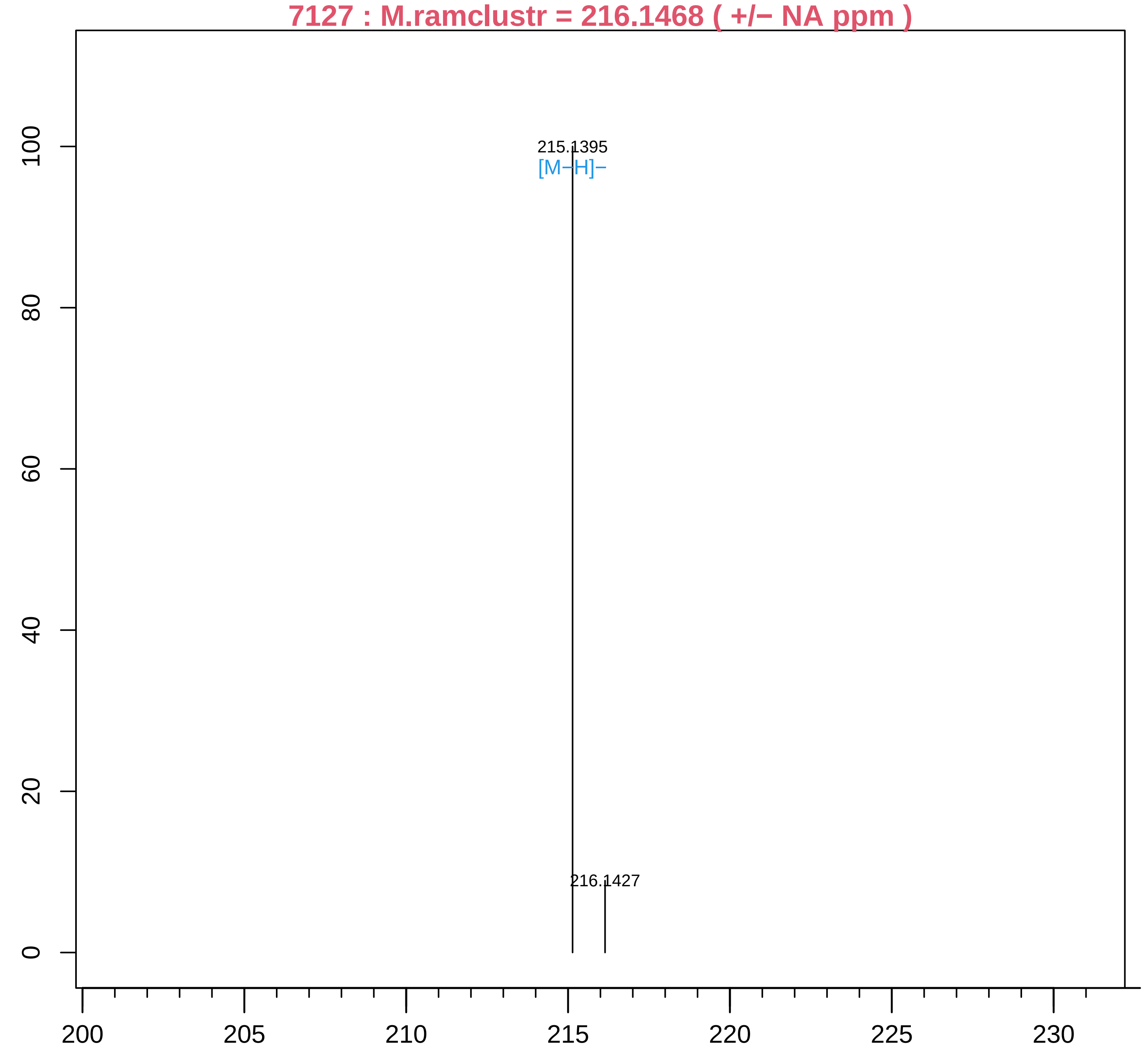
7127 : M.ramclustr = 216.1468 (+/− NA ppm)
215.1395
[M−H]−
216.1427
0
20
40
60
80
100
200
205
210
215
220
225
230

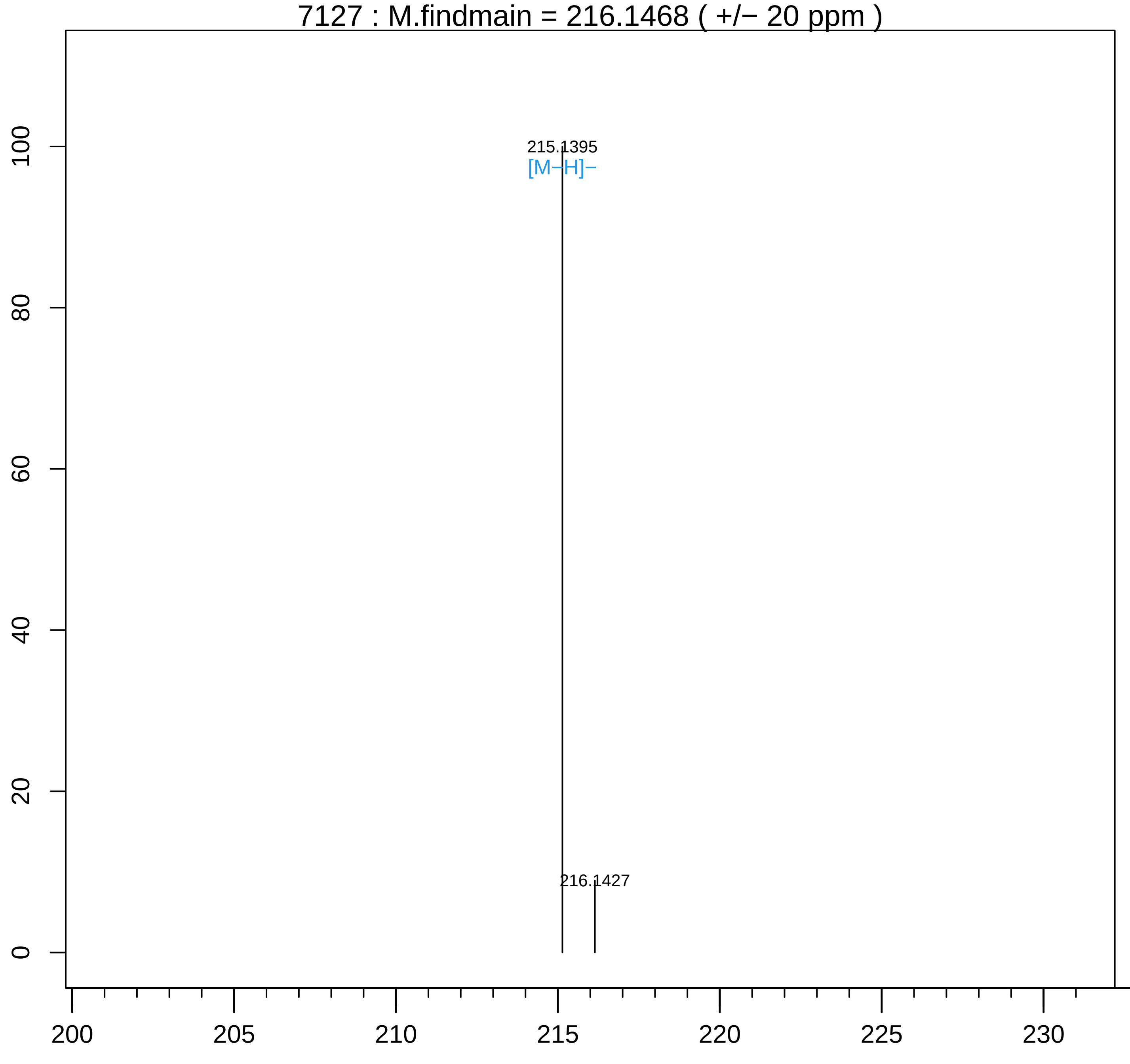
7127 : M.findmain = 216.1468 (+/− 20 ppm)
215.1395
[M−H]−
216.1427
0
20
40
60
80
100
200
205
210
215
220
225
230

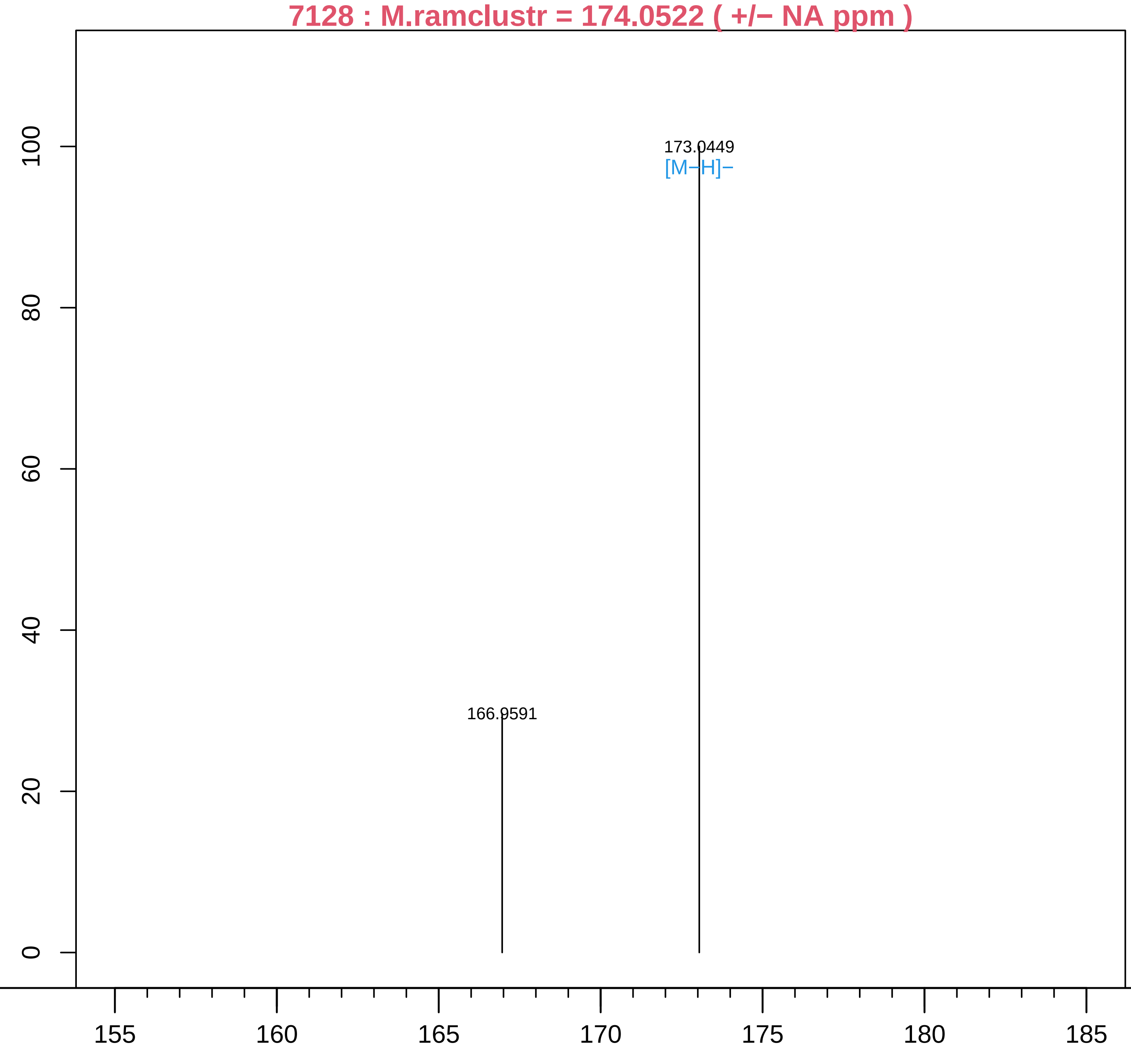
7128 : M.ramclustr = 174.0522 (+/− NA ppm)
173.0449
[M−H]−
166.9591
0
20
40
60
80
100
155
160
165
170
175
180
185

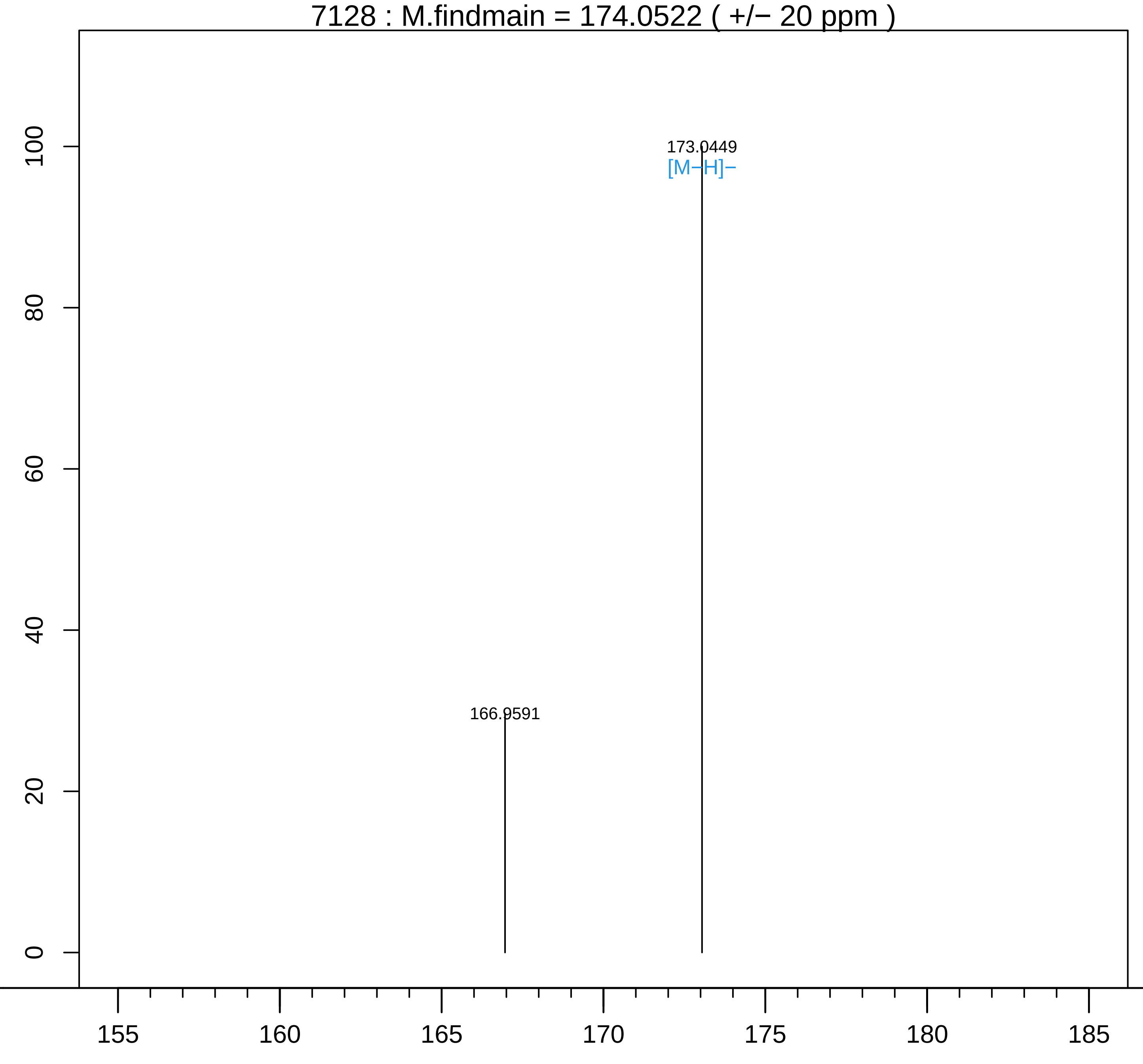
7128 : M.findmain = 174.0522 (+/− 20 ppm)
173.0449
[M−H]−
166.9591
0
20
40
60
80
100
155
160
165
170
175
180
185

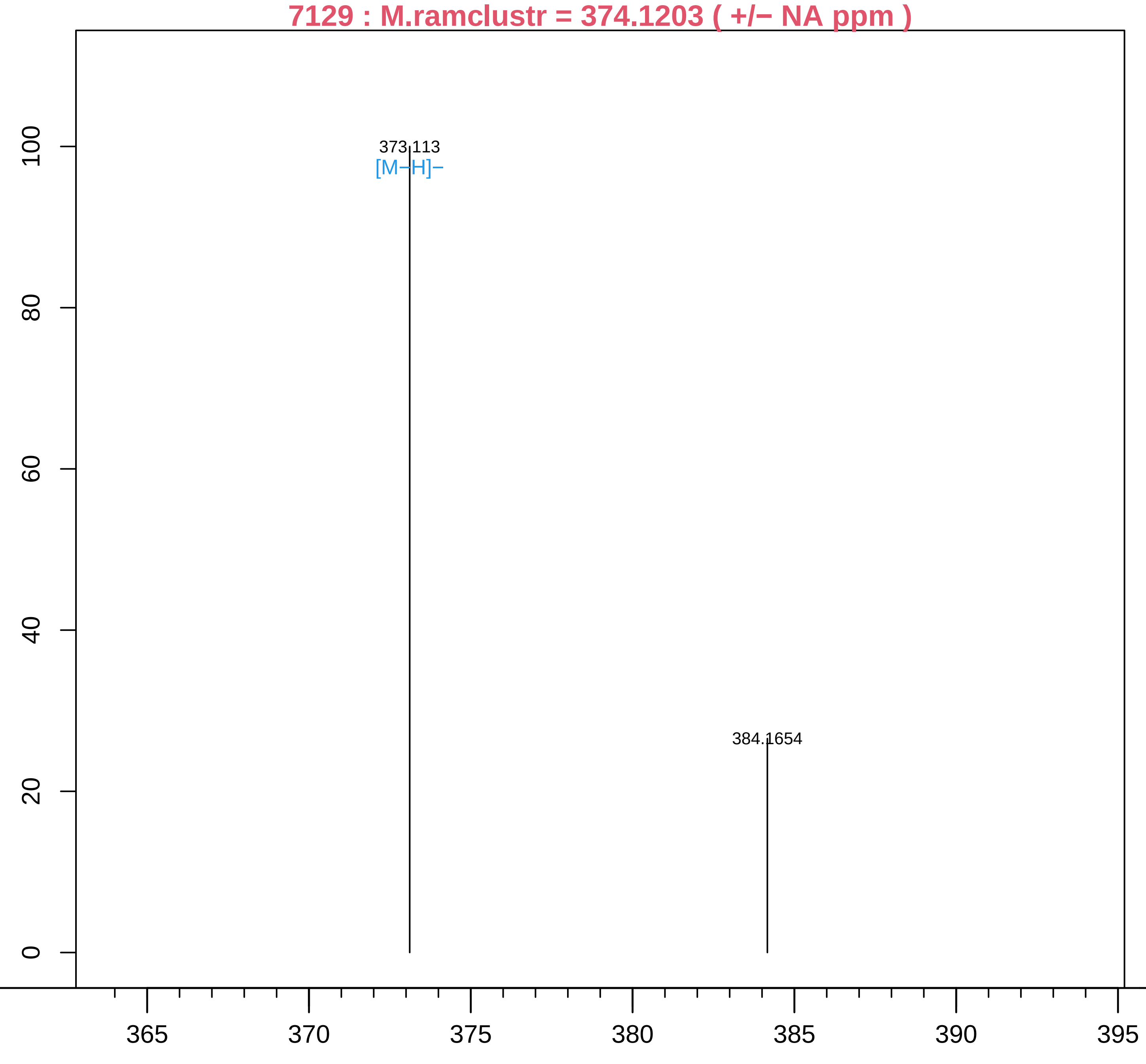

7129 : M.ramclustr = 374.1203 (+/− NA ppm)
373.113
[M−H]−
384.1654
0
20
40
60
80
100
365
370
375
380
385
390
395

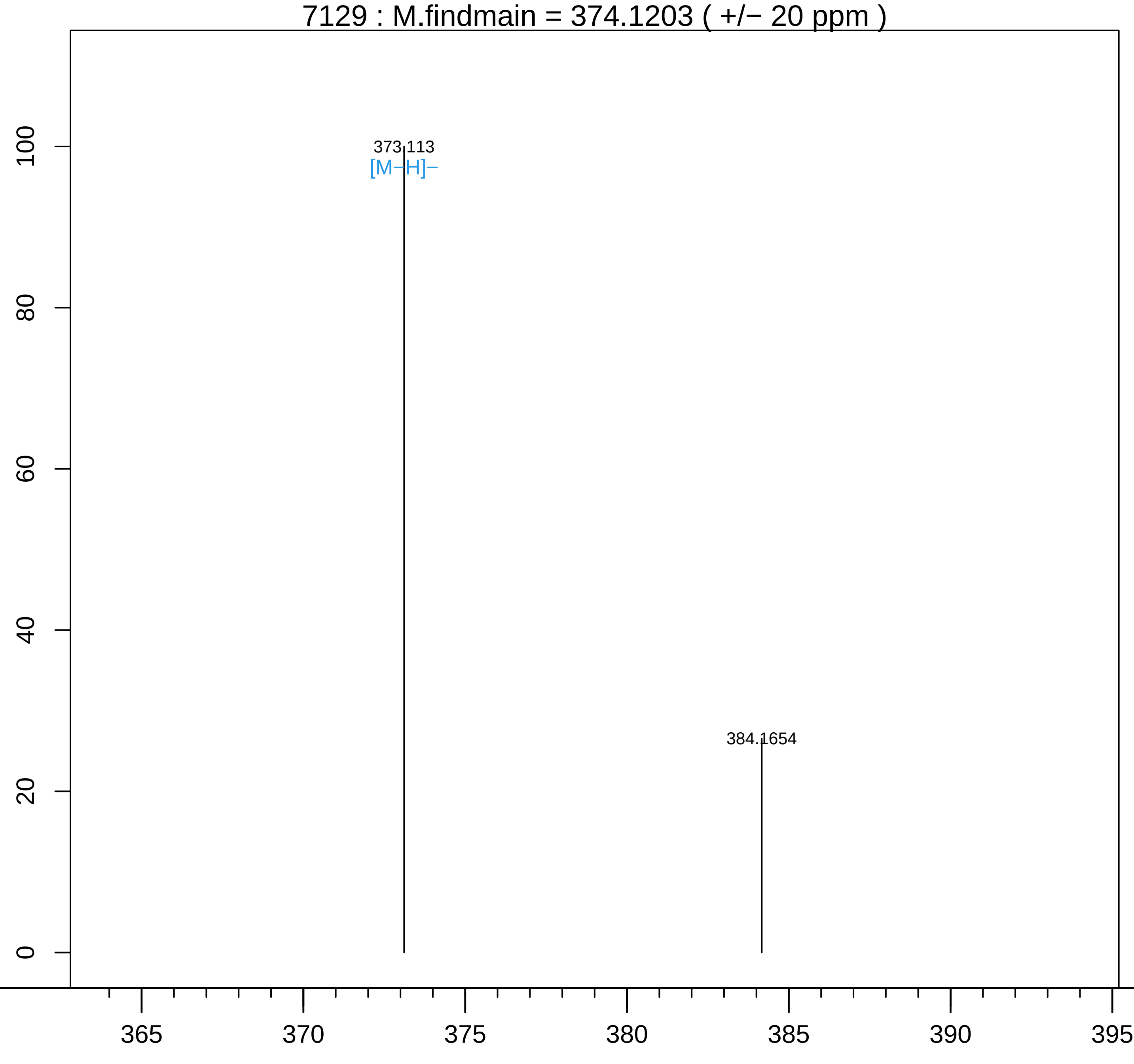

7129 : M.findmain = 374.1203 (+/− 20 ppm)
373.113
[M−H]−
384.1654
0
20
40
60
80
100
365
370
375
380
385
390
395

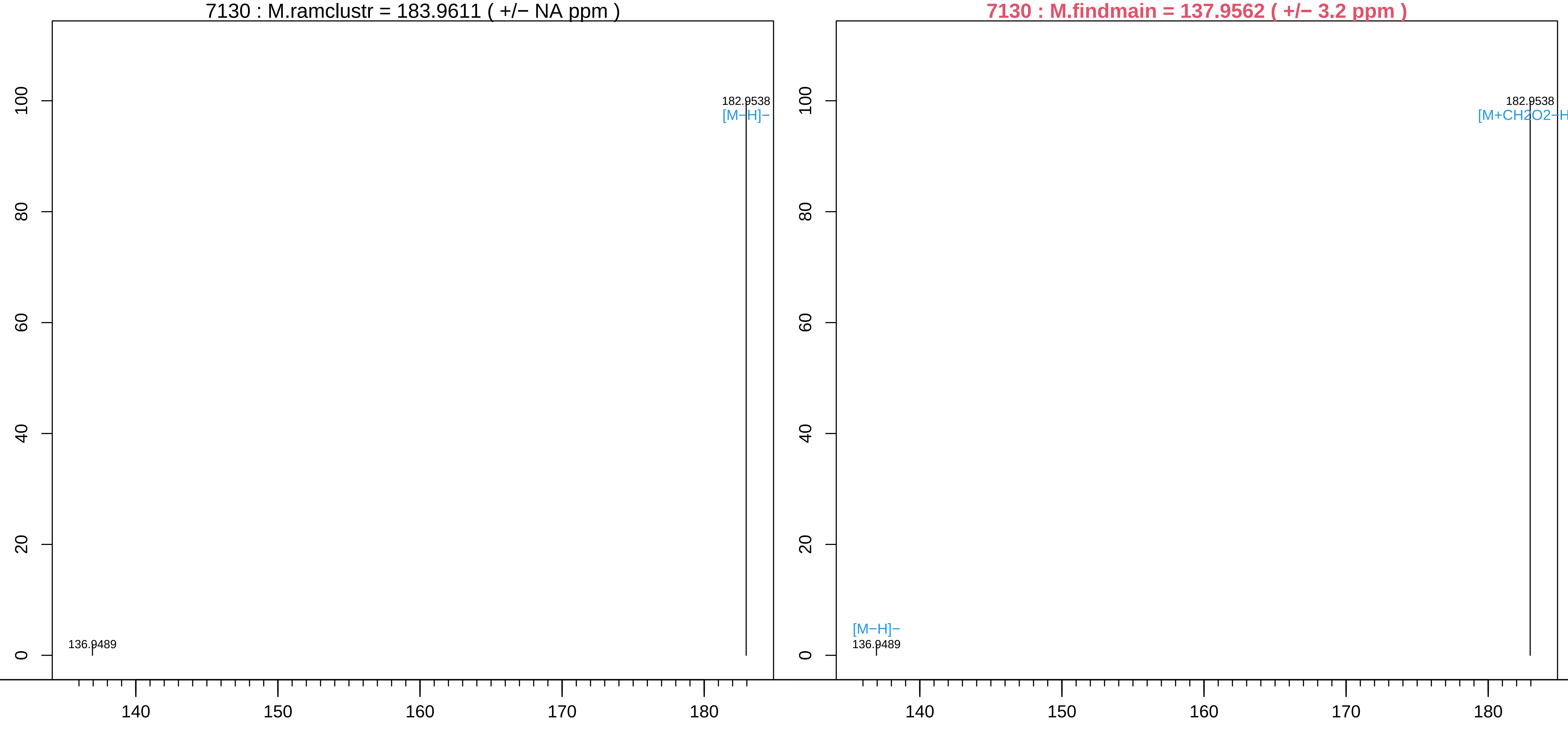
7130 : M.ramclustr = 183.9611 (+/− NA ppm)
182.9538
[M−H]−
136.9489
7130 : M.findmain = 137.9562 (+/− 3.2 ppm)
182.9538
[M+CH2O2−H
[M−H]−
136.9489
0
20
40
60
80
100
140
150
160
170
180

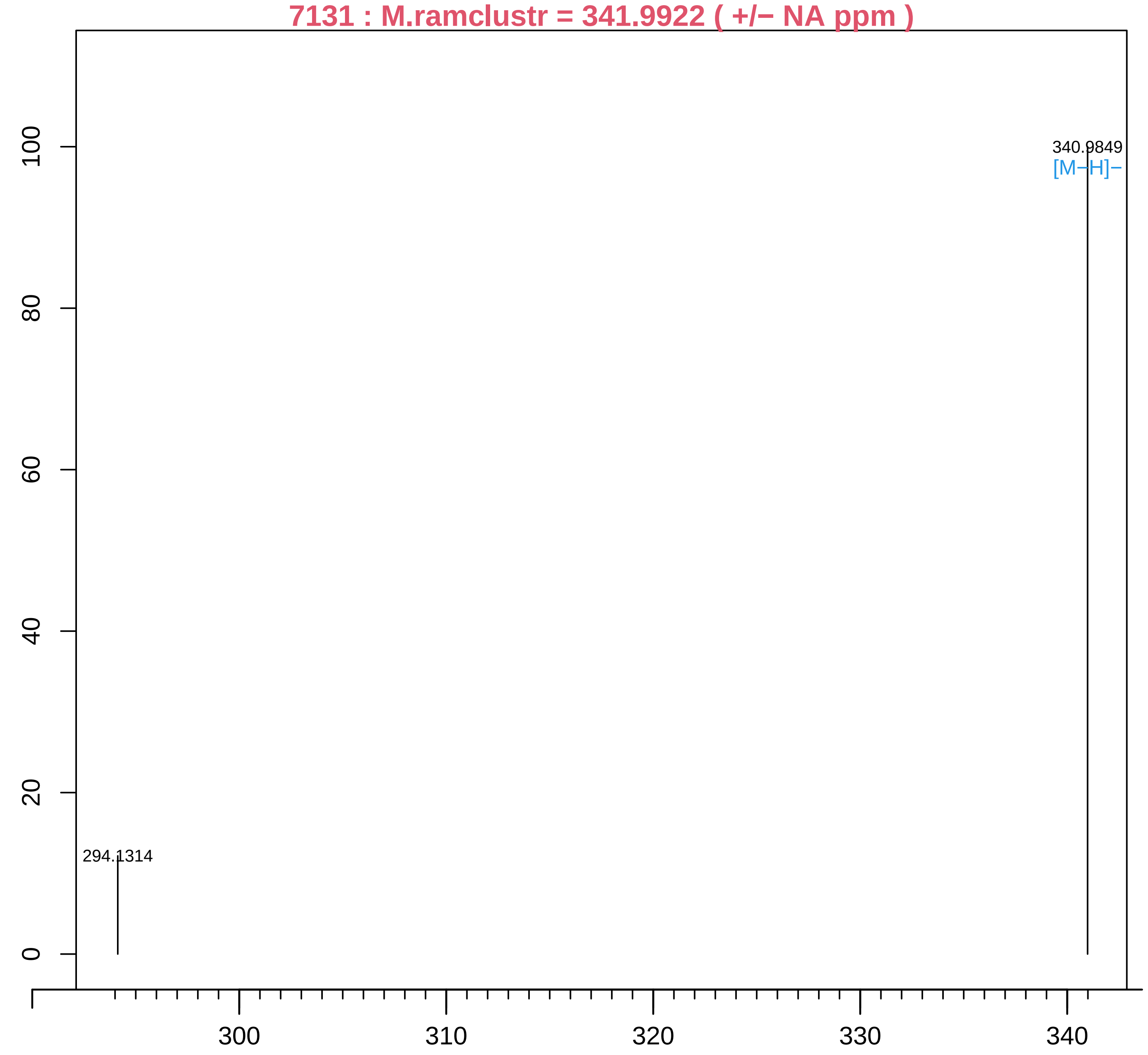

7131 : M.ramclustr = 341.9922 (+/− NA ppm)
340.9849
[M−H]−
294.1314
0
20
40
60
80
100
300
310
320
330
340

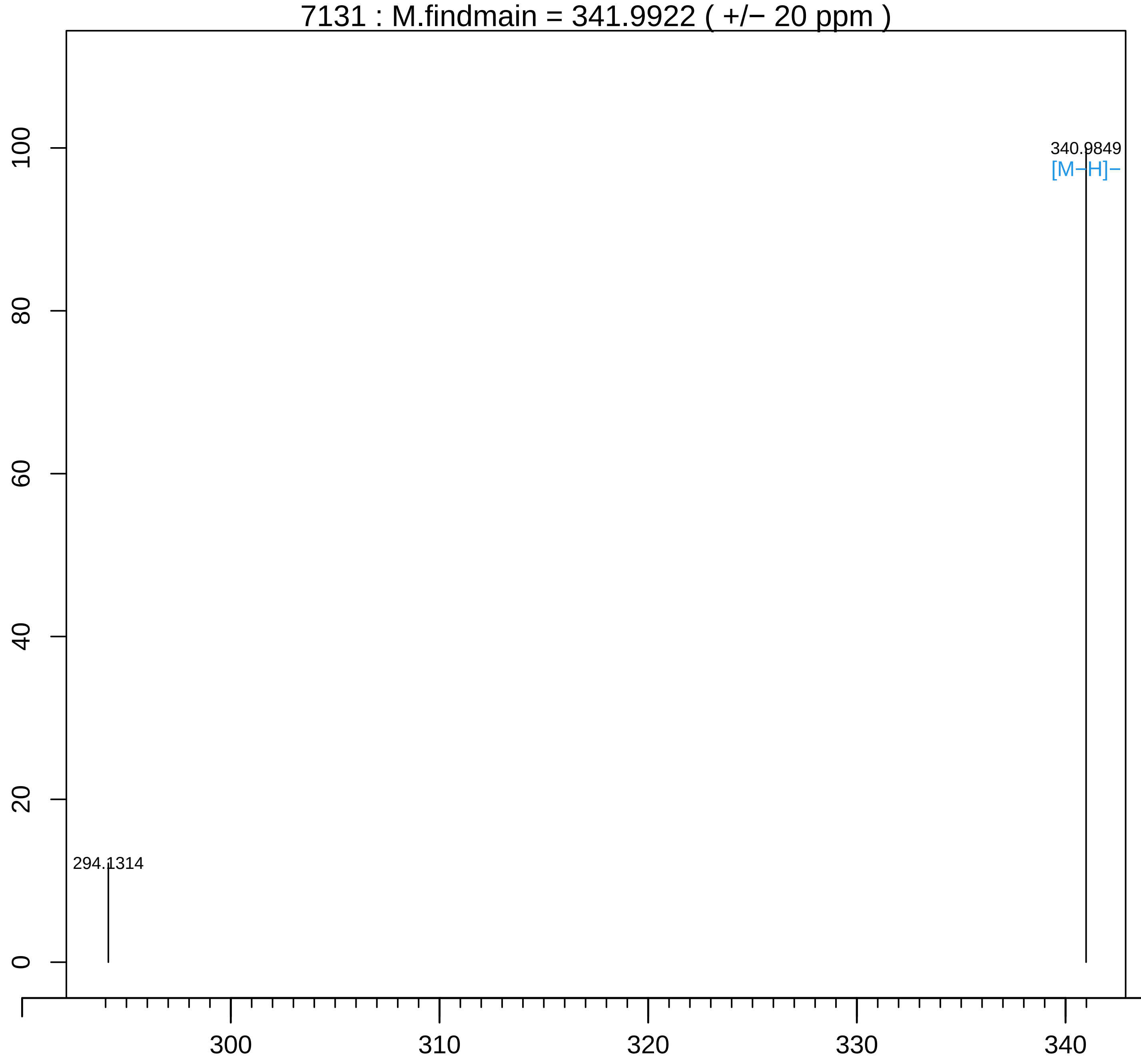

7131 : M.findmain = 341.9922 (+/− 20 ppm)
340.9849
[M−H]−
294.1314
0
20
40
60
80
100
300
310
320
330
340

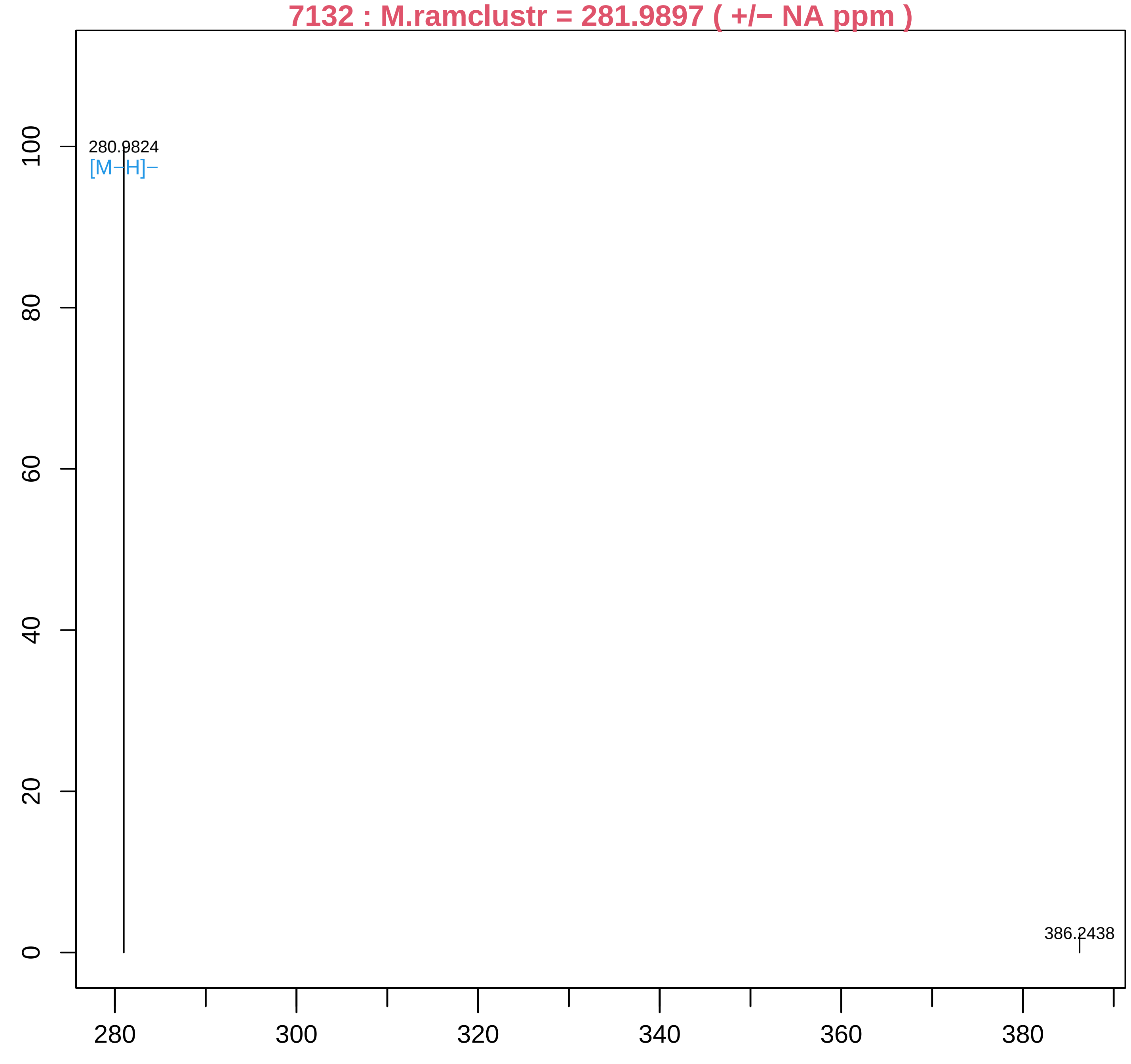
7132 : M.ramclustr = 281.9897 (+/− NA ppm)
280.9824
[M−H]−
386.2438
0
20
40
60
80
100
280
300
320
340
360
380

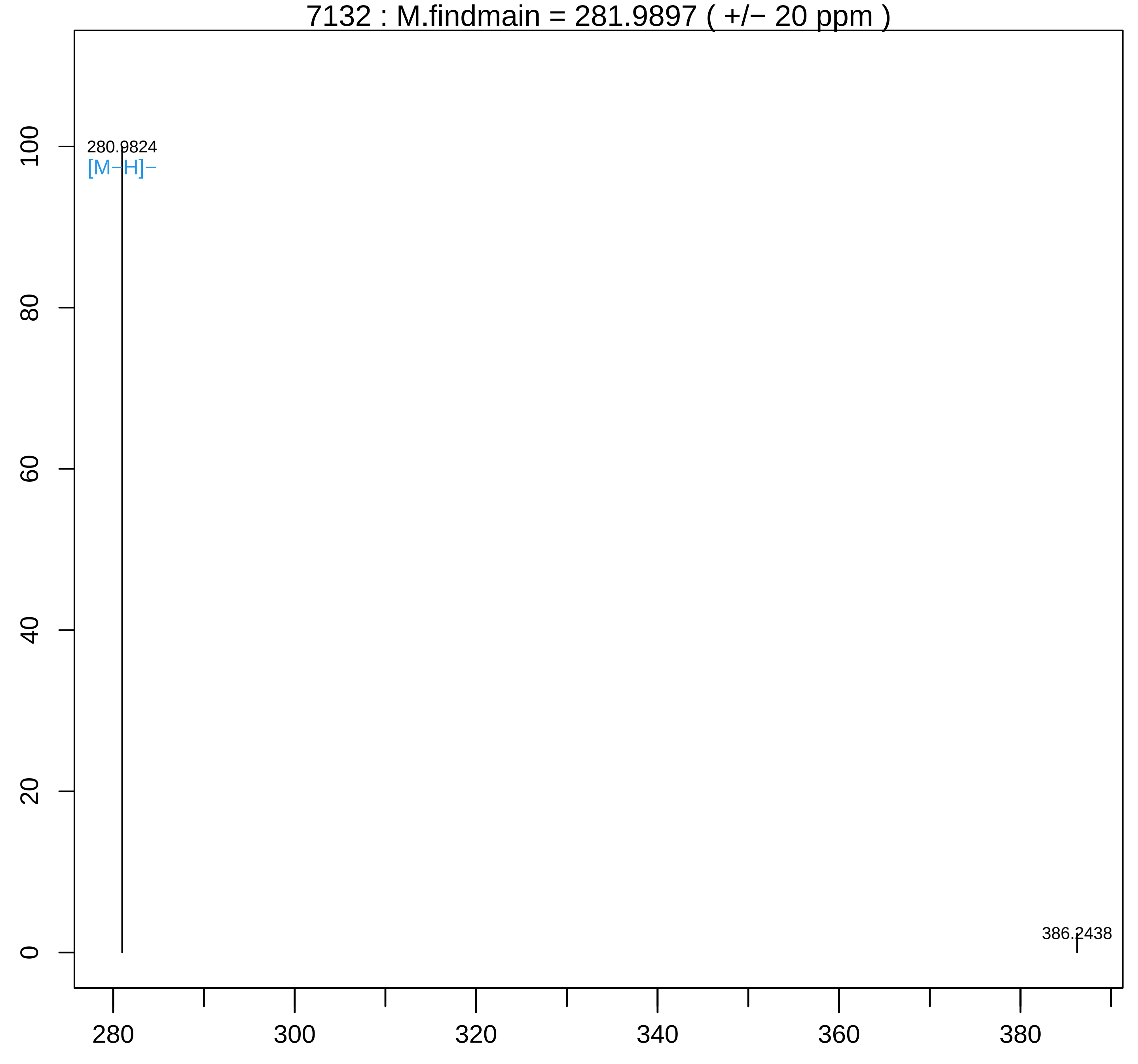
7132 : M.findmain = 281.9897 (+/− 20 ppm)
280.9824
[M−H]−
386.2438
0
20
40
60
80
100
280
300
320
340
360
380

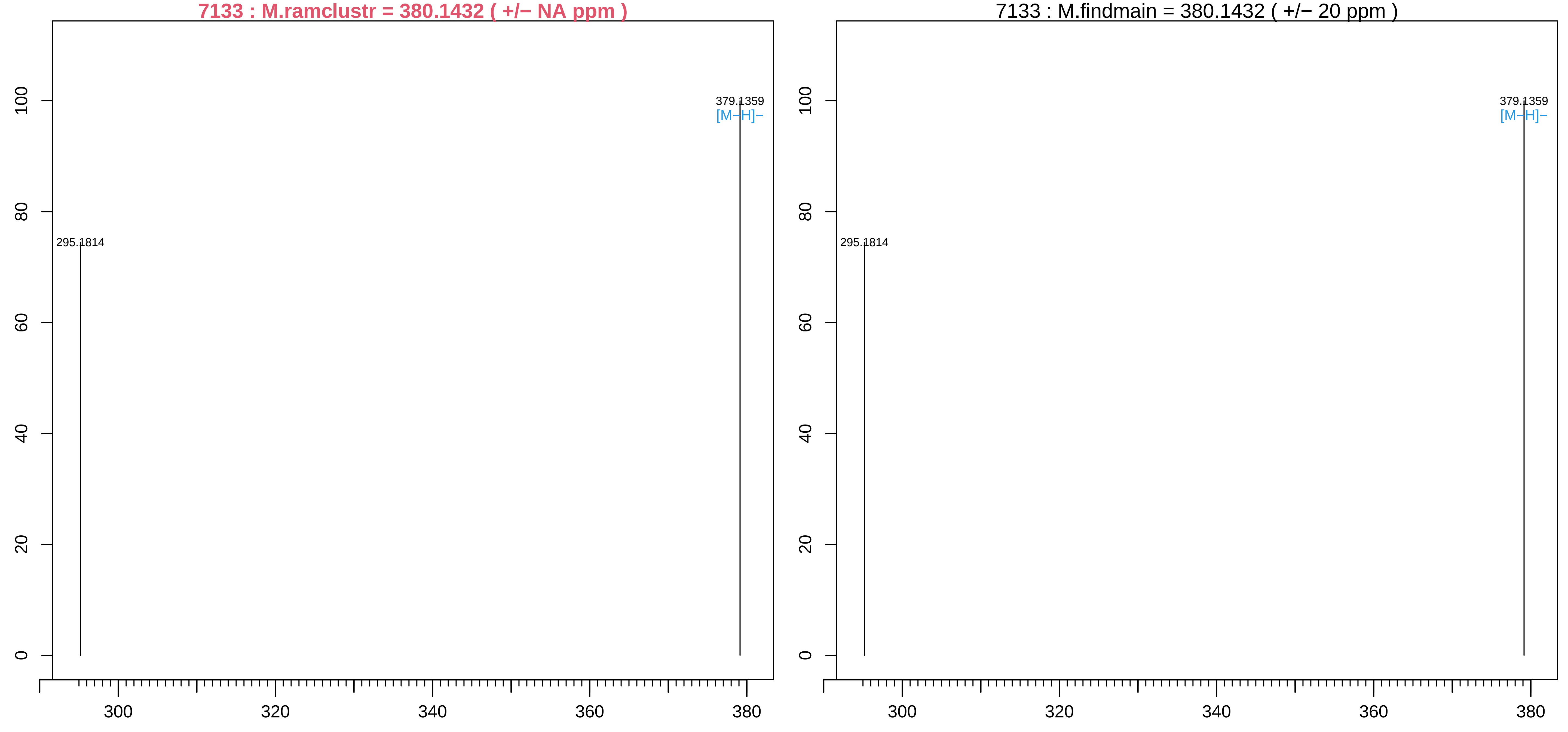

7133 : M.ramclustr = 380.1432 (+/− NA ppm)
7133 : M.findmain = 380.1432 (+/− 20 ppm)
295.1814
379.1359
[M−H]−
0
20
40
60
80
100
300
320
340
360
380

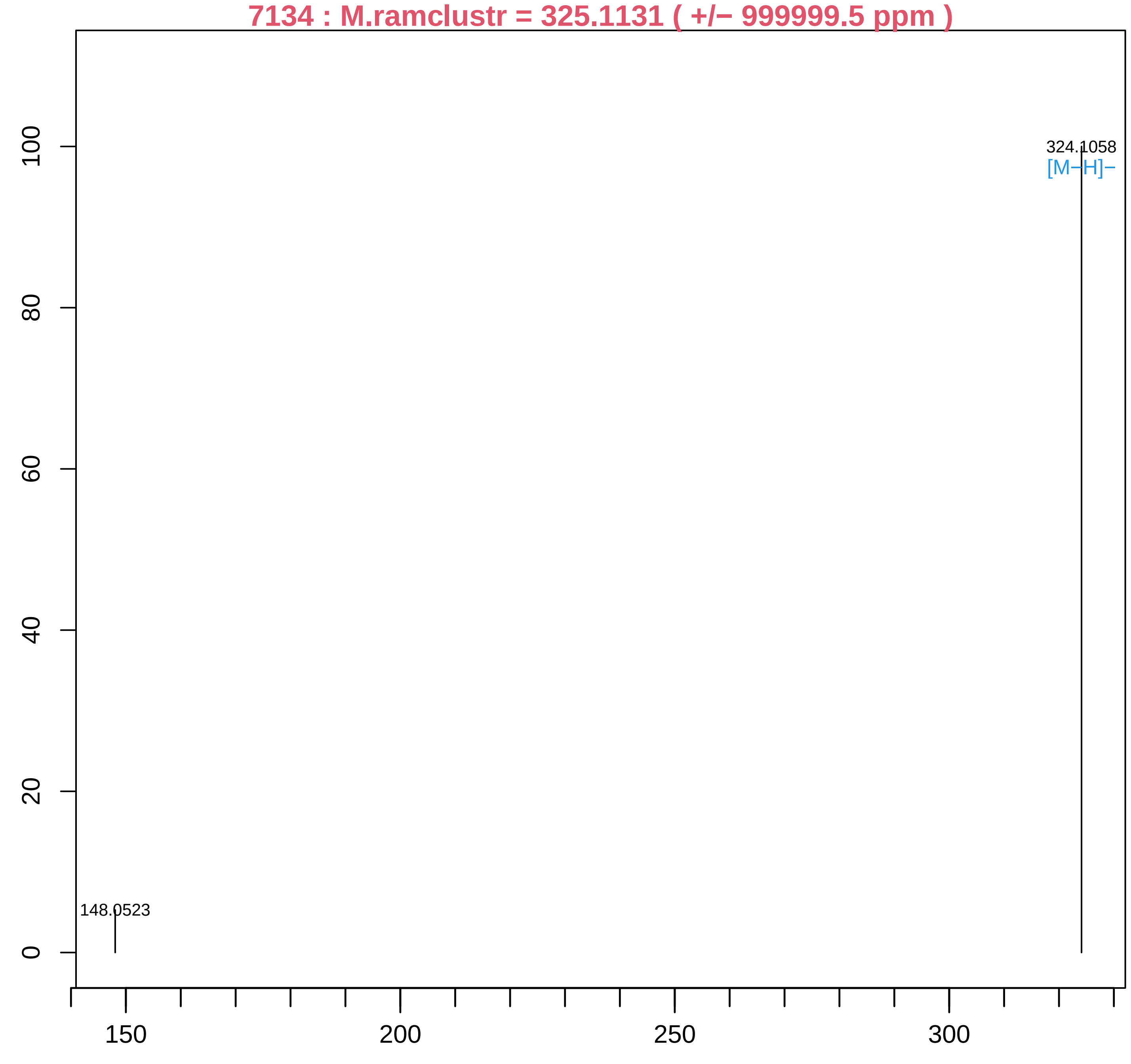
7134 : M.ramclustr = 325.1131 (+/− 999999.5 ppm)
324.1058
[M−H]−
148.0523
0
20
40
60
80
100
150
200
250
300

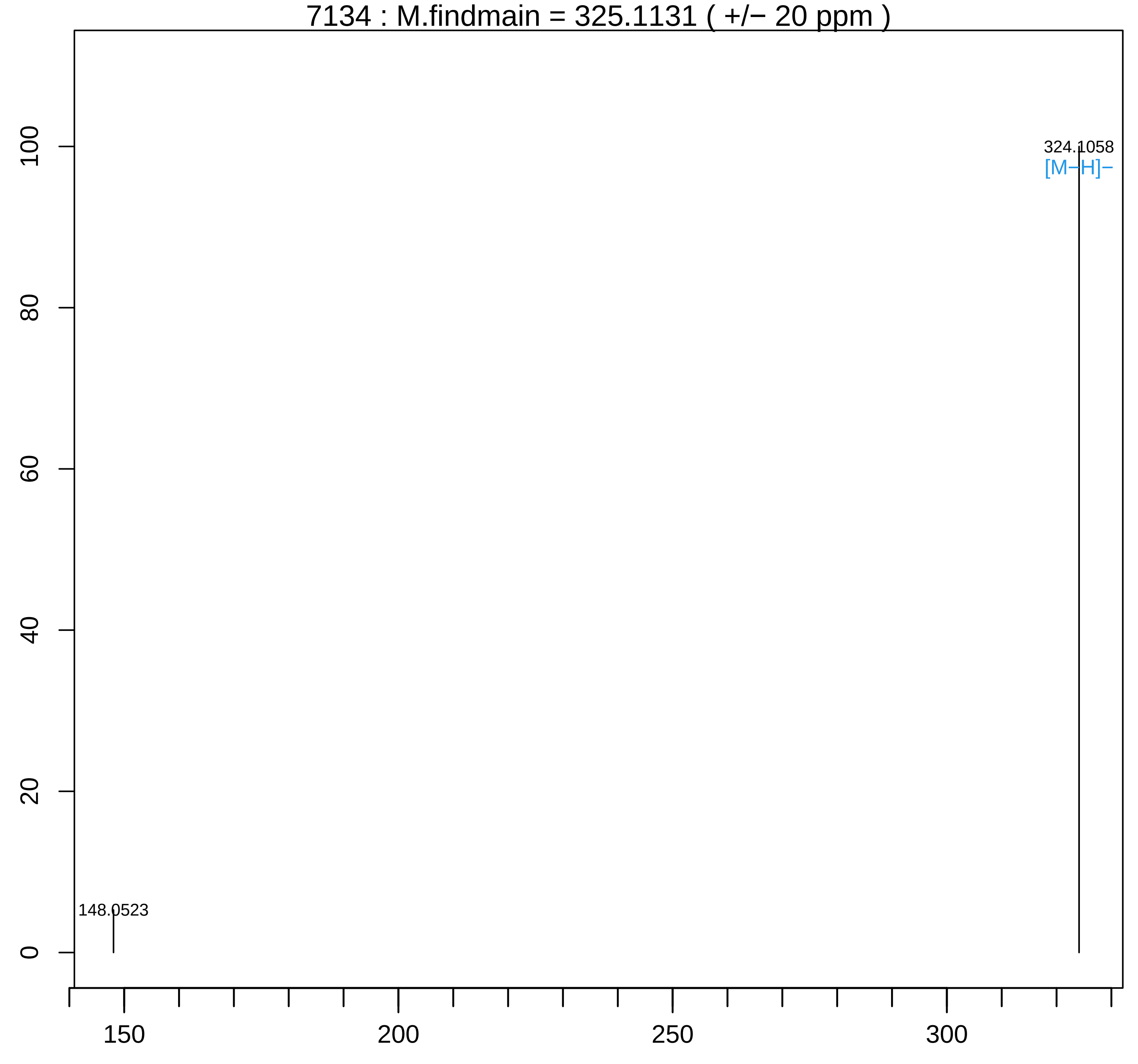
7134 : M.findmain = 325.1131 (+/− 20 ppm)
324.1058
[M−H]−
148.0523
0
20
40
60
80
100
150
200
250
300

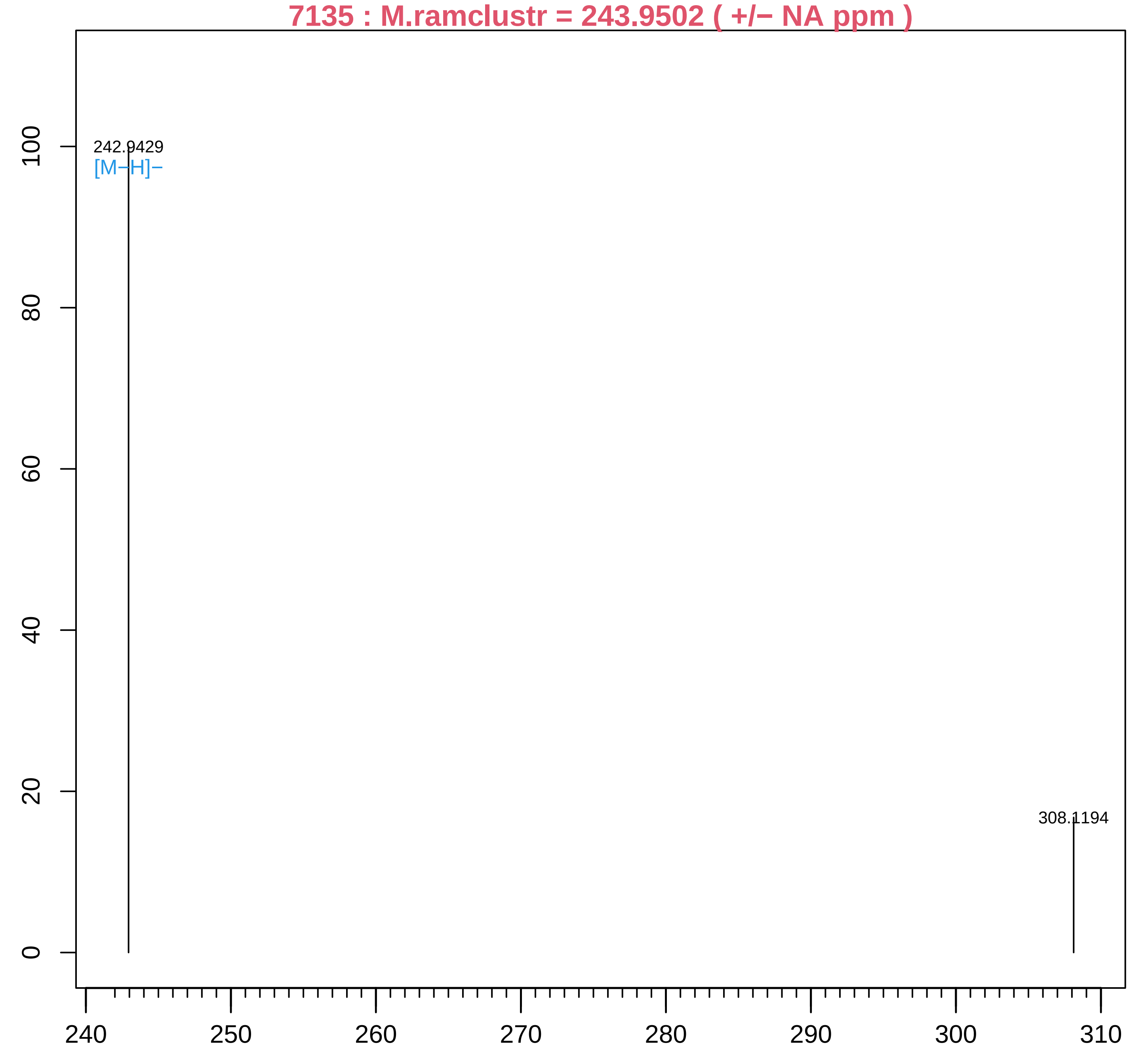
7135 : M.ramclustr = 243.9502 (+/− NA ppm)
242.9429
[M−H]−
308.1194
0
20
40
60
80
100
240
250
260
270
280
290
300
310

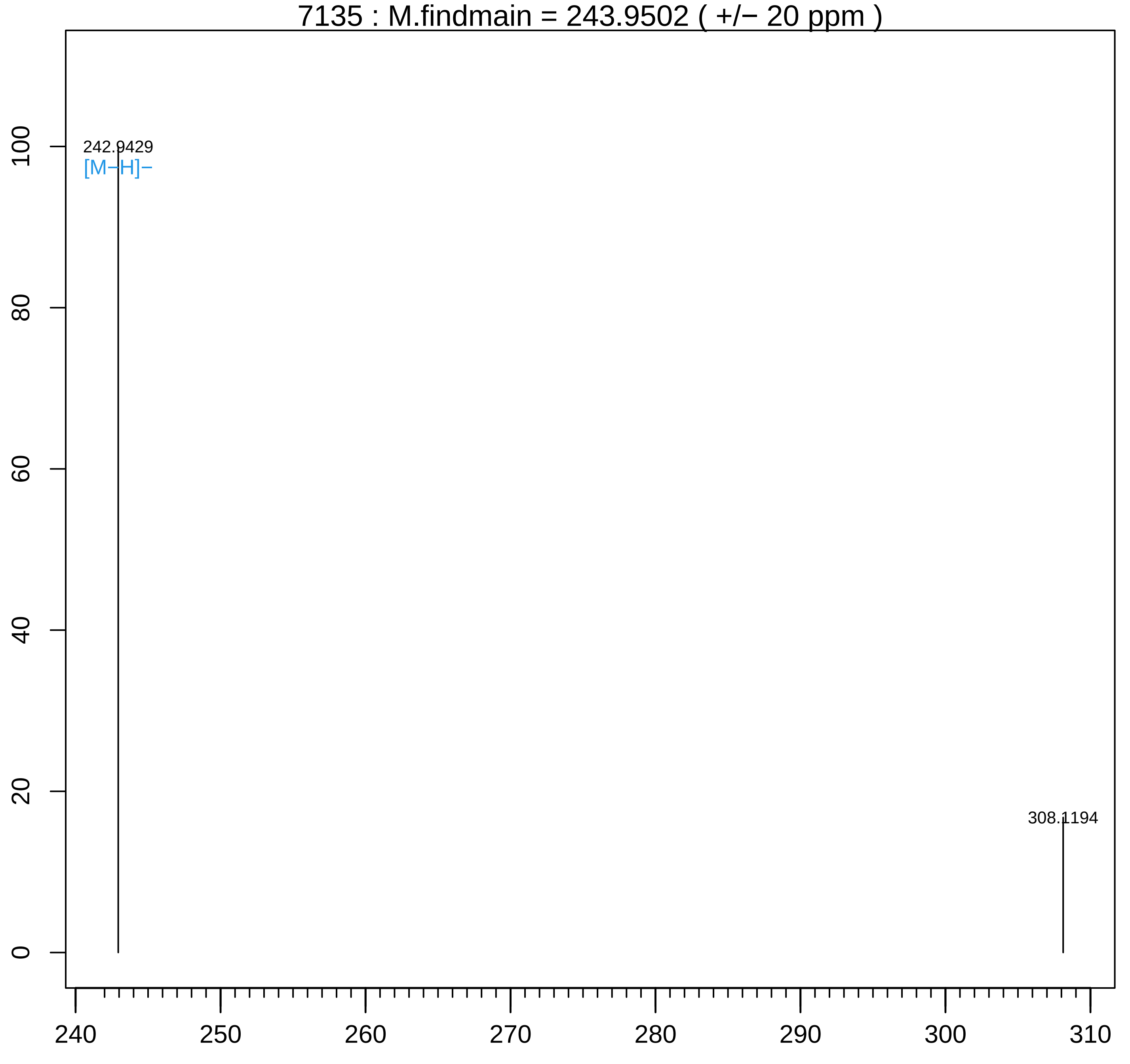
7135 : M.findmain = 243.9502 (+/− 20 ppm)
242.9429
[M−H]−
308.1194
0
20
40
60
80
100
240
250
260
270
280
290
300
310

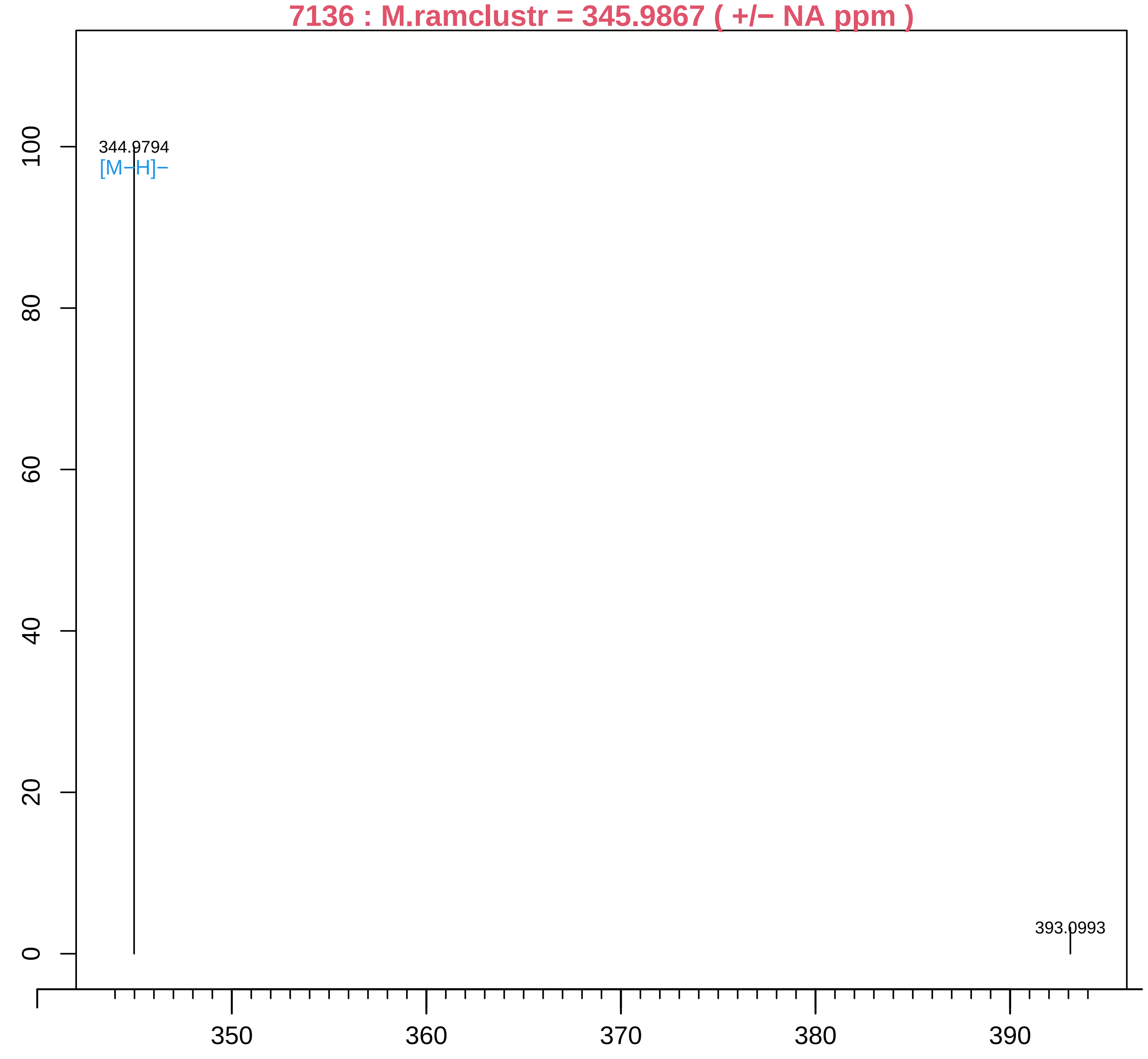
7136 : M.ramclustr = 345.9867 (+/− NA ppm)
344.9794
[M−H]−
393.0993
0
20
40
60
80
100
350
360
370
380
390

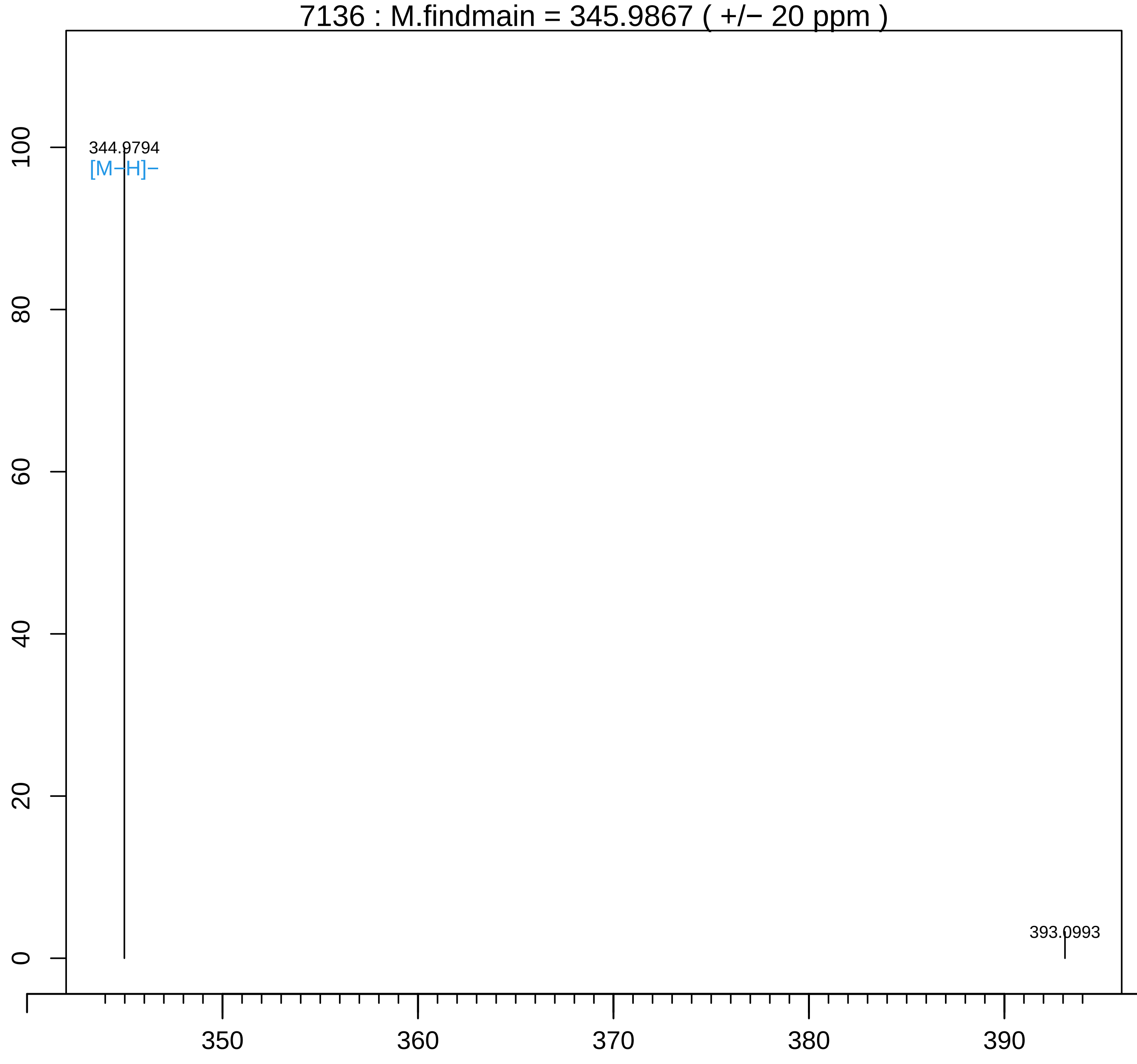
7136 : M.findmain = 345.9867 (+/− 20 ppm)
344.9794
[M−H]−
393.0993
0
20
40
60
80
100
350
360
370
380
390

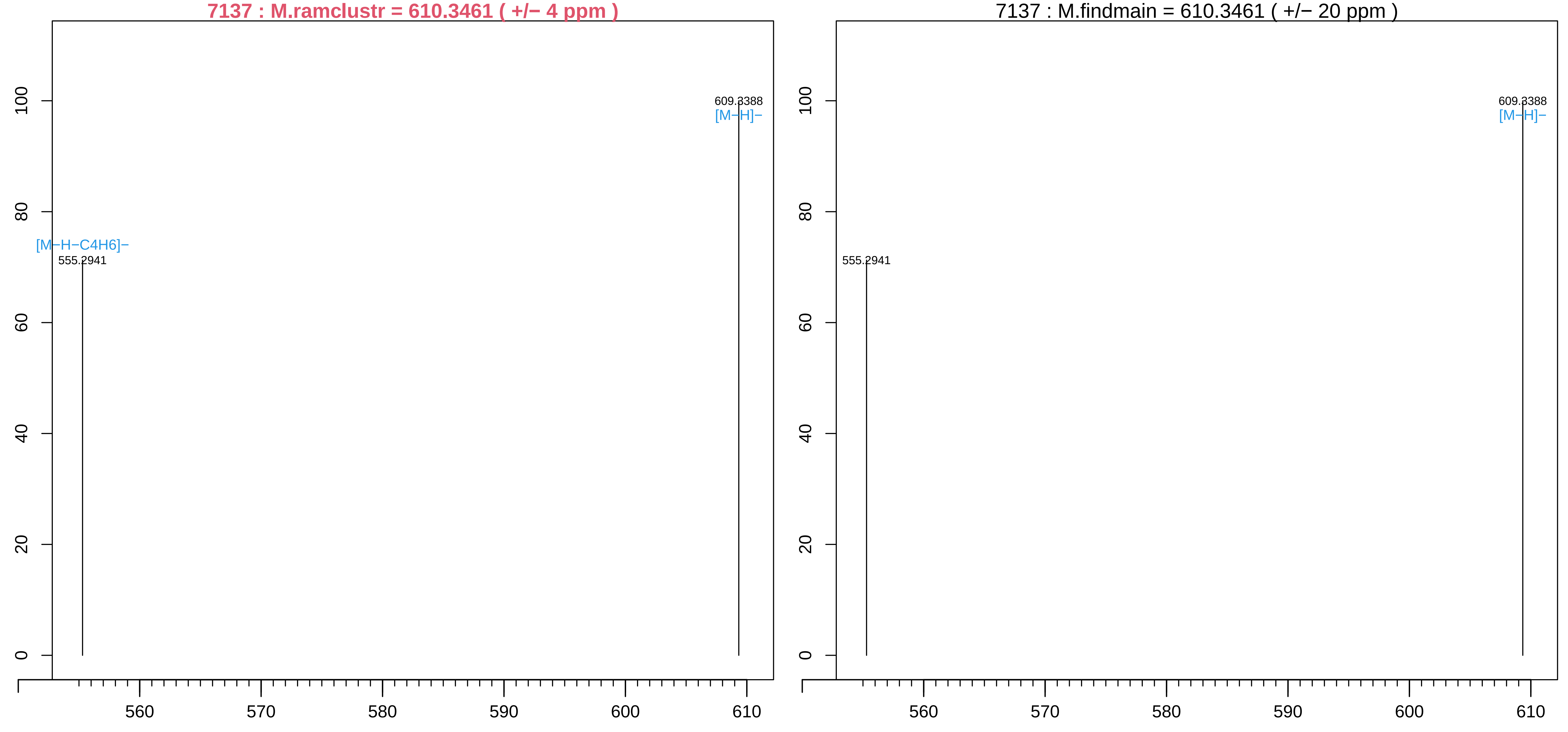
7137 : M.ramclustr = 610.3461 (+/− 4 ppm)
7137 : M.findmain = 610.3461 (+/− 20 ppm)
609.3388
[M−H]−
[M−H−C4H6]−
555.2941
0
20
40
60
80
100
560
570
580
590
600
610

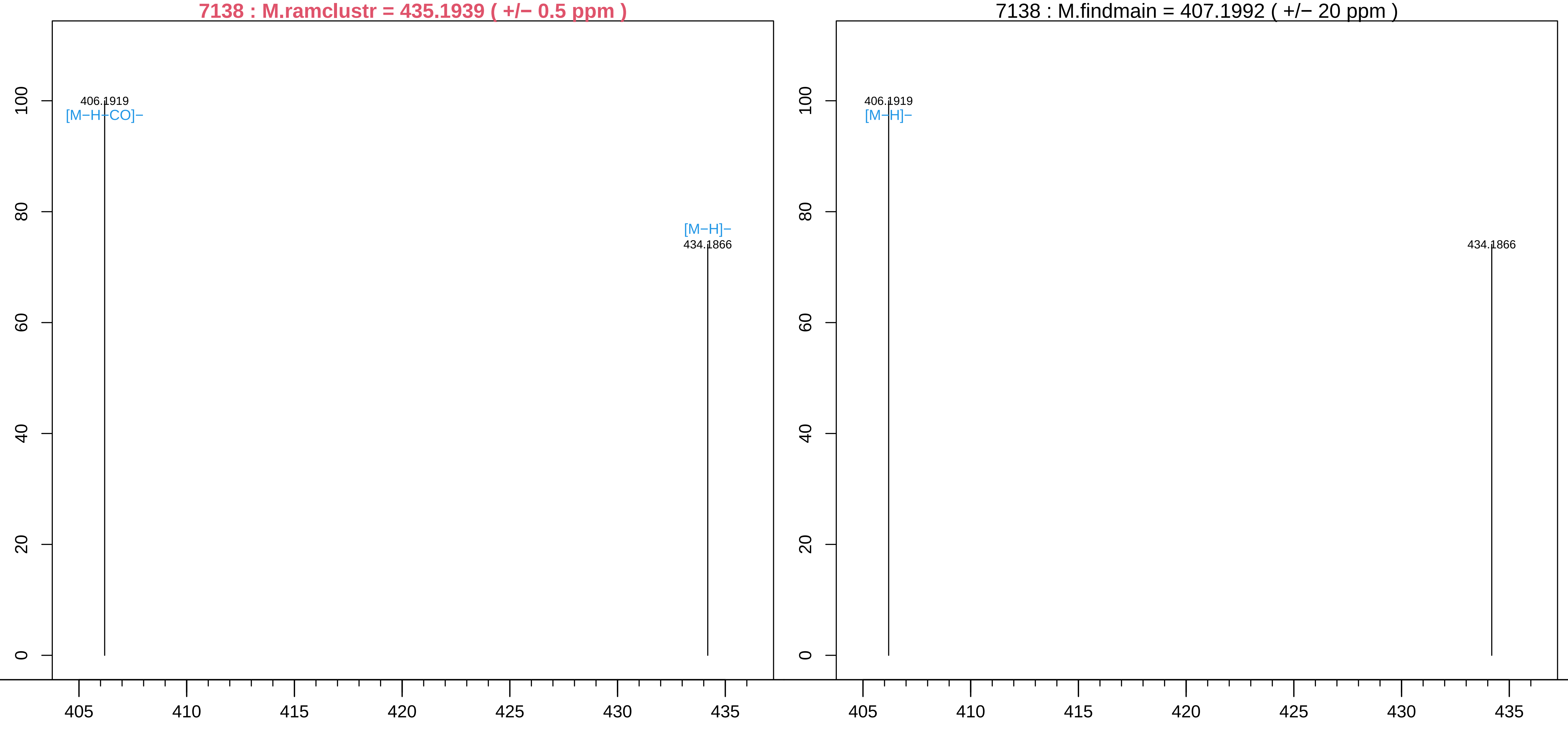
7138 : M.ramclustr = 435.1939 (+/− 0.5 ppm)
406.1919
[M−H−CO]−
[M−H]−
434.1866
7138 : M.findmain = 407.1992 (+/− 20 ppm)
406.1919
[M−H]−
434.1866
0
20
40
60
80
100
405
410
415
420
425
430
435

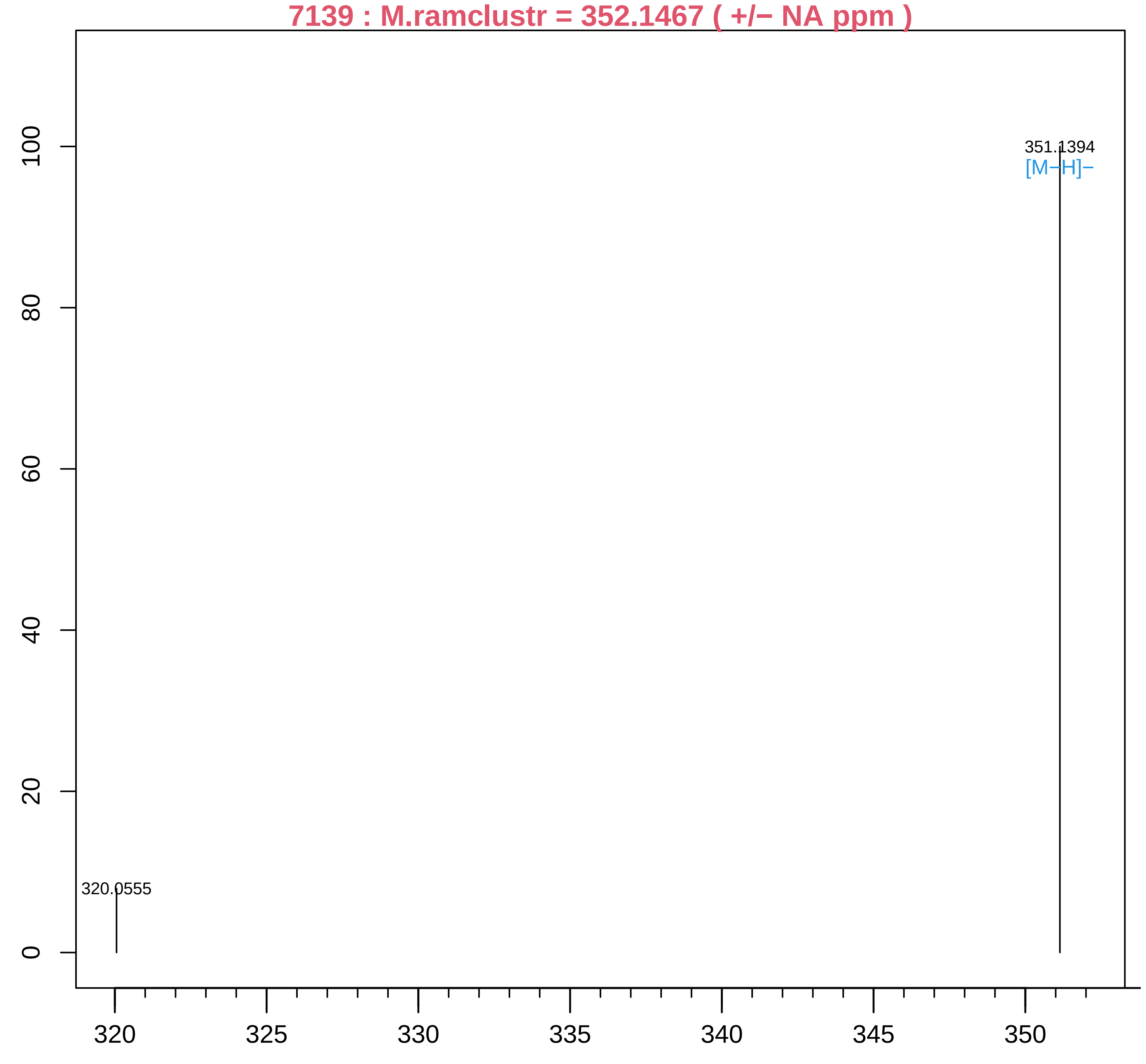
7139 : M.ramclustr = 352.1467 (+/− NA ppm)
351.1394
[M−H]−
320.0555
0
20
40
60
80
100
320
325
330
335
340
345
350

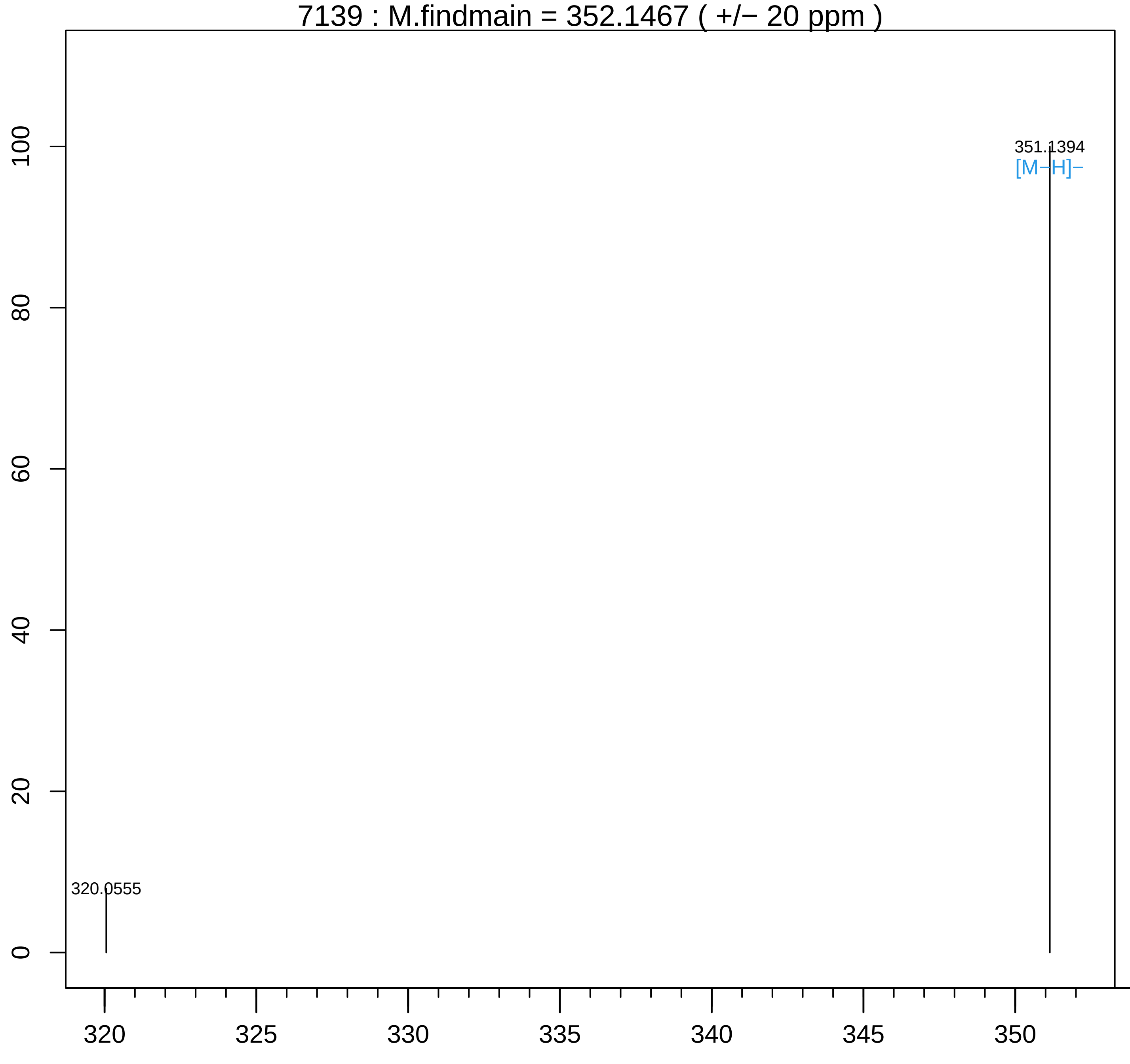
7139 : M.findmain = 352.1467 (+/− 20 ppm)
351.1394
[M−H]−
320.0555
0
20
40
60
80
100
320
325
330
335
340
345
350

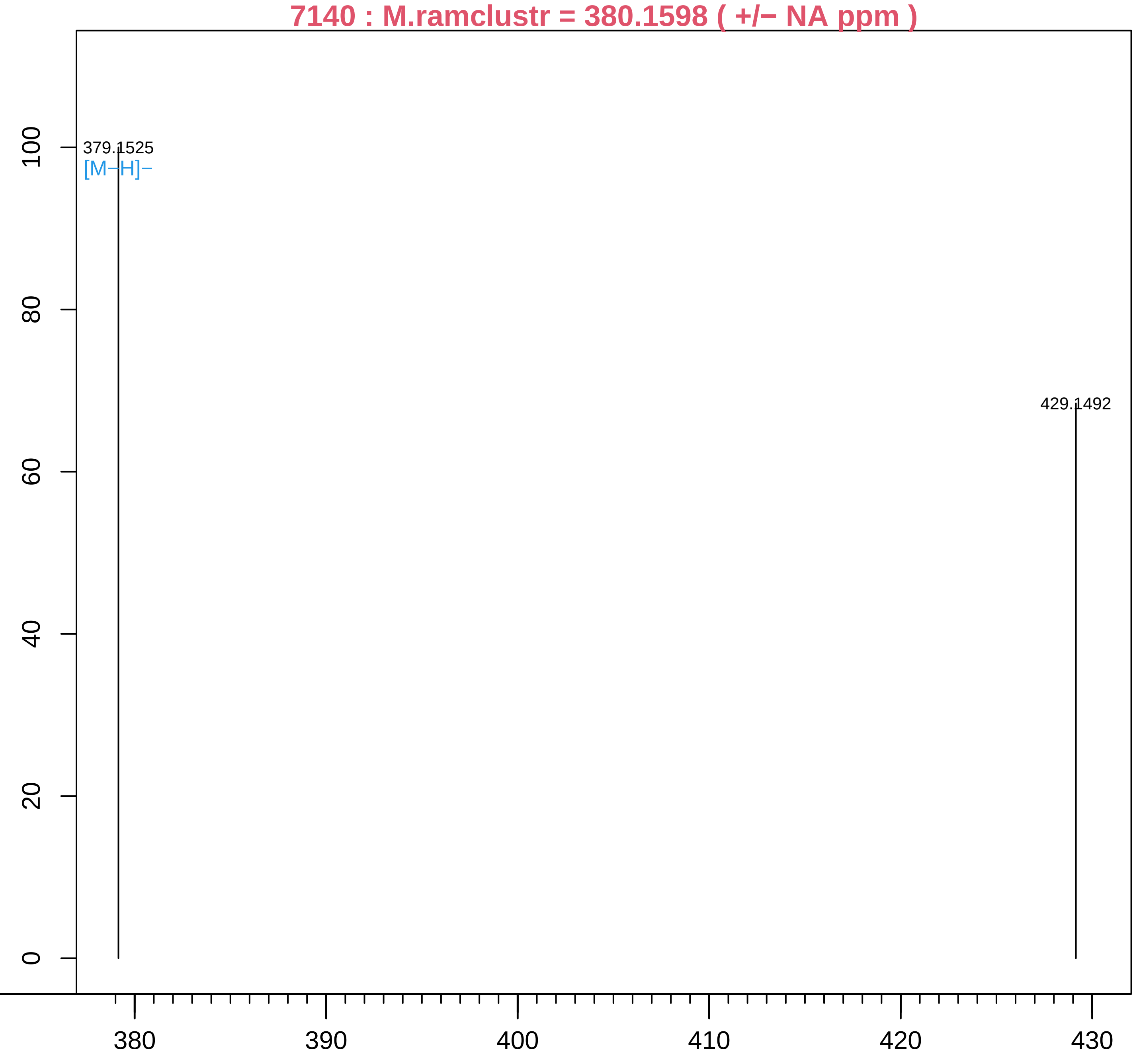
7140 : M.ramclustr = 380.1598 (+/− NA ppm)
379.1525
[M−H]−
429.1492
0
20
40
60
80
100
380
390
400
410
420
430

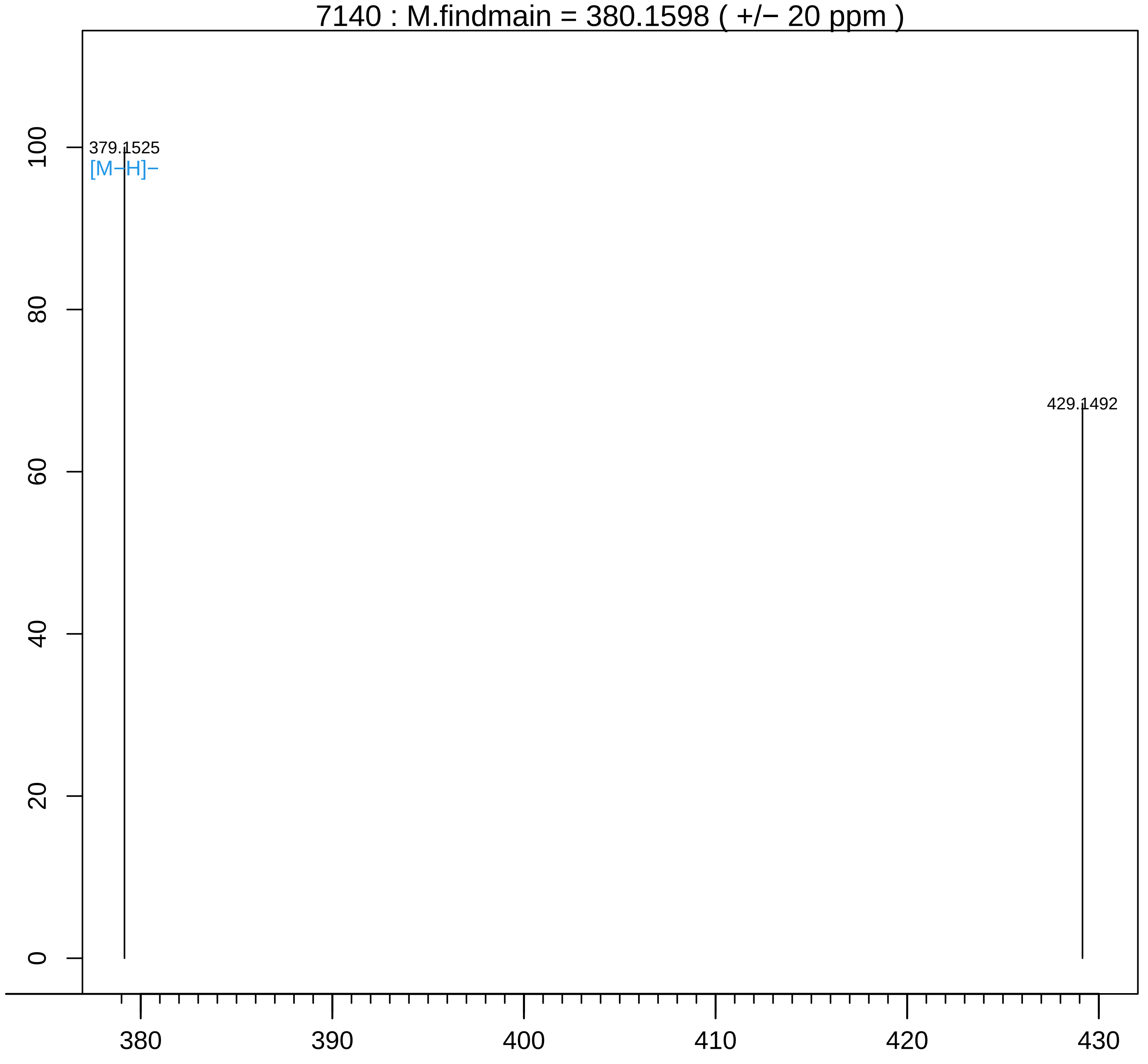
7140 : M.findmain = 380.1598 (+/− 20 ppm)
379.1525
[M−H]−
429.1492
0
20
40
60
80
100
380
390
400
410
420
430

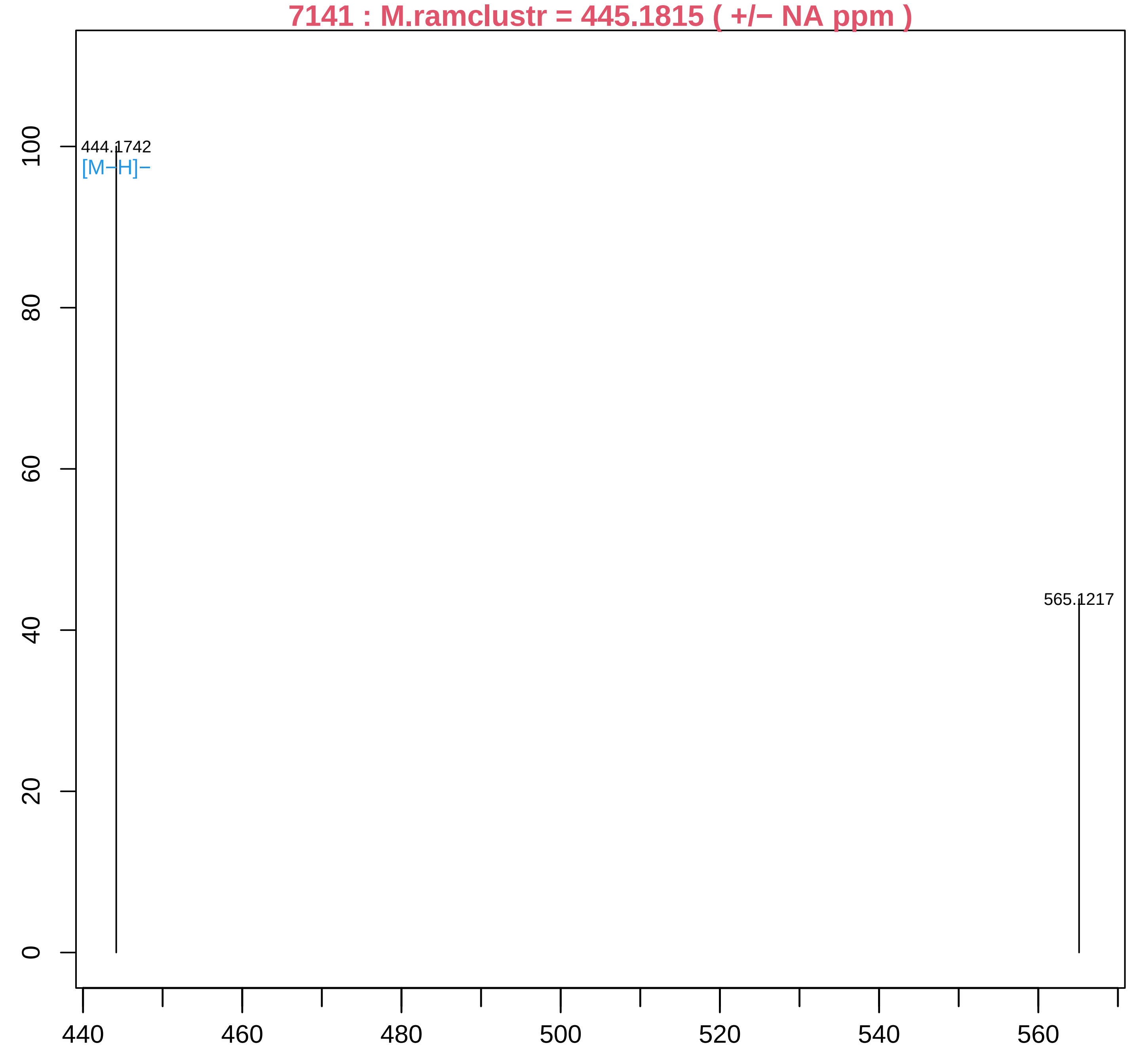

7141 : M.ramclustr = 445.1815 (+/− NA ppm)
444.1742
[M−H]−
565.1217
0
20
40
60
80
100
440
460
480
500
520
540
560

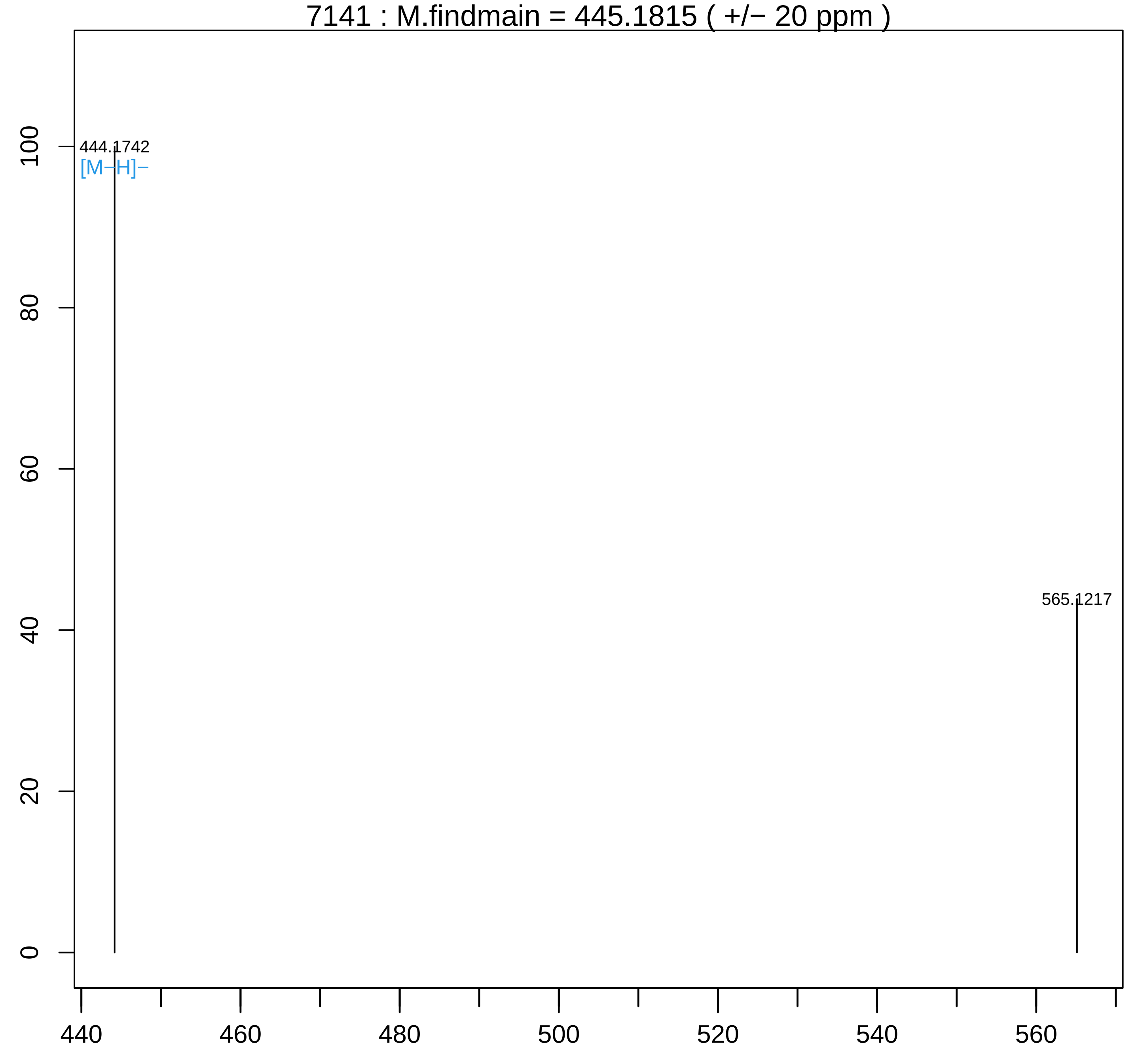

7141 : M.findmain = 445.1815 (+/− 20 ppm)
444.1742
[M−H]−
565.1217
0
20
40
60
80
100
440
460
480
500
520
540
560

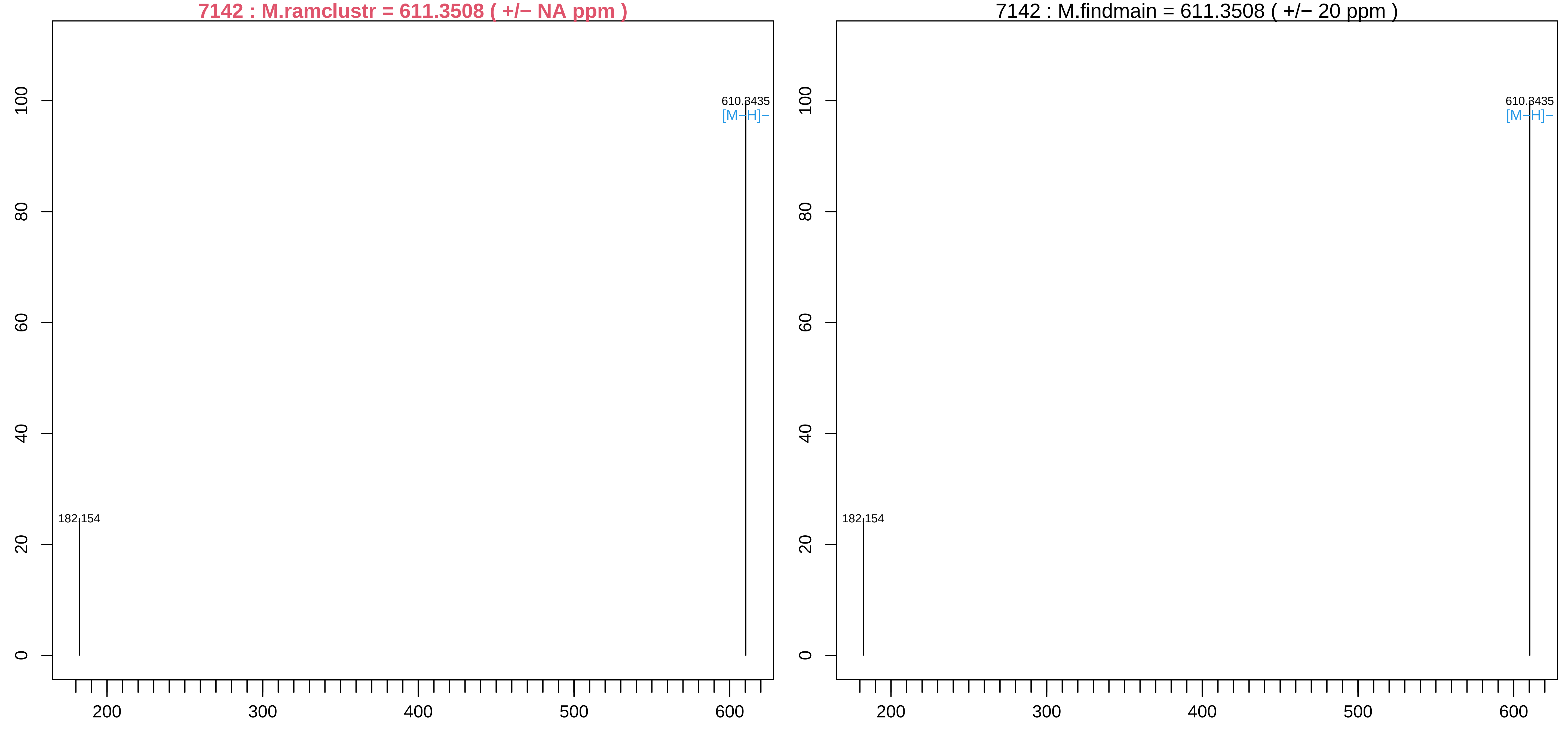

7142 : M.ramclustr = 611.3508 (+/− NA ppm)
610.3435
[M−H]−
182.154
0
20
40
60
80
100
200
300
400
500
600
7142 : M.findmain = 611.3508 (+/− 20 ppm)
610.3435
[M−H]−
182.154
0
20
40
60
80
100
200
300
400
500
600

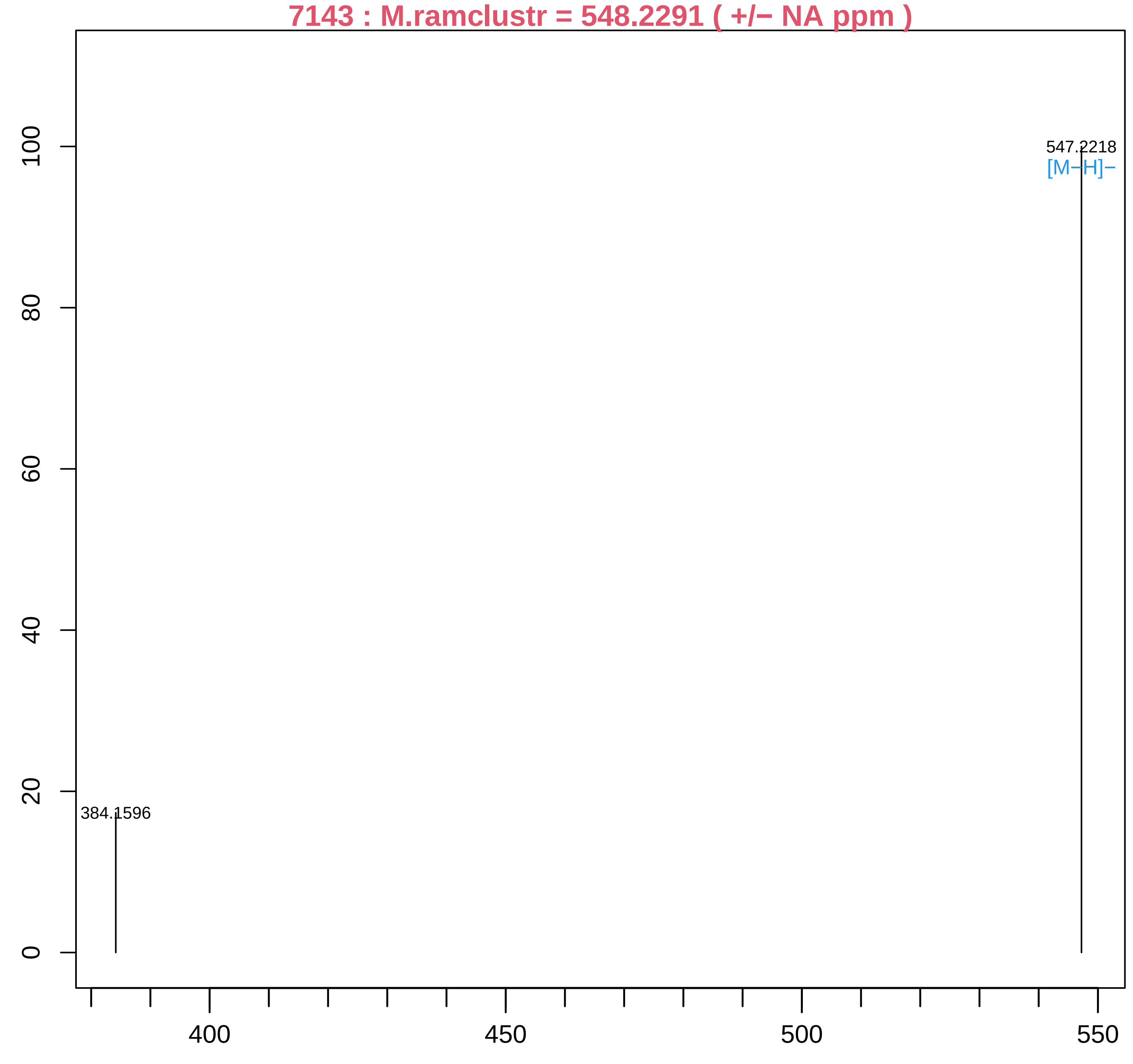
7143 : M.ramclustr = 548.2291 (+/− NA ppm)
547.2218
[M−H]−
384.1596
0
20
40
60
80
100
400
450
500
550

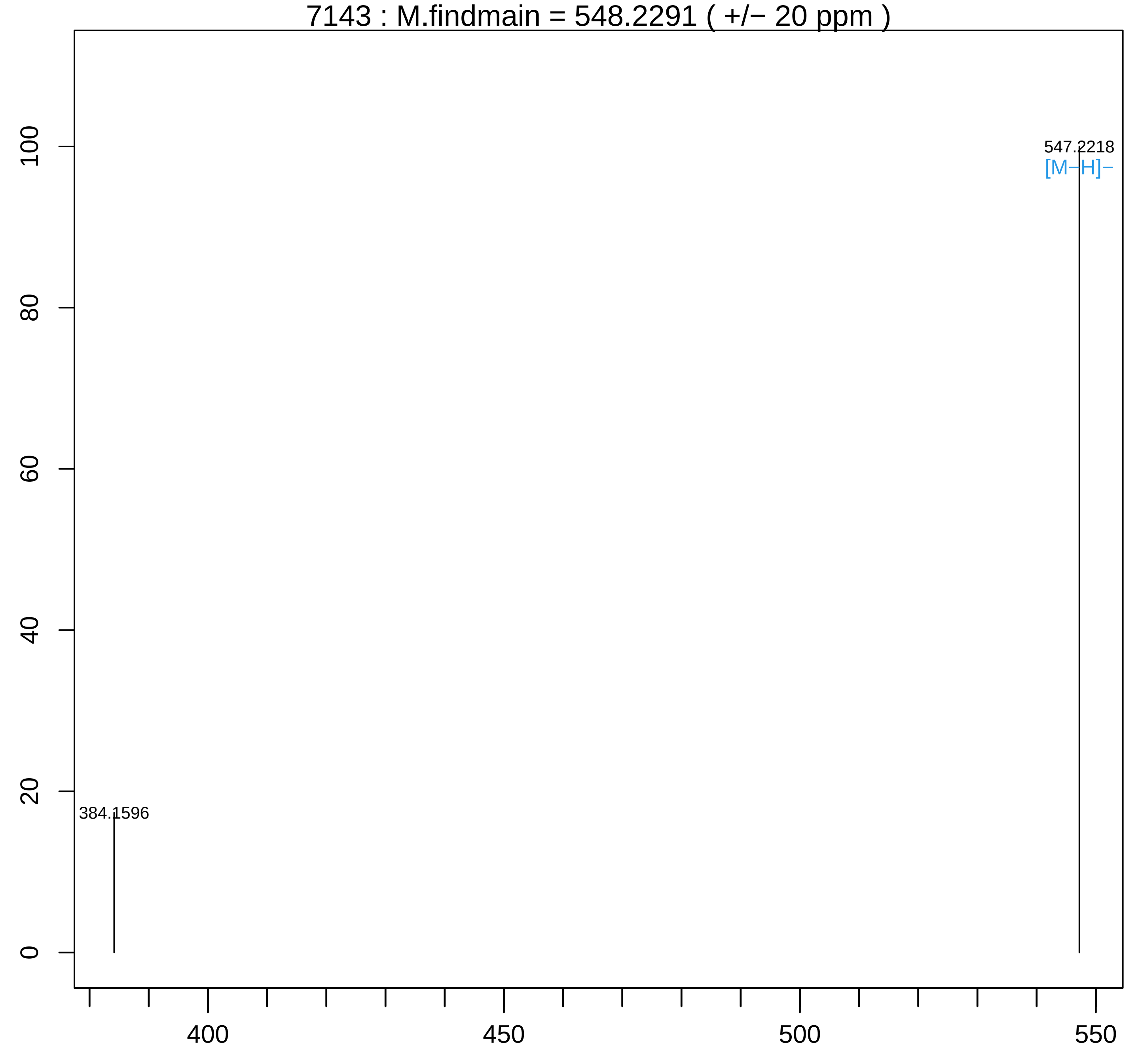
7143 : M.findmain = 548.2291 (+/− 20 ppm)
547.2218
[M−H]−
384.1596
0
20
40
60
80
100
400
450
500
550

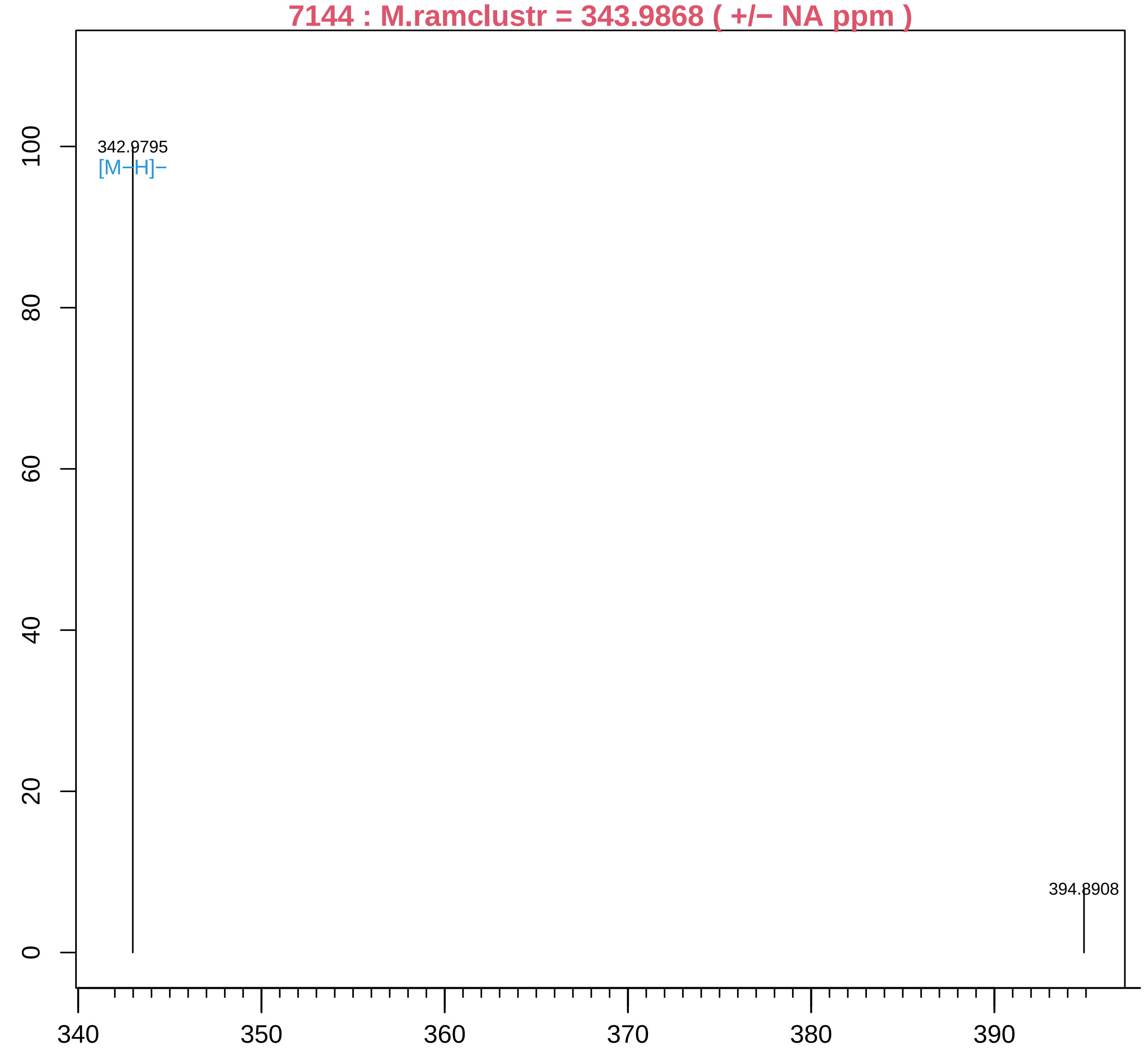
7144 : M.ramclustr = 343.9868 (+/− NA ppm)
342.9795
[M−H]−
394.8908
0
20
40
60
80
100
340
350
360
370
380
390

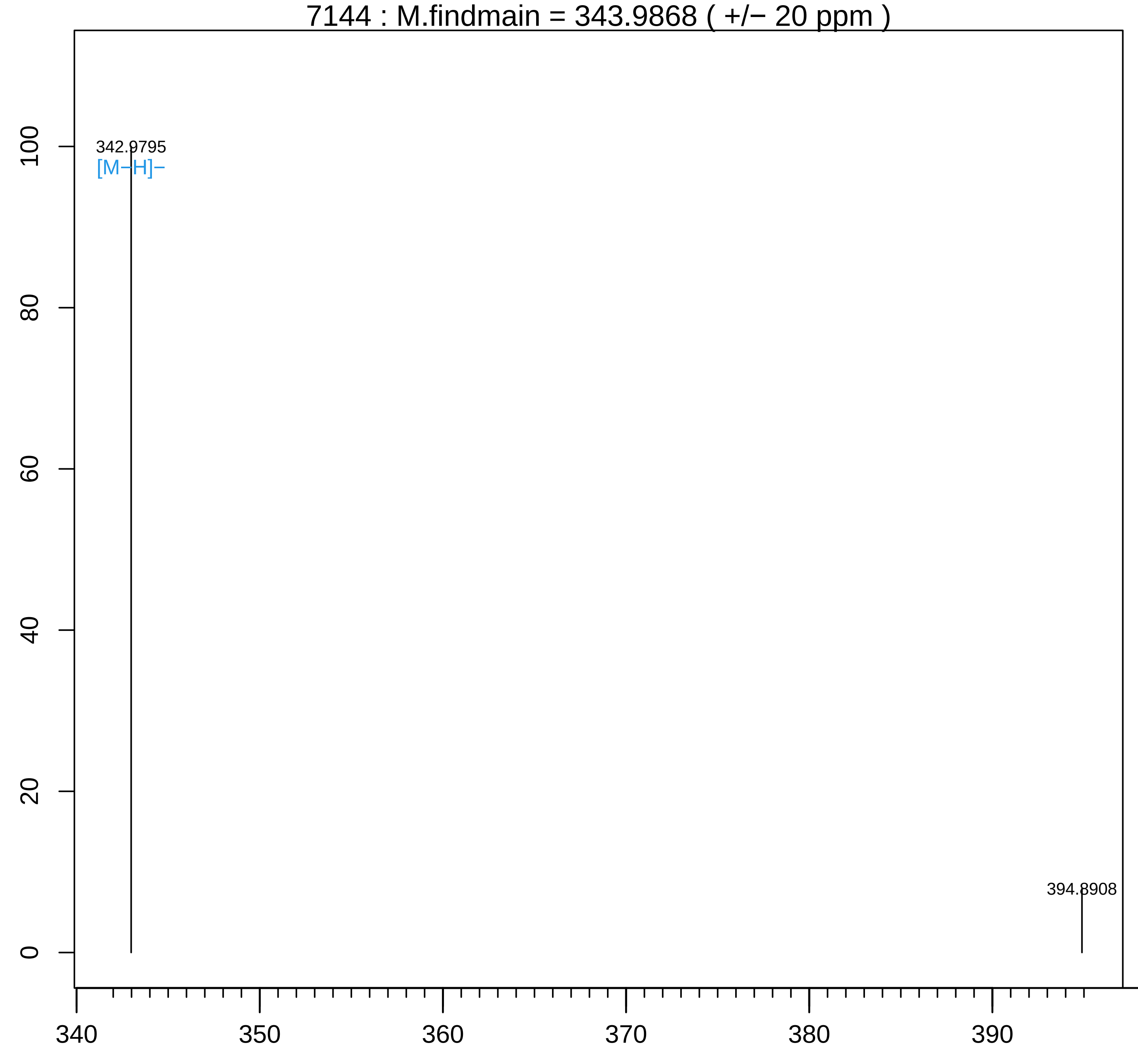
7144 : M.findmain = 343.9868 (+/− 20 ppm)
342.9795
[M−H]−
394.8908
0
20
40
60
80
100
340
350
360
370
380
390

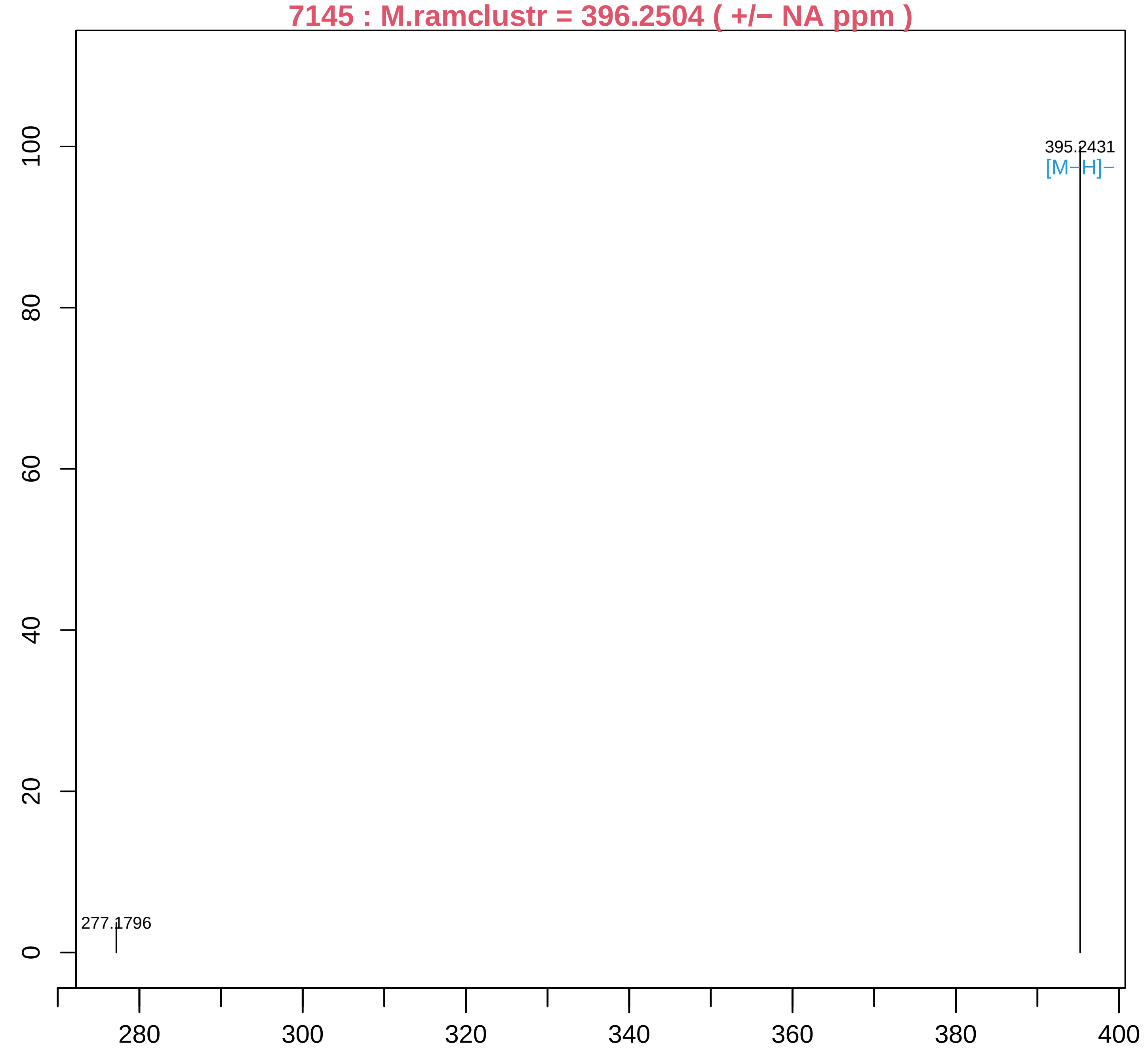
7145 : M.ramclustr = 396.2504 (+/− NA ppm)
395.2431
[M−H]−
277.1796
0
20
40
60
80
100
280
300
320
340
360
380
400

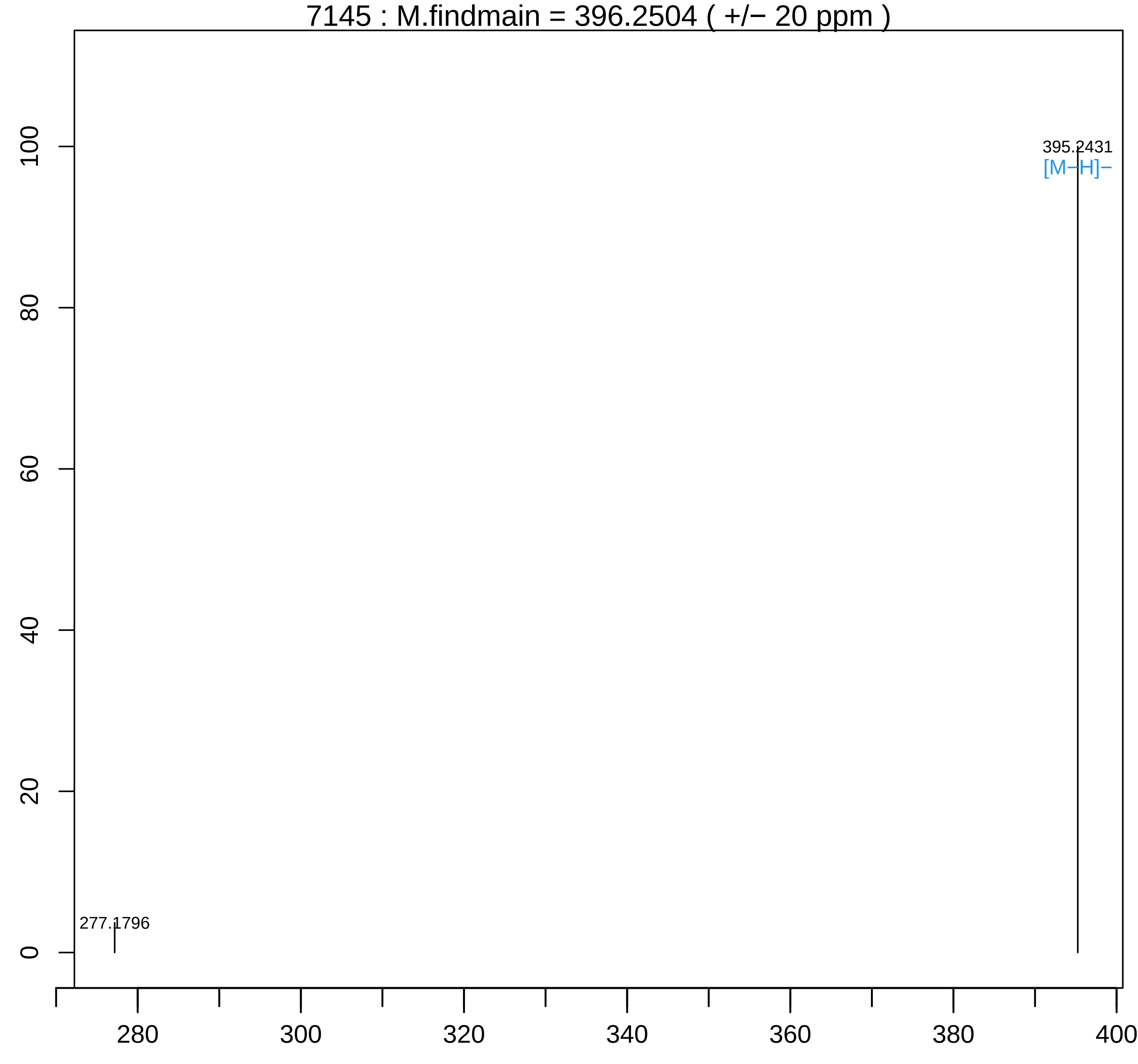
7145 : M.findmain = 396.2504 (+/− 20 ppm)
395.2431
[M−H]−
277.1796
0
20
40
60
80
100
280
300
320
340
360
380
400

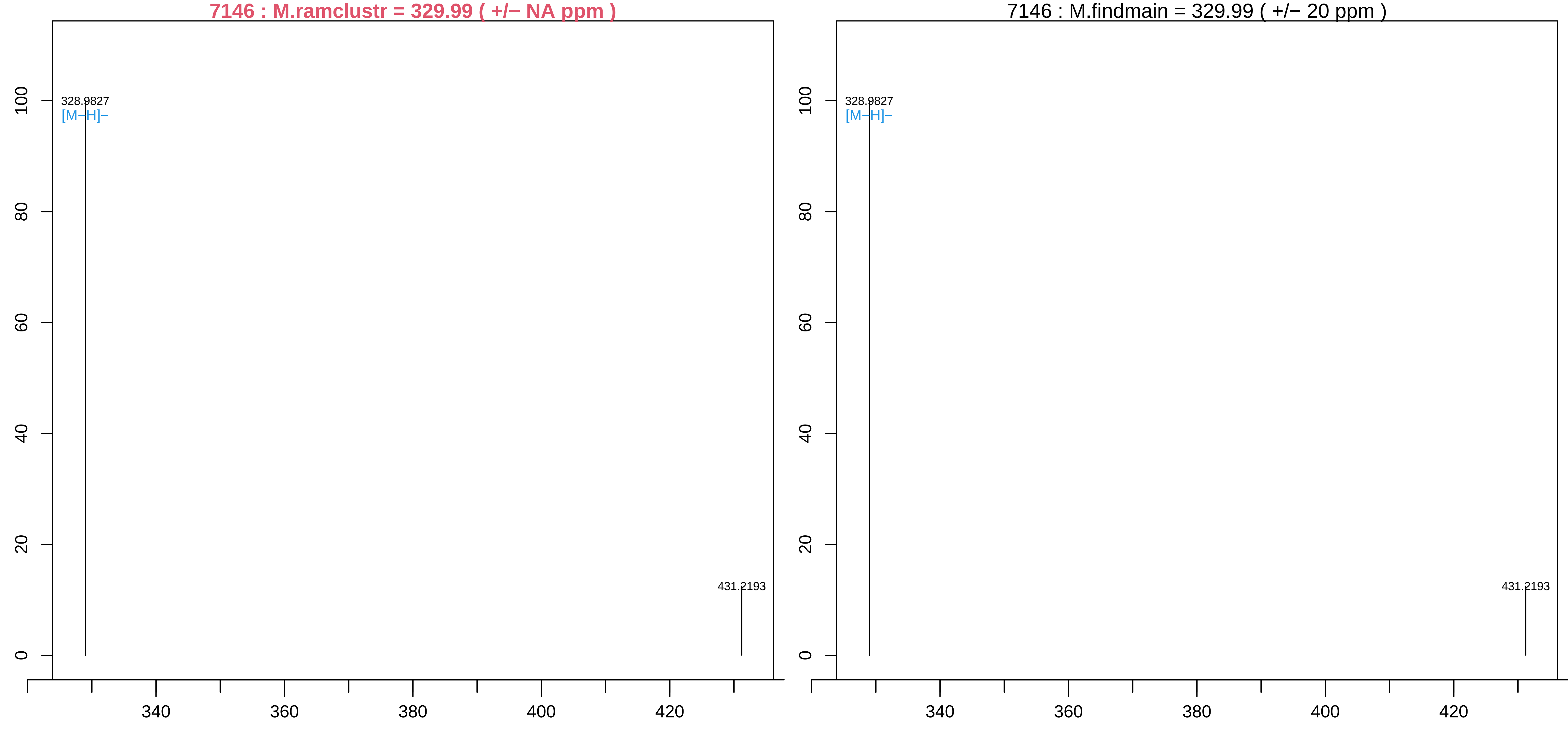
7146 : M.ramclustr = 329.99 (+/− NA ppm)
328.9827
[M−H]−
431.2193
0
20
40
60
80
100
340
360
380
400
420
7146 : M.findmain = 329.99 (+/− 20 ppm)
328.9827
[M−H]−
431.2193
0
20
40
60
80
100
340
360
380
400
420

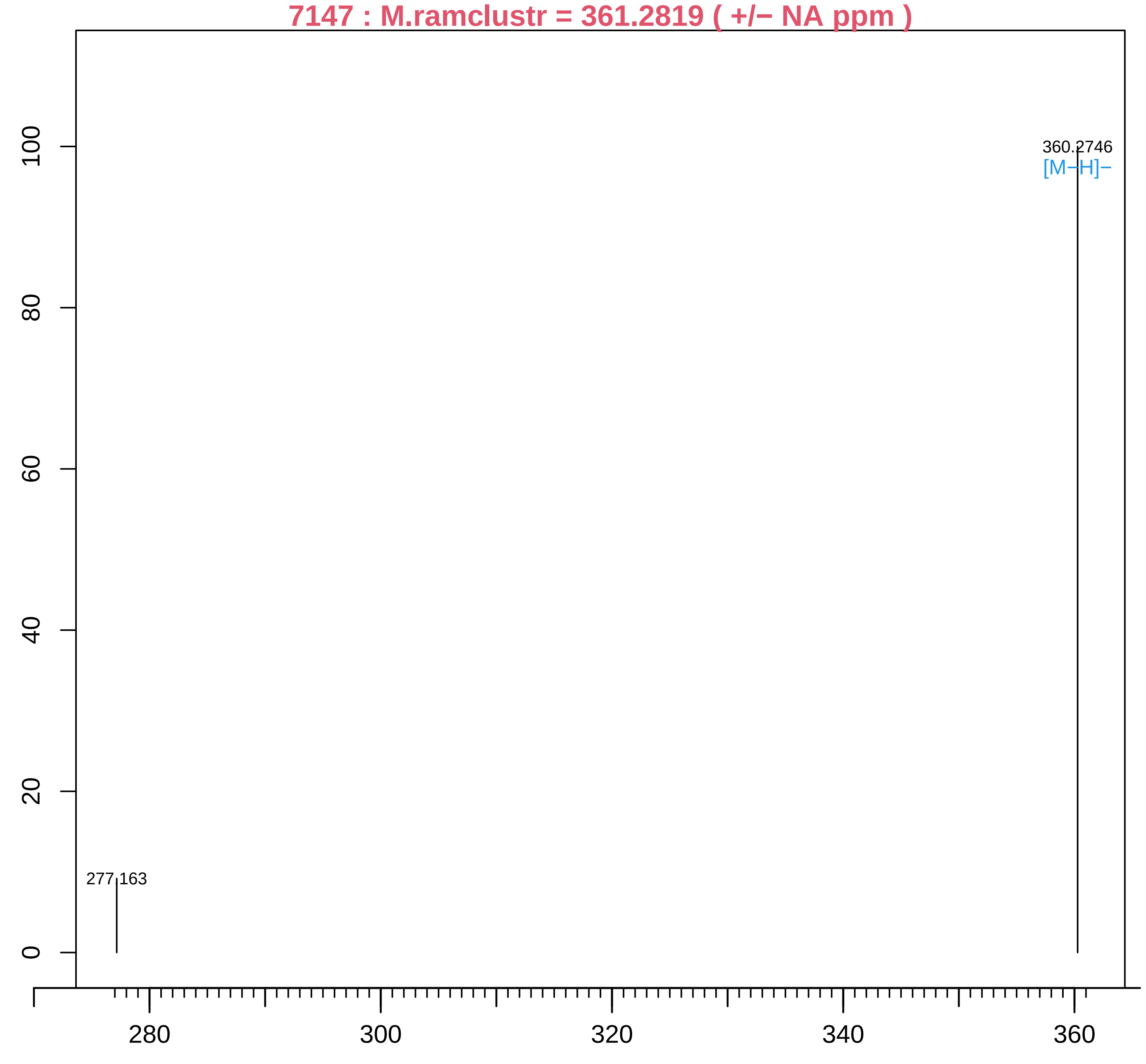
7147 : M.ramclustr = 361.2819 (+/− NA ppm)
360.2746
[M−H]−
277.163
0
20
40
60
80
100
280
300
320
340
360

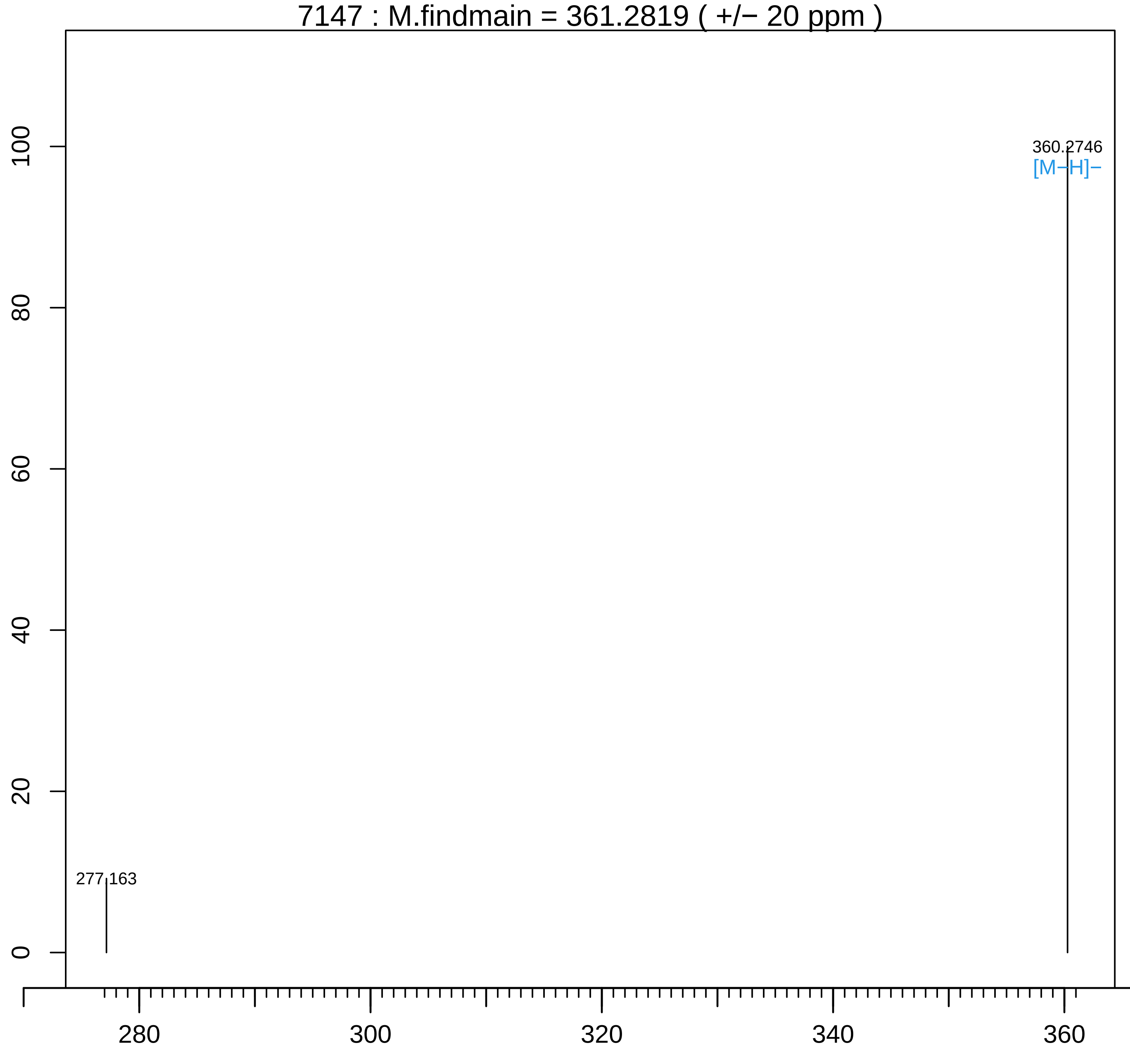
7147 : M.findmain = 361.2819 (+/− 20 ppm)
360.2746
[M−H]−
277.163
0
20
40
60
80
100
280
300
320
340
360

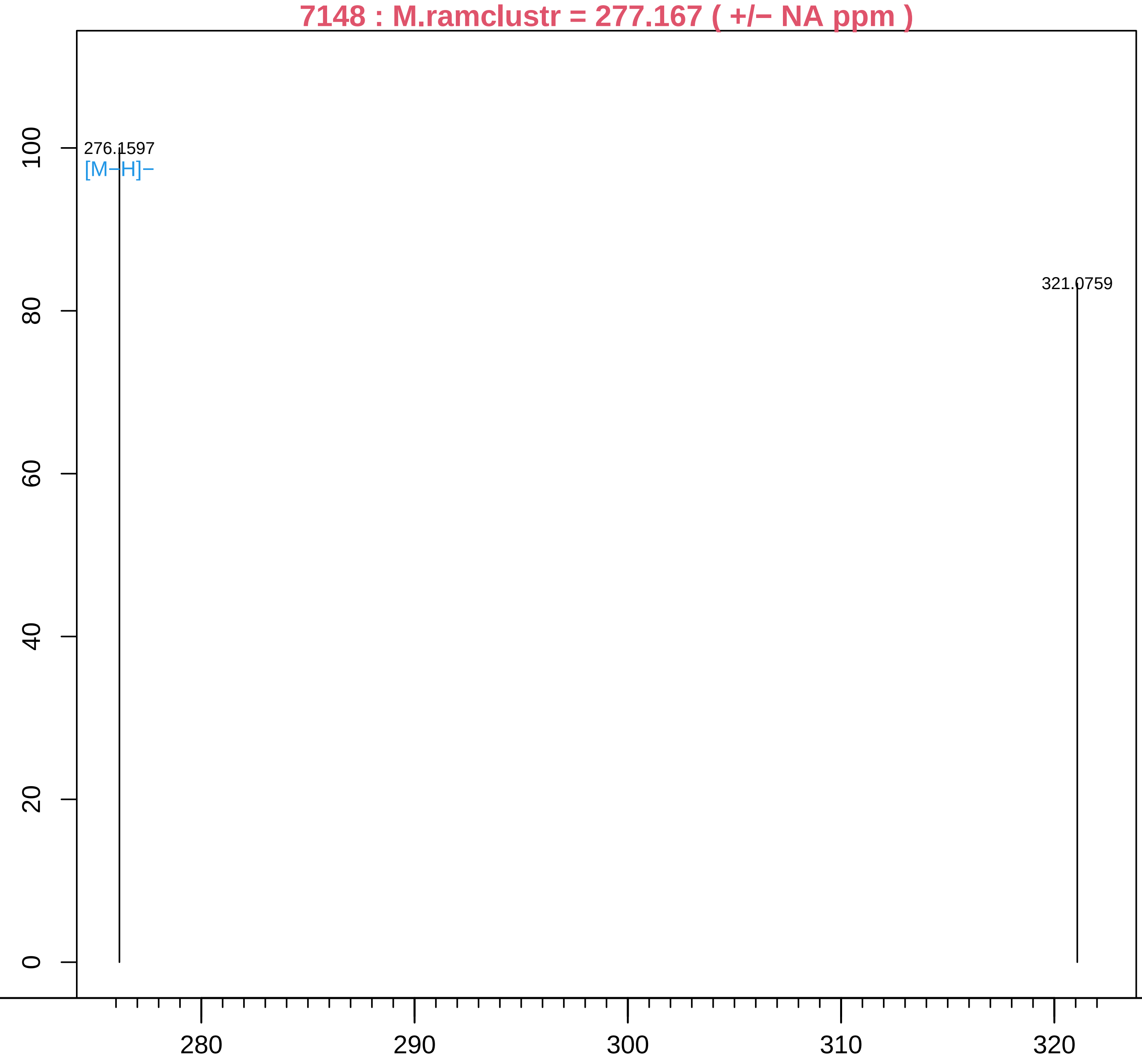
7148 : M.ramclustr = 277.167 (+/− NA ppm)
276.1597
[M−H]−
321.0759
0
20
40
60
80
100
280
290
300
310
320
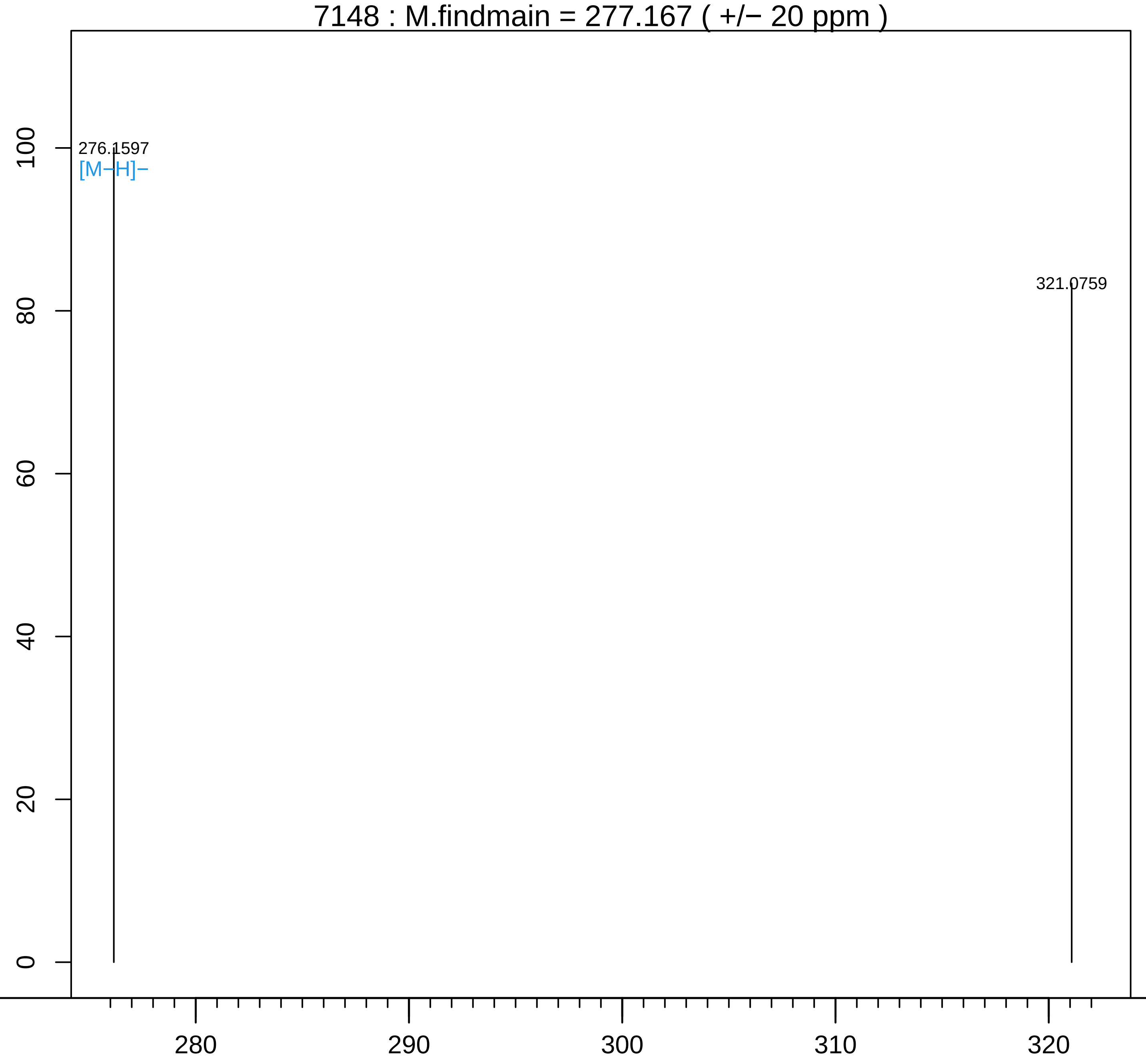
7148 : M.findmain = 277.167 (+/− 20 ppm)
276.1597
[M−H]−
321.0759
0
20
40
60
80
100
280
290
300
310
320

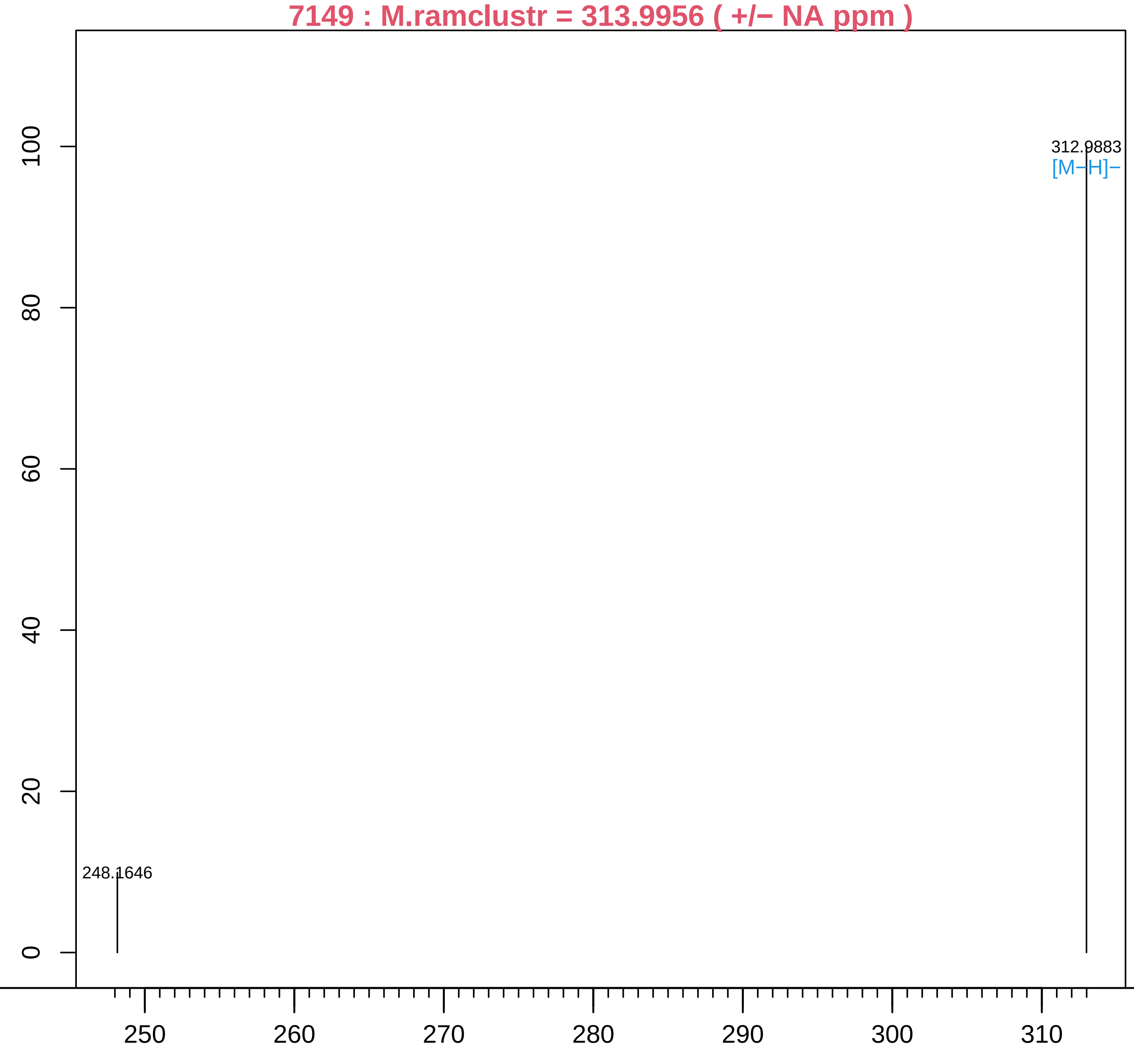
7149 : M.ramclustr = 313.9956 (+/− NA ppm)
312.9883
[M−H]−
248.1646
0
20
40
60
80
100
250
260
270
280
290
300
310
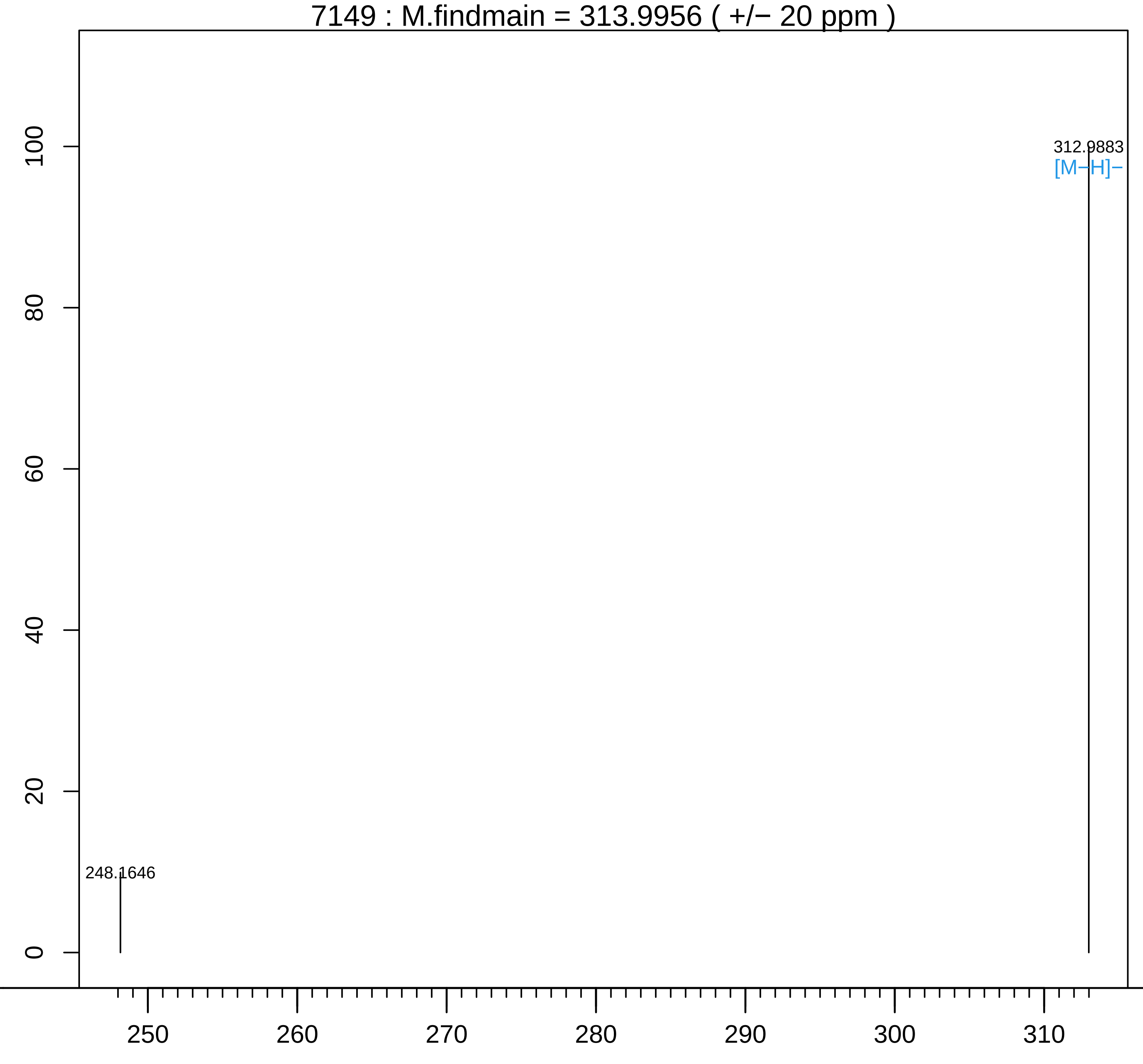
7149 : M.findmain = 313.9956 (+/− 20 ppm)
312.9883
[M−H]−
248.1646
0
20
40
60
80
100
250
260
270
280
290
300
310

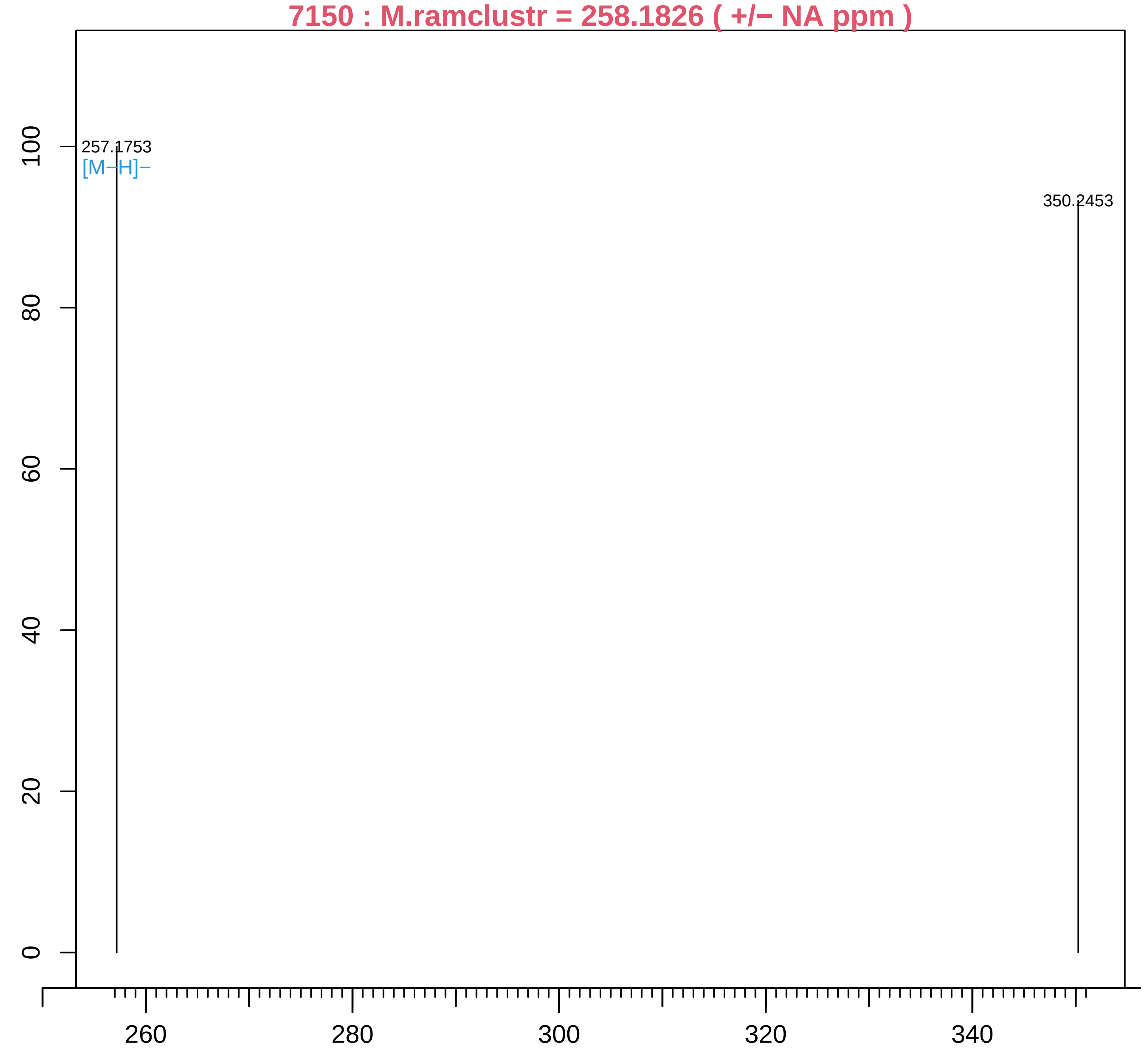

7150 : M.ramclustr = 258.1826 (+/− NA ppm)
257.1753
[M−H]−
350.2453
0
20
40
60
80
100
260
280
300
320
340

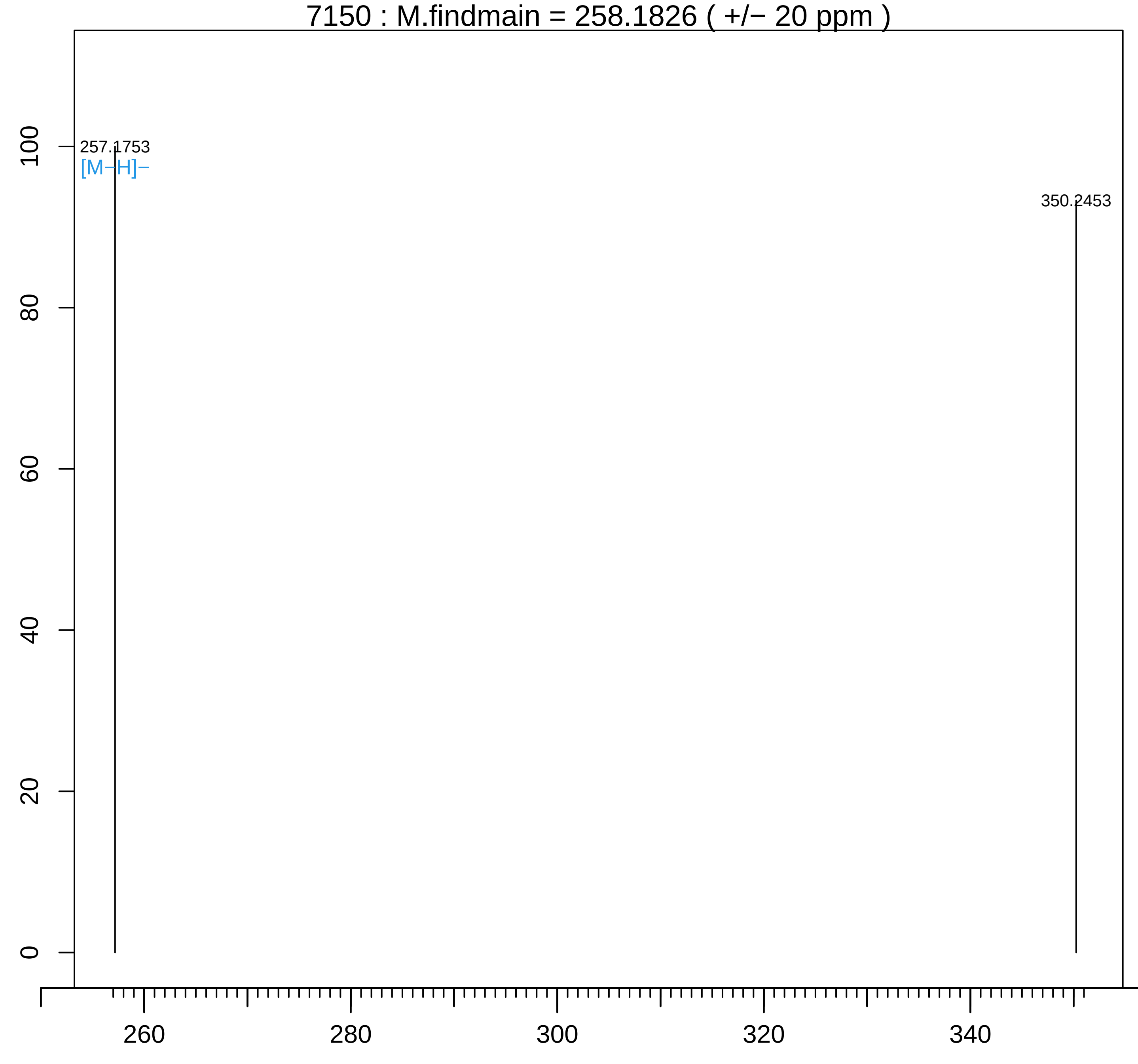

7150 : M.findmain = 258.1826 (+/− 20 ppm)
257.1753
[M−H]−
350.2453
0
20
40
60
80
100
260
280
300
320
340

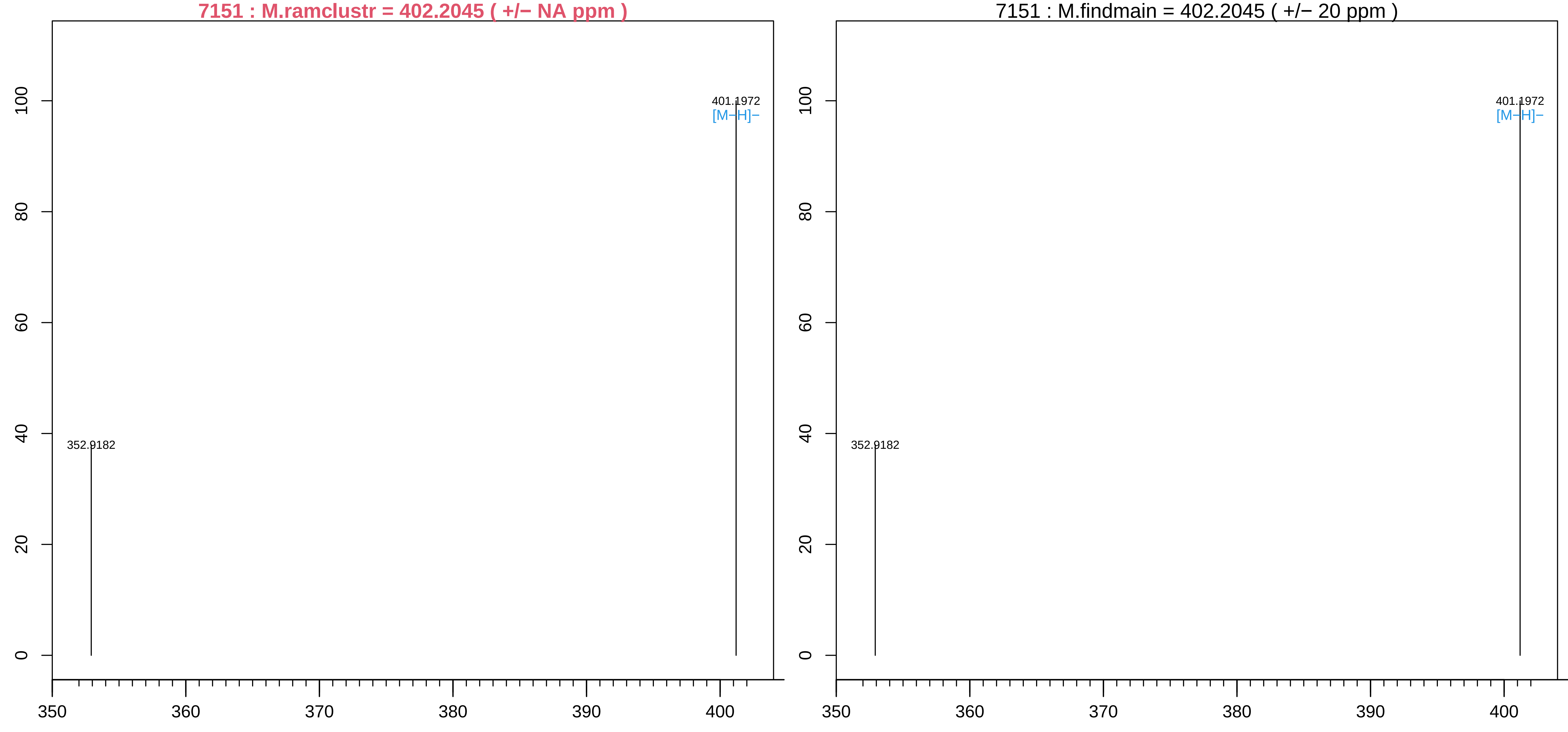
7151 : M.ramclustr = 402.2045 (+/− NA ppm)
401.1972
[M−H]−
352.9182
7151 : M.findmain = 402.2045 (+/− 20 ppm)
401.1972
[M−H]−
352.9182
0
20
40
60
80
100
350
360
370
380
390
400

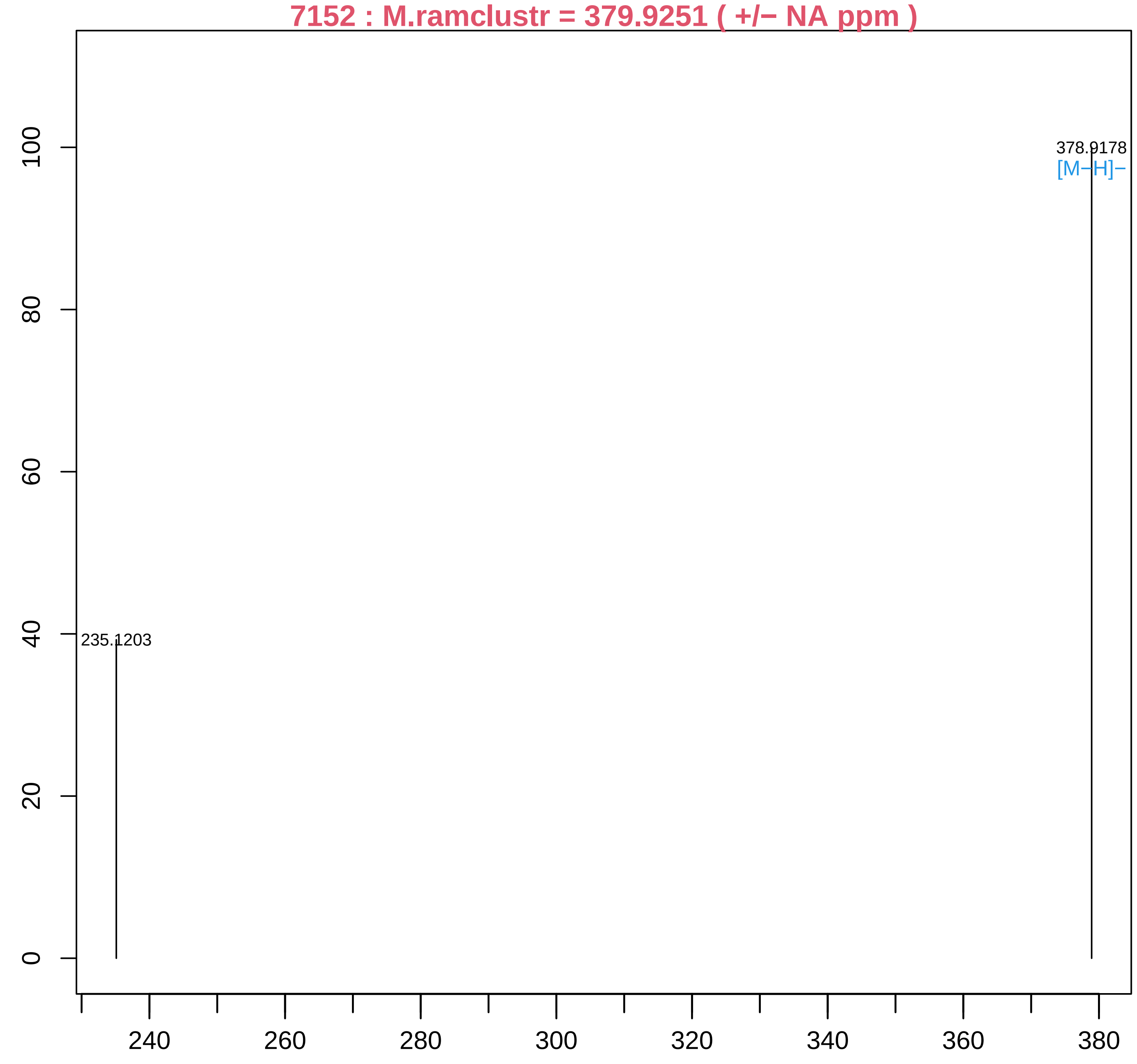
7152 : M.ramclustr = 379.9251 (+/− NA ppm)
378.9178
[M−H]−
235.1203
0
20
40
60
80
100
240
260
280
300
320
340
360
380

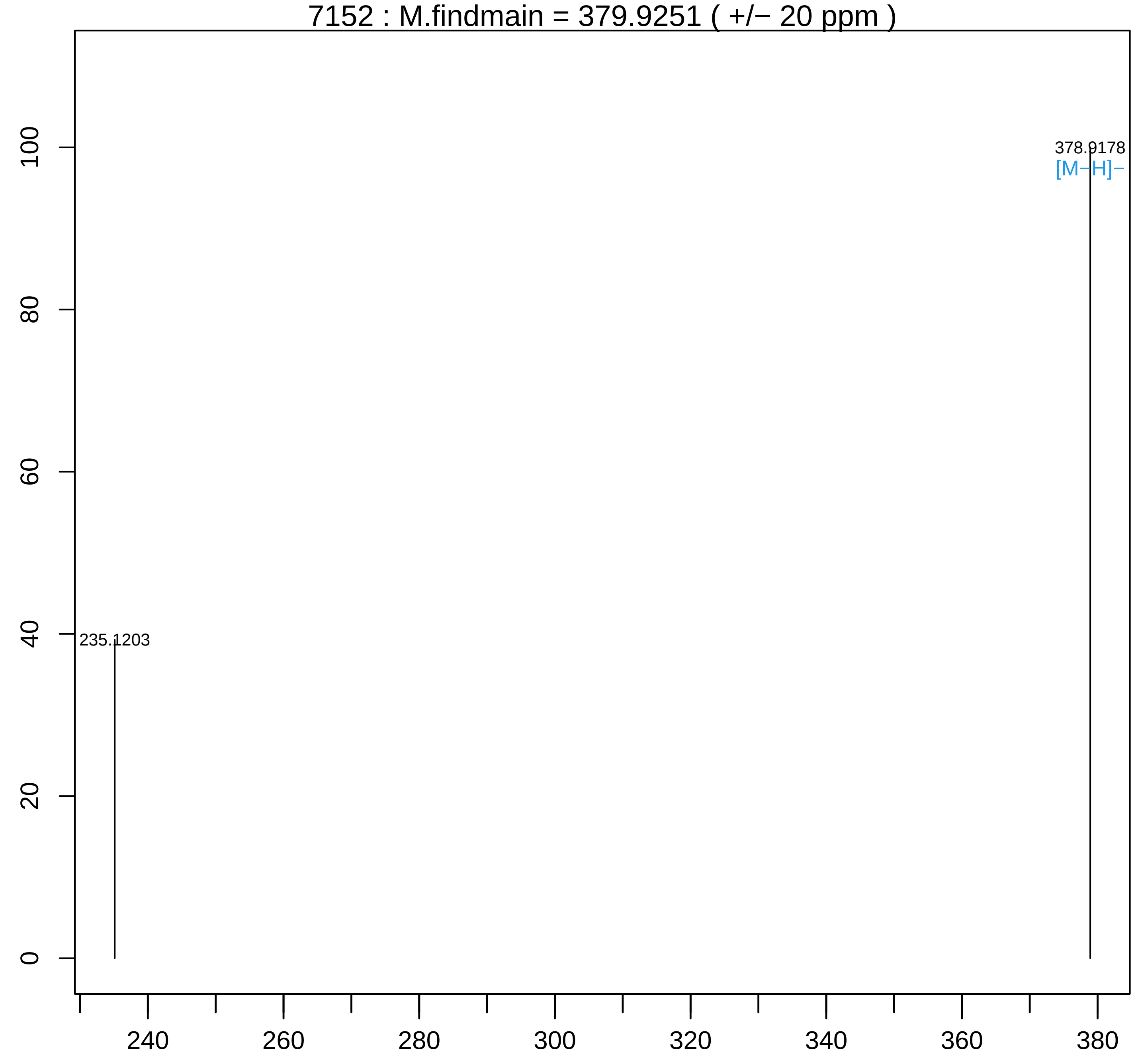
7152 : M.findmain = 379.9251 (+/− 20 ppm)
378.9178
[M−H]−
235.1203
0
20
40
60
80
100
240
260
280
300
320
340
360
380

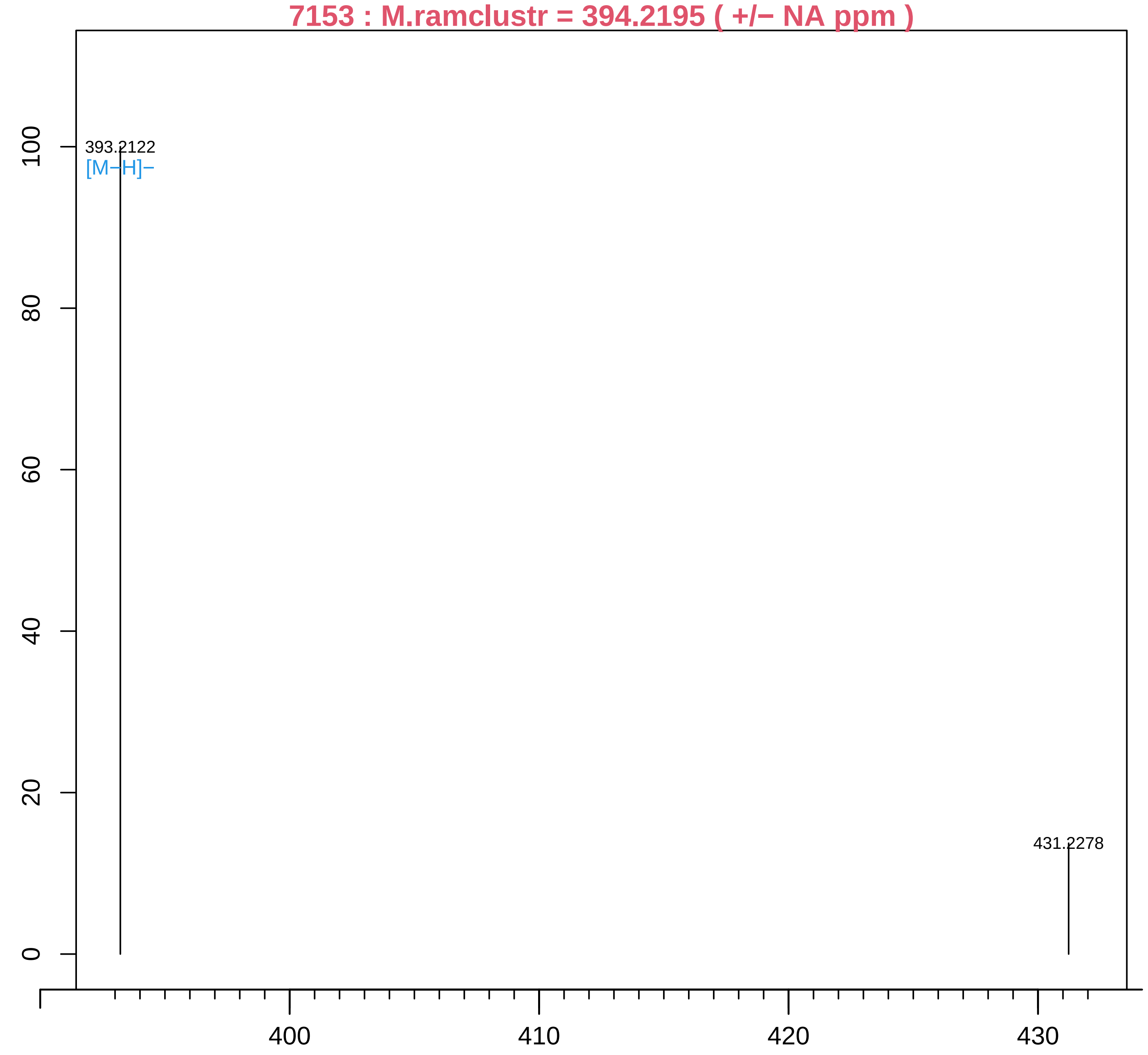
7153 : M.ramclustr = 394.2195 (+/− NA ppm)
393.2122
[M−H]−
431.2278
0
20
40
60
80
100
400
410
420
430

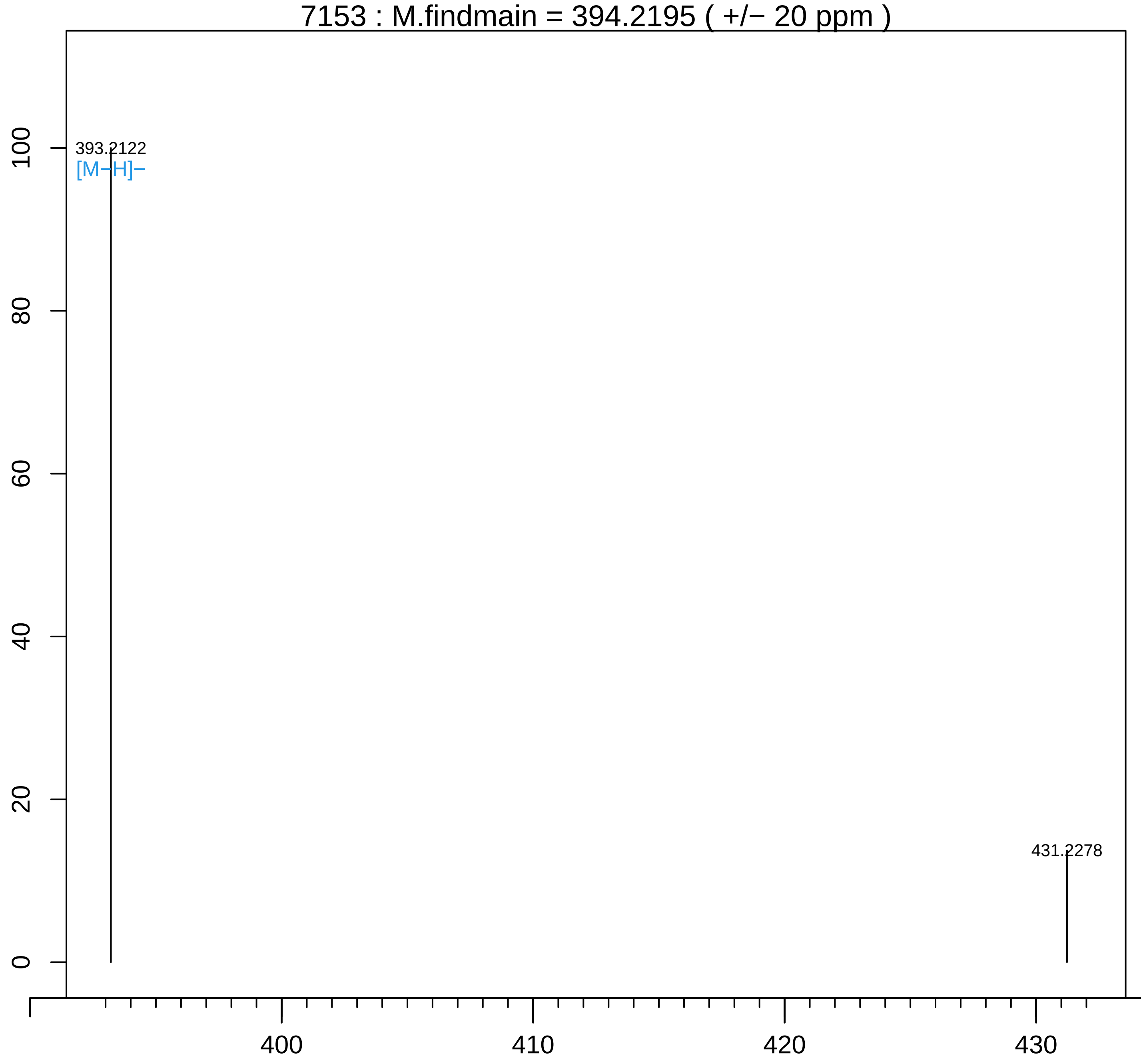
7153 : M.findmain = 394.2195 (+/− 20 ppm)
393.2122
[M−H]−
431.2278
0
20
40
60
80
100
400
410
420
430

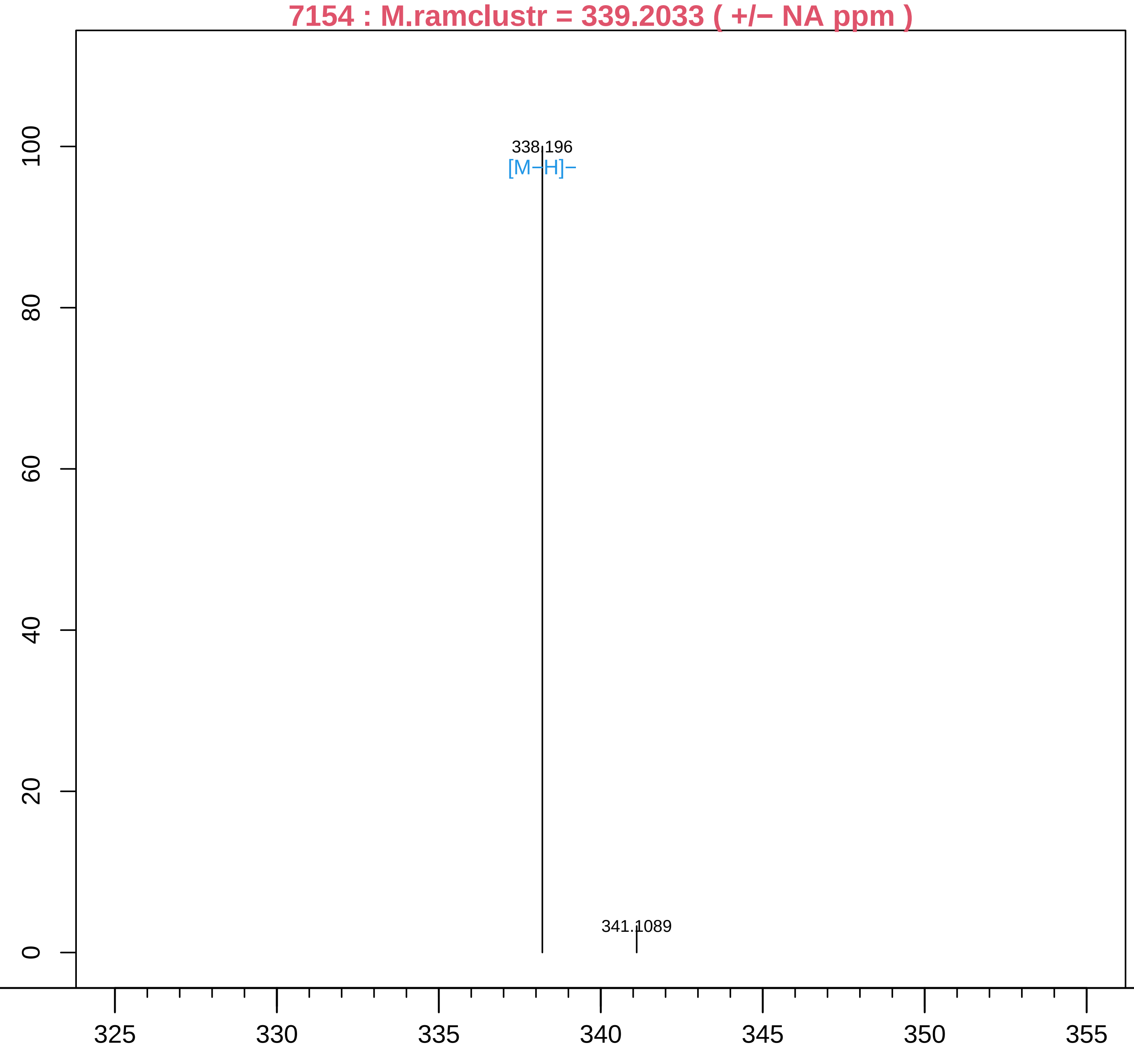
7154 : M.ramclustr = 339.2033 (+/− NA ppm)
338.196
[M−H]−
341.1089
0
20
40
60
80
100
325
330
335
340
345
350
355

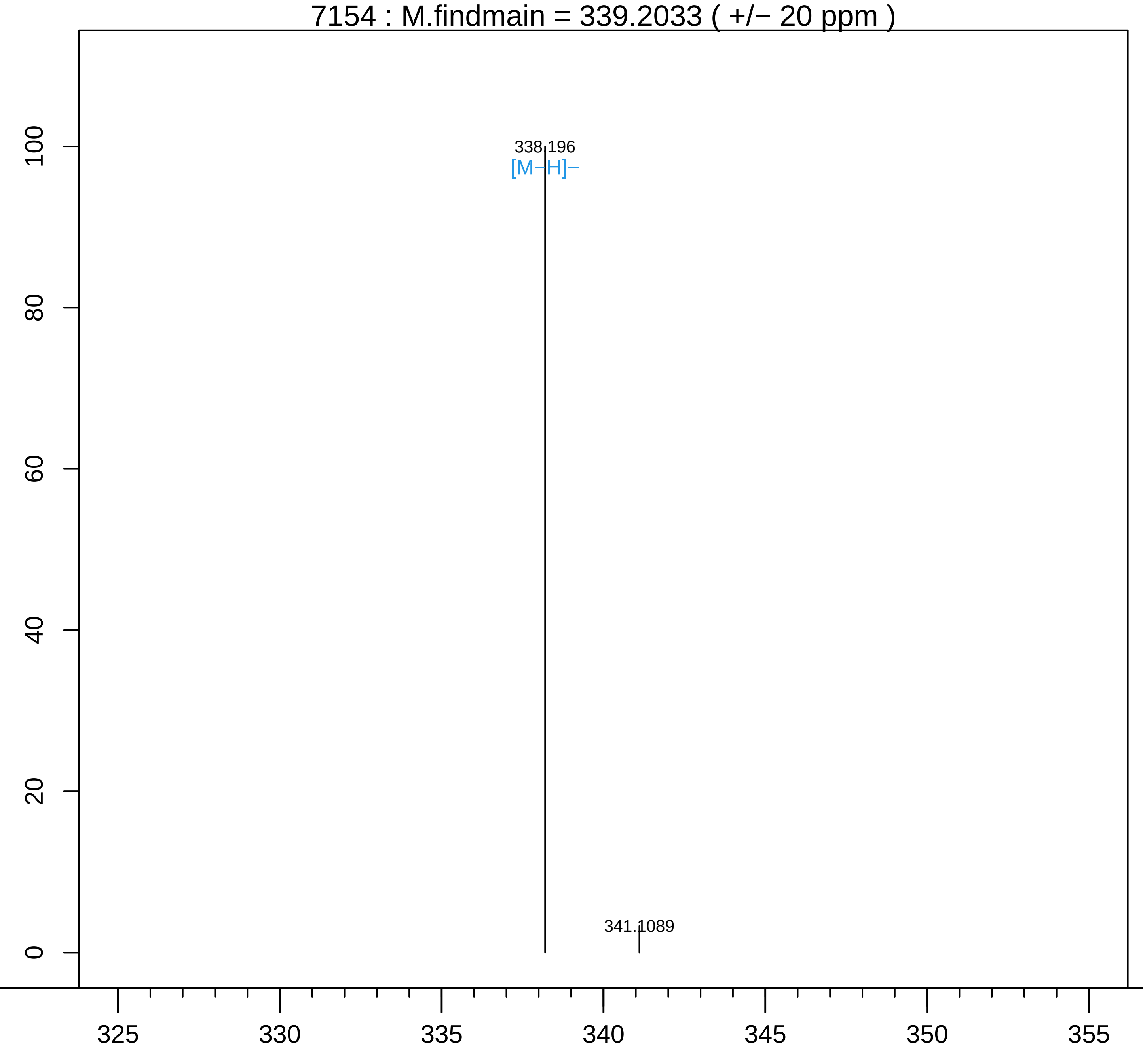
7154 : M.findmain = 339.2033 (+/− 20 ppm)
338.196
[M−H]−
341.1089
0
20
40
60
80
100
325
330
335
340
345
350
355

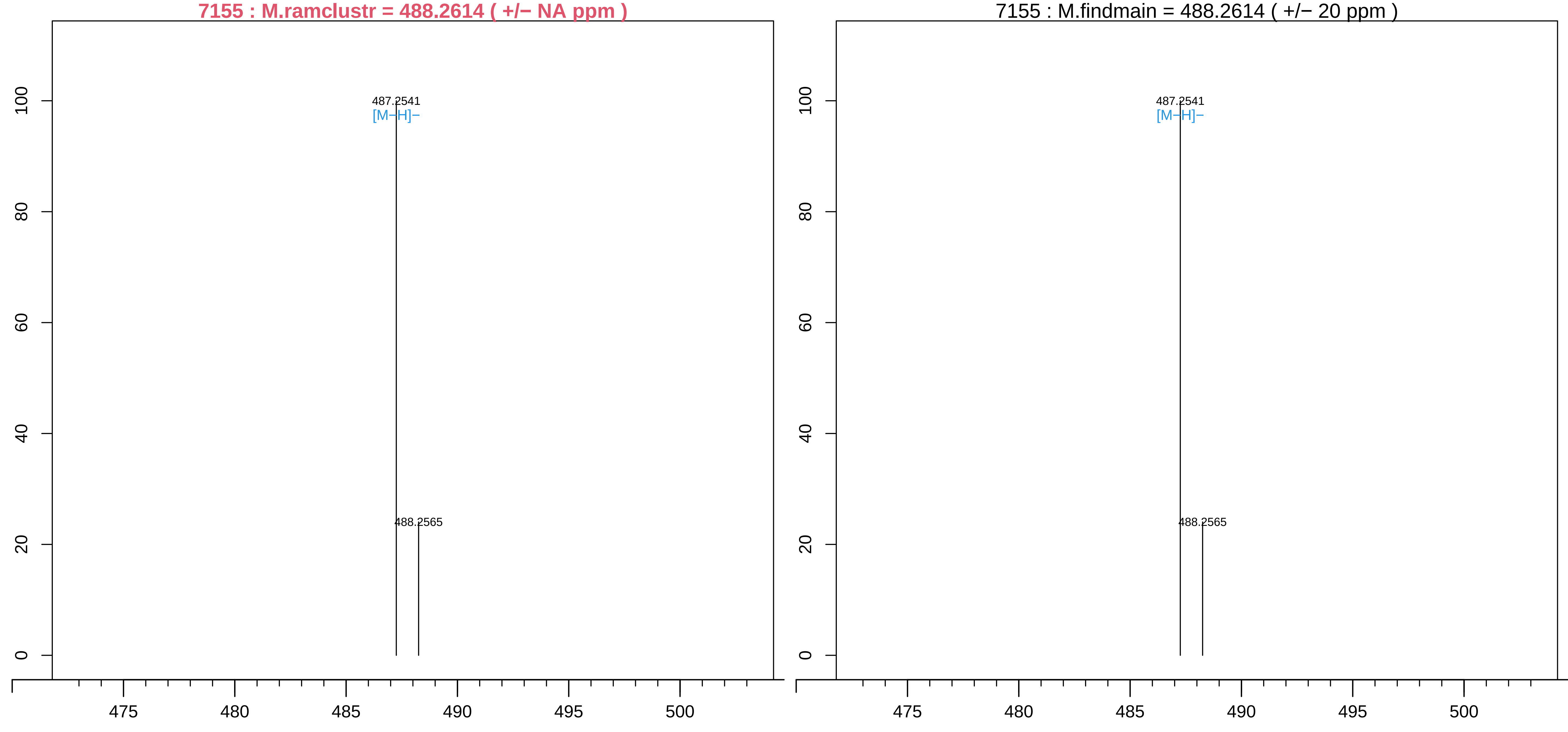
7155 : M.ramclustr = 488.2614 (+/− NA ppm)
487.2541
[M−H]−
488.2565
0
20
40
60
80
100
475
480
485
490
495
500
7155 : M.findmain = 488.2614 (+/− 20 ppm)
487.2541
[M−H]−
488.2565
0
20
40
60
80
100
475
480
485
490
495
500

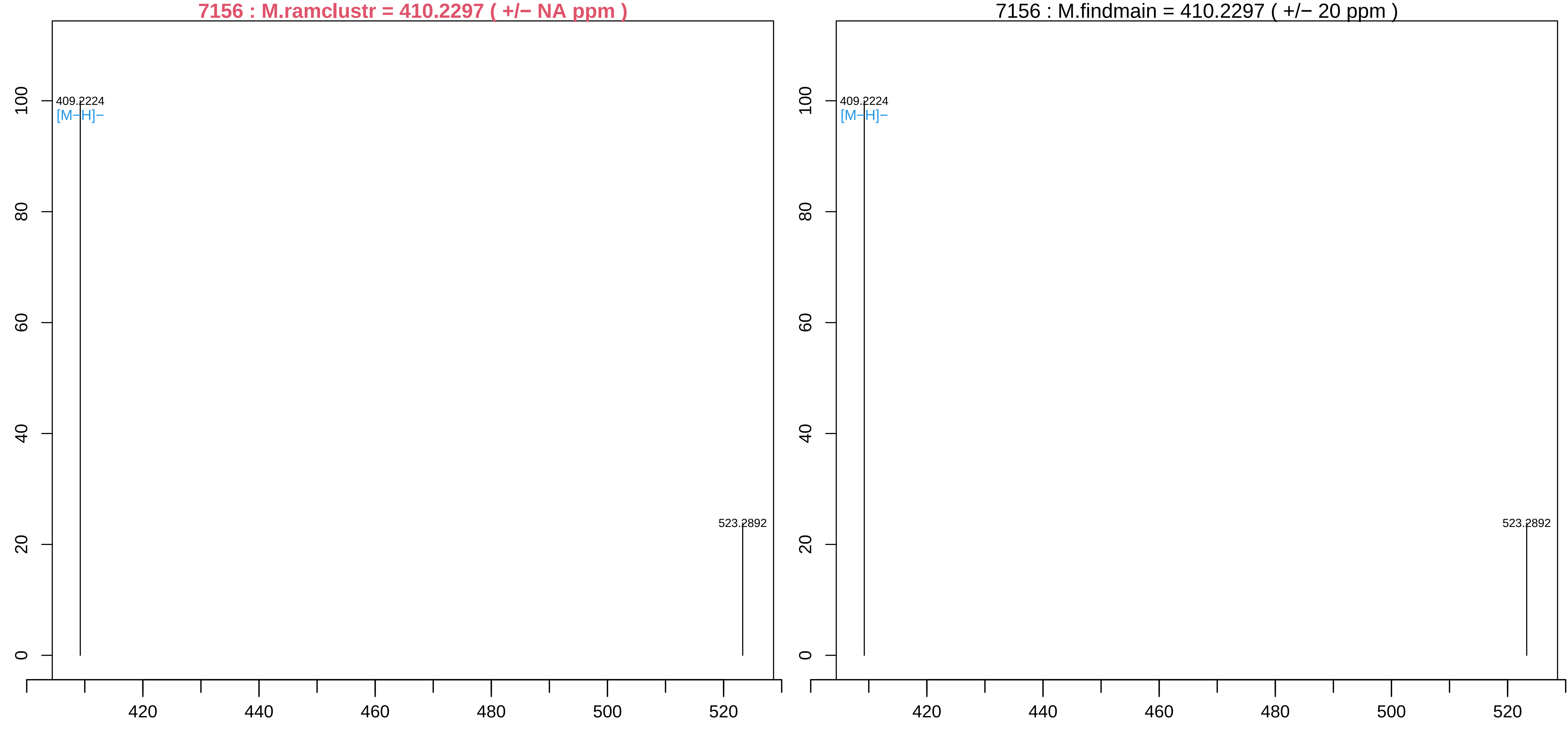
7156 : M.ramclustr = 410.2297 (+/− NA ppm)
409.2224
[M−H]−
523.2892
7156 : M.findmain = 410.2297 (+/− 20 ppm)
409.2224
[M−H]−
523.2892
0
20
40
60
80
100
420
440
460
480
500
520

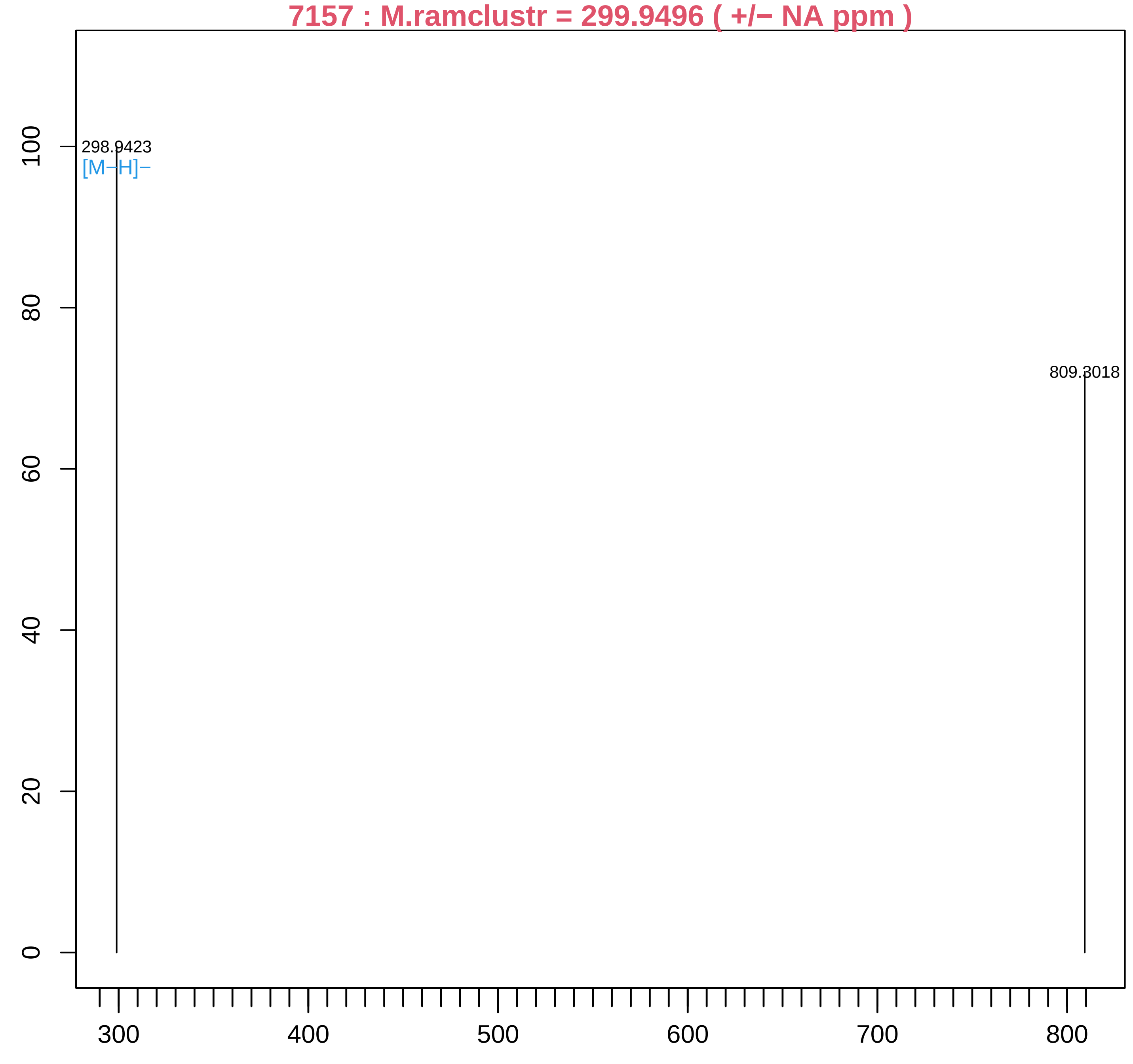
7157 : M.ramclustr = 299.9496 (+/− NA ppm)
298.9423
[M−H]−
809.3018
0
20
40
60
80
100
300
400
500
600
700
800

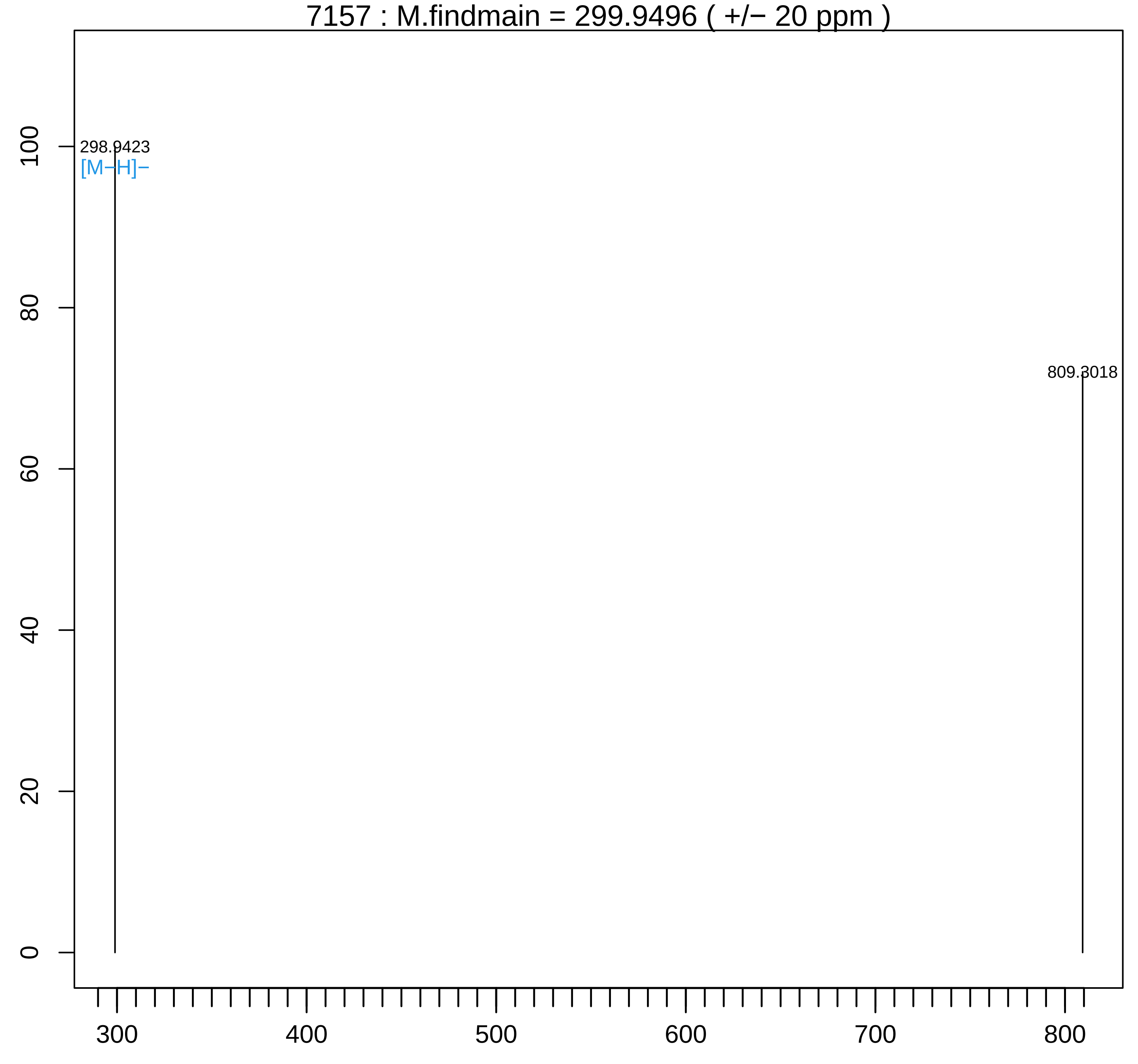
7157 : M.findmain = 299.9496 (+/− 20 ppm)
298.9423
[M−H]−
809.3018
0
20
40
60
80
100
300
400
500
600
700
800

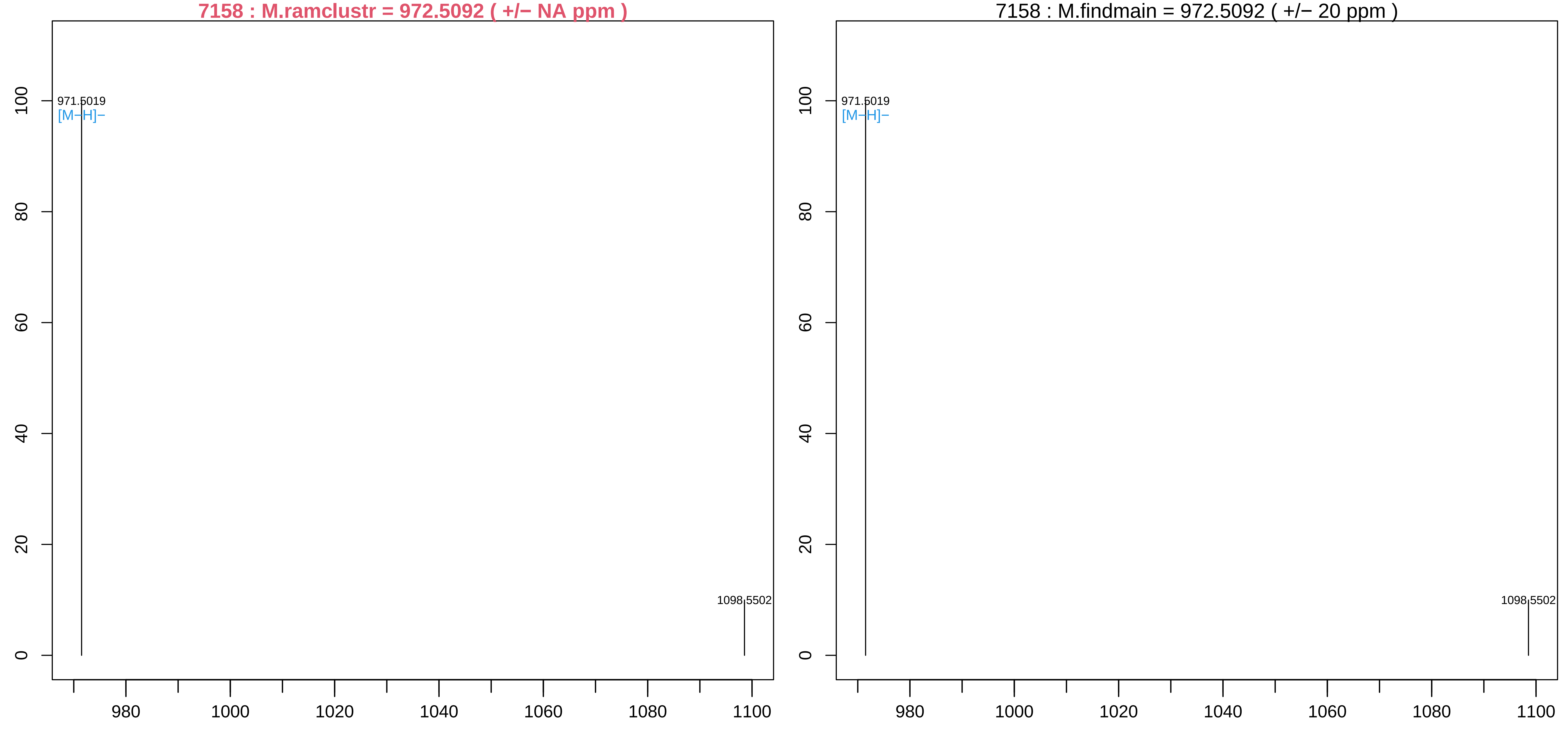
7158 : M.ramclustr = 972.5092 (+/− NA ppm)
971.5019
[M−H]−
1098.5502
0
20
40
60
80
100
980
1000
1020
1040
1060
1080
1100
7158 : M.findmain = 972.5092 (+/− 20 ppm)
971.5019
[M−H]−
1098.5502
0
20
40
60
80
100
980
1000
1020
1040
1060
1080
1100

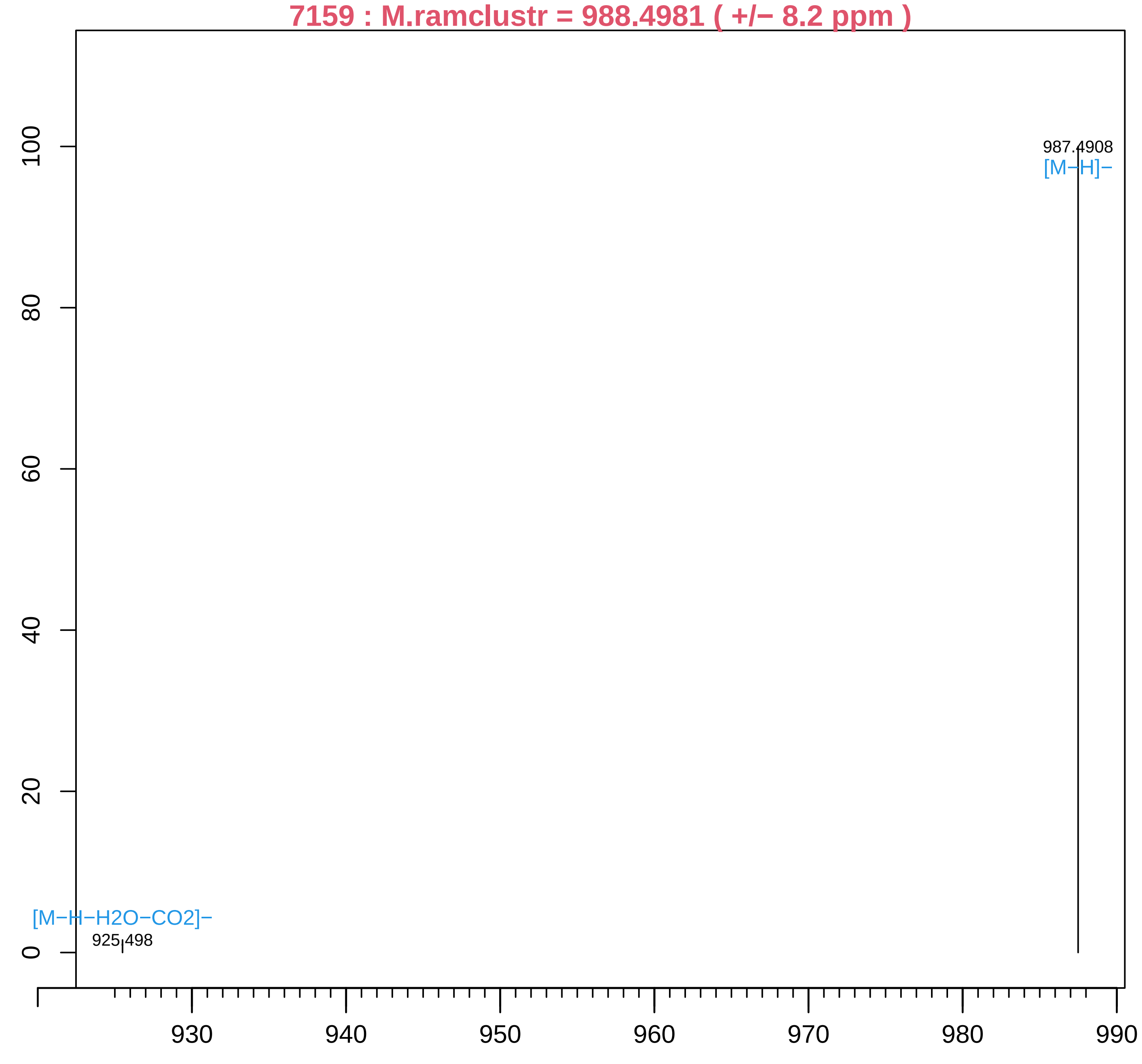

7159 : M.ramclustr = 988.4981 (+/− 8.2 ppm)
987.4908
[M−H]−
[M−H−H2O−CO2]−
925.498

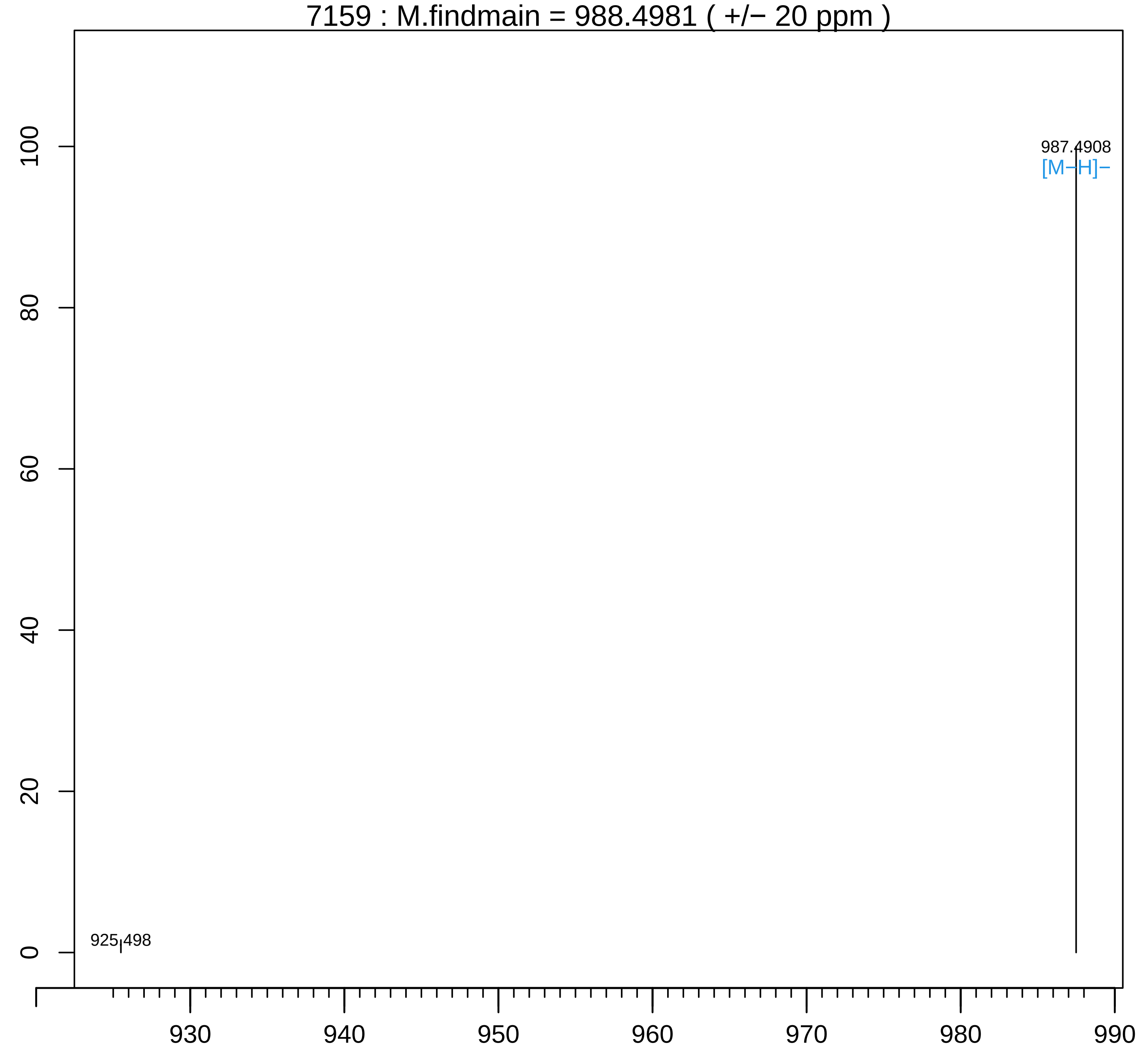

7159 : M.findmain = 988.4981 (+/− 20 ppm)
987.4908
[M−H]−
925.498

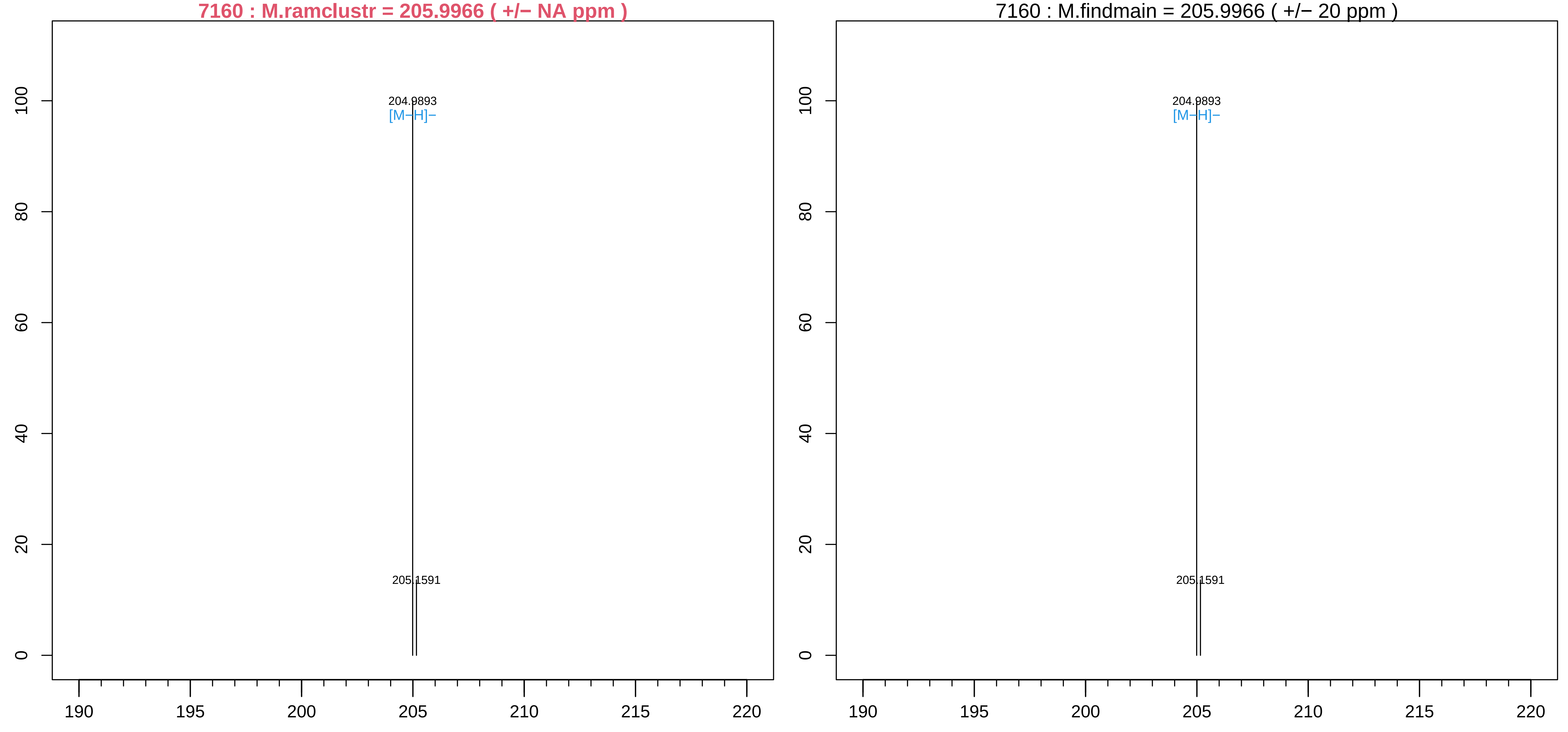
7160 : M.ramclustr = 205.9966 (+/− NA ppm)
204.9893
[M−H]−
205.1591
7160 : M.findmain = 205.9966 (+/− 20 ppm)
204.9893
[M−H]−
205.1591
0
20
40
60
80
100
190
195
200
205
210
215
220

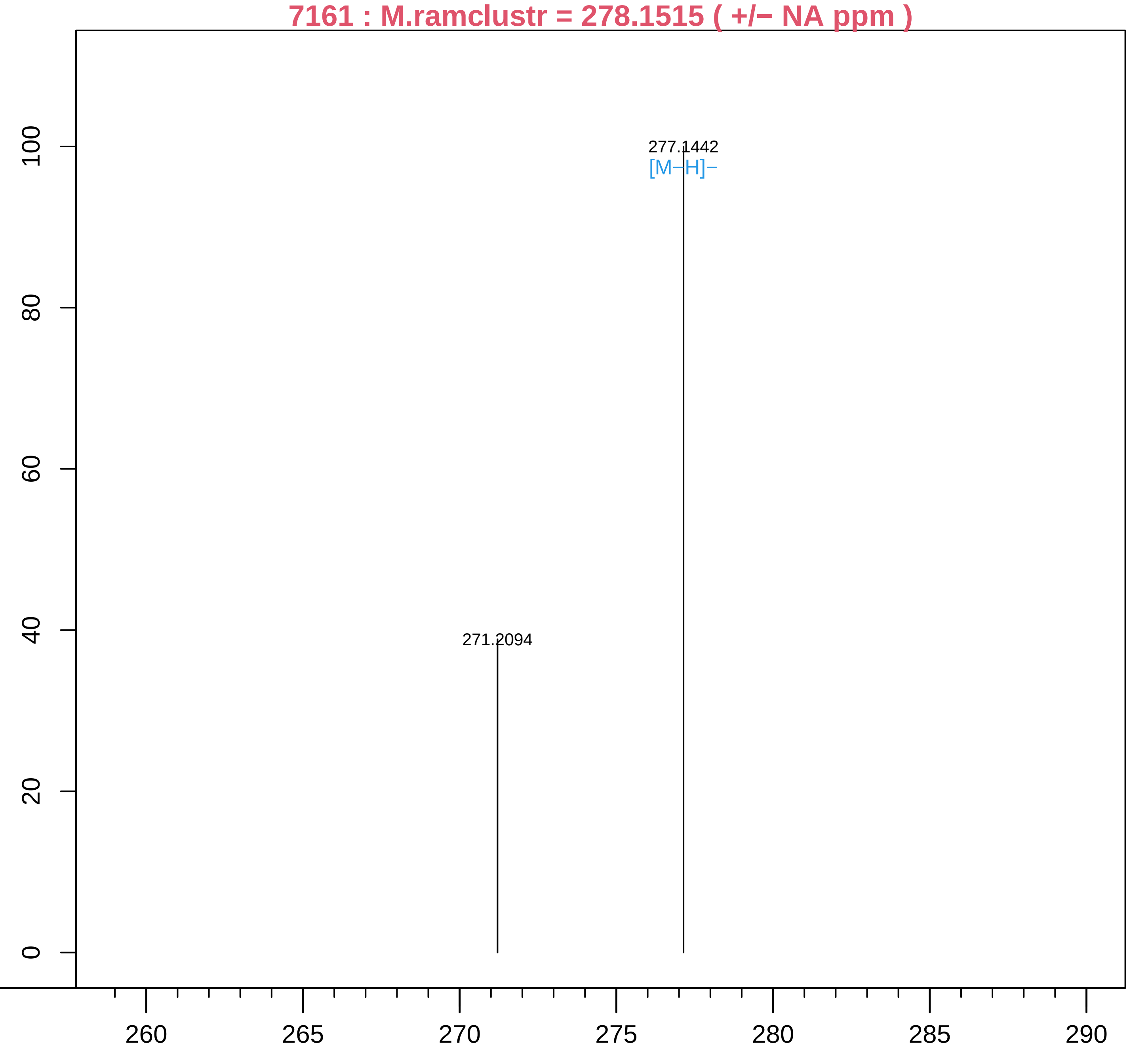

7161 : M.ramclustr = 278.1515 (+/− NA ppm)
277.1442
[M−H]−
271.2094
0
20
40
60
80
100
260
265
270
275
280
285
290

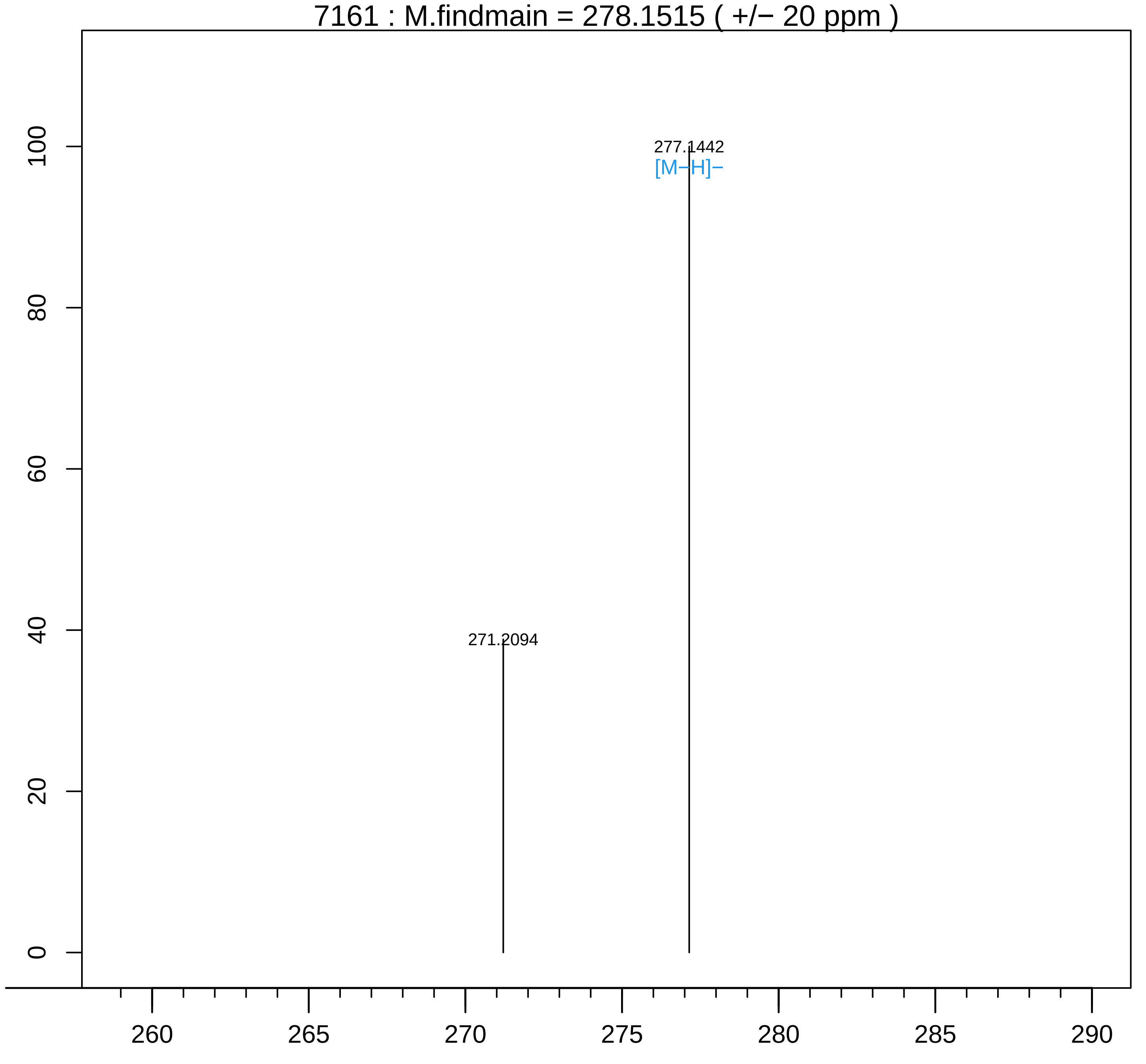

7161 : M.findmain = 278.1515 (+/− 20 ppm)
277.1442
[M−H]−
271.2094
0
20
40
60
80
100
260
265
270
275
280
285
290

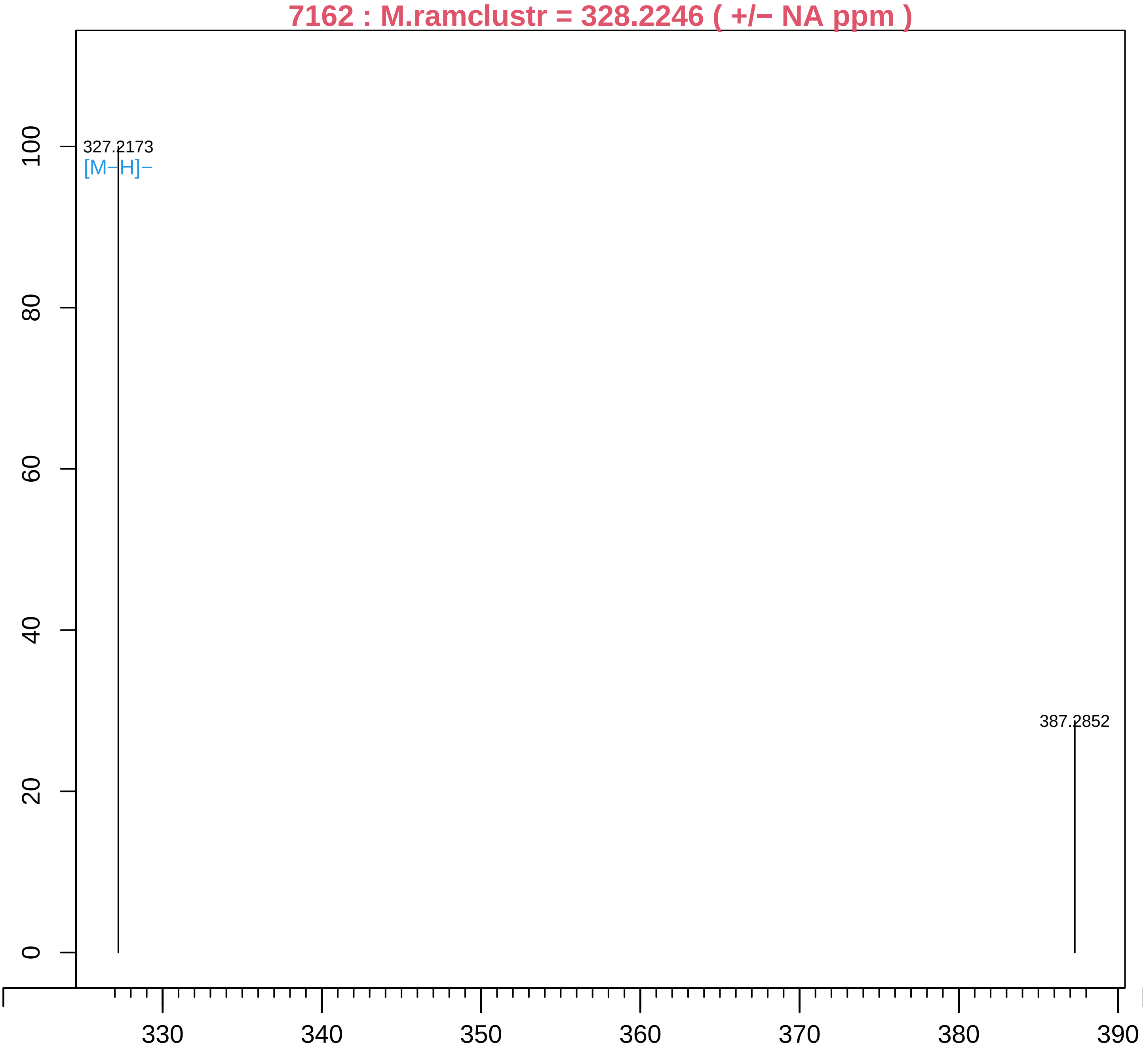
7162 : M.ramclustr = 328.2246 (+/− NA ppm)
327.2173
[M−H]−
387.2852
0
20
40
60
80
100
330
340
350
360
370
380
390
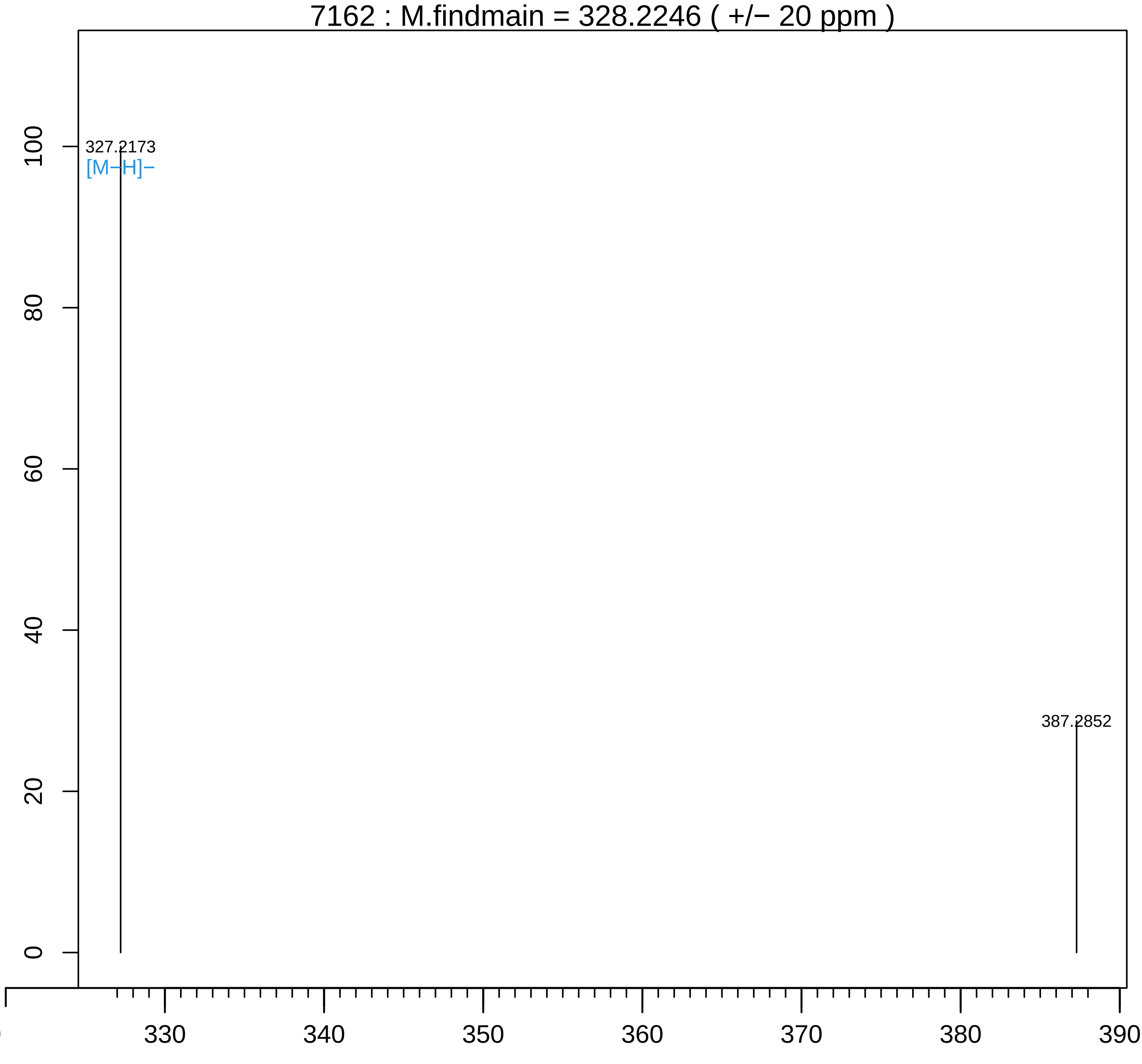
7162 : M.findmain = 328.2246 (+/− 20 ppm)
327.2173
[M−H]−
387.2852
0
20
40
60
80
100
330
340
350
360
370
380
390

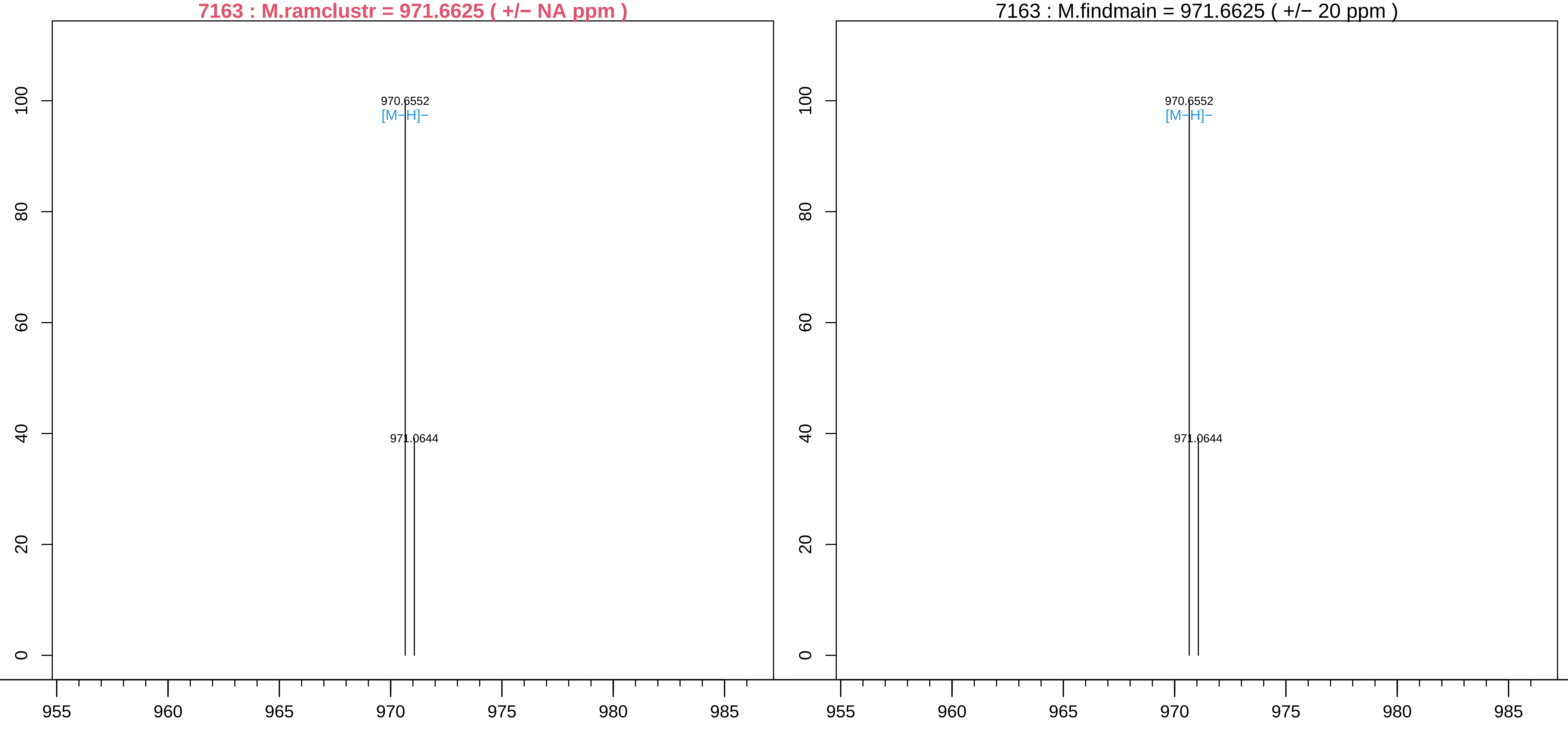

7163 : M.ramclustr = 971.6625 (+/− NA ppm)
970.6552
[M−H]−
971.0644
7163 : M.findmain = 971.6625 (+/− 20 ppm)
970.6552
[M−H]−
971.0644
0
20
40
60
80
100
955
960
965
970
975
980
985

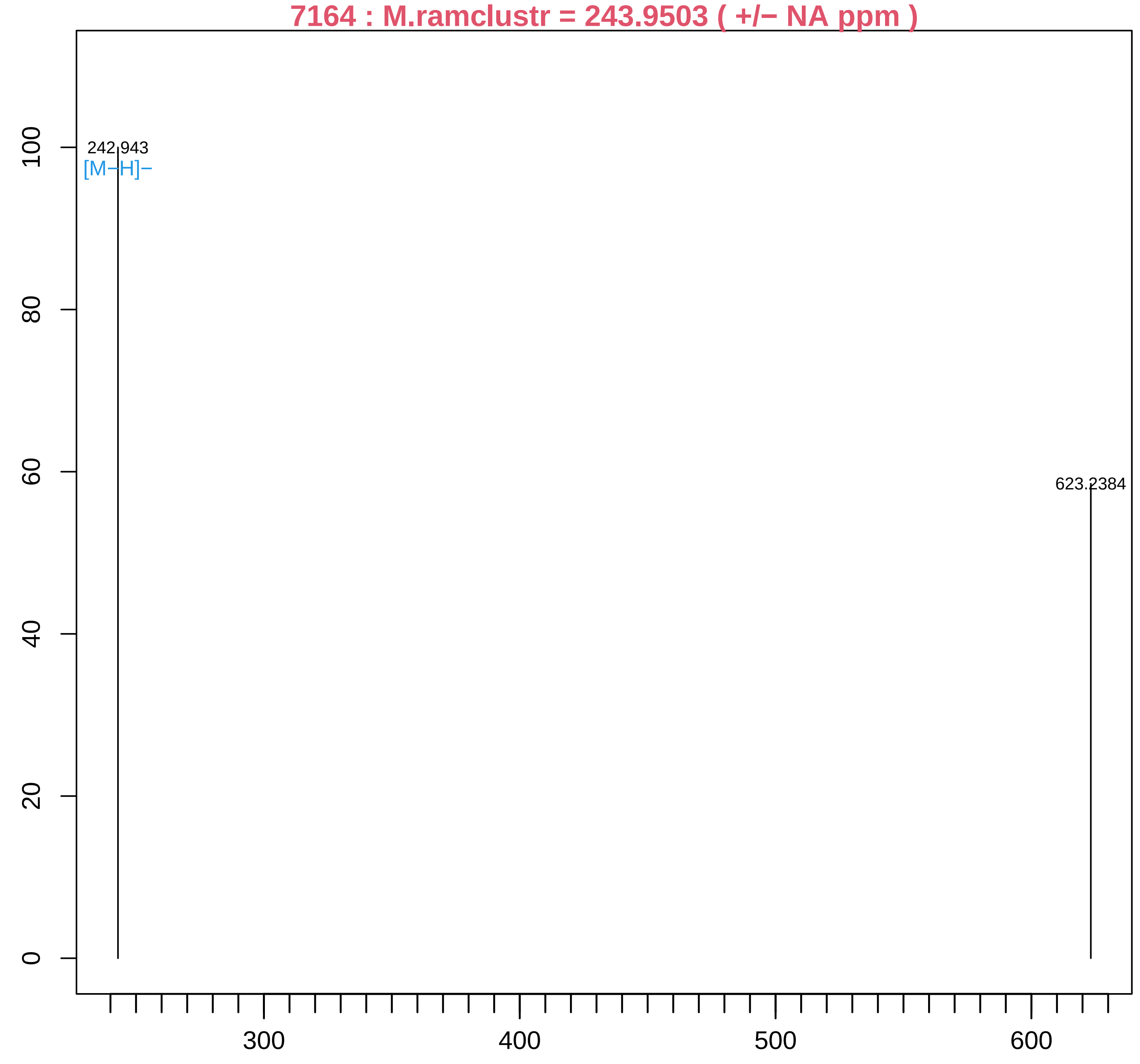

7164 : M.ramclustr = 243.9503 (+/− NA ppm)
242.943
[M−H]−
623.2384
0
20
40
60
80
100
300
400
500
600

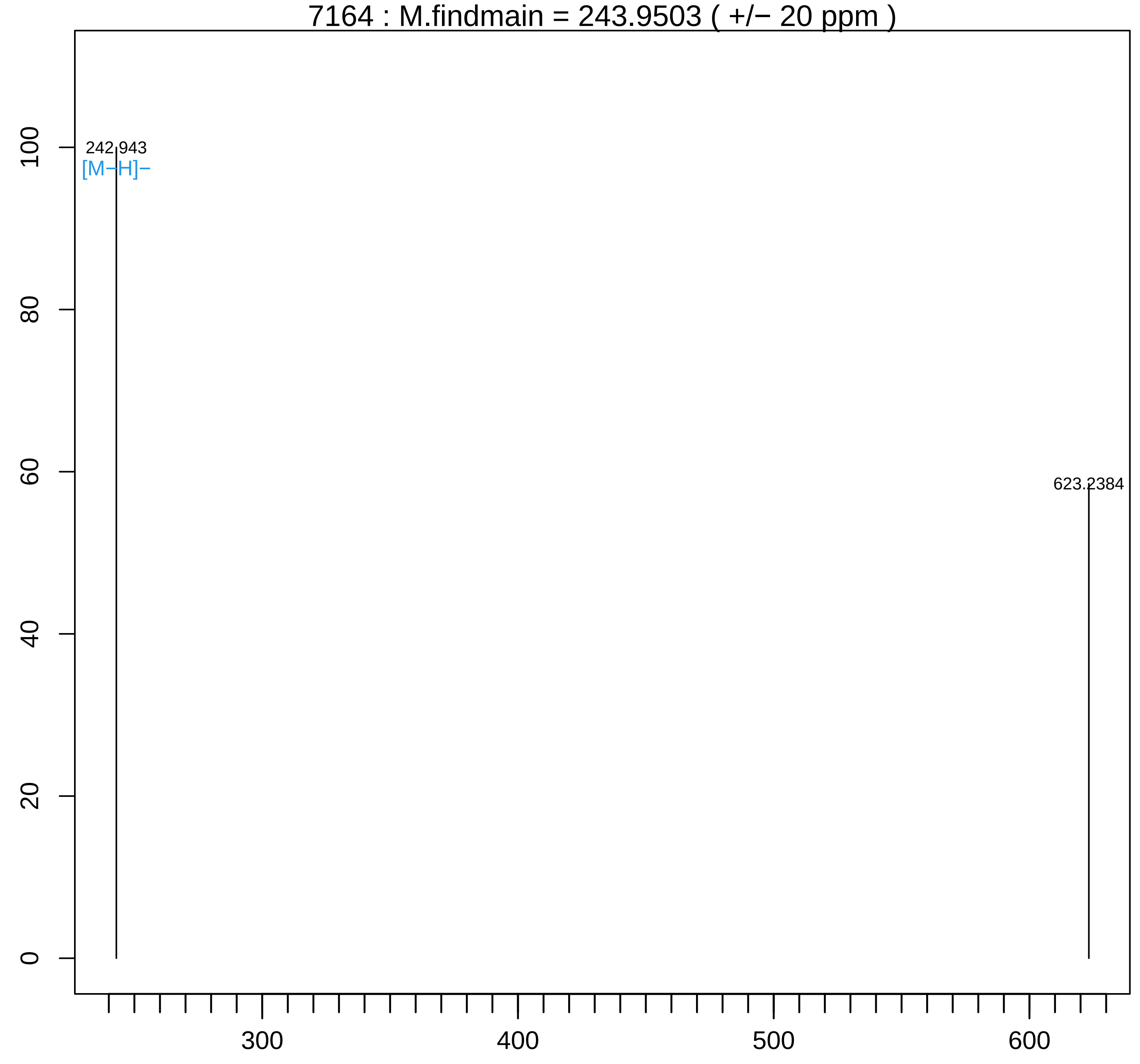

7164 : M.findmain = 243.9503 (+/− 20 ppm)
242.943
[M−H]−
623.2384
0
20
40
60
80
100
300
400
500
600

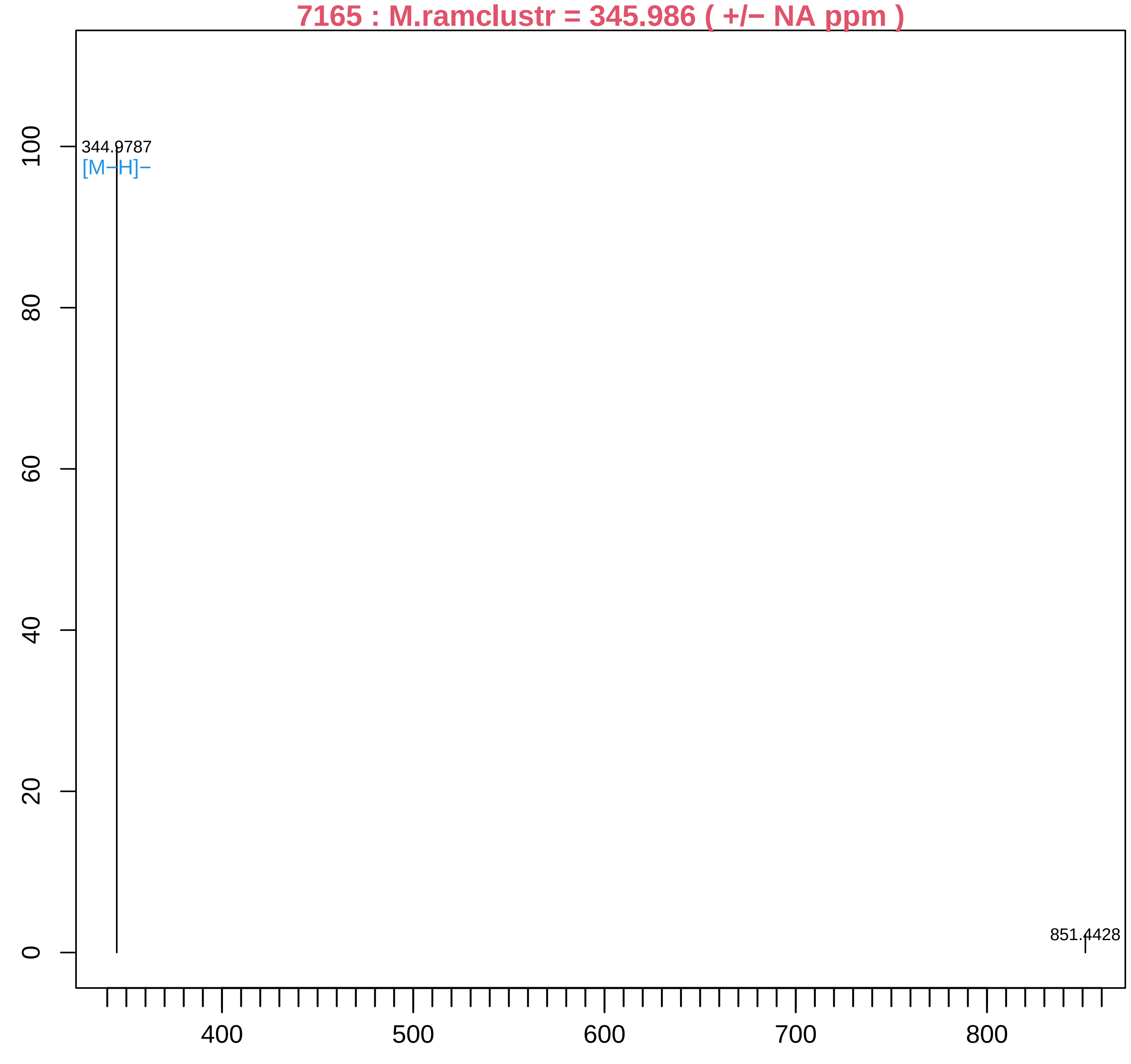
7165 : M.ramclustr = 345.986 (+/− NA ppm)
344.9787
[M−H]−
851.4428
0
20
40
60
80
100
400
500
600
700
800

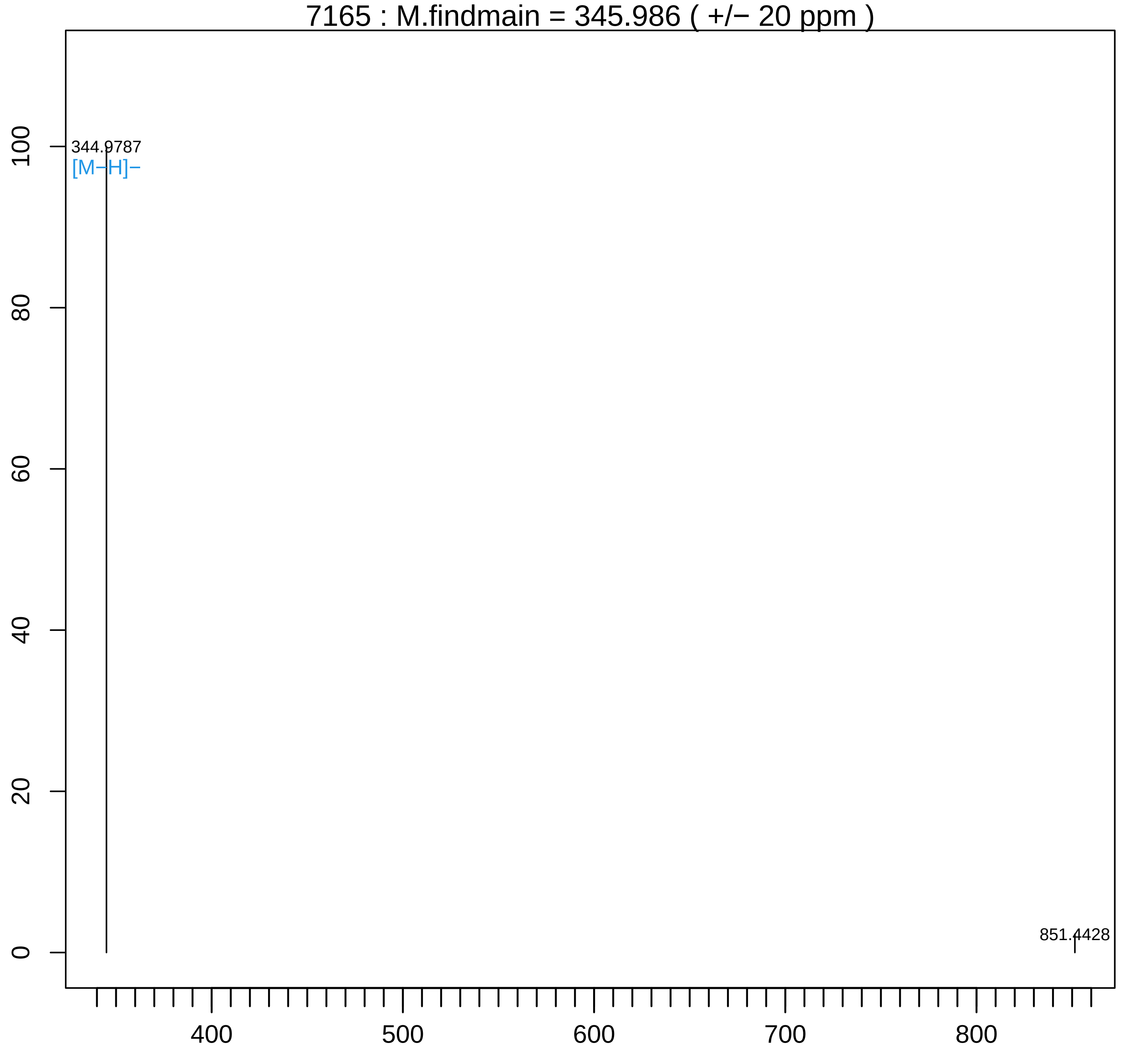
7165 : M.findmain = 345.986 (+/− 20 ppm)
344.9787
[M−H]−
851.4428
0
20
40
60
80
100
400
500
600
700
800

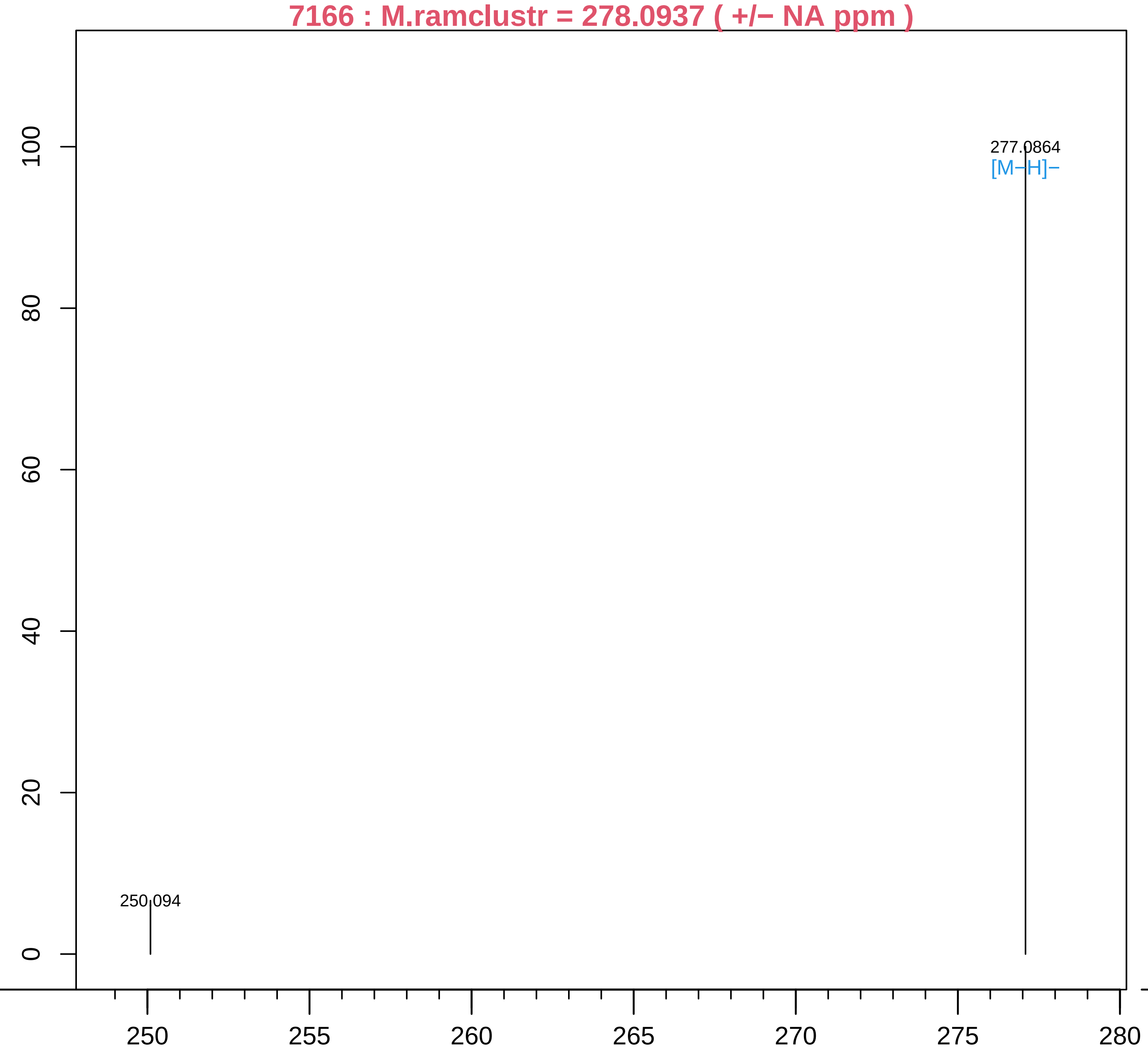
7166 : M.ramclustr = 278.0937 (+/− NA ppm)
277.0864
[M−H]−
250.094
0
20
40
60
80
100
250
255
260
265
270
275
280

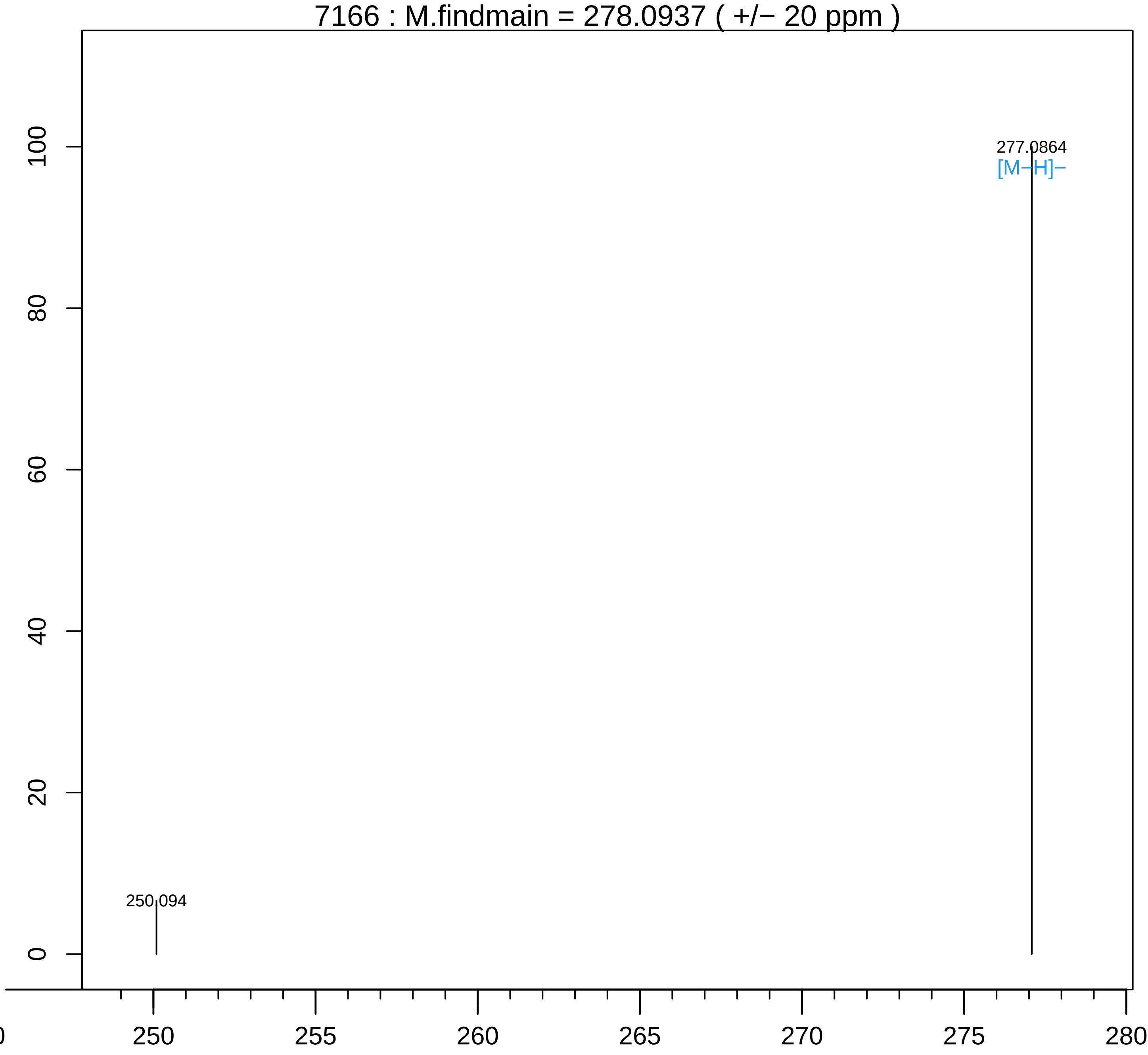
7166 : M.findmain = 278.0937 (+/− 20 ppm)
277.0864
[M−H]−
250.094
0
20
40
60
80
100
250
255
260
265
270
275
280

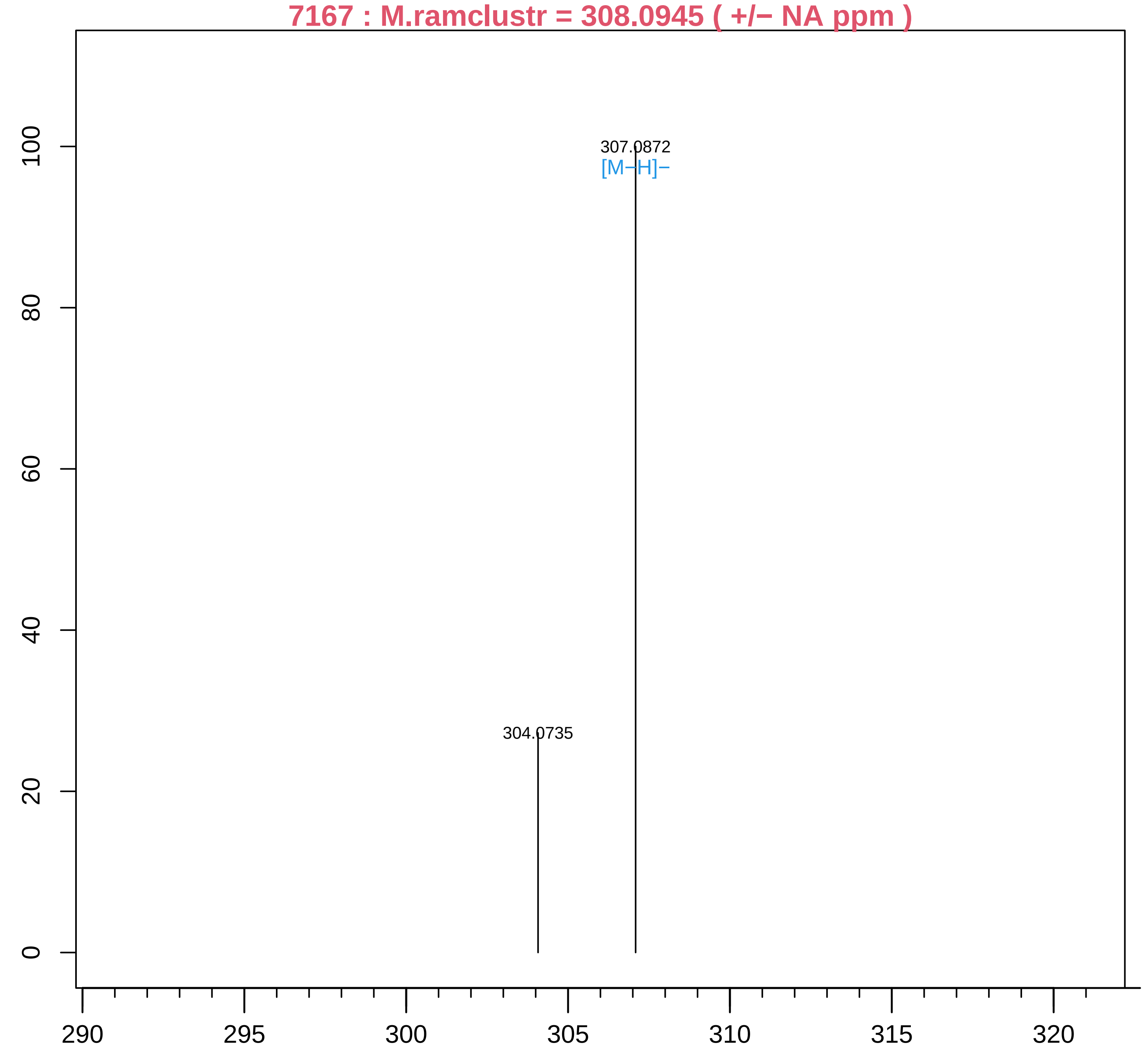

7167 : M.ramclustr = 308.0945 (+/− NA ppm)
307.0872
[M−H]−
304.0735
0
20
40
60
80
100
290
295
300
305
310
315
320

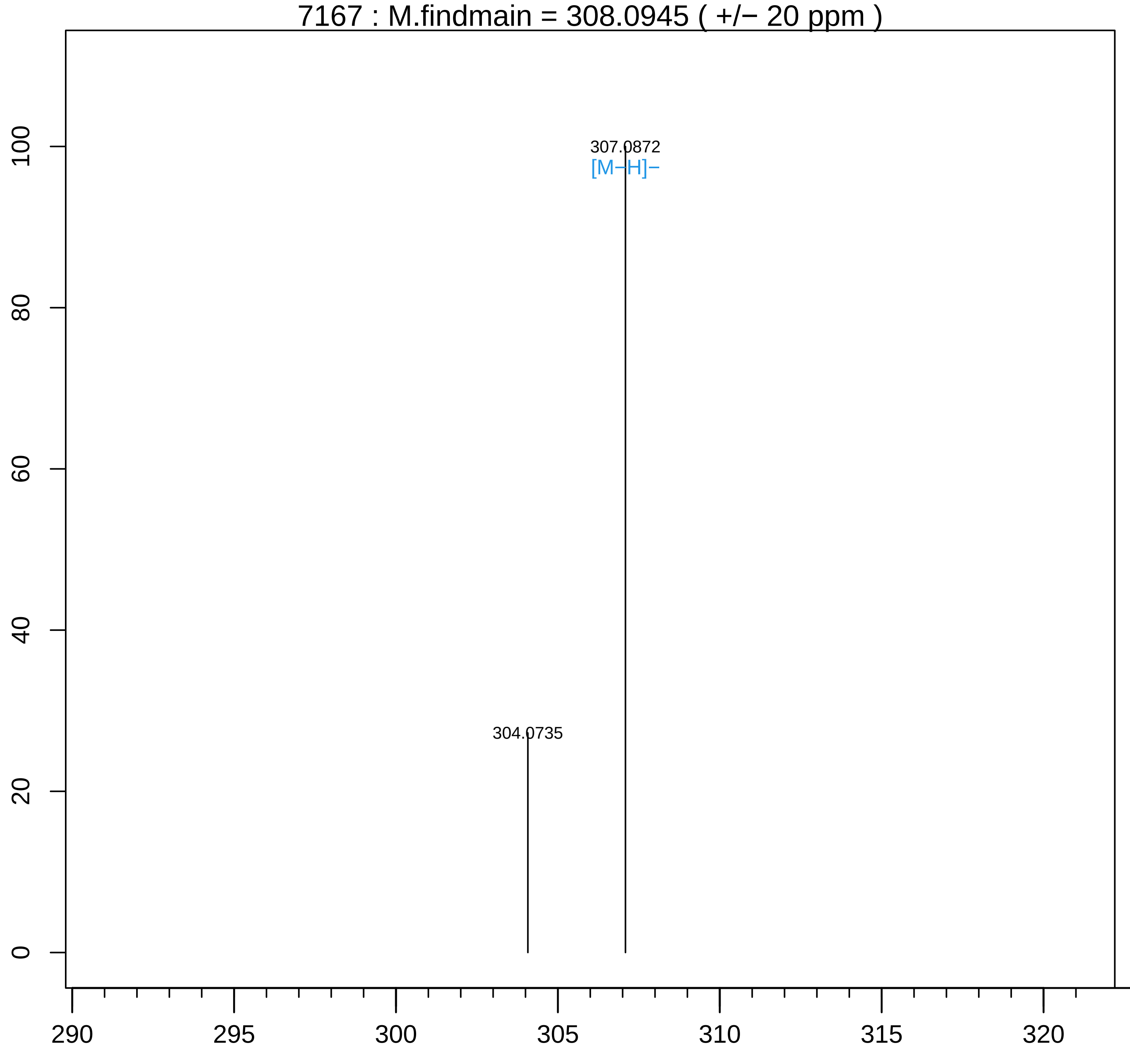

7167 : M.findmain = 308.0945 (+/− 20 ppm)
307.0872
[M−H]−
304.0735
0
20
40
60
80
100
290
295
300
305
310
315
320

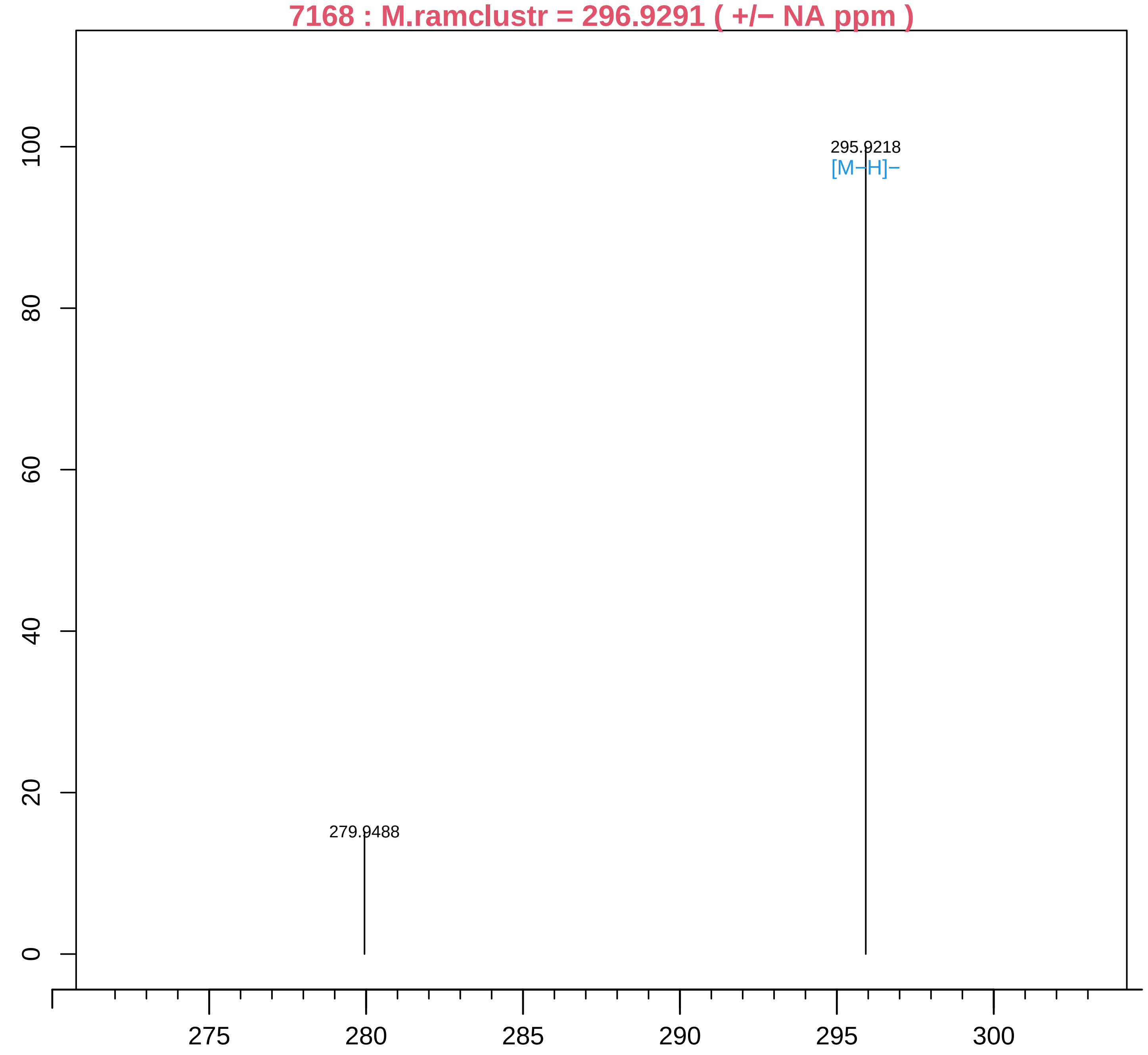
7168 : M.ramclustr = 296.9291 (+/− NA ppm)
295.9218
[M−H]−
279.9488
0
20
40
60
80
100
275
280
285
290
295
300

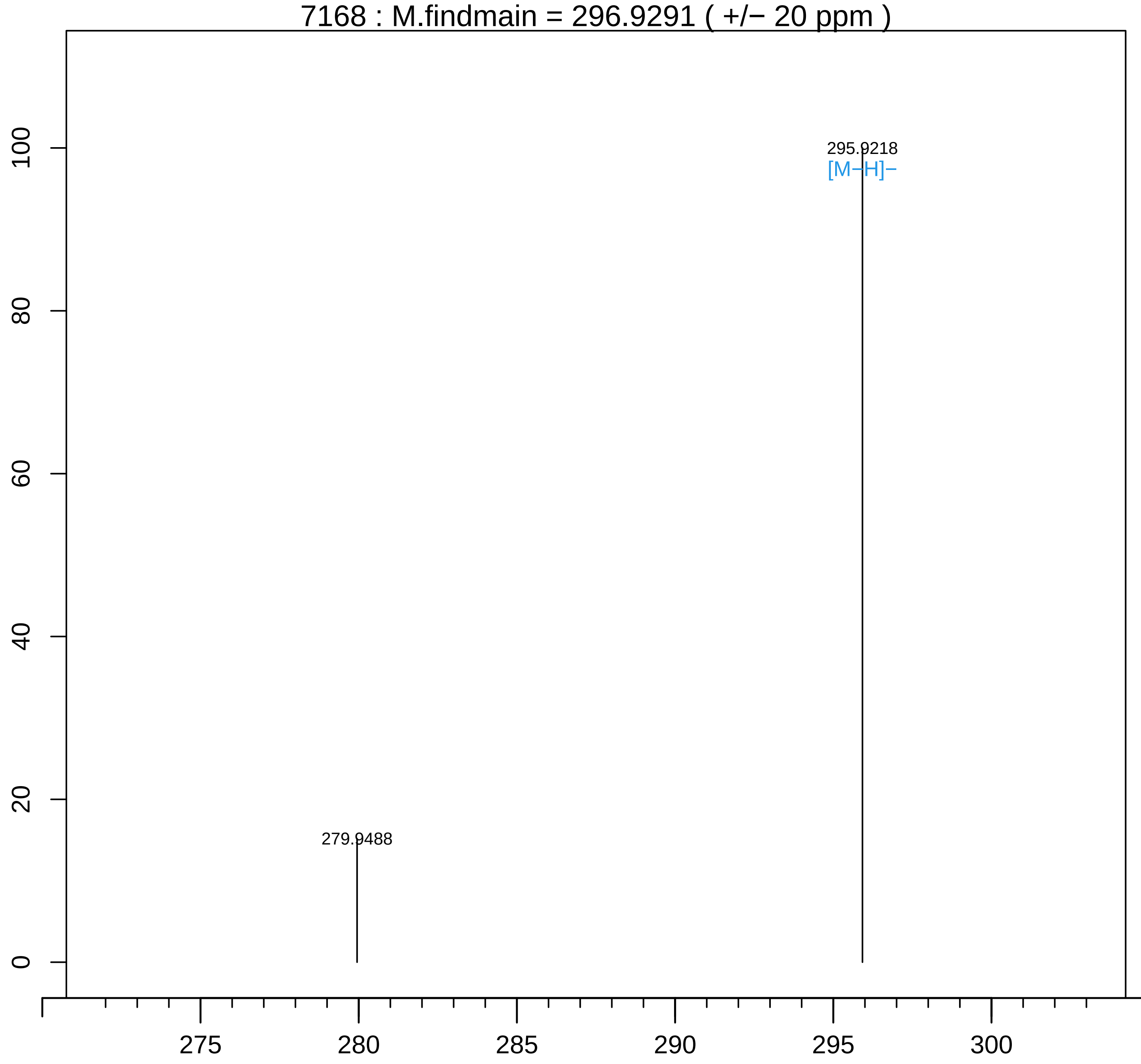
7168 : M.findmain = 296.9291 (+/− 20 ppm)
295.9218
[M−H]−
279.9488
0
20
40
60
80
100
275
280
285
290
295
300

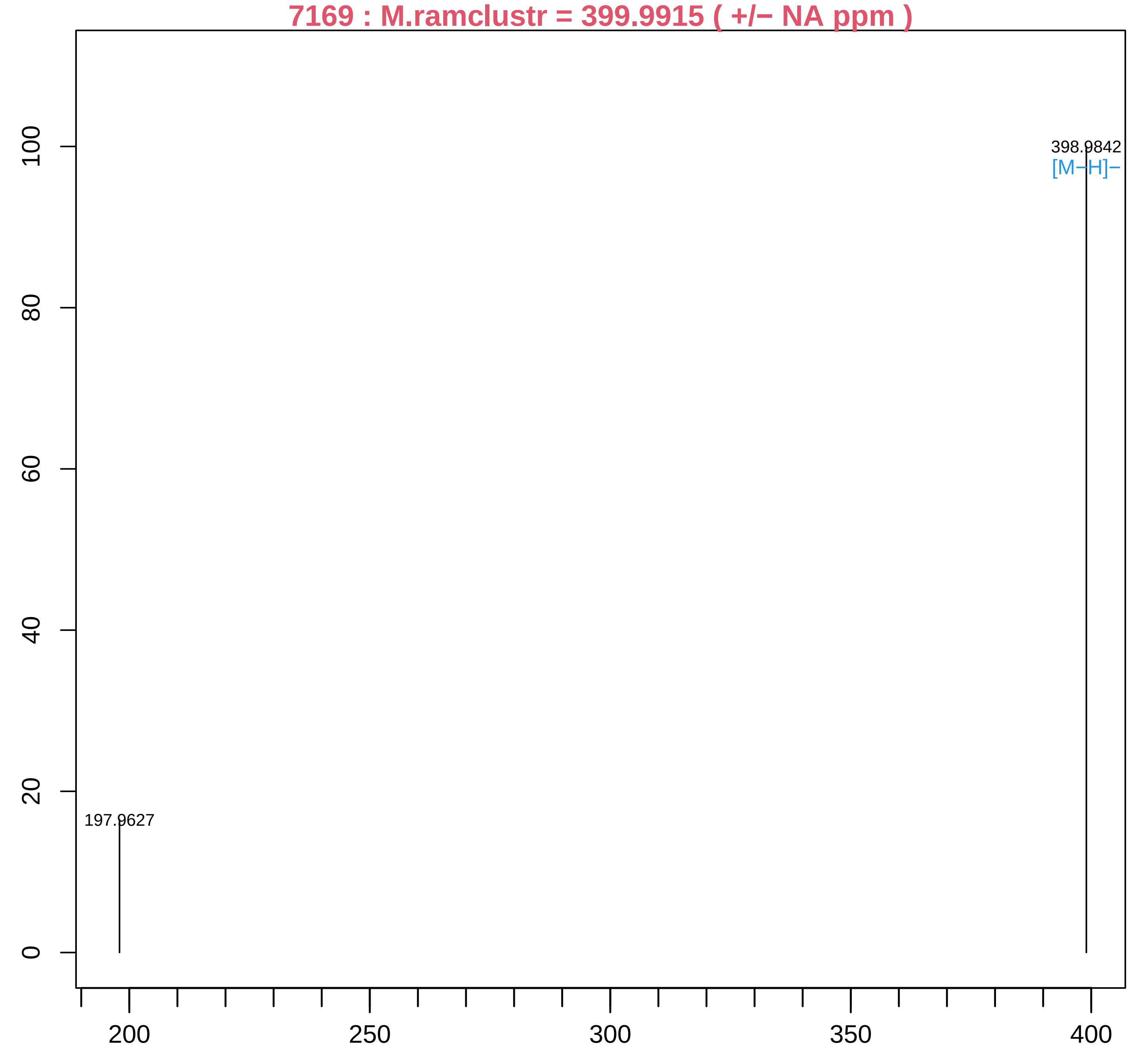
7169 : M.ramclustr = 399.9915 (+/− NA ppm)
398.9842
[M−H]−
197.9627
0
20
40
60
80
100
200
250
300
350
400

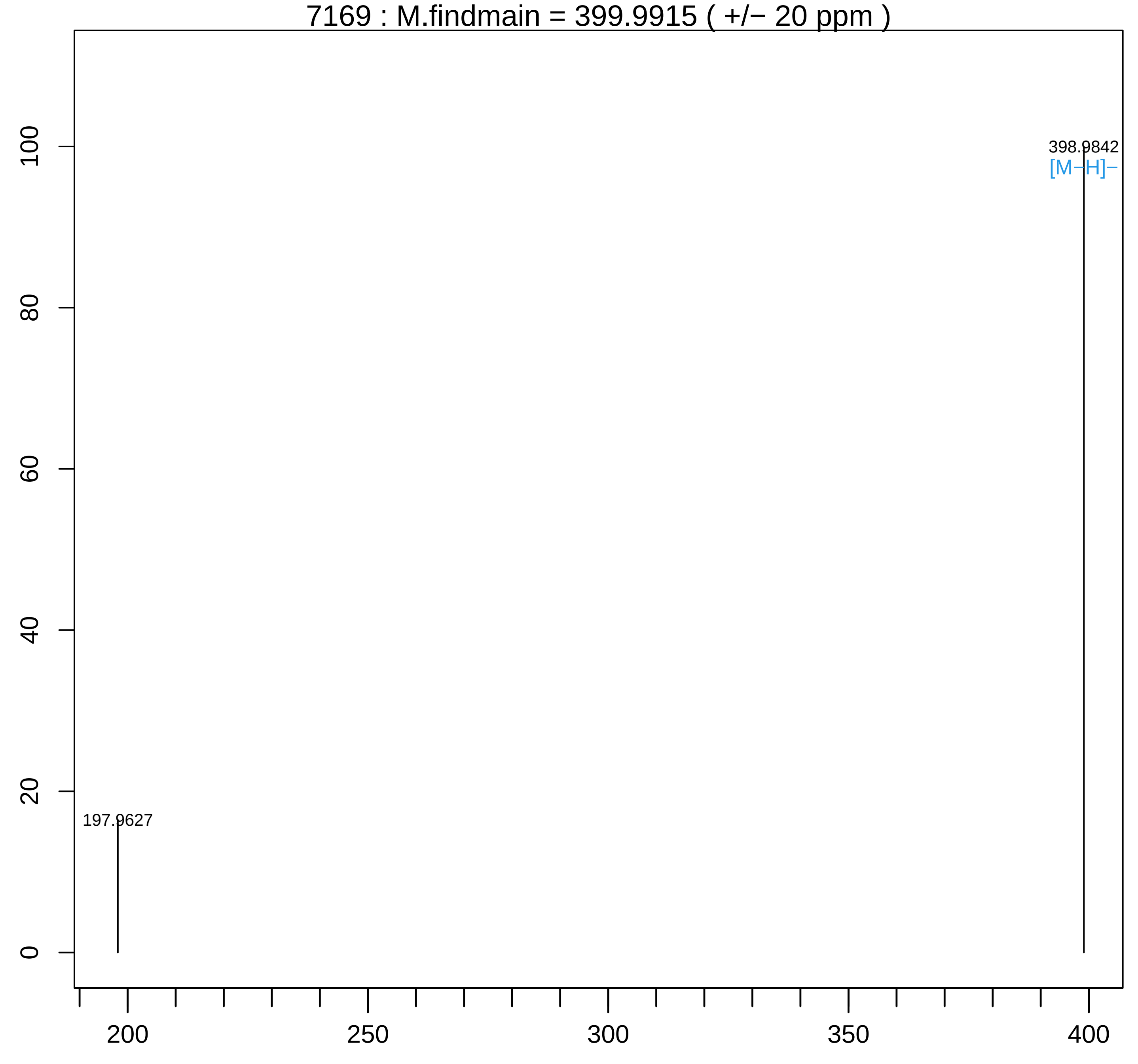
7169 : M.findmain = 399.9915 (+/− 20 ppm)
398.9842
[M−H]−
197.9627
0
20
40
60
80
100
200
250
300
350
400

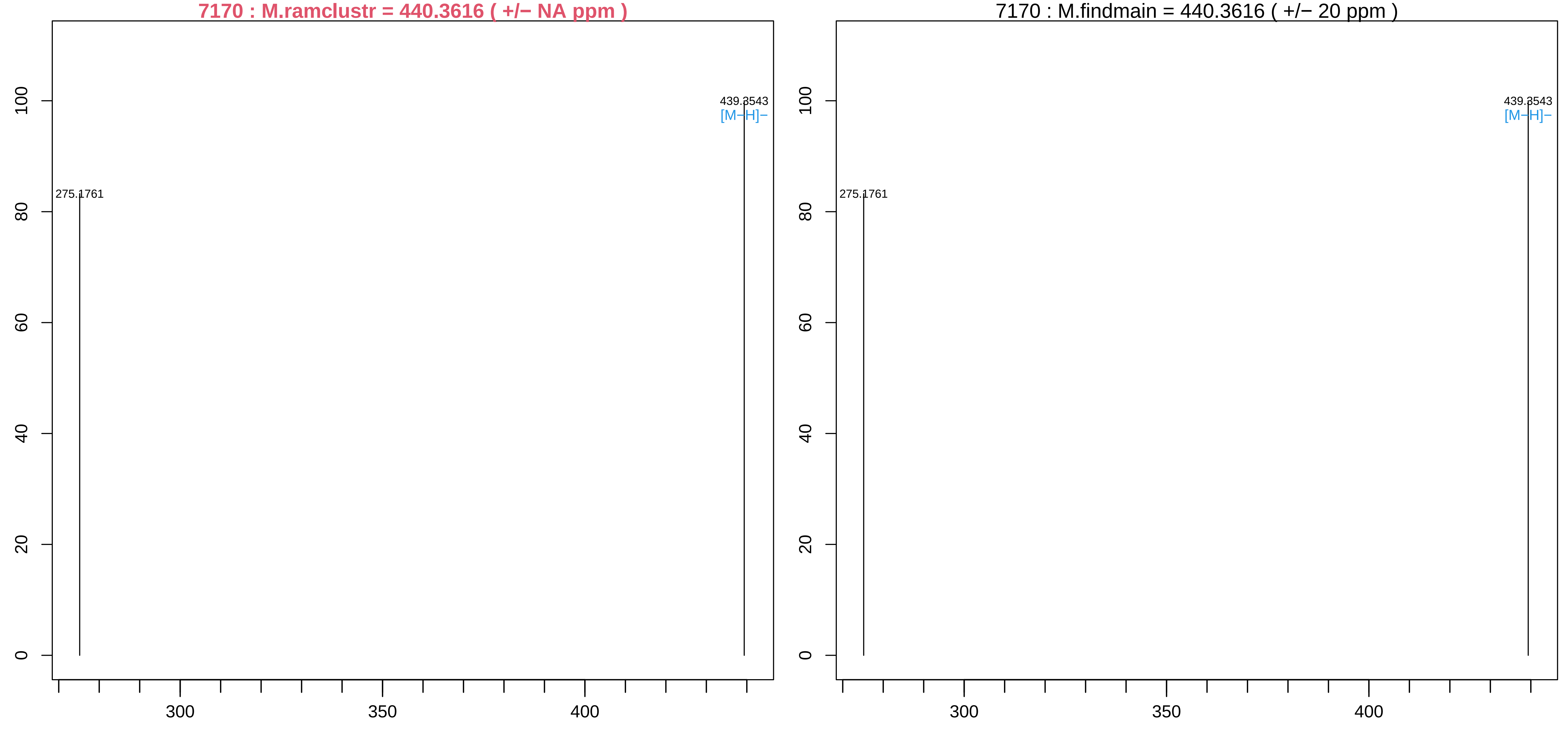

7170 : M.ramclustr = 440.3616 (+/− NA ppm)
439.3543
[M−H]−
275.1761
0
20
40
60
80
100
300
350
400
7170 : M.findmain = 440.3616 (+/− 20 ppm)
439.3543
[M−H]−
275.1761
0
20
40
60
80
100
300
350
400

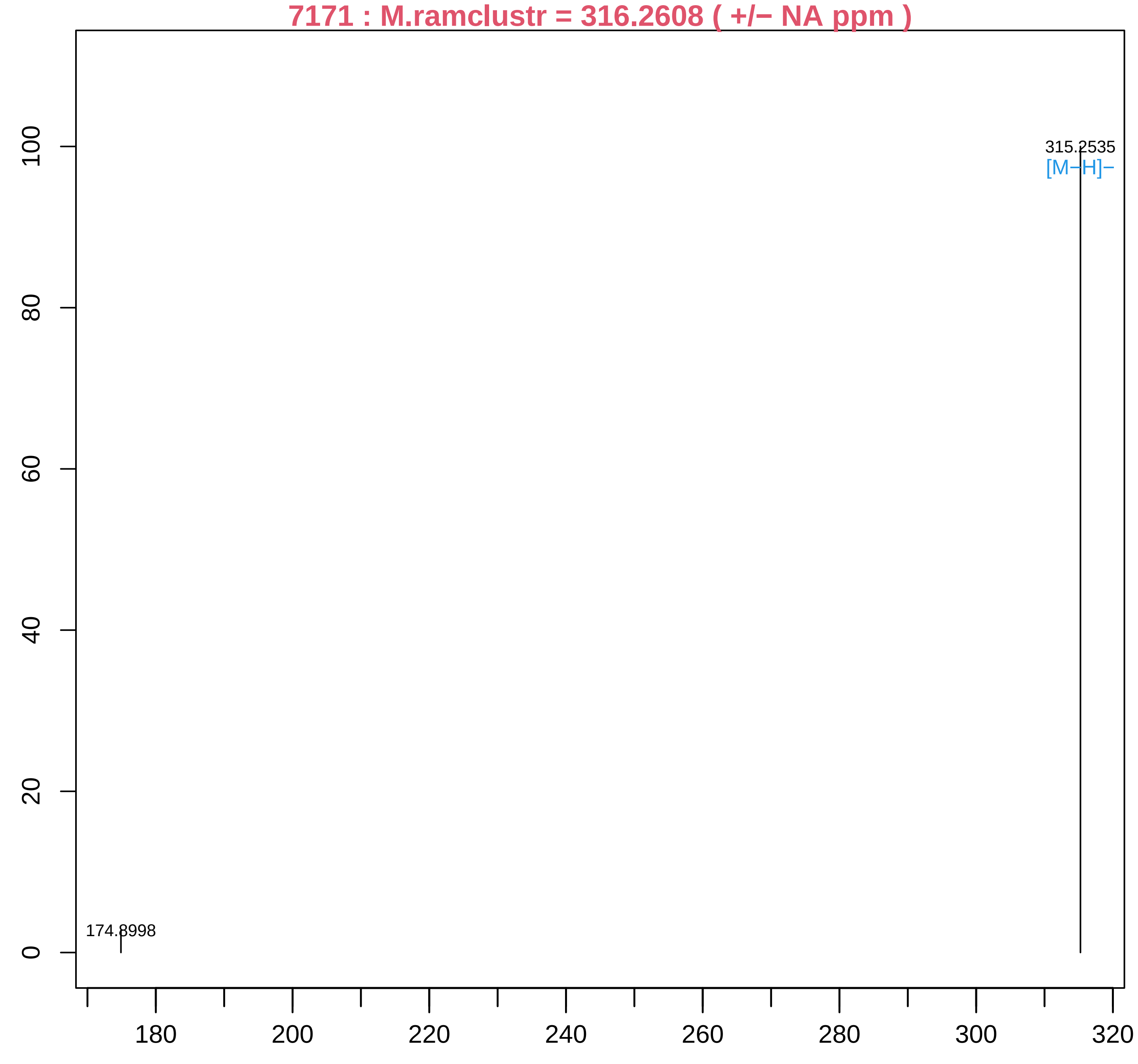
7171 : M.ramclustr = 316.2608 (+/− NA ppm)
315.2535
[M−H]−
174.8998
0
20
40
60
80
100
180
200
220
240
260
280
300
320

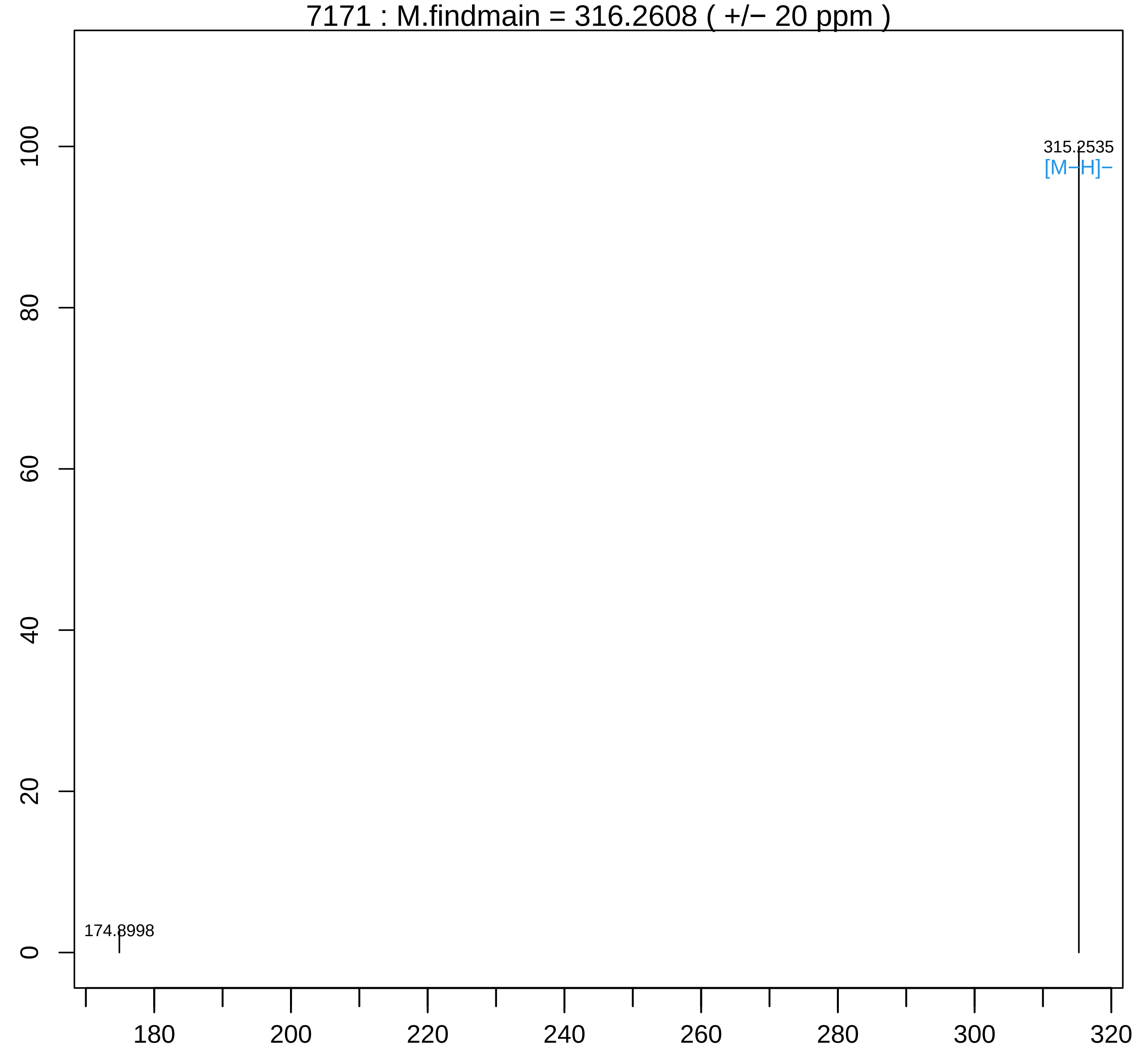
7171 : M.findmain = 316.2608 (+/− 20 ppm)
315.2535
[M−H]−
174.8998
0
20
40
60
80
100
180
200
220
240
260
280
300
320

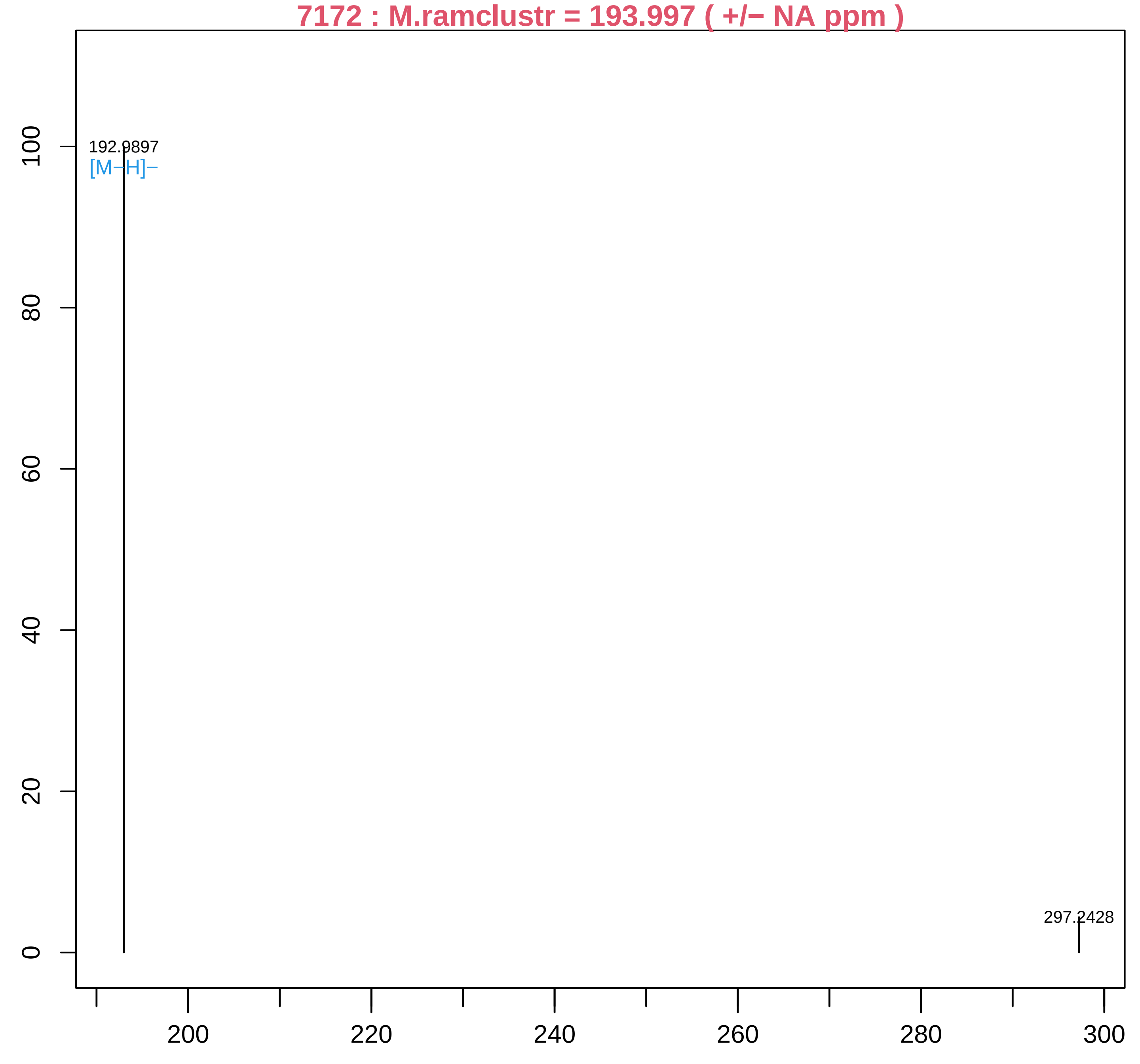
7172 : M.ramclustr = 193.997 (+/− NA ppm)
192.9897
[M−H]−
297.2428
0
20
40
60
80
100
200
220
240
260
280
300

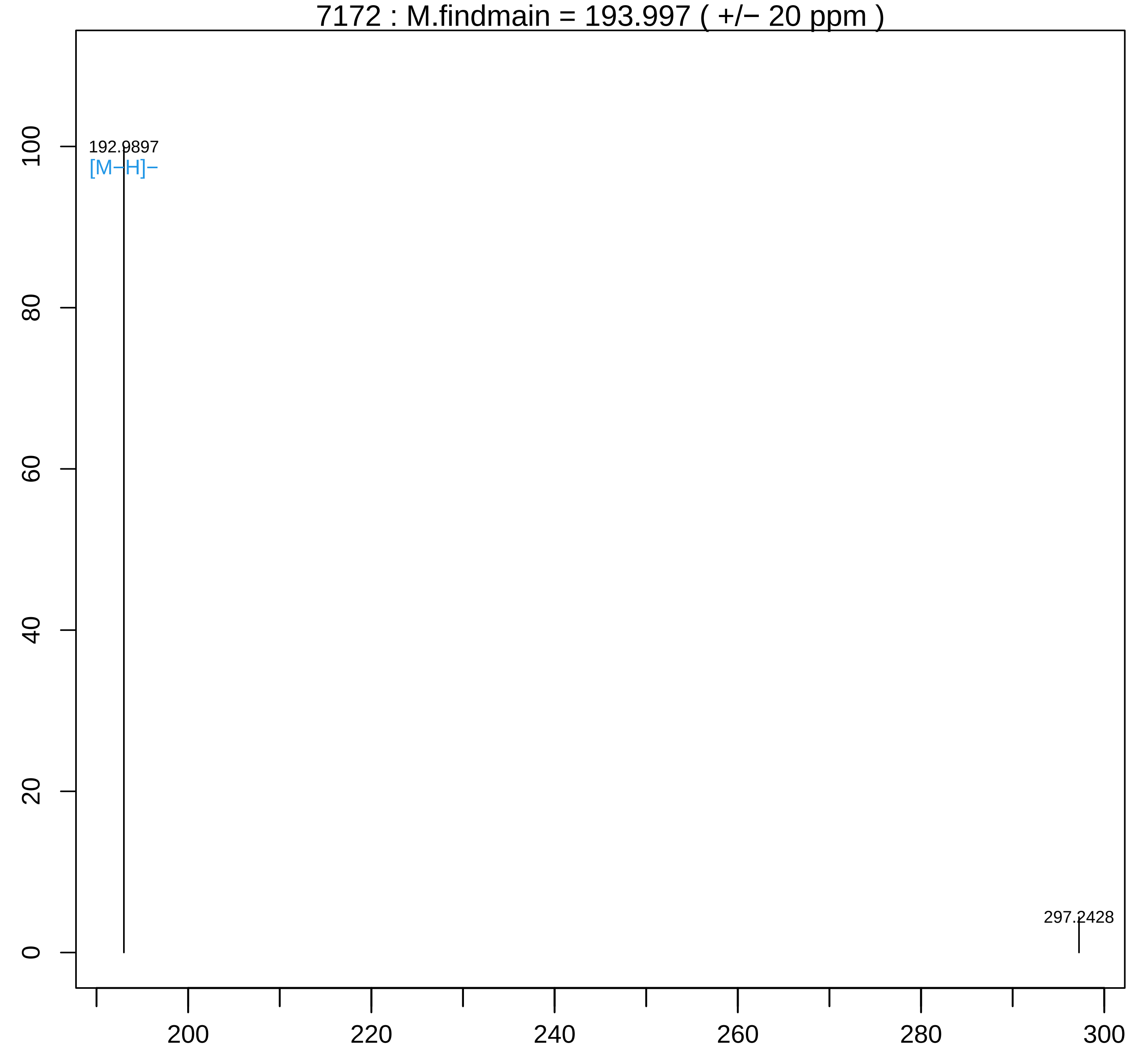
7172 : M.findmain = 193.997 (+/− 20 ppm)
192.9897
[M−H]−
297.2428
0
20
40
60
80
100
200
220
240
260
280
300

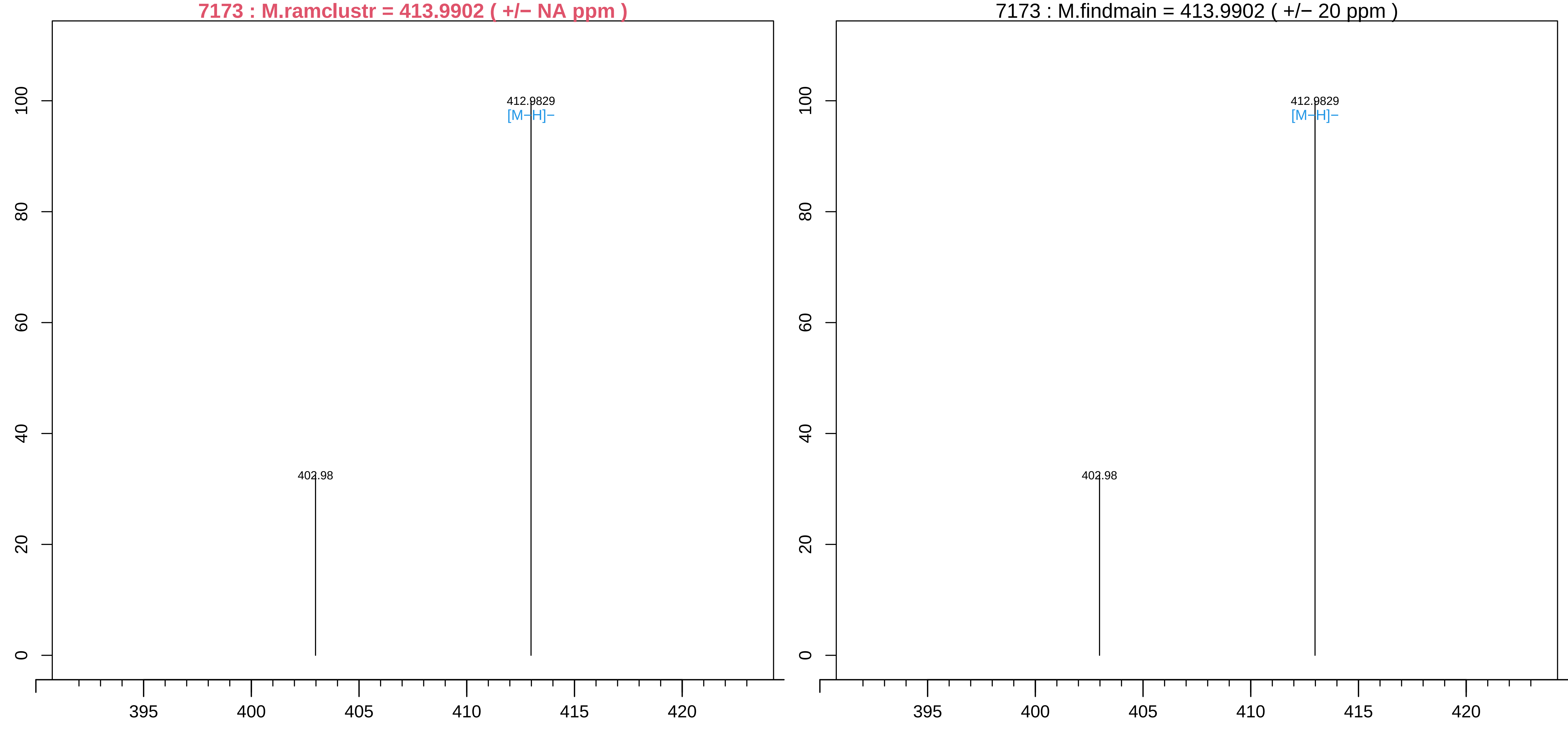
7173 : M.ramclustr = 413.9902 (+/− NA ppm)
412.9829
[M−H]−
402.98
7173 : M.findmain = 413.9902 (+/− 20 ppm)
412.9829
[M−H]−
402.98
0
20
40
60
80
100
395
400
405
410
415
420

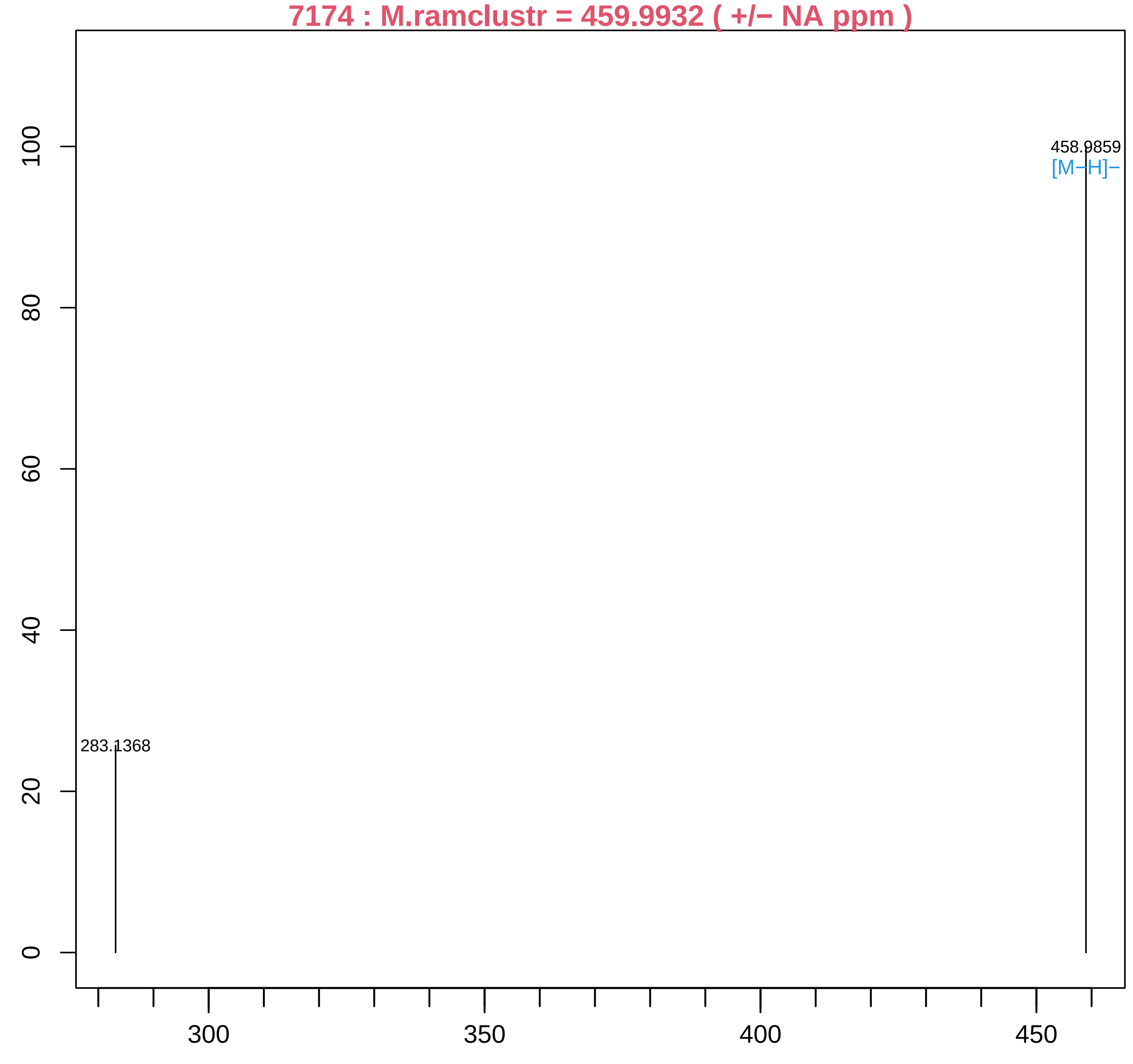
7174 : M.ramclustr = 459.9932 (+/− NA ppm)
458.9859
[M−H]−
283.1368
0
20
40
60
80
100
300
350
400
450

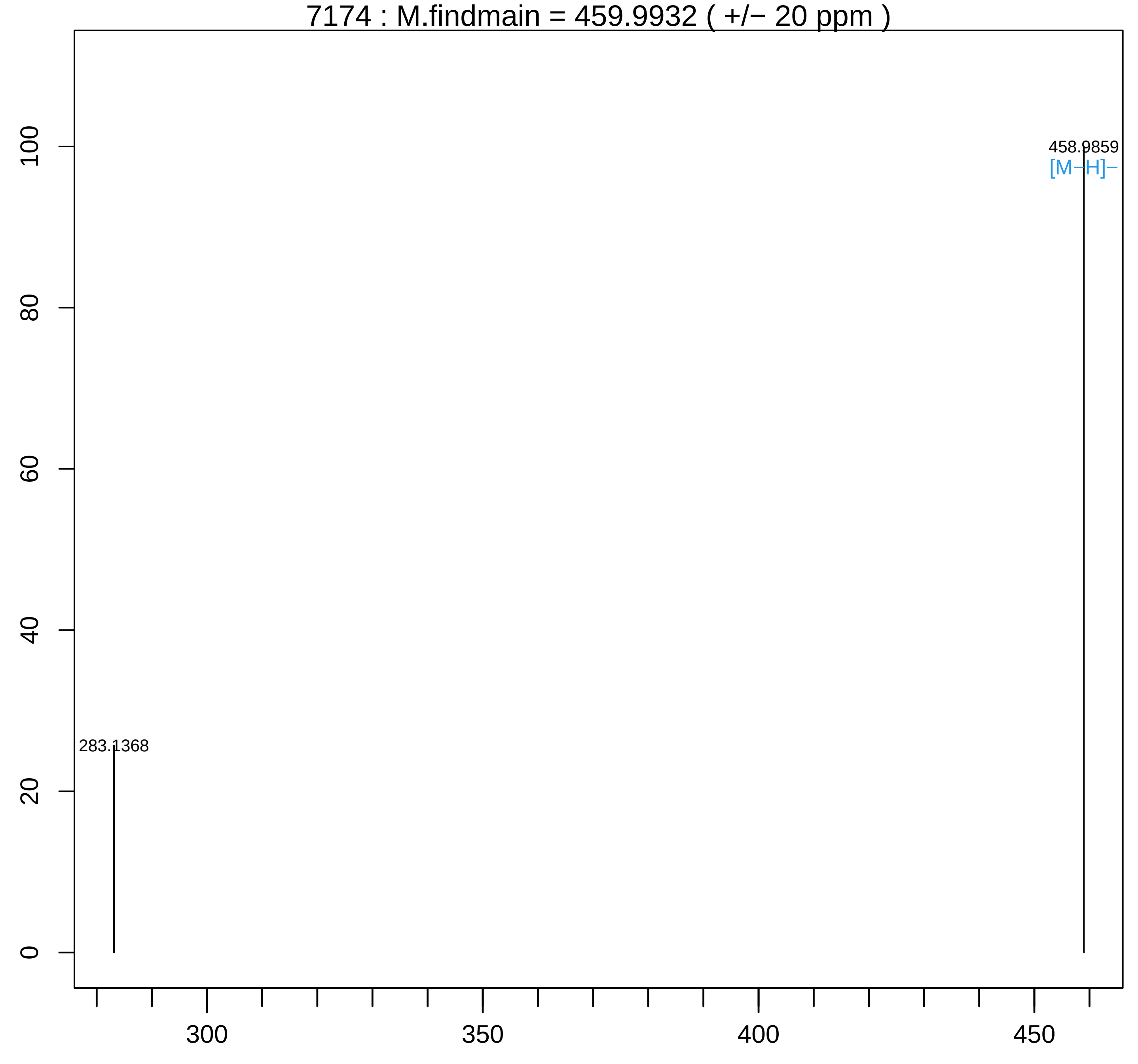
7174 : M.findmain = 459.9932 (+/− 20 ppm)
458.9859
[M−H]−
283.1368
0
20
40
60
80
100
300
350
400
450

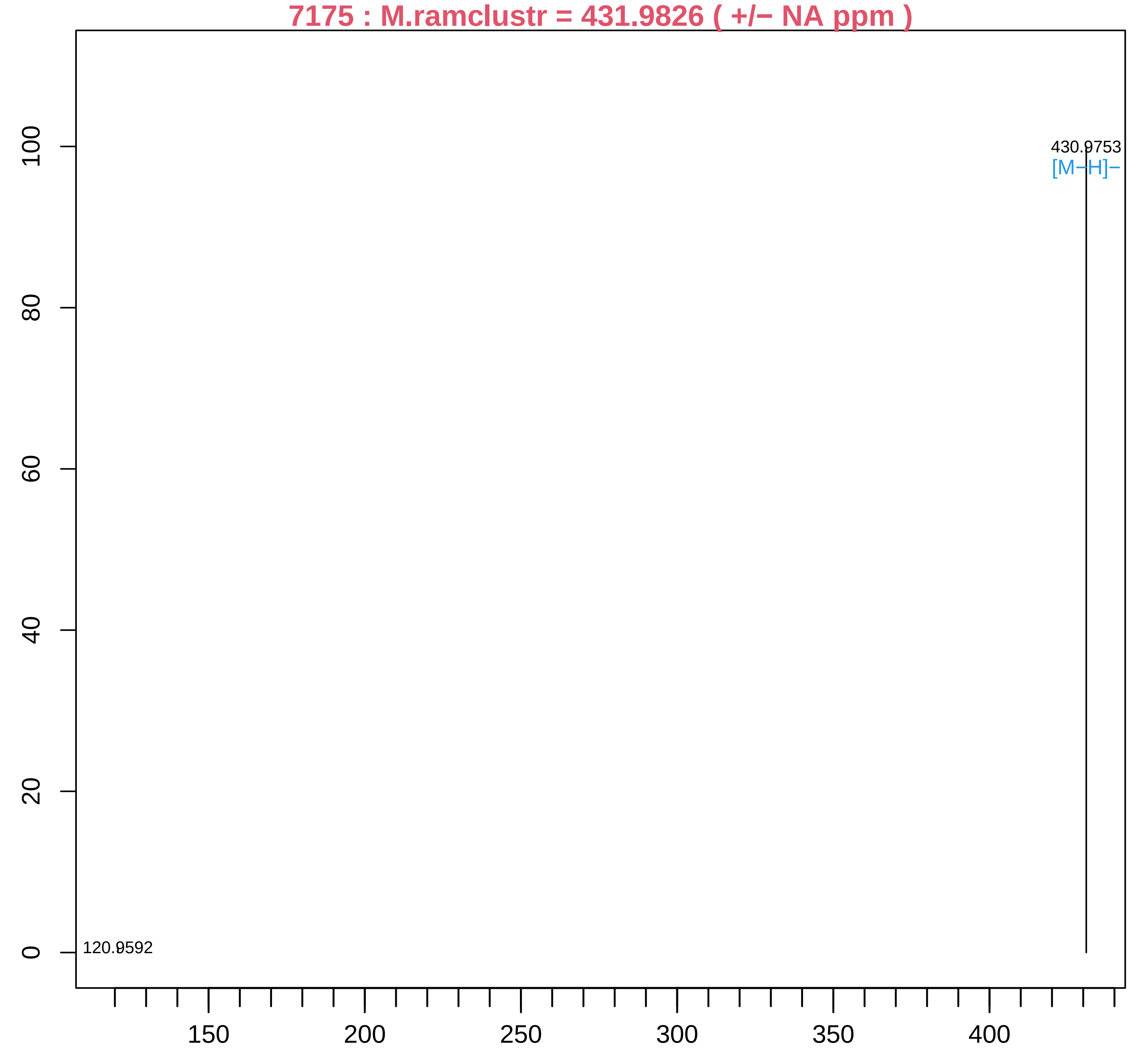
7175 : M.ramclustr = 431.9826 (+/− NA ppm)
430.9753
[M−H]−
120.9592
0
20
40
60
80
100
150
200
250
300
350
400

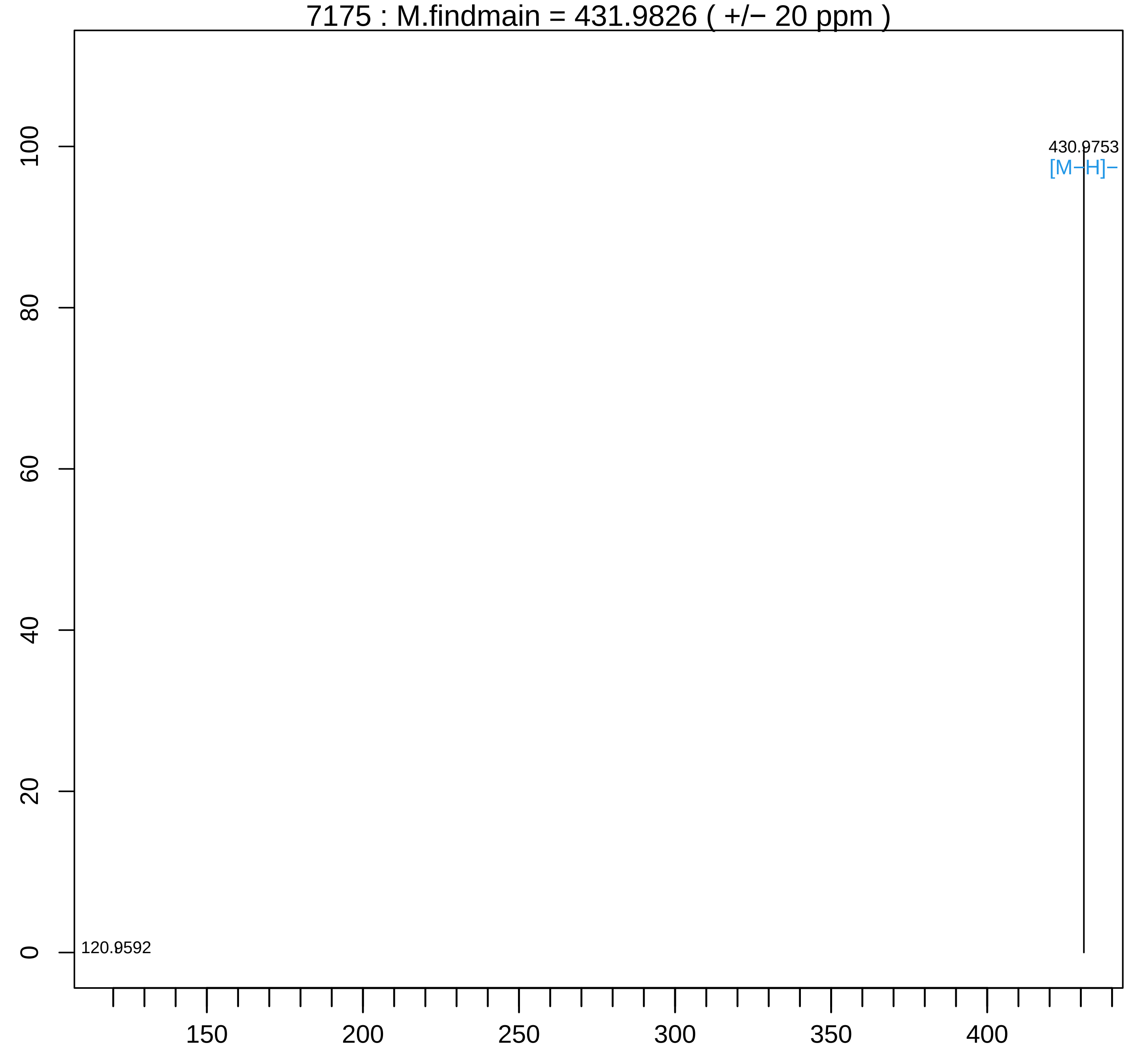
7175 : M.findmain = 431.9826 (+/− 20 ppm)
430.9753
[M−H]−
120.9592
0
20
40
60
80
100
150
200
250
300
350
400

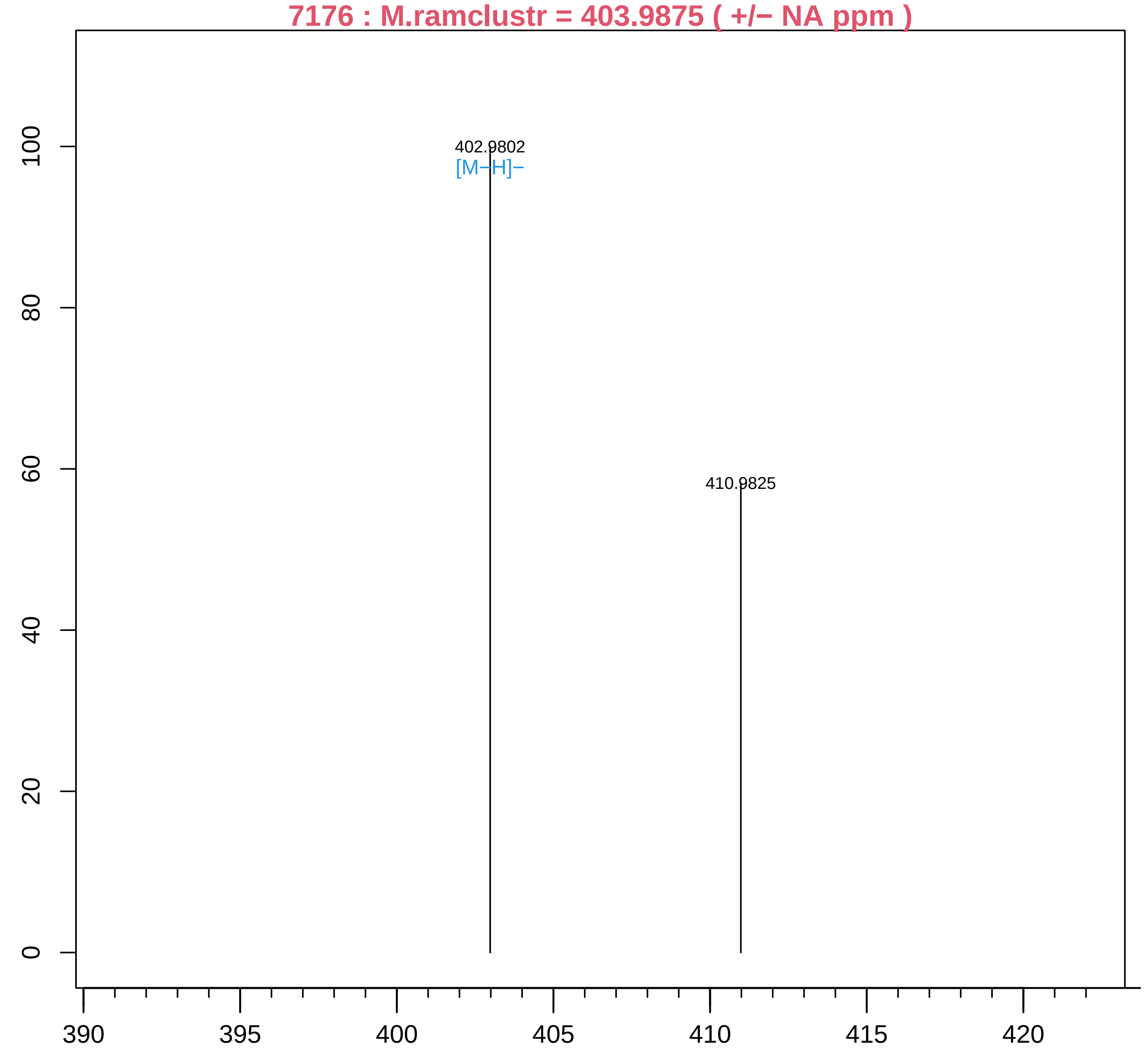

7176 : M.ramclustr = 403.9875 (+/− NA ppm)
402.9802
[M−H]−
410.9825
0
20
40
60
80
100
390
395
400
405
410
415
420

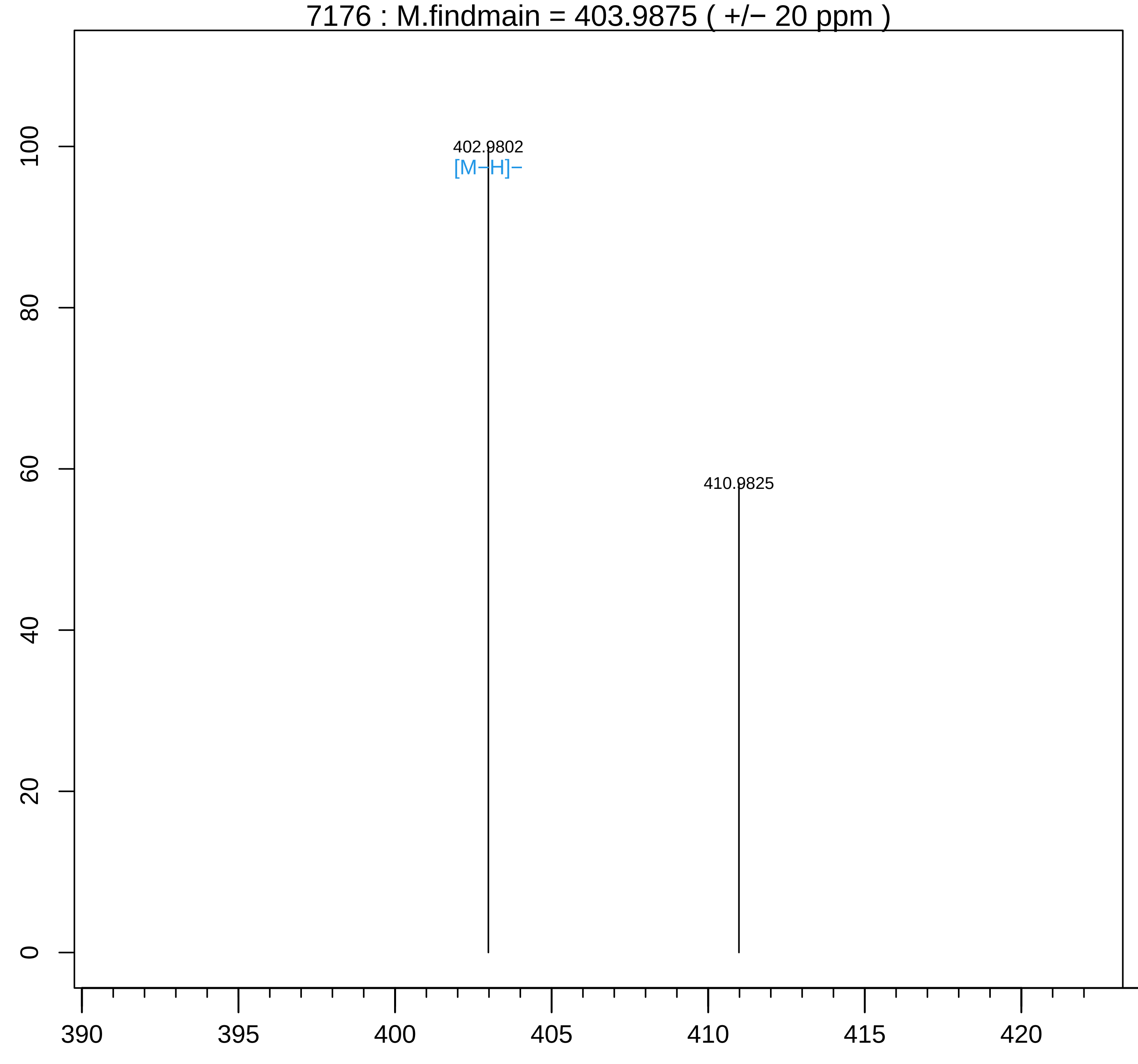

7176 : M.findmain = 403.9875 (+/− 20 ppm)
402.9802
[M−H]−
410.9825
0
20
40
60
80
100
390
395
400
405
410
415
420

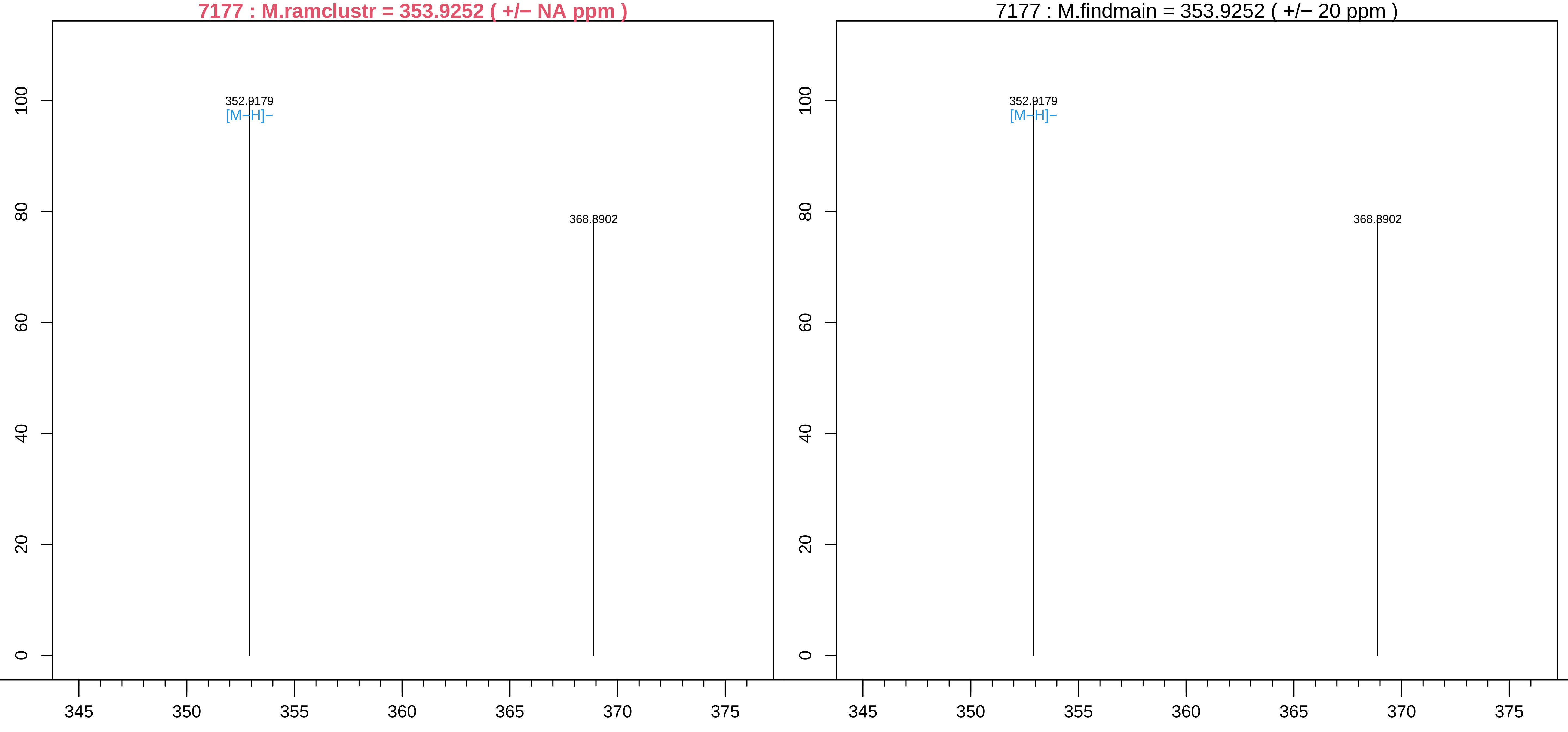
7177 : M.ramclustr = 353.9252 (+/− NA ppm)
352.9179
[M−H]−
368.8902
7177 : M.findmain = 353.9252 (+/− 20 ppm)
352.9179
[M−H]−
368.8902
0
20
40
60
80
100
345
350
355
360
365
370
375

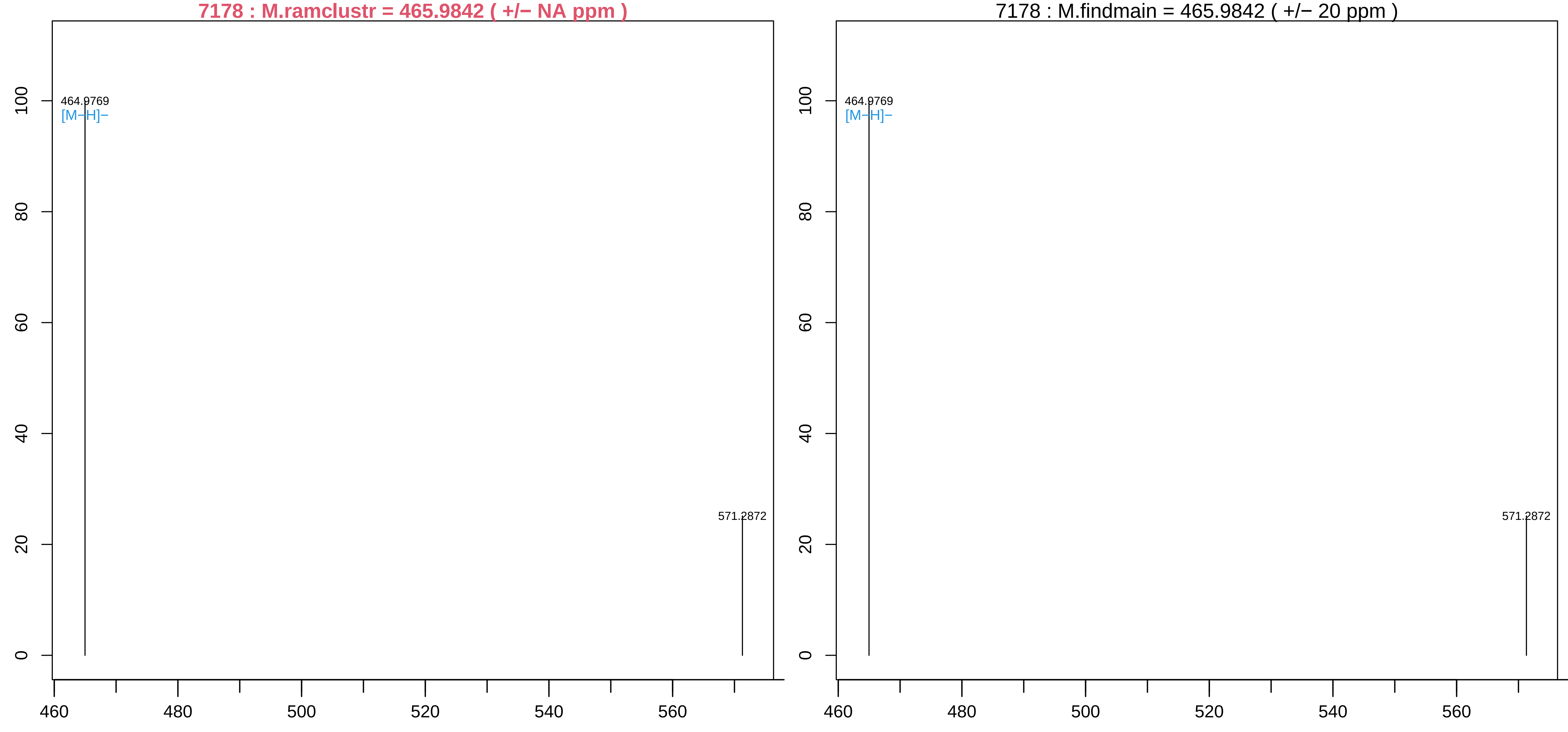

7178 : M.ramclustr = 465.9842 (+/− NA ppm)
464.9769
[M−H]−
571.2872
7178 : M.findmain = 465.9842 (+/− 20 ppm)
464.9769
[M−H]−
571.2872
0
20
40
60
80
100
460
480
500
520
540
560

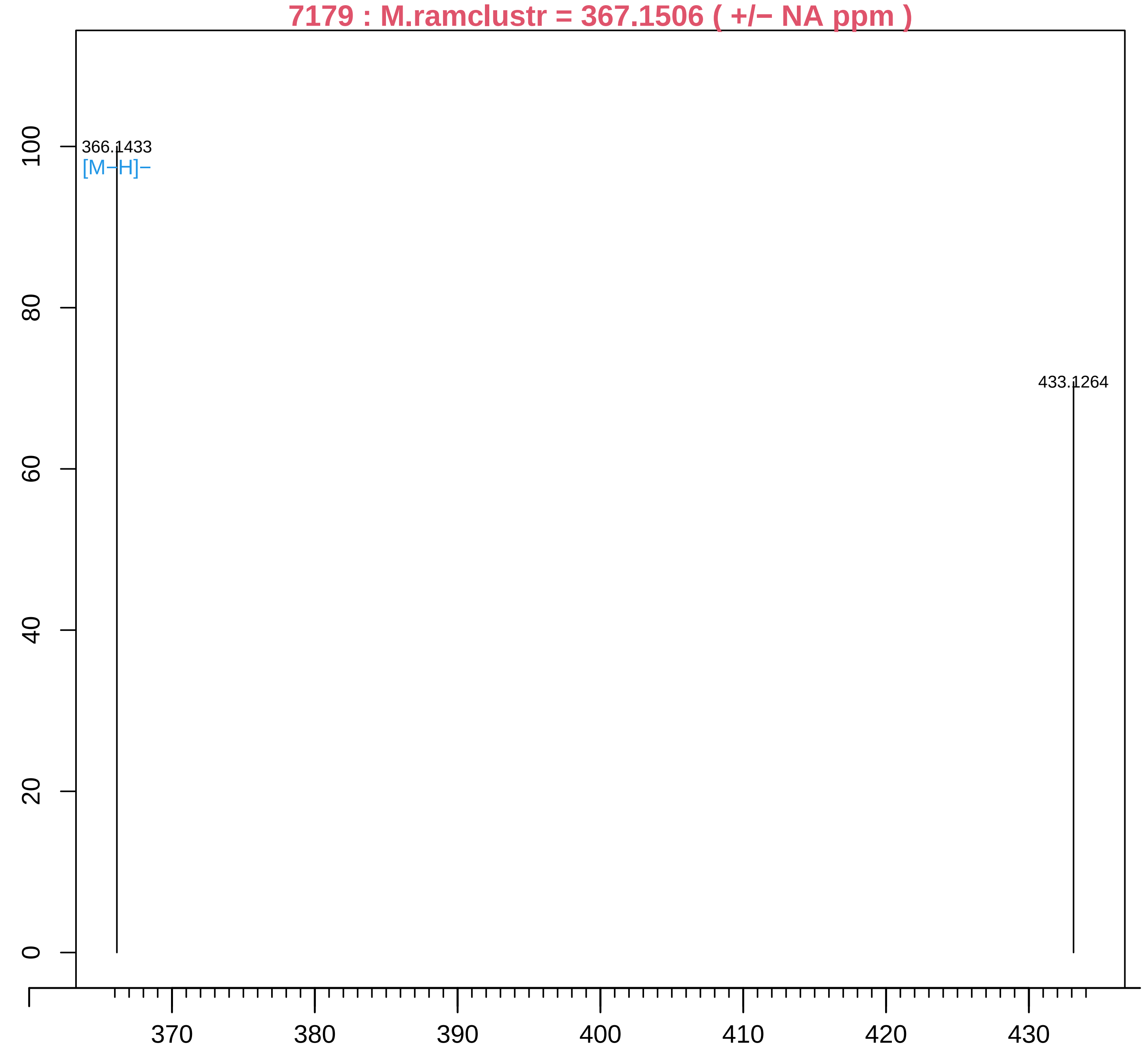
7179 : M.ramclustr = 367.1506 (+/− NA ppm)
366.1433
[M−H]−
433.1264
0
20
40
60
80
100
370
380
390
400
410
420
430

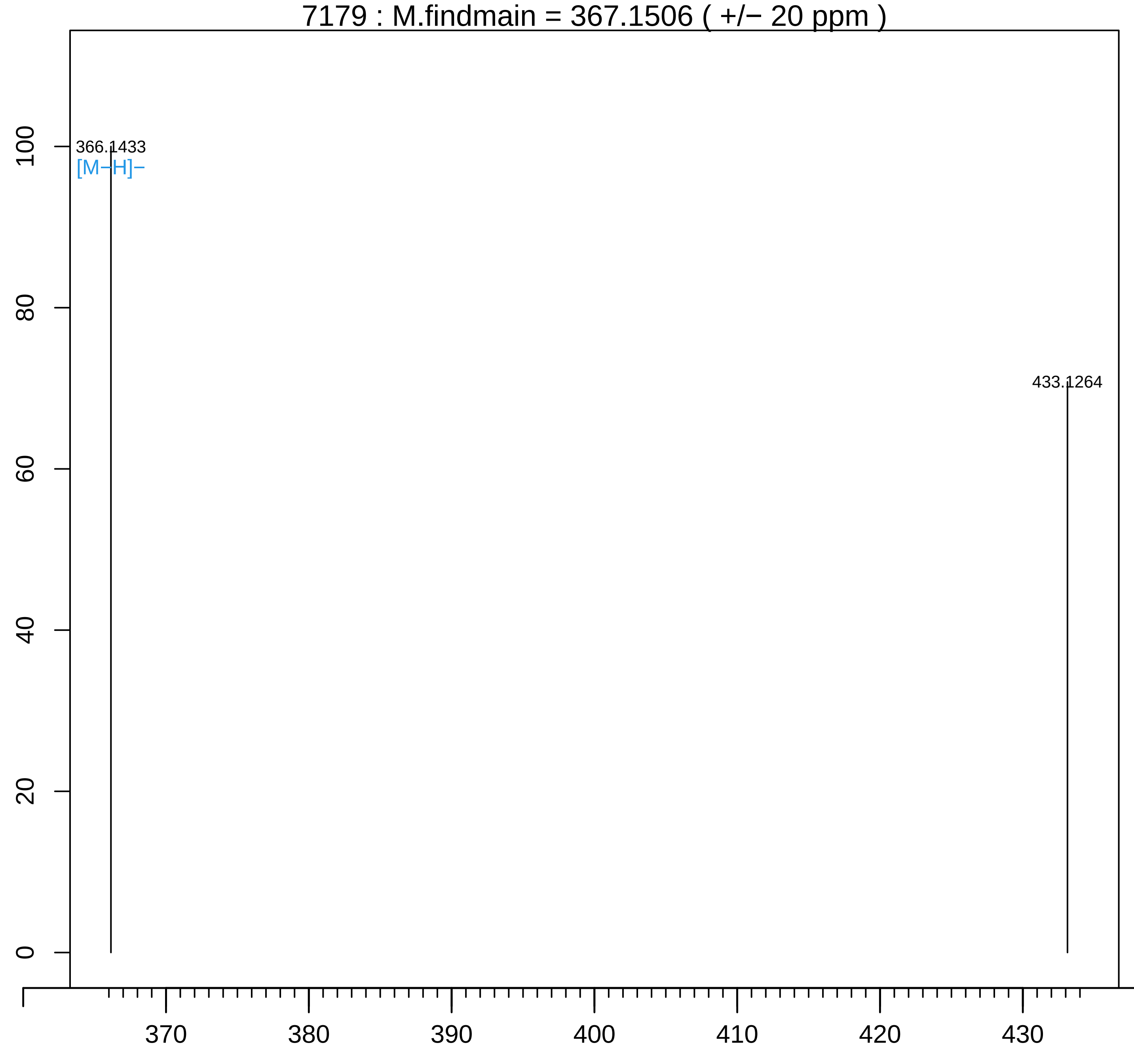
7179 : M.findmain = 367.1506 (+/− 20 ppm)
366.1433
[M−H]−
433.1264
0
20
40
60
80
100
370
380
390
400
410
420
430

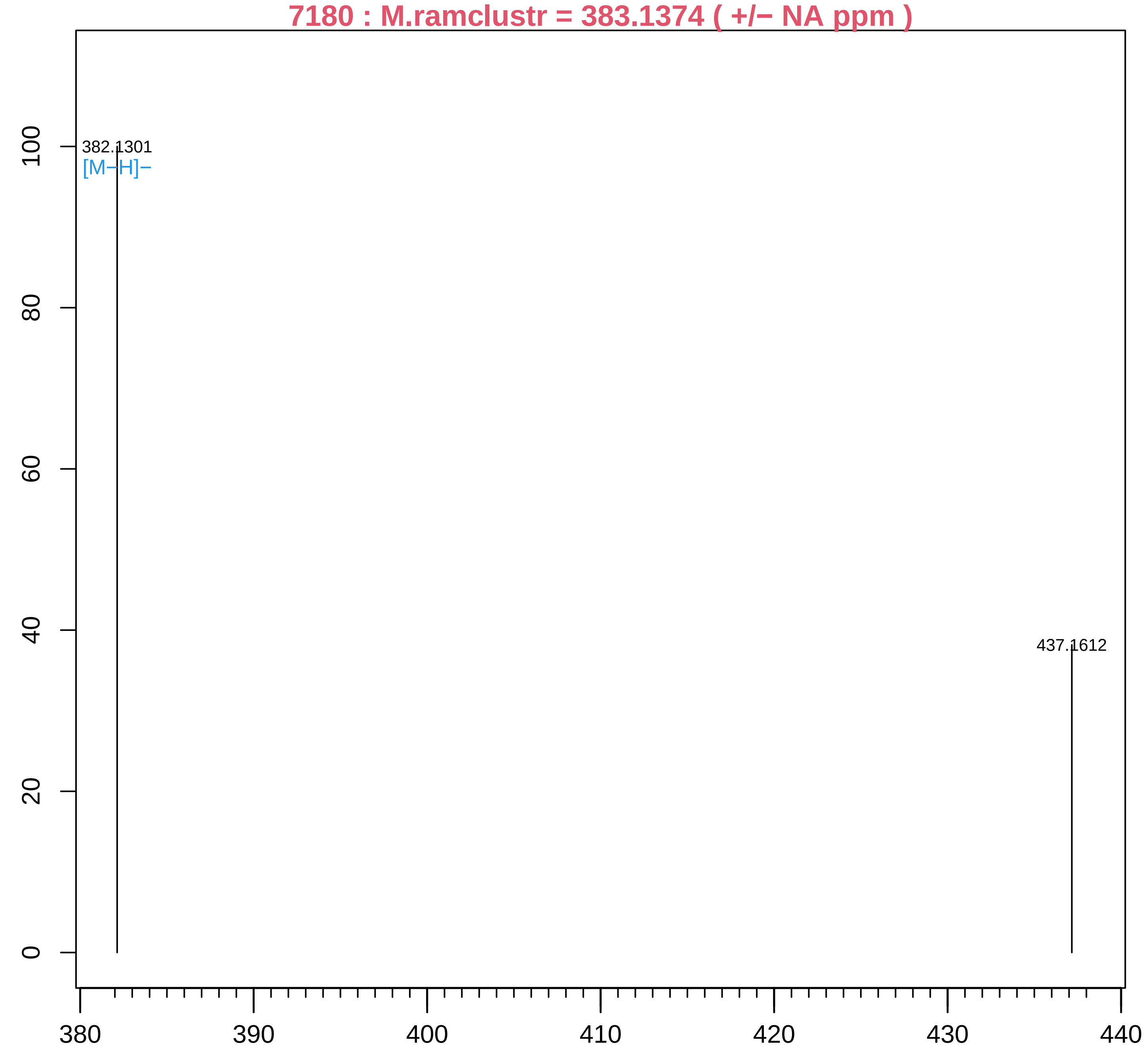
7180 : M.ramclustr = 383.1374 (+/− NA ppm)
382.1301
[M−H]−
437.1612
0
20
40
60
80
100
380
390
400
410
420
430
440

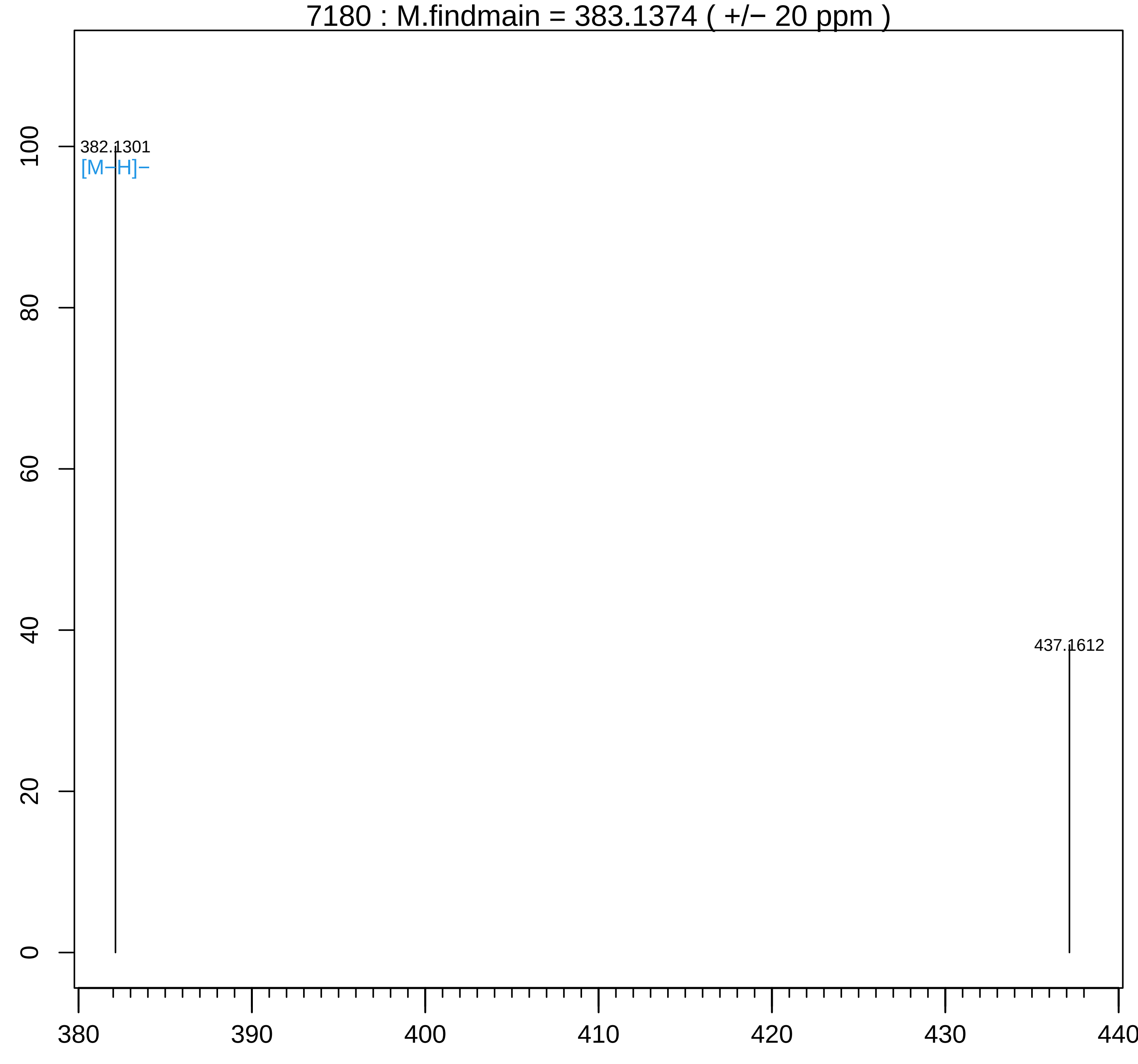
7180 : M.findmain = 383.1374 (+/− 20 ppm)
382.1301
[M−H]−
437.1612
0
20
40
60
80
100
380
390
400
410
420
430
440

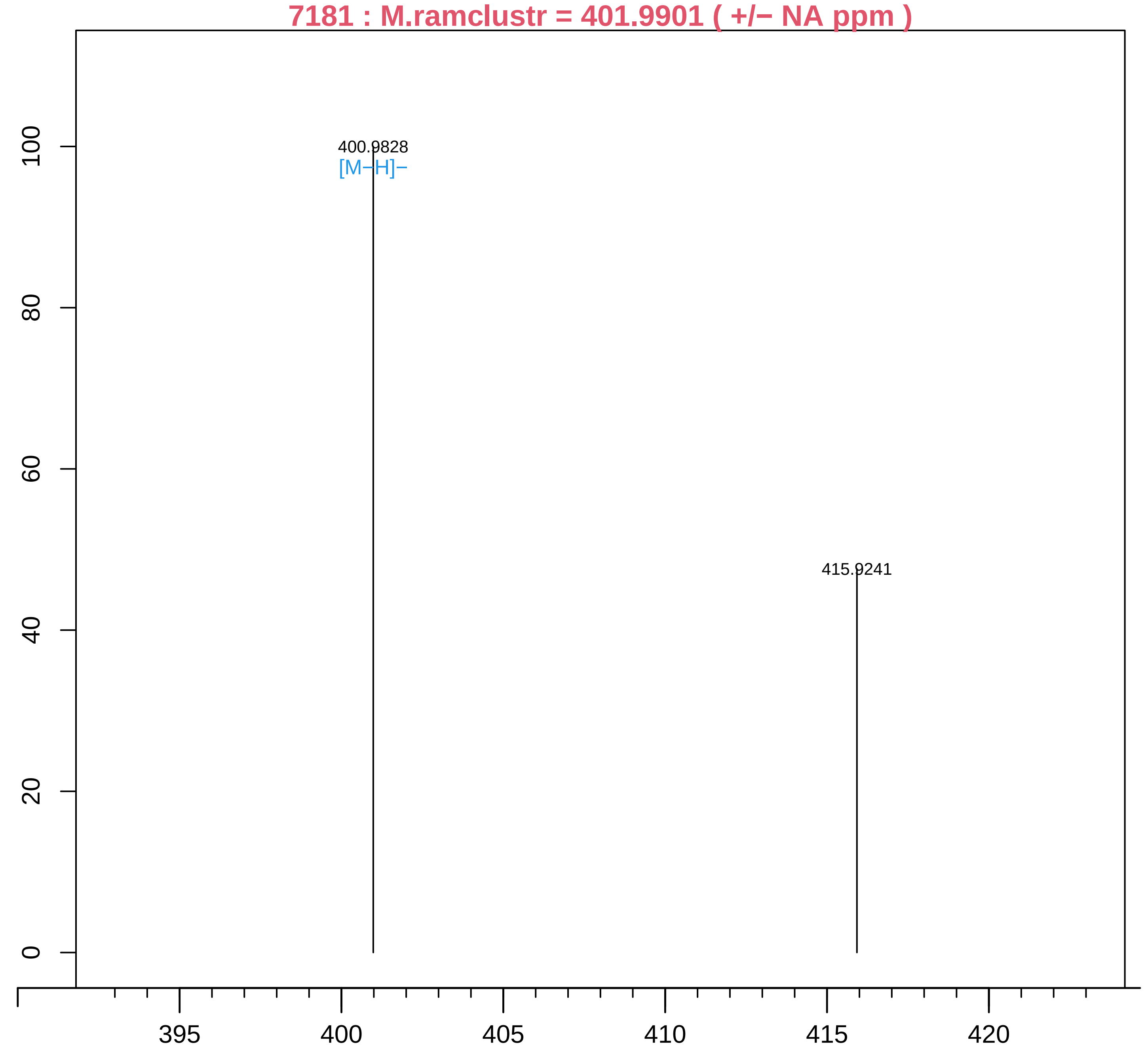
7181 : M.ramclustr = 401.9901 (+/− NA ppm)
400.9828
[M−H]−
415.9241
0
20
40
60
80
100
395
400
405
410
415
420

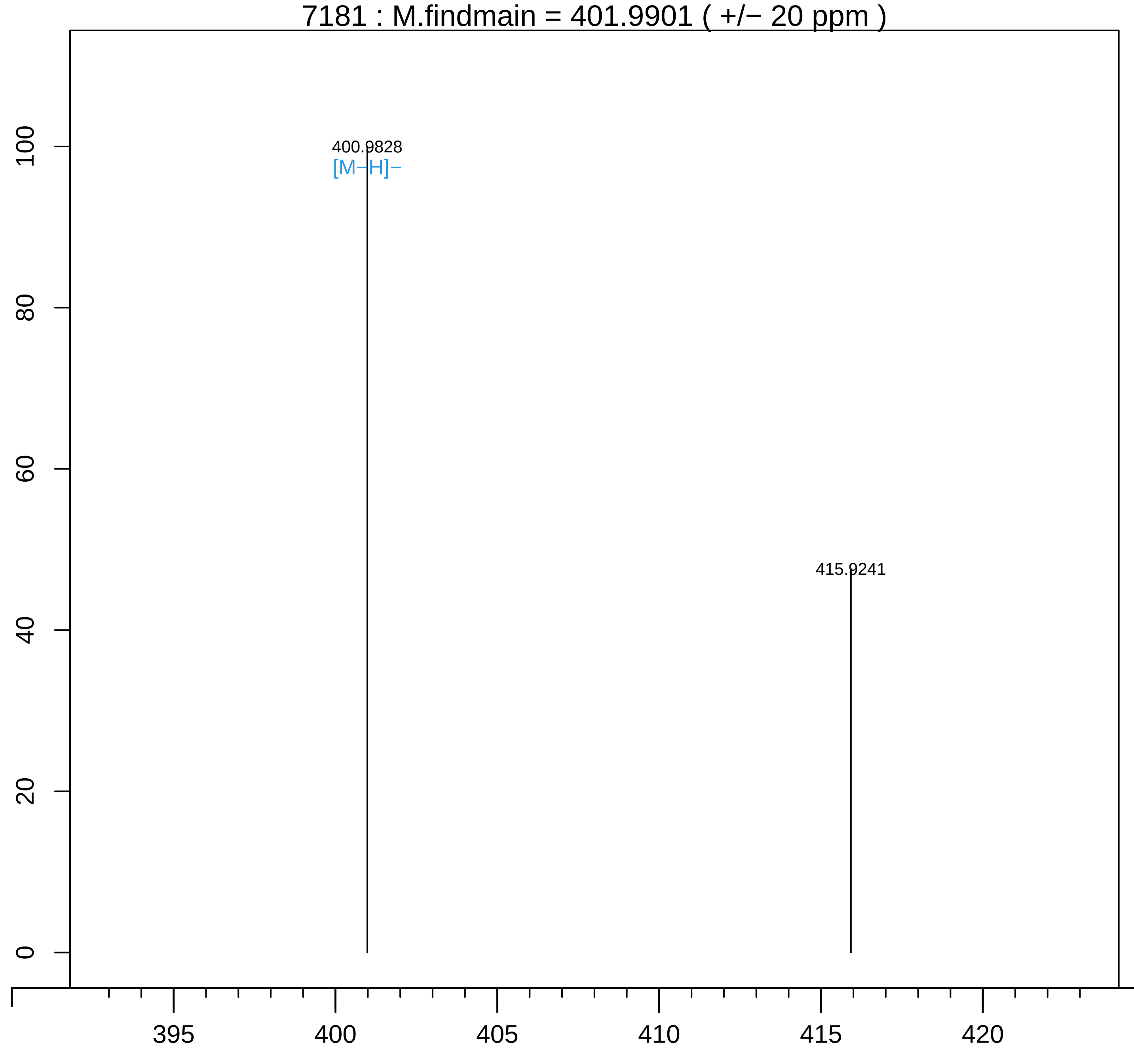
7181 : M.findmain = 401.9901 (+/− 20 ppm)
400.9828
[M−H]−
415.9241
0
20
40
60
80
100
395
400
405
410
415
420

7182 : M.ramclustr = 539.9877 (+/− NA ppm)

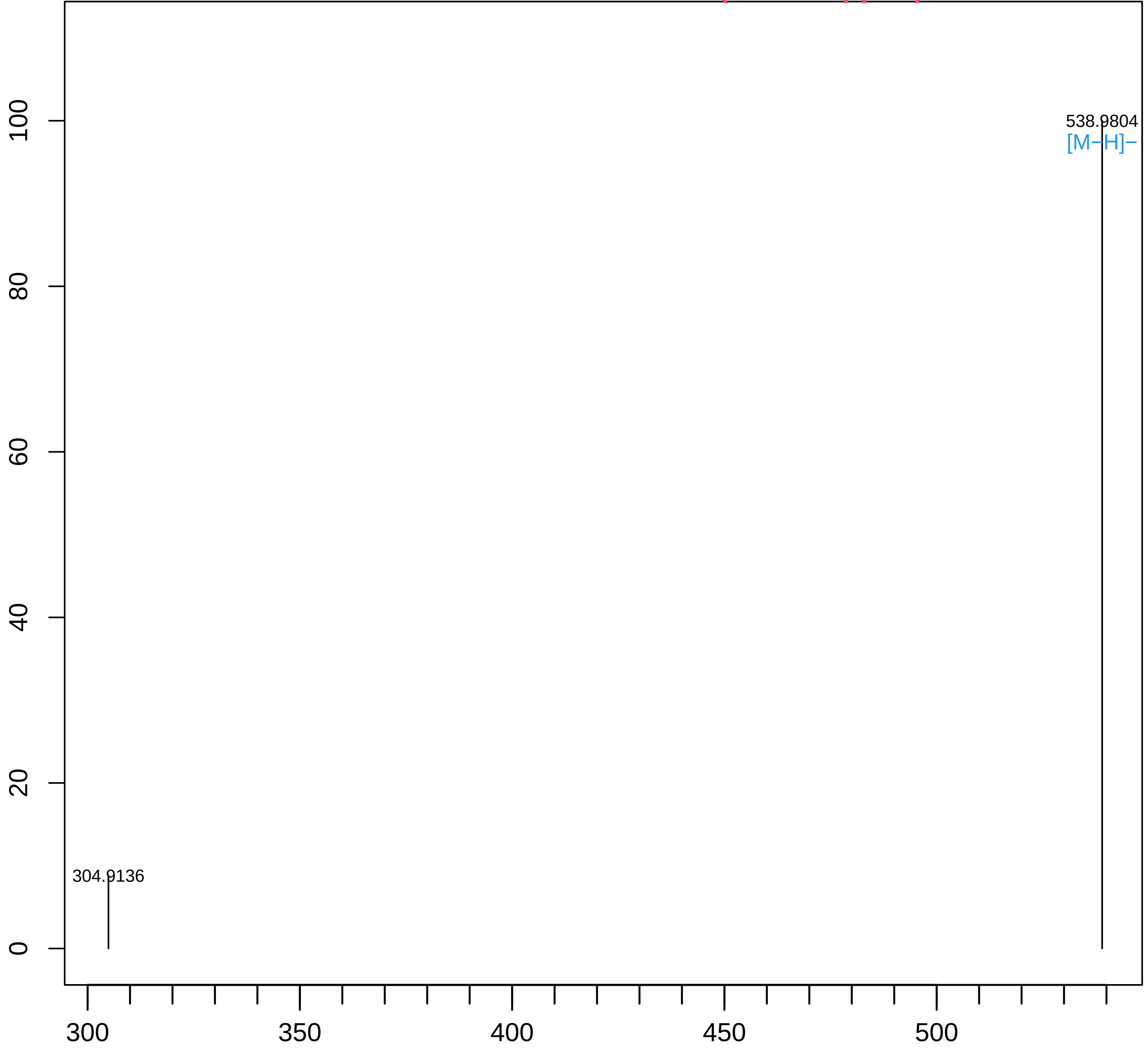

7182 : M.findmain = 539.9877 (+/− 20 ppm)

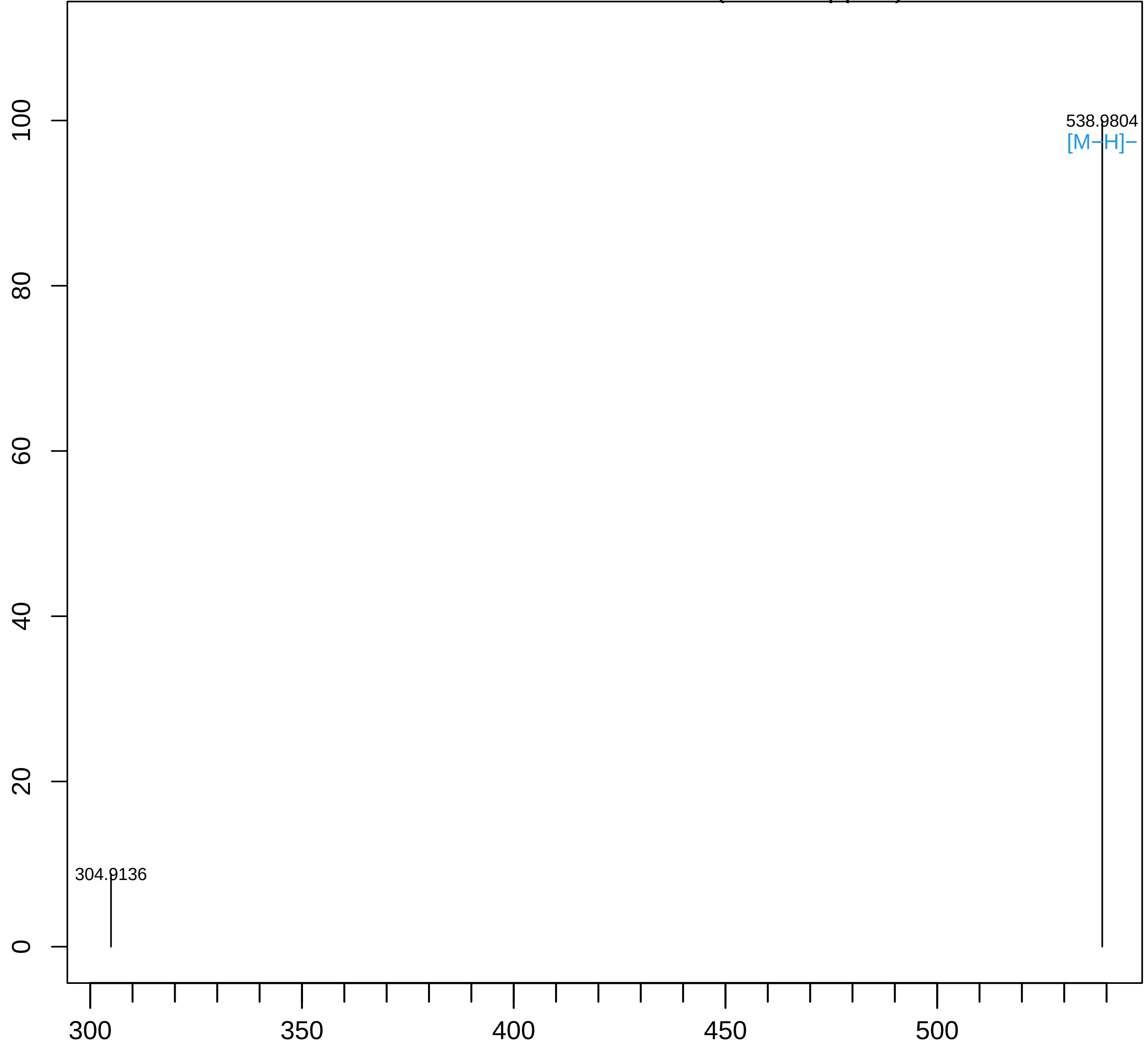

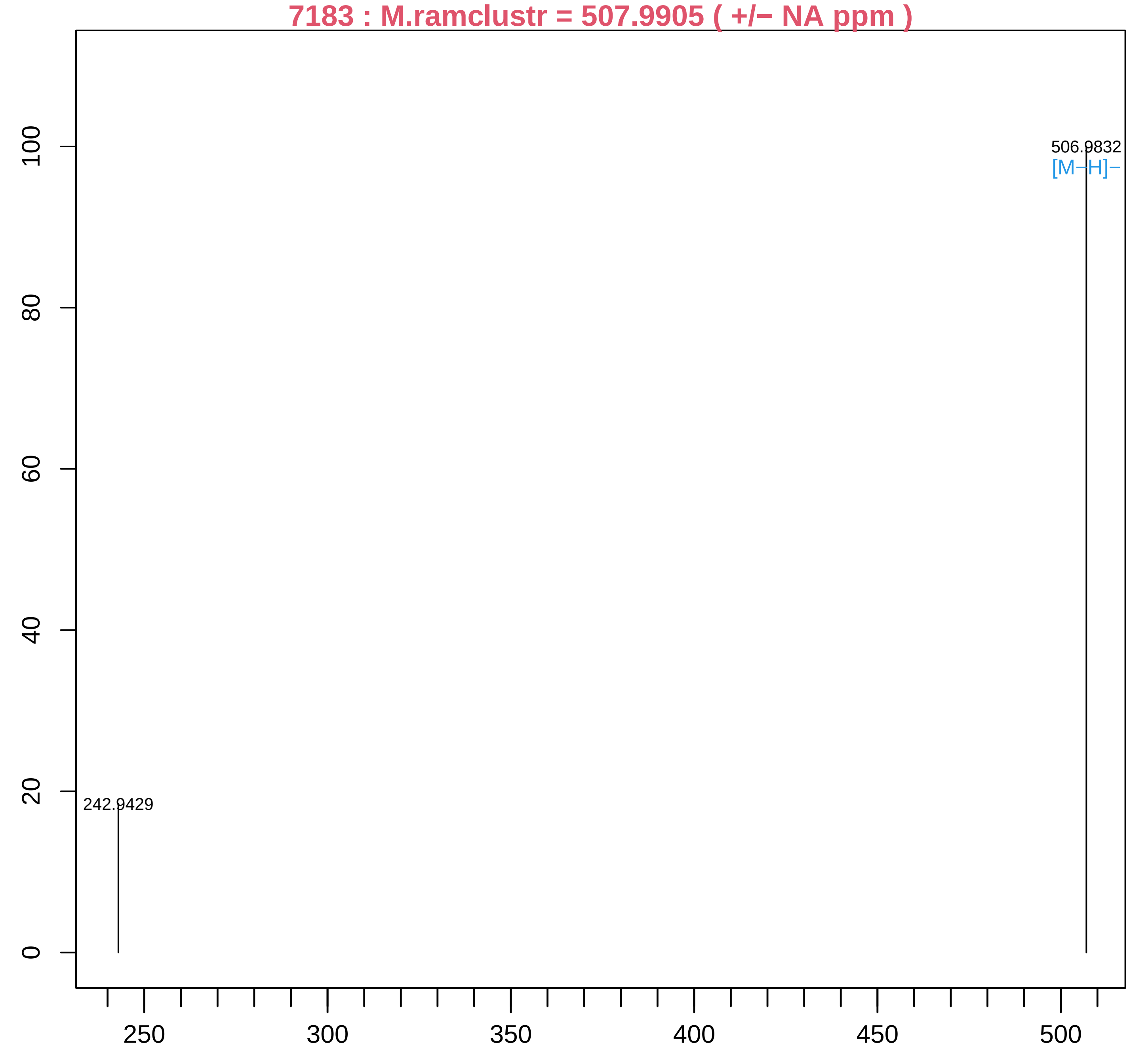
7183 : M.ramclustr = 507.9905 (+/− NA ppm)
506.9832
[M−H]−
242.9429
0
20
40
60
80
100
250
300
350
400
450
500

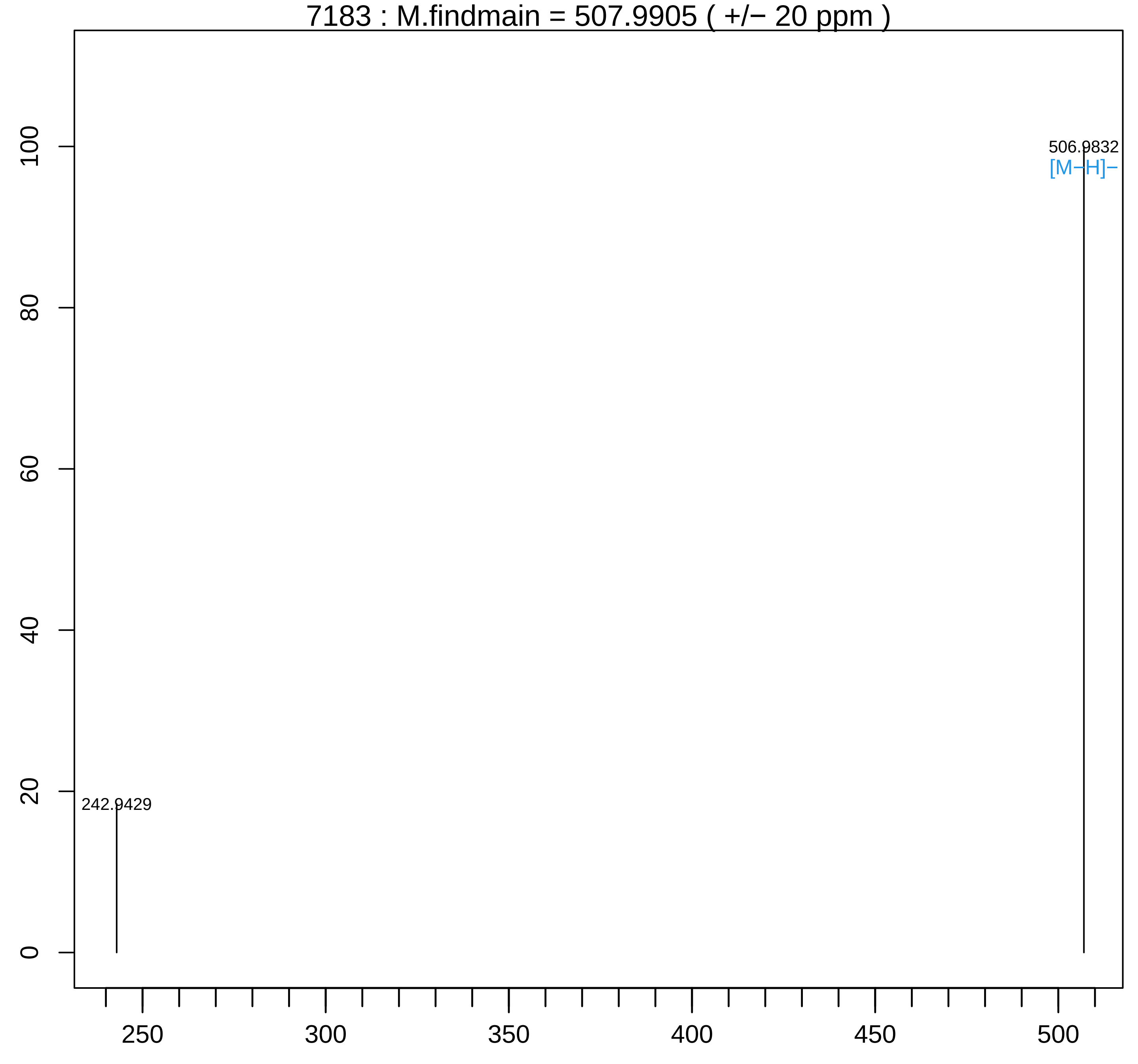
7183 : M.findmain = 507.9905 (+/− 20 ppm)
506.9832
[M−H]−
242.9429
0
20
40
60
80
100
250
300
350
400
450
500

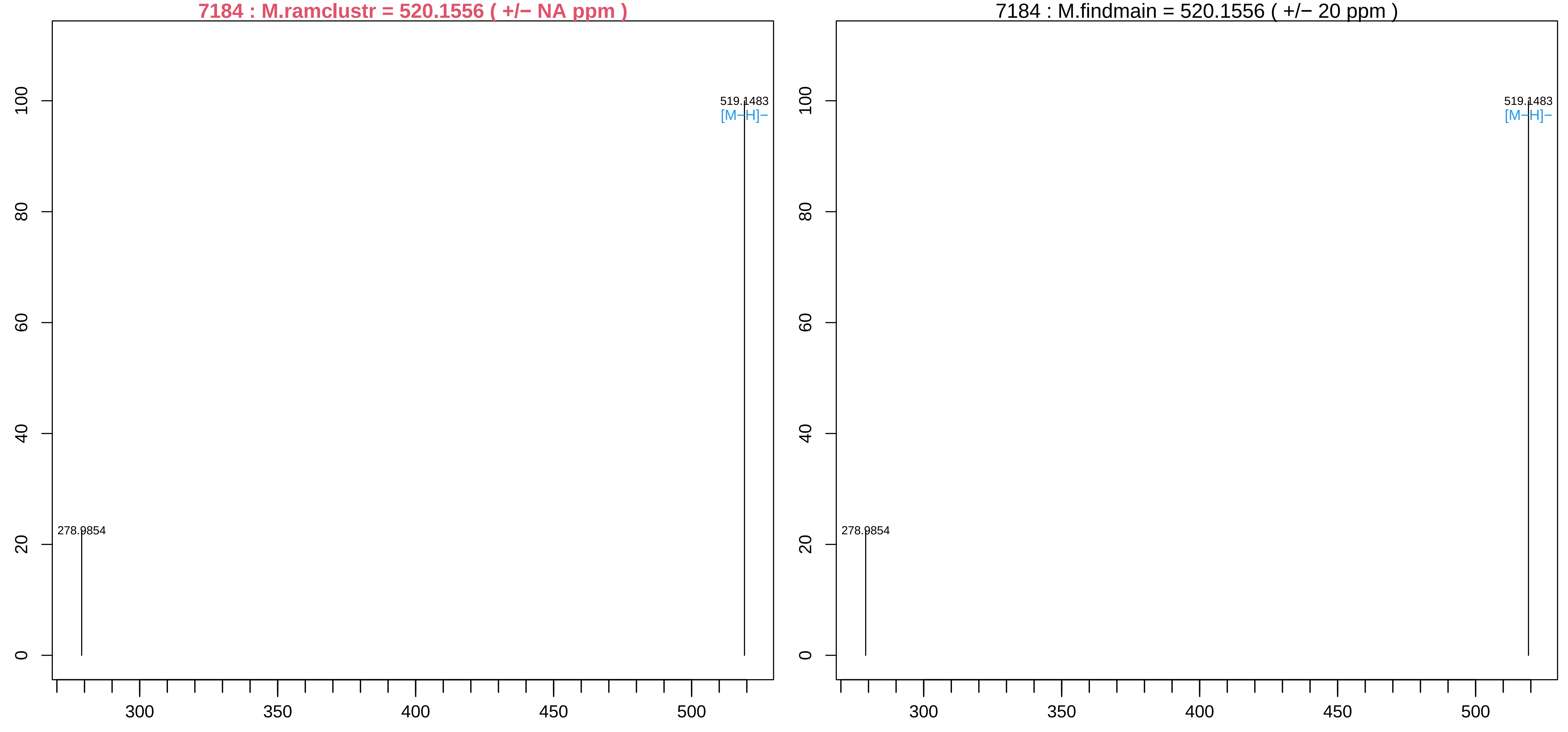
7184 : M.ramclustr = 520.1556 (+/− NA ppm)
519.1483
[M−H]−
278.9854
0
20
40
60
80
100
300
350
400
450
500
7184 : M.findmain = 520.1556 (+/− 20 ppm)
519.1483
[M−H]−
278.9854
0
20
40
60
80
100
300
350
400
450
500

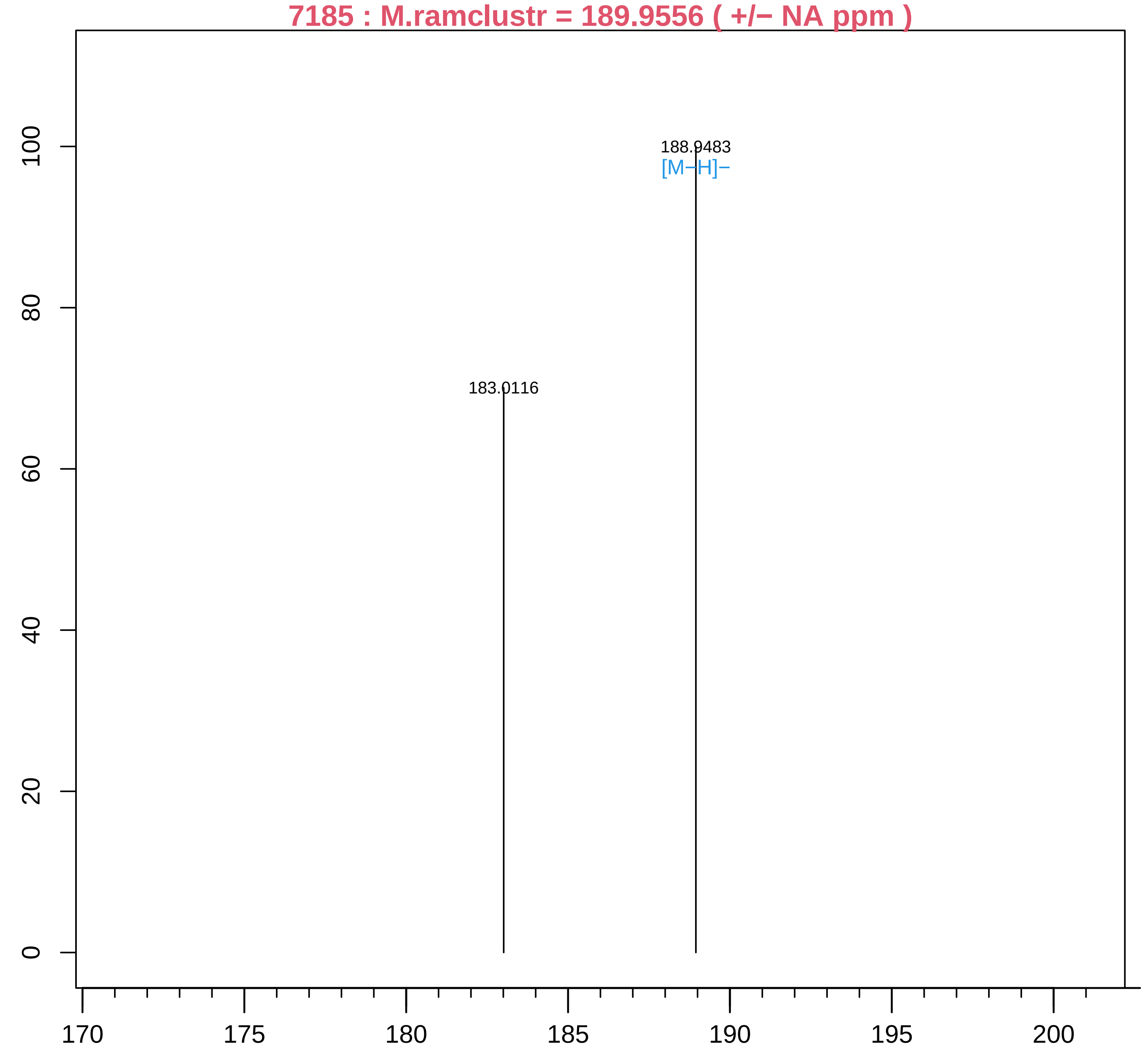
7185 : M.ramclustr = 189.9556 (+/− NA ppm)
188.9483
[M−H]−
183.0116
0
20
40
60
80
100
170
175
180
185
190
195
200

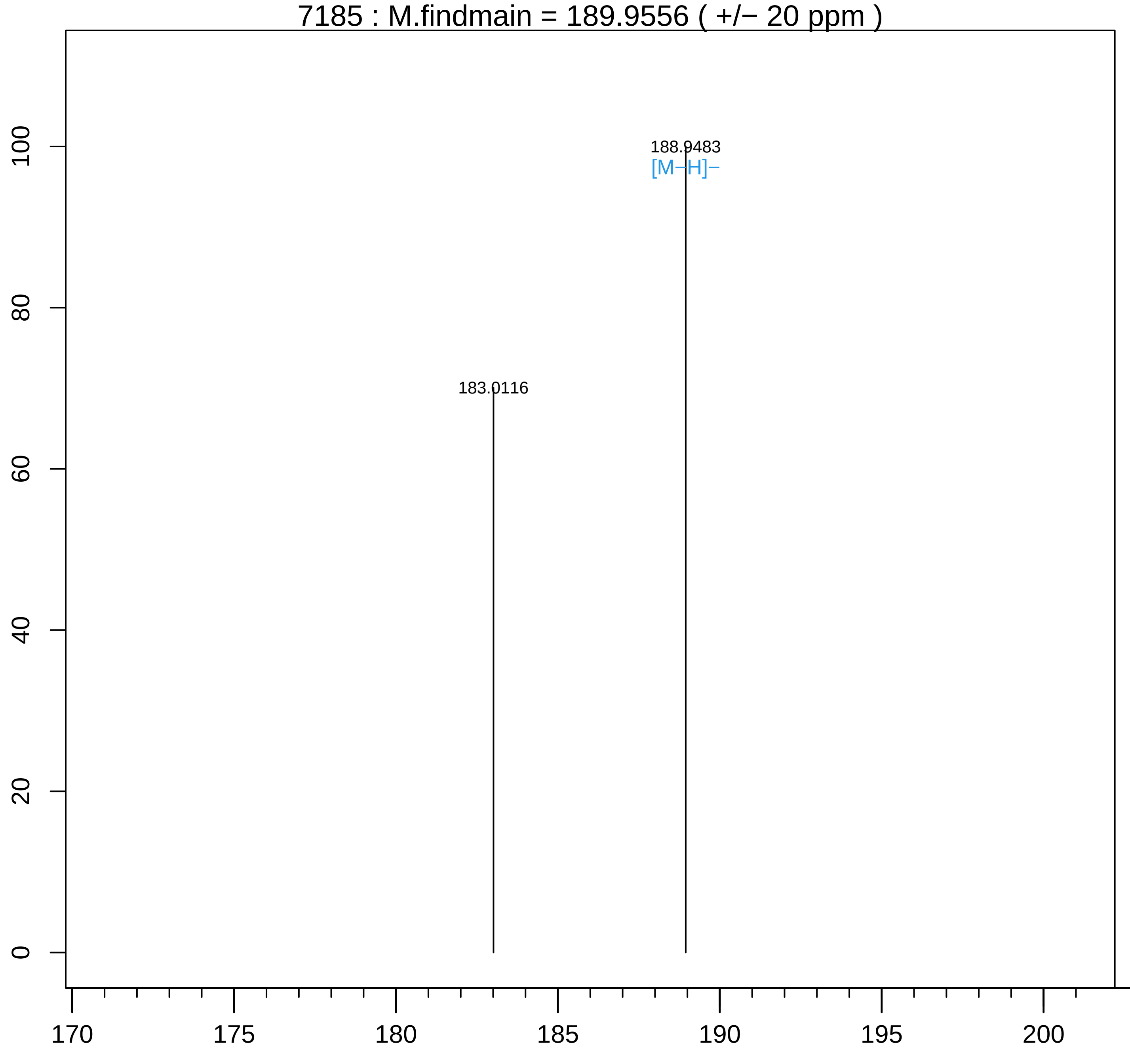
7185 : M.findmain = 189.9556 (+/− 20 ppm)
188.9483
[M−H]−
183.0116
0
20
40
60
80
100
170
175
180
185
190
195
200

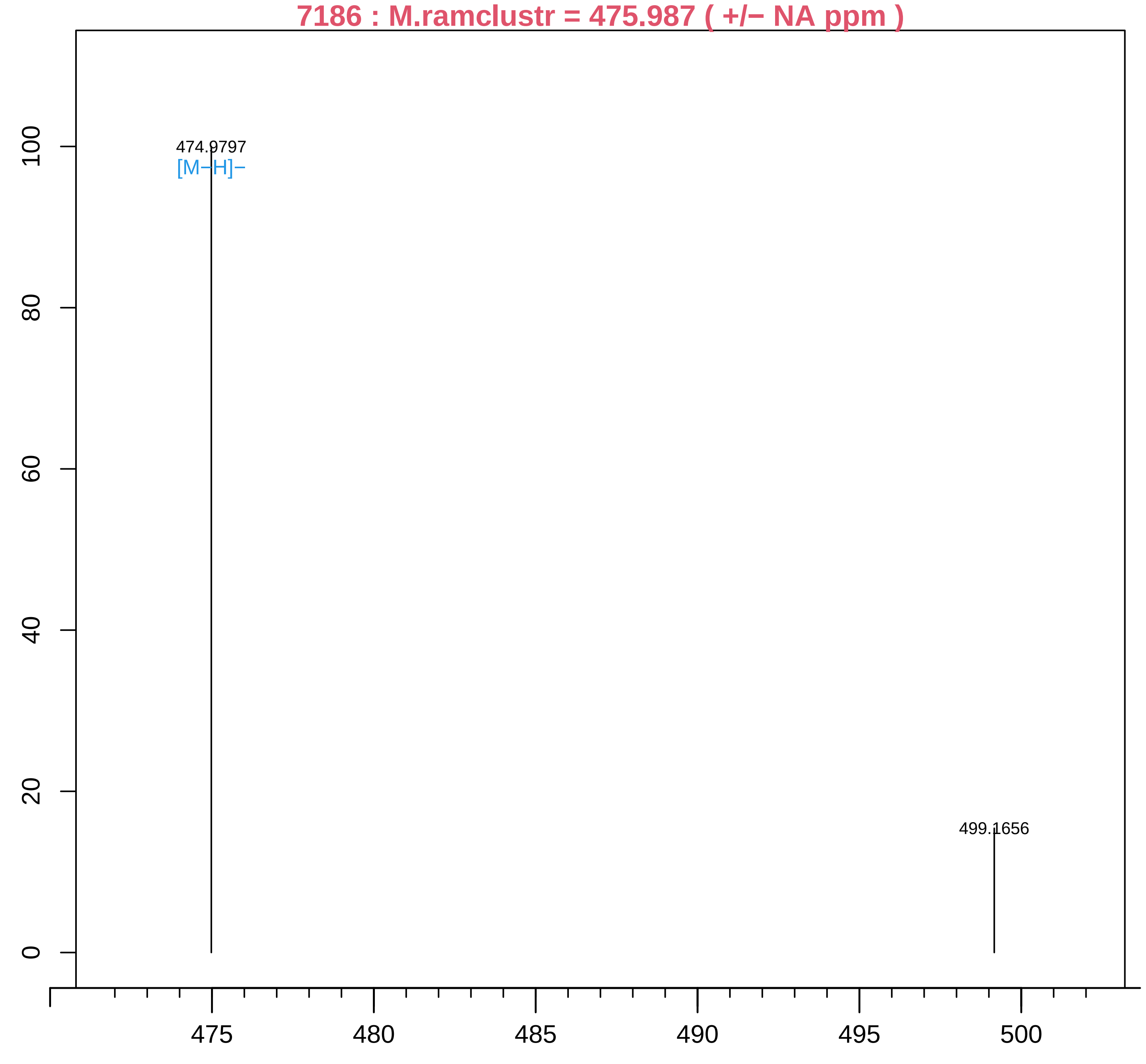
7186 : M.ramclustr = 475.987 (+/− NA ppm)
474.9797
[M−H]−
499.1656
0
20
40
60
80
100
475
480
485
490
495
500

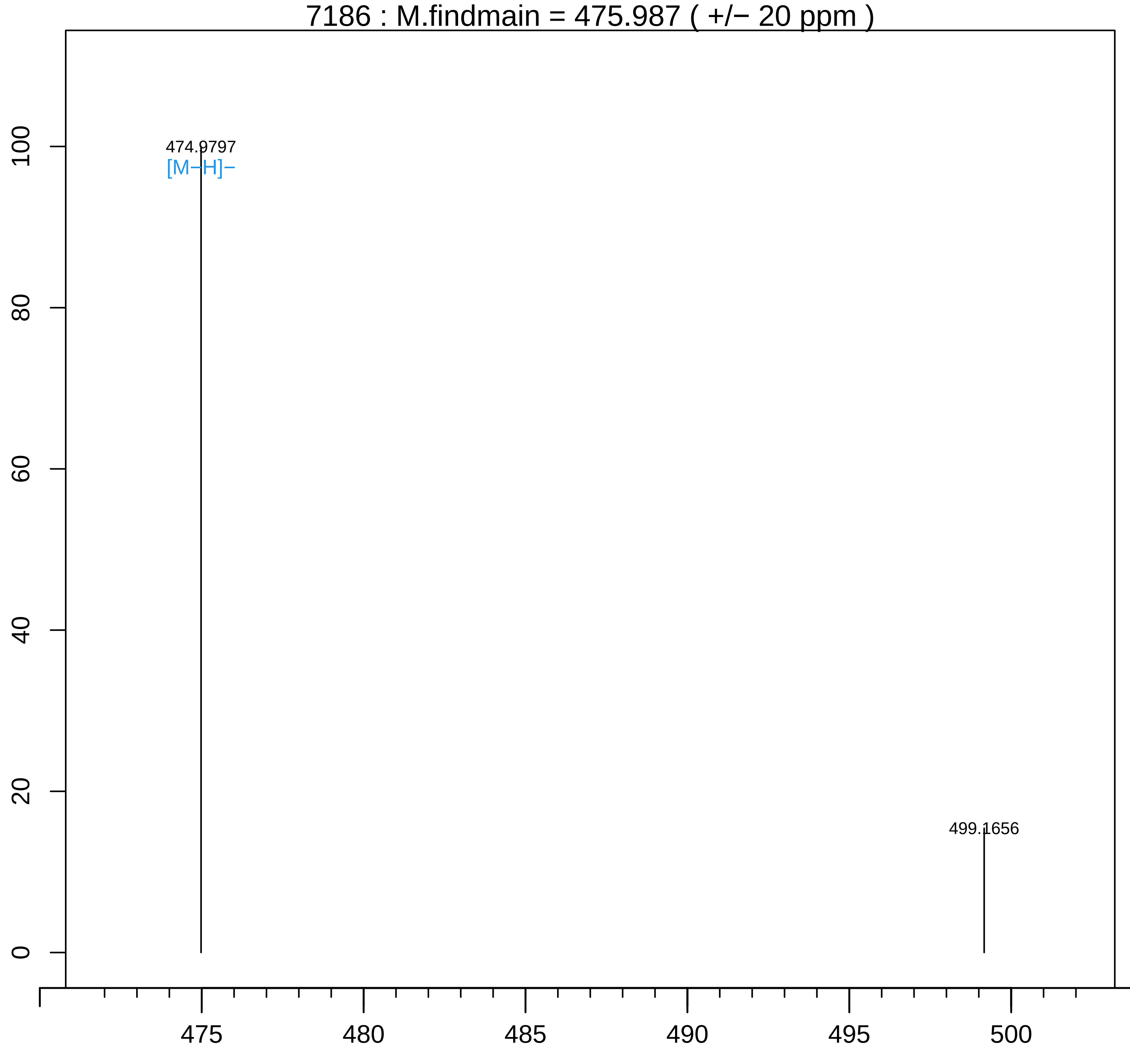
7186 : M.findmain = 475.987 (+/− 20 ppm)
474.9797
[M−H]−
499.1656
0
20
40
60
80
100
475
480
485
490
495
500

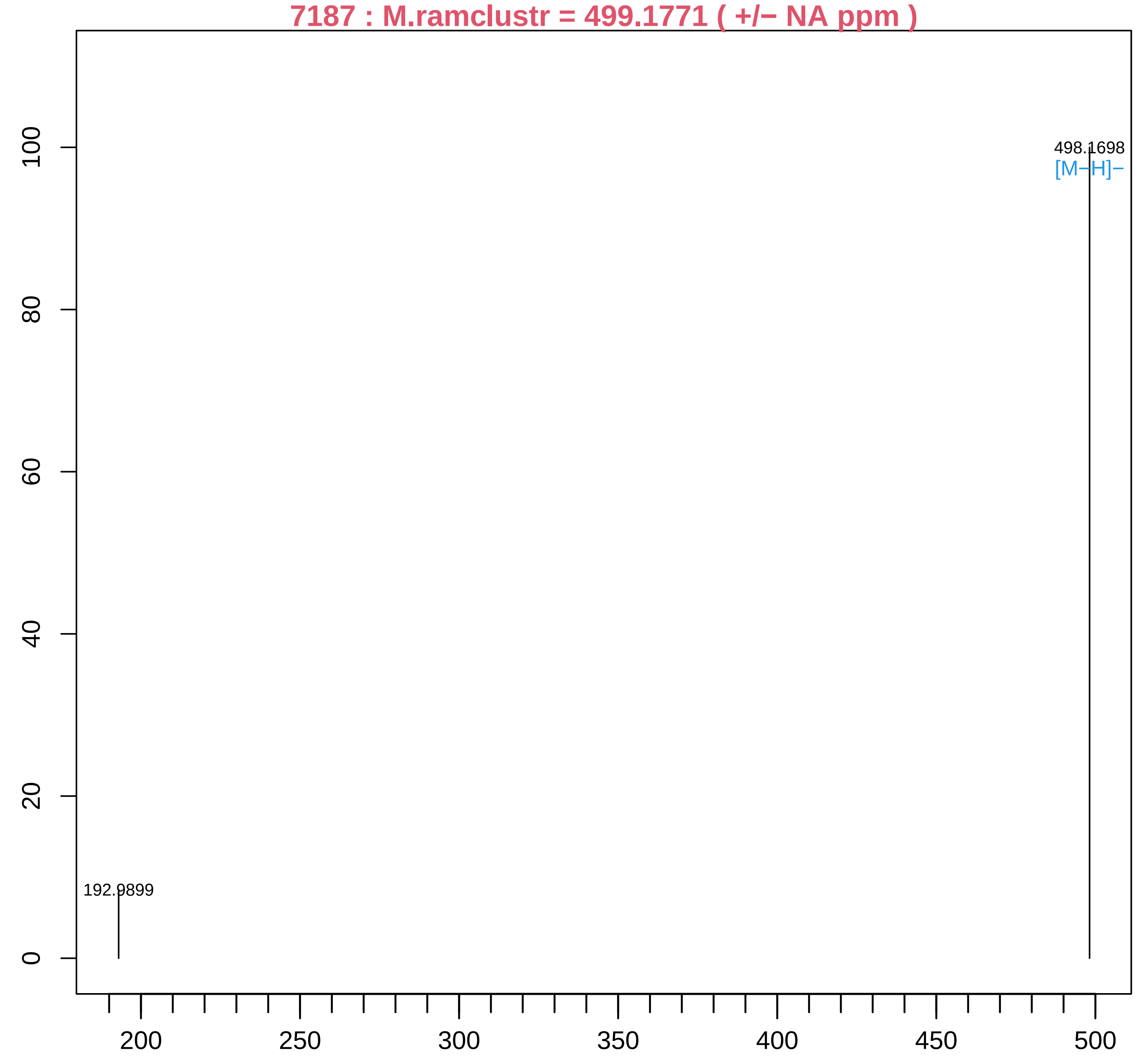
7187 : M.ramclustr = 499.1771 (+/− NA ppm)
498.1698
[M−H]−
192.9899
0
20
40
60
80
100
200
250
300
350
400
450
500

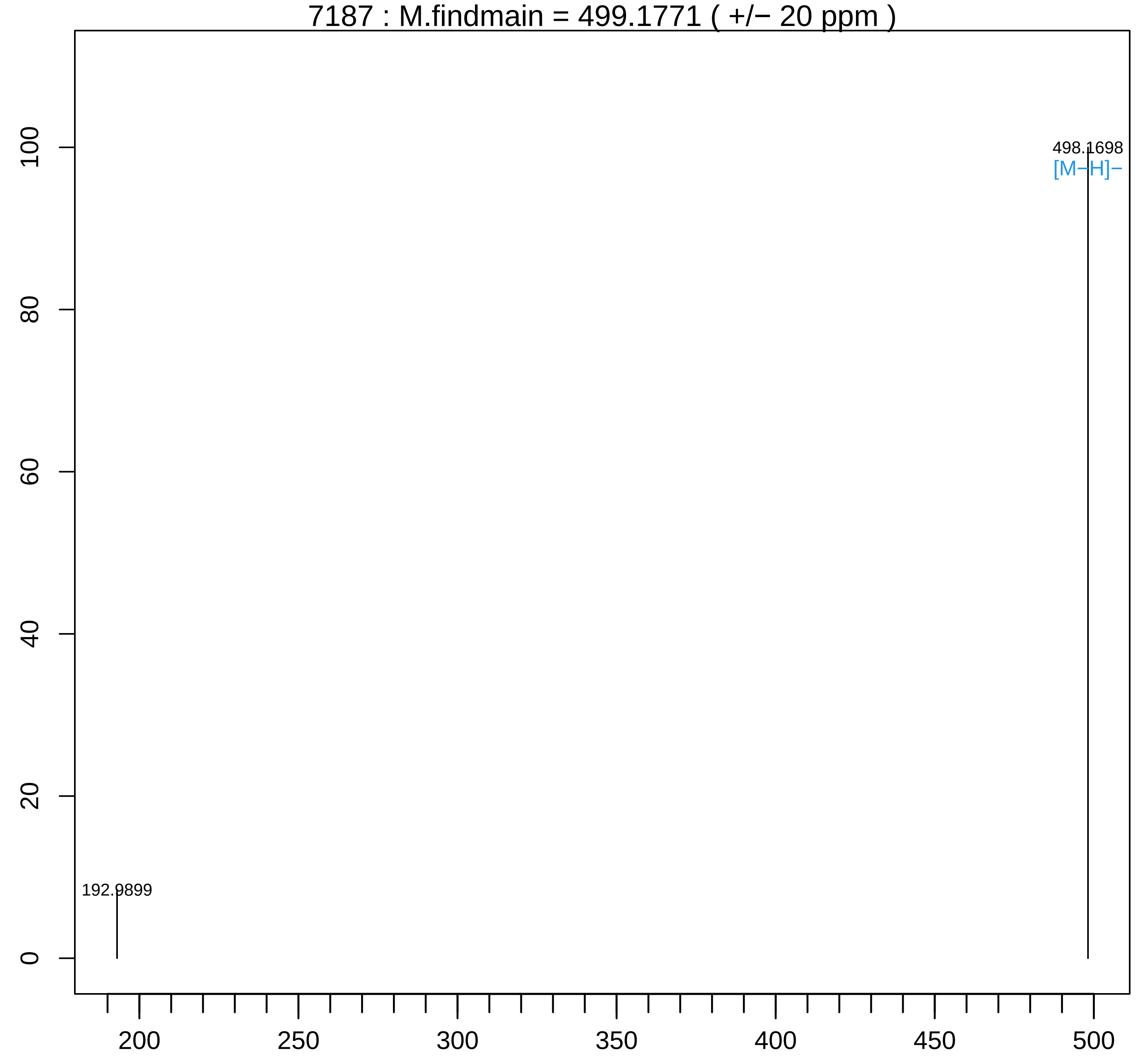
7187 : M.findmain = 499.1771 (+/− 20 ppm)
498.1698
[M−H]−
192.9899
0
20
40
60
80
100
200
250
300
350
400
450
500

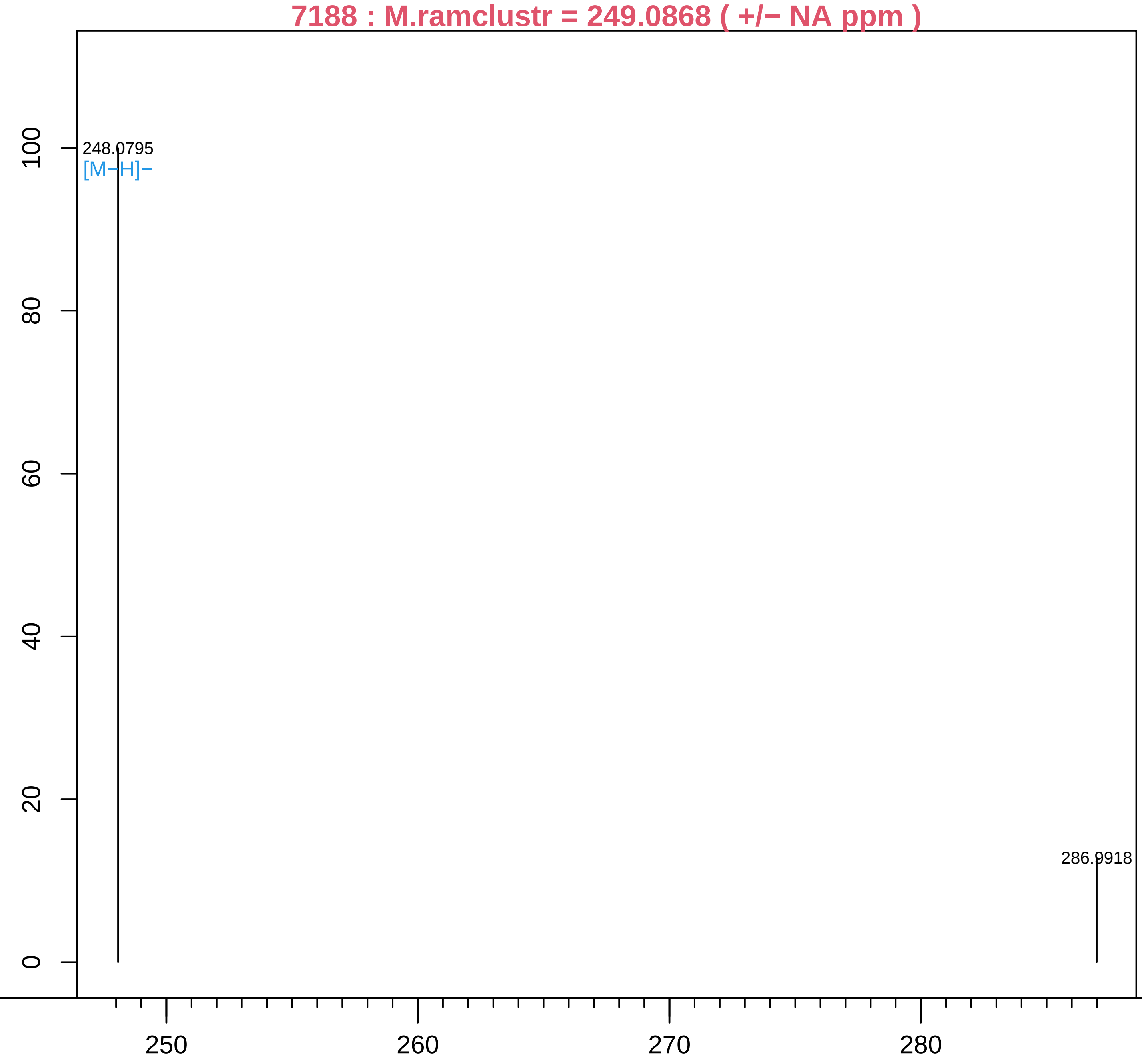
7188 : M.ramclustr = 249.0868 (+/− NA ppm)
248.0795
[M−H]−
286.9918
0
20
40
60
80
100
250
260
270
280

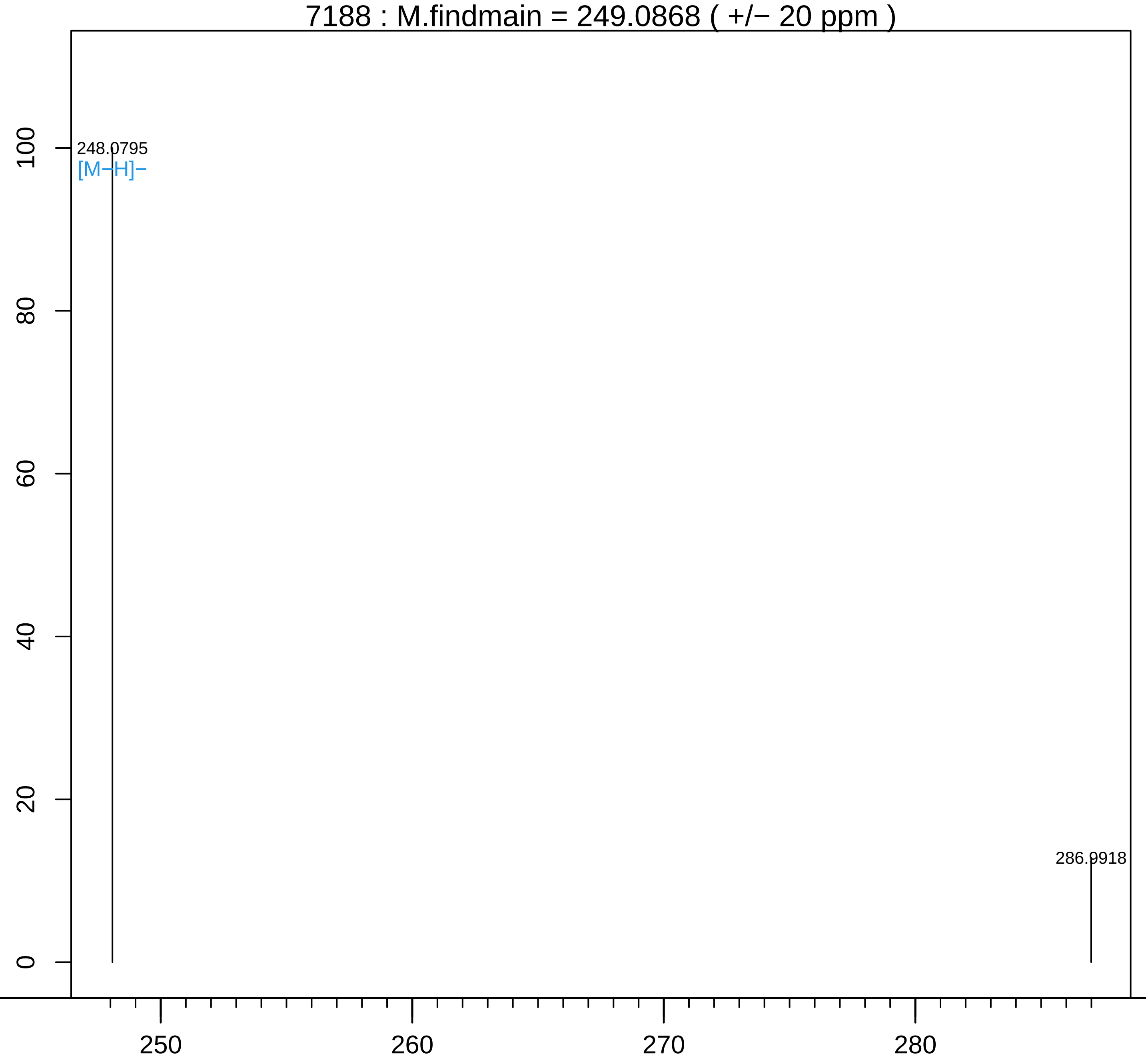
7188 : M.findmain = 249.0868 (+/− 20 ppm)
248.0795
[M−H]−
286.9918
0
20
40
60
80
100
250
260
270
280

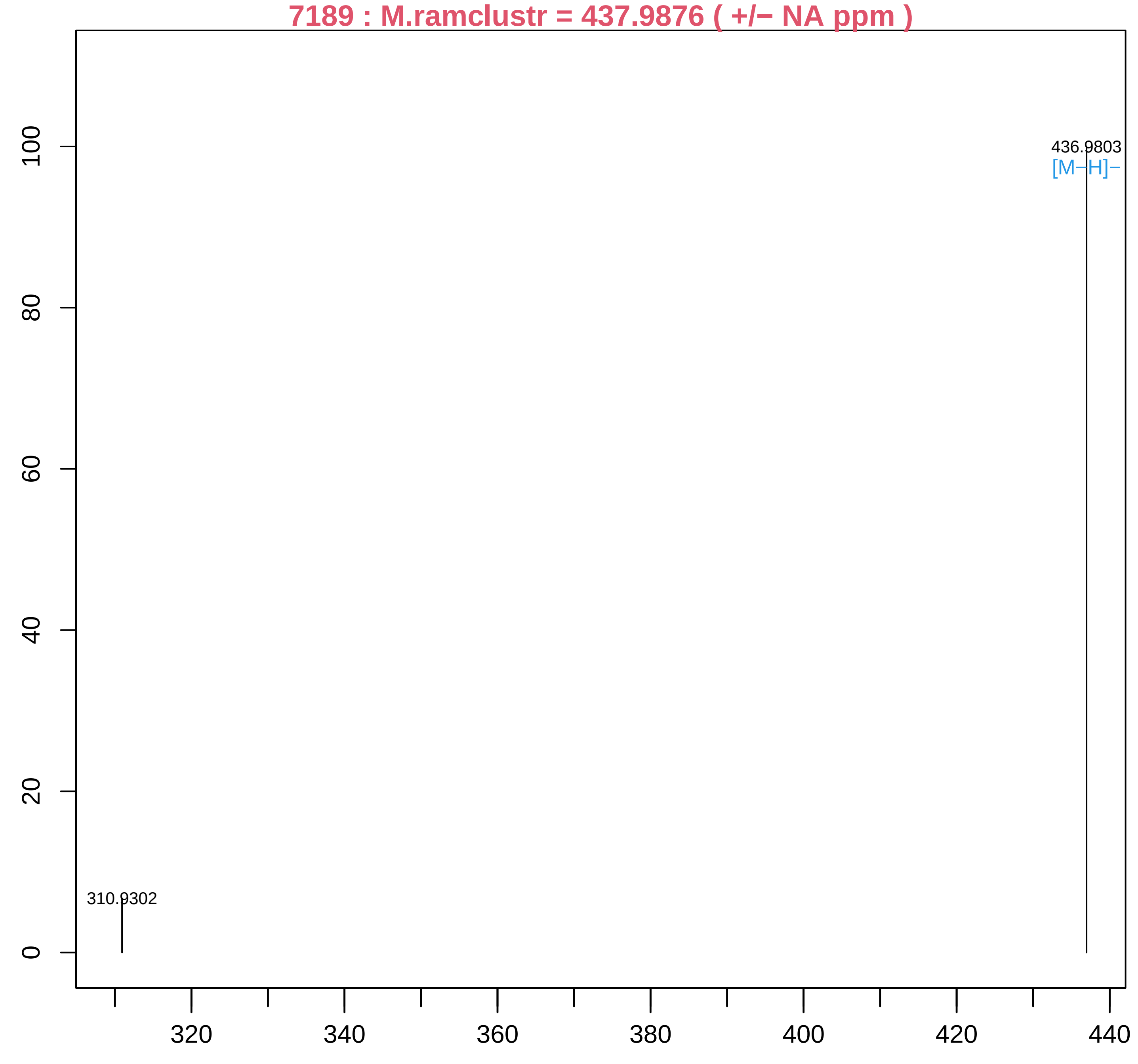

7189 : M.ramclustr = 437.9876 (+/− NA ppm)
436.9803
[M−H]−
310.9302
0
20
40
60
80
100
320
340
360
380
400
420
440

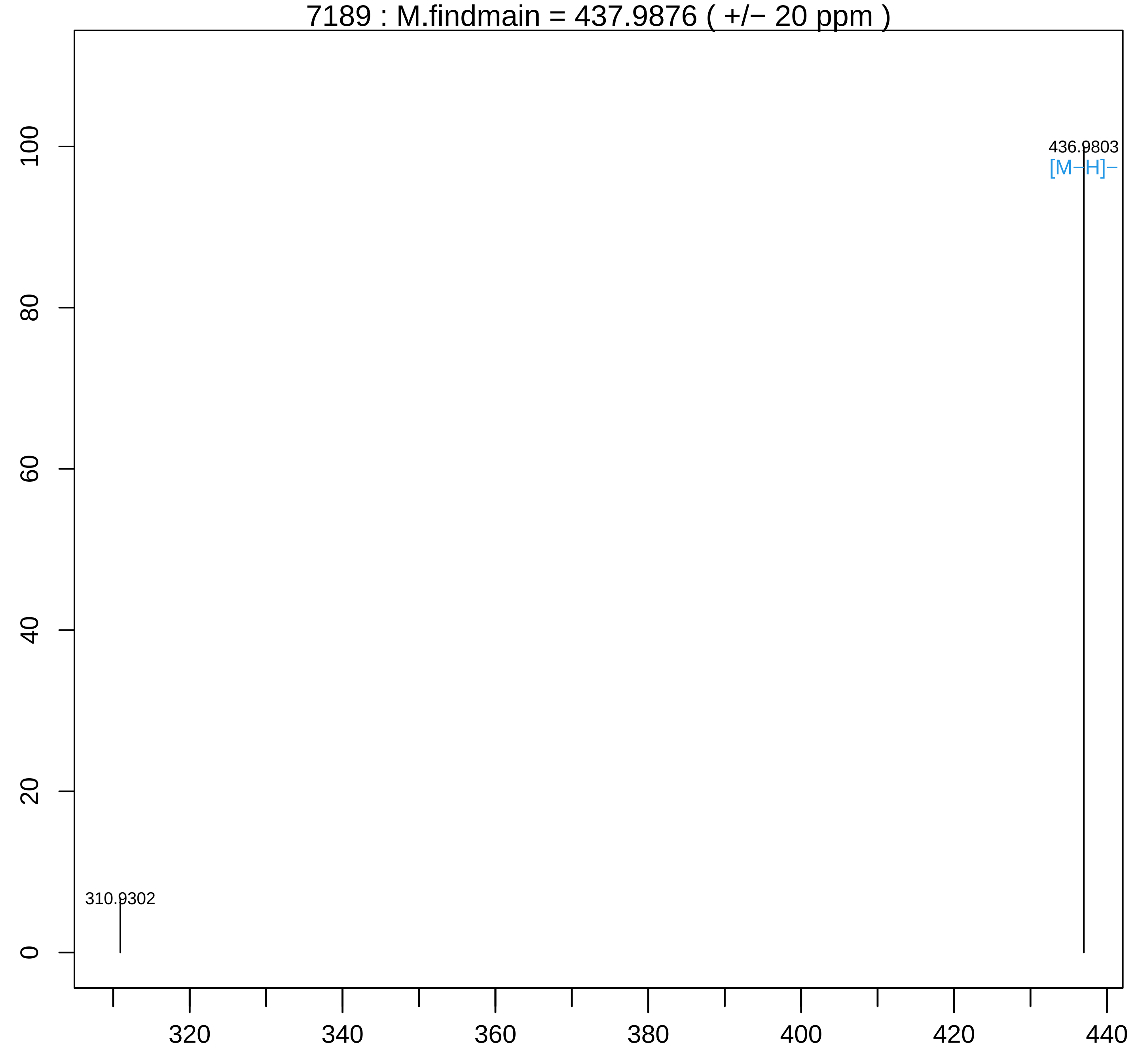

7189 : M.findmain = 437.9876 (+/− 20 ppm)
436.9803
[M−H]−
310.9302
0
20
40
60
80
100
320
340
360
380
400
420
440

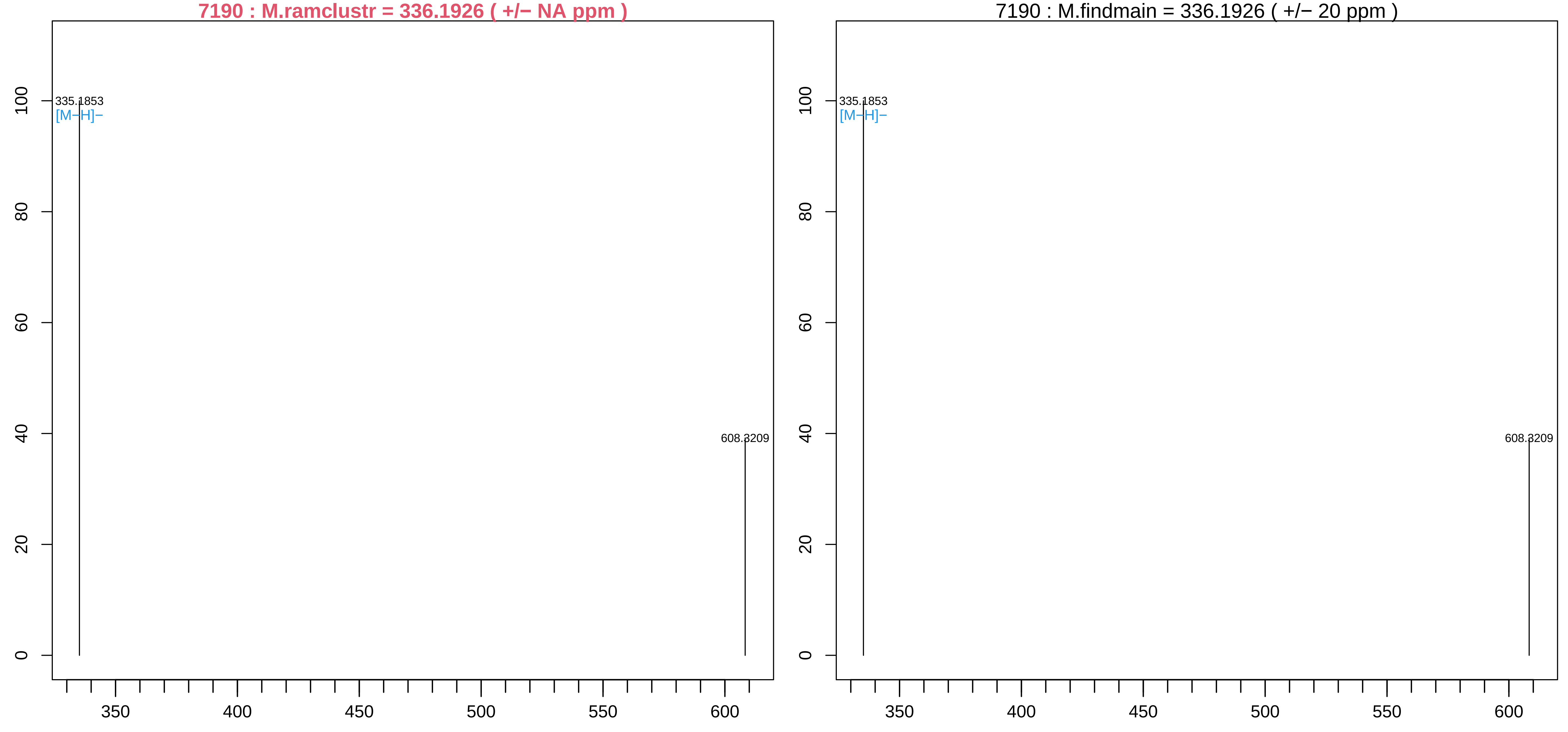
7190 : M.ramclustr = 336.1926 (+/− NA ppm)
335.1853
[M−H]−
608.3209
7190 : M.findmain = 336.1926 (+/− 20 ppm)
335.1853
[M−H]−
608.3209
0
20
40
60
80
100
350
400
450
500
550
600

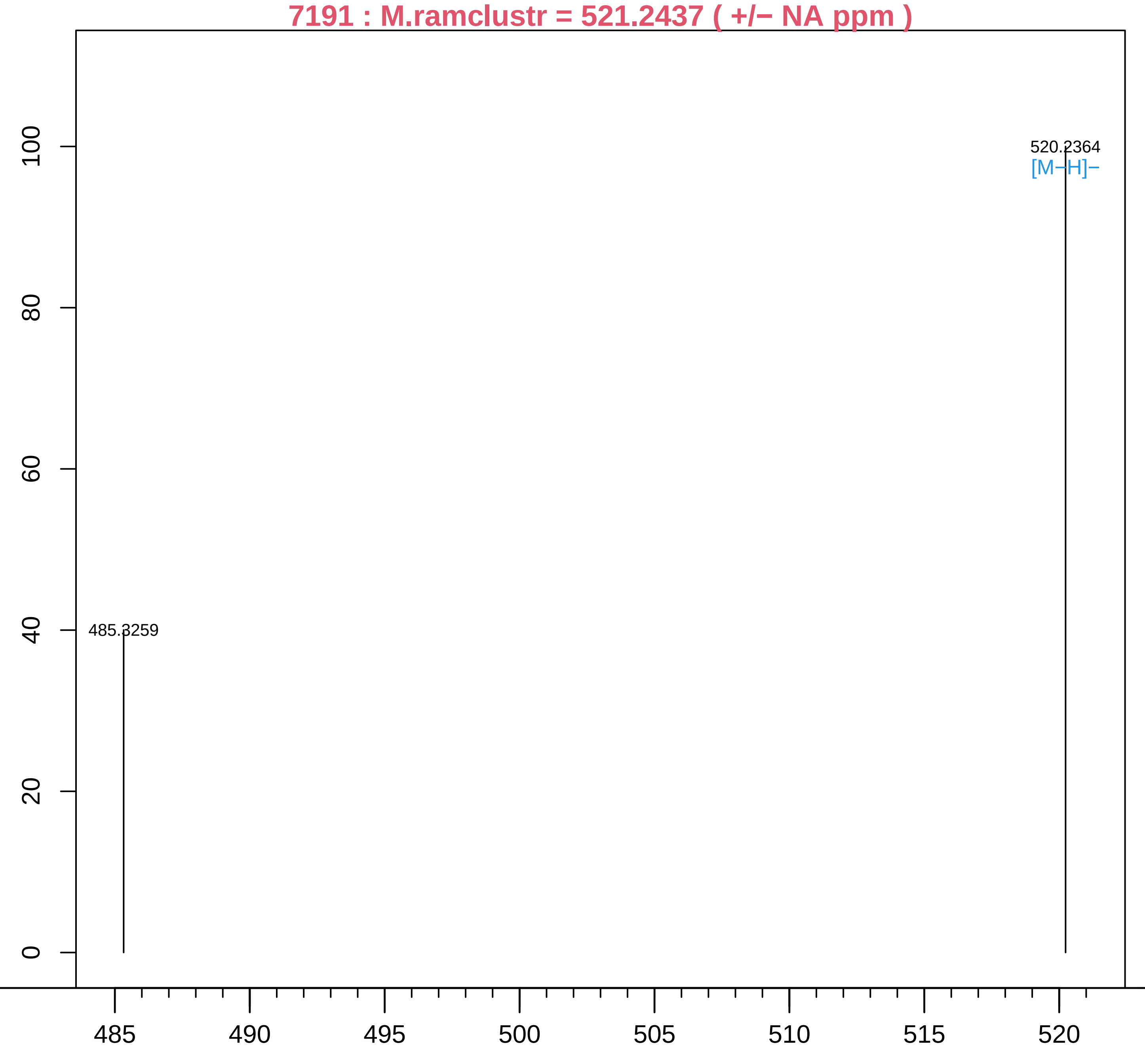
7191 : M.ramclustr = 521.2437 (+/− NA ppm)
520.2364
[M−H]−
485.3259
0
20
40
60
80
100
485
490
495
500
505
510
515
520
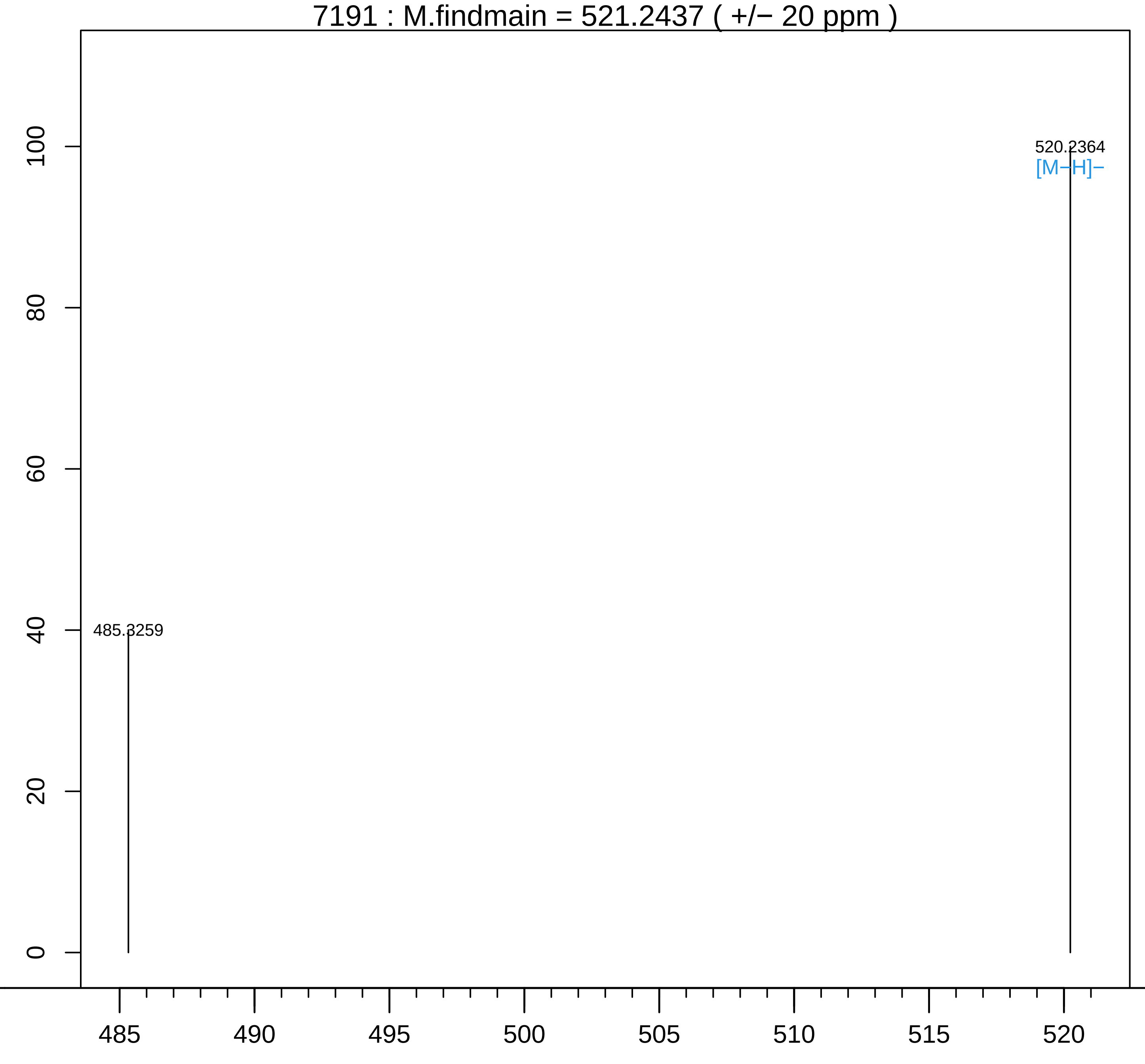
7191 : M.findmain = 521.2437 (+/− 20 ppm)
520.2364
[M−H]−
485.3259
0
20
40
60
80
100
485
490
495
500
505
510
515
520

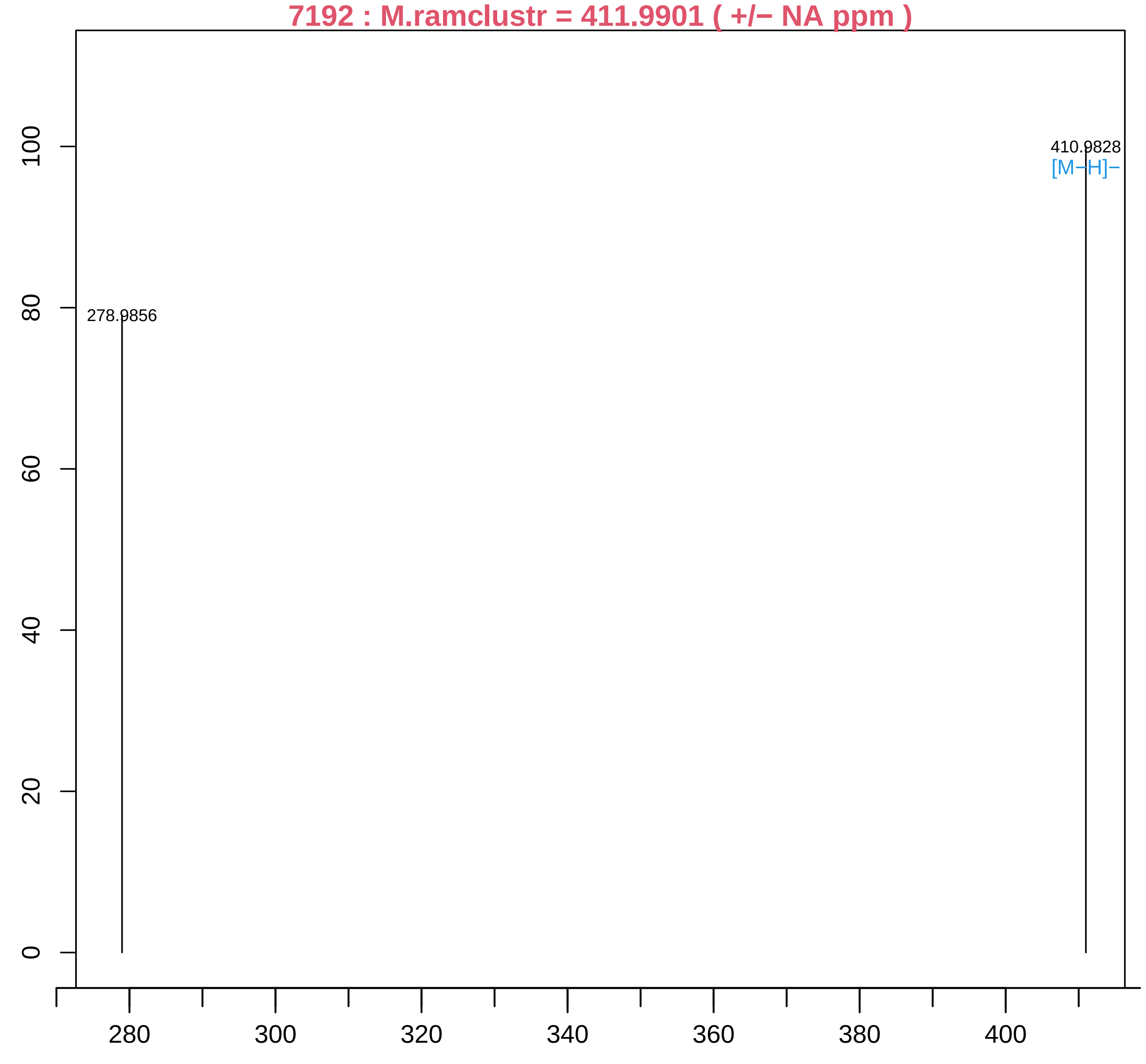
7192 : M.ramclustr = 411.9901 (+/− NA ppm)
410.9828
[M−H]−
278.9856
0
20
40
60
80
100
280
300
320
340
360
380
400

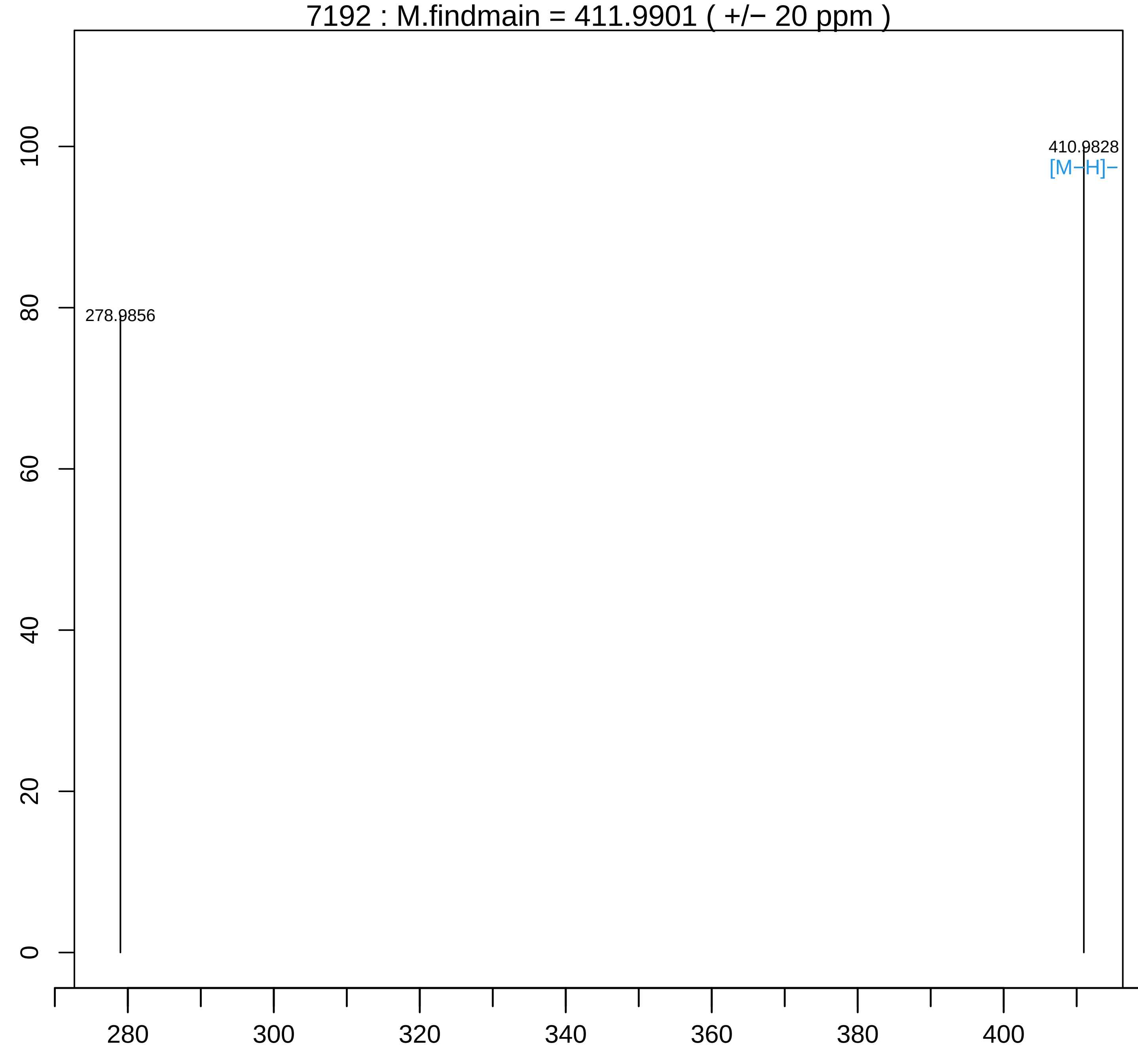
7192 : M.findmain = 411.9901 (+/− 20 ppm)
410.9828
[M−H]−
278.9856
0
20
40
60
80
100
280
300
320
340
360
380
400

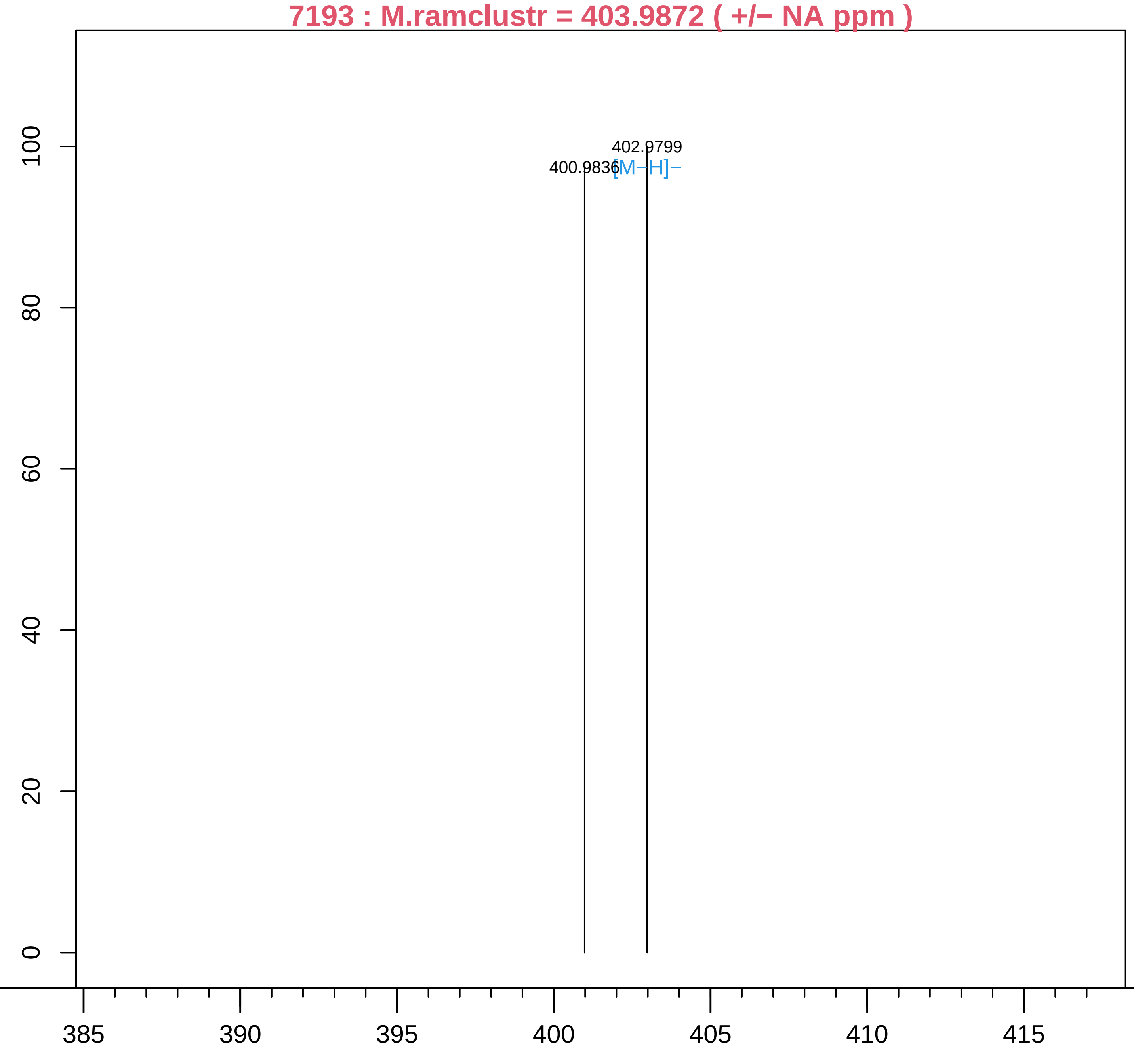

7193 : M.ramclustr = 403.9872 (+/− NA ppm)
402.9799
400.9836
[M−H]−
0
20
40
60
80
100
385
390
395
400
405
410
415

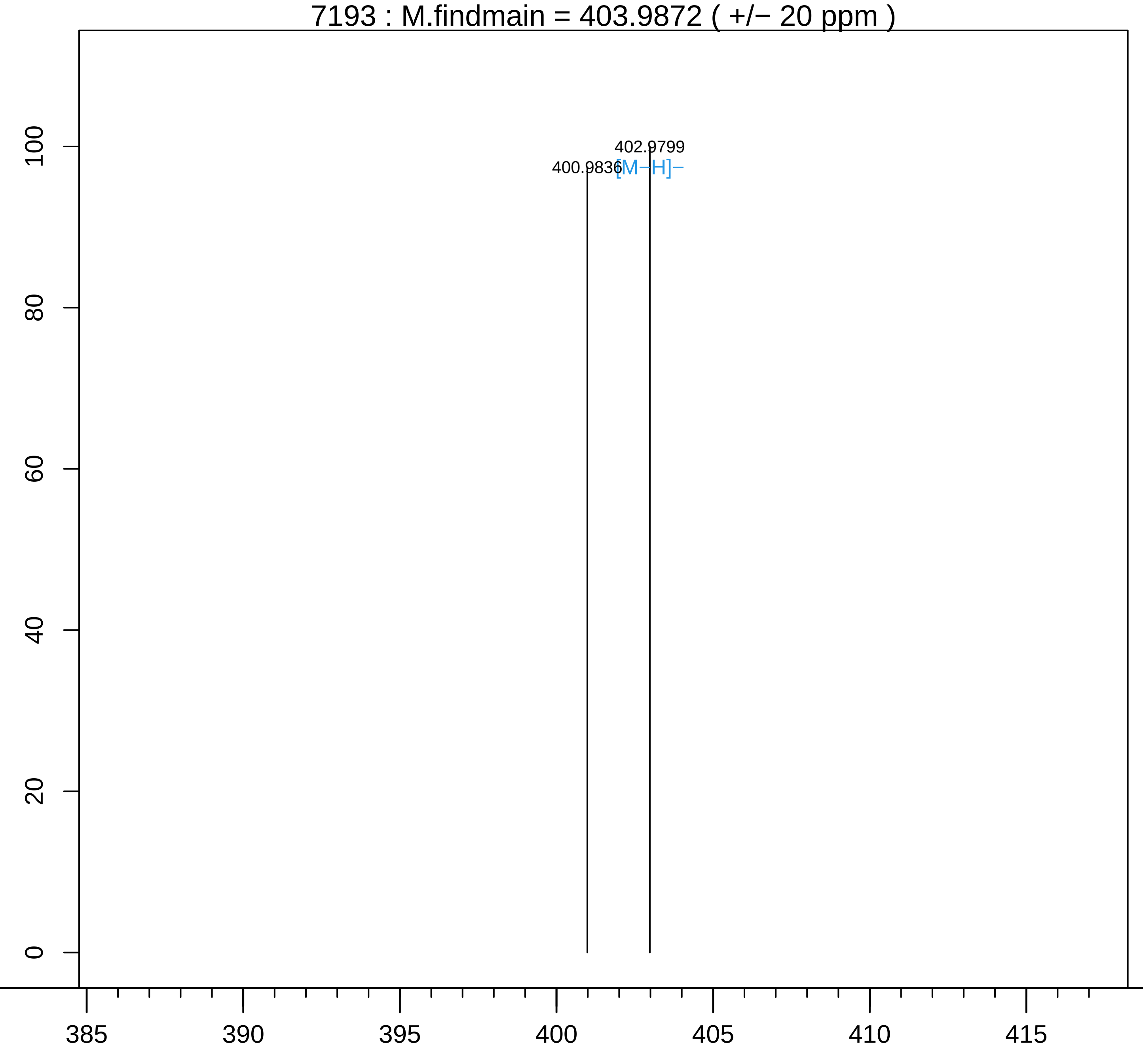

7193 : M.findmain = 403.9872 (+/− 20 ppm)
402.9799
400.9836
[M−H]−
0
20
40
60
80
100
385
390
395
400
405
410
415

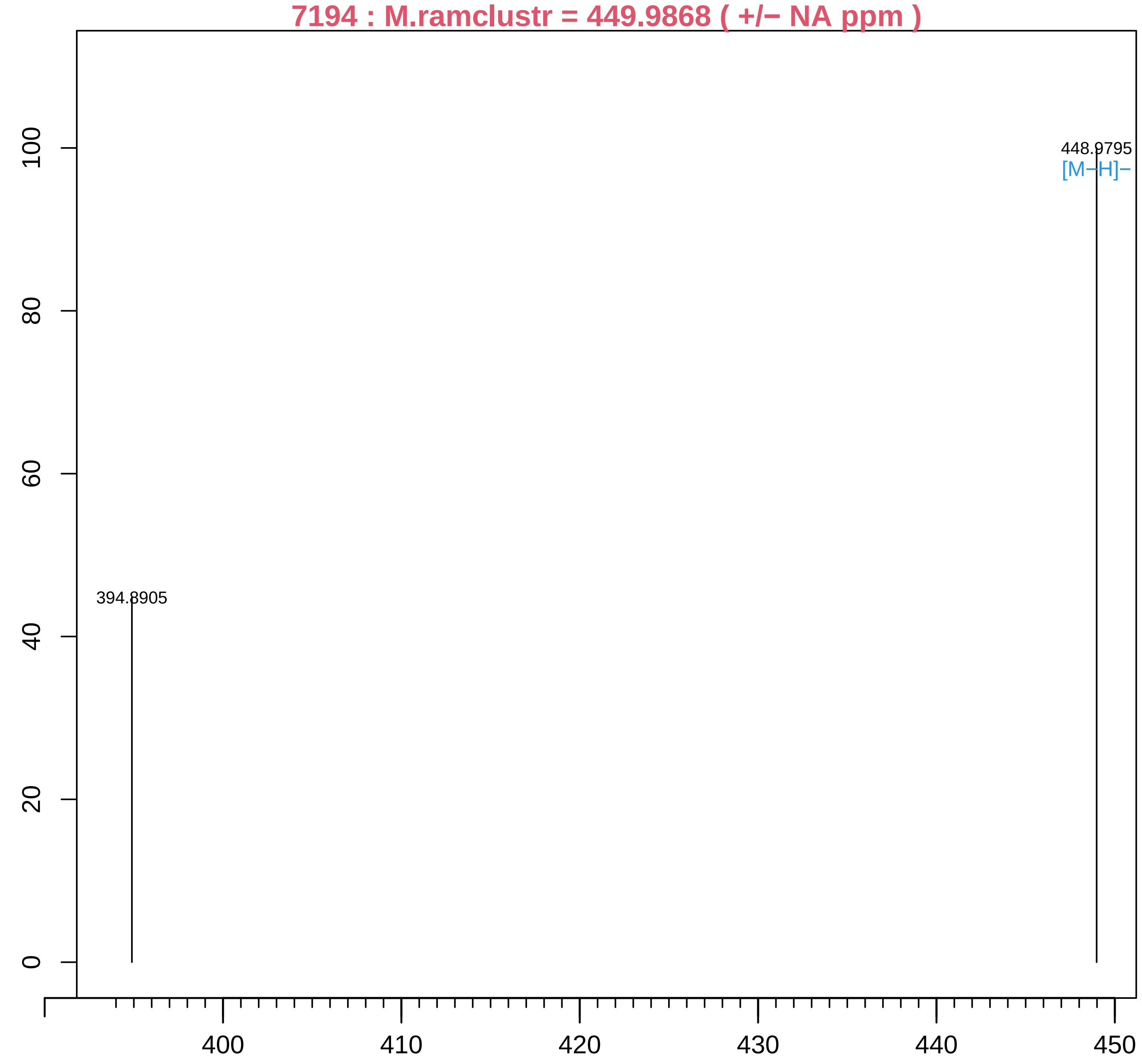
7194 : M.ramclustr = 449.9868 (+/− NA ppm)
448.9795
[M−H]−
394.8905
0
20
40
60
80
100
400
410
420
430
440
450

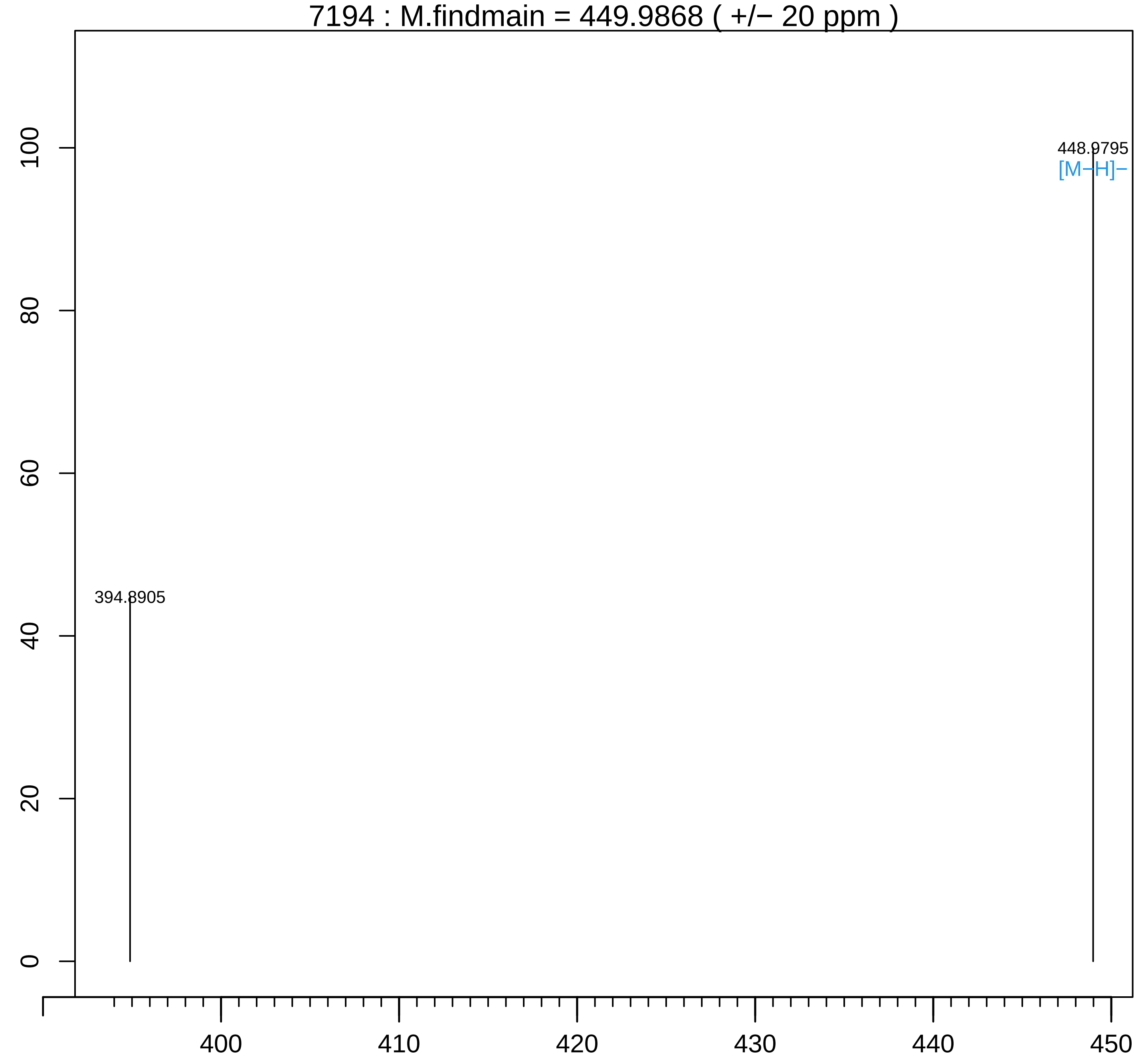
7194 : M.findmain = 449.9868 (+/− 20 ppm)
448.9795
[M−H]−
394.8905
0
20
40
60
80
100
400
410
420
430
440
450

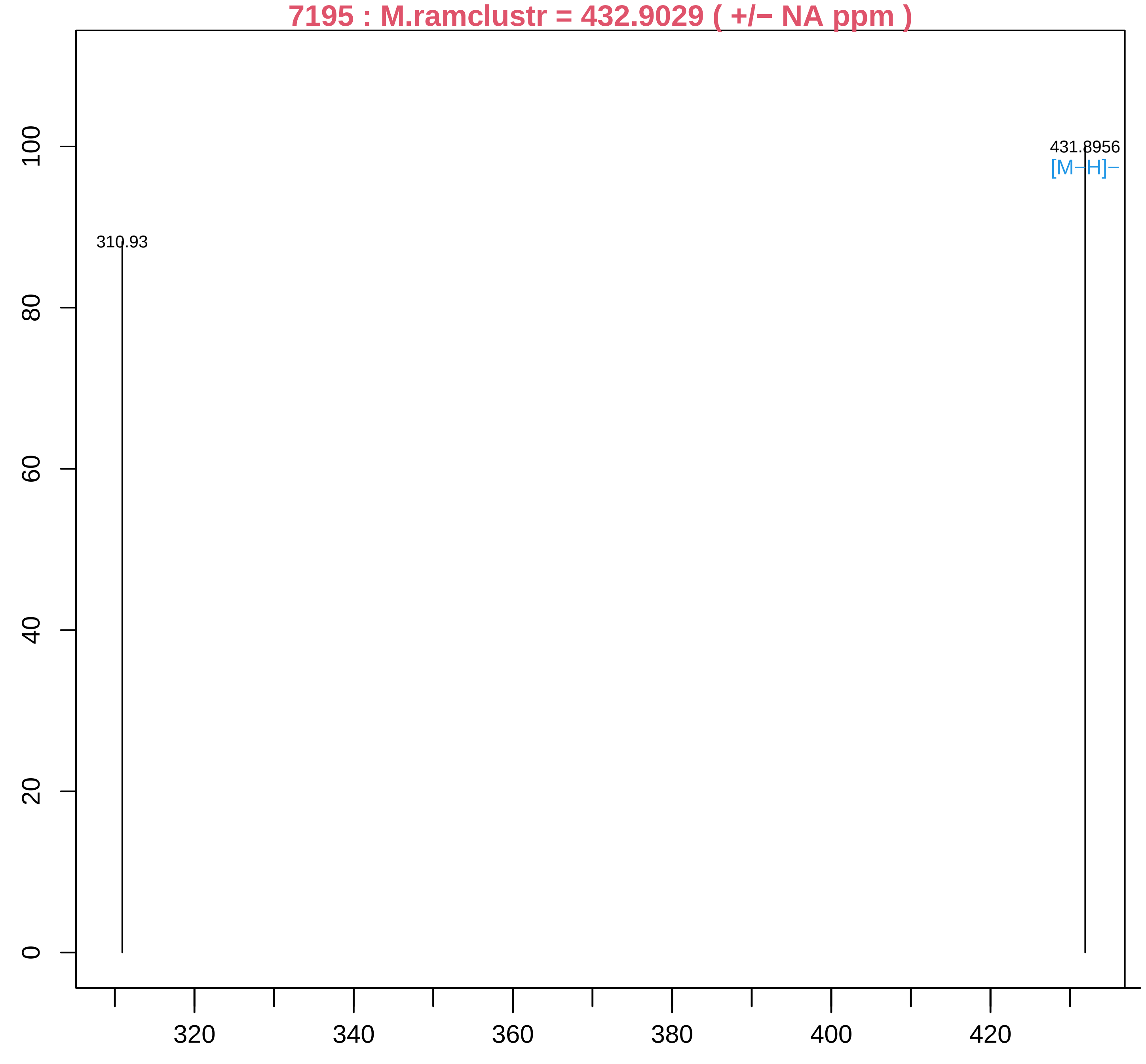
7195 : M.ramclustr = 432.9029 (+/− NA ppm)
431.8956
[M−H]−
310.93
0
20
40
60
80
100
320
340
360
380
400
420

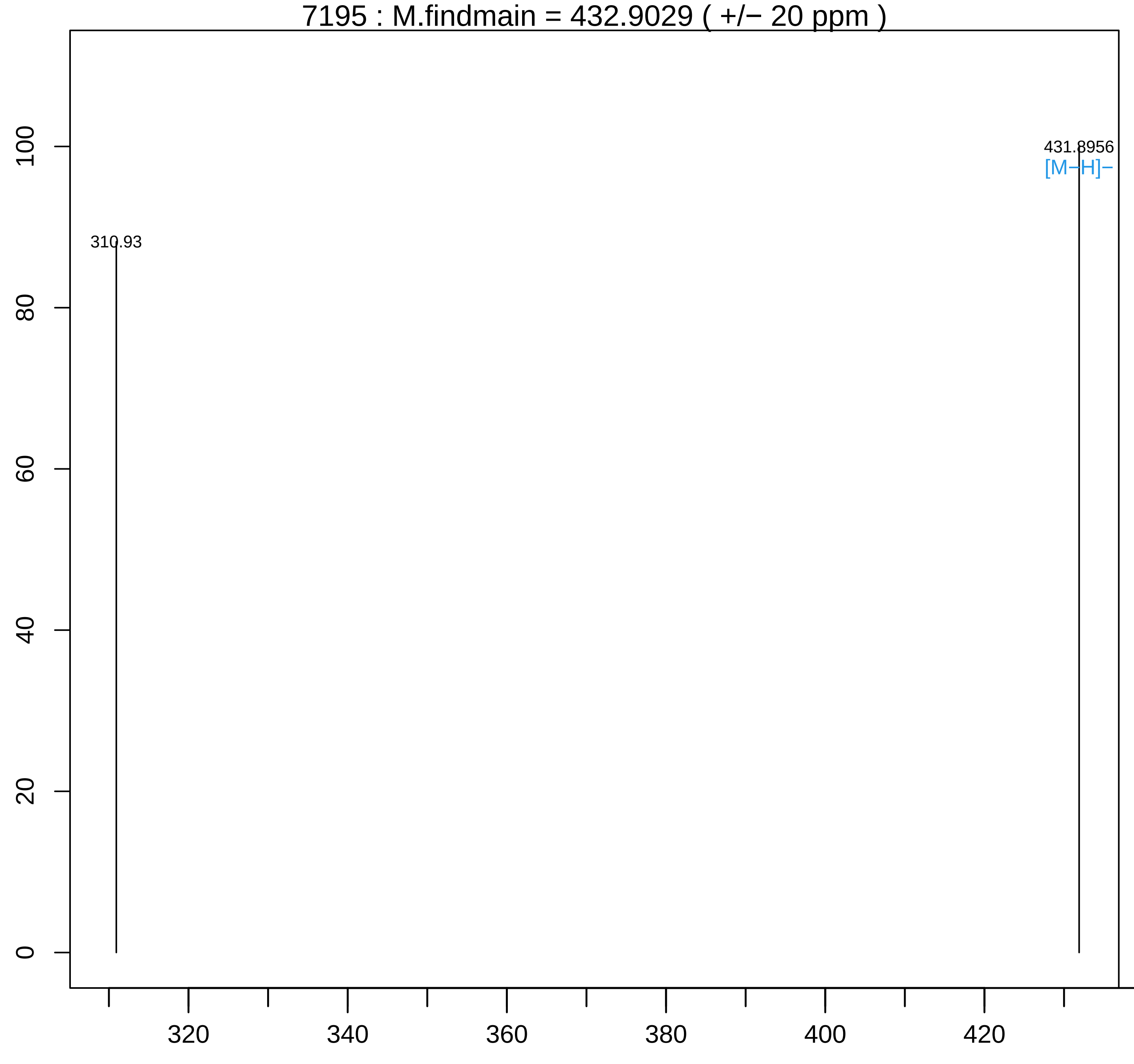
7195 : M.findmain = 432.9029 (+/− 20 ppm)
431.8956
[M−H]−
310.93
0
20
40
60
80
100
320
340
360
380
400
420

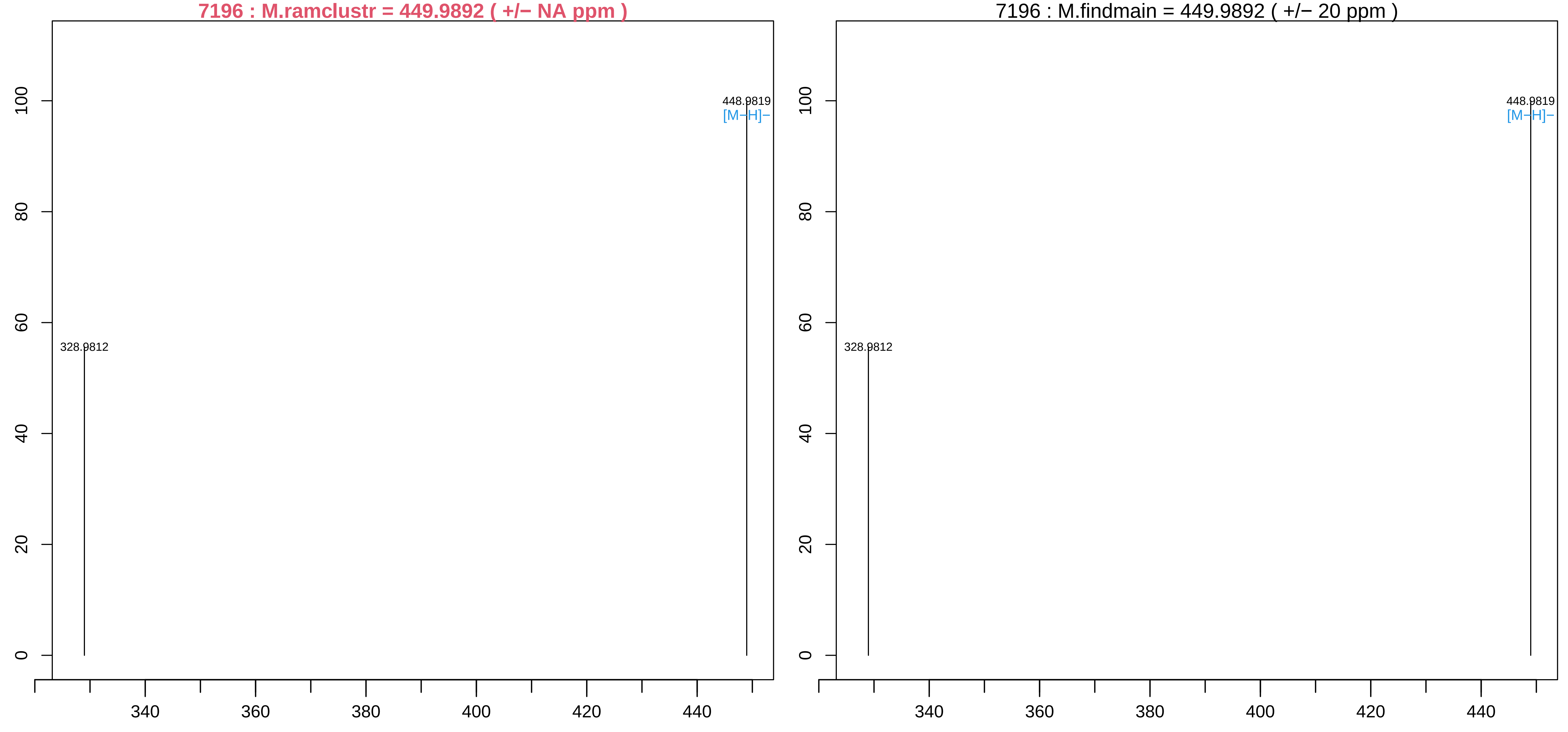

7196 : M.ramclustr = 449.9892 (+/− NA ppm)
448.9819
[M−H]−
328.9812
7196 : M.findmain = 449.9892 (+/− 20 ppm)
448.9819
[M−H]−
328.9812
0
20
40
60
80
100
340
360
380
400
420
440

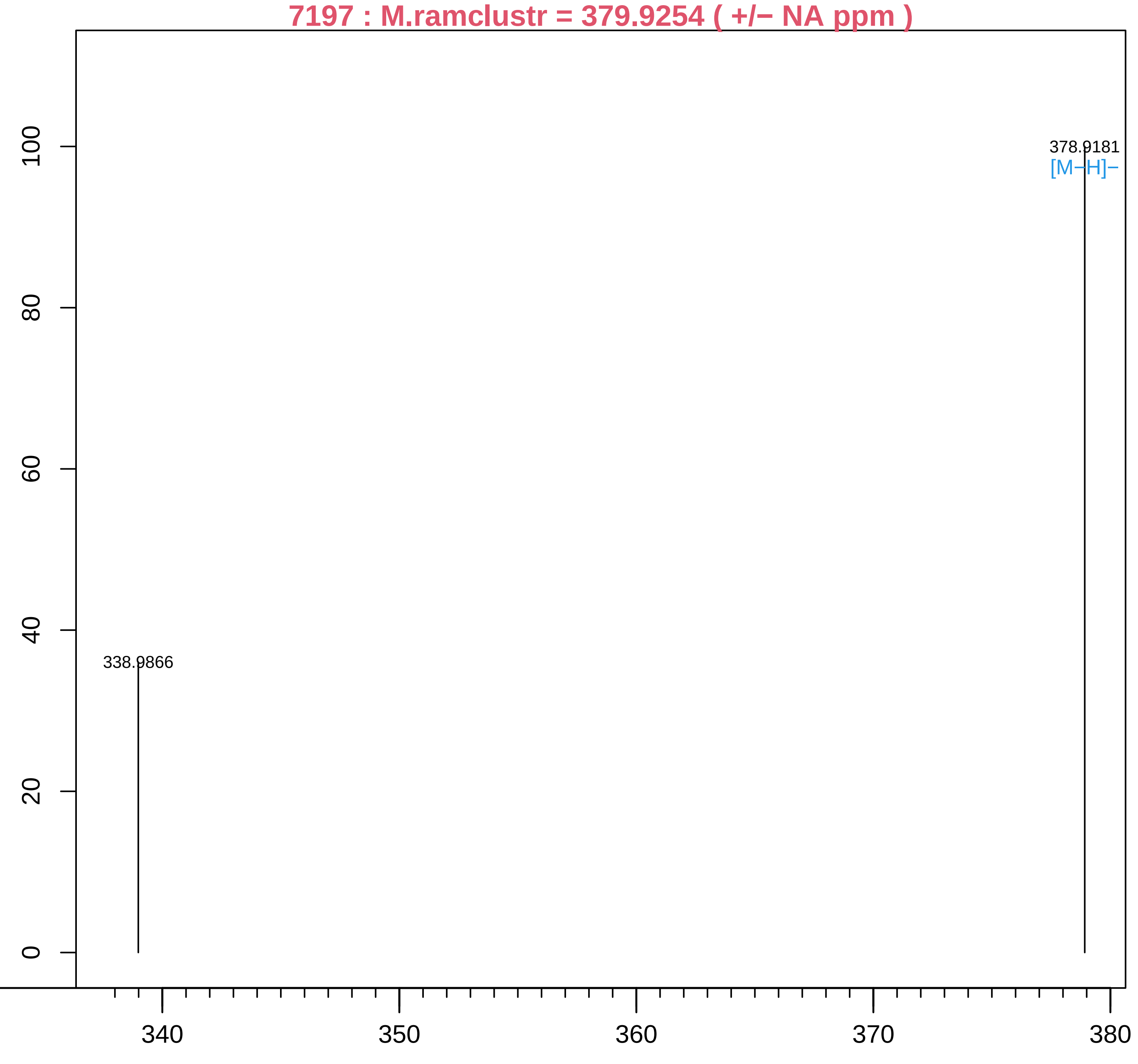
7197 : M.ramclustr = 379.9254 (+/− NA ppm)
378.9181
[M−H]−
338.9866
0
20
40
60
80
100
340
350
360
370
380

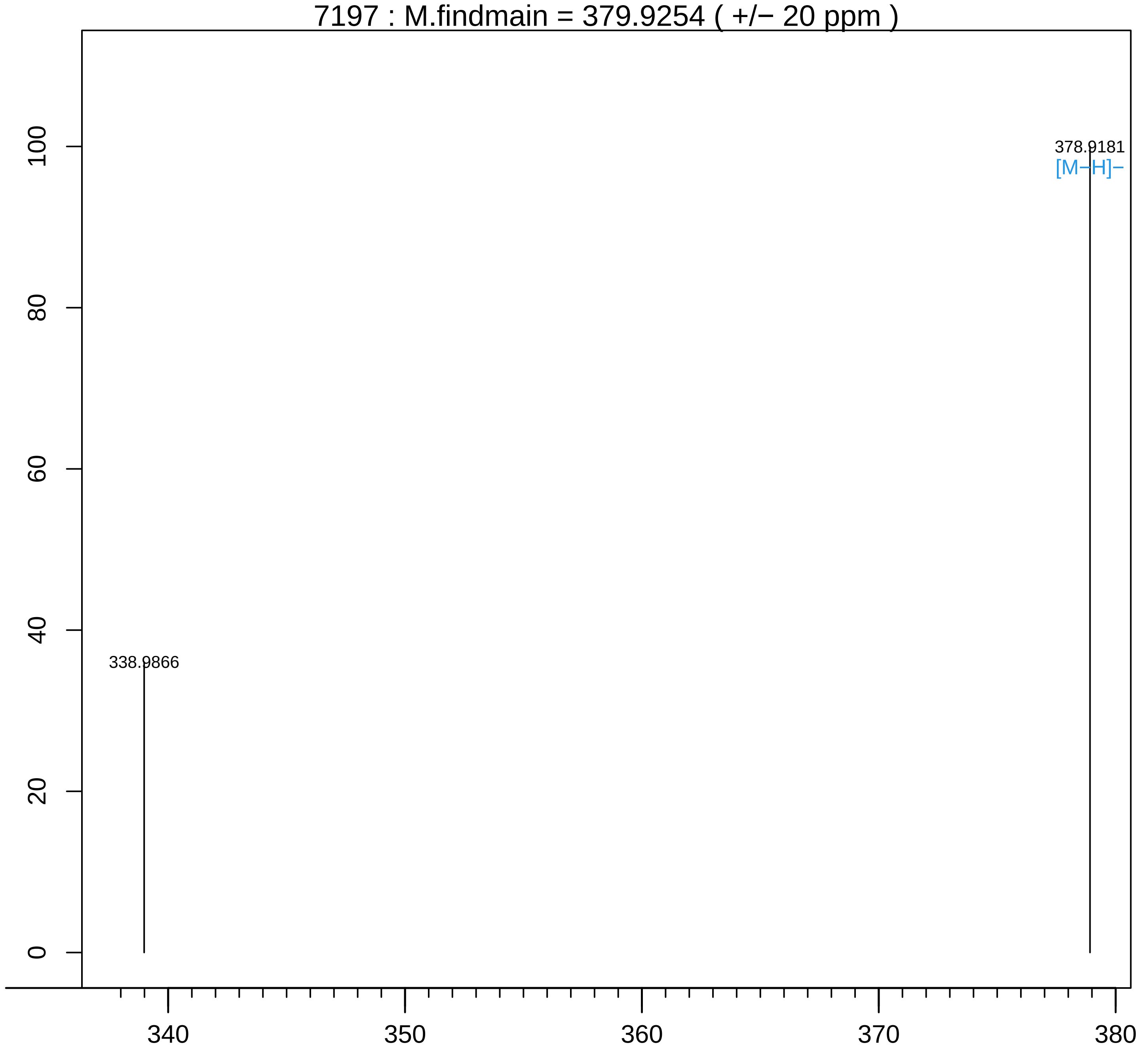
7197 : M.findmain = 379.9254 (+/− 20 ppm)
378.9181
[M−H]−
338.9866
0
20
40
60
80
100
340
350
360
370
380

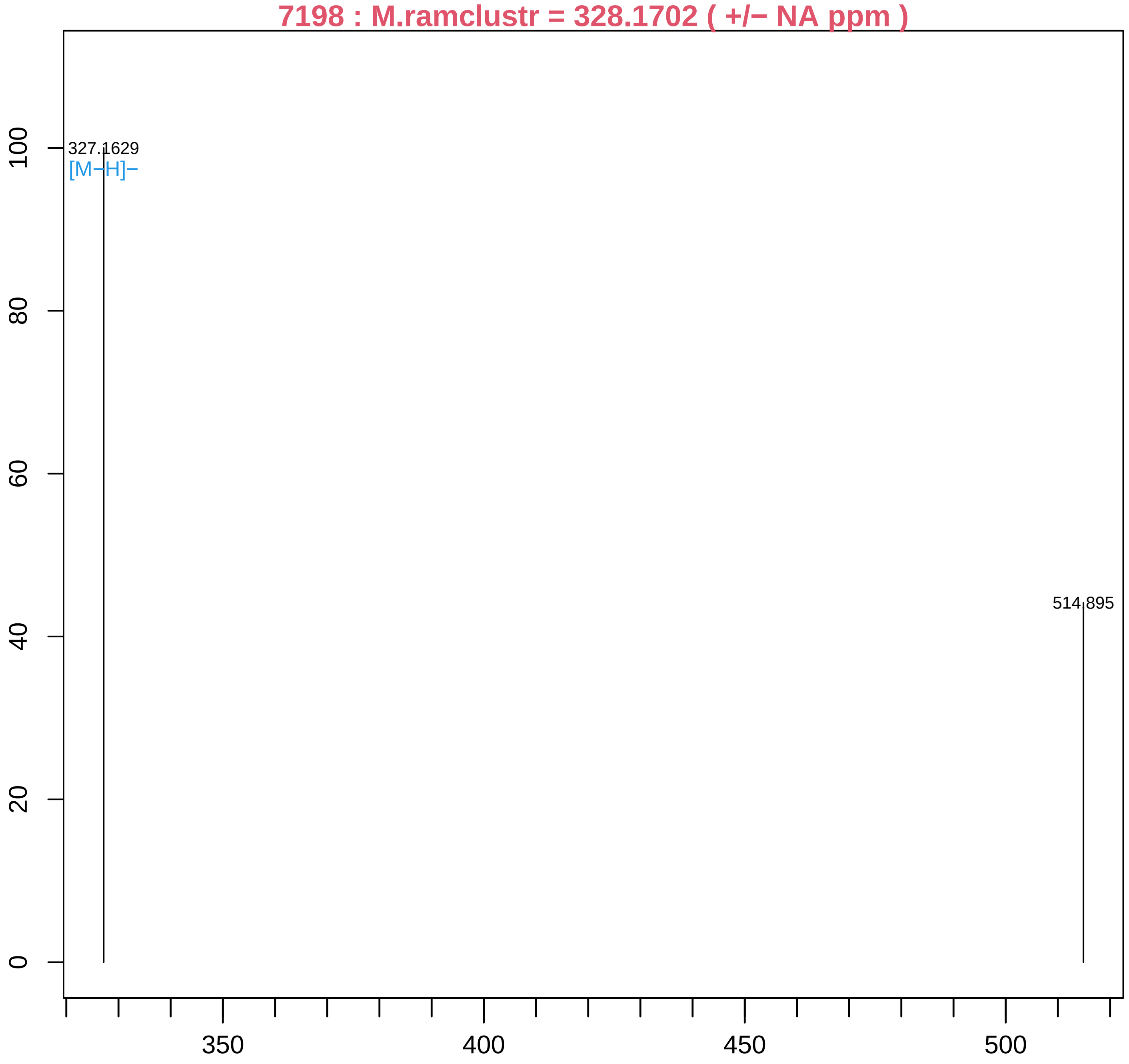
7198 : M.ramclustr = 328.1702 (+/− NA ppm)
327.1629
[M−H]−
514.895
0
20
40
60
80
100
350
400
450
500

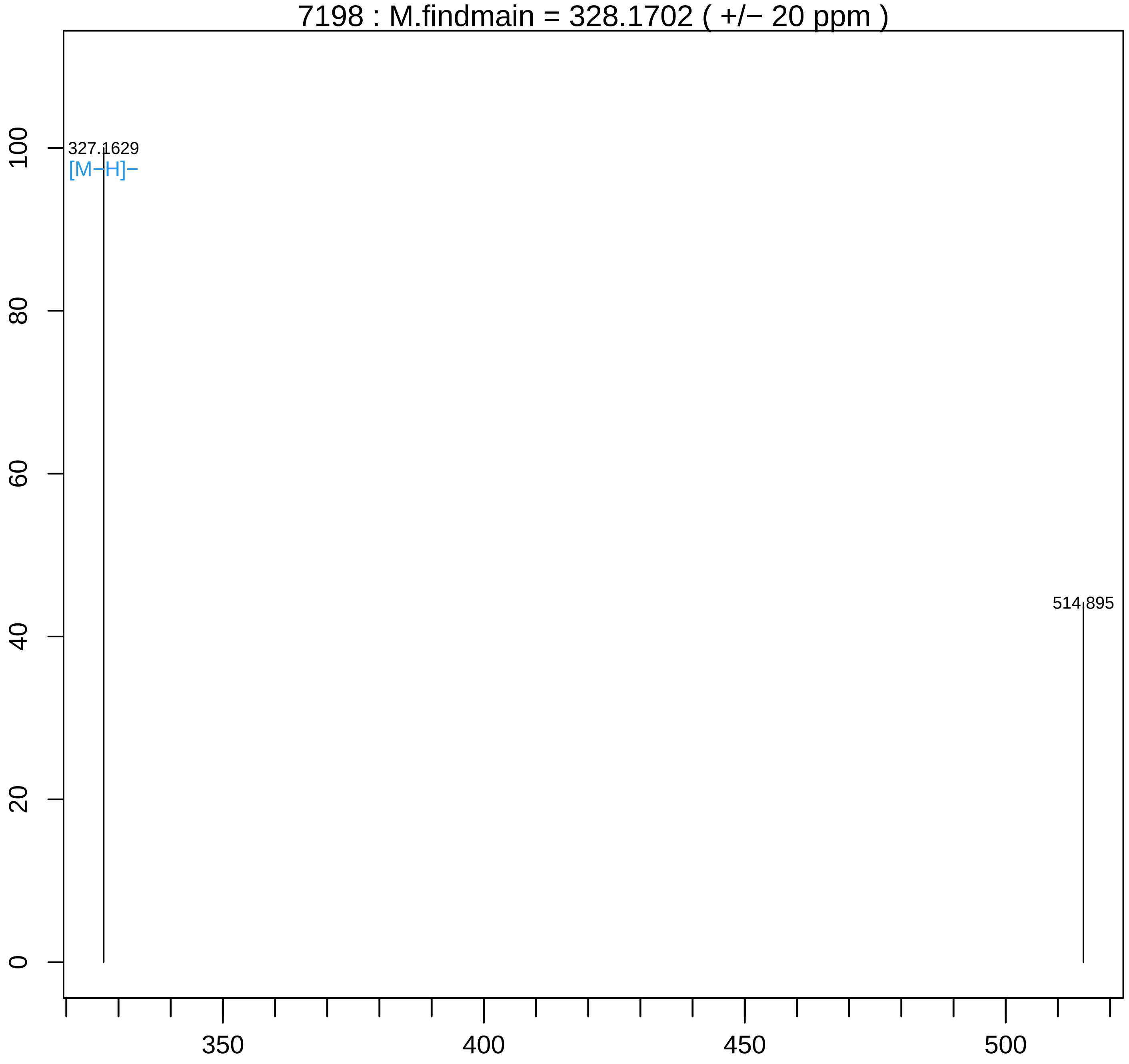
7198 : M.findmain = 328.1702 (+/− 20 ppm)
327.1629
[M−H]−
514.895
0
20
40
60
80
100
350
400
450
500

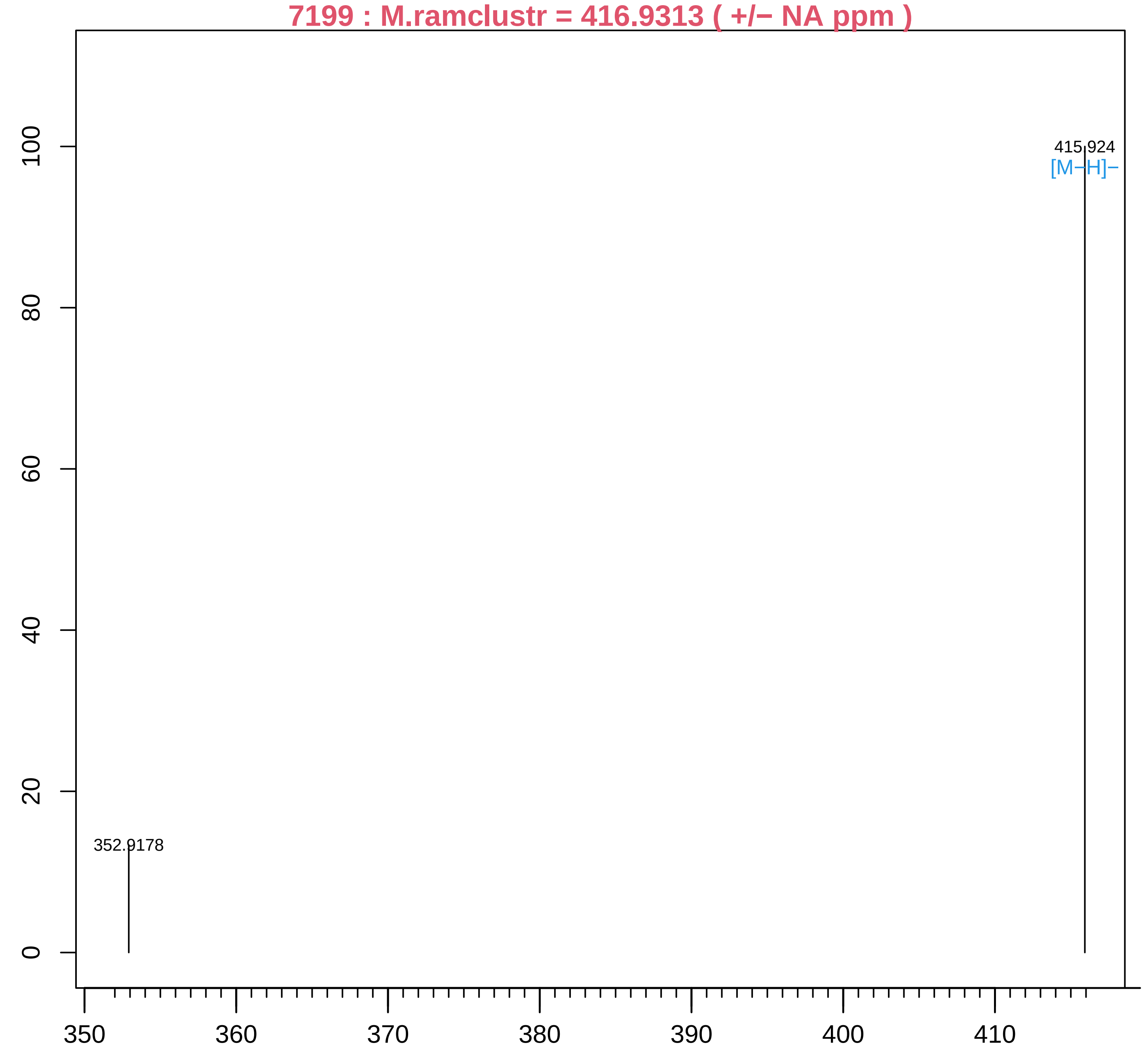
7199 : M.ramclustr = 416.9313 (+/− NA ppm)
415.924
[M−H]−
352.9178
0
20
40
60
80
100
350
360
370
380
390
400
410

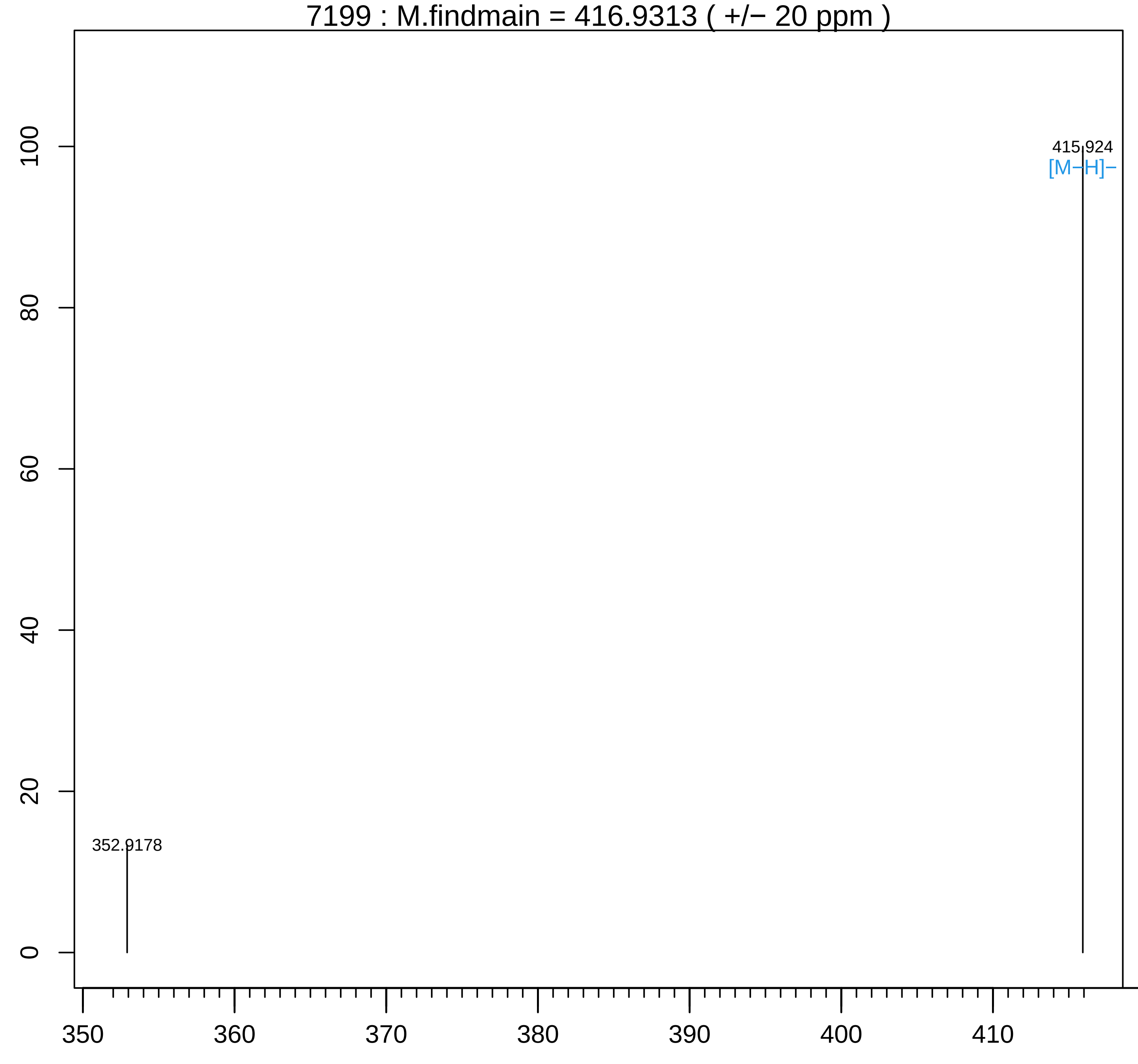
7199 : M.findmain = 416.9313 (+/− 20 ppm)
415.924
[M−H]−
352.9178
0
20
40
60
80
100
350
360
370
380
390
400
410

7200 : M.ramclustr = 432.9033 (+/− NA ppm)

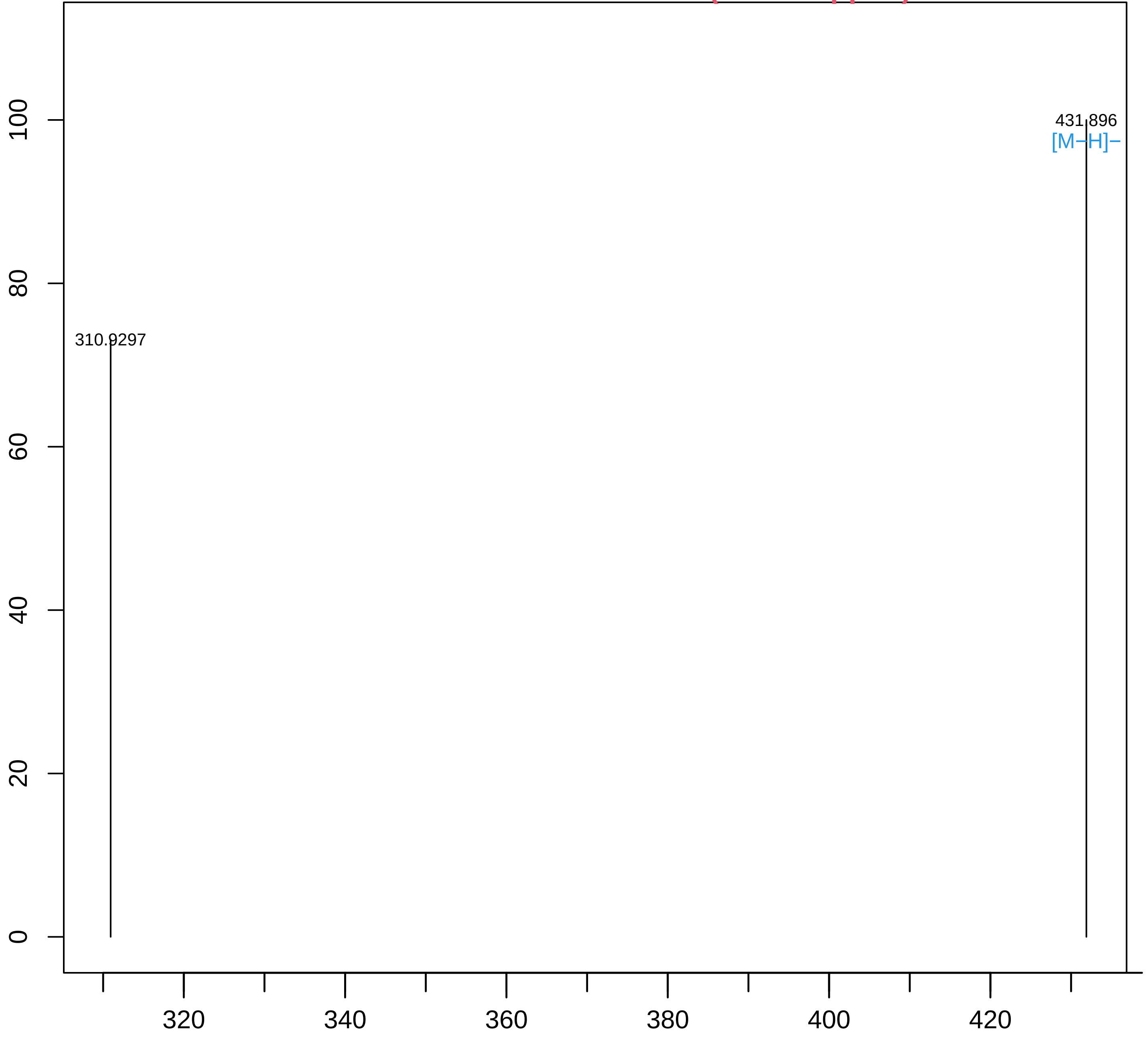

7200 : M.findmain = 432.9033 (+/− 20 ppm)

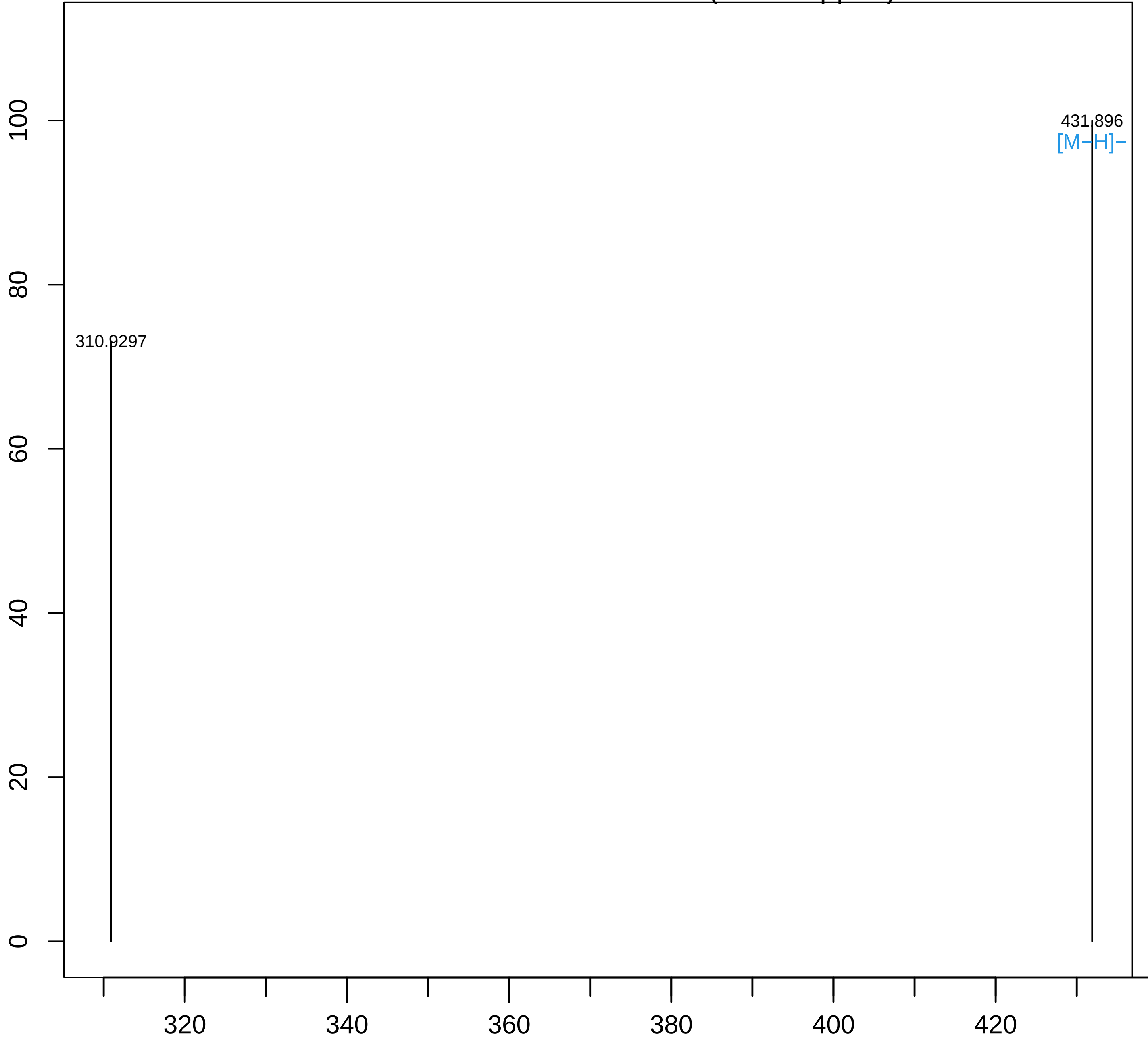

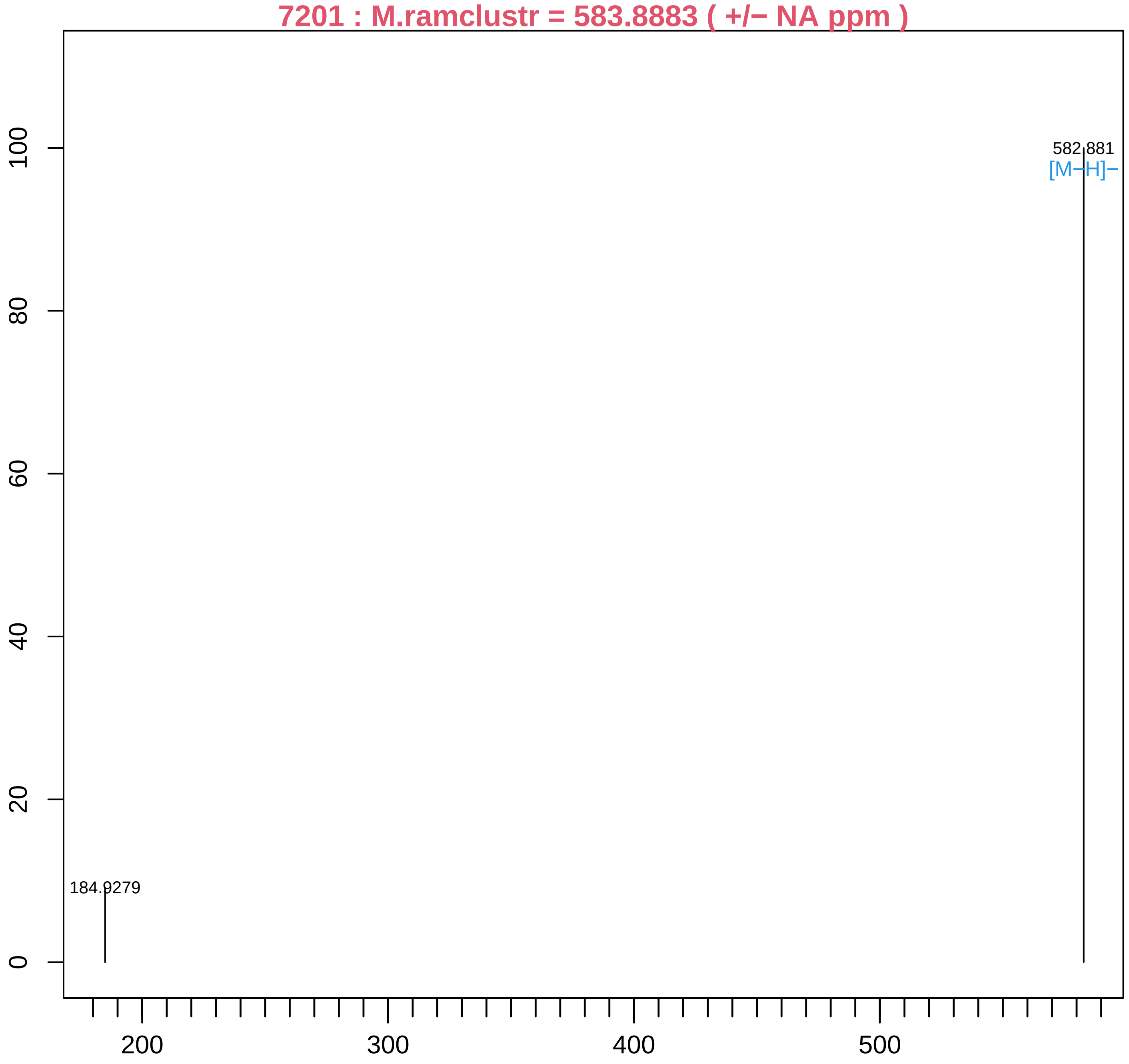
7201 : M.ramclustr = 583.8883 (+/− NA ppm)
582.881
[M−H]−
184.9279
0
20
40
60
80
100
200
300
400
500

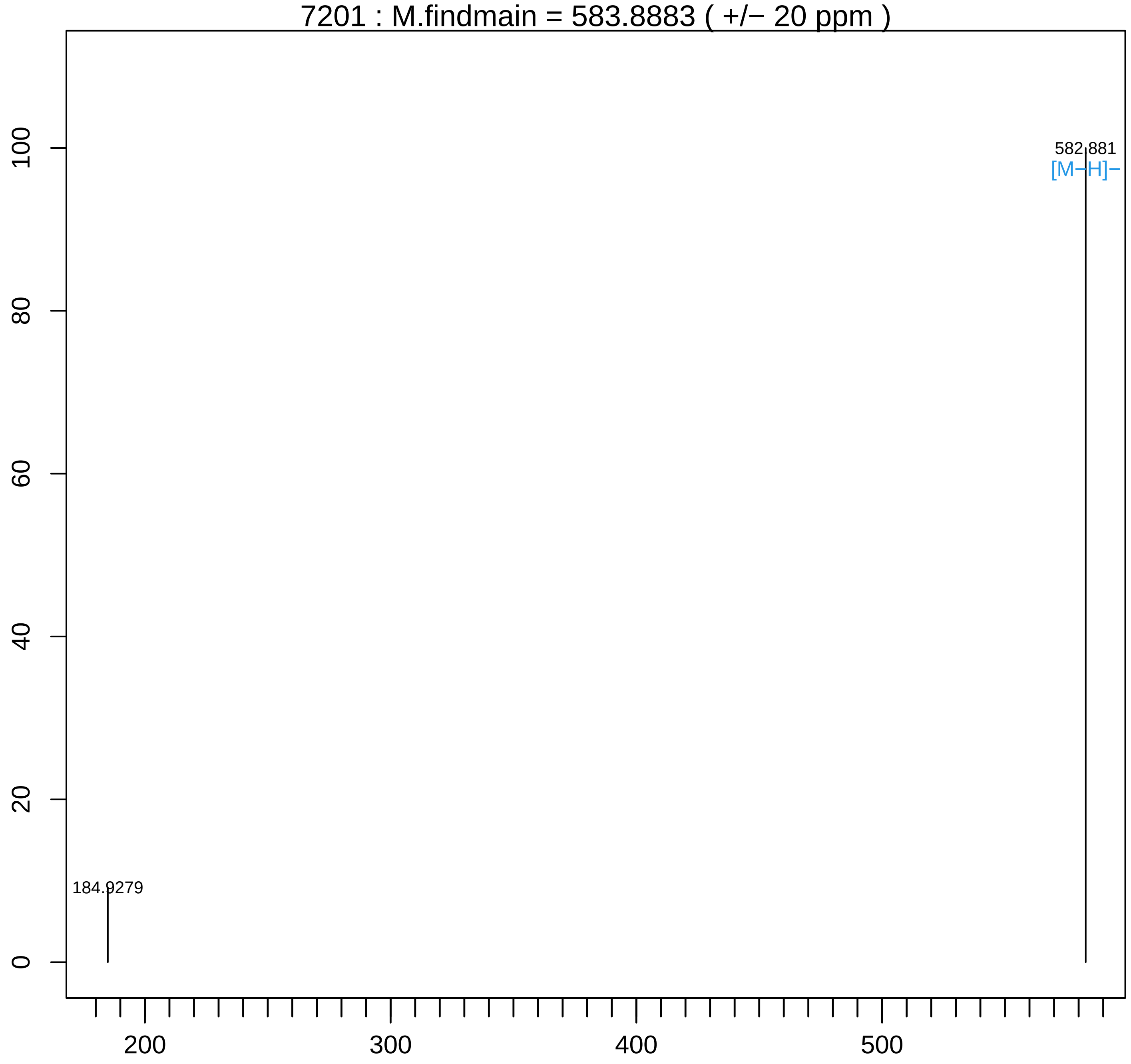
7201 : M.findmain = 583.8883 (+/− 20 ppm)
582.881
[M−H]−
184.9279
0
20
40
60
80
100
200
300
400
500

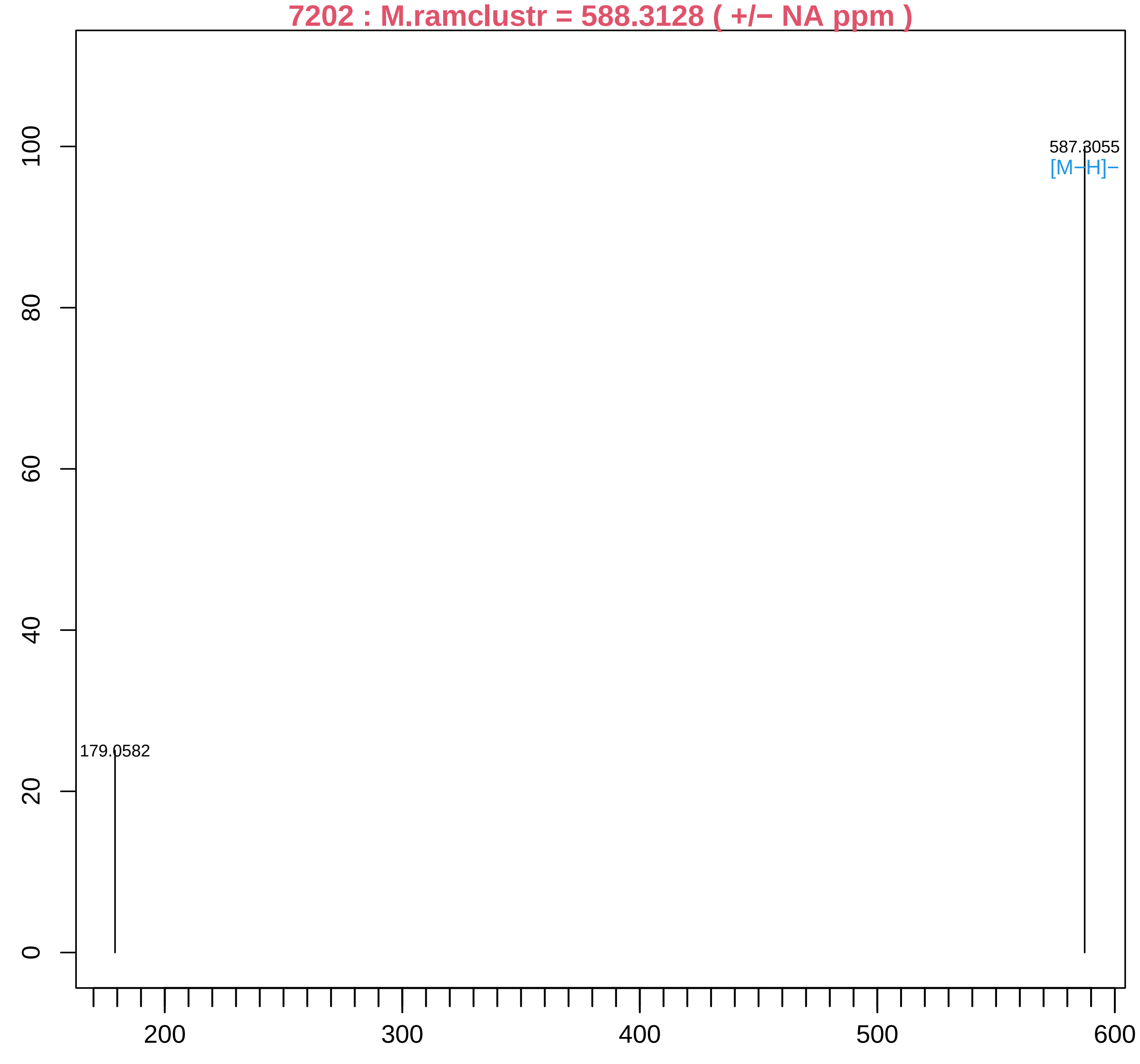
7202 : M.ramclustr = 588.3128 (+/− NA ppm)
587.3055
[M−H]−
179.0582
0
20
40
60
80
100
200
300
400
500
600

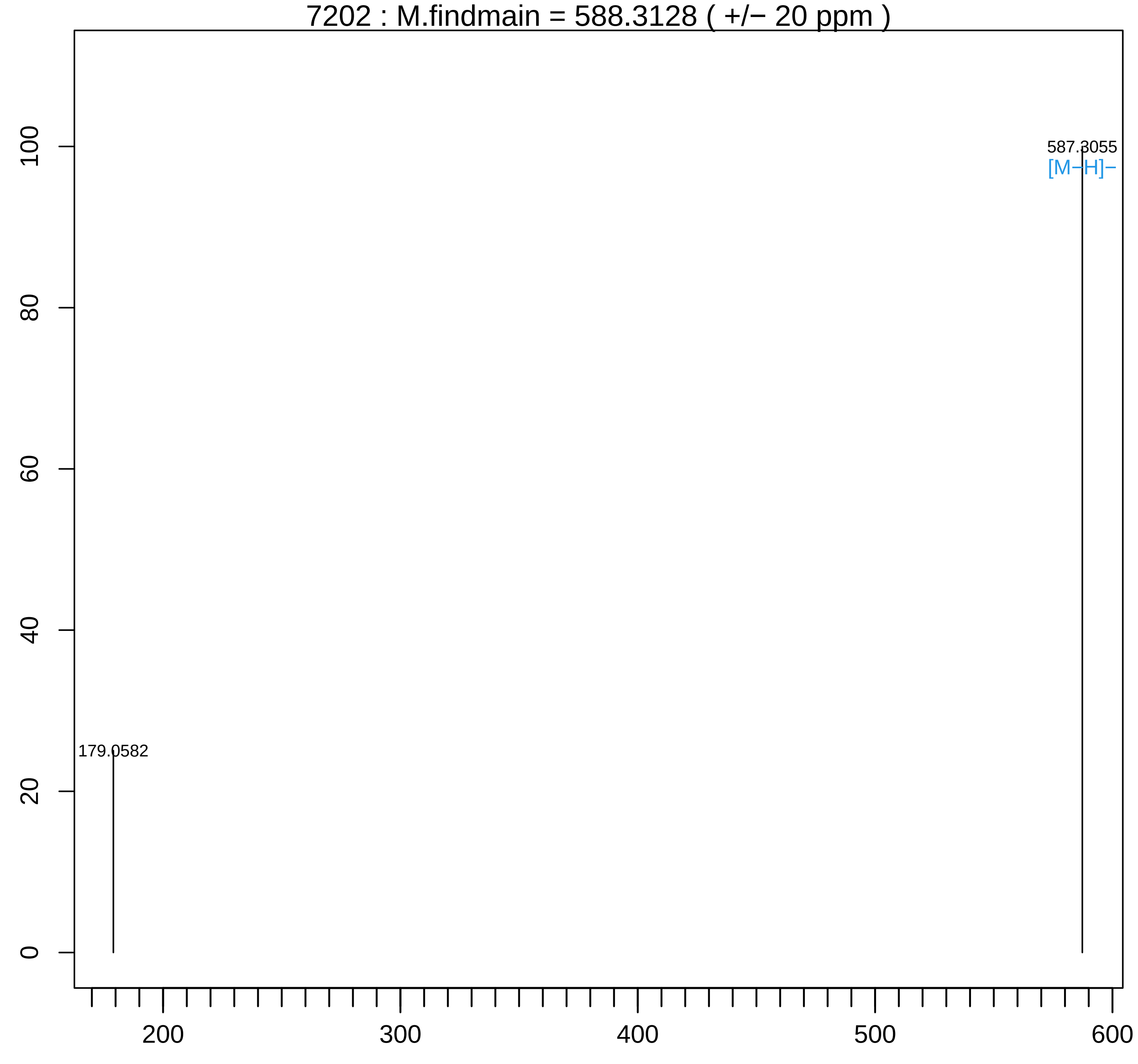
7202 : M.findmain = 588.3128 (+/− 20 ppm)
587.3055
[M−H]−
179.0582
0
20
40
60
80
100
200
300
400
500
600

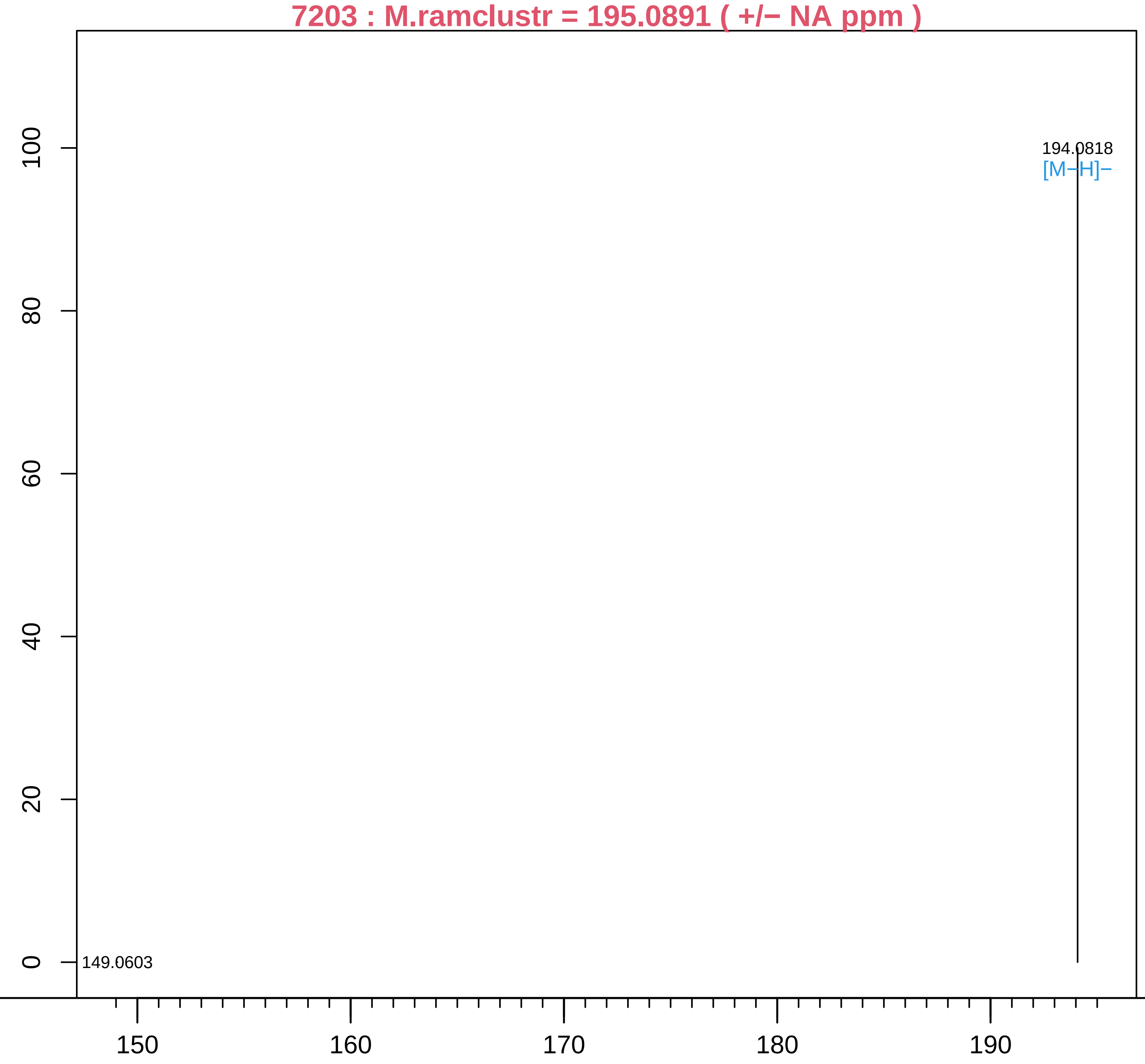
7203 : M.ramclustr = 195.0891 (+/− NA ppm)
194.0818
[M−H]−
149.0603
0
20
40
60
80
100
150
160
170
180
190

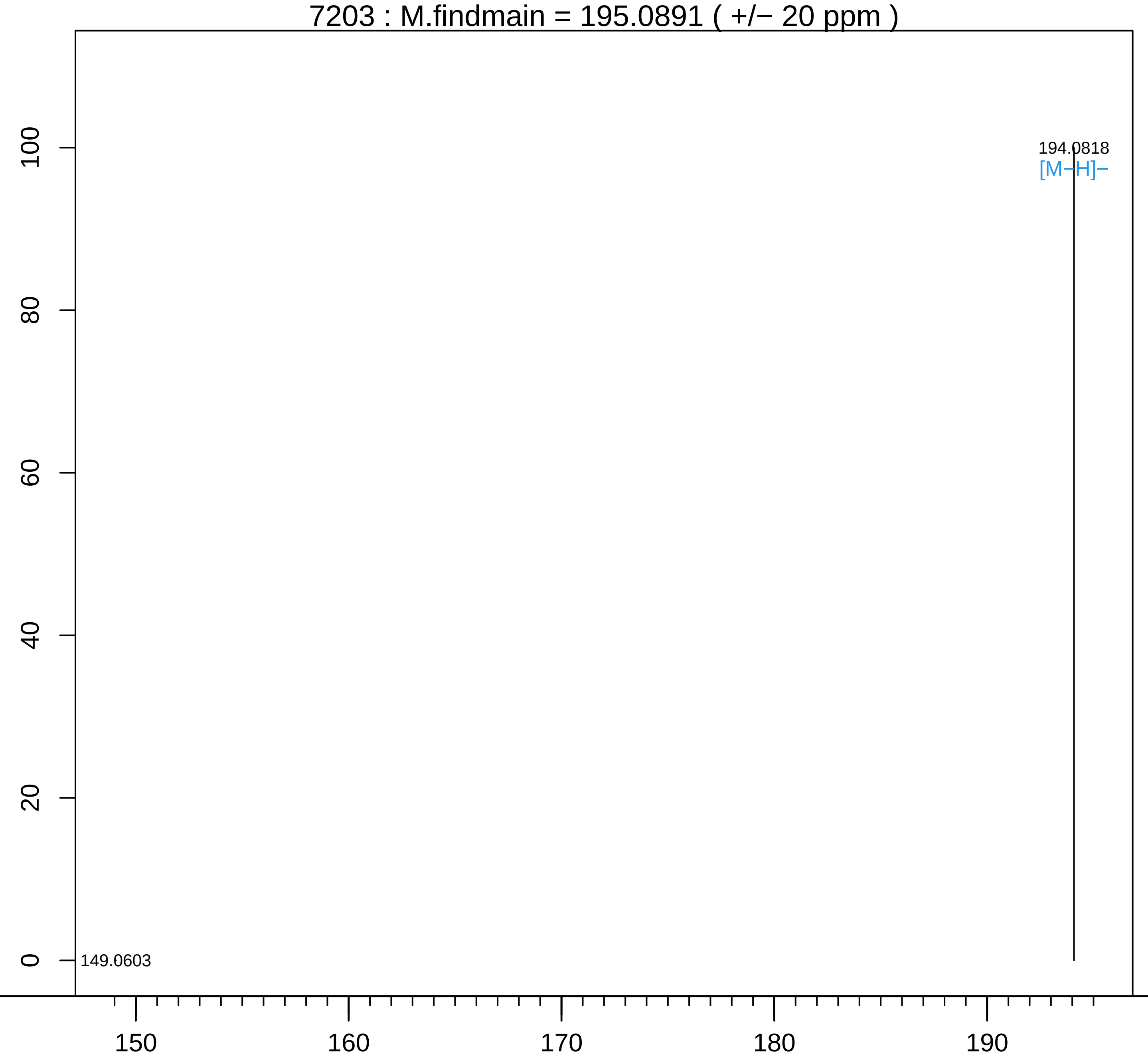
7203 : M.findmain = 195.0891 (+/− 20 ppm)
194.0818
[M−H]−
149.0603
0
20
40
60
80
100
150
160
170
180
190

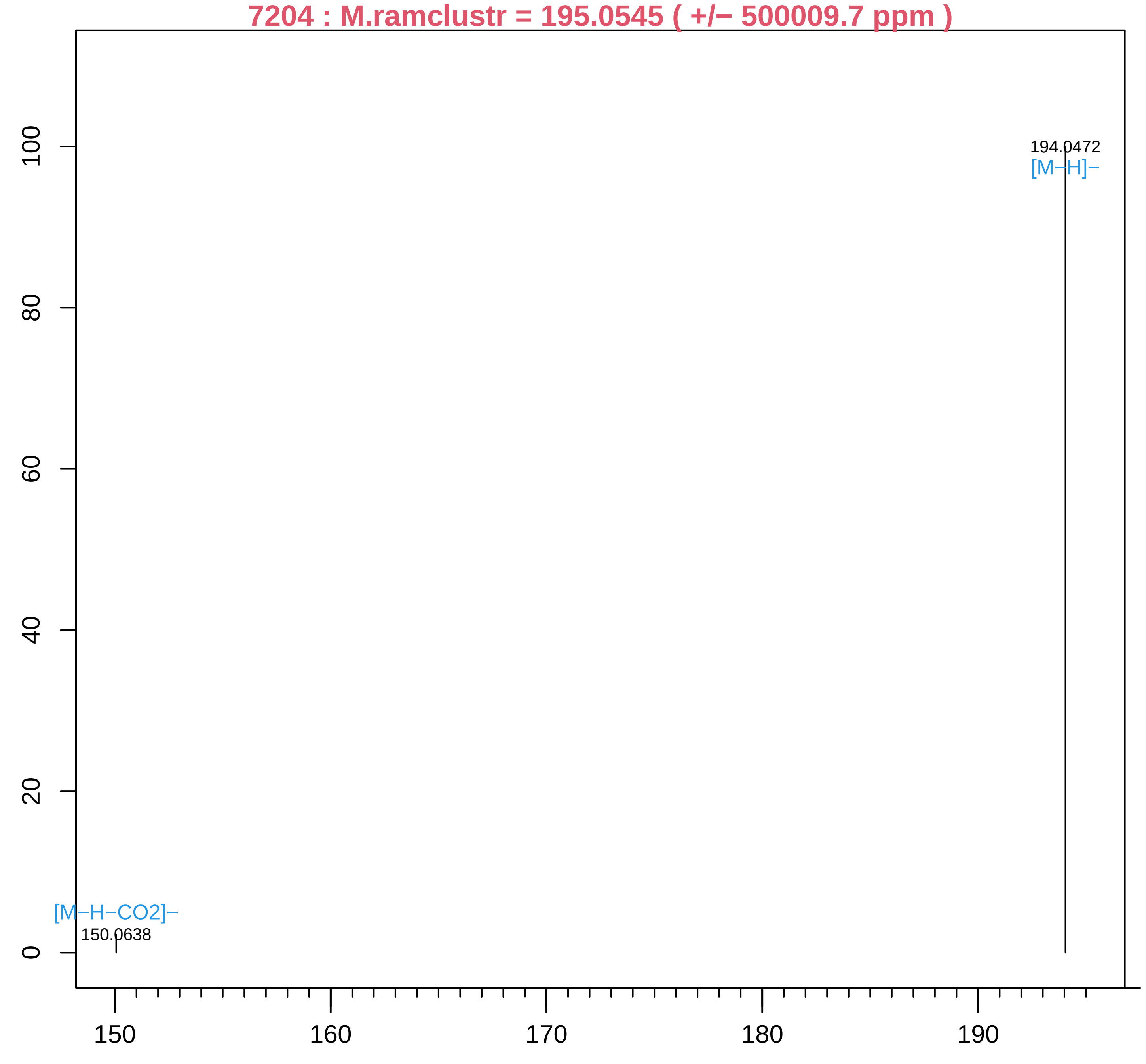

7204 : M.ramclustr = 195.0545 (+/− 500009.7 ppm)
194.0472
[M−H]−
[M−H−CO2]−
150.0638
0
20
40
60
80
100
150
160
170
180
190

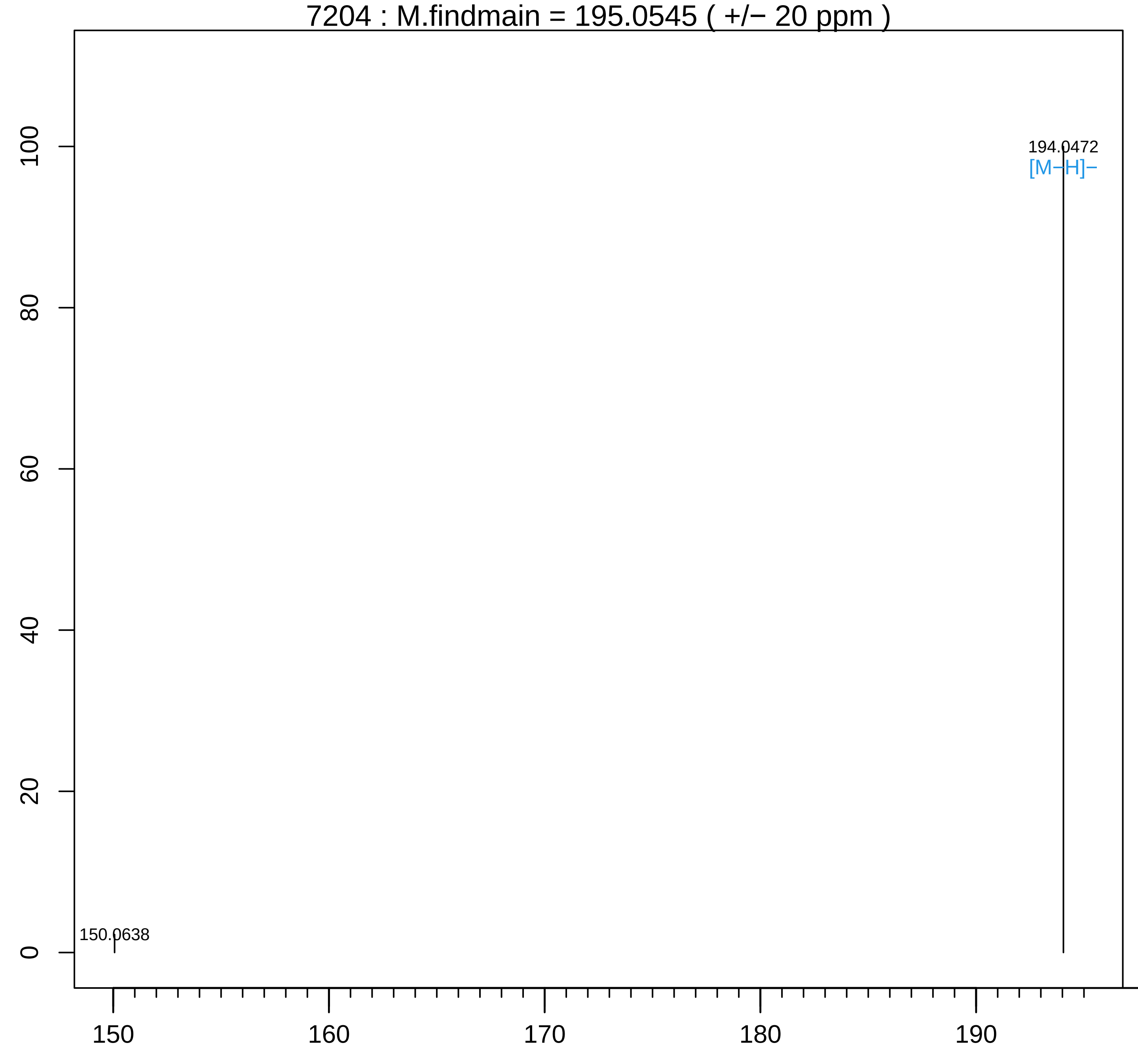

7204 : M.findmain = 195.0545 (+/− 20 ppm)
194.0472
[M−H]−
150.0638
0
20
40
60
80
100
150
160
170
180
190

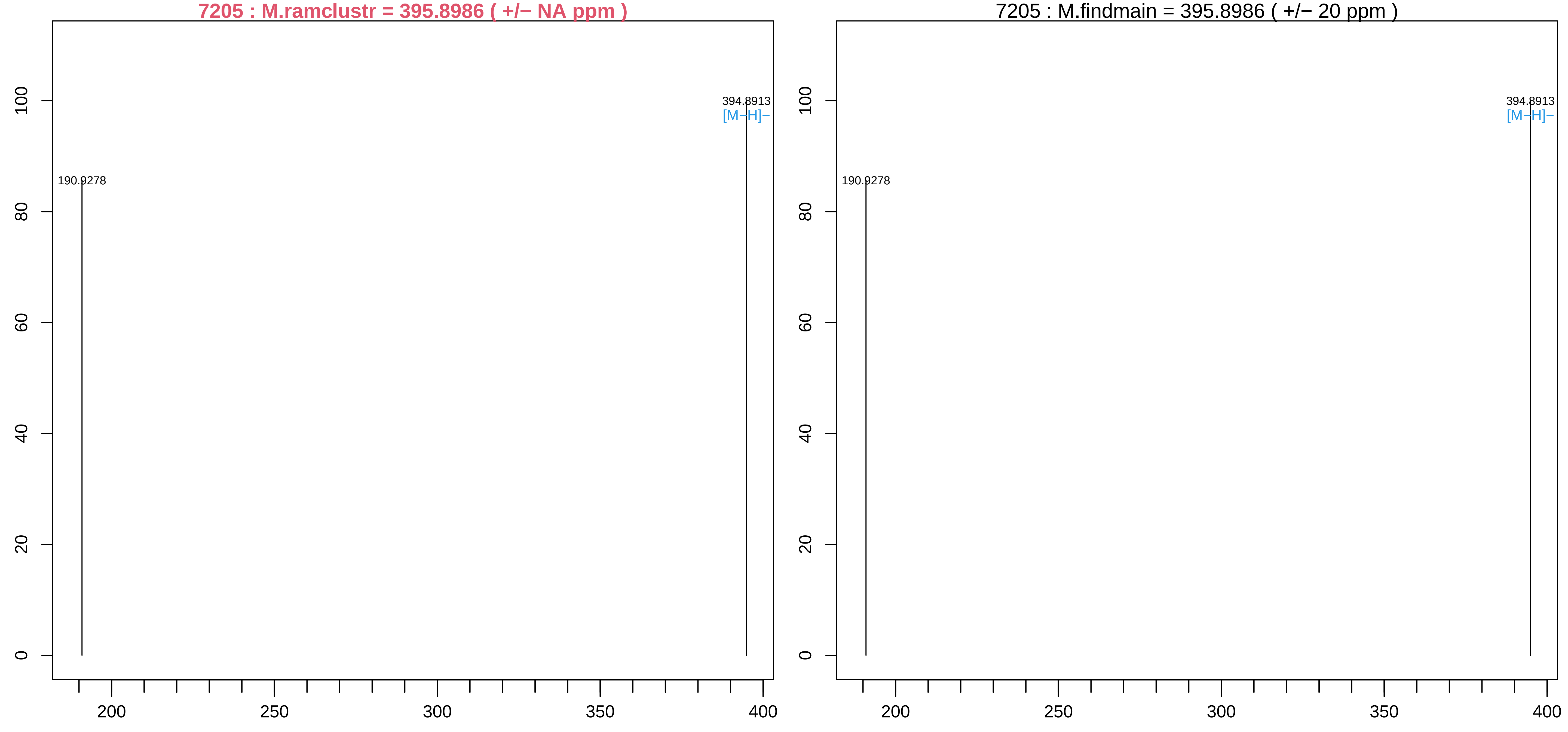
7205 : M.ramclustr = 395.8986 (+/− NA ppm)
394.8913
[M−H]−
190.9278
7205 : M.findmain = 395.8986 (+/− 20 ppm)
394.8913
[M−H]−
190.9278
0
20
40
60
80
100
200
250
300
350
400

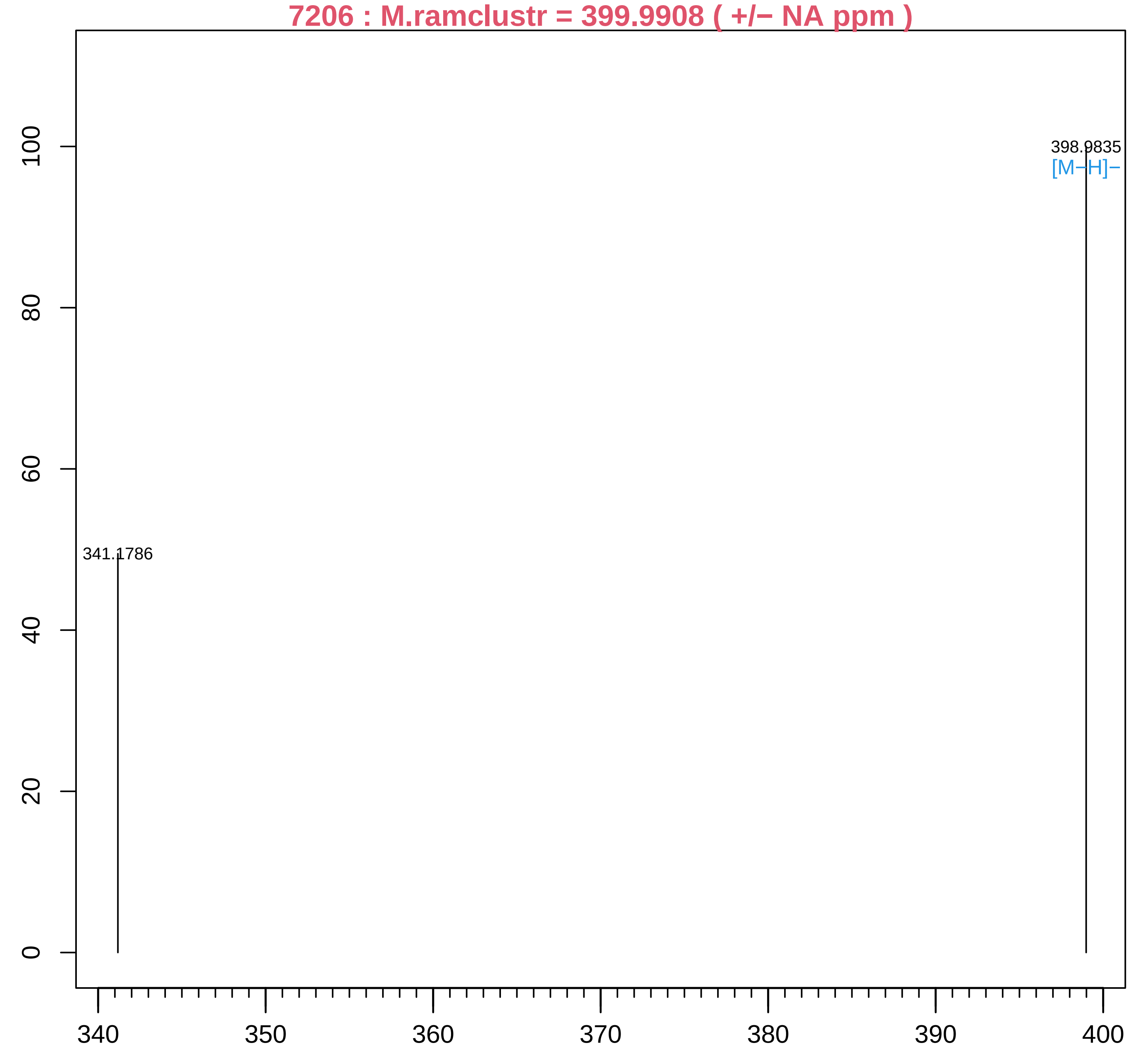
7206 : M.ramclustr = 399.9908 (+/− NA ppm)
398.9835
[M−H]−
341.1786
0
20
40
60
80
100
340
350
360
370
380
390
400

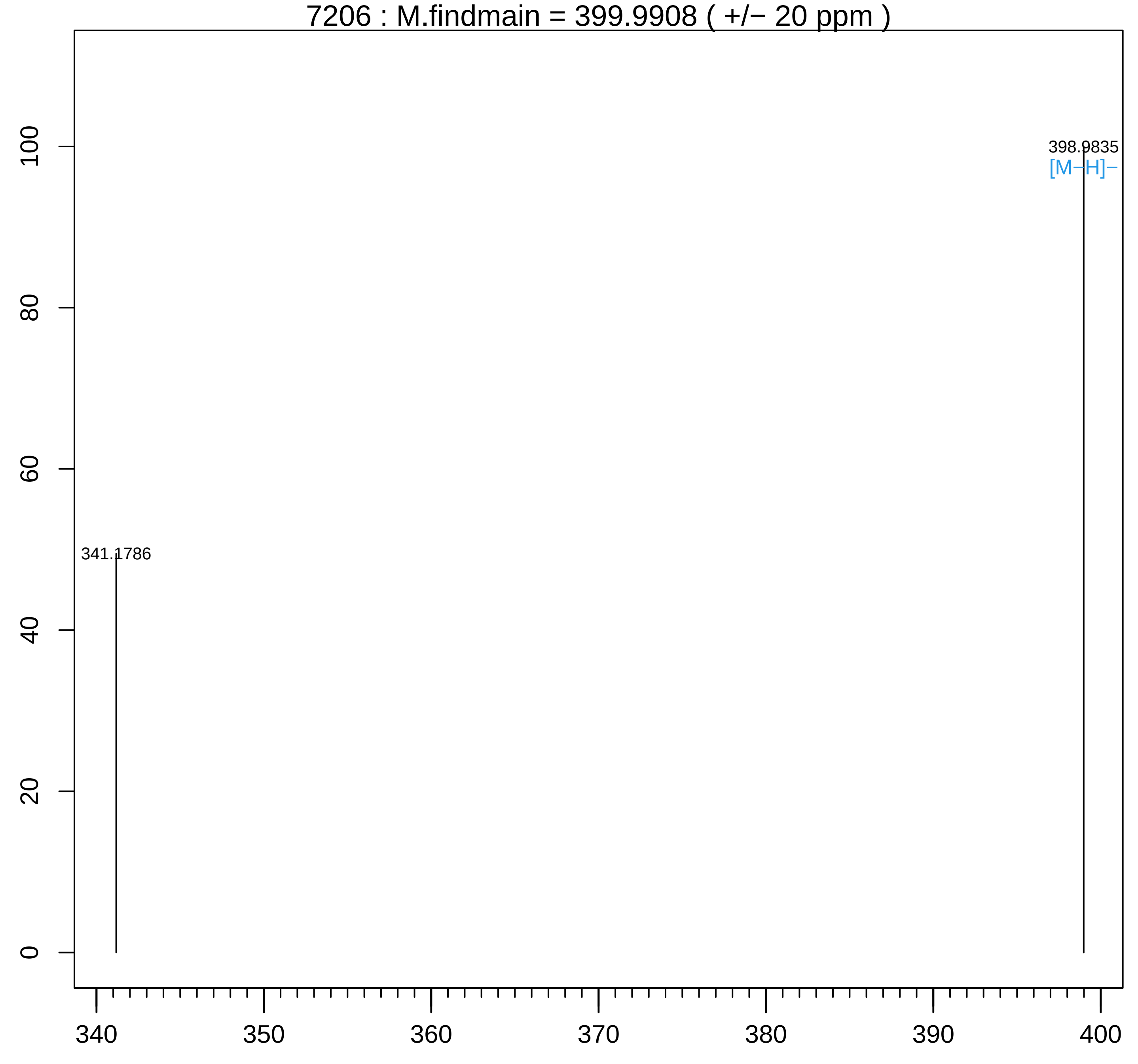
7206 : M.findmain = 399.9908 (+/− 20 ppm)
398.9835
[M−H]−
341.1786
0
20
40
60
80
100
340
350
360
370
380
390
400

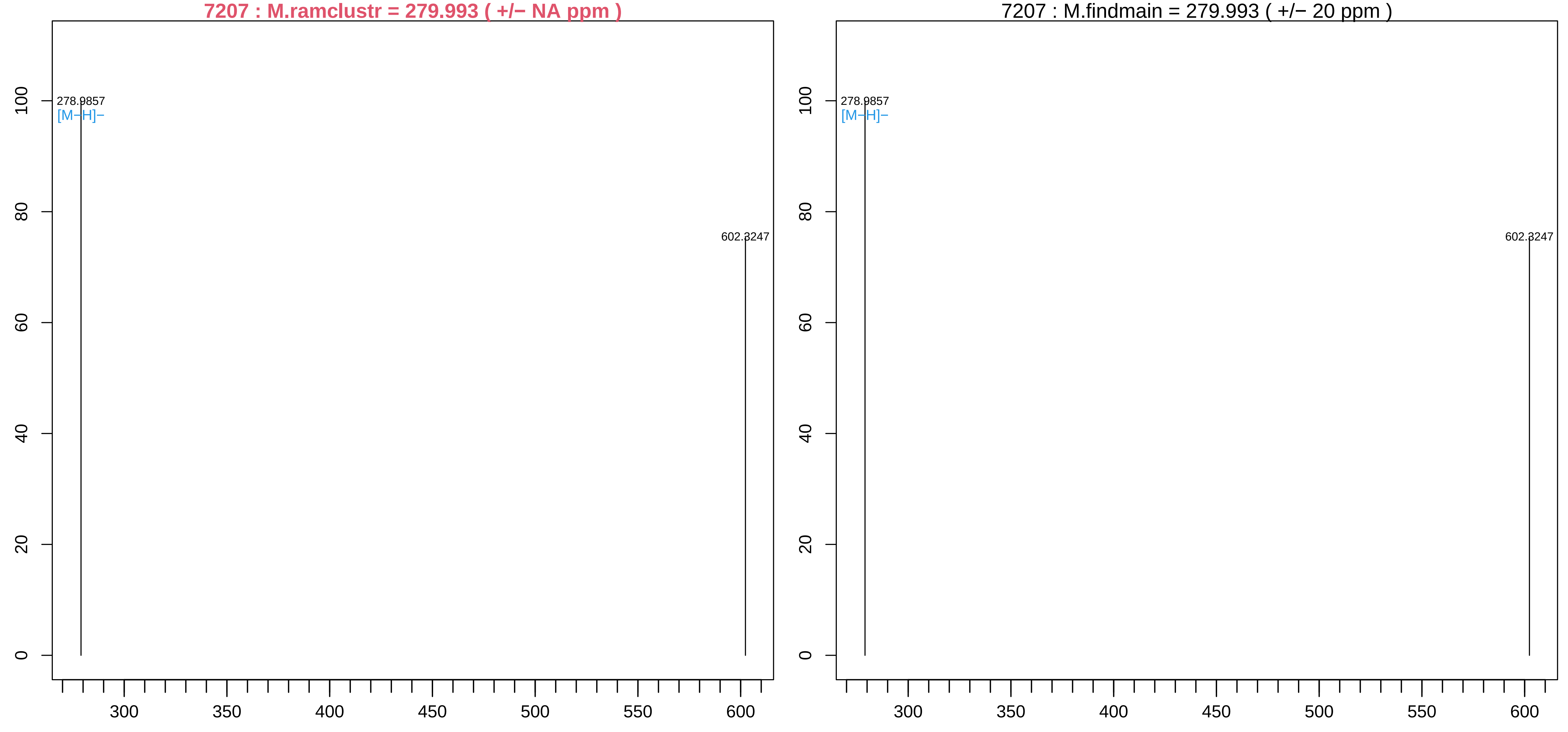
7207 : M.ramclustr = 279.993 (+/− NA ppm)
278.9857
[M−H]−
602.3247
0
20
40
60
80
100
300
350
400
450
500
550
600
7207 : M.findmain = 279.993 (+/− 20 ppm)
278.9857
[M−H]−
602.3247
0
20
40
60
80
100
300
350
400
450
500
550
600

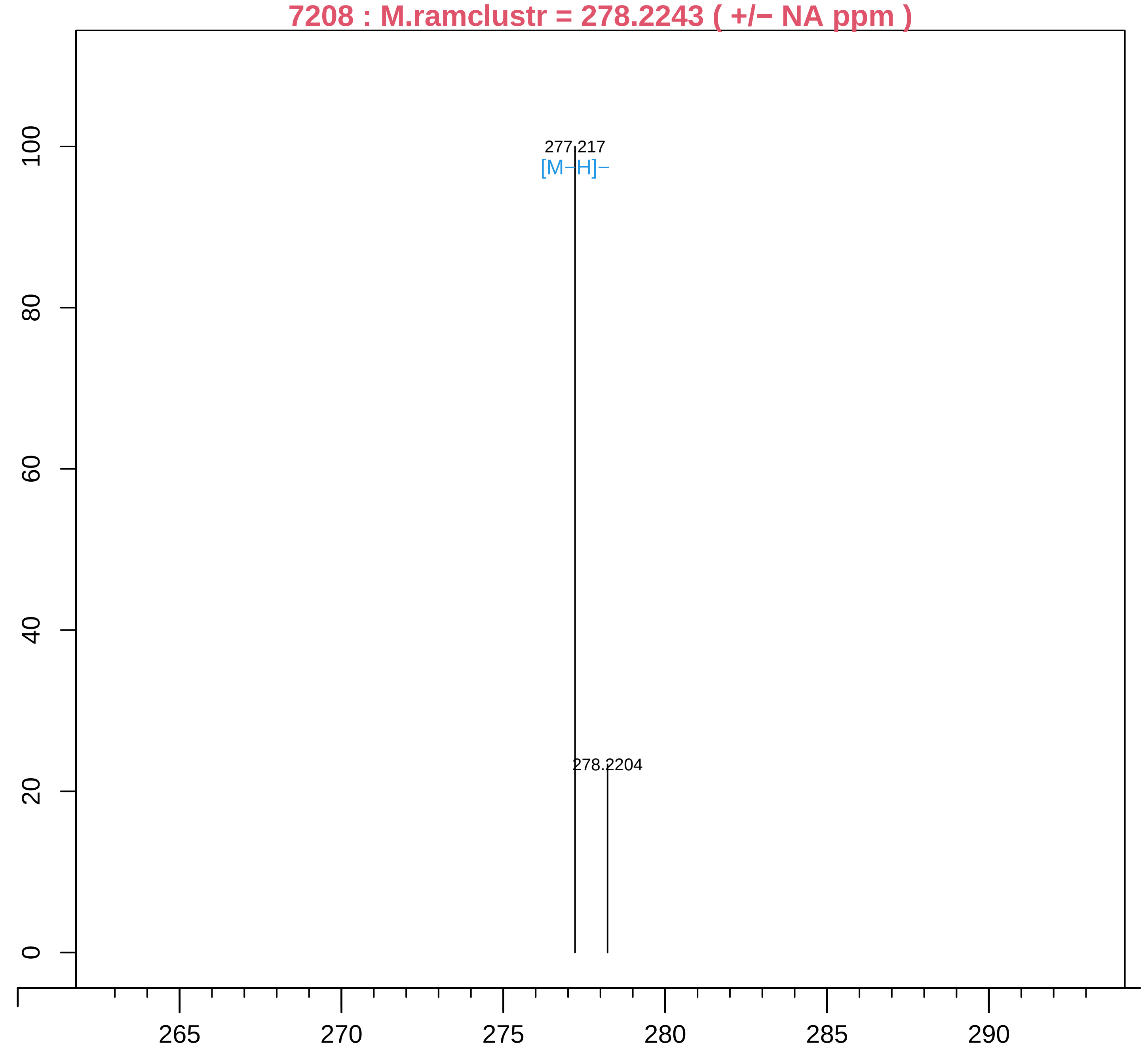
7208 : M.ramclustr = 278.2243 (+/− NA ppm)
277.217
[M−H]−
278.2204
0
20
40
60
80
100
265
270
275
280
285
290

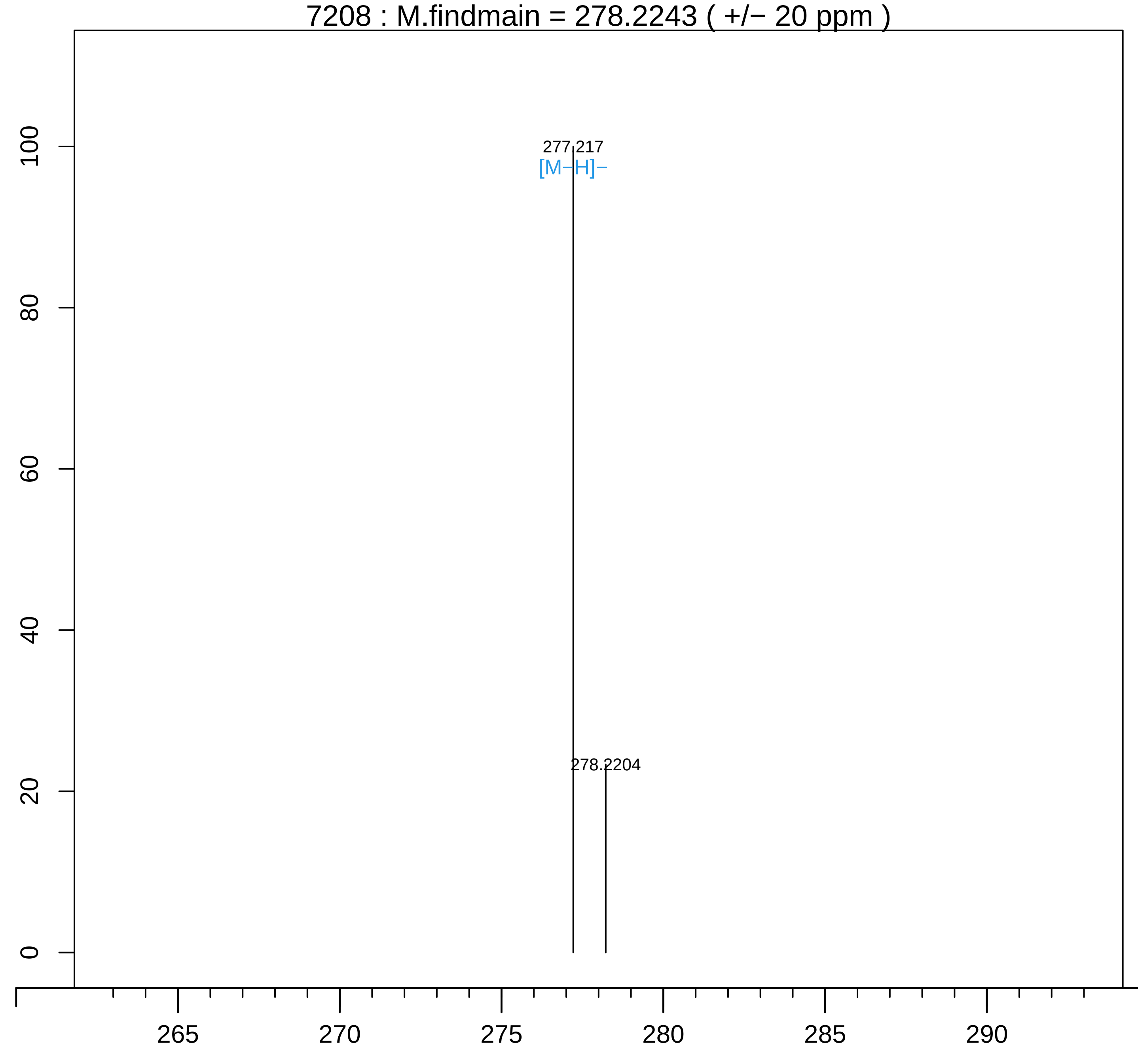
7208 : M.findmain = 278.2243 (+/− 20 ppm)
277.217
[M−H]−
278.2204
0
20
40
60
80
100
265
270
275
280
285
290

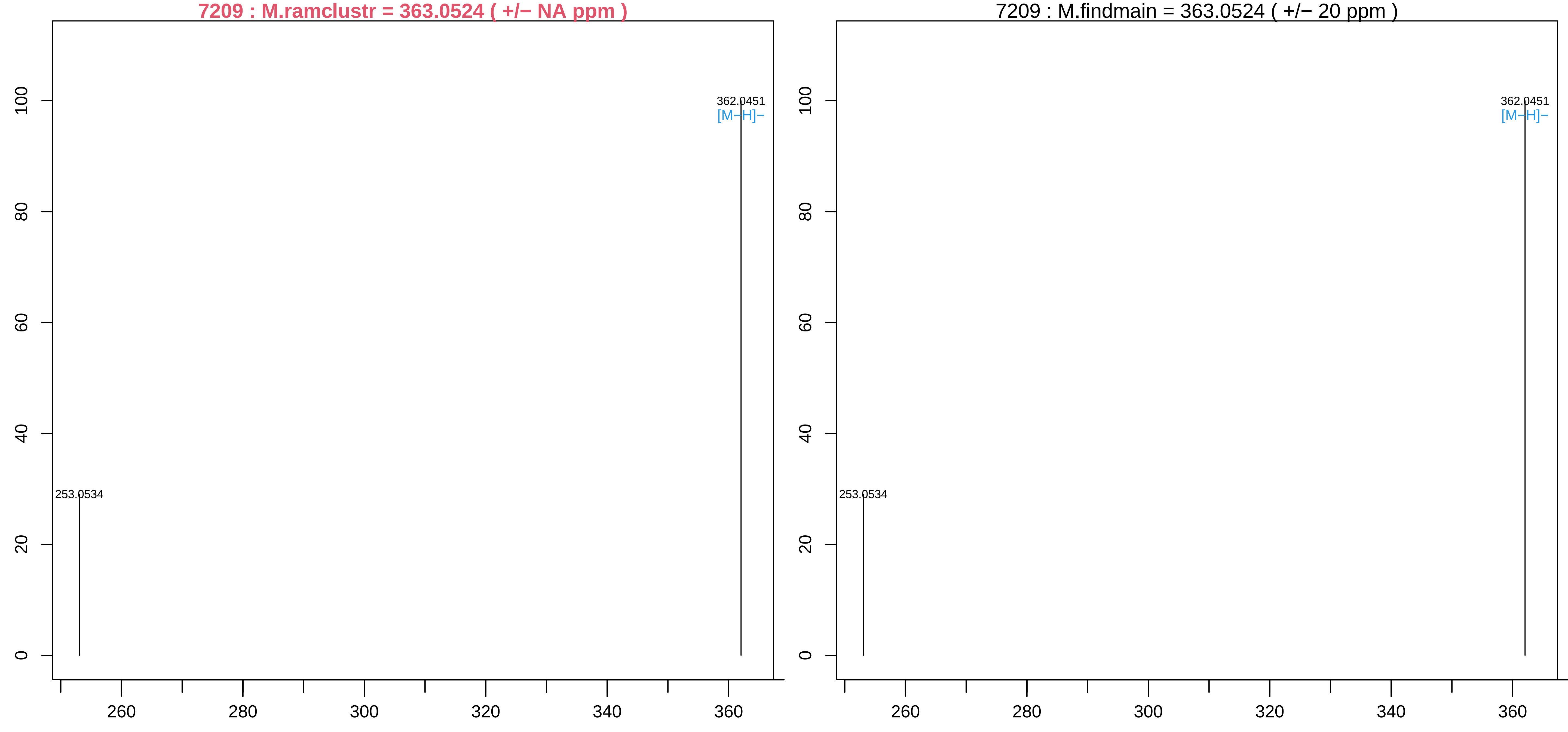
7209 : M.ramclustr = 363.0524 (+/− NA ppm)
362.0451
[M−H]−
253.0534
7209 : M.findmain = 363.0524 (+/− 20 ppm)
362.0451
[M−H]−
253.0534
0
20
40
60
80
100
260
280
300
320
340
360

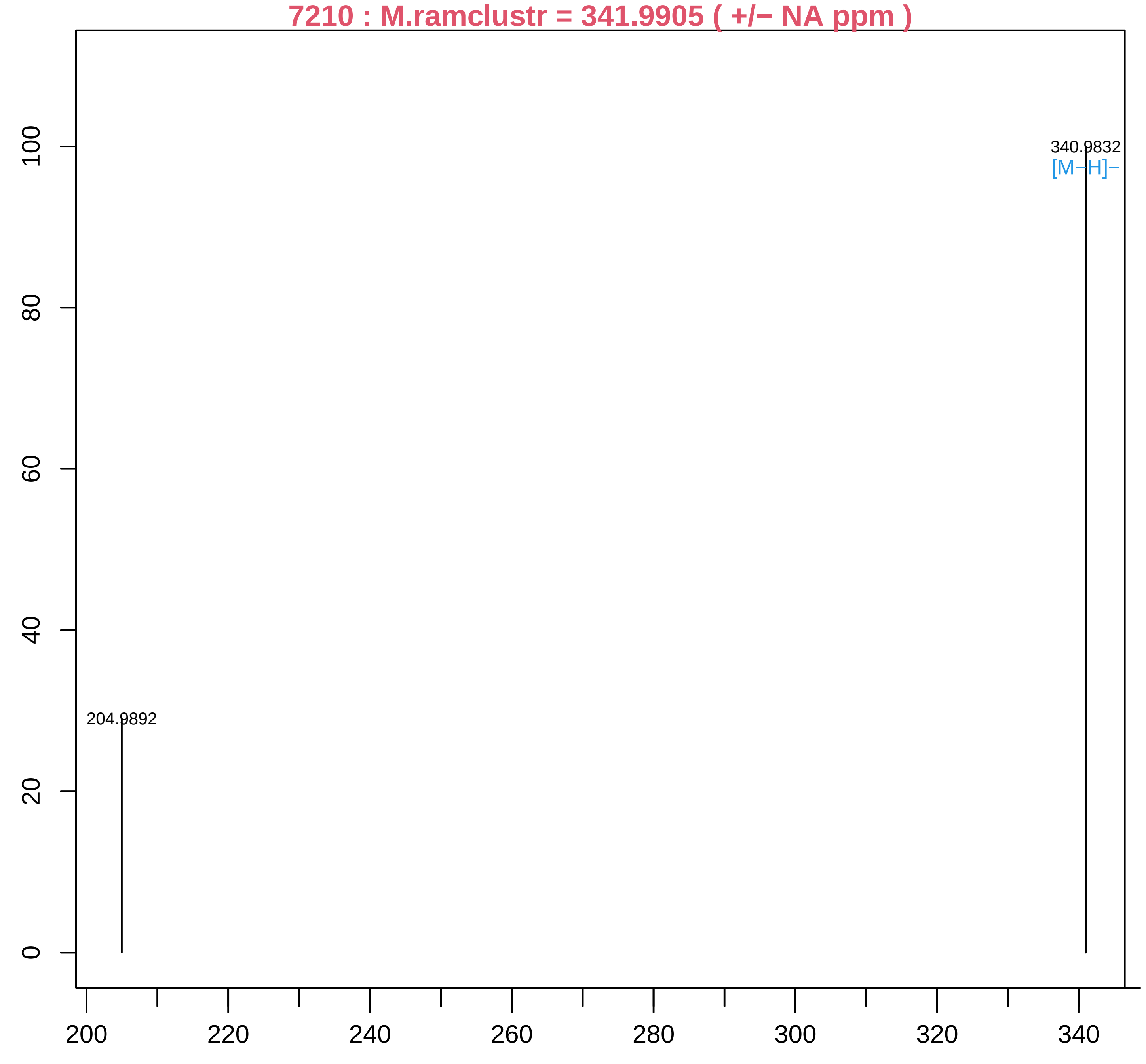
7210 : M.ramclustr = 341.9905 (+/− NA ppm)
340.9832
[M−H]−
204.9892
0
20
40
60
80
100
200
220
240
260
280
300
320
340

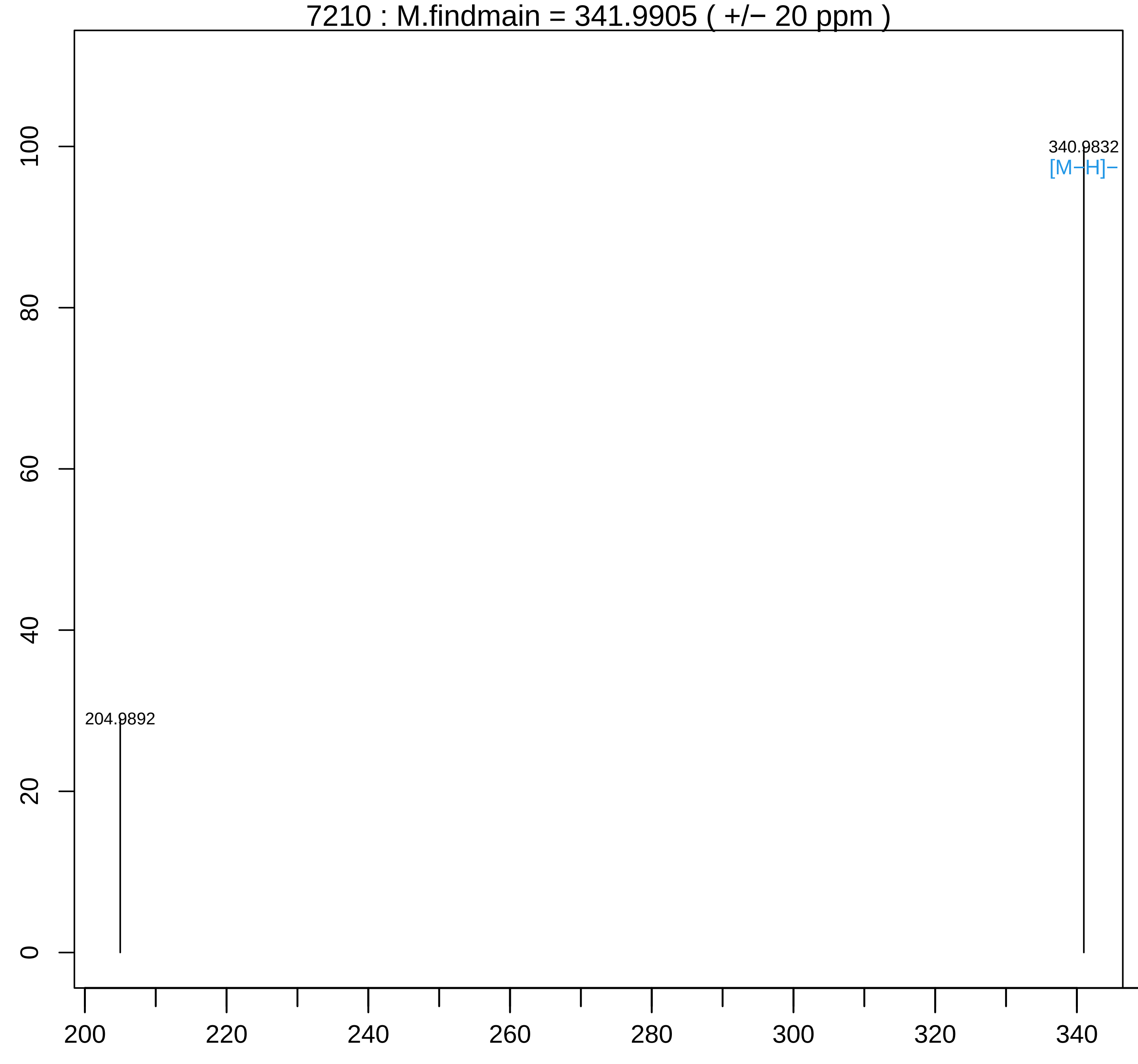
7210 : M.findmain = 341.9905 (+/− 20 ppm)
340.9832
[M−H]−
204.9892
0
20
40
60
80
100
200
220
240
260
280
300
320
340

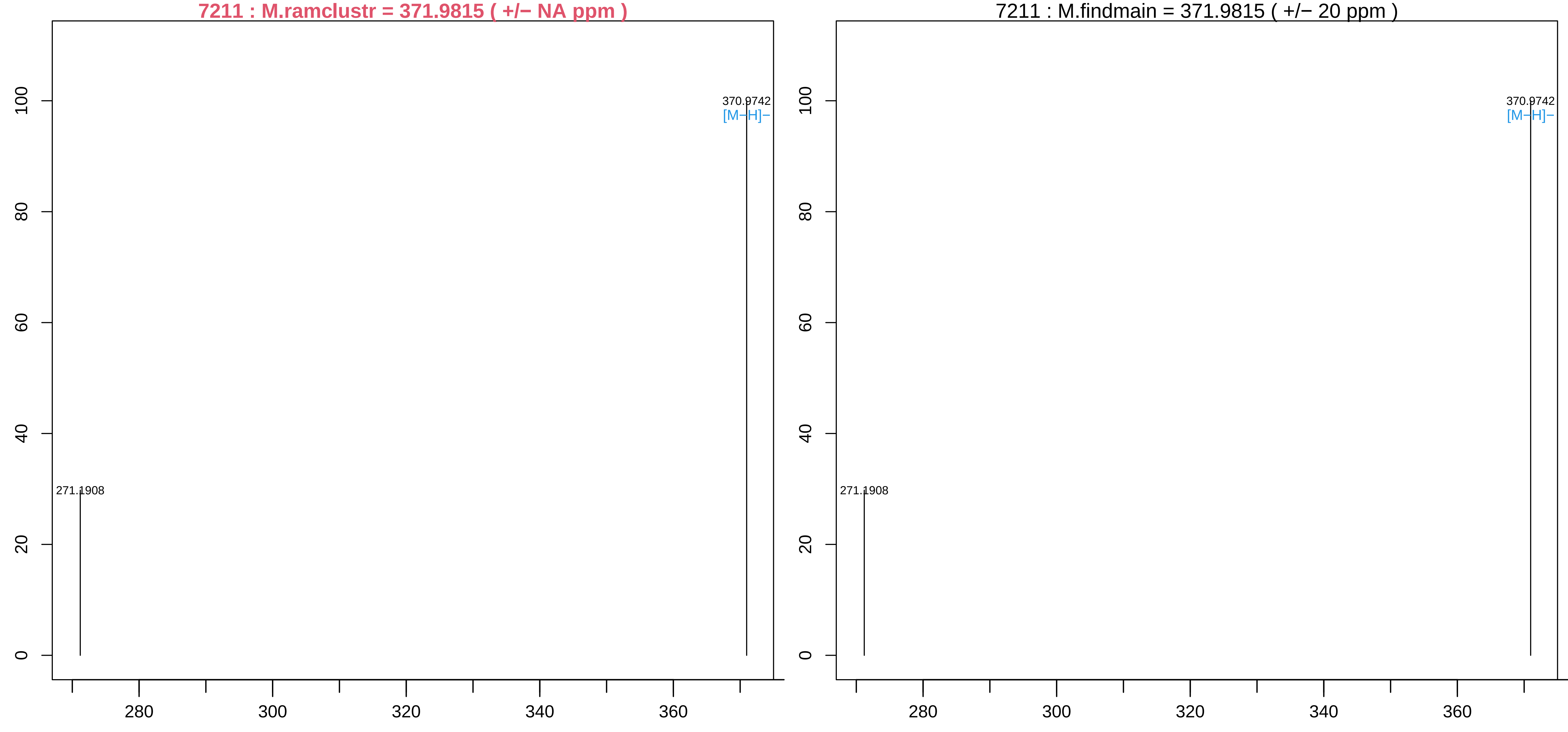
7211 : M.ramclustr = 371.9815 (+/− NA ppm)
7211 : M.findmain = 371.9815 (+/− 20 ppm)
370.9742
[M−H]−
271.1908
0
20
40
60
80
100
280
300
320
340
360

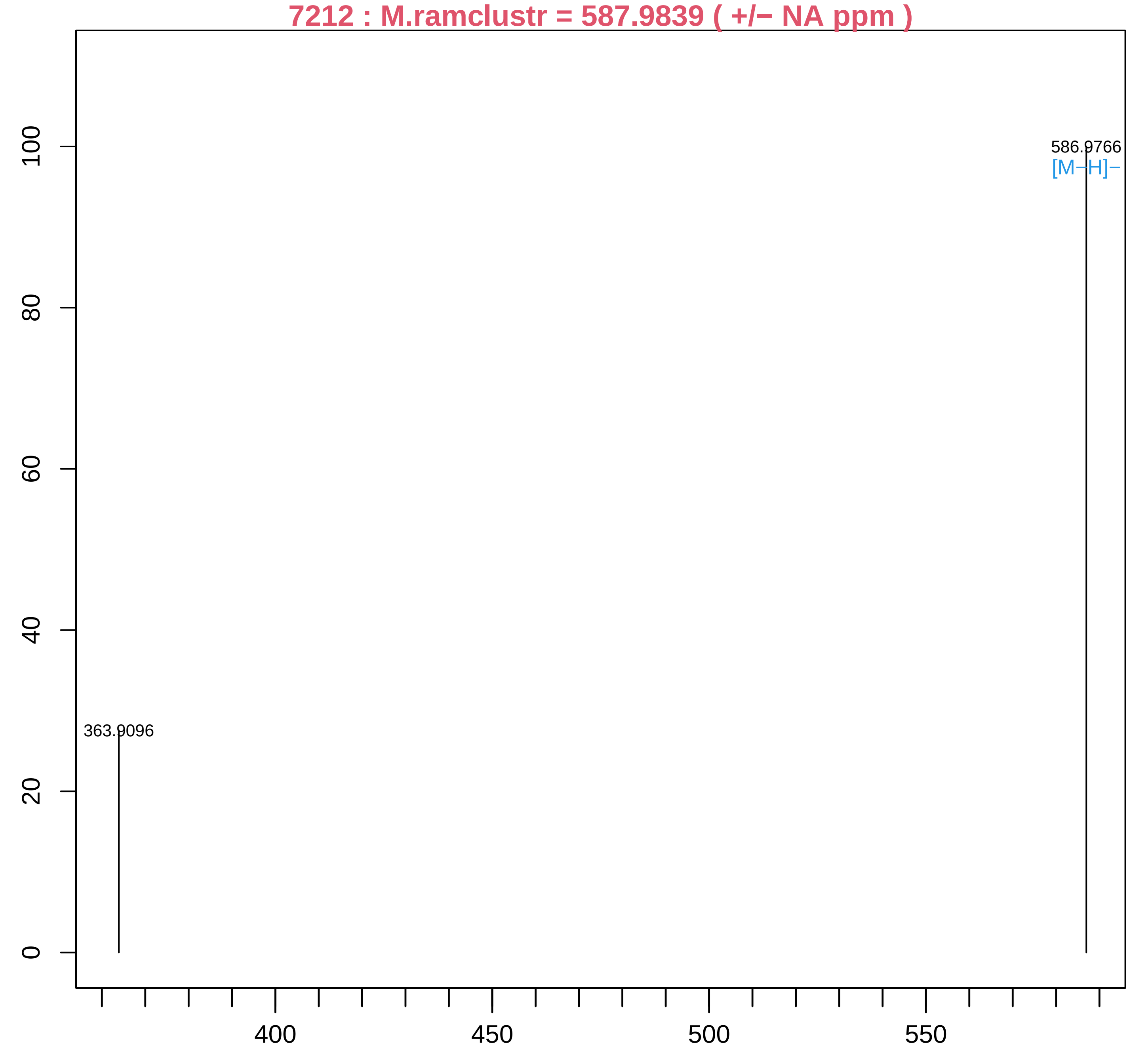
7212 : M.ramclustr = 587.9839 (+/− NA ppm)
586.9766
[M−H]−
363.9096
0
20
40
60
80
100
400
450
500
550

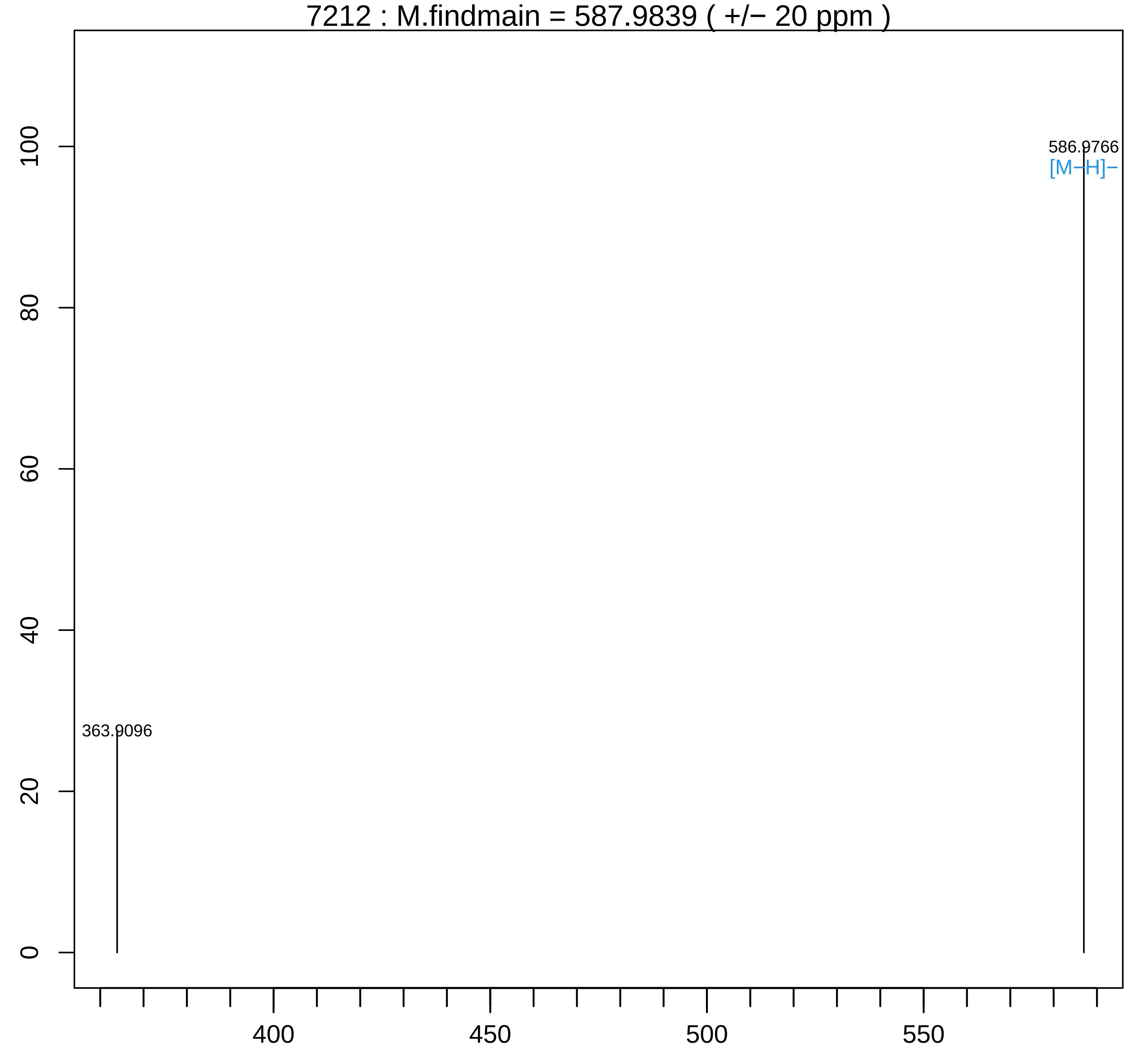
7212 : M.findmain = 587.9839 (+/− 20 ppm)
586.9766
[M−H]−
363.9096
0
20
40
60
80
100
400
450
500
550

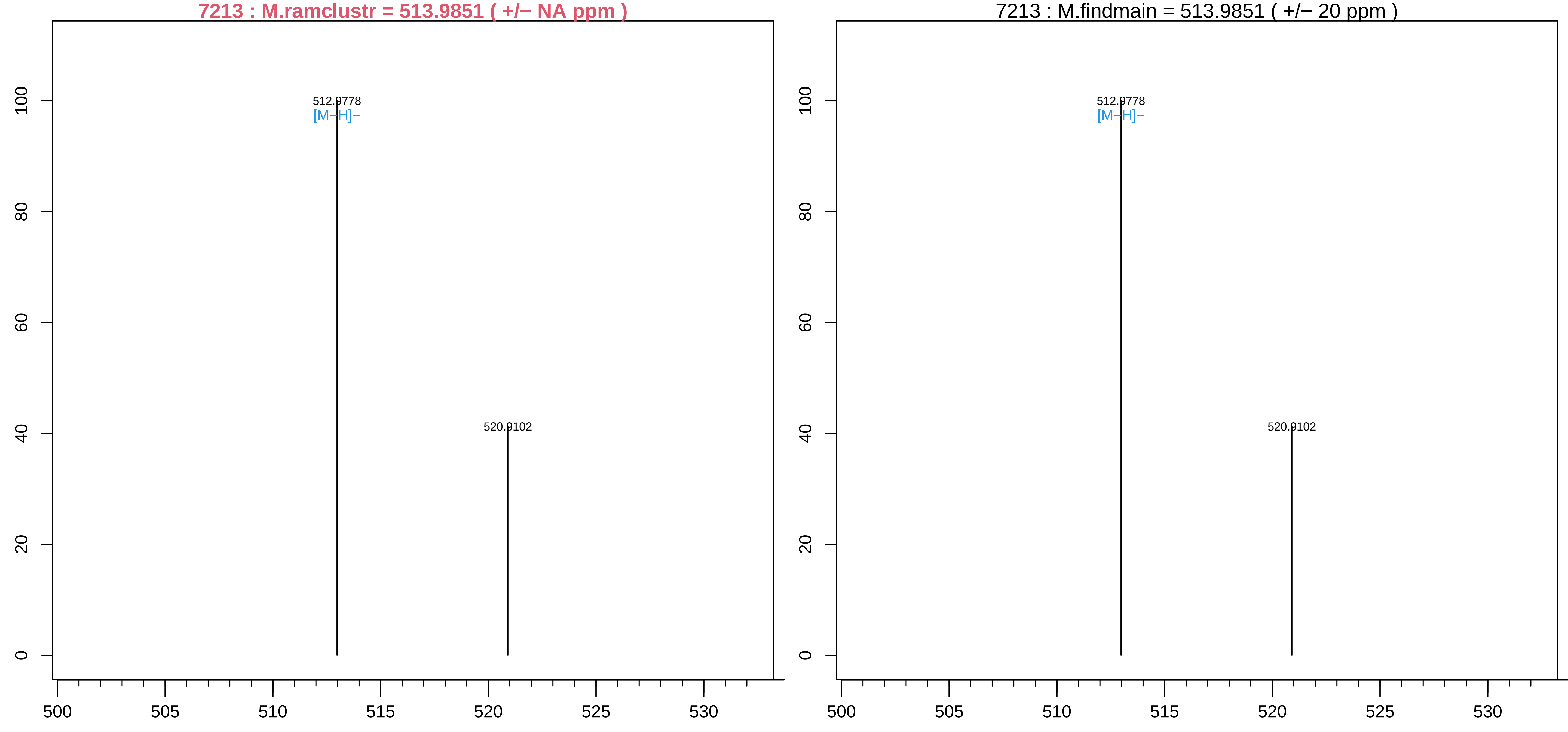
7213 : M.ramclustr = 513.9851 (+/− NA ppm)
512.9778
[M−H]−
520.9102
7213 : M.findmain = 513.9851 (+/− 20 ppm)
512.9778
[M−H]−
520.9102
0
20
40
60
80
100
500
505
510
515
520
525
530

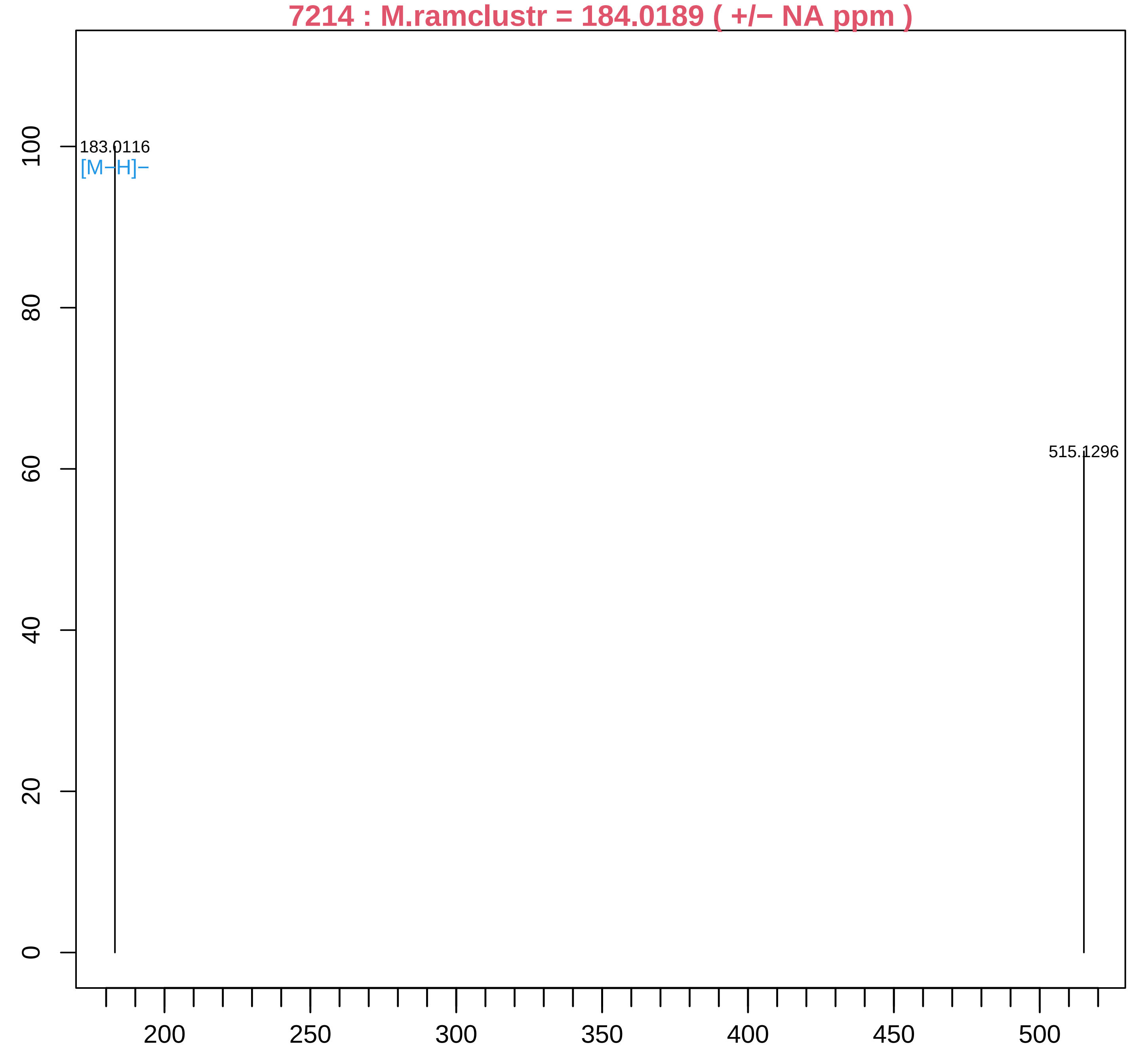
7214 : M.ramclustr = 184.0189 (+/− NA ppm)
183.0116
[M−H]−
515.1296
0
20
40
60
80
100
200
250
300
350
400
450
500

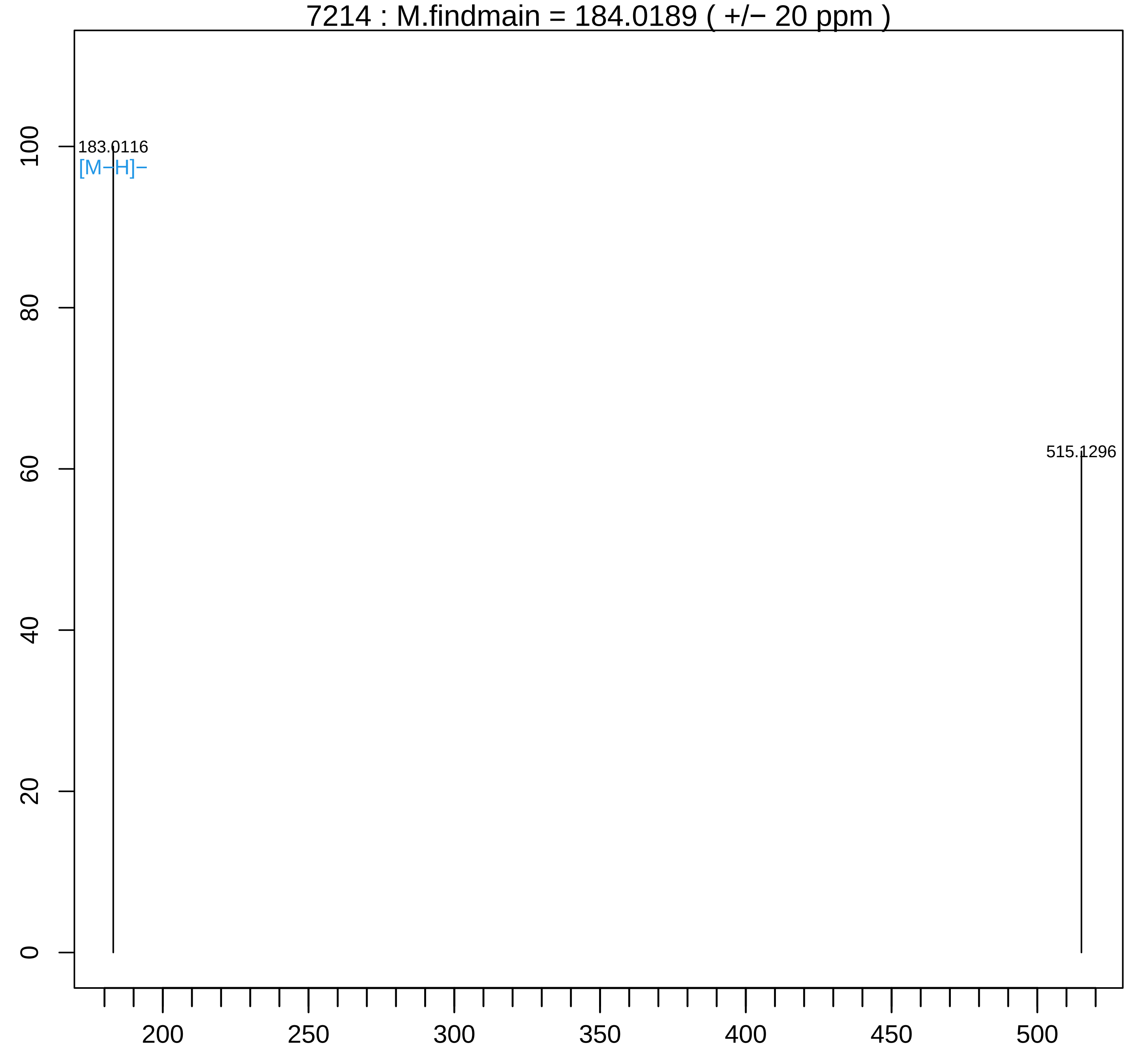
7214 : M.findmain = 184.0189 (+/− 20 ppm)
183.0116
[M−H]−
515.1296
0
20
40
60
80
100
200
250
300
350
400
450
500

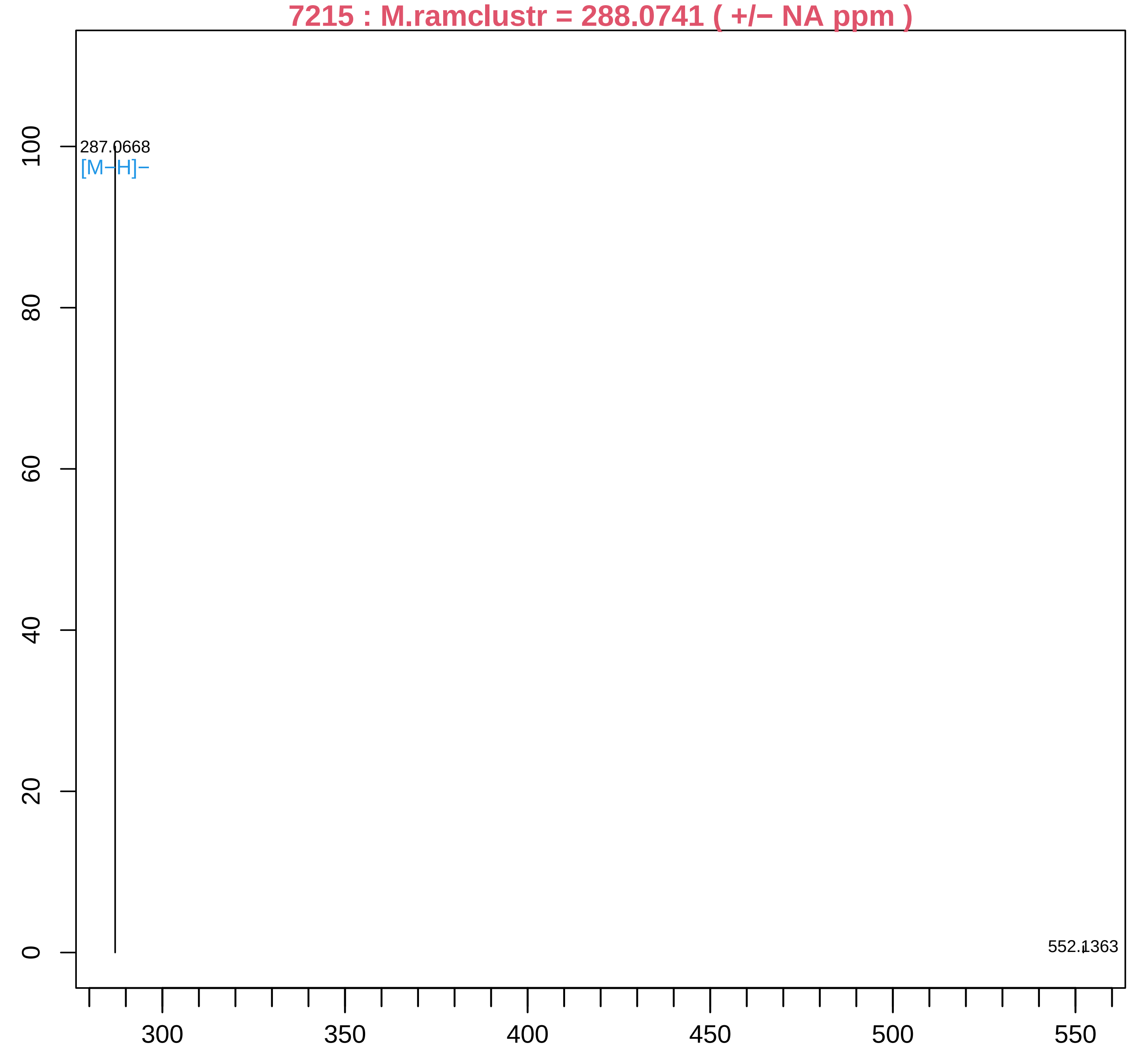
7215 : M.ramclustr = 288.0741 (+/− NA ppm)
287.0668
[M−H]−
552.1363
0
20
40
60
80
100
300
350
400
450
500
550

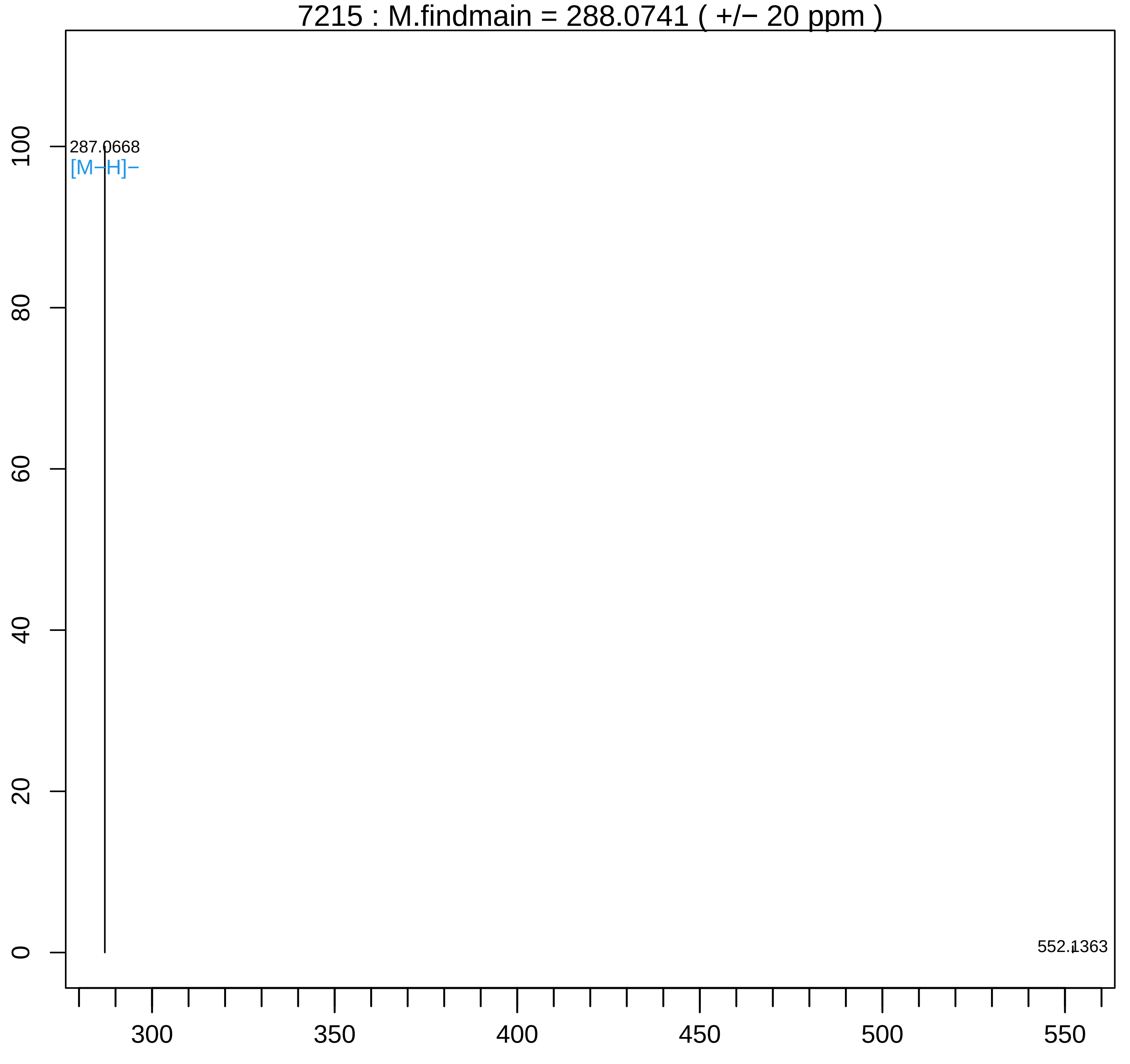
7215 : M.findmain = 288.0741 (+/− 20 ppm)
287.0668
[M−H]−
552.1363
0
20
40
60
80
100
300
350
400
450
500
550

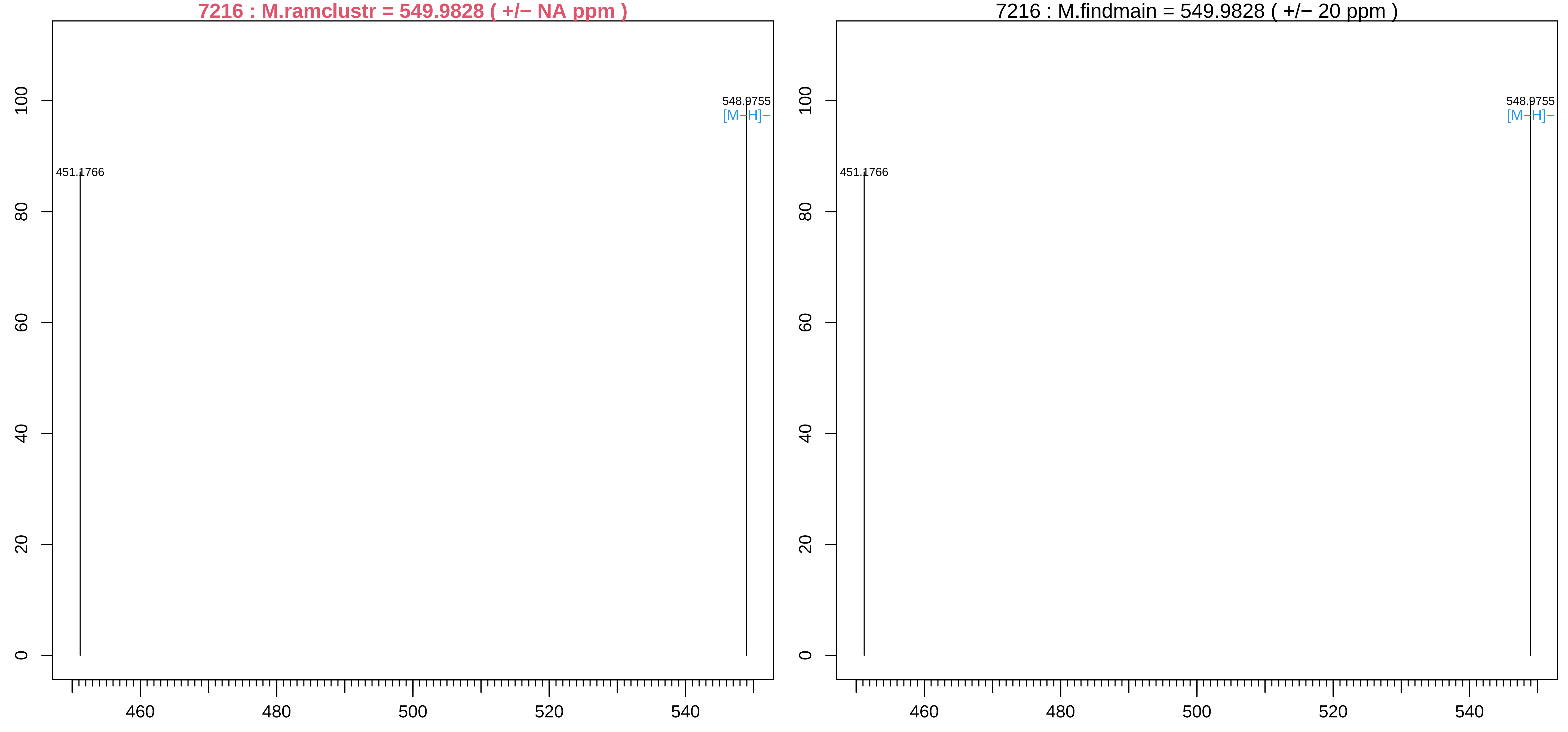
7216 : M.ramclustr = 549.9828 (+/− NA ppm)
548.9755
[M−H]−
451.1766
0
20
40
60
80
100
460
480
500
520
540
7216 : M.findmain = 549.9828 (+/− 20 ppm)
548.9755
[M−H]−
451.1766
0
20
40
60
80
100
460
480
500
520
540

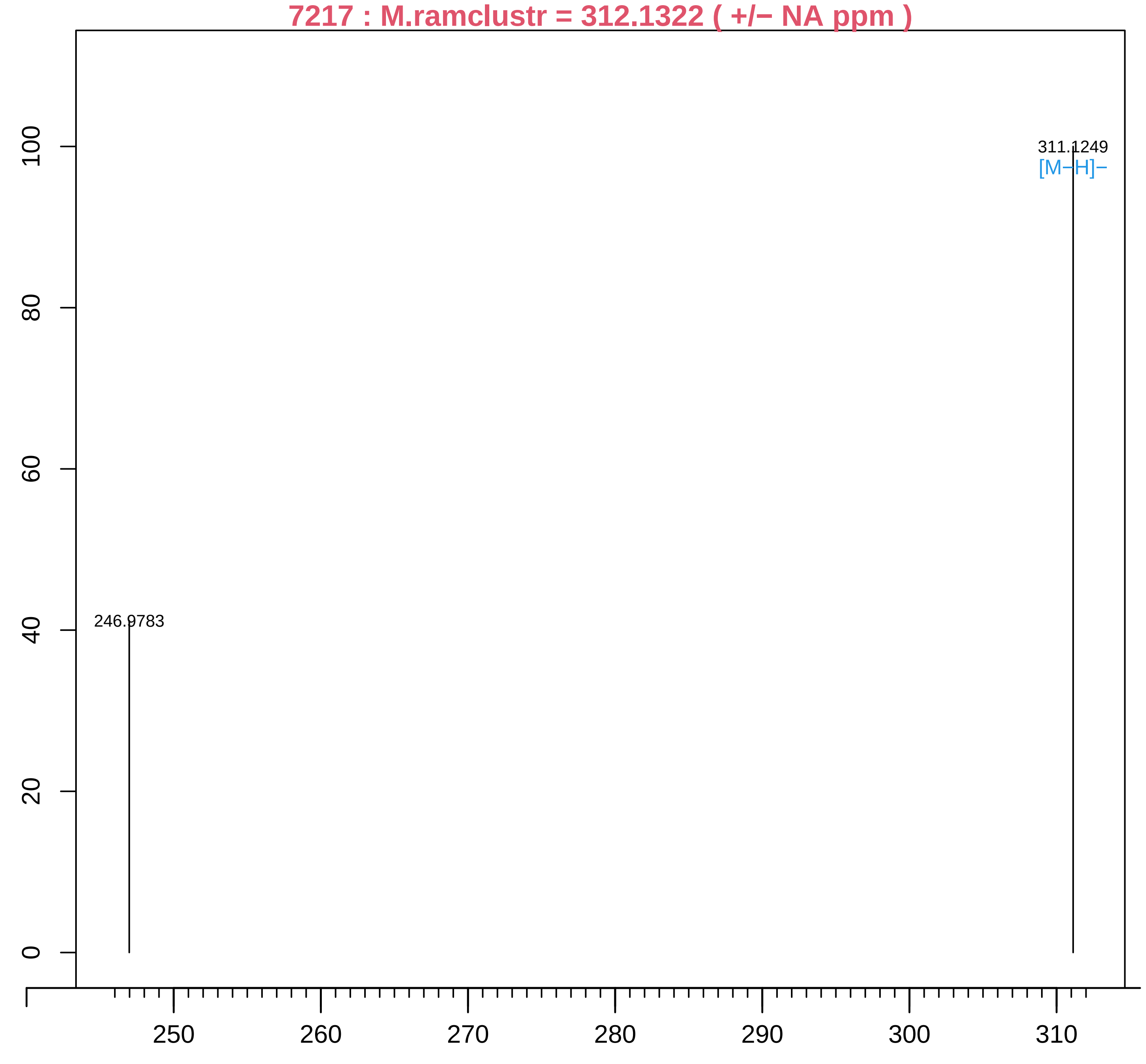
7217 : M.ramclustr = 312.1322 (+/− NA ppm)
311.1249
[M−H]−
246.9783
0
20
40
60
80
100
250
260
270
280
290
300
310

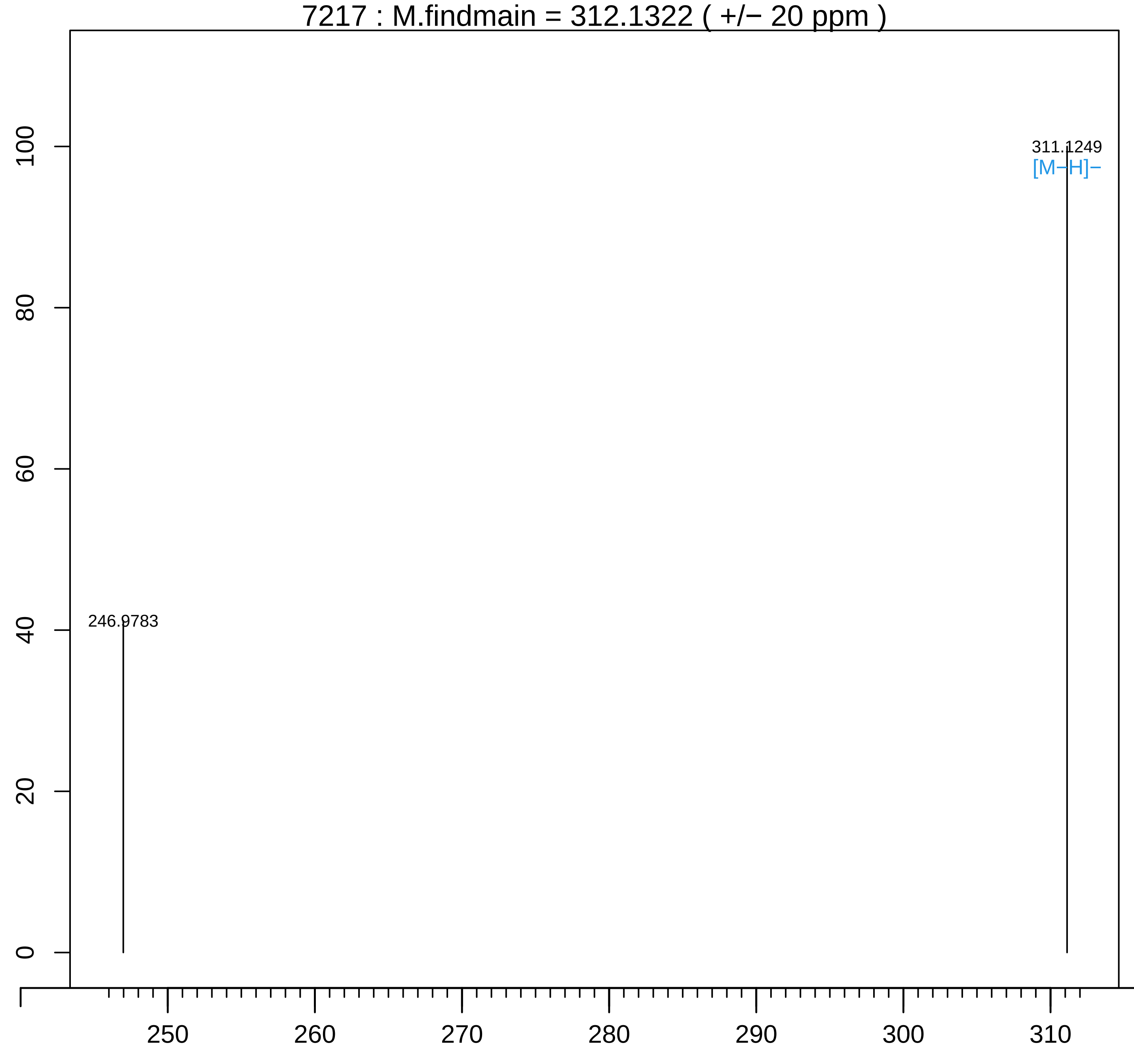
7217 : M.findmain = 312.1322 (+/− 20 ppm)
311.1249
[M−H]−
246.9783
0
20
40
60
80
100
250
260
270
280
290
300
310

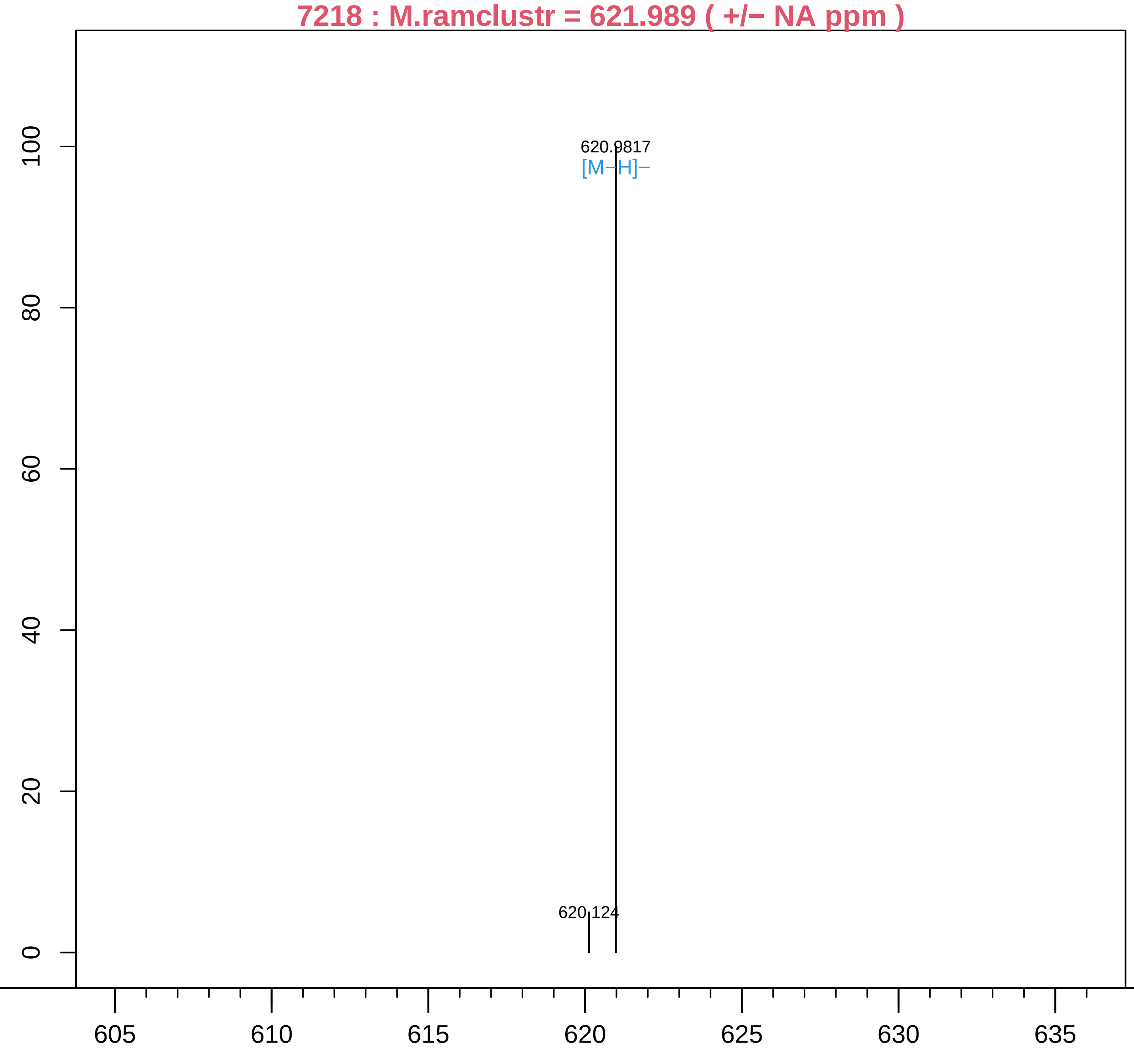

7218 : M.ramclustr = 621.989 (+/− NA ppm)
620.9817
[M−H]−
620.124

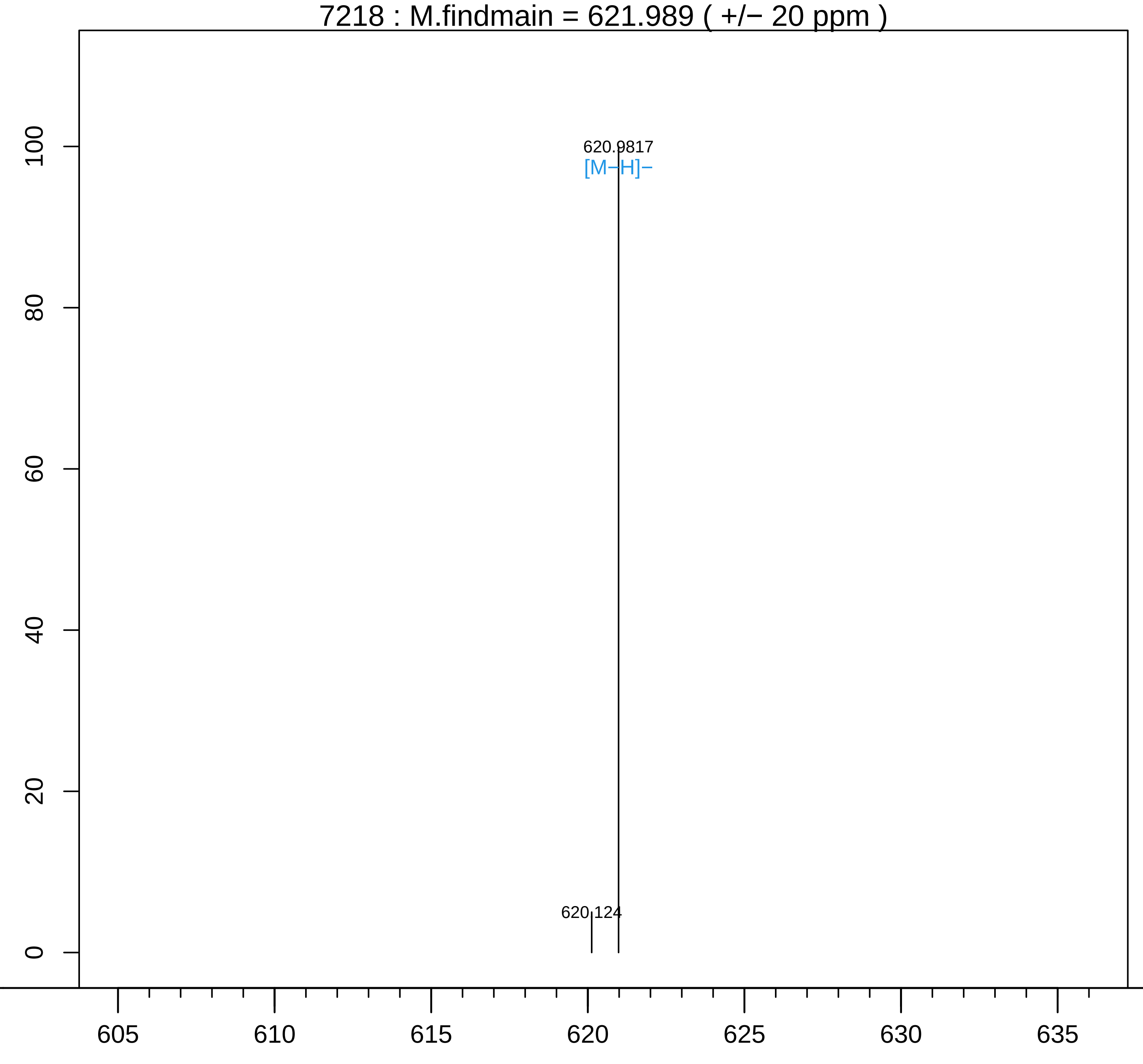

7218 : M.findmain = 621.989 (+/− 20 ppm)
620.9817
[M−H]−
620.124

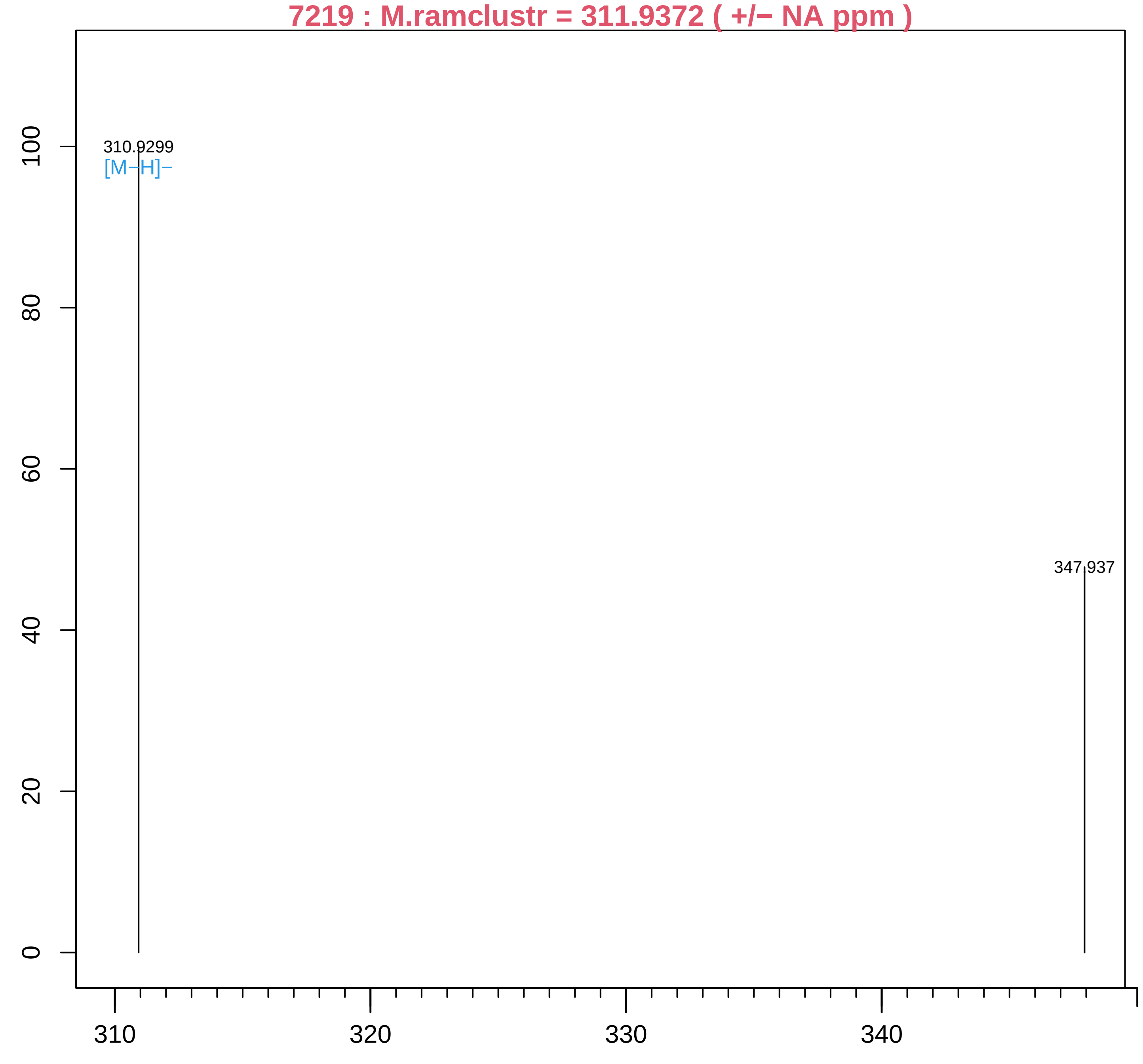
7219 : M.ramclustr = 311.9372 (+/− NA ppm)
310.9299
[M−H]−
347.937
0
20
40
60
80
100
310
320
330
340

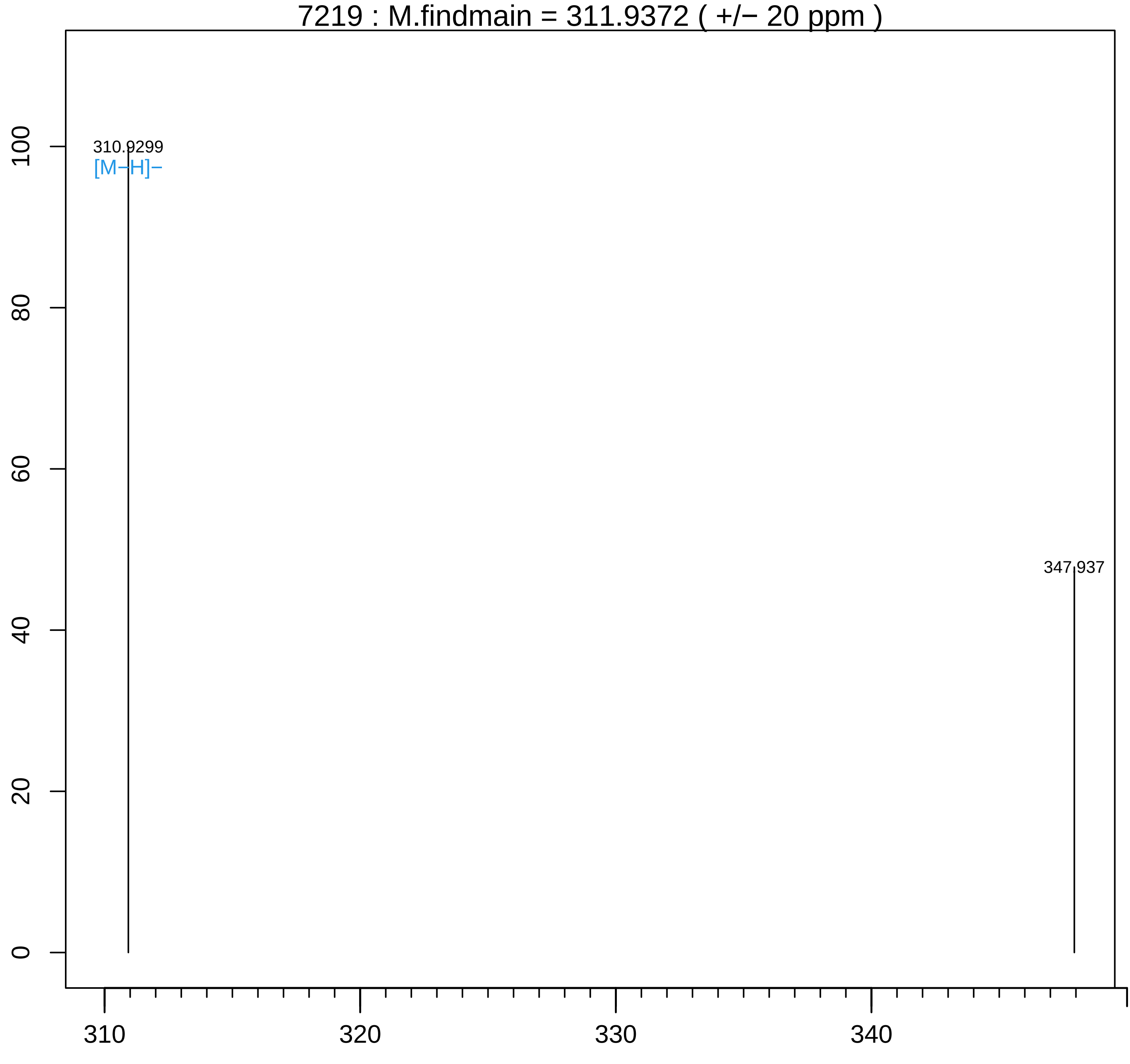
7219 : M.findmain = 311.9372 (+/− 20 ppm)
310.9299
[M−H]−
347.937
0
20
40
60
80
100
310
320
330
340

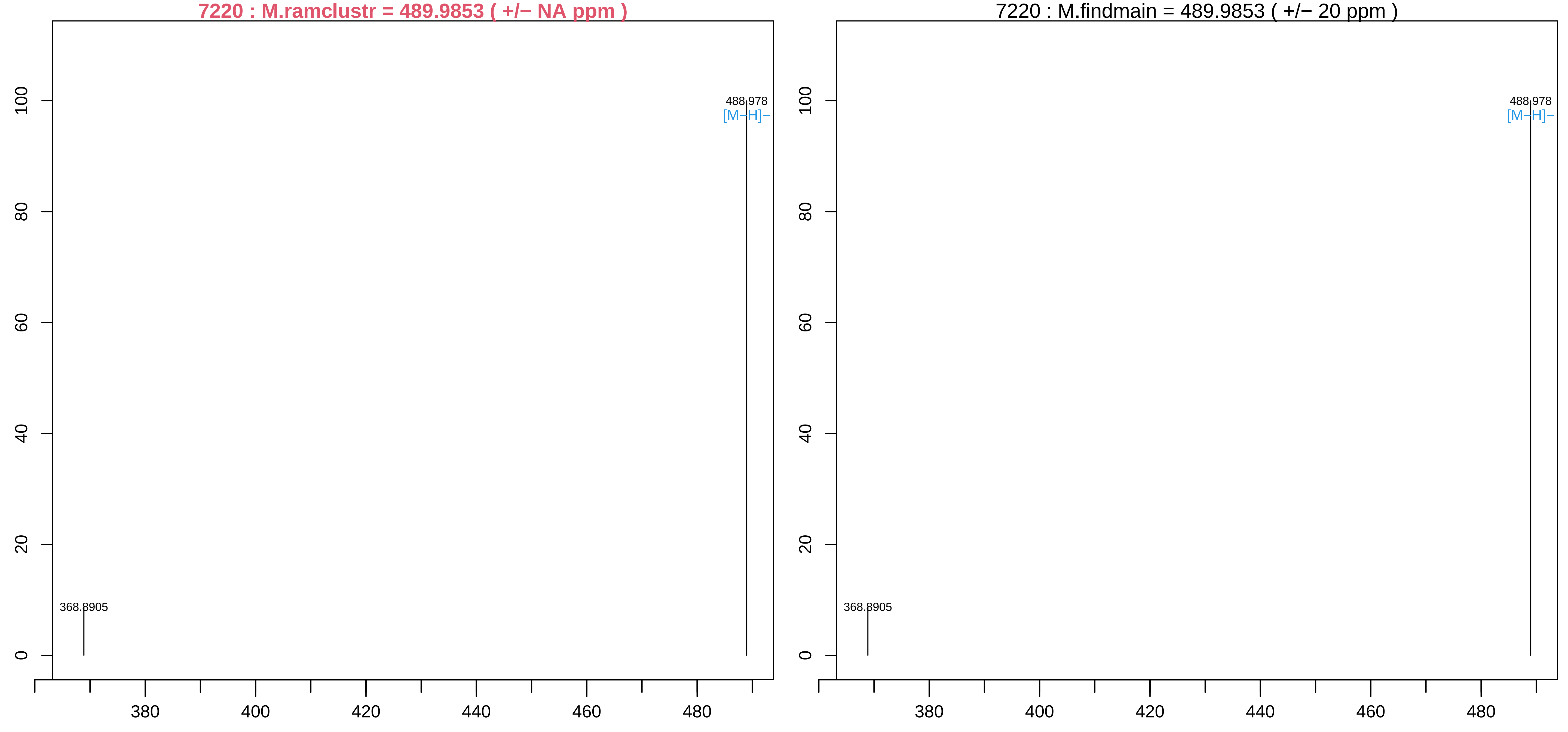
7220 : M.ramclustr = 489.9853 (+/− NA ppm)
488.978
[M−H]−
368.8905
7220 : M.findmain = 489.9853 (+/− 20 ppm)
488.978
[M−H]−
368.8905
0
20
40
60
80
100
380
400
420
440
460
480

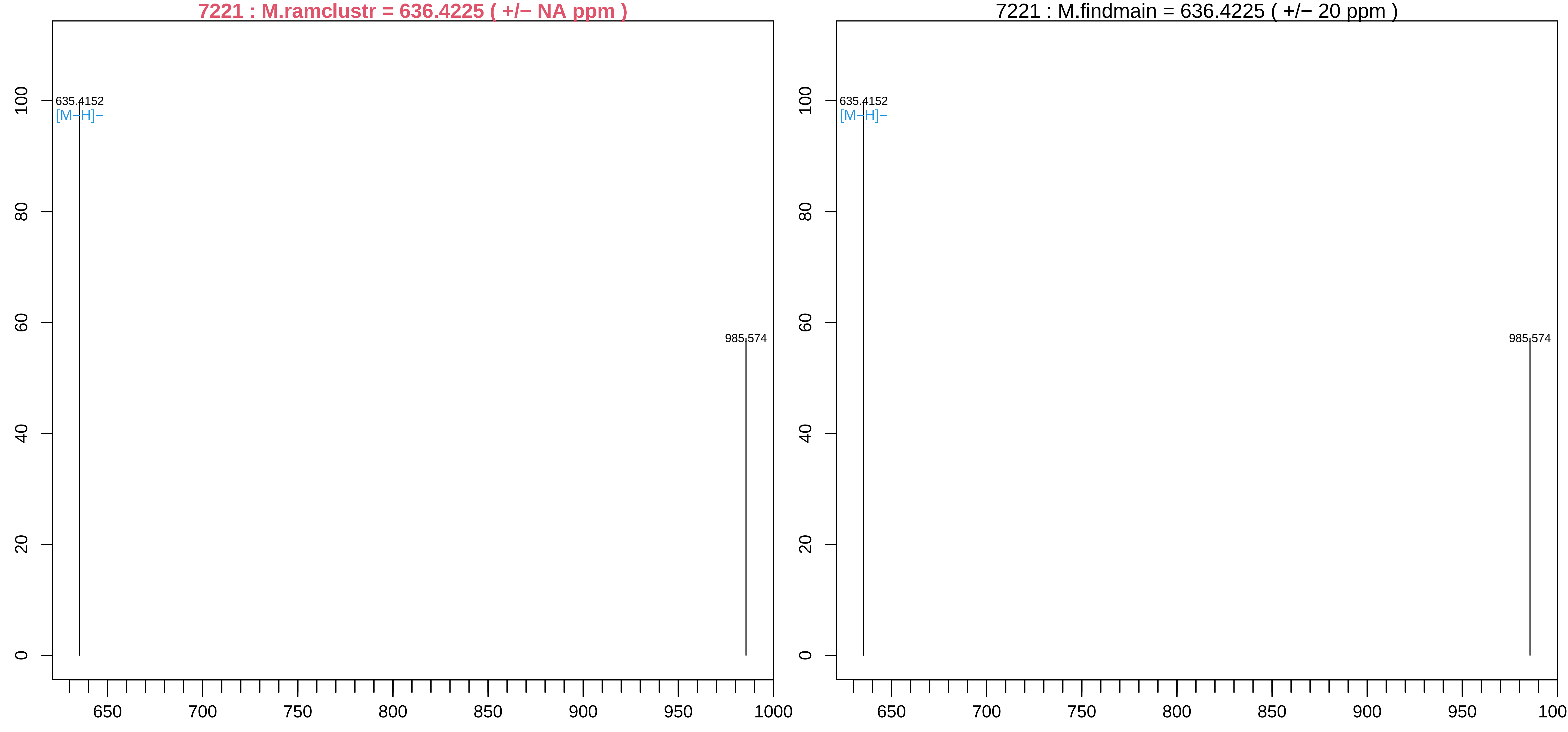
7221 : M.ramclustr = 636.4225 (+/− NA ppm)
635.4152
[M−H]−
985.574
7221 : M.findmain = 636.4225 (+/− 20 ppm)
635.4152
[M−H]−
985.574
0
20
40
60
80
100
650
700
750
800
850
900
950
1000

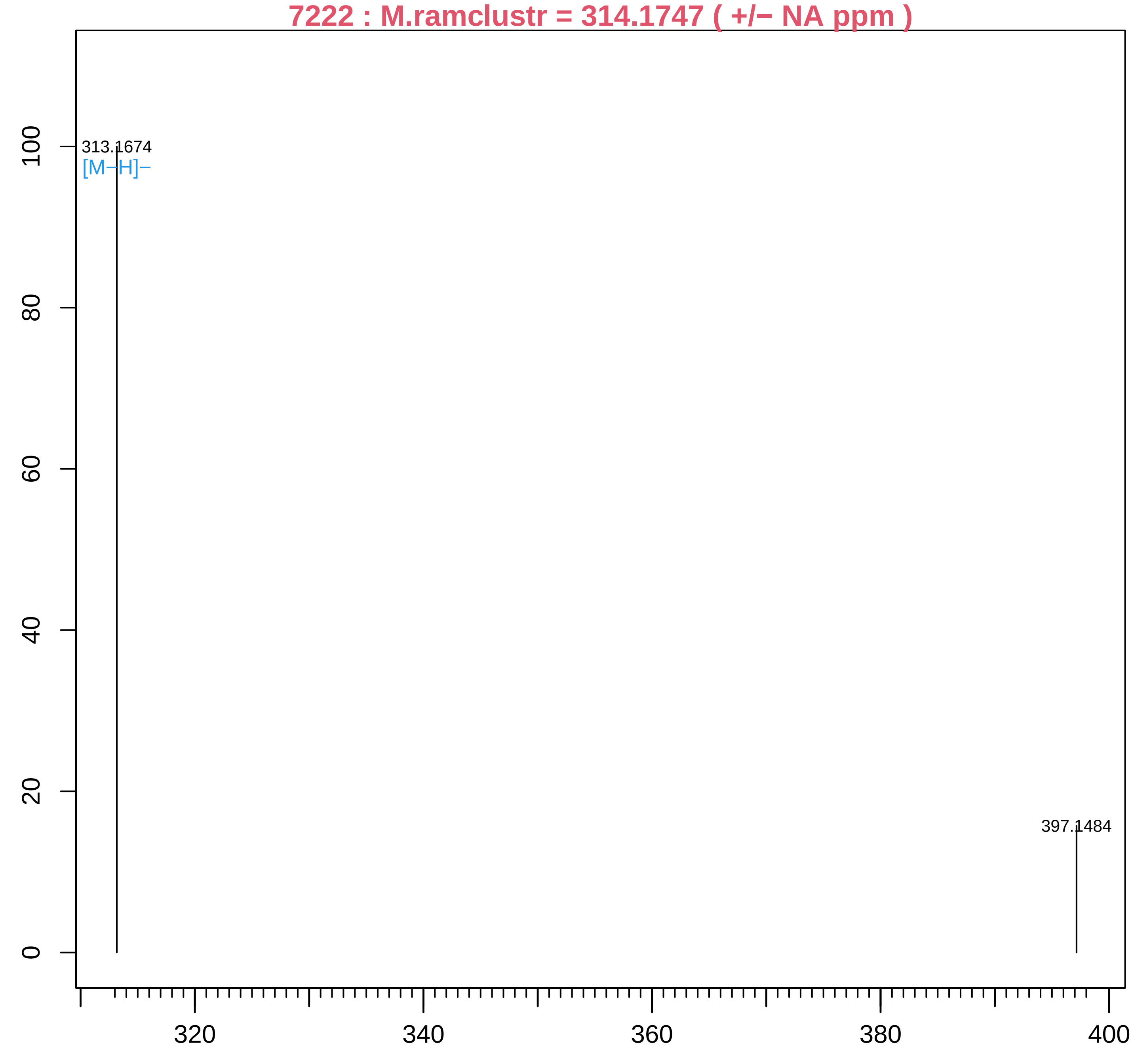
7222 : M.ramclustr = 314.1747 (+/− NA ppm)
313.1674
[M−H]−
397.1484
0
20
40
60
80
100
320
340
360
380
400

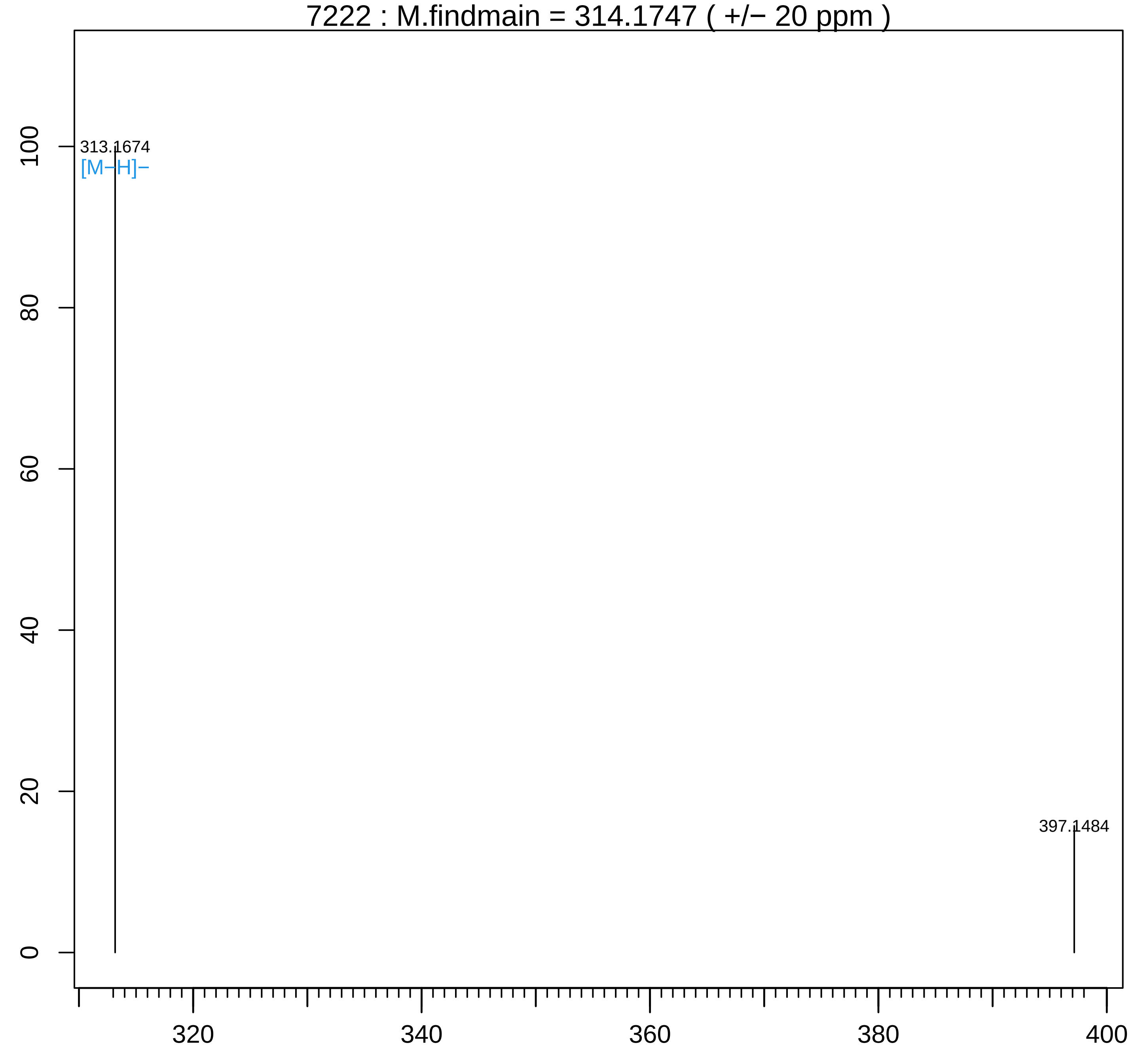
7222 : M.findmain = 314.1747 (+/− 20 ppm)
313.1674
[M−H]−
397.1484
0
20
40
60
80
100
320
340
360
380
400

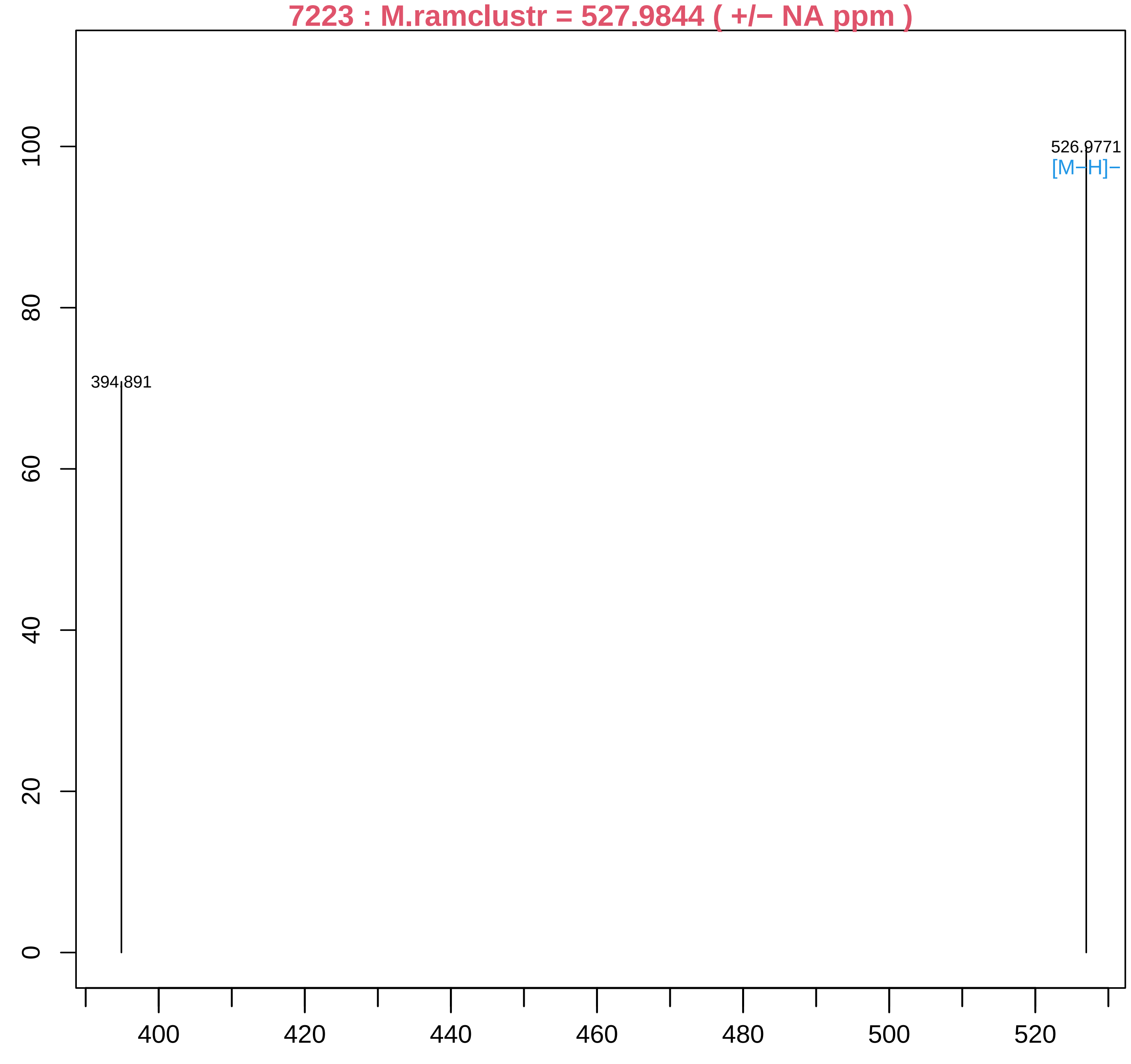
7223 : M.ramclustr = 527.9844 (+/− NA ppm)
526.9771
[M−H]−
394.891
0
20
40
60
80
100
400
420
440
460
480
500
520
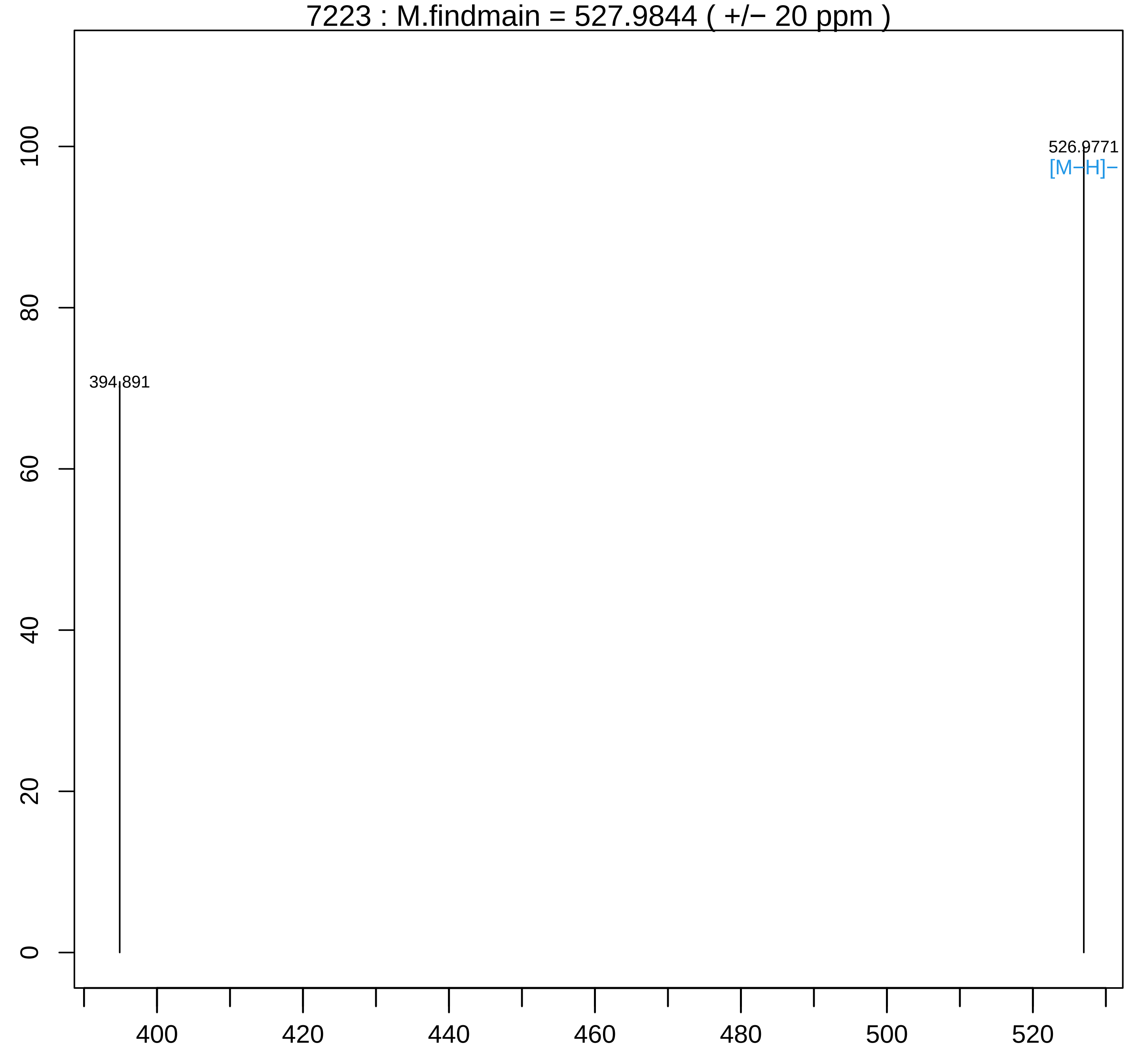
7223 : M.findmain = 527.9844 (+/− 20 ppm)
526.9771
[M−H]−
394.891
0
20
40
60
80
100
400
420
440
460
480
500
520

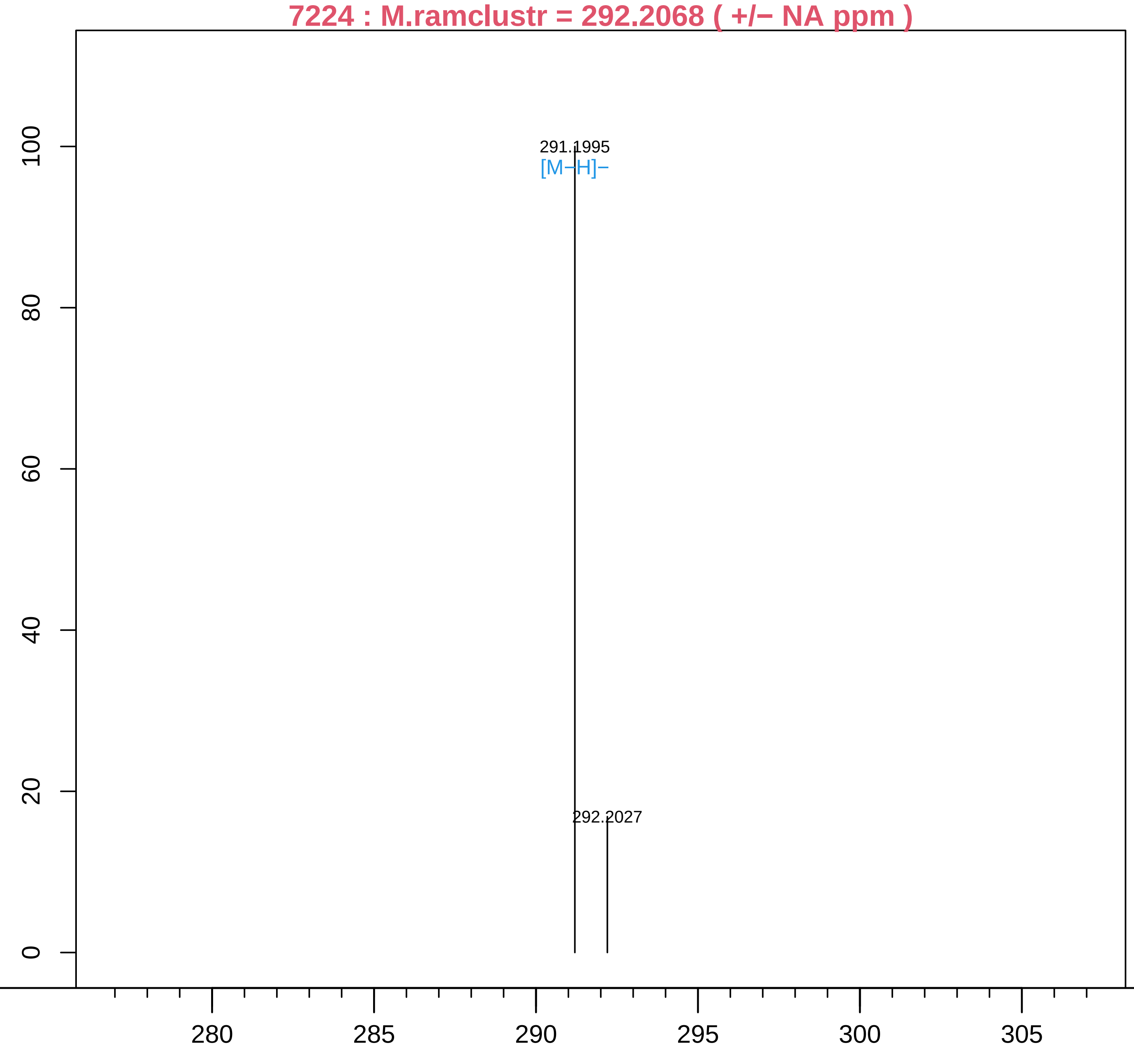
7224 : M.ramclustr = 292.2068 (+/− NA ppm)
291.1995
[M−H]−
292.2027
0
20
40
60
80
100
280
285
290
295
300
305

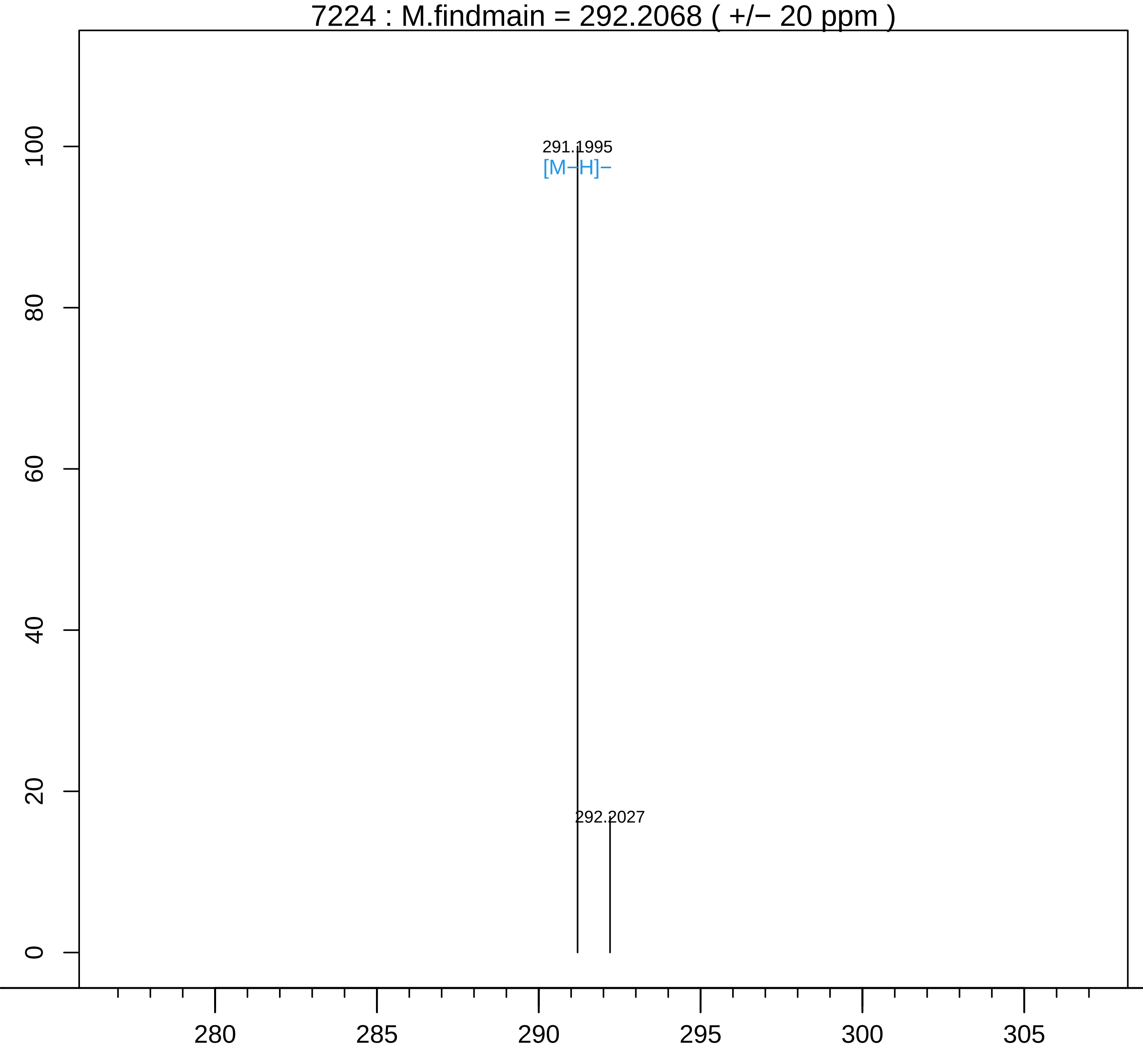
7224 : M.findmain = 292.2068 (+/− 20 ppm)
291.1995
[M−H]−
292.2027
0
20
40
60
80
100
280
285
290
295
300
305

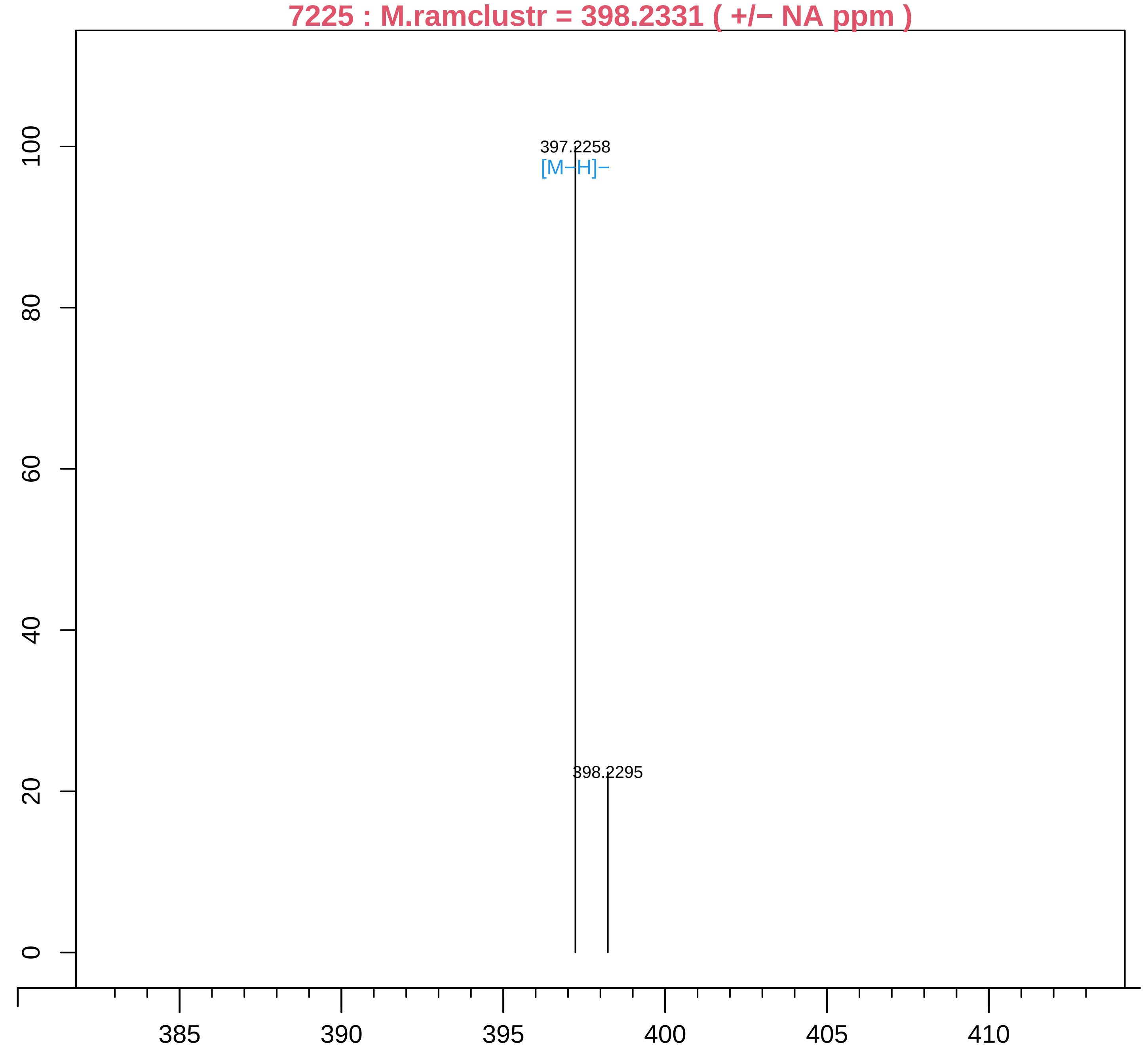
7225 : M.ramclustr = 398.2331 (+/− NA ppm)
397.2258
[M−H]−
398.2295
0
20
40
60
80
100
385
390
395
400
405
410

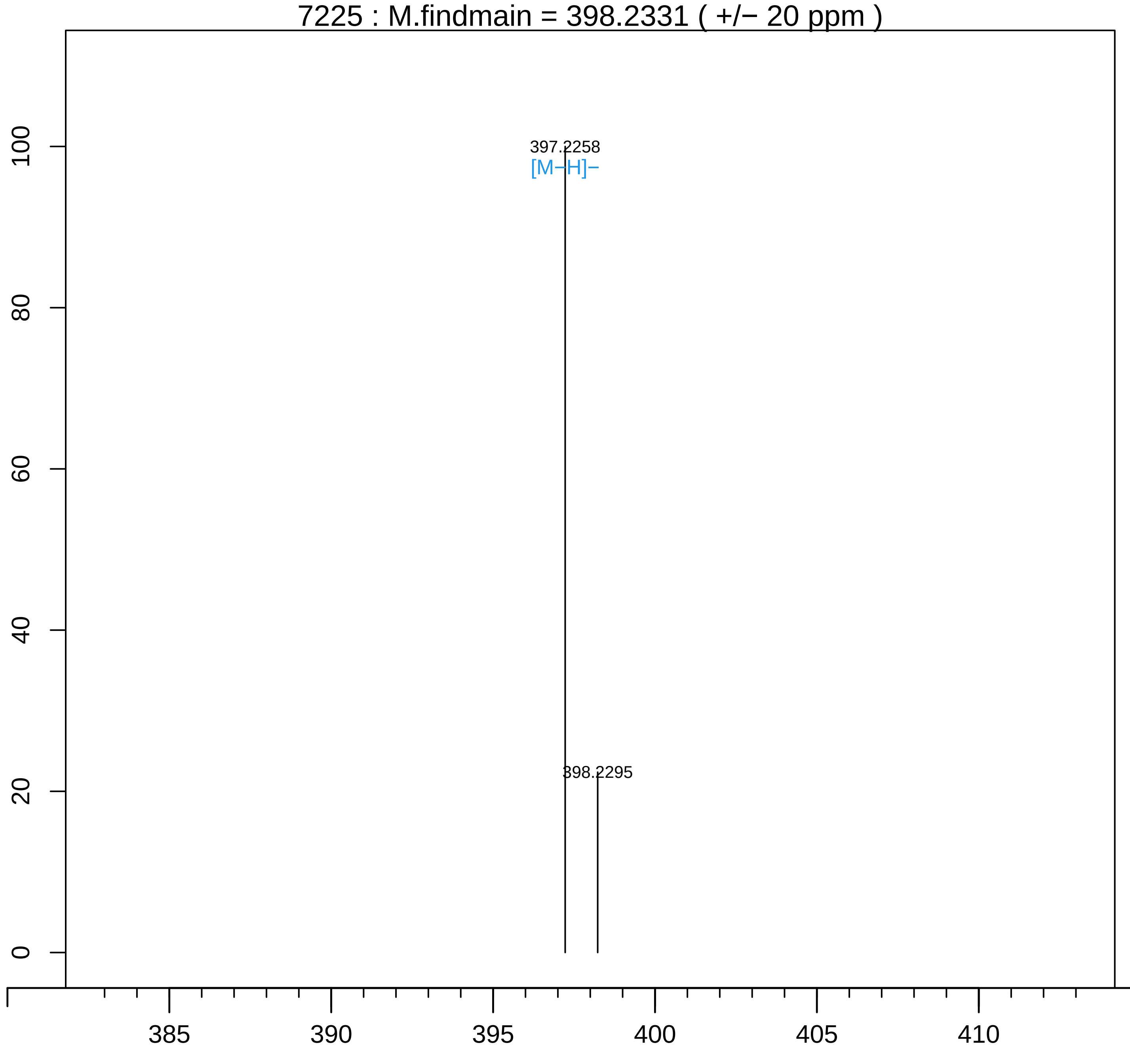
7225 : M.findmain = 398.2331 (+/− 20 ppm)
397.2258
[M−H]−
398.2295
0
20
40
60
80
100
385
390
395
400
405
410

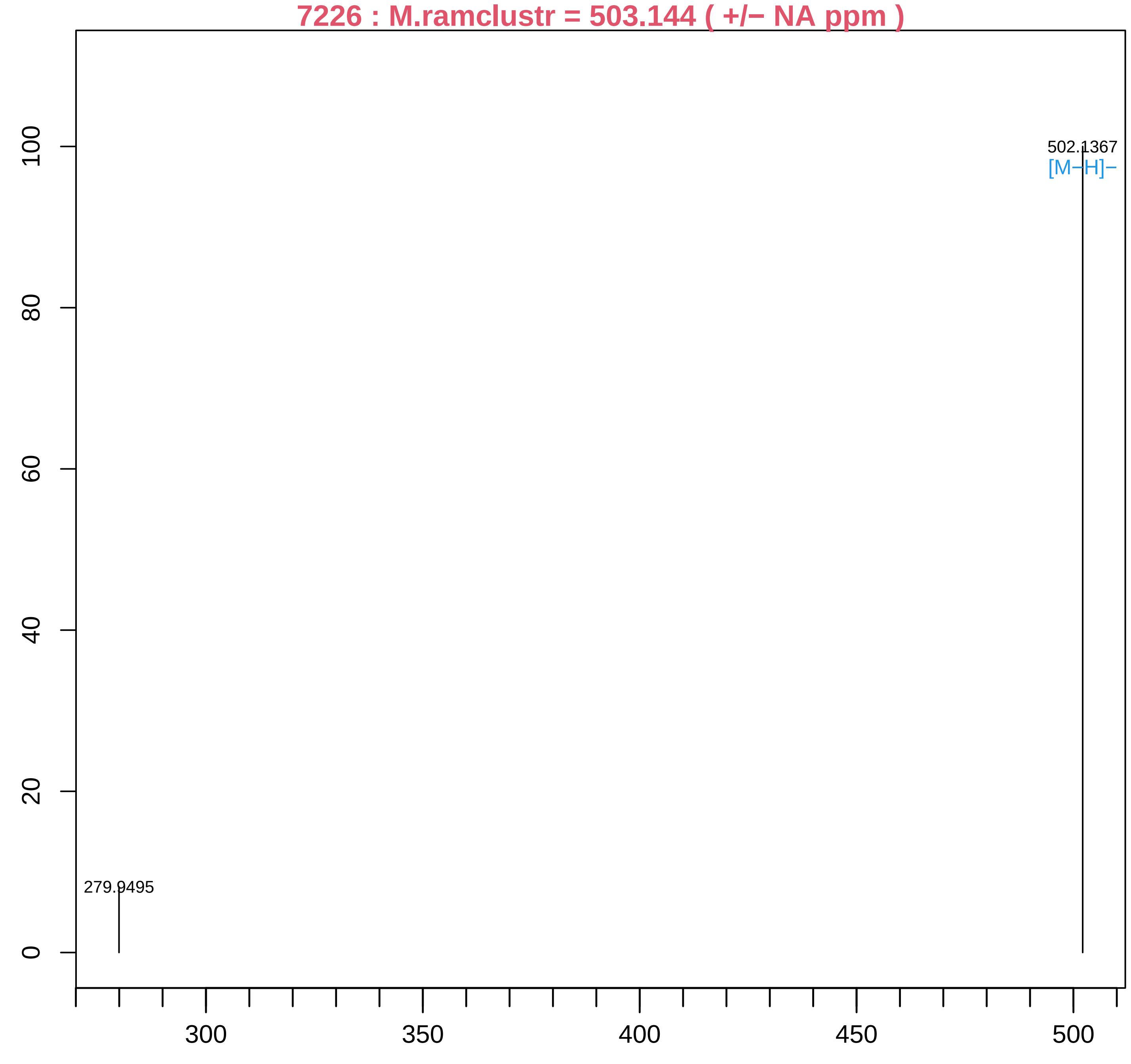
7226 : M.ramclustr = 503.144 (+/− NA ppm)
502.1367
[M−H]−
279.9495
0
20
40
60
80
100
300
350
400
450
500

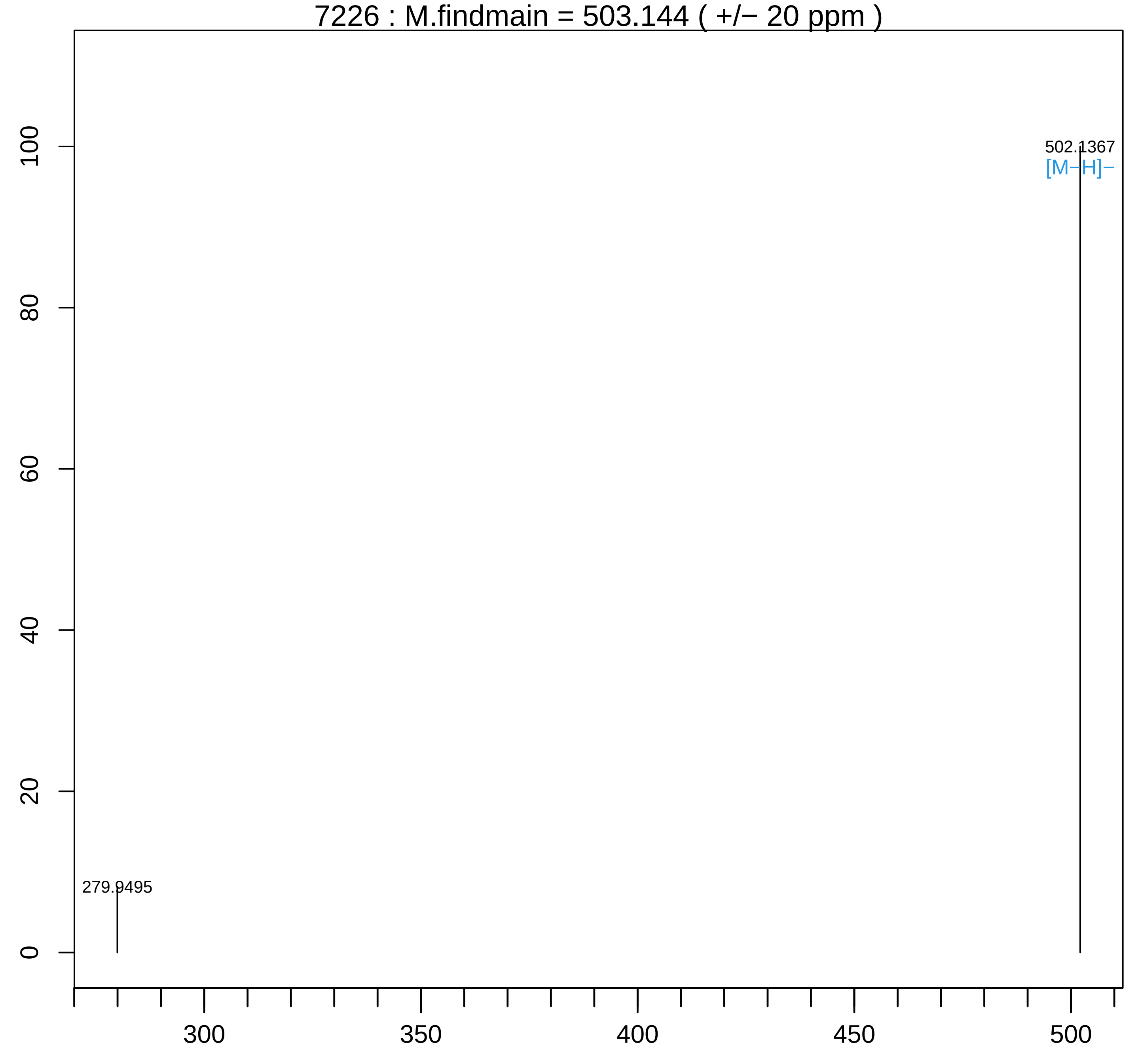
7226 : M.findmain = 503.144 (+/− 20 ppm)
502.1367
[M−H]−
279.9495
0
20
40
60
80
100
300
350
400
450
500

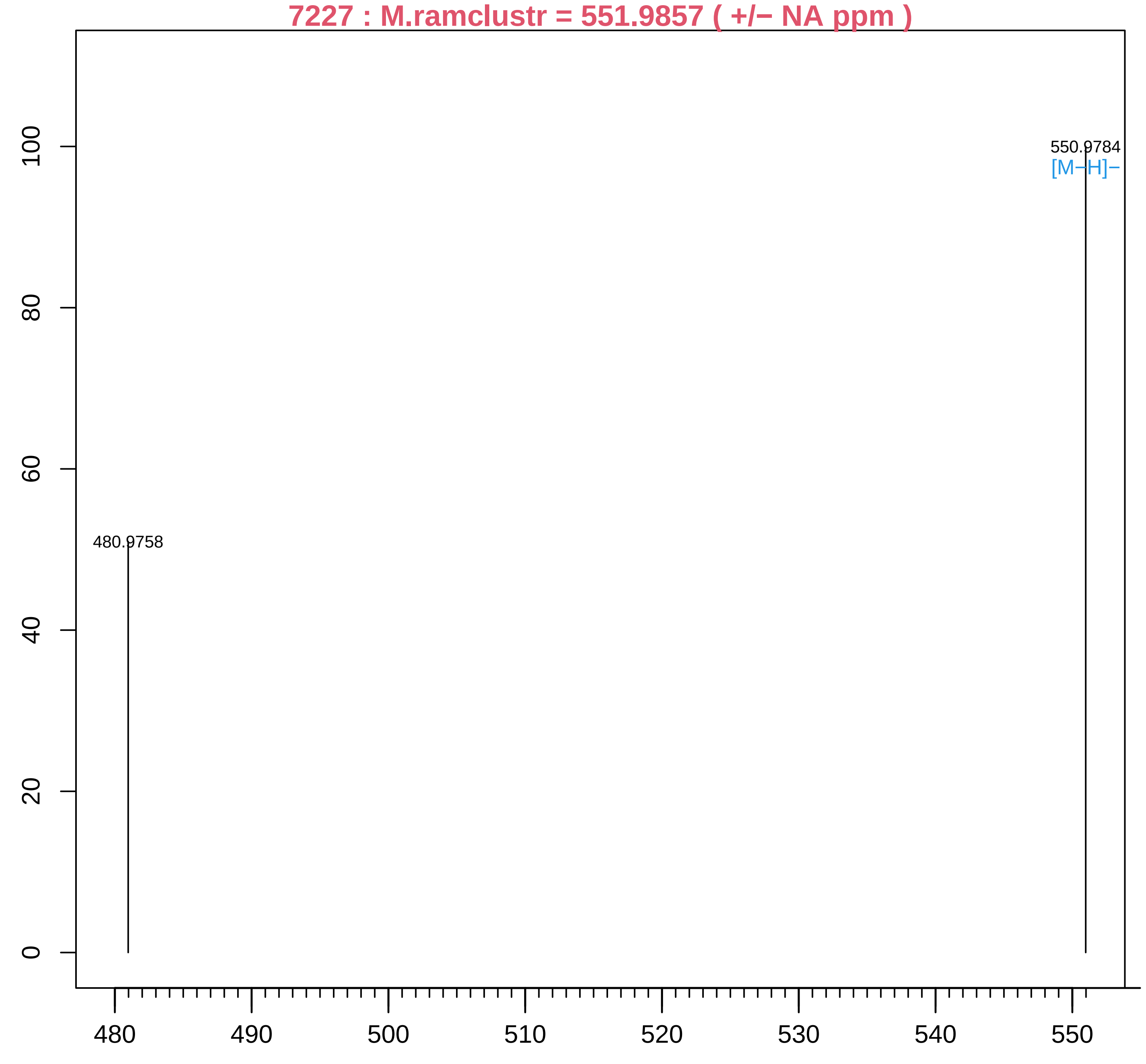

7227 : M.ramclustr = 551.9857 (+/− NA ppm)
550.9784
[M−H]−
480.9758
0
20
40
60
80
100
480
490
500
510
520
530
540
550

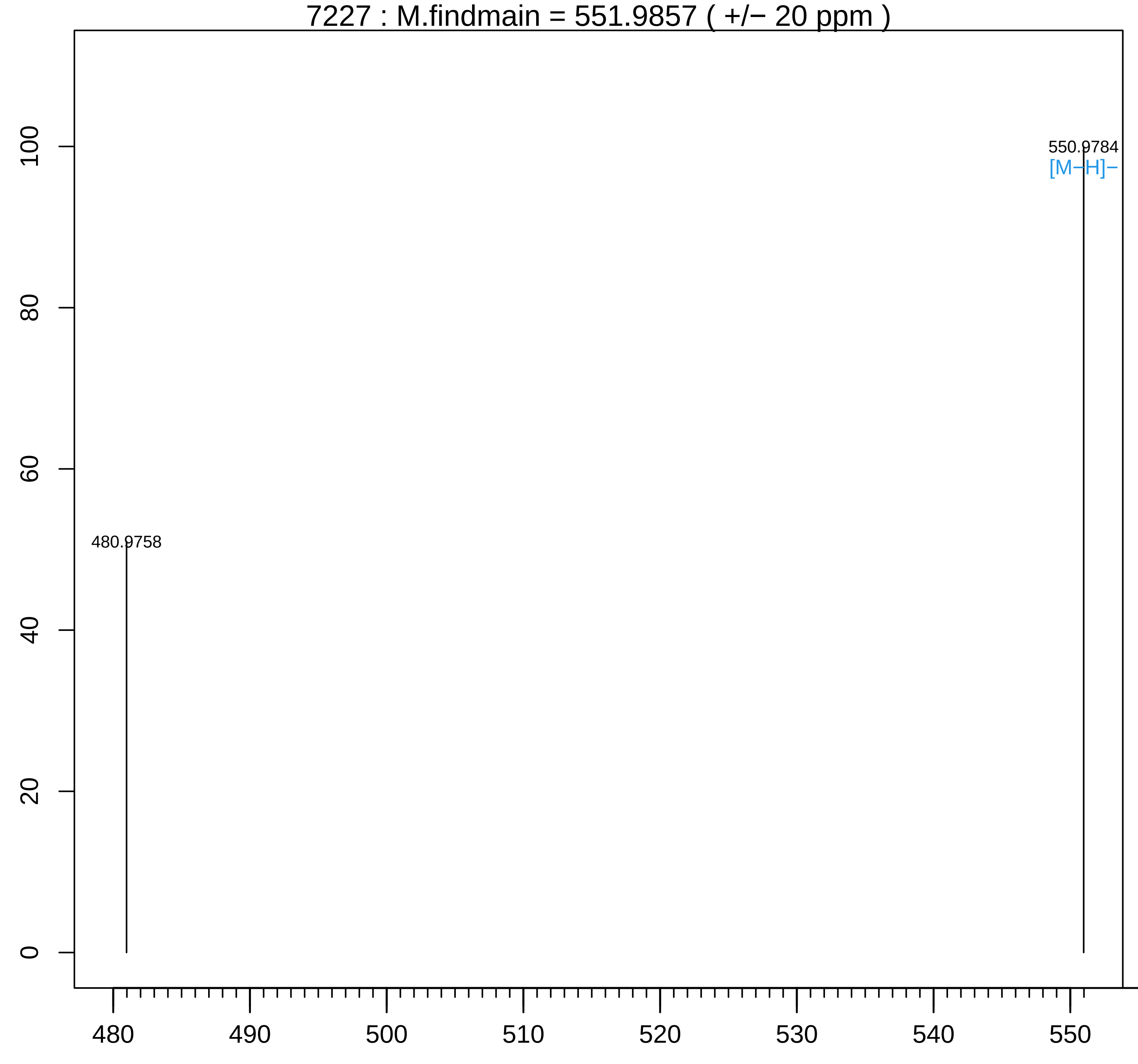

7227 : M.findmain = 551.9857 (+/− 20 ppm)
550.9784
[M−H]−
480.9758
0
20
40
60
80
100
480
490
500
510
520
530
540
550

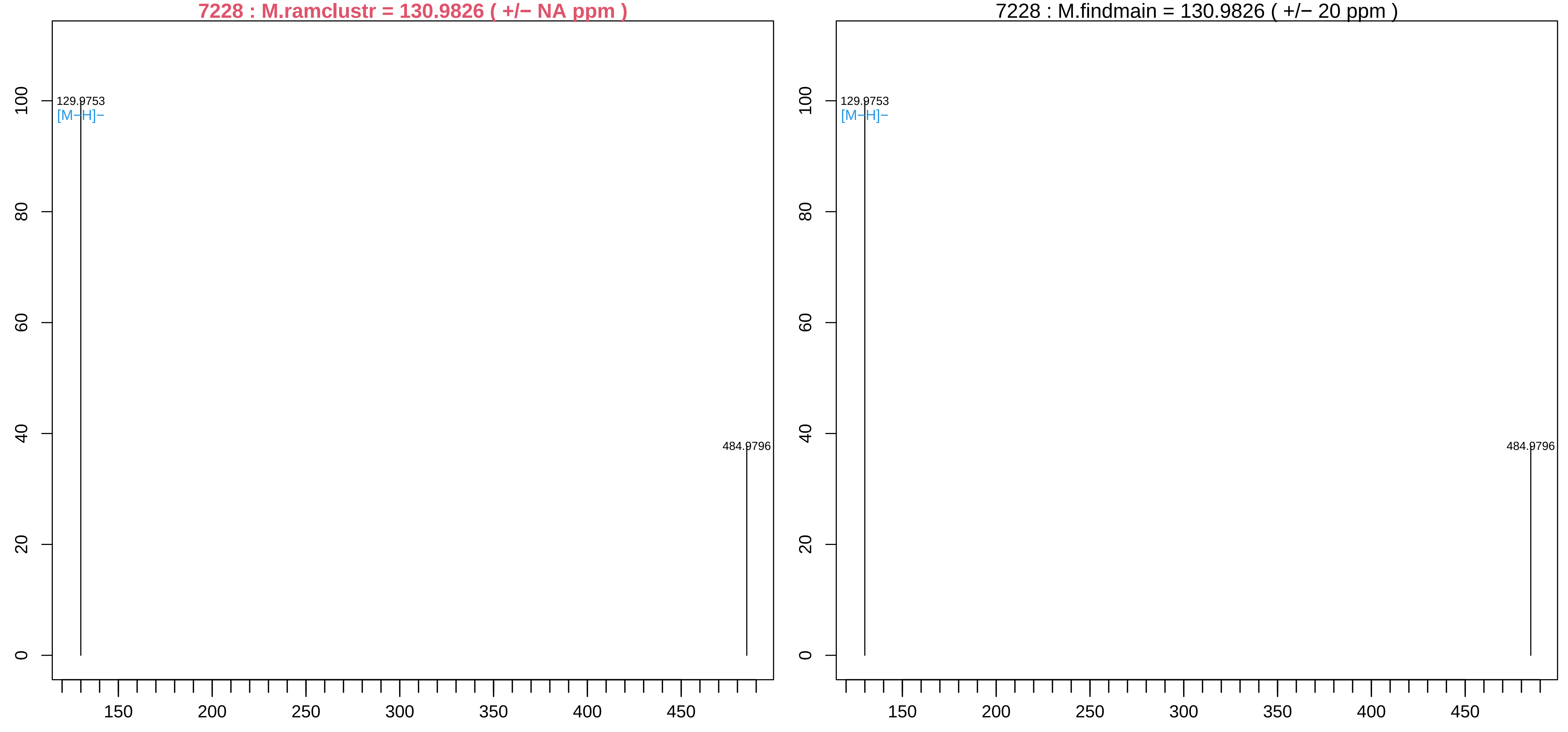
7228 : M.ramclustr = 130.9826 (+/− NA ppm)
129.9753
[M−H]−
484.9796
7228 : M.findmain = 130.9826 (+/− 20 ppm)
129.9753
[M−H]−
484.9796

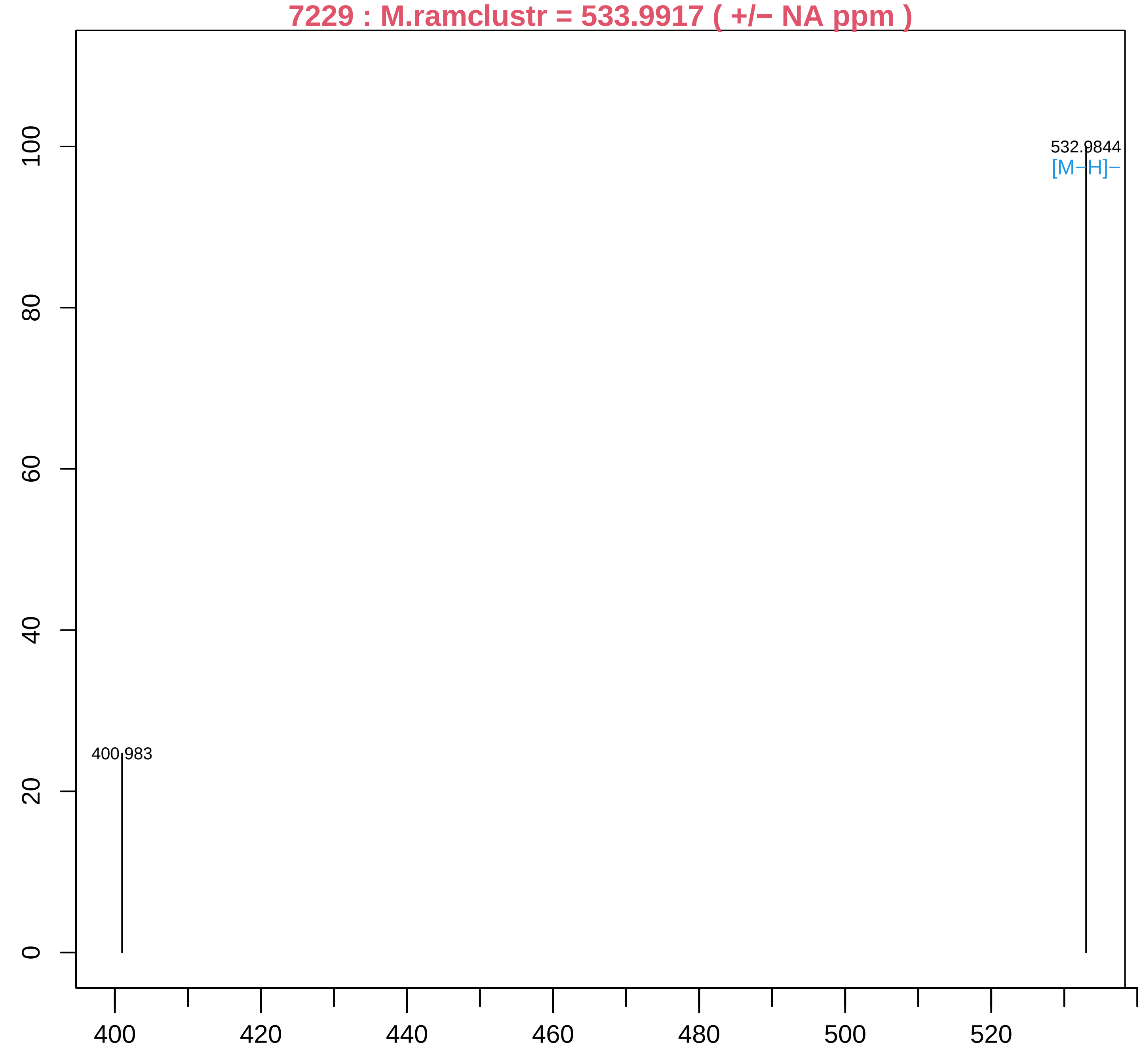
7229 : M.ramclustr = 533.9917 (+/− NA ppm)
532.9844
[M−H]−
400.983
0
20
40
60
80
100
400
420
440
460
480
500
520

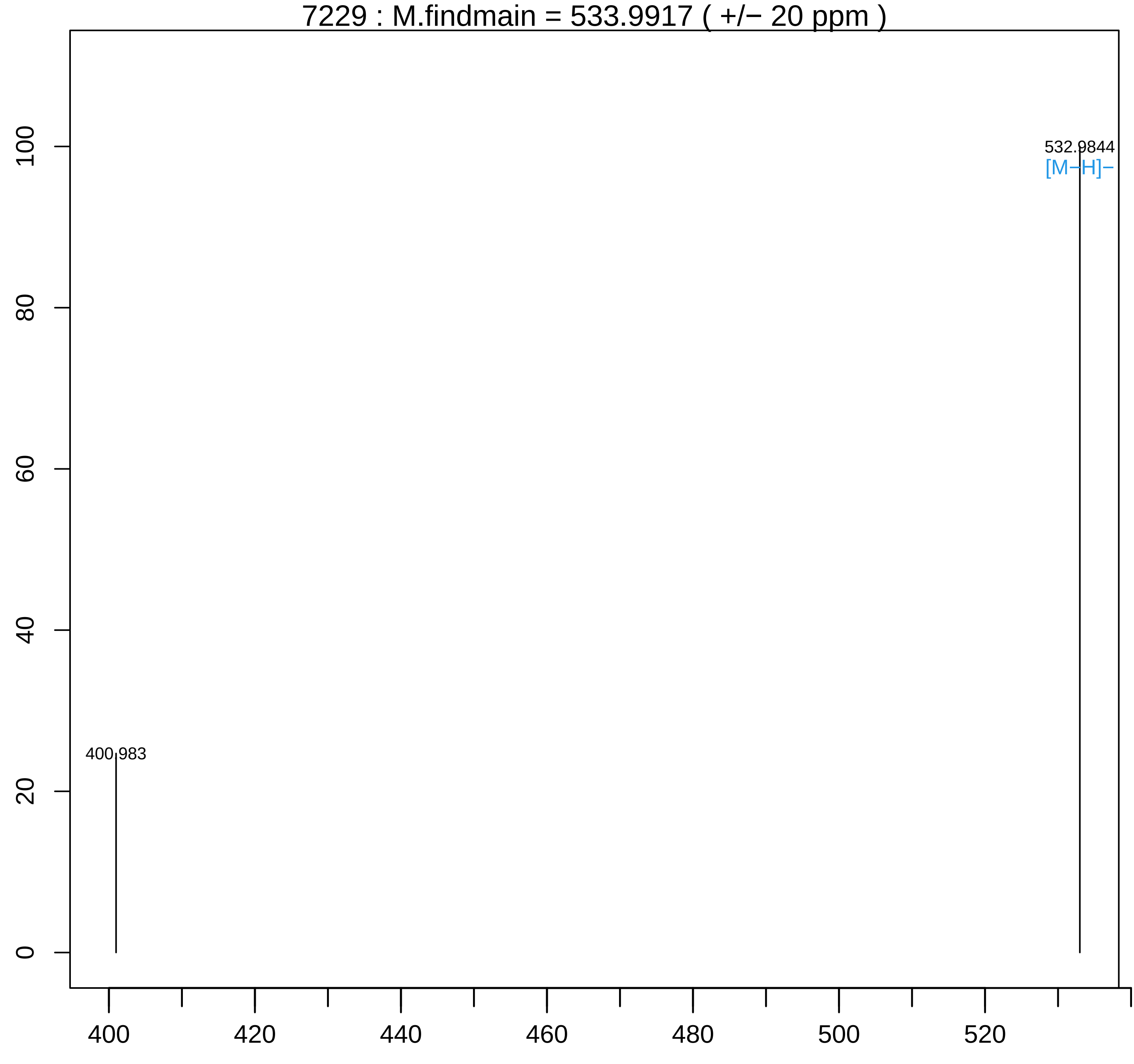
7229 : M.findmain = 533.9917 (+/− 20 ppm)
532.9844
[M−H]−
400.983
0
20
40
60
80
100
400
420
440
460
480
500
520

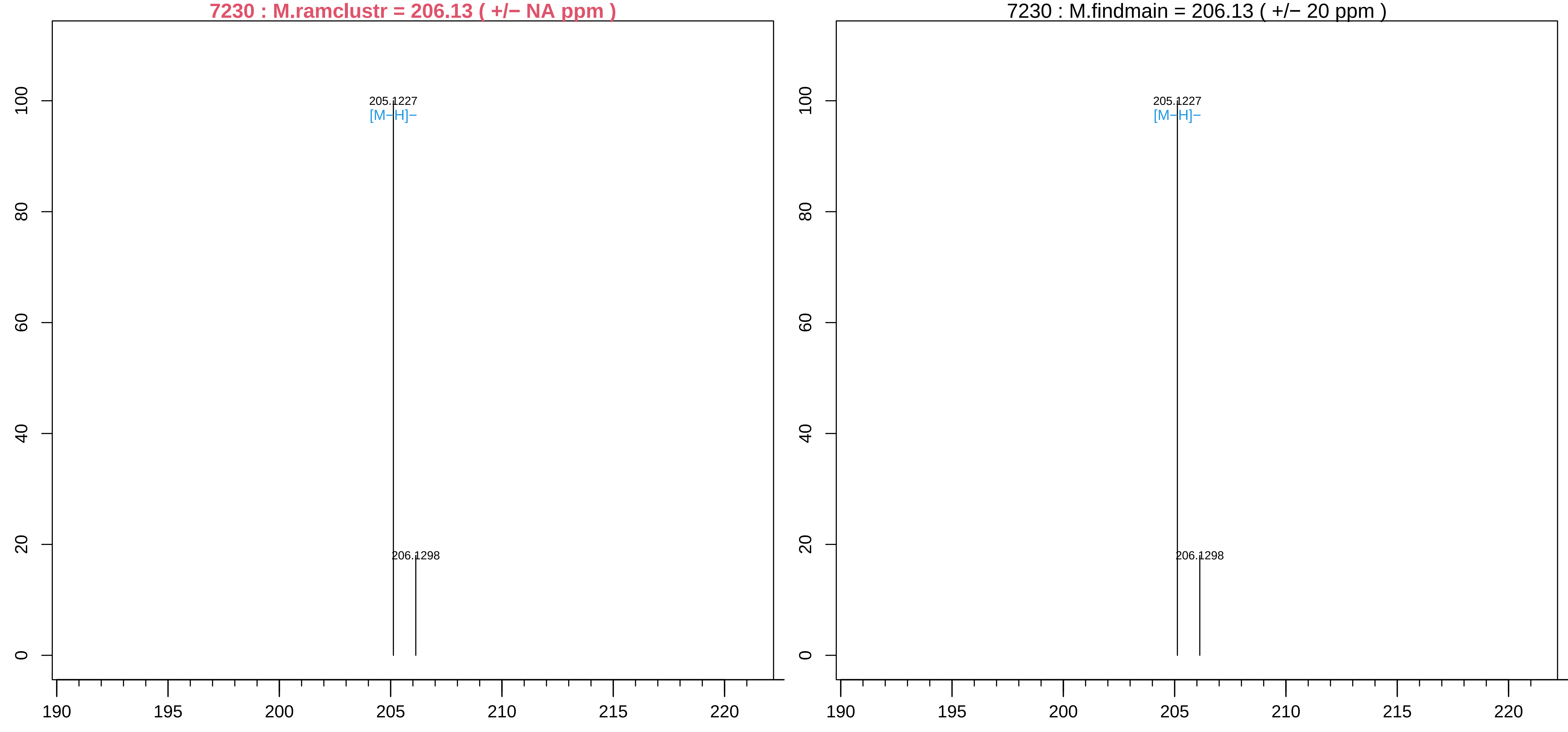
7230 : M.ramclustr = 206.13 (+/− NA ppm)
205.1227
[M−H]−
206.1298
0
20
40
60
80
100
190
195
200
205
210
215
220
7230 : M.findmain = 206.13 (+/− 20 ppm)
205.1227
[M−H]−
206.1298
0
20
40
60
80
100
190
195
200
205
210
215
220

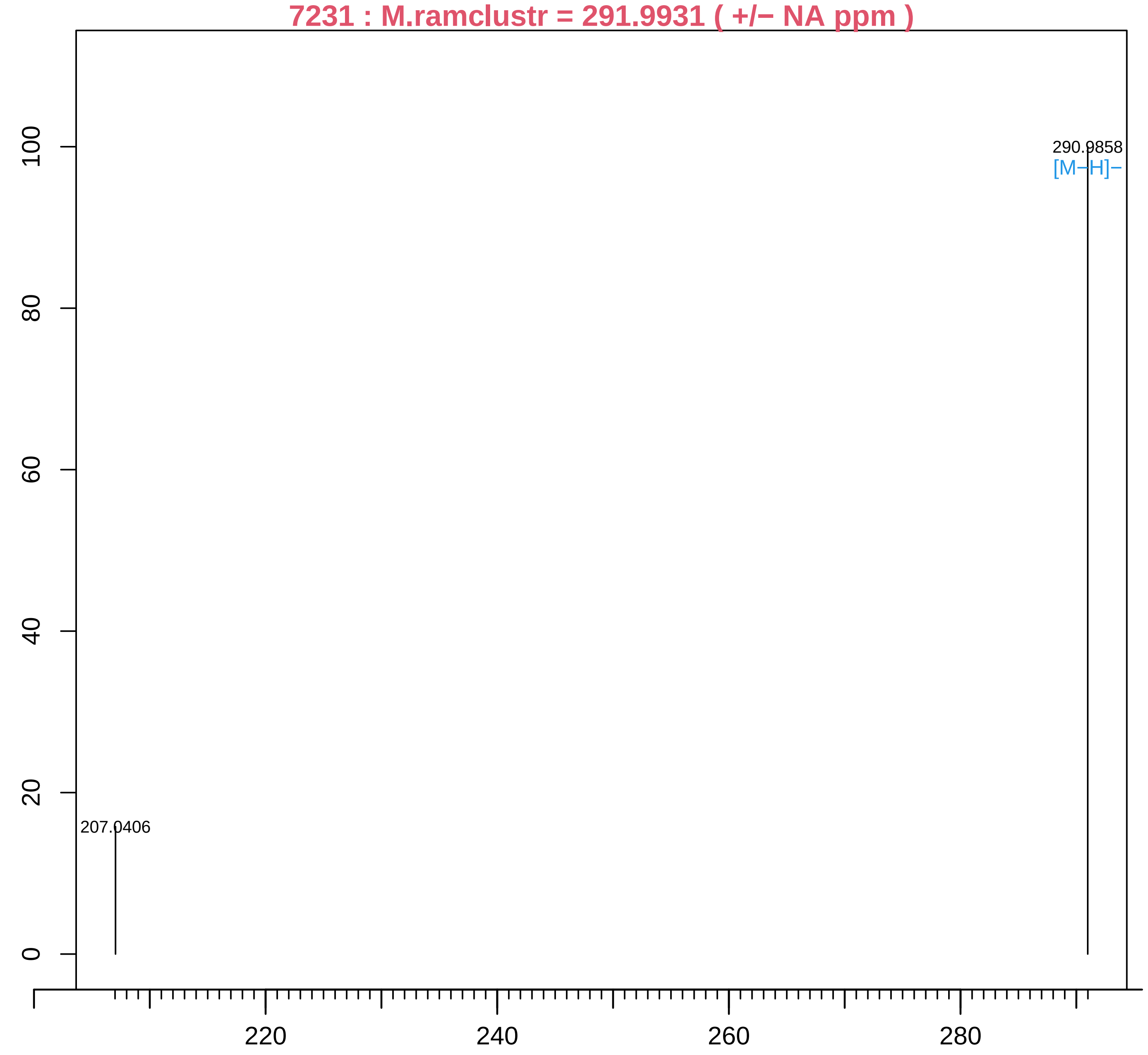
7231 : M.ramclustr = 291.9931 (+/− NA ppm)
290.9858
[M−H]−
207.0406
0
20
40
60
80
100
220
240
260
280

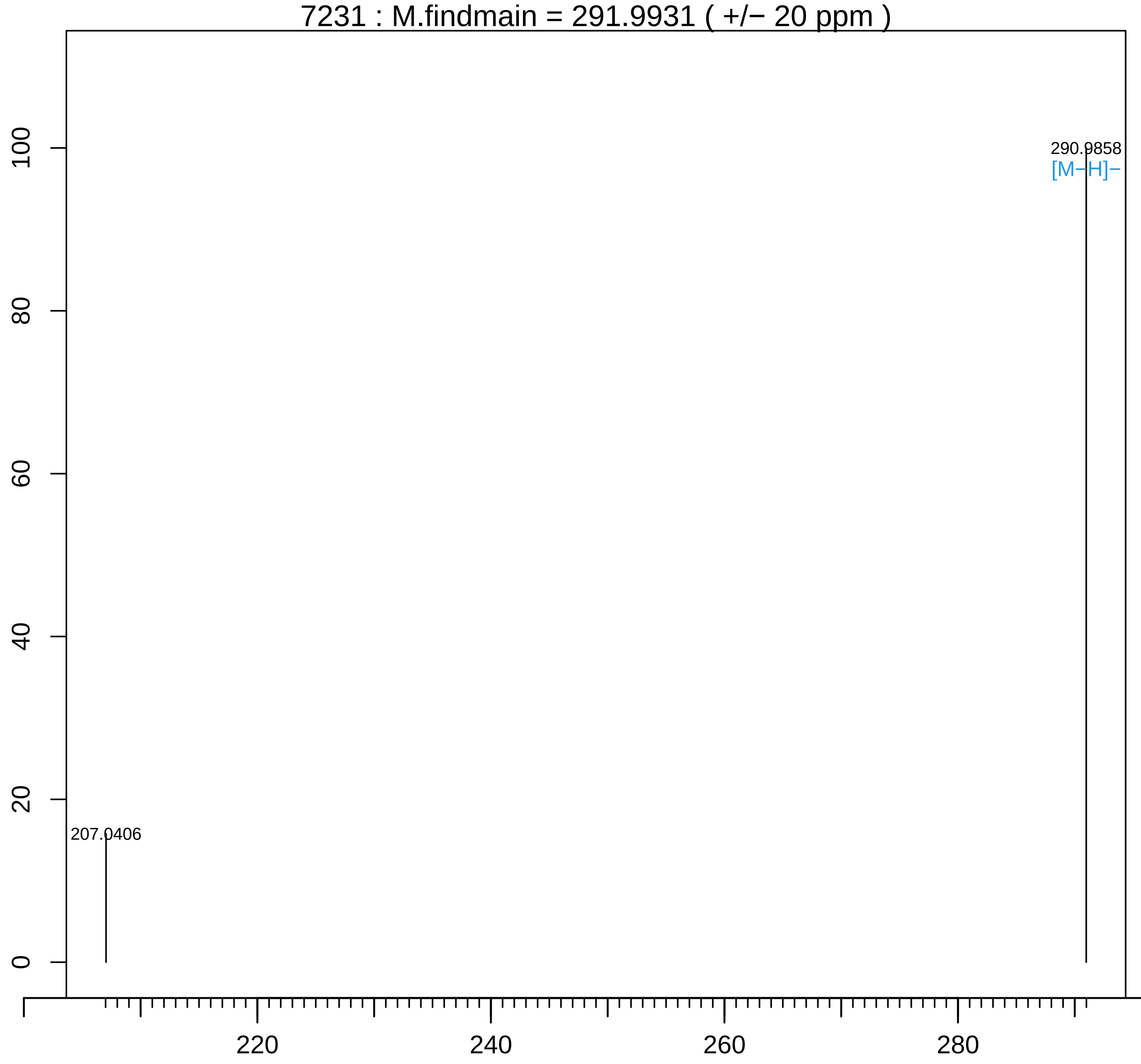
7231 : M.findmain = 291.9931 (+/− 20 ppm)
290.9858
[M−H]−
207.0406
0
20
40
60
80
100
220
240
260
280

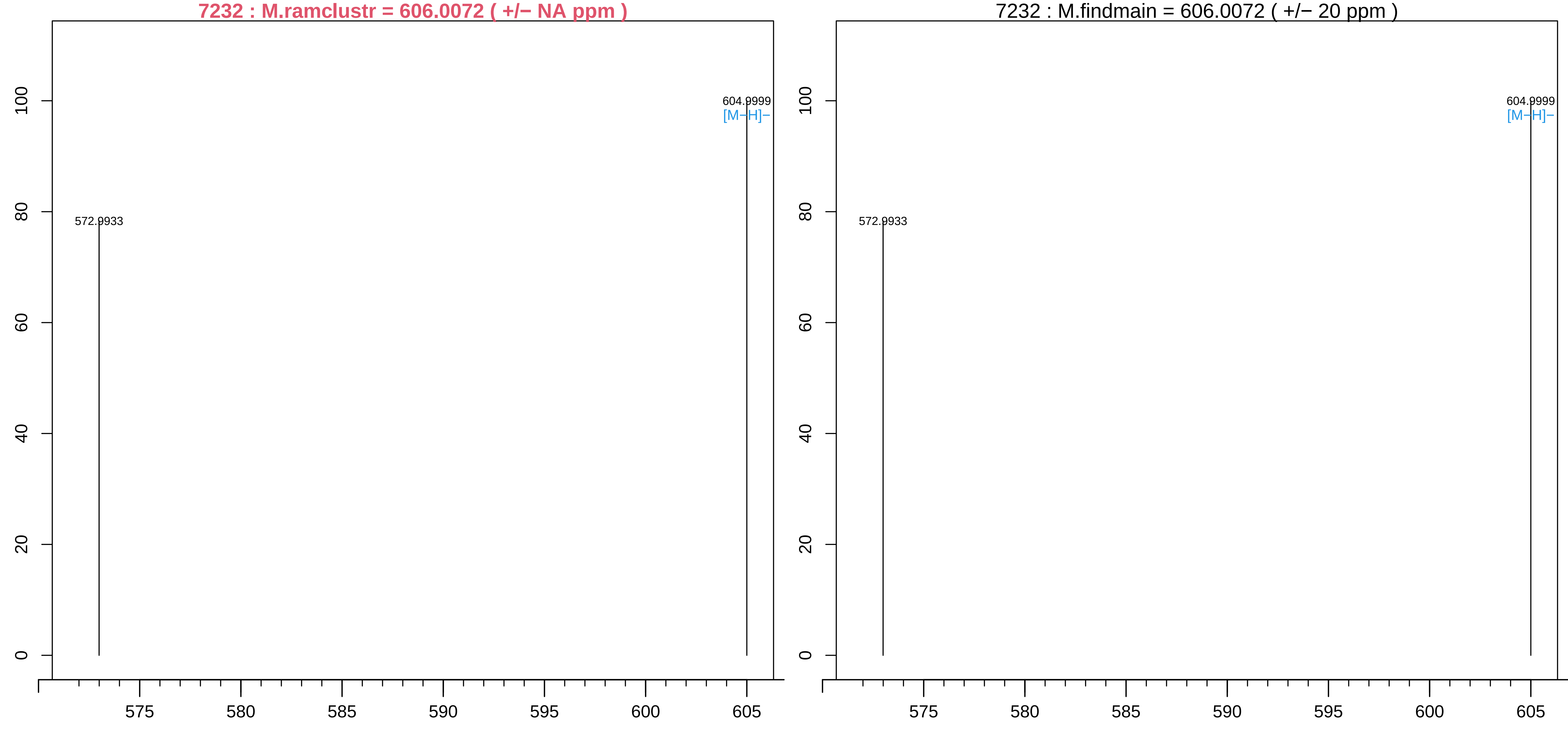
7232 : M.ramclustr = 606.0072 (+/− NA ppm)
7232 : M.findmain = 606.0072 (+/− 20 ppm)
604.9999
[M−H]−
572.9933
604.9999
[M−H]−
572.9933
0
20
40
60
80
100
575
580
585
590
595
600
605

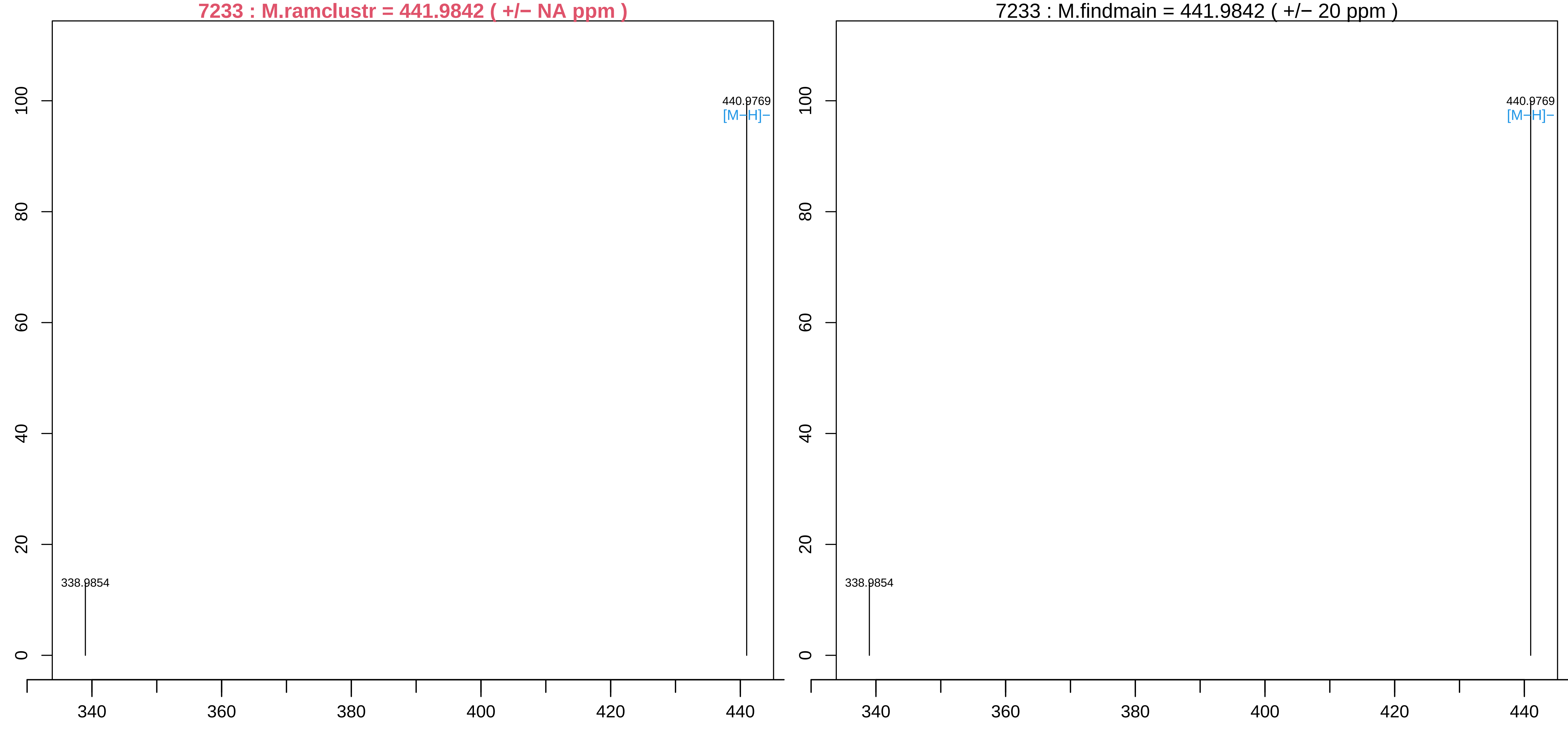
7233 : M.ramclustr = 441.9842 (+/− NA ppm)
440.9769
[M−H]−
338.9854
7233 : M.findmain = 441.9842 (+/− 20 ppm)
440.9769
[M−H]−
338.9854
0
20
40
60
80
100
340
360
380
400
420
440

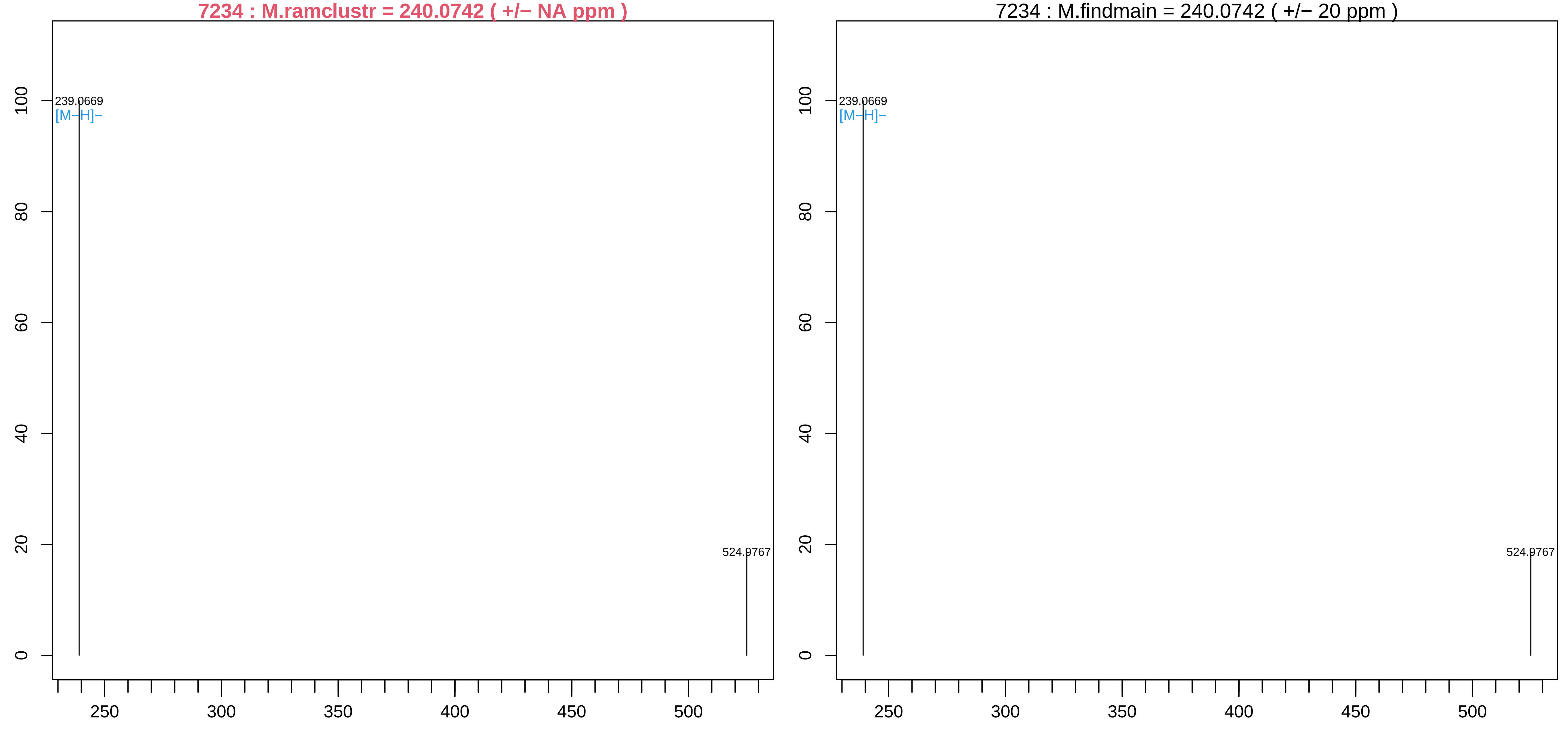

7234 : M.ramclustr = 240.0742 (+/− NA ppm)
239.0669
[M−H]−
524.9767
7234 : M.findmain = 240.0742 (+/− 20 ppm)
239.0669
[M−H]−
524.9767
0
20
40
60
80
100
250
300
350
400
450
500

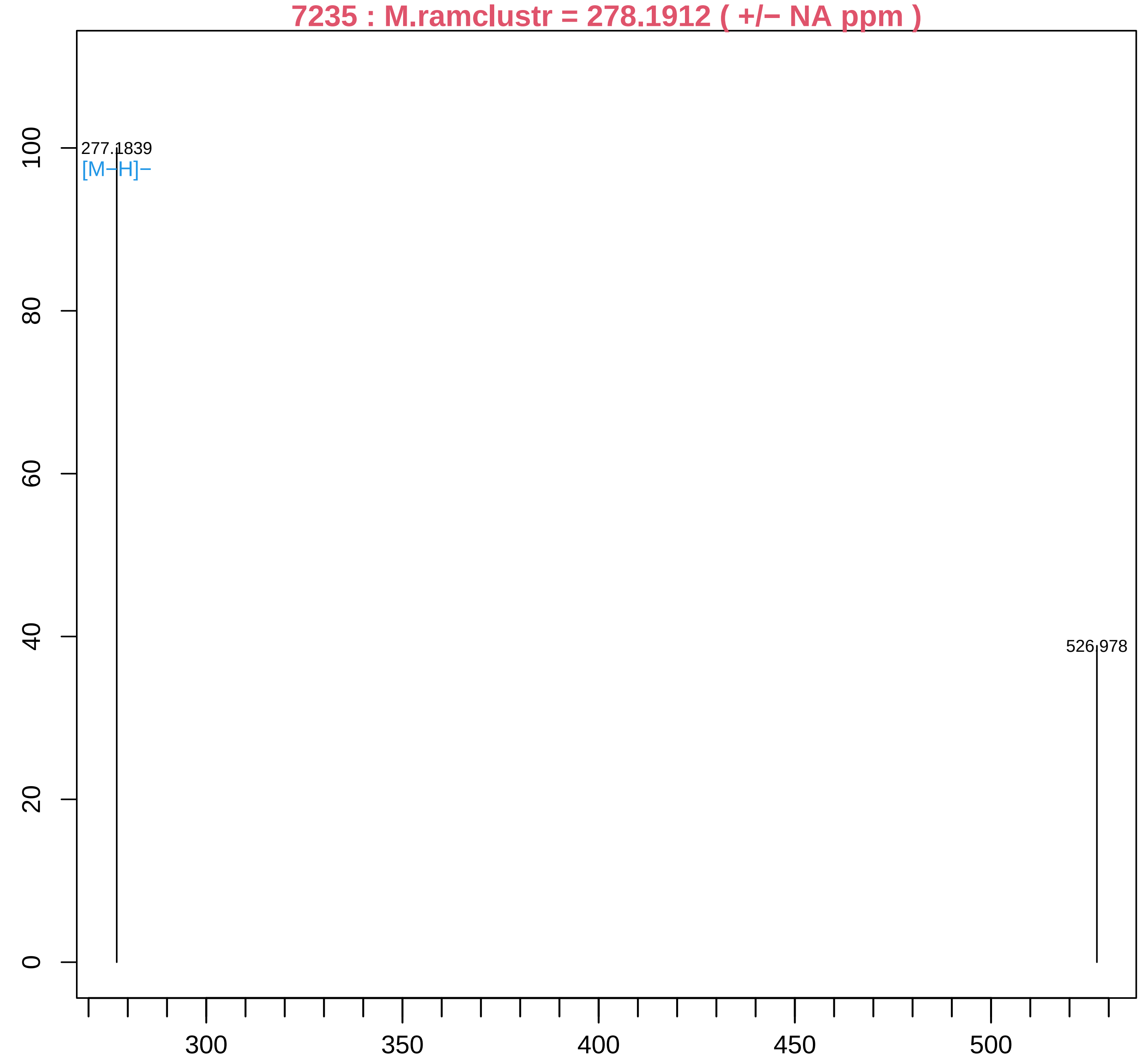
7235 : M.ramclustr = 278.1912 (+/− NA ppm)
277.1839
[M−H]−
526.978
0
20
40
60
80
100
300
350
400
450
500

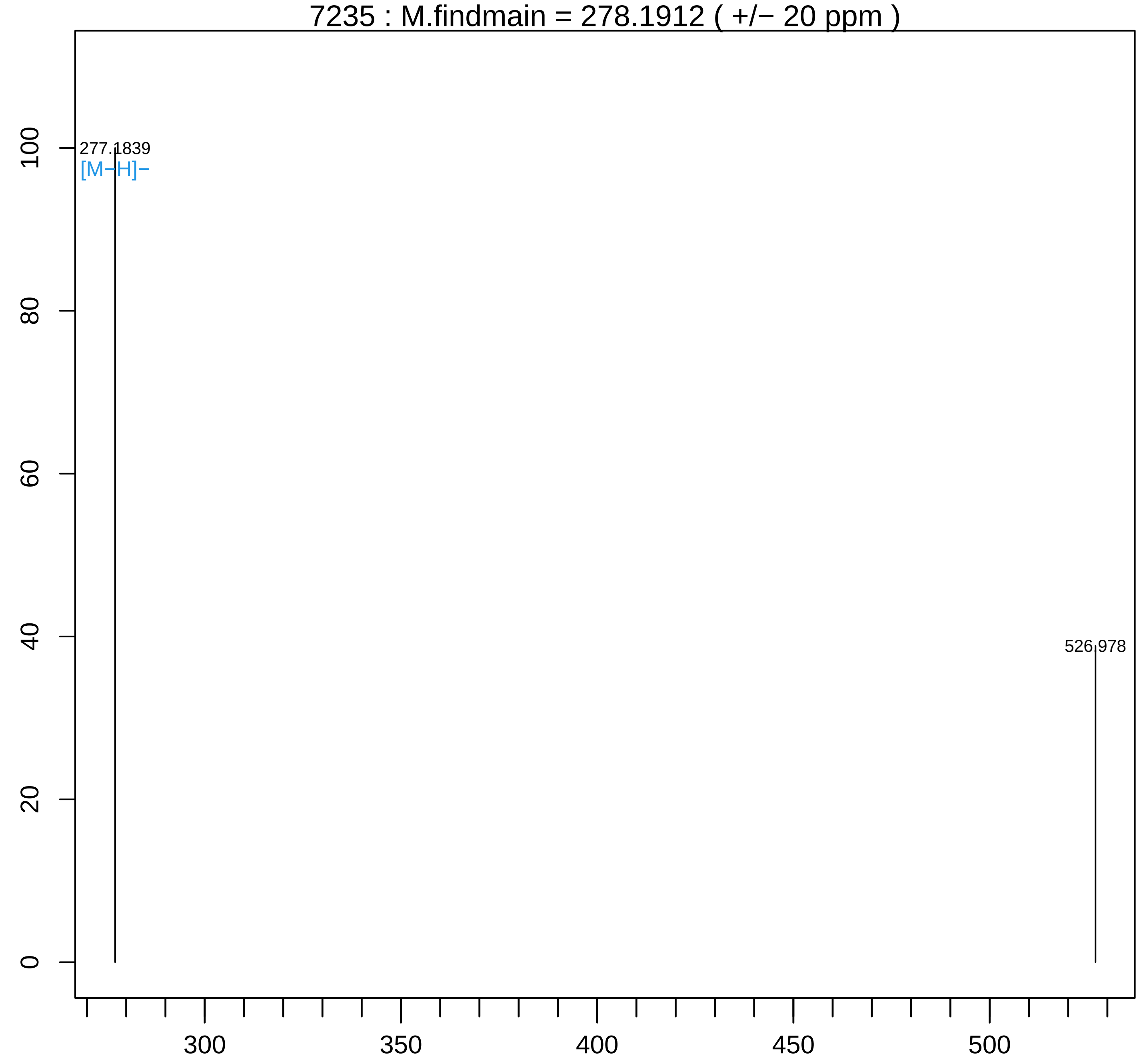
7235 : M.findmain = 278.1912 (+/− 20 ppm)
277.1839
[M−H]−
526.978
0
20
40
60
80
100
300
350
400
450
500

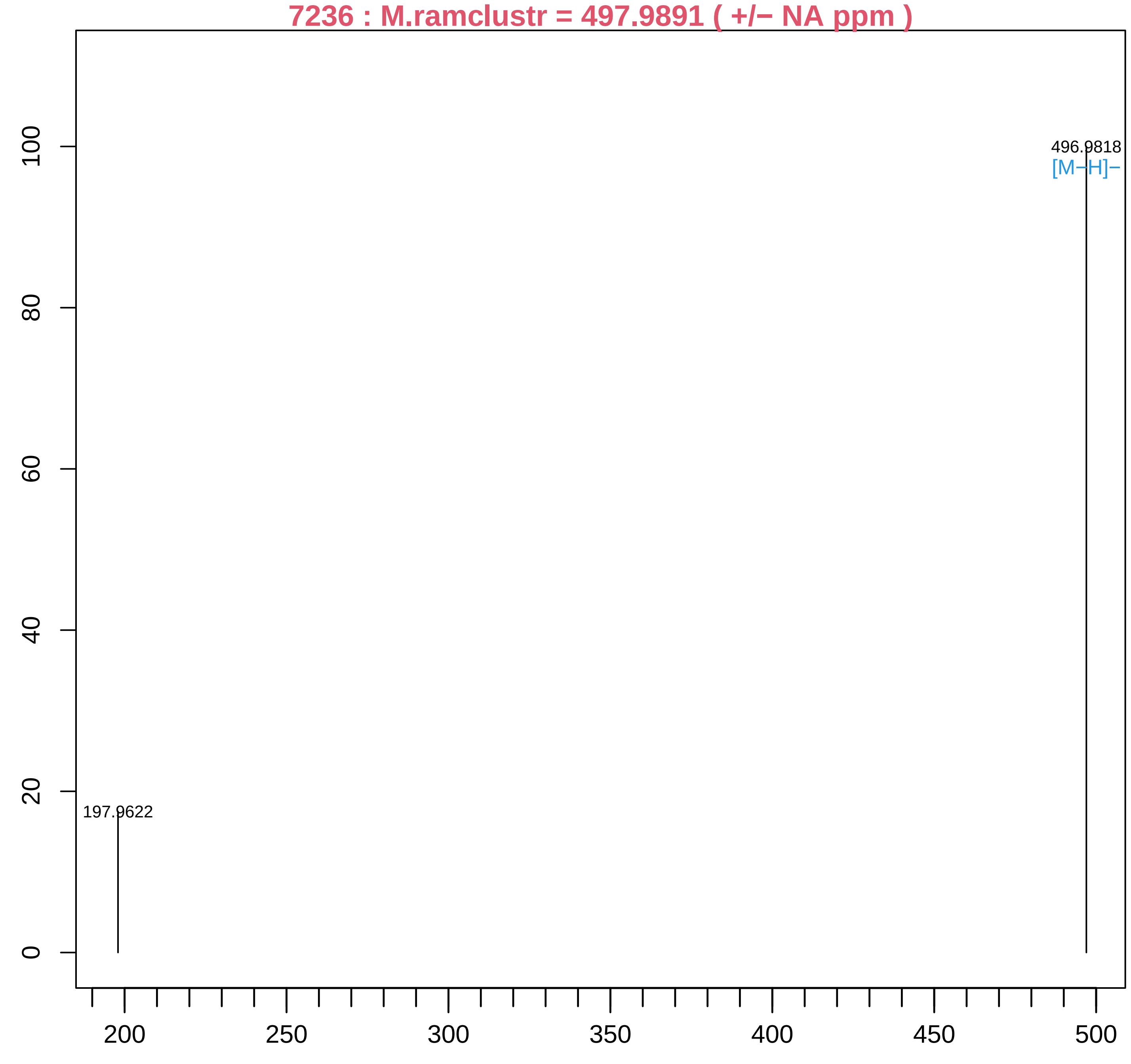
7236 : M.ramclustr = 497.9891 (+/− NA ppm)
496.9818
[M−H]−
197.9622
0
20
40
60
80
100
200
250
300
350
400
450
500

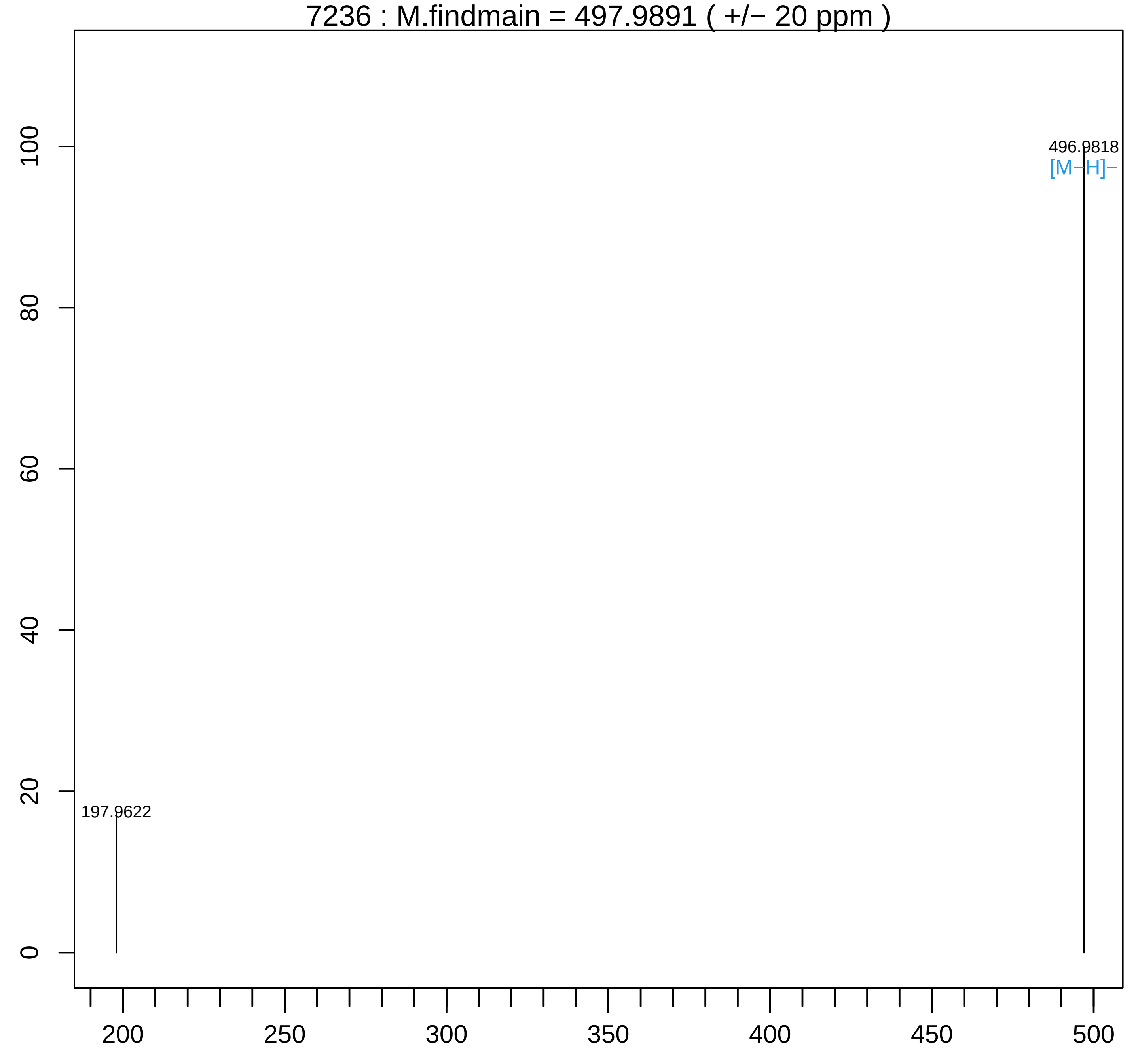
7236 : M.findmain = 497.9891 (+/− 20 ppm)
496.9818
[M−H]−
197.9622
0
20
40
60
80
100
200
250
300
350
400
450
500

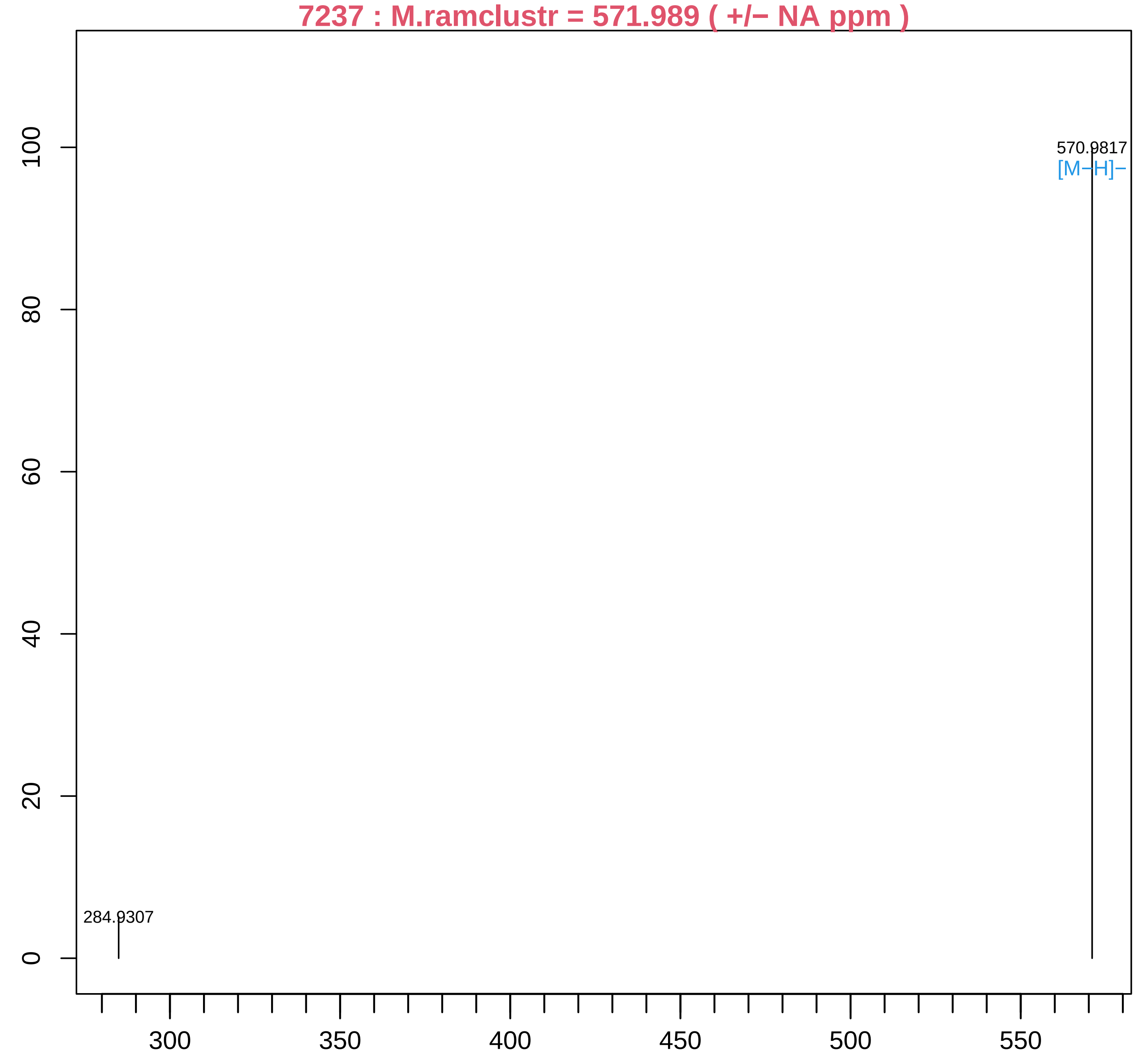
7237 : M.ramclustr = 571.989 (+/− NA ppm)
570.9817
[M−H]−
284.9307
0
20
40
60
80
100
300
350
400
450
500
550

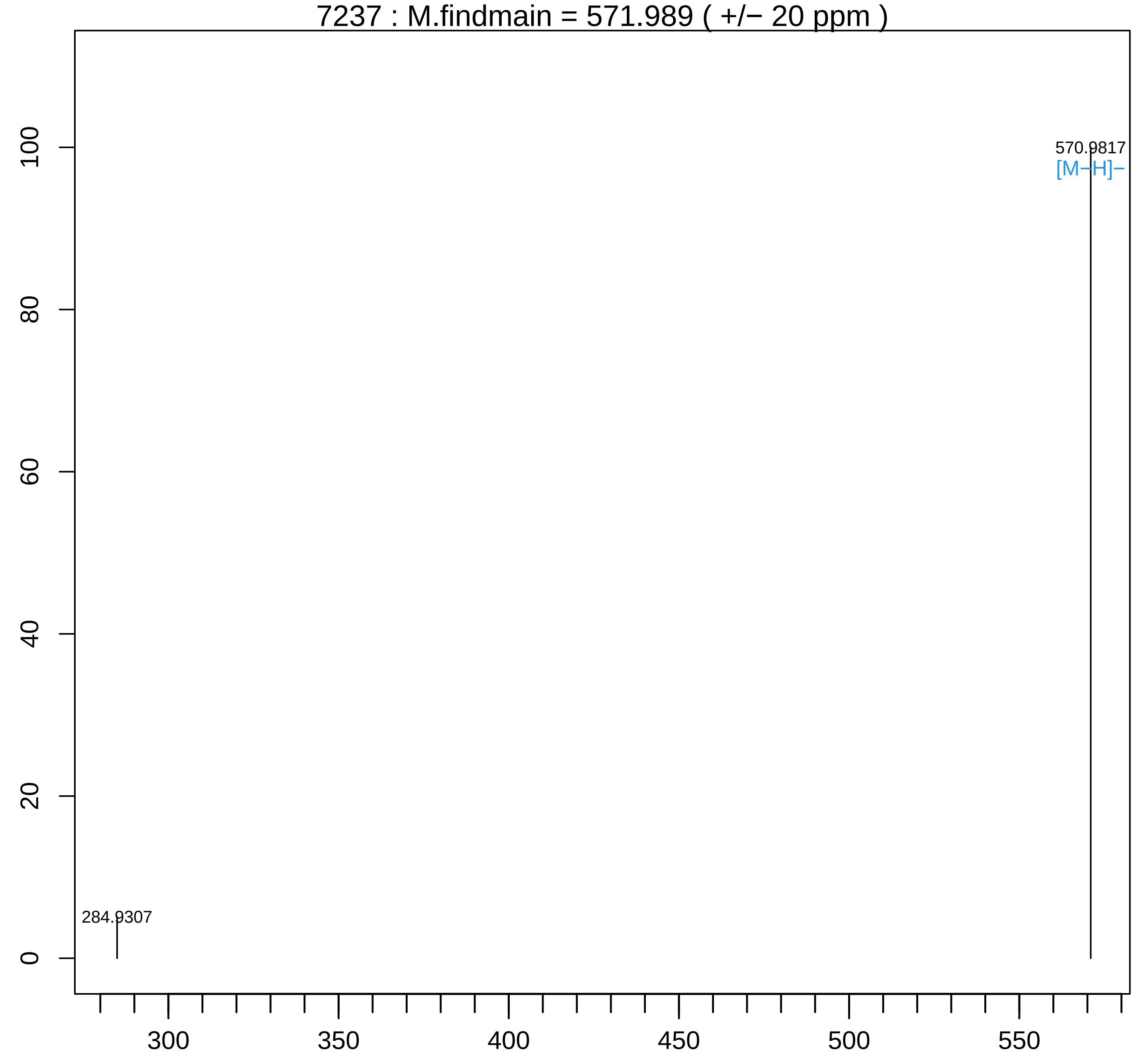
7237 : M.findmain = 571.989 (+/− 20 ppm)
570.9817
[M−H]−
284.9307
0
20
40
60
80
100
300
350
400
450
500
550

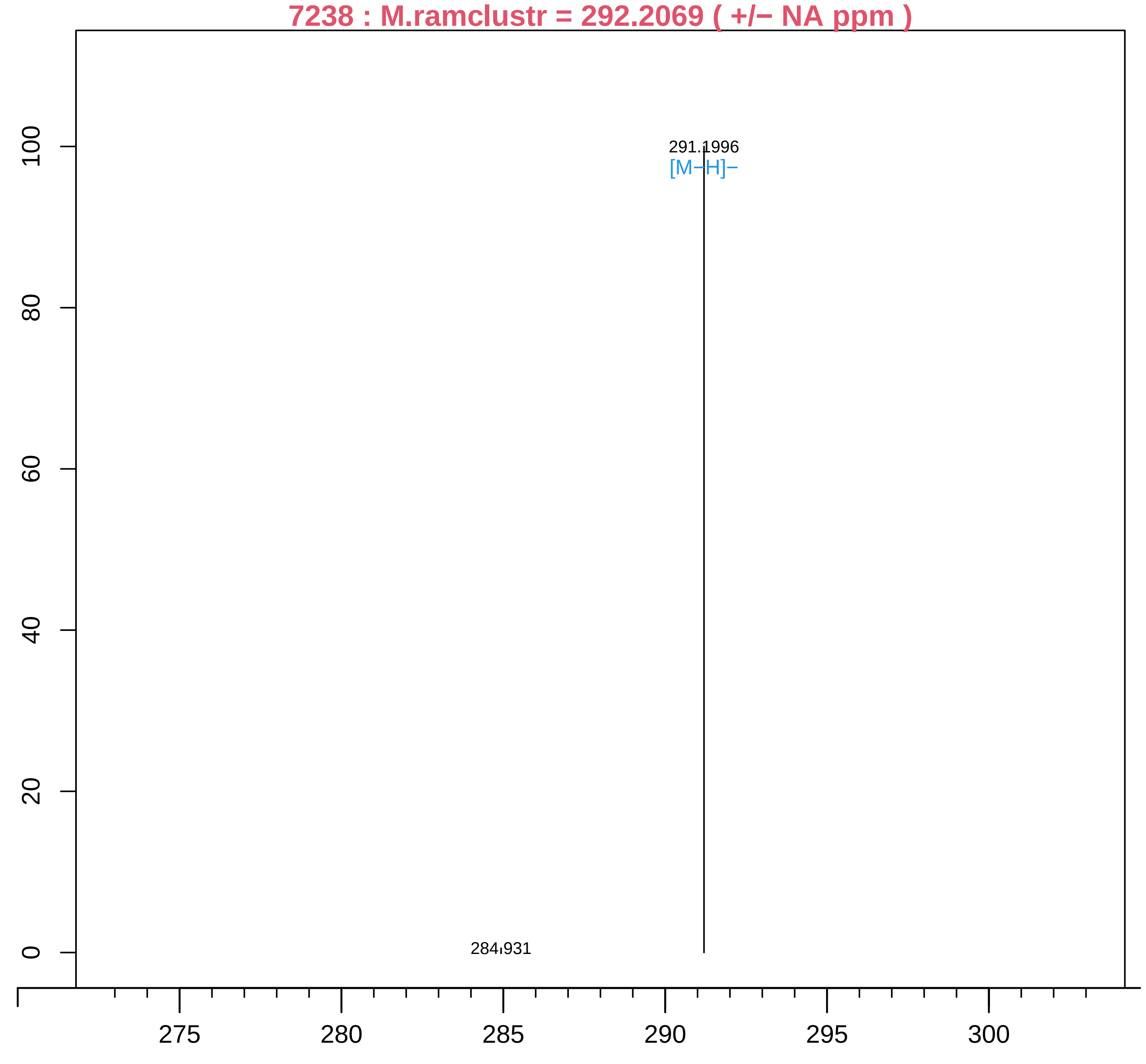

7238 : M.ramclustr = 292.2069 (+/− NA ppm)
291.1996
[M−H]−
284.931
0
20
40
60
80
100
275
280
285
290
295
300

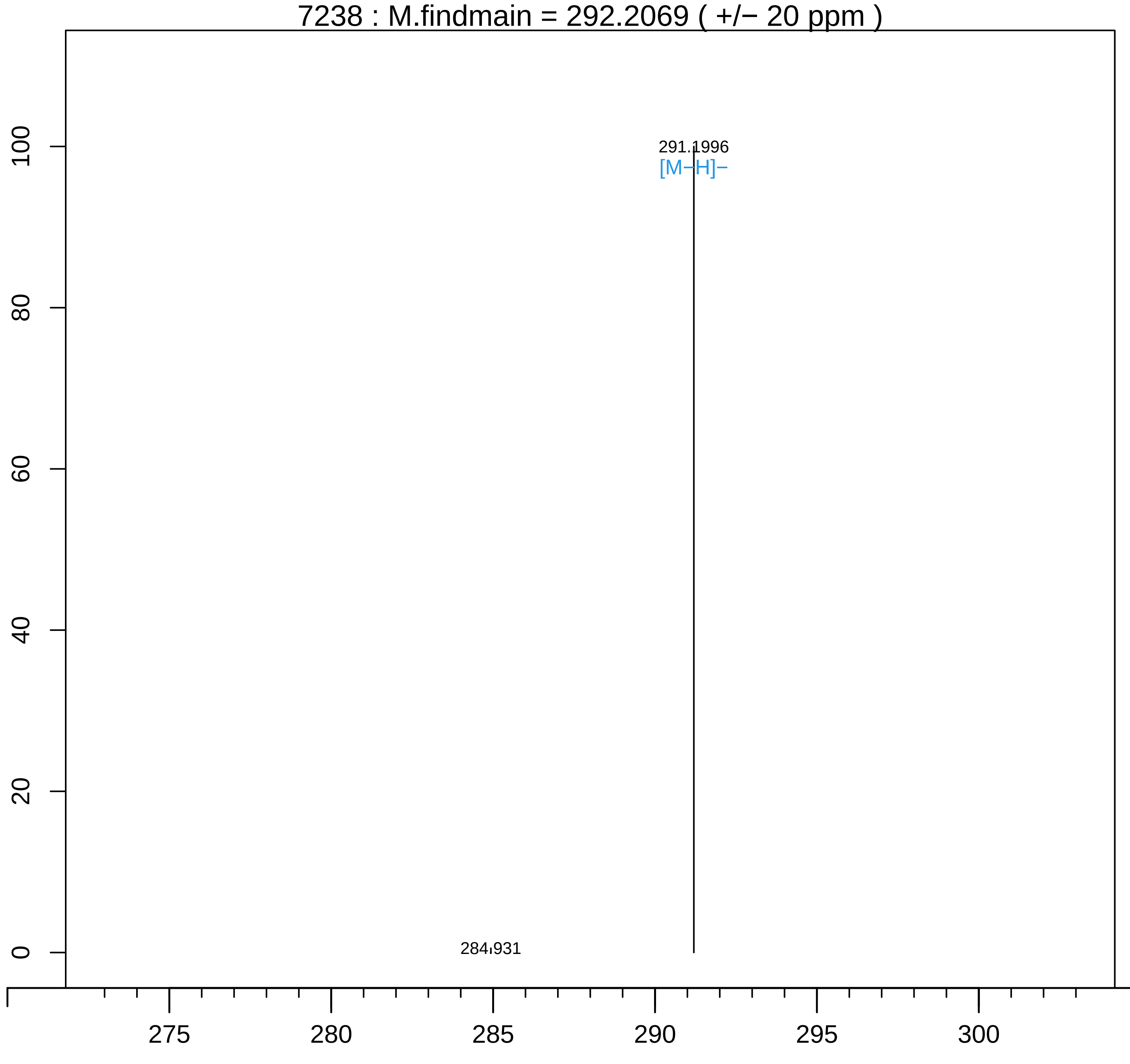

7238 : M.findmain = 292.2069 (+/− 20 ppm)
291.1996
[M−H]−
284.931
0
20
40
60
80
100
275
280
285
290
295
300

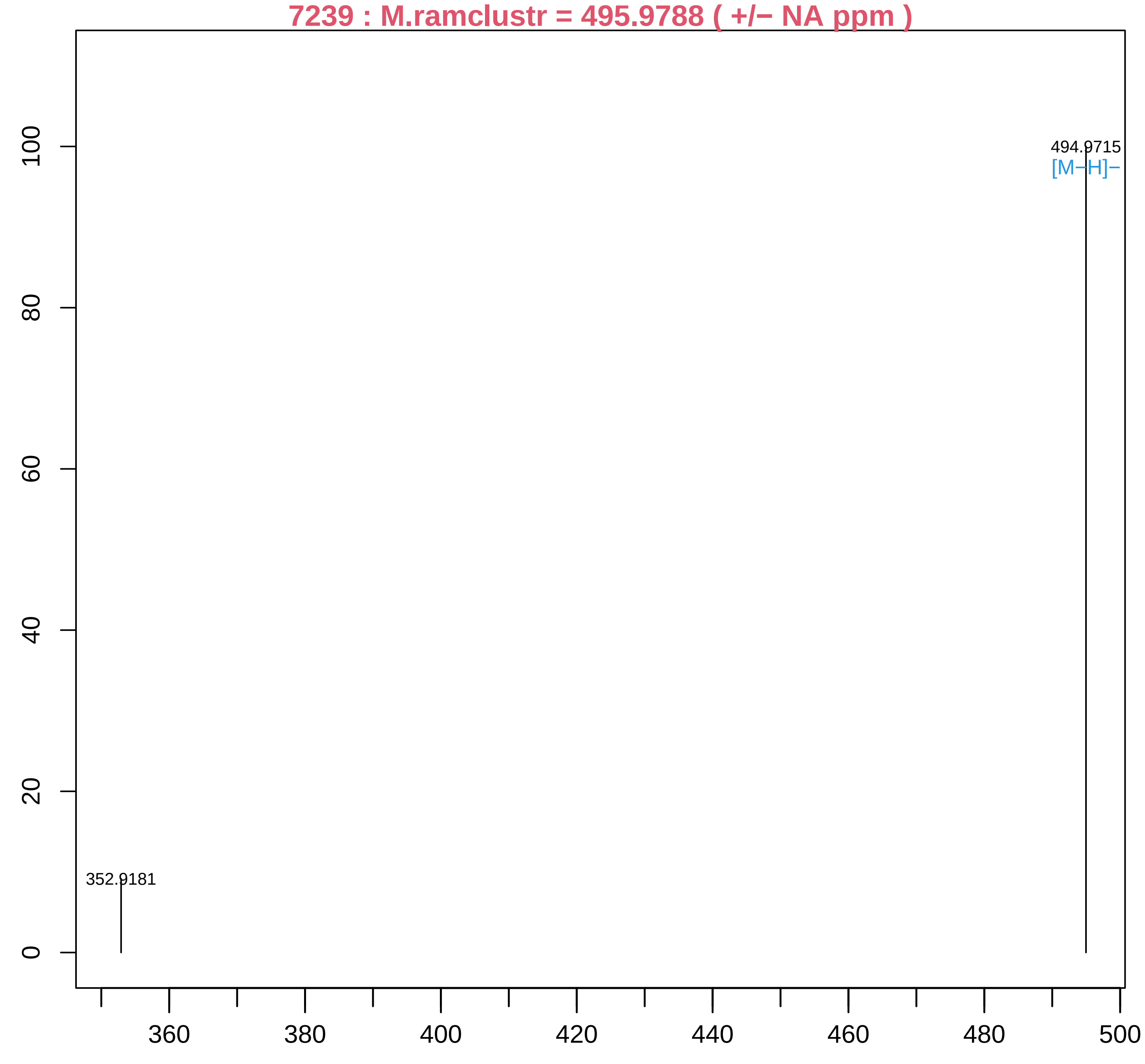
7239 : M.ramclustr = 495.9788 (+/− NA ppm)
494.9715
[M−H]−
352.9181
0
20
40
60
80
100
360
380
400
420
440
460
480
500

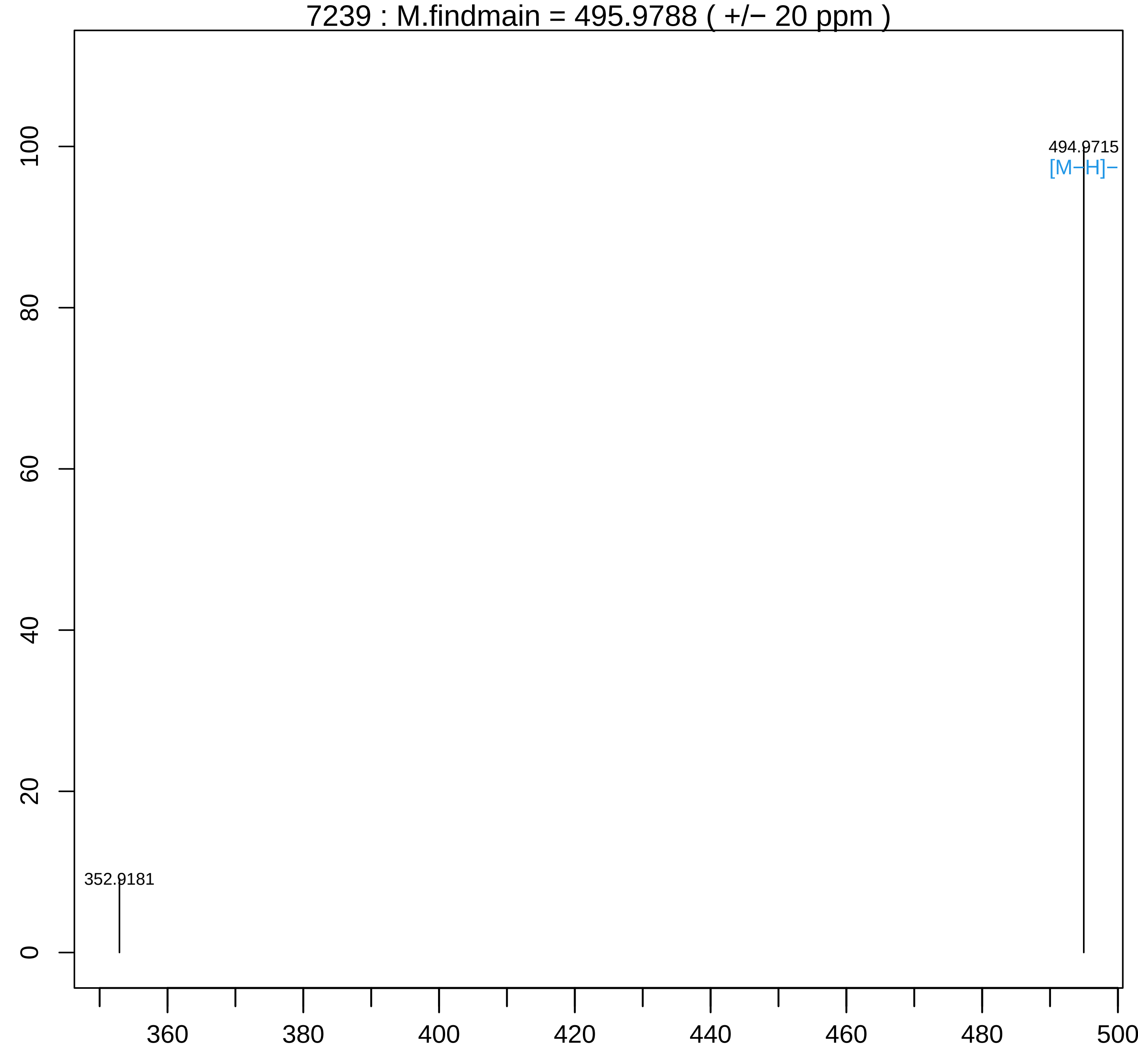
7239 : M.findmain = 495.9788 (+/− 20 ppm)
494.9715
[M−H]−
352.9181
0
20
40
60
80
100
360
380
400
420
440
460
480
500

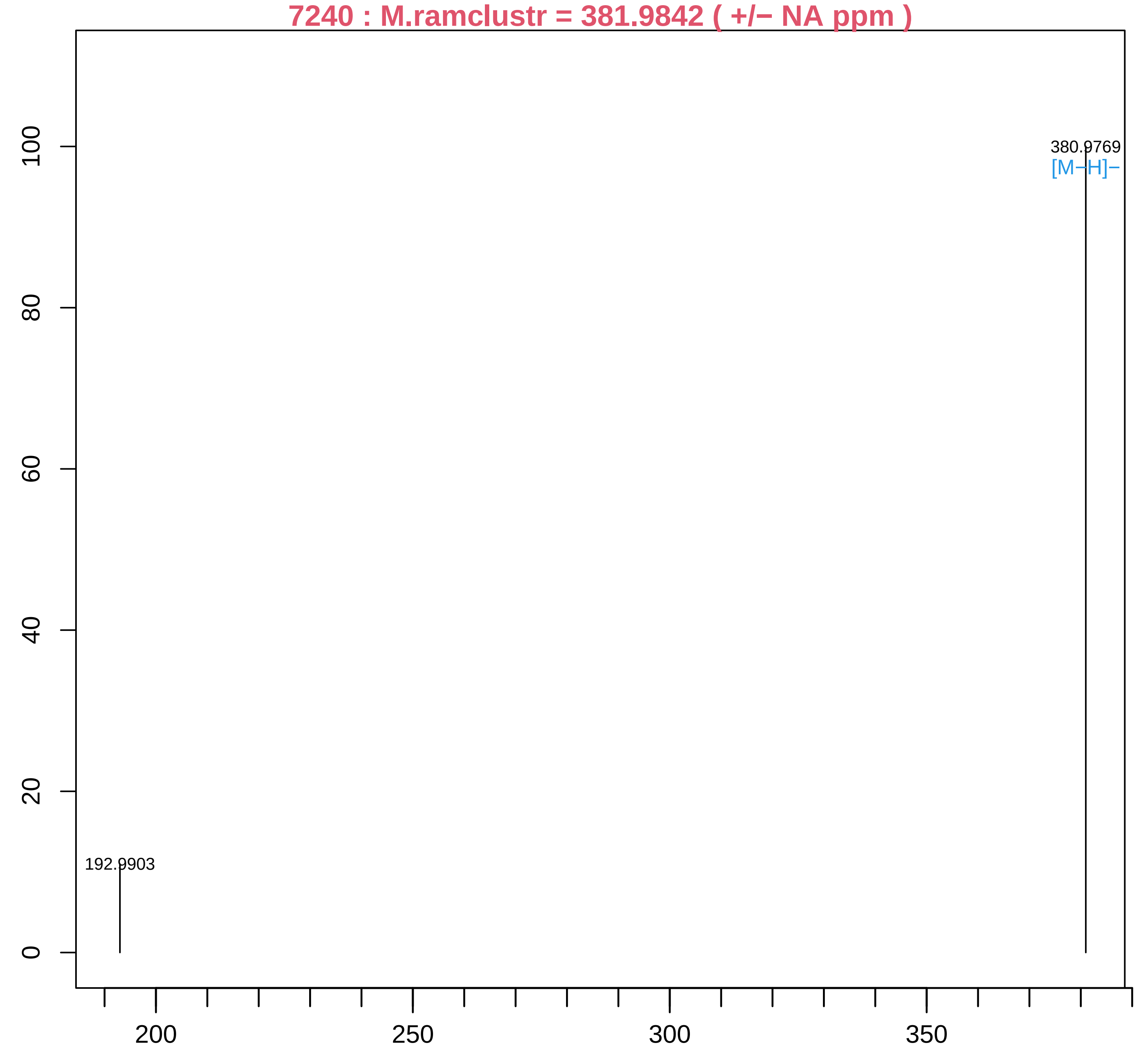
7240 : M.ramclustr = 381.9842 (+/− NA ppm)
380.9769
[M−H]−
192.9903
0
20
40
60
80
100
200
250
300
350

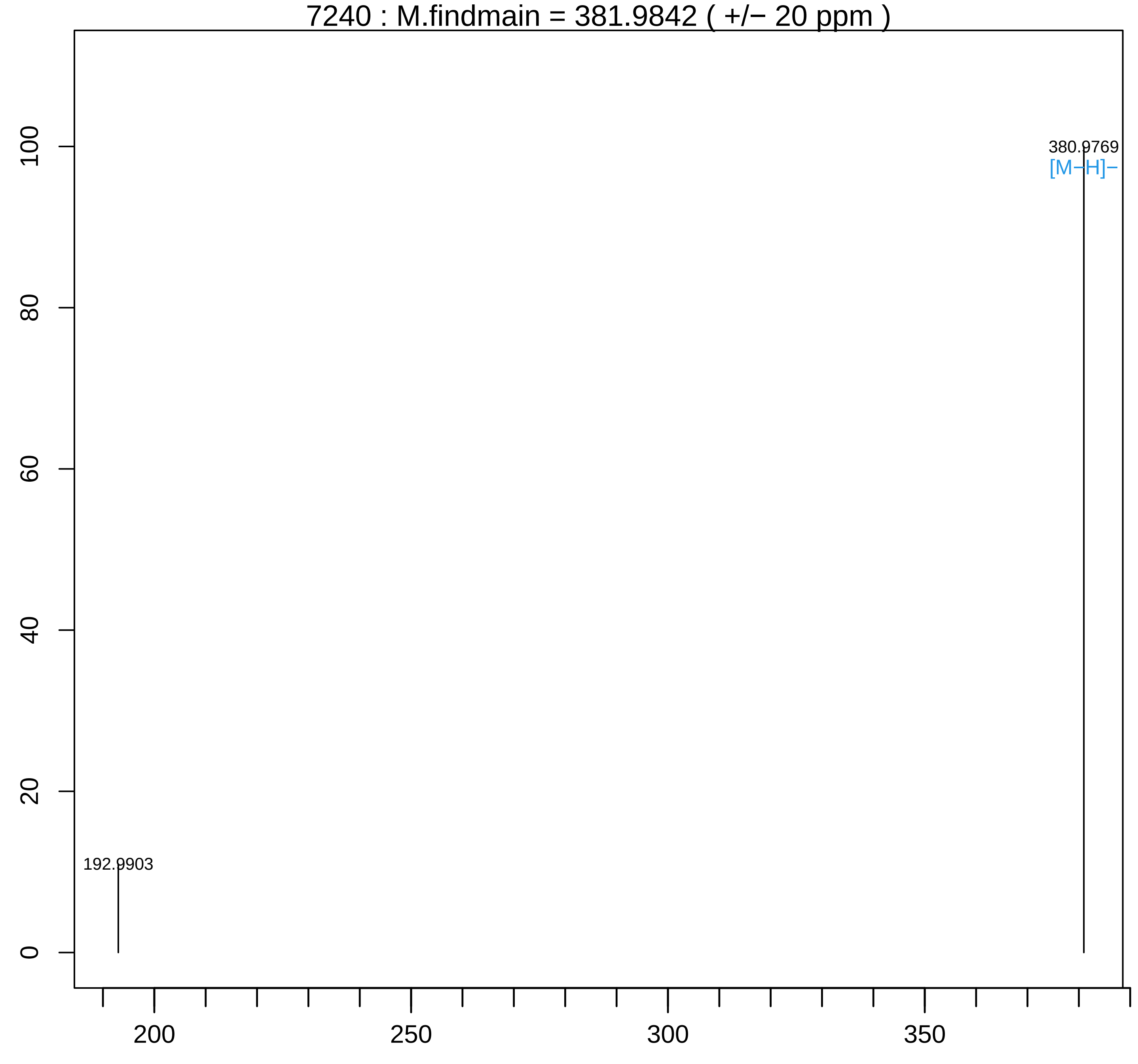
7240 : M.findmain = 381.9842 (+/− 20 ppm)
380.9769
[M−H]−
192.9903
0
20
40
60
80
100
200
250
300
350

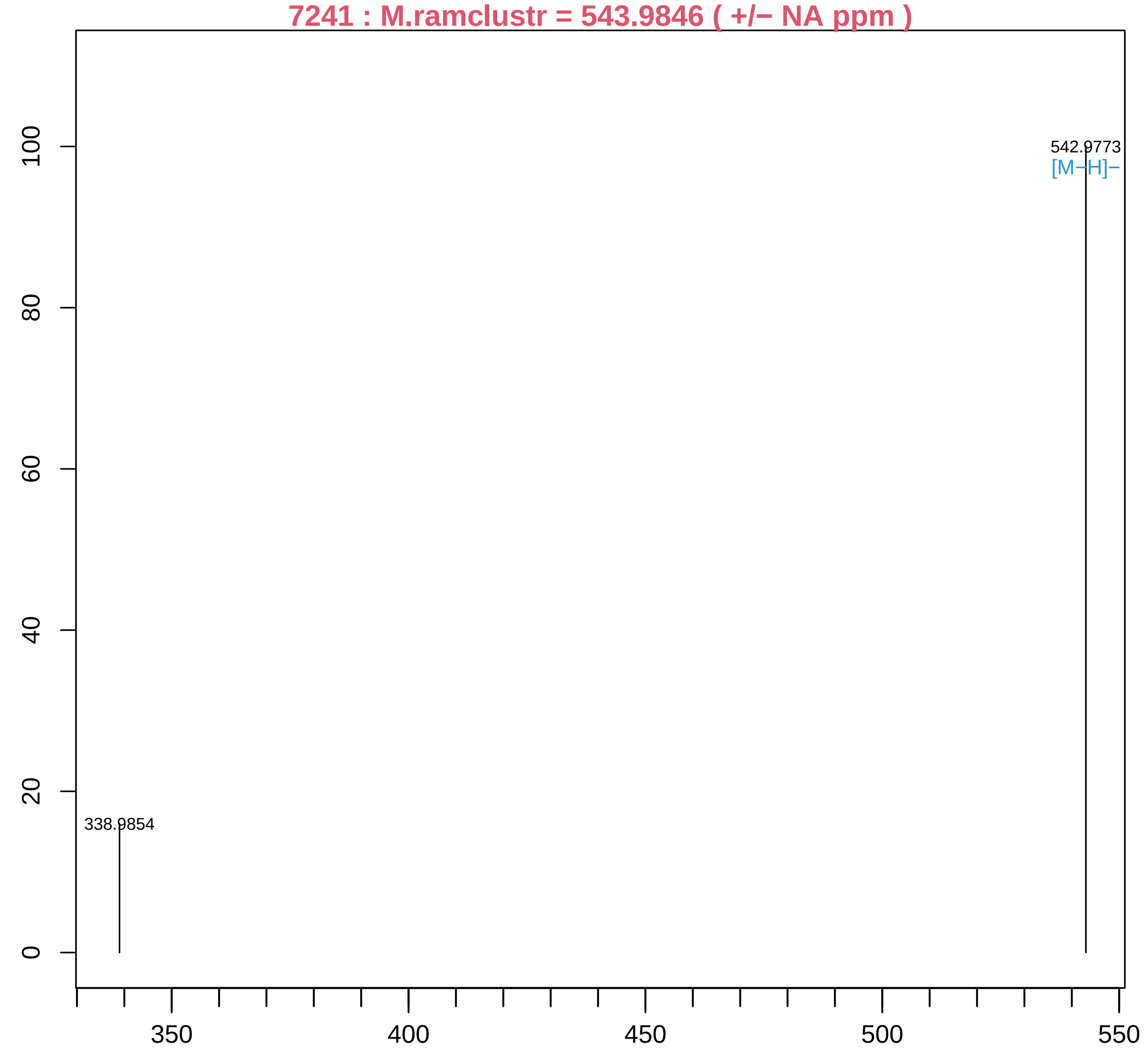
7241 : M.ramclustr = 543.9846 (+/− NA ppm)
542.9773
[M−H]−
338.9854
0
20
40
60
80
100
350
400
450
500
550

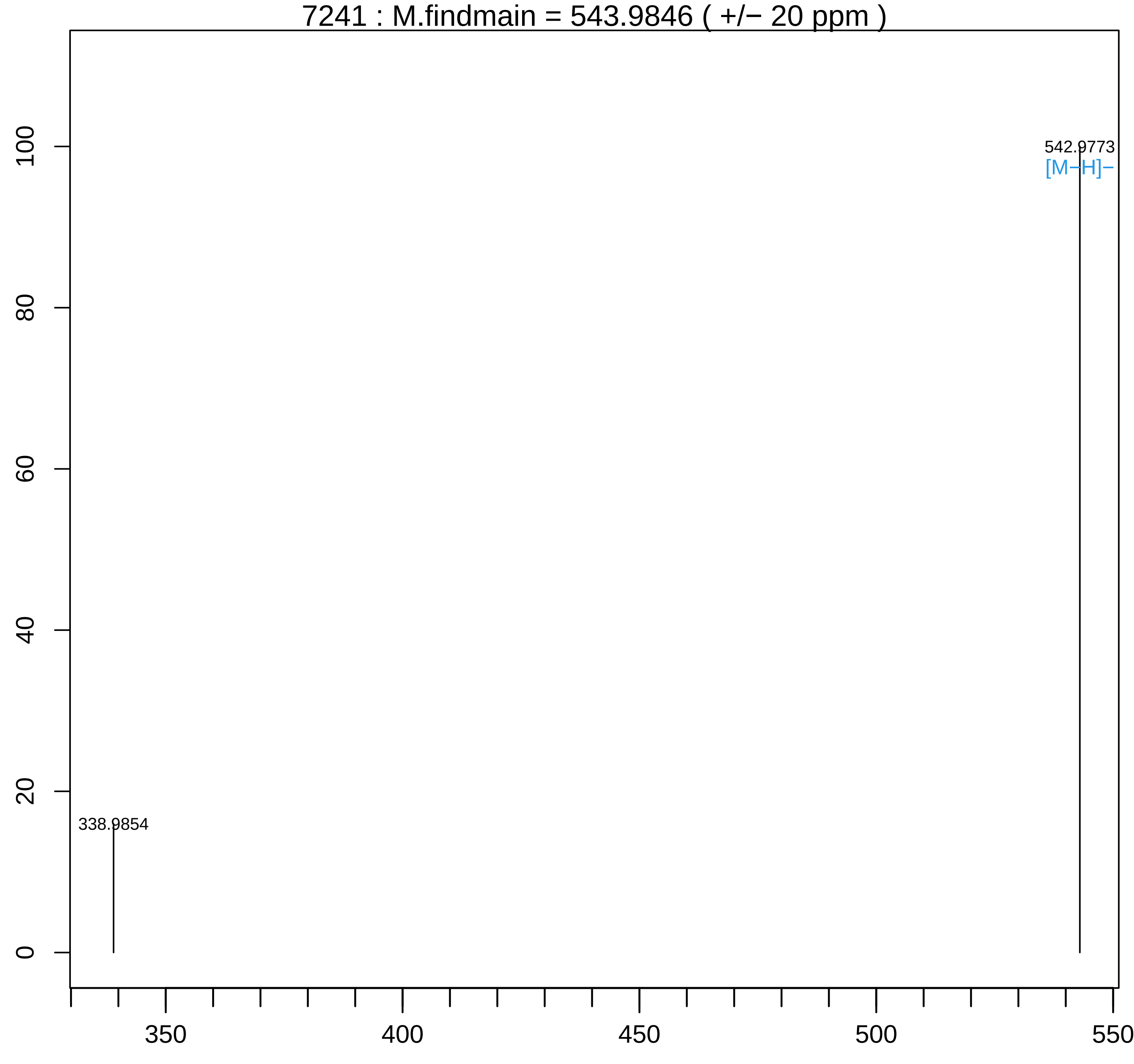
7241 : M.findmain = 543.9846 (+/− 20 ppm)
542.9773
[M−H]−
338.9854
0
20
40
60
80
100
350
400
450
500
550

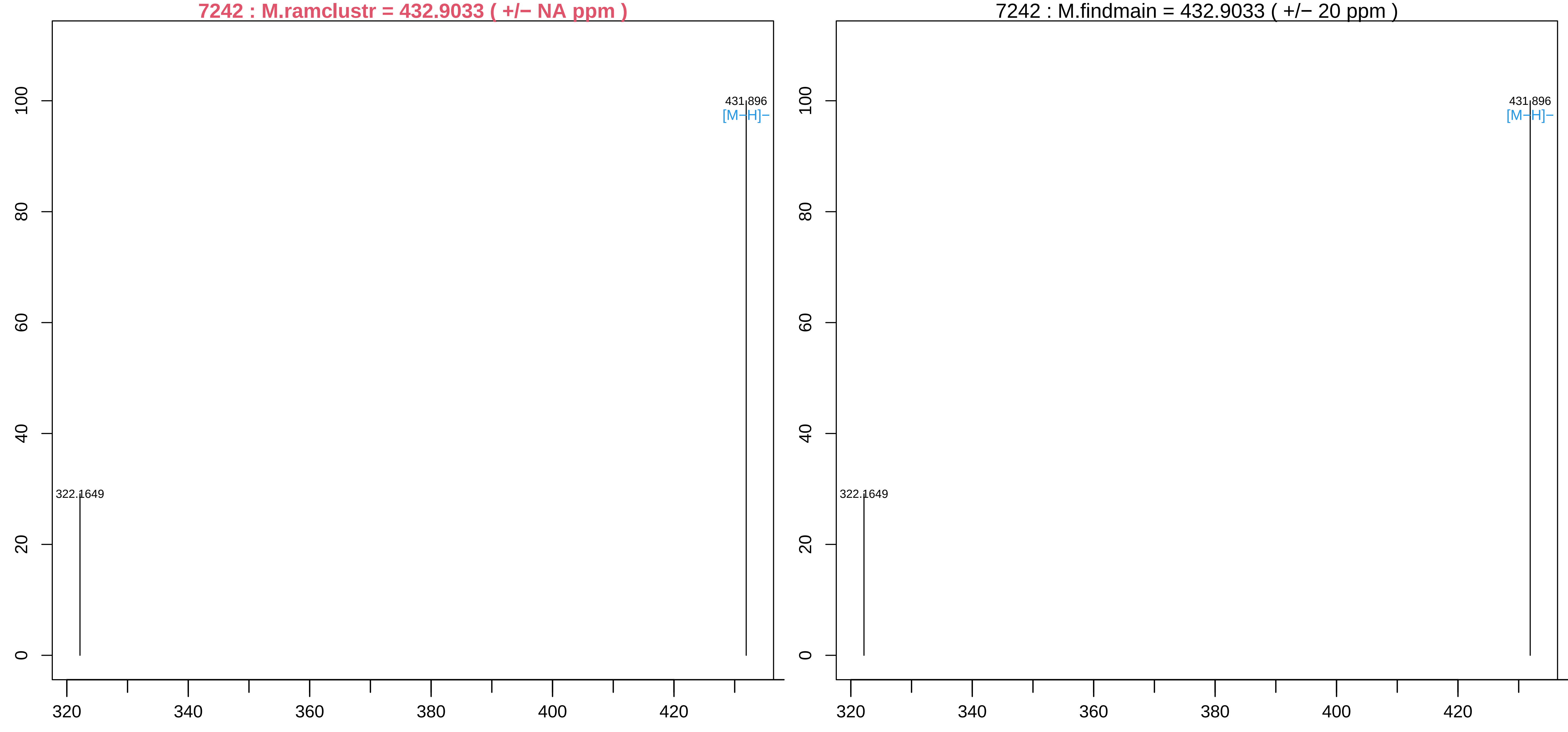
7242 : M.ramclustr = 432.9033 (+/− NA ppm)
7242 : M.findmain = 432.9033 (+/− 20 ppm)
431.896
[M−H]−
322.1649
0
20
40
60
80
100
320
340
360
380
400
420

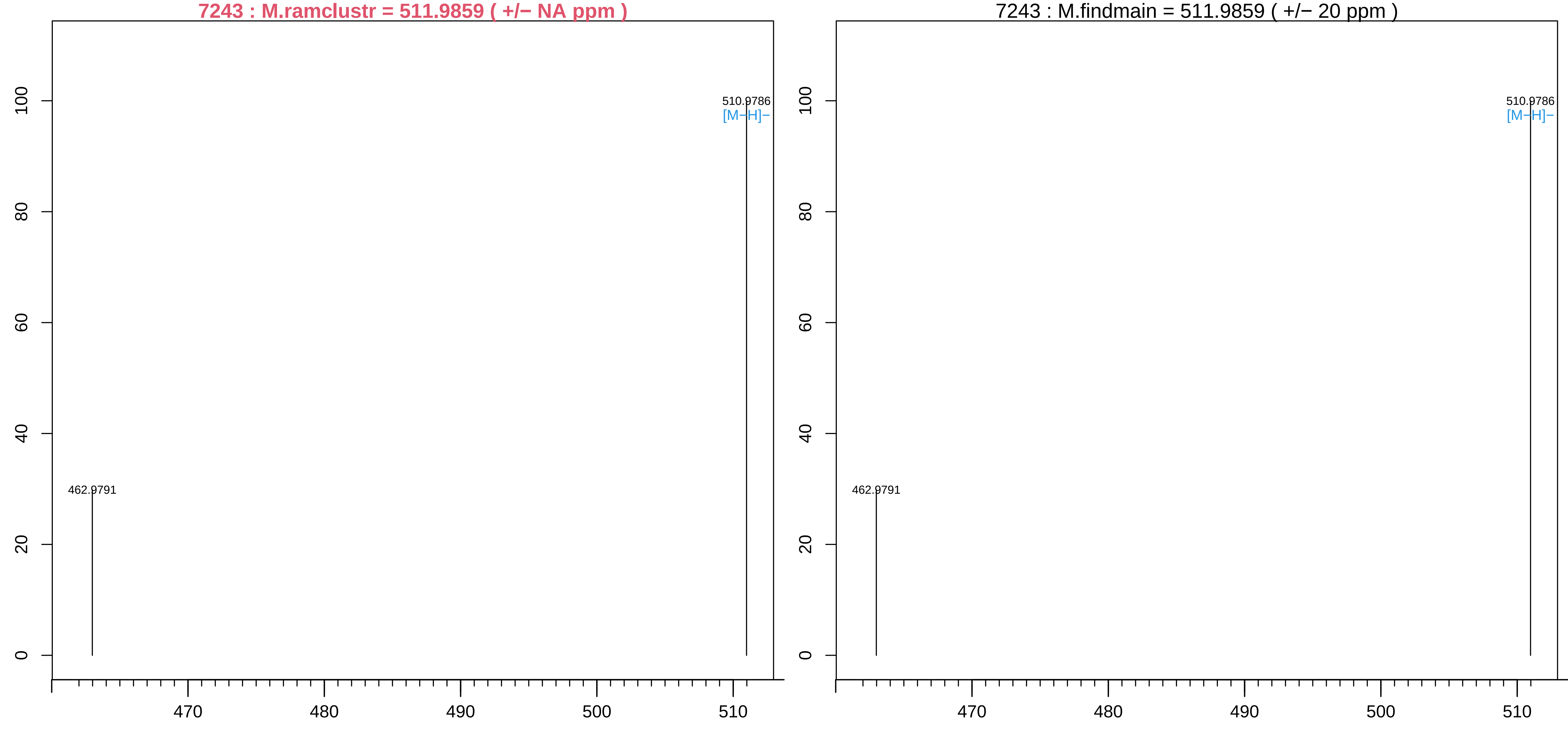
7243 : M.ramclustr = 511.9859 (+/− NA ppm)
510.9786
[M−H]−
462.9791
7243 : M.findmain = 511.9859 (+/− 20 ppm)
510.9786
[M−H]−
462.9791
0
20
40
60
80
100
470
480
490
500
510

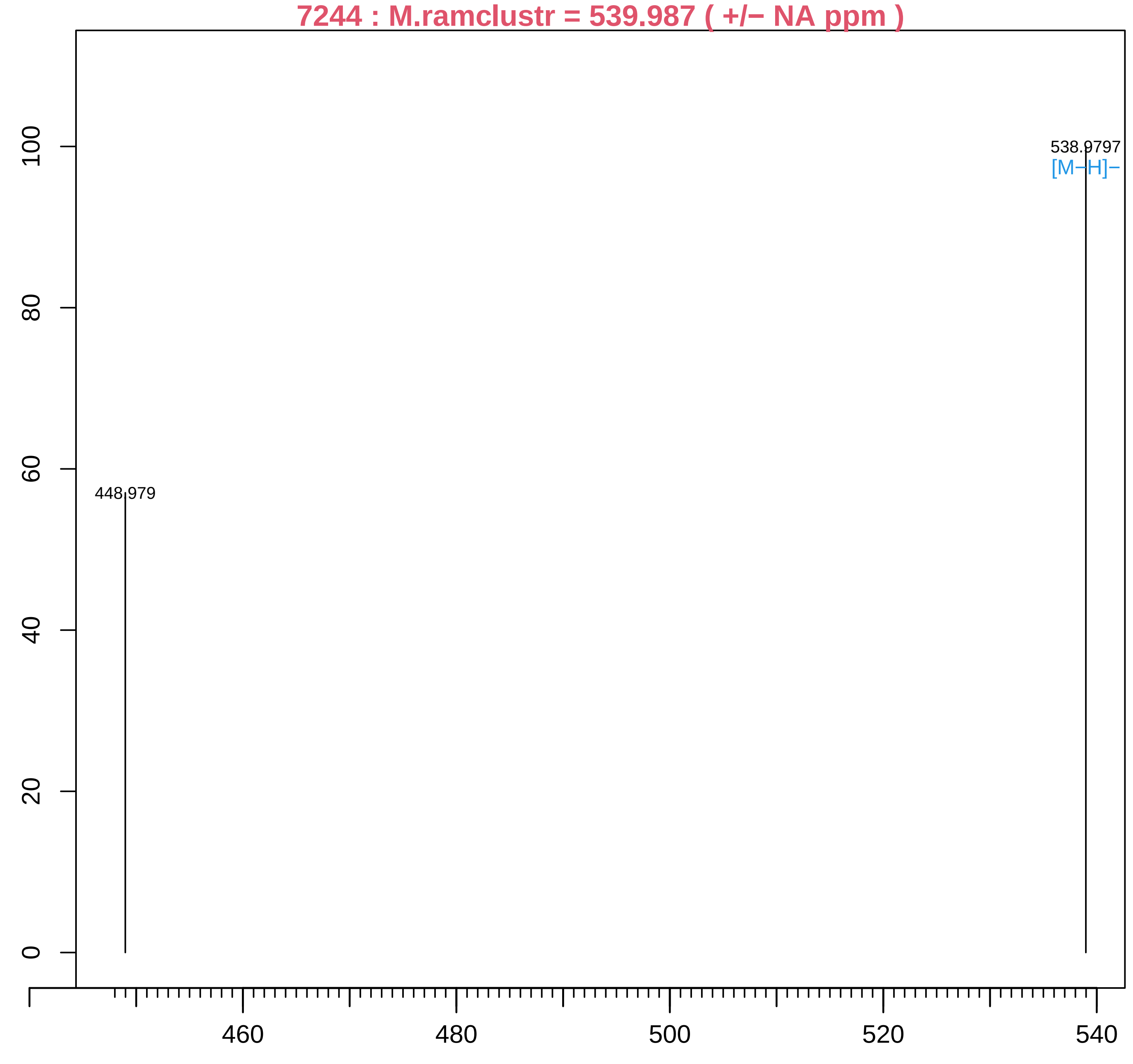
7244 : M.ramclustr = 539.987 (+/− NA ppm)
538.9797
[M−H]−
448.979
0
20
40
60
80
100
460
480
500
520
540

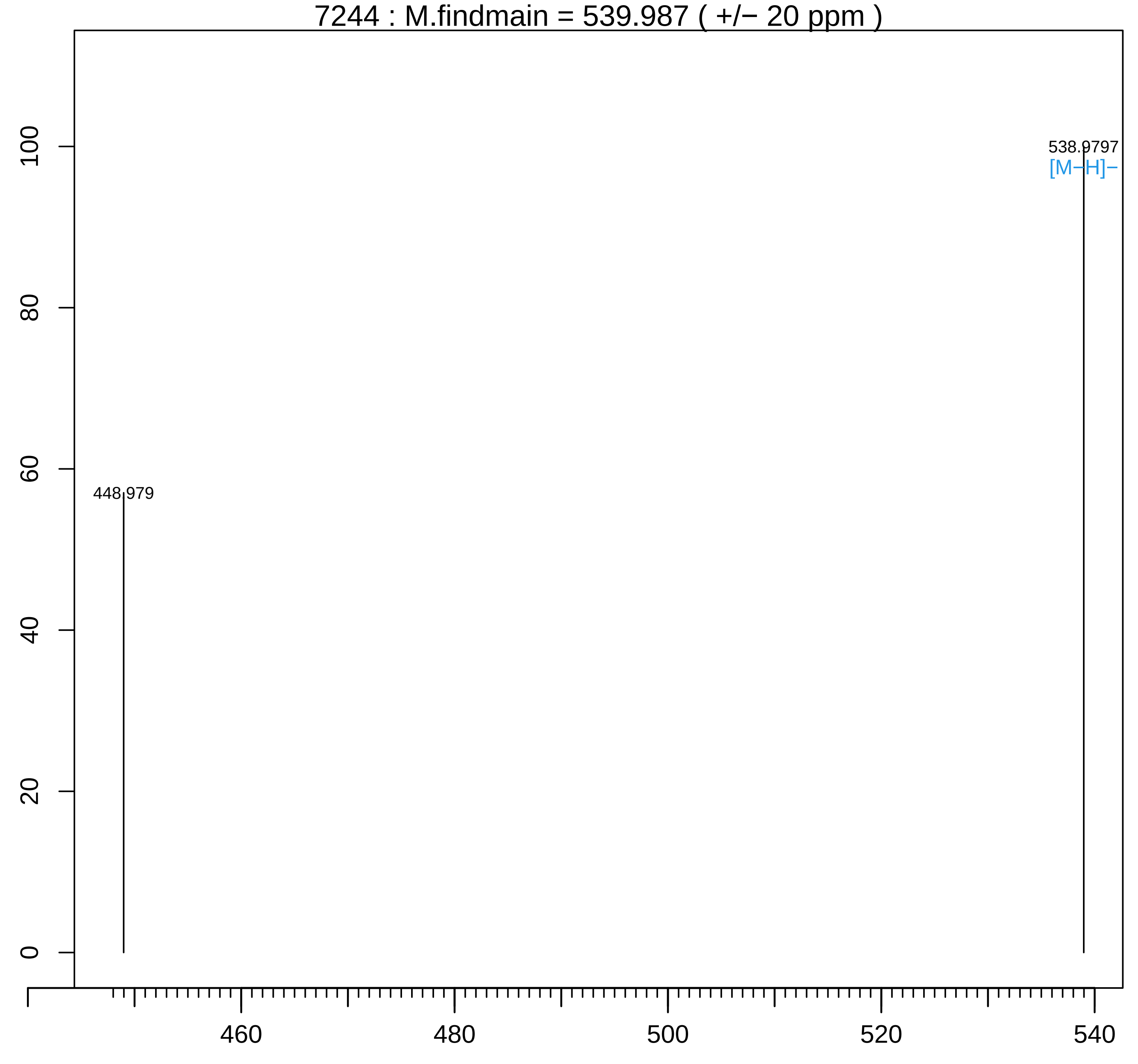
7244 : M.findmain = 539.987 (+/− 20 ppm)
538.9797
[M−H]−
448.979
0
20
40
60
80
100
460
480
500
520
540

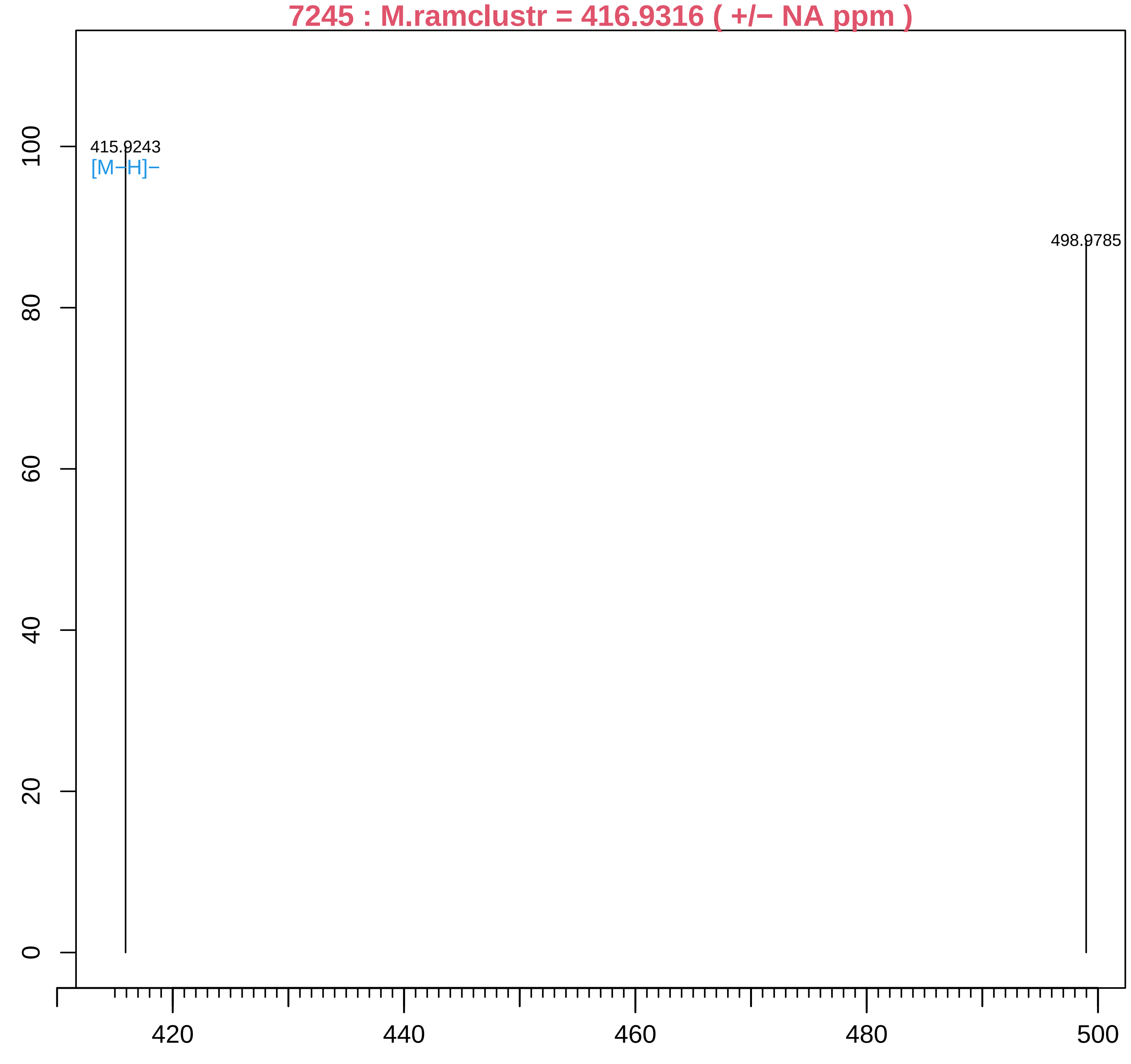
7245 : M.ramclustr = 416.9316 (+/− NA ppm)
415.9243
[M−H]−
498.9785
0
20
40
60
80
100
420
440
460
480
500

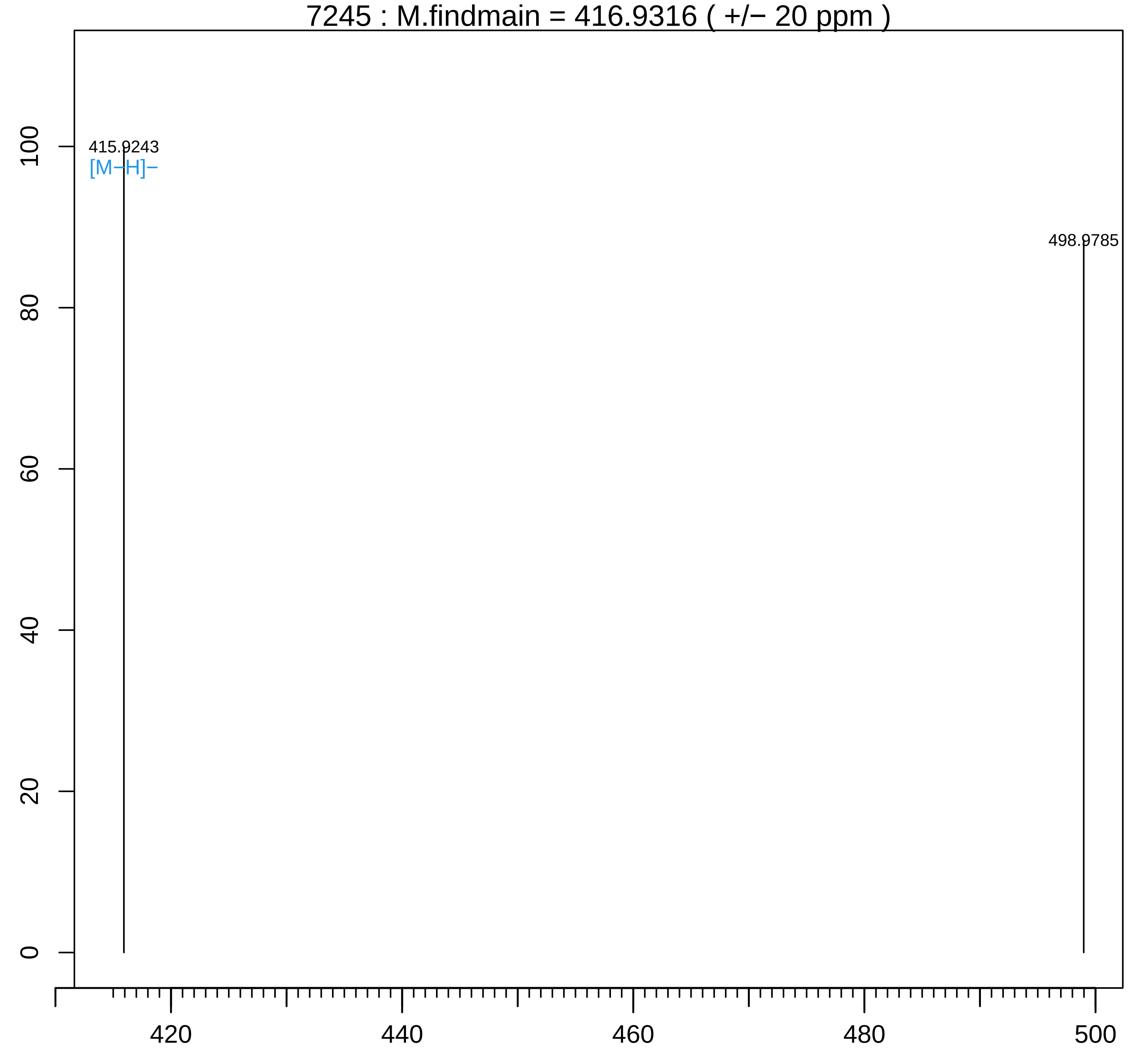
7245 : M.findmain = 416.9316 (+/− 20 ppm)
415.9243
[M−H]−
498.9785
0
20
40
60
80
100
420
440
460
480
500

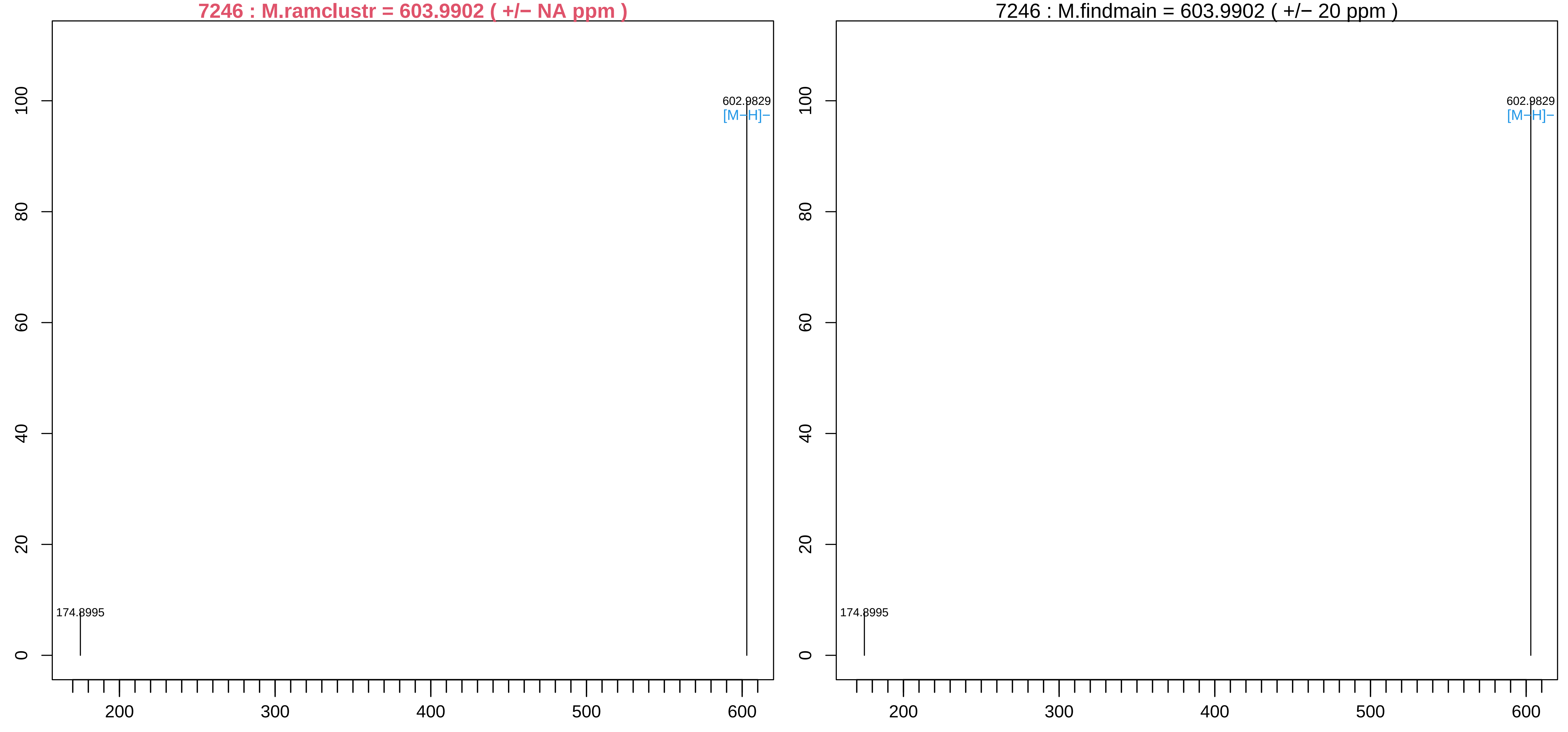
7246 : M.ramclustr = 603.9902 (+/− NA ppm)
602.9829
[M−H]−
174.8995
0
20
40
60
80
100
200
300
400
500
600
7246 : M.findmain = 603.9902 (+/− 20 ppm)
602.9829
[M−H]−
174.8995
0
20
40
60
80
100
200
300
400
500
600

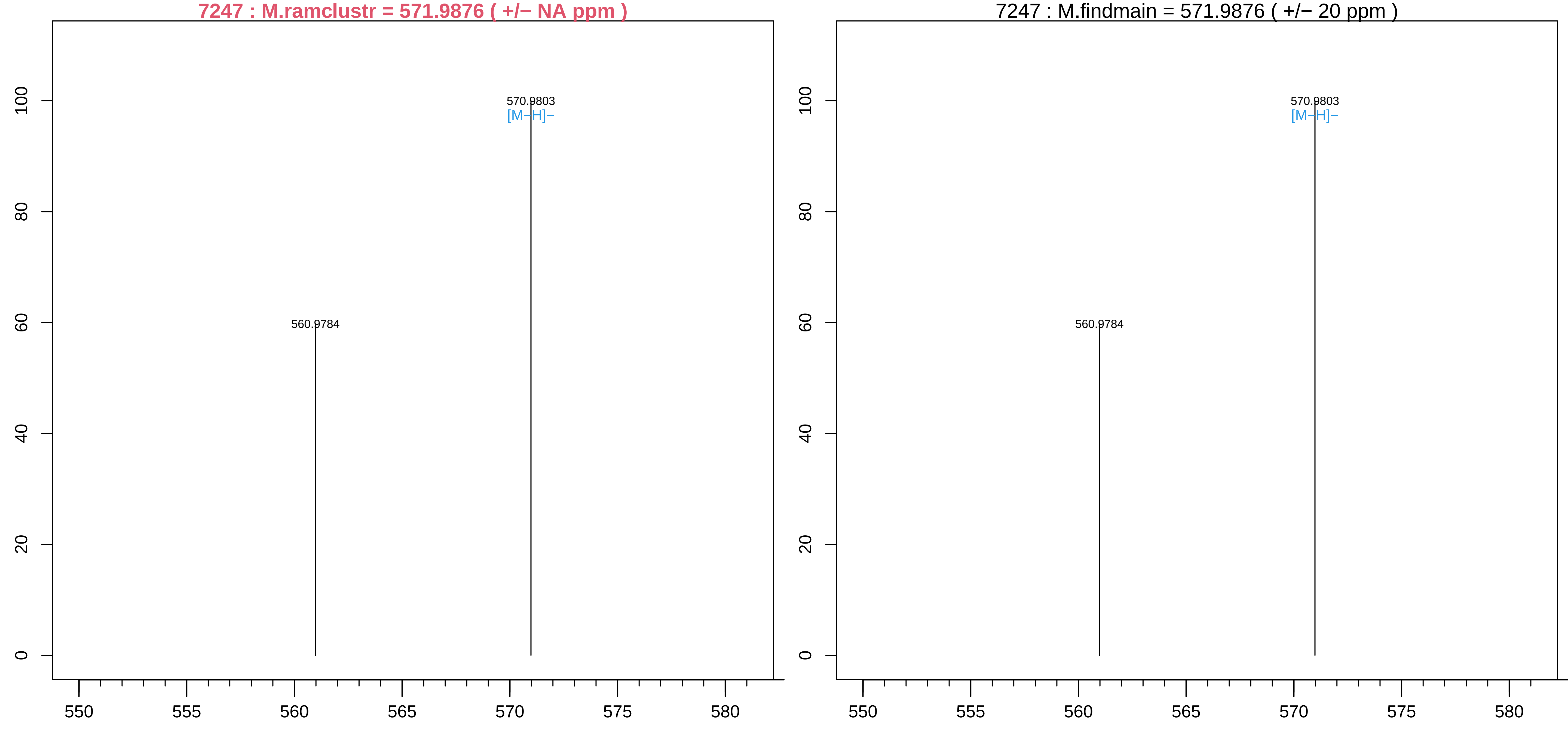
7247 : M.ramclustr = 571.9876 (+/− NA ppm)
570.9803
[M−H]−
560.9784
7247 : M.findmain = 571.9876 (+/− 20 ppm)
570.9803
[M−H]−
560.9784

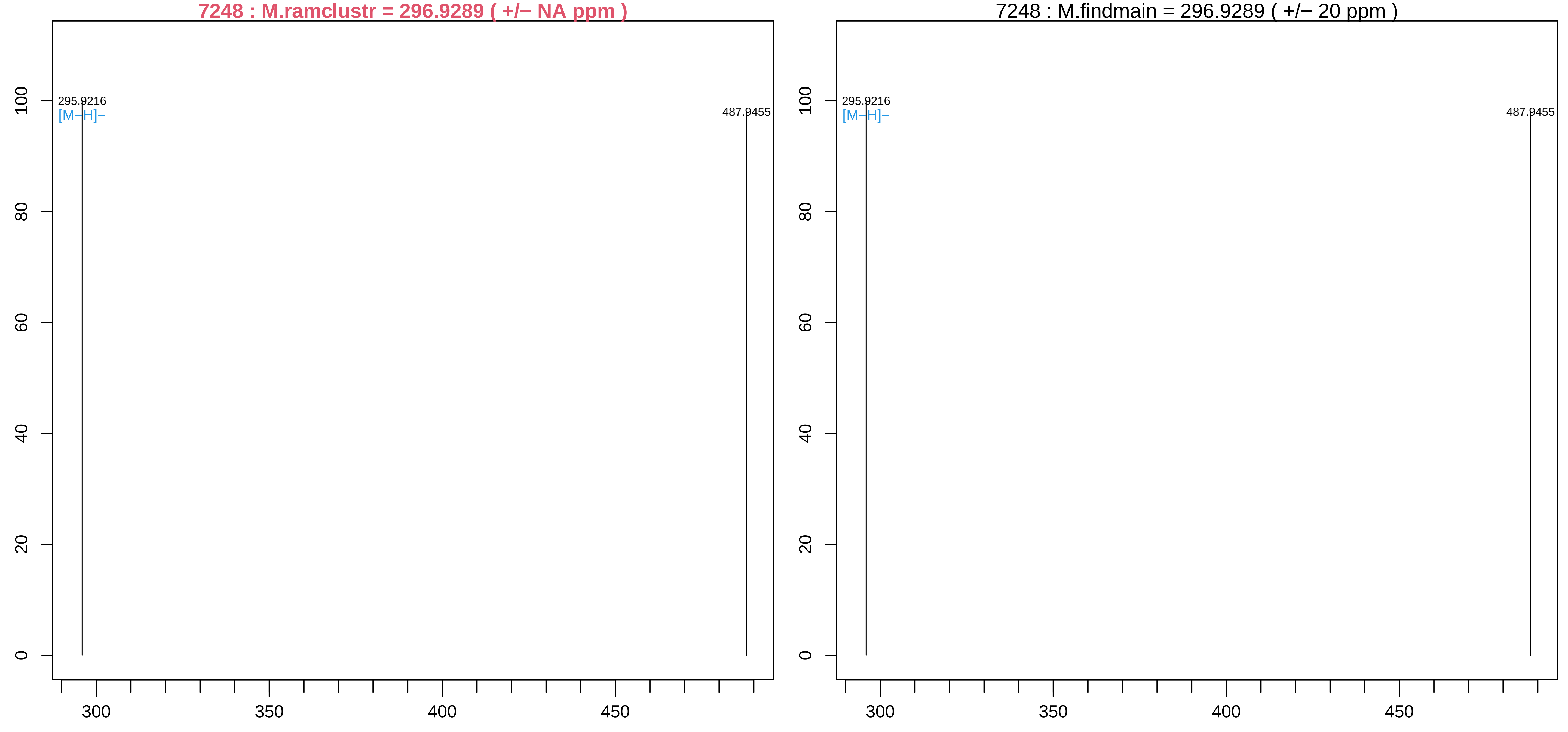
7248 : M.ramclustr = 296.9289 (+/− NA ppm)
295.9216
[M−H]−
487.9455
7248 : M.findmain = 296.9289 (+/− 20 ppm)
295.9216
[M−H]−
487.9455
0
20
40
60
80
100
300
350
400
450

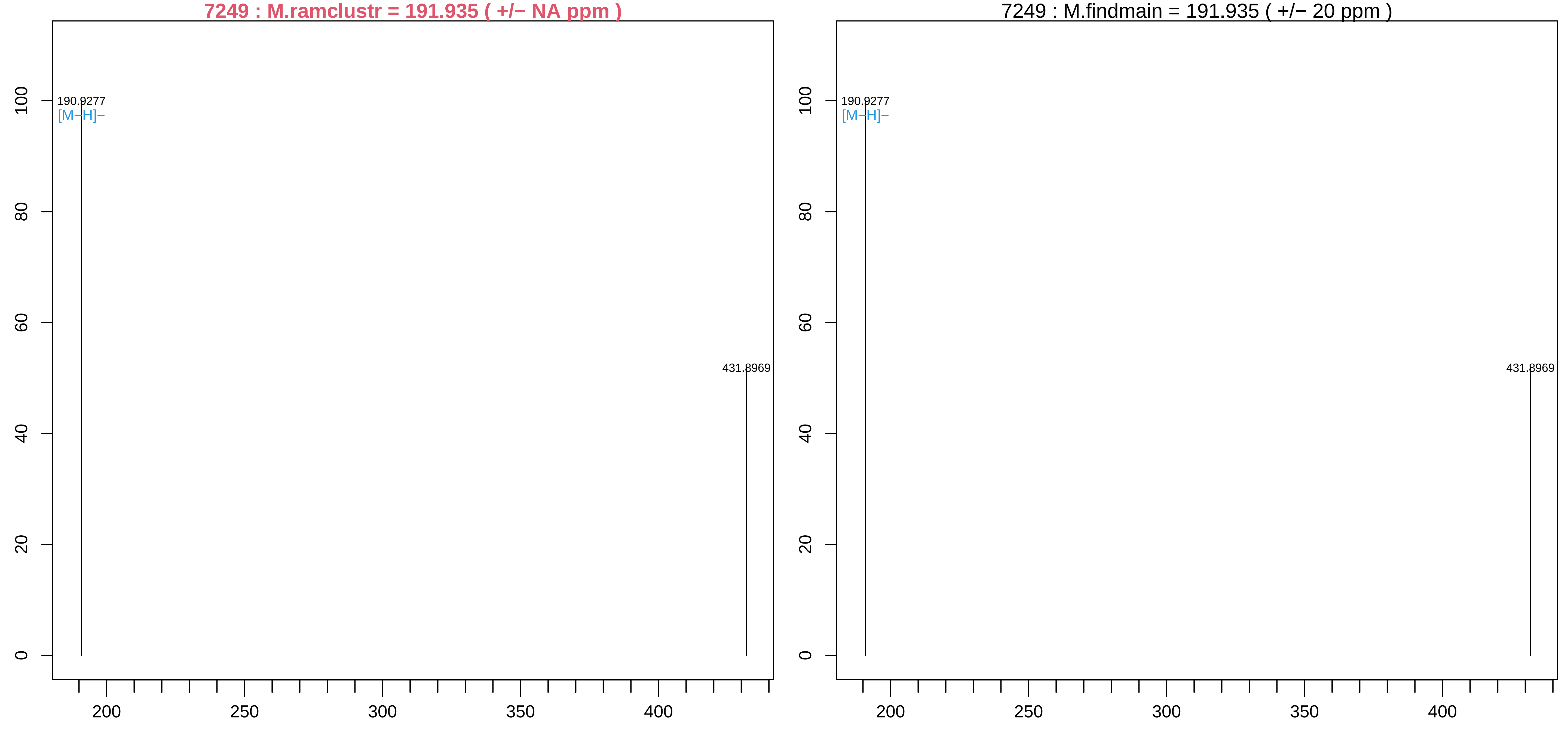

7249 : M.ramclustr = 191.935 (+/− NA ppm)
190.9277
[M−H]−
431.8969
0
20
40
60
80
100
200
250
300
350
400
7249 : M.findmain = 191.935 (+/− 20 ppm)
190.9277
[M−H]−
431.8969
0
20
40
60
80
100
200
250
300
350
400

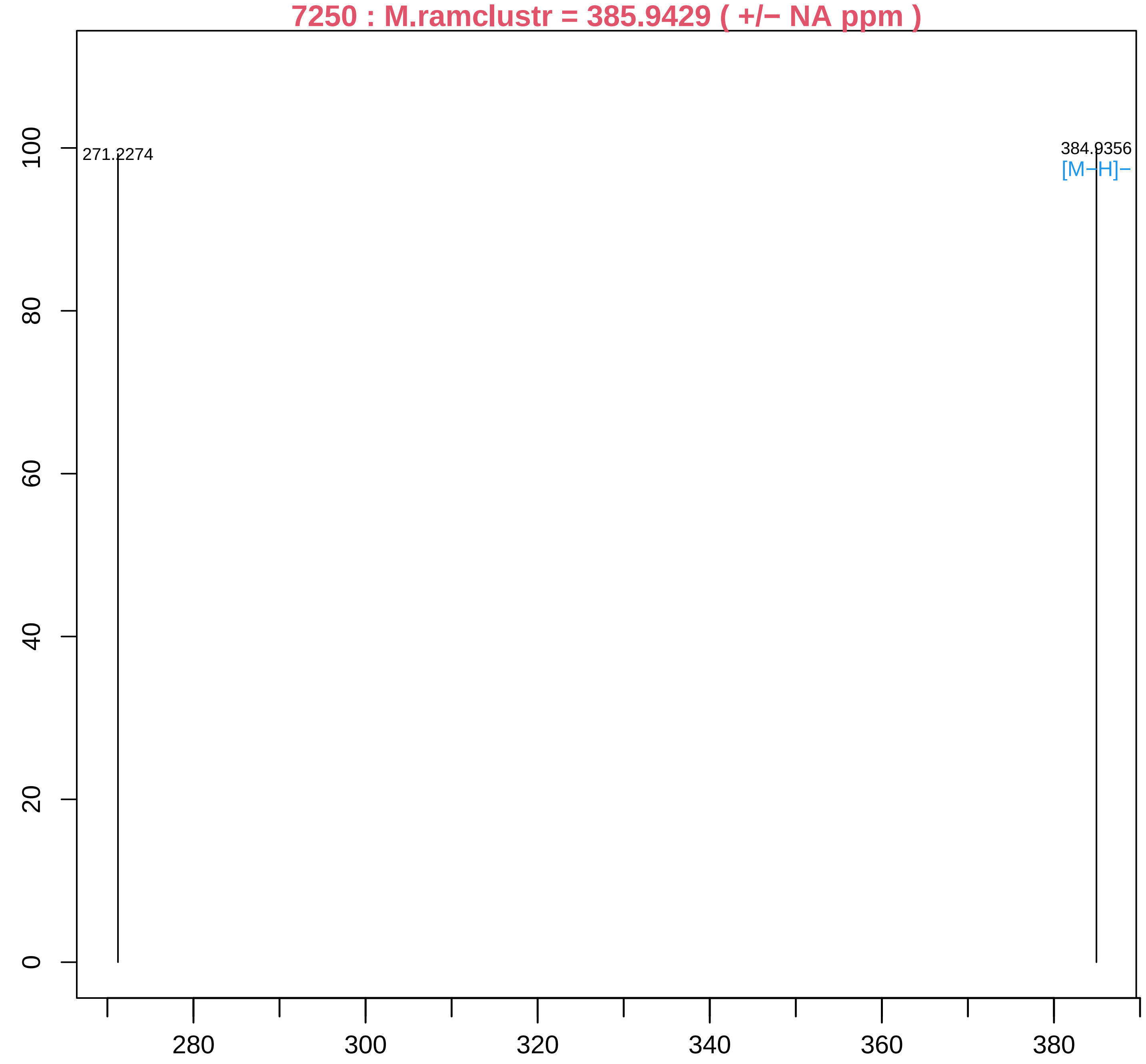
7250 : M.ramclustr = 385.9429 (+/− NA ppm)
271.2274
384.9356
[M−H]−
0
20
40
60
80
100
280
300
320
340
360
380

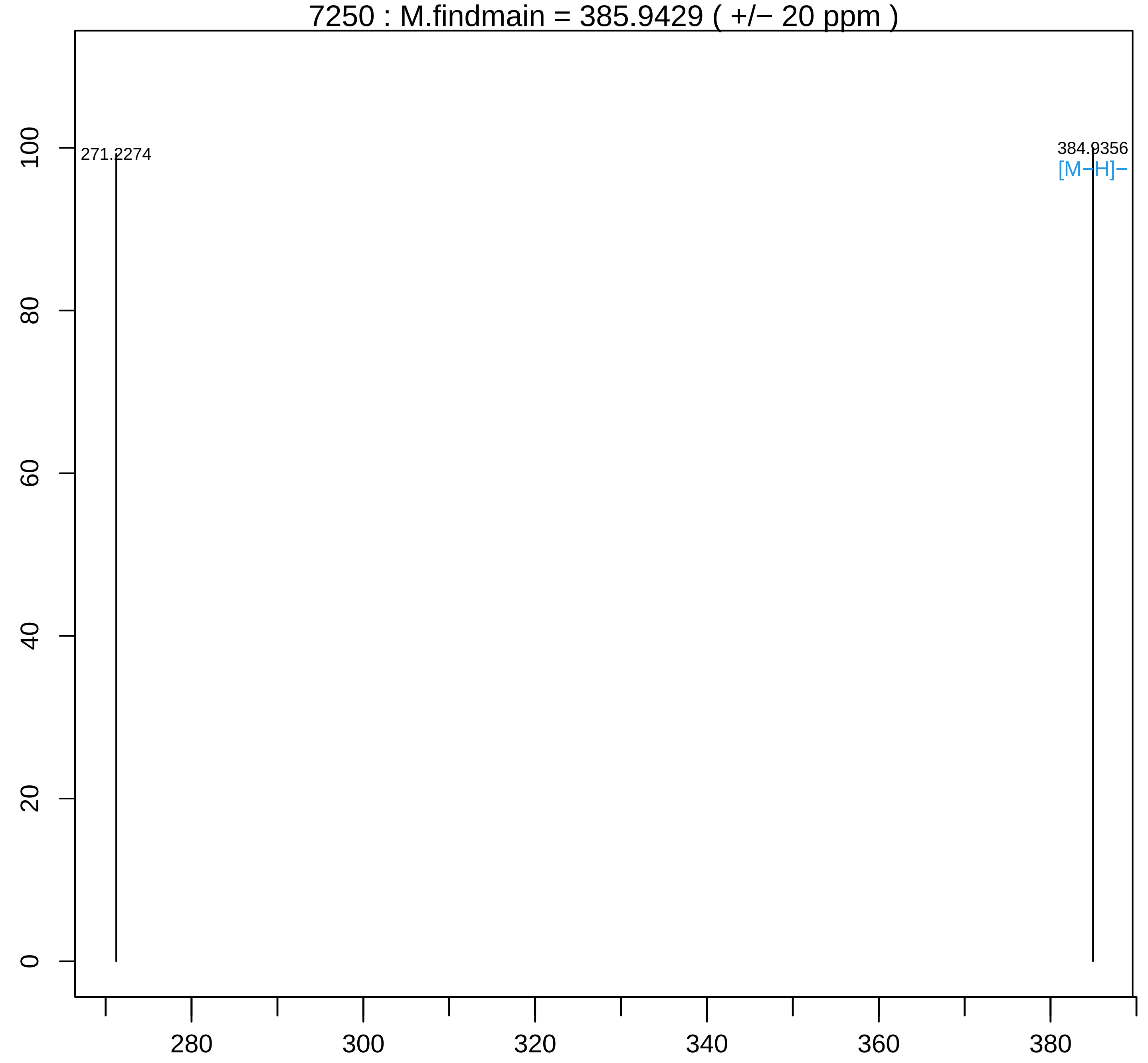
7250 : M.findmain = 385.9429 (+/− 20 ppm)
271.2274
384.9356
[M−H]−
0
20
40
60
80
100
280
300
320
340
360
380

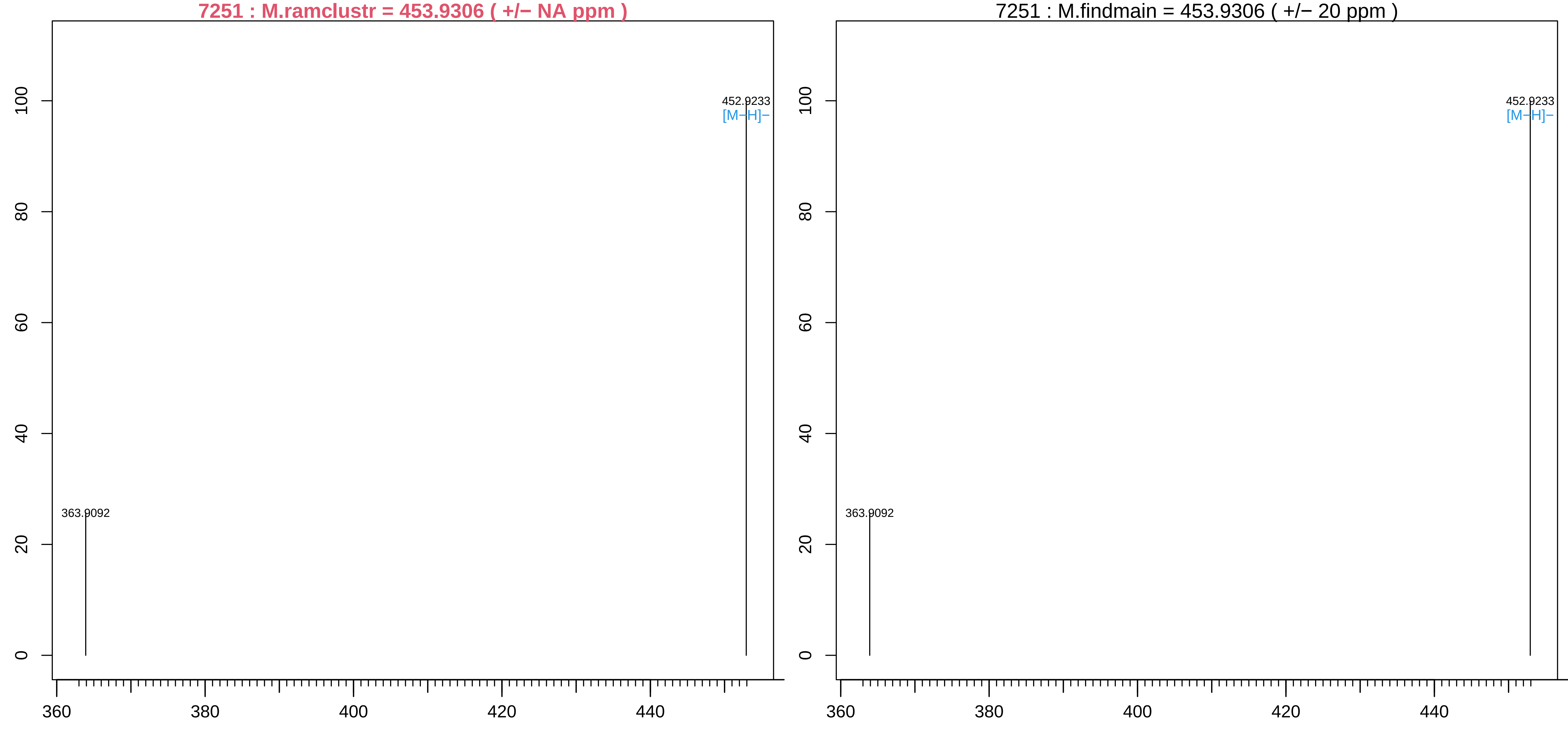
7251 : M.ramclustr = 453.9306 (+/− NA ppm)
452.9233
[M−H]−
363.9092
7251 : M.findmain = 453.9306 (+/− 20 ppm)
452.9233
[M−H]−
363.9092
0
20
40
60
80
100
360
380
400
420
440

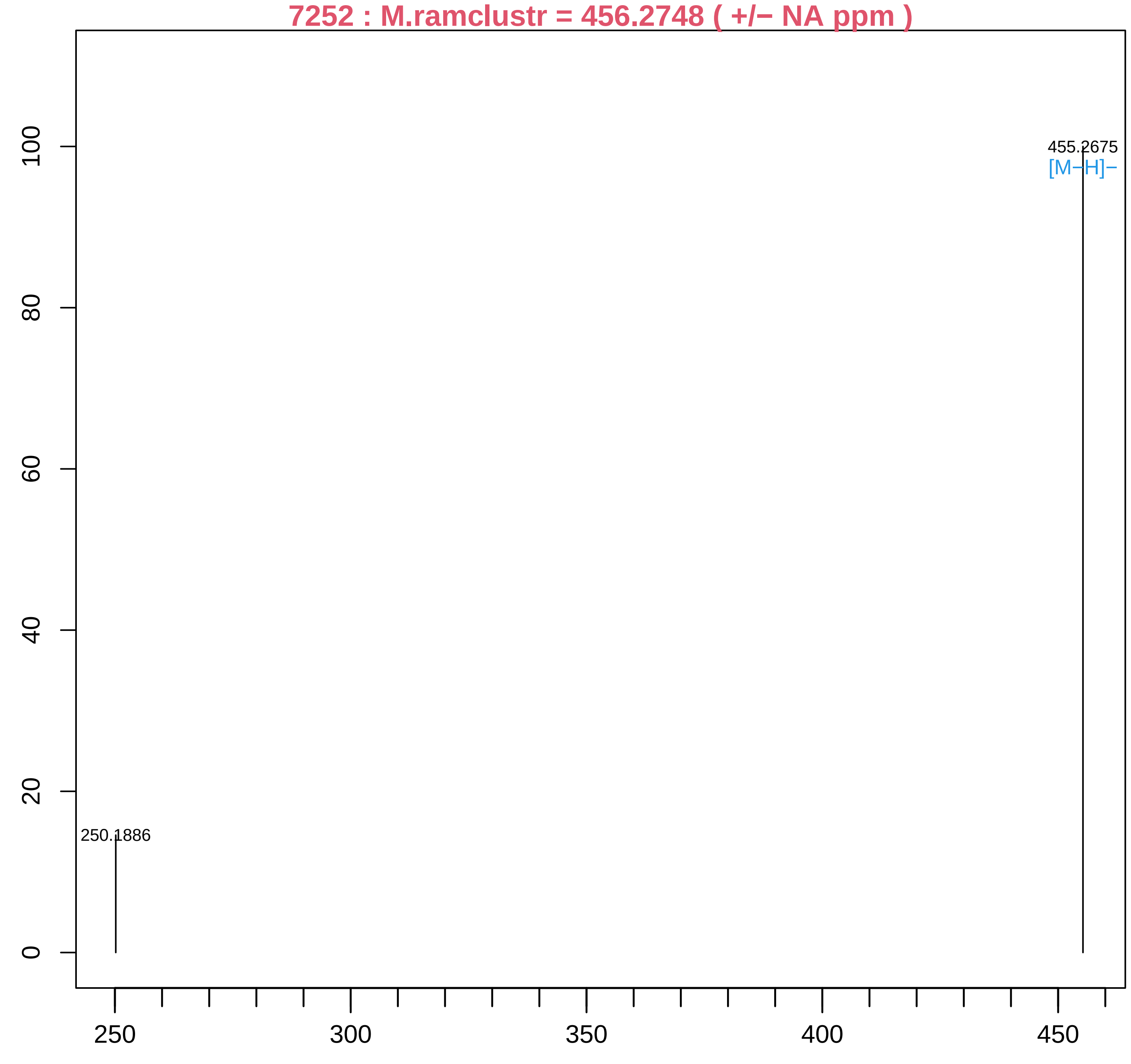
7252 : M.ramclustr = 456.2748 (+/− NA ppm)
455.2675
[M−H]−
250.1886
0
20
40
60
80
100
250
300
350
400
450

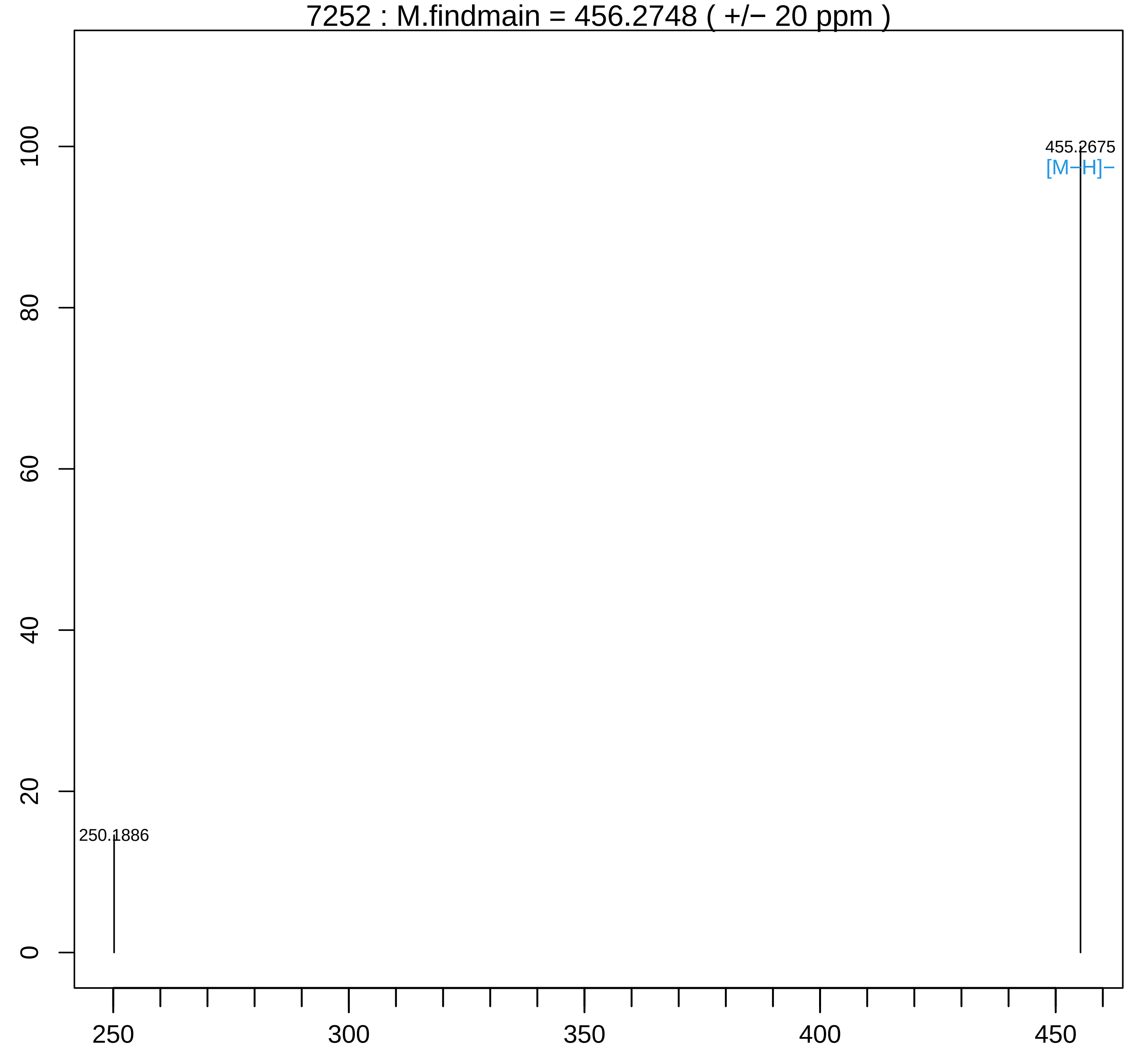
7252 : M.findmain = 456.2748 (+/− 20 ppm)
455.2675
[M−H]−
250.1886
0
20
40
60
80
100
250
300
350
400
450

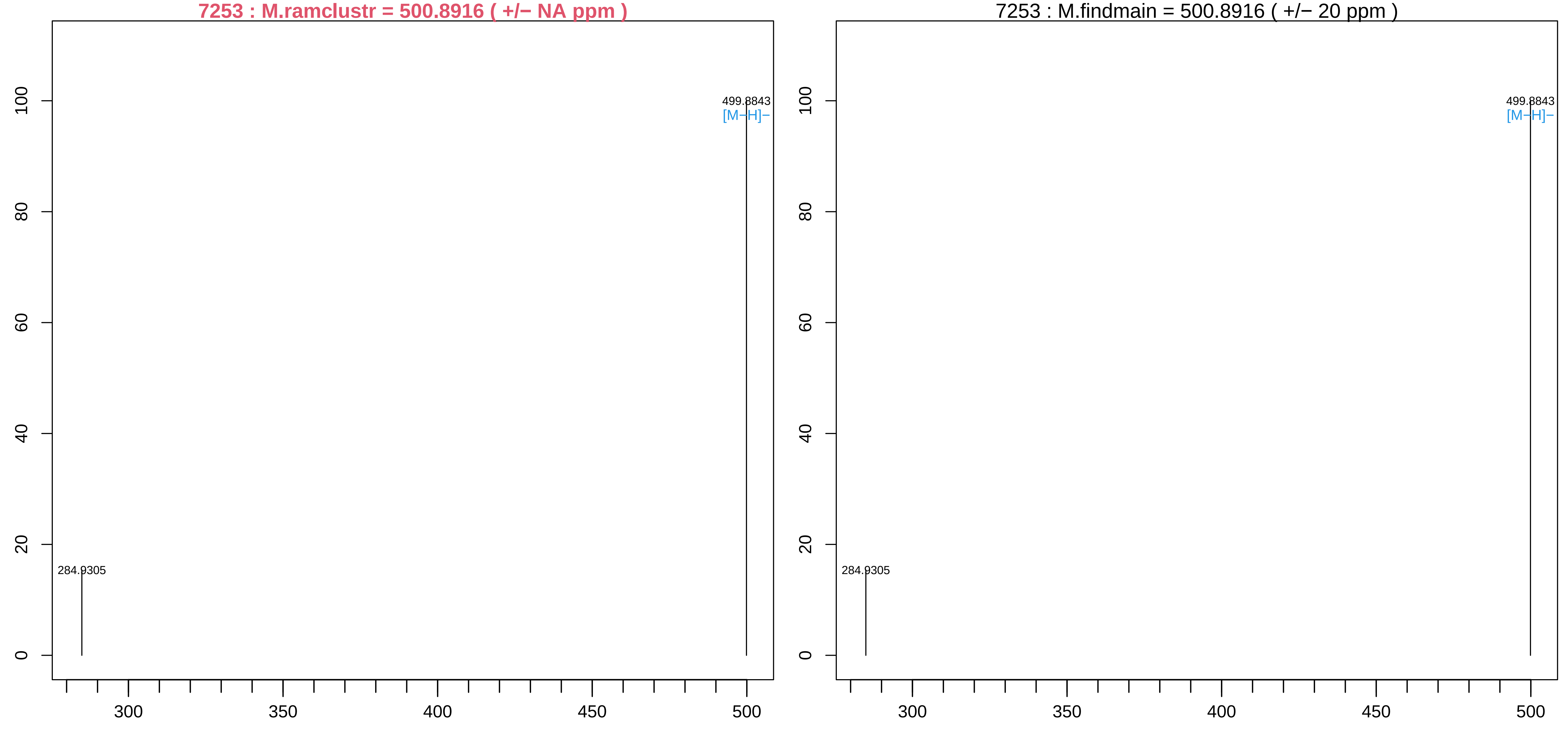
7253 : M.ramclustr = 500.8916 (+/− NA ppm)
7253 : M.findmain = 500.8916 (+/− 20 ppm)
499.8843
[M−H]−
284.9305
0
20
40
60
80
100
300
350
400
450
500

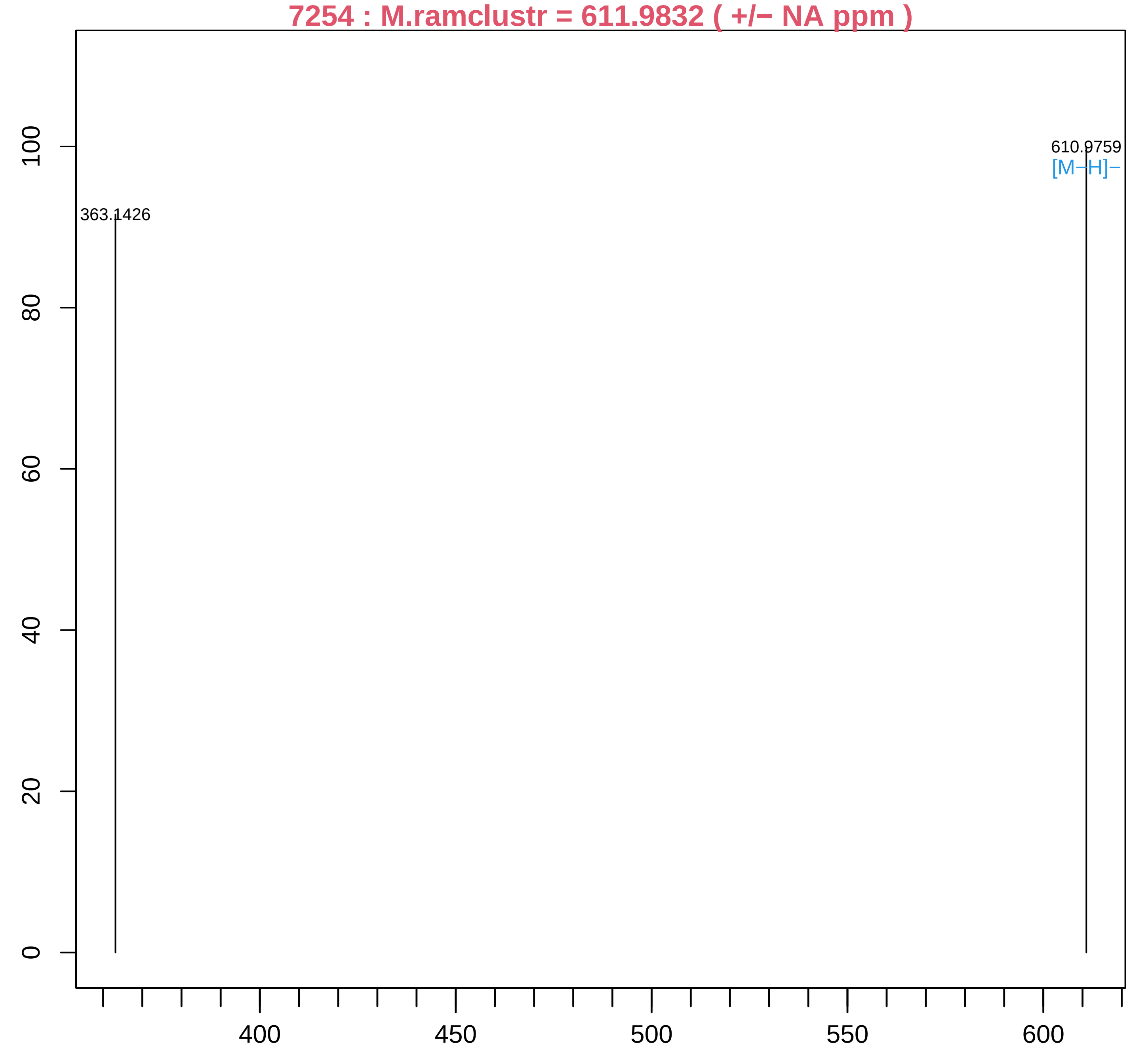
7254 : M.ramclustr = 611.9832 (+/− NA ppm)
610.9759
[M−H]−
363.1426
0
20
40
60
80
100
400
450
500
550
600

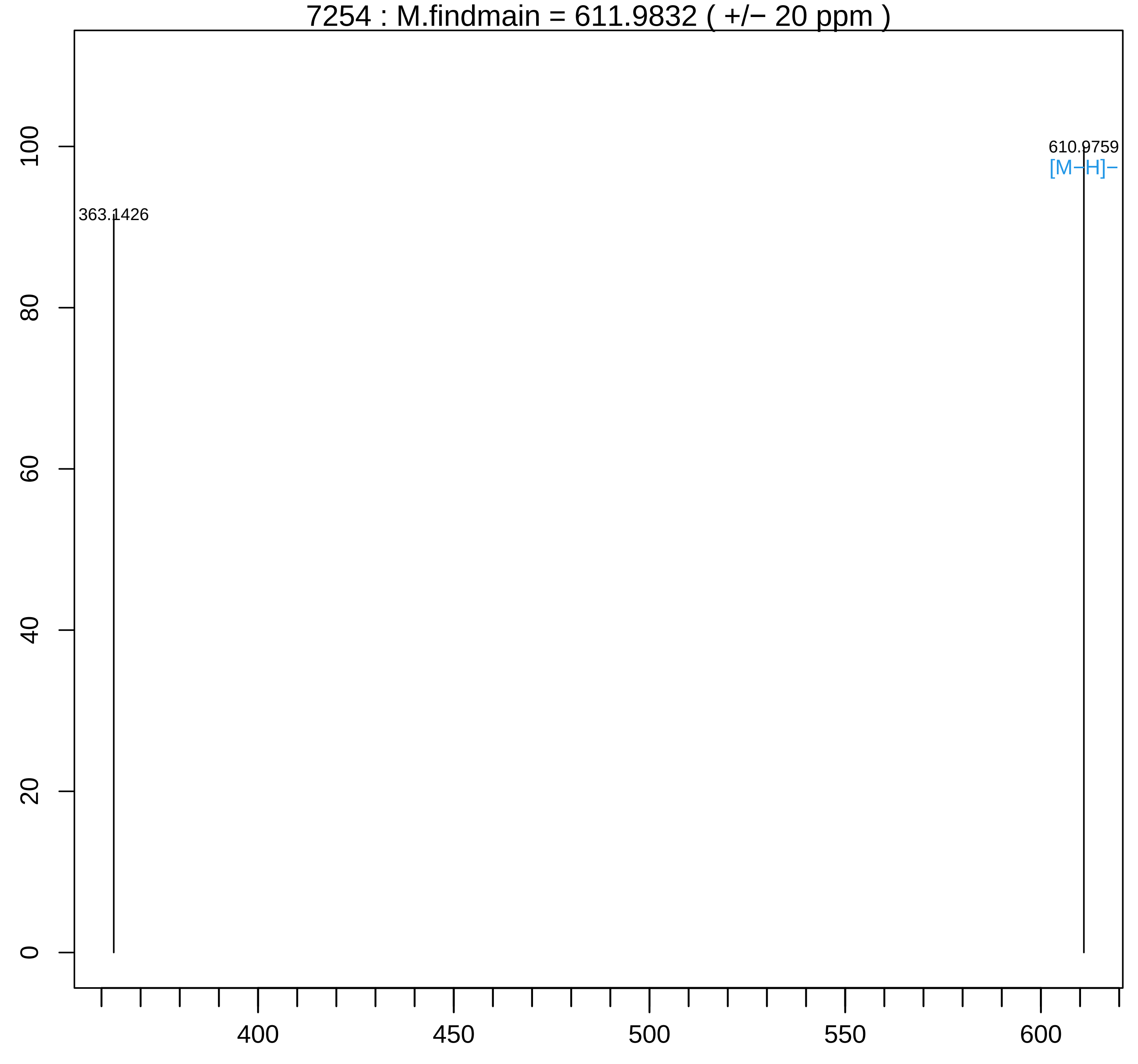
7254 : M.findmain = 611.9832 (+/− 20 ppm)
610.9759
[M−H]−
363.1426
0
20
40
60
80
100
400
450
500
550
600

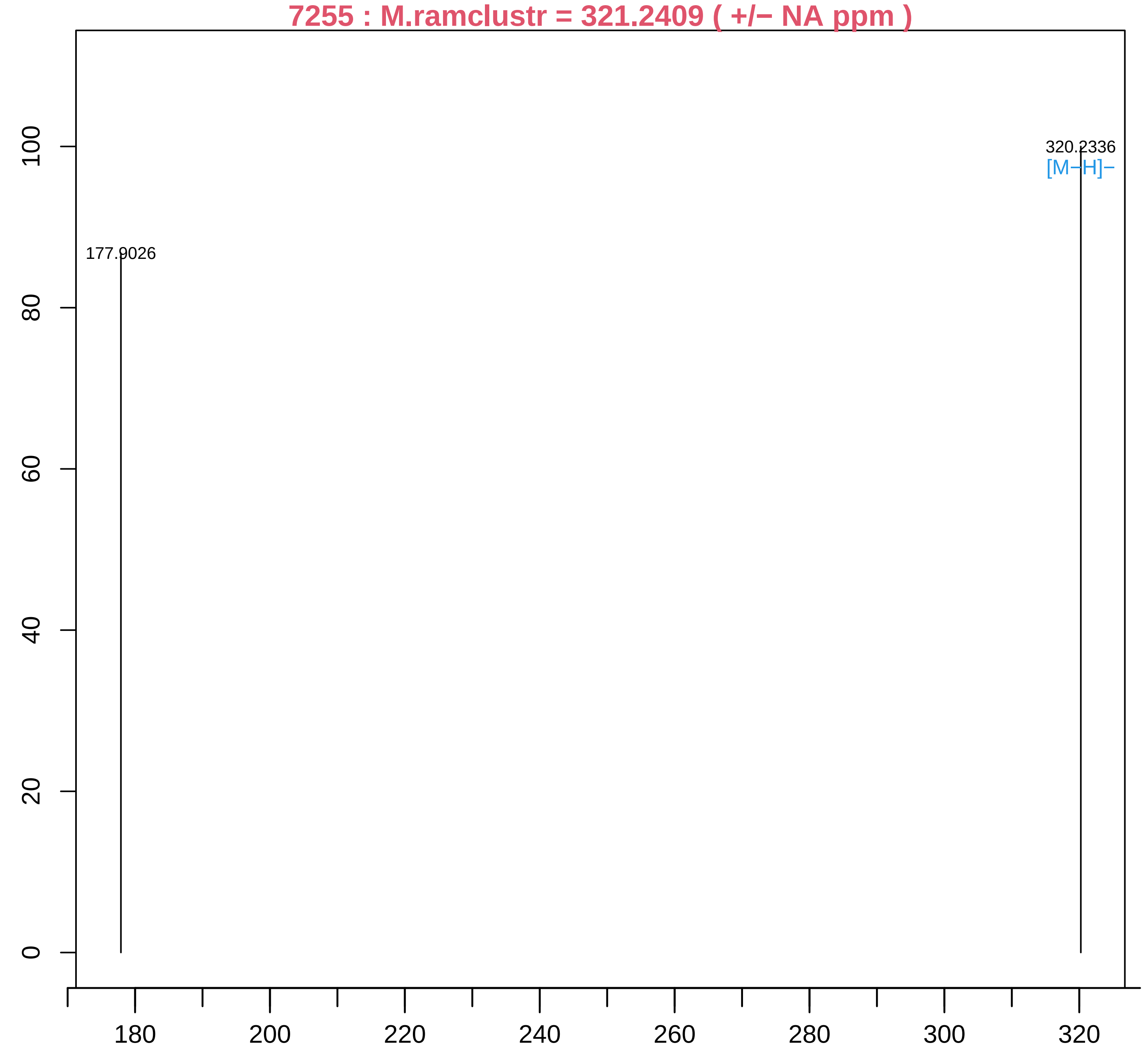
7255 : M.ramclustr = 321.2409 (+/− NA ppm)
177.9026
320.2336
[M−H]−
0
20
40
60
80
100
180
200
220
240
260
280
300
320

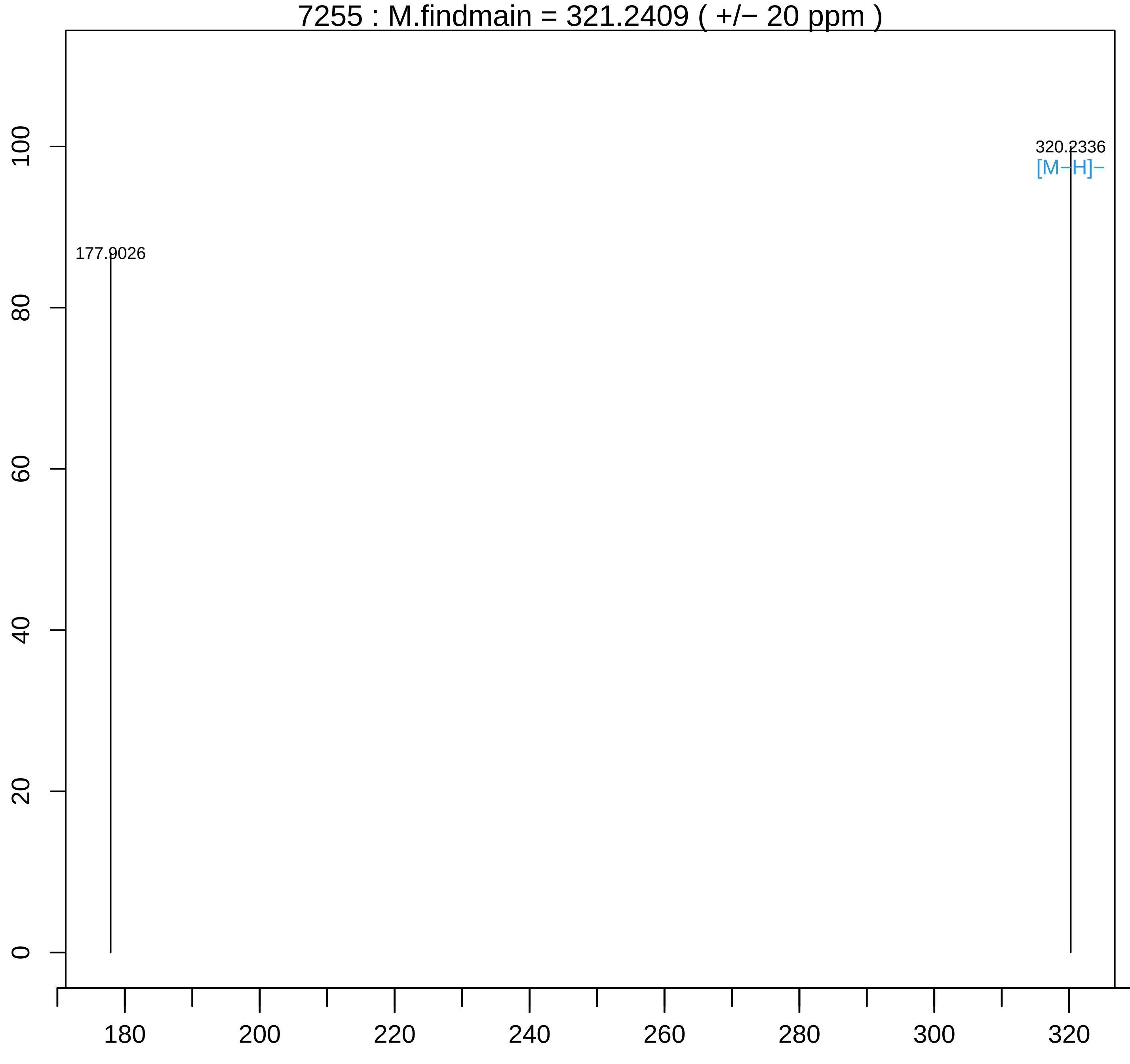
7255 : M.findmain = 321.2409 (+/− 20 ppm)
177.9026
320.2336
[M−H]−
0
20
40
60
80
100
180
200
220
240
260
280
300
320

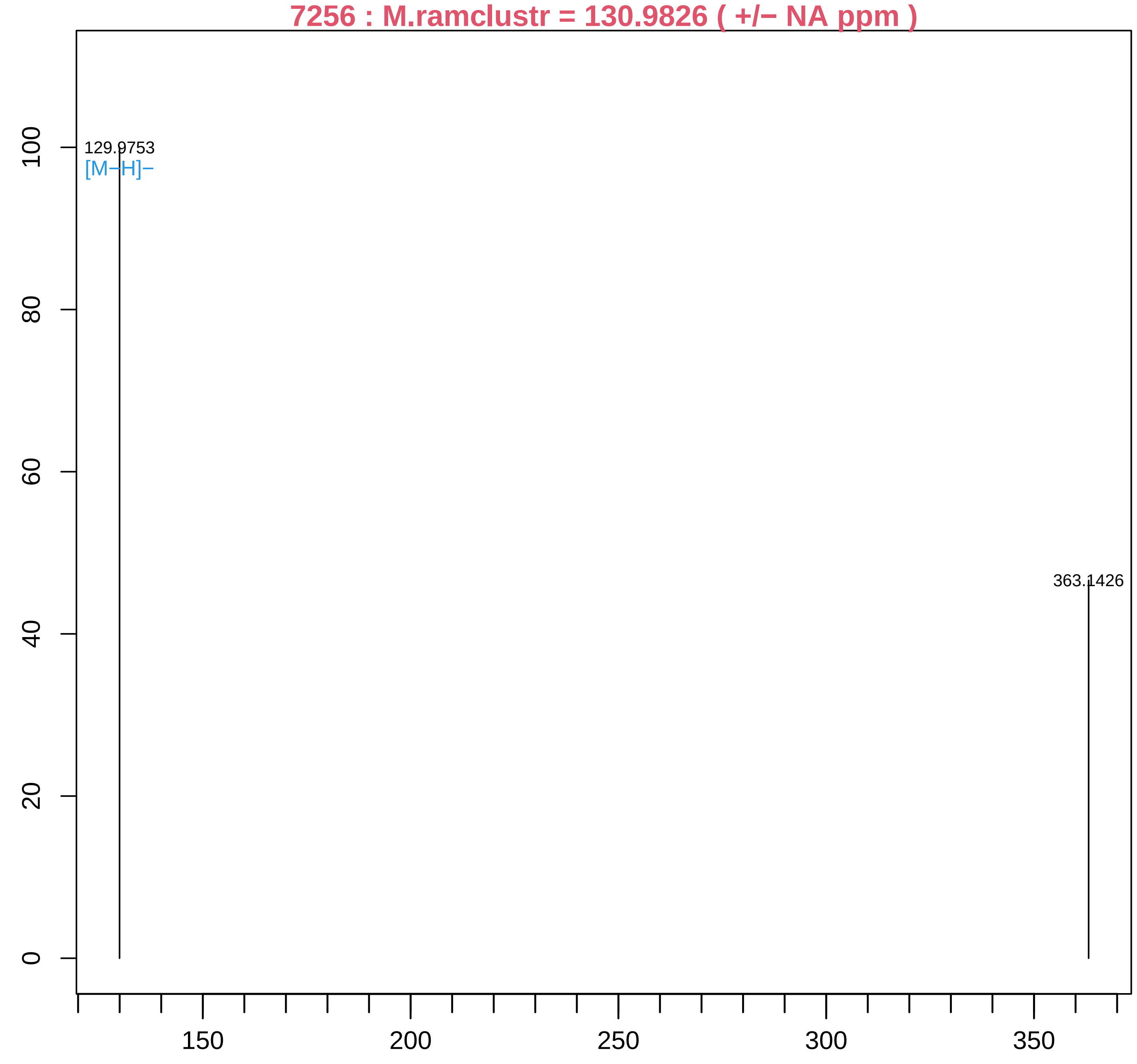
7256 : M.ramclustr = 130.9826 (+/− NA ppm)
129.9753
[M−H]−
363.1426
0
20
40
60
80
100
150
200
250
300
350

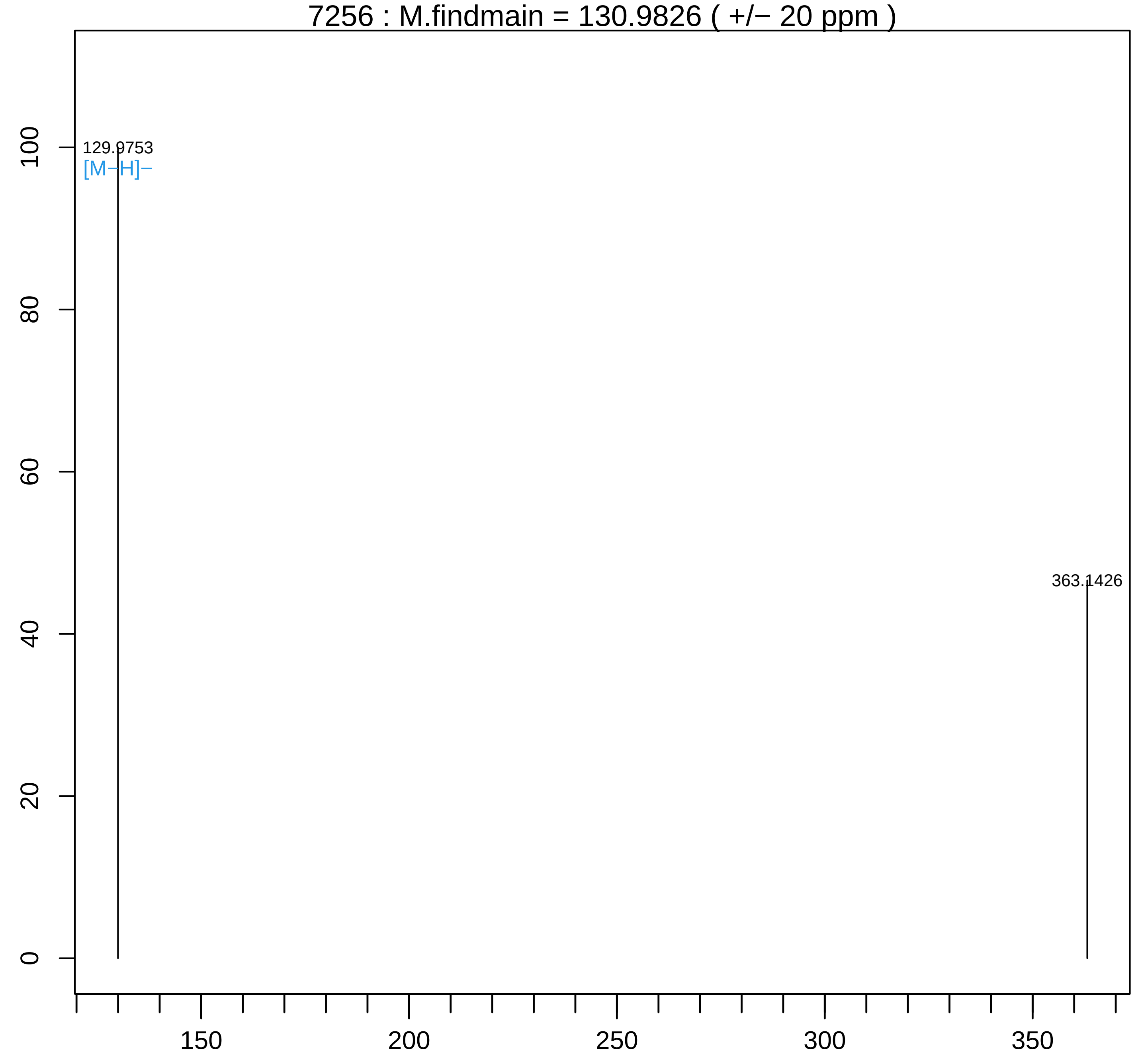
7256 : M.findmain = 130.9826 (+/− 20 ppm)
129.9753
[M−H]−
363.1426
0
20
40
60
80
100
150
200
250
300
350

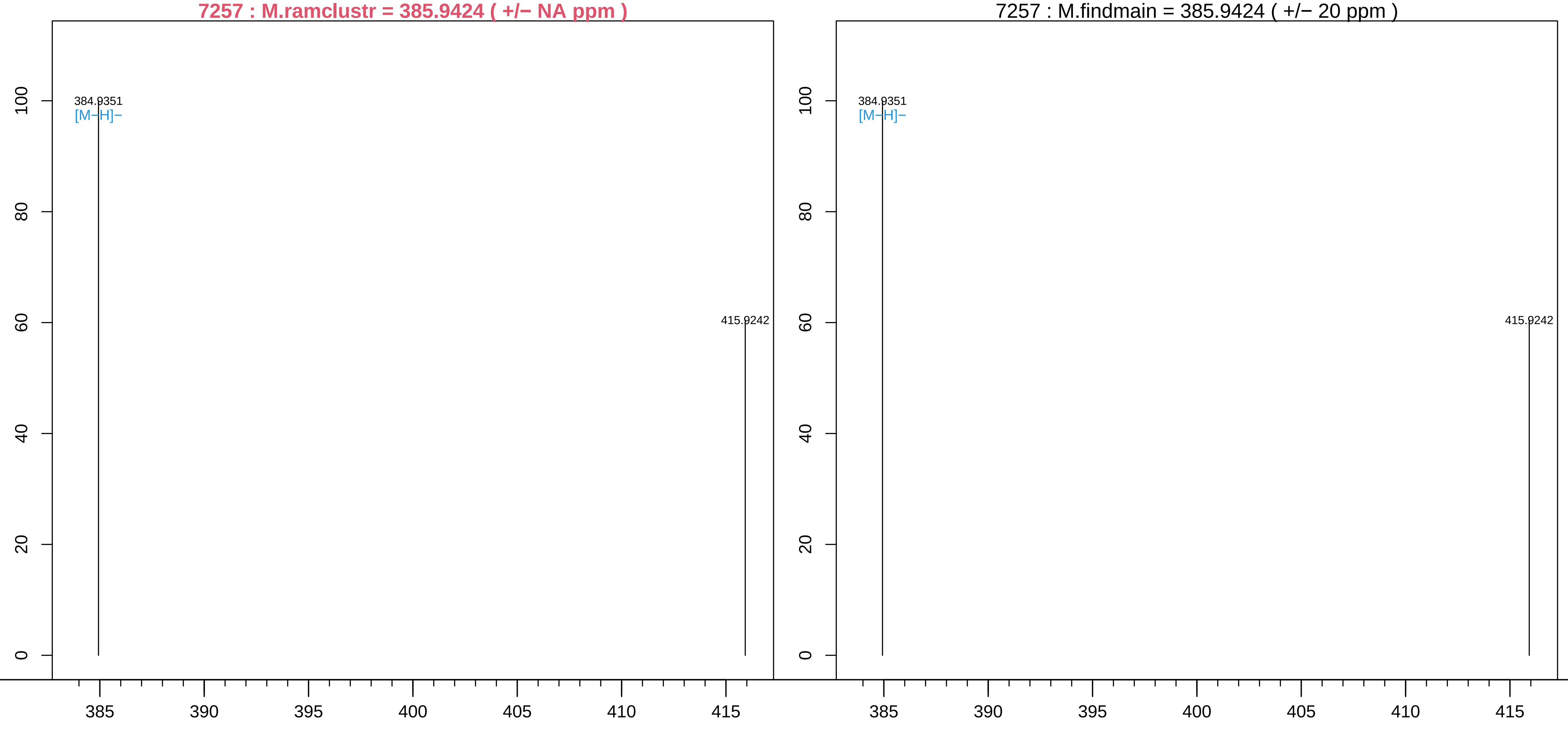
7257 : M.ramclustr = 385.9424 (+/− NA ppm)
384.9351
[M−H]−
415.9242
7257 : M.findmain = 385.9424 (+/− 20 ppm)
384.9351
[M−H]−
415.9242
0
20
40
60
80
100
385
390
395
400
405
410
415

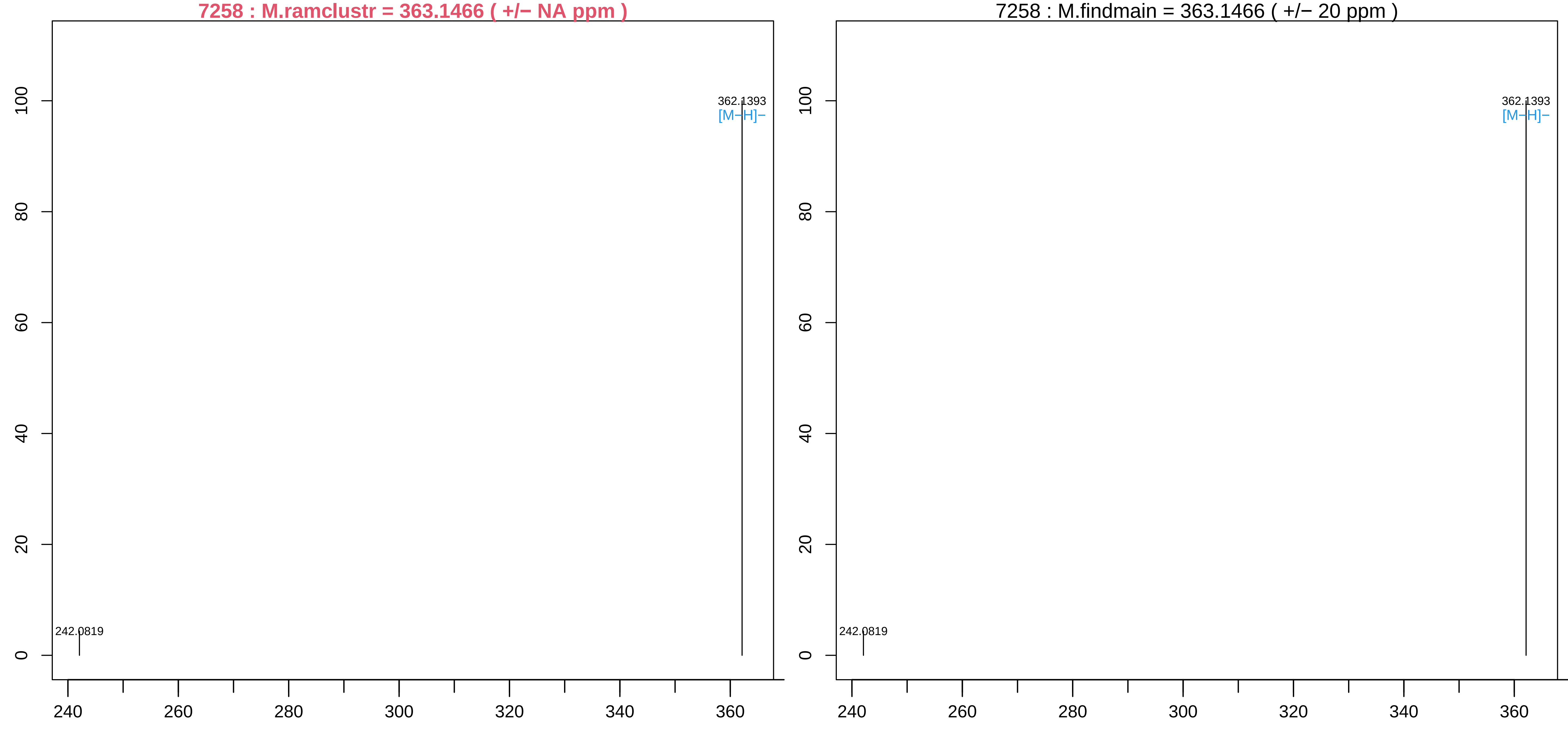
7258 : M.ramclustr = 363.1466 (+/− NA ppm)
7258 : M.findmain = 363.1466 (+/− 20 ppm)
362.1393
[M−H]−
242.0819
0
20
40
60
80
100
240
260
280
300
320
340
360

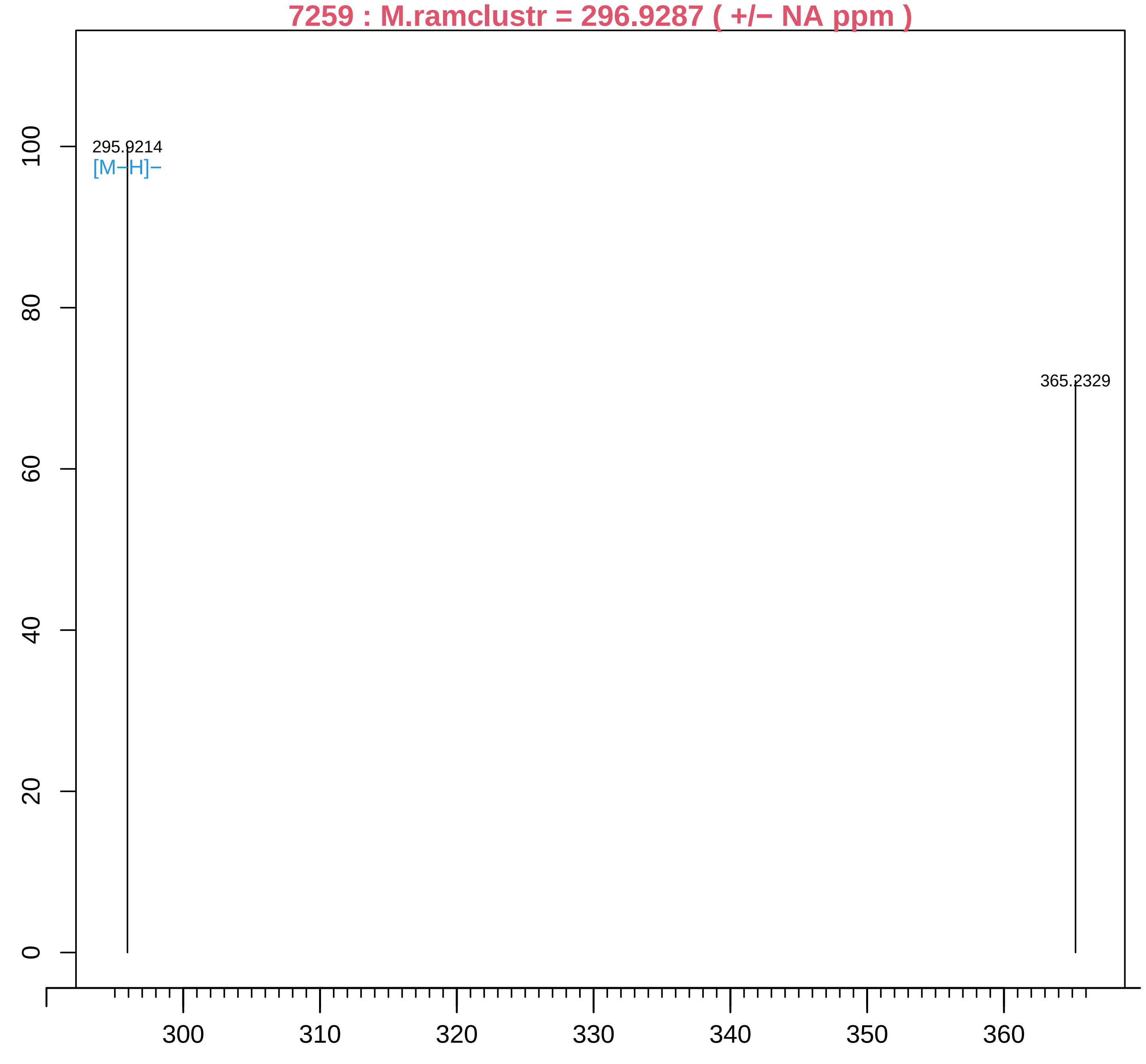
7259 : M.ramclustr = 296.9287 (+/− NA ppm)
295.9214
[M−H]−
365.2329
0
20
40
60
80
100
300
310
320
330
340
350
360

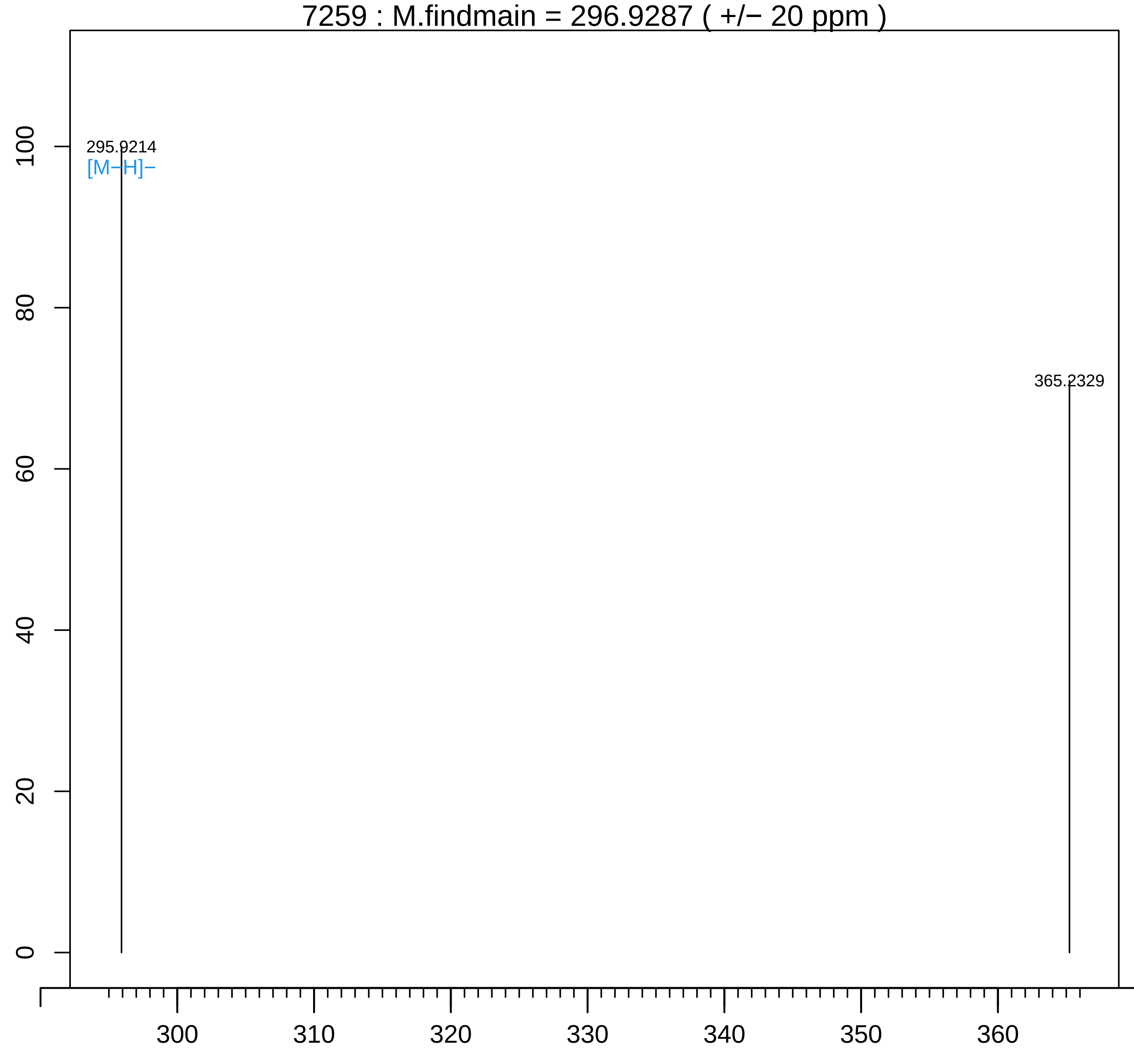
7259 : M.findmain = 296.9287 (+/− 20 ppm)
295.9214
[M−H]−
365.2329
0
20
40
60
80
100
300
310
320
330
340
350
360

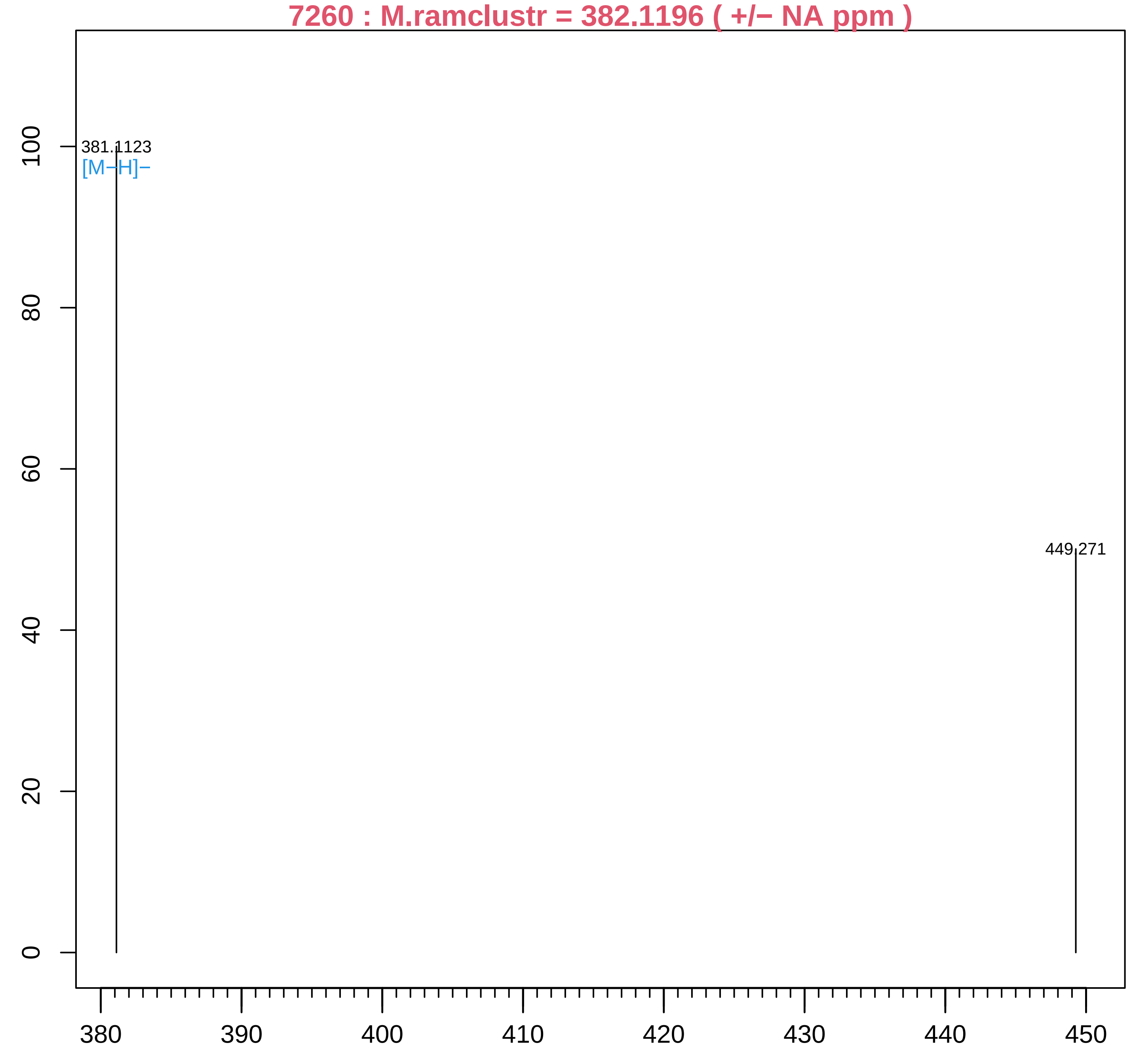
7260 : M.ramclustr = 382.1196 (+/− NA ppm)
381.1123
[M−H]−
449.271
0
20
40
60
80
100
380
390
400
410
420
430
440
450

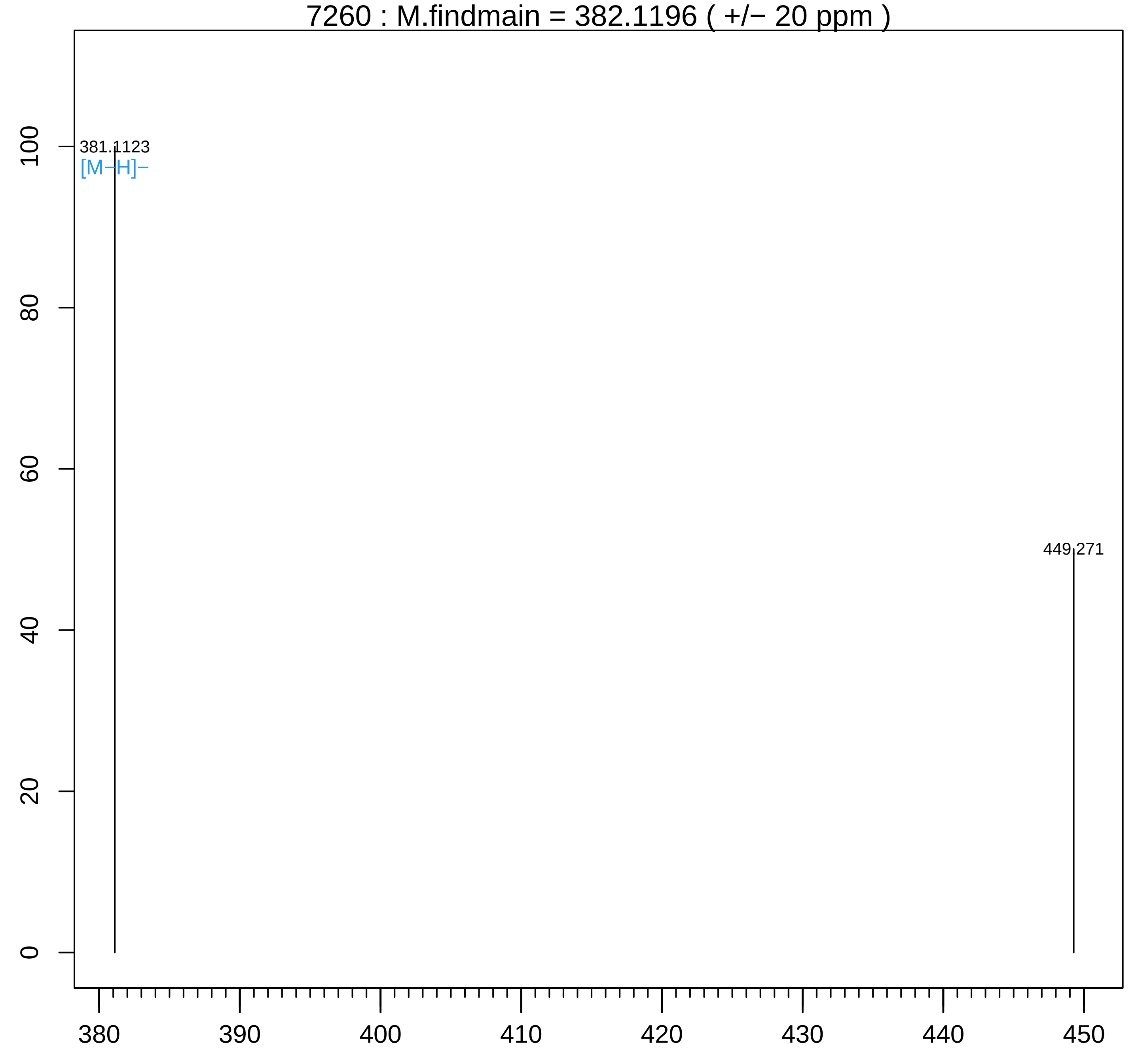
7260 : M.findmain = 382.1196 (+/− 20 ppm)
381.1123
[M−H]−
449.271
0
20
40
60
80
100
380
390
400
410
420
430
440
450

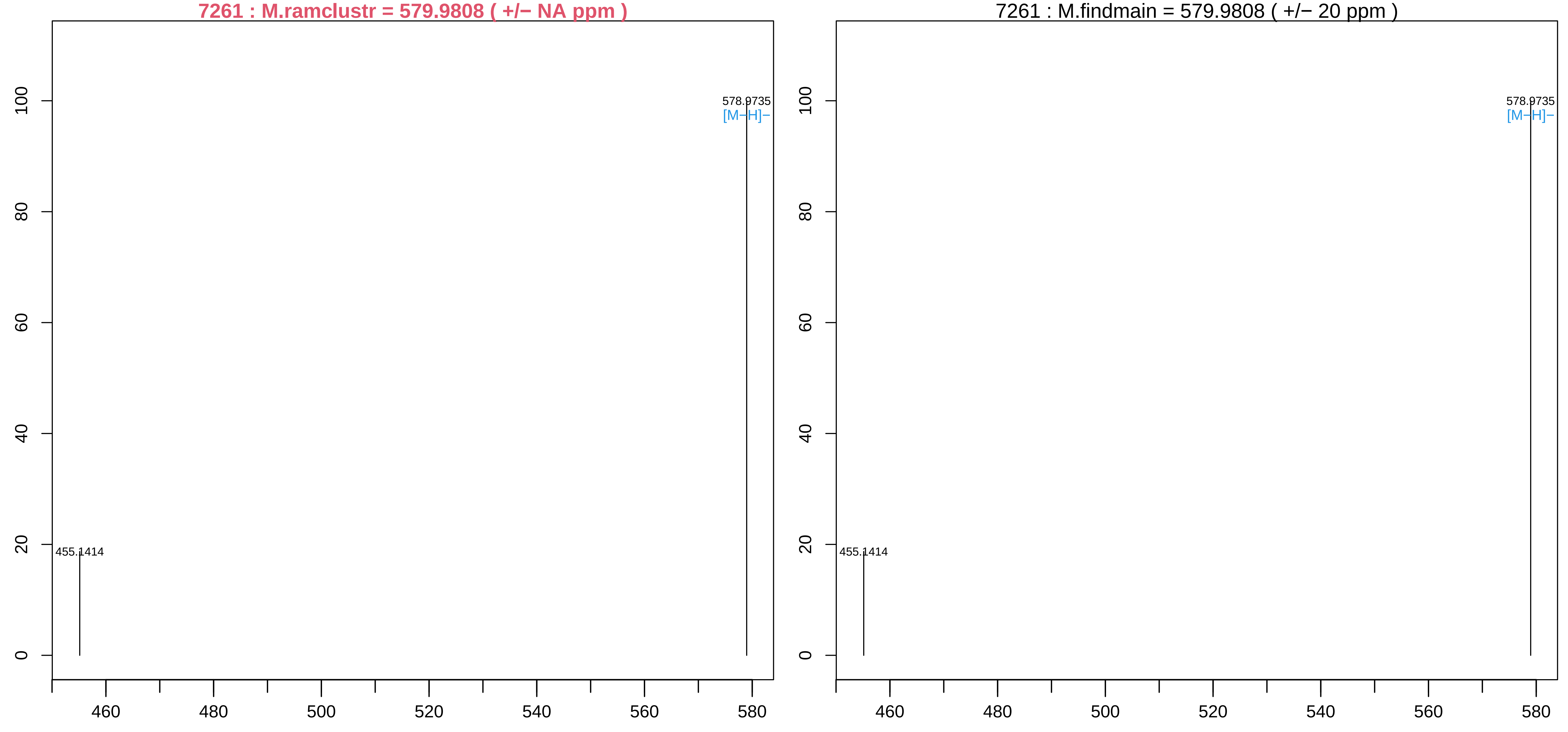
7261 : M.ramclustr = 579.9808 (+/− NA ppm)
7261 : M.findmain = 579.9808 (+/− 20 ppm)
578.9735
[M−H]−
455.1414
0
20
40
60
80
100
460
480
500
520
540
560
580

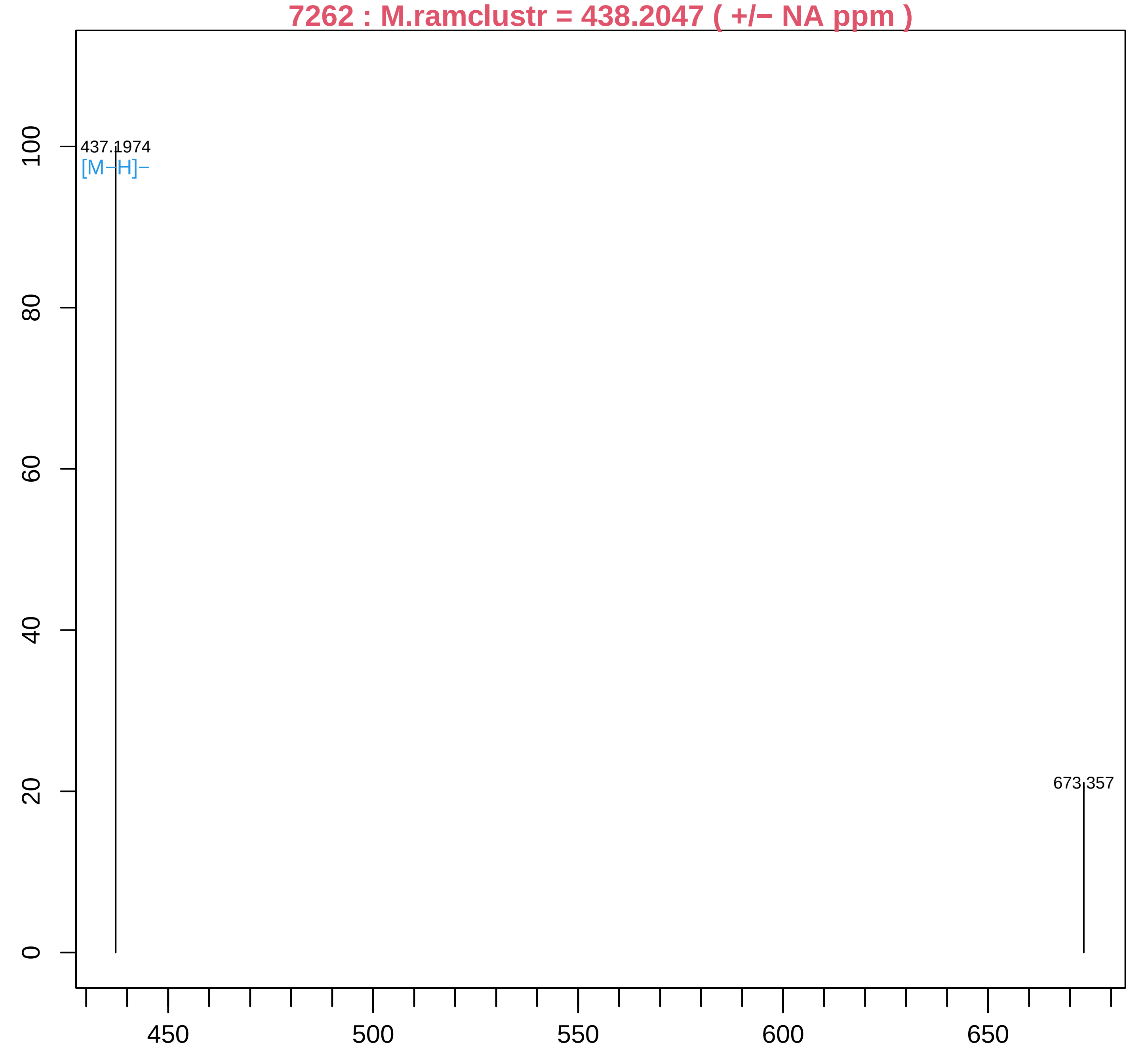
7262 : M.ramclustr = 438.2047 (+/− NA ppm)
437.1974
[M−H]−
673.357
0
20
40
60
80
100
450
500
550
600
650

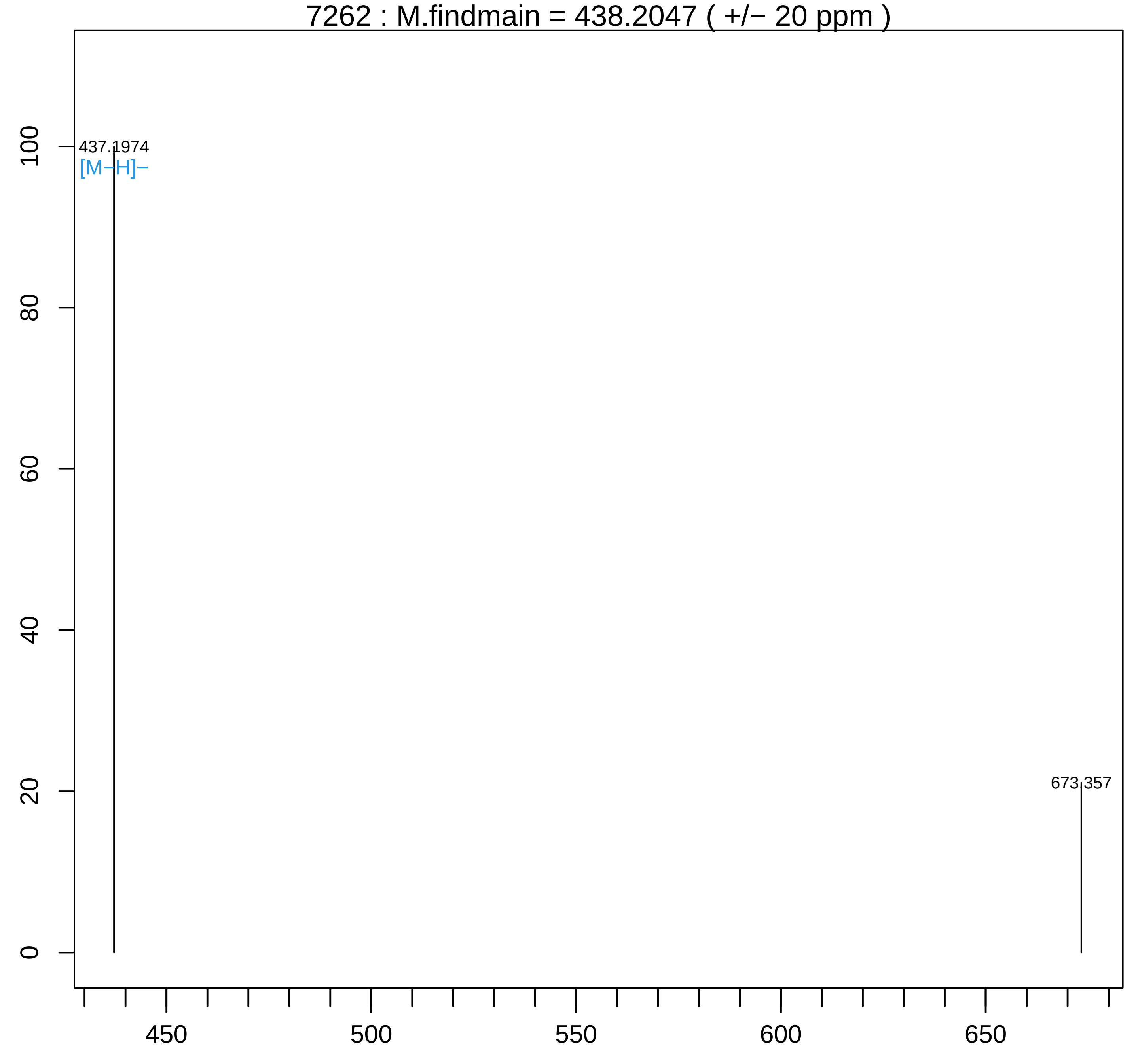
7262 : M.findmain = 438.2047 (+/− 20 ppm)
437.1974
[M−H]−
673.357
0
20
40
60
80
100
450
500
550
600
650

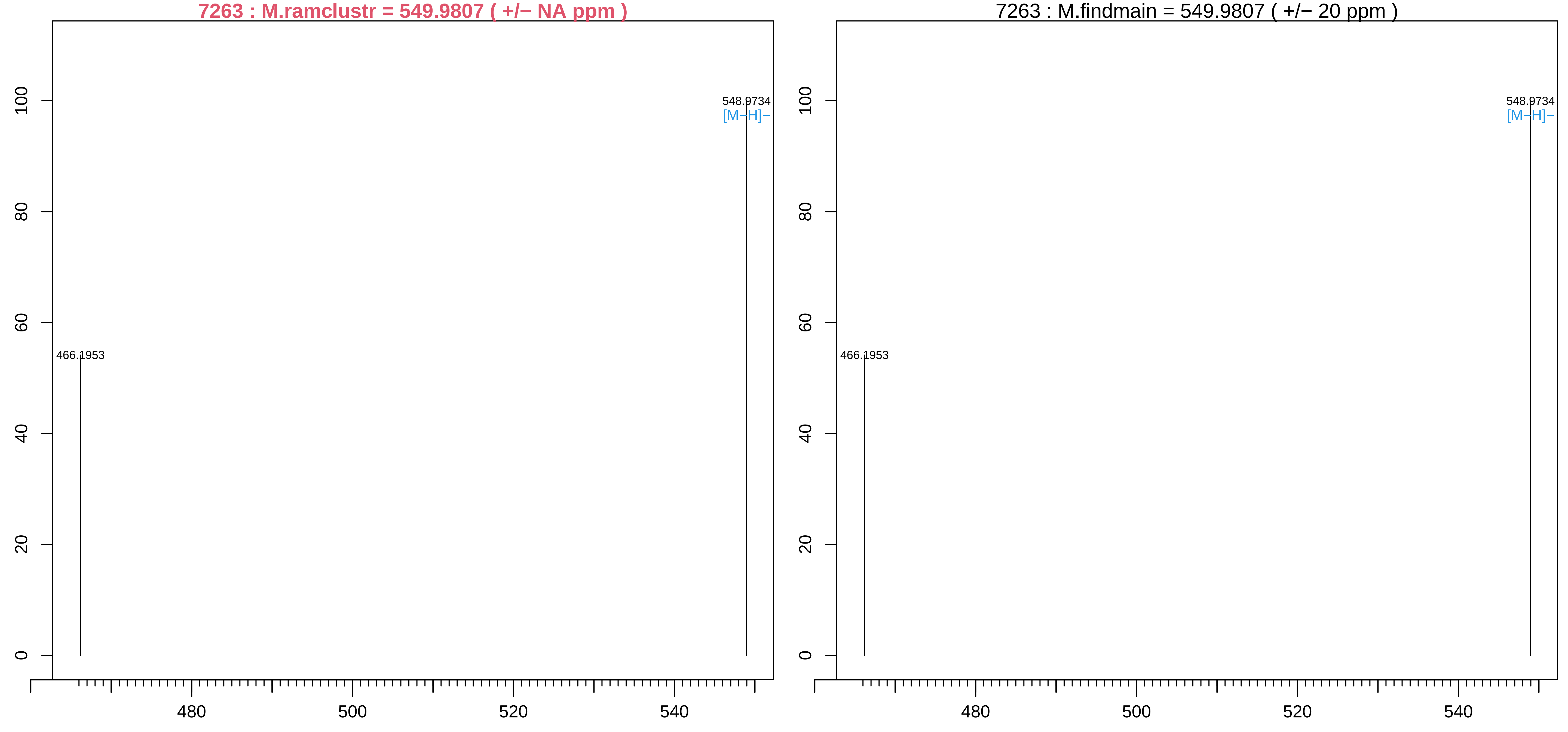
7263 : M.ramclustr = 549.9807 (+/− NA ppm)
548.9734
[M−H]−
466.1953
0
20
40
60
80
100
480
500
520
540
7263 : M.findmain = 549.9807 (+/− 20 ppm)
548.9734
[M−H]−
466.1953
0
20
40
60
80
100
480
500
520
540

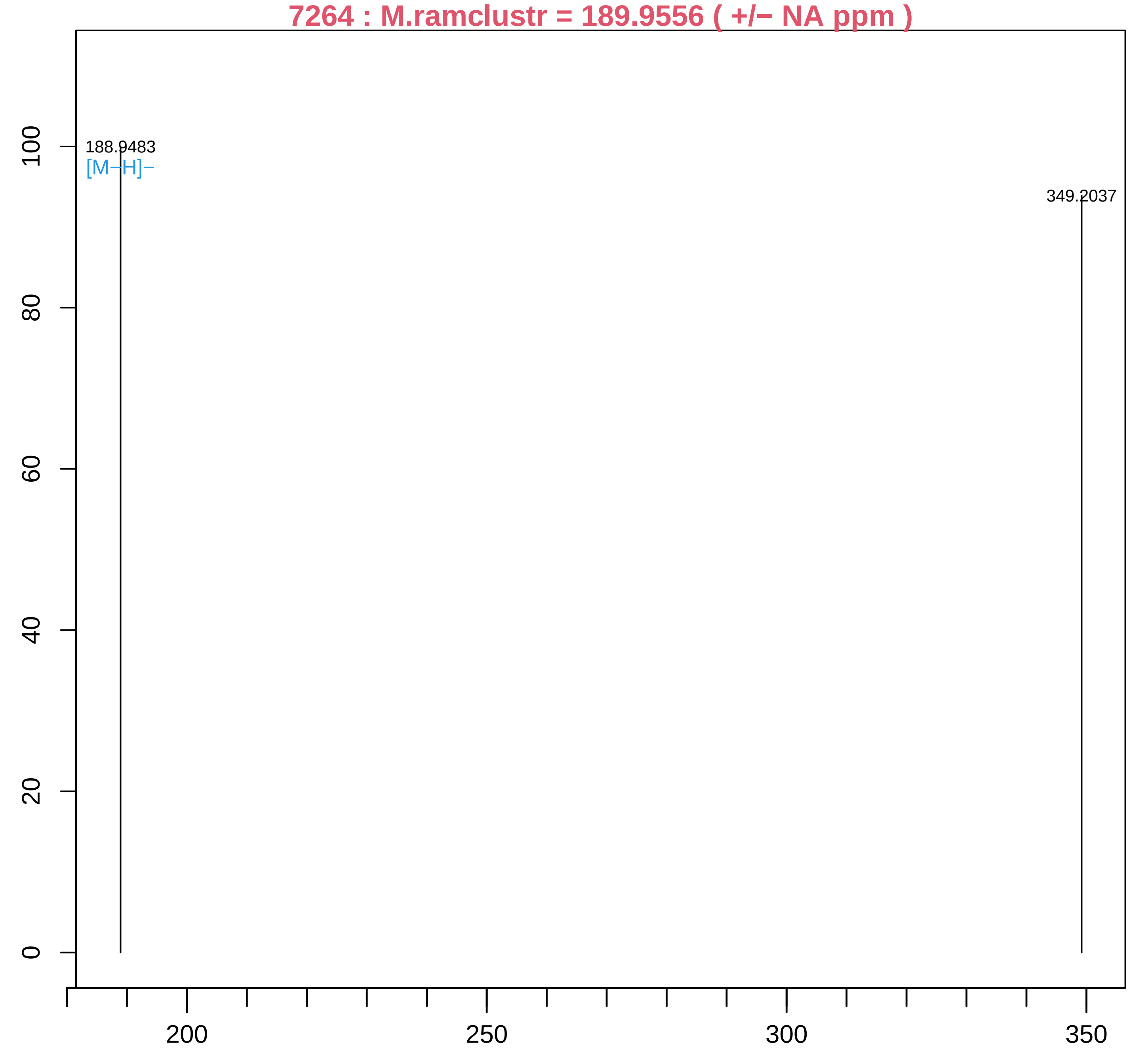
7264 : M.ramclustr = 189.9556 (+/− NA ppm)
188.9483
[M−H]−
349.2037
0
20
40
60
80
100
200
250
300
350

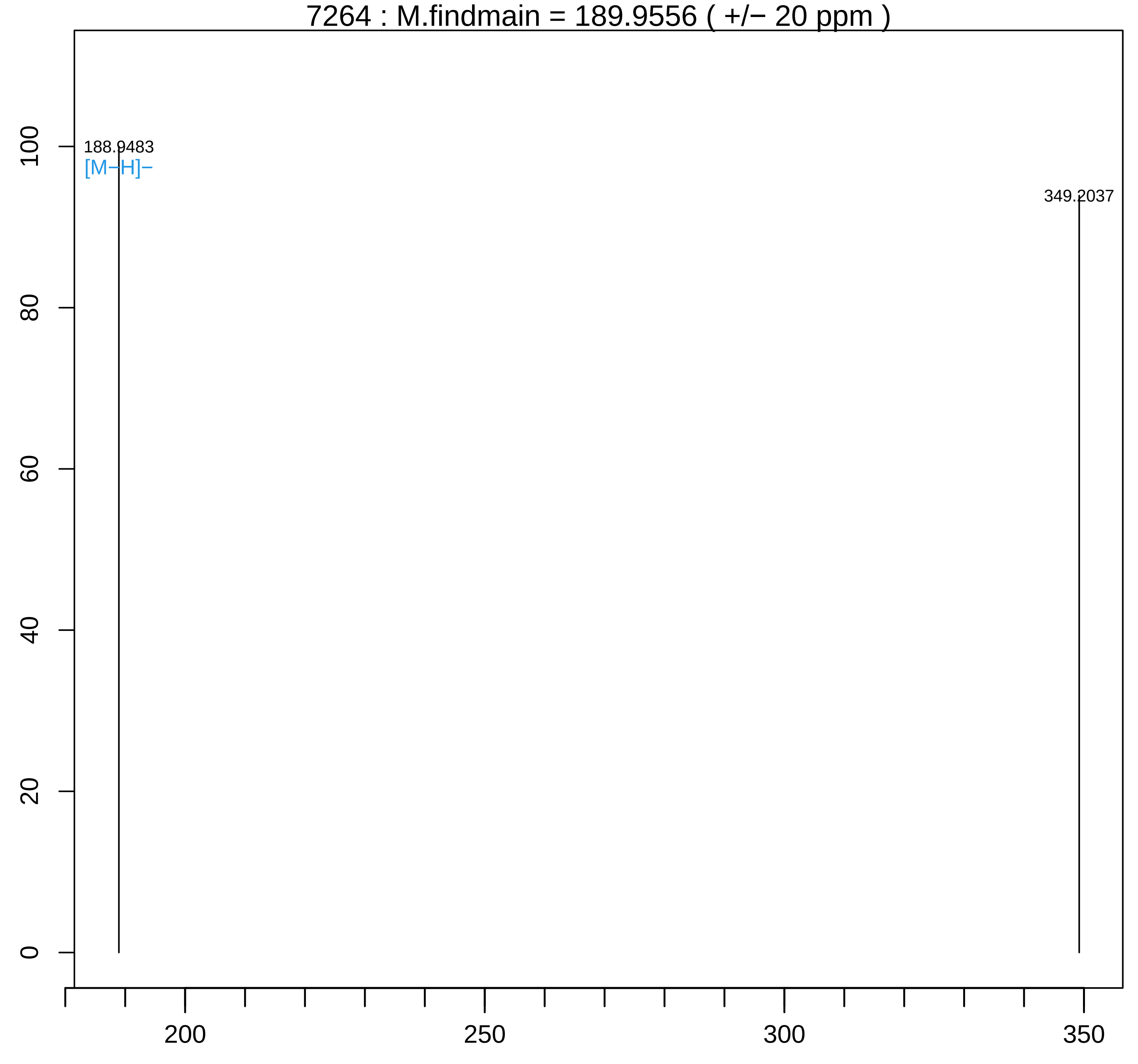
7264 : M.findmain = 189.9556 (+/− 20 ppm)
188.9483
[M−H]−
349.2037
0
20
40
60
80
100
200
250
300
350

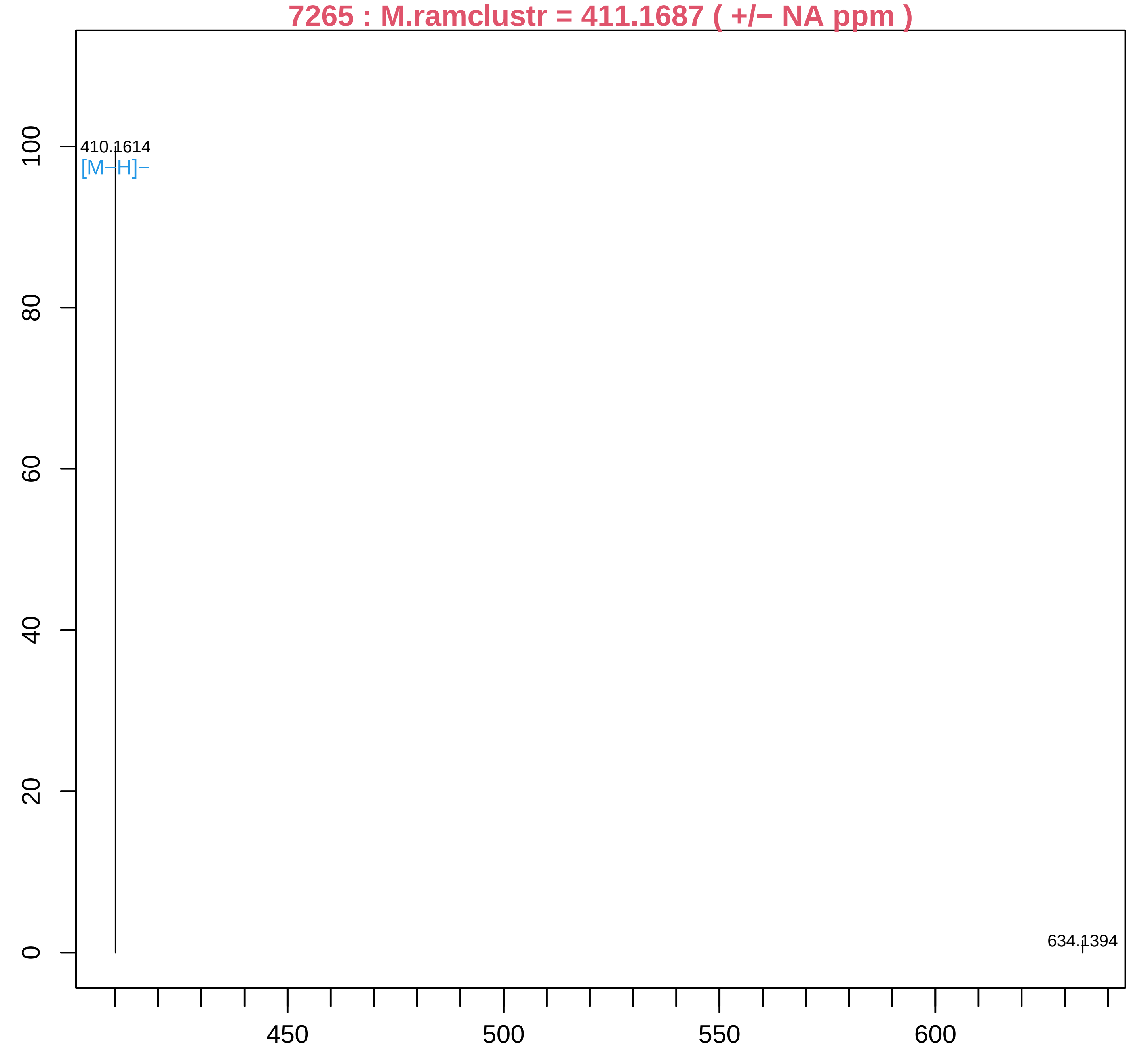
7265 : M.ramclustr = 411.1687 (+/− NA ppm)
410.1614
[M−H]−
634.1394
0
20
40
60
80
100
450
500
550
600

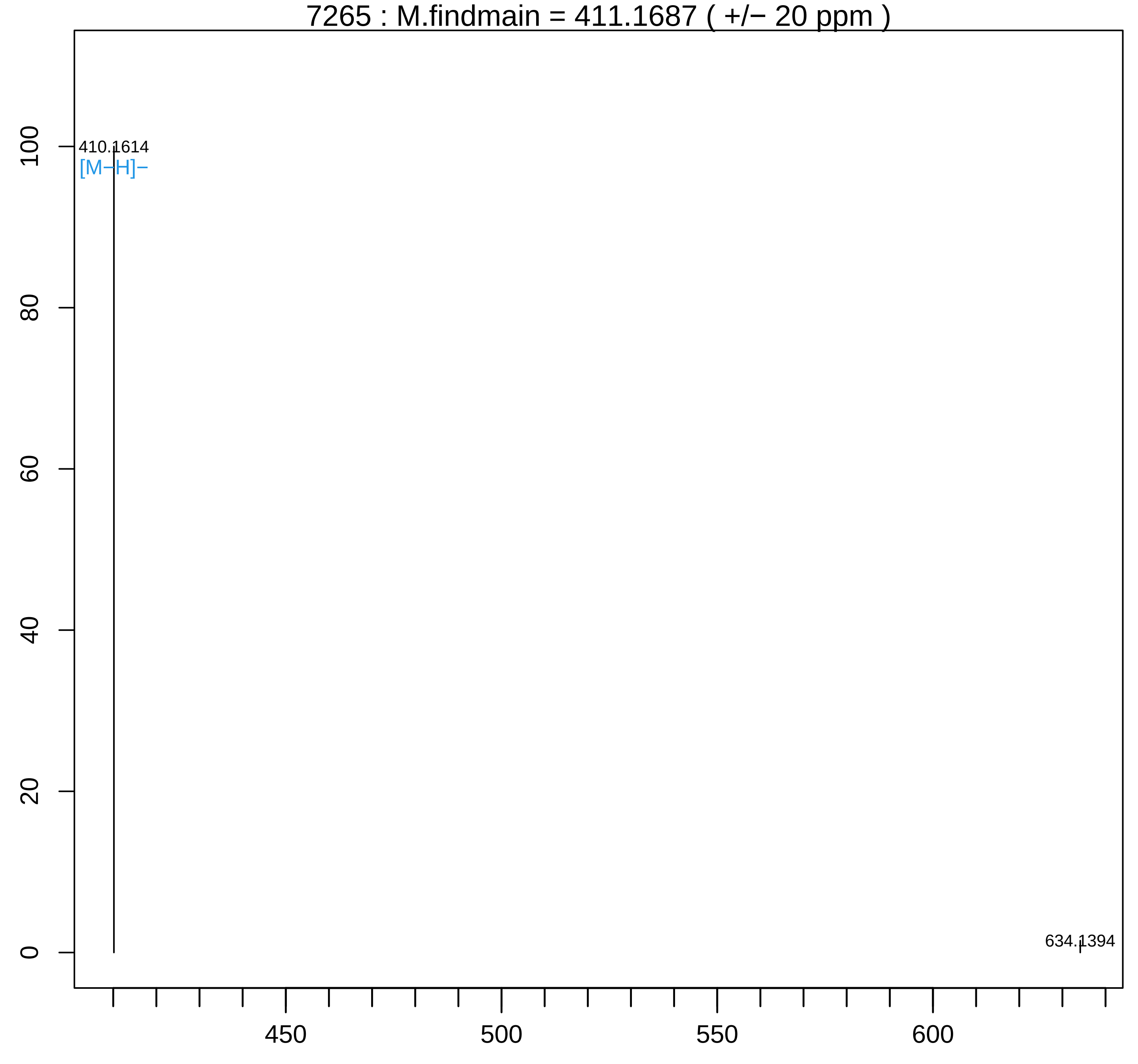
7265 : M.findmain = 411.1687 (+/− 20 ppm)
410.1614
[M−H]−
634.1394
0
20
40
60
80
100
450
500
550
600

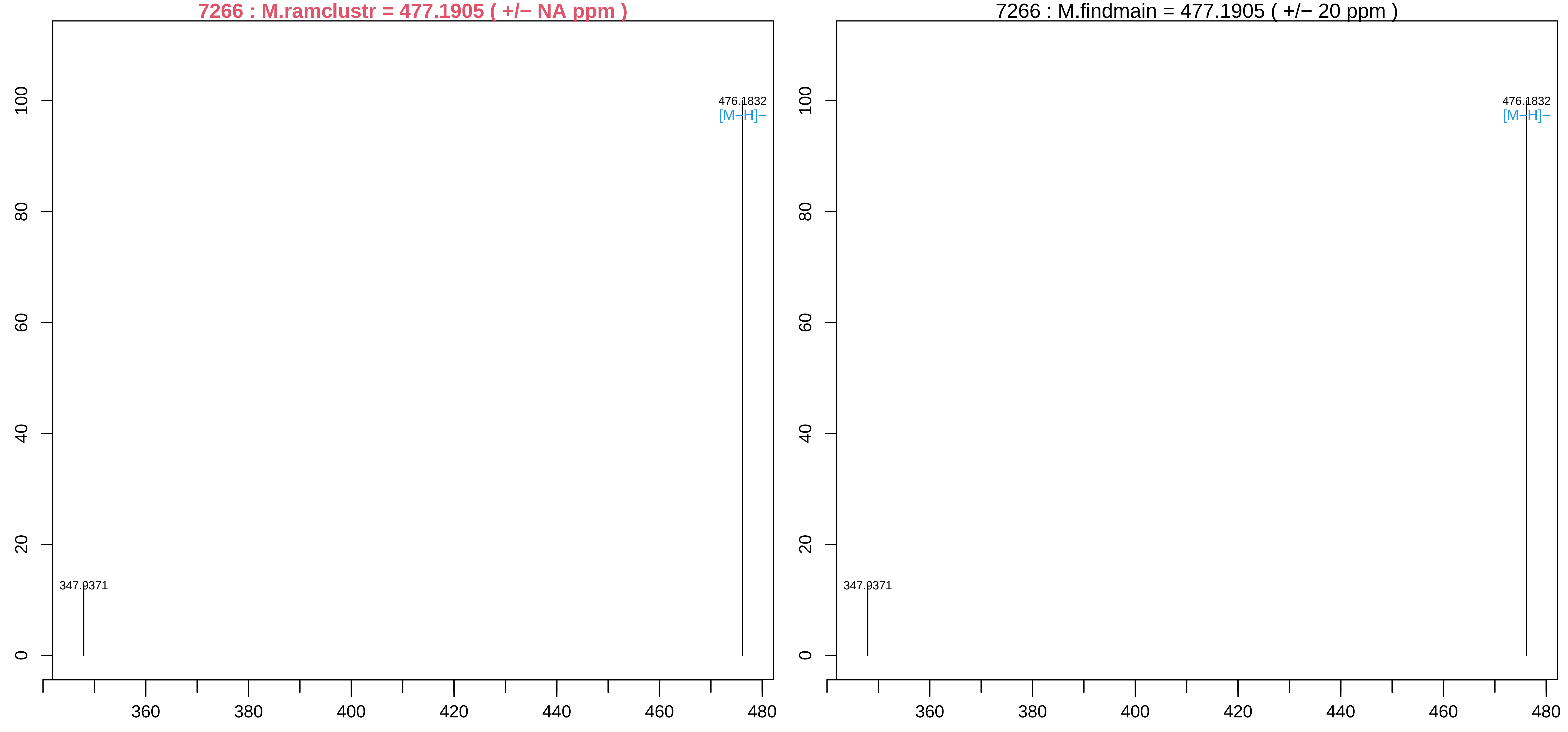
7266 : M.ramclustr = 477.1905 (+/− NA ppm)
7266 : M.findmain = 477.1905 (+/− 20 ppm)
476.1832
[M−H]−
347.9371
0
20
40
60
80
100
360
380
400
420
440
460
480

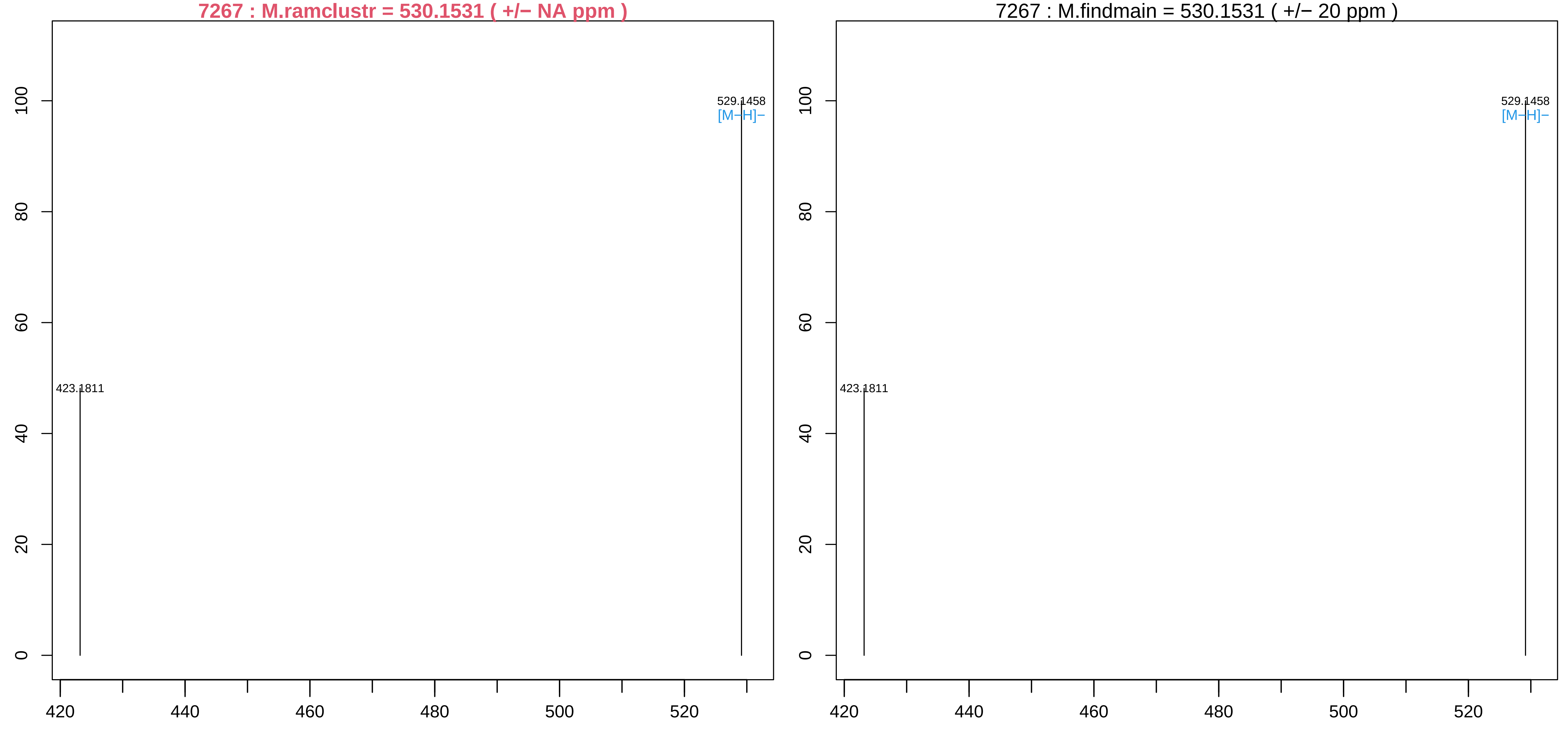
7267 : M.ramclustr = 530.1531 (+/− NA ppm)
529.1458
[M−H]−
423.1811
7267 : M.findmain = 530.1531 (+/− 20 ppm)
529.1458
[M−H]−
423.1811
0
20
40
60
80
100
420
440
460
480
500
520

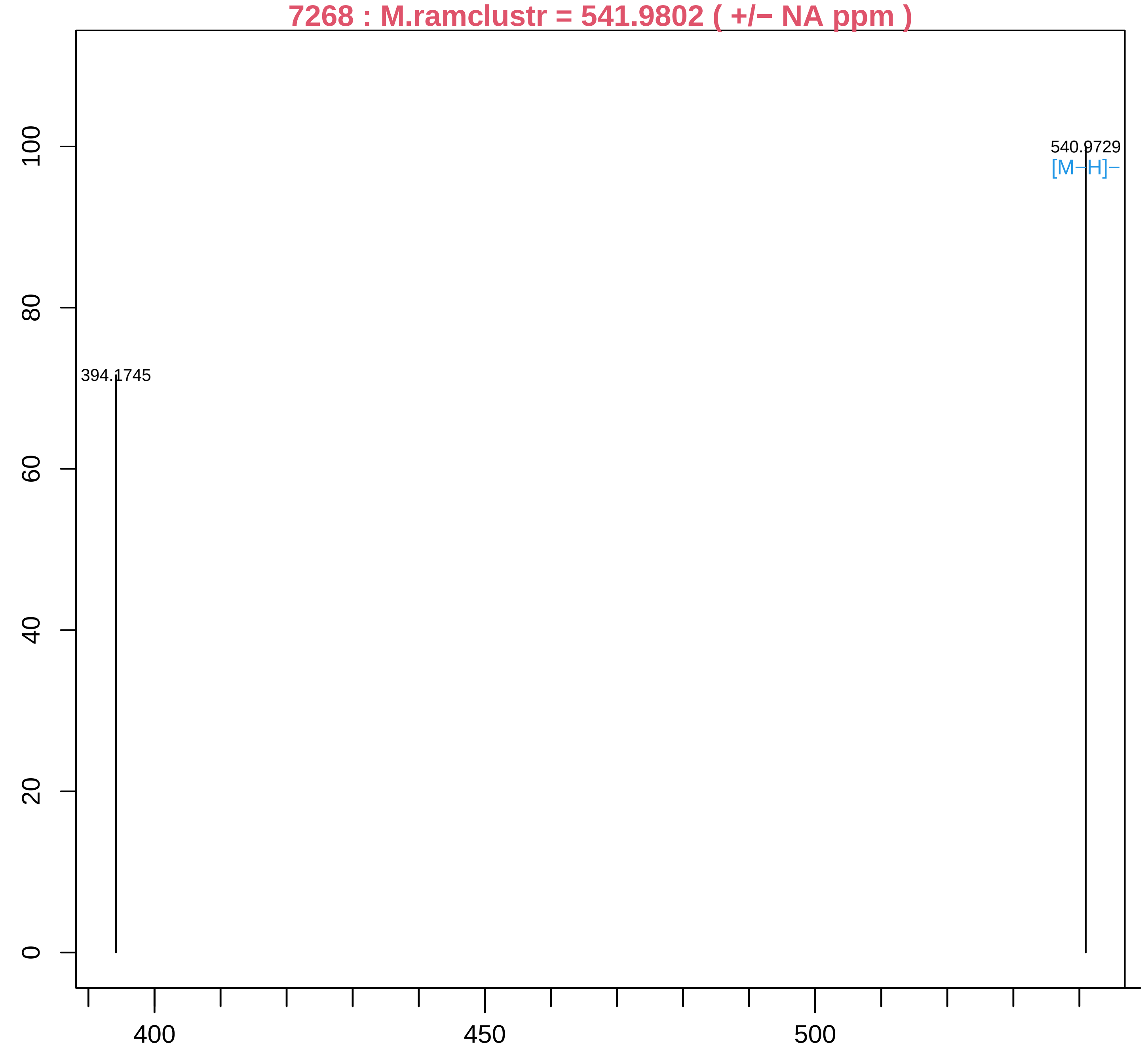
7268 : M.ramclustr = 541.9802 (+/− NA ppm)
540.9729
[M−H]−
394.1745
0
20
40
60
80
100
400
450
500

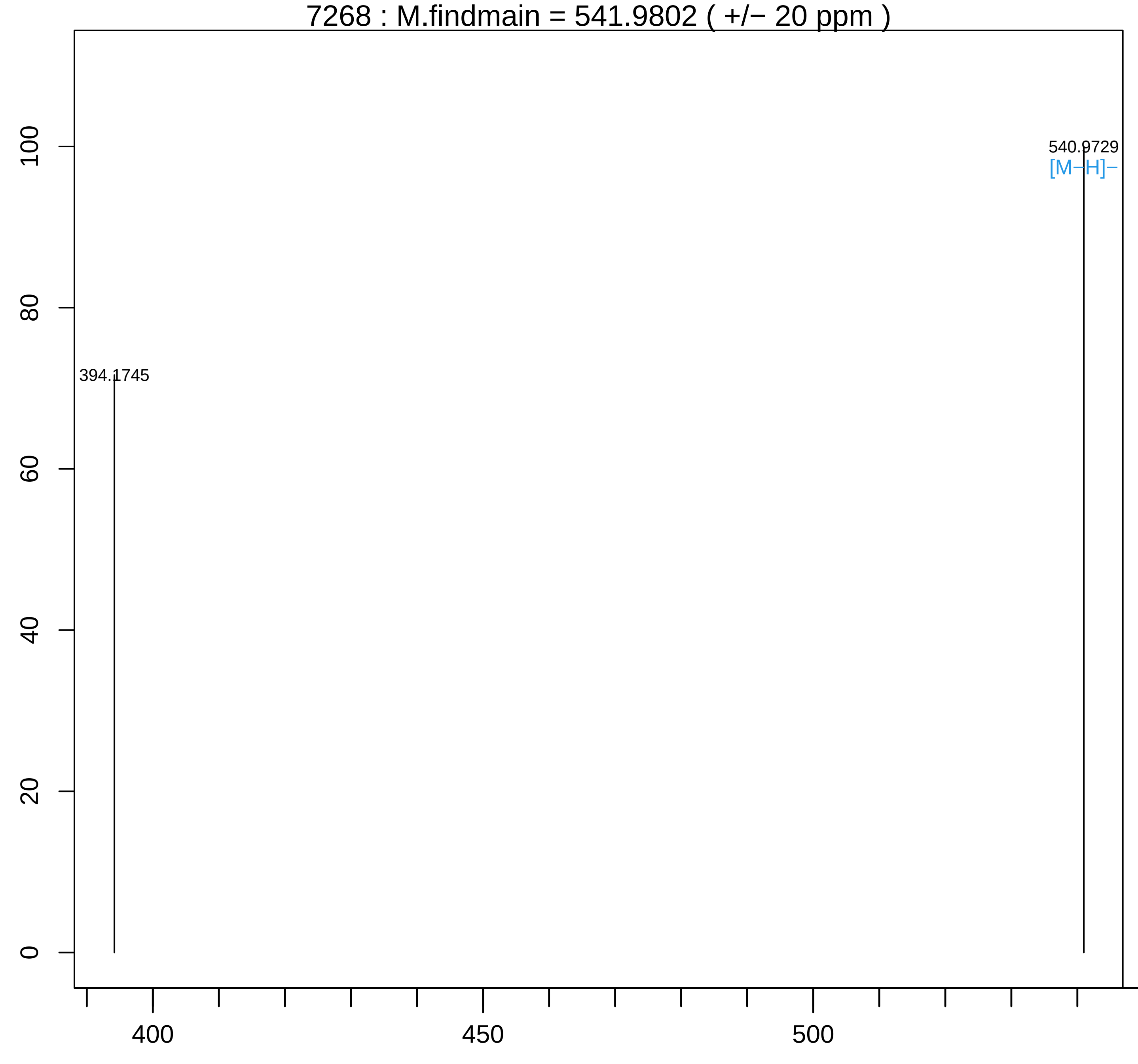
7268 : M.findmain = 541.9802 (+/− 20 ppm)
540.9729
[M−H]−
394.1745
0
20
40
60
80
100
400
450
500

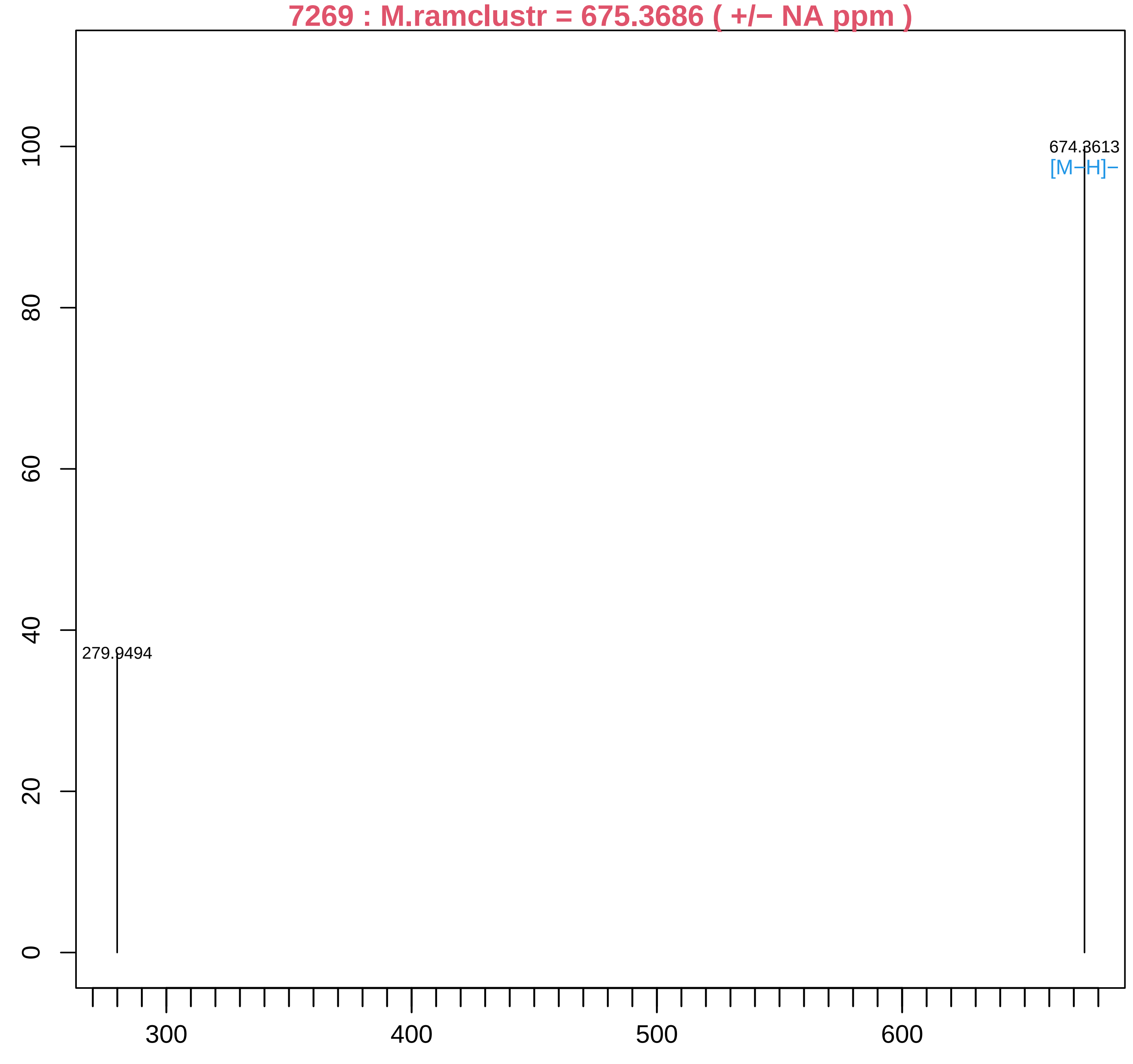
7269 : M.ramclustr = 675.3686 (+/− NA ppm)
674.3613
[M−H]−
279.9494
0
20
40
60
80
100
300
400
500
600

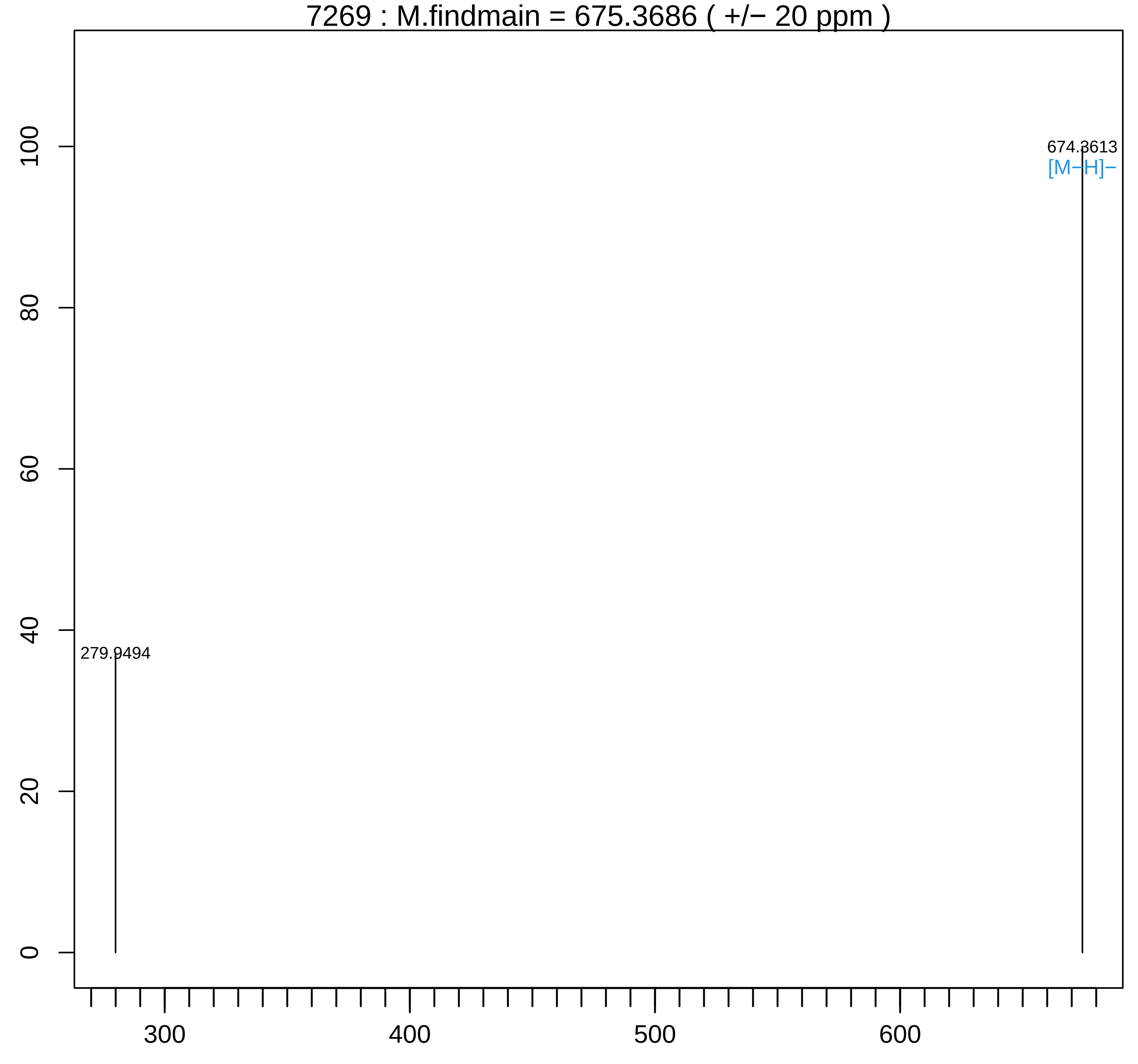
7269 : M.findmain = 675.3686 (+/− 20 ppm)
674.3613
[M−H]−
279.9494
0
20
40
60
80
100
300
400
500
600

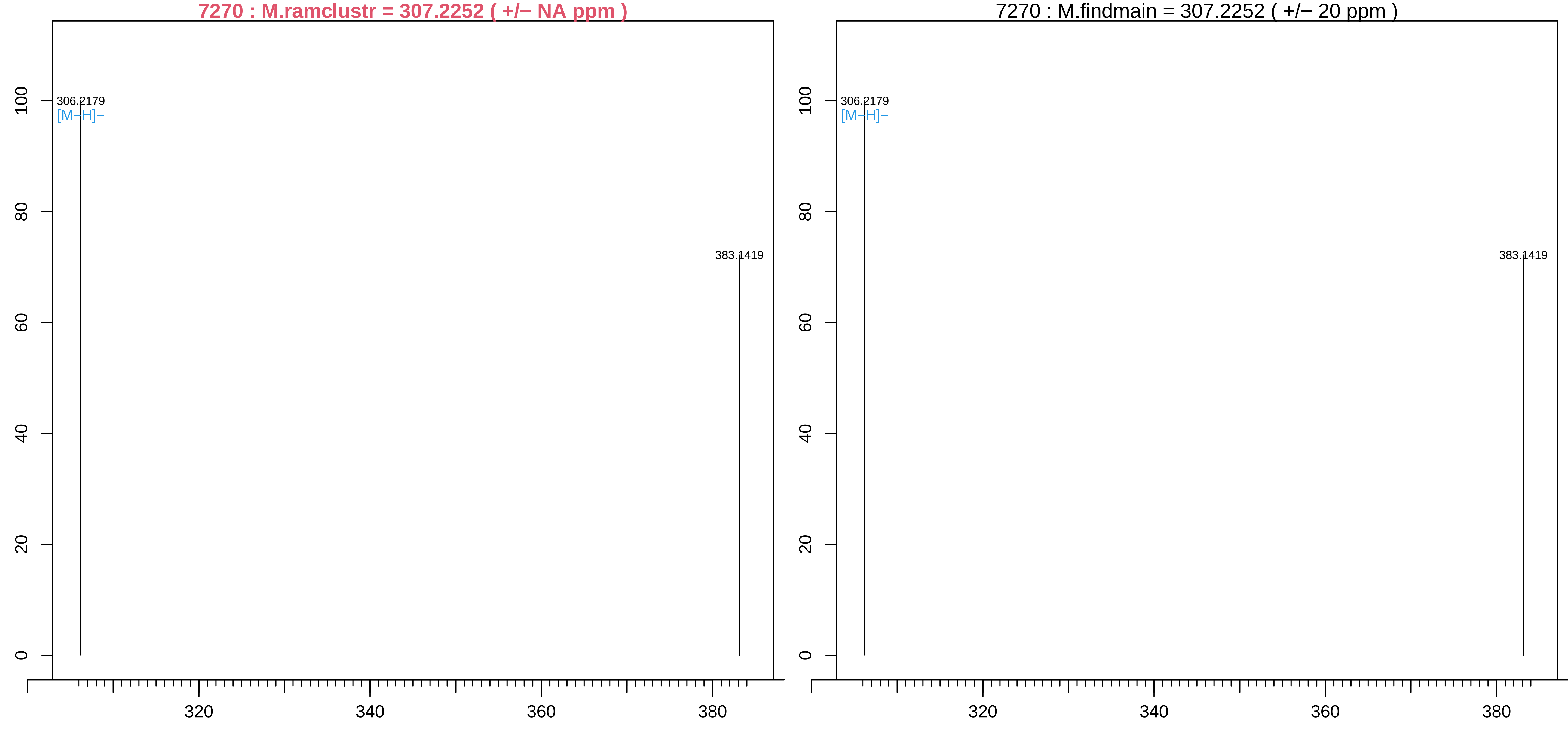
7270 : M.ramclustr = 307.2252 (+/− NA ppm)
306.2179
[M−H]−
383.1419
0
20
40
60
80
100
320
340
360
380
7270 : M.findmain = 307.2252 (+/− 20 ppm)
306.2179
[M−H]−
383.1419
0
20
40
60
80
100
320
340
360
380

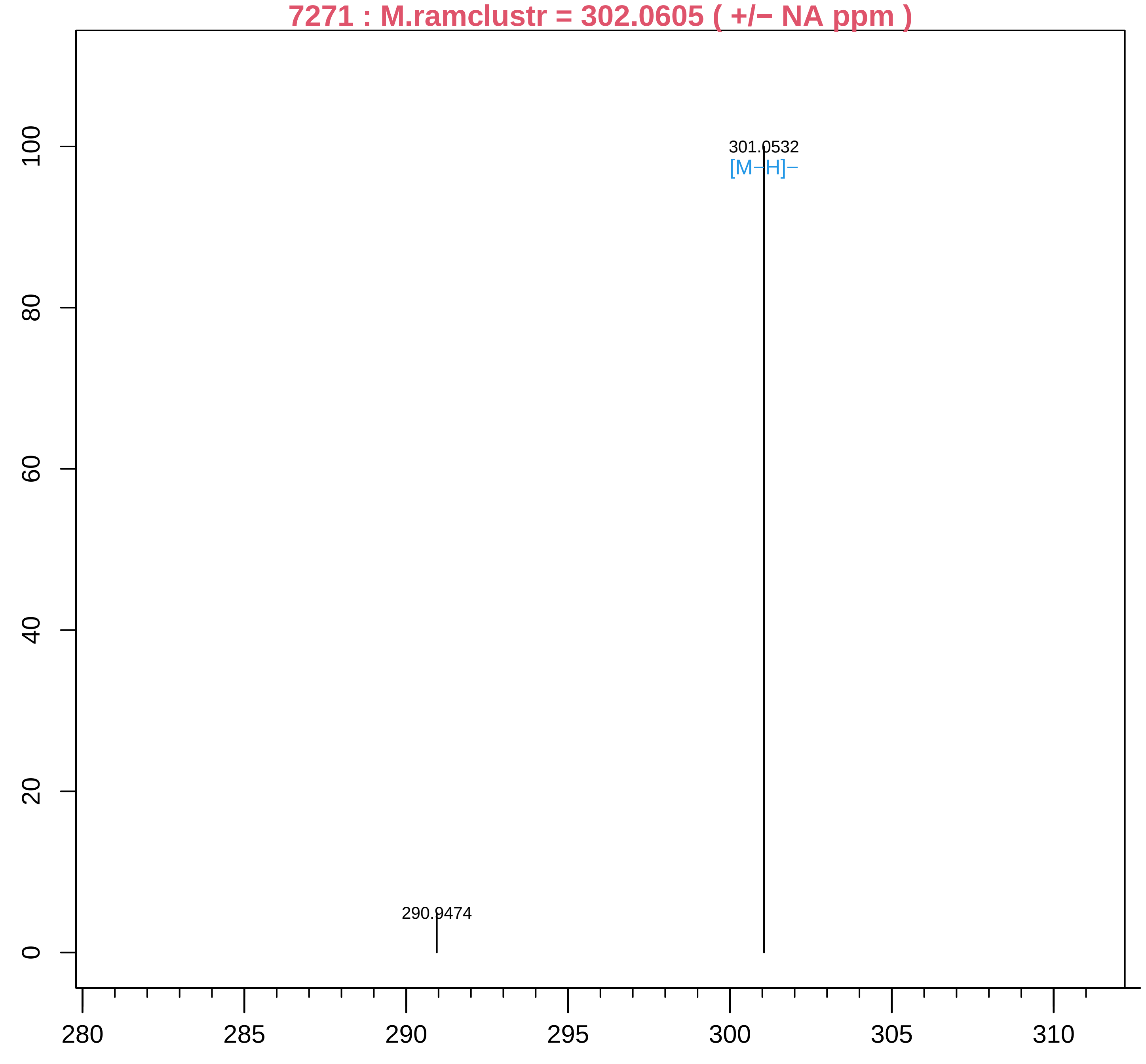
7271 : M.ramclustr = 302.0605 (+/− NA ppm)
301.0532
[M−H]−
290.9474
0
20
40
60
80
100
280
285
290
295
300
305
310

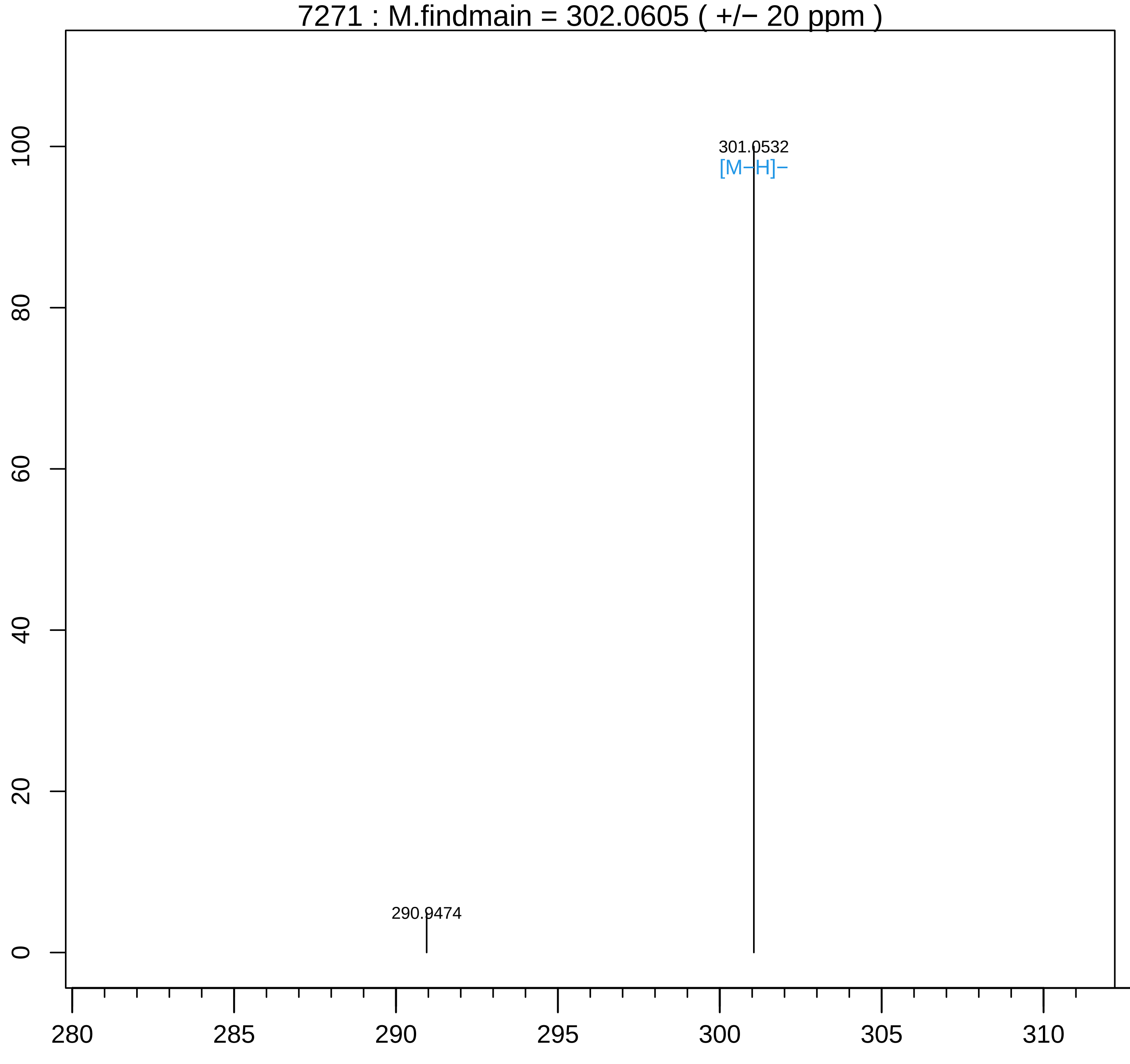
7271 : M.findmain = 302.0605 (+/− 20 ppm)
301.0532
[M−H]−
290.9474
0
20
40
60
80
100
280
285
290
295
300
305
310

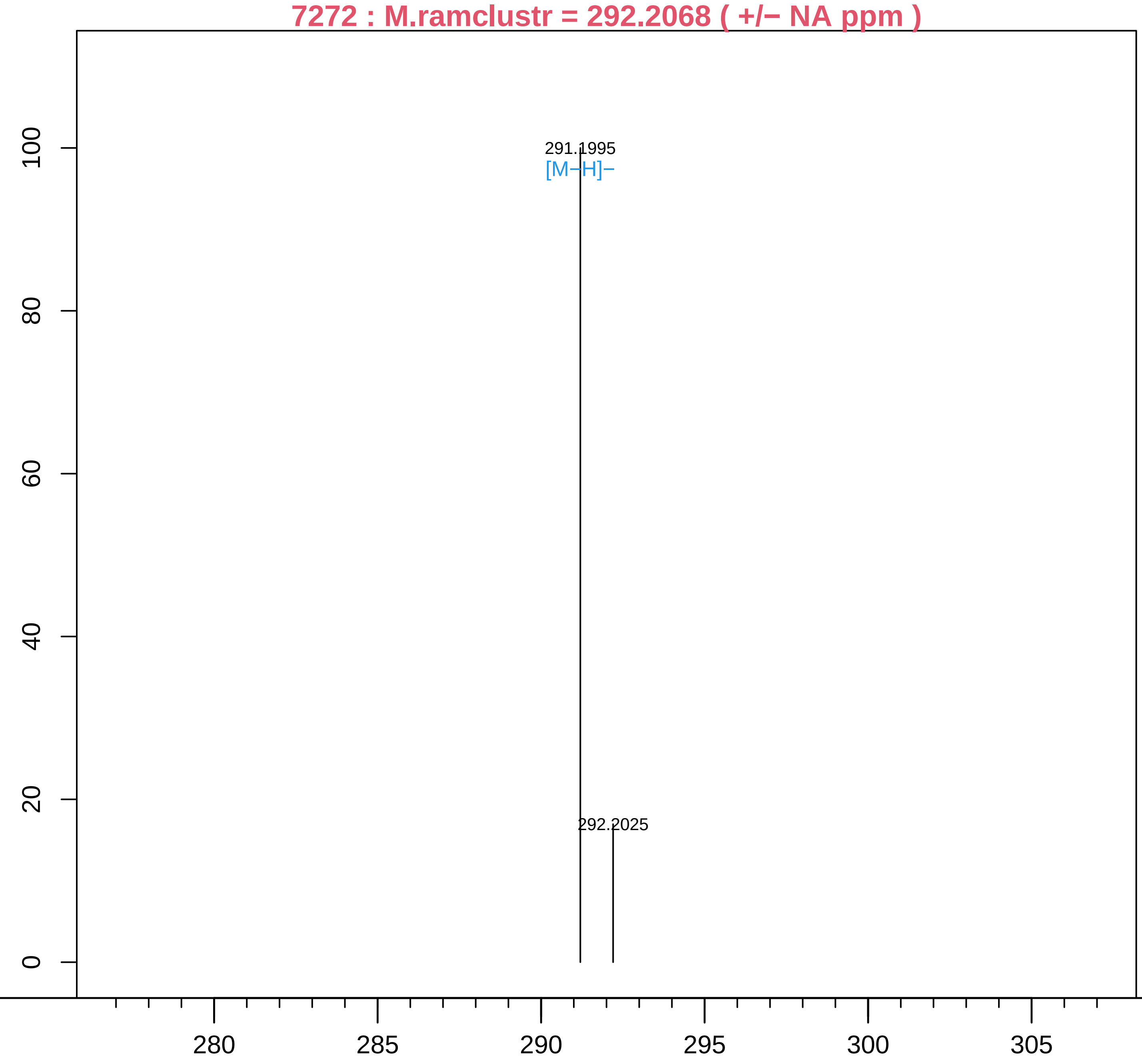
7272 : M.ramclustr = 292.2068 (+/− NA ppm)
291.1995
[M−H]−
292.2025
0
20
40
60
80
100
280
285
290
295
300
305
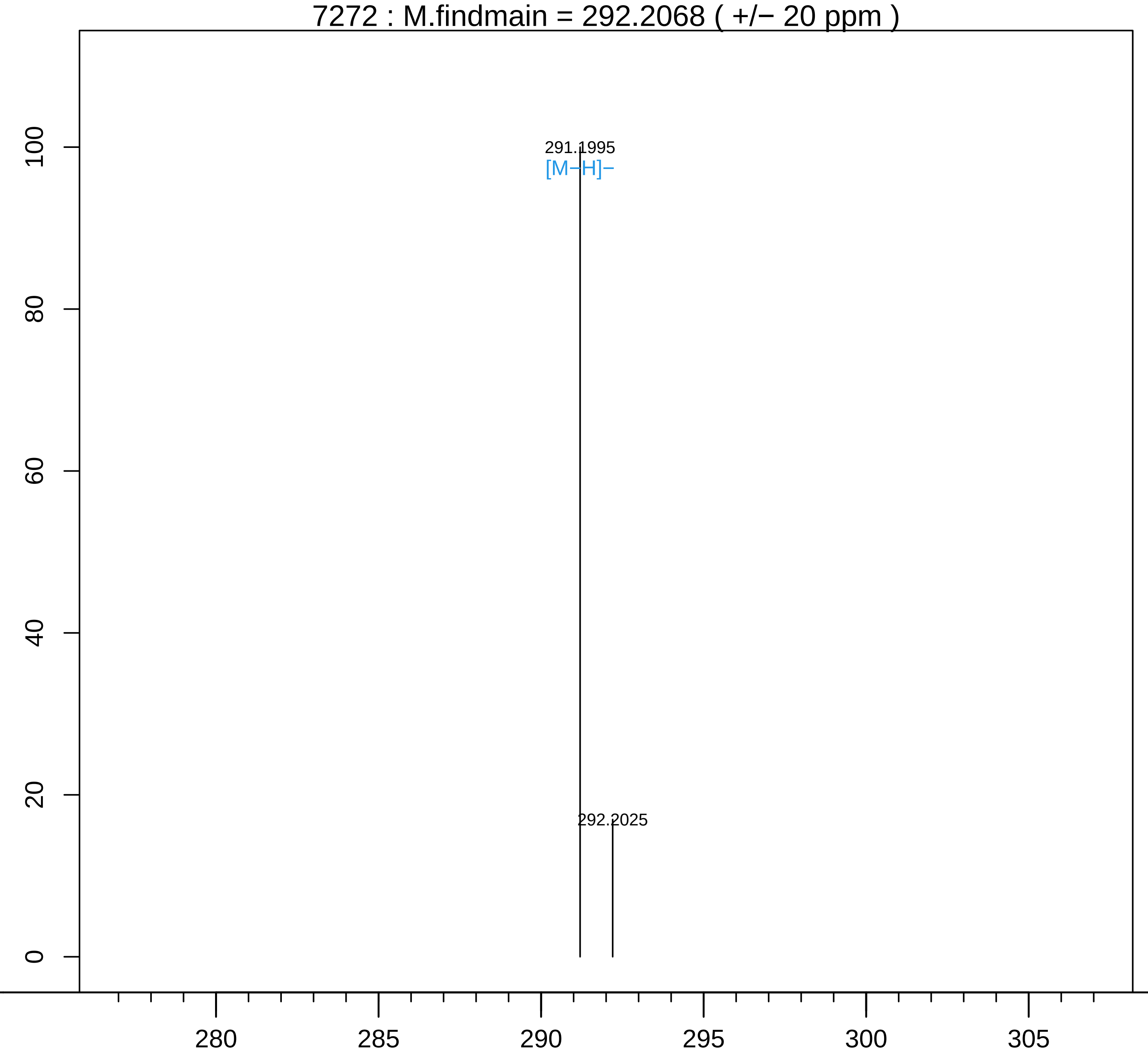
7272 : M.findmain = 292.2068 (+/− 20 ppm)
291.1995
[M−H]−
292.2025
0
20
40
60
80
100
280
285
290
295
300
305

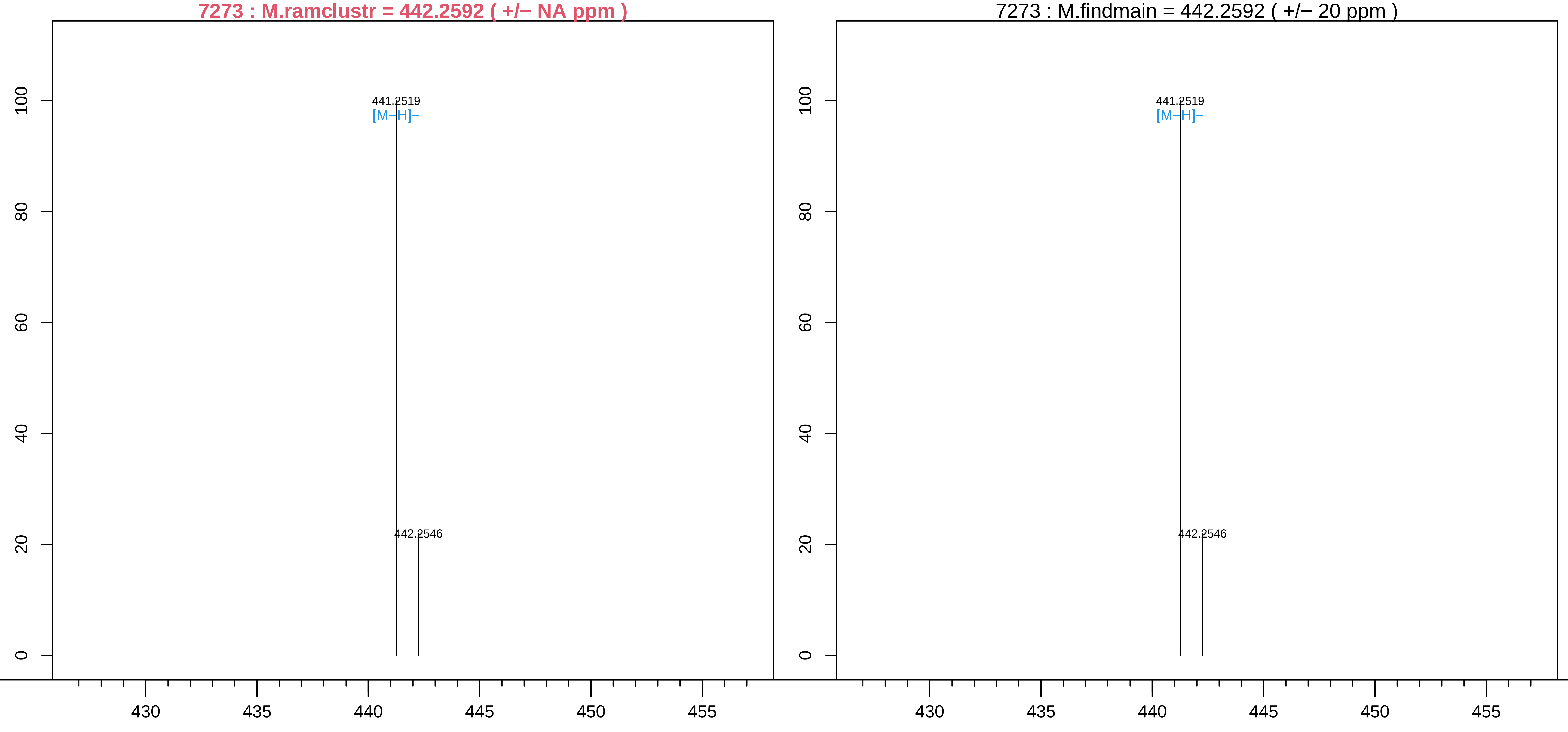
7273 : M.ramclustr = 442.2592 (+/− NA ppm)
7273 : M.findmain = 442.2592 (+/− 20 ppm)
441.2519
[M−H]−
442.2546
0
20
40
60
80
100
430
435
440
445
450
455

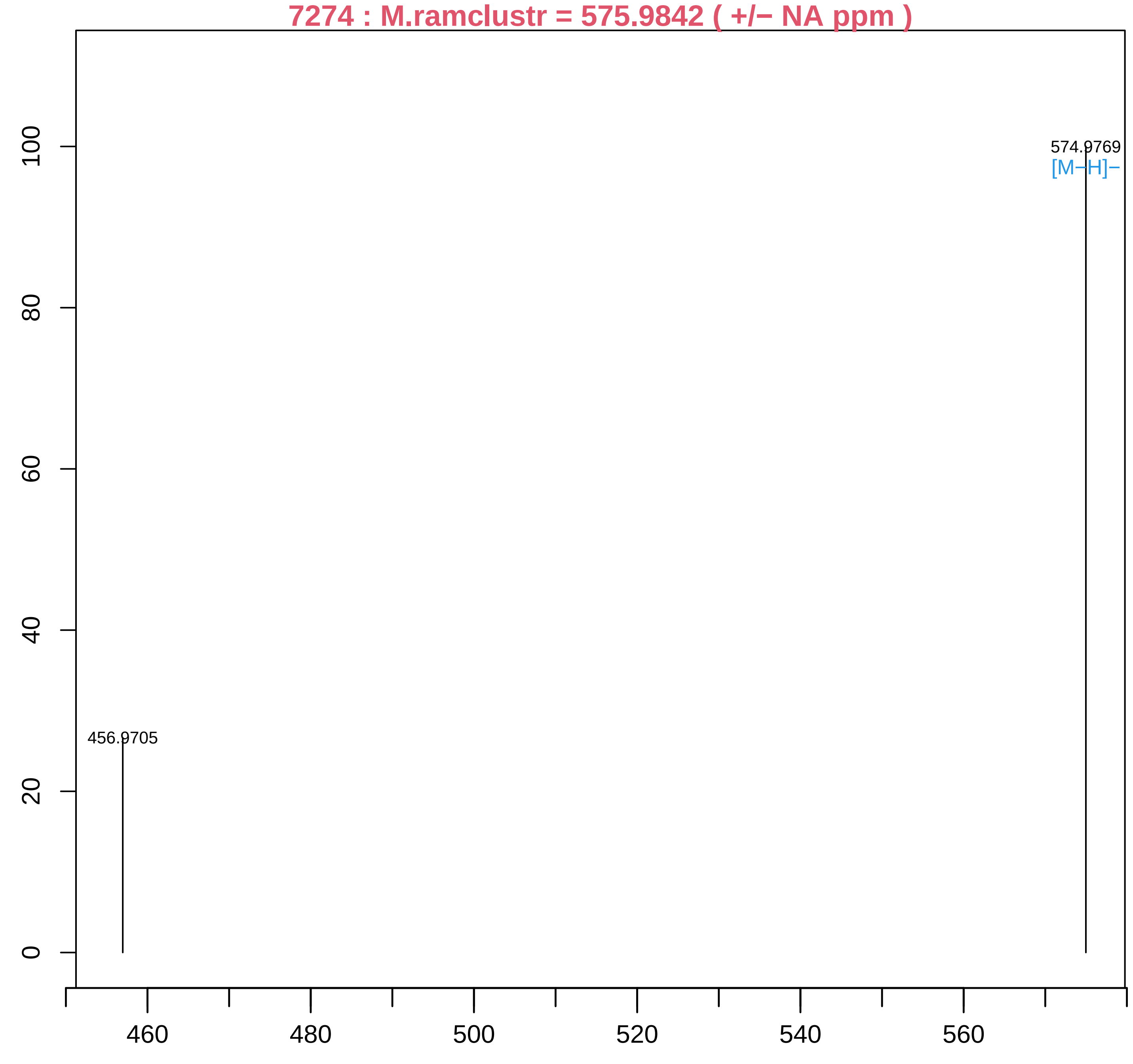
7274 : M.ramclustr = 575.9842 (+/− NA ppm)
574.9769
[M−H]−
456.9705
0
20
40
60
80
100
460
480
500
520
540
560

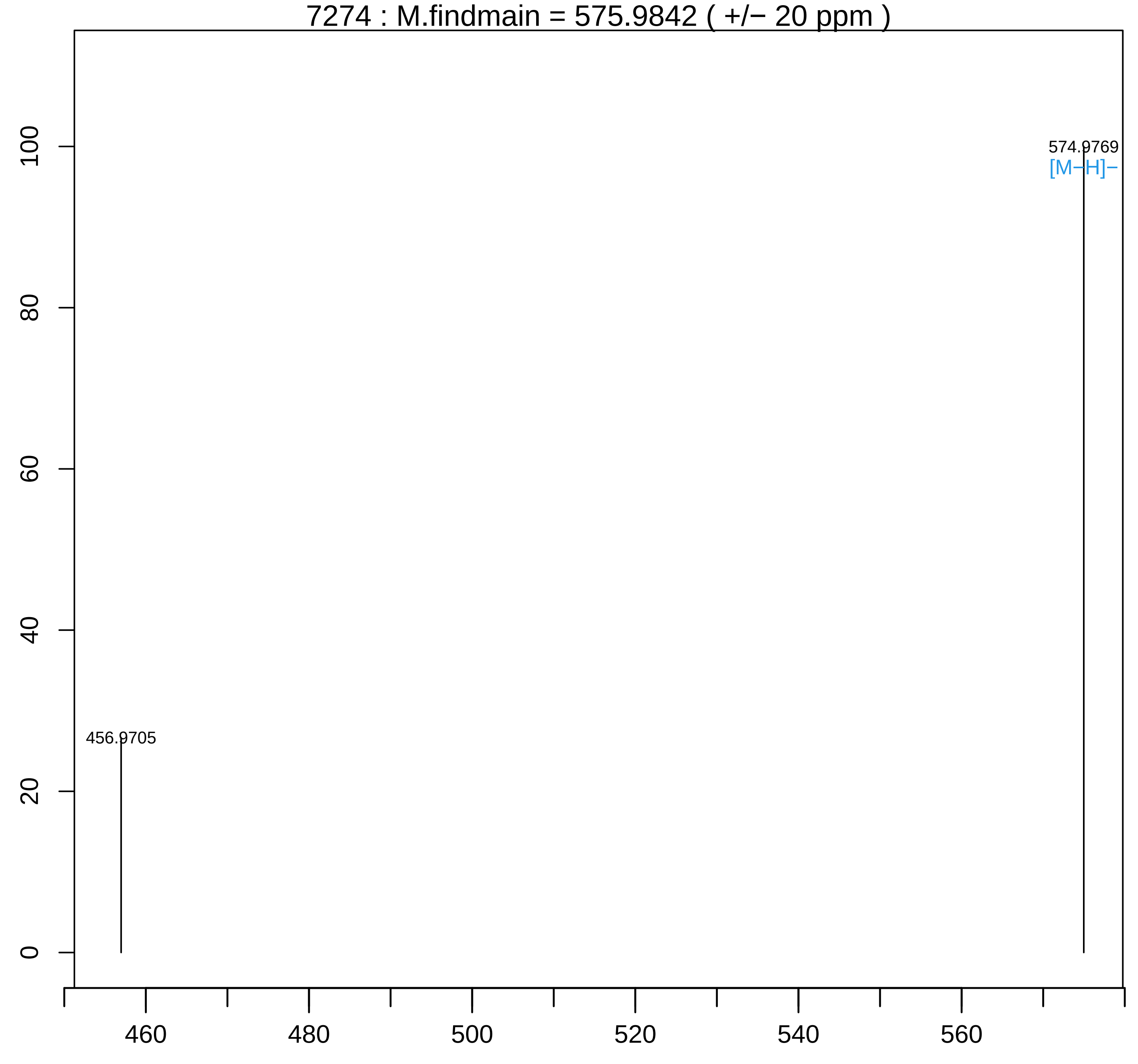
7274 : M.findmain = 575.9842 (+/− 20 ppm)
574.9769
[M−H]−
456.9705
0
20
40
60
80
100
460
480
500
520
540
560

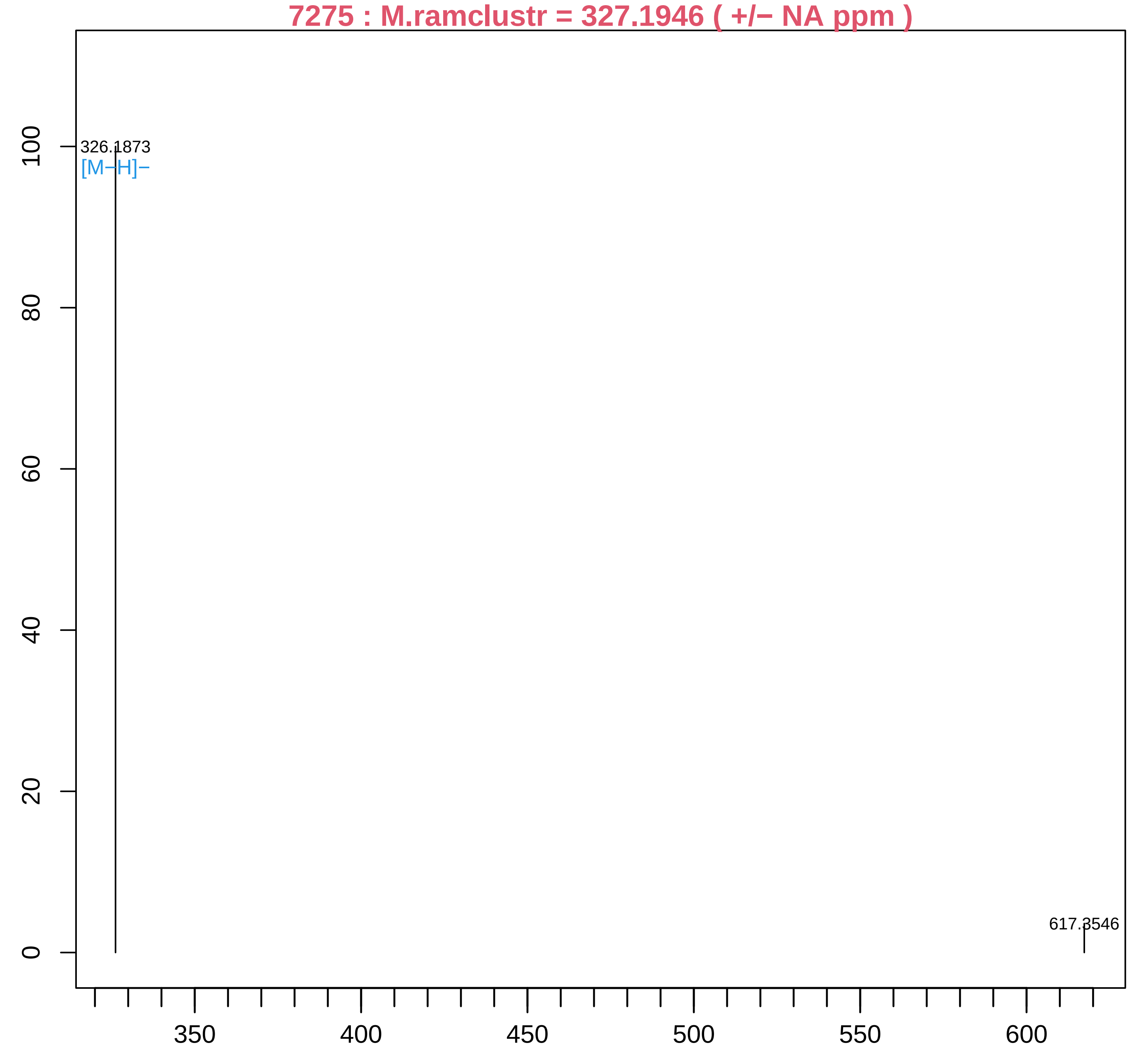
7275 : M.ramclustr = 327.1946 (+/− NA ppm)
326.1873
[M−H]−
617.3546
0
20
40
60
80
100
350
400
450
500
550
600

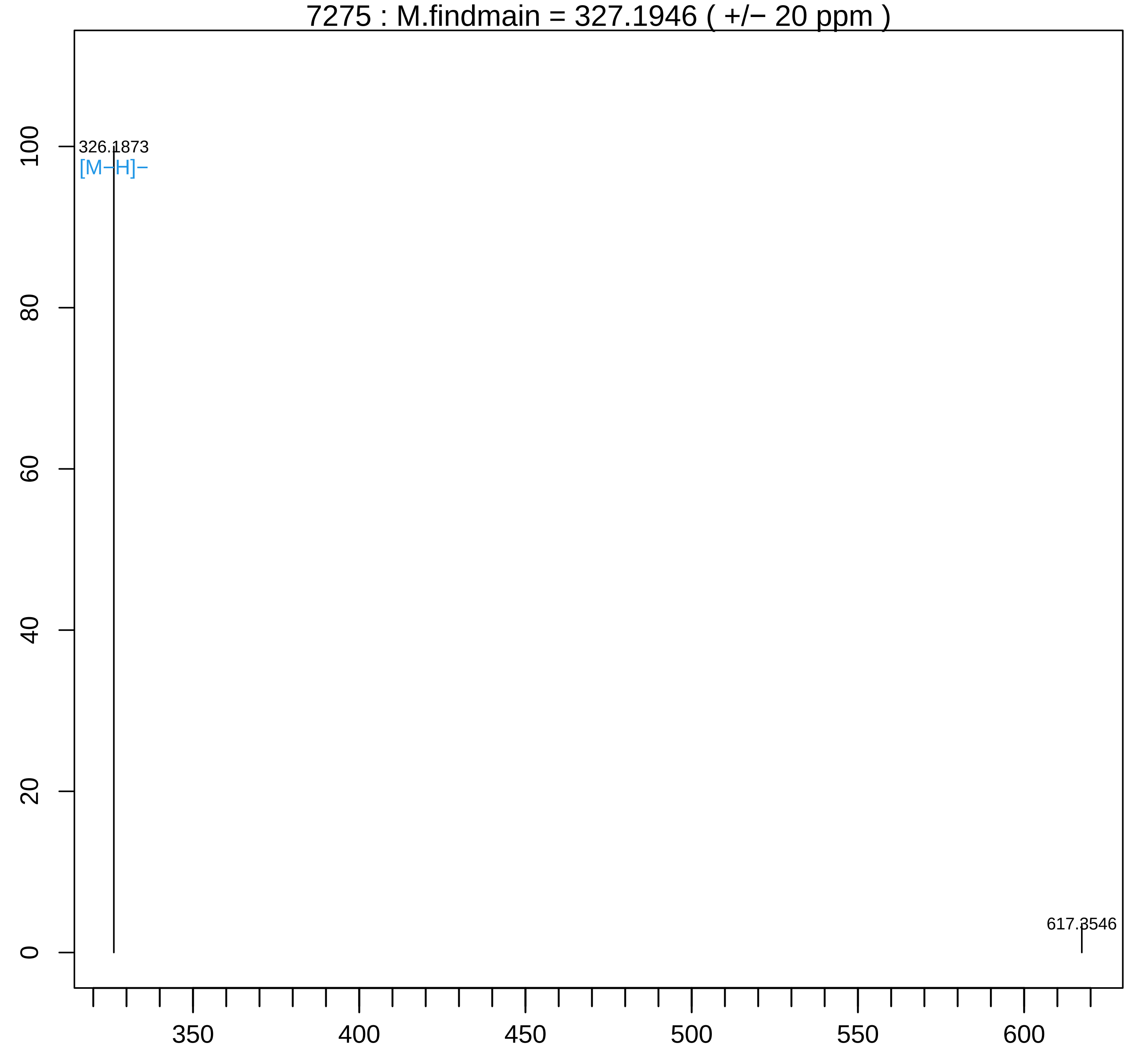
7275 : M.findmain = 327.1946 (+/− 20 ppm)
326.1873
[M−H]−
617.3546
0
20
40
60
80
100
350
400
450
500
550
600

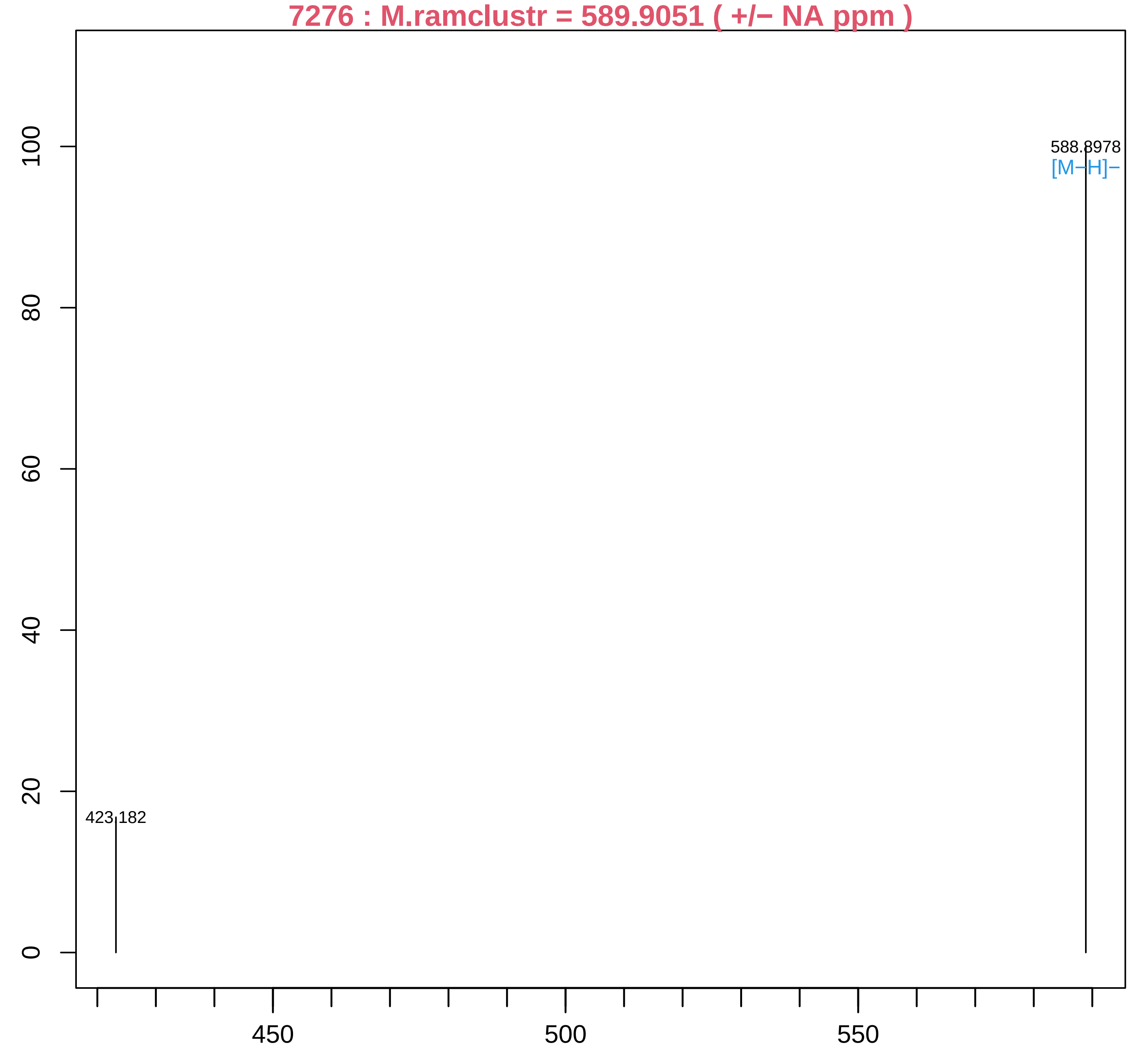
7276 : M.ramclustr = 589.9051 (+/− NA ppm)
588.8978
[M−H]−
423.182
0
20
40
60
80
100
450
500
550

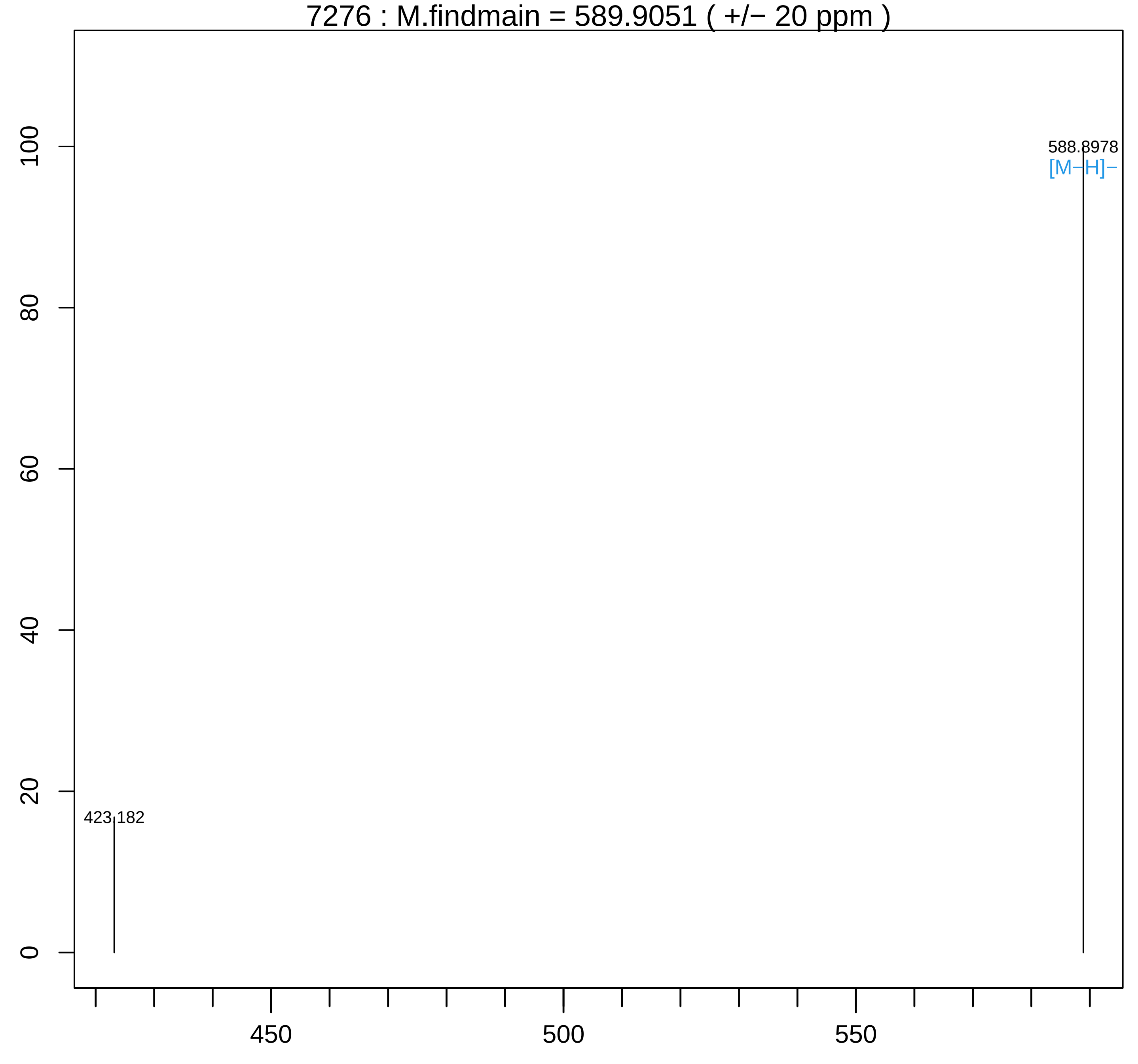
7276 : M.findmain = 589.9051 (+/− 20 ppm)
588.8978
[M−H]−
423.182
0
20
40
60
80
100
450
500
550

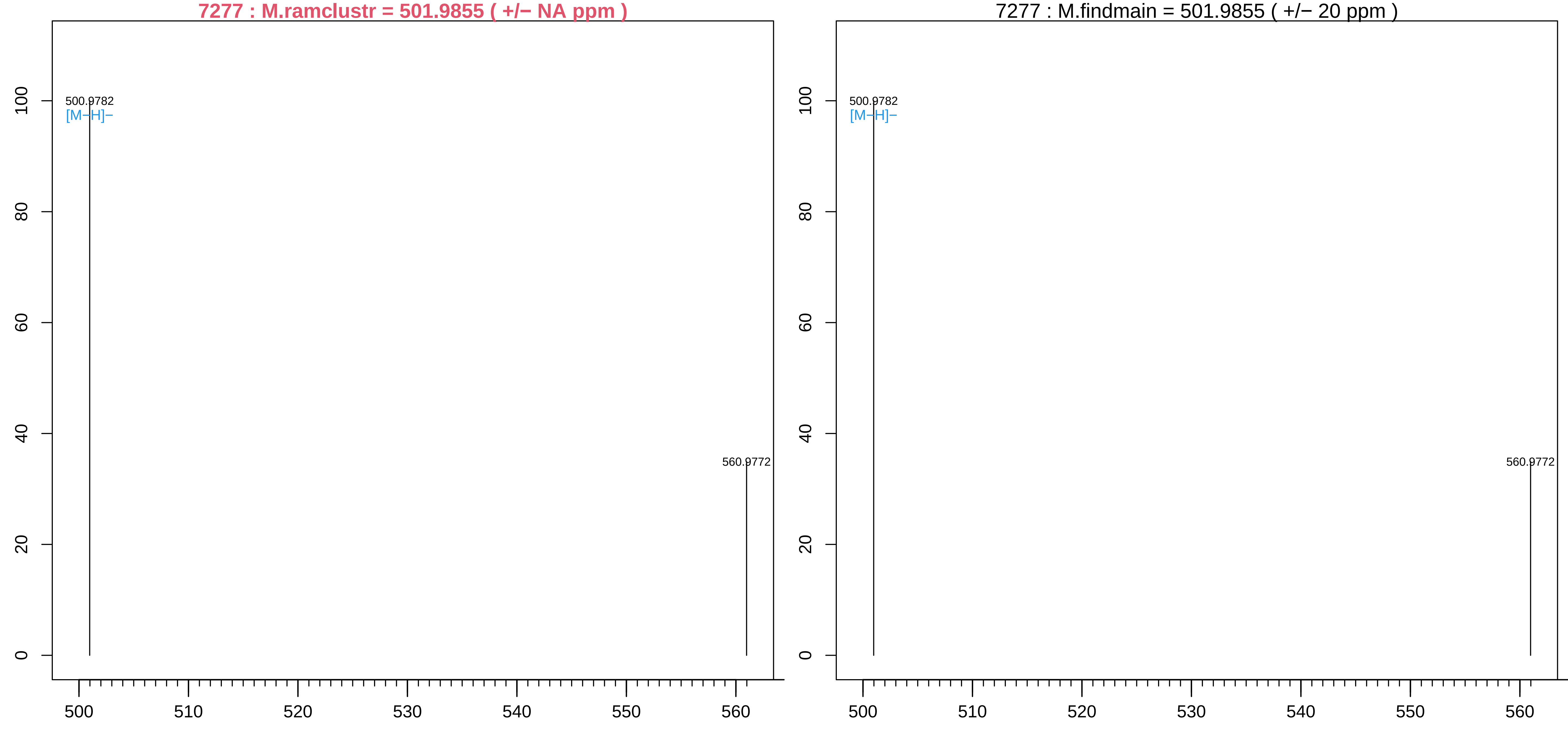
7277 : M.ramclustr = 501.9855 (+/− NA ppm)
500.9782
[M−H]−
560.9772
0
20
40
60
80
100
500
510
520
530
540
550
560
7277 : M.findmain = 501.9855 (+/− 20 ppm)
500.9782
[M−H]−
560.9772
0
20
40
60
80
100
500
510
520
530
540
550
560

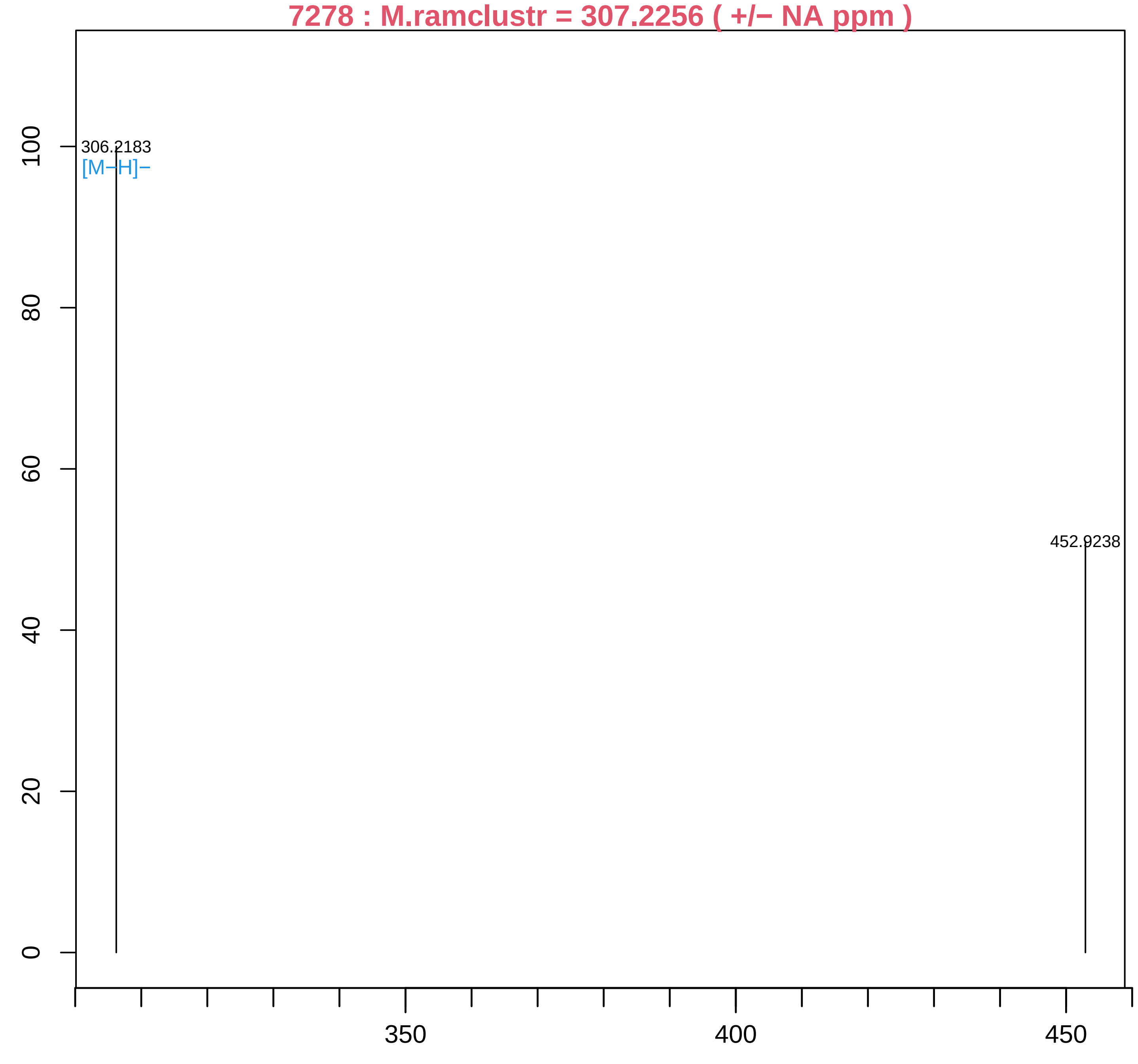
7278 : M.ramclustr = 307.2256 (+/− NA ppm)
306.2183
[M−H]−
452.9238
0
20
40
60
80
100
350
400
450

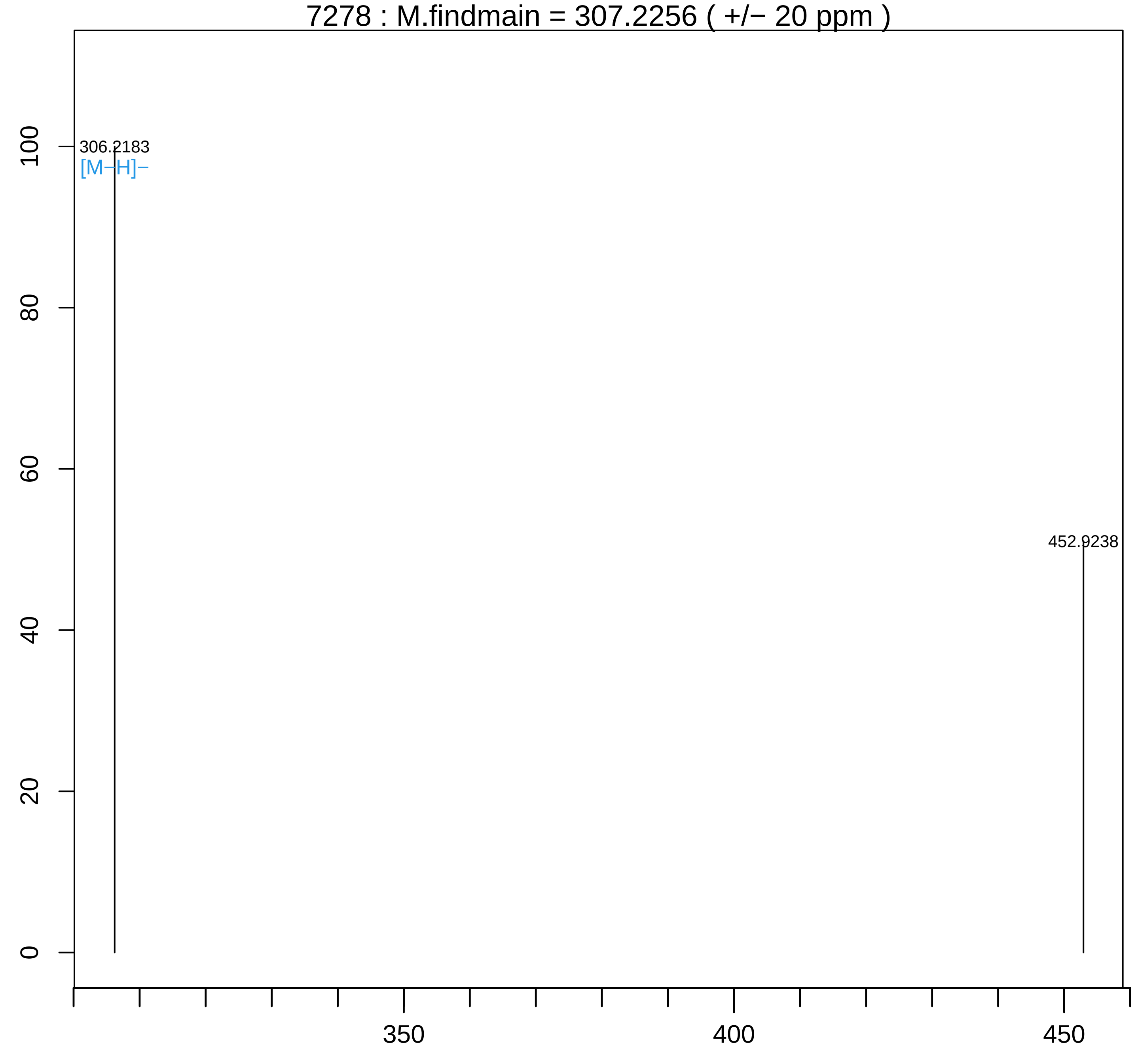
7278 : M.findmain = 307.2256 (+/− 20 ppm)
306.2183
[M−H]−
452.9238
0
20
40
60
80
100
350
400
450

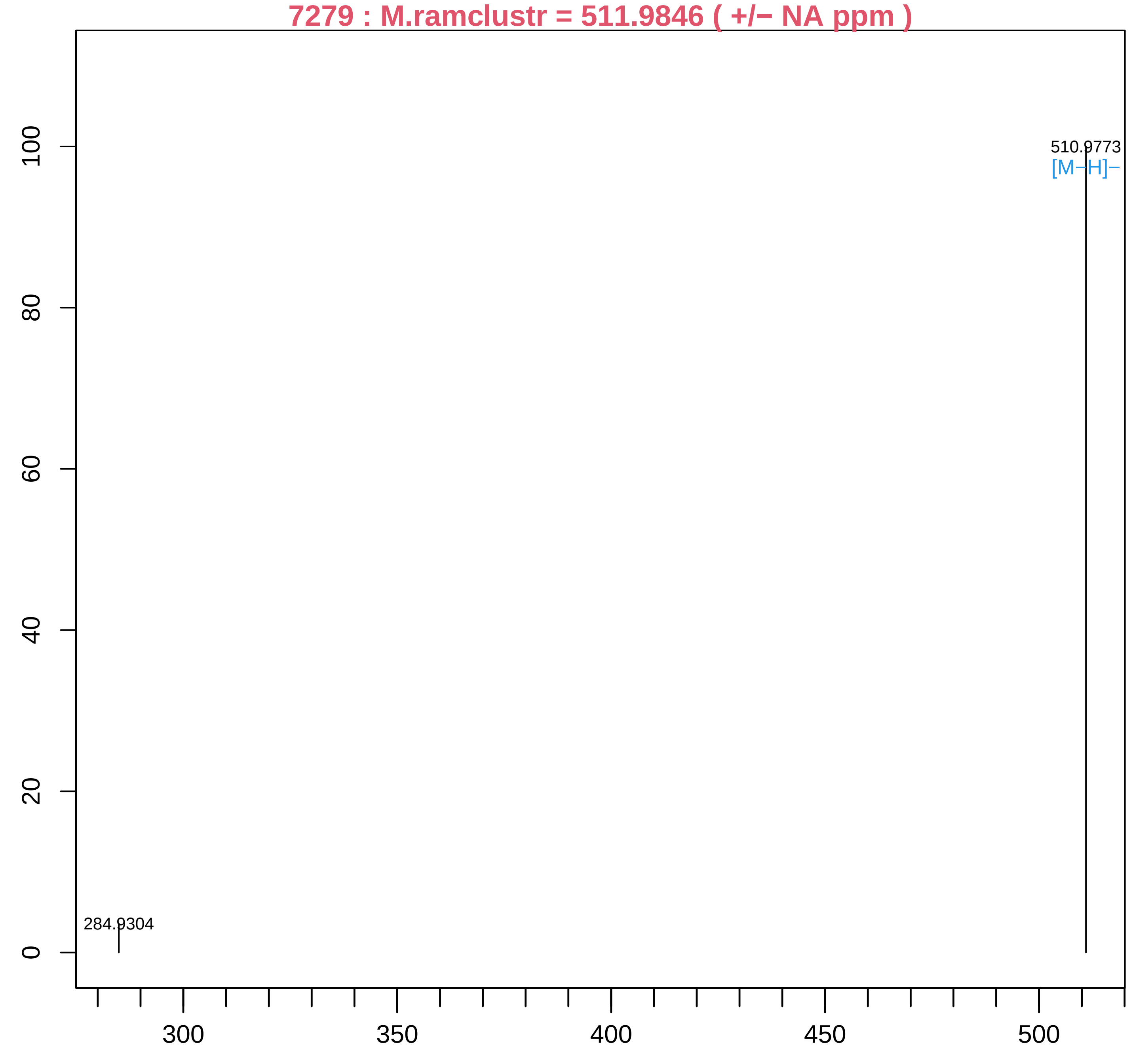

7279 : M.ramclustr = 511.9846 (+/− NA ppm)
510.9773
[M−H]−
284.9304
0
20
40
60
80
100
300
350
400
450
500

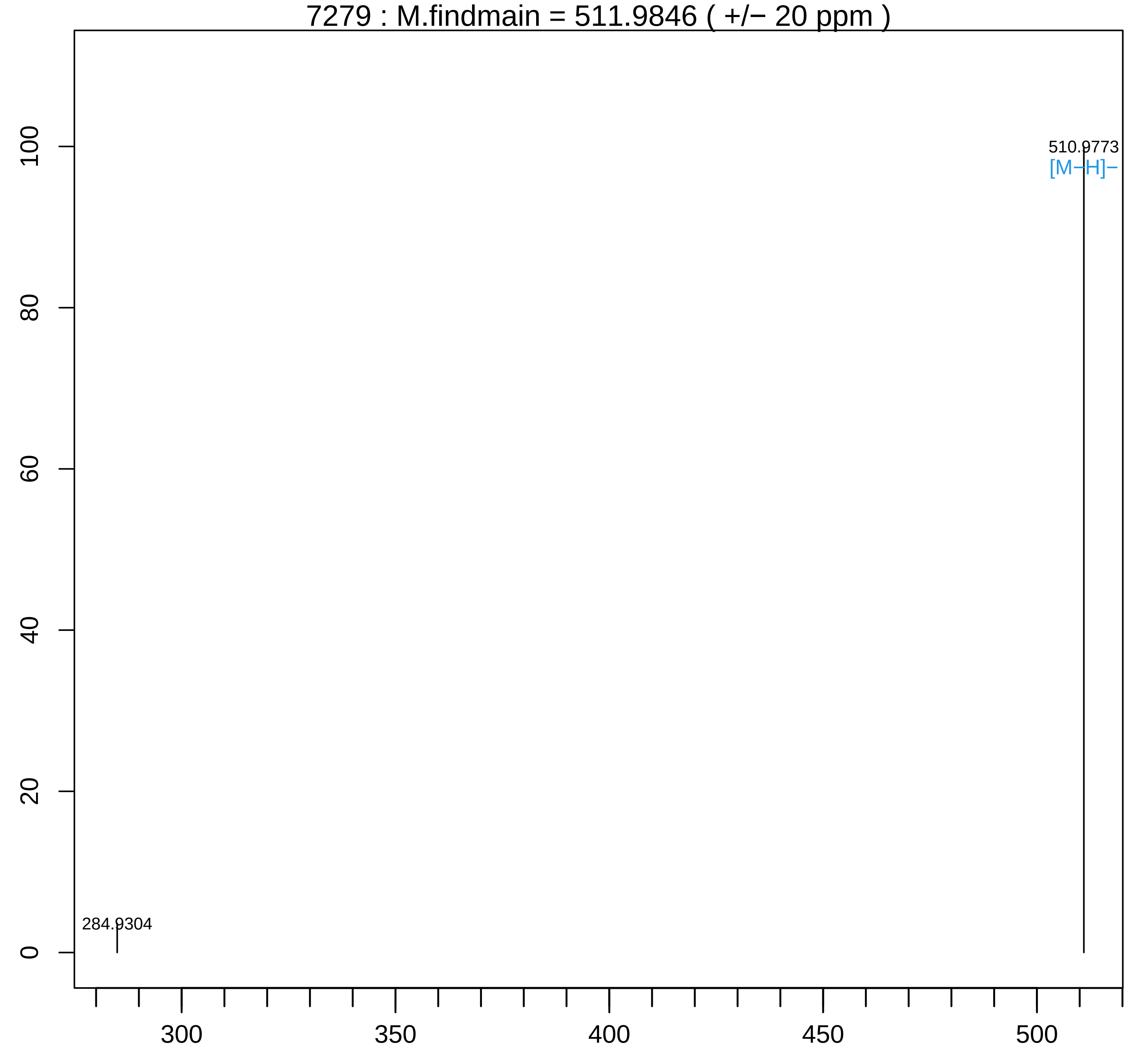

7279 : M.findmain = 511.9846 (+/− 20 ppm)
510.9773
[M−H]−
284.9304
0
20
40
60
80
100
300
350
400
450
500

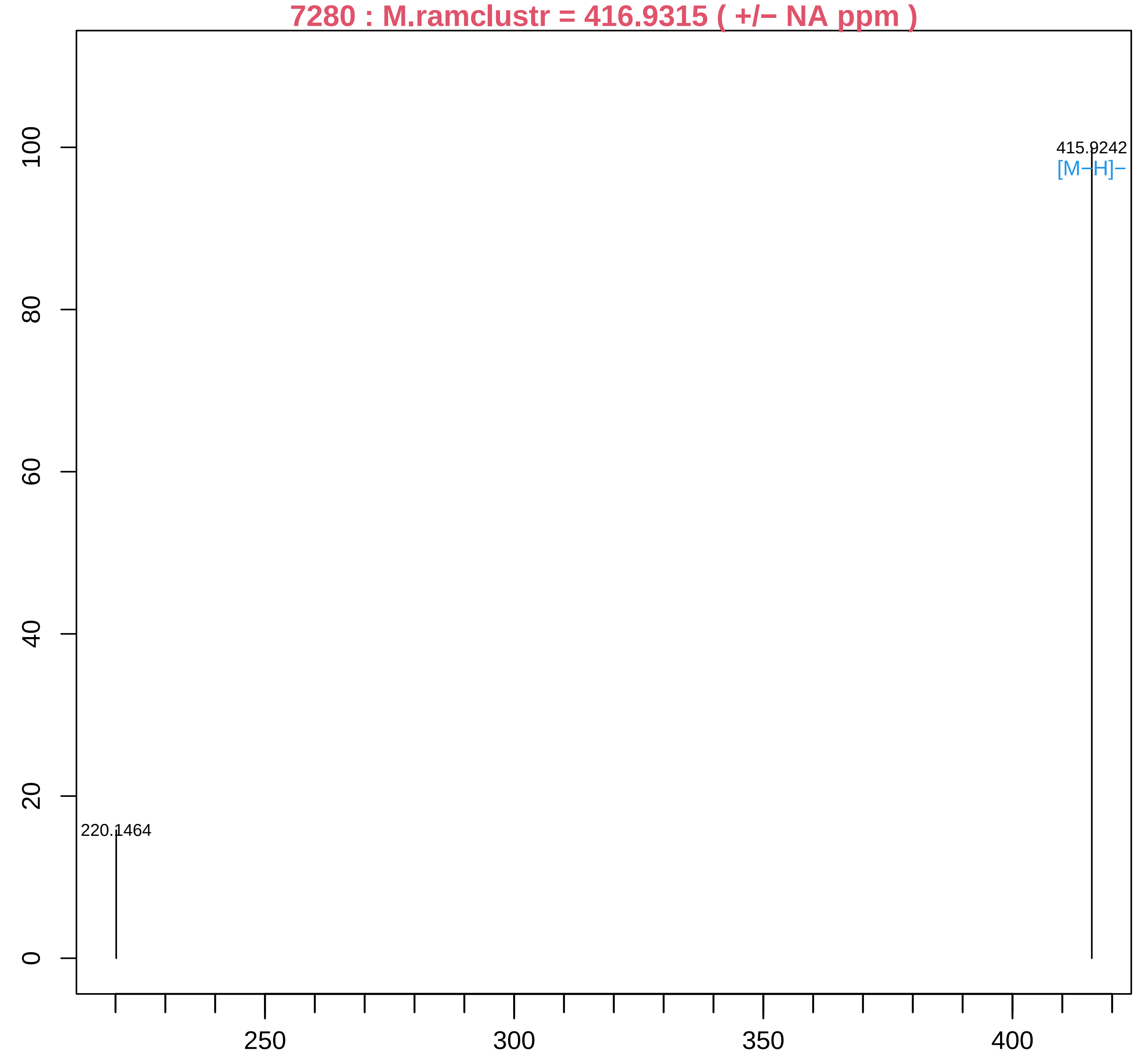
7280 : M.ramclustr = 416.9315 (+/− NA ppm)
415.9242
[M−H]−
220.1464
0
20
40
60
80
100
250
300
350
400

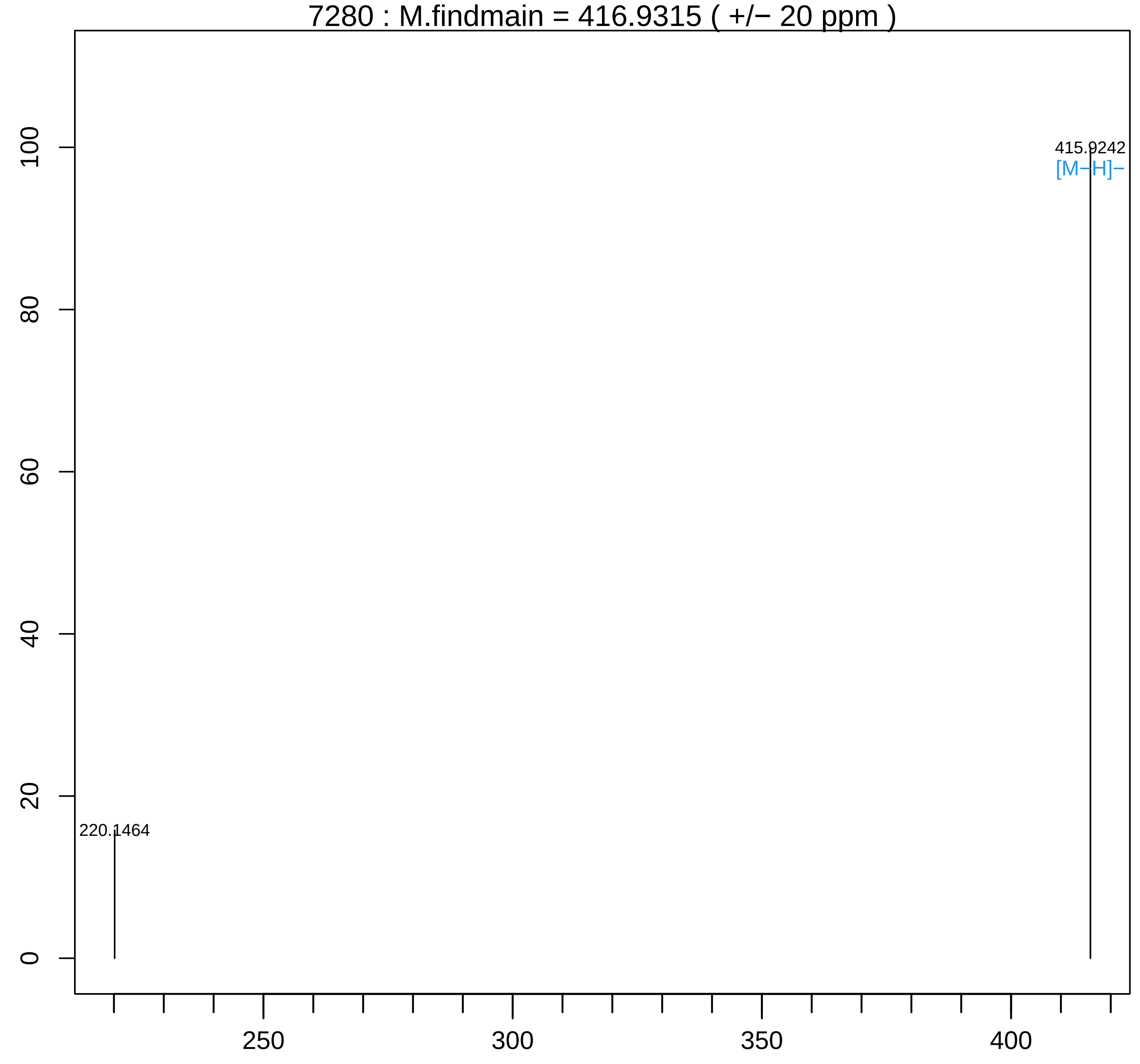
7280 : M.findmain = 416.9315 (+/− 20 ppm)
415.9242
[M−H]−
220.1464
0
20
40
60
80
100
250
300
350
400

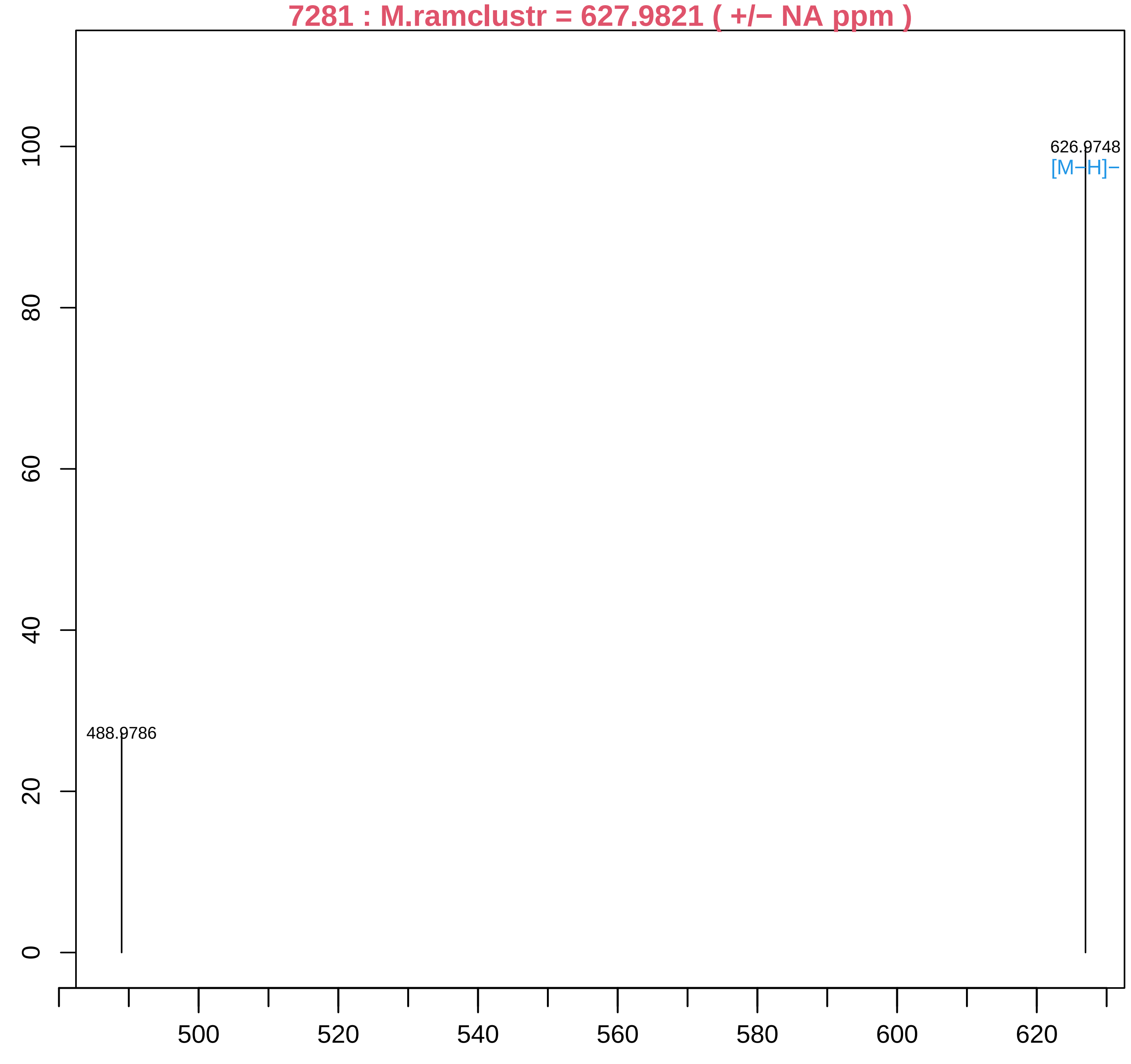
7281 : M.ramclustr = 627.9821 (+/− NA ppm)
626.9748
[M−H]−
488.9786
0
20
40
60
80
100
500
520
540
560
580
600
620

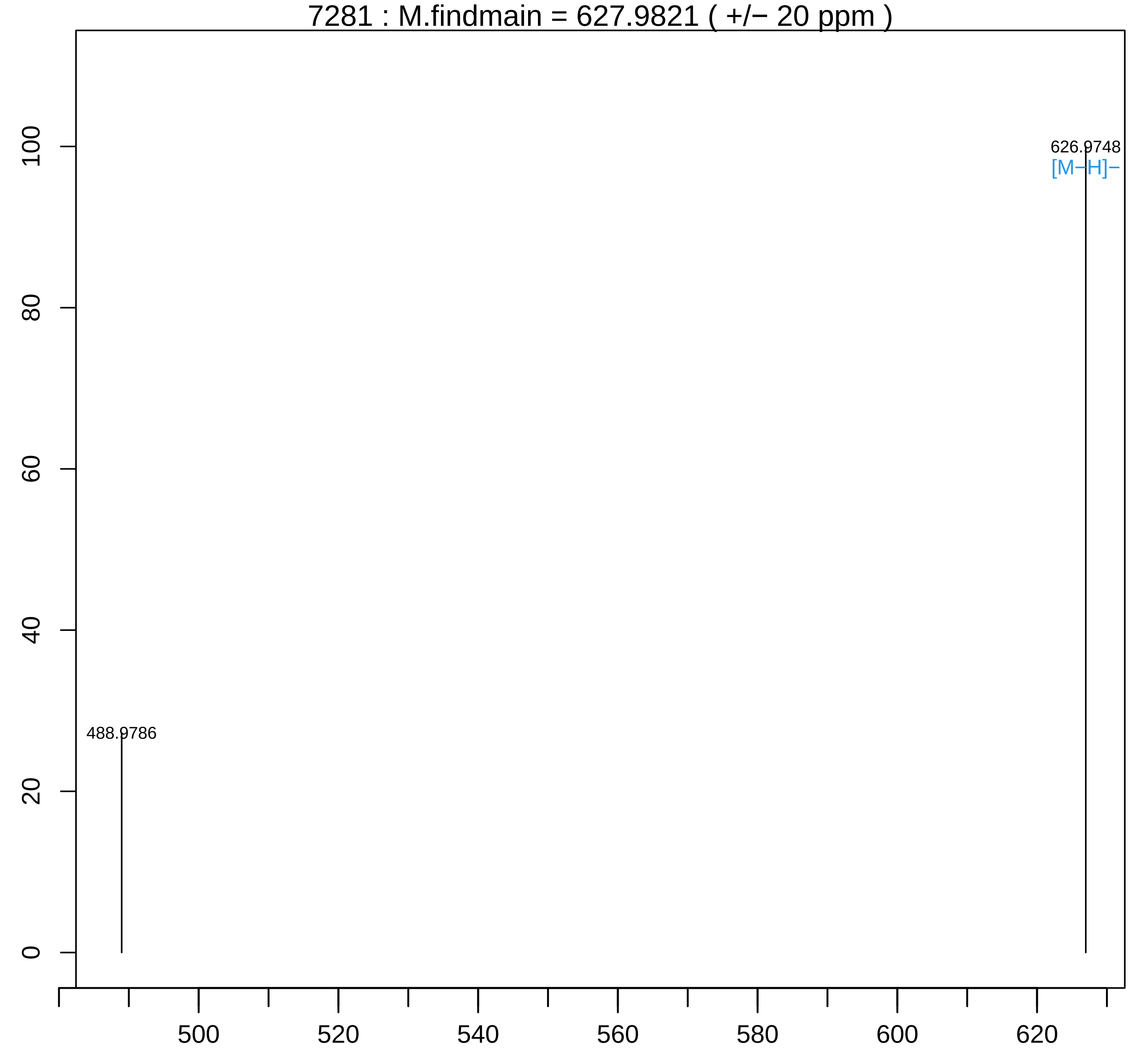
7281 : M.findmain = 627.9821 (+/− 20 ppm)
626.9748
[M−H]−
488.9786
0
20
40
60
80
100
500
520
540
560
580
600
620

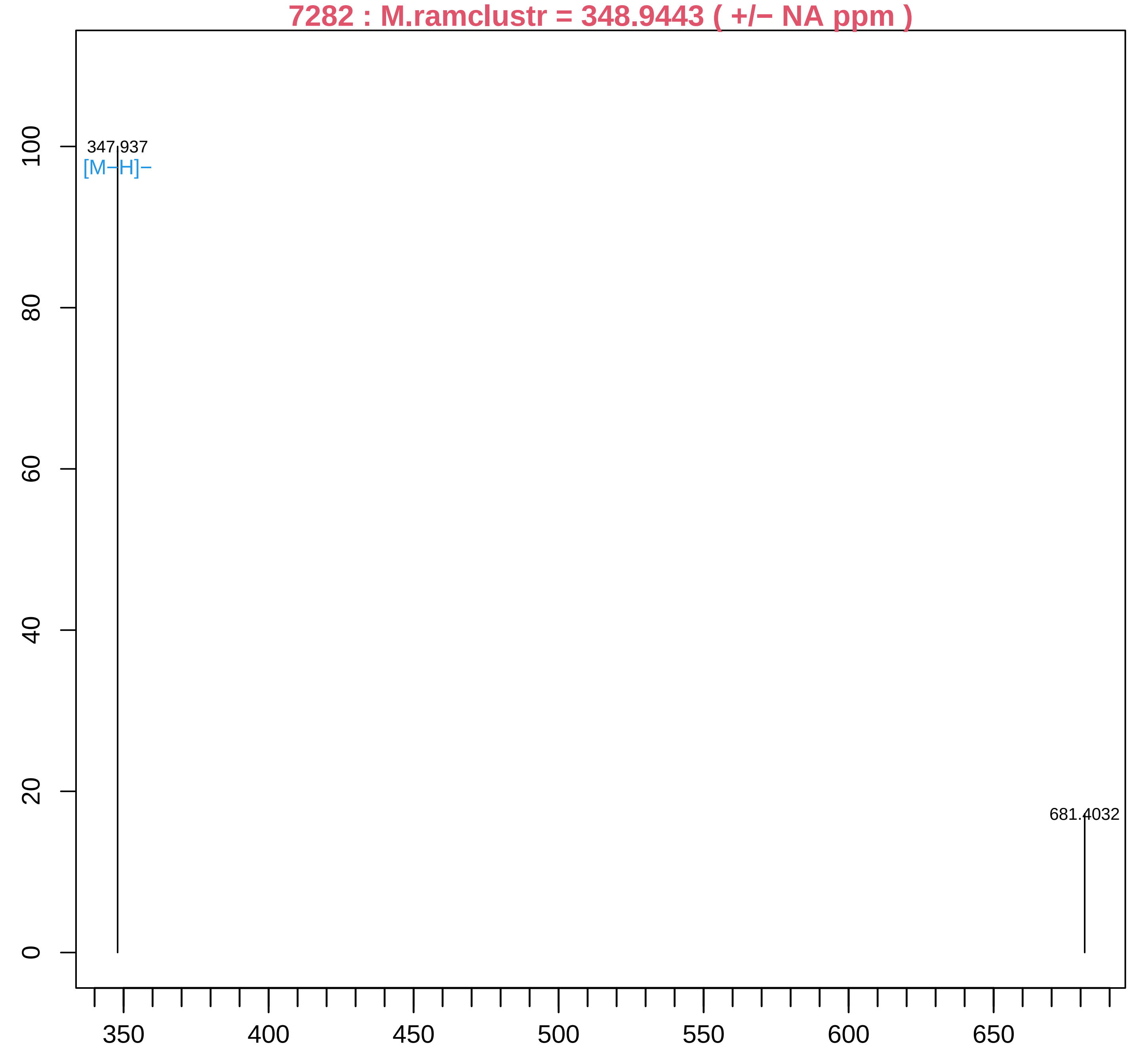
7282 : M.ramclustr = 348.9443 (+/− NA ppm)
347.937
[M−H]−
681.4032
0
20
40
60
80
100
350
400
450
500
550
600
650

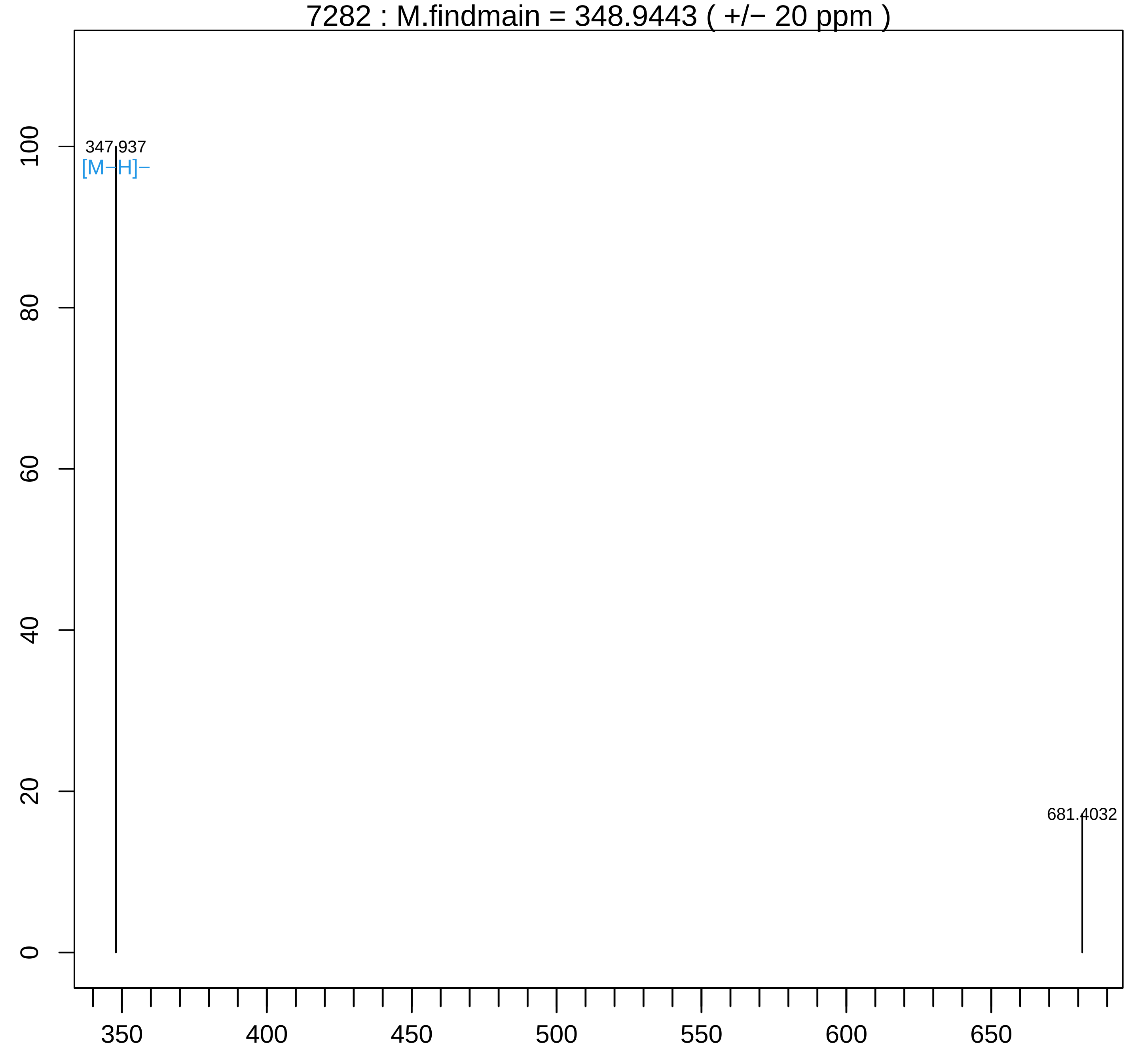
7282 : M.findmain = 348.9443 (+/− 20 ppm)
347.937
[M−H]−
681.4032
0
20
40
60
80
100
350
400
450
500
550
600
650

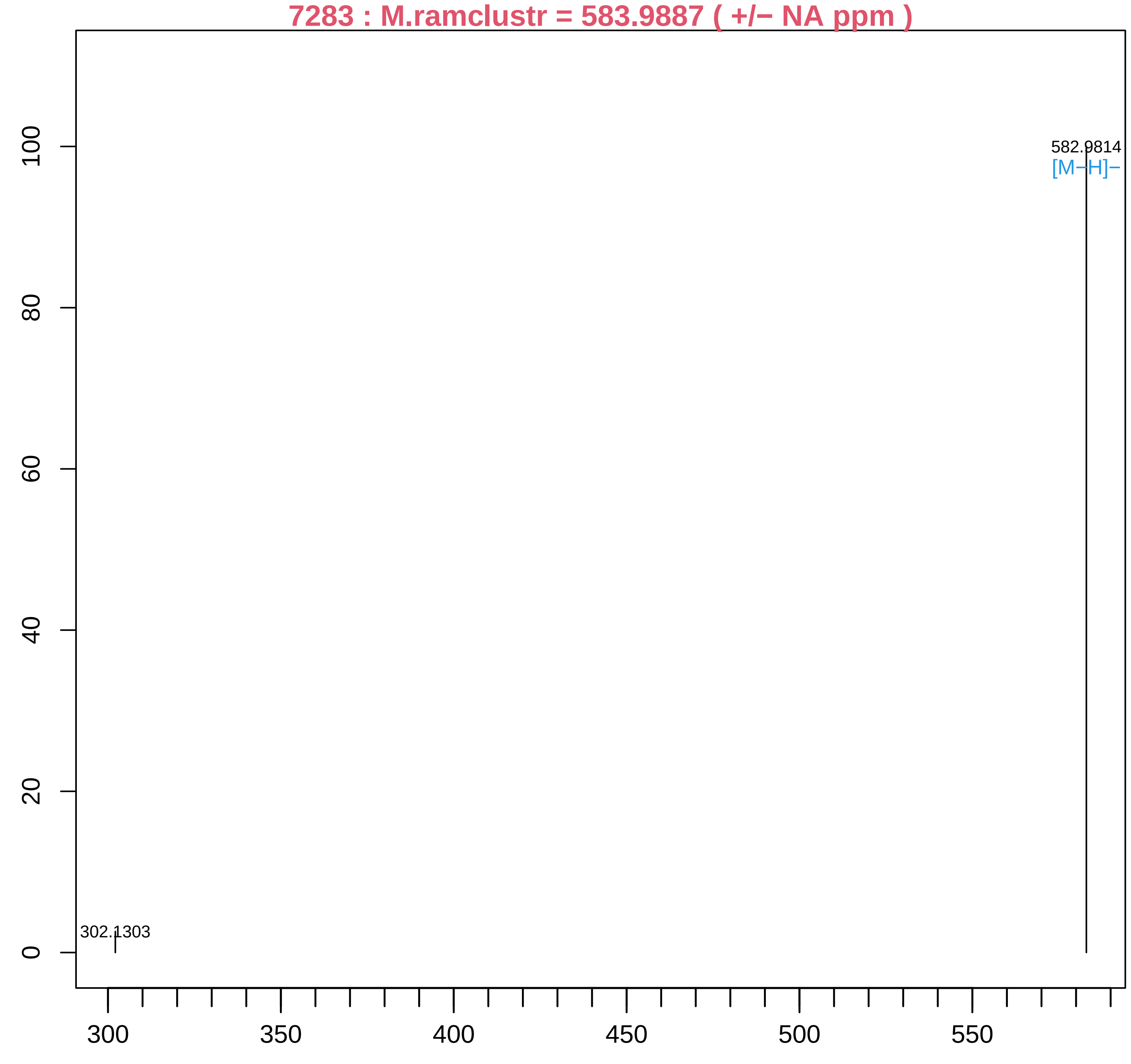
7283 : M.ramclustr = 583.9887 (+/− NA ppm)
582.9814
[M−H]−
302.1303
0
20
40
60
80
100
300
350
400
450
500
550

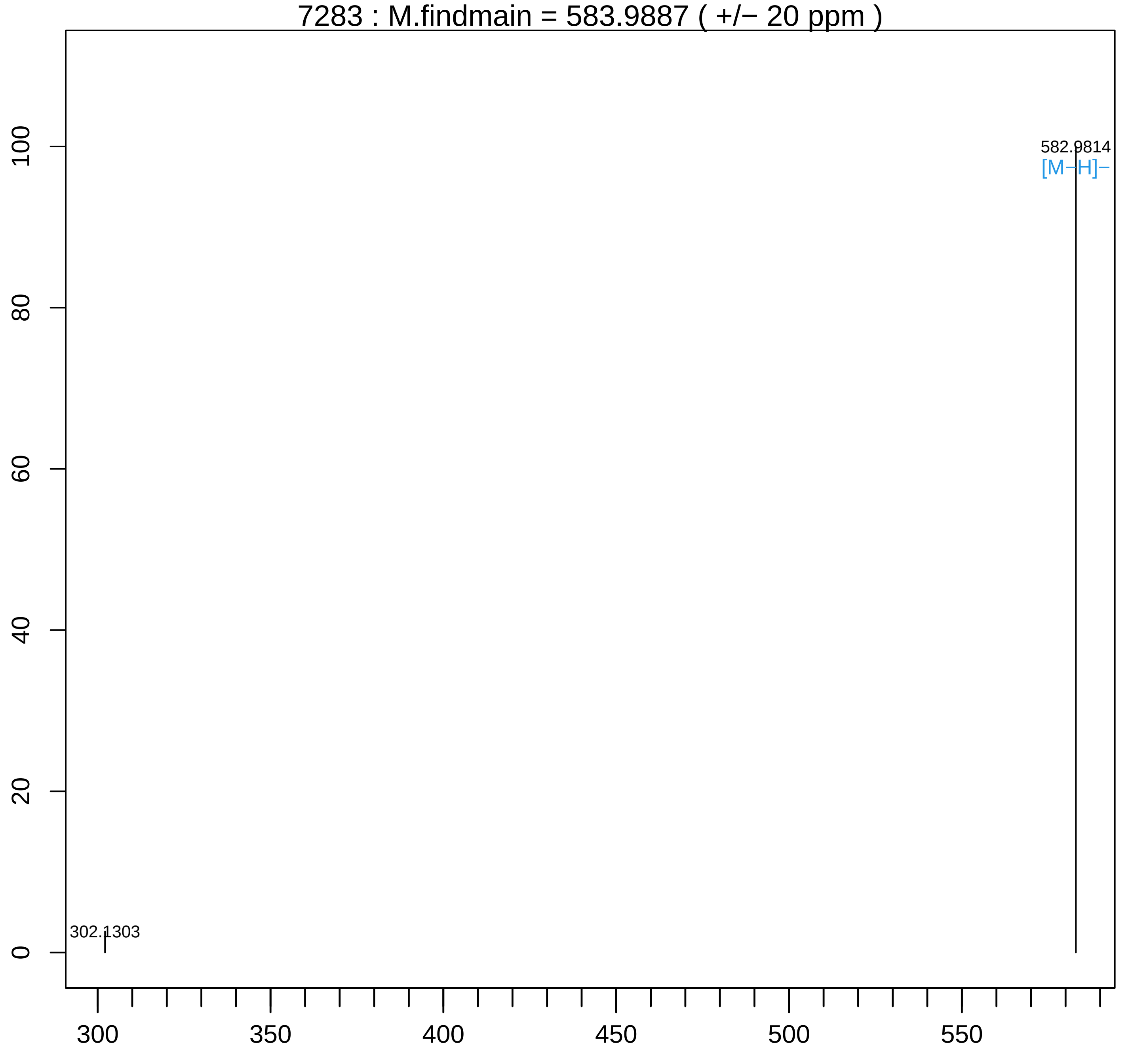
7283 : M.findmain = 583.9887 (+/− 20 ppm)
582.9814
[M−H]−
302.1303
0
20
40
60
80
100
300
350
400
450
500
550

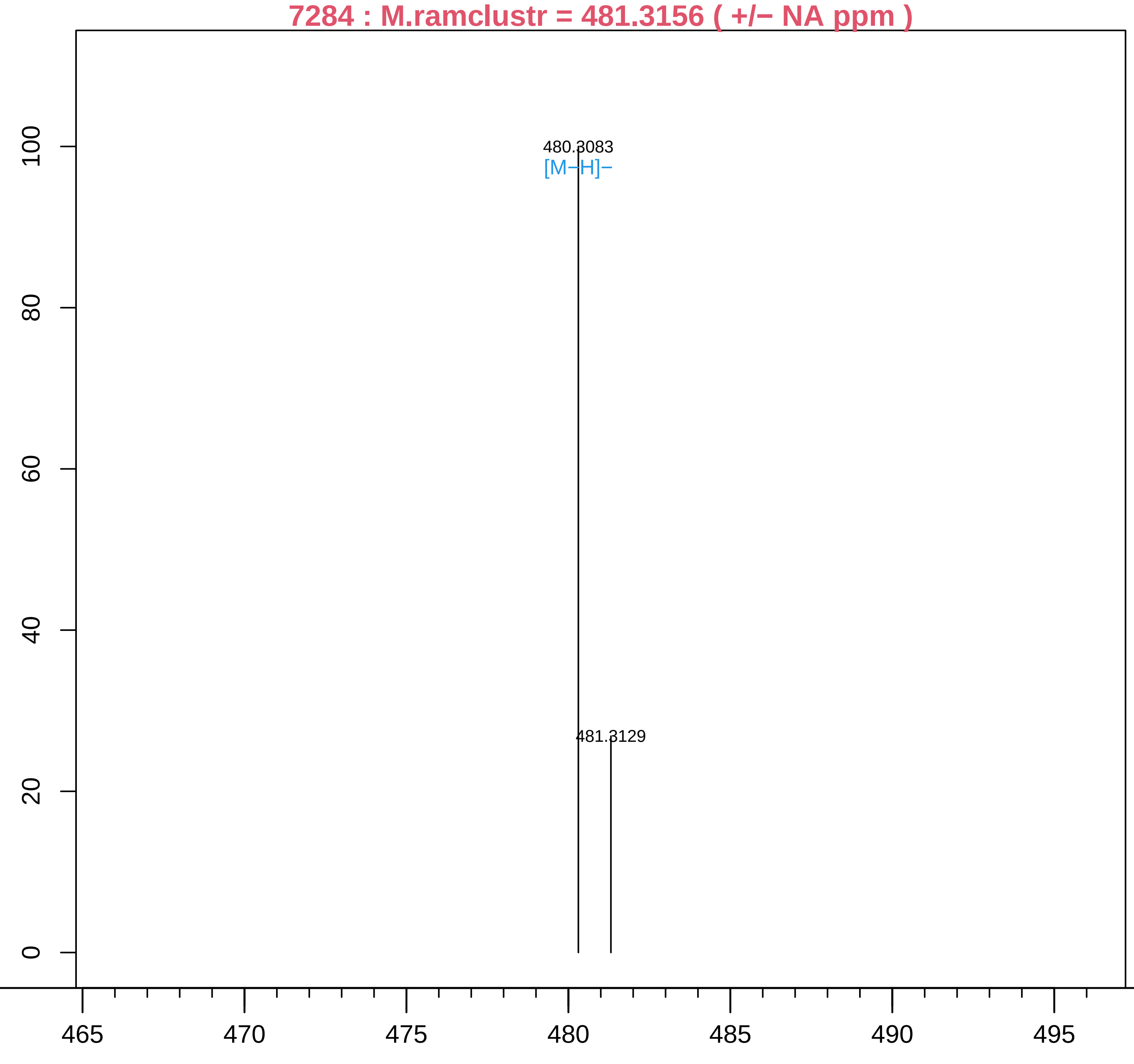
7284 : M.ramclustr = 481.3156 (+/− NA ppm)
480.3083
[M−H]−
481.3129
0
20
40
60
80
100
465
470
475
480
485
490
495

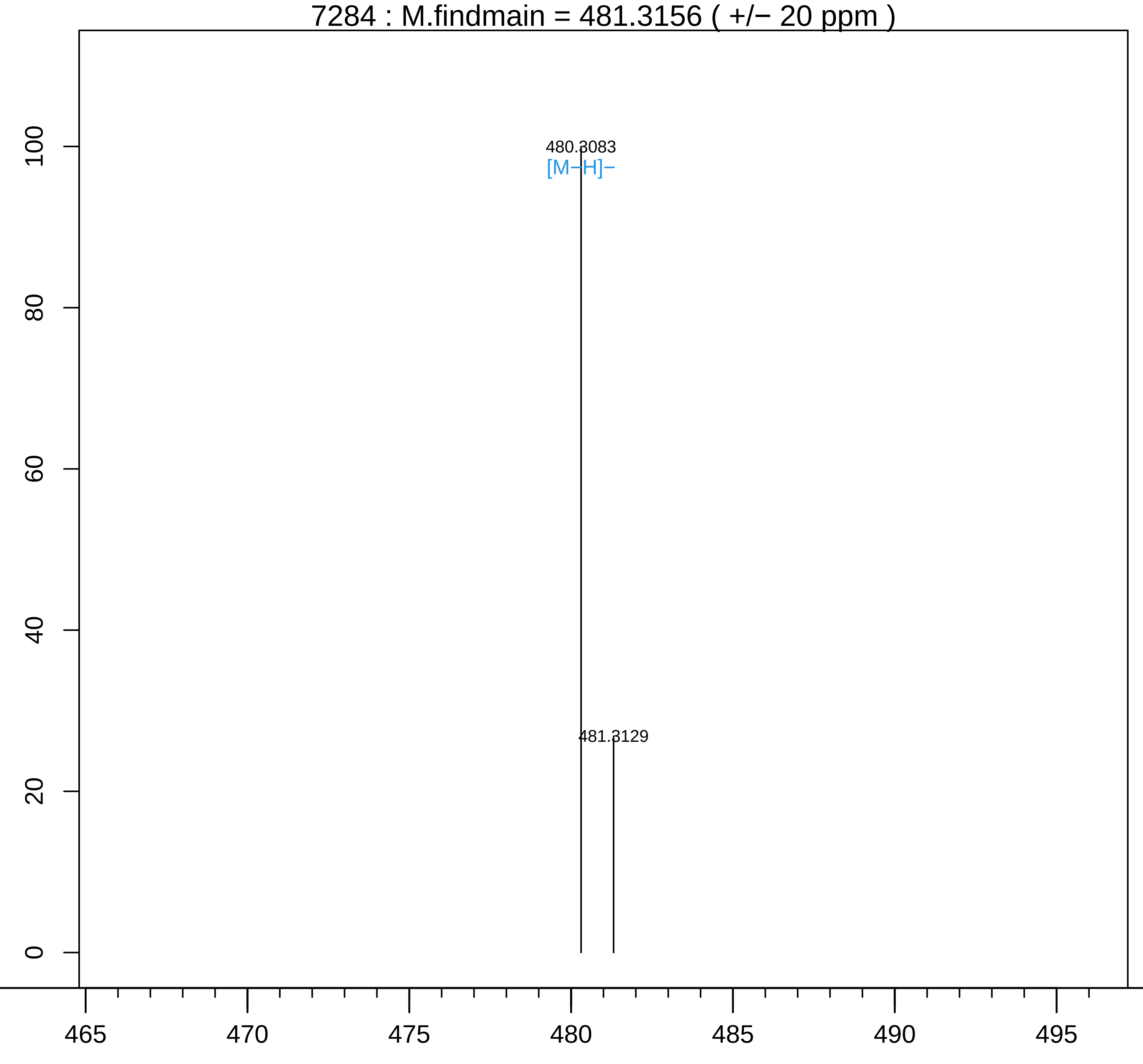
7284 : M.findmain = 481.3156 (+/− 20 ppm)
480.3083
[M−H]−
481.3129
0
20
40
60
80
100
465
470
475
480
485
490
495

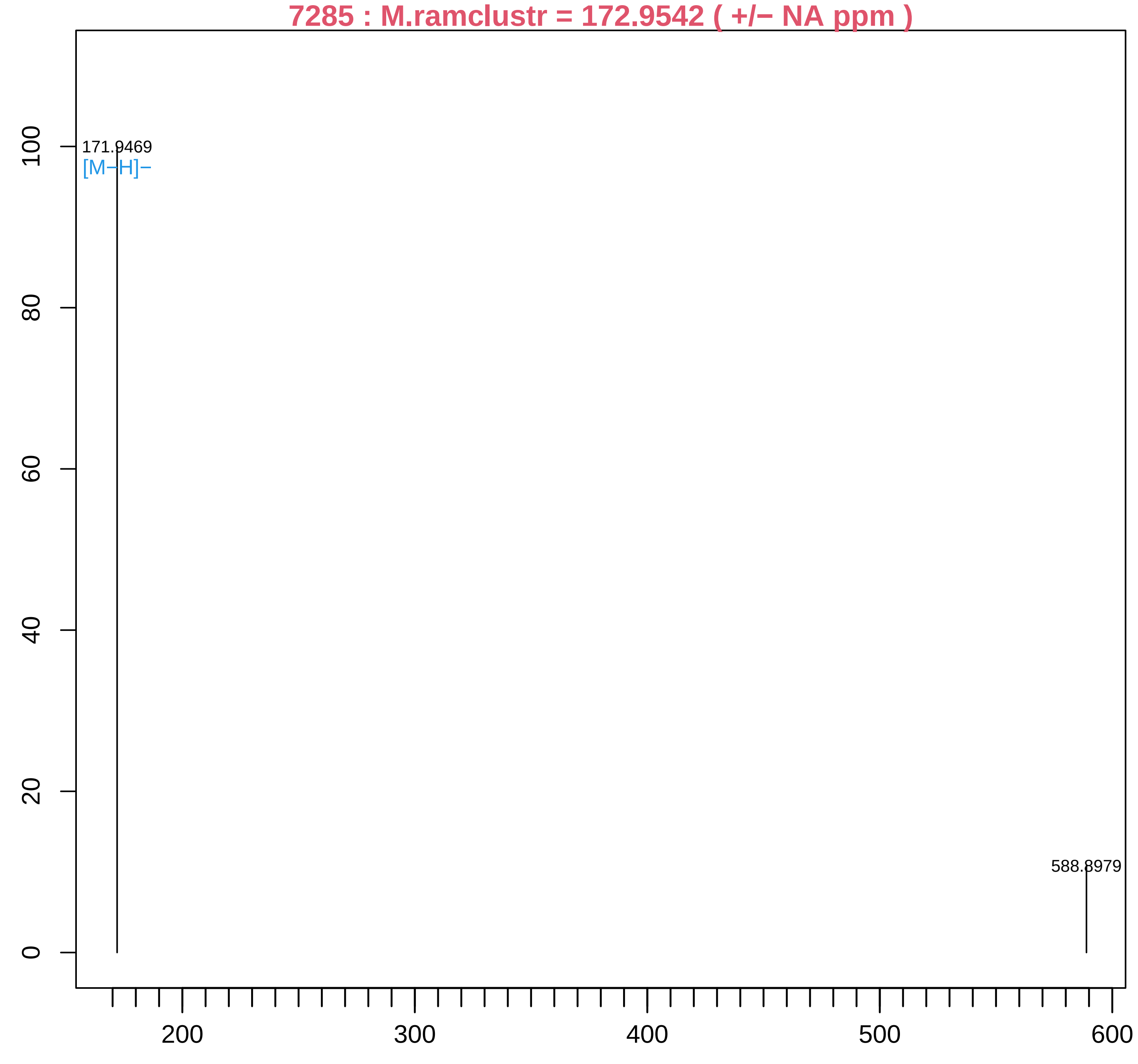
7285 : M.ramclustr = 172.9542 (+/− NA ppm)
171.9469
[M−H]−
588.8979
0
20
40
60
80
100
200
300
400
500
600

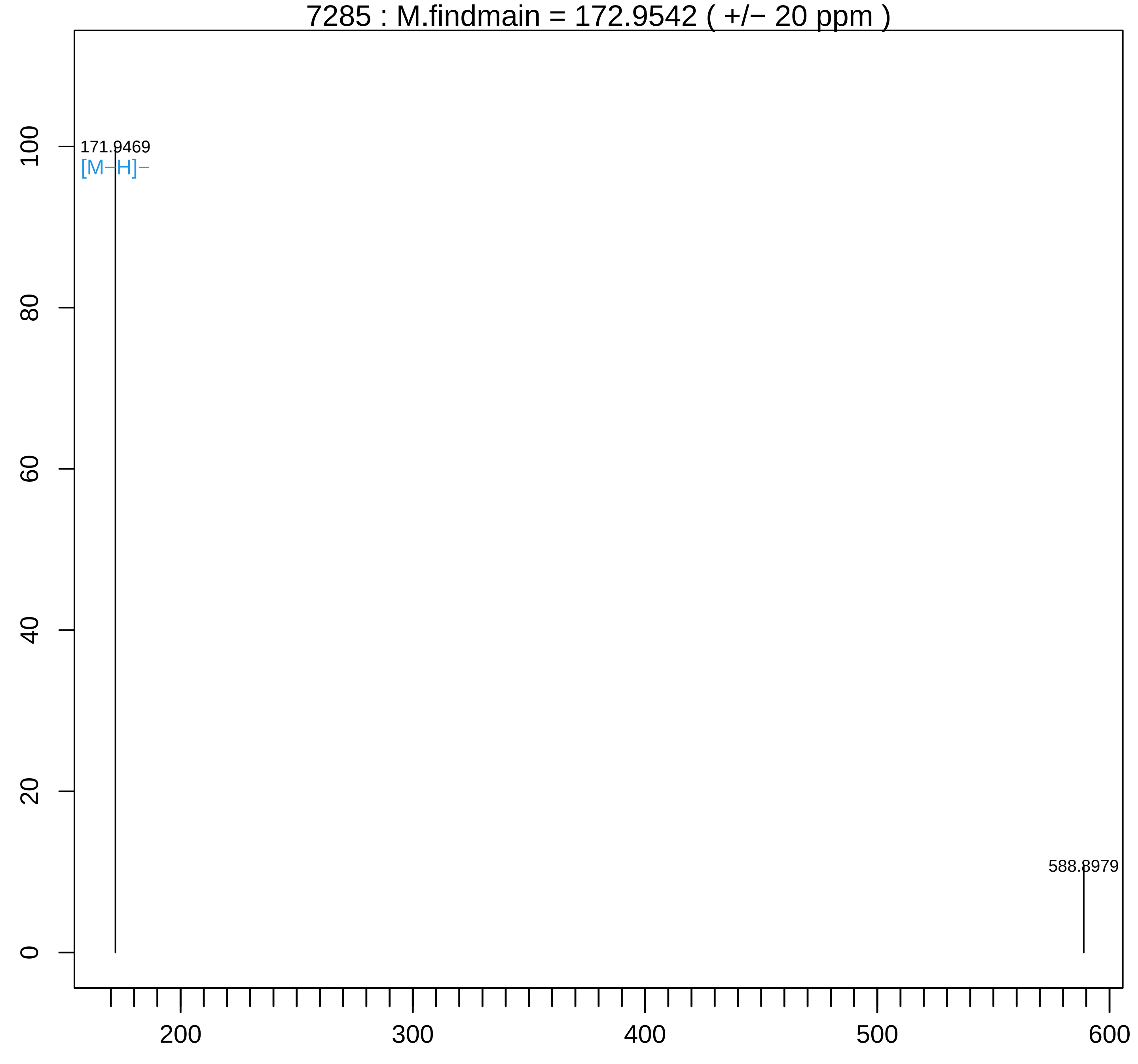
7285 : M.findmain = 172.9542 (+/− 20 ppm)
171.9469
[M−H]−
588.8979
0
20
40
60
80
100
200
300
400
500
600

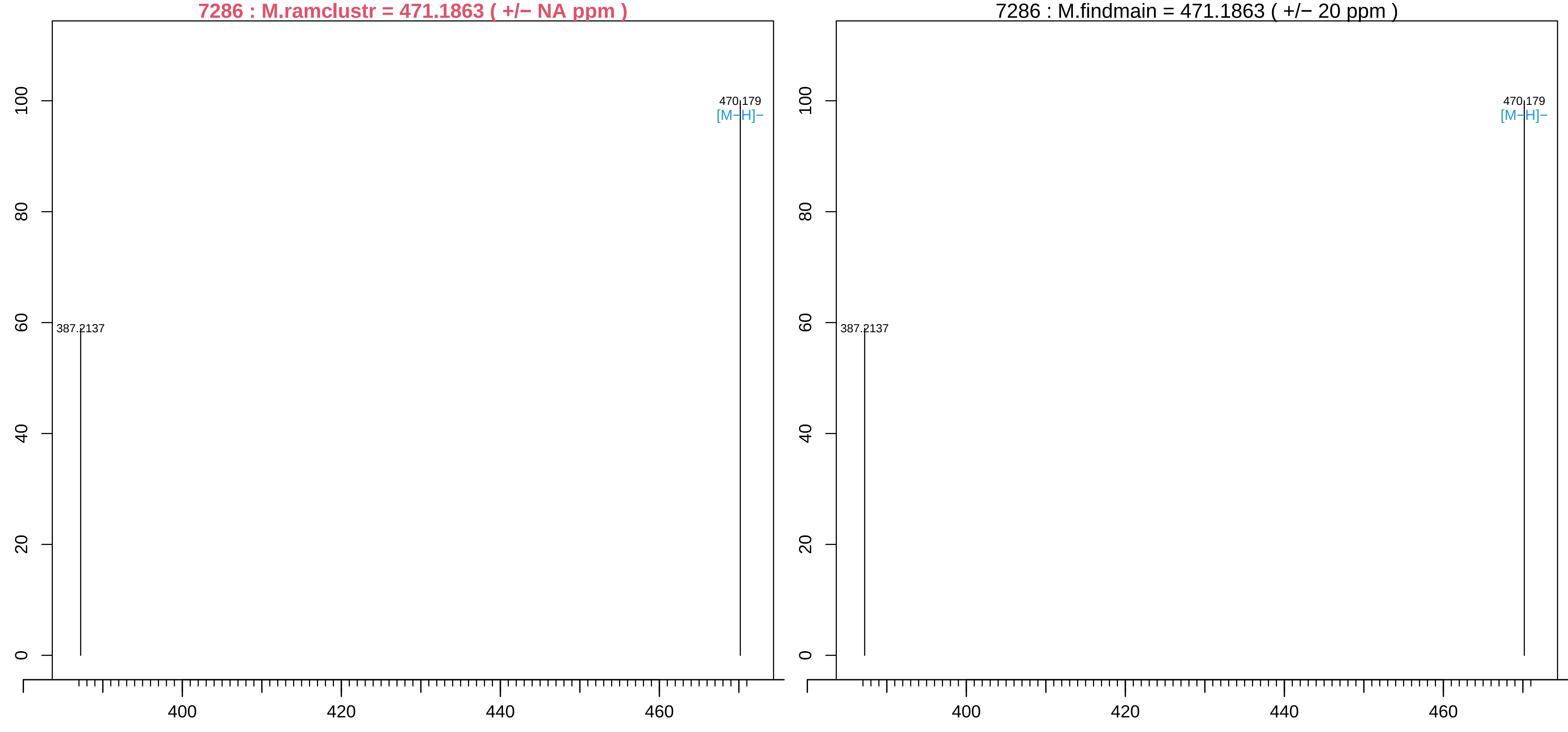
7286 : M.ramclustr = 471.1863 (+/− NA ppm)
7286 : M.findmain = 471.1863 (+/− 20 ppm)
470.179
[M−H]−
387.2137
0
20
40
60
80
100
400
420
440
460

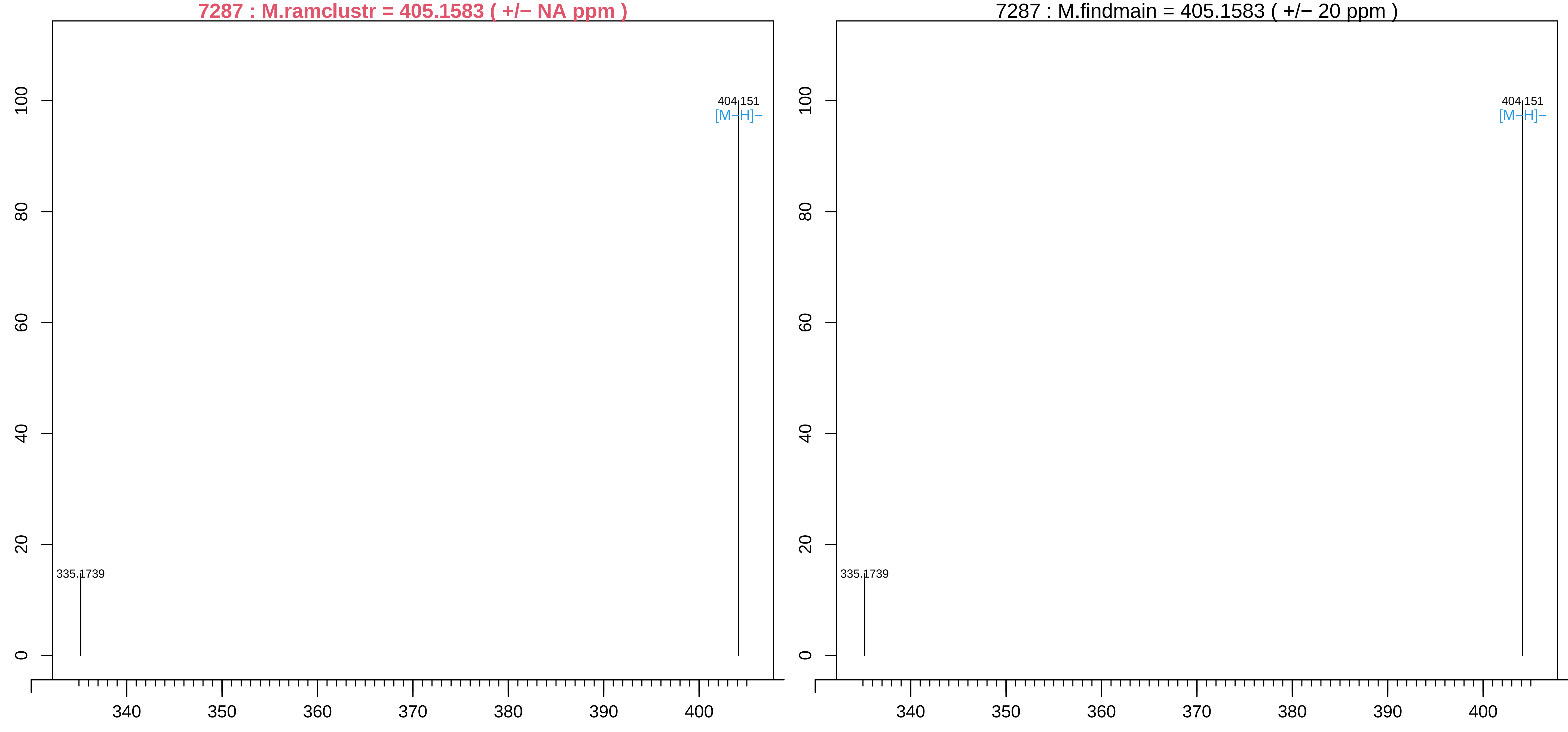
7287 : M.ramclustr = 405.1583 (+/− NA ppm)
7287 : M.findmain = 405.1583 (+/− 20 ppm)
404.151
[M−H]−
335.1739
0
20
40
60
80
100
340
350
360
370
380
390
400

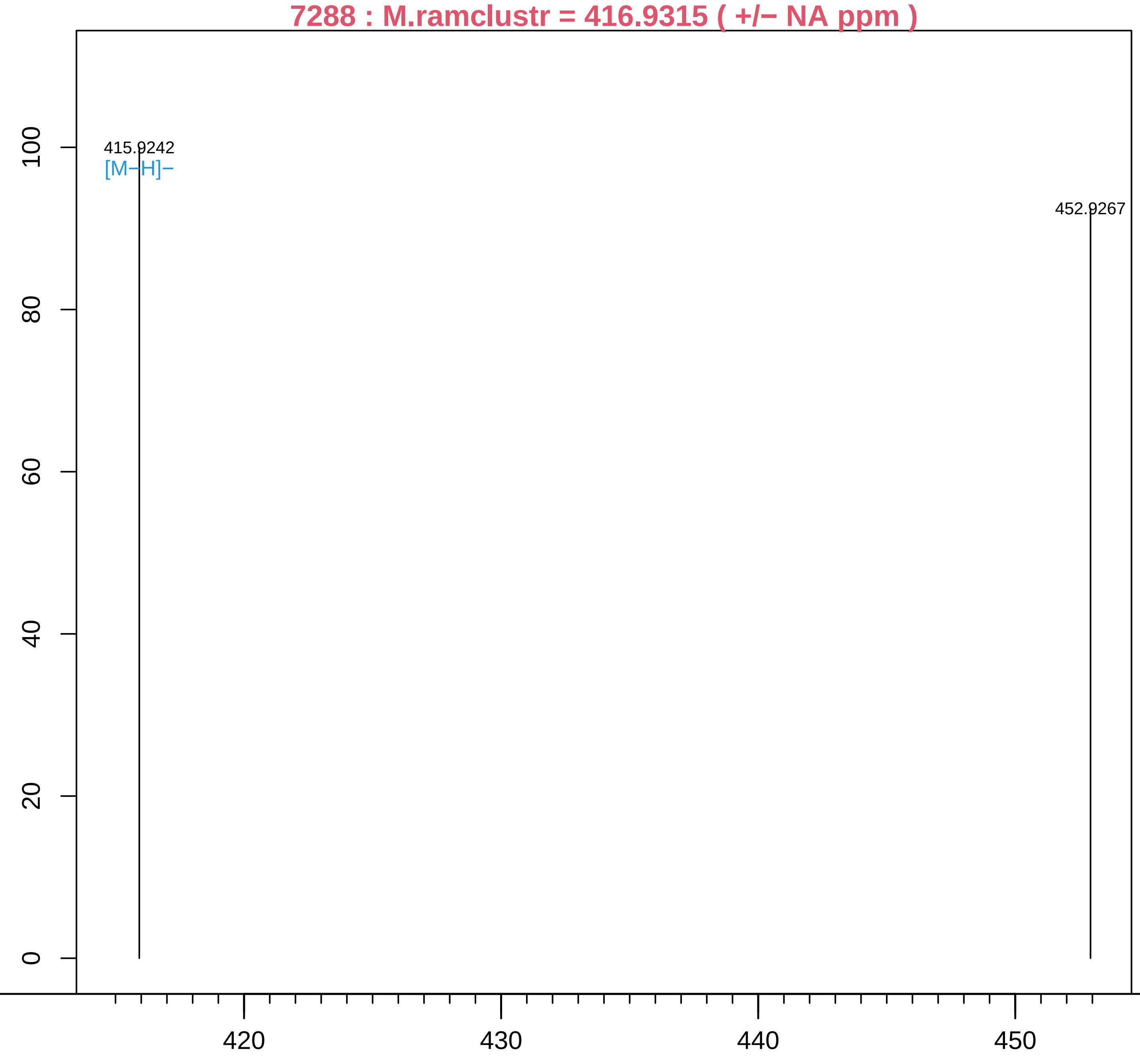
7288 : M.ramclustr = 416.9315 (+/− NA ppm)
415.9242
[M−H]−
452.9267
0
20
40
60
80
100
420
430
440
450

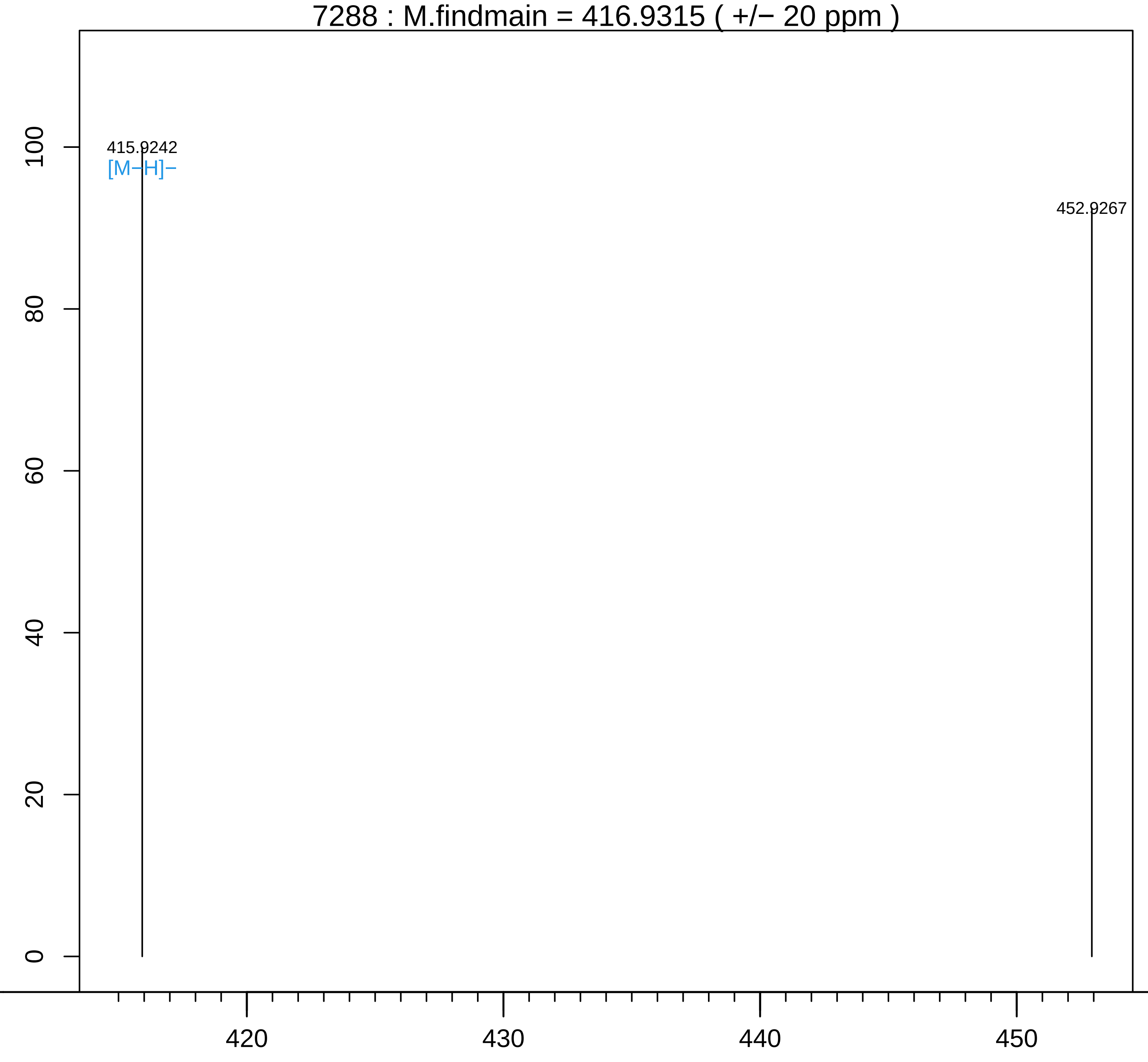
7288 : M.findmain = 416.9315 (+/− 20 ppm)
415.9242
[M−H]−
452.9267
0
20
40
60
80
100
420
430
440
450

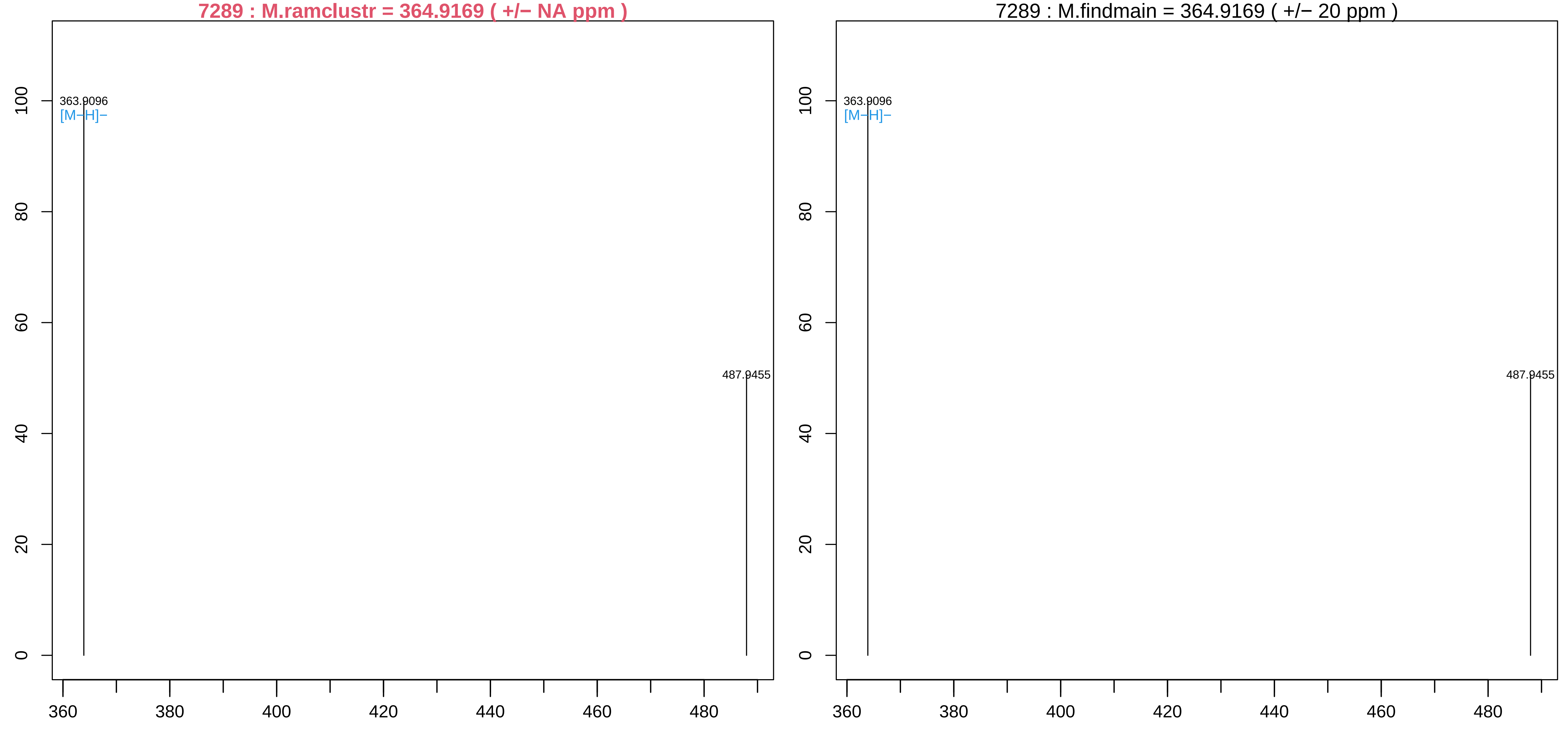

7289 : M.ramclustr = 364.9169 (+/− NA ppm)
363.9096
[M−H]−
487.9455
0
20
40
60
80
100
360
380
400
420
440
460
480
7289 : M.findmain = 364.9169 (+/− 20 ppm)
363.9096
[M−H]−
487.9455
0
20
40
60
80
100
360
380
400
420
440
460
480

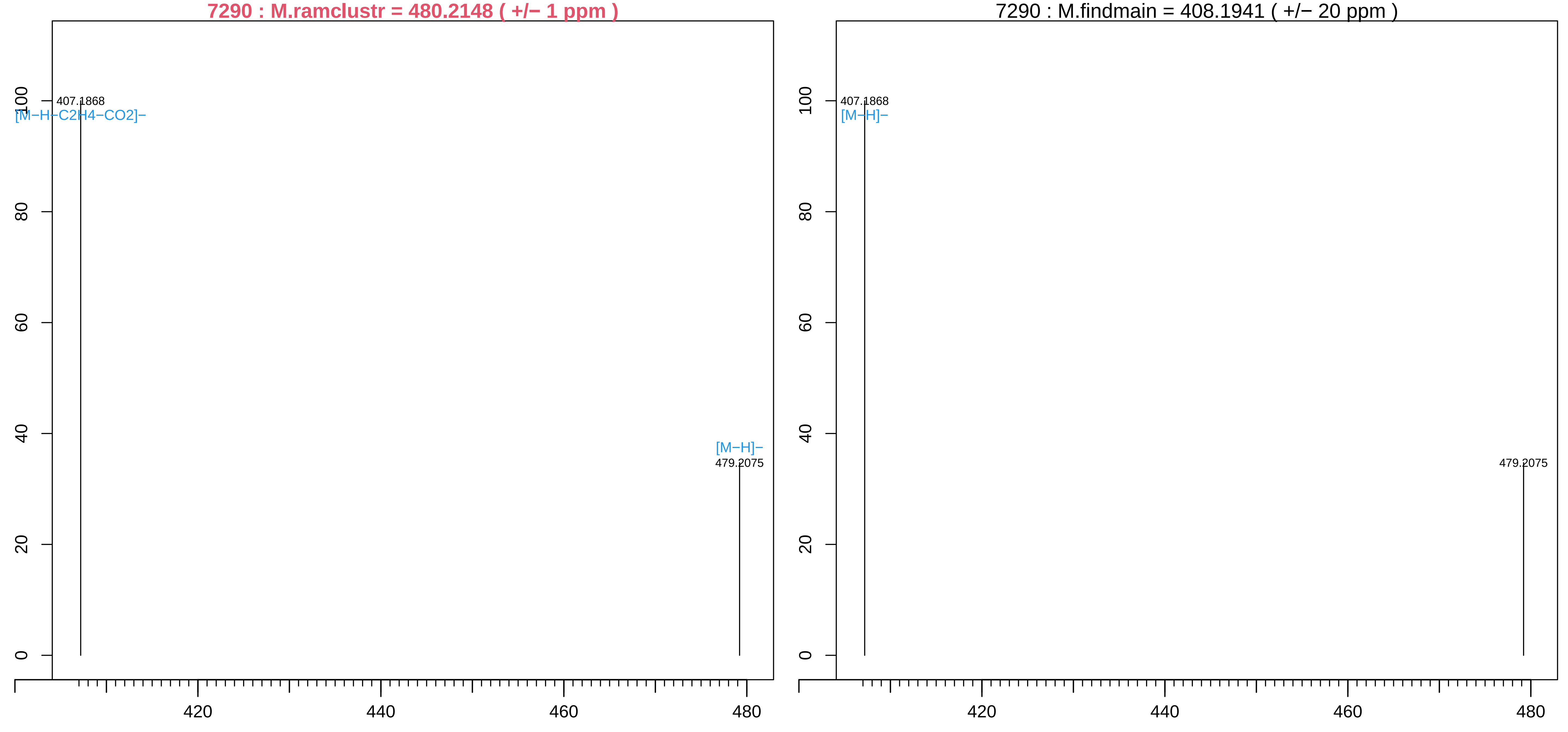
7290 : M.ramclustr = 480.2148 (+/− 1 ppm)
407.1868
[M−H−C2H4−CO2]−
[M−H]−
479.2075
0
20
40
60
80
100
420
440
460
480
7290 : M.findmain = 408.1941 (+/− 20 ppm)
407.1868
[M−H]−
479.2075
0
20
40
60
80
100
420
440
460
480

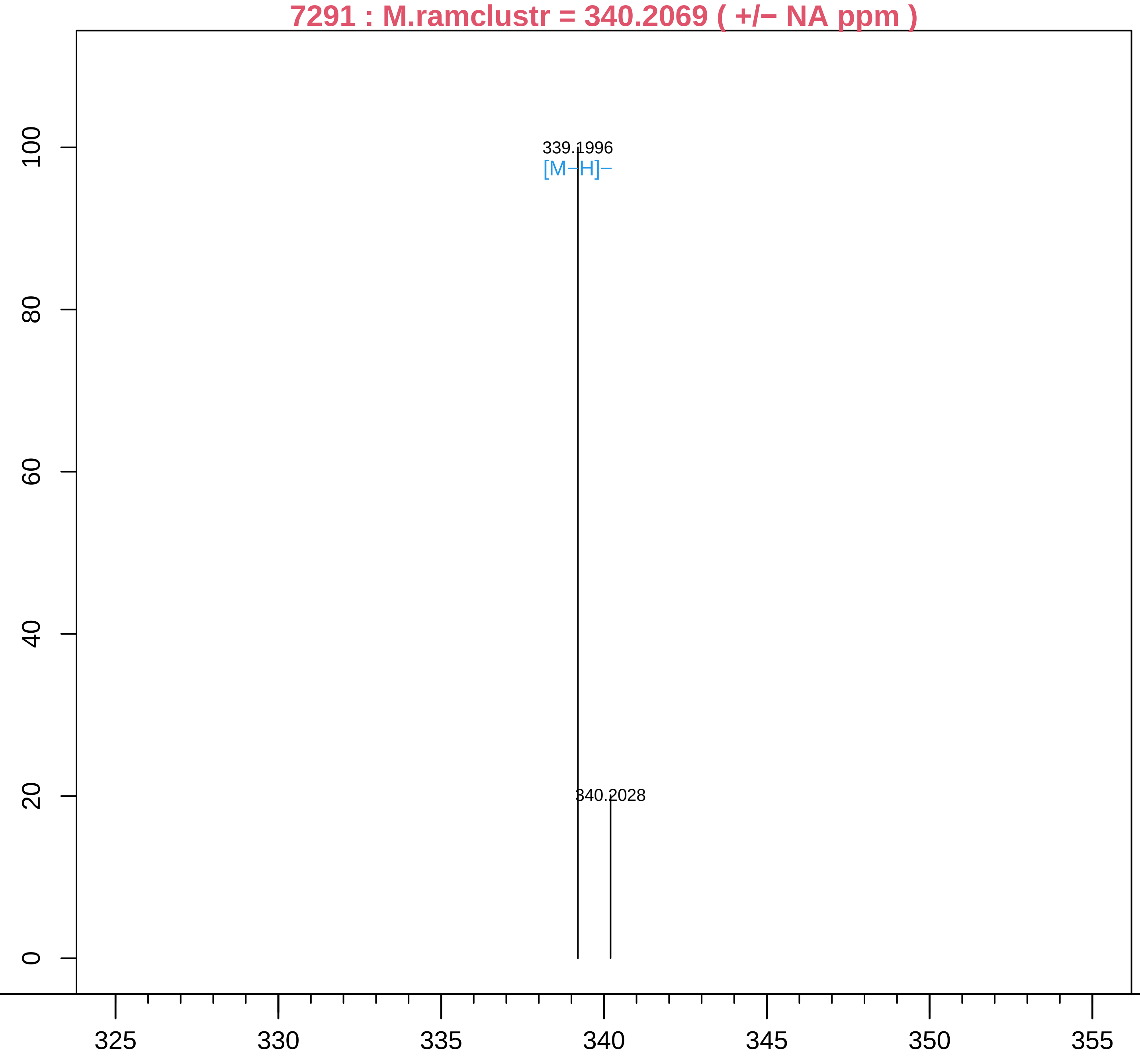
7291 : M.ramclustr = 340.2069 (+/− NA ppm)
339.1996
[M−H]−
340.2028
0
20
40
60
80
100
325
330
335
340
345
350
355

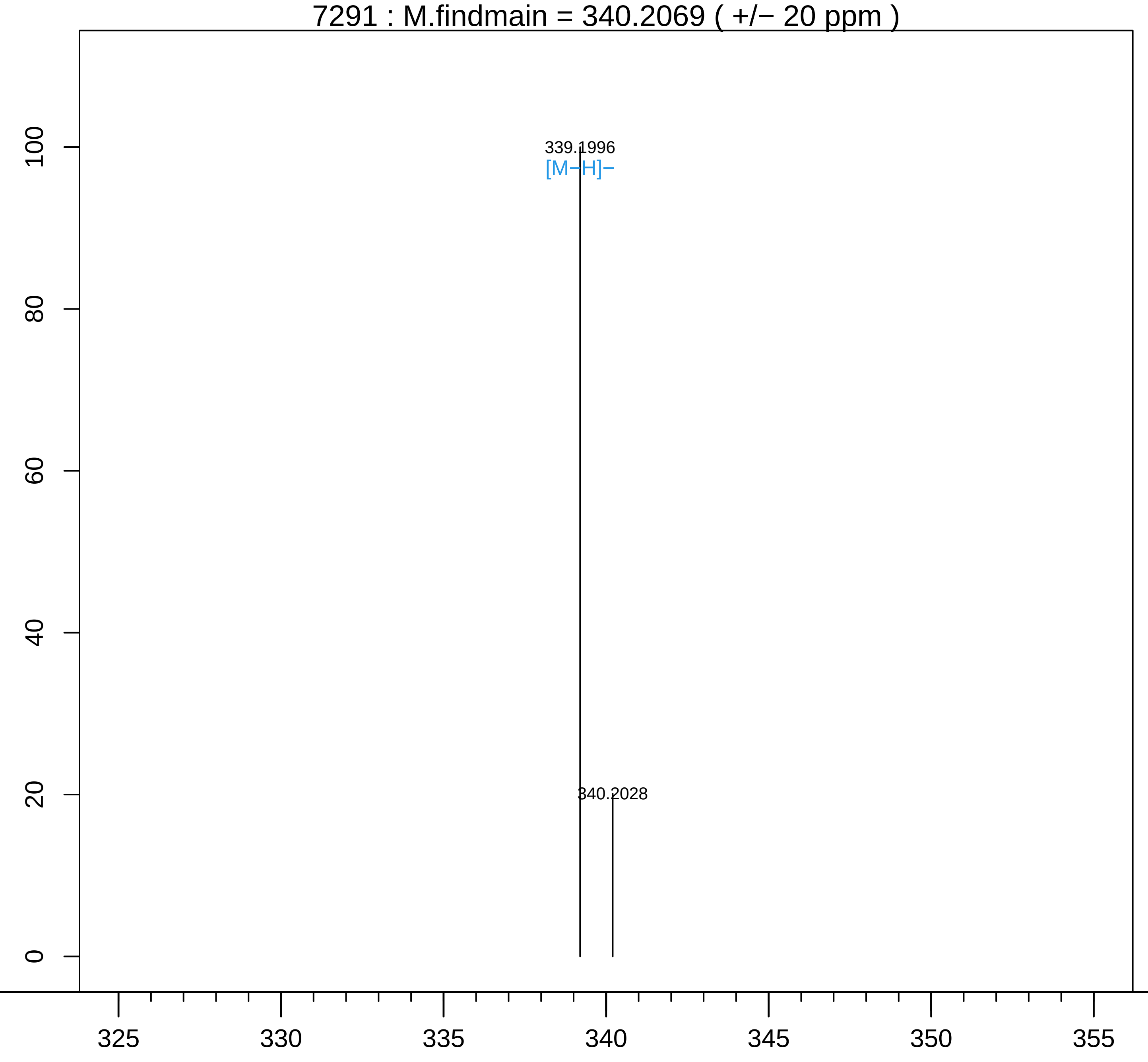
7291 : M.findmain = 340.2069 (+/− 20 ppm)
339.1996
[M−H]−
340.2028
0
20
40
60
80
100
325
330
335
340
345
350
355

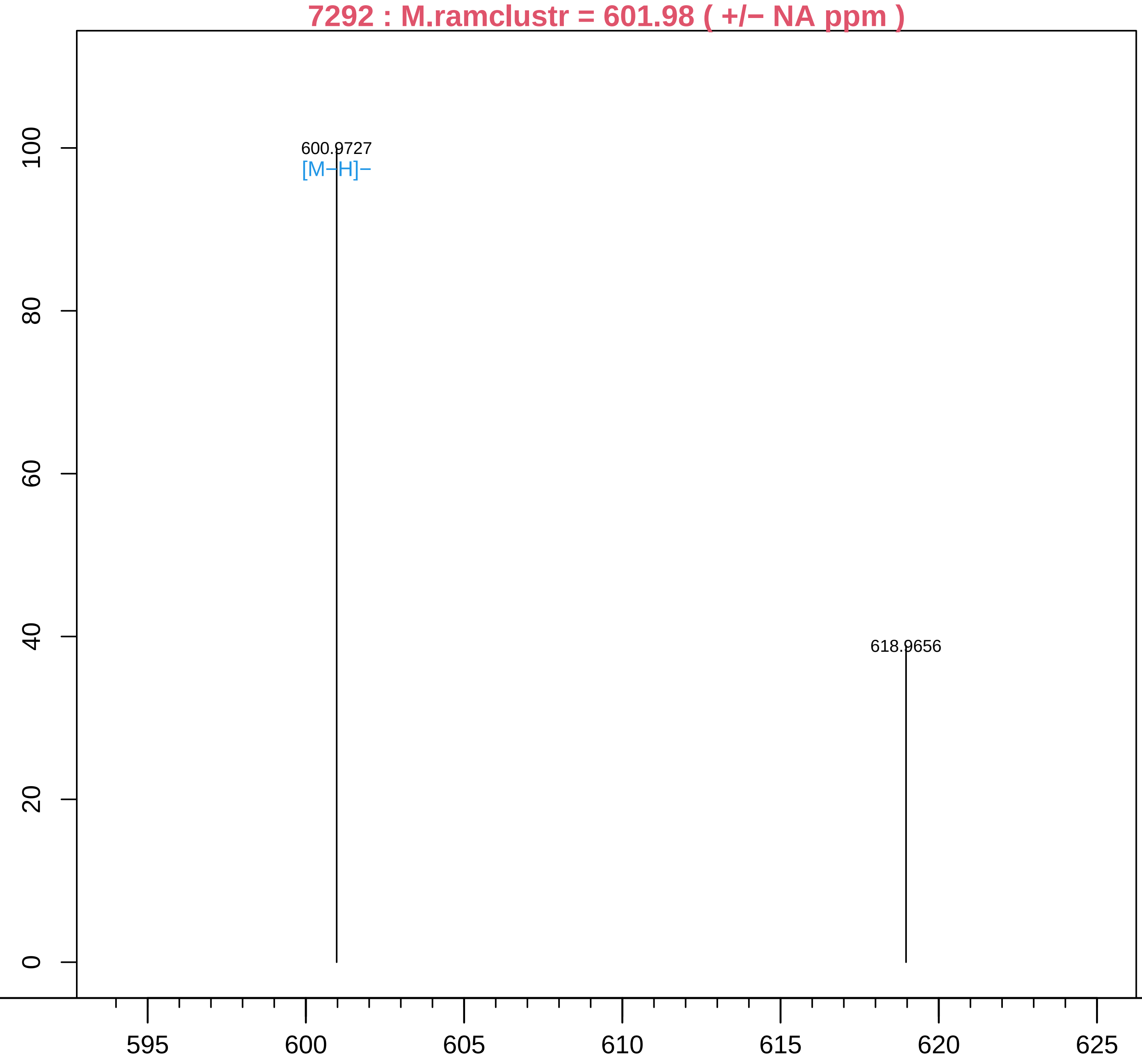
7292 : M.ramclustr = 601.98 (+/− NA ppm)
600.9727
[M−H]−
618.9656
0
20
40
60
80
100
595
600
605
610
615
620
625

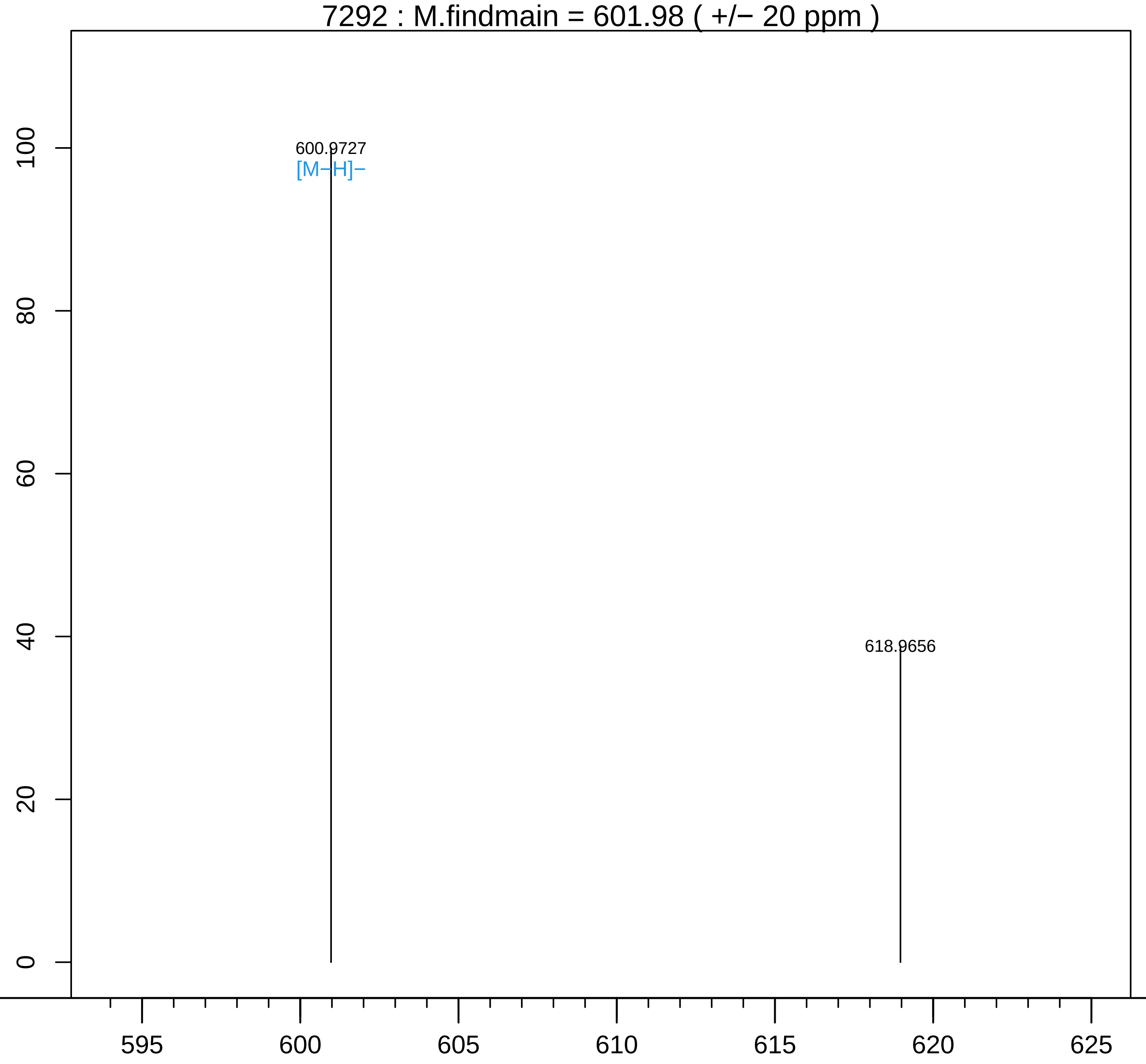
7292 : M.findmain = 601.98 (+/− 20 ppm)
600.9727
[M−H]−
618.9656
0
20
40
60
80
100
595
600
605
610
615
620
625

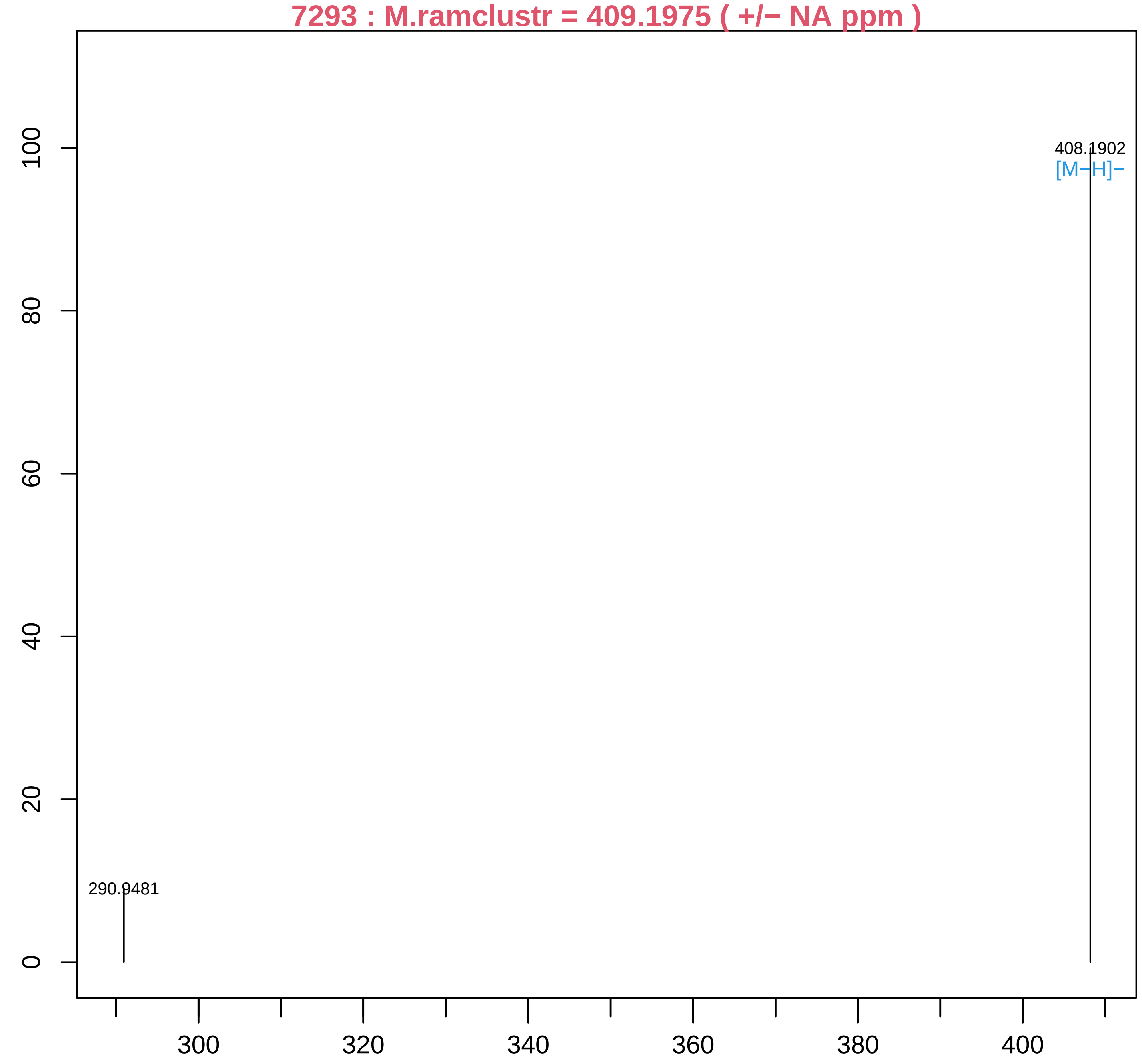
7293 : M.ramclustr = 409.1975 (+/− NA ppm)
408.1902
[M−H]−
290.9481
0
20
40
60
80
100
300
320
340
360
380
400

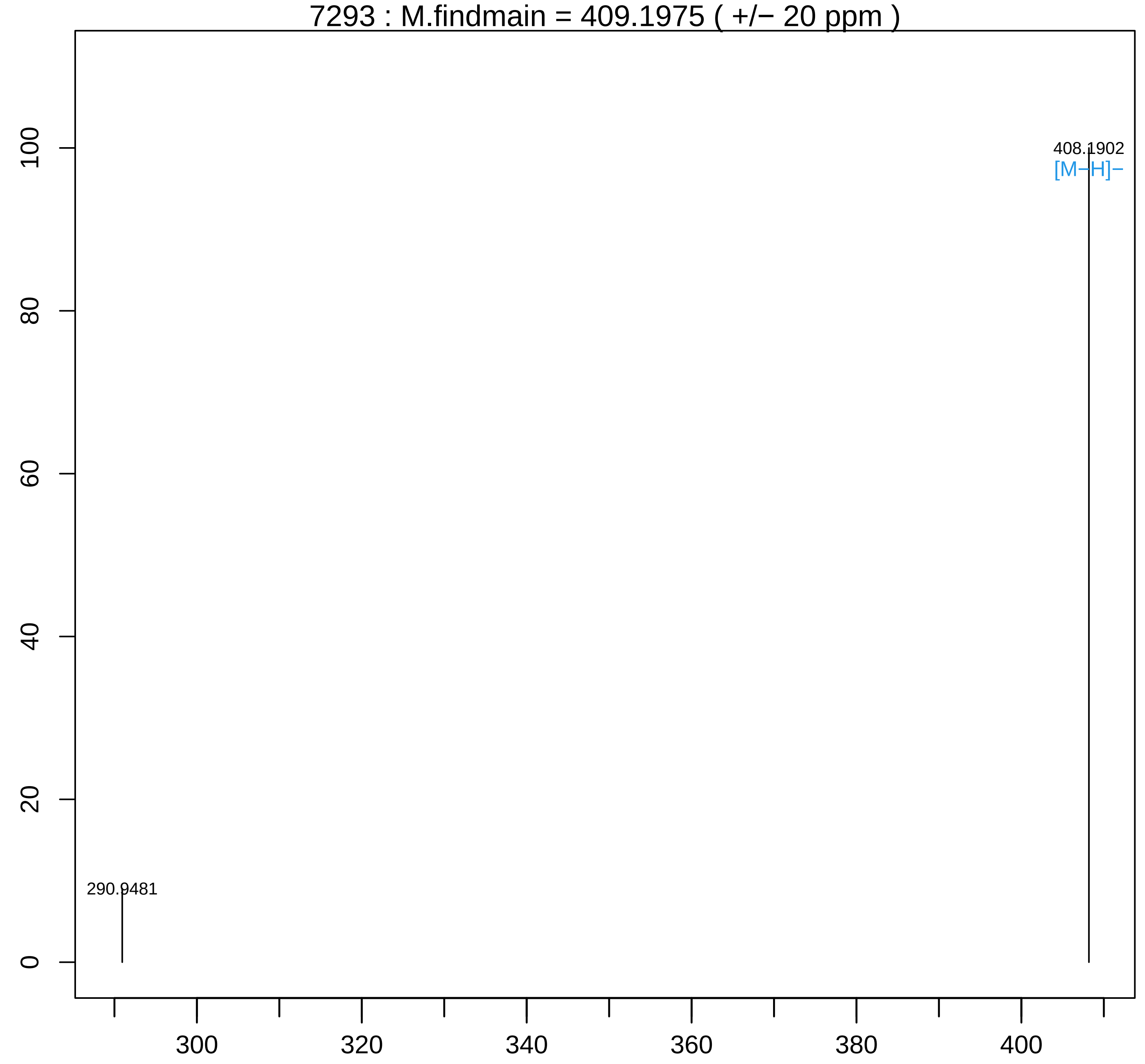
7293 : M.findmain = 409.1975 (+/− 20 ppm)
408.1902
[M−H]−
290.9481
0
20
40
60
80
100
300
320
340
360
380
400

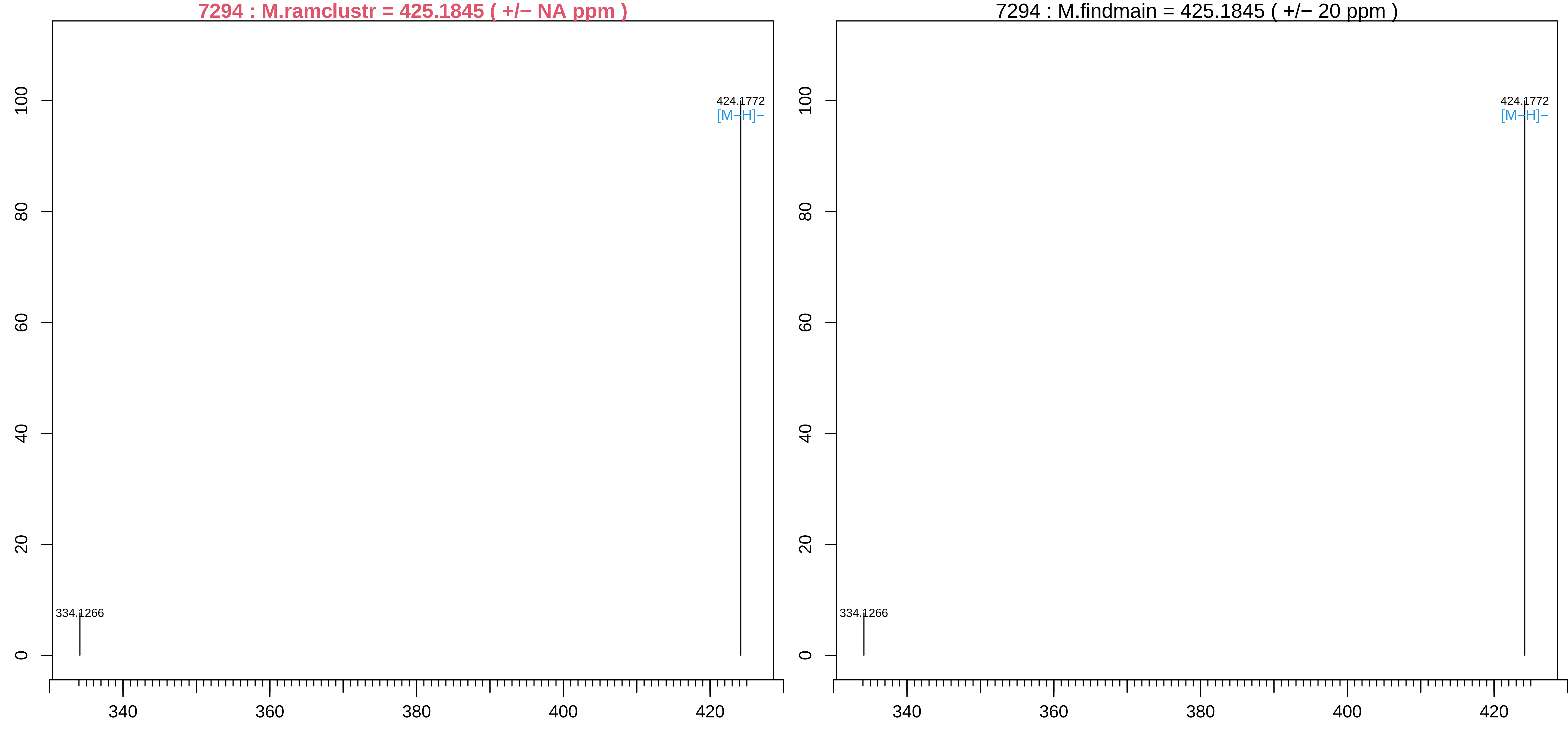
7294 : M.ramclustr = 425.1845 (+/− NA ppm)
424.1772
[M−H]−
334.1266
7294 : M.findmain = 425.1845 (+/− 20 ppm)
424.1772
[M−H]−
334.1266
0
20
40
60
80
100
340
360
380
400
420

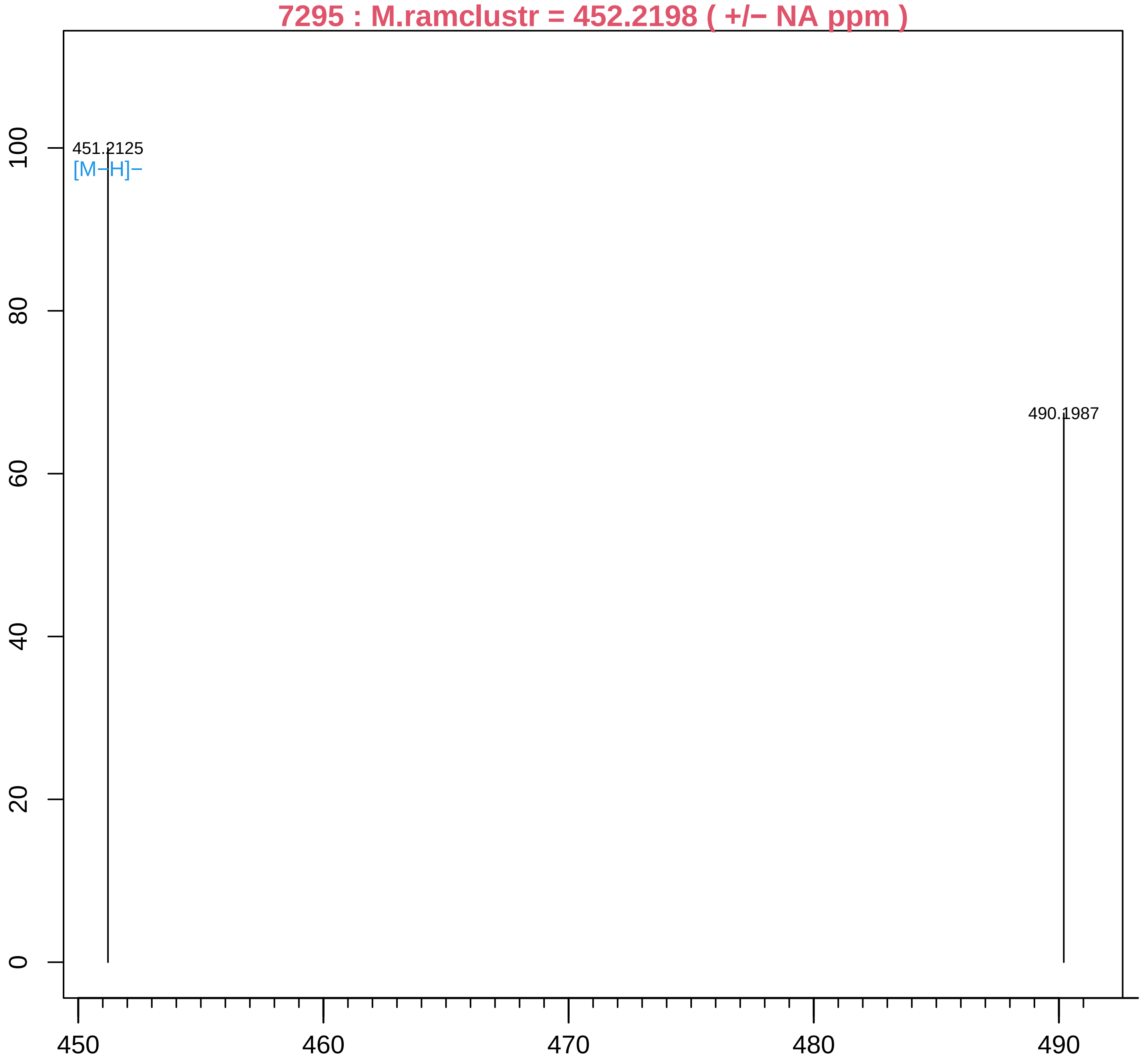
7295 : M.ramclustr = 452.2198 (+/− NA ppm)
451.2125
[M−H]−
490.1987
0
20
40
60
80
100
450
460
470
480
490

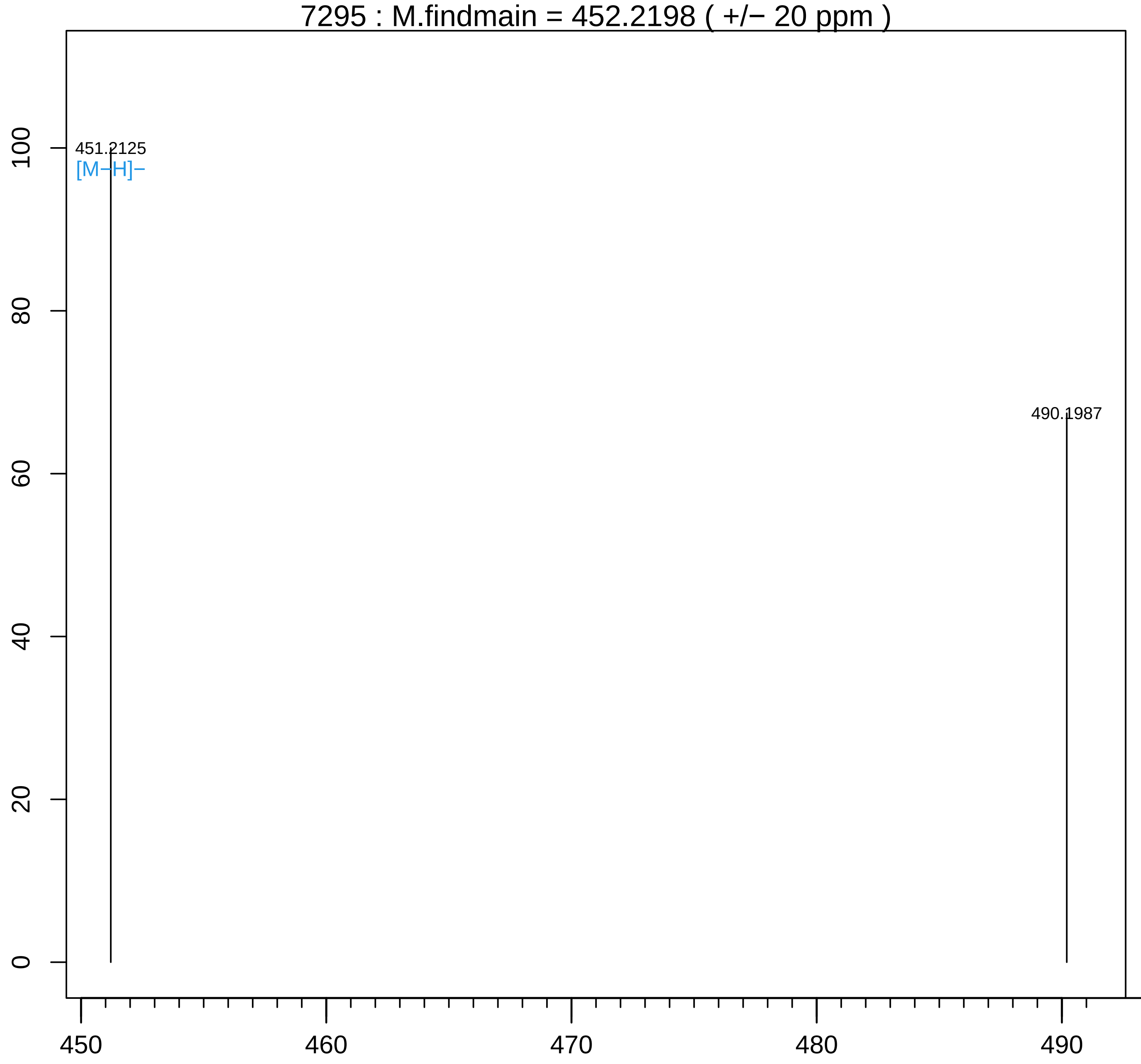
7295 : M.findmain = 452.2198 (+/− 20 ppm)
451.2125
[M−H]−
490.1987
0
20
40
60
80
100
450
460
470
480
490

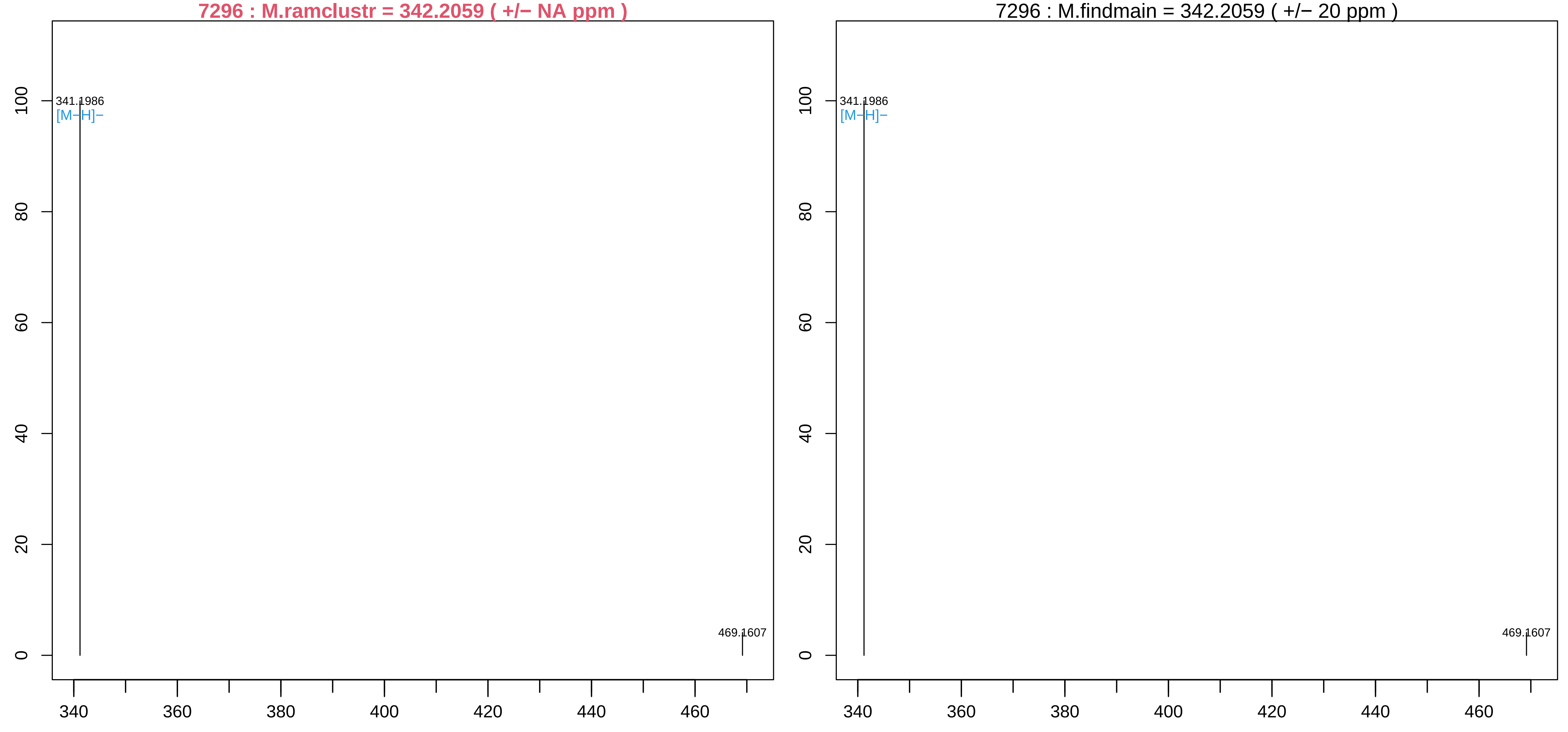
7296 : M.ramclustr = 342.2059 (+/− NA ppm)
341.1986
[M−H]−
469.1607
7296 : M.findmain = 342.2059 (+/− 20 ppm)
341.1986
[M−H]−
469.1607
0
20
40
60
80
100
340
360
380
400
420
440
460

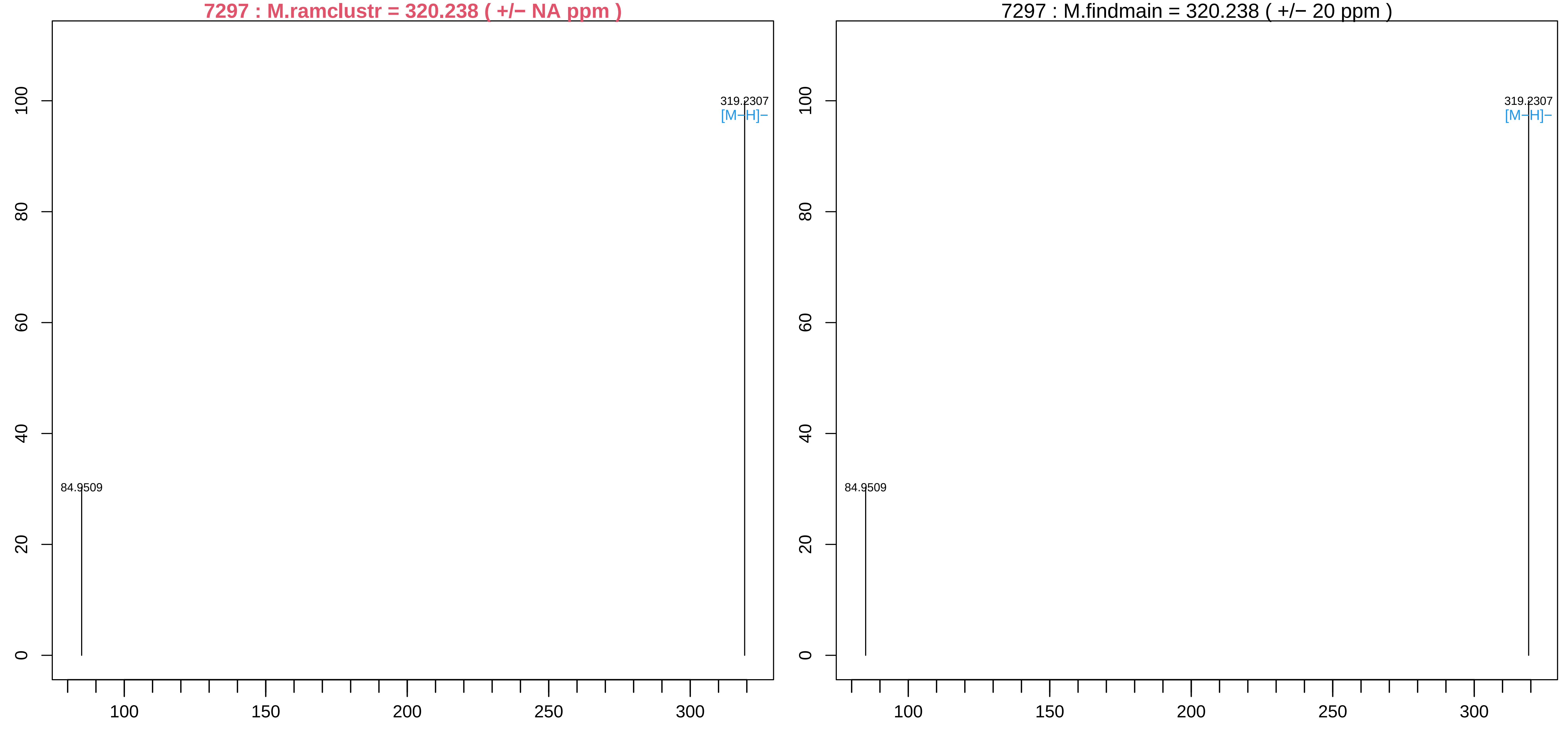
7297 : M.ramclustr = 320.238 (+/− NA ppm)
319.2307
[M−H]−
84.9509
0
20
40
60
80
100
100
150
200
250
300
7297 : M.findmain = 320.238 (+/− 20 ppm)
319.2307
[M−H]−
84.9509
0
20
40
60
80
100
100
150
200
250
300

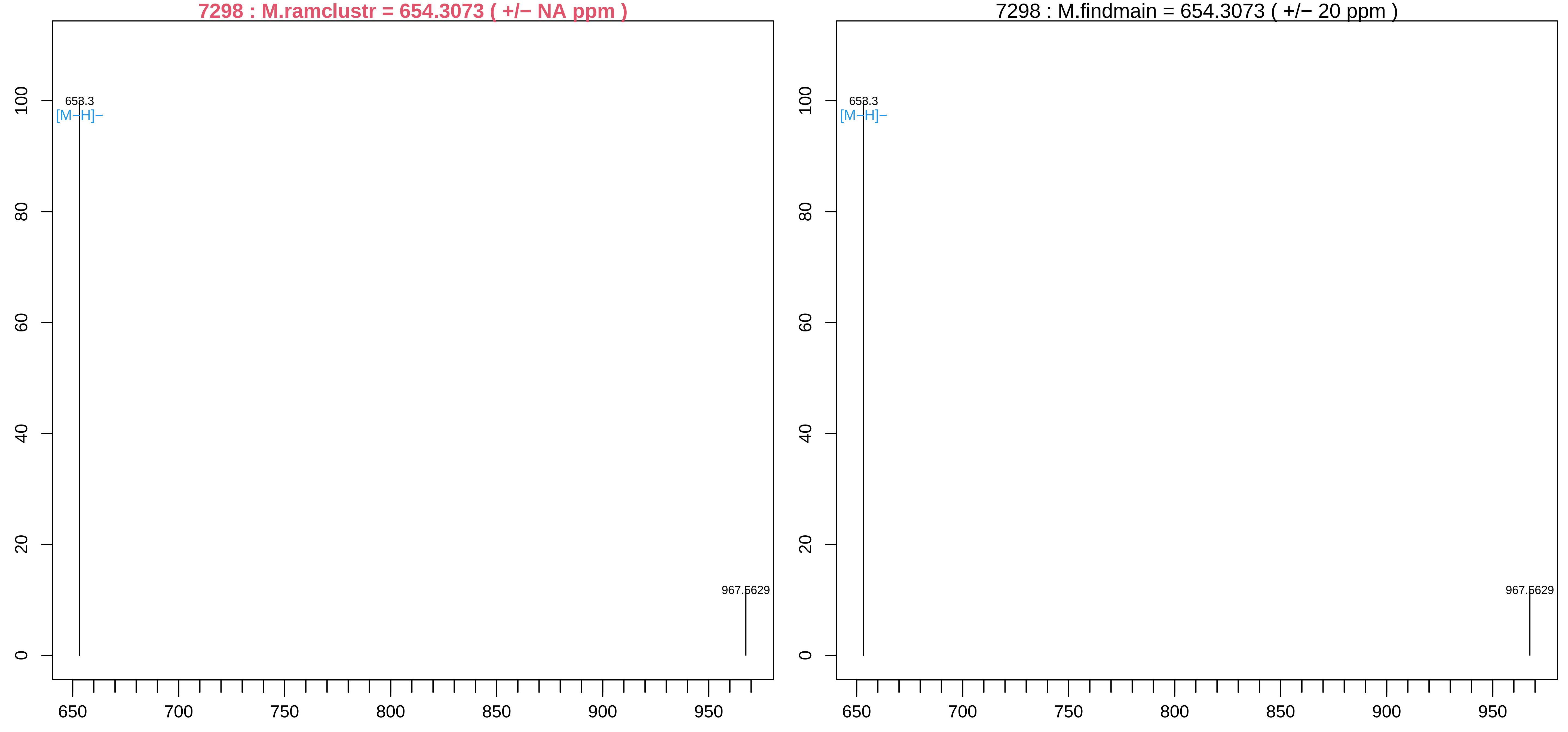
7298 : M.ramclustr = 654.3073 (+/− NA ppm)
653.3
[M−H]−
967.5629
7298 : M.findmain = 654.3073 (+/− 20 ppm)
653.3
[M−H]−
967.5629
0
20
40
60
80
100
650
700
750
800
850
900
950

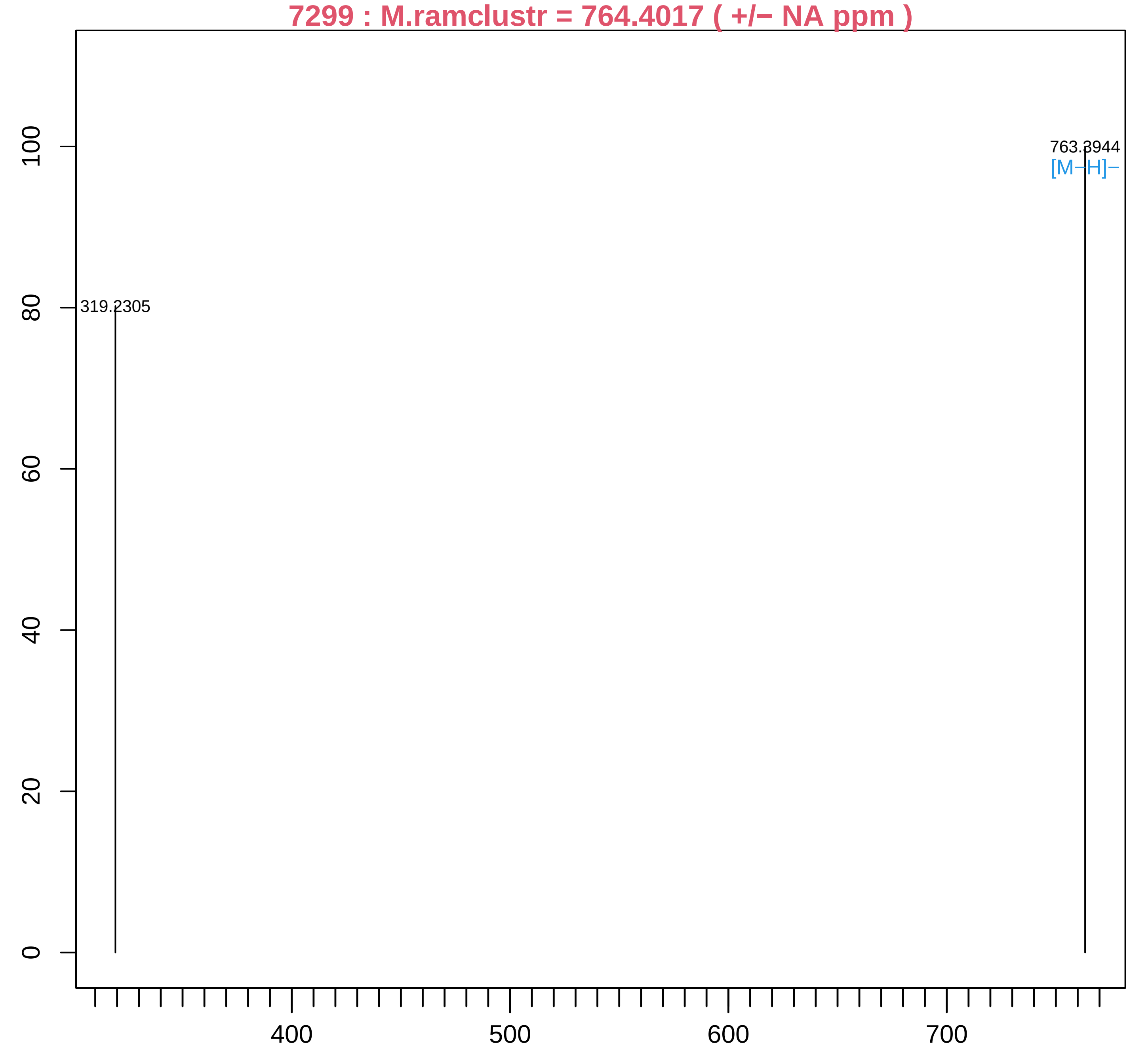
7299 : M.ramclustr = 764.4017 (+/− NA ppm)
763.3944
[M−H]−
319.2305
0
20
40
60
80
100
400
500
600
700

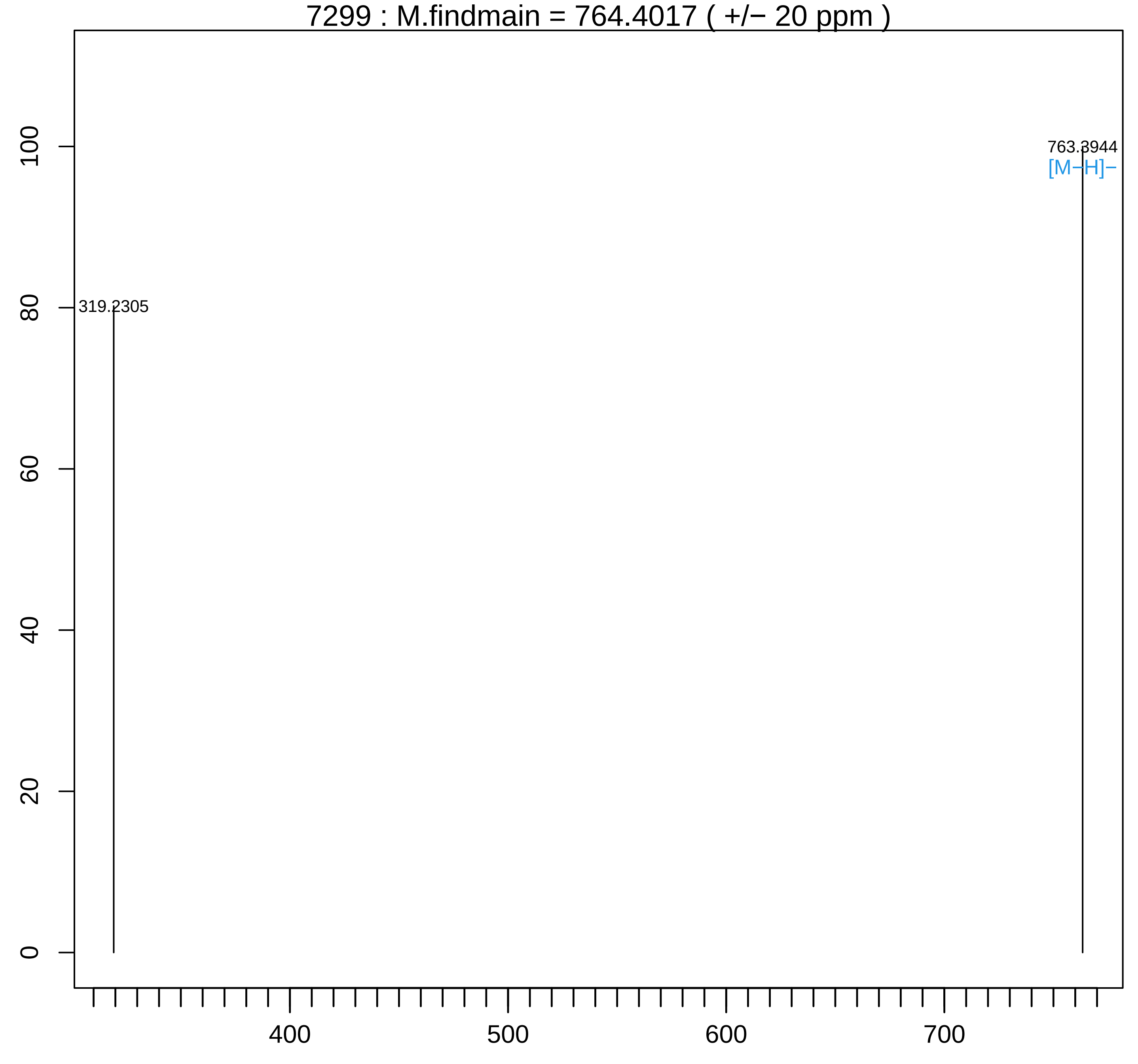
7299 : M.findmain = 764.4017 (+/− 20 ppm)
763.3944
[M−H]−
319.2305
0
20
40
60
80
100
400
500
600
700

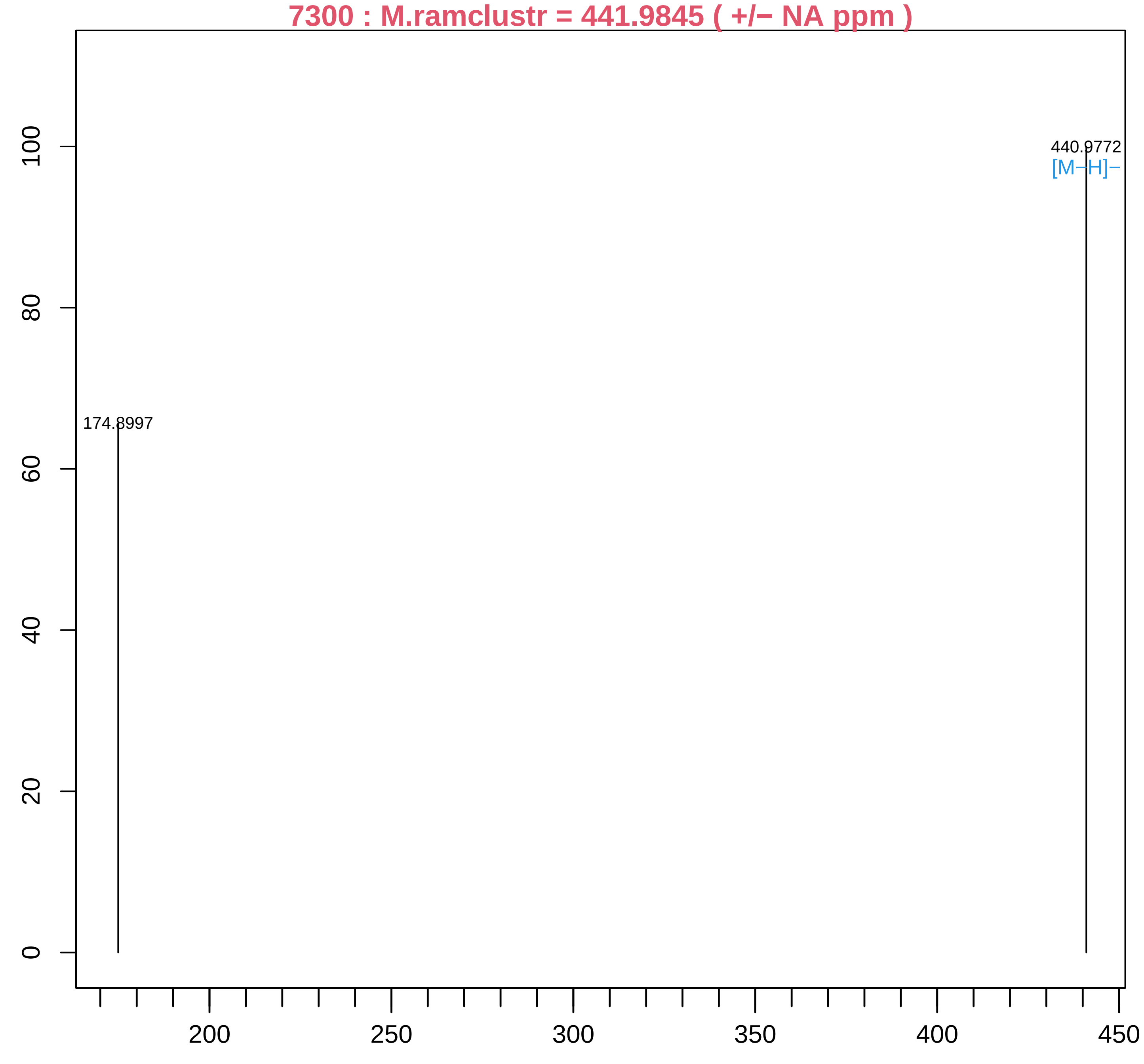
7300 : M.ramclustr = 441.9845 (+/− NA ppm)
440.9772
[M−H]−
174.8997
0
20
40
60
80
100
200
250
300
350
400
450

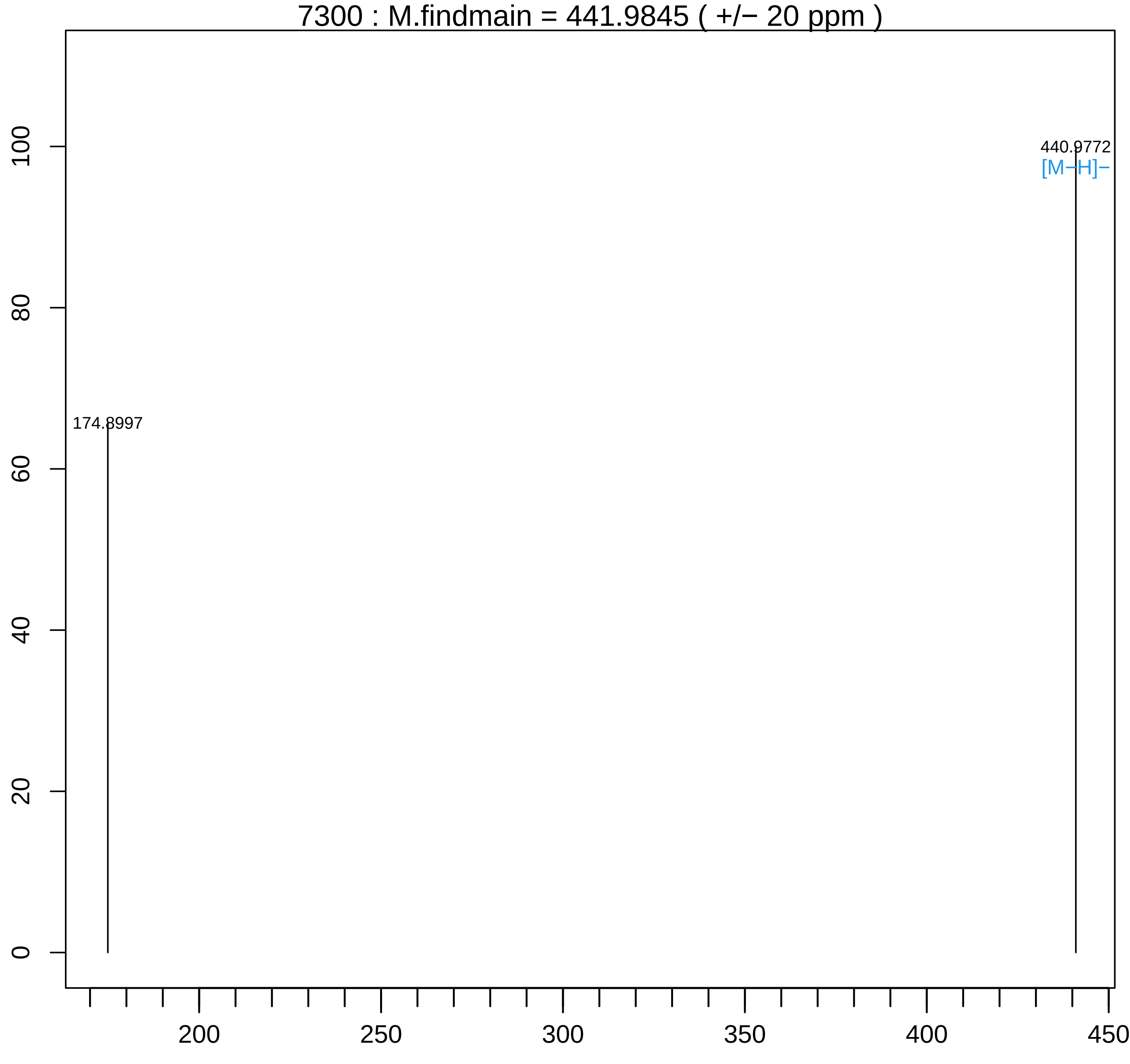
7300 : M.findmain = 441.9845 (+/− 20 ppm)
440.9772
[M−H]−
174.8997
0
20
40
60
80
100
200
250
300
350
400
450

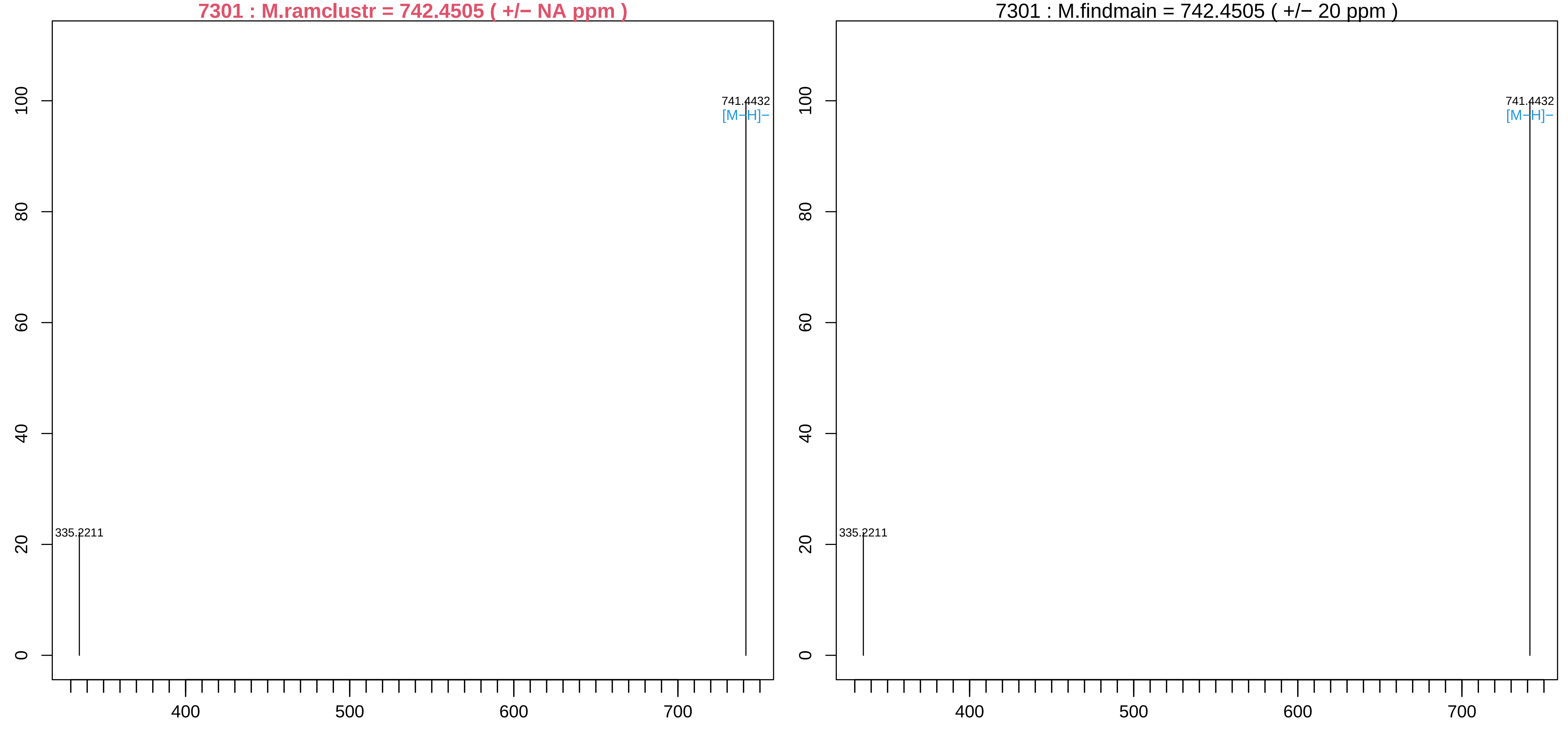

7301 : M.ramclustr = 742.4505 (+/− NA ppm)
741.4432
[M−H]−
335.2211
7301 : M.findmain = 742.4505 (+/− 20 ppm)
741.4432
[M−H]−
335.2211
0
20
40
60
80
100
400
500
600
700

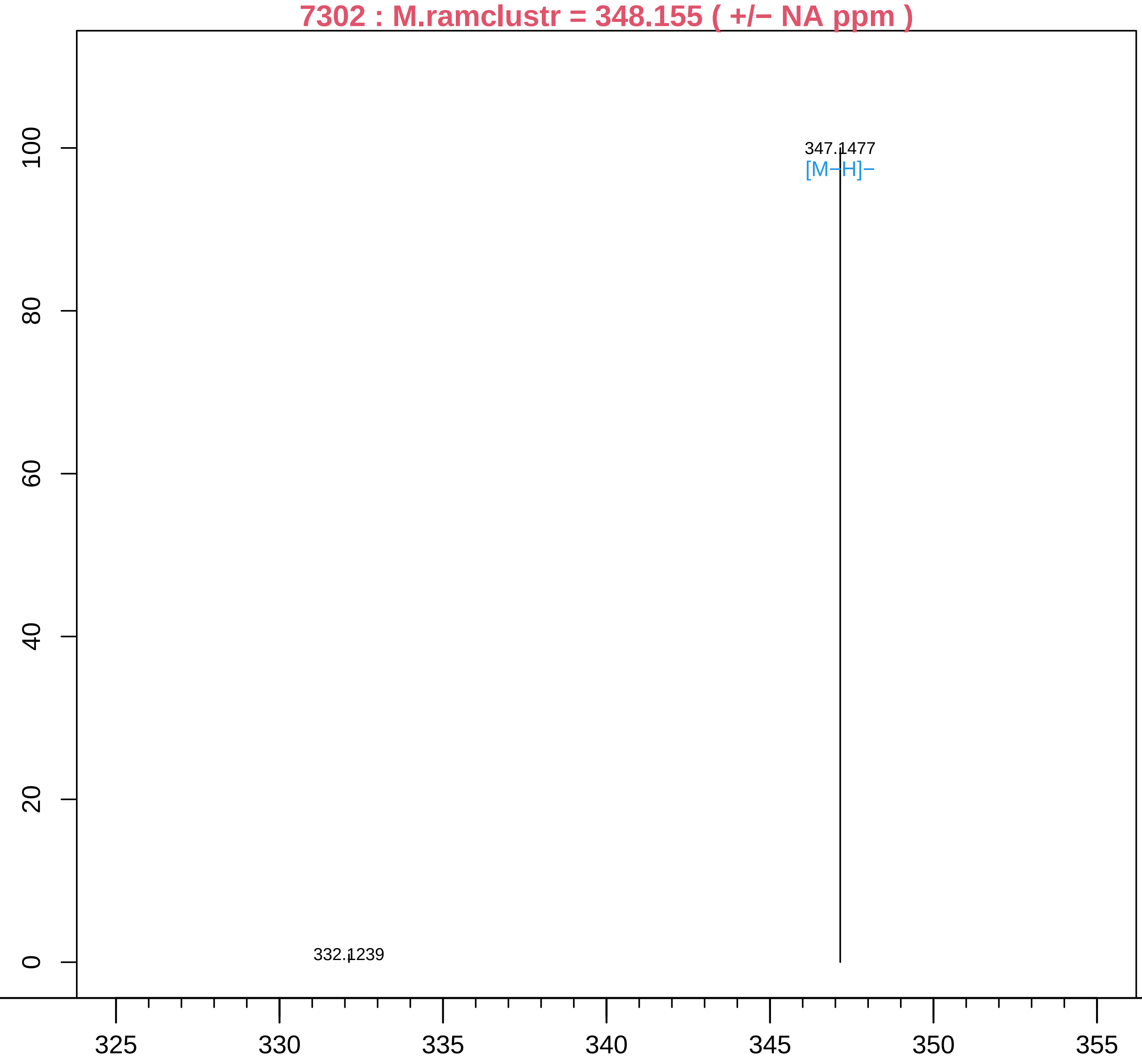
7302 : M.ramclustr = 348.155 (+/− NA ppm)
347.1477
[M−H]−
332.1239
0
20
40
60
80
100
325
330
335
340
345
350
355

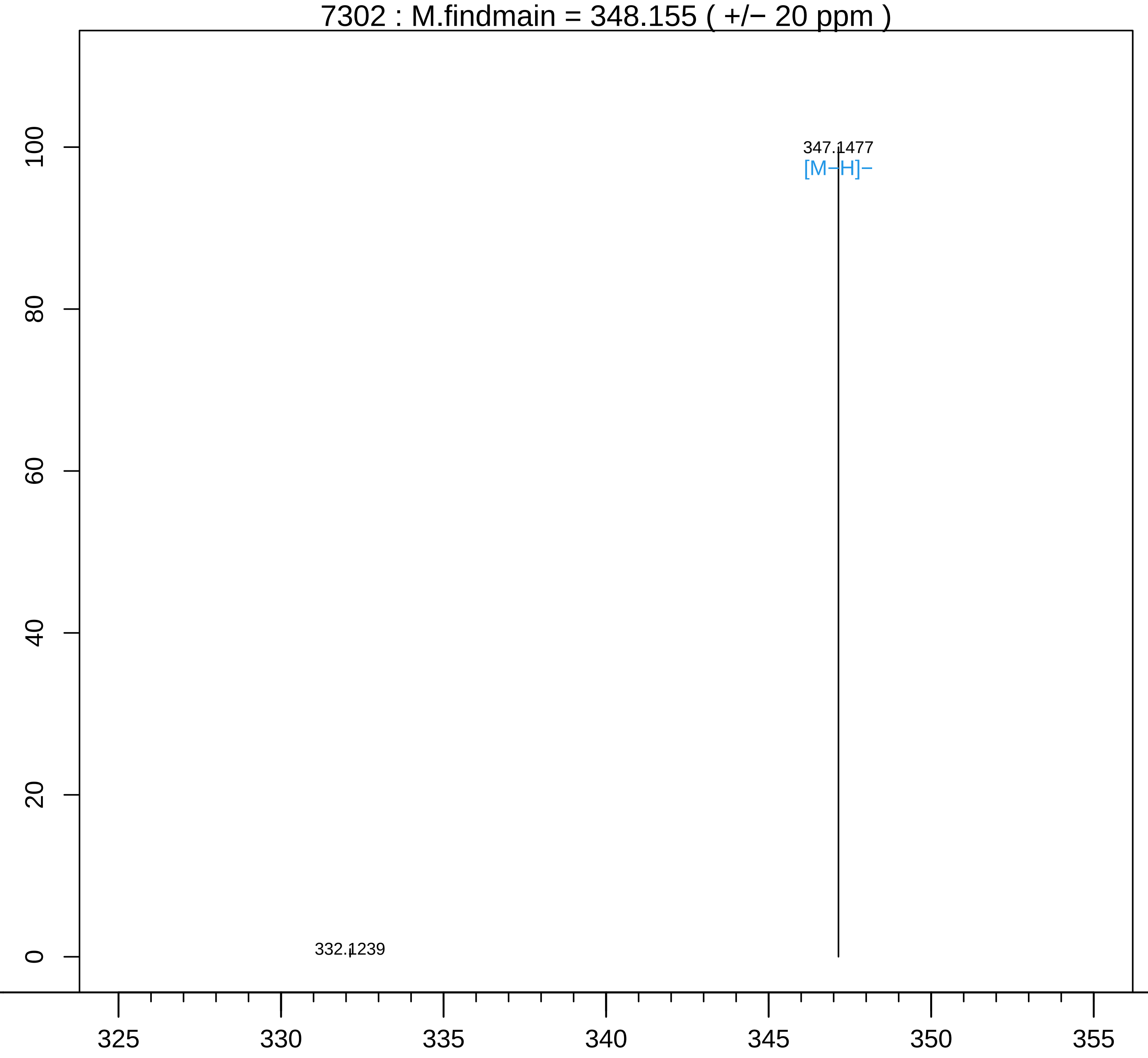
7302 : M.findmain = 348.155 (+/− 20 ppm)
347.1477
[M−H]−
332.1239
0
20
40
60
80
100
325
330
335
340
345
350
355

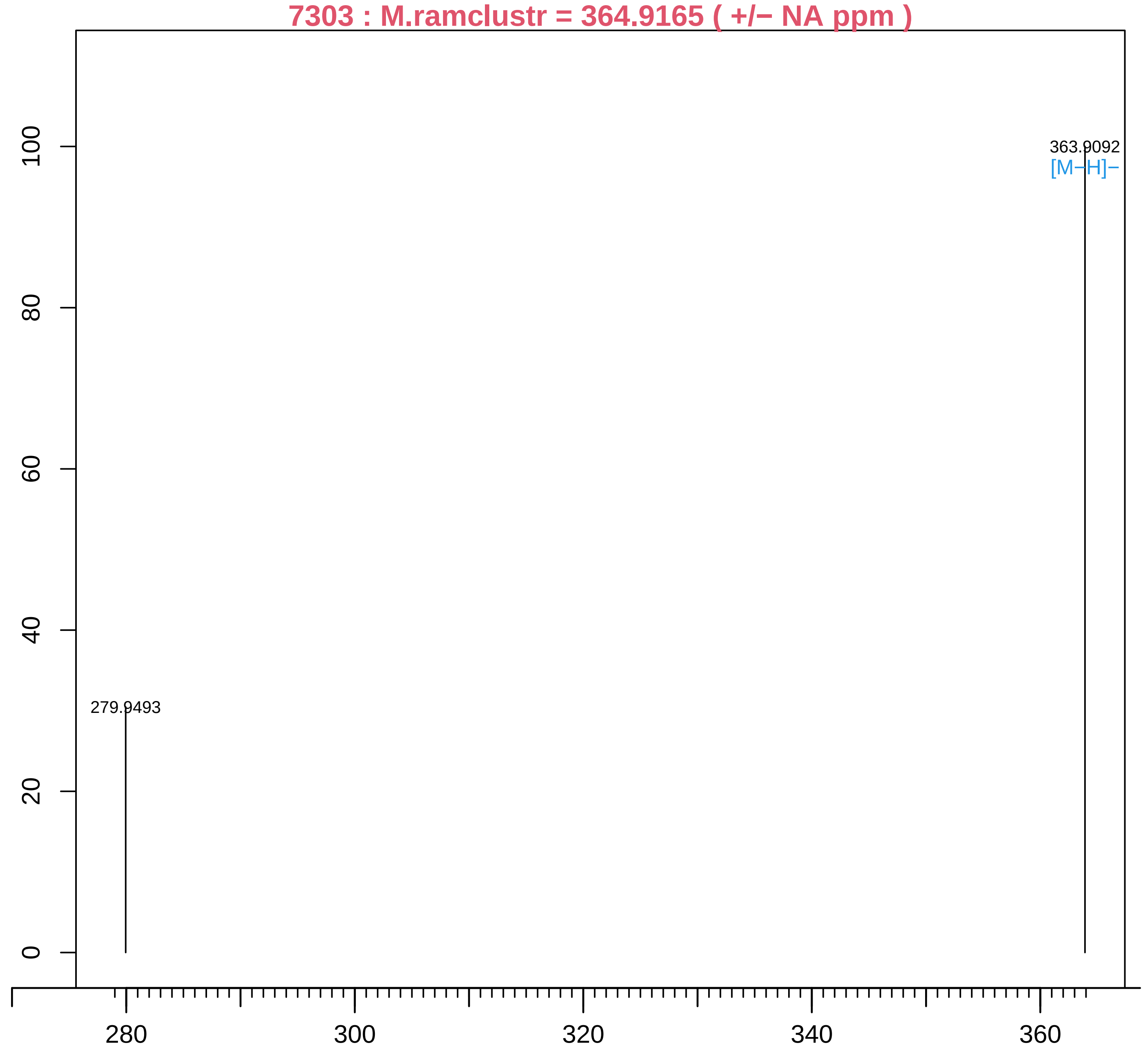
7303 : M.ramclustr = 364.9165 (+/− NA ppm)
363.9092
[M−H]−
279.9493
0
20
40
60
80
100
280
300
320
340
360

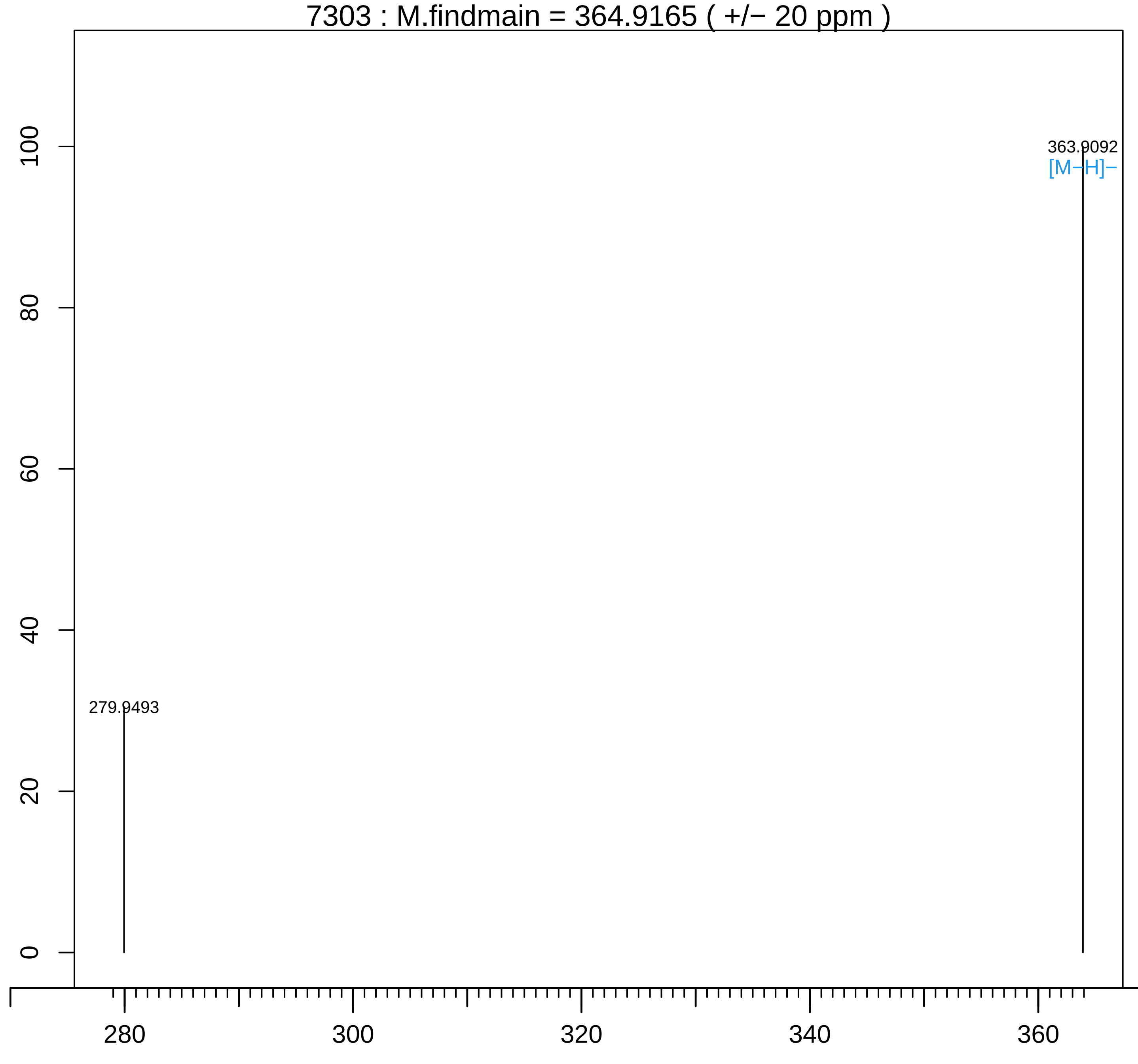
7303 : M.findmain = 364.9165 (+/− 20 ppm)
363.9092
[M−H]−
279.9493
0
20
40
60
80
100
280
300
320
340
360

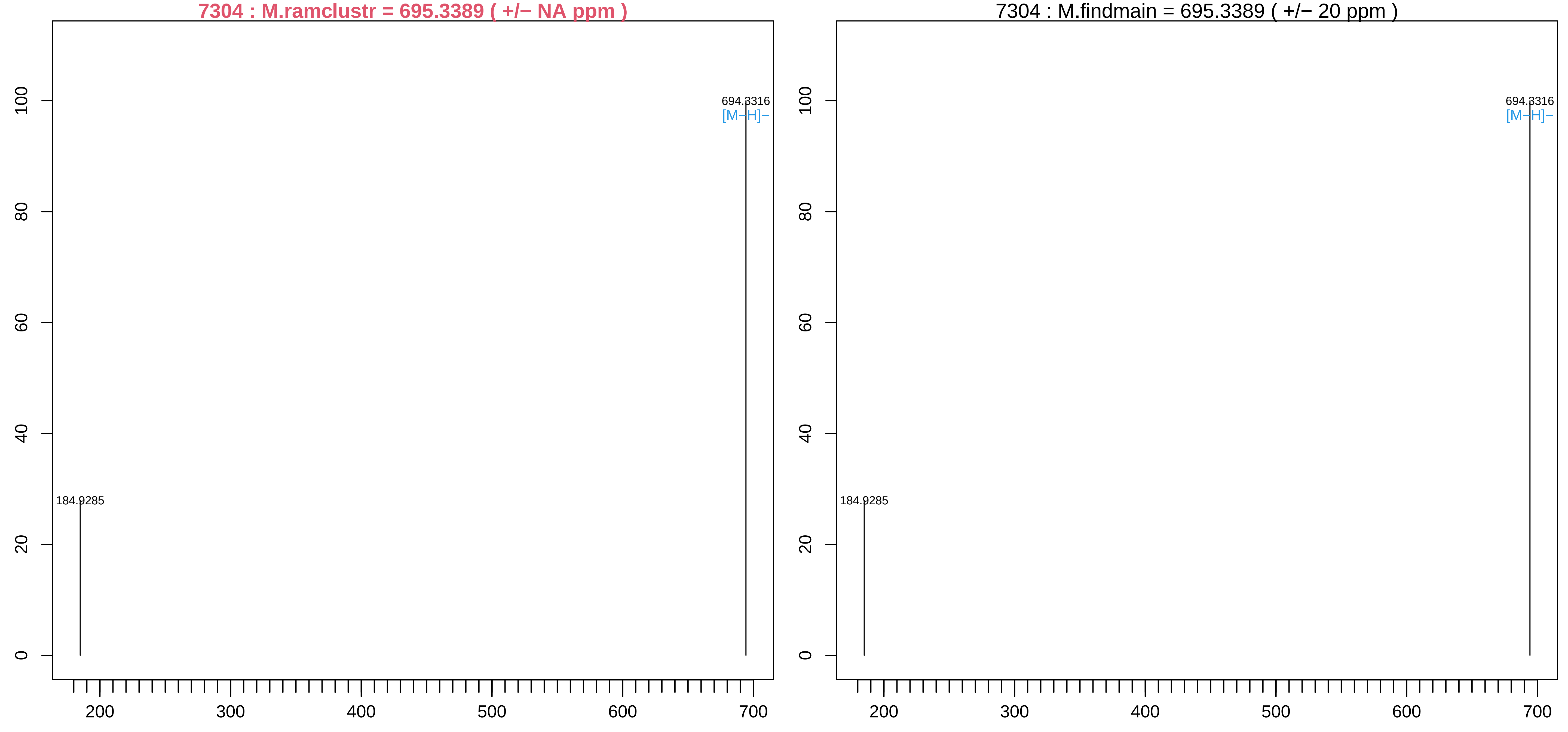
7304 : M.ramclustr = 695.3389 (+/− NA ppm)
694.3316
[M−H]−
184.9285
7304 : M.findmain = 695.3389 (+/− 20 ppm)
694.3316
[M−H]−
184.9285

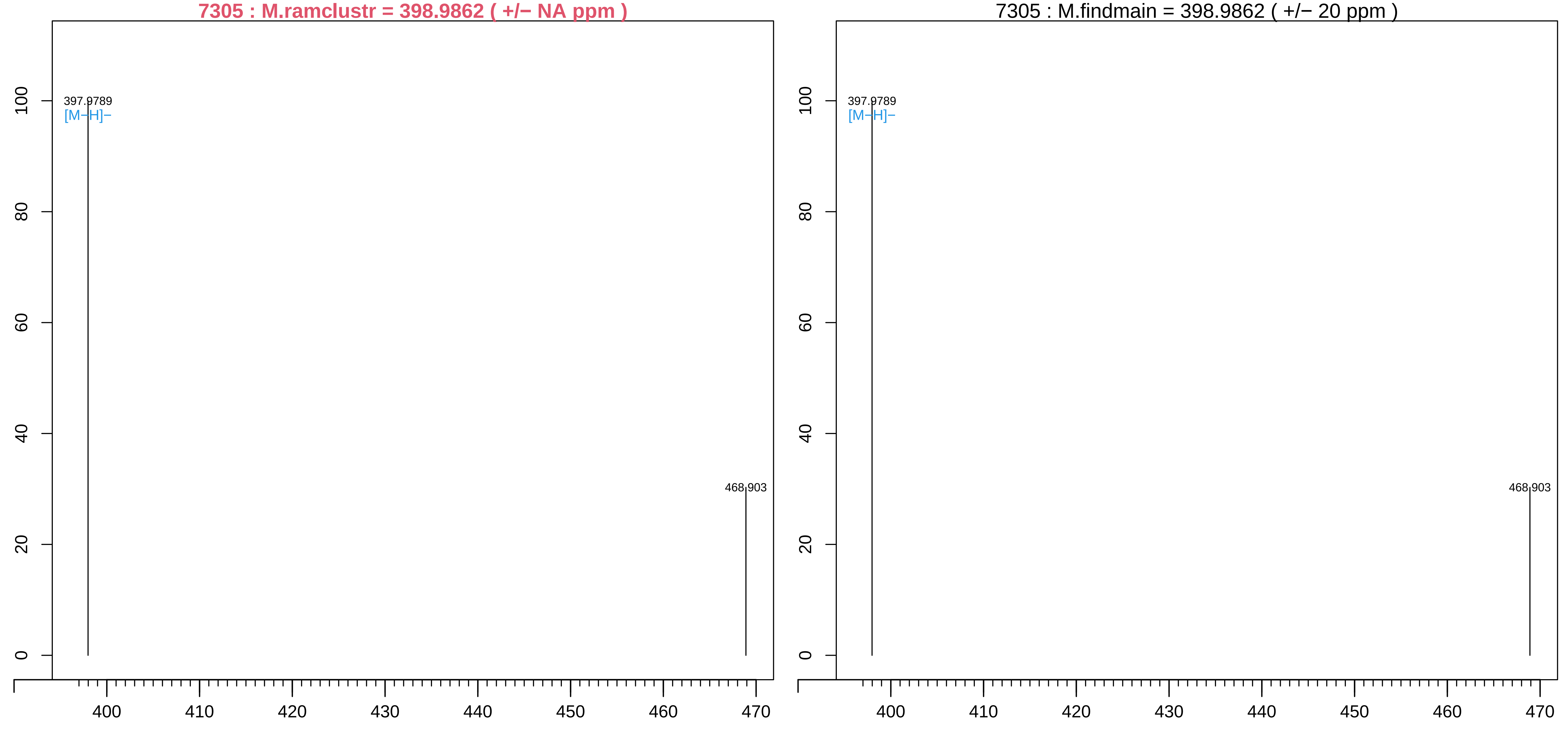
7305 : M.ramclustr = 398.9862 (+/− NA ppm)
397.9789
[M−H]−
468.903
7305 : M.findmain = 398.9862 (+/− 20 ppm)
397.9789
[M−H]−
468.903
0
20
40
60
80
100
400
410
420
430
440
450
460
470

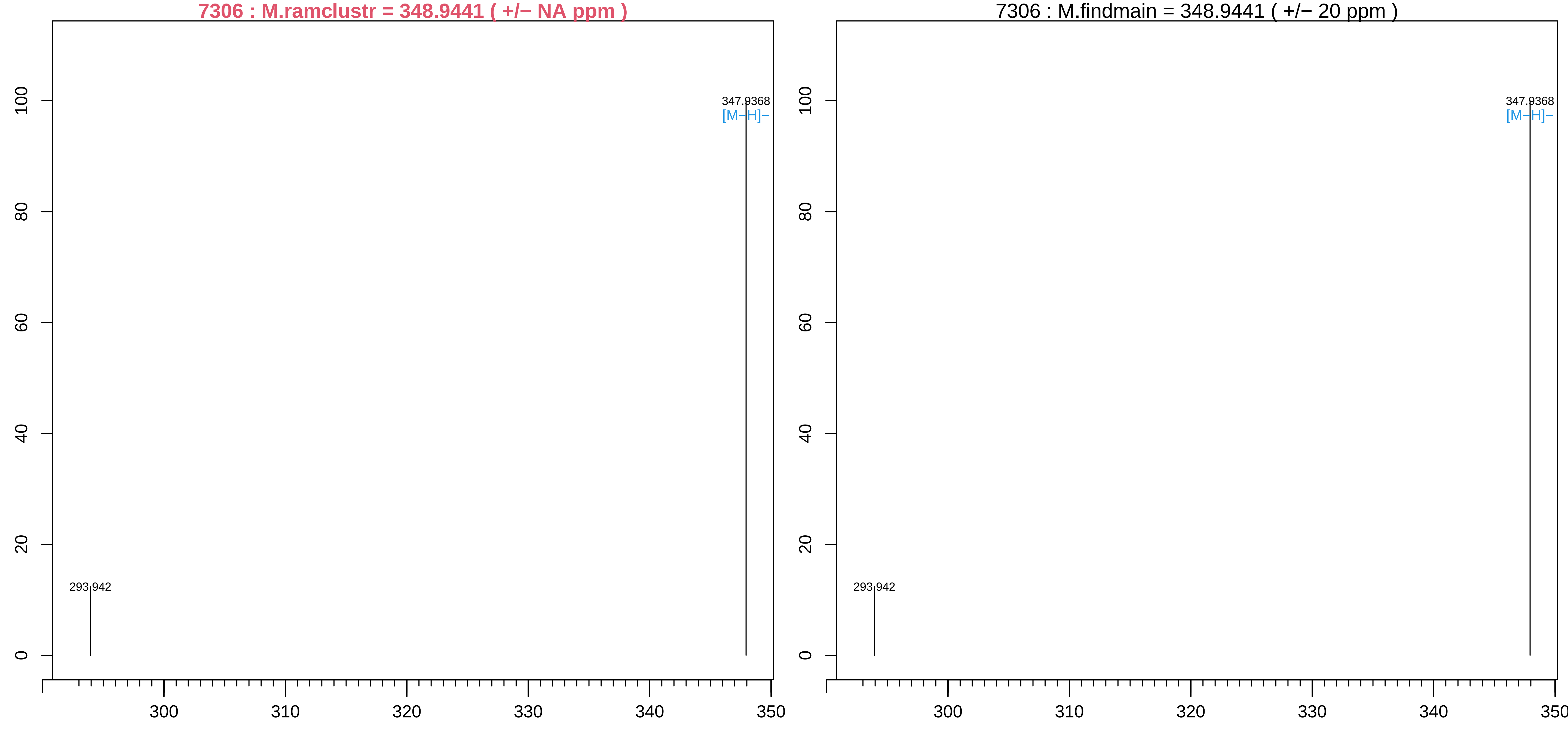
7306 : M.ramclustr = 348.9441 (+/− NA ppm)
347.9368
[M−H]−
293.942
7306 : M.findmain = 348.9441 (+/− 20 ppm)
347.9368
[M−H]−
293.942

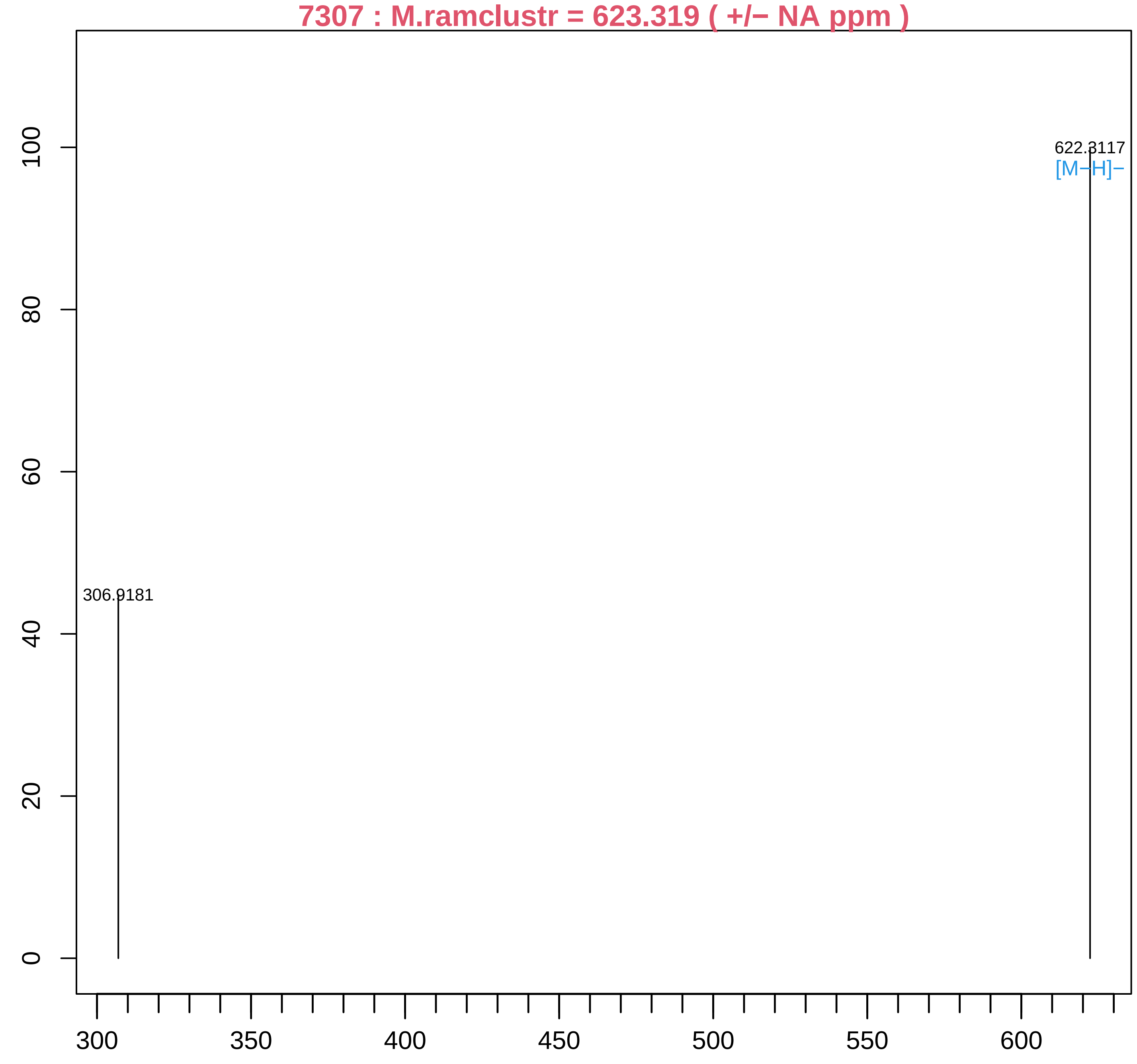
7307 : M.ramclustr = 623.319 (+/− NA ppm)
622.3117
[M−H]−
306.9181
0
20
40
60
80
100
300
350
400
450
500
550
600

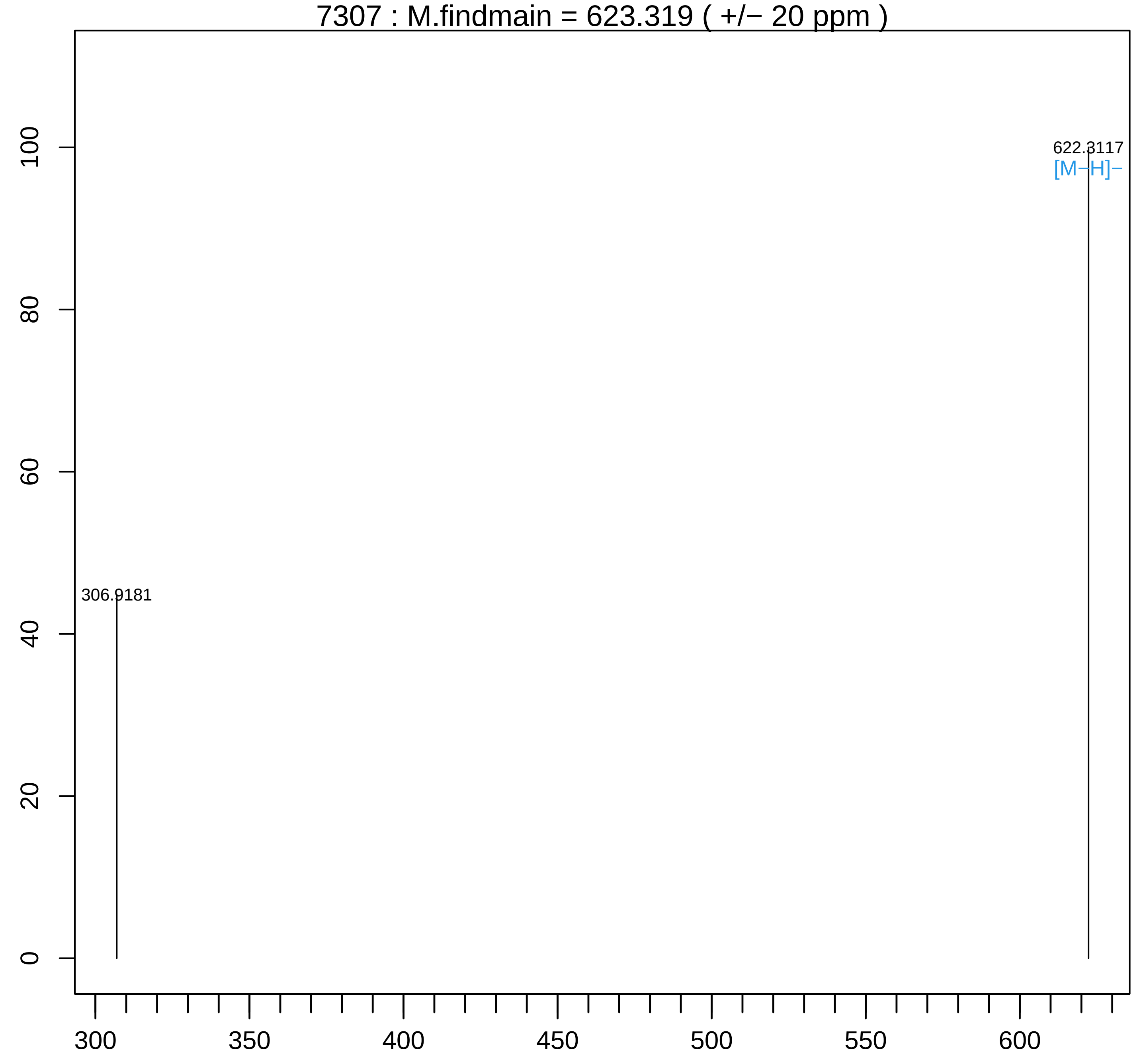
7307 : M.findmain = 623.319 (+/− 20 ppm)
622.3117
[M−H]−
306.9181
0
20
40
60
80
100
300
350
400
450
500
550
600

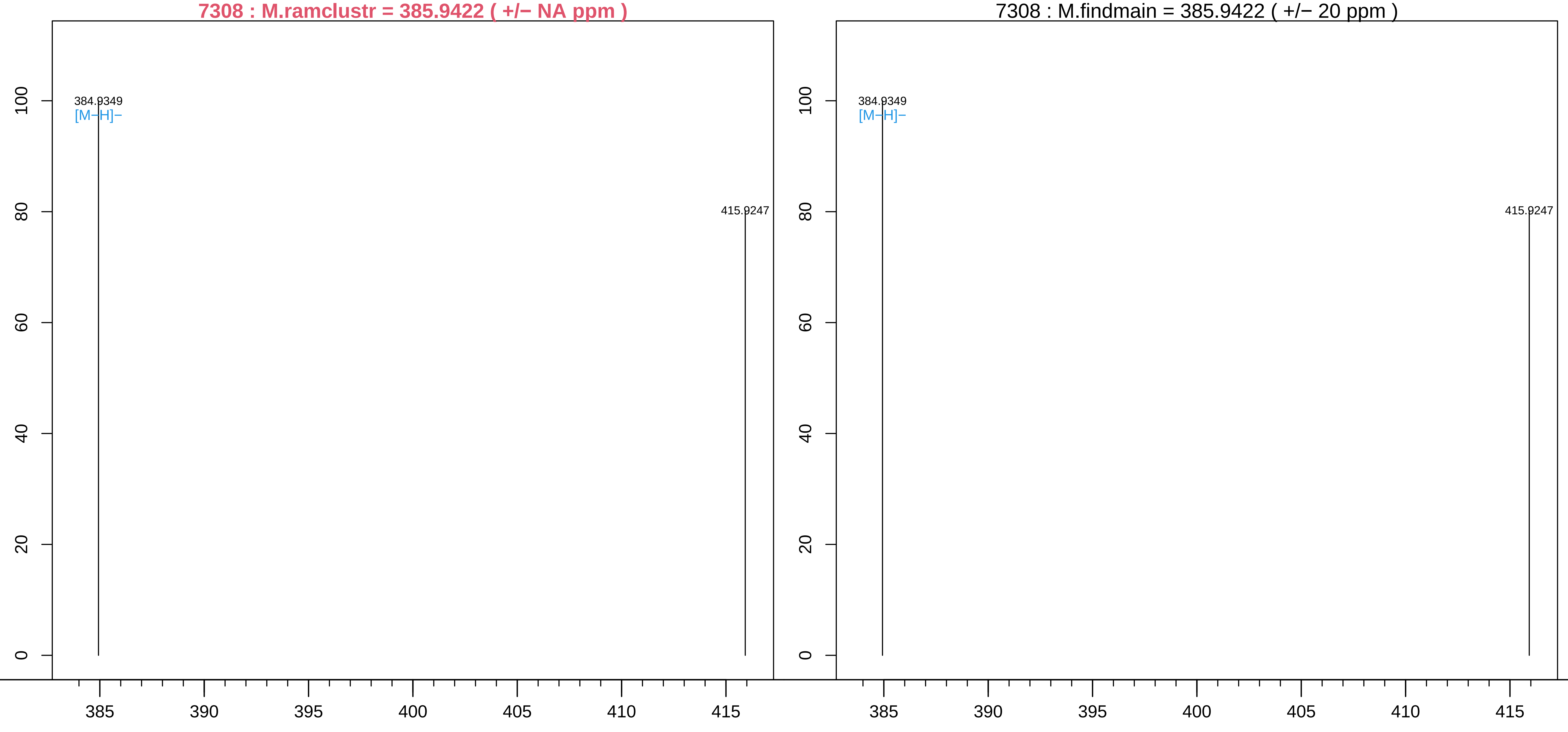
7308 : M.ramclustr = 385.9422 (+/− NA ppm)
384.9349
[M−H]−
415.9247
7308 : M.findmain = 385.9422 (+/− 20 ppm)
384.9349
[M−H]−
415.9247
0
20
40
60
80
100
385
390
395
400
405
410
415

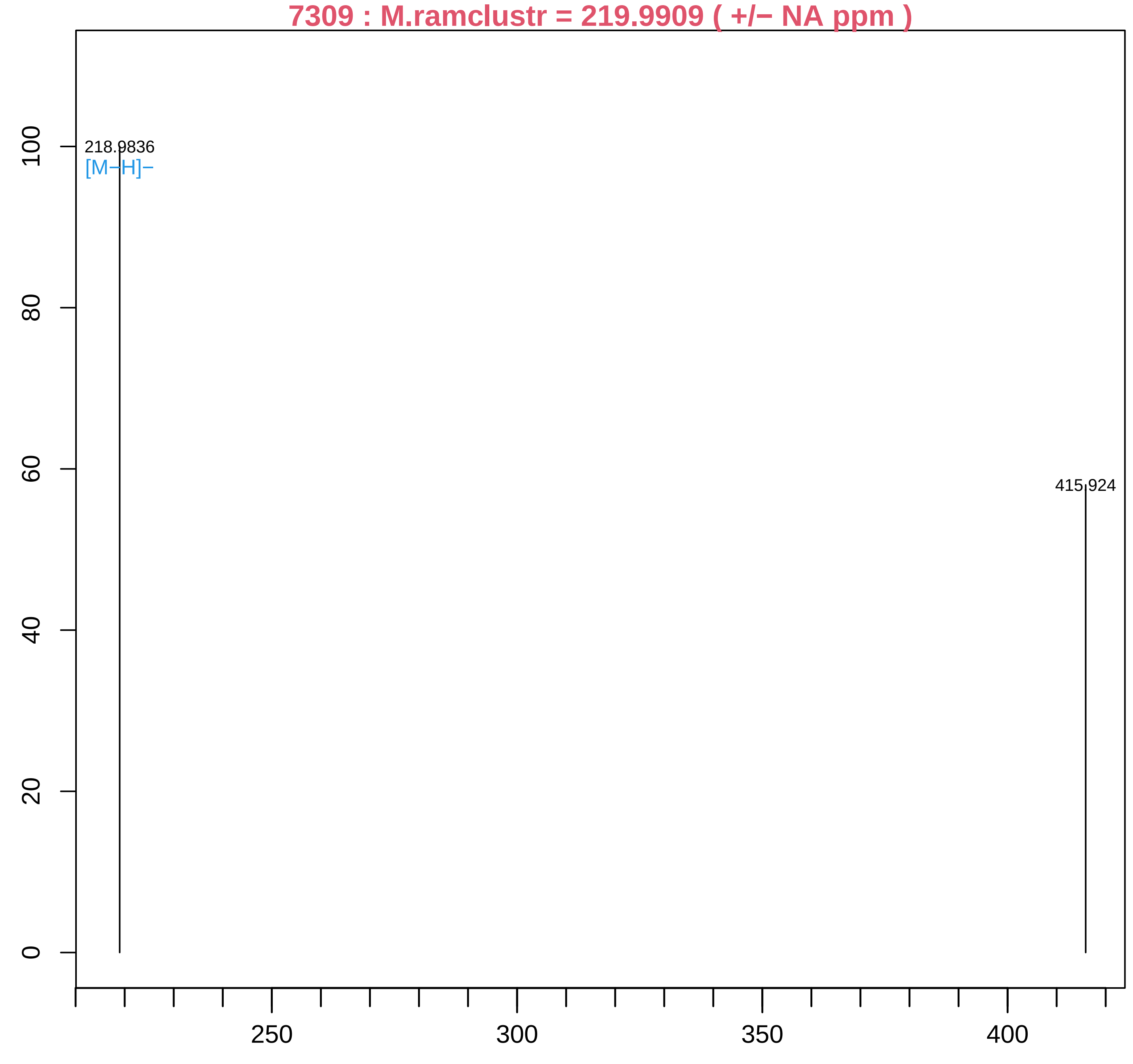
7309 : M.ramclustr = 219.9909 (+/− NA ppm)
218.9836
[M−H]−
415.924
0
20
40
60
80
100
250
300
350
400

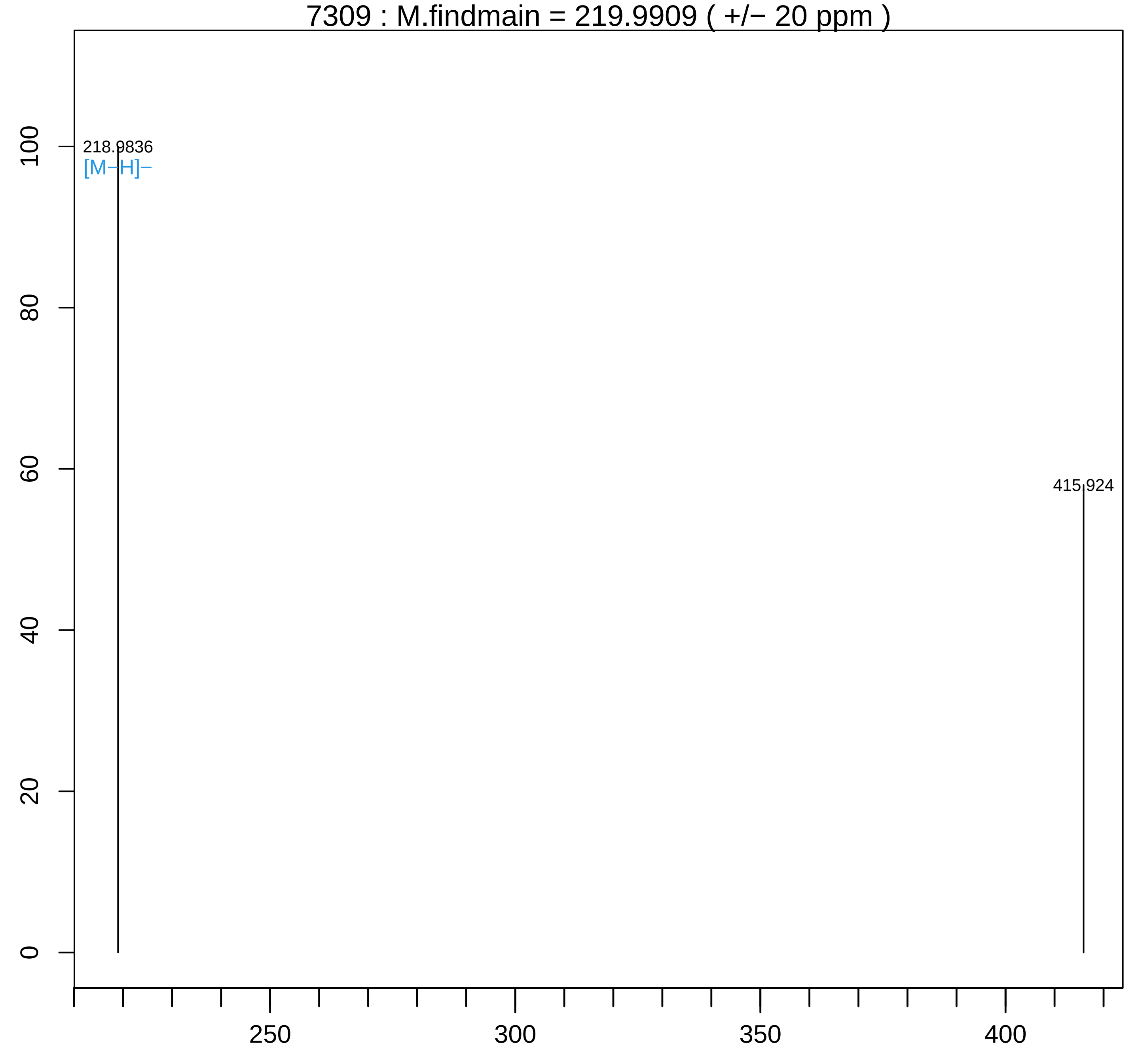
7309 : M.findmain = 219.9909 (+/− 20 ppm)
218.9836
[M−H]−
415.924
0
20
40
60
80
100
250
300
350
400

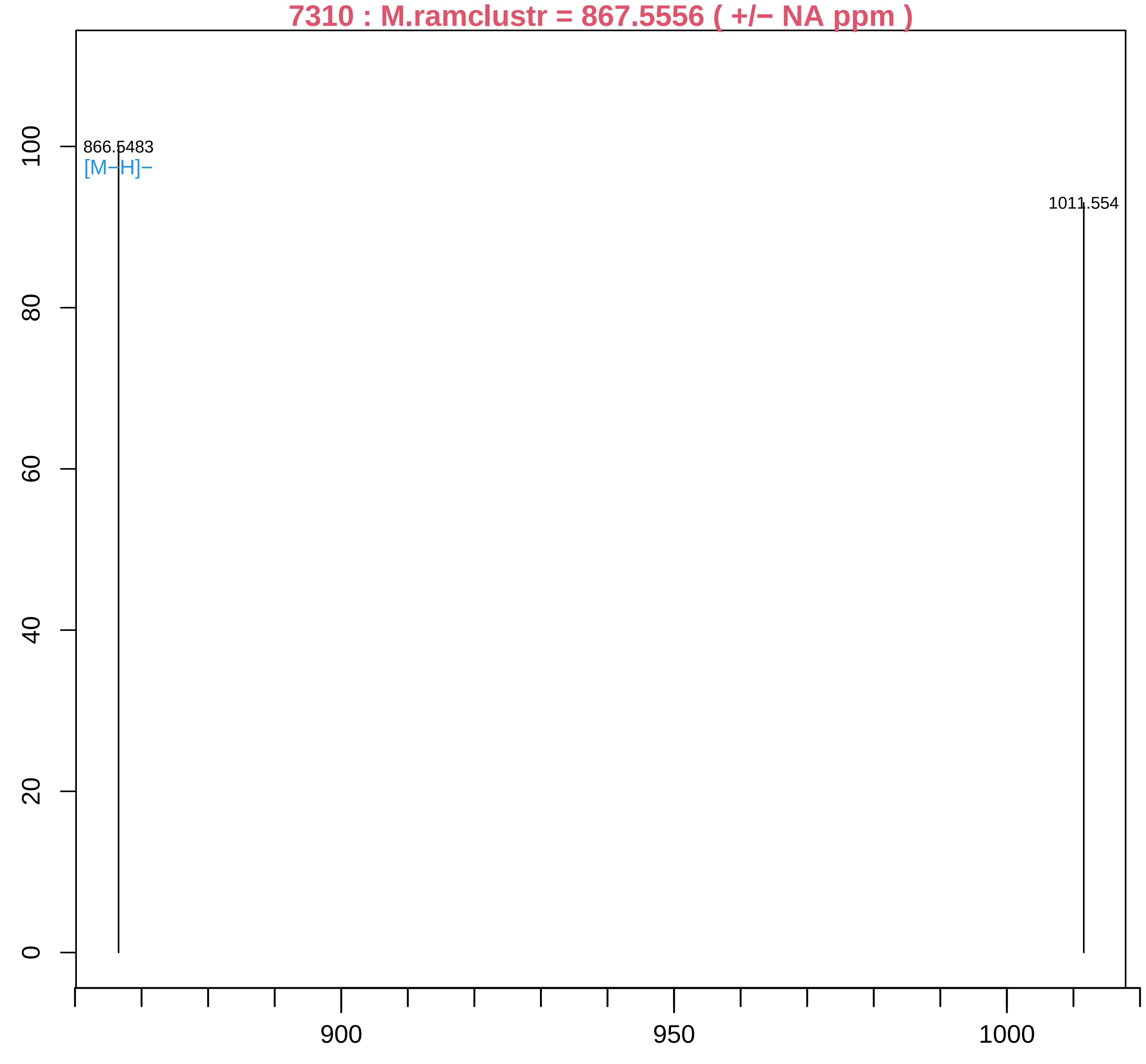
7310 : M.ramclustr = 867.5556 (+/− NA ppm)
866.5483
[M−H]−
1011.554
0
20
40
60
80
100
900
950
1000

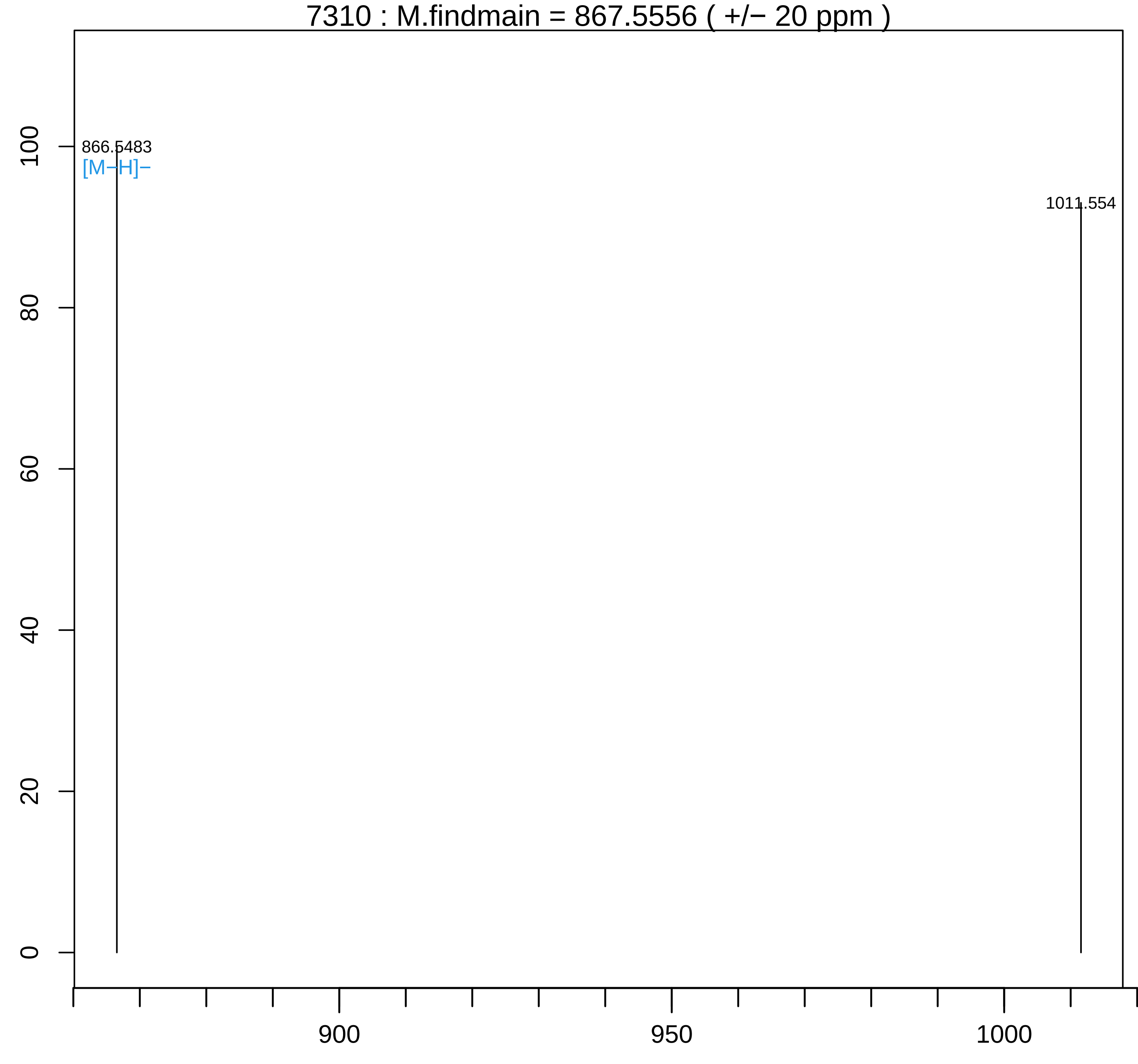
7310 : M.findmain = 867.5556 (+/− 20 ppm)
866.5483
[M−H]−
1011.554
0
20
40
60
80
100
900
950
1000
